# Multiomics analysis of the giant triton snail salivary gland, a crown-of-thorns starfish predator

**DOI:** 10.1038/s41598-017-05974-x

**Published:** 2017-07-20

**Authors:** U. Bose, T. Wang, M. Zhao, C. A. Motti, M. R. Hall, S. F. Cummins

**Affiliations:** 1 Faculty of Science, Health, Education and Engineering, Genecology Research Center, University of the Sunshine Coast, Maroochydore DC, Queensland 4558 Australia; 2 Australian Institute of Marine Science, Townsville, Queensland 4810 Australia

## Abstract

The giant triton snail (*Charonia tritonis*) is one of the few natural predators of the adult Crown-of-Thorns starfish (COTS), a corallivore that has been damaging to many reefs in the Indo-Pacific. *Charonia* species have large salivary glands (SGs) that are suspected to produce either a venom and/or sulphuric acid which can immobilize their prey and neutralize the intrinsic toxic properties of COTS. To date, there is little information on the types of toxins produced by tritons. In this paper, the predatory behaviour of the *C. tritonis* is described. Then, the *C. tritonis* SG, which itself is made up of an anterior lobe (AL) and posterior lobe (PL), was analyzed using an integrated transcriptomics and proteomics approach, to identify putative toxin- and feeding-related proteins. A *de novo* transcriptome database and *in silico* protein analysis predicts that ~3800 proteins have features consistent with being secreted. A gland-specific proteomics analysis confirmed the presence of numerous SG-AL and SG-PL proteins, including those with similarity to cysteine-rich venom proteins. Sulfuric acid biosynthesis enzymes were identified, specific to the SG-PL. Our analysis of the *C. tritonis* SG (AL and PL) has provided a deeper insight into the biomolecular toolkit used for predation and feeding by *C. tritonis*.

## Introduction

If a generalist predator evolves to a more a specialist diet, it is assumed that it would be accompanied by modifications of characters that permit greater efficiency in capturing specific prey species. These likely include foraging behaviors and search strategies with fine-tuned chemosensory systems, physiological processes associated with digestion and waste removal, and other features involved with prey capture^1^. Among predatory taxa the evolution of a specialized diet is likely to be strongly linked to the evolution of venoms used to subdue prey. Although the evolution of many of these characters may be difficult to trace for a species that has evolved a restricted diet, the evolution of venoms can be inferred from analyses of expression of genes encoding venom and digestion components^2, 3^.

Marine organisms from a broad range of phyla, from bacteria and algae to invertebrates and vertebrates, are known to produce toxins with high chemical diversity, divergence of which is likely driven by speciation and diet^4–6^. Some examples include tetrodotoxins produced by bacteria found in the saliva of the blue-ringed octopus and puffer fish^7, 8^, potent pore-forming toxins produced by cnidarians^9^, okadaic acid produced by bacteria in marine sponges^10^, and saponins (steroidal and triterpenoid) that are produced by echinoderms^11^. Venoms, and in particular specific toxins, play multiple roles from foraging to defense and intraspecific conflict^12^. Although there are many species of predatory marine snails, studies of venom toxins have been restricted to species within the superfamily Conoidea^13^. These venomous marine cone snails synthesize a remarkable diversity of pharmacologically active small peptides (conotoxins) to enable prey capture, self-defense and intra-specific competition^14–16^. The potency and selective profiles of the conotoxins vary depending on species targets that may include various subtypes of voltage- and ligand-gated ion channels, G protein-coupled receptors and neurotransmitter transporters^17^.

Gastropods of the superfamily Tonnoidea are effective predators and thought to capture prey through envenomation^18^. Within the Tonnoidea, several species in the Cassidae family of snails are specialist predators of echinoderms. The large paired and mono-lobed salivary gland (SG) of Cassidae family snails are known to be toxic and inject a strong acid or venomous saliva into their prey to paralyse them before consumption^19–21^. For example, Cassidae helmet shell snails secrete an acidic saliva to soften the body wall of their preferred prey, sea urchin^22, 23^, through which they bore a wide hole using their radula apparatus and long proboscis^22^. In *Cassidaria echinophora* and *Tonna galea* sulfuric acid is secreted through their proboscis^24, 25^. The saliva of *Cassis tuberosa* can immobilize the spines of sea urchin^18^. Within the Tonnidae group of marine snails, which also predate crustaceans and bivalve molluscs, large SGs have been identified that produce complex salivary secretions^26^.

The giant triton snail *Charonia tritonis* (superfamily Tonnoidea) is found on reefs throughout the Indo-Pacific where it predates upon echinoderms including starfish, sea cucumbers and sea urchins^27–29^. Giant tritons rely on their highly developed olfactory sense to track and locate prey^30, 31^. Upon contact with prey, they initiate a ‘tapping’ behaviour using their cephalic tentacles. Although the prey attempts to escape, the giant triton immobilizes it initially by mechanical means, positioning its large muscular foot over the aboral surface. This is rapidly followed by insertion of the proboscis and most likely injection of venom(s) that paralyses the prey^27, 30^. At least several species within the Ranellidae (Tonnidea) are known to produce sulphuric acid to access their prey^32^. The marine gastropoda, *Gyrineum natator* uses sulfuric acid to capture their bivalve prey and also use this acid for their defense^32^. The turban shell, *Lunella coronate* possesses sulphuric acid-producing glands which are used for their defense, to externally digest its accessed prey or to attack less accessible prey^33^. It has been proposed that the Atlantic triton snail (*Charonia variegate*) possesses toxins derived from its foot or mouth which may assist to immobilize prey^34^. In the knobbed triton snail (*Charonia lampas rubicunda*), the proboscis delivers a SG secretion that induces instant paralysis of the sea star *Patriella brevispina* ^35^. Recently a peptide venom was identified within the SG of the hairy triton snail, *Monoplex parthenopeus* (Subfamily Cymatiinae)^36, 37^. Conversely, another study on *C. lampas* feeding behavior found no evidence of the injection of either venom or acid^30^, although it should be noted that to date, SGs of *C. lampas* have not been analysed for the presence of such biomolecules^30^.

Based on feeding trials with *C. tritonis*, their preferred prey includes the crown-of-thorns starfish (COTS), *Acanthaster planci*, a corallivore asteroid that has contributed to mass coral loss throughout Indo-Pacific coral reefs^35^. However, *C. tritonis* are either naturally rare or endangered due to unregulated harvesting, with many countries now prohibiting their collection. Whatever the case, it has been proposed that the giant triton, as a major predator of COTS, has a role sin regulating the populations of COTS. For this reason, it is desirable to assess basic processes of their biology to assist in the potential development of captive breeding programs for release of giant tritons onto reefs infested with COTS. Consumption of COTS would require a special digestive strategy especially as COTS not only present a formidable physical defense in the form of sharp aboral spines but also contain and secrete various sulfated steroidal saponins and toxins^38^. Saponins are toxic and can deter their predators. Therefore, it is likely that giant triton snails have evolved specific traits to combat their effects^35^. Although an early study by Endean (1972) indicated that *Charonia* sp. uses venom to paralyze their prey, questions remain as to the chemical nature of these, and what role they play in the capture, immobilization and digestion of COTS^35^.

In this study, we describe the behaviour associated with *C. tritonis* COTS predation. Following an anatomical analysis of the *C. tritonis* SG, we have performed next-generation transcriptome sequencing and annotation of ensuing transcripts in association with proteomic analyses. We report for the first time the existence of numerous secreted proteins, including a diverse array of putative toxin- and feeding-like protein families in *C. tritonis*.

## Materials and Methods

### Triton behaviour in response to COTS

*C. tritonis* (N = 8) were collected from the Great Barrier Reef under special permit (G13/36390.1) and held in a 4,000 L indoor 4 m^2^ diameter holding tank at ambient temperature (26–28 °C) and salinity (32–35 ppt) with simulated natural photoperiod at the Australian Institute of Marine Science (AIMS). Water current in the tank (clockwise) was induced through airlifts via three 5 cm diameter PVC pipes with water intake at the base of the tank and expulsion through a 90 degree elbow at the surface. The giant tritons were periodically presented with live COTS, between 1 to 2 COTS per giant triton per week. General observations were made on the reaction of the giant tritons as well as the COTS and video recorded on GoPro over 8 h.

### Anatomy and tissue collection

For tissue collection, wild *C. tritonis* were collected from Kavieng, Papua New Guinea and temporarily held and fed on echinoderms at the Marine Research Station, Kavieng. Animals were anaesthetized with isotonic MgCl_2_ and the anterior portion removed from the shell. During the dissection the proboscis and SG were photographed using an iPhone 6 (8 MP, phase detection autofocus, dual-LED, Apple Inc. USA). For analysis of SG cell composition, the gland was spread onto a slide, then viewed and photographed using a Leica microscope equipped with a CCD camera. SGs were dissected and separated into the anterior lobe (AL) and posterior lobe (PL). Tissues collected for RNA isolation were stored in RNAlater (Ambion, California). For protein isolation, tissues were processed immediately as described below.

### RNA isolation, sequencing and transcriptome assembly

RNA was extracted from tissue using TRIzol Reagent (Invitrogen Corp., Carlsbad, CA, USA), as per the manufacturers protocol. Following extraction, RNA was assessed for quality by visualisation on a 1.2% agarose gel, and quantified using a Nanodrop spectrophotometer (Thermo scientific). Total RNA samples were sent to Australian Genome Research Facility (Brisbane, Australia) for library construction and sequenced (paired-end) using an Illumina HiSeq 2500 sequencing platform. Raw sequence reads (100 bp) were assembled into contigs (>200 bp) using the CLC genomics software (Qiagen). Protein coding regions were determined using the open reading frame (ORF) predictor^39^. Relative expression of genes in each tissue transcriptome was determined based on RPKM (Reads Per Kilobase of exon per Million mapped reads) values, utilizing the commercially available CLC Genomic Workbench 7 software^40^.

### Gene annotation, protein models and prediction of secreted proteins (exoproteome)

A BLASTp search was used to annotate proteins from each *C. tritonis* transcriptome. Schematic diagrams of protein domain structures were prepared using IBS illustrator (IBS, version 1.0) software^41^. Multiple sequence alignments were performed using the MEGA 6.0 platform with the ClustalW protocol and the Gonnet protein weight matrix^42^. SWISS-MODEL^43^ was used to predict the 3D protein structure of an echotoxin-like protein identified from giant triton SG^44^. First, BLASTp analysis was used to identify a template that shared significant sequence similarity to a *C. tritonis* echotoxin sequence. The best match was selected based on the presence of similar domains and plausibility quality control. Finally, based on the alignment, the coordinates of the model were constructed for the structurally conserved regions of the model. N-terminal signal sequences were predicted using the SignalP 4.1^45^, Predisi^46^ and TMHMM^47^. A protein was designated as secreted only when it met the criteria of both SignalP and Predisi, and did not have a transmembrane domain predicted by TMHMM. Simple Modular Architecture Research Tool (SMART) was used to identify conserved domains in SG proteins^48^. Glycosylation sites were identified by using NetNGlyc 1.0 server^49^.

### Protein isolation from salivary gland lobes and nanoHPLC-ESI-Triple TOF

Frozen samples of SG lobes were homogenized in protein extraction buffer (8 M urea, 4 M thiourea, 0.8 M NH_4_HCO_3_, pH 8.0) in a 1:5 w:v ratio. Crude extracts were then centrifuged for 20 min (12,000 xg, 4 °C), then supernatant was collected, fractionated by 1D SDS-PAGE and stained using Coomassie Blue (GE Healthcare, city). Gel bands were excised and digested with trypsin following the protocol described previously^50^. Before LC-MS analysis, Zip-tip C18 (Merck Millipore, USA) was used to desalt and concentrate peptides and small proteins.

Tryptic peptides were further analysed by liquid chromatography-tandem mass spectrometry (LC-MS/MS) on a Shimadzu Prominance Nano HPLC (Japan) coupled to a Triple-TOF 5600 mass spectrometer (ABSCIEX, Canada) equipped with a nano electrospray ion source. Aliquots (6 µL) of each extract were injected onto a 50 mm × 300 µm C18 trap column (Agilent Technologies, Australia) at 30 µL/min. The samples were desalted on the trap column for 5 min using solvent A [0.1% formic acid (aq)] at 30 µL/min. The trap column was then placed in-line with a 150 mm × 75 µm 300SBC18 3.5 µm analytical nano HPLC column (Agilent Technologies) for mass spectrometry analysis. Peptides were eluted with a linear gradient of 1–40% solvent B [90:10 acetonitrile: 0.1% formic acid (aq)] over 35 min at 300 nL/min flow rate, followed by a steeper gradient from 40% to 80% solvent B over 5 min. Solvent B was then held at 80% for 5 min to wash the column and then returned to 1% solvent B for equilibration before the next sample injection. The ionspray voltage was set to 2400 V, declustering potential (DP) 100 V, curtain gas flow 25, nebuliser gas 1 (GS1) 12 and interface heater at 150 °C. The mass spectrometer acquired 500 ms full scan TOF-MS data followed full scan (20 × 50 ms) product ion data in an Information Dependent Acquisition (IDA) mode. Full scan TOF-MS data was acquired over the mass range 350–1800 m/z and for product ion MS/MS 100–1800 m/z. Ions observed in the TOF-MS scan exceeding a threshold of 100 counts and a charge state of +2 to +5 were set to trigger the acquisition of product ion, MS/MS spectra of the resultant 20 most intense ions. The data was acquired and processed using Analyst TF 1.5.1 software (ABSCIEX, Canada).

Proteins were identified by database searching using PEAKS v7.0 (BSI, Canada) against the protein database built from the *C. tritonis* SG lobe transcriptomes. Search parameters were as follows: precursor ion mass tolerance, 0.1 Da; fragment ion mass tolerance, 0.1 Da; fully tryptic enzyme specificity with two possible missed cleavage sites; monoisotopic precursor mass; a fixed modification of cysteine carbamidomethylation; and variable modifications which included methionine oxidation, conversion of glutamine and glutamic acid to pyroglutamic acid, acetylation of lysine and deamidation of asparagine; false discovery rate (FDR) was set to ≤ 1%, and (−10*lgP) was calculated accordingly where P is the probability that an observed match is a random event.

## Results and Discussion

### *C. tritonis* hunting behavior

Aquatic invertebrates primarily rely on their olfactory sense to detect and locate potential prey^51^. The otherwise mainly sedentary COTS exhibited extreme agitation and movement when placed into the tank holding giant tritons. Similarly, sedentary giant tritons become active within a few minutes of exposure to the scent of COTS exhibiting a sweeping motion of both tentacles and forward movement. In a 4 m diameter tank with a clockwise water flow tritons would track the odour of COTS by moving counter-clockwise into the current even if the prey item was within 0.5 m of it down current. The COTS themselves are known to detect the odour of giant tritons and, rather than being normally sedentary, would exhibit pronounced movement^31^. A previous study had observed that *C. tritonis* hunting behavior (upon various echinoderm species, including COTS) was most prominent in the evening (57.1%), compared to morning (35.7%) and afternoon (7.1%)^52^. We observed that the entire hunt, attack and consumption of COTS could be completed within 4 h.

**Figure 1:**
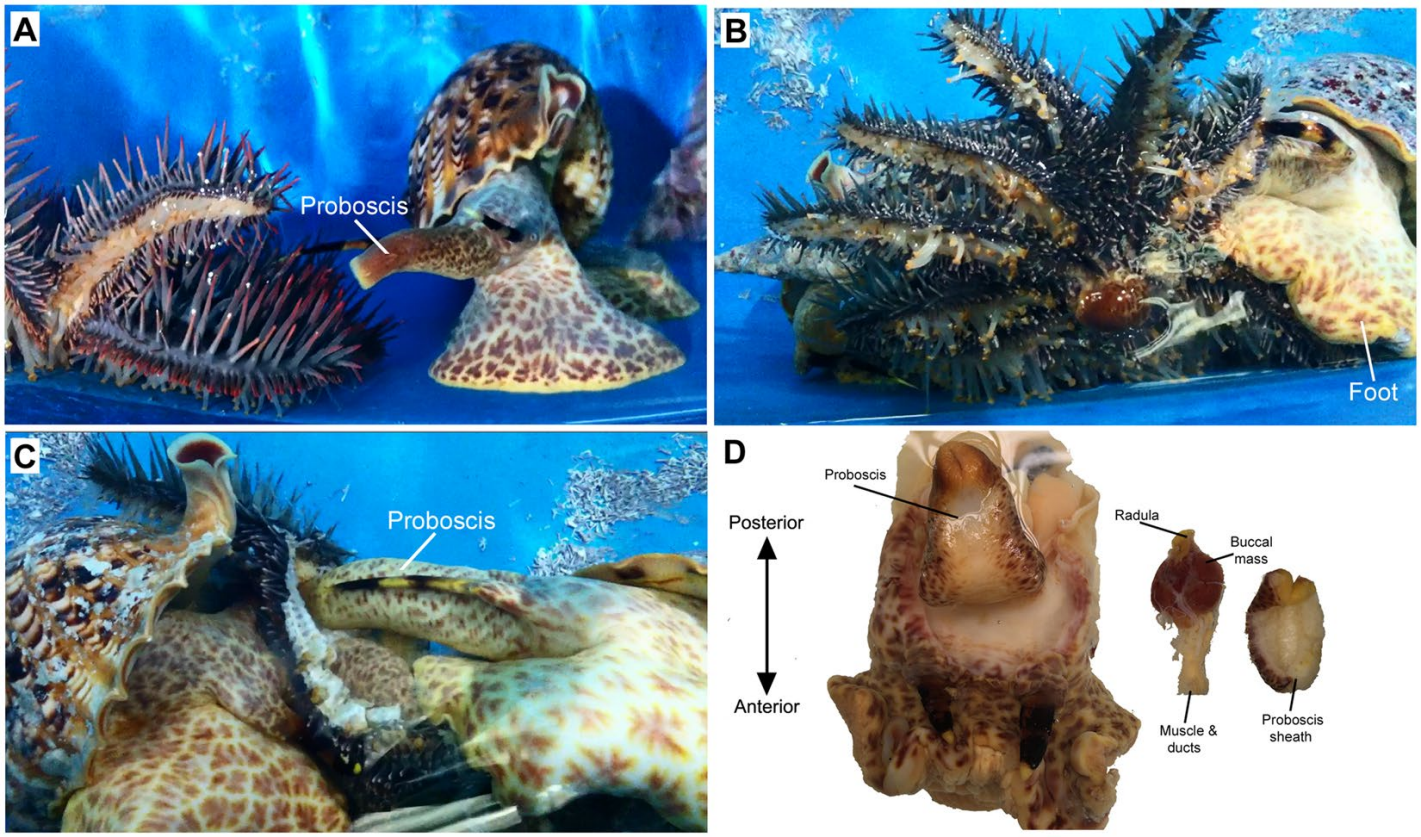
*Charonia tritonis* attack on a Crown-of-Thorns starfish (COTS). (**A**) *C. tritonis* withdraws proboscis in preparation for attack. (**B**) *C. tritonis* uses muscular foot to immobilize COTS. (**C**) Two *C. tritonis* feeding on a COTS, proboscis of the snail on the right penetrating COTS body wall. (**D**) Location of the proboscis, and internal organs including buccal mass, ducts and radula in *C. tritonis*.

Tritons were documented hunting, attacking and feeding on COTS (Supplementary Video S1). Upon initial contact with COTS, the snails’ large muscular foot covers the COTS arms to suppress movement. Simultaneously, *C. tritonis* retract then elongated their proboscis and moves it gradually around the base of the spines until insertion into the central disc area (Fig. 1A–C). It is possible that the proboscis seeks to cut the circumoral neural ring, as the COTS, while highly alarmed at this stage, become uncoordinated with no directional movement. At this point, the *C. tritonis* begin to saw into the COTS flesh using their radula. Closer inspection of the internal anatomy of the *C. tritonis* proboscis reveals the muscle, ducts, and buccal mass housing the radula at the proboscis tip (Fig. 1D). The radulae have been described in significant detail for other triton species, through scanning electron microscopy, showing the presence of cuspless marginal teeth and variations within the shape of central teeth^36^. Giant triton snails produce a prolific amount of mucus during the attack and consumption of the COTS, which may be associated with sequestration and possibly detoxification of saponins released by COTS or to absorb saponins before they reach the interior of the shell and make contact with the gills. Saponins readily cross the gills of fish and lyse red blood cells causing respiratory distress and in high enough concentration can cause death^53^. The terrestrial slug, *Arion lusitanicus*, has been shown to sequester and detoxify alkaloids from a variety of plants^54^. Given that giant tritons are not only exposed to secreted saponins during the attack but also ingest them in high concentrations, it is likely that they can metabolise saponins. Metabolic pathways for saponin detoxification mechanisms have been described for plant-fungi interactions, where the fungi contains genes that encode enzymes that break down plant saponins, leading to disease resistance^55, 56^.

### *C. tritonis* salivary gland anatomy and transcriptome assembly

**Figure 2:**
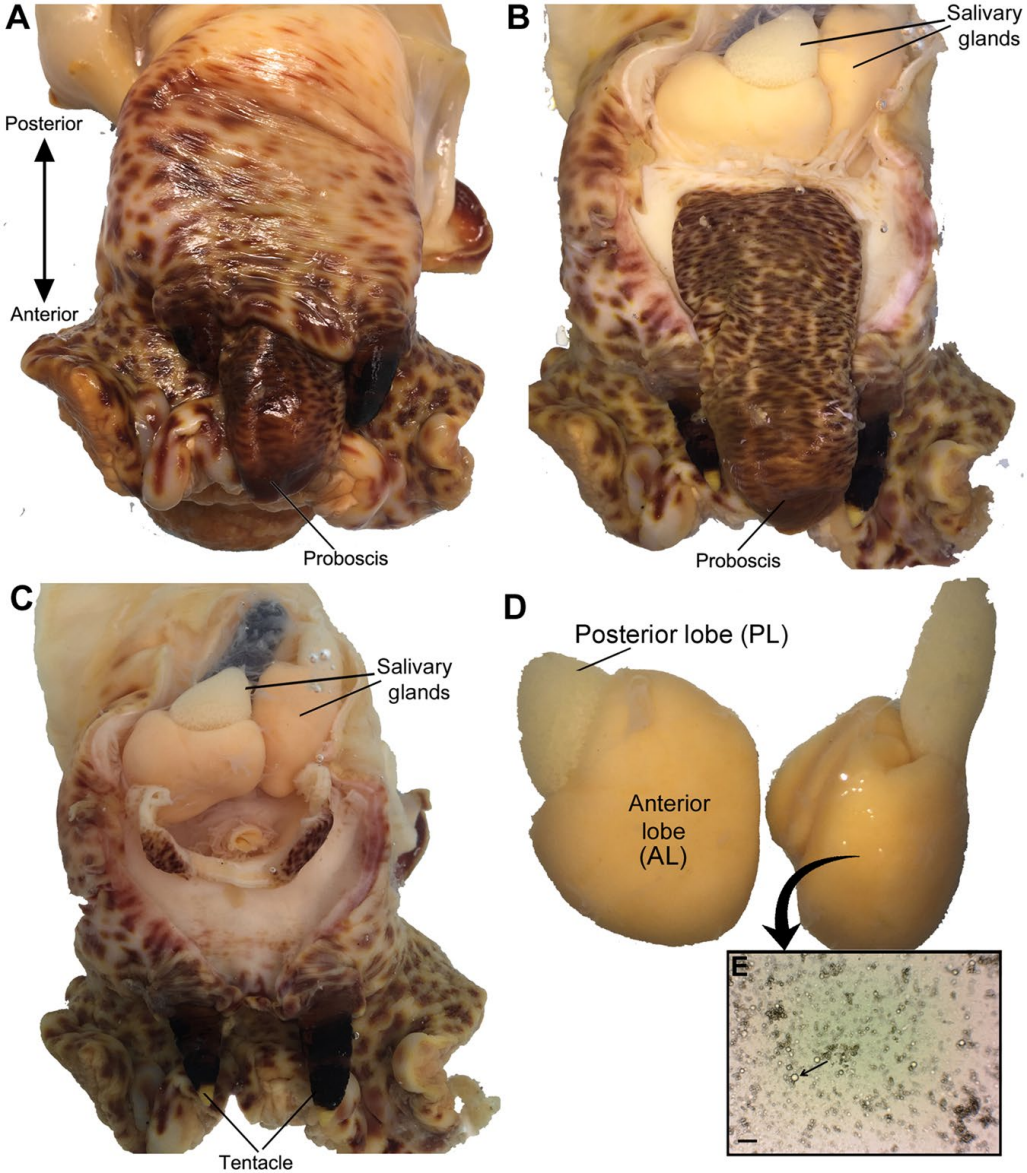
*Charonia tritonis* salivary gland (SG) anatomy and proteomics study. (**A**) Cephalic region of the *C. tritonis*. (**B**) Cephalic region with full proboscis and paired SGs exposed. (**C**) Cephalic region with proboscis removed. (**D**) Isolated SG showing region of anterior lobe (AL) and posterior lobe (PL). (**E**) Cell smear of AL. Arrow shows prominent mucin-like globlet cell. Scale bar represents 200 μm.

In those Tonnoidea investigated to date, SGs are paired, with each gland divided into two parts: the smaller tubular or acinous anterior lobes (AL) and the voluminous posterior lobes (PL) that putatively secrete sulphuric acid^19, 24, 25, 36^. Their size, shape and structure may vary significantly between genera and those species within^36^. The *C. tritonis* paired SGs were identified within the region of the foregut, similarly split morphologically into anterior and posterior lobes; the AL is larger and has an orange appearance, while the PL is smaller and white (Fig. 2A–D). This is in contrast to what is observed in other Tonnoidea, where the PL is the larger lobe. The histology of the SG has been described for two Tonnoidea, the *Argobuccinum pusulosum* and *Monoplex intermedius*, showing a posterior salivary duct entering the inside of the anterior lobe^21, 57^. In this study, no histology was performed, however, a cell smear of the AL revealed a mixture of cells, with large and clear cells supported by mucin-like molecules being the most prominent (Fig. 2E). In *C. intermedius*, the fine structure analysis of the salivary glands has revealed posterior acid secreting and acinous anterior lobes^21^. Transverse sections through the posterior SG of the *C. lampas* show many cells that appear to contain a basophilic mucus^30^.

Raw sequence RNA-seq reads were obtained for both AL and PL of the *C. tritonis* SG using Illumina technologies (NCBI PRJNA383875), then assembled into transcriptome library contigs. In the SG-AL, there were 84,807,390 total filtered reads assembled into 105,955 contigs, 105,297 ORFs (Supplementary Data S1) and 18,729 ORFs that have a BLASTp match to a known protein (Supplementary Data S2a). Likewise, in the SG-PL there were 81,597,308 total filtered reads assembled into 115,171 contigs, 114,289 ORFs (Supplementary Data S3) and 19,005 ORFs that have a BLASTp match to a known protein (Supplementary Data S4a).

#### *In silico* exoproteome prediction and proteomic analysis

**Figure 3:**
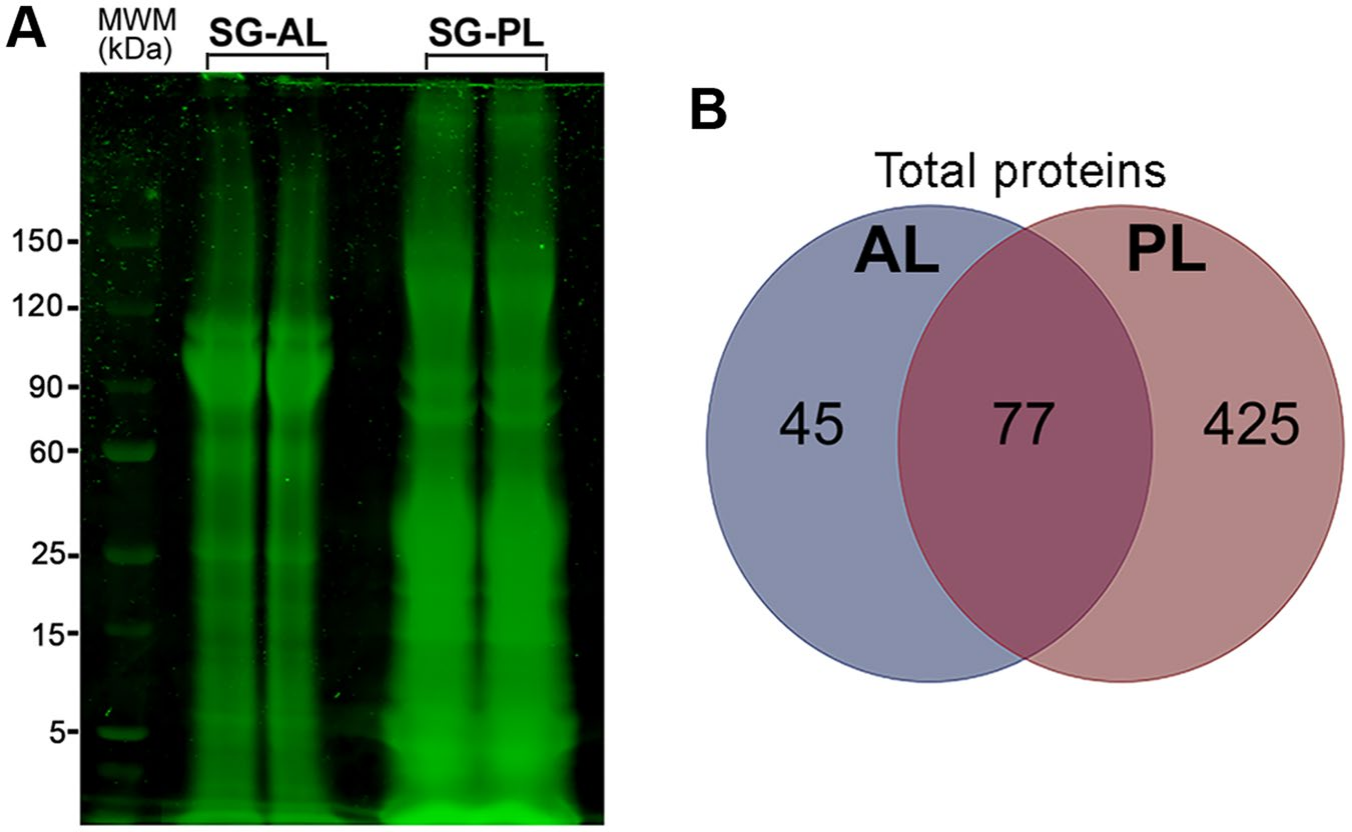
Proteomics analysis of *Charonia tritonis* anterior lobe (AL) and posterior lobe (PL) of the salivary gland (SG). (**A**) SDS-PAGE and Coomassie stain of extracts derived from the SG-AL and SG-PL. (**B**) Comparison of total proteins identified in extracts of SG-AL and SG-PL, based on Pfam analysis.

An *in silico* analysis was performed to identify putative SG secreted proteins, which predicted that the SG-AL and SG-PL secrete 3,805 and 3,860 proteins, respectively; all contain signal peptides and no transmembrane domains (Supplementary Data S2b and S4b). Due to technical limitations, proteins were extracted from crude intact SG-AL and SG-PL, rather than performing gland ‘milking’ which is now recognized as a more efficient method for obtaining cone snail venom^58^. Crude protein extracts were initially separated by SDS-PAGE and viewed by Coomassie staining, demonstrating the presence of a large number of proteins with a high-low molecular weight distribution (Fig. 3A). Bands that were extracted from fractionated SG-AL revealed 191 proteins (Supplementary Data S3c). Further annotation (BLASTp, signal peptide, transmembrane domain) indicated that 27 of these proteins have BLAST matches and features typical of secreted proteins. (Supplementary Data S2d and Supplementary Data S5). All of these proteins have a homolog within the *C. reticulate* salivary transcriptome. Fractionated SG-PL extracts revealed 775 proteins (Supplementary Data S4c), including 59 proteins that have features consistent with being secreted (Supplementary Data S4d and Supplementary Data S6). Four of these proteins do not have any homology with any derived protein within the *C. reticulate* salivary transcriptome. All total proteins identified could be placed into Pfam categories (protein family database): 122 and 502 Pfam families were identified from SG-AL and SG-PL, respectively, with 77 families common to both (Fig. 3B).

Supplementary Data S7 provides a summary of the MS analysis of the SG-AL and SG-PL extracts. Proteins that were highly represented in MS analysis for either or both the SG-AL and SG-PL, are shown in Table 1. In the *C. tritonis* SG-PL, the most highly represented protein was the lectin L6-like protein. Glycan-binding proteins, commonly known as lectins, play a crucial role in innate (and adaptive) immunity. Binding of potential pathogens by lectins leads to phagocytosis, complement activation, and antigen processing but also to regulation of adaptive immune functions^59, 60^. In addition to their role in pathogen recognition, some lectins act as direct defense effectors by intoxicating the antagonist upon binding^61^. In the SG-AL, the most highly represented protein was aminopeptidase N, suggesting high abundance in this lobe. Aminopeptidase N is widely distributed in both plant and animal species where it is known to selectively break down amino acids from the amino-terminus of proteins or oligopeptides^62^. In animals, this enzyme is most abundant along the brush border membrane of the intestines, facilitating the digestion of ingested protein^63^. However, in the midgut of insects, the enzyme can act as a receptor binding to toxin proteins produced by viruses and bacteria^64–66^.

**Table 1:** Proteins identified in the anterior lobe (AL) and posterior lobe (PL) of the *Charonia tritonis* salivary gland (SG). (✓) Represents identified, and () represents not identified. Numbers represent number of peptide sequences from the proteomic analyses that match to the transcriptome.

AL	PL	AL	PL	Best BLAST match	AL	PL	AL	PL	Best BLAST match
Transcriptome		Proteome			Transcriptome		Proteome		
✓	✓		21	4-hydroxyphenylpyruvate dioxygenase	✓	✓		28	Filamin-A-like isoform X1
✓	✓		20	4-hydroxyphenylpyruvate dioxygenase	✓	✓		25	Filamin-C-like isoform X1
✓	✓	14	✓	78 kDa glucose-regulated		✓		51	Fish-egg lectin-like
✓		13	✓	A disintegrin and metalloase with thrombospondin motifs 18	✓	✓		19	Fructose-bisphosphate aldolase 1
✓	✓	29	✓	A disintegrin and metalloase with thrombospondin motifs 20-like	✓	✓		18	Fructose-bisphosphate aldolase 1
✓	✓		42	Actin	✓	✓		17	Fructose-bisphosphate aldolase 1
✓	✓		38	Actin	✓	✓	58		Galactose-1-phosphate uridylyltransferase
✓	✓		36	Actin	✓	✓	21	17	GLIPR1 1
✓	✓	14		ADAM family mig-17-like	✓	✓	20	17	GLIPR1 1
✓	✓		19	Advillin	✓	✓		19	Glyceraldehyde-3-phosphate dehydrogenase
✓	✓		19	Advillin	✓	✓		19	Heat shock 70
✓	✓		17	Alpha-enolase isoform X1	✓	✓		18	Heat shock 70
✓	✓		16	Alpha-enolase isoform X1	✓	✓		57	Hemocyanin isoform 1
✓	✓	54		Aminopeptidase N	✓	✓		38	Hemocyanin isoform 1
✓	✓	51		Aminopeptidase N	✓	✓		33	Hemocyanin isoform 1
✓	✓	28		Aminopeptidase N	✓	✓		38	Hemocyanin isoform 2
✓	✓	26		Aminopeptidase N	✓		33		Hemorrhagic metallo ase-disintegrin-like kaouthiagin isoform X1
✓	✓	20		Aminopeptidase N	✓	✓	25		Hepatic lectin-like
✓	✓	18		Aminopeptidase N	✓		15		Hypothetical protein LOTGIDRAFT_174864
✓	✓	17		Aminopeptidase N	✓	✓		59	Lectin L6-like
✓	✓	16		Aminopeptidase N	✓	✓		18	Lectin L6-like
✓	✓	13		Aminopeptidase N	✓	✓		18	Lectin L6-like
✓	✓	13		Aminopeptidase N	✓	✓	21		Mast cell carboxypeptidase A
✓	✓	100		Aminopeptidase N isoform X2	✓		13		MAX gene-associated
✓	✓	24		Aminopeptidase N-like	✓	✓		20	MAX gene-associated
✓	✓	23		Aminopeptidase N-like	✓	✓	75		Mucin-19-like isoform X3
✓	✓	17		Aminopeptidase N-like	✓	✓		25	Myosin heavy chain
✓	✓	17		Arylsulfatase B	✓	✓		22	Myosin heavy chain
✓	✓	20	44	Arylsulfatase B-like	✓	✓		34	Myosin heavy chain isoform A
✓	✓	15	58	Arylsulfatase B-like	✓	✓		25	Neuroendocrine convertase 1
✓	✓	14		Arylsulfatase B-like	✓	✓		19	Neuroendocrine convertase 1
✓	✓		24	Cartilage matrix -like	✓	✓		22	Paramyosin
✓	✓		21	Cartilage matrix -like	✓	✓		21	Paramyosin
✓	✓		20	Cartilage matrix -like	✓	✓		26	Paramyosin-like isoform X2
✓	✓		15	Cartilage matrix -like	✓	✓	20		Probable palmitoyltransferase ZDHHC4
✓	✓		15	Cartilage matrix -like	✓	✓	26		Probable serine carboxypeptidase CPVL
✓		20		Cholinesterase	✓	✓	19		Probable serine carboxypeptidase CPVL
✓		13		C-type lectin domain family member A isoform X1	✓	✓	40		Retinal degeneration B-like isoform X1
✓	✓	29		Cysteine-rich secretory Mr30	✓	✓	30		Ribonuclease Z
✓	✓	23		Cysteine-rich secretory Mr30	✓	✓	30		Ribosomal S6 kinase 2 alpha
✓	✓		18	Disulfide isomerase	✓		18		Serine protease 33-like isoform X2
✓	✓	18	61	Endoglucanase 4-like	✓	✓		26	Tropomyosin
✓	✓	17	71	Endoglucanase A-like	✓	✓		17	Tubulin beta-4B chain
✓	✓	28		Endoplasmic reticulum aminopeptidase 1	✓	✓		17	Twitchin isoform X18
✓	✓	63		Endoplasmic reticulum aminopeptidase 2	✓	✓	15		Venom serine carboxypeptidase
✓	✓		51	Filamin-A-like isoform X1	✓		24		Venom serine carboxypeptidase CPVL
✓	✓		30	Filamin-A-like isoform X1	✓		23		Venom serine carboxypeptidase CPVL
					✓		26		Zinc metallo ase-disintegrin-like VLAIP-A

The hemorrhagic metalloprotease disintegrin-like proteins and cysteine-rich secretory Mr30 were also highly represented in our MS analysis (Table 1). Hemorrhagic metalloprotease disintegrin-like proteins have been isolated from snake venom where have been shown to inhibit the process of collagen- and ADP-induced platelet aggregation^67^, break-down of coagulation factors and the initiation of apoptosis^68^. The cysteine-rich secretory Mr30 has been identified from *Conus* snails and reported to have similar properties to Tex31, a protease responsible for processing of pro-conotoxins^69, 70^. The *C. tritonis* SG does not contain conotoxins, yet the cysteine-rich secretory Mr30 may help to pre-process other types of pro-toxin proteins, including the venom serine carboxypeptidase, a member of the peptidase S10 family. This enzyme is known to have various physiological functions, including enhancing massive release of histamine, degradation of neurotransmitters, mediation of immunity-related processes, phosphorylation of venom proteins, and influencing the nervous system as a neurotoxic polypeptide^71^. The biological role of this peptidase in *C. tritonis* venom is unclear, but given that members of the peptidase S10 family are active at acidic pH^72^, they may perform their function in the venom before it is injected into the prey.

Arylsulfatase hydrolyses the sulfate group of ingested biomolecules, such as glycosaminoglycans (GAGs), which are large sugar molecules^73, 74^. In molluscs, arylsulfatase activity has been reported in various tissues of ivory barnacles (*Balanus eburneus*), including the mantle, suggesting a probable relationship between arylsulfatase activity and the cyclic formation and hardening of the exoskeleton^75^. In snails of the family Muricidae, arylsulfatase is found in the hypobranchial gland where it plays a role in Tyrian purple biosynthesis^76^; it helps in the conversion of tyrindoxyl sulphate into the biologically active precursors of Tyrian purple^77, 78^.

**Figure 4:**
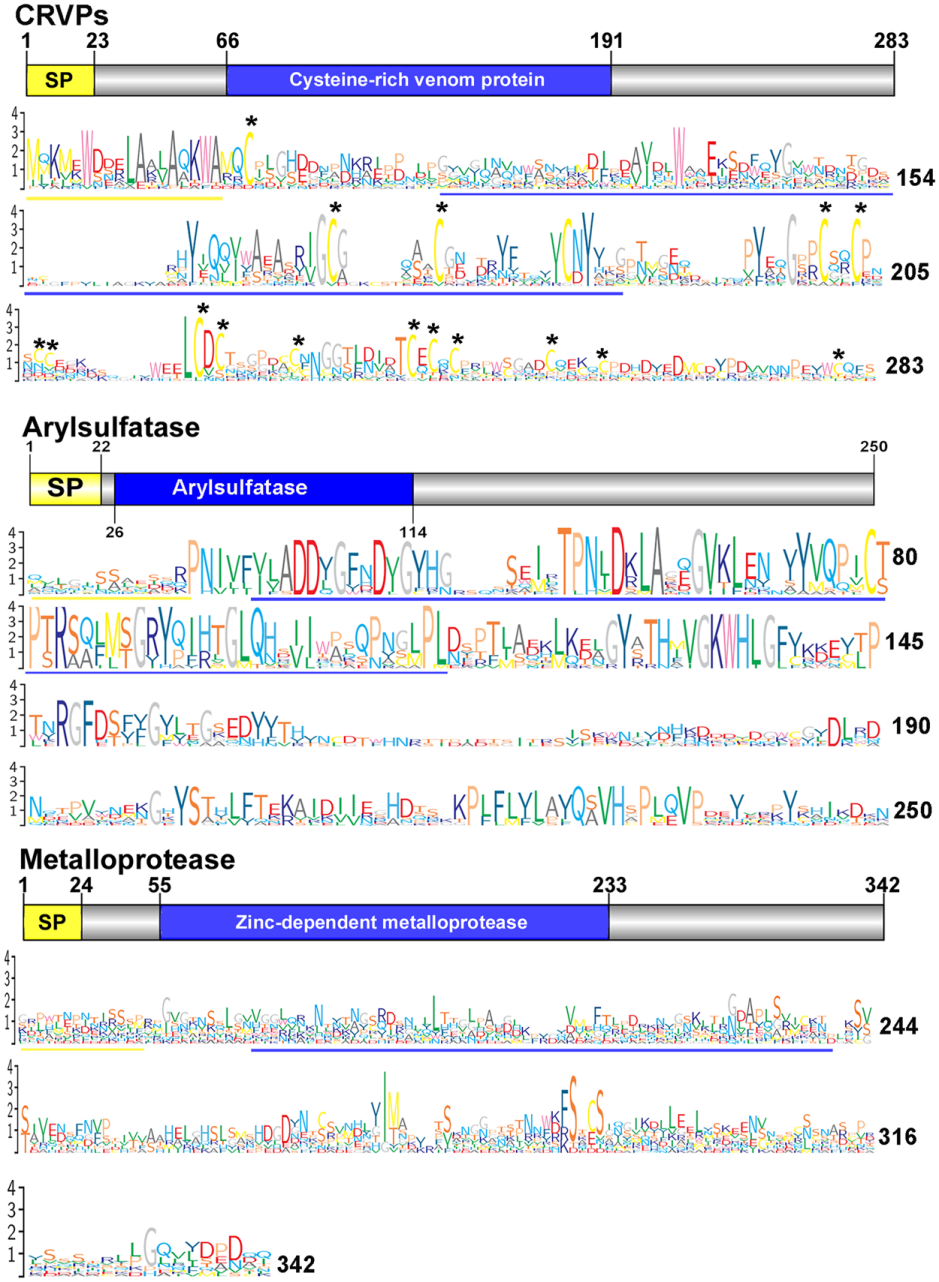
Molecular characterisation of *Charonia tritonis* cysteine-rich venom proteins (CRVPs), arylsulfatase and metalloprotease. Schematic diagram (top) showing the general organization with signal peptide (SP) and conserved domains (blue). Sequence logo representation (below) of multiple sequence alignments for *C. tritonis* with other species (Supplementary Data S8). Asterisks represent site of conserved cysteine (C) residues. Region of signal peptide (yellow) and conserved domain (blue) are shown.

A single arylsulfatase was identified in the *C. tritonis* SG-AL through proteomic analysis, although another 29 transcripts were present in the transcriptomes that encode for arylsulfatases. No arylsulfatase proteins were identified in the SG-PL; however, 36 transcripts were identified that encode for arylsulfatases. This finding provides further evidence that arylsulfatase genes are encoded within the animal’s genome, rather than being obtained from dietary sources or through symbiotic microorganisms, as has been suggested from other molluscan studies^79, 80^. It is speculated that SG arylsulfatase is involved in the breakdown of prey biomolecules during feeding. Echinoderms are well known to contain a diverse array of saponin molecules (to deter predators, parasites and microbes) including sulfated saponins; this subclass may be neutralized or broken down by arylsulfatase-mediated hydrolysis depending on the position of sulfation on either the aglycone or sugar moiety^81^. *C. tritonis* arylsulfatases contain all conserved arylsulfatase domains, and besides a variable signal sequence, the mature enzyme is highly conserved with arylsulfatases from five other species (*Aplysia californica*, *Octopus bimaculosides*, *Helix pomatia*, *Biomphalaria glabrata* and *Strongylocentrotus purpuratus* (Fig. 4).

We report the identification of putative toxin-related proteins from the transcriptome, with some being supported by the proteome MS identification, as shown in Table 2; their gene expression level (RPKM) is provided in Supplementary Data S8.

**Table 2:** Identification of putative toxin-related proteins from the *Charonia tritonis* salivary gland (SG) anterior lobe (AL) and posterior lobe (PL).

Annotation	SG Transcriptome	SG-AL Transcriptome/Proteome	SG-PL Transcriptome/Proteome
Cysteine-rich venom protein	11	8/2	3/2
Snake venom metalloproteinase-like	9	3/1	6/2
Echotoxin	2	2/0	2/0
Venom Carboxylesterase-6	2	2/0	0/0
Venom serine carboxypeptidase	9	4/0	5/2

The cysteine-rich venom proteins (CRVPs) belong to the larger family of proteins known as the cysteine-rich secretory proteins (CRISPs), and are found in the venoms of diverse species, including snakes, cone snails, coleoids, stinging insects, scorpions and spiders^82–88^. Proteins from this family are also commonly found in the mammalian male reproductive tract^89^ and are associated with a broad range of functions, such as fertilization and sperm-egg interaction^86, 88^. In the current study, 8 transcripts that encode venom-like CRISPs were identified within the SG-AL, and 2 were confirmed through proteomic analysis (Table 2). In addition, 3 transcripts that encode venom-like CRISPs were identified from the SG-PL, and an additional 2 confirmed through proteomics analysis (Table 2). Domain analysis predicts regions characteristic of CRISPs, including the first class of pathogenesis-related proteins (Pr-1) and a cysteine-rich domain (CRD) (Fig. 4)^90^. Multiple sequence alignment with CRISPs from 2 other species, *Conus textile* and *Conus marmoreus*, showed that there is a high level of diversity in *C. tritonis* venom-like CRISPs, besides spatial arrangement of cysteine (C) residues (Fig. 4). CRVPs are well known to exert multiple activities through the blocking of L-type Ca^2+^ channels and K^+^ channel inhibitors^87^, which can reduce smooth muscle contraction and cause myonecrosis. Recent studies have revealed that CRISPs in snake venoms inhibit smooth muscle contraction and cyclic nucleotide-gated ion channels^83^. Considering that the CRISPs found in the venom of snakes could function as ion channel blockers^91, 92^, the triton CRISPs might have a similar function by targeting the ion channels of prey.

Metalloproteinases are a family of proteolytic enzymes that are involved in a large number of biological processes. A variety of metalloproteinases are found in the venoms of spiders, scorpions, centipedes, cone snails and the platypus^93–98^. In snakes, these enzymes cause hemorrhaging upon envenomation^99^. The biochemical basis for metalloproteinase activity is through proteolytic destruction of tissue basement membranes and of the extracellular matrix surrounding capillaries and small vessels. They may also interfere with coagulation, thus complementing loss of blood from the vasculature. The variety of hemorrhagic toxins found in snake venoms is due to the presence of structurally related proteins composed of various domains^99^. The type of domains found in each toxin plays a major role in the hemorrhagic potency of the protein.

Three transcripts were identified that encode venom-related metalloproteinases in the *C. tritonis* SG-AL with a single zinc metalloproteinase confirmed by proteome analysis. In the SG-PL, seven transcripts were found which encode for metalloproteinease proteins and two proteins in the *in silico* exoproteome analysis. The zinc-dependent metalloproteinase contains a conserved metalloproteinease domain (Fig. 4). Multiple sequence alignment shows the conservation of cysteines and glycosaminoglycan attachment sites (serine motifs) in *C. tritonis*-derived metalloproteineases with four other species i.e. *Lottia gigantea*, *Crassostrea gigas*, *Biomphalaria glabrata* and *Octopus bimaculoides*. In the parasitic wasp *Chelonus inanitus*, conservation of high number of serine motifs is thought to be involved in substrate or site specific binding of venom protein^100^. This group of metalloproteineases belong to the MEROPS peptidase family M12, subfamily M12B [adamalysin family, clan (MA(M)]. The adamalysins are zinc-dependent endopeptidases also found in snake venom. The ‘A disintegrin and metalloprotease’ (ADAM) family of metalloproteases (also referred to as adamalysin-like metalloproteases) contains proteolytic domains from snake venoms, proteases from the mammalian reproductive tract, and the tumor necrosis factor alpha convertase, TACE. ADAMs are glycoproteins, which are involved in cell signaling, cell fusion, and cell-cell interactions. This supports a role for *C. tritonis* salivary gland metalloproteinase in defense, although further study is required to define its function.

**Figure 5:**
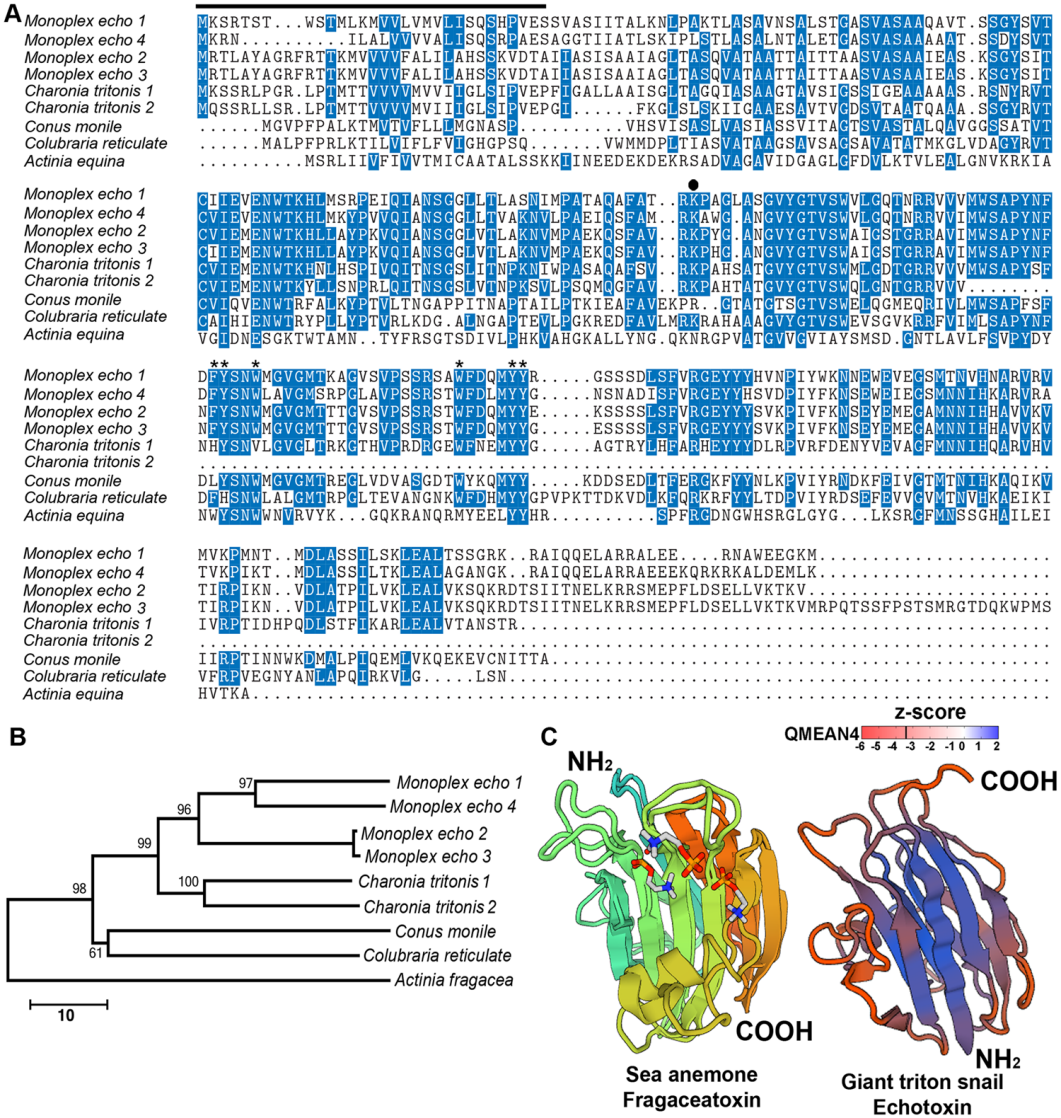
Molecular characterization of *Charonia tritonis* echotoxin proteins. (**A**) Multiple sequence alignment of *C. tritonis* echotoxin and with echotoxins from other species. Genbank accession numbers for all proteins are provided in Supplementary Data S8. Shading represents high amino acid conservation and the line shows region of signal peptide based on *M. echo*. The Lys (K) residue and residues involved in the aromatic patch are indicated by a closed circle and asterisks, respectively. (**B**) Phylogenetic tree of echotoxin proteins using neighbor-joining estimation. Scale bar represents amino acid substitutions. (**C**) 3D structure of fragaceatoxin from sea anemone (SWISS-MODEL id: 4tsn.3.A; identity 22.68% and sequence similarity 0.32) and the predicted *C. tritonis* echotoxin model, shown using ribbon representation in DeepView. Analysis of Z-scores of the template protein (fragaceatoxin) and echotoxin from *C. tritonis* shows the geometrical features responsible for an observed negative value. Large negative values correspond to red regions in the color gradient, and light red and deep blue region represents maximum match with protein used for modelling and the experimental protein. The structure reveals up to one ligand (phosphocholine) bound to a single chain of fragaceatoxin, a single binding site is also present in the echotoxin of *C. tritonis*.

Echotoxins (1, 2 and 3) are lethal and hemolytic proteinaceous toxins of approximately 25 kDa, which were identified from the SG of the Tonnoidea, *Monoplex echo* ^44, 101, 102^. Other echotoxins were subsequently identified in non-Tonnoidea snails through transcriptomics analysis of *Conus consors* and *Conus geographus* (superfamily Conoidea)^103, 104^, as well as the *C. reticulate* (superfamily Buccinoidea)^6^. In this study, two deduced echotoxin-like proteins were identified from *C. tritonis*, present in both the SG-AL and SG-PL, at 275 residues (full-length) and 142 residues (partial-length). Multiple sequence alignment of known species echotoxins shows high conservation throughout the entire precursor besides the N- and C-terminal regions (Fig. 5A). No N-terminal signal peptide was predicted for either *C. tritonis* echotoxin-like proteins (based on SignalP analysis), yet alignment with *M. echo* echotoxins suggests that a signal peptide would be cleaved following V_30_EP. Of the 6 aromatic residues that are thought to form an aromatic patch on the surface of these types of protein^105^, 4 are conserved in the *C. tritonis* echotoxin-like protein. Also, a lysine residue, which is predicted to be involved in the assembly of actinoporin molecules for pore-formation to lipid membranes^105^, is present. A phylogenetic analysis supports, with high confidence, the evolutionary origin of *M. echo* echotoxins being most closely related to  *C. tritonis* (Fig. 5B).

Echotoxins lyse erythrocytes following binding to gangliosides, which is a similar mechanism to that of some bacterial hemolysins^106^, yet dissimilar to the marine hemolysins, for example, sea anemone hemolysins bind to sphingomonnyelin^107^. Sea anemones are a rich source of lethal pore-forming toxins (PFTs) that may include a combination of peptides and proteins, known as cytolysins or actinoporins^108^. PFTs target cell membranes forming water-filled pores across the lipid bilayer, followed by oligomerization and penetration of the protein subunits through the lipid bilayer. The discovery of actinoporin-like hemolysins (echotoxins) within higher eumetazoans is of particular interest in comparative biochemistry. However, it should be noted that echotoxins and actinoporins have distinct modes of action^101, 107^. Interestingly, a sequence homology search using *C. tritonis* echotoxins revealed some similarity (a three-turn alpha helix and beta sheet) with fragaceatoxin, an actinoporin-type of pore forming hemolytic protein from sea anemone (SWISS-MODEL ID: 4tsn.3.A) (Fig. 5C). The crystal structures of two other actinoporin proteins from sea anemone, equinotoxin II and sticholysin II, both revealed a compact beta-sandwich consisting of ten strands in two sheets flanked on each side by two short alpha-helices, which is a similar topology to osmotin, a plant defense protein belonging to the fifth class of the pathogenesis-related proteins (Pr-5)^109, 110^. Studies have reported that the beta sandwich structure attaches to the membrane, while a three-turn alpha helix lying on the surface of the beta sheet may be involved in membrane pore formation, possibly via the penetration of the membrane by the helix^109–112^. Additionally, computer-aided protein structure prediction identified a ligand-binding site for phospohocholine on *C. tritonis* echotoxin (Fig. 5C). In sea anemone, small and basic α-pore forming actionporin proteins have a phosphocholine binding site which facilitates binding to the cell membrane and formation of pores, a feature that they share with toxins such as diphtheria and anthrax^111^. The identification of echotoxins from *C. tritonis* suggests a broad role for these in marine gastropod snails, and are most likely important for prey interaction.

### Analysis of sulfuric acid biosynthesis enzyme genes

Many marine gastropod snails, for the purpose of feeding and defense, release strong acids^20, 21, 113–116^. Although acids can facilitate penetration through the prey calcareous body wall, the acids can also serve as an allomone through their action to deter epibiont fouling, kill/traumatize their prey or avoid predation^112^. Several research studies within the molluscan Pleurobranchoidea family (commonly known as sea slugs) have focused on sulfuric acid production through histochemistry, showing large acid vacuoles in the median buccal gland and the subepithelial glands^113, 116, 117^.

In the Tonnoidea superfamily, acid production in several families, including the Ranellidae and Cassidae, has been investigated^32^. For example, in the family Ranellidae, extracts of the SG-PL of *Cymatium lampas* induced immediate paralysis in the sea star *Patriella brevispina* ^35^. The PL-SGs of *Cymatuim intermedius* and *Gyrineum natator* both contain specialized acid-producing and protein-secreting epithelial cells and secrete strong acids^20, 21, 114^. *G. natator* uses sulfuric acid produced in the salivary glands to make holes in the oyster shells and also uses these secretions to attack prey only when more easily obtained food is not available^32^. These marine gastropods can also use these acid secretions for their defense^30, 32, 33^. In *Charonia* species, saliva secreted from *C. tritonis* putatively immobilizes COTS^35^. However, previous studies were unable to identify the chemical components of secreted saliva from *Charonia* sp. salivary gland; *C. lampas* did not inject venom or acid into its prey but rather used its foot to capture and manipulate the prey and its radula to consume the flesh^30^.

**Figure 6:**
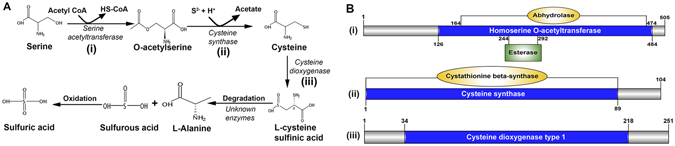
Identification of enzymes associated with the sulfuric acid biosynthesis pathway. (**A**) Pathway for biosynthesis of sulfuric acid. Those enzymes identified within the anterior lobe of the *Charonia tritonis* salivary gland are shown as (i), (ii) and (iii). (**B**) Schematics showing enzymes (i), (ii) and (iii) including characteristic domains. Biosynthetic enzymes derived from *C. tritonis* are listed in Supplementary Data S8.

Cysteine biosynthesis genes were found in the *C. tritonis* SG-AL and the *in silico* exoproteome analysis (Supplementary Data S2a). Proteomics analysis revealed one arylsulfatase B-like protein Arylsulfatase, a sulfur scavenging enzyme, which may play a role in the breakdown of sulphated saponins^81^. Presence of a higher level of arylsulfatase in the digestive organs of the predatory mollusks has been reported^118^. It has also been confirmed that this enzyme catalyzed cleavage of sulfate in the C-4 position of xylose incorporated into carbohydrate chains of saponins from sea cucumbers^81^. In triton snail, after breakdown of saponin by arylsulfatase, released sulfates may be processed in the SG-AL to synthesize sulphuric acid. Three major enzymes required for the biosynthesis of sulfuric acids, serine acetyltransferase, cysteine synthase and cysteine dioxygenase, have been found in *C. tritonis* (Fig. 6A) and domain conservation for those enzymes in SG-AL tissue subsequently identified (Fig. 6B). Although the present study reveals the genetic metabolic tools required for biosynthesis of sulfuric acids in the SG-AL, further studies are necessary to confirm its production.

## Conclusions

We have described through analysis of tank assays the process involved in *C. tritonis* attack on COTS, including proboscis extension, penetration, and the likely secretion of SG-derived feeding and putative toxin-related proteins. Transcriptome and proteome analysis of the SG-AL and SG-PL have identified putative venom- and feeding-related proteins. This work provides insight into the source of bioactive components used by *C. tritonis* to predate on COTS.

### Data Accessibility

Raw sequence data for transcriptome assemblies can be found at NCBI PRJNA383875. Protein sequences for all species proteins used in this investigation are provided in File S8.

## Electronic supplementary material

Supplementary Video S1.

Supplementary Information

Dataset 1

Dataset 2

Dataset 3

Dataset 4

Dataset 5

Dataset 6

Dataset 7

Dataset 8
