# Supplementary material for: Multiomics analysis of the giant triton snail salivary gland, a crown-of-thorns starfish predator
Source: Sci Rep. 2017 Jul 20;7:6000. doi: 10.1038/s41598-017-05974-x (PMC5519703; doi:10.1038/s41598-017-05974-x)
Supplement: Supplementary file 2 — Supplementary Information [file 41598_2017_5974_MOESM2_ESM.doc]

**Multiomics analysis of the Giant triton snail salivary gland, a Crown-of-thorns starfish predator**

U. Bose1,2, T. Wang1, M. Zhao1, C. A. Motti2, M. R. Hall2, S. F. Cummins1*

1 Faculty of Science, Health, Education and Engineering, Genecology Research Center, University of the Sunshine Coast, Maroochydore DC, Queensland, Australia, 4558

2 Australian Institute of Marine Science, Townsville, Queensland, Australia, 4810

*­Corresponding author:

Scott Cummins. scummins@usc.edu.au

Genetic, Ecology and Physiology Centre, Faculty of Science, Health, Education and Engineering, University of the Sunshine Coast, Maroochydore DC, Australia, 4558

**Supplementary Information File**

**Supplementary Video S1.** Time-lapse video showing *C. tritonis* attack on a Crown-of-thorns starfish.

**Supplementary Dataset File S1.** Lists of genes identified from *C. tritonis* SG-AL gland.

**Supplementary Dataset File S2.** Complete lists and summary of proteins identified from transcriptome and proteome analyses of the *C. tritonis* anterior lobe of the salivary gland (SG-AL).

**Supplementary Dataset File S3.** Lists of genes identified from *C. tritonis* SG-PL gland.

**Supplementary Dataset File S4.** Complete lists and summary of proteins identified from transcriptome and proteome analyses of the *C. tritonis* anterior lobe of the salivary gland (SG-PL).

**Supplementary Dataset File S5.** Annotated proteins identified from proteomic analyses of *C. tritonis* anterior lobe salivary gland tissues. Proteins are annotated for signal peptides, number of domains, cysteines, cleavage sites and glycosylation sites.

**Supplementary Dataset File S6.** Annotated proteins identified from proteomic analyses of *C. tritonis* posterior lobe salivary gland tissues. Proteins are annotated for signal peptides, number of domains, cysteines, cleavage sites and glycosylation sites.

**Supplementary Dataset File S7.** Summary of the LC-MS-based proteomics analysis of the SG-AL and SG-PL extracts.

**Supplementary Dataset File S8.** Genbank accession numbers for cysteine-rich secretory protein, arylsulfatase, metalloproteinease and echotoxin-like proteins from *C. tritonis* and other species. Sulfuric acid biosynthesis enzymes identified in *C. tritonis*.
