# Supplementary material for: Multiomics analysis of the giant triton snail salivary gland, a crown-of-thorns starfish predator
Source: Sci Rep. 2017 Jul 20;7:6000. doi: 10.1038/s41598-017-05974-x (PMC5519703; doi:10.1038/s41598-017-05974-x)
Supplement: Supplementary file 7 — Dataset 5 [file 41598_2017_5974_MOESM7_ESM.doc]

Yellow - signal peptide

Green - Cysteines

Red - Cleavage site

Dark blue - Glycosylation site

Grey – Domains as determined by SMART

**>SG-AL_contig_1197_1_-2_14_775**

MSARMACVSTLLACCFFTAVLAGEEACYSYAGGSVYPQETRRTSGHTIQWSQAVISKPAPDWNGTAVVNGEFKDIKLSDFRGKYLVFFFYPLDFTFVCPTEIVAFSDSIGKFKSINTEVVACSVDSQFTHLAWINTPRSQGGLGPLKIPLLSDITHDIAKAYGVYLQDLGHSLRGLFIIDPKGILRQITMNDLPVGRSVDETLRLVQAFQYTDSHGEVCPVGWKPGSDTIIPDPKQSQKYFKKQKDPEGEKEEL

**>SG-AL_contig_65864_1_-3_9_533**

MFTMMKAVCVVLLLCLAFTGGEGKKRKCDLRYKIISSEHTMCLDKKPEAKVVRLTPTKKQAIVDRHNNIRRTVQPEAANMLKMMWSFKLERLALKWALQCSSVSEDHPNKRILPNLGVDYVAQNLAVNPDDIYQAVLTWFQTKRNWQYGNETSLDGDDAAYVNQITATAAYIGCG

**>SG-AL_contig_532_1_-2_8_1396**

MTYTVTTLALLVVVTLWTADVSGFSAQRKTRSTSQTQQTDQSQHYGVLLDAGSSSTKVNIFSYIPGEPPNFVPQVQLTDTERIKPGLSSFMENLDGVSDYISAALNDAKSKIPEDLQASTPVYLMATAGLRTLSASQAKALLQSVRDAMSSTTINPFYFEEDYVSILSGEEEGVYAWLAANYLHGFFSTSMTREDSVGLLEMGGGSLQVAFLPDDPIYQGEFQVYVGRRRFDLYVHSYLGFGSNYIAGQVRRVLSQNSNYRNGNELNDPCMLIEDVASYEAGEVTLTVKGRSKPDECLEVLEQILESSDTALCSPKPCAIRNTYQPAVGSLPFYAISVFLYAPLALGAVEDNDILNISKLTDEALDYCTWNVSEAVAKTGTKAKYASTNCLMGLYIPQLLVDSFGFDQHTDRITVTQEIDDKDINWGLGAMIQHLSMAFMDDLESAVHCRQGDSVLPLDRARA

**>SG-AL_contig_11717_1_-2_125_1768**

MAFMTTALTLLLLISATRGQQADVIQTTLGRVQGLRETVLGKDYDVYWGIPFAKPPLGNLRFKHPEAADPWNGILSADTKPNSCYQGIDTTFEMFSGVNMWNPNTNVSEDCLYLNVWVPRAFGTQPPSTTMVWIFGGGFWAGTSTLDVYNGAKLAVTENVIVASMNYRLGPLGFLYTGTTDAPGNQGLLDQTLALKWIYENIVSFGGQQNSITIFGESAGAVSVGLHLMSNLSQDYFTRAILQSGSPTADWGMMSHATATARAEILSEALNCPVSSSSSMVQCLRGLDAGDITIKQWNVQTTGAWFDTTMSPVVDNYFIMNEPSTLMAEGDVKDTELLIGVNKDEGMYWLLYAFPNMFPLDNSGTISAAEFRDLVQSLTLNAGDDAVNAVINEYVGSTGSVDTHTYRNIADDISGDMVFICSAVEFAKDFAALGGNRDVYLYSFEHNLSNNPWPDWTGVMHGYEIEAVFALPQESTYTQQDRNVAARVTGYWSRFATTGNPNAAGSTLWPKLTSQNDGYLRIKQQGDSVGHGLRQSECSVLSTHDLIM

**>SG-AL_contig_22216_1_-1_19_1992**

MISSPIYRALLLAVAMLLLTNQGQCWKCHKDETVCEIWLELEHGLTMMKGKSLITLKDGLLFPYDTVNLTRENAIPKEQVITGDGFPEPMVVIMVNGTVPGPVIEVYENQTVRVHLHNKMASESTSLHFHGQVQNSTPWMDGVAFVSQCPILPGQTFIHQFKAEPAGTYWYHSHSGGQTSMGMFGGLVVHPRSRPNEPRYHDDFTLLIMDWNHDWSDTMTFMKMQYGMYVKGKKEGAASNLSGAHYSMFRIQSALINGRGRYTDPETSLHNGAPLEVFTVRRWNLYRFRLINSGSVFPFKLSVDGHKLKVVATDGMELDTPVMADSIIITPGERYDFELTASDVAANYWIRAKSLERDVNHAAEAILHYEGAPNNTDPTSRERDCTEDEPCTVINCPFHHYPASAHTKCINVEKLRASEKMPHHIGAGATNVKEYFLNWAFPGTTWTPASVNGIQMELPHVSALTQPLEFATRCPNEGYKCTEDQVCRCTAVLDLHGGDVVQMVLTNQGRGKGWDHPIHMHGHSFFVLKIGYPEYDNVTGKFIRDNMDINCRGNSDRDLSYCNNATWTNSSWSGNSVPGLELEKPMLKDTVNVPTGGYVVIRIQANNPGVWPMHCHVSLHLSDGMFLLLNESYPNHPPPPPGFRQCGDFSVPYPDVHH

**>SG-AL_contig_965_1_-2_98_1321**

MKFLAVLVALVGVALCEPTIYFKEEFGDGWEDRWVDSTSKGGEQGKFKHSAGKFYGDAEKDKGLQTSQDARFYGISAKFDTFSNEGKTLVIQFTVKHEQNIDCGGGYAKVFPSGLDQAGMHGESPYLIMFGPDICGPGTKKVHVIFNYKGKNLLTKKDIRCKDDVYTHLYTLIVRSDNTYEVKIDNEKVESGELEADWDFLPAKKIKDPEAKKPDDWDEREKIDDPEDKKPEDWDKPEHIPDPDAKKPDDWDDEMDGEWEPPMIDNPEYQGEWKPKQIDNPAYKGKWVHPEIDNPDYVADDKLYSYEDIGAIGFDLWQVKSGTIFDNVLITDDEDYAKEFGEGTWGKTKEPEKKMKEEQDEEDRKKREKREEEKKRKEEEDAKKSEEGEDEEEEEEEEEPEDDEEVHDEL

**> SG-AL_contig_1575_1_-1_10_996**

MVSIFSSLLFLACVSAASSQSNGVWGTCSSQTQNSNIYTYVDSNSKCCDFKSSNYLMSVCCQDTSTNTCKCHPSIARSPYCLLTSVPATPPSARLCPAPFWTSGRLLNSSPVTSGCIGSGTVAFVTTTGLICNAALTADVIATLSTCRTLIKSALSNSAIPISLLLGSVSMPLVRGDLQDGKTGGNLTLWKLSPSAVIVLKQVLRNCPSTNCPYNPSTMSNAVDFNSCYVETYGASSVGATSYGKLSKVYLQKTNPTTCPGVSQGSGIECFHSTSGGAACLGDYAGPVFCPLNTGETALYGLTSANTCDPFNNLFAVVPLVQTVEIPLP

**>SG-AL_contig_540_1_-2_71_1516**

MKLLFLIAVLASISHVGWSQCSTISKLPVSDASCCSRLEMYQLYQLFPSVFSNVAKPNYTSEDCCRVRYTDPCTCDPSSPRTPSTCIDFLPITAKNSEGCCGKLYQTNITTTKCYPRYIFENFRIEVKKFCESHFDVTYKYTPTAKEISGMCVRTNAMPYDKCVKAKPTNPGKCYCKPAVTSCFLPTTTTTSTTTTTSPTTTTSTTTTTSPTTTTSPTTTTTTTTTTTTPAPLCPAPPPPRASGSRVLGGWTANPCSASGVLSFSSGKDSAQMQMCNGVIAYNPTEKTNEVYITDFCKNLMAFVKTALKLADSGDYNNAILFNPTDMATTAAASNISYFAPPVNLLTGRCRSVACLYNAATMKDVIDFNSCYMTSYGYDVETSDGTNGRSGSLNNVTMTKQDTCKTKPVSTTAECFIADDPDVRGCWGDNGAPVYCRLKTTKETVLYGLLDVTGNMPATFGAPTNQKNLCSKNNEFHVLPVS

**>SG-AL_contig_304_1_-1_271_4635**

MVGVWVRGGAWLLIVATLVALSAAAPTSEEAEESEGRNCFYNNTEYVHGLSWSPPYPPCLVCTCKDADVLCNFQPNCKTQYASAEVRTAALAETLNSLQGPSGPRGEPGYPGRQGDRGDAGPDGTNGVPGPPGSPGPSEMTADQAYQMYYARNYGGKSAPAYGPQYLQAQVGPMGPRGTPGPPGAGGPQGMEGMRGQPGDSGPPGPPGNRGEPGPLGPQGIEGDLGRNGEPGSSGPPGPKGPPGSPGMPGMPGIKGHRGFTGYPGKRGDPGSSGEMGETGPPGQAGPPGPAGPRGSPGERGQDGPPGQDGLRGVDGLSGPAGPPGPVGNTGPPGVPGVSGAKGEQGSPGPKGGPGPQGARGDNGMPGITGEVGMPGPPGLSGLPGEQGASGDPGIPGPPGFPGPRGQPGLAGSPGMNGPKGSTGLPGQSGYKGIAGEKGQTGPAGVVGKVGSAGSQGRRGQRGRPGPPGPQGVPGERGLPGGRGMPGSPGLLGPKGEDGEPGPRGPRGETGSNGEVGRSGPPGIPGEAGPPGIPGPDGNSGPPGEPGPIGSEGRPGEMGPPGQRGPQGTQGPQGPPGERGPPGKDGTPGTTGPRGAPGDVGSTGEMGPQGQPGAEGEPGDRGPQGTPGPAGFQGPPGLPGAPGEAGRDGEMGPPGPAGVPGAEGYPGERGFPGERGPQGPDGEPGVPGTEGPTGPDGPAGPAGEIGIPGDIGPQGPIGAPGDRGPPGPAGEKGEQGEPGPTGPDGLPGRQGRHGERGPLGPQGIAGPPAIKGGPGDPGFQGQNGPPGTPGPRGEPGIEGEQGYPGLAGQAGSPGAKGETGLSGQKGEQGEAGPPGEPGQAGPPGLSGVPGRRGPTGDRGIQGTPGDPGQPGRPGPQGPTGPSGLLGNPGAPGPRGIKGATGESGPRGRPGSDGAPGVAGNPGMKGQRGEDGSPGGEGPTGPPGPQGQRGENGIPGVPGERGSAGTVGHPGARGSPGRRGADGFPGQQGEPGSPGAPGPNGSPGGHGAPGNNGAPGQQGQAGEPGPVGELGPPGPPGLPGLQGPAGPAGPIGAAGERGLRGEIGPEGPTGPTGPRGTPGPQGPMGELGELGDDGDKGDAGNPGYQGLPGLPGPEGPTGEVGSAGPRGVPGSRGSDGPRGPMGSDGEPGPQGPRGPPGPRGPPGDDGRRGPSGEEGEMGPPGPPGQTVYANAMTSWFQGATKGPGVQGDAPAESRNTLVALKEVANDIEKLKAPAGTRESPGMTCRDIAMVDPSLKNGYYWINPNGGSISDAIRVFCKMKGNKTQTCLEPSTSEYTSQKWEFTKQDQGYWLFAGSFIEKEMFDYGSHKSQIKMLQHHTDNGRQRLTVKCRDVVAVYDGVNKDYNKAISFTSFDEELMTSRSKGPFRYKVLKDGCKKQNGKEGEALLEVGGPKLLKRIPILDVGFVEQPQGHFGLNIGRACFWQKGGKGRKGRKKRRK

**>SG-AL_contig_2037_1_-1_259_12510**

MAASAILLLLVLPGAMAGPVQWQEDNDMCSTRCPDSVKFRYQIGSSYEYRYEAETRTTVAGAAEDTASITIGATARIEVLGPCDMALRLTDVAIKRSHEQNTKRMVQADDMSRARNALEDQSLRFAFYDGAVGDLCPDDDEELWALNVKRGVLTSFQNSMRQMEREEHVQEADVTGQCPTHYKVTQQGWRSLTVTKVKDLLSCTRRHDYRTSLLATPYHAPNTLQSLPLMKSSHECTHDISKELNILQGSTCHEVHVFRPFSKVSSGAVTEASHTLTFRNKASGTRTRNGPITRRTTLVYEHKQGQDKGESFFVDVKNKMKEICDVTKVDIRPQVPHMFGELVHMMRNLDYSDLQDLSDLLERNKLCPENAKKTRKFFRDALPMTATGSSLRMMADLLTSGKVKDTQATFWLTTLAFLPNPTKDMISAVQPLLSNERIQQDALLAVSSLVNNYCEQHADCAQDDEIQNVLDTLTAILGPDCQVHDNNFKQVMLTLRAIGNIGRSEAASLRLQNCFTKSELPMDVRVAALDAYRRMPCSADRNNAMEVFLRHSEDSELRIAAYLTVMKCPSDSVLNQVRQALQNEKVNQVGSFVWTHLTNLLETESPHKQAIRDVLENELLQKEFKKDRRKYSRNTELSFFSDKFSVGGVFESNMIWSANSFIPRSAMVNLTVDMFGQSVNLLEVGGRVQGLEYLLQMYFGSENTGKEGGYGQTNVVKNAKLARTKANYGNVKDDLRGQMYMRVFGNELGYGMLDGSDLKKLKEGKAKSFFEDLGKKLMKDGEVSFTQSAMFLDTTIIIPTVVGLPLNLTLNGSASLELQASQKMDFKKINAKSRAFEVAAVLQPSGAVELASMMSVDAFVTQVGLKMTSRLHTSTSVKGHVELARGQILSVEIDTPRDSLELFDFQSKFFIVHDSYEKEQEMITANRREKTACSGEALAKILGMELCASLAFPNASTALSAPYFPFTGPTSISLTLRKHDVHSGYKLLAKQIRTSSSIITQVALDTPGSKVDRAISLGYVANLKAKTIEADLLSPWKKVSAKASVDTSKKMYKADAAVSVDTGKEYKVHAQVAHDIRSRKEVSIKVKPTLVVTSPDGTLLSVNGNMDYRKNKLIKTNMVVEMKDVLKKPIAVDYTMMMRARGDKTRYRNNLKLSSQFATVKSSLQALVGSFTAKKRSALLTVRSKVDYNLHFWPGIKKNLVNLGLKIHDRSTPALSKYGINMNFDAKRTPGVNMLLNGEVSHKKTLTEVEVKIRHGKNVKLKADKDKELLIAGLVAHKIADSQATVRFDVKAVYPDKAIEYQMKGKHEHNKRMIEDYFTVRYAKGKSVDAHMRLAEVKKKRSATFDLSYPAGKTYPARDVKLNADLIEKSAKHHVLSALASIQKGQKHSFVVSFKRPTDNSVEISSEMGIYKRSPIKLDISGNYGLRDMKGQGKLTVEGKTYRMNYTSDIQKDKYAKFTMNFVHPERRIQGLLEGGRDTREAVKRAKMEVLWDADKDLSQKAGVSMELADKVNADDINIGGMLEVFTPVLEYQHLKGELRFENTNRRILTMAETILGDDSKEYSAKFMLNQPVTPNKFKTQVWLKTPHPTVRHVEVKADHSFGDSGKLSTAVKGIYNKDSVQVGISGSSKGDLVVRVLEGAAFFKSTLRDAENFRLTFNHRDVSGRYNTQAVLDANGDQYSAIMEANFQKVHYQITTDGDINILAPQLTSPVKVLWKHKNSLHEVNSAVTVDFPEDQQVHLEVGGKANIVTNVYEGRAQLRGPWKENGAEHTSSLQVNFAMPRSGHDLTVTLQLDQDTYVFHETQKDTSTSHEVTQSLKMSRFPQYNCQHKCNIQYGNFPYLVSMEVQMADRPMLNVDVEIEKPKRDKYRTTSRVVFILEDINQNIAISTRHEKNRRGQWVTKGDIEYEMGKTLSFSSVVDVEKDRKMINLNVDTPFDEVQNIRLEYSHSGNVEKNFTAACSLAIDPQFDTVSSEIFWSNRDRFYGNLKVDTPFKELRHLEVKSQSWMEGSKRHSILSVDYPPRQVYKLDTVYSCNFPRDLDFTLNVTTPRKELPSLHTQVRYQYTDTETAGNFRLITPAIQYDELSTSFNYRQGPKSFSSHVNNVIGTWNTWATGADFNWDNKIDGTFSIENTFHGRQKMHLLLTHRGHTWEDFRTKAGFKMNGDRLESELAYSNKDGRSGLFMLATPDPDIDFFKTTFFQSLTEDKFEGRYTAQYGESSKPYDLGLATAYSDDKIAFNSNLKTPHTEDVEIEIGFARKRRVEVKLFGMYGSNDKINVAIKYNIKKEFWDVGGEFEYLIQGSGRNIGLTFKRDGPLDNLKCYITGKYMGKQVSVETQYKDGEKKNGRLDVSTNFKDYTNLAASFEHSGDSENFETIVYLKYRDDKEASGTVDFQRNKWKRVAMTMTFNLPYEEFRENKLTYKHTNRKNKLSAEGSLKLGPARAITGELGFVDGRRLTVSVRGPYRQFESFTATGTYDKSNKRLEGDASVKLVRETQPVTATYKVRAGTWPIMINIEAQTPFEGWKNVELTTTHDGDVTDFTSSHSLTAEAVGTVRSELIWTYISPSVLDGLWTLSSSVEGMEDLKLAFKNSESGSEYNSQLIVGWDLLKEIVLDGRVHLDKSFSSNRCKGSISMTTPFPEASQFSVSFDHSHDVRLRQNSAVARFNDKTYLDADFQYTVGDRHVGSMEFREPRPMEFLIGGVYTEKAMEGELKLNWDKRDPEMNIHLKGDYTDHTDQLGTDRNMKVWVIHPARTMGMEYLMKKAAKEFQSDFLLTWDKATSQTLSYIINWTDRSSHYSSAYDAMVKVGVPMRSVQVEGAYSNTGRAKAAEGSIMWDVDRDKNMKVGVKGELDKRGNTNKVRLGLDLPFVDKEYSLTTTAAVNTGSTLLDAKTEFSYSVDPRKLLVVTTKIEDMSYGRDATNYSLAFSVLHPMTDVSIQMMSHVGTSPVLSSMATSVVYLTAEREKKMMELRGQIDKLKRVMGIEFKSPVKDVSLKGELSGGREYNMRLVSNVDGQKSQVTDIFIAPETRTFEMTFRYDKAAPDNSVKLFGHMPSDSILQTELYRMEQGRRVSETLMTVRLNSSHVLHTRLNWRPNMLRDLQQYLGTQLTVFSYGSSEAFQAAAGAVGHELKAKYEVIMQEVKEEAGPLLQQFQKEMKSLQVHLDSMQTDMRRFYYSNALHVQDLGGVATDIMETILTGMQGMVMQFTQLQQQLQQDIAQTMQQLGKYPMAQKYAQLVADFAAGLKNSRVVLEQALDQLSIQVSKFSDQSYRKYLIISRQIERKLKGYTAALRQTPPYQTLAQAYNTAQSSLSQLSRGDYKQLADAANLKLMNALDQVGLKEFYLLMVTKSKRLIADQFHDLVRRPEFQQIYVISNEIYQQGVWAYHYWQVEKNTRMAVGQLLELAKDITLLEITRIKNAVVDLDKSRVIVFDPQNGELQFELYLPVALKSLAEPPDMRVMKYVNRVRSWVATYIPSPQHGLWTSINNYLPNMDTNTWLPPFTAHAYVMGDQHVVTFDQKHYNFAGRCSYILARDMADDRFTVVLNYNNRPRHPQVQSIVILLRGVAIEVDRNSKVKVNNEERELPFFSGIISAVRQQSIVRVQDGQALTVEIDSSRAIYSMALSGWYHGRTAGLLGTYDNEPFNDMMDSQHRITNDIEAFTNSWEVGHRFCRSRNLAISEDPESESEEVQACRKFLTDSSSYFSPCFGQVKASELVNVCVAQARSGLSIQEAKCKAGQHYRYMCARQGVMLGTPAECVSCQLPSGQILGAGESETLKEEVGQGLESADVVFVVEESSCNAWAERSLARLATQLAQSLQARGLRGNRFGLVSYSGNGSQEQQLVHTIEGQVFGKPSNFLKATESLRLIYPSTAGNTGAAVREAVKYPFRVGVSKLLVLLPCSDCTGDMYALGQVLLSHGFQLHMLDAQDFQIDGSSNSSPVRVFGLDNSKAHTQKSAARKSEAIFKKLGTPQGSCAQLALSTNGSVFDVSHMMARSRIQNEFLESFAARGALNALPPTCQVCSCHDDGTGHGHTVCRLCNNNSWLWNHIPTWLQSSSTSIMGVFNDLHHQIITSLDAPVVAVTA

**>SG-AL_contig_12906_1_-3_477_5534**

MLTFCVYVLLALGGGALGANVPLSDGAVDLLQAVEIGEGKEGVIRTEGFCPKRAVRDGEPMARGFPDLGYRINKDAQLRVELQKSFKGGFPKHFSILMTVKPDQGAKGKIFSIYDDKNANEILALKVGRNSELIYLDSKGKPEKPVPLGIDLTDGEWHRIGLSIKGNTLTVIDNCEQTKEPIVLGRSIPVLPFERKGLIFVGYQVFADDLFTGVIQDLIITRDPGDAYEICEVYCPDCEFALPYGAVYSASSSSGRFNASSSSSSSSSSSSSSSSSGSSSSSSSSSSFNRGRPGGVMTLEYGSAEMTDVTYRFGPPGPPGARGVSGPPGGKGEEGPDGRDGIPGTNGLPGPPGHVFVIPLASDGKQSNYAEHFRTMLDQHMLAMRGASGPMGMTGLPGRVGAPGPPGVKGDLGNTGEQGPMGPMGLPGPPGPVGKRGRPGNDGDRGLQGPPGDNGMPGFPGMPGLPGEKGHRGIPGSAGEPGSMGSDGEPGDSGDPGPPGPPGDVGPRGVLGPRGRPGPAGPPGQPGSEGPVGPKGNMGPVGSPGAPGQTGPPGQTGPPGPSGPLGPPGISGSTGKPGLPGLPGSLGPAGLPGPPGDYGAKGDQGPMGLQGVIGYPGARGSKGDMGSRGGQGEKGDKGGPGADGDRGGHGPKGDKGMRGPPGPMGIEGIEGAKGDDGPPGEVGPQGPFGEKGRVGAPGFPGYPGPAGLKGSRGRSGRRGRRGARGKRGLPGPPGEAGEPGVRGQRGERGKQGIPGPFGPKGDPGPPGPPGFTGEQGIQGPPGPVGIIGPPGAPGVPGKDGQMGMSGDRGEPGHPGSPGQPGPVGIMGPPGPNGEVGPDGERGPPGNPGPPGDQGVSGQSGKPGERGPPGPQGNPGPHGPPGLGGFPGGRGFPGGPGPEGPRGDPGPLGPPGPPGDKGGIGDTGPAGPAGRPGTPGAAGSMGSVGPKGDTGPEGPQGQQGTEGAPGPRGYRGPTGPVGPPGMDGDKGDRGPPGEIGMKGDYGPQGPPGPQGPLGLQGPPGPPGPPGESGPRGLQGPSGAKGSEGPRGLLGPTGLNGLQGMPGPPGPKGDQGDPGKKGPPGPIGPLGPAGPAGPSGPTGLPGTPGGEGPMGLKGEQGPPGPPGIPGADADRGPPGRPGPKGEEGRSGPPGLPGPQGERGTDGEKGDPGTFGPPGPQGETGEQGQKGEPGEGGIDGEPGPPGVMGPVGPPGEIGLPGIPGKQGEMGIPGLPGSVGEPGTKGDRGFRGPPGQRGQPGPPGVAGTTGPPGPTGQPGPVGETGSSGPPGTPGEQGPIGPPGPLGKKGSRGLKGVKGHRGPMGNLGPEGQPGMKGEPGPFGPAGPKGDKGQDGPPGPRGPPGNDGAPGIPGPMGPPGPKGEPGQHGAKGEQGNPGPIGAPGLPGDKLHVGPADLATVPEQRRRRRRRRRRRRSPDSSDIDFDVQSVYTRLMSRQQDGHTLQLQEVVQEVSTVMLKHIDQLKAQVDNIKYPQGTRENPALSCREIALGHPEYKTGWYWVDPTQGSIDDALQVWCNMTSSIETCVYPSPKTKMVSEKAWNRPEGRGRWFSQLDGGFQMQYASPVQLKLLRMASEGATQRFTYYCSGSVAWFDQSSGNHKSAIILRGDNRHEFDTSKFSFKQIIHDGCRDRRQNGFTVFEIKTRKLDRLPITNFMAKDYGNPWQKFGFEAGPLCFH

**>SG-AL_contig_21048_1_-3_300_2099**

MAKISPSLRLSNPFLPLTTATVLLVLLSLHPPRCQARLTAGDSRQLDHFILEVMRCADIPTLSVSLVGPDGVSYEQGYDDTSKALPTPRQRTTKDTVFCLGGSTQAFTSVLLSMLLSYNENKTFDTPIHEITGSHYHLPGRFRTGRINLKDILGMRTGLSNMDIMAIAMGRNRYRLMQNLHYAPEVSQFREENVYSEILYSLAEDAASALGGETWSKLIRQYIFNRLGMDNAGFVHIDARENGHVAPPVEAYSGQYTQIPWEAYKGQEIVAAATSACASGSDMSKWIHFILTGGKTLNNEQIVNEETITDTFKSVQSRSNGGDPHITGFTQPTIQMSYTRESNALGWIKGMYRGYAFVSQDGSLPGYESLTTIIPQRSVGVFTAFTGAGGSKAYAAKVLINLFAIDLLLHGTGWVDRNSVCDVLDKMAAHADSADSSQQSRSGYLGRANEPVRPVEEYEGTYRNYGFGDVMVRRNETGPLKLVYGELGHYVLHPTQQNDTFIMQAAQGPLWYTTNADEYRKKGPFLAIFNYRQNDPSHRIETVTIPHFARDMDPEFSKNPREQRRPEYDDDHHCDACAGIVASVSLVLASCLMSGLGLLR

**>SG-AL_contig_22642_1_-1_157_1503**

MNASNTCLTLAALLLCLSHGLAFRLDWTVPNAWLSPPARYSWGLPPPSEWQHTSPSKLPVAMGRNAGNDVSPDKAVLSSVELSKKSADSDFKEWLDSAGTEQMKSGDVHGFDIPGELQEDDPKNQAKKAETELLKQTFDKADRSLVKKYLNNKDVKQRQVQPFEMDMVKQLFSEPGMGLDKNPFPYSDADLQKHPVIKNEKGLVEVTEQNVNTGSIHNPNEKQAALELIKKGLDAIGTGLIKKGLDTVGSGLIKRGLTQLADSLGNGDPTKEVDIDMFEGGLDSVGAGMFTQGLDTAGLKLIKRGMDKIEAGLVGSSSDANRQLDSGGISLMKTGFDHLETGLVKHGVDMDGLGMITEGLEKLGKWLIKQGSDHESYDVVKATGDKKTDTTKETFADYKLIKKGFDKLVAELNTKASDDGEPGIVKTDFNQDEMEFIKKKLHDVGAGLI

**>SG-AL_contig_8755_1_-2_2_4930**

MADGSRTVVCLESLLSCVQVRSRTIMNVRTVILCLCLFALVASAQEDPPRGRSGCYDDAGRAQRCMPEFVNAAFGLPVDASNTCGVSKETEYCLQTGASGARKPCYICDARREGLNHPPEYMTDFNKQDNWTWWQSETMLEGVQFPTTVNLTLNLRKAFDITYVRIRFMSPRPESFAIYKRTKEDGPWIPYQFYSASCESTYSLPRRGIITRSNEAVAICTDEFSDISPLTGGSVAFSTLEGRPSAFEFSESPELQEWVSATDIKIVLTRMNTFGDEVFGDPKVLKSYFYAISDLAVGARCKCNGHGSSCQIIRDQNLEDRLVCKCEHFTAGPDCGECLPFYNDRPWSRATERDANECQPCDCNGLSDRCYFDRELYEQTGHGGHCESCRDNTGGPHCEVCLPNHYRRMPENRCLACGCNEMGSETLQCDDQGQCRCKPGVGGQRCDRCLPNYYDFSDTGCQPCACVVAGSLNNEPRCDSITGECDCKENVEGRRCDRPKPGYFGLSEANPFGALACFCYGHSSVCTSATGYYGRNISTNFATGNERWTAVSRSGQDLDTQYNGITEKLGVSAPSMEVVYFNAPDRYLGDQRFSYNQFLTFELQIGEETARPSVVDIIIESNDQRIATHIFAQSNPVPDVASQNYSFRMHEDPQFQWSPRLKPQEFISILANVTALKIRATYNPDGVGFIDNIHLGTARQAFNDGPEAGWVEQCTCPEGYIGQFCESCAPGYRRDVMNGGPFARCVPCECNSHSDICDVNTGRCICEHNTEGNNCERCARGFFGDPTQGTPNDCQPCPCPNNGPCVQLPNGDVVCTECEEGYGGNLCDICLDGYSGDPAGHHGSRRPCERCTCNGNIDSNAVGNCDTTTGECLKCIYNTGGFYCEKCLPSFYGDALALPKGQCQACNCNPTGTIIQPGVLGCDPVSGKCACLPNVGGRQCNRCDPGYWNLDSGTGCEACDCDRTGSTNYTCNENGGQCQCRSGVTGRRCDTCQPFFFGFSQSGCQACNCDPIGSLDLQCDQYGYCPCRPNVDGRRCDRCQENKYNMSAGCLDCPQCYDLVQEQVTIHRGKLRDLTSLINNIGNNPSLFNDSEFLSVLGQVNKSINVLLDEARGASTGDGTIGQQLQELKDALREVLEKCGQITRSIAAAASESKDSIRDIEVAEAAIRRAEMSLREADDYIGREGRQALAQALEALQQFGKQSQQMTEIAQRATDESIKQMKDANMIDNVAKNAVNTSQEALRLAEETLRMPDTNRAQIERLTREFSDASELYDRTEELAKVALERANEAHKEALDLLREAQSPLPTVDVRQLSEDATKIKEDAARIKLRAEELMQNNKDLLAEVEQQTNLSRSTLTEGNHLQQRVDELLAEVDAARDVARKAVESGERTLREANETLQTLLGFDRLVSENKDAADDALKKVPEIENMIAEAMRTTQQAREALRGAESEAKEGLRIAEMAEFTATEASKEAEKIRDEARDTKNKAVDLKDQTETLAQEVQEANKSVTAYEQQADADEGMIDEARRKAREAQITADAAADKVNKALDQVTSIKNILTYLEDVDTDRLAELEAELDRLETELNQANIDSEIETLTTQNDRIKCWRNKYETELSQFKKDVDNIAAIRDSLPDDCFKTIDIESPLNG

**>SG-AL_contig_57071_1_-3_120_461**

MGTTRRILLVALFATCFGLALCDDRPNIVFVLADDYGFNDVGYHGSEILTPNLDRLAGEGVKLENYYVQPICTPTRSQLMSGRYQIHTGLQHDIIWASQPNGLPLDSPTIAEKL

**>SG-AL_contig_1102_1_-2_356_4330**

MMKMKMMTVVASGVVLALSCFLLSVHAQESVNEVSRGDNAELGNLDQMQAQQAGSKAGPAITNGGYMAPGPPGPRGPPGPPGSPGQPGFQGQRGPNGEPGPAGHPGQRGFPGPPGPHGLDGDEGMPGDPGPPGPVGAAGNAGPPGVPGMPGPKGHRGFSGRPGKEGEVGRPGEKGPGGPSGPVGAVGPSGARGAPGERGRDGSSGSAGQPGNDGTPGNPGGPGPVGPPGPPGFPGAAGAKGDRGPSGASGNTGPPGPAGSDGLPGAPGTVGQPGIRGEDGSSGNKGPDGPVGPAGPPGFPGPQGPPGGVGPPGAPGGKGNNGRDGSNGAPGNPGPQGAPGASGERGLPGLPGPEGKRGPSGPAGPGGAAGPTGEMGAQGLSGLPGRPGFPGPPGQDGARGESGERGSPGPDGSTGAMGPPGPSGPRGSPGLPGEDGTSGEPGPAGNDGLDGNPGEQGPQGQPGPPGLTGAPGNRGESGSEGRPGPQGPNGPPGARGERGPAGPEGPVGRPGNPGRQGEAGPSGPPGEAGFQGLPGPPGPAGQNGNPGATGVPGPPGESGLEGIPGERGFPGEPGPSGQRGVPGDRGNTGPAGSPGRPGPSGPPGARGAPGPPGDVGLGGENGVPGRVGARGPPGPSGEAGREGERGSPGEVGAPGPIGPPGPPGTVAIKGDRGAPGLPGNPGPSGPSGRTGAPGAPGPAGNVGIDGLPGESGASGDAGARGARGDAGPRGYPGERGIDGEAGRPGTDGSKGERGEAGPAGPSGQPGAPGAVGPPGLNGPPGPLGATGNQGQPGERGSPGETGQPGMPGETGNPGQPGSDGSPGERGSPGLGGPPGPPGITGPQGERGSSGFPGAAGEAGPPGPVGIPGEPGPRGENGKDGAPGLRGLPGQPGPAGFPGESGRPGERGKDGPAGPTGRPGAKGSRGNPGTQGPPGLGGPVGAPGQPGPAGPTGERGERGERGNSGPVGTVGPAGAPGAQGPQGAAGSTGSAGAKGDKGWPGMPGPGGPPGPPGPAGDNGLPGPPGPPGSQGSPGARGPPGRNGEPGPQGRPGNHGQRGPSGENGPAGIPGPRGPPGPPGPPGHSPVYSPAPSAGFKGPDPYMQYDQPINTAETYEGLNFADHALNRVRRVTGKATSPGMSCRDIKERNPDFPSGEYWIDPNERSAVDAILVYCRMETMETCIFPSPSTFDRQRWTKTEGSGQFFMEEMVGDREFFYKTDFSQLKFLQMLSEGARQRVTYHCFNSHARGSRLMLHTGEELDTELYKYKKSTRIATSDECAHDNQWHSAVFDIRTNKTDILPITDIRLFDVGRQNQQFGIELGEVCFG

**>SG-AL_contig_5353_1_-2_98_1114**

MQYMVWTLLLVSVMTPHLLVRAQSTNEIFGSDGRLIMDPFELLLFQMIVCSLPPEAGPCETIVPMVFYNRTAGQCQNFTYGGCKGNANRFKTEEECEIACTPSWCSLPAETGPCKAFIPSFFYNITAGKCQFFVYGGCEGNDNRFETMGECESACSLPNTTSVAVLAAAAAAAAAAAAGETVVTEINLDQTQPAISSTGPLQPPAAEAVITEIALGGPPQSVDSSTAAAAAAALPPPPPATAAETGVTEPSVDQPAASVVSSTPGPETSSDTGNASSDHTQSHQEACSLPPVTGPCRSYIRSFFYNAIEGQCQLFIYGGCGGNNNRFRTKDGCENFCNP

**>SG-AL_contig_1240_1_-1_187_2190**

MKWFLLPYCFAALLLSTAWIVQADDDEEEGETKKKDKEGVGTVIGIDLGTTYSCVGVFKNGRVEIIANDQGNRITPSYVAFTSDERLIGDAAKNQLTSNPENTIFDVKRLIGRMWDDRSVQKDISFYPFKVINKNNKPHIQAAVSGETKTFAPEEISAMVLGKMRDIAEAYLGKKIVNAVVTVPAYFNDAQRQATKDAGTIAGLNVMRIINEPTAAAIAYGLDKRDGEKNILVFDLGGGTFDVSLLTIDNGVFEVVATNGDTHLGGEDFDQRVMDHFIKLYKKKKGKDLRKDNRAVQKLRREVEKAKRALSSQHQVRVEVESIFDGEDFSEQLTRAKFEELNMDLFRSTVKPVKQVLDDSDLKKEEVDEIVLVGGSTRIPKVQQLVKEFFNGKEPNRGINPDEAVAYGAAVQAGVLSGEEDTGDLVLLDVNPLTMGIETVGGVMTKLIPRNTVVPTKKSQIFSTAADNQPTVTIQVYEGERSMTKDNHLLGKFDLTGIPPAPRGVPQIEVTFEIDVNGILRVSAEDKGTGNKNDIVIQNDHNRLSPEDIERMINDAEKYADEDKKLRERVDAKNELESYAYSLKNQVNDKEKLGAKLSDEDKEKITETLDDAISWLEGHQDADTEEFKDKKKEVEEVIQPIISKLYEGAGGAPPPTGDEGDEQERDEL

**>SG-AL_contig_21456_1_-3_198_1223**

MEDSHGYCLFVVLIVAMTSSGVFSQSDTPDSSQYEPSPGLFDRLEEPTDKTIDQLITEALGGIDVASNKILAPGAYVLAELDMYLSQEQFLNLYEPPSNHILRGPNGTIIRSPSSPQPPAMANSTSARSKRKATRDVILRWPGAQIPYQFARGDFTDKERYMIKQSMTEWERYTCLKFRPASSADTNVVRFQNGEGCNSQLGMVGGVQLLNLDVAGCRFKGLYLHEIGHAIGLVHEHQLPDRDNYIYVLLQNVAPHFRIFFNKYSTTVVNQFNVPYEYSSVMHYGITAFSHNGKAQTIRTRDRSKESSIGKVYLKELSFSDVKVVNEMYSCNAFCPDVVTCT

**>SG-AL_contig_609_1_-1_94_1560**

MQATMTLMVLVCTSWLLLLGTMMTIVGAAPAGDEIASLPGLKKQPSFKQYSGYLQASGTKKLHYWFVESQNNPKTDPVVLWMNGGPGCSSDLGFLTEHGPFRVQDDGATLLDNPFSWNTITSMLYLEAPAGVGYSYSNDGNYSTNDDLVAHDNYLALKDFFLHKFPELSKNEFFITGESYGGVYVPTLSALVVDDDSFNFQGFAVGNGLSSYELNDDSLIYFSYFHGLIGEQLWDGLVSTCCHGNATNCTFIKNAVKNDTCGYYVSKVQAIVYGGDLNIYNLYGPCVQNSGMKTKYNPLQHRLVTANFGWAFANSPAVKAERQFIEKLQTQSGNRRLSITPPCIDAKGAIIYMNRKDVRTALHIPANLPEWSPCSNLNYKRVYMEMSAQYKKVLGKGKRVFVYNGDVDMACNFLGDEWFTDRLSLPSKKARSEWTYTDATHTSQVAGFFKEFELLTFVTVRGAGHMVPTDRPRPALKMFVHFITNKPLN

**>SG-AL_contig_2339_1_-3_6_596**

MQLCLVLVCCGLMVGPIWAGTVESVAVAKHNQLRAAEVETNSASNIYALKWNSALAAQAATWTRKCRVEHTPGLDRNIGQNLYSAGYIGSEKPKATKVLKDAIDLWMEEKSLNDGTFACCTRLTMTCCHYTQVVSSRSKEVGCAVEFCPGNQIIVICNYTPMGNFNDQAAYRTGPPCSGCKKKDKCNADKLCVSRKG

**>SG-AL_contig_16009_1_-3_45_827**

MFTMMLTAGAALLLCLMFTGVETQTNKKGAKCDLQFSIINDNHTMCLTDKPNAVKISLSENDRSYIVDKHNEFRRNANPIPANMLKMQWSSELAEVAQKWAMQCPTGHEDDKNKRAMPSVGMNIGQNIAIDTDIANAIGFWYGKRSGWIYGELTNLKIRAMPFVQQIWASAAYIGCGSASCGDTKYFVCDYSSGPTVSERPYETGKRCSQCPNNCEGWLCNCENPRTCLNGGTFNIDTCQCDCVAPKCGFKCGDDCSDTDA

**>SG-AL_contig_17334_1_-3_6_596**

MQLCLVLVCCGLVVGPIWAVSVKMATVVKHNGVRKIEVQTDSASNIYELKWNSELAAKAAAWARRCQLGFSPDLDVNIAQNVFMAHYFEFRKPKAAMLIKNAVDSWMEEKSKNNGNFACCKDSSVDCCSYTQVVSSRSKEVGCAVEYCPGNQAYVVCNYKPGGNFNNQDAYLTGPPCSGCEGNDQCNRDKLCVSNKR

**>SG-AL_contig_4768_1_-2_83_1579**

MDKAKMTNYYGRALLIAVFLLALASPVVCNPAGPAERQMSYEGFQVHRLTSDDPLTLSETLEHLEQQSMVDVWSRGSRSADVIVAPKHISYVRKRSADRGIRSDVIVEDLQSELQKDFVQRQRRRKRSVLDVTSRYLAYDEMQAYLYQVAREATNASVTVDSIGLSYENRNVSVIGIRERAYNLKPKQAILIDAGIHAREWIAPAMALNIVNKLAFGRDQEARRMLQLFDWFIVPCVNPDGYVFSMSSATNRMWRKTRTKQYNARCYGVDGNRNFGYEWNPAIGGSTDYCSDVYSGPHAFSEPETRNIRDWLESHRNQAVAYVTMHSYGEYILYPYGSSRNKPVADKALLETLGTSFKQELQKAGHNYKVGNSATLLYPAAGGSDDYAKGTVGIKVSYTLELSPASSTGGGFQLSKNKIRKVVEDTWPGFTAMANKLYTELNPQSSSGAYLGTGDNSVNGRGRIYFNSFVDGYLKRCCEQYSSFQYKCCTALGRSVGSG

**>SG-AL_contig_92890_1_-2_11_625**

MASIYNFAFGLSLSLLFWTFVLANPEQGKWTFALDQEKQFAAVAKHMFNNSKVTVAVSLTCGSSDTGDLEISWLLRFSPCALEYSTITEDVNSNKNKGQIELYMNNPITYRIVPYDRMELYRESLIHPCSEEDFKLEGNPDQKLKILKIPKLSTIPSGVNANGAVPGPSGADTKAAGTKTESGAQSSNDQQTASAAGSAAGSSTG

**>SG-AL_contig_24521_1_-3_339_2282**

MRGTYGLLLLTLSLLHTTTTTTSPFTNDWVAVIPGGAEHAHTVARRHAFEVVRSMPHFTDHYLLRRSDVPQRSRRSSDHHTWSLTEDTQVEWAQQQVAKTRVKRHNAEDHEFNDPLFPDEWYLVNRAQSSRDTQQSVTKMSMRVQGAWGKGYSGYNVVVTILDDGLEHTNKDIQANYDPCASTDLNDHDSDPMPRYDPSNENRHGTRCAGEVAMVANNGLCGVGIAYNSSIGGVRMLDGPVTDAIEGMALCFNHTYIDIYSSSWGPSDDGKTVEGPSKLATQALEKGIKYGRGGKGAIYVWASGNGGMSDDNCNCDGYTSSIYTLSVSSATEHGLAPWYAERCASTMATTYSSGYRGEQQVISADLHNGCTEHHSGTSASAPMAAGIIALVLEANPSLTWRDVQHLVVLTSKGGALSAEKGWYRNGAGLCVNPAFGFGLLDAEALVTQAINWTHVPPVSVCHVNAANTSSLPRAVKSGHMVQIEIETNGCRGQQNEVNYLEHVQFVVTLTYSTRGALSITAISPSGTKTVLMPPRYLDRSSRGFRQWPLMSVHTWGEQPAGVWTFQVQDQSGNRANRGELKEVMMILHGTKTAPDYRNNTAYSCGLPVGSNNSTGLRTEDKRSDNRPAFMSLVEKALDTEKEKETIGV

**>SG-AL_contig_6770_1_-3_108_1574**

MANLCGPLTHFCVLIFLLVAVSLTFAGDEDARAADDVALVRSMRSVSRRGGRRGNEGIAVDSGGRGGRRGNRGNRRNHGPGRRGGRGRGGRGGRGGRGGRGRGRQGRPGRRVFSRREQRLIGRLTQATAIFSLQMYNILKQDRGELIFSPHSIHTALTMTYLGAREKTAKQMMKTLGLKRLRKNKAHLAYAALVNSLTNSGNVTLNVANAAYVKPNLPIEETFRTGLQNMYKAEFDHFDYQAVGGPEAPINAWVELKTENKIQNLLRPGTISISTALIIINAIYFKGNWKDKFNVEDTTQQIFYQDGGSTTSVQMMNRVGRYNYTRSNELAAHIVELPYQDGRFSMYVILPVTRNRLADVEQRLTLERLNDALQAMWTRKLDLYLPKFKTESEFSLRRVLRSMRMIIPFSPRANFTGICSVGGVAISDVIHKAVVEVSEEGTEAAAATAVILERMSMPMQVRADHPFIYAIRDNFSKAWLFMGKFAAQQ
