# Supplementary material for: Multiomics analysis of the giant triton snail salivary gland, a crown-of-thorns starfish predator
Source: Sci Rep. 2017 Jul 20;7:6000. doi: 10.1038/s41598-017-05974-x (PMC5519703; doi:10.1038/s41598-017-05974-x)
Supplement: Supplementary file 8 — Dataset 6 [file 41598_2017_5974_MOESM8_ESM.doc]

Yellow - signal peptide

Green - Cysteines

Red - Cleavage site

Dark blue - Glycosylation site

Grey – Domains as determined by SMART

**> SG-PL_contig_8677_1**

MKPFLLPACFVALLLSSACYVQADDDDGDGKKKDKDGVGTVIGIDLGTTYSCVGVFKNGRVEIIANDQGNRITPSYVAFTDEERLIGDAAKNQLTSNPENTIFDVKRMIGRTWDDRSVQKDIQYYPFKVINKNNKPHVQAKVSGDTKTFAPEEISAMVLGKMRDIAEAYLGKKINNAVVTVPAYFNDAQRQATKDAGTIAGLNVMRIINEPTAAAIAYGLDKRDGEKNILVFDLGGGTFDVSLLTIDNGVFEVVATNGDTHLGGEDFDQRVMDHFIKLYKKKKGKDIRKDNRAVQKLRREVEKAKRALSSQHQVRVEVESLFDGEDFSEQLTRAKFEELNMDLFRSTMKPVKQVLDDSDLKKEEVDEIVLVGGSTRIPKVQQLVKEFFGGKEPNRGINPDEAVAYGAAVQAGVLSGEEDTGDLVLLDVNPLTMGIETVGGVMTKLIPRNTVVPTKKSQIFSTAADNQPTVTIQVFEGERSMTKDNHLLGKFDLTGIPAAPRGVPQIEVTFEIDVNGILRVSAEDKGTGNKNDIVIQNDHNRLSPEDIERMINDAEKYADEDKKLKERVDAKNELESYAYSLKNQVNDKEKLGSKLSEQDKEKVIETLDEAISWLESHQEADAEEFQEKKKEVEAIVQPIVNKVYGGAGGAPPPPSGEEEDSERDEL

**> SG-PL_contig_35849_1**

MGVKVSVTSLGIVLLVLQASLLTHGAVDLDQGPVIGIDLGTTYSCVGVYNKGQVEIIPNDLGNRITPSYVAFTPEGRLVGDAAKNQLTSNPDNTVFDVKRLMGRTWDDPAVQADIHNYPFKVINDHNRPAIQVVVGGEIKQFTPEEISAMVLGKMKSVAEHYLGHNVTRAVVTVPAYFNDAQRRATEDAGRIAGLNVLRILNEPTAASLSYGLEQKGDERTVLVFDLGGGTFDVSVLTVDNGIFEVLATAGDTHLGGEDFDQRLVNHLVKVVKQKTGDDVRSNKRAMTRLRRSAEKAKRALSSLHSTKVDLEGVATDVSDVPLTRAKFEELNMDLFRSTLKTVQQALADSGKSVEEIDDIVLVGGSTRIPRVQTLIKDFFGGKEPHRGINPDEAVAYGAAVQAAVLSGQKMASEVLIMDVNALSLGIETVGGVMTSLIPRGTTIPVRKAKVFSTAADNQNTVTIQVY

**> SG-PL_contig_315_1**

MTGVWRLLTGSAVVMMAVIYSAGTSTQNTIELRVIVDHFAATKWGNTVGGNVDINDYVYSVFKNVNSVFKKLKNSGIKLDVRVVGIEIVTTEEISSEMPPGSGKRNSSEVIEELKNWLASKNYRQTDHTVLFTGLDLLGDDLSTANAGKAYLGKMCDQDFSLSVVEAPHSSAVLTIAHELGHSLNSVHDVGACMNGHIMNSSLNIGQASRNNFKFSSCSIQSIKSILRELSGKDSCLLDTSFEWTEMSPLPLGQLLPPDQTCKLAYGYNSFFSRRVYSENGDDTYDSMCGKIWCKKGQQHSSARASDGFICGNRKLCSLGECISSGSTEVLDTCPQGDSPQANDAGEKCKQVVTADNGECYLEHIKQACCSSCDEVHTRRAGCEYGDKSKSCAEFRDFPGLCDQQPDACCGTCWERSNPTESTCTDKLDDCQERLAKNNARCSKSAFRENCCASCADVSTNPETESTCTDKWDNCQEKLAKNSDKCSKSTFRENCCASCR

**> SG-PL_contig_847_1**

MTGIWRLLTGSAVVMMAVIYSAPVAKAAMNTIQLKVVVDRLANNKWRRILEPEVNGDITDYVYSVFKDVNSIFKTLRSSRIKLNVQVVDVDVTTNDITRQTDSGVVLRNLIRWLGTRQGDNWDHTMLLTGLDLIVDDDKNKTGQTYLEDMCHETRSVSVVEALLGSTVLNAAHELAHSLGAKHDNGTCLNGYIMTDSTNTQFPSLNNFKFSSCSIQEIKEGLKKLSKNGNCLLGGNPNAKQESAKTVGDLLTPDQMCKLEFGSSSYFSRRFYSGDQSESYDRMCLAIKCRNGRLTSRPDTMKSTSARASDGFICGNRKRCSLGECIPSGSTEVLDNCPQGDAPVANIEGQTCGQVKAARFAECYAKYFQKACCGTCQGEYIGQPGCEYGDKSYSCVKYRNFAGLCDQQPETCCGTCWKRSNPTWR

**> SG-PL_contig_517_1**

MAGVWRLLIGSAAMLAVIYAALFDNTIQNSIEIRVIVDSWAFNSWMSKVATSSRELQKVEETKTAITNYVHSVFNDLNSIFKELMSSGINLDARILSIEFVTDEVVFSESEPGSGQRDSREVLGELTSWLATKNYAQADHTMLLTGLNLTQKGSDSTNKVNGGNMCDRINSLSVVEATPSGAAVISIAAALGFSMSANHNVNDPDCKKNYIINSGEGSEPISPKNFRFSDCSIRAIKTSLKKLHKSGDSCLSVANTNTSRVPAPALGQLYTPDEMCKLAVNKSFFFKRSLFGNGAFPYDVICRVIMCTNSEETTRVLASDGFVCGKDKRCSLGECKPSSVLSDVPDTCPQGDTPDAAAR

**> SG-PL_contig_1404_1**

MWPTTLLPVVLAFAVSGLTHGIEDKAFWMNVGQKILDEALKVKLNNRVAKNVILFMGDGMGISTITAARIYKGQLQGNTGEETQLTFEKFPHAAFSKTYCVDRQTPGSASSGTAYHSGVKVNFGTVGFDANVLRRNCSLINDSSVRVETMLDWSYAAGKSVGIVTTTRVTHASPAAAYAHSADRNWERDTDIPTGCGLDIASQLIRENSNITVVMGGGRRNFIGREDGQNLVEEWKTNKQNEGKTARYIQNATQLRNLDASAVDNVLGLFTESHMSWELSRDNDTEPSLTEMTVKAIEILQKNDKGFFLFVEGGRIDHGHHDNWAKRALVETIAMDDAVAAALRVTNQDDTLIVVTADHSHPFSLVGFPKRGNDILGLVDPVDPGQETTDGMPYTTLVYGNGAGPMRETNLTGVNATGNDFRFPVAVPMDKEWETHAGEDVGIFAIGPMAHLFHGVHEQNYIAHAMAYAACVGPNTKHCTTKPPPDN

**> SG-PL_contig_21281_1**

MDRLLVLALLVGLSYGLDNGLARTPPMGWLSWERFRCETDCKKFPDSCVSENLYKAMADKVVELGLKDLGYEYINIDDCWPAMQRDSETGKLIPDPQRFPSGIKALADYIHARGLKLGIYEDFGHKTCAGYPGSEFYMEMDANTFAEWEVDMLKFDGCNADASDMHIGYPIMQFFLNQTRRPILYSCEWPLYEYAHGLKHDYAAIRRTCNMWRNFGDVTDSWDSILSIINYYGANDQNFSAYAGPGGWSDPDMLVVGDFGLSTYQQKAQFGMWAMFAAPLFMSVDLRTISEEALAILKNKEVIAINQDPLGVQAVRLFEMPNSVSIWLKPLSQNCTALAFFNKWNQGRPTPLSVTLGDYGLKNPNGYNVSDVFDGQQSGIFKPATEMNLLVDPTGILLLKAVPLK

**> SG-PL_contig_18727_1**

MKYAVHILPLLCVIAMASAVEFEALNSLRSKTPATVQAATVMSMLQRLVHDRASEFMIKVDSSLAPAGKDAFKVETVSSQIQITGTTGVAAAMGVYYYLKNYCNSQFTWAGEHLDLPTPLHPVPSPVLINVLDKFRYYQNVCTVSYSSVFWNWTRWERHLDWMALNGINLPLAFTGQEAIFQRLYMNMGFSNEDMEEFFGGPAFLAWARMGNIQGWGGPLSQTWIVKKLLLQHQILKRMRNFGMIPVLPGFAGHVPRAITKLYPKANVTRLGTWAGFNTTYSATYLLDFSDPLFQTIGSKFIQLMESEFGVDHIYNADTFNEMTPKSSSTEYLASAGRAVFNAMKKADPDAVWLMQGWLFLNGGFWKSAQINALVNSVPRGRMIILDLMSELMPQYLRTNSYFGQPFIWCMLHNFGGTMELYGALDNINVGPSYGRHFIKNSTMVGLGLTMEGIFQNEVIYEFMMENAWRPNPRNITAWISEYPKQRYGFTSKEIDTAWQLLK

**> SG-PL_contig_14618_1**

MKTICAMLMMVLAIHTAHSWDLSFPGGWGPCPSPPVQEHFDITKYQGVWYEYQRFPTAAEALVTCGQASYILLTNATWANTTVAVNNTGIRTLTIGGRTVFRERTHEEGVATIPDLSVPAKLVVQFDRPFIPSVFTRGEYWVLSTNYTVYSLVYSCTALPFNVAHFDSAWILTRERGVAPDNLQALREELRAGGVNPDNFFTVPQDHCDLPALSVNPPHPSVAHSAKFRINWP

**> SG-PL_contig_1472_1**

MKFLAVLVALVGVALCEPTIYFKEEFGDGWEDRWVDSTSKGGEQGKFKHSAGKFYGDAEKDKGLQTSQDARFYGISAKFDTFSNEGKTLVIQFTVKHEQNIDCGGGYAKVFPSGLDQAGMHGESPYLIMFGPDICGPGTKKVHVIFNYKGKNLLTKKDIRCKDDVYTHLYTLIVRSDNTYEVKIDNEKVESGELEADWDFLPAKKIKDPEAKKPDDWDEREKIDDPEDKKPEDWDKPEHIPDPDAKKPDDWDDEMDGEWEPPMIDNPEYQGEWKPKQIDNPAYKGKWVHPEIDNPDYVADDKLYSYEDIGAIGFDLWQVKSGTIFDNVLITDDEDYAKEFGEGTWGKTKEPEKKMKEEQDEEDRKKREEEEKKRKEEEDAKKSEEGEDEEEEEEEEEPEDDEEVHDEL

**> SG-PL_contig_5875_1**

MMESFGMTTATVVATCLFIGLGFQTCHGASWSYTGSHGVDHWATDYPGQCDGQRQSPIDLESGKTMYDKQLQAFSFTGFNSAVGVSWTLSNNGHTVQVTYTAGDLLVNGGSLGDTYRVAQFHFHWGSDNTKGSEHTVDGGAYPMEVHVVCFNTRYADLSTAVTHEDGLAVLGFFFQISANENPTVQKLVQFLPQVMYAGNQTVISAFPLNDMLAGMGEYYRYKGGLTTPTCNEVVTWTVFKNTIKLSNSQMQAFRALKDGDDAAMVNNFRPVQLLFDRTITASFEDEKFAGGSNGLVFNSLALISSLVLSLWFRL

**> SG-PL_contig_36658_1**

MKQLLVVCVFACFVVFATNGQNVVCRLKADVVFIVDSSRSIWKPHYDLELEFLANLTKELNVGPEGTHVGALSFSKGARKEFYLNEVYDAEELQARILAIPFMSGMTTQTDEAFKLARTEMFKVANGARKGLPRMVFIVTDGNSVDRAGTKKEAQRLRNKGVQVFAVGIGDEVAYDQLAEMATKDTPTYVHNVNSFSSLSTLLPELIPAACRAATTTVKPKWTTTAHPVKKTTAEIPEKVKELCTSKKADVVFVLDKSSSIQTTHFRTQLNFVSDVVGIFSVSRDHTHVGVVTFDTNTTIEFGLQQYFTKSEIRRAILDIQYTAGATNTYKALQTVATHVLTPEQGSRKGVATVVIVVTDGKSSNETLTHYWAEKLKASGAYVFAIGVGPQIDHHELSNIGSQPHSNFVFEVREGYGALATIKDILAARTCDVLPAGDIYARCQRNAVATDVMFITDFTHAGARDVSHTTELVKSLAGGIMASGGKVRVGLVSSACPDVTDVRLNRSTTAEEFADAVTSESAGGLAEVLSRARIQLTKERSGARKGVQRLAVVFVHGPVSDVKEATREVMRAKSSANRPGVKFIFVGMGERAVRGQLQDLAAERRPANARIIMTREKAVKGKITMQPVLTKLCEELAP

**> SG-PL_contig_2700_1**

MWKSISFVALFLVLSSKAMQVDAGRQRRQSADCPRDCTGTPLEISFVVDGSASIWPANFTFGLNFIEDFVNTFQISPSIVRVSLITFGERAYEEDKIDFDEYTDNTSLRQAIANIPYRSGIRTNTSGGIMFMLSKQKPKAREGVRHVVIVMTDGNSQERKETKTAAQAARADGLEVFAIGVGHSVSPEELHNIASDDRHVFAVASYNMLEGIKKRLVYQACDVPSYPECEMDPVDLSFVIDSSTSIGQDNFTVGMTFVKEFVDSFQ

**> SG-PL_contig_2081_1**

MQSALLLAIVALCGVVFGQRPFDRDREDRDNTIPECLRKTADVFFVLDSSSSIYIEDYKEVLKFVSQVITRFDVSRDDTRIGALTFSNDFQLGFGLDRFRSKGEVLASVNERSLPYRTGVTNTDLAIRYVRENNAFRPDITKAMVVITDGGSRSPGSTKREADLARDAGFHMVVVGVGQYLDEQEWRAIASDPDNDYVFNITNFSFLDSLKDVLPRRVCLMPPIILGGECRVTENSDLLFLAAPNGINDALDVIEELADSFQSQNRLQVRYIMEVCQDEADTNFEGPDRFCDRFGDALPQNDETYVSLVTQLRNVATNMREERASNQVAVLFVDDLSMRNNRFGILQEVRNAAQFDGIDIIAVDLGVREYSNFVNGMTSSRENVINYPGQSATTSVQQILDRICYHVNFSFGEEIRPN

**> SG-PL_contig_3515_1**

MLAILALPVLLFAQAVMAKPSYRDLLDLASILDVEKRLTKEEAEKYIEVCQQEPLELGLVIDSSVSIPVKDFEKGQNFLQDFLSDYDIGQGRNQVRVAAVTFGQGVYIQDSFNLDSFNDKEEVKFHVTHMPFRYGRRTDTGDALKYMREKQMVNTRPWAPKVVIVLTDGNSQRTSYTAEQAMKTIKANITVFALGVGTAVKQQELLNIAGGNEDRVIRAKSYTELDDKVKRKLAYKTCVLKPRPTTPPPQQPCYEKYPSDINFIFSPASMGVDTTSWVTQFISHTINNEELKSGFQYGVVSGDCPDDEGFQLDRYKEVTGIRERLAKYDRNNLPALVDRAATVAFTPGYGARDNAKKVAVIFVGEAKVDQSKLNAAIEKLLDEGVRVFVATTSSKVQVSVPKEVQVLTSGGTSHRQSSELVTYICHGEKTK

**> SG-PL_contig_39532_1**

MKLFVVACVLLYSVCCHAATLKYTPLHRIKLHPMQTTRQTLKQFGNSIESLRYKYGMVAFSEGVGPQPEPLTNYMDAQYYGAIGLGTPVQNFKVVFDTGSSNLWVPSKKCKVTDIACLLHNKYDSTKSSTYVANGTSFEIRYGTGSLTGFLSQDTLTIGDLKIKNQTFAEATQQPGITFVAAKFDGILGMAYKTISVDGVTPPFYNAVSQGLVPKSIFSFYLDRDPSAKEGGELILGGSDPAKYSGNFTYLPVTKEGYWQIKMDSVSVGGSSSKQFCKGGCQAIADTGTSLIAGPSSEIKALNEAIGATPLAAGEYTVDCSKIPTLPVITFTLGGKVFSLTGRQYILTVSEAGKSVCLSGFTGLDVPPPAGPLWILGDVFIGRYYTEFDMTNNQVGFAVAKSSKPLWFSPERL

**> SG-PL_contig_64416_1**

MKSVLLFALVLCAVYSHAEQKLHRIKLHRMQSSRQTLTSVGNSIKLLKYKYGLSTFNQPTGSAPEPLTNYLDAQYYGVISLGTPEQNFRVVFDTGSSNLWVPSKKCHLTDIACLLHNKYDSTKSSTYVKNGTKFEIRY

**> SG-PL_contig_296_1**

MKTTACLWLLVLLLVAIDDAAVAALDAEGDSSCTKFASNEDAIAKQINGKCYYGFTTNTTWSDARKLCFKEGMWLVSLQTEAEQELVSTTFQFRKYWTSLRLVDGIWYWYASADIKTPAGDDLGNRLQIRPGLRRYGVAKTLGKPGSYRNVLVETMQEAQHPMVCEDDPLSA

**> SG-PL_contig_14454_1**

MVSIFSSLLFLACVSAASSQSSGVWGTCSSQTQNSNIYTYVDSNSKCCDFKSSNYLMSVCCQDTSTNTCKCHPSIARSPYCLLTSVPATPPSARLCPAPFWTSGRLLNSSPVTSGCIGSGTVAFVTTTGLICNAALTADVIATLSTCRTLIKSALSNSAIPISLLLGSVSMPLVRGDLQDGKTGGNLTLWKLSPSAVIVLKQVLRNCPSTNCPYNPSTMSNAVDFNSCYVETYGASSVGATSYGKLSKVYLQKTNPTTCPGVSQGSGIECFHSTSGGAACLGDYAGPVFCPLNTGETALYGLTSANTCDPFNNLFAVVPLVQTVEIPLP

**> SG-PL_contig_647_1**

MGVWVRDGALILVVLAAAALAAPTEETEGKSCFYNNQEYVHGVSWSPPQQPCLQCMCKHGDIHCSILPDCEKSEQHRGDLGISATQGETVPGDISEGAPDEMMAQQGYGGYYNGGKSAPAYGPQYMQAQVGPMGARGSSGPPGPPGPQGMEGPRGQSGDPGPPGPPGNRGEPGPPGPPGIEGDPGRNGEPGAMGPPGSKGPAGSPGMPGMSGMKGHRGFSGFPGKRGEQGPAGEVGEQGPAGPAGPPGPQGSRGGPGERGQDGSPGQNGLRGNDGLPGTPGPPGPVGNIGSPGIPGMAGAKGEQGAPGPRGGDGPQGPRGENGNPGMTGEIGMPGPPGMPGSSGEDGAPGDPGPAGPPGFAGPQGRPGLAGSPGMAGAKGAAGLNGINGLKGQAGERGSTGLPGDVGKPGPPGSAGRRGQRGPEGPPGPVGPQGERGLPGSRGMPGSPGGPGSKGIDGETGSPGPRGEPGASGEAGHPGPQGAPGEVGLQGSSGPNGNSGAPGEAGPSGSDGRPGDRGAPGQPGPQGPPGPTGPPGERGAPGKEGSPGPAGPRGAAGDVGVPGNAGPAGPRGPEGEAGTQGPAGPPGPNGFQGPPGPPGPQGEAGRDGEQGIPGEAGPSGPAGYPGERGFPGERGPAGPAGESGLPGSEGPPGPDGPRGPPGESGIEGESGPQGPIGPPGERGAPGPPGERGELGDPGPSGADGLAGRPGPRGPRGAAGQPGDVGLAAEKGQPGEQGLTGRPGAPGPMGIRGESGPEGEQGPNGFPGQPGQPGAKGETGATGTKGETGDVGAAGTPGRTGPSGMPGNPGRRGSPGDRGLPGVPGEVGPGGRPGPQGPTGSAGPPGPTGVPGGPGSKGAMGETGRQGRPGSNGAAGQSGIPGNPGVRGEDGLPGGEGPPGPAGPAGMRGESGIPGVPGETGPQGPVGQHGRRGASGRPGNDGFPGKQGEAGPPGPAGPTGSPGAPGAPGSDGSPGQAGAAGEGGQAGEMGLRGPSGPPGPQGAAGAVGPSGSVGERGQRGEMGPDGPRGPAGPRGPAGPQGAMGESGDTGPAGETGAKGVPGYRGMAGLPGPEGPHGEPGRVGPRGPPGPRGVDGARGQMGPDGEPGAEGPRGPPGARGPAGEDGRRGRPGDIGPRGLPGSPGGSAYAGAMNSWYRGSSSYSYNGGYNGYQGDAPEEARATMEALERVSEEIKKLRMPTGKWDAPGVTCKDIAMADPTLKNGMYWIDPNGGSTSDAIRVFCKMRAEKTMTCLQSATHVYPTRRWALTKKDMGYWSFASSFEEREEFGYNSTKSQIRMLQHHTSNGRQRLVMKCKDTVAIYDKAINSYDKAMTLTSFDEELLTARSKKSHRYTVLRDGCQERNGREGQTVLEVSGAKMLKRLPILDVGFAEQSEGEFGLRVGRACFWGR

**> SG-PL_contig_3973_1**

MKPSQVATLATILGVAISVFVDTVSSAECEGCTSTICICAGQKGERGARGLPGLRGPQGLPGFLGPEGRAGNFGLKGRSGEPGARGPKGYRGDTGLPGFTGAPGIPGQPGLEGGLGPQGYPGCNGTKGLAGFPGQSGGPGLPGRPGRDGIPGPKGEPGSYVTDGIAIGEKGSQGSRGLPGIDGLSGNPGFQGERGMRGEDGLPGRDGVSGLKGEKGNKGSDGIGILGQKGQKGMKGEPGVAGREGTPPSNSTRVYGEKGQKGDRGEKGMEGERGFPGPGGPMGANGLSGMKGEKGLIGFPGRRGKRGKDGPPGPFGQKGMFGEKGFPGLPGVDGIKGMGGEPGYVGLRGPQGPPGVDGEYILTGESGTQGPRGRDGPPGPQGPRGPDGIDGEIGREGLPGLPGQPGLPGFSGEYGIPGDSMKGDRGLAGLPGEDGNDGLAGFPGLPGDLGEKGQPGETGVGAQGERGLPGLDGLPGLPGQPGDPGFDGRKGEPGFGVRFPGPRGSSGLPGEIGEPGFPGTPGLPGLKGLMGFDGDDCGICPNGEPGRPGEPGDPGFDGGPGFPGPVGFAGLKGEPGEQGFPGEQGREGQPGIPGRPGPDGLPGSKGYKGEQLPPPDFELYRGTKGAKGNIGYPGMPGMDGPQGDTGFRGLPGLYGPQGIKGNPGQPGDDGLDGLPGLPGEPGRKGQPGQSLVGSQGRKGQSGLPGLEGIPGPEGDKGLQGDPFFVELTQGDPGEPGRPGFDGLPGRDGIPGEPGRRGDDGFSGLSGMKGLPGLPGLPGEQGRRGPKGYPGVMGDPGFDGREGRKGELGPDGLPGLPGRPGLPGQKGESPRSAPRGDPGEPGGPGEYGFPGNDGRPGEAGRSGPDGFPGEKGEPGRSGGPGFPGQPGLPGDDGLPGQPGFRGRDGGPGGRGLPGLPGVRGNDGLPGIPGEAGRLGDDGNPGLPGSPGQQGRDGLPGSRGLDGLPGVPGVKGERGIGGRPGSPGPPGVQGLPGRPGSQGDDGGPGEAGVPGIPGENGAPGLDGIDGLPGLKGRQGEPGRGGPDGFPGQRGEDGQPGLNGLPGQDGLPGLPGQKGNRGPSFNGEPGRPGRDGPNGLPGLDGLPGTKGQPGPVGTPGRSGLPGSQGEFGLPGEDGRMGLPGVKGMRGNNGLPGIPGERGGPGVQGPPGLSGRQGERGNPGSDGLPGLDGRPGEDGEPGFPGRSALPGLDGARGDDGTRGMNGFPGEPGRRGEDGNPGLPGRRGEQGEPGFDGRPGFDGEYGRTGEPGPVGRPGENGPPAPPGRPGPPGEEGIPGLPGIPGQRGFMGDDGLPGLPGRKGERGEGIRTSPGPRGYPGEPGVPGLSGRRGAKGMDGLPGLPGLPGMKGESGRTGGMGLPGRPGEDGFRGENGEPGVPGFTGLPGVNGPEGQPGFPGIKGYKGDAGFPGFGGGIGFKGRRGEQGPPGQFPPWTTIVGPKGEPGFDGLPGYDGRPGQRGEPGEDGLFGPAGLYGRRGDDGVPGRSGDSGLPGNPGRRGQSGLPGLPGEPGRRGDAGFPGGVGSGPGIFFTRHSQTSAPPECPIGTVKMWEGYSLLYVMGNGRASGQDLGEAGSCLARFSTMPFMFCNINNVCNIASRSDYSYWLSTPEPMTPMMQPVEGRQIQPYISRCSVCETPAPVMAVHSQTMTIPDCPVGWSGVWIGYSFVVGTGSGSQGSGQGLHSPGSCLEDFRSSPFIECHGRGTCNYYATSSSFWLSTIERNDQFRMPLQETLKAGDLRRRVSRCQVCIKDYSTGPAFFTYANVK

**> SG-PL_contig_144_1**

MFTMMKAVCVVLLLCLAFTGGEGKKRKCDLRYKIISSEHTMCLDKKPEAKVVRLTPTKKQAIVDRHNNIRRTVQPEAANMLKMMWSFKLERLALKWALQCSSVSEDHPNKRILPNLGVDYVAQNLAVNPDDIYQAVLTWFQTKRNWQYGNETSLDGDDAAYVNQITATAAYIGCGQARCGDDTYFVCNYYVGPSESEAPYEQGSPCSRCPDNCNDKLCDCPEYVSCVNGGTFDYKTCQCRCPSPYCGRICTDQYCGMCDTSNTTYCQRISDIPAKCLDVCGIC

**> SG-PL_contig_3149_1**

MFTMMLTAGAALLLCLMFTGVETQICARKYSRIHREHTMCLTKNSDAVKVYLTEREKDVIVSTHNEIRAAANATNMQKVKWDNELAKLAQKWAMQCPSGSEDNPDKRKTPDLPGIIGQNFASGRADIFEAIFHWWNEHSNFQSGGVARHSPDSRNYEQLVWAPADRIGCAASTCTNTHYYICNYHTGPVVGEQVFEAGPPCQRCPNHCEEKLCDCTGSGICMNGNTRDVATCECN

**> SG-PL_contig_6209_1**

MASHFDTMAILLVAFLAVLTPVFGDTPANCTYEDIKGTWVFSVGPGGNDRSVDCSNFTGPAHKQVEITLYYPDIAQDQYGNLGFWTIIYNQGFEVVVTGRKYFAFSKYTASKQKTVSYCTEILPGWSHDVLGHDWACYSGAKKGSEAQSGIKVSAGLPSKVLTFEPRIFHNDLDLIKRINQAQTSWKAVAYPHLEGVKTEYLLKMAGGAASRTVQRPPVAPLTREVFEAAAKLPADFDWRDVDGVNYVSPVRNQGGCGSCYAFASMAMNEARVRIMTNNTHTPIFSPQDIVECSHYSQGCEGGFPYLIAGKYAEDYGLVLESENPYKGVDGKCSTPASVSRYYSTKYDYVGGYYGACNEALMRLALYENGPLAVGFEVYPDFQGYSGGIYHHTGLDSGFNPFQLTNHAVLVVGYGVEKSTGEKFWITKNSWGEGWGENGYFRIRRGNNECSFESMAMQSTPIISL

**> SG-PL_contig_554_1**

MKFSVFILFTSLAFVFAEDVKTEEDIYVLTEKNFDSFVKDNEFVLVEFYAPWCGHCKALAPEYTKAAKILMEDDSDVKLAKVDATVENELASRFGVRGYPTIKFFRGGSDNPVDYAAGRQAADIVNWLKKKTGPPAHELKDKDSAKTFVEKEDVVVIGFFKDQESTGALALKKAAAGIDDIPFAITSEDDVFKEYKMDKDGIVLFKKFDEGRNDFEGDFEEAAITKHVRDNQLPLVVEFTQESAQKIFGGEVKNHILLFLKKDGGEETINKFKAAAGDFKGKVLFIYLDTADEENGRITEFFGLKDEEIPAVRLIQLAEDMSKFKPETTDLETATIKKFVQDFLDGKLKPHLMTEEVPDDWDAKPVKVLVGKNF

**> SG-PL_contig_1307_1**

MRQAIVLFSCVVLAVQASNVLEFTDSNFKEKVQEHDVILVEFYAPWCGHCKRLAPEYEKAADRLVKSDPPVSLAKVDCTAETSICGEFGVSGYPTLKIFKGGEFSKDYQGAREADGIVKTMQKEAGPSSKELTSVKDFEEFLGREGGSVVGFFTDTASTMAKNFQKVADSYEDLRFAHTSTKAVLDKSKYKDEIVLFRPKAMASKFEDTAVKFSGDTTTSKIKTFLDEESFGLCPHRTADNAGKLKKPTFVAFYGVDYVKNPKGSNYWRNRVMKVGKKLRDEGLKAYFAISNKDDHDHELGECGLGEATGDKPIVCAYDERGRKFNMEKEFSMDTFEQFVRDVLDGKVEPYLKSEPIPTNDQPVKVAVAKNFDSLVNDPEKDVLIEFYAPWCGHCKSLAPKYDELAEKLEAEPEIVIAKMDATANDVPSPYTVTGFPTIYFAPKGSKSSPKKYEGGREVDDFIKYLAKEATNELQGYDRKGKKKGGKKKKTEL

**> SG-PL_contig_787_1**

MTGVRVVLLSLVVVGASAFYGPGDDVVELNPNNFHSKVIQSDELWVVEFYAPWCGHCQSLTPEWKKAASALKGIIKVGAVNADEHGGLGSQYGVKGFPTIKFFGLDKFKPQDYQGQRTASGIVDFSISQAKSAASSRLSGKKSGGDSKSRSGGGGGGKPGDPKDVVELTDSNFDEEVLDYDGLVLVEFFAPWCGHCKNLAPHWAQAATELKGKVKVCALDATVHTVMSNRYGVRGFPTIKAFPAGKKDGEAEDYDGGRTASEIVAWALDKVAANIPPPDVYEITSAQVLKDNCEGHQLCIVSVLPQIYDCQSECRNNYLKILRSLGEKFKKHQWGWLWAEAVAQPALEEALEVGGFGYPALAAVNTRKMKFSLLRGQFSEKGINEFLRDLSYGKGSTSPLKKAELPKIESRDPWDGKDAELPQEEDIDLSDVDLDDMDERKVEL

**> SG-PL_contig_14195_1**

MAVRSISAFFCALFLVIFDHAGGEIVKGSVNLNSGVFDKIIEKHKAVLVKFDETYPYGEKQDQFKKVAEAAITQPDLLVAEVQIADYGDKDNADLAERFDVKKEDFPVYKMFLQGTKDPIPFTGDATSADKIKKFIMEKTGLWLGLPACLEEFDKLVSEFFKAKGDKRSAILQKAEDVAKALSKEAEKESAEVYIKTMQKVLDKGDDFINTEIERVEKLKDGKVSDKKKEQLGRRLNILTSFELRLKDEL

**> SG-PL_contig_3626_1**

MKKLIFLGAIGLLFFAVAVKAEEEAGTVDVDLGKSMEASRTDDETMQKEEEAIKLDGLNVAQMKQMREQAEKFAFQAEVNRMMKLIINSLYKNKEIFLRELISNASDALDKIRFLSLTHKDALKATDELGIKIKADTENHVLHVTDTGIGMTHDDLVKNLGTIAKSGTSDFLAKLGEASPTELNDLIGQFGVGFYSSFLVADRVVVTSKHNDDEQYVWESDAESFSVVKDPRGNTLGRGTTVSLHLKEEAHDFLEPSTLTNLVKKYSQFINFNIYLWSSKTVEEEEPADEDTTEEADTEKKEADEDEEGKVEEEEEEKEEKKKTVSKTVWDWELMNSVRPIWTRKPADVSDEEYTEFYKSISKESEEPMGKIHFTAEGEVTFKSILFLPKRSPFDMFNNYGKKVDSIKLYVRRVFITDNFEDMMPKYLSFVRGVVDSDDLPLNVSRETLQQNKLLKVIKKKLVRKTLDMIKKIGKEEYDNFWKEYSTNIKLGVIEDTSNRTRLAKLLKFYSSNSDTEQTTLADYVERMKEKQEAIYFVAGTSREEVSKSPFVERLLKKGYEVVYLTEPVDEYCIQSLPEFEGKKFQNVAKEGLKLDQSEKAKEYREGLEKDYEPLLKWLKEDALKDKIEKATISERLSDSPCALVASQYGWSGNMERIQRAQAYARANDPSQTFYATQKKTLEINPRHPLMKKLKAQVESGSEDQTAKDLAVVMFETATLRSGYMLPDSAGFADRIDRMLRLSMDIALEEKVDEEPVFEEEEEAAEEGGEEEEEVNAEDDENAEGDSGTTHEEL

**> SG-PL_contig_19459_1**

MASLRNAVAVCAILFVCCLGGQSKADESEEELVKEVLFKPEKCGRLSKTHDMLSMHYTGTLTATGAKFDSSHDRKEPFEFQLGVGQVIQGWEKGLVDMCV

**> SG-PL_contig_4851_1**

MLSLRVLLLLCLLAALLGYLEGQSVFSWMKYDHFDEVDHHLRSVNAENCRSKGREELVLRPDTVAQLPVYNQLLNRIWYPNRTSLIHIHNMALNRAFFFSFILQKMNSTSEKEYLAQPNWLYMYMSVTADVNANPHTLNGSALYFDEHCHYPNWYETIPFNDTIPLFAPKAWRHDDWADADNFLR

**> SG-PL_contig_546_1**

MLLLVAAVGAAIAASDAEDESSCTKFAENSDATAKLIDGKCYYGISTEVPWKEAKKICNQAGTRLVRLETAAEQELVSLAFLYRKYWIGLRLIDNTTWYWFATESDKTPATDDLGERLTEKSRLRRFGVARLQGKEGDYQHGLTVSRQRALHPVVCEDDGVSGPSTDTVTVSVTETEATTEGPTTATDAVSVTETETTTEGTDGDIVTVPVTE

**> SG-PL_contig_351_1**

LLLVAAVGAAIAASGAEAASSCSKFAQNNKATARLIDGKCYYGINTPVTWKEAKGICDEAATHLVRLETAAEQELVSLAFQFEKYWIGLRLLD

**> SG-PL_contig_706_1**

MLAAVVLCMLMTSAGGYHTRQEQLNLRYISLRDLTWVDTEWVLAYRLVAGVKMSAYDYYMDRTRNDDRLLARAFRPLPCFDPSVPSPDCHQHYHSALLNDWPSDFIDQVRVSLIADKKEVAHILFNGTGSTSTSWFSADRVLSSSWTDLKTEPHNHFSIEGDHTPRITRRFFVFRNYGSCPRDSGWLSVQDQQTEVCSWGTLSDSHPFPGILYSKLPTYVNFNTADVGRADFLTVTVKFLTSTTYSGCLVQI

**> SG-PL_contig_34_1**

MTSIWPWLIGFAAMMAVVFSTPIDNTTQNSIEINVFVDRWALKRWKAMVATSSVMSEKINETKAAITDYVYSVFNDVNSIFKELMRYGINLDVRVHSLNFIEENEIAPDAKLGFRHVDPLPVVQNLFRYLWYRNYSYIEHVMLFTGLDVNKSDEHFYSETCGNYPLSVLEATPSGSAALSMAYQQALYMYVELDGQKNLCGNNYIMTAVDGPSSPTNFRFSNCSIASIKDFLESMIDRGNCMSAARTNTPPVSAPALGERYTPDDICKLTFGNTSYFCRSLYGYGGKYTYDSMCLEIYCKRKSGRCAKVQAPDGFVCGK

**> SG-PL_contig_402_1**

MPESRLPTAALVALLLLCFLCNGVNGTGQEKRQIHHIPDPTQGQTTAPPDPLTAWMSSTSSSKPNVLFLIADDYRPKMGAYGESNMVTPNLDFLASKSVRFDQAFAQQALCSPSRTSFLTGRRPETTRVFDLHTYFRTVAGNFTTLPEFFKRHGYMSVGVGKIYHPGVASGNTEDYPYSWSVQPYAGVQPFNAHMRKRSTHQPVNETETGKLQDTTIAEYAVNFLKQHSRNRDQPFFLAVGFHKPHLPWIYPKQFLDLYPRSKIYLAAHRTKPAHIPESTWSHSDEIRSYQDIIALNITGPDGIIPDHYQYTLRQAYSAATSYMDANVGKVLHALTQYGFAGNTIVSFLGDHGWHLGDNAEWCKHTNYEMALRIPMMLYVPGVTSMGHNFSFIDALAPGFDPSHDTLQHFGSQHVTEELVEAVDLFPTLIELAGLPMPHTCPENSLHVPTCTEGTSLVPLIRKVRNLLNGESFTWKGAAYSVYHRKWNSRSPILGYTLRTKTHRYTEWVSYDRTLFRPNFNHTFGQELYRHSDDPFEFNNLAQSAASSATVSQLAQMLRKGWRATLQDYLQSVH

**> SG-PL_contig_4169_1**

MRGDGSCVVLLAIALTMMVGRCPAPVSATVEEDEVNCRPQHVHLSFGEAADQMAVMWATNSTCDSVVRYGTNPWHTQDSATGRALPYTLNNAHGLHVINKVMLKGLKSATT

**> SG-PL_contig_1729_1**

MISSPIYRALLLAVAMLLLTNQGQCWKCHKDETVCEIWLELEHGLTMMKGKSLITLKDGLLFPYDTVNLTRENAIPKEQVITGDGFPEPMVVIMVNGTVPGPVIEVYENQTVRVHLHNKMASESTSLHFHGQVQNSTPWMDGVAFVSQCPILPGQTFIHQFKAEPAGTYWYHSHSGGQTSMGMFGGLVVHPRSRPNEPRYHDDFTLLIMDWNHDWSDTMTFMKMQYGMYVKGKKEGAASNLSGAHYSMFRIQSALINGRGRYTDPETSLHNGAPLEVFTVRRWNLYRFRLINSGSVFPFKLSVDGHKLKVVATDGMELDTPVMADSIIITPGERYDFELTASDVAANYWIRAKSLERDVNHAAEAILHYEGAPNNTDPTSRERDCTEDEPCTVINCPFHHYPASAHTKCINVEKLRASEKMPHHIGAGATNVKEYFLNWAFPGTTWTPASVNGIQMELPHVSALTQPLEFATRCPNEGYKCTEDQVCRCTAVLDLHGGDVVQMVLTNQGRGKGWDHPIHMHGHSFFVLKIGYPEYDNVTGKFIRDNMDINCRGNSDRDLSYCNNATWTNSSWSGNSVPGLELEKPMLKDTVNVPTGGYVVIRIQANNPGVWPMHCHVSLHLSDGMFLLLNESYPNHPPPPPGFRQCGDFSVPYPDVHH

**> SG-PL_contig_6669_1**

MGRTTSIVLLCLAAVCVQFAQCKHWALLVAGSNGYYNYRHQADICHAYHIMRNHGIAAENIVTMMYDDIAYNEENPIKGNIINQPKGPNVYPGVIIDYKKKDVTPEVFLAVLSGDEEKVEKLTGHKGKVIKSGPEDHVFVNFADHGAPGLLAFPDSSLYAKDLEKTIKEMHSKKSYQKLVFYVEACESGSMFAKILPKNIGVFATTAANPHQSSYACYYDTLRNTYLGDVYSVMWMQDSDKENLKVETLQKQYQITKKETNTSQVMEYGDLTIAKMTVAEFQGNQNSGVSMTTGSPPHPTADAVPAPDATMSILQHQLADASTPQERRSIAQKISDLMERNAQIDALFKDIVTEVEESIAESGKESVLRFNSKVSYQILKQERLPITQWECYEQAVDALTFFCRKMKLPQEYRALKQLYMLVNLCEYGHSGKTVALSVLTSAYKNEALCR

**> SG-PL_contig_28071_1**

MDIYCRLILHIGIIITMASGDLSGVSEVKDMAVANTHFTFDLLRGCGSDLKGQNFFFAPYSISTALAMTYIGARDQTAHEMKQVLKWSSSVPTKGFSSYLPLLKEAAGQQTPGYVLMGAQRIYVDKKLSLKPEFSSATQQHFDSQASVADFQRNAEGERKSINSWVSDQTRAKIQELLPAGSIDSLTAMVLVQALYFKGNWKHKFDAKRTQPAEFFSPQGTITVSMMEQTRHFNYGISREVDCAAIELPYASNSTSASTHDLSMLILLPNDYQGLDNLEASLTQVIFEKIRSSMRSLEVDLKLPRFSVESSIALKKVLTSLGMGRAFGEDADFTGMVTGPLAISEVYHKAVLEINEEGSEAAAATTITMTLRSLPVVEPFIADHPFLLFIIDNRADIILFCGRVTSPTPVSQSQQKEEL

**> SG-PL_contig_25446_1**

MASSVPVVAVVCCLFVVLTSGKQLPFRNDDTASCGYSSCNPVKDDMINVHLVPHTHDDVGWLVTVDQYYYERVQYILDSVIPELLKDPSKRFIYVEIAFFERWFREQSDSMRHIVKRLVNEGRLEFILGGWCMNDEAATHYNAIIDQHMLGFEYLRENFGKCGKPRVGWQIDPFGHSRETASLFARFGFDSLFFGRLDYQDKINRELHKTMEMIWRGSPDNLGTAADLFTGALPNGYNPPIGFCFDIFCKDKPIVDDPRLHDVNVETKVDAFLAEMSMQALEYATNHLIVTMGSDFQYQAAHNWYLNLDKLIKYVNARQANSSKINLLYSTPSCYTYHVNRANKTWTTKEDDFFPYAHRPHSFWTGYFTSRPTIKGYVRQTNNFLQAVKQLDALALLEDTDNSTFHLRILAKAMGVLQHHDAITGTEKQVVTYDYAERLNNGVLEGQKVYNDAIKKLGGGMKVDQAFCTLLNISVCSMTENGNQFQVTVYNPIGRPGLSYVRIPVSSTRNVGFSVVGPDGKPVSSELIPVSPDTMRIPERHGDSAKYELVFPVSLVPLGFSVFSVVRSKGLQADVQDRKVTAVYDEDLVIRNEYIALAFDKKTGLLKNMTNLEKKISIDLTQSFDYYMGMAGNNSGSKLQASGAYVFRPNGTSPYGMKPGHAKLTSATYFLKGQLVQEVYQEFTPWMTQTVKLYNQQRFAEFQWTVGPIPVKDKQGKEVISRFQSSLKNSKIFYTDANGREILKRELNHRDTWKFNNTEPVSGNYFPINSRIYIRDEAKKVQLTVLTDRSQGGTSLRDGEIEIMVHRRLLYDDGLGVDEALNETGCDGKGLIARGSHYLMLEPIASAAAGHRDLAERLFMGPQISFSQTKSKQVKAVWSGVRQALPDNVHLLTLEQFKSYGPVPSATQPFLLRLEHFYEKGEDPVLSKPVTVNLQKMFSTFDIVKATELTLGADMALADLKRLKWNYPHKDDHFADKKDSTLTFK

**> SG-PL_contig_92_1**

MTLLVAALFALTCLTVPATSVRQMILGVTPFTEEQMAILLSAIFKTEVEFTPPPEINTQIQALIPTDNSQALREEWEKAGMDVTTIVSDLRELAQVEEQHRRRRDAENVNPDTTLNHNYYRSYDQIIEDIKKLVQKFGPTLIRFDESGKNPARTLTAEGRIIHIVELGTQTEKQKPVILLEVNIHAREWITNAANLFMMDWILTNYRLSGGAAKMLLDNYTMVVVPVANPDGYEFSRTHPDNRMWRKNRHVFEFCTGVDLNRNFDMMWRGDLEGYLWFSRSDLNISPDEERQCPLLYPGPSANSEPETQALISIFHHYQENVVLHFFVHAYSEVVLMPWSYNSIAANKIHQEMGDIVARAMDYGEAKTVAEFARYNASGTSLDWALNEKLTESPDSKFIALGME

**> SG-PL_contig_22703_1**

MAMVLVLVVLCVGITLTNACSCMQENTPDRFCRYNLIFRGVALAEYEEREVPLDPTDPLSEYSRDNDRVYMMRVDHAILMPENLREAKTVTMRTPAQDSLCGTRFSLDTDTVFFAEMNSDGELVTGLCDPNVLWASLSESEQEILTDNIVDYCTTKDNVTPPKA

**> SG-PL_contig_529_1**

MAGVWLLLIGSAAMMAVVYSTPIDNTTQNCIEIRVFVDRHAFKSWKDKVATSSTWLQRVNETKAAITDYVYSVFNDVNSIFKELMRYGINLDVRVHSIHFVKQKLLARGINKTTGQLSPGWALYLLSEWVNPNYPAIDHNMLFTGLTLKKKPTQNYPESAYYGSTCFSDPFSVVEATTSGAAALFMAHQLGLSMTVEAEGSQIFCDNGYIMSAFKTHSSPTNFRFSECSIKRIKRYLEVVRDRKNCLTAANTNTPPVSAPALGERYTPDDICKMTFGNSSYFCRSLYGYGGHYSYDSMCGVIYCKQDSGLCTQVRASDGFVCGK

**> SG-PL_contig_662_1**

MTSGLVIWLVFAVYHGSSSVVKGGQAPHFTNEWAAQIPGGEEHAKDVAKRNGFEIITSVDNFDDYYILKRHDVPHRSRRSSHHHTRRLIEDGQIPWAEQQVARSRVKRLAGFRLRSDGSSDFNDPHFRDEWYIENSIPSSRKERGQAIHLDLQVKGAWKQGYTGNNVTVSVLDDGIEHNNTELAKNYDPYASTDLNDNDPDPMPRYDPTNENKHGTRCAGEIAMVANNGKCGVGIAFNSRIGGVRMLDGRVTDSLEARALSFNYSHIDIYSASWGPNDDGVTVEGPGTMAQQALEKGITYGRNGKGVIYAWASGNGGRLQDNCNCDGYTSSIYTLSVSSASEHGQVPWYGELCSSTFATTYSSGNGGDHQVVSADLHDRCTTRHTGTSAAAPMAAGIFALLLEANPHVTWRDVQHLVAVTSKVGPLASEDGWYQNAAGFCVNLAFGFGLLDAAELVSAAHPDNWVSVPQKSICEISADTGSNLPQTLSSGHFVEVLIDTNGCAGQHNEVNFLEHVQFVFTLSYSKRGAISVTAVSPSGTETTLMMPRQWDKSTDGFRQWPLMSVHTWGEDPKGVWRFRVHDGDANESRHGVLKDLKLVLHGTKDAPHYLRDGPVHCDVAVVSKKHTTDVTPSGGDDWGRPSLQPMGTGLMRGQSNFMTQVIQAWMDRQKASQDDREAAIEEYLYL

**> SG-PL_contig_24648_**1

MRGTYGLLLLTLSLLHTTTTTTSPFTNDWVAVIPGGAEHAHTVARRHAFEVVRSMPHFTDHYLLRRSDVPQRSRRSSDHHTWSLTEDTQVEWAQQQVAKTRVKRHNAEDHEFNDPLFPDEWYLVNRAQSSRDTQQSVTKMSMRVQGAWGKGYSGYNVVVTILDDGLEHTNKDIQANYDPCASTDLNDHDSDPMPRYDPSNENRHGTRCAGEVAMVANNGLCGVGIAYNSSIGGVRMLDGPVTDAIEGMALCFNHTYIDIYSSSWGPSDDGKTVEGPSKLATQALEKGIKYGRGGKGAIYVWASGNGGMSDDNCNCDGYTSSIYTLSVSSATEHGLAPWYAERCASTMATTYSSGYRGEQQVISADLHNGCTEHHSGTSASAPMAAGIIALVLEANPSLTWRDVQHLVVLTSKGGALSAEKGWYRNGAGLCVNPAFGFGLLDAEALVTQAINWTHVPPVSVCHVNAANTSSLPRAVKSGHMVQIEIETNGCRGQQNEVNYLEHVQFVVTLTYSTRGALSITAISPSGTKTVLMPPRYLDRSSRGFRQWPLMSVHTWGEQPAGVWTFQVQDQSGNRANRGELKEVMMILHGTKTAPDYRNNTAYSCGLPVGSNNSTGLRTEDKRSDNRPAFMSLVEKALDTEKEKETIGV

**> SG-PL_contig_2692_1**

MDLKNHKTLCFFSLFAVCLGWTIELGKPKPLRLPEGKLAEFLLGLLEGLGNTRQTTKVTGRPSPPHVTTRMSETQFYQPGQEVKFQCQATGSPPPTYTWQKNGKNVNLTDSETRGNELRINGTLLFPGDKVDADTEGVYQCLAENEYGTSLSSLVHLTMARLEMAPMEQPDRMVTPRIGSSLIIPCTSPHSVPEGHVHWIVEYPIGDMDLLEDTTYSDVKLSDRVTMDYNYSLHITNVQQSDEQGGGGYMCVTRNQVLRAMAMGPKTFIRPQSGRFRPPPMPARLMWSSPSYVVALLGTDLKMKCIFSGNPTPRVRWAKGGVRALPSSARLESAGQELVLTDVKYSDAGDYECWATNSLIHIMNELRSFHVEVQSAPQWVDAPQNVEVEAGQTAEVNCSASGLPQPSTAWFVNGMNLSDVVASEERFQSERFDNTSDRLVFHNVTTDDRMVIQCNVSNRHGNIWADVSLNVLATGVENQATTTIQSTSTNDQETTTNIQTSPTNDTETATTTQTTTTSDDQNTTTNAQTTTTIYDNETTTSTQNTTNDNQTTTSTQTTTTTYDNEATTSTQNTTNDNQTTTSTQTTTTTYDNETTTNTQNTTNDNQTTNTSQTGTSDNPYSTTPTQTTITTTSDGSQVTSANQSSATENPYSNATTQSTTTGIPKPRSSTETGREGATVILTCTNASDLHLSVTTFKDGEEINLTNDRFALQLNGAFAMKKVTRHEG

**> SG-PL_contig_6557_1**

MPSSLTSILLICIVIWGPCLCQRRAPTRGQIEDHFLSTYIDWYNADIRPYQRDDDLPVRVNVSYELLHVTNLDEEKGHLDTVGYLAMEWTDNRLTFNHRINLDSIVLRTDRIWVPDIELYNADAPGLKMLNAHSQKVVLMTGGHVIWVPQVATRSLCFA

**> SG-PL_contig_561_1**

MVATLLTVLAAFTKMADTKGERTFIAVKPDGVHRGLIGEIMKRFEQRGYKLVAAKLCMPGEAHLKQHYSDLSTKPFFEGLVKYMNSGPIFAMVWEGKEVVKMGRAMLGATNPLASNPGTIRGDFCIDVGRNVCHGSDSVESAKKEIGLWFTDKDLITYEANAKPWVYE

**> SG-PL_contig_14037_1**

MLVCGAVTLLLVQTFASAEEKSDKPIVTNKVYLDIKIGGSQAGRIVIGLFGQVVPKTVKNFLSLVEGYQDSHGKLLTYKNSKFHRVIKDFMIQGGDFTRGDGTGGRSIFGEKFPDENFELRHYGAGWLSMANAGKDTNGSQFFITTKKTEWLDGRHVVFGKVLAGMRVVRAIEYAETDGYDRPQKPITIADCGISEVMKPFPVAKIGTTDDS

**> SG-PL_contig_2900_1**

MLVRAAVLLVLMQTLAFAEKAKGPKVTDKVFFDIEIGGKNEGRIVIGLFGKTVPKTATNFKSLAVGDKKHEDGSALTYKGSKFHRVIKDFMIQGGDFTRGDGTGGRSIYGEKFPDENFKLKHYGAGWLSMANSGKDTNGSQFFITTKQTQWLDGRHVVFGKVLEGMDVVRKIESSKTDARDKPEKDVVIADCGALDVEAAFPVTKDDAK

**> SG-PL_contig_10497_1**

MSARMACVSTLLACCFLTAVLAGEEACYSYAGGSVYPQETRRTSGHTIQWSQAVISKPAPDWNGTAVVNGEFKDIKLSDFRGKYLVFFFYPLDFTFVCPTEIVAFSDSIGKFKSINTEVVACSVDSQFTHLAWINTPRSQGGLGPLKIPLLSDITHDIAKAYGVYLQDLGHSLRGLFIIDPKGILRQITMNDLPVGRSVDETLRLVQAFQYTDSHGEVCPVGWKPGSDTIIPDPKQSQKYFKKQKDPEGEKEEL

**> SG-PL_contig_50021_1**

MKLTIMALMTSVWLALCVLGTSHAACNNDKNQAWKGGMKLSFGTTTPLYNQTVALASVNSTQAPIVTVDSSTAQSLQDENLLLVMVDPDAPRPASCPKFYWLHWLATAIVQTDGTVKATTLMEYTPPTPPEGSGLHRYQAMLYRAPANHPFLTAPKTRGNFDLHGLASGMRDLVAATQFKTEAKQA

**> SG-PL_contig_10652_1**

MATKAILGLLCCFAVCYNPLAGTPVAEKVAGTPVDGTSTVTLKYDEESKKLTVDPDGVGATIAFSKLGHKINKTGWAYLEVLTFPVAPDYVQAYAAGFVEGLITKDLMHMHWTNTLAGFCTKPYSKFCAKLSAFLTQNLQWMKEQIKKNKNSPFWHHVNLFLYQAAGLADANNLPTPVEPNIHIDPMGFYLFQVDSDIEDLQSVLGDSKMRKTLGAGSCSALIKLLSGNKDLYASQVTWNAYQSMLRILKFYSFPWKMVADGTETIPGQNQTFSSYPATLMSGDDYYSLSSGMVSMETTIGNNNSSRWQYVTSSSVLEGIRSVVANRVAKSGQEWAKTFSLYNSGTYNNEWMVVDYKRFKPGMPTLPPGVLTVLDQIPGMIKWADLTDVLTKQGYFPSYNVPYFPEVFNASGNQASVKKYGDWFTYDKTPRALIFKRDHVKVKDLDSMTKLMRYNDFKHDPLSRCNCTPPYSAENAISARSDLNPKDGKYPFGALGHRNHGSTDMKLTSSHLMTDLTFIAVGGPTWDQQPPFQWSKSDFNATSHVGQPDLWKFSPITFNTTEIFSHC

**> SG-PL_contig_89580_1**

MTRNPSSTNFALFLFTISLCYGRLVRSSDSPIVQQFSVPQNDFPFRNVSLPWDERVKDLVSRLTLEEIQLQMGRGGAGTHGGPAPAIPRLGIG

**> SG-PL_contig_564_1**

MASWITLIALLAVTSAVCLAETEDDGAVFLSPLIAAGSLREAKSRSRVRWNFVNETAVTELWSYAGYITVNDAYDSNMFFWFFPARQNANTSPVLLWLNGGPGVSSMVGALIGNGPLKVTTEGKVVKSKWSWTKAFSMLYVDNPVGAGYSFTGNVSGMPLTIEEITPHLYSILVQFFTVFHEYSQNDLYIGGQSFACQYVSALGKYIHDRNHETPPPSLLLRLKGVYVGGGFCNAAVTYSEFYNNMYYAGLMSDYTRRVHNALAKSTIDNALSTGRVDGTDVYNIISGIITVTDAFDIENQLGKKYSIHSLKAALNTLLSSPTARQALHVGHANGNVSFLAANNDVWAHIKTLNFLRSANEESIFLLENYKVLHYSGNLDLLVSVAATEKFLQQLEWTGADRYYNSTRDPWPRGDALLGWVTQIGNFTRVVVRNAGHQVPHDQPEAAFLMMKNFVLDMPFFPPAESTTDEN

**> SG-PL_contig_2041_1**

MASWITLIALLAVTSAMCLAETVDDGAVFLSPLIAAGRLEEAKSLSRVRWDFVNKVNHTELESYAGYITVNETSDSNLFFWFFPTKKNVTGVPVLLWLNGGPGMSSMVGALIENGPLKVTEEGRVVESKWSWTTEFSMLYVDNPVGVGYSFTASS

**> SG-PL_contig_38_1**

MPCRLLLKSLILLMTVTSPGAGDVYTSTSTILTTALSEGKLLEGLNKFIGFEYDNMNKMSQFLWERSKANHLVSKEDLQWLVEHPNGAYFIVKLFTQHWVPLANTYPVLARMMEPVLSEMPTDRDFEGALSSLIRLQRVYLLSVDHMYGGNYTGHMGPPLQPEDTYLLGQRAFEDGYLSESVQWLELALRLMYTYDAFPATSSVFGGNHAAAPLVTLAQVASLLGRVYFFMKNQTKAQEMYELSSALDPQAYDVKNLKTDLDGNGLEYRHAESEEWEHNMTTLCLRPRHHTVAETTP

**> SG-PL_contig_8568_1**

MRCCSFVLNLAVLVLIVQDGWGQTDQTDLRLRRREKQRYRLAELARASTSFGVNLYRELAARDNSNIIFSPFSVFTALSMTLLGTDGITKSQLRAVLVQRPRQGIHQALHAVFASFPSSPDANVTLRVANAVYYDAQQVSITDSFNRNVRRLYGAYPRMFERPNPEIPINNWIYSQTGGKIQDFFNPGDITSDLVLILLNAVYFQGKWKDKFDERSTQDGDFTTASGQVVRVPMMSRTGRYPVKMLGGELGALVLELPYGNDGRFSMFVLLPTQVNGLSDVEARLTSRLLEEAVSNMPQATRVRVQMPKFTLRVKTSMKSVLQAMGLTDLFSGSAQLSRMATSRRPLAVNDVKHEAVIEVDEKGTTAAAVTSVEVIAISLPPSVFVNHPFLLVLRDNKARLNLFMGRVNDPSPQA
