# Supplementary material for: Multiomics analysis of the giant triton snail salivary gland, a crown-of-thorns starfish predator
Source: Sci Rep. 2017 Jul 20;7:6000. doi: 10.1038/s41598-017-05974-x (PMC5519703; doi:10.1038/s41598-017-05974-x)
Supplement: Supplementary file 10 — Dataset 8 [file 41598_2017_5974_MOESM10_ESM.doc]

File S9: Genbank accession numbers for cysteine-rich venom protein, arylsulfatase, metalloproteinease and echotoxin-like proteins from Charonia tritonis and other species.

Sulfuric acid biosynthesis enzymes identified in *C. tritonis*.

**Cysteine rich venom proteins**

>SGAL_contig_35322_1

MMLLRWDDEVAMLAQKWADNCVLTHDKNGQRNIPGRITVDQNIAMRAKDWTMVITLWHSEKSRFIYGNKSMNDIWPNIGHYTSMMWARKTLVGCGMNTCDDITFHVCDYALAGNLMGAIEKPYKQGAPCSDCPDHCVYSNKLCDCHLTCLNLGTLDPKTCTCKCAVGSSLYTPPNCALNCTGTKDAGFCPGKGGKCPAHTPWCP

>SGPL_contig_1954_1

NISEAVNAWHSTKSSWCYHSIRRVRSKSRPYEQLIWGSVRSVGCGYAECANRRYFICNYYGRPVLFEKPYKIGKPCTECFYRTCDDNLCVNCQYPEQQFCKTLGPEKCELFLFYMDCPHMCGVC

>SGAL_contig_8800_1

MMQTVGAALLLCLIFTGGETKICARKYSRIHREHTMCLTKNSDAVKVYLTEREKDVIVSTHNEIRAAANATNMQKVKWDNELAKLAQKWAMQCPSGSEDNPDKRKTPDLPGIIGQNFASGRADIFEAIFHWWNEHSNFQSGGVARHSPDSRNYEQLVWAPADRIGCAASTCTNTHYYICNYHTGPVVGEQVFEAGPPCQRCPNHCEEKLCDCTGSGICMNGNTRDVATCECN

>SGAL_contig_14037_1

MNTYSTWTYGELTNVKISAMPYVQQIWASAGYIGCGYAKCGDKKYFVCDYSAVPTIPEKPYVEGTRCSLCPDNCEGWLCY

>SGAL_contig_44134_1

MTKATRRPGNGEMEERTDLPYTCTAEYRAVKPKHTMCKPFGPDSYNVGLTPETIQEIVDFHNELRSEVRPRAADMQKMVWDETLARTAAKGARDCNDIHDEVRNEPIYPGVVSGQNIARSHPGLKKTFKDDIQSWYDEVTEYNQNGRRYKKGTGHYLQVVFSQANRVGCGEAFCGNEKIYKVQRRCNYANGVNYTNTYTVGRPCSRCPGHCDRQGKLCDCNGKLCFHGGNLSLATCQCECFSPWRGVNCEIADCSVKDESWCRSY

>SGAL_contig_29657_1

MVWAETVRVGCGFADCGTTTVYICNYAPRMNTVDMDSIYQPGDACQQCPDSCMDGLCDCGEKVCENMGTLDPVTCTCKCLLPFHQPPSCS

>SGAL_contig_55539_1

MMLTAGAVLLLCLIFTGVETKTDQDKPNCNPVYSRISPHHTMCLTDNPDASKLSVTEKMIKKIVKRHNQIRRSVFPYAVNMQKMHWSSQLATLAEKWAMQCPT

>SGPL_contig_144_1 (Identified in proteomics analysis)

MFTMMKAVCVVLLLCLAFTGGEGKKRKCDLRYKIISSEHTMCLDKKPEAKVVRLTPTKKQAIVDRHNNIRRTVQPEAANMLKMMWSFKLERLALKWALQCSSVSEDHPNKRILPNLGVDYVAQNLAVNPDDIYQAVLTWFQTKRNWQYGNETSLDGDDAAYVNQITATAAYIGCGQARCGDDTYFVCNYYVGPSESEAPYEQGSPCSRCPDNCNDKLCDCPEYVSCVNGGTFDYKTCQCRCPSPYCGRICTDQYCGMCDTSNTTYCQRISDIPAKCLDVCGIC

>SGPL_contig_6209_1 (Identified in proteomics analysis)

MASHFDTMAILLVAFLAVLTPVFGDTPANCTYEDIKGTWVFSVGPGGNDRSVDCSNFTGPAHKQVEITLYYPDIAQDQYGNLGFWTIIYNQGFEVVVTGRKYFAFSKYTASKQKTVSYCTEILPGWSHDVLGHDWACYSGAKKGSEAQSGIKVSAGLPSKVLTFEPRIFHNDLDLIKRINQAQTSWKAVAYPHLEGVKTEYLLKMAGGAASRTVQRPPVAPLTREVFEAAAKLPADFDWRDVDGVNYVSPVRNQGGCGSCYAFASMAMNEARVRIMTNNTHTPIFSPQDIVECSHYSQGCEGGFPYLIAGKYAEDYGLVLESENPYKGVDGKCSTPASVSRYYSTKYDYVGGYYGACNEALMRLALYENGPLAVGFEVYPDFQGYSGGIYHHTGLDSGFNPFQLTNHAVLVVGYGVEKSTGEKFWITKNSWGEGWGENGYFRIRRGNNECSFESMAMQSTPIISL

>SGAL_contig_2339_1 (Identified in proteomics analysis)

MQLCLVLVCCGLMVGPIWAGTVESVAVAKHNQLRAAEVETNSASNIYALKWNSALAAQAATWTRKCRVEHTPGLDRNIGQNLYSAGYIGSEKPKATKVLKDAIDLWMEEKSLNDGTFACCTRLTMTCCHYTQVVSSRSKEVGCAVEFCPGNQIIVICNYTPMGNFNDQAAYRTGPPCSGCKKKDKCNADKLCVSRKG

>SGAL_contig_17334_1 (Identified in proteomics analysis)

MQLCLVLVCCGLVVGPIWAVSVKMATVVKHNGVRKIEVQTDSASNIYELKWNSELAAKAAAWARRCQLGFSPDLDVNIAQNVFMAHYFEFRKPKAAMLIKNAVDSWMEEKSKNNGNFACCKDSSVDCCSYTQVVSSRSKEVGCAVEYCPGNQAYVVCNYKPGGNFNNQDAYLTGPPCSGCEGNDQCNRDKLCVSNKR

>Conus_textile_Sequence ID-CAD36507.1

MLSTMQTVGAVLMLSIVLVAGRKRHHCDSKYYELTPAHTMCLTDKPNAVAVPLTQETEHEILEMHNKIRADVTDAANMLKMEWDERLATVAQKWAMQCILGHDSGRRGEPDLPGSVGQNVAWSSGDLTFLGAVQMWADEIVDFQYGVWTDGTGHYIQQVFAGASRIGCGQSACGNNKYFVCNYYKGTMGDEPYQLGRPCSQCRSSCQHIRGSQGRWGSLCDCTNGPDACFNGGIFNINTCQCECSGIWGGADCQEKHCPNEDFDDMCRYPDALRRPQHWCQYDNFQSDCPILCGYCPNPN

>Conus_marmoreus_1_Sequence_ID-ABG36845.1

MLSTMQTVGAILMLSIVFVAGTKRHACDSKYSEVTPTHTMCLTDNANAVAVTLTQEVKVQIVRMHNVIRATVNDAANMMKMEWDDRLAAVAQKWAMQCILGHDGFANHAEPDLPGYVGQNVGWSNYHMTFPDVVDLWAAEIEDYEYGVWNDNTGHYIQQIYAEASRIGCGQSACGEDRYFVCNYYKSTMGNTPYAQGSRCGQCPNSCWEELCDCTSGPDACCNGGSLNIDTCECQCPRLWSGADCQEKQCPDHDYEDMCDYPDVVNNPEYWCQFSNIRSDCPIRCGDCP

>Conus_marmoreus_2_Sequence_ID-ABG36844.1

MLSTMQTVGAILMLSIVFAAGTKRHACDSKYSDVTPTHTMCLTDNANAVAVTLTQEVKVQIVRMHNVIRATVNDAANMMKMEWDDRLAAVAQKWAMQCILGHDGFANHAEPDLPGYVGQNVGWSNYHMTFPDVVDLWAAEIEDYEYGVWNDNTGHYIQQIYAEASRIGCGQSACGEDRYFVCNYYESTMGNTPYAQGSRCGQCPNSCWEELCDCTSGPDACCNGGSLNIDTCECQCPRLWSGADCQEKQCPDHDYEDMCDYPDVVNNPEYWCQFSNIRSDCPIRCGDCP

>Conus_marmoreus_3_Sequence_ID-ABP48101.1

MLSTMQTVGAILMLSIVFAAGTKRHACDSKYSDVTPTHTMCLTDNANAVAVTLTQEVKVQIVRMHNVIRATVNDAANMMKMEWDDRLAAVAQKWAMQCILGHDGFANHAEPDLPGYVGQNVGWSNYHMTFPDVVDLWAAEIEDYEYGVWNDNTGHYIQQIYAEASRIGCGQSACGEDRYFVCNYYKSTMGNTPYAQGSRCGQCPNSCWEELCDCTSGPDACCNGGSLNIDTCECQCPRLWSGADCQEKQCPDHDYEDMCDYPDVVNNPEYWCQFSNIRSDCPIRCGDCP

>Conus_marmoreus_4_Sequence_ID-A1BQQ5.1

MLSTMQTVGAILMLSIVFAAGTKRHACDSKYSDVTPTHTMCLTDNANAMAVTLTQEVKVQIVRMHNVIRATVNDAANMMKMEWDDRLAAVAQKWAMQCILGHDGFANHAEPDLPGYVGQNVGWSNYHMTFPDVVDLWAAEIEDYEYGVWNDNTGHYIQQIYAEASRIGCGQSACGEDRYFVCNYYKSTMGNTPYAQGSRCGQCPNSCWEELCDCTSGPDACCNGGSLNIDTCECQCPRLWSGADCQEKQCPDHDYEDMCDYPDVVNNPEYWCQFSNIRSDCPIRCGDCP

>Colubraria_reticulata_comp112437-c0-seq1

MRLPQLQSPFWQLLGQARQGLPSVYGLPSRDCAAA

>Colubraria_reticulata_comp89900-c0-seq1

MSVGQNMAANHQNWQSAIQGWHKEVDMYKYGPHPDTYLGKDGWKKVGHYTQMVQNTTHLVGCG

**Arylsulfatase**

>SGAL_contig_57071_1 (Identified in proteomics analysis)

MGTTRRILLVALFATCFGLALCDDRPNIVFVLADDYGFNDVGYHGSEILTPNLDRLAGEGVKLENYYVQPICTPTRSQLMSGRYQIHTGLQHDIIWASQPNGLPLDSPTIAEKL

>SGAL_contig_87_1

MVLFLHVSMAARPHIVIILADDLGFDDVGYRSKAMYTPNIDKMAKEGVKLKYAYMQQVCTPSRAAFMTGIYPFRMGLQNSVLSALENRSMPMDKTVLSQRLKTLRYATHYVGKWHLGFCNYNMTPTYRGFDTFYGMYTGKEDYYTHISKWNGYDFHDDNGQNFNVDWTAKGKYSTPLFTERAVKIIEGHDTRKPLFLFLAYQAVHGPEQVPDDYLKYCADVTNETRKTHCAMAAAMDEGIGKVNEALEKRNMTDNLITLFLSDNGGAVKKGSSNWQI

>SGAL_contig_123_1

MRAAGDRDGVSGIDGVDSWKSIVKNWDTSSRDCFVYNIDDVKNTSAVRCDQYKLVHNKAGSPDGWWNWPNGTTVVLPKGRYPEYMLFDIENDPSEMIDLGGNSSFTDTIEKLKAELKKQKDLGFKPSK

>SGAL_contig_3480_1

MTTTTIMMVMMMTTTTTMMMITIMMAITMMSDRVQTCQPGGEGTEDRDSMWADRRLALPLLVLLALTSAVTGQRQRPNIILMLADDLGWADVHRHDASMPTPTLDWLETRGMVLNYSYVLPTCAPTRSAILTGRYPFTYGMQVNDITGARRAWLDETLTLLPATLKSLGYTTHMIGKWHLGFCHESLTPTRRGFDTFYGFYTGAQGYFNHSGDSRYAYDFRDNDEVVWSARGHYSTELHTARAQRIIRERDNQTPFFLYMAFQNVHGPFEAKQRYIDQYCSHVTNETRRIHCGMTAAMDESIRNITDTLSEEGELDNTIIVFLSDNGGPTDGASFNYPLRGKKATLWEGGTRVFTLLAYPGLSRRGGYVYPGLIHAIDWYPTLVQAAGGQPPPGLDGMGMWRELRDNANSPRTEFVYNIDDKNKRSAIRWNHWKLAKGRPGRKYNGWYPPPSLLQQGVRWEEVPKRQVAKWHLYNLAADPSERDNLYYDRSYGRIARLMKRKLRMYKRLLVPFQPAPKVAAGNPRRTGGVYSPGWCQP

>SGAL_contig_5894_1

MEILRSPWVFVTSLVLLLHIVLLHTSTASRPHIVVIMADDLGWNDVGYKNPEVHTPNIDKLAEEGVKLNYSYMQQVCTPSRVAFLTGIYPFRMGLQNLVLRALENNSMPVEHRVLPERLKTLGYATHYIGKWHLGFCHYNMTPTRRGFDTFYGMYNGKGGYFNHTSKWSGYDLQDNKGTDPQNFRVAWEDYGKYSTHLFTNRAVKIIDEHDLSTPLFLFLSYQAVHGPLEVPEGYLQLCPNVVDETRKRHCAVTAAMDEGIGVVMQALRARGLSDNLITLFTTDNGGPVRLGSSNWPLRGSKITLWEGGTRAVSILHSKTHLPYAPYEWKGLIHAVDWYPTLLQAAGDKSKIADIDGVDSWKKIVRNSAKSARQGFVYNINDVKNNAAIRYNRFKLIYNKGGNPDGWYNPPGNGASEEPNGSYSEYMLFDVEKDSEERNDLSGVNKEHIVKTMAKLKKQLATFRQGLKPTTRAAVIAEGKPRFHGGAWVSGWC

>SGAL_contig_7941_1

MENVCFLLLLVCGTCSAVNGQYAAVRESNTSVLSTDDDHDASTFRDSGGGPENTADSTETLPITFSTKTEHSSTNDATIKSSPNAGARVVDENVTGFADSPEDFNTTTTRNSADTRKATTITEMAASERNATQDNTTLTLTDATAKENRHPTRTHTVIMTSTQYGLNLTNDTQTKKDEYQTSSRLDVTMMNSTPDNLTLKDIEKKKSEVPSRPHVVMLNSTQDDPTPKDTQTTTNDAPSRPHIVMIVADDLGWNDVDWRDKTMHTPVLTRLATQGVVLNHSYVQPVCSPSRAAFMSGFYPYHVGLQHDSLRATQKAYLPDNITTLPQHLQQLGYRTHMAGKWHLGFCNWRYTPTYRGFDSFVGFYNAVQDYYTHTGHRHGYDFRYNKDVFYSANGTYSAFVFSQYIQHVISQHDVTTPLFVYLPFQSVHGPLQVPQYYIDRYCSHIQNATRRIHCGMVAALDEAVGNVTSTLEERGMLDNTLLFFTTDNGGPVHKGANNWPLRGSKATLWEGGTRAMAFLHGPNILKHSGTTYDGVIHAVDWLPTLVEAAGGSAVTGLDGVSQWQSVQGNKVPSAREEFVYNIDELKKNAALRQGKYKLLQGDPGHLSGWYPPPQLESVFQPPAYRDTQSAAVDGISNQGNVHLMHILSDRYVSGETSRQYDIFFDTQLLNSSSKHPSTLRDGEGTAGSDATGNPVLAMVSENEVSDKGYQLYDLQADPNEHYDVKKKYPDVFKKMRALLDEYRKSLVPANFPPHDPDSYPSNFNGVWSPGWC

>SGAL_contig_57071_1

MGTTRRILLVALFATCFGLALCDDRPNIVFVLADDYGFNDVGYHGSEILTPNLDRLAGEGVKLENYYVQPICTPTRSQLMSGRYQIHTGLQHDIIWASQPNGLPLDSPTIAEKL

>SGAL_contig_81699_1

MHTPNIDKLAEQGVTFNYSYMQQVCTPSRAAFLSGYYPFRLGLQNSVLTALSNASMPLNYRFLSEHLQDLGYRTHFVGKWHLG

>SGPL_contig_99346_1

MELLFGPILLAALATICSAAVSPRPHIVFIIADDLGWDDVGFHGSEITTLNIDALAYSGIILNKYYVSPICSPTRSAIMTGRHPIHTGMQVGVIGGATPYGLPLNETTMAQHLQTLGYRRHIVGKWHLGFFADPYLPTKRGFETPFGY

>SGPL_contig_921_1

MPSTSVQRGHVGSVVLLLALLGISSAEITKPNIVFVLSDDQGFHDVGYHNSWIRTPNMDSLASQGVKLENYYVQPMCTPTRSQFMSGRYQIHTGLQHKVIFAKQPNALPLDTPTLASTLKDAGYSTHIVGKWHLGFYKPEYLPTNRGFDSFYGFLLGSGNHYTHTSLGHTDLHDNTDDVLEEKGHYSTHLFTEKAIDIVRSHDSSKGPLFLYLAYQSVHSPLQVPSRYKEQYQEIGDDKRRTYAG

>SGPL_contig_922_1

MPSTSVLRGHVGSGVLLLALLGISSAEISKPNIVFVFADDQGFHDVGYHNSWIRTPNIDSLADQGVKLENYYVQPSCTPTRSQFMSGRYQIHTGLQHRIIQPKQPNGLPLDTPTLASTLKDAGYSTHIVGKWHLGFY

>SGPL_contig_12938_1

MITPNVDRLAKNGVILDQAYVQPLCSPSRSAFMTGYYPYRVGLQHMVILPQQPVCAPLNATFLPVEMKKLGYATHMVGKWHLGFCSWQCTPTFRGFDSFLGYYNAAEDYYVRVFERYYDFRDNQTVSVKENGTYSTYVFQERVRKIIESHDTNTPFFLYLPLQSVHPPLEVSMLHVCHQSVFLS

>SGPL_contig_81466_1

MHTPNIDKLAEQGVTFNYSYMQQVCTPSRAAFLSGYYPFRLGLQNSVLTALSNASMPLNYRFLSEHLQDLGYRTHFVGKWHLGFCRKEMTPTYRGFHSFYGMYTGKSDYFTHISKWNGYDFHDDKGDNFTVAWDAKGKYSTTLFTDRALEIIEDHDQSKPLFLFLSYQAVHGPLQVPNEYVTKYCANVTTGGRDRKLHCAMTAAMDEGIGKVMDKLKAKGLHDN

>SGPL_contig_74944_1

MEGWLCPVSLLLAICGMTMGVGASSKTASRPNVLLIVADDLGYGDLGCFGNTTIRTPHLDKLATQGVKLIHNVAAATMCTPSRAAFLTGRYAIRSGMTSP

>Aplysia_californica_1_Sequence_ID-XP_012941568.1

MMKLWLPLLFAVWLSAVCGRDNNQKRPNIIFVLADDYGFNDIGYHGSQIFTPNLDKLASDGVKLENYYVQPICTPTRSQLMSGRYQIHTGLQHAIIWPEQPNGLPLDSPTMADKMKEAGYATHMVGKWHLGFYKDEYLPWNRGFDSYFGYLTGSENYNNHKRTFMDGKWYLDLCDQDGPAHNESGHYSGHLFTEKAIDVVQAHDPSKPLFMYLAYQSVHSPLQVPKPYERKYKHIKDRNRRKYAGMVSVLDEGVGNLTQALKDKGLWDNTILVFSTDNGGQVYEGGNNFPLRGWKASLWEGGFHGVGFVSGGRLPVSGVVNRELIHVSDWFPTLVGLAQGSLNGTKPLDGVDQWDTISQGAPSKRTVLLHNIDPLHPRVGVPLYNDTFDTTVRAAVRVGHMKLITGNPSNGSWVPPPGESQALKFVSDRRTSPGKNVWLFNITADPTEHHDLSDQMPDMVRTLLGYLQRFNETAVPVRYPPYDPRSNPALRGGVWGPWE

>Aplysia_californica_2_Sequence_ID_XP_005090307.1

MMKLWLPLLLAVWLSSASGSNNNNNNNNNNNNSKQPNIIFILADDYGFNDIGYHGSQIATPNLDKLASDGVKLENYYVQPICTPTRSQLMSGRYQIHTGLQHEIIWSEQPNGLPLDSPTMADKMKEAGYATHMVGKWHLGFYKDEYLPWNRGFDSYFGYLTGSEQYFNHKRKYVDGKEYLDLWDNQGPVYNETGHYSANLFTEKAIDLVRGHGQKKPLFLYLAYQSVHCPLQVPEEYERRYSHIKDENRRLYSAMVAAMDEGIGNLTQALKDSGLWEDTLLVFSTVFCVRDICVISYTVYESIRSLQRDVCCFHSRGTPSKRTVLLHNIDPLRPRVGVPLYNDTFDTTVRAAVRVGHMKLITGDPGNGSWVPPPAESQKFIPDRRTSPDKNVWLFNITADPNEHNDLSDQMPDMVRTLLGYLQRFNQTAVPVRYPPYDPRSNPALHGGVWRPWE

>Octopus_bimaculoides_Sequence_ID_XP_014775592.1

MSPTRCVALLLVSLLGASLIAIVSPSTQSQQPNILLILADDYGFNDIGYHGSEIQTPNLDKLAGEGVKLENYYVQPICTPTRSQLMSGRYQIHTGLQHSIIWPQQPNCLPLDSPTLADKMRESGYSTHIVGKWHLGFYKKECLPTNRGFDSYFGYLTGSEDYYTHRHCDGFCGFDLRNNLEPVRNNTEYSAFLFAKKAINVIENHDSSKPLFLYVPFQSVHAPLQVPDKYIAPYSHIKDKNRRVYAGMTACMDEAIGNITNAMKKKGLWNNTVLVFSTDNGGQILEGGNNWPLRGWKGSLWEGGLHGVGFVTSELLKTKGTSSKALIHVSDWFPTLVKLAGGFLNGTKPLDGFDQWSTISEGTPSARKELLHNIDPLHKPKGQSHFPDIFDTRIRAALRYGDWKIITGNPGNDKWIPPPHLNLPTFSKATPLKNIWLFNITADPNEYYDVSLENLNVVEFMLTRLALYNATAVKPRFPNGDPASNPNFHGGFWGPWQ

>Helix_pomatia_Sequence_ID-AAF30403.1

MKEVLAWLSSFLIVNCLRMVICAKQQQPNIVIVVADDYGYRDIGYHGAEFATPNLDKLAAEGVKLENYYVQPICSPTRSQLMTGRYQIHTGLQHDIIWPSQPYGLPLQFPTIADMLKSVGYSTHAIGKWHLGLYKKEYTPLYRGFDSYYGYLEGGEDYYTYYNCDTFHNRTTPADTSILESYSPKNILLGKHEDENKWCGYDLRDMNEPVTDMNGTYSTHLYTKKAIDIINGASTGGKPFLLYLAYQAVHSPMEVPAEYTKPYTFI

>Biomphalaria_glabrata_Sequence_ID-XP_013081934.1

MSVNKVFSELFYVVTFFHLATLAAAQRKPNIVFVVADDYGFHDIGYHGSEIFTPHLDKLAAQGVKLENYYVQPICSPTRSQLMTGRYQIHTGLQHNIIWPSQPYGMPLEFPTIANFLKENGYTTHAVGKWHLGLYKKEYTPLNRGFDTFYGYWEGGEDYYTYYNCDTWHNKSIDIESGITRSYSEKDIQSHQNSDKTQWCGYDLRDMDKPVTNMNGSYSTYVYTSKSIDIINKANPEKPFFLYLAYQAVHSPMEVPDIYLEPYKHIQDKNRRIYAGMTSCMDEGVANVTNALKAKGLWDNTIFIFTTDNGGEVLAGGNNWPLRGYKHTLWEGGVRGISFVTSPLIQQKGVISEELMHVSDWFPTIADIIGVHMNSSLKLDGTNQWPMISKGETSKRKEILHNIDILTPLNGDRMYNDTFDTRIRAAIRMGNYKLITGMPGDGNWYPPPHDSHLKNHKFNSTEASNKNIWLFNVKVDPCEVIDLSGVLLDQVRLMLNRLLYYNATAVSPLYPESDPNCDPTLHGGYWGPWQ

>Strongylocentrotus_purpuratus_Sequence_ID-XP_011667457.1

MAVRHLGEFVVIVYLTLVTSGSSYAEQLPNVVFILADDYGFNDVGYHGRSQGSAILTPNLDRLAGEGVKLENYYVQPICSPTRSQLMSGRYQIHTGLQHGVIRHAQPNCLPLDEVTLPQKLKENGYATNMVGKWHIGFYLDACTPTERGFDSFFGFLVGAQDHYTHILSCEAFNADDGSTKTLSGYDLHANKTTVYQYKGQYSTHLFTNKTIDVIERHDKTKPLFMYLAYQAVHAPLQVPDSYMDPYKNIADKTRRTYAGMVSCMDEGIGNVTRALKEAGLYDNTIIIFSTDNGGHIDRGGNNWPLRGSKGSLWEGGIHGVGFVHSPLLPDAVKGTVNHELIHVSDWLPTIVAGIAGRALNGTKPLDGYNVWSAISGTASSPRTELLHNIDPKKKRPVNEKFDTSIRAALRVGDWKIKTGADYDVTVNNEWMPPPESGLDTIYPTEKPNQKVWLFT

**Snake venom metalloproteinease**

>SGAL_contig_95028_1

MPPPMNPINIRSIMGMGHGTNGNHGGNNTNTAGSRDSNDTPQGSQGVPDNTSLTGTGTSKNGGYDKSSGYGSEHDLGERLSMEENSHHSRSHSASPPSYSAVIRTGPNQIQLVAP

>SGAL_contig_102105_1

MGWHLGVIFLLLLLFGSREAGRYGRDLHDLVQDYDYNRVLQENIPSPHSVVIPTQLRNKVTYPLSTREHHLRHHGTHEHMKQVVIQLRLNERKIRIKLERNDALLSSGIMVKHFTETNQQVIQKTVEHCYY

>SGPL_contig_62783_1

MKSLNTGIVTIINFGKTVPNRVSQLTFSHEAGHNFGSPHDIGSAECAPFGTTRSDANNGNYIMFPSATSGNKVNNDKFSICSRDNMTRIIQQVVDSRRHMCFVASGQAFCGNKIT

>SGPL_contig_64224_1

MYFKDPVFHTVIYPETHMDLDPYRHRRSAEVGMCGNDLAAAWMKWHTESAIDRNPSHRAKRYSSRGESPHLKYMESSNKESSKGDRQRRSTIGEKNTCYLYLRADPILWEHVKSKKYNMKLTSAQCRGG

>SGPL_contig_54233_1

QNDHIDNFTLQFVAFGKRFLLDLHLTRNLFASSYTRKVLHKKGADLKESLHRKEHCYYQGQIRNNLLSHAALSSCDGFRGYVSDGEETYHIDPVNGTLHRLFRNSDRKKLPLKCGTERHDVFHPPPHVFSHRIRKKRTVRGPYDANKFTRFVELYIVNDYRTFDRLGKNATVVFKRSQDIANIVNSLYRQLNIYIVLVGVEV

>SGPL_contig_84860_1

MPPPMNPINIRSIMGMGHGTNGNHGGNNTNTAGSRDSNDTPQGSQGVPDNTSLTGTGTSKNGGYDKSSGYGSEHDLGERLSMEENS

>SGPL_contig_34_1 (Identified in proteomics analysis)

MTSIWPWLIGFAAMMAVVFSTPIDNTTQNSIEINVFVDRWALKRWKAMVATSSVMSEKINETKAAITDYVYSVFNDVNSIFKELMRYGINLDVRVHSLNFIEENEIAPDAKLGFRHVDPLPVVQNLFRYLWYRNYSYIEHVMLFTGLDVNKSDEHFYSETCGNYPLSVLEATPSGSAALSMAYQQALYMYVELDGQKNLCGNNYIMTAVDGPSSPTNFRFSNCSIASIKDFLESMIDRGNCMSAARTNTPPVSAPALGERYTPDDICKLTFGNTSYFCRSLYGYGGKYTYDSMCLEIYCKRKSGRCAKVQAPDGFVCGK

>SGPL_contig_315_1 (Identified in proteomics analysis)

MTGVWRLLTGSAVVMMAVIYSAGTSTQNTIELRVIVDHFAATKWGNTVGGNVDINDYVYSVFKNVNSVFKKLKNSGIKLDVRVVGIEIVTTEEISSEMPPGSGKRNSSEVIEELKNWLASKNYRQTDHTVLFTGLDLLGDDLSTANAGKAYLGKMCDQDFSLSVVEAPHSSAVLTIAHELGHSLNSVHDVGACMNGHIMNSSLNIGQASRNNFKFSSCSIQSIKSILRELSGKDSCLLDTSFEWTEMSPLPLGQLLPPDQTCKLAYGYNSFFSRRVYSENGDDTYDSMCGKIWCKKGQQHSSARASDGFICGNRKLCSLGECISSGSTEVLDTCPQGDSPQANDAGEKCKQVVTADNGECYLEHIKQACCSSCDEVHTRRAGCEYGDKSKSCAEFRDFPGLCDQQPDACCGTCWERSNPTESTCTDKLDDCQERLAKNNARCSKSAFRENCCASCADVSTNPETESTCTDKWDNCQEKLAKNSDKCSKSTFRENCCASCR

>SGAL_contig_21456_1

MEDSHGYCLFVVLIVAMTSSGVFSQSDTPDSSQYEPSPGLFDRLEEPTDKTIDQLITEALGGIDVASNKILAPGAYVLAELDMYLSQEQFLNLYEPPSNHILRGPNGTIIRSPSSPQPPAMANSTSARSKRKATRDVILRWPGAQIPYQFARGDFTDKERYMIKQSMTEWERYTCLKFRPASSADTNVVRFQNGEGCNSQLGMVGGVQLLNLDVAGCRFKGLYLHEIGHAIGLVHEHQLPDRDNYIYVLLQNVAPHFRIFFNKYSTTVVNQFNVPYEYSSVMHYGITAFSHNGKAQTIRTRDRSKESSIGKVYLKELSFSDVKVVNEMYSCNAFCPDVVTCT

>Lottia_gigantea

MERCGRAITMTHITISKLLITLLLLGFVVHNVYGQSNGSQLFHRSRSRHPGHRRSNPVSSPRTLQMLTKRPVFKPTFRPIQQTIRRVIPNRVIVPRQYTPSKTITPRHRLLVNTPGRNVMHHKQYKVPNQLAKSFKPVTPLVKPSIKLSSPYPVSRANYIGELPNEIELLVVVDYEAYLRWYEREGIKYQNGRDIKTQQAITKYVTSLVHDMNNLYHSLEMLDLKIDITLVDILIIQSKEGGYWLSKLSKKGFPRNIVNAASSLASFYRWVKKHEKVLPPHDHAMLLTGFDLQSANEAPNKLTTGHAYLGTMCKRSSVSVVENQFNFEDVVAASHELAHSLGSEHDGDGNPCPDTEGYIMAPVIEVNATNRWSFSTCSHQYIQDLLEKLDREERNCLSTKNNLGHMPQLHDADVKLGALLSPDQQCALIEGPGSFLCRDFYQEYNYHTLCREMWCNNITSPTPTCNTFLGSDGFSCGNQKVCIQGECVFQQTAKVVPDLCPLGDEPGIVWNNYTCPEIAKNAPEQCYDDVTRMKCCLSCQKIHNGDLECPYGDRSSWCRTDLELPVGCYLNEDLCCATCAKIENKNEPECPYGDRSKWCNTTLSVPIGCYENQDLCCETCKQHIDHNNPECPYGDRSTWCSTTMEAPFGCYKNNELCCGTCAEYHQPQNIGCEYGDKSTWCVNEMNVPSGCYLNSDLCCGTCNQHYNSTNLGCEYGDKHTDCAKVPYPMGCYKNSALCCGSCADQKRHDRPGCEFGDKSTWCTNHLKPEQDCELNKELCCGTCVYKEGQFLL

>Crassostrea_gigas

MEIVFTLNESLVDLHLKRNEEISANVPIYTMNRQGTVEAEHIQERNNVFFYQDEQRFAAFYVETVGSSNCLFGSFEDHNNEFFLEPKDLDCMESTNNNQSVFRVLKIKHEGFQYSDNIKMENRTYQPSTDKNGYLDVKRSVAEYKIEMLLIIDYSIYNYWFTQSTGSSTAAKDTDAKYNIRQYYAWVINGMDVRYKNIQSSAYTISILFGGIFIADTVGKSSFTEPFKDSSSPRPGVEASPVLSNVTNWVQNTAGLPAHDHAMMFTRYDLKSQGSSSTKGLAWVAVVCESQSVSIVEDDFNFVILTVAAHELGHSLSASHDGDGNGCSGNDAYIMAASVGVPVNTNPWKFSTCSINYFTTYIASLISKGSNCMTSLSSNFDPTALKQFTSLPGQVYDVDALCKHLMGPQSSFCKFPYKGDYTTLCLSLWCYKTDGSGSCSGFTGVDGLQCGNKKRCQTGVCTYDVCAPQKDESCLFGDDTGTVVTFNTGVTKTCADVPSEPSLCYYESVHKGCCQTCPRYHTGRAGCEYGDRVSGCTSGHCPKYKDTQCCGLCYSGPVITTTTVQAEDTNPCQNYTLKTSTAATFTGITVSPKNNISSTANTRTMSSTSTDKTTMITISNEVTFKFVLRMDIIVAEDLSNSTQYSIVKDKAQYALTDLYRKRLGNSFKSCSVTNLRKGSLVVDYRVFTKNSPSATENMLSINQDILTGKANVTYDGKDAPVSSMSFFDSSGKAVNLTITTTACALLEASKPCQSGSECVEEGNGPVCRAVSTEKGNDLLLMMAVIIGAAIVFVVVVAVIMYFVCRRKETGQKQQEGSLPY

>Biomphalaria_glabrata_1

MTSFNYFLLFLASGSVTLFTAAQSYVAEGYFVIDNKVSLNYREDKKISKPEKELQNDMDYILTQVNELFKSLVPHGITLEIRVKRFNILPVDIFPKNSTKSSSPFDIQPKVSIKLFEKWLLATNSYKNISYDFAFLWTGFNLADDDKAKTAGFSETSQMCDSVKSIGIAEFSRTYYTAISTAHEIAHILGAHHCKPNSLHIMSPVTNLTSPRKWSFDKCSALEIKKYLGTLKTNCLLKTDKNSSKPDVTYASYKGQIFDPDIICHRVKGHRSYMCKMWNFYNDSAPGGDLICSRLQCSEPGTELCVDIVAPNGMVCAVGKRCNAGKCTPDSSVKSKVDPQCLYGDQKVAKAPAKKLDSTCEELIRKLGPASCYKPVYFQQCCSTCARRRINRPGCEYGDRVSICKMFNKNVLCEKEDNTKKCCNSCYGYKPKRSVADNFDSLFSITELSLP

>Biomphalaria_glabrata_2

MKTFSVQLNFTCSPEVIPSEVNLTLNSEIFSVDLHLKRQTNVNPDLPVYTLNVDSTGKHVHQREVLDTSQKIGFYHDLTSGAVFQITNTSDVINHGLKLLFKGQFRYDDKTYFVTHETRQKREATSTDGPLYNLEVLPDPEHRMFDYVKDIPGHLFSKKLTKFHTSVSRRRIAERTRSRRQTPTYHVDLVAVVDFKAYSQFLSAAGNKTLALRDLRQYYSFIIAGVDLLYQGITSTNFITRVHLIKVIVAETAETSDFTERFKQGNKIDADQSLNASYYYVAQSEAVLPYDHVMVFTGFDLVRGGNSGTIGLAYLSTMCEQDGSSVSIIEDLGDYSCISTSAHELGHSFSMSHDGENNSCSLTDRYIMAAYIIAPANATKLNPWRFSACSLAAMKTFVSTLMTKREECLSAQLTVSSDVPQVGSNLPGLNISANDQCKMTYGNQSFVCHKTSDVDLTLTPSAICYYRACYQPLSNNCAVDYETLTGTVCSSGKVAIKLEVLMYSIKYMWAYTS

>Octopus_bimaculoides_1

MQEFYAAMVDRMDTRMRTLTLPNRKLGVTLAGFYIDEDPKSRLWLDKDGRQKPEIKTDQFLKALTHWSKRRNLPEHDHAMFFTILEVFENGQKKLRGYSPQSKICSNESVSIIQERLDFSTITVATHVLAHSLGARHDGDSNPCRPEDHYIMSPKSVTLTEGSILNMWNFSSCSSNSVGQNLKRLNEENSNCLKNTPSVRRSKPHRQLGQMFDADVQCQHIFGETSYVCRSKYWSSYRGLCTGLWCSTNSGHCQKIIPADGTSCGRQKWCMNGKCVTDREAPEVADECPLRDQKNRISGTNLTCEQVSKSHQWECYQTSVAQACCYTCPMKPCDGNTCCKFDDRHSQCRNFSSYACYTPDITKLCCETCKRHCKTNIPGCACGDRRDGCSNITRLDCYTQHKECCYTCSKLRDTTHPDCEFGDRSDQCLASNCIYYDTYRQSRVCCQTCRHLHTLVATTTEVANSPPSPVDSAATGTVPLASLYWLTAQLMLTAITTSTTTTTTTASTTSMMTMMMMMMTALTITMSRPL

>Octopus_bimaculoides_2

MFSSTKCLWITLIQVVYYCQPSFTVPLPPSDIGKNYKISKEGIGIIGPYSNKSIDQLITGALGGIGKAANSIVGANGSIL

AELDMMLNIEQFNDLYEVPNTESEPIRKRRKAVRNEKFRWTNGIIPYEFDPYDFSVKDLYFIKRALTEWERYTCLKFVPARETDKNRILFRHGSGCNSQLGMVGGVQAVNLDANGCRYKGLYLHEVGHAVGLVHEHQLPDRDRHIRIIYQNVLPSMRIWFNKYSTDVINTMNVPYELSSVMHYGITAFSYDGKSQTISALDKSREDEIGRVYQKELAYSDVEIVNKMYNCRARCPDKDSIKCLDGGVLDENCKCICPDGSQDCREGAVVDVSEEDFAKQLYLWLGLQTHLAPEVWKGSSSCKNTYLDAKCDTWSANGDCLTNPKWMNENCAKSCSSCPSDTQPSTCKNSYLNDEKCDQWALEGECQLNAVWMEQYCAKSCRTCDKVTGGGGTDEEYDDCKDSEQYKDKCPGWAQANQCAINPKFMIPHCRMSCKKCQTKGCVNLYKDNICDLKAEGDQCESNREFMRKNCRKSCKLCSEGEIEYDKKTTVYVEPTTAPTTKNNPYNCYDSHTNVEDCKMWRDNNHCNINPDWMARHCRKSCGFCSDTGRTTTTSSSCQNNDPGCDEWAKHDMCTTNARYMLVHCKKSCKVCGGCEDDNQLCAAWAKGHHCNKNAGYMLRHCRKSCNAC

**Echotoxin**

>SG_AL-PL-1 (This sequence was used for 3-D modelling)

MKSSRLPGRLPTMTTVVVVMVVIIGLSIPVEPFIGALLAAISGLTAGQIASAAGTAVSIGSSIGEAAAAASRSNYRVTCVIEMENWTKHNLHSPIVQITNSGSLITNPKNIWPASAQAFSVRKPAHSATGVYGTVSWMLGDTGRRVVVMWSAPYSFNHYSNVLGVGLTR

>SG_AL-PL-2

MQSSRLLSRLPTMTTVVVVMVIIIGLSIPVEPGIFKGLSLSKIIGAAESAVTVGDSVTAATQAAASSGYRVTCVIEMENWTKYLLSNPRLQITNSGSLVTNPKSVLPSQMQGFAVRKPAHTATGVYGTVSWQLGNTGRRVVV

>SG_AL-PL-3

MQLDSRHQGSYISAMKSSRLPGRLPTMTTVVVVMVVIIGLSIPVEPFIGALLAAISGLTAGQIASAAGTAVSIGSSIGEAAAAASRSNYRVTCVIEMENWTKHNLHSPIVQITNSGSLITNPKNIWPASAQAFSVRKPAHSATGVYGTVSWMLGDTGRRVVV

>Monoplex_echo_1_Sequence_ID-BAI78310.1

MKSRTSTWSTMLKMVVLVMVLISQSHPVESSVASIITALKNLPAKTLASAVNSALSTGASVASAAQAVTSSGYSVTCIIEVENWTKHLMSRPEIQIANSGGLLTLASNIMPATAQAFATRKPAGLASGVYGTVSWVLGQTNRRVVVMWSAPYNFDFYSNWMGVGMTKAGVSVPSSRSAWFDQMYYRGSSSDLSFVRGEYYYHVNPIYWKNNEWEVEGSMTNVHNARVRVMVKPMNTMDLASSILSKLEALTSSGRKRAIQQELARRALEERNAWEEGKM

>Monoplex_echo_2_Sequence_ID-BAI78311.1

MRTLAYAGRFRTTKMVVVVFALILAHSSKVDTAIIASISAAIAGLTASQVATAATTAITTAASVASAAIEASKSGYSITCIIEMENWTKHLLAYPKVQIANSGGLVTLAKNVMPAEKQSFAVRKPHGANGVYGTVSWAIGSTGRRAVIMWSAPYNFNFYSNWMGVGMTTTGVSVPSSRSTWFDQMYYGESSSSLSFVRGEYYYSVKPIVFKNSEYEMEGAMNNIHHAVVKVTIRPIKNVDLATPILVKLEALVKSQKRDTSIITNELKRRSMEPFLDSELLVKTKV

>Monoplex_echo_3_Sequence_ID-BAI78312.1

MRTLAYAGRFRTTKMVVVVFALILAHSSKVDTAIIASISAAIAGLTASQVATAATTAITTAASVASAAIEASKSGYSITCVIEMENWTKHLLAYPKVQIANSGGLVTLAKNVMPAEKQSFAVRKPYGANGVYGTVSWAIGSTGRRAVIMWSAPYNFNFYSNWMGVGMTTTGVSVPSSRSTWFDQMYYEKSSSSLSFVRGEYYYSVKPIVFKNSEYEMEGAMNNIHHAVVKVTIRPIKNVDLATPILVKLEALVKSQKRDTSIITNELKRRSMEPFLDSELLVKTKV

>Monoplex_echo_4_Sequence_ID-Q76CA2.2

MRTLAYAGRFRTTKMVVVVFALILAHSSKVDTAIIASISAAIAGLTASQVATAATTAITTAASVASAAIEASKSGYSITCVIEMENWTKHLLAYPKVQIANSGGLVTLAKNVMPAEKQSFAVRKPYGANGVYGTVSWAIGSTGRRAVIMWSAPYNFNFYSNWMGVGMTTTGVSVPSSRSTWFDQMYYEKSSSSLSFVRGEYYYSVKPIVFKNSEYEMEGAMNNIHHAVVKVTIRPIKNVDLATPILVKLEALVKSQKRDTSIITNELKRRSMEPFLDSELLVKTKV

>Conus_monile_1_Sequence_ID-ANC48005.1

MGVPFPALKTMVTVFLLLMGNASPVHSVISASLVASIASSVITAGTSVASTALQAVGGSSATVTCVIQVENWTRFALKYPTVLTNGAPPITNAPTAILPTKIEAFAVEKPRGTATGTSGTVSWELQGMEQRIVLMWSAPFSFDLYSNWMGVGMTREGLVDVASGDTWYKQMYYKDDSEDLTFERGKFYYNLKPVIYRNDKFEIVGTMTNIHKAQIKVIIRPTINNWKDMALPIQEMLVKQEKEVCNITTA

>Actinia_fragacea_2_Swiss-Prot_B9W5G6.2

SADVAGAVIDGAGLGFDVLKTVLEALGNVKRKIAVGIDNESGKTWTAMNTYFRSGTSDIVLPHKVAHGKALLYNGQKNRGPVATGVVGVIAYSMSDGNTLAVLFSVPYDYNWYSNWWNVRVYKGQKRADQRMYEELYYHRSPFRGDNGWHSRGLGYGLKSRGFMNSSGHAILEIHVTKA

> Colubraria _reticulate_ comp89661-c0-seq1

MALPFPRLKTILVIFLFVIGHGPSQVWMMDPLTIASVATAAGSAVSAGSAVATATMKGLVDAGYRVTCAIHIENWTRYPLLYPTVRLKDGALNGAPTEVLPGKREDFAVLMRKRAHAAAGVYGTVSWEVSGVKRRFVIMLSAPYNFDFHSNWLALGMTRPGLTEVANGNKWFDHMYYGPVPKTTDKVDLKFQRKRFYYLTDPVIYRDSEFEVVGVMTNVHKAEIKIVFRPVEGNYANLAPQIRKVLGLSN

Venom Carboxylesterase-6

>SGAL_contig_16433_1

MGKNGVGDVDLMIGFNNKEGGLLAFADILGLVPPEGLYSSAYLGTVLNWCLNYTALNNHPAVKKAIEFFYYGAEAGNSIFESLIPLVDMNSDCMMAMPIIELTHFLAHAPHTAALYLYVMDHGFVFNAQGPVPGAHHGDELILLFDRERLMPEGSIFSTFNKTSLAVEEQPLSAMVIQMWTTFAKTGNPSPPIQ

>SGAL_contig_98437_1

MTWHTSILLLMLTVVQTVRSAPTIQAPWATIQGVSLEGDKGRKMNAYLAIPYALPPVGDRRFEKPEPHPG

**Venom serine carboxypeptidase**

>SGAL_contig_62796_1

MTLQSIVTISSVHAYREMVRKSVASDYSGYGLADAWAGFIWNRFPAEAYPLFLSKLLEQNRIQEAQERSRVRNLTSEDIESYSGYLTVNKLYYSNMFFWFF

>SGAL_contig_72832_1

MFPMTTIPENAFLMDHYKVLQYTGNLDVIVNTAMTEAFLDHVDWSGQEEYRHSDRQKWFVDDELAGWFTTVKNFTRVIVVNAGHMVPYDQPSRALDMMRRFVWDGFQKDTSG

>SGAL_contig_100234_1

MTEAFLQHVPWAGQEMYRQARRRRWFHDGSELAGWFVSINNFTRVIVRNSGHMVPYDQPKWA

>SGAL_contig_63065_1

TSAVCFAETEGDDAVFLSPLIAAGRISEAKSLSRVSWDFVNEVNETDLESYSGYITVDEAYNSNMFFWF

>SGPL_contig_1444_1

MYYAGLISDYTRRTYSAIVNNVIDKAVSAGRLDGEDLYKMLAAVENVTENPDIDNQLGGEHTVHLLKDRLETLLNSPTARQALHVGQANGNVKFTSQSDEVFEFIKTRNYFRSANEDNIFLLENYKVLHYSGNLDVIVSVAATEKFLQQLEWPGTDRYYNSTRDPWF

>SGPL_contig_2042_1

MASWITLIALLAVTSAVCLAETEDDGAVFLSPLIAAERISEAKSLSRVWWDFVNEENGTELESYAGYITVDETYNSNTFFWFFPATKNASTSPVLLWLNGGPGVSSMVGALIENGPLKITSEGKVVKSEWSWTRAFSMLYVDNPVGTGYSFTGN

>SGPL_contig_97782_1

MFFWFFPAFKNKESAPVLLWLNGGPGRSSLVGGLMENGPLQITVNGTVERRNVSWTELFSMLYVDNPV

>SGPL_contig_564_1 (Identified in proteomics analysis)

MASWITLIALLAVTSAVCLAETEDDGAVFLSPLIAAGSLREAKSRSRVRWNFVNETAVTELWSYAGYITVNDAYDSNMFFWFFPARQNANTSPVLLWLNGGPGVSSMVGALIGNGPLKVTTEGKVVKSKWSWTKAFSMLYVDNPVGAGYSFTGNVSGMPLTIEEITPHLYSILVQFFTVFHEYSQNDLYIGGQSFACQYVSALGKYIHDRNHETPPPSLLLRLKGVYVGGGFCNAAVTYSEFYNNMYYAGLMSDYTRRVHNALAKSTIDNALSTGRVDGTDVYNIISGIITVTDAFDIENQLGKKYSIHSLKAALNTLLSSPTARQALHVGHANGNVSFLAANNDVWAHIKTLNFLRSANEESIFLLENYKVLHYSGNLDLLVSVAATEKFLQQLEWTGADRYYNSTRDPWPRGDALLGWVTQIGNFTRVVVRNAGHQVPHDQPEAAFLMMKNFVLDMPFFPPAESTTDEN

>SGPL_contig_2041_1 (Identified in proteomics analysis)

MASWITLIALLAVTSAMCLAETVDDGAVFLSPLIAAGRLEEAKSLSRVRWDFVNKVNHTELESYAGYITVNETSDSNLFFWFFPTKKNVTGVPVLLWLNGGPGMSSMVGALIENGPLKVTEEGRVVESKWSWTTEFSMLYVDNPVGVGYSFTASS

**Enzymes required for sulfuric acid biosynthesis**

>Sulfate_adenylytransferase_SG-AL

MRSAVQRATNVVYQSHHVSRDKRGKALGQRSGFRGCTIWLTGLSGAGKTTISFALEEYLVGQGIPAYSLDGDNVRTGLNKNLSFSPEDREENIRRIAEVAKLFADGGIVCITSFISPYEKDRANARKLHEDEGLHFYECYVDTPLNVCENRDVKGLYKKAREGKIKGFTGVDSAYEPPPKPELLLKAGEQTVDECVQHIVSALMDDEIVPRGAVTTVRELFVPENKKAEAKQEAEALPSVQITKLDLQWTQVLAEGWATPLPGFMREREYLQCQHFGCLLDGGVSNQSIPIVLPIKTEDKERLDACNAFTLKYGDKNIAILRKPEFFPHNKEERCSRQFGTSHTGHPYIKMIFDSGDWLVGGDLEVLDRITWEDGLDEYRLTPRELRARFKKIGADAVFAFQLRNPVHNGHALLMTDTRRRLLDRGYKNPVLLLHPLGGWTKDDDVPLPVRMKQHAAILDEKVLDPKNTVIAIFPSPMMYAGPTEVQWHAKARMATGANFYIVGRDPAGMPHPDGTRDLFDHTHGAKVLTMAPGLTQLEIVPFRVAAYNLKKKAMDFFSPERKDDFLFISGTKMRKMARAGETPPDGFMAPKAWDVMVAYYQSLA

>adenosine_SG-AL

MVKEGVLLAYGNPLLDISVNDDSCIKLLEKYDLKANNAILAEEKHIPLYDELVDQYPNKVEFIPGGATLNAMIAVQWILQKPKVTTYFGCINKDKNGDIIKQKSEEAGVNVQFQYTDKEVTGTCGVICTGSNRSLIANLAAANCFTEAHLDVPANWKLVEKAQYYYSAGFPLTVSPSTMLRMARHAQENKKTYCMNLSAPFLCNFFKEPMTQLLPYVNFLFCNESEADEFAKVHEYGTTDRKEIALRMAAWPKEDKSRPRTVVITQGADPVLVATDGKITEFPVKLVTARDIVDTNGAGDGFVGGFLAQLVQGKPIEECVRCGIYIGNIVVQQPGFTLPGLPNYS

>adenosine_5'-sulfatophosphate_sulfotransferase_SG-AL

MHYSTRPDKKRVKNLMFATYISATIGIVLLTAVGFRFYSRFKKRARGIEELDVPQYGRRSRYLCRYRGYVLPSLLVDTIVNDIPWFEVRSDDVWVISFPKAGTTWLQEIVYLISTDYDYEKATATDIDERFPFLEFPLPGIKEVSKKDSPRFIKSHLPFSLLPKQFEEKKPKVIYLARNPKDTVVSLDAFTRLTNYVTFSGNMDDFARLFVEGTVVYGPWTKHVQEGWERRNDDNVLFVTYEDLHKDFCGTVREIAKFLGKPLSDSQLRELEHHCSFRNMQKNDRVNYSWQKGIWKEGDFMRKGKIGDWKNHLSLEMSAKLDAMATSLSPLGLDIVDS

>Thiosulfonate-reductase_SG-AL

MGTRKTDPYSDHLKSFSMTDPDWPQFMRVNPILDWHYETVWRFLRALHLPYCSLYDQGYTSLGSMNNTHP

>cysteine_dioxygenase_SG-AL

MASDAGHLKSSLSESDQVSVTDGQMEVDLEGDFADPKSIRAPENLQDLIKQLHKVFAKDNININYVKALMESYKSNRDDWKKFAKFDPHRYTRNLVDSGNGQFNLMVLCWNEAQGSSIHSHAGSHCFLKVLEGEVKEEMYEWPEHLLGPHQQTGSGKVRGEQAEMTATEELTFQQDLCTYICDDLGLHRIENPSHVNKAVTLHLYSPPFDECLSFDQRTGSHITATVTFWSKFGKRTPFGKDPKLTRKENN

>Serine-acetyltransferase_SG-AL

MASAFLVLSKNWNSARDATLCGCRLLFAVHEKLSFSTNSQYFQAAWERTGQLDSNRLLSSRSTETKPERTWPQIETPELDKIVECPDGLDLPSSDFNFKGLESSFPCVSRNSLQGPEPDYDKITTGYKTFTSKEPFYLKYNNGVIPELQVAYETWGELNEDRSNAVLVHAGLSASSHAKSHFDNPRPGWWEKFVGPGCAVDTSKFFVICSNNLGGCYGTSGPSSINPLTGKPFATTFPIVSVDDMVQAQFRLLDFLGIERLHASVGSSLGGMLSLMLAALNPERVGRIVSISSCGQSHPSSIAVRYLQRKCIMCDPHWNKGHYYDGQFPKTGMKLAREIATLTYRSGPEWDERFGRNRISEESPSLCPTFVIESYLEHQGESFSTKFDPNSLLYISKAMDMFDVSEGYPSLEAGLERIKCPVMVIGVQTDILFPIWQQRQLAAALQNSGNQAVTYYELNSIYGHDTFLLDLNGVGAGVKGFLETRQREYGWLNKHKRNEKKRKHF

>Cysteine-synthase/Cystathionine_beta-synthase_SG-AL

GGTLTGVARKIKERCPTCKVIGVDPLGSLLAEPEELNKTDVSFYEVEGIGYDFVPTVCERKYVDKWYKCEDKETFVMSRRLIREEGLLCGGSGGSAMYCALQAI
