# Supplementary material for: Multiomics analysis of the giant triton snail salivary gland, a crown-of-thorns starfish predator
Source: Sci Rep. 2017 Jul 20;7:6000. doi: 10.1038/s41598-017-05974-x (PMC5519703; doi:10.1038/s41598-017-05974-x)
Supplement: Supplementary file 3 — Dataset 1 [file 41598_2017_5974_MOESM3_ESM.doc]

**Supplementary dataset file S1:** Lists of genes identified from *C. tritonis* SG-AL gland.

>SG-AL_contig_1_1_-1_289_495

MILHLHLFSYWTRHILRLICQSTHLKYRSYFIIYNHSYSIFRLRFTLRSNVILGSHCNYQ

SFISNPIHW

>SG-AL_contig_3_1_-1_295_468

MVETKNKQKNRQTSEQKDLLDRINCLVCNNTKETDTFEADTAELKKMGWGKEKERTYV

>SG-AL_contig_4_1_-3_804_1001

MFALDRIVFCASFCTSRLLIRHYIWSLVSRMKWVIFCSASTGPYSFSRRLMAPARSLPSV

TNGSRG

>SG-AL_contig_5_1_-3_2613_3170

MNLIILFKDTVSSQIFRSFGLNIGGFINVITALFLFLIFINLRGLIPYVFSPTRHLAVSL

SLGLPLWLSLIISAVFFNPSSVIAGLLPMGAPAPLNPFLVIIETVRIIVRPITLSVRLTA

NISAGHIVLTLIGNYLTASFFISSIFSIIILLSIQVLYTIFEFGISLIQAYIFCLLITLY

SDEHPH

>SG-AL_contig_6_1_-2_74_628

MAGFGSRSSSYGRSRDDYEGGSSLDMFVPAGDCGKIIGRGGSKIREMQDRTGCKINVCRD

EEGNGTRRVELSGTDDAVERAKDMINQIMEGGGDDRGGRRQNYGSSYGGGGSGSKDIINI

DSSFVGRIIGKGGSRIRELQDETGCRINVSRDGGRYGQTEVELIGSWSAIQQAKDRIDDI

TSQAY

>SG-AL_contig_7_1_-1_2770_3108

MGIPHLALFHLYTHALFKALLFLCAGTIIHNRCNTQDIRHIGLISRQAPLTVACINIANL

SLCGAPFLSGFYSKDLILELSLTSPTRLIIIILIFLATGITAAYSFRLSFCSL

>SG-AL_contig_8_1_-2_2_148

CIKNLSDIVSETHTDVVKEIRQQWKSFILMLDNYDLQTAVYAWPSVVCG

>SG-AL_contig_9_1_-1_64_567

MVATLLTVLAAFTKMADTKGERTFIAVKPDGVHRGLIGEIMKRFEQRGYKLVAAKLCMPG

EAHLKQHYSDLSTKPFFEGLVKYMNSGPIFAMVWEGKEVVKMGRAMLGATNPLASNPGTI

RGDFCIDVGRNVCHGSDSVESAKKEIGLWFTDKDLITYEANAKPWVYE

>SG-AL_contig_10_1_-1_1_93

TPSSQNFPYSLRKLVTISSFNQNAEVLFASL

>SG-AL_contig_11_1_-2_38_1726

MCSLVRRHAARCLSYKTMARHMSSLKDYVAVNEPMFEYRPGSAESKELEEALKRYENKVV

DVPIVIGDEEIRPGDVKTQVRPFDHHKKVSTFYYATPDLLKKAIESSMKARKDWERRPQE

ERSQIFLDAADLLAKKYRMDILATTMLGQAKNIWQAEIDAAAELVDFLRFNVMFAERVTA

YQPLSPDSIITNTMSYRGMEGFWAAITPFNFTAIGAHLPMAPALMGNVALWKPSDTAILS

NYIAYQAYREAGLPAGVINFLPADGPVFGDTVLASPDLCGINFTGSVRTFKRLWRQVAEN

LDIYKTYPRLIGECGGKNMHFIHKSADVQSVVNGTLLSAFEFNGQKCSACSRLYVPQSIW

PKVKEGLLTALKDVKVGSPLERENLVTAVIDDKAFDRIKTYLEKAKSDPSLTVVAGGTCD

NSKGYFVQPTIVETSDPNNTLMQEEIFGPILTVYPYPDEQYLQMADMANQTSPFGLTGAI

YVQDPEAREQLCKTFRDVAGNMYINDKSTGSVVGQQPFGGARLSGTNDKAGGPHYLTKFV

SQQSIKERLQPMTSWWYPSMDKK

>SG-AL_contig_12_1_-2_164_424

MYLSLLSCLFSYICILLAVAFFTLLERKGLRYIQIRKGPNKVGLAGLPQPIADAAKLLTK

EIAKPTIANYSPYFIAPVFRFILALLL

>SG-AL_contig_13_1_-2_47_475

MTQIMFETFNSPAMYVAIQAVLSLYASGRTTGIVLDSGDGVTHTVPIYEGYALPHAIMRL

DLAGRDLTDYLMKILTERGYSFTTTAEREIVRDIKEKLCYVALDFEQEMQTAASSSSLEK

SYELPDGQVITIGNERFRCPEAM

>SG-AL_contig_14_1_+2_185_415

MLSSQLVPDSCVLLTTRHTSLPVATRQKALHVECTFLSLGLFTTVVYLRHSLWSLFQRIF

QRISTDPDVVWSRSSLH

>SG-AL_contig_15_1_-1_43_654

MGAYKYMQEMWRKKQSDVMRFLLRVRCWQFRQLSSVHRAPRPTRPDKARRLGYRAKQGYV

IYRVRVRRGGRKRPVPKGCTYGKPVTHGVNQLKFQRSLRSVAEERVGRRCGGLRVLNSYW

VCEDSTYKFFEIILVDPFHKAIRCDPKANWICNSVHKHREMRGLTSAGRSSRGLGKGHLF

NKTTGGSRHANWKRRNTLSLRRKR

>SG-AL_contig_16_1_-2_329_958

MSGLRAPKAGSNRDAQAKVEASFDAEDAKKILRWIQAATGIQFPIEESNDRYKVSENFYK

VLKDGHLLCRLVDAFLPEHMKMDFNSKTFEKRANPAFEAARERERIELFIQRIQDFGVGE

QDTFQTDCLYERTNLPKVMGCIRVFGIEVESHPSYTGQKIWPRKSEANPRNFSEEKLRAS

ENVISLQYGTNKGASQAGMNFGKKRMILKE

>SG-AL_contig_17_1_+3_3_257

ERGYSLYSVTIPFVSNQEILAHTTDAHSQINEHTHTYSLIPADIKPPPPYFLKQRTKRQT

NIQIYSQQLTKQIDYSTKTVCQKSK

>SG-AL_contig_18_1_-3_60_932

MKMKLQLRRLPVHVRAEEEAEMRKRPQRRQKRWIPAVLTGRQRKRSKDVGRKRRSDAVRK

RKYGESKRRRGVGRRKNAERRRDREKKRKRGWQLKEQNKTGSREKMEERRREEEEKKKKK

KRKGLGGLSPEKKKLLKKLIMQKAAEDLRNEAKAKALEKEQFINERVTPLKAEGLGQADL

QNLVKELHAKVAELEEEVYDWEIKIRKQDFEINELTLKVNDTKGKFIKPVLRKVNKTESK

LNKLQTKESRDFRGNLKSTGQNKFALKEDEDTNKPDWREALKHGEGEEAES

>SG-AL_contig_19_1_+2_122_409

MCYIHTHTHMHSAATHQACNVQNDPQLLHKTKSEPRQILYTSSDNICNTRQTKSTSGYHH

LVLPITVSFIPTVLLPVTVSFIPTVLLLVTVQQLAC

>SG-AL_contig_20_1_-3_2790_3032

MIKKVINIYKFWRGRGLTVIFLEMEIMLFFFLQFAACYLQIYSVSLYLLVLEFYFYCYSF

LLFLLFIVWLLACFLIQTCIL

>SG-AL_contig_21_1_+1_367_576

MAVEIRKCMEIRNVQKLSEFRHLQIQNKFANIHDQSKFITLLTVGLQQLQKSGSFQRGTV

HKCKSPIFKL

>SG-AL_contig_22_1_-3_459_1283

MDTNTTTAAVCLFLLTWNFFASSLAWDFLLRCEDRPCLNGGTCIIDEFFSSCRCREGFIG

SRCQTAFHPSVTTSHPVTTPTPTPASFDDNDDGVFDINSTMTITGSVLGFGFLGLLCFLC

VHKMPRSDSREDLCDTPPIARRDANIARLGASPALLRDPRYDPPPPPPTGEDNSAYAREL

NNMADIPPPPMADLPPPYSSLEFPPAYSTVVKEEMTERNDEDCGQRSINRSEDCEERNVN

AVAVTTDTDSPVILSSIESAEEPSHGEHQRRDAAD

>SG-AL_contig_23_1_-3_66_869

MMSIPKSPLAYAVNQVVANAAKPVGKAVVSKADKIVVAPQKLAQTSYALSKLTPTGADRV

SFSLCSPAQVRFAHTDVQVPDFSAYRRDGTKDNHASSQSSAGGRRAFTYLLAGGGTVTGA

YAAKTVVNEFVSSLSASADVLALAKIEIKLGDIPEGKNMTFKWRGKPLFVRHRSAEEIEI

EQGVDISQLRHQETDAERVQDPQWLVLLGICTHLGCVPLANAGDYGGYYCPCHGSHYDAS

GRIRKGPAPLNLEVPAYTFQEEGLLVVG

>SG-AL_contig_24_1_-3_3_128

KLCEHIYGTVLAKRMPLEQVLVTITILSSKAVCIEMRESMIH

>SG-AL_contig_25_1_+3_417_677

MAFQILWNVDKRYNKEIHSDIIICLSPIIYTLIRQIKTKLHPASLILQSSSESSSKKRTI

TSDFVNESNIIRVNVAFPVSLKINREG

>SG-AL_contig_26_1_-3_78_731

MTGGTDDPISMSPPHIYVERTLAIIKPDALHKAEEIEDIILRSGFAILQKRRVHLTPEQA

SDLYAEHYGKLFFPSLVAYMSSGPIIAMVISRHQAITYWRELIGPTNTIKARQTHPDCLR

AIYGTDDQRNALHGSDGLPSSQREIRFFFPDSVVEPVATGQAAKDYLSKAVNPTLLKGLT

ELCKQKPEDPVIWLADWLLKNNPNKPKVGDNAIVEEPK

>SG-AL_contig_27_1_-1_1_174

IVFISYFDRQTDIPVLKVKCIGTNLVMILDVQRFLFKILFHIPKDTYMFRGLSFFPMS

>SG-AL_contig_28_1_-3_111_1538

MGLKFLEFVKPFCAVLPEIAKPDRKIQFREKVLWTAITLFIFLICCQIPLFGIVSSDSAD

PFYWIRVILASNRGTLMELGISPIVTSSLIMQLLAGAKIIEVGDTPKDRALFNGAQKLFG

MVMTVGQAIVYVMTGMYGDPADIGPGVCLLIVIQLFVAGIVVLLLDELLQKGYGLGSGIS

LFIATNICETIVWKAFSPATVNTGRGTEFEGALIALFHLLATRQDKVRGLREAFYRQNLP

NLMNLLATVLVFAIVIYFQGFRVDLPIKSARYRGQQTSYPIKLFYTSNIPIILQSALVSN

LYIISQMLATKFSGNLFVNLIGVWADVGGGGPARSYPIGGLCYYLSPPETLGHIVEDPIH

AVLYIIFMLGSCAFFSKTWIDVSGSSAKDVAKQLKEQQMVMRGHREKSMIKELNRYIPTA

AAFGGLCIGALSVLADFLGAIGSGTGILLAVTIIYQYFEYFVKEQSEMGGMSTLLF

>SG-AL_contig_29_1_-2_587_808

MFTRFITLLSICQCICLNTVHKISDRNVYKTDKRNVGMALQFYITEWRCHHVSEQFTGTM

FKLSSLILKLTGAF

>SG-AL_contig_30_1_+3_168_1355

MVQAGPGLGDGCGVAQHANSTLHLGQVTTRYHSGGLVVDTHLETCGTPVHKLDGTLGLDG

GDGSVDILGDHITTVQHAAGHVLAMTGVTLHQLVGRLKTGVGHFSNSQLLVVGLFSGDDR

GIGGQRKVDTWVGHQVGLELCQIHVQGTIKAQGGSDGGHNLTDQTVEVGVGGTLNVKVAT

AYVVDGLIVHHEGTVRVLQGGVGGQDGIIGLHHSSRHLRSRVDGELQLGLLAVVHRQTFH

EKRGEARASATTKGVEDEETLKSCALVSQLPDAVQHQIDQFLTNCVVTTSIVVGGVLFAC

DELFWVEKLSVCAGPHLINNCGFQVDKHSSWYMFARPSFTEKSVEGVISPSNRFVGGHLT

VGLDAVLQTVQLPAGIAHLDTSLTHVHGDTFTHDDE

>SG-AL_contig_31_1_-3_138_2705

MSAEGLRVIRGPGWSQGDKDGGEGHVGTVSKVKDDGTVEVTWDGGQVVEVKAASKAIRVL

DSAPVGVRHMDFACDECQESGICGMRWRCSQCEDFDLCSLCYFADRHDCTHQFDRYETPF

GSPERMEKRSKSTKIRVMGIFPDATVTRGRDWEWQDQDGGIGSDGRVLDIVTAAAHSTRS

MVKVEWKNKFCNVYRMGFKGKVDLKYLEEAPGGECYPHHLPVLDPKNYTEETLKISEEDE

DSAISEGDHVVITVAADELQHLQKSRSGWAVGMAQCIGKVGDVKGFAANGDVIVSFGAKK

YRLVPAALKKVASISVGDHVRVSTDKAKVTTLQEGHGGYNDDMDVALGKVGEVVKVDSDG

DVVVQFGQQKWLFNPACLAQAQGEDVDTVTVDVSRRRVLQRGDSTLGALNQLLGAVFLAA

AAEQQQGVGAKLMIKAVIDNEVSAVKSLIEKDKDLVHAKEQGVSALHLAANQGNFQIVQV

LVENGAKINDTDSDGDTPILAGMKHAVVADYLISKGCDLTIANDNGQTVAHKAALFNHHQ

LLRELIKKGVDLNVMDGEGDTPLHDAIHQGSHTAAEVLINWPQINVRRANKKGFTPLHYA

ALKDTPTIAEVLLKKDKTIVDEQKEDGFTPLHVAAINNHTDVMKVLLEKGKAKVDMRTQK

KQTALHLAAEQAYMDAVQLLLQYGADVNARDGDGDRPLHIVMCSFTQGGEELRLLMTLLG

QTNKLKEEERLRIACYLLQAGADPEARNDQGKTAVTVCGSPLIVEGVKRFLDANPELSRP

QRPKTPALVRQMSRSAMELCRKCCTKKAAILFVPCGHRSACRDCCSKHKLRACPQCGTPA

KSRYDENGKEMGCVVM

>SG-AL_contig_32_1_-3_174_848

MACQQVQQIQQVQQINVPTFKLVLVGDGGVGKTTFVKRHVTGEFEKKYVATLGVEVHPLC

FHTNRGEIKFNVWDTAGQEKFGGLRDGYYIQGHCAIIMFDVTSRVTYKNVPNWHRDLVRV

CENIPIVLCGNKVDIKDRKVKAKAIVFHRKKNLQYYDISAKSNYNFEKPFLWLARKLVGD

PNLEFVEMPALVPPEVTMDPSLAKKYEEELKVAQEVALPDDDEDI

>SG-AL_contig_33_1_-3_2787_3005

MNEEFPVSASHKLALITSLPFVHTARRYYRLNGLVRASDWSRPALHRAAPLVEKTLELDR

LEEVKVVDRKSVV

>SG-AL_contig_34_1_-2_101_1273

MASIAAKGASQVRFLGQAQQQLPRIFAAVVVSKRNASYDLPSMKRGRGGRSSFSGIVATV

FGASGFVGRYVCNRLGKIGSQIIVPYRGDPYEVERLRLCGDLGQVLFFPFFLNDEEAVRK

VMKYSNVVINLVGREWETRNYNFNAVHVDGARLIARLAHESGVEKLIHFSSLNASPTPQP

IYNKAGSKFLRSKYEGELAVREEFPEAIIFRPADIYGSEDRFLRYYANFWRRAHGTVPLW

KKGDATIKQPVFVQDVAQGVIEAIRNPDIAGQTYELVGPESYYLSDLVDYFYRVMRFRNV

KRSYLSPAFRMKAYAMSFAPAEPLYSLHKLEKEFVTDVTTGCPTLEDLGVSLTRIENQVP

WELRPFRQGAYYEEYLGEFADPVPPPAASAH

>SG-AL_contig_35_1_+2_20_190

MTSQNRDHILETHTNKSHIQISPPVSVVLGLYIVMRHIWPLPSLFTTVTQSCTIIFS

>SG-AL_contig_36_1_-2_89_673

MAMLAVRFTCLFLVFGLGSVFGQTVPCDTTRDVCMRNGFSSLSIFNNQIRCCLPGYSMAM

VNNNCQCTKTADAVLCSKGPDVCLNAPMRSWDGQGNMLCCPSNYPSMNSQSMMLNGMMFS

SCTCSRSGGASNVQVISGNGQGTVVFGGNRNFNNYFQKWNQRFRQTMQNRMQALQDQLRA

MRNRISVASNSWLQG

>SG-AL_contig_37_1_+2_2_2764

GLRCGKIGLQCESIVKFPRLFEKYPFPILINAALLKIADVYLSGNNFFRLCILRVVQQSE

KHLEKILNVDEFVHRIYGVYHCNDPVARAITIRTLGSIASVIPEKKNIHHSIILALDSHN

AVEVEAAIFASDKFSEISKTFSTTICSKMIAMLSGLDTPLEMKMKLIPSLRHMHHDADTA

AKALAVCESLLEGYPAQNVVMLTLNTMSRLAVHSLVNMSIHLSRLLTVLTSDLRWGVHLT

CLCNLQLLAKNGPHLFQASSVEAIGEFLMKTSFPQLKVQAVCVLNTICSSSAIRQLPWST

VNKVLTISRRLCYDYDAILASKSVKLIAEIAQSKRCDKDISSEAVAAIQTFLLLSADGSP

QSIGPLKCVLKAVIDLSATDSSAADVFVTTLSSLLSASSGETAAAVCEALSAIGDRNAES

IHPAMPTVIACLDRWLTTGLSNPSLQVQLCTLVFQATRECVLPMDVLASLRVVVDKSGLW

QIYKISRQAMRYGQYSFAEFLFTHLAYKVASEHYYNWLHGLQFICSAQLKLGSLMTDGSN

VQPCLAAAISGYQRALVSFKAASSVAFPLEFQCGYVRIRTAWLSALRLLLLTCNTFRSQP

PPALASAHANTSNKESERWTQVIKQLDRCVKKFQEIGQQISALYWSSFDADPATLTNITL

LQQGCDCIRFAISAVITTIQTVEGNMGERQYLGHLTVNSNDRSMQNVLNSISKELDILLP

QNSGLIEKNYKLIDFLTGSAKSLVQASLPFPRFFFQMLQSTDVKLAISPQASISGEPVLV

RNDVSLGLKVEGVIQRGERPSLFRSVQSVKVTVSTSLTARAAAPNMPPPPVMENPGFKLS

QTVEPHNEYFSTNFILPFPELGIHSIVVEAAIVDEKGACWKTRPRSTLTVKSYNDTIQHA

ARAGARQHGSAMGSHVLPTGH

>SG-AL_contig_38_1_-1_1291_2394

MEIFNESSDDEPFLGFRLEDIELHEVQARSDIDVSSVSSVHTSDLSDWDDRDQDFDSDID

DRGDHRPRDRHGWSPVTSRVDIVNFTEETGPNVSVGEHPTQVDFFFQLFEPETIQKIVNN

TNKYAREKIALKENGDPLWAETSIPEIKAYFGILILMGIHNLPRDEMYWSSDDRLGVAGV

NKVMPLKRFKKLQEYIHVCDNDNQPDPADPNRDRLFKVRELIDMANRTFLQKYKPSCQLS

IDEAMVPYKGRSCLRQYMPIKPIKWGMKVWCAADSNSGYLLQFDVYCGKRADGTHRGLGY

DVVMKLATHFLNKYHHLYFDNFFSSVALMEELLRQKTYACATVRTNRVGLPGAMRSHRRL

GRGQHIVH

>SG-AL_contig_39_1_-1_94_822

MSAGNMMPLWVLVNIMMALTSYTVMPASLSQTQSPGYRAAVYAHKLVLPTGLPVSRAMAV

EDMMKNVDIYREQARLAASQGAQIIVFPEDGLYGIGLHRSVIRAYLEPVPDPRTTAPWVP

CSDPHRVTPSDILTQLSCIARNNSIYLTANMGGIQPCDDVTMLCPPDGHYQYNTDVVFDP

TGQLIARYHKHTMFDEAEFDTAPAELVTFDTPFGRFGVFTCFDILFHDPAVVLAEQGVHN

IVF

>SG-AL_contig_40_1_-3_3_110

RKNEEGEKKTVGKKKGKNEKDWLMVSKDGERNGRNE

>SG-AL_contig_41_1_+3_3_170

FFSFYVFLSLPTTSDTGMGFGPQQKMYNTAYTTDYQCHMTTYVHQNYQYNPKTNNK

>SG-AL_contig_42_1_-3_108_548

MSSGVTVADETKSAYQAIKMHQKGLKKRRCSVFKLSDDLKEIVVDSVLTDLFSEDSDEKH

FETLISQLPKDDGRYLIYDFNYTGSFGPSSKMIMVLWCPSSIPVKKKMVYASSKDSLKKA

CPECVEFQADCCEDLCEKEIQLKLCKV

>SG-AL_contig_43_1_-3_228_839

MFDLYRKVNSREVIVGWYSTGFEVSVHSVLIHEYYSREAKSPIHLTVDTVMRGGKMNMKA

FISTAIGVPGKTTGTMFTPVQVDIHSYDPEQTGVEVLSQGKYKQKRTVTVQSDLSKVESS

TSTLRSMLQQVNEYVDNVLAGKVPADPTIGRFLLELVSSVPKIEKDEFENMLNTNMKDLL

MVMYLSNLTRTQLLLNDKINNLPS

>SG-AL_contig_44_1_-2_98_364

MDDRDAVLPTSCVPKHQCNTDASTWLDLKGKPLPKVGEEIEAHACAHWNRNCCKWKQPIR

VHNCGDFYLYHITPFSDCTQAFCLEMLDK

>SG-AL_contig_45_1_-3_108_959

MDAIKKKMLAMKMDKENALDRAEQMEQKLRDTEEQKAKLEEDLNTLQKKYSNLENEYDKA

NEGLIEANTKLEASEKRVNELESDLAGLNRRIQLLEEDLERSEERLQTATEKLEEASKAA

DESERGRKVLESKSLASDESIDGLEAQLKEAKYIAEDAERKYDEAARKLAITEVDLERAE

ARLEAAEAKILELEEELKVVGNNMKSLEISEQEASQREDSYEETIRDLTQRLKDAENRAQ

EAERTVSKLQKEVDRLEDELLAEKERYKNISDELDQTFAELAGY

>SG-AL_contig_46_1_+1_1_228

LSFSANSSSEVKSNTAAWGVKGQTTRPADPRCTVPFQPVAVGTFRALPPNRLPCVQAFSA

DGRDLAHWSHCDAHNP

>SG-AL_contig_47_1_-1_121_861

MAASTSISSHSSFAVLAAIFLLVSFCVVLSAGERVLGEETDEFDFDGLPGAQHEFKVDVP

GGIEDCFFQRVVRGAKLHVSFEVLRGGDRSVNVILRNSRWQVINAHHWKSQGVFEFEVPY

DDTYAVCVDNSYGRFSAKLVYLYIVSFVMADWAAYVKEFEDIHSIADNVTQLLANVDLSV

QDMRKAQTQSRFHLVRDWYLIWANNRYVQNWSIAQCVVIMTTSIIQVVFVRRLFKTTNVT

PTAKPRA

>SG-AL_contig_48_1_-1_85_270

MYVLVNLNGARKTENKVVYWKNMAEEWVSSGEGIMFVMYCLRRLMVTLMIILVSPVNFYI

LL

>SG-AL_contig_50_1_+2_305_415

MVNASRRNMVTSVYEFNLTSVLQVSSRMVNYRRKLSK

>SG-AL_contig_51_1_-3_1077_1481

MQGMHACSHTQHADGQIATHIHTNTLVHPMPPPSLLPSTHREKDKHLELLIGGGGAGLSP

MFMAPFRLGRPCHVSIITTNVGENSPRTYAMPSSFKAWVNFHPGWPQKSKHFLFYKSTPF

FFLTMWRQSRKILLT

>SG-AL_contig_52_1_-1_76_2601

MVNFTVDQIRGIMDNKRNIRNMSVIAHVDHGKSTLTDSLVSKAGIIASAKAGETRFTDTR

KDEQERCITIKSTAISLYYELNKDDMKYIKQDYDPENNAFLINLIDSPGHVDFSSEVTAA

LRVTDGALVVVDCVSGVCVQTETVLRQAISERIRPVLLMNKMDLALLTLQLEAEDLYQTF

QRIIENVNVIVGTYSEEEGPMGNIAVDPKDGTVGFGSGLHGWAFTLKDFAKMYCTKFGME

EGKLMKRLWGDQFYNAKEKKWLKEQRDGAERGFTTYILKPIYQIFKTAMSGKSEKVLELA

EKLNIKLAADDKELHEKQLLKTIMRKWLPAGDAMLQMIVIHLPSPVTAQKYRMELLYEGP

HDDPCGVGIKTCDPKGELMMYVSKMVPTSDKGRFFAFGRVFSGTVATGQKVRIMGPNYVP

GKKEDLYDKSIQRTILMMGRYTEAIEDVPCGNICGLVGVDQYLVKTGTISTYKEAHNLKV

MKFSVSPVVRVAVECRNPSELPKLVEGLKRLAKSDPMVQCAIEESGEHIISGAGELHLEI

CLKDLEEDHAGIPIRVSDPVVSYRESVREESSQVCLSKSPNKHNRIFMKAAPLNEGIAED

IDKGNITTRQDIKERGRYLVDRYGWDATEARKIWCFGPEGTGPNLLVDCTKAVQYLNEIK

DSVVAGFQWATKEGVLCEENMRSVRFNIQDVTLHADAIHRGGGQIIPTARRVLYASVLTA

QPCLMEPIYLVEIMCPEQAQGGIYGVMNRRRGIVLENSQIGSTPQTIIKANMPVNESFGF

TADLRSATGGQAFPQCVFDHWALLQGDVFVDGSKPSQIVIDTRKRKGLKEGIPPLDNYMD

KL

>SG-AL_contig_53_1_+2_2204_2596

MYSSHTLMHSHTLMRSRTNTCMYTLRTRACKSPAFMENIQYYIFLYISDAHSKLQTAQNN

CRQQSVNNILQTTHNQTNPLGYRCKNQENQHLQIEQVCTSNILQTTTQLFPFGYIQIITT

KTYTDKSATQA

>SG-AL_contig_54_1_-1_187_438

MAGVVENSRTEPAPPTSLMATADAINYEEPSQDPYKKAISYLEKHNILQIFQTMTASIIY

QKPDNPLDFMIAEIGEMKKRRGTQ

>SG-AL_contig_55_1_-2_236_1075

MDGIKLTVAMLVVLSVCGLCQAQNWRWWRQQRKEQQLQQEQEHQKRLEQERLEREHQRGL

EQQWKERLERERLKQQKLEAERKRQELAQRRRERSRLKQRLERLRLEQQQLEARRQREEA

LQEEMEQLIGNYELEDLALMIGMEALGRILRPLREIPTEAPIDDCASRTTHVELEEGLMG

DFVYNVCGTGDDASIRIRFTFPGETTPVIDTTTPLQTTVATTGPPMRLCPIIPTTTPAAD

KETTTDNANVEDTTEIQDVEETTEAEELDEDTTTPFVMLF

>SG-AL_contig_56_1_+1_34_543

MIHLLLLGNTNSATSAACGLGVLTAHTQTPVVSQTAMGTDLLESLQILTQLVVQLVRQHL

SKTTVLDVLLTIEEPVGDLVLARIAHHGDDALHLLLTQFSSTLGDIHIGLFANNVAESST

NTLDGSDGEHNLDPAINVGVEYTQDVLKLLWNHERHDCSSPQRKRKEVSL

>SG-AL_contig_57_1_+1_229_1173

MAKNDQSMHVVTIDLQAVLQAPCGQVSQLYYKRKLCVYNFTVYSLADANGTCYTWDETEG

KRGSCEIATCVYRYLSSLPRCIQKVTIFSDCCAEQNRNQYLAAALIHAVSHIDNLNVITQ

KYLESGHTQMECDLMHAAIAFSKKNNPVYTPSGWDIILRMARRGNPYTVVPLKHTNILDF

KRLASSVVRNTKTDTKGRRVLWRDIKCLQFRKGQRELMFFKSSFDQEDFNEVKLIGTSKR

NRPALVEKIPFLYSSKLPISEAKKRDLQFPCCSGRNTTPSTVLSPHLPLPLTDFLNQTPK

KMSKIQTKLLCVTTA

>SG-AL_contig_58_1_-2_842_1018

MQIYRLTTVKNVPLTVRPYFFCLIKQSYLQRVPAKVYGVCQLFFVDHKLSQPHPEASIK

>SG-AL_contig_59_1_-1_1_153

DNTNNNGLISIMNSVINIASSQHFTNVSTHMKVVQHVLFFFLICCLTFGYL

>SG-AL_contig_60_1_+3_90_938

MPPRRKLSELDRGRAVAWVQDGVPKREVARRLQMSISVIVRLFQRFTATGRVHERCRSGR

PKMTNPREDRLIERQALQTRTASSSIIRRQLRVATNINISQQTIRNRLHGFNLRSRRPAV

WPRLTPAHRAARRDFCRRHVRWTRLQWSQVLFSDESRFTMNHNDGRLRVWRRPGQRFIDA

TVQEKVAFGGGSVMVWGAFSLHHITPLYHVQGNLTGFRYRDEILRPLAVPTLQLMGPTAV

YQDDNARPHRARVVNDFLQQFGVNRMEWPACSPDLNPIENLWD

>SG-AL_contig_61_1_-2_8_985

MATRVLSSFPLTMGKRLCRSYAQIGMGGLPKSVKIVEVGPRDGLQNEKATVPTSVKVEFI

NKLSEAGLPVIEVTSFVSPKWVPQMADHSEVMNTISRNPNVIYTALTPNVKGLEAALAAK

ASEVAIFGAASESFSKKNINCTITESLARFQAVMDRAKEANIRVRGYVSCVLGCPYEGEI

KPEQVAEVARQMYDMGCYEISLGDTIGRGTPGAMRSLISTVSREVPLECLAVHCHDTYGQ

ALANILVALQMGISVVDSSAAGLGGCPYAKGASGNVSTEDVVYMMNGLNINTGVDIHKLI

EAGQYISNAIGRKTLSKVGQATHSSL

>SG-AL_contig_62_1_-3_132_1202

MITGTSQADCAVLIVAAGTGEFEAGISKEGQTREHALLAYTLGVKQLIIGVNKMDSTTPP

YSEGRFNEIQKEVSNYIKKIGYNPKAVAFVPISGWNGDNMLEPSEKMGWYKGWAIERKEG

SASGKTLLQALDSILPPTRPTDKALRLPLQDVYKIGGIGTVPVGRVETGILKPGMVVTFA

PNPITTEVKSVEMHHEALSEALPGDNVGFNVKSISVKDIRRGNVAGDSKNDPPKECKQFY

AQVIILNHPGEIKAGYAPVLDCHTAHIACKFAELKEKCDRRSGKKLEDNPKMVKSGDAAM

VDLIPSKPMCVEAFSDYPPLGRFAVRDMKQTVAVGVIKSVEKVEVTGKVTKSAAKKK

>SG-AL_contig_63_1_-3_108_773

MGFGDLKTDGGLKSLNDFLADRSYIEGYTASQADSVVFAALSGAPKASFPHALRWYNHIK

SMDRSALPGVKKPLTSYGGATPAAAAAGGGDADDDDDDIDLFGSDDDDDEAAEQLKQQRL

KAYADKKAKKPALIPKSNIILEVKPWDDETDMKQLEQSVRSVSTDGLVWGASKLVPVGYG

INKLQISCVVEDDKVGTDYLEEEITAFEDYVQSVDILAFNKV

>SG-AL_contig_64_1_+2_1307_1846

MPADVNGRGRHLAEAVHGKVEPAIRTRPPHLPVKGGEGLAQPAAVPKPASHSSPTDPVLR

ANPFPEVTDPICRLPLPTLFYRLEAVHLGDLLRIWVRPGTRITPSPWDFQGPTETHRTPQ

EPRCFTGTPCPYLRASRFQGASPLQRKENSSRGLGRRLPLRLRCRTWPQRTNLRVQVREY

>SG-AL_contig_65_1_-2_281_796

MVVSARSVCCCFLLLFVLLFLLFVVVGCFPTYSRSFRHLRERRTDRKTDKETQNSRKVKR

TFGLRVCRRNARRQNTRRLNARRRNARRRNMVSGAEIICTDCRSFSFNFAQEGLKSRRAS

VSICAVRWSSEINHGGHQRRTAHKFIGAARTRSKRGKCTDIIGCKIRRKWTH

>SG-AL_contig_66_1_-1_7_327

MYITIISVNANGQDDGYIYRTIINVNASSPDDSYIIEPLSVSMPAVQMMEIYIYIYIYIN

AIISVNANGQDDGCIHRTIISVNANGQADGYIEPLSMLTPMVKPMAM

>SG-AL_contig_67_1_-1_223_1473

MADLPRNDLETDEAPLRLRRVQGRVPQLPYSSTDDKQLSIMDERVDSGVGYSLESQEFSA

LSIEGVRDFLATESGKDCYKQVMDNRCVSGSRSDIITASLQNQLRNLSLKDGEQTRCDSG

ICDSGNFSVEEPFPVVESAFPECGLTSSLPFESTFSQQELEELFSQDEDGDSYLHMSIIH

LLPDVSLKVISVAPSPDYINIPNMLHQTPLHLAVATRQLWVARRLMVAGASMDAPDHCGN

TPLHIACREGMMDMVQLLLSPVKYQETLANQYPIPMQRLPQDLSIRNYEGYTCTHLALQG

GHLHILQLLLSKGANINEPDGKSGRTLLHMAADMGYMQALELLMQHQGLDLDARTYGGLT

ALSLAHGRRLNHVVELLYRAGADCTQLTEDAGESTDEDMNESVYDDICINGQPIQIS

>SG-AL_contig_68_1_+2_4760_5038

MSQLSHIVTSIYVKQKRRWDVCVCVCVCVCVCACMGGGVCERERENAHVHARACVRMSSS

LFPKQKSLHHCFRSVSTILSQNKLYLLYKNGKE

>SG-AL_contig_69_1_+3_2682_3050

MDIQYSKCLVLFVYTMDIQYSKYLVCLHSEHSILKMFSFVCLYNRHSILKMFSLFTQWTF

NTLKKKFSFVCLHNRHSIFKMFSFVCLHNGHSIFKMFSFVCLHNGLLIFKMHDTVKLSAG

ISW

>SG-AL_contig_70_1_-1_115_1590

MRFSTVLALSLLAFAAADDVKQDEGVYVLTEKNFDDFVKDNEFVLVEFYAPWCGHCKALA

PEYSKAAKKLSDEDSNVKLAKVDATEEGALSTKYEVRGYPTLKFFRSGTASEYAGGRQAA

DIVNWLKKKTGPPAKDLKTKDEAKAFVDNDQVVVLGFFKDQESAGAVTFKKVALGFDSIP

FGITSEDSVFKEYKMDKDGVVLLKKFDEGRNDFEGELEEEAITKHIKNNQLPLVVEFSQD

TAQKVFGGDTKNHVILFLKKEEGDDLLKNFKAAAPDYKGKVLFIYMDTETVEHERIMEFF

GLKKEETPAVRLIKLGEEMAKYKPESKDLSTATITKFVQDFLDGKLKPHLKSEDVPDDWD

AKPVKVLVGKNFKEVALDKSKAVFVEFYAPWCGHCKQLAPIWDELGEKFKDKDDVVIAKM

DSTANEVEEVKIQSFPTLKYFPKDGGDITEYNGERTLKAMAKFIENGGKEEPAKAEETEE

EEGEKKKHTDEL

>SG-AL_contig_71_1_+3_1197_1811

MSAESTFGRQAAGLLGFFGSLLLGISRPTTTTSHMLDLRGRGLLCQLLEKVFTFHVGPVV

RVTLVLAAQLDLGWSTSEFVCQAVKRLIHLHCRLLLALVHLPGALCQFNHVILLSFVALF

QVVLVAHQTAHVLVQLAGLLQKLGGVHAVDVEGLDALGEDGTLLLFQLLLGLLQLLFAHH

TVQLTKPQRPPNQPFKRLALVTLHY

>SG-AL_contig_72_1_+2_632_781

MGLVLTSTPTCCDSIEHFWWNYSCVRNFWNSVEHLINKECENTFNVTISR

>SG-AL_contig_73_1_-2_131_1084

MSQEQMTLRGTLQGHGGWVTQIATNPQFPETILSASRDKTLILWKLTRDESNYGIPLKSL

TGHGHFVSDVVLSSDGQFALSSSWDRTLRLWDLLECKTTRRFVGHDKDVMSVAFSADNRQ

IVSGSRDKTIKLWNTLGVCKYTVQEDQHKDWVSCVRFSPNAQNPIIVSCGWDKQVKVWNL

TNCKLKTNHHGHTQPLDTVTVSPDGSLCASGGMDGQAMLWDLNEGKHLYTLDGGDSIHAM

TFSPNRYWLCAATGPSIKIWDLEGKVIVDELKPDVMTAGCGEPPLCLSLAWSSDGQTLFA

GYSDKLIRVWQVSMAQRC

>SG-AL_contig_74_1_-2_326_2380

MTSGLVIWLVFAVYHGSSSVVKGGQAPHFTNEWAAQIPGGEEHAKDVAKRNGFEIITSVD

NFDDYYILKRHDVPHRSRRSSHHHTRRLIEDGQIPWAEQQVARSRVKRLAGFRLRSDGSS

DFNDPHFRDEWYIENSIPSSRKERGQAIHLDLQVKGAWKQGYTGNNVTVSVLDDGIEHNN

TELAKNYDPYASTDLNDNDPDPMPRYDPTNENKHGTRCAGEIAMVANNGKCGVGIAFNSR

IGGVRMLDGRVTDSLEARALSFNYSHIDIYSASWGPNDDGVTVEGPGTMAQQALEKGITY

GRNGKGVIYAWASGNGGRLQDNCNCDGYTSSIYTLSVSSASEHGQVPWYGELCSSTFATT

YSSGNGGDHQVVSADLHDRCTTRHTGTSAAAPMAAGIFALLLEANPHVTWRDVQHLVAVT

SKVGPLASEDGWYQNAAGFCVNLAFGFGLLDAAELVSAAHPDNWVSVPQKSICEISADTG

SNLPQTLSSGHFVEVLIDTNGCAGQHNEVNFLEHVQFVFTLSYSKRGAISVTAVSPSGTE

TTLMMPRQWDKSTDGFRQWPLMSVHTWGEDPKGVWRFRVHDGDANESRHGVLKDLKLVLH

GTKDAPHYLRDGPVHCDVAVVSKKHTTDVTPSGGDDWGRPSLQPMGTGLMRGQSNFMTQV

IQAWMDRQKASQDDREAAIEEYLYL

>SG-AL_contig_75_1_-1_1_291

GIACSSSSLLVHYSLVEFWIQHASPFMCREREAYGEVMGCLLDAAKSCRPQRKRIHFPDR

EMVKRGIHYICDVAEDEQFDVNITCARSHFQKVYDCI

>SG-AL_contig_76_1_+2_1301_1537

MFLLDDNFDNQLESDNSLEDAVCMNVHPSGRFILAYINVHQVFSVGSEQTWQQRSPVPSA

ASSAPPLAHRLQVYRVKMT

>SG-AL_contig_77_1_+1_1_174

CIEEYTMFPQVKIFVLAHRSVMYTSLLDVSSMVNIAVLQHGIVYHILSVVCCLYCLLV

>SG-AL_contig_78_1_-1_400_1194

MPKPKKGKKKVAAAPFLGKKVEPKKVVNPLFEKRPKNFGIGQDIQPKRDLSRFVRWPKYI

RLQRQKAILYQRLKVPPPINQFTQAADKQTATQIFRLLDKYKPETKQAKKERLRKRAEER

ASGKQDRPTKRLPVARSGINTVTALVESKRAQLVVIAHDVDPIEIVMFLPALCRKMGVPY

AIVKGKARLGRVVHRKTATCLALANVNPEDKPALSKLVEAVKTNFNDRSEEIRRHWGGGV

MGAKSQARIAKLEKVKARELAQKMG

>SG-AL_contig_79_1_-2_782_2065

MYCNTTTTTTTSSFYVSRHRNLSGPCTGSGRSTLDNRALATPPPGVPGCRAEREGHSSRD

KENHHPRGEGRSGTHRKLDRSLSDSEDRINGNGRGVNSSRYKTELCRPFDESGHCKYGDK

CQFAHGAHELRNLTRHPKYKTERCRTYHTIGFCPYGPRCHFIHNEDDNSVTQRKNVTNAT

NVTRLSSQKYILPPSAPTSVLPHRPSNLSGLKSLGSSFESVSATSSPGNLSPSHFDDQFP

AVGTFSPSAVNDTYTDAIRNGESLRTSPVFNFPGPDPTSVGSVTAPLDVQTQQLYYNKMV

DMAVLDSFSTVLNLSQNQHNNQSNYSTNLNVCQMQQQQAAFLESLSPAERSWPLTEAMGD

TGAVLPPSKSSSPFSDDSMEGSGSLEMTSKPPAISASMVDNREFLVHRPMDMGRSMRLPI

FNQLSSEL

>SG-AL_contig_80_1_-1_2380_3003

MGGQQSCTAQLSDFQKKKLLHEFHTFFDLNKNGTLEWKDFDMARQRICQMSGWKAGSEQF

TSTQQLFTDIWRRLEDEGDSDMDGKITAEEWTAMWQRIHCEGVQQEKKGETPADHMPEWL

SRYVKYKFALYDRTGDGLIDADEYEYVLSDFGVPAKDARNCFLIFSNNAERQIDLEYFTC

LCTQYYRSDQPADLGNFITGRLHFDDCT

>SG-AL_contig_81_1_+2_290_655

MNMHACTRINTHSHTVHAHTHTHTHTHTHALVRTYTHTHTHTHTQSHFGFAPCAVLNDSG

VGNWLVIDTVPVFDALNWSLFEANRFHHRSSCFPPFPFHTPSSPHRLPRIILAFSPHRNP

TA

>SG-AL_contig_82_1_-2_137_679

MCENRATKSGIARDVQQKIAGKYDQEIAAHCMAWLARSTGEGLDTSGDIDSVYTQLKDGY

VLCKLMNTINPGCIPAGKMKRQTMAFKQMEMLEMFNTKIKDQPLLIPEHSVFASTDLFEK

QNMVQVITCIESVARRCQSINCPVQGFGPKEAEANKREFSDEQLAQGKNIIGLQMGSNKG

A

>SG-AL_contig_83_1_-1_202_1638

MPGAHDQLSKYNKIEAFVKGFKVSTSSYKRVMHLLNQEMNHGLHKHSHPVADIKMFPTFV

RSLPDGSERGDFLALDLGGTNFRVLLVRLQGHDIDIQSKTYLIPQRIMLGTGTQLFDHIA

DCIGKFVRDHNLSGQTLPLGFTFSFPCRQEGLAKARLSKWTKGFRCEGVVGEDICNLLHE

ALKRRTNCDVEIVAIVNDAVGTLMSAAHVDRNCEIGLILGTGCNACYMENLGNVGTWDDE

KNHPDQVIINTEWGAFGDNGCLDFLRTEYDRELDTFSINPGKQIMEKMISGMYMGEIVRL

VLERLTMEGLLFRSVDTECDLFERGRFFTKYVSEIESDTDEFFRNTKQVLEEIGIENYST

EDCKIVKYVCTLISARAAFLAASGLATLINRMNKSEMTIAVDGSLYRFHPRFHNLMCLKI

QELVKPGLKFKLSLSHDGSGKGAALTAAVSLRLRNMKYTKENVASKNETPVRISIPQEA

>SG-AL_contig_84_1_-1_46_555

MAPKKATKAPAKAGAKKTESAAKESAAVGKAKDKAQRAKKAVLRGRHDKRSRKIRTSVHF

RRPKTLVLPRAPRYPRKSAPRVNRLDAFSIVKFPLTTESAMKKIEDNNTLVFIVDKRANK

PLVKQAVKKLYDIEVARVNTLIRPDGEKKAYVRLMPDYDALDVANKIGII

>SG-AL_contig_85_1_-1_190_375

MSLDAYSVGSSQNSGKHFCMNVIGVKHNLANFTYFSECECNLCKTSVHGGTLSLCMDVAR

LI

>SG-AL_contig_86_1_+2_152_262

MYTSLLDVSSLVNIAVLQHGIAYHILSVVCCLYCLLV

>SG-AL_contig_87_1_-1_31_861

MVLFLHVSMAARPHIVIILADDLGFDDVGYRSKAMYTPNIDKMAKEGVKLKYAYMQQVCT

PSRAAFMTGIYPFRMGLQNSVLSALENRSMPMDKTVLSQRLKTLRYATHYVGKWHLGFCN

YNMTPTYRGFDTFYGMYTGKEDYYTHISKWNGYDFHDDNGQNFNVDWTAKGKYSTPLFTE

RAVKIIEGHDTRKPLFLFLAYQAVHGPEQVPDDYLKYCADVTNETRKTHCAMAAAMDEGI

GKVNEALEKRNMTDNLITLFLSDNGGAVKKGSSNWQI

>SG-AL_contig_88_1_-2_707_952

MVKASSLWYFDSSRTQEFGKIGKGVGDVPKMDKTKNGQIMVVLVMTVMCSSEVGVWLPIW

RGTKKQPYTQSSHPNVLVVSLS

>SG-AL_contig_89_1_-2_2_289

CGRWVGVRSSVTPTATRSINTPVQKGGIRRQRRSLFVAFCRVNSGLNSSCAPSSTREVVS

GHNPVRLSHQKTLTRCGKIDCFVDKKSYRIIIFVLF

>SG-AL_contig_90_1_-2_395_619

MFESSTGVLHMKTFLETCVSFYRSDLLQFFFFSFSFILFSVQCFFFYFFFLFILFFFINP

FFLFFFLVGFLCFFF

>SG-AL_contig_91_1_-3_456_638

MENTPQDSTPKFNCKQAGSANFKANLRRTRTYLSDTHNIDNIHAIFCNPLFKILLTFQYQ

N

>SG-AL_contig_92_1_+1_286_552

MRCSFRTSSFFFQSFARSEADDLTSCSICVAVNHQKIQMTGPLQNESMDWLMNRFPPENG

LLPGLLLNIDFEKAFDSLGWQFVFKVFKA

>SG-AL_contig_93_1_+2_1169_1519

MLWTSFCLYFMNSNLFWAHTFKAYMSFWNHITPKNSTKLIAEPVFYNENIKVGNVIIKDR

DRLIEKGIYSIRHFLTENGRFKTYEEFRLTLIAASILSLIMVGYLQYNSTKEQAEYN

>SG-AL_contig_94_1_-3_771_971

MFIISRKIFGSFSFFQIKAFEKLNRKSRKCGEHTPPTLTNQLAFELLKLKSPPPPKGFFP

FKFLIHY

>SG-AL_contig_95_1_-1_76_1197

MDSVRSGPFGQIFRPDNFVFGQSGAGNNWAKGHYTEGAELVDSVLDVVRKESESCDCLQG

FQLTHSLGGGTGSGMGTLLISKIREEYPDRIMNTFSVVPSPKVSDTVVEPYNATLSVHQL

VENTDETYCIDNEALYDICFRTLKLTTPTYGDLNHLVSATMSGVTTCLRFPGQLNADLRK

LAVNMVPFPRLHFFMPGFAPLTSRGSQQYRALTVPELTQQMFDAKNMMAACDPRHGRYLT

VAAIFRGRMSMKEVDEQMLNVQNKNSSYFVEWIPNNVKTAVCDIPPRGLKMSATFIGNST

AIQELFKRISEQFTAMFRRKAFLHWYTGEGMDEMEFTEAESNMNDLVSEYQQYQDATAEE

EGEFDEEEGEEELA

>SG-AL_contig_96_1_-2_2_220

FDIHHTDLKKEDNTNLLASLVKCHIASSYSDHLKRYTDQKKGRFSVFNTCFNLEKSFHTG

STKEYLQLNSMQN

>SG-AL_contig_97_1_+1_1_255

FFFFVAIRINFLLRKRGYRKASHCSLKTESTLTYRAIVQLRRLWGNKVHLGNMLHAIDSA

NTSSLSSYTKKEIASHSYGYLYMAQ

>SG-AL_contig_98_1_-1_208_351

MNTKIVIHTFFFSFFLHITITFGYKLQCASHMPQCFVFLMSLLLFFTH

>SG-AL_contig_99_1_-2_2_70

ELKASTCFLFHINILLCKIYVVF

>SG-AL_contig_100_1_-1_82_756

MLSAARNAARSLLKPGVAAPAAAQLKHTLPDLPYDYNALEPYISADIMKLHHQKHHATYV

NNLNIAEEKVAEALAKNDVQAVISLQPALKFNGGGHINHSIFWEVLAPQGGGEPTGDLLT

QIRHDYGSFENMKKELVATTVAIQGSGWGWLGFNTMSGRLRVAACANQDPLQATTGLIPL

LGIDVWEHAYYLQYKNVRPDYVNAIFNIVNWQNVADRFHNAKMAN

>SG-AL_contig_101_1_+1_766_1065

MFITLFFSVLQCIKSWLMLASKDPLCLSLEPSIRCISSQPQAHMITTWINHNPVYNTPKN

CGQSDTHLHYPHDARQKCSQQSFQFFSPNSERDCKFFPFL

>SG-AL_contig_102_1_+2_74_163

MTPSSAMKTSSINDYLLKKPRLLINDYLLV

>SG-AL_contig_103_1_-2_71_1549

MRQAIVLFSCVVLAVQASNVLEFTDSNFKEKVQEHDVILVEFYAPWCGHCKRLAPEYEKA

ADRLVKSDPPVSLAKVDCTAETSICGEFGVSGYPTLKIFKGGEFSKDYQGAREADGIVKT

MQKEAGPSSKELTSVKDFEEFLGREGGSVVGFFTDTASTMAKNFQKVADSYEDLRFAHTS

TKAVLDKSKYKDEIVLFRPKAMASKFEDTAVKFSGDTTTSKIKTFLDEESFGLCPHRTAD

NAGKLKKPTFVAFYGVDYVKNPKGSNYWRNRVMKVGKKLRDEGLKAYFAISNKDDHDHEL

GECGLGEATGDKPIVCAYDERGRKFNMEKEFSMDTFEQFVRDVLDGKVEPYLKSEPIPTN

DQPVKVAVAKNFDSLVNDPEKDVLIEFYAPWCGHCKSLAPKYDELAEKLEAEPEIVIAKM

DATANDVPSPYTVTGFPTIYFAPKGSKSSPKKYEGGREVDDFIKYLAKEATNELQGYDRK

GKKKGGKKKKTEL

>SG-AL_contig_104_1_-1_259_2172

MNNAGPSYPMASLYVGDLHPDVTEAMLFEKFSTAGPVLSIRVCRDMITRRSLGYAYVNFQ

QPADAERALDTMNFDTVKGRPIRIMWSQRDPSVRKSGVGNVFIKNLDKTIDNKALYDTFS

AFGNILSCKIACDDQGSKGYGFVHFETEEAAKQAIEKVNGMLLNGKKVYVGRFIPRKERM

MIMGDKMKRFNNVYVKNFAEELDDDKLRELFAQYGKIISAKVMVSEDGKSRGFGFVSFEE

PEGAEKAVEHINGTEVGGRIVYCGRAQKRAERQAELKERFEKIKLERMNRYQGVNLYVKN

LDDTIDDERLRKEFSAFGTITSAKIMMEGGRSKGFGFVCFSSPEEATKAVTEMNGRIIVA

KPLYVALAQRKEDRRAQLASRYMQHMANLRQQQSAQFSQMFQAAPGNFFIPAMAQTQNRF

YTPHGGQMPMRANPRWNTPVRPAQQAAGFQGGMPPAQMRQRAPTQSAAARAAMNARPITG

QAAVPPSAGGQPGPRPMGPGVSRPPTAVQAAPAQQNRYKFTNNMRNAPAAVQPGVMPQAQ

QQSVIIPGQDPLTATMLAAAPPQEQKQMLGERLFPLIQRMYPELAGKITGMLLEIDNSEL

LHMLESQESLKAKVEEAVAVLQAHQAKEAAAAAAAKDK

>SG-AL_contig_105_1_+2_782_1021

MGHSLTQTHSITIFNISLQFLPLSLSFVQGNLISNIVMKMAFSLSLSLSPSLSHTHYIYT

LKHQNLSDSRRDFDTFRFKT

>SG-AL_contig_106_1_-1_1_174

MNDTLKLLVMMYFITFILCCCQIIHIYTVRALLVCQTLVFCHGFLFFHLDMLCLDTVE

>SG-AL_contig_107_1_-2_83_1171

MPEFACYLTREQQDELRGIAQAIVAPGKGILAADESVGSMGKRLQGIGVENTEENRRQYR

EVLFTTENALANHISGVILFHETLYQKDSNGKPFVDILKEKGIIPGIKVDKGVVPLAGSH

NECTTQGLDGLSERCAEYKKQGAQFAKWRCVLKISKNTPSYQAMMENANVLARYASICQQ

NGLVPIVEPEVLPDGEHDLETAQKVTEQVLAFQYKALADHHIFLEGTLLKPNMVTAGQAC

TKKYTHEQIAIATVTALSRAVPPAVPGICFLSGGQSEEDATINLDAINKYKGKNPWAVTF

SYGRALQASVLAAWKGQKGNVSAAQAELMKRAKANGEAAVGKYAGGVSGAAGGQSLFVAG

HAY

>SG-AL_contig_108_1_-3_60_908

MEECYHKEHLACWNCDKQLIACRYIMRDEHPHCVPCYQQLFANNCEDCGKPIGPDYKDLS

YKDKHWHEFCFKCSQCEKSLVEQPFAPKNEKIFCSECYDNAFAARCDGCGNPFKGGMKKF

EYKGKQWHDECFICAECDQPIGNRSFIPRDDRVICVPCYENNYAQRCSKCSGVINKGGIS

YKNTPWHRECFTCTKCDKMLAGEKFTSKDEQPYCGDCFGDLFAKKCCRCVKPITGIGGTK

FISFEERHWHSDCFICYKCETSMVGRGFLMNGEEIMCPDCGRT

>SG-AL_contig_109_1_-3_60_2873

MKLQVQMNNLRVSQLERNKEIMLIKRQLLLQEVNNVLLQAHITRRETELYQYREARRYGP

AVRRWNTFSGQGDNEARPPLLRNISLQDGDDPLHPAAHPPASSSKHSSKHDDIPYITDED

VSIPTAGVAEGAEEGRRGLSLSPTRPLIRTKAPSHETCTDHQEQHLASPIAATTTTTTPS

SQVTAATTGHPLQSAMKSTRMVEQGKPRVVSPAVEPPGTASVSVLPAQQTPSSVPSSKEK

ETMKTSPTTATTMMTTTSEVSKVETGSPTLLSQTPTSIPSAGAPGAEGRIVSTETETPTH

PIPERSDMGLRTSSSPAQSASPDDDKYSQTQSVMECTKSVLEPEASSLHQAEPSPGVSAP

SPSGAAAGGVDITSKGQPPKAIRSPVSRQKYLRRQKEEREKRKERASSPRSSHSSDSGKP

PLPRQSTPHQSPSATHRSKSVGDMEGIESDSTQTVAPLREVIQKFEKRATVCETEERKIE

FRKTPSPTLHLPRVGLVSRVRRLKPAAELLQESQRYRSGHSIYATRIMQRYLPARTTPPG

ATGPSQDKENISGNSYVQAIVKRLSRENTPVKSPGSSSASSSLSLHRTDSPGRTHSDLVS

HIVQKLSSSSGPTDTNHKTSLSPVSSSSLPLKDLTNEGRVKKLTETSAGEEAHGPTITDQ

TSTLLDLAASDRTRATEDASLQPESVTEVAVSPPEAKPDSTISPTSHMSMPALASDQPEV

PPEDSARERAATYCVSEGDRPRQLQLQQLHGPDNGSASIEHISEEILDPALLSIRERKGE

KISPTSSTKSKNALCCQSSTCTKSKSSSSSSSQQTSGAHKIPSSPKTQERRSGREKMETI

GVLCKQSMSFDLGVSLQAQPLEAMPQRPQSAESLRTPTEGEGRTPTEGEGKERAKVRPSS

TSSEGEAPASSEEKKKSRSRLLDSSWLQKPRKFFKVSK

>SG-AL_contig_110_1_+1_526_822

MVTGSDLEAGDVVAMEFLVLSAGVDSTSLLKVVPVIIVVLRQFLTKSPTVRDIHIKQQSM

THAALFSSSDQVWVFEEGARNSSSRDLPSSSNTLDATER

>SG-AL_contig_111_1_+1_592_1032

MIMLSPYKYINEITNTNEIIILSQGLSSLFIIILQWNTLAKPSLLVHQFCRNTHTKPSLF

IHHTAIEYSHKTITVHTSYCTGSLTKPLFIHHTAMKYSHKTITVHTSYCTGTHKTITGHT

SYCNRTLTKPSLVIHHTAIELSQNHHW

>SG-AL_contig_112_1_+1_1_381

EIKEKNQKNPPPPPPKKKKNPIKTPHTFGHIFLNIYSSNIFNLKTNKKVLEPKQLISETI

RNEPREFQTISLKASRKIIGHEVCFLNTTNFSVDRPICRKYKFTWINQGNSRCAATETSM

STGKILP

>SG-AL_contig_113_1_-1_277_1980

MSEEVQRWRQECSSVESMRKNALDALRLLETEVTQLQDADIQLKQMKDEYNTLRGQYDEV

DVQKTKAVAAVHTLRAKVARLSKFCQEKDELLRRLGEEVRGGRGRSSSLLEELTRMETLA

GGEEYNRPLSPLDLITGPAMSDTERLLSERIPHTSFTRWGSSSSLGDPRDISASADLLHS

GDRSSVQRPKSAEQTRRGQHTAYPLKQSAGVGSILGYGGESLCVAVLDYVPDAASRSGHR

HLELPLKEGDHVHVRGPVDRHGYCEAEVGGRTGLVPASHLFPLSDSGATYRPTSQTYGRH

KKSGDSAEQIVQMFDELQDARLVSGLAHTHSSYSSQPGHSTATQTRQRRSAPKSHGAPQA

PSNLHVQEVDGNSLVLAWQPPILDQHGYSNGCKVVGYRIYLNGRVCQQPHNPKLSRTRVE

GLRHDRPQQLAIQTLAASGQLSSRVELAYQGASSLAHDHRGEDGETDTDLSSILNSIQYK

TGKKRTVMGLYDYDPEKQSPGDYTACELAFHAGDIMLVYGDERQDGFFHGERNGQRGLVP

ACFVEPVAQGSKSMQKPSAANGSASPRS

>SG-AL_contig_114_1_-2_17_601

MADKSGSDDAGRSFVSNLATELFTSPLNLALLGICGFLIYKIIASRRRTEPSEPKEPELP

PLKKQDFTLEQLREYDGKGKNGRILIAVNGKVFDVTKGKRFYGPGGPYGLFSGRDASRAL

ATFSLNEDSLREEYDDLSDLNSMQMESVREWEMQFTEKYVYVGKLLKPGEQPADYTDTED

EGGDDKKQPTEKKTD

>SG-AL_contig_115_1_-1_178_498

MKRTDQLMNILALFSRVPDSVSLNTGRPWILLPYCILFLSLVSWPLASLAASILLSLYTY

RHISFPYNSPLLRSWIVFSWLIISCLPREQCLVFSDKPFCDLPACAA

>SG-AL_contig_116_1_-3_3_167

ALIAILLLLALTTVTLATGEDEAMDITKLLKRSAEFQTVERPARCVCFRAPCDCP

>SG-AL_contig_117_1_+2_368_592

MHSITVKNMSLKFQPIQHKGIIIILLRTQTQKCHTSHCTNSTRFSLPFTLIWSCITFNIY

TNRSRLSNTNNSSRW

>SG-AL_contig_118_1_-2_182_346

MFIACCSFVTLCHYVFVGYFLHSTRFILLVSAVLHASVSGIISMAVLASEKCQNM

>SG-AL_contig_119_1_+1_1288_1491

MKDATEHVTKNTCFQYEHQMTSFRCRKRIQSKYNITGVNRQSHRKANSPMQTIISFSVQS

GHHSYEFY

>SG-AL_contig_120_1_-2_218_5596

MTECCQGVSLGTGSPSVSRRCVHRQATSKPGIMSLWFLLATFLVVLTAFAEIAAAQELIP

PDPRCGAGCTTVGNCRCIGEKGSRGAPGLIGPEGPQGNQGFPGPEGLPGPKGLKGEPGMP

GQQGEKGNRGKMGAPGFPGINGVPGIPGPVGPRGPPGLDGCNGTQGDPGSPGLPGLPGLE

GQPGIPGLKGAKGDAALGAPLVKGQKGEPGPAGFPGLPGAPGVDGPTGPKGEPGLPGFAG

NPGSLGMKGDKGSTGISYRGGKGFQGQKGEPGPPGLPGPYTLEPSTVIRGVPGLPGEKGE

KGDIGMKGPEGPRGYPGEPGRTGLPGEKGMLGEKGVPGIAGPRGKQGFPGNPGLPGMKGD

RGMDGLPGLPGLIGMKGERGIPGLTGPSGPQGPPGRPGGKGTVGEPGPRGFPGPVGLPGD

PGLPGLPGQDGPRGLPGSEGGRGIPGPPGPQGRDGPKGESGDSGPPGLPGLDGRPGLPGR

AGPKGEPGPYGEPGLTIPGARGPPGLPGLDGIPGQKGERGPTGLPGPPGNSSQGLPGLPG

PKGEKGQQGFSGLPGRDGQPGTPGDKGNSGGPCDRCLPGVKGEKGSQGLDGLPGLPGERG

GSGFPGPKGEPGDDGSTGQPGLPGLPGNEGLPGLPGERGLKGEPARPDVIQGPEGLKGDL

GSPGLPGEPGRPGRSGPQGPPGSPGLPGMKGERGLKGFPGLDGRPGPAGQPGIPGEAGLP

GIGIAGPPGERGDPGMNGLPGLPGLPGSKGEPGKVEGRDFKGQKGERGFDGVPGKEGLPG

LPGLSGAKGEPGLPGLPGLKGDPGEPGLSGVTGPRGLPGPRGEKGLPGLPGAPGLTGMKG

DSGLPGLPGMDGEPGLPGLPGEALQGLPGPRGNPGPPGLPGRDGEPGAPGPDGLPGLPGL

AGEVGEDGLPGLPGRDGSKGESGLPGRPGPPGEDGLPGLPGQPGDPGLKGDSGLPGLPGL

SGPKGEPGLDGRPGEPGTPGLDGRPGEPGAPGLDGLPGLPGMKGEIGVMGFPGEPGRDGQ

PGLPGLPGAQGLKGQRGFPGEPGLPGLRGPKGEDGLPGLPGQDGDSGQPGLPGLPGTKGD

SGLPGLPGGPGEPGRDGLPGLKGELGEPGIGEPGLPGLPGEPGRDGEPGRPGLPGIIGEK

GLPGFPGERGQKGESGLPGSPGLPGLDGLPGMKGNSGLPGLPGTSGEKGEPGLPGLDGLP

GLKGEPGFSGRDGLPGLPGLKGDMGRPGLDGEPCQSGVKGEPGLPGSPGLPGLPGAKGES

GLPGLPGLNGEKGGPGLPGLDGLPGAPGSKGNPGPAGPSGPPGLPGLPSVGLKGEPGRDG

GPGRDGGFGVKGEPGLPGLDGLPGMKGEPCTDSGVGPRGFKGDPGLPGIPGEDGPPGLPG

NDGLPGLPGLKGDIGEPGFSGEPGRDGLPGLPGQKGLPGLPGTPGRDGPPGEPGLPGLSG

PPGLKGDRGDDGLPGLRGDPGQKGEPGPRGLSPVINSTLLPKGEPGQPGDRGLPGQPGQP

GEKGFPGRDGLPGHDGAKGDPGLPGLPGMDGPPGPPGLDGLPGTDGGPGNPGPKGEPGPG

GLPGPTPPTGYLLVRHSQSTEIPTCPLDQVKLWDGYSLLYIEGNEKSHHQDLGWAGSCLP

RFSTMPFLFCNMNNVCNYASRNDKSYWLSTNAPIPMMPVGERDIVPYISRCVVCDVPANV

IAVHSQTLQLPPCPRGWKSLWIGYSFAMHTAAGPGGGGQSLSSPGSCLEDFRATPFIECN

GARGTCHYFANKYSFWLTTIENNEQFIRPRSQTLKAGNLRTRVSRCQVCMKDV

>SG-AL_contig_121_1_-2_53_580

MQLSSQESIDMAEKLTSHDAYNGVSQDFNNSSQNYNGQNEASQFSHLPAPPPYPDRSSPA

LTVAPTQSSPMIISEQSPFSGKYANLRRTLMTDFTKPSEMLTETTNLMNHVNSPSQLSVS

TQQQSISSPEGSQQNSPSPQALTSHLSPQISQASPQEERSSLSQQLSPQQQQQQQQ

>SG-AL_contig_122_1_-3_33_335

MTKGTSSFGKRHNKTHTHCRRCGRRSYHIQKKTCSSCGYPSAKIRKFNWSVKSKRRRTTG

TGRMRHLKVVYRRFRNGFREGTVPKPRRKIAAPASSAVAAK

>SG-AL_contig_123_1_-1_136_519

MRAAGDRDGVSGIDGVDSWKSIVKNWDTSSRDCFVYNIDDVKNTSAVRCDQYKLVHNKAG

SPDGWWNWPNGTTVVLPKGRYPEYMLFDIENDPSEMIDLGGNSSFTDTIEKLKAELKKQK

DLGFKPSK

>SG-AL_contig_124_1_-1_232_693

MASGADKGIIVVLAVDNSTHSEYAFNYYATEVHRPENKVHVIHSSEAWMHCHHLKDNPTE

GHVQDMKSKEDTKVEEIKNKFTKLLQEHNMVGEFKVVGGKDTWHQIIEYQESNGAQLIVV

GTRGSNVIRRTLLGSVSDSIVHHAHCPVLVCRHS

>SG-AL_contig_125_1_+2_2_202

FKEKSIHIKSIQNPFHLMSLSMKHYLDITFPRKLERQQYFCFAIHDMHHEMRLYTCSRGS

PFVYYSL

>SG-AL_contig_126_1_-1_16_882

MAFFAVTNSLLFFLLTFAAMLKQSSQQLNPPDGLPPGPQSMTSPYQGTQVDAARPNSLPG

VSKNQASAMELLSSMTPQHMASLSPQQRLQLQQLMIRQQQEQMQMMQQLQQQVRLTQQKT

PTAKSKDSILGAFTGSGKQQKQPPYLLSAANTQQGSILQENLHQKASQSPSSPFPLPPPP

GAALVSGPPSQPGPQIYPEGGSIPQLTGSSVARGQPVPPQQKSPRLEDFLSVAGGMSSAG

LNPDQKTSAGKQSPLPQSPLPPAFMAMLPPQQQQQVMKEYQLQQQQQQQ

>SG-AL_contig_127_1_-2_65_550

MKNYTLLGQRFYEVVKIRDMSSAHIKMASTLLRTSAGVLRRTVAYNTARAASGGGPPIDH

QKGKVASDKVTMPDTLGHAVGIERYELLAKLAGNEDPFEMNVKKRAKGTVDEPTLIPSLY

EKRLVGCICEEDAVSINWMHLHKGEPRRCECGFWFKLVDLPH

>SG-AL_contig_128_1_+2_1304_1585

MQPNHQGVLKQKTNKQKNRQKERIQQHGSFRNLLSPHKRKSQGCKNIVYDHSSYSSMFES

SIMNRYLVTGMMGEHFFFLDKRSDYTQYVQKQLT

>SG-AL_contig_129_1_-3_3_86

ACIGFTTEFMVSALKKYKLQSFVSSNEG

>SG-AL_contig_130_1_-2_2_238

FLFSSRPVVEARRTTDRRSAVTQHLQAPGLQKIFFKFWASTPALLGPCVCVSASKGMLCG

HIAPHSPVRRVFSPFLCCF

>SG-AL_contig_131_1_-2_2_244

FLFFSSRPVVEARRTTDRRSAVTQHLQAPGLQKFFFKFWASTPALLGSCVCVSASKGLLF

TSCGHITPYTPVRRVFSPFLC

>SG-AL_contig_132_1_+1_1_183

PQQQQQKQQWTNNKFKRKFFFPLSFSDHTLSPINTETQLVRFLLLFYVMQILVSHIRNIH

V

>SG-AL_contig_133_1_-3_51_299

MQNEAGEFVDMYIPRKCSASNSIIAAKDYASIQINFAEVDPTTGRMTGTNKTYAICGSIR

RMGESDDSLLRLAKRDGIVAKNS

>SG-AL_contig_134_1_-2_224_544

MCDDEVAALVVDNGSGMCKAGFAGDDAPRAVFPSIVGRPRHQGVMVGMGQKDSYVGDEAQ

SKRGILTLKYPIEHGIVTNWDDMEKIWHHTFYNELRVAPEEHPVLLT

>SG-AL_contig_135_1_+2_662_970

MNSAAGSNWSALVWTHPKILKHNLYRDRKREGERGWERDYSSPVDDSGPIQDCTTKRQKK

ISIFIIIVINRDKLQYDTSSPHLRLLSRLGHRPSTNALHPPRS

>SG-AL_contig_136_1_-1_88_393

MARAFKRFLPLFDRILVERNMPEVKTKGGIMIPEKAQGKVQEAVVVAVGGGAKTEKGDVV

PVSVNVGDKVLLPEYGGTKLVLEEKEYFLFRDGDLLGKFESQ

>SG-AL_contig_137_1_-2_203_1531

MADNPTGVLIKGGKVVNDDQSFDADVYIENGLIKQVGSNLKVPDEGVHTIDATGKLVMPG

GIDTHTHMQLPFMGTVACDDFYLGTRAALAGGTTMIMDFVLDQRGMSLLEAYNKWRDWAD

EKVCCDYALHVAVTWWSNQVAKEMETLTKEKGVNSFKMFMAYKDVFLLEDADLYSAFRRC

KELGAVPMVHAENGHLIALKSKEMIDNGITGPEGHEMCRPEDVEAEATQRAITIADQAGA

PIYVVHVMSKSSADVVSAARRKGKVVFGEPIAACLGTDGTNYWNKCWRHAAGHVMGPPLR

PDPTTPAYLMDLLANNDLQLTGTDNCTFKGEQKALGKDDFRGIPNGVNGVEDRMSVIWEK

GVHSGKMDPCRFVAVTSTNAAKIFNIYPRKGRIAEGSDADIVIWDPNQSRKISAETHHQA

VDFNIFEGMECHGVPLYVISNGK

>SG-AL_contig_138_1_-1_109_492

MQIFVKTLTGKTITLEVEPSDTIENVKAKIQDKEGIPPDQQRLIFAGKQLEDGRTLSDYN

IQKESTLHLVLRLRGGIIEPSLRILASKYNCDKMICRKCYARLHPRATNCRKRKCGHTSN

IRPKKKLK

>SG-AL_contig_139_1_+2_548_1093

MHDLHHPRHSLTAWCALSTTLMFVEVDKTRDASHDVGTLVHDDDSCCTQCCLCLNQAVEV

HWYIVTHTFRDEGGGGATRYNSQQVVPAPDDSTSMSLDQLPQGDGHLFLHCGRVVHLARD

VEQLRPPIAFTPHTSKPFTSSATDNRCHSNGLHISNSGGATKDTHICREWGFESGLALLA

LQ

>SG-AL_contig_140_1_-2_149_1435

MGLFKGKESEKGGAVPVALADVSGSDSSMSSSSSLDGKDDRGTWSGRMDFLLSCIGFAVG

LGNIWRFPYLCYKSGGGAFLVPYVIFLVLCGMPLFFMEITYGQFASLSPITVWRMSPLFK

GVGYGMVIISGIVCVYYNIIITWTFYYLFMSMRKILPWADCDNAWNDERCVLNKFASEPD

TNSTTANYTISLNGTDLNSTVIMAANATNATLNSSHLMTASEQFWERNVLEITDGIEDLG

SIRWPLFGCLALAWIVVFLCLCKGVKSSGKVVYVTATFPYLVLIILLVRGATLPGALEGI

KFYLIPDWEKLLEFKVWGDAAIQIFYSVGMAWGGLITMASYNKFHNNVYRDAIIVPLMNC

GTSVFAGLVIFSVLGFMAHETGAEIKDVVTQGPGLTFVVYPEAVAKLPISPLWALLFFLM

LFTIGLDSQ

>SG-AL_contig_141_1_-3_144_533

MVRMNVLADALRNISRAEQGGKRQVLIRPCSKVVIKFLTVMMRHGYIGEFEVVDDHRSGK

IVVNLTGRLNKCAVISPRFDVSVRDMETWTNNLLPSRQFGYIVLTTSGGIMDHEEARRKH

LGGKILGYFF

>SG-AL_contig_142_1_+3_51_1109

MTAVDNVIIYLLTVSFSIMIIRHTESSLLITPLFHFLNEGSLSLLDTCLPLGLGQFSTVP

PLLLHSLLWRRLGLWVITDQGMAFLVHFLHPAGRDAQLDEARELFLVQGLVLSLQVVHVL

GHMLAVDVVTVNLRNVLALLAVVPREALGAVWDVQTTINSALHGTKDASTSGGAVETHVE

VATEGSRLVILILHVVLLSVSHLLALVDGIHAKLLQQSSGQKQSSAVSSRIVSQPDLHAI

SGQLVSVCSTYNHVTLDPGVGYLTDDVLVGEANNQPVLGGIVLVLVLESQPLASIVVGAT

LTSPAELDLKPLEVGLVLDNFHKPHVCKFEVLHFDHSNSIGWGLTISHRNFIG

>SG-AL_contig_143_1_-3_3_194

SHTMVHVCVCVCVCVCVYTHTYILVLRCYVMICLSSVYIYFCMWSYSKSAIISDAMLFDC

IISV

>SG-AL_contig_144_1_+3_3_203

STFSVLWQTDSVSVYFLCPMADSVSVYFLWQLFSNLSFDRRQRRQESTSAQNIQHAEDPQ

TGTKASG

>SG-AL_contig_145_1_-1_28_1131

MCCLIGRANEYVLSAFIFDGEGRGVDSVFENQKILNMPGFRGGRGGGRRDYGGGDMDPNA

EQFRKLFIGGLSYETDEDSLRSHFETWGEIVDCVVMRDPVSKRSRGFGFITYKDAESIDQ

AQANRPHKLDNREVDTKRAMPREESGRSESQQSVKKMFVGGVKDDTTEDMIRETFGSFGE

ITGIDLITDKNTGKTRGFCFVTFDDHDSVDKAVLKKRHDLNGRQVEVKKAQSKSDMGSGP

GGGRGGGRGGGRGGRGGRQEYGSGGGGYGGGGYGSGNSYGGGSYGGGGGYGSQGGYGGGG

YDSYGGQGGYGNQGGGWNNNFGSGYGESYGGGAMRSGGYGGRSQGPYGGGYGSGGGGGGS

YGGGYGRR

>SG-AL_contig_146_1_-2_17_574

MADNEWGEVQSSLSKISAAETSDLCHALAEQICHNRVPAPDTYLKSLSLEEWWTVTGVLR

NFFTSSIKNDSTKDQMLESMKGVPEEYRKVIAEAASVHRDQLRQRLLDDTTAISHCVLRD

FDWQLKLVMSSDKLATLNEPLLNVDLDLQDGAGDSQQVAIELDHEDLKKLLNSLEGCSKA

VQQLTV

>SG-AL_contig_147_1_+2_809_1384

MCGRLFEVLVEHVLSQAGAHSLEVGDVVTQLLDGADLFLQVLALDEVRHLGVLVAVSHLM

QVQQGLVHALLQTQGHLHGVQTCAPLVTIRLLDVLQNDTSSTHVLELHQFLRVLTFLVRH

LLEELGEPWQRHVITVEVERHGLVAVRSIELHVDLAVDAGLTLGMVVLATLTQSHICKSV

VFRLLWSFLKAA

>SG-AL_contig_148_1_+2_1472_2017

MHTFTLLMHYNPKLPSNEGDLKTKIFFFYYGLQVQILFKNVLSISFMLFPFHSPALRLIR

SRWVYRLLRQNKPTTLCTELPTTLQPSSYRLVHVQCDYTKVSRGLKYPVKNKTKTQISCF

EIGSPATTLRSNTLSFSLINSCIHSPLPSSKLNHKYTCMYSNKIKQKERQYLVFDTLFSI

PQ

>SG-AL_contig_149_1_+1_1483_5160

MYSSTGRRIKGRGSIRFRSRSRSRSITPPHWRREMERTMKSKEPMEPQVEEKWAKGDFLQ

QQERPSAKSRLGARVDIEKESAPLGKRFELEKEEKEEGEETDDEEEEEDKQKKDKKESKK

HKKHKKESKHKKKHKKSKSSSDDSDSEKEEKKKKSSKKKDHKEDSKKKKEVKKKNYRKKN

ISSSSDSEEEKEKENREKEKRKAEKNKEGKRDDSRSKHRNRSGSFDRRDQSDYRRKSRDN

SRRSREKESRRKDSSKGNRSPSQERARNGQVKRFGKRDRDDSTDQDRSRERRRSKSRSKS

RSEERRTSGSKAKDKRKSKSRSAERRRSRSRSKSKDGRRARGDESRRRSGSRDAVQKRRS

RSRSEDRRVSQRQDRSPDRRRARDDRRRSRSRSADRRRRFSPIRRYGGRRGASPFRRRSP

PRRDSRRSRTPPYKRRRGRSSSSSDSESDRDQRRRAAAEKKLKMFGMPLIRKSESPPPTH

WKPGQKPLSAKTTTTSNVEEAAKVPAEVPVLVGQALQDISLPDGSSPLVSTKPNSEKPDV

VEKPKIMYNLDKEESITTLPVKPPQTKEKERKSRSSSKSSRSSHSSRSSSGSKSPSPKKK

TASSDSDSEKKKEKKVVEERMKWQPPPEPEDMEVDEESVPKPPNTVVPVEKGVDSPTVRI

KTPPLPPLDQVAVAAAELARKREELNRNSASNIPLPVEGVKPPSAPVLEPHSPSSPPPPK

PSADDKPADQRGRYDRSSSSSHSGSSSSRSGSESPVKSVPSENVPKPLLDLANQIPLPPT

SVPPEPEQKTSAPEGRSRSLSSSSSSSRSQSPSKEKNTIPMDIQEKAKDEEESAKMSQES

TAPVKVPSRWDFKPVPLPVPAPPKEEEPAESKVPEAKKPSPLEEKAKKAATPDKPRGRSS

SSSSSSDVDSSSSGSSSDSSRSSVDKKPTKKKRAPAVKAKSPEPKKKSSRSKTRSPSTRR

RSPVGSRDRTRTDRSLSRSGRRGSRSPRRNGRSPRRSPYRSSRRSPIRRSPIRRRSPHPR

RSLSPVRSLSWRSKMVTPRMLSPTRRRRSISPRRSRSRRRTRSRSPRSKRSRSRSPRKRK

SRSKSPRKRKSRSKSPRKKKSRSKSPRQRKSKSPTPRQRKSRSKSPRQRKSKSKSPPPQR

KSRSESTQRKVKSKSPVTRSSRTKSPTEKSGRGSQKPEEIAAKRVHKRSTSSSSDSSSSS

SDASPEKKRAKRASSSSSSSSSSSSD

>SG-AL_contig_150_1_-1_94_2754

MQRRSLRSRYTRSANSDTESQRLPSDLQPLHYDLVIEPDIYQDDSSMFSFEGRVTITMKC

ISPTTTIVLNAKELSIDQSDIVVSGPAGNLTVVNLVLNDEMETLTISLGEELRRDENYTV

QIDYSGPLRDDPEGLYFSAYERDGQTVYLAASFFQYESARKAFPCMDEPALKATFTIHVI

RRSFNLAVSNMPVETTRNLAGGKVVDDFETSVPMGTNRVAFIVCEFTRKTKMTNRNVKME

VWSRQEVVDQMDFALDIGVKTIEFLEEYFQIDYPLPKFSMVGTTGHASGWTWGLVMETDE

TFLLNDHTATLIDKMITAGNVANRIYHSWLGNLVMPASWNDYWLNEALASFMGIMVFDHV

YPDRRMMDFQVGELQKAMIDDGFVFSYPLSDDIYDQIADSKGAFVLRMLHFAIGDDQFRK

GMKKYLSTWSHSYAHNTDLWNTLEDDQLDVSIADFMNTWTQQMNYPVVTVTTEGQTVTLT

QERFLQDPRAEDPGDYNSPFDYKWDIPFTFTSSDQQNFDITASDVIWFTKNEEQKTITVP

SLSEDSWVLGNIRLKGYYRVNYNDNNWRKLMAQLRADPTVIDPLNRVQIITDTFAMTSAG

LVNDTRALEVLEYLDKETEYAPWIKATYKLTQMSKIFSNTDIFPAFKAFMVEKTRPLFVN

LRMTPVDDSMETELRTLIAKTACEYDGPECIEDAVRYLHNWKNNPDNNRIPKAMHATVYC

GSLHKGNTGDWDFVYERFLAERDPLEKRILMASLSCTNNSNDLDRLLDRALNSQDFTTYD

FMTVLQYTILNPVGSKRAWTFISNTWDSQNEKYRADGFPFTISHFGDVFSTQDQLQKLQD

FVDEQEHLGEDTEAFSAVVDDITKNVAWMERNAGTMRNWLQKQGFSA

>SG-AL_contig_151_1_-1_1_1110

SAEGTDSGSVSCDDALRSLCPDFKPDALLHLLEEYWGESGVFSFQPETQTDQTSTSAFGQ

LGVIDAVGGELSPETSLVGSAATNVDSINELIQSDHQYHKEEPKPPSVHTSLSNSSVSTN

LPVGRQVFTTSSSNILPDLGTQLIFPSGQNMVSTSTVKCSPSRATLTRGNTSSVNTSTSA

SASDKLSLATMVDPNMVYSPRPRTVQPSPVDTKDSVPDMDNSSFLSPDMLESLDGLLSFD

DLTDFSLLDQKPQQQQQTVITSSSSPSGATTEGKVSQRRPSPTSTTVKHPVSTFDSSNKI

QALKPLPSKTEYQDGHLSPDSGFTSDGTSYLSDSSASPVSLTSPDMEDLSWQEEFVRVNM

ELFPDVGLGV

>SG-AL_contig_152_1_-1_112_1110

MTIKIFIGNLNEDTEADDLRILFEKYGTVEECDVLKNYGFVHMSSKSDANTAITNLNGYN

VKGQRMRVELSTGKRGNPRGDRRGQERGGRPRPYPPGPPRRGGLPPPPPDYDRYDPYYRY

AYPERDPYYRLHPADRYLPPRLPPADRYALSRERLLPEERAAYALPPDPRDRLLVRERLP

LPYAEERAAYDRLRAAERAPLPAEPYYRERSPPPARPPPEYYDSRKPPLMRNPPPAAESP

ASSRLSSAACPPSNGYDYYRKAAASAERPSFGGVGSSQDYYATPPRAATGYERQALQKQQ

GTSGGGGYGGLQQQQQRKPYDSQAALQTQPIFF

>SG-AL_contig_153_1_-1_73_519

MGISVKDVNQHEFTKALAAFLKKSGKLKVPEWADIVKLGRFNELSPYDEDWFYTRAASTC

RHLYIRSPAGIGAFTKIYGAKKRNGTAPSHSCRANRSVARRVLQSLETLKLVEKDPNSGG

RRLTAQGRRDLDRIAAQVKSKKAEAPKSV

>SG-AL_contig_154_1_+2_638_1000

MVGTLDFRLEGLEGWGSNLPGNLTWWVKGGDFSNLPDVHTCKCLNPFHVYVQPLPTYACC

PAWDMGRQQMLSIHHDPEPVISVVCMCKQNINMHVKDHVSHVSVRWLMETQKHPACTKKR

K

>SG-AL_contig_155_1_-2_491_739

MAFSVQCTCMYLLTHLLFTSLVCCYQLHSVLRTCILYIIAGFEKCKTAIISKRQQTQSTH

THTHTHTHTHTHTHTHTHTHTHT

>SG-AL_contig_156_1_+2_2_202

HCVKVTDLVSHVSVWGLMETQKHPAYMPTKDGVCRLRSNTTPAPCWTNSMHIQENNAQTN

KINKHMN

>SG-AL_contig_157_1_-3_27_365

MAQRLTLRRRLSYNTRSNRRRVSKTPGGKLVYLYTKKFGTTPKCGDCKCKLRGLTAARPR

ELSAMSKRHKTVSRTYGGSICHKCVRSRIVRAFLIEEQKIVVRVLKAQEASKK

>SG-AL_contig_158_1_-3_24_695

MPEITMSKGKSWAEQVDEGEGEGDEGVLPPNTEEFDEKKGLKKITEYKYNEEGKIVKTVK

TYQTKTLKVSKSIAQRKTWKKFGAAEMDPPGPNPANTVISEEIFMQFVNSSEQKQPEEDD

DPLGKLKNQKIVQCRICKGDHWTTKCPYKDTLAPLQEKLTEEQRKPAETPPPSSGASAGP

KPAGTKYVPPSLRDGASKGRGESMSNQRKDEAATIRVTNLSEDT

>SG-AL_contig_159_1_-1_79_1023

MVKEDKATWKSNYFMKITKLFDDYPKCFIVNADNVGSKQMQQIRQGLREKAVVLMGKNTM

MRKAIRGHLENNPSLERLLPCIRNNVGFVFTREDLVEVRELLLANKVKAPARAGALAPLD

VKIPAQTTTLGPEKTSFFQALQIPTKITKGTIEILNEVNLIKEGEKVGQSESTLLNMLNI

SPFTYGLVIENVYDSGTIFSPAVLDITDDDIRVSFMAGVRNVAALSLAINYPTTASAPHS

IVNGFKRLLAVAVETDIDFAEAEQTKEYLKDPSKFAAAVAAAAPETSASAEAPKEEAKKE

ESEESDDDMGFGLFD

>SG-AL_contig_160_1_+1_79_252

MKKLIPSRRSLVREKTLTKRFCSHMRKFVDSGVSIRPKMTRRCMEMEQFRDVNWSLID

>SG-AL_contig_161_1_-2_515_763

MNRLQRISLSCPHLYASSDHLRSSADTNSYNFPHVRTKAYGQRFFSYPAPSTWNQLLHSV

RHAITLSSFRSALKISFFQQLLD

>SG-AL_contig_162_1_-1_301_441

MKEEKTKQNRTSVILQCQLHTLPGKSDPNLLLQVIVSFFLYFLFSLP

>SG-AL_contig_163_1_+2_2_187

FINPLKETPCLPIIHDMAQTNHHTPPSPPTHVCVCICSTPNSTHTPKNMKALLQLPIYSF

CQ

>SG-AL_contig_164_1_-3_192_911

MIVSTRVRVGRSHDSFGFPPVLTKEDRVNMEKMTVEALNMLEGDLQGTYYPLTGMSKETQ

KQLTEDHFLFNDSDRFLRAAGGYDDWPTGRGIFHTPDKKFLAWVNEEDHMRFISMQMGGD

LKEVYLRLVKAIKQLEKKLSFARREGLGYLTFCPSNLGTTLRASVHIKIPKLSATPEFKE

FCDKYNVQARGIHGEHTESVGGVYDISNKRRLGLTEYQAILEMKTGVEAVIQKEKQLAGK

>SG-AL_contig_165_1_+1_241_675

MDTISFSRKTEFSPMEICIFADNSHACKSSGGWCPVLYLSAHNEFLVCIHFKMETPAQIG

SQCGKATGPAPSTSGMFTSTSSSTHQQELPAMRVEGQGPPVPWPAFRPLPSTLHLHEGSQ

GASVSDPRGSHPSLLLPSRLAHPSR

>SG-AL_contig_166_1_+1_568_705

MYCEQIMSKCVQCPLHELTFTPTPTGKTTTRFDTTDRSNSGKGHQE

>SG-AL_contig_167_1_+2_605_793

MVCLREISEFKHCVRNCKSTASVPIQYIRSYPSEWCLHIKVNFFIIQPDDLKLKITKYTY

IPH

>SG-AL_contig_168_1_-3_72_3872

MLVLFETAAGYAVFKVLKEKKLKQSENLFHDFQSPESANEIVKLKRFHKFDDMEVALSAE

TAAIEGQLSDSLKKLLKKLVSKEAQEELAVADAKLGNLIKTEFGIQCVSNTSINELMRCI

RSQMESLLGNREHTAMALGLAHSLSRYKVKFSPDKVDTMIVQAISSLDEIDKEINNYQMK

AREWYSWHFPELYKLVNDSETFLRIVMRLGNKQNAASTDLSDILPEDVETDVKEAAEISM

GTNITPEDIGIILTLCKQIAGFVQYRAELFEYLKNRMSTIAPNLTVLVGEQVGARLISHA

GSLVNLAKHASSTVQILGAEKALFRALKSKHDTPKYGLIYHASLVGQATPKNKGKLSRML

AAKCALSARVDALGDDDTTDREIGLKHRAKLEMRIRQLENKGVQRISGRGKEARAAARYE

NVSDVRTYNTAADSSLPSVPVQQKKRKREEQEQEQEQEAGTSEGNKPKKAKKEHKMKIEP

VSDGDTAATSPSEPEKKKKKKKKRKHEDEEEERPAKVKKEPDVDAAASDSPSASGEKKKK

KKKKSKGKKTKDGHKTKGKVKRKKDKRSKNKSEVNGDLPFLSSTNSATAQGNQDAVCKDK

DTNSSVDSSSSSEEEVTSDDDEKKAKVTGGSTTQPDSEQPKRVEAIKVKIPRNLATNPDP

QSSSESSSSSESSSDDDSDDDSSNEEEKDNTGVVPFDPASLNEPLFPDLNTSVTMKKPLT

PVLTKKESSSEDDEPLANINRKQPTKKDPTTEDEENQNIQQNANITNSATDCETKQDISN

IVQEEMQTEPKVPESSTPMDTGMPDQILSETDGRVVVPEEILVQKERTANLAEEKDNNNP

AGIEFNDVDMSHGSDIKLDASETEPQEKVGESAAEPMPAETENKQPTLMEVDDAAPQQPF

LFFTPLFTNTNAASLSKDGPTRTEDPIAKPTECETNETKTAQDFNIEPQITETGITATPI

TETTTMEPATTVTASTETAITTETATTAIVTAETTTTETAKPIMETTTANVCNDPQEGIS

EERGLDNAQQEHSDNAFNQEPTKDVVTIAEQVSVIVQDTASGGPLATEGESDGGGGECGD

GSGDHGSGDYGGVTGDHQDGGAVDGSGDTNAQGEDNPATPNPVSIVKQDRKQGGNKLSRL

SNIIDNLRVTKEKQVLTGEGEKEDSPEQRASPPPPAPKPAFLPPPLVTPPEDKVSAGSSF

TPPPPVPARHASPHLTPLHHHQQQQQQQQHQQQQQQQQQHQQHQQHHQQQHQQQQQQQQQ

QQQQQQQ

>SG-AL_contig_169_1_+2_227_733

MIILKSRPTICSLDPIPMSLLPECLDEILPSLTSIVNDCLLSGFFPTLFKNAVVKPLLKK

SNLDCNNLNNYHPVLSNLPFLLKAIEKIALQHLFAYLTTNNLLCSSQTANNLLCSSQSAY

LPYHSTKTALLRVINDILLALDSGNVSVLTLPDLSAHLILQTMVFFFTD

>SG-AL_contig_170_1_-2_1193_1465

MYKQNSPSLSIPKMDRFVSCFSFLNVCARNKFHKNQRNVKETIQNNHSISRKPAEKSDAL

VSSTRLDPGQPTNQHTFEWPTSLKNNQQMDK

>SG-AL_contig_171_1_-2_17_1405

MATFILRHALSSQSFVLPSLTRATIDALKKNNKSLFTSVHRYYASPATKEIFKRDKPHVN

IGTVGHVDHGKTTLTAAITKVLAEDAKADFKRYEDIDKAPEEKARGITINAALVEYETDE

RHYGHVDCPGHADYIKNMITGTAQMDGAILVVAATDGTMPQTREHLLLAKQIGITKLVVF

VNKADAADSEMLELVEMEVRELLTEFGYDGDNTPVVIGSALCALEDRQPEIGAQKVRELL

KAVDSYIPLPVRDLDKPFYMPIETIFSIQGRGTVVSGKLERGIVKKGDDCEIMGYDKTWK

STVTGIEMFKKQLERAEAGDQLGALIRGIKRDDLRRGVVLGKPGSMSMHNHFEAQIYLLS

KEEGGRSKPFTPFFQTQMFCTTWDAPALLEIPDKDLIMPGEDARVIMNLRKRMVIDKGIR

FTLRDGTGTLGYGVITNLLSDRNVEELEKERLAARDKKRSQQH

>SG-AL_contig_172_1_+2_2_199

PFPSPLHVSVSICRSIFFPYRSTPMYNSHSHTNTQTIFHIYLFFFFLKKPKFFFFFFSLF

DATADT

>SG-AL_contig_173_1_-3_738_968

MKYICLYNGSINSAHFLLNGLCQVYRIEQRNMIPRSGCKDTQLMKLFLHLNMVVVFACPT

LERDHLAENLHLSCSVH

>SG-AL_contig_174_1_+2_578_706

MDWPPSVPKDYGICEAGELQDTSQDKIKENLLLPMQKAHPFHD

>SG-AL_contig_175_1_-3_540_749

MKHEYHHCVYLLQLFHLVSIVSHFYSDHIIIIIIILIIFDHPESRVGSQMLYIDSKLCSF

NFKYNNNDNI

>SG-AL_contig_176_1_-1_133_309

MRQLAQACGSPVALTSANRSAAQSTLTLDEFEYLWPELDLLVDGGSLSDTEGTFGIHCR

>SG-AL_contig_177_1_+3_999_1589

MASHDTQKSCMDVFPICLHNDNLNGPQHLDGFGEANIIKVEMDETSKSQYADDVPQVLDP

GDKFHKVICQNTSHTPVSMEVDKGSEDVIHDGSLDEQSCWHDAHHADSHHTLHLPVSGCH

HHMTRAPFLLWCEGTTAGTKVVQGSCTGRAGGEDFTVHFMVQCSWCVAHFDQQTSGPSTT

GPKQREGSHVFKVSCGR

>SG-AL_contig_178_1_-3_15_707

MMKTGDCVVKHGGTFTTCLGMVGGICDFCEAWVCHGRKCLQTHACTCPLQDAACFECKRG

VWEHGGRIFKCSFCTNFLCEDDQFEHQASCQVVEAENLKCLSCNRHGQYSCLRCKTCYCE

DHVRRKGVKYTKDQVIPCPKCGNDTSEVKELSMSTRRYAYGRQQMGDDDDDDDYGGAYGG

FSFSGYGISTSANGGYNDDDSEEEDSEDEEEEEEEETNDVSKDLQAASLTY

>SG-AL_contig_179_1_+3_102_314

MITFHQHITKPGPKISVMLRVSNITLSLLLLLCLFTSPCLLFWHFWGIGFSLLSRLCESF

PRAWDLTGKNF

>SG-AL_contig_180_1_+3_3_317

WVKKKATEGKGIQWHKEGGGGGLSDLDFADDITALTSDTQDLQSLVTDISEDASSTGLTI

SAKKQKKICSLDHTHHHQQCTLGRTTLRSSKTSPTWAVQSATQAI

>SG-AL_contig_181_1_+1_106_459

MTKDLNYQIGKATTAFNQLSKIWNSKKISLNLKLRFYNSNVLSTLLYGCETWHLKTSQEK

KLDSFDLKCLCKVLGIKWTDFVTNEEVRSRSKQNPVTSIICKRQIGRASCRERVLRLV

>SG-AL_contig_182_1_-1_274_2484

MSDQIDQLQKVKSRVEKEKTALKSEVDDLQAQIQHIGKNKGMSEKMTKQVEAQMSELNAK

VDEANRNVNELQSQKNRLQQDSADLTRQLEEAEHRVGALTKEKSSLASQLEEAKRSLEDE

TRARQKLSSEVRNLTSDLEALREQLEEEQDSKSDLQRQLSKANSEAQQWRSKFEGEGAAR

AEELEESKRKLQNKLSEAEQAAEGALAKVAGLEKAKARLQGELEDLMVDVERANANANSL

EKKQRAFDKTIAEWQAKVNDLQSELENAQKEARGYSAELFRVKAQLEEAHDSTEALRREN

KNLADEIHDLTDQLSDGGRSVHEAEKARKRLELEKEELQAALEEAEAALEQEEAKVMRSQ

LEISQVRSEIDRRIQDKEEEFENTRRNHQRALDSMQASLEAEAKGKAEAMRVKKKLESDI

NELEIALDQANRGKAEMEKNVKKMQQQVQEEQAKVEEEQRARDEARESYNMAERRCTALS

GEAEELRNALESAERARKGAENELTDANDRVNELSAQVSSFSGQKRKLEADIQAMQTDLD

EMNGELRGSEERSKKAVADATRLADELRQEQEHSMNVEKLRKGLEGQVKDMQARLDEAEA

QALKGGKKIIQKLEQRVRELEMELDSEQRRHAETQKNMRKADRRLKELAFQSDEDRKNQE

RLQDLVDKLQAKIKTYKRQVEEAEEIAAVNLAKFRKVQNELEDAEERADTAEGTLQKLRA

KNRSSVSVARGVSASPA

>SG-AL_contig_183_1_-3_228_977

MKTSLVFCGFVFLPAAAIATFGLVGGIGDTTVSPTYEPVVFAVEAAKAKLAQNYANSGSL

TLQKIVHARTQVVAGQKLFLTLLLSNDFVCNVTVWYRAWLQGENRTIVTDGPNCEQPQVP

ARVARTGGISHHKPLSLAGNDEPLLAALRFGACAASKGVNSPFAYVLGDDTSGVTYTKQV

TAGTTYKFYNVSLLPTMCRKGPGDCASLNTAACANSRSPGLHPQRCHFTVQSRLWMPTPH

SLSSVVCMDN

>SG-AL_contig_184_1_+3_177_518

MISHVKSSDRSVHNTYKQLQILPFHLLIRYNTLLLMHKIVHNACPQYLKSMFCFQFQRES

GRAIVPKPNIDLFRMSLSFNGSKEWNTLPAICRQIPVISVFKAKIKTFLFDTFD

>SG-AL_contig_185_1_-1_1_798

CTTSQGPTPVRPAAGRVFSLVLAPSSGPSPGWLPQAPPSLCQIPHDQDVALASEVSDLIH

KGAVEVLSHPPGPGFYGQLFVVPKSSEGWRPFLVRIHFKMETPALIRGSIQEGDWASSID

LKDAYFHILIHPSCRKYLRCVWKGQVLQFRALPFGLSLAPYVFTRVVRELASLAQEEAIL

LSCYLDNWLILAESSTLCCLQISPQKCNLTPSQSFDFLGMRFNTLSLRVFPTPTRLQALR

LKLLDTECDPGLLPGKFPRSSASRSP

>SG-AL_contig_186_1_-2_107_370

MGWGKEKERTYVTSQTFLRSSTRKVQNASKADDDRFGLWRPVFNSFFFFSLHLLPLSLLL

SYASLMELTNSKFQPKTLKKSKKKLSLP

>SG-AL_contig_187_1_-1_259_1335

MKMASREQPILQLGGDWVVDQSYDFSHFDDLEWPEAQGLPEVSFKPETRADTQIDELLES

YRSHNGGSLEEEWMEQPDFIGSLPSDKLFLGEDFAFTPSPPPLTTLLGFTGDPEPEVGNH

GFVFADEFLKDLNKELTLQVDTPPSSPTPTDQVAPEIVLEDTPIQIVFEDGFLQSPLSPD

DVESVLSSASSQSPPPPAVSPSTSTVDVSCLHTDSSSWSMASSELYKVVENTEKARATPY

SRPHKSSKSNKSKGRKQTASIDPNPSELELELMSKKDRKKLQNKNAAIRYRMKKKAESEV

KRTEVEDLDNINMQLREKCESLEREVKCMRDLINDIRKARGQPPLPLSQSALALKIEQL

>SG-AL_contig_188_1_+1_244_708

MPVCTTSCGKINDVSFLFPPLTAALAADILKTSNTSQQKLKIQIMINFDFTQIFYFPHFS

HTAVNHFSTHSSHCIQLIFIFSFNLKILFKHCSRLKSKAEQCILFFTTSQIYTFYTDGKN

QWLPSANISTITQCNKTGPLLYDRRLVKLTIAGHF

>SG-AL_contig_189_1_-1_304_732

MAAGQQFLGHWKLADSENWDDFMKACGVGFVLRKVGNSITNYEEWKNEGDQWVLHITSTF

KSKLVNFKLGEEFDEETMDGRKVKSVITIEGDKIVHKQKATKEGEVDCTITREILEDGRL

QVTFVADNKGGLSAKRFFTPHKP

>SG-AL_contig_190_1_+1_1_162

LTWEIGKISTLYPPGEVSVVRTQAFHTLQFGSQASSPFDHRASSAYRECGFKFP

>SG-AL_contig_191_1_-2_2_265

PSLPWRPHFLDRKPPRFLASFDHTCICMFHELNHEICHTRYQVVTVTMQLYLKALISFFL

LCCSFLLKESFPPCSEGFFFSSVVLYYV

>SG-AL_contig_192_1_+3_3_122

PATRDPGHRRPQSPGREGEKQAGRHLHHKVDVVVRQTCRE

>SG-AL_contig_193_1_-1_235_1287

MNSAVLLLLCIVACSLTQALASSIIRFEQKRAPVRLRFDPYEITWNKFKTQYGRDYKTQD

EEDKRQRVFLENVKRIEQHNWQYFQMKKSYYLDINQFADMTAEEYQQYNKLLKRNSTAVT

FTKCSQFMPPLNWFLPSSVDWRTKGFVTPVKNQEACGSCWAFSTTGSLEGQHFRATGVLK

SLSEQQLVDCSESFGNEGCNGGLMDNAFEYINSVGGLETEGDYPYTAETGSCHFDKSEVV

ADLKNCVDIPSEDEHALMEAVASVGPVSVAIDAGHQSFQMYAGGVYDEPECSSTQLDHGV

LTVGYGSENGKEYWLVKNSWGATWGEEGYIKMSRNKDNQCGIATQASYPIV

>SG-AL_contig_194_1_-2_26_814

MASRLCRAVRPIANIARKQLFQQPVAATQLRFQSTADGVPPESRPTVRPQNPLLKQQAKD

FGQYVAECLPRFVQKVDVTYTDELEIMVHPEGIIPVLTFLKDHTNAQFLCITDIAGLDVP

TREYRFEVVYNLLSLRYNTRIRVKTYTDELTPLDSACEVYAGANWYEREIWDMYGVFFAN

HPDLRRILTDYGFEGHPFRKDFPLSGYVEVRYDDEVKRVVVEPIELQQEFRKFEYSTPWE

TFPTYRRIESGTEDTPTPPEPQK

>SG-AL_contig_195_1_+1_202_468

MPGQSVFGLEALGIFQRVINQAKSSGLATTKLGAEAEAEDDIRGGFVQLRQLISDLCLWH

SGPSWMEYIHNHLLPVEQTVGQELSSTNS

>SG-AL_contig_196_1_-2_14_577

MKTINSSQTVKAKKGVSISVKARKVTVKGPRGSLTRNFRHLPVDMLMTSKDVLKVEKWFG

NRSELAAVNTVCSHVENMQKGVTLGFLYKMKAVFAHFPINVVISDGGTLVEVRNFLGEKF

TRRVEMLPGVTCSPSSNKDEFLIQGNDIELVSRSAALIQQSTTVKRKDIRKFLDGIYVSQ

KTTIQEDE

>SG-AL_contig_197_1_-1_70_294

MTDVLTLLSLSSCLCVFLFTPHFCYHYSLISGFLKKKKMFQCRTKPNFTKDWDGKSSTPT

DDCNGQILELCFADF

>SG-AL_contig_198_1_-3_69_1250

MAVAAARPLISVYNEKGETTESNVTLPAVFRAPIRPDIVNFVHFEMKRNGRQPYAVSEKA

GHQTSAESWGTGRAVARIPRVRGGGTHRSGQGAFGNMCRGGRMFAPTKTWRRWHRKINVN

QRRYAICSAIAATGVPALVMSKGHRIEELPEVPLVVADKVEELKKTKEAVALLKKLRAWK

DIEKVYSSKRFRAGKGKMRNRRRIQRRGPLVIYNQDNGICRAFRNIPGITLISVDRLNLL

KVAPGGHVGRFCIWTQSAFSRLDKIYGTWRKASEEKSGYNLPSLKMTQTDLTKLLKSEEI

RAALRAPRKEVKRRILKKNPLKNKRVMVRLNPYAKAAAKAAQSVEQNRLRKKEELLNKKR

GIKTPLPALPKGKASAKKVAQRKKAKGGKAKGKK

>SG-AL_contig_199_1_+2_23_490

MEKMGLKVSTRERVQNLNNQQQTAVATRHRRQAVQNRRRTGQDKTHSKDRAEDTRGQTAG

ANGRSNDIHKENTQQWTWVQEMGLGGERRQGGIRSSRAMTMKSGWGGSFAVFECAEDGMR

RWNLAFTYGSQQGKGMLCRFCSANKAGGLPLVPAKG

>SG-AL_contig_200_1_-2_512_2116

MSDREGSHSPLSSSPPPLKLDTSSLHPPQQQPPLLSPNSSSAPSSAATRPGSSNSNGGGP

VGDLNDPFNQQIQDAVSQVLDGYDWSLVTTPSRNQNGEKRKPHIKRPMNAFMVWAQAARR

KLADQYPHLHNAELSKTLGKLWRLLNEGEKKPFVDEAERLRLQHKKDYPDYKYQPRRRKP

LKGSNQNNDNSAPLPQGVMLKAPGGRDTSSPSDDCSSECSSQPGGSNGPPTPPATPNQHE

ALSMKCLYDHRKRGYMLGPNGHPIDFSRVDLRDLSPDVMEPLDDQDLDQYLPPPGMGLPN

HHGHPHPHPQLGGRGEGQYSQCYLGPGTNGVATSSGSWVSPYRMNAAASCLPPHYMVNNN

NNSNNNSASSLSNGASSNITPPFDLSDQTARSPSSQTSPPSFLGPSPSGSDCKYRDDEPS

CQQVKIEQMNAQQQQQQQQSQVQHQQRYCDPKYDGFDVSSTSSGRYGSGGLDSGVHFPPP

PPPMHGPYLSHSQPASSGSGSSGPTNYQYGVGLHRQMFNPIAAAVPGEQSWERYT

>SG-AL_contig_201_1_+1_1_207

RENCVKDCNQIRCSKEKVAAVRLYAEHGRPLTDKRETEMEQEGRKETDRQRGEGCIFFFL

FLFGCVCVC

>SG-AL_contig_202_1_-1_61_1023

MDVPSEYRQPSQKRRGKGSRGGGGTSRPQLFEDTSNNHPPPAPPQGYGMGYGQPQYGYEF

DQPGMYAPQQGQFAGQQFFQDPMASMAMQYGTTLADQGKEYVHKNLEKYVATSKLKYYFA

VDTTYVGKKLGLLFFPYAHTDWTVQYNEEDPVTPRYDINAPDLYIPVMAFVTYILVAGVV

LGTQERFTPEQIGIQASTALVWIIIELLATVFSLYVMNLTADLKYTDCLAYSGYKFVGMI

FSLLGGLAVQGTGYYIVLLWFSLALAFFLVRSLKHKVQPHSGNEEYRRGTKRSLYFTICV

AVAQPLLMWWLTSHIMFGRKA

>SG-AL_contig_203_1_-3_267_2843

MDYDSDVHVVRKVSRTYNVYRGSSPATQNRLEARIRELEDALDAERDGRVRAEKMVTELS

FRLDQVQDSLDEQGSSTSAQMELNKKRELEISKLRKDLDMANAQFEASEASLRKRHTEAV

NNLSDQVDYLTKHKNRVEKEKQTLVVELDGLSGELDSVSKAKIALEGRVDGAEQSAARLK

GQVDDLTRQLNELTMLKARLTQENFDLQHQVQELDSSNAALAKAKNSLQYSNDDLKKQLD

DESRQRQNLQVSLSGLRSDYDNLNARLEEETETSSTLRNTLSRVSADYAALKTRFDKEIA

AKTEELEETRRRLNLRITELEDSCEQLRIRNSGLEKAKAKLANELKEVTIELENTQIIVQ

DLTKRNRQLENENGALLRRVDELTAENGQLRSEKAALEQDNQRLRVANAELTERNDNLQR

ENKTLSDQLREANQALKDANRELADLRQLRAQLEAERDSLAAALKDTEDALRDAEAKLAN

AQAALNQLRIDMENRLREKDEEIENIRRSSARAIEELQRTLVEVETRYKTEISRLKKKYE

TDIRELEGALDNANRANAEYLKQIKSLQMRVKELEALLDDERRIADDLRNQLAVSERRRV

QLGQELEDARSMLEAAERGRKNAENELGETTVRLSEVTIQVTSLTNDKRRLEGDMAAMQS

DLDDAFNRMRAAEERAERLAAENARLADELRQEQENYKNAEGLRKQLDIEIREITIRLEE

AEAFAVKEGKRLVAKLQGRVRDLEGELENEARRGREAMAGMRKAERLYKDLIAQTEEERR

QLAELSSLNDQLSLRIKTLKRQIEESEDVVNVTMNKYRKAQALIEEADQRADNAEKNLQS

VRRARSMSVTREITRVINV

>SG-AL_contig_204_1_-1_481_687

MGTRKHPACTPTEDGGCRVCSNITPAQFRLSNRIYTHPKKCCSERVKKAVNDYLTLFRAR

EDKAARERI

>SG-AL_contig_205_1_-3_3_227

DFQDLTLVRHIRKLEQTPNNRDFEADDHEDWSSEVGGGGGGSGDIDRDDFLHVGYEFPTN

KQCLDFFHPLFFGFF

>SG-AL_contig_206_1_+3_63_272

MMWTDWDLNSQPSKFQSIAAQLFFTAGYIIPNNMISIFHLSFPEVKVYLEYYCAYIMCLL

CVVTVIYQNE

>SG-AL_contig_207_1_-1_655_1689

MLLKNVLLYTVLIGWIPSVWSFEPFTSVLGVGAVGAAFYGVGYMLKCRFYECCNDNRWVI

NNITALHGELHRRLYGQHLVKKAVANHIQGHVRKHHPVKALALSFHGPTGTGKNHVSHII

ANALYKKGLQSENVRFIAATKNFPHQHKVSKYKDQLKDVVEKQVKKCPQSLFIFDEIDKM

PAGLIDTLKPYLDYVDHLDGVDYRHAIFIFLSNAGSESITEYVLQQLREGVKREEIGLVG

AEKILAKAAVNSKSKEGYWHSELISKHLITAFLPFLPLERKHIRQCVQDLMVLRKYYKSQ

RSVPDEKVEDVLAELTFYPEKEQMFSVTGCKRVAEKVDYVMFGSR

>SG-AL_contig_208_1_-1_61_897

MADSAGGGRGGFRGGFGGEGRGRGRGRGRGRGRGRGRGRGAKEGEKEWVPVTKLGRLVKD

GKIKSLEEIYMFSLPIKECEIIDTFLPTGTLKDEVLKIMPVQKQTRAGQRTRFKAFVAVG

DYNGHVGLGVKCSKEVATAIRGAIILAKLSVVPVRRGYWGNKIGKPHTVPCKVTGKCGSV

VVRLIPAPRGTGIVSAPVPKKLLTMAGIDDCYTSSRGATATLGNFAKATYAAISKTYSYL

TPDLWKETVFTKAPYQEFTDYLMKNHSRVAVQKQEQKVY

>SG-AL_contig_209_1_-3_336_1853

MTTPATIPVFTVTTKEEEPYIMRELDAKGGARYVGFIKDLMDVLADKVGFQYNFKVVDDG

MYGQQSPAGWTGMIGEIVNKKADIAAGALTVTGPREEVVDFTKPFMTTAVNLLVKKPERM

DMGLGYLVRPFSTDFWILLLVSVVIVGFVFFLIGRFSPYEWVKVAGEKDQRGARNAFSLR

NSYLFVLSSLTWQGFREPPRSLSGRFLAAFWWIFILFTIVAYTANLTAYLLARPEQIPLM

PFKTYEDLVESNDIKAGSLAFGATQASLRKSRDPTLQSLWNKMSQENSWVNSYSEGLQRV

QKSDGHFVVFMESTTAEYLARKNCDVMLYGNNIFHKGYALATQKGSVIGSMLNKAMLELK

ETNELHMLIQRWWRFSGECSGIDGREYSQKGDSLSSLPIYPVTLRDMAVAILILFLGFIV

AVVGLILEIIYHTISTGGKIEKPKVLDSKIFKAPKFTKGKSSKPAAATSEDPEAGMSSEP

LSGEALGEGTSGEGASGEGVEEAREA

>SG-AL_contig_210_1_-1_1_123

FFFFSHEGGGGGKFISLWATFPDLSLTHQHVEFKSLMLTKL

>SG-AL_contig_211_1_-3_30_431

MASATTRSLLCVLVLSSVTVFSSQAACTNIELGEIFTCITPYGQQFEDAIGLTSFDNYVC

NTSRITTDICRLFRKIVICVNGLDVSRENCLLSGTRQVNVRLGIPCSVEDFYAVCKDAPP

IGGLDTTSVADTTV

>SG-AL_contig_212_1_-2_197_2785

MDRSQLRKSELKTTDPPKTDFRSVLSKPQATSAHSKFSAQSSTDSKPSWVKTESPREQKK

NGHDMDMKQSSSSKTDFRSVLKNRQVEDSRTSSVSRDSKPKVQSTKSETDFRSVLAKMKH

GGEDSKTTQPKASTSKVTPDRTLNKALSKDLNSIRSKFETPAEDVKSVQSKPPRPTTTAT

TTTVSNVSELSSTDTNSTATASWRREWRDRSAGKQTDGGKITIKTTDNNSSIEKTTDRWA

EKNTENIRTEKTTDRWKDKTPDSGRSAALTTDKERWTDKSTDRWTDKSTNRWTEKTTNGE

KPAEKDTDNNSSTVRTTDSERWTDKGRDRWTEKTTDRDKSAEKGAEDNRPAVKATENDRP

AAKTTDRERPEEKCDDTNRLAAKVKTTDNERWAEKTTDKWTKKTRDRWTDKTTDRWKERY

ADRDGPAEKSTDDKPTLKASDNNRSTAKTTDSSRSAEKFTDINRPTVKTTDTSRSALRAA

ERERISVKTMDNTADDKVEVISSLGDTSRFSDDAKCSDSSQLSDSAISSAFSKLSELSDL

SDSGSAVSEDVPEPHGRKRLERLKLRLGGGESGESEGAEELSEPPSIRQRLADRSVTYGG

QAVLQCTVTGVPVPDINWSVNKKTIKPSRFFQLSYRENVAQLVVAEAFSEDEGEYTCTAT

NTAGTDSCSCYLTVEASSSRSSQSGSHTFEGPVSTPPHVTTLTPSTLTVRRGQPASFQLT

FTGEPPPAVRWFLAKTPIQPDDKISIVTEGGVSTLTIGEASANNSGRYTVTVTNDLGSDL

QSATLSVEDVPECPVGTPHVYDVSKQSVSLSWCGPAYDGGSAITHYVIEVKDSNTKEWTS

LVSDCKSTSYHALTLKADTNYQF

>SG-AL_contig_213_1_-3_63_5507

MERLAFLIQAVLCLFCCGSNASPTLIPPILLEELNAETQEFGYLMTMPQTLQAGSKEQYC

LTLHGVTRDTTVTMELLRVVEEGEDEEEEEESQTYMNTYINGESQCNIFRVPDEPGRYEV

NLQNETGTFASSEVQVLGNNLITFVQTDKPIYKPGQKVKFRILTMMRNLRSRVGQIETVF

IKDPNDFKVKKFSNVQSKGMASLEFQLINEAKLGTWTIEVTVDGETTSQKFKVEEYVLPR

FEVIIEPPAYFLVTSETFDGRVCAKYTYGKPVQGFLKLQLCPSGYGTFGAPCPLLQTEID

GCTPFSFNSSELLSSGRYFYGRSFNIDASVTEMGTGVQVNGTAPGPLVSYDPLRIEFLDD

TNGFFKPSLPVYGRVKVSKPDGSPAVNESVRVSASDYNNNIYLDKVFVTDPMGEIHYSIC

HGLQENTSSLSMFAEAINFKEKTDVPFGRRLYQPTGHGSFRQWFSPSFSYIQIPAIHKPL

KCGDDLNLNVPFTHNGLKPYVKFYYQVMSRGRIVHSGTTEAPTLPDPLSFLEPAINNCLE

VKERPTPPPLPTIPPPVNDTDSGEIAPEEPPEPPMIDEPPMTIERSSRLMESAVREPPEP

PMMEPPRIPALQPVQIEPLSETFGQFDADLRRKRSIMPHYGRGEEEEIVRSEHISDQLSN

VQLTIRVTAEMSPSFSVLLFYIRDDGETVADSMEFKVEPCFENEVKMKFVDETVLPGAET

TIELKAASGSVCGIGVVDKSVTILGGDHQITPAKVFEKLAQYSLSSRGRFRYSYYRSEYD

YCKDRLSKLEEEEEDSEDDGEREPGEWSYMRRSYFSKYVDAVEAFKQMGMLVLTNLDVET

RPCGWNQPVYFDGLAMAGPLNSVPGPGVPGAPGPVPVVARLGSAEEEKSPRTNFPETWLW

DLHEVGASGEVDLQLTVPHTITKWVGNTMCINDKAGFGLSPVTSITTFQPFFLAFNLPYN

AVRGERLPITVTVYNYLDKCLNMLLMVDGMKGFKVYSARSNRQPFCLCGGKSKTTRMYFT

AQDIGKLPIFARAEIVPGQCGNAVEMDTQYIGLSDSVQRKVLVKAEGVTQRYSHSLYICP

KDGERYINNFTLPLPKDMVRDSSRGDVTVIGDIMGPALDNLDNLVRMPTGCGEQNMVGFT

PNIYVLKYLTATGRLTEVMQDKAKNFMEIGYQRELTYRHSDGSFSAFGPKGDKPGSTWLT

AFVVKSFAQARPYIFIDEEDLELSVNYLKSMQNPEEGPVNGCFFERGKVFSSYMQGGLGK

NPNDAALSAYVLIALLQADLSTEDQVAVNGINCINRQLQNDPELDVYTLSLIAYVNVLYD

PNSPTTANIMQRLDRRATVEGDLKYWKREGELPKPVNSWYYYAAPSAEVEMTSYALLATL

LFYNRDAIARAQPSVFWLSKQRNAFGGYASTQDTVLGLNALSEFAARTYSSETTDLRLRV

DYESSRVNFEVSNDNSLELQRQAFDVKDNGTMWVSATGTGCALIQANIHYNKMPTKLGID

DPKFNLRVDPRNYQHDRNRCNKRTIYVTFGQRNSGQFSTGMTMLTVRMVTGWSVIPGSLK

ALRSRYPMLGIQREEIKEEEGMINFYLDELDTRTRRFALDVEQDQSLLVRAPKPAEVQIY

QYYEKDVTVIRSYQLRTRCGTKEELPYDQPESGTDDSFVMQRRLPLGGPDSGRGRPADLN

VQPSREGAGAPAARSCPVCNLQKAPPNYNEIVCNSTAVYKAHAGRNKRFSLKIKANLRPR

KKQRDLNLFANIRMAEGCQCALLDPPMRAVIIVTQAANLRDKTLTVDSRTYVFNLRRDRT

LEKKARKAQRKCRMN

>SG-AL_contig_214_1_-2_158_352

MQVAMCFTIVLCLVTVVNTLAADTHGVLSDGSLDRLKKENMAESRLSSFERMKKTCCSFE

RWCCG

>SG-AL_contig_215_1_-2_224_1351

MCDEDVAALVIDNGSGMCKAGFAGDDAPRAVFPSIVGRPRHQGVMVGMGQKDSYVGDEAQ

SKRGILTLKYPIEHGIVTNWDDMEKIWHHTFYNELRVAPEEHPVLLTEAPLNPKANREKM

TQIMFETFNAPAMYVAIQAVLSLYASGRTTGIVLDSGDGVTHTVPIYEGYALPHAIMRLD

LAGRDLTDYLMKILTERGYSFTTTAEREIVRDIKEKLCYVALDFEQEMQTAASSSSLEKS

YELPDGQVITIGNERFRCPEAMFQPSFLGMESAGVHETTYNSIMKCDVDIRKDLYANTVL

SGGSTMYPGIADRMQKEITALAPPTMKIKIIAPPERKYSVWIGGSILASLSTFQQMWISK

QEYDESGPSIVHRKCF

>SG-AL_contig_216_1_+1_730_1056

MQKNNKHQKIDSQYILIKISPLILGQEKYIMLFNITVMKIFSTVVMTLTQRERERERERE

REREREQNKNQTQNNKMEKRLVFIFTPHENLFHYHTPCTSIWYLSLFQF

>SG-AL_contig_217_1_-1_220_1074

MADAEAAQEEKPQEVEKPKEKKTIAEKIKGTVKWFNVKSGYGFINRNDTNEDVFVHQTAI

IKNNPKKYLRSVGDNEEVEFDVVEGEKGNEAANVTGPNGTNVQGSKYAADRRRYRNGGRG

GWYRGGYRRGGRRNGEQENDDGEIEENEDQDKGQRGGRRRRGGRGYYPRRYFGGPVRRYG

YQGGPMMPPDYYMEQAPFPRRRPFQRYNYGMNRRDYYEPGYYQPRGYGRGGFRGGRGRGR

GGRGQGGNRRNNKAEDTKQAVEQKEVEKGSGDAKADATTEDADKE

>SG-AL_contig_218_1_+2_308_583

MPAAIYSKSTPFTAYDILYIHKHFYTHQLNPVNGSGSLLYCLRRKKKRKFHFYVQFFHPL

FQQSNTHNSFLNMCFYKPCMLPHSFSLSFAPS

>SG-AL_contig_219_1_+1_1_129

TLILTLFPYTTLFRSVRCENEHSHYDAKRSGVKMNMPTMMLLL

>SG-AL_contig_220_1_-2_1430_1657

MAWAEAASCYANVPFCLVCRYENENFQSADLDKMFFSRCLKKGGKTFFMDSWCSLYVCVR

FEDNFFSAKILGSFSI

>SG-AL_contig_221_1_-1_73_534

MFRIVHRASSALYGITRQSLQRPIAAATVTVRGKHGGEKETDEAFDARYENYFSRPDIDG

WEIRKAMNDLQGEDLVPEPKIIIAAMKACRRINDYALAVRYLEAVKDKCGGRLNEVWPYI

RQEIKPTLDDLGILTPEEMGYDKPELALKDVYDM

>SG-AL_contig_222_1_-2_1112_1408

MYPTCFYLLCSRHGVLRPKLLPAKTVAGDNFGRPRSQNCHQSEICCPLSQIWEGHDIFQK

MNYWAEFASVPNLPRHSFFLLLWCMYLSLSLYICLSLSL

>SG-AL_contig_223_1_-1_1_222

GKRTHIQARKLVARVFVLSSQRRISPQTLAPFRVPAVCCRSAKFVDCSAHNPASSALSGL

ALRSLAAYRPTLST

>SG-AL_contig_224_1_+1_61_222

MTRIALKQRMFKLCVVSILCLVIKTVHMSASSDYRRPSLACISIIAPFHQLTSS

>SG-AL_contig_225_1_+3_1350_1673

MGHYDNKKACLSNGICFVGYTITVNTVLHRITIKQTCRNKSTKRVYPVSEQWVMCLFSCK

LLYPTNKRVEKRSSKLLHALVRGAYQLLIKPMCCKQINATFFFILYAR

>SG-AL_contig_226_1_+2_149_358

MVQYQHLCDYRQFWHYSHILLSILKTKSVLPHLLSADHISTIYFLWSSFRLIINSIRLSF

LAVNTLWVKF

>SG-AL_contig_227_1_-2_83_1825

MFGQNPGGFSFGQPTSGNTTAGGSFPFGATSTGTTAPAPAAAAGSFTFGTPASTMAAGAT

NQATLGFSFGAQATAAASSTNPAGGFTFGTPASTAAASATAQPATTGGFSFGSTAPATGQ

PGGFSFGGATAATTQPAAGGGGSGFSFGGAGITPASGAKTTPAVGFSFGGMSTTPTTQTT

GGATGAGTGAGFSFGLGGGTTASTTTAGLFGGQGMLPTPQPTAATAATSAPSTTASSLAS

LLAAPAPTSTQPTLQALGVTVRPHAASGGFFSTSVAASSTTATTSTTTAAPSFAFTTPTP

ATASSAGGFSLSGGAPTLGTSAPSLGLGAPKLGLTATATAASTSATGFSLGLTSAAPSLS

TATTTTTATAAATAATSDGAAKPGRQMTYKQLEEETNKWMSELEEQERNFLRQATQVNAW

DRLVIDNGEKIVQLNSDLERVKLEQQKLDHELDFVHSQQRELADLLTPLEAALEAMPAIS

YQQHADLEREHTYQLAENLDAQLKRMGQDLKEIIDRLNSSNTKPDPDDPVQQITKILNAH

MDSLLWVDRQTGTLNRRVEEMGKLMENERKEQERNFRLAYS

>SG-AL_contig_228_1_-1_43_696

MGRRPARCYRYCKNKPYPKSRFCRGVPDPKIRIFDLGRKKARVDDFPLCIHLISDEYEQL

SSEALEAGRICANKYLVKHCGKDAFHLRVRLHPFHINRINKMLSCAGADRLQTGMRGAYG

KPQGTVARVHIGQPIMSVRAREQHEAAIIEALRRAKFKYPGRQKIVVSKKWGFTKWTRDD

YTRMRAEGHLIPDGVGAQYKPNKGPLDAWKHRVAVAHQ

>SG-AL_contig_229_1_+3_564_755

MPTSSAMSEASMSMSVWQIQSLQCQWHPWFKHFYDKLSLCYFKDFILCYKMFVKVNLCNI

RDIY

>SG-AL_contig_230_1_+2_2_310

LFLSLLHTHTHTHTHTHAHICRVDEIKCKPFISSIYWSGHWKKICSTTFNAVLGGNNYIA

YNGLPSMKCWDFLPYTTASQRLSVNKSDTFPVHFSLHQFLCIC

>SG-AL_contig_231_1_-3_21_629

MAAKKLGKLVVKNSCLFLCDMQEKFRPTIQYFPQIVNVSARMLQAARILDMPVVVTEQYP

KGLGHVVPELGVKDEKVFPKTCFSMVLPEVEEYMKQRSDVKSVVLCGIETQACILQTALD

LIEKGYDVHVIADACSSRSMVDRMYALERMRKAGAYLTTSESMMLLLCRDAAHPKFRDIQ

KIIWEPAPDSGLLTGMPQDGSAL

>SG-AL_contig_232_1_-3_918_1112

MKIGLFAGLLQSQRTMEFVPLKRLQDTSQGKVLRRNYCCPCSRPTLSTTPVKSKEEAKAN

AAWSW

>SG-AL_contig_233_1_-1_262_5217

MLLEDYSKYKYLSHGHIPVAGVDDAQEFRSLVESMTIMGFSPDDQAALMRVISAELLMGN

MQFKQERNSDQATLPDNTVAQKACHLLGVPVTALTQAFLKPKIKVGRDFVTKAQTKAQVE

FAVEAISKALYERMFKWIVQRINKSLDRTKRQGASFIGILDIAGFEIFKMNSFEQLCINY

TNEKLQQLFNHTMFILEQEEYQREGIEWKFIDFGLDLQPTIDLLEKPMGIFALVDEECWF

PKATDKTFVDKLLAQHTSHPKFQKPDFRADAHFSVIHYADKVDYSADQWLMKNMDPLNEN

VVAILQSSTDNFVASIWKDADIVGMGAAAAVDTMFGSRTRKGMFRTVSQLYKEQLSKLMA

TLRNTNPNFVRCIIPNHEKKAGKIDSPLVLDQLRCNGVLEGIRICRQGFPNRILFQEFRQ

RYELLTPNAIPRGFMDGKKAVEKMIASLELDTNLYRIGQSKIFFRAGVLAHLEEERDLKL

TDIIVQFQALVRGLLARRNYQKRLQQINAIRVIQRNCAAYLKLRNWQWWRLFTKVKPLLS

VTGQEEKLHTKEEELKKMKETYEKQKQDVEQLESRYAQIIEEKNILAEQLQAETELCAEA

EEARSHLAKRKEELEEILHEMELRMEEEEERMVSMSDEKKKLNQTVQDLEEQLEEEEQAR

QKLHLEKVAVDSKLKKLEEDLALNEDSNGKLIKEKKSLEERLSEANAQIVEEEEKSKQLG

KLKNKYEAIIADLEERLRKEQQARQELEKIRRRLETELNDLRDQLNEKRQQVEELQAQLS

KREEEVQIALQKADEEGVSRQTTNKQMRELSTQLQELQEDLETERESRNKAEKLKRDLNE

ELEALKTELEDSLDTTAAVQDLRNKREQELQDLKRTLETNQQQHETQIQEVRSRYNQQIE

TLSDEVETTRKAKATLEKTKANLEAENQEMASDLKSVQMAKTESERKRKQAEQQLAELSI

RLQEVEQGRGESTDKASRLQQELDQANSQLEQVDTKAIQLGQKVSSLEAQLADSQDICQE

ETRQKLAVQSKLRQAEDEKESAMERLEEEEESKRALEKQVSDLNQKMVELKRKAEEDLAN

NEALEEYKKKAAREMDQLQAQVEEARTNADRMDKSRRKLQAEVEDLTIALDSQRSNVSNM

EKKQRKFDQTLAEAKAESERMAQEKDSIEKESREKETKILNLTRNLDEMQERVEQLERVR

QQQARELDDLVSSKDDVGKNVHELDKARRALEATVAEQRVQIEELEDELQAAEDAKLRLE

VNMNALKEKYKNEVAGREEQEEESKRSLLRQLREMESELEDERKQRAMAVNARNKLQGDI

QGMEQQLEMANKVKEDAVKQYKKITTQMKDFQRELDDARASREEMAGQAKDNEKKVKNLE

AELLKLQEDLASAERQRRNAEAERDELADEIGSSASSKQSLIDEKRRLEARISELEDELE

DEHTNLELASDKARKSLMQMEQLNTDLAAERSVSQKLENQRLALERQNKEMRDKLQELEG

QNRARTKATIAALEGKIVNLEEQLDQEAKDRAALARTNRKMDKRLKELQIQAEDERRSAD

QYKEQLEKMNNRVKALKRQVDEAEEEVARINAQKRKVQRELDDQMEQNDTLVRETQSLRK

YRPSAQVRTSRVTVKLGEDEGSEEGPDEQTTT

>SG-AL_contig_234_1_-1_121_588

MSSLSKSDIEDITEVFELFDFWDGRDGDVDAFKVGDMCFCLGLNPTQEIIHKNGGTLKLG

EKAYKLNEFLPIYETISKEKDTGTFADFNEAFKTFDREGQGFISAAELRHLLTGLGEKLT

DGEVDELIKATDTREDLEGNVKYDDFIEKVLKGPEM

>SG-AL_contig_235_1_-1_19_528

MAPKKSTKATAKGEMKKVYSDPKESMLVGKAKDKAQRAKKAVLHGRHDKRSRKIRTSVHF

RRPKTMSLPRAPRYPRQSASKQNPLDAFRIVKFPLTTESTMKAIEDNNTMVFIVDKRANK

PLIKQAVKQLYNIQVARVNTLIRPDGEKKAYVRLQPDYDALDVASKIGII

>SG-AL_contig_236_1_-2_2_271

CYHFYVISPTIKKRTGSSLIRPQDYVQHRSGNYSRHNANATQLQRVFQEKVKMQVAMCFT

IVLCLVTVVNTLAADTHGVLSDGSLDRLKK

>SG-AL_contig_237_1_-3_1842_2096

MFSAVNFSTRFSCVVASQKTYGSNLMIADFPHHSHYYCNMYRQVDTVLLRMTIQCLSYKF

VGCTVYSFCTLKNDPSVWLTNLSPK

>SG-AL_contig_238_1_-2_62_5245

MLDGTVNDRIFNWMKNFVRNFASQLDVDKGVYRVGAMTFTSRPSGQFQLNRYNFQDEVMG

ALDGLSGTGRGRVDTARAFDYVRDNMFTQPNGDRPLARNNIVMLTGNEKSLSTDRAVAAA

RRLKDLGTDIYTVGINLRNTDEIDAVSSKPLDYYQTLINSEGELEQIPGIYKYRIETATT

TTTTTTPKPTTTPRRVPVFSDTGYDLVFMLDGTVNDRIFNWMKNFVRNFASQLDVDKGVY

RVGAMTFTSRPSGQFQLNRYNFQDEVMGALDGLSGTGRGRVDTARAFDYVRDTMFTRRNG

DRPLARNNIVMLTGNEKSLSTDRTVAAARRLKDLGTDIYTVGINLRNTNEIDAVSSKPLD

YYQTLINSEGELEEIPGIYKYRIETATTTTTTTTPRPTTTPRRKPVFSETGTDLIFMLDG

TVNDQIFGWMKNFVRNFASLLDIDNGEYRVGAMTFTSRPSVQFHLNKHNFQDEVMGAIDE

FRGTRRGRVDTARAFDYVRDTMFTRRNGDRPRARNYVVMLTGNEKSTSTDRAISSARRLK

DLGTDIYTVGMNLRNTDEIDAVSSKPLEDFETLINSEPELEEVPGIYKYRMERAPPPFTR

STTTTTTTTTTTTPRPRRPTTPYTGECESTGDVTFVLDSSGSVGELNFDRLRNFTASAVR

DLEIDIGFFRIAVVTFSDSANIEFFLNTYSSKQEIIDAIRRIPYVYGYTHTAEALRRTRT

EVYNSVNGDRANVPNLVVIVTDGESNINPFETLPEANTLKLSGTSIITVAVGLSSNVELQ

GLTSPPIQDNLIFVDDYESLHSLSDQIVGPLCSDPNLCKEGPCLNEGVCLDGLRSYTCVC

QPGYYGVNCEKQCGPKTDVVFILDSSTSVGSDNFNRIKTYAEMLMRDMNPDSCDVNIGAM

KYSSAAMIQFRLGEYDTEEDLTRAINAIGYTRGRANMADALRVLRTQMFNGRNGDRPDAK

NVAYLLTDGGIEINRDITLSEADLIIDAGVRLIPLAVGLRQRNEIELIANAQGLPLLEID

SEAKLEQMSDQVLEAVMDTGEAANQCSSAPCQNGGRCFNEALGFRCECAEGWTGDVCDKE

CSGQADVVFVLDTSRYNSRKDVRSIKRFARDVVKRMAFRRGNFRVGVVDFGETARTRLTF

AEGNNKRTVKSVIGGIRPRKEDPKPANGIRRVEADIVGRLGDREAVPNYLVFVTTSMGGS

STLQAINNLKRVGTRVIGVGINLSSSDERLLESAVSQPTRNMMFTVTDSVDLDNIAEDFV

DFVCEEKNMCAENPCRNGGYCTNGAKDYGCRCLPGFAGKNCERSCADIADVAFLLDMSGS

VGFDNFRRMKEYVYYMVENFSIGPDGTHVGLATFSTNARTQFYLNQFQDKRDLQNAISAI

SYEYGNTNTAAGLKLLRSSMFSERRGNRLEVPDYAILITDGLSNVNAEQTIPEAELARKQ

GIHVFAIGIGVSDNWELRAIASEPADNNAYVIQEYANLWNISDKLLDATCKDAGVCADSP

CLNGGTCIPGVGTYSCKCMQGFVGPHCEQDCQETKDIVFVLDSSTSVGPDNFELMLNYMQ

LLVKDLTGAGQNHRFSLITYSTEVKTIFSFSRYNQPEQVVQAISTTRYTPGSTNTAGGLR

QARQMFQPGFGERFTAKDVVILLTDGQSNINFYDTIPAATDLKSDGTRVIGIGIGLTNAD

ELNAIASGPSDVFQVATFDALKDIKTEIVAGSCRPMDGERKAPKELEG

>SG-AL_contig_239_1_-1_766_954

MLVFVLSCYCCGIGDSFLFISLYLFMFVVSLLIFPVIWMAIAFILPGIILCFLISYFQKA

GNF

>SG-AL_contig_240_1_-3_27_230

MLTAEGSQGPLAPVHKQLGPGGAPRRLVSGGDTGVGFSLRPGGWSPHRSPSPTSDPIHGR

LVQRLGSP

>SG-AL_contig_241_1_+1_1_249

LEVDLGGPVAIPDRAPGQGRGLHSGVDADHELMKGDRSRTGCRHALRIWGLQRVGHRIQG

QGGGAKLPQPLCRSGRPPLRPGL

>SG-AL_contig_242_1_+1_2323_2658

MLGLNRLPHYERENKNKWENIKPPLLPTKCFSDALPRSTTEKKFIKMEFIHRKDKIKTGN

MTREKWKMITTMWWQIKRLDLPISPTTVTHVYYPDTQLQETSCAKSMTAANH

>SG-AL_contig_243_1_-3_60_458

MGRVRTKTVKKAARVIIEKYYTKLTHDFHTNKRVCEEIAIIPSKKLRNKIAGFVTHLMKR

IQRGPVRGISIKLQEEERERRDNYVPDVSALDLDIIEVDPDTKEMLKDLDLGNISGLQVT

QPINTVSFRAPRN

>SG-AL_contig_244_1_-1_67_3297

MRVLPGVDVSKVKVTGPGVQGTVPASLPAQFTVDTRETESADLECVVKRPDGTFIRPYIQ

DNADGTFDVMYTPEDLGPYDVIVKYAGQDVPGAPFAVKATPTGDASKCKITDGADKMVPV

NKETVITVDAAEAGNGKVTCRIRQPNGSDIDIDIIDKGDGTFSISFTPQFPGDYTIEIKF

GGNLVPDGEYTIQVGATEEMISPDQVDKLRAGAPGGAPFQTVDFRFQVGPIFNFVSAAVV

TPSGKTAYPKIEDNQDGTVTVRYQPTETGLHELHVKYNNESIEGSPFLFHVDAVNSGYVT

AYGPGLSHGVADEPAFFTINTKNAGPGGLSLAVEGPSKTEIQCVDNQDGTCTVSYLPTVP

GEYLITVKFADKNIMGSPFTAKITPSSREPRKRAQFSVGSSSEVSLKVTETDISDLTATV

RSPSGHEEPCLLKRLANGHLGMTFTPREKGEHLVNVFRNGKHIPNSPFKIMVGESEMGNA

SKVKITGKGITEGMANEVNEFFVDTREAGYGNMSMSIEGPSKADIECHDTPDGTCRVTYK

PTEPGTYVLNFKFADQHVPGSPFTVNVGGEPSAKITERITRERVAADVSHVGSECELSLK

IPGTSPLDMKASVKSPSGLTEVCDVTSLDDSHYSVKFVPKEMGIHTVSVKHKDMHIPGSP

FEFTVGPIAGGGSHKVHAAGPGLERGEVKQPCDFNIYTREAGAGGLSIAVEGPSKAEIDF

QDRKDGSCGVTYNVTEPGEYFVSIKFNDEHIPDSPFRVPITGPIGDARKISVSPLQEKTL

LVGDTATFVVNLNGAQGTLQARIQPPTGPMQEALVQEMENGQYAIRFIPRENGLHLVHVL

FNGHHIPGSPFRIMVGKLVADAGNVTAYGDGLHTGKTGQISKFIVNTVNAGAGALAVTVE

GPSKVKLECREVEEGYEFTYCPKAPGDYLITIRYAGVNIAGSPFKARVEGAGQPSGVQEH

ASVVVETVTKTSTTTKFSGLQQFVSDATKCVASGPGLTKGVRGQTNTFTVDTTGAGNNMM

FVGMMGPECPVDELVVKFVGNKKYSVSYKVKMQGRYILIVRWGEQDIPGSPFHVEVV

>SG-AL_contig_245_1_-3_165_4073

MEEGPQTLDVTYEGIPIPGSPFNVNAVPGSDANRVRAYGPGLEGGLTQEPAIFTVDMKGA

GKGSLGLGIEGPVEAKMKCDDNRDGTCRVEYLPTKPGNYDITITFGEKPIKNSPFRVKIN

NPVDARKVKCYGPGLDTEEVSANAPAVFTIDAREAGEAPLDVTYTDPKTGAKKSADIVPV

EEGVYQATYVPTAEGKCKVDVKYGGDHVPNSPFNMSVVPGVDPQKVKVKGPGTDKTTLAS

LPAEFMVDTRNAGAGDLECQVKRPDGTFIRPFLNDNGDGTYDVIYTPEDLGIYDIIVKYD

GQNVAEAPFQVKALPTGDASKCKILDGADKVIPVNKETVITVDAAEAGKGKVTCRVRSPS

DSDIDIDIIDKGDGTFAITFIPQYPGEYTIEIKFGGNLIPNGKYKAEAVPEDEFEQYLLQ

RPVVDDLISADQVDRLQSAAPGSGLYQPVDFCFQVGPIFNFVSASVIMPSGKTSYPKIED

NRDGTVTVRYQPTETGLHELHVKYNNECIEGSPFLFHVDAVNSGYVTAYGPGLSHGIVYE

PAIFTINTKEAGAGGLALAVEGPSKTEIKCVDNQDGTCTVSYLPTVPGEYHITVKFDKEN

IVGSPFTAKVTPGEPRKRAQLSVGSSSEVSLKVTEKDITDLTATIRSPSGREEPCLLKRL

ANGHLGITFTPRETGEHLVNVLRNGKHIANSPFKIMVGESELGNANKVKVSGKGLTEGMA

NEVNEFVVDTREAGYGGMSLSIEGPSKADIECHDNEDGTCRVTYKPTEPGTYIINIKFAD

QHVPGSPYSVKIGGEPSAKITERITRHKAAADITHVGSQCELSLKIPGTSPFDMTASVKS

PSGLTELCDVASLDDNHYSIKFVPKEMGVHTVSVKHRDMHIPGSPFQFTVGPISGGGSHK

VHAAGPGLERGEVNQPCDFNIYTREAGAGALSIAVEGPSKAEIEFEDRKDGSCGVTYNIS

EPGEYYVSVKFNDEHIPESPFRVQVTPSIGDARKISISALQEKGLQVNKPAAFVVKFNGA

RGKLTAHVISPSGAQEEALVQEVDDGEYAVRFIPRENGLHLVHVMLDNYHIPDSPFRIMI

GKKDAADAGKVSASGDGLQTGIAGEISKFMVNTLNAGAGALAVTVEGPSKVKLECNEMDE

GYAFTYCPKAPGDYLITIRYAGVNIAGSPFKARVEGSGEASSSTEHASVVVETVTKTSTT

TKFSGIQKFSSDASKCVAKGNGLTKAFRGKTTSFTVDVTGSGNNILYVGMAGPKSPVDEL

TVKYVGNNQYTINYTVKQQGQYMLIVRWGEEDIPGSPFAVEVI

>SG-AL_contig_246_1_-2_53_2167

MSSSAEQRPEAAEEEILPTKEQTLLAEAPVTTEVSDSACTDEPSAHQEKPLPTTQTPAEQ

PPSSAQEQGCPAEKELSSAQEQTAQEQPASVQEQIMLTEQASVQEQTSAGQSAAVQEQLT

PEEEQPAAVREQITPEEEQPASVREQITPEEQPASVREQITPVVEQPASVREQVTTEVEQ

PASVQEQVTPEVEQPASVQEQVTHEEEQPASVQEQITHEKEQPASVQEQITHEKEQPASV

QERITHEEEQPASVEEQSFIQEQPPSGQPASVQAQIPAEEQPESVQKQATPAEEETPQEQ

TTPAEQPPASIQEQKAAEEPKSIEEKMSDEQEYQSKTDLPAEEKITAEEQSVSVEKRESP

TEKSSASAEEPNSTEEKMSSDEQEYQSKMDLPAEEQISAEEQSVSVEKRESPTEKSSAST

EQATPSSEEQSSSIEQAISSKEQHVSIQAQTSSQEQSTEEHTTTEEQLVIVDEQATSSAE

ETPASVEEQVFETSSPEQSTEEYTATEEEQSASVEKRAAEEQSSYVQGQATPAEGQSDSA

EQVSPSTEQSGPVEEQETHSTKQSASTEEQATYAEEQQILAAAEERPTEECTTTEEELAS

VQEQSTPSTEVPSGSAEEEATPAEEQQVSVEVASPRKQSSEEYTSAEEQSIESEPQASES

EEAMFPPHSIPTEGPPDETGISALPGAEDDREMDDENNTEPALSQ

>SG-AL_contig_247_1_-1_61_1251

MAEPESKAPNVEEEVEEMEDELEEAAGEAGTDQAADSTKIAAQLMKNPQVLAALQDKLGS

IVGTPSGYIESLPKVVKRRIKALKKLQNDAIQLESQFYREVHTLECKYAAQYCPLFEKRH

EILTGKMEPTDEECDWPSDEEKEEEDKLADELKEKAAVEDKKEEEKKGEDMKGIPSFWLT

IFKNVDMLAELVQDDDEPILNHLQDVKVKFSDSEPQGFTLEFHFEPNEYFTETLLTKQYT

MRFEQDPTDPLSYEGPEIVKCVGCPITWNKGKNVTVQLVKKKQKHKGKGITRTVTKTVKK

TSFFNFFSPPQVPEGDNEDDLDEETEALLAADFEIGHFIRERVVPRAVLYFTGEALENDD

YDEDDGEEEDEEGDYDEENDPDFQPKEGDKPAECKQQ

>SG-AL_contig_248_1_+3_255_539

MGNPVLSIIPHNSNKYTGFAWLCLNFAVLLQERMAGCLCRQVQGERAKKMHMYVHTHNVI

ISNTIILRCQVILIFSLYQFCIIMILKRAMAIHSK

>SG-AL_contig_249_1_-1_1_318

RSPAPDAAPGTQEPPRGPGGDINLGPFSVLDHSQPAHARTRESGTQSDATRSSPGGRGPP

GFPPPAWINPSFDPLGSWPSSEHARRDRARPSSSRSPAHDGVEGPW

>SG-AL_contig_250_1_-2_968_5008

MRRVLCMVIVFSTLIYKELCACQVYLQLFYESSVQGNCILTMGNRQGGKTITRHDSVYQQ

KTKKSLGKSVDGSTVDVDLLTPQFQLADFHSLSLHPAVGPTTLISSAFRPKGGGGSTYDN

DNSPSLLSKILHADEHFDSGLPADPSTVDICEGVTPANDNYVSQMTCPPSSTDTKDETVK

STCSQNHQDVSKEQFAVSLVSSNESHSFDTSQDCSLETDSDPGTRDCRDNWLAGITHTTT

HCIDRLEELKRNIEQSRTEKFVHFSNTGSAQLQSGADYDNAEYLKEPGTCSHQRNKSMSS

KTVVPADVSQKNIKTKRKAVRKLSEPWQFVHKKDTNLSKNIISGAWSRSAENSPVIVRKA

LPHPGSPVIVQRTPSQSPSVERRFPANASSSCLLSTREAESFDNPKSGTHAYHKVSSENK

GSFGATEAAHSISRQKPAEISLPLFSKSPVLFRKMPESPILLSSDHKAKSSERERSSDRT

TGDYPMFPSSSVQSSSTYHTQAPKLSCPSSSPKSQRRNLPLQEDPILPSIPLCASPTSSF

SAALSYSSPTPIQVCSQPRGTPVKDCILVSQPSTAVVLATGESQDSLQTTEEVAVQHSVN

TETNGKMVQTENCTHSQDICTTSSKHPSNTEVTEDTIKQNSESAVFGSVSPKLMRHKRLS

KNNPNSSPNEQALPNQLPATQTATSKISRNWENTGTETGGTPHADKCVHIHMENKEKTTR

VSQFLPSVSMSDNISTSGACIGIKNLGQRIRDVINESTVRTAPHGKLSSSSFGKYERLLS

KENTLLNTSAEATPTSHSFPRLSTKTNERPFYDRPLPSPTTERASSAGGRRGEKLLEELG

VKTSTSPVKDFHVTLSVGYSYHLNSFTDACSSHVSNSYQHNSVEKPGGQRQVAELTQANL

RQQSEMFSEGTESKLHEAVTARLGVPGRRLVPSDTTDTLSTDRETSGSSKRESSTSTATD

DTLTSFKTRSTADSADFEEGFVLRESLVFIRSANKEDMHSDVGAFDSQSTTVKRKLAEGL

PPNPRAGYLPPKTRKKRRKQILSQTIIARTDPPDGLLKRNRNARDIYCDDISDESDLFSS

RDDISDFPSHRDIYSDPPSSRDDIYSDLPSSRDDISDIPNSRDTISAFPSTKTNISNLSS

SIDDMSDHGTIQKRVFDRAQLLSPASHKRSFGDKSSRPMARKATIREDRSKDLAVNNEDL

VFKGHKSSSHDSSDTCVDINETSADVTDSGTIPDKPVIKSLTFEPLPKATRPDVLLPPRS

RSTGQGRRLTKASFERSGHREDGGDRDSYEQSEDTSVYCPYDDDIDKGKQLLLQRFCSPE

STKKVVLSCIIYNSSCQSFDAHGSQEP

>SG-AL_contig_251_1_-3_60_284

MASRGAMKGVSLIKRGWHEIPEIMCCLGMIGYSTALLGLGYLRYDRDSNHRYKFEYTVVR

DTDVKEEWVKKKIYN

>SG-AL_contig_252_1_+3_2238_2519

MKHASPAQFALKTNTMQLYLLEILGPTQIIYLNAAIGVPTTKSSALHCVMKRWMGNESTA

QMSLGLITQASHTMSMNDAESRAEDSSNTDLYPT

>SG-AL_contig_253_1_-2_65_418

MYLEKILLLKTHTYTLNISQLLNVPLFYHYVVINCWVLRAKASFMLLISSFGLLSLCVHC

VFVKTVIIFFPADDSRVLHLDGGGVYIMLSNLLVTLYHSNSEQRTEEHFRKLATSGAR

>SG-AL_contig_254_1_-2_2_298

HAETEEKTVLPNSDDIGKEKTEAELRKSIGEFDKHNLKHQDVAEKNPLPSTDAIAAEKKE

EEFKHSIEGFNRSSLKKAETDVKNPLPTKEAIQQEKQGK

>SG-AL_contig_255_1_+2_734_892

MNTITHEFLQSICQYCAVQNKLHKIEGTKNCLGNTCSYLLNDNFLNRKLFFRL

>SG-AL_contig_256_1_-1_76_567

MATQQKAGESEVRNVVIAMDESDFANFGFEYYMKNVHKPNDHVVLLHVPEYHTVIQSPMV

MTDVSVVTDLLQDEEKRIKAFLDQLGSKLKSHGIGGKVKSVGGNAGEVICKVSNDEKAGL

IVIGSRGMGTIRRTLLGSVSDYVMHHAHVPVLVCKLEHHHQHAH

>SG-AL_contig_257_1_+1_1234_1602

MAFIPASLLGSNTFIFFGVFVFVVVVVVVVVVVVVLFWFWFFVCLFVLLSHLLRRFSGLV

PRRSFRLLQTPPNPNSFRRNLSVRKPVEASDVELPQGYPPEEPLHRCWVISAEFNSACSD

PAY

>SG-AL_contig_258_1_-1_304_6666

MDKGRWEKVAEVQGHKCTVPKLQEGHQYKFRVVAENRNGESEALETESPITAKNPFDEPH

PPSQPEVVDRDRNFIALKWEPPSNDGGSPITGYDIERKDPKSNRWVKINKAPLLDCEYRD

EKVKESNEYEYRVVANNEAGPSDPSIPTKPIPARPMKETPKVNLDNLFGAKEIRVRAGEP

LNIQLGISGTPDPTVEWLKDGVPVGNRAQTQNDEKEARLKVAKAERGDTGNYTIKVANVH

GEDTADVKVVVLDRPSPPEGPLEASDIWAEQMKLSWKPPKDNGGAEVSGYILEKQEEGSN

IWEKVPGMISDLSQVAKSLKEGKTYKFRVKAENIYGISDPLEGVKVTAKNPFDTPDAPRD

LEIPKYDKYSASLTWREPLSDGGNKIKGYIVEKRERNKDWVKACPYPIPETNFTVLNLKE

GNIVEFRVCAVNDGGPGKPSKATPPHTVRDQIFPAGAPAAPSVDKITKNSVDLSWVRPSS

DGGAKLTGYVVEKKKKGDDWIECAHLPATATTATVQGLLEGEEYQFRIRADNAAGPGEPS

KPTKSITAADQPAKPKLDMGRVKDVTVKAGQEFHITIPYTGVPTPTAKWDNNGTDLEESP

RVSAKLTEDTVILAVAKAKRDDTGKYTVTLKNPSGVETGTVNVNVLDKPMPPKGPIEADE

IDGESMTLSWQPPKDDGGEPIANYVVEKRKAGSSRWTKVSSFCHSPKCQVRNLEPGVKYE

FRVMAENGQGVSEPLETEDSILAKLPFDAPRAPGQPKCAGTTEDSISLEWTTPKNDGGSA

IYGYVLEKREKGDKRWTKACVQEIPDNEYTVKGLMEGKEYEFRVAAVNAAGIGDFSDCTA

GIKAAPPPVAPKINRDLMGSAKDIKAKVGDEFKIAVPFSANPTPTAMWNQAGLTVHATER

IKLDTQPDQALLICKRAEKSDAGKYTLTLSNDKGQDSTFVNVVVYDTPARPEGPLGISEV

TPDSCVLKWNAPLEDGGSNITNYVVEKQDVRTGKWEPVSKFVRNTTFEVMGLVEGHEYNF

RVMAENEFGVGEPLESTMPTVAQHPYTPPDPPGNLRVDDVDESSVSLMWTKPRNDGGKKI

QGYVIEYKEPTSNRWKTYNDTLIKDITATVEGLDKDTDYEFRIKAKNAAGLGNASESTGA

VHVKPKYNKPNAPSVPEAVKVGKTYVDLRWEKPRNDGGSRITGYLVEKREKGMSMWTKAN

DYTVNDCNFTIHNLPENSEYEFRVAAINAAGTGDASLPCAMVKVKEKLAGSAPDFTKRLQ

NGKGPLGGETSFNVEFTGKPKPDVIWYKNGTEVGNAGRTRIKSDDTTSTLYIGELYEKDA

GDISCELVNSFGRDSCKAHLAVQVPPKLEREIRDQKVDKGDQFKIKIPFSGSGPFDFKVK

ANGRELRESDRIKISPFEDYVTLIIKETEVKDSGKYEVEISNDSGSVTAAFGLKVCSPPG

EPTGPLNVTDITKNTCRLSWKPPKEDGGARVTSYQIERQEVGKPYWVTVSSHCKDTMVDV

QGLYEHNQYLFRVAAINENGVGEFLTVENPIIAKMPFDAPETPGTPEVTEVGGDFVSLTW

DKPKSDGGGRITGYWIEKREHGTENWSRVNTNPCITNMINIPNLIEDRKYEFRIFAENEA

GMSKPSMASNSVKIKDPNAAIVPEFSSGLRKTAAVEGKTARFECEVSGQPKPDIQWYKGS

REIYDNDKFEILTEGDKQILIVHDVYGEDADEYCIRASNKGGSRVSRAQLEINSPPKINV

PPRFRDVCTFEKGENVVLKIPFTGSPKPTVKWIRDGEELRGSRYHSEVTERHAILTIKDA

SKEDDGPYRLSLENTLGSDSAVLKIQVNDRPDPPRFPVIENIRDESVVMSWKPPLNDGGS

FITAYIIEKREAPSNNWIRVASTRFAFHNITGLSPSKEYQFRVIAENFYGRSDPCETTEA

VKTEEPEEMKKKKQLEDEFGRKVRGKYDGPKINDYDRFFDDIWKKFVPQPVEIKQGSVYD

YYDILEELGSGAFGVVHRCVEKATGRVFVAKFINTPYPLDKYTVKNEINIMNQLHHPKLI

NLHDAFEDKYEMVLILEFLAGGELFDRIAAEDYRMSEAEVINYMRQACEGLKHMHENSIV

HLDIKPEIGRASCRERVLRLV

>SG-AL_contig_259_1_+1_1_120

HGQSQTLGADSVTTFPVTDSWCRCRQCDNISSHRLLVQTV

>SG-AL_contig_260_1_+2_638_787

MVDSDSEFRKHSVANYLFQLGLTNKYILSFNLLARLHEYCALDLKKINKK

>SG-AL_contig_261_1_-1_187_357

MGTIFMQTSKQIKTLRMYLLVKSNWNKSFAILYFLNSESLSTIPLSNSYRRKCKIKQ

>SG-AL_contig_262_1_+2_2_181

NLILINHSCIALFSKLIWAHCALHSYRTQSVIHAEVLISHTQTHNQNARTHRCTRINTYS

>SG-AL_contig_263_1_-3_3_425

EAKQTCTHLWLHHFNHNFPLFSGRTILSPDVICPSLSLRRRFYRAVSLHAASRDNLSLCW

SVLCPCILQVFSAFLSSTAASSTRSLLSLTPWPAWDLGFMCSFTVYVCWSVCSPCSSFIL

LLAADLLLSRMNECLGLYVCL

>SG-AL_contig_264_1_-3_3_359

SKGFVREQFAWRNYYWYLTNEGIQYLRDYLHLPAEIVPATLKRQTRPEAQRPRPKGPEGG

PRGPAPDNRDREYRKAGGPPGSDKKADVGAGANESFQFRGGYGRGRPGGGFGAPPPSQE

>SG-AL_contig_265_1_-1_310_5223

MPVPGVDDSQEFRSLVESMTIMGFAPEDQSALLRVISAVLLMGSLQFKQDRNSDQATLPD

NTVAQKACHLLGVPVTALTQAFLKPRIKVGRDFVTKAQTKAQVEFAVEAISKALYERMFK

WIVQRINRSLDRTKRQGASFIGILDIAGFEIFKMNSFEQLCINYTNEKLQQLFNHTMFIL

EQEEYQREGIEWKFIDFGLDLQPTIDLLEKPMGIFALLDEECWFPKATDKTLVDKLLAQH

TSHPKFQKPDFRADAHFSIIHYADKVDYSADQWLMKNMDPLNENVVSILQSSTDNFVAGI

WKDAEIVGMGAAAAVETMFGSRTRKGMFRTVGQLYKEQLSRLMATLRNTNPNFVRCIIPN

HEKKAGKIDSPLVLDQLRCNGVLEGIRICRQGFPNRILFQEFRQRYEILTPNAIPRGFMD

GKKAVEKMIAALELDNNLFRIGQSKIFFRAGVLAHLEEERDLKLTDIIVQFQALARGLLA

RRNYQKRLQQINAIRVIQRNCAAYLKLRNWQWWRLFTKVKPLLSVTGQEEKLHSKEEELK

KVKETYEKQKQDVEQLESRYAQIIEEKNILAEQLQAETELCAEAEESRARLSKRKEELEE

ILHEMELRMEEEEERIITMADEKKKMQQTVQDLEEQLEEEEQARQKLQLEKVAMDSKLKK

LEEDLAVSDDSNAKFIKEKKSLEERLSEANSQIVEEEEKSKQLGKLKNKYEAIIADLEER

LRKEQQARQELEKIRRRLETELNDLRDQLNEKRQQVEDLQAQLSRREEEVQIALQKADEE

GVSRQNVNKQMRELTNQLQELQEDLETEREARNKAEKLKRDLNEELEALKTELEDSLDTT

AAVQDLRNKRESELQDLKRTLETTQQQHEAQVQDVRGRYNQQIETLTEEVENTRKAKATL

EKTKATLEAENQEMASDLKSVQMAKTESERKRKQAEQQLAEMSIRLQELEQGRGESTDKV

NKLQQELEQANSILEQTDTKAIQLSQRVSSLEAQLADSQDISQEETRQKLALQSKLRQAE

DEKESAMERLEEEEEAKKALERHLADLQQKMVDLKRKAEEDLANNEALEEYKKKAAREMD

QLHTQVEEARSNADRMDKSRRKLQAEVEDLTIALDSQRSNVSNMEKKQRKFDQTLAEAKG

ESERLTLEKDNLEKESRDKETKILNLTRNLDEVQERVEQLERLRQQQARELEDLVSSKDD

VGKNVHELDKAKRALEATVAEQRVQIEELEDELQAAEDAKLRLEVNMNALRDKYKSEVAG

REEQEEESKRSLLRQLREMEAELEDERKQRALAVNARNKLQGDIQGMEQQLEMATKVKED

AVKQYKKIFAQMKDFQHELDDARMAREEMAAQAKDNEKKVKNLEAELVRLQEDLASAERQ

RRNAEAERDELAEEIGSSTSGKQALLDDKRRLEARISELEDELEDEHTNLEMANDKARKS

MIQMEQIATDLAAERSVSQKLENQRLALERQNKEMREKLQELEGQSRARTKATIAALEGK

ITNLEEQLDQEAKERAALARTNRKMDKRLKELQIQAEDERRSADQYKEQLDKMNNRVKAL

KRQVDEAEEECARLSAQKRKVQRDLDDQMEQNDVLMRETQSLRKYRPSAQARSRAAVSLD

KLADEDGSEDGHDAQTET

>SG-AL_contig_266_1_-3_3_200

FYMQPWTSRVRGGKKFLIQFISGNSFLFRVGKVNRPAAIKKEGGNTEMELSCGHNLHWVV

QREIFF

>SG-AL_contig_267_1_-2_860_1081

MTASCIKNDVLFYIFNYYFDLCEKEIGLFLNALVFALFPTRTENSGIILYMTVLNCCVAY

FLFFVVFCVCVFFR

>SG-AL_contig_268_1_+1_46_237

MLKSGGECVVHWMRTLCNQVWNSGEVPENWKNGAIVCIPKKSNLTECDNWRGGLLSCPYQ

ASSS

>SG-AL_contig_269_1_-3_147_833

MSKPTMPCGYLGPQEKWEAMGLSTVTHNSPDIRCHTCRLEIKFKTTKPTKVTAVLLDANS

DEKYPELVFTQTVQELVYLVVAFPAAGFFKLQVHALPAEDESLSLPNIYNYLIQNTVPPS

GPVTPFPKQFAQWKDGCYLFEPFCLKGMDAAQIKVIVPGARAVALTINEEWFPLKQRPST

GKDDVKHRPWSGHLEGLSKFKGKAEKAVLTANFDEKDETKYTSLLSYNL

>SG-AL_contig_270_1_+1_1573_1866

MIFHSRSLKYLGVKTDQNLSMQEHISNICCGSYLELRRITSIRPYLSKTACKQPVSATIT

SRLDYCNSVLVGLPAEQISRLQRIQNNAACLIMKKRKH

>SG-AL_contig_271_1_+1_40_216

MNNNKLKLNTDKTEAIPVGTTSQFQLVGSECANIGGTDSSFQTSVTYLRVKTDQNLSMQ

>SG-AL_contig_272_1_+3_3_260

TKICLCKNTLATSAVVLIYSLGGSLPSDCVHLSKTACKRLVSATITSRLDYCNSVLAGLP

AEQISRLQRVQNNAARLIMKKRKHDH

>SG-AL_contig_273_1_+3_1881_2186

MTQKLNMWTRPRIHFHITLSPFDSVQSNAVYLKMYPTVIGGNMLKKNKCNLGFSFPSHSC

LSIHDSTAIYFCSSSQSLDDGFDHQKMVIMASTTIYSPNELY

>SG-AL_contig_274_1_+2_1028_1321

MHTHTSLHFLNSPFGKSKCFYLHYHHYQQEQYQQLQQQYKRYTTITREERSRTLIVLTET

EAGNKIRTGESYIMYFFFYHGFQFCETVEQNWEAEDVH

>SG-AL_contig_275_1_-2_59_1519

MTEVDLREASSAIDVFGYPLVSKAYSKTWSYREDALLAVYKQMQELPVRDKEEAKVMMRA

AVFLIKRGIDDKVYAVFKAALNLLRMMLTEFVPKHKIPKQETSAAVERTLQNLFHKSGDT

AARNRDDAKRFIFDMAEFPDVKPTQTVPHECVKPIKLNMAPRLAQSRAEILESLYKDLKL

NNGLTLDNMMGFCTVALNHNAGEVREVTERIIKQLYTDHGAPVKNYLPPDDEKTRKNTLW

RMLFEYFDKVDGKPSKNDLKKQKAEEEAKKQAEINALQKQLQELRQLNNAKEAEKENAKP

AKKKPEPKKPPSKKPKRPDDDEVSTFNTDKICIFCSERNEAFTEEGLDMHYWKNCPMLKR

CANCKQVVEIATYTDHVLTECEAKSNFGKCPRCSEAIPRAELDQHIADKTCNPSKPNMNH

CPLCHENLSTGEDGWKDHLMSREGCKQNPRRLLALNKQPGAGTKAGGRQPPSKIAKPTTK

PSSKTPR

>SG-AL_contig_276_1_-3_255_758

MEGGRLRVGAAVAIVVGLVVMDLAVFPTSGQQTARDFFKPGVLDTCCTIGMQWSNSSGRC

SGYPAPVPGVDAGSQIACLSIVEVCCLKQVQLDMCESGKRTALDSQLCAVRDSDAGAEQF

RECCQCCQMGVAARRQSSSCQVPGLGEPCDSKFAECCTGRPSANFTIP

>SG-AL_contig_277_1_-1_7_156

MYISSFISIYVCLIFISLYLFFFFCYEYISLVYLFSQHHELTSPQEPGHF

>SG-AL_contig_278_1_+1_757_1065

MKLQAHHQGLRTFWRLYYVMSCSIQRYRPGESNICLKKNSTHVHKHKHTHKLYTHFCQRL

FIDQCHTDCSAKRRSHDKAHAARVMCQACAVSLHINIIFLLGA

>SG-AL_contig_279_1_-3_195_794

MLENANVLARYASICQQNGLVPIVEPEVLPDGDHDLETAQKVTEQVLAYTYKALSDHHVY

LEGSLLKPNMVTAGQACSKKYSHEQIAVATVTALSRAVPGAVPGICFLSGGQSEEDATIN

LDAINKYKGKNPWALTFSYGRALQASVLAAWKGKKENMPAAQGELLKRAKANGEACVGKY

AGGVKGSAGGQSLFVAGHAY

>SG-AL_contig_280_1_-1_1_210

RRLYRQVLFTSDKSLGNNISGVILFHETLYQKDDTGKPFVQSLKEKGIIPGIKVDKGVVP

LASTHNECTT

>SG-AL_contig_281_1_-1_1_210

RRLYRQVLFTSDKSLGNNISGVILFHETLYQKDDTGKPFVQSLKEKGIIPGIKVDKGVVP

LAGTHNECTT

>SG-AL_contig_282_1_-2_473_817

MTNTENEMTRKVKEKRQQNVNTKRRNTHTHTKPNKKALQQNPQKADNENTFNKRETLCPK

SHSDYHDVLYKQTCSQSTIYVHVWSLFKGASSHWDKFHMRSAQIRQQPSGRLLEP

>SG-AL_contig_283_1_-1_1_135

STQTTTQVLVRWREKFDQSGIKPLHFQTSCFLEQKLYPLGHTPTH

>SG-AL_contig_284_1_+3_3_200

LSLHDALPIHARTQTHTHTHTHIHTRARARTQAHAQTYNHTYTGVRNIDNVLQILNSKLK

TKFQYF

>SG-AL_contig_285_1_-2_146_2107

MADSEIERGNWGRQLDFLLSCVGYAVGLGNVWRFPYLAYRNGGASFLFPYIIMLGIAGLP

LFFLELAVGQFASEGPITVWKMSPFFAGIGFAMCTISGVVSIYYNVIITYAIYYMFVAFV

NLDGDLPWARCHNETWATVKCRDKPFPFFKDLNETARMDAIFNDVYNVSCINDKMMDDNF

VGNLTKKFNETTDLTFSTLNSTHILLKVFNPCKKMFVSPASDYLSQYVLRLQEADDMANL

GGISLKIILTLALAWTLIFFCLMKGVKSSGKVVYFTATFPYVILIVLLVRGVTLDGYIDG

IKFYVIPQWSKLAEAKVWGDAATQIFYSLGVGFGGLLTMASYNKFKNNCYRDAIMVALIN

CGTSIFAGFAIFSLLGHMAFVTNQDVGDVADSGPGLAFVAYPEGITKLPAQSLWAFLFFF

MILTLGLDSQFAMMETVISGISDMFPHVLRKRKTLFTFLTCLVGFLLGIPQATKGGMWVL

TLMDWYSGSYNLMFVALAEIICLMYVYGFRNFCSDVEMMVGHKPNIYWLSTWIVITPLAV

AFIIIIGAVQYTKAGYAGYMFEDWVQGLGWIMVTVPIAMIVVVGLVQMCRYGVPGCFKPL

PSWGPADPENRTGRYAPTTALPFTVQDEKGAPINGPELGYENGGYVKTVESHRL

>SG-AL_contig_286_1_+1_1_72

VHTSSSKLIEHTDHEHIKSTMGNR

>SG-AL_contig_287_1_+2_569_982

MYSLKSTYYTELINVGNNRQGGKRKNNPEARNNRKANIDTNTHKNSLSLSLSLSLSLSRS

RSLSLSLSLSLSLSLSLSVCLSLSLSLSLSLSVSVSLSLSLSLCSVCVCVCVCVCVCGGG

GGGGRGGGEKKKVGGGGG

>SG-AL_contig_288_1_-3_513_758

MTIIIECQNCHCQAHFFHYFPKFSSWLTISLPHRILQEAGRFDEGVKELIIAIGRSEASP

PSILKFITIASLNSVIIFMIIR

>SG-AL_contig_289_1_+3_111_251

MTSVQVIQHQQHSNAVDTCHEIHSCCPLGVKLRSVISLLCHDIEPVH

>SG-AL_contig_290_1_-2_20_631

MGAYKYMQEMWRKKQSDVMRFLLRVRCWQFRQLSSVHRAPRPTRPDKARRLGYRAKQGYV

IYRVRVRRGGRKRPVPKGCTYGKPVTHGVNQLKFQRSLRSVAEERVGRRCGGLRVLNSYW

VCEDSTYKFFEIILVDPFHKAIRCDPKANWICNPVHKHREMRGLTSAGRSSRGLGKGHLF

NKTTGGSRRANWKRRNTLSLRRKR

>SG-AL_contig_291_1_-2_2_280

SSSVTVNILLFLLYLNFCCIAVDRLGMRKNNYQYYSFKKTKQMQLLKCTSQNTPSLLTFS

FALYKASNCALTSCICLVFQDLFKKKEKKRCMY

>SG-AL_contig_292_1_-1_31_363

MVEDFQKAEPKWQKEIFENLQKVGNWWEEVVEDIQKSEPKWRKEIYEDLQKAGNWWEEMV

EDLGKSKNWLEEMVEDFKKSEKNWWEELAEDFVKFEKNWWEELTEDYEKSK

>SG-AL_contig_293_1_-1_1_213

RHWQVCTHVDLRDRKNLHPLPTRQRCLGGELNTGPSVWKSGVYPHEYRAAKWGNLRLLGS

SRLHRTSEKPL

>SG-AL_contig_294_1_+1_154_372

MFSIDKKSKNSLNYKSVFFFGFLVFFLNRLQTLPHVLTSELYKRVKKRTQSGFTANISYI

HMTFELQSFTGRS

>SG-AL_contig_295_1_-3_39_467

MIYQVKCLNTVLVQYADDLCMWMQMNMKRKLFCTYTRKIIRLELDIIGQFMNENGIHLST

EKTKIVLGNSGSNRENMPTFKLGNAILRYNQSIKFLGVFLTPKLSWDLHIEYLFTKTRKS

LNFLKIVVQTTIGTRPINTRASL

>SG-AL_contig_296_1_-3_60_269

MKILLNQSTAIITSIITIQKRERSFVCACAHERERERERERERERERECALVSVCVPVSV

RTRVCVCVCV

>SG-AL_contig_297_1_+2_2_502

FFFFFSTINSLLHCKLSATEEHLHTGPSVADCVDGLCDLEACDVAEVHGFQHFLSVRINL

NDVLVQSRHLRYIVVSPLSLLLLQLDGNATHRATLDSLHEMGHKPRDFVTEFLAGDNGYL

LADPFVGVEVERQSGVILLNDHPGCFFYCLCPYTCHVGCSILKIGRA

>SG-AL_contig_298_1_-2_95_1081

MSDAKRVARSTLDQLKNVTTVVADTGDFEAMKKYKPTDATTNPSLILAASGMPAYQSLID

DAVDWAKDQGGSEAEQLSKAMDKVCINFGCKILGIIPGRVSTEVDARLSFDKEAMVKKAR

QLIELYKAEGVGKERVLIKLSSTWEGIQAASELESKYGIHCNMTLIFNFYQAVACGEVGA

TLISPFVGRILDWYVKNTEQKSYEPLNDPGVRSVTTIYNYYKKFGFKTVVMGASFRNTGQ

ILGLAGCDLLTISPTLLEKLSQSTECIDVHLDKEKASDCDLAKVSVNEKVFRWQMNEDEM

ADNKLSEGIRKFAADAVKLENLLRQRLVQ

>SG-AL_contig_299_1_-1_208_372

MHSLWYVQTKLTETQCEEKLILLVVYHLNPQQSQNPWLSVVVFFLCVCIVQVNDS

>SG-AL_contig_300_1_+2_3311_3619

MTSQHRQTAFVSHHKLHPVITNCTRLLLTNCFQSSQPVFTSHHRTALLSLLQYLHSVSAT

FTHKYRQVFVSQLHNTLHHKLQSNMVHHTFSHGCIHHISQQPK

>SG-AL_contig_301_1_-2_698_892

MFIIFSTLSAHHFLKNASVPPLDFVCVCSAHNPTLSWSLSELKQTDELHHRVSVSMTHHT

VYVTI

>SG-AL_contig_302_1_-3_1545_1754

MHGRQIAVIVNSRAVVHNIAACAHVKCGIVCSFTDPRYECLLMLVIVIIILTRNGTMWTQ

LRAAYVSIKH

>SG-AL_contig_303_1_-3_12_2186

MATQTTGNAICLLIVAFFSSMHCFMDIEELQSINYGINILKDPIVIPETHPESAILVTSR

HGQQYQCNYPSHQEQKQQKEEEEKIALETGVVELLKPLSQQPCLLKTKDWWTYEVCYGKY

VRQYHVEDGHIRGNIMYLGHFQSDFDWNNETLRESRVRSKIASGRYHSQYYTNGSKCDLT

GQGRQTEVRYLCEDAEDDYIVRVDEPETCVYVITVHSPRICSHPHLKSPTPSQPIPIVCV

PLLTDQQYVQYQAHMEAVRQKEEEARARAKAEQEQREREKALAEAQEEDDVIEDGLADSV

NSILKKSLGKEFKSVGSTTDQPTSGIEDKPSLGWKVVKDLDDIEKLLDPQGILSNMDMSK

KGQGAKTSKTTAKEDTLKNVDKKFDEVLEQLNKKDRELQERQRTGQVEADDTVDASVASD

LDEFEQEVQHLKDKQAMTQKRLQYIRERVRKAVTSQFKDIIEEAEAEIGSEVNEDLAFKQ

LASSLNMLMEKLEHTEKEIKNMDQEIAKLGDKEASADIDEGEELEEEEDDNEEEDEEDES

KEKEQESSVADGEEPKSEGVQAPPTTKQATEKQMKDGGRWKMKVRTVTQEEIDKDGSAKL

PPTKQQKHLEKSLKESLQKAGVDPNSVKVHVKIIGGANSNSDDDTTLHVLSDEESASFQN

MIVAILGGSNEAMKEQKRQKTLEDSYNFVFDQAITQDSGDDAKSSDSGDSTDSDDEALSD

SVLGL

>SG-AL_contig_304_1_-1_271_4635

MVGVWVRGGAWLLIVATLVALSAAAPTSEEAEESEGRNCFYNNTEYVHGLSWSPPYPPCL

VCTCKDADVLCNFQPNCKTQYASAEVRTAALAETLNSLQGPSGPRGEPGYPGRQGDRGDA

GPDGTNGVPGPPGSPGPSEMTADQAYQMYYARNYGGKSAPAYGPQYLQAQVGPMGPRGTP

GPPGAGGPQGMEGMRGQPGDSGPPGPPGNRGEPGPLGPQGIEGDLGRNGEPGSSGPPGPK

GPPGSPGMPGMPGIKGHRGFTGYPGKRGDPGSSGEMGETGPPGQAGPPGPAGPRGSPGER

GQDGPPGQDGLRGVDGLSGPAGPPGPVGNTGPPGVPGVSGAKGEQGSPGPKGGPGPQGAR

GDNGMPGITGEVGMPGPPGLSGLPGEQGASGDPGIPGPPGFPGPRGQPGLAGSPGMNGPK

GSTGLPGQSGYKGIAGEKGQTGPAGVVGKVGSAGSQGRRGQRGRPGPPGPQGVPGERGLP

GGRGMPGSPGLLGPKGEDGEPGPRGPRGETGSNGEVGRSGPPGIPGEAGPPGIPGPDGNS

GPPGEPGPIGSEGRPGEMGPPGQRGPQGTQGPQGPPGERGPPGKDGTPGTTGPRGAPGDV

GSTGEMGPQGQPGAEGEPGDRGPQGTPGPAGFQGPPGLPGAPGEAGRDGEMGPPGPAGVP

GAEGYPGERGFPGERGPQGPDGEPGVPGTEGPTGPDGPAGPAGEIGIPGDIGPQGPIGAP

GDRGPPGPAGEKGEQGEPGPTGPDGLPGRQGRHGERGPLGPQGIAGPPAIKGGPGDPGFQ

GQNGPPGTPGPRGEPGIEGEQGYPGLAGQAGSPGAKGETGLSGQKGEQGEAGPPGEPGQA

GPPGLSGVPGRRGPTGDRGIQGTPGDPGQPGRPGPQGPTGPSGLLGNPGAPGPRGIKGAT

GESGPRGRPGSDGAPGVAGNPGMKGQRGEDGSPGGEGPTGPPGPQGQRGENGIPGVPGER

GSAGTVGHPGARGSPGRRGADGFPGQQGEPGSPGAPGPNGSPGGHGAPGNNGAPGQQGQA

GEPGPVGELGPPGPPGLPGLQGPAGPAGPIGAAGERGLRGEIGPEGPTGPTGPRGTPGPQ

GPMGELGELGDDGDKGDAGNPGYQGLPGLPGPEGPTGEVGSAGPRGVPGSRGSDGPRGPM

GSDGEPGPQGPRGPPGPRGPPGDDGRRGPSGEEGEMGPPGPPGQTVYANAMTSWFQGATK

GPGVQGDAPAESRNTLVALKEVANDIEKLKAPAGTRESPGMTCRDIAMVDPSLKNGYYWI

NPNGGSISDAIRVFCKMKGNKTQTCLEPSTSEYTSQKWEFTKQDQGYWLFAGSFIEKEMF

DYGSHKSQIKMLQHHTDNGRQRLTVKCRDVVAVYDGVNKDYNKAISFTSFDEELMTSRSK

GPFRYKVLKDGCKKQNGKEGEALLEVGGPKLLKRIPILDVGFVEQPQGHFGLNIGRACFW

QKGGKGRKGRKKRRK

>SG-AL_contig_305_1_+1_1_183

KKKKKQKIPKQKKKKKKYNKKQQKKKIDNIFDVNCSYRIRYTGLSSMWSNLFISFRSHSR

I

>SG-AL_contig_306_1_-2_137_1795

MLTARLASTLARQLPRATPQICKNAFGAGFVAARNLQTSQQSYAAAGGAEVSSILEDRIL

GQAPQANLEETGRVLSIGDGIARVYGLKNIQAEEMVEFSSGLKGMALNLEPDNVGVVVFG

NDKLIKEGDIVKRTGAIVDVPVGKELLGRVVDALGNPIDGKGPVTSPDRQRVGIKAPGII

PRTSVKEPMQTGIKAVDSLVPIGRGQRELIIGDRQTGKTAVAIDTIINQKRFNEGSNEKQ

KLYCIYVAIGQKRSTVAQLVKRLTNADAMKYTIVVSATASDAAPLQYLAPYSGCAMGEYF

RDNGMHALIIYDDLSKQAVAYRQMSLLLRRPPGREAYPGDVFYLHSRLLERAAKMNDDNG

GGSLTALPVIETQAGDVSAYIPTNVISITDGQIFLETELFYKGIRPAINVGLSVSRVGSA

AQTRAMKQVAGSMKLELAQYREVAAFAQFGSDLDQATQNLLNRGVRLTELLKQGQYVPMA

IEEQVAVIYAGVRGHLDKMDPSKIVAFEAEFLKHLQSSQQDMLKIIANEGKISETTEEKL

KQVVTSFLAGFNA

>SG-AL_contig_307_1_-2_65_2209

MGQAPPAAPRDFQLSTVTQDKGEAPPPGPASTPQQKPTLEAIDRQVLQSVDDHAIQVSKS

DHNSFRDLVWDLIYSKNITNEIEKVRVIFRWLATKNLKEMNFDKVEKGSPEEVLMGLKTG

TTTYAMVFDTMCTYAGLHSKIISGYAKGADYKPGMKFTPGQNQHSWNAIYIYGTWCLIDA

HWAARRIIGKQASSDEEFHYQLDEYFFLPDPHQLIYTHFPDDPRWQLLERSVTLEEFENM

PHMKPQFFKYGLEFVTHRTAVIYSRGEIHIRLRYPAHKIAVAFNFTIQFEDGIEEFHGIK

LNRFGLQESVGGIASFSLRLPVTGSYMLFIYAKEDTPETKENVYAQVCEYKIEHESVSDT

TMEPYPPCSSLTWGPGSAFYRYGLTTYQQSSTICTREGKAELQIRQPRSMQFMAKLKHNN

ISDSELEAYVIHRIVGNTAYFNITAPGRGEYGLEIYANDPASEGSTLYHIAQFLVLCQED

VKATPMPKLPPGYLGAQPKFSEFGMTAASHQDPIIHLDCNTVTVLLKCAQSMRVTANLIS

VEDEKDYPDYVFSNTIGTSVSFIVNLPQTGFFKLQIYGIPTCDKRQELPGIYNYLINCRQ

VTQAVFPFPKQYAQWKEGCFMTEPGVIPSPKGPFAGQVQVDCLPEKVNFRVTIPGAEAVA

VVANQDWTHLERSDGDEWYGQCKIGHCYNTNSKVTVNANFGGDKTSYATLLEYVL

>SG-AL_contig_308_1_+1_205_363

MYNYYITIHDNVQLLCKNVWSAWVRILGHLAWWVKVGHFPNLPGQHMCRPASA

>SG-AL_contig_309_1_-3_57_278

MKTKYCDALERPISSTDTMTIKAKENTLAHNFKRLRGESFESPVSKCFLVLCFQQMIHKI

NTLFCLDFSFLICK

>SG-AL_contig_310_1_-1_376_3423

MFWKFNLLTSSHVDTLLEKEDVTLRELMDEDDILQECKAQNRKLIDFLTQSDNMEDMVKM

ITKEPEEDIDEKLRFKYPNTACELLTSDVSQINDKLASDRELVDQLYAFLESERPLNPLL

ASFFSKVMGLLITRKSEMIFEYLKSQKDFVGSLLHHIGTSAIMDLLLRLITCIESPETRR

AVIEWLNENQIVQRLVDCIVPSNDEDIHCNAAQSLCDIVRLGREQMMQLQDTSDPDPLLT

TMEMQETVSKMLSNMFELEQNESVIVNGLFVIHTLLEVRRQGQEGLNEQVADTDRLSQGI

SNVMAAIIPRLKNFHNLLITPPKQKYSTMPTSVGALEPPLGNARLQVTKLITALVATNMA

AVNMELVNLNTLQALLDLYFRYSWNNFLHTQVEQCITLVLNNPPVEVDGATEQPLLQQLF

TDVRLPQRLLDEWEENDQQQTKEGGKRKGFMGHLTKMANALTGSKERGCNCEMIKQLWTE

VPEECRQKWDAFVLGTLADINKRNRVEFRGHPLASSSEDDDADFKDIPFPQDTAMQQAFS

DYQLQQMTSNFIDQFGFNEEEFAEQEEKIDAPFSDRISSINFNIQANEVTQPNNTMFDQA

CNDRIQQFDDDDSDEDIWVEKPLTFDKNAQHMRSGRLQGQTARANSSDDSTDSEEELDSP

HSIPQQSASPSSSSSVPFSHMSSATTTSTATPTVTVPTVMAAAAAAEKMDVDSSEGPWAA

NGEGSTSSEPTDMETNSPWDTETVPPTAPREGWASFDKSEATSEDNWADFSSFSDMGSAG

NGPRSSSPVAMDTSDSSRASLYMVGGDSTMAESSSTSVTSGTQGTVQPSSAGTDLKVESL

EQASEDQPLPDSTSMSSPRSPVQSGAISSSPPTTQTPPVDVTVSSTSSEPETQEAVAGEV

SMEAKEPATGATEPVSTTPNPMTVDSSTGAGNQDSSPHFLTGSGLMRKRTGSGEESAANT

RSGDQSHKDAEAAQEPDTTDSIDLEAARARAKEAMERYDAAASMMSAETTVRNGPV

>SG-AL_contig_311_1_+1_31_966

MANVVHSVLNMYSLQLSQVRVPQASVIISCMLYSSPGVSSSTSPLIGSRGSCLGSPVFLG

HGPVLCGSRGLWGRCSSRCGTKRLDGRGNRLAGPVAHLLHFTTGPLVLHRGSFLLLHDGS

LLGFLFLLLNLLRVPVEVQIHHDFPRVITTDGTTQTQNLASQHPPHGANSHLTLVVARDG

NVNMSQRGVSVAESNDWDVDIRGFRHGLMVNAWVGHHQQTWLTVSSLDLVGEGTRRETSS

DGGSSSSRSKLENSTLAIGTSRYDTHISWVLNSSNGTGSQHQLFPCLFDVDNVHTIRLSL

VDVLFHLIILIC

>SG-AL_contig_312_1_+1_1_72

THTHRHKEKTLNFFFSLEYDIINV

>SG-AL_contig_313_1_-2_137_496

MPGEKQEVEVESTETAKTTPEIKEKTVEDVKAQKEAADNKAEAEAESTEETTEKEEAEGD

KNDNASTEEDQKEAEEKTETAAEKRSADEENGEDESPTKKAKTTEKEESDKKEEEQTESA

>SG-AL_contig_314_1_-2_2_9430

DKDILEQALKEKVEFLEKVEAQSSQRINELEALEKSFREQLESRDTGKTDSLNHSLKEKL

AVAEQEIRKLQESNRELEENEEILRENWRRVADEDANRLLCLEEKVRMLDLMNQDLKSKL

AEAQDFVVINSAPSQGSLADELSASSKRGQARSHSDSPAPGGSDEESGDDSPKKVVTKLK

RQLSILEEEKNGKIANLQERLADMRENEIKLSETLAEMEMTERELRAKLALYESSEVTVE

KMLKYQDKIHELRTSQESLLDQLETMESQEETLQERLEETERRLKGKIVTLEVEMKNLKQ

KELKGNAKIRELEKHEEELTEKVQKQAEKENGLYVRISGLMDQVKEHKTQITEQEKKLAE

KDERCRKVASLETKFLQIEGAYRDKVTSLEASLAEAQSLLAEEEKRHEETRQTHDEALHS

RDKEHNQRIFILEEMFAQKEKGLNENFAALESSIAERDMTISQQDKMLGEKDDQIAKLEE

ELSGKDSQIRQLEDATSSRQSEEQELEKNLSLKDECICDLEKCLNESNSRIGTLEKDLSE

KESHVSELEKVVSDLETDKLDKQGVIDNLEKDLSEKVVCIRALEADLGRKECRIAEMERD

INENDTLKGSLAEKDSHISQLEDNLSEKVSCISQLEQDVGGKVSQISRLEEDMTGKDVQI

TKLEQELNDRVSQVSELERDSARKGEDVSRMVKDLSERDTHVKRLETEVLHKESQVSQLQ

TELEYRDGQIADLEAQLEAKGNEISDRLEEAQQDITNKDDHIAQLEERLIDMSSTIVQLE

QKVCEKDICISGLEGQLTDKDTIISEHVTQLQSKTETEQSSTTKLSLLEVEFAGIKSKLS

QSDARCEEYSRKLTLVENELDEERANARSLKLDSDALLAENGHLQSQMTAKVSELETQLK

EQDCDFQSRMSVRESDFHQACASLDAECESLRGELLEKDSVIEKLESTKNDLLSKIENLK

DKEKELANKLSVLELDINTLHTQKENELQDVVMMWESRLKNTEHEKETAVERSSSLEADL

LSRNQTIQQHLEEISKLESSLNKAKAEIGTLQGTLSQLQLQEEESGMKAEVFLQQETMLK

TKVSQVEAQLEASSKEQEVQKASVSELLLQKQNLEDTLRKVEVDLSDSHVLNAQLQETVS

QLNKDTKSLQEEVTDLQRLVADLEIYQMKEQEIHKLLEQEKGRMQELESQISCLKDEKQS

ALEESGKLHKELDLLKGTVKDLETQITRLSQAEESLKSHILAEQSSKLEADKRLNQVSEE

ATVSQERVDQLLKEAREKDEQIDQFRVELLSVRQELDTHQRFTVCIEQKDQTLHSLQENE

KALQEREALLQEKLTSMEIQMGRHQEDHSQMCQKLQELEKREKELLEQITDIEQSVIVPL

EAENAELKEEVSSMKQQNEALNLQVDKQEERTREDSEELQDLQKSVNDLQMSRTEIQSKL

DSVMQELNKQESENEKLQSKVVDLEADNQNLDDQLENDRVELNKLQMELNKVQMELNKDF

CDRVAQTDSDAQVSETELRLQQKLTVLQDAESTFMSRIIDLEEHEQQLCEELESTKRLLQ

EKEQYVEQSQTKEFLSQSQVSHDTVDASVDTEGLQEPSITKVPELFARVQELESVNQRLS

CSLEQATAGEVDRRQLTDKLRLLEQAEDRLMERVMELEENDIALRTQLERSRHRARVADE

LEEEVSCLLQQEDEWKEKLASLTSENNTLSQKIESLENEFGCEQSRNENLAFQIETEQRQ

LQDAQAEVKLVQTKLQAVRSEYEEKLSALEESEKKIENQLDSQSQRECDLRREIQVLREE

MEDVCERYKELEQENSRLKQEHSKVLVELKDSESQCLQLAGGVSREPTMQAVQAQRHQTV

LPHGASVSSSSMSSPGQQFSSPPSVCSNVTVHMVQSSREGAKSSKTSDSEASLLSRIAQL

EKELREARSRPEGVGGPLQDKQSSVDSSEEPCDSDSSGVQSSSVQSVKIFTLHDALEENA

SLKAQLQDLQFRLVSQQVSSSFASGDGSSRRVSGDRQSAETLQLQAEISRLETRIAELEQ

SLKEAEGAVQEVVTVVRTRSVHTLEQKIETIQHTIHTQSPTWSLHQELLELLTSQADLQK

QVSHLMETEHNLRSALKQTEDELARAKNGSGTTTLGARRLEADRRLSLQSRLSRFGGGIS

SSKVQSERVQVDSSQKAKEQWLERQVADLQLAKANLERRLHEAERRLAFRPRPTAISRDE

LLFRIEQLEGEQSEWRKKVEDLEALNSHYLKEKLELASERDSLEQKLQVRAQSPGAGKEV

GVVFARGRREEPLGSSGSVSEGEPSIDPDSDAAMLEKPEDVLQKRIKELENLEGYLRQQL

TEVESDREQLHEIARKDKAMIHEQNVRIRELMLQERNLRSQVSGLEDSERSLYNRCSELE

DTVSRLEDRVHELEVHERRLRELVRKLKLDEEVWMSKSGDLTASMEQLSSSEVSLRKMVQ

DLQIEKGGLSDRTEYLEVRVKELEALETTLVQRLKNYENVEASLQNRLQHTENSEAAAHS

KASELDVINMDLSSRLQRAVEENAVLSQHVAQQQGQIHEMDRKLSAATDAQMGLNQHVES

LQKNEGSLQKKVREYELRDIDAQARVRELEQTEEILKDKVNQLQRSENRLKARLVEFETV

GSQVLENMPSAQKQQLPQKLEDCQKRVVVLQRQLEVTQAELHRQQQVEDDATSVKVPTAA

LTQMRSKVALLEETEYQMSELEKLNAQLNENILQLRQGKGSCGHEVELEVLKRRLHSYQN

TVQKLKHHLSEGQVPSLWAAGQGQGPESEDLPARHLQWLESERVLQSVRSLTQPPGLPHT

QEEIIQAHSGTLQTGRAQGVASVPSEPEESHWRTVELRRRSQLEAEQRGASASMPHASAT

TSTALPHQEELMIGLSLPSESERGSFKSQNDTDSDMSGISHPHLRMVGSTTHRGSAPTPG

VVPASRGGGRTETRTPPMPTSAWPPTSTSAPSAYQQQRSNAGFVTDDSFYLPSSAQSESE

SQEEGEAPPLPSSAPPGMAVSAPPSHPQPPPSTSADKTFVSAVALSSVGQKGGESGGVGE

GVIGQKERTQTTPLQPSLQTTSAVGLSIRQRIAQIERQLKEKPSPKAGSKAEEEDTFTWK

AKATENARLLGAAERESKQLKDD

>SG-AL_contig_315_1_-1_94_3042

MDEHKVPIEELYDRLGTDPNSGLSPERARDILLRDGPNELTPPKTTPEWVKFCKQLFGGF

SLLLWIGATLCFIAYSIQASAYENPPGDNLYLGVVLAAVVIVTGVFSYYQEAKSSRIMDS

FKNLVPQFAVVCRGDQKLSVKAEELVVGDIVECKFGDRVPADLRVISAHGFKVDNSSLTG

ESEPQTRTAEFTHENPLETRNICFFSTNAVEGTCRGIVIKTGDNTVMGRIANLASGLEVG

DTPIAKEIAHFIHIITGVAVFLGVSFFIIAFILGYFWLDAVIFLIGIIVANVPEGLLATV

TVCLTLTAKRMARKNCLVKNLEAVETLGSTSTICSDKTGTLTQNRMTVAHMWFDGRIVEA

DTSDDQSNATYSSSDPTWMALARISMLCNRAEFKGNQDNIPILKRECNGDASESALLKCV

ELSIGQVTEYRRRNKKIVEIPFNSTNKYQVSIHETEDPNDPRYLLVMKGAPERIMDRCST

ILLNGKEQPLDDNFREAFNAAYLELGGLGERVLGFCDYFLPIDQYPPGYEFDPDGPNFPL

TGLRFVGLMSMIDPPRAAVPDAVGKCRSAGIKVIMVTGDHPITAKAIAKGVGIISEGSKT

VEDIAAERGIPVEEVDPREAKAAVIHGGDLRDMTPAQIDEILRNHSEIVFARTSPQQKLI

IVEGCQRQGQIVAVTGDGVNDSPALKKADIGVAMGIAGSDVSKQAADMILLDDNFASIVT

GVEEGRLIFDNLKKSIAYTLTSNIPEISPFLVFILADIPLPLGTITILCIDLGTDMVPAI

SLAYEKAENDIMKRQPRDPIKDKLVNERLISMAYGQIGMIQASAGFFTYFVIMGENGFWY

NRLLGIRMEWDSRGVNDLEDSYGQEWTYSQRKKLEYTCHTAFFVSIVIVQWADLIICKTR

RLSLFQQGMKNHHLTFGLFFETALAAFLCYCPGLDKGLRMFPLRFTWWLVALPFSLTIFI

YDECRKLILRRNPGGFVERETYY

>SG-AL_contig_316_1_-1_1603_1989

MFVRLWLRDSGMIKSLDNLRTSNFAQLNIILILLSLGGLPPMLGFISKWLVVTSRVPGPF

ILFLFFLILGSLIRLFYYLSLFFSVTLRGIKKFSIFESNINYRGFLIIIVLLNMLGGLLI

LLINSLSLA

>SG-AL_contig_317_1_+3_3_332

FKSALKIKISLSAALRLITSLSITSVQVFQHQHPSDAIDTCHEIHSCCALGIKFLPANGL

SAMTLSFSTTSSLSSYHSSFFQICSKNNSLSAALRLFTILSISMQVFQHQ

>SG-AL_contig_318_1_+2_2_175

KKKKTITKKTFKSIFHVNVVFRVFFFFFFKIHDDFFFFFFQLFFFLFPPPTSWKYVKK

>SG-AL_contig_319_1_+2_2_271

QSSLTTARCLPHPSLILFYLNINLNVIQIHSISFLVSNKQMNKQANKQTILFVFLFIYFL

LQRIRRVKFYCSPWGERVAVVQSHPYFFFL

>SG-AL_contig_320_1_+1_1_231

LPICRVKFYCSPWEERVAVVQCHPFFFFRLLLFLLTYKSGFFSTEFCQGQLFSFHGIFNM

CVLPTGPRFRNVSSEKK

>SG-AL_contig_321_1_+1_631_936

MCSHTITKNTVTRAHTHICTHTNLHMLTHTHVCANMQSSFHICMYCILSALTSILSGWSC

LQYTGHETGSLKIKVLWKLVSCDSCVCWKILWISLLWESYSE

>SG-AL_contig_322_1_+1_1_204

FFFFDCQHHSLILEKKVYTTNITHAQFYNYEINELNISTKSLNSYIHVRKPGNFVSEQGE

RQKNNPTT

>SG-AL_contig_323_1_-3_45_770

MKLNIAFPATGCQKLVEIDDEKKLRPFYEKRISAEVSAESLGDEWKGYVFRITGGNDKQG

FPMKQGVLTNGRVRLLLSKGHSCYRPRRTGERRRKSVHGCIVDANLSVLSLVIVRKGEGE

IAGLTDTTVPRRLGPKRASKIRRLFNLSKEDDVRQYVVRRPLAEKEGKKARSKAPKIQRL

VTPVVLQRKRARMALKRKRAEKKRDDAAEYAKLLAMRLKEAKERKLERRRSSSRKDSMRK

SQ

>SG-AL_contig_324_1_-2_428_547

MAIIVQLASVALKNVVKMARPVQRQNGIEAKGEGLTFHLI

>SG-AL_contig_325_1_-3_1365_1628

MYPLFLLYVFLFPCFKMCVTCFQKTIYYFGVGITFVAVSWHLSDVSVLLKKLSVIFKHGY

FFFRLHNMAHTIKLSNVSLIMIRHRQEL

>SG-AL_contig_326_1_-2_830_1285

MRIPLFAAFPANKPCDFFLQQIHLWSPGALRQFLALRVGSPILFAHCSDWKDMTRFVCSR

DREGRSAHAPIAPSVCYVDAKTHKWSVHIFRCKGRHLCTPSVFFCLFLFVFLDQNLHSDL

RISVHSFCCMFLLLDVILLVSLGCLVLTITVG

>SG-AL_contig_327_1_-2_206_373

MCLPPNLSHPHFYVILTQLNCVPKVIKDEIIVVWLHFLCFVCVYFFVLPLLMSWNV

>SG-AL_contig_328_1_-2_320_2248

MRSLPMTSRHMTTFHMTGQQKTCWKRSRRLHRHLFTMKLLVLLAFVAIGQSALLGTKKCT

YGPSYWCSHIHHAKECHSMQHCMTTVWKNRHQASIGSTESCYDVIKILGDVRHSGAPDLR

EQMAKSCTQMPDEKHSMCKDLMMNDQNEVLLLLQSDLPSINVAAALGLCGEFSDTVPHPL

VEDSVKQPHVEKDQVAPLSGDYCTDCLNFFGDLQEIFNNTVLKLSEQFKEKICSKLGPFE

KFCNQTTDAYAELITDLGVQQLNPQDMCFLLQMCDSETPADVMKKVKSIIAKVPNSVDCD

LCKKLIKDVQNVLREPTLQNDIINIVNNDLCPLLPGDLAAQCKSYVSQYGPLLFQVLIAE

LDPVMFCETLGFCNATETKHLTVVPLGEQMSSVHVLAPEHKEQAHFGPFCYICKEVMEKL

EGMLDPNATKQEIDWALEEVCSLLPEKLKTECDDFVKAYGSLIEEALAEMAPALVCTALG

LCEGNKPVNVEHHMPVKDDEKCLICETAVAYIKSLINNPDNVKTIEQLLSEVCGFLPSSY

KLMCEEIVNVYTPDLIKLLNKYDDPQTVCSKLGLCDSTKTAINKSDQSAHPLLFQPRVVQ

QAPTSSQVLCSKGPKFFCATVETARPCKAVDFCVKYFNLKRDA

>SG-AL_contig_329_1_-3_705_2018

MASLAGQNGPCESSTDDSTQKKQGTRVFKKSSPNGKITTYLGKRDFVDHLTHIEPVDGVV

LVDPEYLKDRKVYAHILAAFRYGREDLDVLGLTFRKDLYLSSMQVYPPLKDQAEQKPLTR

LQERLIKKLGPNAYPFFFELPPNAPASVTLQPAPGDTGKPCGVDYELKTFVADSMDEKPH

KRNSVRLAIRKLTYAPEEPAPQPSAEAVKDFMMSPGSIRLEASLDKEKYYHGESIAINVL

VDNNTNKTVKKIKISVRQFADICLFSTAQYKCTVAELDSEEGFPIQPSQTGFCKVFHLTP

LLANNRDKRGLALDGKLKHEDTNLASSTIMTKDSQKENLGIVVSYRVKVKLILGFGSGDL

AVELPFTLTHPKPVESPPPSRTPSMVPAEAGEGAPVDTNLIQFDTNGEPFYADKDDDFIF

EDFARLRLKGHEGDDTEA

>SG-AL_contig_330_1_-1_1_930

MPQSGVLILSEEEKRTLISEGYPIPSKLPLTKQEEKNLKKVRRKIKNKISAQESRRKKKE

YVEQLEKRVEAANSENSDLKKKVESLENNNRTLLGQLHKLQAMLSKVPKTAAASATQTGT

VLMVLVLCFAVFLGGSGTGPSSLNVGYSTVGQAPLVFQQPGPLRAQPNMGPGPARAADTA

DDYTTPNLKSRVLMAFSKEEPDDWCDEYRPLVPWEREESCPTGMEPEPQANDPSGPADPG

VDNVMPVVPHTLGKEDGEQQSMAVVTIAVGAKNNDSQTTDIEDYQAMMHPDIAVAPTLGR

GNFSAELETA

>SG-AL_contig_331_1_-3_222_443

MWVFVLYYYFVRMDLPCEIRAALPVRAHRCSAAPSFFFLVPAGCVRVSISHQTLTWLTGS

LTCVFDVVLRAYTH

>SG-AL_contig_332_1_+2_2_187

RIVYKPVRLLLIAACGTAGHVYIKPALASPFCEKVSGFSGFISSGGFPHFLLHLSEQHGR

RC

>SG-AL_contig_333_1_-1_46_273

MHLYQNRLEQTHQSNTLEHITEQKVYTFFIETCYNNNTYILRNLTKHNGLFFETLYNIYS

VETTKCMLEKELRVLT

>SG-AL_contig_334_1_+3_3_215

NSKIGGGEGSPSLVCPPHGGVYNGIGCYEGDARQTFRAGNPEVAQVVVGEGGFTPPVGCL

QDVPDGKGVME

>SG-AL_contig_335_1_+2_407_643

MINRIGFIYTFFPIISHLHHLVFLSSDCTTSLVKSTRVYLISHTLHLTKRFPEMACSEEN

SRKTKQKRVINRTHETVTL

>SG-AL_contig_336_1_-2_2_73

KKKKKKKKKKGRDRFYIYIYIYII

>SG-AL_contig_337_1_-2_197_823

MVKFYSGTVTYKWSWDQMAQAFWQRYPNPYSKHVLTEDILSRHVEGNCLFTRRLLTKTNS

VPRWGERFIPNGARKMCIIEESVIDAESRTITTYTRNTAMQHVMNVQEKCVYKINPDNDK

WTVCEKNAWVSSAVFGFSMAIQAFGIDRFKKNAAKANKGYEYVLEKLFTPVLHHQVHCTE

NLKDKAIKVAQAAKAKAPASIIAARPQPS

>SG-AL_contig_338_1_-1_817_1047

MGRVSCGHCCQICPLCLCTCVHVVSLAPISGSLVLDLFVHYIFVNIASLFSLSSDHRAFF

FFSFVCLSLCSLSLRFS

>SG-AL_contig_339_1_-2_14_529

MTAPPPPPPPPQVLNITASFHDDMQGVVSYVRDTPEPFSIKSGVKQRCILVLTLFGIFSM

LLNFAFTDSAEGIHLYTRNNGKLCNLTRLRAKTKVHTVLIREMLFMDDAALTSHTEEDLQ

RLINRFAHACREFGLTISIKKTNVMGQDVPAPPSINIDNEVLEITDHFTYLG

>SG-AL_contig_340_1_+3_399_890

MFCPDHVFLQLNQIVFSSPQPQPQPEPIMHIFLWHDLSILHPQPLNYLHWWFLHQKNRTS

RLNPRYPKAWFSRIHAKERLEAITFSNWNNSAGAFLKPEPDLQRSQTSADRRSDLFTVSQ

SEKTCSLSANQKRPNKQKLTLSLPHPVEQLSGSERVNFLGQLRE

>SG-AL_contig_341_1_+1_1_972

TTVQHAAGHVLAVTGVTLHHLVGRLEAGVGDLSHRQLLVVGLLGGDDRGVGGQREVDTGV

GHQVGLELGQIHVQGTIKAQGGSDGGHDLADQTVEVGVGGTLNVQVAAADVVDGLVVHHE

GAVRVLQGGVGGQDGVVGLHHSGGHLGGGVDGELQLRLLAIVHRQTLHQQGGEARAGATA

EGVEDEEALQTGALVSQLTDSVEDEVDDLLADGVVTTGIVVGGIFLACHQLLGVEELAVG

SGADLIDDSGLQVDEDGTGHVFAGAGLAEEGVERVVTTADGLVRGHLAVRLDAVLQAVQL

PAGIADLDSGLAHVHGDTFTHDDE

>SG-AL_contig_342_1_-2_1415_1639

MLLIHISVSYSQQHDTIYMSQTLVLSAMQNVFISCLNFHEFLQVIVLYANVECPIIEGLC

DKIVFATMSSIVNLW

>SG-AL_contig_343_1_-3_3_287

KSNCGLVGFVGVCVVSLDLLGVHVCSNTGRVCVRACLCVCCNIGLVGCVCVVTLNLLGCG

GVCVCCDIVLKSNNGLAGRVCVVTLDLLDMILCSF

>SG-AL_contig_344_1_+2_77_202

MVSDVLINLLSLPYHTTGGFFVCLFFFLQTFQFALQSMDTFV

>SG-AL_contig_345_1_-3_1791_2045

MLWPLWFMIFSLLALCTVQNPREQKPVVQHMNNMHAFYIMFFVMKFCKTFQLFVCMCIAV

TLSSVKILYEHVLLMNVESVIHAYA

>SG-AL_contig_346_1_-1_262_2214

MYTVIVHFGGKEVPQSPIKVQIESSMDTSGITVEGLEPKIPVGVQKTASIKIPPKLPKAA

VKMVITSPTGKATNVPVNDLVDRKVAPFTLQEPGPHTVDITYGGVPVKDSPFKVEAVPDL

GPPKVKAYGPGLTSGEAMKPAQFTIDAREETRPGNLGVTVEGPKESKIDVKDNGDGTCSV

TYMPEVPGDYKVNVTHAERPIVGSPFTAKIKPAVAEAPKVKAYGPGLTGGEANKPAQFTI

DAREETKPGNLGVTVEGPKESKIDVKDNGDGTCGVTYMPEVPGDYKVNVTHADRPIPGSP

FTAKIKPSTAVPAGDAPRVKAYGPGLTGGEAQKPAQFTIDTREETRPGGLGVTVEGPKEA

KIECMDNGDGTCSVSYLPEVPGDYKVNVTHAEKPIPGSPFNANIKPSTAGPDLSGVKAYG

PGLSPDGVFMEAPTEFTVDAKSVTPSGEGQVQAILTTPSGRKMATTVTNKKDGTYPVLYT

PMEKGPQTLDVTYNNAPIPGSPFKVEAVPGNDASRVRAYGPGLEGGVSNRPNKFTVDMKG

AGKGGLGLGIQGPVEAKMNCKDNHDGTCEVEYFPTKPGDYDISVTFADKPIPDSPFRVRV

TSEVDASKVKCYGPGLDAKGVTACAPARFTVDTRDAGEAPLQVTYTDQTGA

>SG-AL_contig_347_1_+2_2_256

HLHNKKKTGRKLQEINPYLFSLLNDNTENVIAIGFTHPQIFFHVHKIIDAICFNQLLFLS

PSDTLSLIISVARTGTKCRKAPKIK

>SG-AL_contig_348_1_+3_1344_1670

MINLFFTLHSSVAIFIGVQLYFSFFKKIQLKIPPFYSRNYLFYVQEKPVFKLKMKSGVHF

NFQYQLFFDRSILLSVFVQNGEAWGRNSRYCSICSLCPWNVFRYVFSLF

>SG-AL_contig_349_1_-2_176_1153

MQLGILDTCIITEELAFGCTGIQTAIEANSLGQMPVIIAGNEAQQKKYLGRLTEEPMMCS

YCVTEPGAGSDVAAVRMKAVKKGDKYILNGQKMWITNGGHANWFFVLARTGSDPKVPASK

AFTGFIVDGDSPGLTREPKEWNMGQRASDTRGITFEDVEVPAENVLGQEGDGFKVAMGAF

DKTRPPVAAGAVGLAQRALEEATRYSMERKTMGKAICEHQAVAFMLADMAIGIESARLVT

MRSAWEVDQGRRNTYYASIAKALAGDVANKCATDAVQIFGGNGFNSEYPVEKLMRDAKIY

QIYEGTAQIQRMIVSREIVTKAKQRM

>SG-AL_contig_350_1_-1_712_1107

MLLTFQNSILVLLLVFRISPLLSVASFQLSLLLFIRVFDDGSGSLSLRVIMVSFVAVSIT

SLFFRDWVVSPVPSSQPGGPVDCALLGHYPLTCLAWVTLLVAEATAGIALGVPVACKLPS

HNKVAILQAPVL

>SG-AL_contig_351_1_+2_2_154

FFFFLMPVKGSHTEKLCLSSFVNQLSILIQSCHVLGLNFVNDSICFESDHA

>SG-AL_contig_352_1_+2_107_241

MCRCLELCARCMRLVSIAKESAQVQNGNLAQKAVVLSIQIKWIQW

>SG-AL_contig_353_1_-1_88_1284

MPPKKKDEGACKPSPLIGRMGTNLKIGILGLPNVGKSTFFNVLTKNSAPAENFPFCTIDP

SESRVPVPDKRWEFLCDFYQPASKVPAYLSVVDIAGLVKGAHEGKGLGNAFLSNINACDA

MFHVVRAFSDEAIVHVEGEVDPSRDLEIIHDELRLKDMEYLRKHLEPLERSVVRGGGDKK

KKEEYDVLKKVECMLEEKKWVRLAHWDAKEIEVLNQHLFLTSKPVIYLVNLSEKDYIRKK

NKWLVKIKEWVDTNDPGALVIPLSCALELSLTDLSTDEERQNHLKELGTTSALEKIIVNG

YKALNLVYFFTTGKDEVKAWTIQMGTKAPGAAGRIHTDFEKGFIMAEVMNFEDFKEAGSE

AACKAAGKYKQKGRDYVVNDGDIIFFKFNAGAGLTGKKK

>SG-AL_contig_354_1_-1_184_675

MADEGKKQRATSNVFAMFRQAQVQEFKEAFTMIDQNRDGVIDIEDLKDMYSNLGRIPPDA

ELKEMLAEAPGALNFTMFLNLFGEKLSGTDPEDAIRNAFAMFDDEGKGFLPEEYLKDLLQ

NMGDNFTADEIKQTWKEAPLEKGKFNYQEFVSILKGKQEDESAA

>SG-AL_contig_355_1_-2_197_280

MEISEKGYLSRIYRELSYYPAVHEDIYI

>SG-AL_contig_356_1_-3_3_92

FYVDIRCRMCLVSACPFLPVVIEVQSIMDA

>SG-AL_contig_357_1_-3_90_380

MRAMGAHLQQEQGCSSPSQQDLASDLASLGTEAVGAIVESAGATVKLSYNLTLLRRDSIL

AASNLPAEDFPALRSVPMQPSSFFGPFAEPFVSARAA

>SG-AL_contig_358_1_-3_1650_2120

MYWCDVSSYHITFSLVIHYWVTLTLFLWFLFHIKDICLPLLFTHIEADWIAMFSIPFNIF

DWPSSVPKDYGRSCTAELMFVGHKPMLCVMENLLLQSFIVRLTTVVLTQWFHPLMFRQCM

NAEILYLFKTVVLLPIWRTVNTLTIWRAVSTLRFGEL

>SG-AL_contig_359_1_-1_397_2436

MDSGSCLQRILLLLVFLGAGFLQDSVVAFHWNHRLRNASTLSRCVGDDVTFRWSYVTGID

EKVLATFLFSDSNGTLVTGFANRVTTSSDRVTFLPPTGMTLTGLTSDDAGMYGVHVRLFG

SGHLHTQVVNLVVLEPPKLLDGKLEVEQLQPKATAPTGPNATCASTSELQCGHFLTIGNP

PVSVKWSDPDKQEHVSTVLENGRFLFDLPAYKGGNYTCTLVLAPEVSKCLQSDPPFVTTA

SIFLETTATKVEEQAANLQELKETVEQVRTQAFAVLKTHNPHFTQEMEDRLETLENSTES

LALDMDTQRRSTQMLSDSNTEAITHLRNSFQRALTESEATAKQSLQDHAKDLLTKLKAAT

DAISIVRESSAAQQQSMQTIQQQLKALQSQFKDQQNVNSQLHSELATARNSLTRQKAATA

DLEDKVLKHQEENDQQKKINSDQKKVNSDQLRTITDLQSNLSAMAKRIEHLETVSAILQS

KWAKQQQELETQKATSAEQERISAEFLPSLAAVQNGLEIQKNATDHLASQQTEYASVFDR

KLSAVQKTVGGIQAKLDDVSTHTKSELESLGQNVNNTQINLTTALTTITSIRQRVGTNKN

NLVQITERLTSLVERVNESDGNQQRSLSNQVALLQQNVSSIHRQYEVMLPRLATLESGLE

TVNGRIHHIRLVGGRNALEG

>SG-AL_contig_360_1_+1_106_498

MPDLLHVIPVGDDAMFNGVLEGQDTTLALCLISNITVLLAHAHHHTLMSGATNDGGEDSS

WSIISSETGLAHTGPIVDDQSGNVVVTHVEKFSKVCKRTKGKVSCSLGESLVCSGEFYFV

RMLYELRRGQR

>SG-AL_contig_361_1_-1_58_267

MFPHLYVPTSLYSHVSMFPHLYVPTSLRSHISMFPRLYVPTSLCSHVSMFPHLYVPTSLC

SHISMFPHLY

>SG-AL_contig_362_1_-3_30_614

MAARSTVCKLLLSSNAISKFLTCNQPALRGITMTAVRTQCSAVDDEGEEKPTRAKHLVVD

PETSMRYMESNAYAEAYGDKPVWFHYRRNFKGQRAPETRKMCIRGGVISTGSPCPVCRDE

YLVLDHKNVKLLEQFISPYTGEVLETKKTGICQKQHRKLLVAVRKAKDLGYLEKNVPFRV

YDYSQYWDETKGKVL

>SG-AL_contig_363_1_+2_11_457

MNNLHSCWIDHRKNNLDNCIQMSSLLHSCWIDHRKQNLHSCWIDHRKNNLHSCWIDDRKN

NLGNCIQMSSLLHSCWIDHRKNNLYSCWIDSTLKNLHNFLMDDRKKSLHNFLMDDRKKSQ

QACTRTWDSPWSRSQNRWDSTYCIRVHTL

>SG-AL_contig_364_1_-1_1_261

ATSEGRAASTVRVTGPLSALPFEPLDVVPFSLLSYKTAFLLALATCGRRSEIHALSGAKD

DIGFHSNGDLALTVLPEFRAKTQQPGS

>SG-AL_contig_365_1_-1_1_216

SPVELANFLAFLATSEGRAASTIRGYRSAICTTLRAPRRGSLLAALLQDGLPPGLVHLLP

SQRDPHPEWSQR

>SG-AL_contig_366_1_-3_93_1373

MATGTLYTFPKNFRAYKALIAARYSGADLKVDPSFKFGETNKSDSFLKKFPLGKVPAFSS

NNGLNLTESNAIAQYVGNKQLQGESAEAAAQVQQWINFADSEILPAACTWVFPCLGITQF

NKQETERAKAQVTKALSVVNNHLLTRTYLVGECITQADISMACNLLMLYEQVMDPEFRKP

FQNVNRWFTTLVNQPNFKAVTGEIALATKMAQFDAKKYAELHGGQGDNKKKEKKEKAPKQ

QEPKKEKAKPKAKEPEDEEEDIPKPKESKDPFMAFPKGTFDMDEWKRTYSNMETETEALP

YFWSKFDKEHYSVWLGEYTEDLSHSLTFQTCNLVAGMFQRLDKMRKHAFGSVVIFGEDKK

NQIGGIWIWRSHELAFTLSPDWQIDYESYKWTKLNPDDAATKKMIDEYLLWEGDFGGKKV

NQGKVFK

>SG-AL_contig_367_1_-3_1179_1529

MSESVNIKQPGNISSSFPFVWNCGTFLPQWSLGTTFSYNCTHLFVFGCIHVCNNIIFICI

ICLSLPLKITRFLTPSLSCLFLFFVFLFLSVFLVSHQFSHPHPHPHFPPPPPHPKTK

>SG-AL_contig_368_1_+3_525_725

MINKHAGLSWLCCVLVVLYPFLLFSGDILTNQTDEATKRQNLTPKPFLQDQKNQVIFRHE

CPVVICS

>SG-AL_contig_369_1_+1_349_609

MSTLMLKRPHIIKRKRSSALTSTQRSNYNGRKAECLSSQTLIPVFYQIHFSSLAFNNDLS

FSVCVLKNTFFHAPASSFSICKKSIRN

>SG-AL_contig_370_1_+1_1_147

QPLRSAPDTIYRFLDTDVPQSVAVHFLCPAHITGMSFPCLFDSHKLSSF

>SG-AL_contig_371_1_+2_245_460

MTTETKTYCYYYYYILFIAQPTTMAISGLSPCKVHQSQEDDCVEHKTSSNLKTKTLQKIT

IHDIFTVHDTLF

>SG-AL_contig_372_1_+3_378_671

MLSAIQSIETQHIYRTDLMLSAIQSIKADTCSVSTDGLCHVRYNHAWCSVWPSTSRQTHV

RYNHAWCMQCVAKSSRQTHVHQVLMAHSHVQHGAVCGQ

>SG-AL_contig_373_1_+2_2_268

GKQGCLPCNGRAETNLGTLKKKGRGVNECVCVCVCVCVNDVRKSLRERERERGRERCLCP

AVISAFAQPPLHPTPLLSSPSQYACALHP

>SG-AL_contig_374_1_-2_347_1681

MREIVHIQAGQCGNQIGSKFWECISDEHGIDTTGNYHGDADVQLERINVYYNQASGGKYV

PRCVLIDLEPGTMDAVRSGPFGQIFRPDNFVFGQSGAGNNWAKGHYTEGAELVDSVLDVV

RKEAESCDCLQGFQLTHSLGGGTGSGMGTLLISKIREEYPDRIMNTFSVVPSPKVSDTVV

EPYNATLSVHQLVENTDETYCIDNEALYDICFRTLKLTTPTYGDLNHLVSATMSGVTTCL

RFPGQLNADLRKLAVNMVPFPRLHFFMPGFAPLTSRGSQQYRALTVPELTQQMFDAKNMM

AACDPRHGRYLTVAAMFRGRMSMKEVDEQMLNVQNKNSSYFVEWIPNNVKTAVCDIPPRG

LKMSSTFIGNSTAIQELFKRISEQFTAMFRRKAFLHWYTGEGMDEMEFTEAESNMNDLVS

EYQQYQDATAEEEGEFDEEEGEEEA

>SG-AL_contig_375_1_+3_990_1154

MHLHNKYKGISWFLQSARLSKCNMISLFELTCRLRQFPMPTIFHEIKIKIEHINP

>SG-AL_contig_376_1_-1_67_1905

MEETQNMEIEATGGDLDDVIILDDVDEENKTEVDEENKIEVPRENIKPPVRDQGVYWQVR

VCPILFDDFCKLNDIFTHLVKLSVIFEYRMSKYLSSIFSTSRFYRKDVDKEIPWLPNEKT

YTKPQNAATAGMLEMKVADSFLLDVMKRLRGPNMSRKSRMRFEIRKVPPLPERKKKQREP

RRGKKTEAEEKTEGEEKTEEEEKTEEEEKIEGDEETGGDEETGGDEKTGEEETGGDEETG

GEEKTGEERMEEEEKTEEADFDQMLINFSVRLRPVKVPAFKHEEGHVDTREDNQIFLTKV

PDDVPDNILQAILMLEMVERKMGENDTGTATFNVRRATGAQAYMELFDGILVNNCKDIEM

ATTLGTLYDAKKKHSEKQQENREANEKQLQGQKRKPDSAPAEVQEAKRPRGDPLNPTATM

WMLGRKMRGMRGSLTSSGGRAPQRRMQGATARGKGGVALKGQTKGARGQPASRAGLRRQG

ARVGTPRGRGGMETGAQRGRRAAKAKSRRPVPRGREPPRALLDLPMERPPLRGRASPRAE

RGLLEMDDMWARRGMDIPRRGEFDVLDRAAGFRDEPLFSTRRSLDRRLEGLDDRYMRDLE

FRGDFAPPQRRRW

>SG-AL_contig_377_1_-1_1_234

ADRRLAVFLELWSWIRLSAAFTFRVLRHCLIQFVTSAARIVYDVTLAWPHTKQVCKGEKK

AARLRECKREREKINIFS

>SG-AL_contig_378_1_-1_19_2409

MAGMQFEYDEEGGTFFYFLLSFWALGLFPATYFCWPRAKASEEDPKKKGICNCLPCLKKN

IIRKKQEATVKPMIIKGVLVIGWIIFFLLAYKVSMIQLDYVEYDPFLELEIDRGASASEI

RRAYRKLSLIYHPDKETGDEKRFMRIAKAYAALTDEESRKNWEEYGNPDGPEATRFGIAL

PKWIVERENSMWVLAVYGLVFMIILPIVVGVWWYRSIKFSSDQVLLDTTRLYYYFINKTP

NMILKRAVMILAASCEFDRMHNAEVQDRPSDNEELPQLIKILGNIQEKNRERPLCFPYSV

KTRVLLHSHFQRLLLNEDTLGADQSYILQKCPYLINEMINICASLVAMAMSGRVHQPPRL

ETVENLMKLSQMIVQALDEKGSPLQQLPHITPDMLRHFTTRKRNIRHIRDFVAMKDEDRR

QMLRSLGEGEYRDIIQVCGTMPYVDMTIRSEVLDEEDTTITVGSIVTVTVNLVRKDMSAL

FDKEISAIEQIDEDDAPLNGNGDEEGDEDDEHDERQGNNVTGENKPKVWEKQKKGKKKGG

KAKKKVKQPYQWKVAAQAKIDKENAAAASKSAAEGTKSKSSGASAVGASAAENEEHEEEE

VATESGEENNASDEEQETSGGGGGGGEAASEEKEEKVEKKKDEDEDEDEDWDTFKMESKK

ENSLETKKKESHCVHCPFFPADKQEAWWLYVADKKRHLLITAPVHVCSLKEEEEVKLKFS

APAKQGVYHYTVVLRSDSFFSDFDQQQNIKLDVKEAKIVKDHPQWDISDDEEDKANNDDE

TEEEEDSDYSSEDDGSD

>SG-AL_contig_379_1_-2_83_2986

MSRSMIPSQQKLAEKLTILNDRGIGMLTRIYNIKKMLGSPDNKPAFLTDKALEPATKALL

RRFPQYDSKASQLTVVNNMKNEIMKALSLYYYTFLDIMYFKDHVVDLLTTIDACQAFLDI

RLNFDLTKMYLELVATYVSIMILVSKVDDRKAVIALFNVAHEFLQGKGDQVYPRLGQMFL

DYDPPMKKLAEEFVPHSRILIPALLSLTQIYPEHNRTAEQLRQMQVLSVLAEPSKMSSVP

TTEVIQCEYLSMNTMERWIIFGGMLCYQGLTDPNMYALWKASLESGYILQLCRDEVLHIH

SYVISFFESLKNQHGQGKRVAEIKEFQQHALSTSSGIHRERRKFLRTTLRQLTLVFTEEP

GLLGPKALHVFKALSMAQDEVHWVLRHVANPPSRRHNVKLPQEDFHDRQLPELLFYMEEL

RGLVKKYNQVIQRYYVQYLSNYDAVCLNSIIQSIAMVPEDESVILSSFYNTMVSLSVKQV

EDNELFDFRGFRLDWFRLQAYTSVSKAGLVLRDHQDLGKHMNTVIFHTKMVDYLDEMINE

CGDLSVYCFYTNLFEHQFKQCMEFPAQHRFSIIFPMICAHFMNATHELCPEERHSIGTTS

VQYAHWFLREMSEEVNQVITAICEEQCMLSYKLLPKHSAAIILAQVQKKKKKEREKKDKE

PEKPGLESVRKNREVFTRMDKLHMALTELCYAINYCNVIQVWEHGFIPREFFLQHLEARF

NKALVGMMMYNPETTEIAKPSELLNGVRAYMNVLQGLENYVHIDIARVFNNVLPQQTQNT

DATGEKTITANYTNWYLEVLLRRATCNAGQIVYSPRQKAFVTVSTSDGQQNFAAEEYADL

TELRALAELIGPYGMKYLGERLMLSIASQVDELKKIVVANKETLVQLRTSFDKPEMMREL

SKKLHMPHKNAPSDVDIVLMRMTIIGVLLSFRALAQEALNDVLENRIPFLMGSIRDIHHH

MPDNRDSM

>SG-AL_contig_380_1_-2_224_1822

MQIFVKTLTGKTITLEVEPSDTIENVKAKIQDKEGIPPDQQRLIFAGKQLEDGRTLSDYN

IQKESTLHLVLRLRGGMQIFVKTLTGKTITLEVEPSDTIENVKAKIQDKEGIPPDQQRLI

FAGKQLEDGRTLSDYNIQKESTLHLVLRLRGGMQIFVKTLTGKTITLEVEPSDTIENVKA

KIQDKEGIPPDQQRLIFAGKQLEDGRTLSDYNIQKESTLHLVLRLRGGMQIFVKTLTGKT

ITLEVEPSDTIENVKAKIQDKEGIPPDQQRLIFAGKQLEDGRTLSDYNIQKESTLHLVLR

LRGGMQIFVKTLTGKTITLEVEPSDTIENVKAKIQDKEGIPPDQQRLIFAGKQLEDGRTL

SDYNIQKESTLHLVLRLRGGMQIFVKTLTGKTITLEVEPSDTIENVKAKIQDKEGIPPDQ

QRLIFAGKQLEDGRTLSDYNIQKESTLHLVLRLRGGMQIFVKTLTGKTITLEVEPSDTIE

NVKAKIQDKEGIPPDQQRLIFAGKQLEDGRTLSDYNIQKESTLHLVLRLRGGN

>SG-AL_contig_381_1_+2_2_631

PSTPVDGQCSVESLAHHASAWRQLHLSDWVVEAIEEGFRPPWERGKPPIRPLPPSRARFP

TIRNSPGLGAGLPDPQRGRGSFTPPPWTWLLWQTLGGPQILRALAPGAGPVSPQRVPGPH

HFLTETPTPIRASIREGDWASSIVPMDACLHGLIHPSCRKHPRCVWKAQALQFQALPFGL

SLAPLQLTRVSREMASWAQDVAISFSCHLD

>SG-AL_contig_382_1_-1_1759_2097

MNGGCGGQHLQPENLASDKCGTCVTLLCIHMFSWMYIKSFSIQKSLDLLTVYTCLWACEL

NLDNILIVCCVLVVIFCFVLVFVCVCFKQCFSMLSCFSAFFNFFGLFFWLPVH

>SG-AL_contig_383_1_-2_2_226

VCWLCVCVCVCVCVCVCVCVCVCVCVWIYPPPPPPLRVLTTLAFQRYLWFNFLRCLQDYL

VTTTTTPPPPFLLLF

>SG-AL_contig_384_1_+3_726_830

MVLTISTEPHLNFSHEKSLSFLTLHFLHFPSDACF

>SG-AL_contig_385_1_-2_152_736

MATPAKVSKTDSKNNHNPTDEQPDHQEAIEHIDEVQNDIDRLNEQASEEILTVEQKYNKL

RQPFFKKREDLISKIPNFWVTSFMNHPQVSALLTEDDEEALQYLSVVDVEEFDDIKSGYT

VKFHFEANPYFENKEISKEFHLNESGEPSSKSTKINWKEGKDLTKKSTPAQKGKKRTHED

QESFFSWFSDHGDAG

>SG-AL_contig_386_1_-1_79_714

MDNTTPPYSEARFNEIQKEVSTYIKKIGYNPKCVAFVPISGWNGDNMLEDGGNMNWFKGW

TIERKEGNTSGKTLLQALDSILPPSRPVDKPLRLPLQDVYKIGGIGTVPVGRVETGILKP

GMVVTFAPNPITTEVKSVEMHHESLSEALPGDNVGFNVKNISVKDIRRGNVAGDSKNDPP

KECKQFYAQVIILNHPGEIQAGYAPVLDCHTD

>SG-AL_contig_387_1_-2_2_73

KCTHRVIANSLHEMYTQSHSKQFT

>SG-AL_contig_388_1_-2_1196_1462

MRVSPFHALSLTSMYSWRKLTAALLVAAELDVGIQTFSTTFCTTFCTYTSHWGDVQTIEY

HWFAWMWRHCFECKIAVRNKIGCEIKKKK

>SG-AL_contig_389_1_-2_371_1327

MPGTVAPAEDFDAGAVAEKLRNAMSGFGTDEETVLQVMGNHSNEQRLEIAEAFKVAYGKD

LIDDIKSELGGHFEDLCVAMLTPPRLYDAKQLHDCVCGAGTDETSLAEIMATRSNGEIAE

IKEKYKEEYDCELEEDLQSDTSGYFGRLMVSLCVGGRESDEWEWADEDKAAEDAQKFLDA

GEGAWGTEEAEMNAVLCLTSPGQLRKTIEKYEELTGGTTMEEAIRNECSGSLQEGYLAIV

ESVKSKPKYFARRLHDCMAGLGTSDCDLIRIIVSRSEIDMEEIMAAYEEMYEKPLVEAVD

SECSGDYRKLLIAILNPPC

>SG-AL_contig_390_1_-2_2_529

VKGVTSRWHTKKLPRKTHKGLRKVACIGAWHPSRVQYSVARAGQKGYHHRTEINKKIYRI

GAGIHTKDGKLVQNNAATEFDPTDKSITPMGGFPHYGEVKQDYIMIKGCVVGPRKRVLTL

RKSLLTTFRRRHMEKITLKFIDTSSKWGHGRFQTPEEKHNFMGPLKKELLNREAAA

>SG-AL_contig_391_1_+3_315_599

MQFINIFLSKECCVLAVKSCTTGKTTKHRTNQCDCESNIKTVLFKSKSLAQQFFIQIVYL

SSTSLSNQASSIPYPHISTEDMIQVYKVQVITTDK

>SG-AL_contig_392_1_-1_169_1038

MPGANDQLNKYNKIEAFVKGFKLSTSSYKRVMHLLHQEMTNGLTKHTHHIADLKMFPTFV

RSLPDGSEKGEFLALDLGGTNFRVLLVRLQGHDIDIQSKTYLIPQRIMLGTGTQLFDHIA

DCIGKFVRDHNLSGHTLPLGFTFSFPCKQEGLAKARLSKWTKGFRCEGVVGEDICGLLHE

ALKRRTNCNVEIVAIVNDAVGTLMSAAHMDRNCEIGLILGTGCNACYMEKLGNVETWDDE

KNHPDQVIINTEWGAFGDNGCLDFLRTEYDRELDTFSINPGKQIMEKMIS

>SG-AL_contig_393_1_-2_1238_1408

MVRIICHEFCVQRGYLVDFDVLNVVTAPIWPYWSAGLLAIYDNNTDNSTGDKSYQIP

>SG-AL_contig_394_1_-1_1_594

CDSNFLDLNVTKTKELIIDFRTSKTDVEPVIIKGQPVNIVESYKYLGTITDNKLNWSSNI

DTCCKKANKRLFFLRKLKKFQVNKRILALFYQSIIQSAMLYNQVCFFRSAKKADTKRMDR

IAKIVGDKIDPPSAIYEQLAIKKVRRILSDNDHPLNSVLSSCVSARDTSHRLRCLKARTN

RMRDSFLPTAIRLYNATI

>SG-AL_contig_395_1_-2_335_496

MNDHEKSNELENSKNRRMTAAATTVLISPYFTSKMGLQPKHDVQVECFFYISEF

>SG-AL_contig_396_1_-3_69_1304

MTLGAAINVWGRKFILCDCDDFTKEYYRTKFGITEFKPITCNMDGGVRQQREFPPYNGFG

SEEDSLCSCMGLLPKPPRRDFIKFMERDRRGLESNVLRFLARMNTEKPIDKDRRFIISYF

LSDDTILVFEPPVRNSGIIGGKFLERDRVKKPDQAPYGTQLSEYYMASDLFVGASVSFNR

HKFILIDADEYAFRYMEEHPMEFPKSNLQMIVEKIRARLSQNMDEVRSVFTRHDASGRGT

VGYEQLRQLLMQVDPSLAEQEVMTVARHFCSQAQQGALDGNKLIAIAQEQLRKRNYESFN

NLLETLQHQDRTRCGFVAPEMLRTVFLSHHVPLPDDMLRAVLASAPANEKGEIAYMELLK

SINWRDYPVPPMQYQPVHPDEDWKGTAATNAVTSVNYFALLEAVFGQAVAAE

>SG-AL_contig_397_1_-2_164_1783

MPCRLLLKSLILLMTVTSPGAGDVYTSTSTILTTALSEGKLLEGLNKFIGFEYDNMNKMS

QFLWERSKANHLVSKEDLQWLVEHPNGAYFIVKLFTQHWVPLANTYPVLARMMEPVLSEM

PTDRDFEGALSSLIRLQRVYLLSVDHMYGGNYTGHMGPPLQPEDTYLLGQRAFEDGYLSE

SVQWLELALRLMYTYDAFPATSSVFGGNHAAAPLVTLAQVASLLGRVYFFMKNQTKAQEM

YELSSALDPQAYDVKNLKTDLDGNGLEYRHAESEEWEHNMTTLCLRPRHHTVAETTPMHV

CRYKRAFGLSYLTFKEEILSLQPYTSLFYDVINDEQIDAIKGYAKGKMMRGKVGQRGLTS

DIRTGDLSWIHDADLTTAAAVSQKVKAITGLEVDQRDPDGPSSAEPIQVVNYGLGGHYDA

HTDPFGMVPPDNAMLNRSGERIATFLIYLSDVEKGGSTVFLHTGISVPPRKGMVLFWYNF

TPALDNELTLHAGCPVVIGQKWIANKWIWTYGNTFRRPCGPHANSTQQHVDNMLASRLLV

>SG-AL_contig_398_1_-1_19_1494

MTVFARKLYRVYGAALTWCQCKRAVKQCLSSIQTCQQTRPSLVSIARFQCTQASSDILNN

TQTPSSESVSSDEVHSQITQRGIGKSYDEQDLKLLLKPDQPPDARILRVAIIGLPNSGKS

TLTNSLMGWRVSSVSKKVHTTRKNTVAICTEDNKQIIFLDTPGILSATSRKRHNLEKSLV

IDPERSLHQADLVCVLVDMSDRWRRNELDPLILQVLHLHQHIPAVLVLNKVDLLHSKELL

LQITRSLTQGIVDNQQVAVITKTTRQQGKLLDKEALFDAMGNSQPQNMSDTGSHQVSSLP

SDQPNDMALEQQPSPGDSVPDSSSSDTELWTSYFQRMREASHAVRGMKGWPHFKRVFMVS

ALSGDGVDDVKDYLLQSARPGDWEYHSSLVTSQDPREVVLMCVREKLLEHLKQEMPYNMA

LDLVLWEVDEDGMFNIIINITAQNRRQMSFLVGPKGKMIQKIASEAKQEMMNAFRCEMRL

KLIAKAKLTHKR

>SG-AL_contig_399_1_+1_208_306

MLLVNIFLIVSILHKDYFSYSIKIQQIIETKLL

>SG-AL_contig_400_1_-2_80_856

MGRVIRAQRKGAGSVFKAHTRNRKGKPALRPVDYAERHGYIKGVIKDIIHDPGRGAPLAK

VAFRDPYKYKTKIETFVAAEGMYTGQFLYCGKKATLQIGNILPVGAMPEGTIVCCIEEKT

GDRGKLVRTSGNSGIVISHNPDTNRTRIKLPSGSKKVVPSANRAMVGIVAGGGRIDKPML

KAGRAYHKYKAKRNCWPKVRGVAMNPVEHPHGGGNHQHIGKASTVRRDTSAGRKVGLIAA

RRTGRLRGTREVIQKGEKE

>SG-AL_contig_401_1_+2_2_412

PPPLLSPNTHIQTNVHSVTSTRSRHRLDVTTRADAVPSAGTSWSTRQFEIKSKWKPISPQ

TKPIQFKRFAVQYSSGLQQLVFTSYEHKFDSKRMIYINMCVYVIFTLVPPSQGRGRKGLV

ILLMLQKKSLKALDLIF

>SG-AL_contig_402_1_-2_203_556

MNATPTTARTCSGKIFSRSWPRNPHRPKSSTWGEGKVRGVRRGVKKKSRRPPARKSLVSS

GRDTFDWSPFPRTTCNDWLRQIPALMIRPISRIVAALMNIRPPPHWTESNYIYPIVWQ

>SG-AL_contig_403_1_-2_1154_1423

MEFMFGRFDCMYVCVCLIIACAGGLLFQPHLCLSNGLICLFLQTGYMNMLHTCAHAYVYT

HARMHARTQTHIHFIFHMYLVYAIFCRKLD

>SG-AL_contig_404_1_+1_154_258

MTQKDDNSLQRIFNNNNNNNNNNNNNNNNKKSFLY

>SG-AL_contig_405_1_-2_74_2260

MLRLWPLRQALSSAGARFIPACRKSTAADPAPELVEVFVNDRQVFVEPGTTVLQACAAAG

IEIPRFCYHERLSIAGNCRMCLVEVERSPKPIASCAMPVMKGMKVKTDSPMTRKAREGVM

EFLLMNHPLDCPICDQGGECDLQDQSMNFGSDRSRFTDNAFSGKRAVEDKNVGPLVKTIM

TRCIHCTRCIRFASEVAGVEDLGTTGRGNEMQVGTYVEKMLQSELSGNIIDLCPVGALTS

KPYAFTARPWETRKIESIDVMDALGSNIVVSMRTNEVMRILPRMNEDINEEWISDKTRFA

YDGLKRQRLTTPFVKNAKGELVQSDWETAFFTIVDKLKGVEGSQISAVVGGLVDAEALTA

LKDLMNRLGCETLCTEEIFPMDGSGTDFRSNYLLNTTISGVEEADLVLFVGTNPRFEAPI

FNARVRKSWTHNELRVAMVGAPVDLTYEYDHLGDTPAVLEQLADGSHPFSKVLELAKRPM

VVVGSTSLQRGDGAAVHSAVSTLANRLRTTSGADGDWRVLNVLHRVASQVAALDLGYAAG

VDGVRQTKPQVLFLLGADEGTITRSDLPADAFVIYQGHHGDQGAQMADVILPGAAYTEKD

TTYVNTEGRAQLTRQAVTPPGLARNDWKILRALSELLGVTLPYEKLIEIRQRMAEVSPTL

VRVGDVEEANFFQQSADMAKSVKSALGSEPLSPPQHVLKDFYMTDTISRASPTMAKCVQA

AKEAQTAKH

>SG-AL_contig_406_1_-3_147_683

MPVNCYELIARRQPQNYTLFSDLDAGISVTLSMRKRFMTALHIRHHQSYATVEVNSQGSF

TFTKPPGPSPADSIVHTRPDSQSLQVELGQYVVTVNITKKACRATIITVDMDVGANGANP

ALLRKYSGVVTKGYGSTKGKSRSKSKKGKERGSKACKANSFPADHSFVVKGKRLLRYKA

>SG-AL_contig_407_1_+3_1809_2072

MSRLTTSPNKDLYTVSINVSLSKGSLSFTHKMTFCWHVFLCNYVLPFNRARNTHLSDCPG

QLSLQADKWKHSLHLSVGNQFLTVSETD

>SG-AL_contig_408_1_-2_2_79

KKKPTCPSNFYLPVLSKDIVNKGNYR

>SG-AL_contig_409_1_-2_29_358

MTTTLKPYLTCVKETLTAAICVQNFDSQVVERHNKPEVEVKSSKELLLTPVVISRNDKEK

VLIEGSINSIRISIAVKQADEIERILCHKFMRFMMMRAENFVVLRRKPVE

>SG-AL_contig_410_1_+2_2_340

FFFFFEADNILFLNHKTSDNPTPSHTASCSGRRLLVSSGHAANDVRVTRIGDGECADSEV

FATRCTQLNVVAIVVVHSGFGQHSVILDFTLPEGRGVVGNDDKLGFTLPQCLQ

>SG-AL_contig_411_1_-2_119_847

MKDAVDPQLRDQQAGFRMNRSCTDQIATMRIILEQSLEWNSPLYVNFIDYEKAFDSVDRQ

TLWKLLRHYGVPEKITNIIRNSYEGMTCKVVHDRQLTDAFQVKTGVRQGCLLSPFLFLLA

IDWVMKTSTAQTRNGIQWTLWTQLDDLDFADDLALLSHTQQQMQEKTDAVAETSACLGLN

IHRGKSKVLKVNAANTTPIRLEIESLEEVESLTYLGSNVDKQGGTDWQGKDGFPPAEERL

GIH

>SG-AL_contig_412_1_-3_3_227

IDSALQTSERDKSPWQSTTSCENRSSVVNCNLETTFMLFINCKKKLYRGMVQVSGKKYLH

SRFLNFQEIEINMLP

>SG-AL_contig_413_1_-3_117_1010

MAVADLCRKFGSLLLQSSGRLMTQPSVGQLSANSHLTRSLHSSGRLDGKMKLIKQGIDLD

KYTLRPVPLPRSGGRGANGRVQTHGVGGGHRKNYRMVDFKRKGPTEGEPLVEEVQTIRYD

PCRTADIAIVAGGNDKRYILASQNMKVGDLIKTSGKLTRMAVRAAEGDAHPVGSLPVGSV

IHNVELFAGEGGTIARAAGTSAQLVRKVGDRCIIRMPSKREINVSQECMATVGRVSNIDH

NKKPIGKAGRNRWFGVRPQSGWWHRKTGYNGRKIRPIKPTKTYLKPLPPKPALHKFTV

>SG-AL_contig_414_1_+2_536_718

MVPHITFHPMKYTVGVQVQVHTYIYIYIYIYIFSTQCSTVVCKQTPARHFIFANFSTKAV

P

>SG-AL_contig_415_1_-1_202_381

MTGSSKKRKICTHGSVQYTYEVLNIFFYFFVQNLFFESCQYCVSFIKFSCRQGVMICFFC

>SG-AL_contig_416_1_+1_100_456

MVRTLDFQLEGLEGLGSNLLGYLAWWVRVEIFSIFQVNICTTYDDTSYHWNSASVAGRVE

LSLCNGSSSLGAFYCTGFCAAFFLSDGCTDVILVLDGLAISQSITQPNLSVVLTMTHSH

>SG-AL_contig_417_1_-2_2_721

KALSRIPESLLKKRKQRSEAKEKALKAAEINKKKQKAKKGIIFRRAEKYVREYRARENDL

LRLKRQARKTGNFYVEPEPKLAFVLRIRGINGVHPRPRKVLQLFRLRQINNGVFVKLNKA

TLNMLKIAEPYITWGVPNLKTVRELIYKRGYGKVERQRVPLTDNAIVEKQLGEKDILCME

DLIHEVFSVGPNFKAAANFLWPFKLNTPNGGWRRKYNHFNDGGDFGFRDDKINALLRRMI

>SG-AL_contig_418_1_-3_117_2108

MDDEHCGMDTLEIQRIVGFSGCVPNGLQVHPDCKHLVYPLGCNVIIEDLSCGDQNILSKH

TNNVSCIAISPDGRFIATGQINYMGFKAPIILWDFQTKQPVLQRELHKVKVEALAFSSDG

AYLISLGGRDCGAVVVWDLCKGEALCGSPAQVESAGITRCLAASKKQFDIFVTAGANTLR

VWQLQVDNRKIRPTDVKLGGGLKREVTCLEMPDKCAEFPYMLCGTTTGDILGIHLGSNQL

QFVLPQKDLFSLGVTALSIVKFHDNCIDVLVGTGDGLVGRYEMKLSLDKRNYVQGSFKHH

AGVKPWQDNKLKKRSAVTSIAKRGAGHMFFVGTENSQIYRFNYAELSAALIKTCHSSPVN

DLIFPFGFSELLVTCSFEEIRVWNTCSGQELCRYTVPNMTCNAVCITRDGRSIFSAWDDG

KIRVIGFRQEKDGTSKLVEKYTVRDTHNKGVTAIAITSCGTRLVTGGGEGQVRVWQICED

NIISRSRGDLVANMKEHKGKVTSIQIHPSDRSCVSASTDGSTIIWDLESNSRSQIVLANT

LFKCVCFGAKGVHILTSGTDHKIGYWEVQDGSALRELEGSKTGAINTMDISADGQAYITG

GEDRLLKLWKYDEGQVTHVGIGHSDQITKARICPNRHLIVSGGEDGAIHMWRFPNCMLQQ

QQRM

>SG-AL_contig_419_1_+1_235_330

MLTVKPYCSLQKGDLSMTYIYICTEVKSSPSC

>SG-AL_contig_420_1_-3_48_275

MVNTAAMEVTSHLYTVTQIFPPIINPAAMEIIHPIINPAAMKNIHPITHPVAKKAKPVDW

GKEERFWTVNCKQKPP

>SG-AL_contig_421_1_-1_169_366

MVYKMNKPSESESLPLPLPLPLPLSLPHHSLSVSPPLSPSFPLPPSLPSSLSDSGIGAIQ

WKKFSQ

>SG-AL_contig_422_1_-2_122_865

MTDYEEEQRNEIEALEAIYPDEIDILQTSPHHVFTVKVASQDPDDSFQENIDGTPEKVGC

TVQFTYTPTYPDEAPVMELTSIESLEDDQVEMIQEFLTEQAKENLGMVMVFTIISALQEK

LTVLVEDMKKFAEEKRIQKQKEEEEAAQKRFEGTRVTVETFMAWKTKFDAERNERKRMEK

DRDVLSKKPTGKDLFMTDATLNDSDINFLQDEGDNVEVDESLFEDMDDLDLDEDLDDEDD

EDDPDYVP

>SG-AL_contig_423_1_-1_1543_1836

MHKIFKVEPHNRFERQDKSLWQKRLFSDASISELTTHFGTRKLGCFLCQCLDSGLLYDIQ

FLLAVLSDRQCQIFSKFFNFTSRQEAHSCLASLKRFAG

>SG-AL_contig_424_1_-3_387_878

MPYLTADSAIKLSYNLVLIRRDAILSASILPPEDFAALRSVPVQPSSFFGPFAEPFVSAQ

AAANRDSATLSSWPRPPGHPSAQVRRPAQPRKPPAPPRAWRPVGSQTDSRSRQDFRRGPA

AKPKQKPQPAAPGPDSCLPPHHPQVTRAWWAACPIMPRRGGGCT

>SG-AL_contig_425_1_-2_629_1009

MLTKVSSRHGGNDPSPFGAYQGAGRGKRSSLKFWSSAMLQRLFDKAATAARSSPLFPSLE

TLTCQLRAPFPRVGSSRTTAARGASGDTVNLMPSPSTLQRCRRLHRSASASATVPFSALA

ELDSLAR

>SG-AL_contig_426_1_+2_2_295

GGGHPGGGGGASESGEEPHTSCIRLPRARMQRLEKSAWSWAVQARLSSSARAEKGTVADA

GADLCKRRRSCANTSGGLMNRALASPDTPLTAVVPEEP

>SG-AL_contig_427_1_+2_143_328

MNTHQGTNLRWNSSSNTYKTIKYREREQNSNNEIFAAKTLHVIIPFAFFALLLSVFFPLF

HR

>SG-AL_contig_428_1_+1_178_378

MRLPSEPNACFKPYGSPPKGDLSMRLLSDILKETISFFVCLAATRVPMLVHLIRWHKNLL

VKKTQHS

>SG-AL_contig_429_1_-3_60_1229

MSNPVFIEIAVPDQTLELRVYLKSLGAEISEENADSGILTDLKNIIEASSICWKELNSDS

DVEMVFNGIITLILVVPESDAESVVTLFCEKVGKAPHGDKRGVSRLRMLNNLFNNLDDGS

RNRAVVLTSMVRLAGQTDLLNMVPTDVEKIRAWTTKWELSTAKLQNLYRTLYDALVECKA

SEQATKVMIELLGTYTEDNASQARDDAHKCIVTCLADPNTFLMDHLLTLKPVKFLEGELI

HDLLSIFVSGRLSQYQQFYKNNTDFVQSLGLNHEHNMRKMRLLTFMQMAETKKEMDFALI

QEELVLGPQAVEEFVIDVLKTRAVNAKIDQMQKKVMVTRTTHRTFGKHHWQQIRQRLAEC

DENLSAVEGQLGTLANIKLQQQQMMVAQQT

>SG-AL_contig_430_1_-3_3_155

FSFLYCHLLFSAFPVHSTFFFPASLHTVFSPPPTPLQFMMYYLCCFALATR

>SG-AL_contig_431_1_+2_1094_1426

MQTTEYIFVMCFPKEGQRFGLDCIFFGRNGKKFHMKCGLSPWREHVTKERHHSFKKKKKK

SSVCMYFCLPMTVDFSTEFCQGQLFFIAVGSLHALSACCTQDLGLSSHLKD

>SG-AL_contig_432_1_-2_776_1141

MVRTLHHAEVLSSDASTKKMECKHVCRTLRNNGYPRWMFKVPRPKKPKPSEAEISVGISY

VKRLSEKLSCVFQQYGARVFHKPFGSLRGRLMHVKDRTDKSKVCGAIYHIQCSDYPGDYS

EA

>SG-AL_contig_433_1_-2_497_1183

MGSIFMWCCRRFRRGSSPDAGKTDGEDSGKKGGKGRQSRSKSLPFDGSSKSSKSHKKKGA

AGEEEKIEDLSLVKREHKVLQSYQKPPPPRNRARASRVPRALKTQASTESLDIFQEVEES

TTAILENVSKKTGKLDDNNSPSTSKPQSDKDEGIQFSTPSAHENLPNLSTKVSAKTEDAT

VETTKDSTGEAEEAKGETEGATRETVKDAHAEDASGETTKEATGETTKE

>SG-AL_contig_434_1_+2_1775_2011

MNISDQTKQTVKRRLQKNVQLEAVRLAPAFPCGTDGMGCHPCLGRLLVQGKIIFIQSSPS

EGKSLSCENSPPRKVANIN

>SG-AL_contig_435_1_+3_390_689

MTGALRWAVNRPVFMLKAGIELQTSHKKTNTSTTWRMLLDKSGRSGPQLGHNHSNLRFLL

LPSSFVCPLVLYYHGSIFSSYKNKKRTTESNLAGMWHMNR

>SG-AL_contig_436_1_-2_2_190

IPACNIKVGLFAAHLDAPVILVVSVFHRSCSLPILMLQSFWWCQCFIGPIPHPTSISRAL

DFA

>SG-AL_contig_437_1_+2_2_343

QRRLQVALYPRSPSSCARTYAASPSFFPATSSQQVALLRAPASTPPLAFPGVFVLRSPVF

WFFACSFLEFRVISLPGCVLPLPTAQSRGETYLFPLLFSLSGASKFLFLKFLHF

>SG-AL_contig_438_1_+3_249_584

MSAYIPLGIQFYIRHTAKSLETKTFATINIMIPVLPSTVYQALESERDRKGEREGGGRER

EKDGQRKRGRKRDREREGGEREKERERETKREGVRGKNRKGERGGGGGRERG

>SG-AL_contig_439_1_+2_2_454

FFFPVIYNHKFISDRLFFDSIAANWVQSLFALLLHCLQTLLVWCQKAAHRPRLLRPQVLW

TVFLGAVEFPQVLLLGLVHHGHQTSDGFAHGMDFCQLGGSSTSHLGNPKGLKFRFEFLQL

LEEFILLLAPKVLCLNLTHVSCDYRGYMGWE

>SG-AL_contig_440_1_+3_3_176

PICSYFLHFVGYITACESLGHFHSNSNTEDSLTGCTFNKSHCLFICLFVSFFLYYKSI

>SG-AL_contig_441_1_+3_1416_1676

MLTMLSDHQKLKMLIAEKDETLQGIVQKEMTTHNYLPITETVKQPHAYQQKCKLWQDFVD

TTLQSEGEIRQQQAFQVLSGIRKLYAI

>SG-AL_contig_442_1_+2_2_139

RQGGKAPVTKWLTWRRNKWPCEEYRSNFLSHTKCQNKFSHSKTEIY

>SG-AL_contig_443_1_-2_1970_2200

MAWRLFPDSAIQHCMIMRIKRYLQNLMKATVFVIFKLNLLVYLLSGFISFMIYFIYMFLI

WHLHLLFSSFPCLVIIR

>SG-AL_contig_444_1_-2_737_940

MFLPRERKKSTSLSPIPQENRKMSYRCFYCIYTLRLNVKFFFFCFCVTLFFFDCAIFVRD

DVCVGDAQ

>SG-AL_contig_445_1_-1_589_960

MGSAHISGSWTPDKARLDINMLEMEAMSHSLLAFAHLLWNKSVRLFMDNTTVACYMNKRG

VHSSALSTRAESLLRWCRSNGIALSAAHIVGKSNILADSLSRGHSIIHTEWTLKWQTLQR

VWDK

>SG-AL_contig_446_1_-2_398_631

MFLSAVFYELLMAVFLSLVTIRSTHLFTDVFTLLNTAKHLEICSATLLVVNLDLYIDCDG

LWEANWMTSLRVIFVCVQ

>SG-AL_contig_447_1_-1_334_648

MVQYNQKEQYPQKAFSYFFSELRQAIYPLAVFCIWQWQHLGISNCVVKNSPDKALFRGVV

GVFWKTRHEIDCAMTGTTYQDALWCKDTGHVKCKSAFCPLSLSAK

>SG-AL_contig_448_1_-1_94_354

MFCEFWLHCLIYSDSIAILGLLSPSWLQLYFKIKPLKLIKSYFLYTIINHMFISPAHTHT

HTLTHLHATHTHTHTHTHTHTHTHTHM

>SG-AL_contig_449_1_+3_1929_2150

MPFIACNAFWCIPFPLDESKSETSMSKKSSNFAHTHTWTFLLQPISNILIPGYFFTADVM

QIKTKTFLHSYFHT

>SG-AL_contig_450_1_-2_17_1555

MAVVIESADVVRLIEQYLKENNLLRTLSVLQDESGISLNTVDSVESFTSDIQAGHWDTVL

HAIQTLKLPDNKLIDLYEQIVLELIELRELGAARSLLRQTDPMIMLKHQQADRYIHLENM

LARSYFDPREAYIDGQTKEKRRQAIAQALAGEVNVVPPSRLLALLGQALKWQQHQGLLPP

GTTIDVFRGKAAVKEQEEEKYPTQLSKFIKFGSKAHVECARFSPDGQYLVTGSVDGFIEV

WNFTTGKIRKDLRYQAQDNFMMMDDAVLCLAFSRDSEMLASGSQDGKMKVWKITTGQCLR

KFEKAHSKGVTCISFSKDNSQLLSASFDMNIRIHGLKSGKQLKEFRGHTSFVNDAVFNQD

GHQIISASSDGTIKVWSMKTSECVNTFKSLGGTVGTEITVHSVHPMVKNPDQFVVCNRSN

TVVISNFQGQIVRSFSSGKREGGDFVCATLSPRGEWIYCVGEDMVLYCFSISTGKLERTL

TVHEKDVIGICHHPHQNLIGTYSEDGLLKLWKP

>SG-AL_contig_451_1_+2_2_157

GGESSLGKGRGWGFNSGTKTGWERLFTNTGRFNQTVQPFTFSFLKLPVVNVL

>SG-AL_contig_452_1_-1_64_339

MDGGPSPGHVKELKEKDDKAMKELENKYTRFMKDNGVVGEFKLVPGGKDAWHQIIKYQEK

VGGVLIVVGTRGAGAIRRTMMGSVSDSVVHHA

>SG-AL_contig_453_1_-3_372_728

MKVVLLFFNFFFNVKCWIRSTQLYFLNRKKKECGHNHSCKRSNIHTVEIFIQNILASSSH

LFYCLTYYSDFMYITFKTHLVLTIFSPTFAPHTGHLYGLETISQKASYSQLDMNIFKSD

>SG-AL_contig_454_1_+2_2_550

FFFSSFHSIYSVINSYTSIPTLTQASQIKKSGSHESFSILYWTESDSSLILLRAKASVGV

CHFNGQLLCTLHNGLAFAAGHTVRNLSREAAVLHQQHLQLLNVSDDELPEAIGTKVTRVF

GTSVTNIGHQILALETTPHSVVNTLGFAPVWLDPQVSIRLVTDKFFGPFLDNVRLDKRSK

SHD

>SG-AL_contig_455_1_+1_889_1206

MITSEAKSPGHVPLMNFLISISSNVRSAARAANPSWVRWAVCQEFHPLVTPILYGACACS

YVSGLSWLMVALGTPARMFFKAHGVVCKPRNTSKATGGTVGSLTAA

>SG-AL_contig_456_1_-3_1758_1964

MLLNFVCQCCCCFTWVGAVFHQYANDEIGNTSLHLLLHFFFSSNNTGDRKLIWLFVDTVT

NWMCSGNGN

>SG-AL_contig_457_1_-1_1711_2010

MLCQCHSLFEGLQQQFVCVCVCKQYWFCGPQSIHICCLLGWGMIIVIFFFFSAYVSFLGW

CFYRYMCTSLLYVQPMGWYLYRNICTCIAAVSTTHGLLFT

>SG-AL_contig_458_1_-1_181_966

MGYRGRRKSVKRVTAPKSWMLDKLGGNFAPKPSSGPHKSRECLPMAVLLRNRLKYALTYD

ECKKIVMQRLIKVDGKVRTDKTYPCGFMDVITIDRTNEFFRILYDVKGRFVVHRIKGEEA

KYKLCRVQRVQVGPKGVPFIITHDGRTIRYPDPLVKVNDSVVLDISTGKMKDFIKFDSGN

LVMVTGGHNLGRVGTITHRERHPGSFDIVHIKDTLGHMFATRLSYVFVIGKGSKQWISLP

KGKGVRLTIAEERDRRLAAKAQ

>SG-AL_contig_459_1_-1_55_219

MVVVCSVLFLYTWNCQLHRMVISSKYTPIICQHFHVVFVQSVGDEVTQVQSVLSV

>SG-AL_contig_460_1_+2_2_367

VLPLYQIQSCFAAGSTFISNTVIFCTWFYLYITYSHILHLVLPLYHLQPYFAAGSTFISP

SHILHLILHTPLMGFISPTQSHFASDSTHTHIQPYFAPDSTYIFISPNHILQLILHTPLY

HL

>SG-AL_contig_461_1_-1_202_486

MKKLSENVFYVFFGSEWVCPSLFCYEENFCQLCNIAVDFVFTRSSVDVIIKTQVLEIVDL

KKKKKCLQTASTTTKMIWSELSWLVFKSLPSLGVS

>SG-AL_contig_462_1_+3_93_200

MLFTAQLTMKSTSELNKIESNTKETTNELYIHYIYV

>SG-AL_contig_463_1_-3_207_3584

MHGYFVTPPSYEGSVEKGQEGMDTEPQKIMAVNGWVMGDDPLRNFAEGSNVYLRRELVAW

GDSVKLRYGQKPSDNQYLWNHMKTYAETSAKIFHAIRLDNCHSTPIHVAQYMLDAARKVR

PDLYVVAELFTGSEALDNLFINKLGINSLIRESMTANDAHEEGRLVYRFGGFPVGSFAQP

RVRPLVPTVAHALFYDQTHDNPSPIEKRSVYDLWPTAALISMANCAIGSNRGYDQLVPHH

IHVVDEKRQYPAWGNQIQKCESVNLSIGITNGKRILNNLHYELAIGGYKEVFVDQLHSEV

TAVTRHNPLTHQSVILVACTSFQNPYDPAVNYQFRSVKVEGKVDEVLFEGSMRCTQEYQY

NKDDHFVNGLPNYNLDIREHIPLSSSQFCHENVLDNNGLREVCFKNITPGTVVAIRTSLS

DNAKEAVLELRRGMGQFGYMMRSYSGNTTFDDTWDKSNFRAVVSRLTLGDLNRVMYRVDQ

EERDDNFGFSTYHIPNHGNLCYSGLRGVMSVLGELRPGNDLGHPLCNNLREGNWLMDYTS

NRLKVVGNTLDLGEWFEGMFHHLKKIPRYLIPCYFDAVVTGAYIVLREMALQQYSDFVKD

GSTFVQALALGSTQFCGFVRSAKLPPLSPQLDPRARSELNIKTGEEEEAPVSMAAGLPHF

ASGVWRCWGRDTFIALRGMLLVTGRFDCARGMILGFAGTLRHGLIPNLLFEGKRARYNCR

DAVWWWLHGIKAYTMMAPNGLKILMEPVARLFPTDDSPCLKPGECDQPLHVVMQEALTRH

VTGVKFRERGAGPGLDEHMSDAGFDVEIGVKWPEGYVYGGSVHNSGTWMDKMGSSRKAGN

WGKPATPRDGSAVELVGLCSSVVHWLAELSKRDEYPYTGVTVIQPDYSDELLTFEHWGYT

LRQNFEKDFYISTQPNPANEPNPELIVRRGMYKDCLNATQYWSDYQLRPNFCVAMVVAPE

LFTEENAWTALELAQEVLLGPLGMKTLDPSDWAYDGNYNVTNDGDDPKLAHGFNYHNGPE

WVWPVAYFLRAKLYFAIKLEKKRKGILKETAEFVDSVLCRHYQELMHSPWKSLPELTNTN

GSYCADSCRAQAWSDGCILEVLYDLERIETASRPSIINFQASMGKA

>SG-AL_contig_464_1_+1_1345_1581

MRPSGQDTPYRKAQRLLCKGSKNINKRTRLSKDEGRRRFTQCQPTTDGEDSRTVASKRLF

VRWRYLFLGHFMQIGRAHV

>SG-AL_contig_465_1_-2_2_103

TLPSTSIRRGHGHVVSGHSRGLIVSYLILSTLNI

>SG-AL_contig_466_1_-2_467_733

MISLFFPIPLIHKSMTLNLIHWYQNSISLSAFLLCYIFQWEHGCQVLCKARKYVRPFVKM

RISLTFLFVFLFSFLFSIFIYLFRNEEDF

>SG-AL_contig_467_1_-3_153_356

MDSKAPMIKLARVTKVLGRTGSQGQCTQVRVEFLDDSNRSIIRNVKGPVREGDILTLLES

EREARRLR

>SG-AL_contig_468_1_-3_105_215

MGGDSSSSSLQFYLPFHIFTRHLPFLPSSHLPLHPSH

>SG-AL_contig_469_1_-2_35_7861

MGAEARPEGSDLVGRDDDDDKLSSSSSDAAQLEDVEDQDAEILERSEEKTEETDVEEQFS

PLSKGSPSKQVFSFSEALEMDLHSQICSEQTSHVPQAAEEFDTFGKIQTVDYSSRISAES

SRKHESLVYATESFEQFEDEMAQIPEKSHIIEERVSYHVTEFSQIQSITTTEISSDRTDI

QETQEPSIPEAETERQEQFLSKEKETTVVSSVIEEDTTVELDQQIISETETDRTEQFIVE

EKETTTEELLSKTSGAVAEPESDTDQPQTSKVSRLTYMAFDNMGFTEEDMSEESHRDGHH

KLSKQETREISPEDLEEKPYTFDFPDDYKQELEAQKQYEAAFHPEEWVASEHIDEGDSGE

EEEEEDETVDREQESDVVLSYEATTEDSGAYLIEVEEKDVPPEDFPQHSKSVSDQTEPDA

QDVEPEQPVPIGVETSGEVAETSSESSSDNDVEVFDEFTGTYIKMPWEIAHQYKRQFSES

YIHQEKKISSLHQNQSIDLGSFYRREDSTDYPSVSFDSEGQEPQEQQPKRTKVTFDLSYD

ETCEYKQYEMEEETVLEEDVSKPEKVFEPHDQIEHTHEEEVITSIQESRQQVKEELTKDT

GFESDYLDSQLTAAGATASHFESTLFQSAAEEVISSYSTAESSLEQSLEMRPSSEAEEDR

APVKLQPTEPLMSVVSMESRLAQQELLQEMADEKDRSVSPSSEESPSAFTAQVELQSAIV

EEPEPEPLTSAAQVPTTFPMAVHSLGKSLADNEVYESSETERDSTEEKLSPIDEGPGVDD

IEDDDAPAKSVPSEKRVTFQTDTLSTPRASEQSADEFKMSTSSSEMSVEPTLLAASYELD

TGRVCHVVSAFDISPDSVEKKLTPNAPAKSILSSPEDDVFETDASLLLHSEMADTPTKDD

SEDLTVTKQLSSDFVPSPPTSTPRDGGIMHVHTASDLAEASSVLEHIKTEVTEDEEQEAG

HTLDESTVHEEDELSSPFEMMSPSELKETTYMAADTCFGQMVTSAESAASGSSFADMLSS

DLKEDIITNGPTEVEYVAEYDTEAVEGQMSATDAEVKEQLEEAMVSETECEEQEEESGAV

AAVWATEEEKAPSGAEATLSELQLPPGTDVNTMSTSEQTLYGLEMPSEETVTMSTSLQRD

LMSKSEVDQDSDQTQPPTEVTFKTDEQVSDQVETSAEMRASALFESDDMTSAEATPVGAL

PEQQQVVEGREDEEEEGAVEDFEEQALPHAEALIPEETEDEPYQLDPGSVYPGKRALEIQ

IPIDIDIKEDPQQYIEEEEGQETYEEEPVPERRLEDDFDLDDDNLLGEAAGGEEEFMEQE

AEEMEFMEKPEEELLRLKDSKPESEDEDEPQQETESVQATGLLGVQGYDDLERPLSPTPD

AFRQMFFGRDDDKETESPAQSGAEEDIEKTASHFVESILEDVKEKVQSLSPEKPVEEKEY

EEQMKEEDTEDAAVEEDRKADLEESEEDLPEAEQQKEDTEELDDQVLPMAPTRSQQTKTT

VLMKQVSEDIPGITITEHLSADTEETEDEEADETDFLTSLEKDVPVQCVPEEEEEKKKKD

DIDGSVFPASSEGDFHVQCIPEEEEEKKKEMDETDFPISSGKDLPVQCVPEEEEEKQKEV

DETDFPISSGKDLPVQCVPEEEEEKEVDETDFPISSGKDLPVQCVPEEEEEKKKEVDETD

FPMSSEKDFTVQCVPEEEEEKKKDDVDGSGFPMSFEKVFHVQCVPDEEEEEKKKDDVDGR

EEELYFVETSVDAEMQFEMTFENEPQSKTDTKKESFLVASDPASAHTFASVAPLPLRDVF

SSSKVEAGAGSDEEELELMEADIKMHDYAEPLSPEDHGDSSSVDSFATVVAADAEEEDEE

EDERDVEDRLAEIASMSSSMHSDILITQAEMEERFEETEDDEVKFPQEWPKPKELAKEPD

LSSSTESDRYDSKDNELSSSVESDRYAGKEELSSSVESDRYGSNVEMSSSVESDRYEFVD

RAALSVITELSEEEQFEMIEKDDLGNETGPESDPQEQFHCSPPEGPSRSPGFGSTRYFTK

PLHDKDDASVSSSLLEFERLEKQMGNSGSSSIDTSDKESIGGSYDERKHHHYLVSSFDDK

KFEYGSFDEKKLIRYKREMERDDISTTSSLAEFEKLERQIDQQGSSSSVEEKFTVESKSS

GGKSGSGSGSGSVAGSVSSLAEFEKLEAELATDSDDRRSSMDSSLQRRSETSSLASLNEF

ERLERDMVANNELEAEAQKIVSYLEAGSLDSGRMFYYSSSVGYSSSDLSEGEAAKQEHDK

DSLEGRDAVEQDSLSEGEDKEELDSLDGDTSEMTEMTSSVIFAGPAEPAASTAGTSHDLD

ADSLAGECIMQLSTDSLVLTQQMRTSDSSKFDTDSLQEQEDIMVRSVDSLEQEKHSDKCD

QDSLQEDIMHTSADSLELVQVEEQIPENLMLMSVESAHWSMGSSGGTMCRSEESHTDSHD

FMQVSAESIEELKSSHTEQTLKSQMSKSSEYVKEGASIEMMKIQMTKSSEYVKEGASIEV

MHKTHATNPFLDDDGNVMESSGYSQEEEDEEEEKESPFMHWGPYKEEKKVYTMAEWEAMK

KQKLREKEERSSSPQTAEGEPGGDSIDRE

>SG-AL_contig_470_1_-1_1_255

RQNKVFEGRQLGRHLINPVLQKLSVLMGKLHSLKMSVSSRAERSGHTGTYILQDTLDMMQ

LFFQIQMCISAILCCHTNKTGEFIC

>SG-AL_contig_471_1_-2_1028_1294

MFHRSCSTSPTSHPPPQPPCALPAHGISCSLWSLPLYQRQIWVKRIWNCPSAMNPSRTNS

EQSLALEGCLKLGHMALILSFPSCSKRKK

>SG-AL_contig_472_1_-3_234_437

MLDAFADKNLALRMRLLENETNSWQMLLSKQCTKETDTNRLMACGKTQNYWILSLKAFPH

LTTGICDV

>SG-AL_contig_473_1_-3_174_683

MATGTTTQGPTTAGMIQNGAQGTDNQGPEVEEPEEEVILEGEELFMTLTPEEQDIWQKMM

QERENALQAGKEHFEQWKDRFNRRRMFAYSRARKLHESYRQHSSFYADLSVYLASVQSKS

RRNSKVKMSTRKSEESGQTPSNTQPQQQEQLNQQQQQQVQQGEQQQQQQQ

>SG-AL_contig_474_1_+1_121_558

MYTRKHQIQAEGRFIVATKDNTDYFLDDSSSLAKDFSISCFFSAILSSRLLRASLTLVRR

ASAWSLSSLVLAFSAFLLWMNSIRMRLFLKPLPLLFRYNSWYRCLSIFLASRYLRSRFLR

TRMRRIHRILTGIRALAVPLRFPWPM

>SG-AL_contig_475_1_-1_103_468

MGHGKRKGTANARMPVKVLWIRRMRVLRNLLRKYREAKKIDKHLYHELYLKSKGNGFKNK

RILMEFIHKRKAEKARTKLLSDQAEARRAKVKEARRRREERIAQKKQEMLKSFAKDDEAS

KK

>SG-AL_contig_476_1_-3_6_683

MSGGGQWGGQQDPFYSPVKTESSLGGGLASLTACSLEASLPGFTSSTSTTTTSPSHHHHL

ASSSTSSSTAACKMEYEGAGMMEGGHYSGGGMGAMVVSPSRSMRTLSESSTATTSTMPDS

PGRGSGRGSGSSLGKGGSKRRQVPKGSEEYKEKRARNNVAVRKSRAKAKEKQRMTETRVK

ELTDLNESLQKKVEVLTKELTVLRRLFVDVGATLPDDLSKLLGPQS

>SG-AL_contig_477_1_-3_267_1241

MAPVPRIGIGMDCSITPLRHGGLSLVQTTDFFYPLVDDPFMQGRIACANALSDLYAMGVT

DCDNMLMLLAASNKMSDKERDTVMPLIMRGFKDTAEQAGTQVTGGQTVINPWLTIGGVAT

TVCQPNEFILPDNAVVGDVLVLTKPLGTQVAANAYQWLDQPERWNRIKLVVSEDEVRKAY

QRAMFSMARLNRVAARLMHKYNAHGATDVTGFGLLGHANNLAKVQKNEVGFVIHNLPIIA

KMAAISKACGNMFGLLQGHSAETSGGLLIALPREQAAAFCKDIEKQEGYQSWIIGIVEKG

SRTARIIDKPRVIEVPAKDREGELW

>SG-AL_contig_478_1_+3_180_329

MHGMLPSVYFLSALESRAINSMLFQENLAILSKNQDISFSTIQFFLNHVE

>SG-AL_contig_479_1_+3_855_1154

MCSNDSRRGSHSSQCVVMMTVGEGSTVHRAPQFMMYSNDDSRRGPQFTMCSSDDSRRGST

VHRAPQFMMYSNDDSRRGSHSSQCKVMMTVGESPTVHNVK

>SG-AL_contig_480_1_-3_36_227

MGCSSTPFLSRRTHKLGRTSRLCQRIGIMCRHTTYAMIILDLFPSLGFAAFVHKEFPSSV

ETLS

>SG-AL_contig_481_1_+1_1_141

NDHGKSISPCRDPTCKTVAHMGDQTHKLCVISTAFLPTELAGLTEVM

>SG-AL_contig_482_1_+1_460_750

MTFQFTAKTYTTERCHSPRFCSMLSKTYFCILSADVLWIQLTPEDMGNITQTPTHDETRT

QTENKPMKHCKTYLPLVMHTTVKSNVNKKLGCLFYVV

>SG-AL_contig_483_1_+2_554_817

MAECVAFTMSQASSSVSESSSLTLHVGRHSLTGSTRRFHVPCLVMKVTRVMLSERTTGIK

EHLSMKKDSLDCNRGTLCTHWTHILMAK

>SG-AL_contig_484_1_-1_79_2235

MASGNTPGTGETQPREIKVLETAEDIQQRRDEVLQRYAAFKQACSDRKALLEDSRRYQYF

KRDADELESWILEKLQTASDESYKDPTNLQAKIQKHQAFEAEVAAHANAIQLLDETGNEM

ISQEHFASEIIKERLEELHRLWELLLSKLTEKGTRLQQALRLVQFMRECREVMFWIADKE

AFVTSDEFGQDLEHVEVLQKKFDEFQKDLQNHEDRVSEVNTLAEQLISDCHPEEETIRQK

QDEVNEAWKRLRQLSLLRQERLYGAHEIQRFNRDADETIAWIQEKDVVLSSDDYGRDLAS

VQTLQRKHEGVERDLAALEEKVNALGTESVRLQGIHGNQADQIHSKQEEMEQNWEQLRNK

ASDRKSRLDDSYFLHRFLADFRDLVSWISDIKAIISADDLAKDVAGAEALLERHQEHKGE

IDAREDSFVSCAEAGERLVSSNHYAADEVREKLGILASEKKGLLELWEERRIQYEQCMDL

QLFVRDTEQADAWMTKQEAFLVNEDLGDSLDSVEALSKKHDDFEKSLAAQEEKIKALDEF

ATKLIEGEHYASDDVAQRRDLLLHRRDLLYEKSARRRDQLELSYQFQLFERDCDETKGWI

NEKLKTASDESYLDPTNLQTKLQKHQNFEAEVEANRNRIESIQASGNELIENDHYAKDTI

RKRVEEIERLWETLLAQTEKKGAKLTEACQQQQFNRNVDDVERWLNDIESSLISEDYGK

>SG-AL_contig_485_1_-3_30_446

MTRSNCHMTTLKFFPQKEARNKRTRTTYTVENNNKLVKMMCGGTTEARDAAGQKDIQDLC

DKFRKEAEEKTKKTFAQYAAIKYKTQVVAGTNYFVKIQVDGDEHIHVRIFQPLPCNTDQS

SELVAIQTDKSLDDELEYF

>SG-AL_contig_486_1_-1_1_114

FSSLLFSAPFGTWLVQNWEAERKDEMFFKLSAKLLLNF

>SG-AL_contig_487_1_-1_34_1677

MSENDVKRSEEMTLPQETCISAESSTMVPNKLVTDGIPTGADANITEMGTTAETSAMAAR

IPNEGSRTPSGPNGGYKDQEKSNRTPGRKRTTPNATGLKTPVSPIKEPKGSKRQSQSSSG

RQKKKVRRSLTFSSPIKQEETLMATENIANALTNSGPSEPESAAGSTNSTADYLSSSSST

STTLPSISNLLHSSTNAGISINIHVTNDANNKVRSVGHTNSEDVVAGRMLQNTLDQSSKG

TVRADVATRPGEGTPQLHYSVPRYTVEWHGDYHHPEPEEGPVMDRAPCRYQEHVMPVPAA

PNTTASRFRYPYRDPNNIHGYYSQHHPYPSPSSPLDRYYTADYAKEWSEMYTKYPSEHYP

SGYEAQLPDVSTAYQAAQWSSGWGRNTDMFIDPYQKPASSRPNMELEMRDIPYSFYHKAT

APYPRVQPGFPEEVYSQHPPHYGGYGGTSAMVSHHHAKERLVAAVPEVMQEARQPENNCE

SHRLHQPWAMASCTQGPQVLQEGSVGDLGPRAHSPQQLQPPVECGVQESNHQETGESTSQ

LPEEQSQM

>SG-AL_contig_488_1_-2_2_145

SDTVSCNVRILFDSRNSQDKEAKLYFVSKTNLYSFYTSGCDALCGAEA

>SG-AL_contig_489_1_-3_3_203

TACCTKTLFNGHVSCNLKCQLYTHGLIQELAILTKFFILEEYWWFKQSNFHCQTLFHVMK

KADLCHS

>SG-AL_contig_490_1_+2_1217_1621

MPKDHVSSATNHSPLSYHFHQTSFESSSPHTTQMRISLVQQNFASTVQNPCFTTNHTSIF

QHPAYLPCPTFDAQKADQSQKGKKKNNQIGTQRGWVVDHTRRKLFAQLQSTHIFNTDMAY

DYSTHLSIRELIAQL

>SG-AL_contig_491_1_-2_137_3871

MALRAMEARWRTVYLCVLCALGIQTSGVSSQRLDSASSDAVREIDLLSAIGIPEDTKVGV

TYTAGLDGYPTFLITSQAFIREPAQLFFRQPLFSEFAITMTVQPYRREGGFVFAVVNPYQ

TIISFGVQIMEYDSTQQNVALYFTPNTRIAEQSDVIANFTVPSMVGRWNKLSFKVLRDRI

VLYYNCQEYEQAYWFRTVENLDFEPGSSLYVGQAGPNFQRTIPSFEGGLQELKISSNPND

AEDEDCDFEISGSGSGSVVIEQPTGPTYTPPPWERPDEKLQGQKGERGLPGLKGDKGDQG

PQGLPGATHPPPITPPPPDIELLRGPKGVPGDPGFPGEPGVRGQKGEPGEPGLNGIPGAD

GRDGEKGEKGDMGESGQPGLTVRGPPGPPGPPGRPGSLDVIPELEQGPQGEKGEPGEPGE

AGVGLPGIPGPPGERGGPGLPGPRGVKGDAVLGPPGVPGISGQPGPPGPPGPPGTPGFVL

DTGSGDGEEVPGPPGPKGEPGRPGLMGRQGPVGPEGPSGLMGTPGVPGLPGPPGMPGTPG

LNITGPTGPMGPPGRDGLPGSTGAPGMPGVSGAPGEPGLPGREGPKGDAGQPGLPGSPGI

DGERGLPGRDGTDGLPGRPGNDGRAGPPGPPGPPGSGSGGGIIFGSGMGDTEDIVGQPGP

RGPPGPQGAPGIPGLRGLTGEPGMRGPIGPAGEQGPRGERGFDGEPGIPGVRGEAGVPGR

PGEAGAAGTPGLPGTKGEPGVPGISLPSEPGIPGTPGSDGRPGERGFQGPPGPPGPPGPA

GPAGRPGSGTGGGFSIPEGSGDFEIIGGKGSPGEPGIPGLPGPKGNMGLAGAPGLPGDVG

RPGAPGIPGDPGEPGLVGLPGSKGSKGDLGGVGPAGPVGPSGPPGPPGKPGVSSSGGEVE

VIPGEKGEKGERGIVGPSGPRGFPGPAGEMGKPGFPGRQGLRGRKGDMGIGMKGAKGEPG

GSGVFIPGPKGSKGDSGVPGLDGRQGPPGPPGPAGGPGGSEPGIVLAEKGQKGEPGLSGR

HGRNGEDGRSGPQGLPGLPGDEGPRGPPGPPGPAGVPAIGSIGSGDGDIVLRGPPGLPGP

PGPSGERGLRGLPGIGQEGPRGRVGPQGQPGPPGPPGLGIPGPQGPPGPPGYSGDFSING

RGPPGPPGPPGPPGRPASGLGISVGGGPVVFPNMDALLKVSYNMPVGTMAYVADQEYAYL

RVSQGWRYIQLGQLIPLPSPKPPSEITTTTTPAPIIPGIPPPDYI

>SG-AL_contig_492_1_-3_66_653

MAGFVSKSPVGSSHSGSSNYSGTNNSPNSRSTGSSISGPITKIREKNVYPTPWRQRVKTS

PDGEVTQIMSRTDADGLKVTKTKVTRDLRSPRSVNSPRSVGSPRNINSPASLSSSRAGST

TGSRPTSRAGSEAGSDDRGDAEVYTTMQTESRNVGGRKDTTLTYRTHVVETRTVTLPSAT

QTKISRIPKMSQKPRK

>SG-AL_contig_493_1_-3_69_380

MGIDKTQTGPAFAMERKTTDSHTQNWEIFLIHHFAQWNEQVSECGLLKSTLISEHLGIIC

GRMSFHGFIFRIFIFSANIYSFDVVGCLSKSLVADYSWVCAFSM

>SG-AL_contig_494_1_-1_1_228

SSDLQDASQGKVLKRTCCCPCSRPTLSATPVKFRERLKPVLLGASEGRIECACWICSCFH

KPPNADMAYRVFNVHI

>SG-AL_contig_495_1_-3_30_251

MRAVLSPLPHCFTFPSPEEITVFWMQRKKTNESCLLFLLYSLHPNFSSVLYLFVCLLLLL

VMVVAVMVVLLLLF

>SG-AL_contig_496_1_-3_102_1160

MDEDQQQFDKDDYTGLDGQNGENFEQQESQEQAVEDGGDQAMETGGDQAEDDNEDDRKIF

VGGLSWETTTKDLREYFEKYGEVSSCTLKTDLETRKSRGFGFVLFKDAETVQTVLKTKEH

KLHGRTIDPKPANPRQGIKKIFVGRLDPGVPEDDVKSYFGKFGNVAKIDFPFDKMKDQRR

AFCFVEFESDKAVKKVLDQATHKLGGQEVDVKKATPNASQMPGGRGGRGWGGGPGFGGRG

GRGGAMRGRGGWNQGGNWNQGGGYGYGGGNYSGYNGYGQNNYGNYGGYGNYGGYGNDYYG

YGQGGWGGYDQGYGGYGSYDGYDYGGWGYGQDEGGYGKTQKRGGHSGYHPYNR

>SG-AL_contig_497_1_-3_30_335

MSPMRWQGLKVLPSLRILYSHCLCLYDPAVETKIGYLEFPLKPPKIAPMAPTCIKKIKIS

KKSHSKQACSYHQYTQRRKYNINFSVIHSTISNLCNTHPVLL

>SG-AL_contig_498_1_-3_1173_1451

MCALKIGKVYILLYVLIACFVCLCLKVVKYAHVCIMPFCALVWGKLHVHIALIYTLIFKS

GTNFTCILPLFVHLCLKVGQNSRGYCPCLYTCV

>SG-AL_contig_499_1_+2_2828_3088

MEKSFASFLTLNIPLCKIPQWEAGIEALSQKVKITDRYWTSVVGTTAACGATCPCHVTSS

QQHRLHEYIFLQVPEQIATCFAEYMYV

>SG-AL_contig_500_1_-1_13_540

MKVKGELREYKVVGRGLPSAKVRTPTLYYMRIFAPDQATAKSRFWYFLSQLRKLKKATGE

IVSCSRVYEKRPLKIKNFGVWLRYNSRSGTHNMYREYRDLTAAKAVTACYTDMAARHRAR

ASSIHIIRVEEVAAAKCCRANILQFHNNQIKFPLPHRVNQKLHAPRFCASRPHTTF

>SG-AL_contig_501_1_+2_53_310

MQQTSSMLQMVTAKQSSLQMPCQFWKQTLQANCHICKKHFKTSDAPDRYCSRSLHTVASQ

EMKRRIEWRSWEQSMYRKTTASAPQR

>SG-AL_contig_502_1_-2_488_751

MCFVSVCGSVCGWVQGGCFVGFLFCFIVVDGGGGGDLCVCVCMHACACVPLGKRNRDRPT

GTWHCTVQHNMKGNELTWLTQDHRGWRR

>SG-AL_contig_503_1_+2_2_70

PLDYHARLTIITENKIHKHNKTE

>SG-AL_contig_504_1_+3_150_332

MFNGEGRMESVAAISVNLRHFQLPHTHTKTTNQLPHHFTKNDIKINSPVLRSNIKTNFPV

L

>SG-AL_contig_505_1_-3_3_257

KLKRPSPFKKARAFRYVSFKKDVTRNKKALSLPPGTRLLCFVLWRNGSHSVRQVVKLTSW

LAEGHNVVQLPSFPLSTHLLPPPPP

>SG-AL_contig_506_1_-3_42_545

MNMIDLNSVNSQEISILATQIDFMKQRQVMLNSTYNPKSGTSKGVNKVVIDYAKSMLYSV

PADYPDRCFKMSYPISFSRCIPDDAEYLGPSYLGPLGIGMLPYDGWRFRSNNGSLMITIA

VTKNCVPLVEGVTPSVDPRLNGQLYMISHFQLGLKDPSAFDLPPSCFQ

>SG-AL_contig_507_1_+1_1_189

QQQKNHKTVWYQRTLCTQRIVYKSRKIKRSRNRIMAIKRCNHKKLTSLLFDRPHNSSHHN

QLN

>SG-AL_contig_508_1_-2_98_232

MYHSSLNTVHIQSEQFRELASTICFASWKMGVIHDMYTFPHLEFD

>SG-AL_contig_509_1_+3_3_197

LFFFFLRGNNKILVVNAANKTPIRLESEALDEVGSFTDLGSTVDKQGGTDADIRTRTGKA

RAAFH

>SG-AL_contig_510_1_-1_1_300

AATAPVTASEPQPSTSKQTDDEPHTVASEQKIREFLARKRKLESDSDSDLSSEMIDVAKS

DDPGPYGAQQAPVQGMNIIINLMCLQSLFARCLCPDCEKP

>SG-AL_contig_511_1_-2_527_724

MTTFVDMFICTVYYALYMYNYYASFSVILANSLYISHRTACVMTYIMSMCSYGVYHEHCL

VARTRI

>SG-AL_contig_512_1_-1_511_645

MIIHAHAHAHTQKEEMAHCTLSSLFIFTILRWYTAFLRIFELFKD

>SG-AL_contig_513_1_-1_1_135

VSCVWFILKTVSSIHTNQIKFSCSFFCFYRMEILDSGSRVYNNTV

>SG-AL_contig_514_1_+2_26_235

MIKNKDLSVLTQRRKSGVFNFCCAVRVRPPTYLGLRQNSIIVRFTPQIQYFHPIKTSGEQ

SNLIWLACGI

>SG-AL_contig_515_1_+1_517_654

MMRYVDFDNLVVMTLKLCQGHPNFCKFESDSRHYLSYESNYGHFNL

>SG-AL_contig_516_1_+1_1888_2211

MFDPTGLDTGFIVSIMFDPTGLDTGFIVSVMYDPIGLDTGVIVSVMYDPTGLDTGCIVSV

VFDPTDLDTGFIVSVMFDPTGLDTGFIVSIIFDPTGLDTDFIVWQQMR

>SG-AL_contig_517_1_-2_56_298

MLHGAPYWYQRAGIFFLITLNLKSTKHQIDIHFYCHFFSRTGQLVSQNSRSSFIFYQFFV

SVCSRRHHQVGNFMRYATNTM

>SG-AL_contig_518_1_+2_878_1126

MSVKTFVQIKVVTLCAGKWNQGVSTVILPRIWSLFRRDSSHSDSSISAPPKYASSYLADS

LSLCWQGLEIGKGGRDETYETLS

>SG-AL_contig_519_1_-3_624_875

MIAGFPPLITSLNLELKANLTNELTKCYLLSVFPFLKDKLVNFFKTNRQCSHVGYNLVDG

IILLTVRLSSAVFLFSGCSHAFSV

>SG-AL_contig_520_1_+3_252_392

MYLAKLGNFILSTLVVLLVLLLLEFLPFVQRKDINNSVLLTPEVKSG

>SG-AL_contig_521_1_+3_900_1130

MFYRCHKSQTSCKLSIPICVFKTTFWTQGLHYESRWVIIFIYNKRIPVTCFFKTIPALLV

VFFGLFYFFFCCHDKYT

>SG-AL_contig_522_1_-1_118_1230

MSEKHNFGISPVTCHAISGDKAGLALSLNDHEVKIYKKSGAKWQETESLGEHGQRVTGID

WAPKSNRIVTCGADRNAYVWTFEDGKWKPHLVILRINRAATCVKWSPEENKFAVGSGARL

ISVSYYEDEHTWWVSKHIKKPIRSTVTCIDWHPNNSLIAAGSTDFKTRVFSGYVKEVESK

PSSNSWGSKMTFANLMAEFSNGGGGWVHAVSFSPSGDKIAWVGHDSSVSVADSSQDNKLV

VVKDNFLPFMGITWVTENSMVAAGFDCNPTLFTYDAGKGTLSLVSKLDIPQEKAAGTVSA

MSHFRNMDKKASSGEKSAELKTQHQNTITQVSVFEGTKDKCKRIATSGVDGSIIIWDLVS

LEKSIQALKIA

>SG-AL_contig_523_1_+1_1_216

SIQSHFQCFFHAGIVKIHASVHARMVNLTALVHNRSVRHFDQGCFSKWVIIRFWYLCVWS

RIMQNKNTHKKR

>SG-AL_contig_524_1_-3_105_380

MAKRTKKVGIVGKYGTRYGASLRKTVKKMEITQHSKYTCGFCGKDSMKRRATGIWGCTKC

KKVVAGGAWVYVTTAATSVRSAVRRLRESKEQ

>SG-AL_contig_525_1_-1_1_663

ASTLRNEITQARQQIQNALKQPGNQGSGGGTNPQVQKRIDDLEKTVADLRNFVHKLENRI

AVLEKGGKGDAPAASSAKQPAPAAAVEDDEDDDDIDLFGDDDDDDDGAAKLNEARAKALE

QYAAKKAKKPAVVAKSSVILDCKPWDDETDMKEMEKLVRKIEKDGLTWGAAKLVDLAYGI

KKLTIICVVEDEKISVEDLKEEIEGIEDLVQSVDIAAFNKI

>SG-AL_contig_526_1_+2_512_742

MFFPVVVYTYVLVWVWVCITACFGNCLFSSLYIQHIVQTHLPISSDTIFRASFMVSLGFL

CFRINCNINTVNLKIVS

>SG-AL_contig_527_1_+3_90_206

MIYTQVHGNREGGCLVKHLRLFYNSETTKRKPSCHRANI

>SG-AL_contig_528_1_-2_2_322

AKENEEAFKGWLKTKQTQIKREKLLKKREEEEIKDGYYVRSRDECDQAYKEWLKRKYAEM

RRQRAAEKQRARSFRSYSRKSRKQQMMARAVRQSQAFRYVDYYGYRF

>SG-AL_contig_529_1_+2_608_844

MSTEKVTAQTPINFKEMTHTYANCFSLDSRKKTSVIQIKAATHQILHTWENPFFCLLQRY

KVAEDNENYLIKFSYIQFS

>SG-AL_contig_530_1_+3_39_263

MLCDVKVSVTPLIQVFLGLPFTLNQILFVISLLSQLAGLHSGHMTNPLNSLQMNMMVDWS

CFHHFSLSPSPRHGR

>SG-AL_contig_531_1_-3_60_398

MKILTERGYSFTTTAEREIVRDIKEKLCYVALDFEQEMATAASSSSLEKSYELPDGQVIT

IGNERFRCPESLFQPSFLGMESAGIHETTYNSIMKCDVDIRKDLYANTVLSGG

>SG-AL_contig_532_1_-2_8_1396

MTYTVTTLALLVVVTLWTADVSGFSAQRKTRSTSQTQQTDQSQHYGVLLDAGSSSTKVNI

FSYIPGEPPNFVPQVQLTDTERIKPGLSSFMENLDGVSDYISAALNDAKSKIPEDLQAST

PVYLMATAGLRTLSASQAKALLQSVRDAMSSTTINPFYFEEDYVSILSGEEEGVYAWLAA

NYLHGFFSTSMTREDSVGLLEMGGGSLQVAFLPDDPIYQGEFQVYVGRRRFDLYVHSYLG

FGSNYIAGQVRRVLSQNSNYRNGNELNDPCMLIEDVASYEAGEVTLTVKGRSKPDECLEV

LEQILESSDTALCSPKPCAIRNTYQPAVGSLPFYAISVFLYAPLALGAVEDNDILNISKL

TDEALDYCTWNVSEAVAKTGTKAKYASTNCLMGLYIPQLLVDSFGFDQHTDRITVTQEID

DKDINWGLGAMIQHLSMAFMDDLESAVHCRQGDSVLPLDRARA

>SG-AL_contig_533_1_-3_282_3275

MVRKNKLHPSQRVEEKLEQLEENMNVAKKDKLFADPPEEEIQQNQLQNPIKNFHHLDSLP

SRETGAWMGLNEKEKSYETDKEYIGEEDQFFRGEKLYGYKDPAHKIGPLVAPDYSPMIDQ

VESGKDEAIKLMSQIFKLMGRDDIDRSFGVTGYLPTIYQSAILASLLHPQLSPGPSVKLE

DTDNLNLLPVSDIHTPHKLMHYHKPGGTNRSQIKDIPQQNGSQKKTQLGSGDVPKNYSGE

QSLPFVKNLSFYDSRDTDIASKKNLSYAAEDSDFNTSHENGHGDDLGAYEMVVLYQNQRH

RVQLSRTREGQPREILEIAHLVFNSKTSKHSVDSQEVRIRSFDGGHIIDKNRHAWTDSGG

RGIPDHSGRQPGLAPKDEEMEKSGGNTINKNFPRLSGLKRKNSLDVFLHGDKRHKDHPEE

NWTNIDVDPMLKIDTAFLPLWPDTSSGTKKSQKEQIDRDQGDQQDIFSSLSESSNPAVTS

SGANGRSDGSSSAASLNSLHHDTKEYEDSSTGEGQTDDSDQKSGEDAAVSQSDSLKQNKD

NDTSEDDSHGHVASKEKNSSHSEVNQGRTVSDNKAEKGELPRERKKAKQSTDEEVSSSTN

SPDSRGMATATVCSSNNCEHLNDMEIDGSSVAPISHVLDQAVPISHVLDQAVPISHVLDQ

AVPDCPSSLLLSQGSSVEGTPTLTFHTPVQISSPADSGLTHTTFRDYYKEPFEWSIFSPP

DKDKAFVYSKDNPLTTQETQSQSSLGACGGHTSVIAQEDACYFHLGSHPDDDWGPSVAEE

SVFSFMPYHHGEQHPVQSDSHSAAHLHHSVPQEPSFQQNRVQAVPPPASHLDPLLSESTS

YSHHTHFQPISVFPSHPTPVHLTPVIVHAAAAIPPWATSTTRINLAQATNPEFVELRQRR

ATFSDSWHCHSGIDAANMALAGFFNRGRGHGVRCFYCGLELYHLTSAHNIWDEHVRHNPH

CPYLVAIMGHDFVQQTLRRLRDDNNDSAKSNDKDNTRH

>SG-AL_contig_534_1_+2_2_688

VLSILKTWPSHLHCLFFILRNTYLIFPQETLPFTLLNTYLIFPQETLPFTLLNIYLIFPQ

ETLPSTLLNTYPIFPQETLPSTLLNTYLIFPQETLPFTLLNTYLMFPQETLPFTLLNTYL

IFPQETLPFTLLNTYPIFPQETLPFTLLNTYPIFPQKTLPSKSTVQFAGLSLMNYHADSV

HLTQTNIDIITKYLLQKYYDYEKQIIFHKASHHVTLWEWNPNSREPSVP

>SG-AL_contig_535_1_-2_455_2089

MRKKKRGLLVIFVVGSVVLGLVYLLTSDSDFWTRVLLLDSTVKFDPKSIDVARYIGTVES

PDQENPTSRYGFNIVASNELPPDRPVPDARPAKCAELYPEADKSLPKTSIVIAFHNEARS

ALLRTLVSILTRSPLQIVTEVILVDDFSDNKEDGILLASIPLVKLIRNEKREGVARSRVR

GSEVARGEVLVFVDSHCEVTPGWLQPLLTTVKKVPHSAVSPVIDLIDSQTLEYKPIVDLV

RGGFDWSLHFRWENLPFGLSRRDPTDVFKTPVLGGSVVVIRKDWFNNLGTFDTQLSTWGG

DNFDLSFRVWMCGGEARIVPCSRVGHVTRKHQPFTFPQGGISNTYIRNTRRVAEVWMDEY

KRFFYAARPTARMQDFGDISDRKRLRERLKCKTFRWYMDTVYPELKLPVSDEVAYGKIQQ

GTGCVDLDVGQLPVIAKLRPCVQNKDSQEWSWRRKGSIVSNGMCLSVNPMETQMYVVVTF

CDSSDSQRWMRQRQQIVHHVTGQCLDSRWAKTGLQVSQCDEQLDAQRWSITMETMSDLEK

DYIDT

>SG-AL_contig_536_1_-2_143_412

MGVPYAIVKGKARLGRVVHRKTATCLALATVNPEDKPALSKLVEAVKTNFNDRSEEIRRH

WGGGVMGAKSQARIAKLEKVKARELAQKMG

>SG-AL_contig_537_1_-1_172_5493

MKPSQVATLATILGVAISVFVDTVSSAECEGCTSTICICAGQKGERGARGLPGLRGPQGL

PGFLGPEGRAGNFGLKGRSGEPGARGPKGYRGDTGLPGFTGAPGIPGQPGLEGGLGPQGY

PGCNGTKGLAGFPGQSGGPGLPGRPGRDGIPGPKGEPGSYVTDGIAIGEKGSQGSRGLPG

IDGLSGNPGFQGERGMRGEDGLPGRDGVSGLKGEKGNKGSDGIGILGQKGQKGMKGEPGV

AGREGTPPSNSTRVYGEKGQKGDRGEKGMEGERGFPGPGGPMGANGLSGMKGEKGLIGFP

GRRGKRGKDGPPGPFGQKGMFGEKGFPGLPGVDGIKGMGGEPGYVGLRGPQGPPGVDGEY

ILTGESGTQGPRGRDGPPGPQGPRGPDGIDGEIGREGLPGLPGQPGLPGFSGEYGIPGDS

MKGDRGLAGLPGEDGNDGLAGFPGLPGDLGEKGQPGETGVGAQGERGLPGLDGLPGLPGQ

PGDPGFDGRKGEPGFGVRFPGPRGSSGLPGEIGEPGFPGTPGLPGLKGLMGFDGDDCGIC

PNGEPGRPGEPGDPGFDGGPGFPGPVGFAGLKGEPGEQGFPGEQGREGQPGIPGRPGPDG

LPGSKGYKGEQLPPPDFELYRGTKGAKGNIGYPGMPGMDGPQGDTGFRGLPGLYGPQGIK

GNPGQPGDDGLDGLPGLPGEPGRKGQPGQSLVGSQGRKGQSGLPGLEGIPGPEGDKGLQG

DPFFVELTQGDPGEPGRPGFDGLPGRDGIPGEPGRRGDDGFSGLSGMKGLPGLPGLPGEQ

GRRGPKGYPGVMGDPGFDGREGRKGELGPDGLPGLPGRPGLPGQKGESPRSAPRGDPGEP

GGPGEYGFPGNDGRPGEAGRSGPDGFPGEKGEPGRSGGPGFPGQPGLPGDDGLPGQPGFR

GRDGGPGGRGLPGLPGVRGNDGLPGIPGEAGRLGDDGNPGLPGSPGQQGRDGLPGSRGLD

GLPGVPGVKGERGIGGRPGSPGPPGVQGLPGRPGSQGDDGGPGEAGVPGIPGENGAPGLD

GIDGLPGLKGRQGEPGRGGPDGFPGQRGEDGQPGLNGLPGQDGLPGLPGQKGNRGPSFNG

EPGRPGRDGPNGLPGLDGLPGTKGQPGPVGTPGRSGLPGSQGEFGLPGEDGRMGLPGVKG

MRGNNGLPGIPGERGGPGVQGPPGLSGRQGERGNPGSDGLPGLDGRPGEDGEPGFPGRSA

LPGLDGARGDDGTRGMNGFPGEPGRRGEDGNPGLPGRRGEQGEPGFDGRPGFDGEYGRTG

EPGPVGRPGENGPPAPPGRPGPPGEEGIPGLPGIPGQRGFMGDDGLPGLPGRKGERGEGI

RTSPGPRGYPGEPGVPGLSGRRGAKGMDGLPGLPGLPGMKGESGRTGGMGLPGRPGEDGF

RGENGEPGVPGFTGLPGVNGPEGQPGFPGIKGYKGDAGFPGFGGGIGFKGRRGEQGPPGQ

FPPWTTIVGPKGEPGFDGLPGYDGRPGQRGEPGEDGLFGPAGLYGRRGDDGVPGRSGDSG

LPGNPGRRGQSGLPGLPGEPGRRGDAGFPGGVGSGPGIFFTRHSQTSAPPECPIGTVKMW

EGYSLLYVMGNGRASGQDLGEAGSCLARFSTMPFMFCNINNVCNIASRSDYSYWLSTPEP

MTPMMQPVEGRQIQPYISRCSVCETPAPVMAVHSQTMTIPDCPVGWSGVWIGYSFVVGTG

SGSQGSGQGLHSPGSCLEDFRSSPFIECHGRGTCNYYATSSSFWLSTIERNDQFRMPLQE

TLKAGDLRRRVSRCQVCIKDYSTGPAFFTYANVK

>SG-AL_contig_538_1_+2_2189_2503

MAKNLTDYKTISANFNENWTDIYLFAICFLVLCTSPALFNKPLTLICSHRKLQEKETDHI

NYSTKKKKRYVRYNKKRTNLRLTHKHHLHCQENLNQSECMDSSTR

>SG-AL_contig_539_1_-2_2_238

LSCLLVGKFSAHTGHLGHCLDQTRWNTRSSSKIHQVLSLTCTFFFSSPKLRSVVLFAADK

MAALERHAAFCGNFRRSRS

>SG-AL_contig_540_1_-2_71_1516

MKLLFLIAVLASISHVGWSQCSTISKLPVSDASCCSRLEMYQLYQLFPSVFSNVAKPNYT

SEDCCRVRYTDPCTCDPSSPRTPSTCIDFLPITAKNSEGCCGKLYQTNITTTKCYPRYIF

ENFRIEVKKFCESHFDVTYKYTPTAKEISGMCVRTNAMPYDKCVKAKPTNPGKCYCKPAV

TSCFLPTTTTTSTTTTTSPTTTTSTTTTTSPTTTTSPTTTTTTTTTTTTPAPLCPAPPPP

RASGSRVLGGWTANPCSASGVLSFSSGKDSAQMQMCNGVIAYNPTEKTNEVYITDFCKNL

MAFVKTALKLADSGDYNNAILFNPTDMATTAAASNISYFAPPVNLLTGRCRSVACLYNAA

TMKDVIDFNSCYMTSYGYDVETSDGTNGRSGSLNNVTMTKQDTCKTKPVSTTAECFIADD

PDVRGCWGDNGAPVYCRLKTTKETVLYGLLDVTGNMPATFGAPTNQKNLCSKNNEFHVLP

VS

>SG-AL_contig_541_1_-1_76_786

MLRYRRMLGGTTANSCSANGVLKFNSGTDQMCNGVLAYNPETNANQIYITDFCKGLMIFV

KNVLKLNDFGTYNGANIPNPTSLPFTAIANNISYFASPASALSSAQCQSVACLYNGATMK

DAIDFTNCYISSFGDDVKGSDGKVGNSGMLNNVAVKQGTCQTKPITRTAKCFVAVDPNVR

GCWGDNGAPVYCRLKSTRETVLYGMLDVTGSPPPKFDMNSNNILCADNNEFHVLPVS

>SG-AL_contig_542_1_-3_3_302

KVKSNPNEFEWVGQHIRKIDVAGSARCVLCSADIVYTKFLSAFVWRSEHLITKSLSLSLS

LSLSHIHYLPLSQTLSLFPFPCVLVFFMPKYCFMFLSFNL

>SG-AL_contig_543_1_-1_154_720

MSGYGGRGGGFGRSRGGDDYSEGGSTDDMFVPARDCGKIIGRGGSKIRELQDQSRCRIKV

FHDDEGDGTRRVELNGSDDAIDRARDLIYQILEAGDDRGGGGRQYQQQSYGGGGGGGGSK

EVIQVDSGFVGRIIGKGGSRVRELQDESGCRINVSRDGGSYGQTDVELIGTRSQIQRAKD

AIDDLVNQA

>SG-AL_contig_544_1_-1_1_309

WHGTVYDAKTGGCWRKKKKKRKEKILVIKRLLFFLHEILKENVQTFSYLVHVCLLHFLFD

VLLLHLPPPPPPFSPHLSSCLSSLISLSIFWVLLAILVLTPEF

>SG-AL_contig_545_1_+2_2_106

HCSFVKCAFICLFLSLLDWGAGHFKRKKKGQKRKK

>SG-AL_contig_546_1_-2_344_1321

MFDEMASGLDQLRTMTTVVADTGDFEAMKKYKPTDATTNPSLIFAASGMPSYQKLIDDAV

SWAKEQGGSEDQQLTNAMDKVCINFGCKILDIIPGRVSTEVDARLSFDKDAMVSKAKRLI

ELYKAAGISKERILIKLSSTWEGVQAARELESKYGIHCNMTLIFNFHQAVACGDAGATLI

SPFVGRIFDWFVKNTDQKSFEPLDDPGVRSVTKIYNYYKKFGFKTVVMGASFRNTGQILG

LAGCDLLTISPGLLEKLSQSTENIVRHLKKDKAACSDQEKVTVDEKMFRWEMNEDEMASD

KLSEGIRKFAVDAVKLENVLRQRLLK

>SG-AL_contig_547_1_-2_83_820

MGRKSAKSKEKKMKRKEESAKLALANKKVAEANAVEDPIAPFPVFRKYDRNGISVTLETT

RVDDLDKDTVDWIFQLTSSNMKTLYEQSDWGWSDREKREELTDSRAWYLLARTLEGRPVA

FSHFRFDLEEGDEVLYCYEIQLEKEVRRKGLGKFMMQILELMAHRYQMLKVMLTTFKHNP

QAQNFFKTKLKYEVDEISPEASMYEEDYTYIILSKTIPQKSSSAPAKTSTSPLKDSNAAN

TNDVTN

>SG-AL_contig_548_1_-3_216_380

MISHHPALNFTEDEPWAPLTFDPWRQQYLKNERTLPSSAALIASSSGFAQSSREP

>SG-AL_contig_549_1_-1_82_744

MPTNPDEPYYCSQQINIPPELMDILKQFTKAAIRTQPKDVLAWSAAYFRAMAKGEMPPVK

ERLEMPMATQKTDTGLTAGLLKVLNKQLGPKKTVGLNMIEEKWLDLALPKEQFDDLVRVG

SFSGQVEWNRFFALAASALGDSITDAMKVVCEVLTADPEGGPARVPFPLFKDLYQYLAKI

DGEISQQQVNDVINHLQYDVDKQDGYVMPRNFLSPDCPQLS

>SG-AL_contig_550_1_+1_151_726

MFKNTFQSGFLSILYSIGSKPLQIWDKKVRNGHIKRITDNDIQSLVLEIVGTNVSTTFIT

CPADPKKTLGIKLPFLVMIIKNLKKYFTFEVQVLDDKNVRRRFRASNYQSTTRVKPFICT

MPMRLDDGWNQIQFNLSDFTRRAYGTNYIETLRVQIHANCRIRRVYFSDRLYSEDELPAE

FKLYLPVQNKKP

>SG-AL_contig_551_1_-3_3_674

ARTFFYTFKTGSCIQHGEDSHLVCPSFLLLLHDTLQQHAFTSVCPLGIAPRCSSPQRSAG

VYHAPEGLRCSQAVMGRGLRGYTRVYFPWGGSAGSTGSHASSTWEGSEGSPRTRAVHGGS

IFCCYRGSTNRGLRVSAFWEGGCTHSAHHIPGRDHVMGLEGLGSYHGIHHTIPVTLRQQV

DAPHHHQYYHQSPPYLLTLHCPKLSTIPMQVLHAPDHHHTAHRR

>SG-AL_contig_552_1_+2_764_1081

MDRERRKKQMTVKERKTAETKRAIEKRKQSTEFPEEGILFILKEPLASPRRGNGFTSLRQ

GLLSLINDSIWNVSATAKYVLMWRLSTAMISYQHNHHHRMQTCKIP

>SG-AL_contig_553_1_+1_388_888

MVFVHCSCWKFVCPHHLATAPINTRFIINIPSKTSDVKPGTVTPARFVLRLAVQNGHVGS

VGRVCDDHSGLREGVVDGTVGVGDVFVVEVEIDVFVDEENERLRKTFGHTTPFQSLVGVQ

VGKAHFAHDFSLKPAVLIHVTVAEAEKQQQANNTCPDHACDPDSTFT

>SG-AL_contig_554_1_+2_299_484

MLLKSIIKKNILYKKKAVAILGVFIITQKRTQNMGEKAQGRRGNCYVIIILGMSLTLQRI

QN

>SG-AL_contig_555_1_+1_1_249

RSSGSGSGIVITVVSPCSLHFHIFCLLGLGWFLLLFGFGFFSSAGTLRIRVVCWLDQLIH

LLADLLRHCCPLLLPRLGLLVLS

>SG-AL_contig_556_1_-1_1_273

DYSFVAIGLSLLSFFNFFYYYLCVCLIIHNSGFFFLPNFTRDYSFVAIGSFTCAVCMLQT

GPPFIASSERLAHRPLPEVWWRGEEKSLPDS

>SG-AL_contig_557_1_+3_3_101

GLNSRHEILSPWLYQKSQPVHVKCCERWDSKNR

>SG-AL_contig_558_1_-1_115_1011

MVQGMLLGLVVDNRLEITNCFPFPRHPEEDDFDEVNYPIEMMRNLRHMNIDHLHVGWYQS

TYFGSFINRLLLESQFSYQHSIEESVVLIYDPLKTTQGFLSLKAYRLTPAMMKFFKEGDF

TPDAIASHSISFETMFEEIPVVLKNSHLVNVLLCELSGPTKRDHKFNFLDLGTSAVLEKN

LRQLMECVDDVAMDTNKYLNFQRQNFRQNQAKIQYQQKRNQENQQRQMRGEPPLPEEDIN

KLFKPLQPPPRLDCLLVANQIDQYCNSICEFSSQSIAKLLMAESLQENAGASGASGSSS

>SG-AL_contig_559_1_+3_3_209

VSAARAVDSRSDVSSSVTACNIKIGPFAECATGPPIPPISRPCQCAGSRSLPDLGGAHIW

NCPSVMDSS

>SG-AL_contig_560_1_-3_570_803

MWTLKLFILQNCVLKYWSECNAREWKGHGGERMCWEERVEVAVSIHNAARFALFHYLQHS

MSHFMYTLCQLDHSLSII

>SG-AL_contig_561_1_-3_36_467

MSYLQTNVISHAQRVRRLYKEATRQLQALYGYQRHVFRYHAVVLRARFDEHKDEVDMRVA

KKLVLEGEKELFLKQHPQPLKFPLSPGGVAYAREPHVPDWILDVWHPYEKAQYPEYFARR

EIRKREFIERWEKKYGKPDPDAAH

>SG-AL_contig_562_1_+1_166_417

MMHRLHHRHHRRICHRYHHHRNDHHNYHHNRRRYRFCYHHYHNHHTHITISIIIMIPVVV

ILLRLRYSRVVRRMRQKEHSNGRG

>SG-AL_contig_563_1_-3_354_1346

MMIAIFLAIIPVLVASEGCKLPEQLRGHWNSNRGGWNAMTITNTTIRDVRANGHNLPFTC

YMQNGSTYILQSEVQEVATVQFNYYTCVDIIPSLSNPDIVFFHVLTERNQMHQNDSVISI

LSTAEKSFDIICQKNHTQLPIGYNMFFRPDHEASLVNTTCPGILQAEFSANFTTEADYNK

CPEGFELNGCDRTHFTFNYTMCGSDTEGHVASGAPNYTCLTTVRDGNVTYTLIFMQQEVS

PEQPNHYQFACLAETMKENGELKITKSAGTCGATPSVTMLASETVSCRAPTTAAPEQTTQ

SGSSTASQITMSGLFTMLVSALLCVLVAPAL

>SG-AL_contig_564_1_+2_326_802

MFKKSKFTARFTFNTRQYTNSLHYNYPTKFLRIQSSATQFTTVNRVYIFSKAVQHLILRP

LWRGSTRLKHFFHDVELDTSTGFIFCHSKVVKHVIVAKIGSVGISVLVGQPFPLVGVCMT

STNVLGLQMFQLTVNIVAVTHYMLVDLRKTDQTEEIGRA

>SG-AL_contig_565_1_-1_49_435

MAMGAVRHIADIRADDASKEGAGIIGLQAGSNKGASQAGMSMGAVRHIADIRADDASKAS

AGVIGLQAGSNKGASQAGMSMGAQRHIADIQVDDMSQASKASINLQYGSNMGANQSGMSY

GGRRDIMGN

>SG-AL_contig_566_1_+3_3_206

GLRSSTITDTVSVASEVYGQSLYKGNRKSKTTIHYFSPRVSDTKEQSKRSDKARLYSSTL

ISIKQWVN

>SG-AL_contig_567_1_-3_174_440

MADAAVLQPKSMVRPKVWDEEVEEAYRFQLAGYRDAVEYKQIQKTEHIDRWPHNGYVKKL

VRRDGCFYYYNKTRECPDKEIHKTKVYSY

>SG-AL_contig_568_1_-2_29_463

MASLVNHRVLALSSVVRCMLRSVSTTAVRTAEGSNVGTPTHTGQVWDQNDYRRIRFMDKD

KLVNHQFAIDLIAEDPIVVVNSNHVFSDSGGALGHPKVYINLDKPEVGICGYSGRKFIQT

KFYDAAKHGPSITYEEYLKQVQPSE

>SG-AL_contig_569_1_+2_2_184

CTRGLTVVRQSAYILGDPLSVKLGNVNNKQQLWQQLKNCKTKPQQNLECTVHEYITQLRN

M

>SG-AL_contig_570_1_+3_906_1199

MPLQHSFAMDTGLTGRTAQPVYTTLTLRSQRRRNTTTPSTISGLINLAQLATQPTQRQAL

NTNSLSIKCLYTLQRGLPLCYNTHHTMSSACSLLHHTE

>SG-AL_contig_571_1_-2_335_742

MASADLTWLIVRNSSSFLLKRNRQNFTLEPNNLKGRNSFRYSGLVRKRTVGVEPSKDGKG

VVLVTKKVDGCRRPGKNQNSVELKRDSRRTFRAIRKITKKGGYRKDLSMAAVRRAAAIIK

SQRPVVPRKTGRVKKD

>SG-AL_contig_572_1_-2_11_436

MAAETELKNKAEQATHAAREFSNIFYECFDKKRHAIKNLYHDSATLVWNGRAVSGKEAIM

KFLEDIPTSSTYLSSLDTQPVDESANENQTTIVVTTLGKMQFSDSKMRSFYQTFLLSSQD

NVWKIVSDVYRFMEVPPSSAGL

>SG-AL_contig_573_1_+3_132_608

MINTASVVPFPGLNSDCMLLTGIIFLCSRIFSSIFIPCSSSLMPLKFPHSSGSPFCLYMG

TISLFSHFSGICFSSQTLFSNSSIKSTPLSPRCLIRSIGRPEHPVTFPFFILTMASSPSF

LDISWTGPSTAGTSPNCSLAFSTFSRLLKYSLHLSLIFC

>SG-AL_contig_574_1_-1_1_165

ISSSPPTLFLFSFFMASLSSDNLKGPVSMSSVVLSSPSPYLPASYIISHGSNAKT

>SG-AL_contig_575_1_-2_215_451

MPLACRAATEMCHSYLFWASFCIFCCCIHNKVVFFFRDWVVSPVPNPQLGGPVDCALSGL

YTSAWVVLSGLEAPAFARQ

>SG-AL_contig_576_1_-2_56_673

MDNTVRYELLENEEVPAQPVTLAMIVPPAPPPYESETVTNASSTAQPAKLPNYVEATTLP

SYEEAERSKMEEVLHHSNDNVTDSSLESGQSREVFSDMTIGTDGMFICTFLISFLFNWLG

FLLSLCLSNTVAGRCGALSGLGLSIIKWVAIVKHKQWASEYADGESWLWWLLFFCGFLLF

IRGSVQYVRVKYEWAQLNRLHRYRLI

>SG-AL_contig_577_1_+1_1_252

AQVAILLTPAHNVGQGPDSDRRQGVSRNLGLSKEFWEKPEGDRTVPVKAISRSAPLRARI

SLLRLLEANAKRKVVFKVSRSKDA

>SG-AL_contig_578_1_+2_968_1153

MHKFSPKIKASNRHRKPAVSIMHKVHYKNRTSDSYSYCSDYCFISILVLFFVLLIQTLDL

AC

>SG-AL_contig_579_1_-1_2593_2844

MYCHQSCQVLSFASSPECTSLFWHGQFFFLTIDEKETPEGAGEDPEKHCLHLPLKQSCTT

LGGLCCLHNRCRHAQDTPLRIQGT

>SG-AL_contig_580_1_-3_18_1463

MAASLLKVTSSVAKVLATAQKAPCVSAIIAQRWRSTRAATFEQTILNVPETRVSTLENGL

RVATEDSGIPTCTVGLWIDAGSRFENEKNNGVAHFLEHMAFKGTKTRSQTDLELEVENMG

AHLNAYTSREQTVYYAKCFQNDLPKAVDILSDIIQNSLLGEQEIERERGVILREMQEVET

NLQEVVFDHLHATAFQGTALGRTILGPTQNIQSINRNDLLEYIGTHYKGPRMVLAAAGGV

NHEELVQLANKHFGSLSAGYEGEIPVLPQCRFTGSEIRVRDDSMPLAHVAIAVEGCGWQN

PDNIALMVANTLIGSWDRSYGGGANHASPLAAASAEAGLCHSFQSFNTCYTDTGLWGLYF

VSDGLQIEDMLHNAQAEWMRLCSSVTEFEVTRAKNLLKTNMLLQLDGSTPIAEDVGRQML

CYGRRVPLPELEARIEAVNAKVVRDVCTKYIYDKCPAVAGVGPVEQLPDYNRIRSAMYWL

RL

>SG-AL_contig_581_1_-2_290_2974

MASPSAMFEVVSGSGSTSSDSIVNISRSGASSIVTPATTTTTNPNEDELKTEVDQLRKDK

KVLEGRIRGFKTLSDLLVDFRKENDQLRCQLKALNAEPLIMPGSDFKAASVSSPFSLVSL

QAAQQNQSSSTMSQFDMSQLTLQDQKPSSTSSVARSTSSLDPKGPPVSYPPVSGPPFSLQ

ETVGPPGDLKETKLISLLSPTESGTKLKLNTAEPQGSVSKQESLEPTSGQHPDIGTTQTS

KTHHDSSMASENSSPELLQLDSDKYDEATYEKVGLQLGMGLAGLTNSMYAVGGSTRESRP

TDFPSLSSGISNSFVQPSSHDSTSNPPFMGMMHQSLTGLQQNLTGMQQSLTGSATNKHNS

DLATEVENITERLNSEPGDSMPAIAFQMHQIAERISKLEQKERNQKTLNDTLMRKNQELK

RLVKVTETTHQTEIVALREQNMRLIQQLKGQHHEDQTKSKAVKSSGSHSDWVHVEKPPQM

GESLGNTDHSLVGRVRSLEQEKAELLRANSNWKSQWDQMELDHQTQINELRPKLVLLNQE

LVSLKTRLAEQTSEFEGRMLEMKRRTGEEENLKEEAQHQQRLAERRCSELQDQLNEMRTQ

VADLARDKHVLSTELNILKAHTTNQEMRTGSAIGAADAGSRSALEAEIAMLKEQLKVFAE

DFEQERIDRTTAQAARDSAKKSVDTMARQLHSLSSQVKHLQKQIKDRDDTISWTLRQNET

LQRQATDFKKKLASEWEEKQSLQRRVLALEQQLQQQPIRNPYLTSGTTYNPAPMHQPSPL

TRTTAPYFGGQGPAGGPGRNRPITTAMSPEHLPGAWTCTACTYINYPGRTVCESCGLVNT

ARSHPDQFGFASHDMYGSEHALLMSRGGDHHPGGGIMPTNGDLVVDSGVCATTPY

>SG-AL_contig_582_1_+1_2509_2796

MYTHMVTYIHLCSSVHMVTYVHLCSSVHINKYLNKCIPVVRRMFKIKTHPTHDNFVYLLT

HQPLNNKNKRKCQIIHTLSSCQLYNNSNTHTHSLVH

>SG-AL_contig_583_1_-1_94_432

MQAVEDVYNAFNGFLSGKGPKRLLSELKKISDFLESEDECKFLVDNVLSFADCYVLPRLQ

HIRVAGKTYMDFDIPKEFTAIWKYLAEAYKTEAFRSTMPSDQDIVFHYEKKVG

>SG-AL_contig_584_1_-2_8_268

MPLLPHPPTVCTPPPLPRMAMRHPPTRATNLLRPMTKQPRSTTNFHVYSLTISAILILGG

STELVVGFLCQSGLNYDYWAFSAVVNR

>SG-AL_contig_585_1_-1_2047_2427

MVVLLTVIEIGYVLAVSTIVMCETTSVDRCLIHSCTIWYGFHFPVFTRSSIESPEPELTV

EDSQLALKHSLFSLFNYCHSAAWGKWSSRVHIVLRQGELERCIESSQGSLEFKLLGHCTC

LTFLFSL

>SG-AL_contig_586_1_-3_132_341

MDDGDDDDSHHHPDPSDRGGLPVRKRKVFGSAGNKNKVTVPIMPASTSSRHAALTGRDGK

RGEGHHQLVN

>SG-AL_contig_587_1_+1_1_540

FFFFFSRQQILFHQKCLSLATDTWGLSLHILGGLGLGLAALGGCRLSRLLDSFLLLLLLL

VCPDSLFTLGLAHFGFLVTLGDDLAEGHTSDSTLELVGATGSLLDHLLCLALLVLATVQN

SPCHLTGVTLHGMGPLTFLAQEVENSSVHFDHSFPMARVNFDTAVRTQFDLHGAGEMRSE

>SG-AL_contig_588_1_+2_278_457

MLCISYFSFNLSLFQHNSSSKLIQMTYKVHVWVFRQINYVSAKISWQFKGGPFTKCGSQV

>SG-AL_contig_589_1_-1_91_2094

MGGAVSTGDDNDELVDNLVDADYIKTNKIERIFRAVDRASYFLPDHVESAYKDLAWKHGH

LHLSAPCIYSEVLESLQLEAGQSFLNLGSGTGYLSTMSGLILGAYGANHGVEVHDDVVEY

AREKLEEFKSKSKAFEEFDFCEPQFVVGNCLQLNSACRLYDRVYCGAACPAEHENYMRNL

IKVGGILVMPLSDQLLQIRRVSETEWQSKNVLPVSFATLQSPSKEKPDVVDLPECQVLSL

QEICRHNIRAIIRNNICEETPEILRPHRKQRSSKPKRNVGRRGRLVNLVPMQMGMMILNS

FNDSSDDIDDDEDEDDDIKLGSESGPLKTSDDEINDAEEDDDDDEDEGNVSENEEESTAS

VSTTESSTHGRQSDRSEQNVKRKLRQDSIKEETEREAAAEALNSGLSMEERIVLESKPST

STTSTDNKRSSPSSFCQDWEGEAGNGSKRMRCVDMQDGGDRDVEEAKETDVSNLLSDQAE

QRAQRPQRELSSEDSDVDSMDVDPVRVAFQAPPTPTSVLHALHLLSIPRKVRCSSSNSAD

TSETSGFGSLGDEPLDIPGMSGDRDSPPSMTADRNFSRSISIDGESPPSVNANGDSSPRC

MESALDAFASLGKKVSAGKTSSDSDTSGEGATSPDQYKGPTLGQLMKERIQGLPLPLALR

GYLTYYRN

>SG-AL_contig_590_1_-3_78_914

MADAPGGGRGGFRGGFGGEGRGRGRGRGRGRGRGRGRGRGAKEGEKEWVPVTKLGRLVKD

GKIKSLEEIYMFSLPIKECEIIDMFLPSGTLKDEVLKIMPVQKQTRAGQRTRFKAFVAVG

DYNGHVGLGVKCSKEVATAIRGAIILAKLSVIPVRRGYWGNKIGKPHTVPCKVTGKCGSV

VVRLIPAPRGTGIVSAPVPKKLLTMAGIDDCYTSSRGATATLGNFAKATYQAISKTYAYL

TPDLWKETVFTKSPYQEFTDYLMKNHSRVAVQKQEQKIY

>SG-AL_contig_591_1_-1_139_633

MPPKFDPNAIFTVYCRAVGGEVPATSSLAPKIGPLGLSPKKIGDDIAKTTSDWKGLKITV

KLVVQNRQARVEVVPSAASLIIKALKEPPRDRKKVKNVKHSGNLALDDIIKIARTMRPRS

MAKGLDGTVKEILGTAQSVGCTIEKSNPHAIIEKIDAGEIEIPEE

>SG-AL_contig_592_1_-3_33_662

MASFRFSQLVRQFGTSAVQHGKLVSTPLQVFGIEGRYATALFSAASKQNKLDAVEKEMKT

FQGLLQTDKKLAEFVANPSLKRVEKKAVLEEVMKKLKYSDLSVNLVAAMADNGRIAKIGG

VLSTFAQLMSAHRGEVICTVKTAKALDNASLNELKVALQGFLKKGETLQLTTEVDPSLIG

GMVVSIGDKYVDMSMATKIKNYTNIIKQAV

>SG-AL_contig_593_1_-3_60_2003

MDTQSIRSSSSSTTSMDKIKGGNDERGGWGGKLDFILTCIGYAVGLGNIWRFPYLCYKSG

GGAFFIPYCIFLILCGLPLFFMEVSYGQFSSLSPLAIWRICPMFKGVGIGMVMVSSIVCI

YYNVIIAWTIYYLFMSFRATLPWSHCDNDWNSENCMDTGKMLEAESAANATPASTVAMNF

SNVMGNTVYNATSNVTSVIADTSKHRSMSASEEFWERHVLQLSSGIDEVGNVRWQLLITL

AVSWLCVFLCLFKGVKVLGKVMHFAAPFPYLVLLVLLIRGVTLPGAIDGISFYIIPRWEK

LLEFQVWGDAALQIFYSVGMAWGGIITMASYNKFNNNVYRDALIVPFINCGTSIFAGFVI

FSILGFMAREAGSTVDQIVTQGPGLTFVAYPEAVSKLPISPLWAVLFFLMLFVIGLDSQF

GMFETMLSGLIDEFPKQLRNKKTIVTAVTCFAEFLLGLPSITEGGIYVLQIMDWYCASFS

LMLISLAECMAIAWVYGLDQFFKDIALMIGYEPHRMFWTIMWKFVTPSAILFIWLFSVVT

LGPVTYGDYQYPEWAIAMGWSIGICSILPIPGYAIYKTSTTEGPLCQRITSLLKPTPEWG

PSQPEDRKRYLASRGLDDPECVMSLTEKPSPIYKPSSIEHDFMPKSEV

>SG-AL_contig_594_1_-1_1_225

DWHSVLESCPKFRIIVNSYPLFLRRRRSQETNNNLNRVSLFYVDGHFICGYILELCSKFR

IIYNPLQPLQAFVQE

>SG-AL_contig_595_1_-1_130_426

MIKEDGQVIHFNNPKVQASLAANTFAVTGHAEMKQIAEMLPGILNQLGTESLSSLRKLAS

SVANAAGDSSKQNSGDIDDDDEVPELVGNFDEASKGEGV

>SG-AL_contig_596_1_+1_118_219

MGTGDNEGRTDTESISRSIPEPFMVSDVYVAIFT

>SG-AL_contig_597_1_-2_1742_1975

MLKLKFSPKCKSQKLGRKKSNQYNCYKQKCNCTMNIRRMTSIETGSHSQSNTQMKRADYT

TLKLPCKNQVDTYQKEHR

>SG-AL_contig_598_1_+3_1095_1316

MYRWKLDAFKTKYTRNIVCLRGQKITTSRILSCIKIKRFIPILSQKTVHEIFESTSLTYK

FFVSLSKSPIANYL

>SG-AL_contig_599_1_+3_3_158

QKITTSHILSCIKIKRFIPILSQKTVYEIFESTSLTYEFFVSLSKSPIANYL

>SG-AL_contig_600_1_-1_46_1881

MESRYNFKRITVVPPSKDLINIVLSKTQRKTPTVIHKHYSIVRIRGFYMRKIKFCQQTIR

DKLTAILTEFPNIQDLHPFYCDLLSLLYNRDHYKLALGQLNTARHLIDNVGKDYVRLMKY

GDTMYRCKQLKIAALGRMVKILKRQSQNLTYLEEVRQHVSRLPSIDPNTRTIIICGFPNV

GKSSFINKITNADVEVQPYAFTTKSLYVGHTDYKFNRYQVLDTPGILDHPMEEMNTIEMQ

AITALTHLRAMVIFMMDLSEQCQHTVEEQVSLFQSIMPLFKNKPLMVVCNKMDITTLDDL

PSDKQQLVRDLKTAGTPLLQMSTVTQDGVMSVRNEACQRLLDFRVESKLNTKRVQDIANR

LFVAYPKKRDDLERPPTIPPGVRQKREKQAKRRKLERDIELEQGDDYTLDLRKEWDLPNP

EEKYDKIPEILNGKNVADFIDPDIDAKLARLMEEERLREEAGFYNLPSEDEDDKEMEATL

AKIQEKRKLKRLEAIDKRNNNRSILPRSASLAVTRGRKRKREESAGPSSEPMESRSMSRA

RTVSASRTRSQTRDQSGLRDQTMVAKVKKMAKKAQKPRNHQAKKGEGDRVILNMKPKHLF

AGKRKGGKTDRR

>SG-AL_contig_601_1_-2_134_1234

MPRGLVKAKKYDWKDSNLALFGSDTEKEVKKESAESEPAWQGAGQEPGLKIWRIEQFEVK

DWPESDYGEFYNGDSYIILNTYKDEETEELNYDVHFWIGKNSTQDEYGTAAYKTVELDTL

LDDKPVQHREVMAHESALFKSYFKTGISTMEGGASSGFRSVPPEEYTPRLMRFAKDKGGR

VITREIPRCRSLVTSGDVFMLDMGLKLYQFNGAESSPMERSKAMQYIGELKSKRGEAESD

VLDGDSTPESHDFFKALDEEPNDGNDSDDEDGDCDKKLLRINTDSHDFEDVKEGSITIGD

FNSDDVFIFDSGSTVFVWVGSNASRAEKKNGLPIAHNYLRTSGAPWRSVTVIREGTKSAQ

FLSALEA

>SG-AL_contig_602_1_-1_1_66

FFFKTNFRYVSVLSLQSLIMKD

>SG-AL_contig_603_1_-1_76_1746

MAYRRTNVRAGDLIDIPGGLNTGSQVIVRGRVLVHETRFCINLRCAEDSDSDIALHFNPR

NDQGDVVVRNTFQGGSWQEEERDTPRFPFDNGRKFTIRIMTFPDYYRIMVNNQDFVRFDH

RIPAADVRCLQLGDGAEYYEASVQNNCRAPFMGHFCGGLKPGQSVRVRGMVKGEADRFHV

NFQCDHDGDSVAFHFNPRQNQGEVVLNAKLDGSWGDEQKSDDFPFKCEEFFDLFFIALDD

KFNVYVNEEYYTSFDHRCNFRDVCFLVIKGDVELMDVEILDPLPDDFIKEIPQGMEKNDL

VVVKGFFYSEGNRFQVNLIKGESCDDDIALHFNPRRDQDEVVINSRDGGSWEEEERHPLP

SSMSKMVPFEVEILAKKNKFKIYTNGKKVCKFRARDDIEQIRSINVCGDAYMYEVKLLRR

VDEPFTDSLPGTLEPGNWVTITGAPDDDDERFAINLQCGPDCQEDDIAFHFNPRFPEEVV

VRNTREGGGWGEEERDQPCFPFEHDDRYELAMVMLHHGIRVFVNHRHFTDYKFRMDPNRI

THIHLSGGSDYFEPEFY

>SG-AL_contig_604_1_+3_132_305

MLNCSKYSNVLNWGTDYYYYILFIAQPTIKTISGLSPHKVHQSQEDNRVDHKTSVNLK

>SG-AL_contig_605_1_-3_1578_3704

MKTTKMTISSPRLLILLMLVMMMMTNARAQAPTPPTITKPAYYEEIYTRENLNQHQKCEA

TGYPVPDIEWFYSKTKDDFGAIITKTNPEGGLASFDGKTGDLWIGNLSASAIGYYSCRAI

NTFTGGLKVTALSPRLYIGIARVGQWNPSAATSTVTVTVGQYAELPCQDVPAFFGPTGYR

WVNVSTSNSDQLPPIQMGNRKFIDDKGNLHFTYMEQSDLQLNYNYACGLTNQIAGQSPSG

GTIRLEMTLGSLAPTNPVLRYKTSKNADGSTFELGYPAKIKCFFSGYPTPVVTWKDNGGN

EITEGNGYTFENDKRTLVINIAGTEHDTTYTCVGTNPQDSSKSVTEDVWVNITSPPIWVK

PLQSQTVVQGKNATFYCDSRSMVDENSPEKPKWFKNGSPMSNTHDPKKFSFSNEGKTMTV

ISLNKRTDISCFQCLVKNSVGESFGDGCLNVILPIEILTQPDPKQVISKGDVVNLTVVAI

TDPDYTLSYRWIFKNKTYDSVDAPPYVNYDFISGLAYINTSGMSDEQLRNITGVYQRIVF

HRFESRTVPVEVELYVPPGPAVAGFPFWIIGLIIAILLILLIIIICCCVYCRRKMQEGDY

PVDRKETAAGLDPEKEVKESSFPALSRADDPDQLKKKGSHLDIDDEYSNPDDSFGSADYG

EDEPSAFNEDGSFIGVYKNKPKSAVHNPGFQPNPQLHRDYPTYRPESNV

>SG-AL_contig_606_1_+3_3_119

FLLPHLLHHSCVTTPTLLISALQHISTTSKNEVTMRTGQ

>SG-AL_contig_607_1_-1_256_1032

MFKRVCVLFALAILMANCSVAVNWTSISGALKSVSIGPSGIWGANNADNIYYRASTYDLN

NVQGTKWITVPGKLKQVDVGTNIVWGVNSDDNIYYRHGISEDNPTGSAWVHVCGKLKHVT

VSNEGHVWGVNNVDQIFIRVGANNCNPYGVSWKRIPGSLKQISAGFSGVWGVNSNNQIYY

KTGTYGDNGSEGGAWKQVAGSLKYISSGNGAVIGVNSNDDIYIRNGVNFKNPTGTSWTKL

NGRLDVVDGVDDSLYVGVN

>SG-AL_contig_608_1_+1_121_396

MGISRCHFFPDFFMPPPPIGPFPAALAAASFNAFCSSFCFCLKATSSSSISDFCFFGALR

GFFFPPSLPDMMKIFSPRQMPVRLARAWEKAD

>SG-AL_contig_609_1_-1_94_1560

MQATMTLMVLVCTSWLLLLGTMMTIVGAAPAGDEIASLPGLKKQPSFKQYSGYLQASGTK

KLHYWFVESQNNPKTDPVVLWMNGGPGCSSDLGFLTEHGPFRVQDDGATLLDNPFSWNTI

TSMLYLEAPAGVGYSYSNDGNYSTNDDLVAHDNYLALKDFFLHKFPELSKNEFFITGESY

GGVYVPTLSALVVDDDSFNFQGFAVGNGLSSYELNDDSLIYFSYFHGLIGEQLWDGLVST

CCHGNATNCTFIKNAVKNDTCGYYVSKVQAIVYGGDLNIYNLYGPCVQNSGMKTKYNPLQ

HRLVTANFGWAFANSPAVKAERQFIEKLQTQSGNRRLSITPPCIDAKGAIIYMNRKDVRT

ALHIPANLPEWSPCSNLNYKRVYMEMSAQYKKVLGKGKRVFVYNGDVDMACNFLGDEWFT

DRLSLPSKKARSEWTYTDATHTSQVAGFFKEFELLTFVTVRGAGHMVPTDRPRPALKMFV

HFITNKPLN

>SG-AL_contig_610_1_+3_3_113

QHKKKHKPHKNLLIKKIIILLFFLYFFFFSFHFHFIQ

>SG-AL_contig_611_1_-3_15_554

MAGIGMLGPGSFLIGTGFMQCHQQMEAVIMLTISVGLCGFHFSGYFINHGDIAPAFAGTL

FGISNTAATVPGILAPYVVAALTPNGSQQEWQLAFYVAAGIYFFGAACYVLLGQGEVQPW

AEFEDHHGDPKDQDDEASTGFGLHELQQVHEEEEEDEEEAKAAEKMLNRVNGDTSNAERV

>SG-AL_contig_612_1_-2_95_1774

MWFSEAGEIVEMFRNSFPLSFLFFVPIFILISPVSPVYAAHEFNVYRMQQFDLQGSSYGC

KNSLVNMEARPIDSKMLTRRCVVARLRDVTMPKFRDLVTNNAGALLVLLPQDLSQLSPEE

QEHLQSLERDLMQEETSLPVFFAHETEDLKDIYEDLEHGAAGDQASSAWEALLSSVTANG

FQMVVNGAQAKALQDFQITNIQGRLSGHGIEEQLPTIVITAHYDATGVAPGLAYGADSNG

SGVVALLELARLFSKLYTNSRTHAKYNLVFLLSGGGKFNYQGTKRWIEDNMESTDSNLLS

DVAFVVCVDSVGGGDSLHLHVSKPPREDSNGGIFLKNLEAAAQAQDPPVEFSMVHKKINL

ADEMLAWEHERFSIKRLLAFTLSRFNTTRALDRTSILDTQTKVDDEKLSTNIRVIGEALA

RHVYNISSQGAFSLFTDAMDTQEDAETAWLQFLTAQPRAAQLLPPESVVLGTLENTLSRY

LKDVRRTTFKADKRDPEFVFYTGAVYAMNAFNVKPAVFDLFLALGIGAYLAVIYVCATNF

HRVYGRLRRSMASSQKEKSN

>SG-AL_contig_613_1_+3_3_101

SFFFLIHNCFNNELCMRMEYTNSLQTVAASDIP

>SG-AL_contig_614_1_-3_3_416

FYQSDCLQLSFFLQFFSFILLTSLFFFFFAYCAVKSLSVSVSVSVSVCLSVCLSLSLSLS

LSVLCLSLCLCLSLSVCLSLSLSGAQGSIKGLARSKIFRIHICILSFPLALPLSLSPSLY

PLSLPPLSLSPSLSVSSL

>SG-AL_contig_615_1_-3_33_431

MSEEDRAMLQMCTLRIVQAPKCLDPLNDLLRKAADFLEMNIEKTSTIQSLRNKIPCLDDC

LLHFLSLAGFQQQEDETIAYPLCSTEIFESCELLVSLLNELDDDDLDVTACCLQDTPTSQ

AIITALRPVWEND

>SG-AL_contig_616_1_-3_303_3551

MMCDVMPTISEDGSNDRESQGSGDESNFEQLMVNMLDERDKLMETLRETQEALASTQVKL

QEVEKDRNSLQKQMSATTPQELATVTKELNQIRDHLLEREEEIAELKAERNNTRLLLEHL

ECLVSRHERSLRMTVVKRQASSPAGVSSEVEVLKALKSLFEHHKALDEKVRERLRVALER

VQQLEEELATANQELFTLREVQSKSRHSSGSVDESRLSKAGSVDNAPAGEGETQSAPLAH

PKRLSNGSIEPDSDVTRVIELQDTVEKQNTELTSSRTRVLELTNRCTELEESFGTAQKEL

NKAQEQLVKLQRDLRESQAQKEDQEERIATLEKRYLNAQRESTSVHDLNDKLENDVAMKD

AQIKAMEEKLHAMTERLDMSEQRLQQSLKKAAVLPTMEAELQQRLEALSQAEERHGSIEE

RLRTLEQNLQDKDAELQRSRQREKMNEEHNARLSATVDRLLSESNERLQLHLKERMAALE

EKNNLSQELETVRKMLDEIQQEKERSNDDVEQYRREVETLRMQIEQLMRSGYVFEDTVSR

RHESAREQALLDDPGKVNTLNEQEWEKLQQAHVLANVQQAFDTSDTEGTDDNESLFGTVD

LLSPSGGHADAQTLALMLQEQLDAINNEIRLIQEEKESTEQRADELESRAPAGMTMSQLY

TYSSSSPQLATSTDNTATQQQYSRDDSAIGAKEESRQIKCESSPPTPRSLRLERVAAALA

KSESDTASRFQEMARSSGSPVGSLHSSQDSLHKQAKKKGLKSSLGRFFSKKDKKAKEAYA

REMAAAGFDGADATGSELAVGLGQSDFDRRMKKKHELLEEAMKAATPFALWNGPTVVAWL

EMWVGMPPWYVAACRANVKSGAIMSALSDTEIQREIGISNPVHRLKLRLAIQEMVSLTSP

SAPKTSRTTLAFGEMNHEWIGKEWLPSLGLPQYRSHFMECLVDARMLDHLTKKDLRGQLK

MVDSFHRTSLQYGINCLKKINYDRKELERRREESAAEVKDVLVWSNERVIRWVQSINLRE

YANNLIESGVHGALIALDESFDHNSLALALQIPTQSTQARMVLEREFNNLLARGTDRRLE

EAR

>SG-AL_contig_617_1_+2_134_418

MQQVHVPNTSSSSGVATHRNIRLHRFHAECNTRKYNAFANYFQAVQNKRMIFHTHTHTHT

HTPKSVAFTYTSNMKHIMADGLAILTLFSSIFYKH

>SG-AL_contig_618_1_+3_3_314

GDVVLAGLELPQSDVDGDTTFTLGLQLVQHPGVFEGTLAHLLGFFLELFNCSLVNTAALV

DQVTSGSGLARVHMADHHNVDVNLFLTHIAEFRSSTTCSSGAQA

>SG-AL_contig_619_1_+3_180_389

MLHRSIPKGCPCINTVQMSVSLKEEEYMSTLDQAFQDLPEHDATAKWNSTKEAIYNTAMS

TFGQDWFNAY

>SG-AL_contig_620_1_+1_598_915

MPSMPGDLFFSNLMAVLTSSAKSSMSVRSIDKGSSGWAEVEGSGLLSTSQKYSTLFASFS

SPLDRSVPPLDLLGPGLDEVVPVTHLAIVYTDLISLQAAASSVFSA

>SG-AL_contig_621_1_+3_396_671

MLITCSWYQCIPSMGLMVSYTCPLQFYEKGQETISYCPSNFQNVPLVSFDHFNSPFSLYA

CVHIITCVNEIFMSIHFRYAPLSACTHASIHL

>SG-AL_contig_622_1_+3_3_71

PEIMFPQSNIREVATTFIMIVED

>SG-AL_contig_623_1_-1_1_318

MFEVAEGGFLDIDVKIYGPDNKVIHQGERESNGKYTFAAHMDGVYKYCFSNQMSTMTPKI

VMFTMDVGEPKPKDQEGDKDADGNHNKLEEMVNQLSTALTGAKHEQ

>SG-AL_contig_624_1_-2_83_1681

MTELNTAILLLFVSATIAAGCNETQSFPCDGDRCIPISWRCDGHTDCLDETDETSCEDIQ

QCLDVSQFRCVNRRCIHQAWVCDGENDCGDSSDEANCSINTCEPGEFQCQTDQKCIPGRW

RCDNAVDCLDRSDEKNCSTTVAPTITTTTSSTSGSDCSGLWCESDAVCINTSWICDGYMN

CADGSDEKHCSTTTTSTTTTFSTTTTITTKVPTTQSDCEGFQCESDDMCVHSSWVCDNDA

DCPDGSDEENCSTPTSETAPTTTTTAVNELTTTAAFRCADSEFQCESDPSLCIYKAWRCD

NDPDCPDQSDEKNCKNTTTSTTTTTSAVTTPTSIHVSSSPASLCSDGEFQCESDPDMCIH

WSWQCDGDSDCSDSSDEKNCITTTSAAVSTTFTPTPASPTPASITPVEASGIPGNVEAQA

TSSPNQAINTTSSGGTRTTISTALPNADQTSSTEQTSPIQQTSPTEQTSPTATSSSSVSP

SADGSRKKGRVFYMLLMGFRAESEKKAISMFEMMKNLAKNATRGEIAFVGNAS

>SG-AL_contig_625_1_+1_94_270

MLHGKTTCERLIGKECKLVSYRKCSMTGYKAHRLETVHYPSTTTKRICDTAYQYMTKSC

>SG-AL_contig_626_1_-3_93_500

MRIKTIQSKTYTTPRRPFEKERLDQELKLIGEYGLRNKREVWRVKYTLGKIRTAARELLT

LEEKDPRRLFEGNALLRRLVRIGVLDENKMKLDYVLGLRLEDFLERRLQTQVFKLGLAKS

IHHARVLIRQRHIRDI

>SG-AL_contig_627_1_-2_176_400

MTGTLFSNSRILAITAKTIFQTCRERVDYQDRFFFSKFFDCAVGCFCVTLCFPVWELLWK

FRRRYQRTVIFVLLV

>SG-AL_contig_628_1_-3_144_1214

MLDNELNHNAEVQLMNQPNSAAVAHPLSEMCSSASLTVPETPKEGVVIKVDYVGACFAEG

QIRRKRIRPRFPGLEVAGTIHTIGHNLPNCNFSTGDKVVLFPDDSLADSGYAEYLAIDDT

CQLMQIPQDLSLEVATMLPGDALSAYAAVIAAKPHVEKLQQVKSCVNVLVVGSGGLGLWT

IRLAQLLMGSLCNSVRLFIADNSIDKLLTAQDHGCYDIVYWNEEDHEEYIVERTLDACRG

GVDVIIDYVSNPRTMQRSLKVLNREGLILVAGNSKTEVSISLSTLAAKQQSIAGIPRGTM

NQLQDLVTFLAEGKLNPPCYAVFPIEDMKQVFDDLCECRITGRAIFRLGSTATVTDN

>SG-AL_contig_629_1_-3_174_272

MTTIPYSQTAEATSQKPPLIPAVAFANLYICLE

>SG-AL_contig_630_1_+3_111_317

MLILQNFVTIFQVENRHLHRKSCNFCWISCKKTKTYHTLARRLTKSLHQKLVNVMHCHLI

WAAKVQFPS

>SG-AL_contig_631_1_+2_491_913

MLSTCTAWRVSLASLRKHAHPKPHMKEEESFPCKLTPFSCKMSSLLGSYLPHERCISYTS

ALSFLLTIYIINDKQYISHNLSAHVIVLVTELAITLCTVLLQSESLGVSTSISYCPSVCP

QHSSVSLSVSPVSLSVSPASL

>SG-AL_contig_632_1_-3_192_371

MILPENQITVHETAEVSLGTVDSVADTYRLSTMRFRQSLSSDLPFSTVIGENVGDSVQYS

>SG-AL_contig_633_1_-3_45_284

MVDHFTALSRYSDNTPLFSETLNPQVCISGRFSDYHELKKSKKIIGCANLYHSQFQLDWL

QEFKNKNHYTRHAFGLELMI

>SG-AL_contig_634_1_-2_2_136

LLYHENSVYRQLYGYHLTSLLLILINIDNVLRHAALEDEVNLGFS

>SG-AL_contig_635_1_+1_160_312

MFTFNSRRFNHHIQQHKLLKPVYKPKKVALPVKVVCFLIIKQWLRLCYLYH

>SG-AL_contig_636_1_-2_176_880

MRRSPERAKSVPPALYFREEQRRKLQAVPTFADWRRVARATSVPADLLPVSRPWYMPYFE

RRYTPYYERRGWPYYGRRGLPYYERRGLPYYERGWPYYGRRYARSLPAPVEAFVPLPSEG

LRAHKIPRRYLLRGVARAGFISPWDYYDDVPGVRRRQEEEEAEEERRSGIRPRVLASPLA

RASLARAWRHSFPRVAPSRAEQAYYRETAYGFPLLFVPCKGSKKSDRQKAAIIAS

>SG-AL_contig_637_1_-2_44_265

MKPCRKGDTNAFCELCSNNFNVKSTGKTMFCDTRVVIRLASIKPTQRFENEGCRTKQLPD

FSRVKLKRQMQRQM

>SG-AL_contig_638_1_+1_1_210

LRLKFQLYLEKSSNFFVLLPSFSNLCVGFVLVSLHVSPNIVSPARLNTEVTGANFTECIR

IAFFARLDER

>SG-AL_contig_639_1_-3_2571_2810

MCSPSFQRVCVHCADACENQQSVCVSIVQMPVKTNRVCVCVSIVQMPVKTNRLCVSIVQM

PVKTRRVCLSIVQMPVKTNR

>SG-AL_contig_640_1_-3_69_1016

MAEACSKEDLEQCFDTLAQEARELYLDRSVPVLDSTPTPLQFYRSYVAPNKPVVLSNFTS

SWPALQKWNHQYFRQVIGDEEVTVAVTPNGYADAVNQGYFVMPEERRMTVSAFLDILEDL

ESVDGVFYVQKQNSNLTEEFQSVMGDVETEPEWGTEVFGKPPDAVNMWIGDGRAITSMHR

DHYENLYSVIVGSKTFLLIPPSDLPFVPYETYPAAHYKEKNGQFSIEPDPETGEVPWIAV

DPLSPDLSRYPEYSRCRPIEVTVHAGQTLYLPSLWFHHVRQSHGCVAVNLWYDMEFDIKY

NYYKFVENVSNLVRRK

>SG-AL_contig_641_1_+1_2449_2775

MMRATPVHHHIHQQRIKWFGHLTRMQPHQLAQQAYNKKISGFKVRGRPRKTWTDGVKETL

TLHGISPLQAFQFIADHQLFLPATPMMVQTLTSHGISPLQAFQLAADRQ

>SG-AL_contig_642_1_+3_129_980

MRQATTGRRTGIQWTFTKQLEDLDFADDISLLSHRQQDAQEKLCRVAEEAEKTGLEINIG

KTEFMRVNNKKQDPVRLHQENIKEVDKFVYLGSIVSKDGGTDEDIKCRINKARHAFNTLR

PIWRSTALSLHNKIRIFNTNVKSVLLYGSETWRVTKTNTHKLQTFTNRCLRNILNVRWPE

VVSNEQLWDRTRHTPIESEIKKRKWGWIGHTLRKPASNVTRQALDWNPQGKRKVGRPKQT

WRRSIDAEAKAAGMTWAELKGVSRNRVRWRGVVAALCSPRNQEA

>SG-AL_contig_643_1_+2_2_223

NKIQYYPHRDLSLGVLASAQFGHDFAQHLVAQSSIWVQCHQHLFGKLISQLSCSFEIFQG

TLFISIQTFDESSL

>SG-AL_contig_644_1_+3_825_1322

MVDKAEPGCIPMSPSCICLLGFRWTCHDFVDEICIFEVAFQIDVNVCQLHDVVHIFVSES

LTHGCKKMTALGSRDVALSLTVERLEGFLEVPESAPFLHAVHLLEDGQELLEGVLLLSRL

GSSSHAFDGGESGIQIHTSQQVSHFDSIDQPVTTVPEIEQVEDILD

>SG-AL_contig_645_1_-1_34_480

MYACARFVPAVRSVAAASGRGLLRPLGSTVVTSRQEVPQPCHSLTTFSSINFSSPSVSAL

LNQVRSFQTSTSWKDIDQAAKYIGAGAATVGVAGSGAGIGSVFGSLVIGYARNPSLKQQL

FSYAILGFALSEAMGLFCLMMAFLLLFAF

>SG-AL_contig_646_1_-3_393_536

MISVKTRLCSNCQRDQVYVNVLFIVSCDYALNYYFKKKKKKKKKNKNK

>SG-AL_contig_647_1_-3_48_671

MAPKRNNIIPNGHFHKDWQRMVKTWFNQPMRKKRRQAKRVKKALEIAPRPVAGLVRPVVR

CPTFKYNTRVRAGRGFTLDELKGAGVNKKQARSIGISVDYRRRNSSMESLQANVQRLKEY

KSKLILFPRHASKPKKGDATAEEMNLATQLKRRPVMPISDPVRREKARAITDEEKNFKAF

IVLRQARINKKLYGKREKKRREAEEKDK

>SG-AL_contig_648_1_-2_38_346

MEQAAANQVGAGGVPAGAQTQDYYRTENIPARFDNPDWFQGYGGKPQHPMYTTSASRYGA

KPPSVHTMPTQFYAKSQKFSEHLGQCGMYRNYSLNTEMDKSKV

>SG-AL_contig_649_1_-1_202_390

MDTTMSGLPFLLLLYPTALELSSLAHQTLLFSSCLQVQPQNISLSAVLSYTINSNFYRFL

SYD

>SG-AL_contig_650_1_+3_1041_1229

MAMLCTSYIGTKLPNIDNFFLLHVTGCSWVFFVTFKCNMMLILSFKMFYSHENKIHTHLS

TIK

>SG-AL_contig_651_1_-1_19_333

MAGPGSVKETTMSDEDFSYLPPARENLYREEGVKEKFIRKTKENPFVPIGVLVTTFALSF

GLWQMKTGNRRMSQNMMRLRIFGQGFTVAAVLAGVAYGASGKSKK

>SG-AL_contig_652_1_+3_21_242

MLVYSMIPYKFTMANNDSKSKHSLIISEILNTHCHCMHPHTSRSFPRTHTCTRVDTHTHT

HYPRKHLGNFQMSI

>SG-AL_contig_653_1_-1_1_105

LFLFIILFLFCFFFVVFFFLHHLVEILKCFYSRLL

>SG-AL_contig_654_1_-1_124_285

MCLSSSGNIIHTHTKANTIHFEGIMWPKKEQKKMQKNHLSVFMDSERLFCLKSL

>SG-AL_contig_655_1_+1_139_462

MLNFYLNIFFPCEVLDALPRESTVHCTAPSFFLSEFCSFLCVSFWHCCLYICSCNKIRNT

PSCPQSLEIHVVNSTILRYMTFYSSCLQLLSLPCTHTHTHTHTHTHTE

>SG-AL_contig_656_1_+3_966_1298

MRGCVQGWDVEGVGCGHSSFCSVGDVAERDLSLEHNAGNIAGADSVGVVARGAGGGDGFG

GGVQAALHTISSEGSIFKVEGLQANLVSIEGGAGLVEMQRLFRLTHVECLC

>SG-AL_contig_657_1_+2_2_130

IMRASRAGCAAPWRRLCTEFFGNELFPAQSEAKRDPCCISWIW

>SG-AL_contig_658_1_+1_43_210

MNINHISQSMRLISFSMKYRIKTKTPLDKTKESIFRYCQHSEQKKTGWGTFHHKSL

>SG-AL_contig_659_1_-2_1040_1381

MYSGIGMVMVLFSPFQKRVKFCSFLNFLFTLNDFKRSAKVCLRNIERKLSGVGDSALAIL

KYILPFNYEFVWKCSFLWQREFSTNNMHDKSGSFRLWVALPCCAPQGSDGAAIV

>SG-AL_contig_660_1_+3_3_413

DLGHLLTVGLGVEGSLCQQDGVLLRGHTQLVVEGVVPDLLHVIPVGDNAVLDGVLEGQDT

TLALGFVSHVAVFLAHTHHHTLVTGSAHNGGKDSPRSVVPSKPGLAHAGAVVHNQGSNLV

VAHLDGWMGFESLRKKN

>SG-AL_contig_661_1_+1_1_249

DGVLEGQDTTLALGLVTYIAVLLTHTHHHTLVSGTAHNGGEDGSGGIIPSKTSLAHSGPI

VHNKSGNVFVTHFRSLKLPKRTD

>SG-AL_contig_662_1_-1_478_636

MLTYYITFGSGRTVAESHTYGSVIVPFVWTVAGSFAYTVWLSGHTLTTLLWFR

>SG-AL_contig_663_1_-1_1_4977

KKPAAEEKPAEEKKPAAEEKKPAAEELVVPEVKKVKEETPVEPEPTPVVEEKKPIVEEKK

PAVEEKPAVKEPVVPESKEVEKDKPVEPEAAPVVEEKKPAAEEKPAVVPEVEKVEEKAPA

EPEPKPAVQEKKPVVEEPVVPQAKKVEEEKPVLEKEPAKTESKFKPIPETAEGEIPIRAP

HYLADMNDVVVELGQLARFDCRVAAYPDPEITWLKDGNKVVPTERYILQNFHDDIFSLLI

KEVKREDSGCYTCLARNEYGEAKTEALLNVMALKAEAEKGDMAPSFATKFGNVECLEGVP

VEFTCQIKGRPPPTITWLLNGREVAPSLDILTRQQEDSIMLAFRSVQLEHSGDIVCKLKN

HLGEATCRAKLLVKEDLSKKGDIPLFEERPSDMEVTEGGEAKFECVITGTPEPEVLWYFN

GRELFDSRRRVIRKKGDKYQLILREVIPDNAGVYTCKAVNAAGDASCAVELSVKEIPKEP

KRRKTADDEEVEYSFPPTFTRRILDQRVSPGRTARFECLMLGIPLPDVEWQKDGKSLKPS

DKLRIGREGSTAILEIDGAEPEDAGEYTCILTNDAGKATSRAGLTVQEEKKVRYTTPRLE

ARVPDVTTRAPSRGREDLYQPVTQPVRELTPEEKVPSLPFDKPHLLEVRDESVKLSWLPA

RMSGLPDNARHITYTIEARELPSNRWTRLATGIESTTYVARHLRPEKEYMFRVYAENKYG

QSEPTLPATMVARQEPEERKRYYAREESPAPIQFPSARPFVMDIGAETLRLGWHPAARAH

RLETPVSYRVEAQKLPSEEWIPLASRVRDTSLYLSDLEPDRDYNVRVRAQGPFGVSQPTA

PVWLPRAAGFTGVPVSRPQITELEPDTVRLQWQRVDVPAFTHEQDPLTYMIEMQEPPSVH

WREVASYVPHTHYIVHDLEPGTDYRFRVRAQTLEGLRSEPSPATSIFRTLALTRTPIDYV

EVTDYEPDYGTVGLSWRRVEVPPFDADDEPLLYMIECQEPLSDTWRSLVSGVPTTRYRVP

DISSTDDYRFRVRALTPYGISPPSHAAGLYRSPTPSRPLASDLFISSMEPESMRLSWQPQ

SLPSSHATTPSYQPHSYQIEALEYPSMQWRPLASDVRDTSYQLTGLAPTRDYSFRVRAMT

PSGGLTEPTTPVTVTSLPVRPRMPSKEPVVKEMGVDSVRLQWPRAELPYYVRQTTPITYS

IEMQEVPGYTWTPVRRGIPETSYVVKGLRSDRDYRFRIRPETEYGPGEYSLPVHAYRKPT

VSLPSREPFISDIGPSRITLTWLPAELPTGAQTTRPVTYRIEMREPPSGHWIPVAERVSS

NSFTVNFLNPDKDYAFRVFAELDGVESEPTAIAYLPHRMGPPKMLQEPPYISSVHPDSVW

LSWRSVDIPARITDYAPVTYRIEAQEPPLMEWRPVARHIPTTHHHLTGLRPGQQYNFRVR

AENDIGLSEPTPSVLLTTRSVPPNMPSHEPLISDVSPGSLRLSWRPAEVPSFLLDSLPVT

YAIYYQHLPDVEWHPLARRVAGTSYYITGLSPDNNYAFRVQAENQFGSSKPTHPARMPRL

AEIEAPIYSPEIEEVEPSSLRLAWKTPRIGPYKKKKPTYAIETLEPSTWTWRPLASNLTT

PSYKVSTLSPTQDYIFRVRAEADKVLSEPSFPISYSRTR

>SG-AL_contig_664_1_-1_1_357

QQEAPPVRVAGSRSRGVGGRRPLPRLERSDRLRLSSLSDPLQGGEEGQDRASSAHPDCTH

VASTIVVSRSLGSYPHFASVTPSKADLLQQVRSGVLHLNPQLFRLQAWLLHRPKGVHQS

>SG-AL_contig_665_1_+3_657_965

MYTHEFYLADVTRPILGADFFITHGLVIDRKGKRLLSLDKTSVFLKSTDSPLTLAGLSLH

PHNTYSDLLQQFPELLTPRFDSTVNKHGVEHHIITHGPPVHAH

>SG-AL_contig_666_1_-3_102_761

MKTVLATVLAFAFLQVVWVSFSTGEAVDCSALNASSTECCEGGNGSCSFYNCSNSSGVCM

TASATTIFCPDKEAFTQVCIGSTPTPSDPCKNYNNHTECCKNDCGFFTCTNATANFTVCG

QKNATDVCTEKETQSNDTCESHPTTTTVTPPPATTTLKPETCTSHSGNKIACCDRSCLFY

NCTDPTKHTTKKTVCVEKTAKKICPAEDKAVSVSCNVEPT

>SG-AL_contig_667_1_-1_856_1071

MLFSFLPIPICVCIMYACQFDLAKTNVMRMFHLRKSRITGFHFSLSSLFCCRCWYILLLS

LSYVNFTDFLFD

>SG-AL_contig_668_1_-3_4032_4301

MILTHMLVLCYSFFFLNKCQRFFPLRFLVKTVNFTNTLIVLLLLLLLLLLLLLLLLLLLL

LLMLLFYTADFTGRISSYFLRINTTLNCID

>SG-AL_contig_669_1_-3_66_1325

MSNKVEKPTLSGTRLKSRKRDEKEKYDPNSFRDTIIQGLQKHAGDLEKVSTFLDVTGSRL

KYRRYADVLFDILFAGGILAPGGSVVVDADQTKVSRTDVCVFACDARPEKIKGFYEVFYK

LIRRYKYLEKPFEEAVHRVLVFQKGFQAEERQKLAIVTGQILANNLASPRVLEALFKDHL

VKEGLSLECASQVFTVWLKEKEVREVFAALKKVQVDSRLQELFPVSKQSPQTLCTYFRDN

GLDAIADIYTSTQATKAKKEAQKALSEMMKDESPMSEMIEFIKDLVSKQGMTQKEVTITI

WNTIMQGVEWNKKEDLVAEQALKHLAKYTPLLVLAAQSTRSQLALLLRIQEYCYENMNFL

KSFQKIIVMLYKRDVVGEDAILCWYKQSHSPKGKNIFVEQMKTFVEWLQNAEEESEVEEE

>SG-AL_contig_670_1_+2_710_940

MTSVITSGDPNHDISNHVMTLQLLSGHSSLNGEKSFHIVICLPCNKYTHAALCSQIHSPD

WWQQTQCSCDPSYIIHS

>SG-AL_contig_671_1_-2_68_220

MGAHKTFRVKMKLAKKIKQNRPIPQWVRMRTGNTIRYNAKRRHWRRTKLKM

>SG-AL_contig_672_1_-3_324_2294

MWTDMRGTVMVLVVMLVVTTVGRKPRAPGPNMCMVQEVYGTGEKFFTHCVAKHLKMICER

PTFVSWDCCSGFERKEGQRGCTQEKPLGNLLETVEELDLLQFAQQIVRTNMTDMLVNQGA

FTVFAPSDVAFRETTGYERNQLQTASGSQALMLYHIAPGKLPLSTFRARNQKFVTMYRDK

KIRVNKYAYGVATVNCARIIRPDEKGTNGVVHVIDKVIKPLEAEGNLAESIFSNDRYSQF

QMALFVSDMVNQLRQSKNRGYTVLAPTDRAFSRLPSHLLDRILTDAYTAERVIRHHVVKG

VYCGDAIVVSVGLKTLDEGRVLFRCTRHGLTANRARVVDQDIVASNGVIHGIDKVLLPDS

VREPSDLLDEMHVRKFMELTRRAGLEHKLNSVTNATIFAPTDKAFDDLPSSARAALTNEP

LAMTRLLDYHIAKGKLIEDKLISDMELETGLSALIKVTVFRNGVAIDTAKSVGPLRQCDK

AAIQKIDKVLMPSEDTIMDLINQDKDLSMFQQAMETSGVSEMLLREGRYTVLAPSNKAFS

YLNQWRLDEMMNDPKRLKQFVDRHIINRMIAKCEVPDSSTYGIKSRQGDLTIFNYDTKGS

LLVNDFINVKEVDKLAVNGVLHKIDDVLKCTCERGITMSSRYQHRTRSRRHFHRLKH

>SG-AL_contig_673_1_+2_266_550

MTGRLEVQTPISSYYHCGIFVFNQLLPGVERATGSLGRLGPHSRVSSTLWMCHRGCCSKF

PDQHCRCLQLSGLADARIYYDRTVQYTVSTRNGTP

>SG-AL_contig_674_1_-1_1882_2028

MLCQVCIFELCEGCFSIPFKLLFAFLFDIGDGCGCSSNSYSHGIMHEYK

>SG-AL_contig_675_1_-3_72_2309

MFTFPKAVCLSLKHTQRAVPVCSSYRLVHRQPIDEEWKKAAAQELKGKDVDKTLMWKTAE

GINIKPLYTKEDVKEIQKEIPGQFPYTRGPYSTMYTHRPWTIRQYSGFSTVEESNKFYKA

NIAAGQQGLSVAFDLPTHRGYDSDNPRVTGDVGMAGVAIDSVEDTKLLFDSIPLNKLSTS

MTMNGAVLPVMAMYVVAAEEQGAKQEELAGTIQNDILKEFMVRNTYIFPPEPSMRVIGDI

FEYTSKYMPKFNSISISGYHLQEAGADTALELAFTVADGLEYVRTGVTRGLDVDKFAPRL

SFFWGIGMNFYLEIAKLRAARQLWAHLIKDKFAAKTDKSLLLRCHCQTSGWSLTEQDPYN

NIVRTAIEAMAAVFGGTQSLHTNSFDEALGLPTPFSARIARNTQIILQEESGIPKVADPW

GGSYMMESLTEEIYEAAMEIIKEVEDLGGMAKAVASGMPKLRIEECAARRQARIDTANEV

IVGVNKYRLPEEEGVEVLSIDNTLVRQKQIARLKQVRETRDSAKVQSVLKAITECCRSGD

GNLLALSIEAARARASVGEITDAMEEVFGRHVASDRMVSGAYKSEFGQEDEISNVMKRVE

SFHEREGRRPRILVAKVGQDGHDRGGKVIATGFADLGFDVDIGPLFATPKEAAQQAIDAD

VHIVGVSSLAAGHKTLIPQLIEELKAMGRPDIIVVAGGVIPPQDYNELYEHGCAAIFGPG

TRIPDAALKVLDMVENGADNKKSQAM

>SG-AL_contig_676_1_-1_208_1932

MNKLAHPKLLMLWDAFEEPRTMVLVMEFVGAGELFERVIREDFVLTERDCVHFVRQICSG

VGYMHSQSILHLDLKPENVLCVAENSNQIKIIDFGLARFYQPGDSLQVLFGTPEFIAPEV

VNYDAINFATDMWSIGVICYILLSGLSPFQGDNDAETLSNVTSGEYDFDDDSFDDISDNA

KEFISGLLVKRKDKRNTIAQCEKHPWLAQQDQRIRCKRLNTDRLRCFMARRKWQKTGNAI

RALGRMASLQKLLAGGSSSHSSSSSLSSIPDSSAVQTASGANTPVFSPPVKQSASSHPLA

FAPVKEDDKVSTPGADTATDTETGTGRVEADREKKRAEDTNDGSLKTVTWRTTTTTTTES

SPAAVDTQSSPSSRRKEHQDNTLVNTSSSALSGRSKLSKEDAVTVSEVKVTNNSAPEDRA

DTSSPTPPSHLPEACNETTTSNNNNNKSISTKTNTFSPGPSSEEAPSTKCPSGQAECPPS

FLKEMVDCTAFKGDVVRFDVTVTGMPPPRLMWMVEEEDLEEDERHVVELGENGHCSLIVR

HVTEDDEGEYSCRAINSQGEATCMADLNVYRMGSV

>SG-AL_contig_677_1_-1_1_858

VEQSECCKRGCRLFSILSFVHDHSDSNATITDCAANCNEAYGNQREKDRACQLGCDIQLQ

ASTQAAAQVEGKHRQMEDDMRDTVHMLYPLLYMHSVYSNMLDKVHHHMEVSWSIFTQANS

GELVVLRSEPRIIDFVSSDEEDIPDGVPWLDDKTSSYVETNIVGQDNSATPLLNFRASQM

GAAHSPDDGLRLDTDDRAGRSDWLTCVAKKTGIARIVLCILILATAVVMIWFCLSAATTA

PEQHLSISGDLEYLRAMGEKDGFKLLHPQDRVEARPLPIKIRVEQI

>SG-AL_contig_678_1_-2_14_6187

MPDYFVTFPMDTRIYQRPVTTHGTKQIRVLLQPVENLGDLHTCLNAHLTRYNEEFGNVHL

DIMLSDFTIAHVVRMHRILSFHHGGNMLLIGAVGSHLSRLCRLALHVADIPIHKVDTSKQ

SNFFDGLRSAIRLSGSEGKIITLMFTSRDLTDCVYLDAINSLLVSGEYPHLFSNDEMDGL

LQAIHPAMKREFPSLSMDPMKFFVSRVKSNLHIILCMQPSSVLLRDAYNKYPGLLSGCQI

NWLCDWPQETLLGEASYFIAKHQLTQEFEDLRDSITTCLANIHSFVLRDCKQLPWAGDLN

PEVTLSTVKVHDKKKDQLKVQTVKVPNLPYSKTILQERIKQRHRDRNTSAKNEVYVGPTT

YHRFMESFKYLYTSKAQQRTESVEQLKKVLATLDKTRTDAKVMKKAIKTINGNFEDACKN

TADLLTRLTNKATALEKLKAKIGLSKSLDAYLHLTEFESEEEEEDELLKEEEYDDYDAEF

DKMREATLKTRQVQAKEEYAEAQKRVEECRQQLEHAKSQVLVWKSKVDRQCIERLKAFQS

PPLLVGQVMEMVMILIGKRLPSQRIQEARDYGGKEDLSSRMSSSSSSTKLIVKKTERQRS

AKQTKDGERFDRAQWKSMVQTMTESTKFVDMLHNVSWEDGLPSDVLQAVESYLAVSKEGQ

LGVTGEGSMLENASEKQFVAVKRSASPQGRNSGISISGAKYSSEDCATLVQYTIAIVEYT

RRCGPLKAAIERMHELEREIEENERMQKQAEAEPKEEEEKKEEEPEEELSEADLPRLQEE

VNALQVEFDQAVVEKHSLHMELQSMNERLKAASEMVESLKDQEVSWRQHVKDNSSNELLI

ANCLTAAGFLTYSGAVNVDTRLRMGEFFMQVCEHHGLPLHKNLLFRNIELIDFLYTPIDI

MKLEMMRLPTTRLMLENSCFLMQEASTTAWPLICDPTSRVLQWLSGLYQGRELVVVKYHE

MRSQLENCLADGTPLLVTDCDVQQLVCDGRFIDTIASCVSFINGKSRFKVIVTDHEVECD

PKFRLFLHTTGEPQTLPHYLAAYTSVLFFQQNRHCVEEELLNIFMAHEKSRLENESSTLR

QEKEENMLYLDKLERQMREQLSSDVRLMNDLQATRRLAELKKQFDETQETLTRVAISTSS

IQKAREGFRVIAQRAAVCFDTTQYMREINPLYQTSFSQFRALFEAAISHSDRSAVKALID

KLTHSAFSATVRSVFERDRQVYTLLLATEVEESQGNIGPGEREYIVSPNYSSVVMSALGY

IQPADSRIVQAKKPFDWMTDDQFHNLQVLATHFEWFQEMFDRMPKDGRETQWRNLCESDT

PELQPLPDKMDEVYKPMQRLCVVRAVRSDRLIHAAVIFINSVLGKKYTADVPPDFSTLLR

QSSPTIPIIMIFDTEADTAVKLFTDFANRKQNRHVEIYLTDNSSAPEKVVRKQITKGMVE

GQWVLLHNAHNCPHLLNAVETLLAESSPSASSDQTFRLWITSKAVPKALPVRLLQNSVKI

IINSPKVMKDSLVRSMSWMEPDILKQTSRSEWPAMLHNLCYLHAALQLRARYGQGGWNLP

EDFMFIGFRELQEAVGFTVGEFKDPMYVLAGDGNQVPRTTSWTGIRYILAEVVYGSYITD

PGDQQSLSAMVDYWMSPTAVKKEFEVARLKYRLPQVFFNPNVRLNTVIQALDSLNHLFLD

VPEGCHLHPNVETLLGDDQYVFTRLNKIFDVMPSTSTLSHKLFPRPPTPFIGPPLAGVSS

QSNNPAIVEQGVFSTASYATYKMRKDVELWEICYNMLQRVPKAYNKDYIIEKVKKIGGFT

TFNNFIMKELEIMYRLLNEIKTTLTAIKIATESNLLGDQMSEHTLSVADDLHHLRIPQLW

CDMSGNSAPPLTWGLGQWLTELQSRCHHFERILSLGREKMPAYWLGAFFNPRGLLALVKQ

ESIRGFSGDRSGNFEQFVFQTEVTSRDKDHLRDPPQEGMFVFGIYVWGCAWEKTTGELQD

SPPRIGCAALPVVHLTCWPASDKPTGQDSTRASETYQCPVYHSRIAPREVVMELDVRREG

IPATRWALRGLSATIRPY

>SG-AL_contig_679_1_-1_34_135

MITRDIPAGVLQNLVCAHEVLLQTKVTSVDHSLD

>SG-AL_contig_680_1_-1_28_330

MGSLGRLGTTQLCLIHFMDASLRVLFKVHDQYCRCLHLLGLINARIYYERKYKVQPWHAV

SILDGIPLHHDPSPHYHEICHQSIVKIVPDSVTFHLKQHRR

>SG-AL_contig_681_1_-2_2_229

ICTTHQFTLLSLSHSQLLQSSKTENRHHKQTQKQDSGKSVLHSKIKKYAHFLLLQRKAIT

EEPLEEKQQQRTRIWQ

>SG-AL_contig_682_1_+1_733_978

MIIIRHCLLLPFVFFSTAVALCTNSIHGHTVMLLFFLWNIEHLCVLDFFFFFYKRIWILF

WWGEIPTVYIYVTSLCYRPWPW

>SG-AL_contig_683_1_-1_58_1653

MSADQVAEQFVVDLLHYLKSSKLYTDLFNAAQNEAFKPSIRVSELRKGVQAGLINGGWDR

KLRNAIYHYMHSHPRQPNHPDPEHNKEPILYIRKAQQAWEKKILKSLNSMCTELNIPLAR

RRAEREQKEMSSKWTELGADAPDDKPWISLNLSQIRPVYAPKDFLEVVAGLQNNNYQNSI

SNGGSFYNLWGIIQVPIKLKSINQLRVQYSEMSISQCQTGVDDMSDIPSEVFDLERWKLG

NKVLGACHAPVAREFSKKGCPACMRAELWCHMLGVEVDDVDILYYEQLKNCVLHHDLLVD

NLLYKDVKLTATNDDQYFVFEDYLYQVLLPFSRDTVVLKHFSHNSATPPKSYIRGKLGVE

EFAVVYPPNGVIPFHGFAMYVAPLCYVYKDVVTLYYVFRRMYTQYFFRLHSVSSHPQGIV

ALSLLFETQLQTHEPELFCHLKTVGCQPLKIAFKWLMRAFSGFLASDQVLLLWDRVLAFD

SLEILPVFAVAIFSFRKTNLMKVQTYNAAEAVLADVTTLQVVPLLQLALFSK

>SG-AL_contig_684_1_-2_23_235

MSRCRPDFTHDFLFRPRLLVLARKLCLSVYNLNCIINFEKWPSPRTTPIITRTGRPTEMV

SRGPRLSDILP

>SG-AL_contig_685_1_+1_247_423

MKFTCIHNDNSRQPHAHEVTSFISCIYQCTSLSLSLSLSLSLSLSLSLILFSVFSVKLF

>SG-AL_contig_686_1_+1_574_720

MSELIKRNPILLWPLVKSLGNGKPSYMLLQKHHPLWFSLTYFQPQILSN

>SG-AL_contig_687_1_+1_2662_3036

MSLLLGLNIMLTCFSSSRHGKLTKHYCIISLSLPLENKDQSSGHLPVNKRKLCSSRVRIV

SWGLGDQLSYYRSRRRGQWIMMMMVVVMLKMTMTATVMMMMMIMMVVEMVVVLLLLITMA

IIMMT

>SG-AL_contig_688_1_-3_60_659

MEGQNKSQSIFSPYSSSDPSMNVPVTAGSAGAVISPYLNFDPSYINPGSDSQFIFPEGAS

RQRGRLELSFSQIGGSVFAGAAVGGLNGLYTGFRETSANQLTGAVRRTQMLNFITKQGAS

SAQTLGVVALMYSVFGVVLSYGRGVDDELNTVVAGTATGLLYKSSAGWKKCVRGGAIGLT

LATAYVLFTSRDRLKSLMDN

>SG-AL_contig_689_1_-3_483_635

MVSSLASPLKLPDLSCRCHYLPHKNQTFDKKSLIFFLYLATGRHQRLFLFL

>SG-AL_contig_690_1_-2_1628_1954

MNPIVSSNKYSGLHSGAVLFPTSFEFDTCKAVLFVILSSFPEGPRVHRRSPIRVLTGPTL

LNFRDRTTTGVFIVVRPSTILSRSQTSSHYKCPTLFLRLLYCLFQSDSA

>SG-AL_contig_691_1_-2_104_562

MAKNTGASKFRKVDVDQYDDDKFVDENEADADSSDSQGANDAEVSNLLAQGKHQDALKLV

LTSAPVASKNQAVKDRAANTVVRVLTSFKTADIEKAVKGLDARDVDMLMKYIYRGFESPT

DGSSAVLLTWHEKTFAAGGLGCIVRVLTDRKRV

>SG-AL_contig_692_1_+2_425_607

MTLVIIMNFYSKCRRVSTQGKFKEKDREMGRDKKILGEGGGGNRERKRERKERDARVPSS

F

>SG-AL_contig_693_1_-3_123_305

MRKREELRTNIKKEKEEEKTRKKERKKGRKEECRRRRDEHEQESNLSDSAKSSVGVDQST

A

>SG-AL_contig_694_1_-1_1_123

ESESKLHKAKSVDGIRSFGGNCEENGIKYAAMTQEGNYRIK

>SG-AL_contig_695_1_-2_104_709

MTVGKSSKMLQHINYRMRCTLQDGRTFIGTFKAFDKHMNLILADCDEFRRVKPKTAKSGE

REEKRTLGLVLVRGEHLVSMTVEGPPSADEGIARVPLGGAAGGPGIGRAAGRGVGGASGS

GAAPGLQGPIRGVGGPSPQMMAPGRGATMSAPARPNMPRMPMGGPGGPRGPPMHMMGGPA

GRGGPPPRGPMPGMMRGMGPPM

>SG-AL_contig_696_1_-1_1_3936

QRYGSAANSTTTATAAVVVEDRNNRGSDSAVSRSVHSPVSHPGSPPVDRERDRSAAEHRN

GADKVSASRKEKDKNSNHSSPKDKSSAREKEKSVQRTSSRDSVAHSPDKSLSRDGSREGS

NGKGRRDSSSSKHESPPVFKDFSAYHQQEIYSLADHGNIRDPGFVYPTKTTTATAGRDNK

LSNSKNSNNGKDKTSTHSDVKRKEKALEKQKSRDSGIGRTLDRPQSREATREQTLEKQIS

RDSGTGRSVEGHSPRESSRVDITGRQVSVSGVSREGDSVELRDPARPEDRTGGVHTPSRD

SGTYAWDRSHSKHSSADSVTARPRKVSRERTLERQQSVDDSRLKDPNDRRGNPSRSGGKD

STVEDQTSRSNPHRSFSRERTLERQTSVENPTRHDHGDREDTSQRRFSKGRTLDKQATVD

ETGKPDPGHKAENPFRDNSRDRSQDRRTSTDEIQLRDLSKEKRRAHRPSSRDEAQFSRQT

SIDDGSSSHIEPDSARSQNSGRPHSRDFTKTEDSVRNSDNDRQPQRHVSRDRTLDRQWSV

DETRRVVRDEHDYEPSRIPRRTSEQPNRETVFNGERLSARQAEGEKRRSQDISRVHVHNP

EAGQNVAVGRSRAGDSAGDGVSVREEEHFRGSERDRRGGNVQRDRCDGQGRSRNIRDRSG

RDSEISGVDIRDSDVTSRDRRDSGNRNTGTRDSEGRTRSRRDSEPTSVDLRDSDVTSRDR

RDSGNRNTGTRDSEGRTRSRHDSELTSVDLRDSNARSRDRRDSGVRGESDVRSRERSDSG

DARNRERRESGDVTNRERRDSGDVRNRERRNRGDVRNGERRESGDVRNKERRESGDFRNR

ELLDSGDVKNRGRRDSGDVRNRERRESGDVRNREILDSGDVRNRERRESGDVRNREILDR

GDVRNRERRERGDRIRDKQQSDSRLSKISEGRGAGTSRTQHGVVTDTGASNRHSRGHGIE

LVEVHRSQDDYDQQTDRPGEHEPRREKSRQKTSDDQKPEDFKRKPVSKHLAEAVTTTKGR

PERSVGQTTGEGIVEEEKIKERGRSVSYSADNTGRSRSSREIIGEEHSRQHPDRSLSDKQ

GRNRDSGAGGVRHDTARPVHSGVQRVKAEERAGVHTTGRSRDREWERERQQYESSRTGRG

GGTDHSPIAPERRVASEERNRLGGTEREDRSRLGTIAREEISRLGTGKVSEGRNQQQPAT

SDRKLRTSDRDLDERDGDALDRRERVTSVGSDRRGGNQGRERDRQEGRDKHPGDRERFAA

RQQREENRHRQSDRERQYSDRERQHSDRDRQRGDRERRDSDRDRYSDRERHH

>SG-AL_contig_697_1_-2_53_1402

MSGDKDWPGRWSHIKRLLERTGPFAHPDFEPSPEVLGFMKETCKLLVIGAGGLGCELLKD

LALMGFHDIHVIDMDTIDVSNLNRQFLFRPKDVGSSKAEVAAAFINTRVPGCKVTPHFSK

IQDFDESFYRDFHVVVCGLDSIVARRWINGMLISLLNFEEGEVDQSSVIPIVDGGTEGFK

GNARVVLPGITACIECTLDLYPPQINFPLCTIAHTPRLPEHCIEYVRILLWPKEQPFGAG

VAIDGDNPHHLTWILQKSQERANEYGIAGVNYRLTQGVVKRIIPAVASTNAVIAAACATE

VFKVASSCCQPLNNYMNFNDTEGVYTYTFEAMKREDCLICSSQPQSLSFSENDKLQDVID

FLIESTTYQMRSPGITTSVNGKNKTLYMQTVKSIEERTRENLKKTLKELGLKTGQELYVA

DVTTPTSLTFTLTIATNMEMGAENEVNAAN

>SG-AL_contig_698_1_-2_146_241

MVCQLKLVFSVDVEVCVFVCKCHMEMLRSATE

>SG-AL_contig_699_1_-2_110_493

MMALKDKVCTRELQVHDKELCVGEFGHNDFTSALMNPKCLHWARDLARKLRKGYANSTQI

LLRSLPQENSTIIRLSCLDPDACSEESTKLSMKYLAKSLPNVVPDAELGSLDSEVRDYVI

DRDVEKLA

>SG-AL_contig_700_1_-2_476_766

MVLYLLTRLMHRDLDGAAVRASELKSYECHCIYNGNKMLLGLLLLLFVCLFVVFVVVVVL

VWVLGCWFFCFLRWSHSCPLLLYLPNPEPEFDTKNPF

>SG-AL_contig_701_1_-2_854_1087

MSNKLILFVCLFVCLFVCLFVYLFVCFLQFEFAQEGFISFIPLLFPTKPVISEYINKSVT

LLWKKNQQIHTGSILPNV

>SG-AL_contig_702_1_-3_387_605

MLFSFHLFRKINKCVCLFVYLFVYLLICLFIYSSFYLFVCLCLLIVYLFLCFVLGGCRLR

KKIMWGYVYFCFF

>SG-AL_contig_703_1_-1_82_594

MSSRKTKGKTTKKRAQRATSNVFAMFDQAQIQEFKEAFNMIDQNRDGFIDKEDLGDMLAS

LGKNPTEAQLDEMMNAAPGPINFTMFLTMFGDKLNGTDPEDVIKNAFACFDEDGKGFIQE

DRLRELLTTMGDRFTDDEVDEMFRDAPIKRGNFDYLEFTRILKYGKKENDE

>SG-AL_contig_704_1_+1_73_231

MFCLSNNNSLNSNNTFYFKFRPRYNSVISFKLNLLWDSRASMAWLPTKLNMTT

>SG-AL_contig_705_1_-1_298_915

MASGDVEQVELRRYDTSQPWGFRLQGGSEFNMPLYVAQVSPKSIAGKAGMKVGDAILQIS

GASTQGWSHSQAKQAMIRAGNDVDLVLQRGMINPQDIPQEDTGEKRVELDEGSINPGMNE

GSKYRNVMPKSYRILEQQLGGESGGGSPAPSVFGKKGGAAAAAPQPAATAEQGPKPASIF

DRKKQDRSDYLQAQGQTIQKAYGQKY

>SG-AL_contig_706_1_+3_3_104

ASSGSTRRSLVVGGSPACGWAARRTLVLRLKHSG

>SG-AL_contig_707_1_-2_80_304

MTGVRVMALPLHYFILFILPFLLYFYISILFQLDNCITPKHPDFDLIQVTVTPRPHGDAR

GSFSINIILTFWKFC

>SG-AL_contig_708_1_+2_515_709

MCCLFIYTLFVCDFGMVIKSCYIQETVQLKKRKCLRFRVFSYGNKKSSFLLVLLFAFIFF

FNKKC

>SG-AL_contig_709_1_+1_166_399

MYIIICREGVESGGKCDGLMEKRKWMDREGRKKSRNKENNRKEEIFSTEFPEGRDAIYTE

RAPGIPMGGSKTKTCIMS

>SG-AL_contig_710_1_+3_3_446

IKRPYRQITLQNGHPVPIPVFTISLPSRQSKIAQTVHHKLRREVEGVRERKLCEGLLEKK

VEWTGKAEKKQTTVKEGRGAETKRTIEKRKFLAQNFQKVGILFILKEPLASPRGRGSPTD

TNKQQKNNKNTTTTTTNKQTNKTNKTEQ

>SG-AL_contig_711_1_-1_664_861

MNVHTGRCWITSVLPRTKHTKPKETNNQLLVPSGVVPEHFIKVSCCKIIGCLVQVPFRVH

LLKLFL

>SG-AL_contig_712_1_+1_115_333

MAECLHVAYGGVSTCKFMLMNGNKATENIPFTPSPYPPASCTRKLVAVVKEFKGYFAENS

LIPYTVTALSLFP

>SG-AL_contig_713_1_-2_545_1486

MVSYFVLALVLVVGVSSFPQQSENVVKWVDLIPSKTVGPKKGCLVRSTPKHLRKYPARAP

RIYEEPGYDFSKLPKEHDWRNVNGVNYASTTRNQHIPQYCGSCWAMGSTSALADRINIKR

KGAWPSAYLSVQDVIDCGNAGSCEGGDDIGVYEYAHSNGIPDETCNNYQAKDQDCKGFNR

CGTCSPDSCYAIKNYTLWKVGVHDDASGRNKMMAEIYHNGPISCGIEATDGFEQYKGGIY

TEYIPVPGINHIISVAGWGVENGTEYWVVRNSWGEPWGEQGWAKLVTSLYKNDTGNLYNL

GVEEDCHFGDIIIN

>SG-AL_contig_714_1_-2_29_298

MASMDAIKKKMLSMKMDKENALDRAEQMEQKLRDTEDQKSKLEEDLHLLQKKYSNLENEF

DKVNEGLQEANAKLEASDKRLNELETDMAG

>SG-AL_contig_715_1_+1_1_198

FFFFKMKSLLLPKEHLKSLVIFEKKNQNVPKINAQYNKQDTRGKHSTDLFKTAQDIASWD

FNISQQ

>SG-AL_contig_716_1_+3_3_245

SFLDTTDPGLSWSASGLVSSHLHVVCTMGRVGDQHSGEASKPFYSLVFYVMEDWCCPHHF

SDWYMLRSPDLQTFITSVCQY

>SG-AL_contig_717_1_-2_128_622

MRLEKCYFCSSTIYPGHGMQFVRNDCKTFRFCRSKCYKAFQKKRNPRKTRWTKAYRKATG

KDLTVDPVYEFEKRRNEPVKYTQELMQKTMKAMAAIEKIRVKRAGLHIQQRLTKGRKLRL

EHGAKILEKNISLLEPHKVAPKKKMEERLVEVIEEKDEEEEMLLN

>SG-AL_contig_718_1_+3_3_146

NHPVYIHIYVFNSPITYSTNKSSLVFLFLNYIVNNTHAIYSVCFRLIL

>SG-AL_contig_719_1_-3_2205_3146

MDDQTVQGMGEESPTNLIINYLPQTLTDEEYRSMFLSIGPIKSAKIIRDKATGYSYGFGF

VDYQHPADADRAIQTLNGLQLQNKRIKVAKARMGGESIKGANLYVRNLPVPWTESELNKL

FEPYGKIIQSRVLVDLQTGVSKRVGFVLYNTRSEADAAIRSLSGSTPDGCTEPILIKFAD

DNSKKMKAPNVQYVPSPMFPHHGPGPMRNQGNRFRYNPMSGNYNMPNSGLNVNQGGYAIF

AYNIGNNATERTLWQLFSPFGTVQKVNVVMEPGKGICKGYGFVTMTNFQEAQNAINHLNG

FYFQGRPLQVSFKT

>SG-AL_contig_720_1_+1_1618_1887

MHTHQSMQTDANYISLISMVTLAMKYTIVVNLALKSNQIFVSLTLLAPSTDLSVPQFLPL

DRVRIHLYRNTHLFFHFLIQLYSDLALLAC

>SG-AL_contig_721_1_-3_189_323

MANTQFATVAAAKTRQKQADSLSFRCGFKAEQICTNTGSSLRLQM

>SG-AL_contig_722_1_-3_285_527

MVFMSALSVFFGLYVCFCVCVCAQIFFFLGHRNKRAIVLFILWSVHADCVFVDFLQSPIL

LGGKNIKRWLSFNLYCVCTYI

>SG-AL_contig_723_1_-1_52_273

MADVGKDLGNTADPKNIQDLTTFVQNLLQQMQDKFQTMSDQIISRIDDMGGRIDDLEKNI

ADLMTQAGVDDGRV

>SG-AL_contig_724_1_-2_182_340

MCTSLVLKLNNICTSLDILKYLHLETNNMCTSLQLNKIRNVITNASAALKKKT

>SG-AL_contig_725_1_+1_1_255

RRARPSEEPPPRPSTDGLHETLPRSSRFYSCNSTVKPNYVCSSLHAGSSPFCLEAYVTHT

TRHCSSQRRPSFHPLSPADKHTTHI

>SG-AL_contig_726_1_+1_1171_1488

MGLKFTWQLCMIGKYQAGSRTSHPEKKKISAKNRQSQESINSAVCNRHMSIKPCAQTKVH

QMHVSDGVGISDLCPVLTKGSKSHLFYNFYNGLKNHLGPMTSILSF

>SG-AL_contig_727_1_-2_23_262

MKKDVSKADALRTQYFCFEVPKRGEYACFCFLFTILTSLSVGYKLKKDPKGIVCLFLFPV

VCVFFLRNPILNGAKVTRCY

>SG-AL_contig_728_1_-1_37_939

MSTTNGPASSEQQVPNGSSTPQSRRNQVMLGDLPDDFLRSGRSQMQEDERIARVLQAQQQ

AGFVQPIPSNIIGRLSLTIVQAKLNKNYGLTKMDPYCRVRVGHTVFETPTAVNGAKNPRW

NKTVQLYFPHGVDSMYLEVFDERSFTVDDRVAWGVINVPQSVMNGETVDEWFSLSGRLGD

GLEGSINIVMSLLPVSSLPPQVSYVSAYTTPMVMPMYFPAPVVGQQVGQPVAYPPQPGMM

PQQQQQQQQQQQQQPQMTEEDLKQVKDMFPDMEDEVIKSVFEANRGNKDATINSLLSMTA

E

>SG-AL_contig_729_1_+1_1528_1767

MRSMSASLRRLHPLAYSSAEGLGQHISRIFVAVRHTVHSQTGCSHRSSKAVAVIASKIMQ

LPISLFVSLGASVCGTWCST

>SG-AL_contig_730_1_-2_275_1600

MRAAILREKAELERQLQELRHEGSHDEMSHRDKRLSRREAGEVKRQCVEDSEAIGESSED

GRRYSRRSGQVLDYVDSDDSVAEHWREVSHFLRAKRKRSKMCKRDHRLRKYPRSRSPSSQ

DQRSLSTNFVDLPPKAVGTYAHRPERLSSSRTSRSRQLPSPVTSAKPRRPGNSQARLLPS

EEEDDNFVESTCTTRQKSEPIHQPDDSYADEQDDNPQSDDDSAGPGEESQIQDSNHSETK

SPGHQKFCAALGAGDRGKMLECLFMSMEAMQQEIATVKSGVRDEIAALRLMVLQNLNQSS

AAHAGMIFDLPEEFTFPLSTVEEVAELEEKLTNSSFKRDMVNALAATGGLDIKTALWGIM

KRLFTSNLAMKYNYIGSKGKLCFRDLILRRVVFEAVRRLCPKATEYDIYLKLSMWLTSAR

HRDGGRKRRFLACAEAEERLLA

>SG-AL_contig_731_1_+1_1_210

FFFLNKSMYYQPKHNHTFITWDSKCTTISRSHTKVSMHLVGTRTHPLQITKITSNKHTLS

TQLKSFWHCM

>SG-AL_contig_732_1_-3_177_767

MSRYSSSIEGKIYVGDLPPDASEKEIERAFSYYGHLRNVWVARNPPGFAFVEFEDSRDAE

DSVRSLDGTVICGNRVRVEHSTGRTRPKFGMRGGRGPPRRAFRPEDRCYECGERGHYAYD

CYKYRRGGGRSRRSYSRSRSRSRSRSRGRRYSRSRSHSRSRSPSKSRSRSPVERKRSRSR

SGSRRRDSPEDGHRSDN

>SG-AL_contig_733_1_+2_104_217

MSDDRTRLTAAHTVTASISGTAHTFYFSSTAQSINLDP

>SG-AL_contig_734_1_-2_80_736

MADEKEEVPESPEIHFEPVVQLKPVETKTLEEEENVLLLLRAKLFRFDSEAEPPEWKERG

TGEVKILEHRVSNRIRILMRRDKTLKICANHYITPIMQLKPNCGSDRAWVWSTLADFADE

EAKEELLAIRFANAENAQKFKTVFEEAQAKEESLKKLKKSGEREEKKPNGDVEAAEKKEE

SEDKLAEQLGDLSVKDSAGGDTPATESNAKDTEKQEEKD

>SG-AL_contig_735_1_-3_69_1460

MVWTAVSFVFFAIFLTIRAEPAVLAPQQNRSLYSEPVSEGELMQLYDQMKQGDTLLHERL

KDSYNALRELLKEGDAHLKEQLVQGMAQAKEQQKEGDARLKEELVEDTTWLKDKLVKAES

RIKEELAEGEAKTKEELTNQKEAFEKLRLSVKEWTQTITAEMDHLKQQMSDVQVELQYSA

TTLKSTKDQLHTTQTQLQATQETLQSTQETLQSTQEALQVTQDKVQSTNEELLRTRQQLQ

NEVQNFTAVKTQLQAAQDYVYSLKVKVNSLQSTPNLGELGSSGKWDELDYRDVGSTFTRW

GRKSCPDNTSLVYSGVVGGAWYDHTGGASNYLCLSLDPQLDNKTREGWHALLYGAEYTRV

TGHGGQDVVCSVCRAPQATTLMIPATLTCPQGWTTQYKGQLMSSYQDYKGRTEYVCMDEV

KEHRDGGNERKTGALFYPVASQCGSLPCPPFVQGKVVTCVVCSL

>SG-AL_contig_736_1_+2_125_247

MIIIMTIFKKMIINSIHNSTSNHTTSLVKQDTEVNRALKHA

>SG-AL_contig_737_1_-1_160_612

MRDAKLDKPAINEIVLVGGSTRIPKIQKLLQDFFNGKDLCKSINPDEAVAYGAAVQAAIL

HGDKSEEVQDLLLLDVAPLSMGIETAGGVMTALIKRNTTIPTKQTQTFTTYSDNQPGVLI

QVYEGERAMTRDNNLLGKFELTGIPPAPRGV

>SG-AL_contig_738_1_+3_5001_5846

MQLKVGLDLSGLLGLAGLAKVGRLAHVVAHQLVQESLVRGLGEHALFFQDGQQTHGLLNK

LNAGLQVHTEVNELPVDAFLLVLLLLQHEHVVVEELLQALVGVVDTQLFKAVEIEDFETS

DIQNTDEELALVLGIQGLVDTLDQPLKHTVVQRLGQGSYGVGHLVHVATLGHVLASDLDL

GLQQRLEEISSIDTQKEGHLLSLSSAVSLSLLFTGPLLELHLSEMHDGSGTLVHAGLFLI

CEAQHIKGSISQLHFLDVVNTVHNQVTLVDERVQAGIGLDQT

>SG-AL_contig_739_1_+1_130_339

MFSRKPPYQVLEIHAKLLCSSCLDIVIETTQKGVLLCYLSIFMAFLYGSFVVLSFLSISF

LFGVCNLEVI

>SG-AL_contig_740_1_+1_1_216

GLQCVLGGGGCQGLWLRSGHGWLCRPRPSYSKKNFFFFFLIYMPLVFFLLAVSLVSSSPP

SLVKVKAGCCSQ

>SG-AL_contig_741_1_-1_31_1680

MTQIIVLNQNTRRESGRKVQLGNIAAGKTIADVIRTCLGPKSMLKMLMDPMGGIVMTNDG

NAILREINVQHPAAKSMIEVARTQDEETGDGTKSVIILAGEVLSVAEQFLEQQMHPTVII

AAYRQALEDILEVLRNKICVSVDTSDRTQMLNIVRSCLGTKFISKWMDLACQIALDATST

VFIEENGRKEIDIKRYAKVEKVPGDTIEDSQVLKGVMINKDVVHPKMRRRIENPRIMLLD

CNLEYKKGESATNIEISKEEDFAKILQMEEEYIRKMCDEVIAFKPDLIITEKGVSDLAQH

FLVKAGISVIRRVRKSDNNRIARACGATIANRTDELKEEDIGTGCGLFEVRKIGDDYFTF

LVDCKDPKACTILLRGASKDILAEVERNLQDAMNVARNVMIDPYLCPGGGASEMAVAQAL

NEKAKSITGVHQWPYRAISRALEIIPRTLIQNCGANTIRTLTALRAKHAQGQKNTWGIDG

HTGNIVDMTEFGVWEPLSVKLQVYKTACETAILLLRIDDIVSGTKKMDAAMSGASRQETQ

QAPPPAGGDE

>SG-AL_contig_742_1_+3_3_227

NGPGRQTNADDRKGKKNGRNEASNRKEEIANIEFPEGGDTIYTERVPGIPTDANSTGEGE

RGSTWLELHQASQSD

>SG-AL_contig_743_1_-2_89_391

MSVFHQREINLCRKWSVFHQRGINLDNIFHQREVNLCRKWSVFHQREIVLYRKWSVFHQR

EIDLYRKWSVFHQREINLCRKCPFSTRGKQILLKMVCFPPE

>SG-AL_contig_744_1_-1_79_1137

MSTSLISAWKKNLFSNPFFATCENDEQAPALKVNEERTIAAAATEEYSCEFGSAKYYGLC

AFGGLLSCGLTHTAVVPLDLVKCRIQVDAAKYGNIINGFRVTLAEEGFRALGKGWAPTFF

GYSMQGVGKFGFYEAFKILYSGLLGEENTYLWRTSLYLAASASAEFFADIALCPMEAVKV

RIQTQPGWSTTLREGFPRIMQEEGVSGFYKGLVPLWARQIPYTMMKFACFERTVEALYKY

VVPKPRSECSKPEQLVVTFAAGYIAGVFCAIVSHPADTIVSKLNQDKGSNFMQVASKLGL

MGMWKGLFPRIIMIGTLTALQWFIYDSVKVACRLPRPPPPEMPESLKKKMGLA

>SG-AL_contig_745_1_+1_97_309

MTVFGGLWKHKHTQRAHFILLSSAPSCIGCSLFWILPVSQKGWACCMANSRFSLTLCSGL

CPGVSPAVQFP

>SG-AL_contig_746_1_-2_2015_2485

MTGATGFQEPQSCHKTLGSTVALTNANVRPSPVTQRQDLQSHHIKMPSLLIVKNCSGLLS

VFLGCVWVYFCFVCCWRVFVCFVLFLFVCCCCFYTCTACFFCLPLALFLSSLTCTHLSIR

LRVSRPSPFALESPQRWHWDVKTPFPVVPLFAQTSVL

>SG-AL_contig_747_1_-1_811_933

MADNAGSWWIGRGAQGFKYFVHTRAFIHPVLCSHTGIHSSC

>SG-AL_contig_748_1_-1_151_408

MDRPFLVHCQQQDCISSSSASLLILHSCLLSSSCILLVMSSSVCPVIPLSVCSVKVNLEL

FCNPFQKKKKMFAHWVCDFPCASKKR

>SG-AL_contig_749_1_-3_27_971

MASGGRSRTSRVCCPEHPDAPLVEDYHAGDLICPECGLVVGDRVIDVGSEWRTFNNEKST

KDNSRVGAAENPLLDGGELSTMIAAGPGTECRDEFGKPMYRNRRSLNSSDRTLMNAFHEV

NQMADRLQLPKMIADRASTLFKQVHEGKALKGRSNDAIASACMYIACRQEGVPRTFKEIC

AVSKISKKEIGRVFKLILKNLETNVEVITTGDFMSRFCSNLSLPSTVQKAATHIARKAVE

MDLLPGRSPISVAAAAIYMASQASEEKKSQREIGEIAGVAEVTIRQAYKSMYPRAPDLFP

TDFKFAIPIENLPTQ

>SG-AL_contig_750_1_-2_77_322

MRLPLYLYVGSTGNIPRLFIQIRQYIAIVLLVVCLKKNLRREYQHILFVCFLFNLFCHIS

QFVFHHLKGLFRYVYKKQLREC

>SG-AL_contig_751_1_-1_1_81

VCEMERKEKNSSLSGPLIAFIWLSRPR

>SG-AL_contig_752_1_-1_847_1122

MPSNIVILGCSVLGQLKGLCIPTVLIVASLLILCFSDITIPFQNVLLVPYSLVDETQVKK

YKVCLHSLCLLFHIHSCALIGPMQSIPPCQLV

>SG-AL_contig_753_1_+2_2_211

DEDSAQLLSLAALPFPNFEESQGPIHSWADWMPHVHMKFPDHDSRKHMQPDWKASALPLH

QPGLKQIYHI

>SG-AL_contig_754_1_+3_1074_1505

MMTAGIKLEIRSDSKALLTEPNNPLCPIPSVVTHTTHLANTICHHPHNPPYQYHLSSPTQ

PALPIPFVITHTTHLAGTICHHPHELLCQYHLSSHIQPALPILSVITRTTCFANTICHHP

QHHISQHMNHAFISTTTMLTRLSP

>SG-AL_contig_755_1_+2_344_613

MDYLQCCAKRNTKCTFVADTKGVQYIHIQRNHTKTVHYIHMQVLCTVLKSAGTYSSKATT

KTTTNTTTSISKSYTTKGKASPLTLNWSAF

>SG-AL_contig_756_1_+2_1088_1333

MACCAAGSIASLDWWSCGLRALQRMSCFFTMLSHCCQWGSCGVLFFNSALATLPSIYRCI

HHRLCDKSLHIFWQFVLFLPRA

>SG-AL_contig_757_1_+2_452_1090

MKQKVSLAGEKCLAVSCSECLLICYCFRHKSEPCLPPFSLPSSDIQHHCINFISFINVNL

YTCTRLTQLWVVALLIKPCPHIPAVSAARNETDSLESTTQYVQSHSLGLRHTLCQRHKTM

MHVLSVSKSVHRCMWDPNLKIWRGNILWQICICIYLYDRTPAFPSGGACDCCHHFKLPFQ

ARCIHVYFKLHGETLQNMKISMVTLFSQNIPSS

>SG-AL_contig_758_1_-3_306_515

MLLYSMRIAMVCSSSSSSSSSSSSSSSSSSSSSSSRSSSIAQSTTMAISGLSSRKIHQSQ

EDNRVDHSRV

>SG-AL_contig_759_1_+3_1767_2003

MSQGGSLATVWQLWHSSVVRVLDLSLLMLDTGNSQGDKDWVKTDGSTIKFFCHCRFTQSV

PSMALGWSQGLQGQELQVG

>SG-AL_contig_760_1_+1_592_891

MDKERVSSKGEDFPTCSSKQMRGYSACRNKWVLDVGYGEHHHEFHPDLWVKISTQLRPAK

KLNIPRFPLPNTKYIHFYAVSIQLCHASSFLSLSKGFPNN

>SG-AL_contig_761_1_+3_90_299

MTMIFIKISVHLRNSRYQTSVIFQLKKKKMKGQKHQCTHIQTEMYCVTTGYCLFWVSGRE

TNTITICRMT

>SG-AL_contig_762_1_-3_87_329

MKYSPSQRLITSNSLSASCLLTSIPKEREPLPGRHCKQLIISHPAAPGLGQINIIVFKQQ

FCLVLLKFSSASFAFVVFSTL

>SG-AL_contig_763_1_-3_39_527

MPTGNGAAGHGGHGALGGHTGHPCGGCGRLIQDRFLLHALDRYWHTGCLKCTCCQAQLGE

IGKSCFTKAGMILCRNDYIRLFGSDGSCMGCGQKIPASELVYRVQATVYHIKCFVCVTCR

QPLQQGDRYGLVNGSLVCEQDYPKLAKSQGHVPLPARTSHKVC

>SG-AL_contig_764_1_+3_3_197

FFFCFFQAFIKKKVTEVCKSQHSPALSENLSCCCQPKTSDHVPFFLSHTCDSACQNHRYC

TILTK

>SG-AL_contig_765_1_-2_155_427

MGTALIMLAPRKVEVSTTSLPYDLRVTRKPLPLYIYIYIYIYILADRKQTTVLLFCHLFF

RLEVSEVMPVTLWYVMFSRPIHKHTRTEKGR

>SG-AL_contig_766_1_+3_729_962

MGQVYQTLLHGDDNNNKCTENRFEISKPFTCMDLHNVQEVQGHFKSFQGVKLEVLIMPYL

DTFPSIASPKSQPEGFVQ

>SG-AL_contig_767_1_-3_126_314

MRVVKYHPPHVALYVLLAFVFSYDFKYGFHFFSVLNFFIFLFRVFNLYFMKREIDYSCFD

FET

>SG-AL_contig_768_1_+3_1635_2000

MQCPLPVAQCTNSDVTQKKTRSHTADSRTEVAVTGLQLQSVHHKCPGGRRTSAGRATMEE

HDYNVGYHSVTHSDIPILKPCRGFTHQPISNYLTRHFSSAFQMSLTAARQFSSACEKCHL

SK

>SG-AL_contig_769_1_-1_1_171

IKRFKIVLTGPLMYREICCDEDVQIVSIVLPQGLYSSPSLLLIRIRTFSFTDFPVAF

>SG-AL_contig_770_1_-1_1_210

WLAWCDTNHVDPRAPSPVDLANFLAFLSTSEGLAASTIKGYRSAICTTLHQLGGPSLSDN

FLLREVSRGL

>SG-AL_contig_771_1_-2_5_757

MAGTRGEEVYLPESIELEAKEKAKFKMDVKKISKKSKEDISTRGVVYLGHIPAGFYEPQM

KKFFGQFGRVTRLRLSRSKKTGRSKGYAFIEFFNQEVAKVVADTMNNYLMFMRLLKCEYI

EPERLHPDTFKGCNKKFAAPKVAEVTAVRHNKTKTSEQEKRARGRQAKKLKTLKSKLTSL

GIDYALDLCEEVASPSVPEVVKTPKVLKEDSSDAEVTFKTPPGTFKSSRLTTTPKAKTSS

AKKKKGVKRLK

>SG-AL_contig_772_1_+3_1779_2111

MKENTGTQITFTNHDTTWNKPVRKETRMLIFTTYCDFFQLSVKHEYLSFSVSVLFSGMGI

AQDKKPLKLRGVWGHTSLEIFFSPQIEVCAIRVFLGIKSKITSKIFNILCG

>SG-AL_contig_773_1_-3_177_488

MHFLFDYQGGGVGLILWQFTLRSVIGPSYLLWQFTLRSVTGPSYLLWQFTLRSVIGPSYL

LRQGREDSLRAHLIYCMYTCTHTHTHTHTHTHTHIYIYIYIGLE

>SG-AL_contig_774_1_-3_3_146

LNRRMAYAMIILDSFPSHGICSVGAYGVCSEHGDTVLRKLKLKHENPH

>SG-AL_contig_775_1_-3_375_512

MYISHHNCKIYELNIILSGLCSGMTTFYDLTLNHFEGHLYVLISST

>SG-AL_contig_776_1_+3_6_869

MAAAVIPKIEQLSSRVIRILGCNPGAHRLQGTNTYLVGTGKRRILVDTGDAGVPEYIGSL

KTALEQFSVSLQEIVVTHWHPDHVGGVDDICHAFSQKFAVSKIKSLSGKDAPLEETDYSF

ISDQHVFHTEGATLKVHFNPGHSDDHIILHMLEDNVVFSGDTILGGTTTTVEDLHSYMKS

LEDLLNINPALIYPGHGYVIDNPQETVQGYIKHRQMREDQIVKCLNTNKDRLMEPMDIVK

IIYVGVAETLYPSAANNVKQHLLKLQRENKVESPDGEKWKLVENQSNL

>SG-AL_contig_777_1_-3_27_1556

MEYERQYQSSYIVPEVIKNFLIFFQKSVNEQVLFEVQNCYENGFNKLTDRFFKNTPWPEA

DVVAPIVGNDQVFLILYKELYYRHVYARVQNGPTLEQRFESYYNYCNLFNYILNADGPVP

LELPNQWLWDIIDEFIYQFQSFSQYRNKISKKSDDEIEVLRTNPKIWNVHSVLNVLHSLV

EKSNINRQLEIYASGGDPDSVSGEFGQNSLYKMLGYFSLIGLLRLHSLLGDYYQALKVLE

NIDFNKKNLYSRVPTCQITTFYYVGFAYMMMRRYQDAIRTFANILLYIQRTKQMFQNRAS

LYDQISKINDKMYTLLAICLVLHPMRIDESVHSHLREKYADKQLRMQKCDKQEFEQCFAF

ACPKFLSPVPPNYDTMTDTYHKEPFKLQLAVFLDEVQQQIQIPTIRSYLKLYTTLPISKL

AIFMDEPEDRLRTQLMCFKHKMKNLVWTKGTSCLEGEFQSASEVDFYVDGDMIHIADTKV

ARRYGDFFIRQIHKFEEITRNLQQIGTGHK

>SG-AL_contig_778_1_+2_140_343

MINQGLMCSMHSAHVKEPTATKHLSLAKFCRKIYSYISKYTRIKKKYLGGTALWRRALPR

ESNPKFTW

>SG-AL_contig_779_1_-3_51_1901

MASRNLPKVRDAEKESLYGYVHAVSGPVVVAQNMSGAAMYELVRVGHGELVGEIIRLEGD

MATIQVYEETSGVTVGDPVLRTGKPLSVQLGPGIMGSIFDGIQRPLEAISDLTQSIYIPK

GINTSALDFNKQWEFLPSKDVRVGSHITGGDIYGVVPENILIKHRIMLPPNARGTVTYIA

PNGNYSVNDVVLETEFDGKKSQYSMLQVWPVRQMRPVSEKLAANYPLLTGQRVLDSLFPC

VQGGTTAIPGAFGCGKTVISQSLSKYSNSDVIIYVGCGERGNEMSEVLRDFPELTMEVEG

KPESIMKRTALVANTSNMPVAAREASIYTGITLSEYFRDMGLNVSMMADSTSRWAEALRE

ISGRLAEMPADSGYPAYLGARLASFYERAGRVICLGNPHREGSVSIVGAVSPPGGDFSDP

VTSATLGIVQVFWGLDKKLAQRKHFPSINWLISYSKYMRALDDFYDKNHPEFVPLRTKCK

EILQEEEDLSEIVQLVGKGSLAESDKITLEVAKLIKDDYLQQNGYTPYDRFCPFYKTVGM

LRNIISFYDMSRHAVESTAQSENKITWAIIRERLSDVLYKLSSMKFKDPVKDGEAKIKAD

FDELMEEMQTKFRNLED

>SG-AL_contig_780_1_-3_87_437

MVVQKKSKKAAENINARLALVMKSGKYFLGYKQTLKTLRQGKAKLVIIANNTPPLRKSEI

EYYAMLAKTGVHHYNGNNIELGTACGKYFRVCTLSITDPGDSDIIRSMPTGDQQPAA

>SG-AL_contig_781_1_-2_56_1090

MVKEGVLLAYGNPLLDISVNDDSCIKLLEKYDLKANNAILAEEKHIPLYDELVDQYPNKV

EFIPGGATLNAMIAVQWILQKPKVTTYFGCINKDKNGDIIKQKSEEAGVNVQFQYTDKEV

TGTCGVICTGSNRSLIANLAAANCFTEAHLDVPANWKLVEKAQYYYSAGFPLTVSPSTML

RMARHAQENKKTYCMNLSAPFLCNFFKEPMTQLLPYVNFLFCNESEADEFAKVHEYGTTD

RKEIALRMAAWPKEDKSRPRTVVITQGADPVLVATDGKITEFPVKLVTARDIVDTNGAGD

GFVGGFLAQLVQGKPIEECVRCGIYIGNIVVQQPGFTLPGLPNYS

>SG-AL_contig_782_1_+2_521_685

MKDNVYTHTQLPYSSLSTYHLIFKPRLSHTGLITGRVCVQIPTSPKSSHRFFSPN

>SG-AL_contig_783_1_-3_159_587

MQSCGETVDCVNNGICSSDKKCECKTNYKATHDNGACKLAVGQDCTDDANACGVNAQCNA

EKNCACKTNFVSVDSRCAKKVETACSANSDCVMNAECKDKKCACSDGYSANNDKTCDKGG

AQTVVASLLLMLAAFVTSRLISA

>SG-AL_contig_784_1_-1_88_987

MTKILGRYLLVLLLLLPTTFLFVGPKISRNVALADDSMEGEDEEAIVETDEGPAGSEEDV

VEETDTGVTEQEEEEEEEQALKPSPDADTYILFTKPTGNGHELPAGKNVRLLVGFTNKGN

GEFVVESMEASFRYPQDYSFYLQNFTVVQLNREVESKRQATFEYGFIPNEAFSARPFGLT

VNINYKDGQGNLFQNAVFNETIQVVEPDEGLDGETFFMYLFLAALGILFFLAVHHGVSSL

GKKHRSKSRLVVEMGTQNADIDYDWIPKETIKEMNRSPRRTPKQSPRQRRSKRSSGSGEE

>SG-AL_contig_785_1_-2_26_889

MADEYDEPISDQEKLKVASNFILHAPPGEFNEVFNDVRILLNDDNLLREGASGAFAQYNR

DQFTPCKVEGCDLQALVTEHGDQGYGRFLDPRTKQTFKYDHLRKEGSDYQPAEVDKRAEP

WRLEVESAMTAYVQSHYPHGVVTVYGNTADGNITLIACIESHQFQPRNFWNGRWRSQWSV

TFSESGGSAEVTGVLKVQVHYYEDGNVQLVSSKEIKANLKVTKEQQTAKAFVNIVSEAES

EYQRALSENYQTMSDTTFKALRRQLPVTRTKIDWNKILGYKIGGELKN

>SG-AL_contig_786_1_-1_124_360

MDIESMNRYVSPINPAVFPHLTVVLLGIGIFFLAWFFVYEVTSTKFTRNLYKEMSVACVA

SIFMGFGSLFLLLWVGIFV

>SG-AL_contig_787_1_-3_576_1220

MPTLGYWKIRGLAQPARLMLHYAGEEFEDVMYEQGDAPEFSREAWTKVKNTLGLDFPNLP

YYIDGDIKITQSNAILRRIARKHDLLGRTEAEKVNVDMMLENAMDFRNGVVRMVYNPDYN

KLIKQYEPRCKELLESYEKWLCNKKWFAGDIVTVADFPMYELLDQHRLMIPGCLDAFPKL

SAFMQAFEALPRIKTYMASSKFIKRPINNKVAQFK

>SG-AL_contig_788_1_-2_254_1585

MADNTDNESKPETKEEKERSKRDDRDRKRSRSPRRRSRSRDRDRKRSRDKESRRKKSRSR

SKERTKKPRKKKPYKYWDIPPPGFEHVTPMQYKAMQAAGQIPQAMPISASINPTATVTLP

GSAMSRQARRLYVGNIPFGVTEESMMDFFNHQMKITGLAQAEGNPVIAVHINLDKNFAFL

ELRSVDETTQALAFDGITYENQPLKIRRPSDYKPLPGMAENPTLAVPGVVSTVVQDSAHK

IFVGGVPSYLQEDQLKELLLSFGPLKAFNLVKDNATGLSKGFAFCEYVDPNITDQACAGL

NGMQLGDKKLIVQRASVGAKGAQNQAPVQLQVPGLNLNQGAGMATEVLCLMNMIMPEELM

DEEEYDDILEDVKEECGKYGIVRSLEIPRPIKNVEVPGVGKIFVEFNSVIDCQKAQQALA

GRKFSNRVVVTSYFDPDAYHRREF

>SG-AL_contig_789_1_-3_144_1019

MDTDKVKVFGSRVRVDAVSKVAELELAEKEKMKDKVEKIIRHGCNVFINRQLIYNYPEQL

FADSGVMAIEHADFEGVERLALVTGGEIASTFDCPEKAKLGHCDLIEEVMIGEDKLIKFS

GVALGEACTIVLRGATQQILDEAERSLHDALCVLAQTVKETRTVYGGGCSEMLMAEAVSQ

LAAKTPGKEAIAMEAFARALRMLPTIIADNAGYDSSDLVSRMRAAHTEGQKTVGLDMERG

DTGKMDELGITESFQVKRQVLLSGAEAAEMIIRVDNIIRSAPRQRPRDDRCH

>SG-AL_contig_790_1_-3_3_257

KLQVFINTCLRQILRIRWPERIANEELWRRTNQRPIGDTIRNRKWKWIGHTLRRGPNSIT

RQALDWNPQGKRKRGRPATTWRRTL

>SG-AL_contig_791_1_+3_1506_2099

MYWKALFSSFPTWNDFLISSTTVFSKCFVLPQMKSSTWHAKMPSTSFPSIQKKTTGSTFD

TLPPSFCRTSFTFLYHCKEASFRPYTHFYRCHNFPLLFASFGGLIKMSLSISFPCRKAVL

MSMEHRVHFLCVKIERITFNDSLVQVGEPFCMSSFPISSNPLATSLAFGMTPSFDSLSVQ

IHRVETHFCPVFSTSVKT

>SG-AL_contig_792_1_-3_3_161

PPPPLSQQGDNHLGPPVTAPVNQRHQRSQVEFTSGLEAVEETSVFLIIFYAFS

>SG-AL_contig_793_1_+3_3_548

FFFFLKLSHLFTNFSLFSSWFTENKHPLACDSLGSPDYNSLRAGVGAFPAAEGCHNVDKA

TVVLDASLGTAGLLLLLLLRLHLWCLASHFSSTSEGAVNFSSQNADLNFQRGQVLKGGQE

GAILQRFAGAEDSKLFCRNSLPLLYQSLNIFNSFSPLDIKGVSLLGVYEQLHRGQVSSFL

NT

>SG-AL_contig_794_1_-2_554_1330

MTSEHSNLISRGSETAERYEKYVRPILAEFLGVTLFVCMVCMSVLNSGAASPDGTVLFSA

PAIALAQGLTIVILVMVFADVSGAHLNPAVTAGILMAGGIGPLMALFYICAQLLGGVTGA

ALARGILSDETYEAIGGGAQTLGSGVTVGQGILCELVLTAVLVLTVLMTAVDPQTKTTLF

PLAVGMAVIVGVLTGVFISGASMNPARSFGPAVVISYATSNFWAYHYVYWVGPFLGAIVA

AVWYRLFLASSENRLVLKE

>SG-AL_contig_795_1_-2_14_1240

MAASVKQQLAQLASQSHSGGSHKDVSEKYKVILRGIIQTGGSERVSSLQTFVEALVNENV

SLVISRQILGDFASQLEHLSDPDAKVVSHFTLDKIHTRVISFEEQVAAIRQYLASIYERE

QSWREAANVLVGIPLETGQKQYSTDYKLETYLKIARLYLEDDDAVQAEAFINRASILQAD

TKNEELQIHYKACYARVLDFRRKFIEAAQRYNELSYKTIIAEGERLTALKNALICTILAS

AGQQRSRMLATLFKDERCQQLSAFNILEKMYLDRIIRSSDLQEFEGMLLPHQKAITADGS

TIVDRAVIEHNLLSASKLYNNISFTELGALLEIPPAKAEKIASQMITEGRMNGYVDQIDS

IVHFEARETLPMWDRQIQSLCFQVNNIIEKISQTAPDWFGKAMEKEQTQ

>SG-AL_contig_796_1_-1_106_2886

MGIFILLIAIIAAFARPGSYLCQAVTGGQSESHPDTPTPTTPPPTKDYITTDGRQFPWKD

IRLPRLVLPDSYEIFLHPNISQSVFSGSVRIHSSVVTSTDFFVFHVKELNVTKVTVLRGE

VEVEVADTMEYKTNEQFHVRVKQKLAADSKIIIVVDFQAALVNKLAGFYKSSYKTAAGKK

REIATTHFEPTDARAAFPCFDEPDLKANFSLSIVREPQHITLFNMPLKSTSPFQGGLLKD

VYEMSVKMSTYLVAFVVCDFKNISSHTKQNTLVRVFAPEDQIDQAHYALGVAVKVLDYYN

EFFGVPFPLPKQDMIAIPDFAAGAMENWGLITYRMTSILYDPLKTSPFSKQWITIVISHE

LAHQWFGDIVTMKWWDDLWLNEGFASFVEYIGANISEPSFGMMDQFVASTLQRALYLDSF

TNSHPIQAEVSDPAQINEIFDTISYDKGASLIAMLQGFIGEEAFKKGLHEYLRSHAYGNA

RTADLWQALQEASNMPPTMTVKDVMDTWTLQMGYPVITVKRRPGTQHLTLSQNRFLLDNS

GTVIDSKFKSPYNYIWKVPLTYRTQTNPDKKLVWLKDKEMTLPVTLSDTDWIKFNLESRG

FYRVNYEPDLWRRIIRQLKDDVNTFSTRDRAGLLDDAFSLARAGLLSYDIVFELLAYFDK

ETEYVPISSGITGYGFIKNRLYVTDVFPKLQKYMLRLISTRLGSVGWQNQTQHLDKLLQE

VLLSFSISLNDKDNIEKAKQLFQSWSNIPDNLRGLVYCMGVKYGDDTTWQLMYNNFTTSN

VPTEKKQFLKALTYTTDPRRIQLFLRDSMNDKIVRSQDTSTVISGIADQAGGHIFAWRFI

QQHWETLYKRYGTTSFILGRILKSVVGPFSSQFDYDEVTAFFKDRNLGSGSMALKQSLET

VKSHMDWTARNQDSIVQWFQTNIKDTP

>SG-AL_contig_797_1_-1_1_150

AYEKAHIDISYLASSSISDVSCTKSVSPLLSSGYTMKYQVVYLFSLLFYH

>SG-AL_contig_798_1_+2_818_1120

MFLKKKSEKKRKRKEQRERGERERERERERERRRRRRGEERERERQRDRHRREQQRETER

QTERQQKIKPNLLGTKTCICLMSRNWNKTINEKRCQNLDCS

>SG-AL_contig_799_1_+1_148_201

MLFNKCYLKSETVRKHHE

>SG-AL_contig_800_1_-3_75_458

MKLVRFLMKLSHETVTIELKNGTQVHGTVTGVDVSMNTHLKAVKMTVKNRDPQQLDSLSI

RGNNIRYFILPDTLPLDTLLIDDAPRAKQRKQQGGRPQMGGRGRGRGGRGRGRGGPRGGG

RGGPRGRR

>SG-AL_contig_801_1_+3_105_227

MNNVCANTLKERTVHITNYKKTTTIIIILHTNIFHKIIIAE

>SG-AL_contig_802_1_+3_3_83

FFFYHLKNLFKISEQRGLQTTVQRCIQ

>SG-AL_contig_803_1_+2_572_1675

MTSPCYDIIAGLLEADLVQSDVTTDLKISGGAHGQAVTELQQVLVVDGNLEKVMLNQNQD

VKAGVGHKGEVGHFAFFSPPSPALAMVADNGVHAAVIGTETHVKVERYVTSFISIRLQVD

GEPGGIGEGHAADGDHAARAQAAWNAGQEWNRRVEDWLRTIAWLGWGYSSRGVIKGWRCE

GSPGRRPWRETVGWAAAASAMESDEVDGSFTLNHDELGSLLGQAVGEVPDVASVHFQLVH

LRLDDDADVKRSAFVELNPVLAVLIAPAVVLHVVQHHAVLVHPHVEVQGRVGLQLGPLAQ

IDAESMVRVVGDDTLHLYLHPIMYSSRDTPRVGHHHRGDCCVHVEEEPSLVLRVDFKSSR

LNAPCLAG

>SG-AL_contig_804_1_-1_208_861

MVDLLSDMETLLASDRHFLLGNWIHDAIGYAADFQETFLLQNNARNQITIWGPDGEIVDY

ASKQWSGVFKDYYVPRWQFFEDFLVQLIQNKSMWDGGKFNRQILEKVELPFTFAMSLPPT

EPTGNSVAIVKELHTKYRNGTHRDFFRTLAVSAFKVKNRKPFSPFSWKQGSQKQIKTNDI

EEDIMVEKENAKWLSFISGKRQDLLFREVHTKKTFVVI

>SG-AL_contig_805_1_-1_121_717

MACRRYMFLLVAAAFVAVALSEEVPCMTSREDCTQGIGYSTLIIINDNFHCCAAGYSMRW

DDSSDKCFCDKTSEVVACTSSACGKNDSVVSFTNKNNVTECCQAGMTMTVFQGTLNGTKM

DSCSCSSSSTWGLEVVSYSGANTSHIHFNNYLGMPHFSETSFNNWISHSLSEMREAFRRL

FDDYFYDEDNYDDDYYDDK

>SG-AL_contig_806_1_-1_1_168

PRTRTGQDTATRVSCLGAARKACGRAGCSLLEPCDFMCSTSGLLCSNVLAEWFVTM

>SG-AL_contig_807_1_-2_2_361

EVIKDNLNPEFAKKFVLKYFFEELQKLKFDIYDVDSPSKQLDRHDFLGSVELTLGEIVGS

PGSKTEKALRGPVPNSGKIILRVEEVSSCKEIATMLFKGTSLDKKDFFGKSDPFLVFHRA

>SG-AL_contig_808_1_-1_1186_1644

MLLRQLPTGDKFICELMLFLWLMWDNGHVALASFLCVAVSVVMIDLMPSLCISLPVIKAV

KKFIYIHLTYKSHATISKDVMPVVQLVVVRSEESGYMKGMLMFRFTADFQRQYILIEYKY

PVLQKLSHVLVCSTVRSLPFYCVWKKRLGFVFF

>SG-AL_contig_809_1_-3_117_3584

MGKKTVSVPASASTSLAPSTTAVSVSVVDCAKFDKGDQHDSGIDVSDGQPNSAASSTRSS

PSADNKLKDSEAKVTEKMLADKLKVAENSSVKMDAPKPPRQAKIVSGSSEKTANKTAEAS

ANVKVIQKPETSKEKTSSLQHVSDMSNKPERIQLPPSFKDPIFGKGTEIQLTFGFDTSLE

PMDSPDSGSSPAEARDERSDVNYVSPAGSSITSPTSPATQDLNSKIASCKNCWELPTVFE

QKSNSLSSASSLGTSLSVSATSTITESKGASTVPSFTTFTTTAQVSVTNAASPSPMMHVA

MDKPSLSPVASDNMPFASADRKESMVYTPSAERKDSLVFTPSTERKDSLVFTPPAERKDN

IFTSAAERKDNIFTPAAERKDDIFAGAAERTDNIFTPAAERKDNMFTGAAERKDSIFAGA

AERKDNIFTPAGERKDSMVFVPSAERKGESMVFPPASDRNTESNMFVSSGERKGDNMVFA

PAVDRKGDVLDNKPQNLDTITSHHGISEPSNVCKVRPQQVRPVENVSELNQNMSNLGSSM

SASASSLMSSMPAVPSPPILMAQSQAFQPYLPAAASQLLPQETRFPQQPSYGFGMSQQPQ

PSSQQAAQPGLTPQQAMSQPSLFLPAQQDLFTTFQRSHGPAPAPAPTQQGGMGGPQAPAF

GQQTNLNTVMVSSANSTLMSTSIKPGPNPNAAPFNSAVTKNIGPSQMPFNQMSQMVFLEL

TQPLGQLFNTSQLLGNAVQTSQSLNNSQMLPSQLVQQPRSGVQNVQAVQPSSSFYQQPQP

ALTQTGFFPTQQPSSTLQGALQPPQGTAQFSMQPFSSQPHGLGLTIHSPAPGASLNQPLG

LGPAHQQHQAPPQHQHQAPPQQQTNKGSQFGNIMQQHSSQSAPLKSPPNSLGHGQGNQGH

GQAGQGHSQGSFSANNKTMSASQQQQQQQQQQSMAAGTKNFNAQMNSRNAAQRFPPPVAQ

PGPKFGPSPFIQQTVQTGGPMVRPQLMMGNMVRSNMGPPAPRPNPIQRPAGPPPSAPAPQ

HNPNLGMKPGGGAGLGPRPPSQTQSHLAGGVHMLKAQHVKERQDLLANLRSYMGTNKQAA

SNKISTSTPPAPTDLAPPSAPKADASGDGKKTMTAPLLKSASTSSTSGSSPSPAAVTSGP

PGSVAPKGEEGGSPQK

>SG-AL_contig_810_1_-3_393_755

MVRGVSSSSAVHTSFHYSVIIGCGVIITPSSAVCCLVWQVSRYSQSCWSVPVCQYLSPSS

SSSAGSFMKNTCAQNFSLVIRINGCNVCHCVCVRERDTYSGTFMILLKVNGGVPPVSLLH

H

>SG-AL_contig_811_1_+2_461_658

MKSHRIWDNCSHCYDDENGDDADDAMEFHLVLRLHDRVVCYPYFHHKPWHQPGPRDTDTC

GVGQEI

>SG-AL_contig_812_1_+3_3_164

KVPVPGSPQEPLMSSRHGATHQRRLCTAAGPLLCSSTGPVLIHPTKPPANDCNR

>SG-AL_contig_813_1_+1_79_219

MNGHRIWDNFSQCYDDENGDDSDDAVDFQLGLRLCDRAVLYAFFHNI

>SG-AL_contig_814_1_-2_455_3511

MHRIGKLLTKGPSSRILPQILRTTRGYASSVEDGSGARTGKGADRAFATQASAEPFLSGS

SSTYVEEMYLAWQRDPNSVHKSWDTFFRQTSKGAPPGQAYVRPPTLTPFPSAPMPLPLHA

VMQGAPVDEKTIEDHLSVQSIIRSYQIRGHHLAELDPLQINRGDLDSSIPAELLMSHYQL

DDNDLERVFRLPATTYIGGNEAALPLKEIIRRLEDVYCRHIGVEFMFINNLDQTDWIRRK

FESPGIMQFGIEDKRLILARLIRSTRFEEFLARKWSSEKRFGLEGCEVLIPCMKTIIDSS

SALGVDSFVIGMPHRGRLNVLANVCRKPLEKIICQFDSKLEASDEGSGDVKYHLGMSHER

LNRVTNKNIRIAVVANPSHLEAVDPVVQGKVKAEQFYRGDTEGKKVMSILLHGDAAFAGQ

GIVYETMHLSDLPAFTTHGTIHVVVNNQIGFTTDPRMSRSSPYCTDVARVVNAPIFHVNA

DDPEAVTYVCKVAAEWRATFSKDVIIDLVCYRRNGHNEMDEPMFTQPLMYKKIAKEPSVL

TKYAESLIRNNFVTQQEYEEEIAKYDRICEEAYMLAKKETAVSNAEWLDSPWTDFFTTHR

TDQMKNPKTSVPEETLLDIGQKTCKEPEDFVVHGGLKRVLRARGEMVKKRMADWALGEFF

AFGSLLKEGIHVRLSGQDVERGTFSHRHHVLHHQEIDKKTIVPLNHLYEDQAMYTVCNSS

LSEYGVLGFELGYSMTNPNALVIWEAQFGDFSNTAQCIIDQFISSGQAKWVRQSGLVLLL

PHGYEGMGPEHSSCRIERFLQMSNDDPEHFPVEDENFEIQQLHEINWFVCNLTTPANFFH

AMRRQIALPFRKPLIVATPKSLLRLPEARSSFDDMIEGSRFQRIIPEAGAAKDNADTVKK

LLFCSGKVYYELVKEREKKDLASDIAITRIEQLTPFPFDLVKAELQKYPKAKVCWVQEEH

KNMGPYQYVEPRVRTIFKKMEDSREMEYAGRFPSAAAATGNKQMHLIELSSFLEAALTF

>SG-AL_contig_815_1_-2_659_841

MFLVKISKLQVIVLPYHEYTDKQGFMCSEKVIERSRYNIKKTFFLDVTRLSKAMNTFYDY

E

>SG-AL_contig_816_1_-3_54_266

MGRVWQNFIFRLRKAVDGYSLTCPAGARRLKQLVAGLFLNIRLGFTLRNNNRQSATTSKR

QKRNVLRFAHL

>SG-AL_contig_817_1_-1_220_651

MAVEKRLCEKNAVCSECQAMLIGKLKGVRNELFSQNKQLPGLMRDGLTVPSQMLGDAVQG

MENVFQQSKRQLYMDRVWQRFIFRLKKAVDGNSLTCSAGVCRLKQLVAGLFLNIRLKFAL

PNSNRQNSATSKRKNRHVLKFAHL

>SG-AL_contig_818_1_-3_3_200

CEKVAAAKRLSEKNAFCSDCQVMLIGKLEGVRNELFLQNKQLPGLTGNGLTIPSQMLVDA

VQGMEN

>SG-AL_contig_819_1_+2_5_244

MHIFIDSHRFTGTFHSKQALTISCMLSGSHYCFQHSEKNSINSIHTPFVLHHIYGSITLD

VTGFENLNTFRHSQDNIHGT

>SG-AL_contig_820_1_-3_84_779

MATQDISPLSTADTLSSSNFINNKNLFAKVSQRFGLLPGAKGLNEIAQVIEEGTQTGGSG

SPLDQVRREAEVVADDVILNFGKEDLKKPPNKFCKTMRRTVKELSGRHDIAFKGMVNKLN

LDSNNPFQTFVNVADEIFDDGQINWGRIVAVYAFASRLATRDTDKSDFPKKIALFVGKYV

ANKLGKWIQDNGGWEAFAEYFPDQGEIEDTMWKGLLFTAVGLGALASVVATR

>SG-AL_contig_821_1_-1_55_228

MEAKMKDGTIKYHNSNVIDADIVDSLGKPDKSLDGSFYKVKLKKKSSDTPDLQEFFST

>SG-AL_contig_822_1_-2_53_217

MKIVTFGDNVIKRCGHFTKEYLDSFHLNEMKSVTRQIYILYVTFALTTHTVLNTR

>SG-AL_contig_823_1_+3_3_167

SLIHFTDMSLRVLLKFPDQHHRCLYLSGLVDTRIYYERKCRVQPYHTASVLDGTP

>SG-AL_contig_824_1_+1_37_411

MSLHVTTLSHLKSCIHLCISDGDLHFHTWFNADGSNLLDDFGGAVQVDDTLVDAHLVAVP

GLGALTTRCFTGGDTQDLCGHAYGSLHLQLFVLCSLDQISTHLFQTLHIAASQGDTNAVD

GRLLC

>SG-AL_contig_825_1_+3_3_239

FFFLNFIIFFLFCSNTHDFEEQKIDIIQPTELDAKPMFTSHHNDTRLSKPDTHNFHDVYH

SKLLQYNMDLFYLKLLQTE

>SG-AL_contig_826_1_-2_140_244

MQFSVCALLQIKSEISKKEKKFMHSPQHTKVTLLV

>SG-AL_contig_827_1_-3_120_512

MSAPFKQMENIEVFLKAAENYGVPSNSLFPTADLFEGRNMAMVISTILQFGSEAQRHGFN

GPTCGAKPSEKHEVHFTEEQLRAGQGIIGLQAGTNKCASQSGMKMGASRHIADIRTDDMS

KEGQGIIGLQA

>SG-AL_contig_828_1_-2_107_1615

MVVFSLTLVRHGETVANKDGVIQGHQDVALSEIGLKQARLVGIRLKNDRFTHIFASDLGR

AAQTAQAIAEANSMCRCSVQLDHRLRERRFGIFEGKTFKELFEAAQKSNKKWSDFTPDGA

ETYNEVRFRAQAFFRELCEKMAVSGLSIERTYIPTSRKDTHSSDVGLSNGRTMVSSVDTP

SKVLLATDNKTENGATNTKCMNSQAGSNNPQFVEEGVAAKRPRIEEPQLHRLSLDGVGPL

SDLDTDCVVGDVEGAGATTMARSNSSSSSGCSSLHDSVECPEADVDLPTPTEDEERDTDS

SDVGISRSKKDSTPSTSQSGSLRASADGVKQQPPAGRTWFSEASQSTSKWHWDGLQPWVR

DTDPSPISGFSVDPSLQTCPNVSLSPLLEHRFTSVSTVSSGRNSSFDDADIVPSAAGDVL

VVSHGGLIKELVTHFIEDLNCKVPGGKGHALRVCPNTGVSRFMVTLSDLDLCPTVTCLLI

HDKDHLRSFQEPLAPPQHNTPAV

>SG-AL_contig_829_1_+2_986_1342

MTSHSAQAGCLCVSTSHQTQTWLMGFLTCIFDVLQNEQHRGSDTRAVQKTGQQKNPNKQK

KVFPYPEWLLESETPASLRDNIKRVVPISLPLLYLPHHYQGTGTHLQLGGLGWCVQARA

>SG-AL_contig_830_1_-2_2_199

FSQCCDDENEDDSDDAMEFHFRVRLPGRAVLCASFYNISWRQQEVKLTCMFFWCMYGISY

IFTFAD

>SG-AL_contig_831_1_-1_151_1839

MPCCRKKPKLEKQASQLQGSSDSIEELEPFETEPMQRVLAAQTLKIANIDGLAEGRKLET

EIEDDIQWEERAPPKEVIGRARHKIESILHCRKMLLVIVVLNIIDCLLVVAELTLDFNAV

TNHFVATTDHNDHFLRKLKVYNAPFLADLPDVEDDNLTQLFDRLLQSYIVWNITHMSYSG

PNLAADCVPIQDSYDTQSSGSGSNVLTRLLLFTNEASKQPVHMEPLDLEHRKQEEVGHIL

HYFSISILSFLLLETILKVACVGTRIFKKKMEMFDATVIVVSFILDLIFTGGLTSHNLKR

FVIILSFLLPWRIIRVLNSLIVALLNKHRFHLKLLYKQKRKITKQYQDASETMKSVQKQV

EALKGLCSNKGVHEWEVHKAIAQSVQPNAQGGLKALGSLVFRAAESFAPFHHRDHSKKGH

GSPERTNSQPNDHFERSTSSPNGDTQAYTEEVDIEVDVDLDSDEAEEFTNKYRPRSPAKR

KHRGDSGSPRKKDARSPKHSPPSDKGERRQSSGDTPDKTSPKRRSMTQSTGSLQCPRADP

EDIDIPREEEGRGPRRNSKTTIV

>SG-AL_contig_832_1_+2_2_166

LPTWRGARGLPCTQSSHPKCTRGLTVVRQSAYILGDPQSVKLGNANKQQQRVTKC

>SG-AL_contig_833_1_+1_817_1071

MKIRPKYTDEKKKTKQNKNKTGQENAIGVPRTSGVIHFANHCTPLALWSAKSGTTRTTRK

TDTTYEIGILFAVSFLTNIKTKLDD

>SG-AL_contig_834_1_-1_1_186

HLCLVPCLRWYLDRSWTRRSSQGLFISMNESNHKDIKAGMVSRWSRGTILKAYTQAQKIP

AV

>SG-AL_contig_835_1_+3_18_164

MVPLIQRETVPAEMSLRYDSFIEMKSHLWEERLVRDLSRYHRRQRTRPK

>SG-AL_contig_836_1_+3_2130_3482

MHIPTNTSLFLRSHKWCHWGDLDLVVGHVARSGTELGVGLDDLVDGVQEVLLGGHLPTSA

DGEHARFCAHAANLCACTVGAEAGQQLVSYIFLHTHGTCVDFEDVGATLQIRQAELDLSV

ETSRSHEGRVQRVWTVRGHQNLDVATGIKPVQLIDQLQHGPLDLVVTPCAIVKPGTSDSV

DFIKENDACLLGSRHFKQLSHHPGTLSHVFLHQLGSNDADEARVSTVCHRPGTQGFASTW

RTKQQHPFGRLDAQVHETFRMQKRSFHHLPQFLNLFLAATDIAVRHIRLLLHLHHGHCGI

NLGGQGDVDLVLVTINTHTHALLNVSGSHLIPKVHHKLGKLFDIDDVFSIIRICVDDLCA

ASHLKRLFTLQSLLVCCQIPQSWRSQSCVRLLDACQFVDILQNFLHVLLNTLDGTAVLTY

AISFQKSNISFVQRLNRFFFFIGIIFDFSLI

>SG-AL_contig_837_1_+3_3_212

THTSSLYERESCLLLKTTFCSVSRPPDKDGHHGGHKFTLQEGTTPHTRMAQIQGKKGFDV

LRVLSTPLSL

>SG-AL_contig_838_1_-3_2364_2582

MHYFVLPFVFCSLPVFLVILLYLCVCVCTYDVLCYLVQLVVYLIYIGTMGSGACYHYKEV

FWQVHFVFADFVS

>SG-AL_contig_839_1_-1_337_729

MSLFLEILLHKNVTNINTLESSYRTIAGGVATLTTLYIKVNYHIDYIVHKGELSVNCLCS

FKHKLTRRVLETMHKSFILPVFDYADVVWDGCTDYQSNILENLHLDALCVITCFVRGTSH

EQLTKNLAFVH

>SG-AL_contig_840_1_-1_73_339

MPKPKKGKKKVAAAPFLAKKVEAKKVTNPLFEKRPKNFGIGQDIQPKRDLSRFVRWPKYI

RLQRQKAILYQRLKVPPPINQFTQAADKQ

>SG-AL_contig_841_1_-2_227_532

MFVCLSASVSLSVSVCLFLSLSFAHEYTLTFALSLSLSLSPPPLLSLSPSLPLSPPHSFP

PLPSTLSPLLSPCFPSPFLPPSPSLPLPPPPPFPFPPPPPPP

>SG-AL_contig_842_1_+2_2_304

CYILIFVSLFITLALNGVHSNLLVVLLQGCQILTGLRELTFLHTLTHVPVHKGTLGVHEV

KLMVQTGPGFGDSCGVAQHTHSTLHLGQVTPRHHSGGLVVD

>SG-AL_contig_843_1_+3_3_116

ACRSICHCYCSIFCQKQLNQPTGFFRHDTQKLEHGTVE

>SG-AL_contig_844_1_-2_2_148

IKWNGWRCQDSLVLFALRNLMVKIMCYMFFCRIIVFRFDKILCLMRLSS

>SG-AL_contig_845_1_-2_1247_1429

MTLVTEYPSLLVLVTEHPSILVFTVRFFKINLFPRQSSHGSLEVFEGLGILRGHFQVFES

L

>SG-AL_contig_846_1_-2_113_607

MAGLPRVFFDMTADGQSIGRIVMELRSDVVPQTAENFRALCTGEKGFGYKGSTFHRVIKD

FMCQGGDFTRHNGTGGKSIYGEKFADENFILKHTGPGVLSMANAGPGTNGSQFFLCTVKT

TWLDGKHVVFGSVVEGMDVVKQIEGCGSQSGKTSKKIIISNCGQL

>SG-AL_contig_847_1_-1_2914_3159

MYYVVMQVISTLKIQYCSNNRSQTSKTVVFFYTIKSSLRPKSKLKVFNKPRYDAHNIFCF

LIINYNKVFKQTRIQCPEYPAS

>SG-AL_contig_848_1_-3_159_3611

MSTVKAEDQSSEEWLDSHSVQHLQLTLQDLVDKGKIGKLSPDQQTGKVTQHIQFDAYDVN

EFEYKLNMAIEMYTKRIKWLLQGSKRVFGHVKGERVAVIIDSSDANLGFGRQTAMQESLI

HLLDEQLSRKKGMYLVSFGTDLDPLWPVVRDVNDRIVEEGKEWVVRFKQRGGSNLLKAMK

HVMRLPDIDAIVIILGSVPDQASELLCDYILQMGLGRSIPLHTVAYDCSNALTNSTLRNI

AEASGGRHHCYTASSEEQIYTGTDISLLLKEINSAQDIINKIKDMRKGMMGSALISIMNE

IKTEVAKLPQSRFLPRPPGHDLPLRIDVPKFQPRGSVEWLKQHGLKAKHLDLYQVLAPNA

YSYREEFIPVIKRAVQSQVHEKAMVQFAWHDGSTKNIHVDMTQLFEYQKQLKEIVRLYER

RVDWLASGSRRIFGTLVEKNVVLLVDNSVSNINYLVHIQHSLRLLLEQQVANKEYFNIIA

FGGKAAAWRPTMVRPTAENLQEAWKWVLELQCGGSRNFLSALRLTMENDEEQKHHIYVQG

VYVFTSGVPDQDQDVVISYAEETASGWNAIIHSILFNVDDYDEYGAIPGRYANITRTSEC

LRQLAHCTGGRFMWFRETGIIENDDIQLVNSEIERALNFSRKCSMLVEAVKRKYKNRFHY

PEMEEVRALPPPAGLESGRMKRALPSPSPQYYNYGSSSGQREESEVRHERHAISWRPSSA

SRVRDPKLTPKAPAQRSRPTSAKDPMQCTRSSRKTVSSSHFFLEGKKHETGSVVREYPNA

KSVRKGIASPVLPDAEDPVSSKEWMRVYSLSKLKLDLNRLISGPDCRHLEKPVKALRKQV

AAKYCDIFPTINIKGTMKHLQLLPHELNEYEAQVERVLRRYLKRLQWLLSGSRRVFGTLT

HKKVVLLVDTSGSMEPRLNELKKELAALMWDQIYKLDIWLNIVQFSGSCHKWRDSIQPPS

EDNCHSAVQWTSNFVANGNTCTLEALQLAFEDPDVEAIYLLTDGKPDTSTSLVLREVARM

NLDRNITVNTISFNCSDSTANSFLHLLATETGGRYHRCPDDFNADMFVHKLLSEGFGDSE

YPHLPQFEGDDLRRLGMEVSMARRYLQQARVYKDMYKAKTTFPERPSSAKGNAAPPQQTS

FVVGRSKAIVR

>SG-AL_contig_849_1_+1_430_696

MCVKDPVSHVHVGWVMETKISSRHTHRRRVSRLCSNIIACTVWAHIAAMSPLSSGATRGV

ANYNSAVHQAPCQPAKTAKKWPCSSPDVA

>SG-AL_contig_850_1_-3_999_1301

MISFFVNHYSLLHYIYTSTIQGYRSAISTTILQLGGPSLLDNFLLRDTIRGAKYTCIHVC

LDFIAKFCQGHLCCCGIFFTCTKLVSSCCTRDLSLPSHLKD

>SG-AL_contig_851_1_-3_165_314

MDRYSTITVLVSLVHCQKNGKKYEEMKIYMNYSEFFFFLLIKGISQISSH

>SG-AL_contig_852_1_-3_72_1202

METVSKISHPASVLLVIGDLTAEFKHKGHRAALSAVRVTDSVESLHQHPQSWSTQVLDLL

KQLDISVVLVRGMVNADLHIACMATGIALVDHVPFRALKVLQSACQVDLITYILDAHEGS

VCCGVSLKPLQEDWCDTFCTNRKHLMVTPPVFVQTMVISEPSAAGKGVKEEEFWHCAARL

RQAAEDGKLLQGGGLVEEFCARHLTAHLNHPGKASVGMRWPGELSLYRPVVRAALVDVFH

TFTRTVHRNRQTAGCSTHSQQNVCDGPNPHRCDHVSSCSVGQNTGHYSHCSLFNKLPNSH

CCESLSSSSVCQSTARDPNHPLLDLPMLEACPNHAESPSHEPLDLQMLKTDQEFSSAKLP

LHICETQNACKNSHLLP

>SG-AL_contig_853_1_+1_202_363

MSEVTHKCCTYSSITQCLRMFILFHYNIQCLRRMLSKKLHPLPTLTPILFLFFF

>SG-AL_contig_854_1_+3_978_1355

MSHNNSLSSCISLYHVISHHKPHKIYIKLYVRASSIFHHISHNGYITLNLTLLFPITCLA

TSLFSSVCLTIYKAVSHYIYVFDVSPVSPYLTFYQAVFRYIITHRLSHYNYVTAVSRDII

SHLMSG

>SG-AL_contig_855_1_-1_1855_2418

MYYLLPPVLPHLFISTVELAQLNCVAVSPLLFNFLMHLYHILDLPVSCDVSPLGLTYPLV

LFVKGLCLCIFERALWLTSQNITKKDLAQKLLCARQHVFVNDDNGNVVENAGLGKYDIGP

FTEAVLSHAFQKVFKLPRHLRYVDFFVVDDKTLISLSMSSGVIPFMISDRPALGSFIGIT

VILASFTL

>SG-AL_contig_856_1_-2_1547_1786

MKIIKRSGAVCLVSFSLNQRAVTFCLTWIESHSIWCIILSQRGLKKVGSRQWAVFNLVGI

KRWLVVEFELRGSLLHYNFS

>SG-AL_contig_857_1_-1_1_219

FFFFLVLCLFFNCTVSLVHIIHCFILFFFSFSFSSLPIHEANLVLWKRALIDLCSLRYFT

VCTRCKRFLFLVF

>SG-AL_contig_858_1_-2_1760_2053

MLKVICNRKGFLFRNFLFTVFMWAKLMIYGTTHYHHLDPRLSSVVLLVYSAFSYFTWNSA

ELNCRQYMPFVDYTKWSVFILLILLQQRLNKTPTIYGK

>SG-AL_contig_859_1_-1_190_312

MELCQKIFPVSATSVLIQLESVYNTCNPWPTYCQRHGERDR

>SG-AL_contig_860_1_-1_76_555

MADDHEEQFEQATSGASKTFPKQCSALRKNEFVMIKSRPCKIVEMSTSKTGKHGHAKVHI

VALDIFTNKKYEDICPSTHNMLCPHVTRCDYMAMSVDITDGFLSLMDENGGTRDDLKLPE

DADLRREITNRLDNDETFSVTVLSALGEEAVIAVKKTESK

>SG-AL_contig_861_1_-1_58_2547

MSLFFIRHSALCGSGIVLSKLHRTLPNARVQLSERRQFPKSSCQHYSNLFSARHDDDQRT

LKLSVKTTSDTNKRISFQGFGHEHQRHICCTMAASSSSGSGSSQNHLALEKSPYLLQHAS

NPVQWYPWGEEAIQKAKAEDKPIFLSVGYSTCHWCHVMERESFESPEIGDILNKFFISIK

VDREERPDVDRVYMTFVQATTGSGGWPMSVWLTPDLKPIFGGTYFPPDDRYYGRPGFRSL

LMLIAEKWKSSRSQIEEQGTAILDALMRGTHVAEHGETAVPSLQVMDKLHIMLEDSYDEE

YGGFGEAPKFPQPVNFSALFRLYSMEKDTGRGKSALSMCLHTLRMMAKGGMHDHIAQGFH

RYSTDKVWHVPHFEKMLYDQGQLAVSYLDAYQISKEAIFAEMARDIFEYVNRDLSHPEGG

FYSAEDADSLPTADSKEKKEGAFCVWTWEELHSLLADKVNETGDGPTLAELFCFHYGCQK

EGNVDPMQDPHEELKKQNVLMVQGSVEQTASKFDMQPDEVDAALQKCRQNLFDVRQKRPK

PHLDNKMVSAWNGLMISGMARGGQVLGDSALTERAVKAAQFLQTHMYHSEDNTLLRSCYT

ALDGGVTQVSKPIEGFVDDYAFVIRGLLDLYEASLDDCWLEWCQNLQTKQDELFWDEEGG

GYFSSQDQDASIVLRMKEVQDGAEPSSNSVSCMNLLRLASYLDQPGLRLKAERILRLCAK

HLISMPVAVPELVCGLMLHLRSPKQIIIMGSPDSEDTKAMLKAVHNHFIPNKILILADET

EQGFLYKKVELLKSLQKLEGKSTAYVCENFACSLPTNSLEIFEQQLESCL

>SG-AL_contig_862_1_-3_420_872

MLFNELKATAAFVILVLTSLLALPSADILLPKYVNSSTISRSSPLSLTVPFPAFTFINLF

FDVLMRSPTAPAALANASVSSCWLSYEWQRKAKVVREVQILESCCLRPLDASAVMCAFHV

CISSLPSLGQDKQKWREDTSLLDTCVDSKSF

>SG-AL_contig_863_1_+3_198_482

MLKSFIRCKYREKERRHLKLQKSYVIITRCQSAYNMYGSYQTLYNIIKYSITLSHLFLFV

CCCFFFRADFLWHCARNNRSIAVGSCNAVSACCHT

>SG-AL_contig_864_1_+2_128_484

MLTWEIGKISTLYPPGEVSDEGSNPCLPNNQVDSKLLWSDRKSAKNKTKQNKTPLWMTTA

KEVSSRDDSSYHWNPASEAKRVELSLCNSSSTLKKKKKEKKFFHRFSCSLLIIRQLHRR

>SG-AL_contig_865_1_-2_128_328

MFILVFCSVLLMLLAAFRVFFHGWWSDYLNCRYYTEERRKKCDQMQCGRGGREGAVNFNV

HILQNAD

>SG-AL_contig_866_1_-1_148_2103

MIMDGLDTLSQVALADVSSVCFDTALALTGASRHASESLEDSLNTPVTTGSESTFFGPDT

LEPPPITATANIIASINIPDAKTMEGMLPSYRPQMSTGYNNSNDSSNSNNNGSNGMTHTI

TYTGTLMTAPMPSAGCNMNESGTGNPNQPNLAALFSPLSPLFSALMNSPASLTPGQLLQQ

QQGMSLPNDYQQQASCEFSAPVCTTSDSSSTTTHFPSSSPSPASIHSPVSQMSQPEHYSQ

GFGSPLTPHSVHSPESQLGDLQEPVKMEDNMGGSYSSPPPPPPYSQSVPMPVSMDMDGMG

VMKQPPTYSSCPQQQQPMPPQQEVNNFLGMPPCSLPETILQMQISDLGNNNNNKQTLNAD

IRWTVAPTSNMPVMPQLPNFHALQVNTPQFPMTGPIKTEPGVDPMEQNCYMMGDSSSNCM

NFNSSGSCKAASMAMLNQPYQQNHNNLKLLPVKPRKYPNRPSKTPPHERPYACPVDNCDR

RFSRSDELTRHIRIHTGQKPFHCKVCGRSFSRSDHLTTHVRTHTGEKPFSCDICGRKFAR

SDEKKRHAKVHMKQRVKKDAKLLATTASMPTSSSSAASSSHSHTPCTVMTSMVTPSLQAS

AMSASAHSAAGTLPLVVDSTGLCSVSGLESGLGVSDASHVVPSLPLVVTSSL

>SG-AL_contig_867_1_+2_113_295

MIYVVGCKYEYILMIFCRYGSASTFTSRVYRKYTIHSAESLVMLTISPYYMRWINVILKI

N

>SG-AL_contig_868_1_-3_153_2432

MPYTASVGYTPSVVQKDVQVQPQKAVVSSVAFIPASTKPLQVQKRSVGQAPPTAPREFQL

MTVDQDKGEAPPPAPAPEPQQKPTLQAIDQIVLQSVDDHAIQVSKCDHNSFRDLVWDLIY

SKNITNEIEKVRVVFRWLASKNLKEMNFDHVEKGSPEEVLMGLKTGATTYAMLFDIMCNY

AGLHSKIIGGYAKGADYIPGMKFNSGQNQHTWNAIYIYGTWCLIDAHWGARRLVGKQTSS

EDFHYQLDEYFFLPDPHQLIYTHFPDDPRWQLLDRCVTLEEFENMPHMKPQFFKFGMEFV

THRNAIIRSRGEVQVRLRYPAKQVAVAFNFTIQFENGDEEYKGTRLSRYGMQDNEDGVAT

FTLRLPTSASYILFIYAKEDTADKKENVYSQVCEYKVEHETQPGSAPEPFPPCSSLTWGP

CSTFYRYGLSTTQQSPVIHTCEGKAQLQIQQPRPMQLMARLKHNNISDSDLESYVMQRTV

GNTIIFDINAPGRGEYGLEVFANDPATEGQTLYHNAQYLLVCQEDVKTTPLPKLPPGYLG

PQPRFSEFGMSTMSHQDPVIHVDTNVLTVVFKCQQPMRLLANLIAVEGDKDFSNFTFSNT

KGSNTIFSINIPQTGFFKLQIYGTPVSDKGQQIPGIYNYLVNCRQVTQAVYPFPKQYAQW

KEGCFMAEPVVIPSRQGPFADQVQKERLPDTITFRVEIPGAKSVAVVVDTDWTHLEKAGV

NEWSGNVSVGHCYGTNTRVTLNANYGADKTNYATLLEYFL

>SG-AL_contig_869_1_-3_1731_1970

MQVPLLMSVCTYSSRNCSIYSFCFNVNPCSFSLVVICVSVYSGRNPYMWSLCLMVKPTIQ

VLVFVSVFTLKIPSTFSFTN

>SG-AL_contig_870_1_+2_596_838

MKSKTWLEMVKQSHQWNKHSGRWTVQTQLCLGIGPSPVKVCTSTFDGHMVKFSFFPVSFI

CRDCSQYLKEMFGFIYCTMSD

>SG-AL_contig_871_1_+2_269_460

MHTHTLSNAYMLTEPHNCNTLTGRSSQITPPNNCRYLHALTHTHNITNFVSDAHNLYANH

TVYM

>SG-AL_contig_872_1_-3_852_1115

MVMIKEAVVCTSIFQLDMIKEAVVCIRSGKLLSVYDQGSCCLFPNFSVGYDQGSCCPFPN

FLIGYDQGSRCLFLNFSVGYDQGNLSVP

>SG-AL_contig_873_1_-3_225_3266

MLQVDGSCFNDSMINETDLNWNSPVMEFNASWFTLSEDFLFNLTISAPGSPTAHSTQVIQ

IESDMHVIPLSVECKQCRDNWINTNDKVSWEARCQHFSKCSQSRLRYKWKLSVVEGDDPE

EERDASNYAHKGCNKAGRSFYRLMFRNTTAAKTTTEKPITVTTPAAKVLVPSDPPPHGNS

GLGMSEPMFEFFWGELDLKEHPGPSSRKEISGRHSSSGMMNPAGALIPIEEVSVDSRYRD

TPFSMDMREGDPYHKSEVKSSSKRKYHTGSKAYLEPFRGIDDGCDELCKAFKEMRSLPPH

ITMRKSSQVLLEPRIMASEPDGPSLVLKPGTLIEGLTYLVQVSVSDPDEPEWRGTAITYF

KVNKSPWGGGCSIVPTSGTEMDTVFSMFCKEWRDDQRPIWFEIGYSLEKTEVDPASSPSS

SSSSSSFPSPSIHRHIIYQGLRHDVLFQLPAGLPSYNREVFLDISIRDNWGASTNLCTIP

VRVTPRPQLYQDDGDDLTTTNGTNNHHITSLQADVNSLSHHLETSLYHGDIRGVQDRSEV

FRLLATRLNLLATAAAPVDDSSNSSVTTTNTLRLLADIRYQLLQALRHMPNRDLVEVGKT

LRCLLYVTTVVTQIDGRAFAMGIQLLEKISDRSRQLEGGWRSPLSHDNLPMTLQAVSNLL

QAATHSTLMMEVEDIDHKRFLLTQRSMDIVNNMLTLALKDHALGEVPLHHRTEFISVTAS

HYPAHSSPSINISQATFHLPHNMSHVLKSVHSGHADTYNLDDVECFQVRATSFQQNPFVY

KKEIPTNEIEIQSEVVSLDMFSCGGEERLVVEELEEDNNVRIIIPLASSVKSEGEYQLSR

THMNIHEFNISEPQENLYLTLLVNLNPIPVGSQPFPVTVVISQDWPPHPERNIYRQDLSR

EDNQLRIFFTPQEIKESLDRAGTYYFGLVETSLNSGRRRKTEALTRNYTLSAWFGSCLYW

NPAESLWSEQGCQVSPQTTTTSAHCRCSHLTTFGSHFELVPNDLSFTDIEHFFG

>SG-AL_contig_874_1_+1_472_717

MVTSVSVGLEVCWRAEMSSVGVVTQKWCRRWGSKNKTNQKTKNNTMQPFKQEHCKVKKAT

EQASTSVGSVTFLYMSRISFCK

>SG-AL_contig_875_1_-1_2137_2718

MFLGLLGGCRLGSSRWSLGPLPRGPRVSTLWGPITGSLHYGSRHANGFPRSLSRSRNGRV

IGGSHEAYKFPTLLGLRGRSKVYTLDTFNRVCSAISRYPNVLGRGVCSPPLSSRQILLFY

LPTPVPTPPSHQQSALPCATTAYHSILLPPRQHLIHRVCLCLSLLVFFAPLLVLTSTQQG

VPKSFFFFFLFRKV

>SG-AL_contig_876_1_+3_1488_1625

MQNQQTSKQNNSNITTIESDKKQTKTTITTKYIQKTNKQTKKPIGA

>SG-AL_contig_877_1_-3_24_275

MCTVHTHVHTHTHTHLPLPAPFPVPSAWITSQIMTSVVCVLLLHRLTALCEGTRLYMFVC

CSCLCSYYVHMCVFTSFGWEEGRV

>SG-AL_contig_878_1_-2_140_433

MQIFVKTLTGKTITLEVEPSDTIENVKAKIQDKEGIPPDQQRLIFAGKQLEDGRTLSDYN

IQKESTLHLVLRLRGGMQIFVKTLTGKTITLEVEPSDT

>SG-AL_contig_879_1_-2_104_856

METSIYALLPEEEKKEEKQKRYTSKFRQTVKEEYASGRGNYRTMGQAKVPVPTTESFLKK

REKEPVLPRKEKFTYPDDDQRRPPVPDRQDQPLMGLKTNKNFITTNAVENITSVPKKPEK

KFADTRNGDTHNLIPSGLEPVYVHRKDFGETPVYLNKRKEEMKRAQEEYDAYVAEHFRRG

ALRCLTVDERRCIIDGLKANWEDIHEQYQGLSVVTDTTPKKNRKERMEAQMKQLEKDIEM

FEKFSTIYIGN

>SG-AL_contig_880_1_-3_219_446

MILDEFCLYYMCSYVFMLCLEIPLTADSYQFFSLPQINLFNQMNNETYAEYYKTIIYSLF

LPLVLSFLCFISFIVI

>SG-AL_contig_881_1_-2_533_706

MCMRKQFFFMFSAAELISLFRFTHKKESLGFCPILLIKLGLKLAFIKFHNFTRVKLTL

>SG-AL_contig_882_1_-1_103_588

MRGIRGADYECHRQARRSGMSGTFRAFLSTNTQNLDSIIYYSRDRQIPIVNKKGQLLFNS

WEDLVNGAGGYINDNAALYSFDGKDVMNDSMWPQKIVWHGSSTKGSKMDGKLCNQWHSRN

GDSMGLGSSLNKHMLLDQDEYSCNNRFIVLCIQNTARSNLRK

>SG-AL_contig_883_1_-3_78_224

MCVFLFLCLQQCFCTFLSTLFLVIKKSAFKVLSTNFCLCQYAVVSFILE

>SG-AL_contig_884_1_+2_176_448

MKLFTSEPVSYKYFSTIVTISTESLQTLPPPPVNTSSKGAQTTKMDENMKMKNLSSILYT

VSAFLFFFVIHACCSISSLCFSEQSDFFLKT

>SG-AL_contig_885_1_-3_3_191

FSYFLAVLNSSHLMPRIFLRRLFSKPFNVFSKAFVQHQLSDPYVRTVRTLDLKGFSFVLL

LMF

>SG-AL_contig_886_1_+3_1038_1280

MFLLQVLSPSLVLILQCHARHIGQGPGVDSFLYRLPSVQLLSGRCCLPLFLQTGVLLRVV

FGLPLLLCPFGLQNSAAFGSL

>SG-AL_contig_887_1_+1_766_1077

MVSHTTSNPATGHTLKHRLTAAGPWLGSPTKSTPGMVRRCRAAVTLLTVGSGEPLLLLGA

ATHGDVTDDEARREPLATKPSALTGGAFKTPTPLPSVTNDTWDS

>SG-AL_contig_888_1_+1_226_342

MFYCNLNNNVIIHTIYNCSDGSALWVCTLPRESCLNFTR

>SG-AL_contig_889_1_+2_47_478

MSSASVGAREISASMSSGVGFSGISKTASSSSCSEPSASSKTSSTSLGPRDISASVSFGA

GFSVTSKTASSSAGFTISASLGMSSASVGAREISASVSSGAGFSVISNMASSSSGLVTSI

SSIISSASLGPREISASTSSSVGF

>SG-AL_contig_890_1_-1_43_1614

MHALRQACSGLVRATKCALTTQAVPQAASRTLPAYFNNRNYATEAAKPAAASGGAVGRIV

AVIGAVVDVQFDDNLPPILNALEVEKRSPRLILEVAQHLGENTVRTIAMDGTEGLIRGQA

CKDTGTPIRIPVGPVTLGRIINVIGEPIDERGPVETDKFAAIHQEAPDYVDMSVEQEILE

TGIKVVDMLAPYAKGGKIGLFGGAGVGKTVLIMELINNVAKAHGGYSVFAGVGERTREGN

DLYHEMITSKVISLTDDTSKVSLVYGQMNEPPGARARVALTGLTVAEYFRDEEGQDVLLF

IDNIFRFTQAGSEVSALLGRIPSAVGYQPTLATDMGTMQERITTTKKGSITSVQAIYVPA

DDLTDPAPATTFAHLDATTVLSRGIAELGIYPAVDPLDSTSRILDPNIVGEEHYKTARAV

QKILQDFKSLQDIIAILGMDELSEDDKLTVARARKMQRFLSQPFQVAEVFTGSEGKYVHL

KDTISGFQKILNGELDHVPEVAFFMVGGIEEVISKAERLAAEQH

>SG-AL_contig_891_1_-1_58_276

MLRIKDIKLKWFQIQIIYRILAANVILKEMKIMDTNQCTFCIEAKDTIEHFMWRYKYTKH

FWLDLEENDKRKV

>SG-AL_contig_892_1_+3_3_194

TSSYFSLPLTPFLSSFQLHGPAQNAERHRSVCRWHQKSNKEREREREREREREREIKQTH

TDNR

>SG-AL_contig_893_1_-3_180_1445

MQRLTTVFHHLNPSQLSVGMTDVAALPNYQRKADDIVVVSALRTPITKAKRGSFKNTYLD

DMLSAVFQGVLAETGLSASDVEDICVGNVNDPNSPLTARVAQFFSGMPETVPIYSTNRQC

ASGLQAFMNVAGSLKTGQCNIGIAAGVESMTKSPLGAGGFDFNPKISEFQKAQDSLIPMG

ITSENVAERFGISRKQQDEFALASQQKAAKAAASGLFDAEIIPVTTMVEDAGGQAQQVTV

TKDEGIRPTTMEGLAKLRAAFKEGGSTTAGNSSQVSDGAAAVVVTTRSEAARRGLPVLGV

LRSFAVVGCPPDIMGIGPAVAIPEALKKAGLTVSDIDIFEINEAFASQARYCVEKLGIPF

ERVNPKGGAIALGHPLGCTGARMIATLLHEMKRRGKRGMGVVSMCMGTGMGAAAVFEYQG

AN

>SG-AL_contig_894_1_+2_2_211

LLYQIHPKSLYLKDLTTLCKAPGLSLNHFKPSAGVSNTSIHPLPNTVRTRASSSTKCCTQ

LGFNVLCYYV

>SG-AL_contig_895_1_-2_20_478

MFWFVLHLSTQPTTDRANAGYLSDHQQAVSNMGRVFLQHIGGSRIFSCINCDTQLTNRSE

LISTRFTGATGRAFLFNRVVNLNFSEVQDRVMLTGRHMVRDVSCKKCDTKLGWIYEFATE

ENQRYKEGRVILERALVSESESFDEPAQNQNDS

>SG-AL_contig_896_1_-3_75_221

MYMFTLLLIFFFSLVTLILCLNICVYLYSVSRKRFLIVIGLMDKFWCHV

>SG-AL_contig_897_1_-1_1_111

ELKNLIGAGNRKIKNASVKKVISRLLLSSMNMITKGS

>SG-AL_contig_898_1_-2_2_211

VWQFFSTLPCRQPYSDFRGYPGPVNGCLASCFCYRYVILVVDATFGSCFIYMLFYLSFYL

YFGELQGFFQ

>SG-AL_contig_899_1_-2_443_691

MFLQMAAACEEFISGDLTITELKDLMVTITYFNSLQKNNLNHMHTLIIKANIVFMPIIFT

TSSHPTLYTTKRIQYKANVSEKC

>SG-AL_contig_900_1_-3_108_1247

MPLISGKTNLCPNSSTGSDLNVLPASLLAYLSAEPKSRKASAFLEQVLSCMTGSQGMSLG

NAVCRRCGDGFGMQEQIINSNGEVWHPHCFVCAQCFQPFPEGTFYEFEGRKYCEHDFRVL

FAPSCGKCGEFISGRVIKAMNASWHPQCFLCQLCGICMADSGFVKNAGRALCRECNAKEK

AKGTGKYVCHKCHDVIEINGHIKFRGEAYHPYHFNCHSCGVELNTEAREKDSELYCLRCH

DKMGIPICGACRRPIEERVVHALGKAWHVEHFVCAKCEKPFLGTRHYEKKGLAYCEIHYH

QLFGNICFVCNNIINGDVFSAFNKAWCVHHFACSICDRKMSQKTKFFEFDQKPVCKNCYD

KFPGELKKRLKKAYDEQGRK

>SG-AL_contig_901_1_+2_872_1099

MAVNRPVYTLQAGTELETLLYQKNQPAREKNKQKNCTCGPTCGPPEGQTHDPDYQHGSLS

SSANGPLKYDTTECYA

>SG-AL_contig_902_1_-1_1_90

CVCVCETTEFFCAKSNIPDCETWKVLTVRP

>SG-AL_contig_903_1_-2_263_3439

MSHSDDDQHRVSGEAKSSEGKQSKPLDSLSEDTLAKMSEREKRWQEADAVVQRAQGGLEI

DEALEDKRREEAEILRHQDQRRQEVGQTLEEQFRKDADIVIRTLEGQADINTEPSVSVAL

SSGAEDQIAQGKKELGKAVADKRKEEMELERRRLEAQIAYERETRQAMQLAREKEILEDQ

ARREQVLAAAEARREAALQQERELQMEALREQEAAAKRRREAELRHEEEIRREAEKRREA

ELLREAEIQREAQRQEMEMIKEAERLREIALQKEAEMRRQAELERDAARASEEALQKEVE

LAREMARRRHDTQLEASRQREAEEAIERAGGGRLHADSVSIRDVEEEADKEVLRRGHELV

RRKEMEEAVDLVPMSHKAQLLQQALEQETAYQRPDLHILWERFMAGSNNGPEPDSSLNSE

KVNMLADLLRDPTQHLVHNFLTQRQTQHQEVQQRAAAYRQVVRQKQEEDNRINEEEKAAL

ERELRRQKARQQQQRLSSEEESNGSYGEILQQRERDRLKRREVRTQRKKLGKSPSPSPSF

SKLDQHNEGAQGDTMETLYSIPEDMSHEVSPQKSRSPHRSSYQRRADIIDPNMDKLRNRI

EQQRKKISKERVKEMKREQKLDKLKALLGAKQAGLLDDKAISAHLASISSTSAASSDDSA

TLQSQLTALSDNTTTAKDSSTEMRLAKLARGSAGGMHHRDRHRANYHSMNLSMDTEDFTH

LQEDSSSQTENEYSFHHGNRYHSVRKSSKHSRKRLSPSGQPSKHGGKFMKLHQMYSPYKR

EDFDLPQRTVDRRKLISPFEKSLSMRTKDISTMYPSPKHFSPPHRKQHHGSRLISQGVQT

SPNSRKSPQRSRSFSPPYKDKPAVPTMSQRVLQPRGKDTFQARQNSKKAGTNRLPRQSRS

ISPAFDSSQVAACLSPERDQPSSASLARSDRHRRQSPSPPRHKHRSPSPVELRLKAWTER

EARSPLRDPRFHRKLSPNGHQDKFSPPRRVKNYNFDGENQPPVRQLRRMKPPAPSAMTWF

IPMKEDKPWRQPLKERQAYSSCEDWRPMALSQKQWRELA

>SG-AL_contig_904_1_-1_1_375

QCSVGSLARHAQEWKRLHLSDWVVGVTEDGYKLPWEGAKPPLRPLPPSRRQIPHDQDAAL

ASEVADLIHKGAAEALPSPLWAWRLWQTPCSPQILGWLAPGAGPVCPQRVPGPHPLQRET

PALIR

>SG-AL_contig_905_1_-2_2_265

QGKCNTNIINHRPVALTSLVCKLMEKIILCRLMHYCEKNNVLPVNQAGFRKGRSAEELLT

KLTTSIKRQFARRKCVLATFFDISKAYD

>SG-AL_contig_906_1_+1_1_288

GYIHVSISHRTLTWLTGSLTCIFDVLHVYTHKGGSGTSRSALYMVTLEIGKISILYPPGR

VELRPDILINFGMAYVFKEFVIWLKVSGCQLLVFKS

>SG-AL_contig_907_1_+3_2370_2807

MDLKQKKQCTHYFTGQIAPPPYKNQTNKNNSSPRKINKKTDALYYIHMIMARGNIPMTLL

PSFTLKQSSEQSVPSQFTVLVFFKTLTRNNHPLLHYPHFFPKTASCTHKNHGSTLGTEHW

YFVILLSNKPTTNFLRCSYDQLVLRK

>SG-AL_contig_908_1_-1_193_393

MRQINYFKIMNFRGFLFSAHIKSVNIACCIHVSFYRHLFPTALRFYAQLQFSIHNKALLC

LCSLEKK

>SG-AL_contig_909_1_-1_142_669

MKAYRPPWTGNVKTPCYFLFLSPFIREGNKMPAKGPIKAVQVFGRKKTATAVAHCKQGNG

LIKVNGRPLDQVEPIILRAKLEEPLLLLGAERFAGVDIRVRVKGGGHVAQVYAARQAISK

SLIAYYQKFVDEQSKKEIRDILISYDRSLLVADPRRCEPKKFGGPGARARYQKSYR

>SG-AL_contig_910_1_+1_223_315

MPFQQYLYKSNWHQICVQNFIKGLNPFTARK

>SG-AL_contig_911_1_+1_157_414

MVSPVVWVKNAVFRKSRGQVNMAKWLAQWATDLKVMGLNPLQSIKRKIIVEFLSPDWLLP

TVRHAMGSMGGLRPHSRVSFTLRMRH

>SG-AL_contig_912_1_-3_54_272

MVKSHFYNEVVKSCHYIPDVRFDLETELHCFNLLLEVVPECFGGQRLRRNLSEIALGEQL

TQIRIRINSHLPV

>SG-AL_contig_913_1_-2_296_1618

MASNGIDGESSADRNVEIWKIKKLIKSLEAARGNGTSMISLIIPPKDQIARVNKMLADEF

GTASNIKSRVNRLSVLGAITSVQQRLKLYNKVPPNGLVIYCGTIITEEGKEKKVNIDFEP

FRPINTSLYLCDNKFHTEALTALLADDNKFGFIVMDGNGALFGTLSGNTREVLHKFTVDL

PKKHGRGGQSALRFARLRMEKRHNYVRKVAEVAVQLFITNDKPNISGLVLAGSADFKTEL

SQSDMFDPRLQGKVIKIVDVSYGGENGFNQAIELASDALANVKFIQEKKLIGRYFDEISQ

DTGKYCFGVDDTLRALEMGAVDILIVWENIDIQRYVLKNHTTDEERILFLRPEQEKDKTH

FTDKDTGVEMELMEQMPLLEWFANNYKNFGATLEIITDKSQEGAQFVKGFGGIGGILRYQ

VDFQLMEPMINDFDDFDLDDY

>SG-AL_contig_914_1_-3_2487_2963

MKSDMCHGICNLKSVHLHPPSTHTHILTCRHTHTHIHTHIHNTRTHTTHTHAYNTHTRIY

THMHTTPPPHTHTPHTQHPPTHTHTPPPHTHTHTHTPTHTHTTPMHTHTTHTYNTHKHTH

HTQTTRTHTHARTRTHTHTHIHTHTHTHTHKHIHIHINT

>SG-AL_contig_915_1_-1_682_3915

MARIVVLVSCTVFVFLTVGHYSYAEETRDLADAFSRSEVFLTGEFFTPRDTRRRFVMVRA

QNRRKGTLFEISVDFRQPQVILRLRSSDRRDYQLAFPTRALHQDDRKRFLLHFYDLDLPS

NSVRLFVDCEDIGVDKTEIPIRSILSNGVNVRQTPSFKFHVQTTLPEILMSQGCGRESYL

QPPTTPAPKLELPAWVSERVRPERPEPDPPRREGHAPSDYDSLNGRKQQIPDRLDQGPRA

VPDEFSRVINSGGDVNMLMTAALRDLTTTIRQLRADLNAQTQETRELRQTLATCAICKEG

VPAEQPGPSGPSRQRCESNPCFRGVRCVDTEEGFRCGRCPRGYYGDGVRCERYTTCADSP

CFRGVECRDTERGFRCGPCPAGMTGDGTRSGCRPLLSTCADRPCFPGVECKDTPGGATCG

ACPTGYAGNGTHCEDIDECLYSRPCDRLATCRNLSPGFTCTACPPGYTSNRVEGVGIAEA

QRQKQVCEDIDECAENNGGCVENSKCENTPGSYRCGECVEGYEGDQRQGCSPEGQLCQDG

KTRCSNHAKCVQPQGRKGYSCQCNVGYAGDGKQCFRDTDLDGIPDEELPCADRRCRKDNC

VLVPNSGQEDADADGIGNACDDDMDNDGIINNPDNCPLVANPDQSDTEYEPDKRGDACDN

CPTVPNPDQTDTDVDGMGDNCDPDIDNDGIPNREDNCIRTINPGQLDTDKDGVGDACDNC

IYIPNENQFDSDNDLVGDICDTNDDIDRDGIQNNFDNCEVNPNPEQLDTDKDGQGDACDE

DDDDDDIPDIRDNCVLIPNPDQRDTDGDGRGDICQDDYDGDGHDDLYDVCPDNGEIFATD

FRAFQTVILDPEGDSQIDPNWIVLNQGAEIVQTMNSDPGLAISYNAFSGVDFSGTFFINT

EVDDDYAGFIFSYQDSSSFYVIMWKKSTQTYWHPTPFRAVAEPGIQMKLVKSDSGPGEIL

RNALWHTGNTTHQVKLLWKDPRNVGWKDKTAYRWELIHRPAYGLLRVLFFEETELVADSG

NVYDYELKGGRLGVFCFSQEMVIWSDLVYRCNDYIPHGLLEDGSDSEDYEYDYDYEDN

>SG-AL_contig_916_1_-1_31_444

MNLLGVLYCGIPLLLVYGVEAAEKKLQIGIKKRVDPTDCTIKSRKGDTLHMHYTGKLEDG

TEFDSSLTRKPFVFTLGAGQVIKGWDQGLLGMCEGEKRKLVIPSEMGYGEQGAPPKIPGG

ATLVFEVELLQVERKEEL

>SG-AL_contig_917_1_-1_91_303

MVFGTVSRSLVRKFSTSAVARSQGWQQYGIPGANLPFNINNRYKLTLLFAVFFSSGFTGP

FLLVRHQILKK

>SG-AL_contig_918_1_+1_283_420

MSWLKPYLSFLINKFSLCFHSTQLKKQQQKNMSWMKSYLAFLINQA

>SG-AL_contig_919_1_+1_10_249

MFMYMSLHSGLVLNKDVLIFLFFFKDCTVLIFGLFHFCEALQLHALHLQQSRSNDRDKTL

FSDTFIHILFYHLSCVDPSN

>SG-AL_contig_920_1_+2_428_730

MAMQVEAFILVHSKDSGHKTHNSNGKLYVPLGVALVVVFLPGPLWHRQVVHVCMLLNGEL

FVCLPVCWSMAAHAACPLQPGSSADGSCHWNKFSQHFVEAT

>SG-AL_contig_921_1_+2_1313_1498

MVSVVVWRASHGNHPNFTMYNSTFGETLHTPLISTTTTQSTSCPDQLLHEVVTATTSLPR

WH

>SG-AL_contig_922_1_+1_406_609

METETERQRETERERERERGQFANMNIDVQITNSISESFNKTIRLQLSIQVKQGKLMKKA

KREVVVSL

>SG-AL_contig_923_1_-2_485_2905

MQPVPAANADLMMTDDNRLAGTPEALPEPVQYGSSVDSVPQQAASSAAAEQQNLRDLGIY

DDDDEGVDNGFDDREGGAMFGTQGSDPSDEIQPQSLPEPAAAIETPSSAEPDLIQDTFCQ

ASAMEQSQLFEQEIEPDYSADVKETSPVSDHQADMMMGAGEQSYSDKEPSPDPQEKESSP

ALEEPSFADDVVESQRSNAISQNIQYGMTSDNGFQMGGIREEEEEEDAAERENFERDAQG

QMNQGNDMNKQSMEEMGIYDDDDDDEGQDEDEETAGDHGKDAYAYENDVNLEDEEIRMAG

SSRGEMLDQHAADQDPQRSNIPEAFERDESPVPVEKQMDAGPEEGAEVDSGQEDTDSIDG

GTPDRDKDIDETAVHQQSDLAQRQDSLEDLRGEQEAGDGQQQFNPFIGLGAQNQGMAAGV

DNLHGNFEHDDLDDSHQRGGSAEFDPLAKWGPPTGLPAPSPLQNDTEQDDVGQQQTTMVE

SHEGLVDGGVVYGQDGVYHQEQPCGQDRAFHGFGTAAMDEFGIVGAGHNVDTSASDKGSE

VDPSEFDPLAEWGQPMGLPSPPPANSTNKTTGAKGGAGDTKKSEEKKHGASPMKGKTGAD

RRPASATKAPAKPANGTAEPPKSARSKPDPKRASLDTSRSTRPASGKSLDSSRMESKTRT

TTSASAARKPASASKARASPDVIKMPPLPAFTPFYMDLTYIPNHGDTSYVDVEFFKRIRA

RNYVLSARNPNPKVLEMLIDAKATWDKANDQEVTVIPTYETQILQHWMAVHKEKLAESKI

NIAPAASRCTVRLQDHQDSCYTYRLEF

>SG-AL_contig_924_1_-1_2806_3006

MGIIVAWLFDLAQRWIMLTTYLNWFWIWYLNILGFFVVLYLNLKSTSCAECESSEYFVIK

DFRFFSY

>SG-AL_contig_925_1_-2_467_1615

MDKSTVILLLCLAGCCAASNMQIFGPKERTMKVGQSLQLICQVDLGLEVILNNVVQWTHV

SQVDNTETVLTQSQYPKEEGTRLGYNVSVQNSQSETNFTLSVTSLTDPMDGKYICSDVNK

EHNPADVEVVIASEVQTVQLTFDEEVVKNVDKVTVQREEGEFEVKCTAKGFSPSAAVNIF

IGEQQLETAAPEIKIDTELSQAVKARRYMATVSIDSLKLEGHHDGQILKCEAAASFDEAI

SKVASMPLKVIVLEPEIMCSENTSAMSGDKYIEISCLVTHTNVNVKAYHWEIDGKDVTLD

NNSYNHVKTEPVKDEEEQDKTTLELYEVTQADFDKTFVLVVELENGEKFEKQISLLRKGK

TSASATRPAGYFLGFLLSLLLSL

>SG-AL_contig_926_1_-3_93_1268

MDLQYGIAVTNKYSLFCDDEVDPLEILRQQEESKNAAPSKKDKKKSAKKAVATENKPKQP

EQQFKREDTNQPRQNGRGGRGGRGRELRENDGSRPPRRQGPRDNRDYKGDENVPPEFRDR

GGDMANRRDREGNVSGFERGGRGSRGSRGGRGRGRGGPRPESARGNFGGPIGDRRREFDR

HSGTDRTGVKPTDKRDGSGAHNWGSYRDDVEEGQSQAPADVSGEWAAPPAENAENDVNES

GEAAAPPKEEEEPKEMTLDEWKALQDQKRMKTQFNIRRPGEGVDNNQWKKGVAYKKKCME

DDEESEEEEEEEEDRHGHKKQLVDIRITFADNPRRGGRGGRGGRGGRGGPRGGRGGFGGG

DRGDRPSRGGPRESAPRMDDETDFPRLVKSEA

>SG-AL_contig_927_1_-1_373_897

MSQYLNNMEIGDYMDFRGPNGLLVYDGKGSFKVRPDKKEEPETKVVKKVGMIAGGTGITP

MLQFIRQVFKDPEDNTDLWLLFANQTENDILLRTELEDIQQQYPSRFKLWYTLDRPEAGW

KYSSGFINDTMIKEHLPPPDDDTLILMCGPPPMIQFACQPNLDKLGYAASSRFAY

>SG-AL_contig_928_1_-3_21_1196

MHAVRQACTGFVRATKCALTSPAVPQTATRALPAYFNNRNYASEAAQPAAGLAVGRIVAV

IGAVVDVQFDDNLPPILNALEVEKRSPRLILEVAQHLGENTVRTIAMDGTEGLIRGQACK

DTGTPIRIPVGPKTLGRIINVIGEPIDERGPVETDNYSAIHQEAPDYVDMSVEQEILETG

IKVVDMLAPYAKGGKIGLFGGAGVGKTVLIMELINNVAKAHGGYSVFAGVGERTREGNDL

YHEMITSKVISLTDDTSKVSLVYGQMNEPPGARARVALTGLTVAEYFRDEEGQDVLLFID

NIFRFTQAGSEVSALLGRIPSAVGYQPTLATDMGTMQERITTTKKGSITSVQAIYVPADD

LTDPAPATTFAHLDATTVLSRGIAELGIYPAV

>SG-AL_contig_929_1_-3_129_461

MASKDELACVYAALILADDQVAVTADKLNTILKAAGVSVEPYWPSLFAKALEGMSIKELV

SNVGSSAGVAPAPAAGGAPAAGDAAPAEAKKEEKKEESEESDEDMGFGLFD

>SG-AL_contig_930_1_-3_165_251

MYCSTDCALFSSFLFCLKSVIQSSVMHYK

>SG-AL_contig_931_1_-3_3_236

SSAFTPPSPSALLLLLCLSVSVFFFYSVHLSYTFIHRIDRLNTGLVSTGLDECLLGFFTP

YLPLLYERVVYWASLDMT

>SG-AL_contig_932_1_-2_836_1066

MVFCLKHNQVFCLKTLTCAIIVCSCSFCTWRISFTSCFVLFCFVLFSRRDSSFVAVFLKM

VCCFKPQYVKNIHVFYL

>SG-AL_contig_933_1_-1_1_219

IKKKNLKYFFIFFYKNDKIFKKKKKKKQQKKKPQKQTTGDIPVKKKSVNNKTVMVIVMKN

SVEDKLAGNERRE

>SG-AL_contig_934_1_+1_1_222

GGREKKKKKKKKKKRRKGGGGGGGGLLSSTARKLAQCGQICSKLFSKGMTWTVKNMGGGT

SLGPRCGCHGYLTM

>SG-AL_contig_935_1_+1_898_1176

MICNKYVLNPGTSGKYSEIHRLKLLSEYNSTGMEEQDNNKSLDSNYHTVLSSAYCVCVSY

TCANKLNTDFKKRKKEQNYLKVCFVWQHYLLDR

>SG-AL_contig_936_1_-2_56_586

MPAYHSQYTNPPKLVGNMALLPLKTTYKGPASKEMGDADIIDEAIYYFKANIFFRNYEIK

SEADRTLIYITLYISECLKKLQKCSSRNQGEKEMYTLGISNFPIPGEAGFPLNAMYAKPA

NRAEDDTMRAYLQQLRQETGNRLLDKVFDSQTDKPSKWWLCFAKRKFMDKSLSAPGY

>SG-AL_contig_937_1_-2_131_2632

MVLSPPYSDRQPISTGELLQIVGLTVLSDANMKLSMRYPHFRPEIQEMAAAGIMRKGDMA

EKNEVFDNQPSGGTVAAANTVTVRKDFPDTWIWKTAVTDASGQLSMVETLPDSITSWVIS

AFAVRDDTELALTPNPFELSAFNPFFASLKLPYSIRRGETFQMRCTIFNYQTFSVTAEVT

LASSPDYKISGNPKQKIEVKSNSADSVTFLIVATTVKTIDIQVSAKGQKSDGSGGTFTDV

VTKKLIVKPEGVKKMKVVSFPVLLSSEMPSHTVTVYRDIAEEIVPDSLTTIVKITGDMMG

NVMSNIDDLVTQPTGCGEQNMITTVPNIYAWTYLKAINELTAEFKEVAKNFMKTGYLRET

QDYRWDSGGYSAFGMISDEKYGASTWLTAFVVRSFAQAKQFIPETVSMDLLNGDITFLLK

LQNPDGTFMEKGRVLHDEMKGETGEGKGLSVYITMCLLEAKKYGFEFEASKLTSAVNNVK

KIANGKEVKTNPFLAAITAYTLALHNANDQTTVNNLIGYIEAEGKAPWKKPKPGPSDVEI

TAYSSLALNTIGSFGAVRDMVMWLQKQQNANGGFSSTQDTVVALQAVSEYAVSFTGTASS

TVSITTLDPVMPTKTVTIDSNNKALLQIMELPDETKSLNVTVQGQKNSLSIVKIVWYYYT

IPNKSSEGSDKLKISTNVIMLGPQRFKVRMCMNKDRTQDYKNMMVVLFNAPSGAVMENIL

EAKQQNSKVMRLEQTENENQYNFYLNEPTDECLQFRMEQSVVVEDQKPGTAILYAYYKTD

FRAESEVVMPLECPPSGCCIEDNCGTSHAAHYASLSRLLLSACVCLLLSLLIAV

>SG-AL_contig_938_1_-3_27_224

MCSHGRGGVRGGGVDGGGGGGGKRGGGGGGEQTLDCLNDMEVTSVFIQAAAEDKQRWDES

GHIEMG

>SG-AL_contig_939_1_+1_241_396

MQIRQKQMMRMVLRSELSEGWTKFLLYFSCNTDDLHMITGLLQPPRTMELHH

>SG-AL_contig_940_1_+2_179_400

MARDRTNGEGMKIQAATSQTPNRHSFTFPFYSQDLTTWRHGLSSLASYPPPPPPPPPPPT

QPKCPPPPPTTLAS

>SG-AL_contig_941_1_-1_286_1041

MSSDAKCAAAKDAEKDAVPDNHLPQHHQPQGFGVKGEEAGATPGDPNENPDVGPLSPGLD

VHAVLGVPRPKRPSERSEKAEEEEGDSDTDDLIDSDDDTEEWQEGEYFVIEEDDEELKEL

QTQMLSENASLSRDMLVPIFAVCCTAYDKWIRENVTTVEEEEMRVRRTQLQLYQKVLELY

GSDAEWQGLTHAQRVNRNKEAHETSLRAKELGAPPETVTEASVAAQKKVAEDTFSTSAIE

KRIADSMNNLHV

>SG-AL_contig_942_1_+3_69_203

MTYEQNSNRPYTGVKFEYNAMVNALPDTWKRQLNSNLYNTPVNRK

>SG-AL_contig_943_1_-2_287_427

MSMAKLALIGLIVAGINPFTYLNLPTPSIYNWATQNKVYACLMLFFI

>SG-AL_contig_944_1_-1_79_1458

MQGQKLYNDLCDLKNEWERWTDKKIVLNNVRREVSHRLKDHEFSIEERRERLREMLRQED

QEYMAAIEAAEETTLERQAKMRERAKSLKERREQERLQFVQDKYDQQFRAQCEELRSTLS

KREQDIVCAERLEQLRLKQEEAREKKAHDDLYAQLWEKDRQEKMAREEREAQAAHERNRE

TLDVLRKQMAALETQKEEAKRLKEEELQLMREQAALRKMEEAAAAEEKRRRQQENRDMLD

LSLKMKMRKKAKEEQEQLAFDLKMLEELLEESRNEAKEQLQRKIELREEDRRYREYLARM

VQEEKQRERELEHLIHQEVEAAWQKRIAQWRTERLMRRKLLDEVMAARAVQIQERLAEND

RRKAENERERVALIRSIEENRQYEAEQTAKRMAENAKYQSDLRDQIHYNTHMREERRRND

DFEHLMGMQAEKEYQEKLKTALDNPTFDRLHPMRRAMLNK

>SG-AL_contig_945_1_-3_18_329

MASRLASTFARRFYSVQGPSAVGGEHHGGAKRWKLLSFLVALPGVAVCWINAFVLNASHP

ERPEFVAYPHLRLRNKKYPWGDGNHTLFHNSHMNPLPDGYEEHH

>SG-AL_contig_946_1_+3_159_1511

MSSTEITDSQPSSASSRLSPNASVTVDITTADNTSDTAVTEETIKPADNKTPNCSCCHSD

CVTMEDFQRTTQTLQAEISALRSTLHESRQTFKIEIDALHTTITHLKSISSSQPSKSTSS

NQPPKSTSSRQHPKSTQSHNKSYNCHQPSTIATHSQQEPTPTSTKTSTQPTHQPPPPSPK

SSTQRNSNTSPHPLQSVYIANETTVVLIGDSTVRGIDLSRSIQPHDTPQKICTSGLTITH

VTEWLTQQTPTNKVKQATLHVGVNDCIKSTSETGVSKTQWENLITQCQRVFPKAQIQASS

IIPARGRNPLNTTIRPSNNNLQDVCDNVGIKYVDHCTTFTTYNGAPRMALYADSIHPSKK

GTARMAECLFPPKSTTLLKPPPQHQRATSAGKTVHTTLLKSPPQHQRANSAGRIVHRNPT

NRNMTHSITKLPTHSHPPPLIQSEYPELIQQ

>SG-AL_contig_947_1_-3_84_938

MFFLVGLGLGDAKDITEKGLEVVRRAKRVYLEAYTSILTVGKEALEKHYGREVLLADRDM

VEQSSDELMADADTEDIAFLVVGDPLGATTHTDLILRAVEKGIQYRIIHNASIMSAVGCC

GLQLYNFGEAVSIVFWTDTWRPDSFYDKIAVNLERGMHTLCLLDIKVKEQSLENLMRGRK

IYEPPRFMTVSLAAEQLLEIIERKKAEGSVEKLALSPNTVCVGLARIGAEDQKIVTGTLQ

QLASVDIGGPLHSLVIPGHMHPLEMDMLRLFAIDDTVKNLLISSE

>SG-AL_contig_948_1_+3_1419_1769

MGGVTSRTSINPVGHQNKKWWQYFFHSLVNPSALHKLSSSWRSHLTTPHPRRTTITCGFE

LTTLTSFAQDTRPENAKLAEARQYLSPQSLSLSLTTTRLRRLKADQRCVVSVSKWAG

>SG-AL_contig_949_1_-3_3_149

YTRARTHTHTRARARAHTHTHTHTHTHTHASFASGGDLNDAFAFPENVG

>SG-AL_contig_950_1_+2_2_247

LTKSVYFCKTNSKKKKKKKKKKKKKKREQRGPGGWASFKAIRHDKRNYTYKINMPCSMTS

DHMPTEKQKYQLNRCVYKQLVK

>SG-AL_contig_951_1_-3_123_638

MATALGFRLTGFGYSHLIRNLTKSEQNLLKKIFAEADEDDKGFLTRADLKVAVISLLGYK

PSKYEANQILEKYGHAVEDGIFVLNLGQFIEAMTPKLINRDEDDEIRQTFMAFDTHCKGF

LTTDDVKKVFSQLAPHMGQHRLESIFKELDRDRDGRVSYKDFDFMMKFDPLS

>SG-AL_contig_952_1_+1_1_204

HQFDMVYDAKQELLDENSPSSVTCSLFPFLAALPLINTNRKFSLENDYRMLHWFIFSFHL

TAVLVKYK

>SG-AL_contig_953_1_-2_107_520

MIKTKNGKTVTGFATVARYLSQGGNIPCDLTLEEQAAVDQWLEYHVTQVVHCGQSQHCAQ

VLQELNSYLSDKVYFVTHHPTVADIIIYHGLYAVFMKLAYYEKEKYVHLSRWFDNIQQDK

RIRQSLNLLPFSRTSLYS

>SG-AL_contig_954_1_-1_142_354

MVIKMLRSYFVELHGAGICGGKYSFKWRYNLPFIHAFVVKQNEKFSLKLVDKTAIHEMQG

LFKYSPVTINL

>SG-AL_contig_955_1_-1_1_1839

MGGGGGGRGGGGGGMGRNRGDQQNPNFQNNFRRGNKENDSHSPQNQRRNSPQNQRRNSPQ

NQRRTNQTQQQQHNNPQHNQSPLNRPGSGTGQRDNIPPRFQRMAQQQQTESGGKASGPVL

QPGGGMGGPGSGPGGARGMMSASPPLSGGGGGLLNGKEEQLSLRPQKNFMFKPNTPAMLP

RSAQAAPSAPLVPPSNSLSTLGTVSPKKMTVDVANPPINPILNKQPQITIKPAKEEKAKP

PKKETLTPEELKEKVPDLLNEFFNQQDVTKTVAAIRELGIPKRMIPDFLSQFLIGSVNRT

EMEQDEVMKLVAALKTEKMVTSDQFVESFKMVADQMSALEQETPLIKSHAARFGAQAVSC

EVASLAELAEPFQNGVHYPLFLLCLQAAHRIRDKQWLVRVFNDSKINLQGMLPEIDQKKE

RMLEILEDRGLSFMFPLMRVQSELGRQIAAEPSAGAIFKWIKERVDSELFTNHNFINILV

TSMVKYITGESTLAENADPTVTPDKPLMDKEKELLDKLKGVLQKFLNEHIDLQVAALYAV

QVFCHSHQFPKGMLLRFFDNLYDQEIIEEEAFLHWKEEVNDQYPGKGMALFQVNQWLTWL

EQAEEESEDEDDE

>SG-AL_contig_956_1_-2_425_544

MFLVQRHIHWLQINHSIVIELHFISQYISVYVIVIIFVII

>SG-AL_contig_957_1_-2_2_184

FFSPPPPPFSPPPPPPPPPPPPPLPFTFHFPVLSSLQLSFILKACALKIEKGKKSFLWYK

C

>SG-AL_contig_958_1_-2_353_1207

MRFVQFEWNGRLAVGVETKEGGDIVDVTEVDPTVPHNMRDFIQGGEPNLAAARNAINSGH

FVLKRENIRVLAPITNPEKILCVGMNYKDHCEEQGAPVPEEPVIFSKFNSAIIGPNDDLR

YPEVTQQLDWEVELVVVIGKTAKNVPVENAMDYVFGFTVAHDVSARDWQFKNGGQFLLGK

TMDGFCPLGPAILMKESIKDPHNLKLWTRVNGVTKQDSNTNQLVFKTPELVAFISRFMTL

KPGDIILTGTPPGVGVFRKPPEFLKKGDVVEVGIEGIGQLTNKVA

>SG-AL_contig_959_1_-3_120_1028

MGSKKLGFAENFALSGAAAIISKTAAAPIERIKLLVQNQDEMIKAGRLSEPYKGVIDCTV

RTFRTEGILPFWRGNLANCLRYFPTQALNFAFKDKVKALFKTSPNESYVLKFSKNIFSGG

VAGALSLCFVYSLDYARTRLANDAKAAGKGSGERQFNGLIDVYTKTLKSDGVVGLYRGFV

ISCVGIVVYRGFYFGLYDSLKPLMLGDDANVFVSFCLGYAVTVSSGLLSYPIDTIRRRMM

MTSGQAVKYKGSMDCAMQIIKNEGFMSMMKGAGANILRGVAGAGVLAGFDSFKAAYIQWR

LKQ

>SG-AL_contig_960_1_+1_181_516

MCKLAAWPGKIIDKCMELNRVLISAQIQSDLTTSQWPTKPWETLLRTKYLCTFHVQGSQL

QNVTGMDYPGSRVNRGFPYWNSAMQPTGLHRQVAYLSPLTPSSQALANCIET

>SG-AL_contig_961_1_-1_1_186

WVLFLLHGLVKKKREGREGGGEGGGGGGGYEGKDIHFSVLICQNRFCFVVVVVFVFICSE

FE

>SG-AL_contig_962_1_+2_443_739

MLGVCMRERERERERERERESYTHCVCVCVCVCVCVCVCVCMHASQVEKHQTCEVLSVFS

ATRTPQNNDIKVDNKMQVSTTGHITPCIYTVLAIGFKLQ

>SG-AL_contig_963_1_+1_352_594

MHADNGGEKTKTKNKQNRRKDGQVKPYYQHSSSIFIIVTLSKNQSCSHIRRIVFIKSQSC

SYIRRTVFIKCQICPNFAQIF

>SG-AL_contig_964_1_-3_204_452

MKKGTILVSVCVFLSLNIWQIDFTVRKWCLTSGLISALLFSVSLILVKWKVWLTCSCLHP

IHQLPTESRRIGEGVTVSVSLLL

>SG-AL_contig_965_1_-2_98_1321

MKFLAVLVALVGVALCEPTIYFKEEFGDGWEDRWVDSTSKGGEQGKFKHSAGKFYGDAEK

DKGLQTSQDARFYGISAKFDTFSNEGKTLVIQFTVKHEQNIDCGGGYAKVFPSGLDQAGM

HGESPYLIMFGPDICGPGTKKVHVIFNYKGKNLLTKKDIRCKDDVYTHLYTLIVRSDNTY

EVKIDNEKVESGELEADWDFLPAKKIKDPEAKKPDDWDEREKIDDPEDKKPEDWDKPEHI

PDPDAKKPDDWDDEMDGEWEPPMIDNPEYQGEWKPKQIDNPAYKGKWVHPEIDNPDYVAD

DKLYSYEDIGAIGFDLWQVKSGTIFDNVLITDDEDYAKEFGEGTWGKTKEPEKKMKEEQD

EEDRKKREEEEKKRKEEEDAKKSEEGEDEEEEEEEEEPEDDEEVHDEL

>SG-AL_contig_966_1_-2_368_2113

MNVPHRILTPEEPGKSLGKSLVDRDISCGDSNFLSPGVPYADGTSQGSQESLDKRSSLPR

LKSYDAVVFDVLRVSPEDFASQITLMDLPVFKSIQPDELTSCAWTTKEKLIKAPNVVAFT

RRFNHVNFWVQKEILNCQTLKTRADVLSHFIKIAKKLLELNNLHAVMAVISALQSAAIFR

LSGTWMMLSKKDKMMYEKMADMFSECNNRQKLRDVMDNVKLPCIPYLGLYLTDLIYIDVA

HPHSGGLESPPRRLQMNNILRVIADFQQSTYDHLPVLDHVQNYLRSVRYIEELQKFVEDD

NYKASLKLEAPVSGQSSANATSREDVSVLPPSPATEPRQQFSAGKLCASSAHRKTKSLSA

NFMSCTHPQVERTYSLPSRATAPYVQGIRHLLDDSVLEESPCASSDGSVCSKIQQAFSGE

GREPLESTSSDHETFWPAHNNFQWQETEEAVYTANFTCEGCVRRKTLIKDGKKPTVSSWT

RYWMALWGTNLLLYPAKSLRGSDRSNFKSNPSKMSSIVGWMVVMGDNPLQPDAFQLTDPT

RAGNVYKFRAGSQSQALIWCRHLSEASKRYAPRQPANLMSFD

>SG-AL_contig_967_1_-3_21_1124

MASAGNPRVFFDVTIGGEELGRIAFELYADKVPKTAENFRALCTGEKGASSKPDINLHFK

GSIFHRIIKDFMIHGGDFTNHNGTGGESIYGERFDDESFDLKHDRPMLLSMANAGPNTNG

SQFFITTVPTPHLDGKHVVFGEVIAGGSVVRELENVEKEGETPTQTCTIADCGELPADFD

LTTLNCDDSGDIYADYPNNSGIDFTSESCREEVRKLVLEVKLVGNNLFKAGKFGPARRKY

KKALRYLENLDKEQKFSDEEQKAMEKELHIPVSLNLAACSLKLHDYTEAMQYCQEVLRCD

EDNGKAYYRQGQAYSGNKDYELALASFQKALALNPDDKGIKQEIQKVKRTIEDDKKKEKQ

MYSRMFAS

>SG-AL_contig_968_1_-3_135_6569

MADATARSLQYEYKANSNLVLSVDRSLIDRRARDEATGEVVTLVGKLGGTRMGDKAQKTK

PPLMEERKNKRRKRDEAQHDMVKMKGTTLLTSGIDEMVGIMYRPKTVETRQTYEVLLSFI

QAALGDQPRDILCGAADEVLAVLKNDRMKEKEKKKDVEAMLGPTPDERFALLVNLGRKIT

DWGQEDRAPTTDNIDETYGVNVQFEESDDDDAADMYGEVQEDEEDEENEGEEANIDATLH

ANLSEEARNKSEGGLHPREIDAFWIQRKLSKYYPDDPTTAQTKAREVLDILKAAGDDREC

ENKLVMLLGVTQFEFIKVLRQYRQMILFCTLLASAQTASERSKIEEQMEADTDLSRILHS

LQETDQDDLVTEERARRQQARQSRVAADLESMDVDEDVEVGPVQALDLDDLTFTQGSHFM

ANKRCQLPDGSFRKQRKGYEEVHVPALKPKPFDDDESLVPIDRLPKYAQPAFDSFKSLNR

IQSRLFKAATETDQNLLLCAPTGAGKTNVALLCILREIGKHVNSDGTINTEDFKVIYVAP

MRSLVQEMVGNFSMRLQSYGIKVAELTGDHQLTREQVNETQIIVCTPEKWDIITRKGGER

TYTQFVKLMIFDEIHLLHDDRGPVLEALVARAIRNIETTQEDIRLVGLSATLPNYEDIAT

FLRVDPKEGLFFFDNSFRPVPLEQQFIGITEKKAVKRFQLMNDIVYEKVIEHAGKNQVLV

FVHSRKETGKTARAIRDMCLEKDTLGLFLKEGSASTEVLRREAEQVKNHELKDLLPYGFA

IHHAGMTKVDRTLVEDLYSDRHVQVLVSTSTLAWGVNLPAHTVIIRGTQVYSPEKGRWIE

LSALDVMQMLGRGGRPQYDTKGEGIMITNHGELQYYLSLMNQQLPIESQFISKLADNLNA

EVVLGTVQNVREAVDWLGYTYLYIRMLRNPTLYGISHDQAREDPFLQQRRKDLIHTAACS

LDRNNLVKYDRKTGHLQVTELGRIASHYYCTYESMATYNQLLKPTLSEIELFRVFSLSSE

FKYITVREEEKLELLKLLERVPIPIKESIEEPSAKVNVLLQAYISQLKLEGFALMVDMVF

ITQSAGRLVRALFEIVLYRGWAQLADKAVALCKMVNKRMWQSMSPLRQFRRIPEEVVRKI

EKKNFPFERLYDLNHNEIGELIRMPKMGKTIHKYVHQFPKLELSVHVQPITRSTLKVELT

ITPDFQWEEKVHGNSEAFWILVEDVDSEVILHHEYFLLKSKFAHDEHTVKFFVPVFEPLP

PQYFIRVVSDTWIGSETQLPVSFRHLILPERYPPPTELLDLQPLPVSALRNPAYEALYSE

QFSFFNPIQTQVFNSVYNSDDNVFLGAPSGSGKTICAEFGILRMFTQSADARCVYIAPLE

ALAQQVYMEMYNKFASQLGKKVVLLTGETGTDLKLLAKGNIVISTPEKWDVLSRRWKQRK

NVQNVSLFIIDELHLIGGEVGPVLEVICSRMRYISSQLERNIRIVALSSSLSNARDVAQW

LGCGTTAFFNFHPNVRPVPLELHIQGFNISHNASRIIAMGKPAYQAIVRHSPRKPAILFV

PSRKQTRITAIDVLTYAAAELQAGTDDKVQSRFLHVTEQDLEPFLEKISDMTLRETLASG

VGYLHEGLSEAERRIVEQLFNSGAVQIIVATRGLAWGMAAAAHLVVVMDTQYYDGKTHSY

EDYPVTDVLQMIGRANRPLHDQEGKAVIMCQSSKKEFFKKFLFEPLPVESHLDHCLHDHF

SAEIVTKTIENKQDAVDYLTWTFLYRRMTQNPNYYNLQGVSHRHLSDHLSELVENTISDL

EQSKCISVEDDMDVAPLNLGMIAAYYYINYTTIELFSMSLNNKTKIKGLLEIVANAAEYE

NLPIRHGETEILRQLGARLPNKQAVPKFNDPHTKTNLLLQAHLSRMQLSAELQSDTEEIL

SKAIRLIQACVDVLSSNGWLSPALAAMELAQMTTQALWNKDSYLKQLPHFSQDTIKRCQD

KNLETIFDIMEMEDDDRNALLQLTEAQMADVARFCNRYPNIELTYEVQDKDSIHSGSTVN

VVVTLEREDEVTGPVIAPFFPQKREEGWWVVIGDAKNNSLLSIKRLTLQQKAKVKLDFLA

PSIGRHSYTVYFMSDSYMGCDQEYKISIDVKEGGETSDESGSESN

>SG-AL_contig_969_1_-1_139_468

MADIPSGDPEKGKKVFVLKCAQCHTVEAGGKHKVGPNLHGLIGRKTGQASGFSYTDANKS

KGITWGKETLFTYLENPKKYIPGTKMVFAGLKKKNERADLIAYLESATKA

>SG-AL_contig_970_1_+1_1_189

GLTYRCWTGTLTTPLEHKMPTCKMRCLRRAVNKTRRHMVRNTRIREMVETPPVHHHFYQQ

KNR

>SG-AL_contig_971_1_-1_91_1611

MAVALPPINSGNGNKKVPAYAETGPRGPPSGRLRPLRIGNKKERELQDQRMQLRSLQKDL

NKADTAKFRNSYKHSLCLDMLKDGFHLSFKELFNLIQQQLQEREAAGPESLMWTQTMLQD

KHEELDTLKYYLTGAETAQRKGDYLAVYMARYELACYFQPTKDKWLADHFFQTCLDTAAC

IDADGGQTKAEAHCNLGIVLEDNFDYFGAAEHFEAYYELSKASLDGWVSDVVNKGKVSPF

IDACVHLYRIYTSIGDQLEREDEPEKMLEFLTKAFDMTKQAEDKKLEGNAAYRLGLAYEK

SGDVDTALVHLNNFYEVSKAADRADGMGKACDAIAKAYARIGQKEKSIAYLKEFVETAQS

CGMDAELSQACHNLGNVLNSLGNYKEAGEYFNRAYNISRAMGESEAIQVNRVQSGIAQAH

AMLNLYSHIVVEGVHSRDYLERTIDWKSVRANIQESRDERMRKSVQAEPVLSTKDEPAPA

EEPADPVNEATAEPAQEPTEDAEQPAE

>SG-AL_contig_972_1_-3_288_2744

MGLGVDEAFQGAGAAPGMEIWRIEKLKVVKQVPESFGCFYSGDSYIVLVTKQVPNSSRLD

WDIHFWLGKDTSQDEKGVAAYKTVELDEGLGGGPVQHREVQDHESKQFLSYFKAGIRYLD

GGVDSGFKKVERDKYETRLMQIKGKRNVRVRQVQCHSSSLNQGDVFILDCGLVIYVWSGP

QSSRMERIKGAEVAKRINDEERGGRAAIKIIEKTWDTDAAFFKALGSHEPIKTADDAGDD

TDFERISAEEVKLYRVSDASGSLEVSEVGEKPLKREHLDSSDCFILDSGPSGIYAWIGKG

CTKNEKKSAWQNATEFLTIRGYADWTPVTQVVEGGETPLFKQYFSKWSDPNAQTGLGRVN

KVEKVAKQSNEKFETASLHSNKTPAKKEETLADDGTGKVQVWRVENGDLVPQPEDMHGIF

YTGDCYLVLYTYTKGNSPKEYYILYFWQGAKSSIDEKGASAIFAQRMDDNELQGAAVQVR

VVQGREPEHFLLIFKGKMVIFLGGINNKEPDSSIRLFQVRGTKTVNTKAVQVQPRAGSLN

SNDVFIVDTEKEVFLWVGKGASEEEKNVTNYVVKHICPDRNSSDVVTVEESQEPPEFWDV

LGGHEPYATGARLEEQGSDMPSRLFQCSNSSGRFKVEEVVDFSQEDLCEDDVMLLDTFSE

VFVWVGKGANETEKKEALTTALEYIRTDPAGRNEDNTLILNIKQGFEPTSFTGHFHGWDP

EKWSQGKTFEDLKREMGQENVAVTSVKEVGKNKELANYYKTHPIDVLKKKTLPKGVDPTC

KERYLSDADFEEVFSMSRSDYQNLKPWKQQDLKKKVGLF

>SG-AL_contig_973_1_+2_362_538

MEGIQNCLRSMNPSGTNSKLWGEKNTALLVSLPFSFHPPVGVKLDILTSCSLRFACFFQ

>SG-AL_contig_974_1_+1_1060_1374

MCILKRFPNRVLQHSENVSLRVTKYESTKISKTPRRTLTTFVKPSAFQVSVTLAPIREKG

SHTYCTVLCVVLNCGLYCIVLYCNNVFAVLVLCCTSMADTVPTSD

>SG-AL_contig_975_1_-1_7_267

MVNLATTVGVVTAVWAFIGIVLPILVQFLMWKSPNKGIIQICLILTAVCCYLFWISTYLC

QLNPMIGPQLETNVIRMMQLEWPADSN

>SG-AL_contig_976_1_-3_1485_1841

MRWTEGAISVDWFKLPAAVGLFEEMQIVSQLFPGLHMFRLSYLCNYKNTSQLQATAEFVC

CDPIPSPARQETLDFMISAHSFTESWYVCICACCFLPSGWLLSFTRVPVVLYGHLHIVL

>SG-AL_contig_977_1_+3_3_116

IKQNPLWYVYVQADIVNNLISSTCDNFIHLNKLSRTLW

>SG-AL_contig_978_1_+1_118_3021

MPMDSSLTGNQIRQTFIDFFKEKEHVYVHSSSTVPLDDPTLLFANAGMNQFKSIFLGTVD

PNSDLAKYTRVANTQKCIRAGGKHNDLDDVGKDVYHHTFFEMLGNWSFGDYYKKEICTWA

WELLTERLGLEKERLYITYFGGNPAGGLEPDEEARQIWMNLGVPSDRVMPFDMKDNFWEM

GEVGPCGPCSEIHYDRIGGRDARKMVNMDDPDVLEIWNLVFIQYNRETDGKLTLLPKKHI

DCGMGLERLVSVIQRKTSNYDTDFFVPLFDAIQQATGSPKYQGRVGKEDVDGIDMAYRVL

ADHARTITIALSDGGRPDNTGRGYVLRRILRRAVRYATEKLNAKPGMFASLVDTVIQLLG

DAFPEVKKDPEMVKEIINEEEAQFLKTLSRGQKMLLRTIEKIGTSSTVLPGDVAWRLYDT

YGFPVDLTLLMAEEKGLTIDDEGFEKAKQHSLLMSQGGGGTMDDQIALEVHSINELQNRG

IAPTDDSPKYNYTADVEGHYKFEPTTGKVIALRYNKAFVDEVTTGQECGVLLDRTSFYAE

QGGQIYDEGFLVKEGTEDVEFKVKNVQVRGGYVLHVGVIEGTLKVGDTMKLLIDESRRCA

IMKNHTGTHMLNFGLREVLKEADQRGSLVAPDRLRFDFTAKGAMSVKEVKQTEEAVNKMA

AKNEEVYDGNMKLALAKSIQGLRAVFDETYPDPVRVLSIGIPVTQLEADPMGPAASVTSV

EFCGGTHLKRAGHLGQFYIVSEEAISKGIRRIIAVSGTEAHKAQHRVEMLEKSVGELKQK

LDKGKGDACFSDRELNRQIVALDDEVAQSVICYWRKDELRKELKALKKVVDDMDKARKAA

ITTEVVEESKKMIEASPNQKMVVHQFREGAHAKALDGAMKQYKSLSPGTSAMFFSVDPEA

KKIVCMCAVPKEVATNGLTAKAWIDHVVGVINGKGGGSDVSAQATGSNPAGVSEAMKLAT

DFANLKLS

>SG-AL_contig_979_1_-2_191_961

MASLSKAIAKNVLFSHLDDNERSDIFDAMFPVHRHAGEVIIQQGDEGDNFYVIDQGEVDV

YVNNDHVTTIGEGGSFGELALIYGIPRAATVKAKNDVKLWGIDRDSYRRILMGSTIRKRK

IYEDFLSKVSILENLDKWERLTVADALEPVQFEDGQEIVKQGEPGEDFFIILEGSAAVLQ

RRADNEESVEVGRLGTSDYFGEIALLLDRPRAATVVARGPLKCVKLDRARFERVLGPCSD

ILKRNIGQYNSYVSLSV

>SG-AL_contig_980_1_-2_233_967

MSSLSSLDTSASVYQPAQLLNWVYLTLADQQSVAGTTSQGHLNNPLNGQSPASAYSNGNN

NELWTPGGTLNNVNNLLKSLNNQQTSPLGNLTTNPFQSPGSQPLSTSPFGSMVDLADTFN

ELSLDRKPCKRPPSSYLCHLCFNKGHYIKDCPQARPKGEGLTPYQGKKRCFGEYKCPKCK

RKWMSGNSWANMGQECIKCHIMVYPHKQRPLEKPDGLDVSDQSKVHPQHLCEKCKMLGYY

CRRMN

>SG-AL_contig_981_1_-1_232_369

MVCCWVLILYVLNKSIFKSCLLCSLSTCFVCHIACHLFCVFCSDGL

>SG-AL_contig_982_1_-3_3_284

EGTRGQVTHTHTHTHTHTHTHTHTHTHTHVLNQQNYTSFQGNRRDCTKMNLCVCVCVCVC

LRERESVCVCVWVHAACERKVCIQSFLNKSKPAE

>SG-AL_contig_983_1_-2_326_451

MIIIIIMLFTLLFTFLSLSLSLSLSLSLCYSSGSSNTCSSLI

>SG-AL_contig_984_1_+2_1442_1615

MPFIRFEPVSYKGLATLDSLWLSMCVVYMCVCIYIFICIYIQREIDMCVCINVYSVCV

>SG-AL_contig_985_1_-1_55_285

MLHLLFPWWNCAISSILCVEHRRLLLRLNDIGEELLIVAFCVKGSEVEKIYLFIYLLINL

HFLLFYFLSSLSLILMA

>SG-AL_contig_986_1_+3_1323_1619

MAEMYVQSVRQVWTTSWIQGNYSTDWSLGGQEASIPHGLVVCSPCFGSVILHLQWNTRKE

GTCTAMLGSVTFSLHWKYKTPGLCAATSTCHVHHHNFIL

>SG-AL_contig_987_1_-1_1_276

KTATQKKTDALDQEFLTQKNMKPHAKGPKAVSGYVRTEALKQVGVSQGQDFSSEASSPHS

SQSSLVGKPWKKHNNRNKKEKLRRVYKDLDQN

>SG-AL_contig_988_1_-2_44_235

MHCVTSEHPSSLSVCIFITLVMPFLSYLICNMKVMLSFHFINFSFHFTSSLKLQKINVWY

MFMR

>SG-AL_contig_989_1_-2_1550_2155

MLSKWMSLLSCLECRDHLSLQLDETGKINLVIHARSRLTGASSTWTITFWATKTSSSTRS

TTTTTSSTTATSSPTRVIRKCDPDVCQQLLAVTMSVIILFFLLLTFLLWVFNLFLVCNTL

LPLGVIHSFSSLAYVEGCLHTLLVLESLPFIKRHFFRFLLLLGWGYSLTTFVQVSRFWSW

FNGRRWSPFPRHIRRIGGCTWL

>SG-AL_contig_990_1_+1_79_252

MMCKILQKHADRQTSTGHDAMDYQSSAHHRSAILKFIIKTQSHATAPSHLFLKIKTHT

>SG-AL_contig_991_1_+3_207_650

MSPVGSPPHLGSSVNLDGLNHQVVSVQTLELSVALSILQQLQQELCALLGPSALGTLKLL

GLGFASSTSTEATEWNNFLLGHHVLQVTHGTLQVHLLDGLSCLTRVLEMNTHVRASSLAR

LRSIFWFLRVTSHFFFLTGKGSSIPIDV

>SG-AL_contig_992_1_+3_225_467

MSFPAVYNKNVVTKPELISDLTLNLYIKLSIKSKPHYCSTEQLFQHLKDQKIVYITSVSL

ANIYIPIMDMITSYKTNFYKI

>SG-AL_contig_993_1_-1_49_933

MEAIMSRSVALFTPQLTQVRGMATLKDIRLRLKSVTNIQKITKSMKMVSAAKYARAEREL

KPARSYGAGAKAFFDAAELEQDKTQPSQMIVVMSSDRGLCGGVHSGICRELRSLLGERAT

GGSIETKLVVIGDKAKGILQRTYKDHFLMSFNEYGRKPPVFADAALVANAILDADYKFDA

AHLYFNTFRSVVSYRVTELPLFNQDQIAKSEKINLYDSIDEEVLRSYSEFALTSLIYFAM

KEGACSEQSSRMTAMDAASKNAGEMIDKLTLTFNRTRQAVITKELIEIISGAAAL

>SG-AL_contig_994_1_-1_1066_1260

MKDSGFFLSFFLVCLDFLLLLLVVVGIFVCVCFCVCFTCGALIFRYHAIIWRWKTYNTFF

VRFRN

>SG-AL_contig_995_1_+3_615_812

MLRSGKSSIHASADQHQSIELQITSIITSSCFHQHPTMSIYIHYFQQWISHHISLVLFSA

QHPHKH

>SG-AL_contig_996_1_-3_3_146

DYQSNQSQSIHCACSIVLLLVKYYCHSVSVLIVDCSIIVFHCTLCSGL

>SG-AL_contig_997_1_+1_97_261

MTYYLIVMFLKANKNRLRRGCQPFQLIYQPQITKKCKKIIKKIILRSHKKGHTGK

>SG-AL_contig_998_1_-3_39_425

MTQKRRNNGRSKKGRGHVKHVRCTNCARCVPKDKAIKKFVIRNIVEAAAVRDISDASVYE

VYALPKLYAKLLYCVSCAIHSKVVRNRSREARKDRTPPIRFRPVRQEGTGPRAGGQGGGQ

GMGSGPGPR

>SG-AL_contig_999_1_+3_1248_1553

MGPQITVQFHTPVHKMTTGHEIYNTDGGGENLMKKSIHNLFLKILSPFSHYHYSQTFPSL

IHLFPCCLLKKNSSVQVSQDNPVVTSRHRSSTESGPPLNTKY

>SG-AL_contig_1000_1_-3_174_1178

MALHCSVLRGLPRLLPLFSAHSLKALSAPSCSACQHRHYMHKGEEKKSTMLKKMLLSPET

EFIMEAHSGISARIAEEAGFKGIWASGLSMSAQLGVRDSNEASWTQVLEVLEFMNDACDI

PILVDADTGYGNFNNARRLVRKLEDRGIAGAAIEDKLFPKTNSLLDGRQQPLADIEEFSL

KIKAMKDTARDPDFCVVARVEALIAGWPMEEALKRADAFLEAGADAILMHSKRKDSSEIE

EFMRLWNNKGPVVLVPTTYHTTPTQTLRDWGTALVIWANHSLRASVKAMQALTNQIYREQ

SITNVEKEIVPVKELFRLTKDDELKEAEDKYLPKA

>SG-AL_contig_1001_1_+3_117_392

METTYTDYKTVDNNNYYSIIIIIIHYHHHSRKDQAFCLFLGGQCYYHKTTKKATVKEMTQ

INERTVQDKIIDQSTKTKELTKVKSKDFQTCQ

>SG-AL_contig_1002_1_-2_155_349

MYTSVCMYTSQLFLFSKHKYMSLEKTVSRNMTNIAPSSHPLAPLKKKGTDDKHYTKYIHY

QNTNR

>SG-AL_contig_1003_1_+3_3_170

CWLCVCIYMCVCVCVCVCVCVCVCVVLCASERAFICSNNKLNLTKTLHDKCLNQNF

>SG-AL_contig_1004_1_-3_219_2060

MEVQVLVTCALLAVVPCLTHVARASGQPQQRTGWQDLKDLETEFWDWRMQDAPVFASTVG

DSTHNDMLESYQPDVYANRQSTVQNFFNRAQAIDSTHMNKSQKLDYDIFTDHLQTFLDGA

RWNDYFTVNPVNFLEGITYDPGRMATFMPFNTVGDFENYLQRLIKLPVQVDQQIDLLKIA

VSAGHTLNNMSVAAVPDQIDSLVNETTRDTAFYQPTFTSVLDKSHIGMSKRRDLRTRAET

AINNIKQALLRLKTYLIQSYMPAARPTWGIWGWRDGRAIYEQCLKYHTSTNMTAQEIHEL

GLREVARIRASMDQALERLQFTGSIKQFYDSLRSDPRFHKNSSEALLDTYKDYIYNRVTP

KLDALFKDIPTLPLKVEASESDGPLGIYLSGTPDGTVPGTFYANIMHPNDTVTISMMSLV

LHEAVPGHHLQAIYSLTSSIPKYRAMYEDDHYYRMPATFPIYTAYIEGWALYAEYLGEEL

NLYTDDYMLMGRYSEEIFRACRLVVDTGLHYFGWERERAIQYMLNNTASSRTELTTEIDR

YMTWPGQATAYKIGEIKIKSLRQKATKALGSRFQLKEFHHVLLQNGGMPLSLLERLVDQW

IAEAQVRMDTDLVG

>SG-AL_contig_1005_1_-2_140_1831

MPPKKKKSGKKGKKSGKKSGRASPSKFQGESVNETSREYFLVQIRDLENRLTRYQKKCDE

LEVANGQFNDKFNQMETDKREVVAFWKRQVESKTDEIADLNDRYIGLQQTRDNEREQYEL

QIQQQRNEYQEMKDQLTSENMILGGKLASLEEFKVQKEDLMAKFAMMEEELKKKDQDHKE

NIYNLEKKAVMDKDRLKKEMILRVNQVAAEFRKVSNKQMAETTKRTIRENVSINAQLAKM

SDKTMELIQENDELKQKEKKQKQQIELLEANEKELAKKNHSSQKIIRMLTEKCRNQEEII

ADLELREQEHQELEAESELLRQQVSATREEIQNLARENEQLDDKLSTTQRDRDTEQGQKQ

QVERILARAAESLRAALRKTSPEDGEEEEDGMNDIERRDHMLEQLLILLNSAAAVGLGPD

PGDLGKQFEETPQRITRLPGSGKGIRKGEMPMSPMTKGGTLPHYSLGDLGLIPRPKQNIP

TFLERTQTLSATTRLGGLRKVLTRSVATQTVSAPKALFYADQLLSRAPASTQDTVMKEMH

QTQVPKSSPPLGPITSKQLLPSVH

>SG-AL_contig_1006_1_+3_210_458

MTSHATRNSTSSFSSIVYCENFFPLNGSNPGVREGGRERGRGGRRERGERENESGERGER

EGEDRGRRRQRGRGRDSDSDGLY

>SG-AL_contig_1007_1_+1_169_603

MPKNKGKGGKNRRRGKNENENEKRELVFKEEGQEYAQVTKMLGNGRLEAMCFDGVKRLCH

IRGKLRKKVWINGGDIILLGLRDYQDTKADVILKYTADEARNLKAYGELPESAKINENAG

MEDDFDENITFDNFDDDDEDDIDGI

>SG-AL_contig_1008_1_+1_61_159

MDYINCLDYELCKLFGLQTVNCSDYDLYKLLGL

>SG-AL_contig_1009_1_+3_153_293

MDHCDTVNGPHLFSLVYVDSLYWILTSICTKPVAHLRGLCGAEVELP

>SG-AL_contig_1010_1_+1_292_630

MTQSQRPMPFRCIHFHAYVDGNFIYICKSTDCRHLVFVCLFIFKHTSFRCIHFHYPNPNT

LMEICKSTDCRHLLLFVCFSFSNTHSQSLYPCQPSFFLKGRRGNKMPGMMKPY

>SG-AL_contig_1011_1_-2_44_967

MPTGRGTMLPGFELLFSSVQSQLRSPQDAIVSAIHWNIINAGCKLTGVGENPRGEKQCTE

VLLNGWNSDEIYVLHYETTGDPKHAYLLKVLPVDGSLLLHFQRENETVTTLELSVQEFAS

DDLSSVSTAFHGMDHLNSRVRKSLVEPAMGNSSSNGQGQAGNSRSTSSKEKEKKDKKKSS

LLVEERRPHVSHQPRPMQPGWYREEGGMGLDPFSVGRADLDPLAGLGGMGGGGGMIMDPR

HSGGPRFGPEGGGGRFGGPSRGGGIHPGALPPGAVPPGARFDPFGPPGTRPGPDPDHERP

PGYDDMFM

>SG-AL_contig_1012_1_-2_32_1741

MAANVQSMCNFWKNFDLQELQRELDTTATDLANRQDESDASRKRLVDQSREFKKNTPEDI

RKIVAPLLKSFQAEVDSLSKRSKAAEGAFLSVYKKLIDLPDPVAVLEQALQTQKRAQRVS

DLEIENKQLRETLDDYNNEFAEVKNQEVTIKQLKEKLRDYEDRLESTAEARAKEKERELQ

RLFAEKERQLQETQLSVARKLGEAEQKIISLQAALESIQSELFEVKSKYDEATSAKSDEM

DMVMADLERANERAASSEREVERLRQQLQQVAAQSLQQQHQSSSDPMRQTPDMEHALDIL

SRSNLEVELAAKEKEVAQLVEDVQKLQASLSKLRESAAAQVSKLEEELSMKNKALAILED

KLSTQEDYEEIKRELGVLKSMEFASTLEGEGDKGKGRSLEVLLLEKNKALQNENTQLKVS

CTSLSEQVKQLTGELTAASTTVTEQKSLIAQLEDDLRNVNALSSMFRGDAEGEPGPTNPA

TEVVADIVRDVMPSGVVTESSSSSKSAADSLLPIVQSQRERYRLRAQELEAQTLSQQQQL

MLFQNEVDKLRLDNVKLYEKIRFLQSYPTR

>SG-AL_contig_1013_1_-1_109_8247

MEEVMEEGPERDNVEVKEESEILPDGTVHTTQRVSHQKLKHIHRSFHSQSGEEEVHDEDV

TVPGKQEVIEMFDQPPKLVQESEEIEQVLEDGTKVKKQVVMNRMVHLIKMRHESFDEEGR

KMEEDFEFEEVIPGTESAFVAGVDSDYEEEMEMKRTAAMGEMEQVMDDGTVVAQGILTTE

TTTTVRSRSGSIEEISESALVTEERVSPSPGRISPKHVSYVHEGNLDDGSGELYDTSEDA

YDYSSKTVVQSKLVTGHLEETTSHGVVESTIDVVQDMITKGQLTLDNSEAVAQDNRQAPD

SDTPLITITEDLSTPPDEDEDVASYFGVKYEASSEGDQQKQEEATKAAAEITEPKLTIAP

SQMEPVEQEPGSLSEDVDQRTTKDIEQEHIPYTEDTKQAVSEHVTQVVERTVKDEYGRTG

IEITKKETVFTESLVHKEEYSVAVSETHIEEETETVPEDVRFVEKFKVAQETSEEARQVD

VVEEQVKPLSTALSMAAVCVVPEEEEVEEETVESTQVPETKAAVCLVPEEEAVEETIKPE

KAAPAPQADVCLVPAEDEIEEETTEISTTPVSEAATCLVPKDDFVVEKTVKISQTPLSEA

GTCLVSEEEIAEETMEISQAPLSEVGTCLVPEDEDIVEETVEVSQASLSEAGTCLVSREE

EIEDELVLPGQSSVRETAVFVPEERVKEETIEISQAPLSKASTCLVPGEEEIEEVVVQPQ

LLSVHETAVCLVPEEEVQKETIEVKEAPSAPETGICLEPCEEPIKPEEASPAPQADVCLV

PAEDEIDEETVEISQAHVIEAGTCIVPEEEEVKEETVEVSQAPVSEAGTCLVPEEEEVEK

EIAQPRQLFIHETSVCLVPEDVQEETIEISQAPLSEAGTCLVPEEEEVEEEVVQPQQTSV

HETAVCLVPEEGVQEETIEISQAPVSEAGTCFVPEEEEVEEEVMQPQQSSVHETAICLVP

EEEVQKETTEISQVPVSEAGICFVSEEKEAEEEVLQPQQLSVHETAVFLVPEEEIKEETI

DVSQAPVSKAGTCLVPEEEAEEEVVQPQQSSVQDTAVCLVPEEEIQEETIESTKAPSASE

AGVCIVPEEEDMEMQDKQEKDDFVYTETRVSVVDEDLKEEAVQEAVDTRAEAERSIVPKG

DRTDGGPVVRPDITEYQSREDIEYEEEDDEDWEILEKEDLTEEEQVAAMNEALMQVSDSK

DTQGETEDYIDSSGQEDAQLHVVEAQAYQQAEEHYDSQFSDYYQYEQITEQEFQVTKFET

EQSVDQDVAESKDISGERPVEKSEQLVSALPVSEPSAVEMVETEEQKEQADSFEGMAEED

KEVEDSDVKLIGSKPSETSLDMSEVVDIRDDTDRTYENKIDEVIMIEAREEMEGEALEVC

VARLSPESDKEAEELDAHFQEPYTQVQPEMIHDVQYKVDELYEQIESEEKETYQEIPDQT

EQENISTEDQIHVTAQVSMEVVEGVAAEQELASSPGSKISDISPVHEEQMEPVHVTEYAQ

SIQQESYTVRGESQESYEQMSYAEYKTETVTTTFEEVRHETISESVTQLQEEKHSAAESQ

QREGEPEPFTEIPSDEPARTDLLIAAMPEVPQRSPHVSSSDDDGEKDSGEEDTSRMPSGQ

QIKQQQIFAQQQEPDTQQYDSDISSEKFEPESKVVETTDAEAEQMAEQLQEKKSDYESED

DFVETSERFDQELAELDASPVKESPPDVAELDQEIEEQDALTLALMSQYHVDVTKEGQHM

THFEMAEEAECAVLDFREGESDYESEEEGDVALETFDVSVDDKDEEDDQYVIAEDGTGQL

VDVESTEKYEEELAEKLREEEQPEDFTQLETTQELQQSESEMHISKEEVVVSSRDFYGTD

VQGAEGFTQEFPEDRPDIDMSQSNESTEVQMASSVEGLLVEPYPQEYDIMAISGATEGDQ

FREPDFTDMFEAHVEGEISDHDDLYEGYSDESEEELKPLKIPVEDSPTEQPTTDFLQDTM

EQAERPDIEAGDSRQSPVYDRSPEEEVYRSPTEEHPEDRPFDSPLQEELVEEDKHGMPSS

EPQLEVKGRAEQVAVSGILAELQDIEQYAGQELSDEELEDDLEQYEKEPEQEQLAKSEQS

LSEAIIVTDQFVDRKEIFTTEERDASVPTKTIDQQEDVTTEPEQEQLAKSEQSLAEAIIV

TDQFVDRKEIFTTEQREESVPKETVDQQEDVITEPEQEQLAKSEQSLSVAIIVTDQFVDR

KEIFTTEQSEEPVPTETIDQQEDVTTEQREESVPTEMTELQEEVDEIAANVYVDRMERFI

EESVETLAPGGESTVNGFISGTTDIDSSGAGAAEPVIEERHLSAQSSLSEHEAQFPEYMD

EHHLSLHPPLYPSSSTEVQDRSRTPSDIETEEPAQNGMTTAAGATGSYTSEITSGSQSYL

SESTVSRMSESDFTSHSETWAASECTMTGSEFEHEGRITVDTSEQIFIEQKIEDSYQEFD

ETKPQMDTLDRPMSPSEYTLVTSHDQDRMMQILNLQAEKPAEEQSEEEEDEEEGEDEMDR

DSLLSEESDEVMSDDEQGSERLPSPTEYTLIASQDQEALTKVLGLHAESQPPEALHVDTQ

EPFSTAVTTGGKEEENSMYQTEELRVETELELQSSGSSEQAVIQLLDSVDNTVSNISSDR

EFEKDSDIHKDFEAPLEPSAPYMPSSPGSENLYQEMFTKGDLTEAHLMGEHASSFEDLFA

VGMVPQTAAFIGE

>SG-AL_contig_1014_1_-2_1181_1561

MIYFDFCLFFYLFIFVLVLSSTFIYVCYFLCLIVGVCVWLSCLHSIHLFVYVCMCYVCSH

FTYVQYVLVFVCVCYLFVHVCVHLFVSLCMFVVHVHLVLTELVLDWMAVQPVICQVYFDV

IVVVVYM

>SG-AL_contig_1015_1_-2_2_193

CKMVFPFGLCIVFRQNGRKQGPFSQVSAFPFAAKLSSRESRAIFALTQHRLLTAEQEFIT

IWLD

>SG-AL_contig_1016_1_-1_139_2091

MAKAPAIGIDLGTTYSCVGVFQHGKVEIIANDQGNRTTPSYVAFTDTERLIGDAAKNQVA

MNPENTIFDAKRLIGRRFDDPAVQSDKKHWPFDVNNESGKPKLKVDYKGESKTFFPEEIS

SMVLVKMKETAEAYLGKTVTDAVVTVPAYFNDSQRQATKDAGTISGLNVLRIINEPTAAA

IAYGLDKKHGVERNVLIFDLGGGTFDVSILTIEDGIFEVKSTAGDTHLGGEDFDNRMVNH

FIQEFKRKHKKDISDNKRAVRRLRTACERAKRTLSSSTQASIEIDSLFEGIDFYTSITRA

RFEELNADLFRGTLEPVEKAMRDAKLDKAAINEIVLVGGSTRIPKIQKLLQDFFNGKELC

KSINPDEAVAYGAAVQAAILHGDKSEEVQDLLLLDVAPLSMGIETAGGVMTALIKRNTTI

PTKQTQTFTTYSDNQPGVLIQVYEGERAMTRDNNLLGKFELTGIPPAPRGVPQIEVTFDI

DANGILNVSACDKSTGKENKITITNDKGRLSKEEIERMVNDAEKYKDDDDKQKNRIQAKN

SLESYAFHMKSTIEDEKIKDKIGEDDKKTIIDKCTEVIAWLDANQLAEKEEFDDKQKELE

KVCNPIITRLYQAAGGSAGMPNFDPSGAGAPGAGGAAPGGGSSGPTIEEVD

>SG-AL_contig_1017_1_+3_3138_3434

MKSCEGSVRFNCVPGCFTSFQFRSVPFSFLASSHSCCTSWLTDTPPHPGLLHCYPNIRVH

NELSTSRCCEATHQKNSALLLIHFCGWVPHICLLSISLS

>SG-AL_contig_1018_1_-1_142_381

MVFAGGQVMKYPYTVTGKLAHFPYRWHWKNGRFVRFLGFTLLAVFPLIVKIHSAVHAPGN

VRQWEEIRKKREHTHFDPVH

>SG-AL_contig_1019_1_-1_55_192

MSVSVVTHTHIPTLELWVFLLFCFVLVLFVCFFLFFFFVLIFVIYI

>SG-AL_contig_1020_1_+2_386_604

MSAENIIFTLLFMPEYPPSIADDAESGAVKTSKVLIGNEASINKSLNMADIFLCIRHKKG

RQQQVLMCAGDPC

>SG-AL_contig_1021_1_+1_304_516

MKTRSMPLGSVLPRFWWLWWRYDLLCFFYKNSLFCFSLQYCFCSCCRLHHSGLWFRSAAG

VAYWLCVRERE

>SG-AL_contig_1022_1_-1_1_210

SADERGFPGQKSRRKVDAWSLGSSRGGLTTDSEMYDTGDDEILEACVRTAAAPTNKPHRE

RRRSRNHRKS

>SG-AL_contig_1023_1_+3_3_128

VMGSTLSRATVGVCVCVHESVCVCLVHVGWLFGQSCGLKISH

>SG-AL_contig_1024_1_-2_845_979

MDPFLDIEKYVVCHEYFVFTFTSDICMNVLQASMLNILFMSLVCF

>SG-AL_contig_1025_1_+2_632_991

MDLLCKSCELWKKNMSHFLCSIFQPPLFWLVIFFFRCIAMMHYNDTVVHILSSTCCCNIS

TFTSPYTFSPSLFSLFCCLVHYLKSSCNCSEIIPNKRHLATKIQPPKSTCLHCKSYQSNK

>SG-AL_contig_1026_1_-3_81_1028

MADVKRLVFSILTFLEDQKKTGGLDDEQVESLEVSIQCLETVYGLSSSDQNQAAHYHVQR

NLLDIFNSQLSEADSDLSSLSLHEPTAEEREEAEKLKNKGNEFMKSEKFSDALESYTQAI

KRDGKNAVYFCNRAAAYSKLNKHQQAIEDCNRALSIDPSYSKAYGRMGIAFTALNDHESA

RECYRKALELDPDNQSYQNNLEIAEQKLREAAAGAGFAAGPAGGLGGMDIGSLLSNPQLM

NMATNMMSNPQMQQMLSSIMSNQSGGEGGLSSLLQAGQQLASQIQQQNPELVQQLRTQLQ

RPPNQSDQDEQPGGGQ

>SG-AL_contig_1027_1_-2_20_382

MSAYPAKGDQKPAADESPIHRIRITLTSRNVKSLEKVCADLIKGAKDKQLKVKGPVRMPT

KILRITTRKTPCGEGSKTWDRYQMRIHKRIIDLHSPSEIVKQITSISIEPGVEVEVTIAD

A

>SG-AL_contig_1028_1_+3_888_1082

MNTTAFMHPLHVLVRNSSLSLFINSLFNILVNISLISSVILRQIACSLPDIKYLKKEKRL

QAVYW

>SG-AL_contig_1029_1_+1_775_1053

MQSASHADLPAQLALSLFLNSRTTLHLTFPHTCSSLTQLYSKLNLRIYTHRGNNNEWKSL

TDIFKTELLHSCTHKQHICAHTHTHALSLCHTH

>SG-AL_contig_1030_1_-2_617_853

MGRNISTQSMFHICVMYPHNLWFLDIRSNLSLHLMIPVCIIISTQCKVPRYVFEYIHVLN

GSYMYHMCRQGVVCEYPCR

>SG-AL_contig_1031_1_-1_64_1014

MGVDYYSILNLTRSAADADIKKHYRKLALKFHPEKNPDDQAAADKFVQLAEAYDVLSDPR

KRAVYDQFGEEGLKNGVPSGSGESGAWTKGYVFHGNPNLVFRNFFGGNNPFQEFYDRVDG

DLSMGFGGLNGRGAKKQDPPIERDLYLSLEEVYHGCTKKMKISRRVMNEDGHTSSIRDKI

LTITVKKGWKPGTKITFPKEGDQGPNNIPADIVFIVKDKSHPRFCREGLNLIHTASVALG

YALTGTTVDIHTLDERILHIPINDIIKPGYKKIVPGEGMPMSTDPKQKGDLIIEFDIKFP

TSLNPDRKDLVRTALLF

>SG-AL_contig_1032_1_-1_28_834

MATPAKAAKTDGKNNHDAADEQADKEQQEAIEQIDEVQNQIDRLNENASEEILKVEQKFN

KLRQPYFKKRSDLIGKIPNFWVTSFVNHPQVSALLTEDDEEALQFLSKVDVQEFDDIKSG

YTISFTFESNPYFENDVISKEFHLNEAGEPSSKSTAIKWKEGKDLTKKTGTPQKGKKRTH

DEQDSFFCWFSDHGDAGADELGEVIKDDIWPNPLQYYLASEIDEETGAGDEDDLDEENLD

GEGEEEEEEDFEGEEEDGAEEEEGDEEEG

>SG-AL_contig_1033_1_-2_395_2620

MQAIVLTRHRSVSAPLMPENLHDPKDKEGAGGVGLGYGAGSLSHLKNIQRPSQLACLRGL

HSPNLDRTAFRFDVENGAGPGRSPLDGGSPSPSGLVLQHLPQRRESFLYRSESDYDLSPK

TGMPRHSASLGATDRCSEPQHAEDLIVTPFAQILASLRSVRLNYLSLTNVPAKDRPRRGS

GTAPHAHSLLSNAQGLSSPSPHHLHNTHDTPSQLHPQDEAYMKLAIETLEELDWCMEQLE

TIQTHRSVSDMASSKFKRMLNRELSHFAESSKSGNQIAEYICSTYLDKQQDVDLPTVKVE

QVEPTTKERPATEKNRPMSQISGVHKLKHTNSFSGTIPSHGVETPHAQQLTQCLADIDVW

GIDLFKVAEYSNRRPLTSVCYTILQKRELVKTFKIPPSTLVTYLMHLEDHYRRDIPYHNS

THAADVVQSTHVLLSAPALENVFTSLEILAAIFACAIHDVDHPGLTNQYLINTSSELALM

YNDESVLENHHLAVAFKLLQEENCDIFVNLSKKQRQSLRKMVIDMVLATDMSKHMSLLAD

LKTMVETKKVAGSGVLLLNNYTDRIQVLQNMVHCSDLSNPTKPLDVYRKWVDCVMNEFFR

QGDMERQHGIDISPMCDRHTATVEKSQVGFIDYIVHPLWETWADLVYPDAVEILDTLEDN

RDWFNSKIPISPSSSFCSKRPPGEESSVENSPPTRFQFDIEEEEEGEAESEQSTPQSQPA

STTSSSAASPKVKSKQQPAGKK

>SG-AL_contig_1034_1_+3_111_320

MNSLPIQCRKTETDKTNQKRDITKREDLTAMPPNVCVHIINDALFTQNLKILLPKQYNCS

NSSFQELVIY

>SG-AL_contig_1035_1_-3_1242_7841

MHYKKQLLLAGGNNSPSASTTHSSATSISASIDVNRSVTDQLTKLAKAEKIALGSSDGKT

DSEHNTDTATAGTFSNSLTSASLQRSERDHRVNDLMTDRNRESSAFTSVRVGALLNNTSS

SSSEERAESRASDSQVSSGLGDDRRPGSQLDDLRSAKSSPTSSPLILDRSEPINIYRDPE

LMSKNPVRSGLSVTTIHKTMSGPGYPTVPGGVPAPPPGLTTSTTLASHPSSLSRSLLPGL

PYSPHQLPPAALSHGLPLTHFSQLEVLSARELAAAQQHQQQQQQIVALQQQYQKAAQLMH

PMPYGGHRSLSQLELLWQQKLQPGPLPPAWVLAKHPEGAYSDSAVIPREHLERMEQDRIE

RERLEKRLEIERMERDRAERAERERREKREKMERERLEREKAEKEKAERERAERERLERE

KLEKERKEREWEHQRHQERERILRESAETSAQAAVDEHFVESFNRVAKLRGVAPWVKPSI

TKGTGKGLIKMETEKAPEEERMKREVDQQRAMMSDDKRYMNAVMEQQRSDTKPEVMMGKD

GKPIFGYPPYHYSSNFHPSCTNVPTSATKPETIFSLYGYPSQQHSTITTEQLQKKGLAPG

GRPLKEEKSIYPGQVGNPSPPRGVVHMVGHPPKEMKMERSVIVENKKMEKQPMAAHSHHP

SSLSSSASSSPHPQPRPAHTPEQRPDRPLSSSTSLSNPAALPPHLMGQAMGSSPSNFGAQ

SSHPTAFRSLDPKAMSRSQSPYKVAGSHQTSVSGQPQSSSGQPQPINFSSKSKGQSGSSS

PYPTPVIAQPPVSLPASSLAFSYSLIQQGLVPNPMYSQGSLAPAMQMARSQQPNTTAART

SSPASLTQNSHPPAVAGMPPASQAPKPQLSPSGSSKRKSNGTGRDNGTLQSKRAKNVAGS

MSDNGRTGSAVSVPVTTPQVLTNPSPYTTSSSSSMAGRMSGPLTANPSSGAPPTGFLDSF

RSFVENTVQNAFLQDEKTSREKEERDSLQKQQQAQQQQQLAQHQQQQNQLQQPVQQQQQQ

QHAVGSVVEKMDTSVVGSGSGETESPSPVLPVSGQPASSSSMASIMDTINRVANGQDTDS

DTLSAPSPPPQTRPENASPQHRGSSSSAPKLKKAWLMRHADDEKPSTSPLIPSPLVAQDD

SNSSQSTSKEVKQYLKTSSVVDPVAATINGDVSPSPATTNSSNSTTNGQNLPNGNISSDL

QGTNYESTSSASETELQTTASTKKRSKSKKTNSASKKVKMAEKAPSPVLPLQPTNVGSSA

RSKKTSNKRMKNEKDSSKGKEKTSQPETRSVSPSPSSTSRKSPAPSTSTPSSVSGGGKKE

DKSSKSSSSKQSGDSSGPPSPPSQASGSHDGEKSSSPEAVAHPKDKKKTRRSKEPPKDTP

SRNGKQGSSFNEPLVKCSVATLKRTGAPFLQNGPCSEVTPKLSKCRECKMTPNQRSKKIP

NIFCRFYAFRRLKYSAKGVVTNAGFSELSDADPDDIEPWLPRYPIEEPTLDEDMGKFILS

KVGDKFCELVEQEKEAKSLSGMDPPIVWKRAVTGVREMCDVCDTTLFNMHWVCHKCGFVV

CLDCYKAKIKATEGEDEDDCKWLTCSSNRQAHDARRLMLTQIIPSDALWELGRLIHDIRR

KWSIPAKCPCVQNSLESKPAGKNGMNPQLLKKMVNGMGGQEGSHKKSRKAAAAAAALQAE

EEKLEGQEYNQDKLGLLAEVALGGGDKSSKHSKLESLSEEEFGEDGDKKSGCSTLRELLT

KTAGKGKVPNEKKSKPKSSGSTLDDIIQSVVEKSCRDLDQPNQTFKFLHYIPRLGQWNRE

LPIITHNLTETSVLYPDVPHSWLCDGRLLRLHDPRHKGNLRIFQEQWNRGQPVLVSCVHK

LVNKDLWKPETFNKEFGHQDNDLVNCRNSNVIMGQPMSDFWEGFESIENRLLDGDDEPML

LKLKDWPPGDDFSELMPAHFHDLMQALPLPEYTRRSGKLNLASRLPDFLVRPDLGPKMYN

AYGSAKFPKEGTTNLHLDISDAVNVLVHVGVATDGPGGQKEHEEAAVRAIDEAGCDSITK

RRVREVHELPGALWQIYDAHDADKIRDFLNKVAKERGEIIEPDHDSIHDQSWYLDEELRD

RLSKEYGVQGYTIVQCMGDAIFIPAGAPHQVRNLHSCIKVAEDFVSPEHINHCFRLTQEF

RQLSNTHSNHEDKLQVKNIIYHAVKDSIAVLRELDPDDKK

>SG-AL_contig_1036_1_-2_2_175

FLVSSELRCHYSTHVYILPFEIKEGDFCHVLAWVMDMPVDQRRCIEHTFDRKMASLCM

>SG-AL_contig_1037_1_+2_2_274

SRPQELSLPECDVFGRAFYEYYPSRSLSLYSYLLNIEPFFTAFRCSTLPRHFGTNFQTRG

SVPHAMTSQRFLAGERDFIAADETRADCVAF

>SG-AL_contig_1038_1_+2_497_613

MIIISTFLNLLLKMVINKILSIKLNFNFWGTNCRVGQGV

>SG-AL_contig_1039_1_-3_42_1571

MDYVPSPESFYGPDFAKSTFAASPNSLYMYDQERHDMQNTLNNGILGQTCSAVGMEPEFD

LNPQYENSNSLEGYFGNVLPSVSEGSVSTQSARCSEKQGSNGMLGFLEATQPELKQGGPT

LAELNMDPYLFEDIYSIIGEEEKVQASSTVKQAASDVRKQATATNPVKMETSPKMYTSLG

PYQTHQPQVGMTPQTQPQSTTSSLSNLFSIKPQNIAVVPPISIATTIKTEPMDTYDSGSK

SCLHKMLSSPPLKAVLSAATTPVQISSPPRQVMSVVGQSRAPSLAQQPVKSESVEEKWKE

IEKFIHDPELSASRKRKRHASGGSAIMSDDESHMGNLSDDEDSDSDADSDFSDTPFEESL

TELAKKSKQYFWQYNVQAKGPKGTRLKLQINDADPHHPSTFEDPVFDATSTSLVGIRHGG

KARKGDGNEVSPNPRKLCQIGQQLYKLNRQINNCQVSNDLPANIRNKSRKEKNKLASRAC

RLKKKAQHEANKIKLTGLDTEQNELLDVIR

>SG-AL_contig_1040_1_-3_192_602

MRAVPISSSLLLYLSLSLKKIGYPFTTWLGLIRTQEENINITCTQKYMCVNIYLHTHTHA

HTHTQNKHTLHNTHYTTHTHTHTHTHTRTRCLGCHVLLMLMLCGNFIMSSILEYMCTVIS

VEYVCNVLCSGVHVLCN

>SG-AL_contig_1041_1_-1_1_426

MSEFKITEKQYNDANQTFSLFDKKGNGTVSTKELGNVFKSLGLQVEDEKLKEWADEADEE

AAGFITWEQFKPVFELKLREDEDERELREAFRVLDKGNKGVIPVEDLRWLLKSLGDDLTE

DEIEDMIMETDTDGSGTVDYEE

>SG-AL_contig_1042_1_-1_1_288

PKVTPENTTCETFTGSDASDFVPVSASTPKKTTMAVHSQTSPSPRKEKTPQKIVTSTQTK

TPSPQKRVSPRKRVLVDTATSPFHSKAKQLRTTSDL

>SG-AL_contig_1043_1_+1_487_1191

MVEVTSPLRDQSGATSLGDVASACASRSTHAESSSSLPETVAAGPKVLSVAVLAVDLLFV

HGHGGRVQGLVAALAVEAALMPRGVDADSPLGIVHSLAAAWALGSGAAAASSCSYGRILG

LGVHLALLVAEALSLVHAEGASAAAKAISLGSVLAAMTLLAVQLQLVVSHGGAVQHLVAH

PTLEAELVVLGPSGHSLLGGVHRLAALGALGLVWRLEGHLDCWSLYCSRSDMQPG

>SG-AL_contig_1044_1_+3_3_128

RLPVPYLQLRNDVYWYTTETNRYDKLSPHTPEHKKANSQQLT

>SG-AL_contig_1045_1_-1_1024_1254

MLRYTLVPVSYAISVTEVNSCRGLWCPWFVGVVVGRTWWWSLYQRYAHLVWLCNSAITGI

NWGISVSRSKHTGSEQA

>SG-AL_contig_1046_1_-3_513_836

MTFQNRSVRPWNSNSYPNTYTYCYLVYVDFTSVQICFIGFLYAIKFVKPLISSHLRCGDS

SRFDTKHFSAVMALLAVVTCQTLTIIPPLCLIIPCHEQTDAPLITLTG

>SG-AL_contig_1047_1_-3_3_125

FLLLLKTFIDFSNSETRPILHNFVFKHLDRLKTCTCIVQSN

>SG-AL_contig_1048_1_-2_2_187

FVLFFNIVWFVKSSLSVMYSFHVQRSWLLRFTSKFQHFSSVCHHTIILVPFVQLYENACW

YL

>SG-AL_contig_1049_1_+3_564_767

MELMLLVFSIFPGQRTHTHTHTHTHTHARHKQNTTVTTCNKDKNRNTTTSVKNYEILCIS

GYHYSQRT

>SG-AL_contig_1050_1_-2_104_430

MYITVDLGSNKIYSHHCQYVCGIIQNSCGLSMEISSQILHEGFPVRVTLPRNTCISLSAF

ILYRLIFKTHRQPFCVTFDVLHVVTVCISASIKKLLLQSIQFTFSFCKV

>SG-AL_contig_1051_1_+3_84_230

MNFVSPISSAVHGVSQKIPFWQYLEFNLFVISFKFYSTECCRIKENYHD

>SG-AL_contig_1052_1_-1_4_2472

MIRKNLGLLELTTKNDLKSSPLLLAASSGGLSAVKCLISLGADIKRQDGQGNNMVTLAAL

RFHTNVLEFLIEWDNPNVPVWKVLVDMLRDSSMGKKDSAVKCLEVLSTCKPDHWKAILDA

GAVPALVALMKLDNEEIQSVAASVLCNISENDEIRHALTAAQAGPILIQLLASSVDDIQS

RAAIILSDLACVDGNQEMIATSGGVEPLVNLLDSELEDVLVNAVNAIRVLCSGNHANQTA

AAASGALEPLVEFLTVASDDLQAGTAAAIAAVCSGHKENQDSVMVEGAAKPLVDLIRSSR

STTVQVKAASALEALAQNNPNSQRIFLELDAPKALIRLLKNIYTEVREQGSCALWSLAGH

TKTQQKYIAQRITIPHIIQMLLEPTEKLLYVGCMTAIALGRENMENQNKLAEADAFQQLV

RLLRTNKTSDSVLLMVIKVLGILCVGVAYRNNKVTQRRIAEEGGIHILVNTLHRATSEEV

QVEVAISLASVVLSNRQNQEKLQEEPHFKFDILLDLLRSKNEEIRLHAGMALTIFAFNNT

PQQFAIREAGGIKFSVFEPFLEAEEEFHQCYAAFQVVVLARVIVDQDQVTLTARGITQLV

DKLRSGDDKVLVLACSLLSSLAHTRAGIPDAMITTGAIDLLVNHLNSANDQVRNAAAVSL

GYLTFNKTAARTLLSICRNTPGLFNKLTDNIGVDPRISQPFVREFERAKMIGLPSQCLEI

NGGPPVQEPVKSGQPRPQTSQNRRHNALAPLSRAVSAPSTRAASRRSQIPNIVVQDSENK

MPNINTKQQHSAGSHRPQSSSTALSSKPESFKTRLNAWKTDKK

>SG-AL_contig_1053_1_-1_334_1839

MVFIKSGSSWCVVVKVLIGVLLLLPAIVMGRTVSVVAVKVAPYIIENKDPATGKVTYDGF

IKELLDELGLMTGMTFKIHVTSQFGENLDDGSWTGLMGDLISGKAEIGAAPLTITAQRNK

VVDFSIPFQHFGPVIVLKRITVPPPTFFERVRFVLDPMEDSVWLMSLVAYFVISVVLYVI

CYVNPYEWRHLCADHQATRREAESFTCVNTFWFVLSTWTWQGYIRAPRSLGGRVVVTVWW

MYVVVFLLTYTASLTNLLRVGPQENQVDTKILSLEDLAAQDNVDFGFVQGGSTAAFLKKS

QLPYLRQLWSTVRRKQSYVKNVEEGIERVRNVNHTKPFAFLTESAMAHHMLRQEPCDLYM

VGDLTITGSYGLAYRMDLDLRDQMDMALLLLQENGNLQMLEEKWFGGKCHAHVLDPKSRD

SIKVTPFYAVNMGSFSGVLVILSAGIVVGAIITIFEIIIFRIAEKNPDSGHKEPPKTKGK

EVQAKKGNTPPNVANARDVTSV

>SG-AL_contig_1054_1_-3_138_2987

MTSVPKVFIKKTKRGRIAKIVREHYLRDDVTCGSRVCGFCQHPRKEKPPLQEAPESHSSL

CEKQHYLLPDTNVILHQIDVIEDPSLTNVIILQTVLDEVKHRSLPAFKRLKDMLVNPDKH

FYTYANEFSKETYVVREPGESANDRNDRAIRVAAEWYREHLLGGIDVILITNDVENRNRA

VDDGLQAFTVHDYVKSLENAGSLLDRLTNPLSKIAIQGNKVLFPEHLPLSEIQMGIKSGR

YKQGGFLASRENYLEANISVEGMDKMVFVQGMINMNRAVDGDTVAIEILPKSEWSCPSSM

VLVDREEQQKEEDDLDENEKEVKNPVPEELRQPTGRVIGIIKRKWRQYCGILQPSLLKEA

TRHLFIPAEKKIPKVRIETRQAATLTGKRIIVAVDSWPRTSRYPLGHFVRELGMVGEKDA

ENEVLLLEHDVPHSNFSQAVLNCLPKMPWLITEEDEAKRTNLRHLDICSVDPPGCTDIDD

ALHCRRLDNGNLEVGVHIADVSHFIRPNTALDKEAATRGTTVYLTDQRIDMVPELLSSNL

CSLRSNVDRFAFSVLWEVTDEAEMVGKPQFMKSIIHSKASLTYAEAQMKIDDPNKHDSLT

VSLRNLNKLAKILKKRRIDAGALSLASTEVRFYVDSETHDPIDVQVKELRDTNSMVEEFM

LLANITVAQKIKDEFPHCACLRRHPAPPPSNFEPLIKAAKSKGLKINVSSGKELSESLDQ

AVFPKNPFFNTMLRIMTTRCMMQAVYFCSGMLPEEEFVHYGLAAAIYTHYTSPIRRYSDI

IVHRLLAAAINADVSYPELMDKHKTQIVCNNLNYRHKMAQYAGRASVNLHTQLFFKKRVQ

DEEGYVLFVRKNALQILIPKYGLEGTLFLHALEKGAKSLFTYDEEENTQTAGDVVFHVFD

RVIVQLSIDRTNVQHMKLSLCLVEPAVPGFSVPPINTDHSMEPPRKKRRN

>SG-AL_contig_1055_1_-1_52_1503

MPLPSDFQQQISCDFTVPVCTISDTSAPFPSTSPSPASVHSPVSQVSQAEQDTYSQGFGS

PLTPCSPTSPEAQLGLQDSSVKAEDSLGGSYSSPPPPPPYTQSLPMPVSMDLGAVSALKQ

PPSYTSCTIQQAPQPQQPQPQQQQQQQQQPQETTNFLTLPAACTLPETILQMQISELGPN

KQTLNADLRWTITPSASGQLAQLPDFSALQVNPPQFPITSTIKTEPGAEAMEQDRFMMPT

QMDFGPSSSGTSGKAASMAVLNQPYAQSPSNLKLLPVKPRKYPNRPSKTPPHERPYACPV

DNCDRRFSRSDELTRHIRIHTGQKPFHCKVCGRSFSRSDHLTTHVRTHTGEKPFSCDVCG

RKFARSDEKKRHAKVHMKQRVKKDAKLLATTASLPTTATAASSTVSLPSSTHTHTLTSSA

LSSSVVSQTLQDSSMISPSAHSASGTLPLVVDSTGLCSVSGLETVGVSCSSVSPSLPLVV

TTSL

>SG-AL_contig_1056_1_-2_71_628

MVRYAREPDNASKSAKARGSYLRVHFKNTRETAQAIKNMHLKRAVRYLKNVMAKKEIVPF

RRFSGCAGRKAQSKQFKCTQGRWPKKSAEFLLQLLKNAESNAEFKGLDTDHLVIEAIQVN

AAPKMRRRTYRAHGRINPYMSSPSHIEVILAEKEQAVLRPTAGEEEPVKKKVSQKKLKRQ

KMMQRD

>SG-AL_contig_1057_1_+1_1_129

NVTGEKYSICCCCCIFKVFRLVVNIVVDKHLCAKGRLKLDATF

>SG-AL_contig_1058_1_+3_3_107

QTLNSKLVRIHISLSTSCVTSYFASPVFRPSFDLR

>SG-AL_contig_1059_1_-2_269_1243

MAAVPRLGIGMDSSITPLRHGGLSLVQTTDFFYPLVDDPFMQGKIACANVLSDLYAMGVT

DCDNMLMLVAASNKMSEKERDTIMPLIMRGFKDCAEEAGTQVTGGQTVINPWLTIGGVAT

TVCQPNEFILPENAVVGDVLVLTKPLGTQVAVNAYQWLDQPERWNRIKLVVSEDDVRKAY

QRAMFSMARLNRIAARLMHKYNAHGATDVTGFGLLGHANNLAKIQKNEVGFVIHNLPIIA

KMAAISKACGNMFGLLQGHSAETSGGLLIALPREQAAAFCKDIEKQEGYQSWIIGIVEKG

TRTARIIDKPRVIEVPAKDREGELW

>SG-AL_contig_1060_1_+2_119_784

MILGPLHFVCNGTSIQLDLHNVGLLLAFAQQLHLGVGQHAHHLTVLLDLVQITADLLLAR

LILPLLAVLSEGLLLGARPILVEATAALFIQMFSPHGSEGAEATRGVDVANQTHTYHWGG

LHNCHGLYHLLFVGFRSWAIHLTKDVGHASLVADEGGWVHRLGAIVFGECLAFATMAATA

FFGQEALGSVTRCRKFSVRHDRRSEEHTSELQSPAMISYAVF

>SG-AL_contig_1061_1_-1_19_255

MKSVCMIVLKAYYHGFACHSQQWKDLVVPFKVISLRLLIILFEYIIYFVSKYSVQRYTDL

RKVHVLILCDVRILILIFI

>SG-AL_contig_1062_1_+1_907_1095

MGQSLHRTAIVHPSPSFSPTLQSTFNHIHNQTKDSYSIHITHCKSKFKKRTNENLLLIQS

HNP

>SG-AL_contig_1063_1_+3_192_539

MKYRGRSPRRVKGGGGRGGEGERWREHVATVRRQPCLFFFPPCMRVIVARARARVCVCVC

VCVCVYLTKVEFFFYIENFARADNSFVAMKSFTHVKCMLHTVYCLVRKTSTQTTTT

>SG-AL_contig_1064_1_-2_584_907

MEMGGKCNKSTNKVTDQTVTQQTMGFIMAVKSNKICRLSVYSNARSLLITVYLNARSCLQ

AEQSMEEQIRLNTTTPASHNPTFYATYILYSTSQSMPKHVPKLHSILH

>SG-AL_contig_1065_1_+2_1055_1528

MYTYLKLTFFLVCAALSCALVYADMACVPHKALCNLSHNRQMDQSSMKENNKRAKYAKTQ

VQTPPLPQTLDMQNLKGQINRKRLCTCRIWKDRFTSLDMQNLEDRGTEKNPNQNNIWICR

IWKVKTRFFSAPSSAPLLCHYPHVAHFGSPTYLQTYRN

>SG-AL_contig_1066_1_-2_3029_4642

MRLLLLIMTGIGFTLGLLCGLLLQLPAEGLPASTHPDSSTSNNHNDDVKGRWRRSVGDQN

VVKKEQEPGRAQHSPSEDEGSDSRSALKPSFQSGPNPGIDSNPFVSSGRDTDNRHQPATK

NGLSQAEPIALTVKTGQHKGEAERFEDNRHPHLSPATARPVTSFTIRLKNTGGRFVVDSA

VQRQEDGGGRGRSVQSQSDGDRQTSHPRWNNTDMKTSFNNKNLSDIVQGVLWSRKLDDSC

PRGLDPHDISSWRRKAAGMAVVKMEEGCGRMQNRLVTFKDGSKACARYRLNTDQIQGEVY

SYYLAHLLHITNLPPAILLPVDTLSPQWRNIHLEMSLAQWADGRLVVLTRWLDDLLPSHI

PQELREDDRKLHPTVEILGKKTVPQLCDLLQWSDLIVFDYLTANLDRMVNNMFNRQWNAE

MMNNPAHNLERTKSGSLVFLDNESGLFHGYRLLEKYHSYHESLLSSLCVFRESTVKTVKT

LHSSNNVGQDLQRLFDEGELHHKHLPSIPEKNVKILQDRLANVYDQIVRCEALYGGHR

>SG-AL_contig_1067_1_-1_241_363

MVFPTDSIVTHFPEYALSSVDVAVAFRIYRSEVKYRSRLFI

>SG-AL_contig_1068_1_+3_939_1157

MQSYYNCVNISVRYLFVFARNSAKDLMCSSLNITIATKSCRFAISALNISYILRISLPEH

LNTTCISPSLSTV

>SG-AL_contig_1069_1_-3_3_335

TPTPAYILRGCGDWIAKENKSKRVSSKTLLTDFEKRIKSKESIKIDLDSSVLLSFPVHAV

GGSVVGSIVCMLCLPKFLKEAGLVSGGHCCSFAQCNNRFEQFQSFSLKNSG

>SG-AL_contig_1070_1_+3_1134_1574

MQAAVDTQLGSVIAPCNHTASSSACLVQGKGPTWCVSEEQVMRTQSSLTDTTVCVILYRL

DNNGKASDQITKSCPTTLDFPSTANKKVKFQFATFTHIHQSQKFWTLSIDIPQIHTKKIH

SQNNRHRICNTSWFEVINNVSLAPMAD

>SG-AL_contig_1071_1_-1_280_459

MKFVWTPWLTDKTKNKHIHRCHIYVSLQFVRLKVGVGKCFKFEHRLKLEAEQQEFNDNVQ

>SG-AL_contig_1072_1_+1_190_801

MFINRWRRTASVSVQRQHFKKLSRSRFEISFSSSLCRFHFPHTQTFSLSNIHARTPTHTR

SLSHCIWRCILRFLIILLFTEEMVLYEHKAFFKLRKNVTIWVNRPVFAALYLKQRSVPCV

AVHKKILHFSYLISLLGERIRYDFILLLWCYSIHRPDLSTEFSVSSNVSILVFTLLQLVS

SPRHMIQIVFITLHSLLLTLFCTV

>SG-AL_contig_1073_1_+2_50_208

MKQYTKNTKKELNTSTKKIHYKHCGPLITYIISITKYPNIKCTLHSYLTVNSE

>SG-AL_contig_1074_1_-2_29_478

MELVYSVPFKVLELPNLKLKKPSWVRMPSPMVVFSGVLLSYFLVTGGLIYDVINEPPSIG

STTDEKGNSKPVAFMPYRVNGQYIMEGLASSFMFTLGGLGFIVLDQVNKPLIPRLNRIIM

LGVGFTAVTIAFFLCRVFMRIKLPGYLLDY

>SG-AL_contig_1075_1_-3_897_1184

MLQKVWVCKTYNEGMRSVDIFTVYKLWVCETHDKGMRSFSLKAWVCITHSTGMGTAKSNT

DLLTNKQKQMKISTKDSKDVSDAYLKFLVILTKTDF

>SG-AL_contig_1076_1_+3_45_236

MCMHACVSVRMTRQRERVRKQGCLFTDDVYQIKFIAQWNTANHGIFLKIHPVHGSAIVFF

LPTF

>SG-AL_contig_1077_1_-2_479_3145

MMGDPYNDGYMEEEEEWDREGLLDPAWEKQQKKTFTAWCNSHLRKAGTGIEDIEEDFRNG

LKLMLLLEVISGEQLPKPDRGKMRFHKIANVNKALDFIASKGVKLVSIGAEEIVDGNVKM

TLGMIWTIILRFAIQDITVEEMTAKEGLLLWCQRKTAPYKNVNVQNFHLSWKDGLAFCAL

IHRHRPELIDYYKLSRDNPLENLNTAFDIAEKHLDIPRMLDPEDMVNSAKPDERSVMAYV

SSYYHAFSGAQQAETAANRICKVLKVNQENERLMEEYERLASDLLEWIRRTRPWLENRTT

DNTLPGTRRKLDEFRDYRRKQKPPKLEDKAKLENSFNTLQTRLRLSNRPAYLPTEGKMVS

DIANAWKGLEHAEKGFEEWLLSELQRLERLDHLAQKFRHKCEIHEEWAKGKEDMLQSRDF

KQCRLNELKAMKKKHEAFESDLAAHQDRVEQIAAIAQELNALDYHDVAAVNARCQHICDQ

WDRLGQLTVKRREGLEEAERILERIDQLHLEFAKRAAPFNNWMDGAKEDLMDMFIVHTTE

EVQGLVDAHEQFKATLGEADKEFSTIVSLAQEVIRLGQQYGLTPPDNPYTTLHAQDITNK

WSEVRDLVPRRDTTLQQEMMRQQNNERLRRQFAAKANVVGQWIETQLDSVASIGVQMKGS

LEDQLRKLQAFDKAIIAYKPNMDELERYNQEVQEAMIFENRYTQYTMETLRVGWEQLLTA

VARNINEVENQILTRDSKGISEDQMNEFRMSFNHFDRNRTRRLEPKEFKACLVSLGYKIR

DDRQGEADFQRIMSIVDPNGTGYVTFDAFLDFMTRETADTDTAEQVMQSFKILAGDKPYI

TAQILRQELPPDQAEYCIQRMAPYSGRDAVPGALDYMSFSTALYGESDL

>SG-AL_contig_1078_1_-2_164_322

MTHLGVTVMLKLKVPAGGGGFIMKQPQTEELSVWKLCANHAGSFWVPAKHIFK

>SG-AL_contig_1079_1_-2_20_733

MATRNEAFSPSWSSVFNFDSLERPVQLHLKNVYTCLGVGMLAAAAGGYMHLFTNIMQSGF

LTTIIALGLMLWLNTTRHCKENFNKRIAIFTGFTFCSGVSLGPLLDMAIMIEPSIIPTAL

LGTAVIFISFTVAALYNNNRSFLYMGGLLMSALSWLMILSFANIFFGSRMLFDVSLYLGL

LVFCAFVLYDTQLIVEKRRRGDDDFIWHSVDLFLDFINIFRKLMIILSKKESEKKRKD

>SG-AL_contig_1080_1_-3_1008_1478

MGQAITDIVMYHAKRLAQSKGIGQSEWSTQVINFWPHHYIAYTNDRDPAKSAIVFISVIS

IFKYNKVNNISNVITSIIQILDRSVADLEKVQCSVLSTMLSPESELGLFHRRMLQHKSKW

LWPIVKFMRVQRRWRNRGNILQAYVVNKIDVCITSHH

>SG-AL_contig_1081_1_-2_473_3262

MSFPVCKFFKSEQGCKFGNKCRYKHSDPLNLPAFNQSTDHQNQNDGSVRKSYSSQTSALS

QEHLTDSGEYEASVRNPRILHTDHLTDYYSYTVPGNNEDPLRFAPSFRTAETHLEKKFQE

LEATDHNEEAVISNFHPAFQQRSHRKLCHYFARSGNCCFGSKCRFEHAQPHKILHTQPCQ

ISPRFQEDRGGSRSWATENWTDGYQSSMSQDGKTEEGKGVRHSWYSQNQRSFSSYPGGNP

FPRTGPATFVSADKSAQNGSPADQPTQSGPGREGTENIDEKNQNFNQNFMSDKICQFFVR

GRCWKGRRCRFLHPRHQAPVVVGEQGETESNTKNEDKSSTSLAQNQKEETLKFSPATSAE

GTHTESVSSSKPSVMRKPRTPHVAQGTRKYCREEVDDVEGSRLRRTEIEQLVKRFPKSKM

KVNRDCAEYFQCIISFNPTDPDWPFDVKVFQLQVDIPSDYPFEMLSVTLPAEQDLPETVR

RYLEVSIEEWLEEKERQLADSGQVELVFRPFLRWLDRNIEDVVTEGLRQLKREMVAKAAG

LEFVSAKQLQKQYPGQSANSYDDGDNEDDDGSDDEDETASESQDEDTSSDYDESDEEESC

QERVDEDEDGRKKGRKSQQDKAKLMMAEPDRKGTEVTLKNLQLKESAATLFFEKMCLLIQ

CERCKATSEFMASPGRVNMVQCSQCSQTQMATYRPAMVHHFSSVIGYLDLDACLPYDVIL

QNCSAVISCLNCSKDTKAGVVAPGQPQDHWCRVCHHKLRIATDGVRFTQLEPSTTIQSLK

GSVKVVVPTRKRIPKDPAIQEGKPLPNFGTCKHYKKSYRWLRFPCCGKCYPCDICHDSKE

DHEMKFANRMICGFCCKEQMYAAERPCKGCDMNMTKVHTKHWEGGQGCRDKISMSRNDKQ

KHRNANKTVSKHSQKVKDMQSKKRTKLRHT

>SG-AL_contig_1082_1_-2_194_1588

MSLVDLGKRLLEAAKRGDTDSVRALMSNGAPFTTDWLGTSPLHFAAQFGHLQTAEVLLRS

GISRDARTKVDRTPLHVAAQAGHLDIVELLLTNSADIEAKDMLKMTPLHWATEKGHVDII

DLLVRHGADLNCEDKFDRTPLDIAMANSRTDIAQMLQLAHHTDIGGVTEGDTITIETTEP

IITETVTTSGVKTEVSSDSEQSSTSVLATLAALAEATAPISAANVTSAGAVHWLEAQGIT

MISSPDGGLITSAVESGQTITLTEAGKIALNYIRQDGLGEGSAEEAGLETVVGEDGQKVI

TIVANQSGEELTEEGPVLVTVADDGTEMPVGTQIVEEAEGEVVAEVESDLQQAMESSGTL

IIEEGDGTTITVTHTNDDETEETEGDSENVELRRQLQALKRQTEEYKEQLRVKAEEAAKL

SQRLHEIQTSTTGESELVVEEEAQKIRQQLASLEGTEESVTETSS

>SG-AL_contig_1083_1_-1_733_1008

MTRFLCVCVCVCVCVCASLNGCLSMYILHVHVHSLISDIILHHQKHSQSIFSGTKSCIIK

SIHNQYFLATNLASSKAFTINIFWHQNTRRFT

>SG-AL_contig_1084_1_-1_442_771

MGFRRLNFFSMFPAIFSKASRAQYNKAKADQLGGNLKGDGMQNGGTLVVEKGGKTLLSFK

QESPSDQVDNAEVLKALGIEEKPPQPPAEGAAAAAGVTCEDDVCQMPKKE

>SG-AL_contig_1085_1_-1_133_363

MSIQASQFERNRHGRENSFGHVKGKISHMFHCHCIHDVFIVCVLWDTHCLALFRLLQLVW

SPTNNITVPLAGINWVA

>SG-AL_contig_1086_1_+1_841_1083

MCYAHAYILFVFITLSLILYTFLFICLLFGWFVSLFCLYFPYVCDFLFTFRLCSFIPHCC

SFIAFLTDIVHPQSRMLKTSQ

>SG-AL_contig_1087_1_+3_1299_1562

MCSLSLSLSLSLSLSLSAHPLSPSLINIKQHTHTNTHAHKHTLQSKHTHTHTHTHTHTHT

HTHTFILDHRLRTQQTTWTGRQTSSQKT

>SG-AL_contig_1088_1_-2_500_1069

MKGLVLATLAVLIACTIGVPSVVRYDVDREYQKTVRLQAFNFTNCLPVAREIAVVNSLIL

LPDPLHFPGALSVSFDITFRAEVSDPLKAVVLFKKRVGNTWIKIPCIGNIGSCTYEDICE

LLQNIPQCPDSFIAAGVPCKCPFKTGEYKLSKAEFEVSAAVLPSGEYHVQANLTTGSATV

GCYDIYAAFY

>SG-AL_contig_1089_1_+1_118_378

MSRYLYFILLFLFTQDANLCLHCRQKFRPANQDVRRKFHFPFVSIMLTYNRPGNHDSPSF

HNGTRYKATHTCPVWSQLQLSTHLQLF

>SG-AL_contig_1090_1_+1_85_177

MYSLGLLPAFISHSRKLNSRLNHSDRSETTF

>SG-AL_contig_1091_1_+3_150_296

MVKRTHKSVKRPWCTVLEDLFLNLQISLFLFLTHQSMKCFYFLYTDAHI

>SG-AL_contig_1092_1_+2_740_895

MLYYKKVNTHILKERRKRKKKRKKQNKHQKKIMTHKTYKLKQNTTSEHNVTH

>SG-AL_contig_1093_1_+3_450_719

MNLEDFGRAALPPIMFGTGGERVKTCGFVLSAKNLKHHPFPIPSLYNTQNNPRIFFFFNS

YVCLKFQFFFFLFFFFFLIFFLLFFFFLIP

>SG-AL_contig_1094_1_-3_1485_1742

MLLPKPVGLEHNMNFSMLFFCLFVVCRVVIYILTSFFYIYFFTPPPPPPPLFFSLFFFFI

IIFIIKCRHCPYMLEIIPMLFVGKVE

>SG-AL_contig_1095_1_-1_799_1185

MHIRYIYIYNIYITLMTSACLFTLHRYRLIKYINIFSVFTSGQGMSDLWQSTSAMFHIVC

CKTVKTPRVCNIYELLFKFLGWGIAVSESFSKIELTLKKKKNSPAGIFLSKSYWCCCESF

WLTPNYLRL

>SG-AL_contig_1096_1_-3_66_1058

MTEPTRVCITGAAGQIAYSLLYSVAKGDVFGKDQPLILSLLDIPPMMEVLNGVCMELMDC

AMPLLKEVIPTADPKVAFKNIDAAFLVGAMPRREGMERKDLLRSNIKIFKDQGEALNAVA

KKTVKVVVVGNPANTNALMCSQFAPDIPKKNFTCLTRLDQNRAQSQIADHLKLPNEAVKN

IIIWGNHSSTQFPDVSHATALIDGKTVPVCEAIKDEEYLRKKFVKTVQTRGAEVIKARKL

SSAMSAAKATADHMRDWWFGTEEGRWVSMGVISSGHYGVAKDIIYSFPVRIKDKEWSIVD

GLSISEFAREMMDKTANELLEEKSMALSVKE

>SG-AL_contig_1097_1_-1_1_354

YFPLNPLRPHCCLLATVVLPFPPAPSVVFPLENRSTFQPVSCETRQHQQSQDWAEAGTVH

TVHAGAHPGPAAGWTCGKKSQECFLLARAIFLFKFVSRHLLSSFTGINSLCLFFSSHM

>SG-AL_contig_1098_1_-3_780_974

MHQMVRISYEACEQKCNKHECQDSSVVHKVYELKCNIKSCIWIVFSLAGYKVISLIRKQI

VCLIY

>SG-AL_contig_1099_1_-2_86_691

MQDPNEDTEWNDALRRHNIIPQREKEVTEDDIINMVEQTVQQKQSGKSLEDMTLHELDEN

EDDIDEEEERLFEEYRRNRIAEMMAKQSRAKFGEVKEITKADWVDEVNKAGDGVWVVVHV

YKQGIPLCSLINQYLSNLARKFPETKFLKSISSVCIPNYPDRNLPTIFVYFENDLKKQFV

GPIAMGGMNLKQDELEWMLSKM

>SG-AL_contig_1100_1_-1_127_1830

MARDSTSRSPTPDRHRKKRKPSLSSESASSRSRSRSPKAQAAKNVPRKNQRYSSESPERS

PPRSKPSSSKSSRKRSDSPRARSGSGDGRGHRKRSPERKKRARSPQAYDSTKRKKHRSDS

VSSASPPSRSHYQKNSSKSRKRHDSHSPMRRKKRSRSVDRRSCSRSPNHSGSGSSSQSHK

DSRSRRQHNGSRYTSRSISRSRSPVNKSRSSSRSQSRSPVRKKSSKSQSKSRSRSRGKEA

PAVITGEKKEPSSSNFSRIAPKEFGNRQQHQNGDHYHSGSRPAENGHRGYQRHGGHGGHH

RRPHHISHMMDRRRTERERIAEAGVPEVWAGSPPPPEHSSDEEETVKGKKKDVSAKGDKD

RSHSDSGSSSGKKKSKKHKKKSSKQKKEKKKSKKSRKSSKKKKRKSEESSADDDDDDEDS

EVSEDEEGEPMWIERNIDNDEDGELVGPKPYVESASGVKPLDYGKALLPGEGAAMAAYIA

EGKRIPRRGEIGLSSEEIAAYEDQGYVMSGSRHRRMEAVRLRKENQIYSADEKRALAKFN

HEERSKREVKILSQLRDIVHRKTKQHQS

>SG-AL_contig_1101_1_-2_2_82

THTHTSDVLKRVDLFPTLSGMCEPTSS

>SG-AL_contig_1102_1_-2_356_4330

MMKMKMMTVVASGVVLALSCFLLSVHAQESVNEVSRGDNAELGNLDQMQAQQAGSKAGPA

ITNGGYMAPGPPGPRGPPGPPGSPGQPGFQGQRGPNGEPGPAGHPGQRGFPGPPGPHGLD

GDEGMPGDPGPPGPVGAAGNAGPPGVPGMPGPKGHRGFSGRPGKEGEVGRPGEKGPGGPS

GPVGAVGPSGARGAPGERGRDGSSGSAGQPGNDGTPGNPGGPGPVGPPGPPGFPGAAGAK

GDRGPSGASGNTGPPGPAGSDGLPGAPGTVGQPGIRGEDGSSGNKGPDGPVGPAGPPGFP

GPQGPPGGVGPPGAPGGKGNNGRDGSNGAPGNPGPQGAPGASGERGLPGLPGPEGKRGPS

GPAGPGGAAGPTGEMGAQGLSGLPGRPGFPGPPGQDGARGESGERGSPGPDGSTGAMGPP

GPSGPRGSPGLPGEDGTSGEPGPAGNDGLDGNPGEQGPQGQPGPPGLTGAPGNRGESGSE

GRPGPQGPNGPPGARGERGPAGPEGPVGRPGNPGRQGEAGPSGPPGEAGFQGLPGPPGPA

GQNGNPGATGVPGPPGESGLEGIPGERGFPGEPGPSGQRGVPGDRGNTGPAGSPGRPGPS

GPPGARGAPGPPGDVGLGGENGVPGRVGARGPPGPSGEAGREGERGSPGEVGAPGPIGPP

GPPGTVAIKGDRGAPGLPGNPGPSGPSGRTGAPGAPGPAGNVGIDGLPGESGASGDAGAR

GARGDAGPRGYPGERGIDGEAGRPGTDGSKGERGEAGPAGPSGQPGAPGAVGPPGLNGPP

GPLGATGNQGQPGERGSPGETGQPGMPGETGNPGQPGSDGSPGERGSPGLGGPPGPPGIT

GPQGERGSSGFPGAAGEAGPPGPVGIPGEPGPRGENGKDGAPGLRGLPGQPGPAGFPGES

GRPGERGKDGPAGPTGRPGAKGSRGNPGTQGPPGLGGPVGAPGQPGPAGPTGERGERGER

GNSGPVGTVGPAGAPGAQGPQGAAGSTGSAGAKGDKGWPGMPGPGGPPGPPGPAGDNGLP

GPPGPPGSQGSPGARGPPGRNGEPGPQGRPGNHGQRGPSGENGPAGIPGPRGPPGPPGPP

GHSPVYSPAPSAGFKGPDPYMQYDQPINTAETYEGLNFADHALNRVRRVTGKATSPGMSC

RDIKERNPDFPSGEYWIDPNERSAVDAILVYCRMETMETCIFPSPSTFDRQRWTKTEGSG

QFFMEEMVGDREFFYKTDFSQLKFLQMLSEGARQRVTYHCFNSHARGSRLMLHTGEELDT

ELYKYKKSTRIATSDECAHDNQWHSAVFDIRTNKTDILPITDIRLFDVGRQNQQFGIELG

EVCFG

>SG-AL_contig_1103_1_+3_462_851

MGCVILYYHMHNAVRILHVNENKTKISGFSSPLPPFAAWEDQLSESCSVSLKFLSTTKLK

AQSDNTVAMRVHLQLLLTEQFQVCLYICYSETICNIFNRQINPYLRKQRLLKKPNMMQTA

ITSPMHYFFI

>SG-AL_contig_1104_1_-1_37_516

MILYNFPLPGNHCRRHTTTMAEKEHVLHVNTVADNYTLYGRPCEISSFSFIPTERNEPAE

RTVFNSAKKERNGYATYDRLFRKVDGYNNKLHRDDREHAKSRGLRVNDEETSKEVPTLAS

SDYGHRLENDLDLPDRAHVRIAHVQTEFYRRNGTMSTENC

>SG-AL_contig_1105_1_+3_807_1061

MLRILLLSFTCPINASSPKVTRLLMKHSLFWTFTLPRRWRSVEQSDTLTPTNKYTRSLTK

AMHGILNHSTVVFDSTLQVIIFNQS

>SG-AL_contig_1106_1_-2_830_1084

MSFPIYILNWCVLFVYLVMNGYCILPLVEGLFLCSIYSITWNCLPFPFKSFYLLTFHCSL

KVFFCQQLVLTFCCSILFEIGLESA

>SG-AL_contig_1107_1_-2_335_472

MTRMMNIHFTGKKSELIKDYKLSPDCTYINLFIFSPCPPVFLQEAG

>SG-AL_contig_1108_1_-3_66_5642

MPQAPSSSYPPGEEGRLRYSRRSSASSSSSQQSLSVDNSVPSSSRRGDMGAASSRLMDTS

PVHSTGRDSVVQNRLPPSGSGMLLGTGMGMPEPRVVLENINVSDSSRWLGHSHHLPPPPP

PPQHQHHQQQQHHQRSALESKKKSSASSSTSSSSSSSGPVVMLEMLDPATLKSIASGGSV

KAVCDNESGRRKRYDPGYSIMRSEDGSMSFQASRRRAERGRGTKRKSQYLEDSEEESSEF

VEMEEDEETYMARKRKKEKQRQRRSMVPTLSNEELMDSPTFKKFTSCMDYVFDTAEDANI

AALDINDEDVECPPELLVAKGIMGDLCGESAKLKSMNVMNQIPPGKLVRLMTVMSWNIRD

GTKVTPFVNQDEDEEESRLWREVTMERVIRSMDASLTCLSIMTSSTMHKMVYLEDVIERM

ILYSKFQLQNTIYPEFDPVYRVDPDAKDGYHGSLKAKRARAHTVKHKSTINLYNKMCEMI

NWFAELLIIQEMTDTIILQMSSVGVSAFFVENISELQLNAMKLVTTIFSRYDKHRQLILE

DIFASLARLPSSKRNLRNFRLNSEESIQMVTALALQLIQCVVRLPSPDSSSPSTEEPEPK

RQHKAGSDNDVVIVTSYETAMRTAYNFLTVFLKKCTTKGEEDYRPLFENFVQDLLSTVNK

PEWPSSELLLSLLGRILVQQFSSKSTDTSLRVASLEYLGIVAARLRKDAISSHLNQEVID

DIVLKVNEDDEEEHSSTRSKKKTKQAADTTQSLQESILEYLAYNGQSEPALQFARQFYIA

QWYRDTTVEAEKKIRKQQNTSGSDDEDFNETEDERTTEVMQNVEKRKNFMLSQIASAQTT

LATYRPSPSKLDYDSACLVARYLSSKRPFAQSFDVYLTQILKVLRETAVAVRTKAMKCLT

AVVESDPGILARNDMQRGVHARFLDQSTSVREAAVELVGKFILIRPELIPKYYDMLSERI

LDTGISVRKRVIKIFKDICIEHQDFNKIPEMCVKMIRRVNDEEGIKKLVNEVFQTMWFSP

VSTRERDSSRVLQKVMNITDVVAASKDTGYEFFEQMLENLLKKDEEGNYNKTAILACTQI

VDCLVENVLRIEEKSVEMSEGRNTSSRLVACLSTLYLFSKIKADLMVRHATTLQPYLDIK

CSNQSDYLVLHYVARILELVVPLMDHPSENFLMALEEDMIKLILKHGMMVVQSCVSCLGS

VVNNVSHNYTLVKDCFGKFFSVLTKLMLDHKKDCANPTLKGRKPTLLRSLFTIGQLTRHF

NFDSKELGEDKVCMRQKVFDVLYYFIHHEEEDVQHKALTALGFFLCRHYEYMLGKLLKDM

YHDFLTNDNAPIKLRCQVLKNLQLYLVEEEISMAKSDAEWNKHAKQEDLKEMGDVQSGMA

STVVQLYLKQVLEAFYHDHSQVRITALNVVQLILRQGLVHPVQCMPYLICMSTDPENTIR

IKADQQLEEIEKKYPGFTHMKALQGIKNSYRLQQVLHKNNPFEPIRGFRQSEEGQYQAFN

GFLYSVLRSNRSHRRGLLTSLLNLFDDTGLQKISMGEQVYVADNLAFFPFQVQDEPLFVI

HQIDIIVSVAGSNLMQSFKENLIPRNHGEQNGTHPEALTEEQRQKLAAEDDDDDDPESLI

SRLPDDISALQEVMQQSQGCILLLVMKQHLKDMFGITDSKIHRYSPTEAAKVFDKPLNRK

NIIRFNPQHTLDQLRSVYDADLEEEEKRQKVVDEYLEFRDLMMSIDPTDEDDSGDEQRST

GAATPIRKSPAHDGDGGDADNAIVVDGVQPPDGTAGETVDGMEKPVNSVVNSTPKPAKLP

YYYQRRERSTPHHHRNRTPKLLMQSSTPKPPVPKKKRKKRKRMDGSDDDSDDDDPDFVL

>SG-AL_contig_1109_1_-3_27_1253

MFHVQTGTKVQDIQNSLKPFIKNIFHYADIDSGNVRVSLSYFRKNPTLLFHLQKHKTKAA

VTRAVDAFNSRRVRGKRNDAGAALENARTTIFDERRGDRRGVPNAVILITDNKVNISPKN

LQQQADALKAAGARVITVGVGKANRNELRSAASDPQAENAYYFDSYASLRNDASAAAVRS

RIYALQDLPPPTRAPPPPTTTTTLAPVVPAVDLTGSSEADLVFALHFNPRSSDADFQRLV

RWLQSLVRSADVDSGKVRIGLYVDDQYMPFHLNAFQSGQEMEEILGSLPKAVARSRRFNL

VSTLRTVREKMFTQANGDRPNVPNGLVIITDTTSTDNRQRVQQETTRMAQAGIGIYTVGV

GLSDKGEMETVASSPRNVFAASDYRELEINVHSLRGEIKALGPAGAVQP

>SG-AL_contig_1110_1_-3_30_668

MDGRLPLRLQSPPPRQQPDDEAAADARGSRSSPDLGDSDLLYDEASLSRNPPEVPAQNCR

HLPTPTPPRRSWTSWSRPLSFRGSTPASTDGPWAPGCSRSPSLPGPPPSYEGRQGAEISD

PRESVRPPATWKTGSSWRSLAPLAAITPRGLSTYAPPRGPQICDPTPPQSLDFLGMRFVT

RARRVSPTPARLQALQLKPSSLRARTSAPSERD

>SG-AL_contig_1111_1_+3_159_380

MGHLTELVDSLELSIKLHPGKLYTQSLSSGKKSSDYKPAQYSLFIISRTILLLSDTYQSN

FYFLTKFLPCVTKD

>SG-AL_contig_1112_1_-1_322_4542

MGVWVRDGALILVVLAAAALAAPTEETEGKSCFYNNQEYVHGVSWSPPQQPCLQCMCKHG

DIHCSILPDCEKSEQHRGDLGISATQGETVPGDISEGAPDEMMAQQGYGGYYNGGKSAPA

YGPQYMQAQVGPMGARGSSGPPGPPGPQGMEGPRGQSGDPGPPGPPGNRGEPGPPGPPGI

EGDPGRNGEPGAMGPPGSKGPAGSPGMPGMSGMKGHRGFSGFPGKRGEQGPAGEVGEQGP

AGPAGPPGPQGSRGGPGERGQDGSPGQNGLRGNDGLPGTPGPPGPVGNIGSPGIPGMAGA

KGEQGAPGPRGGDGPQGPRGENGNPGMTGEIGMPGPPGMPGSSGEDGAPGDPGPAGPPGF

AGPQGRPGLAGSPGMAGAKGAAGLNGINGLKGQAGERGSTGLPGDVGKPGPPGSAGRRGQ

RGPEGPPGPVGPQGERGLPGSRGMPGSPGGPGSKGIDGETGSPGPRGEPGASGEAGHPGP

QGAPGEVGLQGSSGPNGNSGAPGEAGPSGSDGRPGDRGAPGQPGPQGPPGPTGPPGERGA

PGKEGSPGPAGPRGAAGDVGVPGNAGPAGPRGPEGEAGTQGPAGPPGPNGFQGPPGPPGP

QGEAGRDGEQGIPGEAGPSGPAGYPGERGFPGERGPAGPAGESGLPGSEGPPGPDGPRGP

PGESGIEGESGPQGPIGPPGERGAPGPPGERGELGDPGPSGADGLAGRPGPRGPRGAAGQ

PGDVGLAAEKGQPGEQGLTGRPGAPGPMGIRGESGPEGEQGPNGFPGQPGQPGAKGETGA

TGTKGETGDVGAAGTPGRTGPSGMPGNPGRRGSPGDRGLPGVPGEVGPGGRPGPQGPTGS

AGPPGPTGVPGGPGSKGAMGETGRQGRPGSNGAAGQSGIPGNPGVRGEDGLPGGEGPPGP

AGPAGMRGESGIPGVPGETGPQGPVGQHGRRGASGRPGNDGFPGKQGEAGPPGPAGPTGS

PGAPGAPGSDGSPGQAGAAGEGGQAGEMGLRGPSGPPGPQGAAGAVGPSGSVGERGQRGE

MGPDGPRGPAGPRGPAGPQGAMGESGDTGPAGETGAKGVPGYRGMAGLPGPEGPHGEPGR

VGPRGPPGPRGVDGARGQMGPDGEPGAEGPRGPPGARGPAGEDGRRGRPGDIGPRGLPGS

PGGSAYAGAMNSWYRGSSSYSYNGGYNGYQGDAPEEARATMEALERVSEEIKKLRMPTGK

WDAPGVTCKDIAMADPTLKNGMYWIDPNGGSTSDAIRVFCKMRAEKTMTCLQSATHVYPT

RRWALTKKDMGYWSFASSFEEREEFGYNSTKSQIRMLQHHTSNGRQRLVMKCKDTVAIYD

KAINSYDKAMTLTSFDEELLTARSKKSHRYTVLRDGCQERNGREGQTVLEVSGAKMLKRL

PILDVGFAEQSEGEFGLRVGRACFWGR

>SG-AL_contig_1113_1_-1_82_969

MSGLGDRLRSFCTFVWNGEEKTFLGRGGRSWVEIGVFYLIYYACLAGFFVGMLAVFYQTV

DEKRPKLTGDSSLLKANPGMGFQPMPDVEYTLVRFRVKPESYEPHVKSISKILKPYLEAE

LDHKQRGIDCTPDNLPQEGEACRVNVSALTQECNIDNNFGYGGSPKPCILLRLNKIFDWM

PEPYTEKNIKDSGASPELQERMLEDPNMIYVDCVGENPADRDNIGPIRYFPEQGFRHYYY

PFTNQPEYMSPLVFVQFTKPPKNIALMVECRALAPNIRYDRTEKEGGVHFELLVDP

>SG-AL_contig_1114_1_-3_561_746

MFQSFWCQCFIGPVPLSISRPCQRVGFCTGSMSLPDNYWQIWVERIWNCLSVMNIPLEQT

PN

>SG-AL_contig_1115_1_+2_131_280

MVSMKENLWKTPWFFHYSSVISVSLHLKLTVIYHALCLTLLRLRARARAR

>SG-AL_contig_1116_1_-2_389_604

MTQYLCSDTICNQASQSFYSFHPLKMFFCYVLPKTVFMFLRKVNILYFIWLVLCFLVGLG

SYTFLCAFDLEF

>SG-AL_contig_1117_1_-2_287_472

MNLPENRRLSGLPDHQGCAWLPVSSFACWLAGWLAGWLGLDLIVIIIIIIIIIIITMKIY

IA

>SG-AL_contig_1118_1_-2_2_610

IFKKSFTAVKTEPLGAVSRSAAANTEPGEADRRQAGDILPYPTYAIRCPFCSILGQKVPL

LLSDLLTKTCTLYNILNAHTHTHTHSKIAFVTCYITYSSGYPAMLFIFRVTQQNDVKLSG

WLSCIRLRQCSIFFSYLQNNCCVFNMYNSGLSICLRYRPCFLCLKLVVFDYNYVKTSFTI

QQISCMTQESVPKLLPPCHENSS

>SG-AL_contig_1119_1_-2_2_202

ESRQSHLEQYHEVQRPTPNLVKPTGDRRHCNIQFTAASECRKPRDERQQDKHLKKYRMTC

GKQSISV

>SG-AL_contig_1120_1_-1_22_2727

MANPSTPTTIHRGIVKQVLSGDTIVIRGQPKGGPPPEKTLCLSNIQAPKLARRANPNIEG

SVETKDEVFAWEAREFLRKKLIGKEISFYLEYKVPGSGREYGAVYLGKDNEGENMTESLI

ADGLVDVRRMGLRKDDADQQRLVQLEEAARAAGKGKHAADASTHVRDITWIIENPRHFVD

SHHSKPIEGVIEHVRDGCTVRAFLLPTFDYVTVMLSGIKCPMFKTDETGNQVPEPFAEEC

RYFTESRLLQREVKIILEGVSNQNLLGTVLHPNGNITELLLKEGFARCVDWSMGVVTQGA

DKYRAAEKVAKEGKRRIWKNYAPPTANLDIKDKSYIGKVVEVVNGDGLVVKPADNTFKKI

FLASIRPPRLPTETSENGAPAPSGKRIRPLYDIPYMYEAREFLRKKLIGKKVNVEVDYIQ

PPNQGFPEKTCCSVSIGGINIAEALVSKGLARVVRYRQDDDQRSSHYDELLAAEARAEKK

GVGLFSKKESPLHRVADIAGDVPKAKQFLPFLQRAGRSDAVVEFVASGSRLRLFLPRETC

LITFLISGIDCPKGKRQLPSGQVFEADPYGEEALLFTKEMCMQREVEVEVEAIDKGGNFI

GWLYVDNTNLSVALVEAGLAKVHFTAERSSHYKTLLSAEERAKTAKLKVWSNFEEPKEEV

VTEEPVERKLNYSKVVVTEVTDDFTFFAQKVDNGSQLERLMEQMRQELESNPPLQGAYTA

KRGDICAAKFSADGMWYRAKVLKVEPGKANILFIDYGNKESVTSSSLATLPATFQSMSAQ

ASEYSLAGVALPPDVVEDAKQDAVDALYRDILNHEMRLNVEYRSNGIDHVTLLTAESEGG

NDIGLGLISDGYVLVEERKEKRLAKLMSDYKEAQDEALKKRKNIWHYGDFREDDTREFGV

SR

>SG-AL_contig_1121_1_+2_107_370

MSQYYTPNHTLFTVPITPSITLYLQYTKIAACLHMSFTPFIFSLIRLFTPVLSLTQDLQA

FIKFTQDPSTDTHISTSAAFLTWTDMVW

>SG-AL_contig_1122_1_-2_11_961

MSKCIQTTRSIKHRLTLVNRYYCLTRATASKASTMSALQEVSNNKMFGGFQKVFSHQSSE

LKCTMKFAVFLPPSAEGGKVPVLYWLSGLTCTEQNFVTKAGAQKYAAEHGLIIVAPDTSP

RGAGIEGEDDSYDFGTGAGFYVDATQEKWKQNYRMYSYITKELPALINANFPTMPDRQSI

FGHSMGGHGALICALKNPGMYRSVSAFAPICNPTQCPWGTKAFSGYLGLDTATWQEYDAT

CLLRKYNGPPLELLVDQGKGDNFLTDKQLLPENLMEVCASTKTPLILRMQEDYDHSYFFI

ATFIEDHIKHHAKFLKV

>SG-AL_contig_1123_1_-3_3_176

KKQMMVVFQNPVVRLPQSCTGNTKNTKPAAFETFCGIVPHHQRCHFDYVHVTMLSIAS

>SG-AL_contig_1124_1_+3_957_1205

MVHPTHSLHTNMQNIWPTILTGNSQYALKKNKPEYPVTIYNLLCDLEFKDFTICPYPSLS

SITTFKRFKQSKHRSCLCNNVGD

>SG-AL_contig_1125_1_+3_3_221

HEHTHEIASALHCYFLPGLKKKKRVIQTGNSLYTVTPRQQLNESKYSKKFSSTLIQRYFH

DSAMIKQITHFTL

>SG-AL_contig_1126_1_+3_27_3764

MEKENSKNTQEETLSCAKGAETRGVGPSGAPTKPRHHVPQPIMIFGDQINLNGARPVRIH

QAGAVPGSVPQRNPPKSTGGPFVSSCGVRRRARTATDQRKKEPPSINIMHWNAEGVSTKR

EDLQQFLYENNISICCIQETHLQEGKTFKIRGYQVFRSDRQGRRKGGVLTLVRNNLNASE

STRYMDEAEYLQVKVSTPNRILNIVNYYCPDDKLLALDSIQVPDSDFLITGDFNSRSQSW

GYNILDTRGEEVENWQDDHHLTLVNDPTDPPTFYSRRWHTTTTPDLAFCTDDIHRKISRK

VEHQLGGSDHRPVILTINGVEMPGHAQHPRWNYKKGKWGLFSIRTNELTRSIKTEGRNIN

NIVKDFNNSILQAAKECIPRGARKDYKPYWSEALQQTHDALNEAREKAETQPSQENNIKL

KECAAKHLRTKIESKRAGWKEKTASLNMEKDTSKVYRLMKAMNDEGTKGQKITLEENGQT

LTGKAAANTFASTYAEESDTTIPKHSRREVRTEEKERLAGNRPTHEAMKQDLSMAELKQA

IQKLKEKKSPGPDNITNEMLQHLGNSALQTLLGIFNLSWKEGQVPQCWKEATMIPILKRG

KNKDKPASYRPISLTSCVCKTMERIINQRLQLYLESESIIAPEQAGFRQYRSTEDQTTHL

SQVIEDAFQTRKVTLAVFIDLQKAFDKVWKDGLLVKLLRNGIQGNMYQWTKSYLHNRRAR

VLVDGHCGRKVLLRQGVPQGGVLSPTLFILFINDVVAELPRGVHAALYADDLVIWSNEEH

ATTATYRIQLALESIAAWAESWCITINREKSTATLFTLSPKAKPGKLTLGNTPLKFEDQQ

TYLGVTFDKRMTWKAHIHEAENKARRKLNIMRKLAGTNWGANAKLLKSVYQGAVRPHLEY

GSNSWMTAAKTHHQTLDKVQNQALRIITGAMKSTPINKMEEITGIPPLSTRREQKVLIQS

KKYECTTDHPMKSRIQQLSSGRLKRSSFAHETRGLARKYQEKLPEQVVPPSFSLDTPPWE

ERRTNIKVNTSVPHVTTKDEQSDTVKKTLTLAMLEERYPQENWIQIYTDGSATDAVKCGG

AGVYIQYPSGEWQAEATPTGLHCTNYMAEVKALIHAAHVISSRVDNNTQVVFLTDALSVL

QSYNAGQLKDLEKALFNLNALRIELQWIPSHCGIKGNEQADKMAKMGAKEEQENNPVSLK

EMNTIIKSLFRSPPKPDSYHQLSRQAQVVIFRLRTGHNRQNKHLHK

>SG-AL_contig_1127_1_-3_3_752

PLEPELPLPASPSWSSFGRLLEQGSRSRRLDDPRAGTPPSPPACRSGVPSSFAGRKVGWT

EDFPSGCNHLHRGNSQTMRLQQTPGAPVPRQTWATRTSFTTRPPCHATHQRCPHRATATS

PCRPLLGGAGQAGAGRSAFGGLRQPPQTGPGHLAAAGARRSLALLRPTKVVREPESRTQG

SPFALLLPGRPAHPGGARPPLPPLHPEGSRLMRHPGAPRFATSRLHSPRLPGHEVRHPGT

EGVPNAGPAP

>SG-AL_contig_1128_1_+1_1_516

HLFNICTNNESTYYSVCQLHNHHSYQRRIHFLFCLSIRQSITLLITRPSLQATCSPCRLT

HLRSLFSTVQSKNTFQSLPPSPPQLNEGSISASGFFAKTVKVSLSLHSILSILSVAGEIA

QFGFCFSAAQSGDITDTSLHSFAYSTHKLRTSPRYQRITHFRFPCFFSGLLS

>SG-AL_contig_1129_1_-1_247_375

MLRIMRKFLFVFILVLSQRTCKIQAALPRESVSPQSSATHFFF

>SG-AL_contig_1130_1_+3_258_470

MAAKILHKATRLKVKHSVLVKTDYFYFCLVRCIYDMSIINCTSKQEVCCTLRCCMTFEKL

PVTCITPSSHF

>SG-AL_contig_1131_1_-2_23_1030

MATLVRRLLGLLTPISVRTTCMRCLSTRLAEVGVLEPPREQGKAYETGQLFLHKIFGYRG

IVLFPWLARVYDRDIATKTEDRSLDEQTTTVGKEVKGRNHLYYQVLIDARDCPHIRAQTE

AVTFLGNQENSRALYAIPGLDYVAHEDLMPYSSAEQSPIQHELFDKFLMHDSESSPPFMA

RETLRAWQEKNHPWLQLSDVHRETTENIRVTVIPFYMGCREAQQTNVFWWRYCVRLENMG

EDTVQLRERHWRIFSLSGTLETVRGRGVVGQEPILSVEQPAFQYSSHVSLQAPSGHMWGT

YKMERRDGTSFDCKIPPFSLESKSDDNNSPHGFLGS

>SG-AL_contig_1132_1_-2_23_226

MLCGDCLYSWQAPSITIFILLKRRKNALKSEFGCLHDKVLKNGHTHNLLIQMYSWSHCGK

TECNIYIG

>SG-AL_contig_1133_1_-2_2_184

KHGGRGDGDSCHNADPEKSWSLPPHSPIISGLGDNLYFFFYSDPFSARGPELGTQYKPAH

N

>SG-AL_contig_1134_1_-1_10_1746

MNLITKGSGQRSSATPANLSANKFNDHFLSVSELLVHPCINTDQYIPSENLINFCKKRTS

STTSFSIPSIAVHETGMYISKMNNKKSSGLDEISPKILKLSLPYITESLTYIYNISIEHN

VFPRALKEAKVIPLPKTKDLSKITNYRPISLLSVLSKPLEKHVLTYLLDYLENRSLLHPL

QSGFRQKHSCHTALTRLADTWLLAMNESKLTGTIFLDFKKAFDLIDHDILLTKLDRYLVN

HTAVSFFRSYLNERQQKVLLNGSYSSGGQVKCGVPQGSILGPVLFSIFINDLPLHISDPT

VFCDMFADDGTIHTQEKNIVTVNTRLQSSLNEISEWCTKNAMILNPKKTECMVICTRQKR

QLGLLSLNLTMGGNSITQVPEHRLLGVIIDHNLTWQNHIDYICKTISKNMFILSKLRYLT

DRNARQLFYNAHIKSHIDYASTVWDGSSEVHLKRMNTLHRRAAKLILPDPTLTTDEKLHQ

LEILPLSSHFMFNKGIMMFKVSRKSVPQYLLNFFNQQVRSYSSSRQSHFKIARPRIDIYK

SSLSFSGALLWNSLPQSIVNAGSLASFRKSLFKHLMSIS

>SG-AL_contig_1135_1_-3_9_242

MNLITKRSGQRSSDTPPALSANKVHNHFLSVSELLAHPCANTGQHILSENLIDFCTKIQI

AQLYLAFQAEQSKNLACT

>SG-AL_contig_1136_1_-2_44_871

MALADVCGFKYQDGKSKLSLGFGEGFNDPSAFDALAASQSFTVPQGIDIAQNLAHFSDEK

SNVKIHFDHGTTTLAFKFQGGVIVAVDSRATAGPYIASQTVKKVIEINPYLLGTMAGGAA

DCSFWERVLAQQCRVYELRNKTRISVAAASKLLANIVYDYKGMGLSMGTMIVGWDKKGPG

LYYVDSDGERLSGSVFSVGSGSVYAYGVLDSGYKWDLSVDDAYDLGKRAIYHATHRDAYS

GGVVNLYHMRETGWIKVSQTDVQELHYKYKEEKENK

>SG-AL_contig_1137_1_-3_3_266

PAKRQTHRRQGERPSGRAQQIQRRSSDRVDPEPRRSPSSLGCLGSQTHGGPLCDQVQQEA

SRVRVAGSRSGGVGGGRPLPRLERSDRL

>SG-AL_contig_1138_1_-3_78_683

MSVLNDGRNEIRVNNSDGKCLLENWVEERSVKHIDPEIKLEGAVTSKAQILRDGHKGVLT

TDFDARADNKTTVRLTYTPPEPDATRKVGSKKELMERMLFAKVCNEMKVEEDGGPEKTDF

RSVKMADYDFEFTPQKIAPTRDHNYRSEQPVTFWSEHKDKIHGVTQVKTRDTAFRRNDAF

SKPIGEYWEEPKPYEQEDYPKM

>SG-AL_contig_1139_1_+1_568_804

MNFIINSCIKIKLEEKIHKISFVLFFIIIFFPDNKFILQFHISPKPSAPSQVAPIYTTFH

TRKHPSNKIFRRKAGTCAN

>SG-AL_contig_1140_1_-3_126_359

MSAACIVDRRESYDDSCVRWEWTEKSVTATSIPDEFEQVGQHIRKIDVAAGKSSTCNLTD

TFSMISYPPEVNTSSVTH

>SG-AL_contig_1141_1_+1_82_318

MSAEHRHIAHSLQHLFSEYVALHAQTHWDLISLSPIFLSTPTSTYSSRNCRHNFLRYRRC

VLPTFCVSKSNRARWQLTN

>SG-AL_contig_1142_1_-2_311_613

MNTCRRNHKWHECDGKGFCARCIGGSGHRLMYGSSLILLLLFPHPDCSISLSLSLSLSLS

LSALFLIISFCLSLRLSPSSPSHAFWCFLCQNIFIFLSFNL

>SG-AL_contig_1143_1_-3_81_203

MSAACIINCGGSYDGSCVRNKWRWEWTIKSVKVKSNPGKFE

>SG-AL_contig_1144_1_-2_62_253

MSAARIIDRGESYDDSCVRNKWEWTEKSVKVKSNPIEFERVGQHIRKIDVAGSARCVLCS

ADIV

>SG-AL_contig_1145_1_+1_4_393

MLVGPHPSPSAVYIGQNEIEVVQDFTYLGSSISNSSDMTKELNCRIGKAATAFNQLSKIW

NSKKISLKLKLRFYNSNVLSTLLYGCESWHLKTSQEKKLDSFDLRCLRKILGIKWTDFVT

NEEVRSRSKQ

>SG-AL_contig_1146_1_-1_103_786

MSLTDSEVARQIKHMMAFIEQEANEKAEEIDAKAEEEFNIEKGRLVQQQRVKIMEHYERR

EKQVELQKKIQSSNLLNQARLRVLKAREDHIKALLEEARGRLSKVAADKQRYREILVGLI

AQGLYQLLEPVAILRCRAQDVELVKEALAAAIDNYKQATKGMKEVKVNLDTEHFLGNEVS

GGVELVSRSEKIKVTNTLDSRLDLIYQQMLPELRDILFGRNPNRRFTD

>SG-AL_contig_1147_1_-3_105_272

MACLAVKNGLLAYLYFILFVSISRKKSMLLFVVCVPQLNTLGVTQYICTLSYESET

>SG-AL_contig_1148_1_+2_176_316

MNHEMKEMLSKYKMNRHICLRLLLLMYCHTVTWFYNFSCHIQLHFRS

>SG-AL_contig_1149_1_+3_18_452

MLKFTSKDHLSHFHIPCAQTQCTENTGCQTDQSTRMLPRDTTLLFKDLPAMKRECRVEHI

ATKLKLWIQTFCWTLGHHFWEQLKDFSASMTFSQPFHDISQPLSEFSLTLATFQRLENGI

PILKDLKRLCEPCKMFRHTSCHAFK

>SG-AL_contig_1150_1_-3_3_212

RRDACVCLGTAVGREFLGRRSGPAGVGLHWFDMVQYMTPTIRLKTTRSKKGKNKDRLLWF

GVKNHKSGGK

>SG-AL_contig_1151_1_-3_486_800

MCLFIYLNFEAILCVGLYICCFCIYHFKVLIVGLITFICICVNLWTIILFSPHPYCLIFG

YIHVSHIWTFWFSSADIFGIIVTKTNVFIVFCSTFFVDFFFFVSE

>SG-AL_contig_1152_1_-3_3_152

ENGDDSDYVMEFHPGLRLHDRAVLYASFHDISWRQPGIRGTDTCDVGEET

>SG-AL_contig_1153_1_-1_913_1221

MGLNSKQHFLSPRQNQLSQPTGLWSSMILKVMLSLSLSLSLSLSLSSPREQFLSPRQNQL

NQSTLYSSMIHKVMLSLSLSLSLSLSLSLSLSPQHTFLLPPTK

>SG-AL_contig_1154_1_-3_33_257

MVLLLLCFCLFVCCCCLLFLICRFSGSFSTCGAGVLFDEISACLLLFGVAAFVFNYRTSN

CYTQSLALSTFMPFN

>SG-AL_contig_1155_1_-2_137_1978

MTTKIRCHYEVLGVERTANNDDMKKAYRKLALKFHPDKNPDSVEEATEQFRLVQQAYEVL

TDPQERAWYDRHREAILRGGQGKGDKYEDNSVDLFQYFTSSCYSGFEDDEKGFYTVYREV

FKTVSAEDYEFMDDKDSDFSFPEFGDSQSDYDEVVKPFYDFWHAFCTCKSYVWMEEYDTR

EAPDRRTRRMMEAENKKKRDAAKKERNEEIRALVAFVRKRDKRVQENKKKLEQRAAEIEK

KTKEKREKELQERHKLRENYKETGWSAMSGLEDDIQRLEADLAKQFGDHSDDYTDDDAAA

DAEEGAANENAAIPELEEEDLFDDLFCPACNKAFKSEKAMSNHEKSKKHKEMVAILRADL

EMDDELMEEQEGEEDRGGGGGSADEQIEGVDRPTTDGEKETQSKGQVVEEEEEEEEEYVS

RAKAKKQKKKKKQKFQEQETSEDMDGLTSSVNHLHVTASKQGDDDDYGKRNAKSKKNKKK

DRKAANASSSVDQATKEPAAANEAESEAVGDGETTGEGQTTEGDGETQSEGAPTEQPSQE

TGNVKGSKAGSSKPFKVKDSQKCLVCHEAFPSRSKLFDHIKSTGHAVPLSAKQDAAAAES

AETGKKKGRRKGKQ

>SG-AL_contig_1156_1_-2_80_703

MGISRDRWHKRRKTGGRMAQLRKKRKFELGRPAAMTKLGPKRIHTVRTMGGNKKYRALRL

DHGNFSWGSESVTRRTRVIDVIYNASNNELVRTKTLVKSCIVQVDSTPFRQWYEAHYATP

LGRKKGVKLTAQEEGVINKERSKKVMKKYEARKKVAKVETALEEQFSAGRVLAKIASRPG

KHGRADGYVLEGKELDFYLRKVKSKKGK

>SG-AL_contig_1157_1_-2_854_1072

MDTFQIWVMYSIHSVLRILQFMHIAYLQRDCYAKCEQSQHIFFPLCHYYFPCVSMNVLLL

AAFNGRRKMARIP

>SG-AL_contig_1158_1_+2_755_955

MYTCPIRFFYLTRIFSPMLMQRKLTKSQMPSHKEKKKRRKKKISITEIINYVHPFNQVFQ

VDPFLYT

>SG-AL_contig_1159_1_+3_477_695

MQCLSVHVINCKHFIYVTSCTQILDFYSIWIFLHIIVQINTLKIFLIKRSGKRLKLKTED

NARKNLTCFAFLF

>SG-AL_contig_1160_1_-2_842_1132

MLCSISYFQSFCLFGGFFVVIVLFVWFVFLVIVMLSMFYLALKLLIWNSFAQLKRRRVFV

YLKRCKLLKTLWSLLCHAVSLLIYFYPVDCVHKCLQN

>SG-AL_contig_1161_1_-3_15_260

MMTCFRWFAGELESQSEQHRSRDIEEVQKMGQQEEKKMWIVFFEVRFYSAQHKCRLSQSQ

DYAKENCPRNGTKRLHIPVTMV

>SG-AL_contig_1162_1_-1_151_1113

MATAAMLKGLKAQADQAENLISQLKLQISVLQTATAVSAGKGEEERLRVENDALRKEIEK

VKLGLIVAEIRNGVRQVPVPQQRDLTASAKQTQVESVQDKTAQAPAPAKVEEKAAASEPK

AKKAKKDDQPAKNKPAKEESKDKGKKAADAGASAGDSDKLDVSRLDFRIGKIVAVEKHPD

ADTLYVETVDLGEEKPRTIVSGLVNHVPIEQMRERLAVFMCNLKASKMRGISSEGMIMCA

KDGKVEILEVPPGVVPGDRITFDGYPGTPDKQLNPKKKVWETLKPDIRTNGERVATYKGV

PFKVEGKGLVKAPTLPDAEIS

>SG-AL_contig_1163_1_+3_162_338

MTTSLPHYHSSRPRSNLMYNQDSYCFERNAEDTNMHVHEINTSDRYFAAHKIFLTNKLE

>SG-AL_contig_1164_1_+2_488_634

MVEIHSLRKCKIFRKNMSWFAVSSFNKDIKHIQELLLLLLYIVYSPADH

>SG-AL_contig_1165_1_-1_1_126

NRKKCLLYKKKKKKKKKKSKNLNRNGYCRINMKLVSGNQVIT

>SG-AL_contig_1166_1_+3_768_929

MWSTSLIVFTRPNKLVFVCFSPIAKCSKSPETAYRFHVITEKQAKKHKFVSVLQ

>SG-AL_contig_1167_1_-2_986_3097

MAFIGDAGRVDGSWELTIYVTDLQEECRLRVKGDLHIGGVMLNLVQSLDIAQDWSDHALW

WPDHNMWLLRPKSTLDQCGVQADALLHFTPMHKNLRVQLPDLQVHTLRVDFSVKCFSAVV

QLSKELGLRHPEELSLLRKWDRDDLKRVAKEATKQKKKKESGELASGPLSANGTLNSSSG

SIDGRLSPYPSRSPQGPSPTSTLNRSGGTSLSISAYNTLNGTLTPGSSHSLSFESSLETS

LANSPPVPSKEAFQYLFRPKNFAEKARINCAWLDSSRSLYEQGVQENDFIMLKFKFFNFY

DLNMKYDAVRINQIYEQAKWALISEEIDCTEEEMMVFAALQFQVQLQTNMPQHDSVDGNG

PGPLPVEQDEVGAALADLQSSLEGTSVSTSGDITHIPELADYIKFFKPKKLTLKSYKTYW

ATFKDTHLAFYRNQEESAGPPVIKISVRGCEIHPDVNLATKKYGIKLFVPDSDTADGMSE

IYLRFETEDQYCRWMAACKLASKGRTMADSSYESEVAALQSFLGMQHPSSAPVLEPSQVD

IHPEDFIAPRFFRKLKSKSLIHRIMEAHANVRNLALLEAKMAYIKAWQSLPEFGITYFRI

RMSNGKKEELIGVASNRVIRMDLNTGDSLKTWRYNVLQSWHVNWENETVILDFENQKVTF

APLECSCKTVHEYIGGYIFLSTRSQDKNQSLNEELFHKLTGGWS

>SG-AL_contig_1168_1_+1_661_909

MTSTSTELQDLTSRLTNRLTISAAVYGMEISNEKSEAMAKSTTTVVQNYMNGQKLDEPTR

YSVQRWQSTKEIRTRTAAAMARK

>SG-AL_contig_1169_1_+1_625_846

MLFSSSELFHTHQESKQSHNYLTTIQICTWTLCHRVSDTLHQDVVYKIIHILTSKVPLHR

QCTNASYQIADFIK

>SG-AL_contig_1170_1_+1_1_252

QNGIEVIQDFTYLGSSISNSSDMTKELNCRIGKATTAFNQLSKIWNSKKISLNLKLRFYN

SQTSCPLCCTAVKMAPENKPGEKA

>SG-AL_contig_1171_1_+3_3_185

LSTLLYGCETWHLKTSQEKKLDSFDLKCLRKILGIKWTDFVTNEEVRSRSKQNPVTRLCN

K

>SG-AL_contig_1172_1_-1_406_888

MIRFQPSCTYYFNKKNQGTESFVNIYQGLGAQTRILHTSDEERSEDCSRGDDDEQRRLLK

RSKATTSPFQRKLHSPSTSEQHTRYHRRRPQRTDSPLLGTGPFDTHKQWTTSVSINHHQQ

HFSKPRKELRISPLNFNLLSLSSSSSSSLPVTSTYSEYSFF

>SG-AL_contig_1173_1_-1_2656_2853

MCIVLCSLSSFVLRYFIVDCIISLVCHCVSVKFIHPPSIRVVILTIHHVAGIQILGVRVC

AGCLYL

>SG-AL_contig_1174_1_-1_970_1170

MSWNQFNIVSLSILVFSAVMWFGILSVKETTRLARIVKQASKTVGVPQRQQCDLYSFALK

KKSILHC

>SG-AL_contig_1175_1_+1_1_501

GNYSCLSSIHTHTHWKLLLSFIHSLTHIYKLEITLVFHPFSYTHTQQKIILVFHPFTHKH

THWGLLSSFIYSYTHTGNYSSFIHFLSLTHMRTHTLKIILVSHPFSLTHTYWKKLVFHPF

SHIHTHTHWKLLLSFIPSLTDTPTHTRARTCTHIGNYSLSFIHSLTH

>SG-AL_contig_1176_1_+2_11_187

MLITHITQRCGFDPQARHCFLKTLPARRPYAQAYGICHYHFGFLSSLGICSVGAYDTCI

>SG-AL_contig_1177_1_-3_78_356

MEEPLHSVCSQPLKRSSSGTKNRRAWRILLQYCCNSGLLFGVGLSQYRSLVGCRLWFLYS

ANFRLKTICMDEQFVLPQYRCFRNWMDVNCILV

>SG-AL_contig_1178_1_-2_2_199

RGQPFKFKLGVGQVIKGWDEGVAQMKKGEVAKLVIPPDMGYGASGVQNVIPPNATLHFEV

ELIDFK

>SG-AL_contig_1179_1_+3_66_440

MVMPVCSNNSEIIFNIDWSFQLSRLTQDCKDKWGVTPRVNWIPLQFWGKDILSASNIIFS

NGLLDPWSGGGVLESLSPSLIAIKIPNAAQHLDLRANNPDDPAEVRAAREQEKNIMKTWL

FRPGY

>SG-AL_contig_1180_1_-2_2_139

PWTPAIFQRKLKQVVLHPGAASLNPLTPSVYPPSSCIISHDSKTNT

>SG-AL_contig_1181_1_+2_2_187

MNVQYFFFFFNDTATTEIYTNLNTLSLHDALPIRLPSSVCLRFSLLHTYSQVMHDRLKTR

PA

>SG-AL_contig_1182_1_-1_19_930

MEIQIVHARNGELITSLGDLQASSTIYEVKKAFCKEKPKYYPDRQSFRTEARGKMLKDNL

TLGSLGLKNGSRLYFKDLGPQVGWTTVFLTEYAGPLVIYLVFFTRPAIIYGAEAAKLPYA

DVVKYAAGCWTFHFAKRLLETVFVHRFSHATMPITNIFKNSMYYWGFAAFVSYFVNHPLY

TPPAYGDVQVYGGLAAFAFGELGNFSIHFALRNLRPAGSTERKIPYSTSNPFTWLFGLVS

CPNYSYEFIAWTAFTVMTQCLPAGLFTLAGLYQMAVWAKGKHRNYLKEFKDYPRRRTAII

PFLV

>SG-AL_contig_1183_1_+2_377_529

MWVSTPIPKHRLYSIVPFGGFCDTVYYQDQELFHMPKIQVLYFCAFHSNAS

>SG-AL_contig_1184_1_-1_1816_2376

MVSWLRPTLKCLLGSLEWSGFCLLFFNHSCPVFCLLFLCFVHFSHLYQRKPQQCFLFFLH

FTVPVFSRGSHNSVSCSSFTSLFPSLSEEATTVFLVLPSLHCSRLYQRKPQQCCHGLVVA

NTLTVSMWSERVNNCHEQNWTQIFPFGIGTCSRRIHHTQTISYAFYPNTPVMIWRLLEPV

QNRMHWQ

>SG-AL_contig_1185_1_+1_370_546

MPSASHPIRITSTICFFRKQKWQSPSPVTFNPDGAVPGYPLQSLSAVPRLFWESRVEVT

>SG-AL_contig_1186_1_-1_184_357

MSSRLNQTLYQSLDTSASQVCFFFVQIVYQVVNYSYIISYESLGNKSSLVLIYERCKD

>SG-AL_contig_1187_1_-3_126_299

MSFAQHLSSDYCTDSELMARASGNTKMPCVCWRSVREIRRVVVFLFTLLVLAVFMLDT

>SG-AL_contig_1188_1_+3_51_206

MTLNSRHAVGSIHTKSTSLQHRAYYKQYVSVSYATPSTNTYVEVYKVFWKMI

>SG-AL_contig_1189_1_-3_261_560

MDGDQQSQKKIEDCKNESEHINLKVTGQDGSVVHFKIKKNTPLRKLMGAYCNRAGITTGA

VRFRFDGNPINDEDTPNTLEMEDGDSIDVFQQQTGGCHHL

>SG-AL_contig_1190_1_-3_126_2375

MSVGEDTNECPLCMEPLEIDDVNFFPCTCGYQICRFCWHRIRTDENGLCPACRKQYPEDP

AEFKPLTEDEVQNIKKERKQKECLRKQKAAENRKHLANVRVVQKNLVFVVGLSPRLADPE

VLKKQEYFGKFGKIHKVVINQSTSYAGSQGPSASAYVTYVRVEDALRAILSVNNVHVDGR

TLKTSLGTTKYCSNFLRSSQCTKNDCMYLHELGEDAASFTKEEMQMGKHQEYEQKLIEQF

HNSQNSTNNHISNNKQQSSSKKSSAPVGQQQAQQHSPPLPDPGPTNSTKPPRLMALGPQS

GEPFQTSSVSEAWPSLQPESGQSVGSSVGSKPELLTNGSRSSSSNSLNRTNTPPMASSVS

SKAVGNGRHRSISENHLQISDTVGSEADLGSSDSPLTLPGGQSVHTPLPKVQTLSSSPLT

PPVPIGPPPTNSHSPTHESGAQSLPGSTLRQLPLSLPQSCEPLTPIGDSRRLANFQQANN

SLSFFSNSILGSKPVLPVVPQPQTQTEGFPPTEMSDSISVRSSVDWQAAFGFKHNEDFKQ

NSEEMKESEDDLEFDPCAESFKGLADLMMKENENDRHWNTKDRQWPLNHSSKGQDQHAYA

SHMPQPLPSVPAASAPSQHNLPPGFSLSHIQHQQQHQQQQLQQHFYRQEMSGSRMMDFMN

FGARPRFPPAMHNNFQGVPEMGSSGPLHHPSVSQPNPLSSQVSQNDIYNVQGMQEALRSM

LPNVNISFGSPPQQLQPPPPPPPGVSHPPS

>SG-AL_contig_1191_1_+3_3_137

TDIQAAVVILEGDGLILCHKLSQPHFGVVYNTFSKHHTHLAKFTH

>SG-AL_contig_1192_1_+2_422_724

MCVVGNIVISNILVHKVFEWKYINLTAHIKIYVCVIYLENYTHVTFFYIHVCIFWYLGQN

KFVIPVLFDAFVCMDVCACMSVQPVSSLQHSCVCGIFHDFG

>SG-AL_contig_1193_1_-1_241_363

MKGCSRFVCFPSHSLTHSLSLSLSLSLSLSLPPSSSLSLSP

>SG-AL_contig_1194_1_+3_3294_3854

MLELVTLSFQNLHLMTKFRDICCQSLIVSSVAFQRSIDCLELCLQVANGQLVVLLPLSCL

LQETLFMMYSSLVMSSQVYVVSGQCLFSTVQFNLYRFELRFQIIILCLQLLLFFENDQTI

LSCLNFFACSFGKLISEPLNFLFYINRRIHFLPWLLTHRLRKEVRALQCQRRATETFTAN

GRHVWTK

>SG-AL_contig_1195_1_+2_119_304

MATKLHKLEKLLHAIMCSKLSGLQAFSLTLHTNFTELAKCEHTHKQRLYHMYNCQHYRMK

KK

>SG-AL_contig_1196_1_+1_199_1713

MLNFRTSGATETDSGKRNDMALIYIRSTECDDYILATLDSGGNILIETNQGVGIYRMKVS

GNFANGELHELVYKREGTNMYLTLDGVREASIVYPDYDLEKIDTIIIGGLLMDDDNTFGN

TANFTGCISNTVFIPEANLDLKIRSLKEFHLHKPQVTVHGPDVVSCSEAMLTGKVVTEAP

ESVTVTAQTFEILTMPSWEVGPARLVTLSPPRVFTEAPGTTSSPATTTSNKTKVLMARDQ

EAGVDDLTIIMAVSVIAAILVIALLVALLLCYKRRQRYEAKKDPDIELKQPLNYQSVAAV

QSSPASGDHLARLDEFSTISVTLGPKPNTNGNSIEVTKRHSQPRPEDGTTFEHPIYNRQK

QRPASSISEVLEELERRHTSRNSTDSTDNIVGRPHGEGELEWDPQADRTPLTYKPEDMNT

SATDSPGNQNSLLDGNGDSGYEAESRPEITEDDITPGGDDEDLDEDDSTDAKLYMYNISN

IDSEDSPILTSPSRQRLLGRNSRDT

>SG-AL_contig_1197_1_-2_14_775

MSARMACVSTLLACCFFTAVLAGEEACYSYAGGSVYPQETRRTSGHTIQWSQAVISKPAP

DWNGTAVVNGEFKDIKLSDFRGKYLVFFFYPLDFTFVCPTEIVAFSDSIGKFKSINTEVV

ACSVDSQFTHLAWINTPRSQGGLGPLKIPLLSDITHDIAKAYGVYLQDLGHSLRGLFIID

PKGILRQITMNDLPVGRSVDETLRLVQAFQYTDSHGEVCPVGWKPGSDTIIPDPKQSQKY

FKKQKDPEGEKEEL

>SG-AL_contig_1198_1_+1_373_537

MPDGTSFVYLYAHTTSNTHVKEPVSHVSVWWLMETRTYSACTLYSPFTGSKLYWL

>SG-AL_contig_1199_1_-1_7_858

MAEEGEEVEKLALKKELKWLLGGEVHHSLDSIELTLKECCKCFPLLIHGNSEGTGSNNEA

HGQESTQYPAKRMLLSSATSTSPGTMKCMITLRGDSILEADIMFKHKEKGKDHQNYRTTI

MQQKVWKIQQIQDAANHLEEALRIVGERQQHYKFKTAQEVLMLLEKLKKSVQACRHCLST

PKRRSLHDLVLSKTREIFSPPIPQNVALSFYIHGSKIILAIYLLHMNGQQKMDVSQRLQM

EAAVQWLNEAVALFTLALQQCQQLADKVTTVCQCGGLDPQMSIT

>SG-AL_contig_1200_1_-1_13_2424

MAASSDSEEESFVELGKPLPFVEGEGKKKPITLQDASSRSAKDAKGRPRFHGAFTGGFSA

GYFNTVGSKEGWAPSTFVSSRQQKQDATLQQPEDFMDEEDLGEHGIAPRQFATSDAFSSD

AQSRKRRAEVMLSTRDSILPASAALADLIVPERLPIGIKLLRKMGWKEGQGIGPRVKTRK

KKKKGNKAPGVKTYGCFLPSQSDEENEIDPGDLDLQNVTFAPKDVTPISLTSKDNVHGLG

YRGLDPRSALPTTHVNLFEAPAVRSFRGRKGIRGQAFGVGALEDEDADIYATDAMSNYDQ

TMELDDSEQFFGWTAPGARKGKGKQDQPKVPVSYVGKLLEGFTLSAKPLPPKKVFPAPTL

PKGFQPFHKFKKTAEQLVQEAIGEKPATAGHQPAQQKLNAVERGLMLGETPIATSVFDLI

PKEDIRKLEAVKDAAKMAASRFHSAGTTSENSDDPAPSVTAATITEQTSSQQAAKPCSSG

GEKVNPTPLPAGSVGVMPFHKDPPKQDRYERYLALVKEGVKEPYVEVAASHMTEWERHRE

REEFSKAAHLYRPLSNMMASRFTRGTFIDDVEKQQSGLVEAVTEKSEQMKAAEMKMYGKL

TREEVEWHPHNLLCKRFNVPNPYPDSDIVGVPGLKRDKFSIFSFLSVPTQHPTTTQHTDA

SRDAPKAKETTSSLTRELNASKRKKGAMSIFSVLEEEEKTPRKPPPALSLSELGDSPNDT

DDKAAEGGDENDKNEEENNEDADDGHPKDLFRAIFKNSDSESSSASDKSDSEKSDEDDDM

DSKEKEEMDKDDMDSKEKEEMDKD

>SG-AL_contig_1201_1_-3_84_1688

MGKKVEKALSKDKNHSSASHDTTDDDPNKTPEPYKCTGNFQNDFTEMCRRLGLAVIPPVV

LRPRAPGASASHDTKPDRGGAKGKQAAPEPDPEPELNEDGELIEPPPKTYITKEKFEYFK

PCVQVEMDHPDKGDTVTEIFIKGWKIDMSMMGIFQMCWPAMDKLNTINLWGVGLTGDTLQ

LLASFLPLCVNIKNLILDGNTVKEENFSSLMGEESTIVNLSLRYCGITNKGAHGISLALG

TAKAANAKLMSLNLSGNLIDDVGAEYIATGLKMNRSLLSLSLNSNAIGDKGAAKIAEALS

RFPLSHDEVVERRRQLSDRGSPDRNGKSPPPSRRDGSKDRPGSVRSLGTHADKDKKGTRD

KPSAKKKDPKGKDGKEDTKGPKKEDTRGATKTKGASMAADSGKTIGGKTKNSKPGPKNKP

PQDHEMDVHEVINPLLEVADLIDGQLWVAGNRILINLNLARNQIGESGLNSLLKAVQYQT

TLTMDSRHGGSGLMRLCLDRNAVPRDNLTLKKINEHMLPKDPFYKPPVTPEAETA

>SG-AL_contig_1202_1_+3_267_452

MHLAHVKDSTATKELSLAKFGRKIHCHRSTKIQAYKKIVAPYFGDVLSLGRVACISRRKS

LL

>SG-AL_contig_1203_1_-3_114_1400

MPRIPPKNKEKGIGDRLIRFKCGLQNTILDVLRARPGWQETAGDGDWDFYWCEAYWMKET

FDHAYLEEHVRINHFRNHYELTRKNLMVKNLKRLRRQLEREAGKLDAQKCDFFPSTYELP

SEYHIFVDEYRRNPGFIWIMKPIAKSQGKGIFLFKRLKDITDWKKGTEYQPMAEQAKDGP

ETYVVQRYIENPYLIGGRKFDLRIYVLVTSYTPLRVWLYRSGFARFSNTRFSLESIADNY

VHLTNVAVQKTAPDYDPEKGCKWSLQQLRQYLTAKHGINSVEELFRQVNEIFIYSLQSCQ

RVIINDKHCFELYGYDILIDANLKPWLIEVNASPSLTASNQEDYELKFGLLNDVLNVLDM

ENRLSGKERRVGLFDLMWDDGAVILDDSITSDYINNFATSLNAFLGCNTDRKDNLRQMYD

TAQALKKVS

>SG-AL_contig_1204_1_-1_1_900

TTTTSTTTTTTSTTTTTTTLNPNRCGVSSDLTSVTNVADRSLVFSVARIVGGTVTGSCSL

VPWQVRLNINNTQMCGGSIIDATHILTAAHCVNNMPPDLLQVIAGDYNLAMYDPNEQVRA

VSQVQVHPDYKTVPTPLTSYLPHTLVNDIAVLTLSSPLKMSPDNPCAVPICLDPSFAPDG

KTCTVSGWGATQSGSKQASLLLQRGNVPTYSGDKCMSTFSIPDKYYMGDTSSELCAGISE

GGVDSCQGDSGGPLVCLNPATDSYVQVGIVSYGYLCAQANFPGIYTNVAAYNDWINNAIA

>SG-AL_contig_1205_1_+2_182_358

MKSPIYLKKAIPRNQYFYRFCIQHLQFLYLHIHAFVCISIFISNYYFVSTETDSYLFLS

>SG-AL_contig_1206_1_-2_362_667

MWCMQYLTAVYIPLSSAYHVTSPTLCMHNTTSSPVCCRQLVFFTFIPKPLSCMSCNVARK

GEWGLCLQQCCFLPFGLLHECLKGVGGSEYFLRNVKQKNGRL

>SG-AL_contig_1207_1_-1_43_495

MGRMHNPGKGISQSALPYRRSVPTWLKLSSDDVVDQIIKLAKKGLMPSQIGVILRDSHGV

AQVRHVTGNKILRVLKSKGLAPDLPEDLYCLIKKAVSIRKHLERNRKDKDAKFRLILVES

RIHRLARYYKKKRILPPVWKYESSTASALVA

>SG-AL_contig_1208_1_+1_1_399

LVVLLQGSQILTGLRELTLLHTLTHIPVDEGTLGVHQIELVIQTGPGLGDGGGVAQHAHG

TLHLSQVATRHHSGGLVVDAHLETSGTPVHELDGALGLDGGDGSIDILGHHITTVQHAAG

HVLAVTGVTLHHL

>SG-AL_contig_1209_1_+2_2_316

CQILTGLGELTLLHTLTHIPVDEGTLGVHQIKLVVQTGPGLGDGGGVTQHAHGTLHLGQV

TTGHHSGGLVVDAHLEASRAPVHELDGALGLDGGDGSVDILGHHI

>SG-AL_contig_1210_1_-1_61_837

MDPSNIKWGGAGADYVVESTGVFTTIEKASAHLKGGAKKVVISAPSADAPMFVMGVNEDT

YKSDLTVVSNASCTTNCLAPLAKVVHDKFGILEGLMTTVHACTATQKTVDGPSAKDWRGG

RGAAQNIIPSSTGAAKAVGKVIPALNGKLTGMAFRVPTPDVSVVDLTVRLEKAATYDDIK

AAVKEAANGPMKGILFYTEDDVVSTDFVGDAHSSIFDAKAGIQLSPNFVKLVSWYDNEMG

YSNRVVELLAFMAKKDGSK

>SG-AL_contig_1211_1_+2_2_418

GSIDIQVNLLHKPAAGAVRISSKQVLTLLIFNLQLAVCSRQIQRTPTIPYELHPSATNCL

ADSLSLRKNHIHSKLLLKVLTPWNLLPSVSETLPFPSTLFRHSTRPATCISHPLPQIPQH

SWPDDLESIRTSMKHTEDY

>SG-AL_contig_1212_1_-3_126_545

MDSGSSDLDKLSASVGLAVTSTHDHDYSSKRPRMSTVTEELSFEEIFLSPAPSPAPSPAP

TPSTSYVITEPVEDKSRNRREKNNVASKRSREIRKQKFVGMEEEAKRLIVENARLEKRVV

ELERMAKQMREILVAKMARK

>SG-AL_contig_1213_1_+1_358_486

MFHQGRRDTISHMKKKGFNEMTLELHITPMIQENIVEFHKSDS

>SG-AL_contig_1214_1_+1_1_237

RSTCVQTCQCLNPLRVYTHAEYQICTLKDPVSHVSGWLTETRKHPACTQQGLAVRKIQQD

LMLRSEEHTSELQSPAMIS

>SG-AL_contig_1215_1_+1_1_303

GVGGGGWGGGEGGGGGGFGSSVHFLYAVLTSASYHLKDFNSPRNTTLSCTWFFCSFVFPF

FPSCQEGSFLHQDKNCTYLSIHCSFFLNASIHTSVQIIMLY

>SG-AL_contig_1216_1_-3_2880_3188

MVSHASSHKSCLPNIHTHTVCLSLRPISFVTLSAVCAMCVIVIDLQPWSASIMTVSMFHA

SVHMIVNNDTRLLSVRCWVSCTLICLDFTLSLPHPIHQLPVGS

>SG-AL_contig_1217_1_+3_3438_3713

MDELLAYQPMIHNLQLKKEIKNQNGPLNFSLNIHTLLIARQNRIAFTFSQHTASTLAANN

TLLSHMLQECFGRRSMICQPVQTASIIAADSF

>SG-AL_contig_1218_1_-1_55_399

MVNEAEKFKEADEKQRQRVESRNKLESYVFSVRQALGDAGDKLTTEDREVVSKACEDTLA

WLDSNSLAEKDEYEHKLQELQKLCSPLMAKIHSAGQNCNSSSSHCGNGPTVEEMD

>SG-AL_contig_1220_1_-1_1_150

FLPSVSGCRKEESVLCRLHTGHTFLTHSHLLKGDVPPWCFPCRFFLAVLP

>SG-AL_contig_1221_1_+1_919_1050

MIHKQGKQRDIGFSKTIKSAAMSGNNDASYWLKLIIYARHTLTQ

>SG-AL_contig_1222_1_+1_163_408

MHFKAFLHSKTMFTGPLNRGTGDFRLRYILGVFALKNLMFRIICRELCSFAFFFPGFEEG

LLLFGCVIDFDGLNVVTALIWL

>SG-AL_contig_1223_1_+3_3_95

GGRFEARKSADSTVFWGYKCMSVYLTSKIVT

>SG-AL_contig_1224_1_-3_24_842

MADEIPVIPKVSVLEVKTGNIVNWNRLKRKPNYTPTAEVAECVTATVKKYLDVSDKTGLQ

YVKELVCADTEQQQSLHQDERGELKVIIKVFVCSADQPAEVVLEAIEKVMGELQVTFVET

VLLSLSMFESEEEISVEQIKVYWQALEELVAQERVLSIGVSDLDKNKLEQLYSWAKVKPT

IDQVNLASCCVMPKDLTEYAKQVNIQLQTHNDPPELLPRSQLQEALRQCGTEQDSFGWAA

LWVARYSVLVKCRGIIKSKGYILRALRDPHRRM

>SG-AL_contig_1225_1_-2_2_196

DNPAFPLQRLRTATYTVTVLDNAALGIHFLLLTSYHYVIDRHNDDDRRMCPHSNFGICCF

NLCCI

>SG-AL_contig_1226_1_-3_228_494

MTNLAPPSSTASASKLDNTSSLSSSVSVQNLLSLDSNSSFSLHLTVQTLPSQLDNTSSLS

LYLHVQRVLFDILISTWTVLNSFCSKCFL

>SG-AL_contig_1227_1_-1_178_429

MVIPLFLFLLSYIVVAENHWLNVLVVGSLCGWCLFVICFVVDVLVCVWCFGVCVCVILFC

FVLKMKNLRFYVHENTLDTFLHLL

>SG-AL_contig_1228_1_-2_1172_1441

MSHLTPILFLYGCQEEQQFSVETRCVEDVYYILFPVYLTHTDLDRGGNGHLGAFSSFVFL

SFDTCVHLFSFALFYHHHTSQSTDIHTMHY

>SG-AL_contig_1229_1_+3_546_884

METPSVVLHHEFVELTKIHFPVLVLVDVIDDLVHLLVGHLHLLLDLVQQPLHLVIGDEAL

PLVVQDPEHLFVTCLFVLVCQQVLYLAVELVEKVHVVLSLHIVRHLATELEKS

>SG-AL_contig_1230_1_-3_24_3164

MSSDSAAPPAKRARIEENTSGNCFGVTGSTSNNSVDMEKNGAVDEIDEGLYSRQLYVLGH

EAMRKMAHSNVLISGMGGLGIEIAKNVVLGGVKSVTIHDPDKACYSDLSSQFFLREEDVG

KNRAEVSQPRLAELNNYVPVNTSTAELDEAFLSQFQVVVLTSAKYDDQIRVGEYCHSHNI

KFIVAETHGLFGKIFCDFGTNFVIMDTDGEQPISCMIADIEKSESSVVATVDETKHGLED

GDYVTFSEVQGMTELNGCDPIKIKVLGPYTFSIGDTTKYSDYIRGGNAIQVKMPKTINFK

SIKAAMDAPEILPSDFAKFDRPGQLHIAFKALSDYEKKHGSLPKPRCLADGEEFVKVVTQ

VNETSPAKVDELDPDLMKEFAYSACGDICPVQAFIGGIAAQEVMKACSGKFTPLQQFLYF

DALECLPEDKSVLTADACKPRNCRYDGQIAVFGADFQEKLKGQKYFLVGAGAIGCEMLKS

WAMMGVGCGPNGMIHVTDMDRIEKSNLNRQFLFRPDDVQKPKSGTAAAAVRVMNPEINVK

AKEDRVGPETESIFTDEFFEALDGVTNALDNVDARMYMDRRCVYYRKPLLESGTLGTKCN

VQVVIPNVTESYSSSQDPPEKSIPICTLKNFPNAIEHTLQWAREEFEDKFAQPSANASQY

VLDPKFMERIMKQQGSQPIETLESLRRVVDTEFYMSFEKCVEWARNLFQANYSNQIKQLL

FNFPPDQMTSSGAPFWSGPKRCPHPLEFDADKELHLDYIEAAANLKAFMCGLPPVRDRAT

IKEMVLKVKVPEFVPKSGVKIAVTDAEVVNSDSTDKDSFESIKNALPSPDNLKGRQLNIV

DFEKDDDANFHMDFITAASNLRAENYSIPPADRHKSKLIAGRIIPAIATTTAMVVGLVGL

EMLKLVQGHTNVERFKNGFANLALPFFAFSEPILAPKSKYYDKEFTLWDRFTVQGEMTLQ

VFLDYFQKEHGLEITMLSQGVCLLYSNFMQSGKQQERKSLPLSELVKKVSKKKIPSHVRA

LVLEICCDDKNGEDVEVPFVKYVLPRS

>SG-AL_contig_1231_1_-1_31_348

MAFLLALATCSRRSKIHALSGAKDDIGFHSNGDLALTVLPEFIAKTQQPGSRPRSFRIPS

LVSCLDPRDARSEERLLCPVCCVKYYLKCSAPRRSSQQSLLISLNE

>SG-AL_contig_1232_1_-3_3_227

AKTQQPGSRPQSFRIPSLVSCLDPRDAGSKERLLCPVRCVKYYLKRSGLGIPLSRDSSSP

SARTTRGTSQRPPSP

>SG-AL_contig_1233_1_+3_204_347

MHRKSQKIINSFSLSHSRRGSRYFVIYLREMWTCFFSQTYFFFSFLTR

>SG-AL_contig_1234_1_+3_93_422

MQVYGIPPKVTMAIENIYNNSRCCVRTEDGLSDWFEVLTGVHQGCLLSPLFFALSRDWVL

KKATEGKGIQWHEGERLSNLDFADDIAALTSDTQDLQSLVTDISEDASSI

>SG-AL_contig_1235_1_+1_301_579

MPQAHVLSRTYSCAHMLPTPCSLCPPPPSFPHTCGLEHAYVHIHTHTHTKWIQHNHGICH

NSTHDHPPTLHPSHTHTQKQVNLETKLKVFMIL

>SG-AL_contig_1236_1_-1_1_246

CHGFFNVHKSNAAQGSQFIISSERLASRPSIKVLWRREKNPCLNRNQTWVSCILGWCFIH

WATHNTIHVLPKLYICKNLKTD

>SG-AL_contig_1237_1_-1_1150_1338

MNAVGDEFHLILNVQNTQKLPCGNDGGSFSINSIPTFWKFCARNFLFSIAFFVSAFFPSF

TVV

>SG-AL_contig_1238_1_-2_68_1834

MPRPVNVRVTTMDAELEFAIQPNTTGKQLFDQVVKTIGLREIWFFGLQYLDSKGYSTWLK

LNKKVLSQDVKKESPLQFRFRAKFFPEDVSEELIQEITQRMFFLQVKEGILSDEIYCPPE

TSVLLASYACQAKYNDFNPNETLTNDRLLPQRVFDQHKLSKDQWEDRIRSWWGEHKGMMR

DDSMMEYLKIAQDLEMYGVNYFDIKNKRGTDLLLGVDALGLNVYEKDDRLTPKIGFPWSE

IRNISFNDKKFVIKPIDKKAPDFVFYAPRLRINKRILALCMGNHELYMRRRKPDTIEVQQ

MKAQAREEKLAKQQERAQLERMRLEAEESERMRKDLEEKLKRMQEEQDRTTKDMQDLQAR

IREKDDKVAELLTLREELEAERCRAEELRLEMERSHEMEQSERQKLADELESARSSLQEK

LSDIQTKEQNVSELNTELEAARREAEERNEELSRVRVEKLQREQELQEKLQEQLQLKSEM

QSTHDENDEPEEEENKTEYGGELQIDEELASKPRAEEERLTEAEKNARLQKQLDMLKADL

GKERLDDAKVTKNDELHAENVRQGRDKYKTLKQIRQGNTKRRVDEFESM

>SG-AL_contig_1239_1_-2_2_247

RTKRHRQYIINWGERKLCEGLLEKRKWNGPGRQKKTDDSKGGEKDRNKRAIEGEISSTEF

PEGEDTIYTERAPGIPTGENQR

>SG-AL_contig_1240_1_-1_187_2190

MKWFLLPYCFAALLLSTAWIVQADDDEEEGETKKKDKEGVGTVIGIDLGTTYSCVGVFKN

GRVEIIANDQGNRITPSYVAFTSDERLIGDAAKNQLTSNPENTIFDVKRLIGRMWDDRSV

QKDISFYPFKVINKNNKPHIQAAVSGETKTFAPEEISAMVLGKMRDIAEAYLGKKIVNAV

VTVPAYFNDAQRQATKDAGTIAGLNVMRIINEPTAAAIAYGLDKRDGEKNILVFDLGGGT

FDVSLLTIDNGVFEVVATNGDTHLGGEDFDQRVMDHFIKLYKKKKGKDLRKDNRAVQKLR

REVEKAKRALSSQHQVRVEVESIFDGEDFSEQLTRAKFEELNMDLFRSTVKPVKQVLDDS

DLKKEEVDEIVLVGGSTRIPKVQQLVKEFFNGKEPNRGINPDEAVAYGAAVQAGVLSGEE

DTGDLVLLDVNPLTMGIETVGGVMTKLIPRNTVVPTKKSQIFSTAADNQPTVTIQVYEGE

RSMTKDNHLLGKFDLTGIPPAPRGVPQIEVTFEIDVNGILRVSAEDKGTGNKNDIVIQND

HNRLSPEDIERMINDAEKYADEDKKLRERVDAKNELESYAYSLKNQVNDKEKLGAKLSDE

DKEKITETLDDAISWLEGHQDADTEEFKDKKKEVEEVIQPIISKLYEGAGGAPPPTGDEG

DEQERDEL

>SG-AL_contig_1241_1_-1_1_345

LPVSERINYKVAYTCFSIRTGSASTYLSELVTLYIPPCSLCSSSDSRLLCQRRYKRKCHG

YRSFSYYAPQLWNSLPLHIRHSCSLAVFKSNLKTFLSAVPSMAIFTDFCHMTDTC

>SG-AL_contig_1242_1_-1_190_588

MLKPLQKIKTQLLALFLSHTINSQALLKKVHWLRISERISYKVVCICFNMITGSAPTYLS

ELITLYKPTHSLLSSSDSRLLCQGCNNRKCHGYHSFSYYAPQLWNSLPLHIRHSCSLAAF

KSNLKTFLFQQCY

>SG-AL_contig_1243_1_-2_143_349

MSDLLCAFSILDQWLLQLLLPFSEEKSLYAVTHNTQKDQQSYVCIYFGFVGCCWVSSALH

HCNGEIFYF

>SG-AL_contig_1244_1_-3_135_614

MADKKAPKQLLAEIGQADVNKMKHVQTSEGNTLPTAQAIAQEKTEAELRTNIGAFDKTRL

KQTQTTEKTVLPNSEDIGKEKTEAELRKSIGEFDKKRLKQQQVAEKNPLPSPDAIAAEKK

EEEFKQSITGFNRRSLKETKTEEKNPLPTKEAIQQEKAAK

>SG-AL_contig_1245_1_+1_1_381

DILCASFTTGNRVTGKTTDGGWVFVFLIGGGGGGVCFLGRHAQFYCFKQQNSTDCKIKSL

KQGLEQINYAHLPHPCQFAGPPKKHFHRLSELFSDLEFLNNTLHCQSARPQKKKFIAFLH

FHYSEIP

>SG-AL_contig_1246_1_-1_169_954

MRKLEAGVHIVVGTPGRVYDMINRRALDPTNIKMFVLDEADEMLSRGFKDQIYDVFRFMP

AEIQVILLSATMPADVLEVTHRFMRDPVRILVKKEELTLEGIRQFYVQVEREDWKLDTLC

DLYETLTITQAVIFCNTRRKVDWLTEKMHARDFTVSAMHGDMDQKERDVIMREFRTGSSR

VLITTDLLARGIDVQQVSLVINYDLPANRENYIHRIGRGGRFGRKGVAINFVTTEDIRTL

HDIEQFYNTTIEEMPMNVADLL

>SG-AL_contig_1247_1_-3_480_716

MHTYIHTYVHTFVQTCTHTYIKTYIHTHMHTSIDTCVMKGVCSRRIHLHSVPYLPYTNLP

AIIWQTLWACARPYALARS

>SG-AL_contig_1248_1_+1_304_501

MHAHLKFPEYNSHPCKLVQTDWKGSLYLCINRRNKCLKATSDTFTILMSFASSTALHINN

IKQDLY

>SG-AL_contig_1249_1_-3_213_344

MTGTEREITFKPSFGAKVYSPSTSLLCIEIQPSPFRPFFSPQFV

>SG-AL_contig_1250_1_-1_325_501

MSQIIHDAIRLVFDLILAGCQFSQVIHMSALHTCATSAWLHFYKWTKLDFFFLLKSRVG

>SG-AL_contig_1251_1_+3_1296_1535

MLAMGEAEIFNNSLPTERSGYGKTFITLNKGDTVLRTGNKHNDYPDNVAKCLVQQSTDGT

GAVLVHGIYSEHGDAVLRKF

>SG-AL_contig_1252_1_-3_6_449

MAAPKEKSVTNPLPEDIWMMRPCEIYLEEYTDCTSYKGRFHQYYTQGSYADCERWKEDYK

NCLQFRNMGDVAAAESIIASETERRAARLEGARGNKVWEYRQSPPVDWNSPMADWTKQRQ

ESVLAKAQAQKDSTGTLSPPESNRCVIS

>SG-AL_contig_1253_1_-1_136_1941

MPNNRHQRNHRQREEDAHNNNQQAPAIPAVHHPREVAAGAGNAMAHHAQRNDNAGALRKC

CTPGTCRSPDDTIDPEDPFDAVRVLCNNENCGEGCWMHKDCFEDFEQSVLAYLRSCGRAR

SWSEKQRLQNLWTKKGYDLAFKACDCKCGRGHLRKDLDYFPAPFNENSKKQKKHKKKNDK

PMPVVSTTHKSNSHSNQVGHQNGSGPAATAVATTTIAHPPTGTRAHQNHVTTNGNSSRTE

KKGKKTTTTMRTQDAHSNDNSHVQQLGVLDNHSKVATTTTTTNPHIITNSNNNVIHGVLP

GSGNPQLRLRTNSVSSTGSAGSHSSSSSIPSSAGSASPISSSPVSGDMFFKSRKSSDASC

DSGPTSLMGFRQRLDLSAFASLPRNKQNPYHIRMEDSSTLAYSFSTCSIAATKVYPLAEV

AAEAGSCDVETRNFVLGNLLNKKMCSSRCVLCKTVLPVFDRFPLLDGTFFLSPQSYDEAA

IQVIWNGRLQFLNAACLSCLEGHSSVKCLACKRLWDGCSLVIGSMYTYDIFAAAPCCQKR

LTCKCCRRAVVDITKGLEFYSQYSQMIICPYCKANDYHFIRSLLETFTISEEGAGQSEPI

CH

>SG-AL_contig_1254_1_-2_128_304

MINPVVEWWVFFFLGIVEYWNIYYVESGLQRLMLITHITCDRIVLYSNFICFCIRKKLF

>SG-AL_contig_1255_1_-1_1_171

ALHCGDMLSLRTRTQPFLPTSDGSAYSLLLAVDCFHVQTPVCDRLNLLQYNLILFQY

>SG-AL_contig_1256_1_-3_243_1946

MKKTYIEVAEERLGRKKSKPSKPYISEEVVELAKQKSQARKSGRKEEYKKLKRMIKNKIR

RDKKNWLEEECAKINEHNEYRRSRELFQQIKKIQSTKFQSRNECINSKDGITLTEPEHIM

NRWNEYGASLFSINGPQDNLYPDPNCPEPEPEPLLSEIEAAVKQLKPRKSPGLDNVPGEL

LKHAGEGGLKAIHHLCCKIWNTHQWPTDWKLQEFVMLYKNGNVKDCNNYRTIALISHASK

ILLIIILNRLKPKIEEELSDCQAGYRSNRGTTDMLFVLQILTEKIKSTDEEVFITFIDYS

KAFDSVIHLHLFNTMVKMGFPKHLISLIASLYKDQKATIRWNSRKCELFNIHKGVRQGCI

LSPYLFNIYTEQVMRDADIEDMGIKIGGKNLTNLRYADDTALLADNITSMKRILYRVDAA

GQKAGLNLNAKKTKVLHIGGRVTENNQNILINKVPLENVKEFKYLGSIKTNDGTCAKDIK

TRIGMAKQKMVQLNNIWKDRGIPNDLKIKLLKCLIWPVMIYGCEAWTLRKADCKRIEAAE

MWFYRRLLRVKWTDRRTNESVLKELDTT

>SG-AL_contig_1257_1_-3_177_479

MAASPVISELSSSFNCLLYSAPVADMQAMCFTLALFAETPGMTPGYKMACEMALNFYGCK

ANTSLPVTAVPIPPSTTTTPPTTSTTAATTTTTPTTTTPTT

>SG-AL_contig_1258_1_+3_3_182

LSPIIMPSFRPICLQIKSQSAFCPTTILTNFSRQPQAKSAAGMRQQCPERVNKPKSSLFF

>SG-AL_contig_1259_1_+2_509_706

MASTSTRIRRRMTAEQIAELTMTYDSHNKEIGGSFTTESEDFEPDSESESDFDTDVNVHV

CLCLHV

>SG-AL_contig_1260_1_-2_239_451

MLQCENTRNWNSALVQVYWYLVYTAVSVVRVISYLGQIEVIYLALLILGPTFICACLLLQ

VFLFCFLFQTN

>SG-AL_contig_1261_1_-2_2_154

PLPSPLYPLTLHGKGMDNFKGWRAFFSSFLFLFFQFYLNELLFKLLTFLIH

>SG-AL_contig_1262_1_-2_191_319

MWWPLFSVSVCFVHVVLLPVVSSLALTVSCRRHVSERQTNQQP

>SG-AL_contig_1263_1_-3_447_1565

MGTVVKVVLVVLLAVCVHRALRIYTLLDGFAPYHYKHHPGPCKQVEGVEFGSEDFQTLSN

GQTFITSGLRYSFMSPKSNEHWTRHSVRGRIYLLNLRNLTARAQELKISSTSNFQTDTFV

PHGISVWEDKRTGRHVLFVVNHPFGGLLADRVEKFIYDPSKQELVHQTSFSDPSMLIVND

VQATGENSFYFTNCQYFRTPLGIMLELLLELPLTNVVYYDGDSYTVVDSGLRSPNGVAMS

PDEKHIYVASFLSQEILVYSRNPNNTLTRVQEYPVYTFPDNIQVDQKTGQLYVGCHPSFY

TFFAYTDDIAARQAPSQVLDLRVENGVITSSEEVLYDHGELISSSSSALVFDKGTLLVGS

VFHRLVTCQIHVH

>SG-AL_contig_1264_1_-3_3_278

HLLSLSLSLSLSLSLSLSLSLSLSLSLSPRNTVKFLQFLLQLQRRCSYDITCLSMDGNVI

HLAYTLPFRGIPQSCPLIVLLFVCSIDYDIFN

>SG-AL_contig_1265_1_+2_2_211

FFEMSMCKCNVAFICISEDGSNTFKSNILHLYTYHKVLQSPKMHLHEFFLLFHSSFHSCH

VHEGKQIYKA

>SG-AL_contig_1266_1_-1_52_567

MLKRMEETTEHLRALTSSMGELDRELKEAILMDGMTNDIAKRSSARNDDGVNCRLHLRED

ICQVWELMKDRADFIHSPSSAGKSSVDESVPNYYQFGSGPEEYAGSDSHPERRLQQSWTH

PGTRKKIDVLQGEHGFQLQDRRIRQHRRLLRLPQFSTESRQETLHFRHLDES

>SG-AL_contig_1267_1_-2_2_226

HYHHHRHHHNHRRRRRYCSLPHYRCCHIISNRFVSSSHHYHIIFFIVIMTSILSDLHPSP

HPFTPPPPHHHRRAL

>SG-AL_contig_1268_1_+1_331_633

MFSFFAGFLSTFYSTSSQVAVGLPFCTFIAAFCLFTFSFSSHSWSFILYLHCCASVFFSS

CALFFFSQLLDSFSFLIFLLSFCFFISANLSFPASCCLFLN

>SG-AL_contig_1269_1_+1_2503_2718

MLTVNFLNMRMRHPDTERKEKKCVFKDAKNTQPKLKSNLAFFDKNQYKLLQIMNKNQYKL

LQTMNKKRGILP

>SG-AL_contig_1270_1_+2_908_1132

MFFQNFLSQICYNTPLPPTSSIRSFALILLLSQKTFDLQSNVPTATVFEFIIFSNAALHI

LFCTHLWKSGLPGVV

>SG-AL_contig_1271_1_-3_69_245

MNCDFLQVFFSGLTKFCFQRGRLLDFDGTLRGDSPDMAIAVGWAINNVIIIIIIIITGI

>SG-AL_contig_1272_1_-1_1_129

LYFVDVCLFCVLPPVHPAVNGYLTFFKVREGKAARERRWAPPS

>SG-AL_contig_1273_1_-2_1349_1615

MQACFCVHICIRHFIIDHHSPPLPENPLFFNLQACFSSFTFLWLYICNSVVIFCCCLAYM

CVAVSLKSKVSLSLSLSLSLSLSLSDTIQ

>SG-AL_contig_1274_1_-1_1_243

EGDENEEGEWQRLTPPHAQVLGVNQSAPPHMAVPGLGLPWGGLPPPLRPPLLLSLAPRRR

PPLPADHQGPSVSPQTPAGAL

>SG-AL_contig_1275_1_+3_114_407

MGQWIKHTKIMMNLISTNFNMAAGAPINVEATLPGTGQTIPLAPGTRLGDREAERYPEGL

VESCPEPSRIPDLDAIMAKHQTGKKEEKIRLTMTLGKG

>SG-AL_contig_1276_1_+2_116_583

MKNNQLCSSVHKVPGAILVLHFPSTELCFSYSLIPYCEVAHQPNRQDYEKKAQPCGFWCY

PEVIKVCGSHCHEALHCWVKNVTAIMCTTMRICHFLLPFNLQRVSHFCPFSYGTHFVLRN

IVHFPWIPFWFRRARHVYDLYALEQKTFERKFNRRC

>SG-AL_contig_1277_1_+3_6_215

MRLGEFYFLFHRAPGGVLGEGDRKSSPLLAFKTSSNSIHFCFFVLLYLADGVSSVEKDPE

HRASVLQMAV

>SG-AL_contig_1278_1_+3_1215_1415

MLANLNLVLLTKVLLILNVQHSWVNLCGNYLETPAYHHDMKIYSLYSFKQNKTPQLSTCI

IIFMFTT

>SG-AL_contig_1279_1_-2_32_1147

MGDEEMSSGYEGHMSKRQKTDDGMHGMGGRDRFDDPHKPNPSPVLHVRGLSEAAIEADLV

EAVQHFGAVRDVIMMPRRKQALIEFEDINGARNCVEYVQSSAIYVAGQPAFFNYSTSQKI

QRPGDPEDNRPANNVLLFTILNPQYPITVEVMHTICSPYGQVLRIVIFKKNGVQTMVEFD

NIESAKRAKQSLNGADVYSGCCTLKIEYAKPTRLNVVKNDAESWDYTNPSLGKDPHHQQR

GQPLLQEPRFGNAPAPYNGPADMRGGPAGAYGGGGFGGGYYDDYDGYGGRGPFGGPPDRF

GGPRQDRFGPPGRYGEQSMGPQASGQGCVVMVYGLNMEKMNCDKLFNLFCLYGNVVRIKF

LKSKEGAAMVQL

>SG-AL_contig_1280_1_-1_97_213

MDILYRLLIIIIMHVGLKCEKYLKKKSCLFHTKFCALVV

>SG-AL_contig_1281_1_-3_1569_1871

MSMNRQADDQTCSFFKVLVTDCARFVDTFSFNIIILFHHLSYRHFRSLVSLEVTTAFDLF

HNQLRIMYTLEKKSPKYTHVLNIIISVMFFKACAAELSRVE

>SG-AL_contig_1282_1_+1_85_333

MSVLSFALCTGCLFQTELNINWIPCATNLFISLLHPTFVTTSIFIRQLAACAQLQTLFPF

RFLDLNYQLLASVLLLLQDHLPG

>SG-AL_contig_1283_1_-2_74_760

MSTAGVFSDFQQYLDQDHELKEEIRTVVRELEQTAREIQSVMQAIHQTAAPEQVLLACEK

ATPLFAKVREQFSELASKIPVNQYYRFNDMWRFVIQRLAYLSALIVYLTQDQLVSQQETA

DMLGVKVRREDGFHIDLDDFLMGLLTMASELSRFAVNAVVSGDPGRPIKVSRFLAELDSG

FRLLNLKNDMLRKKFDSLKYSVQKVEGVVYDLTIRSLVPSGDGDVPSKD

>SG-AL_contig_1284_1_-1_85_2502

MADAQNEEDLATAILKKKERPNRLLVEEAINEDNSVVSLSQAKMDELQLFRGDTVLLKGK

RRKETVCIVLSDDTTGDDKIRINRCVRSNLRVRLGDVVSIQACPDVKYGKRIHVLPIDDT

VEGITGNLFEVYLKPYFLEAYRPIRKGDIFLVRGGMRAVEFKVIETDPAPHCIVAPDTVI

HCEGEPVKREEEEEALNEVGYDDIGGCRKQLAQIKEMVELPLRHPQLFKAIGVKPPRGIL

LYGPPGTGKTLIARAVANETGAFFFLINGPEIMSKLAGESESNLRKAFEEAEKNAPAIIF

IDELDAIAPKREKTHGEVERRIVSQLLTLMDGLKQRSHVIVMAATNRPNSVDAALRRFGR

FDREVDIGIPDATGRLEILRIHTKNMKLGTDVDLEQVASETHGHVGADVAALCSEAALQQ

IREKMDLIDLEDDTIDAEVLDSLAVTMDNFRWALGKSNPSALRETSVEVPNVTWEDVGGL

ENVKKELQELVQYPVEHPEKFLKFGMTPSKGVLFYGPPGCGKTLLAKAIANECQANFISI

KGPELLTMWFGESEANVRDIFDKARSAAPCVLFFDELDSIAKSRGGNVGDGGGAADRVIN

QLLTEMDGMSAKKNVFIIGATNRPDIIDPAILRPGRLDQLIYIPLPDEKSRVSILKANLR

KSPVAKDVDLDYLAKVTQGFSGADLTEICQRACKLAIRLAIEAEIRFERERAQNPDADME

VEDYDPVPEISRAHFEEAMKFARRSVSDNDIRKYEMFAQTLQQSRGFGGNFRFPGQTGGS

GGQGSSQGGSGGGNFGGEDGDDDLYS

>SG-AL_contig_1285_1_+2_179_448

MTIHPFYSLVCLVLAAWKITPYNGLNNNLVWECTLIADSTIQKTSDVRIFITQGSQRSDG

RGHRMVKTLDFQTGGFGEPGFESSGTSPGG

>SG-AL_contig_1286_1_-3_21_506

MSGIALARLAEERKSWRKDHPFGFVAKPTKNPDGSLNLYNWECKIPGKKGTMWEEGLFSL

RMLFKEDYPSTPPKCKFEPPLFHPNVYPSGTVCLSLLDEEKDWRPAVTIKQILLGIQELL

NECNVKDPAQAEAYTIYCQNSAEYERRVRAQAIKFRDPSIAH

>SG-AL_contig_1287_1_+1_748_1149

MWCLDHISSQQTNISSQHINRISGQKVLDGIEISQFIVNKQKVHGDKRHINDRSTFSKHT

YSHSSENLHITHLHKYQTFLHPVLILMMHSNNHVLILMMLSDSGWITLLSITAIPCLMSN

AFMEKVKKNQKTIT

>SG-AL_contig_1288_1_-3_3_368

TAFLLAWSTCCRRSEIHALSGAKDDIGFHSNGDPALVVLPEFRAKTRQPGSRPRASRIPR

LVSCLDARGEERLLCPVRCIKYYLKRSAPRRHSQRRLLISINEFYSRDISVTTISRWLPT

TF

>SG-AL_contig_1289_1_-1_43_213

MSIQGGCSRSPIPRNLNHRKWKFWWASHSQGTLTEAGDGLPATRCQLPEFFTSHQAL

>SG-AL_contig_1290_1_+2_2_295

TFQLFFFCHDHLFRIWSPSFPIHFSQPQLFQNTPPRMLTFIHSENASFTITHHLYNSSYT

ITYITVLSTQSHILSFSPSKHKIKTSNNNTCHFNTFKK

>SG-AL_contig_1291_1_+2_2_487

TLSLHDALPICFPLSFPAVLSDVCTSRPTLSLPSDAIPPWSSPPLRLSVPISWFALLSRC

EFSQAPLSLCLAERWPSTCLKVRLSVPERKQHCPHPQTDPSSLQQAPELLPKSQTPHLGF

VQHQWLRNLQDLLILIVHEPEIPLQDLLQWFGQMRTRQSLPC

>SG-AL_contig_1292_1_+3_402_539

MCVSPHYNADLLCAVYTEFTVRASFKQIIKNKGVTLGQICIFFCVF

>SG-AL_contig_1293_1_+3_81_206

MCVSPHDNADLLCAVYTEFTVRASFKQIITNKGVNLGQICII

>SG-AL_contig_1294_1_-3_711_1370

MSRPELPHGYLGPQGSWQEWSMSTVSHNSPEICTEKNSVEIKIRTPQAMKVTANLITCGS

EQEHKEQVFTQTQGDIVSLILEMPDAGYFKLQIYALPSKDQSKTLPNVYNYLIHNSAAPC

GPVAAFPKQFAQWKEGCYLWEPLHLDNKDCADFKVTIPGAKAVALTVDTEWFHLQKGSDG

VWQGKVTGLQKFKGKVNKASLNANYGQDETKYTSLLEYNL

>SG-AL_contig_1295_1_-2_2_166

SFITLDNLDMEIEKLLNLRQDYNFAIDKQGNKISGSISNVDSTKMKDSLEQQDLV

>SG-AL_contig_1296_1_-1_2662_3111

MRRRRFKLPLPSVILGNAQSLGNKCDELTACCQYMHEFREANVLCFSETWLKEDDADPDI

PGFNVFCCDRSAGNTGKRRGGGVCVFVNNRWCTNVTVKEAFCHEDIELLTVALWPFYLPR

EFNQVFITVVYTHPRANVNTAAHMLSDVTH

>SG-AL_contig_1297_1_+1_157_375

MYCVRKKLITTKYTKQITIRNFSKKLCLLMVIYICLIWHITKYNNSTLSYFKNFISLHHI

LSCGNFKTEAQAL

>SG-AL_contig_1298_1_+1_274_402

MGPIGIKYSKMIEITPKWKVDISLRTAQAITTPSYTANFFSTQ

>SG-AL_contig_1299_1_+1_2449_2688

MLNDLDYAWISFQAEMTYLQQKYCAVHLSMDPSCLQEFSADLVTTNASVTRLRTTNPSVT

SPNKTLGYLKLLPFLTSLIR

>SG-AL_contig_1300_1_-2_224_457

MWYHPFVMPCGQKQNTNRKTQTLAYTMNQKTLSSTLDQKTVSSILDPNPPTFVMHHGPKL

SCTLDQRLHDNNSIYCSS

>SG-AL_contig_1301_1_+3_2100_2339

MVKHKSPVIFMTFLPIAFFCTKPFFFSHLLLSLSSPFHSSIRTMPLSAMLPDGFCSWTSK

STRRYYVQVRAVKCTQYIYR

>SG-AL_contig_1302_1_-2_113_538

MAGNFYFVIVGHHDNPVFEMEFCPPNKAADPKRDDHRHLNQFIAHAALDLVDEQMWSTNN

MFLKVVDKFNEWFVSAFVTAGRMRFLILHDVKNEDGIKNFFTEVYETYIKFAMNPFYEIN

TPIKSQPFEKKVQFFGRKYLTG

>SG-AL_contig_1303_1_-1_22_5100

MQKKHGLLEADVVAHQDRMEALNSQATLFASGDHFDKDTIGKKAVEVKERYEKLKAPLVA

RKQKLTEAQSLQQFLRDVEDEEDWIREKEPIAASTNRGRDLIGVQNLMKKHQALQAELAG

HEPRIQNVCATGQTMVKEGHFAHDTIKGKINDLQDRWQQLKDKAGQRKQDLEDSLQAQQY

FADANEAETWMREKEPIAANTDYGKDEDSAEALLKKHEAFMADLEAYRSVIEGLRDQAQS

CKQQEAPVIDDLATEKVVALYDYTEKSPREISMKKGDVLTLLNSANKDWWKVEVNDRQGF

VPAAYVKKLDPSLSASRNNLMDEFTISVRQNQIETQYANLLDLANQRRENVKESCKAYQL

VREAAELAAWITEKENMMIGEEVGEDLEQVEEMQKKFDDFQKDLKANEARLQELNNIADR

LTAMGHTEAAEKIREQITQLNQRWVTLQEVSAERAQTLGSAFEVQRYHREVDETKDWINE

KDAALNNDNYGHDLASVQALQRKHEGLERDLAALGEKVRDLDSNAQRLMQTHPDQAESIY

EHQTEINEMWNSLTIKADARKGKLLDSYDYQRFLSDYRDLTSWINSMMALVSSDELAKDV

TGAEALLERHQEHRTEIDARAGTFQAFELLGQQLLQNQHYASDDVQDKLEQLAKAREELE

QAWIVRRMKLDQCLELQLFYRDCEQAESWMASREAFLAGDSVDGDNVETLIKKHEDFDRA

INSQEEKINALQQFADQLVTAKHYDSPAINDKRDSVLDRWSNLKDALIDNRSKLGEAQTL

QQFSRDADEMENWLQEKLQIAMDESYKDPSNIQSKFQKHQAFEAELAANADRLQGLLGTG

QNLIDRRQCAGSEDAVQARLESLASQWEDLVAKSTEKSDKLKDASRQQTYNAGVKDMEFW

LGEVEQMLSTEDYGKDLASVTNLLKKHQLMEADITAHDDRIKDLNDQADQFVEAQVWEAE

SINDRKRTINERYEKIKELAIHRRNRLNEANTMHQFLRDIDDEEAWIKEKKLLVGSDDYG

RDLTGVQNLRKKHKRLEAEIENHEPSIQAVHETGESLMAESEIGASDIRARLDQLTQSWE

ELKEMASTRGQKLEQSYAFQQFSANVEEEESWISEKQHLLSAGDFGDTMAAVQGLLKKHE

AFETDFQVHRDRCTEIKKEGQRLIDEGNHNADSITERIAGLEQKLSELEAVASQRRSSLA

DNSAFLQFMWKTDVVDSWIADKETQVRSEDFGRDLSSVQTLLTKQETFDAGLQAFEKEGI

QTITTLKDQLVNARHAQTTTIERRYNDVIARWQKLLADSEGRRQKLLLLQEQYRQIEDLY

LTFAKKASAFNSWFENAEEDLTDPVRCNSVEEIRALREAHDQFKASLSAAQADFNQLAAL

DKQIKAFEVGPNPYTWFTMDALEETWRNLQKIIKERDTDLEREERRQEENDQLRKQFAQA

ANAFHGWLTDTRSSLMESSGSLEEQLEATKRKATEVRSQKGQLKKIEDLGAAMEERLILD

NRYTEHSTVGLAQQWDQLDQLCMRMQHNLEQQIEARNRSGVSEDALREFSMMFKHFDKDK

SGKLDHHEFKSCLRALGYDLPMVEEGQTDPEFQAICDIVDPNRDGYVSLQEYMAFMISRE

TENVQSSSEVEQAFRALTSGEKPYITAQELYANLTKEQADYCISRMKPYVDKSGRAIQDS

YDYVDFTRQLFVN

>SG-AL_contig_1304_1_-1_76_5100

MESLNSQATLFASGGHFDKDTIGKKAVEVKERYEKLKAPMTERKQKLTEAQSLQQFLRDM

EDEEDWIREKEPIAASTNRGRDLIGVQNLMKKHQALQAELAGHEPRIQNVCGTGQAMLKE

GHFAQDTIKEKIQDLQDRWQQLKDKASQRKQDLEDSLQAQQYFADANEAETWMREKEPIA

ANTDYGKDEDSAEALLKKHEAFMADLDAYRSVIEGLREQAQACKQQEAPVIDDLGSERVM

ALYDYTEKSPREISMKKGDVLTLLNSANKDWWKVEVNDRQGFVPAAYVKKLDPSLSASRN

NLMDEFTISVRQNQIETQYANLLDLANQRRENVKESCKAYQLVREAAELAAWITEKENLM

IGEDVGDDLEQVEEMQKKFDDFQKDLKANEARLQELNNIADRLTAMGHTEAAEKIREQIT

QLNQRWVTLQEVSAERAQTLGSAFEVQRYHREVDETKDWITEKDAALNTDNYGHDLASVQ

ALQRKHEGLERDLAALGQKVRDLDSNAQRLMQTHPDQAEGIYEHQTEVNEMWNSLTVKAD

ARKGKLLDSYDLQRFLSDYRDLTSWINSMMALVSSDELAKDVTGAEALLERHQEHRTEID

ARAGTFQAFESLGQQLLQNQHYASDDVQDKLEELAKSREELEQAWIVRRMKLDQCLELQL

FYRDCEQAESWMASREAFLAGDSVDGDSVETLIKKHEDFDRAINSQEEKINALQQFADQL

IGSNHYDAPAITDKRDQVLDRWSQLKEALIDNRSKLGEAQTLQQFSRDADEMENWLQEKL

QIATDESYKDPSNIQSKFQKHQAFEAELAANADRLQGLLGTGQNLIDRRQCAGSEDAVQA

RLESLASQWEDLVTKSTEKSEKLKEASRQQTYNAGVKDMEFWLGEVEQMLCTEDHGKDLA

SVTNLLKKHQLMEADITAHDDRIKDLNNQADQFVEAQVWEAESINDRKRTINERYEKIKE

LAIHRRNRLNEANTMHQFLRDIDDEEAWIKEKKLLVGSDDYGRDLTGVQNLRKKHKRLEA

EIENHEPSIQAVHETGETLMAESNIGVSDIRSRLDQLSQSWEELKEMAANRGQKLEQSYA

FQQFSANVEEEESWISEKQHLLSGGDFGDTMAAVQGLLKKHEAFETDFQVHRDRCSEIKK

EGQRLIDEGNHNADSIRERIAGLQQKLGELENAASLRHGALVDNSAFLQFMWKTDVVDSW

IADKETQVRSEDFGRDLSSVQTLLTKQETFDAGLQAFEKEGIQTITTLKDQLVNAQHAQT

PAIERRYNDVIARWQKLLADSEGRRQKLLLLQEQYRQIEDLYLTFAKKASAFNSWFENAE

EDLTDPVRCNSVEEIKALREAHDQFKASLCAALADFNQLAALDRQIKAFEVGPNPYTWFT

MDALEETWRNLQKIIKERDADLDREERRQEENDQLRKQFAQAANAFHAWLTETRSAMMEG

SGSLEEQLEATKLKATEVRSQKGHLKKVEDLGAAMEERLILDNRYTEHSTVGLAQQWDQL

DQLCMRMQHNLEQQIEARNRSGVSEDALREFSMMFKHFDKDKSGKLDHQEFKSCLRALGY

DLPMVEEGQVDPEFQAILDMVDPNRDGHVSLQEYMAFMISRETENVQSSSEVEQAFRALT

SGEKPYITAQELYSNLTKEQADYCIGRMQPYMDKTGRVIPDTFDYVDFTHQLFVN

>SG-AL_contig_1305_1_-1_508_780

MTQRIKNLFELYNGRKRNSVATLLLGYACCQCKRVCVILTDWVFKFRFRRELGGRLSLSN

SITEKQLKYKNTAFCFCKIFRPSGDEKTLIL

>SG-AL_contig_1306_1_-1_1636_1791

MKSLLKLVTIGKVMFVKMNEIDLISMLVVLCLKPYNKIHIKLGFKMMSLSVK

>SG-AL_contig_1307_1_+1_475_726

MPEYILSSPKSKSGTGFTGHLLTITRLCTSTLLLTIKQPVQSFSIQKKPYNLITKPINHN

YMRQFSIISNFRTAMNPVCHSVPS

>SG-AL_contig_1308_1_+2_2_115

LLGEHVFINPSLSLSLSLSLSLSHTHTHTHTITHSINQ

>SG-AL_contig_1309_1_-2_2_319

FYVFGGKAIYFRRNLLFGGQFPSLVASKVGEEVLFPHLPQKLLILGTFLACVTLTLALFI

PNSCISDSSMVHTFGPNLIKICPETAEKILLTVTHFINTFQPRSRS

>SG-AL_contig_1310_1_+1_1_168

MKCVSNIFSAVSGQILTKFGPKVWTVEESLIISLEWQRPRSQGHPKCLKLAQWEGV

>SG-AL_contig_1311_1_-2_2_250

FFLVYPRGDAGGSFSVNSIPTFWKFCARNALFSVALFVSVLFLSFTVVCFFLPSRSIPLS

FFQQALHPPSSLCVLSVLFCSGD

>SG-AL_contig_1312_1_-1_130_2235

MRDFEDEVKFQVNGPDAEIDFLLDGLKLVEVPVTPNWLEKANNDTEKYRKSQFDIKFRLP

ADVDRNDLDVEVKLTKHQFAFGAKIQTDCILTPKAARYNNFSYSVFNWATIGAYKWRMGQ

AQPGPPDYYDAVDTNNVLLQHGFKVRAHCILWGVPDNVPNWVKDITDPTELRKTALDHVD

YMVNLTKSGLAEQWDVQNEWIHGHFYEERLQDPNFTMDVFKYVRSLDPDVKLYLNDFMAV

TTGAQTQDLVDIVKRFRKYGAPIDGVGIQGHTKDYIKPDPTMIWRRLDKFMTLKGLDVMV

TEFDVGSNDDVIRADWLEDAMRAFYAHPVLEGVILWGFWNIRMRYENKYLVYGDRFTLAE

GGIRYACLVKNEWTTHQNFNLSHYPDLTVSLNGYEGEYDLVVRKKGVPVQRKTFLIKTPK

TLKVTITGSDKPIALPEEYPDYVTECISHRNQHSLGNVTASTTATKASCVTRKQSRSLEH

VSVTCNEGELLLGCSGSYVSGSGYKEYSTSRQGERITMEAGVPTCHAYGKDLGQTFTVKA

IARCCQVPSLQCDYRQAGPSLQMAEALAETTCLDGASALGCAAYSEHAELSGVVYRANTT

GSCVGKNGPSRSPVFKSGVVTTAPCCTKSGMKCVAVRSTGTKSTSSGDRSQVKCPDSGQV

MTSCAGEGLSYRAEVLTGSDSNDVCTVFGVSALAIATCCSLA

>SG-AL_contig_1313_1_+2_1568_1849

MCPSRHDSSWYEHGQVGRKLLMTLLETLKSKAVQGGGGVISRSGRRKRRPKKARYLGNRR

IFRNSLCNNDYHSPGSIFSHSPTCSTSTTPRLTL

>SG-AL_contig_1314_1_-3_834_1823

MKMENTRFILVSVLLAACVVISECTEGSTECTTGAACFPKGMPDDLTPSDPAYYCCLQKN

MTRHSDDLPDGTKNCSCKPDFTLDSKIQNWADNTMHEGRMCSREEDCEGSKSSGSINFRF

KTCCWTNSYNGNNVYTATTIFDGDKEEYIGCRCVNHTIPTPDPAAYTVPPVLSYNSSGEA

CLYDKACKDKPYLKCQSSDVTFCCYGSRGIVTDNEGQVTRCTCTNQLFLEHCHPNPVSIA

MTTMPCQSDCAGRADGDKCTDAHNAERCCPNNMVVVASKTAGNFTSCTCKAQGQKNCAED

SSSAARLCCHLCDLIVFVVTALSLAQIARH

>SG-AL_contig_1315_1_-3_687_1598

MSLYPTLEDMKVDQMVQAQVQHEQRQAITQGYPPAQSDYPPAYNPAASASAALYPSLDDY

MGLHLTPQFVQENMPVVAASYPQNMQVAQRQSPSQSMVAPVTGNANKELMRAEIKQGIRE

VVLCKDEKGMLGVRVRHVNNGLFVALVTKNSPAALAGLRFGDQILTINGQTMAGWDNDKA

HNFIKKLDPQRIAMAVRDRPFERTVSMQKDSNGYIGFVFKEGKIKSLVKDSSAARNGLLT

DHHLVEVNGQNVVGLKDSEIGEIIDAGGRTITVTVMPSFLYEHVVKCMGNSLIKKLMDSS

IPDL

>SG-AL_contig_1316_1_+1_793_1125

MNKRTQNDRSGKYRQKKWEYKAEKEKKVFTAGHSPESSMIRFWRTVTAESSGRTVAADRA

QRVFTVGITRLGLKILLQNCSNIIFFFSNRQLIFENSFPQIVHLEPGEETL

>SG-AL_contig_1317_1_-1_706_1032

MCHTMCLGRHVISFGGLIKYYCIVLYCIVLYCIVLYRIILCCVVLHCIVLHCTVLYCIAL

HCIVLRLLHCIVLHSIVLHCNVLHCILLHCIALYCIVLCCIVLHCTVLL

>SG-AL_contig_1318_1_-1_145_3903

MASNMEEELVPSVDDQAFDPEPTPTYDAAFPALTSEHRELNPALVNTGAWQPKFQPRTSK

CTQVFTVPMVERRFKDPNEHKFGDETEQAKICKDIMSKYGVAIEISLARDQSMTVVINGR

SDNVMKARREVVSKLQTQAMYQVKIPREHHRFILGQKGKKLAELELATATKITVPRAEEN

LDLIKITGTKEGIEKARHEIECISNEQAKLAFERLPVQKAFHPFVCGPFNQNIKQLIDET

GARINVPPASVDKDEIVVSGEKEGVAKCKHVIMGIFEEKKRKCQTVSVEVKKSQHRYVIG

PRGNGIQDILQNTEVAVDVPPLDSPSETITLRGEQDKLGPALTMVYSKANSVATSEVTAP

AWLHRFIIGRKGANIRKITEEHPKVHIEFTEGQDKITVEGSPEEVQEAIMVLEGIITDLK

NRMDYAEIEVDPKFHKHIIGKNGVNITRIKNETGVSVRIPSDGDNSNLIRIEGDPAGVQK

AKQELLEMSQRMENEKTRDIIIDQRFHGTIIGARGENIREIREKFNQVQITFPDQGKKSD

IVTLRGPKQDVDKCFKHLQVLHKEMLESNYQAKVKIFKDFHKNIIGKGGANIRKIRDETN

TRIDLPGEHAESDVITITGKKEDVEMAKNKIEVIQKELANVREVTLEIPHRLHNSIIGSK

GRLIRAIMEDNGGVIIRFPQEGNTSDKVVIRGPKDDVENAKKQLLELVNEKKESSFTAEV

KAKPQYHKFLIGRGGTNIKKVRESTGARIIFPGSGDPDQEIITLMGKEEEVKMAKQELEN

SIKELDNIVESEMNVDMKHHRYFVARRGEVLRNIAEEYGGVTVSFPRSGVKSERVVIKGA

KDCVEGAKKKIAGIVADLEAQVTVECIIPQQYHRTVMGAQGMHVREITRMHEVGIKFPDK

PMPNGRRAEGEEEPVANGDVSSEDGSEKGPRKSDIIIITGKPEKCEAAKKALLDLVPITE

EVPVPFDYHRFIIGQKGRDVRRMMQDFDVNISIPPPSENSDLVRVTGPPDNVQRARKALE

ERVFQLDTEKEDRALKSFQLQVEVDVSHHPKIIGRKGAVISKIRSDHDVNVQFPDRNEVN

QSVIIITGYKHNAQSACDEILSLVKDLEEMKSVKVFIDHRVHPRIIGSKGRAVRRIMDDY

NVDIRFPRSDSAEPDIVCITGSDDAVQDCTDHLLNLQAEYLQDLMDQEYLQGFKHPPSKS

DDEPSHPADSHGFVVRNAPWHAAPDTASTEEFPSFGSVVRPKSGHAWGPMKKN

>SG-AL_contig_1319_1_+3_60_218

MSNVCKVAYLELRRISSIYHYLSTEATKHLCVLLSSQGWITVILFWPAFLNIC

>SG-AL_contig_1320_1_+3_2343_2627

MHRNITVTRRTNNASISKTNPQPPLQTYTFFFPSFIVHVSSCVQSSFSIFCYKICTNTTL

HFYWQQKSDSFDANTPVVGKQIYKWRSSPIKSHPF

>SG-AL_contig_1321_1_-2_2_1198

MATRTMVEHVVVDAGGFIRQAPIREIGEQVYSVPQVVQEITDKETKRRLQVLPYTLNLQK

PTPEAIIFVTDFAKKTGDYKHLSCTDIGVLALTYMLEKQHVGDEHIIKEPSKKPAEYIVP

SKGSSGQQIAGFYVEGETKIERKENSNKEVEEKEEEAKAEIGGDVLPEDLLEEETEENEE

EAEEENDDDDEGWITPSNINDIKRQMGIGLAAAEKTKVSVACLTTDFAMQNVLIQMGLNV

VSVDGMLIKQARSYVLRCFACRKITYNTMKVFCPNCGNKTMKRVAVMIDEEGKVHYYFSK

RPLNHRGTKYPLPTPKGGKHSNNPHLCEDQPMPQQRARKGKQVDALDPDYVAGSSPFAFI

DVTSRAATIGISGKHDFRTGAYWNKRNPNEARKRHGRKR

>SG-AL_contig_1322_1_-1_385_2055

MAVLFFLAFSAAVASVAQTASGSTRPNVLFLVVDDLRPRLGCYGDSNMVTPNIDQLASRS

VRFDRAYVQQAVCSPSRTSFLTGRRPDSTRIFDLEHYFRKLAGNYTTLPQHFKNNGYITQ

SVGKIFHPGSASGFTDDYPYSWTFPAFHAPTRKYTGKKICPRPDGKLYHDAVCPVDLKTV

PGGSLPDIQNTDYTIEFLKNRSMDHKPFFVGMGYHRPHLPFAYPKEYLNLYPMSKIHYAV

NPYRPPWLPDVAWTPWDELRHYEDISVMNISYPFGSVPLDYQLKLRQSYSAAASYTDAQV

GRVLHTLDQLGLANNTIVTFHGDHGWQLGEHGEWCKHTNFDIATRIPMMVYVPGVTSKET

HPGKTFPFQDALDKTLTFRPEKQILSPDIVSDAFVEAVDLYATISELAGLDVPPTCPDSP

FNVTFCTEGSSLVPVIKNLTSDSYDVSKPMTWKPAVFSQYPRPSTYPQHDSINPDLSDIH

IMGYTMRTDQYRYTQWIAYNHTTFFADWNTVYARELYIYASDPYEDLNVAQVPGFQGLVK

TLSTQLRAGWRNALPKP

>SG-AL_contig_1323_1_+2_242_457

MTLIMDKSQSHLHYTVLTLVRATIGPSLVKNCTIFSYTVLYNYLVTMDFPCQICPAPLKE

SVSPQLLVFFKI

>SG-AL_contig_1324_1_-3_90_428

MPETLRSVVAKFYDEYMQSTPKKLKVVDAYLFYILLTGVVQFGYCCLVGTYPFNSFLAGF

ISTVGSFVLGVCLRLQSNPQNKNDFTGISPERSFADFIFAHIILHLVVVNFIG

>SG-AL_contig_1325_1_+2_2_97

RLLVKGLNGGRLDGRINDKHIHLQKLPFCLPK

>SG-AL_contig_1326_1_-2_179_445

MFRQARFSRPVILPPILCTRRAYIQVFLLLLVGVFILVVRMMSSLRGTFLHYHLESDTDG

ELVILPTEFAQRHRAACLDGSPPAYYYRQ

>SG-AL_contig_1327_1_-2_5_1108

MQAIYYPPQRLSVKYHQRLGLSSHHDEYSLWFDVTAPPQPSEAEEPPGCDCGLHIATGRS

RLGMLSDETRNKVFLQVLKKNITSSTVCLCICDGSLLPIMAAKLGAKKVFVYDSSPPSRW

LLKSIIEENQLEERIVVLAKYTTEAEQEDFQGFEVDLVIGEPFFQGVSLPWDTLHFWYAL

NHLRCHRIGLSPSAKVFPATITIYAMAMHFKDLWKIRAPVGCCEGFKLHLFDNLIQDASE

LVDCSVEPQPLWEYPGRAVSQPVKVLSIDMNEDSKMGKTALDLEFKTEVEDRMCGVALWC

EMDFGEGLTISTGPRKTPVMGQPVEWNVYWKQAVYLIREPHDVHLGQRLKFGFEFLPSDA

SVILNTRC

>SG-AL_contig_1328_1_+3_3_137

FFFFFLFFCFKIFFFFFFFFFHNTKMYLGHCCEYVPVLLSGSDDI

>SG-AL_contig_1329_1_-2_452_598

MKLRQIRCSNDDFEYSDRNRNVNLNQNVIPTLLVPGPNWPSIGAFAWLQ

>SG-AL_contig_1330_1_-1_1_201

LILGTFGVPATLTLTLSIPNSCISDSSMVHTFGPNLLKIRPETAEIILLAFFQHTHTHCS

CVIRNLF

>SG-AL_contig_1331_1_+1_172_351

MCVHLCSGSKVPAQNERICVQEVNSPKTGLQAYNQWTSDVQKIFNPTKYIFLRCIKNTAE

>SG-AL_contig_1332_1_-3_48_470

MALTVDASSFETAPDAHCHLLPCTINKNGEANVQKFFETSVRNEGDGLTAAFRGRPLNGE

EVVLPSGYAGVVLREVGSAVTEDQDRLLRVDQRFTSFTAWNLDKPPSGDDKLNKAMQWID

IAKALHQPVDEENSQKSITGK

>SG-AL_contig_1333_1_+2_59_259

MYCIVLYYFSSQWIYHANLGLLSKGEHVTAVQHHPLFFFFFSACWVCSCFHKPPNTDMAY

RIFNVRI

>SG-AL_contig_1334_1_+2_971_1219

MEVEIRMMILPFQMGLSCVSTAVVCTILARASGFELSSEMIAPKHWKMEIVSSFCSPTDV

STDVIGANLLMISFVYSALLSMP

>SG-AL_contig_1335_1_+1_1_135

FFFFKDRNVFIVQDLFFNTESTYRKTMLNFIQTFCCSNGMSVYLL

>SG-AL_contig_1336_1_-1_385_498

MDELNKICEFTLITVLTLAGLRVQGHNCARSVEVCRKL

>SG-AL_contig_1337_1_-3_21_902

MEEAAAPGSSQNRPRFYCHKCSAEITPSIPDYTCPGCQNGFVEELAASNGSGASRDSSTA

TPPAEPEDVSSEIREVVHAVLSSAFGPEAPPTPSVARRRRHRRHLPYVEIRRRDQLDAEA

ASPVASLRVLRPARRQTEVGGLMHHFLSRLGQQATFVPLNVMHIHGNLGDYAWGAGAMDA

IVSQLLNQLENHGPPPAEGEKIDALPTVTISQEQVDRTLQCSVCMDDFVLGESVRQLPCD

HHYHSPCIVPWLKLHGTCPVCRKDLNGQDTSTTENSFQVPDDLHDSPSMDDPRT

>SG-AL_contig_1338_1_-1_406_870

MKTRHIYEKQNLINWASKPLSPVSVRWLLVREYTHHAQNVRIFKMMVDFKRKNKSTWSQP

CKEQTVAIMLDCLGLTETCEEVPSGRLGRGQVGYINISLKLSNSLYSDLMASAMIIWGLP

SCGICGVRVASSAHNGRHEKLTKVFYCVIVFILGK

>SG-AL_contig_1339_1_-1_136_486

MKWQYKEEHPFEKRRAEGEKIRKKYPDRVPVIVEKAPKARVGDLDKKKYLVPSDLTVGQF

YFLIRKRIHLRPEDALFFFVNNVIPPTSATMGALYQEHHEEDFFLYIAYSDESVYGA

>SG-AL_contig_1340_1_+3_204_395

MLMFQYYFTTQTLGTKTKCLFATENRTSRQQIVQGTCYYIIKKKRRKYQLFLVMCQKTFN

ISVP

>SG-AL_contig_1341_1_-1_1075_1254

MTKRLGMTRRAIAWKFARLGSRSRGHGSSWKFPSRAANVGFPLTCREVTQRVGESCLLIG

>SG-AL_contig_1342_1_+2_95_346

MDREGRKKQTTVKEGRAAETKRAIEKRTFPAQNVQKVGILFILNEPPASPQGGIKRAFLK

FRRRTNISTGDTVRKLHFSTLLRH

>SG-AL_contig_1343_1_+1_928_1224

MDIKTLCRKRRIPILLKQFKATRDSLMLLFKNWHTQQIQDGKKNNTLNCTRPLYFEHLAM

YLQKCKTSERFKDYWLRLQTVQRKQAPVTKTCESLPYQP

>SG-AL_contig_1344_1_-3_192_1445

MQSALLLAIVALCGVVFGQRPFDRDREDRDNTIPECLRKTADVFFVLDSSSSIYIEDYKE

VLKFVSQVITRFDVSRDDTRIGALTFSNDFQLGFGLDRFRSKGEVLASVNERSLPYRTGV

TNTDLAIRYVRENNAFRPDITKAMVVITDGGSRSPGSTKREADLARDAGFHMVVVGVGQY

LDEQEWRAIASDPDNDYVFNITNFSFLDSLKDVLPRRVCLMPPIILGGECRVTENSDLLF

LAAPNGINDALDVIEELADSFQSQNRLQVRYIMEVCQDEADTNFEGPDRFCDRFGDALPQ

NDETYVSLVTQLRNVATNMREERASNQVAVLFVDDLSMRNNRFGILQEVRNAAQFDGIDI

IAVDLGVREYSNFVNGMTSSRENVINYPGQSATTSVQQILDRICYHVNFSFGEEIRPN

>SG-AL_contig_1345_1_-3_936_1211

MLFSLLCHHFPPTPIPAIWVLLTFPSNISLQPLFPRFGFCCRFPPIPSHHPFPLLSAICL

PFAAIWILLPFALCGFIYFNFFFFLTSLVWLN

>SG-AL_contig_1346_1_+2_2_295

YVDLGDRKNLHPLPTRRGDRGGFEPRSSKPSSLKVKCLHHSTTALVIYLSLLYKQKVSLF

SENPKLETNSTTKTLWYTKPLDLILSYASVSAQSETFR

>SG-AL_contig_1347_1_-2_2_307

ESPPGSPAGSTASRSDIQSPVASPVGGSQKGSPPGSPAHSGSERGSAPASPTGSNKASSH

GSPPGSPAHEGSPPGSPAGSAHRSDRGSEAGSRKSSPAHSPA

>SG-AL_contig_1348_1_+3_3_161

RVVEIKFRWPSLNRDSGSDLPSIYDQIIQPMRGTVNVSPQWAPCTARIGKPVC

>SG-AL_contig_1349_1_+3_234_449

MYDAKYNQIKLLKLLLLLQLRERERERERERERERERERERVCVCVCVCVCVCVCVWGGV

GGGGGGVCVVRG

>SG-AL_contig_1350_1_+1_685_909

MIISNINKELKFYVHIYSGMFSHTHTHTHTLTRTGQHYRGLGDRNTRGKQIFLNLQFDLL

RKLHYLNTTHMLRIK

>SG-AL_contig_1351_1_-3_3_263

FSALSCQSTFFFKIPLSLSLSLSLSLSLTTQYSTHTAIHFLTTQGLCMKNCPCIYMSCFF

YLLRSGNVVLDDCIQIVVAVAHTVQTT

>SG-AL_contig_1352_1_+1_1171_1380

MHHTMFSLLRSHIRVIKNFIPSRIIFALCQKRFGRHWLITQFRTEHVLNSHMFIFRTEHV

LDSHMFILIY

>SG-AL_contig_1353_1_-3_177_1469

MSIKKIHARQIFDSRGNPTVEVDLTTDKGMFRAAVPSGASTGIYEALEMRDKESSYHGKG

VLKAVGNVNNVIAPALLQAGLNVADQNAVDEFMLKLDGTENKSKLGANAILGVSMAACKA

GAAEKGVPLYRHIADLAGNKNLILPVPAFNVINGGSHAGNKLAMQEFMILPTGAKTFTEA

MQMGTETYHHLKAVIKKKYGQDACNVGDEGGFAPNILENKEGLDLLKNAIEKAGYTGKIE

IAMDVAASEFCKEKKYDLDFKNPNSDASKWLLPDQLADLYKTFIKDYPIVSIEDPFDQDD

WEAYTNFTADVTCQVVGDDLLVTNPKRVQKGIDIKACNALLLKVNQIGSVTESIKAWKMS

RDAGWGVMVSHRSGETEDTFIADLVVGLCTGQIKTGAPCRSERLAKYNQILRIEEELGAS

AKYAGKNFRKP

>SG-AL_contig_1354_1_-1_169_333

MMFVLLFMILLMEMLLCSFFPPFPPLSCTCARAYTNDRSLPYHYHHCHCCCGCCC

>SG-AL_contig_1355_1_+2_2_142

PVCFSILRIQLKMLKPYSNEIATNIRNTPNAITVLVHVHVLSIFHSL

>SG-AL_contig_1356_1_+1_652_801

MSYYYIKNPMGNEDGKLKFYKMPHYWNCLFASKPRFHNFIIGVHITPNNA

>SG-AL_contig_1357_1_-1_460_1581

MKFSVFILFTSLAFVFAEDVKTEEDIYVLTEKNFDSFVKDNEFVLVEFYAPWCGHCKALA

PEYTKAAKILMEDDSDVKLAKVDATVESELASRFGVRGYPTIKFFRGGSDNPVDYAAGRQ

AADIVNWLKKKTGPPAHELKDKDSAKTFVEKEDVVVIGFFKDQESTGALALKKAAAGIDD

IPFAITSEDDVFKEYKMDKDGIVLFKKFDEGRNDFEGDFEEASITKHVRDNQLPLVVEFT

QESAQKIFGGEVKNHILLFLKKDGGEETINKFKAAAGDFKGKVLFIYLDTADEENGRITE

FFGLKDEEIPAVRLIQLAEDMSKFKPETTDLETATIKKFVQDFLDGKLKPHLMTEEVPDD

WDAKPVKVLVGKNF

>SG-AL_contig_1358_1_+2_1880_2224

MFLSLLIKIAQTLNTMKLTLTLVVATQVAPLYVYGQLVVALLGRSVWSVSYLSWIRKAIV

SSAFIRIFLSWAQEPAFVSFQAHLSKHVAKIQVIGRSFLTLSKMGHGCCSNHLKR

>SG-AL_contig_1359_1_+3_69_215

MLFNKQINYSEEKLIDKRARQNTTDLLFWHALPSAKSSFEHSVECRNFL

>SG-AL_contig_1360_1_-2_2_160

FDSLTIFCPCTFVMCYLRSETFRPCDRNERNGFTIISILTRLELERHMLTSSV

>SG-AL_contig_1361_1_-3_3_362

LFRSVQHSTFALFQRRSQKDSHHGVRFNLVLSPEKLRSGLTLLPRLQQHSWTHPQVWFKH

LQKVFPAVCRFHRLQEAELKMTVGSYVWTPSGLRVTDGSRLLNRLKSTDWTWASPHETIP

>SG-AL_contig_1362_1_+1_1579_1887

MRILTVFDGCCLSRDRIPLPLPAVCQPLHTCSFSLDSRWCCPTSLSASGQNSSEIILKHV

TVRPLPCCPLRFPLVVTAGSPVVSPSGSSHIASRRQQRKRHRC

>SG-AL_contig_1363_1_-3_198_341

MPLPCCFPALQEVRKRPCCAKERVNGKKNEEKWSYFKLLSYFVCIFEN

>SG-AL_contig_1364_1_-2_38_220

MGQALDDWEKKHLQCLSVTLGNKDLSCLIFSFCQIRSEVTLQHCWSARTPLEQFSNPMLY

K

>SG-AL_contig_1365_1_-1_88_303

MSERVTFTFESHNDPNTIIFIQHILSVTYKQSLPNFCLRCDLTQKNCLQSCSELIIEKIP

RQFLRACLSDSV

>SG-AL_contig_1366_1_-3_405_641

MMSLQPSDWKKSTQKPVQEKVLKWKSPCTATLPFSWLQKLGCSVTKSHHDIRLLSTIYNT

VQCCTTQYNTTQRNTIILY

>SG-AL_contig_1367_1_-2_2_199

FDIHHTDLKKEDNTNLLASLVKCHIANNYSNHLKIYTDGSPIEEEAGSAYLIPGLKLEKK

IISHRT

>SG-AL_contig_1368_1_-1_61_966

MASVFGRTSRQALLVAKSGTFSPKANFVSFKGKHGKKWVVAMVGAATVGALGVGAALATP

VLAEGLELHPTKMPWSHNGMLSALDAASVRRGYQVYKQVCAACHSMEYLCYRNLVGAIMD

EAEAKAEAEDIMVDDGPDEEGNMFKRPGKLSDRFPAPYPNAEAARAANEGAYPPDLSYIV

QARHGGEDYVFALLTGYCEPPAGVSLREGLHYNPYFPGGAIAMAQALYNEIIEYEDGTPA

TASQCAKDVCVFLRWAAEPEHDTRKQMGLKAIMVFSFMLAVSYYLKRHKWSVLKSRKIAF

KN

>SG-AL_contig_1369_1_+2_2_115

SLSHTHTTYYFIQICAQLQTNNKYILFIINTNRTSTNN

>SG-AL_contig_1370_1_-2_20_1432

MKNLYSTHPHFVRCIIPNEFKEPGLIDSKLVLHQLQCNGVLEGIRICRKGFPNRIIYSEF

KQRYSILAPNAIPQGFVDGKQVTDKILSALQLDTAEYRLGTTKVFFKAGVLGMLEDMRDE

RLSKIISMFQAFIRGYLMRKAYKKLQDQRVGLSVIQRNIRKWLALRNWMWWKLYCKVKPL

LNIARAEDEMKKKEEELEKTKQELEKTEKMRKELEEQNVTLLQAKNDLYLQLQAEQDNLA

DAEEKIEKLVGQKADYEQQLKELEERLLDQEDEAGEMGEKNKKLNDEVKDLKADVEDLEN

SLAKAEQEKQTKDNQIKTLQDEMAQQDEQIAKLNKDKKSMEELQKKTLEDLQAEEDKVNH

LSKLKTKLEQTLDELEDNLEREKKQRGDVEKAKRKLEQDLKATQENVEDLERVKRELEEN

VRRKDMEINGLNSRLEDEQNLVAQLQKKIKELQARIEELEEELEAERAARA

>SG-AL_contig_1371_1_-3_339_4994

MDSIRLGYDSKVYHMEQGEPCGDSPPPPPQPLSLQSTPPPPQPILGTESGGKNSVEAPPA

WTSRHGDQLAECQGCIAEQRIEHTCTDANPNHNHHQHHHPSRCPDHDHQFVDVQRKTDNI

DAHQLDVQETAQDLELESEIALSEQKPRLGRNTWPRKKVRQLRHAADIFDCDEDGGEDPG

EIMVESGSNYFVSVDDSVCDTGGRLVVEYSLSRTGSVHRIPRNSKNPLPPELETVKKGTP

LYGQPSWWGEDTDICEKDTMDLPQKADSKSPRPSSLVVQSDDSQRHHHQHKDSKAIYMEI

PHTATTPERDKPLSGHHRDKVTPARSISSLSKDSLVVSPDSARERSVDKDSDPGKAGSSE

MPSNPALSFTVEFGDEKKPKLKSGASLSQFVPPKLRSSLSSHARSDKASSRGRDSDGSCK

NSEERELSPHTSVRRRSEEQIGGDIISKGKSARRSSGQGTGVGSLRSADFASDAAPVSAK

SRKDAEKRTRDAEKDVEKKPPKAQDPRKPSTRATSSSTKSSKPKPQPTAAAIAASVQAAQ

YSDPTAYLIDRMFAGTTESNADSPRSDFSDRERSDSPTAPEHIMYKEAKDYDRANQRGGL

RSSSKPVQKQKSLSKSPASSGKSPASNGKSPAGNGKSPAGAATPKSPAAAVASPRSTASK

TGKCKTPEKLSKPLKEKNTEPDMVAPPMLPVAPSPLEGGGDQEDKVSEAGTYTIEAEEDG

EEELIARQQIDEVFGVDMNSLSFERPIIDSLEPFRTSEDDEEGETTLLEDEGDSLEAGCQ

TPCEEGESIFEDEEGMMSRSIGEEEVPSWISQLNALTAASRQTPPTPKEGGAKGTDDASK

KPPQGGLSRKRPGTGRKLPSIPAASDQSPVSSEHSARLLDNSPDSPANSLNCTAPARMEN

GLERSADDSPSRNVRFSPIVSSSGAVSGKSRVEGSPCGSENSSTNINSGTKPKGTSICSQ

GSIDTELLLRDTETVMAAMEARMGFCSEDREDEGGESDTDLTSTVAMVNGDEDFVKPTKY

HSPRDTMTGKSSKEKDNSNIRIASASSSKKSLTRSYSEQKPPLRGDRSPRSAAGVSVVSD

VFSRSESLDQSFQSDASDGGSDRTDTSFSRSGSRGKGTITMTKPNRAFALRRARADGSEV

TDTARSRTTAASSAATPSTRRSSSIHGTSRTACSDRTGTGRSEASLGAQIVKKARENTGA

SKARDSSITRQDGVPRNSLRAADRRSSAGSSSASSTISSSKKERDIRTLKSQLKSAPGNK

DLAVTGTSSSRSQSQPGSRSNSPKAAERMAWKRRKEYDPRKAVAEAKANKAKEPRPKPKV

QSSSLYKQRMTRSASFTNSVELSGRVRHDTYNVDSVSSVEDLSSAATASDSYGEVGFRRA

FIPFHNPLRSDRLSHSADEDEAGTFPTSQSYDSLIVSSIYQLSLKLKTTMDKALNKLREQ

DRAGITPSPIDDFLGHPAKSEIPAWKSANQELAGVLKNLRKMEHHLHLMERALFPDEDTD

TDVSGMSSVEKHKYFQEIERIRSELAGFQPIDSPHAQDPGSPELAELSKHTH

>SG-AL_contig_1372_1_+1_1_192

TRRSLDLHTHTHTHTHTQNACTLVKKTKATYNPIQTHNDHDIYIKNKIKQHYSHHHSKHH

DSEK

>SG-AL_contig_1373_1_-1_1_105

TLRCMQNNTVELNCFCVSLCEITLVLALFQASNFP

>SG-AL_contig_1374_1_+1_3625_4038

MVACFFAKSGRVATIQLEDRKTVTADCYINHCLPKIFQAWCKQRPRTGVRGLLLHQGNDG

TRTAAATLDFLAQNNVQMVTHPPYFPDLIPWDGFFIPFRQAAVEGKAVSKCRKCSSIFRG

RHFGPTPVNVVRCHGQLV

>SG-AL_contig_1375_1_+2_2_379

FFLLFNQFNNHVIKIAIQSIRSIVIPIIISKVFQTEWYISLLDSICGTSIWNPSIFKQIN

QFTTHHHCKTCCRAIKSILPVIILKDIHKSIYNPLSPIANCQTYNSRSIVPIIILKHTGV

DMNLLP

>SG-AL_contig_1376_1_+1_511_684

MTEYPFVGDCGVPYHHLQLPPETTIIPYLFQICQLQIATNPLKLCLCCTLLISVQLSF

>SG-AL_contig_1377_1_+2_95_295

MGRNEVFTIHINFTAKQDFFFVLLNTFFFPFTVDPTDEWAETGYLPSTLILQQNRMLFFF

VCFFFFV

>SG-AL_contig_1378_1_+1_1_198

QNIMTSINEISNTAVQSASLSYKKAKILKKTGTHLSPTPTLLANLRTSTFHIFLSTEEYI

FKTILT

>SG-AL_contig_1379_1_+3_921_1208

MTRRYKKSDRETEIWDCFSAMPPSNSVREDRRLTSVLQEQQELPHSSGQSFTTLSVSGRE

FLGIGPKTKFWSTQLTCDIFHSTGCSQISQHFPSLH

>SG-AL_contig_1380_1_+2_2_211

LWQTSANMSANASALSSYLEKGYFLALLDMLIFCVSLGSFLSDLQIVVQYCSLACSMILL

LFEWAFFIAI

>SG-AL_contig_1381_1_+3_1428_2042

MAANLESAARLGYEQLKQAALAAQKRLEQFPVKLSPHLDAKDNLEDKNASGLFPTDGPNL

TPVLICGDGNCLPQTASLLALGTESQHLEMRVHIITELRLNEDFCLTDTNLNSSTPGSAE

PYAQYSDQYEMELMSEAVKGVFEKEMRSTVVPNSYMGIWQLHAVPNILQSSLYSIYSEYR

GYNTYVPICIRFYNQELELLHLKQL

>SG-AL_contig_1382_1_-1_79_324

MATLGSPRQVSALIRAGRYAALLSGFVYGSYHLRSLTKKEEVIQEHENKLKAARDAKLKA

DKDASSKLEMDALGKECGVVKA

>SG-AL_contig_1383_1_+3_1902_2363

MTPDGLLAKASFCGRSRNFINHRFLHPPAGQTHGTHTYTHSVSNTTPQMHALHIHTVCLE

SSKKMCELCLFHSPNALTVLFCSICLPTLSDTHPNKYKQICTLLSPSHTHTHTHTHTPCG

KKMNKSRSGGTHTHTHTLSLADDHMSLYTNSQSP

>SG-AL_contig_1384_1_+3_2322_2675

MLASISAMNAFQKNNIGATTSRNCHQPQQTAVERYTCTGSFMPSRVILHECCTDTLSHYE

IIIMYFCILSYLWKDTLHVQMFGCIDFHKTRGISCLFKALLNTTKVTGWTQTQATWHQ

>SG-AL_contig_1385_1_+3_3_116

EKAMVTWFYSATCSKKRQGLCVNVIGVNNIRVGRFFQI

>SG-AL_contig_1386_1_-2_2_169

ESTNKIYNRLEVAQPVETAIENTHQFKVPACCKERSRMFSRKTDFTFFSCKGEEFG

>SG-AL_contig_1387_1_-2_227_1048

MVAGCGMTCIKYIVFAVNFLFFVMGIAAVALGIWALVDKNDMDALTKIDSDGKLEEFNAV

GLLQSGAIVLIVGGACLLILGFLGCCGAVKEVKCLLATYAAIVVIIIIIQIAAAAIAIAF

RGRIEDYLQEGLIKAVKENYDGNVTSDNAFSRAFDFAQVYFECCGVNSTANFEDSPWYQK

EHTKTNPHTVPDTCCKLKNRDAFFDDNKVELDNKDCPTKKPAEDNPNTAKHCYTSIEDWI

KQRAGIIIGIAVGIIVIEILAVIFACCLYRSIED

>SG-AL_contig_1388_1_-3_84_182

MYTSVIDCSESSILHPGDLLTKIAYSGIWQNTS

>SG-AL_contig_1389_1_-1_19_213

MEDTRYANFPQVKCYHHYIKLIHDEITTIPKKVGKCTVCGVTEDELYFLDNSTRFKNKQT

TPPPP

>SG-AL_contig_1390_1_-1_79_258

MNHIWNKQSALNKLKYAIKKTTRIQIHLFLESTKSTNTSKLLFYNKITTNYCLEPYMMNI

>SG-AL_contig_1391_1_-1_49_219

MTYKLKEADTQTLQEKTENVQFLVRLKTNFMSWIILLDAKKQDCNRLKRLKKNVYLQ

>SG-AL_contig_1392_1_-2_215_3073

MARTLKRTGSSFEPVSKCMWCCVLLLFLSLTFRVDGVQANSNSSSVVSSDGQTNETESTV

EKGMRPFFDMVKSFLGVIQPHRLADQTWLNLSDIVRDGEFQTEQLTNWQNWAKYLVGFVV

CVAVGVVFFIAMPITGLVFCCCRCGGKCGGRSKPHDPKHAGCKRKSYCTLLIVFSTIALA

GVVCVFMSNQILYDKLQNENDAGPVGKLVYSVDEVNRFTDSTVHDLTVAVLDTFVATSAG

IITDIRDTASDAIEDVLDTLNTTSLLDQSKAVSSDVDKTKQELSNVATQLGILSSLGSML

TGNLTDVRIQIDNVCTTASQCPGYDASKYNTIADFSSLDSLSVETNNVARSLNLSQYVSQ

AEDTINTTKANATQSVASGINDATVSVDNVKQEIENGVNKFSSEVADNITAVIKRIRSDL

YDAQDDVSKYSQYVWYGGIALSCVLLLVVVLYYVGILFGLCGERPGYGAQCCNTGTGANF

LMAGVGITFLLSWLIMLVCIVLFVTGGPIYTELCRYFDDHQPQALKPFSDAVMNAVDFKD

TFFDGKDVHLDLVDILEGCKNDSAIYSVLHLDNMVNISQYTDLSSFRTALDNLKQENFNI

PNVTILTSDMNQSITNFANSGLDSISFASYYNEVNKNLTAGGLPAMAAELRSIAQISSLP

PATRQTLNTSADKLDVLQTNLVEPMKQAKKSLNDSLTYVQTQANSDLKISTDALLEGLQR

AQDNFNSQKNTLVQDQVSRVIDRVMKLAEDTVQDIITDMNENVGKCRPVYNSFYSASDSV

CVTALEPSNGLWFGIGWCLFFFVPALIFAVKLAGLYRKEHDERDFDDPNISLYEGGKQDG

IPLTSQRQPGPTSPSRQGHDGGVANHGYQGESHMGPVDYPGYKGGAVNSGYQPDNYPGRM

DRHAPPPYGSHHNGSGEIKLPAYPGGDWKYPGASAPYCSTNTQGVVGVPSSAY

>SG-AL_contig_1393_1_+3_528_758

MQATYLHMYIHAGHSYHCLHSNAHTRLQLLTVDYRYTDTYHLHCNGFFQSITMHTPRTLD

IATTAHLLCLSVTVPNI

>SG-AL_contig_1394_1_-1_172_537

MAPEIHDAYIESADMLVKADPLAAVDVYMKFPVSETPSFDDAYIFGEIIRILMKFEKYDD

ERLASNMITYGKILGLGVLDRYVKVLEEKFKNKLLQKVYAGVNGKAVDDPDMQTFFKFKC

WI

>SG-AL_contig_1395_1_-3_258_1163

MKLTDTAFDVLGFTEEEKTSMYKCTAAIMHFGEMKFKQRPREEQAEADGTAEAEKVAFLM

GVNAGDLLKGLLKPKIKVGNEYVTQGRNKDQVVYSVAALAKSLYNRMFEWLVKRVNKTLD

TKNKRQFFIGVLDIAGFEIFDFNSFEQLCINYTNERLQQFFNHHMFILEQEEYKKEGIEW

EFIDFGMDLQACIDLIEKPMGLLSILEEECMFPKASDKTFLDKLMGNHMGKSPNFGKAGK

PKKPGQVQAHFELHHYAGSVPYNITGWLEKNKDPLNETVVEILSHSKESLVSALFSAPDA

AE

>SG-AL_contig_1396_1_-1_160_1431

MADSRTYDLVIFGATGFTGQFVVDEVARVEDEEGGLTWAVAGRSMEKLQKVLAQSSQRTG

KNLEEKPIIIADSESHISLLEMCKQTRVVLNCVGPYRFYGEQVVKACLEGGASHVDISGE

PQYLEKMQLMYNEKAREAGIYIVGACGFDSIPADLGILFTRSKFKGDLNAVEGFLQITPG

PDGMVGNYATFESAVHGFAHQGELAPMRKVMFPNPLPRSAHRPAKRPVIFHSPEVDKWCL

PFPGSDNSVVYRTQRYNFDVNKQRPVQFIPYFCRSSRFAVYAMAFFSIIFAILAKFSLTR

MLLLKFPGIFTAGMFKKGGPTLAQIKGTTFSWTFLATGYSQKLEDPEAQHEEAPDQTIKT

KISGPEAGYVTTPICMVQSSVALIKEKARLPKEGGVYTPGVAFGNTSLIERLDKHNVKFQ

VLEE

>SG-AL_contig_1397_1_-1_61_2619

MGSSKKHKEKDRDREHKRKKHRRSRSRSKSRERKSRRHHSPENSDRFAYRDAGGTSSGYA

YGRDAYTGGREGRDRERERYAGEYDDYFHPPMPQEPSFKEEPDNVDLGKEQLSLSIEDTN

KLRAKLGLKPLDVGGAGDSDSKPKEEQDVHVPAINLSEKLNTEKLREKMLLMKEKRKVKE

KLRAVKGLGESDSDEDDVFSWVKKNRTVSKEKEMANKRAQMLEEMDAEFGIGNLVDQEFQ

AGRKDAYSSQDLSGLRVEHNMEKFKEGSNVILTLKDKGVLDEEDDDVLVNVNIADDERAE

KNVENKKKKPDYKPYDEPESDEYGMFQVKDVLDKYDEEIHGEKKESFVLGSGGKYDAEHE

RNMKQIRRQLKQQGQTLMGPAPTIASEFMTREEVEAKAKVKAKAKFKKIKKKVRKIRKKE

VLKADDLLPLPDQIAEPTDYGSRHRGRGVNMEVKEEVPEEEEDIREAVAPQLEPMDDDAA

FYTGILPIKEEPDFKLQIPPSGIPPPPPPPAINDDEDVIGPDEDLTGVAVEEESLRQELQ

SMLHRTRRLNQRHERKVGVQKVMESMVKKEEDMEASTSSAKLSRGLSASTGGKKGTNIIL

NSMSEFCRSLGEIPTYGLSGNREEEREEIMDLELELMEQRKQEQEMEEQASGWAEVDIDT

NPVNIMGEEKSVLEEEPIVTESIGSALKLAMKKGYLEGEGKQKAVMNPKHSHLQAQNYSI

EDKRYDDLDEKYRKRDRYGGGMITDFKDKDGYRPEIRLEYVDDSGRVLNPKEAFRQLSHR

FHGKGSGKKKTEKRQKKVEEEQLMKQMSSTDTPLNTLSMLQDKQRTEKSPYVILSGNKTF

SSTSQTLSKPHPH

>SG-AL_contig_1398_1_+2_581_928

MCVCAFVCMCVEKSVCGCRCVCMHTSVLYSCVGICAWASAYAYACKHAYTCVHVVVCMYV

SMLRMVTFICMYAQMCVCVCVCVCVCISVCIHMCLCICKQLTLIICDHQPKFLRMC

>SG-AL_contig_1399_1_-3_921_1502

MDEQTSEETSVPEDVTGSTMITHANATGVEVNTSLTTSEESLTTPTQPTDSASVTQSGVT

SFNATTATVQTPSQTTVTNDVISRRVRPPKLSLSQKSCRCVPTITAAKQPAMMQQKRDEM

AVIVKSLIIKKDNISATRRRLVSASDDRSSSKVMGAMGIVLCVLPVLFMLLTDLLGLFTR

HQEHSCDVYVHHQR

>SG-AL_contig_1400_1_-1_202_390

MVNVLVPCFWCCCSESFHCVDRHQMVCTEYQQCSTMIKYTILQKVKCLCCGWQSETCNLG

LCC

>SG-AL_contig_1401_1_+3_1581_1913

MLRNWALSRPYRKGLTAELDSSSQRQTTTAQRETQGGHRARRRVRQAKGNQHRVQAASSR

ARQQATFWSCFTSSSCTTSSPPTATPGGRFPPPAPPTPPSLSQTGFSEGGS

>SG-AL_contig_1402_1_+2_155_262

MAISGLSPHKVHQSCHHIKSIKVNKTTALNTKPLQT

>SG-AL_contig_1403_1_-2_32_250

MPILQRAVSQPATRAEVEEDVTSIWGSTPPSRGWADSQPSSVWDLDNVPTVPPARPPLEK

TAHVRSQENETAK

>SG-AL_contig_1404_1_-2_158_1573

MSNENWRDKGGEEEEEDWRKPTNKERWATRTLWRDSSNRGGMDLDRRNSAFSRGRGFESD

ERGRRQGEADDVSEWADDGAVISPRDDQREHAHRPDHHQSSEDQLQKWPRRPPLAAYENE

DDAERRKPSQRSPPPFSTQAGSSSIPPLAKPEMKQPPRKQNEEVASSATAAMTPASVMPS

MPQGMTLTNIMSDEKTEAKNATATSMTDENALKWFYTDPQDEIQGPFTGEEMAHWFSAGF

FNMHLLVKRGCDHQFQPLGDLINQCGYVPFLPGLSRPPVAQMTTLPTNPTNTNTTAAATT

AAATAAAAVAAATTQPAMSAQVLPPSLSFPLSMGGVGVDRGRGADNFTAQQLIIQNQIVQ

HHTGMKELHAQVLSQLQKQKSFTQLDPVQQEKLSTQMIQNNPLMLQHMQQLQQLQLLQQG

LSAIAPLGVAAQPSLQRGLSTPLGTTVTVAAQLPPLSSLPLDKALTTTNVTL

>SG-AL_contig_1405_1_+2_1325_1564

MASSVEVTEFVFELTMQERYDLDMAEELGLDQEVLPANDDFGNEKGEGSASDSNHSPRLG

KVFVIFCLAWILECCFHQAS

>SG-AL_contig_1406_1_+1_1327_2079

MCTCVCLPAALSMPKYSSACITYTRDWGDCLLRSWKVISHSYRFALCSSAIAIHTIHTTY

QPNIHPLLCCENHDRNKQEREPPRNTKTLQTATKHHLHTEWFCHINTSIIRDTVSIFRFV

GRSVTIKIIDEYIIFVKRNIPNTKGHRQCQTEWQKHMHHKNLNNNNHKPRATIGASNEEI

KMAAGSASHAETGSAVQDLSSSSDRMQAMNSSKSMRPSPSISTSAIISSISSSVKSSPMF

CSTALRSEGVM

>SG-AL_contig_1407_1_-3_3_224

KTRTKKKTNKKKKKKKKKKKKTTKKKEKGGEEEEGKKKTITSNNNFKTSPAHRVFFAGSR

SLPDNHWQVWVKCI

>SG-AL_contig_1408_1_+2_776_1033

MHTHTNLLQIQHLINLTGPCGIYMDARTHTHTPTSNSAFNKSYWAKRHIHGCTHTHTHTP

TSNSAFNKFCWAMRHNKYINTQQINT

>SG-AL_contig_1409_1_-2_134_1114

MADTDKLNIDSIIARLLEVRGSRPGKNVQLTESEIRGLCLKSREIFLSQPILLELEAPLK

ICGDIHGQYYDLLRLFEYGGFPPESNYLFLGDYVDRGKQSLETICLLLAYKIKYPENFFL

LRGNHECASINRIYGFYDECKRRYNIKLWKTFTDCFNCLPIAAIIDEKIFCCHGGLSPDL

QSMEQIRRIMRPTDVPDQGLLCDLLWSDPDKETMGWGENDRGVSFTFGAEVVAKFLHKHD

LDLICRAHQVVEDGYEFFAKRQLVTLFSAPNYCGEFDNAGAMMSVDETLMCSFQILKPAD

KKKFPYGGLNQGRPVTPPRGNAKAKGK

>SG-AL_contig_1410_1_-3_21_1883

MDPEMNLERGWLYGSLNGIVGLFPEEYVRPLARHEVETPNMKPKLYQPPQVGRPLSREGA

PGPDDRSETSQGTVVNDGKFSMMEFALLNFRESLDKYEMLRTEDGSIRGTVKMIENIKLH

NLQKDADSKRRGSGGDWSWKEHAEMIKWTRSPIQASLLKLSTAEMNKLALECFIAIMRFM

GDYPMGSNQSDFDCVMKILRSCHKFPELRDEVFCQLCKQTTNNRSMRGKSKIKGWRLFAL

VAAYCDCSDTLRPYLFKYLETTATDVNRTYNHAAALCLQNLRKTFKYGGRKNVPLREEVQ

ALADGRNCKRFPFFYSGTEIQGGLLQVKSCTVVHDCILDVCVGLNINDPVEMEEYTLFIR

TGDGVFSKLSQEEYVLDVTAEFVRKKSHYDLIFQRTVWIYPLSHMDNEIYVDMMFHQCIF

DYMDGLMIVRPGGQPSAQTMEDIALMAALLHKANNFVGLPTLKQLEVLVPKTVRSSPWMR

GQQWLNRIQERQKDVGGRSSLAAKALFLEILSKWPLFGSTFFLIKSIPNMTGECKLAVNK

KGLSFLHRDTHEIIHAHPFSEILSTRRYHSDSGGNYLDMKIGNLMVQKILRIETDQGSDI

SNLIGQYMQVINRRHKQSERL

>SG-AL_contig_1411_1_-1_115_369

MNNQLFLHLRMDIIVLCWTQCMGPICNEYQTSFFFFLIEDFPQLFLIGAHVHHKLFVGVW

LVYDWRTSEEAHNAAYYYYCVLFCQ

>SG-AL_contig_1412_1_+3_165_317

MLIDYVCLVLCISKSFQQNAKLICQSALVRHSREYGAETMTYFPFCFVNYK

>SG-AL_contig_1413_1_+3_255_479

MFYMLLGADWLQKKSPITAITHSTLRGLTCMERKCHGIHMFSLFVSACFWFGWIWFGLIC

LFVCLFICLFVGWLV

>SG-AL_contig_1414_1_-3_1140_1520

MYWHTFLFQGKIDRFPFELFLYMYFGYVLFQKCARWLYFVFVFIDLDGRSAPAYFFVLFS

FVCVCVVVQNIHHYPLCSHDFQIDTYCNNIVIFFKISFCYFSICSRYYKIYRVLTLASYT

VPCRTGA

>SG-AL_contig_1415_1_+3_1488_1655

MHNLQLFILTISVYKKAQWFQNCVGLIATHDFCVVLNKNVVKLLTDTDTYNRFYLF

>SG-AL_contig_1416_1_-1_889_1155

MLSAHEKVKTMQIAVVNWRTALTPVTTDAVGSECIMNLSSISPCLPCRLSINRRDKRFPE

SGMAWFPAVLVQLTCSVCVCVCVCMTILS

>SG-AL_contig_1417_1_-2_104_730

MPIEAVEKPVEETKPDESASSDSDDDDSPPELEEGDAAAMQQQSQVAAAAGLTEELVSKA

KQSRSEKKARKAMSKLGLKSVAGVTRVTIRKSKNILFVINRPDVYKSPASDTYIVFGEAK

IEDLNQQAQMAAAEKFRDKPPAGPGDMPSTVSQPIQEESEEEEEEDDTGVEQKDIELVMS

QANVSKSKAIRALKNNNNDIVNAIMELTM

>SG-AL_contig_1418_1_-3_243_1463

MSVLNLEAKLERIDPATPWGFRMQGGKDFNSPISIQRVNPGSLAAKCGLQTGDMIVKIGN

TDTSTLRHKEAQNSILAAGNRLDLLLQRGGAPMRPGGYETFATPVINTGGFNAPQSFKPA

YDAGLPHQTQANLSMSPGSSAPPAPLFVPKPAGPPPTTSITFTPKPVPQAPAAKPWTPAP

PPSAPAGGPRPFGAGGGGGRGGAAPRPSGSGARRVRVGEAITKPPSTDRIPICASCNIAI

RGPFVTALGNCWCPDHFVCANPQCGVKLIDIGFVEEGGHLYCERDYEKYMAPHCAKCSAA

IVGECVNALQTTWHPTCFLCTQCKQPIGGNQFHIEEGKPYCEKDWAAMFQTMCAGCDFPI

EPGDRWLEAIGKNFHAECFNCSTCQVSLEGVPFVAKGGKPFCKKHAR

>SG-AL_contig_1419_1_+2_2_298

FFFCRQHWFITVKCSSCNNSTRKPRMDGYDAGLHSFHGSTLHLGVQLSGVQQGNIVRYHL

NLLASRSDSSNVRMSPSLTGPFTLRMMERLLSSRNSTRT

>SG-AL_contig_1420_1_+1_805_1047

MQKVLHALHQTIMINISAQLPPPTRFGISSDPSSDPSSDSGNHYMKLITISFYQPVAVPV

RPVLHYQPVAVLVHQSYITAC

>SG-AL_contig_1421_1_-3_57_695

MAGRGGAQRPNGATQGKICQFKLVLLGESAVGKSSLVLRFVKGQFHEYQESTIGAAFLTQ

TVCLDDTTVKFEIWDTAGQERYHSLAPMYYRGAQAAIVVYDITNQDTFGRAKTWVKELQR

QASPNIVIALAGNKADLANKRMVEFEEAQAYAEENSLLFMETSAKTAMNVNDIFLAIAKK

LPKNEGGSGSQQRQQGVRLNNEQNQQAEGGCCK

>SG-AL_contig_1422_1_-1_1_138

FVQEGFITLGQFHIYSTQTCQQLFVRLINLCKTHCSGKVLKWGGLK

>SG-AL_contig_1423_1_-3_30_482

MVTHDAVRSRGVLFRLTQSFCFQRHVNHADDKIALNHIAATRTRNRETDLYLKSACIKKY

LEIIAVILSYFCFTEFNLFADKTCHSLGEFLRALRQTAYLYKFARKRKGRDGPCSRRDWR

YLQSLDPHNQLPSTWLTSPTLRKSPNLTRAS

>SG-AL_contig_1424_1_-2_2_211

KGDHFLSLSQTSLHIDCSACCASSKPALEAQCNRCAAILSRQWSHWAHRLGFQTRCACAV

WRVITTRSAV

>SG-AL_contig_1425_1_+3_1242_1454

MTSYQHRVLHKRVAAKKLIHKKLICKINTTKQSTIPPPHTPNQTKKKRKFKKKIRIIWIN

IHVFNSNVHSK

>SG-AL_contig_1426_1_+2_860_1096

MAQICDFILDTQTNKQTNNTRKQSYTSKDVLAHMFSCIQTDSRNCSALLDMQYQNPQRLI

RFNRLFNQPSKSPVFASAF

>SG-AL_contig_1427_1_-2_74_325

MLSAYVTLFQLNRLFQLFFTNRCGTQTKHITNISVKHYMPIISHTLAFILLFIVLESDVS

LPILNPNYLLETGIPEAAIYFVFY

>SG-AL_contig_1428_1_+2_2_181

ANSLSLHKTLLTGKILKMRRCKGTIRHTGETTRMIGTLRAAANRPVLLLGSGIELERERF

>SG-AL_contig_1429_1_-2_2_223

NLYIYVYLFLYFWYYRSSNTSAGLVSINLIVNVCCTYSYALLVIVYLHCILFLGKLLWLS

VGIQYAILSVYFYR

>SG-AL_contig_1430_1_+1_1_1242

ASVATRLACVSSRQTLLPSRCLLSVQPDRRRHRVVSRRRLPARAKTFQQRLDEVSAKDSN

LNRSVNIGFPLISSAEREHREQKKQHQTKKTRGKQEFIARHRQLKVDLKAVEEQWKQEQG

PYHIHSITEHYGLYNDLFGSAFFYNTTPMSICYDYDEEFVTPVYYGNKISPSEAAVAPFV

DYESGGDTLWSLVMTSPDGHLQHNDKECLHWMVMNIRGSDVQGGETLCDYLQPFPVRGTG

YLRYAFILFQQHTHLNFSGHLQLSDSKSLSERTFSTGDFYRKHQKEITPASICFFQSRWD

SSVTSAFHDVLKMKEPSYGYIRPPEYFQEEEQKQLQDSFHWYLDFSVDENECVEEDDVQI

QEFQQQENPLGVLIEGSVFPSILSPTQPVPSWMKLRMKNLRLPEHRWKSLNSEK

>SG-AL_contig_1431_1_-2_92_205

MCLQTTRQYVGLLLAVPGPKIWSRKEFKLCKFAGDWIV

>SG-AL_contig_1432_1_-1_28_546

MNMPPLFRAALALIGLSLVLFLAGFASNFWFVSSQPNSSFHFGLWLICTSSYGCVNYDRI

LGTTPAHAYVARFCTIYEMIAVIVGLVLLCLFVCKSAQQRWARSSYSLLLSAGITGFLGA

IIFIGREKSLDSTSSVSVSWSGILVIFAAIMQIVSGVLILLPIRRGQVEFEQV

>SG-AL_contig_1433_1_+3_906_1043

MFQPNACHNHTRNVLLFIHCIGFRARVAKTCTQQTSTWNGELRKLV

>SG-AL_contig_1434_1_-2_50_352

MLIPKKNRVVIYEYLFKEGVLVAKKDFFVPKHPDIDVPNLQVIKAMTSLKSKGFVREQFA

WRNYYWYLTNEGIQYLRDYLHLPAEIVPATLKRQTRPEAQR

>SG-AL_contig_1435_1_+3_75_314

MGRSNNNDTNTKIGDNKERSDDDHYIIIVIYSLANRCCHFKSENSLKLELIKINPSSSKI

TTQTESTVVALPINYAKLNI

>SG-AL_contig_1436_1_-2_131_253

MYIMCHFYVYIKHCFIFRFQDCVAVLLLLRNNITFEKQKKK

>SG-AL_contig_1437_1_-1_49_249

MPFGTLSGDAGLFFIGYAASPENHEYMLSRMVGAGSDGHCDDIMRLSKNVKGTYWYFPGA

AELKKLE

>SG-AL_contig_1438_1_-3_159_683

MVRFSSCHSPSLTQMAARKQLRKHIRRIREREYSKLRALVPAVATKKKVTKVQVIEEAVR

YIEELHAALIQRFRQKHGCGAESMAQETVRSFITQMMPPLRPHPHPHSHPHPQPRSHQPH

PFQSPTHPRAHSHSVSQPSVYPHPPCPVPPFLSACSFEKQRKRASFLLRKRRRDC

>SG-AL_contig_1439_1_-3_3_239

SSCPQLGPHRSSDGLDPELSVLSSVVHWYLLLKDLCDLFSKRLPFYSFSVLDPEAFAFPA

FSLDWGGLRACAFPLFPIL

>SG-AL_contig_1440_1_+3_3_77

VVPLVMRMRWRNHHSSSFLMAQPKL

>SG-AL_contig_1441_1_-2_731_3913

MGQKPTALLSEVRHRSRSFTEGRATSDLKDPHPSHHILLPCPHPAWKSSPTTPRDSHLDD

SDDAITPGRHPVPSPRQRDVSESGDAGKSDDVASPRESSSSSVVRHPRQRIVSDDDSCVP

GETSRDGGDVIGLNVDVNRDFTDGRDYLLSGDDFRNDDDVDEGDRGKFSGHGGGVEEKRI

QVTTEHSRTYSAAWDEYDSEEESNQFDNHLNNSSVKDTAKFSDRDSASDGTWVTSGPETG

SCTIEAAARSNTIPRHRPPTLFSAQTAIFGSKQDVKSAVVAADSRDEEQLSRFSSSETLG

RQPRGPILRYLQKTRAESSSPLASPRAFSLDSPREKKFVFPGEFPGGSSVEGEHDSKKTP

VEQTQSSSSTKAPVEQAPRSSSTKAPVEQTRSSSSTSSETSKITSRTRATQRPSHLPTAS

SKNSSTGIPKPTKKSSSIPQRARSSSQGPPQNQRKSTSGIPSKISSTSGPAQSSRGGTSS

GIPTRPRSASTGPNYSTRQPLEKPPESSRIPRVGAKSKKEKSGGRRPHPMAVMELAAKDL

QSPSLHGFLCRKSFNDQWIRYWYVLQEDSLYCYLTPDDHVTVDVTDLDGYSVTSLEDKFP

GQRFVLHLDHEDYIPVYLSASSREEMEEWNASLTSAIKVIPRVNGLHLPPQHEEGEGARA

AHVGEDRVLGIKQKLLAEVLRQTQELEKKQAARLATKHKQTSLDENELHTPPSTPPPPSV

TPTGEQICTLTRLRQRRMSTQIKMDTLQRQMQTNGHAKKSRFTFGKKKSAEETPTPQNPD

IVQQLKTLSDHLSAIDRDLIQNGGVMTLDRKTSSLTSSSSSRGRKTMSRHSQELEGSAVE

GGGKKSSSKGVDSLMTEESVINRSNSLKSSVQKLAHRTFGKTGWYRSKKSSQGDSLEHEE

SEDRNEDPDHHHHHHHNNNNTITTTNSNNNNDSPDVIDLSHRLMRTWPARLVSQDSEEDK

FGGDTGGDVFLPPPSSFFDGCGEDSPSSPVSSPRSVRSAGSAGSVDVREEGGGEGAARRS

GSSLGQESRIPVRRRRQVDPDAMAHIEAFEEMSRQILGEPT

>SG-AL_contig_1442_1_+1_1426_1647

MFLALVSIYRQQTTMNRLSFNQSFLWTPWLTTASLLFTILDMFCCCYCLFLSLFVCFLLL

FFICHSDLTKENRH

>SG-AL_contig_1443_1_+1_61_267

MSGALYVCLFVCLFFSVLNVLLPVRVFGWFRQIGILSNPTFSLLGLNFFLILLFKTQPLA

VISFHNYMN

>SG-AL_contig_1444_1_-3_207_428

MCLKGKKKKSNKNKFKHDSAFIMGVVMMKTKFPLLRSSIFRYFQTRFTHTIFFFFFLLFC

LPKTAYKSHLNVKL

>SG-AL_contig_1445_1_-3_3_689

MLRRVLTYHVVPEGELSSRQLYNDRLLDTLDADHKIRINEYSSFPFRQQHVLTAQCVPLV

NGNLAACNGVVHVLDKVLLPPSGNVVDVLAADSQYSTLVRLLKKAGLADSLQEEGPFTVF

APTNQAFKDLGKERLRSLEDDPEELASVLKMHVFKEHLCCAGIFNNPWWYQQKLRSLNGR

TVFLTRDRRDRPLVNDMVIGLCDNTATNGVVHTIDSVLLPIQDPWYMFP

>SG-AL_contig_1446_1_-3_795_1154

MAVPKKTNNNRKQPTTTKKSLAQVSVHPSSCQTAAQTSSSNSRDYLPISQYCTNDNVYKK

RKEIFQIKNLRLKILTFLLYRWGIFPLLRLFLNCKVLMFVPYKETGTISTGRRKKRQSYF

>SG-AL_contig_1447_1_+2_953_1135

MIKVGNSSHVYHCMEHLMNTATFCKDLFRFSMFFIISLIFLAHPHKVQACFCIGLQPKSL

K

>SG-AL_contig_1448_1_-1_1_219

SSNLFHEIHGCSHVWLLRKDCWYFAEDYLHHQLMRLPLKYLSPFPTFFPFMFAMHSVPSP

SICKSSSVKYLYQ

>SG-AL_contig_1449_1_-2_1262_1555

MDSRLIRVVPIAVFNQEGWCMDYISYINSRLFPLDKQQMGITKPHATKLQCTTCNGLPEQ

FHSLHLQLTCLYSNSDIRSKFITDTSLCGIEKSENNLQ

>SG-AL_contig_1450_1_+1_1_315

GGRGGGVWGGAHHPFFLCGGGGGGGRPQGLLPRVECTLYIDSLVILRPHSRVYFTDASIR

VLLKFPDQYRKFLYLLGLVDAKIHHDIKCKVQPIVESIITKELMD

>SG-AL_contig_1451_1_+2_257_652

MIYLKTARLIPQIWKHSVVVPVSKQGKPQSDKNNYMPIALTFHTGKLMEKVVLNRLTYYC

DKNKIIPINQAGFRKGRSITDNLVKLTTQLRRQFVRKKNLLATFLDVEKAYGCVLYGRLS

YNLKSTGLPENI

>SG-AL_contig_1452_1_-3_90_4079

MTFKLFIRLADREDFDQPAPFDLVKSLDWRTLKDLLGSYYYSDVTEHLDVRYWDEEDFVN

VSSQEELEEAIRLAEEKSYLQLYVRLTGADGDATPDHQPVADSSDHSAGGEEGPVGKTLQ

KSDPESNTETDRDCIPSETLHTSNCLFDTSQQPLAVDSPEPSLDDLKKDPFKLVSFIKAN

ALKKGMIRKLTESSADDLSSSDHLLKGQSPVDAVLTQQIFANAVPATPKQRFQQAIFSAA

ATPNAQRYQQLAWLQEVEAEAEGVSYDEGNAEGDEKNNAVDQAHTMLPGALSGANVIAIE

MPEVEHQGTETAEPAEMAETGEVSTRSSRDAETGFWVQAQQGAVKEGQRRFTTTAIAREQ

TPLDLSQKHHARRSIHTDRVQKKKKKDLVDDSSPEDSLLRYMAKKGKSSTTTTTTSSLLD

TPSEGLSSETPLPKKHERRSTKSDGTQKKKKHSDLLTEDAFVHYMTKFETTLKKTMAKEI

AKKTARTVIKHMPKQTEGSEGHDSQPPLVLSNRSGLVHEDIICDCCDREIVGTRYICGNC

PNYDLCEECENIQPSFHERSHVFIKLRRPCLKAGTHHGVRKPLLKRLVYEHMEEERKVDP

GRVQVIMDRINAQGDKSGKRESRNNNNSEDGGREVNGQQKETEEEQQEEECRQEQYSAEF

ADERTFENDAIVQSAEKKCLNTGPDAWSTIAVDGECTSSLPVNPSDTDKDKPGAQFLTKH

EEAAAKVDESATEKDVAALEIWIESSTAPAILSNTSSPLLDFFEYPLTPMQLKEEAHAAT

SEILDGDDLDSILELTDDGYVIYPTTKDGSTTSSPACLVKSSQPCQEIPETVLTPSQAAV

ADQEAVSEPSSLRAESPFTVASMDVEMLKASYHSSFLWKLVDADLQPPLTVHPGWSSGSE

PDYQKEVLEKVSDGVSSLSSWSEESSSEEDFCVIVPDCFDVNKPLDPSSLAKLASQGTEK

PHHSEHKRTKDSCTDIICDVVPDQLMKAETVNITSPAAGQQPQDSNHESQAETESQENED

KTSATNEENALLVQVEDSGKQPQDSSHESQAQTERHENVDETWVEDYTVDESAKVQEEAF

APVLGDARNEKEPTAADARNETTCASAASEMDRPTNQEQALDVADPSQQPSSCTSLKSAE

FESEDEDQTNLKHPTDSGEEGAAAAADSISDPNQQLNQMMTNTIKKASSGLQQLFQTGKD

IFKTWQTGDSYEYKESTYEYKPSEYKLPENTWQPSNKEYIPPKSNFQFVPSEYQLPKCNY

SGPKSQVSAGQGFTGEYAGPLQRIREMGYFNNEHTLDLLSKHNGDIQKVVNEILEEADND

WAEKRHGDHF

>SG-AL_contig_1453_1_-1_1_195

AISSLLLLSLFLPFFFPFAVVCFFSAFLVHSTFFSPASLHTIFSPPTHTLLLVCDVLSML

FCFGD

>SG-AL_contig_1454_1_+1_163_483

MDREGRKKQDNSKGKKKAETKTAIEKRKFPAQNFQKVGILFILKEPLASLQRVVFFYSSV

VYRLYSSHLNTMPSLTSRFFFLSLFVCLSIKWFALYLGVCPEHVYHK

>SG-AL_contig_1455_1_-2_326_3058

MGRYLVSAVMWDYEWVSNVPSRAMAWLDLLIEKGLSLDTLFDRPLPDVLLFGARLDTPCP

DYFTTKGPESISPLILAIRHYNLGLARYLLKKGASPNFKDSMGYTPLIHAVKMNDMTMVK

LLFDYDYIHDKETKASQIVGASNKPVLVKKYSRNIFLIKTDKRAEEMEKDSSVDEDAGMK

DTEGDDIEEGISENDMEEDGGELEEDEDVLEDDLEEEDMDVNGDLGEDIEEDLDEEEEGD

DSDHDTDVVEDDEDEEPPVPVVEPPQRLGSKQKSLVKITKEELYSRPHVSKTSSINLNDG

DRQCQTAFHHAVYSLDYGTFDNGDIVEVLIGAGGLQDKPDIKRQLPRDVALMYGATKIYN

ALVKNNGAKEKPTVERFTVTDGLLADGPKVEFASDADAVLTDLDKDGTGDSVAGKQPKVD

SNCEMRNTGEVYQDETKNVTYSITLTKVDVASGPWGMYNFYKLQVVRQKAKDMYVLFTRW

GRIGDRGQWQHTPFPTAEDAVKEFCKVFRQKTGNDFNHLDRFVEHKKKYRLMPTMDEPKK

AKEVKFNFQSPVPSKLNPAVQDLLQELTSVTMLQAATKSKGLDEDFMPFGRIMQCSLDKA

KNVLLELEKLITTVELQRKNMTTTPMEEYQANCEKVATLTNEFYHLVPVGGFEYEAIRPV

RDKKVLKEHFRLVSDLMDMEVACKILLGSQRRLTEMNPYDYVYRSMGCKIQPLSENEHET

QYILTYINASGRGCKVNAVYKVARPGEADAMNSLNLPNNRLLWHGTSMANIVSILNRGLL

VAPVDAPKTGNLFGEGVYLADCFTKSRSYCYNAVGSCKVMLLCQVALGNSIDDFSHEEED

HHDEDVHSLKVLGKMAPDPDFDIVLPYGATMPLGQLKPMTYEGKTPLVQYNEYVIHNPQQ

ICIRYIIMFDG

>SG-AL_contig_1456_1_+1_460_648

MLTKSNVVARKRSEHNMCKPERKDQISRQRKARAWKTDESCHFCGFRFTMTSTVSFDILL

FHF

>SG-AL_contig_1457_1_-2_2_127

ESKKIGETIDMIFRFRCSRVVWGMTSLVIFSVFTCHCVSDQT

>SG-AL_contig_1458_1_-1_229_636

MALQFGKVTQNLCVAATSYCRIFAAKYAKKVAGRGGKVAGGKKGGPAPVKKAILEVETDA

AKLTQFCCGANIYTTGTDPELKPDAEYPEWLWSLRLDRQPPDLSELDQENPQYWRRLRKL

TLRRNLQLMKVKKLGK

>SG-AL_contig_1459_1_-2_125_1552

MGLKFLEFVKPFCSVLPEIAKPDRKIQFREKVLWTAITLFIFLICCQIPLFGIVSSDSAD

PFYWIRVILASNRGTLMELGISPIVTSSLIMQLLAGAKIIEVGDTPKDRALFNGAQKLFG

MVMTVGQAIVYVMTGMYGDPADIGPGVCLLIVIQLFVAGIVVLLLDELLQKGYGLGSGIS

LFIATNICETIVWKAFSPATVNTGRGTEFEGALIALFHLLATRQDKVRGLREAFYRQNLP

NLMNLLATVLVFAIVIYFQGFRVDLPIKSARYRGQQTSYPIKLFYTSNIPIILQSALVSN

LYIISQMLAAKFSGNFFVNLIGVWADVGGGGPARSYPIGGLCYYLSPPETLGHILEDPIH

AVLYIVFMLGSCAFFSKTWIDVSGSSAKDVAKQLKEQQMVMRGHREKSMIKELNRYIPTA

AAFGGLCIGALSVLADFLGAIGSGTGILLAVTIIYQYFEYFVKEQSEMGGMSTLLF

>SG-AL_contig_1460_1_-3_399_566

MFQKFCVFTCYNSNGEFRLCPFYYVIKCLWFIKSHMCSCRHEMRLHQETMKGFMSS

>SG-AL_contig_1461_1_+1_40_423

MLHTGTCFIVSSERLAPRPSPELEWKGKRWSEKNLCLSLESNRGSFTQKKCNLTLRPQGH

LGSFMCQTKRNISATLSFAVAVVVVLLFCFVSFCFVLFVSATLSRRSLDHSENTKMAVLD

STMDGFES

>SG-AL_contig_1462_1_-3_84_632

MADTLLFKRSQTMYPYWTNYPPTVPTYPASQTMFRRQEPEKYTKPSGRDSYQNWTDKWYD

WAPPVRREEELPKVADRQEPCSRPWQYRPGTSAIPKWSKENKDWPVDYSTFMRMGRPHSE

TTRARGIQRLGYRDENLYPYTSMMTSNYRRPVYSVYGPMLRRQVYGLAHQHTRHGNMPFE

DYY

>SG-AL_contig_1463_1_+2_191_313

MKQGYSMNLHQFAACIASIIFVRPSSRQKVGCVHGSVLIGI

>SG-AL_contig_1464_1_+2_47_976

MAQSWPDAVLEFITLLFSVVYFWCEAVVKCILPSKFFRKDVQGQRVLITGAGSGLGKLMA

YRFMQLGCNLVLWDVNTQAIEEMEKDMGSQVCCKAYTVDLSSRDQIYKAADKVKEELGTV

DILVNNAGIVTGRKFLDCPDSLIEKTMEVNTSAHFWMVKAFLPAMVAQNRGHVVSIASSA

GLFGVSGLADYCASKFAAVGFDESLRFELEAMGKTGVHTTVVCPYFINTGMFDGVQTRFP

CVLPILDPVKTVDSIIDAVLCDQAVLILPRFLYIIYALKGLLPVQVALRLSTYMGVSRSM

DTFTGRSKNQ

>SG-AL_contig_1465_1_+3_3_200

PPNPHTHWHAHLLNNTTACPKIATTLISYLQVKKLQQLQHKKLLPSIQNSLSLSLSLSLS

LDLFDD

>SG-AL_contig_1466_1_-1_439_561

MLAKRERNPYTPSRCPPASFIGSHDSTANPYPCPAGSGWIG

>SG-AL_contig_1467_1_-3_105_1445

MILMEDFMNANEADVRIVPVDTENEEKQMVFIESFLQNNRLEHLHQVMGCHPRYLEAFVK

TQNYLLREDGPLPFTYRHYIAIMAAARHQCLYLVRLHAHDFLLQGGEPAWLKGLDSAPAK

LRNLCEINNILAHRPWLIKKEHIEKLTRGNDTWSLSEVMQALIIIAHFHTLCSFVYGCGI

APEIDQGGCTMRPASSSGDDESEDYASDSSTGGELNANVLEDSSLEALMERMRQLTESSQ

EEGPTQEEMLKSFQSVENQSAEISVPTKEKTTSRKEEVVRFVTNSDFIYSDFAKRAADLD

IPTFRVQDYSWEDHGFSLANRLYSDIGNLLEERFNIAFNLTYYTMGDNCEVDTSAFRQAI

WYYIHCMYGIRHDDYDYAQVNQMLERNLKTYIKTVTCYPERITKKDYESVMREFKHSERV

HVNFMMTEARMQAELLYALRAVNRYMT

>SG-AL_contig_1468_1_-3_843_1169

MLVHTFFSHAHALIHMLNNLTSICTDTHPYKYARLYTHLPTCTDTYVHWSYMFLYSFIRC

LCFTFSGTENTLICRKQSWCQSQLGKDCEGGGGGGGGMVFLSILVGLIL

>SG-AL_contig_1469_1_-2_503_649

MHARTHACMQTGPVALKLSLFCITEKNVFCGRCAVPNRTNTLSFTHTLN

>SG-AL_contig_1470_1_-2_929_1303

MAWHFLCQKLFRVLVLSIIKKEKNPSCHHSFLFVLFVCVLLFFCLHSPHFFMLDLSTLEF

CPMFSAAFDTLSTDFTVSLTLKTFIICSENAFVQELFLFTVCDMFSLMNSLDPVLMNYKD

VLLSH

>SG-AL_contig_1471_1_+1_154_393

MCIHMKRVSGTCRSAHMLTWETGKINALYPPGEVPEVRTQAFQTLQFGSPVSSPFDYRAL

SNFKGKKYNNFQTPEDLKVH

>SG-AL_contig_1472_1_-3_123_2003

MEMENVDMDDGEELRPAFDRSKLPTAPKAARGSDVDIDQIPDRPPFTAYLGNLAYEVNEE

KLQQLFEGRQMEVVNVRLPNESGRVRGFGYVEFGRKEDLITALTFAGEMFHGRKLRVDLA

GGDRERGDNAGRSDRNDWRGGGGDPDRTEGDWRRRDPPAWNNDRGATSGFDDRSGYNDRG

GDRYGDRYNDRGGGFGNRDRGFNDRGGDRDSFRDRRGFGSDRDGDRGYGGGRYGDRDGDR

GGRYDRGGGRYNDNGGRDGDRYGRDDGFGGRDNGDRYDRRDGGRYDDRRGAAGGYDDRRG

GGRYDDRGGGRYDDRRGDRYGGRSGGGFEDRGFGGRRGGERDYRRDDDGGRYGGYREDRG

GSRDGSQEGPRERPRLNLQPRTKPVETAPAEPSRQSSIFGSAKPVDTAAKEREIEEKLRK

LDTNAHYTSEELGPRDRDGREGGRPRRDSDRSDGGRGGGRERRLSSSSSKAGASRRDSET

SRTSDDAHPPERDSRPPARDERKMVPAPPPKENAWSRPRTEAISRSADVSRQVSSSSTGS

QDSRDRDQDYYRRDNRRSSDEGRRTGYGRGNPGRGDRHEPPKNSKREKALPKSIDDMPKW

NEGPGKNWADVNKYAVIMDDDYRSDED

>SG-AL_contig_1473_1_-3_87_296

MSDSNSHPSFPSLQVGLQFGWAIKNELEWWLRQRMRVTRGTTRGPSYQLISYFWFRPHVL

AAKFGRDRAT

>SG-AL_contig_1474_1_-2_74_316

METDISAGLVLVLSLGLSLAYGQASQVLGSLSPRALSLYYRLFSSLQQKAPWAILEAAHW

CSDNLFFNFSRMSERPDLMVP

>SG-AL_contig_1475_1_+2_113_232

MPHWSQLLVECDLVKCDSHCILRHLGLKGLNYSLPSEIQS

>SG-AL_contig_1476_1_+3_174_395

MATCICIFMCMIIYSSLGYIIFKQSDVNKITKLNQSSTKMSTMQVPHFNFVVVVVFCGGV

FFFERQVQETTFIL

>SG-AL_contig_1477_1_-3_51_440

MELEHYTKGEVKEVEDPYPFSVDDTVQMRITQGSKIRNVMGFAMKKMADKEVRQIAWNGS

GPSINKTITCAEIMKRKIKTLHQITKVRFKRIEEYWVPKVEGLETLKVNRDIPAISILLS

KDPLDTAEPG

>SG-AL_contig_1478_1_-3_6_581

MAASFRRGLACLFQNGQALSKRAATLTRVVPTFAANTICSTSKPSILSSFLGKSSFGSWS

PAWQTWNSAHQCLPIQPAIPLLEQVRTKVRYSRNRGKPQTVHAVPNRFYRLNWGIWIRTR

AGRGKRLWKKTESRKYRLRQHVFCNRTQSKLLDKMVTRFWRRPKYWVDDPYEPYQKRENF

DLYFPNKRRFYP

>SG-AL_contig_1479_1_+1_160_582

MCIPIKCPNPKDLEERKYSLAKSWFRVERTPTSCVWHIGTIVKLKYSSSFNQNNRLSKLF

CRERIAPVCHLRQGNNTCTVTARHDDNVYSYNKTSHTNNEEKYEIFIDNTENSICKKKPK

KHHHLSTCLMELSVSVITAND

>SG-AL_contig_1480_1_+1_247_459

MDGLHNFGQNTFYIISRGKSRLFKQNINRYRALGAEENGDKSLLTLKTRSQYNCFFVISA

NQISVQLFFCH

>SG-AL_contig_1481_1_+2_794_1078

MGLRFYFSVSAKNASFPLQFNENKGNCHCPRNLPLYCPSLLLGQFQKNSLKRLMLMIQLS

HTFQLIHFLFHGSIKDFIVYSPDLMTEFHCSFHHQ

>SG-AL_contig_1482_1_-2_59_2629

MSRSQGLESIYRIAPYYFVHVLDQNSNVTKLEIGPQTYIRQDNERVVFGPEKMIVVPPRH

YCIVENPVVRDADNDVVMDSAGQAKLQHADQEIRLAQEPFPLYPGEVLKQPVSPLKVVPA

NSALRLRAVLDFEDAHSGSKRTAGDEWLFEGPGTYIPRKEVVVEETIRATVIRPNQAIRL

RARKECIDREGVAHVTGEEWLVKKTGAYLPGAYEEVVDIVNAYVLTDKKALHMRALRTYK

DDFNKTRKNGEEWLITMHDTEAHIPNVYEEVVGVVQITTLSNRQYCVILDPCDATGRPQL

GQKKQVKGEKAFFLMPGERLEKGIQNVYILGEDEGLILRANEQFKDGAVERKPGDRWMIR

GPVEYVPPVEMEVLMKRQAIPLDENEGIYVRDIKTGKVRAITGNTYMLNQDEELWEKVLP

PVVEELLMGAKDPLADRSDRSKSRDDAPKIRDKTRVINFRVPHNAAVQIYDYKEKKARVM

FGPELVMLGPDEQFTQLSLSGGKPKKPNQIKALCLLLGPDFATDIITVETADHARLSLQL

SYNWHFDTGDRSDATKAKLFSVPDFTGDSCKAIASRVRGAVAQVQFDDFHKNSAKIIRSS

VFGLDANNKVKESFRFSQNNLVITSIDIQSVEPVDQRTRDALQKSVQLAIEITTNSQEAA

ARHEAERLEQEAKGRLERQKIMDEAEAEKSRRNLLELQAQSAAVESTGQAKAEAQSGAEA

ARIEGEAAVEQAKLKAQAMKIAAESELDRLTSAREAELKYLSEQNEMELTKAKELATIET

TKFKNMVDAIGTNTLAAIANAGPEMQVKMLQALGLKSTLITDGSSPINLFNTASGLIGGL

VPTKRRRQEEEDDDEVA

>SG-AL_contig_1483_1_-2_167_1972

MERVTCFLKKITRVKTISGDIQQTALKRCLTTLEMTLLGIGHMVGAGIYVLTGTVVREKA

GPSAILSYLFAGFAALLSALCYAEFGAQMPKAGSAYSYTYVTAGEIWGFLVGWNIVLESM

IGVASVARAWSGTVDSLFDGAIRNGTVTYVGYLVQDSQWISEYPDLLAAAITLVIFSIVA

TGAKPSIIFNSVFTVLSGIVVLFIMGCGFSHANLSYWTSSSFGGFFPFGFSGILSAAASC

FFAYVGFEGIAVASEEAKTPAKSVPLATVVSMMVVTLMYMGATSALTLMVPYYDVSLSAG

FPAAFRDVGDRWAQYPVAIGTLLGISCSLLGFAYSLPRSVYAMADDGLLFGFLAYVYPKT

QTPIFSILVFGTLSTITTILFDIATLVEFLSIGTLFSYTFVAANVIILRYLPRDRFPFKL

KAAEETCCCDANATEETRSSGMEATSEEHTSRTKELDGRCPTERESPEEGCPVELHTDEN

CLELSQQDCEHCSCFCSCENIPLISHYEGFGKLRSRFRDVPVLSRLRPGKGVEIAVVCLV

VVLAGFSVLLLFRFDLFADGVWWSFIVLILFVVCIVFLISVIVAHEQNTGVATFQVPLVP

FL

>SG-AL_contig_1484_1_-2_2_127

RHTTDGTSNTNTFKDISDKKVLLFQTTKERHHVDSMLILIRN

>SG-AL_contig_1485_1_-3_111_224

MVKIRCHELYCFAGFLISGLQKFVINAVVFLTLMDFVW

>SG-AL_contig_1486_1_-3_279_467

MRVCIFIEFLWKGGWFGCNGCFCQDVLLKVETLGHFTYLFSICTVTFLHVFCLVIMPHPP

LSE

>SG-AL_contig_1487_1_-1_55_594

MAVNAEGQSAPLEGKDTAKPAKKITPPGPPKALKTSRIGPDHVTLEWKPPSEDGGSKVTG

YRVEKCEDTSEEWIKVEDIKSFDTAYKVTGLKEGVGYYFSVSAKNQVGYGEPCETDTAVK

PKRPEGKPGPPTGPLRTEDVEKTSVTLSWKPPKDDGGSPLTEYVFERRESTRSTWTKVDT

>SG-AL_contig_1488_1_-2_257_1675

MAVNKEGSSLPLECQEEVKPKKPPETPGKPVGPIKFSQILADSVTLTWSPPKKDGGSPIV

SYTIELTDDGGRNWRTVDSVEAPSTVYTAKGLTEDQEYKFRVSAVNKVGQGEPLVSDSVT

PHRKIEKPSKPEGPLEPIDVQRNNVTLRWKPPKDDGGSPLTGYIIEKKDAKRGNWMKAGK

VDGVTTEFCVPDLVEKSDYLFRVMAVNKVGQSESLDTDTATMAKSPFDVPGKPKALEISN

VTDKTADLQWKAPDSDGGSPLTSYIIEARPDSRSTWTKAGSVDGATTNFTVPDLREGIQY

HFRVSAVNAEGQSEPLEGKETAKPTKKILPPGPPRNLTTSHLGPDHVTLQWKPPSEDGGS

KVTGYRVEKCEDTSEEWVKVEDIKSYDTTYKVTGLEEEVGYYFAVSAKNEMGYGEPCETE

AVVKPKKPEGPPGPPTGPLRTEDVEKTSVTLSWKPPKDDGGSPLTEYVFERRE

>SG-AL_contig_1489_1_-2_17_265

MQNEAGEFVDMYIPRKCSASNNIIAAKDYASIQINFAEVDPATGRMTGTNKTYAICGSIR

RMGESDDSLLRLAKRDGIVSKNI

>SG-AL_contig_1490_1_-1_1912_2190

MKSSTQITCWAAGTVTKSGTVVLVGGLQTLQPNIVVTCYTEFQCVSVLHALNFWIFFILL

LCNVLLFFTHDTGKKPECIKTLLIVHCMLQSAL

>SG-AL_contig_1491_1_-3_18_545

MSGRRRHKWAIDFAGSSRSRGDRGALVPAHSDVHVPSPVGCVDPANIQERVKEIDPNLIV

KRSWDIALGPIKQVPMNMFIMWMAGSSISIFPIMMVGMMFFRPIQAILAIQNTFKTIEGN

QAAFQKLVYVLGNLACLAMAVYKCQVMGLLPTHASDWLAFAQPQQAMEWSGGGTYL

>SG-AL_contig_1492_1_-2_17_511

MATKLKGLQLLRLWQRRIPRYPVYWHGDPQLRVVMPQFWMKLVRPKRDIPNNQVRFIVHP

QMSVIDIKNYLEKIYNVPVLDVRTRVMAGEDIKHPTRGHVVAKEDDYKVAFVHLGDGVTF

EFPDLFEENKPATEKQTKELKRQERTTLEQERKDWDKLTIPSWFR

>SG-AL_contig_1493_1_+3_3_113

NGSLFISSIFACGQESREMSIQSCDKFSGDQVFLGGN

>SG-AL_contig_1494_1_-2_203_430

MKNEVYIIFFQSGWHVSVCVYQKLMHCIYIFASEMERREVVIKKQFCTYCIYTCFWLHVV

VCTVEVIGCVLKLYRA

>SG-AL_contig_1495_1_-1_118_2172

MGDGLARILTKQSFDPEAFIAKLSASGDGEDALLDLRQKVQALNDDTAQALKKNVYKNYS

QFIETAREISILEGEMYQLSNMLTEQRSLMSSMMELSLVSDKAVVEDPKEEKKEESPEEE

TRRNLAFLLEKVEGCSSVTEVPGRHLVHDTDLVELDPETFVQMQRTHAFLLSDSLMMATW

VPTRRGPMRYRFQGLYELDSLAVVNVRDAGPVKNAFKILMFPETRMYQADSAKSKRQWLD

ILEDTKKKKALKDKQKKEATNRLSDQLSPSPLLSPSGEVGFPSSGSKDAVAEEPVTMDTD

FLQADWLQELPEDLDMLIAQRNFEGAVDTVDKVTDFLKDCPKGPALKEFKARIEHRVKQL

TDGLMSELQVSPERSLRGGPRAARHAVTQLIRLGKSAQACELFLKNRSAVIRYNIRQLKF

EGATTLYIRCLCDVFFSSLSDTANEFLRAFPDHYGCYSAFVIWGKQELKSFVSIFRRQVF

ESKASLTTIAECVQLAKTHCAELSKMGVDMEFSLAIMMENPIHQVIEETRDQMVEGIRYR

AQDELWRPMNLGTRDECEKFVAEMKNLGFDIKQFVFDGCFVSLTPTVVQFSKSSLTLADD

LLRLYTIELESLIAAALVTVFSAQMSQFQTALKTDKFASEKNTMVKCAHFVGEKVMGRIF

TLYQQNGVTLPQQLQTVQMNFRQIK

>SG-AL_contig_1496_1_+1_946_1158

MALVHDQSVLHFSQECLLKRVVICSGIFACGQKSHEIRIHTSGDKFSEDFFRRKLVGLFS

AENVPPNGGPF

>SG-AL_contig_1497_1_-1_16_1587

MALSWKHVVPWTNRSEFVQTYLDLYSKNAERQRNAVETISLWKCRATNKLSVAIESSAAL

TDVYLQYQTALEKKKLRQKSNNLRSIMTLAMIRFVNHVTEKGQNKHFAQPVHKIAQEFGI

PNWIVQLRHDGTHGSIPCLDVLTKAVQWALTYLQDVFWSQQVKEADECTDPDTRKRKRPI

KSLYNSAEIKDLLFRFQKASHKSVDEGPDSEAAREEKRTLRRIEHALNSQQRNNVLACLV

EPGHLVLTEQQLHAYHVNLDETLRAEHPRVPGVIISIWKELLLLIHGKQLTSLLLQHMVK

KVTGEGDRESLQSLMLAAWLRLVLLYETSNYSKTADERASQPALYSGAIDFAKDLLFYTC

LESKNFLTCRLLSLAGDLSPETERELDELDKVYSGRRGRTTAASKDHSDDETAVREEEEA

QSPVKKLKLNVPAEEESIWKLCTDPVDWEKLPIGLLPSQANLEDFGSEEEHRSHDDYSST

DEDGRSTEDELDTSKQGPPTSQNPRERSSENTEKDDLVKHIIVH

>SG-AL_contig_1498_1_-1_139_1389

MAAQGGDKRGKVVLAYSGGLDTSCILVWLVEQGYQVVAYLANIGQEEDYEAVKAKALKFG

AVKMVVPDLRKEFVSEFIWPAIQANAMYEDRYLLGTSLARPLIARAMVKVAKDEGAQFVS

HGATGKGNDQIRFELGAYALHPSIKVISPWRMPEFYQRFPGRTDLFEYAKKHGIPLPVTP

KSPWSMDGNLMHISYESGVLEDPSKPGPPGVFNMTVDPEKAPDKAEIIEIDFEKGVPVCV

RNQSDGTVKKEALALFIYLNQLGGKHGIGRVDLVENRFIGMKSRGLYETPAGTILIAAHT

DIEVFTMDRELRKVKRGLDIQFSEQVYRGFWFSPECEFTRFCIIKSQEWVTGTVILKLYK

GAIYVQGRKSPYSLYNEELVSMNVQGEYEPTDAGGFIKINALRLQEYARLRSKTSTK

>SG-AL_contig_1499_1_+2_563_784

MRSDWKAEAVPLCLPLHLVNLGKEVDQVNCKGRKLPSCIMTNIELLGLFLTVTHDCSATF

AFLSCTQNLIFNVP

>SG-AL_contig_1500_1_-3_690_920

MTISELCNELNYPGLCLRRGNLRRLWCFRKCVTGSTIVPYSSYMVWSIFVLDAGILWFNP

FHCLRSSNVRVEWIELK

>SG-AL_contig_1501_1_-3_156_1172

MLLSQAVRCSTVPLTAFVTTLGRSSVKVPKQPWLTTRFQTYASESRHAVRRQAQKAKSLK

EKLMAPAGDTAFNLGRGLVAGASAVGIGALCYYGLGLSNEVGAIDRSVMWSQEVRDRIRS

TYMYFGGSIVLTALSAVAVSRSPAMMNLMMRNSWMAIGATFAAMIGSGMVVRSIPYKEGF

GGKQMAWMVHAGIMGAVVAPLCLLGGPILTRAAWYTAGVVGGLSTVAMCAPSEKFLYMGG

PLAIGLGIVLASSIGSMFLPPTTALGAGLYSVSMYGGLVLFGMFLLYDTQRIIKMAEHYP

TYTDRPYDPVNMSIGIYLDTLNIFIRIAMILAGGGGRRK

>SG-AL_contig_1502_1_-1_49_273

MFCMRTHTKGMVSKSGLSQCVEYPVKKEICVCVCVFIFLLAGYTCSCLNIITDTCGHSVI

FHVDRSLIPDGRGSH

>SG-AL_contig_1503_1_+1_109_321

MGFKEKSQYTERPEEHHQTFTQEIIIYLFYSVSMGIANTLQHHQNHSFMQSFFPKANGTC

APLQFKTKIIH

>SG-AL_contig_1504_1_-2_107_1726

MASPMGQIAFDEYGRPFIILRDQERKSRLTGIEAHKSHILAAKSVANILKTSLGPKGLDK

MMVSPDGDVTVTNDGATILDMMDVDHQIAKLMVQLSKSQDDEIGDGTTGVVVLAGALLEY

AEKLLDHGIHPIRIADGYEIAAKMAVDTLDKISDVYEVDLKNPEPLIKLAMTTLGSKIVN

RCHRQMAEIAVNAILAVADLKRKDVDFELIKVEGKVGGKLEDTMLVKGVVVDKDMSHPQM

PKVVKDAKIAILTCPFEPPKPKTKHKLDITSVEDYRKLREYELNKFESMVQSVKDKGANL

VICQWGFDDEANHLLLQKELPAVRWVGGPEIELIAIATGGRIVPRFEELTTEKLGKAGIV

RELTFGTTKDRMLVIEDCLNTRAVTIFIRGGNKMIVEEAKRSLHDALCVIRNLVRDHRVV

YGGGAPEIAASISVAEAADKISTLEQYAVRAFSEALEAVPLALAENSGHAPIQTLAEVKS

RQVKENNPRLGIDCLLKGTNDMKEQNVIETLMAKKQQILLATQLVRMILKIDDIRAPSDM

>SG-AL_contig_1505_1_+2_113_340

MAIYSWLCSHVRPSAGFSQPGLSALHCFYTSPQNEVQIARRVDRVFNPYHFLHLHNCSVD

STGRHAVKSHPLYNAR

>SG-AL_contig_1506_1_-3_1059_1370

MHACARTQTHTSTYASIYQQAHTHTRTSDHSSASSQYFVFLSSHACVCTGTHNYTCTSTC

FDKCIINILRAHFFPFKNHKCNSIGFGFNLIYSQKVGGTCGASF

>SG-AL_contig_1507_1_-1_43_198

MSTAFIMLAPRKVEVSTTSFLYDLWDMGQPSFYRYVFSVGDKHYGNGRGKKK

>SG-AL_contig_1508_1_+1_1477_1902

MPASSSWLSPPPPSSSADFTRPLSSATCVLQLDSLFFRPVMVVMVASWKRDCVSSWERWS

RFMAVSCCWREESSSLKFLRFSSSVFNSISRSLIFRSCAAMVSLKPLSWLKSMTLALRAS

IRSSGLSLQAISSAFSRILASR

>SG-AL_contig_1509_1_-1_133_354

MYNGEEIDTVESVYKRVTRLSTHDECAHDNQWHSAVFDVRTNRTDILPITDVLLFDVGRE

NQQFGIELGEVCFS

>SG-AL_contig_1510_1_+3_387_821

MCETLSVCMSVNVLHCFISYYMFCKIYNIYCASCYVQDFPQVSISSVHLDRILFKQDLPR

DNFTVAMDYFTRAKCMLPTQSTTQTSVEGQKIPCPKLGLSLWHITSLTSVLITQTCAVTF

ITCHWANILANVFQWWSLLMEGEIC

>SG-AL_contig_1511_1_+1_193_441

MVSRIGTFMVVTTCVAVASHGSIFIVVTTHVVASRGSTLIVFTTNVAITSRLVVTTCAVV

TSRGSTFSSSVLPCLVLWLTAAP

>SG-AL_contig_1512_1_+1_2014_2277

MSHRLPNLSDCVNSCGLFLKGVYNIIRANIMWTVGKHKEHQHSKFKPHYMDKNQRLQWRN

ITLKFVSGLKATNACCCMTSTYLFTMSS

>SG-AL_contig_1513_1_-3_3_689

PGFHASHSHNDHVECKWSPDGSHVAVSSSNGQLFLVNRARSVKIASIFSDFVDGNLSTAG

SFDFDPRVRFSAMAVGTSENILAHINIDCSRQDQVFQQQVETEDMIDCVQYNQDGTRLAV

SFRTFQVAVYSTGDLSIIHTIDMSGLCSHHKSMYENQRVPTVHRLSFSYDGRHLATSSCD

GYVRVWAVPRLLTLQDWCRKRILQSVPILSIRKFSLPEKVKQFLLSEFF

>SG-AL_contig_1514_1_-2_1226_2191

MSNFKRILFLIVTCLACYCGFLCRNYLFSTNQTHQGNDVVSIQDSPAHQPNNAKRHGLNS

SKAAVFQDFALFLNAKDKDIKRSIDLKILKRRLGNISNDTNPKRELVLNLAHPRQRNVDF

VKVGNLYVFGTYLDLRQRGNATVRILTFKHKNHNDSLELRFTDRKQQHALHANVFQMCEN

HGKLYEGWILSYHLPQTLHALPRQISLSPINPRNQNHSHISISFNLKTVPSQTDSDAKQI

GVCVPPLFGKLRTSSIINFILMCKILGADMIFLYLGNISLELKTFLRRFSQDDDGLVSLV

SWNLPENVSDSEDSIWYHGQLL

>SG-AL_contig_1515_1_-2_248_694

MYACARFVPTVRSIAVAGSRSLMRPLGGTMVTTRQEVQQPSHSLATWNGVNFNSSSVSTL

VNQIRSFQTSVTRRDIDQAAKYIGAGAATVGVAGSGAGIGSVFGSLVIGYARNPSLKQQL

FSYAILGFALSEAMGLFCLMMAFLLLFAF

>SG-AL_contig_1516_1_+1_1_318

LYGCETWHLKTCQEKRLDSFDLKCFCKILSIKWTRDFVTNEEVRSRSKQNPVTSIICKRR

LSWLGHTTRLPSSRPANQVLNWNPGGQRRRGRPKMNWRQTVDRDLQ

>SG-AL_contig_1517_1_-2_2384_2578

MEQEAFHQLLQESICHAVLLSSADLVKFSKPHNFLVIERKSYSYLQVCCILCGLCIVFKC

RLLQS

>SG-AL_contig_1518_1_-1_382_621

MRVIHLCIFKIYPSIYLIMRVIHVCIYLFKKLLFCFVYGGSADCQSWDKGGNIKHDPRER

ITVAVVCMLFLCSVVLCKSV

>SG-AL_contig_1519_1_-2_2_148

QTNPQKFKTKLTAWSVTPPPKKKKQNNKKQPFKTINCLVCNPKRPLRQN

>SG-AL_contig_1520_1_-1_787_1047

MLYCSTCEKKKIIKCRHACVVIEMLLLTHQVICDFFHMIYSILMTSELSWKIVLFSYYKT

DQVFRNIPTKGLLELSVTTQEHEFLTK

>SG-AL_contig_1521_1_-2_29_889

MVSKFRLALVQLAVGASKRDNIAHAVSMVKQAASAGASMIALPECFNSPYGTSYFPEYAE

SIPGPSTEALAKAAKDNKVFLIGGSIPEKHSDGKLYNTSTIFSPEGKMIGVHRKIHLFDI

DVPGKIRFQESEVLSPGNHLTCFDTPFCKVGVAICYDIRFAELAQLYAQRGCKLLVYPGA

FNMTTGPAHWELLQRGRALDNQLYVATVSPARDEGASYIAWGHSTVVSPWGEVIAKTDHK

EEIVYADIDMDKVEEVRTMVPLRNQRRPDLYKIIDISKSDDEQEKQK

>SG-AL_contig_1522_1_-1_133_5982

MKELPYGSWTTVLPHTSKWSHEVTGLRPNMDYSFRVSAVTDAGTSPPSNAAYLHRRPVIP

RTPLEFPELRDVADDAVSLHWKRVDVPAYEADEEPLSFMIESQRLPSYDWEPVARGITDT

SYRISGLQPSQDYVFRVRGEMPSGVTTPSIPVPYYRRPERTGVPINYVTVDDHNVPPYSA

RIQWKPVYIPRYTSPSSTLYNLEMRETPQQDWQTLARDVKGLEYTVPDLSPRKTYMFRVR

AKSPAGDLSEPSIPVPFYPLHLRAYDSQRVREYVPINEDFSLPNRPYISTLIVKVPPRMH

IEKPDMYSINPTTAHLTWNAARVPSATTQLSPTTYRVEVREDGTFNWVEKASNISGLGCD

ISGLNPMLDYAFRVRAVNDFGWSEATLPTFLHRPINLDYEEPDIDWEKISPTVPPLVAPR

LPIDSPRMTPVSDDSLRLSWHPARIPAYARKSPISYTVEIKDPSAAAWSPVASKLSDTTY

QVMGLEPKRPYLFRVRAESEFGSSEPTLPAELSRTKPTHTRSQSVERQKLSADYDLSTRR

GSFIDSITTGVPPRVPTGRPTISNATETSLTLSWPAARLPAYLKNTKLDYIIEYREASNR

LWTNLAENIPDTSYKVTGLDPEQDYMFRVRAHNDAGTSEPTLPATLERPKKKAATPQRRG

SSVERRSLDRRSMDRMSERRSLSRASRDRKSESRLSRRESAKKDGEDGDTLPGMSPEDSP

HAPEFSSLAEEAVQYGVESHPMTISLQLRGYPPPEVNWYHQGKKLELGERYDTYVTPTGQ

VFLEFLNMGWEDIGDYKCVAENELGTAEKTVKIDVADPPTFIEPLRDIMVVERGQGTLSC

SVDGIPYPSVRFMKDWRPLTDTSRLVVTNDPEKAEHWTMTISDAIPADAGSYMCVAENVA

GKIFSTGRVTVEDDTVASLLPGYKDTNLEDNYYVLEEIGRGRHGRVRRVIEKSSGKEFAA

KFIQVREAVDKEFFHAELDNLLKQKYKNIVSVNDAYETPRQLIIVTELLGGGELLDAVVK

QGQWTEGKAAGVVKQLLETLQHVHSLGIVHMDIKPSNLLLSGNNVDSAKLIDFGLARKLR

PSQDNFLNHGTPGFTSPEALDVKPISTASDVWGVGVLAYILLGGQSPFQADSVADSLKKT

QACDWSFDVCKSFSDVSQDAKDFISKILQLSADDRPTVDECLNHPWLKKTEGGAKIDVAP

HKAFQDRDKKQRQSTAVRTVAYLRTLQRQMTGGDVLEKGLRPAVDPKTGLVSFPDIEAYG

EFLDSESWYEWQSRYGQGPDSGLLPLKDPEYTARIRGYKRTVGSADDSAFLLEDMDAMSA

ADREKYKMMKERRQMADIDKEEIPPSLEKELQWLEERKQARKARSPQRQGSVDSRRSSIS

TEGEAGGPVPVFRQRLQDLTFETGDTITMYCALEGPPPYSTVWYKNEELLSDGSRVKQEL

FEDGQATLTISAAKPYDVGVYKCVARSKSGRASSRMRLLLGDVPERPGQPAVLQTAAQEL

VLIWEAPRFDGNCPILYYRVDYKAAGDRRWTTALYTPLETCLVTGLKPNTSYRLRVSAAN

LLGRGPFSWSTVEVTTKKQGSISSLSIEGLNLEQLKQRQEEGKEIANKPRETEIAEEGPE

DTSSPILQTTEPDKEYALGDTIYVGQFGEVKEGVNKKTSKRCLVKCVPTTLKDSQHEFDI

LKTVRHETILRLLSAYSTKSTLFLVFEDAPGIHVAHRLSLRRRYSEDCVAYVIRQALFAL

QYLHRNSVIHLNLQPSALLLSSSQGLNVKLADFSLAQVLTNGVDGVKVPRKGYPDFIVPE

VVVKDKASPASDVWGVGTLTFLLLSGLSPFAGHSDEETLLSVAYSRYDAGDLLDGVSNEA

LKFLFKVMKRLPRNRPSVQECLEHKWLQLTDAMMKTRESNLFRSNRLRQFVLNYDKKRQS

KDCRILFDDLVLPPELVASLQPDVLTSDKK

>SG-AL_contig_1523_1_-1_103_738

MITCKMQPVPEEHRTEMNLNPCGREKSNCTIFLSYTSNLISAGTREVIRYLVQHNMVDCL

VTTAGGIEEDFIKCLGPTYLGDFHLSGRELRNKGINRIGNLLVPNDNYCMFEDWLTPILD

RMLHEQREQGLMWTPSKMIHRLGKEINNPDSVYYWAYRNNIPVFCPALTDGSLGDIMFFH

SYKNPGLIVDIVEDLRRMNSQAMYSVHSGMII

>SG-AL_contig_1524_1_-3_312_2591

MWLKLCLLLIYIIVLFLLARLVEALTWYETGSLSRRLLDPMTLSVAKLKALLEQRGVSYD

TVVEKTELTQLVDGSGQVHEDEVELLQPTEDTPVSANTNFTSGAHFYEQVEDAKDSVWLV

QVIARRYDSSRVHPLPLSEVTWKSLRKKITKFGIHTGVLDCELDPWWCERRGWHFSHLLL

ALPQVYQQKANVATYTYQGPLRQNAVSQWIRQKLHEKIETIQDFRTFKDEWLTFKNAWQE

PEVRALLFTDQDSIPMFFSALSVKFPGRVRFGVVNNNSGSTLNWAESVLPGQNFSLPLYL

LLTSEGPYVYGKNQGDCLTFNSMETLLKFLHPCLNDIFIFSFLMANLVSVLEFFIAQGGV

GRRLRRCLWCVVKYNAAVIMLWLPLIAIFQLPYLDHLPLFGLKVSRLLATSPFGEAFRRD

SFFFFQHPYILAASFLLACATFAAIAYKVTGGQFEEDNEPWFNFAQMRTLTHLRSNEFFE

PMFMSGYELSGLGSRLYLPTMSLQPVVSQQYITLLPTWQYCSKFTSTHQYGCTASSSPSG

EESSEMTAGLESVGACCQDGSRQMPPSYSSHQDNFGQLYPSTASANDLLDGNYHCECQQH

ARFHQASSSTNTQCIPSSASKPVLSEQSSRAPINDDTARCINCNQCPIKQESAPADTVPS

VSAFKEVDGFEKETANSSGCVMEPPPTEWLDFPEGYLPCSQCVICLEDYSVGITLCGLPC

GHVFHHSCILTWLKRDHHFCPVCRWPSFRPKPSLVHSHSE

>SG-AL_contig_1525_1_+1_1_267

FFCSCRCSCSSTLTLTGKRKRRKIQYVNDHVITGTIPNMFNMLFSCWIINVSKTRENVAD

DDDDNGKDDDDVDEDDDKSSIIAVHTFVI

>SG-AL_contig_1526_1_+1_259_516

MFYIHEKRNKECWQLMLHFKFNVQFAEEIDLTIHVLLKNQHYTFKNKPSLPLWAKVTFSC

QIWNDFNELLIQAVNLTQILPPHKNK

>SG-AL_contig_1527_1_-2_2_184

AKGRSLTSRPAPRKLSMLNRLFRPWPRMPWPRRRIYTLMTFLNQLYSTTIKTNPRKDVHA

S

>SG-AL_contig_1528_1_+2_476_652

MQRIIGTQQLSVNRIQKKFTTYHDQKRNRPQNPTNKILKTCKITNTNPSCCVIVYAYFV

>SG-AL_contig_1529_1_-1_1948_2124

MRVCFLTLPFIIVNLFSPAAVVCLVSYKLSASSWGLPDPVLLNIVDIQSSCGHFIPRND

>SG-AL_contig_1530_1_+2_158_283

METRKHPVCLSRKWSMTACMAGFPTEDGIRRLCSNITPAQCG

>SG-AL_contig_1531_1_+1_676_927

MSTHQHLDPSHQVITIMFFLNAILLIVCLFKYFPLGHFFVSCNVVQAPAERTMFASSTTC

LTMIGLFQRVNALLFHVIIFFLTF

>SG-AL_contig_1532_1_-1_46_291

MGRHFGKLTKVRNIITYSLSPFEQRAFAGAISKGVPNMVRRFTGQVFRVVPPLFLCYLVF

DWGRKEHARLQRKAAHAAVTES

>SG-AL_contig_1533_1_-2_2_133

HRKSHKIKKNKKTSTDKNNISSTKLPLSLSPLSLHLSSFCLALL

>SG-AL_contig_1534_1_-1_64_1683

MHQYRSEYPQIRFSTLYRGCRKYFILHLLVVCVVLYLLIAIGGGLIPKFLIPRLPFLEIN

SDINALMYFDVQNDASKVTWSRATNSKEALENAANSNVHMIEGDVILQGQGTQHQKLIPI

MAKPPRSESDIQLVDWLNRIKHLNKGIKLDFAIIDALEISLQRLQEFDFSFPIRYPVWIH

ADVLEGPHGGKVKVDPSRFFQTLKRVFPKCTVSLGWTTGTHTDLSQSGYSWDMVLDMYEL

VRKWRIDDQPVVFEARLSLIRNSVPQLKWLCDNIPHSALNVLHVEGDAVVSEDLMYIAYR

FPPEGVFFDLNHRQFETMVAQYRHFSKEKVSHFVSKRDEVVFKPKAWVKMGFYTEEDSIL

PSTEALVLTSPIVYIVTKSTYKPTSQIYIQGRVQFLNRNDASSEAFATGLNIYLRPVAYA

SFDHIVGIKCFLGVDGDIEVKGSELSKDIPDFRKTAQITPSAIHCFRFHLTDAGDQVIFK

VSTVHDCHTLESVVPKGDLHLTLTVDIPHRLTHDQNPFILHMEDDKRQAVVDELSVKHVL

>SG-AL_contig_1535_1_+3_1062_1349

MLPLGMPTMLCCLAASVLREMFTSVTEFPPTRIMPSLRDQIEGGRSVRDGLICSGKQLQY

THTKQRHYTPNSNTTHILHTKQQHYTHTTHQTATLH

>SG-AL_contig_1536_1_-1_118_1380

MTSKAEKPTLSGTRLKTRKRDEKEKYDPNAFRDAIIQGLNETEGDLEKVSKFLDVSGSRL

NYRRYAEVLFDVLFAGGILAPGGFLVEDSDPSKPSRTDVCVFNCEAAPEKIRGFYEVFYK

LIRRYKYLEKAFEDEVRKVLVFQKGFTDEQRQKLGIVTGQILANGLSSPRVLDALFEDHL

VKEVISLECAAQIFGIWLQEKDVKEVFSALKKVQIDTRLQELFPANKRNPETFCTYFREK

GLGAIADMQMSTQATKAKKEAQKALSEMITNESPVADMLEYTQDLIQKQGMTETEVTITI

WNTVMMAVEWNKKEDLVAEQAMKHLRAYTPLLAATAKAQRAELALMLRVQEYCYENMNFL

KSFQKIIIMLYKTDVLSEDTILRWYKQAHSSKGKSVFLEQMKTFVEWLENAEEESDEEDE

G

>SG-AL_contig_1537_1_+1_259_399

MHALHDIISTTWSHNLLICFLLENLYQGTLPLNSSRTHACLLHKHTH

>SG-AL_contig_1538_1_+3_552_773

MRYKNHNRITTRYRFSFLKNGTKHLPSNYFHRRSTHTNHKDTIYYYNTQRLINIMTRTTY

RLQVTDITGHSYLP

>SG-AL_contig_1539_1_-1_958_1515

MYTTPLTQLINNHSVHHEIFADDMQLEYSDIPENYDSLINKLQNCISDVKQWMSNNKLKL

NNDKTEAIRFVKHTWISDVSLPSTIALGSTEIPFSQDVRDRGLYIGSDLTLKHHIAKTCQ

ASYLKLWRISSIRPFSQKTSYTSADIFLCTVQIKLLQCLQPLQRVQNSAACLIFKAPKRQ

QSSDLL

>SG-AL_contig_1540_1_-2_2_268

ANIISVQQNGTEFRTDDRHRDGCVGLRRNHHTDSCPVFDVEITKQGCGADVLYPHGSMLL

LVLDIHLPVPVEPTDWPTAGDTSHQDDAT

>SG-AL_contig_1541_1_-2_5_1216

MAEAMAINSGEHIKSTQEAKNPGMETGIPSSGEQHAPPMQQKPVCVLVLGMAGSGKTTFV

QRITAHLHAKKTPPYVINLDPAVMNLPYPANIDIRDTVNYKEVMKQYALGPNGGIVTSLN

LFATRFDQVMNLIEKKATDSRHVILDTPGQIEVFTWSASGTIVTESLASSFPTVVVYVMD

TPRSVSPVTFMSNMLYACSILYKTKLPFVVVMNKVDIVSNSFAVEWMRDFESFQEALEHE

TSYVSNLTRSMSLVLDEFYTNLRCVGVSAVTGEGIEEFFQKVDEAAEEYEREYRPVYEQL

RQEKEEAVRKKEEKQMGRFRIDRHAGDSTKDLLFQKPESISLRAAVEAEDDDDEDDEEGN

FRVPSSDDDEPSEVESFSRYLQQHKQRQAEKIKKNTQRSNQPSS

>SG-AL_contig_1542_1_-3_3_158

QAQFMTRQKILSSAVLSIVPDDPTHYSVDYLVSVKIQLLKIVALFLRCSCFC

>SG-AL_contig_1543_1_+3_177_350

MALLRFKPCEEHAHTLQFSLLFNWTYTKTSFSNAWTNYILTTMLTYRVTNDITNYGQT

>SG-AL_contig_1544_1_+1_196_453

MATIPLSRPQKPPVVQRVITTGDFFGRNHSAHAGCLCVSIRHRTQIWLTGSLTCVFDVLC

DLMFSAQCSHILHVMFSVMSNRNSKR

>SG-AL_contig_1545_1_+1_166_306

MTSCGVRCISYYTIFLVKKLHWIVKALRKEMYFLNKGKEKEHFCHCD

>SG-AL_contig_1546_1_-1_67_453

MRGYDFVVLESYAKYVHREADRLGIESTAWAVPARSFEIRTFKENSSVVDNKYHLKTYER

TVQIENVPSPSLLVLLDKMMIHLAEGIDVTIKEPDADEEEFRYIPDKEMEELKSQIKAIE

DAREERRKK

>SG-AL_contig_1547_1_+2_14_601

MGGVLADAVCSLVPCLVQLLFVEFLYNRLSFISGRRRHDGAADLTDGLPFPVLLLQFVTL

HHLPSIQSDRQLGLGAIDGGVLEGLSQLEGVGLCGHVGLEVHRGRVGVATHAEFHSVGQL

PVGLDPLLAEPNSGTKLFKTSGGSQIDLPFSDERFNHLDYCFYACWEERKDGILNLWLAV

CLTETVQKLLVQERPP

>SG-AL_contig_1548_1_+1_1900_2226

MSINQQSSSDRQSQCFLFFFKSKKWHITLLVCCYLIATLDHHIRRCDQDSLLFDCKMRKL

DITTTCRIQFSYQFKNSNTNLFWSTVLSLQIAYSPSIILLTASYITTAH

>SG-AL_contig_1549_1_-3_57_179

MGRGSSIPSLGIFSSEGWQQYMTNPQEISNLWTYRLIFQNS

>SG-AL_contig_1550_1_+3_3_239

FFCIESSFIERIHLQIIQVQADIHKAISSRRHRLIYSQIISEFIAYNLQLTPELILYNTV

NKHDNVCLGSQTQNMTEQI

>SG-AL_contig_1551_1_-1_16_201

MGTYPSSRTEKVKQGGNGDGYRWPQEKLRSLPPHSPATTQVMGKPSSYYSQGRQSPLEVL

QY

>SG-AL_contig_1552_1_-3_12_377

MNEKNIRRGGGRDGGGRDDRRRGGGGGGGGGGGYAEDMRYLQSQLAMNTFELQRQIASLR

GRDRGYGGGGFRDFDGPMGGGGYGGGYGRDDWGGYGGGGGRGGSRYGGGGGGGRKQQRGG

WR

>SG-AL_contig_1553_1_-2_2_286

LSLYIAYRISHCGVVVSEQTGRLRIRIPISPIRIIVEFFGPNQLLCAHWEDWAHTVESHP

LHGCVIEGVAQISCPTSQVSVTLGPGWHQDVRQGT

>SG-AL_contig_1554_1_-1_124_345

MDMGQNVPYFTQRLHPELMSRMAVPQSSQSMETAKSQCETANNAEVTSAAALSVAAVKCD

ETFINLYMKLLILA

>SG-AL_contig_1555_1_+2_1319_1576

MKSTKQDENVHICAKISTKQDENVQRCNTSSYLSKYCSIKVSYTVQNKKHCCPETMCKKS

TSGIYDAKCLASCHFHTTSTLTMVIY

>SG-AL_contig_1556_1_-1_1_450

CCLAVFASSQYFHQYYDQDECCLALFASSQYFYYYNQDECCLAVFASSQYFYYYHQDECC

LAVPASSQYFHQDYAQDECCLAVFAWSQYFHQYYDQDECCLAVLASSNIFILMIKTSVVW

LCLPRPNIFINIIIKMSVVWLFSSSQYFHQ

>SG-AL_contig_1557_1_-1_163_450

MIKMSAVWLFASSQYFYYYDQDECCLAVPASSNIFILMINMSVVWLCLPRSNIFINIMIK

MCVVWLFASSQYFHQYYDQDECCLAVFVIPIFSSIL

>SG-AL_contig_1558_1_-1_1_282

LLADLGGAHVELPECLQFLLNTLQTPRCLVAWHAYEVMLTDCCWKRLDSLLQCKRLFSVK

LCLKSKKTTFIARGGRRRGLLFGPVCLIPIFSFL

>SG-AL_contig_1559_1_-2_1337_1528

MWLQSVVLGQFYCLLIRINHHLLQLFIPVKISHLSNKPSLNYVLINVDDNDYGMIISYNN

NMQE

>SG-AL_contig_1560_1_-1_277_492

MPTLPATGILLLRPREWNCRCAIVSVSQDSISMLGISSICTNAANAMGWKRIQNDNGISH

MPAHKPYVLARS

>SG-AL_contig_1561_1_-2_767_1066

MNGLEAALLQSMHLPTTARNGVSWYDTHWYSTMVMAPVSRKPHISASNDLKVYLTLCKTS

SCTAKVLTWKGDGIWILPQTMTFYLQYLCCCHVISQHANL

>SG-AL_contig_1562_1_+1_136_267

MYNTEHDLHNSDESAFNKNNTENYCGEGICHFSKNKPKTSQDIN

>SG-AL_contig_1563_1_+1_259_570

MQNVWRRRLLVKYGWAFVKFLSSCPKWDMCVCVCIQAYFCSDVSPVLSCLFFMFLHKPHD

VWFAFCDLSSHSNKFHAFTVCNTRLRKSVQSIKFAFSLSEHNEQ

>SG-AL_contig_1564_1_-1_25_2655

METETDSNKIPEPEIKTDPEDASADVEKTDEEKVEGEEGEEDEKAEGEEEVLSEDAEALK

EELDKIVVSPPEPQKPEEVFDPADFPPSYKDNLPKELLVLQYADNFRRQYVHLYRDRKPL

FLNPVNECLVEKFVCTTIRPTQLPYKELYNWDGAAEFVADYLDFETLDPAYEVPTRMLSP

ATVLQRQKGNCFEYSTLLCSLLIASGYDAYVVNGYATRETCLADETREICPLLKKKEEAI

RQERTKEMKKYTVKPPKDLRSRFILKMEARKVAEEKSAEDKRKAEEEQRLLELEKPPPDP

LHGLRVHAWVLVLSGKREVPESFFIEPFTGLSHPVSSPNYLGIESVWNNVNYWVNMQDCS

EGVGVIGYDLGDCTKWEFMFPGLDKPILEIPEAEDDLMLDDDDEDKDQDVEKHLDMPPSW

VEPLHLSQRDFEMRCPLGKKVKLYKRAKLEKFAHYLMKDGLVSRLSVFENRELTDLVMVK

EYYQHREDKLYSRIHNHRTGWVTEHFYEGRSHCLKEHQYKAGTPGVENERTMAFFNVARV

DGLVKRVETPTEMTEHYNDRDDFLYYKHVIFGRRPKKFGPQDEVNSVNPRPIVKMVMKFK

RNPGKDANEDISEMVFLVSEDKLQLTYHTEQSNIAASTRDFVKPTNWDEKGATLSWANEM

HNTFQVDPSVKSDKQVRLYQLLLDLLKEEERARERVRESEDEVREILADREKEEMASDLE

ISVYDTERNEKAKKNRKELEQRLLEEKLRKQEMEIDYLAPFLAQMSDPEKISRQQAYKLK

EDCLADLKQRLIDKANLIQARFEKETQELQKKQAWYQQNQVSMSKDDEEDYLNYCSEAMF

RIHILELRLNRHKEMAPHKYMALEQKLRNDARLAEYF

>SG-AL_contig_1565_1_-1_43_279

MLMLFYIGKVRETIFTLIVIEFVCTLPMDILHFIVLTWSQPTIPLLLHRTARCKDICRQF

LVFLLFLFCFFQFVLYIRW

>SG-AL_contig_1566_1_-2_152_259

MCTTHVSVMLTCVVFVSQSVLKSVVVFKVSTINHFN

>SG-AL_contig_1567_1_-2_302_529

MPFGDGIHLAASCSVLSLCTATHLSLSLYMTVQSFHPVKKWVQCLYFGLLGASNKQVMQV

FEFELAVCPGRIHHCH

>SG-AL_contig_1568_1_-3_141_962

MMEVVSQRRMLLLLPVLVSLSAGLSSHNLDEHTQVRRLVESLLMTDSIDGYDLATVHALD

PATNTEYGQTKRDIEAHASDFFSKKRSFSLQLTSGRRLRMSLVRRSVHTSKTTFTELTEE

GKRETIPLTFGDCFFSGSLDGEDGFASLAMCHGKLMGSVSTPKRHFEIRTLPHLNSKRSD

DDVMHVLVTWRDRMTRFVADGIDIPDEDDIDVTTSRRRPRRIVRDPEATVELSDYVDPLF

LQDMEDRFNITSNEDILTLILWKWASAAAVYAKK

>SG-AL_contig_1569_1_+1_28_246

MAYRIFNMCTRCSASLYTRRWSDKRSVQMLTWEVGKFTPNPPDAAQAELNSRPSKWKPDV

LTNLAIASVKNSS

>SG-AL_contig_1570_1_+3_3_188

DIKSKTTHTHTHTHTHTHTHTHKFIINILKLLFKHSTIVEKSPQITKYYHFLKIVINICT

MS

>SG-AL_contig_1571_1_+2_2_151

VSVCVCMRACACVGVCVCVFVVVVVLFNIQFHFEENYVCMKPTPCDLYFH

>SG-AL_contig_1572_1_+3_201_539

MTMLVCLSLSPIMNDNACLSVSVSLTVCPILVTFARLFPQEHPALINRKRPEHSVPGVHV

YPYHSFFVWTKYGYFRTAPHTRFSWLWPKALSVNDTSEMLRPTLNCCYALQCY

>SG-AL_contig_1573_1_-3_1275_1451

MKTKHICNVNECVNVGLVLQDKFFPLFVTLKWTCTYVSERAHLEFFPFPPVGNDNLTLY

>SG-AL_contig_1574_1_-2_386_5416

MATQAGVCVFKEKAFWHSDHGVQDSMERFVRELLIVLDTTRYPALGVIYFGHDTEATILF

QMIQDRRSRWHDQDKVNQLYWLLSDGAGTSPSFASLAAPYNLIAIAPATSHLDDINNYFF

HVLDNPEELGPYWQSLVSEYLHDVEGCFADRSPVSSCRLRDKIQPYDYLSAALDSFYSLA

ALLGRAHKEVCGGTGSPGVCSNLSDALKNGLLSLVQPQAMNYSLHFVEERLPKQFRHDRM

LVPDPYGDFMPANSPVYFLNSASSGQWTKIGEYCNGSLDFVPKLDLPFVYRSECGTDCKQ

CRKSDTIRFVYHPGDILLLGLYSISSPVATASFSCGDYRKQSSSVAAMMAFLRAIETMMS

QIGSGNGSRFGALVLDDCYNKLTASALLADLFSGQITLYDYNTDQPIPMDKVKAVIGSQS

SSVTLSALSLLTPLQIPTISYSATSPDLDDRVSYPYFLRPVPSDSLQARALVDVALLLNV

TYVGALYQDNNYGRRGMQEFVRMAEEEGICVETPVAVQVSNNEVDFHKAVRDIRNKDAMV

VVVFLVESSVIKLLDAIEGMNLDLIFLASEAWSLSTDTLQDQRGVTARGSLVLTVDTSRE

AGNVGMANYFKELTTPALTGHELPWMRDFWENEKQCNLAGGFTNSYEKMCDPGDKLSNEV

VRELSEDQKVVQTINAVASIITALEQQCGHHRDPVACMTDLHVDTAASLIRDVQLRDQRG

QSFIPYLNDSNGNVGFTLHNVQKLAGNTYRYVEVGQYNPVQGLQITDDIKFYNPVGQPMS

SGIVSTCLYKLQCKSVCGKGKTPTTPYPPVTPTSPIQQGDGEKNGGLVAAVVVMSLLCIA

LAAVLVVGVCCAKRRGRIPGLGVVKKDVSGPATRASGFGEVNMGFSPTSPRVSLPLPAIP

QPSGRDTVEQYNLPDMDCRVSISRSSEASSSHCGGPSPSRFDEVSHYDYSEGEGRGRRPR

RHHFHSSSQHQDLQRQAQELSLPQGLLQQHTAPSTVSLPSAVSGKDRPRARSVPRADRPN

FLPVQGPRPNTTRSVDMVGAQDHQHLTGRPRAYSHDAAFRERLGTLSDDDQQILLKYLTT

ARQHSKSPRASQVPSMSPVMQNREGGGRPTFSLPPVQSQMSEVEGRGVVSPNIMFENPPY

FCSNKTSLSTRPEDDPSLFYLHAIPAEKDLYLEVIGDPSDILPSSVSPHSPATVSPHSEP

ATSPCSPLSASSHFPSPVTWPLLAVTSSPQSLLFSSPVGIPAGPVPKTSPSTSGSSNITS

VTSLLSPQITSPLSLPTSVRDSTASESSGAVFVSPDHECPPLKLSGPTDSLSPPVFHSPL

HFNINPGFTPSPSVPQEQTRTTPVMQGKDISMSPTQHVALSPERLQQLINRNSKSQAAKH

STPQTSPQSAVPGVVVRDHTGALFWVPNVVVPLSQHSLPVSPTSTSSVSNSPTVAGSSHS

LNHSDFGHSPTEDVHARNSFPQGSGHVTQSALPLQGNGMAQFPLYTHSPTNGLSQDCRQQ

STANSSPKLQAKSRKPKMGVDALSAQRKLTDIGDVNLNMQRELGTPAALSSSGEHLNCVS

STPKTSTHDVLTDHPGSPQATEAGPVTVVAPPLSALGPSTLGPPSSLSAVTLHGDSDSGA

YVRSCQLVPSPSPCKPLSVPQVSGSGDSTHTSGDSGTGEDLNTDADGNVHAMFEVCV

>SG-AL_contig_1575_1_-1_10_996

MVSIFSSLLFLACVSAASSQSNGVWGTCSSQTQNSNIYTYVDSNSKCCDFKSSNYLMSVC

CQDTSTNTCKCHPSIARSPYCLLTSVPATPPSARLCPAPFWTSGRLLNSSPVTSGCIGSG

TVAFVTTTGLICNAALTADVIATLSTCRTLIKSALSNSAIPISLLLGSVSMPLVRGDLQD

GKTGGNLTLWKLSPSAVIVLKQVLRNCPSTNCPYNPSTMSNAVDFNSCYVETYGASSVGA

TSYGKLSKVYLQKTNPTTCPGVSQGSGIECFHSTSGGAACLGDYAGPVFCPLNTGETALY

GLTSANTCDPFNNLFAVVPLVQTVEIPLP

>SG-AL_contig_1576_1_+1_2842_3225

MTVTLTPQRKDSLLKGCKQLSRSKSTSVCNLARIIGMIMASFPAIKYGSLHYRHLERDKT

RALEASKGNSDHVMQLSTEAISELTWWIDSVCGANNDIYCSDPDIIVSSDASLLGWGCRC

DGISSGGH

>SG-AL_contig_1577_1_-1_85_345

MSECFPARKKAYTVYITWLETIKKNLTFIYLSGSVVIVDVHNMYVDTSLSFLAILSYCTR

MLFHMDLFHVTSTIVYIFFCTALFLLF

>SG-AL_contig_1578_1_+1_634_834

MHQLQIQIYTLNVHGITYNKNTVKPHLERNPCRYACCLQTHTCATGSKSFSCCCLFAFPS

LTLWGSP

>SG-AL_contig_1579_1_+2_278_457

MRHWGCCSNFLTNAAGVCTSQAWLTPGYIMKVSAEYSSITQSLSEMELHSIITIFIIIAF

>SG-AL_contig_1580_1_-3_258_605

MRSLSYSPTREEISKYFKKYVSSDGQLDFASFLDVMHDHSTVENCQRELQAALMAQDRTH

SGYVSANDIRQILTSLGEKLSRNEVDTLFREAGISSTGQVKILDFVQTVMTPSPDY

>SG-AL_contig_1581_1_-1_40_330

MPTPLSLIEFAGIVGGVGFAAGLNFMQKKPVLTGIYKHMAFGVVGYLAGQRLERFNEENG

RKKLLVIEDYVRLHPEDFVEEAPKKYGDVLMKWFPVR

>SG-AL_contig_1582_1_-2_2795_3055

MLFIDIHFSCLCITTYDFPRGDAGGSFRINNIPTFWKFCASNFLFYCSLCLCRFSFLYCR

LLFSVFPVHSTFFFPASLHTIFSRPSL

>SG-AL_contig_1583_1_+1_1_186

FFFLISVSFTSKFIYTQILKQLILQTTLKLLNGITNGFYSIKPKYFYKLELSYYQHHCHY

CL

>SG-AL_contig_1584_1_+3_1689_1961

MVYHNSNCKFYLMIYHYSDCKFYLMIYHNSNCKFYLMIYHNSDCKFYLMIYHNSDCKFYL

MVYHKFQLNFLMVNFLMVYYNSNCKFSYGLP

>SG-AL_contig_1585_1_-3_87_1199

MADSVTVDAAANATLNETAANATAKVPATIEGMSIAYLSLFFMAILPIFIGSFRSVKHHQ

EQKNSGEKPETMSSKDAAMFPLIASGTLLGIYIVFKIFSKEYINLLLAFYFFFLGVLALA

HIAGPIIEKLLPASFPNLDYHLIFTQGKGENKEEMMNYEFDRKDLVCLAVSTLIGIWYLW

KKHWIANNLFGLAFAINGVELLSLNRISTGCILLGGLFVYDIFWVFGTDVMVTVAKSFEA

PIKLVFPQDLLEKGLAANNFAMLGLGDIVIPGIFIALLLRFDISLKNNKKTYFYSGFVAY

CLGLLTTIVVMHVFKHAQPALLYLVPACIGLPVLVALVKGDIKTMFSYEDHSEEKEKADS

KDKDTSVKKEK

>SG-AL_contig_1586_1_+2_965_1204

MIAHTYPSISICPGICVHSTTRQNHSYLHPLPNHVCILCVNIIFKLRATRHHNYLRQYHL

SNYIPDILITFKIFTNFSAI

>SG-AL_contig_1587_1_-3_63_677

MLKEGEKPSPSQPPAQPSRPFFGDIFSSWHEAFIGTPGLNRIPPEERLDSDLDSKRPNSE

EIADLFEQQKKFFPFSYGSKNMDIETVTTEADGVMEKHHTIHDSSGYERTTVARRMGDSG

HVHIVTTIKDENGQEKKHEMWIQNFDGKISEFDKQWQGEFDKQWQGKRDTADRVLINPKP

VMPVPSFPGGILSYTRGFFRKVFGF

>SG-AL_contig_1588_1_-1_406_705

MNRCTSYAMDPFLSSSVYGIPEDLTCPSIYVTFGSYAMDPFPSSSVYGMHQDLTSPSIYV

TFGSYAMDPLPSYSICGGSGYGLPEDLASPSIYVTYAMII

>SG-AL_contig_1589_1_+3_315_3368

MTPGHPDDPFSLPRIHCLFCGTTADVDTCRKGHEAYPVKTKPHEGFQATILGVCKERNDS

WAKAVSDRIQSTCREDLFAWDAVYHHACSVSFRTHRNIPARFADAKNPKPTPSSEDPQQA

RQEAFLKVCQYLEENDDEQATVSDLVSKMQEFCRDGVDAYTTKHMKNKLLEHFGADILIS

TRGKKTNVVTLRTAAWKILSQYFDTPQTEDPDAKKLNLIETAAKLIKSDIKGVFATKEVY

PGSHDIKSVPRNLNFVPELLQLFLRTIFCGKENDLKIAAIGQAIMQAARPRMLLAPLQVG

LGIQMHHQFGSQFLIDILFQLGFCTSYSEVKKFEVSAAMTQGTNIPGITKDHFVQYVADN

VDHNVCTLDGFSTFHGMGTIAVITPGTRRQQAIRRISGTLEDVTAVGKINIHFYNHLRQG

FDSFRYTQLHDFYCEDPQSTQDLLWKLSWPLHSPRPGWSGFMQLLSRGEHPGRSSVFFLP

MIDLNPNSMSCIYSTLLFVCAEARRHRIKPVLTFDQPLWWKFQLIVQKEPEHSDLKPIVL

CLGGFHTQMSFLGCIGRIMSGSGLQQVLEIVYATNTVGHMLTGRAMVRAFRGHMLVDAAL

NAMITARAFDVYLPVSPAVITDEDLSGGDAMAQKDAQLYESDESQTTQVPEDLQQALEQF

DNLIQGETLSVPKEVLDRLEKQLKEEKLKLDGHRTARLWLQCMEMVDILRRFVRGERTGN

WHLHLRALREMLPFLAAAGHNLYTKSLYIYLLQMQDLPFSHPDVYESFCKGHHVIRRSER

HWAGLSPDLVIEQVLMRSVKTTGGLTRGRGIGESQRTQWLLSMPDCAEINSAMQEVTGTQ

FCSSEQHKEASESRQLRDDKDTLAILQYLISRNPFGSYSSSLCNIANGVTSGKNVNADKA

KEIGHQIMETMLGKSVLEHTFKRKDQVVTMDVKNCVTTGDESVSVDPQLLFQRLVTAAKD

ITEDMRDLFQFELCGHPSSLFEPSGLMQLAVKAALADALWTMAGCTDIPLPKLDENTL

>SG-AL_contig_1590_1_-3_81_1265

MDSKGRKVIVCDNGTGFVKCGYAGSNFPAHIFPSLVGRPIIRSSAKIGDIEVKDLMVGDE

ASQLRSMLEVNYPMDNGIVRNWEDMIHVYDHTFGKDRLNVDTKNSRILLTEPPMNPMKNR

EKMVEVMFEKYQFDSVHVAIQAVLTLYAQGLLTGVVVDSGDGVTHICPVYDGFSLPHLTR

RLDIAGRDITRYLIKLLLLRGYAFNHSADFETVRMLKEKLCYTAYDVEQEQKLALETTVL

VEPYMLPDGRVIKVGGERFEAPEALFQPHLINVDGVGVAELLFNTIQAADMDIRPDFYKH

IVLSGGSTMYPGLPSRLEREIKQLYLERVLRGDTTRLSKFKIRIEDPPRRKHMVFLGGSV

LADIMKDKDDFWMMLSEYQEMGVKVLDKLMKPTKG

>SG-AL_contig_1591_1_-3_378_2294

MDKKTNTIKMAVYIPGNCVVGEWELKVKTTTEGKKGTATEGEKEDLVYEYPQPVIILFNP

WSKADQVYMADTALLQEYVLNPSGSVYKGTSCFISSSPWCYGQFDKGILSICLSILRRAF

GFKMSPCMGDPVTVSRALTKIVNDAGDQGVLVGNWSGNYDGGKSPGAWTSSTAILRDYQD

SGKPVKYGQCWVFSHVLTAVCRALGLPCRSVTNFGSAHDTDNTCTIDYYFDENGEEKNDM

NDSIWNFHVWNEVWILRPDLEKMKLGNFDGWHVIDATPQEQSYGVYQCGPCPVKAVKQGQ

ITVGFDTAFVFAEVNADVVHWEVNAEGEAEKVSKRDKERVGKKISTKPPDGKPHTINLSP

WMNDKGLRLDLTEEYKCREGTPEEREAVRRAVGYTRHGSKLYSECCDLEVTFDKDTKTMV

GQDMVVKAHIKNCTKEDKEVSVLMAARTCNYWNRSQDDKALAKEKREPKTLRPGQVETIQ

VTVKCCDYMAKCDKANNVYAYVCARDVKANMPYTAQEPFAIGLPDLQFAGPQKAKSGEMV

TVKVTFTNPLDVPLSKCGMALEGGLKVGPDDKNPLDNFGWIYVMECGEIPKKKKMEWTFQ

MFAEDDPDEEEETIRTEKFSFYCDQLDTITGCHSITVVK

>SG-AL_contig_1592_1_+1_1204_1485

MPSTNPTHRSCTCLPGLAKTTFILPFTNPKHSPVYVPSVQSYPEHESCTCVFGLVTIFTW

LLTNLSHHHHSAVYFTSNMIYSLSEAKRKVTIYK

>SG-AL_contig_1593_1_+2_17_217

MACMMFVSCSSPPLSIKWLSWLSNVGNCILGICSINIFHPKRKIHTLLTDLCLIPLLYFD

FPGAVNT

>SG-AL_contig_1594_1_+1_421_555

MFYATLTTTHSLVDCRRSSCKCQSQDIQRVTKKRSDQISHTLKKK

>SG-AL_contig_1595_1_+1_1_126

FFFFPCQRLFIQPLHSLYGLISTANILKALANRPNHQTNHNG

>SG-AL_contig_1596_1_-2_20_580

MGIDICHKNDRKVHRTEPKSQDVYLRLLVKLYRFLARRTNAKFNKIILKRLFMSKTNRPP

LSLSKIIRLMKKPGREGKTVVVIGTITDDVRIHKIPKLKICALRVTAGARARILKSGGEL

MTLDQLALRSPKGQNTVLVQGPRKAREAYRHFGAPGVPNSKAAPYVRSKGRKFERARGRR

KSRGYKN

>SG-AL_contig_1597_1_-1_52_1728

MAFRRTNVRAHDMIHVPGGLNTGGQIVVRGRVLPHETQFVINLRCGEEDDSDIALHFNPR

NNDGDTVVRNSFQGGSWQNEERDIPRFPFDNGRKFTIRIMTFPDYYRILVNNWEFVRFDH

RIPPSEVRYVQLGDGAEYYEASVQNNCRAPFFGHFPGGLKIGQAVRVRGMLTDDSDGFAV

NFQCDSDGDNIGMHFNPRPNEEDVVLNSCEGGCWGDEQRGFENDFHFRQGEFFDIFFIAV

EGRFNVYVNEKYFTTFDYRCSYWDICHLVVKGDVELMDVELLDPLPDDFIKEIPQGMEKN

DLVVIKGFFFPEGNTFSVNLIKGSCCDDDIAFHFNPRRNEGEVVLNSKEGGDWEEEERHP

LPICMQELVPFELQILAKSHKFKVYSNDRKLCKFRARDDIEDVRAINVKGEAYIYEVKLL

RRLEEPFTDSVPGVLEPGNWVLMYGSPDDDVEGFAINLQCGPDKDSDDIAFHFNPRFNEG

ETVRNSREGGDWGGEERDQPYFPFEPEDRFELAFVMLSHGIRVFVNRRRYIDYNFRMNPD

RICHIYLSGGCDYFEPEFF

>SG-AL_contig_1598_1_-3_105_293

MYCRKRNIRHSVAVHCINPSSRENYSVYCHFLSLFCSPTFSRHVYFLPSQRHWAKDGVPV

FFF

>SG-AL_contig_1599_1_+2_584_838

MLQFTKTCQIKDKNDWKKVSKKTMRNIKLLNTRIKFESNKNTYTRKAMQLMYSVRQRTKL

CYNALVNTNVAHVCYKNGKIHQCAI

>SG-AL_contig_1600_1_-2_224_1189

MDSLAGKVILITGASTGIGGFTARYMSKFKPKLVLIARSPGKLKDVQQLCEEGGCGKGNV

LTITADVAKPEDLTRIISETITAFGQLDVLVNNAASFVVNDVQTTTLEGYERTMAVNVRA

PFFLSQAALPHLKKTKGNIINISSVAAHRPFLFTAAYCISKAALEHFTRTLAFEVARDGV

RVNAVTVGVTVTENIEEKFKVLGKEMDQNEVFPGVTDYQSLGGACNMEDVAKVVTFLASD

LSSAMTGTNIPVERGYMLTSACSLPAVMNALSSSQWANSGDQNAKPTEESLKDLIAMKDL

LGKGDGATKSSESEGPTKPSEG

>SG-AL_contig_1601_1_-3_564_719

MTGLKTNKNKKYTRAHTYYFETITFSISDGFSFFFHSSVLRLCCLSFLPRKC

>SG-AL_contig_1602_1_+1_1_243

CLEQHKVCVVADQTVWNNTKFVLSRTKRFILSKMSFLFEFCPQDLFCLLVFLFVYSLPHC

KADNRAFPLLELKTTTCSYSI

>SG-AL_contig_1603_1_+3_1065_1271

MHVCEHCVCVCVCECECVCVCAGVHNIRQLFINKNLTTNANSTFCLLGMKISRSPVHHHF

PSDICPDME

>SG-AL_contig_1604_1_-3_147_632

MADTPFKYNPESPPYTSFFGIVGVTAAMSFCALGAAYGTAKSGTGISAMAVMRPELIMKS

VIPVVMAGIIAIYGLVVAVVITQNMSGDESYTLYKSFLHLGAGLSVGLSGLAAGFAIGIV

GDAGVRGTAQQPRLFVGMILILIFAEVLGLYGLIVALILSVK

>SG-AL_contig_1605_1_+2_875_1276

MKMDTMYTESSSQTEFTCSAQGEMTVCDNTTIKKHFPHCCSVTKITKFTEGEEQRNSALS

CTNKLMLNDKYHLFRAALAKKQYLFNQSPMMKKRAQRTGWGQITEKRADDLLIRRNTAHW

SPTQHSPLLTMRTC

>SG-AL_contig_1606_1_+2_503_799

MTHSCDKLLLKPHETSCIKNSSLCPFNNLFGSIFQSAYTQHIMFNFSNRNTWGKNLKTFL

ILKTKSILQMYIQSEIFLLKKISVTACETLAMVWQLLQS

>SG-AL_contig_1607_1_+3_18_269

MKFIEKSARIILQSLVTTVQCLQTPTYLMSTQILMLQLFLVIQIKRPTQICIPTIGQTRK

RNTRVTSFLFRYDYAIWQGCGEHT

>SG-AL_contig_1608_1_+2_191_472

MEIQQKAQAHSLCLIHFHLSSLPVLTCKSSPLSYIKKIMIENTFLRIHTGYVCVCVCVCV

CVCVRVHGGLRGDGGLRDRAMDGYGTRFDKLCEY

>SG-AL_contig_1609_1_-2_2_1015

EVVITDPHGRKDAVKPTIQPVVGKEGTFLVEYTPNDQGVHTVSIFFTGIQIPKSPYTVQV

SPPSNAKACYATGRGIQPRGIRVKENAVFYVHTEGAGEGDVAVKIIGPGGAEEKCNIRKV

PDKPGVWECIYIPSKPGNYVITVTFGGEPIPKSPFRVEVGPIWLSKIVAYGPGLEGGVVN

QPAIFTVETNGEVGALGFSIEGPSEAKIDCKDNGDGSADVTYWPTVPGEYAVHILCNDKD

IPKSPYMADIKPATNEFNPGRVIAKGPGLQKTGVQVNKWAEFTVDTKLAGKAPLQITCMD

AEYKMVEVQIKDNRDGTYSCRYMPTRNIKHTITITYGA

>SG-AL_contig_1610_1_+3_84_488

MNSNLFWKHIFTAYNSFCRNVKSTSPIGLLSEPILYNEKIIIGDDMIKFTSWFEKGVYCI

AHFLNGNGSFMIYDEFNRKFNTDVNFLVYYGCKVSIKKHIQETELSTPNGCAHDISMALK

IIDSVLTGVGRYYDI

>SG-AL_contig_1611_1_-2_2_361

PPTLSLSLSLSLSLSLSLSLSLSLCQAALACPPKNSPKATLSQDNLSLLLLGIFGTRFPR

LLIVRKDGKEEQSYIICHKFFCQHYLACSGLINITATTFPVMFCRLSLEFVTCSVHILPK

>SG-AL_contig_1612_1_-1_895_1137

MARPVNNLNQFLLDSVNFLKGKTFKILMQLCRLGNWIRLYSCKLCLIKGEHIGAFQCDGC

VLCTVIFMPISPIPMANTGFT

>SG-AL_contig_1613_1_+3_66_359

MICLGLSHEPRDKQRRCCANKHKSAASRLRQSRFACIWRLVSLRFVGRVYKRMCVPEWWR

SSFGLVKKRLNKPRKTSALPSLVFSCLAAVCTLLSWPF

>SG-AL_contig_1614_1_+2_1061_1219

MTVYVKMDTAFFFTFITELLKLQSSVVPQSLRTKVKKRLNKPRKTSALPSLVF

>SG-AL_contig_1615_1_+2_3479_3709

MVSTDPFGLTTPSLPTGILDLNAPTFPPHFILSFTIQTPPCLSHGTASALLHMFLSLLLT

RQLMKINTTWPMNLGKP

>SG-AL_contig_1616_1_-3_3_236

VSVLAKREFTWQNSSFRLLDISPNPSSFSQNPCARSPWETAMPNRLRPPTRGDSALLLGH

QWNSTVPVPIQLTKDTAC

>SG-AL_contig_1617_1_-2_2_118

AGGNERVKGCFGVFFSICSLLLLLTQLCVVSFSLFGPFS

>SG-AL_contig_1618_1_+1_331_684

MICARLELLVFLCLQSADQRATNNADIRLSSLFLSLSLLSSIQHLLQPSTAETVRVITGP

RVQGSTCSVHVPGMQARVTSRMQSRTATRDFFFLMTTASPVMRSMSSSLRLTRPCWHW

>SG-AL_contig_1619_1_-1_103_558

MSSDVEYLLLKEKGPYHEVYVRRRLNTNPGACKFYTIIISVISISLVVLLLSLVYVFVLV

PSWQGSVLNWSRRTMQKAHTHVLTHTQCGPVEGVMEDDVFIFKGIPYSLPPVGARRWEKP

QKLENKTCWQGIYQAKEFGSMCVQPAGISNFS

>SG-AL_contig_1620_1_+1_1_267

RSDQIRSDQIKSNQITLCHIKSNQVKSNHINRSTNIESNQIKVIINQFRPNQINQIRPNQ

ITLNQIKSSHIKPHQIKSDQIRSDRNESN

>SG-AL_contig_1621_1_-3_837_1166

MIEHCLEKRSFSSNTFAVFRHKIYLIKHFVCIVHYMRCVEIIDWIVDSECLEKGNLVLEI

KASFIMFIKVCSIPLTYHISLMIFIYLFLNLLICLAFKLLGTFFSVHFRK

>SG-AL_contig_1622_1_-3_285_467

MCVAFACCALQGFFHHIPAPSHCAKLTNIACPSCCCIFPFQGLLRLCVYPFFVLTCIPTE

V

>SG-AL_contig_1623_1_+2_95_199

MFSALGYSLYTTEIRHYFNPYHYSSIITKTKSLPL

>SG-AL_contig_1624_1_-3_3_86

KKRKKTSIPIRIVSQVRVHIILSEISCH

>SG-AL_contig_1625_1_-2_8_439

MNNQLPAYEEKAFTTAEIRTAAEESYGFETVERAQKIGGLWRLYPCSTEVRHKLLIEGFT

PRGLLIEVKDKNPYVVVSPDGEEREAAATKLIISNVPLSFSDEEILKTIKQIGVSVHSKL

IAERDRDKDGKLTRWKTGRRFVYI

>SG-AL_contig_1626_1_-2_47_355

MAGVVPVFIMNNQLPAYEEKAFTTAEICSAAEKRCGFETIEGAQRIGGLWRLYPCSTKVR

HKLLVEGFLLREGTNRSQRQKPLRSGFTGWRRERGSCNKTDNQ

>SG-AL_contig_1627_1_+3_1980_2477

MKSSFVLTVKESSHSILTHLILTIIVYHKLMTFSYLSLTLSYCFPTNLFYSGILTSPKYL

YSTKYTAVYEYCCQHSCSCICAFGKASPIQIQCSQHEFSGNRKGLPNFPHTKRLTCLLLP

KNITIIKTNRKCTKIIHFSHVKSHSKINQQCHAHIHTVACFLSIHA

>SG-AL_contig_1628_1_-1_79_216

MTSVQMVSHTGSQNRDNDVVHINSVASYMSNSSIVAVHEKNHKRPH

>SG-AL_contig_1629_1_+3_3_125

SLVFVLQRMPSFLVSTETAHYALYPAFQVQLSTLIHTSSLC

>SG-AL_contig_1630_1_+1_613_807

MQVYMDHLWNISQQTVFCSMHVGIKASTIGMATSDFLLAKREWIKRDSTVSAQVPVTGVN

SDSPL

>SG-AL_contig_1631_1_+1_1_207

LVCVVKLASVHRFSHRFFFFFVFFLNDIGIKMDRKETDCRGFLFLNDSDIKMDRKETDCR

KGRKADQKL

>SG-AL_contig_1632_1_-2_2_253

SGQYSCQTNADSVQVHSVWLTVAEEENETESSQDASEGTPRVGLSGVFDDETEERAAAAG

KTTSSCSTSSLIVLVLVIVFSSWG

>SG-AL_contig_1633_1_-2_2513_2761

MVNCSLISAVLFYCSLISAVLFCLPHTYFISLVFQLLDTLFSWFSSYLVTCDPMCRTQEM

FVLFVRFLLPVLGTSCLSLVNWI

>SG-AL_contig_1634_1_-2_2465_2743

MNGSPPSVCLSLFHTYTHTFKVQSSKFKHQHDFSIYVQSTCFQFNCYPSMLSKLDDKRWS

FPHGFVLSPSPSSTENIGNRNSFQLAVYLKKVV

>SG-AL_contig_1635_1_-1_76_477

MSLDFERMGRTFVQEYYQRFDVKQMRESVICLYYPSALLTFEGDQFFGTDEIRKKLTEGF

PMNNINRCVTKVDCQPTAEGYVIIFVIGQLQASDCEGDKPMGFSHMFMLKPCEGGSWFIS

NEIFRLTLHDFAAH

>SG-AL_contig_1636_1_+2_65_526

MLEALLAILGHRSAHSLATGPVIALPFISPLLLTITPALSSNTMNVPSFLLMAFLCRITT

AGITFLRSSGFPFLTVATNMSPEPAAGRRFRRPLMPWTAITYKFLAPVLSAQLITAPTGR

PSAMRNLPPAEPPLPLFDMLYLHLAQKGIGRAHV

>SG-AL_contig_1637_1_-1_181_333

MLFCYLNIYSSFCLFAVDNIYCWLVLYVGLEITLFYFPVKVVLVNVSFVPE

>SG-AL_contig_1638_1_+2_308_796

MNIKKIACPVTHTHTHTHTHTHTHTHQTQTTWSTIIHINDKHSNTKHKFTQHGNARSTSL

KKVILFFKFIKVSVCEHSALFFLCLLCLLVPPVDFLLVILQHFFRFLQITWIRFNRIQSR

ITLSLSLFRHQFIFSVFLNTRFKIRLFFTFAFHSFLFLFLKPA

>SG-AL_contig_1639_1_+2_1094_1432

MLAPRNGEVSTTSLPYDLRVMGQPLHTHTHTHTHTHTHTHKSGKWFPSECDASTISWMSP

FGGLLKQAFGPEGNLMFPVGYPQRSSDLFILSLSLSWTDFVFFKLGQVIFDWR

>SG-AL_contig_1640_1_-1_517_765

MDSSSKRNPQHKNWPVHCLPQCSNHSGGVNLNVSKVPLSTPIPPISKPCRHIGFCAGSRS

LPDHYWQISVKHIWNCPSVMNPS

>SG-AL_contig_1641_1_+2_143_274

MNRSQKTNNKSEQKDQQQNRSKNTQVKTEGKDNTPDLEPAVGGV

>SG-AL_contig_1642_1_-3_843_1094

MTLLMHASIHVCACCVRVCLTIKWLCPTLETCLLYLCSPCLLSHCTKVHWRCHVVCRTLN

RDLSVILGCNRLDLLCLGGHHLPF

>SG-AL_contig_1643_1_-2_410_694

MERDFTSQSLQQTPKYRRTWLFIITAIIQSCIRTHHLISLLDVKLKNERTYTHTKSVSKK

DHTLIKKQNKNKPKKKPQPQDGQAILILSLFTEFQ

>SG-AL_contig_1644_1_+2_83_187

MHVRVYVCVCVLKYLIYTCQVFAIEFKVCFIQFNV

>SG-AL_contig_1645_1_-3_3_293

IPDHVSSGLVDGSSQADSKCTSTKAYAPFTVSKSHEMHPLPPTNSVHPLTQISGIPFKYT

VNRLKILTCPGKLNSTFLLSPSQQPSHSVPHFLLLPG

>SG-AL_contig_1646_1_-3_603_818

MAETLQFSLVFTCCPNTYFFKSYNCYLVCRVEKNQVFHLFSQGNTVVDQVLSICSFCETP

SVLCDCHCRLFS

>SG-AL_contig_1647_1_+3_993_1208

MDTQKYNGVHWSVSPQIKICSTGMNSKVYSVINYESSLSLLLPVFASFIKIYQSPVLVFA

ELFCLKIGVVHF

>SG-AL_contig_1648_1_-1_19_999

MACTGKVLTRLTALGGRQSLRSIYTATRPNLMIGSHTKLICQGFTGKQGTFHSQQAIEYG

SNLVGGVSPGKGGSTHLDRPVFNSVKEAVDKTGADASVIFVPPAFAAAAILEAIDAEVGL

VVCITEGIPQQDMVRVKHRLVRQNKTRLIGPNCPGIIKPGECKIGIMPGHIHKQGKIGIV

SRSGTLTYEAVHQTTQVGLGQSLCVGIGGDPFNGTNFIDCLEIFLADPQTEGIVLIGEIG

GQAEENAADYLKNHNTGPKAKPVVSFIAGLTAPPGRRMGHAGAIIAGGKGGANEKIDSLK

SAGVEVSMSPAQLGTTMLQAMENAGHA

>SG-AL_contig_1649_1_-3_147_347

MCDACMHTEHNKCMFTTASMHTHVVHFCLVGKHIIYQPPVRLCLSLSPSPVLNATSKQLT

MLSIFPI

>SG-AL_contig_1650_1_+3_618_746

MIIIIMIVGLHQLGLTLADAFYLSALGQRLLWLKRSHLCVLTL

>SG-AL_contig_1651_1_+1_991_1242

MLFHAKSCPDLPCCFVNLSPVNPCCVLSISCEIVMKICTESCLRYLCFHPAHPEMCALVT

IQPGQLPFVKTLPSVENNCALSAL

>SG-AL_contig_1652_1_-1_2053_2454

MFIVPTKLKVPTGIIYIYIYIYIYPKQNKNENTPKQHSMAKPFLPLLILKFINQRHFILC

IECNNQHIHSCIQIQLIYTTGTILVILEKSINISTGYTVAGLQYSRQVYNMHITTKSPCY

CMYKAGTNVLCVCL

>SG-AL_contig_1653_1_-1_64_282

MYDLCNETSSNIVVNHSPNEIPPTLQETDLNFPLKNNGIINKKQIKIYNCKNDTNMRLLL

TSSIRLIILLLKV

>SG-AL_contig_1654_1_+2_221_400

MNSLIPAQSDTASHSVQFLLPRLYQDFSGFLQHTWGAIGHLLSSNGWNSCPHHSPPAGVN

>SG-AL_contig_1655_1_-3_84_1415

MKFLGLGLALGILCFCLGSSYGSVCSDKAAMEAAQRRCFSKFGLEMDMSTQEIQNVVQKD

PAFACRKPIEYKAGLRCAMTLAKTCLAEAGQSNDILPDVDRMIHGVTYLCNNIRSVDTTC

VQTAQDGLRSCAMNKAMAIFSGSSNTDITYITCKAFEITVECLQSSLTGCAMRTKSILVR

FTQHYLTPPACRSGSDSDVGYGFTTVAPMTTAQLVTVVPMLPHVSCYTPSSMQEGQRQCF

ENEGISIHLPQVNLRNGMQAKQSYLDAVLANNHDIHQLCSEVTRYQRAMGCAMEVARKCL

PAGYKDYLPGTHNVQRMLSELCNNVDNVNQACVTSKASALFDCGYNKGVEHRSYDAKVLL

CNAFKWNVACQKEVLPSCGCRTTALYVKIAKEYLNPPACSPITEADPVCIAGSDRFASSA

PTQSVLSTFLLLAAIAACLSFWKL

>SG-AL_contig_1656_1_-1_163_321

MHAGCFRVSINHHDRTQTRHMGSLACVFDVLRVYAYEGGSSTCRPKHMLTWEI

>SG-AL_contig_1657_1_+1_79_1623

MGKKNRKGGGGGATAKAPSPSGSGSKDSAESGSSLSKSARREVTELVNQLLEVCSKPSSP

GAKELEDYPEIHGLVEKIRKIQSGLKPVEVDRVQAFPAFMDWLHKNGVDTSAVEIHDFPG

YGCGLRATKDVKESELLVKIPRKVMMTVDSAFKSQIGDLIAEDKILQVMPNVTLAMHLLV

ESKTPDSFWKPYIDTLPTSYSTPLYYSLEELDYLRGSPTLGNSITQYRSVARQYAYFYRM

LRKHASASKLKMLENFTFDDYRWAVSAVMTRQNLIPKEDNADKKTYGLIPLWDMCNHASG

QYTTDFDTENNCSMCYSQQDAAAGEQIFMFYGPRSNGEFLVHNGFVALDNEHDRLCIKLG

ISKGDPLFEAKTELLGKISLQMSRFFFLHCGDCPVDGETLAFLRIFSMDEDTLKSRYTGD

VSEETQNNLLELESPVSPDNEEKVWTFLGTRAALLLKAYPTTVEDDENQLKKTDLPNTLV

MALELRLREKKILQQTIAYASRRKEAAVEGSVQCD

>SG-AL_contig_1658_1_-3_54_329

MILKFGISPFPCLPPCLRFPVCLRFPLSPPPSLSLSSFTFPPLSGLSLGCFLFTQQLTIL

RTKDRYREYRFRKEELKNKTKQNSISARQNYQ

>SG-AL_contig_1659_1_+1_253_522

MFWKHSKLKRVADDVVVVLIHLLLLIVVFLLMLLVIVVVVLLIGHLTLTWFTGSLNSYLI

FCMHINTKEVQALVGLHIMLTLEIRKMSIP

>SG-AL_contig_1660_1_-2_2_352

KLPAAHEKETVDDLSAGDSSAQGSAGATVPTIPISTALTPNEDVTPPASAKGTESSSPSQ

SAESADPSVSSKVDQSDAGSGGATTNSATTSNSEENPTENREAESTVTENTDNEKPV

>SG-AL_contig_1661_1_-2_131_316

MQVIQLPLLELYHRAQILMKTDPDKQEHQKYSWAELLQRVVSGNLVYPWVNPYSTNSKEK

EL

>SG-AL_contig_1662_1_-1_3418_3696

MQWGHTCLREKPGQKRSKFELVFIFFWWSIKPFVSPSPLSLSSLSSSADVELQKYSLITS

LNSGESKSEGSFSSAASLADNAFFEPAIFVYFQ

>SG-AL_contig_1663_1_-2_137_331

MDLYCNIYKMEFFFLFNTHGHSLSHPHNFKHSPTSTFYSFSLWTLLRAKDLSVGMISERT

VVTET

>SG-AL_contig_1664_1_-2_38_1165

MPTKFLFQYLDLLLPAITNIMNESLTSGIFPLEFKTAIVKPLLKKSSLDPNNLKNYRPIS

NLPYLSKLLERLVLHQLFSHLTTHNLLSAHQSAYRPGHSTETVLLRIVNDLLNSLDQDKV

SVLLLLDLSAAFDTVDHKILLTRLSQNFGITSTALNWFRSYLSERKQTVLVSGNRSSESV

LDFGVPQGSVLGPVLFILYTTPLTPLIDMYSVHHEMYADDTQLNHSESQSNYNHLIQTLQ

DCVTEVKTWMSENRLKLNDEKTEALRLLPPSIDSTFLPSTVALGNSIIPFSDHVRNLGFF

LDKDLSMQQHITKTCQAAYIEVRRISSIRHYLTLDATKTLVSTSILSRLDYCNSLLIGCP

RKLLRPLQQVQNSAAK

>SG-AL_contig_1665_1_+1_1534_4005

MDDYLDSDTSAYTEKHMRQKIESHFGDEIMITCIRGKQNVVTFRSVAEKILKQFGNSKKE

TDSEKEKLRIIQTATRLILSDIQRIDTSKQEYPSTAEISDASKNIEQLTPSVAPFLGTLL

EGKKEPELKIASLGQALMQAARPNVFIQPLQLALGVSLRLTGSRHVVDQLNRLGFSLSYN

EVKLYEMCDAVEQGTELVGISPQNSFIQFAADNVDHQINTLDGTGTFHGMGIIGAATPGT

KDSRIIRRDTSVTPEKVSALGRVPIRFYDTSTLNISMAYEILKEISADDGSKKLDLLWKV

SWPLHSPRPGWSGMMQAVCNEVYPGQSTFTFLPMIDIDPTNMSCIFSTLHFVASLAKQYN

CTPVLTFDQPLWWKSMIIVDSEPPNSSLRSIVLRLGGFHCEMSFLGCIGKLMSGSGLEQV

LEVAFARHSVNHMLSGKAVSRAVWGHFLVDSVLNSLFVSTAFRIPLNRHSIADTEQGEND

SKTGEPSSSQENLSADDPMDESQHQQVDEDGVSSEQSADSSMQRKLSSVLQVFDNVIDKQ

MAVDELDEEANKDVEAVEQQLLKTRSQLEGRTSKLWLQYMMMVDILRQFLKAERTGNWML

HLQALQRMLPFLAAAGHSQYTKSVHIYLQHMQKLEDHHPDVFDAFMQGHHVLRRSDRFWA

GLSADLVIEQVLMRSIKSAGGLTRGRGMADSQRTQWLLSMPACADMHNALQEVTGLEYCT

SEQHVEAGENRMARDQQDMQALLNFLIDRSPFEDDNSLRNISTGVTADTSVNVDKAEMVG

KAILASMTGVHVKDFKLSKKQQAGTMDVRTCARVDGESFQADAQ

>SG-AL_contig_1666_1_-2_569_1234

MPVCLSVSTCLYVCLFVSFYVSACLSVCQFLRVCVSVSFYVSVCLSVSFYVSVCLSVCQF

LRVCMPVCQFLRVCMSVCLSVSTCLYACLFVSFYVSVCMSVYQFLHVCLSVSVCPSVCQF

LRVCMSVCLSVSSCLYVCLFVSFYVSVCLSVCQFLRVCMYVCLSVPTCLSVSFCMSVCLS

VSTCLYVCLFISFYVSVCMSVYQFLHVCMSVSFYMSVCLSVS

>SG-AL_contig_1667_1_-3_3_227

IFFIFFYFFFSASSIHHLLYILFSCQIYSFFIKPLLFLLAFNVCHVLYDLFVSVKIFISF

LLNLCFICCIICCQT

>SG-AL_contig_1668_1_-3_3_149

TVMTLGPRSIETVPRFKFDWKSTTRKFVCSKLLRRAECACVNCAFFLSC

>SG-AL_contig_1669_1_-3_3_182

GEGGGKGLASFQIVCTVHRKARGSLSHNCKLHHWGVRWEILTILRSAFRGQVSSLSSLPD

>SG-AL_contig_1670_1_-3_24_1064

MDGTENGDYLGLDLGGTNYRVVLVRFTKGVADITIQNYVIPTHVLSGPSSGAFDFIADGI

RQFFEKYNMTGKTDKIPLGFTFSFPSRQKTLKSQTLVTWTKSFKCPDGPGLDPVQLLEEA

LQRNAPNLPIEVVVVMSDTTSTLMAGNYVDKKARVGLILGTGCNAAFVESLDNIERWAGD

RKDPQHVIVNVEWGSTGDRGCLDFCRTRYEKQLDQLSNHPKSFTFEKSVGGLYLGELVRL

TLVKLAEEGLVFGCHLPEALQPAWSLTASQITQIESDTGRDQCSHTMNVLHELGVSQPEP

DDVAVLREVAEMVCRRGAYLVCCGLAALVKRMDLPAVTVAIDGSVYE

>SG-AL_contig_1671_1_-3_3_173

PPPLLPSSIGPIQNKQPAISKYHLSCIFYNLFDCFIIMMPSFSLTKWTMLNACVSMS

>SG-AL_contig_1672_1_-3_42_149

MSFARHPIMVGSVCYNVNCTVVPIINTSCSHAKFCK

>SG-AL_contig_1673_1_+2_2_238

SDLFFLHENDNLLKHIHFFHKHINSHSSAINNKSSLTHSPQIIHVLSKSSYGCVNGEKAV

LLFRQAFMGTISFSEKNAQ

>SG-AL_contig_1674_1_+1_70_216

MSKQQFSINKNEVWPPTSNNSHNHNARTDYETSLPSKISNNNFMKNMSC

>SG-AL_contig_1675_1_+1_73_396

MGSTTCPTVPAPSTSTATETVTEDASDGEDDCGEGKTRISSKDALDCVQKIDVYCLQSGC

DDAIMKSFHVFTDLLEKHMASAKTQTQITDFFQVGTKSGESTAKDQGK

>SG-AL_contig_1676_1_+1_61_402

MMVFATMGSTTCPTVPAPLTSTVTETVTEDASDGEDDCGEGKTRISSKDALDCVQKIDAY

CLQSGCDDAIMKSFHVFTDHLEKHMASAKTQTQITDFFQVGTKSGECTAKDQGK

>SG-AL_contig_1677_1_+3_267_533

MYTCNRHSAIMYNWTMKVFMYNRTIREDGVCTQAQYQQKDCTQKKWEGLNVHTKVRTKPY

GTVYADFYSCQSAGINTIYIIAQTVHTQH

>SG-AL_contig_1678_1_-2_218_409

MIGAYHFTVYIKKSVLCSNEHFVCGLTMRTSSVFGERLLLVKKTCEPPFLLLKRDYKTPL

QWNT

>SG-AL_contig_1679_1_+3_1158_1481

MGICPYTHSPYCGDGACAFSLVLILSLLFVSSTSEKDHLPSNDYLFWSLLHNNLYHKRME

VGNKTNSPFQIAIEAKQKKTPQTKPPPQEHPINLPPTSFLFSLWKKMR

>SG-AL_contig_1680_1_-2_119_361

MKCACWVFSWFHKPLDSDMDHNTCISNAYLMFCMPYSQRGFRHNQVCIYVDLADRKNLHL

LPTRHSQAGPNSDLPVGNLMS

>SG-AL_contig_1681_1_+1_1924_2184

MATLHHHQYTALDWNSLVSFSPLRRYTVNLLLARQSRRDQRHTIKLMATSDCIKFTALLK

NYPQLSLEGTALCVITAVLSYKTVIWT

>SG-AL_contig_1682_1_-2_2_445

PSLSHLHLPPPPIFTPSLSHLHLPSPPIFTSFPLLSSPPSLSHPHPPPSPIFTSLSHLHL

LRLPSSPPSLSHPHPSPIFISLPLPLVSSSLSPSLPPPPHTHTHSKRTLVSDIVAHLLSQ

PFALFFHQVFICNSSVTSLSVSTAHTHT

>SG-AL_contig_1683_1_-3_30_380

MGAKKVDPKGGKKAAPTKAKKEGSGGGKAKKKKWSKGKVRDKLNNLILFDKATYDKMLKE

VPSYKLITPSVVSERLKVRGSLAKQALRELHSKGMIRLVSKHSSQLIYTRTTKSSDE

>SG-AL_contig_1684_1_-2_35_316

MYFISRLRTAAAVLKPKPIIQTAVRNGGTWYYRRPAPDPPKYIMYHAEILGFLMWYWILY

HIWKEPEHITGHFPYPDPSQWTDEELGIPPDEEG

>SG-AL_contig_1685_1_-3_15_1034

MNKMDVQNAQKNWQLANNIESVSSADDIYRFDAKQQQDILTAKPWDKDPHYFKHIKISAL

ALLKMVMHARSGGNLEVMGLLLGKVDGNTMIVMDGVALPVEGTETRVNAQAQAYEYMASY

TEAVKMVGRLENACGWYHSHPGYGCWLSGIDVSTQMLNQQFQEPFVAIVIDPVRTISAGK

VNIGAFRTYPKGYKPPDEPPSEYQSIPLSKIEDFGVHCKNYYSLEISYFKSSMDRRLLDS

LWNKYWVNTLSSSSLLTNADYTTGQINDLALKLEQSESQLGRGGFIIGVDTQDKKTEDKL

SKATKDGCKTTIEAIHGLMSQVIKDRLFNQVHCGKAGPSS

>SG-AL_contig_1686_1_+1_67_264

MFSRSHKSVLVLHILAFSCVGTIATTTFHIYSTLLHTSFSIILIVGLAYVILVCCKLCES

LQVINL

>SG-AL_contig_1687_1_-1_127_378

MAAALMQDNVWLQKQRFEEAEAHYYAIQAGTATPGASSQGGASTLRNEITQARQQIQNAL

KQPGNQGSGGGTNPQVQKRIDDLE

>SG-AL_contig_1688_1_-1_136_327

MRENNPHPTMMMRLTTMMTLMCIYNTFIYIKYYIHNIIHISCCCYLLWSQCRIRNQLHNI

HAHE

>SG-AL_contig_1689_1_-1_502_720

MSLFTFLEVIKICEIKSDQAVTFILFFLFIVIHHSCTNGVTLRLLLFHSEICDLLQFNQH

SSCKEVKSVIAST

>SG-AL_contig_1690_1_+3_2013_2606

MYIPCKYFELHTLHTTNHTHIINTHPHYFIFNQGNIHTVPSSLMYVYFILTHPKNNLRPE

MCFTPPQWTTITTLFYLVHLKLTSHLSSCQCNNQNNLPISDGICACVPDEDEWFHSEFTN

MEHHSWSVQSRTPAVFTQNTATVSIVRLYLLAFTQSQCHSHGILLTWFLLLLLLMLLLLL

YSAAVHHWDPALVFYQLK

>SG-AL_contig_1691_1_-3_84_488

MLLQAASRLVGRQVVQLTRRNIGLSAVAAQKAATVSDPIQQLFLDKVREYRTKSVGGKLV

DATPQVEQSLKDELEKLDRVYNAVGQDMTQFPSFNFTDPQLESVGLGDTKDVEVVTAEEE

VEEEEEDNKPYFILP

>SG-AL_contig_1692_1_-2_599_817

MYIVYLHCKCCSLFILCSVLYLWGCHIVLRLKFAFKQIAVNLVLMSVIMSLSHQSTSSYF

RLSVHLNTSACNM

>SG-AL_contig_1693_1_+1_22_186

MRVNLLVIMLQRLLQCSKLGLHDKMKILTFQMRLQAEHFYIQHNYNPFPYPRCKL

>SG-AL_contig_1694_1_-2_80_382

MFHATGCHFSRIFHSLGCHFSRIFHAMGCHFSRIFHAVGCHFSRMFHAMGCFFLWNVPCD

GVLFLRMFLVMGCGFSVLLVGILLVVTCPPDLFRYHSILWR

>SG-AL_contig_1695_1_+2_2_205

GVWYIFFYNLSSKNHTIFSVNASFLLEILSVYSAILTNKPDSLTLFCNINIQIRFTHYFF

FISFIFSP

>SG-AL_contig_1696_1_-2_8_481

MMSRRKQALIEFEDISGAKNCVEYAQSSAIYVAGQPTFFNYSTSQKIQRPGEGDDARPAN

HVLLFTILNPQYPITVDVMHTICSPYGQVQRIVIFKKNGVQAMIEFDNLESAKRAKQSLN

GADIYSGCCTLKIEFAKPTRLNVVKNDGESWDYTNPSL

>SG-AL_contig_1697_1_+3_489_782

MVPFTNPVKNWSHNTNTYLHSHFHIAIKTCLFIPGKGDTFSNNKKIADNKPIFMWPNFIQ

TKNGVDFLNISTYYVLIKEFSLYICTHKLQLNFTLNVT

>SG-AL_contig_1698_1_-3_15_1385

MVSPTGTLSSQSMVQQQQQYHQQFQQHGQQQQQQPQQQLQQQQLQQQQLQQQQLQQQQLQ

LQQQQQQQQQHQLQHQVSDGSLHIDTSHRMLSPGSQARSPSSAGSPPSPGNLNTLRQELS

TAHSMSGSVVHSHSLTGQSSPSVYFGLSRRGSLSSLADSDMVHTTPKFVKDTSKYWYKPN

ITREEAITLLRDRAPGTFVIRDSNSFPGAFGLALKVAQVPANVQSKPSSDPQSELVRHFL

IEPTPKGVRLRGCSNEPAFGSLAALVYQHSITPLALPCKLSLPDAGDAVDGPASPDMVSP

TELNSAADLLAKGAACNVVYINTIDTESLTGPQAIAKAIKATMDTQPVPKTTVVHFKVSS

QGITLTDNNRKLFFRRHYPVSAVTYCGMDPQDRRWKRDTDTPGVQVDAKIFGFVARKQGG

VSDNSCHLFAELDPEQPASAIVNFVTKVMIGQSKVKS

>SG-AL_contig_1699_1_-3_117_3548

MTTCVPQVSNDLSNEALNKPQSDRSVGQSSQGSRGIMSSLRDMLNRSQEGTGTEELLFDM

TYVTERIISMSFPAEGHDTTYAFNLKDVVRMLRTKHGDNYLVLNLSEKRQDLVKANPQVK

DYGWPDHLAPPLERLCSVCKAIDAWLNADPRNVAVLHCKGGRARIAIIIAAYMHYSNICA

SADQALDRFAMKRFYDDKLGGLPLPSQRRYVHYFAGLLSGAIKINSNALYLHHILIHGVP

NFDTRGGCRPFIKVYQGMQPIFTSGVYNVTDNMQKVCISISPGIPLRGDILIKCYHKKAR

SNQREVIWQCQFHTCAIQDGNVVFSKQELDDGIHDPRFPDNGKVEFVFGPSSDVAVSVSG

FKSDVTVPVDDNEESLTRSDSYENFHKALAAQGLEKNANGNALNGVTTQQKTQVQVQHTQ

GPRDGSLYATVNKQHKTETETTSLAHNGPSHLPNGSLTSSVDSGYAGPRDTSYGSAHQQQ

QAEQLSSSYTTQVNSSRTNYNTNTLPRAGGAVVEDKLNYNSIPHQQPPPPTFDECSQLDE

LLSDLLSDQVIVKSPTSPRSPSGPRSPSGPRSPLSPASPQQNLSGAKTMTTATHTYSTFS

STDSGSGRTGPADSMFVQRSEVAYKVPVHVEERHNYSISGQPPESEKSRVPKGAFTYTPT

SPSQTDQSRPFQSLERDMNRTERVIGLQQSPPSRTHTLPYRADYENRYNASETQHYNTMD

SDQMSWLQQQQQKLQRIRDGRDASGRTEQEKQLVNELKFAQNRYFKQRAHSEAEEQAVMD

SYRQPPMHNGPITPASAPAAYAPPPREFGGETGHFNTFSQSQSQFVESHYSGGGERSSNK

PPPSPTMQRSAQPVSVPVQPPVRTSSKDYMRSRSHSSSSNAWQASPPPTSRPLARQYSET

LYDREHGSNLQSRHTPSSHSTPPRSPRARSPTHMGPTATTTYTTYRTIHKEVHDDAIGRP

QQQAQQKQMVAAPVVQSQAPPPQQQPPQPQSSANQQTHHNYITEVFVHRVGAGDKASSDR

TDRTDGGEDTRLDELTRSLQAATDSIMEERMSNTQQQQQQQQQQQQQQQQQQSFQQQSWE

EHYQTSQVQKSHEAPAPKPVGVVEEDRIMLKPIGPGVQMLEPEASPTATNTLNRATTPSF

PTSP

>SG-AL_contig_1700_1_-3_15_350

MLGSVVTSRLTTTLHHLADALHARSVDLSLGTVTSKVCLQYQSTPLSSRQVWGKYGQDMA

HSQLPAVLKRARHYAFSPANGHRPSASKVMLLVLDDATTPVLPKLLKEASRL

>SG-AL_contig_1701_1_-3_3_119

FFQLTFVTRNTCMVHNIHVRDVVHVVLLCLYNIVLALVH

>SG-AL_contig_1702_1_+1_1_729

CLALSLACSAVCLALSLACSAVCLALSLVCSAVCLALSLACSAVCLALSLACSAVCLALS

LTCSAVCLTLSLACSAVCLALSLMVNTIQLGDSIIPLSDSVKNLGVTLDSTLSMQKFLTQ

TCQSCYYQIRRIGYIRKYLSTEAASKLVTSLILSRLDYCNALLSGLPASSLLCLQRIQNT

AARLVLRKKKFDHVSPLLRSLHWLPVSNRTEYELDTLCYKSIHQSAPSYLNIYMPTRTLC

SAS

>SG-AL_contig_1703_1_-3_237_1256

MYDTSPSSPFKGEIRQLWPENFSTKAFAESLLELDPALEETLDLSEFETNTNPVGSPSGF

PDRASGSPPVTTRDLLTLTPAVTTQYLAQLEGQSPIFTADHGVAVNHPQQHPAPSPPHLA

LAADDVVQTFPDDVLMPEFQSLVAMTTAPSTSTCQQGGAEWAQTPFYSTVKTEDLTNLTA

CSVEASLPGFTSTTTTSSICKSEPFGQDDLSCSQFTDLTDLSPSPRSTRSMSESSTATSV

MSDTPRRIGKGGSRRRQVPKGSEEYKEKRARNNVAVRKSRAKAKEKQRMTEARVKELADN

NDALQKRVEMLTKELNVLKGLFINVGAKLPDDLSKLLEQS

>SG-AL_contig_1704_1_-1_103_1308

MSGDRIVRVGSRKSELALIQTRTVIELLKRKDAHLKFETITMHTTGDKILDSALSKIGEK

SLFTRELEDALLKKKVDFVVHSLKDLPTEIPPGLVIGAVLKRDNPHDAVVMHPSHHGKKL

SDLPAGSVIGTSALRRAAQLQRKYPHFKIENIRGNLNTRFRKLSEDGSTYDAIILAVSGL

ERMNWHHLISEVLSPEDCMHAVSQGALAVECRAGDEATLKLLRELNDIDTALSVVAERAF

LRTLEGGCSVPVSVYTEKTEEKLMMRGGVFSICGTQAVKEEMTCDLQEMTDKQSHEVDPK

SLQDGYITIICHEANNHLRYHNARHLGQKLASSMIKDGADVILKAAKNKTKEEILAEHAK

RKLKAEAAEQEGAKSAKVRKLEGASDVVSAAAVASTSTVSSA

>SG-AL_contig_1705_1_+1_1660_2091

MHALYMDIYIYSHGLFWSNLNIIHMKQKDENKSDLYFMHQLAIKMLMIFGSPTNSKICYY

THTHTHTHTGPLIILAHHMRNEGIFLINTFIISSFIKSVSFSNLFPPHLHACLHFNQAHF

KKSEVTGIILTSEGHQYSTDHIIY

>SG-AL_contig_1706_1_+1_301_645

MTCVLCVWQCDLVVGCFLLLSFHLSALSLSGLVVHHSVCNKSPSGTQILLVSDKGRMTSH

THEQKVGPHTHGRHTLAVCRIVRQPTRPGQPWLFLQPAVSSETVGSEYQYKMCKQ

>SG-AL_contig_1707_1_-1_112_1128

MDKNTEIKEEKVLRQQLIDTQQVLKEREGQFEKSLNYIGELEQRVEKLSSGQNNIEELQR

QNEKLSQELHVYRNAAQHHQDTECQVGENKVEQLPETPIDYKGEIQLLQQHVEKLNSELY

LYRSAAQHRKDAGCQVGEDQMLERQLEKSQRYNEDLRQQIEKLSSELHSYRSAAQHRLEA

ECQVGEKDIAEAYGDKPFPVWDIPDESAQQTVEGQTEDGISIADSLTAAAQKVVWEQAGY

VYDSASGLYYDSNTGFYYDTTSGLTYDPSTQAYYYYDQTEEQYKFHSYTYRQHGEQGSVE

TDTSQNKATTSEQTENMCNHSDSGGESRRNRHREKRKVQ

>SG-AL_contig_1708_1_-1_115_2898

MSVQSLVSKKKKTFIGLPAPLGYVPGLGRGATGFTTRSDIGPARDADDIPEDRHAPPNKK

MKPKDEEEDEDLNDANYDEFSGYGGSLCSKDPYDKDDEEADAIYEAIDKRMDEKRKERRE

EKLKQDIEKFRQERPKIQQQFSDLKRDLADVSVDEWNNLPEVGDARNRKQRVGRAEKFTP

VPDSVLARAAASGSVNSSVSEQEQKYGGLTTPFGSITPIGSATDIDMKKIGQARNTLMDI

KLTQVSDSVSGQTVVDPKGYLTDLQSMLPSHGGDINDVKKARLLLKSVRETNPKHPPAWI

ASARLEEVTGKVQAARNLIMKGCEECPKSEDVWLEAARLMPGDQAKAVVAQAVRHLPQSV

RIWIKAANLEVELKAQKRVFRKALEHIPNSVRLWKAAVELEDEEDARIMLSRAVECCPTS

VELWLALARLENYENARKVLNKARENIPTDRQIWITAAKLEEAHGNITMVEKIVDRALNS

LRANMVEINREQWIKDAEDCERSGSLNTCQAIIRAVISVGVEEEDKKHTWMEDAEACAVH

GAYECARAVYAYALTVFPSKKSIWLRAAHFEKNHGTRESLEALLQRAVAHCPKAEVLWLM

GAKSKWLAGDVPAARSILALAFQANPNSEEIWLAAVKLESENNEFERARRLLHKARNSAP

TARVFMKSVKLEWCLGEVTSALRLLEEAVKHYPDFPKLWMMCGQIQEQQGDHEAARTAYN

KGLKKCPTSWPLWLLLSRLEERTGQLTRARSILEKARLKNPKTPQLWLEAVRVESRAGLK

NIALTLMAKALQECPSSGILWAEAIFMEPRPQRKTKSVDALRKCEHDPHVLLAASKLFWS

ERKLSKARDWFNRTVKIDPGLGDAWAAFHKFESQHGDQKVADEIVSRCVAAEPHQGEQWC

QVSKAIPNWRLKTGEILLLASSAMAMPT

>SG-AL_contig_1709_1_+2_203_481

MTRQTYKKRQQNGPCGMIHPQKCPYNLQGVADIIPFQSIYQNQVYKTSKKVRLQEILLKP

EKHTLRASTLLKLRAIYTPSKAQTWHGLLILVS

>SG-AL_contig_1710_1_+1_490_816

MTILHSQTVASQSTLRSLPHDTVKSISRFNLSPLHVIQHHTSSIYSSKEILSRHTKKRIS

KAVSLQTFTSVGPICTLTHLVAIQHNHNNNVHDNNYVKTKDRSYVIVKT

>SG-AL_contig_1711_1_-1_280_2694

MDYDDVVVRKVSRTYNVYRGTSPTTQNRLESRIRELEDALDSERDGRVRAEKMVAELNFR

VDQLQDSLDEQGSSTSAQIELNKKRESEIIKMRKDLELANAQFEATESSMRKRHTEAIND

LSDQVDYLTKNKNRVEKEKQTLVVELDGLSGQLDSVAKAKVTLEGRVDAAEQSAARLKGQ

VDDLTRQLNDLTGLKAKLTQENFDLQHQVQELDSSNAALAKAKNQMQMSNDDLKRQLDDE

SRQRQNLQVNLSALQSDYDNLNARLEEEAETSTSLRNQLSKVNADFTSLKSRFDKELAAK

TEELEEMRRRMQVRITELEDSCEQLRVRNSSLEKAKAKLANEIKEITIELENTQIIVQDL

TKRNRQLENENAGLLRRVDELTAENGTLRNEKMALEQEVQRLRVANAELTERNDNLQREN

KNLSDQLREANLALKDANRELADLRQLRAQLEAERDSLAAALKDTEDALRDAEAKLANAQ

AALNQLRSEMENRLREKDEEIENIRKSSARVIEELQRTLVEVETRYKTEISRLKKKFEAD

IRELEAALDNANRANAEYLKQIKSLQMRVKELETIVEEERRTTDDLRNQLAISERKRVAI

AQELEDARALLEGAERARKNAETELADTSARLSEITLQVTTLTNDKRRLEADLGAMQSDL

DDAINNQRSAEERAERLSAENARLADELRQEQENYKNAESLRKQLEIEIREITIRLEEAE

AFAVKEGKRLVAKLQSRVRDLEAELENEARRVRESVANQRKAERFFKELQAQTEEERRQL

AELSSLNDQLQLRIKTYKRQLEEAE

>SG-AL_contig_1712_1_-1_1_282

HTHTHFLLVYILLLRSVRTSLYAPHFIEISFEMCMIFVVIVIVCLNLTKPGHSCAVCCKI

HIDSEGFELKIIGKNGLSCIVCSSLYNLFLVKRI

>SG-AL_contig_1713_1_-2_2_226

QPGLRDKDTYGVCQEILATPSVMYLCGRSLSNKPIAFSHIYKPTKIAGHERPQNLNFSQC

YDDENGDDSDDTMKF

>SG-AL_contig_1714_1_+1_949_1272

MSFIRGNNIISRNQYTYKHIKHKVLISRKEIIMQTNTQSALQIAVKRNQQVPSPACSHQD

VECITPPQYIHTEASDSLTFQSGTFILRHSKHMRKLSADGIFATDNVL

>SG-AL_contig_1715_1_-2_2411_2662

MFILLYTHIHSHLPFRFPPRTQVSYVFPSVCSMYTSSSAFEHGHSPNGHIHLRHVQLGLD

AYMHVVLVGWPMPCSQHVSAQCIL

>SG-AL_contig_1716_1_-1_1_138

SLSLSTYPLLFPFLQQYTHYRFDLSKLQEVGGGVSVIMIKRNVINM

>SG-AL_contig_1717_1_-3_1299_1469

MSMLVTSAVGVFYCACVCFICVLMTIQVCPVVVTTTVDIQWYKKREKTQIIKNFQKS

>SG-AL_contig_1718_1_+2_2_88

LRNGCYSCSLFDYLHTISNLFRIIMPLLL

>SG-AL_contig_1719_1_+2_119_298

MNECVENNTCLIMTSYCCITVLHVSMLTKSLQGFLLKNTFKYCAGVHDKLVYVLHCLAEA

>SG-AL_contig_1720_1_-2_119_1342

MAAVLPQRPDAGTYTFASQPRALKQRKKYRDPVQQTEDPEGLVNYGNIMYDRRIVRGNTY

AQHTLPAHAQPDPIEIQRQQENRRRAIARKRAKDQLRPRSPDPVDGRKHIDVQTELYLEE

LSDRVEEADVECQTDGFLDRPPSPLFIPAKTGVDIATQILAGDLFDFDVEVKPILEVIVG

KTIEQSLLEVMEEEELANLRQQQRAFEELRNAELVEQQRLEEQERRHREEKERRMKQQRE

VVRKEKETAEKIAARAFAQSYLADLVPSVFGTLSDNGYFFDPVERDVEQGFLPWLMDQVV

DQLERNEQGRMVLDGMLREVVARRMEMYNKLDDTASSVGQASRVVSSMQREQSSAEQPAP

ATTTESQEAAAAEKPAEEQAAAPETAEEAPDSGEAAEAGGEEEAAADE

>SG-AL_contig_1721_1_-3_3_242

TSVTPINAQSHIYTKHRQPFLTTFKAYIYIYIYIYSFVHTFSGIRSKYTWYSIRNAVVED

VKIRALLISRLFVKLFKSNK

>SG-AL_contig_1722_1_-3_117_1022

MSKKLGFAENFALSGAAAIISKTAAAPIERIKLLVQNQDEMIKAGRLSEPYKGVIDCTVR

TFRTEGILPFWRGNLANCLRYFPTQALNFAFKDKVKALFKTSPNESYVLKFSKNIFSGGV

AGALSLCFVYSLDYARTRLANDAKAAGKGVGERQFNGLIDVYTKTLKSDGIVGLYRGFVI

SCVGIVVYRGFYFGLYDSLKPLMLGEDANVFVSFCLGYAVTVSSGLLSYPIDTIRRRMMM

TSGQAVKYKGSMDCAMQIIKNEGFMSMMKGAGANILRGVAGAGVLAGFDSFKAAYIEWRI

KQ

>SG-AL_contig_1723_1_-3_165_320

MKSANTFLPQNFYLNIYLPDSETCRLNYYGGGVVVVALLIQSRSYLLKPVLS

>SG-AL_contig_1724_1_-2_104_343

MVILCTCVCVCEWKWVSLVINMLSLLHFYLFMCHHTKHLLVTKGQNKVSCLMSCLTYINF

RVNIIRPMCNIYHLCVCVCV

>SG-AL_contig_1725_1_+1_46_222

MPDRPIARLSPPKCSQRTEFTLFWCSLIFTYFYMNARQKKTTKNPKFTPVKANSLQCLY

>SG-AL_contig_1726_1_-3_3_164

TLKLKLTLFPFIVYCTNLPKLVNSQILLHCNHLIHIHDSLTLFTFIPYCMNEPY

>SG-AL_contig_1727_1_+3_174_335

MVTQLESCHVASQTRSGSHITGLTNGRQLLPLQHPSFTKRLVTNLYCDQKFMKF

>SG-AL_contig_1728_1_+1_1603_2160

MDETEAACTWIGRMVLYLYTNQVRNRPQKHDKTINQSNKQTKNHPSFCGSSARRSFNVTV

HVPHKSGNGGTTVQALKKRKTISKWQCNVAQNMDVKNKAIYCSQQKFTLIKYKIYTLYLH

YCFMYIPWTIMYKTGGLSMHGEITVFTNYRSSVCGMKYCIYKLLSFTGHAYSYLNKHTWH

DLRCTC

>SG-AL_contig_1729_1_+3_156_293

MFNSFIFLYCFSPQVKLFPSQQTSFHVLQKSCSLTLMNKDGSRFPR

>SG-AL_contig_1730_1_-2_122_220

MKELNVTGPAHLAPYLPLPAVKAAQQFDQNRFI

>SG-AL_contig_1731_1_-2_2_325

RLPPSGLSHPFLGLLPGPQGRQGRGTPPLPGPLRQVLPETLGSEASFPAEAPHLHQRVLL

EGHLSDHHLSLAADHLLRAYKQAGSSPLLDSPRAHELRALSSSAAFHH

>SG-AL_contig_1732_1_-1_1_258

SHPWSPPWTQERRLCPVRHVKYYLKRSAPRCSSQWRLLISLENYLRDISVTTISRWLSAT

VMRAYKESGSSSLPDSPRAHELRALA

>SG-AL_contig_1733_1_-1_1_153

SLSCQSLSLRPNNLALARGLFRSHPWSPPWTPGMLGARNASFARSAASNTN

>SG-AL_contig_1734_1_-3_57_188

MEGKTTNIMMSDIINTAGATLKTNEKKKTMFSYNVLYNRVIRKT

>SG-AL_contig_1735_1_+1_1_369

KKKKEKKKKKKKKKKKRGGGVNPETDEESPLVFGAVSLCGGENQESDVCSELSTTCTQAV

CVCVCGSLVFWSKPRPSPQRHNVKHTYTHPNPLSPKPGHHHHHHRRMVEGGGPRAGHPLF

LSP

>SG-AL_contig_1736_1_-2_2_163

FFTCITPFNKEPWPIVKTSFVSIFSASVSGLPVFVVVATLLTKPDIVANASDHY

>SG-AL_contig_1737_1_-3_174_314

MSNSQAHPSFPALTAVVLLGSNLNHGSALSYALMGGRSADWLADGGT

>SG-AL_contig_1738_1_+2_1604_1894

MVKKTGIIRHVCTAHVLIACDCQGQVVSWSEKLWGLCNQVSCHALFRTLKYILTNMLYMS

IYYHTQHVIFIIIIHTISITIIIVMLASSVYYFCNYD

>SG-AL_contig_1739_1_-1_304_2550

MSEGTRVNGPSSPTGKYIDTIDPDDPEYIRNLQRPAEVKEDMKQMEGRSRVSVVLNSQAF

KEELEAVIHEQIRQGGPASIFALQQISDLLLPQSRGSYSSFGRSSPVIPINDIRGVETLG

YSKQEKVLRCKLASCYRLVDLFGWTHGIFNHISARVSQDQEHFLVNPFGMLYSEITASTL

VKVDVRGEIVDTGSTGLGINKFSFCLHSAIHQARPDLRCVIHLHTPAAVAVSATKHGLLP

ISQEALICGKTSFHGYGGMLMDQEARDHLSRNLGPFNKVMFLRNHGVVACGQSIEEAFHF

AFNVMAACEVQVKAMPLGVENLVLVDEETQEKTFKMGSQGGSGTDSSGKKWRCGELEFEA

LMRTLDNSGYRTGHMYKQPLMKQEKRERINSDVEVPPASSSFTYVFDGDYEHSKYVSPIK

MAMERQKQAYKAGWLTSPNTYKKQEIEEIGTTTPKKITKWVPGGAETQEQATAIKMENPN

QFAPQGSNPKEFKEKQKSIKKDYYEEKVSAGPQSKILEGISAWDEAAKRKDGEQTVIVGA

ASKGIIQRDHQHNAVVYRSQYAANPFENMSEEEVDAYKQGMLQQEQEEDAEDVPGPEGRL

ISSQERMQNIQHSQEDVQLDTEENTVQETNSDSHVNDKNERRPVHSPRPPISPKESMPPV

PARRTHVDAKRRVQREVQEREGDGDKPGSPTRSDPMDSASGGETLEERSSKEGSPTKEAP

SPTKDKKKKKKFRMPSFSKKKKDSKESTI

>SG-AL_contig_1740_1_-2_1253_1591

MFVFSLLFLTWKICTVLRPPLISLQCFRSALAYGEFYIRHTHTHTHTHRHTTHKRTLRHA

RTHAHTHTHTHTTPHHTTPHARARVRMHAHTHTHTLPPPHHTTTPHHTTHSLS

>SG-AL_contig_1741_1_-2_32_190

MCVFECNIDANREELIWLTVYHCGPFTSLLRPCLFGLHLLSRKVVFYCVNLVK

>SG-AL_contig_1742_1_-2_827_1042

MKLKYNASTFYSCQFVKKATTKILVWSQNSIPGRGDKIYTFFFFYSFIFYFFFCLICFLS

FIIFYFFFVFML

>SG-AL_contig_1743_1_-2_98_496

MAKENVMELVRPGFEVDEDDDDDLDETLAERLWGLTEMFPEPLQKVVGNAVSLTFSALVA

GFSLGRSALWIAASSATILVLPVIFESERAQQQEQQLQQQRQILLGPNAAVSAAGSNLLP

GMGMMPHGMTPPK

>SG-AL_contig_1744_1_-1_124_1137

MPRGLVKAKKYDWKDSNLANFGSDEEKQVKKESAEQEPAWQGAGQEPCLKIWRIEQFEVK

DWPVEDYGEFYNGDSYIILNTYKDEDTEELNYDVHFWIGKNSTQDEYGTAAYKTVELDTL

LDDKPVQHREVQGHESTLFKSYFPKGIQTLEGGASSGFRSIPDKEEYEARLMRFAKDKKG

NICVREIPRARDLVTSGDVFIMDVGMTVYQFNGKNCSPMEKSKAMEFIGTIKSKRGEVVS

EVLDEDLTPSSHPFFKLLDEEPDDDGNASDDEDGDSDKKLCRVNTETRTYEDVKEGDVTI

NDFASDDVFIYDSGDIVFVWIGSKASPAEKKNGLPLAH

>SG-AL_contig_1745_1_+1_739_918

MCMCVGQEGGGRGIYMCMRHTKRMCVCVCVCARARAHVQAHVRLYMCLMSIILVCTLTLW

>SG-AL_contig_1746_1_-1_430_642

MKQKSYTNTHTHTHTQRMEHARNLQYSEVQYLLKCSLCFTLICNLVELFYFFICYSVVVL

IFANNLYSGLN

>SG-AL_contig_1747_1_-1_49_246

MFCIKLFCLQYNVLEEKHLHFNGKKSNMKYKLLTQKRGILDTYKKYYSCNKGPFLISAPF

GRQEKK

>SG-AL_contig_1748_1_+3_3_122

TGEILTKQNDQNNRNKIFSFVLYVLSKSGYTGVRKIKMTR

>SG-AL_contig_1749_1_+3_186_293

MFVCFLIYLFIHLLLLFVCVPQLNTLGVTQYINSVL

>SG-AL_contig_1750_1_+3_462_683

MSYLSDLCLHLGLLYIQNGPKWPLVFKKNNFIKALKMYYLSDLCLRGGRMVKILDCQAGG

FGRPGFQPWAPCLV

>SG-AL_contig_1751_1_+3_123_230

MQISRSHNHVTQTAILLVSVVVWRRVQGLENEEVIL

>SG-AL_contig_1752_1_-1_664_888

MKIENDTKKRYKQESKIQHFTFIQNSTNKCHLTKLHKQMSFTHFLPFSPSSSFHEKLFLN

LQYYSDTFFLFLKVG

>SG-AL_contig_1753_1_+3_168_938

MEALTKAISKNVLFAHLDDSEKTDIFDAMFPVEHKDGEVIIQQGDEGDNFYVIEQGEVVI

YVGNEPVVTIGEGGSFGELALIYGTPRAATVKAKGEVKLWGIDRDSYRRILMGSTIRKRK

MYEDFLGKVSLLENLDKWERLTVADALEPVQFEDGVEIIRQGDPGDDFYIIIEGNAVVLQ

TKEGTDKPVEVGSLGTSDYFGEIALLLDRPRAATVVSRGPLKCVKLDRSRFERVLGPCSD

ILKRNIRQYNSYVALSV

>SG-AL_contig_1754_1_-1_1_153

HVSALRMACHTSVIDCSCLSSEDVCCLSQIQGLGNCVAVVLSNHIGTVMKF

>SG-AL_contig_1755_1_+1_1_195

QSNTHHANSPTLIMPSLTTQCDNSPTLIMPTVQHSSCQHSSCQQSNTHHANSPTLIMPSL

TGHTT

>SG-AL_contig_1756_1_+1_1702_2043

MVSIWSSASFLLMGWCLFWPQGISRFLVVVFFGVYILSSSSFLFIGWCLFWSQVVSRFLV

VVFFGGVYVEQGFFSLYGMVLVLEKGCRMVQTFTCQADTEVLWFNSQTHPFSSV

>SG-AL_contig_1757_1_+3_39_185

MWQKCNDMNTEKSKCTLLLHYPSWGKTITTRQSNHTVPFSCFCTVKNPS

>SG-AL_contig_1758_1_+1_2539_2946

MTTSAFCSSFFSVFSLLSPSSAFSLSFSSPSVSPVFSLLSSASEGPVFCPSLPPVFMFFC

SISGLKSSCLACDAASSCLASAATSSFFFSSLFPVTVSSFLLSVLSSACFTAALTCSSLT

SMLALFSPPSLALTSA

>SG-AL_contig_1759_1_-3_3_335

KKNLSLHSIPFLYNTKSGNNGWGKIHFYPEIVQESSYNPFTQRKHREKLVPPLCLTDILL

SGKIQYWLLCFTAARGHQVWCQSIQTRVYNKLRHPKANYRGYSPKTMYWKL

>SG-AL_contig_1760_1_-1_31_768

MSRGSSAGFDRFITIFSPEGRLYQVEYAFKAINQGGLTSVAVRGKDSAVVVTQKKVPDKL

LDPATVTHLYSLTERIGCVMTGMIADSQSQVQRARYEAAKWKYTYGYEIPVDILCKRIAN

INQVYTQSAEMRPLGCCMILIGYDDENGPQVFKTDPAGYYCGFKATSAGSKQLEANGFLE

KKIKKKQDFAYNEAVEIAITCLSTVLSADFKASEVEVGVVTKDNPNFKVLTEPEIDVHLT

AIAEKD

>SG-AL_contig_1761_1_-3_363_1007

MSRLYKIDNNSLSIFIKLFDAHIQLVVLYGAELWGLQPGSFVTEKVHLFALKRYLGVNMK

TPNDLVYGELGRFPMYINAVVCCVRYWLRLIQMDDHRLSFKAYVLLKNVDERDKTNWVTR

IQKILCINGFSYVWESQSVGCVKSFIKLFSQKLIDIRWQCWKEHVDTSDRFQLYRRFKVN

AGAETYITLNVNRYVRNALTKLRLGISGLNVHTSR

>SG-AL_contig_1762_1_-1_91_534

MLKCADDTFFNFWVRLFTVLFDNGVYPDNWSESIILPLFKKGNQNGPNNYKGISLGDISS

KVYSSIINRRLQEWVEQNNITGEHQAGFKKDYSTIDHMFTLLAIIQKQFSLHRKLYVAFI

DFEKAFDSISQKLLWLIILKNGVKGKLY

>SG-AL_contig_1763_1_-2_455_592

MQENFLFSSFSTDVQIAEFSCLPFSKYFIGLTQKCYIERKKGCKKI

>SG-AL_contig_1764_1_+1_574_762

MTEKAPDTGSNLRLGQGFFSFFYKTSRAWSWCKSFVSEGKPRFCVQHRLCKHKRTHGNKG

LLW

>SG-AL_contig_1765_1_+3_3_71

FQSHKINVKQCHITYKTHRRVFH

>SG-AL_contig_1766_1_+1_337_531

MNLPILAELDRFPISMQIISNTEILVTYNRIRQYILYQIDIQYVTKQTNTKSWFELSKRF

SMKLG

>SG-AL_contig_1767_1_+2_2_175

IFHKNGNFMYAQEHLAKQASKAIHALKRSTKGSEFRLGIMMHLYDTIITQSYDINVWR

>SG-AL_contig_1768_1_-2_596_841

MAMPDASCLCVMMTVVFSFMATVSVIVVVDVSRCQTLMLYVFTSSVTLLSGSLFSFTRRL

VQMEKCRLPVSRSIQQCTNTGF

>SG-AL_contig_1769_1_-3_345_6614

MPVVSPTPHCRHGSEQGLYEPAYESDACGVGYVVNIDGIESHRVLRDAQVMLQRMEHRGA

CGCDNDSGDGAGVLTGIPHRFYAKTLHSTGYNGVLPEPGQYATGMLFLDRATAAQAEETL

AKFADKYGLQVVAWRTVPVDNSCIGQVAKSMEPFIRQVFVTKPDGVSDIRQFDRQVYLLR

KYASHHVPETGVRFYICSLSAHTVVYKGQLNTAQLWKYFPDLNDPDYESHIALIHSRFST

NTFPSWERAHPQRYLAHNGEINTLRGNHNLMKAREGVMSSVVYGDKLSDLYPVVEEGMSD

SGCVDNVLEFLCMAGGRELPEAVMTMVPEAWQNDKNMSEEKKAFYRWAAFGMEPWDGPAL

LTFTDGRYIGAILDRNGLRPSRYYVTNDNFMYMSSEVGVTDRLPEEVVRKGRLKPGRMLL

VDTKEKVFMKDEILKSNIASLRPVSNWLEETMTMSELRDNHDQTAVHKLSSYRQMSAVGS

YLETDPRLSLYNYTMDGINLLVLPMVKEKKEALGSMGNDAPLACLSNFNPLVFSYFKQLF

AQVTNPPIDPFREKIVMSLACPVGPEHNILEPAPQQCQRLWLEQPIISLYDMEVLRHTSH

KGWKAKQINIVYPEEDGEDGLLPALKRICQEAAEAAQSGYKLIILTDRKADPNNVPVSSL

LAVGAVHHHLIDEKLRMKVGLIVETGEAREIHHMCLLLGYGADAICPFLVLQLIRRLRDQ

GLLDPPLSNEEIYFNFRAACARGISKVMAKMGISTLHSYKGAQIFEAVGLNQEVVDLCFE

GTASRIGGVTFHILGKEAMNRHRKAYEKRDCDNQLIMSPGQYHWRDGGERHMNDPLSIAN

LQDASRNNSRDTYARFTEAAWGSAKKCTLRGQLDFHYQDIALDINEVEDAKEIVKRFATG

AMSFGSISLEAHTALAVGMNRIGAKSNTGEGGENSDRYLNQDPDFNTRSKIKQVASGRFG

VTSSYLSHADELQIKMAQGAKPGEGGELPGYKVSKEIAKTRHSIPGVGLISPPPHHDIYS

IEDLAQLIYDLKSANPQARISVKLVSEVGVGIVASGVAKGKAEHITISGHDGGTGASSWT

GIKNAGLPWELGISETHQTLVMNNLRSRIVLQADGQIRCGRDVVVAALLGADEFGFSTAP

LIVLGCTMMRKCHLNTCPVGVATQDPVLREKFAGKPEHLVNYLFLLAEEVREHMAKMGFR

TFQEMIGRTDKLRVASDAHNPKAQLLNFDRILKNALEIRPGTNIEGGSVQQLFDMDQKLD

LELIEKCRAVVEGKSKQVVIDLIVKNTDRTFGATLSNFISNQKAEEGLPEESIIINCKGS

GGQSFCAFLAKGVHVTLEGDANDYVGKGLSGGEVVIYPPKDMHKDFKSVDNIIVGNVCLY

GATSGKAFFCGQTAERFCVRNSGAIAVCEGCGDHGCEYMTGGRVLVLGLTGRNFAAGMSG

GITYVYDVANRFHSLCNLETVALEPLKKNIDVDFVRQLLEEFHKKTGSQVASNILNNWDS

EREKFVKVFPNEYRRALREMAEEAAEIERLKKLERSVSSESITEVHIRKMESLENMYDED

IINDLGEDGKEASDEEEKVAVVDIEETVPDANKNVELDKTRGFVKYKRATYQYRNAKDRQ

KDWDEIYNHKTVIEGLRMQAARCMDCGVPFCQSNNGCPLSNLIPKWNDLVFKNKWKEALD

NLHQTNNFPEFTGRVCPAPCEGACVLGINSPAVTIKNIENSIIDNGFKQGWVRPTPPSIR

TGKKVAVVGSGPAGLAAAAQLNKAGHKVTVYERNDRVGGLLRYGIPTMKLSKEVVQRRVD

ILTIEGIEFKVSVEVGKDIQASELIRNNDAVLLAMGATWPRDLPIPGRQLEGIHFAMNFL

ETWQKQQQGNSSPYISAKDKDVIVIGGGDTGCDCIATSLRQGAKTITTFEILPKPPPSRE

TNNPWPTWPKIFRVDYGHEEVQLKWGRDPRIFEIMSTEFLDDGNGHVSGVRTKQIKWEKN

EAGRFVMKDVEGSEKVFKADLVLLAMGFLGPERNIVEEISLELDQRGNVSTPNSKYNTSV

PRLYAAGDCRRGQSLVVWAISEGRQAARQIDMDLMGYTSLAGPGGIVKVE

>SG-AL_contig_1770_1_-2_935_1171

MCVKMDLSVQHPYGNGCISWHSINFAVIGTGLSQHCVPSLNVHGVRGRMIEISFFFFFCL

FVLHCLLVAGAVTILSPVL

>SG-AL_contig_1771_1_+2_287_562

MYHTKCCHVEKEEREKERVREGRMIKKTILLTHFINRHTQTDTHPSQEYEIISLPHLIFF

FLKGKLEKKDLHILAFVTHTYRGIFLKMITQL

>SG-AL_contig_1772_1_+1_1_147

LYVCCVCVCVCVCVCVCVCVCVCVCVCACMNIRETYLPGHSHNTLCEQA

>SG-AL_contig_1773_1_+2_2_211

SSCSCGSWYGWRWRWRWSGGSYCTSGCWCSCDSWCSCGSSWCSCGGSWCSSGSCCSSCGS

SCNSGSWCSG

>SG-AL_contig_1774_1_+2_2_406

AVVVVGAAVVVAAVVAVGAVGGAGAAVVLGAVVVVGAVVVVGAVVVVAAVVVVDAVVVVA

AAIVVVLAVVVVGPVVVVAAGVVFGAVVVVGAVVVVGIVVVVAAVVVGAVVVVVAAVVVG

AAVVVVAVVVVGAAV

>SG-AL_contig_1775_1_-3_669_911

MCNLRSKCAISEDVQVYRPVTANTTVQYAPVYIIMIYRLFLLCTFYSNFLIVHREKCVRH

RLKVSVKHCTHVCKTTEKYIH

>SG-AL_contig_1776_1_-3_282_476

MQVDLKPNTTGPNFFMVGDQYLCIMLNFIGGELVLLMVCEMCDTVLYVVSAVVNMSVIVC

ECNKM

>SG-AL_contig_1777_1_-3_3_323

PISQLSVCSVHLPVLLSVLSISQFSVCPVHLHFCLSCPSPRLSLFCPSPLLSLFCPSPTS

VYVLSISLLSVVSAHQGWTDQCLLYALSCLYVLIGGFSPFEKKSRFL

>SG-AL_contig_1778_1_+3_156_341

MNRWGNNKRSYLKLRIQLLQEQNRKGHLSFQTDIFTWTYSKSNLALFLCNFMNQKHVPSA

QH

>SG-AL_contig_1779_1_-2_833_1027

MNRNYSHECEWIQQFWGCFFFILQCSSDGHSLSVSTFVSSFISVCVGFCCCVGGHVSFKF

LLFVQ

>SG-AL_contig_1780_1_+3_3_179

CGRKVWLQPRVKKTKPTNKGTKSELERDLPVVLAANCNILKSPRDIWSGFLHRLWMDCS

>SG-AL_contig_1781_1_-1_34_267

MFLAWLKWLLMSLPAPPAGLTVLPRQMNCSVVGRSAPSAWVGEGRGMFTVITYFLLADLL

ATCCAELLRCDVFSCMCA

>SG-AL_contig_1782_1_+1_1_135

GSDFTILQFLAVPPPSPYRITTSSRKDLQQKSNLFHVFSTADEQG

>SG-AL_contig_1783_1_-1_457_624

MSVLLHITVFSITIVGRTEICQTLFLILIVVMLVYRFCNFVWFLHQHQTWKFTKMH

>SG-AL_contig_1784_1_+3_84_581

MNSGSSKVKVHISANMDIVLLETRLDFAILFACLRGKRSIKGQLKALCQLVFNGHLRVQY

IVCVPLFCECQTIFRPLVLGLQVATDFSSVTAGVATGGEFHPTVSLRLGFQLEEPIVVAF

GEEISGGFAKVRIARRSHFLFFQLVSPLTRSLTQQKQLKCRKERTR

>SG-AL_contig_1785_1_-2_62_556

MAPPSYSDLGKAARDIFSKGFNYGFFKLEAKTKTDSGVEFTTSGNSSNDTGKVSGNLETK

YKCSEYGVTFTEKWTTDNVLDTEITIEDQLTKGLKLAFDTSFAPQTGKKSGKIKTGYKQD

YVNVNADVDFDFAGPTVHAAAVLGYNGWLAGYQMAFDTAKSKLAK

>SG-AL_contig_1786_1_-3_1227_1574

MSSLRHCFTFPNSAGYIYSLADLRSHAQFFFLSRNKHVQIDWKAVVLLLRQPGLHKTLGK

KSLISKGTVAFIILVITLAVIIIKMYCLLQFPSKAGSDCYCHFFIRFTTTLLVKTS

>SG-AL_contig_1787_1_-3_618_770

MICIRDITHTLSAHDFDFYLGIIQLSLASSGMKGRVECSRCIDVIAICFSS

>SG-AL_contig_1788_1_+3_3_284

FGESYQQHKNQIKRLLIGYSLKEYLSARVVVHTHHTRTTPSLRSKARPRTIWSFASDLHH

TFSLANHIFVGQCSHISDHCSAQHCHERTQSYVI

>SG-AL_contig_1789_1_-3_9_1475

MNQRFLFPQTPQPSTDNDPLGPLPDGWEKRADPNGRVYFVNHKNRTTQWEDPRTQGIMQE

DPLPEGWEMRYTQEGVRYFVDHNTRTTTFQDPRGGPSKGPKGAYGVPIQYERSFRWKLGQ

FRYLCHSNALPGHVKINVSRDNLFEDSFAQIMRLQPFDLRRRLYIIFKGEEGLDYGGVAR

EWFFHLSHDVLNPMYCLFEYASNSNYSLQINPASSVNPDHLLYFRFIGRFIAMALYHGKF

IDSGFTMPFYKRMLSKKLTMKDLESIDPEYYQSLQWVKENNIEECGLELYFTTDFEILGK

IEQHDLKPGGGDIRVTEENKEEYLNLMTQWRVTRGVEDQTKAFLEGFNEVVPLQWLQYFD

ERELELMLCGMQEIDVDDWERNTIYRHYQRNTKQVAWFWKFVREIDNEKRTRLLQFVTGT

CRLPVGGFAELMGSNGPQRFCIEKVGKETWLPRSHTCFNRLDLPPYRSYDQLVEKLNLAI

EETEGFGQE

>SG-AL_contig_1790_1_-3_60_638

MYSASAKIIKPQGEKPDDFESSVSQALLELEMNSDLKASLHELFITGAKEIDVQGKKAII

IMVPVPQLRAYQKIQTRLVRELEKKFSGKHVVFIAQRRILPKPTRKSRRKNKQKRPRSRT

LTMVHDNILEDLCFPAEIVGKRIRIKLDGSRLIKVQLDKNQQTTVEHKLETFSAVYKKLT

GKDVNFEFPECVV

>SG-AL_contig_1791_1_+1_2467_2778

MFCFYLQLKMLRMRGREIYTSQFMVCVTIKQNSNRYMKDLHALHTESTVSIITKTNTARH

NHHQHRHSQTQSSPTQTQPDNQHRPSNIHITTHQLLSTHYVHAQ

>SG-AL_contig_1792_1_-1_133_2322

MKDKRRNSEKRKEKSRDAARCRRSKETEVFFELSNSLPLPSSITSQLDKASVMRLAISFL

KMCNVLEQHNWSELEKEKGKGDGKVRADQLFPKALDGFVFILSHEGDIVYVNESIVKYLG

IQQIELMGQSIFEFAHPRDEDEIREALSSRPHGPKAPITEERVFFLRMKCTLTAKGRNVN

LKSASYKVIKCTGRLVTERGRKQGTGVGVFSPYLLAIGEPIPHPANIEMPIDHNTFLSKH

DMNMRFTFCDDRVKELIKYDSDDLEGKSLYDYHHAVDSEIVEKAYKDLLARGKGQTMTGQ

YRFLAQGGGWVWVVTQATVIYNSRNQKAQWVVCVHYVLSHVEHSDMILSEVQLPIAKCSP

SPSTSLQLSTDTVFTHKTEDMDQGYLIPVEFRDKLKLTKDNEPEDLTCLAPTAGDECVSL

GFPSFSQVAMANLRDDDCTMSSPPALLTVNLKKEPSTSPALCRIRDQANLNTLSPASSSS

SSRINSPSDYLKAASPDDVLSMDKFFQEMDTKADGNMENESIDFDARAPYIPMTGDEDLT

LLPPSSESLITLQNEVDPGLFGKTEFVFKPKTEDIFGETTSQPRQSMRDMICGSTTVASI

ERPPDIMFQQIKRPLDMNSLEKGPPAPKIQRVLGPKQINKVPTVKGSKDSVLLNLLLTGE

DPNGYKVLSPSTSVNMPHTKNIVPPRNVQSQLLRLTTRDCEVNAPTHNGGLLQGAELLKA

LEIDVPQTPR

>SG-AL_contig_1793_1_+3_600_782

MTPTEKNWGSRFHKLNTQCNKDIFDFSLSNLLSEFTKQTKKEHNKLSNKMKFIYVPLLGL

I

>SG-AL_contig_1794_1_+3_3_266

WCARAQYLPLSFRPIEEASRMPASSAPTPDFGVFQAPRSFFQALGLTPALPWSSVRFRFQ

GALFPPVYPALPHLVRRIFSSFSRSWTL

>SG-AL_contig_1795_1_+3_798_947

MALKCLSTFFVFFGNTCIIWEILPILQNPRHLSDTPHYDRWSTVNEKYNF

>SG-AL_contig_1796_1_+2_848_1093

MNRKRYMTGEEVLRLGYVSVLSTILAKFQLIHSFYTLVGYPLVVNSTTGLFSVKNKRRGV

QYKIYSKHIIIPLFQMVAKNNS

>SG-AL_contig_1797_1_+2_2_208

FNHLFCSKQPQFKSFRKNVKLVYPTSNFETQHHIQRTMLNAMSPQTTLILISKQMSKATR

YPRPSTLLK

>SG-AL_contig_1798_1_+1_1789_2223

MKWFCTAKNMRLEMEHHTLSNTLPEQHSSLTSCDHTLKTGTFSFLLRSEYKNIPYAPKPQ

MPCDGKSTQHNHSKCYKSMHKPYAPRFYLGYILLSVLTHIWKQNYIVKRISISNPTLCRP

PVIPQKATVSTVNLARHFRQCQSTK

>SG-AL_contig_1799_1_-3_2391_2681

MGKTLCVQHSVHPAMTGYLTLFRVREDQVVRENRWCITIIMLAARKVEVSAISLPYNLSQ

SYGKTLTFPYHLMWDSVLTVRCDREEQFTSEDGLVKR

>SG-AL_contig_1800_1_+2_185_445

MWVFLKTEYTVLSLKNTTLALLGTGECMGTDDTSHQILLVIVYTNVFCHQNQTDFSFVLC

KYVKQKSGKSLKTKLRGGNSVHFQIDD

>SG-AL_contig_1801_1_-1_85_3867

MKLFPKTITGPLHAQNKGACDWGWQGVLAYGCHNLVTVVDPKSVQVIQVLDKHKGIVTQV

KWAHENYHHDLNSPYSLRLASADNAGVIVSWDVSQGTSRTEFSDGNKPVQALEWLKNQDA

SRDLLLALHPPYSLILWNADTGTRLWKKSYTETLLSFTFDPFNGKNVAFLGQDCIIFVND

FVITKTPSSNGKKYYISNPSTQGMSRSSSSGSLDRKGSASKTLAKRMSRILVGESKPSKP

TGAEEESVALNECLQLSFHQSCRHHILLLYAREILILDLQINQTVGIIAMDRTSSPFMQV

VSCWQRDMLLCLHDNGSLSVRVRRKTNTISTPAPEGHGAFDDTPPNVSLDVAYDLRCQSD

PLRVTRHNRVYGASMCPMSENNVALIMSDSRVIFWELQTAEIMANHNTQQVSSPLHPPEP

INTEGLTADDLNKIAFPIISEIPRSSIGLSDMLASSVGNSVGRMGPGVQLKFLMTGLLNG

ISSHITVVRMCPPLTNKNWDIYEPVMALGTYSGAVQLANAASGQIEKEYSLHSSIVRGIE

WASLKHFVSYAFPNPGPSGLVRNELLYVDTVSGRQTQLRTNKDEESPIEQLQVSYLKQYF

LITFKDKPLELWDLRSHTIIRELPRNFPRPTAVTWSPSHSLKSLKKKLLDSGAEEKPAAQ

KLSGEGDTTVSSFATNSDPSAGKDKKNQDKVSVREHFVMTDKDCTVFHFIVEGNAISDVT

KIPPESGLTRVTSLAWKGDFLVFGDAEGIQCLWDLKAKISRTTPTNRGWIKKIRFAPGRE

NLKFFTLHNDGVLIWDIGAEGKSEILHSIKSPKEIPKVVDCEWAGSDRPAIVTSTSCLHI

MNLDMRHSSSPVDNWQLAETVQCPYLLSSKASALLKCVLQHQPWQDTFSFTVEGFREEDK

ELQKAVNIHLALMDSTLKIYLPKCQFGTAERCLTAARLFGDESEIHFWTVALHYLRTNKS

KPSWKEAGSPTSPVEGGDLVELAQSDEEQQNQRLRSLFRDQPLERCYDSLCDNLSYKTYQ

LDRVSLHDSKRATYEHTQKCVENYILLGQTDRAVQLLLETEAENDSYYIDSLRACLVASV

RSSGASQSTMKLVATNLIANGRLAEGVQLLCLIDKGLDACRYLLANGAWDQSVWLAKSTL

EFGECSEVMKRWADHLCTAQVNQKSKALLVMLSLGQFNKVVEMLYSMRCFDRAACFIEAC

LQFDALTNTEETSPLFEAVFLEYARFLINLGHGKAASYYCSLAGDKGAQLYKEVCILFNY

N

>SG-AL_contig_1802_1_+3_741_1028

MCLLKAWQHYIRRPGLSLVDVIKAWQLNVRRPSLSLVSALKVWQHYISRLSLSLVDVIKV

WQHYIRRPSLSLVSVLKVWQHYIRRPSLSLVSVLKA

>SG-AL_contig_1803_1_-3_3_125

PFVFDNSRSTMYARFVEERFISWNRSICAPSQSVIHNSLRF

>SG-AL_contig_1804_1_-3_570_815

MGPWSLISLERVVLFLHHRRSLYGDTSRLHEHGGPLKCLWVPVERVSLRPAFQLWWRDEN

IPYQSEYQTLDRLLGWVLYSNQ

>SG-AL_contig_1805_1_+3_3_248

FFFCLIAKQHNDGYTLAQIIHHHHHHHHHRRHRRRRRHRHPPHLHHPPPPLHLEGQHLHH

HYPPPHHHLHHHRHPHPHPPLL

>SG-AL_contig_1806_1_-1_34_1086

MAGDNDREKMEIHVWKGDWGLPSIDPNSLSVLAYCKFSGVPVDVKVTGNPWRSPTGQLPS

LRHKDVKENKVHDIFTYLRKQNWGCDFELTTKQSADVLAFSAMLEEKLLPALLHLWWIDG

KTYADFTRPWYTKIIPFPFKFFLPAWKQKAASLRVFLTKGGENINDSDVEAKIYKEAKEC

LNLLSYKLGAKEYMFGRQPSSLDALVFGYLAPMLKAPLPSNQLQTHLRHCDNLCSLVNKI

MQRFFPPSRAEVEEKRKKEEAARKAGVSPDDTEFPNKRRNMILAAIFAASAMVGYSFMTG

LINLRMMDYEPSSSSSSSSSSSSSKPQAPDLTASGFDPLFQGRNNTEQSQE

>SG-AL_contig_1807_1_-1_115_963

MGASNSTPLPPPAASAATAPPSGCPMHQQNTAAPQPPSECPMHQQTMASISECPMRQGQA

TDGGFNTDNMMPPPNQRPAPDQPFPLPTERQKSTIPKAGTDETWVYPSQQMFWNAMLRKG

WRWKDDDMKPADMDHIIKIHNANNEQAWQEILKWEAMHATEGEAMPRLKKFGGKAKEYSP

RARIRHWLGYELPFDRHDWIVDRNGRDVRYIIDYYDGGKVDMSNYQFTILDVRPALDSFQ

AFSDRMKVWYWRMTASSSPETTPSNGQAVPHMPSSSDSGQRSS

>SG-AL_contig_1808_1_-1_1_288

LPLFFIRSVLHSFFAHSFVSSAPPAIISTSPHPFLLLLRSVFQVSLLSVFPTPFCVSASF

KVSGSTLILRISERTSGFVVGVQSLCFFLQILEGWC

>SG-AL_contig_1809_1_+1_1_402

GSRCSGGGRCSGGGKCSGGGRCSGGGRCSGGGSRCSFGSRCSGGSRCSGGSRCSGSGRYN

GCGGCSGSGRCSCGRCSGGSRCSGSSRCSGGCRCSGGSRCSGGSRCSGGSGCSGGSRCSG

GSRCSGGCRCSGSG

>SG-AL_contig_1810_1_+2_2_208

TSIQSAAGGGDCSNMSQYEPNYTLKTSKTKTVKPYRHSLFQVCYPLYCKCSLMLNTKQNV

NCEHCKVLS

>SG-AL_contig_1811_1_-3_171_974

MHNLRDLCTMISETFGEHRNSEQVFLPKLLRTDCHNMDSNDIKIDTSPTGNHRFSFSRTN

STGSIPAGGCTSSASSAHNTDDEDSDSSKTLSYKDRRREAHTVAEQKRRDAIKKGYDELM

SIVPTCHQQDSITSQKMSKATVLQRSIDYMQFLNQQKKKQEEDLDSLRKEVMALKIMKAN

YEHIVKTHQNTPQHGQNQVSDEVKFQVFQQIMDTLFQSFNKSISVANFAELSACVFSWLE

EYCKPQTLREAVMTILTQLNSQLTSSQQ

>SG-AL_contig_1812_1_-3_2406_2681

MQIPNALTFTHCVIITVAVFVSVAQPCHSFAAFIPVSLVLHSLSVLWYLCAYLTFVITVY

THAHAHIFYHLLLIIIRFRITQNRCSDDQILF

>SG-AL_contig_1813_1_+2_719_940

MYWSSNLNKQTKHSIKKNKKPQSFVCLTGFSSAVNTSHQESLQNFAFFTSHCYLFAVISV

QKMITRVIMMMMML

>SG-AL_contig_1814_1_+1_190_666

MGRHLSLPLASVLVHDALCVDGKAPVGVDGHAEQAGIGVDHSGRVTGLQVVEHRGLVEIG

HVGHVLDLLELGRVHLLDVILLEGLGVVVFDELDGNLFTLCALDLGSKEPILLVGYPTVL

LTVERLGLLLHHLLAVHRKVLEVLVIGIVGSHGELLECG

>SG-AL_contig_1815_1_-1_493_6885

MELMGSGTFDANFLMKNADAIDGILAGGGSIQRIVDSLNSLVGTLKYVIPAKNHNIITDI

QTVISGLLGLDLIQSYLIKAIQVSDLQQNAGAVRQYLISELGFTPDAATAILKSTYSVRL

FLNASELMTTDEPCDVRLLKFILMNTTQQLKDEVVRRFCDFNDRQVTDFMRVMVPQLDIG

KLLQKYISYSGQGLLDSINMTRSDVENVFDSLSDGSDALEQVADYLLADNSSFSPLVSGL

DQSTEGGFDAMVGLQPALCGLSGPEVSDQFNLNSKVQGAPSQQDLAEEKSIQDEFCKDMY

RSIKSSSMGAVIWAFVKPLLSGKILFTPNTPLTRQVISSANEVFDTLSEVKRVSKKWSEK

IPSLVALSESASKMSGLQSTLNNRFIQGILQSTLGMDLDDLSNGLKALGNSSVNASSIKT

LQRVAELVYNYTSCLDLERFEAVDSEEELERRAFQLYNNNTFLAGIVFKNMGNVSSSGGR

TKRQANNNQALPQHVIYKIRMDIENTPTTNRIKDRLWIPYARDDFANDLRYMRGFIQLQD

MVENAILRLQTDSSYNRTGVTMQQMPFPCHTEDDYIHYLGSYLLPILMTFAWLAAIGIAT

HNLVYDRQSGQEESLRIMGMMSGLNVLAWLLVTMLIMALVAVFITIIFKYAGIFQHSSLL

LLFLYVLDFCFSTTMMCYFISAFFSRVTLAILSVLIIYFLSFLPYIVLVSAEVYMTMWQK

SLACLSSTTAFSFGAIFITRFEEETVGLQWSKVNDKMDDSVDFAWMCYMMLIDSAIYLVL

GWYIRNVKPGKYGVKQPWYFPVCPSYWCTCSCFSGSTKQLPPPQDANQDLFEPAPVGLDI

GISVLGLRKTFRGGQEVVHGVDMDIFHDQITTLLGHNGAAKTTTLHMLVGVLNPSGGVVL

VEGKKTQGRTGLLGLCPQHNTLFEYMTVKEHMEFYGGVKGHLSTLELKREIRRLLHDVDL

WHLKDVPAMDLSGGMQRRLCVALAFVGGSKTIVLDEPTSGVDPHARKNIWNLILKNRPGR

TILMSTHHLDEADMLSDRILIMHCGRLLSVGSPAFLKNQLGGGYKLTINTNPTLDIQNEL

EATDGNGSTEDISDQHTKVMTFVKVCLPSARFVESVGSDLTVSIPCHTDDGATFSQFFRQ

LETQKMELGVRSYGISNTTLEEIFLELTSKADDQYSSTSHDRSASDSTDSESVSMDSDNA

ILRPSSGRPQRSVKGVSLKCQQLGALLLKRIHHYRRNWRMIISTLFLPLLAFLCAMGFSA

VRPTEENMRNLIMSPALYAMDKPVSYAFVKDNRDNTASHHFMDTLMAADPGIGTTCMEGF

HPSDGFKCVAAGSMRQSTKKDQVCHCNDYKYQCKDATQSEGVEQMLTRPDITLQNLALKD

VYRYLVDSYMNYIDQRFGGWSLEPPSDNGSLAKVWFNNKGYHTMPSYFNALSNNLLRSAV

SSTDAHKYGITLYNHPLMLNSKQLSRESIEAAATDVGIALVILLALSFIPSAFMVYMVNE

RISQEKHLQSISGVGTVLYWSASFLWDLMVYSITLGVMVVIIVIFKAEAFYDRNNLGAFA

LLVFLYGWAVMPLMYCTLRCFRSGSSAYLTLFCTNMLLGLLSTMCLFVLGLMDYTSEVKD

AADVLKYVFLVFPQFCLGMGLVDLAANQFQYEILRRFGEDTYVDPFDFELLAWNFVALAI

QGFVFFFLTFFIHSRKERTLSMTSNMHYSASKEDEDVFAEHDRVHAGGNGDSLAVKDLCK

VYRRGLKRFCAVNHLSFGVNRGECFGLLGVNGAGKTTTFRMLTGATTPSDGDAIIHGKQL

STLRSRLGDEIGYCPQEDALDHFLSGKETLKFHAKLRGFGSDQIEAVVSEQLKRLHLESL

ADKAVHTYSGGNKRKLALAVAMLGDPPLLLLDEPTTGMDAATRRLAWKCITMATQRGQSV

LLTSHSMDECDSLCSRLAIMVNGQLKCIGSPQHLKYKFGEGYTVVLHLQGLSTSRYNVVE

AFSAQFPGSVIKDQHHSVIEVSVPRGAGSVADIFTAVQRTQTEHHIMYYSVSQTTLDTVF

VNFAREQTDDSEQDSDMDSSSGGSSSGNSGDVKDSSFQKSMIGKTYAYMNPEYSSGPLDS

FDPSAPANVPPVGVMAFDNKAYDDEMFVTRL

>SG-AL_contig_1816_1_+2_1253_1621

MNKQTPHSPKPINHSTNQFNIRLTNHSKSKYKNKEQTSKQINKYPNEQNNEQTSKPINKY

PHKQNNEANITKQTNIFKHGMGETSLTIQIKLFNPTQSQLLSNPNSSATSSNQRSTQNHS

MRF

>SG-AL_contig_1817_1_-1_34_990

MRECISIHVGQAGVQIGNACWELYCLEHGIQPDGQMPSDKTIGGGDDSFNTFFSETGAGK

HVPRAVFVDLEPTVVDEVRTGTYRQLFHPEQLVTGKEDAANNYARGHYTIGKEIVDLVLD

RIRKLADQCTGLQGFLIFHSFGGGTGSGFTSLLMERLSVDYGKKSKLEFAIYPAPQVSTA

VVEPYNSILTTHTTLEHSDCAFMVDNEAIYDICRRNLDIERPTYTNLNRLIGQIVSSITA

SLRFDGALNVDLTEFQTNLVPYPRIHFPLATYAPVISAEKAYHEQLSVAEITNACFEPAN

QMVKCDPRHGKYMACCMLY

>SG-AL_contig_1818_1_-1_1306_1557

MFLCISTNLEHMFCQIGSHEFLELKYFSTYRLTSGVWLRQQLNVFIQKYTDNDNDYCDDA

DRVFYGGLLHAHARTAPPPPPPPH

>SG-AL_contig_1819_1_+2_2_163

VPKRNLKSAGERAFSFIAPSVWNSLPADLRDVPTLSHFKSQLKTCLFTQAFHCQ

>SG-AL_contig_1820_1_-2_1031_1306

MVTQKTNQNSYADIFVHNTISKKERIAHILYFMVLRKGHTCNQKHWVGQCHKKQTGKVKM

EAEMTRDDTIYTVEQIDLNMTDMYTSCALTDN

>SG-AL_contig_1821_1_+2_2_196

KKKKKRGGSFCMCVDVPFQTTSRNVPHLLWEHTGPSLWRTKRLTEHSERVAVNPSLSSFP

LFPLL

>SG-AL_contig_1822_1_-1_13_1743

MYRLPSLLRPVVARKFGPALVRCYAKDLKFGADARALMLQGVDLLADAVAVTMGPKGRNV

ILEQSWGSPKITKDGVTVAKSIELKEKYQNIGAKLVQDVANNTNEEAGDGTTAATVLARA

IAKEGFEKISKGANPVEIRRGVMLAVEAVVDQLKSVSKPVTTPEEIAQVATISANGDATV

GDLISGAMKKIGRDGVITVKDGKTLKDELEIIEGMKFDRGYISPYFINTSKGAKCEFQDA

LVLLSDKKISSAQSIIPALELANTARKPLVIIAEDVDGEALSTLVLNRIKVGLQVVAVKA

PGFGDNRKNTLQDMACATGAVVFGDDGNLYNLEDIQMQDFGQIGEVTVTKDDTLLMKGKG

DKADIEKRVQQIKEDIELSTSEYEKEKMGERLAKLSNGVAVLKVGGTSEVEVNEKKDRIN

DALNATRAAIEEGIVAGGGTALLRCVTMLEGIETENDDQKIGLEIVRKALRMPALTIAQN

AGVDAHVVVEKVLNAEGDMGYDALNDQYTNLIERGIIDPTKVVRTALMDAAGVASLLTTA

EAVVTEIPKDDKADMGGMGGMGGMGGMGGMGGMGGMM

>SG-AL_contig_1823_1_-3_366_1238

MATPRSHTTSLTAGTGKDPTETDQFLGPVSFQQPHQQQARNSDSATSRHDQSEDQDRSQQ

GGQEECQSQRQNETQSQDSHHLQHNEDGQGQSYELQRQNKGQQSQQTEEGQGYGYQQTRQ

NEEGPTRDCQQTAHNEEGQSQSFQHLTQNENSQDQNCYPNITRAEEEKNKVIQKERVTAS

SHRESGTKNMPQPSPLLSDTAQSSTAAATDDDDKIRLDRDSSITSQPAVLQPYRRPHDHR

LTEKKQRKEEEEEEGGGGGGRGRIQVCCDCLQACLCLFCCCSVRVLNKFLY

>SG-AL_contig_1824_1_-1_1231_1473

MTSNNTFNQYVESCTKIVRLNTFFCHLVEFSLLYNKHNIDREFGGIHTHNSNKTFCQDVE

NHSKVGRLTALLCHLAEFKPL

>SG-AL_contig_1825_1_-2_248_838

MMQWVLSTVLALSCAHFAQSAGKAAQIQVSVMNMNTGSAINTTYIFGIYGRSSLTNPVSG

HLVHVRSEGGSNHGCSDYNHDLPSHDWVALIERGSCYFTEKVRVATKQYNASAVIVYDNQ

PTSQLVAMQSFVKDEVYLFVTQAYGRRLALLVDSSLHVHVTITASDAVPSSSFPSLPSTA

IVVSLQALCCCLFRLWL

>SG-AL_contig_1826_1_+2_2_118

IEKTKLLAQNFQKVGILFMLKEPRHPHRGHKHTNCWLLV

>SG-AL_contig_1827_1_-1_193_309

MLSKGHRATALFCIDVIDIYMHCDLHCLSQNVKYTQRNL

>SG-AL_contig_1828_1_-1_139_627

MTFTEMCSTCETTTEKSSLTIGQALERCLCRAVASQRVSVGIQECAKLLGCAPERVMLCV

IPDMASSPDVSLHIQHTLIRSFCWENDIRLLTVKNCDKLQNILGFASCSSAVAGSESETP

VPSCLLVEVPKDDLSAEEDFVCNYHDTIIYSDIYPKPIIQLPV

>SG-AL_contig_1829_1_+2_446_691

MCNTHTRTHTHTHVRTHTHTRIRDIFTDPKANTELLYSTRNMAITYLQSFDQDLFQIHIR

MNNKNHRGKIKPRLTNSSSKQD

>SG-AL_contig_1830_1_-1_187_1167

MAEGSSNGQGNRPSRQPKDLQGLLNFCVENTKSEDAPSSAHREMSPERKEWLSQVIDEMT

GDPIQEVKEHIRTVGIFLGSQHPTDQARSNALDSLDELFDWCESLDMAIDFVKIGGFAIL

HELITHSDSEFRRQGFHLVGVLVQNNPMCQEAVIKAELLPTMLNVLNAEQIPEVQNQAVH

AISCLVRDSNLEAAQLQFVAHKGFEVLKKVLLSPGLFKCVVKIAFFLETMCQKDPQYKDA

VCEAGIMELLVQELKGEHEQMHEQVMSALLSIARDHAPTQRVLARPDLNMAPFLTQRIEL

LTDKPENEEERHYAEELLQVIQSSDSS

>SG-AL_contig_1831_1_-1_703_942

MNLGLMVCGRAGISSVGSLPLKRLTLQGVWYINPPPKQEKSKQTNLSLTVTHMRDHVHCP

NPQFYNQTQTPFLSLTHTHT

>SG-AL_contig_1832_1_-1_181_330

MRLGPSPLRQHSNTLSPDCLQTPSFPIIFGISELMVPGEFPLLRLTGQSR

>SG-AL_contig_1833_1_-1_97_315

MADRLIVNQSCYVYRPCVNRGTNFNDSSVKNRWKWDWMEAAVEEERLGKHLRKITTSGHA

VCVLCQHDINKIN

>SG-AL_contig_1834_1_-2_32_214

MSGPMPQMVSGQNSDQTFFPEATLHLYERVLLQGHLSRHGLSLDQRLYSELTLGLRRTTA

P

>SG-AL_contig_1835_1_+2_725_958

MEGDVSLGTAEGMDLASALTSGQSETEGSFQGKQVKGDVSDGLKGRIPEEIQHQEKVPLR

GPSGVVGSAKNCPSNCVS

>SG-AL_contig_1836_1_-2_2_223

LSEVLEAASWRSANPFISHYLRNFRLARADGSRGISFVLANKPISLARGLSGTTRAMPSS

FSSDFCTSLTWHNI

>SG-AL_contig_1837_1_+3_1668_2249

MTPKDTHEWKIGVSMRVEGDERGGWQQLKKPAFKSHVSTKENTLTRKPRRNKLDKMKIPF

FSSTLCKYVMITTLARSARDNIQRGGTLSHTYTQTLFSFISHIQLNAYKCHIHPDTYKVK

YTTLFCCSFVVFCWCCFLFCFFVVGVLVFFAARDATLIIKSKELFFFTAFPLCHAIHKIE

RNHHNRNPSSHPNP

>SG-AL_contig_1838_1_+1_16_213

MAFKSRTTNVHNLHAPGRFHMLTHIGQSAMRHLKDKSAHTSENMVSTNCQSLITRIFRCS

IMNVAQ

>SG-AL_contig_1839_1_+1_37_276

MAFESSITNIRNLHVPGKFQFAMRHLEVESAHTTENMVSTDQRSLNTRIFGRVHLTVTLC

GWPFFSFLLQVHRWLFVWTT

>SG-AL_contig_1840_1_+2_257_673

MPCISICGYMHESLWVGVYACMPACTLSCVCRPANLNLALGVAFLLSEHSKMAFESSTTN

IHDLHEPSRFRFAMRHPEVELAHNIENMVSTDRRSLNTRIFGHVHFMVTLRSWSFLHFCF

MFIAGFLFGRHDFLFSQLN

>SG-AL_contig_1841_1_-3_165_659

MAGSALKRLMAEYKQLTLNPPEGIIAGPINEDNFFEWEALIMGPKDTVFDGGVFPARLTF

PADYPHSPPKMRFTCELFHPNIYPDGRVCISILHAPGDDPMGYEKSDERWSPVQSVEKIL

LSVVSMLAEPNDESAANVDAAKKWREDRHQFEEIAKATVKKSLGL

>SG-AL_contig_1842_1_-1_25_309

MSAEDRALRKQWIQDQHLSPNEPRYVKEVTPRNFFRRLYMGPADAIFAKLIPVIGRTPAS

MARVAVPRMLLILGGVYWAYYQLKYHPKDWTREGG

>SG-AL_contig_1843_1_-2_2813_3199

MAFTVQMYLSVMHVRKKLQQSDSAALRPARYQDRAVASQATWVGVWLKKTCMGPLFHPAV

NGYLILYTAVEGRAAREGGGICPGKASLRVMRCNVSVSYCPNVIYVYIHASTYIMLSESE

LNICLSRID

>SG-AL_contig_1844_1_-3_84_1451

MKIEEVKSTTKAQRIASHSHVKGLGLDENGYAIQSSAGLVGQEGAREAAGVVVELIRSKK

MAGRAVLLAGPPGTGKTAIALAIAQELGNKVPFCPMVGSEVYSSEIKKTEVLMENFRRAI

GLRIKETKEVYEGEVVELTPVETENPMGGYGKTVSHVIIGLKTAKGTKQLKLDPSIYESL

QKEKAEVGDVIYIEANSGAVKRQGRSDSYATEFDLEAEEYVPLPKGDVHKKKEVIQDVTL

HDLDIANARPQGGQDIMSMMGQLMKPKKTEITDKLRKEINKVVNKYIDQGVAELVPGVLF

IDEVHMLDIECFTYLHRALESTIAPIVIFATNRGRCTIKGTEDVVSPHGMPMDLLDRVMI

VRTLPYSPEEMTQILQIRSQTEGIQVEEESLQQLGDIGTRTTLRYAVQLLTPGSILARIN

GKDGVGKEEVEEIDKLFYDAKASAKILQEQDDKFLK

>SG-AL_contig_1845_1_+2_182_397

MSGHTFNTRVEQNINRAVKNQSNGEHKSTQTGYKLTQFTHKKIDERNLSIGLTQRIVNQQ

IYNLKKLFHKNK

>SG-AL_contig_1846_1_+1_2593_2889

MLRTNLRQLQVPRFERTYKDKCFQNLLSRIVNNLFRYCKDAQMRTKGHNVVSHFPVHNGP

FLSYELTILSCASLFNYFSRSFKIGVNIGVNSVYYGTIF

>SG-AL_contig_1847_1_-2_38_1393

MRSLETLIPTLLLAAAITCSKALDFGEWPRDDGVMYACVGNSVTFSWKYEQEDDEELVDV

HWEFGSAFSMDIIASFTREQFLPNPLYSHRLSHVPNAGMTLNDVTLEDTGNYSVEVNGYD

SARTFFSHRRTVFLQVADDVMIKGHSLHIRQDPIALWNPEREEWAIRLLCGTFTFRGHPP

VGVTWTSPTGDETSSTSFDSGTFSLTLPSPVTGGNYSCHLPNHLLPEVCAKRRPRHDDDE

TLVKAKLFVDELKARMSVMEAEQKDLKGKHRKLLKDNALLSGQLQSVEDFHEEMVANLST

QLRQDLQQGLAPLKKGLVSDKRPAVAFHARLSASVTYDASNDLSLTDVITNLGEGYDAKS

GVFTAPLSGVYFVTGVASVGGSDNRAYLKLIVEDDEVAYSHAFQESHHYQTGSCNAVLQL

EKGQRVWLKSDGKGFFWGKRTSFSGFLIAADP

>SG-AL_contig_1848_1_+2_185_349

MRLLKCQILKENMFARARVCVCVCVCSLDPCFLLVLLIRRHGNGRNLLVKKTQHS

>SG-AL_contig_1849_1_+3_57_161

MRFNKQINYSEEKLIKNRARQNTTDRVVLARVAVG

>SG-AL_contig_1850_1_-2_512_631

MSCPLLPKVSFSLFPSSSSIETITWRLSCLFLKARNKRID

>SG-AL_contig_1851_1_-3_813_1091

MDDETPFSTTTEMCLLCHFFSVQTTDVSNSESSREIMTITQKRLDYAKSGFLPLTTPYLN

HEFLNTVTTGVVTQTEQLLVASQLCLLHGFNYC

>SG-AL_contig_1852_1_-2_809_1024

MSFFPDLGIQSNKTILTARAYRVSDHFADSLVSRVVSKLKTLQVIFFFFFFVLYNIFFFF

FKIKTPPPPNGF

>SG-AL_contig_1853_1_-3_372_713

MCARVCLCSSVLVCLCARVCVCVRTCCFLKITRTCGVSVIAIVVWRQKHVGLAGACIGRC

SHVLRLELLRTQTVCLLSIVGGTFLVKTLFCAFVVACKFYEKRKNFNQEVLKSS

>SG-AL_contig_1854_1_+1_1_126

LETLDFCCCWYCIYTCYNLSNQQTHQFKQAMTNSPDLQYGSN

>SG-AL_contig_1855_1_+3_426_608

MVNMIRYKHQLLVVHCIRPLDSCPGNVDSILFTSHFSSQHTHTHTHTHTHTHAREDHTYT

Q

>SG-AL_contig_1856_1_-1_1_264

FAYCAGVFETELDVLTGEFLIRRVDIMYDCGESLNPLIDIGQIEGAFVMGLGHHLMEDMI

YDTTTGRLLNGSAGEYMVPTTKDIPIDW

>SG-AL_contig_1857_1_+2_194_307

MNNIYLCTYFHRQLYKFSVKRSRHTLRLTTHRQGCVCV

>SG-AL_contig_1858_1_+2_113_358

MSYFPQYRTQWVLVSLWKTLFNLLGKSKSQNLVFFIGWGKSQLVSVTRNRLRMTNVCYIF

VIQDQFSLMRFQIHQKEIIRNG

>SG-AL_contig_1859_1_-3_3_386

SAPGLPGRLAPSSSVPDPDHDPYLQDVGTVFPPGVCDQPGEVRSLPQSVVRLLRDEIRHA

IHDSLSIIRPLGQTDGPPGEASAIPPRHRPGSGVASGLDGVSGDPRAPGQSPQTPSAAGA

SASLVSGL

>SG-AL_contig_1860_1_-2_2_259

HSIKKKKSNKKINAQNTHTHTHTHTHTHTQNANNRTNENLYCRFCVRKSMKLYEKWRFAS

HMYQDGIGCAYSSILILLSWRFGSLY

>SG-AL_contig_1861_1_-2_50_2731

MKSNKVYLCALAGLAVLVVVGLCDDTPFEWMRPDRFDEINDKFYSITGENCRSKRQEDLQ

FQDATVAQKPRYNMLLSTIIYSNRSSLLHAHNMALNRAFYYSFIYQQMNNSWDFSTQPGL

MYIYMGHAADVSANPGFINGSSIFFDNNCSYPNWYRKMDFNTTLPLFGPRAWREDDYNEP

TNWLREPSNNTIDIEDYGSGRIRNYTHPQYKGNPWYNPWLPDLFEKDDSVRKFTYSVGIK

YSNTTGIFTKDVFTDYSFFGPPQPGQQSGTESLPVRISAPYFDCDHSNRWIVTASSPVVE

YMPRYSPWIHLRRARFVAVTTMDVEFERLDMNQCPLAEGNPAPNYFAGTARCRSQTTMCE

PLMGFGFRRGGYQCVCLPGYYYPWWHDGPFQGMEIEQATWDEWQYGFDCLSVEQRMLTPD

MAPEIVTRKRRSVYTAKDKFMDALPKQQSVRLTARRKRSVIDAVKDYREKTKKLEAAVSA

SEAAHPKIVKKRFVPKQPAKVTMPSAHHNRQKRAATDEEAAARMQRIMDRKYQTNKVNCH

DKKAYQLFLPGDVNYGVERQFEAQGRTALRLAHFLSDFLQNVDEYEEFGDLKGDRRLNET

QIFAEVIANVMGDFRILGSGVFFDRYKFRMSPPVNNTDPRFVNGITREFFGPYAWRHASD

EVGLDYFNALDYSGFKDFYTDLPWFQNLKSRWATNFYDLKKFTVKPMIRSDYNGTSSIRF

EYYPLTYRAATYEDGEWLRPEFKCEGRVDDWVITYLAPFFGKNDLKTRIEFKGVVTVDVS

LKYLDINQCPSNFYIANAFKNTARCDYQSQYCVPLEGKRFTTGGYKCECRQGYEYSFNDL

AWFFDGQTMEQEYSKLQRGEPNRYHTLKCRIGAASTLTSSLLLVVVLAVLQLLW

>SG-AL_contig_1862_1_+3_909_1355

MESMYKPLNEKKKFNKRKTYSFLNFITYMFEKLQNTHTHTRVHVHMCACVHMCACVRLID

TGVTVGAIRPLIVCYSIFISRICRIVVIFWNISHKIFIHDTFITTFIMINSNIVVGTHLQ

YQFCHLSGNENHPTLRHYFEVCTMRAELE

>SG-AL_contig_1863_1_-2_908_1159

MPVNNASNAYHAKILLVVVIVLGSLCDFSNFMFLRGGRNRVRAVIAFETVLLQKAQTLNC

YSSYLSLKINHHGLQKYWTHFACR

>SG-AL_contig_1864_1_-1_31_399

MNYRYVVAIISRTLETFYKLHVVSLCSLQSRACLHVNKNTDNFDSFSLNLKKSRLHCLCF

NNSEFYESSGRNMENKHIQEITEGRAKVLLPTSVFYNPVQEFNRDLTTCIISQFAEEHLA

QQP

>SG-AL_contig_1865_1_+3_456_737

MDFYSHILGTILWQNDSKAWNVKTDFILVYRESSCSKFKHNNHQTSQTFTSATNNKINTF

TYFTFVLSIVPINSVEMRFIHNSSQHFYYEKKLY

>SG-AL_contig_1866_1_+1_70_366

MWHQSSVVRVTLRSFFSGAPTCQGESELSPLWSDPVAVTTVVWALDTVRVTCLVPVKDVS

MTTESLPPCGDSFAGPEGLLLELGGEGWGGLDARAPRGD

>SG-AL_contig_1867_1_-1_1_198

WDTGCLRKNMCLQDWSSVPHEDPHQALKTSIPTHPSERRENPSSSSVVNINPILTDTACT

YTTLPA

>SG-AL_contig_1868_1_-3_135_1067

MMVEDRAAAKELDQWIEQLNNCTQLSENQVKTLCEKAKEILSKESNVQEVKCPVTVCGDV

HGQFHDLMELFRIGGKSPDTNYLFMGDYVDRGYYSVETVSLLVALKVRFRDRITILRGNH

ESRQITQVYGFYDECLRKYGNANVWKYFTDLFDFLPLTALVDGQIFCLHGGLSPSIDTLD

HIRALDRIQEVPHEGPMCDLLWSDPDDRGGWGISPRGAGYTFGQDISETFNHSNGLTLIS

RAHQLVMEGYNWCHDRNVVTIFSAPNYCYRCGNQAAIMELDDALKYSFLQFDPAPRRGEP

HVTRRTPDYFL

>SG-AL_contig_1869_1_-3_90_761

MVKNPCEEKTCRRGEMCIVDKKYRARCVCYSKCLPALPGDDRFQVCSKRNVTYESECALD

RDHCLCRRNLDGCLNSKYKKIRLDYYGPCQQLTPCSEREFKEFPRRTREWLFIIMKELAD

RDNMPSEYQDLLEQARQEINHSHAVLWKFCDLDVSPHDRFVSRRELMYTIQSLKALEHCL

IPFLDSCDADTDGQITLWEWGRCLELKEDEIKDVCKAARRGRGN

>SG-AL_contig_1870_1_+2_1553_1834

MLTEERETVCVLYIASKHNTRFFQLACMHICQACMNLCTCMTAYVVFTSTILLCCAVTSN

ALYLLKNFFGGDRKKKSTALNSIIKRRFYFQFVS

>SG-AL_contig_1871_1_-2_44_319

MLAGSFGFIINPLILGHLMENQGAIWLAYLLIGEATLCVLIFVLLLLFSRLYLQTTYFLP

NSSHDVSINVKTDMAAPECIQHILKMDHQLVS

>SG-AL_contig_1872_1_-1_1_504

LSNNFLLREVSRGLTAAEDRTPRRLPRWDLALVLGALRSPPFEPLDVVPFSLLSYKTAFL

LALATCGRRSEIHALSGAKDDIGFHANGDLALSFLPEFRAKTQLPGSRPQSFRIPILVSC

LNPRDTRGEEHLLCPVRCVKYYLKHSAPRRSSQRRLLISISENYSRDI

>SG-AL_contig_1873_1_+3_3_116

WRLGSVIVNTGMDTRCPFCSVICLYGLFMCFVASKYFL

>SG-AL_contig_1874_1_+3_3_185

LSRSIHHSQSIPYEFRPNLPAIICHSFKPAQNPMHWQSFETGTGKTGPMKHQNTQLQFCN

Y

>SG-AL_contig_1875_1_-3_444_656

MLLASWLVHLKELWTQDLMTRVQSQPVSAHLNASVSLVVLVFHRSCFPRPRFKTLPMHWV

LRRFKEWQIIA

>SG-AL_contig_1876_1_+3_438_647

MHSSRITLGPKPTHTSICIHTHIHTHALFTSNTGPSVLAITFVIYFFFIQLLLFVYLDHA

CLICLKIFLV

>SG-AL_contig_1877_1_+3_273_455

MATAHFTKTSSLAFRKYEPQEPATDEKKTEERLSTDKARATFCTHSKCIANTLISLFVKR

C

>SG-AL_contig_1878_1_-1_1591_1842

MLDIWNMFVSQNTLILSDSHSVSVSLCACLSSLFTSPAPPFPTPRSSSPFSSCIAEPYLK

KKCLSPLVLQSHLRNIAIFFLWYL

>SG-AL_contig_1879_1_+1_832_1044

MSKNMEKTFRNNLTCLKNAEKKTYRNDVTCQTFCPNVIAVLKEAKWSTPQCKKLSFKCRR

RNNGRILDHIP

>SG-AL_contig_1880_1_+3_3_194

FIYFCNTYFIPTPSGSHTHTHTHTHTHTQRTATLFLDVWIYTLYAKPHHALKRSVDKYMN

AGHC

>SG-AL_contig_1881_1_+3_51_218

MYMYTYWCFFSDMNTQCLVMHAPYNQARGSDCRVHKTELMSCAHLISRQEAVSVVF

>SG-AL_contig_1882_1_-2_2_223

NTTVTACYLIIRCAHDISSVQNTTVTASYLIIRCVHYQALCVHVREETPVYVRIHVCVSS

ASHAQTGQKKVVRN

>SG-AL_contig_1883_1_-3_1335_1535

MIFFYLFVTSLSKRKTYSLQFLSLSFLICELFIADAIGKFHHYIVNIVYVSSSNSERKIT

LFSSTWL

>SG-AL_contig_1884_1_-1_61_168

MLFLCKNMITFHAESIKTASASGNPAKNILAPSGLT

>SG-AL_contig_1885_1_+2_1481_1708

MASPVEAMVINALELIMQEGSDLDSAEESGVESFLDSDQEVLPSNDFGCENGEGSVSDRL

SEGDTDKHSSGLKHFL

>SG-AL_contig_1886_1_-2_2_298

STGTPCRPETTSNHLFGERDQTFTRKPTMSAPLRGALRSLQVVNKAVAHQKATVVSGPPR

YRVSMGEKVGLGLVMVAAVCAPAAYILANLDNYRGGRPE

>SG-AL_contig_1887_1_+3_3_200

VCARACERDLQTFKLLLYHFLFQFSPAFRHFELLHFLDAVRHMLSLQYTDRKNFFQQPKR

FQRNNQ

>SG-AL_contig_1888_1_+3_948_1175

MLMNKTWLLILSLHTKRTVNIATMVIYNNYINIYHIYRYRPLPRMSFHNITICVSIFFFF

LVLKFYEINHSLKSES

>SG-AL_contig_1889_1_-1_313_555

MTKPNGSVYPCFAPYIFYCIKGGIGISLVKSLSLSLSLSLSLSLSFSPCLSLPLSLSLSL

SLPLNLSFSLSPCLSLPLSLS

>SG-AL_contig_1890_1_+3_3_221

GGGGGGSAGSVNRTRAVTHTKQWQFCSHGSVGQYARSKEKKRKKKEPVLACLLFRLAAVS

TYQRVTDWHVDLH

>SG-AL_contig_1891_1_-1_61_177

MPMYSQFLFVYFVYICNLHILSCCYLPGALANNKTFKIL

>SG-AL_contig_1892_1_-2_29_2401

MGGSKKEKKEERKAKRARVEEEEYEQEEEDDFIDTGEVSKDVPAAALRNVDEEEASKTDD

FGAQDFRKTLELKTDHNSRPLWIAPNGHIFLESFSPVYRHAHDFLIAISEPVCRPEHIHE

YKLTAYSLYAAVSVGLQTGDIIEYLRRLSKTSLPEGIVEFIKLCTVSYGKVKLVLKHNRY

FVESQHPEVLQKLLKDPGIQESRKREEGEEENTDGFIAGELSGKSALQLKKPANGKTEGQ

PGTSSEEPQNGEETKEEVPDDIFNFYEKMDREEEDEEEEGIMKTVSFEIKQESVEMVQKR

CIELEYPLLAEYDFRNDTINPDINIDLKPSTVLRPYQEKSLRKMFGNGRARSGVIVLPCG

AGKTLVGVTAACTVRKRCLCLATSGVAVEQWRSQFKMWSTIDDSLICRFTSDAKDKPMGC

AVCISTYSMLAHSTKRSYEAEKMMEWMQNQEWGLMVLDEVQTIPAKMFRRVLTIVNAHCK

LGLTATLVREDDKIADLNFLIGPKLYEANWMELQNHGFIARVQCAEVWCPMTPEFYREYL

TTKSQKKLLLYGMNPNKFRACQFLIRYHERRNDKIIVFSDNVFALQHYAVMLNKPYLYGP

TSQGERLQILQNFQHNPKVNTIFVSKVADTSFDLPEANVLIQISAHGGSRRQEAQRLGRI

LRAKKGSAAEEYNAYFYSLVSQDTIEMAYSLKRQRFLVNQGYSYKVITRLEGLDEEDLMY

STKEDQIQLLQKVLAATDKDAEEERGVGEMGKGGIQRKSGTMSSMSGADDTIYMEYRKKA

QHPLFKKFKQK

>SG-AL_contig_1893_1_-3_87_320

MPHERCLVISFACIYCHNEQDVIILCLLDNTLEKNIISFIDDAISYFFIMIFLFNCEQQI

IQCFFSAVKTVFHGKKKK

>SG-AL_contig_1894_1_+3_201_632

MTGSTDSRYFVLVLFGEDAVHIHVPDWTHTPPSWNLVHVGNTFSQQQEYEIKSSPNTSIQ

QSQRFISQLKQNRLLSCVDSLVHVSEDGTTKPSTENPPTQTSIRTPNQTTIHNTHTKWSA

HPSTELTRSVKKESTKSQIKNMPN

>SG-AL_contig_1895_1_-1_85_414

MKTNLPVFKLRDSSVRRRYSDFEWLRNELERDSKIVVPPLPGKALKRQLPFRGDDGIFEE

EFIEERRKGLEQFINKVAGHPLAQNEKCLHMFLQEPVIDKNYVPGKIRTN

>SG-AL_contig_1896_1_+2_2_145

EVGEITRFTQHTQIKITLSFQTKSRQNLKISTQLGSLVLSARYQMMTG

>SG-AL_contig_1897_1_-2_74_667

MYLLRRIVVFYHVGVFFMWLSVTLVYPTSDQQPNSDAALKSSDSCDGLLMGQYLCNDTEI

DPDTQQAAGCDKTTRLAKVWCQLAPGVECAEQFPNNTFQKNVPCKWTNGHSFEKALLLSV

FLGMFGVDRFYLGYPAIGLVKFATLGFMFLGQLVDILLIATQVVKPSDGSEYVIDYYGAA

VQRIVRDNYTYLKPPDYG

>SG-AL_contig_1898_1_-3_108_1286

MTRNQRFNPHPGHPHPAALKKQQERAVREAYRRERKIDSYLADDENFPSFAIQLKKLGLQ

LRDIPGDGNCLFRALGDQLEGHCRNHYQHRVDTVSYMLEHRTDFEPFVEDDVPFDEHINI

LKKLGTHAGNDAIVAFAKLHNVNVMIHQLNGKPLMIQGPSIGGKVRQLHIAYHNGDHYSS

IRRDGDNTESPANICLQEDAKLSGPVYGGGSDHGISHGNTGVDHLESEVMMATGCQSVEQ

VRDALDECDYDVDAAIATLMQTMELQDDGFCEEAGPQHHIDGGLWAADGTASRIFGGGGG

VSGAYREDTSSDVSPLGSNRGGGARPKHSLAAAPSSQQHGSGRKGRETKKMEKKRRAEER

HRQRLLGSDPQRQLQREMQQEDAVVIKQNVTRI

>SG-AL_contig_1899_1_-1_49_1233

MYDEMMEKKLPVDRLTFNELIRAATFHQDTYDTKWKQIQSLLTDMQLCDVEPNVSTFNSV

LFSLARIPQYRQAPFITLQTLNEMKNLGIEPSLASWKHVLTIFYPNENSESNMLYKIMDY

LEGKEFALQEPDDMEFFVGAMKRCCQNLHDVELALRIDSLVNTGDNALLLGDSRLENMYY

SFFLRIVCTFETLDKIMEVYQRVTPHLWTPNFSILDEVLKALELQDGYKYLPLLWTDLLL

FEFLRNSELVEKLLQLMAKKKQETELQSQYCHIARQLLTRWNDEREEGKRPSLSTGAMIS

HLITIAENSDDYDLAWSIFETFQNNRNMFTGLPDEGAMKVLVTCCLEHGDLEHAMTVLKV

MSSLEMSLTSDTAQLILDSDKVVLSSSQRDVLSNI

>SG-AL_contig_1900_1_+1_1_129

LSFLPPLLKSTPVKQILSSQRQSPSGLIFTAAIDKQFFAVQNV

>SG-AL_contig_1901_1_+1_1_177

EALRILLPFINRSQFASTAGFHSVIVHLATRKSFMKIVLKRNCTDVVSNGSWGKKRKSG

>SG-AL_contig_1902_1_-1_409_1623

MQADDFEEEEALTSERELADDAQWKIIQKNTFTRWANEHLKTVNKAIAELESDFSDGLRL

IALIEVLSSKKFKHVNRRPNFRTQKLENVTMTLKFLEEDEGIRIVNIDSTDIVDKKLKLI

LGLIWTLILHYSISMPMWEGEEATPQEGGPTPKQRLLNWVQSKVPDMPIKNFTTDWNDGR

AIGALVDAVAPGLCPDWEDWDPKKAKDNAKEAMDAAEKWLDVPQLIKPEEMTNPKVDDLS

MMTYLSQFPKAKLKPGAPIRPRVSPGINPSRVRAFGPGLEPKGNKVGAPARFTVETFSAG

KGTVEVVILNPKGAKEPCEVVFNKDKKETYSCVYVPSMEGQYRVIIKFASKEIPKSPFNV

TVEGALGDPTKVTAKGPGIEKTGVVATKKTYFEVYTKDAIKIHGA

>SG-AL_contig_1903_1_+1_922_1263

MQTEVVKCWNPFQSQTQPLSGKWHGIQTSHVPKGVQIREDCSICMNNILYHKEHANNNHI

ASQGCVCNISEYSNVFTHTHTHTHTHTHTHSDLNHLWEYSYFHSNACDRLNMCS

>SG-AL_contig_1904_1_-1_1_207

ETNHLCKWALLFTITITLWCTSRLCPRPHPVCSVHPQPLPEIVSSHSLSHHTFSDDHQFY

KPGNISDLD

>SG-AL_contig_1905_1_-3_210_725

MPPKRDPEMEAQALDWIEANMGEPVDRKKPYDDLLKDGIVLCKLMNKLMPGCVKKIDTKG

GGFALMQNIERFQNAAKAYGVPPAEVFQTVDLWEKKNIPQVTLCIHALGRTAQTRDDYKG

PMLGPKMAEKNERQFTEAQMLEAKNVVSLQYGSNQGASQAGINMGKPRSIMD

>SG-AL_contig_1906_1_-3_3_149

ALQPFLKSKVLNLLTEEICGLQPCFGRVEIENCNKSKFLHAVFCVFIAF

>SG-AL_contig_1907_1_+2_347_490

MSHSLNLFILIPCKYQAWELLKTKNSETRGVRGHAPPGKCFFLDRNLC

>SG-AL_contig_1908_1_+3_1260_1589

MKPLQLTLQRNSTQKEWYDRVDINRPNENSATQINMSYLKSIHLQFSLFSLQDFTFSFTQ

KQITCFSSHHFYMCEAMPLRNVDKIPGTDMLTITDGLYLRQKFAEVRKVF

>SG-AL_contig_1909_1_-2_251_751

MAGEGTNVIIAYDGSEHSEYALEWYIKNMHKAGNHVTLVHVPDLNDMLHSTRWSNSLYVF

DREVLEAMLKDEQQRVQKDLEKFAEKLKALGVGGKVKSVAAAKPGEGIAKECENAHLVVV

GSRGMGAVRRTLMGSVSDYLVHHCHCPVLVVKHPEHFHLHGMPQKMD

>SG-AL_contig_1910_1_+1_1_189

DDSKGKKGAETKRAIEKRKFLAQNFQKVGMLFILKEPPASPQGESTANAQRLASNLIFTS

LIH

>SG-AL_contig_1911_1_-1_64_699

MATSSEDITTMKEEAEEGIEKAVVCPDDLIKHPLQNTWAMWFFKNDKARNWCENLRLVTT

FSTVEDFWALYNHIQKASKLPSGCDYSVFKDGIEPMWEDNQNKKGGRWLVNMNKSQRHND

LDNVWQEILLCLIGEAFDEQSDEICGAVVNIRNKGDKLGIWTRDFNRQDAILKIGRTLKE

RLNMPKNCIMGYQAHTDTISKTGSTVKNRYQL

>SG-AL_contig_1912_1_-3_129_1577

MSHRTFEKDRPGKLFVGGLAEDIDEKTLEREFSKFGRITESMVIRDKVRQVSRGFGFVTY

ENPMDAEEAVKHMDAKEIHGKKLHVEDAVRGGILNHDSSRFSRDGGGFRDDGFRGRPRGG

PPRGRGGPPMRGGRGFPPRDGPPRGGGPGYGGRGESFSRGGRGRGGMMSRFDSRSSPPPM

RRDDSPSRSGLMSRPISGRGRNGFDSGRGRPMSGFDSRRDDRYDSGFSDRGPPPRSRDPY

GSPPRDTGRGSGLLDSRLGPSRSSRDYPPSSRDYPPSRDYPSQRDYSPDRGADRYSYRDN

YGQSRGEDRGDGYSRGGASRGDYMTSRSDLRDSPRDSYTPRGGDGYSSLSSRDYRDPMSS

RGGDRFDRDYEGGSSSMRFSRDLDSRGPPRSDNYMSRESGPPSRSYGGSGSMSGSRGPPP

FSSSRGPYPSGGSDRRSSGDRDSFAPGARRPAGPPPSRGGPPPSKRPRSDGPASRPPPPS

FRR

>SG-AL_contig_1913_1_-2_56_2866

MDYFRNLLPLGPQAGGEQPSGAETIERLCDRVQTSSLLEDRRDAVRALKSLSKKFRLEIG

TQAMDILVKVLETDRGDAEITGYALDTLHHVMSNEPSEDDEMPTQASVDLGEQFTEIFIK

KHENVVLLVSLLEEYDFHVRWPTVRLLTVLLTNRPKDLQECLLVVPMAVSKLMDLLSDSR

EIIRNDALLLLNQLTKGHANIQKIVAFENAFERILDIIVEEGSSDGGIVVEDCLLLLLNL

LKNNASNQNFFKEGSYIQRLTYFFELDDATQNKQTGWSAQKVTNIHLMLQLVRTLVSPSN

PQNQTVACQKVMNQCGLLQQLCAILMASGVPADVLTETINTVSEVIRGNQPNQEYFASVL

APSNPPRPAIVVLLMSMVNEKQPFVLRCAVLYCFQSFLFKNEVGQSQIIQTLLPTSADAN

TISAGQLLCGGLFSADSLSNWFAAVAMLHAVLGNTNQREQLLRVQLATSLGNPPVSLLQQ

CCNMLAQGAKHQTRVGLLMLLSTWLADCPVSVTHFLSNSANVPYLISQVSSSEGDELELI

VQGLCAFVLGLCMVSNDDQNPAFTKASLKEIIQNRIGLEMFTDKLTQVAKNESYTRAAKK

PHLNYKHPSEIVFDFEFTSLFKHLENEVLVAVSGAENMGLKAASRREHECIVENYKELIK

EQDQRLNDMSQQVLELQSKHTSATATIEELRTQVQQLKDQNALLKAQKGGAAQGTVNGEG

VESLQREMQTLRIQLSEKEGQVEKLKKDLTVSEARLLSQNGSGGEDGKENGPSESALLQA

SVSRLTADVEELRAQCAHKDNEIANLRSQLDRAQADLLARNSQESNSGAGQTTEEMQRLL

EEKTQLQERLKKSNEENLNLSNKIRALEEAKEAECGEKAGLREELDTLKKEQDDLLVLLA

DQDAKIGKFKDKLKELGQEVEEDDDEEDFEDEEDDDA

>SG-AL_contig_1914_1_-1_109_876

MADREDQVYKAKLAEQAERYDEMVEAMKKVASMEVELTVEERNLLSVAYKNVIGARRASW

RIISSVEQKDEKSEDKMAINKDYRAMIESELKNICNDVLNIIDKFLIPNATVGESKVFYY

KMKGDYHRYLAEFATGADRKNAAEQSLVAYKSASDISMTDLPPTHPIRLGLALNFSVFYY

EILNSPDRACRLAKAAFDDAIAELDTLSEESYKDSTLIMQLLRDNLTLWTSDMQGEGGDE

QQQQQVQDIEGEQEGS

>SG-AL_contig_1915_1_-3_102_4427

MSCQLSVLSRFQDVTAVRRFLFPHLKDNSPKPAGEQQTPDGDAEWSEDWGWGAEEDEGAG

LSSAAQPNVETVDDGIHKWLQNSIVSLSPNNDIIAVANKDKICVLTQKWDPQNKGEEIDT

RLTTVWEGSIAHEDGELITDIICLPLASQKRSTQGAPDWTCVVVGFTSGYVRMYTETGVL

LLSQLLHLEPVQKLKCRSYGPPRFLGLGEKHEELIILYKKAVVSIDGFSLIQSLRACRNQ

VARATASGGDSYVQPPPLAYKKWALGDQERVTDLVSCGVSTPSTFDQLKKASMEHGFNAQ

PRVSPPPASVYITSGTSPYVGFYYAIEGNMPPILSEVAMAMAHKLKSALMSAASGWLGFG

GKQKEEAKEKPKIEPAAPLNVKCGLPDLRRCGDSIVLSPCNNYIATTDSFGRVILIDVQK

GLAVRMWKGYRDAQLGWVQVKEDEGSHRHSPSQSRVAQFLIIYAPRRGILEVWTAVHGPR

VAAFNVSKHGVLVCPSYGLMGLNNVTYRTVKSRAFQCALIDPEGSVKTLDIPFHLALSDK

SSKRARDLHLLKKLKTSLKEASTETENLRNTVKETFLDIRIASISHQALERILAIRYLSV

GFMQEVVQACISQLSAKGEEHLDIDSKMLLRYAHMQAGLLDTYDAIYKLSTQTTYIDSAA

ESSYTEVLQSGLGINKEEATHISGQMEKFLSALETNNSAAPTVTISKRPSLLGVTSFQHC

FSSRSHTTSISEDSGTLTKAMGTICVSKDIREERKLALAHLLFSACISGKCTAEDIGVVL

QKSNLAPDQLMKLLLLHCLSMECLGPSAIPSLKALVKTITAMTDTSEVLVDSNKKSPWWQ

RVRDACSQSQQNCAAYITALVCRKVAADFITSQTEAKDPDADTASDKEDDDKAGENAEKD

NNSPTHTEEWEDVSVDMAHWELLLKQLEDTVALTTLLHIHIPSAEKETVDVEGSKESIKV

SVSKILEGGKGSISEVIARYVASQGVGPKSLYKLPAEGEGQSQGQEGSVLSEEDEKSVQN

ISSLERLQCSLEDLRVRFPNSLDNDILISNCGWEYAVLWNKDPEEVQHLQSSVEYLKLVQ

NAVLRQGVSSMLWHMFVTKRMSAAAQLMEKVGKAPKDRLCRKETNMSDKSMTTFVGLVVE

LLNIIMEANCEANEVPVFNVEHNWQSMRGPASLGELAVDAKPTNYGLVRLHWHLALLMHA

VMVFNMKNVKVLSMFDRKGKAALFKDLHSHPLLPSKNIDDALSFDRCKLLTRIVTHAIET

LGDPIHQSKVTRIGPNAGPGLIRRPSMHTFYWPRTCMDLAREMSTDEENIRRHFIIELYN

FGHDDLAQTIHGFVGEQDKFGKQLLLVAGRRIAYRAIHENPKNTPRLLGEMTPSLSYFIQ

TMEMDKLMYKNALLADTTQLVHEAAKRLPEHSREYDYALELIHFIDLLSSLKISAEPEVI

GQ

>SG-AL_contig_1916_1_-3_24_449

MRGKSTELLCANPGSKVRNHMKSYPNFEAKTLEQFFLFLFFLSFFLSVYYCFCTRSVKCQ

CTLRVLHAKCHVRTHILALCKYTCTMSDKSVHFCMCAYALVHLLAIFFFFFTCWLIIREY

MSLMYKFALIHTLYILILHYCG

>SG-AL_contig_1917_1_-2_2_220

PSRSLSLYSYLLNIEPFFTAFRCSTLPRHFGTNFQTRGSVPHAMTSQRFLTGERDFIAAD

ETRADCVAFFCKI

>SG-AL_contig_1918_1_+2_215_454

MLFGGKTDVFHSLQLQLSKSNHDNTYSSNQDIIQSIHAKIYWKMIILLFHLITSLLNEIK

LCRREVRTSKHDWNIIVFGR

>SG-AL_contig_1919_1_+3_621_1001

MSEFKTMASNDDLLYCIIFDRQAIFKRCKILLLAKSFHYRKCNSTKITIIQLCWLPGMKM

KKRKEKWRRDSFPFQLKDVSLLFQGGFKFYFSRDFLSDVCHVHVNMQYIAWFAGKKHIHK

SNDYHQM

>SG-AL_contig_1920_1_-2_230_418

MTVSVQCFKNSAPSCSVQRESYVLTKRCKQRLNNNNNNNNNNNNNNNNNNNNNNNNNKKT

ILL

>SG-AL_contig_1921_1_-1_31_543

MSSRKTKSKTTKKRAQRATSNVFAMFDQAQIQEFKEAFNMIDQNRDGFIDKEDLGDMLAS

LGKNPTETQLDEMMNAAPGPINFTMFLTMFGDKLNGTDPEDVIKNAFACFDEDGKGFIQE

DRLRELLTTMGDRFTDDEVDEMFRDAPIKRGNFDYLEFTRILKYGKKEADE

>SG-AL_contig_1922_1_+1_523_738

MYVTPETQEPTQHCTMAMLTSEKSLSFFLRHPHHANNHLYHQLVHCKCIICKYPLKQEKK

LKRLRQPNDQHL

>SG-AL_contig_1923_1_+1_1_246

RSTLILTLFPYTTLFRSLTKTAGVCISQAWLMPDNHKRNRRVQPCNAVSIQNSIITIPIL

MYHFSIMKIVPDSVALHALLLW

>SG-AL_contig_1924_1_+2_485_1708

MGDFSGRVGPRQTPWENYLGPHSDTSTECNYNGQILALCAEFSLWITNTFYQQRLSQTQT

WYKWNDLNVSSQIDFILTRTDQKKNITDARAIPDINLDIGHRPVMITSRQQKRNPPRNKH

KRPDRISLRKLRQEEVKEEVENEIMKKLAEVDPVEFSTEEAWQFFKNALIEVLNKACGTK

KVGKGPVKRTPWWNETVKEATAEKKKLYKTWLQSKMEEDYIKCRLASRHCKRVVREAKEQ

SWKKYREELSELCRHSPREFYRSIKAMRLRDDPYGPTSVINDKGGNPLNEGNKIKERWKE

YFQELLNSHQQQTTQGPFQPSHPDETEPTILRSEVQRALKTSPRYKAARIDGITTEGIQA

CGETVITWLTTIFQKAWSERKVPEDWQRAVVVPIWKKKGSKRDCGMYR

>SG-AL_contig_1925_1_-3_1488_1775

MCLQDFFEFCEWILTRIEPHIFSIQKLPMCSKIILSPVIWVTASRNVRMFEQVCLCFYGV

FLYSILVKCLACWYHVCLTEIGLFSRHCYAKICLET

>SG-AL_contig_1926_1_+1_1723_1956

MVQYKSSFQKTVMCWHSKTYIMWDSLQGKVGLEFTTTVCVCQEWGVRKGEGVCVCVCVCV

CVCVCVFCCCCCWCQMIT

>SG-AL_contig_1927_1_+1_325_552

MILAIKVYVKLSIRANNDNINPSWFVSFSSKRYIYWCLHARPFNKYLITVKATTGDSNCG

HTDLFTIYLTRQFCGI

>SG-AL_contig_1928_1_+2_2918_3208

MPGHMKRSIEYRPITKFLDGTPQHHLYYPHSHYSLVKKIVPHSVALHCPALMLTSVPVGL

ETCWRAEMSSVGLVTGACRRWASTTTTTTTTTKWALT

>SG-AL_contig_1929_1_+1_3394_3876

MKLLIVWISLQKCTLKKGEHNLLPPEKQTNKWPHLQFTQCVSVKCHNHFSQERLEVLHYS

SQHSQLTAIKAKSCSSYSMIYKLHRFLQQYVNTDTKTTTTFPRKDLKFHIINASQPSQLT

GSRPFHVQAIQQFTGLLNRLLKTVCKHRHQNTGIYAMSNIV

>SG-AL_contig_1930_1_-3_27_743

MAPNKYSVLLPTYEERENLPFIVWLLVKYFSESNINFEIIIIDDGSPDGTLEVATELQRI

YGEDKIVLRPRGKKLGLGTAYVHGIKHATGNFIIIMDADMSHHPKFIPEFIKKQMEGNYD

VVTGTRYSGDGGVFGWDLKRKLISRVANFITQVLLRPGVSDLTGSFRLYRKEVLQHLIDQ

CVSKGYVFQMEMIVRARQFNYSIGEVPISFVDRVYGESKLGGQEVVDFLKGVLYLFATT

>SG-AL_contig_1931_1_-1_127_681

MREYKLVVLGSGGVGKSALTVQFVQGIFVEKYDPTIEDSYRKQVEVDGQQCMLEILDTAG

TEQFTAMRDLYMKNGQGFLLVYSITAQSTFNDLQELREQILRVKDTDDVPMLLVGNKSDL

EDERVVGKDQGINLARTFACAFLETSAKSKLNVNEIFMDLVRQINKKSPERSSKGTKSTK

KCTVL

>SG-AL_contig_1932_1_-1_1_129

HLQLAIRGDEELDSLIKATIAGGGVIPHIHKSLIGKKGSQKAA

>SG-AL_contig_1933_1_-1_1_882

SAILQAIPVGNREGVSPGCIDRCEGESRCRNNGRCVNLYTDFHCDCFGTDFEGRSCEQQG

PTVLTLSGYEWVTYQIYQGEDNRPFSDRNRIALEFKTQRPSGILLYAVGGSPHHNHITAA

LHSGVLHVSVAFGDDDLDFPLGIRLDDNRWHNLTIVHNGQQVDVYLDGDMTSKVKNGDFF

LSLDPLAYIGGGDNFVITRGLPVTRNFVGCLKNVYFNDHSILYKLSNGSHLTTYHGGERP

TFGCSPVRDIPISFPNAASALRWRLGARDENLTVEFQFRTVREDSIMFYVELLS

>SG-AL_contig_1934_1_+1_598_816

MELDRKLNSTLNPSIATNLSMSSGTFLNVMHPCLNRSWSPNAEVKSTPNNYVLSHSKCGS

VSLLDLVTTYLAE

>SG-AL_contig_1935_1_+2_1205_1546

MNLMKKHVKLDEHASTFKIIPVKLDHAITFKITPVKLDHAITFRIIPKRHPYPRTKNSLL

PKAVCGQIIFNYLLFPKHRFRLEGFSKLLLTKTVKTSAGCSLLFIITPSPEKAS

>SG-AL_contig_1936_1_+3_594_956

MIAWLLHGAHDQTQVNSVAVSLSFASVQFRDHFLLLEKQKTDTQLTYKVFRTERFAMKKK

TNIEREKEKDLRKSFHLINDTQTGANLTTYIQYLPSNNLLTCLNGILKYQGVNLTKQVGI

D

>SG-AL_contig_1937_1_+1_139_279

MRAVPVSIDALRSPNLNGVRYPFTAGLTGGRTHKVFLQPDSNPCSQT

>SG-AL_contig_1938_1_+1_1318_1518

MLSLFKQNVNSKQFGGRTGKKNQAGCFVDYFSIGMHTICLYVYACIHTNTHIAHSCSLSL

SLSLTHT

>SG-AL_contig_1940_1_-3_90_350

MFYVGVFRSKSKSGVFLFSNAVKRKKNWWSLMFYVGVFRSKSKSGVFLFSNAVKSWINKI

LENKLHPFHFPVQHGSCAKCLEMSVST

>SG-AL_contig_1941_1_-2_2_97

VSVARRESNKCSSKRSNRKEIEEYRATDKCAD

>SG-AL_contig_1942_1_-3_336_857

MQTVSSLEFCCDLCVPSLSLLPMWIDRLQRTEQGMAVEAAILIFLTLSMTSAWARDGDLL

HSACEEACQQWESIREECFKAKCQSYVDEGKDWFPCFLSCWDLSRPCLSRCADSFWNEYS

SCSLQCVGRTGRQRWRCRRACAIQRFVESEKKLVFPSIPRIKVDVAKRISPKRK

>SG-AL_contig_1943_1_+1_1_252

WKIKYHCHCLSPLLNICKLFFFHQFGIPVDFWGFLFVCLFCLFVCLFIYLFIYLFVCLFI

HYLFVCLLPSSGKSQMLKFSGQIH

>SG-AL_contig_1944_1_-1_1_201

YGQDLKKKNNFNFAKLFFAFQFLFLEEKILTAFLVFFFRLSFFRLFLEVMSSKSGSHTSK

RRRSSVP

>SG-AL_contig_1945_1_-2_584_937

MPVQEKDCKMYSSELLYHEFMNTYIAMCLCSCKMYSSELLCHEFMNTYIAMCLCSCKMYG

SELLCHEFMNTYIVMCLCSCKMYGSELLCHEFMNTYIAMCLCSLPFALSVVHLDLKKK

>SG-AL_contig_1946_1_-2_80_7297

MFSAAGSSSQQGSDLPASSRQYQRASRFHSAAGYGQTDSGPGAGNSHGHSNVQARASYSQ

PSSGFRFEAGSSSSQSSENNMSNRNSSSSSSSSSSSSSSSSSSSSSVSFQGRGMENSGSS

RREVSDSRVSQAGRQSVDRSSRDEGSKTLSAHSHLAHARASSHGSPNQPSDPENGQKVKA

ADRSPGERRHSVPAQSQADHARSAADIKPTSHASSRASASRNATSRDSHRRRQSVASSSR

GISDLDKVSALASDIEQRTYFRELFSRRCKDCKVKGETYRGHSRFEYSEGCQRYRCICSC

DGSHTCPPRYTINVCRDGKNGESARNVADARQRSDQRRRRCKTCEALGQQFEGNAYFDLQ

DGCTKFRGCICYCNGTWVCPSERAINTCQQETPTPTPSVCRACNAKGKMVAANSLFELEE

PCRRYRYCRCHCNGSWECPSRFAENTCHSPTMAPEEQEHPGCQKCSTPDKQQVPGNSYFQ

MTDGCMHYKNCVCRCNGSWTCPAQHARNTCADPDSAEGRSNASLVPVSSAECQSCLAHGR

YFRGNTYFSTRQGCISYQRCVCRCDGSWDCPNRYVSDLCADADPTNDVDNPNATCSFCDA

DGKIIPSNSFFNRTQACVQSTQCLCHCDGTWTCPERFTRNVCVQPAKHVPSQEPRGCSSC

LVNSRVVRGNSYFDFISDCFRYRDCLCKCDGSYHCQEERAENICNYNTASTASPASDSSS

ESSSSESSSSESSSASSSSSSSASSSSSSSSQASSLASSCVRCFAFGLLHEPDSRFVHEE

DCVDYECQCHCNGSYTCPPERARRTCEPGCRECELGGQFFEGNTYFQYRQGCINYLCTCF

CNGSYTCPPQRANNTCVSEEGGVDSGSLVTDISRLSGDRDSCQQCVTNNGQSHQPNTDFE

LTEGCIHYQCRCHCDGSWNCPGEAARNACRGELLGGCRSCVISESQIYRGAEDFQMRDGC

IHYMCHCNCDGSWQCPGEQARDICLGEVPGGCRVCRVSKNETYRGGSQFQLRQGCNHYQC

NCHCNGSWECPGETARDVCIGEVPGGCRVCRLEGGETYLGESDFQMVKNCIHYNCRCNCD

GSWNCPGERARRVCNLDGSPLTGQAVTTTQAPSVCRRCLVSDREQFTPNQDFSLRRGCHV

YTCQCECDGSWDCPANLTRNICARRDNPQLRAQTCSSCRVSSYTYPGRSSFLLTRGCAQY

NCQCDCGGRWTCNHTVARDICKDRGSQPYSAGRERFRSRQTAVSSVSSVSSSSSQPQAQN

SLVSDTRPRAVSTTRGSSVSAANLGSFPAASRSSASCRPCRVDGHDYDPESDFIINKKCK

RFHCFCSCNGRWRCPRDRTVDLCREDDQNPGDRRRTPARHPPVVPAQVVAGGQRYGTANI

RVIGANTAASSSSSSSSSMNTRRGEQGHGQLNVRGYYVNSASQADNRQAGQQAFYQAGRQ

AGQRAHSQADKQTGQQAYNQAYRQAGQQVYGQANNRQTDSAQEAGYSASQRLQQSGRQQA

GHQSRQAGQRYEERKDQSGIFYQQAGAQTFGQRSQQSHAARAQSHSLQGRDQQSASHASS

AMASSDQDASSASSVVSVQDRGEIKGDMGNSCMPCQVDDKRYNPGAKFDWRRGCIIYKCF

CMCSGSYRCHLSVDSDCSKEDPTPGAGGIPTGPAAGQVPANKPGGSCGNCYVQGYVYPGN

MSFTLRQGCQELSCQCQCDGKHECADQKPIPGCIAMSTPLGGAVYPYRAASSVFPLGGGG

YAVKPGRVINTFQQTIFSSGNCPSCQDGQGGAGGGGLVLPAILPAHMTSGLLPGQYNPEQ

PSGQQPSGQQPTGQYSPQQSPGQYSPDQASGQHRYPVAAQPQSGVQTPPRGNNVQPRMPY

SGQAAAFPMQPQPLPAGSHRRTSVQHVAMRPVHPADSAMQDRNDQKCAFCFVNGQSHRGS

FEYTKDCYHVTCYCDCLGRLRCSVQQAMGEDCPPQYGPISGRCRSCVVQSHEYPPSLQFA

LRVGCYGYDCTCGCDGLWMCPTQQPSNYCVRPASPPAGVVDERQDVLAAESGQGFVSKSH

HHHHGDRAGLEDTTFSRTAELSEDSLLNCQDCEVRGTTYPSRTRFLLRDDCLQHTCDCAC

DGSWACPKALTLDVCQEGKGGEVVQEMEQQEKRIQEEQEQRQQNEEREMDDHKRALAEVA

AHQARLAAMNRDCRSCQVHDKHYLSNADFHYRQDCHLYTCHCFCNGSWDCPAALTRNMCH

EADFQPEGCRHCRLGNNTYPGGRNFSYNEGCWEFRCECGCNGLAVCPPEQSRNRCQAKQV

QALTQPGSDDMTPPLNCKPCMVDGQMIRSRTAFSRRRGCQEYLCQCECNGTWTCPQDRSI

NVCDLDPSSDPSLTPDPAHPPKGCKVGRRTYFTRLFAFREGCVQHMCICYPNGAWRCPAG

KERRLC

>SG-AL_contig_1947_1_+1_1126_1359

MRVQNEKQKLHEPFCFDGYTKMLLNINVDNDFKHYYLQPTQQTNKLRKHKNMYNTETGLH

STFLPILQESRHFSETTG

>SG-AL_contig_1948_1_+3_54_230

MYHYGCCTNFVTNTTGVFISQALLTPQYIYYERKCSVKPSHSVSIQNGISQQQQHHIPS

>SG-AL_contig_1949_1_+1_1_198

VWWSQVSIYIPRYLNNVKKTYTIYENKKSESYSLSTSSLPSSRPRWVLFEKQTQLGIQLY

TRSTLT

>SG-AL_contig_1950_1_+3_147_353

MRMCCSHTFLQNANFRHSFPSVTMTLTLAFSIPNSCISDCFGQNLVKIRPKTNEKILLRH

FIQTHIAPV

>SG-AL_contig_1951_1_-1_1_210

FFNVMFFQSTTGFKKINSVHFFFFFGLFWVFLVGQTGLPSIILPFFCVCVFFFQLTLQLG

PEYYSVCTVT

>SG-AL_contig_1952_1_-3_582_926

MDLIGQLPDLNLGVHAGVVESRIDSKPEPQDQRFAAVRGVPSNQGSATKLSMNGAKMEIQ

HILLPLLQILTCQPLPNQGKQMLQFSSTMQVLVFRRQKFIRCPLNLRIKCLEEAG

>SG-AL_contig_1953_1_+3_3_158

EQRRADMIKSQLKVREIFTTALSAQMGAAFVLAVNTFCWFCNIIFFGIKRLT

>SG-AL_contig_1954_1_-2_212_1744

MAVQNDSTPNGSASPPVSPTVMNEVTITEEGGEKGKEEEMGKRDVFKECLAIAAGLFHRY

RKYTVVLGGIFVYLPIGVPWYFGNLATYLASYFRHKEAVVDDVVDPQWIFSAFFVAFSLA

IVLSGFLANTLGTRLTVLIAMLVHSGATFLSYFAIQHSMPALIVTFGAVGGLGAGLAYGP

PMPVVSKWLPRHIGMASGVLLTGFGGGAVFYNELITFYINPNNIQPDVTTERTSYFSQPE

VLNKIPEIFLVNGALTLGLQLVGLLLIRDPQDGDQIDEPESTELKPIVPKDHNQTGELTE

VDLQSDTVSTQSTVEMEIENKELSPLDVLKQKDFYILWMCLGLNHYGYVIKNNYYKEFGA

GRVDNDHLLTSIGTVSTIAVAVSRLFWGVITDVIGIKMTVMVLLGSTAVFASFWFFTLLA

GPGLYFLWTVIVTWILAGAFITFPLAALSVFGQQHYASNYGLISTVQIVVNLVSPPLIRA

LLDTYGWFGVFCSIAVSNLIGTVAALFLSTM

>SG-AL_contig_1955_1_+3_45_179

MSQSSEYVYLAIYIAYLLFFPAFSFGESILSTDPFNLKQLVESMV

>SG-AL_contig_1956_1_-3_237_422

MVDSKRVVRTFFTIDTCIIVIITPTVRNCSYSKIRSNTVSCKITVTVPYRATQYHAKLQL

QY

>SG-AL_contig_1957_1_-2_2_205

IQSTQYHAKLQLQYHTEHTVSCKIAVTVSYRAHSITQNCSYSTIQSTQFHAKLQLQYHTE

HTVSCKLA

>SG-AL_contig_1958_1_-1_856_1167

MCSDANVSFCQEQRFRQFFCHSNIVITITLLICKFWYCAKMVVPVCFRCSLSLSLSLSLS

LSLSHTHTHTHTHMHTELSGSKAAWLVFKDSHKCPLQIGTTGPL

>SG-AL_contig_1959_1_-2_89_370

MPQGKLKTKVQLPKGVKQKQKSSKKDVLRKGHRTIAPKKSRLIETAKVKKGLEKQLKASI

EQDAMSRAAHAEPKTLTILKPSTSTDSGNANKKT

>SG-AL_contig_1960_1_+1_1375_1629

MQENGTFLNFNEFKPKYSINKNITKCHGHAEAVKKYIKSFDFKIHSNRAFDLKKSLKIVY

SVHKGTKPYYKVLNDNRNKQNICNK

>SG-AL_contig_1961_1_-3_141_2090

MIITFERPFNTAEDSVVLKFVTGSRPLQSKGSVISVRKTSDALPGDWTYEVSSVMERSVT

LKVTSAADAIVGRYEMFIDSIHQPPNGGELDKHRYKHPDDIYLVFNPWNEADGVYLENST

EREEHVLRETGRIWLGTVGKFCVRPWNFGQFDDVCLFAALALLEKSELGDQARNNPVQVV

RAICRVINSTERDSGVLCGNWSGKYDDGVAPYAWNGSSAILEEFLKKRKGVKYGQCWTFA

AVATTLLRALGIPTRCVTCFRSSHDSDFSNPVNAHWSTDNRPRKSMDDAIWDYHVWGESW

FKRPDLPHGYDGWQAFDPTPQECNEGVFTCGPASVKAVRNGELYFGFHSKFLFAEAHGER

VHWLVDSDGNMTAFQMEKRVVGRQISTKAAGTISRLDITDLYKPPDGSSEEKEILEKASR

LCFRSMPRQIANGTEDMEFNFQGEGHKAGDVSACLKMKNDSGQPRTVDVYLCATSAYNTG

VPAADLRDHSESPIIEPHAERDVVLTLKCTDYINNKDTDSHVNIYVIAHVRETRQRYASR

DTIWIEKPHLEVKTEGRTSLSQPFQVVLKVVNTLSVPLTNGIFNVEGPGIQRVMGVKLKK

PIGPGEECRETVTLKARRGGRQEVVANFYCRQICDVASAAELDITDDAKS

>SG-AL_contig_1962_1_-2_167_1942

MPAARSEQEIAEEEELQLALALSRSEAESKEKERERLRKNYGVYGGGSNSQSSTHAEPAP

QPAMIPAIDTSDMDPELARYLNRNYWQKRSEDTTKTATTMPSAPSVTTAPQNSVGKISEA

SVTASEQPLQNGEQAESDKAQFLAALQSSLEIFVNRMRSNSQRGRPIANDSSVQSLFTVI

SNMHPQLLKYQQEMEDSRAYYEGLQDKLTQLRDAREALDSLREEHREKRRREMEEMERQR

QIQMMQKLEVMRQKKHEYLEMQRQLALQRLQEQEREMAIRLEQQKQLTHMRQMQAYGYPQ

MYSQQQMGTQSMMAGGPGMMPPQGMPPSMGPAGPQSMVPGYPAGPQSTEGSPVHQLSGYG

QPVGPQSLQPGIEVPQGAPPGQLQGSQTQGVYMQPGVSQGTFSQAPAGVAVPAGTGAPYN

TQMTTIPSSYQPQTQLPTQPQMDGGQMPPASGTATNYTMPPASMGYTDPGLPSMGHHSMA

YTSMPPTMDYGSFNMQSLASTLPQPPNQPGGMYSTPNQPPPPQVQQAAPTQQYAMGMGYP

ANLPNNQMAPPPSQGQQPPPPAPPQMQQQPPPPQQQHPQQESLLDAQLISFD

>SG-AL_contig_1963_1_-1_13_1788

MASSDPGDDSLYPIAVLIDELRNEDVQLRLNSIKKLSTIALALGVERTRTELIHFLTDTI

YDEDEVLLALAEQLGNFTPLVGGPDHVHCLLPPLESLATVEETVVRDKAVESLRNIAAQH

NQSDLENHFVPLVKRLSTGDWFTSRTSACGLFACCYARVATNVKAELRQHFRNLCGDDTP

MVRRAAAGKLGEFAKVVEAEYLKSDLIPLFTVLAQDEQDSVRLLAVEACVSIASLLPSED

TEQLVMPTLRQAAEDKSWRVRYMVADKFTELQKAVGPEITKNDLVPAFNSLLKDCEAEVR

AASANKVKDFCQNLSTDVRETIIMSHLLPCVKDLVADANQHVKSALASVIMGLSPILGKD

NTIEHLLPLFLSQLKDECPDVRLNIISNLDCVNEVIGIKQLSQSLLPAIVELAEDTKWRV

RLAIIEYMPLLAGQLGVEFFDEKLNSLCMTWLVDHVFAIREAATLNLKKLVEKFGMDWAQ

QTVIPKVLYMAQDTNYLHRMTCLFCINLLAETLGQELTTKLLLPTVIAMGDDGVANVRFN

VAKTLQKIAPLLDQSTVQSQVKPLLEKLKTDSDVDVQFFATEALDAVTSGTK

>SG-AL_contig_1964_1_-1_1_156

CFLTDIFCSYNDTSCLHLSLCLNQLWMEWITVVTIFGAILKKQLFIFNTLCI

>SG-AL_contig_1965_1_-3_3_209

FPTKFVSPPLLSTSSLSLLTYHPPHPLSTSSSDVCFIDLLSHIPGELASVLSLVSETLLV

LFIASLLDG

>SG-AL_contig_1966_1_-1_1_243

ISQLFVDGFSSDFVSLGQSVVGSADVWSGLVQYLDRTQVQNAKRFTCEGVVCFEDRPLLI

FKSSVWCGMKIYSRRKIHQDQ

>SG-AL_contig_1967_1_-1_7_354

MQQIEVICCWNERVSRNRGIDCLPASTPQNGLAPIHLKRQKLSENLSGRRSKVERDPNFS

QGQHYESSGQCPSLLPCCFTFLIPQEGQAQRKLCAFVYVCEENVSTKISQLFVDGF

>SG-AL_contig_1968_1_-1_22_1305

MSTLEDRAIWEDGEESLGEEVLRMSNDEIVSRTRLLDNEIKIMKSEIMRISHELQAQKEK

IKENTEKIKVNKTLPYLVSNVIELLDMDPQDAGEEDGANIDLDAQRKGKCAVIKTSTRQT

YFLPVIGLVDAEQLKPGDLVGVNKDSYLILETLPQEYDSRVKAMEVDERPTEQYSDIGGL

DKQIQELIEAVVLPMTHKERFEALGIQPPKGVLLYGAPGTGKTLLARACAAQTKSTFLKL

AGPQLVQMFIGDGAKLVRDAFALAKEKAPAIVFIDELDAIGTKRFDSEKAGDREVQRTML

ELLNQMDGFQPNAMIKVIAATNRVDILDPALLRSGRLDRKIEFPHPNEEARARILQIHSR

KMNVSKDVNYEELARCTDDFNGAQLKAVCVEAGMIAMRREGTELSHEDYMDAIIEVQAKK

KTNLQYYA

>SG-AL_contig_1969_1_+3_3_164

EQYYNRYDCFFNIKSLLGEEGGGRSTVMKTTMRMTAQSTQKIAENYIFKSNLQK

>SG-AL_contig_1970_1_+1_481_654

MTKDETMGGGVYSVNIQQWLLLLKFVFCCTDVEAPFIIQTTGIEISLGFQVAVVNVLI

>SG-AL_contig_1971_1_-2_356_1675

MAVNIDRSNMDQFYRYKMPRLLAKVEGKGNGIKTVIVNMAEIAKALSRPPTYPTKYFGCE

LGAQTQFDTKNDRYIVNGSHDADKLQNLLDGFIKKFVLCDECSNPETNLIVSVKKQMISQ

RCIACGHNGNVDMRHKLTTFILKNPPDQDLQTTTPSKGKKSKKDKDKKEENDRHSPEANA

QQQMAAQRASGGTIDVPPEQNDGDDDDWGEDFSEEARKQRMDELSGAAKGLALTDDLEKT

DKERMDIVYKLIKSKRDSGELGRCVKEVLGEAERLEVKEKVPLVLVELLLTDKILMQAKE

HRILFLGFCKDSPKAQKYLIGGLEMLIGQEYSSALMPRVPHILKLFYDLDILDEEVILDW

AKKVSKKYVSKEIAQQMREKAKPFIDWLEQASEEEEDEEDEEEEQEVVYSTTGQVGEQPI

EKPQENGDGDDDDDFDIDAI

>SG-AL_contig_1972_1_-1_1_165

FLHKFEIIHFQFKVPIKQNIKRHLKTDYCYGMTICLTVFFFFHSYHPPHYTLFCE

>SG-AL_contig_1973_1_-3_996_1391

MPSTRRVWQTTQHPHYCHGSITLEILLHYLPEAVERTTNSPEARSLSLQCLPWRRMGPWC

PFGSGSPILSRQPWSGLSPDLPQCSSVIFGSLAVMLRFKRYLQNNTEHAHILDCTLTFTE

RKNEDLKTVIET

>SG-AL_contig_1974_1_+1_1_66

FFFFLNILHISADSRTNNTSKY

>SG-AL_contig_1975_1_-3_756_905

MRVLRRKILLLIFPKMMEIESFKMTKRHHQVSTVNHCFPYMVFISELFQA

>SG-AL_contig_1976_1_+2_1169_1519

MILTKCNWLYNHDLDLEKRGHTHSDTPNFLFCTKICTHMSSTPEIFGDQFFICLASVISI

HFKVTNSGLFPQPILCKHDTDLSITVALFSDPFQLYDDCDPNKWVMFIYIYSVSCVK

>SG-AL_contig_1977_1_-1_1_120

IFFFFFRLDSASVLQYASSGRLPLSTEVLLSDLDFFFPFL

>SG-AL_contig_1978_1_-3_3_197

IQKKVKFTLCFVVLHCMNFKNKSFDQNITSNLSYFTLLISQRELMYTICLCIGKKHSNLE

VNQFH

>SG-AL_contig_1979_1_-3_78_749

MASGSGSDKLAKLREELTDLTAEEMQSLKEKMGLKAFRRVFDHIEETKVRGPAPKANKHR

PIEVSSKKPFDPLSKLPATKKRVVRDPRFDDISGTFREEIFNRDYSFVKEIKDEEKKKLK

KQLKNEKNQTKKQKLKETLNKMSQEEQTKDAKQKKKKLIQDWKEKERERVKEGKNPYFLK

KSDLHKLELADKFRELKKKGQVAKQIEKKRKRNAAKERKKGIIR

>SG-AL_contig_1980_1_-1_568_795

MLWDFVKISLLPACLMESNIFKDTEDETLCVYLSCTHSLCTFHFPFVLKISYFIAFTNYC

SLSGARCKILSAVLTK

>SG-AL_contig_1981_1_+3_1149_1364

MLITVLNTEQFVPVKILNMPNTWMLSGKIYKSFTRSILSEPSVPKMQGGSTVKGRGNLQY

CWSSDNTPFHET

>SG-AL_contig_1982_1_-3_27_512

MQKAAEDLRNEAKAKAEEKERYINERVTPLKADGLGQAELQSLVKELHSKVAVLEEEVYD

WEIKIRKQDFEINELTLKVNDTKGKFIKPVLRKVNKTESKLTKLQTKESRDFRGNLKSTG

QNKFALKEEDETSKQPDWRKDLKHGGEGEGEGNPGEEVPAES

>SG-AL_contig_1983_1_-1_127_1155

MCPYFLINVNTPLPFVFQYVGWDVARYIIAIGAMCGLSTSLLGSMFPLPRVLYAMGADGV

IFRFFAEISPRFKTPFVATVTSGVFAGVLAMIFSLKELIEMMSIGTLLAYTLVAICVLIL

RYETDENMPQHATLERPPENTGEDPPIKLRYLLFPPVTDKPSSVTSAVTKTCVAAMCLSI

TAFNAVVIFCEYELLAGECWAILMAAVLGILIIGFLFCITRQPRSHCKVTFLVPFVPFVP

AFSCLVNVYLMFKLDRLTWIRFGVWMVLGFLVYFLYGIENANMSIFYGHKKAEAEEGGTG

KDVSDGKGCRGRGCCGLLGSSSKKGNTEDSDADRYDSIGENTP

>SG-AL_contig_1984_1_+2_2_127

NEFFIVTLDGQALLIFFLLCHVDDKKTTKKHDLKNDSRSSQF

>SG-AL_contig_1985_1_+2_137_244

MVQQLKCQITHHKTKLNSCIYYSTHSLKVIITELLL

>SG-AL_contig_1986_1_-1_310_579

MADTLAWRPSSVSVACVLHLVCVGDKASSSFHCVTCLQLINLQLPFSPIGCGRETILPLS

IFPSAVFFFSSGIVILLLSNRRTRFLYRNT

>SG-AL_contig_1987_1_-2_2_268

GPDGANLFIYHLPQEFGDQDLMQMFMPFGNVVSAKVFIDKQTNLSKCFGFVSYDSPMAAQ

AAITSMNGFQIGMKRLKVQLKRPKNDKPY

>SG-AL_contig_1988_1_+1_1_75

QKLNPPTPSPYPPAYCQKLVDRVRE

>SG-AL_contig_1989_1_-2_2_127

DLFLHFKLFIVVTQTIYNLNSTGSVVCSDLECFTPVVLLFSH

>SG-AL_contig_1990_1_-3_60_578

MEEYARDPCPWRIVDDCGGAFSMGAIGGAFFHSIKGYRNAPSGQYRRLIGSMIAMKERAP

IVGGNFAVWGGLFSAIDCSLVYIRKKEDPWNSISSGAMTGAILSVRNGAGAMVGSAIIGG

VLLALIEGMGIMFTRFTAEQFNPANRLAEDPSQMPPQQSNSSPFFTNQSPQYQ

>SG-AL_contig_1991_1_-2_122_1708

MTGIICTIGPVTRDVPQLQKMIIEGMNIARMNFSHGTHEYHKGTIENVRKAVASFSEPRP

IAIALDTKGPEIRTGLLEGGASAEVTLKTGGSIKITTDDAFMEKCSAEVLWVDYKNITKV

MEVGKRIFIDDGLISVIVKEKGDNFLMCDVENGGDLGSKKGCNLPGIPVDLPAVSEKDKS

DLLFGVEMQVDMVFASFIRSGEGVREIRSILGEKGKNIKIIAKLENHQGIKNFDEILEEV

DGVMVARGDMGIEIPPEKVFLAQKMMIGKCNRAGKPVICATQMLESMTKKPRATRAESSD

VANAVLDGADCVMLSGETAKGSYPLESVRTMHHICREAESAIFHNQQFEELRRETPVFTD

PTHTIAIAAVEASFTCMAAAIIVITTSGRSAHLVAAYRPRCPILAITRNPQTARQCHLYR

GIFPVLYVDNKMNQWTADVDRRIWKAVHLAMERNFVRAGDPIVVLTGWKPGSGSTNTMRI

ITATDVRDKELLPPITGISSVPSFNRVGDDMTSDASLVSVPTDENVKFF

>SG-AL_contig_1992_1_-2_53_358

MGKAEHRFSVHHTLSVVWEDCYCNLQRNFHWKKQIDILYASYIISHMWIKENKSVCVDVC

CPSRFCSYGLEILSSVCLIVIFYTRPTPFVFCVQLATAVVVE

>SG-AL_contig_1993_1_-1_247_417

MVVLCGFSLCVDQYINHLPADSVVVSFPTATPWLDFLSVDIVVFNTHKIMIKKIKKV

>SG-AL_contig_1994_1_-1_1_1734

KVEDVRCDATYACSSGQTCCKLTSGQWGCCPLPQAVCCSDHIHCCPHGTTCDGASGICTQ

GPITLPWLKKQPAIVKTLLKEPEKVEDVQCDATHACPNGETCCKLPSGQWGCCPLPQAVC

CSDHIHCCPHDTTCDVAAGTCKQGPITLPWLKKQPAIVKTLPQEPEKVEYVQCDATHACP

DAQTCCKLPSGQWGCCPLPQAVCCSDHIHCCPHGATCDVAAGRCDQGPITLPLLKKQPAI

VKTRPQEPEKVEDVQCDATYACPSGQTCCKLTSGQWGCCPMPQAVCCSDHIHCCPHGTTC

DVAAGTCEQGPITLPWLKKQPAIVKTLPQEPEKVEDVRCDATHACPSGQTCCKLTSGQWG

CCPLPQAVCCLDHIHCCPHGTTCDVATGSCNKGLITLPWLKKQSAIVKTLPQEPEKVEDV

QCDATHACPSGQTCCKLPSGQWGCCPLPQAVCCSDRIHCCPHGTTCDVAAGTCDQGPISL

SWLKKQPAIVKTLPQEPEKVEDVQCDATHACPDAQTCCKMTSGQWGCCPMPQAVCCSDHI

HCCPQGYTCDLAAGSCEKGSTILPWMTKQSTIVKTLPQ

>SG-AL_contig_1995_1_-3_1626_1886

MNEGMTQENKPQSNTFQSLHIFVSKQNNGGRLLGLSEQGQMAYLKKKKKSLWDRLPCKQV

KYSFLSCFCLPVGDEIKAKISGKCAVY

>SG-AL_contig_1996_1_+3_570_800

MHPITNKDKGDPKNHGSCFKSAHKPYNNYKKLSHTVFDVTSYMLQFFVYISVLHTLTIYL

KKQTQTNKKTNSQKISS

>SG-AL_contig_1997_1_+2_1028_1582

MGFCVLAGCIVHEHSIKAFMTLVHLLQQMFTQSGQLLPSSCLLAFLVHPHTWVVPVSQPQ

SVHLDIIAGVSESCKNSPPSSATCYCLVENLRLLLEWNICKSFNRFLSAFEHAAKSRHYH

KAQTKKLPPQEHRRHWHWQSRQFFVSLHFKTPPSKRGLIVVCFATSHFLTLSSKERPPMA

TTGIE

>SG-AL_contig_1998_1_-1_1_186

LSVVCVYMCVCVCVCERERERERERERNCASILELHGNVLFFCFIIEFPGLCLYFILLLI

GL

>SG-AL_contig_1999_1_+2_2_244

LYFVTEYCRHTKCFCTLLRNTATDITFGLKKPTKINMKQEHMIKGIKQYIKKKRLNKKRQ

YQQQHEKSEIRQHHEKKRKKF

>SG-AL_contig_2000_1_-2_2_280

REKNREREREREREREREREREREREREREAPFQSNFHLIIKCTKTFYFLLFLVCTDCVC

LLKWFTMVEIEETVNNILLSYHPPDFLYVQLLY

>SG-AL_contig_2001_1_-1_1_225

THTHTHTHTHTQSKHKAHTHTHTHTHTHIHTHTHTHINYYSHPYVSPSSSLRFISLKRQQ

TLLLSDFLKYYVLYY

>SG-AL_contig_2002_1_-3_42_1280

MEPPRQPTVHKAPRPLAEIVSQVEGTFSFLQESEIDVDSPHIDPAVVAAQPMMRPPSVPS

QQPPQSNIMPAGFPAPPHVDQSSGSEAQDPLSLSQQSLTQSNLDYSGGSQSFAQAGLSHA

QNLSSGVVADAMFQPQVEETPAPPSQQQHSHPMMGQALASGESVAQYDLPPSIPLPPSQA

SQDGTQMTGQEKKFQLNASATEFQSRTMYSQMPLNLQNLSSQPPPPQNMSSQAPSQPQPP

HQPNSLPESRPIPPPTTVNTEFPSNANFAQAAGDFSQQGGGNFQGNYQRGGRGGPGYRGG

PRGGRGGNNNGNAMQNGFNNRAQNGNARPGNRGGASFQGYPPRPDYRPDGFQGFNSNGFG

GAPYQKRGGAGGPGYNRGGNNRGAGGMRGGVPRGGPPRGAGGNRGGMNRPGNQ

>SG-AL_contig_2003_1_-2_2_175

LSLYKCEEELLFHSFASCSLKNWPVASLHSLGSSDMTYISMQLAGYALAARQIHRPKK

>SG-AL_contig_2004_1_-1_196_381

MILLTFSYFGNEICTYPLGQYGADQCCQQFCSTYIIVCLPLTKLVQATVPCLINYLFHQV

SE

>SG-AL_contig_2005_1_-2_608_1630

MPSLPASPYFRDEVACFKFVGVILLMYYCVMDIIMFMTFMKTDLSQSARPYEKGELFSPF

THLSVKGLMNDHTSQYVIWPAAEYFDTFTHFSTVFSFISPNMISFFHLFLAAVVCRLMSS

ESLQSRRLAVLLFEFRTFLDALDGVVYRSHSNTKGIYQSVRSTTGYYVDIMCDLIGGVFM

MFGVLFYLFKRFDPARANQLPLKSTEAGIAVNGGPSGQTLTYSKKMLFWKCWCYGLAVAL

AGKFWDNIVGDLKSLFQTPMTDPAMSALQFETCHSFSSILIMWVWRLVEGQALLQYVLLA

VFLDKIWEFLNWVQYLASVIIISLFVISLLYVRHLHVVLHV

>SG-AL_contig_2006_1_-2_107_310

MIFSLRQLQEKSREQGQLLYMMFIDLTKAFDLVSRKGLFELLRKIGCPPKLHSMVISFHA

DMKGTVLY

>SG-AL_contig_2007_1_+2_2_103

DHLPCTDEDLNESLTILPDAIRPYLGWIVCVILW

>SG-AL_contig_2008_1_-3_87_341

MESLLVCIVNFVNYVTQGLEIILYTFMTFVKWHMCTHVRSCTAFVLVYVKVMQATVDVLN

RLHCVFIWGFIFLWKINSSICKKNK

>SG-AL_contig_2009_1_-3_774_983

MCTLRMLPLLLFQLACWVFMPVKETMHNLFVGQFFFKVGISCSCKFLNCTEYQICRSNNF

LSFKSHSELY

>SG-AL_contig_2010_1_+2_2_154

KNDAVNMANDEHVKQGEDNGISPILLHSNQCVPTAINSSTDTQCWYWRLAT

>SG-AL_contig_2011_1_+3_1557_2045

MHSDPTARMQAQVMFWGCISYSGVATLVDVNRNINSQKYAEILDQTLWPVVAKYFSDKSW

IFQDDNCPVHNSRYTLQWKRENSIPSSLWPLQSPDLNIIENVWRKIKSALNRRTQEVNCR

ADLVRVVQEIWVSLSPAYIQSLCNSVPARIRHVYWANGCISHW

>SG-AL_contig_2012_1_-2_59_400

MCVRTRAHTHTHTHTEQTHTSCLTCVHVHTHTLTHTQTLAHKSHTHTRVCTHTHKAGLNV

RACTHACMHGIHIQANRSHTHTDTGKNSLCVSLSHTHAHWQTNHTYTLTAHTHT

>SG-AL_contig_2013_1_-3_72_461

MRACACAHAHTHTYTHTHKLPNVCACAHTYTDTHTDTGTQITHTYTRVHTHKAGLNVRAC

THAWHTHTGKQITHTHRHWQKLTVCVSLTHTCTLANKSHIHTDRAHTHTHRENAQRTRCA

QCQHKYCLSC

>SG-AL_contig_2014_1_-1_100_411

MHKAWDRFTNKQLRNYHREHLLLFCPFVDSDPNYALYPIIPRYGVPDGLNSVHMWCVCVH

PHTCACVHMPDQSILISGEVYGVNPSSFKCTGLRQPWTNSFHIC

>SG-AL_contig_2015_1_-2_2_184

LSESLSLSLSLSLSPSLSPPSPPSPPPSLSHLFFVGLYRILCDICQVGICDVRYINVATL

C

>SG-AL_contig_2016_1_+3_351_521

MQRQTLSNYLLLTILFSPISENGLTTEFALSNLNNHFSKDKLPTKFYLWGGGEKKKF

>SG-AL_contig_2017_1_+1_7_474

MHARMHVHTHTHTHTHTHTYMHTCISKCTPFHVGMLTCMCTHTHKHAHNVTDKFKRHTHT

QAVRDEYKTCARAHTQTVRDEYNTHTHIHTESVRDKYKSDTNRETQTSKIEAKTMFIIYF

VYQPIKRILAQYQLTLMNTYYFENNVLLQYPLPIIC

>SG-AL_contig_2018_1_-2_134_250

MQLYTIILLECFTQVTSPLCLVLLQGLNLRFSVTPSLLV

>SG-AL_contig_2019_1_-3_144_1127

MDTDTPAESKKDEIGKEEEKKEEDKEKKEDVDKVKEEKEEKTEEEKKDKEEAGPSDDKKE

DKPEGVDGDATKGTEEEKKEDGEEKKDGESKMETEESTVVTPKDDASANIATAAAAALSA

AAVKAKHLAAVEERKIKSLVALLVETQMKKLEIKLRHFEELETIMDREREALEYQRQQLL

QERQQFHLEQIRAAEYRARQIAISQLAAEQRQQPATASPDQAGASGQPTTTTSASSAASQ

NPALAAAVSQGSYAMHGSPLPPQGPQVQATAISQGSPAPPPASTTPTNSAPSSQPPQQQP

PTPAGSTGSATPSEGTEAAPAQSPAPAK

>SG-AL_contig_2020_1_-2_215_496

MSSFLHSTVFQGYLNSLRFTHLHDAEHNTKRSTDITRMYYNIQSCAYTCANIYIHTKPNH

TLAYSRGDAGGSFSINSIPTLRKFCASNFLFTVA

>SG-AL_contig_2021_1_-1_151_453

MTCVYPVQCQGHSSVVLLLAHVLSKCQGHSAVSIAVCENVSGNLLADVWLVHCLDLSARH

DGLCLPCPSDKDHSVGTLVHIVSVCCCGCGLLTFSSPRSCT

>SG-AL_contig_2022_1_-1_1096_1389

MQPSVHPAVNRCLSQGMYVRQMPIPKISFCNFMEDKSARERSTSHTSWPESREVYPSSFP

KLPYPYTFCIVGGGFFFTFLSYCVLLFLFSICVTYLYV

>SG-AL_contig_2023_1_-2_62_739

MADEGNFTSKVQGRQLASRWEQRSPHLQLDPVGIWDDIVTNRNVFLDKISERLMSSDTME

TDEGGMFADEKLDLRLSMARSCQLQNNFNLTLNILKETFSQCKQDNSGFQLVEWSHLYAS

THHRKALSAVSSWTENTVYNVVSTLDQLGKVSETEPLRERPDLKLRQSVLMGDACDTLIT

GLMETDLWKSLSDKTADKLKTYANITRVNRDEVLGKLVMKGFQCMR

>SG-AL_contig_2024_1_-2_131_1480

MGRTTSIVLLCLAAVCVQFAQCKHWALLVAGSNGYYNYRHQADICHAYHIMRNHGIAAEN

IVTMMYDDIAYNEENPIKGNIINQPKGPNVYPGVIIDYKKKDVTPEVFLAVLSGDEEKVE

KLTGHKGKVIKSGPEDHVFVNFADHGAPGLLAFPDSSLYAKDLEKTIKEMHSKKSYQKLV

FYVEACESGSMFAKILPKNIGVFATTAANPHQSSYACYYDTLRNTYLGDVYSVMWMQDSD

KENLKVETLQKQYQITKKETNTSQVMEYGDLTIAKMTVAEFQGNQNSGVSMTTGSPPHPT

ADAVPAPDATMSILQHQLADASTPQERRSIAQKISDLMERNAQIDALFKDIVTEVEESIA

ESGKESVLRFNSKVSYQILKQERLPITQWECYEQAVDALTFFCRKMKLPQEYRALKQLYM

LVNLCEYGHSGKTVALSVLTSAYKNEALCR

>SG-AL_contig_2025_1_+2_164_397

MTAHNFSLKQITDFYWKKHRSQITQHTEDKTTRKSHIDSCVGVNFSIKTLQGPISKYRSV

DVATSFTINRPMSNTDVN

>SG-AL_contig_2026_1_-1_49_216

MDLHLEELEAGVPCINPFVDSGCQTQETEVAEGAEALEKFQQETKPLLRGNCIVTS

>SG-AL_contig_2027_1_-3_792_1109

MKTTLLSECMAAFSKSARNFGVFLDEFLSLELIEIMCAKSLETHRNYVCKVLYIQLQARV

TCLSLLMPQTSYHSFLFCLDKIIFNSVFAEMTNYPNSNKSKTVQLD

>SG-AL_contig_2028_1_-1_85_2151

MSREIDDPHKRLLTNLLQLQNLIKRDPESYKHEFTIHWKRYQELLDVFQMDPSTFLKEFD

DLTSFLAQVSHCYSDELKEYPGLIHEVLQAHGTVLDPTIRMTMVKGLIMLRNKNLIEATS

ILELFFKLFRCQDKLLRKTIFNYIVQDVKNINAKHKNVRLNTTLQNFMYRMLRDNNATAA

KMSLDVMIELYKRNIWKDAKTVNVIVTACTSRVTKILVTALKFFLGKDPTEEEDSDSEDE

KQPSQKTAKEIILAHRVGKKSRKRQKKLSRALQQAQKRKKNKQKSEVFDFSALRLIHDPQ

GFSETLLHRVEHGTENERFEVKLMMIDLISRLIGVHQLQLERFYPFLKRFLQPHQKEVTK

LLLFAAQASHDLVPPEDLEQVLKTIADNFISERNSSEVMAVGLNSVREICARCPLAIGET

LLQDLAQYKTHKAKAVMMAARSLIQVYRHSNPELLHKKDRGKPTEATKERKLAYGEVATH

EFIPGAETLLKMDQEQQEQEDWESASDDEDMDDDSDGWIDVHHSSDEEEVKEETPQEAED

KKKMAQEVCSTRIMTQEDFRRLQQAQLAKEAGLTTTTKSRKRKRPAHTSQDRSEMPNLGD

IENVHKKRAHDKDSRLSTVMAGREGRGKFSGGPQRMNPHASTTNKEKKKNKAFMMIKHKP

KLKEKKKRSFRDKQIALRDYLLKQQKKRR

>SG-AL_contig_2029_1_+2_50_184

MDYFAGQLWMDYFAGRLWFDNIISIHSNVSNNESDRAIKVTGRSV

>SG-AL_contig_2030_1_+1_415_684

MVIDIHSLSKHAVKLYLPMLNNLVLFIFILQDCPILYTHSLNTHMHNRKQGGASLLKQDD

AHICSQTEAHTHRHTHKCKAYTNIAITLQR

>SG-AL_contig_2031_1_-1_145_1896

MARLVVCLGFASLIFGTLLLTTEAYRWQESEEADRTLHVCAGDYFKLVWTLIPEEGETIV

DVQWLHQPLGGENISIAGFIDNNFVADKDRFVFIPNVGIIVRDVMRNDTGDYYVKVRLHD

VSCESLLVIERSISVVVEGVAPKTEDGQLHVSERLIHDDTILFAEDGEPLVQLQCGRFTD

LGQPSVDPVWTMPSGEEVSSSFEEDDGMFVLALPAPIQPGNYTCRLPASSPSSSSPSSTH

VCLDDNSPLKRPAFIYLDEKDTRLAVLETRQIALIDAVTELRSLYKNVSSRHIKRSDYEN

DQEEMMSRLGELNKALNDNLDQQTKKDRSGRQSCRDHVAWKGTDGLLKSVSVGQAGVWGA

NKDNRIYFRAGSYSQQTNAGSNWINVAGLLKQVDVGNDVMWGVNHGNSVFYRVGISASSP

IGTSWVHVSSEIKHVTVSPNGYVWAVGTDGSIKYRTGASNTNRKGSGWKRIAGSLKQVSA

GPSGVWGVNDNDKIYYRTGTFGDNGSTGTDWTLVAGGLKYISSGDGYVIGVNSQDMIYRR

EGITANNPTGISWLKLDGRLKVIDSYGCYVWGVNSKDRIYIGAM

>SG-AL_contig_2032_1_+2_1553_1900

MNIIHITTKLIVYQDTATNVMNSATFLAVATHLKIKFYVNIIQFQKRNSSGNHSTFSVKC

EQKPGTTSDQPCCHSLLTYYIVSWAGLGKSLNLERRKSGGWHRPPSGVQRQLSWWE

>SG-AL_contig_2033_1_-2_941_1243

MNPEILVDIFSFHTHSLSFSFSLSLTHTFLSLILSLSNTYSCFILSLSLSVSVSLSHTHT

HTLTHSCGRIHTHTCTENPLPSSSDFFKQYFDNYDDLPDVL

>SG-AL_contig_2034_1_-2_182_613

MSEFKVAVGKELLSKKGVQTTESFKLGDQDVVGIYFSAHWCPPCRGFTPVLAECYEQLKK

KGKKFEVIFVSLDRNKEAFEEYYNDMPWLAVVHDEKEIKESLQAEFKVNGIPALVLVKGC

GKVITTDGRTIVSGDPNGTNFPWN

>SG-AL_contig_2035_1_-1_1_555

EPDEPTPLPVVPTSCSCDQVWTEVKDLRETLLVENRKLKDQLKSYNDSCQVSFTARLSER

AGHKFWSDHPLVFDSVLTNKGEAYDINKGIFTAPCNGQYFFRLSMRSHQENDSGYVDGAI

EVNGEETARTSVFAADMMDHYEQASNGLVLTLKEGDEVQVRIQTTSSGKIYGEVFSVFTG

FFISA

>SG-AL_contig_2036_1_+2_104_529

MQYLGPSIMYLTSPHKKYCIQTQDAMQTTSHSPKRQQQQTKQNKKHTKTSNTLQNNNSKY

SAHSHSLQTNQPTNFPLPFHGKKVLQNQIKKKSTPKCTNKARFILSQYKRFYKNYQVTIR

PSTRGDSPLLSGHVSGIHYYLF

>SG-AL_contig_2037_1_-1_259_12510

MAASAILLLLVLPGAMAGPVQWQEDNDMCSTRCPDSVKFRYQIGSSYEYRYEAETRTTVA

GAAEDTASITIGATARIEVLGPCDMALRLTDVAIKRSHEQNTKRMVQADDMSRARNALED

QSLRFAFYDGAVGDLCPDDDEELWALNVKRGVLTSFQNSMRQMEREEHVQEADVTGQCPT

HYKVTQQGWRSLTVTKVKDLLSCTRRHDYRTSLLATPYHAPNTLQSLPLMKSSHECTHDI

SKELNILQGSTCHEVHVFRPFSKVSSGAVTEASHTLTFRNKASGTRTRNGPITRRTTLVY

EHKQGQDKGESFFVDVKNKMKEICDVTKVDIRPQVPHMFGELVHMMRNLDYSDLQDLSDL

LERNKLCPENAKKTRKFFRDALPMTATGSSLRMMADLLTSGKVKDTQATFWLTTLAFLPN

PTKDMISAVQPLLSNERIQQDALLAVSSLVNNYCEQHADCAQDDEIQNVLDTLTAILGPD

CQVHDNNFKQVMLTLRAIGNIGRSEAASLRLQNCFTKSELPMDVRVAALDAYRRMPCSAD

RNNAMEVFLRHSEDSELRIAAYLTVMKCPSDSVLNQVRQALQNEKVNQVGSFVWTHLTNL

LETESPHKQAIRDVLENELLQKEFKKDRRKYSRNTELSFFSDKFSVGGVFESNMIWSANS

FIPRSAMVNLTVDMFGQSVNLLEVGGRVQGLEYLLQMYFGSENTGKEGGYGQTNVVKNAK

LARTKANYGNVKDDLRGQMYMRVFGNELGYGMLDGSDLKKLKEGKAKSFFEDLGKKLMKD

GEVSFTQSAMFLDTTIIIPTVVGLPLNLTLNGSASLELQASQKMDFKKINAKSRAFEVAA

VLQPSGAVELASMMSVDAFVTQVGLKMTSRLHTSTSVKGHVELARGQILSVEIDTPRDSL

ELFDFQSKFFIVHDSYEKEQEMITANRREKTACSGEALAKILGMELCASLAFPNASTALS

APYFPFTGPTSISLTLRKHDVHSGYKLLAKQIRTSSSIITQVALDTPGSKVDRAISLGYV

ANLKAKTIEADLLSPWKKVSAKASVDTSKKMYKADAAVSVDTGKEYKVHAQVAHDIRSRK

EVSIKVKPTLVVTSPDGTLLSVNGNMDYRKNKLIKTNMVVEMKDVLKKPIAVDYTMMMRA

RGDKTRYRNNLKLSSQFATVKSSLQALVGSFTAKKRSALLTVRSKVDYNLHFWPGIKKNL

VNLGLKIHDRSTPALSKYGINMNFDAKRTPGVNMLLNGEVSHKKTLTEVEVKIRHGKNVK

LKADKDKELLIAGLVAHKIADSQATVRFDVKAVYPDKAIEYQMKGKHEHNKRMIEDYFTV

RYAKGKSVDAHMRLAEVKKKRSATFDLSYPAGKTYPARDVKLNADLIEKSAKHHVLSALA

SIQKGQKHSFVVSFKRPTDNSVEISSEMGIYKRSPIKLDISGNYGLRDMKGQGKLTVEGK

TYRMNYTSDIQKDKYAKFTMNFVHPERRIQGLLEGGRDTREAVKRAKMEVLWDADKDLSQ

KAGVSMELADKVNADDINIGGMLEVFTPVLEYQHLKGELRFENTNRRILTMAETILGDDS

KEYSAKFMLNQPVTPNKFKTQVWLKTPHPTVRHVEVKADHSFGDSGKLSTAVKGIYNKDS

VQVGISGSSKGDLVVRVLEGAAFFKSTLRDAENFRLTFNHRDVSGRYNTQAVLDANGDQY

SAIMEANFQKVHYQITTDGDINILAPQLTSPVKVLWKHKNSLHEVNSAVTVDFPEDQQVH

LEVGGKANIVTNVYEGRAQLRGPWKENGAEHTSSLQVNFAMPRSGHDLTVTLQLDQDTYV

FHETQKDTSTSHEVTQSLKMSRFPQYNCQHKCNIQYGNFPYLVSMEVQMADRPMLNVDVE

IEKPKRDKYRTTSRVVFILEDINQNIAISTRHEKNRRGQWVTKGDIEYEMGKTLSFSSVV

DVEKDRKMINLNVDTPFDEVQNIRLEYSHSGNVEKNFTAACSLAIDPQFDTVSSEIFWSN

RDRFYGNLKVDTPFKELRHLEVKSQSWMEGSKRHSILSVDYPPRQVYKLDTVYSCNFPRD

LDFTLNVTTPRKELPSLHTQVRYQYTDTETAGNFRLITPAIQYDELSTSFNYRQGPKSFS

SHVNNVIGTWNTWATGADFNWDNKIDGTFSIENTFHGRQKMHLLLTHRGHTWEDFRTKAG

FKMNGDRLESELAYSNKDGRSGLFMLATPDPDIDFFKTTFFQSLTEDKFEGRYTAQYGES

SKPYDLGLATAYSDDKIAFNSNLKTPHTEDVEIEIGFARKRRVEVKLFGMYGSNDKINVA

IKYNIKKEFWDVGGEFEYLIQGSGRNIGLTFKRDGPLDNLKCYITGKYMGKQVSVETQYK

DGEKKNGRLDVSTNFKDYTNLAASFEHSGDSENFETIVYLKYRDDKEASGTVDFQRNKWK

RVAMTMTFNLPYEEFRENKLTYKHTNRKNKLSAEGSLKLGPARAITGELGFVDGRRLTVS

VRGPYRQFESFTATGTYDKSNKRLEGDASVKLVRETQPVTATYKVRAGTWPIMINIEAQT

PFEGWKNVELTTTHDGDVTDFTSSHSLTAEAVGTVRSELIWTYISPSVLDGLWTLSSSVE

GMEDLKLAFKNSESGSEYNSQLIVGWDLLKEIVLDGRVHLDKSFSSNRCKGSISMTTPFP

EASQFSVSFDHSHDVRLRQNSAVARFNDKTYLDADFQYTVGDRHVGSMEFREPRPMEFLI

GGVYTEKAMEGELKLNWDKRDPEMNIHLKGDYTDHTDQLGTDRNMKVWVIHPARTMGMEY

LMKKAAKEFQSDFLLTWDKATSQTLSYIINWTDRSSHYSSAYDAMVKVGVPMRSVQVEGA

YSNTGRAKAAEGSIMWDVDRDKNMKVGVKGELDKRGNTNKVRLGLDLPFVDKEYSLTTTA

AVNTGSTLLDAKTEFSYSVDPRKLLVVTTKIEDMSYGRDATNYSLAFSVLHPMTDVSIQM

MSHVGTSPVLSSMATSVVYLTAEREKKMMELRGQIDKLKRVMGIEFKSPVKDVSLKGELS

GGREYNMRLVSNVDGQKSQVTDIFIAPETRTFEMTFRYDKAAPDNSVKLFGHMPSDSILQ

TELYRMEQGRRVSETLMTVRLNSSHVLHTRLNWRPNMLRDLQQYLGTQLTVFSYGSSEAF

QAAAGAVGHELKAKYEVIMQEVKEEAGPLLQQFQKEMKSLQVHLDSMQTDMRRFYYSNAL

HVQDLGGVATDIMETILTGMQGMVMQFTQLQQQLQQDIAQTMQQLGKYPMAQKYAQLVAD

FAAGLKNSRVVLEQALDQLSIQVSKFSDQSYRKYLIISRQIERKLKGYTAALRQTPPYQT

LAQAYNTAQSSLSQLSRGDYKQLADAANLKLMNALDQVGLKEFYLLMVTKSKRLIADQFH

DLVRRPEFQQIYVISNEIYQQGVWAYHYWQVEKNTRMAVGQLLELAKDITLLEITRIKNA

VVDLDKSRVIVFDPQNGELQFELYLPVALKSLAEPPDMRVMKYVNRVRSWVATYIPSPQH

GLWTSINNYLPNMDTNTWLPPFTAHAYVMGDQHVVTFDQKHYNFAGRCSYILARDMADDR

FTVVLNYNNRPRHPQVQSIVILLRGVAIEVDRNSKVKVNNEERELPFFSGIISAVRQQSI

VRVQDGQALTVEIDSSRAIYSMALSGWYHGRTAGLLGTYDNEPFNDMMDSQHRITNDIEA

FTNSWEVGHRFCRSRNLAISEDPESESEEVQACRKFLTDSSSYFSPCFGQVKASELVNVC

VAQARSGLSIQEAKCKAGQHYRYMCARQGVMLGTPAECVSCQLPSGQILGAGESETLKEE

VGQGLESADVVFVVEESSCNAWAERSLARLATQLAQSLQARGLRGNRFGLVSYSGNGSQE

QQLVHTIEGQVFGKPSNFLKATESLRLIYPSTAGNTGAAVREAVKYPFRVGVSKLLVLLP

CSDCTGDMYALGQVLLSHGFQLHMLDAQDFQIDGSSNSSPVRVFGLDNSKAHTQKSAARK

SEAIFKKLGTPQGSCAQLALSTNGSVFDVSHMMARSRIQNEFLESFAARGALNALPPTCQ

VCSCHDDGTGHGHTVCRLCNNNSWLWNHIPTWLQSSSTSIMGVFNDLHHQIITSLDAPVV

AVTA

>SG-AL_contig_2038_1_+3_3_179

HARERAHTHTHTHTHTHTHTHTHVCAHINTHYRIIPQKSKIYTYINTHMHTRNKRNQNI

>SG-AL_contig_2039_1_-1_1102_1293

MCVCVCARVYVQVHVSDFDKDHFLLYLIIINVVRPFQSRMQISIIIVLNMCILDFQTGSF

GRPG

>SG-AL_contig_2040_1_+3_3_227

ILQIYTLYSQKHGSNRHISATRLWLCRSALQECMIQSANTKNTLPSDQPLLNALLVLWDT

RLKTFQAVRNTCYHY

>SG-AL_contig_2041_1_+3_1056_1379

MLLTRASSSFSLVTHTPSDQHFQFDYLTHRTAERPPAPPKSPYGLTVQRLLAPAKHVLTM

DAAIVVVINEILARLIASFSPFPARHSALFRVKIGYVFAKLRLIKVVR

>SG-AL_contig_2042_1_-3_252_548

MKVTSVPFYLKKNCKNFSLCTTVLYIMIIVVIIVMIKFLIFLFLLNAVVVAVINYCCVRT

ITCINKYPPMQWLNYIELCKLQPMLVTVLGRERSQPEHA

>SG-AL_contig_2043_1_-2_14_1120

MAEQMESMQATPSVTGSGDGTEAGTSSAEGKTNGAIPAEEMTSKDYYFDSYAHFGIHEEM

LKDEVRTRTYRNSMYYNKHLFKGKVVLDVGSGTGILCMFAAKAGAAKVIGIECSSIIDYS

QKIVKANKLDDTITLIRGKVEEVSLPDGIEKVDIIISEWMGYCLFYESMLKTVLYARDKW

LRPETGLIFPDRATLYITAIEDRQYKDEKINWWHSVYGFDMSVIGEVAVCEPLVDVVDPK

QVVTNSCLVKEVDMYTVKEDDLSFSAPFHLSVRRNDYLHALVTFFNIEFTKCHKLTGFST

APEAPYTHWKQTVFYLGDHDLTVKKGEVMDGVFTMKPNKANVRDLDFNVTFSYNGELSDA

NASLNFKMR

>SG-AL_contig_2044_1_-3_36_203

MISVVFFFTLSMYRAFYTIDPLVLSCLYHTTSFVNTVFRIVFKLFEFILHVENYKH

>SG-AL_contig_2045_1_-3_36_203

MISVVFFFTLSMYRAFYTIDPLDLSCLYHTTSFVNTVFRIVFKLFEFIPHVENYKH

>SG-AL_contig_2046_1_-2_41_256

MMMHVSSRIVMNIVTQFLKMNESETCHDRQLRLNGRWVLKVCLFVRVGMGERRNHCRCLL

LMLYLQKVKKFT

>SG-AL_contig_2047_1_+2_356_730

MEFERGRESEVRQRARERERERERETERERAKERESDTHTHNIVACICDHMRLHMYQDSH

NGLQIVEMALHLSPEDENNKNQQVEEESTNLSGTLISQLLTTKTKVDTFVSTPSQDLFLV

RLTFF

>SG-AL_contig_2048_1_+2_17_310

MVKTRTSKLEGLEGLGSNLLGHLAWRAKGGYFFQSPRSKINICADLLVPEPPSCLYARET

SNTHVKDPVSLVNVRWLMETRKHPACAKNRRVYASGGL

>SG-AL_contig_2049_1_+3_1692_1925

MHTLFSLHRLQAVLALASPLDFTGVVERVGLLQHNFHSPSGLKEVNSFDFSSGDLASVSS

FIKQRVQICRHCLPPQAE

>SG-AL_contig_2050_1_-3_843_1094

MTILTDKTRPLPDGISSGQSDRSVRFRAPRARPSRYMNSFIPTAVPASNQDVQYSHRQDN

GAVPPQTPLADECMCADLCFMCYV

>SG-AL_contig_2051_1_-2_2_163

LPTRRGARGFKLRPSKPPVWKSSVLEVQCLHHSTTAPITINFASQLNENVVVWE

>SG-AL_contig_2052_1_-1_244_579

MVGLMVLLHLTCISLTLNCAVVFPLLTFFLPVELECCISFHHHCIRKMKDRTIKFCFFFN

KSCLISAEMRLSQCRNEDDITPVVCSPQVTPDSFNQVSGQFYVTALLCLVVM

>SG-AL_contig_2053_1_+2_473_742

MQCEMLKMYRWNQFNFQFSHILSYTNTHPSDACNYCIAKQRNEMLDQVYPDNPLCLHRSS

VKIKPSSILFVLSTVLKTLTFNYVCTKSTF

>SG-AL_contig_2054_1_+3_171_554

MTLILALSVPNLCISDSSTVHFFGLNLVKIHSEIAEKILLTHFVHTHTHTHTHTHRPLGN

MKNLPFFKKRKIKKQMDSDSEEKVYKRTGCWPSWLVMEGADSDTDKPSTEPPLQLPRALR

GFLENPSQ

>SG-AL_contig_2055_1_-1_436_558

MKTNEGKMILSKFVLTKYLLSLKWKPVPQISLSKMRLKHKA

>SG-AL_contig_2056_1_+3_3_404

FLFPSGHVSAASRTRTSSLSQLRTLVFPGAPVLCLHSTWVPRLSTLLFCAISLPGCALPS

PIAQFHGDHHLVRRIFSFIFPVWCLAIMASKPGSRLGVGHDSTDPSGYRSASQSPDRRTD

RSPSRIRSKRDRST

>SG-AL_contig_2057_1_+2_416_508

MLHSCFECQHAVYILCVFRSYPYEQGLCNSV

>SG-AL_contig_2058_1_+2_122_337

MTRGGGNAAQSFLNSSVASITGLHAAHNPSCTRQTSMLLILVLHSNSPQKPAILRFTTVQ

SLGSKSRNPVHV

>SG-AL_contig_2059_1_+3_1572_1781

MLCLGGGGGGGSCGVCVCVCVCVCVCECTVYFDNLAAVRSDVSHIHSLLIQIAHKSYDKP

STSLISKRWF

>SG-AL_contig_2060_1_-2_2_244

HQSAIPRGDAGGSFSINSIPTFWKFCASNFLFSITLFVSASFFFPLVLSAFFCLPGPFHF

LFSSKSSHKFLSPQFIDVLSV

>SG-AL_contig_2061_1_+1_196_495

MLSEKINTLGNSVKLPEDSNRQFLFFLKNCIHRSSVHENRVENKKSTRSQKNDYYLKLTT

FLSQYTQINVKLNSLSHKQLINCISILSTDLDETKPHDPQ

>SG-AL_contig_2062_1_+3_789_1091

MEIEDEYHVFFVCKAYDKLRADVVLLDKGQTRCATDYVEVMSAEDKQSVLQLSKFLYKVF

QTRKQICKVKMIRIIGQAVIWSGLTGRLSKDTRLNERNNFD

>SG-AL_contig_2063_1_+3_81_230

MDRGCGSRLTAGCSRLTAGCSGATASSSSWSWIGLGLGQKLCQLMTQAKC

>SG-AL_contig_2064_1_-2_320_448

MGCGFNCQVDHSLFAQAYGSCHDQFGYFSIVQHFACWFKDAVL

>SG-AL_contig_2065_1_+2_1394_1684

MLLHSLHPCRFEIRPSNWKSDIPTTRLSCPPKGFNGLSDLQTPVALTSFYGRARGTTFIC

CMQCLRAFTATPEALKLSLNIKFEGCDSQVIRTPDLQ

>SG-AL_contig_2066_1_+2_77_583

MHTSYGSTRVSAQRLAEFKGQSSVTFDLAKMASSAESDRDFDFESLLNNVAEASFEISGI

DHDSDISISEVSSVHTSDLSDFSDKLLNFSDTRSDDVENVMRKKFTRTVGPACSLPFDVK

PINYFLQVKVDSLFKKMADEANPPLNKEVITKQSNQPFFSSKMSSSLQD

>SG-AL_contig_2067_1_+2_2_202

NKILKTSIKVLLSVKRFFISPRHSLNYMPSLFIIHTQTLAEFRSYPLPPFTKIKYEGKMK

EKLKKTK

>SG-AL_contig_2068_1_+3_3_131

PIMNSFHFHRKQFLQTSTLSKSKVSGYTHDKRHVPSTLILNII

>SG-AL_contig_2069_1_-3_57_1052

MAAAGDWCLIESDPGVFTELIKGFGCTGVQVEEIWSMDKDSFEQLKPVHGLIFLFKWVPD

KEPDGSVVQDSRLEKIFFAKQMINNACATQAILSILLNCTHSDLFLGETLSSFKDFSKNF

DPTLRGLSLSNSDVIREVHNSFSRQQMFEFDEKLAKKDDDVFHFVGYIPFEGRLYELDGL

KDGPVDLGAIAADSEWTDVVRPVIEKRMQKYSADEIHFNLMAVVSDRKTAFEKKLTDLQT

QLDQGAMDTDDLQGEISRLNFLLQEEDRKRKQYKVENVRRRHNYLPFIMELLKILATEKK

LLPLIEKAKEIAAQKRNEKEKAQQKMESQASQ

>SG-AL_contig_2070_1_+2_74_835

MSWNPFSRLAASKKPVISRTVEREFEKEVKKLEELDEVTKRMYKDGKRLVEANTALTKIE

RRLTQELLGTLMFQSEQELRWQMEEWDHALATLDTHMQDVNAVVQKAVIDPIKKFNTIYP

NVQAAVKRREQSLQELQKCQSKVSKYQERDRTGPNIVKLDAGRRALVQAQNEFSSQNAAL

LEDMPKLHESRIQYFQPSLEALIKAQVQYTTEAFKVYGELSNTLNGQKDCSNQEYASQIQ

QALGDIKALSITVD

>SG-AL_contig_2071_1_+2_1262_1474

MCQYKMNYYIHVAHKCCSITLRLIQERIHSYCIFFHLRPIALRFLNQTNFRADVHVYVLS

DKLRPPSCTFF

>SG-AL_contig_2072_1_-2_230_388

MMRHKWILNVPANEKLLQNYELIGAMLAKLSAVSLFVYYITVYLSLFHKYLCP

>SG-AL_contig_2073_1_+3_360_479

MSTKLIHVYFSLVQTVLMSIFLYYRLCFKSFVFHVTPAVS

>SG-AL_contig_2074_1_-2_2_139

GIGLLRALAMVIDSLLSEAICSASAREVFLAWRRYLKKTETEAACI

>SG-AL_contig_2075_1_+2_212_361

MFRSTSTKLYDATYYSEQFSKCFFLHFFLMNMAKVKKIIKKLNDCYISLV

>SG-AL_contig_2076_1_+1_466_648

MNGPNPVYRCQFARDNVYIMFAPACWQNLEPPCCTQVLRPHLKALPFFPSIPHQNSDLSN

L

>SG-AL_contig_2077_1_-1_40_732

MQIQVVLLVVLVAIGTTMVLSAEVEEDEELDPFDMVNFDHANMKMRRKVPADSKPEPEQP

STKEKLQETERPPPKEDKVPEPPPPTKRDQQAPASGKQDKVQEAVPGSCPALSLFQQLVS

RLLRFISGKVSQMDVNTPTELCVRMRLTSDQVEMLENFKRDNSKDRVFGAHDILNEAIQG

VHVLNGDDPILRFSWVEEYFGVTVDKALQIFQLLACVVVLAVALPKVRLSG

>SG-AL_contig_2078_1_+1_1_195

FFFYFSLFICINNSEIKNKRDSVITHDMNRTTNHPNCNLSADKKHTGYKISYNLVNPCSP

QDIVS

>SG-AL_contig_2079_1_-3_30_1049

MDAKELISQNEVKKSVQALLQVKSLADSKEKKPLFGDESNVNLQFALKKMVTSQQTIKLK

LPNGLEEMNKEVCLFVRDLDKTDREYEKSARHFKQKLKAEGVTCVSQIIPLKALKLEYKP

FEAKRKLSDSFDIFLADARIIRLLPAYLGKAFYGKKRQPVQVRMDSKDLKGEFEAAIGNS

RCILSGKGPVGMAVVGTTSMSAAELVQNISASAEQIAAAVPGGPVNIRSMYIKTKDSLAL

PIYTSIDSANEVQLPKTKKKQEAVVVDEVTTVERGLVKVLPSGIVRLVDNSGKVIKPKVM

KLKKPAKAVKRAGKRGQKPLKGKSKSKKNLKDAAKKKKAK

>SG-AL_contig_2080_1_+3_2085_2489

METEHTSGTYISLCDESSQLGTLTDNASTTPTFPRSAVKLNNIYRLLKQTDSEIDDESND

SDSDIVLDLEQIHGHRIIDVGLLSKKILSQPVGGLCQGEVSLFEVKRDRLGSELAFHCSS

IRCSGSVVTTFSDDA

>SG-AL_contig_2081_1_+2_746_1087

MRRGMGAGDGRGVCSNKALLTRYFCFLRSLQSTSEPSSTSSSSSSSFSSSFSLSSSFPSP

PPPPSLPPPPPLFLLHLLLSSSTFSYPLPPSPILLHLLLSSSTFSYPLPPSPIL

>SG-AL_contig_2082_1_-2_26_1003

MAIGGDQGGSVRGPASFNGIVGLKPTWGLVPYTGAAGMDATVDHLGPMTKTVADCALLLE

VIAGYDEGRDPRQWPSMSVPEYSKLIDQGVAGKKIGLLVEGFEPPFTDDEIKNTVRAAAH

SLSNAGCDVRDISVPMHKDAQAIWTVTTGHAAIKCLLEGNGTGYFHKGFYPTSMQEAIYR

GRTTYPQDMQNIFKLFSMSMRYIDRLYGNRFYGKGQNLIYELSRRYDAALQEVDVLVMPT

SPHAASRIPPADCSFADFAKSCMPSGANTTAFNSTGHPALSINAGFTAPDQDRGRLPIGL

SIVGKKFDEVTVLQVARALEKLTSEQ

>SG-AL_contig_2083_1_-1_22_480

MQEAVYRGRTTYPQDMQPLLKLFSMFFGYIDRLYGNRFYGKGQNLVYELSRRYDAALQEV

DVLVMPTLSHVASRIPPADCSFADFAQSCLDSTFNTMPFNITGHPALTINAGFTAPDQDR

GRLPIGMMIVGKKFDEVTVLQVARALEKLTSEQ

>SG-AL_contig_2084_1_+2_2_235

FFFFNISHIIYSATHDRANDDNMKHSLSTEHSFYSINTYKYISKFCLWENAMKQKNNDCK

LKETRTFIFGINNLATYC

>SG-AL_contig_2085_1_+3_3_221

LPIGSLTSQPPGCCTHQTVFHILTINILHYVFLLPRIFRMGTRVLSECFVHLKFSRLPKS

FLCSEVFSDHNVL

>SG-AL_contig_2086_1_-2_20_118

MFSTHLMSTIKKSTSKFQQMADTQNMHVRNQLQ

>SG-AL_contig_2087_1_-2_74_2563

MPQLYLKEVEPFLVAPQNGKQTEDGNNPHSSIGGQKVYSRHTTKRSTSLIVTDALLLCLE

FFQVLAILQAMSLKWIWPETWIKSTSFLFFFNMDVWEFIKVSCGAFIGARLYDTPSATVS

TQFYYILIGWAAFFMLLALIWAAIRVTLHVKQPAYLLVHNARLKRAMIIILQIVALPFGT

SAFKVFHCTSTGEMSVDNSVECWSGLHWAYIVPVLVAVLVVMMVFPAWLVWHIRHEAMAS

ADEHHQDFLKLKEVEYMSGLDVVWSVHGFHLFSSFCLRAIYYRPVMHVIKLLILILYAAT

FQSSSAQAISMLVLLLLVALLTMVIRPFRLTVFNVIMVMGYFCLTGDALFGAVITQFDPT

QVRSPWLVEPYSLAIMIAVNAVFLLGIVIFFLIYLLAYRFCCQGRCVLEPLWPVMTAYEY

DVEGVETQKYLAAVLRGRAVIERCRRLPSMFAPVHEMSHQICVLNAYMRESELMHDGLHP

TLWAVLDEMIDMHHRLEPKSLFGEMVQESIRKNAAHFMTLMPMFAQRLAQRDYDFMLVPP

YKKRLLLKMYIIALWLDGSRRRKTHQELTQKVLISIWEESPEVVQVTDEREYREDLYPET

IQGPETEALYMTGTLDLGRGLYDLEAVEEEEESLSTFLKRAPPTLVLKDEDFSLQDAQAG

ELTGADNPAFIPDDDKDTSEYHGHSNQQLTTTEVHDTSEAPPDVVVDLEALVKGDNHSEG

EATVTFVDSHTGETNTDMPSEGELEQEDEEPDDGTTRRHNTSVSVISTKSASSAKSGASA

RSKTSVKSESAGKSGDVTAASAKSAKSQASTKGSRSKGGKTFKGKQAAKQ

>SG-AL_contig_2088_1_-2_116_2272

MAMVSEQPMPAFGAFERQLKTLCIQEFPCGLGEWREPDAKLQKPKKIQLKSIYGRVQKLP

PPDLNLSREPGAQTERTEALQELVGSKFSPWHLDYSWSDEAKELREIAVKSPWLVDNHFV

LNHFRTLKIVDKGVTEVDEKLLQLQNLIELTLTANFIHTVNSKHLPQSLQVLELCANEIS

DISALCVRPPLLLHLGLGYNCISFIDDYITGDYWPALLSLDLCNNNLTDLLDIIRKLSTL

PKLRNLILLGNPLSLIPGYRGYTIDSLRKLSVLDDIRISADERHHFKGLARRREYILDEA

KVALEVTYLKGVPMPDEVKNPDDLPEYPVIERNYFVQFMFPADLSARSSRLHNITQDDFE

DLGTIPDAEHQDMTSDVQSARLEGSQQSGSHTEKNVIFNEQPEMKMMESMPLSLIEEAEE

PPRSISNLASQAGEGEEFKDTDETQVPDAQTKPNIPLAPISSVGKPWAEEIDLDWKADVT

RFDLIALRDFFKQGMDFSVVEEMVLCYPMEEEENVDTPTGSKKSKDGSKDKKDAKGKQTK

GAPAKQEKIKSAGGKDKPKAKKDDKGKKKKKGEPEQELRREAPQITTLATFHIPLAEFLE

GEFKFSSVYTQGGVEIPLSTARSAGSADPKKDKKDAKDKKKGAPSPRAKKDDKKDAKGGK

DDKKDAKGKKGAHPPPPPEDSDETPPPPPPLEVKVSVRLHNWTTAMDSVREEDKMPKEE

>SG-AL_contig_2089_1_-2_629_934

MMIPSINPVQTMNPSINQSNKKSTKKPTKPMYILYFIEIASCCHSSQNLQFTIHCIDLSM

VGTETQSLMQIAKPLEFRVCSRGIHGTWTIPYAFHSNLLAII

>SG-AL_contig_2090_1_-3_558_812

MVSLCHVLVLFSSYHLYLSLPHPIHQLLALSAMIAKQKLTLFTPHPIHQPPILSAMIVQQ

KLNLFTPTPYPPASCIVNMIAQQKL

>SG-AL_contig_2091_1_-2_68_331

MADNVRSEDVLGQKWDRCVSDTMIKFGCGLGLGIVFSVVFFRRRPWPVAFGAGVGLGMGY

ANCQHDFQNPFLQHGKKIKVTESSSSKP

>SG-AL_contig_2092_1_-3_21_407

MAQQPSVPEQARPWNPKFTYADRERWVCIYPAYINSRKTLKEGRRIPKDKAVDNPSYTEI

RDVCAAAGMVLGVENKTYPRELDHRDLKYRGRIRVQLKKEDGSLMMDQFPSKQSILIYLG

DTIPKLKSR

>SG-AL_contig_2093_1_+3_3255_3671

MSCGLDTSPGFVQTFHVKIHKLSKIFSRTIYPIFKDLARQLPGIWGVNLDQNNQHTDVVK

GRNSLLFKDLHSMKRECHFEHIAAELKLRVQTLCWTHDHQSGEQLKDFSASMTFSQPLSE

FSQTPVIFQRLENGTPFLK

>SG-AL_contig_2094_1_+2_2_193

ACWVDVQAYGLCHDHFGSFSITWHLRRLCIWNYFEHGNTVLRKLKTWVQMQHKVLILNCD

IHGT

>SG-AL_contig_2095_1_-3_984_1220

MIYNFTLQVAIALTIKNHSSYPVTYCFIMLFCCCLPSSPSSSVISSVWAMLFVVCVPQLN

TLGVIQYICTLSYESETTS

>SG-AL_contig_2096_1_+1_1_192

FPSEHCRPCSAKHVLILTLHGFLGKIQQWFPVAFCRFFFGFLRFWPISYNKAFTFYFHGF

LVLV

>SG-AL_contig_2097_1_+1_1_237

FPSEHCRPCSAKHVLILTLHGFSWEDTAVVSRCLLPVFLRFSPLLAHYNRACRTVYSGII

FVQVHFQNKQGRSLTYFIL

>SG-AL_contig_2098_1_+2_53_259

MTPLHTDRSPIKTKNKKVKDTVLHFTIKDQIMNTCTQYTNALIEELHSHYVRGEWGNLRY

AKRMNDENF

>SG-AL_contig_2099_1_-3_3_197

VCTSYKYVIICTSYKYIIICTSHKYIIICTTCHMIEIQSIFFSIAFIMPPGFFYFSVKYC

PTFIS

>SG-AL_contig_2100_1_+2_1382_1672

MVTGRTPTSFDLLGLLRTQTLKNAGIHCHTGVHCSVSILRKYSTISHINLHLTCGHSFFF

PPYKFYCFVIILQSDTIDVSESDRQVNNTHELCNWNQ

>SG-AL_contig_2101_1_-1_52_924

MASEKKKRAVEPLETEDTFDEESDGDVTDESENEFMDQEVQVEFEARPPDDGDFHGIKTL

LSQLFLKANINLGDLTDIIISQNYVGGVIKQVDVDEDDDDDAMDDDIGDSVFGLITAINL

TDKQGKECIKQLISMVTAHCQQSAPDVAGTVTEMLHNPDNHVGLLLNERYINIPPQIAVP

SFQSLKKDLEKACRKKMNYSFKHMLMLCKTYRIKGEPKNHDSRSLFYTNGEEELIAEMSD

LQATWSVADERDGVADGRWDEDEMEATRTLLVFSADKLDSIIHKLETELAT

>SG-AL_contig_2102_1_-3_255_410

MCKYVCVCVCENCCFYHGIENNFVNYCVIGCVCERQILYLHIVHSCVIQSLC

>SG-AL_contig_2103_1_+1_1_192

HLLFLHALPSLTLKNGIYSQLGRLEAAYTTFCLSERWSKQFLKQNTLGGLSTSSQLLISF

LKKY

>SG-AL_contig_2104_1_-3_96_338

MVCIPCIVIPFVLWFFHKYIQPYITKIWNPWKKIEGKNEGGGDSAAPLKCPLRTTEKNGA

VPNGVGNGHTEVSSGGDKKHD

>SG-AL_contig_2105_1_-3_270_554

MSILIIVTSFGMRFVSSFVFVGFFLFKFVCLFVCLFRFYFVFIRNLRSVRVGSCVFDVIL

FADCYLFLNLPFSLKKEIISYYLGAICVFVCMCVC

>SG-AL_contig_2106_1_-2_215_343

MFVLLDMFCVTLLVSSRSSTSLVVSQPKFNHFTPSPFPPAVKA

>SG-AL_contig_2107_1_+3_618_854

MLAIYDTMYHTFPSLRHFITHRASTFVAQKDENEHFKCLGQNHLTFIIQCENVDEKVWLD

GHHFYLKFLYHRNKTTQHL

>SG-AL_contig_2108_1_+3_531_815

MLTVSAGTPETFGKTNGCLKAVLHSLVTMRSQQYFICRVPTKRSNFDFISTDFPASSKCL

DTSTPVELLKYKQLQTVTLKILWQGRNCSYLCGTV

>SG-AL_contig_2109_1_+3_3_80

ALPIWFSLLKTVSQCIDRSIQLCSKP

>SG-AL_contig_2110_1_-2_77_307

MLIPKKNLVMIYEYLFKEGVLVARKDFFAPRHPDIEVPNLQVIKAMTSLKSKGYVREQFA

WRTYYWYLTNEGIQYLR

>SG-AL_contig_2111_1_-3_111_1913

MKGKAFCLLLLPYFVSVLCKVNQDTIDQNIVVSHVERKIDIASHLVKTYTSITIENKGSS

AVRSFLYVVDPSLKTKLSFIGAVTKDNKDDKLDVAPTTVTGQSDKEFYRVTLPSSLDAGK

TVTIDVEAVFAHVLTPYPSQITQAEKQYVVFEGNLYFYSPYKTVSQKTSVTASSTSLESY

TKTKPVSLSENVISYGPYDEKAPFSEAVLKVHEENNSPFLTVTDLERVIEVSHWGNIAVE

EHVDIRHSGATLKGPFSRFDYQRSQDGVSSIKSFKTSLPASARDVYYRDEIGNISTSHMR

ELDDAVDLELRPRFPLFGGWKTRYFLGYNVPSYEYLYYKGDNYALKMRFVDHVYDDMIVD

DAVVRVILPEGSKNIEVRAPFDVKRESDSVHHTYLDTSGRPVVVLRKSNLVEQHIQDFEV

HYTFMRMQLLQEPLLVVGAFYLLFVAVIIYVRLDFSITKDEAKESRMRVASLVEEVQSTQ

DRRSALYQSYDDAINRFKSSKDVNAFVASRKKVDADYRQLTTQIQNLQQQLKAEGADIAE

KVAELQRLDGQYKDQINLAITMAEKLVAGKTNRQQYLESETGVSSKRDDVYQKMETLRAM

L

>SG-AL_contig_2112_1_+1_1_168

FFFFYNFQVFIHQSSMVQYSLIHKTKQYLHMRYIFLVIPFKLQFPQRMTSNNNITT

>SG-AL_contig_2113_1_+1_286_522

MMMLSTHLFHRPLDSRPAHVPNTSTLRPQQSNLLHRHSTKGQTGKKTINKNRPGMHSQKL

FQPSSLLYLQASTGNANET

>SG-AL_contig_2114_1_-1_508_807

MAQFVHGKREIMRTVVMLQEYFSLMLSFCYFAVVNIKEYLCCFLVFYLNNNVEKGLCVCL

YISNTHKNTPLQLHTHTHTHTLSLSFSCGCTHRHTHHHTL

>SG-AL_contig_2115_1_+3_168_353

MKGKPKVSQMTCIPSIIFHYTIFQYCTIFHYTIFQYCKIFHYTIFHYHDIFTPFALGKPS

WD

>SG-AL_contig_2116_1_-1_28_174

MPPAPLSYLRESFTQLKDIPCGLRLGRNQAVTVSLLSVGCVLCFLLGIL

>SG-AL_contig_2117_1_-1_208_1023

MATQAFYRMGVICAVVAFVLMFVSFASPFWYKSWTRVHSPFASIGLWHVCLSGFIMPRDP

AMKSYVGCWWIHSTEFELVTNQLMPAWFRIIQMLCVFTLLLDLFGMIFTLVYLLDGARAK

LYGNDKGMMFIIDSILMLVSAFLVFLIALIFAEMSNDSDWMPRPWMNYLSWSYGLCVLSG

FFSAFGGMVLFILGLIFKDKDRRELDNLDSAAELRRHQQVAREKEEEAMAPRHIAPAHIP

PTRFSPPTEMAVTHAPPTIAAKPQPRVGESFV

>SG-AL_contig_2118_1_-3_57_227

MYTARAIILMKSYIETSRIITLFNCTQGRLATTQFFSHKIMYVLINFYCTNFYHVSG

>SG-AL_contig_2119_1_-2_158_673

MTVTEVYNALCHLKQTGTRGLDGIDGKILKLSAPIISDTLTYIYNLCIDKNHFPTQLKQA

KVIPLFKSGDNSQPSNYRPISILPVLSKPLEKHINKHIYTHLDTFDLIHKNQSGFRKNHS

CHTALANLIDNWLCNINENKFCGALFVDFAKAFDVIDHTLLCKKLSLYGITS

>SG-AL_contig_2120_1_-1_304_930

MDTMENAPSDSNIVKISLPWKWRPVKRRMLPGCELFMDGDAAMQIARKLIPLEVDGKPAE

DTEANVTKEAIPCCAVGCLQTFTSLLSYENHYNSVHRHTCQKCHRTFPSPFLLDIHIQEN

HDPLFALLVSRQNMYQCLVESCGQKFRSSDKRRQHMIKIHCYPANFRFHRLTGADAQHRK

KPTQPSPAARGMEVDNPRSPPIQTEKWQA

>SG-AL_contig_2121_1_+1_1_207

RRGLVFHADKATSIKHYYSSRRLVGTSDRVVDSRSHVSSSIPPRNIKTGLLAAHLNAPTI

LVVSEFHKH

>SG-AL_contig_2122_1_-1_64_1092

MKTIMNQAKMSGKVQPMQKQQKDAKVQKQPQTLAKKEVQKQPQSAAKKEAAADKQKQQKQ

PQALAKKEAEAGKQKQQQKKEKKPQQQQPANKKQEQKGKQAAKKDAPDDSGDAERREDRD

KCTLFVKNLAAGTTVKDLKALSKDIQEVRLRITVKGKKEKRRTMGFAFLEFASEAQADKH

FKSLQGTKVKDAELVVDYVGQKSNYLADKPKKEAANPDPLRLYITGFGTTTKEEDLYKLF

PKAQEIVLPVRRRDGHPIGFAFAAFATAEQCKAAHDKVQGAKVHDRSVVVLYAKKRSHEQ

QKKDAKKAEKSKVQVTKKEEKKNQKNQPPAKKARLEVKEEDED

>SG-AL_contig_2123_1_-1_1759_2061

MTDLSDEVLRLWCVLCVVCVHTAIDANAGITSSVDPSPLPPASVILFNYVVILLIPALCI

YVTVLTVVEERLCLSLMMLRHPDRVTVCQVDFFVICGHCMK

>SG-AL_contig_2124_1_+1_1630_1971

MRGGDKIQPSIFIETWYKRYKFHTLQNWTDPSSSNLPLIQLAHTQLHMQAENDMLSCEQN

IFSHAFCHHKRISKICAFHVYIKSKQIHVDTLNQKRYRDNFSLNQKQAQHFYHC

>SG-AL_contig_2125_1_-3_3_164

ICAHTHTPARMHAHIHTQPSNTNITQEHKRILQKSSSPASFKGLGTFFCVLSSP

>SG-AL_contig_2126_1_-3_81_341

MPAMKGLTISSLKGHPSLIPLFVCVGAGVVGAGYYLTRLSTRCPDVSWNKRDPVEPWNKL

SPTYQYKFYSPKRDYAKEAAPAERPDI

>SG-AL_contig_2127_1_-2_32_337

MHQVILNQEKFSGHLETYILRSCVVSRSTKIVWLDLGVTSSWWVIQPDKIIMGLCCIHLD

EAVLCIAPLPVTLFHCHFLFFSRSLLTVEMQTYYPLYVCGYQ

>SG-AL_contig_2128_1_+1_1_132

VSVLIIGHRFSRGYFLENGYSQLQKKKTYYPKTLTLISFVTVKV

>SG-AL_contig_2129_1_-2_212_454

MKLMKSLKLKTPHLCLASKDPEAMAFDVFTLAWSSLLASAFLSFPDPPEITQEAKTRDSD

YDSDSANFASSTLVPGPDLGP

>SG-AL_contig_2130_1_-3_3_188

QPSLSSRWCFGQSPVFFQQSRKVTQSPCTSLNGLSGKSGVILTTSPPIILFQLRSLFLAH

LF

>SG-AL_contig_2131_1_+2_176_583

MNAVQPIACRHIISQVYGRHMVAWLREENTYLAEKISGNQHFLSTSKAKNKARRLQNVDK

NRVADPRSAEPRCHYQKLLCMHRYAQRCIIVCVCVCCTFIRTSVCERERGRERGRGGERG

RGGGGGKKKKNRKKKT

>SG-AL_contig_2132_1_+3_45_386

MYWWVCFFFVICGLFGVFFTSSPCRKRNTHCSKIMEEKKQISHQPRNTYQRKLNLACILV

TYYKHSFLSTGLFKRRERTLYKENIVRLKQVYWMSILVSYSCNSQSVFQQKSVL

>SG-AL_contig_2133_1_-2_155_283

MVKIKCYEQCCFAGFLFSSLKRFCDQRGCLLDSDGLYVKTALI

>SG-AL_contig_2134_1_-3_12_1016

MAISCGRFALLRRLLVPPKLSGCCVHTDVRVTNAHVQEKRSKAFEMEKQRQRSLIPRIEK

IEVKFKGQPEDCTLIMNKNLSTPYHCAMHIQELLMSRSALALVNGQAWDMHRPLAESCEL

QFLHFEDPDPTLANMAFWRACSFILGYVLETAFKDDHYVELCSFPKPNIRSGSFVYDADL

RVGDWKPSSRELNILGRTGYKVFFNNYSFERLDVDASVALKMFEDNRFKTAQIPSIAAKS

ETGSKVTVYRMGDHVDITSGPLISNTSQLGRFVVTAFHDIHSSTYGPLTRVQGVGLPVQL

NMHFWTFENILYNRAQKLNDAPLPELKPSQPPSRT

>SG-AL_contig_2135_1_-1_283_5226

MEESTMTSSGIQTSPSEVHHAAKRQTSYLSNRITSEMKNASKHEKKFTTLDFSQEQNMSG

FDFNHSTLSPRDSQTTSSETEFPIFGTAQPETIQPRRFHESSLEDSNKGKVTTERNGIFS

LREMDLNGTPQTEVSAPTEMNNTFTKGHTLTESDDATATDKDGMFTSKWLDSQSVYLSSD

KTEAAMFSRAPTKQFTTTDGMFTTQVEMSQTAKSDSDSKVTSDGFYVSGMLRTRRQTSST

NGLTLQHTTADFSSTVPTKPQSTSAGVSTLFPTSSSDSQMTSEVTSLSATPTSLSTSTRT

STTTTTTATTTTTVTSTTAASVGSQCQSNESCTAVLNSVCLQAVCTCLPDFHNNPSNDTC

LQEPFDLRVWKHNATCVVFSWRGGGDEYNVSVTSSMIYDVSKSKMTTQTRTLMCGFSPGS

NLTVSVVLSSGGEPAITSTVLKPKPPKNGRILLLNETCIAADWQPGEGLTESYIVKLVAV

YEGFSETTDTTNQTRWTKCGLNPGTQFSIKIFSVSYGTKSEAYAFIGSNSTLPSRPVDGS

SERDQSDPRHCVDLRWTAGAGHSDLFLLSWQGAASQQINKNVSGSTTRVCRLTPGTMTLF

YVKAVSFSSYSDPLKVEACTDPRDVHRLGVAQVDATWVTLVWAPPAEGNVEHYIVKGNHS

DDSRVVPENRMSSTIEGLSPGTSFTFSVTAMFCNRTSSQTFVNVMTNFSALTSVNVTAMG

TILSIFWESDQDTDIPVWLTVAYRQQTVYRQTVQSSPYLVQTNRSGVCFTVTLQVVNSTQ

SQRIVHATEPAAPEEVYHLQGRRLTNNRRALNITWSPPAVTNGIIEFYRVTARGLRGLQQ

TNRTRDCDHECTLDCHSPTHRDTRTSGKLAPQQSGDSITTYSMVVDELSEYENYIVEVTA

YTSAGGGPSSSVRVDGQLLDSVSEMKVVALNSTCLNLTWAYPQNANYNNNNTDLIYFLIE

YQTIDVTDDDEVNETAIINSTNTGALLCGLQYWTRYEVNITAIDQQGPGRTISCHHRTAE

HVPGVVSNMTVSVVTTIYPPRAMNVTWAPPIHVYGVLNSYFVTVRDTNTSRLIEVRQISG

TNEKNAEGYKETFLLVGDHHYHVEVFAATNAGNGTKAQESVLMPAGALPPTLDTVSIDSS

STDMTSALVHIPTSLLCDFTYGVPVHAGYIVYPHGRRLQGIRNGISEGSLPVLTDTWAQS

DTAFFVQLDVFSQCERATGNYLSVRVGDRKGCDSGRGDTASGGKNACGGRLRANTQYHFL

VVFCTSQACTYSLPDPVFRTLPEPVVKTDSSAAGLGAGLAVVFLVMIVGAAAVTCKMYKK

RPGWKPFTRTPAPPVTKPLEVISKNRPVALDHFIEHVQQLHRDSNLGFSEEYKTIKDISP

VYATRAAELLPCKPKNRYTNILPWDRSRVKLIPTDDEEGSDYINANYLPGENHQREFIAA

QGPLPGTIDDFWRMVWENRVCVIVMLTQCIEKGKTKCEKYWPDEGVQTIYGDLMVQVRSQ

SVLPDYIIRVVDIKLGNQSRMIKHFHFMRWPDHGCPERTSMLLNFVSSVRAHMPHSGAGP

TLVHCSAGVGRTGTFIALDRLLLQIRQRNEVDIFNLVFSMREHRMQMVQTENQYVYIHDC

LADALTKDKESDDDQSEDDEEEHIYGNC

>SG-AL_contig_2136_1_-1_1_297

IYNIYIYIYIYIYILYIHIYTYIQQFCLICLFTCVQIQFTSMHSCHECFYHICLKRLIKI

LENRIEILQCVEIQEFIVCMRTITCSTFICEHKEEQDQD

>SG-AL_contig_2137_1_-2_140_3313

MTTAEEKRIFIGGLVKDVTEKELVEKFSRFGQVSQVEIKTRTTDTDSKTFAYLNLAAEDS

GFKKCFTAYNNTKWKGGQLKLQYAKESFLSKLKKEQTEAKISSKKSEGKPSTVHRSPALE

MDGMKKAVPGTPMDDKGNWVMGKYSRVLPVLRLRNPSTRKIVTMDPSKMTHCLKKLKSDN

FDSEKIALSDLSWPVEENNNDTNKRKREWVPPKYESTPKERSTFKLKQESIFSRLKELAA

EECVSDIEMEIVPVGKSVAVSKLHASGACDSDDSAGSADTDEIVSKISHKKTSEVVATCT

SANANGSIKKKKQLKSSVRCDAQPGASKFETPSKLCGGEKVPLSLQTKKQSPKKTAKPDL

KVTTCTNTEEKHRPQFQFVKTPDLEGRVLKKSKKLSPSKVAPNGNSSTDEASEENEDMTM

QVSRTSCKEKTARKTPEFKGLSTLSSDSEGNLSKAKSAENKNSPTPNRQKKSGTTHKDVK

ATISCIEQKRQPKDVSSEYEDRDDMSSIISQSGEKVNGSVNSRTLDTSSKKTPTTVQTPK

YENLGVLPSDSSVAVKKRNGSVSWPVREDTSKVESGLQIPSSVSGQQKRRKRSKPVSSDD

SDLEDNDFEKVGKKLAARALQKQQRNGIAGKGQQQAFSATAASTTTDSNTERKRKTSQGD

KGHLTSMQRSHQAYSKIQQLQKKKENLITKTLREGTGSAHIHFSDSDLEEEEKSLKLIEK

TPKMSVFTDSASDSGSESEVDFSIRPQFKGSEGMKLLELQSKIHDERFRFDDRFQNEGES

EDEQVEQGSEDEMAEEKKRNMEILEKVLGHKPRQRTKIVYADMNKRRYIPSQTTHLDLSS

TVTPKKRKETNSSVELPKSPDHESLPKSPDQESEEETNSSDDGEGEEGESPMQISSPTTL

SDSGAHLKGLFSGGSAFSFFGANAEESDHESQPVTFSQHVDMEEEDEEEEEEEEEEEEEE

EKEKMPELRVGETDRASFPVQQQPSFMVARDDPRLTAAVKFFKRHSQEDYEDLCEHWSEE

RQKIMQLVRERHKRACRKDRKMKQGVKSQAFVKTKKVL

>SG-AL_contig_2138_1_+1_1378_1590

MSIERAWHELWRQSIFKHIFPILDIVETKTTWLKPRRSICVVFLTNTTAKRTVMGEKRSV

LPLYSTIKYFV

>SG-AL_contig_2139_1_-2_44_214

MLLVVTEANSSTAVIHRSKPLLLTVIFFNLAPDCCVLRQYCCLFTKHAHRNCWFSQC

>SG-AL_contig_2140_1_+2_146_436

MYNAYCIDVPLYIVLNYNAYRTKRTLYTVLNVQTGVTLTEKYSHWDRYKHQRHLFSKQYP

QQHTNTCSNDGSAHTHTQTHVYNGLTQNILVKKCIIM

>SG-AL_contig_2141_1_-2_170_376

MLSPFVNCCSCIVSVGCWRVQSVPLFRVRGVAFSWGVSLVCFSLFLFFFHFVCSFLVVNF

VIFLCQFLL

>SG-AL_contig_2142_1_-2_416_661

MYVVMHSQGGVFWRAGSVQGCMTCVYGRRGCVLVLMSIIYKSVLPLFFIFSYSSVFRVSF

FPSLRSRMFKMFTFVSDLAKFN

>SG-AL_contig_2143_1_+2_80_1075

MSYLQCPECPMTFKARNALNRHAKTHSHSREHGCWCGAAFNRLYNLRRHMRLVHGSDEAL

PPLRKVEVLDGNQSVKQAVSTPPAKKMLVKSRKVKADCMQKEQEAAAAAVVVGGEDEGGD

GAGDGGNRGMEREEVQVLPQVGMTPLVGQNQQIGLQNVNNYSAMSAAAASAGGVMAQANV

IHPFSHPHPHLTMEQSYAMVSATLHHHHHNLHHDPQQTIIASAGGEPSSITPHPFPHLSH

PSLDYSQLPNLSTVVPYSSNIATTNAATTTTAANSSAPRQDYYPAAGGAGMEGLQQPAAD

YTTSPFSFIQNFLLPSVGVANLLDLAHASGAK

>SG-AL_contig_2144_1_+2_2_145

DLSFSGFKGALATADLSCLGQRHRGSAEDEAEKKQNKKETHSQANTLA

>SG-AL_contig_2145_1_+2_2_196

GTSRKRVLKESAVKCLCRTSCNSLQSIRRCFTVTGARQGKHQGGTSLVDRVRFLVKGLNI

TETDY

>SG-AL_contig_2146_1_+3_798_1016

MHTYSDTQPPNSCKPIHILFLCTMWFYDEKNTGIRIKTWLGPKALSLNDTSEMVRPILNC

CYVLQCYVGIVFI

>SG-AL_contig_2147_1_-2_104_541

MTDEIKDRFVGKWKLDRSENFDEFLCAMGLNFFIRKMAGLSKPENEIAVEDDGTIVIRKN

STFMKNEQRFKLDEEFEETNSYTKTKFRNKPTFENGKLRIAPTPVEPVDVTYPQYSEREI

TRDGELLMTIKVNDVLCKQWFKKVET

>SG-AL_contig_2148_1_+1_85_465

MLWTFVICPAAHKFLKSHTMFILIQLVQMYSWVACQHECSSNLFRCVCDKLALQSMTPST

RMFVTLIQQVQKTYDKVALVCQTGFRCLWVANNSIHNITSRNVHHSFNRYRMCVCVKIAL

LVSITPT

>SG-AL_contig_2149_1_-3_105_1667

MASSWLLLMLTVVIITMTTVKSACNPDIGPAGSYDCFASHYYDKGQWGTCVNKNYLDRKS

KGRYSCRNSLATFCYFQCMLEVHGLDSGNVTSDCACDPHNPVTPAPSEVTTLPPRCFSPD

GTDCSWYRDCLEVKYQCASGEDDYGLKYGETFCELYSRHNHNNKLSSQAWEWVNNVRKCL

LVELVPLLRKFESNTSCADIKTLAFRSHLGCYVHPFKNTSSICDLSLKDYWKVFWTIRKR

FHEDFVESVLRLLEVRRDCRARYVRQVDAEMRSLLVRVNIPTHKLTKRSASGGPSSFEPG

YEAIEQNREQLARKLTEEELPRELSARRVPDSVPRELSAGIVADFEPDLDKERPESSYGR

FRRQALSSEEEWDTNSQALLYHQKAADVCEDFARQLHWAEKNVLWFTYGVYMGQGQLAVR

ILLADKNITITNTDGSSANLTAVLEETMEAFEQGKIMVEVDGKKISPGVLSSCEDLDCQQ

LFWNVTAPPLSTSRSSSLLTTWPVGWLIPFLVVMALLREEF

>SG-AL_contig_2150_1_-2_14_2806

MTPEDLTAIGITKPAHRKRLKAEIARLHIHDGIPDFCPHDLIEWLHLLGLGMYHETLAGQ

GYDNIEYVTDITWEDLEEIGIKKLGHQKKIMLAIDRLKRITSASKRLSTVEPRRSSIEML

EPPPPAPPIGRWSGEISALPPHMYEGGLGVGAKPKKSPSGDSISTTGSGGSGSNSSQSSG

EMRAIPLPREDAGISGGMPYNRHPSISSSSSGGHSDVIAIQVKRQRSSTSEDSKETGGQQ

GLYQSFQGPVGGKGGSGEFPVTYAMSTSDPGVGIQMAPRVQPKPVAKIIAKTKRSSRENS

PDAEMEKHECENNSDGNKGGPVLSSSMPTVTEDGGDAYSGGTLKRGQGAGGQLRVEIRNH

IYDQPQVSPSGSNAPSSPKPQLVAKPASPLHVATHHNPSVPSSGSPTGRSKKAPPPPPKR

THSIRNDSAPPTVSKVETSNSFSVTTTASVGSANITTPSSNSGKNASNMLASSAAAVKQE

PTVTEKTQPQTEAFASCVKSLSERFGSKKSEIDGSQESLSSDSDDFPPPPPPIAMDIITP

KIHNYGIPSKSDMHHSPLHVREYGMHGHHPTPQKDYASGFQSRLKTLQASDGGSTRSDPH

CLLASVPPLRRISPGRDIPATSMHHDRSKDMEAHSLSSNLGDKRSESTTSFESTASTSST

DSNTLPFANENVGTIKQRAAAMKPSIVQTLDSGDGQRNVDLNVAMFDSSVGEKMNVDVHR

TKMCGASTEGVGASKGANGHGVGQPRTVSATSGGGVVNNRCGASAVPHNTHTQTVASASS

KPTETVPSNNNRMSQKNCGASSEGPVRPPVPGKKPTLVPVSPQPQQQQQQAQQQQYVQQQ

QQPKPEVQGSQPQKQQYVQSQAQQQQQPPSQTSQQPPKPAPASQKPSAELSVPIDTEAPQ

RKSSDVLSDIDNMLQGLTDELDAMLLEETEG

>SG-AL_contig_2151_1_+1_1_318

GWQQLAVLASTHPNPTLTTVYGRHSQSNNQLTGNSQSEKTKQQKPATPLLDNNICIHLKS

NQQANVIHVNINIYICISWQKLINKNIIATTKCDNGNTHDIHHTKL

>SG-AL_contig_2152_1_-1_1693_1953

MHSVVRMQCSLTCTYGFQCLFLLVVRIFVYLFVCVCVYVYSLLCLSLSVCSQLDAFHSVL

LNIPRPKRLLLFFQFDKNITCCFCYAL

>SG-AL_contig_2153_1_-3_1494_1751

MSLKIYYKNIAQEGKDNAINDASLQWESVCHCRITFLGGKGCVLMFWGLGGLLTVCLQTT

MLAETHITCSHMPQHRFLQLHVMSVC

>SG-AL_contig_2154_1_-3_3_110

VEPDCVNEPHPTSPSPLPLHPSNNSSHESLTAETFK

>SG-AL_contig_2155_1_-2_314_850

MDMRRHSSVTNRKEFIARAVKLATSTASGMRCRIQPRVGTAAGTGCRTRFRVRHSPGEWG

LRRWVAGRRKTNPPRAPGFMFEVRAETRTQRNGKEPVMVAVPVMVRKGEDLEAVQVVKGQ

DVVVIVVVVIKAKGATTENHCHIYRLGSCLLLVLNVSWTLYYDLALNVSWIFLLCIIVV

>SG-AL_contig_2156_1_-3_3_245

MALYHILYYIILYYIILYYIILYYIILYYQFGLWECFVYVRPLLTLLLLGPQLCDNCIVL

FVIVMVIVEIRIQLFPTEWER

>SG-AL_contig_2157_1_-2_5_2503

MPGNDGLGPRVRDNNDGTICVEYQPKCEGRHEVLMTHNGTAVKGSPFSCVVDSVNGGFVT

AFGVGLVGGNSGEEETFTVVAKQGTAKDIDINIDGPAKVDIKRHDNPDGSTNINYLPMTP

GAYNIHIKYKGTPVKGSPFAAKVSGEGRKRSQLSLGNSAEYSLKIIEAEIVDLVGTVQTP

SGSIEPCILKKMGDGEIGIASFNPRSKGEYKVNVMRNDKHINHSPFKVKVGDKEMGHAAG

CKVTGITDKVTANKPNDLIVDTTGAGYGGLSVAIEGPHRSDIECFEVNERKYTIHYSPHE

PGIYILNVLFADDHVPGSPFLLNVGGAPSGRIRETVTRQMEAAEASVPNKMCEFILQIPG

TNPFDMEASVSDPDGTTELCDVMDLEDFHYKIMFTPKVNGTHTLSVRHKAMHISGSPFQY

SVGQLNLGGTHKVQVGGPGLEKGEVGIWNACNIYTREAGPGTLSMAVEGPGKAEIRLEER

PNGFLGATYKVPKPGMYGLHMKFDEEHIPGSPFMVNIAPDSGSARQVTVHSLRDRNLAVD

KPNTFTVNFNGATGRLHAAVRTPSGGHMDCFVQEMDTGMYGVRFIPKENGVHYVDVKLND

AHIPDSPFAMMVGCSHADPAMVSASGEALEHAKCGVKNKFVVHTAGAGSGLLGVFVDGPS

KVALSCREVDDGYEFTFNPFCPGDYLIAIKYGNIPIAGSPYKTNVSGSGRRASAVKEQSS

MVVETVEKKAGAGGKIRKFRGDASKVVAKGQGLKKAFTNRLQNFILDVKDAGEALLTVGM

IAPSGHPEAELTVKKTTNTSYTVTYKVTEIGDHILHVKWGDDDIPGSPFLLST

>SG-AL_contig_2158_1_-1_319_498

MLIGVVRATQMERVVNSTLCLFGVWGLFLFCFFADYIFLTFQFYSGNTREMPTFQFHSGN

>SG-AL_contig_2159_1_+1_1_240

SLSHISLSLSLSLSLSLSLSLSLSLSLSLWDNAVMSTKQVTFNVCLYRNQVCMGQPVPIN

LKKDKVKFRYVQRRERANPN

>SG-AL_contig_2160_1_-1_919_1140

MQCAGSTDRCFLPILIKSMQCVGSSDRCFLPIWIKYVQCAGSSDLCFTPTGECLKMLYLA

HGFTESGRQHATHF

>SG-AL_contig_2161_1_-2_122_274

MCSRGRRCETRSARSGFEPLSVQPSFIHRFLLFISIISLSSVLLHSSVCAH

>SG-AL_contig_2162_1_+1_1_234

HGGRQVETDRQQDLTINKGIIFIYYQFDMLHLCAQAKMHIQQKELFSFIANMICCIRAQK

LKVKCLFNKRKYFHSLPI

>SG-AL_contig_2163_1_+1_457_684

MALSFLVDFLETMYHVLFCVDPESVFNAIRLLKYKSRSEIIFEISQLIHTFTTTSSRFRH

YLPLGSTSQWFLQLTS

>SG-AL_contig_2164_1_-2_110_520

MMASIVRRVIGSPPAPTPGGPYSPAVIVDKTMYISGMVGVVPATKQLVPGGVGPETEQTL

KNIGKVLTAAGISYKNVVKTTVLLTDINDFPAMNEAYAKFFTESKPARAAYQVVSLPNKQ

AKVEIEAVAVIGDIVEG

>SG-AL_contig_2165_1_-2_2_337

KKPGSAGASGQSGKGHNEIKEPKKPGSAGASGRSGKGDKEAKGPKKPFEQPKTSGVPAES

KPPPPPPPEPPFDNSKYQVQELYQYNPMSYYDIECSLSPFRCPVPSSIKKPA

>SG-AL_contig_2166_1_-2_95_721

MLVRAAVLLVLMQTLAFAEKAKGPKVTDKVFFDIEIGGKNEGRIVIGLFGKTVPKTATNF

KSLAVGDKKHEDGSALTYKGSKFHRVIKDFMIQGGDFTRGDGTGGRSIYGEKFPDENFKL

KHYGAGWLSMANSGKDTNGSQFFITTKQTQWLDGRHVVFGKVLEGMDVVRKIESSKTDAR

DKPEKDVVIADCGALDVEAAFPVTKDDAK

>SG-AL_contig_2167_1_+3_231_614

MQLVSQETYKFCLSYMQKKSECKQTFLRKLKKTKKNPLKNKKLQKDITNSNTNSTKLQLR

AKASMCTIYCCFDFFKQFYIRNDCQSCHYQTSFISLWLKPTSTGLDMLNLNSQNSAPILK

PLDCFENL

>SG-AL_contig_2168_1_+1_478_729

MPTSCKNMQTIPAFKRKQLTRVHKNPFIVLSLENNTIHTTLHFFNKTIIYHVTCPRSAVD

DFKNRCLKKAKHNTFCSILGEDFL

>SG-AL_contig_2169_1_+1_310_459

MGLTDTEIVKKKKTPTTETPYSYTAHFTAPTFSEALLSQNAQVRNMTCIS

>SG-AL_contig_2170_1_+2_371_502

MCMYNRHIQKETKTLDSFSLHCPLFQFAHSMHKTSAGFTIKSFT

>SG-AL_contig_2171_1_+1_841_1071

MQQPDPHPFLSPLKVNKNSKDCPVTIDWPPSVPKDYRICAAGDTPGHKPGQSVKENLLLP

MQQAHPFHDTGKIQGRG

>SG-AL_contig_2172_1_+1_1_204

RERERVNNTSFNHYTILDMYIFYHFWTHKFHQFSTYRATISVTVSVLMSTGHQFCQPWTC

SSRVQCHF

>SG-AL_contig_2173_1_-2_254_544

MVKSTHSYFVAGMFQYSLTLSIHTLTYTHSVYSSQVGECDAKMAYLFNMLLIHDKLLFGC

NKVLEHNKLHLAMMKLCQELHKGEQVLNYLLRKWLEV

>SG-AL_contig_2174_1_-3_3_194

CFVWGGGDCLWFLEFHSWWTLEMCILALLFNGAITNYYLCESEQEVLYNDGVDFVFMLSM

HRIE

>SG-AL_contig_2175_1_+2_149_388

MNKGSNTFPITTGPCLTVMCQTSGLDSHQTSGLDSHQTSGLDSHRTSKLDSHQTSGLDSH

QTSGLDSHQTSGLDSRQSAL

>SG-AL_contig_2176_1_-2_5_250

MYVLSCPFEIFIKTSLVSFPPLQCALSRFLKYPHEKRLVSSFYETVADVVQLPSMAFQSW

LAHSFLMGLNDDIFSHCFCGII

>SG-AL_contig_2177_1_-2_92_358

MMLCQYVTMCEYMFMYVCNKVCLERLLAFIVGSFQKFGSFCDGELQTFKTDWTWSTYECL

NCAEKKLHICGTSFFPVCEQKLTKLNPQK

>SG-AL_contig_2178_1_+2_338_463

MTKYCLHCQPNISQFKGTTSGAETCFGCRMNSTKVSNNVFHM

>SG-AL_contig_2179_1_+2_2_226

FFFLVSHSLYFRHGHHRDQTVQQNHMICYTVTTLGKEKVTHHIPFSHPLRNNHPHLSGNN

IIKRICPELVSTGQP

>SG-AL_contig_2180_1_-1_124_339

MLQEVQNKIQQNYANMGFEYSALVNAIPDTWKQLIIKRDVELNLYNEKSWSDGGALDTKP

AAFLKMVSNKGT

>SG-AL_contig_2181_1_+2_80_238

MNFTTLSSSSTAFWFLNEYSFTTTNQKPAVSSHTRHGSRSSQRNFQHALKSPD

>SG-AL_contig_2182_1_+1_604_1053

MKTAMLPFETTHVSGSSQACDILLSSLSLNITHCDILKDFVVLPPLPPPIIQSQVWHLPN

SYCSAPCNLFLSSFCLPILTCYIPLMSEYGCLHSEVLKKCHTCNLLIQMYSWSHCGKTAC

IYWVTPRVLSWGTQTNNNNIILGNVKGLSQ

>SG-AL_contig_2183_1_+2_116_379

MRMPAGAAETNIRNKYGELVGKSWITRLDQLFPKAYHLKDSEGRTRQDALQFVVEGVIHA

TQISNQRIQLDSKPLRQTGISYGDEEHH

>SG-AL_contig_2184_1_-3_240_455

MFSFYCTHHVTYAFVNVGLVMTKAWFRRASDFQSDCKSCPRESLCVQPPVHPAVNGYLTL

FTVREGKAARER

>SG-AL_contig_2185_1_-2_2_64

HNFLSPQFMMYYLCCFALATR

>SG-AL_contig_2186_1_-2_2_208

KVKGHFMHFKREMMLILLLFLYLGVVIYPDITFNTQIIVLILYVQKYFRVFEPNFIVLSY

LGRLVQLLL

>SG-AL_contig_2187_1_-2_53_220

MRRHMAYAIIILDSFPSYGIWSFGACGIYSKHGDAVFRKLKLKWEVLNTTLTHQND

>SG-AL_contig_2188_1_-3_138_800

MSSSEEVSWISWFCGLRGNEFFCEVDEDYIQDKFNLTGLNEQVPHYRQALDMILDLEPDD

ELEDNPNQSDLIEQAAEMLYGLIHARYIMTNRGIAQMIEKWQQGDFGYCPRVYCENQPML

PIGLSDVPGEAMVKLYCPKCQDAYTPKSSRHHHTDGAYFGTGFPHMLFMVHPEYRPKRPA

NQFVPRLYGFKIHPLAYQLQYQAAANFKMPMRGTSHPNSKR

>SG-AL_contig_2189_1_+1_1_72

AHTSAAPFCHSQKDSMFLNRNDVI

>SG-AL_contig_2190_1_+1_1_276

CVCACVCECVCESVCVCVCETERDCGHVHMYVHLHLCTRLYTYPTSCINTHPDTHERPNT

HTHAEEAKIPKLHMYTLAKLQAKIMHVLNAAL

>SG-AL_contig_2191_1_+3_159_395

MQKTEHSLSFPVAIFYLRAETLSWLAIQTRHVNETNQSQPFLSRRQPTSESNKELCCGSP

DGTFLRAVHVRVCRAVEGL

>SG-AL_contig_2192_1_-1_52_1743

MATDAAQPMVEVIPPPEASMDPATTPEETLFAVNKAPETDSAPVEDRLSPEPSEGSGATV

GSAKAAGGSGSESNGASAAATGGCENKSEQDGKTVAAKTKKKEDKSIEYILRALSSLNSP

EEKLAALCKKYADLHEEHRVLQSSFKQQQRKMTVGQREKDQLQAEHTKAVIAKSKLESLC

RELQKQNKAVKEESILRQKEEEERRKEISAKFQTTIAEIQTQMTENHEKNVKLREENHDL

ACRLKTFLEQQDLREQQLDKLHKHRELENQLSEAKLQQATAVMKEHEEKHLKEKELLLLQ

TAEAQKKNAMLEAQLNMYKDRYEEFQGTINKSNDMFQKLKAEMDKMGKRIKKLEKEGAQW

RAKWEASNKALLDMAEEKTRSDKEKQLLQTKIIKLESLCRALQGEMHGKKSASPQPTSSE

ESSKNTLASAPVENAAASNAPASTTTTPPNTPEENTSRASPNPDSPASLPQSLPTGQNGP

ISASASTPVLDSSTEVESESPAEACHAAEAAALVEENAATVGSESPSSCQEVSVPSAAAQ

EPDEAETSSSTADLPGSADQEEGV

>SG-AL_contig_2193_1_+3_84_341

MRWQGLEMGWGRRGGEPMKQQADTIRMIGALKWAEHRADFMLWAGTELETSDLESTALST

EPASPLKNVAHLRVECTTRCVISLAL

>SG-AL_contig_2194_1_-1_13_1104

MAALSTSRQLTRVARGSTWSALKRQFSASSSRLATQMAVRDALNTAMDEEIERDEKVFLM

GEEVAQYDGAYKVSRGLWKKYGDKRIIDTPITEMGFAGIAVGAAMAGLRPICEFMTFNFS

MQAIDQVINSAAKTFYMSAGAVPVPIVFRGPNGAALGVAAQHSQCFASWYSSCPGLKVFS

PYSSEDCRGLLKTAIRDPDPVVFLENEIMYGTPFDISDEALSPDFLIPIGKAKIEREGSH

ITLVAHSIGVGRSLEAAKELAGLGIECEVINLRSLRPIDDEAIVKSVMKTNHLVTVEGGW

PQCGIGSEITARVMESQAFNYLDAPVYRVTGADVPMPYAKSLEELALPQSFNVITAVKKS

LNVS

>SG-AL_contig_2195_1_-2_2_214

VCVCVRALKASFVCARRVCVCESVCVHHSILYDGSVDHRLLFSFLQKKKKNKMRSLTLQN

DVKTAAKMTSK

>SG-AL_contig_2196_1_+2_2_217

HYCQNNLITREHLAIQDSSVKGLTNDFKDNRTLGITFIYQLVHLFTYLLSVHKPKTVSMT

NLDVHCTTDIKD

>SG-AL_contig_2197_1_+2_131_286

MLLVVCVCVFCVYMLYVCVCVLRQHTLILFYSTVFLLCGDVPVTCQICRHSL

>SG-AL_contig_2198_1_+3_3_98

SFNLKLCMLLVCVCVETACRNFVLFYCFSTVW

>SG-AL_contig_2199_1_-3_120_434

MIKSLLAHGNFSFHLASGWLHLWPDPGQCCTLFRLALPLLSAHVTRSIGLEVIGLTTLLP

SPKFCLRQTCCKADHFIWFLYLPWCIHFGFASMVESFCIFLFMTF

>SG-AL_contig_2200_1_-1_31_651

MAMGAVRHIADIRADDASREGAGIIGLQAGSNKGASQAGMSMGAVRHIADIRADDASKEG

AGIIGLQAGSNKGASQSGMSMGAVRHIADIRADDASKEGAGIIGLQAGSNKGATQAGMSM

GAVRHIADIRADDASQASAGVIGLQAGSNKGATQAGMAMGAQRHIADIKVDDMSQASKAS

INLQYGSNVGANQSGMSYGGRRDIMGN

>SG-AL_contig_2201_1_-1_121_534

MFVKPAVKFTERGIHSGGIICLIRNSLVSYVPEVHVDSGNFLLFIIDNTLFRFSKNVLYL

CAYVPPEHSPYYAVCDVDNGISQLEDCLVDYLVNREECVVLMNGDLNARTAEVFPECCDE

SGMYSCFDKEKTDSIEVF

>SG-AL_contig_2202_1_-2_1076_1372

MNWTFNLLLAKLHLELGEEEKKIHPQRNPCIQGLSERLIKVSHKFHIRVYAVVYQMTCDC

GSEDMVETAKMLDTRSKNIINSSSRTLKKQDTNFQEKKI

>SG-AL_contig_2203_1_-3_3_179

CLLLLFFGVGGVRVGICCFLCVKVFKTMIVKVGSLCLDNVCSACVKGFFVRMLFIQYEK

>SG-AL_contig_2204_1_+2_2_292

SICLSVYLSIYLSIYLSIYLSIYLSIYLSICLSVYLYMYLSFSACRVSNMHVKDPVSYIS

DWFMETPKHPACNQQGLAARKVQRDRMLLSLEDIVTS

>SG-AL_contig_2205_1_-2_65_514

MPLRKIPKVLSPDLLHALCSMGHGDEIVLADLHFPSSSVCKHGPKEIRADGIGAPEMLKA

VMEVFPLDQYVDMPAAVMELVPSDKAKNFPVPVWDTYQEILNTAEKKQIKLEKVERFAFY

ERAKKAYAVVHTGETAQYANIILKKGCVFD

>SG-AL_contig_2206_1_+2_50_394

MLIYHSGFFYTIYGGITPLLPWVLLHSLAHFARMTSIYHLLCRVVLFFQTLLPPVVTVYG

WFNKHSIQAVLSLVISKADFDPVISPCNLCILIHYYTCLYIYVMYTLNLYPIPVY

>SG-AL_contig_2207_1_+1_1_120

KIKRQKDPPNWHNHTIHLGIQLLNLSRTAKQSMSKFTLVI

>SG-AL_contig_2208_1_-2_2_136

SLLNKLQTPTIKLYEVSFRFPSQFCVLTFWCPKKKEYKFWIQACF

>SG-AL_contig_2209_1_+2_176_307

MQMWGSHQCGDELSLACICSVTSIFTDQSQTEEAAKFYTTMATN

>SG-AL_contig_2210_1_-1_1_264

ISSAVFKKDGVHTTNKPNDALKHILAYCFVLSICCLKLAVQLCMIKMVKTGKVWKSYLLN

RLTTFCHYSFLIVAVLYHPSSVESRCSS

>SG-AL_contig_2211_1_+2_278_592

MAMSYKQHFLNHIDILHSNQNTKLCYIHVTFTFRKILSGMSDGTALLVDLVDCSLFQTWP

FFLLCITESPVLSDSYNLRTKEKLSMFLGIVRLICSANSIFFKDC

>SG-AL_contig_2212_1_-2_92_289

MFHHQISIVRRLVSISVTATNYVRGDYKDDDDIDIIGTYIIRLENVTSPHCLVGNFPPKY

PQQSLK

>SG-AL_contig_2213_1_-3_288_779

MAAASMGAFGTYKTQYHRLVEERERRAALGQGGQTVWVTKRYDPLDLRILAPVPRHKLVS

GQLPTPAEKHWVPVTDPPLRSFERGRAGVFANFPSTRDENWSTLRAMLPSLGSAHKVRPP

SWGTGQGPPPFILERSPKRLPKVNSPMTRYADEMHSADRLFRLH

>SG-AL_contig_2214_1_-1_40_1017

MIVHQSQAGCIIGRAGFKVKELREKTGAGIKVYSTCCPNSTDRVVGISGKRKVVVGCVEE

IIELLQTAPPKGPIQPYDPTYFDEFAAPEYGGFISSESRSSKGLPRGSVAPVPPMRGGMR

GGRLGGMDDYGMGMGGGFGGGMRGGRGGFGARGGMGSMRGGLSGMGGGFGGMGDGGMGDS

MGGMNRGSFGRGGMRGNMSSGMGRGGSMLGGGSSFGGGNDHSSGMQQNFSGDSTNMSGGQ

GMSFGEDGSETTQVTIPKDMAGAIIGKGGARITEIRRRSGAQIVIDEALPGSNERIISIT

GSQEQIQNAQYMLQLSVKQQTEGGNY

>SG-AL_contig_2215_1_-3_288_428

MMILSHECTTQLQMVSKTIKKNSELTRVKKKNLKSLFFVLARITCKK

>SG-AL_contig_2216_1_-1_1_129

VHVQVFFFLFLLVIPVNKIILNRKDICANHSTVISEKIYSAKI

>SG-AL_contig_2217_1_+3_309_575

MYVIKNTNVSSISGNFSLNITPQNSKYLYNTINLYQQSLKLVISHSSNFFFTEYYQRNSF

VAASSLRALGACCTQDLVLSSHPKILAFE

>SG-AL_contig_2218_1_+1_619_903

MGKQKQKIQKEATKTTTTKTEGRWKGECQLQKTKKTTVKYISYTHQQSSTHLTLQTNASH

ISHQYYNTHTHTHTHTHTHTHTHKNYDTNQTINLL

>SG-AL_contig_2219_1_+1_1_297

RRPLKGSPPGARLPCVRCGLLKMEERCRQLLALKRENGGKVRTPTGCRRPGADPGGLSIL

WMSSPWRGKGPSWGRGAKRSSQTETGLNQGPNSKGESQG

>SG-AL_contig_2220_1_-1_1_369

SIATAGAILQTNTLLAQRDALLASSLLPKEDHAALRSLPPQGPSFFGSFAQTALSLKAES

LRDALALSLVQSRPSLPPSKRPSSASWGPQPRRKVPRPSSDFHPALSRPPTPVAQALWPQ

AYL

>SG-AL_contig_2221_1_+2_149_325

MSTKTAYNMYISYTDFHKETTHNISNDSTENCIRNTEVYIYVNSHNPFFMLADIKPFLF

>SG-AL_contig_2222_1_-2_68_1084

MTRRNRRAVLTGSTADLGLPCDVDAALTWWNGTSYVIKGHAIWAMSSTNISAEYRTSLDE

WNLCSWKICELYDQTGSWQALLPDRGFSCNGDPSFCGLRFDQVTLAGSHNAGSGFDGGFG

FFNCWVRNQYMTILEQLEFGVRFLDVDTSWEHCGLLGAYHNFMCGGPVCKMIKQIRQFLR

TNRQEVIGINFNHEMRDKSRVLPTLLRQLTSQLVFYANTRYRNDGEWPLLEDAVRTNQRL

FVFIDNDVMSLMEADYQIHHWIHEERMLKSTWQGSVSVNDDCLQVIESSQERCQYHQNAP

LLEVSILGYSTGCVTTVADLCLPHVHNISRQCEGPRHAQ

>SG-AL_contig_2223_1_+1_292_501

MSTAVKTSKVLIELARKQRSINAWLTFSCVYVTSRDFLTMSTASPNDEELHTDVYPVKYW

FLSKQAGDQC

>SG-AL_contig_2224_1_+3_537_1097

MLLNTVILPTIYSIFPEIISKVHISHISCSPLSILSHKTVHCFACNVSPSDPEAGSCCSI

EFCTSSESSCCFEVHLHLVQLTLGISKCSVRESRCLLKSIILSLCDFEALSGNCAANFFH

FLFSKAISALVLLETGTAVGDVCCSSSTASFSFSFCLSFFFFFFEPWSETPLSLRLPFCT

ALYRSEE

>SG-AL_contig_2225_1_+2_632_1135

MNFIEKDNEKVFSDEKKILKQVKKFQENLYFYNVMDVDLNNNLLDVPVLLREDSDSIERL

ITRDEALSPLPKIKNNKSPGSDSFTMEFFKFFFINICHLYSAFNQLQSRQGIISCIPKES

RFKQYIKNWNPISLINTSYEVDLSVYSQQLKTVLPKIIHEDKQGVHER

>SG-AL_contig_2226_1_-1_1_153

LHITQIKKKRGVGSSHLTLMLQRKHIIHSNKKKIHHRTVRSSATTTEIWLF

>SG-AL_contig_2227_1_+2_128_433

MYEQLSVGIVSQSKGTIFIIKGTVVRGKDRKRLMPRINIYRPVEVEGAFPKGASSHTHFY

ESSIILSTFICSECLFLAFFFTVFLHFPSFFHFLFFHFFPQS

>SG-AL_contig_2228_1_-1_1_216

LLYEETEDSSYASDVEGFIKDFSSGSVVHTTCGLAFRQQTRPNRYAGGAAWIALEAAEAG

IDADASRKFAVE

>SG-AL_contig_2229_1_-1_229_951

MGLQWEKFVVLLEERLGRGAAYSTPAEGEEEHRESVVRPRPPFPRPIHKTSSADAAMMSR

DEFGNITCPTKTKKSLAKTMKRKLQFLRRRHTDSTLSAVIKGDNKSLANIHPDDALQWSR

SFESLLLDKTGLELFRGFLRSEFSDENLEFWIAVEEFKLVKPRKVSSVAQKIYSDFVEAQ

APREINVDSETRVRTLANMNTPSKSVFEEAQRKVQALMEKDSYPRFLESDIYQRIIRSQK

S

>SG-AL_contig_2230_1_-3_1947_2429

MIWSFYKYCVYILSFSLPCCVLTLCVCVCVCVCVLAHVCACVCVKYNLVLPLCVHCAIWQ

GVIWLFHYLCTVEVVCKMRYLAQSSVIWCELSFHCQNTHFVKKWVFRRGIFPVCTYIELR

VCVNACMMVCLLPILVCVYVCVHLYVHVLCLDPNCQCHFGI

>SG-AL_contig_2231_1_-3_3_278

ITNTFYQQRFSQTQICYKWNNLNVSSQIDFILTRTDQRKNITDARAIPDINLDTDHRPVM

ITSRQQKRNPPRNKHKRPDQINLRKLRQEEVK

>SG-AL_contig_2232_1_+3_273_497

MEQSECDGHLLSSFAAVQYKPVCCLLSKHPLVKWLEHWTSTGQAWVRICLGTTWWVKGGD

FSDLPGQHRCRPASA

>SG-AL_contig_2233_1_-3_9_689

MAASLRSQFSRLLSVTTTHQPVFVRWKKPPKWRDPANTSWAAPLLEKMKQHETSGSDDVE

RPPAMLHMVWRVKSLYGRPYWEKNMIKALGLDKVKKFEPVVHKNTYYVNKKLESVKHLVQ

IVPITFPHGLPSDESDFHHCVLHRDGQLEVKKTLHPLEERFAEEEEDQKKVWKMDEETVE

RATRKVLEQFALSREYFPAKYVYKYNQDGKEHRYTGNHNILGDRDWY

>SG-AL_contig_2234_1_-1_1957_3369

MVTVARPVKLTNKLSSMIHTAVHNHHKDRGNGGEIQWCFSQVKGSVEEEVADADIISCVE

FNSTGELLATGDKGGRVVIFQRDKASKNAVPSRGEYNVYSTFQSHEPEFDYLKSLEIEEK

INKIRWLKQTNRNHFLLSTNDKTIKLWKVAERDKRPEGYNLKDEAGLSRDPTNITSLRVP

VFQPMELMVEASPKRVFANAHTYHINSISVNSDQETYLSADDLRINLWNLEITDQSFNIV

DIKPANMEELTEVITAAEFHPQQCSTFVYSSSKGTIRLCDMRDQALCDRHAKMFEEPEDP

TNRSFFSEIISSISDVKFSHNGRYMMTRDYLSVKVWDLAMESRPVETFQVHEYLRSKLCS

LYENDCIFDKFECSWSGDDRHIMTGSYNNFFRMFDRDSKRDSTLEACREITKPRTVLKPR

KVCTGGKRKKDEISVDCLDFNKKILHTGWHPLENIIAIAATNNLYIFCSKD

>SG-AL_contig_2235_1_+2_206_415

MTIELGIYIITSEHSHHRTQKVTIHFTMPELTMEKIKVIAFTSIENEWNPSLNRRMTWTL

VTSVGTYQHR

>SG-AL_contig_2236_1_-1_103_1065

MGGREEPGKIFVGGLSWETDQDSLQGYFSQFGEITDCIVMMNQQTGKSRGFGFVSFKDPS

TVQVILSSKNHVIDGRTVDAKACNVRGTTPKPQNRYGGGGGMTGRTTKIFIGGLPNNVGE

DTIRESFSRFGNVTEVVIMYDHEKGRSRGFGFLTFDNEDSVEQACAEHYVDVNGKQVECK

KAEPRGAGGRGGGSVGRGGGRGGGNPGGFGGGPGPQGWGQGGPQQGNWNQGWNQDGGYQQ

QGWNQQQGWNQGGYNNGWQGPGGPQGMGGPQGMGGPQGMGGPQGMGGPQGMGGPQGMGGP

QGMGGPQGMEIGRASCRERVL

>SG-AL_contig_2237_1_-3_33_440

MEEQRKREEKVEEASSFFSFHQAVPPTSSKSSALFPQVPSAFFKSSGFSLPADDTQQPGR

PYVTGSETAAAQQGGRDGGWGGAPRNTSPVLLGSNQLVTTPIFPPSYFASAPYGSLCSAE

PYSAPSPPSPPPPYLK

>SG-AL_contig_2238_1_-2_671_931

MYVDDEMVCEEPGYPLFSVLPYSRARHRRRVTGYIFSCTTCFWSSHRHPVSWKLIDKVFI

PVIMCWFLNTIVIFSVLSELTLHYIIK

>SG-AL_contig_2239_1_+3_3_458

GFRKGRAAVDHLVKITTQIKYQFARRKNVLATFFDIRNAYDQVWHLRLLHKLKCIGLSGC

VYDYFKAFLSNRSIQIKIGNAYSNLRLIDMGIPQGSVIAPILFNILRYDLPQALSNKVEL

VQYADVLCMWMKVTMKRHSSKRSINYIKNVYQ

>SG-AL_contig_2240_1_+1_196_384

MGIPQGSVIAPMLFNILTYDLPQALSNKVELVQYADDLCMWMKVTMKRHSSKRSINYIKN

VYQ

>SG-AL_contig_2241_1_+1_580_753

MLVLKYFVQVAFLFFLTLPFLLLLLLLLLLLFVLRWSLKTQQTVPVHLVSLPSSLCTV

>SG-AL_contig_2242_1_-1_496_1965

MTSCKMMTYLVTLVCALCCCMVTWCEGIRSDVFLSTAETSELLASEKGLLTFVHHCLTQT

ATSTGNTAFNRLAGELQSELDAIPQSQDAKHPVDAYVGLRRFTRYWAKYISVLAPVVAHS

VERSLAENRLVYPDHQVLHHARLAIVRLQAAYHLNTSSLLNSAHVNLSAEDAASMAMEAQ

LSGFPALAEDWKHLASSLFPSEQDIPLDESLAWKRSSENDREAEETHAIRRRYAQFFSLC

RHFQDSHDVTVLRSERCEYSAGSMPLYRWKREVLSTDPYVALLYDFVTDREADVIVSKAM

NRLTQAHTGFAPDKSILQTQRRLGKIAWLRGAGSTVLSRITRRIQDVTGLHTAFRPVASF

GEDFQVVNYGIGGHYIPHYDFFRVKNAFSAADMYLRDSGDRAATFMIYLGNVTRGGATVF

PHLGIGAPPVKNAALFWYNFDPDGIPDPRSQHGGCPVVIGEKWVTNKWIHEISTAMSKTC

QTSPWKSYNT

>SG-AL_contig_2243_1_-2_146_517

MMEVYHYTSKEGLKNIKRYRYIKESQLSAADAFFGQGVYLTTMSPEEGRERIANNNWGAQ

GPRMEESGRVECAIKLRIPQSNLKRAHSKGRDIWVHPGNVYLDEYHHSFIQLGGDDGAWW

CAIL

>SG-AL_contig_2244_1_-2_2_295

PHGVPSTGSPPGQSLQTPTAEEGVPSTGSPPGQSLQTPTAEGTQGPLAPVHEQLGPGGSP

GELVPGGDAGVGLSLRPGGRSPHRPPSPTGDPIHGRLV

>SG-AL_contig_2245_1_-1_274_369

MAYAMIISDYPFSVTWHSPWNLFRVRKHCLEK

>SG-AL_contig_2246_1_-3_3_173

AKLRLTGRKQRYLFAKSHLSKFKKNNNTNNNNNNKETNHHQLLVCIFSQWCQCELHS

>SG-AL_contig_2247_1_-3_3_173

PEHTHTPACTHACTHPHTHNAIPVTVRILWWREKECVCVFDRMYECMLVCVFSVCIY

>SG-AL_contig_2248_1_-1_274_2088

MRSAVQRATNVVYQSHHVSRDKRGKALGQRSGFRGCTIWLTGLSGAGKTTISFALEEYLV

GQGIPAYSLDGDNVRTGLNKNLSFSPEDREENIRRIAEVAKLFADGGIVCITSFISPYEK

DRANARKLHEDEGLHFYECYVDTPLNVCENRDVKGLYKKAREGKIKGFTGVDSAYEPPPK

PELLLKAGEQTVDECVQHIVSALMDDEIVPRGAVTTVRELFVPENKKAEAKQEAEALPSV

QITKLDLQWTQVLAEGWATPLPGFMREREYLQCQHFGCLLDGGVSNQSIPIVLPIKTEDK

ERLDACNAFTLKYGDKNIAILRKPEFFPHNKEERCSRQFGTSHTGHPYIKMIFDSGDWLV

GGDLEVLDRITWEDGLDEYRLTPRELRARFKKIGADAVFAFQLRNPVHNGHALLMTDTRR

RLLDRGYKNPVLLLHPLGGWTKDDDVPLPVRMKQHAAILDEKVLDPKNTVIAIFPSPMMY

AGPTEVQWHAKARMATGANFYIVGRDPAGMPHPDGTRDLFDHTHGAKVLTMAPGLTQLEI

VPFRVAAYNLKKKAMDFFSPERKDDFLFISGTKMRKMARAGETPPDGFMAPKAWDVMVAY

YQSLA

>SG-AL_contig_2249_1_+2_170_358

MHPSKHADMPCVHTHTHTHTRAHTHTHTHTQCNNLKSFLLSLVTKDNNIKRNPILTSVTV

KEK

>SG-AL_contig_2250_1_+2_206_388

MEDVQHAKRVLHALHAMKPRSLKLKSKVTEIIILKKTDIAQHPRKQAPKNFALMEYKTIL

Q

>SG-AL_contig_2251_1_-1_1306_1473

MVWFCPNRSVGLSHCVGWSSVSYRVDVDRSLAHAHTVAVPYLYSRSGDSVPHRFVL

>SG-AL_contig_2252_1_-1_82_540

MAHSGVKVDDQCIDEYNQMKLDKSRQRRCIIYKISDDKSKIEIEKVIYKNLEKDQKTEYE

DIIRDLPDSEGRYIVWDMKVPAKSCGDSETVFRDDLVFMAKCFDDCKVTVKMLHASSEDA

LRRKLEGVSARLDCKDSGDMCYDHMICKCKHKK

>SG-AL_contig_2253_1_-2_2_112

YLFVYLFNTDCSINPKKICIWIYYYVNPDISRYALNE

>SG-AL_contig_2254_1_+3_3_221

TLFRFFFFSKFSQVYFKTKTKQNSHSPFFDFSSLQTLEMHQKTRWSSHTINLSLLAPLDK

NCVYMILKILSQI

>SG-AL_contig_2255_1_+1_517_729

MQCLLVGGLMATLLTSVDSSTVCVGGGWGLRVYEDGVCHLVCKSTCAIMQMQQLAFVTVL

TSCLGLSCVCV

>SG-AL_contig_2256_1_-2_2_85

FLTMKSFYQVTTYHAICRSITAKYGEIG

>SG-AL_contig_2257_1_-3_3_128

SVPGCRLSVNLSIVMVTNLSFLFLYMYPRTQCDHESKLDLGD

>SG-AL_contig_2258_1_-1_34_552

MAAPTKSGSGDSVALPPVLEWAQRRDRVFITVALENCANPTHDVKGNIFFFKGKGGTENL

EHEVTLELFGEIDPEKSRCETNDRHSMFTLIKKDQDASYWPRLTKDKKKFHFVKTDFNRW

KDESDSEEEEKEDFNLDEMMNSMGGLNSAQFGGGMAAPEEEGDSDDEDLPDLQ

>SG-AL_contig_2259_1_-3_870_1055

MKISVLRDFDAYINLTSWQVVLPFLAYHTIHTRCHEARQRGEMLWRKQLSVIGISLGFGR

SL

>SG-AL_contig_2260_1_-2_2_220

MQTHDTNAQKHTIFILKHVSRSLTLSPPTLSLSHTQTRTRVHTHSYAHTCTQTHNTHAHT

QGTANEKCGGRLI

>SG-AL_contig_2261_1_-2_2_214

SPPSGNFVLEISSFLLLSLFLPFFLPLLLSCFFLPFRSIPLSFFKQGFTYFSLPPTPTPR

QIMCCVPLVTR

>SG-AL_contig_2262_1_-1_49_336

MSYNSDEYEQRSQTKGPAQQECGEDYMPDADGVIESNYDNVCDSFDDMNLREHLLRGIYA

YGFEKPSAIQQRAIIPCIMGRDVIAQAQSVRTAFPA

>SG-AL_contig_2263_1_+2_542_814

MKLVSARTSELSCTLYNGVSKIYSFLVCSLYLRLISQMHALTCGYFCRTTLPRFKIDISH

FTVIGQLDNLKLLQSAHSTHPSKTLWIFQSL

>SG-AL_contig_2264_1_-1_565_5841

MAQTQQKGYRDEALAVSRHRSGSTQLSHEAETQPQPLSAPPTQSAFKNLRTMFEDSAQQQ

GPGRSWFGPKVNRIKEMFQVGGGGGSFGMGEPSHSVSGPLPSRSSSAGLPETDRSPESKR

KKLRDAALSGSRESLSNMSPRSLPASSNVSLSAPDLLPQHPPPNLMEATSHVQRFNYTRM

LFAKMEEESRLAQEREKVMRRKSSPTRSPSSPPPALSPRSPPPMSPTRKVFSHDDVWRLT

ENAGEQSHMHGSRRDSSGSKTGSCTDIAAKQSITQQPATQPNVEVIRASSSSQVSSHLGS

HHMPASSSSVTIRSHSVRSQPDVIGDNVPGDRTGKYIKNFDLIKPAENIDKKERDNDELQ

AVRARVTGGFKRQPDTGMDEPHIGEHSMMRGRPPPSKSLSSSSTHSSSAPAHENGVDTSM

VIMRQKKSPTTPDTAARRLSREEIDAALVRADNYLSTLHAPAEDVQNVKRRSWEVKSRKE

DVRTKRYSLDEVNSNRTRLTKQTAVSHTDSPATTSDARDEVDHMPVPAVSDISALKRQGF

HEDRQEHKQSSPFDIPPPSYESSTSGHVRSSRLVSPEDKPKLHKLPPPPNKPIPPPNRPN

TAAKRAAPPPPLPATPPKGNPHAFRAQPPPFASPIPWPVKETELYMQDTVTHRDHGRADG

TVSTSFGAPSSSLDSSESGMDRLLLTEEDAASQEEAASLWKQHVLREAGDGREVLPLSSD

LPLSDGERADESEPFIPYIEIPPLSSTDEDSSSDEEIEVKKPSRVRFSRKPIKVFPTFST

IDYDRRNEDVDPVSASAEYELEKRVEKMDVFQVDLQKGSSGLGLSIIGMGVGADAGLEKL

GIFIKTLTEGGAAQQDGRIQVNDQIIEVDGKSLVGVTQAYAASVLRNTSGEVKFMIGREK

DPSKSEVARLIQQSLAQDRAMREREQERLMQLEEQFHPRDDIMEHQHMHEQFQQQQHQQQ

QMNGHPPFPKEREEGRLDEEEELAEVEDMVDAEVEDEEEEREEQEMLMTRQEIMRLQEQE

KGVVDSQEKEHQDRHWDVESTSRETTHMAALPSATSGSGIVSGDVVDKMSVEVMEQESSS

DGGSPGTELEKLFVQLKETQYKMALADAELAKLRAKVILLETTEAQKKQAEKKHEDALHR

LREVEKQLESARKENSQYQDMLEGSQGQYILLEKKMKADFGALEKKYHKAKKLIKEYQQR

EKDFIQERESLLEQQNEKDQQYNDLVKFLKDRVFQLESELTEARQAAGLPPPPSQPVDDV

QLPVTRATPEPVQAVLSVDEEDPLSPLAHDVLDVSTSSEISDSFLSPDFADGDDDGLERS

LDRDEELKLLSAPDAELLDASGHHVRPQLPSSSPPSLRRQFMEQEQDLTEEEVVISSASA

STISSSPVSPIARQDSESGLETWIKHDSDSTVRKSDAKKRRAQQAGGASVQVTAHGGSAQ

ITTPSSSAQASSLPSSIAPSGGGDTDSQSDSQSLQNTSASCSKDDSQSDTSSSVSHTSYD

PSKPHFHGMGSEIPESAAYEGEGGVALVSAKPPTSGKGFGLPKFSMKPKSPFGGGGGNRK

GGEGIVLLSSKRIGEGSSVPSHPSSSGITLISKRSLDASSSDLDAISTGSDLSTSLMVSE

PDDDATRSGFTLNISGTPAAVENVSPNKRSQNQFQSCCITEWSCEHVCHWLIALEMEKYT

SVFTDKNITGSQLLLLDSSRLKMLGIVSTKDREVLKKKIKELRVAAEKERKEKEKEQKLQ

EKERKAREKEQKKQQQKKK

>SG-AL_contig_2265_1_-1_1420_1701

MFIIWQFCNVPGWLVSCLAGCVQSGMGCMLSLFPISLLIRLAGLGFPPSSLFPPSAYSSS

LGYLGKEGKQRSQQPDIGEFLGRILSCIPHQATC

>SG-AL_contig_2266_1_+1_181_789

MGKGGFSEKIFHCMKKHPKNTQKSWTSFIKFVWVFLFCFFNSWTGNIHMYRTEISLPLFF

FFFKQKAQPTLTETGLWKGVGLGSCLNYLNHAIGNVIICHTLCSVLCATHLGHSNHEGWG

GCGKERGREFQRRALFWKRYKLRFVLSPISMVFVSSLHTIHACTDSILAVSGFGIKKVCR

VLCIYIFLQILDTMLVYSYQLVT

>SG-AL_contig_2267_1_-1_220_513

MMCVIARILACDRCANDRGVIMQYKQVFWMCLCVGRIGKTNIFKRLNIFLLKTIPFLSLF

LFFFLMEVFLICLWYIFNVMMVNCSSGCTFCDFFHFVF

>SG-AL_contig_2268_1_-1_16_360

MLIGVKSTALRLGDQTKPWLAGFSSFMIGSLALTGLMCDQTWPYYCGVAGVAAHLAHQLY

TVDLDNADDCAAKFRSNSKLGFFLFLSIVASTLLRTASDDKDDKASASDKDDTME

>SG-AL_contig_2269_1_-2_338_1669

MPIMPQTILPDTTNMSYYGVERIGDYMCDVWRKVVVNGKKTSTYSLYVDQYRGNPIRYEM

MGFDSLLGSHYDKYVLDYHDYIGGISISSDIFNVSAIANLSCSGFPGPGDAKEHRLVFNP

MHEFVHGDDSHVPEMFHRFKTQHSKVYKDNMEHEIRQNHFRQNVRYVHSKNRAGLSYKLA

INHLADRSDEELKLMNGFRRSMNYDGIQGKPFNMKEFENASIPDQMDWRLYGAVTPVKDQ

GVCGSCWSFGTTGTLEGALFMKTGNLIRLSQQELVDCSWGEGNNGCDGGEDFRAYNWIMH

PGIDGLTSEAHYGPYEAVDGKCGFPKKLPAVVQLAGYVNVTAYDQTALKTAIAMKGPVSV

AIDASHKSLSFYANGVYYEPACGSKPEDLDHAVLAVGYGEMNGEPYWLIKNSWSTYWGNA

GYVLMSQKNNNCGVATSPTYPILK

>SG-AL_contig_2270_1_-3_405_692

MTLCGNKEQHGQGQQKTEKVGGFWRRAASCSGRTQPRQGKARLIVNRGTNFDDSSVKNHW

KWDWMEAVVKEETGKIRESFEKDYNAGTCSMCTMPT

>SG-AL_contig_2271_1_-2_1973_2392

MDSTELGHKMADDCSQMCVHQKRHVPLVVYHKVVCRVKEIYNLTLLEAVFVFILSFTACP

DCPTANCFVRIMLSTVCIVREHNHLVAKRSCCFWLHSFFMDFFVWLWFLFVGFFINYFHF

VYIFNAFNYCTNWNKDLIFV

>SG-AL_contig_2272_1_-3_327_674

MSRFLNNYCLLHSDAWQRNVNSSRCSVAMRCNRGQILTVVTPLQVSHKLCLRVVYFINFK

LIFLWMWLEYYRSPECNISIKYIYLFFWFVLINDARLRLCLLYSYLLYRSAYVSFL

>SG-AL_contig_2273_1_-3_1278_1463

MSLVLYLSSEVKLLLCIHYFKLNFKSSKVCDGYNICSCWQSERSPKSFYTKETIVISFLT

GS

>SG-AL_contig_2274_1_-2_260_709

MIPIAPFWQAQPWFPDLRSLSHVPPLEPSPSPDLLRLPRSGTTHLNPEMPRLRAWPFAAT

PAYTDQFPSLGRESRQTHARPREAATALAASGAYPEPRPLDTRFRRKGKESAYDSKWSKR

LACAVPAKSTHDRPPPIRPGEFPDVPHHLR

>SG-AL_contig_2275_1_-2_35_379

MATQKLTTGLTGLAVARHPHQTLKVLYEKILRTLQKFPEEAAYRRYTEQIVTDRLGLVKA

ESDAQKLEKKLNAGHIEEVILQAERELLLSRKMLQWRAWEPLIAEAPKNQWKWPL

>SG-AL_contig_2276_1_-1_178_393

MEECRTRRKRKQYCIVGRLKVKVKLVDLVSRGRYRPYRDPYKAFFSAAIGRCGRHWVKIR

HKKLSEQVQDDN

>SG-AL_contig_2277_1_+2_2_199

VALFRNAFDSISSLMLSLCSASSAFSFAELFKLSTSSWSRLTQCLPQRPKKSLIGIPVRP

IAAARY

>SG-AL_contig_2278_1_+2_359_529

MHSLFCGSRKITYDSSSIDIMAKIHQYNNDSFPTKIHQRLISNLDPPMHSGVCGLVD

>SG-AL_contig_2279_1_-2_125_2047

MEGLPFLPGNSFTDPTQNKYHLSHTLGYKNGYALQKRPEVGIGGKPIEYNQLDQADLDHL

ANFNPTLTYGQAKQAPPEDFIPAHVAWDKKVLKFSAYYKQTVHESQNEFYRVRPVDIYYY

LEDDSIAVIEPHVENSGMPQGKLIKRQRLPKNDRGDNWHWKDINNGLNVTFYGKVFRVVN

CDKFTCDFLESEGIIVNPPECQPKDPYIERRKEAAALRTYTTPPAFDKLKQFLELDRKVL

RFFCMWDDRSNMFGEMRPFVIHYYLVDDTLEIRECHQPNDGRDPFPVLIGRHKVPRDRYN

VPSSFPAVVMELSDNEIKDYFTPKDFMIGQTVTLYGRRFLVYDCDNFTKAFYYHNFGVTD

FPSVDVDGSGKKLPKMEIPPYNNFGSLEDSLQSCLSLIPQPPKKDFIKMLENDHKVLRFE

AVMDSLRPEDSGRRFIISYRLADDMLTVYEPPVRNSGIIGGKFLERTRVCKPSSTPDAPD

YYGPQDFYIGAVIEIFNHRFVITSADAFVLSYMEEHANQFPPATISSLKETLGQTQAAKE

MVKGGPMKVKRTQGDLDQLVKQVRAQLKKIAITDKCRVDEMFLRYDRDRTGYISVENMRD

LCRRLQLPVDDDVILALVQQCTMDQEGRISLEDFRRFVESS

>SG-AL_contig_2280_1_+2_2_184

FFFKKHFYFNRSRTLSNEHLLGNTIYHNPMHHGNKAVVLGNKDFVKLVVSLKANKMHAVT

K

>SG-AL_contig_2281_1_+2_242_373

MSRVSKSTKHGIQQHQKRYSINIFNPFIPSPTASYRKLVDRMKE

>SG-AL_contig_2282_1_-2_2_148

KFYKVTNSRKLKASLLETQESIKKGCTRAHGKCYVAKWLVPIPVWRLTK

>SG-AL_contig_2283_1_+2_2_157

FFFFRKKSTFTFISFLYKNKAPDTNILLESKRTIIKLKIATSLIILETNQTI

>SG-AL_contig_2284_1_-3_123_425

MLVGLDMLAVLLHFGGCAYAGCRVSIRHRVLTWFTRFLMCIFDLLRSCLHEGGSGTGRSA

QMLTWEIGKMFFFFCFSPLLWKNLHPLPTRQIGTLTFQLGI

>SG-AL_contig_2285_1_-3_126_545

MSKRGRGGSAGGKFRIALGLPVGAVINCADNTGAKNLYVIAVHGIKGRLNRLPAAGSGDM

FVATVKKGKPELRKKVMPAVVIRQRKAIRRKDGTFIVFEDNAGVIVNNKGEMKGSAITGP

VAKECADLWPRIASNASSIA

>SG-AL_contig_2286_1_-2_2_220

LLASVNHHQYSLTFRIRALKETHQWVSTVICVARPCQLSTVTATRNLSSESVVAAVLVSS

PITQDVSTVTNLR

>SG-AL_contig_2287_1_-1_1_303

CSLGECISSGSTEVLDTCPQGDSPQANDAGEKCEQIVTADNGKCYQEHIEQACCSSCDEV

HTRRAGCEYGDKSKSCAEFRDFPGLCDQQPDACCGTCWERS

>SG-AL_contig_2288_1_-1_52_222

MATTLSFTDVCILRMEMTPTTACAERFGVRRDNTIFQHGHQMVLSAEIGSDVLLASV

>SG-AL_contig_2289_1_-3_45_1304

MMGVWRLLTGSAVVMMAVIYSAPVAKAAMNTIQLKVVVDRLANNKWRRILEPEVNGDITD

YVYSVFKDVNSIFKTLRSSRIKLNVQVVDVDVTTNDITRQTDSGVVLRNLIRWLGTRQGD

NWDHTMLLTGLDLIVDDDKNKTGQTYLEDMCHETRSVSVVEALLGSTVLNAAHELAHSLG

AKHDNGTCLNGYIMTDSTNTQFPSLNNFKFSSCSIQEIKEGLKKLSKNGNCLLGGNPNAK

QESAKTVGDLLTPDQMCKLEFGSSSYFSRRFYSGDQSESYDRMCLAIKCRNGRLTSRPDT

MKSTSARASDGFICGNRKRCSLGECISSGSTEVLDTCPQGDSPVANIEGQTCGQVKAARF

AECYAKYFQKACCGTCQGEYIGQPGCEYGDKSYSCVKYRNFAGLCDQQPETCCGTCSERS

>SG-AL_contig_2290_1_-1_52_225

MATTLSFPDVCILRMEMTPTTACAGRFGVRRDNNILQHGHQMVLSAEIGSYVLLASVY

>SG-AL_contig_2291_1_-2_47_382

MDSAHIAGTWTPDEAHLHINVLEMEAVSCALLAFAHHLRNKSVKLFTDNTTVTRGVHFSA

LSMRAEDLLRWCGTKRISLSAAHIVGKSNILADALSRGHSIIHMEWTLKRQT

>SG-AL_contig_2292_1_-1_1_534

STSLRPDSTRGFRSRSLPGLGSPDQLRLPSFPDSPQGHQEGERGESAYDPRSALSAGTAL

VSGPETPLSCPITGADPEPGSALTSLLRDPSFKPGYAPPARLASVWQPLPALTDTPLSVS

GPSSSHPRTRAVSSALSASGALPATHSLVSLSRRKGMVSVYNSKRVSPDMALVWSAGL

>SG-AL_contig_2293_1_+1_2368_2670

MAYLEGHRMTVAKSEGQLKVVKLWKENRKERRVQLVFFSERFPIFVMLFVFTLNFHGRRF

NCQKKKKKKAALPALPPGGGHPHMRNTFLFRGNSIKSCQNK

>SG-AL_contig_2294_1_-1_133_495

MQEKTDAVAETSACLGLNIHRGKSKVLKVNAANTTPIRLESEALEEVESFTYLGSTVDKQ

GGTDADIQTRIGKARTAFHQLKNVWGSTDLTTNIKIRIFNTIVKPVLLYGAETWRTTVTT

M

>SG-AL_contig_2295_1_-1_76_681

MRAGVDVMVRLFCGGFGSLDTNNRLAILLFASDMHLIHQFSDDQSPETLRDVIANLTQPN

GNTCTGDALQFAHDVIFSSELGSRPDVVHDTLLVTDGHSNCGEITVQEGARLLQDVSNVF

ALGVGVANDTAARWELNSVVSENDPRHIFSLARFQDFKDMLDSIEQRQQTEPCLPILDVT

SAPDSARAGGGENEAVDRLGGR

>SG-AL_contig_2296_1_+3_3_197

GEPAVALTMPVLTVPSFLLPRKCLFQRKETSDCRKLINLYADYNLSSKDCKQIRFASIWW

LKFIS

>SG-AL_contig_2297_1_-1_1_429

VCDRVQQTSLDLCITSPRPGGLGNRHPINRLGSPPELRLPPISDPFQSDLESEIQEHKDD

LIAHLWPAQLLFPDLQSLSHKAPLELPLLWDLVRQPRSGLTHSNPQLLRLHAWLLCGKTC

PHNNQGHLRTWKSDHTAFILTTL

>SG-AL_contig_2298_1_-3_3_116

PHPPPPTLTTLGVIPPFPSQFSKTHTLWVSEWCTFLTF

>SG-AL_contig_2299_1_+2_86_217

MYKCLVPFYGMYLCLLLNADVSVLCPFLECTLFCFKMESAQQPE

>SG-AL_contig_2300_1_-2_152_247

MHSVCSPSCKLMKSCNIYYTMVKISSPLSLSL

>SG-AL_contig_2301_1_-2_263_499

MTQYNTERKRAPRYFTFPIPKEGQVPFTAGWTGAVCSSEGTNLVTRAFANHSRLCLTLAF

VNMSEFVLRPLTMLSLLTM

>SG-AL_contig_2302_1_-2_2_421

KRPLQRALRNHWRQSASSWDQVVPLGSWFREVTQEWASASNLAEGVPIVPSSPGDPLHGR

LLPGLGCTRGLSTRLRDVDSGRSPPRHQLSRDRRPPQGQDGQAVHRQHDSQLLHEQGWGG

HSSALSSRAETLQRWCESNG

>SG-AL_contig_2303_1_+1_1507_1830

MKKKKKKWIKKKEERKKKKKKKKKIGVEKKKKKKKQKRGGKKKKRTNQPTNKDSYEYTSQ

CIIHAQTRSLGQSKGHCTYDWVKFRAWRGAVGLTLSPLLFQPDSITVH

>SG-AL_contig_2304_1_-1_595_849

MPLAATEVTVCTRKIKVSDTFIMPLHFRTSCFSVSVYFQRFALSFIHPWAFFSVFLIQNN

KKKGCGAGDSFSQHDARLYVTSIYA

>SG-AL_contig_2305_1_-1_163_303

MLDFHIWHMKTVIVPLPRNESVTSPRANVGLGSVMVDLEFCIKLKLN

>SG-AL_contig_2306_1_-2_1889_2542

MPSAYTRSDGNLFNLLRLRAKTKVQKVLIREMLFADDTALTAHTEEALQQLISCFARACR

EFGLIINLKKNSIIGQDVSSIHSVSIGNYTLEVVKDFTTISSNLTLDAELNTQIGKVAIT

MTRLVKRVWDKPILTTNTKMKVYQACVPSTLLYGCEAGTLYSCQECRFNTFHLSCLKTIL

GIAWQGGVPNKNVLAQAGIPSMFALLIQRHLRWLGHVS

>SG-AL_contig_2307_1_-3_30_455

MASTRPNHKPEDVREISRHDKAILSRIFNPGLPSSELQEEIEHPELSENVPEDARVVQAK

ALEVEGVNAAEEGNVQMAINFFSQAIQVAPHWPSAYNNRAQALRLMGDAKGALDDLNMAV

TLSKGEGNSACQAYTQRALIKR

>SG-AL_contig_2308_1_-3_150_530

MSTSEVFRPSDGDLLIRDNIAEGAAAGTDLFRQRPATQGGYRFGQLSHNSFFTRHNPHPH

RVRHLKGLLDVPICSVNDDGYFASPRYSLQFPPNTFNNTKLNKWKGRIPVNAINVNSQLH

PINTITG

>SG-AL_contig_2309_1_+1_1_264

VCVYVSVRVYVRTHSVTDHKHKRNNTYDCLHLFWTKMHPPLSCQLFKQPYTYTYILNLFK

KNNHQPTTQNHQRSTGTINVFNQEKITY

>SG-AL_contig_2310_1_-1_16_309

MAAAVRTLKTVRPHIPLIKFPTRGPAAAFNPAASAPTVNSASPTSNPPGSPPASAATSKP

IGTTPRGSGIDDLNLPMKYQRKLIDVDEMEFIERGGPA

>SG-AL_contig_2311_1_+3_225_440

MLQKENSIYNLFLSYTLILLMLQLSFLTVSQNNKNQKNSTYNLVVIYTLLLLTALQKKTA

CTIKFQHIHCFY

>SG-AL_contig_2312_1_-3_3_218

AVLCVLVARLKILLITILSTGDSVQLGQIWSSCCVTFVCVVFCFYNKNLSHRFEVGLFSK

FHVQDDCNIFFF

>SG-AL_contig_2313_1_+3_3_140

ANITKQLYGVLGVCSTGFKVILKNWIKHTFNLLCPFLCSYICSFFL

>SG-AL_contig_2314_1_+3_540_1016

MTVSIPTHIHTHTHTVTHTHSRTQFPNIIFRNTTFLPNYLNKAIFLKTPLKQRIHKFQQR

SHNFIYIVFFSNTQSYNTVLFCLLMFTHKTCFITVVKGSKHARILTYIHIYIIIYTHMHV

QPMLTHIQKHTQIPKLYHTHNLPTSLGTSSPLSILVHNN

>SG-AL_contig_2315_1_-2_410_685

MHDVNIDLCVCACDVHRMALCDDAVSSVLLNKNIVMWFSYMCVWGWVLFVPVLLIIRLSR

MIFMYAFHLKLGTALVLYSPPLPLSCFMRIFF

>SG-AL_contig_2316_1_-3_18_866

MAASSCLKKFSPAAKAVIQLFQTRFTQTAAAAAPQAAPRIKKFEIYRFDPDKSGDKPHVQ

TYEVDLNHCGPMVLDGLIKIKNEMDPTLTFRRSCREGICGSCSMNIDGTNTLACLYKINS

NPRKPVKVYPLPHMYVVKDLVPDMSNFYAQYRSIEPYLKKKEDGSYTPGDKQYLQSVEDR

AKLDGLYECILCACCSTSCPSYWWNSDKYLGPAVLMQAYRWMIDSRDEFTDERLAMMEDG

FTVYRCHTIMNCTKTCPKGLNPGQAIGEIKKLLMNYNKKAAAH

>SG-AL_contig_2317_1_+2_107_340

MLIKVKTLTGKEIEIDIEPTDKVERIKERVEEKEGIPPQQQRLIFSGKQMNDEKTAQDYK

VMGGSVLHLVLALRGGFS

>SG-AL_contig_2318_1_-3_222_587

MEHQLNTVNEESLKVGLKIHSGKTKFMTNFDTPDQKQIERTETEKVTNYKYLGQTIAMEN

RTRNEVLIRIKAGWSVFGKYREIFLDKHLPISLKRKVFNQCFLPTMTYEWQTWSLTKAIV

QK

>SG-AL_contig_2319_1_+2_1136_1669

MVCLSWLHWCESHSRKTLRVQPPVVGHQPCTHTCQLLRFTRHCYVFSLRFKCYGVQTKRY

AKPQNCIINMFTTFLHTTAGPHSEFADGIVFEYCPILTLSKRGDHSSCAHASQLSFPGRF

CKLFSEMSKREDKAPPPKKQKVRHGRYLSSWATEKTFQGMVKESTDSREYAYCIPCAK

>SG-AL_contig_2320_1_+2_113_739

MQDTSLIGGTDMENHHHACRVGLRPEVCPIPLPWNSSMQQHKAHGEMQPGADGDSLTHEA

LATEHRVSPGAGIQAAARTETITVIVRIIRGEDQTSQTHTLTVTRVGVTEIERRMSAIEV

TKKILLETVKATEAGIGIKEMLMTTAVSEDQDGAGGSVHTKEIPEAATSMNIQEGIEVEV

IGVGRWGMHKVDGTMSMKGHTTLTTGTAD

>SG-AL_contig_2321_1_-2_2_202

QQGTTVGGTTQSEPVWDDITDISLPLDDAETTQRISLSDDTITTYSLMGGPATDTCSSSV

PASTSMP

>SG-AL_contig_2323_1_+2_2_169

KKNKKNKKKKKTKTPQKKKKKKHQKKKKKKKRENWRERMRQRDRDRGGERGIKYND

>SG-AL_contig_2324_1_-3_1794_2219

MVVLRWCGGGARSKLEKKRAEQTWQRLLMESCLCLSSDCLLMHFVEACLCLLSDCVLMHF

MKACLCLLSDFLDVCVDGVVYTIFLSITLTDDSLKQRNQEKSLFYGSCRTVLLFVLFESC

GILALMFVLTWFVFSFLWLSRV

>SG-AL_contig_2325_1_-3_3_281

LINNNFVFASHLPALFFFLCIPLTIFLSVIFLPSQPFGCLFSPRACSCLHLLFLIAHSDR

LLSWCPGVKIQDISHVSYCSTLRFVCLLYCALS

>SG-AL_contig_2326_1_+3_3_179

THTCQHSHLYIQYSLISSSLIQIRFDTFLINNLRTTKLGKNVTASNGIYVMKIICIHCC

>SG-AL_contig_2327_1_-1_46_315

MECWKNPQLKMAIMTCGHIVSRGDRKECQFWDTMRICLSCSVELMCSERAATFLTDFLFH

LYHPVAEHMCGSQGRSTRGILDMFPFEWRK

>SG-AL_contig_2328_1_+2_473_592

MSLTLQPPCLHPVCYTGSVQAANNWLRQLSRDTHCLPACI

>SG-AL_contig_2329_1_-3_3_152

KEESKTCICWCVNAVCFNIKSSCKHKLFLQKQLSLICLYCGVHRSTVLSS

>SG-AL_contig_2330_1_+3_96_428

MTSTLNMCEEIESDNEFYAQHLKTHQIHAKNQYDAFSSQPIEKQCQLYTRTGNQSMMVSI

TSNVVPKQTHGYCWLVRQSRHEVVPLHWSHTTRTQPVRMMTTGHMISQCLC

>SG-AL_contig_2331_1_-1_475_726

MYDFISIITLTEEHYNLHTTHREVHIHFVNAMIFTCNTYFPYHTKHTPTLHRSFLCMIQP

FCVSNCSYKPKNSLCSDVHSRKSL

>SG-AL_contig_2332_1_-1_214_864

MSRIQCMLLLLAVLVACSFAVPTEKKQRSLDKSLSDLDHDENGEHNADYDHDAFLGHEDK

KTFDQLSPEESKDRLAIIIKKIDKDNDGFVSENELQEWIQYVQKKYIINDTDRMWKDHNP

DEDKILSWQAYQKRTFGYEDDPNVKGSRTEAYAEMINRDRRRWDKADKNKDNRLSKEEFM

DFLHPEDAEHMRDIVVMETIEDIDKDKDGMIDLEEYI

>SG-AL_contig_2333_1_-1_934_1353

MSTDGNFSHQGGRAWVISPNNPPLFAQAVLLRTASLQETSRWGEGVKWSVFALDYTAWNT

PQQICESRQFPRSLFFDVDYPAAGKSSHQVCIVKAIPFTVVSYSSTRLNKSAQCWQFPVV

SLAGGCTGATSPMPTRSRLT

>SG-AL_contig_2334_1_+1_244_525

MRRMTGEEILHLLDLEDPADSDSDESFSPQENENSSDSDEENSVVTSVTVLELAVVASTS

QQTDRGRGVRQLSRTRPGVRGRGRGIRDTDNNSG

>SG-AL_contig_2335_1_-1_1_414

GPSGGPPTCRRAANDEAAAGATGAGSPGADATTTPQTGTPLPQTSGPRLQQVVGPWARPL

PARRYGPSLRRTSCPSRPGLASPWDATPAPATHQATAAVASWARCSRRRRGPGRGPPWTS

HRRGTTPPLPHTRRPLRH

>SG-AL_contig_2336_1_-1_430_585

MVFLKLPDQHRGCLYLSGLVDAGIVCERECGGQPCHAVSVQKWNSIALYFLC

>SG-AL_contig_2337_1_+2_89_340

MLWSSIFGLRLCSGLPRFLSHTILGSTGPERYRHLQFWSGNLSNTVNFASRKWMRRDCVV

PVFPMSPHHAQLWVGAGQGRKIPQ

>SG-AL_contig_2338_1_+1_685_1041

MMIWTVYHSEVYRGVRGGICYRSQLLTTQKGKKGWDGKYVDDMENNCYQTVTSISKYVDQ

TESNCYQTATSISKYVDQMENNCYQTATSISVLSLMSIIQTLYHSSYSADIIFRKWCLL

>SG-AL_contig_2339_1_-3_6_596

MQLCLVLVCCGLMVGPIWAGTVESVAVAKHNQLRAAEVETNSASNIYALKWNSALAAQAA

TWTRKCRVEHTPGLDRNIGQNLYSAGYIGSEKPKATKVLKDAIDLWMEEKSLNDGTFACC

TRLTMTCCHYTQVVSSRSKEVGCAVEFCPGNQIIVICNYTPMGNFNDQAAYRTGPPCSGC

KKKDKCNADKLCVSRKG

>SG-AL_contig_2340_1_-3_2154_2480

MSHSIFFKSCLTCTSIIILRILTVVWFLVVIGIQCCMHLLIVHLHIVDMFTRMFQMSEEL

FLKRAGVGWVGDRTPGGIVLTKLPQTLQLLVWVEDKIIPVPFLNCIEAI

>SG-AL_contig_2341_1_-3_6_1931

MASDENKPEINGTEEPGQNAARTSQNQGTAAEMARPNNFQKIAQKKMMFKRFPRTKKLEK

LAVYSACKSEGCTCNGWKNPNPPPTQTRVDISQPLASLTDPCRSCGHSLGIHVSHLQNVE

MDELDRLLRLVVDVENLFLCVHREEDVDTKQIYFHLFKLLRKCIHNQAIPVVEGHIGKPP

FERPSIAKGVMNFVMFKFGHMSQTDWQTMYDLAKMFLHCLNHWKLETPASRANRYGEDDM

KEYKVHYMRWLCFCHAPAFCDSLQRNETTLIFGRTLLRQVFKTMSTQLLEKFRAEKDRMP

PDKRTLVLTHFPRFLRMLEEQVYLKASPIWDPEFSHSQSGPNLPLSPTLGTPGQAAGVSG

LNRFTSSTVTAASNGDGFTSINISGGVITPRRSSRSQEAAALAEKRKAEEELAEMESSAK

VPRLEVEGDVPNDIIAEILATITDPNEMIGPDSSLFPAHAARDEAARIEEEKGRIKFVVI

SNSLSNKTSKQVYMWLIGLQTVFSHQLPRMPKEYITRLVFDPKHKCLALVKDNKVIGGIC

FRMFPRQGFTEIVFCAVTSNEQVKGYGTHMMNHLKDYHIQHNVCHFLTFADEFAIGYFKK

QGFSKEIKLAKAAYLGYIKDYEGATLMGCELNPRIKYTEFSH

>SG-AL_contig_2342_1_-1_67_1113

MAQINGYKEELQDELSKTDPNSHASRLLTQEIERLEKGDDRKDSRSIAFIDVHPQEPQNA

GLKVRIPVQEFPRFNFVGKLLGPKGNTLKRLQEETGCKMAILGRGSMRDKTKEEEMRKEG

GKKYAHLNDELHVYVEAQAEMTDAYRRLAHAVSELKKFLVPEANDDIRQQQLQEMMYLGE

DAPRGGRGGFRGGRGGPAGRGGPAPVRGSRGGAPPRGAPSRGAAAPPMRGASRGRGGGAP

PPPAQSSPYDDYGYSGSGYGSSSRGGAGSSGGYDDGYDSRGYGSAQSDSQYYGSSGYGSG

AQSSAASYDDYGSYDYGDSYGNGAGPMKSSSRGGAKPRSHPYSGGRAAY

>SG-AL_contig_2343_1_-3_3_161

LKGNPSSGLEILLTVRHGTDMKPKGVFFSKYIFSKIYLPCRTQTPTHPSPLLP

>SG-AL_contig_2344_1_-1_1_207

YNPFFDCRVKRSQKEYFFLNTCLAKYTYHVRLKLPPILPLFCLNMQWVYCRVWAIKSDLE

GKFPKRMRI

>SG-AL_contig_2345_1_+1_1_273

GKKNGRDNESNRREEIASTEFPEGGDTIYSERAPGIPTGEFSLRKKSIDTFQKKVCSFSF

LRGGYQSSSFRHSIICVPSADPFLLSGTCSV

>SG-AL_contig_2346_1_-3_1077_1385

MLLGSEGVETSLLSGQHAKAIVSHLCSFFHPLPLSIVLLTYFYFVCLVPPLTPLSRIVSH

LCSLFHPLPLSIVLLTYFYFVCLVPPLTPLSRMSPLSSISTCF

>SG-AL_contig_2347_1_-3_171_281

MCRCMVIFWIFHHMVYMVPFCVQKSDNFSRHYWLIAN

>SG-AL_contig_2348_1_-2_32_1114

MPVSSSTYGFSPKKVTLSRLGGGGPDFGSDFTRSRPTTYSMQPVHVHTAARSPQQSSDYM

MNRMQDSMDNISLSPRTPDFGYQAPPFSGQYIPTGIRLDMEREEELRQQQQQQQQQQQQQ

QQQYQSAPQQEYQPPSQQYYFEPQQPYVPTAVRLDHQMGEEEPQGELQPVRERRKTFMNA

PSRPVAAPTGKSTAPKVLRPKPGSSSFTFGVDYTKAPPKTSSVTWRPQQQRPVNVPPPQP

EERPAALWSQHDEPDQPSVPPAWRTSLRTTGVKPWDKDLDYTGQQQAPRASTQVQNPDKP

GDRDWTQSAVYRMIYEEEHRGRGGPQQQQQKPAPPPPAPKPQVQSFQQAFTPTDDFGTSD

F

>SG-AL_contig_2349_1_-3_162_326

MILPSCFILTNWSSVSLQVTLCMSKLFLLFPCLCLAFEVQGQKDIFPSYVFNLKR

>SG-AL_contig_2350_1_-3_195_1850

MASVGPAPNRSWRRYGRARSTSGARSYFGDDDASPDTSAYPSRSSSVGPPLPSRSSILSS

SPLSSPHTSRRRLAPFPMTRMSESRVLDSGSSKSWGQSLSPSRLSNYSSPTRPDYDPPYV

EYEPDIKSTMLPRRRQKVLGSSRRPAGNYYERLLKKRSKRQGRLGPYYDVESIEGSDLSG

VLSDVDSFDGTYDNDSDNESSSVSPSSYRHLSTALSPSSPYYVDQDTEFVPFKPSRPLQT

KGLLEFSSYDDDDNNDESSNSQPLPSPTTDLYLSRYLPSSLVSAATSASSPTLSRLPYHS

VVPYTSSLSDDSLGVVPSSTPTASQQLLDSLVSTSVQRAKNALNNLAIPEYSGASLLLSV

EGAGLSPEGKRVDFTYTPPPIPLYGRTLALDDKILGIRPSSTIKPLDNFISGYVERMGAL

RAALSDRLDKDKEKRIQYGLKPLDTGPRPSRTYSSYTPSYSSSYVPSYTQSYEYVPVKHV

TSQKLERLPVTTFRRRQDDGHTQRLDVFSLGEFRPRLSSSTAREISIPASATGGHQLSVL

DKINIKAAIIAS

>SG-AL_contig_2351_1_+3_225_371

MSLTHSTNRFYAFTITYTCSISYEKACQMNTENKRQESYPVRNERLIAY

>SG-AL_contig_2352_1_+3_186_335

MCVHLSFLFFLFIFLFHADTNLEGSQFWAATPGLEEVTVSARTPSPVVGT

>SG-AL_contig_2353_1_+2_4241_4750

MIHPQRITTKSTKKGHELQDWLPHTRTRQLLFTLFAATTIMPFLILTDQFTPPACLPICL

PNRGHMSTTGRGEGNNYHFLHVCTNSIAFLVCKNPVLFFHDVQILHYNISKKTRQCDKHT

VHTAHYHRPHTNTVNHFQAPHSPLPSSSKADIYKYTFHRVEMLHEQTDYK

>SG-AL_contig_2354_1_+2_1646_1948

MSHTPNSTGENTPLSLSMVIAMHCCLFLLLFSRKGNVIYLFVKGYSYASLLVSFFLLLSV

HLYHHGCDRLVFTYVINQNICTHINAESLTLCFLLFFFLSS

>SG-AL_contig_2355_1_-1_10_267

MKNRGVDHKEILRLAGEEWRNMSAEQKKPYEKRSVEAGKKYEQEMVEYRKKEKAGAAAVQ

QDNGADDDDEEEDDDEEEVDDDDDDE

>SG-AL_contig_2356_1_-2_2228_2479

MSGYATYCWGPDYFTHSVCLCTPSPSFFLLCFLRCMYFLSTPNVCVCVCEREREREQRNL

EETCFASSVSIPPALPIFSPLYLN

>SG-AL_contig_2357_1_+1_133_264

MFLLLRNSKWNVLPCVYFPIQNYLIFKPYNEKQAVVKHYLYINT

>SG-AL_contig_2358_1_-2_221_409

MSSSQSDVITNISVIADQNVNSLKADVPSKDCIILNAAVVFLPYFCFPPQISVQRSQVLS

RCH

>SG-AL_contig_2359_1_+2_2_265

KPLTHKHTNNFSRLCLFFFFKKKNSLELSEKGRFLKWLDHLVFFYFSIWQCSMQRGEYWF

YTDTSLTSLRDKSRYSSTNQIPRSHCGI

>SG-AL_contig_2360_1_-2_2_205

EYMLFDIENDPSEMIDLGGNSSFTDTIEKLKAELKKQKDLGFKPSKPAPKPGVGKPKFHN

GTWVSGWC

>SG-AL_contig_2361_1_-3_3_143

SDNIPILFALLWQPTKYSNSVCSVVTDYHYPHLMSSFISSEAFPRSR

>SG-AL_contig_2362_1_+3_1494_1691

MTITLCSAGYSSPAFTCIMVPNLRDQGNLLDISATSRLIFHTLASRGGQCRGPTVSWHLL

FLSLAR

>SG-AL_contig_2363_1_-2_119_814

MPPRIMILRCSICQRCLNNWYFEKDGQLYCRHDYWSKFGTDCNRCSHLITGPLMVAGEHR

YHPECFQCQECQSFIGDGDTYALVERSKLFCGSCYKEVTRPLLTAAPHRRKPHCIQLVEI

PPTPEHAQRGTVHLGTEQLPGTTYRYSFTTLDRLGQPLITITELDLSPELEGLKIGDRIL

EVNGSTIKDKSLEEVDALLNSKDSVQITVERDTSPLRVNTEADQCSSPNPVP

>SG-AL_contig_2364_1_-3_93_896

MADSYSFVKGGKLTLKGNKNKKLKKHKHKKRKHEESSMGESSSATSHNKEDTHSHGGWWE

VKKFDDISGNIALEIGNSTYISALDNGLLILGAPRSHGEPPDPTEVFTVLKLSETKVAFK

SGYGKYLSVDMERRVVGRSEAIGNREQFEPVFQDGEMALNGCNNCFVSVDDDGDVVCQSS

TAGKEHMIKLRSNLSIEEDPLEKVPREERGSVKDAEVNYVKKFQSFQDRRLRINQNDRAE

LKHAKKEGSFHETLLDRREKMKADRYCK

>SG-AL_contig_2365_1_+3_3_152

KEKQKPQNQKEKKNIRQNTENESTVSMHFVVSIFFYPVDKVMKRGMDFKS

>SG-AL_contig_2366_1_-2_125_817

MGPPSTKIFIGNLPDDCDIDRLKSLFETIGTIAEMDVIKNYGFVHYETEKDAKKAVEELN

EAEFDGNTLKVEQSRSTVRHKPGMGSMTECYRCGKSGHWSKDCPSVGGRGGRGRGRGRGD

RGPYRDPYDDPYLDPYYRRRYLPPPPAYDRYSPYDPYERRRLALARDPYYLERERDYYDQ

LAARDAAYYEYYARRRAFAAEAGRPLSARDGYAPPPRSDLAASRSRVPGPY

>SG-AL_contig_2367_1_-3_237_368

MINCFSVCASVCLFVCMYVSREKCDSKPFLVVHILITNSNHRSG

>SG-AL_contig_2368_1_-2_2_2356

MFILASRADYFRKQVPGLSIEPDPSGEKPVMVVSDVQPDVMEQLLKFIYTDTCDILQVGS

KVAFAHLEKKGENNGMGPSFQEMDTIAFDGCVTSAFAVEQQRKKGKGGKESKKEEKGGPK

GRDPVKVLQEAARKFGVKGLSKRLEAVKCVNGVIHSCGKKLQPPRIRYERTKLGELYDVC

IRANDDTTIQCHKCVLAARLEYFHSMLGSGWIETSDTESLSLPIPGDILEILLDFIYHDD

SPVVEESQDLELLCNVLVVADQLLVQRLKEVCEVTLTSLMTLRNVGELLEIATAYNAAQL

VQACRQFIILNLPALLETRNLDMVGDDTMQELTDYYRNSVGRMSCRVITPWSEGPNHDFL

AALTAEFDTELTPRTRDTSSKKPKNKRRQARTKSMGEEAATSAGLLTERQSSVASDVSEN

KASLMAEHQISVASDVSAKSDEDLEMEDVAVDTGMELAGSARPSSRPIAIQKPGKPQSWP

PLQSESRIIDTTSNNLPLQSPLSPFPGTGVSLRSIMQEQQVTLQSVKPKGGGRISWKEVR

KQQNKAAAAQAQKAPSHETGHAPVTPTSPTAASPPPCPWGSVNKVVSSFRDLMLQDKTSQ

QAHTQAKVTTASATHSHANVKRATSPTTQVATAARNSAAFLGKSPPASVNKSSAPSTTSF

SWGLPIRILSQPQATLTSPTEASAASSPPENPWQRLVPTSPAAGATANMCFSDIVQDEIH

QKHTLERASQKPLHLIQMEEEAISQLLQHYHAHERFDEHITVERVSGPMAAPLWNRKRTT

SSSHP

>SG-AL_contig_2369_1_+1_1_87

FFLICMFFLLSLLSEMHEPSSLKNHHYFQ

>SG-AL_contig_2370_1_-1_28_1545

MLASSFSRFSLALKGNAVASGRQGLAALYVSARNYSSDDNDLVVIGSGPGGYVAAIKAAQ

LGMKTVCVEKNDTLGGTCLNVGCIPSKALLNNSHLYHMAKDKDLEARGIEMDGVRLNLDK

MMEVKQNAVKALTGGIAHLFKKNSITHVEGFGRISGPNEVTVKRNDGSEEKISTKNILIA

TGSEVTPFPGIEIDEKTVVSSTGALSLEKVPEKMIVIGAGVIGVELGSVWHRLGAQVTCV

EFLGHVGGLGIDMEVAKNFSRSLTKQGIKFKLNTKVTSATRDGGVIKVATESVKDGKKEE

LDCDVLLVCIGRRPYTEGLGLDSVGLQPDNRGRVQVNSRFQTSVPSIYAIGDVIQGPMLA

HKAEDEGIICVEGMAGGAVHIDYNCVPSVIYTHPEVAWVGKSEEQLKEEGVEYNVGTFPL

LANSRAKCNNDTDGMVKILGDKKSDRLLGAHIIASVAGELINEAALALEYGASCEDIARV

CHAHPTVAEAFREANIAASFGKPINF

>SG-AL_contig_2371_1_+1_1_138

RTFLLKTISTTITFHSKQQSTDSRVNYAIQRISLMAVCHHMRKIIK

>SG-AL_contig_2372_1_+2_50_226

MLVTSATKTFCCKIQNQGYCLLFAFPSLTLWGSSNIYALCLMRVRPRVHLDEVACMAVF

>SG-AL_contig_2373_1_-1_1_195

SSKMYSWSHCGKTECIYWMTPRVLSWGTQTNNKLSYISLCELLLIFLMNNLNIIFLSFFC

AVTLY

>SG-AL_contig_2374_1_-1_7_579

MSVGSATGRSRYTEYLVIVYRSDKLSLLASLQYPDHDDVFEREPYSVLFHTTDSRVTKFG

FMGVHLRPDDVRVEIDALIKAYEDASAAFHTQNFLIAGDFNADCRYLSNRAYHNSAIFQD

DRFEFLVDITADSTVNSNTDCAYDRFVATGSVRPAVVSGSVRVYDYQTSMDLSYDQALEI

SDHFPIEMQLQ

>SG-AL_contig_2375_1_+2_791_1108

MLKIIAHWIKTTKTKHGGQNELACCIQSRFSMCACLINYIQLLKRCRQRLQASILFEHGI

LKFWISRVTIFFSLSLFLRIQGTHIFSCETRSVSTENSGSEENSHA

>SG-AL_contig_2376_1_-3_1956_2204

MECVAFCESTALPALLPLMENAESTDVDFLTSWTGQTALEWISEVIEQNCSYAVNVLNHK

QYSQTILAVSFFLLCLQIHFLQL

>SG-AL_contig_2377_1_-3_93_365

MLAKEERALLLEQLDFPFNLHAGREKETESCRRLDEKAVESRYVHLSCIYKSRLGTACLF

MKKLFLFISTSVKEIITIYYYCSMLPTKLTL

>SG-AL_contig_2378_1_-1_856_1074

MVQPISCGHSGNMLVTSNGTGSIAKSCMSMWLTLHICALHITLTNFFAMIFNCQMARVLM

NTCLLSINQYGNW

>SG-AL_contig_2379_1_+2_2_196

LAERSWGATDENQGEGGHLEIFRHVAKLHMTYMRTCFNITAWSLKTRFPSSREDRVRYMN

KETGN

>SG-AL_contig_2380_1_-2_1496_2092

MVQFFPTFRLYPKRPLKACGIKTCDLDPLPACLLIHYLDALLPYFTSVINNPFLLVISLH

HSRLPLSKPLLKKPSLDPEILKNYHPVSNLPFLSKITERMVLQLSEHLHNKNLLYPLQSA

YRSSHSTEAAVLKIVNDLLLALDNSQVSLRLLDLSAAFDTIDHAILLSHLQHTLWFSGNA

HFLFKSSLSDRKQTITMTL

>SG-AL_contig_2381_1_+1_1_219

GAHLLSLAALPFPNFEKNRVPIHGWVDWRSHTHIKLPAMIQTCTNLCRQTGRQVLYLCTN

QASYMTHHDHLLQ

>SG-AL_contig_2382_1_+3_3_101

NSARNKGRFHFMELLKLTLTFLSIIIIMHYFLI

>SG-AL_contig_2383_1_+3_1602_1958

MAECPSSQYTPYSIADLTVTGLSNCLLGFIPKPKTKATNNIQSLSNNFERTKQQIYEQIL

NVRIPTPNITQIYTSLSLNKIRLFGKKKIVYKYTYCQEYNCKGGKKEERHFSCIRSNHI

>SG-AL_contig_2384_1_+1_1504_1710

MHTQHKHFLSENLKYYLLYVDLTTPFAYLPQFINMYPELFVSLSLSLSLSLFVCKTNGLC

YTILNTECS

>SG-AL_contig_2385_1_-3_126_617

MAARKFIGREPIFRGKTLYELCCNLRDFGEGRVVYRHIYARFPEKSFYRLTKVEPDLSDP

TRQGGTAWGEKVYRGQKFGECRIDAGYKTDWHLVPKDQESEFCKITSVLAAKEKPPTHFR

CPPLLELLTKIEMEKSGVTPNPELFQLELTPNKAKQPLSKDLDS

>SG-AL_contig_2386_1_+1_1_150

GGGLTQPFISPFTRTIADNNLSPHLTMLEIYCWSNPKFRQNKNKSNLFVI

>SG-AL_contig_2387_1_-1_13_2820

MDKIPSVVVPGDGPFFKNKPSNQVVMEGQTMRMDVEVALDGKPMPNVHFLRAGRELKEDS

RVSFYTDKAMGIHTLEIKKTRLTDEAKYTVNLEQDGIITDSATFSVFIKDPKDSNLDFRA

LLKHRESKQKKEEEEELDFGSLKPVDKKGRRLSQIEVMKMNLKKVEKTEGSDSEEEKRDS

SRRQSLEPDIPVIKVSADKLEAMEQQRRASMQTRRTSLAEVISDWPMLQKRIIQKEEPDK

FVRELEDIKVLEGQGVEFSAEFCKPNAKLRWFKNKLEVFHGHKYHFVNDDVEYRLVISNV

KPEDGGKFTVECNGISSSAWLYVEAKEPEYYFTQKLPENYEVKRKKNGMLECFVSDPRAF

IKWYRNGEPMEYVPGKYEIQRRENRCILRIINAQADDEAQYTCKCGNATTSATVKVGEPE

WEFMKMLDDVEAVEREKALMECDVSDPEADVTWWRGDKELKAGGKYEFIKEGLKRRLQIK

NCSTKDDGKYTCKMLEQQTTGELYVEPDVKFIKKLADKNCKETETIVLDVKATNPHKHPV

KWLKDGKPIPDSIRFEEGQKGELYKLTIKNTEFDDGGEYTLQIGERSTRCQVHVEPLPKP

PKIDLDKIPKEIFVKEGENINLEIPYTGAPLPRAQWSKNGQLLNEANLDTETVPGCTTLR

IPGAQRGDTGEYELTLSNEAGTDKVPIKITIMGPPSPPMGPLDVMDIYSDRCALLWDKPK

DDGGAPITHYVVEKCDLEKGEWEPVCETDDLEIDVSDLTPGHKYQFRVAAVNSEGQSEFL

GTDGAILAKDPWDPTSPPGDPNILDYDKDYVEVEFEPPEKENGAPVQGYVIEYREKGDED

WNMGVETNTPDNKGTVSGLKEGKEYEFRVMARNKAGLSEPSAVSPSIKCKARKVKPRIDH

QSLPQNIKIKVGQKFSLPVSYIGEPNPTVSWTAKNL

>SG-AL_contig_2388_1_+2_413_628

MYCNLPTVQHPTLGTSPTVNATKSSAVISILKLTTLPLKQQQRKKTNQPQPTSTEEAVKL

KKPKNTKTKQKK

>SG-AL_contig_2389_1_+2_329_652

METLDLNLPPCTNKFRHHMTTKSRATSRSRKNPVTHLKKKVTNSLKMGKLDQVWLVIYMP

RQKYTDKCRYITHIQTHTCTHTHTHTHTHSHTHVYANAHVKTNTQAHT

>SG-AL_contig_2390_1_-3_3_125

KKKKVNSETHHLGQLSHPFQIENPSHIYFVIKLITHTYPAL

>SG-AL_contig_2391_1_-1_64_2748

MAVVSTETKAQGLWKKKVLSTSRPSTLDLFKPSPKPAPESEPHASTQTSTTPGQPHPTSP

RASAKDVGRHNFQRLGRSATRVSIFLASEDSALSERNAHKSVSDSDLREGANGTSSRVTR

KFLYSLAAISMENRRGSKSTAKGGPRRESGSGINRLLGIVGAMRRGSANTEQRATTFGGV

NKMASLVNLMGLKHRAQVRLKEAKSKILWRKLRLWAGIIYALSRLLRRHAFSAITDPASP

FYSGHTADTASMPMDNMDDSAEQDSDMLFDPSRFKANTQRRMSQEVQRILTKPAEDRTPE

EIHFAMIGLRNIDQLAIYPVRMQRNLAQYAEFQSFQAKRRILRQGHVAEGFYVILYGNVI

VAVKNEDTGFAKIVVELPRGETFGDLAIVNGGTRNSSVLTKTYTELLYVSKSVYEAIFLT

GGMKYLNDPDHLEFLSSLAFLQGWPIEKISNKESEKSVRFMYIQRNGVIMRDSNHTNWLV

VVKTGSVKVLKKLVRSKPTVSKRSGLYRHQTCASGFESFVTDAREYDRYLSCKGITLPPP

LQVDDDDQEEGEEREEETGEEEEEEYYEEDVMGEEDEAIKEVAEENKNSHFHQRLHQHHY

RQHRHHHHHQHDHHEHDDDNDDDNHHHNYHQEHHSQDHQHQQQENRHHYYHHHHHHHHQH

HRQRKRGMAYQEEKERGSTVQGKVRPHTGVKGCVTGPHTTPGARKAWSAKHPSPAPPSVS

VSRSSHKSSFLPKRSKLSTPKIRFDVEHRSRDEEQEIPQEAKFALTKRTILDDLDATYLS

RTHTVTEADRKPQFVYVQTLTKGDVFGLAPLVFPDQPGLCLVSNGAECMFIDRRFYLDNC

SPELSRRLKTEVSPYPSDSKLQQDLVNRINWDVYKQVVKRQVYQQAPSRVTLTSL

>SG-AL_contig_2392_1_-2_17_1645

MARMQAPVILLKEGTDTSQGIPQIISNINACQAIADAVRTTLGPRGMDKLIVDDRGSVVI

SNDGATILKTLDIVHPAAKTLVDIAKSQDAEVGDGTTSVTLLAGELLKETKCFVEDGVHP

QIIIRAIRKAAALAVEKIKNISVSIKKDNPKEMRELLLKCAATALSSKLVAAQKDFFAAM

VVDAVMMLDDLLPLNMIGIKKVQGGALEDSMLVAGVAFKKTFSYAGFEMQPKTYSKPRIS

LLNIELELKSERDNAEVRVDSVEEYQSIVDAEWKILYDKLDKIHQSGAKVVLSKLPIGDV

ATQYFADRDMFCAGRVPEEDLRRTMKACGGSIQTTVQNLTPDVLGTCETFEEVQIGGERY

NVFKGCPQARTCTIILRGGAEQFMEETERSLHDAIMIVRRAIKNDSVVAGGGAVEMEVSY

HLREHSHTIAGKEQLLISAYAKALEVIPRQLCDNAGFDATNILNKLRQKHAQGGRWYGVN

ILREDIADNFDDCVWEPAIVKINALTAASEATCLILSVDETIKNPRSGQGDAPGAGRGRG

RPM

>SG-AL_contig_2393_1_+2_23_322

MMMITTMMMMMIMMMMMMIIMIIMTIMMMIIIKEEEEEDDDDDDDDDNIIIMIIITMMMM

MMMTMMMMIMVLILFFRICLTCVLSCRCRAVKTSRLTVQA

>SG-AL_contig_2394_1_-2_155_1270

MSKSVQTQQKQEDRPKASLEDLWAKLKKQDSKSLLKQNLTEDLYKKLKDKSTKVGGTLAD

CIRSGCEHPESGVGIYACDPEGYTVFADVLDKVIKQYHKVGDLNHPEVDFGEDKIDESFS

NLDPSGKFIVSTRVRVGRSHDGYGFPPVLNKEQRIDLEKKTVDALKTLKHELEGTYYPLQ

GMSKETEKQLVEDHFLFKDDDRFLRDAGGYDDWPVGRGIFHNAKKTFLAWVNEEDHMRFI

SMQMGGDLKEVYLRLVKALKHLEGQNLQFAKKAGLGFLTFCPSNLGTTLRASVHIKIPHF

SAAPEFKAFCDKANIQARGIHGEHTESKGGVYDISNKRRLGLTEFQAIEEMKKGVLALIE

EEKKLACGGVKS

>SG-AL_contig_2395_1_-2_170_760

MSENRATKSGLALDAQKKIQSKYDQAKAAECMAWIADTLNLKLDTSGTCESVYDQLKDGK

ILISLLQAICPEIKLSESVKKDQTLSFKIREKIDVFLSKIKEPPLEINDLCVFATVDLFE

CQNICQVITCLEAVGRRCYSVNTPVKGFGPKEAVANKREFSKEQLDQGKTVIGLQMGSNK

GATQSGQNFGKGRQITD

>SG-AL_contig_2396_1_-2_2_241

KVLLKWNSLYVIYTVVVLFPVERSFVKMEQFVYTLWLHFSLLERRNPFCIACTVNMKTII

CSCIFHYFRHLGLSVVALFH

>SG-AL_contig_2397_1_-3_21_266

MWQQQCLNPYIRCGTAAFRLFCFDAPASLRTKRQKCSAACTVGANRRRHTHIFLCGTSAA

AAESLLHPNKKIPRRYRSATGA

>SG-AL_contig_2398_1_-2_353_634

MYVQTALLVKPLSVVCCILFIRFVFCHILSVLFRWGFFCFPSQNIFTFKPFMIFMQRRCF

MQHFHVYSISMDLREPRCDCGATLICIFYMTDRL

>SG-AL_contig_2399_1_-3_132_746

MSTMNPEYDYLFKLLLIGDSGVGKSCLLLRFADDTYTESYISTIGVDFKIRTIELDGKTI

KLQIWDTAGQERFRTITSSYYRGAHGIIVVYDVTDQESFNNVKQWLQEIDRYASENVNKL

LVGNKCDLTTKKVVDYTTAKEYADQLGIPFLETSAKNATNVEQAFMTMAAEIKNRMGPVT

AASENKPSVKINSSTPVKQSGGGCC

>SG-AL_contig_2400_1_+2_770_937

MFNTDTTNIKNEHYFFYQLTSFVAFNLCADCFLKMPTSKLQPTKVSEATNVKWTAS

>SG-AL_contig_2401_1_-2_257_496

MYVYDLCFCKYHVMITACFIYECFYANHFSTFCPTIDRSNIFIMGTQDIQFSVFRINCLK

DEPRTTVCFCWTLLETFIMV

>SG-AL_contig_2402_1_+2_728_1054

MSHTKFNIEDCRFCRTQVQRRSKFTRKQTKTLCQCIFCSYFRYFYHNKDGHAQLSIIKTV

HLLLSRERDCMAQNCCDEDRFLTAKSKIICRIDARMYTIRCDSVFLLLK

>SG-AL_contig_2403_1_+2_59_247

MHRHKHIHAGIQVCMHACTHHHTNVHTHTRTLKLCSPSYQADNTDRSIQNSITQECNGKY

THQ

>SG-AL_contig_2404_1_+1_1_231

QSVFVTNLHTDDSTRQAGRQDNNPEVKQRDRAADFSSDQSHRRRPTDCQQSRISKPKGPA

LSDVYKETETRISKPER

>SG-AL_contig_2405_1_-2_80_1360

MTSEKEGGLDDAVEERVINEEYKIWKKNTPFLYDLVMTHALEWPSLTAQWLPDITRPEGK

DYSLHRLILGTHTSDEQNHLLIASVQLPNDNAQFDAAQYDSEKGEFGGFGSVSGKIEIEI

KINHEGEVNRARYMPQNPCIIATKTPSSDVLVFDYTKHPSKPDPSGECIPELRLKGHQKE

GYGLSWNPNLNGHLLSASDDHTICLWDINSNQRETRVVDAHTIFTGHTSVVEDVAWHLLH

ESLFGSVADDQKLMIWDTRSNTTSKPSHVVDAHTAEVNCLSFNPYSEFILATGSADKTVA

LWDLRNLKLKLHSFESHKDEIFQVQWSPHNETILASSGTDRRLHVWDLSKIGEEQLPEDA

EDGPPELLFIHGGHTAKISDFSWNPNEPWIVCSVSEDNIMQVWQMAENIYTDEEPDAPGG

GGDLETS

>SG-AL_contig_2406_1_-2_29_514

MQLDSRHQGSYISAMKSSRLPGRLPTMTTVVVVMVVIIGLSIPVEPFIGALLAAISGLTA

GQIASAAGTAVSIGSSIGEAAAAASRSNYRVTCVIEMENWTKHNLHSPIVQITNSGSLIT

NPKNIWPASAQAFSVRKPAHSATGVYGTVSWMLGDTGRRVVV

>SG-AL_contig_2407_1_-3_93_518

MQSSRLLSRLPTMTTVVVVMVIIIGLSIPVEPGIFKGLSLSKIIGAAESAVTVGDSVTAA

TQAAASSGYRVTCVIEMENWTKYLLSNPRLQITNSGSLVTNPKSVLPSQMQGFAVRKPAH

TATGVYGTVSWQLGNTGRRVVV

>SG-AL_contig_2408_1_+2_77_409

MVSQAHRPSTQGVYSSHWSPWLQWCSDNQIDPHNPSQTQVTNFLAFLSTDLSLSASSVKV

HQATVCTTLHQIRGPSFSDNPCSGILSIPLLSRTPGTPDGHQLGTSSWLSQ

>SG-AL_contig_2409_1_+2_1400_1630

MDSPTSWEEGDSAVCWGITRAGALPVPDLSARPVHTGEGQTGVSTQGKLVRHSTTNSSDG

HHGPGQWSADSLAHDTE

>SG-AL_contig_2410_1_+2_20_157

MCVCVCVCVCVSRQILKPKMQSSAPVHVPFLQLFDKKMRSPMKDIK

>SG-AL_contig_2411_1_-3_3_230

LIFCARALSLSLSLSLSLSLSLSLSLSLCLNETQRPKPQFIISLYGKNILAREKDSSSKM

FYLYSQSQFRKEENCI

>SG-AL_contig_2412_1_-3_66_1022

MERVATGASPKEKSKDKNLKAEMRHLHEEVMSVKVKEAKSLADLKETKQKVMELETQNQI

CQNQIRRMDEENKTLKHKLEEAAERERDLRTNIKDLHRKLDDTESKRKEEAMMGRIKEAE

KVQVIAELRHKIAEIDIQREELLTAGQLQDKTDKEDLETQLYDLQDEMIQLKMATPHAGI

VTTTDNVMTASFLTTHDSDEEWDVEVRDSENLDRTLSDIIEGKSPFTTPSKTIPRFGSLD

GAGDVTEEGERLSEQRTDGHVLDETLYACSNSSVMGNGEINSESLQGATKPGRESSQGKE

QTGCGSSTERDSHMEKSFR

>SG-AL_contig_2413_1_-3_87_1121

MLSRIARPSQAVCILRHQFSTSSKVKKDAKVAVLGAAGGIGQPLSLLLKQMPIISHLNLY

DIAHTPGVAADLSHIETRAKVTGFLGPEELHKCLEGANVVLIPAGVPRKPGMTRDDLFNT

NAGIVRDLTDAVASVCPNAMVGVITNPVNSTVPIASEVLKKRGVYDERRVFGVTTLDVVR

SNTFISEAKGLDVSKTNVPVIGGHSGITIIPLISQCTPPVSFPMEERTKISTRIQNAGTE

VVDAKAGAGSATLSMAYAAAEFCKSLLEAISGGEGVVQCAYVRSDETEARYFATPVLLGK

KGVEKNLGIGKLIEFEVNLLKAAMPELMANIQKGEEFVEKTYSVK

>SG-AL_contig_2414_1_-2_86_403

MNAVAPNNNDVRRPNKVTRYKAQSTGGPLDDPTPEYMNLLGMVFSMCGLMMKMKWSAWVA

VYCSFISFANSRTSEDTKQMLSSFMLSISAVVMSYLQSPQPMMPPW

>SG-AL_contig_2415_1_-3_3345_3572

MLCACTCVYGPPTVQCTLYCCFGVDVLHLVRCTSHYCVGLDVPVYLTFLVYSVLCTVVHL

CTDCVLSLCLLFLWTK

>SG-AL_contig_2416_1_+3_3_107

LLRIHISKYAVECDSSLHHNKHSVSTRVTIIIKST

>SG-AL_contig_2417_1_+1_52_288

MFETGKTAQVAAEMRCYNLAILGISESRWTGSGQRRLATGELLLFSGHEEEDAPHTKGVA

LMLSRAAQGALVGWEAHGP

>SG-AL_contig_2418_1_+3_69_260

MVLIQHTASPFCQSTNYNRDSLTCLRFFFPKHKCETTIISLHSEQTKQHHHHKQFLHHCK

TNIV

>SG-AL_contig_2419_1_-1_82_807

MKLNIAFPATGCQKLVEIDDERKLRPFYEKRISAEVSAEPLGDEWKGYMFRITGGNDKQG

FPMKQGVLTNGRVRLLLSKGHSCYRPRRTGERRRKSVHGCIVDANLSVLSLVIIKKGEGE

IAGLTDTTVPRRLGPKRASKIRHLFNLAKEDDVRQYVVRRPLQEKEGKKARSKAPKIQRL

VTPVVLQRKRARLAVKRKRAEKKRVDAADYAKLLAMRLKEAKERKMERRRSSSRKDSMRK

SQ

>SG-AL_contig_2420_1_-1_7_1095

MANAASLLAVVLYCLVASSIGQKLVLTHAPSYVSIQQQQQVKPLSASDVPNVITDTLGLS

DSPKSTWSGLKSGSLFRRPKANVLLTVVTAKKSNLDVSPQHMASFPVDADLPLLDLSPVM

NRIQSTFLDHEPLMLDLMADANSFDLNTPNDVFRKLPSTMRSVADRLLDPDSVLSRVNAG

SLNRSDAPDLSLMAELQMVHNVLDTLKTGENKKTGKAPDLFSFIISGLPAVSDAHGADSQ

KTKDAHNMVSQFIDRVTKDFRQLYKDNVVVEVLSVPAPEKTLVRKSRSLMQNSPTTTPAP

EKMPDYNLAWDYHDNFPAMFNIILWMTVVLSITVFVIAYGMWNIDPGRDSIIYRMTSQRL

KKD

>SG-AL_contig_2421_1_-1_1_237

PPPPPPYRPLLKACGVCFPDCILIFVMALGLIQVTQLSPVHSCVDMYINYIIRITKTFLF

LFIPAFQCSVVNSVCNFIH

>SG-AL_contig_2422_1_+2_863_1051

MQLCDQQVLKEYSTVILNPLQLSTVANSLFSFRPIIKIWIQGMNMIMCASLSLTERKQTS

SYL

>SG-AL_contig_2423_1_-2_2_136

LCNCFMLVHLCKLFCSSCGLSRFAKLPAVERLLPCVLMTNLDSCQ

>SG-AL_contig_2424_1_-3_222_473

MAAVFQIAVHFCAPSALFWVFLDMKTCIIWALPSVTFVQLLYASTSAAGSSAQVVHCLEI

CIAKLPAVERLLPCVLMTNLDSCQ

>SG-AL_contig_2425_1_+2_407_751

MTVKCQLLNCTLKPVAKVVNYFWSPNFESHVLKMTTPTVPNFNSNFKITVSPVWKRLQLY

ILYRKKKKSSETVVFSVVRISKTVRLDLLTLSFPELYAIFAKKSPASSKKSQKFK

>SG-AL_contig_2426_1_-2_2_178

VIVLFCFFVFFVRLVIFCAKTANSSRSERIEIICHELCSFAGLFVFRFDEVLCSMRLSS

>SG-AL_contig_2427_1_-3_108_257

MLCILRTNRKQRTIEVVSVNFSTSRCMVTVLNSFLDTSFRCIQSSVLHIL

>SG-AL_contig_2428_1_+3_405_659

MQSVWTSCKKDPMGPNATILKNKCLASQLTTSTFCATMTSHFSIFLHLRQLELWPQRNKY

PNIHRKKNRQPLQTWTFQQTEAMSH

>SG-AL_contig_2429_1_+1_1_315

CENKNVWSVFLVSPASFYLESASTFSSSCYHSSFFQICSKNISLSVALSLIKILSITSVQ

VFHHQQHSDGVDICHDIHSCYSLGIKLRLAIDLLVMTLSLSMSSL

>SG-AL_contig_2430_1_-2_74_202

MALNLDNSNRSALTHQTYACNQRRLHTICKMTYTNWGKEVVVE

>SG-AL_contig_2431_1_-3_45_479

MMSMGNGLGIVPALQSVGAAGLGAKGTVVAGLMSYTAADKMVHGESADAKTVGCVTAGLT

AAAAAPYVIPAAAATVGFTKAGVAAGSTAATMMSMGNGLGIVPALQSVGAAGLSAKGAVV

AGLMGYTAADKMVHSESDPEVKSKL

>SG-AL_contig_2432_1_+1_1_405

KIYNKNRSKEQKTVNDPRTQKDYRRESEEFSLYLHQKKEDGLGGDPPSTNQTGHYTTQVS

TTDTVVTALFTVSPPHPQSGTTTTTARRRRGKRVGCYIQVNMAVVSIFFFFFFFSVKASG

SKGHALKFFFFFPII

>SG-AL_contig_2433_1_+3_3_293

LNTPNGCAHDIPTALKIIYSVPKGVRRYYDILTTVDALPSSCSKWLEKLGTNIPRKRVFT

KIHKIQKFKLKWLQIRIVHSILGTNIILKEMGLVNNF

>SG-AL_contig_2434_1_-1_1_123

CFYKNTFSLQGLLNQIMNLCVYFHQHQTRLRFQLICTSDTF

>SG-AL_contig_2435_1_-3_1578_1781

MKGLSCPLQFESSSTNHLSFFVVHVSTASAWHLFLYHHLMNTALLYSSPTTLHGGFSVLN

NTGGFFVS

>SG-AL_contig_2436_1_-3_3_491

PTPTPHSRQCVLLYIAAIVNHQWPVSTLIAVVVSPVLWVCSVSLACGPPAVPCSILFVGC

CTWVSLLKSANPWHVSLEGSGRWHTLSVRVTWIGGAVLCSLQAAALDMRASLYIVYTSNC

GHKVCSCHRVAVPLLCFAVVQHPLHEDTQFQKKLCRATSIPSI

>SG-AL_contig_2437_1_+1_196_294

MDLVSFSLAQYFSAVVYIQAVQSSLNCRCFFFL

>SG-AL_contig_2438_1_+1_145_243

MDLMSFSLTQYFSAVVYIQAVQSSLNRRCFVFL

>SG-AL_contig_2439_1_-3_426_584

MVRLCNTSYHCRDGLRLWSFLLIVSTVTHFPVYALSSVYVAVNVGVGCFIPYL

>SG-AL_contig_2440_1_+2_2_91

ERDGQTDCFQLSVTQVIFNTNVQFMLNIKC

>SG-AL_contig_2441_1_+2_2696_2938

MCRRTNEWCSGSDTQTVQKIAGKRKRTGIILIAYRIICDTQHQQWLMPSPQTTAPKVHSY

KTIKDTHNVQKNGQRWGEKRQ

>SG-AL_contig_2442_1_-1_151_444

MRSQVRVGQPVSLLGLAQVVCTDMSPRDAVGSAWSAGLGQLQMGARSASTVDPGPQLDEM

SPISMRWPIAHHRQTYYQLGLTLMANPLMTGGMRGVPL

>SG-AL_contig_2443_1_-2_2_163

ASEYFAKIPTTREVNNSVVSFWNKGVTATFSTLADAPEYGKKAVETTKKLLSDK

>SG-AL_contig_2444_1_-3_57_569

MMLVLLSLAFFSLMSVMAYLSLAVLTVTLSFRVYKNIMAAVQKTNDGHPFKQYLDLDITL

KEERVSRVTQTVLQHVNRSFLELRRLFLIEDLVDSIKFGLLLWVLTYIGSWFNGMTLIIL

LVVAVFSLPKVYETYKVQIDNYVNMATTQLNTILTQVQSKVPFLKKKAKAQ

>SG-AL_contig_2445_1_-2_416_1060

MNLFTQENLHRLKLGLYGGILFFSFFVIIAVPVTTNNLDFCLLYTDRKSLGSSSNCNYCV

AMAVIFGLLYALFRLVSLLLLVLGKLGPDFVLFTDLFQLIFTAVDTVAWFLVFISACILS

AGMNSFCGNYNVDCGDVSIPNDDSAVSRMRTAEAGAWISFVLWLVLMVVGIFWLFRQGKL

PFLSRPAQAGDGTSAGATGPGPQMETPPASEEPKY

>SG-AL_contig_2446_1_-3_51_620

MKKALQYSATPGMPDLVQWVKKLQKELHNPPTLGIEDPQRQLDVIITSGSLDGICKTMEA

CLQEGHNVLLESPTYSGTLSAVKPIRPNILGVRIDKDGLDPSHLREVLSQWQPSDVKNDT

NNIPKLLYVIPNGGNPTGTGLTLDRKKEIYEIARTYDLLILEDDPYYFLQFNKPYVPSFL

SLDEDGRVIR

>SG-AL_contig_2447_1_-1_1_102

LKDISVFSSSTFLSACTCKERKLNVTDLLWCLYC

>SG-AL_contig_2448_1_-3_186_302

MFNPIYFQNTSSELLFLAMDVSYKLYPALCKFKFKRTMT

>SG-AL_contig_2449_1_+1_766_1089

MTGNATSSHLSFKPATTNTDDRNITSSIPLLTVNCSILSRTLLFNNYSSFNFSPNCSFLI

FYLPNIPCSSISPNQCIVQDPLSITVHHQVFLSSKRSLLNPFPNLFTI

>SG-AL_contig_2450_1_+1_1105_1395

MKLILVKKNAVQTLEGQTGETDSKTCTNVLPFTKQHENPARDTDRTTRQSAASKTLGLNS

SVTFCEMQFAFLYHYTRQAYIYIYTHHLYFKTLIPYQ

>SG-AL_contig_2451_1_+1_1_213

FYQCQKCFILQHRQCSPSSPISPKQPTPKTRISQCCLQLQNILITNWNSSLLQQRNLKAF

STLNTVKTGQL

>SG-AL_contig_2452_1_+3_3_188

RYKKVDQLPGQLSGNYVSRNVKVGCLLLGILTYNPVSRYRKLRYKTVLLVVQETFIYLSK

LA

>SG-AL_contig_2453_1_-3_3_167

QQRGVWHNIQNSLSHPMSFQQKIKLNTCFEVDSSKIVKGVRFFPAISHRTSSSRV

>SG-AL_contig_2454_1_-2_353_3049

MWKQLWNHPFSSVVLLGICLTVATAFGTDGVCSSTQFQCANKRCISLAWHCDGEDDCHDK

SDEVNCTSTTCGLSEFKCLSDNVCIPLRWTCDDANDCVDFSDESSELCKEVTCDEDEFRC

SDKKTCIQKAWLCDGENDCEHGEDEEKCGKTTCKPDTEFQCKKNNQCISSRWVCDRNKDC

VDGSDEDDCPESLSVCEEYEFNCTNSQCIQKSWVCDGDEDCLDGTDELGCEDKEETCRKS

EFQCKTLDRMCIHISWQCDGDFDCEDHSDEKHCNITCNSDQFLCEENYCIHESLKCDGQA

DCLNGSDEINCPTVAPACANNTFDCFGDGTKCIPYSLVCDKWNDCGNFEDERTENHDPCS

VENPCNINNGGCQHICYRSPAGAICDCHTGFELANNSRTECVNVNECAVPGTCSQICIDT

KGSYKCDCHKGYELMHQHSCKATKGEPELLLADRKDLRRYHLLTKKYTLLIDDKTVEGAI

AMDFHFVSKYVFWTDVSKEQIMKVSTTNNEIEMVVSQNVSVPDGLAVDWVHGNLYWTDTG

LDHIEVATMDGSMRKILIKDGLDEPRAIALHPKHRLMFWTDWGQEPKIESCGMNGKHRKT

IISEAITWPNGLTIDYVDDRLYWIDAKLHQIGSSDLHGQNRRMILRGHQYLGHPFAVTVF

EDFLYWSDWPSESIRRYNKFQKGSVETIAQGLDTPMDIHIFHPYRQVAYENSCGSDNRGC

EHFCLPDPVTSYTCECMTGYKRDPADSTKCVEGEEPVTTTSSTTTTTTTAVPTHKPSSTS

AQTPRPNTTPKQPVVTTPQPTEPTTTTEGQPINKEDNEGEGQVAVIVIAVVALIAILVLA

VGIFVFRRYRNRNVKSMNFDNPVYRKTTMEDEKVFVDKSGSRQHLPSSMQPLHTDNELV

>SG-AL_contig_2455_1_-1_370_648

MPPPHPPHPPKKPKHTHKKTTKHAAAVQKKTVLWESVCEYVDSSSLKNMYFNIGSLYHLC

VQGPLIVWIHVKMLGRYLYLLLIYLFLLAHIYK

>SG-AL_contig_2456_1_+3_3_134

PPQQNQYITVKHKEVHVKLSYDRKLKPSENYTTSPNLTKFANFI

>SG-AL_contig_2457_1_+2_1799_2200

MSCRNSQLINQMCMSCRNSKYTLQNITERCFMKRYQIWKRITLAPSLSKSLGFFGFFVFC

FVLYFAHFEEANPNQMNTRRKHVPSFSPFNFLHEETMTDQCFEIKLANLYKQLLPPKTVL

DRIQSKLVNMQTVK

>SG-AL_contig_2458_1_-3_3_131

VQLLLLFIIIILLLLLLLLLLFMIPETACGFASAVFSVELELS

>SG-AL_contig_2459_1_+1_58_255

MGALTAHLIYSKNYKSLLSSPFFSFYKIKSLSYMFNDNDNHSTETGFPGTGMMEMFLQLL

ETQVFM

>SG-AL_contig_2460_1_+1_907_1050

MYNDIYFNKCFLTKILQLINHSRYYRYKLLHAYQVNVLNMMYELKIMK

>SG-AL_contig_2461_1_+1_586_834

MPQAMNESWVKLSLTVTHLISKFVTIHSFIARKFLCIYFIYRDTGLALCVCVCVCVCVLE

WDTWEKCGESKKKQLADNKNDKR

>SG-AL_contig_2462_1_-2_65_346

MLAADDTRRLIIGASFGGVIVLFILIAIIVKCRRRVKRRSVRRWDVMDYKYGKTKLYAPT

NDDNDDDDDFQDDHDFEMDIPDGSMQTKKLINGK

>SG-AL_contig_2463_1_-1_16_1035

MPKKVTMNRLGGGGPDYGADFSRGFVPMTQPMHVHTGGKGPGHNVEMLSKVQESLDNISL

SPRTPEYGGYQAPSFPAPTSPGGQYVPTAVRLDMERENEARRQQQMRDNPPYVPTAVRID

QEMAAAGKSLPPAHKPHAQAPWKGHHQDEEDSGPAWKTSLKPTSTKPWDADLNYVPGQQQ

APRSSGYQPGGHQGPTAAPYNPAPAPAQERSEQDGPKVVHLQYNSPMGLYSRQNVQETYA

GQTKAMAAAGSLSPGGTQMQQNHAGGAPGDRDWNQSAVFRMIHEDDHRGGGRQAHGYAPQ

PMQPQTYYKAPAPQPAPKPQVQTFQQSFRPAIDEFGTSDF

>SG-AL_contig_2464_1_-2_125_334

MLPVGQQRTVTVRLQSDGRPQRKDGQPLPLLQPGCSGCEHGRILRGHAPVRRSECTAEGL

QELSHPGLPL

>SG-AL_contig_2465_1_+3_3_116

IMIIFAVTVQASITVISYFNVNNITVVVTTATTSFCLP

>SG-AL_contig_2466_1_-2_104_316

MPQAGSGTQDPPPKPESLEGRTGVGFFCLVGIRGSLLTLPCARESWSGTSPFRGRWVGPW

DQGVGATRVSC

>SG-AL_contig_2467_1_-3_1326_1430

MKIMPCAFEAQLGVWFTLSACVQIIDIFFRFKCVV

>SG-AL_contig_2468_1_+3_189_392

MGPITSGGCRRSTLFIKYSLDHGLYHLWGVGGCRRSTLFLKHSWLRSAAGGTVSQTLAGS

VFSLAFVC

>SG-AL_contig_2469_1_-1_922_1173

MMTVTNDITTRNQSQGLRFGSCRWPPVYRAVLQWSSLWARSKCQLILLIIYFCHCRILLN

IPILHFSVNLIIIIIIIIMIKKMC

>SG-AL_contig_2470_1_-2_80_988

MSKLLRKYLLVLLLLLPTTILFVGQSKVSSNVAFADDSMEGEDDEAVVETDDGPASSDED

VTEDADTGVTDQEQEEEEGEDRALKPSPDADTVIHFVKPTGNGQELPAGKVVRVLVGFTN

KGYGEFTVESMDASFRYPQDYSYYLQNYTTQTLNRLVESKRQATFEYSFIPNEAFSGRPF

GLTVNVNYKDGQDNQFQTAVFNETIQVVESDEGLDGETFFLYVFLAALVILALLGIHQLV

TTFGKKRLSKPRPAVEMGTQNADIDYDWIPKETIKEMNRSPRRSPKQSPRQRRNQRSSGS

AEE

>SG-AL_contig_2471_1_-2_104_1609

MESASFHHVVFFVFTALFATLSRGQNVLHVSSNCHGVYYDRLGCFTRDYPFNNTPQLPQN

PNFVDTTFYLYTRSNPHQGQLLSARNESQDVGGSNFNSRKPVEVLVHGFLQNDRSPWVLE

MTHTFLEHKDVNVITVSWGAGAGFPYSQAAANTRVAGAELARLIIKLGEQKRVSPRKFHL

IGHSLGAHVAGHAGNTVRSMNSHVKIGRITGLDPADPNFSFQSPEVRLDPSDARWVDVIH

TDGAPYNTYGGYGTFQAMGHIDFYPNGGKNQPGCHTDTSVYNFVRDAYQQGVTNAEYAMA

CSHDRSILLFIKSIASQCPFTAYPCASEDEFRAGLCLRCGNRPCPSMGYGAQLYRRTGRF

YLHTLAQSPFCGYHYLVRMTLSDNMRSTSGQLRVKISGDQGQTAPLLLHDGDMTASQEIS

SVMLSTEEVGHVTNVTFTFTRGEGYLSWLSMSSSEVRMATVMVEGVNCRNILYFCPESSV

VQPGQPLQLSNGTRRPGNCFQH

>SG-AL_contig_2472_1_+1_682_948

MKYTQVFYFIWTPNKKKNIGEGKRKILRTRARTHARARAHTHTHTHMTIAQITHAYIHIQ

TLIQYTDTSMHSNLTWVFATLHVPAFIFF

>SG-AL_contig_2473_1_+1_1_240

SNIKLIFVPDVTLSKKHSSALASTWIKYAMQYSTLKRDNRTTVKRGSQIILNGKINNKLL

KTADEHVSRWQAMPRDRKST

>SG-AL_contig_2474_1_-1_1_1248

GEDGGADVASRGAGGGGGGSQSLTGDEEPVVQVFPSERPEINPQASREEQIRQQELERRR

ILQQRRRQQQQSRAGGSPQQRLSSGRQRQVYTGSQQLGSISGQPTQQYSGQRYRQRYGGQ

QTSYGGQVQPVFNGQIQPTYGQLQPVSRGQGQGVYGGGGRRFPYGYPLPDQQAGYDSECP

NTGIRIQINGIPCEQAVEYYGSYLCYRHEYTSRECCLKCQTLKNPARVGCEYGDHSGRCS

ELQPYDCYDLRNRQSCCQSCERMRIAGARSGCEYGDMTPNCDRVRQNPGLCYIPENRYLC

CSTCSTIRVTEDPECPWGDFNTELCQPFDQVGNIRINCYVQSIKRLCCQSCTRLEEWIPM

NQPGCEYGDRPVTFNTGAHRSLNCTSFMRVYGYEQCSLNQDVASNCCYTCHRYSQG

>SG-AL_contig_2475_1_+2_2_130

RERERILKGRLYIDQNRPLPVLVKCLLTNIFFSSLYVYPFSLI

>SG-AL_contig_2476_1_-3_213_806

MRGNMDRGSYRRMDRPPRGGRFGGGPRYDGFSNGGYQGPPPGYDGGFQPYHKPPGFGSGP

PGFKKPYGDYQRDYSQQMRQPPPQPMPSYAPPPPTFYEPPPSSYSNYNERSHDHSGQDYN

SSSTSSRSRRDSKRSYEQDVDDFLRRTTFGVSKYRERERRDRDRDHDRDREHRDRDRDRD

RERDRDYDRDRHRHRERR

>SG-AL_contig_2477_1_-1_160_2940

MQKIEKNQQQPKKEEGGSTAAVEREQVEAEGSREATPAAPEIGEEGEADGEGEKEGKKKK

KKKGEKEKEEKKPKNAALVAKIKEQQAKLKEEEEKRKRAEEEAERLEAERIRKIEEEKRL

EEERRLKKKQKKKEREERLKKEGKFLTEKQKQDRMRLNQMLDVIKEQGGNVPNKDEVPKK

RPFYGKKKSKDSQQKDDSEKSPSPEDERAPELPTEESVLSEEQEPKAVEEKMEIAEEKEE

VKEAWDEESEEEKEGDEEKTKGSKAKLKKQVSKESKGSEEDEEESDEEESSEEEESSEES

ESEDERTPVERGRDKVLKRQQAAEEKRTSDVLRAPVVCVLGHVDTGKTKILDKLRRTNVQ

DGEAGGITQQIGATNVPKEAILEKTKMCKEFAKGDLKLPGLLIIDTPGHESFSNLRSRGS

SLCDIAILVIDIMHGLEPQTLESINLLKSRKTPFIVALNKIDRLYGWKSYPQNDVANTIK

AQAGHTKSEFDERTKLVITQLAEEGLNATLFYENKNPKEYVSLVPTSAHSGDGMGNLVAL

ICILTQTLLAKRLAYSEELQATVMEVKALQGMGTTIDVILVNGKVIEGQTMVLVGTEGPI

VTPIRGLLMPQPMKELRVKTNAYIHQKEVLAAQGVKIIAKDLEKSLAGLPMYIAHDKDEI

EYFKREAERGLEDALQAIKLSDRGVFVQASTLGSLEALLEYLRSEKIKYAGINIGPVHKK

DIMKASIMLEHDPQYAVILAFDVEVKRDVQELADSTGVTIFTADIIYHLFDKFMQYRDDL

LKKKQEEFKHVAVFPCKLKILPEFVFNSRDPIIMGVLVHSGFLKDGTPLCVLEKEGEGVI

ELGRVGSIESNHKPVDIARQGMEVCIKILPQPGEAPRMYGRHFTADDLLVSKISRESIDA

VKDYFRKEMTKSDWVLIMELKKEFRIL

>SG-AL_contig_2478_1_-2_5_367

MSYQQTPNAPPPYQADPYHQGGPPPPGFAAPYPGAPPPGYGPPPAAGYPAGYAGPPQQAG

YGPPPGSYPPPAGSCHSHQPQTTVVYVREEDRRRDHDDSLAESCCLLACCWAALCCCLAS

D

>SG-AL_contig_2479_1_+3_2079_2588

MYRDSSHKALNTVCSFSLNYCSALTTKNSHPLRGCWELFQYKKYPPPSENLWEKFSVLSL

SLFLPFSFLSITSSSIFFCLTTLSPIFCSRQSLVPFYFLFLFYFFNFYLPVFRVCRLCSN

TCRLHSISPPTRTMSLCTYEPTQHASDTDYVRHNQAPTQKKKNSQLALSS

>SG-AL_contig_2480_1_-1_163_390

MQTMQATYNIQKHLHKQGERGSCTIHCQEKRWVFKLDLKAERVGVWRRESGSLFQADGPE

TAKERWPTVDSLNLGT

>SG-AL_contig_2481_1_+2_1457_1708

MCIFLLHPTFVTTSIFIRQLAVCAQLQTLFPFRFLDLNYQLLASVLLLFQDHLPLSLCQT

PTLSAFKSNFPTMNSTTTSFPQFV

>SG-AL_contig_2482_1_+2_146_403

MNSKTLLDGISLCRILVIRRKPCWMCRDCGPAIWVNPLKINDPLIKPLLSLVREKHSTPL

PTLPVPRFHDDELYECQSLCYSLNTM

>SG-AL_contig_2483_1_-3_153_359

MEEARHLAFLDTLVRNIFLRWYYQVNSLQESNTHRSVNFESNHNLQHKCWVVRTLSHKAY

TISTETVDK

>SG-AL_contig_2484_1_-2_2_184

AMCDFIFLSPASRCTRLLHLSGNNSEICTGGVRVLISCAVRSTVAVQLLRSLNLTNDNQV

N

>SG-AL_contig_2485_1_-2_161_1504

MPKNNKKRGAQKKGAGAGATGSHPPTDEENSVAGDNWSTASVLSDNSSAYDCGSAEEQAQ

QDGEREDNVEQENFEDRMCECIEGLLVKSGPQRKKYLEALKQCFCKKYCVDFLDKRKVTV

TDSLLKCLKKGKADEQKLAAKCLGLLALQLGGEVSQVYDEIQATLATLIADNTAAVTTRA

QCASALAMCCFINAVTSDDDVHELSNKMGLMEDVFNKLSGRNKDNGSPQATQITSELCVC

CLDAWCLLLTRTPDHFIQKKARSHISLLGDLLQNSDTELRIAAGQAIALLYDLNRYIDED

FDESVEDTEQLCEQLKELSTESTKHTARNALREQRARFRDILQTVQDKEVDGMYVKMGTK

EILMLEDWSSKVQYNAFCKALSSGILTHLQENIVLRDFFDLGAPLPAGGQTGHKMSKIQR

KYYNSQAEKARTISRGKNRDKRSVVMGY

>SG-AL_contig_2486_1_-3_3_203

VVCRLHSCPIIAAPHTVPVYSVCELSVLYVLMFGLSAERNAAFLQGLEETNPCFMQHSIF

LMMSYKL

>SG-AL_contig_2487_1_-2_41_1330

MLQQQDNIEATVSAKAAMAAGTQQAGGSRFALGDIGNIGAKVSSLTIDPSKKTGAVKKEI

LSQQPIITRQKSIRRAAASAMAPPAASTTSGATKLSSVSTSIKATALKTEIYCDIKQEPA

VIVEPVPSVAALPTAPMDISEDRSEAFSCGVLPVEDIDANDHDNPQLVSEYVNDIYDYMR

QMERQLSVRPSYLEGAEITGKMRAILVDWLCQVHHRFHLLQETLYLTVTIMDRYLQKKPV

TRNKLQLVGVTAMLVASKYEEMFAPEVADFVYITDNAYTKADIREMERDILRTLNFSFGR

PLCLHFLRRNSKAGQVDATKHTLAKYLMELTITEYDMVHVLPSHIAAAALYLSIKLLDNS

SWTPTLQHYSTYSEDELLPTAAKLASLVVKAENSKQTAIRNKYSTSKLMKISQTAELKSA

FIRQLAAQAT

>SG-AL_contig_2488_1_+3_1725_1988

MECSGEGQPFNNLRTKLCSLHDILFKNRFSDLYTNFLSFYGNNFYKSELYRKPFSFFFFS

PIIMIMNCIFHQQHSSLKYANNPKKTKP

>SG-AL_contig_2489_1_-2_23_316

MADDQVAPVGTTDIVDQQDNTTEIPDNIQKLITSEKEGGESPAKRAKVDLQSLPTKAYLD

QTVVPILLQGMSVLSKERPPNPVEFLAAFLLKNKGHYE

>SG-AL_contig_2490_1_+3_516_731

MLKTNNVVSPQCFQSDRNNVGIASRFYHKIFLYLQCVAKRNQMTSCIHLLQLIVDQTCNC

WSKGFFFCVVFS

>SG-AL_contig_2491_1_+1_1_132

FLAHELYVCAASILKKKCLRVKKKWSFTDFNQVQLLCFFVYLSK

>SG-AL_contig_2492_1_-1_1_279

LEVDTSSCCPRGDAGGSFSINSVPTFWKFCAGNFLFSVALFVSAVFVFFYFLYCRLVFFC

LPSLFHFLFSSIPSQFSLAPVYDVLSVLFCSGD

>SG-AL_contig_2493_1_-3_153_317

MVNLGDIFPDFEADTSAGEIKFHEAIKGSWAILFSHPADYTPVCTTELGRVVKLM

>SG-AL_contig_2494_1_-3_3_152

AICRQIVSKRQRFGACSKCTTTHCLSSPGVIACCSLQTSCHYSHSLLKIH

>SG-AL_contig_2495_1_+3_261_470

MPNNNNNNNTTNNYNNNNNINYYYYYHSSSSSNKNSNSFAQIRQIILMTPHKFTHNQLTS

TLSHFNHNQL

>SG-AL_contig_2496_1_-3_3_131

VLFCFSFPLLSSMYYFFTSLVTAFIVMLQTIVTYDFLLLTILQ

>SG-AL_contig_2497_1_+3_123_482

MYGRLQVCLHLVNAALKVLILLQQLLNLHLSLLDALLQVGADGHGHKLVPEACPESILLV

NLLQDHRLRVLHLLCQLQGLVLVQQAGALLRVCGRFRCVLQPFLFIPDSFSQLLIQSELS

>SG-AL_contig_2498_1_-1_241_1941

MANRPLGIENMAVSSDSSASQHNLKDSEKDESDLRTTQSNPKVDAPKKKTTFKITSVTKS

SQRGGSGDLTQDNDLDSQDDLDETVDSHTEDLSSEIYDTNSKATDTDVQDPLLTPEEVVR

ESEPKEKPARFKVVKVETKEPFKRGRWLCYDYLDAPEKPENKQEEINVNSGSSSAANSVH

YVHGVDDPSKNPLLAGATGTLQSQQQPAPGDVYLPKLQGEGLQQVQPASSSQPSLTPVSQ

SNVSFSNHVAMSSVPAPYGVQPVVSGQTMGQSMRSMGSTTNVQPLQLGQTTTSQTVSGNI

PYSSGQPQPIPTPSQGSSIPASVHSQPASVPGSVAVSQPLPPGAMQTGEYMNSMGPGQVN

VPGQPGLPPQQGLHDMSSQQPAYSLNTGAVAGMHQQQQNHRSSLPPPHSQLADGFGSDVD

IASLSGPPLGNLGILRGDGDDSSTTLGLAPLEIAVGGITTGTSADDEITEETGSSTVAID

NKIEQAMDLVKSHLMYAVREEVEVLKEQIKELMERMHKLEHENKLLRSEASPETLAKLPA

PLPPLQNPLLQQLMTSLHNPASSSSSS

>SG-AL_contig_2499_1_+1_208_369

MDILGLKSITKMNRQYRLLPKFYPSCNRTRTYTYQHAICKIFSYSSKCLDYVTA

>SG-AL_contig_2500_1_+3_1038_1268

MYPNGVLCHTVHTVFHFYRSTALYLQKCSSPTGLNISASKAHVSKWSFMSHHTHSISFLQ

VNSALPSEMFFTRHFKM

>SG-AL_contig_2501_1_+1_1618_1851

MRVNPTLSRRDRSTISHHCYHHYTAHLCFPGFFFRLFFMCAYTAHGWEMPKILRETITIF

ANDSLCFKGGSLDYSPCI

>SG-AL_contig_2502_1_-1_82_1041

MWLRPFVVQITKNACLLQRSPVLLGQKGCLFCGRPNKQGVDLESHQQQKEDTVVSQSERS

LPIITSRKLQFPSTYNPPRQAWLETLQTLEDQKLGLIDLHPDIFGMYPRLDVVHQNILWQ

RAYKRISYAKTKSRAEMPGGGRKPWPQKGTGRARHGSIRSPIWHKGGAAMGPRGPKSYFY

MLPVHYRVLGLRSMISIKMAQDDLHVVENLDIPVDDAEYLTELADVRFWGDSVLFIDDND

TLPRNTAIALSEIPHFNAMPVYGLNVYSMLKHHTLVLTLAAVEKIEERLLHHMYSMAREK

KYVPNARSPRPFIRDPVIQD

>SG-AL_contig_2503_1_+1_1_450

HHHQHQPINTITIIIITPTTATIIIITSTSLSIPSSSSSSLSSLSPAPLSSPAPASQYHH

HHHHLYHRRRHHHHQHNHHRHQYYHHHYHHRHHHHPYHCHYHHHHYHHHRRRPHQQPPPL

SPYSFPKSEMFFSLVLGVNSLGSVSSSHQP

>SG-AL_contig_2504_1_-3_201_2591

MRTKLLWGSCLVVFLFIFDVECRPREQLCRLGMFPEDGMYTVLPRHDATFVCNATCLGPL

RRGQLYWRNPRGERVPSLGANRQMERIHALGYNRWMLNRLVILRALPEDNGVYTCEIDHD

GHVMSYTAFLSVKDDLPRTDDGPRRGDIHVEADPDFGHIRPVREASQDSHRRYRTRQMGR

GHLSSLRIRYSGSVPDDQKTPQDGMGKGVIDHLEKMEAPAQVAKDNIRNDVYKAKARVLT

HDIKNDVRNTEVRVLSNDDVGKVGVRVRRASGVSSQPLMETVTMADSPEKGTDYSKIVPY

SKILTEPPSLMADSSEKGTDYSKISTESPSFDKSEPTMMMETSSQSSMPSDDGADTVIID

KYNVSLSSTQANNEKMQGSKGTTTEEPRAWTTSSSGEVEMTPVSSNITSKTTPISSNITN

KTSESSEDEKSTPVDMGSGSGTNIGGGPGSGSSSGSVSGSNSGSTASTGSDSSSASSSSG

SGSSSSAGSSSGSSGSSGSSGASGESGVQSGGGQAIPKSGSVDQSKEGEEESDFGRYGVL

AFCLALAVIFLLLVALLLCFILPRFCKQKKLHILNLGSRDDSHKERSDKSNDNSNVMLLG

ERPHKPLAPMTSATGLNGEYITSVDLDAAQSTPEGEGKEEGKEERDKKKEETAPNGTLAD

AKMAEGGEPDGAKPQESYVSTFKIGRLSSSKQKEEETPLVATHAPLEDNVFAAAPTSIPE

EMNDDGKKKTNTSSSSSSDDEDDSKSSSSDSSKDPEDGVASGQTKLPDDKDGDLMAAIHL

QISPTGGISVETAQTDV

>SG-AL_contig_2505_1_-3_69_305

MAGTDKVMKYPYTVSGKLAHFPYRWHWKNGRFVRFLGFTLLGVFPLICKIHSAVHAPGNV

RQWEEIRKKREHTHFDPVH

>SG-AL_contig_2506_1_-1_1_282

FKERDFLEGIFKFWRDAKPEAYDPKKCWELRVRQNLGTRYDSRFNVFDWDYSMHLVERGG

QIVNSREYKNWRDCGQAFTVREGTYDVPNKTLAS

>SG-AL_contig_2507_1_+1_64_342

MVSILSLTSSPSARNQLPHSVRHAITLPSFKSASVRHAITLPSFKSALKTSLFSATLRLI

TILSISSVQAFQHQQHSDAVDTCYEMRSCCSLG

>SG-AL_contig_2508_1_+1_274_519

MATMWTFNLSCPRYGLILLVVPVTLMRDGFHVQLVTASWLFSFKTKAYLVHIINLVTGCV

SSTLPSCGINFQPFQNICINFL

>SG-AL_contig_2509_1_+3_3_263

SLHWLPVQQTNYKLSLLYFKSLNDLLYLCASSRHLRSSADTRIYKLPHVRTKTYGQHSFS

YQAPSTWNQSASTFSSSCYHSSFFQSY

>SG-AL_contig_2510_1_+3_3_236

RSSHISPVLHSLHWLIEQRINYKLSLSCFKFLNDQALSCSHLYAPSCHLHSFADTRIYKL

PHVRTKTYSEHSYQAPST

>SG-AL_contig_2511_1_+3_618_932

MYNSVCSAHTPHKLCRNVTLGIIYQNEKHSAFIQSYLKHKGNLEIAQLTTQSQVARMDVN

TSRKLHYIHYLWFAIYLNNQQQTAVATRHRRQAVQNRRRTGQDKT

>SG-AL_contig_2512_1_-2_332_3904

MGEQQQQPHVTMKERLIHGGRSMFNFFKKQPPKEGEEREKLKKELFDFQKCADHGFPSKP

SALAYDPKLKLLAIGTKSGMVKVHGAPGVVFSGVHKEDVSVTNLFFLPEEGRLVSVCSDN

TLHLWEINLRSGNSVLEEVKEFSMENNKMKAVSTCCLTTDSQNLLLGTEGGNIHILNIPS

FTLADQIIYQDIVMQNVPDDFKVSPGAVEAIAVHPTDSDKFLIGYNRGLIVLWDNKESKT

EQTYNGSQQLESLTWNRNGEEFYSAHADGCYITWDAKDSSEPKEASVTPYGPFPCKGINK

LQRKSSKGGRYVVFSGGMPRASYGDRHTVTAMQGDDTHVVFDFTSRVVDFILLSRADETD

TPDIRAENDEPHSLVILAEEELVVIDLETVDWPAFRLPYLQSVHSSAITCTQHVLDVPDQ

FWQKLVDAGDAQSKSYSAREWPITGGKNLAKEPSSKDLLITGHEDGTVRFWDASGTSLSL

LYRVATSPIFGIVDHACETPSGDEEEWPPFRKVGTFDPYSDDPRLAIQKTWLCALSETLV

VAGTAGQVLVMQMEREPRTQELQAYTVSIVGDKDNFVWKGHEALTPTVGDTRFDAGYQPT

CIMQLYPPAACTALAFHSEWQLVAAGTAHGFGLFDFAQKKQVAYRCTLNVEGMSGNAGTA

VSRRRSFRKSLRESFRRLRKGQTPKQRTEQTKEKSETQHIEQTPAEGETAAATGSTTEPA

APAAEEATTEPAATTEATPAPEATPAAESTPATEPTPAAEATPEPAPEATPAPEATPAPE

ATPAPETSPAPESTPSPQATPAPPQTPTATESRQGERVVEAPADDSMTSMVRCLYFANTF

IINGGSNTPSLWVGTNGGHVYVYSLTVPASDKRSTEDVGCVLAKEIKLRHHAPVISIAVI

DSKNRVLPESLEVVHERAKAPEMEGQHSVIICSEEQLKAFTLPAMKPRWKTKVTALDGSR

LRRVAFVSFRSNSGDNYSEYDIVSLNNQGELGVYSVMSLRRQILSAALKKCDVNGISSFV

FTKNGQGYFLQSPSEFSHVSLSTSNSSRPVCMVELKEGMRPEPPEPVVEVTPPSPTEEEQ

QTVAAEQPQETEAHESIETGAEAAAYDTADISTGLNDSQVNDSRADTSTADTTIDSVREI

VTSAAGTTIDSVHEIVTETVSSINQTTTMVTTDTTVVTVTAPSPLPPEVSA

>SG-AL_contig_2513_1_-1_172_390

MKCVNDHFHWSPPIISFFICLFVSVTVPIFSLSVYLFVSVTVRVHLISSTCSRSLDTGCL

QNCKSKFCRFDIK

>SG-AL_contig_2514_1_-3_132_848

MPSSLTSILLICIVIWGPCLCQRRAPTRGQIEDHFLSTYIDWYNADIRPYQRDDDLPVRV

NVSYELLHVTNLDEEKGHLDTVGYLAMEWTDNRLTFNHRINLDSIVLRTDRIWVPDIELY

NADAPGLKMLNAHSQKVVLMTGGHVIWVPQVATRSLCFAGLEQGGQDTINCTMTFGSWVY

NANQLNLQPQRVITDDLVPNARWTVTAATMERINKTYPCCPDTPYGTVDVTLTLQRRQS

>SG-AL_contig_2515_1_+2_2_73

KKKKKISQIPILQVASKINLIIKS

>SG-AL_contig_2516_1_+1_754_963

MASTSSRAKKRLTAEQVAELTMNHDSNKKEIGGSFTTESEDFEPDSESESDSDTDVNVRA

CLRLHVCFML

>SG-AL_contig_2517_1_-2_2_112

LCLSLSHNAQTVVMQTYNGQSFVNLVFGSPEKNEYKK

>SG-AL_contig_2518_1_-3_636_2021

MYESLCSCIWANCELSEMFKCPVGVRQGCVLSPTLFSVFINQLANHMDGRGRHGVQLLPG

TMELFILLFADDVALLSTTPRGLQNQLNVLQSCCDNMKLNVNIEKTKVMVFRKRGFLGKH

EKWFYNDIRLEVVNRYCYLGFNFTTKLSAKVGTEHLVTKGKKAAVLLNRAFQKYKEMSTD

TFFKIFDAKVQPILLYSSEIWGLNRLEHIEKVHMLACKRFLGVPTRTPNKLVYGDLGRFP

VYVNSAVNCLRYWFKVLHMDPDRLPFKAYQMLMELDRSGTDCWTSKIREILCETGFNIVW

LQQGVGNVKDFLHAFKQRLVDIFIQEWSSTLKDRDRYEIYRSFKTIFEREKYVYCMDTYC

FRVAIAQARFNVLLLNNNIYRYSESDQDKACSFCRTQPENEEHLLFHCSTYTDLRLKFLG

NLIHMPLHILLNTTHVQHRHSVSRFIFHAINRRKRLLTGDEH

>SG-AL_contig_2519_1_+3_51_392

MQNSKIGGGERSPPLVCLLHGGVYNGIGRYKRDVRQTICMRDPKVSKVVVSEHGFPPPVH

CCQDIPDREGVVEGGRGCQGSEFVSSGTIWEGGGIRLLLSSNDHSREPARNGSR

>SG-AL_contig_2520_1_-1_1_159

FVRPLPFDLSSLSGPTSSRIYHRHRSRGPCGTQAPRPQQGGDLSGTCFTDGLR

>SG-AL_contig_2521_1_+1_100_603

MPLHEIVVITLQSGSDDEGGKTDVEKYTIKILEVIVCNCKRSACYNSYQFTSFIHVSTCD

VYRCLFWGFFTYVYIVVCIICDSENVYSSALSIKSLVRNYKKFCDRGLYKNVYKVLLDYL

MHGSSSQDSLLHNFKVRMFISLLHNLKVRMFIVYPFCSIFLYTVMTET

>SG-AL_contig_2522_1_+3_2055_2267

MYKRIQHIVQDVFSFKAGQFTAKRKARLESKACIQIIIEFLAGQSKTCTKPHNMYTPFSS

LAVKCIMTCIK

>SG-AL_contig_2523_1_-1_1_204

SLSLSLLLVCVVCVGSTQQLPPPGWGGTCRTLIQNCDSLTGASSIFISTMALSHPLPAHQ

LLLQVAGG

>SG-AL_contig_2524_1_-1_94_273

MNPSLTSLPIFNNKAFSEEGDCHFRFPNLSPSNVCVVLEGRYIVFASFIDSLSQGKWMYN

>SG-AL_contig_2525_1_-2_47_988

MVLRPLMDLLEGSLSMFAQVCEKTVLKRLLKELWKIVMHTMEKTVVLPPLSDPRQDMSKE

AEKNLSPKQCAVLDMALDTIKQYFHADGRGLKKTFLDKSAELQSLRYALSLYTQTTDTLI

KTFVSTQTSQDQGSNEEPVGEVSIQVDLFTHPGTGEHKVTVKVVAANGLKWQTTGMFWPL

VEVHLIGPHLSDKKRKHSTKSKSNNWSPKYNETFHFILGNEDEPDAYELHIRVKDYCFAR

EDRLIGIAVMQLRDIVEQGSCACWCTLGRRVHLDDTGFTILRILSQRTNDEVAKEFVKLK

SECRHVEEVLVQQS

>SG-AL_contig_2526_1_-2_59_352

MGCPICGPLCSRYYMIISVWGIIMLALLGIFFWVRSPALFEDVPMAKWDENGFSLDYAKK

KYEQNAINCWIAAAIYVAVFVFSFVQYKMNNRSNYEMS

>SG-AL_contig_2527_1_-1_22_3873

MKTKYLANPDYNYEKVNRASLACGPMVKWAIAQIQYADMLKRVEPLRNELNGLETQAEEN

RLKGEEVNKLIQGLEQSISKYKEEYAVLISQAQAIKADLAAVEAKVERSVALLNSLGNEK

QRWEAGSETFKQQMATIIGDVLLSAAFMAYAGYFDQQMRQNLFGNWCTHLHQAMIQFRPD

LARTEYLSNADERLNWQANALPTDELCTENAIMLKRFNRYPLIVDPSGQATEFVMNEYRE

RKITKTSFLDDAFRKNLESALRFGNPLLVQDVESYDPILNPVLNRELRRTGGRVLITLGD

QDIDLSPSFTIFLTTRDPNTDFPPDLCSRVTFVNFTVTRSSLQSQCLNQVLKAERPDVDE

KRSDLLKLQGEFQLRLRHLEKSLLQSLNDAKGNILDDDSILTHLETLKLEAAEVAKKVEE

TDVVMAEVETTSQQYIPLAQSCSSIYFTLEALQQVHFLYQYSLQFFLEIFHAALNTQQLG

SMKEYAARLSIITSSLFTETYGRVGRGMLYQDRITFAIQLCRIHLESMASEGTYDAEFNH

FLRSQEGLLLDKSGPVSAGLSQQQNSALLRLSKLPAFRNMLNEVKNNPDFQPWLDSAKPE

VDFPPLWSEEKAQTPIAKAVHGLLVIQALRPDRLIAMARIFVEKTLGSDFVHITEKELNL

SQIVETEIKGNTPVLMCSAPGYDPSSRVDDLAAELSKQMTSIAIGSAEGFSQAEKAINSS

SKSGRWVLLKNVHLAPQWLVQLEKKLHNLAPHPSFRLFLTMEINPKLPTNLLRAGRVFVF

EPPPGVSANLMRTFSTVPTSRMCKAPNERARLYFLLAWFHAIVQERMRYTPLGWAKKYEF

TESDLKVACDMLDTWIDSVAMGRTNLPPEKVPWEALRTLLAECIYGGKIDNDFDQRLLNT

FVNKLFTPESYDSSFVLVRNVDDIAGKHIGMPDGVRREQFVQFVDNMEGPQTPSWLGLPN

NAEKVLLTTLGSDMIAKLMRMQLLEEADVDIGGGISPEEEQRLADGRPAWMRTLHSSVTT

WLKLVPQSLGTMKRTVENIKDPLFRYFEREVNTGSRLLLDVRRDLNDVMQVCQGEKKPTN

HHRSMMADLVKGIIPGFWRRYTVPAGLTVIQWITDFSLRIKQLQSIVQASLASSSRELKN

LNVWLGGLFIPEAYITATRQFVAQANNWSLEELYLDVLVQDGKPQALDDCSFGVTGLKLQ

GAVCISNKLQLSTTISTDLKLTVLRWIKVDGSKNPAGRVTLPVYLNATRGQLLFTLDFDC

ADKPAGKDHGFYERGVALICSDLS

>SG-AL_contig_2528_1_-2_23_571

MSNNAIPRAFVKNAMQNGMGRYICQLQRVTLNFCKSTAASVGMRDFIEKHLIDFTRGNPG

VVVYVKPRRHRAPTLAAEYLNGRREVIKTSNMPAEEVCKWLEHLRTRSGVQIVNMPKKWH

TDTPSIQGVWHPFLNKNPSLGTTSFPAPELYSASTEEKTATDIVLEESGMKKLGSVEMIK

SVE

>SG-AL_contig_2529_1_+1_331_477

MRNNRLKECFKDKGACVSTCPMNTAWRNTYCSLEQTRRTGSTCGTVYAA

>SG-AL_contig_2530_1_-2_50_2881

MEDDKEQAPHQTTSNEPVTEREETLQEPNQHEKHGSVTEQEQPESTMREQTAEVACYTHQ

SILEIQEQNVAREPASAPGIEPERTTTTQSTTEEELGTYPTSALGEQTTAEKEDKHSPST

SVLQEPRKKVKRFPSIYSRQMSASLSGVQDNPKAWRMSFERMSFEQRMSFEQSQSQNTGD

EEDYNHDDDDEFDVFEHVEETITEQDVEEFESSAPPEPQIHGGVVLPEESRQKSEPETER

EPAQDAGKFSDIRPKLDQSLQEEWCTSAQQAKECSMSEQQEKEQEQELRTSEQEQEKEQD

HLPKDDDVSDDESSCSLSLPLSNPFAALTQQDSLSGVTSDVMSGTDQPEQEVNLKAIRKR

QIERMKNVAGDQMDEYVTSPEPGSFTLTQTQTERGQFEKQQQLHHHQQQQEQQHQQQQEQ

KQQQKQEQEQRLSSIGTTTSQFFNRSPISDAYLELYGGRLRQPATESHTPQTTPEIEPQP

QRQIPGSLTSETDILEETQKKKRKKKKEKIHEDTEQLPAVEDDNVERTVTSADDAESALT

SKPWLKIEDDEQNVYTSDNIVPESRSKKRKKKRTKKTKKMEETEDSVADDSCSHKVSTVI

SVEALETADEVAELPDDDNEKRKRKRKKKKKKGTQEEQNNSEAASEDADLAGASLAWSAT

SQQAFTTSSSNLVMGDHPAVPVSQPGLSASYSSLFFAPPLEITVPPSIATAAPPPATSLS

APVIDACPSGEDVDQLDQDTKKRRKKKKKSCSRDRGASASADLYNKPGHSVVCPPTSLPF

IHLSTEPDATQSSGEEPPLNMPLAEGEGETRLPRERTKRREQDLSSVTSPLLTPPSPVAT

RLKSRSSSRRRRRWRQRRELGEEAERSSASRSRSRQRQEEPDEPHFHLALTSEGEGGGND

EDDDVEGAMTSRGHVMHGLSALGGGRKKRKKRNNIVVVNQPKGH

>SG-AL_contig_2531_1_-1_226_585

MEKTLLFFAYSFYQLIFYLFVYIFICLDLARLIWFCLWNYKVVPQGGQRFRLPALRRFET

SVDCDTAPVSAIYSYCVVDDNRNTTALFGERVEILGLALLHVGFYVCCPYLILCNVSQNI

>SG-AL_contig_2532_1_+3_120_221

MTQIQLKKSKCMCYHSKNVITNFLFNTSIIKSKS

>SG-AL_contig_2533_1_-2_2_295

SLYFQLFGILFPPFFLWLRFSVYYFPLFSVFQCVLSLCFQCIISFYFQLFRIISLCFQCI

ISLCFQLFSMLFSLKLFSNRHIFSVLTFVFSVSAFFPH

>SG-AL_contig_2534_1_-2_125_2929

MTDSKYFTTTKKGEIFELKAELNSDKKEKKKEAVKKVIASMTVGKDVSALFTDVVNCMQT

ENLELKKLVYLYLMNYAKSQSDTAIISVNTFVKDCEDSNPLIRALAVRTMGCIRVDKITE

YLCEPLRKCLKDEDPYVRKTAAVCVAKMHDINAQLVEDQGFLDLLKDLLSDSNPMVVANG

VAAISEILETSPTAQQSLEMNIGTINKLLTALNECSEWGQVFILDAISNYNPKDDKEAQS

ICERVTPRLAHANAAVVLSAVKVIMKLMEMLDPHSDYITMLVKKLSPPLVTLLSAEPEIQ

YVALRNINLIVQKRSDVLKAEMKVFFVKYNDPIYVKLEKLDIMIRLTNQGNIAQVLAELK

EYATEVDVDFVRKSVRAIGRCAIKVEQAAERCVSTLLDLIQTKVNYVVQEAIVVIKDIFR

KYPNKYESIIATLCENLDTLDEPEARASMIWIIGEYAERIDNADELLESFLEGFQDENAQ

VQLQLLTAIVKLFLKRPTDTQELVQQVLSLATQDSDNPDLRDRGYIYWRLLSTDPAAAKE

VVLAEKPLISEETDLLEPTLLDELICHISSLASVYHKPPNAFVEGKASLRRSLPPRSQSD

NLGAEPASTVESSPGGAPAAPQATVIPGADSLIGDLLDMDLGGPTPFQQQQIYQQMPQSS

QPVQTGVVDFLGGEGLDSLLGGGGGGMDSLGGLGDIFGAPSSNSYVPPAEVWLPAAKGKG

LEINGTFARKNGQIFMELTFTNKAMQPMSGFAIQFNKNSFGLTPAAPLQIQSPLIPNSSA

SASLQLNTSGQVQKMDPLLMLQVAVKNNIDVFYFSSAVPMHVLFMEDGEMDKKEFLASWK

EIPTTNEVQYVINDVQHNADTVSQKLRNNNIFTIAKRNVEGQDMLYQSLKLSNGIKVLAE

LKIAPSNPNFTLSMKCRVLDIYTGVQGAYETILHN

>SG-AL_contig_2535_1_+3_3_212

LRKLKSFPLIQFLICLAKNIPILPVYVRTIKCSGSTSQHKSEMYPGCIKQLVPIPLIIWN

NLNILTNAIA

>SG-AL_contig_2536_1_-2_95_1594

MVLDIELFRADKGGDPAKVRENQAKRFKDVTLVDRVVEKDTAWRKLRFQGDHLNKLKNVC

SKEIGEKMKKKEPVGDNETIPEEIESNIDELTQDKLKDLTVSQIKQIRLLIDQQMKDNEQ

ALQEREAERNESLREIGNMLHESCIISNNEDDNGIERLFGDVETKKRYSHVDLVVMVDGV

DSDRGTVAAGARGYYLKGPLVFLEQALINHAMMMLYGKGYQPLYTPFFMRKEVMQEVAQL

SQFDEELYKVIGKGSEKKEEGDVVDEKYLIATSEQPIAAYHRDEWIPTESLPLRYLGYST

CFRQEVGSHGRDTRGIFRVHQFEKVEQFCITAPTDGKSWEMFNEMIGNAENFTQSLGIPY

RVVNIVSGELNNAAAKKLDLEAWFPGSGAFRELVSCSNCTDYQSRRLRIRYGQTKKMGQE

VPYVHMLNATMCATTRVICAILENNQTEDGVIVPEVLRRFMPEGLCEKIPFVKPAPIDEQ

ELKKQKKHKEAQSKKNKTEQ

>SG-AL_contig_2537_1_+2_635_1015

MGSYSLSNIQQLSKHVSSRYMGFYSLSNILWQTKGILLFVKHTLANKIRIYSVMFIHVNG

SKIDQFRSGLGSTVHNKIFFFFSKIIREHCKFLIFLLCFFFLLKCVCDRVVFQNNRFVQI

YSTDVNL

>SG-AL_contig_2538_1_-1_1213_1530

MFLIVYIRCLFGLIIFLSVCKKSFDMLIYLVLYPLQNYFMFSLRIVRLFCAIQKVKPPRA

PPWGCRGVFQNKQYPPPSGNSVLAICSILLFSLSLPFFFPLLSCFF

>SG-AL_contig_2539_1_+3_3_320

FFFFFFFFLLDFFFFFWYFCFIYNVGSCSAVLHFLFISVTSCRHPLGYFLPIFGSSCKGA

ALLSRSFFLCVLLFCVFLKQHVRLVEKNLCSSSVPNSSLPWLSKRI

>SG-AL_contig_2540_1_+2_1946_2293

MLLSFTGSNLLFKRNKKDFKYLILVILFVVVVWLFVIMTSAFSYRLEVVKSKQSKTRKFP

IFNFGRIFFRVIETSSIINDAKTVYTFTGRFSFTGLTMTFNQRKENLITFNVVRIF

>SG-AL_contig_2541_1_-1_1363_1791

MLSVVLLCFIWLVLFEMKSAWFCIEVFVLSIEMLHDINRNNFNFKKKEGSFQTAIVCTLS

FCVLYKFQLSNVCTSGEKDRPAQKGTYAPSLGSCIISLQRFRERTRQTKDVSQTGFFNGV

KKQNKTKNKCLSLSQGCNTSVIT

>SG-AL_contig_2542_1_-3_195_473

MLLLLLLLGSSSRLHRHHHHHRCHDQPAEPRKMCDQIGRQVVVSCSARIGRQLVSCSART

NHCPLAQGFCCMCACMSGVVMVELNTPVSPEHS

>SG-AL_contig_2543_1_-2_122_1558

MAVNAFSHIQDPSAKVRALADYGSHVEVDKLISPKLYFRSGREMLRMANMYCDEGNLESA

FILYSKFITLFVERLPSHPDYKTSPPTDIQDFKRKVKKVFPLAEEIKSKLKKRYTEEEEK

RQAEEEKRQAQLAIEEEKRRKEAEEQMREDEALAQKLQSEAEQQWMQEQEDKYKRLQQQE

EERLAAENKRREEEASSSGKGGSILLIPADVSGNVPSPAAISPGVPTPAQAQVPPGGNSA

MSHISSIPSVPDRELKKNLYISEQGSSSLDEIPSVPPPPYSPPIPDRSTKPADHFMSTGL

FGGSGAIRDVIVPADLMQKFINAAQSNTLRKTETMGILCGKVSGHAFKITHMFIPNQTGT

PDSCDMQNEEELIFYQDKHDLITLGWIHTHPTQTAFLSSVDMHTHFAYQKMLPEAVAIVC

SPKYQETGLFSLTDLGLDVIGRCREKSFHYHAKEPPLFESSAHAKIVSTESITMTDMRK

>SG-AL_contig_2544_1_-1_163_360

MCWAVSHFLQSSGCIQNETRADVTCANGHNFICGLPQQRDENRLPSRILKREYLSMVWQF

QSQPGT

>SG-AL_contig_2545_1_+3_108_305

MHIHERLCSSITPAQLGLNNHVGILKFFKKSQGYINCVQDFFSPLSFLSFCHFLFFSSCW

SFHIFN

>SG-AL_contig_2546_1_-1_1_162

FMLKYNQVLINGARNAHPSDCPGQLSLQADKWKCSLHLSIGNQFLTLLTVFLLS

>SG-AL_contig_2547_1_+1_34_1527

MFTSESLEPVEFKSSWKKERQMSEASAQTGDIKTEEVGCQSWRVHEEGTQTEEDSDHRLT

MAKVDEGHLLEALLSVEAMVIRCLNNNTKTRAFEDYDVSTGEEATSVTCVHTLTHSELSG

ELQVTGLSWNSTGSTLAVSFGRFDHEDWCTHRAAVATWNLDRRSVKEDKPDTVLDSPCCI

MCLEFHPENPAWIVGGNFNGEVLVWDLSQEDDLLLATSGIGDDAHREPVARVHWVKDTSS

KKKRYNAISIGGDGKILLWKVDPKRFRLKLLDGFVVMAQSLPRGMKVRGVRGDKEIGVTS

LAFSHEDPDTFLVGSESGCIFRCSMNAKGSPAGSHIMSSIPLRSPVTFTFNPHHGPVHAV

DCSRFHRHAFVSSGQDQCLRVYNLLQAQPVVTIEPGKGYLYAASWSPVCPTIFAATTESG

HLLLYDLSSPNLVPAYTVEASPGKQPVFCLQFNLKQKQLLATGDAGGNVRVFKLSKDFIT

SSAHKLDQLESLVSTGTE

>SG-AL_contig_2548_1_+1_151_348

MVNHIIYEASLIIILCLLKTKFVLMSTGHSPESASIYFWQIVIFFSKLSLDRLACCTADS

VSGDHP

>SG-AL_contig_2549_1_+2_32_886

MLSRNCEWVFVFPSTDNLRRRTNVLLLLLLDLTLSGAHYTLTMAEGGHAQPHHTARVSPP

PRLNVRKGSADWKLFRQMWDNYVIAKLDNESDAYMKALFLHTLGPDRLLIYNGKNFRDDH

KVADIIDAFENPFIGKTNETYERYVFNKRDQKMDEPFEDYVATLRTLMKTCNFCDHLKDS

LLRDCIVLGIRDQTTRERLLQEVDLTLQSCIDNELSAETPAISSQPRRNLTLKKATCKFC

AQTHILKKEECPAWGKNLQNLQEEEPHRCEMPVKEETSCCSRIRI

>SG-AL_contig_2550_1_-3_306_2294

MGSLNGYFPLFLVCAFAQMMVLATQNDHEPMVAFICGKPAMHRGVNEWISDPYTKECWLD

KKPILNYCQKMYPSHNITNVVEASYLVTIPGWPSLKGNSLHTHRVRPFRCIVDDFQSDAL

LVPQHCKFDHKHDQKVCKGFSHWEVEARDACAKDGMHCESFGMLLNCGLGTFSGVEYVCC

PNKTPTRVQPTPEVDGDDDDDDSDSDEDFDEDEEDEDDEEDDDEEDEDSWESEEDTEDDD

EEEEEEDEEEGDEDEEDDEEENDVDEGVNLYELYLKGETMPANLRNEHLKFVAARQYMHK

LQEAKNTQLMKEWTAARDHVNEVRKTDPRTAEKLNKEIADRFQRLYSAYEQEDVAEKQQI

VALHQQHVQADLNQRKRVAMDKYMRSLEKGDVMRIRKNLRNYIKAEEKDTMHTVNHYSHV

KATNAAEATRIHPHVIQHLKLCQQRMEQALEMLERYPDIERQVRPEIEEFMRKFDDMAMR

IRNVVLPDPTTSNIHSDAEDQDSAENESESSEDNNVVQPQKIDDVLDDVSTVGELHDDLS

DSFEESSEEREDEHDYERPRFRPVHQTHNRMHVQQKGLQSASSNNMAQLGTTFAIAIGSV

AIFVVLVVAVVMLRRRSSHRTNVTHGYVEVDPAASPEERHVANMQMNGYENPTYRYFEVQ

APK

>SG-AL_contig_2551_1_-2_857_1105

MIISVLRNGVLIQNFYSHQSSTKYFHCTIDKVSVHINCVVVENFKQRLMSLCNIHFAHKM

CLSGKYEKEVDVFVQHPYCFCIN

>SG-AL_contig_2552_1_-2_2426_2815

MRSVVCIRFMCSEACVLSSFGQHLSWVRKPCVFASALHFIDQIYFYAFCTPRVVETMPRL

HMKCVSSSPSDRMHSVSIDLWTGAQSCVMSYVVCACRTVVTCSPLVHVVCTGRYHDPTVL

VFILRTTWDE

>SG-AL_contig_2553_1_+2_2_145

YKRRRFFFQAFLQLEWKFKIERHIAYVNMCYTAFSIKWSYYIPLVNTI

>SG-AL_contig_2554_1_-2_62_430

MSSLGRLGLHSRVSTQSSLKHCMDASLRVLFKFPDQHHNCLQLSGLVDARIYYERKSRVQ

PCHAVSILDGTPQHHHYHPHSHHQSIVKNCPRFCGPSLPCCHGELIFVGLEVCWRAEMSS

VGE

>SG-AL_contig_2555_1_-1_97_810

MVEGGMKCVKYLLFGFNLIFVIAAIALIAVGAYVQVKLADYFDFFGNQYTGPGILLIAVG

TFIFIIAFFGCMGAVKENYCLTMSFAVLLAVVFVLMIAGGVAAYVLRNKVEDQAVTVLTE

AEKKYKINAGVTQTWDKLQRQFKCCGVKSYTEWEQLNLTTVYPQSCCVDESETECSSDAP

IPASAIKKTACEDAFITWVKDKVAIIGGVGIGLAFILVVGVLFACCLARAIRKEYEVV

>SG-AL_contig_2556_1_+3_1815_2024

MQYCFKMIQIKKRNNTVFKQNELINAEKDSRCLQYALESVCERTPQEFKREKKSARFSKQ

KSDNGLRRIL

>SG-AL_contig_2557_1_-3_12_1229

MADPKYANLPGIDVNSPDVYETSDLPEDDQSQARLSEELDSESVETVAMDTKAAYTTFKG

KGVATAGKVDFSDSVSKARKSGYYSDRTEYELGDGSAPETPLQKFQRLQHEVRELTEEVS

KIKDSMKGESGGDKLSPVSMGQQLQYLQHQLTDLHLEKLLGPDASIDLSDPQGALRQRLL

QQLGSYSPAPQGKDKAAKTPPATGADGEYVTYELYYRPEQAQFSKNARMANLEERLERLE

NTIGQNMDKVGVLTADTENKSLLGAVSVLNTKLSLLDQPNVDAVEARLQSVMHKLSQIAD

KKAAQDSPERQLKISELYELVKKWESVGDTLPQVVDRMVALKDLHEQALQFSQALTYLDT

AQQEVKASLGSHSDMLKQLQASFKENMDGIKSNFDTLDKRMQVVKK

>SG-AL_contig_2558_1_+1_253_369

MTMNKADFTFHPSDKSLLLLLYIVYSPADHYGHIRAVTT

>SG-AL_contig_2559_1_+1_112_312

MLSFPQNPFSFKTAMSHITEMPFPDNRKLYRSVSTCTKGRAGKPNTQLPALLPLITLSIA

NGTILSK

>SG-AL_contig_2560_1_-1_1336_1521

MVCLFCFPKSTLSFIYGLTEKLLNFHPILPQVERGEGRKSDVKVDILQHNLIMSHVLFHA

VV

>SG-AL_contig_2561_1_-1_52_234

MVPVSVLHSLKNFEMVPISLHSQTLKGVVYYPQLGGCWPLISLGFVLFFSNEPDSNFTCA

I

>SG-AL_contig_2562_1_-2_2_142

KSVKRDFRGEIFLSKLQVPSSLYNSSYKDPLIKEAALLVGWCCLCLN

>SG-AL_contig_2563_1_-1_154_1440

MTGETLALILSCGIAALLLLVVVVVVWTRRRRRKPFDLSLEEINTDAVTVKSAPSSRSPS

PKLPKRLSCPDALSYPKRNMLVRSMTVDKIPDFTLPPERIQPRANGERGNNGTALQYQQS

LIGSLQPDLYSHSSSYSEDEDNYLPMSTYGRLWYSLVYDAAVEQLNVTLIKVKDLPGRGT

HRYPRDPFVKLFLLPDDRSCRTSKVRKKTLSPIFNENFVFQVTSEEVKERVLRFSVYDVD

KRKVRHSLGHVMVPLRDVDLGHVDTYWSDLESMSQALSSSLGELQLTLSYLPQTEKIKVG

VIRARNLKHTDMDLDTRLYLKLQLFYGRKVYRTKRTGSRNGSLDVEINESLSFSVSGKQL

ESCRLEIALMMTSQRGAGMLTHDVEYGRVVIGPFMYARGEELIHWQEMMAQPRSVITRWH

SLTATATHD

>SG-AL_contig_2564_1_+3_861_1187

MHALAEKLLSCFTSWGTVTLKNLPYFHSYPPNIVLLSLTNLLNLQNPHPRGADFVMRPTD

TCPHTHTHTHARTHTHSRLPTHAGAHTHTPCQYKSFYSLGQGREQTGNV

>SG-AL_contig_2565_1_-3_207_575

MSIGVPIKVLHEAEGHTVTCETSTGEVYRGKLIEAEDNMNCQFSNITVTFRDGRTSQLEN

VYIRGSKIRFLILPDMLKNAPMFKRVQGRGGGAGRGKSAILRAQAARGRGRGGRGGNVFQ

KRR

>SG-AL_contig_2566_1_-2_1136_1339

MTSAKQNLLHHIFKIQNTYAIHPHLQQRIYTRYMHGIPSPPTTQKTIYTHEKTCINVMNK

EEEIVNFT

>SG-AL_contig_2567_1_-1_94_1371

MWAIGVLAYVLLSGLSPFAGEDDLDTLQNVKQCDWEFDDDAFSNVSQEARDFIRALLQKQ

PQKRMTVHDALEHPWLKGDHSDLTKVIPSSRYSKIRQKIKDRYADWPQPMPAIGRIANFS

SLRKHRPKEFQIYDSYFDRKEAVPRFVRRLRNQVAAEGQTAKFDCKIIAASAPIITWFKD

DGALTQSYKHMQKYSGQEYELRISRIKMEDKGEYCIRAENSFGRKEEKANLKVEPGVDAR

VTPSREMTPRRKRIFEELKVEKPKEDAPPNFGFELRPRFIQAGTEFKLLCEVQAHPVPKV

TWTKDGREIHDGDSYNITYSHGVCSLEVVFARPEHSGRYVCRAVNTLGEHETGCKVEVEE

RAFYKPKEITSNVSRMVRRTTTGATVDQSSSSHESSSYSSTTTTRHSSRVEESSYTSSSS

SRTERK

>SG-AL_contig_2568_1_+2_302_577

MMMMWSVEELERVFFDVHPWPPSVTFDIHGARRRGIPADSAIVDATGTAVGCGDGVLCHS

RVTFARCPITLQHRDTLPVAKVVSCNTPRTSD

>SG-AL_contig_2569_1_-2_110_1441

MTGVRVVLLSLVVVGASAFYGPGDDVVELNPNNFHSKVIQSDELWVVEFYAPWCGHCQSL

TPEWKKAASALKGIIKVGAVNADEHGGLGSQYGVKGFPTIKFFGLDKFKPQDYQGQRTAS

GIVDFSISQAKSAASSRLSGKKSGGDSKSRSGGGGGGKPGDPKDVVELTDSNFDEEVLDY

DGLVLVEFFAPWCGHCKNLAPHWAQAATELKGKVKVCALDATVHTVMSNRYGVRGFPTIK

AFPAGKKDGEAEDYDGGRTASEIVAWALDKVAANIPPPDVYEITSAQVLKDNCEGHQLCI

VSVLPQIYDCQSECRNNYLKILRSLGEKFKKHQWGWLWAEAVAQPALEEALEVGGFGYPA

LAAVNTRKMKFSLLRGQFSEKGINEFLRDLSYGKGSTSPLKKAELPKIESRDPWDGKDAE

LPQEEDIDLSDVDLDDMDERKVEL

>SG-AL_contig_2570_1_-2_17_2323

MAWSPEESGLHQILQLLKESQSPDTATQRAVQQRLEELNRYPDFNNYLIFVLTKLKTEDD

PTRSLSGLILKNNVRAHFEKFPPEVTNFIKQECLNSIGDLSPLIRATIGILVTTIVAKGE

LRNWPELLPHLCQCLDSDDYNVCEGAFGALQKICEDNAETLDSDAVNRPLNILIPKFLQF

FRHQSPKIRSHAIACVNQFIISRTQALMVHIDTFIQNLFFLAKDEDTEVRKNVCRALVML

VEVRMDRLVPHINQIIEYMMERTQDSDDGVALESCEFWLSVAEQPVCREVLAPYIERLLP

ILVKGMKYSEIDIILLKGDVEEDEMVPDKDSDIKPRFHKSRTHSQKYQEGAEEDGEGSED

GFDDDDSLSDWNLRKCSAAALDVLANVFHEDVLPILLPILKETLFHAEWEIKESGILVLG

AIAEGCMNGMVPHLPELVPYLISCLSDRKALVRSITCWTLSRYAHWVVGQPHDLYLKPLM

TELLKRVLDGNKRVQEAACSAFATLEEEACTELVPYLGFILETLVYAFSKYQHKNLLILY

DAIGTLADSVGHHLNKPEYVNLLMPPLIQKWNQLKDEDKDLFPLLECLSSVATALQSGFL

PYCEPVYRRCVHLVEQTLNQNYANLSQPDQYDPPDKDFMIVALDLLSGLAEGLEQHIDSL

VSNSTILKLLYQCMQDPMPEVRQSSFALLGDLTKACFQHVRPCIGDFMPILAANLNPEFI

SVCNNATWAIGEISIKMGVDMQSSVNMVLGPLVEIINRSNTPKTLLENT

>SG-AL_contig_2571_1_+3_801_1100

MLACCSRCYTEIHEPHQNISGYKYRNRILWSGYTPVPYHQVKPFGHKTRLAFSSTQMNKS

AETDQHIICPNVSVKSKNQNVKYSHAIPLSTDNNFYTEYN

>SG-AL_contig_2572_1_-1_505_741

MEVYGVNFYCCVISFFDVCFNKSLFPCLFLLKKTQTGSKLPMLMVQKRIIMCAFFICFAA

ILITCYMFLMSLWLIFHVV

>SG-AL_contig_2573_1_-3_291_1007

MVEGGMKCVKYLLFSFNLIFVIAAIALIAVGAYVQIKLVDYYDFFGNDYTGPGILLICVG

VFIFIIAFFGCMGALKENYCLTMSFAVLLSVIFILMIAGGIAAYVLRNDVEDKVVDILLT

AEKNYKKETGVTETWDRLQREFTCCGVENYTEWQSFNLSTTYPRSCCADESDTAKCPKDS

PIDVAELNRTPCEDAFVSWLKDKVAIIGGVGIGLAFIMVVGVLFACCLARAIRKEYEVV

>SG-AL_contig_2574_1_+3_3_131

KTWTDGVKETLTSHGVSLLQTFQLAADHQLFLTTTLMMVKADR

>SG-AL_contig_2575_1_-3_3_116

VFFTFFFFLSQRGLGFGGEKLETATTNIYLRKDHNHRG

>SG-AL_contig_2576_1_+1_28_3984

MAALMRSARFFSPAVQIPKLLRVVSSGTPHPSIGLSLKKRSTVDQRSWRSTVVEGMETET

NTKLETMVSTVERYVRRFGRISRKQLDPIVSCLDKEGSISVSNSLSLLRLYGSSLFDEPP

ANRLKLADELWEKFKKHGITLEVMHYNTLLAVYNMNKLRFSPTEFLASMEANGVTPNRIT

YQRLITRFCEAGDIEGATQVLEHMKSASLPINDHVFNSFITGHLRSNDPDNARNIVEVMR

QTGLEPTSATYTVLAMGFAERGEIEEVKKYINQAEMENVHLRPGQLLDVYVALAVSGHSQ

HLAEVVSFLHSGTPFNQDAIRVCNQLIAAGYDDAAYQLFLEMPKPSRLDVDQTTLGRFFL

RALAKHERPVEKVVTMVKDMQERDFNRDDLSAVAQLAYLEGKPAYALTVLEKMKEEGLPV

RAHYFWPALCQFRDAKNKEGIYEVMRKMSVLCNTRDDFIQTLHGYTIPALLAADETDFIG

NLEKCGFDVKDIHFSFFLFLLEQLKLQEALEFLEKHEVSVYMASVRACLLSLVGRDKDWH

PMLQILTKLKEQSGVWRAPALECTALVLKQILMKQKKADWADTQDVFNIMLDNDLKILKR

SQVDLLEYFDGAPSEVQQGLKDVVSTDRATNTAEKEPSEFAVPADLSLKELKNLAEQKPN

NKAVLKRLLIKACAVGDAEEAERVRSVLEGEGFVFPPIMLAQLAYLYATFVKDLAKASAY

LQQLEETFPEHENYQTVVLKMAALHAEAGDTDAVVDRLKSFAEKHAAVVERQDVKREENF

SDILRSAADVDTAKRLMAVLTDCGYLPSDSPFLLDTYMEKVLNSGDAERILQEFEEIVKT

HKRSPQVDKVLQFFINKEDPDSLQKAVDLLTSVHGLMNVLHHLIADFIECGHVKKARKIM

ETAGLRANMQRLNYYCQSFIERNMIKELEMLVEVTKDMFAVDRDNMLFHLIRGYVKQKEM

DKAKDVLLKYEEEMIQPSARTLRFLARSLERAGLPVPFEVPDFQPPVNTQREARAKTLEV

KGESVATEVRVTTLATALQVKDSLEKGDVDQVLTMLKSDKTLLAQAMSVMRKPWPSAALQ

SLFESLVQQGEVDILMAINEYILPFHLKNSLRAHIFTAAVNSGRGEEVIERMEGVTSNTA

KFMTFPALDTLSDNAPHLMDKLESLCRRLESEKKYTEPLSVLWCYYKYRDSPRADVIMKE

TPGFNKTVLTGALCQTAVKENKVDVLESLLKLKKDAGEKQGMAVVYSNLLHLYVEQNNVE

AVEKTCEKIVEEGLLRSNLRRTPVIRVENLLKENNKPLPAWGDDSSSDSSSSTSDEEVR

>SG-AL_contig_2577_1_+3_792_1118

MNQSSVKLRWFWTCVASKTIQYCKILVMESVVECAELTPHPTRPVQNVCSRSCEARRNSA

AQFLVKIPEIRKTTLEIIFHVSKGPAIILLRILPQTKKKSVTSNLTGSS

>SG-AL_contig_2578_1_-2_2_475

RRHRSPRGLSPRRRDWDSGRSPPPHLFSRRRQRPTPSLPSNPPPPPPGPAGGGGGGGGAG

QLGPAECGGLAPPAPGGVGLDSTSPGGGTGRGERLGGCRAPAGACEETEGPGGSRGRGAA

VAEPVREGERVSVGGVGLNPDEDAVPGMETCSGGGGGG

>SG-AL_contig_2579_1_-2_17_295

MASSFAKMAGLRTSRMLLSVLKSNTSHILVPVRTKVCTETGAILEKPVRVRFGLVKVACT

VTPFLITGGLLSREFAALLEEHELFVPDDDDDD

>SG-AL_contig_2580_1_-3_3_113

ISAGIIIFVEEKEKNDKEILRRRNARGGIILFFYTLS

>SG-AL_contig_2581_1_-3_93_209

MSSHSVSRSVISFDKDKNYFGVHIHCIICSLLICTYHFS

>SG-AL_contig_2582_1_+2_2_193

LLKPTQLARESRSQTNRRESRLVRRWIGSESNVDAGINLFISHLRLLPRLGHRPSTNALH

PPRS

>SG-AL_contig_2583_1_+1_55_270

MFTTTANFGPSLARDRETGEHTTSFQRFCFVLFETVRDRSHPLQVKQLNIVRSASTNNLQ

WIQMHFTSCKSS

>SG-AL_contig_2584_1_-2_701_1021

MHIFAHSLRSACDCDTGVLSVVSCQQAVEQGQGVLQFCQLSVSCWTGTGSSSILAVVSKL

LDREFSIVSVVSKLLDRVFNCQLSVSCWTGSFQLLVVSKLLDREFNC

>SG-AL_contig_2585_1_-1_3037_6618

MHERALNQEQVGLRQQMQEKAKAGELHVVSGSSSSSSSAADAQKTAGKRRRWDQAADSGA

PAAKKKSSAWDAAESSSATPSNSRWDETPGRPKGSETPGATPSTRQWDATPGHATPGAAT

PGRDNTPGHQASSVRKNRWDETPKADRETPGHGAGWAETPRTDRGGGDLIQETPTPGASK

RRSRWDETPGGATPSGLTPSFTPGGLTPSGMTPSGGMTPSGMTPSGAMTPSGMTPSGMTP

SGVTPTGQKAMGMVTPTPGHLMSMTPEQLQAYTWQREIDERNRPLSDDDLDTMFPPGYKV

LQPPAGYVPIRTPARKLIATPTPLVGVAGTPTGFRMQTPDNKTGVVDMQPKGNLPMMKPD

DMQYFDKLLVEVDEETLSPEEQKERKIMKLLLKIKNGTPPMRKAALRQITDKAREFGAGP

LFNQILPLLMSPTLEDQERHLLVKVIDRILYKLDDLVRPYVHKILVVIEPLLIDEDYYAR

VEGREIISNLAKAAGLATMISTMRPDIDNMDEYVRNTTARAFAVVASALGIPSLLPFLKA

VCKSKKSWQARHTGIKIVQQISILMGCAILPHLKNLVEIIEHGLVDEQQKVRTITALALA

ALAEAATPYGIESFDSVLKPLWKGIRQHRGKGLAAFLKAIGYLIPLMDAEYANYYTREVM

LILIREFQSPDEEMKKIVLKVVKQCCATDGVEPQYIKDEILPSFFKHFWNQRMALDRRNY

RQLVDTTVEIANKVGAAEIVSRVVDDLKDEAEQYRKMVMETIEKIMSNLGASDIDSRLEE

QLIDGILYAFQEQTTEDVVMLNGFGTVVNALGKRVKPYLPQICGTILWRLNNKSAKVRQQ

AADLISRIAVVMKTCQEEKLMGHLGVVLYEYLGEEYPEVLGSILGALKSIVNVIGMTKMT

PPIKDLLPRLTPILKNRHEKVQENCIDLVGRIADRGAEFVSAREWMRICFELLELLKAHK

KAIRRATVNTFGYIAKAIGPHDVLATLLNNLKVQERQNRVCTTVAIAIVAETCSPFTVLP

ALMNEYRVPELNVQNGVLKSLSFMFEYIGEMGKDYIYPITPLLEDALMDRDLVHRQTAMA

AIQHMALGVAGFGCEDALIHLLNYVWPNVFETSPHVVQAFMGAIEGLRVAIGPSKILQYT

LQGLFHPARKVRDVYWKVYNTVYIGAQDGMIPSYPRVPNDTKNQYIRYELDYTL

>SG-AL_contig_2586_1_+3_654_851

MSIRHNIQCCLRTTVLTKDDNAGDVFIRLIMIRLPQQDHSHLWYVVRSHSSLFHSAKFGR

GIQVVP

>SG-AL_contig_2587_1_-2_131_802

MAANPVVEQTLLQATQMIEQQVDAEIAKLENMEEDDFEALRRRRMEALKKQQQQKQEWLN

SGHGDYTEISTEKEFFEQSKKSKNMVCHFYRDTTFRCKIVDKHLALLAPKHLETKFIRFN

VEKCPFLVERLRIKVMPTICIAKDGKTTDYIVGFDDLGGRDEFPTEMLAWRLGRSDVIQY

SGDLLTPPWDPTKEGGQKKIFGYAKSSKTIRDDGDDDSDADDDW

>SG-AL_contig_2588_1_-1_301_519

MHSTTALASSQSKMDMQHTSALASPQSICPLWPAHSAMWLCSACPKPTASLNFGPSFYSL

TYGFEIFVLFPLI

>SG-AL_contig_2589_1_+2_125_364

MYTDTSKYIMHLKSGKGKLHNQPTYFCVTHCACYKPDENTMQPFQFAEKKVCHSGCTSCY

FTRSIPVYIVHTLKNKIPTT

>SG-AL_contig_2590_1_-1_637_786

MGTVLLVEGFGKHSVLGAMIYAVSILIDIEYYSLNVCVRQMVKKKIIKVP

>SG-AL_contig_2591_1_-3_3_236

QDHNNNNNNDNDLFDLRRSEALNCSERAISSRAETCGEPLGPLGRNIARGNYEDIETRAG

LDIACQLLGDFTACMGDL

>SG-AL_contig_2592_1_-1_715_900

MFNLFVIICLQFSFFRFIDTFIMFLRRDIEEDSLLSVCFHCISNVNGVDLCVFFSSNIEL

DE

>SG-AL_contig_2593_1_+2_2_253

RLSSDLFFFITRKHLLATCLFTFSHHITLALSISPNKQNPLNKQKISSLGSFTQEQVTHT

HIHVHTNVRMAHTHSPHTHSKNSV

>SG-AL_contig_2594_1_+1_211_345

MVLCMTLSLISTPRCTHKHKHSLEYDARIGTHNTMHTHFRCVTHT

>SG-AL_contig_2595_1_-1_13_7791

MNPGYAGRSELPDNLKALFRTVAMMVPDYALIAEISLYSCGFVQARPLSVKIVAVYRLCS

EQLSSQHHYDYGMRAVKSVLTAAGNLKLKYPEEDENILMLRSIIDVNLPKFLNHDLPLFD

GITSDLFPGVKLPAPDYDVLNEAVKENCEKMNLQCTTFFLEKIQQIYEMMIVRHGFMIVG

EPLGGKTSAYRTLAGALGDIHEKGLMDENKVQMTVINPKAITMGQLYGCFDPVSHEWSDG

VLAVSYRAFATSTTSDRKWLLFDGPVDAIWIENMNTVLDDNKKLCLMSGEIIQLAPTTNL

MFEPMDLEAASPATVSRCGMIYMEPSTLGWRPLVKSWMNKLPGTLNEMHKSILTDLFERF

VDPCLCLVRKQLKELSPTTDSNLVKALMSLIDCLMDEFHDEGKIAQLEEREIITWLESIF

FFALTWSCGSSTDNNGHAKFDKLLRELISGGMTEETRVMLNMAELTEPPMKPYLNPFPPK

GRVYDYKFVKEGSGKWVSWSEDIKDTEPIPKDAMFNEIIVPTVDTVRYTYLMDVLVRHEK

PCLFVGPTGTGKSCYINDFLLKRLDTNVYKPNIINFSAQTSANQTQNIIMSKLDKRRKGV

YGPPLNKKAIAFVDDLNMPMRETYGAQPPIELLRQWLDHWNWYDLKDTTPIKLVDLQFMG

AMGPPGGGRNPVTPRFLRHLNTITINEFDDDTMTTIFRSIMDWHISAKGFSNEFKPCVDQ

IVQGTLMVYKEAMLNLLPTPMKSHYLFNLRDFARVIQGILLSSPDTTEDPASLKRLWVHE

AFRVFYDRLVDDSDRDWLFNNSRAVVKEKMREDYDQLFSHLDFDNDGKVCEDDIRSLIYC

DFADPKSDAKHYVEVRDLEQLRVVAEGYLEEFNNLSKKPMNLVLFRFAIEHVSRIARIIK

QPRSHALLVGVGGSGRQSLTRLASHMADYDLFQVEISKSYTSFEWREDLKRILRKATETD

NHAVFLFSDTQIKQESFLEDINNLLNAGEVPNLYALDEKQEICEKMRQIDRQRDKTKQTD

GSPVALFNLFIQRVRDQLHIVLAMSPIGDAFRNRLRKFPSLVNCCTIDWFQSWPEDALQA

VASRFLEEIEMSEDERNGCITMCKHFHTSTRLLSDRFALELERHNYVTPTSYLELISTFK

TLLDRKRGEVLKQKKRYEVGLEKLESAAGQVAVMQQELTELQPQLVVASKEVSEIMVVIE

KDSIEAAKVEKVVRADEVVANEQARVAQSIKDECDADLSEALPALNAALAALDTLTPADI

TVVKSMKSPPNGVRLVMEAICVLKGVKPDRIPDPSGSGKKIEDFWGPSKRILGDMKFLEG

LHTFDKDNVPAPIMNAIRKKYITNPDFDPEKVKNASTACEGLCKWVRAMEVYDRVAKVVA

PKKQSLKAANDELAVAMKSLEVKRASLREVQDKLNKLQETLEANKAKKAELENQVDLCTK

KLDRAEQLIGGLGGEKTRWHAAAQDLSRKYINLTGDVLVSSGIVAYLGAFTSAFRQDGAH

EWLKTVVDNGLPCSPDFSLVNTLGEPVKIRAWNIAGLPTDSFSIENGIIISNSRRWPLLI

DPQGQANKWIKNMEKANNLHVIKLSDADFVRTLENCIQFGTPVLLENVAEELDPILESLL

LKQTFKQGGSLCIKLGDSTIEYSQDFKFYITTKLRNPHYLPETSVKVTLLNFMITPEGLQ

DQLLGIVVAKERPELEEEKNALILQSAENKRQLKEIEDKILEVLSASEGNILEDETAIKV

LSSSKKLANEISEKQAVAEETEKKIDEARMGYTKIAVHSTILFFSIADLANIEPMYQYSL

TWFINLFNLSIENAEKSDDLETRLSHLHNHFTYSLYTNVCMSLFEKDKLLFSFLLCVNIL

KHRKEVDEDEWRFLLTGGIGLDNPHSNPTTWLPIKSWDELCRLDNLPNFKDIRKKFISQK

DQWKVIYDSTEPHHDEFPGEWQTRLLAFQRLLVLRCLRPDKVVPAIQEFVTEKIGKKFIE

PPPFDLPKAFGDSNCCAPLLFVLSPGGDPMAALLKFADDQGFGGAKFDSLSLGQGQGPIA

LKMIEKGVKEGTWVMLQNCHLAASWMPTLEKICEDFNPEQTHPDFRLWLTSYPSTTFPVS

ILQNGVKMTNEPPKGLRFNVIRSYYNDPINDPEFFGACKQPVIFHKMLYGLCFFHALVQE

RRKFGPLGWNIPYEFNETDLRISVQQLQMFLNEYEEVQFDAVRYLTGECNYGGRVTDDWD

RRTLRTILSKFYCTDIVEKDTYYFDDSKLYYAPPEGELESYVEYTRSLPINPSPNIFGMH

DNADITKDQKETQQLFDNILLTQARASSGGGKSTDDVVDEVAADIVGKLPPNFDTDSALR

KYPTSYKQSMNTVLVQEMVRFNRLLTTVRGSLENIRKAIKGLVVMSSDLEEVVLSILKGK

IPAMWMKVSYPSLKPLGSYVNDFLARLKFLQDWYDVGAPPVFWVSGFFFTQAFLTGAQQN

FARKYTIPIDLLTFDYEVLDEKEYDTSPDDGVYVQGLFLEGARWDRKQKILAESQPKKLF

DVMPTMWLKPCKRSDLPESNSYLCPVYKTSERRGTLSTTGHSTNFVMSVNLPSEQPPDFW

ILRGVAMLCQLDD

>SG-AL_contig_2596_1_-2_1280_1528

MLRYNEKMEKLRWDKILPDFVNSMFFRFFDKNNSFLFMLFQRFCTAFPLFKFFDENGCFL

FVCFVHDTSAFLVVIILLTVLVV

>SG-AL_contig_2597_1_+1_121_228

MHRNNTKFKDPILSTPYFQNTCNSNIPLSPPLTCIQ

>SG-AL_contig_2598_1_-1_154_1494

MAAATLTSREIPPERVLQPPVNPNPQMEITYTGNTGNDMGISTMGFRAAKYNPSEWHESN

YAKYYQSFADRDQAERVRHESKRTRNETLATTNDTQADVTKKLAERCKDIDFWKAELARE

IRDVIDETDLLLAQKKRLENALRATEVPLHISTDNLNCRQRRQGLDLVQDDVEISLLKEV

EIINNVRDLLQNTIAQSEKQIRLNRDAKQNLEMDWSDKKEALEIDTGCGVLRNHHTNKQF

HDGAAKFEEIQSTPESWAQYSHDNIARGEHERMASIQLRTLIDNVLEDTSRDMREQCDTV

DVNFQKRVTEMEDAKAKLEENLKKVCTEIAEQERNIDDLKAAIRAKEDPMKVAQTRLNSR

TYRPNVELCRDPVQYALVSEVNEICQSIDALKNQLNQAENSLKDLQDNRMSLEKEISVKK

NSIFIDRDKCMTVRTRYPSTIKLQGYQ

>SG-AL_contig_2599_1_+2_2_268

LCLAQSLFPTACLQPRNYSLYQHWNTKLHNDKRLLKRISLQHITRTHIISAVQWCLQGES

CLRKWWNALRNHFFLRQCLTLSNHSLHHH

>SG-AL_contig_2600_1_+2_701_865

MQLIIRCTFIQSHWSNGGDTGLPNPPASNPPWAPCLWVKGGDFYQYPRSTHVWTC

>SG-AL_contig_2601_1_-3_534_1637

MPSRESFNVNTMSCDTHDCFSPERLREEIRTHAKTIVVDVRSQAEFTRGHIRQAINISLP

SLMQKRLKRGSLNISSVIHNNEAKERFSRCCKSHLLVLYDECCSDLNANPSSVLCLLVKK

LRQDGSKVAFLVGGFSTFQQRFPEYCQTQEVTDNTILGLCNLRISDDFTHVSGDGSADLE

NTPHMSPFPVKVLPYLYLGNAKNSADLNQLKKNGIHYILNVTPNVPNMFEDDGDFKYLQI

PINDHWSQNLSSFFPDAIAFIDEARQSKQGVLVHCLAGISRSVTVTVAYLMSRQSMCLNE

AYDFVKKCKPNISPNFTFMGQLLDFEKALHHATSCPCTSKPSSAGDDSLPASDCDSDKAS

PPFEVSPV

>SG-AL_contig_2602_1_-2_2_193

KTTTTHTHTHTHTHTHTHTHTLTQCITPFPLFFPHFCCCLITEVYRRSVKDHRHDTHAHM

YNSS

>SG-AL_contig_2603_1_+1_2599_2892

MIDGYFPNLDSHVQHKGSSLMGNTYFQGHNGNKKCSLHLNMDTLCMVNNLALNLFFEGLI

YIYLTISFTMYDRLVYKFYFTYKPALPYFINNIQQCMI

>SG-AL_contig_2604_1_+2_2_280

SFAYNLIPYEAIIPQRYIGPGLLESDSSGYNNQSEHDLVRELLCSIQHCLKIMSATWNLF

GPYRIASSIQHCLKIMSTTWNLFGPYRIACTGL

>SG-AL_contig_2605_1_-2_1301_1516

MFCISGYNYYIYLLLLLLLLHPAIQYCMYYCQHSSCFISFLCRQYCTMNNNIYTFFSFFG

PILYLFSRQQDK

>SG-AL_contig_2606_1_+3_3_134

LISQSCGLVRILYATNITIVWCWSEFYILLISQSCGVGQNFIYY

>SG-AL_contig_2607_1_+2_98_196

MTIVWWLEFYTLLISQSCGIRILYTTNITIVWY

>SG-AL_contig_2608_1_-1_13_2868

MTRPLELVAFKPFFAQVRLPYKAVRLEEVTLEIGIYNYQHFDINVTVTVRGDLGLCFSDA

AATGQQRFVYGAQLRARRTNSETVKVIPLVADSIKLTVDVRSNNGRERDVVEKTLYVVAE

GRRVQKSITFPLDPSGKYTSHHPQVISELQMVQRTDTATISNLISRYHKKQLTDIRLYMP

QEVIPGSERCQISACGDLMGDIISVSVMNGDNLFESSVLDAEEAIGNLAPSVQALLYLNT

SQFMTPLVQAKGRAHVIHGISRLLTYRKQDGSFSLTSDSPSATWLTALVLKTLCHAEQIT

FIDHESLIRTGFAWLLDQGRQDGKLEERDRRLARPSHEYDLMLSAETLIALHECDRSRNL

TDDDLDLMGRQELLLGDHLEEIVSPLVMAKTAYALTLSDPHDELTQRAVQRLSDMKRSNI

EGLHYWSVSSSENDPPNRPFWYHSGSKASSIEATAYALLVFSRQAQAYQDYAEYADYVVE

VELPSWSVNLDSIAGWLIQKRSSRGAFIGAMDTAAATQALSEYSVGKLGIQEVNLHCNVS

TNPTRNYSHAFDFNTENAITPKSLSNVPVGRQLRVETRGTGLGQMQVRVEYNVPVEANAD

CRYNVTITRRAVSYNNLNRDGDNVCRYCQMGCDDNDDDDNNSDVNRDRAAPARARQSRRR

HQRRQRRQIFSSSVSICIEVCIRSSEDMYADRTILEIQMLSGFQPIAEDLTQIYAKREQL

SLSSLIYVPERTSVEMKFNQVPHNCSTCVSFRAIERRDVKRRNPAIVTVRAHEDPEPTCV

HTYDPVVSEVNLAVYCANSEHNNQGQCQCFSGQCGQCHGRTADVSLNDIQNLVCQSQYIY

KIQQTSHNITDDWVDMRANVIATPKSGPEEVESQIQLVTPRSCTCPYRAGFRAPHYFFGS

HVNIFVDRNNREKKTYLIDQSTTFVSATTVRHRDPDRTGDFINIALGYNCRN

>SG-AL_contig_2609_1_-3_438_716

MHALLNMYYIIILCYKRVHTQGAHILHSMKIGWHVMKLSLGFEFRRGHQMISSFSIQNSY

LQGRICGVGSTKQATRLQVRILCRFRWQVQKVI

>SG-AL_contig_2610_1_+2_32_343

MMSQVDGHHTMHVSVLTLSLYHITGRWTSQLFIVCMCVCVCVCVHVCEIDREDHNLGLGS

HTLQAECILVLYPCACLVSVHACVEDLFAFILFCSPFILLTRGS

>SG-AL_contig_2611_1_+3_456_662

MKTDPAFAKAKCDFELKFEMSRFFFPQNISLSCSPKLSMQTVQSGPVCQNAHACITVDMN

TPQILVIKF

>SG-AL_contig_2612_1_-3_2574_2837

MPLGSFNLLIECKSPISFLLSVCVPFCLSLSLSLSLSVSLCLCLSLFVSLSLFLSFVDFI

SQPKLCVCVCVCVCVCVCVCEGEEGERE

>SG-AL_contig_2613_1_-2_248_550

MFLLQCFCYELWQLYTSSAVFLLRTLAVVHVFCSFCYELCQLYMSSAVFLLRTLPVLHTF

CSVSAANSGSCTHLLYRFCMFSLEVLYLLKTDQTPGITRLC

>SG-AL_contig_2614_1_+3_3_101

HTHTHTHTHTRAHARAHTHTESTYNTHTRTVNI

>SG-AL_contig_2615_1_+2_44_247

MPCDVAKPRQLPPLDSGEERFLRAHKDLDLTPHIVICSVLQEGDAEQPPQAFVFPRGGNF

AFFQDTAG

>SG-AL_contig_2616_1_-2_2_94

SLKHTDCHTTRPAYVSLKHTKRHILGLRMLV

>SG-AL_contig_2617_1_-1_124_402

MGATHQRRLCTAAGHFRGGQQCLFYSTLLRTLPTKPPTNDSNCSVTEPDQVNDPCRKGNR

HHVLTTTSPMNHRHQRPRPEIYCRHGSSRGNRN

>SG-AL_contig_2618_1_+1_100_279

MKYFAYITAISYCFKICLLPINFFLCTEKVIAVHRLLHGPSNESKRVTLRITGHLIACSF

>SG-AL_contig_2619_1_-1_1_333

IVKAALERAQSIVRGSSTLPQPGKEALSAPQALPPDSFFKPTFLNKCNWTRLLPGCPEGW

CQTVCHGRRCDQTMQACVVSQEQFLSARKGNLGPYGPIGHMFKIGYVKQFP

>SG-AL_contig_2620_1_+1_1_111

DLVEGGENLWLRIEPRTFRILCSTLRGHHSIMRYEHK

>SG-AL_contig_2621_1_-2_71_2698

MMMLKRDKKEDEEGGGNPFNNLEKSTVLQEARTFNDTPINPRKCGHILTKLLYIINQGEA

IGANEATEAFFAMTKLFQSKDPILRRLVYMGIKEMSKIANDVIIVTSSLMKDMTGKEDQF

RGPAIRALCSITDTNMLQSIERYMKQAIVDKVPAVSSAALVSSLHLSKNSQDVTKRWVNE

AQEAVNSDNIMVQYHALGLLYHIRRSDKLAVTKLVSKFSKHTLKSPYAFCLLIRIATKLI

EEEDAGSDSPMFDFIESCLRHKSEMVIYEAAQAIVQLKNTTAKDLAPAVSVLQLFCSSPK

PTLRFAAVRTLNKVAMKHPAAVTACNLDLENLITDVNRSIATLAITTLLKTGSETSVDRL

MKQISSFMSEISDEFKIVVVQAIRSLCAKFPRKHGVLMNFLASMLRDEGGFDYKKEIVEC

IIITIEENPEAKEAGLAHLCEFIEDCEHTVLATKILHLLGREGPRTPTPSKYIRFIYNRV

ILENAAVRAAAVSALAKFGGHCEELLQSCVVLLERCQMDTDDEVRDRATFYVNVLKQQQR

ALSSAYILNGLTVSVVGLERSLHQYTLEPTEVPFDMKSVPLATQPLVEQKVRDVPGLAGD

VSAKPEKSAATRQDVYQEQLAAVPELANLGALFKSSQPVEITESETEYYVQCIKHTFSNY

IVLQFDCTNTLNDQVLERVTVEVEAGEGFEVVKQIACPSLPYNKPGTTYTLLHLPEDPTS

VTGTFSCTLKFVVRDCDPNTGEPDDDGYDDEYVLEDVEVTVADHVQKVMKPNFAASWEEV

GTENELEDTYALSTMSTLEEAIKNIIQYLGLQPCERSDKVAEGKSSHTLYLAGMYRGGHD

ALVRAKLALDSSGVTMQLTVRSTDPNVSEVLATAIA

>SG-AL_contig_2622_1_+2_2_346

MHVLLSHKIITIKSRVTQPMRQMNKWKGKISSWSAFFGKIYLCLHVHPRTHTATKATEEA

LKTRVSTTQITLCPPFMPPSTSKAQKQLSASIYCMWLSVTEKCNHDNIVSLRSKA

>SG-AL_contig_2623_1_+1_1_168

WAAMWVMVSTEILFQIPLSQTGFEERTRFHGLRCRQDFFARLGHSGSPPHDGLSLL

>SG-AL_contig_2624_1_-2_155_544

MASDQQEVNSKAYPLADPSLTQTILDLVQQAASYKQLRKGANEATKTLNRGICEFIVMAA

DAEPLEIILHLPLLCEDKNVNYVFVRSKQALGRACGVTRPVIACSVTTKDGSQLVNHITD

VKDSIEKLLI

>SG-AL_contig_2625_1_-2_140_1816

MPHPRSARSDMSDGEMEGIAEAELSKLQRQFRIMENDRAAYTEESQNIIRKQKSEMCMLE

SEKQELLKELRLAESRTNQLRDEEHTDTLVTLLEAKDEHEKDIDESKEEQQDLDAKIREW

EMKIKEQHKNMGGVHMSAQHTTQTQKTVRVLENRLDQSKKRFNDCLCRNAQLRDEIESLR

NEHKRFETLYKKLEKELRSLRREMGDVIEKSTSAYDSRDDAQAKMIILKEKADKDMQQHN

TEMKELVRIIDHDRKLKEFMGIKGQERQEDPQLVAWRQKKEAMEAEKKKESQEDSVETYE

AAFHKIKELTGEDDLDLLVNRFISVEDTNFALFNYVNEQNNEIEKLNDEIQGIKDEIEEF

KEQGIELEDQRQVILKQLEEKQNRSQNEADDYEQKYKEVAKILDQLKAGIDSLFNKINCD

RSAIDDMLGAATGVTEGNMLQYLGIIEQRTNELLAIQTYINAKDADKFDPKAPGLLGEGP

SPPQQQIPVFPPAVGDEFESDGSEASEDEMRPFTRNELQNKVMKTVQKRESAARKEGFKY

DLSNAREKAQKQKQKDSKK

>SG-AL_contig_2626_1_+2_2_211

NQFAIVGFHVTLETTMSGVILKHVDHVIQLNERVIYSDHSHVFVIESSSQCEATNSAKTV

DSNLDRHGEL

>SG-AL_contig_2627_1_+2_698_919

MYFDIIYMTQRGEYQYGIIIFCPYVFLILHFSISLFFLLFLFHFVLFHISFYILQLLLRH

LDLFLLFCRSICRM

>SG-AL_contig_2628_1_+2_2_241

NPPRAPRRVGKGRNLFRSPRSTYVQTCQCLNSLRVVHARRAISNTHVKDPVSHVSVWWLT

ETRKHPACAKKVSTRQLRRT

>SG-AL_contig_2629_1_-2_2_292

DRHFRFVTTRFRLGISDLAVYSFRYKACNDHHLCPLCRESKEDEVYFVLSCKALSDIRKQ

FRQPKYYRQLCLFKLSMLMSSTNTNVLCNFSSFLYKP

>SG-AL_contig_2630_1_+3_132_278

MFFDCCVKGSQKEYFFFLNTCLAEYAYHVRLKLPPILPLFCLSMQWVYC

>SG-AL_contig_2631_1_+1_1_270

CTVLYYVLLYCIVLHCIVLYCVVLYCIVLHCIALYYVVLYYAVTCCVVLYCIMLCCVVLH

CIVLYYVVLHCIVLYCVALYCVALYCVEIY

>SG-AL_contig_2632_1_-3_123_461

MQHNTIQHNTMQHNTTQHNTIQYNTTHYSITQHNIIQYNTMQKQYNATQYSTTQHNTIQY

NTTQYNTTHYSIIQHNIIQCNTMQHNAIQHNIIQYNTIQINTIQCNTIQYNKT

>SG-AL_contig_2633_1_-1_22_1242

MSVNGINGSHYREINYHYAEECSQDDIGTFLFTSESVGEGHPDKICDQISDAILDAHLRQ

DPNAKVACETVTKTGMVLVCGEVSSKANVDYQNVIRGTVKQIGYDDSSKGFDHKTCNVLI

ALEEQSPDIAQGVHVDRDEDNIGAGDQGLMFGYATDETEECMPLTVVLSHQLNAKIAQLR

RSGELPWARPDTKTQVTVEYKYMKGAAVPIRVHTVVISAQHDEEVTLERLRKDLMEKVIT

TVIPSKYLDDRTVLHLQPSGKFIIGGPQGDAGLTGRKIIVDTYGGWGAHGGGAFSGKDFS

KVDRSAAYAARWVAKSLVKAGLCKRVLVQVSYAIGIAEPLSITVFSYGTSKKSEAQLLDI

VKNNFDLRPGRIVKDLKLRQPIYQKTACYGHFGRNEFTWEIPKKLKF

>SG-AL_contig_2634_1_+2_278_541

MIMSFVLLLFYGCWGFLFVCLFSYQAFIFYVIVITSTFHPTTHKLIKLFFLVCSCLVQEV

LRIFFWKLLTDLSGVDMRLALTSPGVKC

>SG-AL_contig_2635_1_-1_388_477

MKKRNSMSSDTSCAQCTRYLYHIKVHVPVV

>SG-AL_contig_2636_1_-3_129_305

MVIGLSPQAVKSRIVEIFISVNVELLITQLQVYLKLWRWSTLQYKIKHRCNGLTHTNKV

>SG-AL_contig_2637_1_+3_138_260

MSQQVQMQASTQTHTQYKINYYSKSCTLANFHNSCTHMETT

>SG-AL_contig_2638_1_-2_14_2629

MTMIDAKIKTDKYPNEQALLCDFELMFNNARHYNEEGSGVYQDADTLDRILRTKWRTMSQ

SRTTTSKRAKSKTSSPMSQKLQDLYESVRDFQDKVGRALSTPFIKLPLKSDYPDYYEVIK

KPMDMQRIQQKLLTGQYDNLEDMVADFVQMFDNACKYNEPESVIYKDALTLQRVVFEKKM

ELMEGTNDVPDVKALVQELIRNLFISTYNSQDDEGRCYSDSFGELPDLIERAKAEDDEQP

SERLLTFDQIKRNIDRGRYRRMDRFQEDMFRVFESARRASRTDSQLYEDAVEMQMLFIKI

RDELCKNGELLLTPALSYTEQHLQSDLEDEKKEKLPIEQKEEEEKKRSGGDTEEKGEEWK

ALQEEPSENEIVYKDQTYMVGDFVYIEPRELNLEPHIMMIEKFNTDESGARMIKGFYFYR

PNETYHLATRKFLEKEVFKTEQSHEISMSQIMGRCYVMFVKDYFKMRPEGLQDKDVYVCE

SRYSIRNRCFKKIKIWQSSRNDTVNIVPREVPLVPVRVESVFASKVDSETKEQDDGDVSV

LDKTRSNVLSEGAPEDGNTYYDQFLHSCGCLKLGDCVYVRSDRDYPLIARIDKLWTNTEG

DAYFHGSWFVRPSEIEHAPTRLFYKREVFLSSIEDTNPLGSVINKCCVLHIKDYCSSRPT

EVPEYDIYICESKYHELERSIKKLLGKGLKKHTLSPKVTDDEVFFFRKPIALQKEPSPLL

MKVSDDQSSYMETESIKDTEGDAASEILSASVEEITPVPEKPTKRKSASNRRQPSGYIVF

AGEIRKSIQNENPESSFGDISRIVGMKWRSLSKEDKEVFEERARKIADEMAAKQQEADKA

LNDSLQRSQSPWSETGGHPSPGGPATPRPSTP

>SG-AL_contig_2639_1_-2_2_190

SINYCCMEKDCTTIRKVIDRIFAEVEVTKFLVAHREYVAQNFEQFNEIRRAVTRGIIHYL

EIF

>SG-AL_contig_2640_1_-1_958_1131

MPAANVSVSFVLLTDQTVNSVQKVCLFLFPFVPFDHCNADNSAFPLLGLKTATCSYLT

>SG-AL_contig_2641_1_-1_1_105

TKMCQILSTFSINELCLLFLWTLHLITNGLKICFV

>SG-AL_contig_2642_1_-2_74_889

MLSTFAVRSGVFGSALLRSQAPRVLAVVPAGTRSKSKAAEQADQWDTANKIFYGPERDTK

NFPHPVMPAESGKVRLGFVPEEWFQFFYNKTGVTGPYVFGVGLVTTLLSKEIWVVDHGFA

EVIGFFGAVWFLTSKVGPKMAKWMDNKTDLVTEDRFVKPVLEAKESAQQIVEANEKAIWQ

EEGQKYLWEAKRENVALQLESAYRRRLNEVYKQVKSRLDYQLDVQNVKRRFEQDHMVNWI

VSNVRKSITPQQEQESIKVCIAQLKKMSTATA

>SG-AL_contig_2643_1_+2_707_1078

MHNVLNILVGLLIKSTTSMTCIPFILLVCDSMCFKCTIEYTHIYILYIYICMYMEFQFTM

RLNSNVLIIFYTCLNYRNDLNSLHVIHQRDDEDNRLYFPMVILAHWPIYIFAKIHTLTLS

PPSC

>SG-AL_contig_2644_1_+3_267_491

MSTQSMIVFMKRGTNLRLVNQDPNQSPTKVTARHSAQKQLPLLDFLHWGLGLQHSTCGLC

FQWRWGKRMELKKKK

>SG-AL_contig_2645_1_+3_894_1376

MRLPRLPSHNVPKLLTHILISLIYILISSPPTLISRSVYLSHTYFSLSLSHTHTHLCFSL

TYILISHTHTYSPLSLTRTHPSLSLTHTYFSLSLTHTHLCFFLTYSLIHSSLSHIHILHT

HLSLTHTHWIILFSLSLTHTHWISLSPLSLTHILSLSPSLS

>SG-AL_contig_2646_1_-2_80_841

MSSIGTGYDLSASHFSPDGRVFQVEYAQKAVENSGTAIGIRGKDGVVMGVEKLVTSKLYE

QGANKRLFNIDRHIGGAVAGLLADARQLVERAKEESANYRYNYGSAIPLKHLTDRVSGYV

HAYTLHSAVRPFGTSMIIASYDADGPEMFLIEPSGVSYGYYGCAIGKAKQTAKTEIEKLK

MKDMTMQDLVKEVARIIYVVHDEVKDKSFELELSWVGDVTGGKHQLVPKDIHAAAEKYAK

EALEESDDSDEEDL

>SG-AL_contig_2647_1_-2_77_628

MSEEHRQKYFPPRPRARTLYPFYQVKDGDDCLKKTLTLSAYSGTIGTTFALVAGLTSTTA

DNPALGSNVLIRLTKYGLPMFAAGGMFAATTCVVANLRGGKDDPINHFWGGLATGSVFGT

AFKSQKLGWGLGFAFGFGAMVWKAGKMEGFANINPSEFRREKTLGGHTRSYFTTRPNPEG

DQNI

>SG-AL_contig_2648_1_-3_24_674

MSSEKFESLEDDLLSVLDEIKTKERKITGSSGEERKNAVRQVERKIEEATLIIQEMEGEA

KVAPVSYRTQMISKIRHHRSEVEQSSRNLRKEGASGTRGADNYGFDREDRLDASNRAKLM

QGNQSLQQTSESIARSHQIAAETDQIGVDIIDELGTQRETLLRTKDKLTDTDANLGRSRK

ILKTMARRMMTNKLILAVIILMELAILGGLVYWKFFS

>SG-AL_contig_2649_1_-1_664_2715

MAASTDLPAFTTSKSIILAIFIHATCILNLLLLLSDSASAQRIAGPGVPVKMLSETPACG

TKFVDPSSSALLADIVLKGNVDNLEQASPVTPLLYKAKVSVQKVYKGQELLIAAASSAST

PDPNSSSARQKERVDVIEVERFGSTENRENRDNCVGHVETGQTYLFFLKSKDVALSRLSS

SSSSLSSSSSSSRSGNDTDVLLSDSPSSSIDPPRLVNTGLPYLSDLKTLKKVKKVLCPDC

SSVPIIKRLKCKHCAVKPSMKRAMSANNTKTVRTGKKLSLRCVARGSPRPSYTWIKDGAV

LYRPQMQREENSESRRQSSPSAGRGRERSGGQGGSGVFKSGTLSVRNSKHYSLLVVKKVT

PEDQGTYACLATNAVGEVEQTVRVRVVGSSNSRSNSRSRSRSRPSPSTPNLDSNLGLPPS

SSSSSSSSSSSSSSSSSGSSSSFSSSSSSSSSSSSSASSSNGEDLCALNPCYNGGSCSVA

EEAVAGPRIRCDCLPDYHGSRCQFRRRRAQESADEVGDIGDGGGDIEGNSQAVNIDNKNN

ADNNNSGSNNNNNVDETAAESSSKSTSLISPTITTNGAAATASSAAEGNPATPQTSNPTS

PRNAYLSKKQRRKYEKSSRVRYGGSDQDGAKSSKSGAPKLKVSECPDEGYCLNGGICKMV

VKLQRRFCECPRGFAGRRCERQFL

>SG-AL_contig_2650_1_-1_481_675

MIFFLHLLINGFLPRNNVERSSRWLLDNDFMILITMIVYFSQVSCSVSTLGTFEHKPFCS

ERPIS

>SG-AL_contig_2651_1_+2_2_100

NFPPINKITRYKSTQSQHKPTRLSPGKSTMSRS

>SG-AL_contig_2652_1_+2_626_847

MTVALTLNANMAVALTLNANMIVALTSNANMIVALTLNPQHDETMTVFNKLRERERERER

EKHDQIKRKLIKER

>SG-AL_contig_2653_1_+3_3_233

TVLELPHDGENENAIGNKGDVVLGAVDQSDNESSDSDYPDEDYILPSISTGRGRGHVRSG

RSGRLIVSYLILSTLNI

>SG-AL_contig_2654_1_-3_270_1013

MSFGSGSGSFTVKKFETQEEVDEKKRKRQEHWEKVRKPDDPEECPEEESRSLFEQLEANQ

ASKQQEFEEQFKMRNSVQGLEEEETKFLNYVSNRQIQLEKEREKEESAVLEQMKEAAVRR

AMEKTQPASDKKTAPSASSSSSAAKKSQQSLLMGAIKRKSTETPSEDGTGNKRPRSESEK

SANDNSPTEDRLLKLIDTTKPVAQVAGILPGIGSYDDESSDSDSFSNESDVEDYIQSKKV

IRVHVQQN

>SG-AL_contig_2655_1_-2_212_304

MYILFRIIVNTFYEEIVDRNICHIFNLVLDY

>SG-AL_contig_2656_1_+2_2_199

GSTFMVSGTNYRLYLYGQWYKLQALTLWVNWPPSVPKDYGTCANGETPRHKPGQSGKENL

YGQWYK

>SG-AL_contig_2657_1_-1_130_219

MVSSKLEDPRGAVLVSPSVKFRLDPFSYIC

>SG-AL_contig_2658_1_-2_2_301

ENRGNEGTCVGVTRWPVRDCGHRTTSDMWLYRAYSGNLYHDGELKHALPRYTVGDVITAV

LDMDARTLSFAKNDQEPQVAFEDIEASELFPVVTFYSSSP

>SG-AL_contig_2659_1_-1_724_1062

MSLSESNSMQQHQNGTSSTAVPVRHLFIRTQEQFCPCRRGSLEKYVGVAPSVPANIAKVS

CFSAVHQNKKGGKLFVPQPMYGFKHCSCRIWYHTFCAVDLSLLFHYPVHGLPA

>SG-AL_contig_2660_1_-3_264_524

MWTMPFQTCTSMFTTAGHAQLRSKYLLGCGILTPKQARKRCRHRQFNYDPIPTSSTPHSQ

DVKRFCTHAYRCIGFSWSPPPPPPNRL

>SG-AL_contig_2661_1_-1_1_1815

QKKDDSSQKKDDSSQAQENKPHEKESAAKQSKSVVPKQKPVKRCRLIVRNLSFKCKESHV

RAHFEKCGPISEVTIPLKNGKLSRGFAFVQFTQLQAAQKAVAKINGQEIMGRPVAVDWAV

AKDRFVASSQPEPDSDKDSEGSEEEEKEDMAEESSDSEEEDEEEESEEDETESEESDSEE

AEEDKSESESEESDSDEDEEAAENDRKEKAKLARKSDVDEGRTLFIRNVPFEAEEEELEE

VFEEFGDVAYVRMVMDKVTGQSRGTAFVQFADKESAEQCLAKAEESQRDGGIRLQGRPLL

IAAALNRTQAQNQKEASKTKEQKDNRNLFLAREGMIREGTQAAAGVSQEDMKKRMKIELA

KREKLKNLSIFVSSTRLCVHNLLPRVDEKELKKVIRQIVDDPAMNITECRVMRDLERRKQ

SGVCKSLGFAFINFSTHEHALKALRGLNNSTVFGDSRRPIAEFSLENKKALEAKQKRLEK

SRVQAAKKNKEADNAQPKPKPKPKAKPKAQTLVTDKIQDRMARNMHGGAPKGMPKRLGPK

IRHRKRQQEQQQGKNKKKKNQHQHQPRSKQQPKPKMMKKKRKEVTDRVDKLINKRQGKKS

RWFET

>SG-AL_contig_2662_1_+3_2076_2642

MVGSRTNNFTHSSQKVTFYSLYIILIIHSSRKLLSITSHQNVIHSPHKLFTVCNFNIHSP

CQVTFYSHSPCQITFYRQFAKFRPFCFEVKLLFNICIQFYLSFLSYSPLSVLKYLSILYR

TYFSSSLSRQFHSVILLAFSMVSLHTDRSVCIQTISSVLHINLLFTVCIQKILFSHFPCK

VTFHGQVCV

>SG-AL_contig_2663_1_-3_366_4208

MEVPLVCAVLTVLLISHQSSAYEEIENCKPAAIIRQARKTIFFQQNGDATLACIAKGDPT

LKYIWLKDGEELDLTTADRQQRLSITSGVGTLVFSPAQKQDAGVYQCKVQNPCGTSLSAR

TNLLHAHINIFPKLQGPEKVKAFLGKPLKLSCDPPESIPEAQVSWILESEEDEEEDPLFE

EEDSGMFRSVELGRRITMDYSGNLYFVSVEEEDGQNDLRYVCMASNNIVRSINTGSDKTI

EIVKLSPAEEATLLQWRSPEEVLALEGRKARFKCIFSGNPTPEVRWRRVDGGPFDEQRMV

QSDDEHEFIISDVTAEDAGKYACSGKNPLLREAKEAIFTLSVQSRPRWTKRPDDMTVGVE

DNVTMVCNADADPEASIQWFINGVPIEQAPPAKNRQQRGNALVFTNVSRDDSQVIQCNAS

NIHGYLWADAYLQVEAMAPSIVHKPAEQKVAKEQNITIACGVEGKPKPKVQWYKNDQKLL

GSRYQIMPSGDLHIDSVVERDSGLYHCTAKNRFGTARAEGNLVVREKTEIVVPPSDDQVN

QGQEAIFTCRAITDIEEQKRLRFVWLKDGVLMDTSDPRIEVHQGNLNIRDTTSKDSAFYT

CVASNDLDSDQAVAKLEVIAPPDPPSRVTMLSCMETQVDIEWEFSESQSNFSPLQEFIIE

YSTDHHPDVWREARRLPASRRQMTYNMSPWATYTFRVRARNLMGLSEPSSPTHLECRSPE

ARPYSNPKNVRTVEDRTGWLIIEWEPMNRMEHNSNDFHYIVTWQAKGVDGREEGVMMSHV

VDDWTLGRHEVNVDGIYQPYTVSVKAANAVGDAIVPPLTVTGHSGEAVPSVVPENFELDP

DQSVTSTSAAFRWDPVDTSPEKMNGKFMGYKIRYWKKNRQVDTLHEELIIIPDSKRHRRR

AGYDDKVRGQVSQLPSFSEIEADVVVVNTYFSSPGSNIVHFTTPEGVPSRVKYLEALFRG

SSHFLLEWGPPVETNGVLTGYQLGYQKVDGLILGELVIARDDLPVGQQRTTLDGLEPSSL

YRVFISAKTAQGPGPHYFVDVRTTDETSELAEPTIDQIVPGEKEANVSFSLTEAQRGERT

GSVYYLEYRRLGEHGWDREAEGATDHFWLTLEDLDPATTYEVRVVSVPKTDRNSKEFKPS

AETRFSTTGIGTKQGSFLSAAWFIGMMVAIALLLLILIIVCIVKRNRGKAYTFPPERDTV

DGTPALFSEPPPKSAKNGVNTGGPQEPEAEKIPLGEETDSMAEYGDDPVRFNEDGSFIGQ

YVDSKPPTTEGPIPSGMSHIV

>SG-AL_contig_2664_1_-3_909_2594

MMNGIHALYPLTEKGARKPKCARCRNHGMVSWLKGHKRHCAYKDCTCPKCNLIAERQRVM

AAQVALKRQQAAEDAIALGLRACVSETGLPVMTQGPLWGPGTVTPPRALAGDMVNGGGGE

GDGDGGDDGGEEVDVEDMDDLNETDLAGAVVDSASDSGRESPTTSSPINVSDDTTTAPPP

PPTIPNTPVSPSPSSSAVASTPTVNNVSLESVSPSEVITATTSSSAPHGVSSVPRSSASS

HVSKVVQHGGYNKPVRRHVTGQGDKNGTAQPTAFRPGRLSPLEILERLYPYQRRAVLELV

LQGCNGDLVKAIEHFLSAQDTVVAQQQQQQQQQASQQHKPHQHLSSHRLDTSPQCRGNPF

VNGLHGVGITPSSSSTTSSNSSLSSLRAGLGSAQFGHVTKLPYAGLKSAFTPLAGMGAGG

HSLHSAFSPHLGSLSALPGLHAADAIRSSFLQHSTHPPPHPPSHPHPHSHHHHPGTTDLL

SATPAHLAYAGLGGLGMGPTLPGMLGPPYAFHPYRLPLGRLAASPPSVRTPDKNSDRSVL

TDSDQVSDGWEEGSSPKDLKED

>SG-AL_contig_2665_1_+2_185_445

MVKYMDRTKGHTFLRKKTTANQMNKLQQTKRPQKDKYVGKKIEHTCIHTYSDYTRIQNTH

IKMTRKQKQANYSKTYKQKLKKKKITN

>SG-AL_contig_2666_1_+1_1657_1956

MPYSSMCKPSPPLTNTHAESHKHIFSSQFLKLQVHLIQRQGQYDRQQPMLIQKIKNDTFS

VDQIHSQTISNQDKRHGNYNGQCLTFKPQQMLQHLKDRQN

>SG-AL_contig_2667_1_-1_856_1116

MCWPGLEMGRGREGTYETISCDLTRMIEALRRAANRPVLCCGVELNSRHLMLRPQLYQQS

QSARCKKRGSPEVRTHGLSAWHSNQLS

>SG-AL_contig_2668_1_-1_1_153

FKRDSSHLDNSICAPPKCASNYPANSLILHKTLYAGTILNTDTTRVIGALR

>SG-AL_contig_2669_1_-2_113_1315

MFGSSQGLKRPSGLDMGDWGGGSDSGGEGKKKKKKSRWNTAVEEKTIIPGMPTTIPPNLS

KEQEQQYIVHLQIEEITRKLRSGDLGIPPNPEDRSPSPEPIYSNDGKRMNTREYRTRKKL

EDERHHLVLQAWKHNPNYRPPSDYKAPIIRVSDKVMIPQEEHPDINFVGLLIGPRGNTLK

NLEKETGAKIIIRGKGSVKEGKIGRKDGKPLPGEDEPLHAYITANNAENVQKAVDKIKEI

IRQGIEVPEGQNDLRRQQLRELALLNGTLRENDGLQKLKQIQEAQTIVTNTILCTVCGGA

GHIAQDCKEKRPGDTFRMAQIQTPTDRAKMDSEYMSLMAELGEGPPPKSTPAPTPPVQRP

PLFQNQLPPNPMAFNPPWQQQQQQQQQQQQQQQQQQQQQQM

>SG-AL_contig_2670_1_-3_3_641

FLSIYLSVHLSICLSVCLSVRLSVCLSGCLSGWLAAWLPVCLSACLPACLPACLPACLSG

CLSGCLSGCLPVCLSVWLSVCLSGCPSVCLSVWLSGCLSVCLSACLSVCLPACLPACLSA

CLPVCLSVCLSVCLPVCLPACLPACLSVCLSVCLSVCLPACLSVWLLINDHFPTTPWWSL

LLLLLLLLLLLLLLLLLLLLLLLLLLLLCVCLN

>SG-AL_contig_2671_1_-3_120_209

MFQAVIWALWDVFQNVNSMLGIVGCFSKRK

>SG-AL_contig_2672_1_-3_141_398

MLCCSVKDMLCCFAKDQKGQSPTCHQFFVLKIVGFHMYLQFIYDLFICITFVCLLLCFFY

YCFYVTVTFLLLLLAFNFNACLFHWI

>SG-AL_contig_2673_1_-3_165_311

MGIWSCSGFTKVKQGGSGDGHHLKMLALRKVEVSATSFPCDLRGQLSPP

>SG-AL_contig_2674_1_-3_555_866

MIIIQLMAVCISLFNHGLRCFLGQTPVVLCASACEHAYITKYETIVVCLLLFERLKSCNV

LCIDHVAQQGLSACDCIFCMYCVYVCLQRLSTCDVCVPAMALQW

>SG-AL_contig_2675_1_-3_87_6797

MQNLLGEFKGLYEGKLKRLDEAERSGEDINRTRVRVLQSYVKDLSEQNEVLVSTVEELER

EANDRVVILEDKLHKTSGSVKDYMAKARDLERELRNLTIEKTRAEALLEDLQSRLTRQEE

ITAQLRDDNGNLNHDIDQLISVISVARSSGQWEIDSVRFRELRVDQVFGSMHETVTKLRA

SGSDSTQVQEMRSQLRRRDDRIDALQQELKILRMEQTHERGQGDMGDYSASRLKADLRKL

EQRTEDLQYEIQTRDRKLGMLQSQVNDLNKELAVKEAENNNHVQTIKMLQDKHRSSAGER

RRSPYHEDIIMANKSEDVIRQLRAELSAIRDEHSQSVHDVSHYERRISTLESELRESKDH

RRKALDEIGALQERLRESQLSTHDHHEILKSELRRRDETIQNLRRDVLNLQEKRDTAIAE

SDQLTKKTSIAEDQLRKLKEELQRHESRVKGKDDEAAHLHAQLEDADTELKNSRSKVVRL

EGELQALRTQSSEAQTQVAKLTSSLQRLQLSSKDTRDLLTTEVAERTDALVKLRNDTRKA

EEKLQDAVTNAQHQSELTRQLRDELKQGKTKSQEIQLKLDESEREKQQLQSRYTAQAKEI

DRLQTAIQEHDLTTSQQETLYRSDIADREEEISRLKADLNVLHEKVAQSEGQLCSREETI

SRLQVQVRDMQEDLTRKDTELHKLEVDLVTTDDERKRCDDQLSRREISLQQLEHDLTLVR

EQYKDAVEENGRLEARIQAFTINAQSEHECLSSEVKRREEAFKRLRLEHVSLQETNSKYE

EKISQLQREGDQLKSQLRSAQNEIESRKAATKNLETSVEEGKSRLSAQLEEQGRLEDKMR

TLNIDLTSQQAQVEKLHHDLDNKDELIQQFRGDLECLHRQQKETTAKVLVRDDNIQKLNS

DMGKIRAKYQEKQQELMACSDEIGKQTELIKKLQDTITQCHYDLDMTRRRGEDDILHRDE

NIKHLQEELLESQTQYTACYNEVVRTEEELEKLKEENSHLAEVGNRATQTSSQLQEQLHQ

LELDLTISQEKHRTCQKEVSNRDQVILRLQTDLDTAQQKYSGCVEELNIREEEVQRLNTK

SKELQQEVRSYLEQLDERDNKIAEDDKNIQKLQHEQDMALQEVKNQQRINEQLKGDLAVS

KSNHQQEAEQLQGDLDLVRDELKTTKDELTEAQQRITDAKSTINGLNEDLERFRNLQEES

ALEINRQADTLREQEALLSESRQQGAVLESQLDTARDEAERLQANVSMYKLKYEQNNNQL

QHMEATVNSLQEKLQDSRAKELEKEQRIERLRTEVETLDGAHRDSQGQVSKCEDLIEQLT

QELSNNQNELASANSRISDCEGEIATLKVSLQDSQQEALQNEERARSFENQMLTYQNEHS

FSNQEFRNKEERIGQLEYDLEGSRADLESKLRHISDLEESMNDLQTELAQTKDQKAAAVN

EINRLEQNIQQLQLNLTSAHQRHKSECVRFEEQVDSLQQELTDARNRGQILEQEVDRKED

QIKRNEAEIASCRDDIRSKVDEIERLERGLQDMKQEMNKVQASKRQAENTVVELHSECDN

LKTDLTEARSQYKDCAQELAHHEEKLMLMEQSLQTTQDQLSQRVTEVVRHEQRNRKLQTE

LKTMRERATSFEEELREQKTVLEKARRDLGSAKEEFHGAVQEGQAYKQQAQKLHVELESS

REQERMLSDQVEQQDQILSQLQTELRAERSRHQDTGKQLQASKKACATLEEDVGTYQRQL

REFSDKVRVRDESLLKQRAELEELQQSIRSLKEELSDKEGQLRSALLDLQSSKKQMQQQS

QEIIKYEDAVAQMQSDQERIQEQLRKTQSDLMDSEHTSHELRVQLTTVQGHHKEGMEQLG

DRSRQLAAHKTELARLHQQNTSMSDEIQIYEEKVRKLHLELKKTQELNKTADEELQHFEE

RYTSLQVDLVSSQEKQKQALAELGSKEEELVVVKVELSSLQEKLKATIEESSKIRNELEL

LRNNYRSANEEIQGLRVALEESRANGDRLHRESELVVQNVNTWVQEQKMANEKLGNKIRE

QSKAIMGLTSEKEMLMDQVQKVQKQNNKLRVELEDRQAEGDKLRALQSHSAHQQVLLNQL

KGRLEEHEVEQDQDKKNKTATIEDLHHRLKSNIENTQMLNTQLSSLQKDNVRLRGELERE

MSLRQNMEMQLQTRDQTLASLRNQMDTRALRAESSSTHTDPERLTRAALSKAMEDTGMAD

PQNLDKAYWIQRVGEMH

>SG-AL_contig_2676_1_-2_29_268

MFMLDFFFSTESNSAHIVYSPTIHGCCLRGDCHDNYVENIESGDRLWCKANGNICIIGVL

MNKMKLASDTTLSPTHTHVV

>SG-AL_contig_2677_1_-1_1288_1530

MLFHVAFFVVLFCVCVVCVCVCDGRDVRAFFSMCRCGCVRCVCVSKRAFLYVFACVRKGE

SMCVCVSKRAFLYVFACVRKG

>SG-AL_contig_2678_1_-1_55_2406

MASIDEVRRLAAEFQRVQLTIAKQKLSERNVVELVSKLVELKLIEVLYTSDGKEYLTPQH

LAKEIRDELTVRGGRINLVDLQQALNVDLNHVEAKASDMVKHDRSLTLILGQLIDSSYRD

KLADEINDLLQEQGHVTVAELAKSYDLPPEFIREVVEEKMGKVIKGQVDSQDKDVLFTDS

FVLRMTAKIRGAFSALTVPTPVGSIRIKAGCQERLFHSILDDLMKQGRLAGSVSGGRQDK

TIFYPEIYTRAQNQWLDSFYAQNGYLEYDSLTRLGLGDVKNTIKKRLKGERLIMLGACCV

GPALQDQVSASVEDALANDTWVDVKTMLPSSLSRKDISELLSHILRDHHGAFMCGDTIVA

SQKLITNSGKIFSDLIRQKAEKDVRENPALLKGDGDKSSAAKMAGLEDVGREDKKEQRRK

KATAGSKKEGTGGREVKTKSTKKKGRGRDVEEDSDDDGRAAGGGGSGSRLQEAPFMNVKE

IQQVLHSQDDLADCPDVLLKDLATQLHRPLSRQYQEMAKSIFLQTSGTGTSADRKKAFGE

LQDKVSGLWTNVHLFEKGVKLFKDVDQANLSRHLLKTLCTDITNLLLNAMATEYMLSITE

DTQFTPESRLKLIGNLPKEVQAPLSKMHLALNGQSLEAFFSPMEQFCNSSHVSIMLKKPD

KRKERQLVFAHRQTLSEQLRGETEAAMALHLAVVILFQTFTQTMVHAPGRMVPTILAHLQ

DMMDQQVYKQLTNFQDLVVQRLQAEGCGDQSTVEFLTKEMDTLLPQVKDIALTTKRKTQT

PEDQ

>SG-AL_contig_2679_1_+2_1502_1642

MLYEYCSFIYKKRAFILCTDNFFEEENWNGRFKKTHLFECSTNADVV

>SG-AL_contig_2680_1_+1_439_633

MCFCLCDIVRVSVNVSLLLTRFCVCGIVRVSAVSVFVVPLCFYHFLPVCLCYCFLMFIFG

VPCLV

>SG-AL_contig_2681_1_+2_2_160

KKPRLWTQSENGMIEFPRATVEGRKVESMDGGRSLRASVFSLFNFSRYKKEDS

>SG-AL_contig_2682_1_-1_265_384

MIFSFGIHAPFSLGKQFVITWGKCPCIGITKCFPFYSWTL

>SG-AL_contig_2683_1_-3_138_764

MFEEMMALKQSEKTAAGSDAKTSNTVSGSTPSELNLNERCSYRNTDMCDDTVIDCGSNLN

SACANDKGCASTEESGTVSGTSTQSDRKIIKGPIEFLPVEYIEENRLSLEQIRELPKFTN

YEAGDLSNVLYVKNLSPRCSEHNLVSLFGRFQKETESQIVYRLMTGRMRGQAFVTFDSID

VATQAMQLVNGFVLHGKPVIVHYGKRNTA

>SG-AL_contig_2684_1_-2_164_322

MRKLFMEVNILIKRSFFCTASLMMVMIHGPQYISHSDNKWQLNYQSSLQLQLI

>SG-AL_contig_2685_1_-2_5_850

MPKIGMVPITEAWRDELKRFTDQLGDVALTKDPAKDCLPREEERPRTTMYSQETGRLIPP

PSRAMSRGNSRRGQRLTNFGGFHHIAPAPDLENVVLTMLCQILQTEDVNAVQAWLCSSGD

REKTMVMDMIKAAMAGREEYWKQYPAAEMDQGGDKKSILPPINGAVRPPTGEMNRLVLEE

GGPGAAQPSQPPLKTLMEEKEFAIDPPTNDVFKPSTAAGKPNSQQAPHPPTVLAPQPPPN

DEAGAGKAGSASPEPKLKYTKTPLKEAADTSASKQDDSDNTF

>SG-AL_contig_2686_1_+2_152_319

MLPQQLTLILPSVPWITEVFMTSLKWFQCNALDTAECQKFVTDRRVKLYTSWKELS

>SG-AL_contig_2687_1_+2_2_253

RHHELIEYLITHSVPDAKSKHVFKGGGAGGVVEVIKHVLMGRVMVVVTIVVAGDTVMTAP

HHYSNKQEDRRRLHSHLRQTQSSS

>SG-AL_contig_2688_1_-2_95_1645

MLAFARFAAPRQVFCRVWTRTYASAAPTTKLFINNQFVESKTDKWVDVHNPATNEVVTRV

PEATQAEMQSAVDAAKQAFTTWAETTILTRQQIMFRFQQLIKDNMSRLAANITEEQGKTL

VDAEGDVLRGLQVVEHCCSITSLQLGETLQGIAKDMDTMTYRLPLGVVSGITPFNFPAMI

PLWMFPMASVCGNTCVLKPSERDPGATMILMELLQEAGMPPGVVNVIHGCHDSVNFICDN

PDIKAISFVGSDQAGQYIYERGSKSGKRVQANMGAKNHGVIMPDANRESTLTQLVGAAFG

AAGQRCMALSTAIFVGESKKWIPELVERAKALKVSAGKEKGADLGPLISPESKKRVCELV

QAGVDAGAELLLDGRDIKVPGYEKGNFVGPTILHQVNPDMKCYTEEIFGPVLVSMEVDTL

EDAIKIVNSNPYGNGTAIFTTNGATARKYTMECDVGQVGVNVPIPVPLPMFSFTGSRGSF

RGDTNFYGKQGIQFYTQIKTVTQMWRGEDAQLSTGSA

>SG-AL_contig_2689_1_+1_379_759

MHKHFCLHLYSDVDRASTSQETHSNSIYRGNKTQNTHTHHHLHNFHSCSCFYDKYCTQGG

RILKTTDINITLNTHTNTEIVIPRNANLLRKSLVRLKSRKKNKRCTTQQTDEMKLSAHTI

QYKRTCN

>SG-AL_contig_2690_1_+1_352_615

MGGYVAETFCANKPDTLHPSAPVFSLNITSSSEDYYDIYSYIHLLILQRGIERMVLGKFC

NQNKIWVACTSGELHGTLLIAWFCSITE

>SG-AL_contig_2691_1_-1_1_72

VIFVILMLEIRNSNAGKRNEEMTS

>SG-AL_contig_2692_1_+2_2_181

FFFFFNYRRNARMPLLITDKTRVRLLQHRNCDYDLISSFLFPALELRTRLFLFFLFFFQH

>SG-AL_contig_2693_1_-3_3_1019

GEGQSLFSERPRPPQPDVFTVGRYPEAYEERGLRHPEPSRAERDLSHRHRHADLSLGGIP

VAERHRTEYPSYSHFPGTVRDASRHPEVESVGYPFPEPYRYRQTRWEPSQYKPFHVTERD

TVSGVSSLGKFPVRDSDREAPKLSSTRERHVRIAPYPAEICTGSRASPYPTTERDRSSDS

PSAKTPHSILSKHPKEKAHPRITSPPARDEPDASHPTSPSTIGVKQSTERKLACEGPTSG

TERSGALAEPCITGIESPWVLPHHEKGTKKATGRDSKGREPDKKGRKVGKGKEGEGSGEK

RWKPKLFFSGFWLHGPSRHRIFASKSAHPQPPVMTTCVP

>SG-AL_contig_2694_1_-3_135_1211

MGANASQLEKEIGSEQFPANEHYFGLVNFGNTCYCNSVLQALYFCRGFREKVLQYKQQQK

NQKKESLLTCLADLFYSIATQKKKVGTIAPKKFITRLRKENELFDNYMQQDAHEFLNYLL

NTVADLLQAEKKSGSKTAVTQGQDNTNTNSNANPKPDPTWVHDIFQGTLTNETRCLNCET

VSSKDEDFLDLSVDVEQNTSITSCLRGFSNTETLCAEHKYYCEVCCSKQEAQKRMRVKKL

PQILALHLKRFKYMEHLNRYIKVSYRVVFPLELRLFNTSDDAFNPERLYDLVAVVVHCGS

GPNRGHYISIVKSHGFWLLFDDDIVDKIDASQIEEFYGIPSDMQKNSESGYILFYQSRE

>SG-AL_contig_2695_1_+1_34_228

MNFVLACVTLTLDLSVQNSCLSDSSKVHTFGQIWQRSVQKQVRKSCEHISLTDTHRSRAI

LNSLP

>SG-AL_contig_2696_1_+1_40_177

MNTEFKYFLKLMRKCCSHTFLKMCYLSMRHLSWTSHFQTHALVTPK

>SG-AL_contig_2697_1_-1_1_195

RNPYLDHRNTSNHISEYSVLEFDDIFICVEKDPHYEQISITTRIQCMFDLFYNVFMLSMF

CDVSF

>SG-AL_contig_2698_1_-2_2_472

NEDTSSGVHSSILKQARRSGQLNLSGRNLTEVPDTVWRINIDVPEEAKSVSLDNTDDRWW

DQVDLTKLILASNALTHLGEGLAQLPALTVLDVHDNRLVSLPNALGDLENLQKLDISRNQ

LQELPSCIGRLSNLVSLHAEHNKLTQLCVEVVDCRKL

>SG-AL_contig_2699_1_+1_235_396

MFVCVGVKTKKSVRISQLSMDGYLPNLEVESSLPGSYECTSLKKAWHQNVKMHT

>SG-AL_contig_2700_1_-3_111_875

MWPTLLTRTPKTLISSLSRVTNLQSAVGSVRNFTVQIRHFTVLCPQQLRCRHCCVKTPVV

SLVPCAPKSETSCGFVHVQHRHWCSVSGGILSMGGQQSKSRKGSSSVQGLNFSPRQEAQP

AFSTTTVEGSSVGVVSGGSGSQRLSSTSASDMAGSVEVVACKVDELSDGDTREVQIGESS

ALLVKEKGSYYAVRNKCSHYGAPLSKGAYCNGVVRCPWHGACFNVKTGDIEDFPGLDSLP

VFQVEVRGDEVVVKA

>SG-AL_contig_2701_1_-1_58_393

MRGMFSQAADVWNFENCSRGIHHNITISCQRFLTTVEKGDIHTIVVVNYRHVLHPCLPDV

CLKGNCFLLFISCCQSLGEGCVESTASQKHYENKTLLRLLTGTVYLFAIALI

>SG-AL_contig_2702_1_-1_94_225

MIVYKVRHYEGVTVLYIATANCSYEVPVCPCQPVQTTTAIASDI

>SG-AL_contig_2703_1_-1_76_666

MTCDYSSLECLLKAPSKGEVVKLCRDVFSAVNRNTVQLPPALLIHCQQTLKLTQDQAQTF

VLSLGSILKQAVFQGSTDPHDIVALFPDGFHKNLRELIGKIIVDNMADWKSQAVGANVSL

PKLADFDWRVDVKTASDTVARMSVPTCILSLQVEAGREEGQTQGQQTVNVELSKETLDTM

LDGLSKIRDQLSSVANR

>SG-AL_contig_2704_1_+3_3_170

IIIIYCFQPPPTTMAISGLSPCKVHQSQEDNRIEHKTSSNLKTKTLQKITIHDILS

>SG-AL_contig_2705_1_+2_854_1072

MEQRWVQSQNLRKAVLTEWNATNLPHIVSTIKKQTNLFIILSPLHPISSFLNFFSPSPYP

TASCKKLVDRVRD

>SG-AL_contig_2706_1_+3_201_371

MGACRRQLIFITRMFYVNVYHKGICLCLSKEDCVFIFIIGRSLCLSKDDCHIVCYYL

>SG-AL_contig_2707_1_+1_1_294

KTVCRNIDLLQFSITTHLKHGLSPQVSFVSKELLLFEKSCLAALNYFHMNRGCGSGLTAG

CSWPTAGCSILIAGCSDVTAGCSRLTAGCGKLKLFAVV

>SG-AL_contig_2708_1_-1_1393_1743

MHAVPVHAAVNGYLTLFRLGKVKQRGRGDGHAFIMLVQREDVPTTSFLCDLRTVRQHWPL

RLCLFLLCFLCVCVCVCVCVVSLVSKTVVLLVLFFSMYNFCHFQAFSQPVESFVPSC

>SG-AL_contig_2709_1_-1_28_726

MDEEVPSGSDNIVTEFNTYEDFLDSQITSFDLYYLEDQELARQLVELGYRGSGEVLKREE

FEARKAAAEASRLSKRSQQKTLASSGKELKDPLLRALATREEANRSGKMTTIIFIRDKNA

RGQEISGYIDYAYRLKAEDFEPYFGGRKRLLPRPSDLSFYNWETQTSTSNPTPNYQVIAE

NASGLLFKNKRDRKIINVDPKAPTPGDNSTRTIIETPKYTQVVIYDHITRRKT

>SG-AL_contig_2710_1_+2_548_826

MSQRYTRTHTTTFTQLPWKNGPFSMSSNLPLTHTRCFVLELAPLLVCHAQLLWENQNDTI

CLLHRHALHYTPQDKLQIIQFCTTVSIVILVCV

>SG-AL_contig_2711_1_+2_2_259

MPAYTNTMVAYTNTMVAYTNTMVAYTELAVTLRIFLTVVNFLLLPVVQALRKGSNCNHFL

GKMKTSAPRSYFQGFSFCVCDHGKVR

>SG-AL_contig_2712_1_-1_481_1893

MAALMCRRFYLSQSRFFRDGVRTICASPIQNANAQPAKKTLTKPNVRNVVLVDGVRTPFL

QSGTSYKNLMPHDLARTALQGLLKRTGLPKDAVEYLVYGTVIQEVKTSNVAREASLGAGI

SDRVPAHTVTQACISSNQAITTGIGLITSGQCDAVVAGGVEFMSDVPIRHSRKMRRLMLS

MNKAKTLPARLQLVSQMLSPAALTPELPAVAEFSTNETMGHSGDRLAAAFNVSRLEQDEF

ALRSHTLAHEASEKGLLTDRLVYKVPGVAQPVEKDNGIRPSSMEAMGKLKPAFIKPYGTV

TAANASFLTDGASACLIMSEEKALAMGYKPLAYLRDFLYVAQDPKDQLLLGPAYATPRIL

GKAGLNLSDISVFEYHEAFAGQILANMKALDSDWFGTNMMGRQGKAGAPPMDKLNLWGGS

LSLGHPFGATGVRLVTTAANRLHKEDGQFGLVAACAAGGLGHAMIVERYPR

>SG-AL_contig_2713_1_+3_27_482

MSKTAEKKKIWKDHENAWQTLHQVMEPPANHRTVFAVADTHHSQTTNCQQSTCYNQRSVC

YNQHWHTTTTSWQLTITSSHITTTSNQSATTSSITTTSNQSATTSSWPTTTSSQPTPCPY

EKSSKQHCSLFQKVKVLNLLTEEICGLQPCFG

>SG-AL_contig_2714_1_+1_1_132

VRMCMCQCLCVCVCVCVHACVCVCVCVCMSVRETFTKLIHFKHQ

>SG-AL_contig_2715_1_-2_86_655

MCALLFCVLLLCVLLLCFHSVPSYSMSFYSVPFYSAPFYSMPFYSVPFYSVLFYSMSFYC

PFILCLFIPCPFIPCPFILCPFILCPFIPCPFIPCPFIPCPFILCPLIPCPFILCPFIPC

PFILSFYCAFILCPFIPCPFILCPFIVHPLILCPFIQLYQCMVFKLYGGLLNVDVEKNVC

VCSHQSTSCE

>SG-AL_contig_2716_1_-3_129_746

MGNLFGKKHSKKPSSKVTDQDRAILQLKQQRDKLKQYQKKVLAQLDRDRDFARKLLHDGK

KDKAKLMLRKKKFQESLLEKTDGQLDNLEKLVNDLEFAQVEHEVVKGLEVGNAALKKMHQ

ILSLEDVERIMDETREGIEVQQEIDALLSGGLSQQDEDDVMAELDEILAEDDNVKLPDVP

TDDVGEKERERAKKRRAEEREAVAAT

>SG-AL_contig_2717_1_-1_1_189

LVDFDGLKVDMPALFVIQTIYDSNNNNNYKCMLLIGPQLIISSEELAPDHHPRGGGRKIF

VPT

>SG-AL_contig_2718_1_-3_3_104

VGLLHGQQQVLLNALPRLVSWSLSSGTNSIVLWD

>SG-AL_contig_2719_1_-3_12_551

MARGGTHMKSVILPESSAAVTGAKLKPRLLHEELKDTEASYKNVLQSVMLASTSGVMRGP

TAAAAKRAEVRSSQLMKRLAEKKDFSAARDHFSGITQQEAVVSQSAGDDAGAELRRSQKD

SFVGKAVRHRKVCRNEDDMVSDALSQTQPPCFAAFPSSSPPQPAGAPSPPPPPTAYGAPP

>SG-AL_contig_2720_1_-2_227_415

MLDFSDEHKSITRYLLASSVGMLNCRLRFCLFVCVFVLFLIIFVIVLLFLVCLCFLFLYQ

LLY

>SG-AL_contig_2721_1_-3_1617_1910

MRPSQDRNCDWMQVHQSGHTQRIMLYLVSEIVTVWACFSDTVGTSSLSLVIPCLISVLRH

CLGFILLAPCDSVVDRLSKMFHGVDKFQLVEYYPAVCF

>SG-AL_contig_2722_1_-2_323_607

MFTVLQLTVSQTDPNCIQQISTQFYSYSFIDINLFSRFSLSFTDKSAFSRILTQFYRHKL

HSSSFHSILQTQSAFVCPGVDASVVRLCKECSQTV

>SG-AL_contig_2723_1_-3_51_2054

MEPCRSGSVRVPPFRCSPCPSAKQRSSLQGPATSSSGRHGPSQHHYASEDTLNHLISIIE

QLKSKDKVISQLERELGHPAVVAGPVSVATEVTRTSSSGQHSNKMETPPHSPHNVLSGLN

QVFQRLHGTGMASFPLGGEEKGAVEGFAGLEAEILGLHDRAQALAFDRQILLDKIKDFEA

KNKQLVEKNKRFLEKNIELTAQNRSLKDGSHTMQQQQKKLLEEAEECRRRQCELARELQR

VKGHNLELEAQAQEVQDLRKQIADQTSSITLLRQACTDKDRRIELIQHRKKRRRLIRSQE

KCQGVKETFYGYDDDDNDASIDSETSLSSVSVSTISEEDACDELSREELERSHRQMLREH

LQLERSHALLQAHTGSSQDPYRELQTRSQLQADLFSAQCRIELLEKEIRTLQSSRRRRGS

GMDGVPDDMDVAALLKERDDLLSARQGLQMKLEQVDAECVRLRQELRKAEERAEDLEFRI

LELEENPPCGDPHSDPNTLPPEESDFDQSNLDPHHDDDLDKSDLDVEHGHTDAADEGEAS

QAAVTECFTQTVAPTGLECLPNQSASSPTQYPAPETNASSTMEFDRLRRELKAALERQGV

QADRILKDLGGLKDVEQKMGSMERMAMEMQKKVIALQQERDHLEERLMRGAPLSINAEVE

RRQNLELK

>SG-AL_contig_2724_1_+1_1453_1680

MMAPRTIPQDLTLNHVKGRCLHCIAGRTASLDDVNGQVEGLYNRIHDQVYATGSEPLCMV

DNRSAVLLLEMTPMQS

>SG-AL_contig_2725_1_-2_83_217

MFWFYVAPILFLFNQSISRYNESPAFVCTGQTGPCRMGIALFCVV

>SG-AL_contig_2726_1_+3_759_1283

MNTKINNSHNKQTTVCSVSRRHKTAIIQVNGPLIILSGRWHMDTIILSGRWHMDTPLLQL

CTLLSTHTHTHTPAPTYNGNANRRHHHQTTRQQSPPSTENSIGNNSHTSSPSSAPTHNNN

SHTSSPSSAPTHNNNNNNNNSHTSSPSSTPTHNNNSHTSSPPSTPTHNNNSHTSS

>SG-AL_contig_2727_1_+1_136_546

MNHLLGTQKTSPLPTTHSHEQLPTVLSNYFTTKIQTLREKLDATPCRPCPPDPSFSGSPL

STFTPVSQEELLNTIRSMALKTCELDPLPGSLYSDCLPALLPFITDIINTSLETGTVPDS

FKFAIVHPLLKKHNLDP

>SG-AL_contig_2728_1_+2_2288_2518

MFYRPLRSENQHWSTCLLPLSYGPGLGLISSFSANNPGCDSLTLRTLLWVVVSTGFYAAS

VQGALFSIQCVLVSTGF

>SG-AL_contig_2729_1_+3_708_917

MTNRCQRQKSPLLTIYTAPKEANKYTKFRFSFKFPVPLIQKNQSSKTFHSSFHNHHAEKK

TSNDIELWLC

>SG-AL_contig_2730_1_+1_1_75

FFFDTSVQFNPSPISKDILNASMTK

>SG-AL_contig_2731_1_+3_3_170

PQAEDDHLKLSVMVILFFQETDSNEICSSIEEGNFLGYFAASENIYRCLRVCSQSF

>SG-AL_contig_2732_1_+2_488_901

MGGGTTLYSFSVSSCMLVFLSGLRPGAADLTNTSQSQTALACFADDFDLPVGGPKDGHST

LWWEFGSFSAFPQSHWKHLSHSSTHIPLFRLLALPSLSHALQCVYTDMCSQALTILLKVS

ISFKYYNDLFLYKVYVVY

>SG-AL_contig_2733_1_-2_50_301

MTSGLWENFYPLPSCLCFAFLSLLYYFYTKRYLNITSKFSFHIKLFHHIYTFLSFTLVFQ

PTTCLPRVHLFSMFEPFHQCCVDL

>SG-AL_contig_2734_1_-1_472_813

MQSDTQFVDKYSIVVYSAQEVSSCDDSSYHWNSASETERVELLLCSGSSPLKSSLAQASV

QPNSSYQIAAQMSSLNSRDYQSVVYCSYDSDHPSVFLVVGGGDGTYYLDELWWW

>SG-AL_contig_2735_1_-1_397_3021

MLKRLFGDALLQIAIAISILRTDLNDLAVPNNEMLNYPEVQDILQGQTLAYLPTNFHNHY

NSLHSIHGFPHPKHADDFFFSSIFNDWRFQRVSTNIEAFLVSSDESFSTLPQQAIANQQT

AQVHASGEDAGSNAASSTSSTTPPDTATASAGDVDVNNVNANHNSVFGDGDFFNDPFPGL

GLSKEDLDLIEVLWRQDVDLGVGKEVFDPNLRKELEQERELELRKEQEKRKEAEKQEREK

QQQTVQWLSENFVIDGETGEYMPISGGGNNNFTSSGPANLYSQPQQPVTLEEALSFVGDL

QIGQPCGQQPQQQQQQGVATVPPSNHLVTGAEGFHYMEQNLIGEAHSSNYVQPNQTQKER

IEQSWSEVYQHMFADNLTGFTNSSLVNMPPMINGGALQPELGSHIFQQQQQQQQQQQQYF

DSQMDLLLSECKNYSDASSMNETRRVMQENTTELESDLAGLYGDSNLQDVPLLDNLPLPS

DQSLESLQSSALEGFMTYLHEPGTNNSSYLTGDLNLTELLRTDTFSNNNTADFNLTDSLL

TDLVPDDDFDLPMSQSLGDTASDSAISMGSGSPHQDINETMEMSPFEGLEGATGGSDYDS

GNYGHHRSQMHSGSNAEEENYQYSYSSYDSPASISNCSTGSNDTEHSSGHSSIAAHSHHF

HHNHTYATQPGHTPREVKKYAKKDGPRQKGPHSRDNKRAEDLKLPFSFNRIVESPVEEFT

ELITKTPLTEAQISLMRDIRRRGKNKVAAQNCRKRKLEVLTTLEDENYETVRARDKLEQE

RQALNIQMRQEKERYNFVYNQVFSSLRDEEGRPYNPQEYCLQQASDGNILLVRKSTGSAT

SCVSSASQPSSSSKNNHNDPEQDKDSSQQKKKRRE

>SG-AL_contig_2736_1_-3_3_113

KKKIMYEIKKKKKKKKKKKKKKKKKNHLCQYSGPSSY

>SG-AL_contig_2737_1_-2_56_3217

MAAAQQLQQQQQFQGDMTPRSQVSSSGVTHGTPSTMSAQTPHCVSTSHAYLTDEASMSNR

DSEPEMGGNADKKKKTKVKRKKVIDKAPRLTVLKYEEDEQEVECLLEFANRSTVTFKFAV

ENDEPHEIVVNLKKENLLQEGQAEAVIKLLHELISMVTEDGKEAIGCCVNYIVPISNTGS

ANKTKTGEGTEFEKKLDEPVTERSRPVDDKGQTGVDERTPSKPKVIVSNTKRFIVSTVTD

PQQTDSRIAEGDEEEASTSPDNPPAVTAQPAAVPDTDEQKSVKSVTRPAVPINITDLQDK

LSRLHTGQKPVAVVAQAGSTETSVVSSVTPTGAQLEHLGHVQQEVQVAQPFTSQQSSTPA

CQQTQPQQYQQQLGPGHPQAQPAQSAQPAQSQQMSSQQQQQQLTLQQQPQAVQQQPMPQT

QVVQQLNQHQSQLDHVSQQYDQQHPLPTQQDQLPVQSQFAQQSGQSLPQQSSQQMSQQQP

QPVQQMGQQQQAQPVQQIGQQQQQSFQQMSQQQQQQQQQPQSVQQMGHHQQPVQQTDQQH

SQLLQHGEHQQFVSQMGQQPHIDQQQPQQVQESGQQQIHPTQLVGQQQNQTFQQSQQAQD

LVQQQQESKAPAAVQMNQPATAQPGSLPQQASQLQLLSMGSQPPQHLTSPGKSETSCVQS

AVSQAPQGVLPPHDENPQAKSAHDTGHQGNVHTHHPLPGQLNHQQLLMAMQQMFNSPATG

FHPVPGYYHHSSGLQHMHHMWQMYTMYQQIMQQQNFLHNQHYQQRPTHQVHPHMTQGSLS

SANTPPHSTESGIHSPMRVVSPPRSPSSARRGIQEDGGGNEGSGDSGATQGSPGQSSKNK

PELSNLMNLEQALIKTMHGHRKDMATVPATPVSLPGVSALPPDSMPEPFDQQQTDCDVSA

QAAAGNAVQDIIHESTFVRSGPTESKSEPLLHQSQHFGRSHFEVETVASTINQSLSHVAS

LKTDSEHLPADKVSSEVRVESTKVKGRFTVTTTRDSKSQSDVSEPGTSAFIRQDGEHVTN

PTASAFVQNSYNIPSLSSANKVDGLHSLSRQPSD

>SG-AL_contig_2738_1_-2_212_1288

MMGKDYYKILGVAKGATEEELKKAYRKMALKYHPDKNKSPGAEEKFKEIAEAYDVLSDPK

KREIFDKYGEEGLKGGVPGGGGSGGFSAGQPGSYHYTFTGDPHETFRMFFGDENPFGNFF

SMGGGGGGPRGGMFSFGGGSGGDDHMDIDDDPFAHFGRGPSTSFGMSRGQPSGRRKHQDA

AVVKDLQVSLEEVLNGTTKKLKITRKVLNADGRSTRVEDKILQIDVKPGWKSGTKITFPK

EGDQSPNNIPADVVFVIKDKPHPTFSRDGQDIRYKAKISLRDALCGTTISVPTLGGRKIP

LTLTEVIKPGSQRRIQGEGLPYAKQPTKKGDLIIEFDIRFPDKIPSGAKDILKDCIPSS

>SG-AL_contig_2739_1_-1_151_588

MSAPLPVATLVLERGDTGLGFNIRGGTDIPHVPGDDGIFVTRVKEDGAAFKDGRLQEGDK

ILEVNGRNIEHVPHHDAVNVFVNAGDSVEMKVQHGAYSELMKNVKPSHQETGGGGRRGFV

KVGVALVLLAVAGIGAFIYMRHQQQT

>SG-AL_contig_2740_1_-2_953_1165

MPKFRVNLLKIEEIGLDIMKVSSEHLSVLIYCAMVKSAPAKIELFSKGRDLCFYRNEEGM

CFDQTVECIAV

>SG-AL_contig_2741_1_+1_1_198

VCYNQLSAYYNQQSHHYNQQSVGYSLKLAYYNQKSAYSHTPCPYEKSSKQHCSLFQKVKA

LNLLTE

>SG-AL_contig_2742_1_-1_229_1173

MMESFGMTTATVVATCLFIGLGFQTCHGASWSYTGSHGVDHWATDYPGQCDGQRQSPIDL

ESGKTMYDKQLQAFSFTGFNSAVGVSWTLSNNGHTVQVTYTAGDLLVNGGSLGDTYRVAQ

FHFHWGSDNTKGSEHTVDGGAYPMEVHVVCFNTRYADLSTAVTHEDGLAVLGFFFQISAN

ENPTVQKLVQFLPQVMYAGNQTVISAFPLNDMLAGMGEYYRYKGGLTTPTCNEVVTWTVF

KNTIKLSNSQMQAFRALKDGDDAAMVNNFRPVQLLFDRTITASFEDEKFAGGSNGLVFNS

LALISSLVLSLWFRL

>SG-AL_contig_2743_1_-2_152_322

MPTSLYTSSLCDLLQDIPVFHGINQHELRQHMIIIKIIVLPLTKYTRKATFHDINQH

>SG-AL_contig_2744_1_-3_546_974

MTTYIDKERIAEAYEDVRDDATATTWLVCKYEGNTICLDAKGENFDDLKEHLGADDRAFA

FVRVIMGDELSKRAKFVLITWAGANVGGLKRAKIGPDKTVVKGVLKSFAVEMAGNEHDEV

TMDHIKDTLKRAGGANYGTGVRD

>SG-AL_contig_2745_1_+1_1_297

SGLDGVSGDPRAPGQSPQTPSAAGASAPLVSGLPAVGHPDRSGDLVHRRDERVDEHHSPS

PGCSHSGPHPAADAIHGCVHDGLGSSHGDSARCGVVVSG

>SG-AL_contig_2746_1_-3_33_158

MHVCKKSHSLLKKRSSHINKPPFIIKVVMPFVWTQHNHYRLK

>SG-AL_contig_2747_1_+1_1207_1446

MTMEDCYKSKTEIKYCHTLLRKCCSQIFLKCANFRHFCQCDHGLGLSLPNSCTCHSSMVH

TSGPNLAKIRPETAEKILLT

>SG-AL_contig_2748_1_+3_60_371

MVQDEHLGKRNLYVNDIFPQNTVCGMVQDEHVGKRTLYANGVLPQNAGVFVEWFKVSIWE

KGTVCVKKFPVAELSLPAFGKFITIFAFLIFLGIFFLKTNSYYR

>SG-AL_contig_2749_1_-2_566_775

MKQMMNQVAALREKSGIPKRDVYPVNIDLSAMRSTTALSHQERVLATFTTPHVRLILEIS

FTNQLSCCKC

>SG-AL_contig_2750_1_-2_161_649

MKSHPWYVGNITRSRAEQMLMQGKYPDGTFMVRRSESAPGEFSITVKYGDGVQHFKVLRD

GAGKYFLWVAKFNSLNELVDYHRTSSVSRTHTILFRDMPVQNTYVEAKFDFEPQDSDELG

FRRKDRIKVLEQSNDNWWKGEINGRVGVFPATYVEPIENCDQP

>SG-AL_contig_2751_1_+1_691_996

MCSGCSGLSIMEKSCLQYWQWANELPAADSIIPGSLLYLERNTCSLFFASFGFSEPDFLF

IFEIFCSAHFHKQHCCLEKWHSSLDFKTRYNETSHFHVWKTK

>SG-AL_contig_2752_1_-2_62_1012

MGVDYYSILKLTRSACDADIKKSYRKLALKFHPEKNADDQAAADKFMQVAEAYDVLSDPR

KRAVYDQFGEEGLKNGVPTGSGETGAWTHGYTFHGNPDKVFRDFFGGDNPFQEFYDRVDG

DLSMGFGGLHGRGAKKQDPPIERDLFLSLEEVFHGCTKKMKISRRVMNEDGHTSSIRDKI

LTITVKKGWRPGTKITFPREGDQGPNNVPADIVFIVKDKPHLRFRREGLNLIHTANVLLG

KALTGTTVDIHTLDERILHIPINDIVKPGYKKIVPGEGMPMSCDSKDKGDLIIEFNIEFP

TSLTPERKDLVRTALLF

>SG-AL_contig_2753_1_+2_23_247

MGVYACRTSDTHQIKDSLCHVGVQWLMETQKHPACMPTEDGVCRLSNNINACTTWAKQCA

HMHPRKLRNEKTPKK

>SG-AL_contig_2754_1_+3_3_188

LHSHTHNFLSTFTPTNQNKLMRFFFYIFNQFPHIQSTDRFEPQTLLIRNMQHCKQPMLKT

LN

>SG-AL_contig_2755_1_-1_1_168

CAPKVCHVCAPKVCHECTTLKGHDLFGVILAENVWTPEDCRGNGIQLTQLISQEMS

>SG-AL_contig_2756_1_+1_2026_2481

MKERRYKKDPVNKISQLPLSKGECENSMLQIHELQACMQATPIHTIGSGLVQDCHCQEVF

VSHIDCASVGRISLPECVGQGFEHDTGLDEIIEGQRTPTEVVKLVQHCLHKSWGHVVSHL

LVSCLQLVAFNRTRAIVVKSLKRTPPLLNVVP

>SG-AL_contig_2757_1_-1_1369_1671

MYGCHSKKGIREEFSSPNVQEAKWRLPPSQFVQIVCRVEWVVFFARLCQYVHMHVVSNGF

CTLVNPSIPTSILQLPALSAMIAKHKLYPFSPSPASYWWVG

>SG-AL_contig_2758_1_-3_3_68

TAYEIIAGDWSSDVCSSDLRAS

>SG-AL_contig_2759_1_+2_23_223

MYTSSVSDFHQFLSQTMYIYKCTFKKRIPNKTTFEKYTALQYTTEKYNDILTWNPVRLKE

PTCHIYH

>SG-AL_contig_2760_1_-3_186_275

MLFGFVLHVSLWTMFYLRILIPPCLRPKCY

>SG-AL_contig_2761_1_+2_119_265

MHTGRHACKGTQTHITYINTRIQKITFSHTASHTSTLQRIGWRADRRKR

>SG-AL_contig_2762_1_-1_130_357

MNPHFLYNCSHDLKKTITYFLYKKRMNIHTFTYKRQMNILHIFMYKRQINICPKLVCKAR

QSITLYKIMQVLRSGN

>SG-AL_contig_2763_1_+2_8_781

MSACVHACVLARVCVCVCVCVCVERGRDGDNKLLTCSRKKTQLSGNDEKCQQRRTRHCAK

EECHSPRLVVFYLAERTSTIMEEEDLPDSVYLNLAASFTSAVPDTGTLDVDHPAHAGSCV

GHCSDFNSSDNEPLLTKRWESFSSSDEEPLILKAGHIHHNDSTLADPTFLPSEEMGDSGD

SSASIPKRKHGKKRRRRYKQSQELKEAQTASLYLAKQRQARNTSVDWIIFCLQMDCAGKW

YHLMGTAFFKPFVCIFQV

>SG-AL_contig_2764_1_-3_87_245

MVTSVSVGLEVCWESRNEQHVSSDTEAVQKMGQQKQTNKQKTNKQVNKISQSE

>SG-AL_contig_2765_1_+3_600_809

MLNDHKRDARNRQTYGQIGKQPLAHTHARKHSTLSVHRDVNNRHRPCVNTIPNFWHRCIP

FTKKQIHVYI

>SG-AL_contig_2766_1_+2_176_331

MRVLSTKPPQHISCDTLLDDSLFLFTLLFRRSLSSVMETNDMYRTQTRFSYS

>SG-AL_contig_2767_1_-2_230_889

MAANEERVSGERPVEDMETETDTTKRPCFPPVSAVKQVGVRQMRRVPVPPHRRTPLQHNW

PKIYTPIVDQLKLQIRYNLKSKHVEIKNCKETTMATALQKAADFVKAFVLGFEVNDAMAL

IRLDDLYLETFSVKDVKSLKGDHLGRAIGRLAGKNGKTKFSIENATKTRIVLADTKVHIL

GSFHNIQVARRSICQLIMGTPPAKIYGRLRAIQSSGGSVL

>SG-AL_contig_2768_1_-3_54_725

MDSSDIMECSCKLHDAVLEGNVEMTRNILHSPSCNVNESDEKWNGRTALHIAAMRGKSDL

VSVLLDSGANVSACDNHGNTPLHWCGHTETIELLIEHGANVLQCNKRGLTPRDMAERRGV

SGSAYSLLCQLELEVKEAEDQGHSQCATATGDPPAWSASVLSRKRKRSVSGQPLSGLWQE

CASELGLRNFLLLVLGILGFSLYITYILTGMHYGSHARIPVDVH

>SG-AL_contig_2769_1_+1_1_111

GHDFTLQSFLLFHSALWTHMHIYMPFTRANEQIKNYF

>SG-AL_contig_2770_1_-1_1_159

SSDSGKLSCGPRAHTGTQTGAVVKYGLIGKQHTERRKIKGCPCATSRSAQILC

>SG-AL_contig_2771_1_+2_2_217

VSSVQLCNGLSSALHLHERIKRLEERRPDTTISLSESTSSSTTTTMRRTWMDGWLEVSPG

PRGRDMNDMAET

>SG-AL_contig_2772_1_+3_1335_1634

MHIFCCRFHYFFYEQDLEVLKTKKKEQNVQIVLAVFVTGLGQIVKSQILKYQKTGGWWQG

LGKEEDLGGLRKKLPERSLKDHRIMNNCHSPGDPRKSEKR

>SG-AL_contig_2773_1_+2_191_385

MNTPINMQRHPCTLFKLSWENEYRQIEHKSHTCTYYTYSITKSSYHPKKPQWYIKLLLFK

NHVHS

>SG-AL_contig_2774_1_-2_92_316

MEMLNLICPSGLSFLWKAPLICLLRWVVHKILCISVLQVSVRSRCLPGVCADEDSMGART

QPCFCYLISHIDQHL

>SG-AL_contig_2775_1_-3_1374_1724

MICVMICESYVLFFCAQKCVVLIRGDLWITIMTPMFINSWVWFMAESVSWGCLLSTWSSI

WVICTRCHLCVLDPCHDYLQQVGIPVSMSERGLSRVVLLPGYMFFVHIFELICRWWV

>SG-AL_contig_2776_1_-3_57_1322

MALACQQNSYLREFTSKVVSCKPAQVTVVQNGKKSKMQCQEVVLEDTILFPEGGGQPDDR

GLLSEAEVIKVERRGATAVHYALGELTPGSEVSIKVDWGRRFDHMQQHTAQHLITAVADN

LYGYKTTSWSLGEEIVTIELDTPAVSQEQLEKIEVEVNEKIRQQVPVTPRLYPDKHDPQL

QQYRARGLPDDHEGPVRVLDIEGVDATLCCGTHVSNLSHLQAIKILGSEKGKKNKTNLIF

VAGGRLLNHFGRSFQVEKELTGILKGPLDKHVELADKAIKGFKSFQKIAQTLLKDMALLE

AQLYNSKPDKKSVFCHFRKDADGDYMNILVNQILNDNPETVCMVCVGSEKDSGLFTLAGP

QDLVASIGPKIADVLEAKEAVSGGRYRGKVNKMSQRVKAERLLQEHVTSLQQASGDAHED

ES

>SG-AL_contig_2777_1_-3_177_782

MKVVVVAMVMLVARANAFWFWGRNDETTATADGRPIEQSCIEEGEAGSCEFFNCFEQRLP

CGRQYYMLRFGNHYCNRFQRFLDSFTEPGQEFLINSQQCVTEGLIEFYRREYMNCHDLEH

AAIALIQPCYLRHGFCNIIVDNSDQFLEVFQIGDLFRGGSAKLWREVFGLARECGGQALA

NFARATSSRLTPFLRSIFEGKK

>SG-AL_contig_2778_1_-3_3_197

HDELSSLSLSLSLSLSLSLSLSLSHSCVKHVCVYGGCDCMLIECKVFSAENKSMSYSPTY

CPVHT

>SG-AL_contig_2779_1_-3_579_872

MFAISSLTLSSLARTSLPCTMFLFSSLTLSSLARTSLPCTMFAISSLTLSSLASTSLPCT

MFLFSSFLSTLTHPQLFLPWPQIIFSLPVSLSQSVWKS

>SG-AL_contig_2780_1_-2_26_673

MAEAMRGTVASLLRGIDRYNPENLAKLERYVQMQAQENTYDLEANLAVLKLYQFNPGYFQ

TEVAALILLKALTNLPHADFLLCKCLIDPSRQKEMPISKIIKLAEDLETCDFDHFWAEVR

DDPDLIGSILGFEDHIRKFVCHVVAATFQTIEQDKLQQLLGHVTPMVAMQWATKYGWTID

SNSMIFVSNQEESIKTKNITEKITFDSVAGIMASSR

>SG-AL_contig_2781_1_-3_1788_1976

MIGWWLIFLFINLSFVHMNYLYSILSAIWAKIHFYRGEKLSAGDMTCEMSTQTLVSILYY

RYS

>SG-AL_contig_2782_1_-1_1042_3165

MRREQVLKICCNHYITEELDLKPMPRSEGKAWIWYAMDFSDGEGKMEQFAIRFRDPDIAN

NFKKVFDESKEKMKTPSKTDSGKLDPTLVNSAGKSEVSRRLFHTEEDDDVEFLYEEMPTP

DQVARARMFLLPDTFYLYENRPGCSGCIGCEDYDPSKASASPSEKSKAATATSAAPPSQP

AKQPVQEKVAPAPQGMMFGGGSGMSFSALAASSGDIPGFGAKDPSKPFHWAGAGQKLFGA

GREEDEGQEGEVAPSEDIHFEPVIPLPDLVEVKTGEEDWTALFNQRAKVYRFDGTQWKER

GIGEMKIMRHTSQLLFRVLLRREQVLKVACNHLISTTMELKPMATSETAWCWVATDYTES

EPKMEQFAVKFKKMEVAKEFKAIFEDCQEKLRQGEKNEQVQETLVSSSMHQPAAEGCSVR

VQDIMTASSQPVTGDKGNLYYTQQLNISAQDTMSSSSQAVTGDRGNPYYSQQLGVAAQDA

MVSSSQPTMGDNGNPYFSRALDVAAQDAQISSSTFQALGSGGNPLFSQLLSPEEVEAEGQ

YAEEDYDDDDEDDEDETTEEDEEEERVEFEKRVTLFQREDDGQWKKVGMGNLRVLYDDDL

NANKLMMLTDQGDKVCNHVITMELTVAVDNKKKSCEWQPIDFATDEPIRRHFKACFSSLQ

SAEEFATIFRQGQKLAAESEISANLTEEIEVPEVFSKGDRSEGAMGKK

>SG-AL_contig_2783_1_-1_1_453

EALSLFSLVQHLLVEFLKNFHLCCFNRADSVTVGHDCDTISNFCLIPGEFFKKLICAAST

RLIYLDCTGLLELDKSEDGHRPKCRNGLQVGLFSFMCLCVCLVSSPLVVSGVYCQLLCSV

CLSVSFTFMPTLSLFLSLTHTHTPTTPLCQQ

>SG-AL_contig_2784_1_+1_82_246

MICINLQCCHAIVFLYWVVKAFRDKYIKCNKAAKMYKRPQEVTDNRSMSIFHLNS

>SG-AL_contig_2785_1_-1_67_2358

MELKIILLFCAAWLATVESRPTRWCVTNRAEAVKCDKMRIEMRNLKLRNVTGQAALPQDF

ECVLRNDVFECMEAIEIDYADLMTLDSGQGYFAGRQHNMMPIMVENYHNTPNERPTYYSV

AMVRADSNVQLDNLNSKSVCFPSIGTSAGWLYPVSKLINEGFISIFECNAIVKAVSSFFK

EICLPGALTSFYNPFGNNPTDVCKLCTGQNTEFCTTSDKRAGFDGAFRCVADGAGDLTFL

RHDTIAQMTDPVLNPGTTYNKEDYRLLCPDRTIKLVDDFLACNWGELPSHMIMTSAVRDI

PVIEGYKAFLQRAVEFFGPDGTYSDKFEIFVSNSTYDTDEFDFVFERKNQMFSDQTVMLE

DIGDKTYFQWVGDDFDQVLENMNHCPSDAVRWCVISEAEKLKCESMLLAFKAKDLKPELD

CLYGGNTTTCMEMIQEGDADVINLDSGDIYMAGRRFGLVPIAAEDYGDMSMKFKVVAVAR

KTDKATTLFNMKGKRSCQPGIGRGDGWIVPLNIFIETEQFLPDDCTIFTNIGELFVRSCI

PGSLDREYNPNQHPINLCEACASGGYRKCQRNSEEQYYGASGSFRCLVEKGGDVAFVRHL

TVRDNTDGRNHAFWARNRRSDDYELMCKDGRRLNIDRFEVCHLGEVPSNAIVTSGSKTTR

QKDIIWNLLNYGQQFFSSDIDGDFHMFDSGNWYSDLLFTDPTVRLMRIPEDRQNYKTWLG

EDFIAQIENLQKYTCVNPDSSAHIMPSLFLVLFLSLLAKIMSYL

>SG-AL_contig_2786_1_-3_3_137

YCSCMLSVPVLFLCAQCCTIVLVCSVLKYCSCVLSIEVLFLCAQY

>SG-AL_contig_2787_1_+3_123_314

MLQYKDTRTIPQYRALKNNTAVQGYKNNIAILGYKNSTAVQGYKNNIAILGYKNNTATLI

LRQI

>SG-AL_contig_2788_1_+1_10_276

MHLSHDMAVPHLMHLSHDMAVPHLMHLSHDMAVTYSRSKILLIMHRHTHLRCLKKMLLRI

QPLPKRVHKPGMCQALVAQRERERERERD

>SG-AL_contig_2789_1_+2_2171_2659

MPQAALRFNRGRDRKEDKHKQKSNAESWCIKSTRVRKQFPISGTLQNISRKTLRNILKGK

KDVCHKDGKDCLGETAMYFYTGFPPFISFPRGLHGSRYRNRSILDVFLVVLLQKPFCGPV

LDQIMALNNNKKTKKTVLTSVLEDLSYYPAAQCHLPFLKGGGG

>SG-AL_contig_2790_1_-2_545_676

MLLITNPILLAFYFLYLRFLTFKIEFSEIHCTAILHNKQCIIFI

>SG-AL_contig_2791_1_+1_2017_2205

MHQHKQNSTAITPNHKPKTHHLLNTETHTILKRRICDWLDNSKIGTEQNYEITSMFAAIL

KHQ

>SG-AL_contig_2792_1_+2_83_247

MVMIFYTDIWLIWNFFFSRATSMHSCCSKLKCKNLATKIHTDFKVYLWTKRKCHQ

>SG-AL_contig_2793_1_-3_216_479

MYKKRAMCNHLTSHVTLLCIYEHSVLPFVLPQTMLSPSRSDVFVYKMYTGRVTMPVMVIS

PSCSFQFMSSFNHYLLVQVTSCHHPLLI

>SG-AL_contig_2794_1_+3_1359_1610

MTFPPVSLTFSTKARNSCPILRCFIYFLYDKLYAFTCIPCTCSHTHTHTHTFGEGKPNVI

KRQWWVKGVEREYLRTCVGIDSIG

>SG-AL_contig_2795_1_+3_3_146

FFLRSQCSLYCYKNIIPKGSIHCSDIYFSNKPDESTGSATAHKILAIH

>SG-AL_contig_2796_1_+2_290_628

MNQNCLDFLIYAYSSKETCLCLPGSSNLDQPNQFLQPLILAKRIREGTQLLWTPISSLNK

SGIFCYANYNHIFAILLTTTEQRLCSCSSKHSKKYFIIQTAILTLKVKAIMRN

>SG-AL_contig_2797_1_-3_69_4415

MKSRGCLQRHMDKCNMCHPPADEIYRHGSLSVFEVDGNTSKLYCQNLCLLAKLFLDHKTL

YYDVEPFLFYVLTINDEWGSHIVGYFSKEKNSQQKYNVSCIMTMPQYQRKGYGRLLIDFS

YMLSRIEDVAGSPEKPLSELGRISYTAYWKSVVLDYLHKHSSSAVCIKAISRASGMCTQD

IVDTLYHLGMLDHRDEKLVFVNQPKVMSAYLEKQKNRPVPRLTLDPESLRWTPIITRASV

DEEYEEAKKELHKVSEGASSLVAENEAMEEELSRGEMAESPDNTPHLPHKGEQRSRKRLM

SGGLGSEPELKRSRTEFSSVAVRGMTVQKKTPLAIDCATDRENRMSMGSPIRSPTRPSPH

LGLYIDITTPGPNSPLKTPPIVSPVKTPPIHSPLHLSPSAQRAKLEDDSTMKVTKLKKKV

PGRSPGRPPGSTNKFPRSPNKMSASVKKSDIAAANNGLPKRRGRPPKNAQTLLSDSGTPG

RSVKPGRRPNLVEEAAGDVTPRLKPGRKPGPGRKPGPGRKPGPGRKPGRKPLVSKDTAVN

SGPEAGNTELEPEERPKKRLKKMHWKKRAKLEKLEKEKVERYEQKKKAEAETESDKVEAT

VTDTISATEQEKDAKDEEVDVSMQEENTSEIDKPSTPMAKEAEEKDEEMDASLTENYGEN

EDNEPTEEKSENVENVQAVCKEDINKNHSVEKDAEPSKESDSSSSSDNSSSSSEDEDDDD

ESSSEDDSSQNSKETGKEDEEEEEEERTEDPETAEASQNLAHIVANLEEADRRDHMKGTN

LSDSSAPASPASPDANPQPEPVEEVGETEQPDKPIVNPPDLHSEDSHNSLSSFSSSSDED

VPPPNTPPPAPRPATPQSTSSPIDSPMPEIPAEVSDTNQPVEGEEDTVDSNASLAEVELS

QEPADPVPTMSSVPSQEAGGLSPRLSEMEALQKDLEELQEEQNCTPPALSSAQQHTPQQH

TPQPLSSQQHTPQQHTPQHTPQGHTPQGQAMATPEPMTPCDNRSMGSVEPPTPPQLQQGP

QSQGPPPTPVSAPTPRANRNPSCSSTGSGPGSVYSQQQAAGSIGGMGQSGMSSSNFTNVD

MDHQLGLNSPAHVSAADMSAMTAGGYTDCAQQTMAFARGRFMDTVNMVPGAVSYSPVATS

SVNYPHNSPISSYSPSMIPQQPPSQQQPQQQKSQPPQAQPQPQQAQPQPQPQPQPQQQPT

PQQNSPRILSSHTPTLSLSGVVAGGQIGIGVAGPGYPGPHCSLAKLQQLTNGLMEIVPEN

QMTPPPNLTTPPPVNMTPSPGLMRSMTTPPVVSLHQQMGQAALMGGVTGGTVSGTKYQQR

QRPSSTATSNTVRKSPSVTPNVTVNTPNPNMPFSPNVTIQPAPLPARYQMNAMNMFNGYR

MQQQVMNHGTAYLNAGFLTQQMPVQMGMMNMNVHPQQFTPQMQQHPSQPNVYPSHYGYNI

MNMNMNMRR

>SG-AL_contig_2798_1_-2_329_646

MCIVAFDLKCVCFFWWFFTHVLVHVCVKVALIHLGTALSILQTIETNSDVQCVRAHVAYF

IASITCTSHLSLFVFGVRSQSKFRDANRHSFPQFHHDSGSRNFVIQ

>SG-AL_contig_2799_1_+2_2_73

TSPSPAFCYKTPSLTNATLGIRKR

>SG-AL_contig_2800_1_-1_1735_1944

MPGFSFLNIWQNAYLQVTLCITEILQAVFIVQHTCMHAHTHSYIPTYVYILYNLYLIFNV

RISGLILTFL

>SG-AL_contig_2801_1_-3_39_338

MTTQMTFQRRGAVKPPEKGSFPLDHEGECKEFMTKYMRCLRENKQENTKCRQESMQYLNC

RMEKNLMLKEDWKKLGYSDLAKDRTASSTSSSTNSSPPSS

>SG-AL_contig_2802_1_-2_746_907

MLKIHIYTKNTFGAGRTHTHTQTHTHTHTHTHTHTHGTIPTLLQRGFTRVGMIG

>SG-AL_contig_2803_1_-3_21_422

METISHLVRVSLPDYLRSVPIPTSLGGFAKLSSEEWLSLVPFVGTVSIIVYVTVLAFKPP

PKPKDVVVNMKVQKESPKVVNMVDIEDLGNKISYCRCWRSKKFPLCDGSHDKHNEETNDN

VGPLVLRRKEAVSK

>SG-AL_contig_2804_1_+1_811_930

MRTLALLKIKITRIQTTEKIQFGILAAQRRIQIFNFPRSL

>SG-AL_contig_2805_1_+1_388_816

MMVFDFPSCLSSLTSSERVLLIVDPREVKLSTASRVTLSIPMERSILTSCALTLVFFRLI

VSLNCLQASVKRSPSLWRASSLWVTRVASLAKSTSLLSTNLTLVFQTCLHYVLTIKIYTF

FAKLRSYTFLDVTDKFIIKAALQ

>SG-AL_contig_2806_1_-2_2_1516

MSAAKVLAFAVGLLSVYSAFGQLPKARFEYKYSFKGPHLVQRDNSIPFWEYSGDALAGDD

GIRLTPSLRSKKGSVWSKNALTSPHWMVDVAVRVNGRGRIGADGMAIWFTEQKGSEGSVF

GSNDMWKGLGIFLDSFDNDNQHNNPYIMAVVSDGTMQFDHNNDGGSQQLGGCLRDFRNKP

FPVRLNIEYYKQALTVKVNNGLSNKPEDFELCLRAVNVHLPQTAYLGMSAATGGLADDHD

VNSLLTHTLHEPGAETAKVSEEERRKFEEEFNQYYKDLERAKEDYQKENPNKADPYNIPE

DSLYESESQRELRQIFDGQMNIQSTVREVSRKLDELLGRQELVLAKISSLPATGTGQVPQ

QVPQMQQQQGQVYMDTIKRHEVDRVLNNQNDLLQQIRDLRSVVNDVAQRSMQQPVAVPQG

GGGGGIGDQMALHEIKDRLNTVSNDVSLLVKRPMPALNTAQCPPPPSGSCLTPVVFFVAM

GAQIVILIAYMVYRQNKEASAKKFY

>SG-AL_contig_2807_1_-1_103_342

MADKGEYQMKTAPFDARFPNQNQTRNCWQNYVDFHRCQKIKGDDYEPCNYFQRVFKSLCP

NAWIEKWDEQVESGTFAGKI

>SG-AL_contig_2808_1_-3_1257_1490

MKSFAEHASINMRNTLDDFKSVNMESRHVVEQLEWKAQKQKILFLERPVNHKPVVKCRVF

KSAQVIHATQRRYLPGEA

>SG-AL_contig_2809_1_-2_218_523

MAYTSVFLITLVCMQVCKKTCLLVVILSTSAVLHFVYWWLYFRLAHFFICKLFLHGVSEC

FLLPSLCIVLINYNTVIIITPLSESLFCCFFANTGFVHVLFM

>SG-AL_contig_2810_1_-3_3_884

EGPLDLYNVPLQVRQTLQQERNKTLLRYFQLQDICVLQAFTERLAEVYNQHNATHIIKSF

LIGGKKTSNSTRQRIYSNFAISIAKARPYLTAEETFTLPDTMPSPPQYKYFWFPLHDRQV

RYLDGYDNGWNQYGNQQGYDNDWNQYDNQQGFDNDWNQYGNEQGYNDWNPYDDQNGYDND

WNPFDNQQGYDNDWNQQAYDIMWSVFGQPKNHERWCDRNFVEKRLRGDGVKVLSIEEICR

KIKKHCSSYAIGQSMLKDYCEETTTTTTTTTTTTTTPPTTTTTPTTTTTTTTTT

>SG-AL_contig_2811_1_-3_78_662

MGPDFGPPAMSPDFGPPVMGPVLGPSVTGQVFGPPAMSPDFRPPDYNNHFPHPGWPHNCH

HNMRPVIKTSKPTTTTTKPTTPAPAPAASCTPSQVLAFFQLLATLPLLLNGNVFTNQMTS

TTTEVPLPRLMRRAVVSVDDGYCHGQLTTAQNNSLQMLLYLVGNRDINAEGPLDLYNVPQ

QVRQTLQQERNKTLL

>SG-AL_contig_2812_1_-3_3_230

HYQVILRIILCRFFPVVSRISGYYPTMCFSLTLWSSMKTSLFSVWAFSSAAFHTLAAQHV

VKQRQRIKGIRGKGTF

>SG-AL_contig_2813_1_-3_690_953

MMKWPDAAEILVENSVRFTIYSYFKICLNVCAFIYLASTYIYLFSKHLNILSAFVSINEI

RCYFNIFMVIFHHEIVLHYVIHIHEILG

>SG-AL_contig_2814_1_-2_695_997

MLISAQMHVCTLFWPTLPRQNNVAAQSAYSDFKEEIVTQKSVQGFLGKIPGEWTGEADNR

KEGAIEKRKAETERVIEKEEISRRNGNRKRGNFQNRISMGR

>SG-AL_contig_2815_1_-3_1083_1343

MRVDFSIEFPQNGDTIYIERTSGIPTVRHMSHCEHVDEYIQKRCHCMYVGVFIIVYVSGC

ICHCQCVMYMSVPLCWCMYLLLLMCNG

>SG-AL_contig_2816_1_+1_430_750

MFNSITCHIHQVSYNSFSIQSHVIFIKSATIHFQLNHSTAMIYSQQQLIYNVVRIQQLTT

QQITIPIQHNHNSHSTQSQFTFNTITIPIQHNHNSYSTNHSFYSKQS

>SG-AL_contig_2817_1_-1_1_297

CSSDLYRQYSSYGGDYSSEYSYPDSSSYRQYSSYGDASSYHPSSSYEDSSSYGQSSTYGD

YSSYGQSSSYGDYSSYGQSSSYGDSSSYDQSSRYGGDYS

>SG-AL_contig_2818_1_-2_1694_1882

MIINTDNEFIEFSLTDQQIQLVKLSMNAFVALTPSQLIFYVQCRFLSSFCLSLLLPHEVI

MCL

>SG-AL_contig_2819_1_+2_77_262

MQNSKIGGGEGSPPLVCPLPGGVNNSVGRYEGDVRQTFCAGDPEVAQVVVSEGGFAPPGG

CL

>SG-AL_contig_2820_1_-1_1_261

PDSPRAHELRALSSSAASQHSLHVRDVLDAAYWRSESTFPKALPPGLQCRRSVSHLLRSG

QCRYRRLHAAGRPGEEIPPFLQSLNSA

>SG-AL_contig_2821_1_+3_183_365

MPSRRAIKQPFCTLIPYDFYTDHPHFINSTIPEAHKVFVKNRQNKTQPLNKQNTIFPLFD

N

>SG-AL_contig_2822_1_+3_117_269

MMITPFFSPTLLPDQANGFKECDNCFRLSSRNWCGLFNDYRYTKFENAATS

>SG-AL_contig_2823_1_+1_208_420

MQILSRFLILMLICTKNITKPFACSFSTSQLSLYCCVTFYNATAPGPNKETMKPDSRWLL

LKNYYIYIMTS

>SG-AL_contig_2824_1_-1_394_585

MNSTIKLPICRTPDKSLHVHLPYFRKQCSKVSADLVCCTGSYYSLNQGVSSSSLYVRRLG

SWEC

>SG-AL_contig_2825_1_+1_25_300

MVQYMKPNNVICSGWGERNHQTNNKRKYPQILQYILICASVITQYNLLELVITQYNLLEQ

QQHNIKNRSQHHTEKRSYNSTTENCTCVTKLN

>SG-AL_contig_2826_1_-2_665_805

MSSTQLKKNLLLRTLQGGILEERSSVILPHPQYSFQRLLQSVFIHTH

>SG-AL_contig_2827_1_-1_742_942

MRYERNILPELCPSLVKRLFAYFCRYACSPCKVLSMFICPLPSSCVQRDQSASFSGPYSF

HFKQVNY

>SG-AL_contig_2828_1_+2_56_391

MYSFKCFTHKKIYVRTWKRTLFCFSFKQTCTAIFHHTTNAYHFHSFSSITFKFTQTAAFY

LLVHQQLSVLLKAHSHTCCGDNRDEYLQMVDDATRSPTLSVPCTDRGTGHHQ

>SG-AL_contig_2829_1_-2_224_412

MGKLTTSAIVIVDLHCKAPVLQRIWDHPRASIGALGHPFEQTETDMIENGLLSKVTLKQL

PDL

>SG-AL_contig_2830_1_+3_255_359

MGSIITQGHLVITNSALGQGPTYSANLSVYLTALT

>SG-AL_contig_2831_1_+3_102_671

MMDAKDGNTIPDRPRPGSTTSALSTGSHQGGSDKNATGTKSNIKLPSENITLGTWNICTL

YACGKVKELEHALLHYKWDIIGLCETRWTGFGETTTEDRHKIWFSGDQQKHQHGVAFLVR

KEIAGSIISCTPVSSRIIMIRVAAKPMNFTVIQAYAPTTTYSDDVVEEFYEELEKTISKI

PKKRPASYTR

>SG-AL_contig_2832_1_-3_108_752

MSVAARFRELGKKIICVGRNYGAHAKELGNPIPEKPILFLKPTSSYLSEGSPIQLPKGCT

SLHHEVELGVIIEHCGKNIPESAALDHVGGYILALDMTARDFQDEAKKKGQPWTLAKGFD

TSCPVSNFIPCDKLSDPQNIGLWLKVNGTTKQDGSTKDMIFSIPALIAYISQFFTLEPGD

VILTGTPSGVGPTKPGDTIQCGLADIVTMQFTVSQ

>SG-AL_contig_2833_1_-1_148_2718

MIQVVPAVDLGDAKLRPWSKYSKARLPEKTDTVKTEKQKKAEAKKKKKEEKDAALLGELH

EDPEFKEYLAVYSAKDRKAWDNDAGMKSAGSVVKKDDDDVNNEKSEEENEDASNEESKDK

ETDSEEIKDGKSQSKMSDLEFLKSKMVDSALLSSDEDEENEDGNITKKFSTPKQEERQKA

DGNKETKSSPQKNTEGAKNKEAPKQKFCLKMKGLAATVKESVIRQFFQPMKVKVHLPTNK

NKISIGIAFVECRSEKDLEAALGRNKSFIGKKKVYLSRVFEEIQAVKPVTEKPKPWEQKQ

QDDEEEESIAESGRLFVRNLAYVCTEEDLEALFSKYGPVTDMDLPVDPFTKKIKGFAHVT

YMFPEHAVKARMELDHKGFKGRMLHILPGKDREEKFVSSEKSSYKKKKEEKQKALVKSSH

NWNTLFLSGDAVATAMAARYQMDKSEVLDDSGRGSLGVRMALGETQLVQETRQFLIDNGV

ALDSFSQADGERSKRVIIAKDLPAATDADTLREVFGKYGTVSRVVLPPHGITAIIEFQET

SEAKRAFSKLGYRRFKDRPLYLEWAPMAIFDKEGEQKEGETSGEKEEEEEKEEPMQTEEE

EKAPKEMNKETKAAPARTEKKAQKEEDSDSEEESEPNSTLYVKNVNFDTSETALKEMFSK

AGEVRRVTIAKKKDVKNPGKFLSMGYGFVEFKKAASAMEAMKTLQNKLLDGHQLELKISQ

REILQPKTGQKKQSSKQKGSTKILVRNIPFQCNKREITELFKPFGELKFVRVPKKASGPG

IQGFAFVDFVTKQEAKRAFDYLCHSTHLYGRRLVLEWANTMETLEELQQKTKRKFHGGGG

PPKKLRKQDLMKTLDME

>SG-AL_contig_2834_1_-1_49_216

MPGFASIHNKITLMRHSNGFSSVLWQISGKTQTENGRQSTCKLFRTEKAMIRILRD

>SG-AL_contig_2835_1_-3_573_794

MKLSHLGLKNANFAFAENCHRLFYTSKNTVFAMIISWEAVNEIYLPKMACDCPWVMKVKI

LLLANACVDCQTDA

>SG-AL_contig_2836_1_-3_159_272

MNSMACSLGQLIDIVNRLATARALWPVSLGQHIDINSQ

>SG-AL_contig_2837_1_-3_141_1565

MASQAGIEDIQDLEKGARVATSVLQQRANDVRANVVNWNSYLQGQMITDEVHKFISRFDK

ASPEERANLISPKPIQFAVIFRSLIDKIAKDQTLQYILTMLDDALQEDPSRVDIFKDYAK

KCKESVWQPFFQLLERSDLFIVHQSSHIVAKIACWSKEPMEKKSLQSYLIWLRDQLRLTN

NEYHSTVARCLQMMMRVDSYRHVFVEVDGIAVISSVLVNHEKIGFQLVYQLIFCLWCLSF

NAFLAESMNRMNLVPVLADILGSTQREKVTRIILALFRNMLEKPQENSVTQDYALSMVRC

KVVKQLDLLEARKWEDPDLEEDIKWISAKLESSVHDLSSFDEYVSEVKSGILEWSPVHKS

DRFWRENAIRLNDKNYELLKILVRLLDSSRDALVLSVAAHDIGEYVRYYPHGKHIIEQLG

AKQKVMQYLTHEDPNVRYEALLAVQKLMVHNWEYLGKKITEDKTKAAGAVAEVKG

>SG-AL_contig_2838_1_+3_684_989

MNSFNLIIPCSSHTKAKLYSNKQVSALDIQFCLPLSSKPYLSCLNYTKILLSCLKHIKIT

VSCLRTHKDHSVNVVNAKDHSVNVPNTQRSQSCLKHTKIGLD

>SG-AL_contig_2839_1_+1_1_204

GHEVSKRKAVEDITAEAKRARLQHRESVLKSVAEAAHSGSGKATEPKPSCSKSDQAPESS

GRGLSPDL

>SG-AL_contig_2840_1_-3_3_119

HLTFSCSLIVCCCIFLSIPYIHLTFSCSLIVCCCCCCFY

>SG-AL_contig_2841_1_-2_2_232

RISLEKNIGLAASSSPAVNGYLTPFKFGERKASKERERGREKMSTALIMLRKVEVSTTSL

SYDLRVMGQHVPFIHNF

>SG-AL_contig_2842_1_-1_1066_1392

MFVLNVGEIFSCSRNSYINDYVDNVQEMWTISVSDFGFLIIYSGKETSDKPYSVCMLVPR

NHLGKPLHVCFIKELTNNYFTGGVDICHVSKIYIPRALMNTRLSIVISF

>SG-AL_contig_2843_1_-3_294_3062

MKNSATFLCVLLCTVQCTSATRIHHRHPDDISDSHDYVSQDHTDPEDVSFKERTKMPPAH

YELAYAEQGENGFDSESSDNSHDQNKVHKNKEESPWNSSLFKWMADPSLTHLPLAYDSPH

GKLEDDEIHNEPGSPQPHRLPDLDDEFPESQTSSEHSPQPLTLSPESEDREDLSNEDGRF

YSQQQRQAQQSSHRGRHHRRSQRPRFGAQEAQGRAVGNRRDEDRNHHSTDVVSNSGNNPS

PGHQRKRRKQHKNRANRLKTRKGLRSRSAPQEGRQPISSLPHSGRQIVYKLPSETPSNRQ

PSPILHGASARRTLPVSREFVSLSTLANGNNDRRPKKQRRNFKFDSKLRRKSKRRRDRRP

DRPRNPRSELSPDPTVLPLLSRSFTHQKNLGGLPDNPQPFGKQEIVLMPIGEDPGKTAFF

QELSEVSPQSLSQELGQVDPVTLPEEESQRHQGPQGSGENEAGCSCSAQSDVCHQKEMDT

DVLTQTFQENLAVLKQVLAEALDRVITDPTQTLSRDLCDVIDAASEMTSQRPNRQGIIGQ

KSKGRKADHYFPEHELEQGAEGGGAEQPKERASGRNIHRNRPPNRNSDLNRLSDRRNTDL

NRHPNRNKDLNRESNRHANLNRHTDRHSGDPNRHPGRDEHHPERSHTDPSNDKGEHEDSS

DEAHDLRDLSEEKDSSSNLTVATNVPQSLTNHRGSQLGPGGIGQEEYDPNTAKSGQGRHD

QRTQDREQHHTPIRGQDKIDQNEPRSGQNRENQNARGIHEELLSPSNIRAPQAGMERQGA

GSVDRDLETDADAREESGSDGGRRERENEDSDNSDDNDNDDDDHDDEDDDAEDVDNYDDG

NDLGSEEYDEEEDAEKVWKLWKQQQQQRRQLEESQRADDTEALPDCELGQWTDWSGPLGF

GVIERRRFVVAGDENCKMPESQG

>SG-AL_contig_2844_1_-1_1_447

SVVEDPLTHKQAQISQEDKLLLGHRQAPTAANIPFKVLADWERIARHLLLCCAGADAFLS

GLSARLQFLGSLNPGSACPSMDEDVRLFGRQVSTYLASIATAGAMLQTNTLLARRDAILA

SSQLPKEDFAALRSLPPQGTTFFGGFAQA

>SG-AL_contig_2845_1_-2_272_439

MMFMFNNSAVLMKVVLYIFFLSKTCENVMIVSLKLVSEFCTSSLNLCFCLFVFYAC

>SG-AL_contig_2846_1_-3_1089_1532

MPSKFYSCGVASLQCCYSLLNMHCVFFPHTLGSRLHPVSQCDCQFSLEFRAGLYLASGVL

LPDFQSLFFSSGSNCFCCRIPPICHKNFCPVLTVSSHYGYRGLFVFRCSLMGSAAATKVE

VSSTSLPYDLSVIEKPVPLYLLKTNSRL

>SG-AL_contig_2847_1_+2_212_355

MALVVGWALTIYNNNNFIAKLLMKVCNALLKYELIYTDVTTNHDCKRA

>SG-AL_contig_2848_1_-2_605_913

MDTGVLFSSSKLVCMLFYCIVSPLSGNSVLEISSFLLLFCFCPFPFLYCRLLFSAFLVHS

PFFFPASLHTIFSFPHPPQFMMYYLCCFALVIRYETVNTGMDT

>SG-AL_contig_2849_1_+2_1469_1744

MLCIVCHNSLLENKEHQETHKQNEWIQTNNIFYYCLLLHKNEQSYFYFILFSSSQRHGTL

SVTYFAAGFVCLPVAACSPVRLAALCTQNSVV

>SG-AL_contig_2850_1_+3_876_1061

MLFISYIHNLLFHLGIVNKVHWKISSFLCLHACEHDCVCVCVCVCVCARVHVCLHPCVCV

HA

>SG-AL_contig_2851_1_-2_80_2734

MSRFFQAGSDSETESESEEEVPTQRPAIPARAYQLSDDEEDTRRVVRSEKDKRYEDLRNI

IKTIRNHKKIKDMASVLTDFEGLCKSYEKAKKVVDKEGIPRFYLRCLVELLDFVNECWED

REWRDTISKNNSKGLTTLRQKLRKYCRDFETEMAKYRENPDEGDDEEEKEDRNEGAESDD

SDVAPARSEDKVMKAAKFVKDRDLDREEDEDSDDIWNASSEEESSSSDEDIPAANKLTAE

YFLKTTTEAEEKKRKERKKDKATKRSERRTKEDDEWQEVKGGAPMIMEKPKMFAKDQEIN

HEAVTKKLYEIVAARGKKGTDRAQMIEMLQELKQVSDTNNLGPAMHIKILFNITAAIFDY

NPNIATCMRPEMWEMCMDHLTTLMTMLTEMEELVVGENILEENESLEATPYRVRGCILTV

MERLDEEFTKMLQNCDAHSTEFVDRLKDEQRVCGLLETLQTYVEKNGTSEEICRAYLRRI

EHCYFKFDQTAFETTTDRSKVTSEQLMDKLCKYIYAKDSTDRLRTRAILCQIYHHALHDR

WFKARDLMLMSHLQDNIQHSDVPTQILYNRTLVQLGLCAFRQGHVRDAHNALVDIQSSNR

AKELLAQGLVPQRQHERTPEQEKIEKRRLLPYHMHINLELLECVYLVSAMLLEIPYMAAH

ELDVRRRMISKNFHHVMKMSERQTLTGPPESMREHVVAASKAMKAGDWRSCKNYIINDKM

NAKVWDLFFQRDKVRDLITSKIQEESLRTYLFCYSSVYDSLSLPLLAQMFELDRPVVHSI

ISKMIINEELMASMDEPTQILSMHRTEPTRLQSLALQLAEKVASHVENNERIMEVKQGNF

YFQKSNVQNYQQNQNWGGNKFQGRRNDRRDNQRYDRNRNYNRDRD

>SG-AL_contig_2852_1_-2_656_973

MHRVRDSAIQSGNAPPVVFNQHNEHIQLYICTIGEMISTVNEMAVWPCLSSKAVALTVTH

TQNTYAEFQLTMYVLFAVPLSVFSRQRFCAWNKFHTHLYHKCHVME

>SG-AL_contig_2853_1_-1_1_96

SALGINSICTNGANANNNKQLDMVECTLKISN

>SG-AL_contig_2854_1_+3_75_332

MCRLMAYAIILLDCPSPSHDILRRLCMWNLFQAQKWCLEKTENETLCVCVCVCVCVCTYH

QSRWQVLTSHIHNFIYYNKGIQFPTH

>SG-AL_contig_2855_1_-2_2_151

VLEVLQIFVCFFGAKFLIHKDAKSNRYGKVKDKRIQINKNNCFLSIFALL

>SG-AL_contig_2856_1_+1_256_429

MGKISTHYPPGEGPKEIRTQTFWLEVPCPNHLTTSSLFSDNYLSYGIFSKWLCQQFLA

>SG-AL_contig_2857_1_-3_30_1208

MAALARRLLLNTTYKKTVLPLLSIGTSGSGNRSYVTHATQQQADACAQYGGRHTVTLMTG

DGVGPEILTHVQEIFRYAGAPIDFEIVEINASTTDAEHLRGALLSVQRNGVALKGNIETK

FDDEQFRSMNVALRSKLNLWSNIVWCRTFKGVVTSRHEDIDIILIRENTEGEYSSLEHEN

VPGVVESLKIITAEKSERIACYAFEFAARNGRKKVTAIHKANIMKLADGLFLESCRKVSK

DFPDIEFTDMIIDNASMQMVSRPQQFDVLVMPNLYGNILSNIAAGLVGGAGLVPGMNVGD

RYAVFESGTRNSGRSLKGKNIANPTGMLLASCDMLDYLGHYDHGTMIRESVMSVISRPQL

QTPDMGGHATTTEVIQAVIEEIKPKTQTRLHND

>SG-AL_contig_2858_1_-1_169_1119

MASAGTGKMYRVKPFFDSNAEIISHVNMYKVKSFESDVSGILSGGEVEDRVSKLQQRQQR

VLKELQQLQHSVQMLAQQRGVSLAQAKPASSAANTRTTAASYSHRSPAQGGLQDLVISAD

PSCPPLSLLVLLKQLSDQHQVLTSTFVHSSAVDTPPNLRTFLHNGPSPAPRCNYQLAVTL

VWKKLSSGPRLMVSPTRQTYIQGETNIARYLARLLNPAYDQGDITTATQIDEMLDLVQQQ

LMGGNNKERLAVLRTLNARLGKGSCLVGGDLTLADVVAWSAIQGANMAADAPANVRKWLQ

ACAQRPEFEEAMRFLSS

>SG-AL_contig_2859_1_-2_98_199

MGDVYLENRHCCFVLLNSFSSSPNSGSDVNLQFQ

>SG-AL_contig_2860_1_-1_1_189

RQTERQTNSQLAGRQTDRETESRKNSVRVTEEAIFCYVNPQCSLRCHSIILALNQQHYKQ

IYF

>SG-AL_contig_2861_1_-3_3_161

CVCITVMLNTCHLNVLVQFCIVFRILLYLLSAPPSQCNMARSQPAKPDVGDSS

>SG-AL_contig_2862_1_+3_189_326

MLQNYKVHHHATTSLCNTMYGKLVLNQHLVCVCVCVCVCVFFFFFF

>SG-AL_contig_2863_1_-2_332_1216

MASLRSSFTLLRTAARQGCRSISTRSSASRKAAPLLVGAAAAGAAGYGAYKIWGSNPVTL

EQAVKTVQEAMPSATVSAAGPPEAQPSVYSQGKDHALYLWIYLKPTANSREVARAVAKLQ

KYVDEVSDPKLRDEDDEIWAGVGFGPNFYCQVAGKTKQNFTYPHRKGALGDMPSSGGDVF

VHAKSNQMSKLFELGQVILKNLPKDSVASFEDIYSFVYRNGRDLSGFIDGTENAADDDDR

REIAVEKATGGSYVITQKWIHDLNVIGTEKDKTLEGWVGRSRADSVELARKTISS

>SG-AL_contig_2864_1_-3_6_1181

MAQKHDNGDYSDDTPEYNVKERLKELNAELAMEPDPDNRQLRVGFKEELIDLVAPAPQVS

DDEDDSPRKCLGYDDHTEKTAPEQGGKTENKKQFVVERDGKFDIVTDHELTTSERAMFNI

PDEGSSDTRTDDGDKHSRGGGEGENRTNISGSSARKENVQPLPPSKPRPNTANGYSRRSV

HPLQSPRPQTASSNRSVSNHSSALQDFNYNSPYAMSCSEKERAQERARLREQQRKEEERR

HQEEEEYRRQESEEAFQAWLQRKMDDDRRKRHEDGDGRRSGKEDKARENEEAFRGWLKQK

QAQLKREKLLKRREEQGLKDGYYVRTREECDNAYKEWLKKKYAEFRQHRAAEKERLKSFQ

SMSRRSRKQQMFARAVRQPQVFRYVDFYGYRF

>SG-AL_contig_2865_1_-3_810_1019

MDLLSEPILCNEQIKLGSDMIKFTGSIEKGVYCIALFFFFIFIGWGEVFSKIIFFFYFLI

ISPPPPPPPS

>SG-AL_contig_2866_1_-3_15_230

MVSILAKHVFWIKYSFFKLVKCSNTLHRNASSWWSGKSLFVKMRFPPCSHFHISMLITMD

KKINWPQILNPN

>SG-AL_contig_2867_1_-1_1_174

CLSKICLIILLSLVYQSERCHVIFNTFVLVKMFNTADILIVHYVDGCWCRPSMGEGWM

>SG-AL_contig_2868_1_-1_160_270

MLLNCFHVKYRLYLGIGINIMYVCVCIDMLFIKDLSN

>SG-AL_contig_2869_1_-1_2980_3297

MYCYLEISSFLLLSLFLPLFLPLLSSAFFCHTGPFHFLFSSKPSHNFSPTVIINTGGPVR

SLPFSCEKCFLAVFLFVCLFTSLFILMGFLVEVKYLLRISFHVGIF

>SG-AL_contig_2870_1_+1_34_354

MQGLVLQGLQDQIVATVLQQLFTKVLGWVKVLAGWVFSLTLALDGIKTGDDTPILHLHMG

RGMGELALVFGTRALVPLVLATHLQRQPATVLGVGVQDRVRHHCHGN

>SG-AL_contig_2871_1_+3_162_242

MVNRGQLGIGEDPHHQQYINRESLTLI

>SG-AL_contig_2872_1_-3_3_233

LFVHLFVISFFIHIHLLYVYITYSIVFMCITYLFIWWRGGCYDRVEGVGEIASSLGMKTF

RLKIRSVWGKNPQNLWI

>SG-AL_contig_2873_1_+3_474_770

MHRHNQCIIKTQKLSLPFHSRRSNRLPLPLCTLKYRDYQCTKTKRLSLALQSLSYRLPLP

TQSIRYLDHHHQWTIEDTQVCSCPMFCTTPDSLPPSCSF

>SG-AL_contig_2874_1_+1_100_297

MKGHRIWDNFSQCCDDENGDDSDDAMELHLGLKLSDRAVLYASFHNISWRQPGPRVTDTC

GVGQEI

>SG-AL_contig_2875_1_+3_144_281

MLDHIFWQYDARLLLFPLEYFCCFTDNYGQPKHSCILYTVVIYGAE

>SG-AL_contig_2876_1_+2_203_625

MVHHERNKEGCSAFVLVVAFLLCQFYSLKKQTCSLPGISNGCSQVTNRKPIALAYGHSHV

TMWRKTMIVTGHTCSSTDSLFCGIFNSCSKTYGFIQQMQQKLQLRKVLKMNNSGWKLSSN

QSISSMYLFEKCVTKDFSRVY

>SG-AL_contig_2877_1_+2_896_1234

MLNGKQKGEQYPFKTMTCSGEYKFMKQLNKKAKHQFPFKQRMPRVFTNMDTLPAMSKSPL

VHLCWVNTSAAQARDILNWHVHLLEGRQVFAFGVFCQISPLKLSICSIISDQK

>SG-AL_contig_2878_1_+1_370_669

MHVNQVHAKCQAISVPKQILFLLRSSQCTAMDRYKCRYTTLFCTSDFTYIHTHPYYIKRQ

SCHSILFPIYYKHRTDQPVLNENPAPTDAAHSFATCSLSH

>SG-AL_contig_2879_1_+1_226_402

MWVLFYSTVFMSDITASMKRHHWSIWILFHSIIFITIVIFLHRKQELCQKTTDKGVYQH

>SG-AL_contig_2880_1_-2_479_2743

MPYAQSHYPFANKKDFEDTFPADFIAEGIDQTRGWFYTLLVLSTLLFGKPPFRNLVANGL

VLASDGQKMSKRKKNYPDPMEIAHKFGADALRLYLVNSPAVRAENLRFKEEGVRDVVKDV

FLPWYNAFRFLMQNVERLERDERGKFVFNEAKQELSDNYMDRWILSFTQSLIKFVTKEMA

AYHLYTVMPQLVKFVDQLTNWYVRMNRRRLKGEGGVADCHKALETLFSVLFSMSRVMAPF

TPFLTELMYQSMRPLLAGEAAISDDTRSVHFLQLPKAQENVIHTEIEAAVSHMQTVIELG

RVIRDRKTLPLKYPLKEVVTISNDPKALEDVRSLEKYILEELNVRSLTTTTDKEKYGVKL

RAEPDHKVLGSKLKSQFSAVSEQIRGLSDAHLTTFLQTGTINVDGFDLQTEDLRVMISVG

EQQGKGEGQQYEAHSDGQILVLLDITPDQSMLDEGVAREVINRIQRLRKKAKLMPSDEIV

VYYQASAELNRIIQQFSDFILATTKQPLKPYPVPPSDSVIITDTMPIKSHKLDMTLTGTP

SSSSASSAAGKSTSQPSSKAQCVPSLLPAPVVDGVKPCVKYVNVQLCDNLKPGECQTGTQ

ATVLLENPAGQNILTSDLLLFQVQCVFGLRGRRVQVFEDAKLSKPVSTQNLSALSGKTLY

VSPATSNNSSTTESSVKSPFCHFVNVEYAGKKATLMLENPRGCMCSLTELRDMARAVFGL

NSKPVVLSQSKDKKDAVTVQSINLLTLHSKQLFVL

>SG-AL_contig_2881_1_-1_34_543

MASTLTRRLVGQLSNSFRYGFCRSYAAAASSGSSVTSQPDADSKVTTITVEDKMDITPLT

GVPEEHIKTRTVRIFVPARNAMQSGTYGMRRWKIEFDVRERWENPLMGWTSTGDPLSNME

VTFDSKEDAMSFCEKNGWQFFIEEPKQTSFKKKSYGANFSWDKRTRRSTK

>SG-AL_contig_2882_1_-1_136_1137

MAKDRFTDEEKRSLDRAHNYLYQEMRQAAEAHRQTRKYMQSYIKPGMTMIEICETLEGTS

RKMINENGLDAGLAFPTGCSLNHCAAHYTPNAGDPTVLNYDDVCKIDFGTHINGRIIDCA

FTLAFNPKYDQLLAAVKDATNTGIREAGIDVRLCDVGERIQEVMESYEVELDGKTYQVKP

IRNLNGHSVGQYRIHAGKTVPIVKGGDATRMEEGEVYAIETFGSTGKGVVHDDMEVSHYM

KNFDVGHVPLRLTKSKQLLHVINNNFSTLAFCRRWLDRLGQTKYLMALKNLCDVGIIDPY

PPLCDIKGCYTAQFEHTLVLRPTCKEVLSRGDDY

>SG-AL_contig_2883_1_+2_293_520

MVNSKSDMLTSTQHSLSTSDPHTPSPLAHTPTHSSNTHNIQIKYLYKTYGHTIDLPFTKL

LDAFFSNQQFLTVTFN

>SG-AL_contig_2884_1_+3_231_596

MNYSIFQLWRGYHSVMMFHPTTSPTFLKITLTKRGQPCMDTHTHTHTKCTEHTQSTQPYT

HTHHRALQIYLPCSYTPPTDSTPMLLLCFSHIQTSVSHTHTHTHTHTHTNTHTHTVISLL

TT

>SG-AL_contig_2885_1_+2_380_568

MCTFRQSHNIIVHSKFISIYYDSHVFIFKNPPMILARHNSRKHGSCTDGDQRLTVAESSQ

YWH

>SG-AL_contig_2886_1_-1_1_8061

KGEEVDLKASETIEILKEPGLTTVNCKDGVRKDTGEYTITVSNKHGSDTATIDVVVLGPP

SRPGGPLEAKNITKDGLTLHWTPSPDDGGGDAPTYKVEKYDLMKGKWEKVADVQGNKCNV

PKLQENHEYKFRVTATNKNGDSEPLEIDSPVTAKRPFDEPHPPSQPEVVDRDRNFIEIKW

EPPTKDGGAPITGYDVERKDPKTNKWVKINKSPIQTCQYKDDRVKENQEYQYRVIANNEA

GPSDPSMETKPIPARPTKETPKLNLDSLYGAKDIRVRAGEPINLQLGISGTPDPTVEWLK

DGVPVGNRAQTQNDAKQAKLHVAKAERGDGGRYTVKVENPHGVDTADINIVVLDKPSAPE

GPIEVSDIMADQMKLRWNPPKENGGAEIMGYMLEKQEEGSNIWEKVPGIINGTSHLVKGL

KDGKAYKFRVKAENMYGASEPLEGDRVIAKNPFDRPDAPRDLEIPKFDRRSATLTWKEPL

SDGGNKIKGYLVEKKERNRDWEKVTAYPVTEKQYTVLNLKEGSEVEFRVMAVNDGGPGEP

SKHTPPHLVRDQVFPAGAPSHPNVDKITKNSVDLSWHKPSSDGGGKLTGYVVEKKKKGDD

NEWEEATVVPATATSACVHGLVEGEEYQFRIRAENAAGPGEPSKPTNTITIADQPDKPKM

DLSKVKDITVKAGQDFKIAIPFTGTPKPTAAWVLNDGDLETSPRVAPKLTDNEVILEVSK

AKRDDTGKYKVTLKNDSGVETATVGVNVLDKPQAPEGPIKASEMDGESMTLSWQPPKDEG

GEPVANYIVEKRKEGHPKWVKVSSFCHNPSCQVRNLEPGTKYEFRVSAENAQGISEPLET

SEAILSKLPFDAPNAPSKPTCVATTEDSITLSWEAPRKDGGSPIKGYVLEKREKGDKKWT

KANVQDIADTEYTVKGLKLDKAYEFRVAALNKAGVGDFSDETGAIKPAPPPVAPRVSRDM

MGAGKDIKAKVGEEFKIAIPFSANPTPTATWAQGGITVQPSERFTMDTQPNQALLVCKKA

EKGDTGRYTVTLHNDKGQDSATVNVTVYDSPTSPEGPLDISNVTPDSCVLKWSPPMEDGG

SPITNYVVEKQDLKSGKWEPVSKFVRNPTFEVMGLTEGHEYNFRVMAENEFGLSEPLDSA

FSIAAQYPYTAPDAPDTPKVDDVDESSVTLVWSKPKNDGGKKIQGYVVEYKEPSSNRWKA

YNDIPTADTKATVKGLKKDTEYEFRVRAKNIAGLGNPSDSTGSVHVKPKYTKPGSPGVPE

ATKVGRTYADLRWDKPRNDGGSKLTGYLVEKREKGMSMWTKAKEVGAADSQCTIDNLAEN

VEYEFRVAALNAAGAGEPSLACAPIKIKEKLEGHAPDFLKKLHNVKGALGGEASFTVEFS

GTPKPKVLWFKNGVEVGNAGRTRIKTDDNQCMLTINELYEKDAGEISCELVNPLGRETCK

ARLDVQVPPKLDKDIRDQKVQKGDQFKIKVPFSGTGPFDFKVKVNGKELKESDRIKISPF

DDYMTLIIKDVELEDAGKFEVEVSNESGAATTNFNLKVCSPPGVPTGPLAVADITKSSCR

LAWKPPKVDGGARVTGYQVERQEVGKPYWVTIFSNCKDTSADVQGLYDNHQYLFRVAANN

ENGMGEFLEVEGGPIVAKMPFDAPEAPGMPDVAEVGGDFVSLSWEKPRSDGGGRITSYWV

DKREHGTDNWSRVNTSPCVTNIINIPNLIEDKRYEFRVFAENEAGMSKPSMASNSVKIKD

PNAAVVPEFSSGLRKVAAVEGKSARFECEVSGQPKPEVQWFKGSREVSDGNKYEVLTEGD

KHILIVHNVYGEDADEYSCRASNKGGNRVTRADLEISSPPKINVPPRFRDVCTFEKGENV

VLKIPFTGHPKPAVKWIRDGEELRGSRYHSDVTERHALLTIKDASKDDDGPYRLSLENNL

GNDSAVIKIQVNDRPDPPRFPVIENIRDESVVMSWKPPLNDGGSFITAYIVEKREVPSQN

WLRVASTRFAFHNITGLSPSKEYQFRVIAENFYGRSEPCETTEPIKMEEPEDMKKKKQLE

DEFGRKVRGKYDGPKINDYDKFFDDIWKKYVPQPVDIKEGSVYDYYEILEELGSGAFGVV

HRCIEKATGRVFVAKFINTPYPLDKHTVKNEINIMNNLHHPKLINLHDAFEDKYEMVLIL

EFLAGGELFDRIAAEDYRMSEAEVINYMRQACEGLKHMHENSIVHLDIKPENIMCETKKS

TNVKIIDFGLASKLNPDEIVKVTTATAEFAAPEIVEREPVGFYTDMWAIGVLAYVLLSGL

SPFAGEDDLDTLQNVKRCDWEFDEEAFANVSQEGKEFIRALLLKQPQKRMTVHEALEHAW

LKGDHSNLTNRIPSSRYSKIRQKIKDKYADWPQPMPAIGRIANFSSLRKHRPKEFMIFDL

YFDRKEAAPRFVRKPRNQVAAEGQTAKFDCKIVAASAPIVTWMKSDMQLSQSYKHMQKYM

GKDYELRISRIKMDDKGEYCVRAENSFGRKEERANLQVEPGIEPPRPPSHEVTPRTKRVF

EELPIEVPKEDTPPNFGFGLRPRFIQAGSEFKLICHVQAFPVPKVTWTRDGRDLSDNDHY

NITYSHGVCTLEVAYSKAADTGEYVCRAVNSLGEKETSCTVTVEERAFYKPKDGSHTSSS

VSRMVRRTTSGTKIGESSYESSYSSKTTRRSDRLDDSYSSSSRTERK

>SG-AL_contig_2887_1_-1_1_189

TWDIGKISTLYPSQGVSRQVEPRTFRLEVQRLPTRLPRPSVCSVHRWVERWDVGCLIFPG

QHQ

>SG-AL_contig_2888_1_-1_238_957

MKLECVVAVMAALVAVSMAAKRRCQACDPDACPALNGANCLAGTTKDECGCCDVCAKLEG

QACDTDGHHDLCGDGLQCKKTANTGAICQCKFQEILCGSDGKTYSNLCQLMTTAVREQRT

NTLNVKSVGPCEPGATIVTKPEYVRNHTESNVVLQCEAMGLPTPSMAWVFTRADNQTFNL

PGDDCKMVTSARGGPGKFMITSWLQIEGLQKFHEGDYTCVAYNQHHTDRSKARIKVVERQ

>SG-AL_contig_2889_1_+3_3_158

RQTFGNKCNVRRRGIHKIWHDFNNFTQLPEYTLKYNNLFVETIVAYYMSFRH

>SG-AL_contig_2890_1_-1_1_228

ILRFKRGRRSAWSRYEEAALSRNPPEVSAQDRRLLHRSAPTSISIPVATLAELDKLARAA

QLSADYSCLYMRALGT

>SG-AL_contig_2891_1_+2_428_682

MLTIKLTKILTNVDPLNTEHKENAHNKTRKLSYQLFQLIGTLCSPTKHLWFHNGSQWCIQ

PNQFIFNISVTRRMNKQATSKQAVK

>SG-AL_contig_2892_1_-2_2_136

LPSLAPSLSPLTPLGQQPDEIQRPSHCGRDLQAVPLPRSTDQLSP

>SG-AL_contig_2893_1_+3_138_263

MYNLYNHRLISTMEEYHSGSTRRTRHSTHTQNLKSYKNNTEK

>SG-AL_contig_2894_1_-1_196_921

MKPVMKHEYQPTACSAYTGTLTVIPTSQIPPHHLQNFTHISPPASPEHRDELKCRPHLTM

YASMPQPQPLHQQQPQQQHPQFLMQHHHHAHQLPPQHQPTLLPKLVPGMHQVPHPIITPP

SSPQLVDLLLPQAGMEGAAGQQPKKRGRRTWGRKRQMTHSCTHPGCNKTYTKSSHLKAHL

RTHTGEKPYHCTWKGCGWKFARSDELTRHYRKHTGDRPFQCHLCERAFSRSDHLSLHMKR

HI

>SG-AL_contig_2895_1_-1_1_237

VNDFNGRKRVGRRPRHGVSDKHKHTPSKITTARGKRDCVMCKREGRKTAAGNAIQCSFQC

VKCDVALCKDRGCFRAFHQ

>SG-AL_contig_2896_1_+2_2_148

GIFNTFERVLLYTVPAYYWYSRRERTVQPACLTRKRSRECLGVRLRVQT

>SG-AL_contig_2897_1_-2_875_1198

MVLPWPLLISVPLARCSNRHFLLDACAACLAYEVFLLLACWAATSSFSGVSVRILVFLKC

LFLPTFLRGEYGCGRNISREHAENDGSSTIIFLYSACAVPFCWDLLVS

>SG-AL_contig_2898_1_+2_2_208

HTHCTNSQSHTYTHINTIQTHTYTHTVQTHTYAHHTLYKLVHTQTTHLVTIKIMQIIQLT

ALLAIKIFK

>SG-AL_contig_2899_1_-3_261_398

MASVRTFTHGINFIIHFSLTINCSFFIERKYVCVQIVVLEWRFDRK

>SG-AL_contig_2900_1_+1_889_1059

MGLYPICMSEAQQVENVYRSDQYLNFQSNPCAQTQLSESFSQSIPSIQQLPYDISHF

>SG-AL_contig_2901_1_-2_422_2953

MEPEEEPPACPVCTEFEPQAWRKSLCRNCFKTFEQHTEEGSDTDTTPPDSKAATASASKP

PKDSKQSTVKEPAQKSPVKDSIQKAAEKSPSSSPSSSSKSSSSTSGSKLGSLKSSTTTSS

TSATTTTTTTTATTSSGKSTSTSSKAKDMASKFEDKSSKGSSKPTTTPSSLTKTTTTSTT

TTSASKTGSSTSLTSSSKASATSTSTPSSGKAGAEKKPSAISALAGKIDTKTTSSTGKST

SDSKSTVSDSKTPTPESKPKTPPSTAKKPLSLSPSKTVGGKEKEEVPSSPSPSSPSPSDS

SARAKWEKRTSGAGGGGGTGVKSDVTKGQKSGGDVKGIRGDSANGAIGVGKSRLAELSSA

KKTGGKEIDTVDTSSSSSPSSAKKSPTTASDKTPPSPTTPAGKARSTDLPASKTSPKFSS

SGRTSSSSSTPPSSPSTLSPTKRDASSTLSSSSATSSKTSLTSRADSKKDAAPESVKGSS

TPAADASATSALLEEKEKVLKELEEKTQALEKELSQKDESLRKKLAAKDEELKGLKDVRE

KKEKELRELKGRLTTMEKESREAMKEVEQAKVASVKGQREEWERSLSELRSQVTAMEEQC

NKLENDNQSLMTKLKEQEVEANQVAANHQQHQQDPSTTAVSGLQGDLRTSELEVEELKEE

NVDLRQELKDLRREMDEMYDTFRENELDEFRELQRELDLTSKNCRVLQFKLRKAERRADQ

VETDRQHYEEKLRILQNQFESGDARGHIEALEEELRTAKEVSVRLHDELDLMEDRRNKAL

EDNRHLTELLEQTDKKQFRMEMEIDKLRDQIADLKQQLREKSPTRKEGTPDRRVISSIYT

RSID

>SG-AL_contig_2902_1_-2_65_556

MAGLLRNGLRAATLARQCSRLAVSQQRGYAEMAFTFASPTDVYYNAANVKQVDVPSFSGS

FGILPSHVPSLAVLKPGVVTVFEEDGSHKKFFVSSGSITINDDSSVQVLAEEAHPLDRLD

NQAIREGIAKAQQEVNAATSEKAKAEAQIALECYEALQKATEAS

>SG-AL_contig_2903_1_-2_179_568

MDLGPRQGDRLPPGPHGVPSASAPSGQGTQTPAAESSPAPLASVREQLGPGGPPGRLVPG

GDAGMGLSLRPGRGCPHCSPSSPGGPLHGRLQPGLGRARGLSTRLRDVDSGRSPPPHQRP

RDGGGVTLPP

>SG-AL_contig_2904_1_-2_2_283

PRGSWISAPPWGFRSTPRSAASRLHSLSTSWAPGSSAQAVFPPGSILSPRQGYCLPSGPH

GVSSASAPSGQSSQASIAEGSPDPLAPVPDQLGS

>SG-AL_contig_2905_1_-2_113_241

MSSAKSRSDRRSPLCHCMSFPSVALFSTLSTASAKRRGDRRQP

>SG-AL_contig_2906_1_-3_72_242

MSVTSDWRSRVSLVSAAISSAKSRSHRRSPLCHCIPFLSVVFFSTQSMASTKRSGDR

>SG-AL_contig_2907_1_-1_4_957

MYRNVRVPHNCQQLQSGVRAASTLDISGIYPPIATPFNEDESVAYDKLQFNINRWNSIPL

RGYVVQGSNGEYAFLTNEERVEMVRRVKDMAPKDKLLIAGSGCESTQATIVMTKKMAEAG

ADAVMVVTPCYYKANMTNEAMVAHYTKVADASPVPVILYSVPGNTGLDLSPEVIIKLASH

PNIIGLKESGGDVSKLGLLAHRTRDQDFQLLAGSAGFLLGAYSVGCVGGVCALANMLGEE

VCRLVELYNTGQMKEAQTLQQRLVAPNASVTRQFGVPGLKAAMDDLGYYGGPTRLPMLPL

TPSQRDSQRQVFKASGFM

>SG-AL_contig_2908_1_+1_319_480

MKYDTVCVLYGECYTYSSWLCEECRFQSLLSDYALQVQRENMCVTEQEKEKVNF

>SG-AL_contig_2909_1_-3_63_1196

MPRDVSSYLSEQQRKSTGETASDWAQIEEFYNKKLWHQLTLKLSAFVKTPVFAKGDSLVR

LYENFLTDFEHRINPLALTEICVLVVKQIQDPKQAVEFIEKLKEKVKSSDEAKVLCMTTI

GNIRLRNKDFENVKTVIEECQGLLDGLDGVTSVHSRFYELTSNYHKLLGNHADYYKEALR

FLGCVQLKDIPSGEQAERAFNLGLAAVLGEGVYNFGELLAHDVLLSLKSSDKEWLVDLLY

AFNSGNISKFEDLKKFWGGQPDLAAAELQMRQKISLLCLMEMTFQRPATDRQLTFQIISE

TARIPVIEVELLVMKALSLGLVKGSIDEVEQRVHMTWVQPRVLDLEQVAVIQNRLQQWSE

DVKQMEHLVENSAHDILT

>SG-AL_contig_2910_1_-1_922_1956

MALINTWHHCCSTTSWMKWGHVATNVHGKRVLRMMLTRKSRSMADLLAELKEELVSLTGH

LFGAKWKHGQFEQMKKQIPAFRGTVGMVLGFAENFTCSYQDEVQSAHWHYEQVTVHPIMT

YCPCSSCDQTMSESLVFISDDKNHDCNAVLRFTQIAMQHLRGSRKIDMQHAMQWTDGCAS

QYKSKGPFADISYALADFGCTWECKFFGSRHGKGPSDGESTVVKHHASSAVKAGIATVST

ACQESFDYCQGSKLNKQPGEGCQHFLRTFFFLCAIRGHRRQRQSCKNGQRDTFSACSEMC

GAKQAPHQGAVLCLSALLVQHWHVFYSRSDRAVGDTIPRCRSSLS

>SG-AL_contig_2911_1_-3_9_119

MHVIFIVSGCLHLFLINLDQGTAILRIVCTFGSSYLR

>SG-AL_contig_2912_1_+2_2_91

ETDNSTHQWAHTQNPHTQAHVHIKRALFIT

>SG-AL_contig_2913_1_-2_74_1906

MENWGLITYQKLLLYNSQLVASDRLWLAQVISHELAHMWFGNTVSPKWWTWTWLKEGFAS

YWETYAVEQLHPDWKVRSVYMASDVFFIMKLDATPLAHPLNSPVETPDEIFDQFDDFAYV

KGSAMVGMMHMIMGEHFIPAVNMYLQDNKFGNADSSNLFDSLNKYISTTPDIDLNMTKIM

EPWVTQMGLPLVNVHRIGDQLHLTQEKYWNTPGSHSDTGAYGYKYEIPITFTTSSERNFN

KPRTDIEWMSVDQDSRTIQLPSANDDWVVINLRHQGYYRVNYDKDMWKAIIDQLNTDHTA

IHEINRASLLNDAWNLYKAELLPLETALQTLDYMKKETEYGPWSVLDTETRYVHDIIKQT

ELFADFQRFMQEMVDEPMRILGLDFPPDNVPPVEALTKTLILTVACAYDHVACVTKATSM

VSDWMNGLGNAISVNIRSVFYCTAVRDGGHDVWDFIFQQHQAEQDDLERYDLFDALGCSK

DPTILYRFLDMCLNEEVVQSDLESCVTAVTRNRYGHDVAFNFVMEHFPVLLSSFSSDTVG

IIVGRATKHFHNNFHLAQIQLLMDTYDVSGITRNLGLAKLGTIKRIRWIKNNLDTVKRWL

ASKSEAVHDTE

>SG-AL_contig_2914_1_+3_555_854

MHKLYTGINSKYACIHTYAHIYINTYKHMYTHMQIRTHMQSHTCTQTHNRTHTNTHSEGK

KIENNTLKLLKWDNTAVIFMVFKSLCACLCTSHMHAHLHL

>SG-AL_contig_2915_1_+2_1370_1528

MQGVTISTDYQHNRSSLWKPKQYLPPRKGRRPFACLSHPDKDDMFNARHQIKV

>SG-AL_contig_2916_1_+3_108_221

MHKLNVQKLESLSSNLQDYISQLAYFVCVSIIYCDNTV

>SG-AL_contig_2917_1_+1_238_618

MTLFLSTCYNRLGLMSLGHKSQVRQTRSQDLASAYGLTQANGICHDFYLLFFLLLCRPIF

CATSVALIPRYSFWFLNTPPTLKFAQLKSQCQEAHGSQRCQVAVGPPTRGDPALLLGHFG

GVQQCLF

>SG-AL_contig_2918_1_-3_159_1820

MQLKAGLQGFDALACLLNYCTTELDAFSCPAISAQLKIAQAKLTEQQQSLDRVSQEKGQV

EQQLQEVTAARDAARQEVEKLQQQLKQAEETHNTAQQQLKEEHCAALKTLQEELTSQHQQ

TIQEIQKHQDKQAEYTRHSQKRRLSELTSEHQMATNQLKLAHLEEIQELRNKHELQIAEL

HVQQRKKLDDITQRFESIKLSLTEKVQSLCGECDDLRVRAKASEEALQRDSDVKVQLALT

PYLQLPKEIESLKTVIDMRTEEIQKLRRQNMDLVKQLEELPIAQEKVAALQQKVENLQAI

INIKTDHEKQLHEKCQVLMRKYDRESRANKRLSMDYEQVVWRMSQSADFTGSTESVSPRQ

LSRSPSRGDQHSHGSSPARSLSSSSQGGDSNGVVVRRHRKGSAGDDNSDHRRRANTFSSD

TSKHKRSGEEGDSRINGPSSSGKQRVLSKSGDYSDLVKSAKEHEESFSSGHESGDMSDFY

SSLESNVILETAQGVSFHVVDKTATDGVASQEEVFMEEMLNVTDEGLPADSSLPSDSMDS

QTEVTVVITSPTSK

>SG-AL_contig_2919_1_-3_24_1436

MGGDEEKPETNRQPHLSKQDLNTLDINILNPLTPEVISRQATINIGTIGHVAHGKSTVVK

AISGVQTVRFKNELERNITIKLGYANAKIYKCDNDACPRPGCYRSCGSSKEDSFACDRPG

CGGKFKVLRHVSFVDCPGHDILMATMLNGAAVMDAALLLIAGNESCPQPQTSEHLAAVEI

MKLKHILILQNKIDLVKESQAKEQYEQILGFVKGTVAEGAPVIPVSAQLKYNIDVICEYI

TKRIPVPVRDFTSEPRLIVIRSFDVNKPGCEVDDLQGGVAGGSILRGVLKVGQEIEVRPG

IVTRDQEGKLSCRPIKSKIVSLYAEQNDLQYAVPGGLIGVGTKLDPTMCRADRLVGQVLG

AVGALPEIYTELEISFFLLRRLLGVKTEGDKKGAKVQKLAKNEVLMVNIGSLSTGGRVQA

VRADLAKIGLTNPVCTEIGEKIALSRRVEKHWRLIGWGQIRRGVKVTPEQG

>SG-AL_contig_2920_1_-3_3_215

IDSALQTCERDKSPWQSTTSCENRSSVVNCNLETTFMLSINCKKKIVSGNGAGFGEEIFA

FPISKFPRDRN

>SG-AL_contig_2921_1_-1_1_159

TNEIIAKALKQKHKTKRSQTTNGLISKITIYKWLTSLPVTKYESVSLQNNHCH

>SG-AL_contig_2922_1_+3_3_167

SAPYSKTGFTMVLKILILMLVVRSVDPQTFFSWWKAVLALPIRVRMSASVPPCTD

>SG-AL_contig_2923_1_+1_1_303

NDSTFSKDVFCSSPPNGKVSLLVTDRQCNEEQNQAGCLDTTSCFSSVKKTWIAAGQEMAV

SNVTFFNYNRSHHWIQTSVICICQTWHSIMIMKSALQIFII

>SG-AL_contig_2924_1_-1_1_207

DPNIVGEDHYKTARAVQKILQDFKSLQDIIAILGMDELSEEDKLTVSRARKMQRFLSQPF

QVAEVFTGS

>SG-AL_contig_2925_1_-1_118_462

MYTHTHCTESGAVCRHLYYTNLFTHTHIYTSEPIHIQVTYTCYTCLNAHARTDLQRPVSG

WNAWNGIGQEGVWVSGRSHTHTHTLHRTACIYVVQTCSHTHTHAPKSSLFLLMKH

>SG-AL_contig_2926_1_-1_1_60

KKKTKKTHTQKVSKRPLRWT

>SG-AL_contig_2927_1_-2_185_277

MHSRKHSISYPVPVIRICCPIQPKISDTTFE

>SG-AL_contig_2928_1_-2_8_3649

MDAQVYNMAEHATSAEMELSSGLGLTLMNSTEMDFGCEEDFDLLKYVEQDNLSMFHESND

LHGLPSFVYNEEEFQTQSQTENIFDTAAIPSVLVKEEPGTILPAHMKSETSLTAHTTAMT

ENQVSHSLRSLLESQNTCSGVQQEPSGLPETIGGIVLDTDIDPSLVSLQTVPAQMASVSQ

PALQNEPMKTDSTQSSDVSSKLLKVLQNQMLQEKKRKQLQQLLLEIEQTQQNERQQQLQQ

LIDISDIRQRAQANTVTNKTIDLNQLKLILKQPSTTVSSVPASAQLPSNTTQVAPAQVRL

VNSQPVQVEASCTASSQNTGQLTLQQLQQLLGKKVTSTAGSSDITAATAINANVILGTPT

AHSTPSASTTISSISTSPVQAVITNGNTLFTTSIPVHVGGDKIAINRLSAAESGNKGEKR

TPHNAIEKRYRLSINDKITELRDLVGGEDTKLSKSAILKKAIDRIHHLQKSVQRLRRENM

ILKMALQKESLEGLLRKGENIDVSMLALSPPYSDRSCSSPESGHTHSDSSPPSSPVAFDD

IANDVSNVKRTFALAQSISDRSRLALCMFMFGILAFNPFNVLFGSQLQDKGDNSGHIGRT

LLGIQEGSGQGWTQTLSAWLPNVLLWMLNAVVVGMVLAKLLVYGEPITQRNTSAHVSYWR

HRRQAEVDTERGDYSSAGQQLRHCLQALGRPLPVSFSDILSGILWQLVRQFLHRLYLGRW

LSTRAGGLRSSKIQPLEVKESARDAALAYHMLNQLHLTGHQKGSAMWGLNLALCAVNMGE

TAKETIPCWLLAEIYATSALQAKSALLDRLQFMARYFLTRARRVCARDGDHVSPSIQWLL

HPEGHNFFVYSKPVDTSSSSILSSSSSEVDPLARVTQAFREHLLEKSMFSLVSPQEPSGK

NGRSEAMQYTQILQECSCLSGHSSSSTAFYNTSTTPAIDEVARWWAAVVSVAFYWLTGDD

ENAARSYPLLDTLPPTLQSSDDPLPRAVYLAYKARKVMVLQPDMSRARFVVRQCDRAGRL

LRDSLKLIYPEEERNTVKSIQLLLCDWLLTTRTELWEMSRSGNRGEEHTASQVELIAFQQ

DLGSLRKLSQINKAALPKVFLHEATARLMAGANPARTQQFLDRSIRRRVQLPKNTGDSSS

DSDVADREQATALLMAGRHLPQPLLKSHEERADLVSEASHMYEMLGDHRSVQACHKALQE

LDMSAPTKEIPIQC

>SG-AL_contig_2929_1_-3_267_1112

MKEVVKVHAEDFDCTVQSGVTREELNSYIRATGLTFPIDPGADASLCGMCATSASGTSAV

RYGTMRENTLNLEVVRADGSVFWTAGKGKRCKKTSAGYNLTNLFVGSEGTLGIITKATIR

LHAIPEATLSAVVHFPGVQEAVDTTVQVLQSGVPIARIEFLDEVAVEAFNKFSKVDKFDM

KVAPSLFLEFHGSPTSVKEQVGVVEEIAGSNGGGDFEWATDPDERNQLWKARHEMLYACL

ALVPGSKPFTTDVCVPVSNLPEAILKTKEMIKKANIVAWPWS

>SG-AL_contig_2930_1_+1_184_444

MSFESKLPSAEGADPELQSFIQMETQRAQFQGQVHKLTDLCWDQCVDKPRDKLDSRTETC

MSNCVERFIDTSLSIANRFQKILQGNQ

>SG-AL_contig_2931_1_+2_398_700

MCSLLERDRNKSNRAYPWHLTHKLFRLWMRRDAGSRRRIATLGPSLHVFVISFSSCLLIV

VISVLKKISNETKQKSKHEQWLKYRNSDSGKIQAVRAAHQV

>SG-AL_contig_2932_1_+1_712_960

MQHNVSHNSRNHVSVLYGVLAFVSIIMIWLELFYSEIKSKWHSTITVTRSVQDAPFQIHV

YIYMFNTTVISINIFIKQFPYKI

>SG-AL_contig_2933_1_+2_2_121

HAGCFRVSISHRTLTWLTGSLTCIFDVLHAYEYTRRGFRH

>SG-AL_contig_2934_1_-2_2_148

TKGVQAPTTPHIHRPGRPEKSPSPSNQARYPRRIRTQAFQVSFLYYLNK

>SG-AL_contig_2935_1_+3_3_395

WLYCIVLYSFFFSFFVTLDLPCEIRAAFPGTARRRIVAPPFFSVLVHAGCVRVSISHRAR

TWLTGSLTCIFGVLHAYTREGDSGTSRSAHILTWEIGKISTLYPPGEVPVVRTEAFHTLQ

FGSQVSSPSDH

>SG-AL_contig_2936_1_+2_881_1048

MTDAQLSHHYIALLLSFSFLFFLFMCRKNVCSVVCSNYCYCYCLSYYLLASIKIIS

>SG-AL_contig_2937_1_-3_3_230

GGDCLDTLLLECVHQITINYYQYYTVMHKIVYMLVQPIAYTVHGTVNPFTPSPFPPASFN

PFAPSPYPPDSTSGFL

>SG-AL_contig_2938_1_-1_46_294

MGYVGGLKVILCNKNKFVGNELEETSRHSLSTFPPLNKVPIQLAGMEANRVFLNARGHCQ

IQVDRLQSGQFDSAPSQPCRKSL

>SG-AL_contig_2939_1_-3_78_803

MERLISAAGWQWLRQFTQHRNRMVSTAFRFTSTDSSFIQSRVTGLSYPQNAITTTQLTPL

LQRLRFCSGLQQQWSTPALSAHRRFHASTRATVRKTAYSNFGHKRKPPSKWSYFYCTMLV

TLFVGFFCLNGFQWLVEKLKVDASSVVESDSKEGKEDKESEEEEEEADEGKKKKKKVGFR

DRRIIGYEDRIRAYSTPDKIFRYFATLKVVNSGEVFMTPEDFVRSITPGTKQPEGLGLDS

FK

>SG-AL_contig_2940_1_+2_422_625

MTIFSRKNLRLLMTIPTIKTASKVPCLLMTPSSIKIIATTTNNNKILSINDCLLLQKLRL

LINDYLLV

>SG-AL_contig_2941_1_+2_2_649

QHSINYPRFIFTSPPTQHSIDNPQFIFTSPPTQQSTIRSLSSPLLQYNAQSTIHSSSSPL

LQYNTQSTIHSSSSPLLNTTINYPQFIFTSPPTQQSTIRSSSSPLPQHSTQSAIHSSSSP

LLQYNTQSTIHSSTSVTSPPIHSVVLCFTSILLSSLCPSFLYPVPRQTDSPSKNSSPRSQ

KQSLVSVFMASFIFMINSTILTPSSYAKHLIYNCKP

>SG-AL_contig_2942_1_+2_2_151

FIAQLTTKAISGLSPCKVHQSQEDNCIEHKTSSNLKTKILQKITIHDILS

>SG-AL_contig_2943_1_-2_2_142

TLPQQDQSTDVKNKIKNKIKMPYQKIKIKRTLCNKLLVEIKFLCEYH

>SG-AL_contig_2944_1_-1_880_1362

MDNKTCEFFVVVLFFLKRLSVHVCLLCCVLYFCRCFLWIFEIYRSFWMLDDDSSLFVMLC

CTQLKVYWDFCLCVCCLVLVMVTTAKEVSIRDDSLNHWNLASEAKRVELCLCNGSSIFKK

KLCLVQFSVWPLSCQIPAQTSSLNSMDYQYLMFRCMLLKHS

>SG-AL_contig_2946_1_+1_289_576

MLITCFCCCFFCFLIVCVCVCVCVCVCVWLRPHLLYHSCVTSPSLLISALQLTSKPTETE

VTMRAGQRRATESGIIFHNGMMMRMGMIAMMLWSSI

>SG-AL_contig_2947_1_-2_8_2161

MDPPMSDCQLQQHVEQKIHNITTCLVRQLMTLPAVTHVEVTGHLSVVVNKRLTFVVFLPP

DFNSECISTAAFQCMCTPQRDQCSSCKVTVGQFVGSSVVEKSEENEGVPMFKASIDEDDE

KGLQTPAEHTHEVLQTGTETPVTHDHELKKISTITTSGQNLSLASVETTQDLDDSNHALT

NLETGINDSQDVGSVVSDRQDLRNIANGLEYNSQESLNVSNATEYTGQYSLNALIDTEYT

GLNSQDSLDRSHKATASRKSKLSLSPQNKPARPTRKRGRLKKLPVSLENEMLTEASILRE

NQGKQCKQQIFTKELVQNEEQETQHKQRTLDKKDGPAPSEGVVERPHEVSEETPKKASEL

QCSVCGKTYPAGKHMAFRKHVKTHTQSVQCDICGKWLSSAGSLKNHQRGLHSKEKPYSCK

ECGESFSFYHSFKLHGYKHSGSRPYVCPHCSKAYLTSSHLKAHLISAHTREKDRQPQFQC

ETCGRSFALAASLKTHSFIHSNVKPFHCEVCSKGFGSKSALKLHGKSVHVTDRKFECDVC

HKLYKSQHLLNIHQQRHTAEENGRFICDVCGRQFMVPSSLKAHALVHQPTCPYTCKICGK

GFKLLPSLYSHRYTHTTERPFQCSKCPKVFKTKSACTAHEQRHTATKAFACQRCDARFPD

KGGLDKHTRTIHSLTNMLQCMVCGKVGTRMDNMRTHVKSHGHDKTFEQLNACIKPINS

>SG-AL_contig_2948_1_+1_250_354

MKPGIFCFLCTPQVQWLTHSKQNFTLHSDVNNTVR

>SG-AL_contig_2949_1_-2_530_1369

MKDKVCSEFVFMPLKGDERVPPMATGGSTVAPADSAGSAAVVASDSAEVKSKIVVVGDCQ

CGKTTLIHRYVQSQFKDTYTPTGFDTYSGSYTVSDTYTIHMSIWDTSGDAGYDRVRPLSY

SDANLVIICFSIDNPESMENVVSKWHPEVREHCPTQPIMLVGCKADLRSEAKHLPAGTPR

PNFVSYDQGLKTAKHIGALVYSETSSKTSQRSVNDVMEVAALSSAGTTGMGAAANPAGAA

NGDAAGFGRQRSFIRRKRFSGLNEAKSHLRKEAAKSCVVM

>SG-AL_contig_2950_1_+2_2_196

SLNIRNSPHISICTFTCKQNTHIQICTETVHMFLSVNNTHTFKFVQSQLKHAQGAHMLQF

VLGER

>SG-AL_contig_2951_1_-1_868_1092

MFLIIVFYILQFAFAAFSEIKTTILELYLLSLISVYLNNGVNSCSSLDRYTNISFSNQLL

LLFFFFSPLTILQVI

>SG-AL_contig_2952_1_+3_702_1049

MSCLGIGSSAVEKILHKHLGARKQCAHWVPHALGDEQERGRVERCLKTLEKFDNGSPNQH

VRSSVETKPGFTSPTLKPNNSPQSGCFQVCPLLPNSKEHEAWENKWWHFSSPDQGM

>SG-AL_contig_2953_1_-3_141_668

MVSHFLLSLPLAVAAFAGLGLTLSFKKGKMLSMLLMGMLMVLLCVWLYNLPWALTHNLLG

VISLRSFHILVGVNFTLCVMVIYAACHCTIELFGALLLLQSAAITVCETGLYQAGLYSLP

AFQLTGVIAAYTIHRLYVANKISRGAATVASAIQLTKTTVGAVDYVAKEGERKGEE

>SG-AL_contig_2954_1_+1_1_282

ECLYLLGPVNAGIVCEREHGGQPRHAVSVQKCNSIALYSCVCVCEEEIKKNPKQQDGQVI

LKISLFSPSSNEFFKTIDKCEKLFVCLCVSGYCT

>SG-AL_contig_2955_1_-1_61_159

MVECMTLNIIGILCDKKCDMKCQQTAFVLTDPS

>SG-AL_contig_2956_1_-2_77_406

MTMEEAAVLNSQNAQRSKDASPTGSNDDDWTMLNDAEPTTDGEGASGSESVPCDGLYPNL

KVAQAVDMMKAMGFNDDGGWLTRLLETTDGDIAQALDTLKLGAQQASRLA

>SG-AL_contig_2957_1_-2_650_1066

MMFEKHMVYSRLTVLSVLLCSQVCGDTLFISSPVTVSRWYGKAQRLLCRKILKIIGDLYF

LIKCLFCCCSYGTRQGVNYFSVWTLKKMLFVYLMQFYTKVQTEDLTCSYVFRYIRQMQLD

TSLLFHPAHTRFGGKENHV

>SG-AL_contig_2958_1_-2_2_187

HTHTHACRKKLGRTYQYIYNFSRSNFFPFKWKKEATILEILRLAIKKRNQIYAKKSNLPW

TI

>SG-AL_contig_2959_1_+2_41_250

MQSKECKQRNAINRMQSTECNQGNAIRDDSNKGFTEKSKEEYFRTSQTQGQTASTTSKKY

PIKKNTDYRY

>SG-AL_contig_2960_1_-3_87_623

MIRGRPPQPLPVTQTPASSVQASGIQVVGARPATQVANVRPQEQMVGPRPIPAGLHTQPV

QGVVSAMGGGPQQHPQQVLMGRRGDGKSDTRSLGYPTQPVMTHSHGKGPAPRGPMMVHHS

HLSGVKAHHAPVTVGGQQVVAKTTIGGKVMYLTERPARARGPRNMPPEWAKNHRGGTYL

>SG-AL_contig_2961_1_-3_1014_1304

MVCVFVWVFDHLVFVVFMYVLFSSISHFVACYSPGVYTCKSCTNFCVNLHCQAVFLFMSH

YCVFFSVHFCPTSFYGSRGDFLVLFLLVWYLYFCCCC

>SG-AL_contig_2962_1_-1_40_243

MFLFCLSLSVCLPSSSKNNSTVLFMTTDTKLISQEKNLRLLNMRSVQLLSGSLLQRNCWS

DWIQSTPL

>SG-AL_contig_2963_1_+1_1258_1602

MSSTKQCTCTRLHVLYILFSFPINCIYTLLLSSLPPTHTYIHTHTYVVTHVQLTSDSANF

TETQLTLASMLQRHDWIGLLQSQRTMESVPLERLQDTSLGKALRRTCCCPCSRPT

>SG-AL_contig_2964_1_-3_21_2273

MIYAGRGATCNIVITQPRRISAVSIAERVAEERSEMLGTSVGYSVRFETVMPRPYGAILY

CTVGTLLRKLEGGLRGISHVIVDEIHERDINTDFLLVLLRDMVRTYPDLRVVLMSATVDT

TLFTEYFGTVTVVEVYGRAFPVQEYYLEDCVQMLNFVPPPNDRKKKSRDEEGDDDEQEEN

CNKKVGAGYSDSTRAALAMLSEKELSFELVEALLRYIEGLGIPGAVLVFLPGWNLIFMLQ

RHLQMSPEFGSSKYRVLPLHSQIPREEQRRVFEPVPDGVTKIILSTNIAETSVTINDVVY

VIDSCKAKMKLFTSHNNMTNYATVWASKTNLEQRRGRAGRVRPGFAFHLCSRSRFDKLDQ

HTTPEIFRTPLHELALSIKLLRLGPISGFLAKAVEPPPIDAVVEAEAILRAMHALDSNDE

LTPLGRILARMPIEPHLGKMIIYGCVFYIGDAMCTIAASTTFPEPFITPSDRRRLGWVHK

SLAGSRHSDHVALLNAFQLWEESRFGGEQCEVNFCDQKSLNMQTLRMTCEAKNQLRDILT

NIGFPEECLLPQPFNFTGPDNRLDVAITLLCLGLYPNVCYHKEKRKVLTVESKAALVHKS

SVNCSNFEIHFPSPFFIFGEKIRTRAVSAKQMTMVTPLQLLLFAARTVVVQGPSMVQLDS

WINLKIDIDLAARMVALRPPLEALMIRACKDPEGIEKPGPQEEGLMAVLRSLSRPSAGRY

GLSEGSSGNYPGSAKMARYDYDSNRRGGFGG

>SG-AL_contig_2965_1_+3_1578_1901

MNKLRLAVSDAVCSCRLHIILSSKLYSVFGICGDGGGGGSGGGVCVCMVCVRACVRVCAR

ARARVCVCVCVCVCVFSKTTTYYILGHLVKSHCNLLLLFNTHGNCILY

>SG-AL_contig_2966_1_+1_4_297

MLPVCCVMLPVCCVMLPVCCVMLPVCCVMLPVCCVMWLVCCVMLPVWSAVLCCWSDVLCC

RSAVLCCQSAVLCCWSAVLCCWSAVLCCWSAVLCYVAS

>SG-AL_contig_2967_1_-3_3_1442

PVEHTDLEIWRGLSHKHWPRLFALYNLSLPGRYISILPSIHLSIKIDSETALKLRHPSEQ

EYVKAPVVPPTGIGDIPRTPVDNKNKWGDVEGSQDSLPSYVYNAQKLKVFYPKSRFEFLI

TVCLCFLSVIVVSYFMVALYKCMCSRNYSRWRHSWTRHYNRRQRNKGAYYKQIKESVPLL

LKGHIQEVECFAVDGQFIVSCCYGGQLRIWDSNTGECISVIQRHSITPPRRRRPCLGRNI

EDSDADLYAEYHGGKMTPESTSSSEGSFIERSRAFERHAEKSRLVMHSELFPDLASSINT

NFASIPAQTVSSNSQGFDFQGWFESVNEEHRRMAVDTLPTRDQRVQSASDGAGLTPPDDL

RVRSWSQGDSPATLGSDHLAGEELEMHPASPIWCLTVRDGLIVAGCGNGRIEMWQAETGR

MKYQYNNSTSGVTAICLVGNRVVAARMNGSLEFLELETYHNPLLPTSPASASRTHRGHAR

>SG-AL_contig_2968_1_-2_38_331

MAVPITLEFSGGAELLFANVKKHEIQLPGSHTWDVKSLLPWIRDNLLKERPELFMQGETV

RPGILVLVNDADWELVGELEYEIQPNDRIVFISTLHGG

>SG-AL_contig_2969_1_-1_142_273

MKLDKVVTSIQSRYTRNMFHYAGVDPNSQAAFRLASEGFVRPED

>SG-AL_contig_2970_1_-3_51_470

MPRFSWAPAAYRQLNGLLCVYKPAGLPVISMLERIQYNLARDLNELPCYKVESQMRKTDP

DADLQTPMVVNTSAPMLTDMLEHRLIVGQRYLPSDINVTHIHGLRRHSSGVMLVGVGKYQ

DSLKLLSLSRYLRVYHVKGR

>SG-AL_contig_2971_1_-3_111_713

MDEQEVLTTLKILIIGESGVGKSSLLLRFTDDTFDPEQAATIGVDFKVKTLSVDGNKAKL

AIWDTAGQERFRTLTPSYYRGAQGVILVYDVSSKQSFTKLDAWLNELETFATKHDMVKML

VGNKIDKERRDVSKEEGLKFARKHHMLFIEASAKTKEGVQCAFEELVEKIIQTPGLWETS

GRSLRLDANNGASATSCYCVV

>SG-AL_contig_2972_1_+1_1246_1413

MYSTIHLYHLSNLQTHTRTHTATSHHTHIHNVLHDTLFSMVFRELRTIFILLTTDL

>SG-AL_contig_2973_1_-3_21_2375

MAQVLRAAAQKSLETLSRASTGRWIHTTACAPAAQKVALSRFEQNQFIDYDKQEHVLKIV

RDRLGRPLTLSEKVLYSHLDDPKNEVIERGTSYLKLRPDRVAMQDATAQMALLQFISSGL

PRVSVPSTVHCDHLIEAQIGGEKDLQRAKDINAEVYDFLSSVSAKYGVGFWKPGSGIIHQ

IILENYAYPGVLLIGTDSHTPNGGGLGGLCIGVGGADAVDVMAGMPWELKCPKVIGVKLT

GKLSGWTSPKDIILKVAGILTVKGGTGAIVEYFGPGVDSISCTGMGTICNMGAEIGATTS

VFPFNYRMQDYLKATNRPEIADLANSYKNLLSADPNAQYDQVIEIDLDKLEPHVNGPFTP

DLAHPISKLGETAKKHDWPLDIRVGLIGSCTNSSYEDMGRSVSIAKQALDHGLKAKSAFT

ITPGSEQIRATIERDGMAEVLREIGGVVLANACGPCIGQWDRQDVKKGEKNTIVTSYNRN

FTGRNDANPATHAFVTSPELVTALAIAGDLSFNPETDELTGADGKTFKLNSPYGDELPAK

GFDPGQDTYQAPPPSGESLSVKVSPESNRLQLLEPFDKWDGNDLTDMPILIKVKGKCTTD

HISAAGQWLKYRGHLDNISNNLLIGAVNSENDEINNVQNQLTGDFGAVPDVARQYKAKKV

PWVVVGDENYGEGSSREHAALEPRHLGGRAIIVKSFARIHETNLKKQGLLPLTFANPADY

DKIQPSDRLSLLNLKDLKPGSNVTCEIKHQDGKTEKIQLKHSMNEGQIEWFHAGSALNRM

KTVQK

>SG-AL_contig_2974_1_+3_3_179

FFSTLVLFTKKSLIDLEIDNEIKSCCLLSRWRGSYWFSAEGGLWLDRNGNDVLFSVLSA

>SG-AL_contig_2975_1_-1_361_3300

MRRIIALLAVASTALAMSEPNAHKPEIIASLYNERGRPEIHGIIREDETEVTLQHPLNAT

DSDHIGGASFICAFKIKESKKRPRKFEVPFEVQLKGEGSGEGVIRVKEGVHLDFEKRSHY

RFYIVAEDCGSPPRESDKAVVLIKVKDVDEFAPRFENSSYTVEVEEGKVFTSLIQLRVMD

DDASDTYGQVAGFEILTPDVPFFISKNGVLQNTEALDYSMHRNYILKVVAVDGGQRRSRP

VFVNIIVKEQCRSGWLNIPSLVRYVAGEGQKRVAESAQLRLCDKTCVPEEVKVKVTLTTK

HIGKGCDRDTYSITSQRKLCGASGDSVDLLPSPWQADWTKSLPTDDGHLMDQIFSFDGSH

NAVEVPAGHFDHHLGNHFTISTWMKHDFPGGVPVKSHNAPKEHIVCMSDGDGMNRHHYAL

WVHGEKLVFLLRQEDVAPEDMDKFKPAEFRWHIPQVNDNEWHHYAISVDFPDVRLYLDGN

LLLTDDTNYEEIDDWPLHKSNHVHSTKLVVGACWQGKQMQFKDHFRGYLAGLSMLKGKTE

SERVIKCLHNCKENLDFRGLSDMSAGTVVSFNGDMTEFTVTGGQVTEVENILRQVTYVNA

RSFPTPGRRNLRIDTTVTCNNQKLVVPAVESYVMVQEPPQAIIVINGAANLTRLVYEFQR

GLRIFHDVHIFATSQPAINSAEEEEEAYEEEETASSEDVKATQHAQKVDQVKSEQDSQKK

NSLLIDMCTVVAEPPLNLFVEHLSLPTRLLENRNLEWSETNDGVVISNADKIENYENVIR

NIHYYHNEPSKLSNRTLTLSCSSQNGRFISNKFVVRLVALHDVHDTKSFAHAQVEPAAQL

QQGLLTASGHQNAHATVASSTSNFGMAAIIVVCVGFLLFMIILGVVRIRAAHRRTQVVHV

EENQDMEWDNSALNITVNPVEQEIFEYEDNTQPSFHDDSDTDDDESSMQGDYGESSEDEA

PAPAKVPNNDLEWDHSNLGF

>SG-AL_contig_2976_1_-3_909_1166

MLQMPFFLSALWFCFVLFFLQSSCLLFLPTNPLTCMFSGKAPYDFIGMLLCFFTVDALDG

MGMGCLFCTVICQSSFTKYLSFLGIS

>SG-AL_contig_2977_1_-3_3_179

FILLLLLMLFQYLFVFSVSLCLHALYILMYWEKKCFSKTLKLEDEYLLDCWQLLCGVPV

>SG-AL_contig_2978_1_-2_80_478

MSGLCGCLGSEKKKTCTSLDAPIELNGNCGVSSTKKSTPTIKVLTYPLYGARGPNFDFVS

TDNGENYVATFRAMLDGEVLGCDSKPIPRAVFEKFQILRKEKRGTCYCIDIDSGGVDPFI

YFRVIDEETARKL

>SG-AL_contig_2979_1_-1_91_861

MNKFGGANARDLNAYKCCFCCHVKVGTILYGLFCLMVDIFGLGLVVLMSIHPDILVPQRV

DWVGQGPQYDGVHVDDLDRRLQLPGIQEPHVITKENLCMAYAVALLSILTTVALLYGVIR

TRPGYMVPFLCQHVFFFCLSCLTLVSYFTYMPHFDVKWWIQENGLSHVPGMDYFMKIDSD

YLMFFMVTMLILTLCSLAYLIGMIWACYKYVQQLAVARTMTREYQVDPDTEMLLPPRYED

AIKPENSTQPPPPPYTQ

>SG-AL_contig_2980_1_+2_8_328

MQTTVKERKNGRDKESNRKEEIASTEFPEGGDTIYIERAPGIPTGVNSHQSIKNHCGMIF

SANQLYPELSVLWAHWEEWDHTAKFCPLYGCVIRVMLKFPDQQHRYL

>SG-AL_contig_2981_1_-2_2_217

QCLCLHVCVCTYIAHVCVCVCVCVCVCFAIAAGSNYHLKYSSTFPGYSACFSSFSLINSG

FLLVYLCVFFII

>SG-AL_contig_2982_1_-3_318_809

MAMVLVLVVLCVGITLTNACSCMQENTPDRFCRYNLIFRGVALAEYEEREVPLDPTDPLS

EYSRDNDRVYMMRVDHAILMPENLREAKTVTMRTPAQDSLCGTRFSLDTDTVFFAEMNSD

GELVTGLCDPNVLWASLSESEQEILTDNIVDYCTTKDNVTPPKA

>SG-AL_contig_2983_1_-3_3_155

SRSLSLSLSLSLSLSLSLSLSLSLSVSVCLALALFLCKVLDCDKQKMKCII

>SG-AL_contig_2984_1_-3_6_497

MASFHFLLFMDLDEMLVPQNTTNWQAMLTEIFKIQQKRSVAALSFRSVFLDPTRTPDSSD

AILYFRHLHRTKAASKVRNKLMVQPLKVLELGIHHVSKAVGEEYASVEVSQKVALLHHYR

MCVTNYEPSMKCYPTRHDSTLLKYKDVLTDWTHTLVNNYSELVV

>SG-AL_contig_2985_1_-1_1_126

FKYRIVSSRKQDATFSVLDHSPVAVANTYIKILSVRIAHFCM

>SG-AL_contig_2986_1_-1_1_822

MEVLKQKMEVLKQKMEVLKQILRVNQEVSSCPSAIQSNNSSSKGDSAVHGMNDWRPFVYG

GLASITAECGTFPIDTTKTRLQIQGQKIDARFTDLKYRGMSHAFFRILREEGAIALYSGI

SPALLRQATYGTIKIGVYHTLKRAVISNPKDETLAVNVICGITAGVVSSSIANPTDVLKV

RMQATGSQSKCDNMLCSFVKIYKTEGVGGLWRGVGPTAQRAAVVAGVELPAYDLCKKHII

RSGLLGDTKETHFLASFLAGLAGAIASNPIDVVK

>SG-AL_contig_2987_1_+1_319_639

MKLEKTWVAIQSEEDGNTLDQGVARRGRELGANAGFGATIQRLLHTIDTARFDNSIFLHN

HCLLPVLHHFFGNHFLAVDVFGGGRGGAEVASPCRSPKTGARKLGHP

>SG-AL_contig_2988_1_-3_105_674

MASKFGARPAGSDGSDHWHRESIAWQYKVSSLNKTRLRFDIFLHIVLGLMMLIRLAPGLT

SLFGLNIIALRRWDLPHPRPWEYAWLFSLVSAVIGWRSTPQNNSFLLKQYMLGTVIFGML

PIVFGVIDLRNDMSAYFSERKYTYVMFGMPAVVLWALFLAICMQLHAFGLYFSVVLLKAW

RPRTERLKAR

>SG-AL_contig_2989_1_+1_1_372

CCVQSWTVVCVCVDSACVQSWTVVCVCSVCSHGLLSVCVCVLCVQSWTVVSVCVDSACVQ

SWTVVCVCVVCSHGLLSVCVCCVCSHGLLSLCVCVVCAVMDCSLCVCVCCVCIHGLLSLC

VLTV

>SG-AL_contig_2990_1_-2_137_334

MLNYFYHLQIRTPINKIQYDMKRPHKQDRRCTSQCLYNHINWTSDENSSAYLSHISQRSF

LENIQV

>SG-AL_contig_2991_1_-1_130_360

MERRKRRRILMARRRKEQRMTRGMKRKARSRVIFFYSSSFCAIFMIRQVGCVWVVNFVCW

FFFWKQGGGRWSGSVDE

>SG-AL_contig_2992_1_-2_71_10288

MKLQNKAKQAKMSKMEGARKTQAEETEKAEGEEGQGEDTSASHAGRAKLTLMKKKIEEKE

RIIRERDETIKLREQQIEAKERVLAERDTFVSNLTEQLEEKTKTIESLQSGRDTAGGDVD

TEQAMYAQMVYKDRKILELNNKVLEMDRRVMDLQELAGEKQEVIRGRDRVVEILQAAIKE

KDQSVHDQAMLITKLTTKIEASEEALDITKETLRRTEHQLKTETDHFHVKLSETQKYFEE

MLAELEGEGKRLKEMIHEKDREIIQGQNDLREIIQRHEQDIQRIVSKEEVNIHDEVIRMF

EQKLKDTNDVLEGKAKVIEVLQKESGEKEKELQENKESIKILREKLQVTSEQLMLVQANF

VDQENQWKEEKQRLELKVKDIVEKHEHELSEKEVQVQTLKSSLTELESAYTQAASQYSAL

QERYQQVTETQSSSSPSDSTTTSAVSSSPSEEVSALQAQLSQLQKELEEKSVLLEESRKA

GEESGGAKSETKMLKMKAQMTSKIKALEKELSDLRQGSSLADEVMVLKNRLAEVEEEKGN

LQLKLVDFEETRAAIGELESRIKELEDGQTQLAEERDQVKTDMAAVTDERNKYSEIVADL

EAQTENLTGQIENLKSQVETLESRQHDIEGEKMSLEVRIAEQEEQKEALEKEKGELLKEV

ERWKERVEYEKQPNIAFERENEELRNSMRQLEEELQAEQKLTKEAKEEINSLQAQLDQSK

QQKEAAAEEYTQSEADLKKKLSETQEELEDARKETARLEEETARQGEKMGLLQEEMGDLQ

RTAEEKSNKQEEEMNCLHKEKQQLQEEVSSKASLVGELELQVKTMAREVEVLREGQSEKG

KEFESKTQSLLVQLSELESELSHQQTISGNFEEEIARLNCELQDRQNEISSLQSKILQSE

GVTKEQKEIVLRLENQLKSVTEELKCECEKCAKLQEDLRDVQTQKDRDLSDKDGALSQVT

DKLNSALLSLGESEQVCSQHKVEIDNLQSQLQVRDSSVYELQTKCEELLLNITEKSTVLD

SLEADLEETNKSREALQSGLENSRKQLSELQVLQKDLQQLLELKQKELDEKTLSYDTLVQ

SMSESTSSTQEALLAKDSTIQNQNEELVQLKASLEEQQHRCSQLESSLEEKDQQMLETSG

QLQVMAAELDVRSETCSSLESAVKRTEKQKERISEQVAELMQTTEAYSQQLQMYGKTAED

LQAQLDILQEQKHTAEQQALEKASQITQLESKLSAALSDKASSDDASAAQLADMEAKVSE

ALEAKTQAESQLEMLDSQMSELGTRFSQLLTEKAEVEAKALKIKQQLEELNTMYSKVVSD

KEQFESEKAGVSHQLEELTGKYSEVLTEKGEMESEYSKLTQQLEELGGKLTEVLMGKAEA

DNRGQKLEQELNSANEMLAHLRSVSEQSEGELSQQLQSSLEEKSTLQGQVLDVQQQLQSV

QEQLQASQGQSQALQDQLSSMTTEKDDLQTQLHSLQDHLTSLEREKDSSQSKVDELNQEV

SRHVDRVAELEQTLRDLSDQFANSQADVAAQRVEKEALQTQLAEQSQMAVQTRCAEELET

VVKDLTEKLSESQSLIFHKQEELERLQSIHESQQMEIQTLQSQYKAIRSSQNTSETQNIV

ELENQIKDLAEKLIESQSAVSDKQEEVDKLQSELSQVNASLTSAQTQLQDRESSLLQQLT

MAQTEQETTVSRLSDLENEVVELRNRLATAETASVNGSSVGGGGDSGGGDVQQVLEAMNM

QLLQKADQIVELETQLQVAEAKVHDLGDKLQMKDEKMKSLVESMQEMEERLEQLTAESEQ

STSYAADSSTTHPADKSMEDELQALKTQAAEREESLAKREKFVADKELTLKKALATVKKL

KFQLQQANTQLEELRKEKENLEQQLSQQPASLPLSDADVLESAPKEQERHQGQAAPGGDS

VDSSAQSSATEELQQQLQAYIEYCARLQEQVQQLTDSVQSGESAKAKVTALEEQVELIRQ

EAEKSQMETLSLRQKMAEVQQLSEKSVQELQLQASQSQDKFREHANSLQLQLADAREECN

KSQQQISSLQKDLLQTQEDNKTAQERLAEALQEAETASKEKSSLQKVVEDLQNHVQLLSE

EAKTLQPSIENMKDQLQQLTKERDSLQLAAENHQQELLHVTEEAESSCQQNILEMESLRY

TVEEKTQLVGDLQIMVENLRSAQLETIENKDSLDSQMSSMLGMVESLQGTLSTRDSELEH

MLAQVGSLKDKLALREDEVTGLQSSLASLQDVIAMTKSDLEAVTDDYGERIQDLEAQINE

LEQQKEQLSTDLETEKNRSQEQMEQLARAVSQQEETTASIEASRETFQSQIMSLSAELAD

AQNDIQQLNDTKAALLSELNAKKESSQSASETEDLSAQLLAYQQEIQTLNDQLSAAKDNM

QVSHSDSVEIEQIRLSQETVLREKKDLEAMLSVVQNEKASIEGQLQQSKIDCDQLSVEIQ

QQQTVLAQKDEKMSILQSNQDSTSAHLSHVLQQLDQLKQQKEQMEVDNDTLRGEIEGMEK

IIADAQFREQESFEERQRMQQDLNKTLQDNTSIKARLEQLQAQSPFIKEVVQPAKDDSWD

WGSQVEVEEVAITRQSLQQTSAAPPSTGMSPSGSGGEDLSRELQEVKQRYQALQQEHEMV

RSRLQVSEVKCEKMLIKLKSFKEKNEKLQLTLDEADRHADHRVYEARRGAEEQLHSLHTR

LRDLETHNGKMIHENTTLKEAAVAASQSLREARQEVEQQRSLADKLQREVDRLREELSGQ

KQIAAVEENEARHSLEAEVSSLRSQLSDLRGHHSMLQEDNDQYQQVLDTLTSTNKDLKRQ

LEQLRFELSKAAVPSSSPADTTAASTKEVKILKQEMQRVEREREEVGEELEGVREEVVTL

RREKQVLSKRVEDVTQQIAQLHDQHRVEVTSLTEQIDGLNWKLEEAAGLEEELEDLRKLS

GSATDDRDKLVVEISRLSSVAGEIESLKTYCASMQKSVTDKEQELEEIRLILNVKEDEMQ

KLEEALSRSKSENLTLKEELGLQKQKITELLALEQTIATLTQELSEAQGEAQQYRRQVET

FTSKLDTVRSLEADFESLSEDYSAVLRDNSALAQQIEELHSRIGDLTKQDHQATLGGEQI

QHLIREKEHLTSRVKELEQQMESLLEGNTRLTQELATTRSSAQQLSFHNETLKAQSQLVT

SGGDQMSRFTADYAKLQNQFREVVEQKNSLQEEMNLARHSLQQRESRCQQLASQVSQLVE

DRTYLNTQLATLSHSLREKESEWTTIRQQYATLHQTHISTQAKVGELERRIALDSLQKDV

DVETATEADQGAQERLQSLATEREQLIQSVLSLEGRLARESAALSQAQRALGEAQEQIRL

QAVRALQEIKLDIEEEDDPRIPETSAMLTRHGMGYVQRLCQLLRVK

>SG-AL_contig_2993_1_-2_2_331

MFQKFCILSQCFILLGDYCILPTVDVYTHASPMCLTQVVRLCSLVLSFHVPQCQCFMFVL

QVLFFFSACAGALFSWPVPCVPILSFVRIAVCFHSSALLTASALFCVPFW

>SG-AL_contig_2994_1_-3_117_356

MTTFTFLHPCLKCLYALITHMIASCQYCQALTLLLPREGTFHQQLCAIFRQDIPTTDETF

GTISEKNTIFLWVSHVHEWN

>SG-AL_contig_2995_1_+2_32_250

MCFKDRSCMPCQCSVAFGNTKTPSMHAHQRLSMQQHNTCTVWAKQHACMHPRKCCSELKK

EKVGAWGHNGVTD

>SG-AL_contig_2996_1_-1_1_207

VCVCVCACMICVCVCACMVCVCVCVCVCVYVKETEREREIQKCDFMTQEAENGITLMYVF

WLHFVSSLY

>SG-AL_contig_2997_1_-1_214_435

MMLRLVKKISMMIQKTFLMKKRPTVTVITCCLVRSVLCTADNDSDEEFVPESSDTDSDSG

DSGDQPSTLKCTGM

>SG-AL_contig_2998_1_-1_79_306

MEIQKNPACTQKKKRRKKKGGAALWRRALPGRAARISHGKPAVTKNKYNTIQYKIQITGF

KFLIYFTENPFLTTYH

>SG-AL_contig_2999_1_-1_1_300

GFEPPRAPRLIGKKWRFFPISQVNIFADLLVPEPPSCLYACRTSNMHVKDPVSHANVQWL

METRKHPACTKNKNKNKKNKGGAALQRRTFPRKSNPNFSL

>SG-AL_contig_3000_1_-2_65_1507

MSTQSWHPPLPPGWEARWDPNQRAFFFIDHSTKTTSWEDPRWPNKQKLATPASGDHHSRQ

GESIPMRDMSHASPQRSPSAHRSHSPRHSPAVPRGETTFSNQREAEAEAIAARLRTRFPD

ASPVVRDVLNMCQNDEDEAASQLMQLGFESKETPPRGGASSHASPAHSKGKQSTTTSPQH

ASTTPKKSTSPAHKQSPAKHQISEAEKKKRENKLIGELKTLEPEVVKMALQACQYDENKA

RITLSRVANNYESSTKRSTQRNATSASSSSASAARSSQPTSPVALGGQTSMEPVVFSQDR

PSPSRTSGATAVGKKSTTRTTTTTTPKPKQQQQQQRRAKHVTKITHPSAGGHGTRVVTDQ

PRPGPAARTAPHHAPVHFGGSGLARGPDPTLHMGPDRGLRTGADPFFYAGANPELCDGSD

PDLLLGSSMGAEGPNPEYHHGNVPDLVTGTSGALGPNPSLRCAAEHPSSIRMEQAFLQTA

I

>SG-AL_contig_3001_1_-3_201_371

MGGAALQRRALPRRAAQISHGKSIVTKIIQYSNNTITNPRTTHPSSLTNHHTKPTAV

>SG-AL_contig_3002_1_-1_193_357

MDALRTKFSNNATCICGCKITPTHVLFFCEQLKPFLPSSLTSDSSLSFEDVLGDN

>SG-AL_contig_3003_1_+1_205_459

MVPFLILRSSTIQNSNSKELAIESSTHFPIKSITSLTTIKTYTSNFFEISRIKPKNIAIQ

PSLSTTPRWELSNACFDIDYTDLKK

>SG-AL_contig_3004_1_+2_2_163

GANRALNLTSDYNSNESINHPVRPLKKGTSLKSMGWVKMVSAAHKRKAREEELF

>SG-AL_contig_3005_1_+3_3_158

WFKNFLWVEGMSDKVILADPFLWQKLLNMKQGKAFLLARFAEKPSASGGVAL

>SG-AL_contig_3006_1_-1_220_411

MFCVFMHTHRFLSAVLTWISPDTVQFHFRLRLRDMAVVYTYSHNKFWHQPGLRDTDTCGV

GQEI

>SG-AL_contig_3007_1_-3_552_758

MLRRFFVSLHSGHTDSDPGVHSSFGDSTLGKVLWNGNCLSNPSWHVLNSEKFIIDGATTM

IIKTYTNEN

>SG-AL_contig_3008_1_+1_448_690

MSLMRILGIFWPQISKQNLLARCDQENKIRLLLLHREDGDGQDMSRDEIKIPHKNCSPLE

TSREMQKRRTKENLATHSGDR

>SG-AL_contig_3009_1_-3_120_239

MFATDIYCASAQMLAMMLHSVWNGGLVIQTSVCSDTLFFK

>SG-AL_contig_3010_1_+2_17_244

MRIHTDRVQALAGLPHVDLRDRKISTLHAPGEVSEEVRTKAFQTLQFGSQVSSPFDHRAR

QRERERERERERESVQ

>SG-AL_contig_3011_1_-2_62_208

MNDGVGDMKCFLVLFKQCMVYCFKQDWASKLNGSDHYMTYRSLKLSLQI

>SG-AL_contig_3012_1_-2_41_427

MDRTLNYGSYTREKFEKCPLFRQCKSQFSDAVFKQLSGMKRWEFPQLNSLIQNYGCSVKQ

FLENFSYIWGFETVLMFISQLASLCVCVYCGKFEEELHVLKICSLHNDSRKNILVRTNAE

YQNFFVTLS

>SG-AL_contig_3013_1_-1_58_282

MHTHSETTNKSKLVISLPSPGEICTHLVLLELSNYHRLQAVWVHCLLYHYKLFRADQAVK

GSMYSFVLKDRWIFD

>SG-AL_contig_3014_1_-2_2_169

HKTTPTTTHTHTHTHTHTHTHTHTHTHTHTHVDITENNVQIYVSEHVYDCLHLTKM

>SG-AL_contig_3015_1_+1_961_1194

MCECVCVCVCPHVHIHAHTFTHIHKLICMHIHTYTHAHVHKNYTHTHTHTRARARACTHT

HTHMHTHTRTCTHTHTHA

>SG-AL_contig_3016_1_-1_709_3072

MLGNIKFIGELGKLEMLHEGILHKCIRQLLEKKKNTPIRDMAEDMECLCQIIRTVGPRID

TPKAKTWIDTYFSRITTYATHEELPSRIRFMLQDVIELRENEWQPRRVASENGPKTIAAI

RQEAAQGRGGGKVMHSQMNGMGHSWKGFGDIFNMPVGSMSNIIGTGPGVIQVDNYTPPPF

MGMGNMGGGGGGRGGGGMMGNNRGNQHNQGFQNNLRRGNKENNSNSGGSPQNQRRNNQSQ

HQNQQFNNPQHNQQPYNRQAGGGQRENLPPRFQRMAQQQQYQQQQQQPQQQQQASQGGKP

PGRHTPPLAMGGPAGSHPANMSASQPLAGVGLLNGKEEVSLRPQKNFMFKPNTPAMLPRS

AQAAPSVPLLPQPGHHLSMSPKRMMVDVANPPINPLLSKQPQITIKPAKEEKPKPQKKET

ISEDELKEKLPGLLNDFFTKQDVTETVSAIKELKIPQKMMPDFLAQFLVGSISRTESEQD

QVMKLIATLRTDKVISSDHFMESFQKVADQMSALEQETPLVKSHVARFGAQALAMEVSSL

AELAEPFQNGVHYPLFLLCLQSAHRVKDKQWLVRAFNDSKIDLIHMLPEIDQKKERMLEI

LEDRGLSFLFPLMRVQSELGRQIAAEPTAGAIFKWIKDRVDSDLFTNPKFINILMTSTVK

YITGESTLTESTDTTVTPEKALCDKEKELLGKLKGVLQKFLHERIDLQVSALYAVQVFCY

NHQFPKGMLLRFFLNLYDEEIIEEEAFLHWKEEVNDEYPGKGKALFQVNQWLTWLEQAEE

ESDDEDEE

>SG-AL_contig_3017_1_+3_33_251

MDWEDRKKQTTVKERKTAETKRAIEKRKLLAQNFQKVGILFILKEPPASPGGHAETVFCT

EYTQRSVKMIQYI

>SG-AL_contig_3018_1_+3_3_254

ISSLFKKKKKRKKKKEKLHANTFRTLSTKQINTRPQRQETPILDTPVSSITPIQAWENVR

VKCSCKIHLFSTVKWRRRDDKAYY

>SG-AL_contig_3019_1_+3_702_1061

MVLQVLRGLNIYPVALIYGPPGLKRPQHLSRAQIYGPPGLKRPQHLSSGTNLWSSRSKEA

SIYPVTQIYDPPGLKRPQHLSRAQVYCPPGLKRPQHLSKAQIYGPPGLKRPQHLSSGTNL

>SG-AL_contig_3020_1_+3_3_227

SACTLCSPFTGSRLYWLWLLPWILPLSWKGWACCMGNSRLSLMLCPGLCPGASPAAQIPQ

SFGTEGGQARWRLET

>SG-AL_contig_3021_1_-2_479_700

MMGITGFFCFVLFFILSNISRRACMHVVYPTLCRRYVAAQSTHSIVGGHACWVFSCFHKS

LNTDMFYRIFNMHI

>SG-AL_contig_3022_1_+3_843_1046

MCFHTNNFLEDYFHSNCFQTNYCCSLSITQGDVSLVSSHKTEGKEKKPKQHISLLENGRK

KMVKIVST

>SG-AL_contig_3023_1_-3_3_197

CWLVGWLCFLFWLLRIHQQKDFGDVTLQLEGEIVTSCYTLVTVYCGTVVVKGSSLLALDV

YYNLF

>SG-AL_contig_3024_1_-2_947_1375

MQVLKNFLHAQICMHTCRHALTHWHILEFASSGETVVIAFQYCLNDRNFFHFTLCWKQSV

SKYSLCTLLFVISWQFTLIQICLVKHHAGLVILESVSTILPHNASVCEPATFLNTFLCFE

KGHFQLSGVCDFVKLARFVFMCI

>SG-AL_contig_3025_1_-2_44_301

MPKYGLEDTEEDTKPKLACSGLRDDLKDCLLKSDCVRKDGKTPKECLLLGNDPSFPTECQ

SLRAAFFECKRSLLDMRQRFRGRKGY

>SG-AL_contig_3026_1_-1_1864_2199

MVLEKVSSTEPASTEAIYFLVIFIPWRERYLAQRKLFPSLLSSLKIFLYTVLKNTVTRVY

PFKCMQFDLLEWKHVLNSVKILHVYMFAFVHSLRFPCFLVRLCDLFLCLIHA

>SG-AL_contig_3027_1_-1_1384_1566

MIHIAFTQYNTTHAHSAEKWLDLFTISELRTNLVIIYRSKSSIIKLIHVNLQIIFCSQVY

F

>SG-AL_contig_3028_1_+1_181_474

MTIGGNKAASIFTNMCIENSGFRLHHGSGSILHCRMCIENSGFRLHHGSGAILHCRKDSP

LFSNQNMKMIIFFVCTFCRPLQAAKSICKCPPVILECS

>SG-AL_contig_3029_1_-1_106_390

MGGGEKFQVPDWRSYKIEGIPELEQTQRMLAARGLKDPWLRNEVWRYTKENYGGQWKNAA

VSLGRGLKWAVAAMAITIVVDQFVSKKKDGHGDHH

>SG-AL_contig_3030_1_+2_2_106

TESRLTRVFHGLKSCRYLNVTSSVERSTNTILVDH

>SG-AL_contig_3031_1_-3_3_254

TTVNVFWVFLCIGFIHFLLREYHQRKIWLLILEICPAVARSHTHHYHHKPFIVRLTLLLL

LTCMHTHLVQNMNDVTRGFFLLNK

>SG-AL_contig_3032_1_-1_1_267

IQAALSAKGASRDLKALVADARRPGTENLYGFRWRKWLEWCKEHNVSHTKPSSLQLANFL

AYLHAQAGLSASSVRGYRSAFCSWGVRLL

>SG-AL_contig_3033_1_+1_1_150

GIFCNKIHWSVLLSFYLCKKTTTNKQEKTNKTNPKNPVVTNYKTLPKPIL

>SG-AL_contig_3034_1_-2_62_532

MDNLEREQQSLKEQIRDLKNATQAFDVTKTFPCSPGSYTVNDNPQRNVSKGDGSLCDKSL

AQGWYRFMLNGQNAEVPTTCVPKNHCSTDAPVWIDLQGKNFPKVGETVEVRACANWDSNC

CNWKTDIKVRNCGPFYVYEYTPMSTCSLSVCVQPKGT

>SG-AL_contig_3035_1_-2_59_286

MEVVPFFKVLFQKDIELKIKYHSLSLSLSLSLSLSLSVTNTHTHTHTITRNLGSYDKQMS

LIHTHTHTHTHTHTHI

>SG-AL_contig_3036_1_+1_31_126

MIYSPVTNTRVRFSFSRKHRPLALRKYELCIF

>SG-AL_contig_3037_1_+2_74_169

MIYSPVTNTRIRFSFSRKHRPLALRKYELCVF

>SG-AL_contig_3038_1_-3_15_635

MASVKLFCARASAQSLLRSMIRPAYAAQERSHYKQVGMQDPDMNWKPIMDRASQTVFWTE

LIKGLGITLGHIFKEPATINYPFEKGPLSPRFRGEHALRRYPSGEERCIACKLCEAICPA

QAITIEAEPRADGSRRTTRYDIDMTKCIYCGFCQEACPVDAIVEGPNFEFSTETREELMY

NKEKLLSNGDKWEAEIAANLQADYLYR

>SG-AL_contig_3039_1_-3_3_236

RVSNGGFRCWPPSSQLAAVSMLHALSILSKSVEGRESINECSANPHPHYLLTTKLQNRSV

LVEEYDTACLCCTGEIPL

>SG-AL_contig_3040_1_-2_959_1129

MFGLVWCVCLFVCLTFICLFEVCFFDSIIQEKGVCLFRPVKRSQFRNVLKPTLGQNI

>SG-AL_contig_3041_1_+2_596_907

MSGEKKIHRDSDTSLSYTECCNPCYMITTKQQHANSSSCRHFTIPFTSEVYKTNQPDKKN

VSNHSSTLHFPVFPHPPPHYDFCITTFRESYSKCICGQFSERHL

>SG-AL_contig_3042_1_+3_1701_1928

MYKKKVMYFSPTLFFTLQPFRFLFVHVFGSSFSCLGRILSTDQMLSYSLSHFTSSRQKLC

ATLILIPSHLIFYTHL

>SG-AL_contig_3043_1_-1_1_687

SPSLCLFSFFFLSLFHLLPLLLSVCSISPSFLSPTSPSPSRLFLSLLPFSLPVLASPSLL

FLSVLPFSLPFLHLLLSVFSLSLSSSILPLLAFPSLCLFSFSFFFLSLSHFSPLLLSVLF

LLPFSLPPFASPSLSLLSLFPFSLPLLASPSLCLFSLFFLSLSHFSLSFSLSLLFLFLLP

FSLPLLASLSLLFLLPFSLPLLASPSLCLFFFFFLSLSHFFFLLLSVSS

>SG-AL_contig_3044_1_-1_1_204

KKKTVTFYQICCNMKSKLISFYLHKFYWVSLLYPALKRDTGKYVFEKKSEVSKQICAFSF

SAVQQPKV

>SG-AL_contig_3045_1_-3_3_443

AWGVDALSLDWSGLIAYAFPPFPILSKVVRKARIERPQLILHPGSSLLASSDLVSGPTGA

LTCASSGSPGQEGPAPTAAVGGGAPQPSSVSPARVAAVRAHLCPPGLTGPAPRSAAIQAA

LSAKGASRDLKALVAETWRAGTENLYG

>SG-AL_contig_3046_1_-3_33_1106

MGSYDGAETCELVGLFLLSELQTEHGNNIGLYRDDGLAALNKSPREVETIKKNMCKTFKK

HNMQITIEANKKVVNYLDITLDLKKNTFKPFKKPNDVPLYVNTKSNHPPSVKRNIPKGIN

KRLSTLSATKTVFNEAANEYQQALKNSGHNHKLSYEEQKPEEETGKNKENKQKNKTRGRK

RDIIWFNPPFEGTVKTNVGKEFFKIVEESFPPNHKLHKIFNRNTVKLSYSCMPNIKQIIE

NNNKKKLHQEKRNSTNQESNCNCRKRDECPLQNNCNSQNIVYQATVTEKNTQKTETYIGM

TETTFKTRYANHKQSFKQERYKNQTELSKHIWTLKENKIDYDISWKIVRRARSYSSSS

>SG-AL_contig_3047_1_+1_1192_1392

MSMTSEQTHNTSTQCYITKFMPFQAYIHSTLHMDKVHALTVYRHTLRTSTQDVTWTLPLH

TFKYSQH

>SG-AL_contig_3048_1_-1_4_288

MPGRLTTKSWSHLPSTKQVSVVAGSYHGPITNCQQSACCNQQPVCYNQLWHTTTTSCQFT

TTSSPITTTSDQSTTTSSWPTTTSSRPTTTSSQPT

>SG-AL_contig_3049_1_-2_215_484

MRCFFKARTEKKTTGRKKLSAEQILVPAINTPCLKLQRKGKFGKTMNMLSRLTAKQRTVS

AVAGTHHSPTINCQLTTASSHTTATNSQSA

>SG-AL_contig_3050_1_-1_109_354

MLLQYRTSTPPPQPDRAAFLENYHQSKQADTALLAECEHVSNNFDSTVNWETEMFQFLSV

HSRFPGTGDGFNINICFSRMKF

>SG-AL_contig_3051_1_+2_449_634

MPVYFTSKIMTYNSKTCICCQHRSCINALKHFKAFLHSKTVVFRTFKGLVNSFRVQKHIQ

HP

>SG-AL_contig_3052_1_-3_90_302

MVWPLLTFFLSVLLVLLEESNHSNAPCTLFETRITSERVSLDLILNLFFLHDWKISPGLF

FCHLSVRTIFV

>SG-AL_contig_3053_1_-1_88_1662

MPTIVALDVSLSMSRPVQMPDTTELYQRRHLAVHGINSLLDFICVNAKMEFTSLVCFSYL

YERSVPFTRDYDVLKGALTKVELYNKTCIETALAGISTVVQEEWGSGIPCQVILVTDGSC

GIGHGSLRQSLETYNQREGTDNKFPLPFPFPAKLHILCVSSPGDSDLKAVLPLYQKLIDI

NDQGGDIYIPDSSLSFKSVQEMFQMCAEKIYTPFHAVLKCGNFSCPVQLHPRPEPYDREL

EFQYVHREMSDTLNIVGFLDVGDVASPPAISRHLVLPTSTKERSAKQDTANGEDSGKENG

KAGVVKTEEEEQGPDDGKTPSFAVLLHGSLKVEVMVAIAKIGEDWYGMLYSWADSKKKAN

LMLSIFEPGLDNIPWLGNLENLCPLSLTAVTQMFGAQAQPPPQFPVRPVEKRSYAQSCVV

WIKDSGLQTDMQKVIRHARKLPDKQQQFYKEMNRIRRAALSFGFHELLEAMATLLERECT

QLPGTAHPSASMQLSHAASGLRAPQALDIVHTIMPLQTNFTAGDR

>SG-AL_contig_3054_1_-3_87_1007

MTDISDLDRQIEQLRRCEVIKESEVKALCAKAREILVEESNVQRVDSPVTVCGDIHGQFY

DLKELFKVGGDVPDTNYLFMGDFVDRGFYSVETFLLLLALKVRYPDRITLIRGNHESRQI

TQVYGFYDECLRKYGSITVWRYCTEIFDYLSLSAIIDGKIFCVHGGLSPSIQTLDQIRSI

DRKQEVPHDGPMCDLLWSDPEDMQGWGVSPRGAGYLFGSDVVSQFNAANDIDLICRAHQL

VMEGYKWHFNETVLTVWSAPNYCYRCGNVAAILELDEHLQRDFTIFEAAPQEARGTPSKK

PQPDYFL

>SG-AL_contig_3055_1_+2_563_643

MLRPTLNCCYALQCYVGIVFIHGKLLA

>SG-AL_contig_3056_1_-3_1569_1790

MNIPSMHFILPSPAPSCIDFILSLGYYRCRGKGGHAAWTTADSQHFALACVLGSVQWLKF

HCPSGLKVANQCLV

>SG-AL_contig_3057_1_+3_63_245

MAGGSNPTSLFSDGRPSMISSASESRPSLNRDAAFELPAIYRQVLTSRDRHSPNKDRAKH

T

>SG-AL_contig_3058_1_-1_103_258

MKCIRKCLLKVMAQRFEIFSHYDPLVLQFGKYPSIKPKVCAYIFCSHTHKYK

>SG-AL_contig_3059_1_+3_963_1157

MSVHASTYIKTWSLGIYHAFSVMYTQKNFNKNFKNYTNDNVSVELPCTLDFSKSSAFAIV

IFLLN

>SG-AL_contig_3060_1_-3_42_446

MVLMLQNPILVLLLAFRTSLVLSLASFQLSPLSFIQVSSNGSGSPSHTVVLVSFVAVSVT

SLSLDWVVSLVPNPQPGGPMDRAVCLQVQHGPLPSTCPAWAALSVTEAIVSIALGVSTAH

KLPDHNKMTILRAPV

>SG-AL_contig_3061_1_+1_343_603

MEHFCFFHFQCFTCRLLVLTLPLLPVSFLYPVLFIRKHVGAFALLYAVRLADSTEMFPMV

FCFVFIICDTVFRFLLVEKCIFQNELN

>SG-AL_contig_3062_1_-1_73_297

MLLELVYLFRTWFTFQNPILVLLLAFRTSFILSVASFQLSPLSFIRVSSDGSGSPSHTIL

VSFVAVSITSLFSGT

>SG-AL_contig_3063_1_-2_2_889

AAADEAAPDSPFPSGAAEYEPKNKKEDAAPAAQQEEEVDIDLSDPEVEKAAIKIQAGFKG

FKARKSLSANKGEGGEDAPKEGEETTASEEVMVDEGEGQTEEVDIDLTDPEVEKAALKIQ

AGYKGFKTRQDLKGQTEAESSTAGAEGETGESSVEKTEEGAASDDKAASSEQQTKDETTE

EEKPVEGGSEEPKSGEEQVEDSQEKSEEQQGQGDAQAAAEGEGEHAEAEGAKPEGGSTKA

EGEEAQQEGEDGRGEAGDAQKEGDDAQREGDSVQEEGGETGKEGEGQKVAESEGGD

>SG-AL_contig_3064_1_-1_1051_1272

MCMCVCACVFQVWSSGGRGSKSQVTISLTWIDLCKTCVDFDDLHWCCVCLLLDSKFKTVI

RLLNSDFDQSKNRV

>SG-AL_contig_3065_1_+3_567_800

MIFLIIHSFETSICENLNTNTSISIRNHCFWHPSVSIPTAFADITPKRYTPCQFWALGTL

TVITIRCSGKLQPKALRE

>SG-AL_contig_3066_1_+2_2_193

LRLNQNKCIFFSPMSLVREEDYPPLKHKKHLGRGVGGEEGQRVERVKWNLNTINLESTEQ

QQLD

>SG-AL_contig_3067_1_+3_123_995

MSVRTYNPSVRVGNWNEDIQLEEDTLKNFLEKRENGELLIQKTHNLTAAMGVNIELSVSQ

DHAIHIGDKVMIKCAAAKDQQKYSANVDPRQDCVVALNITDPTTLLQKNVKGPVIATGCQ

AIAANQRTVFCVESVDGSQGGAQLMYGEPFHLTTVGDNTGKLYLCSDKVTFNKCAKKSRH

QEVSLVPEPSFLTRWMVQHRNPLLRMEYEHHPVEANAEVVIVHCKTNSCLAVEDKFNLRT

PFGREYELSVHTYLDSHRAEEDNNRWMLVVGVPGSCVNPVPDQGPGAAPLH

>SG-AL_contig_3068_1_+1_853_1044

MEKPSSPVRKRSVHSGRFKVAAGSRKKCLSVALFMLLAIFHGIFFASFRSQNGCLSFRQP

LTIY

>SG-AL_contig_3069_1_+3_3_137

QQANELPAVDFMIPGSLLYLERNTCSLFFAILGDRFSVHLFEIAC

>SG-AL_contig_3070_1_+2_2_205

TLALSSSPCSLFFISFGFSEPDFLFIFEISCRTRFHKQRGCLEKWRSSLEFKRDTTRLNA

FMCGKLKL

>SG-AL_contig_3071_1_-1_346_564

MKIKAYEFIFETQICVRKEKEKRLSYIDIKCNLFVFMYELSEATITMMFFSALKPLNKLM

NSSEFVCLSIVFN

>SG-AL_contig_3072_1_+1_1_201

GGVWAGRGRCYTISDQIISKYSNDANAQLAEMFPQNVQLLVNPKLKSLSKKVRPPSLTSA

VFFSIYF

>SG-AL_contig_3073_1_-3_576_800

MNLRKSQHSDVGMHAAYSCVSFIFYVFISVSFFFNSYIYLLIVHYCLCTATPQNLAVMIY

KHGPDCVSRPNMLHR

>SG-AL_contig_3074_1_-3_153_329

MSCVVICQDLLLSFCSHLCIFKEWGCIGICRPSSLLLSCIKEKRGGGGGGDDCCFKGGK

>SG-AL_contig_3075_1_-3_87_359

MVQRVSFRLALPHSAPARCSSCLPQMDLYLYTDASNEIWGAHMGTLHALTCGQILTILPL

KRSQVITIHPLKRSRAQSCLPSLTSLRNPTF

>SG-AL_contig_3076_1_-1_430_804

MDPECPVLSWVWGSLVPPNDRSLCDQVQQTTSRLYFAITYPEAIAINAFLLDWSGLLAYA

FPILQIVIRKANGENNHVSDHANLAISTVVPRPSVLVSRSSSSTAASPRPPSSTTVRVLA

RKPSS

>SG-AL_contig_3077_1_-1_1_108

HMKGMPKAFRTQGEVLEVIQEKRSLSRDVDTTCFRS

>SG-AL_contig_3078_1_-3_75_293

MHSYMLKWLWPCFRMAATEITKSVNVLVIIFSCLVCSTLLGTGVQSKINARGWLRLRKSC

MRPTEGSGNLTLF

>SG-AL_contig_3079_1_-3_87_2189

MDEIAELKYDIDKKLEKEANNLAMLKDCLSRSSSNTQNMLGILTSFESRLQRLESTIVPV

YNETENLRRRQENIEKTMHTLDTVLSYYHAAKDIEAIVKEGPAACDLDTYLAYMEKVLKA

QKYFQTYNAGSKELEDINHIFVDGTQALQHEFKSLLNRHGHPVPPVLIVDMLSSEDELPP

VVTEADLPMEHLPEKVVFDLVKISQWLVEQAQSHQHLKSYGQGRAAMLSRSLQGLKDHLR

SSSGTSSQLSFISQNSPSLGGKFRGSKDAPTRKSMMRLVNNFTKKATSALNKSGFEPGHR

RQGSAANDSIREEVVDVETDLYINELSALLRLMQSEMHLMKGIVPQKHERTVFDTIIQQA

LDAVVNEGEMIAGTARKNMQRHEYSAVLSIFPIVRHLRSIKPEFDLILEGCQAPTRAKLA

SLLSTLDSTGAKALEEFTDSIRNDPDKASNMPRDGTVHELTNHTIIFLTQLQDYAETAGA

MLLMHGEQAAPSTMITTDTCKMKVADYMTRVLSALGLNLSNKSETYSDQALRPIFMLNNF

NYILKALKRTSLLELLLMWNKEVTKYYDDQIVEQKRQYSLSWSKVLHYILEMDKPMSVQR

ASNMDSSVKLKDKERQNIKDKFTGFNKELEDIHRIQKGYAIPDQELRQQLIKENKEFLVP

RYQMFYSKYSALNFTKNRSKYVKYDVEAVNKMLDSFFDASA

>SG-AL_contig_3080_1_+3_174_425

MGFSPNSLFGSFPLTHCLAVYLPCQTSDPPVLWTFRCAGGQGSLRLLDPYALGASMRPGG

QGGPTRLPRKGGGARPTLPGTGES

>SG-AL_contig_3081_1_+2_2_130

NIQLRSPTQMCIHHNNITSNLVLLHICSCTLEITGKRPNICSS

>SG-AL_contig_3082_1_-3_132_326

MILKVVIWSVERLPGRNPACSSVIRMSVLSHILLSSILLKTLPATDRRVIPLQLLQSERS

SFFGS

>SG-AL_contig_3083_1_-1_1_393

LSSAVLFLRAQFCSIVLVCSVLRYCSCVLSFAVLFLCAQFCSIVLVCSVLLYCSCGLSSA

VLFCVLSSAVLFLCPQFYNTVLVCSVLQYCSCVLSVPVLFFCVQCSSIVLLCSVLHYCSC

VLSVAVLFLCA

>SG-AL_contig_3084_1_-1_196_1071

MVPSTPTLSMPRRNDGFAEALNSPHVAQRFVFGAGPEGASQPELAQLESQRALGMDDTRM

DLSQFDEGGARSIPTTPVPQQVSAPVSFESVAAGVASSSAAPGASVSSTVTSAASTGPQS

ATPSQEVTTASTPGTSEASGGQGEGKSQTTGETSKQEPSSETTEGQDEEEAEASLLEGGA

GDDDDTDESKSASKPRIQRIVWDSPQTSTTASASTSTQPQPQQQQQQQQQGSRGMRSRGL

RRGAVPTRPNRGTFSSWRGGVRGGVQQPPQHPQRGMGAMRRGGPRGGYRGQF

>SG-AL_contig_3085_1_-3_3_140

RCFIANSIKTFKVFTAPDSASSTIDGGFGMNNCGKIMFSADVVIEN

>SG-AL_contig_3086_1_+1_226_363

MPKYPSIADAAGPDAKLEAVKTLKVLIKLTKKHRSINIQTWLTFSC

>SG-AL_contig_3087_1_-2_2_244

GGEKKKNKIYDGGGGGGQRKKTKKKKKRCKSIMFSVCVLRPIQVQICQLFRIHRILFRPV

ESCSDVPMMSTLFTKVHFRLA

>SG-AL_contig_3088_1_+1_208_375

MFSYTISIYQILSKFSNLSIKIISQQAQPQMIFCFSHIRSQQKCYVSIIIIIIYCL

>SG-AL_contig_3089_1_-2_11_2008

MEQAWDAFKPALLNTLREVCGTKKCGGKNRKATAWWNQEVKEAIKKKKQLYKAWVKTKTE

EDYVKYRIARRHSKRVVKASKEKSWATYGEKLSEICKNSPREFFKSVRTMRVRDEPFNPT

TVVNDANGEALHKEEEILKRWEDYFKDLLNPSGVQASSSQFSPRYKDQLEPQILEAETRK

TIKTSPKGKAAGNDGITTEAILACGETGVKWLTIIFQKAWEERKVPMDWQNAIVVPIWKK

KGSKKDCSTYRGISLLSHVGKMYAKILEQHTRAKAEHLLSNAQFGFRKGRGCTDAIFALR

QLCERTIEYNQDLHTVFVDQEKAFDRVNRDKLWKILEQYDVKGQLLDNIRAIYANSRSAV

RTASGTSDWFSVTSGVRQGCVLSPLLFVIYMDQITKEANPDLEALNELLFADDQSLMNQN

KAALQVHTDQLNTSCEKYDMKISINKTEVMSVSRNPGKLDININGTQLKQTKEFKYLGSL

FTEDGKLDREIETRCQKMNAVSYQLAPLLKHPSIPMSTKAKLINAIFVPTLTYQCQTWAL

TKALERKLVTCEMRCLRKAVNKTRRDKIKNEVIRDMVGLTPILHHVEQQRIRWFGHLTRM

PPDQPALRAYTTRYTGWKARGRPRRRWSDSVADTLRAHDLTLYQASRLAADRHLCLPATP

KGTSGR

>SG-AL_contig_3090_1_-2_113_835

MMYFDSEANAADLRYFNWLVVVAQVFGLLSVVLVAVWMGHYQGGFAWQSDPTHQFNLHPL

FMIIGMIFLYADGILAYRVFRNDKKLYIKILHAIVHIAALIFAAVGLKAVFDFHNLTEKP

NLYSLHSWVGLFVVVAFGLQWLLGFVAFLIPVAGMTVKRFYMPYHRFWGAALLALAGATA

LMGITENAIFKVKPWAGPEGILINFLGVFIVVFVVLVVYLVTKPDYKRPPAPEEDHIQLA

E

>SG-AL_contig_3091_1_-1_187_5871

MDFSSLDLLASTALRDNNPSNGDNSSTPTDAHHPHHNHHDSLESEEDDDDEADGNRDSVK

DRKDSVNETKDSVNETTGESDSPQKDMGSVKCGKQHKRNSKTMSILVRDGKCVGKKIGRD

AVCDKQKEGGQEIGDCSVTLSPQSSSMQAMDVCTNNPQPRSSVPFKCPKQMAETEVKRIN

ERSSHLCNGFSSSNRQISVTTTCEGSTAVSVTDVAGAAVSCSSVTAEASSDSRVRSCGET

SSEAGDGSAENCVEMADRDCVAGDTSLTAASCDSLQTADVAVSECQDQAAASELHQQVKE

DSGCCDSVPDTGRESRASVGQADVDMQPSQDLPISLHEDKRQEADSTPSQLLNFTVTELH

GTSLISENKMETAVESKTTSSCDETFDLFFVPSTSQPTVEGVGEQNRSYRNSLDQDKGSS

GNKQLNPSTSTDSSKVESILSPPPPPPLHPVAVTALVHLDHPYCTFPRGAIPETDDSGES

ADENVSADEIERRPRSLSVDSVYLQYGVSGLSSQQENLSQNDKLALLSPTLSVDSISNDS

SLSEHSTAAGGQCYSILTGGPSSARTSRPTLVSVDSQGGTDSPAAGTDGASRLHSNLLET

GSNSSLLHASSLSLGPRCTKFSIGNLGSVSNSHLEVEKESIKNKPNIGLSSDPVMKTSLS

SPAIASPASPFPLLQSPGMEWDRSDAGSECPDDLNSSSTEDKFLSSSHDSEAFGTTSQVW

RHPVFHDHDYCCKEISEEYLGKPVAPTPPTKSGKRKYVKKKDRQEEEEKLRKGKYLKREL

LKQDNRFNLASNRGVDRTLAARSSLLAESLATKPNPVGRPRKRTEKQLEEYDGEIGTKMK

ITGKYQDQYVYYLNKSSRNRRRKVDEKVPSMDKIILPAPKPGDIVVPHLSDADCEAIRRG

GRMALQNNNYSNPVTSQPLQPHTDHSDMDSSIVNTILSMETEHGNLASPLDSYDGDGHGD

DFADIEGNLTQEQLRLLVDCLDDYPMEETTTTQAALDLFSSAEGVVAPSTTVDSLAATTV

EEEFMVTGEEAATSHITGSSDSAALLAKVEPNNPSALKRDANGLEFCNYTATVTTSFTVP

VPPPTSQPSVEKNLEFLKTPFTKSDFFPTVSPATTSVSQGLFPTVPPVISSGSQGLFVPT

PPTPITPSVLDVRSDDVNETPWIVTVTLYDNDIPAIIINNMPHVRLVDIHRQILPAKDTG

ILKKRCQLMHIQVSNCTEMQRYFLVQYGRAHNSKSTLVISKDEAQRLITYYAHPPPRNLR

GEEGAGLRRASSYAELRSIMERPGNSGRNSSLPFTQSRKRGGFRRRAAPTRSQGPSPEPV

AQSVSPVLEPMLSSPLPVEPAASVKGLRHKKINFREMLKGEDSSPQTSPPLEGVVDPDST

AVLEEVSVAKGRGSKTGVTAGRNKRRASKESGSAPQSKRGRKAWRKKEEGSSHNVLKNLG

EADIMVCSDSSNSAVVNSKNKKRSPGSSVSGSLGSARSPKSKFGFKVNVKSLVSSGGLLS

ADGSQPRKKNLFGRKSSSSTDVTVEVAEDIVPRSHPLRVLRISHQDVEASFPPAPQIVSP

MAEVHLDLYKRASSPCVKCDTCHKFLSVPGFLKHQHVNGQVETQDTRAEMVLRRRMLVPM

NKENISLEEQRLWNEFVALQESLDHSVCVASMGAYPLTPLSPASPVGSSLHSFGAIVSPA

LEESLLTMTPESPHPAQEGLDHLHNGNLLDSETIPNSQGVTVMDERCATSPGNETGSGSD

AVNVGITFPSEAAAHPWETPLSFCSPPTLSPATTHTDNEEKYDVVNSIEPDTEIPSHPKT

VVPANKKQCPYQRPRKVSPTTNVRTSSRKRETKRLYSFEKYEYGGGKKGGRGEAEVPDFE

DDFPGALLEDVNSVVRSDTLLHLASDAVMHEPQNA

>SG-AL_contig_3092_1_+1_1_156

PTPRLLKQVLNKLYTKFKIMCTKQLTQPRVTVIQQKQYLVFSETKRNLCKCA

>SG-AL_contig_3093_1_-2_380_550

MLFCCGHCLYYFCYSFSIVPENIDIFGVSIILLVQQCPRECCRSWGNLFWHCLCPPF

>SG-AL_contig_3094_1_-1_46_513

MTTLENANPVVFRRSKERPILPEEEDDNVIDKIDNREVFDMIREISDPEHPLTLEELNVV

EESKVTVDDDEESVHIYFTPTIPHCSMATLIGLAIRVKLLRALPARFKVEVEITPGSHAT

EEAINKQLADKERVAAALENSHLLGVVNQCLGGRAK

>SG-AL_contig_3095_1_-1_196_1656

MWPTTLLPVVLAFAVSGLTHGIEDKAFWMNVGQKILDEALKVKLNNRVAKNVILFMGDGM

GISTITAARIYKGQLQGNTGEETQLTFEKFPHAAFSKTYCVDRQTPGSASSGTAYHSGVK

VNFGTVGFDANVLRRNCSLINDSSVRVETMLDWSYAAGKSVGIVTTTRVTHASPAAAYAH

SADRNWERDRDIPPGCGLDIASQLIRENSNITVVMGGGRRNFIGRDDGQNLVEEWKTNKQ

NEGKTARYIQNATQLRNLDASAVDNVLGLFTESHMSWELSRDNDTEPSLTEMTVKAIEIL

QKNDKGFFLFVEGGRIDHGHHDNWAKRALVETIAMDDAVAAALRVTNQDDTLIVVTADHS

HPFSLVGFPKRGNDILGLVDPVDPGQETTDGMPYTTLVYGNGAGPMRETNLTGVNATGND

FRFPVAVPMDKEWETHAGEDVGIFAIGPMAHLFHGVHEQNYIAHAMAYAACVGPNTKHCT

TKPPPDN

>SG-AL_contig_3096_1_-2_71_1099

MVDCEWSVDRRTMLEDHSIGLILAGLVSFLVYACGLVLNLCELMLQYRDELLIPNILEEE

TSNATASYVLPVSLAYWSRPVWTVVYVCQSAWLVFSLASYFRRTATGPAYNNPKLLPVPV

YVFFSLAILTQMGWLVMMNRQYVILALLLIVMSTGCSIICVCEAYRAMLSALPILQQQDR

SCDLWATRLLAHNSLAFFATWITLHATLTFALFLTTFDLENHEVMVKVGSLIQLAIAAFV

VLMYVLTDLTVFDKYSRFVLLPYIGFVLFHAAQLNKNWPDLHSAIFILLASLTAFTFISI

FLKAIVLLCRQVLQSDNDVTMMNVVTRPLPSDLEEETYLLHAK

>SG-AL_contig_3097_1_-3_3615_3890

MRSSVHPTVSGYLTLFRVGEGKAWRERGGTSLQKADLRVVRSLPPHSPTTWVRVMELPLS

LKKKIFFSFRTTSQKEKCFKVSASVTVDLIVD

>SG-AL_contig_3098_1_-1_178_2319

MRPHCSHLLRSWSSLPFIHHGALKTEVLQTLKTSRHHARGVSTISPLAAAFNSKPQRKIN

LSLRRDNAGLFLIPELSDYSGFYLLQHQVSAQVELLVEEATSRNRTRKMVIIFDELSDCL

CRVADMADFIRVAHPDRKFSEAAEDTCVKLSSLVEKLNTNTAIHSALSRVLQEGDVVETD

GIDERVAELFMFDFEQSGIHLEQKKRQRFVELNESILMLGSYFQQGAQRGTSVPKDQLPE

NLRHVFGVDGDNVMVTGLFSDHYSGRVREAAYRIFLYPDAHQEELLTALLSARHQLANLV

GFATYSHRANRGTMMESPDSVMAFLDLLSEKLKPRVEQEMKELLRLKQEQVPSGKIVHAW

DPPYYSALGRQRRCSVPTNEIMAYLSLGSVMSGLDNLFQHLYGVSLHSVEVQPGEVWHYD

VLKLAVEHDKEGTLGYIYCDFFERPGKSHQDCHFTIRGGRQCEDGSYQKPVVVLQMNLPA

PAGNTPTLLTPGMMENLFHEFGHAMHSMLGRTKYQHVTGTRCSTDFAEVPSVLMEFFASD

PRVISSFARHYKTGEPLAESVVQDMCAAKHLFAASDLQQQTFYAVLDQVYHGAHPLPGST

TSIVADVQQRYSSISYINDTAWQLRFGHLVGYGARYYAYLISRAVAASLWHSSFREDPFN

WAMGQIYRVQMLQHGGAIHPSTLVEGMIGEKPSVENMVDALISDLDSSKQYLYS

>SG-AL_contig_3099_1_-1_28_1386

MEERADDGQPGFVDHAEALYTAVTANDVASLQRLLSQGVSPDMFYDDDHNISSKYILHIA

CGKGHSDCVKALLDAGAMPDCHDSWGQTPLHYSVSMQFLDCAQVLLDHMGTNVGEIVNNQ

DCFGKSCLHAAAESGHVEAIRMLLQLGAHVDIRNFDGVTPLMVCAGNCGKNISTESMQVL

IEAGALIDLTDYRSKRSALQRAAVAKNVKGVELLLSVGAEVDMLDVTRRTALTNLMWDHV

RNREGVCHIDADVMTVIVLLTQGGANLNLAVCEYSNPLVTASFLQATPLLRFFLDQGALS

NITFSSGVTPLLVATSKKDLNCVKTLLSYNCCMTTKGRVYRSRLDKEFTFDPIEMALDDG

SYRLIQAFLASGYNASSIRTQLVEKLQTDKSSIDPDLDQWLMARISGPPSLQELTIYSIR

SYLDQRLHKVVQLLPLPSRLKDSILLTDLLKVD

>SG-AL_contig_3100_1_+2_356_511

MWENRLNPSLKYRNWCITCKNVKGEPYNLSYKVLSVDSFVYGRLFPALHKHK

>SG-AL_contig_3101_1_-3_72_1199

MDTYDVIANQPVVIDNGSGVVKAGFAGDQVPKYIFPNFIGRPKHVRVMAGALEGDTFIGP

EAEEHRGLMSIRYPMEHGIVKDWNDMERIWQYIYSKDQLQTFSEEHPVLLTEAPLNPRRN

REKAAEIFFETFNVPALFISMQAVLSLYATGRTTGVVLDAGDGVTHAVPVYEGFAMPHSI

MRVDVAGRDVSRYLRLLLRKEGMDLHTTAEFEVVRQIKERTCYLSINPLKEESVDTEKVQ

YTLPDGNTVEIGPARFRAPELLFRPDLIGEECEGIHEVLSYSIQKSDVDLRKTLYSNIVL

SGGSTLFKGFGDRLLSELKKLAPKDIKIRISAPQERLYSTWIGGSILASLDTFRKMWVSK

REYHEEGVKAIHRKTF

>SG-AL_contig_3102_1_-3_627_839

MTMRIKTCFQPLYELLWICMLMSYICMPIVDTAATLAAVVVLASEFHPSQSYNDVSWTLH

LAVPSVFHKHE

>SG-AL_contig_3103_1_+1_70_237

MCDFCWISCKQNIYPTPARKADHVTAPKINKCYELSLDLSNRSSVCIITTPLQKLN

>SG-AL_contig_3104_1_+2_2_124

HGQQQALHITRPWLVPCRHLSVGAIPQALGTEGSRSTLRQH

>SG-AL_contig_3105_1_-2_2_124

LHRKSVVKHMNKYKFKIQHVVLKPGIQYTMYVGSSNTYRSK

>SG-AL_contig_3106_1_-1_199_1260

MNCASFYDGRNYFLACGQEEKCHLYSVKFKVISPKKENAETNDKGDVKQRKGKTDKTGKD

EKTDSDKNKIPNGTKQLTFDLQCLTSVQTDFEKDGGFQKVVRFSRDHSLVVTGGADGFLR

VWKYPDMKMAYETHGHKNDIDDLDISPSGDKIVTVSRDNTGHVWNTKNGTKVCDLVWTHA

DAAQYRYRCCRYGLVEGQKDKYNLYTISIPIKRSSKPSPCFLTMWDKDNFKVKKGTSAGT

EVLSALAVSEDGIYLGVGSISGSVSVYISFSLQKLYHVKEAHSIFVTGVEFLPTSEATRA

IVGEQDFNLISISADNTVRLHQMPERGTISVFWVILACLLLIFLLFWFMAELGV

>SG-AL_contig_3107_1_-1_616_855

MSDAEYKLTVSFLCPGKQFCMKKSTVSFEFLSFSSIFVFSVVCAPIYLLLSFVGNPNVCL

FLPLQSASFRCDFRIHVRVI

>SG-AL_contig_3108_1_-3_3_167

KKKKKGECNCEEPTKLVDVGVNAFPCNIEVEKHVIFPDSEITVASANSSHERFSF

>SG-AL_contig_3109_1_-3_144_848

MAQSPAGGAVKALQLELKKIQAEPVEGFRVKLINDDNIFEWEVAIFGPPGTLYEGGYFKA

HMKFPGDYPYSPPSVRFITKMWHPNVYENGEVCISILHPPVDDPQSGELPSERWNPTQTV

RTILLSIISLLNEPNTFSPANVDASVMYRKWKDTKGKEKEYENIIRKQVQGTKEDALRDG

VTVPTTLQEYCVSSRPTQDNQVDADFYDDDYVDDGEDEEMYQDEDDDIDSGNEEC

>SG-AL_contig_3110_1_+2_209_829

MNSCYWKKNCTLTYSTNSTNFMLKLFKGKMADCVTKQPEYFHIKYECTTKKAKSPLIVRE

PKKGKKSKGKALKAKQGVALSHEDHPWLYRRHQLIDLRKELTLKKPNRRSLYFFFTIRTL

NLMWGDNLTVKWTQDDKTWDSGHVFGSEYDLSKGNTFSVTGPSQVSFVLDTHLHTNGGEG

FVICFKWSRKNKKPSDTCKKLIVPRGC

>SG-AL_contig_3111_1_-2_71_259

MGTGMSILQCYLPIWPFYVFFCCCFDVFYNFVTLFSNLGMVNQAEGLASSAHPSFICLKE

LNG

>SG-AL_contig_3112_1_+2_1142_1366

MDNKNEAKMCVRENFHFTKETGKIPKAFAGKRVEIFPSSQANICADLLVPEPPSCVYACR

TSNMHVKDPVSHVSV

>SG-AL_contig_3113_1_+3_3_230

NNWIFSCLLKTNKDLVLYLLKMTYLCFSHIISDVSICRKQMSMPAKALVTLCCKRNKDHS

SMKKKTLLQAMLFFPY

>SG-AL_contig_3114_1_-2_68_202

MRLNGRFFHLLQSSPTETETPYYPNHFTVSDCHNFQQNVFNVLAS

>SG-AL_contig_3115_1_-2_68_202

MRLNGRFFYLLQSSPTETETPYYPNHFTVSDCHNFQQNMFNVLAS

>SG-AL_contig_3116_1_-1_64_288

MITHRICLRDRSYYITVYFCCIVVVVVRGAVQSSVESVLEKFKVSGQLSLIVKMFSGGVF

VLRCCCSLLQLFDPH

>SG-AL_contig_3117_1_-2_23_967

MEELGLGTPVEKEDTAVTESRPSTAVQTGGSGDPLSLDGEDLYVKYKKLQRQLEFLEVQE

EYIKDEQKNLKKEYLHAQEEVKRIQSVPLVIGQFLEAVDQNTGIVGSTTGSNYYVRILST

IDRELLKPSSSVALHKHSNALVDVLPPEADSSITMLQADEKPNMTYSDIGGMDIQKQEVR

EAVELPLTHFELYKQIGIDPPRGVLMFGPPGCGKTMLAKAVAHHTTAAFIRVVGSEFVQK

YLGEGPRMVRDVFRLAKENAPAIIFIDEIDAIATKRFDAQTGADREVQRILLELLNQMDG

FDQTVNVKVIMATNR

>SG-AL_contig_3118_1_-1_196_399

MTIITGPAKVLCSGPWIAAKTTVGSERLAPGHLGGACSDPDGGKTKKKVTAEGHRGVMTW

IWPGPLRV

>SG-AL_contig_3119_1_-3_21_1181

MADSEEQEPTPAQDIVVTKYKMAGDMVNGILKELISKSCVGASVLELCELGDSRLTEETS

KVFKKDKEMKKGIAFPTCISVNNCICHFSPLKSDPDVILQDGDMVKIDLGAHVDGFIAVA

AHTLIIGASKDNKVTGRKADVMMAAHMAAEAALRLVKPGNENYQVTDGIQKVAESYNCKP

IEGMLSHQLLKNVIDGEKAFIQNPSEAQRKEHDKCEFEVHEVYAIDILVSTGEGKGRELD

TRTTVYKKKDIIYQLKMKASRQFLSETDKKFGLMPFTLRLFDDEKKAKMGVLECVKHELM

QPFGVLYEREGEVVAQFKATVILMPNGPLKITGLPFEPELYQTDFKIEDEEVKALLAASA

SRKAAKKKKKKAGKSLGESAEGGEGSD

>SG-AL_contig_3120_1_+2_125_460

MGTKSKLIMHTAEMLEYSKLRLHTSLGPGDGAVRTVHISGIWGWGKVRTVHISGTWGWGK

VRTVHISGIWGWGKVRTVHICGMLRCWQSPNYAHLWDEAKSKLCTSLGWGKV

>SG-AL_contig_3121_1_+1_1_222

CSSACVCVCVCVCISVCVCVGGGGGTRERERERERERERERERQTETERARQRERDIRII

YSYYRPWSHQGGVY

>SG-AL_contig_3122_1_+3_96_257

MELFFDDDLINHLVIQTNLCADQFKEAHLNLPEYSRTNSISTFTVPITNKIKQL

>SG-AL_contig_3123_1_-3_3_128

ACLPQFSCMKPATVQLYEACHNSAVLSLPQFSCMKPATIQLY

>SG-AL_contig_3124_1_+1_1_99

SSEQISEPTEESKILPWHTAIPKCYTVAGKGLI

>SG-AL_contig_3125_1_-2_47_1903

MAASSPLDPCSFSRPDVCVVKNINLDLEVDFVKKVIAGTAHLRVERKTGDASVLLLDSNE

LNVEKVTDQDTGEELKFTVGEYVKPFGSKLEIVLPGSAGKLITVSIMYSTSPSCSALQWL

RPEQTAGKRQPYLFSQCQAIHARSLYPCQDTPAVKFTYTAKVKAPRGITILMSAVRLGPV

EVEGDTSCLVHNFEQKVPIPSYLLAIVGGDIESREIGTRSKVWSEKEFVDAAAYEFAETE

KMLQIAEDMLGSYEWGQYDLLVLPPSFPYGGMENPCLTFVTPTLLAGDRSLADVVAHEIS

HSWTGNLVTNSTFQDFWLNEGHTKYVERAVVKRLKGGEKLRQHLASDGWRTLKNAVVRMM

KNGEGPYTKLIPDLTGVDPDDAFSVVPYEKGFALLFYLENLLGGPKIFEPFLEKYIKCFR

FQSISSGQWRDFILSYFNSESDQKKLKAVDWNAWFYAEGMPPINPQFDTSLAEPCLELSE

RWLDASDLELGSFTENDLQDFSSLQKREFIALLLDAETTVSVAKLEWMDKLYNFSEVKNS

EIRFRWLRLCIKSRRTSIIEKALQFVTEQGRMKFVRPIYRDLCKWEEARPAAIDNFQKYR

NEMHNTTAKEVARDLHLDH

>SG-AL_contig_3126_1_-3_3_284

LFHVPFHVASFVLGSVAEEDITCSISDCTAVLFSSQCVANCSVLPYGRITRPRCSDKDNS

TYKVWASYSPTVNRQGKFNFELCLLYSNYVNTSI

>SG-AL_contig_3127_1_-3_39_524

MSDYVAPAFAIVLPNLGGIAGGFLTRRAIPGWFEHLKKPDWRPPNRVFGPMWTFLYSTMG

YASYLVWRDGGGFEGDNRLPLAMYGTQLALNWVWSPIFFGAKSLKWGLVDITVLWGAVAG

TIVTFYPVNKVAAALLVPYISWISLATVLNYVIWRDNKDRTD

>SG-AL_contig_3128_1_-1_109_3321

MLKVQTAALLADSIGSHVSKLSLRCCRGLRTSGRQFCQKVDKEVTPRGTPYNKLTIGVPR

EIFPNERRVALVPAAVQTLVKKGFSINVEAGAGEQAKFMNEDYEAAGASIKAEQDVYNSD

IVLKVRGPLSEEIDKFRENGTLISFLYPAQNKELVDQLAQRNLTVFAMDCVPRISRAQVF

DALSSMANIAGYKAVIEAANNFGRFFTGQITAAGKVPPAKVLVIGGGVAGLSAIGTAKNM

GAIVRAFDTRAAVKEQVESFGAEFLEVNVKESGEGTGGYAKEMSKEFIEAEMALFAKQCK

EIDILITTALIPGKPAPKLFSQEMIESMKPGSVVVDLAAEAGGNIATTKPGQLSVHKDVV

HVGYTDLPSRLPTQSSTLYANNISKLLLSFGEKDHYYLDMEDEVVRGSIILQDGKLMWPP

PPPKVDPAAPAAAASTVAAAVAKEPPPPPNYFNITLKDSLMYTTGLGSAIGLGMASPNPA

FTSMVTVFGLAGIVGYHTVWGVTPALHSPLMSVTNAISGITAVGGMLLMGGGYYPDNTVH

SLAALAAFISSVNIGGGFLVTQRMLDMFKRPDDPPEYNKLYAIPAGVFLAGYGYCAQAGT

YPDIHQMAYLAASLCCVGALTGLSSQTTSRLGNTLGMIGVSSGVAATLGLLKPSPELFAQ

MATCMGLGGTIGTVMAKRLEVTDLPQMVALFHSLVGAAAVLTCFANYILEQPHFATDPAA

TITKTFLFLGTYIGGITFTGSLVAFGKLQGLLKSDPLLLPGRHGMNLSMLLGNVGAMGYF

LYDETPTVGLSMLGITTSLSSIMGVTLTAAIGGADMPVVITVLNSYSGWALCAEGFMLDN

NLMTIVGALIGSSGAILSYIMCKAMNRSLPNVILGGYGTSSTGTGKPMEITGTHTEINVE

DTINLVKDAKNIIIVPGYGLCVAKAQYPIAEMVDMLRKKGKHVRFGIHPVAGRMPGQLNV

LLAEAGVPYDIVLEMDELNDDFPETDLVLVIGANDTVNSAAEEDPNSIIAGMPVLRVWLS

QQVIVMKRSLGVGYAAVDNPIFFKENTAMLLGDAKKTCDALLGKLRQDYEE

>SG-AL_contig_3129_1_-1_1_168

HTYIYTYVSLFVPLFEFDIFLLFPQLVLETQFDRNGFILITQLEFFPAEVYSRSPL

>SG-AL_contig_3130_1_-3_519_950

MSFTGVFGLSYPPSQRQERKVKNYNSFFVKPLFPQHIKIECEVSEIGAYLSSEPEKDLQQ

IDITASSEVGSSTTMREGMFKCPVCNKSFTQFGTMNRHLKLHSGGFTFACSVCGSEYNRN

DNLIRHMRTTHGLFEDRLRAPKCP

>SG-AL_contig_3131_1_-1_913_1167

MFKRGSWHLDSFTCAPPQSANNCLVDSFESCRLPRSWNRRGRWDPYRPYNKHEVDLTRIN

DTSRWAGNKPVFLFLGSSSVRCYIN

>SG-AL_contig_3132_1_-1_43_522

MALYVCLFATCHIRSLIHSRDLIQSPFLGCKRNNFPSQEDAAYDDSNTSLNFHLKRMNRT

APSTTLQSASTVVASMAAGNFQLLARPIIFATSLVIGPQSAWHKKTKKKSPQRRHHIAEN

QPTPWMRVRKMRNLVQDRKYDTDKMVRDKNLFDFQGRRLG

>SG-AL_contig_3133_1_+3_615_932

MKALGDVETWATLQLSDRGQLDNCPTPQRIISFYRVWCSQISVHVPKLIHLKKDNKPSLK

TFIFSASYMNTEHHLCVHPHFLQWGLGLPVHEFWTRHYLDTYIIFQ

>SG-AL_contig_3134_1_-2_236_424

MFELFFKIFSPLHECLNASVYFTSTCQSFFTESRLCVCTCTHFYISVSWDYSKCMRLSSC

LSV

>SG-AL_contig_3135_1_-1_1_153

FFFLFSFLNWSRSFSQLFDSNLLKCDLNMFYLIFCRECLGCYINLAGFVFD

>SG-AL_contig_3136_1_-1_223_1740

MEYLGHTQTATYMTGPRLNSSQTFHLPSIGTMHSSLKSFDTYPAPSPMRRSLTTLPWRPS

TYYQTAKVNPGNPITQSMPDPMSSKTALNELDSLKVPPVFAAARNSLYTRYTPNDWSASN

QGNYLASDKVRSGAERLRFDTVRLCRETDDKTKRTQTDVGKRLGERIGDITFWKTELNHE

TDNMISEINALTEAKRILEKALQETENPLHISQECLYNREKRQTIDLVHDNPEKELIKEV

DVIKRCQERMRNTIEKANVQLGLDRAAQHELEKDSIDKQVAESMDTTCHGIRDSSRGIAF

HNGVHRIDNTVSVPETWARFSNDNVQRSQSERAASKQMRNEIESVLNACANEMWNQWNAV

NVALNQRIQESTDARNRLQTHLSKVLQEIFDMEKNIELLKKSIQDKQAPMQVAHTRLDIR

TRRPNVELCRDPVQHRLVEEVGEITDTVEALQLKLRDSENALQQLLRTKAALEADLAVKN

NSLFIDREKCLAMRKTFPMSPRVVSL

>SG-AL_contig_3137_1_-3_87_2279

MTAFEEMGVMSEIAQAVEEMDWSLPTDVQAEAIPMILGGGDVLMAAETGSGKTGAFCLPI

LQIVQETLLDQREGKGARKSMHSSGSNKWKMNAHDRGDAMAIDPEGTLCQARDAQGWHGT

RSNKAVVGGGKWYYEGTVTDEGLCRLGFSTGQARLDLGTCKFGFGFGGTGKKSWGKQFDS

YGEPFGMNDTVGCYLDLDKGEIRWSKNGVDLGKAYDVPPSVKSEQIYAAVVLKNAEMSFN

FGDTPFKFPPKNGFAGLASASGKNVTDSKIISGGAVSAKPAPNAPQAIIIEPSRELAEQT

IVQLQKFKKHLSNPPVRELLIIGGVSARDQVDALERGVDIVCGTPGRLEDLISTGKLALH

QVRFFVLDECDGLLSQGYGELINRLHQQIPKVTSDGKRLQMIVCSATLHSFDVKKMAERL

MYFPTWIDLKGADAVPETVHHCVCLVDPKVDGEWKSMNRHIITDGVHQKDRVGPDSYSAE

TYSEAVKMLKGEYTVRAIQKHNMDRALIFCRTKLDCDNMERYLNQVGQGKLTCVCLHSDR

NPGERKANLQKFKDGKVRFMICTDVAARGIDVSGLPYVINVTLPDEKQNYIHRIGRVGRA

ERMGLAVSIVSTVKEKVWYHSNCNTRGRGCYNTRLTDSGGCCIWYDEPKLLSDIEEHLDI

TIDKIDKTFDVPVNEFDGKVTYGQKRKAAGSIYKGHVEFLAPTVAELAQLEKSAQTSFID

LKFKKKFARRA

>SG-AL_contig_3138_1_+2_1286_1540

MKQQRKHITNKCCFNSRIFIRKAVEYKLRIFTRKAAKSINNIMFILQRKTSLHVVTLQNK

YVTTQGPSNARGAQDNKDHLYCLLN

>SG-AL_contig_3139_1_-2_542_787

MCKVMMHRPRTIKFEPHCNFACVRTVIRYHLSLEGEEMVSSTSPSLTVLCASLAGIRELC

PFLTTPQKNALIAEKQQIQKHT

>SG-AL_contig_3140_1_-3_189_1538

MENGSENRIQDGVVTDVTMNGEDEMIETEDGPETDNVEERQVRKAKRLLTRQTSGGDSSS

NNNAQANKKLLVLSKNSRKSRDGRGRGLPKKGGAGGKGTWGRPGEVIDEDGKGLDAHDPN

YDSDSLDDQNLAFEKVCPELTSEDFEKFIEPVVMEYFEHGDSNDVIEELNDVNFEHLKSS

LVEFLVSKALDHKDHHRELTSILISDLYGKVLSGSDMTDGFDDVLNRLSDLVIDAPDATS

VVGKFIARAVADDCLPPKYILKYKADLVDCPHTRQAVDHADVLLNQNHGIVRLDNIWGTG

GGIRPVKVLIKKIVMLLKEYLSSSDIGEATRCLTELNVPHFHHEVVYEATVIVLEDSHER

AAELMTKFLKSLADAVIITPQQFKQGFLRVFDNLPDICLDVPNAYQLMENFATMCNKEAF

LSDALLKDLPQRGRKRFVSEGDGGRVKDAL

>SG-AL_contig_3141_1_-2_1718_1909

MCVNSHLAQRKHRSRYSAIKKQHTHTHSDRKLLLRTFFSNLFIFVIRIVYMQPKTYHGFN

LNCY

>SG-AL_contig_3142_1_+3_432_695

MLGTEPTPILQMASSSKVVKHSDSEMKYLIELYQADDVLWKSQRKNYKNQHYWERARQSI

HNEFIGTRNDLHLTSVSKVSCSTPSVPH

>SG-AL_contig_3143_1_+2_146_316

MIEALRWAANRPVFMLRAGTELETSDLESRVLSKEPTSPEKKVAHKGVEPTISASSA

>SG-AL_contig_3144_1_-1_1135_1371

MSIVAPVFHILHRYRCLHTLSKSPKGQVCSLAGQFGMMRSHTKTKVSYKPKGINLQTYCL

HECISLTVMLLLFPDMCLN

>SG-AL_contig_3145_1_-1_1_213

KRCSQPIRCSIIITTRSCEGDNCFRLEILSTMATIEVHMDKFGQHSSHLYWSLPGPRDPP

LTPTNGSPINN

>SG-AL_contig_3146_1_+1_178_420

MPGMLLPSLHHTQIKHKFIKMFKLFPDPAMYTMKKESRLTGSVVIQPVSKNQELACKTII

FLFNNLYSAMARSPVQDEKGG

>SG-AL_contig_3147_1_-2_41_1060

MFPNRGNANRLALHDEDGTSKVEGDCRRMNTLAHYQVPDGAYVALHHKQLLPSYNMSIMS

EKSNMTFYNRSPTLPRSVAPQMIHVVDVDNNGIKHFHLVRQHDVEPSKEGDRGSKMVSEI

YLPRLLVTKGTLQQFVDDLFERIFSTAHRGSALPLAIKYMFDFLDDQALLHNIQDPEVVH

TWKSNSLPLRFWVNIIKNPDFAFDVHKTSIADSCLTVVGQTFMDACSQSEHRLGKDSPSS

KLLYAKDIPKYKQWVERYYQDIKMMPAISDQDMTAMLTEESRIHCTEFNANAALLELYKY

AQKYNEELMTALDEDEFARKSKLSYKLEHVHEDMEGSSIC

>SG-AL_contig_3148_1_-3_87_1073

MVVGESGLGKSTLINSLFLTELYKDREIPNVLDTVNRTVSIEKKQLEIEEKGVRVKLTIV

DTPGFNDAVNAENSHAAIVDYVDQQFQQYYRDESGLNRRNIVDNRVHCCLYFISPYGHGL

KPADVEVMKKLHHKVNIVPLLAKSDILTKVEQKRLKQRIIEEIDNHDIHIYEFPDCDSDE

DDDFKQQDRELKAAVPFAVVGCNTVVELAGKKIRGRAYPWGIVEVDNPSHCDFVRLRQML

ISTHMQDLKDVTADVHYENFRASYIQESMAGQQRERSKLKRDSKPSYDDIADTDKLLEQK

DAEIQRMQQMLAMMQEQLGKQQAQVNGKV

>SG-AL_contig_3149_1_+3_102_272

MTAGRMAEENELSTHWGFMSQPLPAWHTHDSLRTKPKATHEQAKQKGPLDCIHTTAS

>SG-AL_contig_3150_1_+1_145_2253

MADKRRLQGEIERCLKKVTEGVEIFEDIWKKVHQPAINSNQKEKYETDLKKEIKKLQRLR

DQIKTWIANSDIKDKQPLVDSRKLIETQMERFKVLERETKTKAYSKDGLGAMAKMDPQTK

EKADIITWLSSRIYTLQVQVDQLECELETMECTSKKKKMDKEAGPRSDELKQRRDKHKFH

IKHLEMAMRALDNDALQVDQIKLIKEDVDYYVEYNQDPNFEENEYLYQDLDLEDIAGGQA

LAFEGEDHDKVDANSTNSNSPSPSPSITANHSTVVEKVEDKERKRHKSQSEEKKIHMTPQ

LISKPQNAPTKQPPQSSTTVTQNATESPSINNHHPLATATPPLTAYAAAASSQQNTAADS

KPLVNSIDHNSINSNSMPVPSVSNSNSVSLTSSGNPSVPSAPSPHINSTGSTIPLGPMSA

STGINSIQNNSSRLEMSSVTNGPHSTGSHYKNQQESLSLQSIAQQAVNSAGLEARVPSQV

QRTDTSSVFTSSQAPPQPTAPSPDDQSVAASVLTSQTLNVSQISASLSSSQQNAAPQTST

IQLNPLLGVAPLGPVPPTKENTYQLAMTEAAFHHLPHPSDSERLRHYLPRNPCPTPNYYQ

QIPPTQADSLDFFSKLSTETLFFIFYYMEGTKAQYLAAKALKKASWRFHTKYMMWFQRHD

EPKSITEDYEQGTYIYFDYEKWGQRKKDGFTFEYRYLEDRDLN

>SG-AL_contig_3151_1_-1_1_273

DPNEVAACAWIDQKISQAIVKESEEASGPVEHDPSLPETLRALLLDNQQNQVQADISLEP

FLQRSRSDVDCERVSTGTKYALEQFLLKTKE

>SG-AL_contig_3152_1_-2_449_745

MADNTKPPQQLLAEIGKADVQKMKHVQTNEENSLPSAQAIAQEKTEVELRDRIGSFDKSN

LKHAETEEKTVLPNSDVLMFSEISHEKTEVELRERIGSF

>SG-AL_contig_3153_1_-3_303_2489

MPAQNETTQMEDAEVETFAFQAEIAQLMSLIINTFYSNKEIFLRELISNSSDALDKIRYE

SLTDPTKLDSGKDLHIRLVPDKENKTLTIEDTGIGMTKADLVNNLGTIAKSGTKAFMEAL

QAGADISMIGQFGVGFYSAYLVADRVTVESKNNDDEQYTWESSAGGSFTVAAATGVPMGR

GTRITLYLKEDQQEYLEERRIKEVIKKHSQFIGYPIKLLVEKERDKEVEDDEEEEEKKEE

DKEEEAKDEEKPKIEDLDEEEEEQDSSKKKKKTIKEKYSEEEELNKTKPLWTRNPDDITS

EEYAEFYKSLTNDWEDHLAVKHFSVEGQLEFRALLFIPKRAPFDMFENKKKKNNIKLYVR

RVFIMDNCEEIIPEYLNFVKGVVDSEDLPLNISREMLQQSKILKVIRKNLVKKCMELFDD

LAEDKDNYKKFYEQFAKNVKLGIHEDSTNRRKLADFLRYHSSQSADEMVTLKEYVSRMKE

NQKDIYYITGESREAVQNSAFVERVKKRGFEVLYMVDPIDEYCVQQLKEYDGKNLVCVTK

EGLVLPDSEEEKKKYEEAKAEFEGLCKAMKEILDKKVEKVTVSQRLVSSPCCIVTSQYGW

SANMERIMKAQALRDTSTMGYMAAKKHFEINADHPIIKTLKVKCDADKNDKSVKDLCMLL

FETALLSSGFTLEDPTSHANRIHRMVKLGLGIDEDDGVPEAASEEATATDDMPPLEGDED

DASRMEEVD

>SG-AL_contig_3154_1_+1_1_279

GGGDREAGSPKQAQYSRGEKKSAEKQPSCMPFLSRRNKSWSNEKKIKRRKIPSISLWQKR

RPASLLTWVRNFVSATVLAIKKNINKVLVTAPS

>SG-AL_contig_3155_1_+3_3_116

SFDGLNNTEISTAHQMVLLTEVSHYLSFRKQWASHEFC

>SG-AL_contig_3156_1_-2_287_2569

MSSHFDLTTGEPYSKIAKRSLCFYVVVAILVGIVAFVIGILIGRFAVCDDESPAATSRWT

QDADPAISDLLINAIDNKNIENNLRRLTLKPHIAGRQADFDLVDYLKQTFRDYGLDSVRS

APYDVLLSYPDKQQQNSVQLLNSSGGIVFDSLSAESNITYEEGVVPPFLAYSPAKEEMAD

LIYVNYGRVEDYDYLAQNTTINVTGKIVLARYGKIFRGSKVDIAARHGAVGIIIYSDPKD

YTWPEEDGRVYPDTIWLPPTAAQRGTIYIGDGDPLTSGYPAIDTAFRYPENDEKVTLPKI

PAHPIGYGAAIHFLQGLQGEEVPEEWRGGLNITYRFGPLTSGRVRMNITTTNSVRRADNI

IAIIRGEVEPDRYVLMGNHRDAWVYGAIDPSSGTAVMMEVARVMGQLVKQGKWRPRRSIM

FCSWGAEEYGLLGSTEWAEQYVKLLGPRAVAYLNIDIAVQGNYSLRAIGTPLLYRSVYAA

AMKVPNPNPSEVDEGRRTVYDTWKHSFPWKDYGIPRVGPLGSGSDYAPLLQIIGITSVDF

RYTYNTNKYKLGSYPLYHTEYETFDAVKSLMDQEFTYHQAVGRVGVELARNMADSLLIPF

NVSDYVWGLELNRQQLHADYGTLLSQHVQNYSSLELVIEEFGQDVKSFEQIVAKTDKNDP

MAIRAVNDQLTLLEKAFLDPAGLPDRPAKKHLLFAENSNDVYAGSSFPGLVDLLFEIQTL

PEPQQTERWEQVRHHFSVILYVIQAAGFTLRDVTSFVEERY

>SG-AL_contig_3157_1_+1_124_357

MQIYMPSMSENVQKSTRHTKWFEMKYLEHKSISVTLFLESRNRQKPSPRKNEHHSNIKTH

TCISVAFFSSHYDSPSDG

>SG-AL_contig_3158_1_+2_2_124

ERQTQALSFNRTHILSCSKINRKANMKTILQSFLTNTYQCH

>SG-AL_contig_3159_1_+3_249_404

MAFISSTSMFVAQKVKMKAQSYPKFYRLVIVAVFCTVYFFTYQTNLGSNHGL

>SG-AL_contig_3160_1_+3_600_899

MSNRAFNQILDNTATPRLCFLQITNLTASASPRADIKNFIINVTKRVMFIKHELVLFSKH

DKNKWTVNLLLQCSCAMKMSAEYPFMFYIKDSHNVCIIMY

>SG-AL_contig_3161_1_+2_245_493

MESNRSFRLDWPFIWFKDLSTKSTKSVSYIYGGEGNRSHPFTFFMTTHLEQSNQVRSPNN

FSNQVQSPKNLFSDFHWAMVNIL

>SG-AL_contig_3162_1_-3_114_3473

MAEALPHWSSTSQASLPLSDEEVFETEDAIWKLLHTNRPPEKEPKEVYVDRPRVKKHENT

QNLDSFADQNQQNVKSFDETVPSFEGVGIRSGLTVSLSLPSENDQHLSGSAQSESSQRLR

SWSDHVHTDSTEMRRRPPRFHQLERQPSIVDGLLMEIYDQWHRNRTDSLDSDTFTEYSSA

SDVFHGRWETGHYDTAVDQQPAKHYHRHFLQTQTREQLQKMVQDLQRSVTQASARLVRQL

KRRDRRVAKMSRHCDVVTAILQAASLKRQIDTRMKFSLGPGPGQSAFEQWKDAMKAVNRL

PMGIPTEFRKKVWLNLADHYISKLKIDWHKTVRLAFNDRSNPDDDKLGLQIVKDLHRTGC

SSFSGQDNEQDRAVLKRVLLAYARWNKRIGYCQGFNVIAALLLNVMDRKEDDAFKVMIYL

IDHVLPESYFDNNLRALSVDMAVFRDLMRLTLPTLSTHLDSLQHAAQDVNTGACYEPPLT

NVFTMQWFLTLFATCLPKEVVLRVWDAILLEGSEVLLRTALCLWGKLSKRIMTVESADEF

YSLMGELSSDMMEGTVFDSDDMIKAIYAVAPVPFPQLTELREKYTYNIRPFSTTPAPTRK

LSRLAKAVLYSDEEEPDEDELRAMAAFPGMSPVSGARGKGTDISVVGPGVFGAEPEVMSG

SRDTVYMERMSTDITALKMQYHKLRQRQRQAHVIIAAVSAKKAVELKQKSQAPVLVPKIE

TPLAMNHLFIGKVQGRNRLVTKGPCITGSATPGNSGQTQPVHTQSLLQLASCTPNCTAIA

PPRTRTSPALSVGRGDVEGEESLQGQLEESLQICERSKTLCTGSSSEDAEGNTGNAIKST

SVLPEGGSLAAEVRPQDQEEQNCIPSSEDNENHGLSSCCSEPEELDAVRESLNLTVIPDK

DRTYNLDNVDEQHLQLATQQKGSGVTVPGSQNGVASNNQSKNLDCENSKITRNSTTAPSV

HFPNGMGESSPKKSVSANDLLDGRAGGAHTVSKTSNDVGNVCGDGVDSKHSHIAISRSLS

LPTPSTKKTRFSPTTTPTHKHPISLTGIKVRHASSPLPPSSQPSPIKTPRPFNPFPVKHI

NTNRARTGLKLGLYTPSTLEHIQGQLLGGLVLKKARNASS

>SG-AL_contig_3163_1_-3_258_4649

MRAVDRIPYQFGSTNTADALLIMRDDMFTPVNGDRANVPNVAIVITDGVSNVNSERTIPE

ARASRLDGIHIYTVGIGLSDTRELDGIASPPARDNSFVVKDFDDLSFLHQRIFASICPED

FTMPPTTPRPTRPPTTTTTTPRPTTPRRRPVYRETGTDLVFMLDGTVNNQIFGWMKTFVK

RFAAQLDIDGGEYRVAALIFTSRPTVQFHLNRYNFQDEVISAVDRFQGIRRGRVDTARAF

EYVRQNIFTARNGDRRRARNYIVLLTGNERSINTQRAFDSAKQLKDLGTDIYTVGINLRT

TEEIDGVSSKPLERYQTLINSEREMDELPGIYLRNMERAPPPFTRRTTTTTTTTTPRPRR

PTTPYTGVCESSGDVTFILDSSGSVGEENFDRLRNFTMSAVRDLEVDNGFFRISVVTFSD

SANVEFYLNTYSYKREVMDAIRRIPYVYGYTNTADALRRTRTQVYTSINGDRANVPNLVV

IVTDGESNINPLQTLPEANRIKRSGTSIITVAVGVNSNEELRGLTSAPVRENLIHVDDFH

ALDRLSHQIVAPLCTDSNLCEEDPCRNEGVCLDGLRSYVCLCQPGFYGDNCERQCGPRTD

VVFILDSSTSVGTQNFQRIKSYAELLMRDMNPDTCDVNIGAMKYSSAAMIQFNVGRYDTT

EDLSTAINAIGYTQGRANMADALRVLRLEMFNGRNGDRADAKNVAYLLTDGGVEINKDIT

LTEADLIIDAGIRLIPLAVGLRQRNEIESIARAQGLPLMVVDSEIRLQEMSDQVLEAVFD

NDNHCSSAPCHNGARCISEPLGFRCECSDGYTGDLCEKDCSAQADVVFVLDVSRYNSRQD

VRHVKRFARDVVKRMAFRQGNFRVAVVDFASNAKTRLTFDEGNRKKAVKGAIGGIRPRSE

DPNLVNGLRRVISDVVGRRGDRESVPNYMVMVTTTMGGNPALQAINDLKRTGTKVIGVGI

SLSNSDERLMESAVSQPAQNMMFTVTDPADLDSIADSFVNFVCEEKNMCAEKPCQNGGRC

TNGAKDYMCTCAPGFAGKNCERSCADRADVAFLLDTSGSVGFDNFRRMKEYVYNMVENFN

IGPDATQVAVATFSTNARAQFYLNQYNNKQQLQDAISAISYEYGNTNTAAGLKLLRSGIF

STRRGNRAEVPDYAIVITDGLSNVNAEQTIPEAELARKQGVHVFAIGISVSDSWELRAIA

SEPADNNAFLLQEYSDLLGVTDRLVDATCRDSGVCAEIPCRNGGTCIPGVGTFTCQCMKG

YLGKFCEQDCQESKDIAFVLDSSTSVGPDNFNMILTYVKALVQELTAGGPNHRFSVVTYS

TEVRTIFSFSRYNTPQQVMDAITTTRYTPGSTNTAGGLRQARQMFQPGFGERSNAKNVVI

LLTDGQSNVNYYDTIPAADDLKSEGVTIIGIGIGLANADELNSIASGRRDVFQVASFDAL

DDVKAEIVAGSCRNSEEVPRKVEG

>SG-AL_contig_3164_1_-2_95_448

MSDTKEEKDEEKVFGSCEGPDAMYVKLISSDHHEFIVKRDHALTSGTIKAMLSGPGQFSE

NETNEINFREIPSHVLQKVCMYFTYKVRYTNSSTEIPEFPIAPEIALELLMAANFLDC

>SG-AL_contig_3165_1_+2_44_154

MYSLCMCVSMYHNYNGERLEEQKLSFTTRHFPQHFHS

>SG-AL_contig_3166_1_-3_237_440

MYNVHTNTVLCYLAINENLVYRFNKSFLLKVFCICNWFCFFYRISFVWGGGVFGGGFWFC

FVIFYSIL

>SG-AL_contig_3167_1_+1_139_381

MRHCTKHDPTLSVNRVIHHGHSTYQPIPRNPSDSCSTIPRFLCPRTGKILDSHLSLNGKN

PVMLLDSCVSLNGKNPLLDTF

>SG-AL_contig_3168_1_-1_208_1128

MQGSFRCVPKECPLGSRFDAGTGECRRIICRRGMRPNRAGTCVDVDECSEGKHRCLRHQR

CINTVGSYRCRSLVSCPPGYEATSNNGCQDINECTTNKHRCSADQECVNRQGSYFCQCPR

GLRHDASGRCVDVNECAYGAAICPATSRCVNTAGSYKCECKDGFISDENGGCLDIDECLT

DNLCQHKCRNVQGSYFCTCNRGYQLREDKRSCEDIDECTQFRGVGGRGGVCGGQCINTPG

SFRCECPEGWRSTRGGRSCQDIDECKENVAFCPQQDSICINTRGNYKCPVVRCPEGFAKT

TSTGPQH

>SG-AL_contig_3169_1_-1_1_15921

TNFTVPDLKDGTEYHFRVIAVNAEGQSAPLEAKDTAKPVKKIVPPGPPTNLRITKVGPDF

ITLEWKPPHEDGGSKVTGYRIEICDEKSSDWVKVVDLKAYDTSYKVTGLKETLGYFFAVS

AQNSAGYGEPCETERAVKPKRPEGPPSKPVGPLKIKDMEKTAVSLEWQPPSDDGGSPLTG

YVLEKRDTARPTWTRVEKLSPNKTSFKVQNLLEGSEYYFRISAENKHGASEPLETEKSVK

PKSPFDKPSKPTGPIKISDIKKDSVTLSWQPPDSDGGLPIKNYVIEYRDARRTNWMRGGS

VPADTTTFTCPKLLEGNEYIFRVMAVNDEGESAPLESKETVTPQKPTTTPGSPGNLKVKD

IGKDSVSLQWTAPKDNGGTKVTGYTVYYREDGSDTWKKAGSVGPLDSDFTVKNLLLDTKY

HFAVSAENKQGLGEKLETESAVSPKKPLQKPAAPEGPITFTDIQRTSVTFTWKPSPDDGG

SPITAYILERRETWKMTWSPLTTVKPDITSYCAQNLKEGTEYFFRVMAQNSVGKSQPLDS

DGVIPKSPYAAPDTPEGPLEVSDITATSLTLTWKPPKSDGGQPLTGYLVECRDVRRVGWA

PLATLPPNTTSFIADKLLTNGDYYFRVMAQNSEGQSAPLENSKPFRPVRTPELPGTPRGH

LQVNNVTANSAALQWQSPEDDGGSDLTHYLVEVSVDDGEWTKLGKVDSYSTKFRCNDLKT

DSKHLFRVSAVNAVGTGKPVESQVVTPSKPPEAPGKPVGPLEAVDIQRTSITVQWKPPTH

DGGSPLTGYVVEQREAKRLMWSKVTTVKPDTTTCQVTRLIEGTDYMFRVTAVNKEGSSKP

LDSDVFTAKSPFDKPSAPGGPLKITEVTEDSADLEWKPSESDGGSPISHYNVEVRDVRRS

TWTKVGEVRPTDTKYTVGKLSIDTEYLFRVVAVNAEGDSPPLSSTAPTAPVKKLSVPSAP

ESIHVRKVGKNFADLEWSAPKSDGGAKVNVYRIYKSTVLPPVWEEVAKVKGFETTYKVGD

LKEGSEYLFGVAAENEVGLSQVKETEMFIKAAKPLEKPSEPEGPLELVDHTRESLTLSWG

ASKSDGGSPILHYVLERRDGWKMSWTNPIKVKPDGDKLTCCVPNLKDGQDYYFRVFAENT

VGASRAIETETSFKPRSPFKPPSVPTGPVRFNDVQQTSLTLDWLPPKDDGGTPITGYKVL

MSEEGGEFLELGQLEGTKTKMKVKDLKTNAHYVFKICAVNKVGVSKPLESDTVVPKRQPE

PPSRPQGPIKVLTTTKTSATIEWRPPEDDGGLPLTAYIIEKREFPRSTWSRVDKISADDT

THCCPNLTTGSDYFFRVMAENKAGVSPPLEMDKSVKIKSPFDVPSAPVGLRVSNVTDKSA

DITWSAPDSDGGTPITNYIVQTRVIPRSTFTTVEQTPELQVTLTGLAPDNQYMLQIFAVN

AEGQSQPLVSREPICPQRILSVPDAPDSLLVKNITKDGLVLEWTPPHNDGGSKVRRYHLE

KSLKDSDRWEKVATVESFRTQHTVSDLELEKDYLFAVSAENDVGVSEKQTTAKPVRLEKP

IFPPSPPVGPIVISDVTKTSAKISWKAPESDGGSPVTHYSVEKRESWKTSWTPVERLPSD

RMTCDLQHLKEGQDIYVRVKAENVAGQSKPLESEQAITPKSPYKKPSAPESLVATDVTES

SVSLKWQPPASDGGLPIKTYTVERRDKRWGSWVKAGTTKGSVTTLDVENLVTGQEYCFRV

SAENEEGQGPAVEMKDTVKPTREPVAPEAPVGPVHFSHIEATSVVLEWRAPRDDGGAPIT

NYRIEVSLTQDTWTEVTIVEGDVTKIKAKDLTTDKKHYFRIFAINKAGTSKPLVSDAVTP

QRALGPPDKPVGPLEAVATTRDSVTLEWKPPKDDGGSPITGYIIEKREAMRMTWNRADKT

TDDTTKRMVKGLVTGDEFYFRVAAVNKQGTSEFLEMPRPVVIKSPFDVPSPPKGPLKILR

VTEDSADLEWGPSEYDGGTPILQYSIEIRESRRTTWGRAGVVDAMTTKYTARNLVVKNEY

TFRIRAINAEGESLPLDGEEPVIPRKKTEVSRPPNKVSITKTTEDGVTLEWRPPSDDGGA

RIKAYHVQAKEDTRGATWQDVGKVDGYDNRFSVTGLDTGKNYKFAVRAENENGLSEPCEA

DKSASPKKPVKPPSPPQDLAVLELNRDSVLIGWKHSEHDGGSPITNYIIEKRESWKTSWA

HVDRVRGSATSAEVIYLQEGTSYNIRVMAENVAGLSEPAELDEAIIPKSPYKVPTAPVGP

LEVTNVDSQSATLQWKPPEKDGGLPVKRYIVERREAKRQAWVKADTVRGTNTTCTVERLV

EGSHYVFRVTAENDEGLSPPLESDSPVTCRRAAEKPASPSGKLRASKITPDTVTLDWLPP

LDDGGSPLTAFIIEAKDSKTKDWLTVAEVNPEASRQTIKNLTEGQEYLFRISAQNAIGRS

KPVEMDSPVTPSRPIETPGAPRGPLVPKNEGRDAITLSWQPPLDDGGSPITGYIVDKLDI

QRGGWMRAARVPADATSHTVAGLIVGHDYNFRVYAENKAGVGQPLDLKTPIKAKSAFNPP

SSPKNIDVTDIQEDSVILHWSPPDNNGGLDLVGYVVERREVGRQQWSRVGQTGPDVTSLK

ARNLLEGRPYTFRVMAENPEGLSEPAMLLRPVTPERPIEPPGPPQELEVTDVDKESVTLS

WKKPAKEGGAPVQAYIVEKRDGKAASWSRVADTGFATHVFNVDNLIEGYEYFFQVRAKNT

AGIGEPATLKESVVPSKRKEKPTTPVGPLESSATTEDSVTLTWQPPEYSGGTPLTGYIVE

KSDSRRPRWVRVGKVPADQTSTTVESLLENVGYFFRVLAENSVGVSPALENEVPFKPKSP

YGPPSRPEAPIKSLQITRDSATIQWQPPLSDGGSPLTGYVVERREDGRRGWMYCGRTDAD

VTILTCAALMENTPYYFRVYAENKFGRSEPLESEFAVVPKRIFGKLSAPENLRAENVDKD

SLTLAWSPPHDDDGKIKRYIIEKRSADSRTWEPVSDVSGRTKWFMVKDLPPGGSFYFRVK

AENPAGLSEPAELSVPVELKAKKEKPKAPKDAPTILELGTDYVDLSWSPPESDGGSPITG

YQLERCDMHGRSWLPVSKEPITEGSFRVRDLKEGVQYEFRVRAVNAMGTGEPSPSSEPFI

CGHSIAMEAPKFIGVFNDQSVPLGQDARFVCQVVALPTAEISWFKNFREVFDGKKVSLET

DGATHTLRLRSVDFADAGDIECQARNRYGAVSIRAKLTVLAPAHIDLPPHYGKGLRFCRG

DTIKVRVPITGTPAPEVEWLKKNKPLTQIKALAPRLDITGTHLHSTLVIDDCVKSDEGVY

TLQVKNELGSAAVDIPIMVVEPPDPPTDVIITDVDKHAVTLTWEPPKYDGGSPITGYVVE

RLDPATNIWRYAQSVPRPVCTVSCLDEHTEHKFRIMAENMFGVSEPSEASVAATTKEPLP

FINYEDFVDSSFLGSPVDVTRIQGDVLGKYIICEELGRGAFGVVYRAIERATMKNWAAKF

VRCRPGDKDLVRREIELMNSLHHPKLLQLHEAFDQTGEMVMILELLSGGELFDRLVAEDY

DLKESDCITYMRQICQGVQHMHRNNIIHLDLKPENVMCVTKDSNDVKIIDFGLAQRLEEG

KQIKVLFGTAEFCAPEIINFEPVSFCTDLWSLGVVTYVLLSGYSPFAGEDQRETFSLVNN

ADYDFDDDVWDRVSDDAKDFIKRLLIKDKKQRMTIDEALAHPWLNGAAEKKLTGQSLSTA

HHKDYRDRTKHKEDEQDALPMGRLSRDSAVFRKEGDEGVLVRKLVMELPEHAPVLYEPLT

DVTGFEGSRVQLACRIIGKPAPHIQWLQDGQEIKEDSRHHLSYKEGVATLQIVDLEPEDG

GQYTCEAINNLGDIDTTALLQVEEVRHRRKRKEETETIPQVKVARQEEEGGVEETMPPEF

TLPLVDQVRQVGEDVEFSVTVTCRPEPEVQWFCGDVRITPSPRHVFEHDKGMYRLTVRKL

TTQDAAEYKCLAINPFGSATCSCDLKVMESVPEDHRRPRFPRELEDMTVTRGSKVQLECE

VIGFPQPEIQWYKDRQRIESDKHYEVRCENNLCVLQINNTDYDDSGAYMCRAVNDAGSAT

TEARVRVQSVRDTMARPDRAADGARMEAAPTSRYKFQVAPDFTARLKNKRVLEGMTVHLN

CSVTGIPEPDIRWFKDGQQVFEGIDYSMKNNYGLLSLEVPCARSKHAGTYTCEASNDEGT

VTCQATLEVEGEEELESMELEFLRPRFLTPLTDMFVDEGEDVTFQCKAAGKPTPSFRWQK

DMLELTSSNRIEVTADKQGGATLTIRKVKMSDAGLYLVQAQSRSGRSKSSATLRVRGKSK

SPPSSPEAVEYGRPFFEGGFASLQQIAVPGRPPSFSIMLPAKLDVHEGEKLRLDCSVQGF

PTPIVSWHKGHRGLMYGQRHRIMFVGSLHTLEIPQTMSLDKGEYIVRATNAFGCVETACT

VNILPPREKGEGIVAGDMMHLDATPKIKSGKEAPRRGKAPFFIKHLPRTIEVMEGTNVRL

DCILRGGVELVQWSDDDMDQARQQLESEGFEGDFVPMTRHGDGSPVTPSTGRSTDDDGAP

PSFKTTPQDLVLPAGSEAVFECVTCGPPSSPGTWFKDGKALAPSDTLHMTSDGNRHRLVI

AAIVKADEGEYTYEVSSYAGTASCSATLAIEVLSEEGRASRTQSVESHGRGSTDFSKPSS

TQSRQFKQKLPPQTSAVIGDQVTLTCAVSDPMTEVKWEKNGKRVSLTSKRISSVQQRDGT

VFLQLSGLLESDAGDYRCTAILADGTEIQTTTHLSLTAPPQSKSKPKEQDSEVAPVFVKA

LKDQKLRDGDTVILTCSVEGRPRPQVTWSRNGVEILDNQDILISTIGSDCKLQILDVYPE

DEGHYSCTAINSAGETTTTCFITVEGGTRGKRSDANGSGNLGLKEVEFKLDIGKRRLPSP

DVKRTSVEKERAPEFVVKPRRQFVDEGQNAKFKVAYEGSPTTRLSWSRNGKPLEASNKFK

MYEEGGYHYLEVISVKQEDSGMYVGTVQNAAGTDTASAELDVFEKPKLLTRGSPPPTLSN

SLQDLDVIIGDRDVKLKCDVTGGDTCTVTWFHNGREVRQGRQHRCAFNGKTASLTLTEVG

EEDGGEYQCVISNSNGEVTTSCTLVTQEGEKMEPPVFIKELQDMEVEDGDKVELIVEVKG

SQPVQVYWMHNNCQVTVDTPGCVLRAVDGVHSLLIPRATEDSGGEYVCEAYNEFGDTDTF

CRLRVKEIVDKPHPPEFLTRPKPLTVT

>SG-AL_contig_3170_1_-1_1_210

FFSFTFYFLSESTSFVRFYYTKTFLPFLRGCFVLVCFFPYILRGLRDMKMLPLKWLLGDI

LTTNNHADVG

>SG-AL_contig_3171_1_-2_44_382

MVVETVEKKAGAGGKTRKFRGDASKVAAKGQGLKKAFTNRLLSFQVDVKDAGEALLSVGM

IAPSGHPEPELSVKKTTNTNYTISYKVVEIGEHILHVKWGDEDIPGSPFVLST

>SG-AL_contig_3172_1_-3_27_233

MLCIHLHWCLFFFLFFFKSSKYKERDRSIQVDSLLVFFQKRLLGYLRVEFGLACGFAQVV

VAVEYKNLN

>SG-AL_contig_3173_1_+1_94_1218

MAAAPVMTLRHAACQLHCRLTGSALHPLGPIGLMTNYHQVRSKKHAVAQYKLQPDWFVNR

KTQFKDEGLTLDNEKFVKEVIRTSYRNESRVLKDDIMESHDYTKLTRRCGVIAIKIGVLP

QWTKEGKKIMVTLLQILDNHVIRYTPPEEFAKTAGWKPWWGHRFGSVVVGSMSSDPRQFS

KAYVSLFDEAGVPPKKILTRFLITPNAAIQPGTPLTVMHYRVGDYVDVQARTIDHGFQGV

VKRWGMKGMPASHGVTKAHRKMGSTGGGGNKAAIWKGKKMPGHMGNQLSTLKGLKIWRIN

TKYNVLYVQGSNIAGHTHGVVRVYDTILPLRRATPEDHPPFPTWFRDDSPDGMPLEYFDK

NLHRMTDASVNYEEE

>SG-AL_contig_3174_1_+2_164_376

MHWRQHRNDQNNQRHQRGVNGSVRFSDYRYLDFATLRCKETCVPQESFELCWRELSLHVS

LPNRTGRRVEA

>SG-AL_contig_3175_1_-1_94_2442

MYHRGGDGKPRGFGFEGFGFVKASSSSSGSGSSSTPQTATQPLTPASGFAAMSMGMGSTG

TYKFGTPGRTLGYHTNLGKRRVRSEEEYFEDDDEDEPANKMAYQPAPGSPGEEGNESDES

EDALDAYMAGLEKEIVETTEGETEEGKKKPDKGVRDDIDKEDEMEEYLRYMEENPNAGVI

ADEEDDIQYDSDGNPIPAERSKVIDPLPPIDHSDFDYGPFERNFYQEHDAIKALTPGQVD

DLRRALGIKVSGLDPPKPVSSFGHFGFDEKLLTAVRKSEYTQPTPIQAQGIPIGMAGRDI

IGIAKTGSGKTAAFVWPMLIHIMDQKELQPGDGPIGLILAPTRELSQQIYQEARKFGKVY

NIKVVCAYGGGSMWEQSKACQEGAEIIVATPGRLIDLVKKKATNLQRVTFLVFDEADRMF

DLGFEPQVRSIANHVRPDRQTLLFSATFRKRVEKLARDILADPIRVVQGEVGEANEDVTQ

YVEILPSSGPAKWTWLVRRLVEFTSTGSVLIFVTRKANSEELATQLRARDFRLGLLHGDI

SQMERNDIISSFKKKEFPVLVATDVAARGLDIPHIRTVVNYDVARDIDTHTHRIGRTGRA

GEKGSAYTLITDKDKEFAPLLVRNLEGANQTPPPALLDLASQCPWFNKSRFKRGKGKFMG

GRALGVKERPGLGSETATQLQDSTSFSSFRPQGGVLGGGPGSDRLSTMRTAFAAQFKSCF

VEASQEEKKLPTGVIIPPMSQSKDGKAQEPIQMQPASARYSKQSSSSSSSSEGGKKKRKS

RWD

>SG-AL_contig_3176_1_-2_56_862

MVQRGFARSTGPRSQEESYDGVTLAPEVTQAPSPSPSSGSSSSTSSSPTHPLPQRDCLSP

SKQERTPPRKTRPSPQKAGKSLPTKSRACRTITLRNLRNTPETKKEEEAEVRGESVGVEV

MVSGYRSPGHFYIQLQEKQSDLLSLMTELAYYETSPPIDWAWKVGEFCMAKYSKDSSWYR

AQITRQVEDDTYEVMLVDYGHVDVVPASELRIMKKTHATKECFSERCHLAGLTPAGSFDP

SKWSATAIDFVADEIRSKRLFLKLEGDSV

>SG-AL_contig_3177_1_-2_1466_1609

MLILLKSMLTAEREGESVCVCTCPQASVSARECIGSFAQIHIDRMHCE

>SG-AL_contig_3178_1_+1_130_447

MIMTERIVLLQHSEHLQNNNLLYPQQSVYHSTGHSTKTALLKIVSDILLALYDNQIFLLS

LLDLSAAFDRTDHAVLLARFQHTFGILGNVFPDSDPTCQTRNKPSL

>SG-AL_contig_3179_1_-1_19_1683

MASTSTALTVGGDRTTGQSVRTQNVLAACSIANIVKSSLGPVGLDKMLVDDIGDVTITND

GATILKLLEVEHPAAKVLVELADLQDQEVGDGTTSVVIIAAELLKNADDLVKCKIHPTSI

ISGYRLACKEAVKYIQDHLTIPVDKLGRDCILNVAKTSMSSKIIGPDSDLFSNMVVDAAM

AIKTSDGKGGHRYPVKAINVLKAHGRSSRESTLVHGYALNCTIASQAMPRRIADARIACL

DFSLQKAKMHLGVQVLIEDPTHLDGVRQRESDITKERIQKIISAGANVILTTGGIDDLCL

KYFVEAGAMAVRRCKKADLKRIAKATGATFLMTLANLEGEESFDASMLGSAEEVVQERIC

DDELILIKGPKSRTAASIILRGANDFMCDEMERSVHDALCVVRRVLESKTVVIGGGAVEA

ALSIYLENFATSLASREQLAIAEFAKSLLVIPKQLAVNAAQDSTDLVAKLRAYHNTSQTK

PEHSELRWVGLDLVAGVVRDNKKAGVLEPTMSKVKSLKFATEAAITILRIDDMIKLVSQE

EGGKSYQDAVRAGEL

>SG-AL_contig_3180_1_-3_3_296

KYNPLIIKKKKKKQKKKKKRGRKKKKKEEKKKRQKCEFLKKKKKKKKKCVTLCDGKICSC

SIYIFMIVFCFFVLFLISMPCIEYDLTHVYLCNSLNVD

>SG-AL_contig_3181_1_+1_493_723

MLISLSTSIITVGTTYDQSTQPTYLRCCPFMVFHKVLANLSQLGNEWYRLDGFQFRITGL

TGIEVELPSDLTAWVLV

>SG-AL_contig_3182_1_-2_173_343

MKTYTTCEIHNLEKSFVIDKQNVLIIYETLISYSTKCFNHPHILLDGILMFFNQSVK

>SG-AL_contig_3183_1_-2_374_466

MEGGFFSPPFPLKTDSFVNRSAFLGCINTIM

>SG-AL_contig_3184_1_-3_3_524

LFLQRVLPAVPFALFLQRVLPTVPFALFLQCVPSACHQLLVVSIPCHTVLITLPAPRSFY

QLFSSFYQLPVYQLHTYFTSCSFLFKNHQFILPAPILLYPLFSSLYQLPIYSTNFCLSLP

TVQFSLPVTNLLYQLPSYFTHCSALLTNYQFTLPTPTLLYPLFSISLPINNLIY

>SG-AL_contig_3185_1_-1_97_1575

MCGRTACTLAPDHVVRACGFRSRKGDYQRPVWKDPPGGQQYYPSYNMAPSAHTPVLLSSK

HYRGELDSLSERVIQPMKWGLVPSWHKGDPYKVEYETNNCRAEGMLTKRTYKTALEKGNR

CVVLADGFFEWKRGPEGKQPYFIYFPQSSSHSKPETKPDYKPETKSQTFPKTLPNPVKTD

PQPVAFSQTKVKQEPVTIKPDPDGDKPGTQLSAAVPVKQEPGSIKPDPDAINSLPVKQGP

VCVKPDPDAVTQPMKREEQKDEETAASSTGDELQERETEVKKERQTKIKRDRQTEMKTEI

KRERQTEGAECGSERGTHFSLGKTPLKSEVTDDVTSLDILKSADQAVDEDEEWQGKRLLT

IAAVFDVWHPPDGSAPLYSYSVITVEASPAMASIHHRMPAILSTEEEINDWLDFAEVPLM

KAVKAIHPTECVTMHMVSPLVNNSRNNSPQCVEVYNPKQVKKTASSNLMMNWLKRAMPTD

QPHTSDVKRQKQH

>SG-AL_contig_3186_1_-3_1113_1439

MQVTYRHCLYQTDIFRLGWGHSGGGALYGKLPFPFLCPVASWGCWGTTDELTTLLHPSRS

SASLKVVARGRLVHSFTLSSHRFFCLPRLLPPGTVPCRMVFASPLDLVM

>SG-AL_contig_3187_1_+1_1_282

DRVSSLLQQLLVAAPGSLGCVIAFVQQRVRKIVDVVDTVRQQVFLCPRIIRHTGRTGHIR

RRRGPGGHRVTPLLQHRVTFRDRKSTRLNSSHRL

>SG-AL_contig_3188_1_+1_82_588

MNRLSPFLTQSIFLPSLTSKDRILSLSHRMNMHAHTHTHTHTHAHTHTHTQTMLNNIIFG

HDNERLNTERKKVRKPFNKADPTEKNLCQQQSHYKQPIIHEVSSRSMTNVTFLCASLSIV

QRLPLAIKMHKLTKISRCHHTSVDLILSLPHYIHQLQKNVSFHSLILST

>SG-AL_contig_3189_1_-3_3_185

PFCQLQPSYIYRKEEKEGSGVSNHPLRKTAYKCQGVLYWLENFVAVFVKTCCVAEGRLQC

G

>SG-AL_contig_3190_1_-3_3_185

PFCQLQPGYIYRKEEKEGSGVSNHPLRKTAYKCRGVLYWLENFVAVFVKTCCVAEGRLQC

G

>SG-AL_contig_3191_1_-2_260_2053

MANAGAHDNGSQFFFTMAATPELQNKHTIFGKVVGDTLFNMLKLQEVEVNEATERPEEPH

KIISTEVLNNPFDDIVPRQIKRVKKDKEEDRKIKSKSKATKNFSLLSFGDEAEDDEEQVD

KATESFRGKSKSSHDLTDDPRLSSIPAVEKEAAEEEKRKKKAEASPEEVKTKKPETEDTE

VKVRTEPAEEESKKKPDTPEDSKKVKTESKEDDKKRKKFDVPEGGEKKKSPGVEEGEVKK

KKAEGEEEGKKSVKEEPKEEGRKKKESDTSHDGRKRKAEDGGHKEEKKKKSKEREEEDAG

HKEEKKKKSKEREEEDRRKRKAESKEEEGRKKRKGEEEKRKGEEEKKKGEEEKRKGEEEK

EEGKKKIKTEPPESSPQAAEEDKPVKDKDDTKRKVKKEKEESPRGKEKEVKSLLGFMEES

KQNRMEELKKEQRKLQRDIKEFKKRPHAEHKDKAKVKVEEAEEAEVKEEDTEENDLLAMV

KKERQKYKLRKKQLGRRENREEATLAMLAKFQTKLANARTLAGDYDDMDDEKEGDDNEDD

PDDMSWMRHKLHFEDESTKTKVIDANVHDDDRYALNDPRNPLTQRRRETSKKAMREKR

>SG-AL_contig_3192_1_-3_783_1025

MKNTPYKGLSERLARSFCKFDVKINFKLFSALRSQMVCPKLKTVDKHCTVVCHMYCGQCN

IIYRGTARALGIRTEENLAHN

>SG-AL_contig_3193_1_-2_74_439

MGDEADKFVWSVKNGELASVKEFVAKEGKCVNTQINGRFPLHYAADYGQKEVIEFLLSKG

AEINAPDKFDITPLLSAVFEGHTGCVQLLLQKGADKSGKSPDGASYEDCAEKQEIKDLLK

QS

>SG-AL_contig_3194_1_+1_94_318

MSRKCLTYILYMRKMEDGPNRRQMKLYIIKTSDNWDLLHKPLIIIPPKLSGHAQTYMQEQ

TCLPQTNVLRFQISV

>SG-AL_contig_3195_1_+2_581_730

MTRRIMGVNRTKGVGLKAHLYGIVLATFSPEGLWNLCHWRDSRTEAWAKC

>SG-AL_contig_3196_1_+1_319_480

MIRQFINQRGISHTQGPHKHTSQQHAQQWHYTKLKYLQQNICMMPKIKVFPIFF

>SG-AL_contig_3197_1_-2_2_130

LPPLPQSTASLLARRFSESWGASMLVHAERLSLVCCAHVPVSF

>SG-AL_contig_3198_1_+3_54_215

MYGFLNHRYNERVIQIFERQQKKTKLSRHTDRLPFLTILGLVLIKDAVTGDGRG

>SG-AL_contig_3199_1_+1_25_144

MVESETNLVLSKQASNHPIQSIAVMLMRNNIAKLEPFESL

>SG-AL_contig_3200_1_-2_422_556

MHGQHHSSIVHEQTGGGVQVDLSLSENRRSTPLVPDPPNLLVAKH

>SG-AL_contig_3201_1_+3_3_131

QQLYSLITRHHKTGSDFCHSRCCTVGQSEGHSPSWSCRLMKQK

>SG-AL_contig_3202_1_+1_1_279

HFGSVRLWPPFVRTGHLCIYIYIYITTYGYCSKKNPEITLQQISQFKQNAFLSFPFLQNE

SLMQVSFFVIFAKYNPYLFTVLSRTQANILTKV

>SG-AL_contig_3203_1_-1_313_513

MTLCRASRLAADRSFISLPQFPVQADGEKKFLKSLILFIVHFRSLFFFLDCLLSIFVCSF

SIRNCLF

>SG-AL_contig_3204_1_+2_215_337

MRHAYRSMKDSHFAPTFFHHNLITFTQKHIFTHTGNVVKLP

>SG-AL_contig_3205_1_-1_31_177

MSVFCAVSIVFVYFMSFYLFFILIEILKSKPRFRSHPQPSLRRKRVTDG

>SG-AL_contig_3206_1_-2_176_1183

MLSRAHLLIEKEIQKIKSSPPWGVEIEPVSDDNFFEWMAKIHGLKETIWEGGVFQIYIKF

DEHFNVRPPEVYFQTIPFHPNVDMRTGRPCMDYLDNFDLWRPTFSLSMMLVTLQVMLSNP

ILANAINQEAIEMMSQSPHTYRQMVVECVTASQRIDAGGSPQLEDTKVKFAQPPDNQIKH

FPVPRTAGILRSTKVSFDDYHMTWRAIATSKPHESVENQLLEMLQNNPELQKVHVALPPE

EVEEQMKNQMEEHKQLMFGRFKNKLTFSEERDAKLARLQRMKKIYLPPRHSPTPAPTEDI

VLDPTSAAVEGGKDWEKEVDDLVSWTNNLDTATIDT

>SG-AL_contig_3207_1_-3_75_1391

MLSTRSVTLDVDSEKSAIHAACEGGDISEVHAALSEGEKTANSFFEGLTPLHTTVLAGHP

ETCEVMQILLEHGADINAQTRGEEDTPLHLVVENGDFPRDFSLVLMLLENNADLSVRNRK

LRTAYDCAIGKGYFELASTLDGSMPPSQAREFYLQKIGEKYGPYIIAAVLRSDANMLQEN

IHLGGDPNMLNKHGAGAIHYAITHCTLPVMDVLQALADAGADVNLCDDEGDTALNLAIKS

KKLRETGDMIPVVTFLVDNGCDPSLQDLDGRDAFMLAEDRGYDDVLEILRAPRHKPPPPK

GPEAAPQVPEEKPEEFPDPNAPNKDGLYPIHVATYRQDPSERHDLIDSLIERGAGVSQVE

EKEGNTALHLCAQQDLGETAKQLLDHHIDYTVKNNEGKTAYDIAKEKGNKSVADAIEAKQ

KGVQDNWKKGKKAGFCVIL

>SG-AL_contig_3208_1_-2_167_4507

MSRSEEFSEVLPLRAQQGESEDSVHVLKAPKKQSSEARHAVTDPSVLLNDSTEDSQQLRR

LDETGESHRLSLSDQQGSDLTSHSKIDESETGVSATTSWIISPSGILVNNQSDETASFGH

VSDPVSAEYGKAERTESMSASLRGPDVISVGIPDSESLQVSDSLTISRNKQEEARGRAEV

RLQGTHQRANQTQQRTVSDIFSEESGSETEEEGEGELSFKRDENIEETPDTIRPDRVMSV

ESGPVIIVRPTSPHVTHPDRVTSVEQDLSDDEMATDRSRSRKFHRGRRGHGGEISYYKRD

AVEGMPRAQLSVPPKFASRLPGSSVPADDLWSSEYQQRSSLAMSDDSGLFADYIGAYGLR

LGPDSGMPETERTQETRRAQARSSHRRDLDDLPPSHPARIMNEIEKLREEHNRMMMLLER

SRYHPSSPMVSRLYRARSASPCLTHAGSWSSSSGSPVTVIAGFGSVRGSPVTFTTTPSPR

HPISGLITGSVEDSGSSPRTRDSAFHGSGPKRHSVATSTTEHSVDITVQDEWQPVSSHSI

LPGIISTTSAPDLRSDTEPLEGAVGFPSAARSSYYRTVVRPLAVYGNVGSWSNQETERFA

DTQTTTKVSATDMVTIDKAKQTKKSDLSQLSNSHTYTQEGLTGPSVTAPSQSLRGWSATQ

DSSQEKVKARADKNRIGQGMEFSSEVNREWSDSPFDEPQEVEVSQPPLDTLEKGNTTGAQ

PSYTIARRDQQTEANTVASPRSDLHHLLHDQHHLISQFVAEFPQDANAISTPHDSFADDD

LSLDVLIQTPRSLVGDSMPPVDHQLLLSRSLISTGVEAIVDSSDDFTQTDAEEDSNLHQS

QLEHSMGSLDSSNNTETLRIRAELDRLHQERVEIIELLSLKYLPGSLTVELLEAKLNYCL

GQTDMLLASLEKAWAEEEPYDLSRHGRSKRHEEFLSEYRQQFEESRKDIKVCVEEARRVQ

NGTRGRRTTRTRDLIAMQRRAQIEAFKQEREREQALYEEHRNLSPDLADSSRPGSKRVFA

PRFMTPKQHKEHLIRLRRSVVAASTEELNELRRRSHSSSFSLSPDRSFSPVSSLSNVYLS

RESSLCSSLSPDRNSAGLSMSFKTSVSFSTPSLVTPASYSSSNLHTSTTSSTPSLHLASS

SQTGTKQVSQSTEPTHFRRQSIPRHPKSYLAPRSSSCGPAQAKHSFHPHFVDPHLGFHEF

KDERASSCSRESLDPEKLLQESYTARQLNRQQISKAQEALRLLEEHRHGFRFKIRGRDSR

QHRQGPRRQESRQLSSSQTRESTVSEQMIQREIASLQQQQQQESRRQSPGESTHLDSGVI

PLSLQDNGNSTVPPESTSTPRSEESYAVYAKDMLVRNERHIQTIIAKSSHITEEAHAHET

SFGHSEVSTSDTTPSSLQSTDLDSFLDLSNSSNQRATPQFTASRPISSQDIKARIARRLQ

EKSKGKK

>SG-AL_contig_3209_1_+3_96_284

MPPHPTLFTRETKVFMNQNAIQYKNATRQNKKNLQQIHKLTISLRPATTNSDCELKVMVI

INL

>SG-AL_contig_3210_1_-3_126_605

MPLKVGDKLPAVDLYEDNPETKVNTSDLKGKVIIFGVPGAFTPTCNNDHAPGFIQNADAL

KGKGVSSIVCLSVNDPFVMCAWGKALKADGKVRFLADTCADFVKKADLSVDLTAALGNVR

SKRFVLVAEDGIVKSINVEPDGTGFTCSRAQDALNFLQST

>SG-AL_contig_3211_1_+3_1794_2132

MPTFQSGHRFFCCFFYKLFPCTLQIHQSNNENTVIKLTDTAICLLNEPQMVGMNKTRSSV

GPLHLIYLMKIKHTEEIETRWSSQTISDSQATLPIFPFQHYTGLTDNSSFKLT

>SG-AL_contig_3212_1_-2_107_454

MATTIAVRTRKSRKADAVAYSELSVRQNSAILEYCRTSMSALSGATAGIMGLTGLLGFIF

YFVSAFILSVFLTVKAGSRWNSFFITRRILFINGLFGGLFTYILFWTFLYGMVHVY

>SG-AL_contig_3213_1_-1_964_1182

MGKHFVRNADQTHQFPHRITFCQEFRQDPQFPHRTSLEKKQEFWFRKRKKNSGPRFGIAS

LMVLDIQDGSVLC

>SG-AL_contig_3214_1_-1_181_408

MDLLCPLCKYRLDDEFHFLFLCQNTEGLQSQYLPVDFRRDPTKLNLSLVSVLNNQKLMTS

IARYIYHSLKLRLTNV

>SG-AL_contig_3215_1_-3_123_794

MQNVEPAHPCVKGDTVWKELVPITLDVEKMEHLFETRTTETKQKRQDVSGKKEITVLDTK

RSNAINIGLTVLPPPRTIKTAILKMDTSIMNKEGIEKILQTMIPTEEEKSKILEAQMANP

DIPLGTAEQFLLTLSSIFELEARLHLWLFKLDYETLEQEVAEPLMDLKKGMEALHGSKTF

RCVLSALLGIGNFLNGSAARAFSVEFLTRVPEVKDTVHKHSLLY

>SG-AL_contig_3216_1_-3_570_710

MRTNKTIEITNEIIPPMASSTVNANESISITCLSTAFYGSLSLCNVH

>SG-AL_contig_3217_1_+2_2_130

QLRNLYRHISFLGTPISCMMMQNIPKNILPFFPCTQMQTQKKV

>SG-AL_contig_3218_1_-1_1_432

GEEDRHLCPVRSLRWYLDRSRTRRSSQRRLFNSVNESYRKDISAGTVSRWIRETILKAYT

QAQKDTSCLNPRAHEVRAIATSAAFQHSLPLREVLEAASWRSANPFIAHYLRDFRLTRAD

GSRGISFVAANMPISLARDPQSNH

>SG-AL_contig_3219_1_-1_187_390

MKVKTTKHSEVCLIQKGKTDLQIIKVLNKQPAKVIACGKEKFQRFVCSVYVCIFGQHQFR

CLFFYSFF

>SG-AL_contig_3220_1_-2_104_322

MVYIRSTLLHANHIFRHKINTRQESHESNRMLKCKSILAVGARSVHSHLFIICFFLCLIW

RIPSHFQSTNFHT

>SG-AL_contig_3221_1_-1_187_2295

MMEGPSRGNRNRKSFLYTKGSTTCPEVVASGTRWPVTRHNVVQSESCRRGSGNMTFFCGG

NQTCWRGEPNVDQCASPAFRNLLKRFSTDDDNDKREAPEPEETLAVTSELANVTKGDDVR

EEDIVVTSKLIKGLVSSGAATKIKDRDDVKSIVANVVKAGSNLVSTRKTAVLWTRMSERE

KIRSATSLLVAMETATVAMAAELKEPTTIVTRDDNIELELRVVKVSPSGQDNEDMVYNAE

DSDTQFSIPLQTLGRFSSGGLAKMVFMTHYTMGNILDDNNSDGNRITATDDKAKDKVDDN

KTPATLASYIISAAAASSENSVNLPQPVTFTITHTKNIPEGFQPLCSFWSISESGLGVWS

QEGCRLVDTNSSHTKCECDHMTNFAVLMTVQDVEISDVHKSLLRMVTIVGCIISCLCLVA

SWITFTCFTSLQGERNSIHKNLVVCLFIAEMLFLTGIDQTQNRVACGIVAGLMHYFFLAA

FLWMMMEGVHIILMLVQVFDGSRSRLPYYYAGAYGPPILIIAISAGLYAEGYGTEEYCWL

TTEKSFIWSFAGPVAVILIVNSVILIYAMTMVCRHSDYVFSTKDKTTAGSVRSWIQGAMA

LEVLLGLTWIIGFFFLTEQSVAMAYIFTILNSTQGLFIFIFHCLLNKKARKEYRRVMRVK

RQPSSTGTGSGTHTASLAKNKLTSGPSFNRHSPCKEDNPPTYL

>SG-AL_contig_3222_1_-3_51_233

MRCPEGKVIRIADAEWGRTSKTICDAASRNRNSKDDLSCKSDVTDKLQKKCGGEKECKFD

A

>SG-AL_contig_3223_1_-2_59_2923

MDANDEKIKAVLLFANRLIEENHFAADKIHKKAENINERRNTNRQRADEQLDRLKDQLTL

QQFLQECDELRDWLADKMAASQDETYRDAKNLHSKYMRHQAFESEIAANKDRLNRVQQEG

EEIMQMKPETREQIEPMLADLNRQWEDLEMTTKDKGEKLFDANRSILYEQSCDDIDGWVT

ALESQIITSEDFGKDLTTVNLQVQKQNQLENQLKMKEQQVQELESQADHLRRIEPDKVDD

IEKRRALVAERFAKIQAPLMLRRSALDKVKRIHQFMRDVEDEKLWIQEMMPRATATNYGN

SLLSVQLLIKKNHSLQVEIENHENRIMSVVQVGQDLIDEGHPQSEEFRALMEDLLRRWQE

LKDAVEARSQRLKLSEIAQQYYFDAAEAEAWMSEQELYMMAEDRAKDELGAQNMMKKHQT

LENNVEDYADVVRQLGERSRRLIEEEHPECDQMAVRQSQVDKLYAGLKDLAGERRGKLEE

VFKLYTLHREIDDLLQWIAEKSTVAGSNDPGQDYEHVSMLKEQFKEFARDTESMGQERVA

SANEICDQLIALDHSDSATIAEWKDNVNEAWADLLELIDTRTQLLHTSWELHKFLYECKD

TLERIYEKQNYIPEDLGRDAQSVAALQRKHANFEHDLITLGTQVEAIQTESVRLQAGYAG

NKAADIQAREAEVVEAWRNLHIQVETRRMTLADASDYYRFMLMAHDLQLWMDDIIRQMNT

QEKPRDVSGVELLMNNHQSLKAEIDAREENFAICINLGRDLLNRKHYRQEEVREKLIQLT

LERLDMTDLWKDRWEHLQLILEVYQFARDAAVAEAWLVAQEQYFLNQDLGDTLDAVENLI

KKHEAFEKAAATQEERFAALEKPTTFEIKDRQRKQEEEYRKQHPEEVAQAKEERRRAYIV

EFLPPEPPKPEPEPVVEEVLPVAAAVAERRPSGEEVDQTDKVDGDQEAVLQKEAE

>SG-AL_contig_3224_1_+1_2572_4632

MHPGPPGWGLCSGLTTHSLKNQTVTETATSSSSNNDMVVGEPSLVEPMTRAGESLREASA

QTTLLTTKSTTRIGTWNIRTLYETGKTAQVCREMHRYNLKILGLCESRWNGTGQTRLASG

DTIIYSGQEEGQPHTHGVALLMTPEATRALLSWEPVSPRILTARFNSKGRKVTILQCYAP

TNTAELEDKENFFEQVQAIMDKTPKRDLKILMGDLNAKVGADNTNRELIMGRHGTGEQNE

NGELFTEFCTFNDLVIGGTIFPHKKIHKTTWTSPDGKTENQIDHITIGRKWRRSLHDVRV

KRGADAASDHHLVVAVLKTKLKAYIDRAGRPSHKFNVQSLKERGKAEEFKLELRNNFSVL

SQLPEETIEEQWHSLCETWKTTCQTVLGKKTRKHKEWLTTETWALITERKQLKDQINQTQ

DQEEKQVLQAHYWDKNRAVKRSARKDKRNFIEELTQEAETAAGQRNMKRLYEITRTLSGK

NSKPSRPVKDKNGNTISGEEDQRARWAEHFKETLNRPAPSVPPDIPPPTELLGINTGPPT

KNEITKAIKSLKSGKAAGPDGIPPEALKADIQTSTDMLHPLLHKIWEQERVPEDWKKGHL

VKLPKKGDLSSCNNWRGIMLLSIPGKVLTRIILERLKTALDKTLRDEQAGFRQDRSCTDH

IATMRIIIEQSLEWQTPLYSVFVDFQK

>SG-AL_contig_3225_1_-2_257_2983

MEEDRDTLSIPGLDVPTTAVVVQQEVSSVSPAVTENHQTGISHFSAERNLEQSCINDGRR

STLEGLTKNGTERTCQPGLESNYDLDAQTVSASQGTHALAHPTTVSQQPSVKVETVPPLS

ASVSERIESKVDGKALMSAGVQSQTDGKILHENMSGVDCQVGAKGESSYNSFLDDIVQAE

RLDSLQDAGERQMHEGGLSATGHAASAEKQVSDKLGTANEVKCGTEYLDGVISSIQSLTC

NQPDADKDTVRISRAELTLMVEQTLAAQTMYNYMDDLVQKNKTLNQQNEVLKDTIVQIRQ

WQGGKLNSLPLISKGTEVQVFRFPGSFPPYIQSSASGYGTSPPACQNNGQHPVEQEATSL

LQADGDMSASLQLSSQISLDGDPDHSKEASAAGQLIHPVHTMNNTEAGAAVLAQLQPLNN

IPNTSLFVEAHCYSQGPQQHEEGVASAGVSQERFKIVTDHLEKCLRFVNKLTKEGGIQPD

CVLELADLRDKVNSLQKENASLKIELSNRCVPGKEDSAGQSVQQLQAEIRNLNAKLQQQK

AREDELAHHLGKLGAEYKLLKSGGTRPPSQPSSSVGPQALPTIPTTTGIRTTVKCQTEES

FVGIRELAAEADSKQKALCQYYQSELARLNGQLIQTQTHETELIKAVEKLHRELTATNED

NAHLQRSFREKIQKLTKNCEELAQQLVGLNSENDRQNRKLSQEAERNNQLEKIIGDERKR

YSRDEKELLDRVKMLQEMTKELPKLKRLLLAVDKARLEGTPLSQDCADQVTALRNQNSRD

TPATTHSSALRTASPRRGASGVSSGSSVTQSARMSSASRVSSRSSALQGNGHGTPGHSSG

RGSSPNLHAQVNTAPSASLPNLSGQHLQQYAGQATSQGNTNRPLQRNNLTNSASSVSSSV

NSSKQRHQY

>SG-AL_contig_3226_1_-1_160_261

MAVGFPYFQCHFRCVQEAVTALGSCHMRNPTNDM

>SG-AL_contig_3227_1_-2_2_154

PLVSRWWQGGGWSTPSRCLPTVFSVFLVSFLPAQSLAGWFLQVLWTYLSNL

>SG-AL_contig_3228_1_+2_20_292

MCVHAYQYMYIWTYICTQAKASAGFIREHARVRQCITVSSTFSTQRPPKFHVQMSLNVNV

FFKTLIVRLVRLLHLKCCFSPIRCLISLTFK

>SG-AL_contig_3229_1_-1_1_780

DKETEYTPWSKAFGELESLGNKLSNTDVFLAFKAFVAKTANPLYAKLAKKTPADYIETGL

RKRIVRLACIFDGPECVNDALTGLQEWKMNPDRNEIFRTMPHIMYCTGLRQGNTDDWDFV

FERFQAESDHYKKKTLLNSLSCTNNSKDLNRLLDWALNSGNTSSKNFFNVLRSTILNPVG

ANTAWTFINSTWDSHPEKYDTNNHGLQFTIKFFGDAFYTQDQLRKLQDFLDRHGDLGRAG

QAFLAAMESIPNRIAWMEKN

>SG-AL_contig_3230_1_-1_76_894

MLHSYHDSRVHACLYFVAPTGHSLKALDLVTMKKLDSKVNIIPVIAKADTITKTELNKFK

AKIMSELMANGVQIYHFPTDDETVADTNNSMNSHLPFAVVGSNDEVKIGNKMVKARQYPW

GTVQVENENHCDFVKLREMLIRTNMEDLRETTHLRHYELYRRHKLEEMGFSDSDTRSLSE

TYEQKRQEHITNLQKKEEEMRQTFVIRVKEKEGELKEAEKELHARFDQLKKQHADEKKKI

EDSRKRLEDEINVFNQRKAAVEAQHSTMGKKKK

>SG-AL_contig_3231_1_+3_36_239

MRICSSEQTNMLLKYTISEKNSAPTDANKHTRHSRHKQSLVMLWGQRELRGARGRETGRD

GVWSVFKR

>SG-AL_contig_3232_1_+1_1_144

LTHCSCSGVCVCVCVCVYIYIYIYIYICVCVCVCVCMNVCMYNYTSQK

>SG-AL_contig_3233_1_+3_63_311

MKTAYTSSLFIVQRGGFQQKCHFHLIIVTKQAQQLHNQKITLSSSCLQTSTLANALKIQT

KPILSKKSFQIHLVINSLTDTKL

>SG-AL_contig_3234_1_-3_138_332

MLISPVEPLTLALQFNLGLIIEPHEDNTTLSTLRGNQDSKEKKEKKKSKSILKINFKKVW

NEIFL

>SG-AL_contig_3235_1_-1_133_315

MVHTFGPNLVKIHPETAEKILLTHFNNTHNTLTHTYTQVILNNLPPPFFFLKKGGDFSYS

L

>SG-AL_contig_3236_1_+1_1_111

LPISVFSVNDSIKPHYSIICKQSRALRKSKLPCGLSE

>SG-AL_contig_3237_1_-1_157_294

MVKIKCHELCSFAGFLFSGLKRSCDQRVCLLDFDGLYVMTALIWPY

>SG-AL_contig_3238_1_-1_169_801

MSHGHSHGPCGGGADHSHDHPDLEMGKMYSLYTKVDTERVQCLNETVDGSGKTVFKPWDQ

RKDKTKFVASDADEELLFNIPFTGSVKLKGIILIGDNDETHPDKMRLFKNKPSMTFDDTG

MEPEQEFELHPDPEGVLEYNTKVARFNNVNHLSIHFPTNFGSDSTRIYYIGLKGDFTEVN

RQEIVICNYEASANPADHKANALDSVGHMVS

>SG-AL_contig_3239_1_+3_3_158

RIVLFYSSHTNREDRVNNLILALKYVVMSFISANILAQALKCFVRGIKPFVL

>SG-AL_contig_3240_1_-1_118_315

MVRFRSAYIMFCHRVIVHHLCFMLVSSWFVPGNDVLSIICVSYCVVLSSTLTVIRRVPFP

DRKSVV

>SG-AL_contig_3241_1_-1_211_4008

MRWVHCPGVWLLMLMLACGLDAVPQQLMYPYGPQHGDRTLPPEDDFSSDEIPLSVPVVFF

DTPFRSVYVNMNGHVSFETDIPNYQGDLVLPIGFKLIAPFLADVDTRVTGTVYYRETWEE

DVLGRAAADVQSHFSGRADFQPLALFLVTWDKVGYYDKQYDKVNTFQLVLVTDGMSSFVF

FHYLDGGMQWVSSQGKLAPTMPDVPGQAGFDRGDGYDHTSPTLPFSGSTAAFVNESNVNI

PGVWMFQIGNTGPDGDVKAADLNTGRVTIYEVGVGQTCLEGARQCHINARCLDFDQGFCC

QCLPPFYGNGKHCLEPEVPQRLNGRVHGTINQVTLNDLDMHTYVVTTDGRTYTAISRVPT

ALGPAMETLSTVGGAIGWLFAVPVGKDSVNGFTYTGGVFNRTAVVNYQRGNHTMHTMRIT

QRFFGQDALNNLRMDTHLEGDLPDITKGAKVTIDDYNENYKRIGPGLVKSFSVRTYRVND

VAFRYTWDQTLSFEECEADPSPRRHTMKLAVSRNFVRHSDKDQVVRYAMSNKVSIMTGSD

PCSDAAQTCHSHADCVPADQSFHCVCQAGYQGDGLDCQDIDECSLDAGVCDENARCFNVY

GSFQCQCQPGYQGDGRICTRERQQCSGQYCGDNARCVFNSELGRPQCECNPGFRGDGQTC

SPIRFGCNEADICDENAECVLDPETNSYSCQCLDDFSGDGITCQPTSRGDCGHCSRDADC

VYDIERLVYRCNCRYGYTGDGITCTPIDRCSECDTNADCSFDPAANRFQCQCRYGFYGDG

SHCEEYDCRESNVCAPDAVCDFDVNFNAFLCRCPAGYAGDGFRCEVKGCDDMNDCDVNAR

CVPDPLRTSRFVCQCSPGFQGDGKVCIQQVTPCNQVNNCDRHAECVYDPDALSYRCRCQR

GFEGNGYTCQQRDAADCRSQPSLCDRHAACVEGASGLFVCVCNRNYRGDGRSCTVAEREG

ERLVYARGSKVFSVSTTPQPGDYGQQLHFSPGQLAVAVDMDCVQGDVYWTDAAAGLVRKA

SLNGSNSEVILTGLGSPEGIAIDSVSRLLYYTDSKLDVLGVALLDGSHRKTLFNTDMVNP

RAIVLDSSRGKIYWTDWNRNRPQIETANMDSSERHVLVNGDLKLPNGLAFDHFSQQLCWA

DAGTKRLECIRSDGMGRRIVSNAPSHPFGLTMLNGLLYWTDWERKNISGVSQTGGDQVSS

VTPAIGGNGRLYGIVALRDHCPRGSNSCAFSNGGCAFLCFPAASGGRTCACPDGVDPRIC

QDSNNF

>SG-AL_contig_3242_1_-1_1_147

CVCVYMCASMCGCMCACLSKQHIRKHVGLRRGEVVKDGGHSNSISYWPD

>SG-AL_contig_3243_1_-3_3468_3671

MKKRSRRNLSATVRREAKRREIILMNACTCVLVSSVSSNRCAPKCQHQSDSVNLTLDLVL

PQLRRKAG

>SG-AL_contig_3244_1_-1_265_435

MPQMVCLPFSRGYSRFFCPALSCLRWSVYLSAEISVGFSVQLCYASDGLFTFQQRLQ

>SG-AL_contig_3245_1_+2_29_274

MRNATSLDINLCTAKDPINTRIAASLHMRCQGRKVPLNLWVLYTNVYQYRPYCLNVFFFT

NLSGFSMEFCQDQPLCCCGFFL

>SG-AL_contig_3246_1_-3_429_704

MSIIFMIFHIVHCVLAFGFDLFDILSECSCLFGFSGFMSWSVENDADRSLVLIQALSDIF

MNNQTCSINPSKSYSASVAKEQACCGQNWKAT

>SG-AL_contig_3247_1_-3_24_272

MIIGCWEIVIGCRFRSSQRWRCITVPSMKNSLNYCSIQAVKTILMVLILKNQIKMSNTFN

FSVSFQIFSGFFSNFPTVPCCVC

>SG-AL_contig_3248_1_+1_187_354

MYISILWSYVDISHTCMHAHTHTHTHTHTHTTEENLINCFRLLVANLQSQVSSFFL

>SG-AL_contig_3249_1_-1_1_168

SSDLCFCLFSLTLLSAFFCLPGRSHFLLSSKPSHNPTPPRQFMMYYLCCFDLRLGR

>SG-AL_contig_3250_1_-3_27_2144

MASTQDDADKPKSKRVFVNQVDHFQSKNIAKYLSKCVVGASLEDAEEEEDNASVTSSVLE

TPKEGCYEIVGTLKDREQNKPDFVKELILFENKDQLYEHLVECDIIIYDVTEDPDQIDEA

VWAVSEIHSDLDKIDKPKMFILISTVMTWAKSKSLDPDDPDIPFTEDDYRRRKPHPNFKE

HISAEKTVIKLGKTNKAKLVTYTVCAGLSYGAEENIFHYLFKSAWHNAPQLQCFGNGMNV

VPTIHIKDLAGVIQNIADSRPKVRYLIAKDDAQNTLDEIVKAVSINLGTGKVKNITKEEA

LLSKDIEQADFDQLLVNLRMDAVFVKENMRIPWTSEAGIVENIQQVIKEYKDSRNLQPLR

AVVLGPPASGKSTVIQQLCEFYKLHHIKIKDVIDEELDSLQAKARRADTEDQDEDDDGQA

QEAQEILDQINESKEQNGGRIDDQFVIRFVRDRLKSTPCQNQGFLLDGYPKTLEQAKELF

ALEDEEAEEEGKASYDTTVMPEFVVALEATDDFLKNRVMNLPEAVVAGTHNTEKELMRRL

VDYRNNNTEDETVLNYFDELEFHPERIDVTKDDSHMMKNTVERVKKIMGSARNYGPTPEE

LEEMRRQEEEARQKKEAEEKAERERREAEENAERKRRQEEWSQRLTEVRKEEYELLEAQS

IPLRNYLMQHVMPTLTQSLIDCCKARPDDPIDYIAEYLFQHNPQVD

>SG-AL_contig_3251_1_-2_2_139

VSMLVWLLLDGFHCESVGLANNILALSYSWYPCSLQNGKVFLFIII

>SG-AL_contig_3252_1_+1_316_564

MKCLRKTAGKTRRDMIRIEEILKMMVGTTPVHHYIIQQQIKWFGHLTRMPVKQQPFEPTT

PDAGASELEEDHRRTEVTNTLHM

>SG-AL_contig_3253_1_-2_137_1312

MSATSAPVTGSQAGSTTSGTDTVQVTPATADLPKTSEQALAYIQSLVPETDFKYVMFDDN

LVTVSDTGKELGEFTVTIEPSKFRGQECFLIHANSHGAIDGVPCGTSITCHVSKNMETLE

QNHHEYVKLENCPLDRKTIIVRQGDNYVINRVITQGEDVQRTSRSVSHETMKGFISEGSN

LLLHRIFIGKGLPEELRFISFDSETNLCPAIYRSLEERTQTIEGVELRVYGVERTINSNV

DLPTTWQSYFINDGHLSNRVQVGSPVTMKLQRIPTMIERDEEPPKPVFEKKELNWEEDMQ

MYSKFLDRKEELKNDHTVYMRHHPELKALLADFLQFLLLRKPDDVIEFAAEYFAAFSTSM

PTTSPYLASATATPFPNSRTNTQIESLRAPTR

>SG-AL_contig_3254_1_-2_152_3547

MANTSVVPSGNGSSRRGGQMHSLLGEFRQLYESKLKHLDEAEQTGEDINRARARLLQSYV

KDLSEQNEVLVTTVEELEQEANDRVFILEEKLQKTSASVKDYMSKARDMERELRNLTIEK

TRAEALLEDVQSRLTRQEEVTAQLRDENANLNHDIDQLISIISVARSTGQWETENIRFRE

INVDQVFGTLQETYAKLRPLGTDSTQVQELRSQLRRRDDRIEALQQEVHSLRTSPQGEGG

HGDEGDYSISRLKSDLHKLEQRTEDLQYEIQTRDRKVAALQTQVNDLTKELAVKEAENNN

HMQTVKMLQDKHRSNSSEASKSEDTIRQLRAELASIRDQHSQSVHDKSHFERRISTLEAE

LRDSKEQRRKALDEIGALQERVRESQISTHDHHEILKSELRRRDDTIQTLRRDVLNLQEK

RDAAVAESDHQLKIADTAEEQLRRSDEEVQRQETRLKTQDDEISHLRAQLEDADTEAKNS

RSKVIRLEGELQTVRNQYSEIQAQVSKLTNSLQRLQLASKDTRDLLTSEVAERTDALVRL

RTDSREAEEKLREAVANAEHQTELVRQLREELKQAKAKTQDTQLKLDEAEREKQQMQSRY

TAQAREIDRLQTDIQEHDLTTSQQETLYRSDLADREEEMSRLKADINVLHEKIAQSDGQL

CSQEETITRQQMQMRDLQDELARKETDLHKLEIDLVTTDEDRKRSEDQLSRQEISLQQLE

HDLKLIREQYKDAVEENGRLEARIQAFTINAQSEQECLNSEVKRREEAIKRLKLEQVSMQ

EANSRYEEKITQLQRETEQLKGQLRSAQSEIESKKAAAKNLESSVEEGKARLAAQLEDHR

QLEDKMRGLNIELTSQQSQVNKLHHDLGNKDELIQQLRGDLDCLHRQQKETTAKVLVRDD

NIQKLNADMGKLRAMHQEKLHEIGKQSELIKKLQDTLTQCHYDLDLTRRRAEDDVFKRDE

NIKQLQEDLLESQTQYSACYNEVMRLEEELEKVKAENSHLAEVGSRASQTSSQLQEQLHQ

IELDLTISQEKHRTCQKEVTNRDQVILRLQTDLDTAHQKFTGCAEELNVKEKEVQRLNAK

SRDMQQEIRTQLQLLEQRDCKIAEDDKQLQRLHHEHEMTLQEPTSEDPHTHS

>SG-AL_contig_3255_1_-2_2_478

SGALPATLSLVSLSWRKGTECVYNSKWTKWLAWCEPNQVDPHAPSTVELANFLAFLATSE

GLASSTVKGYRSAICTTLQQLGGAHSPTTSSSSKSREAWQLEKPKNPEGFPNGTWPWFSE

LSRPLPSSPWMWFPSYCSRTRRFFSWPWPPVAVAARSTL

>SG-AL_contig_3256_1_-2_2_298

LPATCSLVSLSRRKGTESVYNSKWTKWLAWCEPNQVDPHAPSPVELANFLAFLATSEGLA

ASTVKGYRSAICTTLQQLGGARSLRRLPPPGSLERPDSP

>SG-AL_contig_3257_1_-1_1_243

VDCTQHRCIILKFPPINKITRYKSMQSQHKLTRLSPGKSTMLVRKKKKKKTLSRLHTTLC

EMKPSHNTNVLLLDSVMQINL

>SG-AL_contig_3258_1_-2_191_370

MWRKTVVVLRVISIAILFSCIAVAYIRPFSVIVIVLLLISYIAPAFVHMYPFSGGTTAVV

>SG-AL_contig_3259_1_-3_150_470

MGIVEITTADQFEEMVTKSTSGKLVVVDFFAHWCRPCKMMAPKLEAFADEHKDVEFYKVN

VDELPEVAESCEISAMPTLHFYKSGQKVGDVIGFNEQKIKEAIVKWM

>SG-AL_contig_3260_1_-2_704_1222

MDSAFRSCPRPYSQLFAVLGDVFGFVVPLVQVLMKQRTVFSYQNGFRAVKQVVRRTIEHT

VRPQKVICDLEMALHNAVRAELPAARLCGYHLHFAQSVWRHVQQVGLSALSDQPWLAEVI

GKVLSLDHLITARQTRRLMRYPALVRLFGYVWNTYIREGSPFLQQCGTSSRGP

>SG-AL_contig_3261_1_+3_414_674

MNSKLQVFKKLKKTHAFKCCSQIHANSILFFVTRVSDSVGKKQNKQETNKFYEVRGIDST

KYVEQIKYFYICTKESIPFSCVKPLHC

>SG-AL_contig_3262_1_-1_13_1224

MATKASTSRRVMQEDLRAVEFETSEDVDVTPTFDNMGLREELLRGIYAYGFEKPSAIQQR

AVKPIVKGRDVIAQAQSGTGKTATFSISILQCLDIQLRETQALILSPTRELATQIQKVLL

ALGDYMNVQCHSCIGGTNIGEDIRKLDYGQHIVSGTPGRVFDMIKRRNLRTRAIKMLILD

EADEMLNKGFKEQIYDVYRYLPPATQVLLISATLPHEILEMTNKFMTDPIRILVKRDELT

LEGIKQFFVAVEREEWKFDTLCDLYDTLTITQAVIFCNTKRKVDWLTEKMREANFTVSSM

HGDMPQKEREAIMKVFRAGDSRVLITTDVWARGIDVQQVSLVINYDLPNNRELYIHRIGR

SGRFGRKGVAINFVKNDDIRILRDIEQYYSTQIDEMPMNVADLI

>SG-AL_contig_3263_1_+3_3_125

VYIHEGGPGISRSAHMLTWEFSCFHKPPNTNMAYRILNVHI

>SG-AL_contig_3264_1_+3_3_107

HTPEDSQVKCFLFHHLTVVESWGDRGGNSNTGPRL

>SG-AL_contig_3265_1_+1_1000_1407

MPEVVSLPQSPKPTTQEKLSPFPSLLYLQQEKLSPFSQSPKPTTPEEIVMNKNCLKISHT

MRARRRRMQMYRTTTTNTDNTATILTTLHNTHTKRSQSEWLLTTHKGQQCSHPIKTPNPH

TDDKNFTPTTIITKAL

>SG-AL_contig_3266_1_-2_119_214

MLCCSHALTSCISVIGSKCMLCSRASLINLLL

>SG-AL_contig_3267_1_-3_969_2804

MERCEDGAFPMTNTRCCECGGGAANTPRPRRHRLGVRLRSMSFTAAVLVVMLTVVGVVQA

GDQPRRCQEITIPMCKGIGYNYTYMPNQFNHETQEEAGLEVHQFWPLVEIQCSPDLKFFL

CSMYTPICMSNYKKHLPACRSVCERAKAGCAPLMRQYGFAWPEQMNCDKLPKYGDKNQLC

MDANTTDRQTPPPSTKYLPPTARPPTGDRGGAGDEDHRVQPIPPGALPTRPTENKCECKC

NPPLVPVSQNSNSYYNKITTGGIPNCAINCYSPYFGDEEKTFATFWIGLWSILCCISTSM

TVSTFFVDMQRFKYPERPIIFLSACYFMVSIGYIIRLIMGHEGVACEGERVRYETTGPAV

CTVVFLLIYFFGMASSIWWVILSFTWFLAAGLKWGQEAIASYSQYFHLAAWLIPSVKSIA

VLAMSSVDGDPIAGICFVGNQSLENLRGFVLAPLFVYLIIGTSFLLAGFVSLFRIRSVIK

QQGRGKTDKLERLMIRIGVFSVLYTVPATIVIACYFYEQHLRERWERTHTCLCADDPAAP

DYSVFMLKYFMCLVVGITSGFWIWTGKTLDSWRKLFNGRLCHKHSYSHPNRSMTKYANAS

YQPAKPQPLSHV

>SG-AL_contig_3268_1_+2_2_190

KKNYHVKYENYQNNLLTDKSNSFSKRQTKTFIHTNYYVNSTGIKRYHSHNRGECRGKAIK

KDV

>SG-AL_contig_3269_1_+2_401_1003

MKNEQNIHPQLSIFYIQCPSRRLVKIYHILHIKDYHTLFNIFRSWVVHPNDKSEIVNNCA

VVHRMRCNRYGSPYMGETARMPSIRIKDHQNTTRAQQWENFVKKQDTKSHTKRKGIWGHQ

ESHLREKNQHWKSWRARFKLSANYKRVKSWRARFKLSANYKRVKSRRARFKLSANYERVK

SWRARFKLSANYKQVLWPCDH

>SG-AL_contig_3270_1_-1_55_1749

MSFDAAPILRVAIEPVASGEMGQLVEGLRLLNQADPCVRVLVQDTGEHVIVTAGEIHLQR

CVDDLRERYAKVEVNVSKPIVPFRETVIPRPKVDRVNEAIQDQNTILASTRLKDFEDDKE

VLDEGLVEMMTPNQECVVQIRVLPLPREVVTCLMKHQDVIKTLDTLVADLSVPDLEQDPA

IKIKQTALEELSTLREELRSAFTSEGAQWTDTVDRIWSFGPKRSGPNLLVNGVEDYNRLS

VWSVLEEQGTERVGEVRANDSSIVAGFQMATQAGPLCEEPMQGVCFVVERWENTDARNAN

NSRAQSENVNDRTRDSLSPSNGSTVGKTTDSKSTESCEASHQNSETRSATRKGSNATQNC

ATSENDNTVSDSVGHSDRTEGSERPEAGEVVRRKGRDVFGPMSGQLMSVVKDGCRKGFQT

MPQRLMVAMYQCVIQADTDILGKLYAVIGKRNGRVIAEELREGTQVFSIVTEFPVVESFG

MAEEMRKRTSGLASPQLRFSHWEVLDIDPFWVPSTEEEYLHFGEKADSDNQARVYMNEVR

RRKGLKVDEKIVEFAEKQRTLTRNK

>SG-AL_contig_3271_1_-1_76_411

MPCVCIYVHQSVSVFRFDHAVFRFCYPNSNCIHVNSCWNVNILSFPIFSHTRITNTSTAV

GRMCRNRMTSLLLKYSRKKELLLLDYYFTCLFIFFFYCMKLNACKSCTKLTL

>SG-AL_contig_3272_1_-3_36_527

MAALLRNGLRVSALVRQTSRIAFSQQRGYSEMAFTFASPADVYYNKANVKQVDVPSFSGS

FGILPNHVPSLAVLKPGVVTVFEEDGSHKKFFVSSGSITINDDSSVQVLAEEAHPLDRLD

VQAIRDGINKAQQEVSAASSEKAKAEAQIALECYESLQKAMESN

>SG-AL_contig_3273_1_+2_629_916

MKTQGGYGLGFATVMLSEQGRGDGCHPHKVAPRKIKVSTTSLSYDLRVMGKPLLLSQIGQ

AHKYSVHRDVNIIIKVPIHYQELFYDSRRCDVIGSH

>SG-AL_contig_3274_1_+3_666_872

MFYGNITDLDHYANTSTPSSSNRSGIMTYIHYTKTAEKKNNTHSHIKNTHNTQTQLQKQC

TDTSGQGRQ

>SG-AL_contig_3275_1_-3_519_1115

MPIFSNIMEMRSKYKDAFLKKYNLKLGFMSAFVKAAAFALQDQPVVNAVIDEQEIVYRDY

IDISVAVATPKGLVVPVIRSVEGMNYADIEKGIAELGEKARAGSLAIEDMDGGTFTISNG

GVFGSMFGTPIINPPQSAILGMHGIFDRPVAIGGKVEIRPMMYVALTYDHRLVDGREAVT

FLRKIKSAVEDPRVILLDL

>SG-AL_contig_3276_1_+1_250_507

MDHALSTFCTSFVRLMFRPKLVIQYRSIHVQPRPFCRHISVMVVSWPCHYKPYRNLSLAR

SCSWLVLPPGFPHHGTQHTTPTARYK

>SG-AL_contig_3277_1_+3_627_896

MMCLTVILSRGFFFSRSIHFLAPFRIIRNSQHLQFRRSNNKKKSTHEVFLASCMQITRLL

WICTLISTHAILHKKKSLALFLPMPAHSPA

>SG-AL_contig_3278_1_-2_2_265

KFPRDDRSRRVTPFTDLPDYTFLDGRPTPLYKWQLARKEKNYQLAKRIVMLAKEVEDAKA

AHVKRLQDEAARRENIISSKLKPKSEET

>SG-AL_contig_3279_1_-3_54_287

MDSEIQRWQRPGQLMDLIDDKWREDVLPMEDIAVPQMELPELEQDNGPSTETLQEQEQKW

TDLAMQSLHEQPPTSTQS

>SG-AL_contig_3280_1_+3_123_269

MIKLSPHKYIMIYSVTTLNLSQHYFKIKRHQSDKTSWNRCKGYHWPEVC

>SG-AL_contig_3281_1_+3_852_1004

MTVELLLFDSHGNNAHQLTAVNQQHHKKKTNFIIRPITRAKLNQGHARSIE

>SG-AL_contig_3282_1_+2_2_133

GRERGRETEGKRKREEGAGRKKYFENDCPVTITKVILWTREQVL

>SG-AL_contig_3283_1_-3_36_533

MAMAIKEFIVVPVKGSEGAIHNLFLKREHRQQKPTLFVGNTPPWSTEKDFKDTFTKCCGK

VEKVVLQHSPSGGKEEPPKDDRKAVPLPKISHRVGYTTFKDDESVNKALQLPYSKPLMLR

SEKTATLFGLARYREAYMKMASLNIAETKKEIKARLAEYDRKEKEK

>SG-AL_contig_3284_1_+2_656_1090

MRIDISQTANVLEENGDLIMIYQTKVSKAISDHVKHLLLKLQKFLGNKKIYRNFVYMIMD

LCMLNMIMYKSECIVCSVDSMSDKCHVHLKQTRDPEVTKSHTLFTQNQSQVENMTRLLHS

VCISLLQSSSIFFHTLYIIRNLNFT

>SG-AL_contig_3285_1_-1_55_228

MHLMAHFSQSNVNVTWNKKVCECIRACVRACVCVCVCARARAGDVRERYMKRESTSIN

>SG-AL_contig_3286_1_-1_1_204

IIIIVMASADGSPQIPVLCFSHFLECSPLLRYKPLKQKKSIVLYCVLWFVVYNLHCNFYH

SRIIHSPG

>SG-AL_contig_3287_1_-3_534_710

MQNKQLHLLFCLNCSLLPLLRYARPTNNCIYCAVFIEHARRTSSEFSTLALCVIYSIMS

>SG-AL_contig_3288_1_+2_2_199

ALPIFFSKFVQSLYCKSKCRSIQPKQLSNQQFIQRSSCNSSFPILRIYRKFIISSSPSQN

KKLLIS

>SG-AL_contig_3289_1_+2_2_172

FFFLLMTMLLFVYIKISRQKSKIFITANTCINMTHDNRAERKECVCVCMYACIRVCK

>SG-AL_contig_3290_1_+2_2_148

HTCTSALILSLSAQSACPHTLHGEARSIYKNLKCTQNIRTSLTYSIQPM

>SG-AL_contig_3291_1_+3_3255_3551

MFDQVNCTAYLNYPIKTPYPPNVHVHTIHQMKLIYIYIPSDSPHLTMYLPKFNFVPYLNP

CSTQLLYVEWQHQFRKPCNWDSAYCPCCCCPCCCILPHP

>SG-AL_contig_3292_1_-1_202_390

MLTQDWKTTPTKYTHTECLDTGELALLILIQESKAQSFNNSTTTDRQGKKHTPSFFAIFW

GTV

>SG-AL_contig_3293_1_-1_1_249

YSQNVSFNLSLRQSSVSLGLTLWSVSIIHIMFPSTIPLLQSSVSLGLTLWSVSIIHIIRD

LAGEGTELPSSSLIAWVNFDCMG

>SG-AL_contig_3294_1_+1_520_816

MFLGASILRVVHISSPSRFTFPNPEQGRVSFTSGWTGGRTHKGFTKSRSICSCSQIENSS

HLCAIQCLSGHSWPAINLMQTAPHCSRSSLLLHTQSVVT

>SG-AL_contig_3295_1_-2_5_634

MAGRGGMDMAGGRGGMDMGRGGMDAGIAAGRAGMDLGGQGRFDSMGGRGGLDSNNRDGYD

SMRGAGLGGMDMGNRGGMDFDGGRGGYDQGMRGSTFESSGRSGGYDNKGRGGDMSSGRSD

GRGMGGGARPGQARCTVTVRNIPFNLSWQDLRDRFRDIGDVKFAEILMDQGRPSGDAVIR

FGNPEDAKRAINLVDGTRLEGRTVRVVLRN

>SG-AL_contig_3296_1_+1_142_300

MIEMNVQTLLRTKIKRISKIILAYQNCSTVKTKLPRGKVLMTIIKSATETEQK

>SG-AL_contig_3297_1_-1_124_591

MASRNPRARLSVLEALDMIFEEGLVDEHEVGYYEEEDSEEGDRHLKLFEDIPVENKSYGE

SCERDGDELSDADRDRFGDENAGAPVERGDASQMYDADTKVESEESEEEDVDVQQVVVYC

IVLYCIVLSQWIYHVKFGLLSQGERVAAVQRHPLHL

>SG-AL_contig_3298_1_-1_1003_1362

MYTVIDTALQFQVFEILMETAVQLQNFEILMMISNDAPYILLLCPFVITVYVCKQSILKP

NLICYISCYVFNCTGLRFCLSAFFLMFASDLKYVDLTGCLNFRVHVASIDMMLPTTVESA

>SG-AL_contig_3299_1_-3_855_1193

MQSGKDVTDTEKPLLKHFTFGGGQSMVSQNLYVCSIYWVRHLVKLCETWRSLSDTSHFFF

FTANASSQLPSGQNSRHGPFRDFVIVCNESVHFLILTRLKQVKTFYIYHYKIL

>SG-AL_contig_3300_1_+3_699_872

MYVSSCKPTALSRPSYWEPVKRCFPTHITKNMSQPQFLQLNWWKTLSNIININLFKYH

>SG-AL_contig_3301_1_-1_415_702

MFSDLSGGNTAGVDVTNKIVLVCQLVSSLHGSLSFKKQQQQMSCDIVTRLFCTIKAFPDE

NYHMEDCFVLGPMQTFNLSVSKCLFSCCFISCISFG

>SG-AL_contig_3302_1_+1_1_225

ASQSSSARLIKYTIFLPVLINQPSCKPCTTKWLRKHQCLSNVMARQWSLLEKVIRDEHVR

YCKCSLITRFYVLAK

>SG-AL_contig_3303_1_-3_33_191

MLPLFPLGRSETQAIYRQAGDSYKVMGSGELEMWFQASSMVWQEKPCLQVGGC

>SG-AL_contig_3304_1_+1_409_582

MEKQTNINNTHTHTHTHTHTGAGRSLAEAGRGKKQKHEKICSTLEAIQTGFFCCFFHF

>SG-AL_contig_3305_1_+3_3_173

ETGRPNGTIMLTVQHGTVYEAKYQFYTQKQYKRDILAKYNNYYLVQFKVLSPDNNQH

>SG-AL_contig_3306_1_-2_65_1426

MKSDSLKKDLFIMERVINLNTYQAKQALYRGFKIISDMEREPSSLNQIAVSDMGPNLDRL

WSYTCPLTKGRNVSCLAWNKSNPDLVAVGYGQFEFTTQKSGLICCWSLKNPEFPERVYTS

KHGVTALDFSVANSNILAAGYYDGGVAIYNVRSTSDEPILDNQETQGKHLGPVWQIQWIE

KERGSGEERAEVLISISTDGRVTQWSIRKGFESYDIMRLKKLPTRMAGKAREKKGEAFIS

RHAGGLCFDFHSRDLNVYLAGTEEGYIHKCSCSYNEQYLETFSGHTAPVYHIRWSPFVSD

IFLSCSADWSIRLWHQDRTTPILTFHSSTKTVHDVCWSPRSSTVFACVNEGAVEVWDLAQ

STLDAVITITPTSGAKLTSVSFGKNTECILVGDNEGQVTVYELRCMPTPPPREDQADALN

GVIKASLASQLPSVSGDKPSGTPVDEFDEPIDDK

>SG-AL_contig_3307_1_-2_2174_2470

MQVSFPLELHWSCYIPQTRCQTPRADMTSLIKYSKLIPTKHQHPGDGLGRLRIQTGVSFA

MSVEQKKAALISVVFFSRDMEYSQFVSLGSLCLWASVLE

>SG-AL_contig_3308_1_+3_3_305

RKGERERGREREREEGVGGREGGKNEKRKRGNKEILECFAARTEQIDTTKRQTDRQTDRQ

RHRVCVCVCVCVCVCVCVCQRDEERQLVINCTEVCVCVCVC

>SG-AL_contig_3309_1_-2_32_442

MGEKTSFSCVKYVFFAFNVFIWLLGCGVLSLGVWLQVNRSAHISILPTHSFLSASALCIT

AGAIVLFVGFLGCCGAFYENQCMLIGYFIMVVLIFTLEVTAGALGFLHKDELRAMIQKDL

YLSIQQDYRVTSAGVEN

>SG-AL_contig_3310_1_+2_347_577

MHLERVMAIQNNLYYFWSMNKFKTTNNKYNYSDFSLLFKMVTVHVLQPDTFNCRRNPSIK

MLNVYSHLVLQTHYLQV

>SG-AL_contig_3311_1_+1_1084_1302

MRCVQAIAYNFLGTLLSSGCILFLFCLIKLQHFKRAESSRSFYFLINTNGICPCQDFSQI

VFHPFDYFAVLLT

>SG-AL_contig_3312_1_-3_81_3518

MVFPARGVESADIGRIASKHVKVTVSQRSLQTPGRERQTVTGTKANDMNNTMNHVNERDE

TRSEHEDMETDQIDGNNHHHEDDPPGEIVNGEVKEESTDITEAEMEEDDCRAEATFRYTV

ENISKLKETALSPPCMVRNLPWKIMAMPRNSQNADRQKSLGFFLQCNAESDSASWSCHAS

AELRILNARPDTEPFCRKISHLFYCKENDWGFSHFLSWNELLDPEKGYVKDDKVTLEVQV

TADAPHGVCWDSKKHTGFVGLKNQGATCYMNSLLQTLFFTNKLRRAVYMMPTEGDDGSKS

VPLALQRVFYELQFSDKPVGTKKLTRSFGWETLDSFMQHDVQELCRVLLDNMENKMKGTC

VEGTIPQLFEGKMLSYIKCKNVDYGSQREETFFDIQLNVKGKKNVHESFKEYVTVETLEG

DNKYDAGEFGLQDSEKGVIFVNFPPVLHLHLMRFMYDPVTDTNIKINDQFEFPERLNLDD

FLQKKETTQASYVLHAVLVHSGDNHGGHYVVYINPKGDGKWCKFDDDVVSRCPKQEAIDH

NFGGLDDDQSHKHCTNAYMLVYIRESEVAHVLQTVNESDIPESLINRLSEERRLETQKRK

ERNEAHLYMNVQVLLEDDFCGHQGNDLYDNDKVNLRAFKVKKQATLMEFMETLSENLKYP

VRQLRPWPFHCRQNHTFRPTMIDLESDTSKTVMDLADQENPWAVFVETLSPDSGLKELPP

FDKDSDVLLFLKLYNPRSKAISYCGHLYVPISAKAIELVPELNRKAGFPINTPLMLFEEV

KPNLIERLEDLSLPIEKLLDELMDGDIIVFQREDDGHANFTFPTAKDYFRELFYQVEVTF

CDKQVPSDPGFALELSLRMNYDQMAHAVAQHLGCDPYHLQFFKSQGYRDGPGNAVKCTFE

GNLKDLLIYAKPRQPKKVFYQQLNIKISDLENKRQFKCSWVNGKLKDERELVLYPDKNGR

VSDLLAEARKQVELSENGAGKLRLLEIISYKILSVQREDVQLEQLSSGGTRTFRVEEVPS

DEVTIPDDEILIPVAHFHKEIYQTFGIPFLMKIKNNEPFFKVKERIQKRMDVPEKEFEKY

KFALIVMGRLEYITEEEKPINISHFMPQHLHGNNMQARPWLGIDHVNKTAKRTRYSYQEK

AIKIHN

>SG-AL_contig_3313_1_+3_3_236

LFVTENLLIFQICLPLNSENHHVKQLRCSSWPFSNRFLMSLWFLCYKIGHTICMVIMSII

MRTRALRCFKHVFTDILL

>SG-AL_contig_3314_1_-2_818_1048

MSLRWTEKGSCVIVYPSHLKLRLNGGFPSPLIHQTLMKVTFSHRLSCTCERFFHTPDFRL

NANMESYQFLHFCANFK

>SG-AL_contig_3315_1_-3_192_350

MPNYFYYCAESVQSHEKVRGLVWIDYFIYLLWLFFLFLCFFSTLFFYIFFIDV

>SG-AL_contig_3316_1_-3_129_476

MAQQQKPAAQQAPVDKDKEKKKKESILDLAKYLDKQIRVKFSGGREATGILKGFDPLLNL

VLDGTTEFLRDPDDPLKLTEDTRHLGLVVCRGNAVVLICPADGMESIPNPFIQQES

>SG-AL_contig_3317_1_-3_1284_1511

MQSCTLAYSDMHSYKMKHTYKCTSMCACVLTPTHIHSHTLTFKMTWRGGRMVKTLDFQTG

GYGRPGFEPRTPRLVG

>SG-AL_contig_3318_1_+2_2_100

VFTHTQNRVHKLHRALRINNFHDGYLYEHCTLI

>SG-AL_contig_3319_1_+1_1_177

HLQVHRNVSCSLFKGMDSKKGNTIIWITLHYITYHANMNISWHFHRTLWVLYKYCMCTN

>SG-AL_contig_3320_1_-1_1_231

IYIYKTHLSPVLVALNIFCFVSVYGFSLIPYFLFFFPHKLCFSWFSRVSCCPLIVRNLTA

LFGNNREIKIWISLKKK

>SG-AL_contig_3321_1_-3_3_650

LKSSHHQGNTPHQSLYPRGPRDSVRGNGSHSQRYFNNKHPTHHQHKSSHHFSHKSQPTDN

RTLYAGFLHKDSQEGLGQKLTPFAKKMLEPSLMPGPSQKTWKQKKQNKDWKEQDDFHHQH

KSHKRRSGPVERKDPENPSAHKKKRKRRHKKDQECTGGSSTYNFPNTKGKAVMGRQHQAK

NQQKNLSRSQQQANKQKISTKLAGTSSKGRESLVVT

>SG-AL_contig_3322_1_-3_216_2624

MKTVWVVLPLLTVISSVGHAEDLRVGETVSNPCINVNTCGACIRTAKTCAWCTAANYPRF

SSRCDIVEHLRQDCPEDSLVYPQSSVVVSKNDNVRGGDQNREAVQVQPQVMKITARTNEP

ISFKLTFRQAENYPVDLYFLLDLSYTMVEEEEAQKRLIALGQEMPASMVNITSAFKLGFG

TFVDKTAMPFTAWTQRMLSDRCPLADKSCRPPHDFINQLKLSNDGPAFAAKMGDALNAVS

QSFDDSEGGLDGLMQALACEKEIGWRERSRRIIVYSSNSMFHVAGDGLLGGATKNNDGEC

HLNSQGSYTPSDIVYDFPSVSQIASKLQEKSTSVIFAVMPSVFTHYEKFRSLIGSAEIGV

LEEKSSNIIKLIRNNYDKLRSRIHFVPRKADGVEFRFRSKCLGNVLEETAMCDKLEIKDS

VTFDVSMTVSSDVCQGQSGIVKRQVELAAQALNEKLAIDLNIICDCQCEHPPYEETDSEK

CSKGNGTFECGICSCNTGRYGRVCECSESDISSEMSLENCRQGNDTEVCSGHGECICGVC

ECFTLGKDTARRYSKQHCQCNDYSCPRSGPDAALCGGPTHGRCDCGKCDCLEGWKGPSCD

CSTDNSTCFASNGEMCNNKGWCDCGRCRCNETTDYMGPTCEECPNCPSKCSQYRDCAQCR

AFGTGKYTEKQCSELCPEIILVDELDLKSDRVRKCDFRDTDKCLFHFTYEYDKNNDVIIK

VQRTKECPEELNLALILGPVVAGIVLIPLLLLCIFIFIRNRRDAAEFARFMKEKDKARWE

SAANPIYKDPKSTFKNPTYKGGN

>SG-AL_contig_3323_1_-3_1764_2153

MYCLSTPPLDCKKHAWVVDITQMMTAFSKSCIIYNVCVCKCFMLVLMSVCGGLERFARTV

CVYPFTPSPRCSRFSSFEPPLKFFISFLCDCFFFYTAQNKTNGWYTLIIGIYKVIQHNAL

HICQCMELPL

>SG-AL_contig_3324_1_+2_524_778

MDRTSSQMFIDEKDTHKLFLSRRSNCKTFNGEENKPSYLYQKTEPSLFISDENKLSNLNQ

RTVHSQFFINEKNKLSNLTQRTVHS

>SG-AL_contig_3325_1_-1_1504_1800

MNTWRLIFGERAGDMFFSVDPGMLSSSLYTTAHVCSSSFLLSSNMSVCVSLSMSLTLTAP

PPPSLSLFVACFPFPGFFVSLLILLILLCIHTLVKCHFV

>SG-AL_contig_3326_1_-2_65_490

MIRFILIQNRAGKTRLAKWYMHFDDEEKQKLIEEVHAVVTVRDAKHTNFVEFRNFKIVYR

RYAGLYFCICVDVSDNNLAYLEAIHNFVEVLNEFFHNVCELDLVFNFYKVYSVVDEMFLA

GEIRETSQTKVLKQLLMLSSLE

>SG-AL_contig_3327_1_-3_87_314

MNHSCLTLFSILCWTPCVFNYVAETTIGTNVTVFVDAGCVRVSISPRTLTWLTGSLTCVF

NGQAALVRKEEDKTGS

>SG-AL_contig_3328_1_-1_64_216

MGVTQNSQPSNGCHSKLSPVMGVTKSGTVYCTSCSKLNVLLYFFPWSNALA

>SG-AL_contig_3329_1_-2_92_490

MAGWDAWTASMEGQGAHHCAIYGRDGNPWTSKNMNISPQEVQHVEDVLKNKKDEIFACGV

TLHGEKWTPLVLDQDSSVLVVKGKEACNKEKTMTLCLTKQAVVCGCNKDASVDGRTIRKV

VENTKAELEKLDF

>SG-AL_contig_3330_1_+2_110_319

MRNIERTHFQFHTLPPPISQHLTHSVLINSHDFVTADQEFCVIVHSVDVAQSLLILKSLL

ILMLLVKFLL

>SG-AL_contig_3331_1_-1_1549_1776

MLLPARIYPHFMDHYQNLTKGLVGVDGACLPLSGQVFKPGWGNHQRSSVHPAVNGYLTFR

VTEGKAARERRWASPS

>SG-AL_contig_3332_1_-1_94_1698

MQYDMSKRRLDVGDSSSNKRGRDRDDDRRDRMKDEKESKPKSVFEEFNIPRPTLQMNPFT

GLPFTPRYYELFNKRSQLPVWEYKSQFMEMVSKHQIIVLVGETGSGKTTQIPQWCLEWVR

ARYPKKAVACTQPRRVAAMSVAQRVAEEMDVCLGQEVGYSIRFEDCTSAKTVLKYMTDGM

LLREGMSDPLLENYGMVLLDEAHERTLATDILMGLLKEIAKQHSELKIVVMSATLDAGKF

QQYFDNAPLMSVPGRTHPVEIFYTPEPERDYLEAAIRTVLQIHMCETDAGDILLFLTGQE

EIDEACKRIGREVENLGPDIGDLKCIPLYSTLPPNLQQRIFESAPPPKPNGAVGRKVVVS

TNIAETSLTIDGVVFVIDPGFAKQKVYNPRIRVESLLVTAISKASAQQRAGRAGRTRPGK

CFRLYTEKAYLHEMQENTYPEILRSNLGSVVLQLKKLGIDDLVHFDFMDPPAPETLMRAL

ELLNYLAGLDDDGELTELGSMMAEFPLDPQLAKMVIASCDSNCSNEILSITSMLS

>SG-AL_contig_3333_1_-3_3_131

KKRKGKKKKKKKKKTSQKRESRRKKKTERIEEQGHFITSVGLI

>SG-AL_contig_3334_1_+1_550_765

MLRNKLRVHTFLKNTFYKHVHSHFNNGIMMFMFLSIFPHVTQVTITPQFTLYFMLARTHS

HTQITLRIFILN

>SG-AL_contig_3335_1_-1_25_384

MEKLRAKDLQAKKKGELVKQLEDLRIELQSLHVVQVTAGAASKSLNLHMVRKSIARVMTV

MNQAKKDNLREFYYSKQYQPKDLRRKKTRVMRRLLTPHEKSLKQQRKERLYPKRVYAVKR

>SG-AL_contig_3336_1_-2_1268_1777

MTDCPALDSSRFTLTSQNVTGGAEVNVTCSDFGHRVVGARSLVCNATTGMWDPAVPTCEW

TWEFTTHEKVVLGTAVGCAALIIVIVIVTLIACCCCRRQHEEKEYNDLMYGDNPGFAATP

GDTGGVFPDAYFAYQDAGNKHDPNGMTGTMDRAWLGYIPRPKVAEGRYYQ

>SG-AL_contig_3337_1_+3_1044_1361

MEYKKKLTLMGAWHSACQSRTVNRINSSKSYLLLQADPFLHPLCDINTPQHSPFHSPLTC

CLCLNRMQCCFHHSDRLPLLLHPLPHIRTFCLQHFNHFGNGKMLLS

>SG-AL_contig_3338_1_-1_97_342

MHSCWEMRDNSNGTLLHGARIFSVSAKYVCSSGLVCYSNIQPAPVYSVLIHCFEMEDSFT

VISLFGIHSLDCLSLFIDLLVL

>SG-AL_contig_3339_1_-2_41_691

MSSLFQCFGHRKKKGLKMSKAKTQQPAVIHKVIMVGSGGVGKSALTLQFMYDEFVEDYEP

TKADSYRKKVVLDGEEVQIDILDTAGQEDYAAIRDNYFRTGEGFLCVFSIKEQESFQATQ

EFREQILRVKNDENIPFLLVGNMIDLKEQRQVTYEEAMKRAQEWRVPYVETSAKTCVNVD

KVFYDLMREIRSRKMEAIRQNNGKQKKREKKRKCTIL

>SG-AL_contig_3340_1_+1_190_324

MSTVCVSLTIRMSFFQFNLEMDMFVAYLELNRTIVCLSQIISSKG

>SG-AL_contig_3341_1_-1_1_153

LARFSQLLLDESLPNFYQCRPFRSHECISLEVMAPGSRSHRPKSALFEEGL

>SG-AL_contig_3342_1_+1_52_192

MKLTNGSSFRQNIYNFGQTDPANGIQYAIYIIQILLTYQEIRHRHLK

>SG-AL_contig_3343_1_-3_3_176

PGEGRLLVGLDHHLFLENCGYFSCILGLFALKYKCFLFNVTFSVTKLETLCFKHCLHM

>SG-AL_contig_3344_1_+3_1071_1277

MPTFSLFYRPATGAASVASPPHPIPSNVFSDVCMHFVLVVWPTLCRCHIAAVCILCLQWV

TTSCRQSYS

>SG-AL_contig_3345_1_-2_665_913

MVFCLFVCLFCFVFGLLFGIFPCLWTAPPTCTQIYISGLLCVRLLFYTAINFFNLRQPVI

LRFHTGCFPVSISYQTLTWLAGS

>SG-AL_contig_3346_1_-2_2_394

KDVSLVGRFHCVEEFTPRGTYEDWVLYWHWIYVCESDLNEESNESENSEEKDKSEGSEEK

ESSESSEEKESSESSEEKESSEGSEEKESSELEDSTSKEEEDIPSKISTVSGHIFNFFRS

FVIVVFPQSKK

>SG-AL_contig_3347_1_-3_66_236

MYMPKNVGHRKASLRNCIYQVLTTLHTFERWFKYVYMYWKKARGCLVFVYMCVYVCL

>SG-AL_contig_3348_1_-3_15_914

MANQDVDELFDIRTALLIGNYQHCINEAQKLHVSSPETKVQRDVIMYRAYIAQRKYGVVL

DEIRSSQADELQAVRMFADYLANDSKRDSIVHELDSKMAGSVDISNSIFLLMAASVYYHE

QNFDAALRVLHQSDNLECMALMIEILLKMDRVDLAKKELKRMQDTDEDSIITQLAQAWFN

LTVGGDKFQDAYYIFQEMADKHASTPLLLNGQAACYMAQSHFDDAESVLQEALDKDSNNP

ETLINMVVLSQHIGKSPEVSNRYVSQLKDSHRNHPFVRDYLQKESEFERLARNYAPQASA

>SG-AL_contig_3349_1_+3_3_260

FFYVFSVSFISTFVSKSTVVVHTKRESYIQITETERYRKHTSLFVLPTAHLRCTFQTRQY

TGFPKVNNNSLLSTSIRVFIAKFSSH

>SG-AL_contig_3350_1_+1_1546_1911

MDFKKKKEKRPIHSVVSYRNKHRVGVQSKAAPHIKQNMHDTFFHVITCLAQSDKMNTFTP

QTIHQTLKVSITGKHTLLNIYNIPKKGEWLLSDQDTLSQCMVSLWTTPTSRPIYPKIILG

IF

>SG-AL_contig_3351_1_-1_31_234

MAFLLALATCGRRSEIHALSGAKDDIGFHSNGDLALTVLPEFLAKTQQPGSHPRAFRIPS

LVSCLDPR

>SG-AL_contig_3352_1_-3_852_1073

MSAFLFTNENGFFLRFLPGTTLLLHAAYGTCIRFIVSSERLAAKPPLKGRGKKNPCLTRN

QTWVSLVPGMCFIH

>SG-AL_contig_3353_1_+3_3_152

HTKKAPKDTWYSSCLQSCFIHSDYIYFDLWSLKILIRQYIHKYANLKCSM

>SG-AL_contig_3354_1_+3_78_473

MTEPARQTLKLKFSRPCVKGLTMLMVLSAWMKTESQGSYYMENRSKTATTSDARRKDICS

HNQAAKGKDPKTTSRPTSFGRTFSLDILKPPPVTGSAGVCRPGKLWQRLRMTTTSILLLL

VNTDTEQPLTLP

>SG-AL_contig_3355_1_-1_244_543

MAVGSFFFINMFVKYECLSRCHCYSVCVHNRLLRMWISVLYTVFVTAIHCKPIFVIFIIH

LIVKKMEMCTKRWDGSPVCCYKMTFTYKQDGKAILTLFSP

>SG-AL_contig_3356_1_-3_63_242

MVVVFLNNLSSKTCTIFSVNAFFLLEILSVYSAINILTRFTQFFLSFCYFVYPKLHKNHI

>SG-AL_contig_3357_1_-3_33_305

MKGVWEGMKLMSGYKGRKGGKDTVLSSSEEEANKLNAFYARFDCHNFSAERKELSGLLNK

GSNGKKDKNIVFCEEEVLLHLKRKTKQSAWP

>SG-AL_contig_3358_1_-1_1_228

VQLYQILTFVFNQSLSQCKIPSIWKTTCIIPVPKKTPVTCMNDICSVVLMPAVMKVFETV

VLVYLPEFVKPFSDPL

>SG-AL_contig_3359_1_+2_2_202

KYSFWLHILYHVGLEQNSKIGGGNASRERVGLTGKDRSISRDEGDTLGTVGSRSPEIPEV

EVNEGVL

>SG-AL_contig_3360_1_-3_339_1361

MTSELDALRQETEQLKNQIREARKAAGDTTLAQATASYDPVGRIQMRTRRTLRGHLAKIY

AMHWATDCRNLVSASQDGKLIVWDGYTTNKVHAIPLRSSWVMTCAYAPSGGYVACGGLDN

ICSIYSLKTREGNVRVSRELPGHTGYLSCCRFIDDNQIVTSSGDMTCALWDIETGQQTTS

FAGHTGDVMSLSLSPDMRTFVSGACDASAKLWDIRDGMCKQTFSGHESDINAITYFPNGH

AFATGSDDATCRLFDIRADQEIGMYSHDNIICGITSVAFSKSGRLLLGGYDDFNCNVWDV

LKQERAGVLAGHDNRVSCLGVTEDGSAVATGSWDSFLKIWN

>SG-AL_contig_3361_1_+3_3_155

QYQEMTSIRIWLKSAMCHRNYAKPSSTFSSHFVKEHRSYSHLLCFVLKGSF

>SG-AL_contig_3362_1_-1_154_405

MPPLARGSTGAGVHGAGAPGAGTPEAGTPGAGAPFRTWRPPAPARRSTTTPPLARDSTGA

GALGAGAPKAGTPLRTWRPRHPPP

>SG-AL_contig_3363_1_+3_3_257

GSACSGSACSGGTSSQRRRSRSPTRGSRTRSAGTAVVCWEPCLGSVSPSVVTSTDLRGPW

SGDLLGGGGRRTFWLWQVRPGRRCL

>SG-AL_contig_3364_1_-1_1_195

GNANKQQPSAVIVFCLQNLSMLQSFYISSTISVWLKLEFGCKWEGNNRWCFVLFTGELTA

DIKVK

>SG-AL_contig_3365_1_+1_34_297

MCVYVCLCACVFVYVRVCVCLCACACVCLCASVRVFVCVCVCMRVCVHAFVCMCMCACMH

VCVRMCVCDFTSWSFCDIFVFGVYFIMI

>SG-AL_contig_3366_1_-2_2_325

MKCVSKIVSAVSGRIFTKFGPKVWTVEESLVHEFGTERAKVKVTRAKKGRIISIFGGRCM

NNAFSAISDNNQILFSLLWQSAIVLTSRQVLFYLNLFQGDSDLEKLKI

>SG-AL_contig_3367_1_-1_109_330

MKVRLGNLIRFSRELVNYTRLLTKLHSFICLFLFIPSFVSLFVVCCLCFLTCFRCLFLSC

AFLSRLYSLNDARY

>SG-AL_contig_3368_1_-3_3_230

RGDARGSFSINSIPTFWKFCARNFLFSIVLFVSAVFLLLLLFFFAFTDVCFFLPSRSISL

SFFPASLHTVFSHHSL

>SG-AL_contig_3369_1_+1_46_384

MANSYKPYTFSLLNLSPIGYCKRTALLLHENLAVSKCQHTIRINTKQWSKCYLLITTVKR

FILNYSFRKCYTTVSNQCHLLSRTKCKKKLTYNNNSCQKCFATVLYHSQCIND

>SG-AL_contig_3370_1_-3_936_1178

MCMKGIKIVCVCGERGTEQVICVCVRERGCVCVRGTEQFICVCERERERGVCERGTEQVI

CVCVREALSKLFVCVCVREGH

>SG-AL_contig_3371_1_+3_171_404

MGHREVCVWGSTASTQFTVCRPARHKHCLISAWRHGKHGVGKRGTQRVMSSFSSFVSVGV

NGQRKQTCGHNRHHTDSY

>SG-AL_contig_3372_1_-2_95_199

MLLKRQAPGQALKLFTADRSIHDSNSGVSHILGRC

>SG-AL_contig_3373_1_+1_400_618

MSFAVVLAFDILNCVDLEYIKCLLSYETSGFFVVVHFCFIILLWFKNQKGLFLSFAGREV

YEYLKGVRATQVG

>SG-AL_contig_3374_1_+3_195_332

MGVYTQMRVEQVTRGEDKREYKCIHIKIKSLYEQYKDEKIKTDIEC

>SG-AL_contig_3375_1_-1_1_324

YLFILSLSLSLSLSLSLSLSLSLSLSLPLSLFALHVLGQTDVKLNNTIIVCLLQLLLCPL

RFKTGVYVCCTGKDVYRKLLTFFSHLICNNIYCNNQQNLERNRTSKFV

>SG-AL_contig_3376_1_+3_624_785

MLNDMTHLTCCLAGCTVNNMDLICTTKANSLWHSNPPTQSLKHNKTHSWWHMIL

>SG-AL_contig_3377_1_-1_61_744

MADVENGMGGMEQSFEAGDANVTGITEDSGVEDPELEAIKARVREMEEEAEKLKEMQSEV

EKQMNLSSPTPAATSSPFPSQEEKIEADTRSVYVGNVDYGATAEELEQHFHGCGSVNRVT

ILCDKFSGHPKGFAYVEFADKDSVVTAMALDDSLFRGRQIKVSPKRTNRPGISTTNRGRG

RFRGGRGRGGSSAGGFGGGGYGGGGYGGGYVPGGFAGGYAPRPRRGFG

>SG-AL_contig_3378_1_-2_68_1009

MTTSVHVESIQKIGEVLQDKTKPLKDRFRALFTLKNLGGSEAIDYIASCFDDGSALLKHE

LAYCLGQMQDNYAIPTLISVLEDKSQEAMVRHEAGEALGAIGSPEVLDLLAAYSKDPVVE

VAETCQLAMERITWQQNQKRQEEAGLLPNPYSSVDPAPPAPTVEIDTLRNSLLDESLPLF

QRYRAMFTLRNIGSTEAVIALADGLKCKSALFRHEIAYVLGQIQSDVCVSQLSANLTDKK

ESPMVRHECAEALGSIATHKAMTVLKDYLKDPERVVRESCIVALDMSEYENSGQFQYADT

LQNVTDGKVAGVTG

>SG-AL_contig_3379_1_+1_1_162

RRATLDGMKTLQDQNDKNWMECQNKQTKTGRENKKDSLKMKCMGGKVPHPKSRK

>SG-AL_contig_3380_1_-1_199_363

MFTFYVLGEELFPPCISFLVSLSYFLCQFLFVLTFHPIFVILILQSFYSVQSSSS

>SG-AL_contig_3381_1_-2_68_313

MAGVYCSTVPPLSIVYCWPELPINGDCCSLNCSVTDVYFFYSTMNVCCSPGCSMAFVRVD

SRGNQCPLFSTASPSSQCLLFI

>SG-AL_contig_3382_1_-1_1141_1662

MTNTLSAKSNTMDVEDKQKLLGLKASCSLPSLKVEQLSNQYLSEFNKSADSSKEEESKPV

SRMFRDDLYKVPHTELLNKVLIKKQKVRFRPCLDVHAQESESVPKVESTSLKPSFQKKQK

LKGRLKRQSDYCGTDFVEGNHVDDYTDDDDAFDEESDDSEITTAEISPRGSDRR

>SG-AL_contig_3383_1_-2_644_832

MLQQFSSLIFFLYSLTKSAIKMKRLPLCLHDFCAVVNHAWGTLYVHVASNLPSCEWVPDF

LQS

>SG-AL_contig_3384_1_+3_990_1163

MFQNKNESGISTCTMSQLQVPEKQKQPQPTLVRAHTPYQKHTLETTLRREEDGINWVN

>SG-AL_contig_3385_1_+1_298_411

MHHLYFVTAVFCHIQSCCTTCIFSSLLPMEAEMPLPFN

>SG-AL_contig_3386_1_-3_27_1172

MADRAVILQQFSAVTGVDEDRARFHLESAAWNLEVAMANFYEEEHDVDEGSAANPLDLDE

MEEEVDQPEPEPQRIQKKKTTKSKSKNSRFGTIDSLRADEDSSSEEEGQAFYAGGSERSG

QQVLGPKKKNPDEMVTDLFQAAREHGAEEVDISAATASGGRAFAFHGMGYRLGETEGPLE

SVAGPSIAPSRKQVDMTLKMWKKGFSIDSGELRPYDDPVNKDFLDCIRRGEVPQELIRQA

KGGEVNLSMEDHRTEEFVKAKANVKAFSGEGHMLGSPAPAVVSTASSGASSAAAPTPKVD

IDSSKPVTTLQIRLADGQRMQAKFNLSHTVGDIRSHIVASHGQYAGTPFVLMTTFPNKEL

TEESQTLDEAKLANAVIVQRLK

>SG-AL_contig_3387_1_-1_79_579

MGCCFSSEDDKDSGFPEPDERTRLLDPAIGSSNVPTLGNQEDQQYMQENEKGDEQSVMAR

ILNQNARMVIDVASNDTPIEQHEYHDRARAYNTKLKVALANSSRRGTYKLTLPNNITSPQ

SVLAAPPVSPAEIQLICNTAEKMSKAMRDVHIQHREDLVVSFGVPVP

>SG-AL_contig_3388_1_-1_1_249

DLGFHSNGDLALTALPEFLAKTQQPGSHPRTFRIPSLVSCLDPRDARGEERLLAWSAVSS

TTCNARHLGVPPSGDSLSPSTRS

>SG-AL_contig_3389_1_+1_1_405

IQPDRETDRRETQTGRYSQTERQTGRYSQTERQGETDRKIQPARERDRQGDIARETERRE

RHRQGDTARQRDREKRETQTGRYSQTERQRETQTGRYSQTEREREREKVRNTDREIQPDR

EKRETQTGRYSQTER

>SG-AL_contig_3390_1_-3_195_5507

MKVQLVFEIIKTMKNSIAILLICSSIAVTLGQRRQPQCEQGSCYPATGDLLIGREKMLSA

SSTCGMQRPERYCVVSYLEKPTKCAHCNSSEPWVEGQNEESHRVENIVSSFRGRRRRHRW

WQASNGQEKVSIQLKLEAEFHFTHLIMTFKTFRPKAMLIERSFDFGKTWAVYRYFAENCG

QSFPGISLGPVRSLDQVICEERYSGETPSSGGEVIFRVLPPFIPIADPYSEAVQDLLKLT

DLRVNFTELHTLGDTLLDSRSEIKEKYYYSLYDMTVRGSCSCYGHASRCLPVPGFETRPD

MVHGQCECTHNTKGLNCEMCEEFYNDLPWRPARQNQPNACKKCNCNNHATKCHFDAARFK

STGFVSGGVCDDCMHNTIGVNCQECKSLFFQDPMRDIRDPEICQPCDCDTFGSENQGICE

SITDDLLGTVAGRCICKKYVTGKRCDTCIANYWNLREDNPDGCEQCTCNPLGTIGDLGCD

QDTGICRCKRYVTGRNCDECYPGFYGLSEYQYGCHACDCDIGGALRSTCDQASGQCECRP

NIVGKQCKQVESGFCFAHLDHNIFEAEYGKGSGNAKVYVREPIQGSVSYWTGPGYMKIME

GDSVEISINNVPFSTFYDFVIRYDPRMPEVFQDVRVTLVRPDRVDPDGLCGDFNPQDDFK

DVALQPGARYEIVRPPSCLEEGKTYTLRIDFSQYRTGLQTPEATLLIDSVLLVPNVDFIP

IYQGPGLPEYMLNEFLRFRCQQMQYPSIKQQLPEECRRHTFSISAVLNNGALACDCDPMG

SVSLECDPSGCSCPCKVNVVGRRCDKCAPGTYGFGPEGCQPCNCHEIGARDNFCNEQSGQ

CTCIQNVGGRTCDQCREGFWGFPQCRPCQCNGNADTCDPLTGACRSCRDNTAGDFCERCE

DGYYGDPRVGVRIPCRPCMCPGGPGSANQHADTCTFDPRSQEVYCNCFPGYQPPSCDRCI

DNYYGNPLAAGGSCEACVCNNNIDPNVPGSCNSDTGECLKCLFNSEGFNCEHCVAGYYGD

ATQQSCRQCVCNPIGTNRSGGDCDRVSGQCPCLPNVVGQDCGQCAPAHWDLASGDGCQAC

NCDPTGSLSLDCNQLDGQCPCLDGRGGRTCADCEDFFFGDPNNQCFPCDCDPRGSESMQC

DKRTGQCQCVAGVTGYKCDRCARGTTGQLPNCVPCGECFDNWDRVIRDLRDQTVYLVESA

KNISVTGAIQAFDEEFKMMQNHIDEIRKILSSVNFTQVDIQEIENMLETIKRNLTDNSKK

LNSLDEELKHTTARVQKGNTEITSLREGVEKLRKLAKALQDNATDIQARDVEGAFNITKE

AEKRSREAQRIVDGTGRHLAESERIRQRTEDLINQRQADFDDQLQRNKQNLDSLDSDVSD

LSSRLSDINSMVCGAPGAPCDALCGGGGCGRCGGESCDGAVTKADTALKLAQQAEDLLTD

KEREANSLLFDVQGAKLAAEEAKDEAQMAYNAAFEAKNASENARTQLEDLLNEISKVLGT

PRAKPEDVEVVAQEVLNMRVSLDPDEIRRLADKINSTVQGLQNIDQILADTEASRELAQR

LNRSAEQASIEAREILDTAQEVRDALEGARRAQEMAENAINLAKTDIADAEADLSMIGTE

TSAARDLSDESLRLLQELKDRLEKLRTRYFKNDLDIQTAEDTINKAEIAAEAAERKATIL

ENDYEEASISLNRKYNTTADAQERAAKLAAKADRILQDTLRRKKSLQNIQQDIGENEKAL

VELSEDIDELNRRMDQYLQDIKAKHDDYRTC

>SG-AL_contig_3391_1_+3_3_152

KQNKLKQAPPKQTGRWNKNRRLKFCLWYSTSIVIKTTAIKLQVIIISTKF

>SG-AL_contig_3392_1_+3_507_803

MCLKIVHYDLALQILYFSTNFADRYFRTDFARYIYFSTNLNINMPTCHQTDLYPANKFLK

HFSGFQQLCGKLSLNRNKPSKNRFSQHNPKDVYTYSNEF

>SG-AL_contig_3393_1_-1_643_801

MTCFPLRFADWRRVLQPGLAVLLVVRCLISHDHYDVVDKTTAIPKQSFNQDLN

>SG-AL_contig_3394_1_+2_2_184

KKQQQAWQHLPTPISSPPTFSRLDFNIYTPMATQLHFQICISQPLGQTLNLHIHYSFSLL

G

>SG-AL_contig_3395_1_-3_3_242

PPQGQGGRASHGQHDCRLLREQARGGTLFRPLIEGRDSPEAVLEQQDLSECGSLGWESQH

PRRLSERRPLLLTLGVDPRE

>SG-AL_contig_3396_1_+2_2_460

KKQKKHPKTKQNKKTKTKKKTRGRKKVYLIRLQCHLSICSPQRRTSHFCLYLQRCTLIID

YLSTHSHAYTYTTTQVCANKQVCIVYSAHLHSSTITCIYIIYMYKHTALKKTSLMQSEDI

LSSSTIITVLQLVYLMWKNSSKCFINLRVQSFT

>SG-AL_contig_3397_1_+1_3592_3900

MSRHMVKIRQSTEWRVFVVCTVVRSTQLNTVPIIIHTIHTNTFCPIPGPSHLTPITHSIM

LKFMQCYSTANQPSDVNVSHKQTIFLVMSSSSAQAPNTNTTSC

>SG-AL_contig_3398_1_-1_1_150

STFFPASLHTIFFFFYTTTTTTTVICKCDVVGLIFLTKCGRLGWSAYETV

>SG-AL_contig_3399_1_-3_51_4061

MAEEQAELPTEDHEMEEERQTSEKEDESEDEESDDEEYFEVEKIMARKKRDGIFLYRVRW

KGYPPSNDTWEPVDNLDVVADMVQEFDAKEEEKAKARQEERRLRKLRMEGKKLPGEESSD

SSSSDDERLLNEDTKFFKMLEKNKDIVFGQPDLYSKVKSRAATLANKKKTEESQKESSAA

TSKSPQSQPAKIDGSRGRGRSRQKEQAKQVKSANQNKRSRMSVSKDSEEESDDSPVRKKA

AGRGRGRPKKKISSEEDSDDSSCARKGRGRGRPKKKAIIEDSDENETDSDDSVPLRSNIR

RSIGGPRKKFRIESSEENGSDSSLAQKKQSQSANSSHKKQTTEKAQMKKRLPSPVSEKGT

EKSSSESEEDKETDVKPKQKETTEAKVPAKGDERSDRVSPAAGQDKADKTKVKTDSAKPT

KADSAKPASASSRDAPSKSSSPKTSPVQARVIVPVLEKLAMSSELEGTREDATKESLKEE

KALSPCSRSKQGGEKNVCSEKGQSRGESMEIETSEKKEDSKESKKKSSSRKRKQDTAVEH

TKAVNEGEASPVKTSPLPPHSPPDGQPPQGESDPPILSRETRTSEASSEDVAMHILPPVL

QRSTQEKRVPPLRITVPKLKIEPPKLKPESPKPYLHHSPKSQTVSGKSEKVKLKGDIVKP

GKHEHHTSASVLKASHTSKRPPSRENVPEPAPAVTFLSSCSTVTTGTTSSVTKKDVSRSV

TTAGTGRPDLSLKLGRTLAVESASVKQETVSLKIVKSESVDKTGRSDQVDLKTSRIDTLD

SRRKNSSSSDLSSLNVTVTRDSSSSTFTPSSVKDLPSPSRDTLLSGSSRNTSGVFSSAST

SRETSSLYSHVRDSSISGKGGNFTSASSRDLTHTSTSFRDPHLGSGSSRDSHLVSGSSRD

LHPGSGSPGDIRLVAGYIHLSSGSSRDSLFSSSRDSHSTMRGDPKDLGCAGGSRHSLNSS

ISGKENADRQHFFKSLSSVPKKGDTIQPLDTILNSALYAVEGDRSRQYKGLDSDFDLALQ

RIDLEYLEKLLLPPVAPMELSDEELRQAVIDGNCYLVERALACSKPYNLNLPDNKGYTLL

MNAVLRGSDDIIVHLLSRGADAQVQVNGVTALMLAVEHAESSTVFMLLEMGAHVNAADHS

GETALFKAVRRGDKQILKLILDHGANFSLMSRIGLSPLQMAKQFRLADVETTLIDHIHKV

VRSFEEQVNLTVRGTAKLVSCLFPHHCVPLYESDTITIPFKYQPSMPTGPGMGCLLFIAH

ARFNPQEVKCRLYGQCAVERVTLNGVVQPSLTEESNFVLMCHPLITGWNELVIQTKKDPV

SKAKLIVCAYKAQLIYK

>SG-AL_contig_3400_1_-3_147_467

MFCVCIHTKGVSSGTSRSAHMLTWEIEKSPLFTHRARCPGRIRTQALQTLQFGSQVTSPL

DHRTHERRSEEQWQRVWSSKFVQGFPALGQIPYALRPNPPATTWQTP

>SG-AL_contig_3401_1_+2_362_556

MHLANIKDPTAIKESSLAKFCRKIDSPRLTEIHTDRKRKKKGWRCTAATRSPQGEQPEFH

TANPL

>SG-AL_contig_3402_1_+1_1_402

MNTSSQGTSICYRCGSTEHMSSACKAKGPKDQMPYASCYVCGKSGHLARSCPDNPRGLYP

TGGGCRTCGSVEHFQRDCPEYLEKQGIGSKTVKKINVEASVDAVDDDDDDFAEVRTQQVP

QKATKKKKPKVIMF

>SG-AL_contig_3403_1_+3_81_278

MTRAETVMVDLLTELNLPLTAADHFTQAVKVMFPDSVVAKKFSCGRSKATAIVKELARTQ

KEELRN

>SG-AL_contig_3404_1_-2_2_304

TLDLPCEIWAALLGRARRRSAAPPIFFLFFCFIYLISFWLSRAGDIYKLGLKAFFLSASQ

HFRKNDSETVFLPAYMVCVCVRTCVRVCVCVCVCVCVKNQF

>SG-AL_contig_3405_1_-1_1_96

LNLSRGQHDEGDSHLLCITALPSPTLKKVRYP

>SG-AL_contig_3406_1_-3_252_1748

MDITSVQTDKNQADTTATPAAKPKDSPKKQKESYRPPPPPPRVKTPAEKQNLLIPDHMKE

AMERSSSQAQNELENELMKMHKAMDESLGLPTRIAEDGEGLTEEELEQAQQALKERMEEA

QKKMQEAVLPGSVRSKSPSTPRKSPDLKPAAKKGKKVRVEDPIMVEEKAKKEAAKEQRQL

ERQKRLEEAKALQEMISKKEAERKKKEEEAAHKAKEMEEQVELMKAEEDAIQQAEEDARE

QMAALRKIQREEREARRKADLEKKKQDALAKREKEKKMMEDARKKEQEMLERIADAEMRR

AVREEEERKRDEEERLAQERYEEELREAQQQAEEEERRLQELERQAEEEAFQRIIAEREE

AERQREELELQAKRMAEEEERQRQLMLEEEKRLEEERLHHEAEEQKRKDEEKQRLKELQR

LEEAAREKMRAEIERRREWALKRREVNLDRRKNLDGLRQKQGITEPWVFSYFIQWSREMY

ARPMGVSAEAKKKPMKKKK

>SG-AL_contig_3407_1_+1_940_1296

MFITHCSPHCRLQCHRVADALFKPLRLQTRVHTFEIEQFLLFKKQQNRKTCHPFHSTSTY

FPLTALLKTKNPSINSSLSLSLSLSLSHTHTHVHQQFHHGRICSEGTKVAHDGWMADYA

>SG-AL_contig_3408_1_-3_1152_1343

MHTIMRMQMMSMQANNYFNFFLKERRKENLFRSFVISIASSSPVAKVTVVKASRSRMAIR

AGRR

>SG-AL_contig_3409_1_-3_327_437

MCVYYRDQAEAFNLRETHLCLKRALMSHTWLSFLLEF

>SG-AL_contig_3410_1_+3_273_515

MISYEYCSKVHNHNINQSINQSILMFIHEYGSRLFLILLTKNKTKKKTCLKHTCKQKQAG

TSAVYRCACILSSVQIQLTDQ

>SG-AL_contig_3411_1_-3_1152_1298

MADIWPIGLDNRNMEIGDCQTNETQYCSVLEYGFKSRWAKTFSSINDAH

>SG-AL_contig_3412_1_-1_271_1413

MLLRDKSDKRVSGETLTARSDKLLTASWSGTRGTADTRRRGERRRHSRDSTASPRQLSGS

TPSKKKFKNRMSHSRNPKPLPGSRRFSSPGITMSTNISRGKSFELGQKCDSHVVTHNDHE

QTSTDAGRSLHSGQPVSESDKPLESSRARFESESAITADKTECSPCASQVPKSRDPDSET

ATLPSDSTQNRDNGEGLQQTTRTPPPSSTGLTSQQQDNTSSDGRVEKKTATTITTTTQDK

TPIPSDPINQWTTCRALDDSGDDDDTDDDNNNDDNFHEGQAHSESWTQEAHRAGPDSSSV

DGIFTRATSSSHLARRKRPLLLPPIMLPPIYTVKPTPLKLRDEMYFASGEQRARRPISDE

EWDKLRDCRYLRIRTLRTIKV

>SG-AL_contig_3413_1_+2_2_172

SGRFVFVYAASVVCISWFEEQTYHRSYKTVKHKDYCELYHLVYFKFKSELQAIKAIG

>SG-AL_contig_3414_1_+2_2_181

FMTRPNEECTHSDFYTHSVDHKDNNVRLHKNRLQGSREVTDLCNFNSLSFGLWNKPKHIL

>SG-AL_contig_3415_1_-3_1290_1649

MSMNVRYAEIIPACTVCLVFSFSFLRTMPSHLEYLCLTLTLQMFNPVWRVCVTDARLKIW

ALLSLQPLKVLNSTGYYICGHWLLCSVCFGFDDTCFQICNTVCFSLCADLRHSFNPLTPS

>SG-AL_contig_3416_1_-3_78_545

MGQGDEDAPYAKFAMSLFKILDGPVTLVREKIVEPLQSKNSNKYYHRRYPRVKTIDQCEI

DDPVCVYEAQEQYRRDKQVDSNIVKILRQRKLECMQWEGPDARNKCKKVSDDYDQAATNW

FIKYGDIGANGNVIDAYMKQKHRFIWHRRNPDKPLI

>SG-AL_contig_3417_1_+2_2_148

CVCACITQYFNLLITWSTSMHLSSGTSIITVSLYSNGLVIRQKFCPLSA

>SG-AL_contig_3418_1_-1_25_1647

MGDKTENKSPQKEQKQRTGKGFIYEVVLLDGEHVNFELDRNAEGRELFDKVCQHLDLIEK

DYFGLQFVDERISNHLKCWVSPDKKIAKQKKGGPWLFDFALKFYPPDPTQLRESLTRWLV

TLQTRRDILSGKLPATFLTQAMLGSYCVQADVGDYDPNEHRGVEYIRDTPFAPNQTTDLL

EKIVELHRQHKGQTPDEAEINFLENAKKLSMYGVDLHAAQDVNNVDIMLGVCASGLQVYR

EKLRISRFVWPKILKISYKRKHFYIKVRPSDNSPSENTISFKLASHRLAKRLWKICVEHH

AFFRLREAEQSQNAAMFPRLGSKFRYSGRTLYQTRQNSQILDRPDPYFERSQPARNTFAA

PSNRSHSVDELSNRPDYWKDDMDVDRKRLPSDGKNRKDWKDDPNATERADLENMRPLGSE

EGGALEPGANRSMDGKDRLDRMPGEDDATLNREGGEGTMGLTPAELQKKAKEDEKERKKR

EKEEEKRRKKEEEDRKKAEKAAKKGKGKGDSAEDRKDLMVGGDNSEKAPGRTPGSYGDQE

G

>SG-AL_contig_3419_1_-1_175_1383

MVKETGYYDILGVDPSASESELKKAYRKLALKYHPDKNPGAEAEEKFKAISQAYEVLADP

KKRETYDRGGEEAVKGGGTGGHGFDFHSPMDIFDMFFGGGGRRQQGPRRGKDVVHQMKVS

LEDLYNGSTRKLALQKNVICAKCEGRGGKEGAVQKCGSCRGTGVQLRIQQIGPGMVQQIQ

SRCHECRGEGEAIDPKLRCKVCSGQKIVKERKILEVHIDKGMKDGQQIRFSGEGDQEPGL

EPGDIVIVLDEKEHPQFQRRATDLIMQKEIMLVEALCGFQRTIKTLDNRQLIIHAVPGEV

IKDKAIKCVMGEGMPVHRDPFEKGRLIIQFTVKFPEKLDINKLPSLESCLPPRVEEIIPD

DAENVAMINYDPEAESSRGRGRAEAYDSDDDQPGGQRVQCASH

>SG-AL_contig_3420_1_+3_3_104

TSNATSSFPFQPGITKNKNYNETATKNKFISNHV

>SG-AL_contig_3421_1_-2_11_1600

MADKTDGKSQSQAQVKIGHYILGDTLGVGTFGKVKIATHQMTQHKVAVKILNRQKIKSLD

VVGKIRREIQNLKLFRHPHIIKLYQVISTPTDIFMVMEYVSGGELFDYIVKHGRLKEMEA

RRFFQQIISGVSYCHRHMVVHRDLKPENLLLDGQTGVKIADFGLSNMMHDGEFLRTSCGS

PNYAAPEVISGKLYAGPEVDIWSCGVILYALLCGTLPFDDEHVPTLFRKIKSGIFAIPDY

LKKDVVSLLCHMLQVDPLKRATIKDIREHEWFKKDLPEYLFPSPNDQDASIVDLEVIREI

CEKFNVEEQEVHRAILSGDTHDQLNIAYHLIIDNKRHEDSEFRDFYVASSPPPDCFLATS

PLRAHPERMPEMKDVTHTLDPVNTPASQRNINTPAKKAKWHLGIRSQSKPQDIMHEVFRA

MKTLNYEWKIINQFHVRVRRKNPVSGKYMRMSLQLYQVDQKSYLLDFKSLSAVRDPEEEE

DPLSSSPPASHSQSMTSMEERMDTDEDELRPHQTLEFFEMCADLITALAR

>SG-AL_contig_3422_1_-1_40_909

MLFNIFLERIMTDALEDHEGSVNIGGRTITNLRFADDIDGLAGEEQELVNLISRLNNTST

EYGMEISAEKTKVMTNNPIGFANDIMINNKKLEPVNSFKYLGATIADEGSRPEILSRIAQ

TTAALTKLKPIWNNKNISISSKIRLMRSLVMSIFLYACESWTLKADLEKRIKAVEMRCYR

RLLGISYRDHITNEEVKRRIENEIGPYTDLLTSVKRRKLKWYGHVTRSSGLAKTILQGTV

RGGRRRGRQRKRWENNITEWTGLELRDVLRRAECRDEWRGLVARATEVPL

>SG-AL_contig_3423_1_+1_925_1224

MENQQKTSYLQYLMFAHSLCTVYTEIILTLQYLFKADKKLTCRNQQTKSSDGAVQRSTTN

ILLFFKNFKQIVNFFSLFVSETEQLSLFLCDQDHVLSDAM

>SG-AL_contig_3424_1_-1_13_1377

MASEATPDGRLQKMEVDYSTTVDEKIPKCEQLTKQGKLTEALDILLSLEKQTRTASDTHS

TGKILVAVVKNCFECKNWDALNENIVLLTKKRGQIKQAVTKMIQEACTYVEKTPTMDVKL

KLIDTLRTVTAGKIYVEIERARLTRTLAKIKEDAGKVTEAADILQELQVETFGSMEKKEK

VDFILEQMRLCLAKKDYIRTQIISKKVSTKFFDDADSKDLKLKFYQLMIELDEHEGSYLE

ICKHYRAIYDTPQIKENKDKMKEALKCVVLYLVLAPYDNEQSDLIHRVKEDKTLEQIPKY

RDLLKCFTTPELIQWKMLCQNFETELKTGSANNPPTHVFNPKQENGVKRWSDLKIRVVEH

NIRVMAKYYTRIRIKRMSELLDLADGETEEFLSNLVVNGTVEAKMDRLAGVVQFTPHKDP

SDVLNEWAAKLSQLMSLVNKTNHLINKEEMVHVLH

>SG-AL_contig_3425_1_-3_186_308

MKNSREARLSGGRFEARKSANSTVFWGYKCMSVYLTSKIVT

>SG-AL_contig_3426_1_-3_111_578

MSAAPPPYPGPEGTYPPPNPAYPPANPGYPPVDPKMGGPPPAGYAPPPQAGYGQPAGYGQ

PPPAGPTTVVVSQPAVTMVQLFRESPVHTRCPHCQAEVVTATHYDTGTLAWIICVILCLV

GCSLGCCLIPFCVDPCKDVIHTCPNCRQQIARWNRM

>SG-AL_contig_3427_1_-3_960_1133

MSHIQFLNCFRYIIGLETREGVFVLEAKMSHIQFLNCFRYIIGLETPSFYPLCGYIFE

>SG-AL_contig_3428_1_-1_1_252

AYEIIAGDWSSDVCSSDLIIQAVWDAWFVPMINLFVAKFNKRLPIYVSLVQDLKVMAVDA

LSLDWVPLISYAFPPFPILPRVIR

>SG-AL_contig_3429_1_+3_3_200

FFFSSKQYFIFSFISSQKAGSTCSYTRNPMHTTHSIMQYNNVVQSRTRHNYHALHLYKMR

EGSVGR

>SG-AL_contig_3430_1_-1_52_522

MGGSGSTRRVVVEEVDGSGVVKISDSVVKRIRGDEDGAGDASSAPSSPKYARPPSMSDKD

KEAIQEIEAFYTGRLKQLEQRNSELQKITNEQFVKAVQEVEQKFLKHTCSPVCQDMQTRV

YECYLGNSKQTLNCSAEVRAFSTCVERARQNALLRKS

>SG-AL_contig_3431_1_+1_907_1161

MLTANARNNFTNGHTFMFNLTEHVFGFPKTFFFFLPCLLIRFGGSHNSKILTYRTSVHFW

KDDFQSLKELHNCQNSCMTKMEDSF

>SG-AL_contig_3432_1_+3_231_527

MFSTSSPLCGDKKYFCLLFYKNLHVLRQISAHSVAKSTMKLCVLTKHNIIITIALFPLLS

CVLYYFSGINLRDGWFINRAVDLRSDVSSSTPADDVKLA

>SG-AL_contig_3433_1_+1_1_180

EGGSGTSRSAHMLTWEIEKISTLYPPGEVPVVQTQAFQTLQFGSQVPSPFDHRARSCGIV

>SG-AL_contig_3434_1_+2_2_217

GVTHYICTLSYESETTSTFGREDCVYGSFLVPRHVGSTEVKITQTTILSRQKAIKPTSQY

LRYYYPNNNVRT

>SG-AL_contig_3435_1_-2_182_1762

MDQLAAYDSESESETDESCIISEGQKSTGAFWKSFDDNCTSMSEAGPVEQTPESCGKNFF

GFTSCTSQVNISETEEKEEQMPSTRHVNIHGNSVEVPGGDFWTNLSEAEILSADFESDST

KPRKRMFESPTRTPDCSDQLSVSHPSFPKRLQCDTDLSMYNNVSYADQHSATSSFGCVYT

FSGKSDFVQSDPKTKVYTVHPKISPHLNSQQSCHCSCKEMHRWAGHTGVINRIAWCSNSS

FSHLILSASMDSTVRIWNAWGKQQACVRTLTTHTKAVRDAQWSLDGRQVLSCSYDHTAAV

TDVQTGECLVQFEHSAGVSAGKFHPVTPHLVTTGTKDCIQVWDIRHHEVPIRILTHKEQL

GQIQDLMFSHNGQELFSCGDVVARESADRNIIAWDFQTGVILSNQIFQERYMVTRLCLHP

SHAQFLAQTQAGYVAIFSTQRPYKMNKNKRFEGHKSAGYNIGVSLSQDGSFVYSGSSDGK

LYCYSFNTGKKMRTLNCGQILLDIAAHPVLPSVVAGAAWDGDIVLFQ

>SG-AL_contig_3436_1_+1_1_171

TVCIVHGTWLLIDAWGGKSSLPEGNTWSHHPHRSMNVTVVLPKKEDGHPNLDLPWGV

>SG-AL_contig_3437_1_+2_2_154

KSLIKNPHKITRSTHCYLSTIHISVNNTHSINKKTTEQVASRSLLMQTNAS

>SG-AL_contig_3438_1_-2_2_82

TVLRKLKPKFVVVISNDIYTPKWFRNE

>SG-AL_contig_3439_1_+2_2_253

IVCCSTLDNCIPSHNVHDTLKSFFHPDKSFALNISWSYFSAVKTLPPKNLPFNADIAFST

SSCTENSTKILTMTSAFSSSLCFS

>SG-AL_contig_3440_1_+3_234_467

MKYEICLQFVQYMKNEISLTISRVTTFHTLKIQIHEIEIITFINKTIIKVALTTDTSLKY

LNQTINNLFHYHQIMCIN

>SG-AL_contig_3441_1_+1_115_441

MGEKNPCPIHDLNLGFSRLSSCFRPHIPHNEKYSPFTSTTTTTMRTGWRSRAPCFQSECT

GLHLRYQHEKKTLCVRPPVHPAVDGYLTLFSIWEGKAARERKGTAFIPF

>SG-AL_contig_3442_1_+1_1_237

RPSNSRCRSSLAVCWIGRPLRPGPPELFPRRAGTQCPPQVPCRRGNISDSRGGVWGWYPD

NHPAFRVSCPSAQDRVLVP

>SG-AL_contig_3443_1_+3_3_239

FFFVSIFIKYFIQFCPVHKDNNVLTQTWLVLSQMCPPIPSTDLVSHSTLTCLLFHLHQMF

RLHSDEEFRTKTDTHLTTH

>SG-AL_contig_3444_1_+2_446_706

MMSLSLYWTGINFFHTLFTAFCEPVRKKNSAAHKHTDTQSVRTVQKSYFSLARHTDCKTI

QGDRSWRGKVKSARCVDRHETTQPIRA

>SG-AL_contig_3445_1_-2_11_934

MQEMEVDVDGIQRRLGTGRFQILSFFVLGLIYSRGAWHVFGIMFLAGDPGHKCAFPHDSE

SGLLSLSSSPSSSSSPLFDTDSSNLISDDTSRSPASVLPPHQDPNSSSLYSSPGETGVSM

VPVNWTADSCTVSYVDRNGSQVRLISGCPYGWSYGSLFESTVLSEWDLVCGRNFLVELSI

TIFMGGATLGAMVILPLADRFGRKRVMMGCLLSQACVGTATSFVHSYIVFTVLRFILGML

NIGVGLSCFVFMTETFPADKRTVMSIAFQVFWAAGVMTVAGFGYLLRNWRHLQLVLSLPN

LLTLPCWW

>SG-AL_contig_3446_1_-1_16_987

MADANQKLALAEKEKGNAAYKSRDFSAALTHYEKAMELDPINITIRTNRAAVFFEKGEYE

NCIAECEKAVEIGREQRADFTLIAKAFARIGNAYVKMEDYEKALTFFNKSLSEHRTQDVV

KKANEAQTKLKEKERLAYLNPELSLEEKQLGNECFQKGDYPTAIKHYTEAIKRNPDDAKI

YSNRAACYTKLMEFNLALKDSEECIRLDPKFIKGYLRKGGCLMAMKEMSKAAVAYQKALD

VDPNCKEAIDGYRRAMMEEGSNPEAVRQRAMADPEVQAILGDPAMQIILDQMSKDNNAFK

EHLRNPEIAAKIEKLVEMGIVAVR

>SG-AL_contig_3447_1_+3_1032_1214

MKHTKHASICHVSLIFLLFFFFNYNHTENVPYENEYDMSNWWYCGNSFSCHPFTAKLASY

F

>SG-AL_contig_3448_1_-1_1_159

IKMHEIARTYADAVIFCKKNATQSTRVSSAAMKSCSPARKCCDVMLRGTLSCV

>SG-AL_contig_3449_1_+2_2_259

RAFYEYYPSRSLSLYSYLLNIEPFFTASRCSTLPQHFGTNFQTRDSVPHDMTSQRFLAGE

RDFIAVDKTRTDCVAVFFLQNMTASA

>SG-AL_contig_3450_1_-2_239_436

MERPLASASTRVLYGGRHVFYGNGSLVLATLSASCCKKTLENRCIESKDTNFFPTKKFFP

MSASSD

>SG-AL_contig_3451_1_+3_132_230

MNELVKEYDEANKCTLNPNLQQSGYIIGVWSAC

>SG-AL_contig_3452_1_+3_261_362

MSGVNKVCFWQYLENSLLFFKRVDSTRVHTICFN

>SG-AL_contig_3453_1_-1_88_804

MFDYIVESVNNAIPFSTSTSYIGILDIAGFEYFQVNSFEQFCINYCNEKLQQFFNERILK

EEQVLYEKEGLNVKRIEWTDNQDCIELVEAKPNGIFDLLDEESKLPTPKPEHFTSEVHSR

NKNHFRLSHPRKSKLKTHRDVRDDEGFLIRHFAGAVCYHTKLFIEKNNDALHASLEFLIL

DSKNSLLQKVFADVAKAPTGKSNKTAGKLTFISVGSKFRKQLQVLMDKLRSTVSFGYVG

>SG-AL_contig_3454_1_-3_414_545

MKPVFMDQFFLPVVTKGSSFVAVGILYAQSTVYAVVRTLFFILC

>SG-AL_contig_3455_1_-1_31_255

MSRTGHSVHMRGLPFQAQDQDVFDFFSPIQPVRVTFEYTSNGRPSGKANVDFATHQEALE

AMKKHKSNMQHRYIE

>SG-AL_contig_3456_1_+2_2378_2704

MRYLDKAEGRLNPLAHQQTITMCTYINHTTITYCTTPGTRLHNPCGKKIKEHIKQPPSYS

YHAKTQFPIMYNYFAEQKHKFNTLSLFSDFYVQVKTATCVYTQNNNNNN

>SG-AL_contig_3457_1_-1_7_591

MASTNKEDDDAVSVAKTPAELQRMRLEKLMKNPAKPVYIPDSSKAAKMREPPEFIRNVWG

SSAGAGSGDFHVYRGVRRREYARQKFQKEVTMKLTAEEEYHKKVEENKKAAEEKTAKKRA

KRQKKKGKMKNKKMKVEEVKKEESSESEEEAEKEKKQKHKEAEGGEDENNSRESAEQAQD

SNSTSEADNDTKDES

>SG-AL_contig_3458_1_+1_367_588

MAISNSGVLSSKWRGIHRTRTIPHALSPNLPLIILSLRKTLCAAKLLKCWLFVVCVPQLN

TLGVTQCMHSALRQ

>SG-AL_contig_3459_1_+1_280_501

MKSIVPGTLSRPRLNEVFVDSHCQIKEKEMKHLQGCEGDEGRVERGALTTLTHTSSDAVI

GKVPEKMIDFSGSL

>SG-AL_contig_3460_1_-3_87_1316

MKSVLLFALVLCAVYSHAEQKLHRIKLHRMQSSRQTLTSVGNSIKLLKYKYGLSTFNQPT

GSAPEPLTNYLDAQYYGVISLGTPEQNFRVVFDTGSSNLWVPSKKCHLTDIACLLHNKYD

STKSSTYVKNGTKFEIRYGTGSLSGFLSMDTLTIGGLKIKNQTFAEALSQPGITFIAARF

DGILGMAYAQISVDRVTPPFYNAWFQKLVLKNMFSFYLNRDPSAKEGGELILGGSDPAKY

VGNFSYVPVTKKGYWQFKMDGVSVQGGSQFCMGGCAAIADTGTSLLAGPSSEIAKLNEAI

GATPFIEGEYLIDCSKISNLPVITFTLGGKKFSLTGAQYTLKVSEFGYSICLSGFIGLDV

PPPMGPLWILGDVFIGPYYTEFDMENNKVGFAVTKQSNGTQPLLFSPEKH

>SG-AL_contig_3461_1_-2_101_2272

MEKWIGRFGKIHGWSVVVAAAGVAWVVYKAHQNLRADSESNEAQTTTSVTEEQQIDAVST

TTTGASSRSKRRRQPRQRRKLKIFYGSQTGKSKALAERLAAGVGGQGYQVEVVALKDYDP

EDQLSHEAEDLYVCVFIISTYTEGKPPEGSEWFCQWLEDTSGDFRVQKTLLSDLYYSVFG

LGDSVYKEHFCTVGRNIDTWLHQLSATALQSFTTGDENTAESKNGGVEGDFEAWRKTLWP

KLKQALDGRKVKSCSGSTCSEKCGTCNSKGEGDPEGHHHDGKCSSEEEDEVKFETSSEEE

DEKDADSSEVTQGSGIVDLEDLGKVMHLAKVSRQKAEEELTNGEEREMITPMLRESLEKQ

GYRLIGSHSGVKLCRWTKSMLRGRGGCYKHTFYGIESHRCMETTPSLACANKCVFCWRHH

TNPVGTEWRWKMDDPNTVLEGALKNHMNMIKQFRGVPGVKPERLVEGMTPQHCALSLVGE

PIMYPEINAFVKLLHQRDISSFLVTNAQFPEAIRNLVPVTQLYVSVDAATKDSLKKIDRP

LFRDFWERFVDSLKALSEKGQRTVYRLTLVKAWNTDELDNYAQLVSLGEPDFIEIKGVTY

CGESKASNLTMANVPWHEEVVGFVQQLVDRLPDYELASEHEHSNCLLLAHKKFKIHGEWW

TWIDYPEFHRLIQRYNNSNGAETFTAADYMAKTPYWAVYGAREQGFDPEETRHFRKKRKD

ISGC

>SG-AL_contig_3462_1_-2_308_403

MALGVRKLVFHFFGGLLVSSCLSKNYYNWPLV

>SG-AL_contig_3463_1_-1_40_1680

MQTMVPDHFHPDRKIINTFVTDMHEAMITDGLVSSHPLYVPVATPSQIGEIFDSISYLKG

ALVVQMIHFVIGDDRFRQGMQKYLSTLKYGNAQHTDLWRALESDELDVSITDVMNTWTQQ

MNYPVVTVTFEGQTVTLRQERFLQDHSAEDPGTYTSPFGYKWYIPFTFTSSDERNFNVTV

SDIVWMTIDEDKKTFTVPTLSEDSWVLGNIGLKGYYRVNYDDDNWRKLTAQLRNDHTAID

PVNRAQIISDAWNLASAGLVNTTRALEAVDYLDKETEFMPWSEASFELTKVSNMLSTTDT

FPAFKAFMVEKTSPLYLRLGMTTTDDYLETKLRTHIASTACQYGGPECINDASNYLLEWK

QNPDVNPIPKAMHYIVYCARLRQGNTDDWNFVYGRFLEERDPGERGDLLASLCCTSNTND

LDRLLDRTLNSGDIRTQDFFDVLFYTILNPVGANRAWTFINNTWDSHQEKYAAASVALPY

AIKVFGDVFSTEDQLLKMQDFVNKHNDLGAATQAFSAALESITNNIAWMKKNAGIVRTWL

QERGFSA

>SG-AL_contig_3464_1_+3_234_335

MVNCIDSALNNENHKICLTISVQEIMNQTSTVKT

>SG-AL_contig_3465_1_+1_37_303

MCLFFSHPALLMTSRATLSSQSQARNHDRLTHAQCIHTSYNSRLLSLGYWFLIKPITDFS

WTMSVKTETLCLAYQGKEAENPSQTQREM

>SG-AL_contig_3466_1_+3_3_218

ETAPNPNVGHTHRLTLKAYTLVIDITRDWVDGFLSKPSLNFSSSMSVNTETPCLAYQGKE

TENPSQTCMQCE

>SG-AL_contig_3467_1_-2_182_409

MVDSVAKEVEKEKMKAIGARNLLKSIAKQREAQQQQLQALITEKRMQLERLRIQYDALLK

EEGAQNEFLEQLISQK

>SG-AL_contig_3468_1_+2_1601_1888

MSLPPFKHFVFHGQEATADTRWRIWVDKLENRLCGLDITSDQKKKSLLLHHGDDKIIDSM

TDANKVNSRDADGKPNHIRNSKVIDSIFYAKTKNVI

>SG-AL_contig_3469_1_+2_173_379

MMKQLLYKKKYYQSKNVHGQHDIKQQNNKMNTRQQNKKQNKKQIKTKPCQAKVLTIHIFQ

CYYTLFWGV

>SG-AL_contig_3470_1_-2_2_193

CVCVCVCMCAWCVCVCVCVCVCVCVKQEKNQVYSEKKKNLLAIASADMPYLLLLLLLLFI

VIII

>SG-AL_contig_3471_1_+2_2_268

GRQPCFHSPGADDYHLQLLDLKMQLWQSFHFSLPENQTKMTFVTRTENKKMFCVQVFNKQ

TNEQANEQTNKQTNKQNEQTNKERTNKQT

>SG-AL_contig_3472_1_-1_721_999

MTKVHFENVTVTWNLPNVFNLAWVHLTVQSCYKAKGFPYSSTIYNMYETAELSKYRISNK

TYTMSETSQSNFKNIQSRHVFQCATSHRAVWQV

>SG-AL_contig_3473_1_-2_329_469

MTQLRIMGEMPFIVIIITVIMGLQGGFCWFFLFYFFEEVWCSTQLSY

>SG-AL_contig_3474_1_-2_2_184

RFVCLFIHLILLLLLLLLLLLLLLLLLLLYGSFLGLKRICVLRGCLVDVDGLNVVTALIW

P

>SG-AL_contig_3475_1_-2_224_877

MAMYPNATHGYMPFVKPAHKRQAAKRSKADKDSVKTKAAEGTKTYKVTMRDRRGFTYTYI

IQADASKSLPVLCESRVTAPNNSPLLSNSLPNMRRSARFPVSAPESRSCASLADGKEDLT

KVDDGEKKGLFNSLLQYIKKKTSSKSSKTANTTAAKSKRKVKPLPQRKRQRTVSVMSNLD

PIQESPEEDNLDDTDSYSHDSVTSCSESDSGDGHESCP

>SG-AL_contig_3476_1_+3_135_251

MAGHHNSDNSIIILSKVAEKALLTHLPQQVLILGTFWPV

>SG-AL_contig_3477_1_+1_424_741

MDNPALWCQQIPHQNVRLRNSHQEAIFRLQAGQKTLNHHLFTKFHINQRDQCPCQIGSKT

KEHLLQAWLLHKSFGRQLSGRDPRDQEALQRSAGPSAFIQKTGVSI

>SG-AL_contig_3478_1_+1_34_363

MLYVLLCKMNNPDANPNTTTPFLKMKTMPTNTDAKVQTADTNESEQKTPRVQGVFYRCKP

EAVENQCLWNMNSNNTLFHLLQPHIHRRKKISSMEIISEHLYTQTIKMVW

>SG-AL_contig_3479_1_-1_13_1470

MAVELEADQIFQAVLLFSWVVYAWESYLSSRQRKVYQHYTKVPDVLEGVLDKETFEKARL

YQLDKSNFGFWCGLFSQVESTLILVLGGLPYLWSLSGRLIARYGYGPEYEITQSLVFTVL

SFIFSTVTSQPFSVYSTFVIEERHGFNKQTAGFYVKDQIKKFVVMQAILLPITATLIFII

KAGGDYFFIYAWFFTFVVSLLLITIYADYIAPLFDKFTPLPEGELRTEIEQLAASINFPL

TKLFVVEGSKRSSHSNAYFYGFFKNKRIVLFDTLLEDYAPQNKEGEKEGSKVPEEGEEKT

TEDNSEEETGDRETETEGEKPPVETVGSEEKKHKKIGCNNAEVLAVLGHELGHWYLSHTL

KNLIISQVNTFLCFLVFALLLNKTVLYEAFGFNSQPTLIGLFIIFQFVFAPYNELLSFMM

TVLSRRFEFQADAFAKSMHFAEKLKSALVKLNRDNLGFPLTDWLFSAWHYSHPPLLERLK

ALEKTE

>SG-AL_contig_3480_1_-2_5_1618

MTTTTIMMVMMMTTTTTMMMITIMMAITMMSDRVQTCQPGGEGTEDRDSMWADRRLALPL

LVLLALTSAVTGQRQRPNIILMLADDLGWADVHRHDASMPTPTLDWLETRGMVLNYSYVL

PTCAPTRSAILTGRYPFTYGMQVNDITGARRAWLDETLTLLPATLKSLGYTTHMIGKWHL

GFCHESLTPTRRGFDTFYGFYTGAQGYFNHSGDSRYAYDFRDNDEVVWSARGHYSTELHT

ARAQRIIRERDNQTPFFLYMAFQNVHGPFEAKQRYIDQYCSHVTNETRRIHCGMTAAMDE

SIRNITDTLSEEGELDNTIIVFLSDNGGPTDGASFNYPLRGKKATLWEGGTRVFTLLAYP

GLSRRGGYVYPGLIHAIDWYPTLVQAAGGQPPPGLDGMGMWRELRDNANSPRTEFVYNID

DKNKRSAIRWNHWKLAKGRPGRKYNGWYPPPSLLQQGVRWEEVPKRQVAKWHLYNLAADP

SERDNLYYDRSYGRIARLMKRKLRMYKRLLVPFQPAPKVAAGNPRRTGGVYSPGWCQP

>SG-AL_contig_3481_1_+2_2_196

AGTKLLHNVSRSLQCSYREPLMSLSPVHSHLHLGGFDSINSQIVLVKAVLGNKFGSCICV

SYVTR

>SG-AL_contig_3482_1_+2_668_880

MPAKTSQLSDCQIWAINPAHQVPGKASFQSHKQETILANFESHDEPNIPLITFTHVHVHT

TVYIACLCRRF

>SG-AL_contig_3483_1_-2_5_1330

MENMEKYDLTPVLCQYLDRHLVFPLLEFLSVKELYDEKDMLKGKLNLLSNTNMVDFAMDV

HKNLYPDQRDPMSLREKRQEVVGKLKSLQAETDDITKIFEEPEVQRQIQSSRDGRQLFDF

LHKEYRFKQQMLDTLYKYAKFQYECGNYSGTAEYLYFVRVLLPPSDPNYMNACWGKLASE

ILMQSWDVALEDLKKVQEIIDANQFPNALIGLQQRTWMIHWSLFVFFNHAKGRDLIIDLF

LYQQLYLNAIQTMCPHILRYLTTAVITNKGRRQKVLKDLVKVIQQESYTYNDPITEFVMC

LYVNFDFSGAQQKLRECEVVLMNDFFLVACLEDFIENARLLIFETFCRIHECISITMLAE

KLNMTPDDAERWIVNLIRNARLDAKIDSKLGHVVMGTQAVSPYQQVIEKTKNLAFRSQVL

ALTIEKKTVGHNKQDTQWGGQE

>SG-AL_contig_3484_1_-3_81_947

MGSLRKVFVVGVGMTKFEKPGSRENFDYPDMAKEAGTKALQDAGLSFDKIEQACCSYVYG

FTTCGQRALYQLGMTGIPIYNVNNACCSGSTALFMAKQLVEGGMADCVMAIGFEKMERGP

LKAEYTNRTIPVDKHIEVMTRLRGFEPSPVNPQLFGNAAKEHMEKYGTTKDHLVKIAYKN

HKHSTNNPYSQFQKEYSIDEIKQSTTIFDPLTKLQCCPTSDGSAAVILASEDFVRRHNLQ

SQAIEIVGMEMGTDTPSTFGKSSMSLVGYDMTKNTADQLFKKTDRKSVV

>SG-AL_contig_3485_1_+3_54_272

MSFTSKERFPYTLLHVNGDCTSDPYCLLMPGSLKARQNPMHTTFCLEKCSKVPVVLTKAL

QNVDNVKKQQKNP

>SG-AL_contig_3486_1_+1_304_612

MTFSSSFFFFFLPHHTCIYLQEQIFLNKNLLETTSLLLSFYLFNYLLIYLLFNCAPSEFW

TWDVDCGKFKQLVNEKLDFKNINIIDKIVGVPMQHCCCSQEHV

>SG-AL_contig_3487_1_+3_1101_1457

MILQMHVRAKAHTHMHTHIHTNSPIRMTIPNIHTPTHTGTNPLICMTILKSHFASQVNVS

YCLGTGMVEWDFNGLYECTSKERKLCNFNHMNTPLPTPHFFLVLHMSFFMGPFLKMGIS

>SG-AL_contig_3488_1_+1_2185_2598

MKASLSTNLGGCNIMPSQKCHLLCKIFPMKKNAAVLLPGFKIAVIQTILYNYQKLRNVHV

LCNKQKYTNFMEDFILWKRITNHTHDLEHGVGKIGVTLCLSKNVLSFNGLYHKTHAYSPA

HSCKHACTLSHNHDCHSG

>SG-AL_contig_3489_1_-1_100_354

MEMVHHIGLQLRSTAVCTGIRRLRYGHFDVSHSLLRQQWNLEEIDGNLRQNAHMLSSDWL

AGEGSVRGVDSSSNKLESDEKLIEG

>SG-AL_contig_3490_1_+3_303_668

MSIKRWGRAPNCKAKPLREASTLHNIFFLLKGRCAAKTTGGRKEHHRGMLQRLRSLRIEQ

ILQKGWNNLRHVRNETSSKATHLHTSQSWCMSICPRKTFKLCHILHTPKDLLAPCDFFFI

SV

>SG-AL_contig_3491_1_-1_1_837

MIQDDDDDEDDDDEDDDDDDDDDDDEDEVDDDEDDYSEEEVDVAKEEEKDTENERKNPCQ

EKTCRRGEICIVGKRRKARCTCIPQCGEHEAGDDGYQVCSKRNVTYQSECELDRDHCLCR

RQLEGCSTPKAGKIRLDYYGPCQQLTPCSDREFSIFPSRMREWLYVVMQQMAQRAKIPDY

LDLLEEARQEANHSHAVIWKFCDLDVDPHDRFVSRHELLYTVQSLKALEHCLLPFLDHCD

TNSDNRITLWEWGRCLGLDEDKIKDECKNIRKASGREKK

>SG-AL_contig_3492_1_-1_1_288

KWYSLESACFSLVKFQTSPKTLFRSNFLSNMEESGIGLPSQFFLVCLTSTITYAGVLLRF

VILSWFWLSLVSLTSIVFQITNATFKKKNSGVICAR

>SG-AL_contig_3493_1_+1_520_702

MHFITLHLLMSLTIYVDLSVTNLVKVYFRSPMDFTVYHGQLQLVLTLTFPQTLTQWVGMI

P

>SG-AL_contig_3494_1_-3_1611_3176

MRGDGSCVVLLAIALTMMVGRCPAPVSATVEEDEVNCRPQHVHLSFGEAADQMAVMWATN

STCDSVVRYGTNPWHTQDSATGRALPYTLNNAHGLHVINKVMLKGLKSATTYFYRPTSHK

IGRGPFYFRSPAHGTKWSPELLVVGDLGSHSQLLPLLSDEALSGQYNAVLQLGGLSAYLS

DASGTKGDMLMRGLEHIAAFTPIMTTPGHNETAAGQFPGYRHRFSMPGSAWPIPEDKLWY

SFNLGPVHFVSYCTDVYLHGSKAQQTAQYQWLVKDLSEANTKRSDRPWIVAFGHHPLYCS

PSLTHPDCAKNKSLVRSGLEETLYYYGVDIILQSHPHGYERFYPLFKGVVLANNYTNPRA

PVQIITGPASATPTATPKPTPAPSLTTAAMKTTVAPKSVNSSHGGSSGGGGGSSSGSSGS

TKTPRATVAHTAAKKDTAATTTTTSQTMTAFKFFNGTQPTYGRLRIINATHAHWELLAGS

EEGGRKEREVVDSVWLVQEQHGKFQLKDLPDDVEEKIDKNLV

>SG-AL_contig_3495_1_+3_3_215

VCARACVHSCAFAFKHVCCLGAHPYMLKSLSILVYISACAVCMYMIHECVDVCISVHTNI

PKCAFMTLLFF

>SG-AL_contig_3496_1_+1_190_558

MPGPPGSPSGGQRRRGRCPCLVTRQGSACSGGASSREEKAAAFDEGEQDEECLRRRRLLR

ALPRLHLSLCGWLPLSYEDLGRGLVGRQEPAMRMPVKKRQPSVGSSPVCGGVEPRALRSA

GVR

>SG-AL_contig_3497_1_+3_3_152

YAYKNKYRPPLLASNTSWFTGIKMSQKKCLELADVTKGPKRNTVSVIRVV

>SG-AL_contig_3498_1_-1_70_534

MRNMFGDYRAKMKAMEKKSSAAMKKSKLVPVKEPSSDSRFLRRSAGAQLAHNASEKSVTS

ATDNFSHLYVDSSEPQTDTQTQATCAAESDTTVCKTSSNVPSSEGEFKFNFDGTECAADV

SSSSTSVEAVHHNYFVIKPSSEEFRFNFTEDEQEM

>SG-AL_contig_3499_1_-1_148_321

MFSGFICYFSFVLSMIRPRMVSPVVWMKDVVFQQVLFVTTKLLFQSCTVRPLLDSKEM

>SG-AL_contig_3500_1_-2_158_268

MNMEKSDTNLCPLSTTIKQKASMNLQELCLNNLLDFN

>SG-AL_contig_3501_1_+3_3_209

PIKQTNKQKQLDNNTTAKKNKTNETETLFNQVISDAKNLNKLLHKRTDKMRTYLTRRIET

RILRSTNKH

>SG-AL_contig_3502_1_+3_1170_1445

MLHGSHKSNSMRSETHPTGFRNPSLWDRKPNSVTQSQPNDSETKLAGFMSSIHWLRHQLT

RFMNPTGNSEAKKILPHWPQKSNSQSLFFVCF

>SG-AL_contig_3503_1_-3_87_308

MSELVVSQCLSKLVFQKLLLPSVYIFSFVLATFFCFLYCTVLVAFVITAFDENIKVRPNC

FRRKLKQPELCHRL

>SG-AL_contig_3504_1_-3_330_620

MYNGIAKLFNPFTPSPYPPASCINNLDKSQLYTKGDRRTRGAQRLHQEQVTHPALFNSFF

PRMIRQWNKLPPGLTDSPSLEFFQASLRRYTDSVPQY

>SG-AL_contig_3505_1_-2_1040_1342

MFVQEGFIVLGTNPYALHPKLPAIYLAGIHRTCDNSICTPPFSVIHSTISNLCNTADVLL

CFGRKIGTKTPPCCGALPMKPSSHSTLNCQQFIWQGFIALA

>SG-AL_contig_3506_1_+2_683_802

MIHLCTIMTVNLPNIIAHNILTMFLLDSSQRYHMRSSGQR

>SG-AL_contig_3507_1_-1_37_6678

MAESPVFESPIPESLTGTETSDSYYTAQEEVEEEVTEPAVTAKVIDRPKLVALIPEKVPE

SPERELLPIEVDIEEEIPESETPTEEIQEDVIEKTETTRDAKLPEFPEEPREEIPVFLQE

TTVQEVTTESQEVTFVIPAADEEETEVKVVIPESPADEGKTFPDSRTPPEELPTEDTVIE

AVIEVMEEQPQILTVQAPDEIPPEFLEMLQPQIVNEGEPVTMICRLIGSPAPAVTWYRNS

VVIESGPDVKLYYDSQTGQCTLEIAEVFPDDAGEIVCRAVNPFGEAMTSANLLVQVVDEQ

ETVTTSETTVTETVTTTTVLTQVMEGEELPEVRLVDEHHVTSMEIAPTFTKPLLPTTEVE

EGGTVKLEVILDSQPEAEVKWFVNELQVAPTPRTHLLKEGNTHVLVIMDVTPEDSGEYIC

QAENIYGTVTCKTTLIVTPIHRKEVVIHREDLEKEQEVKPIEEVPTEPAEAPKEEAPEGP

AETAPAEEPIAPLFTTLLQPQRVDDGQRVVMTVHFIGRPSPRITWYLNGVELKPSPDFEI

FVDHTKGESTLIIVEVFPEDEGEYTCIAVNEVGESITTCRLTVVTTEEEVEVESDEVHKE

TIEIDVTKQPETMEVIPVEDLTTHVKEVVEHEVQPAKPATFEIQIGLPQVTKTTATTVVQ

ETTTYTQVMREVVEQEETPSISEGPTTRTTSVVMSKGEEVVPIELRVNIPVQPAESHVVE

QVTEATRLVTSEVQQIPMAEKPTEERHTETIEIVQQPQPQEVQITVPVTKTETVEVHRQM

EHRRESAQRIEIEIHETQLQRQPQPQELEIPISKVSKTEIDIDVTDTTVTEASRPTEDVV

EETRQTIVKETMVVQPETEQPQPVMEEAHIEVSLAPEETITSMEETEIKVPIQDMVTTET

QLVAESEEIPSEVLMEVELVPEEQPEEKETVTFEETITLDTQLADIETPITVAVEESKVE

GIDVPTAELTQEVAETEVTIPAEETIVSEREVVDQSDLIPSEVQLQVELVPGEKPSEEET

PSFQETITLDVQAVDTETPTAVTTEESKVEGVIEVEAPEEVTEVEEAEVSAPTEDVIVTE

REIIETVEEVPTEVQMEIELVPEEKPQDEAVIFQETITVDTQPMEAEMPVSVPTEESKVE

DVLEVQTVEITTDVQLTETEAPTEEVIISEREVISEAAEVPSEVQLEIELVPEEKPVEQE

VISLEEPVLMDTETTVIETQITEAVKETKVVDTTEEYVPEVTTEVAEAEVEAPEEEVIIT

ERAMVEKSVVPAEVLMEVELVPEEKPEEQEGPSFQETITIDTTPTDLETQVSAPAEETQV

EMREVFDVTDSAETFVTEKQVIQVEDTPLQEVILDIQVREEEGVKPGLISTEVEQVVVSE

VEEMKESHKEEIELQIAGQQPQQEEVLFTIQMEPTEPSTSEITMQTTTTEKTEVEFPVSE

EVPFETQTFVSETEVTEKVVEAEERHREELTLQIEGKQQELEEVQFTLQVEEKEQPEETP

LTEVAAPSVEQVLEVPEVVEGKAPEFTWGLMSLKVMDGEEVKFRCEVNGVPMPDISWFHD

DKPIAENQDFSLSYDVESGACTLLIAEVFPQDAGEYRCVAVNTHGEAITRAFLEVESYEY

VPDSEEASESMMSSIEPSSPKPTPADSDTEIPTHSREFVEEIHRLQEQISAELTEHEEEA

PKEESAPEYVAQAEAEDQPEEEAVIMISEVPSETVAGTQQPSESVQVSAEETQFQAEPVQ

EVSEVPQVVEVVSEDQEAAVPSVTAELSQPTLQPETEFQVEVVQETAEQPDVAEVPLEST

SAAEEDVVLDISATTEDTVHEYKTEVVEESIEQPAVSEVITGVPDEAQASQITVDLMTPS

QEVPSQFQAESLEEIVDQPLVVDVPVEESQAAIQVEVPGDVVDAAPEKPVELKVETTVEE

IEQPAITEVTEEAGELASESQISVELVDVSQEQPSQYQTETVDEVAEQPEVSLVTEETTE

EVVPSDISVQLVGAEEQQPSEFKIEVVQEFAEQLEVTDVTEETTEEVVPSDISVDLVGAE

QEQPSEFKIEAVEEIPEQPEITDVTQETTEEIVPSEISVELVGAEQEQPSEFKIEAVEEI

AEQPEVTDVTEETTEEIVPSEISVDFVGAEQKQPSEFKIEAVEEVTEQPQVTDVTEETTV

EVIPSEISVDLVGAEQEQPSEFKIEAVEEIAEQPEIMDVTQETTEEIVPSEISV

>SG-AL_contig_3508_1_-1_1369_3357

MAHQDRYQTNIMHFIDWRFTQLQHEMQVHTGCIINNIDEQFKNLRDSVKEVLGELNKLKD

CVTQTQEEIQDLKEDKSIIFERLDKLERTLERVEEDAKACSLRFMNVEETGWEDKTTTSE

LIIEILNYHDPTPNWKQEDIVRAFRIGKPRQHGSAPRPVIVQFSRWSNKMAVLGGRELRD

NLRLSRIKVAADITSRQKEMVDFHRSLGKIAFIYNGKLQVKDRQQSHPWYYGDGHEGQYF

TGSTRDEDWPYIHHNRHIRRGRGGYNNGQNSYNDRGQPYRYPITPCEECTEDDPATVQRT

QHRNYQHQPQHGHQESPIETGRGISHASHLPTRNMDRQRNEDPFHHDPVNRRIRPIIPGD

RSYSEVTRQGRRIEPVESEQRRVVFMKNRDNRPRSNVSDRRRQHTGHTKRQQETAGSRAS

DTYCVQISASDHDRSSIDDGETEPVDQPSSEQTVYKEGTDDRSVIMAEKESQVSESEDKS

DTTYGDMAGDVEDVQDHLTYDKDNAKRKLTNINGTNDLTVANTNICETQRDTELSGVGHD

DSDMTMVQVSSVNNMITDGQTDKHTSRHTADKHAIPDDTQQDYVEKEQASDVQTSKDDQR

PKRAASIKQTRSQRRTAAHKPCIQSSSILDSLQRASGNCTKASEGNEQSQAKNNTCGASG

TKK

>SG-AL_contig_3509_1_-1_133_999

MVDSTDIDLMRDAIESVNIADCGDRSVCDRNPCRHGAVCEDISMTDYRCLCPESYTGRNC

EIEINDCVTRRPCLNGGTCSMASGGGYQCSCPLGYMGQDCEAEIAIGSAFEVQGDGYVEF

ERSLLPHRRRQIGETILFTITTVEANGLVFWQGQRPGEQSGADYLAVELRDGFVEFRYEL

GSGPAVLISDVPVNDGFPHTIEARRLSRDGTLIVDGGREVTGSSKGPLQVLNVQGNIYFG

GVPDLEKHTDGQSLVNFNGCISNIQIQNEPALDVGARAIQGANVRPCLN

>SG-AL_contig_3510_1_+3_39_194

MLTLSVQGSENFQKSVLDHVCECVCVYVCESDFSRLCDCCVILKCELFPHLH

>SG-AL_contig_3511_1_+2_818_1162

MMDCPLATVTTKEKTKALPEKVPLHLCYCSPPVDKQAADYEAWHCRIYQVTSNFVTPRLR

KKGTSKRTVIPQCPHLTRPSSVAGFACPTSDLPTMWLAANMDISIPKTLPAKTLP

>SG-AL_contig_3512_1_-1_94_762

MTGETTEAYLEEADWMMGYDITVGDIPPIRFMDNPEKDNRSIAHADNYTEFLDPHLGSGV

YNRAFYLLAHVYGIPIRDVYGAFLHANRMYWHHMSNYEYAACDVMKSAYDLGLDGSKFRK

AFDEVGIEACDAEDHVLGLKHKEVYTDITVSKAISPTFVFAVPGQFVESITVKASSDNGD

VYIILSDKRWGDVNDKAEVYAEGLHSVNYELPDNSSATYVTIT

>SG-AL_contig_3513_1_-1_55_441

MFENWYGMSPLGDVMTFRVHFGYHYSNAFWNGFECTFGDGDGWYLHPLVSLDIVGHEVAH

GITEKNSGLYYYQQWGGINEAFSDITGEASEAYLDEADWMMGYDITVGDNPPLRFMDNPE

KDNRSIAHA

>SG-AL_contig_3514_1_-1_208_540

MLTTLAPGFDPQAGHRFYFQRLGLMLMHGLTSSSFGIFPITWHLRRWCIWKLFPAQKPKF

CLPMKIDLFWQHFARANSLIAKGPVTHIKCMLHTGLQFILSSERLAPRPTL

>SG-AL_contig_3515_1_+3_456_674

MSASNISSFDMTSVAVTLVMHEPHFTDGTLLIKPNLQPKSASTPSINSSAIPSTSVAPII

HHATFHIIYQTYM

>SG-AL_contig_3516_1_-2_5_292

MASPSTQPSQLRRINKEENSASFRCQKCLEKGHYTYQCTGKRKYVYRPSRTKDMVKRFKQ

DEEDKKMQLVAKTSTILEKKKKLKKRKKKSSGGEAS

>SG-AL_contig_3517_1_-3_135_344

MLSLTLRSNCYYYYNYDSSLYKDKNLVTSNRHLYIINPSLYILLPTAQYVTCFCKCFTFC

QMFVPHLLIF

>SG-AL_contig_3518_1_+2_803_1081

MITNTDFTHDDKGQSHDNANWIKKDNANSNDSNNLSSSNAMTSVVIQGTQGTQQQTGFSF

SVSGCSNKQSRHTDAYEVATQLFLRRGPPSRVP

>SG-AL_contig_3519_1_-1_184_405

MLGRTSRPCQRVELMPKCKTYAMIILDPFPLHDFCDADTYGIYSECGDAVLRRLKLKVHC

CLVFHSIQTDNTFF

>SG-AL_contig_3520_1_-3_12_221

MITLDPFPSHGICDIGAYGIYSKIGNVRKLKVDDACIDAVETDSIMLILYYLVFLFLCYL

VLSFPNKCFI

>SG-AL_contig_3521_1_+3_330_509

MRLNNVNTLYNKEEKSSSESAKIPTNTTLENNTITKAATSLHFHIPGPQLPSTTHTKMCA

>SG-AL_contig_3522_1_+3_3_71

EIQHSKICSLICFKTVENKTKLK

>SG-AL_contig_3523_1_-2_2_355

ACKNTNLPQNDSVRASRRAPLCCCLCDPSSSYIRENANRCGVQCVPGILDCLWLGLWISG

RPLLGWPSWIPLAWRERKMADSTTLPHGHEREHNCPEVSPLVLFVVCVPQLGALGVAQ

>SG-AL_contig_3524_1_-2_110_1744

MMVDEALNKLHAEYDKQRADLMRRHQEELAALQKQALSPEEMAERRAALMNRQQMELSDL

DQRLQEDEGQVQRTAAADWEVAFARDKLALKERHYKEFADALREYNPDHDGVNSAEGAAK

ELEKVKADLEQRRREKEQQLKKEQDEFEKSEEKRLESELSAYAEELEDETKKEKAKHDRN

IEALNKRKEELIEENKKKLQEEMERLKAAGASKEEQDALLKQHEHNMNNLVGKLEADRLR

MKSNLQERLKKRRQDKMQQKEQEMKHDADRQARQMENYQQKELERLKKDESLTLQEALNI

DRLAGQEGMSGPGSFSDTTAPAQPSGPEQSVPSSQEGSVPSSFTQAAPLNEAELLALLMA

SPLYQKLEKVKKAVAEGALQNAPATSDGHIDSRDSQWTSDKDLSPLDVSSLDARSFVIYK

FGSYIAGTVAARCRHAPVTILLADRIPANPHLTRNLYRNSFHFDANNRILYVRSARLDTV

GEFVLVLVHCLAHIQSGDLRDDTKPAFVQEYQKALTVVCDSLFFAHHNRPSGTLQSVAEA

MGTDT

>SG-AL_contig_3525_1_-2_518_661

MNNVGAMLLTWQLKKMRWSSHPNNFLFYLFRCQEKLLASASPVPGNPG

>SG-AL_contig_3526_1_-2_2_115

SFYIHALLLIRVLSHGFDSLCFCLVSDDSLFSPHVLFL

>SG-AL_contig_3527_1_+1_754_981

MIAKHEIRLVYDDEQFHVLTIMYLYTHTYIAQSRFEDQQFQFMVSVSVFSRQGLHAQNKF

HMHQRCKYHGMEKNPK

>SG-AL_contig_3528_1_+2_629_796

MHQPPPSMPRSLHSALTLSCLPFILTLCLFPRHHFHLLMLLSFLNQGKPKNFHSVL

>SG-AL_contig_3529_1_+1_3013_3345

MRHAMAHNDDMLFVKNPDTGHHTDTTLHHLYKQHDCYTSYMERGQTTNISTSKHRHHTTC

IVYTLTTSQFPFLAETNSCFGQNAVTPIKTYSNINHNTDILTKCSFCSSDL

>SG-AL_contig_3530_1_+2_89_235

MVLFGLHVALQSHATFQPVDLDLIYFIYVTLAKHIHCICRRCNLSQMLH

>SG-AL_contig_3531_1_+1_1_114

ATFQPVDLDLDLIYFIYVTLAEHTRYMSQHCNLSQMLP

>SG-AL_contig_3532_1_-2_47_244

MYLDNVNKLKFLPLPPPPSTSLSLSLIPSLHQFMRFLAHPTVVSPPCPPSCISPLSRVVF

RDSRLP

>SG-AL_contig_3533_1_+1_76_537

MRRLPYLATCLRCQHRSAPSSVTLPFAAVMDLDKLACAAQLSADYSGCCMRALGAWLQQV

WGGGSSDQNDVPTDLVTLGREACGAIVESADTAVKLSYNLALLRRDAILAASNLPAEDFP

ALRTVPMQPTSFVPFAEPFVSSWGAARRDSAALS

>SG-AL_contig_3534_1_+1_1_459

PLITARARRLRPLSTRHIAPGPTSHAQARQGQCRSQYDVASLSVILPEVLAQDLASLTDL

DKLACTAQISADFLNLCMRALGARLQVSRATHVSGPSRPAPPHMAVPGAGQAAPPALSMT

VPGAGAPFFSPGRAPARLVTSKARSLAQPRRPA

>SG-AL_contig_3535_1_-1_1_162

EDLLTLGSEAYYALVDSADPFTIRDFDSAWEGSTPPQCIVCMQRLENPLWRIAA

>SG-AL_contig_3536_1_-1_40_1248

MAEEMEVDTAVAGHEGIKQYYVSKIEELQLVVNDKTQNLRRLEAQRNELNAKVRMLREEL

QLLQEQGSYVGEVVKPMDKKKVLVKVHPEGKFVVDIDKNIDMNSVTPNCRVALRNDSYVL

HKILPNKVDPLVSLMMVEKVPDSTYEMVGGLDKQIKEIKEVIELPVKHPELFEALGIAQP

KGVLLYGPPGTGKTLLARAVAHHTECTFIRVSGSELVQKFIGEGSRMVRELFVMAREHAP

SIIFMDEIDSIGSSRLEGGSGGDSEVQRTMLELLNQLDGFEPKQNIKVIMATNRIDILDS

ALLRPGRIDRKIEFPPPNEEARLDILKIHSRKMNLTRGINLRKIAELMPGASGAEVKGVC

TESGMYALRERRVHVTQEDFELAVAKVMQKDSEKNISIKKQWK

>SG-AL_contig_3537_1_-2_341_1636

MGVPHLEGEDTPPLPSPSDDVDSNGQDRQELTANGQAVLGQPYTSTETDATEEPTIAPDE

ATDKFASRYKRYTDVPVKRLSESQPNGTIGGTGGGGFVGGGLSVTWLTLDTDIEPRPHSQ

QNFLPSPRTERDHEMSDLKLIRDFQVRRKYGRRPNHVRPPKRDKNLLDLGLSGRRYFNSR

ETVTSGSASTDSPRIGKTPTLGEESGRLPSGSTPASSSVLGKTRTDEDRSPVESTAKDLQ

SPVPSFPPLSPGPRVGIRSENSFYSSRHSGQSPSLLVNHFGFSTIRPDTFSGMQKHRMQK

LAAATSEDLIKELEQHAQKQILACQNMGYTHRGSRAAESVLSLKHTVSPSAFHPSSIFTR

HVPREERGSRSGTPAGIGEGEERHRKRLTPAGKLSMPRLLVQDRQEETSRRATLPSVSGN

SLQVVLAQSHGK

>SG-AL_contig_3538_1_+3_792_1004

MSLNTATDAAHSREFVTSCTPVKNRIKRMCISSEYTQPTSKRILTQFINIILCPTRMHRS

TQTYPNAQTIQ

>SG-AL_contig_3539_1_-1_1_354

CTTLQQLGGPTLSDNFLLREVLRGLAAAEARTPRRLPRWDLALVLGALRSPPFEPLDVVP

FSLLSYKTAFLLALSTCGRRSEIHALSGAKDDIGFHSNGDPALVVLPEFRAKTQQPGS

>SG-AL_contig_3540_1_-2_2_271

IKLCLSVCLSVSLSLSLSLSLSLSLSLSLSLTRLSVVFPLLSRARCKQIPNILICPLSYI

DFSPFFSLFFFSLFVCVILFFIIFFFIRMD

>SG-AL_contig_3541_1_-2_1640_1951

MTHVGGYRDKRQIYCIIFFSCVFILTCYVLFPFKMAGTAQHFNWLLVFENGQNCPFMLGT

FPHHTVQNNDVRKGKLLVRLDCYHHHLLLLLLLLLLLLLLLLFP

>SG-AL_contig_3542_1_-3_3_68

LPGTLAEEEEDIHVCQLSRLKR

>SG-AL_contig_3543_1_+3_3_122

RYLGSLKKRKEKIKTRLPRPILYLRKTTKGLEKMNKLRSI

>SG-AL_contig_3544_1_-3_3_161

KAKQRGRGDGHHLHKEVEVSSTSPAYNLRLWDNPYLTLPSYLVQQLSEYSTKQ

>SG-AL_contig_3545_1_+1_928_1131

MATRANWVRSGHTALSQSGNTTGPNNLHIISIISQGEQQPSSVRAEVCTATISKVRQRFC

FKHTLTLW

>SG-AL_contig_3546_1_+3_3_203

SILSSLVWTNLKSCSILSPACCCPRKLGFSATFLEVGCHLMLWSDMEVVVGMSATEADKC

DNLVLQW

>SG-AL_contig_3547_1_-2_2_262

AKFLFLADGSFTQFYLGHGQRTDIDNILSSDYCPFKNSDSFQLLCCFKFSSKQSACLTAV

LSMVQFLSHGFFQCSLFVYIFIICTLQ

>SG-AL_contig_3548_1_-2_848_1009

MLSLCQTLSDVHSEKKLVNTLQVHLHDLDIFEKVKWVQKKNVLQEWNNGPKGTF

>SG-AL_contig_3549_1_-3_105_419

MCLLLFSLSDTSTQPVIQQRRDQNDTLIPSSPSPPPTSTIPPFYLNKRYKLMVTRKVSGC

VLLCVFMISMNILFLTIYFLMIIIVLIIATAVLVVVMIIIIYLQQ

>SG-AL_contig_3550_1_+3_3_257

GTHLQLGGLEAAHTTFCEPGCWIPLQSGHSPGLAWIRVRYIICSDFLSLRISDQTKPFNP

RNKICTTIYFNTCCKNMSYTYTKKN

>SG-AL_contig_3551_1_-2_317_640

MGCMGDVFLFVFVCLFVFVLVCYVLVVFLFCVSLPVKVDFFYKILPVTLFIFLTHLLHRS

CVTIPSYSSRLSNTPISARDNSVVAEESLTFVKCMLDTGPQFIVTPER

>SG-AL_contig_3552_1_-3_48_443

MLRLSHLTRQIRSLCRRTAWSPCPPHAQTPQQCQQHLPALQQRSSWYSSKVQLTEGEKAI

IEKLKSSFPGAKAVEVSDISGGCGAMYQISIEADEFRGKRTVMQHRLVNEVLAEEIKLMH

GLQLNTKVPSDS

>SG-AL_contig_3553_1_-3_18_257

MLFFSLTLLVFLLDEYCNTPVLLLHIFFICTHMLADSRFLCMFCACFFHNFLNNFLIITT

QCKFTFLKKQTKEMCSAICM

>SG-AL_contig_3554_1_+1_1_207

FRHWQVCTHVDPRDQKNFHPLPTRQRCLGRELNPGPSIWKSDVYPLDYNKQYNSHFPIYI

QYISITFVQ

>SG-AL_contig_3555_1_+3_492_737

MSSINQMQIIKNEENQFFACFILPSGYITLLYLTYAITLLYLTYAITLLCLTYAITLLYL

TYAITLLCLTYAITLLCLTYAI

>SG-AL_contig_3556_1_+2_2_124

FFFPDIIGLLIQSQTTTNNNKIMSSKTRNHTHQQQCAHMHA

>SG-AL_contig_3557_1_-1_64_2493

MPRIMIKGGVWRNTEDEILKAAVMKYGKNQWARIASLLHRKSAKQCKARWFEWLDPSIKK

TEWSRAEDEKLLHLAKLMPTQWRTIAPIVGRTAAQCLERYELLLDKAQNKETDADDDPRK

LKPGEIDPNPETKPARPDPVDMDEDELEMLSEARARLANTQGKKAKRKAREKQLEEARRL

AALQKRRELRAAGIEVRQKKRKKRGVDYNAEIPFEKKPAPGFYDTSQETYDPLDPNYKRL

RQQNLDGERRSDKEDRERRKDKQKQKKRKENDLPGVIASQTNFQEPVKKRSKLVLPSPQI

SDAELEEVVKLGQASENARQQVEEAGQEDASSRGLLQDYSTTPAMAGLRTPRTPATHDAI

LQEAQNIMALTNVDTPLKGGLNTPLHDSDFSGVTPRPNTVQTPNTVLGTPYRTPGREGSM

TPRGNATPVIGRPGTDTSSAPGGTTPSGQTPFLRDKLNINQMEDEGGMQGKYYQRELKEQ

LRQGLAALPTPKNDYEIVVPEDEAGGEAEAMEVDRVEDQADLDSRKEAEIERRRQEEMKR

RSQAVQRDMPRPVEINSAIMRPSGPSDPPLSELQRAEELIKQEMLIMMHYDALYSPTPQQ

MGITPGTKKPPNQKGVLNQAQHVAYLDENPYVKFEDQDIEQAKELLNQEMQLVKDGMSHG

DLSLDAFTQVWDECYSQVLFMPTQNRYIRASLASKKDRIQSLDKRLENNRNHMTRDAKQA

AKLEKKLKILLGGYQSRAQGLMKQVADLSDQLEQTFMERNTFDNLRQHEIGAIPKRMESL

KEDVQRQMERERELQKRYSELQHKRDISYR

>SG-AL_contig_3558_1_-2_8_661

MAASLVRSFAFGASARNAFGLRTGAVCQTWTRTAFFRRRKDKQEATEVIAEAPDDQLVAP

TSVSSIAASNGLRKRQGLQTVRGYSPPGDVEARVQKIVSEVCGDVTDWASIKFDNNRIKF

KILEKAMSAFDHYIPNSQLINMQTASDVVAFFSTPVRDTSFYEDLSTLDLPPNLHIQLQP

LRFDPETDTMFGGVSTFPNRPTVVSSLKYRRKYGKTEK

>SG-AL_contig_3559_1_+3_27_230

MIGLLQSQRTMEFVLLERLQDTSLGKGLRRTCCCPCSRPNNGGDAGERTRGLFFTVCGVS

ENVKSGNY

>SG-AL_contig_3560_1_+2_545_856

MSQKSLFHKASVTVIIILYLKASVTLIILYFKARVALIILYLKASVTLIIFSYLKASVTL

IIFSYLKASVTLIIFSYLKASITLIIFSYLKASITLDIFSYLKA

>SG-AL_contig_3561_1_-1_232_1617

MLNQTNVGNNNNKYYIIQLLEETGKKAYHVWQRWGRVGYKGQTNLVHTGGDVDKAVNVFT

KKFFDKTKNHWNNKKFFSKVPGKYDLVHLDYSAKEEDQVDAGDAGDEGDSQAPDSKIHKK

VQDLVKLICDVKSMEEAVVEMKYDAKKAPLGKLTKDQIKAGYEALKDIETLINKKDFGRN

LTDACSDFYTRIPHDFGMRTPPVIRTPQEVKEKLQLLEALEDIEYAIKMLKQGDKSENPV

DRHYHSLTCELDPLDHSSDDFKLMEEYLQKTHATTHNQYKMKLLDVFDVEKKGEKEKYLD

HGNRMLLWHGSRLTNWAGILGQGLRIAPPEAPVTGYMFGKGVYFADMSSKSANYCFASKQ

KNVGLLLLCDVSLGTINEKLDADYYADKLPSGKHSVKGCGSIAPDPGTFKTMPDGLVVPV

GKGKNTGVTNPKGYTLNYNEFIVYNTAQIRMKYLLKVQFNFK

>SG-AL_contig_3562_1_+1_328_495

MISTMTAKCSATYTSLSLNTYFCYKQMYFSVLHMHVFTCMHAHFKHTPYTHRSLSL

>SG-AL_contig_3563_1_+1_1_240

VFLGPVLNVSRQVGALPANQASNHHIVKDKAKSCPIIPGKASNGCPRQAIICVGHMVSTA

TCLLGKCNVFLPSAVKNSVK

>SG-AL_contig_3564_1_+2_257_415

MTRADGEGEVKIPKNEHTNKVFHIFCLKLFVVCVPQLNTLGVIQYICTPSYES

>SG-AL_contig_3565_1_+2_2_283

SPSPPLLLSPPPPPLFQKCFFFFFGGGGVLFLLKSQFPLTSLSSIQSDSSGRKPIKERQF

HNNDTHGCEMKTRTRVVQENKFVSAKKLTLLLHA

>SG-AL_contig_3566_1_-2_56_292

MNNADVIFLMNNAGCYGIFLTNVIGGYVIFLMNNAGCYGIFLVNVARGLSLFCLCVDNVH

DKMIVIMGVLVLLLLVASF

>SG-AL_contig_3567_1_-2_65_493

MMDGEPPPLPARRVTTASAAGRKAVSEELPLRCAESHGQTFESRKNRRHSSAVAAAIAHK

DFLKSMKQLKECGWYWGPLNSDEAVVKLTDKCDGSFLVRDSSSDKYLLAISFKYMGQVHH

TRIEYHKGLFSFWEQPDEHSKAH

>SG-AL_contig_3568_1_+1_193_411

MPCMTVKKIIPFINFHYFDIFKAKQGKIQPIKVESLDANLECVSKVCLNFAFVFSHTVAV

IHHLLAPFVNLLL

>SG-AL_contig_3569_1_-1_256_624

MVTALQRPAVYRAVLLEMSKPAEATSWGWLVGWDIVRNPTTSLHLSCRDFFFYGQLATWT

FFLFSFFSSFLCLSSVCVWWQFFFFPSPTEMSVQGTRSVDLLYIVYGFIYWLMISVFDHL

IIV

>SG-AL_contig_3570_1_+3_354_977

MSNFNVPFHSLTLSTSNRQTHEQSRMGMDVSELGASGRLDILKFHGQTHVPPNLQLALEE

RSLWVEFSSHQVHQVFIAHGDGGVSLVFSSKLHSPTAVFNVDDPLAHFTPWTFDLEGKHA

LHLLHDVAAILFQFFGNLFEDNGRFKVAFTGTRTGGHSLDLVTHRLWVSMPVESADDGTP

QSHIVLQSQLGARHLSLPRHTTQLPTQL

>SG-AL_contig_3571_1_+3_408_635

MIHDIMTEVLSLDWMVLTYLGQIIFVCRLTSVRELLLCQTMRTLYGSVTGKGSVNALIHC

QSEILPILVQATAVMP

>SG-AL_contig_3572_1_+2_176_361

MAICNYKFYQNILVTIMDFFLYLIDQILSRQLLPYCPTYLFELLTHNINSFTNLRQQHSI

YQ

>SG-AL_contig_3573_1_+1_25_252

MHKIINEQDQDNITGLFSINPTRTTKKISIPIHRIDLFKFSLVYTGDTLCNTLPTNMQEC

LSFTVFEKTHITHLMN

>SG-AL_contig_3574_1_-3_18_503

MRQAGEVTFADAHKRHRNEGIIEFASYSDLKKALDKLDNTELNGRKIKLVEDRPKKRSRS

GSRSRRRKSRSRSRSPRSRSHSRSRSDSRSRSASKSSSRSVSKERKSKSEDKSKIDAKKR

SKSASHSKSRSPSRSKSPDDHKGVDSPRYSGDVQHKEDEEDD

>SG-AL_contig_3575_1_+2_785_955

MFEFTWDARTHSLFKHVSAESMPKMYASLVSARTGEKEYDLMNLFSLSLSLSLSLSL

>SG-AL_contig_3576_1_-1_199_357

MSRLKKKLTWPSTTMEFSQCQYYIVICPEAAVKLDMPGFLNLIDAFHRKRENK

>SG-AL_contig_3577_1_+2_2_244

PSIPSSLKGKCLHHSTTAPIVIYVFRKCHNWNMPQELAVKLKGLIIPFNVNHDDPLTHTH

THTHKRVHTHTYTHTDLDVTI

>SG-AL_contig_3578_1_+3_384_590

MYMNDRWGKFSWRSENLNRTKTEGGKQTKNSHRKTNSHNYIKNHSSSIIRYIGMQSASKP

INRLAKTSK

>SG-AL_contig_3579_1_-1_1_369

QTFLHLLHHKHFYISCTTNISTSSHSTVSISSASQIFLYLLHQTFLYPLPERFLYLLRHK

HFYIFCITNISVSSASNVSISSVSLHRLLHQTFLYLLHQMFLYLLHQMFLYLLHQVFLYP

LPG

>SG-AL_contig_3580_1_-3_444_1154

MLCLSASPFKQQTALSCTLPLSVSFSLTLVLLPPASLSLSLSLSLSLSFVLVPLLFPHTL

PLSHPSPPSLSLSLSLSLSLCPSLSFSVPPPLSSNHPHFPTLSPHCPIYLWTYTNPILVL

ELCMVDCVSFWHFLSFFLLFKKSKFLFVIITHKVHIVFSNSFHLYDLQQIHTNGEVHVYS

VNHVNCMIVFMICFSHLHSGVRCLCTCFGFVWGCLFGGVFFGGGGPHWEDCFYKYYV

>SG-AL_contig_3581_1_-1_16_258

MVADCNFQRHSGLIPTASKLDFKGKGLSIWSLRDRITAAFKPTNNFVLNLDSLSERADNV

LGGSFARHLVVLKPNKVVENY

>SG-AL_contig_3582_1_+2_560_877

MVRLCLQSHSMVRLQLQHYHGNHSTPLNTFLFLHLLPSAWTNFFEQERQGKNNKKTPVSN

ETCLYNLMVLNTFCDMAMNVYGSFTETGKKEGDHLHKTDMATFLQL

>SG-AL_contig_3583_1_+3_555_746

MVYNPDMQSLLKLLPVNIVQYTAVSHVIHALTLCKMFIFRYFHILFLHKYSVPKSINLRH

RGQY

>SG-AL_contig_3584_1_+2_395_499

MLSENVCLQCFSCTILLHYDSFHSSVLYSYIVSLF

>SG-AL_contig_3585_1_+2_2_160

KEVCSISGYGKTFTSSLTQLECILVTMSKYCQFSVCVRGHHYIQMILYALFHI

>SG-AL_contig_3586_1_-1_736_1929

MTTEMEDSAFYSQEESNGHHDVTTTTKVRRSSVEIIRNLDNFVGRPRSRSLYASMRPLVY

ITRRIPQRGLDVLLSTCNISQWDSEEAVPRGELLQNVQGVHAILCLPSDLIDLQVLIAAG

SNLRAVATLSEDTSHIDVEECARRNIRVLCCPPPDLDGQADLTVALILLTLRSVAEGMDN

PTHDSNCDHFTLTEGKPVDVTSAWWNNLIEHKTFGVYGSTSLGLAVAARLRQLGANDVIL

TDFGQSTKGEKGSADMSASEAPANVNIEDFERVTREQFLERADVICVCDARASTQNEAAF

NHDAFRDMKCGAILVNSDSGNALNYTALYEALRDGQICAAGLNTCNQTPVPFQYPLQGLR

NCVFMPQTQEDRLDVRHKITAAMTTNLLNTLKETVQAV

>SG-AL_contig_3587_1_-2_44_166

MNIVLKLNISSSVSGAGEGKCRFTNPLCLSVVFLCVMNLLV

>SG-AL_contig_3588_1_-3_183_338

MNRSLPDFGYCLAENGHKLILTLVTNSLSEGLKKNRVTLFAILAAVSALPSG

>SG-AL_contig_3589_1_-1_337_816

MTPEKSFSQKAGGAVAKAPMADNLRFVTKSDGNVHVHPSSVNFQVGHYESPYLVYHEKVK

TSKVYIRDCTMVSVYSLLLFAGGHISIDLDHGNLIVSVDDGWIRCRASSGQVAELLRELR

VEMDKLLEDKIQSPNMDLCNCPRGSRIINTIVELITTQGF

>SG-AL_contig_3590_1_+3_381_587

MCTDSGSRWLARYHGNRSRCRLASTCKCMANTQAHACYTHTHTHTHSLSKAVIIIFNFPK

PQQKRYFVF

>SG-AL_contig_3591_1_-1_1_93

FVDHYMETAVTCSKGSSPLKSFQVAVQLVPQ

>SG-AL_contig_3592_1_-3_21_1970

MADLTAKYQKLAGEYAKLKAQVPVLKKAYLDEQTECNAAREQLKEKEQSARKYQQEVDSL

SFRNQQLSKRVMVLQDELETLESLKKKHKHKGGETPSPEKLQHSTSVYGEELQSKIEENA

KLHSKLQEVSSAYERRIVELEERVKTSEQDRSQHEDVVATLQKSSKTQIEHLQQEKAMVE

VRVQSQENEIKEYRGRAEIAEQKLNVVSQDLSSRLSNANKIITDKLPFIDTKHRDVNGFN

LPTHDRRHQLRARELIGQAANLVGELTQGLSNFFTYSEQRSRIYPADGVSEPLSPTNTKY

CKYLHENVAYLRPVDQSLKLFSEELKDDSLTILETATDLQEFSKNFSVLVAYCNKLLPYQ

MLSLEEESQVSSCTSTLSTKNRDLLKSLARLNATFNKLDTYIAILATQSKQLGGHTQASH

PRFFVLLSTALEQMHEAIKEVSKHYNSKVSLEHQLPTATQKLKTTDECVVSSLISLVTST

GKMSAFMSGNLDFFTEGAGYRTRGSSIGTEPSFEGPRSHPAVSEFRHSTASYLGSLNSRP

RPDSVPHRVAVQNRKTLLSSAESKEGLAKQITIFQEKVGKLEQEKEHWVLELQLLKIKYD

NEQQKVKKMEQELEMLRNSSGVTSAATSVMGSIEDNIDRISLPASSDRRT

>SG-AL_contig_3593_1_+1_238_414

MRVGHMFSPSTLPSSALNRVRYPFTAGWTGGRMHFFFEAVSNLYKRVQADWKAAALHLH

>SG-AL_contig_3594_1_-3_3_140

KTNSKVQKKCRSFLHYILLTVCVLISHLNVEITPIKVSKQHSEQKF

>SG-AL_contig_3595_1_-2_2_226

LSLSLSLSLSLSLSLSDCSHTCRSVYSVQSCTCHSVYVGPADIIFAQLVIHLFGVYTYSP

KFLMSKHFEISVDVK

>SG-AL_contig_3596_1_-1_1_294

CIVLLSTILYCAVLYCTVSCYIVLFCTVLYCPALYCTVLYYVVLCRTILYRSVHYCVVLN

YIVLFELYCTVLYCIVLYILFVSIQCLPLCGFLHLKQM

>SG-AL_contig_3597_1_-1_1_294

CIVLLSTILYCAVLYCTVSCYIVLFCTVLYCPALYCTVLYYVVLCRTILYCSVHYCVVLN

YIVLFELYCTVLYCIVLYILLVSIQCLPLCGFLHLKQM

>SG-AL_contig_3598_1_-3_3579_3797

MFHRKKCGILCVLYRSGIFMLFILGIVVSTHICAGCCCTVHFLKWVAVLTAKITHSCYCW

NSLMNKWEVRCYA

>SG-AL_contig_3599_1_+1_238_390

MGSDLKCLSYPNNTIFSLNITEVALSPKLLMAVWSCCQNNCRPIRSRFQNR

>SG-AL_contig_3600_1_+3_15_242

MDQQIVFFFFLFCTEKYLFAYMYMYVTSNKMQSCVRRSHHHQIKRQTSRSHSPLNQLSQH

GTPHSPLRKTTHTHMH

>SG-AL_contig_3601_1_+1_127_297

MLTLETGLISPFTHQARCPKLKPRSFKPSSLEVQCLHHLNSTSAPVKHPGQHNSTQR

>SG-AL_contig_3602_1_-2_179_2803

MKKIPPGAEASNILVGEVDFLNHQIVAFVRLSKAVILGDITEVPVPTRFIFILLGPMGNQ

NKFHEIGRSIATLMSDEIFHDVAYHAHNREDLLAGIDEFLDQVTVLPPGEWDPSIRIEPP

KSVPSQEGRKTAASAPPQPNGKAVVEEETEEHGDPTLTRTGRIFGGLRDDIMRKIPFYLS

DFKDALHIQCLASFIFLYFACLTPIITFGGLLGDATDNYMAALESILAGAICGVVYSLFA

GQPLTILGSTGPVLVFESILNHFCSGNGLNYLEMRLWIGIWTGVMLIIMVAFDLSALVRY

ITRFTEESFAALISLIFIVEAIVKLIRISSDYPVNRDPDAVLNYNCSCNMTGIPLSPNIS

VDSIDTTNCTKFNGTLFGGGCDTPIYFGDIFFFSVLLFIGTFTLAYSLKMSRNAPVFPTV

VRSTVSDFAVLIAILSMVVVDFLVGVNTPKLHVPDDFAPTNPKRGWVINPVGSNPWWTTL

AAVIPAMLAVILIFMDQQITAVIVNRKENKLVKGRGYHLDLFIVAVLIIVCSFLGLPWFV

AATVLSINHVNSLKKESQCSAPGEQPKFLGVREQRVTGIAIFLFIGISVMLTSVLRFIPM

PVLYGVFLYMGVSSLRGMQLVDRISIWFMPAKYQPDYMYLRHVRTNRVHLFTAIQVFCLA

VLWVIKSVKSISIAFPIMVLAMCFVRKGLDKVFDQRELKWLDDIMPEAHKRAKEDEIIKQ

EEQRRSLIEGSTSIPLAMGLNMEHITIQADIDQVNISEEMSRTALWKSIMHNESSSNLIT

PTEESRSSTKQRKGKKKKSREVNGSERSHNSPAIMEEDEPAEGSPKKKNAPVKFYIDDHE

EEGEKENLIKAPEIVIDPPSDISRLSSPDQDDSKV

>SG-AL_contig_3603_1_-3_138_1982

MWTLWTLLCWVGVAMAAYDPNQDNTQCCVCSNLMPINPAPGSQPQVMPSPFKIIVNSKNY

HPDREIVVELVSEDNYAFRDFILVAWPTDNRPRQAVGTIRLIDENKEFAPKYSCYSNEQK

SFAILAGDLKRKKHLRFGWETKQFVGHVEFRATFIVDDNTYWVAEKSALLIDSRPDAPPL

PKSVQPKVLPPISTDGCGITKGCYREPEGCWEPQCEYIVTWQAVRDKVRFELGGLADSSL

DRYVAVGLSNDTYMGEDTVFACAHNSDSDDTGVYLSYNDGTSNIPLDRTNVERLKTALIS

SQGSYHNGRLRCFFTVERDLDFYYPELPHISGAPYHLLMSRGFASRGRLRRHGLGVNELP

IASPVKVDLISNHYNIGDRARYPLVKAHGCLMILAWIFFASLGLLMTKYYKTMWPNKRMF

DQRYWFVAHFNCMAMVFLVTVIGIILIFVEADGWSVAPDLPQRAHPILGIIIFICIIINP

ILALIRPSEDNKCRPVFNWFHWAFGTIANVLAIPQIFIGMDFGKANVPWWATWILVIWVI

FHIVIELSLEVHQCCTYKKNKERRRKYEQQKRENPKLHIDEPEPAGRRFKRFMLFLHLTV

TIIVTLIMIIIIAVA

>SG-AL_contig_3604_1_+3_1218_1583

MSSSKGTLCIFGPVGLSHNRMRHNLIVGRGGHHLWVWIDHHMLFKRGSARLLSFPSSLLR

SWLQGHRSLISAWRFSGTGPGPLHIRRFFFLCFNSAIDTVADNEEDDQSNNGEGDGCNSA

CG

>SG-AL_contig_3605_1_-2_2_205

FLLLSLFLPFLFPSFTVVYFFLPSWSIPLSLCQQVFTQVPPHTWFMMYYLCCLLWQLGSE

TVNTGIGT

>SG-AL_contig_3606_1_+1_1_207

HTPKVNKKKEMQKKKKKKKKKTSHGSHGATKRFHATLILRMSQMKRFAMRSQRPLELMIT

TSLLLKNVN

>SG-AL_contig_3607_1_-1_1_348

GILPNTILPPTLTTAAAAASNTTTTPAPLNSTGWCIFVYNLAPDTEDSVLWQLFGPFGAV

QSVKVIRDYTSSKCKGFGFVTMTNYEEALVAIQNLNGFTLGNRVLQVSFKANSRKP

>SG-AL_contig_3608_1_-2_32_478

MGVGAGDMSGVVGEETWIDNKLTFEPIVPPKKETKDSLKSGIQGIDHTMPFFSPAATSTV

GRPGKVPPPVPPKANSFDDSFSRTDYKNDRDGIQESRVEHKMVLSSADDEDFDYDAALAD

AIRSVTEFNPDMSVERIECVQQIEEVKDK

>SG-AL_contig_3609_1_-3_309_539

MFVKCTVNKFKVPCFEIHSSSVHAAVCYLKLSSIKNAEAWMFQRAFMSVYCVLLCLYVYL

KAWTYYFLFVLNGTRLD

>SG-AL_contig_3610_1_-3_12_221

MFGKCPVQRHMIITGLLLENLMYSLKKTVQVPILELVTDISNQDKKKNKKTYHTCMVAGR

DFSNTLSTKP

>SG-AL_contig_3611_1_+3_246_386

MRAVSISSPSLLYFPLSLKKITAGGWLHAHNCKVLPRGVIHPLVQAD

>SG-AL_contig_3612_1_+2_353_469

MGLNPHQSKKNTIGFFSPNWLLPRVEHSMGSVGRLEPHS

>SG-AL_contig_3613_1_-3_117_212

MRTQNIRYARLRSSKPCQCLVAYGNTKTPSIH

>SG-AL_contig_3614_1_+3_249_1145

MIRRMTTKGSDIARQLRAAIERVNAAAQRRPEHLQGRHPRLVAVSKTKPVEMIVEAYACG

QRHFGENYVQELVDKGSDHEILQKCGDVKWHFIGHLQRNKVNKVINVPGLYMVETVDSEK

LALALSSAWSKQSCTQPLAVMVQVNTSRERNKNGVSPSDAVPLSVYIREQCPHLQLAGLM

TIGSVESSTSDEQNADFQCLIKCRKAVSEALGIGELDLELSMGMSQDFEHAVEAGSTNVR

VGSTIFGARQYPPSQTDASTPLASGLQAAGPGGDSASHKMTKNTVDVTAAQLNDLSLES

>SG-AL_contig_3615_1_-1_133_546

MDVRFIISSERLVQTCLPLRFFRSFYDNLYETTVVWSCIKNYAFHVIKTMCLRYKCCLDS

FEGLAADTVMLCQAFALCTSYRSSSTMQRWRASKTNVAMRPMKVTMTAAKLVHQRRRRYR

LNSVSVENTWKSTSVTEE

>SG-AL_contig_3616_1_-2_80_2263

MSSSGEEEEVGPSVDSNDPEERIAARRQRISRRIEAAKRAALGEDAGDKKEIKEELSKSR

KQMEESRLRLSKLRKDGFELVTNIRVAGDAREANRRGVEEEAKRIRKEKLEQEAKSSAER

FEEITKKWESALQKEVPQSLHEMLLQQKRACDSMNDEKNKLITEFQQELKAKDDQYVKDL

KKMAEDVDLMIERMDEQINNVTKAHREELEEIERAFVAERNELLDLYKKRWEEKMALRGE

KEEEYMSARLKRVDEYEQQLQHLRVQDAEEYNMVKIRLETDVQILEQQLQQMRATYQLNQ

EKLEYNFQVLKKRDEENTITKSQQKRKITRMQDVLNGLRLKLAKQEKQYRDENAQLSEDY

KRITEQFRDLQKKSKHFMATDGKKFHEIWCMNEEECKELTGNILGADRVIHEQQLGLTWK

LPDLSFTENTGPLLGKTAQSRGVSASQIVQEVLSQTENMEGEEEKEGEEEGKPAIFSKIS

AHTIKQILELLCDESGFLVESKLNKLLQPLEKDERSLMKLDAIFTALGVETEEDIHKVST

YFLHLRERQVAAAASEEKEKGAKAESEAGSGEGDELDEEIRILTGKDGDSDSVQSESVDL

VHPNEVLKALRAFVDDNRRPVKEKGSRSQFRITALDERDCSEDSEYWIKYSSLLSEHKER

VWDALLDGLEKYSEVLNSRSGLLTETEALRTQNAELRLLLHQYVNSKVNGELEIPPTRVL

QLELNPGH

>SG-AL_contig_3617_1_-1_181_384

MGKCSGRDVNFSPCRHYEGGAHLLSLNALPSLTLNKVTQSQVGGLKTSYTKIFPKSDSNL

CSQIGRQE

>SG-AL_contig_3618_1_-2_2_79

PNKQTSKRTNEQISLQANKQTKISRF

>SG-AL_contig_3619_1_+2_2_67

FFENKIVAINMANYFISFAGYN

>SG-AL_contig_3620_1_+2_50_214

MSFNKTGTQIHLENNMSTNETCTQIHIIYKLFHLHPHSTVTPHHPSIHSKKLSTF

>SG-AL_contig_3621_1_+2_1067_1297

MFVGKISFPISRFHFKINVQDENATKYPRNHLTSLKPKMARDEKTTVLSQISFPTCGRRP

QRRQAALNMIVNGGHRI

>SG-AL_contig_3622_1_-1_139_540

MTQLKNELLHFALVLVFYLESTTVTVVFHQAKKHDGNYQELTNNRKLSLFKNRHFSLSLC

ILCALMYLFYYFFQENEKPVHCYNEEVLCIVCMCMINWIFCLSVFVCVCVHLAYELVCRG

ANPYSFTVLVSAQI

>SG-AL_contig_3623_1_-3_1548_1793

MMVWAVWSGGGCMTSYIIHRLHSVCVCVCVCVCVCVCVTHDSTGCVEWCWLYDQLHHPQA

ALCVCVCVCVCVHWTGIACCLC

>SG-AL_contig_3624_1_+2_674_889

MGWNGRISMLKSTSSIQYSSTLHSQPTRYQIINLHHHQFSLHYILNAHNIHISQRLCKPA

PNVMSTRYTIMK

>SG-AL_contig_3625_1_+3_39_341

MHSTVNFLLTDVHTLTTSFNGLEAARTVFCFIKFTVIINTFIQDIAIAFFCKQFAHTLNY

CYITSNKFFMYKVISYMNTFMPQHHTGFFLHAQFKFIKTPI

>SG-AL_contig_3626_1_+2_707_916

MSAKTKSPRRQKKVGKTVENNDNNVREISKQKKKKNLVLSQTCKCLNPLQGSCVYACTSS

NSEICMLEIL

>SG-AL_contig_3627_1_-1_70_2067

MASLANISAKVAQAAGLKTLLGTAPKCLAAKRDFHFRIHGRNTDSKVSTSQMSRDYPVVD

HTYDAVVVGAGGAGLRAAFGLAEVGFKTACITKLFPTRSHTVAAQGGINAALGHMEEDDW

RWHFYDTVKGSDWLGDQDAIHYMCEEAPKAVIELENYGMPFSRLENGKIYQRAFGGQSLN

RGKGGQAHRCCAVADRTGHSLLHTLYGRSLKYDTSYFIEYFALDLIMEEGECRGVIALCL

EDGTIHRFHSKNTILATGGYGRAYFSCTSAHTCTGDGTAMLNRAGLANEDMEFVQFHPTG

IYGAGCLITEGSRGEGGYLVNSEGERFMERYAPNAKDLASRDVVSRSMTIEIREGRGVGP

DKDHVFLQLHHLPSEVLKTRLPGISETAMVFAGVDVTRDPIPVLPTVHYNMGGVPTNYKG

QALKYSKDSGDQVVPGLYAAGEAACASVHGANRLGANSLLDLVVFGRACANTIISENTPG

EKIGAIKQNAGEASVANLDRLRHANGAIPTADLRLNMQKTMQNHAGVFRDGPTMQAGCQK

MDEIYKSMDDIKLTDRGLIWNTDLVETLELQNLMINAMQTIYGAEAREESRGAHAREDFT

KRVDEYDYSKPVQGQTKKPEDQHWRKHTLSYVDEATGKVTLEYRPVIDGTLDEKDCPNIE

PAIRSY

>SG-AL_contig_3628_1_-3_3_95

DIFSHNFPYIVSRRSCHVRLYTVGIFSHNFP

>SG-AL_contig_3629_1_-1_52_168

MSILQCYLPMWPFLCIFCCFEVFLIIFYAFCNLGMIDQA

>SG-AL_contig_3630_1_-2_362_634

MGAALVMLVLRKVEVSDTWTPIVAQVIQTPVLTNRAVGPVTVTKWLAQRTYDESLCEFFT

PTGSYPGLNVLWAHWEDWEHSSSLIHFMDAS

>SG-AL_contig_3631_1_+1_1_198

HRSCVTTPLPFILSLQHSSQATETEVTMRAEHERLQNLGQFFHKGMNCMVIRMGIIVMML

WSSISD

>SG-AL_contig_3632_1_+2_2_160

LTHIHIKILNMSTRYRYTTSFKLNNPNCWTSTNDQNQEGTPIKQHFNWIYQQV

>SG-AL_contig_3633_1_-3_54_509

MLKVILNRLNPQAEKIIAEEQAGFRPGRSTTEQIFNLRILCEKYNQHQQDLYHVFIDFKK

AFDRVWHEALWATMKLYNINANLIKVIESLYNKATSAVFFNNNIGEWFRTTVGVRQGCLL

SPTLFNIFLERIMTDALEDHEGSVNIGGRTIT

>SG-AL_contig_3634_1_+3_15_341

MHKNKEKHTKRERKGSYILSLLNIITHSWYLEHERQHFIKMSIQILPKRIHEQNYRVFKF

LKNKGKKTGLVEIIFSPFTQMSWNPIKLTNHICSDDMHDQTKASLILYI

>SG-AL_contig_3635_1_+3_1314_1520

MQYGSRSLCFWPEPTKSLRNFVHNMQFTPIQYGMNFQPHNLIQLAMAIFQISHAKCERQM

KSEVGNKKK

>SG-AL_contig_3636_1_+2_287_919

MSGLRAPKAGSNREAQAKVDASFDPEDAKKILHWIQAVTGAQFPIEENNDRYRMMENFYK

TLKDGRLLCYLVNAFLPEHMKLDLRSKTFQETKNMAFEAARERERIELFIIKIQEFGVPE

QDTFQTDCLYERTNLNKVIGCIRVFGMEVESHPSYTGQRVWPRKSEANPRNFTEEQLRAS

QNVISLQYGTNKGASQAGMNFGKKRMILQNE

>SG-AL_contig_3637_1_-3_735_983

MAKKKPFNLPRRVTMSHAFYLSTSHNSFELRKTEGERKEKKRQKTKEKRKKREKKGSTKM

GSLSQLPVWLVYLPCQTSANPRQ

>SG-AL_contig_3638_1_-3_30_317

MPLSRQVFSVLVLIRLLPLTCASFLRQPFMVHTKYLLPFKVGLNCNVNSRTILCVQNFFM

QFMVTGECYKSLKILLQMSLHSKMKRCSKAERRQKK

>SG-AL_contig_3639_1_-1_412_567

MATMGSFWTWQQCFAGTLDTGPQTQDNLTMGKKVLVKFHFLSKKQNKTKHTH

>SG-AL_contig_3640_1_-1_142_1176

MAKWGEGDPRWIVEDRPDATNVNNWHWKDKNANDWSKDKLKELLVGLKIENDTGSCEIKE

LTKTEGEAYVTNRKGKLAFFYDWDLSGSWQGKLTDGQKNVKGTFEIPNLGEENEIHEVHV

TFIVDKDNEEARAVKEFMRSEGDKAVRQQLEAYVTLLKNEYSQGVILPSKKADSIPKEQS

NKENIVKREMNKVVESAPSSSKTSNNIGVRIPTTTVTMKETFNCEASFLYNALITQDLLR

AYTGGESTVDAAPGGRFALMGGNVLGDFTELVPEKKVCMRWRMKSWPDEHYSNVTINLEG

KEGTTELSMKQTGVPQKELDRTREGWKVNFWERMKGTFSIGNNIA

>SG-AL_contig_3641_1_-3_111_545

METPFKYNPESPPYTSFFGIVGVTAAMSFCALGAAYGTAKSGTGISAMAVMRPELIMKSV

IPVVMAGIIAIYGLVVAVVITQNMSGDESYTLYKSFLHLGAGLSVGLSGLAAGFAIGIVG

DAGVRGTAQQPRLFVGMILILIFAE

>SG-AL_contig_3642_1_+1_37_231

MMLTLAPWKRPLLHTDVDRRPKSMATILLIKLPLCRLYQKPKMDKYLHDQYHRHTHDTCA

MRQKT

>SG-AL_contig_3643_1_-2_2_157

PNQSSFSRGSNARKRTGASDFELPSGHPPEGTLHCFWVTLMEFNNACSDSTY

>SG-AL_contig_3644_1_+2_77_268

MENSKAVINHFPQMTKTEFIPKHYALEFILKHYTLFLTRENSRNTAKRQGKYPASGYFPL

NPLS

>SG-AL_contig_3645_1_+3_150_539

MTSWSETDSAKTGLLTVILSIIYMSGNVVKDGELWHGLKSLGIDPDRNHETFGDVKKLVT

QEFVKQAYLEFSKLPNLEQPVFEVRWGQRAKLETSKLQILRFVSLMYGKDLEQWTTQFQD

AKNDTQQPTT

>SG-AL_contig_3646_1_-3_18_275

MNDVGGPLMCPCIILTYLYTIYLSGCLWMDICGLDSAHSVWLKYEVDGYYLKNNHSCGTY

GICFSTTGLILLKHILMLPAICLKIT

>SG-AL_contig_3648_1_-2_2_211

SHLLYHFNGIDSTPLIPALQDTTNPKFIQSKSQCQEAHGIQQCQAVAGPPTRKDSALLPD

HFGRVQRCLF

>SG-AL_contig_3649_1_+2_14_907

MSVSSRLETWKMFWSSVPMIAYLIIVLELRPSEVHGMKIPGIPPPPPAAAAAARSLDCLW

GRGGGGGFASSSPEPSRSENKGSLLLMIRSNSLAVTTGTVELPPSGSGRRLRVRDFLSPF

SLLEDLVLRGSFTVLLFPSLLQLPKSPGCEQSAPPAPSVLEARGELSLDLGFTFFFFTFL

LPFFFTLPPPSVSVASPGSGWEPWESSSDRVEADSWESWERSAPRQSVLYSSTDGSRVLL

PPPITNTPLCCCCLRGSLADGGVEKARGRVGVRARGGEAAGPGGTGPAPGCSLHSSSL

>SG-AL_contig_3650_1_+3_771_1079

MIILPKQTFHGMNRHTLLVRVTKASKSLPTCTHSFHILCLSLCCCQPSANKCITDFVSLF

IHYVVLSLQLFFLFWVCVCVCVCVCACACVCACVLLFVVCVPH

>SG-AL_contig_3651_1_-2_755_931

MFHSKNLWEKICEFEHREQTVCDSSVFLYLVCGPCELNDGVCKTRTQPVVCGIPCILSL

>SG-AL_contig_3652_1_-1_205_489

MAESARGMSRGRSRWGGGGGGSRFGGGGRGGGRGGMGGGRGGGMGGGRGGGRGGGRGGMG

GKFNSYGGGSSSYGSTNMAGSSYQQKSNSYGQSWS

>SG-AL_contig_3653_1_+2_197_355

MVERQNTIFTLNNHTKPVCWYMDHLSKIMVTQDCFTLDDLPVGANVKMKVKVT

>SG-AL_contig_3654_1_-3_360_521

MREITVYLLGVYDAKPLSCCLMYQNIFHFLLISPPFTVSPTHMRICIHTHVNTH

>SG-AL_contig_3655_1_+3_3_152

NECHDETVRTVNERHDETTSMVIGDNTDAVCKLTTVGSKDWIPWHAQCWA

>SG-AL_contig_3656_1_-1_61_1401

MAGTVSTVSKFVSRFGCSIQNFLAPTAISASQQRNYGVGSRIKVDNPVVELDGDEMTRII

WEKIKDRLIFPYIDVDCKYYDLGLPYRDQTNDQVTIDSAAAIKKYNVGIKCATITPDEER

VEEFKLKKMWLSPNGTIRNILGGTVFREPIICKKVPRLVPGWTQPIVIGRHAHGDQYKAT

DRVVEGSGKLELVFTPEGGQPEKLTVFDFKNGGVGMAMYNTDESITGFAHASFQYALMKG

WPLYMSTKNTILKRYDGRFKDIFQEIYDKQYKAQFEKQGIWYEHRLIDDMVAQALKSSGG

FVWACKNYDGDVQSDVVAQGYGSLGLMTSVLVCPDGKTIEAEAAHGTVTRHYREHQKGNP

TSTNPIASIFAWTRGLEHRGKLDGNKELQRFAQTMEKACVDTVDSGKMTKDLAGCIYGLK

NVKTEHYLYTMDFLDAIGEEFERQWKA

>SG-AL_contig_3657_1_-2_119_4126

MPVQHGYECRCDPGFTGQFCEKSTTFTFSSNGSQVTYSPSSNQVTDEVLSFRFRTTLSHT

VLAVYAFSESNLVSVEISGGKLLLVFRDDISTFRQTVDSQEHVNNAKWHQTFLHLQDNIS

LTLVSDACGTEHCAQQYSYPRGKPQTSTATVYFGELQPSLLAKTATSSIYVGCLEDILFD

GVYLFPGQRDGNYKDVTQGCPRTVQCDPYTCQRHGNCVDLWDRFRCDCERPFWGTRCENK

YTAATFFKDGLPSIATFSLEVLADTFVSGLNLSLFLRTRQENNIIMYLTNNSTSSSSMTF

ITLEMQDGCLATRLELCGNEQYFFENKSIVDLGSLNFVHLTLQNDVLLLWVNESVCINQT

VTRSSCQFRASTVYFGKVPELSVSRRRRQANPPSLRQLPPYNGIITDAELQNHKLDFEPR

AGAHEVNVVLMSDQSNVTLGAKSTISVCNTSQPCENGGNCSDFFFNDFECQCPDGYLGKN

CSQPDFCKPNSCPSGSQCQSLQLGFECISIASFNGISSLITYTATLPNGLPSQRVTFYMR

TRSFYGAIFEVHAPLEQNNFIKIRVRDGSLEVNYLLKSYGFSSSQRMHERVDDGQWYHVE

MEIDNNAMSLRLSRNSTIIEEKGPVTLEDTVTNLSNLITSGTIQVGDVADSKEQVFNGCL

EELRVGGILLPFILDSTMVLNTATQKFSATLSHVRPGCSGSNACQGNLCMNGQCKDTWND

YKCECVTGYQGQFCEREVNECLAGQCSNGALCMDGVASYSCVCPPGFTGTRCETPFDACA

NNSCANGSTCQVNPNISRGYVCACASTNFSGDLCNVNIENKSCADNLCQNPGTCTDDQNT

STFTCTCAPGYTGSLCESPVNYCVDLPCNNGTCVSDAAQQNFTCQCNTGFTGDVCDINKN

NCTTTSCFNGGTCVDGINKFTCDCVQGFTGPRCEENISECARNVCQHGQCVDLSPGYICD

CNNTGYTGENCSLELNECDHNFCLPQTSSNCSNLEGSYVCECRLGFTGQNCSEVTCDDGQ

CKNGSTCGTVEATWGTDWMCHCQEYYGGKQCDMPGPCAAAPCNASNTQQCNQSDDVFTST

DPNVTSNFTCSCHQGWEGDLCSDDVNECSPANICQHGASCSNLPGSFKCNCTVGYTGPLC

EIDIDECAPQPCQNGGMCTQGIGFYTCNCNGTGYEGDTCSQDINECEHLPCENNNTCINT

EGSFTCKCGEQYLGERCQHHNPCSSTACNNGGSCNHTWDQNQGIYSALCTCINGYHGSRC

EKSPSTGDDDIDIAVIIGPVVGVILVIIIVAVIIFVMVARNKRATHGAYSPSRQETAGAR

VELGNVLKKPPEERLI

>SG-AL_contig_3658_1_+3_174_323

MQYEIFSFHCEMVNETLMLHSISPLQAFQLTADWQLFLPTTPKMVQTDRQ

>SG-AL_contig_3659_1_-2_863_2848

MTGIKMGNQLSRQQAARRWSVLREELTFPEPSWELYDGWEIYHIDIELQTLPPHVRDKIL

DFCVDEYTQEIISCEGGECTHKTHLCSKSQPCSQKQDSSDNIRLQDDVNSTEEKCLQDNG

DVCQNICVEGSESPSGLNNRGDHKNELDELLLQWNLCLRESSGSESYSKTDSVQSRSHDV

SSSRVDPDTVENREEVSASNSVLVNAELKSTVQKRESSSFCKMHEESSSSKLSSCHFKSV

HRRCLSEMESIDFTTLCPIKDPGCCSKDNNQCSKSLPQPVPTSKPNGAMNSLQSKLLACT

GNFDDISPFTSSSLPTRRQSTPAVRTRKQSSLPNSPACRKKRKFSLKDEPGWIPNPLDSD

SDQEQSEDSGVFSDYAASPKQAPNLCAELLAHKAVVRLKERRRHNSEPSGTTSTNSSLSE

NHSDSENNKSSILGTRRHSTSDSKWSLVKSPWRKNKSKLLDDSKLSVPTEVEEGDLEKEL

EWLVSDPDCETSENKDSPEEDVPVNDDSVSPTNSSNTDSSVASISPLYRQPAGALQQELE

LDWLCEPEWNNLELETFWNVLRPHLEFLLQQSETEHLPDPRVVVACMDSADQKMLDSCLL

HVQRHIWKGNYRQAVADFRAVKRHFRCCANSRLIQRGIQQDDVESLRLVFLRKRPSRYRL

WI

>SG-AL_contig_3660_1_+3_243_563

MKEYSTKPRFSSTRTYLFIYLFIYLFIFHLNYIYIQYITDIYLKSMYTLVSVIDCLRIYL

FVCLFVCLFIHFLHRSLRKSLSFWWYRPDRSASVIRLMSRNSPESQR

>SG-AL_contig_3661_1_-2_2_244

FCHTNASPAQDPATFLNTYLCFKKGRFQLSGVCDFIKLQDLFSCVYKVCLQLAQGIVFNL

RKADVFIPPVSTIITANSRYV

>SG-AL_contig_3662_1_-1_1042_1476

MSDVCLEWSRYIDALRDVVFHRLYCGNTWFHMMDGHISMKNVEELKCHGKLKSILVMEDH

LFGGDSGFHQSKNEEKLPLGYDLFLLFVLIQSIGLLKSMLCCCLFVHSESRLVWRIALYK

NDLLLLLLFFYYYYYYYYYYYYYSD

>SG-AL_contig_3663_1_-3_54_239

MFGFDAFTRPYASYSSSQFALMSLYSITIRTCFRDQSWHLKNVEWHSSRLNLLSFPLKTA

CV

>SG-AL_contig_3664_1_-1_7_306

MCFVDMVIGLKFVCVCVCVCVCVCVCVCDRLLITDMKYSYFRSAQRMNIKISEWFNSFDL

VAGVLLVLEICFDCLLCYSTIDCVCVCCTCCSFSRDIVYL

>SG-AL_contig_3665_1_-1_1144_1353

MVQGRQGHRPQFQFQFSQDSVSMLGVNSICTNAANATGWKRIQTDNGISRMQWQGLEVTI

QFQQRQGNRS

>SG-AL_contig_3666_1_+3_36_290

MYKQTNKTISNFPHTAKCIRHFRSLAKTSILFFKYMYTPTQTLSVSFPHAHTHPSMHTYT

LSLKHTHQTTSINKCVIRNVTTPNV

>SG-AL_contig_3667_1_-3_87_1241

MNDGTLHIDTSQRMLAPNQARSPSSAGSPHSPGNINTLRQELSAAHAMSGAVVHPQSVTG

QSSPSVYFGLSRRGSLSSIADSDTMHATPKFVKDTSKYWYKPNITRDDAIALLRDKAPGT

FVIRDSNTFPGAYGLALKVAQLPPNVQAKPTNDPQAELVRHFLIEPTPKGVRLRGCSNEP

VFGSLAALVYQHSITPLALPCKLTLPDIGADMVDMGAPISPEVSSPVEISMAPDVLAKGA

ACNVIFINTVDTESLTGPQAITKAIKTTIDSQPALKTTVVHFKVSNQGITLTDNNRKLFF

RRHYPVSAITYCGMDPQDRRWKRDVDNPNVQMDAKIFGFVARKQGGGSDNSCHLFAELDP

EQPASAIVTFITKVMIGQTKVKSYS

>SG-AL_contig_3668_1_+3_192_503

MTTKRTRTQAGHLMMGDCPRNLCLDGVCLWWRVISAGVFCVAESCSLALSWLLPSARGAP

HQQEHWADQCPQVSLFSSCCYSTQSCRDCAYVLVEGEVVPPLLA

>SG-AL_contig_3669_1_-2_2_202

GSERVKLTLNPYSSKPSLLHARVHLMSCLAHHHHGVFLCKLQYASSAVLFGDPCSVVWHV

GRLGSGV

>SG-AL_contig_3670_1_-3_3_269

VNKLRPTNSSRSKLTHVKDVAEKAKKCGVVCHIQCTGCEDYIGETERPLDKRFKEHQIIS

ESSFHKHLVSQSHSTHLDETSVLSTETHY

>SG-AL_contig_3671_1_-2_2_193

ELFIRDGTVFCSSKQNIDGLQMEHQSVKNKLVFNFISNWYIVAHIESWAFCLFFVTDRSK

CFLK

>SG-AL_contig_3672_1_+1_127_357

MPPATPDRGEKKCLIRIPVRLLDTRSTTFTFTLNLPTMQYCFCFLPVLHSSTSGIVFSLA

KWLKIVTWNSIEYVWRQ

>SG-AL_contig_3673_1_-2_842_1147

MEWGLCSSSSSITIVCLLSVARSLQPASPSSAMGQGGMSLTKIDQLPSRPRKLDRSTAFQ

ALQARSLNLLIGLPSIWSEPYPTRTCSAWVALPRVQARLQLT

>SG-AL_contig_3674_1_+3_3_125

FFFQNIYDFIHFNFTTEWTCINCDFESITKTKDWQSLNTIN

>SG-AL_contig_3675_1_-3_3_284

WLPVQAWLDYKLSALCYNFFSETAPAYISDLLTVYTPSRQLQSSSLCIPHVKTKTSGERS

FSFCAPKQWNSLPSVIRHLQCLSSFKTALKHRLF

>SG-AL_contig_3676_1_-2_2_403

RSAYLEIHCISVICPYLTTQAAKTLVCAFVLSKLDYCNSLLSGCPQYILGKLQKVQNSAA

RLVLKARKYDHAQPLLYKLHWLPVQARVDYKLSTLCYNFFSKLPLPTPLIALLYILLLDT

SVHHQMNAFSASLK

>SG-AL_contig_3677_1_-2_2_178

LFRSACVCVCVCVCVCDKTQSKRSRPVLVGLEPRPSVLFRNSFVSNLIYQLSAGSWCIA

>SG-AL_contig_3678_1_+1_64_261

MQIQCGNKSLFIRLHQHNYVVNISTIFQEFKKTHKRHDMQFVISCIILILKPKHILIHQY

TPESLY

>SG-AL_contig_3679_1_+3_315_419

MKTPSYKQITTTNNKKIQNQTRTHSQLINMKDFNI

>SG-AL_contig_3680_1_+1_1201_1464

MAISITTFTWQPPNIYSVHVAMPTIHTHCNFHFPVTCTLLCIKVRRIIIHPVTVILVTLS

ELLMVQCSAHSYTFLCGVNTHAHTHTHT

>SG-AL_contig_3681_1_+3_3_251

PHRAPHPHPPTHKHTHTPPPPPPHTHTHTASPHNLTIQELKVGEPSVLCQSTHLYSLCIC

LSMSMIRAQIYIIHLSIYVYGQG

>SG-AL_contig_3682_1_+1_850_1059

MEPVSQSAITETNQPKSKNIKAFPLLSRFINVFICCHKLSSQVQCFFGRFYWCYKKDMQY

TKSKTLTTSS

>SG-AL_contig_3683_1_-2_98_382

MACTSLIVLPADVISLWLMVDWTTADGTLRQRSTWLQVPYQSRPRPSQTFLQKQPTPEPS

VEETARSETLMVELITEVNMPVTAIRVLGYTSEQL

>SG-AL_contig_3684_1_-2_2_229

QSVYSIFRGVHAGCFPVSLSRQTPIWLMGSLMCVFDVLRAYALKGGSGICRSAHVDLGNG

ENLHPLPTRSDQAWTL

>SG-AL_contig_3685_1_+2_62_286

MDCSLCVCVCCVCIHGLLSLCVLTVRVCNFGLLSVCVATEVCGDLMKYSLSLSLCCLDGS

LSLSLSLSLSLSVLS

>SG-AL_contig_3686_1_-2_686_793

MKPLRLLYDDGFPHFKFNILMCSSIWISQEYSTLLK

>SG-AL_contig_3687_1_-2_44_481

MTAMEQAPSEQQGPAEGQQVPPMEHQMPTQEQGSPVTRAPAEEQAHEQEPSTPAEENVIG

EDEKTPADKPTSAEEPAPTEEPTPAEEESLPVEEPKPDVPESDTMPPLPILAAATVTGFP

AAAVSGATDSSAETAEASEPATLSQE

>SG-AL_contig_3688_1_-1_52_201

MSTHDDAEITTTSSHASTCFSLLKMVSQCLDDSIYASPQMPWDGTSKIIM

>SG-AL_contig_3689_1_+1_223_366

MPKINAYQMVILQVQTETSSHNSSFQSPQVFFHGKQKFPQHRGSRSVL

>SG-AL_contig_3690_1_-3_45_413

MFKDRFKMKDMGKLSYFLGIDFEQGHGFVKMNQKRYTEKMLERYEMSDCKARGTPCEQKL

EWNESEPVDSHRYREIVGSLIYLMMGTRPDISWVVTRLSQFLSKPLQCHWIALKHVLRYL

RGT

>SG-AL_contig_3691_1_-1_1_366

QCSVGSLARHAQKWQRLHLSDWVVGVTEDGYKLPWEGAKPPLRPFPPSRRQIPHDQEAAL

ALELAYLIHKGAAEALPSPLGAWRLRQTPCSPQILGWLAPVLDLSPLNEFLVRTHFKGRP

RP

>SG-AL_contig_3692_1_-3_18_347

MTAVRQPTPSRRPVLGGEPGPPRPGVEAAAPQRLGGGSHRGWVQATLGRSEAPAQAVSSF

PAPDSPRSGCGPGLGGADLIHKGAAEALPSSLWAWRLWRTPCSPQILGWL

>SG-AL_contig_3693_1_-3_3_227

AYCIPHEDQYAYCIPHEDPHQALKTSMPTHPSDRQESPSSYHVFVLIQGFPRQNESCCPP

ETVRRHCTAPPIVFL

>SG-AL_contig_3694_1_+2_80_226

MIGNKGGVVLDAVDLTMNRVINYPEEDYTLPSTLTGRGMVTLEVGVVEN

>SG-AL_contig_3695_1_+2_542_871

MTTFVPWQLADTIHNAICIQNNNATLNTSSMKLNCDQAIKTIQIHTDNTHRPIQYQLSLH

LKLTQETDQYPYTFHSMTIALFFQHTVILSATITLISIHVLSQKMFTFHL

>SG-AL_contig_3696_1_-1_157_1293

MYKLDLPVDYKEAAAIERRRQMEDERKTRIFNAKSRTIGLDRQAVDQQIFDKKQYEEYER

RRHEAFAADAVRNDMIAEMLQKRQEEDVRALARAENEFRSLHQQPSSRREFDLYDPDALK

KDKPARVHDDDPRCGISSLQKFDGEDLNEKARSKFQAEQARQWFNEQMKEKEQAKQNQAM

ADKLYELKMRELDERACDLAKAEEDCRRAIQMATKDYNAAQDRERKEKERLKKQQDLDDN

MTEIANHVYGDILTENPSVAQSAFGPHRVIPDRWKGMTPQQLEKIRQDQERQRLENERLR

EEEELRNREWERQQNANARAGLLLDREMERKRRDLERQQADENRRLALEQKAHQEFLDKE

VYTNPPTAGYFMQFNTTTR

>SG-AL_contig_3697_1_-1_79_294

MEGENSYFSLCAFSASLHCFQCPFLFSGFTCTDVLTSVDRLDTLSTSRGFDLKWVVYVCL

YLFLGVYKRVRE

>SG-AL_contig_3698_1_+3_774_1037

MSNAVVIISVIFNSFNNKFTFNTIFSILFKIHNNYEQKYLTHHTQNEKDRQYSFSCSFTE

ATLGQHSDKAEAAPVTIRVQSVHKPVCR

>SG-AL_contig_3699_1_+3_54_392

MPSTVQSLPFSASNGAHAQEGPHHWVIVPDSDQLVTSISVTKELSSDHFAVLASLNLSKT

PRNKRTVTRRNLKSIDLNVFTSEATELLSQVSDSTDSATLYTSILHRLLDKHA

>SG-AL_contig_3700_1_-1_1_165

IGGKKSPFGKKETYFKKKKKKKKKKERERERIQVHEGVLRFSNTSVVLLMKCEEH

>SG-AL_contig_3701_1_+3_3_293

TTTTVNIDTTSVNSYKQSWRSTCPGEDDKQKRDALKLQSSIIRHVELISHCHFHQHNCVQ

PFATFTNIIVLSLFVIYTNIIMFILLCVIFTYIIVFS

>SG-AL_contig_3702_1_-3_924_1586

MDITNNEEQNEATVESSKAQSIPSYENTKAMDVTNDEKHGDVFTEIESSETQPTASSHDL

QNVDFGDETTTHNENCVLQSTNTDPLKDQKVPEQHTDPKDMILEDEEEEVMEDFDQDVEL

FIREDGDESIQLPAVRKDVVEEAKEEGKEEGKEEDGEQPQYLQNVVNEEEKEEGKEEGKE

EDGEQPQHLQNVVNEEEKEDASSSPDGQSVGLKEEEQLTQD

>SG-AL_contig_3703_1_-1_10_906

MASKVEVVAAVLSVLCFALASSQPVPVVIWHGMGDSCCNPLSMGSIKNLIEKEVKGVYVR

ALEIGNSIVEDMENGFLMNVNDQIDLVCSKIKNDSKLANGYHAIGFSQGGQFWRAVAQRC

PSPPILNLVSVGGQHQGVYGFPRCPGENETLCDIVRKLLNYGAYVSFVQDHLVQAEYWQD

PLNLAEYRQYSVFLADINQERKVNPSYKENLLKLKNLVLVKFLQDTMVDPKESEWFGFYK

PGQAKEVVPMNETQLYIEDRLGLKELQDSGRLHFLASDTDHLQFKETWFIENIINKFLK

>SG-AL_contig_3704_1_+1_394_609

MYISHLKFSIFCAVTPWKIFFLDRNPSVSGHQIHVHFTSKISTFCAIKRQQKHRKQHIDL

LQFSITTRPKHD

>SG-AL_contig_3705_1_-1_202_498

MSKTVVKYDRTSIAMSKTVVSPLPQAGQQTNKNKRITILTFLQHPTTMQKIILSTFFYIY

SIYEVCFINLETCNKNSTFISLIGKQTFHDYIAITNIDI

>SG-AL_contig_3706_1_+2_2_94

PGHPQGSFSPINYIIDDTISIDHLYDMSNPK

>SG-AL_contig_3707_1_-3_72_1022

MGGKSSKQKKSKKENGENVAAENNAENQPAEGQATEAKAEGDNKPADGATPATEEDKAEG

AESAEKAGDGDTAKDTPAEEAKTEDGGGGTETSKEEPPAAEAAAEAAPATEETKEEPAKP

EEKESAPAEEPAVPAEETPAPAEEKPAEETPAPAEETPAPAEETPAPAEEAPAEEPAAPE

PEPEAESAPAPPAPADDATAASPSDTAETGAADSSGAAGEASEPAAPSPEQESGNNVHKA

EFKWEEGGENVLVSGSFNGWGEKIALVKNGEVFSATIDLPKGEYLYKYLVDEQWVINKNQ

PTKADDEGQENNVIMVA

>SG-AL_contig_3708_1_+1_118_267

MQEPFLTSLTLGLKLHTLCKPTEHCRISRTLFKMGHAEETNTRKACIVSF

>SG-AL_contig_3709_1_-2_44_2188

MGLLTDTKQRQVLTRGIVRYHQKLSVLFYVVGVVWFLALAYRPLSAGTYFSENALLPGLV

ESDLPHGFLSLSSYTELLKAEINKDKKVVPKEWIFKQFQDLGLDTYRHNFTIKYPFKMAI

SQDLVGENIFAILRARRAASTEALVMTAPLRSAGNQKTPTDGSVALMLALAAQFRNNPYW

AKDVIFLLTEFEEIGVHAWLDAYHESKTPYVIADDLPGRSGAIQAAINLELPSDKIRYLN

MKLEGLNGQLPNLDLFNLAVRLSRREHVPATLHHRRDPQIQDVETMEGFQHMLGTMLHMM

WSQASGAPSGNHGLFHRFHIEALTIEGVHKKKGQRTYSLEQVARVVEGVFRSLNNLLERF

HQSFFFYLLPSTDSYVSIGVYMPPFGLIAAAGIIKALALWIITHTPEEDKIDQAASDNSK

EEARKADDQKDEKEKDKKQKEHDMTEEDEKAEGDTSLLKETAGVKESHGESEERKEKGED

GQGDTAEEELTAEDFDLDGEDFESMPRTGLLTVLPVVMMAYLLGLMAYSAPYFLTHFTPA

TKMKAADVILYGILAIFCAGLTYPHLMNRKTLPEDRIHRDWRLLKCVGLIMQGLTLFAVS

LMNISLAFFMAAVSVPVTMLVVPTNNFILRTLQQLALLVVSPMGLLFIAATVNTAMQSPD

VPWVWVVWQAIDEAKQMLFLLLVDQYLFYSWTYTLVSFAFLPTWLLFWAVPSSDP

>SG-AL_contig_3710_1_-2_236_421

MTLGRTILDLEQSIIEVYIFLDFFYMSLNLGFFFYLVQLARKRSLSSVTPHTVHFLLECL

CI

>SG-AL_contig_3711_1_-2_26_1750

MLARAVVLAAVVAVCAGDLTYKIISKKLFSFEILLAFTSPRDISGWRMRVTMPSRTKISL

VRTWDGDWTRERKNTGYLQNMDSTGPMKAGETITNIMTVTCDGYCRFPFELQVVEPQEGG

SISWDGVPASGLGPPIGAGQGTKDYADALKKSIMFYNTQRSGRLPDNNPISWRGDSFLDD

CVPGGWFDNGEDFAKYGLPFGFATHALMWGLYQFKDGYERAGQLDEMYDMIKWATDYMLN

AYDDSTKELVAQIGANDNHHSPGEWDRPEGVVMDRRCVKMSESRPAPELYGEWAAALAAG

YLVWTDKGDTDYANEMLAEAVSMYAVGKASNRTEYNDIADVDYFYHSDGYEDELCLAAVW

LYRATGDSQYLKDAKNYHPSGSFARYSWHSKQVACQLLLYEETNNELYRKEVTRYIERYS

TGPVAKTPCGLAWFSIYKPNSLAAGAAWIALVAANAGIDADDSRKFAVEQINFLLGDNNR

TADGCFSYQIGYGNKYPLHPHHRGISMCDGCTLYGALVGGPDVGGFHRDTQADHVMNGVS

LLHNAAFQSALAAIVQLQKEDAVPGTQSLCTCPSQ

>SG-AL_contig_3712_1_+2_2_244

CFTFPNPEGSQVPIHCWVDLRLLQFYPIQHQTCIVHWKAGDLPLRQPGLTALHSLLPVFS

IGIAHNHGKLMKMTDQSRLTR

>SG-AL_contig_3713_1_+2_569_862

MCVYVCVCTEGDTMSLQGWLWTVRFMKRSKFLNLDKCRMLCPLGTKINGSANTLFQSDIC

PGRRQYNHTFSVLIGLCPSLTPGCITRFHCTHVMIFIL

>SG-AL_contig_3714_1_-2_92_235

MPPCACVCVQYFYVSKKPNVLSAIFQHMRFSVLESQSMVTLNVAYFAY

>SG-AL_contig_3715_1_-1_130_306

METWSCCAILILLSVLVFHSLCGCRLTYPWFTSSVCTLMHMTHTHAYDLCVWEPLEDSN

>SG-AL_contig_3716_1_-3_192_1148

MVSAKVVVLILIVCIIIDEIEAQRNRTRGAGRNRNRGNGNRNRNRGGRNRARGNNRRAGG

SRRGGRRCEEIQVPMCKGLIGYTHTKLPNRFNHTTQLQAYRVLEHLWAQIDRGCSQNFRL

LACSLFLPRCAGRGPAREPCRKTCKITKRVCESRLEQFGYSWPDELDCNTLPKRRCLKHG

PHNSCTIDHTLCEGIDLPMCQNLDYSVGMIPNMFGQCNRTEIATEMGQFQPLVESGCSEN

IVFFMCGVYSPFCTLTEGQMSFPCRELCEQVRTDCEPEYTRISGGLPWPNKFQCHRYPSA

DNANYTCVMPNEGGALALT

>SG-AL_contig_3717_1_-2_2_94

LWFSSLNSESDLSLFFSVDGHYVMILSWNRN

>SG-AL_contig_3718_1_+3_537_875

MWKCQITRKKRSHFLHVEVSNHPEKRSHFLHAEVSNYPEKKKPFPTCGRAKSPGKRSHFL

HEVVSSYLEKRSHFLHEVVSNNPGKKGFPTCGSVKLPQQKEAISNMHMRQITF

>SG-AL_contig_3719_1_-2_2_76

LFKDNVLIEEVYSCVCWVGCIFVER

>SG-AL_contig_3720_1_-3_21_1919

MAKVCSVLILLAVAVVGKALTPSTFLTTVDQQRLRSVFESAAPYTDLPSAHYSILGIKLL

GGTLPNSQDTCKFLQSKVEAGSIASIYHASGAAAALGGCKFDVPSAKQLLTDAIAAKASV

QDVSFAFFAMKNLGLTVDEKTVSASLLEALKSDDSPLSHGYAFMVASELKADTSKFFDNI

EDVIAQADEVDDKYLQFEGGLFVTALVVDGAYKLAEKAKKAPTIAEDKVAKLANYFLSRK

HVHQLKAAFQFVSVIKTLTINKFHIPIAVSLDSSVAVSQSEPVVKVRVTNLLGGELGQLT

VTADSARHLGDEAIVLSKKQFTPSSSEKAQYELNFMEAKPARGFYRLTVSVAPKKADSRL

IGTTGAEVEVKVTTKVSVENVEIGVADKDQTAAARTTKLVYPNKAQNTLEADYHQKILMR

FQLKDRLTGQLMTAHQTFVRLTNLKSKQEIIFVSEADGALNNKFDLDVGASAKEFGYQSG

KYSVELIVGDAIIENPFSWTLADAELTFPEGGAAKGDGQNQYSKKPEIKHMFREPEKRPP

APVSMTFTALVLLPLLILLLLWLKIGVNVSNFPFSVSAIGFHICLAGIFGLYYCYWTQLN

MFQTLRYLGILALPTFLFGNRLLSGIASKRKSA

>SG-AL_contig_3721_1_-3_9_3269

MADEENSVPQAAEEPAADTTAPAGDNMEQQPSSRPQSATKGSRPTSARAKPSSRPGSATA

KPASRPTSATAERPASATKPSSRPTSASKPPPQPGSRPASATRPASGRQGSRPGSAAGSG

AGSQRSGGSGGQEAAEEPLMEEEGGEEDVNDAEEAEEAGPGGEATEDFVPIPEPTALVPI

PQGQPGGGDDGDGGDDDDDDDEEDDGEEMEDSEDGSENETEMVVLDPDHPLMKRFQAALK

SHLVKQDEKITLELRELDESLKTRKKEREELGVELYGVQQELARYQMSLEHLHDDFTQKN

QQRQREEQQLTEVRSLYKDSQLTFNKEKKKGADLQGEVENLALRLFYMENAKEDVRSDIA

VMRRAAEKVESEVSKAEAEKQKQDLYVDRLIERVDKLKEEMAMYEAQIIAQSEETRAAKD

ALMEAHMEMEAIGLEKKQLYQQWNSSLIGMRRRDEAHAAMHEALYQQEQKILSLETEMEG

YKKSIQKEQEQNEKLTLILAKTERDIDAVRKQLTQCNARFDALKAEYTTYTRMLHETEQA

LNRATADKTLRENELNALRKQIEREYQEKISLEDQIMERLRTQLTLDKAAQYSKKLTGRT

RDRTKNLEAQMAEVENSIARDTLDTVNVMARVERLGKVRDGLDADIGQKNAIINRSEQEI

HKRNAIIERKQNLIDQYNKKLEAMISAAGGVELGPLEIQINSLLKSLDVRQQEIGELQQL

WLRQQSELVRLSQDKDTQSSDVEKHKRQLTILSQKKIRTENEIDGQRREMYDIEREIRNM

QNDVIKLNTLLHQQKNKETGLEQTNILTENNFIAGLKEAEIESIELSAKLEAVKEEKERL

LNSLVEAERQIMLWEKKTQLARETRAAVDSEVGQGEIKAMKAEIHRMTVRHTQLMKMQEK

MIQDMEKAVSRRDTIVTRGDAQSKMKKKVLTKGTFERQMAELRKKIKSTIQDANECDTEI

RQLREHQGDLSKSLEERQVNCQTLQGSADTLDGDIERMMELKQKNLTELVSKQQRMKFYQ

QAKEGRYTMLCRTDSAADTEQGKQEQRMQALNAIVDRLNQEFPHTQPTLRRATLTLSSRG

IGGEEFA

>SG-AL_contig_3722_1_+1_229_519

MQGIVQYDGSSSDPFPIRSGVKQGCVLAPTFFGIFFSLLLSYAFSQSEDGAYLHIRSNGN

LFNLVRLRAKTKVQKVLMREMLFADDTALTAHTEEAQ

>SG-AL_contig_3723_1_+1_631_1140

MMCTYSDTFPHLLFFAAILYLQPVRRWYTPPHQKRWKPLRPRTSRAKIKVQKVLIRGMLF

ADAAALTAHTEKALERLINCLTCACREFGLTISLKKTNIMGQDVSSIPSISISNYTHPSG

GGGFHLPWFDHLQLPHPGRQAEYTDRQSSNSNSPPSKERLGQPHTDHQHQ

>SG-AL_contig_3724_1_-1_298_3483

MPGDSGPSGAIGPSGTNGPPGMPGMPGMKGHRGFPGRPGRDGDTGRRGEKGPAGPSGPVG

PPGPNGARGAQGERGRDGPPGAPGIRGNDGSPGSSGPPGPIGPPGPGGFPGSPGTKGDKG

ESGPSGSMGPAGPTGSDGRPGSPGPTGQPGVRGEDGIPGPKGGRGAIGSAGGQGFPGPQG

PPGAAGPPGSAGLKGDNGRDGTPGKSGDPGPQGHPGAPGERGQPGLPGNEGSDGPMGPQG

APGPAGHQGESGSPGAAGLPGRPGMPGNPGKDGARGMAGERGVPGNPGLQGQAGPPGPPG

QIGSPGYSGSDGAPGARGPPGNSGLDGTPGEAGAQGQQGAPGQQGPAGSRGEPGEQGREG

AVGNVGSPGARGDRGPPGPAGSPGGAGGAGPEGEMGPAGNPGAPGFQGLPGPAGPPGENG

NVGQNGPTGPAGDAGSPGARGDRGFPGEQGLSGTRGAPGERGPAGPVGEVGLSGPPGPPG

SRGDPGSPGNIGLGGERGQPGRAGPKGPPGPTGESGRDGEMGPSGEQGPPGNLGFPGPSG

VVAAKGDRGSMGPPGEQGLPGTTGRPGPPGNAGPQGLPGVPGPTGTQGVAGEEGAIGEKG

DTGPQGYPGEQGPAGEPGLPGADGEKGDTGEPGPSGPSGQPGHPGAAGPPGQTGPAGGPG

MAGPVGAPGERGSPGEVGPRGVSGEAGFPGTPGLDGSPGERGQPGHQGPAGPAGEAGPQG

ERGSPGFPGAAGEPGPPGPAGNPGEAGGRGENGKDGSPGPSGPVGPPGAVGYPGDEGVMG

ERGQDGLPGLPGRPGSKGNRGNPGPPGPSGPVGQTGSPGATGNAGAQGERGERGERGTAG

PRGPSGAMGSLGATGQKGPGGKAGATGDRGDKGWPGLPGPIGPPGPPGSNGEAGQAGPPG

PPGNMGNSGTRGLPGRPGEPGPQGRPGNTGARGSMGDDGPKGPNGPRGPPGSPGSPGYAP

VWPAERSQQKGPDPYMQYDQPLETIDPYEGLNFAREALIRARNPVGKESNPGRTCREIKE

NNPDFKSGSYFIDPNLGKASDAIEVRCNMESMETCIRPSMAD

>SG-AL_contig_3725_1_-1_1_165

RGRKWTPISLLTTLNGAVRRLLGASTVITMVSYLLPFRFEKRKVKVSHRPAVIGK

>SG-AL_contig_3726_1_-2_1022_1228

MPSKCSYLFEYATGLQQVLHPHSNRNGKIFILGNHALPFMVVFVFQQSFYLQIKINIFRG

YWEVKCKRP

>SG-AL_contig_3727_1_-2_254_487

MMMRRYGLVLTVLLLRINDVVDLIISLVQFSLVSTSTCAMKLYIHHPLVLITKRVRGRKE

VNKRVSEGSTNVQGMRKL

>SG-AL_contig_3728_1_-1_19_393

MPVPGARVPVASPSGVPMGPPPSGWFPLPFAPWASVGAHHPAVGFPIQGGGGGTPSQMTM

TTAGSQASASSTVTPSTQAATSGVSSPPTGFSYPPQWPGVPHGCFRAQVCGPTPLPTGHG

HTSWP

>SG-AL_contig_3729_1_+1_214_411

MLWRGLEILPELRRLSIFFASPFIDTTVSPYTLPLKQSVKVEIHLPSVVLHEASPLGNHR

IQLLST

>SG-AL_contig_3730_1_+2_2579_2968

MTSKCLLEIKYNSRPFLYKFKCTKQFYSKLYYFFPISTHHFNMTLKKYYKIKKTSHSYTI

TFSNHHQYYYLSQEIEQYVTFIYFLIFHLLVHFFENIKQKLQNDGMSLSASKQYFTVMYT

AEITLYTTKQ

>SG-AL_contig_3731_1_+3_3_269

YHQRCPCLVGSRKGENYGHCTLCKRDFSVKHGGMFDCKRHVERQHTHTHTHTHTHTHTHT

HCDQQFHLNGMTCLFWFVYPFNICSLEFR

>SG-AL_contig_3732_1_-3_3_137

PQDGLPEVSTAKNISPSLSLFFAHTHTHIHVLKCGQVKDTDLLPL

>SG-AL_contig_3733_1_-2_125_829

MPYYGGGGYRGGGGNRGGGRDGEIVTDRNAEQFRKLFIGGLSYDTDEKSLRGHFETWGDI

VDCIVMRDPNSKRSRGFGFITYKDADGIDQAQANRPHKIDNREVETKRAMPREETGSQQT

VKKMFVGGMKDDTTEEMVREVFSEFGEIDDVNMITDKSTGKTRGFCFVTFNDYDPVDKCV

LYKRHKLNGRNVEVKKAVSKEELGGGAGGAGRGGGPAGRGGHGDFSGYGRGGGGG

>SG-AL_contig_3734_1_-2_74_1486

MQFYAKLSLVVLCALAVRWTIAKHGYSGYNKPPMFGDFEAQRHWMEITVNLPPGQWYKNS

TDNDLLYWGLDYPPLTAYHSFLCGFVARWINPEWVALNDSRGLQSEHHKLFMRFSVVLAD

LLIYVPACVLCFLIHRKYSDNDKLKGIAVLLLQPGLALIDHGHFQYNSISLGFTLLATWA

LSCSQDLLGAVLFCLALNYKQMELYHAMPFFCYLLGSCFKLGIPNGTVKLMMLALVVIIA

FTLCWLPFLNDLDSAHSVLLRLFPFGRGLYEDKVANFWCSLSVVVKLKQLLTLDKIVLLC

LCSTLMLLLPSSLHLLWKPSIQRFKLALVNASLVFFLFSFQVHEKSILIAVIPACLLVVE

HPLVVTWFIAISTFSMLPLLVKDGLLIPSFAATFLCYEFIRVTLHPWQKRGTLIKLLHAA

SILGAVVLAVISQVLTPPPKLPDLYTLMITTYSFGHFAAFLVYFHYLQFTV

>SG-AL_contig_3735_1_-2_11_3076

MAAIADDGDDCEVIFENSPLYWSMVDAGLAQVGQKDVHKIKPSSSADNPRIRRQVNKQFL

WSGMFAWCSEHLTSLMSLKHAHKGQVREVQSQVFETLARSALLSEEDYLFLQDLKAERQQ

TETPVVSQPPTRKPRAALFAVITGLLLLAFSVWQPFTFEPCMSVTFSILVVLLLPVLVTQ

AANSWRCRSLQRKGQILLGSVRSVLRAASSWSDVGAKCVRFIQESELVARGFTFVGQQGT

SRVVKELQMTSTQRLCPQLRKTLFLASRSVFFTWRRSFVHLVHLCPVRVEVSGIMRYCSE

IPLSQYPMMLQEDGAGDMEEEAAAALHEMSDGFAIDVLKSLQELNNIHVSEFLRRLAVCF

VETRRGVDLWTSLQDLLDAVSNDLERLHKDLLSHLTAHRRGSLNVTLKGRKSAAWQPETS

AGQLRLTYTAVHSLELHLLAALKKIQDLSGEMEDKLAEDKDQSLTDSENQHDSRDAVEEP

LRENWTQALSLIRSELRACSGCLEEGLKRLEKKSCPVTSVAPAASSAPPALALPSPVHVV

KVSAQDNPVILDEVFEAYSEPQDLDSWRGRYELMASEEEAGQKKQKKNLKLCLKELQSII

DVRAAHRQERERLAMDRMKGSSSTDAQQAPELRASDSDREPVAGSLTLGSVEEPGKEQSS

EQNESVDDLTKDIEERVEFWKQFNQKENRVLTDKETDQTKNAAAMDKAFAPREKAVVMNT

EMDSEDGCKTEEGRKRESGADGTEHPKEAQEAESCQYEDQHAKSSRIIEQGSSSSSTESG

FVFVCGQGGDSDITDLVMSSSDNRDNDITDHIVSSGDSRDNDITDHIVSSSDSRDKETQR

LAHSLQNTRPPTQYETVPDTFSQVSHYLPSNMSPLEQNSPKQNSRDSSENSDSLLKQRAH

MYYENDSDDDISHDNETDEDDNWSRDTEEPATSFQRLPAVRQQELAHTNKNEDGNTGEND

GNAPEGVAEHLSLPAAPSPFAFSIASMVAARAQSFAQTEDTFGDSSSEEQEEEEGREEEG

VS

>SG-AL_contig_3736_1_+3_3_140

IQNLTLSPTLSHDCFANSACAKTGIQNLTLCPTLSHDCFANQFYIF

>SG-AL_contig_3737_1_+1_112_333

MNCTCLKDCTMSYLILFCCYCLCLITNVVYPSCLLKELFITLHPPCPHKGLKMKKILRDL

ATSFLSVNLISLST

>SG-AL_contig_3738_1_-3_15_401

MESKTIKRGDPYSLKFGRTLFTHSSSRVPQSSRLHPIIPQLYVQEWKMDMKNRELITQNA

RLGGIPNFEHDENLFLEKREQMHYNVENANRVENKYKIPERAKLLRPDFHNELSRYQSEL

MFRRDEDTV

>SG-AL_contig_3739_1_-2_290_604

MTVMCLWNFKMLCYESSCFTPWYTASVQKAALVEEIAISQICNSVNVYAKRLLVLSLMTI

SRLGFEEKLLQDHFHFLFCLLVFQCLFDRCSVAGYIISTSNIQWI

>SG-AL_contig_3740_1_-2_32_2725

MAVSRPEHIVDDPTSLRLCLVSGTLVSRSSMLDALDFKINLAGHLDAHNGFQFFHDARGN

GNSSIGGGDGGSLGYATVGLAREPINAWLPLYVTPSHWERVKLILKPSLGYLCTLDPLGF

DWRQMDVLFMILGAMIAQLSDTTVGLHQLQLLFAFQRTCVACVEDFKLTERMTTTVRDFL

REPKCRFKHVMPNLYTLVGYIAALPVSTACCLLGYDEEGRDPSQLPTALWMAFLAEALRR

AAGSLFRSCSDRQLDILVDLLLHGRNNSLMMPGKAQEATCIATETSNNTTGTVQEATCSS

ITITTATTSCEKALPSTDKTLADTSAALKAGTLPSTTSESELAGTVQVLESASTTEAAAA

AATEVMTQVLESASTVKASASGPAAVEVAEQSLSSSSPHTSTTSAHFTILCGSLATQDVD

LTIEGVQESQNSNAETSQAAAAAKPAVEFQHCAKMDSSMEIWARAELSSLQHKGQEKKVS

SSKKQVIQWINEGRREAGLPDWDGTENKKEEEEDPLDCDAVDSSVLEVAAKLLTQLSGKS

HLPLSSLPGAIAFLHTWLSTEHSFAQMSDGLPPAQWIEEVKDTVARVYSAMHDYVAQCRQ

QVSHDDLEEVVIVEGGGNEVAGASRSRQGGGKGGRRGNRGDNDDDDDDDEVYSDDEDYGL

DEDDPMKVLRRRQQEHHSLVTLPMILHILDPCENKTKVTRAMLCQAVIHCTNLRAKEAAS

SGQFLDLKNKESVEGTLRSLHSHLVKVSAVDNSYSILWQRANAYLLHASNIFAFLGYLMN

TYKDRDEGFSWLLTEIARLSEGSRIPCLADKVKVMLTGRWEDHHVVSRGNSCIPGKDVAK

KLEKVLGEEAWSEIDLQLRGNLRIHVYRESDIPNRHGHCNSNPYIPPALRLKLGLPPL

>SG-AL_contig_3741_1_-2_5_244

MGGGGSMGGYYDGQGGGQYSSNNMGGGGGYMGGNSSQGSGYGNQGMTGGYNSMGAGNSGA

YSSMGSSTMSNLANPSYTAF

>SG-AL_contig_3742_1_-3_108_899

MSGEKATASSPLEPFLLLMKNAKGAGAVSVLTQALEAPNVYVFGELLDLPNVQELASGPH

APFFNLLNVFAYGTYQEYKAEKANLPELSAVQLKKLRHLTVVSLATKSKCIPYSVLLTEL

EIPNVRSLEDLIIEGIYADIIHGKLDQRNQQLEVDYVIGRDIRASAMTQMMSVLTEWLKG

CDAVLLGIETQIAKANQNRENQLRTVTAMEQEIATIKKNIKNSMSHEGEEQMVTDSRMDL

TDKPKKSARGKGLRGSAATKLWKS

>SG-AL_contig_3743_1_-2_212_379

MFRCVSENQAVVVENDVPCFPGLESVMTKVLSVTDKSACVVSCVCVLYCPSRILSL

>SG-AL_contig_3744_1_-3_3_104

LLEAMSSVGQQRHQQGSLASGSNVISRSTTTLAG

>SG-AL_contig_3745_1_+3_3_188

HTHTRTPEQDVNVFQTIPMIYMQHDQRNKPGVLFSTIILYKHKKNPHKTTVQKKTNNNSH

FN

>SG-AL_contig_3746_1_-1_49_369

MSSSDLEQCEDLQSFELAHHHPVPDVQPFNLCDGRLQAEPHKDTVGNSSLQPSVNGHHRH

MMSFREFEQLPPLPEDCAELPAMWEDGGSLAIGSARDGFGSGGGIAD

>SG-AL_contig_3747_1_-2_89_481

MAVRAQFESSNDIGVFSKLTNTYCLVGIGGSENFYSVFEGELSDANIPVIHTSLAGCRII

GRMCAGNKHGLLVPNSTTDQELQHIRNSLPDSVRLMRVEERLSALGNVIACNDYEALVHP

DLDKETEEIIS

>SG-AL_contig_3748_1_-2_26_247

MFPYPFFPPPFPKKPLMALTPPPPQQNKQRNKLKNPTKVNVKIRSHTGAAILDRHINHFH

WNTKAEESNKQTNK

>SG-AL_contig_3749_1_-3_3_119

GLRDTDAHGVGQKILAPPSKHAQLQLGAGQGQNISIFFN

>SG-AL_contig_3750_1_-2_692_913

MGSIDGIFQFTSVCLFLLLSDNFKVILLLQIILFGQKDVTVFSFAHRLFVLCMMHYIHVY

IHAHMSLLFNVLHA

>SG-AL_contig_3751_1_+3_12_308

MYRAKQLAQSKDIGQSEWSTQVIYLWSCCYIPYTNYKNPARPTFKLYCHQSFPHPLWGLT

GKITTTTTTKERKRKRPQFPIPCGQLIFVAGNVLPVGSE

>SG-AL_contig_3752_1_+2_1337_1654

MLCNYQVYLSLIQRQKHSHVAMCKSNIQRGLMQNSMDIYAKDLNKLHALFQLLKTNKSNM

EHFQTNTQLWKYIIYINKDNLFCITITIYITENVLVCITIIDNCKC

>SG-AL_contig_3753_1_+1_475_642

MAGRSPTFTLEKVLEEIFQDADSGDKDFGPNDIELLEVHVNHPDFNLEGSLLDIVG

>SG-AL_contig_3754_1_-3_3_578

THSHTHTHTHTHTHTHTHTHSHTHTHTHTHNRSVLENTDRHRCFCFRFRLDWFSDIHSDT

LSQVLPNRRNKSSIGINDEDVSSWRLHARNKFHIYTNAAMPGDGKRIPDYHGMCHTEDLT

SQTLHVFSFKPKSSKGQNSPQTFISLLCVHVHLMQGNLLRTDLERDVCSQRFFNTSNRIR

LHLHYSVKIRRL

>SG-AL_contig_3755_1_+3_3_140

PLPFFFTHFHIFATLKKFFIPLTTDFNQIKMLLYKHKNKKTIHKQR

>SG-AL_contig_3756_1_-1_613_1290

MEDYGQGYVHHYHHHLYHHPLSSELSYEGFSPLSTSSASSSSSIASPLPYGVPDIGDHRE

SAGKMKRKKKKGHAQQVQQRQAANMRERRRMQSINDAFEGLRTHIPTLPYEKRLSKVDTL

RLAIGYIGFLAELVQNDAHAKENVQNHVPEQPKKVIIHCHRGMSEEEAYGYPPLAGHSLS

WTDEKRRGLGPNNTMVAKIWTPEDPRKHKIQTEADCSGLLDSTLDF

>SG-AL_contig_3757_1_-2_197_1135

MSLHRRETILVMVVAMLVEVVWSHGTMMKPIQRSSMFRDARFPEAPTNFDDNQLFCGGFQ

RQYEINGGKCGVCGDPYNDPNPPNEAGGKYANGIIVEEYGQGDVIDVTVRITANHLGYFI

FKLCPNNNVTKRATQECMDQHVLQAMGTSDTNLREKYYINEATGDIQLRLQLPADITCTQ

CVLQWTYNAGNNYGCDENGNCCAGCGNQENFVNCADIAIIPSGAPMFTTRAPTTSESQTI

TPPSTATASSTTTQLTTETSTTTTTSTTTIPSLRKTGEGCRPTATYMNVPGMTNWCNINC

AAGFCPTSHCLCP

>SG-AL_contig_3758_1_+3_270_716

MGTKKAVQIMSTFKWKVDENGDATKSDTDFDILIKKITEYFLPKRNIIHDRSMFNSKSQH

EGETVEESECERSELLVKHCSYADLDEQVRDRLVIGLCDRQLKEKLQLQSDLTGESHHHG

GPTRANHTANERADQAVGMTQHRRDYWSP

>SG-AL_contig_3759_1_+1_1_204

GQRPEFLTSRTFCPDILWGDPQFLHVFSDAIHPSPSWSASWSFSRHHHVNNCSHFVVLFH

SLHVTIPA

>SG-AL_contig_3760_1_+3_216_1064

MDLFQEAWKKEEVPKEWKKGYIIKIPKKGDLSECKNWRGIQLLSLPSKIFTRIILERIRT

AVDEKLRDEQAGFRTGRSCVDQIATLRIIIEQSLEWQSPLYINFIDFKKAFDTVDREVAW

KILRHYGIPPKMVKIIQSLYEDTACHVIHNSSVSDPFTVNTGVRQGCLLSPLIFSLVIDW

VMRTAMNPPRGIQWTLIQKLEDLDFADDISLLTHTLHHMQEKTESLRSTASNTGLEINAE

KTKTLRTNTSQAETIRLGEQQIEDVDKFTYLGSVVNKTGGTDD

>SG-AL_contig_3761_1_+2_311_445

MYTSFSNTPKREKHVENGIGHTTIIVNLVMLILSFPHTNHQLPAG

>SG-AL_contig_3762_1_+1_154_414

MRECMYTFEIYIATQHNANTCNTHVRTAVSCDWLDSTNHRKLWLRHWYIMCVRIMPYIHN

NYEHGLEACTFLAAICINLKTSSTPRL

>SG-AL_contig_3763_1_+2_83_310

MKNQSTAIHQETSAPCCFRQQRNNALTERMKQRSLSLDAGDMSAFCTLPLVVSLTTICVI

PRRVAASMHSPRLCQS

>SG-AL_contig_3764_1_-3_141_326

MVQKSDRHRLPEVEEFLSSSSTVHDLPEHTVTITDMSDRLTATQKEHSDDQQDSAAQQKE

SE

>SG-AL_contig_3765_1_+3_405_1022

MTDHDVDVMFLTESWLCKSGDEGKCAVLTPPGYKLFSFPRHLEPTAKCGGGIAIIMKNCL

MPCSCVTSSFPFSHTSFEVIEVLVTLHNQRLCLLRVYYPPPSVRNKLTDSLFFQELPDFL

DYCVLTEGNLLIMGDLNVHYENVCDATAMKFRELLCMFNLSQSTNEPTFHKSGHTLNLAI

SLDSDYYSLLCVVKISRLTMGVLCVV

>SG-AL_contig_3766_1_+2_236_418

MMDTNCKICIHKCAKVTASKVLYNCQILNSDDAESDSGLYDCQSEILISDGAESDSKKQP

V

>SG-AL_contig_3767_1_+2_71_280

MKMNKTLFQTTQAKSAFQKLSLSNRHKRTVKRYNTNERSNKPFRTELRENANGDLRLTHI

TLKEHAYNTV

>SG-AL_contig_3768_1_+1_562_1008

MFMCLVVNLIFFFYYIFPLCVCVFFLFCFVLFFSNVSSRYILVLCYCCCCSLSLSLSLSL

SLSFARWLAQCLYLSLPFLNTPPPSLSLSLSLSLSLSLSLSLSLIKMEAHRLCRLCSKIY

CLCSVGQFTTRAGVHEPGQSERASDGFIY

>SG-AL_contig_3769_1_-3_285_524

MSRVRCALAAGWTGGRTHKVLRARIERGQADQKAGTLPLGQTGLLLSRSHFKDSDWHLLL

MMPGDHEIYKPIVVPLESQE

>SG-AL_contig_3770_1_-3_459_1682

MLDASEKQALFDQHARRESSSVVEEFFSLDSNAKSVKVPGWIKQEPCEEKEEVDVFDISS

TIYDSVMDADLAVSGAPSSSLWDDINASINVVDNFTDSPVPSSSSSSSLPGLDVLSKVKC

EPMDTSDVSTCHYSKTLSNSRTHSGGLVMARNNSLPFTTTPSTTATTHAQLVNVNVSSLS

NTSFQQFKSPLSQQQFTPPPPPPPVSGSNSPANTSHVVPSVLLPPTPPNSQPASPNSGDF

VVRRTPPPPYPGVVSQASTPQGNIPQPVLSCSPSLALPLAPMSAPSPGRPRSTKPQNTHP

GCSTIKYNRKNNPELEKRRIHFCTFPGCRKAYTKSSHLKAHQRIHTGEKPYTCHFTTCQW

RFARSDELTRHIRKHTGAKPFRCKVCERSFARSDHLALHMKRHEPKNK

>SG-AL_contig_3771_1_-2_824_1186

MVMCVCVCVCVCVCVGCIKVKHYVYCIPRCLVLLLRSGRREISHILLLLYIYKYYILWKK

MCYPMIISALFLTFAFIALLSLHNYFSQKSPKSSERRMNRECFSLFLSPAHTHTTDWNKQ

C

>SG-AL_contig_3772_1_-3_3_188

GGSGTSRSAHMLTWEIGKISTLYPPGEVPVVRTQAFHTLQFGSQVSSPLYRCALQSSLRM

NT

>SG-AL_contig_3773_1_-3_3_95

VFFVFFCFFFFISSYLIESYPTGKMMEVMIH

>SG-AL_contig_3774_1_+3_3_209

TEPLASPRGSVNISQFNFSNLFRNTACLKGNHLAQTFIDFCCRSHVQATDWGTATAVGNF

NSVHQTPLT

>SG-AL_contig_3775_1_-1_43_204

MGGAKVSFGMCSLTRQLPLTLTIGIIFKMNDGWSKVLFGVYSLAKLTNESFLLQ

>SG-AL_contig_3776_1_+1_2761_3000

MELFTTDTSEDKFFTHLINFQSYIVMQLPLVRFFKRNLGCEHQNMLVQLPLQGQHCIKNS

PSISKSLVTVLTTSYAHGSF

>SG-AL_contig_3777_1_+2_845_1348

MLKLSMPYLTEILTYVINLSTDQKIFPREFKKKKNFFQKQETKKFYTITGQYYYYLLSLN

HLKECSQAHMKFNENHSLFHPLQSGFHWYHSCNTALSNMTNSWPSAINETNLSEVLFLDF

NETFDLNDFDTLLSKIKIYLNNPHILPFSSHTLRKGHTACICSWLCLF

>SG-AL_contig_3778_1_-2_440_622

MPPVTVMVYLSASACVNQISAVADASIGERDFLRHFLWLCILREDLSFYVCVTCCKSWRG

I

>SG-AL_contig_3779_1_-2_170_412

MKPVGTDVELLPFSLTACVCTQANHAQENKFVPAVASLPSCEWTSPCSVWGKVKQQWRRD

ERRLHNLYACSLQLTQLLMGA

>SG-AL_contig_3780_1_+1_1_738

FGHVFHVLNENLWPLGQVGFKSRYGFHVAPQFLLKREGVKGLGIDSQGSVQEVVQVHVCH

RHRVPRKVACSSIVLQHALKVCKVTREQFFSEHPFQLRLFVWILFKHPVPHVRCPQVIKK

MDQFVLSQGILFCFAIETQFSAEEASDGQTLPIFLALVDNVRQLAVWRLLFCCRLRAPVI

SSFNSNVFKLDTSNSEKHTNGLRSASSSEIVQLDVCLSHACSFSFEVQVRDRYRGYKILV

SCNGAV

>SG-AL_contig_3781_1_-2_107_877

MGSGVSKTVGVPANPRVEYSHQLSKKENSTFRIDAPVLVDMQHGSVRYRQGQDQPSGKEL

KDREIPHEGSTDLEQKQHYFYHTRLNHFGADAHKSKEEPFKFYYVTKGPTRAQGKQLFYV

YGQGWQAEDSLRVKPTLTVEKGNDFAWPSRCGPNDLLYNVTVLGSTSLEHEDSDWEENRL

LEDLESTPNRSQIQLKVRSDEQVSSVKARLAVKLLKAAANIIICHNETHLRNEDVLGDLR

PQKERSPAILDLQLTLL

>SG-AL_contig_3782_1_+1_1_102

FFFFFFTHIIFIDRDGNQSLTEVKRRIRSVKRKH

>SG-AL_contig_3783_1_-1_91_1452

MFSSTGRSCTRAWGTMRLVFRGMRTQASDNQAVKSAAAAAETGGRKDMPANDMRPLYLDA

QATTPLDPRVLDKMLPYMVSLYGNPHSRTHAYGWESETAMENAREKVAELIGAHPKEIIF

TSGATESNNIAVKGVSRFYKSKKKHIITTQTEHKCVLDSCRALEAEGFDVTYLPVQTNGL

IDLKMLEEAIRPDTALVSIMFVNNEIGVKQPIGDIGKLCRSRKVFLHTDAAQAVGKIPVD

VESMNIDLLSISGHKVYGPKGVGALYVRRRPRVRVEAPQSGGGQERGMRSGTVPTPLVVG

LGAACELAAKEMEYDAKRIEELSHRLVDRIMAECPNIVRNGDTEHTYPGCVNLSFAFVEG

ESLLMALKDVALSSGSACTSASLEPSYVLRAIGADEDLAHSSIRFGIGRFTTEEEIDYTA

DKCIKHVKRLREMSPLWEMVQEGVDIKTIKWTQH

>SG-AL_contig_3784_1_+2_275_421

MSNALVMITENERQDDPLMNRNNRNQCLVWCSHSHQNFIIIQKRVALGN

>SG-AL_contig_3785_1_-1_79_363

MLLNLHKKSWVDGLTLQDYKEHCSLNEKTVGDMLELAKNYHKALEEEETMTPEQLAIKNV

GKQDPKRHLEEHVDVMMTKNIVQCLGAMLHTVVFK

>SG-AL_contig_3786_1_-1_388_627

MQVLWRHLPSTPGKLYQGLPQTFQQHWNIVSTCLLASGSLCSKSCSGCRPIKLNLNKDIR

SRPVSTCILKLISLPESGNV

>SG-AL_contig_3787_1_-1_1_1029

RGLILTSGLLSSVASIHCHTPCSLRASLCPLLTQIRHRNKKQTRFKAPPEPKQKEKWTKT

VKEKAILIKNVKSTHSVVFNSRVPVDDVWLRTHYPRPKFSLEECLVRHKELAEPAMLDNM

DGFVYADMQLNMKTNKKTKFLSSVKGTILLPHQFEHSVQQKIAIFCKKREDVEKANSLGA

SYVGCEDLVKQIVEGVVDKDDIDVVLCTPDAITELIPVRSQLRDKFPNKAKGNVESDIEE

MWTLYTKGVTYTSQKEAEAVGRLQVPLGQLKMPNDQLTANFNAYIDTVCKGRTKTLGPLI

TQIFVIAPPSNERFLLPVEDYVPGYEKTEAKEIDSDSEDEAEA

>SG-AL_contig_3788_1_-1_16_243

MKSTERSLHHLTGSCPCRFSVSNQQVSFAALGVGNACNLFVRFCDGYFFPVFVILLVQLC

GTFTLGLETLIIIVIK

>SG-AL_contig_3789_1_-3_3_161

NMHLKKHCLQQNKVFKHLHLHVKYISPDFQHIQFTITLVVGQGPGMDYGEMKH

>SG-AL_contig_3790_1_+3_3_173

HQFNMVQYMTPKGTLPLNLLNAPNYPQMYETLKALPSPTTKKCLITTTQTSQQTQTT

>SG-AL_contig_3791_1_+2_2_139

SKDTTQSGPYWRTLRTPPVCPLLVTVRAPPSLTPTGDSKGTTQSAP

>SG-AL_contig_3792_1_-2_2_85

LTVTSRGADWVVCLLADWVVSLLSPVGG

>SG-AL_contig_3793_1_-2_29_1534

MASGDSGKKKKKSKGEKGYKEELGEIHASESFALKPSEETSRAIAADDWPLLLKGFDKLN

IKTSHYTPLHSGYSPLCRPIKEYMASGFINLDKPANPSSHEVVAWIKRILRVEKTGHSGT

LDPKVTGCLIVCIERATRLVKSQQGAGKEYVCTFRLHTPVKDEDKVCQILQKLTGAMFQR

PPLISAVKRQLRVRTIYSTDMLEYDKEKGLGIFHVKCEAGTYIRTMCVHLGLHLGVGGQM

QELRRTRSGIQSEEEGMVTMHDVKDAVWELDHHKDETYLRRVIRPLEALLTSHKRVFVKD

SAVNAVCYGAKIMLPGVLRYQDGIELNEEVVIVTTKGEAVALGIALMTTATMAACDHGVA

AKIKRVIMERDLYPKKWKLGRIAQIKQRYKQEGKLDKYGRPNEKTPSNFYQYIGAPPPPQ

APVPTTEAPVTPMQSSSSSSSSKRKRETSDSSDDSADTSAAATPSASGDASSEKKKKKKK

KKDKHGEEERSESEPEKKKKKK

>SG-AL_contig_3794_1_-2_521_625

MVLSPLINFRICIPFNLYFFSTIKFDSFSFYLLLC

>SG-AL_contig_3795_1_-2_407_706

MAIYCSVFWHCSVLFLLLHELCHHLCITDVTHALFSSCLCTCGPGIFGFGEGVKLITMTF

FGSQYSFWFTQVQVKICISIHCLAQNANRAHFNLRMYPIF

>SG-AL_contig_3796_1_-2_29_283

MCECSCIAGIHGAVRVYEGLDVSDEEGKTLAGRMNPISVCLSVPGVLLLETRTERYAISR

FHWDHSPAGSTLRTCHSTPATGHHR

>SG-AL_contig_3797_1_-2_1034_1315

MSSLLCKLPHQSTLKLAVSDRKRHKHYQPSRLIKKTKLLQVCLCSLWAVTVRKSSLQASR

SCWPIFHYNHSTSFHLCVCVCVCVCVCVCVCVCV

>SG-AL_contig_3798_1_+1_307_429

MSLRPVMIQNVSIYHSAKFHACYFINVQFYTNFLNYSTPSP

>SG-AL_contig_3799_1_+1_2203_2400

MFIIFKIVAWLLMDHYLLKNSKHAISKASLLFCWVYLWLYINLMHLVLKSFECVYAFHLV

LTAGCF

>SG-AL_contig_3800_1_-2_194_457

MKSKEEIKRSERKKEGKERQIETERNEGRKKERNIKRQWMYRQSQSLNCGRLCLLEGENK

FRFSNRTLICRATKIIKKTKMHFPLCCN

>SG-AL_contig_3801_1_+2_2_133

MFSYTESFNKGLSHLLMLHSKQCKLVGQWQHHIHTHDNSLTGQV

>SG-AL_contig_3802_1_+3_1467_1742

MSKNRRYTVEKVLQELFMDSNSDKEPNDLDFSPSDYDFDEESDYGSNGNVFTISAGFWCL

IPSYSLSKQCKLVCLQNYAPSQHGYHLVALAT

>SG-AL_contig_3803_1_+2_2_163

TTTTTIIPHAQPNTHAHSVLTQKQDYLKISNKTILIISHHMAQNNTYSTLLQTL

>SG-AL_contig_3804_1_+2_887_1252

MAGGTKIGPPAFFNVPWLDLAKVSAAVVGLSGSLESWDGGVLDPSGRSKTKSGVCLGVCF

SLTTGRLMRLSDFCCISRDLRPAITSSRALSMSSDNSSAPLSDVVVDTEEESMIEDEEGE

EV

>SG-AL_contig_3805_1_-1_205_489

MYACINLRLPCNADSFTSSHWISRSLLILIYHFVPMHMLCSVYKFITHTSSPALTTFSCV

HWCCVVFWLLNCLMQCEKHFKQYYLRQLKFDLYEE

>SG-AL_contig_3806_1_-1_250_1029

MPQNEHIELHRKRHGRRFDYEERKRKREAREPHKQSQTAKKLRGIKAKLYNKQRHHEKIQ

MKKTLRMHEQKTKKQKAEEKCPEGAEPGYLLDREEQIRAKVLSNQIKQKRKEKAGKWDVP

LPKVKAMSEAEVFKVVKSGKTKRKAWKRMVTKVTCVGEGFTRKPAKFERFIRPMGLRFKK

AHVTHPELKATFCLPILGVKKNPTSPLYTSLGVITKGTIVEVNISELGLVTQSGKVVWGK

YAQVTNHPENDGCINAVLLV

>SG-AL_contig_3807_1_-2_2_499

LDIALNISNADLPDFLAGISVSFPDINLPEFLLNIAVGLPVKEIVGFVSDVAADIPGINV

PSFLLSIALQLPRVSIPHFVVDVFSHFPDIDTDNFLDNIEAQFPGLSIQLPAPTGDDGIV

DTLPVPKPSIHRDGEVCTCEVKGDSKYKTFDGATVKVDSECRYLMA

>SG-AL_contig_3808_1_+2_833_1171

MKRKGDLYSYRWIFVIRSILFLKKKKKNFFYRALSVQVACQKWVKWTATWKMKTCSTNVN

IPLFSKQWSITLICPHPQRVYRCIVCIGVCVCACVRTRVHVHESIYLYVLYAM

>SG-AL_contig_3809_1_+1_1_363

QHFACVIRWQSFWPKPSHQYISVWDFSAPTCFYSELNVLCAHWEDWDHAVVCHPFNNGCV

TEGVASNFLTKTAGVCNSWAWLMPGYIIGKEAYSTALSHSLNLQQNSIASSPPSPSIILA

L

>SG-AL_contig_3810_1_-2_5582_6061

MEGNVTKVTRTQSMINSVFPAWIKFEKSVLVVFLHSVIVSSVKSKPLRSLLSRMLHYINH

YYYYYYYYYYYYSSSSSSSLLSTSTPTHQAKSRPNNFPSPTQQVKSRSSSPSTGDVMTDN

SDNIIVTDSNANIKKVEINNNSTVVAEYEIDDTGVADNDK

>SG-AL_contig_3811_1_+1_1_153

SHAHTHTHTHTHTHTHSFFLPSLLTAPCTKQIRPTACRATHEDNDIEDYTH

>SG-AL_contig_3812_1_+2_2_190

VIMKAKCVCVCVCVCVCVCVCVCVCDRHQGMFLVYTFALPGAKLLVLLIVHVQYWVHERG

DIR

>SG-AL_contig_3813_1_-1_235_1992

MDERDNAKLVEKTDDGTEPATHDIPDETDDTVELGDEGREGRFVTSVTLVVSPTQKDKPF

LFDSDGSAVPEKDNRRELPAGDSLEEKSAVQYETSAPSSPANGITAASFELDIQDKRPSV

GEVEVDTSHEQQSSTALNQLAADTLRQTSTDSSEKRHEIVVELDTFQRSSTRRSSFVLAI

EGGEGGVVTDTKTEDNAYPHLHTAETASPLQGQSQHRTDGDGASLDVHEMEIFVDDENDS

RQRIWRHCQGTRKWVCCVIVLVLLAVVMTIILIPQSLVYVEYHQMALARSRVTGRVDRER

TLYSGCYLLFPDTELITFQATAHNLHTKLEVTSADGLALDLQLSLQYFINGEELGELHKT

YGEKYTEVVQRVIESEVKNNVVHFTLDQYRLNRTTVEQSVYRHLATALQGDCCPSCCPRR

CAASSQWSGNNCNTCSTSPNCARGLHLTLQPQHLQLGAIHIPREVNERYLRFTLLQVEAE

KEALVQESKVEMKRTELLTQDIRNQAQELREAARSSARRLSLEAEASAEVTRQTAYTQAM

KSLLGDLNFTTTQHRLSVLLMLTYRDISDRLYFEQLDKLSLYGGLT

>SG-AL_contig_3814_1_-3_3_257

RVAQRKRRRAEKKWKTGLTVHRQTYIKERAAVKSFEQSARRTYYSEKTVSSPSCKQLFSV

THELLGKKQRLSQQFSFLQVTPVFQ

>SG-AL_contig_3815_1_+2_2_178

CVCVYTHKCIFCTNSIFAHKSCTRMSKLFHCNKPLIQTIYTLQNLPFEHIRCNISPMKS

>SG-AL_contig_3816_1_+2_491_871

MIDLLNMFSYCQVVNEPTHVHNHTLDWVTHRSDDNIVRSTSVSQHLKSDHCVCCDINVNI

PPLPTPVYKEKCNLCDTDRLSFREEVQIAVFPSVCQTADQLHSVLRSVLDIHTLVSRWKV

RTSKSSP

>SG-AL_contig_3817_1_-2_164_424

MEGYHGDDLDVRTLRRETGVFIQYRTEGRMKFVRGLTDGWRHSSLHFLPEWLSISITQIT

HFTVVNSVGRGTLTFQYITKNTKMARL

>SG-AL_contig_3818_1_-2_14_1486

MDAGTDAMEVLCGRVIPVKLGIIGVVNRSQADINDKKDLTEALKDEASFLQKKYPSIASR

NGTSYLAKTLNRLLMHHIRDCLPDLKTRINVLIAQFQSLLISFGEPVDDKAQLLLQIITK

FASAYCNTIEGTAKNIETTELCGGARICYIFHETFGRTLEGVDPLGGLSTRDVLTAIRNA

TGPRPALFVPEISFELLVKRQVRRLEEPSLRCVELVHEEMQRMIQHCGTQQEMLRFPKLH

ERILDVVTNLLRRRLPPTNEMVENLVAIELAYINTKHPDFSEAYSIHRCMTDASAGDPHR

TTSLAKNMLDRQAEDGKIVPSQDVVNGGTWKLTNLIRGQKLDVGGDKMSDQGTASQPSSG

PSSAAPSPARSRKGGGINLLPEVPEQPVMKLSSREQRDCEVIERLIKSYFMIVRKNIQDS

TPKAIMHFLVNYVKDNLQSELVSHLYKREEIEMLLEESEHIAARRKEASEMLQALQRASH

IIGEIRETHLW

>SG-AL_contig_3819_1_-3_477_671

MYSVNIFLEVIYYNINYIPVNTSPLVLNMKIKMRLCIGIHNKHETLSPYVFKGFQYVMIK

MQRIK

>SG-AL_contig_3820_1_-1_868_1197

MSIIIRFFGFFYSVIRHWRPGWCRGSSSPSSLTVLLREILCTWLQSTQLWMEPDCSGLAK

GNAVKEGSWTCPPNSRHEISEFSYHLTLLQLGISIFFKQTLLPCQSICIV

>SG-AL_contig_3821_1_-1_52_1482

MLQILQKECDRQTRKIVEQFKSRRQYDRLVKQVQQSAMLQQKQTLGERIDPRQLDVVLSE

VALISARAELYSRFMRRRALGDIEVAMPDEESRKEQVKEIEAFWSGCEMSRMVQELIGVY

IMMEEYFMREMILKAVNMDAIEEGQQTSSMVDDTFFIVKKSVRRAISSCSIDGVCAMLNH

ACTILEQDFREVLYQRLRAGFPSGFDLQQAYNLVQASITQGKLQSSDAEKVKARASFLTS

LNNAEVSCDYCKALKNNLEDEVSKLYVQQCTEQSKAKLESCLDDLGTVSGRFKDVVDFGF

SQLSSSAIKPRIKPLTDTFLSTNHNISEEEFSNYEANDPWVQGSIVTLDTMLTTFKSSLT

SANYDNFVTLVTTEITVQLEKAVGKTSFNRLGGLQFDKELRALVGYLSSVTTWTVRDKFA

RLTQMATILNLERVSEIMDYWGQNSGPLTWRLTPTEVRQTLSLRNDFRSDDIKRLKL

>SG-AL_contig_3822_1_-3_372_497

MNLSFVVSDYFMHYCPQFEMDFFERWGRGTLQLEHLFCKYFY

>SG-AL_contig_3823_1_-1_2398_2877

MALMTQYQCLLSVTMLLQLCRITSPYLLLLMIQLLILKLALSLHESKEYLISHPKSCSSE

KEKQSLLYGRKMFEKLYICKERNTYLKKSKVIPAKTVRPTGCSKCRYCCTENVPEEKRQQ

LFEAFDGLFTFNIDGTIHRVCKKFFMATLAVVDTYVDHAM

>SG-AL_contig_3824_1_+1_16_219

MLCCFIRYYNIVIQSFHIYVSHDTGYSACNLQFNSICSSMYSTPENKAARKIRTFPLSFF

NIKQTKQK

>SG-AL_contig_3825_1_+3_3_206

GPQVLQPRPGLPEGCSEDHARALIPGLPDDSWEDREEGKSVADQGSPVEGEGVHRRRLGV

LRRGAIDR

>SG-AL_contig_3826_1_+2_2_124

TLRHHKCLLFSISLSRNLSAVTTRFALDLSQWLQQGKNTIF

>SG-AL_contig_3827_1_-1_1_300

DLRISYVATKVVHLGDQLEGVNTPRARAVEAQRLMNYLAEFLTEQPLTSAVLTDPFQLQL

TADIIQKLHLIALELPVGGRFDRARQKIAEKYDYVEQELI

>SG-AL_contig_3828_1_+1_865_1230

MKRSLTAGRWGQRFSRIHQKHGTHVFLSYLSTRPRTVITHGLFIRRRKTMKITSLKRKTK

NPLILKGLERGMVVMARMRRADGISHKTQQMTEKRPDEHPPLIETESTDRQTTERDKSTA

VS

>SG-AL_contig_3829_1_-2_2_103

RAICFKSGFFSWALTLEVIVFVVRFCFIFLLHDV

>SG-AL_contig_3830_1_+1_2998_3309

MLRMGEEVSNSYIRRQHYIPTLHTTNSIFPVLFAHTHTPVISSMTLQLAHYKTQTSSLLS

IQNSHFLFLSHTLERNMCHHTISAHYIFTLISESQSLHSYSNSC

>SG-AL_contig_3831_1_+3_3_128

RPPISTLILTLFPYTTLFRSVDENTGYKAGEDPESGMQMAAS

>SG-AL_contig_3832_1_-3_117_272

MCNVTVARCMKWHTSQYVCLLHTEVCYIQIFIEHVHFTQRWRFSLGCACLLK

>SG-AL_contig_3833_1_-2_98_1528

MGSYNYEAGGVGGGSSTADYDMATGMANYQSNTLTAGGGSMGGAGGGYSSGTLGGGGGGG

FSSSTMQSSYNYSVKTVRTVSGQQQILGYAGSQTVGVPPGALGEEVRVDCCLMTNDGRSV

VTGSSLGPPQVWDMQSGELLRIMQGETVGSTNLHLACNDRLLVGAINADLEANQSSVPKG

VHNYMLQTWDFTTGRALEMGDVEVCSALTLMSDNDKVVFARSDKFGGGTNLVVWDLLGNQ

PIKHMRYDAPVGNNDYVHFLALSQNDRYVIAGFTNSFDNYAEFVVFDMTLTSYTVSDPSV

LRLDANPECTAVLPRDEAVTGLRNGDLVIWSMRTGQPSRQLLSSSGRHAHSREVNAVARS

EDGKYLVSASADRTLKLWDVETERHINTLNGHTDEVWCCAISPDNEIIVSGSRDKTIRLW

RLKSGQEICSFNAGVDIFHVTMSRDKSTIVALGDKLGARKLIMLQVVRTKVRRQVSS

>SG-AL_contig_3834_1_+2_470_808

MDVYKSNTGYCYGQGLFACQILLMFLLWLQRNEHFGDFDLMTPATLTYVINFDKNRKVIS

CLCVQNFTAIGYQTPRYRCVRTDRQTDTHTSLSPDDHKVFPEIWKNRNYEASV

>SG-AL_contig_3835_1_-1_112_246

MVKMTCHELCCFAGLLFLDLTRFCVECSCLVDFDGLNMVTAPIWP

>SG-AL_contig_3836_1_+2_194_385

MKTPKAHIHTHSWASHGQKSDDFLIFTNSDINRKTYFFTTLFPRSWINTLLFYILSTQNV

LSKK

>SG-AL_contig_3837_1_+1_313_567

MVVPFSPMSHWTKNHMAKSFKHSPFSTCLSFEFQHEYTYSHFRIPNCLQGPYLVQIQSAE

KFLLPAFCSSDQKYRLAITRLCCLA

>SG-AL_contig_3838_1_-2_2_277

EVTKCVLVLVTWRCCVPKIGLFLPPLHTWKGLLSPNAVLTVKYEMSVFRKIKFNTLKSVY

FMFLCCMTDFGHFFKKCLKIMQFEGAATTAEL

>SG-AL_contig_3839_1_-3_3_200

KVTDKFSAASSVHRCARSTRPFEVTTMGSMQTSGLLSAFHEETPKNSEFTDSVMLEVTKC

VLVLVT

>SG-AL_contig_3840_1_+3_3_221

CQFLPQVGHDVSQFSSTDEAVAVLVKHPEGLPDLLFTVCVLHLAGHHRQELGEVDGAVTI

SIHLIDHVLQFSL

>SG-AL_contig_3841_1_+2_2_118

TNTHWSKSTLWSLLIIPLNRYKKNLILIKMIIINLFINK

>SG-AL_contig_3842_1_-1_514_828

MSSPHLCWAKVVRTQGTTLPSYHNYDVIDRFTDAITGAQAMLVCLANLLEYVSLFLVFTS

AFVCKMMYTVWLEVLFGFLSCFVWLIRLCWVFEPLFFTLFVTSVV

>SG-AL_contig_3843_1_-2_2_79

KNLPCLCLFIETSYSCVLVTKLLDST

>SG-AL_contig_3844_1_+1_1192_1485

MLRLQHQRARPCHPCPLQTLQQCSFFEQSFGKVLLDNCWLQTGMPAPSGTRQPFSNKNYA

RDPPQPSISIGGKPVYTLCFADDIDRMVDSNNELPTFV

>SG-AL_contig_3845_1_-3_1416_1808

MKKISPVFSYRFLDNIVCSDSSLNDILMYVLIALNESFVDVILRISGFIFGCSSSLFLHS

VAECFIFSFDVETNCVCVPCCGGCILCVCVLFGCTQSWPLCFVVRAAWFMLVVASVGYIS

IGSYLYRSVFS

>SG-AL_contig_3846_1_-3_51_260

MSVFGGIWKLEHTQHAHFILPSLALSCIGFYHCRRKGGPAAWATAGSPEHFALALCPGLS

SSTDSIILGD

>SG-AL_contig_3847_1_-3_1521_1943

MVVMFKIGRTLAGSQCMRIMLVSDPCLYCTSTESSKMLTVQTATDTVMGRPKYSIKACLT

LSLPHAIHRKLVDKVRERRVNYQYCLKMLLTFQTSMNDNLRRKRGTIEIKDCRLFILLRK

WVLNYSLLILLSWAIKNCSIV

>SG-AL_contig_3848_1_+3_795_1055

MTVSVKHSPVNKSQAINDHHNIIYHTALPPSLHLRHIVKIIQTSDKAQELGEGRSKQTNK

TTSYNINPLTASDYPPDFSSFLQEAGG

>SG-AL_contig_3849_1_-2_368_580

MTQLRVMGSPLPFYFYVATGLVGIEVELPPSSLTAQVCAGANLTQEENFVCMASSLPSYE

WVPNPVHGWGR

>SG-AL_contig_3850_1_-1_196_1974

MSTSLDQRTKGQTNQVSGAPGISGNDAMSEEDKYMPQPHQGTYTSPGQMPSPGSTDSFFQ

SYYTPNPNTMYPFMSQGLSTTEGPWSNGGDTPMFPMSYDYSSMYGGFGFPFGSWDYPGEY

WNAPTQPAARKDPRPYQETDYYTDPSMLPADSYGMMNGAGPDRGDPMGGVEQGLRGMTIQ

GGLEKKMDGGMGSEIVGGMPPQEMVAPPPQSVSSSMHGVSSQGMGVSVGGSVGGMGSQAP

KKTSWAAIASQPARPQTQVRPRMIPRAPANPNKPHSMDIGTWENRGGMSAGGPVGGKPAS

GQRQTWSAPRQRQMGGTSPAGHSSVNSNSNNSGGNGQQSNHQGNPNPSSYGSGAGSPGNV

AGNGAQATQHPVLDKLRQENQYNPKDFNLNPKGARFFIIKSYSEDDIHRSIKYNIWCSTE

HGNKRLDSAFREREGKGPIYLLFSVNGSGHFCGIAQMLSPIDYNKNSSVWAQDKWKGQFE

VKWVYVKDVPNSQLRHIRLENNENKPVTNSRDTQEVPPEKGKQVLKILHSYKHTTSIFDD

FTHYEKRQEEDQAPQAAPVPHGGHSGVGGGDRGDRGNQRGGRDGRRGGGGRHN

>SG-AL_contig_3851_1_-1_421_1398

MLEEHSLGLVTSTVVSVIVYIIGLVINLSDFARQYLSVPLSMIPSILRNNGTNYMLPVSL

ADWSVSIWGVVYVCQSFWLVYAVSGICRKTAAGPAYNNPKLLPVQVHIFFSLAVLLQVGW

LVSMDKLNLGFALLLILVATGCSVATLCESYRALASTQALLVQLERSCDLWSVRILAQNG

LAVLMVWITLHATLTFALFLTFGMKSHYVAATTSSIVEVCCAGFLIIVYVITDLTVLDKY

SRLVVSPYLAFVLFHAAQLNKDWEDTHTPLFALLAFFTAFSFLALFLKVVLLLWRQLSRG

QPETPITMSHPLPTEMEEEAYLLERK

>SG-AL_contig_3852_1_+3_492_767

MKKTEIRNDMIKSTRWIEKGVDWIAHFWNGNGSFMTYDEFNRKYNINVNFLVYNGCKVSI

KKYIQETELNTPNGCAHDIPTALKIIYSVPKG

>SG-AL_contig_3853_1_-3_3_398

SVQVPLQFCIIFFYLVFCGKISCKRSDVWSSFFTFAFIYIFPRRITKSNIESAHIFIKTN

KIKFTLNLIFAQVTHFDLNNSIFPEYFDMQSNINKKYDQCCPLFLNGSRSKIISPISFRM

TLVPRILCTMRI

>SG-AL_contig_3854_1_-1_850_1326

MHIIHVCHRCGLLVSLILSYKHTKLGTSRTNHTAENILLTSITTVIQIIFFSYMYKSVHI

YPRHLDSTIKNTVHKTYHRLYDCIHHCMYVFICVWWYVNEQKWWEKDYVICHPTGHQVLF

TDVLTLIRTAIQHQKEYEHIHRVQKYSSRRQEQSNFPKC

>SG-AL_contig_3855_1_-1_1_288

CVSLSHTHSSSISSKTFFSLSLCLSVSLSLCLCLSVCLSLSLSLSHTHPAYLLRHFSVCL

SVCLSVCLSLSLSLSVCLSVCLSVCLSHSFSPSLSL

>SG-AL_contig_3856_1_+1_643_1290

MYTLSLSCMLTIMIFHHTLSKTHSHKMYMSLLPPSLHCRCSLSFSLTVRGRLCLVLYLVV

IERRFDGILCQHGAMEFYRRQGQFLCNVSILDVQCFIHCLSLHPLCGQRAGSYGRTTAKC

LELGVHNLSTVVHLDLQLHDITAGWRSHQACSHFLVILVHGPHIPWIFIMIQYIFVIQSR

WGNYAGIHGRGNSFSYRSHTSKQRHVYVLSGCSMQA

>SG-AL_contig_3857_1_+2_5_280

MVNEPKSQPVELTHGVPQGSILGPALFTLYTSQFSYVISQYSIKYHFYADDSQLYNSALA

ENLSTLLSEVSDCFIEIQNWMTQNKLLLNGDK

>SG-AL_contig_3858_1_+1_34_288

MTQNKLMLNGDKTEAMLIGTKHKLSTVTVSSIQLGDNTIVLSDSVKNLGVVLGPQFCMQF

INQTCLSCYGQRGASVLFASIFQLK

>SG-AL_contig_3859_1_-2_2_199

PPGDPLHRHLVPGLGSTRGLSACRRDLDSGRSPPPHQYSRDGGGVPLPPCLCPPPQGQTG

QAFHGQ

>SG-AL_contig_3860_1_-2_140_367

MEAVSRSLLTFAHLLRDKLVKLFTDNTIVTCYMNKQGGRGDHSSALSTRAEVLLRWCKDN

GISLSAAHIVGKANIL

>SG-AL_contig_3861_1_-2_2_259

RLFFLSSQSIPLSFFQQAFTQFSLAPVYDVLSVLFCSGDWVVRLQTLGWVLDVHSAVYLP

VWPFLCILLLLQSISYIFLCFFVTWG

>SG-AL_contig_3862_1_-2_2_142

CRPQHFAYVISWQSVWPKPSCVCVCSKIFSADSGWLLYQRCGLLRNA

>SG-AL_contig_3863_1_-1_301_531

MKLKACSPADLRLRFGTVRNFCLRIAACGMFDMYKGDPRGRILMTYLLIRSQFCLNSCLS

DLQAKQFSVLLYYKAWL

>SG-AL_contig_3864_1_+1_1_204

YKIASLAYLHFDQSLPPYFSDYLCTYQTSRTLLSSTQKFLTVPKRNLKSAGERAFSFIAP

SGTHCQLI

>SG-AL_contig_3865_1_+2_710_955

MSRFVLLEVKAFPQLPSRPTKHHQFLFYFGVVLNFALKLNIFLVCGLLSSVGSSLFCDLV

LKSHCVIISFTVQAYHLTSAVV

>SG-AL_contig_3866_1_+1_1_285

GHFTYSVITCKHLKQIIILAISLTQYHHMQTLKTDNNYPILLKSNNYPISSYTLTQPPTI

CTSTATTIHKKTGVKEKFGYFHFYSFLSHNTLIAE

>SG-AL_contig_3867_1_+1_118_633

MTDMLHGHNRHIKDTNGGERERGGGKQKSMWCYVHDVQDGLFICFIADREQGLKRTVWFQ

AKQPCQSCSLSLLYLDTADIYRLLAGQIEHDASTHRYISFRFQCNQKVCISVLNQPQVVL

TYMLYNHLETDFKTVYRLLPCTKLQAQTRKSTDKRLLTWRWFLTLLYGPNKQ

>SG-AL_contig_3868_1_+3_3_173

FRSFFFHGHKMNFIAAIKFNALNTLFFNSLVLIMLVCPRQFFAVNEKLTYDNTKLKN

>SG-AL_contig_3869_1_-1_67_834

MAQPKNKHVHSAKLAEQAERYDDMADCMHKCVIDCYSGNCVEVLDNEERNLLSVAYKNVV

GARRSAWRVVSSIEQKSEGNDKKLEMTKKYKEKIEKELKEICNQVLELLENHLINKAEID

DIADSHVFYLKMKGDYYRYLSEVSQGETRKDYVTKSNDAYDQATKISKEKMPTTHPIRLG

LALNFSVFHYEIMNNPEQACKLAKTAFDDAISSLDQLNEESYKDSTLIMQLLRDNLTLWT

SDSQGECADDHQQENQ

>SG-AL_contig_3870_1_+1_67_303

MNIVVVSLCSWTTRNEQCLQQDNDRALGLQICHSLSFQRKIAQRIIPCCSSYEVETVPSY

IQFLQFSLLPILKVVHHYL

>SG-AL_contig_3871_1_+2_2_481

TVLQTKTKLKIYKSNVRSILLYASETWRTNKKIESRLRGFEGRCLRRILKLHWQQHVTNR

EVSRRTGTNNIVAEVKQRRWRWLGHTLRMGKNRLPHTALRWAPPGKRKRGRPLGTWRRTV

EEEMREAGKTWNELSWLAQDRKGWRDFVGALCPGTWDEED

>SG-AL_contig_3872_1_+3_147_350

MRKRTHAWKNVIIRLYSTDEQLLISKLFEMLFLSYTRTSLLSHAGFLPLGFLPLGSHTLA

TIQTEHRT

>SG-AL_contig_3873_1_-2_1340_1537

MAVSRISLHAQTYSSSVYADVVLTVVGHCNSMGSFVWFAAPYSAPLRWHCSHSALFQLSS

NKNHLT

>SG-AL_contig_3874_1_+1_1_90

WHGVTKWWSHKHGNFLKIRLKSVQLSTFWA

>SG-AL_contig_3875_1_+1_1_285

AAGVVVAAVNSSINSSSDNTSSSSNSSSNSSSSSSCCCSCCCSSCCSSSSIQFLLLLLLL

RHGTGFRLHITCVYVCVCVCVCLLMKCVSRIFSAA

>SG-AL_contig_3876_1_+1_919_1167

MAGADRSIHSPAKSHPDEENVFTEPPGEVKDLFAWSTEDKASSFRHCFAVYCNYTTTVLM

LIMHTTNFSQQITTCQTKTKEKT

>SG-AL_contig_3877_1_+2_2_223

FHTESIRIYWHRTDHADIYLYNVAMYKLRCYHSTTHSNTNALKNVQTSKQTDKHMRTHVN

LFARYRQTNRHTRK

>SG-AL_contig_3878_1_-1_112_1104

MMPAASNKTEPKTSPEATDPTSRSFLTILDKKVRNIEKRKSRLDVIRAKKAAGDALEKEQ

QETLNNYDGVVASLDFARDLQKTFTSNAQDLEKQQKKQLKREKQEKAANDLKRTVDLLQI

QATLDALGSENVREHYKTGKHGAVVLTEDNLNSLDEIFKLITPPPPGEANYMEQLNAAGE

HINNLLEGKERAVAGSTYKELMDLLHLIIGCGYFERAREAEEEETIAPATETEEKVEEEE

ETPSVPEVPPVNTEKFTEAPQPEVQAPSAPVQPPSPEVEEPSIPHPAQVLQAAVATHEPP

ASPFGVDVPLPGSPPLFQPPPPGPPPPPPPP

>SG-AL_contig_3879_1_-3_210_521

MVIVPGRVEVTANFEGGSSSFQISRPSSAPTHTCAEYVEEGTSVTCTCSSADLGQPAGIV

KWSSSETTQLSLSNINRTQHGSQHVCEVVWNGQRVQSVTYTLRV

>SG-AL_contig_3880_1_+1_286_444

MSPWCSDQYYIISLMTTCMNHQHPANVPLVWQPELHNLSNDNLHEPSTPKILL

>SG-AL_contig_3881_1_-2_8_1528

MSRQVLQVFEQYQKSRTQFVQTVAELASRPQNIETLQNAGVMSLLRPLLLDIVPTIQQTA

ALALGRLANYNDDLAEAVVRGDILPQLVYSLAEQNRFYKKAAAFVLRAVAKHSPQLAQSV

VDCGALDALVICLEEFDPGVKESAAWALGYIARHNAELAQAVVDAGAVPLLVLCIQEPEL

SLKRIAASALSDICKHSPELAQTVVDAGAIAHLAQMILNPDAKLKRQVFSALSQIAKHSV

DLAEMVVEAEIFPAILTCLKDPDEYVKKNVATLIREIAKHTPELAQLIVNAGGVAAVVDY

VGECKSNVRLPGIMMLGYVAAHSENLAMAVIVSKGVVQLAIALSEEQEDHIQAASAWALG

QVGRHTPEHAKAVAVANVLPKLLQCFLRADASEDLQTKSKKALKNILQKCVHLPALEPLL

HDAPPNILKHVVGQFSKVLPHDSKARRLFVTSGGLKKIQDIKADTGTALAEYINTINNCY

PEEIVKYYSPGYSDALLERVEQYQPKV

>SG-AL_contig_3882_1_+3_3_416

CVCGGGSYVCTYATVYHQDRTFKTLILTKNSNKNTFCFILRYTVTGSIFFFCLFGWFLFF

FISLTNTQALLIFTSTHPCTHTHSFTHSYTHTHTHTHTHTHTHTHTHTLSLSLSLSLSLS

LSLLFSTCGSSSSCSSLV

>SG-AL_contig_3883_1_+3_216_320

MGWTVKGEGLTLTVLNQILHSHIWKTKTTYFHTIS

>SG-AL_contig_3884_1_+3_3_164

TRVSRVPCRCCIKWATQSTLHIQTKGQKNEKGEHLTLLNGKGRRFNPLGWKKKI

>SG-AL_contig_3885_1_+3_3_95

FVCLFFIQCVLLTTQHTFPVTSHNIPGISTE

>SG-AL_contig_3886_1_-2_584_718

MIKTWVKQIHPAFSILQVINFIQIFIAYTVDMFKNKTHFTRQKKK

>SG-AL_contig_3887_1_+1_214_354

MFTAEVLYNIINRKLMTYQESAGACCGTKPVHSLTLEFGKHVCVHLE

>SG-AL_contig_3888_1_+1_1_213

TYCIVYLQTILYSLFANNLSLRKYNDQRNDIDNILNINWIDQMVIILIDRFCTKKNMQDN

INIISTDVKYS

>SG-AL_contig_3889_1_-1_1315_1539

MHFKFEVLYIRDSFMAKEKNHTDSAICGRSGFHIVFILCQMCVVHFGGGSSFFFFPHFVC

YTDFHLVSFLMELEC

>SG-AL_contig_3890_1_+1_133_351

MLTREISTLYPPGAVPKEVQTLAFQILQFGSPVSSPFNHRAWWQHYHAQKDPISVEQWCL

VAVLPCPKGSYIS

>SG-AL_contig_3891_1_-2_77_724

MFCKTCLDQPQIADQKGNLFVGCSSFRKASLQSHAVSIRHQMCVEAQKAAASSASPADQP

DDNSLCHVEEERREQVMKLFNTAYYVFKLELPFSSYPSLLRLQEKNGLDLGDSWKTDCSR

FLGDIFQALFEPKLAAINKAKAISILVDTVADCRSRDLLCVYVRYLSEGVPTSCFIAIEE

LQNSNAEGYIQAIDRALDGAGIQSWRERTVSVGSDG

>SG-AL_contig_3892_1_-2_302_466

MFCDRMNQIAVDLTSSLVLISFLQTSCWSTYINQSCRLLVIFIKLRACCQVLCVA

>SG-AL_contig_3893_1_+3_1701_2111

MLIQNALRRINDTPTVGQNQSHKQSGIQSPYHTYSVSSSYNFKTFLHTPTPTHTSTTEMY

TQCTLSTQTSTTDTYKQCTLGTQTSKTHTYKQCTLSTQTTKTHIYKQCTLGTQTSYNSSV

SLRQRLLKVNDKFVLTM

>SG-AL_contig_3894_1_-1_52_249

MHSQEIVVWVVLVSTKLVKILLIGKLEGVWARVMSVVTNFQQNKRGKYVLFPVQHGHELS

MFMSGC

>SG-AL_contig_3895_1_+2_2_211

NYNTEISAVTEASVYHLDNNFIQISLFTDFITQYAIKTIHNVRISNKKSKYFTIFIDQAV

DEKLFTKFKM

>SG-AL_contig_3896_1_-2_617_1138

MSPQDAVGSTWSMGLGELHMGASSVSILDPGPQLNEMSPSQAHERPIAPPSADMLDWCSK

LLHLFQSGPTPKADLLMTGSTCGVSLRLVPIVTPPQDLLHPLSKDADEGNRSRQAEESFS

CGAPDQNPILASRVAPSQCKDITTPGEGTPLRQGPAHRGVDPSQHPCSSFDDGR

>SG-AL_contig_3897_1_-2_338_664

MSDSLNTACFIPTSPRVTSREGASFKQNPPFPSRSHSSPQELEGIGIGNSVRIAMRLHLS

RSSLSPSPETLSLSHVVADHHAANFPRSDKETASHPDITLRAYHEARLH

>SG-AL_contig_3898_1_+1_355_615

MKGLRYLIYSRGNREERKGWAEEGGVEVRTKKTKFEPTKLLFLLCLFTTKKLIHDMFGNL

YTFIKYAQSINITRRSVNCLRMEVSSR

>SG-AL_contig_3899_1_+1_1_312

IPKYLLDRLQHIQNNAAHLISRSSKFDYVTPLLHSLHWPPVSKRIDYKLSSLTFSVVHGS

SPKYLSELLHLYTPSRQLRSSSDTRLLRIPSFRTKSYGDRSFKC

>SG-AL_contig_3900_1_-2_266_373

MICLGLSRKPRDKQRQSCTNKHKSAASQLPQSRFTC

>SG-AL_contig_3901_1_-3_153_1106

MFRNPRDCTTYFWCVHEQQLVRHCPEGLAFNAATTNCDWPDNVDCSQSSVISTKATAATT

TAATTSTQMPITTFVQSTTTIVTTARLTTPSATTQPPATTAMPQITGFPGCSRRVCYHTN

WSQYRPGSGKFLPENVDPFLCTHLAYAFAKLNGNRLAAFEWNDESTEWQKGMYERFNDLK

KTNPNLKTLLAVGGWNMGSAPFTSMVATAQSRQEFADHSVTFLRQHGFDGLDLDWEYPAN

RGSPPEDRDRFTLLVKQLRETYKRDAAKTGKQPLLLTAAVAAGKNTIDTAYNIPQIAEHL

DFISLMSFDFHGSWEGVT

>SG-AL_contig_3902_1_-2_2_175

HTLHQYYIFTHPNVPSRMCIQSLMADMTIIRTPQYINIHIYAYTYIQDIFCFITCIFD

>SG-AL_contig_3903_1_-2_1340_1636

MINLFGFGLKVTLYRKIFKTPNWRSLVTCKCHIGAASCLTSPLAWSVWSISGPGFCLYCD

LQNHTVLQFADPHCVTVLPKYVFVVVIPFLVTLDIPEKF

>SG-AL_contig_3904_1_+3_30_266

MCLSEKLPHSLPTEGSVKHLMIRCVCLKSCLSIESKRGSSTRYMIGLVVSCSSNLVHVWS

HLSLKDNRPALPDAEKTKN

>SG-AL_contig_3905_1_-3_6_377

MHVGNETNPGGTCSSAGTAWEGVLRPWVLLLPVSLLLNSSHSVLFKCNCALRALLLPAYP

VAGNVLPVCGDDAAADEVCLQSILVSLDRFYFRVKYLFAEIQKYKIYTIWFSTRTTITIA

REFN

>SG-AL_contig_3906_1_+1_127_249

MSNIPHKNSYAEKCLMAKELHQILIWDFYHLVKWTEITHSI

>SG-AL_contig_3907_1_-3_3_221

FVCVYFEVRIPDFFFSFSFPFFSFQKASLMQNTKILKGNRYSQNKMSVNELLPILLMYIN

KYGTVSILCHIMV

>SG-AL_contig_3908_1_-2_482_760

MHRYAEINKKYKQKQEHRKAERPIFSCVPITTTTTTTISQNSFFFKSLSFSTSLLGFFFV

INFFQNDLLVDCLSSSVYIITEICHKIELKLVE

>SG-AL_contig_3909_1_+1_403_660

MCMCLQMFPKDKYTRWVSSVRNVKTLNDSTQHASPFYLLTFGSSLEMSIIIRDMATNCAT

VFNVIALLCHKACKHTELHNVAVSFN

>SG-AL_contig_3910_1_+2_1586_1783

MGFFCPDQLVGKTETTQLSLIHFTDASLRVLFKFPNQYHRCLYLLGLVDARIYYERKCRI

QPCHTV

>SG-AL_contig_3911_1_+2_1541_1816

MRICISSFTCSAVCWTIVYRFCWVFVSLLLSSMLPLGLKDLLISRMPWNGNFYFWCAQKC

PTTGIRASLIYQLELFTNCRVLWHGKTENKPV

>SG-AL_contig_3912_1_-2_2_343

DTLSSVTIRPFPDIVSRGEEDRHLCPVRSLRWYLDRSRTRRSSQRRLFISVNESYRKDIS

AGTVSRWIRETILKAYTQAQKDTSCLNPRAHEVRAIATSAAFQHSLPLREVLEA

>SG-AL_contig_3913_1_-2_68_202

MCLRYSHVFVLMCHCLRYSHVFVLMCHCNVSWIQSCVCLDESLCL

>SG-AL_contig_3914_1_+3_3_269

VALPIFFFFSKYKWYFISTFPPQHPRSSHVMTTNTSDIWSRSKLKVIVIQRFHTLFSVLP

PMHICKFDQCGYKNEETKPKTNNKIPQNK

>SG-AL_contig_3915_1_+2_38_598

MLPLVWSRHRLGVHASFGVVKSQTGVHASFGVVKSQTGVHASFGRVKSQSDWVHASFGVV

KSQTGVHASFGVVKSQAGVHASFGVAKSQTGGPCFLWCGQITDWGSMLPLVWSNHRLGSM

LPLVWSSQSDWGGPCFLWRGLHSQTGVHASFGVVVTVRLGGPCFLWRGRHSQTGGSMLPL

VWSSLSD

>SG-AL_contig_3916_1_-1_2014_2271

MDLMIQLLVVFENLKDMCVCFFNKKNLSAFISVICITADMCAFICMYAYLILIRINMSVC

MLARMRSHTQKHTLSPTCCFSLSVFL

>SG-AL_contig_3917_1_-1_352_7806

MPRRRREDKSELRKSDLNKASSHPGVSHTGGEDRGRKSDEEVSDIRSRFSGLNNDKSKTE

GAKSTGVARHPSRGKEFSQLLHKFSSSSEASSSERSDSESRQRRRVASFKRPLEREVSSS

SDDTHSARNTPERTQSLKIVRPTLQEGGKEKEVGRSGSFKSDFMRKRFSPVPQGDAPSAE

LSQVLATHQVVERQHQVKVTEGEQTADRRQVHAEHLKSSAFEEMIADKEVASVLKAHREK

SDLMKESHSDSQSGFSTIDQSLAVLARVTHELQEDKEHSPKTQRLRPPVRTSDSDKDRRE

SLEEVEQFISDVETRISSSLHLTDTSVSQKGLTDKTRKESSLSASVSVTSHAADEKRTAD

ISTTSSVQVSEDRQARAGSKNRTVVADTFKSESRERSREHPNIVEKEQESTTVSVTVREE

KEGSKEREKRDQSTEKETRTVTLPTSSAVSADAKSTCESASTSSTGIRRAESMKQPTSVT

RSESFERRKGILKRSPSLPKQESKPTIDSQLFRIMEQRRQKQLEMAKEEPEEGQEPVVPT

THQRALSAAEEIEENIRCMREGKEGKEVAAQHSEEEAALCMPVAERIFKMQTKIKEKGDT

PVTPKQESKPTIDSQLFRIVEQRRQKELEMAKEEPEEGQKPVVPATRQRALSAAEEIEEN

IRYMREGKEGKEGAAQYSEEEAALCMPVAERIFKMQTKIEEKRHTPVTPKSRSGAATPKS

ARSWSSSITPCKQLSVDEGVAEVTAQGPTFTTSTITTGDSISSNTKTEATIEEEVSGLQL

MERLTTLAASNAQFQERRHKFQKRPREDWRTRTQPITLDEIHEADSLDTVSAFRALVRRN

ASKSMFDQLKTQEQHRPLPVKGTEFPQHLTPNKRGRRTRVQRHKTLPVTAEELNAIPEGE

LMEPVPLRDTHELWKRDSRTDSGILSGSDMDSLGSDSLRSLTLEMDVADNDPSRLSVSSK

TSLFRSLDDRARQEREKEKSASGAKRYIDRKKRERSRTQPVTDDEVKTAAEMSDEKKPTP

VKIPQPEVVNRPKAPSVDLEEEEEKDDQLIKLSLAEKVKLFSQKQKEEKEAAAKPPPRAD

APVPKRRQRKLNSRFNTQPVTPEELEKASKISPLAMSLVKPPDPELLMGLKLKDQQELMM

LHAEATLTKCSSRPSSQPGSRRGSVNAPDAPASADQDEARHDVSDKERTSTTTTTSNTAD

SNKQAVSSSHSDLPRTDSEPRSILKKTKSTESVPTDLKHAPSNQVPLASEPPTQVPQDLK

NEEEERDCASPVPEMVLLSGVGGEEEEESTDISFYLADEDLAGESTTDTAVTEEAAIRTP

DSRRRSRGADRKAQAERYLTQPAPQLTPERSPQTKRKYEGRHMTQPITPDEMKEAEYSTT

PSSKSGGSIADRLNQLKTSGEENWKKRVTKDKELTTPVTDVKWREKSGLAAVRPASISDR

LNKLEVSSEQWKERVEDTDAKTFTVANKITKALVSSESPLVAKIRASSKKDLTRLDDSSN

ASSPVNSPSTPTKEFLPKVPLPKEIIADPEAVKNAEKKEKHKVELRGVDSGEEEKGETVR

VEVPRVDEAEIEQFFSTKEITEITDKVDMDVDDFDNIFIESNEILQSVKKIRPTRRKKAT

SRNPLKTVSQFVEIQTEYIQVSTGAAEKHLRKMKTEGLKRDAGFALEALAGLASKENFSK

VELRKTEPSSLPASLAARRNPFTDLMLLHIKGRRNTQTRLVEPSSKSMNAGDCYILVAPD

KVINWVGEYSNIIEKAKAAEVASFIQQKRDLGYKSTAAVQVVDQAKDHLGPGKHFWAALK

GEKECQACGPDEEDELYESSITKTNMVYRLVDGALQPYEKYWGAIPKHAMLDKKEVLVFD

FGSEFYVWQGKEVSSSQRKQGMSLAQKLWEKGYDYSQCAVSPMAPLRSEEDGGIPVQASQ

RPDWVIFGKVNQNMETILFREKFADWPDTSRLIGVKRDKTAEVKYDFADIKAYDAKKMIP

VNNLPVTLLLEGAHVGRGKKWQEDMQGFIKEQDILTLGTKVWHVMEYEHYQMPENSYGQF

HRGDTYVVRWQYMITNAGLKSLKGQAARHSLTGRERCAYFFWQGQDSTINEKGASALMTV

ELDEERGPQVRVVQGKEPPCFLSLFEGSMIVHVGKREDACTNTEGPWRLYCLRGDEAGEL

CLLEIPVSGANLRSCSSMVLLNTRTGITYIWHGVKSPKHKRQLAVTAVQNMKEKRPLELD

LHEDAHIIVTEVEEGEEKAEIWLALDSKDRSLYHSLLTEPARYDYTPRLFHMTSVSMVFE

VIEQMNPARLPGVTTPFPFFQSDLYKASQPALFLLDVGRCVYLWQGWWPVGDEEMENVHT

GSAAARYNVDRRCAMETTLNYCKERGSAGRTPEAFLVCAGVEPLAFQNLFPFWEVDSTVH

SLAVLDGKREGYCEPVEDVYIKLTQTRYTLEELQERPLPDGVDPLKLESYLADEEFEDVL

EMKRDEFYALPTWRQGQIKQSVGLF

>SG-AL_contig_3918_1_-2_2156_2344

MHIITSVTRTNYSHLMEWLFLAQVLTHQHAYTCSLLMQRHDHLRFSRACTYRTCACTHLH

TCM

>SG-AL_contig_3919_1_-1_1_102

IAVVAGCVQTILYCDFFYLYVTKVLKGKKLSLPA

>SG-AL_contig_3920_1_+1_112_291

MILKSVQQFTALKQFHKHFSQLNLPVMIWKLLKPPCRWHLDMEDKHDLNSVSKYGDHQIN

>SG-AL_contig_3921_1_+3_3_260

SCVVKFTKDKNEWNISSFKNTTYLVLFEVSWYIASIHHLTYSVPMQIHYKLHLCCHSCTS

TCLHYHTSFYKILMSFRYGSCLEVQG

>SG-AL_contig_3922_1_+3_657_845

MYTYMHMHAHAHTHRVIFTLLFSFKPSNPLDRNLACRMNYCPCTVEPNLTFQTCKQFSPK

PQA

>SG-AL_contig_3923_1_-2_230_2029

MSIVKRLERQAAVAESLKEMESLEDESAWDRGKGRTGKAAPKRTESKGEAPVKARRRRRE

SKQMREKTPPLSPLETSQKSSLDSAQSEADEMGDNEVFSGVQEEAASSKRPERAWEHEAE

SQKPSKTNSTGVGVSIPLLFGKTVIDLTPASPQYQDPPEIMEALGMGHFFNQSTQTPDRF

YREMCPSPHEFSSMPPQSDKSTQTPAQFYPPIPTQDVAIDAGQFPDTSNSSMYSLDRSVT

DGEEAPHDNGGGGEEEVVVQEIPPVVSVMPRPKVEKAGIFLTETPQEGGAGGKPVKLPWQ

SSMELPESPMFVLPPPTPRKPWLAPHRKPPAPSPATVTLQNPPSSLNSSPQQHPPPVIVQ

QPKAGGGSSKETAFETWVEKQSAFEEVPGASESAVLAQPQLAATASVNLEPPPAASSVTL

QQPPYPAAGSSTSSQPTQAQAAASVTLEQPSDAGGSTSPQTVEGGASEQKRVRAKQRRER

RRRPGPTSESSTESTVTPPSTPTHKAPAGEERLTSPGEQQGKKKAPPPSVSACEGSGQPK

GGNHPPAGHNRGDNPAGTREIGGNNLWGEKKRQQSASLSSQISDADEFVLANESVENNHR

>SG-AL_contig_3924_1_-1_1_351

PSDLKKEEEEKSRIQRRRRRSRRRRRRRRRRNVNKVCQCKQEICSKSFFKNCGIRRSKLS

ASDESAHENDDDDDDDDDDDDDDDDDDDVDDDDDDDDDDDDDDDDNNKKKKKNNNNF

>SG-AL_contig_3925_1_+2_2366_2704

MRIIMERSNISVSNIHTDVCVSVGILSTVCVCVCVCVCAYECNCASVDVYVLAYVHACVR

VCVYACTHASVRTYSVCMCAWCMFACAFVKFVDFAMQHSVQFVWRYASLYLCC

>SG-AL_contig_3926_1_-1_1375_1518

MTRDLPTLKPAVQRRCPRHSFVVLPSSAYNTGPKRVCSWKDRLLLAQI

>SG-AL_contig_3927_1_-2_89_748

MNRLFGRGKPKEPPPNLTDCISNIDGRGESIDKKIARLDAELSKYRDQMKKMRDGPSKNM

VKQKAMRVLKQKRMYESQRDQLNQQSFNLEQQNFAIQTCKDTKTTVDAMKIGVKDFKKAY

KHINIDQIENMQDDMEDLMEQANEVQEVMGRSYGMPEIDDDELEAELDALGDEIGLDEDA

TYLDDAIGAPSAPTSEPGDSMRGDVPVDEFGLPQIPQSAK

>SG-AL_contig_3928_1_-3_117_1271

MLAVFRRSASQGPRTWILGVLQGNGYATEAAFDTQPFKLHRLDQGPSTTLTVTRDQALKY

YTEMQTIRRLETTAANLYKSKAIRGFCHLYSGQEACAVGMEAAMTKDDAVITAYRAHGWT

YMRGVPLAGVLSELTGRASGCAKGKGGSMHMYTHNFYGGNGIVGAQVPLGAGIAFAMKYE

GKQNVCVTLYGDGAANQGQLFEAFNMSKLWDLPVIYVCENNGYGMGTSADRASACTDYYT

RGDFIPGIWVDSMDVISVKSATEFAKQYAISNGPILLECATYRYHGHSMSDPGTSYRTRE

EVQEVRQKRDPIGLWKERIITVGLATEEELKKIDVDIRKQVDEAAERAKMDPELPVEELY

NQIYIDPPSNMKVRGCDPTIMANSR

>SG-AL_contig_3929_1_+2_2_76

RNCSAAALMGHSVKHKHSIFQACFS

>SG-AL_contig_3930_1_+2_2_76

RNCSAAALMGHSVKHKHSIFQACFS

>SG-AL_contig_3931_1_-3_3_371

VFQDGRGSHVVDDTGEGDDRPTSPPLDQAIGVDADSRHVSPSARGSPPPTDDAPPSPAVP

TAVENAETETADHDGPPASPQSPRRSPSPDAAPGTQEPPRSPGGDTNLGSFSVIDSSQPA

HAR

>SG-AL_contig_3932_1_-2_2_241

RLEERGRHDFSGRDEIRNDFCTFDSQQVSQDGNVDDVGEGEDRPASPPLDQATGVDDGPG

HASPAAWGFLPPTDDASPSP

>SG-AL_contig_3933_1_+2_839_1126

MEETLNIFKLADLADPVTFFKCQIMSIVAYFQIVKYRHETSPLTITEYNRKTKLYHFPKR

LTKEKLNNQLECTCLHYKIPLYSRWVEGKKDTQVTG

>SG-AL_contig_3934_1_-3_129_281

MIDLFATKFSKRLPVYVSPVQDPEAFAIDAFTLDWSGLLAYAFPPFPILQR

>SG-AL_contig_3935_1_-2_2_178

LHIDFKMEYYDANFGNHLEENCIFFKVDPQPIMTISSLFRGFMLKISCYERFGRARISM

>SG-AL_contig_3936_1_+2_1091_1321

MKRPQERPIFCYFSPGTLVVCTKEQNQREHDFHISAPSSPPLHDIIFIFLALQDITEPNF

NICSTSVEESQAIMLSF

>SG-AL_contig_3937_1_-1_292_549

MKYYSNGNGSQVVVSVAPQRLDLRAGTDLDHASSNPFCSLSVLIMDKTMAEASLPIREKW

ALVSIVGSTWPHLPPVNRHQNRRSIC

>SG-AL_contig_3938_1_-3_93_647

MREYKLVVLGSGGVGKSALTVQFVQGIFVEKYDPTIEDSYRKQVEVDGQQCMLEILDTAG

TEQFTAMRDLYMKNGQGFLLVYSITAQSTFNDLQELREQILRVKDTDDVPMLLVGNKCDL

EDERVVGKDQGQNLARNFNCAFLETSAKARVNVSEIFMDLVRQINKKSPEQNGKRQKPKK

GCTVL

>SG-AL_contig_3939_1_+3_438_614

MAFPHPTASQEETVVNFTLRLNSKHILCTVTRFQIHDLISYTLTNLPTSSSTTAPKTNL

>SG-AL_contig_3940_1_-1_349_879

MAAAARGECTDSCKSRRRELENDAKQLRRDLKMREDQFRLLDREAQSLRQYKDAQKEAEV

LLKALSDMQDKNLELENLLSTETRFKQDLFSALGEERRVIKVLQNTIQEKDMEIRELKSK

IAEVMALIPSTSSSYQNLSSDRNSSPLFSVANFCQEDLVVASDLNPNASDYTPKTAI

>SG-AL_contig_3941_1_+3_108_350

MEKQDARDLNFTWGQHEEDTLEKRGRWTLQRKGLTWCTTTFEDRTTTYRGTDSSSGSTDC

EMNYEWKRCHYIFKGTFTFTS

>SG-AL_contig_3942_1_-2_2_310

PPLPSSHPEGPGPLLHPGVPDQPPEVRPHAVTVFRLPGHEVRHPCTEGVPDTSPAPDPPA

QAVVSPGMDLGPRQGDRLPPGPHGVPSSSSPTGQSPQAPTAEG

>SG-AL_contig_3943_1_-1_226_810

MACCVAWNCSNRAKKGTGITFHRFPKQPTRRKEWVRALRREGFQATEHSRICSNHFEESC

FDRTGQTNRLKENAVPSLFDFPKHLQKARKEQKAPPNRETTDLSLTLPSTSKIVQDGHAA

TGVSYNHQYVGAEHSYCVMSPHNMKRRLDKNQDALDTYKKKLKFLYQKNRRLQKKITSLT

EIVESQKSTSLISDS

>SG-AL_contig_3944_1_-3_3_416

LLTGPARRHSNRFPVLVHALLWLWPVCRCRCLHKQQLPGQHTGFFEQHRQCFAQHVRCHQ

TAFHSSETPSRSQAWKEIATREHLELHSDTLQRTSCRLGELAGQFVCVKVWPSSISAVTQ

ILISIISFCLLTWPSVAD

>SG-AL_contig_3945_1_+1_937_1251

MCKRNNIRLKSKLADKTPSSRCGQAFPPFPTLYITDYEVVKPSHLFQHFTSMTMKWSSIP

TFSSTLHHYVNMSFQTRLSHFNPYSDLGAEQQIIIKMLQVPSGDL

>SG-AL_contig_3946_1_-2_2_214

KSQVIFPWKFNTGENETVEQIKWIFDSGNNSVMVASYSHKLFLTNPEYSKRVRHVTPGGL

SLRGVKVSDTG

>SG-AL_contig_3947_1_+1_1_111

LSSLVQTIELLWPTVHQHIIVLANLDFLQHFWKLAVT

>SG-AL_contig_3948_1_+1_103_249

MRTTSRQQVSLKLKRSFVYLLHMAHYCTLSANNLSPFSCKNTTNSINKW

>SG-AL_contig_3949_1_+2_2_259

TIGGGEGSPPLVCLPRGGVNNGVGRYEGDARQTFCAGDPEVAQVVVGESGFTPPVGCLQD

VPDGKGEKEGGGRRQGPEIVGSGAVQ

>SG-AL_contig_3950_1_-3_369_599

MATFALYSSILYRLRNIHLKTAMSGGPVFSLHLLDNDNYYYILFIAQPTIKAISGLSPRK

AHQSQEDNRVDHNHLQT

>SG-AL_contig_3951_1_-1_1648_1863

MVWAIFTLYHLVRISIMHGITGVSSSCESLSMCPCKFRELQMCICSFHPASSTCFVNRPG

FKLDFSFVSQNY

>SG-AL_contig_3952_1_-1_49_225

MFYGIISVEKIATYMILLRLQYTVLHMSKLSTDTMTKNPDLPLLIQQPVILPPRVRSCI

>SG-AL_contig_3953_1_+1_1_171

APQRNFSRINCFSRGWVISFSLKSAISRTIPMPDIYSSMCFATGNYYKRKKLNVNKN

>SG-AL_contig_3954_1_-1_76_243

MEPDRRVDLHGDLESIPSFYPLPLCAFSFFFLSFVVVVVINKIVFLFHTQIYVSIC

>SG-AL_contig_3955_1_-1_250_387

MFALGVYNHLMFILGVYNHLMFTLGRGLNELRVYDHLMFTLGRGLT

>SG-AL_contig_3956_1_-1_217_324

MGERNFALFHFLQSFQMFTVLVHVQNSCLAVTISCH

>SG-AL_contig_3957_1_+1_139_303

MKPSFIVPSNQTAHYQNESNNSMTGASECSTILKSHVLHVSTNSHSSRSLSLSIC

>SG-AL_contig_3958_1_-1_82_1128

MFPELSFNIDHGYLEGLVRGFKSGILRQADYLNLVQCETLEDLKLHLQSTDYGNFLANEP

SPLTVSVIDDKLREKLVIEFQHLRNHCLEPLATFLDYMTYGYMIDNIILLITGTLHQRPI

SELISKCHPLGSFEQMEAIHIASTPAELYNAVLVDTPLAPYFIDCILEQDLDEMNIEIIR

NTLYKAYLEDFYNFCKQMGGATAEVMCEALAFEADRRAFIITINSFGTELTKEDREKLYP

RCGSLYPDGLDMLSQCEDYDQVRAVADYYAEYKELFDDAGTNPGDKTLEDKFFEHEVKLM

KNAFMHQFQFGVFYAYVKLKEQEIRNIIWIAECVAQRHRAKIDSYIPVF

>SG-AL_contig_3959_1_-3_162_608

MPTFDPTDKQGGKAFRKFMEEWEGTFDEINKDFAEDEAYKEDEDLLDKLQQYKKKFIECD

QDLSGDLNMMDLKYMLEKLGQAKTHLELKKMIQEVDSTNKGVINYRDFVRMMLGPQSGVL

KMILMFEAFAKPQEKPTGKPPKRDLSSLP

>SG-AL_contig_3960_1_-2_167_301

MNKLHMHTLQRSHNMTLTAQPHSAEEHHSCAFTELGIAFFFSFFI

>SG-AL_contig_3961_1_+2_248_550

MLTRVIQIRSLTYPCSLIYFKTQVNTIYTYSFFFCILFCMLSYPHNLVYFKTQGTHNMYI

LFFSPFKITASLYPADNSTQLEPCVFTRSTHPFMHNWLTQL

>SG-AL_contig_3962_1_+3_525_734

MVSLSIQSTASLGQYLNITKQATGAHYRIAVKMAAYISFFNQVSTVNVCLNFLFRFYCGG

GEKGGGGERL

>SG-AL_contig_3963_1_+2_215_370

MLPTTHTHTTSFSRILFHRALKFYNQSSIPLHHFRHTHTFAQFQRVSDDKFA

>SG-AL_contig_3964_1_-3_801_1010

MLWKVSRIILYLLPLAVVMHLKGLSLPLHDWHIRGSNPCPNNPVSTPLPFLHSPPLSFPN

FNFYVYSCVH

>SG-AL_contig_3965_1_-3_201_758

MATPKLNLVVATCNNGGIGIEGRIPWRIKQDLAFFRKITTTTEDAEKKNAVIMGRKTWFS

IPEKFRPLSDRVNVVLSRDLPEAPKGAYLARSLTDAITMLTTGPLSSQVESLFVIGGSSV

YGEAITSDKFPCRLYLTRVLGDFKCDVFMPEIGDKFRKLPNPNNVPVEVLCEKGIDFKFE

VYEKDL

>SG-AL_contig_3966_1_+2_2_289

RESKENRGRPRLTGSRTDWRLIQIVKNNRRMPLEELRIKFNELNCVKLSASTMKRRLKEF

RYKRRVVKKKTVILKANRIARRAWCRSRLHKTVDQY

>SG-AL_contig_3967_1_+3_1440_1637

MTDRSNYEQLFQDRLKTKRWSEEITPFRYSLPKMINRSSFEQLFQRPRPGETGWEKSLTS

MLQSDL

>SG-AL_contig_3968_1_-2_2_223

ILCTLSCHISCYVTHSPSHVTLHPTRSCHTLHTVICDISCYVTFTLPCHVTSHTFMSYSA

HCHVIEEFCQVNSR

>SG-AL_contig_3969_1_+3_600_734

MRRESEVPCAARTWRTLKIPRQQRCCPWQNSVEKPTVIGQQIYTH

>SG-AL_contig_3970_1_+2_149_358

MKTCWKKESGMDQEGRNKQTTVKEQKMAETKRAIEKRKFLAQNFQKVVIIFMLKEPSASS

RGGYLLLSST

>SG-AL_contig_3971_1_-2_2_199

DDLINHLVIQTNLCTDQFMEAHPNLPEYSRANIISTFTIQITNKIKLSSYHIIAKGTKEK

KKKPKI

>SG-AL_contig_3972_1_-2_14_280

MELFFYDNLINHLVIQTSLYSDQFMEAHPNLPRYSRANIVLYCIVLYCIVLYCIVLYINF

YCTNYQQNQIIMISHHSKGYKKKKNIPKI

>SG-AL_contig_3973_1_+2_2_292

HTPTHTHTRARAHTHTHTHTHTKTHTQTYMHTLTHTHTNMHAHTLKHHHDAIKLPSSHPA

HTVHCNLSRTICKEQNNQQCVPEQNNNDDLVQENHVS

>SG-AL_contig_3974_1_+3_279_431

MPFSLEVKCLHHSTTAPVCLVFSELQIILSLWQACLCELFCCRESRLNILI

>SG-AL_contig_3975_1_-1_3994_4326

MYNITRVVRRPVYSARDSNSQSFAASLSLDQLTLLKLEHRLIYYRLNHTQTLPSRRLDLL

VGFISPQIVEEGESYCLQRWTSREEGERKKERKKERERERGQAQEQEQILL

>SG-AL_contig_3976_1_+2_149_370

MCLGKNTPFGFIYCTMLDCKQNLMEEGLPLWFERGCLDSMVVLAAIKEMFLEQSALVVLT

SRRQKLMKRFSECQ

>SG-AL_contig_3977_1_+3_93_416

MTYINRAVCLPPQDSYRFVTPKFTNFSQTFKTIITNSKTRFAKYDCLFSCFFLLLFFFHS

LTISKMNTECWKQKLDGHISTASLPSQDFVQSIKCKFFVVVFKRNMEL

>SG-AL_contig_3978_1_-2_389_622

MCSNLIIIIRMFCIISLCVCVCVLRRQMCSWLVACLSLFVCVCFLLFLYLLVVCVCLIIC

LFFPLKGLRGSKGHKYIV

>SG-AL_contig_3979_1_+1_1_303

CLQSHYLVARTKRHRQYIINWGERKLCEGLLEKRKWNGPGRQKKPQTTVKERKNGRDKES

NRNEEIASTEFPEGGDTIYTERAPGIPTGVRTDSNQSNQFI

>SG-AL_contig_3980_1_-1_1_303

VRERERESQPDCSEQNGARFFGPWGVFVRADGNRKELRAQPLTCRLLDQPPLSWQPVQKY

ISFGSTILATFRPDLTRRLLLTYSFLRFRTFFSSQVRTPDL

>SG-AL_contig_3981_1_-1_1_309

VSSSSPQLEGELTWHPDSGCWWDFRSPGVRVLASPARQPHPRALPLRGCRPPRHRRVLRT

PALLWAWRWGRRGGPPGPEVEGVRYEGVGEGSSSPRPLQPGFI

>SG-AL_contig_3982_1_-3_81_512

MSSSKTSTEKSQKQIVAGFQELRQQQRSIASMMAEIEMDMKELELVIETLKEVAPDQRCY

RMVGGVLVERAVGDLLPSLSSDKEQMTQLVENMSKKLEAKGREINLYREENNIRVRGEDD

SKPDAVVSDSKSGSSGGVLVANSS

>SG-AL_contig_3983_1_+1_154_396

MRVFPDLSQAPVTPAETGGPAGMALSARQGNLPAGLHGTPSTSLTPGWGAQTSPAEDQMA

PAHGQLGSNRSPGRLVPGSNP

>SG-AL_contig_3984_1_-2_146_337

MHAVFISRYFLWCFYIYDFFQDIETTIFFVVIHFIIIMLLHMLNYYYCMLFVCMFVPKYN

TDCN

>SG-AL_contig_3985_1_+1_139_348

MAQLESGYPPCRLWHSHKQHSIYIHVQNIISLSYSGTSINLQCFSQPCHYHSNHFLTFFS

LLSFDCHHRC

>SG-AL_contig_3986_1_+3_855_1052

MIILKKHHASCPLPAKEIINLPLEKNPEQNEQYSMLTIYNTKSNFLHNWSLVDFHEKKNV

YHKPQN

>SG-AL_contig_3987_1_-3_3_227

LQSLNSALVRHGTVYKILLSFAFAAYMVRPHLAMNMAVFSKVLPAAMELGGQWNCSWSHL

VNQPCWSCGNNYSST

>SG-AL_contig_3988_1_-1_115_321

MRASSSYHITQREVERRQQMLDGLITKEKQIDQAFKNESGDNRQALLGPSTDPFRSGGGD

PWAEPEHLQ

>SG-AL_contig_3989_1_+1_196_402

MNASLHHTHTHPHTHTHTHKDDELEYTQPVRHVTLNPSCYLLSFKHTHTHTLKRRRGVRV

HSACQTCDP

>SG-AL_contig_3990_1_+3_1704_2033

MHMFILFRVFLLCFLLLHICLLWFISFIHYCFFSTVPGPSSSESELAFPAESEPASEVAL

SVELSDAACSSVELSLGSSACEDSVDVEEGSLPRSACRICSMKLCSFFMA

>SG-AL_contig_3991_1_+3_111_371

MYHEINNTPRIAACASITKIVWSVLSTYSNAMEIRPRQNYSAIHFPLKQITAVLFNTSND

REKQNQRYHKRNGSLVAQNSVGLYFLA

>SG-AL_contig_3992_1_-1_187_1059

MAKSGIKPTACSSNGARNKRDFGFYNGGDSNLRLDYGLPVQELCSGDDVTSKLFGDISMS

LVNAIGVQNLDLTGTCQNVQRECRSFTTYGYEEYALYCIGTTTPSTPTTTPAPASINAVL

NNVFPEVLSSLGVGESSLTSVVDNVLAAYPVISQLIPSFKAVLKCAAVPDIQAMCFTLAL

CAETPGMTPGYKTACEMALNFYKCKANTSLPVTAVPIPPPTTTVPPITTTTTTTTTTTPN

PNRCGVSSDLTSVTNVADRSLVFTMARIVGGTVTGSCSLVPWQVRLNINNT

>SG-AL_contig_3993_1_+3_3_218

IQIHTHTYCQQHCFISNNNNKVNTFLLFRTLISFYFNYFFCGTSIKPLNIKYHKQQTQTN

LIIHNSLKVNAC

>SG-AL_contig_3994_1_-2_11_1603

MTVGIINMASKGGALNMRSLNGPLSDAAARQTHKLAVSRDYISQPRITYKTVSGVNGPLV

ILDDVKFPKFAEIVNLTLADGSQRSGQVLEVSGSKAVVQVFEGTSGIDAKHTTCEFTGDI

LRMPTSEDMLGRVFNGSGKPIDKGPPVLAEDYLDIQGQPINPWSRIYPEEMIQTGISAID

TMNSIARGQKIPIFSAAGLPHNEIAAQICRQGGLVKLPGKSVLDDHEDNFAIVFAAMGVN

MEIARFFKQDFEENGSMENVCLFLNLANDPTIERIITPRIALTTAEYMAYQCEKHVLVIL

TDMSSYAEALREVSAAREEVPGRRGFPGYMYTDLATIYERAGRVEGRNGSITQIPILTMP

NDDITHPIPDLTGYITEGQIYVDRQLHNRQIYPPINVLPSLSRLMKSAIGEGMTREDHSD

VSNQLYACYAIGKDVQAMKAVVGEEALTPEDLLYLEFLTKFEKNFISQGHYENRTIFESL

DIGWQLLRIFPKEMLKRIPQSTLAKYYPRDAHRAQTAAAAGAAATKETTAL

>SG-AL_contig_3995_1_-3_63_242

MKRKKYGDHMYGQFESFYMLHFTLWLNKYKQTVLLSIRLLKRAAYVTLFARICSLIQGKE

>SG-AL_contig_3996_1_+3_3_215

MILEICHLIHILSLRGTDITFCWIPSHCSFYYNDKVDRAAKRGAKRSPDSNNIQLPLSLN

EYYTLVDTYAW

>SG-AL_contig_3997_1_-3_120_335

MDCICYCLCLYFLVLIHITGLIDLSVIKCVKRFGDLHNTWMGWEYIPLKCAGILKHDIEH

DQNNYKPTLFFC

>SG-AL_contig_3998_1_-2_2_211

LALTALPEFLVETQQPGSLPQTVRIPCLVPCPDPRDARSEERPLARSDASSTTCNARHLG

VPPSGAYFLP

>SG-AL_contig_3999_1_+2_2_169

PDGQAHRDRLIGKHTETGTRTDLSHRHRSKHRQEQFVHNKYALTVRQQTILSDNAE

>SG-AL_contig_4000_1_-1_1_138

EFSSLSQHCPSNVQTSDPRRIETTSGLYSLYLVVASSVDGSASDPD

>SG-AL_contig_4001_1_+3_51_224

MYNGISSLFPEGMFDQISSFGCREPRRICSSSLYPFIFVIDLHMGKQPINTLQKLVLC

>SG-AL_contig_4002_1_+2_2_181

RSLTADWHRSKNGQSHRSLTTHNFLLLYQTQNRCSPSNTSSFSLSFLAAQELKTTKLESR

>SG-AL_contig_4003_1_+2_419_631

MQHDAVPCDANTWNSFTSGINNSKLPYRNLPPIQFLIGMMNKITVDSECGALFLKLFYFY

YFLSIYFNFFF

>SG-AL_contig_4004_1_+2_2_241

CLIALNSYLIAPGSFQKELFIILNRSYLQSYRCQSIALDILRSSSEEHFNIITGKAREQP

SCFELFIRFLNCLSTTLFLD

>SG-AL_contig_4005_1_+3_126_356

MSVRVCLTFKSKVGIKMMEFEQHLSLYILASASATSEYFQLQKKYMSIHIVSPATHMCVH

MHAQTHARRTCIDIHVM

>SG-AL_contig_4006_1_-1_2035_3201

MWRCGTGLDWWRHLQATEERAASVEDDADGHLIYRPGDVLQARYEVVATLGEGTFGKVVE

CKDRRKNGETIALKIIKNVEKYREAAKLEINVLEKLKEKDPTGKYLCVQMLDWFDYHGHV

CISFDMLGLSVFDFLKDNSYIAYPIEQVRHISYQLCYAVNFLHENQLTHTDLKPENILFV

DSEFETSYNSKKRRDERRIKRTDIRLIDFGSATFDHEHHSTIVSTRHYRAPEVILELGWA

QPCDVWSIGCIMFELYTGYTLFQTHDNKEHLAMMERILGSLPYRMAKKTKKQKYFYHGRL

DWDEKSSAGRYVRDNVKPLRRYVKDSDNEDHQQLFDLMEKLLAYEVSSRYTLRQAMRHPF

FRKYYEEDLLRHESTRSSSSERRSHSLSR

>SG-AL_contig_4007_1_+3_3_209

ITETLKTDHAHLDIFKSCLTPSFSVGPDFKPESIETLSLAFINIIIYIIRKTKTDKGQTD

KLREQNKRE

>SG-AL_contig_4008_1_-1_376_993

MLISHVAYARHTIIMLISHLTYVHHTIIMLISDVTYLSQQSLCSFRCNISMPYNHHAHFP

CNISTPYNHHAHFPCNISMPYNDYAHFPCNISMPYSHHAHFRCNISMPYNHYAHFPCTYL

CHTIITLGIQSLSLFLMKHSCHTIIMLISDVTYYLCHTITMLISVVTYLCHTIITLGIQS

LCLFLMKHSCHTIIILIFDVTYLCHT

>SG-AL_contig_4009_1_-1_172_351

MKFTAIAHFHSLVDFFSQRLTCTGPVDPNSEHFKVCIAVPLLHSQLGIFVHADTYCVHFP

>SG-AL_contig_4010_1_-3_3_188

TLGWALDVHSAVLFTYMALCFCCFKVFLIFLTLFLKAGDDKLSGMAGSSAPPSLICLKEL

DG

>SG-AL_contig_4011_1_+2_1397_1954

MSMKPVRRAITNKTHSTAASCLSTANRKPFHCCVLSVYSKPKTIPLRPVCLQQTKNHSTA

VDCLQQTKNHSTAADCLSTANRKPFHCCGLSTANQTPFHCCGLSAYSKPKTIPLLWTVCL

QQTKNHSIAASCLSTANQKTLHGFSCLSTAKQNPFHCCGLSTANRKPFHCCGLSVYSKTT

IPLLRP

>SG-AL_contig_4012_1_+2_167_370

MFTSTFKMTSIRYETFHLWCEVYDTPEKSNDTTYTLYHGPFTIMHNEHESSQQSSQLYEY

GMKNISSN

>SG-AL_contig_4013_1_-1_1714_2034

MCKYIYAVTVIFRCVCLSFLFGLETGLCLILIIKVLFYSQFNQDCQCVKRKYYVLLYFRD

GEKSLHELSFYCNLFILLPDQFEYYTQIHSPCPLHKFAFVLRCFLVC

>SG-AL_contig_4014_1_+1_1_195

THMNTHTHTHTHTHTHTMFGNFKTSHIIYTPVLHIYINQLSTSISKLLIQQYLINQTHPN

CTDII

>SG-AL_contig_4015_1_-1_94_375

MAVSSTVTAGWMVDRGPALAVSASSFVFCPPAAGCVSPLAHLDTISDSLAPATAIICKLL

PTVWVDARQFQITLAHVFEVQCWAMSCSQLAVHD

>SG-AL_contig_4016_1_+3_6_236

MYGELAAGHRPVGRPALRFKDVCKRDLKLTGIDLDSWEQLADDRSGWRQAVRDGVKRGEE

RRNQQLEDRRQQRKQRQ

>SG-AL_contig_4017_1_+3_84_455

MQDGRIPKDVMYGKLATGHHPVGCSALCFKDVCKRDLKMTDIDPDSREQLADDCSGWHQA

VQDGVKRGEERCNQQLEDRRQRRKPRQQKQALDPPSNFACSNCGRHCHARIGLLSHSRRC

SKQN

>SG-AL_contig_4018_1_-1_175_453

MGNKVHTLAWVKHTTQRVPVPLQGSGKSKAARRGGGHRLHNAGLEAKLRSLTPHFRTTSG

LWENLYHFVRKIVGAVCISRSSIKTSPAYKVCV

>SG-AL_contig_4019_1_-3_90_428

MPETLRSVVAKFYDEYMQSTPKKLKLVDAYLFYILLTGVVQFGYCCLVGTYPFNSFLAGF

ISTVGSFVLGVCLRLQSNPQNKTDFTGISPERSFADFIFAHVILHLVVINFIG

>SG-AL_contig_4020_1_-1_112_336

MLVNVTCVLFLCKRQEKMDQKQCFNYWGPSFLSLKCESVVPVCLKCRVGFIHDLFSFIVC

RIMLVLENKGHVGRA

>SG-AL_contig_4021_1_-3_3_149

WGTAERGVGRADSVKQVRALSACAVSIRLLLPSLSAAVTGLYSCREAAG

>SG-AL_contig_4023_1_-3_330_467

MYRDCLQCFSLILRLLSGCCSVSCVANFAFVGCFDVQSYTCVNACL

>SG-AL_contig_4024_1_+2_392_658

MYINIQHQFINTKKLKALLSREEAGEIYINYETLMQRVLLPAVTLTFISHYRTVVSDTSH

KPNSQGSDPQLDRLYSKWTFCFWLASIHT

>SG-AL_contig_4025_1_-1_364_657

MLQSFWWCQCFIGPIFIRRICVFNYLFCVFTCVCLFTCVCVYLLVVCVFPCLSVYIHVNS

CVIVCLPVECFWLVGWFCWACLLVCFGVCVCVCVCVFV

>SG-AL_contig_4026_1_-1_403_3399

MDQAFTKSTDEVTAYFGVDENVGLSEEQVKKNLEKYGPNELPAEEGKPLWELILEQFDDL

LVKILLLAAVISFVLAWFEEDHDQVTAFVEPFVILTILICNAVVGVWQERNAESAIEALK

EYEPEVAKVMRGGHKGVQRIKASGLVPGDIVEVSVGDKVPADIRILKIHSTTLRIDQAIL

TGESVSVMKHTDPIPDPRAVNQDKKNLLFSGTNVAAGKCKGLVVGTGLATEIGKIRNEMM

DTEQEKTPLQQKLDEFSQQLSKVITIICIAVWAINIGHFNDPAHGGSWIKGAIYYFKIAV

ALAVAAIPEGLPAVITTCLALGTRRMAKKNAIVRSLPSVETLGCTSVICSDKTGTLTTNQ

MSVCKLFIFSQIDTNQIKTTQFEVTGSTYAPDGELYQNGKKVKASDYPGLEELSTISSMC

NDSSVDYNESKDIYEKVGEATETALTVLAEKLNFYNTDKSGLNKREMGTVCNHVIQDMWR

KEFTLEFSRDRKSMSVYCVPNKPTRVPGGARMFAKGAPEGLLDRCTHVRIGNNKVPMSPA

IKNEIMKLTKIYGTGRDTLRCLALATIDNPPRREDMDLEDSRKFIEYETNMTFVGVVGML

DPPREEVMASIKNCRAAGIRVIVITGDNKATAEAICRRIGVFTENENTEGMSFTGREFDE

LPPEDQRAAVMRSRLFARVEPAHKSKIVEYLQSEGEISAMTGDGVNDAPALKKAEIGIAM

GSGTAVAKSASEMVLADDNFSTIVAAVEEGRAIYNNMKQFIRYLISSNIGEVVCIFLTAA

LGIPEALIPVQLLWVNLVTDGFPATALGFNPPDLDIMQKPPRNPREGLITGWLFFRYMAI

GVYVGCATVGAAAWWFMLYDKGPRLNYYQLTHHMQCPAEPRMFKGVDCNIFSAPEPMTMA

LSVLVTIEMLNALNSLSENQSLTVMPPWINKWLVGAICLSMALHFLILYVDVMSVIFQIT

PLSVEEWVAVLKISIPVIILDETLKFIARKFADDYPGFF

>SG-AL_contig_4027_1_+3_3_203

SFKKSKIAHGGTSRKRVLKESAVKCLCRTSCNSLQSIRRCFTVTGARQGKHQGGTSPFSK

CEWVRKV

>SG-AL_contig_4028_1_-1_1_297

LFFFLSLCVCVCLSLSLSLSLSLSLSLSLSLVLAISLLSFFCLVCLSVMCFVVAFLFIFL

SVVIFLCTFGVKLGFERWYIWKCDCFLYNGIASATVGFF

>SG-AL_contig_4029_1_-2_557_1468

MDLRNLQRARDDLRFRGVKGTTGTQASFLTLFDGDDAKVEELDKLVTGMAGFKRHYIICG

QTYSRKVDVDCVSTLASLAASVHKICTDIRLLANFKELEEPFEKDQIGSSAMPYKRNPMR

SERCCALARHLMALIHDPLATAANQWMERTLDDSANRRIAIAEAFLTADALLGTMQNVFE

GLVVYPKVIERRIRQELPFMATENIIMAMVKSGGDRQECHEQIRVLSQQAGNRVKGEGED

NDLVERIKQCQYFAPVHSQLDTLLQPDTFIGRAPQQVEKFIQAEVEPALEPYTRDLQGRS

VLSV

>SG-AL_contig_4030_1_-1_7_225

MMNQKKLKVSPVGAAASSGAMQVVYSSSTDCLLQTVKTEGVRALYKGFIPNWLRLGPWNI

IFFMTYEQLKKTY

>SG-AL_contig_4031_1_-2_2_274

CVCVWRADSFSSHLSVSSFFLIFLSFFVCSGVVPCLLHVHCVCMYAFVSTLITMTRYVRQ

FCDVLGWRLLLSNLSHCTLQQCAEMSRSLHT

>SG-AL_contig_4032_1_-2_53_493

MSKSAQSDTNSNNAGSLTPTQAGARVSSLPPQGAAGGTTGGVIDRKRLQELVKEVDPLEQ

MDDDVEETLLTIADNFIESVVNTACQLAKHRKSNTLEVKDVQLCLERNHSIWVPGFGTED

LRPYKKSVVTEAHRQRQALIRKTLKKS

>SG-AL_contig_4033_1_-1_547_891

MCIHTHAHIDTHLHTHEHTHTYTHTHTHTHTLSLSCAHMHTQTVDMQVKLSSVKIADSCP

CLSLPLRETLIDYWIYFSYSSQAQRGFFTLFSFFHSSSVKIVGLFKLYEIKCGLL

>SG-AL_contig_4034_1_+2_254_484

MGFSVLKKSGRLSLEISVPPQYGTLHFAIQRHVHIFQSSFIDKYVCAVLYVYVWVCLYTC

SEECVFSRQRIVFKGSF

>SG-AL_contig_4035_1_+1_73_279

MRRSLYLSQLLHLTEPTRIVKQQEHHTFGHKCLALYFLLPRLRYNSSTYCNVFCPVSQAN

CPATSCVCP

>SG-AL_contig_4036_1_+3_690_1163

MTTDLYYIRLRNLAGTCEFHDEDREILTQILHGCQSQRLKRHALCDNLTLAQTLAEACGC

ELPEKRASELERSVNVLNISGDDTAYSFFHSRSCRCGSSHSRGSSLRDAYYNSQHLAQHT

LRILGQETTITHAVPEITLANMVEILIMVVKKTVTIVE

>SG-AL_contig_4037_1_+2_131_250

MRYQFCAESELRLFFIFCRQQTVFFLCIHKVLRPNKKQNK

>SG-AL_contig_4038_1_-1_1453_1737

MNSDCWHQHFWILSVPNQKISCWSHNQQSQFGFLLLIISLVLMRIPYTQVCSPFWTSSKW

YQYQLYFLLHVQQDSLFTHLILLMTYIIGLSWSLL

>SG-AL_contig_4039_1_+1_1_162

LVKYVLNDSDSFLRHEEFSQKWNINVNYLHYLSCIEATKEYVRRRKLKVKILKL

>SG-AL_contig_4040_1_-2_2_229

QDLPIGSQRLLLHPSVCHNDYISFLKLCLIPSKISVKNYRTISNGEVNLFLALQKLYLSL

SGIPIAFEILFLTRIL

>SG-AL_contig_4041_1_-2_1712_1867

MLAVMTLDCSTLHLLQEAAMWLHCLCTLCGLCTIRSLCTMCGLCTICGLCTT

>SG-AL_contig_4042_1_-3_33_236

MALLLAVFSQSLICVIGQSKLYMKLLLFPVAFSPNWALLAHFCIQQTPLISFFFIFFLLV

SRWYHSFK

>SG-AL_contig_4043_1_+1_1_174

LDEGGKGLLIRHFFHRAIPKILFSHPHRGRHCKKCSYSQCSDTVARVSAIHQNPHRQQ

>SG-AL_contig_4044_1_+3_6_467

MVTCPLDGGLTLGSWMRSYLLGFRNVPGPFLVIRHKWHLLDVSSFFFLRVLRGADGVCSV

CESADDAQFVFQGLVAVDLQALVCVAQLPVNGCSLSLFQSRPYLLSGWLSGCLAGWLAGW

LSICLSVCLSGWLAVWLAVWLAVWLADCLAGWLA

>SG-AL_contig_4045_1_+2_125_337

MRMLCLAASIGLAGWLAGSGVLLTITCFLFALSLLYQPLMVSDQGDKFTALLNPTPQKHT

PLIPVSHVPEK

>SG-AL_contig_4046_1_-1_166_330

MMHVLHDFSYDFRLHRWSPVTTDEMKAFLALVITTGLVIKPNIEDYWCQDDLTST

>SG-AL_contig_4047_1_+1_199_360

MSVPGLWKHENNQHAHSPKMQTVQQHSGMWSAHLTACVLCAPLKKKSLIMCNVQ

>SG-AL_contig_4048_1_+2_872_1102

MALCNRIVHRILATNVVLMQMGVKDNARSFCRHEHDSLHYMFWRCANVQPFSYQLPTSIN

NKCTNATSLTFTENIFV

>SG-AL_contig_4049_1_-1_1252_1455

MIIIKRNVLFQLLCSVSQTYTVVFFYLFVCCLFLIHRINEYFFFFFSSVIMTFFLCVCVC

VCVCVCVY

>SG-AL_contig_4050_1_-3_3_356

STGAISGNLQQNDEDSAASSGEENSPKSPLSVHSDESSLPTPSLPARVKKSPRCSKKKRT

VVCHRQGSFLQALKEPEKEVDENELFFKSLLPAMKTLNLVQTLELCSEIQRLVLSSVK

>SG-AL_contig_4051_1_-3_180_1802

MDSVKSVSESSVLFEASRDHSSVMSKSQQFLSSSSKSSGNSEASSSSAKPKLEFLSSSVR

SAGSQGSSSPHHSTVHANSESQTASHTAHSSTAVKTPPTADNTSSPSTPSKTSFTSAASP

GESKREGLRQTDSQTSPAFSPKDDYKLRRQSRSKTLPDQPTGKEEPFATKVQRAASHRVE

ATSSQDASPESSPRHKDKVVETSKSKEPEWFALARRKTGRQDSEEKKDSPSPTKEIAPAK

EPVPASEVPVSSAPGPKAEAVSHSVETSGNMSVSSASSKFERFGENKLSEVSRVRGGSVK

TSPTKTHVFANRGSSVKVQSGRADSGITGSSVTASSPLKTTTPVSSASASSTAASAKQEA

IKEESKGSESSPSREVPKWKTQSVKTSAFSSSTPSVVSSSKFSSVKISSQSTPTPLTSTS

ATKTETRPTSSPAKKSPLTKTPPEEKEKASVPAWRANLSQKKPPASDIKIELIESSSSSS

GTKKEDSAKKEEVTIREESPTKASAASKDVPSDPCRSSATPSRASKVMNLLKNFENVQGV

S

>SG-AL_contig_4052_1_+1_850_1263

MNSGSEAENQLNDPLCTVVYTDAKHISIISFQKQRDRIMEDHICTIIAHSLPHPSCITCT

FNYYEIIICKRGKFCIDVQNLNIEELTKALQQEISDAAELILGMYSQINTFTTQAGYGTQ

TGNWYSSIHKHKEILPVT

>SG-AL_contig_4053_1_-1_73_369

MLCHTFTLPCHVTSHMFAMSYSAHCHTFTLPCHITHHTFTIKYSAHGHLPILTMQCSHPD

TTHHSLTTIHQRPPTSTVCRQAFPTSTWESFSNNLTSCR

>SG-AL_contig_4054_1_-1_1_447

GVEIVFDLYSSTLHKIEVLKLEKRLDDELFYLRDAPPEYSTVPFDFEPIQLPKSAAVPVN

PIKVKLNKRPWLERWERYDLKGVEDLMLPERFYRRAQEVAKPWEKYDIMKHHRDAIDDVE

TETIMKDVYTRTLSTRKTRGKQKASLKKK

>SG-AL_contig_4055_1_-1_1360_1674

MRWQTPQLYLGSVSPLLFHLLTLKRVRYPFTAGQTGGYTRHGTGVKVLFPENILRGNNPW

RGQGAEPLLWGCPLKLKILSKIGQKKSLSLNYYYKQFWPDKKASP

>SG-AL_contig_4056_1_+2_2_208

DVISLADRSRSLMGFHSSSVLAAYLSSRRATPPRCIPSCQPEKRMVLFSDLNFPELFQQT

SLSPRMLSL

>SG-AL_contig_4057_1_+1_82_267

MKAVMVTLICSGDVSVHANDIISLIIKGGLLMWIDRFFNNGSGCLCAYTGCAKNQNYQST

NG

>SG-AL_contig_4058_1_-2_248_496

MSSFILFIYLHFSALMCLCRVCVCVCVCVFSVCVFCVCVCVCVFKLYLCYTCIMLWFSCV

SCVHMLAYLSYYSGFVVCMCMLC

>SG-AL_contig_4059_1_-1_82_3048

MEALNRSSERMISHLNTQVDFLQQKNRELERKLTDSRVAAEEAESKFSRAKSRNLELECQ

LQDVDRVAVKLQTDKAFVVRAADREMGEAKSELEKSRHELEDLDSAVAQLKAENKRVAKD

YADMKARLAVREADNRKLEDLLDRIQEEKKRLSQRNNKLTANEKELVLEIERLKRKNGPS

AKKGKIPSKLDAFIRGIEEERDYYKQQTDVLQRMHRGEPATVTLSKPSSRSSSPVRDKST

AASKKALAQQEAVIRVLEEEKDYYKKEYETLKTLRRSATPVRSTPTKSAKDESELFRLTR

ERDELKALLDKFERHMAEIQANVSVLTAERDKLNNMYQETKEELMHVRRELISSPKSPKT

SLAAQAVLRRVENERDDAVSDLRRMTTERDSLRERIKIATESSLSERAKLEQRVEDLENS

LATIGSEREELLVRLSSLKDDNKAYEEQSKDQSLRLNQMDEELARFKSSAAQLRLLVEEA

EKSLDETRHRLTRREKELQMQEDRCKSLEDQLAHTQHNVAIQRDELSAMRTTLATVDREK

DALQVSVDDKTEKVSVLNAELHDRERVVSDLKVRVGELEAQLHHSNDNLSLKDREIKSLR

RNVESVNEDLQEASRGRDISMQENRRLQDDLSVMAKENQKLTAELQDAMAEREALKDQVQ

QYILEVRRVEDLLSNKESERSDLLEQYRKLSMEAEQYQTTSHQLESEGSNMRLEIMTKES

ELRRAHDKIDNLEREIQEHVQSQQSYEQQVVSLTRSITTLEETLRQAEDEKSVLRADYTS

SRENSAQLDSLKEQLQRKQAALLLEKEQLQSLVEDLRQESECLQEQVSQERGTVKNLEGL

LQNGREKEFQSQLVSQERNTEIQMLKDRLSLNENKVQSQAREVASLRTRNVELEGDVERL

RRHLTNERFERERAVQELRRNGISPPIMISEYTSETYSRHSSRERGRSTSRSPSHSPPRY

RPGSSSRQRSPERYDMEYTSVKSYAHDVL

>SG-AL_contig_4060_1_-3_3_281

LWSWGGGVGVGGGQRWVKSRVMSVRNQIMKELNPFFMGNGCSITGLFIFQNGACSILLQG

SDRKKCDCLFILNQSYIKEVLCVASLTISHYSV

>SG-AL_contig_4061_1_-1_121_396

MSAPRKDEAATIRVTNLSEDTRESDLQELFRPFGPISRIYLAKDKVTGQSKGFAFINFHR

REDAARAIAGVSGFGYDHLILNVEWAKPSGQQ

>SG-AL_contig_4062_1_-3_54_2288

MKDSVEEAPSFIGHDKSSFECMQQKHSCSESDKTPDHQNECSDLEPHDIASCSSDIETKP

VQCSEDQPQKGTKLSYSTLELDGGHHSQVQVIDEDVDLPHNTSEETSPENSSCYKWPRVK

VSNTDGEVFVNEEELKVKYREENTNKKLSASSQDESDDSISGPLEIKPYLKNSFRSKIFS

APIYNVFMLYSSSQQSEQSDFLLSDKQPSSNSSLQRHHHTELNRLATYIEYPSTLPVFAI

TLAGDGFIYKHDEHLVECNFCKMCIDLSELSMPEANPVRLHWERSPQCPLKRGQDCGNVP

VPKVDEKSNMSNDPSLGISDDSHEADVSPKSLSSSGYCSASSDFGKSATASYKADPATLL

PSPVVYHQPAQNIITTDSVPFVQQVQPPSLPPRGKSDSTLSQTRPQQPNPSSIKSAAANA

DSTHQQTEPVAGTANRNKPNNSNTANGNEPSSPPSASTQKGAAASAAASQPKNSKSSKKG

KATKKSEASNAKTGAASNGSSTQNSSSKTNASSGDKGEQSKKKLTYADLGILADKPKRPD

MAVVNKRLKSFEEKWKSSFKKTPEELAECGMYYAGYADCARCFFCGGGLKNWETYDEPWV

EHARWFPKCAYIRLCKGPMFVEIVQRKNKDKQKIVMKDVEEEINQKSRSPDSSPTMLPVN

VSTSGHNEGQTPPPNSKLVADLADLEAENEELQAQVTCKICMDEKSSMVFLPCGHMVACT

NCAIALKNCPLCRSEIKGSVRAHMG

>SG-AL_contig_4063_1_-2_44_3979

MTPKVFQWLLSAAALVCFVTAQMDIFEESVYEYEDCRGEYQMSVGIRSGDIPNSAMTASS

SLKKREPYTARLYNPAGAWTPKVQNAYEWLQIDLGKAYIITKLQTQGRRGSDEYVSEYFL

EYSDDGTTWRRYTNRFGIAEMFLGNKDDSTVATRPLLYPIVATYIRFNPQRWNMVISMRV

DVTGCPYTAHTANFFGNDYIEYDLSQKSYQTERDLLELRFKTNSPHGILFYASGNQGDFL

LLQMNRGYLNFTIDLGSTQTERGITEVTAGSLLDDDQWHDVIIRRERQKLKVVVDRLVNE

LETKGLFYRLDLDKYIDIGGVEHFMQDGIKVKYNFNGCIQNVNFDGARLLRDAQSKDNNA

DISIYGDVGTFCGGTSATPVTFPTLESLLKLTATSSTGIQAALDFRSHDEDGLLVYHPMA

QEGTGLTVLIDQDGFIYYKIVSDTGQLIEDVVRNNDVTRASDGFADGLWHHLHIEVTDKK

VNCTVDRNVKVSNRELEVTPANTYYIGGNELFNGFRGCMRNIQVAGRYLDLNEPLGTDAN

VGVNIGTCAIRDRCTPNPCNHGGRCSQSWNTFDCDCEDTGYKGEMCHISAHQVSCEMQKQ

YSNVEGMEVAVIDPDGSGPYPPFEVTCDRDTNTGQIITYIGHDTQNLIAVNGYQEPGSYV

RKVHYAANDMLAMEDIIERADSCRQYIEYRCNNSKLLAPLTNSEDNTNTARTWGWWVGRT

YQPMYYWGGAAPGSGKCSCGLEEKGCVGSSSTCNCDAGQAIQISDRGYLVHKEYLPVLEL

HFGDTGTVSDTKIAHHKLGKLECFGDDLFDNTVTFNMPDGVLEFPTFEANNAGDIWFQFR

TTATDGVMVHCTGITDFIEVRLFQSDTIQFRYDVGNGVNVLSFRSPAPLNDDRWHTVHIE

KNRKQAWMRIDDFTEVTYNEDADLIRLLDLTTPLYIGALVDYNEGYVGCMRGLRVNGKMM

DMLGQLREGEVYGVVRGCVGKCASSPCFNGGTCREGYSRYTCDCSYTPWRGWNCGREVGV

NLKQNYMIRYEFDETQGLSATDFQYATIGFSTKVKQGILMQMKSVETPDYISVEMNNNGG

VKVVFDVGFQREEVNTPKIDVDFANTQVHVVTIRRSNKGRTVKVQVDNYPPLDHTFRLPD

QADTILDKPRFLYVGNNDTSNTNFGFEGCIYRMQVDNIFPLKRAFQDPRPNYITLVPKDA

IREDMCGFEEVTVVPEPIKTRPYRGPSFNVTYPHTPAKELTSEERAIIAVCVTVFFLLVI

IILIFVFRSRFEGGDYETEEATGAEFADDPDTAVVYNQTGLPNMTTKYEFFM

>SG-AL_contig_4064_1_+1_202_384

MMSTQYVTEGKLNTKENILSTQYNYINIQIKTHPGYSISMKLKQDAQVMIIFWHFWGQSV

L

>SG-AL_contig_4065_1_-2_389_529

MSLCECRKQFIDFLKINTVITKVISVLTSCIRMCQKMMCCLLKMSSK

>SG-AL_contig_4066_1_+1_1_99

KKEKKRKHEIEIMRNKRDMENLFIAVYTVILHN

>SG-AL_contig_4067_1_-2_1634_1900

MFWIMERLTLKVKTNLIKGKICVCALSSKTRRISYRTCLCIGLLTPQDPNERFIPRVFLQ

EEPFKKEQKFRCNLKTAFHTWLTRFYSLK

>SG-AL_contig_4068_1_-2_248_706

MAKALCVLVDTSKKVQGTLTFTQDDGKTTKVEGQVTGLTPGKHGFHIHEFGDTTNGCVSA

GPHFNPFGKEHGSPTDENRHAGDLGNIVAEGNGIAKVSITDSQIPLTGPHSIIGRSLVVH

EKEDDLGKGGNEESKKTGNAGGRLACGIIGITK

>SG-AL_contig_4069_1_-1_28_222

MESFQSCPSCHSSVFQVWYDCINNISETKMNFKILFFSFEYIIITLSLFCNMSVMGRSCI

QPRTR

>SG-AL_contig_4070_1_+3_1566_1787

MCVSACMHMSVCAFVCVYVCMRSLTHALTKCCKTQHNWQTDNKINNILQSQSARNSFLRK

EQEQFYDWNKCQMF

>SG-AL_contig_4071_1_-2_116_466

MKWQYKEEHPFERRRAEGEKIRKKYPDRVPVIVEKAPKARVGDLDKKKYLVPSDLTVGQF

YFLIRKRIHLRSEDALFFFVNNVIPPTSATMGALYQEHHEEDFFLYIAYSDESVYGA

>SG-AL_contig_4072_1_-1_1_231

YCAKAKTALQKYLNDGSLSTDDYEVMEIENDPDCSAIQNHLKVLTGASSVPRVFVNHKFI

GGGDEVVALDSQGKLGA

>SG-AL_contig_4073_1_-3_147_683

MGLTISGLFARMFGKKQMRILMVGLDAAGKTTILYKLKLGEIVTTIPTIGFNVETVEYKN

ISFTVWDVGGQDKIRPLWRHYFQNTQGLIFVVDSNDRERIDEARDELHKMLQEDELRDAV

LLVFANKSDLPHSLHPNECTEKLGLNSLRNKKWFIQSACATQGTGLYEGLEWLSNELGQ

>SG-AL_contig_4074_1_-1_115_492

MELGCACRDWLFPPGDAEGSFSMNSIPTFWTFWARTFLYSITLLVAVFSFLSVTSSFLFF

CLICPFTSIIPRNLHTFSSYLQPTKVGVYRLCSNIMLPAQCGPAHQNNMHTHTSGLGQKL

CQLLHK

>SG-AL_contig_4075_1_+1_1_318

ARERRWAPPSSCWPREKLRSLPPHSPTTSGLRENLYLTFTSPGKAPLYIWYSGTHFFLIF

SLFFSVCIYFYLPITVDFSTEFHLFCCRGIFNVRNQITTQGIVEWW

>SG-AL_contig_4076_1_+2_761_994

MSYCTVLQVLSQGMFSASRGGCSPLGEHVFIAKCYLICWLVCLYVCPACMYFSLIINNDF

LQNLARDNTCCCGFLNVC

>SG-AL_contig_4077_1_-2_11_859

MEQTFHKDHLACFKCEKQLVACRYIAKEDNPYCVPCYQQLFAHNCEVCGKAIGPDYKDLA

YKERHWHEFCFKCSQCQNSLVNQPFAPKDEQIFCSECYDNTFAARCDSCQNPFRGGMKKF

EYKGNKWHEECFTCGDCQQPIGNKSFIPRDERIICLPCYENNYAQKCTKCSGVINKGGIS

YKNMPFHRECFTCTNCNKMLAGEKFTSKDEQPYCGDCYGELFAKRCCVCTKPITGIGGTK

FISFEERHWHSDCFACYRCKASMVGRGFLMQGEEILCPDCGRS

>SG-AL_contig_4078_1_+3_555_755

MCVCMYVCMYVCMYVCFCNFSKMIKKKFIQKLETRSRIFHNLNLKKILSQLKNRDKRQRL

GEGGGGR

>SG-AL_contig_4079_1_-3_108_1979

MASENDANGIDQPVQEKVADAEMEGENLENFNKLTEYGISKTVAKELIKIYGTGKLSHSD

LDKRALDALKEYNPNDAILVLKEFYTKNLEHVTNKSAFLCGQMKTFKSKSKSGPTPQAKG

PDPAKLQELLDRTGYTLDVTTGQRKYGGPPPDWDHSAPGPGHEVFCGKVPREVFEDELIP

LFEKCGKIWDLRLMMDPLSGLNRGYCFVTFCEKEAASEAVKQLDGYEIRKAKKLKINISI

ANLRLFVGNIPKNKTKDEIKEEFGKRTEGLNDVIIYGSADNPKLKNRGFAFLEFDNHKNA

SSAKRKLSTGRVKVWQCDIIVDWADPQEEPDEDTMSKVKVLYVRNLTSDVSEEELKEKFG

DFGKVERVKKIKDYGFVHFEERDDAISAMEALNGEKIGNLEINISLAKPPTENKKKEQRK

QRDQERRSLMREGYSAWDDYYGPPPMRPPMPPRSAFRRLPPPHDPYYDDYYYEEDYYYGA

YAPLPPLPPLPRSRARNGPYSPPPIRASRGGGPPLRGPPVQGGPGARGAGARGRVARPGA

GSLRGGRGGPPFKGQPVQPRGGKRKAGSDFGQGTTKRRTMQDNWGNHPIAQQPLDQLGHG

SGGYDDYASNDAQWYQDSFGPNWG

>SG-AL_contig_4080_1_+2_776_1018

MPHSPTALSTQTLTSFASGLKSCLQLSKLHNVQSNKMTETVWILHAQDKFINNPWLYIYT

QSRTKSFNNSWDIHTSKKKLY

>SG-AL_contig_4081_1_-2_2_199

EADRGRWSHQECGESGSHRKDDQVCCQEKMKVTGTLAFSTCIELSFISNSVQRLLPSQST

RKFVAR

>SG-AL_contig_4082_1_-3_717_1055

MENLSNFLARWRIYFSRLDSLGAGVGLQAALGRQASSHASLSSLLFQQGTRGFRDSLSML

HCYLDDWLCFADSSYLCRSMLHCYLDDWLCLAYSSDLCRTHTSRVIDLCTQLG

>SG-AL_contig_4083_1_+3_645_833

MFRFVCVCVCVCVATSARKCCIYVQAASEQGQAIIHLRYCLCNWTVYNCSVYTVKFMNSI

SHK

>SG-AL_contig_4084_1_+1_1_123

LTLSTSFLVGEGVKGLMKHNATNSIILSACIADIASPYKAI

>SG-AL_contig_4085_1_+1_817_1098

MFRLVGDVTTKVPAHNAVPGGVVLFVKFFLDVGGDVLFNVVLFQSLSGAVHRILLHVLSH

VGVLDYCLTFSHLVSRSFWSLFTQTPLKSKTATI

>SG-AL_contig_4086_1_+3_3_137

GAKEGRGLHRDRPQGCEVLSREDGGEDGVPLNQSQVVAQLDGCVC

>SG-AL_contig_4087_1_-1_424_789

MRAFGMQLQTTWASSSPGQDGVPEDLLLGREVCNALVDSADAVVKLRYKLILVRRDTILS

SSILPAEDFPALQTLSPETFLSKETRLPRRKPLEGHISDHFSLAFGNCHEGLQGVGILLP

PR

>SG-AL_contig_4088_1_-1_4_315

MQGRLSWYSIEKKARCGSVHKLPITLNAALAESGHSKQAHCAYLSEFSAPADTTNTNRIR

VPAEAAPALLVTERAAERSLTMASQKKLTAFLRPSTSSASCSNS

>SG-AL_contig_4089_1_+3_3_125

CLQLTVQESFENGCTFYQSLVVIELNNNNNDMALRILMTLK

>SG-AL_contig_4090_1_+1_1_105

LPYTQMNTPINLGYKLLFLKVKLSCKRICLFIFGV

>SG-AL_contig_4091_1_+1_1_276

IQALYTQIRTNTSIPFKTSLFNFQNLTRISYMVFFKHTSNLDYGYCMYNTPELFHNASQH

TQLSTPKSPRRDERKNTFRKGRESNRDSRSET

>SG-AL_contig_4092_1_-2_98_310

MAGHMFFVYFCYDKLSVCLANKRRKIIFCLYTHRDTCPYFIFIIVVFSILCTYLSLIYHH

VFCLRKALMDV

>SG-AL_contig_4093_1_+1_3208_3783

MVLGRFLQSYAPSLSAETELTRRLADSSKKQWVSLSRSLSLSYSLTLSSSHSFSQFLILS

LSLSSLSLLFLSLSPLSLIFFLSPSLLSLSLSLSLLSLSPPSLTLVLSLSPLSLSSSLSH

SLSPLSLPVFIFSVSPTSLSPPLLFSFTHSLSPFLVLSLSCLILSSLSLSLPPTPHSPPS

LSLLFSLTVSPL

>SG-AL_contig_4094_1_-2_2_67

LTHHNTYATTPTLTSTLISDLP

>SG-AL_contig_4095_1_-3_3_80

TVTSTLIPDSPQHTCNNIHLHFHLNI

>SG-AL_contig_4096_1_-2_2_202

FFFHTICIYQQCKRDTNVFNKLENFHPIKLQHRPQHMFKKTTKKPNIFLLKRRQSHSYSF

IHTELSQ

>SG-AL_contig_4097_1_-2_2_151

TFFTVWECKAARERRWAPPSLCWPREKLRSPPPRSPTTPGLWDNLDLTFY

>SG-AL_contig_4098_1_-3_270_1289

MLDIVPNSSSRTRNMPVNLAPYLLSASPPSAGFSFQSLSLTSSYTPYSTPYSFFSNTSNH

RESPVRDTVYGTAFTSSSPRKSIMSVKPNKDSESHTENTHKADAPAEEQSTSLANNTAKK

MKRVSFADHVGMPLAEVRFMLDSSADPPKFTQKKLNEITGGHAGMMLKTSMDPPNPPLIV

PFPQPLTRENFNEELKRKMVAVENVIVEGYTVTCTIFVNKHATVKRVFGRYTLDSWQSHQ

DITAFYSSSLRVNHDTYQFKIQVPLSADRSKSIEFAVCYEADGVQHWDNNEGDNYTIEWG

QEIDLEPKDSSHNDDSNGNPIAFGGMAWWCDDSDLTRPYY

>SG-AL_contig_4099_1_+2_164_283

MRISLTNSGKPIQPLLTILSTDQVHDKTLHIFMFSECFFF

>SG-AL_contig_4100_1_-3_918_1172

MVPPHISSTTTPNQQCTVHLHLSSTTTLNQQCTVHLHSLPTQHSINGAQFISTSLPVHPV

VLCFTSILPPSPCSSFLSPVPRQTL

>SG-AL_contig_4101_1_-2_20_2740

MADKAKGDTSAKQEKKTDASKEPEKKNDEAKEQELSEEDKQLQEELTMLVERLQESDQRL

YKTALESLRVQIRASTTSMTSVPKPLKFLRPHYDTIKGVHEKIRDKEIKEFCADIISVLG

MTVSEGRDSLHYKLQGSKEEIGAWGHEYVRHLAGQVAQEWQDTAEDDKEKRGQLTTLAKE

IIPYHMSHNAEAEACDLMMEIERLDLAMEYVDEQAFPRVCLYLTSCVAYVPDPENTALLR

CAVDLYRKFKQYPQALRFAMQLNDMKLIEDIFLSCKDLVMQKQLSYMLGRQQIFMELNDD

LEEADELTEIMSNAPLNGAFLSLARELDIMEPKVPEDIYKSHLEPNRTSLGPNNLDSARQ

NLASSFVNGFVNAAFGQDKLLMEDGNKWLYKNKEHGMLSATASLGLILLWDVDGGLIQID

KYLYSSEDYIKAGALLACGIVNTGVRNECDPALALLTDYVSHTSNIMRIGAISGLGLAYA

GSNREDVLGLILPVLGDSKSNMEVIGMASLACGMIAVGSCNGEVTSTILQTMMERSEADL

KDSYSRYMALGLALCYLGKQEAVEATLVALEVVGEPLKSMATILVDVCAYAGTGNVLKVQ

NLLHICSEHDDSKDQDNNEKKEDKNKKKDDKKDKEKEPAGPDLSLQQGIAVLGIGLIAMG

EEIGSEMCFRSFGHLLRYGELPIRRAVPLALALISVSNPKLQILDTLSKFSHDSDPEVAH

NAIMAMGIVGAGTNNARLAAMLRQLAVYHAKDPNNLFMVRLAQGLTHLGKGTLTLCPYHS

DRTLMSPVAVAGLMAALVSCLDVKNIILGKSHYVLYNLVAAMQPRMLVTFDEELRPLPVS

VRVGQAVDVVGQAGKPKTITGFQTHTTPVLLAYGERAELATEEYLPLTSVMEGFVILRKN

PDYEADS

>SG-AL_contig_4102_1_+2_2_169

LTSMLNISFFKPRHSVYLQIFFFGHVFMIIWFCLLVWMFVFMSNFYSSLCNFYKQY

>SG-AL_contig_4103_1_-1_46_207

MKLLSGLFPSTECLCTLFLCKSSVCCFKLLNVCTLLKICLQSSVCFCHLCFTYI

>SG-AL_contig_4104_1_-3_54_353

MLFCMCIHTKGFQALSGSARMLTWEIVKNLHPLPTSQSYQGGFELGSSKPSSLEVKCLHH

STTAPVCERQTERHRNPQPSPIKLSSVLCNSLMSQEAVKI

>SG-AL_contig_4105_1_-1_76_474

MASHTHFEEGSAPSEVTETQPDREQGSDSKEKEQTEKTAEQKEEEKLRAKYPNLKKAGPP

AMIHKRLVQKGKYFDSGDYNMAKAAINNPKKPLPPQEKMLLQESIGEGIPTPENLPPRKP

SIVQSKLASGALS

>SG-AL_contig_4106_1_-3_144_488

MVKTLDIQTGGFGRPGLNHGHLAWSVKGGYFSNLPGQHMCSVLCVYMHAKHQIIMPVKDP

ISRVSVRWLMETRKHQHARQKMGGAALRRHALPGRAAQISHGKSIVTKNKHNTTS

>SG-AL_contig_4107_1_+1_412_888

MKLLLYLKPIQVPPNLCQMIQAHGACCDICCPFSASYNTAPSERFMSDHIPRHSTCCEGA

ILEIHAAASYEPFMSKKRNKPTLWGILRVCCLFSHIRDTPRLMSNQIPCNMKHSHCCYLA

LYFFSSSNYWLLTGSLISRGQQSLVRRVGTITMKLTQGK

>SG-AL_contig_4108_1_-3_3_116

CLYQNELSNNNHHDSLQDSEEDLLKTSLSSRPVDCPFE

>SG-AL_contig_4109_1_-1_19_1287

MASALCRCAFRQLCAKGPGGPFPAANQVRWLNLQEYQSKKLMAESGIAVQQFRVASTPDE

AATIASQLTVPEYVIKAQILAGGRGKGTFDTGFKGGVHLTKNPKEVEGFVKNMLGNKLST

KQTPKGGVKVNRVMVAEALDIIRETYFAILMDRGYGGPVMVGSPEGGMDIEEVAEKNPSA

IFKEPIDIVVGMTDEQAAYMATNLGFDGQKGIQAAEEIKKLYKLFLKVDATQVEINPFGE

TPDGRVVCFDAKINFDDNADFRQKEIFAMDDMAESDPREVEASRHQLNYIGMDGNIACLV

NGAGLAMATMDIIKLHGGSPANFLDVGGTVTEKQVFHAFKLLTSDTQVEAILVNIFGGIV

NCATIAKGITNACKSINLQLPLVVRLEGTNVDEAKKILSESQLPIVSAGDLDDAARKAVA

SLR

>SG-AL_contig_4110_1_-3_600_839

MVHVELLFAAMPFINKLQLCIVIIRKEYQSAIRQNCHIFTVLFHAVACLQVCHTWHIYRR

RHAKQQLHSIRMCTCKIVSL

>SG-AL_contig_4111_1_-3_3_98

HTHTHAHVHTSFSIFKSKYYFTVKMLQFLGKC

>SG-AL_contig_4112_1_-1_91_3108

MYWCQPVRSLLLCAAVCSLLFHSASGRLAVMSVDLGGEFMKIAIVKPGVPMEIVLNKDAS

RKTRSIVAFRDGERLFANDAYNTAVRFPDKAFWFLSHIIGRNYDDPQVEQFRERFPYYKH

IMVKDVDRGTVMFREPDGTVYSSEELLGMMLEKAREYAQDFAEQKIQDAVITIPAFYTQA

ERRAVLAAAELVGLNVLQLMTDTAAVALNYGVFRRKNFNTSTQYYMFYDMGATSTVVSVV

GYKMVKSQVGTRVETNPQLTVKGVGYDRNLGGLEMTLRLRDHLAEVFNSKKQRPVRVQDN

PRAMAKLLKEAERVKKVLSANSDHKAQVEGLLEGEDFASKVTREQYLALIKDLLERVTKP

IEDALKSSEITWPEISEIILMGGGSRLPKVHEILMEYSGKTELGKSINTDEAAALGAVYQ

AAYMGRGFKVKTFYVRDANIFPINVEFDKQKTEEDDTSGTKTIKRTLFGRSNYYPQKKVM

TFNKHYVKDFNFDVRYGDLDFLPEEERKVFPSSLLSKVSLKGVDKAFTKRGKESEYKGVK

AHFRMDESGLLHLDMVESVFDKPGSEQKEDDSTWSKLGSAIGSMFGGQKEEEEAATDTPP

AQDARKEGETDEGTEKTDKAGEKKEKEKTESKEKEKSSGKEKAGQKEKKKEEKKTDKSKD

TGKKTNSQKLNATIRMKDLADFTKEKIRKAKKRLSDLKKKDEEKVAVEKAKNELEGYIID

MQDKLSQDRYEACSTETERSSIMQQLSEASDWLYEQPHNAKRLLFTNKRNDLKSLLKNIE

ERVKEKEGRPKALESLKQALNSSAVFLQVARHNNSQSDNPVFTQVELNTLDKLINETKTW

QTEAVKEQKERGDRQNPTLLVDTIQSKVKALEREVRYLTNKVKNNKASAKKKPKAKANKT

EESKEEKEKKKVEKKKEEKKEEDDDDDDDDDGVVEDEPDERFITDDEETDDRKTDSRPHS

HTSSKTPPKRKSDTDSDKKDTNDSDLGDEDIGSTDSDNDSSKHTEL

>SG-AL_contig_4113_1_+3_1155_1340

MIRILAQVMTILMQKVTLNQLACMNHIGMILLFNSISFIVKTVQICLFTKLCPFPTWVRF

DC

>SG-AL_contig_4114_1_+3_276_515

MIKILTNHNMEYLLILLGLRLPDGVIILMYIISKQHGLAKAEQTQLAKKPVQINNKKIHV

YRRQLTLGYIMDVQDTSLLH

>SG-AL_contig_4115_1_-3_12_1004

MAGVKNAQDLAGFDKLLEDAERQLVVVHFWADWATQCAQMDDVMKELASDMQYVNVSFVR

VAAEDLADVSLRYNIIAVPTFIFLKGKEVIDRIDGAKAAELTQKVGKHVSFLPLKGPLPQ

EGAASKLQDLNSRLKQLINSAPVMLFMKGSPDQPRCGFSRQTVQLLNERKVKYSTFDILT

DEHVRQGLKTYSDWPTYPQLYVKGELLGGLDILKEMAESGELEEALLVQEDLDTRLKKLI

NKSPVMLFMKGCPDTPRCGFSKTMIQILNETGVRYETFDILGDEEVRQGLKKFSKWPTYP

QLYVSGELLGGLDIIKEMKENGELQEALTTS

>SG-AL_contig_4116_1_+3_3_221

FLHRYQFLYLSLSLSLSLSLSLSLSLSDTHTFKPISEPNLTPLSDNCDYRILPHKRTADH

QTFNKKPNTSKSQ

>SG-AL_contig_4117_1_-1_85_2655

MSRTSSIGLVGISPLQFIHVLDKNTNITRLEVGPKTFHLQTHEEIVLGPAPMICIPPGFF

CMILNPVSDYEAGVKCELSLGQEAIKFHGEPFALYPGEQLVKAPDFGLKMQDYKAALQRI

PVVPVNCALRLRALQDHIHNKRLIPAGHQWQVEGPMTYVPRPEVEIEGMIQPEIIKVGEV

LRLRAIQDTRDRNGIKRVTGEEWLVSKPGAYLRGVWEEVVGLERKITLTRDCGLHLRATQ

NIQEYAGKERLAGDEWLLTGEEVEEYNPHVGIEMVKKVKRLVVKKGQYAVIVNPVGKDGK

NQLSRKVLMKGLCSFFLKPGETLEDGKIKDASILSADESLVLQAFEEFTDILCKGVKRQP

GDRWMIRGPLSYIPPITVKIIKKRKTIPLSKNEGIYIQNTKTGRVRAVMGPCAYMLTADE

ELWEKILPPEAETLLIQGGGSGTGDIRKMAYFEQSIDPSLIKGRDKTRVITFRCPGSTAV

QVNNFQQKTSRVVFGPDLVVLSPDENFNVLSLSAGKPKREDALKSLCLMLGPDFITDIME

VETSDHARLRLQVAFNNHFEVDKTDEESVKRIFQVPDFIGFACRQLGSRIRASVALTPFD

DFHRHSAAIIQAAVFGRDAKTGRVMPRLKFLVNDLVISSIDIQNIEPVDTKMRDSLMKSV

QLAIEISTRSVEASAEHEANCREQIAKGQLERQRLLNEVEAEKERAKLYELRAEAAAVES

TGQAKAMAQAEAEKTLIECHSQIEAVTLKAKAEEIEQYSKLETQSMLRVSEMDYKRKMMQ

LEINKEKSHAAVEVTKFANMVQTIGSATISKIARAGPQMKLNMLTALGMESVLLTDGSSP

INLFNTAEGLVGGKSSA

>SG-AL_contig_4118_1_-2_704_913

MFCTFPLYFIFQNINYIFLCVYCCCFFSPPKPPSQEKGIFLSFCLSCLFSLCVFFVSPLF

KNFTSIVIFV

>SG-AL_contig_4119_1_+2_635_3277

MTEHLKNVRQELVHTQALVEARKNEIDTEDHMFKIAEREEGRLGQEIRRLKKELDELKEQ

KNIYENNIFRQTQKLEEMKSQMNWDQQALEAWLEESARKDEDSMTLNKYTRQDESKIKEL

SLRMEKLTEDVRVKRRNLDHEMTATLTAQIELDKTAEDFRRAHSERQELISQWENTIEQM

QRRDREMDLLAAQLARVKVEVRRKEEGIREKQQFLDNEIENNQEQEKKISIAERTSSKMR

REYQEAETQRTQFGDELDALKRTVDRTAMDLESTRSTVTQLKKDVVDKTNKLQQAKDLRE

ALAEKLKMATEATLTAEEKSAKMDALLTEEEMNQKLLEQDLKNMRDILFKKQEDLHKART

EERDLEAIIQGSHAAVRNLNSKINRLDHDSLKQQEILYSQDFAIQQLERRIGRMQGERSN

EEKVQLEQRLNELTNDLDEKNNTHNLLTLQLKRLQDDIRRVARELEKSGAEKSDLTSKIE

EITLYNDNSERELRNLIKSKQDLMVDENIMKLEVKRLRDMLNDRSDEVLTQEKRRLQLET

AMKERMQEISIHKEMLWSQFKAADEERQQISAELHERISKIDKLRKRYEILMVSMAPPEG

EEEKSQAYYVIKAAQEKEELQREGDDLDARIRKAEKEIRALENTLKLMNGRNEQYRKSFN

KITETSDEMDEKQQLEEQMRAVMDKYKYKRRQIRELQEDLHTMSTTLDNLNRDEDAYIAM

IEEKKNKVLQLNKELEDQRAKLERVAKQNARYSRELRSAKKAKDDTPEERDFEIRELREF

NKTVMKQIGEAVNLHPEMNEAVSMYFSQANLPPLVASGIPGSARSSLSSTRSSPASSVKS

SGSARGMATPVQVGADIPASPASSVGSMRSRGSQGSLSGRR

>SG-AL_contig_4120_1_-3_240_326

MPFMALTSHYLADINIDVLTLTFSFIHST

>SG-AL_contig_4121_1_-3_1248_1478

MIIYVGNNLSRFSAEVMVFMTQCNQSQSLNHVHFFVCFLLLFLSKCLMFVYISLDLCRVR

NCRCLRFNGHEPFTEWK

>SG-AL_contig_4122_1_-2_1286_1561

MLAGFHVSNPLSSQHLIMYFVAFVFVYGFENVSWYLFLPYVMNNGYSQELVGFSFRKKME

IILVSKLTKEYFKICFSFLLEENGDNSCEYLE

>SG-AL_contig_4123_1_-2_32_196

MTGSHSINRIVGDRPYLLGRPGWSRGRASAFQSDYKDANLTRANLCVCSLQSTQL

>SG-AL_contig_4124_1_-1_1_270

TAFAHTRKETKDNEPVWPTYKLSLRTRVSHLPDLHLNGRVVTDRSHSQVLGVNRLKHQHE

NGNEDTGIHHGNDMRWMCKCCQECSSKNRN

>SG-AL_contig_4125_1_-1_28_441

MSDAGNMASELKVFRAAEVKTHKDSKSTWLIIHNHVYDVTKFLDEHPGGEEVLLEQAGTD

ATEAFEDVGHSTDARDLMKDYLIGELHQDDRSGQSYQTNTGPLGSEQTSSTNWTGWLIPL

GIAVLAAVAYRFLVAPKY

>SG-AL_contig_4126_1_+2_125_442

MLNIWDDFITMMFIVYRDKMYSFHQYTHVLSSLFKNSEKFIVHIFICVPCISSFLFNNTG

QCTSIFSSVSLASQFLHLIVILNNSDNVQVSFQLYPFNLKFFIQQM

>SG-AL_contig_4127_1_+1_514_702

MANSFSALNGSYPELSVLWANWEDWGTQSRLIHFTDASMRVLLKFPDQHHRFCNSQAWLT

KDI

>SG-AL_contig_4128_1_-1_1_201

QQTSRFLCCGLGLNSRYQISSLQFKRTNNEFLSLLSTSHFSPPTHPQTHQNRGHHDSRTM

TGHRIWD

>SG-AL_contig_4129_1_-3_45_926

MESGGMSGQSSRPPGAQQDSQYPDAVRSNPHLPQPRGPRLTPEEVSVLEECNTESFWYRC

VPLAMAFGGTTAYLVKTGHLKPHPRYGATLKTLGAVFVAYVMGKMSYHKTCQQKILEKIP

HSHLAQSVRKSRGIKSDYDEDPIADTSLDSDYIRKEKMRDTYSSIDTSSKGLDDNLRPSL

DRDVKSKVGEESSPVDAERTLTYDELRQRNRQEYERSMATSPNSPFRQRPFPMHRPGAPS

SAPAESSSPSSASEAPSSGSRAIWDTPSPQSSGKVLDSARGQKKNEYGDVWDSN

>SG-AL_contig_4130_1_+3_132_236

MHIYSTAHIIQTKNNNLGRQCWIRHLKSSTYSIFY

>SG-AL_contig_4131_1_+2_2_157

KTRKKKKKKRKKKMKDAKLHAFTLKPFIKSITVTLSTFKHLTIRLCYSLIII

>SG-AL_contig_4132_1_+1_1_213

KLENTLLFKSSRIRSHLISQRERERERERKRIFFIDKGSGISKHTFLRPAFAHGGLHEHK

CNKNNTKLEHQ

>SG-AL_contig_4133_1_-3_1296_1466

MKQIPNVFLYKDVNMWSEYYFVELWMYFPSEDVHFVVWVYRKVQYFAVIKIGPVFVC

>SG-AL_contig_4134_1_-2_179_517

MAKYPRSDAHIICSQVLYWTALASHDTDPPPGCSGQSFIPPDGGLTRRLKACVASGPCRW

VSFSLLLSMVCVCWPVNRVQLQGKWELRVHSWENVSVLSSRNSYTVLVVFVTF

>SG-AL_contig_4135_1_+2_179_481

MKDEDTEMKTMVHTVTITMTTEETSPTHTKNDRNEDITLPPGTMCPLSETASIIHLIIEI

PICTLRKRVICTTTLRPCQATGRDPPRPFIPDKQWKKTLIV

>SG-AL_contig_4136_1_-1_157_285

MNVPKKFQVFLKQFQIVPKMCSKGLASLQVFPQLCIHHLVFLL

>SG-AL_contig_4137_1_+2_2_202

VQGPPRPRWGAGATPCWGAKPPSRKWISHFLEPNSDLSWEKFYGKGRNLNAQLILKKKQQ

QQKKLFF

>SG-AL_contig_4138_1_+3_3_113

PQIHILSFTSEAGRSFLCFEDGLKSRIKAQFRDRFRF

>SG-AL_contig_4139_1_-2_107_979

MLSGIGPRTHLESLGIPVVSDLPVGQNLQDHPNVDFGISINSSVGITKEKLNSAWEYLKY

ALTEESMLRSTNGAEALLFLGSTESHRQRDHPDVQLHVFSRLLSPPASPPEEAERLSPRR

HSEGASCMPTSLQADSRGTITLRTRNPLDPPVIDPNYLHQDQDVNQLLYGIRECQRLMTT

PALRALDAQSIDKPVKACVDVHTYDSDEYWRCHIRHTVHTTFHPVGTCCMGDVKDDTTVV

DSYLRVKGVQGLRVVDASIMPIIVSGNTNAPTIMIAEKAADLIRWESSRFP

>SG-AL_contig_4140_1_-3_948_1124

MQNMSQMCTSGLACVCVCMCAEDQECRICVYQDCIMQNVSQMCTSGLACVCVCLHAEDQ

>SG-AL_contig_4141_1_-1_49_510

MECHGVPLYVISNGKVVVDDREVKVTKGAGRFIPTPCNSEAVYGRVEARNQARMPKKVER

EPYSGPVIDLGAKPKEPDNPIVPTPISPHSSSVTSATSPEHDLAFHSRGRTRGGYRNMQD

SSFSLSGAQIDDDQAKKSSIRTSNPPGGKSSGLW

>SG-AL_contig_4142_1_-3_189_425

MISYFLLKSCIRSVQLKLSASCMVGLDRKCQPVLRRFQSAELFMNEIRIFVNQAVIFDFV

VFYMGVNTTSPFGSDRVWT

>SG-AL_contig_4143_1_-3_72_341

MVYSREGEFLGCPVQETWNYGYDAKPTNVAVMTDGRLLVAMQGHRYCKVHVYRPRKASAE

DESECTCECGLRGCLRGCCCQSSWDYETLD

>SG-AL_contig_4144_1_-1_757_903

MIWIVKIIMDLIQRIHIVGISTDLICKILLVEILTDLIHKTGMVKSMDC

>SG-AL_contig_4145_1_+3_3_311

MIAALRWVVNKPTAVLQGGFNESFERKIWHPQQLPTQTCVTLKKKKKINISVHFLQFLHA

LNDYFFHILFRFFRNSLHCVCVCVCVCVCVCVCVCVHRKLRIG

>SG-AL_contig_4146_1_-3_399_614

MGYVEYHLTFSVLFLNLFHFFLPLPPLPLILLFYPCFSFFFFFFFYVFFFSVFFFFFFVF

LVCFFFLFLFFF

>SG-AL_contig_4147_1_+2_824_1036

MSTCKIQFTIIVRTDNMTDSPVSLTDEQACGSLCGIEDQYRGLTICKIHKISAFTAVPFF

FLSLMRCSMFM

>SG-AL_contig_4148_1_-1_217_882

MPKKKSTKGASKKKGKKGGKKSAVAAKPGDPFAKNEILSPLKPGERLIKLLTTHPADIRD

VHGVKVSTRILEQMTPQEVRDLRAVFEIFDTDSDGLIAAPELRKALRTLGFKLSREEAQQ

VISDVSIKGRGSLDFNEFLETVIDRQGDTRDIYDEILQGFKMFDYDNTGQISLDNLRKAC

QEAGIKFTQKELEEMVEEADVNGDGQIDQSEFIRIMLQTNLF

>SG-AL_contig_4149_1_-2_53_256

MKQAGATIWAIMLRTRIQAKFQNCADKLSNKHIFCITNNKQSKQLYYFTESKWHHHLVAT

TIRKQERQ

>SG-AL_contig_4150_1_-1_1_66

LELTTVVNLYGQLESPEVEKGF

>SG-AL_contig_4151_1_+1_712_1125

MHSYTQDRRQPNYYKCSVGVCMCVCVCVCVCARARTHTCIHMCVILCPCVYVCMHVYTCA

YVHAHSRVSACVFAFMTNAYSKHMIMFRVPLLKYTTVLLIRHTFLGCCYQCISMQNNFKN

ILTQLPYLTLSSSVKHGT

>SG-AL_contig_4152_1_+2_59_355

MKSKVGVNEKKKKKKKKAVKLLATIRDKPYPERSAALGLPNLENGHKRGDVIDVCKFVSV

NGQYSVESRSHSLKLKKSFYRFDTSNFFTESVFLRGTVG

>SG-AL_contig_4153_1_+2_2_205

SCKFMEAVFVCSRQWAKIFIPVFEVHCRHRLRNCRRWDDYANTDASQQSGQESRKYAHNA

GPKTSLAH

>SG-AL_contig_4154_1_-1_163_1296

MRGRGGPPMGRGMMRPNFRPGPPFRAFIPHIPFDLVQCEPTFPRAKPAIDDKPFTEALLK

RNSDLTPTAQEQASVLALVSKIQTILDNLVVTPGAFEAAQIEEVRQVGSFKKGTMMTGHN

VADIVVLLKTLPTKEAVSALGCKVQEELKLLEPVEVLTMLTNDSGFEISSAEASVKCLIT

TIPPNLKKLDSELHLEGKIMQSHLAAIRHARWFEENAQHSSIKVLIRLLKDLRNRFEGLQ

PLTPWIIELLSHFSVVNHPSRQPLSINTAFRRALQLLSAGLFLPGSVGIADPCEQGTVRV

HVVMTLEQQDQVCFTAQTLLRVLSHGGFKQILGLEGNASIATEMSVWEGVVVTPSDKAFE

KVEKKEEEEEMEEDGDDN

>SG-AL_contig_4155_1_-2_119_349

MWGPQSHGRGEEEKVCKGRRSEHPAGALEDRARPKLCLGSRVPNDVGIPRGDCWAQRDLK

ARWRGHFKNPAQRNQGG

>SG-AL_contig_4156_1_+1_232_381

MTMQNQNQCLNIKKASVHSLMKPINGTLFNFQSYLHLGYQNTQTSNDIFS

>SG-AL_contig_4157_1_-1_1_4728

VFNDFLAKQFGSEDAAVASASAPAQSGSDEGKQELKKESASQSTDTNIPDASSDVDETDL

DGDDVQPSPTVVKEDTRKVEAETAEKELKQREEEEDESPYINGVREMEVEDDEEEEEEEA

EEMQENLEKTAEEKTVQDKSLEDRGEENAETLNGQREEEETDTAENRKEEDSTEVDSEDP

TDVAEEEDETGEEVDETAKSSQTFQDSSTPPENSPEKVPPLPPPQGNLVEPDRKPSEQDD

VKQVIEDKESLEDIVKQDTEGKENTVLTDQGDSKVDPQAAVQADTVVKKTLDDSVDSGKQ

GSIAETEKTSTQKDTDVSVLQDGIAKTVETTVQDSTESGPQGGTAKSSPQEDITKTKKAT

AKEEQEESQLKFQPEEEKPKVMENDKSIEVDPSVTDSFGKDAKEDTSKDLPVKSDDQGEK

KEQARIHPDKTVVESTARSGHEPVDIDFVAGQVPDSSIALDSSQAGAEQPVPSRTPSLQD

MPSLQQTAQSTANIGIPSETGAEQSTQSESEDQTETTEIDGTRFYVVDGEIADIVPESVA

VKKGSEPASTTEDVVMSPPPTQATQAANIQPTATTLTPDVSSLQGVTTPSPPVNSPDIAP

ETQQLQNETKETDARAQKDQFGSPEVEDSLLQKADAEQYQTSADFLGRKVLSVNSQVPPK

VPEDVPAEEQGIKNTAEQQANTQDASEQLPSPSDSSIKKETATETDASQTESSASAGGGT

SVVIEGEEPDQSGVGGDPNQKDAKQEPEIDKTITASSTPDTQDSSAHASPPPAEENLVRK

LPPDAETEPEPEKSAGSVTPPTPVAEENQAKDVNANPETADSQESVTPPPSDRLAWDDVP

PPPAEGQTTPGEKQVADEPTVEDEPTRESDPYQMDDFYGRKISDEELLKAEEEAAAYSSG

SAVGSMEAFSRSFIEKLPPSIQSMLEQEPLGLSPGMTVLVTILTFTVFLCISCFSCLCSS

GGKKAGTKDPLMVVRELEEKLLIAVKEKENLEDMLQERQVENKRMKTEVSDLQSKSGTMT

TDFQNVTLHNEVLKKQVSSMEKEVSELQNQLSRKSDDVKVKDKKTKDIEKETKKLQDAKH

KLEQNLQKKENERQRLEDENENLKTAIQSLTDQVQLLDTSKQQLLSEAEDWKEKVDELKE

RMDQMEQENRQAQETISFKDNELEVMKDCYLQLKSFEKGGDGDGEEEEFDTGDQFKDIQE

KIQAMMDVSKVNATLRAVQEEKDNLENRLKIENDARKELEEQLECVRREMESSMADKMKA

ERQCQESQTKLNVLSSYFKDKELQLQRELGEQEALKKQNLSKLDSADENTRTLQKELELS

KQQVESLKRELTASERDFRSQIAANEKKAHENWLNARAAERELKETRHEAAVLRQKLTDT

ERRQMDLIRPLPTRAMPPPGMLNGPPPPGMERSPSRGSMPPGAIPPGPPGPPGPPPPSFR

EGEFQLSPRERERLPPSHPARHMPPPEARSPPPHPRMMPPDSRGSPPRMLPPEARSPPPY

DRRGPPHPMDSRTPPMRLPPPDMVPPLRGPLPPHMGHPPMGSPGPRMDSPRDPRQGPPYG

RQPPPGSDGRRHQSQV

>SG-AL_contig_4158_1_+3_1155_1382

MLMHWIFHRHEMGFRSGIQHIWHYLTVMNPSSANCCFQFQVLCLESNISKIKMRGTKWFG

FMVKRQLFTITLNGFH

>SG-AL_contig_4159_1_-3_42_314

MRKYRFGDPAKAGFFRKYGTFLYFFAAWNLLGFVAWKSMNASKNAGDEDWDQKTSTEKYL

SLIGFGRGNKEVRHVKMHGLQIDSSPEERTK

>SG-AL_contig_4160_1_+2_2_172

TIFIWSKIRQFVYNINIYTTSATFFSTIFIWSKILKFVSDINIHTISVTFLNIIKYM

>SG-AL_contig_4161_1_-1_1_120

SCLKKKKKRFLEDFHFFNHKNCFFFLLYQIYACVVHIVCR

>SG-AL_contig_4162_1_+3_3_116

ILKKKKTLLDKLFTTEKQDGGRKVEEKLAEMPTPLNTI

>SG-AL_contig_4163_1_-2_2_259

FLFFFFCFFFLVFFFLFFFSPPPPFFFSLCLLGFCCFVLFLVSLIICFLVVEEGDGSGTT

VTDDVAVEVFLIPVYKIIYECMHIQV

>SG-AL_contig_4164_1_-3_3_437

LPVTQPTRGARAELPPPPNANPSSAELDKLEQAAQLSGVYASLHRRALVTWLQQDSISPW

PSFVLRRSSGSWPLGPKGVRSPLLLPGRLAHPGGARPLSSSHLEGLGPMRHPGAPDHHPQ

ICDLTPPQSFDFLGMRFVTRALRVF

>SG-AL_contig_4165_1_-3_636_917

MGTPAMYTPFSKAQMAWFSNQGKRCGELEMDYYRCASKVGLNRARADCDKEYSDFHECMF

RTKQFERYCAMQAERKKQGRPYPPTPHPDGVSII

>SG-AL_contig_4166_1_+3_90_242

MAPIRSLQIVHTISAHTSYSTGIIIKKTNKKNTHTNKKYLTLLANISNFQT

>SG-AL_contig_4167_1_+3_186_362

MILSVCTDNKPTHLFRQQGNKQNRPEDTGPFSELFHQAFSWALLFGITGSRCAYIWCSC

>SG-AL_contig_4168_1_+1_1_183

KESGMDQEERKKADNSKGKKKGRNKDSNRKKVIASTEFPEGGDTIYTERAPSIPTRVPGL

L

>SG-AL_contig_4169_1_-1_157_414

MFIFVLSSLKSFEYYVCIYFIMWCMYTFVCLVLKVFFLLLFMVSRFVSSLYCCMHLKYCT

LMLLYISEQISSISFHICWRHLKYCI

>SG-AL_contig_4170_1_-3_39_260

MGLVEVGDGLGGAVVYLLSYGPRILHVSSPLENGFVFIAVKREYCTESKVICLFLPIYTL

VTVKSAIMFEIKQI

>SG-AL_contig_4171_1_-1_43_330

MKSEDKSGCTLCKIHPYLKYTDVLILSQFLRPDGCLLPRRVTGVCWAQQRRLEDLVNKAQ

RAGLLPQFRPALRSGKPRTNHRSNYKWKRYNVYFKD

>SG-AL_contig_4172_1_+3_72_227

MKELCMHYLYTPFIILSLSISISFSIPTDFLINSFTIRKQKCYKCISYSYHT

>SG-AL_contig_4173_1_-1_19_912

MASLGRVVGSVCRNVLLKTGKTNVGMLPVLHQRFLSTGQFQNLIVEKRGEKKNVALIQLN

RPKALNALCDALMTEMTVALEELQQDKDIGCIVITGSEKAFAAGADIKEMQQMKTFSEAY

NSNFLTDWDVVSRCRKPIIAAVNGYALGGGCELAMMCDIIYAGDKAQFGQPEITLGTIPG

SGGTQRLVHAIGKSKAMELVLTGDRISAVDAEKAGLVSKVVPAAQLVDEAIKTAEKIAGF

SKITVALCKEAINASEEMSLSEGLHFEKRLFHTTFGTSDRMEGMTAFVEKRKPNFTDS

>SG-AL_contig_4174_1_+3_276_422

MEIIHQQSVFSVCIFTSKLRHSHTNLPLHRRPFMLFFSMTLIYIFRSLV

>SG-AL_contig_4175_1_+2_2_463

TFSGHSFTSSGEPFTYSGHLFTFSGHSFTSSGEPFTSSGHLFTFSGHSFTSSSEPFTYSG

RLFTFSGHSFTSSGESFTYSGHLFLHFQVTHLHLQVNHLHIQATYLHFQVTRLHLQVDHL

HIQVTCLHLQVNHLHIQVTYLHFQVTHLHLQVNH

>SG-AL_contig_4176_1_-2_164_934

MMGKIHVVTCGKGAEKVVQHVMLGESVIGLACTGVNVGPKGQLSLVVLTTEDGDAFAFDV

KKYPDIMYQGLGRLLQSVHVLKVVHDSGPVAANLCAQFGIFLQNIFDTQVAYSVVMEKQG

LCGRRVPLQVLCDKCCIQQYTPSPQFQKLLSEDVNAWARRPLNRDMLNTAAADALPLVPT

LYKQLLNVVEKDSWDWFCVLNDKARLSFINPKPTNQSSKQKESHKSNKETYPQLPVFRLS

LTQRALLRDTLPPAVQS

>SG-AL_contig_4177_1_-3_3_242

RHIAFDAHLTDTEQYYSTLDSGIGDIATSSSEKDKHKVTTSRKQRNLNTKRRSVDRDSEL

IKTFTITASSSSSPKSKPKF

>SG-AL_contig_4178_1_+2_2_229

ERKKEKAEKEIEKAGHVIHRYLQFVSLISESCKTDPNIPCKKHQSIKFFDCCIFRQKLRI

IISEELRKLWILYCIV

>SG-AL_contig_4179_1_+3_828_1082

MFSSVPNSTMFSVNSIFAHALRSNFNNSSVLSQNHLVYKHFQNIWLMLSLTFISKQKSNK

NYSKVLYGIIFPSVKNCLVSKMFYV

>SG-AL_contig_4180_1_-1_70_1224

MVLESTFVCVDNSDYMRNGDFVPTRMQAQQDAVNLVCMSKTRSNPENNVGLLKMSSVEVL

VTLTTDVGRILARLHQVQPRGNISFLNAVRVAHLALKHRQGRNHKMRIVVFVGSPIEEDE

KEVVKTAKKLKKEKVNVDIINFGEEGLNTDKLTTFVNTLNGKDGSSSHLVTVPPGPMLSD

ALLSSPVIVGEDGAGAPMSGGFEFGVDPNEDPELALALRVSMEEQRARQEKDSREAVTAS

VAESTNLSTASAEEALLEKALAVSTTGDGASTTVTPSMDFNAMTEDEQIAYALRLSMENT

GAEDTGAAPMEVDTEGATGGGSEEKKEEEDYSEVMQDPAFLQSVLENLPGVDPNSEAIKD

AMKTLTTDSAKDGKDKDSSKKDGKK

>SG-AL_contig_4181_1_-3_3_680

ENGVCLQSRFFGYQRREIKNFVSFLNTLTSTLTFNVQKLYVEERDLERTVGINTGHVHTT

DFVLEDPDREFVVQRGYNSMKAFLKYYVVRHPDKVILRSQDKSLFPDIPEMTAAVSVPPP

ASDTPAGTSDPAKRVSFPPEPATPTTLSPEPAPPLTPGKKSPEPSLIFSREKVKLFRYGS

SGSGGGVGGGADCGVDEVDDVGGVDESSARDCTVAVSKNGRKSGEM

>SG-AL_contig_4182_1_+1_244_318

MKEKTIPFKPFALSPYPPASCKKLV

>SG-AL_contig_4183_1_-1_1_390

DRPLCDQVQQEAPDLCLSSPGPGSPGSGCLLTRLDSPDQLLLLSFSDSPLGDQEGEGRKD

THDPYSILLASPAVVPRPEAPIPQTSAGTIPKPGPSQATTLGDPPCEPGYALPARLAAVP

SLTDTTLLVT

>SG-AL_contig_4184_1_-1_1_657

IITASLLVKYKLEVLVFLYTRFRVRPFDLYKYDKVHPSQFDVFVSFSQHNYRWVVEKLVD

QLENRARPYKLCIHLRDFPVGNPIADSVAWAVDNSRCTLLVLTKDFVASEWCRHEFRAAH

ARLLKDRNAKLLIVVHGPVDARNLDRELLAYLRTNTYLKTEDKWFWAKLEYALPDPQENV

VVLGAARDDESWGQLPPPPIEMDAIVPVALAAGDVHPKV

>SG-AL_contig_4185_1_-3_15_221

MGRSRLKYAHQFFVNSFGREYVHTSKLILNSYVYFSTVNFSGRYVIYKSDRTLLFFSLNF

LFKKTPNCD

>SG-AL_contig_4186_1_+2_188_388

MALFNPQVLGNGKEKRSALSYFIHDHNVDVFAICETWFHPVGNEAKSLTLLLLATSVAGR

WHCFCCQ

>SG-AL_contig_4187_1_-2_20_475

MPGGSLQTGEGPAGDAGAQKEEGMATEELPEHVANKVTNVGTVVPIVEYAAQEWKGDTPK

SKTIKQGYLAISELFHCQHLRRVRGDNYCALRSTLFQVLVAGHRVTHRWPGLISIIDRLH

ELNADMNSGLHLWSFAGRLPWEGEEEKFSKIA

>SG-AL_contig_4188_1_-1_205_408

MKWIDRATGVFKIENSTRVARRWGERKNHPAMNYDKLSRSIRQYYKKNIIKKTQSTKRLV

YQFYSPYL

>SG-AL_contig_4189_1_-1_1_372

AKTVPPKCLRLNKAVSTIYWQDPPGNTKAAESSIKGRTGEKLTDDSADDRTIPDHSARIE

CKDGEKFYADHVIVTPSVGFLRENHSFFTPPLPDRHQKAVDTMGFGNVAKMFLFWSRETG

KCLH

>SG-AL_contig_4190_1_-3_33_1697

MTLLRRCRVNAALTIQLFSQLFHFINMWLFNILVMEPQLQLCTRLWGMRLKRRLGRVELW

AEKQGLELAADCHLCRIIQAAHLLQAPKMTSEDLNNITSTCFKLNSMQLRALLTRYIPDS

KEPPISPAFTDRVVAIAEDMTDELIHTEGGEVRLEEDPDLHLPFLLPEDGYSCDTVRGIP

DGLPEFVSQLSGAGLCHMVANNLASGSWTVYMGDQPLPLETPSSQPEIITVSFKKLMGSM

GLSIVAAVGDGLGERGIYIKSVVPGGAAAQDGRLKAGDQLLEVDGKNLVGLSQEKAAELM

TQTGNVVTLRVYKEGAVHHGLATLLNEPSPTMQRHATKVGPMGSAEEAPPPYSDPQQHSP

DDSGRPRPVPQPRGQNQAPRGGGLDRRRSPSVDNLSSQPQQRQQTQHPPPSNFHRREVDP

RSKSTSNLHMDTSFDHDEPQSGMRGATSVGILHPGAPSSGRHSPYQAHKQSPDVQHNNYE

NQPQRSPQSGHYNQNTSQNFHGGGGVGPNHQFHPASHRPTDRSSVSSRSSSNSGRDPRPQ

SAYYDAHPPQHDEAN

>SG-AL_contig_4191_1_-1_1_153

SFSPPLLSSVFFCLAGPFLFLFSSKPSHNSFSPPPQQFMMYCLCCFALATR

>SG-AL_contig_4192_1_+2_1208_1474

MCSVQTCMFFSLFNILQIQVITDEWHNTGCTLPDKSHKWPMETKLDVFRFGITRLPLGFK

EILTETPKLVIAVTTKDSAVQVKAFLFLF

>SG-AL_contig_4193_1_-1_127_3489

MDRAILKVHLPNGGFNMVKYGDATDVKDIIRLVVGRLAAGERYFARCFALKLMHIGTRRG

YWLHNDLTMYQVRQKYEGKHNPEELRYELRVRYLPRSFQELYSRDKVTFYYLYDQVRSDY

MRDLAEHVDIDVAIRLGCIEMRRFFKDMPQVALDKKSNFEYLEKEVGLKRFLPKNIIESM

KPKALRKLIQNHFRQYAQLSESECVYKFFETLFTVCNFNQERFKCALGSGWSIDVEIVIG

PDVGISYLTGKASSPTHMAEFDQVQSIQTVSGEDGKGLLQLKIAGAAEPLTVTCMSLDTA

EDMADLIDGYCRLIHNMAKTLWSRKDESIPRIPKPGGGRQKQELIPSNERISDYAEIVEE

EGDYSTPDGIRNSRVLNREKSRDYEIQRDNIKLVEILGEGQFGDVYKGMYFDREGMQVPV

AVKTCKETSEETMTEKFLEEAYIMQQFDHPHIVKLIGICSGSRPVWIVMELAKHGEMRAY

LQNNKPRLDLVMLIKYAHQLSTALSYLESKKFVHRDIAARNVLVSSHEMVKLGDFGLSRW

VEEQSYYKASKGKLPIKWMAPESINFRRFTTASDAWMFGVCIWEILMYGVKPFQGVKNND

VIGKIEAGERLPLPAGCPPSLYNLMCVCWQYEPSKRPSFADLRTWLSEIKEEEEYRKAEE

KTRDNRRVQRISWTGSNSSDEEAPPPKPARPQFPTGSTVNLSGNTSIGSVGSQWNSSSLL

PQGHSTPAGPSPNTESVKRQVGKASLSQEHRPPQSTSTTTNLFQGYFPSAAGYMPVGSSH

SQSQAVSSASSQHPMMSPYFSTGGVSLSSSGAAPMFQSNAPVRNGIGRQADVPSQMTGIY

QQAGAGRETVDMRRGQPGPPSPCSSVVLDYAHHRHLADVDEGNIEARLRQQQQQSAADAK

WLQAEEKSLKRDKWLAQQTEESDNTKENSPPFHHPSLPQHTPQVPPSVMQQIQQNQAMAV

RRTSTDSRSSSASDSTEGGMGPLQQQQSQPNRINDRVYESTTSVVRAVMTMTRDAPSVEP

EGYLTMVKTIGQELRSLLASVDEIVSQLPQDSMKPIEMAQKVLSNDMGSLVSSMKLAQQY

STTPLDGEYRKRMLKAAHALAMDSKNLLDSVDTARKASEMS

>SG-AL_contig_4194_1_-2_1349_1690

MNTAYLTMYKANFYQRLSNELHFCPPTIGNLQNLQYQHHCFTNQYETSLFKNILSVELNV

FTWRNLLWCVSVTSLLCLSIMCTLLPYALWILKEKEQPLLFEISISLKNNNNTL

>SG-AL_contig_4195_1_+3_3_284

ISSFSHTLRTVPVRAEGPIFTVNWVRCRALKFTSCLSTPLQGPSIKALFWSMTSTMTASL

PLSSPWAMKATRPISTKRSKTMMCQKGSANEVTE

>SG-AL_contig_4196_1_-2_1958_2221

MLLLLGLFSTYFQIDNFTKEILSCWLVCLSHAKAYAFSFLTHQTHAMHACVHPLTAPYLP

PPPTCTHTLEKDTHTHTHTHTHTHKHTH

>SG-AL_contig_4197_1_-1_1_159

EMGTALIMLAPKEVEVSTISFSYDLGLCDKSFERRNCIKLVSRYRKREIVSVF

>SG-AL_contig_4199_1_-1_418_672

MVLRLSAFVSQFHLSLWTVSIFTVSAKSALLCVSERQYFVTLCSWPSKVKRIKKKNMKKI

TTLESVVICRRDTGIQLKLRLKIHS

>SG-AL_contig_4200_1_+2_1589_2065

MCTNQSITFLQKDELQEPKHSKQYMQSDGIHLTTEGLSIFLRKIKRFLVENISIRQTEPN

KEKQSIPILIINRPDDKNVTENEFPTLSEAYLNKRSTGRWPFALSKNQQYHTDSDIHTHR

KKQRQTNSDSNTQLQYRPSVVVVNMYKTIVPLIHLLHRT

>SG-AL_contig_4201_1_-1_1_183

VCILPSLVGVHAGCFCVSISHQTLTWLTLTGSLTCAFDVLCANTHRWFKHRQVCTYVDLG

D

>SG-AL_contig_4202_1_-3_3_242

YASQTGSKENSPYLFTEIKCALLIMCRDGWMDVYKQTNADTCHVHITIIAHSYTHTFSLS

LSLSHTHTHTQSPILLSLLV

>SG-AL_contig_4203_1_-3_3_242

FKVANKSVPQYLLNLFNQRVLSYSSSRQGHFQIAHPRIDMYKCSLSFSGASLWNSLPQNI

ITTKSLPSFKENLFKYLMSI

>SG-AL_contig_4204_1_-2_2_115

ELKRKFRFVSWDCVCGFISKSLPSVSKVSFSVDVQHYN

>SG-AL_contig_4205_1_-1_49_435

MRVDFLPNFARDNSFVAMGSLTCTKCMLHTGPQFIVSSKRLAPRPLLKVWWRERKSLSDQ

ESDPGFSHPRLVLYPLGHTLQCACALLDMLALIRQRIILLKAIFCCCCLFIFCFVLFSFV

GGIKFGKIV

>SG-AL_contig_4206_1_-1_1_162

AAVSGENASLQCAPPFFFFCFFCLHVFLLTYKSRFFYRILAGQFFCCHGIFNIC

>SG-AL_contig_4207_1_-3_3_215

LFWWGGGVFFFFYCYGGGVYLFFCCWGGGGGVVFVITEFFHVKCRLLSLGKARRHSTAPP

FFLYVCIFVYL

>SG-AL_contig_4208_1_+2_488_688

MNNIVDPYLLLPAEPFIPLHTFPSLRSHLTVPFPHFIHKYYSGTQTHGMTRQSESYLNKY

SMTRVSQ

>SG-AL_contig_4209_1_+2_2_280

FIISLPSHQSKTAHIVHHKLQKGGGRGGGAERKLCEGLLEKKENGMDREGRKKQMIVKER

KRAETKSATEKRTLLAQSFQKVGDACAVPLSDG

>SG-AL_contig_4210_1_+3_381_548

MAWEGRKKQMIVKEEKGAETKRISRRLGYYGKRERRALLSTYNFILCHRHHTVTSK

>SG-AL_contig_4211_1_-1_1_213

YEIIAGDWSSDVCSSDLFLIDRGGTSLIKHNGCPEEIQKGSREHQCQTGSGDEIWQIFSG

IQADIEDTAAG

>SG-AL_contig_4212_1_+3_36_164

MIKRWQHFKSKSIRMVVAELYSVMPYLVCEGGCTEFKKYHESC

>SG-AL_contig_4213_1_-2_65_1738

MITHTTFKTAELSARNSAKDERQSPESLVLLEIVPWDRKTIMACQGQLLLTILLFVFCQT

AVCTEKPNFIIMLMDDMGWGDLGVFGEPSKETPNLDWMASQGMLLPDFYSANPICSPSRA

SLLSGRLPIRNGFYTTNEHAHDAYTPQDIMGGIPEKEILLPEILKKQGYRSKIIGKWHLG

QQPQYLPLKHGFDEWFGAPNCHFGPYNDKTTPNIPVYRNADMIGRYYEDYKIDKKTGESN

LTQIFLEEAITFIENQTQHKRPFFLYWAVDATHEPVYASRKFLGTSLRGRYGDAVRELDH

GVGQIVAKLKELDITNNTLVIFSSDNGAATYAKEMGGSNGPFLCGKQTTFEGGFREPTIA

YWPGKIPAGVVSHQVGSLMDLYTTLAKLSGGSIPTDRIVDGIDLSKTLIQQTVTDRPLFY

YRGNTMMAVRWQQYKAHYWTWTNSLQEFNRGVNFCPGEEVDGVTTHEQMNHTAEPLLFHL

GRDPGEKYIIKSYRPEYKAVMPEIFKIVAKHKANLVPGVPQLNMCDRSMMNWAPPGCEKI

HRCLPIPPSHPFKCYWPH

>SG-AL_contig_4214_1_+2_368_493

MNYQNFKMLCLSNTHTHTHTHTHTHTHKIATHCSESLNMILP

>SG-AL_contig_4215_1_+2_929_1186

MMFSTEQYLNHKQTRLHSSFNRVWDEGPLLDRFYDMSFAVSSYSSTSQSINSKQIKHSIN

NQNHLKQFQPRKMDKLSTLFLNSFTM

>SG-AL_contig_4216_1_+2_2_343

KRKWNEPGRQKKADNSKGKKRGRNKENNRKEEITSTEFPEGGDTIHTGRAPGIPTGDFNC

FKDRWDTTRHDYWCINRIKSENGQAILINCLFTKKKKTPAAQKRIQIFNFSRSL

>SG-AL_contig_4217_1_-2_2_337

VISTDVCYDLNLVVSTDVCYDLTSDHVSVLCKLSVPKPVRHRPIKRISSIHKINNSKFKS

DLDFITTSDMSLLDLNSSLHNLFDRHAPSISLLSGRRESHLGTTRSPPNSLS

>SG-AL_contig_4218_1_-2_2_142

KSCWFCCVHTIVSHIGPLMVLFLITRCTIHLLLYHEDILKSEVLPDY

>SG-AL_contig_4219_1_+1_2077_2697

MLRKLQDDSSHAHAHAAFGLAIDLKKTNIMGQDVSSIPSISISNYTLEVVEDFAYLGSTI

VSNLSQDAKLNTQISRAAIAMARLVKRVRDNPILTTNMKVYQACVLSMLLYGPYTHVKND

DSMPSTYAVSKEFWASLAGCPKQERSGTGGIPSMFALLTQRRLRWLGHVGHMQDGRIPKD

MLYGELAIASRPAGRPALRYKDVHTQI

>SG-AL_contig_4220_1_-1_1_228

FPNPEQDQVPIYSWVNWRQHTQSSPGWHQPGAHLCKQIGKWKLYLCANANQVPVVHTLMY

TVAPPQKKKVVQDILV

>SG-AL_contig_4221_1_-1_865_1191

MLFVHRGGMRTGLNCSSQSCYRHPPPLVACHLVLVTVSSHGWLGLLTSPRNHFVRCILYD

SSENIYTQNNTHLVFPVFCSAPLAMSVCPFSVHCCVVIVCTDMYRAIWR

>SG-AL_contig_4222_1_+2_173_277

MYTRQVRFSPFCCSLRHSGITPPKGTMSHPKHWQR

>SG-AL_contig_4223_1_-2_95_205

MIHAEQMCTGFLFCLSYLCLAQALPWLTFQFSFACSH

>SG-AL_contig_4224_1_-3_27_344

MLNRHVQFSLLPQLSLSAASVALIDLSFLICSFSSTLINHGNSNEDCNPTSPRNDKNSDL

IAASSPRPEVESTIQNSREHMPQNLRRSYLLWNPLMKAADWFDAHD

>SG-AL_contig_4225_1_+3_3_164

LQRREHEMFELGLNMISPCKTARVYAWSVTKSCDHIGIFIIFRLEDVGSQSPFK

>SG-AL_contig_4226_1_+1_52_258

MPFVCVCVCVCLCGETACLNFVLFYFLVTCLSPVRCVFRVCSSATQLYRFKIFFSNKRMY

SILEEQRIT

>SG-AL_contig_4227_1_-1_58_687

MVKSGLEEKPGPNDIPRVSQYRLASHLGSAFLLYALFLWQGLSHLTKPQALPDTKLISRL

RGISHGVMSLVFITALSGAFVAGLDAGLVYNTWPKMADKWIPDDLLAFDPKWKNMFENST

TVQFNHRNLGETTAVAIAGYWWMCRKAMLPPRARLAVNCLLGMAMVQATLGVTTLLTFVP

THWAATHQSGSLVLLSIAVWLAHELRRLPK

>SG-AL_contig_4228_1_+1_1_300

ISQVNICADLLVPEPPSCVYACRTSNTHIKDLISHVSICWLMETGKYQGCTPSEDGSMQT

VQQRDTCTVWAKQQVNTAQVPMLCCALASSFSPSSKGKLS

>SG-AL_contig_4229_1_+3_99_353

MGHSSKSSLQNLQKFSEVDKILYTKFPKSLFNRIHRHTGGYGGQMVETLDFQTGGLVRPG

FESSSGNGWRFFQSPRSNICRPANV

>SG-AL_contig_4230_1_-2_143_1258

MMDSDSSTKFIGSLTKFLQSLCNGYVEFQRGVELVGHIYLSIDTGEKVDYILHEKVSKND

ENSVTFVSNSFHALPLDKLVAKKDASAKPGAGENDSDSDDDIMIVDQTSKEETPGSTNFG

TIPSRNAKQMASSRTEGPLQQGSTSTSSPQRNRQQSGRPSATVTSQSQGTSQENINIGEV

KLEQISSDELLSLASQVDEGDMQSRAGGTGRIGRRAFSDTAGQPVWIKQEAADDGHGGPD

TGWSQGGEGHSDSSNSGSNLYPIMMHQNPAGFSSSPVFPGFSSTSGPSASHGFPHSRHGP

SGTTNPYGNPTPGTSQSMSSMDPAESTLARDSQGFRNRYDVTGYGDQREKTRQYKRQYRQ

RVKENPEWLRLY

>SG-AL_contig_4231_1_+2_515_736

MTNLVHLQQTTFTSTYMQKKTKSLKTTFIIVINLLCGIYFNNYTSTTLPFSNLPEVYERG

GHSTCHTTGSSPIR

>SG-AL_contig_4232_1_+1_61_345

MHCHDSSSRSAYCPMHCHDSSSQSAYCPMHCHHYQLIVLCTVITISLLSYALSAQSAYCP

MHCHHYQLIVLCTVSRISLLSYALSSLSDYCPMHC

>SG-AL_contig_4233_1_+1_1_270

QLIVLCTVSTISLLSYALSAQSAYCPMHCQHNQLIVLCTVMNSSSLSAYCPMHCHHNQLT

VLCTVMIAHHNQLIVLCTVSTISLLSYALS

>SG-AL_contig_4234_1_-1_283_474

MALTGIDFVYCDCVTSILLMTKCDWLLTYLYAALCLCQLHRLQRTFHSVDEINKRYQKEY

MENV

>SG-AL_contig_4235_1_+2_104_208

MTTGQNVGPVLFYNQINIHNTLLFIHSPSLFNLLP

>SG-AL_contig_4236_1_-1_1030_2241

MTSYTGLGLTSGTSLDGLDVCCAEFTGDMGTDIWSFRILEATTVPYDDVWRERLANAPSL

SGLELIKLHYDYAHFMGKAIQEFMKKMTEAGEGGGGTSEPRLDFVATHGHTVFHQPDQKL

TFQLGEGEILATYLRCPLITNFRVKDVCLGGQGAPLAGSSDRFLFSNVALCLNLGGIANI

HVREDQRGWDVCACNIILNRLAKLGDPTTKYDKDGQLASKGKVQQELLVSLEELGYYQLD

SPKSMGTEWIDKHLWPLLDNKKYSVEDLARTYVEHIASQISSACLDGNPIPGSPNRRKKY

GDNVVLVTGGGAHNTFLMQNVKEKLMQRGIELEETVEAETIDYKEAVCFAFLGMRCMLGR

VNVFADSTGGCLDSVSGSIHQPVNPTTTGSYPRGYRFLFRQMSS

>SG-AL_contig_4237_1_-1_1_195

SNICLMSTWQRMVFHNATLWLSSTLCVCIVMYVYCLCSVLLSLYSCFVHNVFIISLFCAL

CTSNF

>SG-AL_contig_4238_1_-3_192_455

MYHVLMLNGILTVCFRVEVAVCSGLTLNDYSCFLCTNQCLTSLFPFLFSSPPVCLLVAVV

SQHFYLDTSISSSLPSLFIWPGNTIRFP

>SG-AL_contig_4239_1_+1_1_96

VHTHTHRLLLEQLSAISCKQTFQTLVETNFIN

>SG-AL_contig_4240_1_+1_361_882

MVMVVMRIMITTHTTQRRPLCKLVQFCVLIIVSPRVLLSFIVKSSEKGDPDLMMVWGRNG

PVLWTPRCPCYALARQPMCHKNHSVVVQCNAMYITQCNEYYINPFTPSLCRLILYNNSFI

PAMQCNAMAYYINPFTPSLCRLIHYNNPFIPAMQCNAMTYYINPFTPSLCRLIF

>SG-AL_contig_4241_1_-3_540_698

MFVGLSLCFSSCGTYFKSLYHTSLDTYLLICWKTSINQRVFKMTLSGPFGCSC

>SG-AL_contig_4242_1_+3_3_239

FFFLTIFTTFISHSDVENNTFIQHMKSHFSSHTIQILATADASHSCTHTQSFTSVFFSSY

LLFRVRIFLTSLYSTFICT

>SG-AL_contig_4243_1_+2_218_445

MFFSILIQTCANPYSQIGRQDPSMWLSGYHNRLLTESLKVQIPNSPKRLSWDFLAPTTQL

SHPRYGCVIEGVAHVS

>SG-AL_contig_4244_1_-3_39_3005

MQQPGGPPPPPHMHPNISAGAPQPPPGQPMVRPPQGHPGMPGSQPPPGPPPMHMGPPHGQ

GPPHGQGPPPMSQAPPQGPPPVSQGQQGGPPGQMMPQGHMPPQGPPQPPQGQPPHMMFQQ

GQGPPRGQGPPPGQGPPGGPPTSHHGMGPPGSQPPLSQPSGMHPGMGQDHGFREGGPSMD

HGPGGPDRSSEMGSRGPMDQDMRRGPVDQDMRRGPVDQDMRRGPVDQDMRRVPVDQDMRR

GPMDRGPDRGPPMGMDRGPDRGPMGMDRGPDRGPPMGMDQNRMGMDQGPDRGPPMGNDRG

PMDQGPDRGPPGRVEPGRGPQPLMQMGPRGPGPQGMEPRPPQPGPPGPREIRLPQALEKV

LEFKSVRAQEMGVDDEDPEADVENEETEPKGPVEEDEFFPGDEEVTIENEEEEEEKESTA

KPAGKESKNKKKKKKKKTKKNRFQQQKSSQQNKTPEKKSEEDVNIEYVQEQLELDPTDPN

YHTFAKIFETFKITEVEKPKDKLEEKREAEKLAEKAPVKPRTMSDGAMDSDDDMDENAQN

EDDANKISKKKLKKLTRLSVAQLKQLVTRPDVVEMHDVTAKDPRLLVHLKATRNTVPVPR

HWCYKRKYLQGKRGIEKQPFELPEFIRATGIEDMRAALQEKEEQKNLKAKMREKVRPKLG

KIDIDYQKLHDAFFRFQTKPKMTIHGDLYYESKEFETRLKEKKPGNLTDDLRVALGMPVG

ANAERVPPPWLIAMQRYGPPPSYPNLKIPGLNAPIPEGCAFGYHAGGWGKPPVDEMGKPL

YGDVFGTQGGDSQDAAEEEELDKAQWGELESESESEEESEEEEEDKDESGLVTPAEGLVT

PSGLTSIPVGVETPDMIELRKRRIEDAMDQGGDTPALYTVLPEKKTAVGGAMMGSAHIYD

MAGVQAGKKGAEKGGTAEGVEVTLNPEELDLDTAAMQAKYDETMREQQSQLEKEDLSDMV

AAHAAKQRKRKKQQADSGKSAKRYKDFKF

>SG-AL_contig_4245_1_-3_84_1022

MACEDAVMATNDDAAQCKRFAVDKGYWGDPYISLLVPKGKTSHAPEINKGYYARVTSIRI

LTEKFIQATNRECQVVSFGAGFDTTYWCLKEQDLNPKSYIEMDFSAVTVRKCQQIKSKKP

LLDKLATEDGDVMWSKSEVHAGDYHLVSADLRKLPEVEAKLLDCGLDKNLPTLFVTECVL

VYIDMKYTENLLKWISDNVPTAMFINYEQVNMGDRFGQVMIDNLKTRDCYLQGVAACMSL

DTQKQRFLSQGWAGATALDMMTVYKHLPQADVQRIEKIEFLDERELLEQLYSHYCLVWAY

QDEASVGLKNIHL

>SG-AL_contig_4246_1_-1_238_642

MSVSSEGQSNNTSKCYQDQFFCLILVSAPNFVFRFTPHCVIVQGISCCVLLLLVVGITLL

PRSKLNRPQWRVAWKSVVRSMSGPAWHYIISNAQSKGYRQGCATALQLKRFFFNSCSNNL

KKNVFPLVTELCTFF

>SG-AL_contig_4247_1_+1_352_552

MSCVASLQLLKSLNDSQQAASVTLADVRIQVASELLPAVYTPAVPLISVKITAIKNRWVR

RWTFREN

>SG-AL_contig_4248_1_-1_235_1062

MSTPSNLLLCLGWSVFLLNASLAVGHAGRGGGGRRGGRGNSDGDNSTGMNHHGGVPMGLV

ENCDDGETNIEIDWDPSSKEEYTCKGKTLQPLNIDVYSHCERETLQAIHVCYPQKIFYEY

DLPPTSGAHRPNWPEYGEYVYVPPQRWLHSLEHGAIVMVYHPCADPVDVVMLKNIVKNCL

RRHVITPYPLMPQDKPLGLLSWGCKVLMSQTITSVARNFIKNHACEGPEGNVAAGGLYKA

GLKGLSHIVSDFYDTQLCLDHYHGHEQEEEGQLATN

>SG-AL_contig_4249_1_+3_1674_1997

MHTPDNAKITSQSGVLALYFILIFFPPSKCMCLLVLLMVPIFHQSHNSIMTQVMKTCIIL

HHVLKLNTSIAFYCIISYVLCFTCSRNTLFHFLWKIKVVSCILYIHQL

>SG-AL_contig_4250_1_-1_490_867

MFPTFMEFTLNQGSTEAYTISLSTFLYIKRKRKKEKKTPPVPHDSPKPRFNKSPRYFKIN

VSVHCKLCAKYAVPFCLVLPTVNIYSHTRCSYTVSLLYSLPTLVIQLIYVFVVGLVCLLI

CLFLCF

>SG-AL_contig_4251_1_+3_204_338

MITFQFPSSVPQAAIHCHIDDTNSPFSFISFSPLTIDASSLHTSP

>SG-AL_contig_4252_1_-1_1_195

IDEVNRPFLTVIPDKEVNRCLKIYMSKIIVEIKQSARLNISINKIINYIHCCSVQSMVNY

DFSSL

>SG-AL_contig_4253_1_+3_3_176

HQQQQQQKHHQHHHQFQNFKHNNHSDISSFNNSRTLNVYVKTEITKNREQILKYIFSK

>SG-AL_contig_4254_1_-3_147_521

MLKPKALTLVLSQANTGGVSCTLLLNNEGALLAYSGFPDHDASSTAAIASNIWNAYVKSA

NQALENSKLECVLVECREGKVAITRVANLLLCVCSKENVGFGMLKAKVEALADYLEGPLS

QVAAS

>SG-AL_contig_4255_1_-3_3_275

AVRSLDRLTIELDGNPVTKFTLWRLFTIHRLSHFNLKKINGVEVSTADMINAERLFGPLS

QLVTTQLPQFRLHMLVGDTRRKQMASLGEEG

>SG-AL_contig_4256_1_-3_3_9689

CRQDEFTCASDGRCIPIIRKCNGVPECQDGSDEGDCGCTDDEFECTSSGECIPTNRRCDR

RSDCEDGSDELNCPCQSNEFTCESDGRCIPDNRVCDRRRDCADGSDERECACGSDEFTCE

SDGRCISSLRRCDRRRDCPDGSDEENCPCRSDQFTCETSGECVPNNRKCDRRMDCADGSD

ERDCGPVCREGELECDGRCLPPQYICNGRAECRDGRDEQNCPSCDDETEFQCVSDGRCIP

IVYRCNGRAECTDRSDEDNCVPRIEVVVTPQSLRVRTGQTVVFFCEAVGVGSTGVRVRWT

RAGNQRLPASASDDGRGRLTLRNVALEDAGDYVCSVMDVSGDFQTTARLQVDYIGPPTSI

PPDGRCRQDEATCSSGQCIPRDYLCDSEQDCSDGSDELDCDSPLPCEPNEFQCSNGRCAM

KIWRCDGDNDCGDGSDESYCPTQDPGEKCRMDQFMCQRGDQCVPASYQCDGEFDCQDRSD

EIGCSVPSIIKPPVPEITVEINGTFTIYCEAVGVPTPLIVWRLNWGNIPTGNRVTVTSVD

GRGTLTIRYARPEDAGAYTCEALNNRGSIFATPDALIIVRRTVGVCQPPRFNADALRQDE

CVRCFCFGHTETCYSSNLQISQITLGSRLELMQRATLLPVEEGFIQYVPSSGDFVIQDFD

RSFPSGSYYWSLPRQYLGNRLTSYGGDLSYRVFYDVDGFDIPTNDPDVIIEGNGITLFYR

RDSNVPPSTTTTVRVPLVETDWEKSEGLIRDDLAISQLATRQDFMLALQNISRILIRATY

DSRQNLIRIGNVLLSTAVTQSPGQGRAVYVEECSCPTGYTGLSCESCAPGFYRVQRGRYL

GECIPCTCNGHSNNCDPETGVCQSCLHNTEGIYCERCAEGYVGDARRGTPNDCEPCPCPL

TIPSNQFSRTCMRQRDGEITCTACLPGHTGRRCETCLPDYRGNPMVPGDRCTPISQADIC

DPRGSLTAVPNVNTGRCDCKANVEGQLCERCRADTFYLSSDYPFGCIPCFCMGVTQMCQS

TSWNRAQVVVSFTNDRSGISLTNMMQTREVTQGFYVDRSSRELVYRRFNNLPQDVYYWKL

PERFLGDKVTAYGGSLRFTIRYRPGADSSPISLGEPIVELGGNDIILVYRGDRQVSPNSA

ESFSIPFYETDWLRIDGEPANREHLLMTLADLDFILIRATYTETTDEASLSDVSLDIAEN

RVTGQDRAFPVEQCICPPGYRGLSCEDCDTGYTRSGGGLYLGLCMPCQCNGHSNECDPET

GICRNCRHNTQGSHCELCVPGYYGNATRGSVGDCQKCPCPLTEPPNQFSPTCFLDADGQV

TCNSCPIGHTGRRCERCESGYQGNPLQPGDFCKRIVDRCDCDRRGTVPNTQCDPVTSQCQ

CKAYVQGRRCASCQEGYFNLEESNQQGCLSCFCMGITNDCSSSSYSREVLRPQFNPDGSH

SFSLTNRRLSRTITDGFVMDASRNEIIFNNFEGIQREQESLFMQLPAKFRGDKVSAYGGY

LRFTLSYSTAFDAGQSYMDVDLEIISNDQRMYLLFNPSPKPQETEDYEILLRESSFRMLD

GSEPTREAFLTMLADIDAILIRATYNSIMSSLSLRNLEMEIAVPRVTGLRRTPEVESCRC

PEGYTGLSCQVCAPGYLRVPDPNTALGRCTRCNCNGHATSCDPISGRCMGCQHNTVGDRC

EKCAPGYYGDPTAGTPNDCRPCPCPLTSPGNQFSATCYLDRDQNVTCDQCRPGYTGRDCG

SCAPGYVGNPREPGSRCTRDDVDRRPTVVVSPITLQEPVGSRARFQCQVGGRGPFNVVWS

RLDGRPLTSRATTGIGPSYELVINRVEYGDAGRYVCTATNAYGTNRGIAELNVQRPDQPL

RVMIEEPRNIVATIGTTVRLVCVAVSYSSEANYVLSWAKEGGILPPRAIDQNGVLVIPNL

QEGDAGQYICTGSDPNSVDRATAVISIQDIDRSPTVRIEPRYLEVNEGDLIEFRCMAEGT

PRPTVRWTRGPEGALPEYASVRDGVLRIPNVRKADEAEYYCTATNQAGTASVRTIIYVTR

REPPGPAITIIIRRAQITATIGSDVQLECYAQDNQNVQLVWSRQGGLPPGSSQVGGVLSI

PNVQPSYSGNYICTGTAPTGTIGRATAVVTVISGEPREAPTARVEPDMQTVSQGTTGTLR

CIVTGQPRPTITWSRARGELTANHNVQGEILRITQASMEDRGVYICSVSNVAGRAQASGI

VDIERRQSPQIELFPQEQQTIQPGGSALFQCRVTGGMPSPTVTWTRVGGKEFTSTTKLMN

GNGVIMFERVTGEEQGGYICTATNSAGTVTLTASLIVEGPPTITISPGKRITAIVGERVD

IECVGEGEPTPTVFWRSDTRRRSDVLPESYEPAPGTAGLIFNPASESDSGRYTCVARNDK

GVTEESVDLSVMPTGLAPLVDIEGPDRMTLSEGESVELSCTARGLRNPLVRWRRPNGQPL

PPGHSVTGGTLYIPRITSEYQGKYECIVTSERGDYSASVILIVSVTPRLTVSPGQITARM

GDSVTMRCQPTGQGPFQIEWQKVGGVLPPGVREFDGVLEIRQVTAADAGQYRCIATNNAG

TSEGYATLRVAVAPSVSVTPREESLQVGSEVEFRCEVTGDPPPTIRWEKDGGSLPPQHQI

RNGLLRLYNIQEEDEGRYICIATSESGVNRDYAYLRVSNIGNIGSKIGQSVQNVDVGDRV

EFECIVTGTPRPTVQWSKLEGPIPASAIIGEGILIIPEAKPEDAGTYRCTATNVAGSVQS

QVQLFVQSKPIISQQQDLQAAPLGSPTTLSCDVYGSPQPQITWTKREGELPVEHRIEQNG

GLYIPRVREEDAGTYECRASNKQGDAVLPIVLVVGAFVPYFPQNPNSYMSFQPLNDVYID

LDILLSFRPEATDGVLLYNGQYRNDRQDSADDRGDFVCFGLNGAYPEFRFDVGSGPAIIR

GQTPLQLNRWHTVHLKRQKKNGTLLVNDEPPYYGEAPGRFVGMDIVQPMYLGGVPNYAEI

PRAAGFNQGFVGSIGQVQLKGTPLNLGASAVELYGVEQYDACRDAPCYNGGRCQVHNNAY

GFRCLCPRGFAGQRCETAGYRCYPGACGEGRCYDLSDGSGFQCICPTGYSGEGCRVGLTV

VDPAFNKTSFISYPTIENGLLSVSLKLMFKPRSRDDGIILYNAQKEDGRGDFVAVIIKDG

FVEFRFDTGKGPAILRSRQQIPINEWAMVMADRKGRDGMLIVNNEDPVN

>SG-AL_contig_4257_1_-1_1_303

ASERKYCQLFPFLCSHGLCQFGQRNLYNSSPGRRKSSRPVPDSVVYHKWAASSVTSTFCQ

NAAVCSMALHQQNRPRVRPGQNISPVSQLPQGPDIPGALTH

>SG-AL_contig_4258_1_+3_1866_2126

MYHVVGINGLLINLADITGSAFEDEAGNNLFLHIPAILSSREDFKHYSEKKKSDECVRAR

ACVCVCVCVCVCVCVCARARARARARM

>SG-AL_contig_4259_1_-2_29_547

MSEIKLVYFDLRARGEPIRLVLEAAGKKYEDVRLSFDQWPDEKPNSPYGQLPYVVYNGNK

YGQSAALASFFAKEYGLYGKSNLDALRVDEVVALCQDFIDAMYKAKFENDETRKEELTQK

LHDEAVPKYLGFFQTLLKDNGDSGFFVGSTVSLADLFTYSTLDSLIAEKGDAA

>SG-AL_contig_4260_1_-1_31_348

MLSKKLNENDVRMLFAPFGTIEDCTVLRDANSTSRGCAFITFATRQCAMNAIKGMHHSQT

MEGCSSPLVVKMADTQKEKEQKKMQQVTSNLFMQFSNLGALGPQYL

>SG-AL_contig_4261_1_-1_457_1296

MASAASSWYTDLDFDSYVLDILLMAWCGGAAMVVMSVNSIKNALGPVTRQPATDRYREPT

DSTLASVAALVHKQGESCLWFNTAMSWLYLHYYNQPVYLEQWVKSLNMQLTKLGGPVQNK

VEKVQPASLPPRILNVKCQTPPDGSVVLLTKLESQDLLLSVFASQQTSEGVWLTNCVAHV

LKLNGTLRLKIYREKKDVKVRATFDSRPEVKVEVTPTNPYQDPTTLVDLGVVEQNVRNAL

ALAVTTFTITKLLMPGDLSTIPGECPEEESKAPTKPGEIR

>SG-AL_contig_4262_1_-1_142_1530

MGCILGSLACCFGSAACSCCCAACPSCKNSTAARIGYSIMLLVGTIVACVMLAPDIREEL

DKIPWLCKDFVGLKVYETSQPRSELCDNVVGFLAVYRVCFAMAAFFVLLALIMIKVKSSR

DPRAKFQNGFWAIKVLMMLALCVAAFFIPRGDFGKAWMVIGLIGAFLFIIIQLILLIDFA

HGWADSWVGHYEETESRAYYVGLFFFTAVFYILSITGIVLFYVFYADGSTCKLHKFFISF

NMILCVVVSIISILPKVQEHQPRSGLLQASIISAYITYLTWSAMTNNPDKACNPSLSQIM

NPSSHNSTGSPTDNGDYATTQFDAQSILALIIWMLAVLYSSIRTSSNSQVGRLTMSERTI

LQSDTGSEGSAEGDEEEQRSQKVWDNEEDAVAYSYSFFHFMLCLASLYIMMTLTNWFSPS

SDFRKLNANMASVWVKISSAWLCVALYVWTLLAPIVLPNRDFN

>SG-AL_contig_4263_1_-2_50_832

MTTSSPAVERLENSPEQVGTALPLPAFEPLQASFREPQAVRGGGKKKPERSPGQARATEQ

SQKMLKLQREQRQKTLQPSKHGFHPRSNSPTFKRLVDAFEKLHDVAIDSVDYRHVDRCHL

CHHVGRTSPVGVVLPAVAGVWDGEEAGEVDDESDSEVNYLHTHPFLSSLPHSSYRSKYIF

GVNSKNKLTRTIGTTMCSNPKLHDAFNRKGCEAPPADPVNDWGAWEVDSVSVESGVDPGR

MTVSLGLPSSDTPTELHLFLP

>SG-AL_contig_4264_1_-3_144_725

MKAKKLSSAGYVFRKDRENDKIQKTTWRCTRRGCPGRAYTQWCDGDQRMAVHHTQAHIHG

PDPDIPDSVEWVENAEVRRTAPISLSLTQDVPREVPWVRPSRPRVDAATQLSVHSSQGHR

YSSGECSATTSAAATPKRKSHDEPEVDIDFDVDLDVHFDSVGRQHSSSQFLNLPGTSSGS

VQQTTKVPRIMAEG

>SG-AL_contig_4265_1_-1_115_345

MHSYRKKRIRKKWVTLHCSNTLSPGRAARISHGKSIVTEKKEIPLKYIVIVLQMYIGPEI

DCLLHSSFVSETILFFR

>SG-AL_contig_4266_1_-1_1_171

ATKELSPAKITEKSTFIGKQNYMHTEERKEWVVRHCSNALSPGQLPKFHIVSPHAAT

>SG-AL_contig_4267_1_+2_2_232

DCVSVCVCVCVCVCVCVCVCVCVTLCVCVYVCIVLYCIVLSQWIYHVKFGLLSLERTHCH

SATPPIFFISFSACLYF

>SG-AL_contig_4268_1_-3_609_911

MERSESIINPRFLALYEKETVLGPTEIDEGRGQSEGTEPEKRAPVVSSFNLSMLSVIHVL

MSAVHVLMFGECVSAWECPLHCMIVEARCHLQRDDGQLCAA

>SG-AL_contig_4269_1_-3_81_1172

MTFCLIVVLVLSEIQYYTSTELQFDYEVDTNFSRKVKINIDMTVAMKCMHIGADILDQTG

QDVFSYGKLKEDPAYFELSPKQKEYLDFTQQVNDYLRQEYHALQELMWRSDKPTFRGGMP

PREDIKSGTPDACRVHGSLEVNKVAGNFHVTAGKSVPVIPKGHAHIAFMIESSDYNFSHR

IDHFSFGDPVTGVVYPLDGTLSVTDGNYHIFQYFIQVVPTEVRTYSNNIDTYQFAVTERN

RTINHKKGSHGVPGIFVKYDLSSLLIRVREEHKPYWQFLVRMCGIIGGVFSVSGMLQSLT

NFLYDAVCCRLQVWKYGRQGSFDHAVEGLPPMSLPTEIPATPLLTSAEEQDAQDGFLLQN

SGYR

>SG-AL_contig_4270_1_+1_631_900

MCTDSREIQSDFFMRSSPRYRTCSRPYSQATLNHLYRCGRKRIVPQAFFDLCVCCRCSFV

STVYTSHQNLAELFFLTQHFVAPTCKARLV

>SG-AL_contig_4271_1_+2_128_238

MSYAFSFSLRFMHISMCAFIFMCTHTSTHIIIINYCL

>SG-AL_contig_4272_1_-3_84_1112

MADEETYDEEEATEDQDEELEHLGDDEQEHENEDDEEKPPPNPLTEDIIAESLSLLCKTG

DGLSHAYVRLDIHERAVTDINILKSFVHLRYVDISKNSLKDISSLSALTHMLTLKADENL

LTSIQIEEMPFLQMASFSHNRLNTTEGLNHPMLEHLILSNNSITQVTGLDATKLTRLHTL

ELRGNKLETTDGIYLPNIKNLFLAANVIKRVEGLTRCANLTTLHLRDNQIEGLDGFTEDL

KELQYINTRGNLISSVKETIKLKDLPLLRALVLSENPVADEDDYRMEILIALRRLERLDK

DEFTDEERHEAEEIYESRKAEAEAAETTEEVANSEADEEGGEI

>SG-AL_contig_4273_1_+1_913_1194

MLRFSVHVCAWGHTLTKRMIEILKFRIVATAKKRSCRCTHSILPLKFVCANERQFGVYER

EDPENAMPRLCERTVVCSVFCLLPLGLVFICNVL

>SG-AL_contig_4274_1_+1_1_120

AQKMIKKIQHKQVSMRENSSLFLKRINIKFNAFHTMAQKV

>SG-AL_contig_4275_1_+2_2_127

FFLNRGVNIFIHSNNRKLIPQIIRKSAYPCKPLTEILIINKV

>SG-AL_contig_4276_1_+1_1_105

LMLFILFFFFLMIRRPPRSTLILTLFPYTTLFRSK

>SG-AL_contig_4277_1_-1_409_657

MCFEMAHRQNTLACEHISDIFPTLWAGFCFPPLSMFTSNAHTLNIQQESGLTVIVISVVR

FYSHGFNLSLDLPTQVVGTPISK

>SG-AL_contig_4278_1_-1_193_315

MCSHLPGTSDLFYRRERGSCGVNVYVELSECSDAVLRKLEL

>SG-AL_contig_4279_1_-3_120_272

MVQRNISAIKQCLPKNHQQLVFVLLTGTLTTKKWESAEYLKKNPHLCPVNT

>SG-AL_contig_4280_1_-3_3_200

FFFTLFFLWEPEEMHVVCGMVEGYGWVIADSIGLSCTGHRQINIMQNSCWCIQNLHLLVS

LDGCRL

>SG-AL_contig_4281_1_-2_2_139

RERVFVCMFVLPILILCVLYFLIMQYIYLCVHDCVLVYKCVSYCFF

>SG-AL_contig_4282_1_+3_3_206

ARNSLLTNLIPPPPHPTHTHTHTHTCIHDLNQYNFFKYMKLKKEKKRIIKPIVLLTVGMG

LITITDNS

>SG-AL_contig_4283_1_+1_262_579

MFKLEDRFKIYTEYHVINGVCKYMTIYLKQNIHYTQTKPKQQQQKNNIKTRHVIHKKDTH

IFNKTYQKWKYSFCPQHNAFQKQVLITSTVNPTNQVPVTAIIMLCQ

>SG-AL_contig_4284_1_-2_242_346

MEVGILSRKSLQMEIFMDRVLQKTDQQNFHILRVS

>SG-AL_contig_4285_1_-2_818_1063

MVWPKGAQLVHVGSHLTVCIALTKWPIASPCEITPDSLCCLAKWLIASPCEITDNLHAWP

NVHINAAGLLLTDIQESDRRKS

>SG-AL_contig_4286_1_-3_894_1175

MADCQNSENRIRIFSEVAETVLFIHLPPKVLIWGTFLAFVTLTVAVSLPNACISYYSTVH

TFGPNLVKIRPETAEKILLTHFIHTHTHTHTHTH

>SG-AL_contig_4287_1_+1_1_138

SLERRELRSRSHRPKKVPKISTFGGRCVNSTFSATSDNIQILFPLL

>SG-AL_contig_4288_1_+1_1_240

VNNEKCTFCGTEKESIEHVFWRCNIIQQFWKSLENLMNGGENMHNFKFTENLILFGVARN

TKTDSIFDFIMLFVKYHIYK

>SG-AL_contig_4289_1_+1_1_216

RHTYSLSLSLSLSLSLSLSLSLSLSLSLRRNLLRSHLAWLWHMIRKHMSNVLQYIFIQHS

NSVAYMFLDYFT

>SG-AL_contig_4290_1_-3_642_815

MHIVLMGWPTLCTQYTLLLHGFHTPILVDWGKRPKKYTLLLHGLHSLTGGSGRTKKMY

>SG-AL_contig_4291_1_+3_3_209

YNISLLIKIWKLKRASTGARTTHTSFSQQLQTQTQIFIIKRLNISNTTFRQIKSRNHITP

VGAHLRKIS

>SG-AL_contig_4292_1_-2_728_1348

MLCSCSKSLRLSEQDCIKYENGRDVCGSSIIVEWAKGSPRRPLSYDECYRCHRSGHWARD

CPDDRWGGGGFRSGRSGRRGYSPPRRRRSYSRSRSRDRRRRSRSRSRDRRRRSRSRDRRS

RSRSKSHDRKSSRRKSDDRKESKSRDRKSGSKSRSRSHSRSKSKSRSRSKSRSRSPEQQN

GDGDGGDTGQQEEEGGSPARSHSGSAN

>SG-AL_contig_4293_1_-3_57_161

MCISPTSFCQELIHSKYSLLSDEVECLVYFPVIEN

>SG-AL_contig_4294_1_+1_1627_1914

MVNDSDQTEMCVCVPAKCINNKKPTLWVDHYCITEPVTDAIHKDVMYVKVFNFKNLVIGK

RILISKEVKTPKVGRENIFITNQCVLMSVFMSMENE

>SG-AL_contig_4295_1_-1_268_1611

MTTLNFWKNSKPCQVVLHSSSVQVSYLKKSNKQVTADISFVYLLAVDSHYNSNADAKDDN

HKHSVSVHYVRPVVNHRLRVDTLTLHSSDLDTCNLLVKALEQELTGTHRPQRLAVIINPN

SGKKQAEKVFSKKVQPIFKLCGIDTTVVVTKAPKQARDILQTMKLDDVDGIITVGGDGLY

CEVMNGLILRTQKDHGVNVHDPDTKIVPCRLPIGIIPAGTGDMVVQYLHGTRDVMTAVVR

VILGGTVPTNAVSVHQGDQLSAYSGLVLGFGLQGDMMADCERFRWMGQSRYNVIPIGTVL

SRRSLDLEVEYVDEDTQQTKKTTDAMYSVDTYVVSKEEAGDRLCNVFGSHSMSVHMTKKC

TLGKHVQQLTKLKENTSGAFDYEFMTTARVQGYTVRIRNAPTVQEDGKKVLKDAYNINCD

GEVLRLTNPEYHVRLHRHAFCVFGEMIV

>SG-AL_contig_4296_1_-1_400_669

MHSSRGKKLNHSTLLANISILKIKSIIIITKYKLRHKELFSLGTSLKIGDDKGGIGNNVS

QVYCNMNPTSGIFIKGTSNSECFQNKYITA

>SG-AL_contig_4297_1_+2_161_322

MKSVFYKYISISNQTANPMVYNPADYRGHWRPEVSQNSKWYFPIEVKKKSNTCR

>SG-AL_contig_4298_1_+3_498_722

MCTAEYRQIDSFDISSQMQLMCTVEMFLLKVCKTQKQLLLRMPYYCTRPLLLKNVTMKWC

LPPSHLNYQHTSTST

>SG-AL_contig_4299_1_-2_71_1303

MEAPKFSRQRALGKNKKKSWRGIDITETEDFLEDERFQLRTTGLVAEKTDDQLFVVERQC

SDKTDEDLAPKKRAKRELNCYSNLINQSRIQCPRKEYPTDESYNTRSLRAFEKKGDAQNK

TTVTARKRPQSAKGDVADLWTEEKTEGEGDDFFNQVTKRKRVQPPKHIRRKCSERAVVEI

PHPGASINPAYDDHQDLLLQAHVVEMKKAKAERKVYNALDAQFPSASEAPTVATWIKEMS

AGLCEEPDTGKEEVDDSHISINPPVNREKKKTPRERRKEKAAKLKKQVMEQKKVKKGTEN

KVYSIIKIRSELSKDKKEATRRAQVKARKQEQKKYTPKKLGKTKVVVPDMELKLSGELVA

NLRSLKPEGHLLEDRYKSLQMRNLLEPRAKAHKKKTKTKKFEKKSFREVTI

>SG-AL_contig_4300_1_+1_250_432

MFVASCGHLSHKFVNNTAVQLSVVSCKFAFTGKYILISILLLYLDQLREDAVTVALQQGC

Q

>SG-AL_contig_4302_1_+3_207_503

MTVSGIGLHSKTCKEYSCSCFSIQLLWDAKMNCAEVSFTSGQVQSRSQSPNGVGKWVGTV

DKHREFCDDIIPLFLAAHHSADNILLLAIMNFPIFMRKS

>SG-AL_contig_4303_1_+3_3_200

MDQEGRKQQIVKERKRAEINSATEKRKLLAQNFQKVGILFILKEPPASPQGMFINFSHML

LFAFPS

>SG-AL_contig_4304_1_-1_1_225

IVGGNHSIDRLSRVFVKQRNVFDSALIQTLNSTNLTFIGISLTVLSFREIKDLNECFVVV

VNDDVVVVVSVCVCV

>SG-AL_contig_4305_1_-1_253_2121

MGSLSGYLVLTVICGIVQVFGNQNDHEPMVAFICGKPAMHRGVDRWISDKDTKECWDDKR

AILDYCKKMYPNHNITNVVEASYLVTIPGWPSLSGNTLHSHRVRPFRCIVDNFQSDALLV

PQHCKFDHEHNDQVCKGFSHWEVKARDACAKDGMHCESFGMLLNCGLGTFSGVEYVCCPN

KTPTHVHPTPTATPTDDDSKRKGKDDWDSKESVDLDVDSSKVNLYELYLKGEEMPSNLVN

EHQRFEAARQYMHKLQEAKNTQLMKEWSAARDHVAEVRKNDPRTAEKLGEEIAQRFRRLY

SAYEQEDIAEKQQIVALHQQRVQSDLNQRKRVAMDKYMRSLEKGDVMRINKNLRNYVKAE

EKDVMHTVNHYSHVKATNAAEATRIHPHVIQHLQLCQQRMEQALQMLERYPDIERQVRPQ

IEDFMKKFDDMATRIRNVVLPEPATSNIQTDNDDSDKTDESASNNVAAVQPQKIDDIDDD

DVEEKVKDKEAADTDNKSTEKADDDDGDDEREDEHDYERKMFRPAHQQHDAVQVEQSLDG

SAVTSSAQVGTTFGIAIGSVAIFVVLVVAVVMLRRRSTSGTNVTHGYVEVDPAASPEERH

VANMQMNGYENPTYRYFEVQTAK

>SG-AL_contig_4306_1_-1_79_435

MVVTRPEDNVNKNETMAASFTEDEDASVFHRLNKPSASHGSRKYHISSQNFQLPQSAVLH

SSAHFVSRDMTSSGYPMGPASPTQSHHSLPWLQMRRGDPMGPASPTQSHSLPWLHMKRG

>SG-AL_contig_4307_1_-2_2_403

EGPGPLLHPGAPDQPPEVRPLAVTVFRLPGREVRHPYIVSVPASGLAPAQAVSPAGTDLD

TSKESASPLGLRESLSPILPPGRARKRPLQGDLLRGKAVELLTVNMTVACYVSKQGAAHS

SALSLRAETRRRRR

>SG-AL_contig_4308_1_+1_160_294

MTACVFVCKLPTMNNRTFDVSYGIAGFRFTQKSVKFKKIKAYIRK

>SG-AL_contig_4309_1_+3_180_398

MTACVFVWKVPTMSNRTLDAAYGIAGFRLTQKLVKFKKVKAYIRKLLHCVSDAVRFSCFF

AIINLNFSNLTAS

>SG-AL_contig_4310_1_-3_30_1943

MASAGQWEVVGKTKKGKGQPPALTKSQKKTFIDRMPKIETRDPVKEDHTIYDAFYANEGK

KTTSAASDIGKQRGTHSQSTTKKKKPEQNGKRETSSRSLALDVALAQIEHAQLESVVSQS

QLRFPENQDVWLKDLASYINLKLDKVTDSDLTFKDKPKDYPQNQLSGACQKTINAVFKKC

SKQSLDHVFYHSVQSMITQSTKGLSTAGYKVFLQMLAFHKPNVVQPKLQQYLELIKTNVN

RPAQCLSLLWAIGQCGVVDLRSGLRVWLDLMLPCLGVRSVAAFPVEYLENLFIWHKQPQA

AYGEVGLREYFHLLDTIFSPTISIQGDLRKRLQAVYPDVKNIAYGANPSTNLRNFFASYL

RRAEPNCSAAMKQEVLLCLVLCLGTDKHSWSTWCQMYTKHLPQSGVLLQHLMENWELLSK

SERHMLRETLRSFAVTNDEMVAQRNTSMEGFDICNTTTKELLQKMNQSHFPWGLLVFFFT

TVFAAVIIYDIVSSPNIKASRTVRFLEDYGILTVLEQALGRITSIAYGAMSFCENNFPRY

YHWLKEMVGPSMRWAWQASKEMFVSLVEWARPHCHWAFSKTQQGLVWVYERSPETWDQYG

AWLWLSWDFIKDYTHWIWKHVLHWLLLTQHWLQDHVIP

>SG-AL_contig_4311_1_+2_347_478

MHYKLKAIQKPSLCFSDWSKRIRNCETHSPPAKLPVTPGFLSRM

>SG-AL_contig_4312_1_+2_494_658

MADGDNHTWEVGGGGLPNSTVTPPEQHWGGGLPNSTVTPPEQHYVKTGSKRASLM

>SG-AL_contig_4313_1_+3_3_161

TSSKTFNNEMESLTGKYVICNFIHRIFNNRWKHLILSPYSTACCRKPVNKVWE

>SG-AL_contig_4314_1_-2_638_946

MPRSRVSAPVLGEGLGVVYVHVFLHFKFYFIVISVLFRDCHHFMPLLPQTMHCQFSVLTK

VFVFEHGLTKNDLTVHKEIISCFNAALFRNFELSESYAEYCVI

>SG-AL_contig_4315_1_-2_20_826

MLSRSASVLFTVLLRVAFLSKHMAKYMWVSTGMVIVGLGLIGMTDFVHKTPPGYDKYGLA

AGCLLMVMSQIMIAMQIVYEEKFMDKHSIHPLRVIGLEGCFGSIVAVILLLPFSYVSSGP

FSALYMSHLVDIKDAFLQMRGSYVLALCFVGRIVSKTVNYYSGLRFVANQHDAAKRITID

VHHDLIIWGISLGVGWQNYFYPQIIGYILVPIGMLVYCDVIPLPAFFRPDPIPPPGGPLL

EHEQLQLDEQRRDLRNGDAQIEGDDNNQQ

>SG-AL_contig_4316_1_-3_3_155

NTLFHENGQSYQLSLIIICVITCFWFLSYLEVILCFLIYPSHYIDLAFCFS

>SG-AL_contig_4318_1_+3_3_272

RESQREEATLVWSNAENGSEQRSVDMVVPGKKAKRQTKRETDGVYLKRHAGTVDHPRRCL

GQSSGGQEFRPLTPPSGKRRGRRRLDLLSL

>SG-AL_contig_4319_1_-1_241_588

MAVPRCASYRCFLADYFPHGTQVTNKTLKVHGIILHFKIIAKTCIALSGPVHVIHLHMYA

HTHILMHFRVTHTHPLPPPPPLQQQQLQADFFYIYKIQHIKYLMLSTSVCKGWKGS

>SG-AL_contig_4320_1_+1_139_402

MQELWITPDDAWDKVLEVKNSGHLTHLVGKSEEEEDWTSCLSNYRTSYYVFQLVWQREAG

HLVLLATGLKGFLRMLYFSLSGRGRLDI

>SG-AL_contig_4321_1_-3_876_1148

MPHCLIIYIFKQVIFLEAVWCVYFKTLFAEAIYLVYLVVASAVLYSSRMKTHHCCGLFLR

QFGTVFKHCWSYLNCSHLRSFSVNVTILATA

>SG-AL_contig_4322_1_+1_58_225

MKCVLKTHYCLFVRHVNKIITLGSKIPILHVLQMVHRNRQALSDQAAVSCTQSCYV

>SG-AL_contig_4323_1_-2_83_2098

MHFHPNMAAINYDHLYHRAHSDTISTPRSDSPVSRTTNQSPLSMMSTTSSPSTNSDIPDK

PPPPYPYGLHSNPRQPPLPPRVPLQNKPPPPPPPSVQGVTLHLPVKTPIVSQSCQRTTTG

PSTNTQHPTLPSLPPASQNGQGAGSNETASEQGEDTDTETVSSSETGATGTGGGGGEKHR

CMSPMPQRKPEAWERDRLRRDTKVRNYSPQAFKFYMEQHVENVLKIHQQRLRRRTQLENE

MAKVNLSEEAARQMRRMLHQKESNYIRLKRAKMDKSMFEKIKTLGVGAFGEVALCRKKDT

GMLYAMKTLRKSDVLKRNQVAHVKAERDILAEADNEWVVKLYYSFQDHNYLYFVMDYVPG

GDLMGLLIRMEILDEPLARFYVAELVQAIESVHRMGFIHRDIKPDNILIDRNGHIKLTDF

GLCTGFRWTHNSKYYQRDGTHARQDSMEVKCQLDEQCQCEILKPLERRRKREQRCLAHSL

VGTPNYIAPEVLLRSSGYTSVCDWWSVGVILFEMLVGHPPFYANTPAETQYKVINWKETL

KIPRESNLSQPAKDLIVRLCCGPEDRLGVNGAGEIKAHPFFSGLNFETLRKQQPPYVPNI

RYATDTSNFDPVDPDKLRNSDSEENKKVEKLENGKHPEHAFFEFTFRRFFDDGGHPMAST

LEDPDSNGPVYV

>SG-AL_contig_4324_1_+1_1_363

THTNTLSLTHTHGGAALQQRPLPRTAARISHGKSIVIKNNYNTKQDQIRYYHAAAKFTSL

LKYSSHCSFSKTTLNFRKTNKQTNKKKPLSLSLLFSHAEDVSPFPQFGKSVQRHYSKCWS

L

>SG-AL_contig_4325_1_-3_204_293

MSPVVCAFMVMFILSVDSWTVWVDFNLTSF

>SG-AL_contig_4326_1_+2_2_274

FIYRVFAFSASTDQIQPVKEIDYGRDILNGTTIDNTVHLHYTFTSSLLVYLKVNKQGGKN

WLFAQYLFCYFDALQHGTGNCSTQEELVERG

>SG-AL_contig_4327_1_+2_200_313

MVKLSAVNINPLTTLPSDVSSQHHPLTTLPSDVSSQHH

>SG-AL_contig_4328_1_-1_1_318

TFIHYLYPFIFSFDYFECQLKKIVSFIHAILFQQILIHSILSPWIYVFSVVALLLVDFSD

ANWQWCPFRDLLDFCASFRVGNVLLFNSFSVTCCTSNKVFTSKKKK

>SG-AL_contig_4329_1_-1_106_2040

MATLSANMYPDINEQDLVTEVHEIVVQRVGHVPVAKGDFSKLPKAVKIFISHCIQLMKPR

GVYICDGSQSEADEIIHKLLERGTLTKLTKYENSYLCRTDPQDVARVEAKTFICTPERYE

TVPHVREGVAGILGHWLGPKDMQKELNDRFPGCMNGRMMYVIPFSMGPVGSKLSKIGVQV

TDSNYVLLCMRIMTRVTPKVWDVLGDGEFVKCIHSMGCPRPVPRRVKNHWPCNPEKVLIT

HFPHERKIKSYGSGYGGNSLLGKKCFALRIASVIARDEGWLAEHMLISGITPPDGKEMFI

TAAFPSACGKTNLAMTTPTLPGYKVQCVGDDIAWMRFNDQGELRAINPEAGFFGVAPGTN

MQTNPNAMKSFQKNSIFTNVGETIDGGYYWEGLEEETPKGIAITTWLNQPWAVGMPGGPA

SHPNARFTCPASQCPIIHPKWEDPDGVPVSAIIFGGRRPKGVPLVFEAKSWEHGVMIGAC

VKSESTAAAEFKGKKIMHDPMAMRPFMGYNFGHYLQHWLDIAKVPGRKLPKIFHVNWFRT

DESGQFLWPGFGENIRVIDWILRRCKGEDIAVETPIGFVPKPGTINMQGLSKINEKELFS

LPKAYWLDDTQESRRFLEDQVGCDTPPIIVELLDKQEAAFKAMAK

>SG-AL_contig_4330_1_+1_25_213

MVMVKTEISLVVCQKCVNSWGLEEGVGVSVWRREGHQIKGTLPFQPWYSAFHQLNLSLCL

AKC

>SG-AL_contig_4331_1_-1_157_456

MKVPTVRNLVYQGKNGGFVNILQNTLDRVFIVSEQPPSPDTFQSSTLFDRSNISFLAWDV

SNAGEALYLIRRMKQRGPAFFQTYRQLVVDIGANDGFLSS

>SG-AL_contig_4332_1_+2_2_172

FFFVCKNKALFTLSASTLYMHPTCVQKKSKECVQADYTSISIEGKIKTRKQKLIQDF

>SG-AL_contig_4333_1_+2_2_154

GLHQFDMVSILAKPGVRKNSAFFFSSGDKISSHLKTYTWPLVPKFFSGHRS

>SG-AL_contig_4334_1_-3_3_65

VYRSPNLTPCIAFVSFNLCIF

>SG-AL_contig_4335_1_+1_1_297

TVVLFVCIHFYLPVTLDFSTLPRTTPSLPCAKYMLNMGLTVCHFIRKTSTQTNTQCQVDG

GRGVGEKSLSNLRTELTACVFAGALLINHWATAPPNMNK

>SG-AL_contig_4336_1_+1_1_111

PFSRKKRSLFYIKFHITKYLIEIFSSVTIIISIIMIR

>SG-AL_contig_4337_1_+1_1342_1578

MKSEYGSVKTIHTTWTKSPFNYYYYYILFTAQPTIEAISGLSPCKVQSQEDNCIDHKTTL

NLKTKILQTTQFMTFYLNH

>SG-AL_contig_4338_1_+1_154_270

MSLVTNGNLIAKGNPISSKIANYCSASGTEEEKYSIYCL

>SG-AL_contig_4340_1_+3_351_494

MYYAQIFHCLYILCLLYILCMTTVVGSIILNGINIHRHVSKLEDLLSD

>SG-AL_contig_4341_1_+2_2_127

HHSWSKVVSHTQECQSKVITNRDITVHSKILLNIMTMKHDNV

>SG-AL_contig_4342_1_-1_634_822

MAPPFFPSVCVYFCLPVIVDFSTEFCQGHLFCCCGIFNMRFVHDSYRTLVYCLIRKPNTQ

TTT

>SG-AL_contig_4343_1_-3_1290_1859

MSVSLCISCLQTVRLWNNDQLKLHNLCLVYFADVSLFVSVQLLGFFSFTNAFCSVMSCLF

FTNTFCFIMSCHVCFFTNALLHHVMSCLFFTNILCSIMPCHVCFFTNALLHHVMSCLFFT

YALLHHVMSCLFFTVALLHHVMSCLFFTNSFCSIMSCHVCFSPIAFAPSCLVMFVFRPPC

APSCLVMFVF

>SG-AL_contig_4344_1_-1_1_198

NDFLRTDGDSNPRPSGQLSVALTTTPRRPVGSIFFIIIIIIIIIVVVVVVVVNCLFSICL

NKLIKT

>SG-AL_contig_4345_1_-3_24_1121

MYHVDQGGFHPKAVDERASGPGWSRQYEVERMAALPHLLHKDQWQERQMLKRKLGPGSYN

IKDFVQVMGDKPRSTRGICEGRAARFGRENSNATPGPGTYGKGGIPHSAIEEKAQRSTST

KGMLDAHSGSRSQPSVGSHLGPGTYNFRCSSDELVNKVTSLRGPYDLFSADRNKPISDGH

FAAPRLANLGPGQYEIKTSLEEWNGEHKKRKGKFGTVAQYPHPPTERIYNSVLSQCPREE

NFPGPGTYDPGQLSKPHNHKQPGFLSSAERVDRRAQRFFTGNFNPVGAGRYDVQKFDEAQ

HRNGHQSDFKSKTGRPSYAFEKFLKERIRGKDIPVAERSYLVTPQGLPSEEKPRHMYSFN

RSITVA

>SG-AL_contig_4346_1_-1_1_108

YTTHLISLIMFRTRYTTHLISLTMFRTRYTTHLCVN

>SG-AL_contig_4347_1_-2_2_124

KERVNDDFSLGMLLKNVSIMKIGTVVGIIIIMCVRTLIIHI

>SG-AL_contig_4348_1_-1_1_252

QNYLRAEPTSHLNNDIRALTMQACATLVKLEPPMSEAEVFDLVKCAIDCVFSLPVDGMPH

KKGREESYEEAMEMEKLLGETLDA

>SG-AL_contig_4349_1_+1_1591_1704

MKKDGLQLLLYGMVAFISCCRRFRRSSISLISADRSKN

>SG-AL_contig_4350_1_-3_57_1430

MMETPTDVFIKQEAMNTNHELITQRQADVCDGEGSDSLTDESSCSKHVTAEKLPPTTNCT

FGMSTAQNTMMPGNQNLLNNSSSSQRNASSDGESRSSLQATDAGHGCQESRQQDDDKTRQ

NREFSASGLVEKRPAQNPNFIQLSNETENNTRWEMMVLLHAEQHRQKSANRMQSSTPSPR

LIQQTASQFQQRTSSPYQNVPNRRVGTKNQGVSTQAYKQATSHLKQVAKNRALGMFTSPR

PDGETNTAYELNLCQRGGSSSSATFNKRDRAGSQTSQKIKPSRASFIPREPLTRSSSSPV

HPKTYSIQTQGSPRKGVPNQHPQGSLVLVYQDMDTMMVVGNECRHRPHTSPSHVTSTLRC

SPRRQLNQQTRLLPNPRQGGVRVIMHPHSASPTPPLMRTQTEDMKAEGVVAVMETPRYNS

SGPNHDNVGGQDQTVSIPLQSCLLGVGGKKCTFSPRPD

>SG-AL_contig_4351_1_+2_125_334

MTACVFLFVCFFLWKVPTMNNGTFDVSYGIAGCRFTQKSVKFKKGLHQKTSILCFRCCSF

FMFLAIINLN

>SG-AL_contig_4352_1_-1_160_282

MFLFSWPSLNNDIINFFLSSLRSLPHFPLVTNTEENPRSKH

>SG-AL_contig_4353_1_-3_642_911

MIRHHIMVTLCSVMIVFVVFDCQCNDSLCCVVYVIIHRFQYHHPLCVEVSGSPVIFHLLL

FKRGIYHLAVNIGFVIFCYSSVLALFELDM

>SG-AL_contig_4354_1_-2_20_1324

MAWRQITGLLAAFAIVNVAFASKRTLILVDNWSIRESHSIFFKNLRDAGFDLTFKTADDA

SLALVKYGEFLYDNLIIFSPSVEEFGGTVDVLAITNFIDGGGNVLVAASSEIGDPLRELA

TECGVEFDEEKTAVIDHLNYDITDQGKHTLIVAEPSSLIDAPMIVGKKGKSPFLFRGVGM

VSDPENPLVLNVLHASSTAYSHSPVDKIEEFPHAVGTNTLLIAGLQARNNARVVFVGSLD

FFSDEFFQSSVQNANKGQKFPQSGNQELAQNLAEWVFHQKGVLRVGKVAHHRTGQKEPDA

AYIINQDIDYSIQIEELVNGEWRSFNGDDVQLEFVRIDPFIRTTLKRKNGVFFTSFTLPD

VYGVFQFRVDYNRVGYTRLFSTTQVSVWPLQHTQYERYIPSAFPYYTSAFSMMVGVVIFS

FVFLHFREEPKEKKE

>SG-AL_contig_4355_1_-1_1_144

FCWSVTNWPSARPVSGINIMCIHRKTCMVQGTELKTFRAVPTVEEGKS

>SG-AL_contig_4356_1_-2_776_946

MLVHLLILLICIDCTYGQHTCTHVLTHARTHAHTHTHTRKATQTDRYKNLLVKDNIG

>SG-AL_contig_4357_1_-1_34_594

MGGAINKLLISLSSMFGKKEMRILMVGLDAAGKTTILYKLKLGEIVTTIPTIGFNVETVE

YKNISFTVWDVGGQDKIRPLWRHYFQNTQGLIFVVDSNDRERVGEAREELTRMLNEDELR

DAVLLVFANKQDLPNAMNAAEITDKLALHSLRNRNWYIQSTCATSGDGLYEGLDWLSNTL

KNSCHKS

>SG-AL_contig_4358_1_+1_1_282

FFFFWGESVFINKCITINHSILCLQTDHTTFVLLKLDKPCLRSTHLPCSSRQQILTTQQI

HHNYTNTCPDGFQLVNHHHQPPFYFEGINLFYIK

>SG-AL_contig_4359_1_+3_291_467

MAFQGAVRLGSLKKNNKRGSERGISAYWGPTCPRYWKDGRGYRVRDQQGPGSRSETPEE

>SG-AL_contig_4360_1_-1_1_294

QHYPGLPRGFGTAFLLSLASSGRRVEIYALSGNPSDLAFLADSSVCLSFFRIFRPGTGTL

LILRLISSFTVPFSTFLDKSDPVGTSAPFAASSFLVRT

>SG-AL_contig_4361_1_+1_238_366

MPTASDLSKKVEKGTEEERARDQQGTGSRSENPEEAEADGNVC

>SG-AL_contig_4362_1_+3_3_140

GWAGVCVRHGKYISQTCILDKILLLASYTVPCRTVSRIQRLEEGFP

>SG-AL_contig_4363_1_-1_1_198

YSDFRGSYAVITSPKWETTIGACTHIFCSGLECLLFFIFPGHFAWGWNVVWPECLQLKCP

PFLKKE

>SG-AL_contig_4364_1_+1_1_363

LSTLPVLTAHQNCVKKPLPSFKTLSTLPVLTAHQNCVKKETLPSFKTLSTLPVLTAHQNC

VKTPPAIIQNPFNPACTNITAHQNCVKMDCNCKESNNNNKKVHPSYFPCILISQVTTPIM

R

>SG-AL_contig_4365_1_-2_116_1012

MVPRHNKILTSLVAGALAGATAKTVIAPLDRTKINFQIANKPFSFRSALRFLYHSLLTEG

FFALWRGNSATMARIVPYAAIQYCAHEQYKIILNPSKSKKHLPPAHRFLAGSLAGVTSVA

CTYPLDLVRARMAVTAKDMYHNIFEVFLKIYREEGLRTVYRGFTPTVLGAIPYSGTSFFT

YETLKKLHAEKFKGEDPSAIERLVFGACAGLLGQSASYPLDIVRRRMQTAGVTGHALDYT

SILGTARQVVAEEGFIQGLYKGLSMNWIKGPIAVGVSFMTFDLALRWLRRLEVFHQDGD

>SG-AL_contig_4366_1_-3_363_509

MGHFVYFMMPSCDFTPFMYIDGTVILPWLFFPFGCGFTVIYQVYQGLMF

>SG-AL_contig_4367_1_+3_3_200

KHFFSFAITLENKKKKFSLSLLFSLSFFFFLLLFFFFFFVGHFLINRLFFCYFFFIFLFF

FIKKNS

>SG-AL_contig_4368_1_+3_597_848

MLQNLNEDTKLALLDIMNEILESEQAPEEWKHGVIIKLPKKGDLSDCNNWRGITLLSVAG

KVFNRMLLSRMRDKIDILLREEQA

>SG-AL_contig_4369_1_-3_174_359

MFCFVFAIVIFYPLFLIVVVFIFVVAVIYPFISVISDLITLTKHSFSIVFFLCCNHLFTE

AY

>SG-AL_contig_4370_1_-2_269_472

MVLYKRAIDFTHTYTNIFLYIHTQKENYNLVVSNSFCDASSHLLPGQRSSIIMAHRHHAA

LVVYTALK

>SG-AL_contig_4371_1_-1_172_360

MKFPQTKMNALNLAPEYQFATINRIKLVNVTCAHTFLTHSGTGVHKSNSADLRFDASQKR

TLS

>SG-AL_contig_4372_1_-2_647_805

MPEPPSCVYARRTSDTHIKDPVSRVGVRWHMEAQKHLACTKIVAFMPIVVSAT

>SG-AL_contig_4373_1_+1_1408_1731

MECYGPIDSIQFCSCFLFVFLNKVMIVYIPVLYCQEHFGLLLLMCSCHFGSLKLFLYYHS

NYEQIKYIKSTSTETFLTVMIFLNCTTFRNGLKTTNNKRKRPKLAQKL

>SG-AL_contig_4374_1_-1_1_150

VTTFLSWCHILLRWSYVSWKCFVLLLCVYVICEHIVFTQLFCQGFFGFSF

>SG-AL_contig_4375_1_-1_169_381

MKLLSGVKILQEITVGMSRYRSHQLRGGHKNLKVDIQDKAATKETFLMYMLSGSGAENPR

NIDRCLCVCYH

>SG-AL_contig_4376_1_-3_63_2003

MAPVRWKVIALIGTPALLLAILGSVSGQCQIPEDYTGDWYWMDNDGSDKVEIVNLNDWGD

YNCEQIKIHDNPYANVRGVNSTMLMKKKGDDICYICLDVLYRTENILQVRRSDCYSDNDN

FARKCPGYYKIPTVDQVKTYFRQTLRTVNCITTFEGMYQFTYEVAWGGGGICDNRDSQIQ

ACQDPGSAYVDNEVFFMTYARCPEVQTSFSQTKRYQCMGSWFAMVGQTGYTFASIADTVE

MDKREKYKCLMTLKNQKSADNTIRWVMSRFAKCDTLNSMHSGPVRLVLRRIPPRSEHLAP

QCNLPRNISGEWYTQGIQFKSKVIINDTHIKYFTRKNEFEYEETYLSCQQTLGTRYLMTK

VIVGKCEVDFVCYDIVPRHHGIVRYRVGKPSKLTEDEKAQQNYMERKFREACSWMSFTFN

RNQTHWKYEVLILDPPSPVPCPVGGRYQFGQWANGELEKYATRIRGVTDRPRVQVDCRIQ

VSEFKSCTQDRSRIEVDAEYCEAVDYRGRPIGEYDEPDHILTCVGFWMEDMKSYMITYDE

EDAISKFRCWVYERISWTEMQLSRSQTARCQPGQTSDDFMMEGTGLKLYLTENERLFDDC

PQRFDPGMDPYRKATVIYVLSAAPSPFPATVIFTTAICLLVSSLLLL

>SG-AL_contig_4377_1_-1_1_300

SASGPTNAHPRPRAVSSALSASGALPATRSLSFLSQRKSTESVYNSKWTKWLAWCGPNQV

DPHAPSPVELANFLAFLATSEDLASSTVKGYRSAICTTLQ

>SG-AL_contig_4378_1_-2_2_292

SLASLSRRKGTESAYNSKWTKWLAWCGPNQVDAHAPSPVELASFLAFLTTSEGLASSTIK

GYRSAICTTLQQLGGGGGGVLSLTTPSPGSLERPDGL

>SG-AL_contig_4379_1_-1_529_819

MQCHVLSVYEFLYVCACCLPLFPLCVCKFACMLVCMYVCVCVCMYVCMYVCMLSVHLCIL

FAPFPTMYVCTHALYMCVCVCVCLCVCMCVCMYVCIY

>SG-AL_contig_4380_1_-2_170_1177

MPPRKKAAAKGKAAAEEETTTPQKETKKRGRAKKEDKEENGSEEATSGTSEPPPKLAKKV

SEALASMDFSSAAKTEDGKPWNLKIASWNINGVRAWLNKDGLAYLNQEKPDVLCIQETKC

SKEKMPPELKVDGYKAHWVSAEKEGYAGTGMYTKTDPISVTYGIGIAKHDNEGRVITAEF

EKFYLVTSYVPNSGQGLVRLKYRTEEWDKDFTAYLKGLDAKKPVILCGDLNVAHLDIDLK

NPKGNKRNAGFTKEEREGFGKLLENGFIDSFRTLYPDKEEAYTFWSNFRQAREKNIGWRL

DYFVMSERLKKNLCDSVIRPQVMGSDHCPIVLLLAL

>SG-AL_contig_4381_1_-1_100_1332

MIVTLKTLQQQTFKIEVEPECTVKLLKERIEAEKGKDYPAGGQKLIYAGKILDDEKTVDE

YKIEEKNFVVVMVTKPKPASAPAAETKPSETPAAANITPAATAPAPTAAAAAPTPAPVSQ

PETEGKGDSNTTSANVAPTPVTPATEASEAGATGTAVQSAESTLVTGSEYETMVTEIMMM

GFERDQVVRALRASFNNPDRAVEYCLCGIPEVNVPEESPAVPVSGNPPAANPPAATTGTP

ASTGASTGAGTGQNPTGVNPSAAGSGTGSFPSEDQLAFLRSQPQFQRMRNAIRENPSLLP

ALLQQIGSTNPALLSLISQNQERFVQMLNEADSAESGGQGGGGSGSGQEQEAGGGGYIQV

TPQEKEAIERLKALGFPEGMCIQAYFACEKNEDLAANFLLSQGFDDDEDQS

>SG-AL_contig_4382_1_+2_2_163

KRRAPRGRGPTRPPVPRFPLTLCDIGQDRGRPITRGPSGEGGRVFVVRHGKYAY

>SG-AL_contig_4383_1_+3_600_953

MIWVTSSVSWRMISSPCSLLVGIRRMLVWHPFCFLCVWQLLHVDSSHCFLIFLPGCGLRN

HSVLHLACFLKSGDRSNHLHVVFWSPSHLHPVNGPVSCLCVLCPLVLCHQNLSLKVDS

>SG-AL_contig_4384_1_+2_2_211

NTLSLHDALPISLTHLITTNNNNNNNNRKTDTDLFTYKLNTLSTQLCKFVAGVLFYFDFN

HTILTLTRCP

>SG-AL_contig_4385_1_-3_72_1178

MWLKICILALCAVYSAQTVQIQPFRSVFTQAEGSTLQVNCTSQNSEAWVADPPGAMPTDA

VRAQADSGDNISTLMLENIQKSNEGTFRCKDKTTGDEVSFELKVFSVMTQNATFEYKVDA

MLHCGVTGDSNVYKYTWRKGSTPVADIVVEEKQAYIQHENGSLEIVNPSRSFAGLYTCMA

VFEDSTGTIRTIDIDVPYRATPKVLPFDKSKNLVQDENLVVECKVLGYPKPYITWLKGNQ

EIKVDSGSRYKLLAANTYDGMSIDNARLEIDMVDFDDAAEYKCMARSGQWPSFNSSQIIL

VRVKDKLAALWPFLGIVAEVVILCTIIFIYEKKRNKDAMLDEDDDQDGAAPEKKSENVRH

RGNTNNPRA

>SG-AL_contig_4386_1_-2_2_205

WGGGERGGEGGGGGGGGGGGSFREIGILLEWLDHCFSLTGRGDMTCLRVREWISSQCFQS

HLSKHFSQ

>SG-AL_contig_4387_1_-1_1132_1461

MTTSVVLRVHFLQKLFHSLLLACRVSDHWKLRLWSCLPDWLLQYATLLFYLLEYSWSSVS

LVHLFYILFLCLLGYTSHLSTHTHTHTHTHIPILSYTHTHTYPFSLSLSH

>SG-AL_contig_4388_1_+3_3_227

RETTATWHNSDHFYTFPLRLLSLVLDILGSFNLHTAISETCVKKFASKHNPRHVFTAAQV

LEQIHESGSEDDGEG

>SG-AL_contig_4389_1_+1_1_315

FFTFPLHSLHLLSPTLDIFGNFNLHTAISEACARKFASKRSQRHVFTALQVLEQIYESGS

EDNAEVDHNESPSETEDDSDNEVFSDVPDENLIKADNDSELCLHV

>SG-AL_contig_4390_1_-3_3_701

PRPLRSEVDSSQVATETSTTAATVSQAPPSPRAKRPIIVLPAPAAVAGRGDRATSVPPVV

VFREDKRQKAQVVPVSAREEWRNRAKARSVPPPPVAAPVRRRPYRAPEKPVVPVQAAVRE

PLPTAGLRAHKVPRRFLTRSGMRTGFSSLHGYYADEPRDRNRQADTEGFRKSVTRARVLG

SPVTKSSALPPYYVPPKPKPAPITESLSEFPKVYVPTKGPKPSNRSKAAIIAS

>SG-AL_contig_4391_1_+1_178_558

MFCMRIHRRGSGTSRSAHMLTWKIGKISTLYPPGKVPVVRTQAFHTPSLEVKCLHHSTTA

PVNAMMYTRKEAAYGHAYSHTQHPTSIQSWPLKMITVFKNLAFKVSVQKQMSRKMNLVVI

VGHLSTL

>SG-AL_contig_4392_1_+2_170_457

MAYRIFNVHICSACVYTRRGFRHYVDLGDRKNLHPLPTRRGGRGGFEPRSSKPSRLKVKC

LHHSTTAPVCVCVCVCVCVCVCVCVYARAVVFITVS

>SG-AL_contig_4393_1_-3_60_179

MMLLLHELSSKPACNKTNLMMLHELSLKPGHNKIKLSDVT

>SG-AL_contig_4394_1_-2_2_67

MMLHELSCLKLGRNKTKLNDVT

>SG-AL_contig_4395_1_-2_53_1399

MATNKQEGQEQLEHKEKSEDGENEHQKQKQTEEPKVKQVTFKETTDVCKEKSPKKVRTRK

KAVMNKLRSQMEFYFGDSNLSKDRFMKKQIDSDPEGYVPLSIFLDFSLIKALTTDVNLLV

KAVQLSQVLQVNADETKVRRTTQIKLMSPEEIDVRTVYVEGFHKFTSRDWIMRKFSKCGK

VNYISMPRFKTTKEIKGYAFVEFETPGEAKKAIRTLNRVPDMMDEPIEQKIASSVKQCKN

LTHWKKRASKEGIDLSALDAKLKEERKQGDEGGPKEGTKSRKRKKKKPRQSVQVEGTTQE

GTSLEKTAVTKDESKENTSKPEVSSETDDAKAKRSKKSKRKNKRKAALNKQISEEHITAV

SQDTGDSKGTVLTSKTSEKSIDTKLKVSKESEASASTTEMIKTQLPEESSESESQSSRKR

PRPNEATSDPCPAKVPRVDPNADQDTKTV

>SG-AL_contig_4396_1_+2_1502_1711

MENLCPYSLAVHTHTGNSTAKEETHLPTFSNQLCARAHTLSLSTPMTVQMIVLPREDKHA

FKYLEKFCFI

>SG-AL_contig_4397_1_-3_39_236

MQYDRITKIIQYSTIQYDSIQYNTMCRLMTFPMIILDSFPSHGIYGVYSLNFPQGDNLPE

KNKIFN

>SG-AL_contig_4398_1_+2_2_163

FPNCEESQVPIHSWIDWRPHTQSFARARFTPKQTRAVRLAGECSTSTPTKPLQY

>SG-AL_contig_4399_1_-2_2_247

LCLCVCVCVCVCVCVCVLQFNVFSLKVILDDHVCVCVCHSLSGRVLSLSSLCDAAAIYRY

WFKPLTPTPTPSAYPPASCKKG

>SG-AL_contig_4400_1_-1_112_1686

MSRLYDTVEPSVIDEEMLMRAVEEQGPKEEAGKIAKTEGIDFGDVTTLRLDFKNILKIDG

LWEFVNLTKLQLDNNIIEKIEGIDVLVNLVWLDLSFNNIEVVEGLDNLIKLEDLTLYNNR

VSRIENMDMLLNLQVLSIGNNDLKELENITYLRRFRKLKTLNLSNNPFCEFDNYKQYVAA

FLPHLEFLDYRLLDDQTKAAAYERHMIQIEEMLDNEKKAQAREEEAEKQATELELHKKAF

VEHMNGPHLFESMYSEDAEGQKLKEMPGVDEILVGYSEKFTGICQQIFEYGLKEHTKREA

EVAMFWECVEEAKTENKDMGMKAVDDFMMEKKRMFVELSHVTDPKMLEQRVSEYNTRVTE

LWDKLMGLELQLVDQLEEVIQNFDRNMQDLVTTFIEHLQGFLAVARDAENVHNERMMEIA

QVTLDKVAKNEMDDELSEELRMLFVDKDTIMNAVSTSHDSHLLKIDNKEDDIVTRINNWL

KSMIETIHDEQEIKRNRSRVTEINHLIDHLREEIDNLEIGSGHVF

>SG-AL_contig_4401_1_-3_141_248

MNFQTRGSVPHAMTSQSFLAGERDFIQQTKLTWTVL

>SG-AL_contig_4402_1_-2_2_136

TNLNTLSLHDALPMRRSILAGPMLVTDTNFIFIPYLLSHEICYMR

>SG-AL_contig_4403_1_-1_490_666

MNKSNISVFRKGGYLGARARWVYDGAAVPVVNVCKYLGISFTTRSSFVTACKDLAKNEF

>SG-AL_contig_4404_1_+1_1_156

ILGTCLVCVTFTLALSLPNSCTRHSLMVHTFGPNLVKICQEIAEKISLANIF

>SG-AL_contig_4405_1_-2_2_103

LLEMVFSTVSVVGGHLGLFFSLPSGSSSLRTPSP

>SG-AL_contig_4406_1_-2_59_175

MCVCMSLCDVCVLTRTFMAVIVERCKSCFILAQCGFSTL

>SG-AL_contig_4407_1_-2_341_493

MSLCDVCVCVIKWCVCMSLCDVCVCPYVMCQCHYVKCMSICDVCVCVCVTM

>SG-AL_contig_4408_1_-3_117_428

MVRWALDDNDGQLSSLCMVPICQIETLVMETIKIQIGGFCSCRLVLSPNVHFLDLTLFFT

DCFTVLYCEKGPLILLSLVSAFLVGFVEQEGYYGVWSCHLDEWE

>SG-AL_contig_4409_1_-2_329_3259

MADECNIKVVCRVRPLNSSEEKAGSKFMLKFPTDDSTSVGGKVFMFDKVLKPNVTQEYVY

TVTAKPIVADVLSGYNGTIFAYGQTSSGKTHTMEGVLGDDAMQGIIPRIVQDIFNYIYGM

DENLEFHIKVSYFEIYMDKIRDLLDVTKTNLSVHEDKNRVPYVKGCTERFVSSPEEVMEV

IDEGKSNRHVAVTNMNEHSSRSHSVFLIHVKQENVDSEKKLHGKLYLVDLAGSEKVSKTG

AEGSVLDEAKNINRSLSALGNVIAALAEGNKSHVPYRDSKLTRILQESLGGNARTTMVIC

CSPASFNESETKSTLMFGQRAKTIKNVVTVNEELTAEEWKRRYEKEKEKNNKLKLIILKL

EAELQSWRAGNAVPSEEQVDVKTVALEGEQVKEGTPPGPGGLHMTTSFVGLAGGGTSAIS

EAERQQLEAEKMNLYQQLDDKDEEINNQSQLIEKLKEQMLEQEELILQSRRDYENVQSDM

GRIQQDNESAKEEVKEVLQALEELAMNYDQKSQEVDNKNHDLETLSEELNQKTSKLNSLQ

SELETLKDTSLHQKKRVTDMMVSLLRDLSDIGTAIGGSAAENKPNPGAGEKLEEEFTVAR

LYISKMKSEVKTLSGRAATLETQHVENTKKLGDSETELSDCKLKIQQYEAKMSTLSETIK

TVEQKKRQLEENIDSLNEECAQLKAQQSMATAVTEKEKETSTKLQAEIEMKETLEKQMMA

HREQHQKQLSTLREEIADKQALIDGLKETNQKLSLAMEKVQGDYEKLKAEDVEKSAKLSE

LSVQLSRREQAKQDLKGLEETVAKELQTLHNLRKLFVQDLQNRVKKSANKTEEEEEESGG

NVAQKQKISFLENNLEQLTKVHKQLVRDNADLRCELPKLEKRLRATMDRVKSLETALKEA

KEGAMRDRKRYQMEVDRIKEAVRQRNLARRGHAAQIAKPIRPGQHPGSGTTQGTGIRGGG

GGMAANGPALIKPESQA

>SG-AL_contig_4410_1_-1_205_3135

MADECNIKVVCRIRPLNNSEEKAGSKFMLKFPTEESISAGGKVFLFDKVLKPNVTQEYVY

TVAAKPIVADVLGGYNGTIFAYGQTSSGKTHTMEGVLGNEHLQGIIPRIVQDIFNYIYGM

DENLEFHIKVSYFEIYMDKIRDLLDVTKTNLSVHEDKNRVPYVKGCTERFVSSPEEVMEV

IDEGKSNRHVAVTNMNEHSSRSHSVFLIHVKQENVDSEKKLHGKLYLVDLAGSEKVSKTG

AEGAVLDEAKNINRSLSALGNVIAALAEGNKAHVPYRDSKLTRILQESLGGNARTTMVIC

CSPASFNEAETKSTLMFGQRAKTIKNVVTVNEELTAEEWKRRYEKEKEKNNKLKLIILKL

EAELQSWRAGQAVPSEEQVDVKTATQEGEPAKEGTPPGPGGLAMATSFVGLAGGGTSAIS

EAERQQMEAEKMQLYQQLDDKDEEINNQSQLIEKLKEQMLEQEELIAQSRRDYETVQSDM

SRIQQDNESAKEEVKEVLQALEELAMNYDQKSQEVDNKNHDIEALNEELNQKVSKFNSLQ

SELESLKDLSLHQKKRVTDMMVSLLKDLSDIGTVIGGSAAENKPNAGAGEKLEEEFTVAR

LYISKMKSEVKTLAGRATTLETQFSENSKKLEDAETELSDNKLKLQQYEAKMSTLSETIK

TVEQKKRQLEENVDSLNEECAQLRAQQSMHMTAVSEKEKETTTKLQAEIEMKETLEQQMM

AHREQHQKQLSALREEIADKQAIIDSLKETNQKLSLAMEKVQGDYDKLKADDVEKSSKLS

ELSTQMSRREQATQDLKGLEETVAKELQTLHNLRKLFVQDLQNRVKKSANKNEDEDEDAG

GTVAQKQKISFLENNLEQLTKVHKQLVRDNADLRCELPKLEKRLRATMDRVKSLESALKE

AKEGAMRDRKRYQMEVDRIKEAVRQRNLARRGHTAQIAKPIRPGQHPGGVSGQGTGIRGG

AGMAANGPATFKVESQA

>SG-AL_contig_4411_1_-3_36_305

MHLCYQMCYSLWTCYRCHAENPENVSRRARSPLQQGHVVLNRCSGQFHCVTSNVFSFFPQ

KYSAGLPIFSCPTTPIVSSLKERKNSAPST

>SG-AL_contig_4412_1_-1_91_636

MFKKFVAKEMVTNVNNAKSSVQKGIRCQVLEQFPYIENYIDQILPKKEALKLVKCQDHIE

ILASQSGELLFFRQRDGPLYPTLKLLHKYPFMLPHMQVDKGAIGFVLSGANIMCPGLTSP

MARMTKVEKDTMVAVMAEGKDTALAIGFTKLSTDDIISKNKGVGVDNIHFLNDGLWQMKA

VK

>SG-AL_contig_4413_1_-2_746_2557

MPSLEQKRVFAALPPAERGKSVKIYGDPKGQNILYCHDKSVIIRNMEDPSQADIYTEHAC

KTTVARYSPSGFYIASGDISGKIRIWDTVNKEHILKAQYPVLGGPIKDIDWSPDSSKIVV

CGEGREKFAHVINMETGTSVGTIDGHSSSINTCSFRPVRPFRCVTASEDRCVGFYVGPPF

KFNKTQRLHSNFVNTARFSPDGSVIISGGADGMLFKYKGDDCSACEEQGEIGKTKAHKGG

IYEICFKKDDSNQVLTVSGDKTAKILDLESGKCLVEFTNNMKEWQGMQLGCLWQGNEIIT

VNGYGHLVYQDTENPSEPKRIVMGHNKPVTSLALSADKTTLFTGGQEGIICWDIATGHSI

VVGGPGHTSEVTDMVTDGDNLISVGVDDCLRFSSVSAQQFGGGEVKLASQPKAVDARNGI

IVVACVQHVTVLEGNRQLSTMPVRSEAWAVSIHPGGVAVAVGYKDKNVKIYSLQCGTLKE

EMTIDAPGEVKCLAYSPDGAFLALGADRTVMVLSTSDYKETSKGETHTARVHCLAWSPDS

SRFASAALDSKVVLWDDVCAHAFKTRPEIKAHTMSHANQLVWLDTQNIMTAGQDSNVKHF

VVNN

>SG-AL_contig_4414_1_-3_270_443

MIVREAKNCKFRNERIGLFCYCWLLFVCLLVFGVFFVVMEFFCKFLKCGLHSLRKKIL

>SG-AL_contig_4415_1_-2_551_1057

MARTASPIRAKHKMSRTIVRVSNRSVIRCVLTPVIVLTVFVMNVFLMLCVMEIILVFLVR

KWRMYVKTCMTMEVATEKKYVMTLIFHVSWNIEGLLNKLSDSDFVAYLSNFDSFCLLETF

VETFISSLFPKHTVFCSPAQKLSVQRRRSGGVIVMVKTELIKFVHERKS

>SG-AL_contig_4416_1_-3_138_2144

MSSKLDTRVIQVTNVAPATTKDQLKTLFQFIGRIRDCKLFPPEGSDQQVAAKVCYVEFEE

STSAGVALHLTNTVFIDRALIVVPVMDGKIPDERVAMQLTPQTVARMAPGQPSWPTNVVS

QLTSTGTGQVITTYDPRLNALGLPQYPPLPSSLDAAKVEEIRRTVYVGNLDPKTTPESLI

SFFSQAGEVKYVRMAGEDTSVARAAYLELTDQRSVATYLGYNGINFNGRSITVSHAKSSI

NKPLSKSQEASRREVEEAMKKVQEAQSLISAAAAEKFTTRSRSRSRRRSRSRSRSRSRRR

SRSRSKGRRRRSRSRSRRKSKSPRRVRRSRSRSPRRRRSRSRSGFVVIPVRERRRSRSTS

RTRRRSRSRSKRRSRTPPRAYTARRSRSRSRGPRRKSRSRTPRRSRSHSRGKGKDKKERG

RDGEKKKKEKKASVSRSPSPPTLQREGSPAPHSSDDRSPARLDHSSKEETVKQPETEAAS

DAALTSGAKKSRSDPAIGKSSQKRDRSESPVVRRRGKSVSRSPMRGGRRRSGSRSPMKSA

RRRSISPRKRRSKSRSPRRSRTPPKKKKTRSPRRSRSGSRHRSKKDKKKERSRSRDRKEK

KEKKKSKDKREEKEESLSSKVARDYDEEEKGFNSGSDHEEMMRSPGDDYEEDRRSIGADR

QLVDMDTSD

>SG-AL_contig_4417_1_-1_67_1491

MGNSSSKKAEQSNVAAQNNAADQPANENQAENVASKDEPAPEAISVGKAANGNIVTHKPY

KEESQSAGKKDGPSDGVAAPSTEGQAGDEVQSEDKAVEDSDVSKGSVAEEPAAPVSEEKT

ESDSVGGEMEESVEVTEATEEHSAEGTDVVPAENGIPETSPETKPEVVEEQVQATAIVAT

QSAAPVAEPVPEPEVVPKEKSDVPKEEVTKASEETSGVDVKDPSDETEEAPPVTKEQAVI

EESVESAEAHLQSAQPPDEPASSSTEPADEASTGISNEAPVETAQDEPVSEDTTPEESTP

KTTEEAVEDNEVASESSPPNTEEPAAVQEETSGQEGSLKDAPLPEPPQDITAEENAPVED

SSVEEVPPSPTKELASTEATASSLAGEEPEQMPGPSDEEKPAETSAPANEQPKEDKTPVE

EPASEEDKEISKQESATEQQSAADKEVSLPEEQKSSEEAAPEMPEQAEAVTEEQQ

>SG-AL_contig_4418_1_-1_37_264

METIRHSYKKKMGGAVLWRCAFPRESNPHLCVCVCVCVCARVHAQCLYSHSNTPCGEVQS

HPWFLYGSQLRQQRDG

>SG-AL_contig_4419_1_-3_780_944

MYCSADPTVKTSFKIQGVASFSVPADLHFMFCLIYLRFSCLQSSHEIRTFLFIRL

>SG-AL_contig_4420_1_-1_1_615

SLQFTFLLNITTFLQSNYLESENTLLTTLSPAKGIVSKFAIGLLSDLIIHRVPRVAIVLV

TTAVQTALLAICIFKGDSFVVLLITTLGVGISNGATWCLTPVMTSEYFGTRYFGRNWGSM

MLGLAFMGLALQKIFGALYQMEITVPGSTECYGLRCFRWSFVVLAGLSLCSCILYVGLLE

RRLKLRKWRRLKKEMKKEQDCQLQQ

>SG-AL_contig_4421_1_+3_6_284

MDIRGPDKDGVDGQGDAFGTWCQKTTTAGVARCIVYLIDIKYGTSGVPTLKKYAKYRKHL

HSTKILSLSLYVCAEGLGKSQSLQVVIPFSKLY

>SG-AL_contig_4422_1_+3_3_152

AFHFFLVSHDRLYIYMCECMRVCVCVCERETEREEHFRTIINHQTNQMQI

>SG-AL_contig_4423_1_-1_1_237

RKFTSHEYTASANSKLTSRCCTVCSNRHFYSSLHWAHLVAISLLFGNMQIVKSLKMTSYP

VPISFQSHLDPFMGETRKK

>SG-AL_contig_4424_1_-3_438_938

MNHVKIILVSTLALLAVVNCQDIQLSANPSNVVSGSTENLEIICKAPSLDEVVNLIILLQ

VDQLDEDTVVPVASERGTGTVLHVEGSRTSAEGKLRGGEAYLKVTIASPEIGNTGKYQCR

FAYLNMTEFRFTVLSKTIDITFTEPDEPTPLPVVPTSCSCDQVWTEV

>SG-AL_contig_4425_1_+3_3_290

PQPIHQLPAGRVRVDRVLAGSWWIGSGSQRVINLDDDIIIIIIYCLWPSQPLRPYQGCHH

NKSIKVKKTNALNKTSSNLKTVFLRKNTIHDILSKP

>SG-AL_contig_4426_1_-1_106_1359

MANRLPAVVVDTGTGYTKMGYAGNTEPQFILLSAIAIKETASVGDQAIRRLGKGVEDLDF

YIGDEALNAPSYAVKWPIRHGIIEDWDLMERFYEQIIFKYLRAEPEDHYFLLTEPPLNTP

ENREYTAEIMFESFNVPGLYIAVQAVLALAASWTSRQVGERTLTGTVIDSGDGVTHVIPV

AEGYVIGSCIKHIPIAGRDITYFIQQLLREREVGIPPEQSLETAKAIKERFSYVCPDVAK

EFAKYDTDPSKWLKKYEGRNAINKQLFSVDVGYERFLGPEIFFHPEFCNPDFTMPISEVL

DDVIQNCPIDVRRGLYKNIVLSGGSTMFRDFGRKIQRDVKRVVDARLKASEELSQGRIKP

KPIEVQVITHHMQRYAVWFGGSMLASTPEFYQVCHTKADYDEHGPSICRHNPVFGTMA

>SG-AL_contig_4427_1_-3_249_608

MIRYKTVQHYITQYNTIQHKPNYKQPNPTHLNTAHITQLPHSRKYSFTFLICYDPTLTLS

IKSVRLDVCKIYSTAQHSTAQDSTAQHSTAQPSPAQHNTTQHNTTQHNTTQHNTTQHNAT

>SG-AL_contig_4428_1_-2_2_127

SLILPGYCLVEIFLICQVYICEDLLMPEPYYKHAEHHMYIKL

>SG-AL_contig_4429_1_-3_789_1001

MCADLLVPEPPSYVYTHRTPNMHVKDPVSHIQHTHPPKMGVWRLCSNIMLARCGPNNIHT

VTQKRENKICL

>SG-AL_contig_4430_1_-2_179_334

MWVTNEVKGCQEINYSLKLSEARALFKIWVKRSITTLGRVAVLKSLILSKLI

>SG-AL_contig_4431_1_-2_2_250

HRGGVQATLGTREAPTQASPSFPAPDSSRSGRSPGLGGGRPDPQRGGGSFIPPPGPGFYG

QLFVVPKSSGGTPRPSQHEARTS

>SG-AL_contig_4432_1_+3_3_167

KVKVGEREKEREREREREREREREREREREHLSNHWNISKACYPKEDQRKYILVM

>SG-AL_contig_4433_1_-1_103_1254

MQKGQHSQHSHMTKTSDMAVLRFRKKRVLLVFLIACLFWLMYLLSSFPSKRKDGAGTDSL

PDVDKSISRKHFTSTASDRRNQKIEPVNNVIAAPQLHPVDVNAGVQGQSNISANGSKSEQ

HLKDIHPSDSHVGVLDIGSLIKNKTYELGVKLKNKTVEVGEKMKNKTLEQLAMMGVVSPR

RMPQLIFVPTKWNLTVSFEDIGDIMKETQFLNKVDPLNLNGPILVSSRNRTTNFTYTMPF

KFGFCDCFERYCFCCSKITNRRLHLNATACSNFTFVSKTHELDLQFSLDGKPVHQNLISA

DKSPLLCMGSHPKVADICVHFFNMTFKVNEHNAHQTQLLGCTDLSLNLYNRTIGAFPVDC

FQIPGDPNQVHKERKFNMMFNWVP

>SG-AL_contig_4434_1_+3_3_419

RVTRLAVEGVLSPPSSFAQGAKGTGSSRREEGAWAARSGGERLLRECLPRECLLCGRLLR

WNLEPEEASSSTDEGEQEGTWATRSGRECLLWVCLLWACLLWACLLQERLFHGRLLRWNL

EPGEASSSADEGEQDEGCL

>SG-AL_contig_4435_1_-1_739_924

MYICRSSCAVCAIYSTYGLFCFMCTAFQKKTLGDELEGGHWVLVRDSHLNPVGLRPPPPH

KK

>SG-AL_contig_4436_1_+3_3_290

IKKKIFPWEKKGKKKKKKKIYKKKKIYIYIYIYIKKNNINSNHKQECISHVRGKCDLYNI

TVKSLVCEQLKIKGHKNQHMHRAFHNRLYSDNQIPA

>SG-AL_contig_4437_1_-1_1_270

RAHSVVQIEWTLNAQVLRQVWDHWYRLMINLFVTRFSKSPHFHLTSTRPSSHHDQCFHTG

LEQSSGLRLPTFLKHTESNQEDKTRENDSD

>SG-AL_contig_4438_1_+2_623_955

MQNITTEFLKSHISTVSKNKIYTENTECYNRTSQITHSSTYQTCYLSNHAFQYLSNTAHL

FTKFVVILKKVKSARSITPLLMFLSIARACACQRLVYITGNVKGKANSFCL

>SG-AL_contig_4439_1_+3_3_212

VLLRPGFKQLKSCNIIKFGFYYDQVLNNSIHVTSLSWFYYDQVLNLIHVTSSSLVLLRPG

FKQLNSCHII

>SG-AL_contig_4440_1_-3_984_1265

MEHPVQAPVSYCIVVFCCWFVCFFLFMGCYSHMLWNIPGVFTCVGVFSSAINLKVVILCF

QGCVAFSCFHKSPNADMAYRIFNVCIWCSVCLYT

>SG-AL_contig_4441_1_-2_107_472

MCILKEILLLHISMHTCRHAYTCMYSHAYTHTHTHTHTHICSHTHRFCFDLLISLPQTVF

ASFYPCFMKCFTVFYIIFVWKMTFVKQQFLMLISLFHTAPPPHTHTHTHTQIISVKWSRF

YR

>SG-AL_contig_4442_1_-1_16_936

MPRRQAEANKTAAATANNANSAMANPESALISSHSSSQSESQFLLAKLPDEGPSSGSGVS

SEPERMERNMFVQELLLSTLTEQLQLATHADDPLLLLDSLRSPEPESQQKSSSSSDSGLG

GSGTAGPKGATGTGSSPPSTAYDGAGGGGKSVATEGLFRSKSASSAPTVNLVAGKSSSGV

SAIASNSSGRPLPGLSSSSLQVEGRGMMMMGGASASGSGLGSPPSPHSLLSSRPPLLPTV

AQPPSPSQPHGSTVNLNEHAMGLSPRQAAASMSTRDTARLNPAAAKRSLLKHLPASRGAT

DMDPPPH

>SG-AL_contig_4443_1_-1_583_1335

MLVVTYLRQTDSQELPSFASYYSSSGLQARTDGSRKPRMSWISQPGQCQKLKWMSHRCRR

RRLVSVLAVCGAISCLFLLYCLSNPDSFFRSADEDGADMYRFFNQNDIGGSLGQTFHYPH

DLTGKNRLESDYEGRYTTHNKFDSHKNYYNEEESSDSYADRVDDDINEKTKSLQEIMDGV

NRAVGTASSRTQSRKHFVKEKTDLFSTDGDESDYLFPPCQEDGTFQSNSMYGFVGPFRYL

ANYKNPCWMEP

>SG-AL_contig_4444_1_-2_170_2740

MASLSTSWIQCAIVFAVVCSFTEGFQWESEDPLVVNGCVGSSVTIPWKFTLSKIESLQSM

HWFRFSTETVASFAGDTTTNPDPRFKPSQGNAELGLESLKLADTGHYQVDVKLLTKDRKG

EWNDPSVRLATALLFVRDPPSLSSKELTIKEVRSPEVCALKRQVTLTCGHFSKRGLPPVD

VVWIDPAGQQLNSTRFTNGNFELDLPRRPLAGNYTCRVRCDNPVLQCLEPGSPVLGSPQM

HVQAPTSEGCTDAHISEPEPTTSPISHPQALERLQANVTELREGLSSLEREQNKLKEENE

QKKVDFTTKVEDLQQLVLRSLQSSSGAQFQVLQQMLTSLQRTVEDVQQNVTALQQERRDN

LNDEAERQQQLLQQQQQQQQQQLQDLSNEVNDLKNKLVLNRETTTSVEDKVGMLKQDLLE

TQATLSTLEDSLNTNMGEREGRRRGDLQRQSESVAELWQNLNRLGDLSNDTAEEMRELQE

AQRICERRQESLTQALNSMSSQVAGMAQDLPARVDQAVSQCNATHAELANLDQRLHTMES

GQPLIVEEKRTSGRDPLVSKALPTLTITSPWDGRENSLFQDFLITSEGRVVIADRYHGGV

IMAHNSSSNDVTSRPLRHGTQIRRLTNMVDGKVAATVDGHYLYIFDTTTTDPEKRDRWVR

TQTKYHGIAARSPQTLFVSVDSEDASSVARIDVISLVEDTAVVMETLLNSSVTADLKRPG

GLTVHEDHLYICDWDSSKIIRLSLQTLQVTVFSGPRSEPNLQFFQPRDAVFDVSGNMYVT

TGGDLCGGLGSHTYLKGFCVVVVSQEGWWDRLILKGQHIYPYSLSLTPTGVAVSWVSWLQ

SPVRSLVQWYDLVKPHD

>SG-AL_contig_4445_1_+2_284_538

MNREMKEKTEKTVQENALKQSIRAMCSNRLCCKGNEQKFGGLTCTLDQEALEIFCGNIFS

IFLIRARPRYFFKRCDRVLPPCSTD

>SG-AL_contig_4446_1_-3_72_512

MSIMNFVGKQILRGQGLTFCRLLAARLSSQTGTKEHIDSLVKGKPVVVFMKGTPDQPRCG

FSNAVVQILRFHGVDKFSSHNILEDEDLRQGIKEYTEWPTIPQVFIDGEFVGGCDIMMDM

HKNGELIEELKKAGIRSALLDNPDAKA

>SG-AL_contig_4447_1_-3_204_1631

MSVKFTLVIIGCMLLGCSYGRNVFRGMFPESYPPMLQNGVDPGQPLFLTPYLQKGQPQIA

HQLSQVTSLPGWSGKSYSGFLTVNATHQSNMFFWFFPAQRDPANAPVLMWLQGGPGGSSL

FGLFVEQGPFYINKDTKLMPRNITWNEKYSLLYVDNPVGTGFSFTKDDAGYATNEVDVAR

DLYSLLEQFFQIYKEFQPNDFYITGESYAGKYVPAISYKIHTENQGNPKVRINFKGMAIG

DGLCDPVTMMSKYADFMYNLGLLDESQSAYFQNQTMKAVQLIKQKQYLEAFKIFDMLLNG

DMSKQEPYFKAKSGLSDYYNFLRTQEPEEYNYYGKYLADPLVRRAIHVGNLTYNDGVAVE

KHLLNDIMASVKPWIAVILDNYKVMIYNGQLDIIIAVPLTEAWLQTVQWKGLPAYKKAER

FVWKIKPSDTEVAGWVRQVNNFYQVIVRSAGHILPHDQPARGYDMIQRFVENRSFH

>SG-AL_contig_4448_1_+2_2_202

ITLILITNCQIVKIVLPSSSKALSWASESDWEKKVTVLFSATSVSLFTMRVDHTGSEVFI

SFGLFSI

>SG-AL_contig_4449_1_+2_149_307

MYNSISSVLIYQNNHTNTLLQHYTWQREREREREREREREREEEEKDKEKGGE

>SG-AL_contig_4450_1_-3_465_731

MLCHVCCCLCECFAIVFSFILPSLCFVVSIVVCVSVLSSVVSLSDCQALFYILLSLCFVM

SIVVCVIVLRLSSVLYCQVYVLSCRLLSV

>SG-AL_contig_4451_1_+3_57_353

MIQRNMNVKMNDTTSMPMAHITQNSRSNLFKEHTLKLMQSHQKQRSSRSVVRKFYEQGGV

TLKKKKKKSGKHENFKLSDIFICNVPFEIFSTNVRAHSF

>SG-AL_contig_4452_1_+3_882_1274

MHQDETSAQSFVQVQGICQFAGRFGRSITTECDEAPPEQIPQFPSLVTMATEGTTVVCHY

SLHSEFGGLIYFLSISFNLNKDHFAGNLHLSCITEVFMFNLILFWPLPLGRSQITESVLL

ILSQSRTKGNH

>SG-AL_contig_4453_1_-2_29_238

MPAYSRDSDNIRKLSGEVERLRIHAQQPHLKLSETIREMINFCNSNMAGDHLINPEKENP

FKPKKTCQIL

>SG-AL_contig_4454_1_+1_1975_2214

MKGKIIALMCHIQCCSYSVLSCHHVVLCATVYSMPLVRTLSTVIMEKPDKTSQQLYDACC

LDCLFCVNGSAYFQAWELLK

>SG-AL_contig_4455_1_+3_3_242

VTDRNNNADESKTKLSNTIPLLIHSFNKNKEDNADISKQKTLLIDQIMTNFQFYFFLWPV

CLIFMVRAFKNKWLKIFFNA

>SG-AL_contig_4456_1_+1_1033_1320

MCNNDISMSRHTVLVCLHTIHTVKKDQIHDLTTATNCQQRQQLSTHTDNCKHEDNSCQHT

ETRNCQHTDNSLSTQRQQLLTHRDNCQQTTVVKTRQ

>SG-AL_contig_4457_1_-2_47_220

MALLFLGSPPISDRFQSVYVNGSQSEPVRLSCGVFRVLCWGLPVHPLYLTPSQHHQPA

>SG-AL_contig_4458_1_-2_122_325

MRAYGIPPKVTTAIENIYSNSRCCVRTEDGLSDWSEVLMGVRQGCLLSPLLFALAIDWVL

KKATEGKG

>SG-AL_contig_4459_1_-2_353_520

MDNHSLLDLDDLVEYVTETINGINAKDKGIALVVLVGQGTDHRFGRSCEPMAFHQK

>SG-AL_contig_4460_1_-1_949_1293

MSKFTLCASAELLKVCKLSEVFSKKIHNSQCNASMFDIVPFLPSTRFMIHVCQNILSFLS

LVMCKPFISKHDHLATAAEFACKCKDHFLLSCCYLYFHAVIHDPLIIQFAHFSHS

>SG-AL_contig_4461_1_-3_123_2144

MLDDRRTRRDPHDDTADRSDSPGRHRRKGRSRQGSREDVLDDRGSNSNRQSGGDYRGRPN

QLNVDKVYDEGTAHNKMAPSVSQHSLGSAASTGTLPDLVSDAGAKDPKQQQQYQGARSGY

ISRVQDIDSLLKEERHRPRSQNHILDAPVPPTSHNPLKPSPVQSPPPARPSSYAFENSAQ

GKSRSGPNNMRPSKSLGDTLIDIEDEKMQRQQAPQPLNAPPPATPTGKRGKVKLSPTAQQ

MWGILQSKKGLVTISDFLTLCEKPPPQRKLIQVGGTDHEDNFKCFSSANEMLDYLGVDVQ

KLEDCALQIYRYHSGAQAEFGTYLDLESALDEQAEELEGFTDQRKNTLILRTQLTVRVHA

IIEKLLNSSGRELRRALFSLKQIFQDDKDLVHEFVNNDGLDCLIKVGAEADQNYQNYILR

ALGQVMLYVDGMEGVIRHNATIQWLYTLLASKGRLVIKTALKLLLVFVEYTESNAMQLLR

AINTVDARRGLKPWTNVMAILSEKDGADSELLVYAMTLVNKVLNAIPEQDSFYDVADALE

EQGMDRITQRHMERQGADLDLLTQFQIYESALKHEDGEDFSEIPQLENLRQIPRMKSEGE

ARKSKRHMPPKMAPSQNLPSMPSMPEESPAEQFQRRRQKHIEHNILPEEEQVFQKGTFRR

SVPGECQNGSSSPS

>SG-AL_contig_4462_1_+1_112_303

MAEQFNLFTLKRETSYIFFHCVLQSHVQNVCITMLNNIDVTCHYYTRSLALSVQIGYLSK

HTKC

>SG-AL_contig_4463_1_-1_1_240

VPLNSCVFELGLKGVCIFVLFCFLRCLDFFMSLCTLSEGSYLNSMCHISHIPTFHTCVSD

VEPSDRKWYSVGYTSRIWCI

>SG-AL_contig_4464_1_+1_727_876

MCICVQLCVVHACVCVCMHMCMMHTHLCMHVRVHVHLWMHYLFGKLVSSM

>SG-AL_contig_4465_1_+3_741_1085

MSCDLYHHALSVDSFLIFLLVHPGNGCLQLSMLSIILNSLQHLCCHFEGKAVTGVCVWDC

VLWLCMRLCVWLCVRLCVCVCACVCACVCVSVCVSVCMCACLCTGCMIKKLRQHL

>SG-AL_contig_4466_1_-1_544_807

MSSAIGHCMKSRLMETIHVVMAAQACCSEFFFFLIMCVFHIYTVMWSSFFSFCLFLFVFS

LRCPCYVHLLSQVYIVSVLQTAWYLAHS

>SG-AL_contig_4467_1_-3_471_935

MSLGHKLGHSDVENLLLPMQQTPPSRNTCGTRERLKLLKNVCFEKHWERNLDDMRQELKL

EPENFCCFFFVTMEFFCMKCKLLPHPGKALFSCMHVFLLTYESLIFQNFASQLLLPRDLL

CALSACCHMECRFVVSTERLVVVHRPPLKVYIYIY

>SG-AL_contig_4468_1_+3_48_182

MGISLIVLASRKADGLDNLTPLRSQCYGKTSTFLFIYDDDDMFPR

>SG-AL_contig_4469_1_-2_110_241

MDFVPVCYRFILHAKCLQSLFSKMNRLYGVLFYQILQTAECLNR

>SG-AL_contig_4470_1_+3_858_1058

MTIHKGVSTTTTILFCTFCSFSRTYLWNLQKMNCSNPILIRNQVLSIQWLCLAPKQSTAM

SNHQRSV

>SG-AL_contig_4471_1_-3_3_242

RERESLKHETLFSNSVDISTQEAGRLQEDNSMLRSRVQHLEAEVQKKTEPGVTQSTQTET

CKSDMIDNVRNSKLRKLFQF

>SG-AL_contig_4472_1_-1_100_1305

MAKLVQPPPRFNHEDWTRSNLTKYANAEVERQGAERLVEESKRLDDETRKRSDKTQADVH

KKLEQRLDDIRYWKKELDDKLAGLTTEIDNLLAFKTRVEKALQATDEPLHIARQCLSNRE

RRLKIDLVHDDVQKELLKEVETLEGVQALLQRTLEEATEQVRLNRKAKYLLEKDLKDKFQ

AQSIDEYCENLRNNSAALKLKPGAAKIEANSTSPEEWQDFSNENIENAERERQNSKNLRS

NIDGILQQTCNDQRKQVEATNLAFSKRIGECKDTKGKLEDHLNKVIGQIKEIEENISRLQ

KAIADKDAPMKLAHTRLDTRTNRPNVELCRDAVQYRLIEEVGELEMSLAQLQSRLADTQD

SLKGLTRNQLDLEDDIQVKSNTLFIDEVECMGMRKSINIQQF

>SG-AL_contig_4473_1_-2_311_505

MDICSYPCCVLWIIPFYRPRVCDMSMYKLLLQYRGCGLFGYCFLSVLFFPLNYTHTHTHD

VFSIC

>SG-AL_contig_4474_1_+3_117_392

MFKPGHKSGCPSIWQPFGLEAVCTKSDRKAVALPLHLQALVNHCNTTYMESGQSTHNILN

YRKNLYIHMHKHTNIFITTQFLGKTPFTVVVT

>SG-AL_contig_4475_1_-3_24_632

MDGFSGNIDWLGSLQIAKRRSFEGGSNSILATVIEVHNHTSKTVASDRLKKLHPSHYIAG

EINPERPNKSWLTLTLINMDEDMNGGYVCMINYMDHPADIKQKTWTAELTVNASRQETVQ

RKGFSVVPGRGVSASCVAELYSRDLVSHIMIVSEDGKSSASWSKDTGYVTKSLVSPVFRP

QEGTFQAHLLHTDCTAVSKYQCK

>SG-AL_contig_4476_1_-2_458_670

MFSGAFLRYNYNIIILSGSESFKSVAPILRQSICLHALGIWRRCSLALYLNVIPLICAHT

HTHTHAHTDQL

>SG-AL_contig_4477_1_-1_1_189

PFTPSPYPSASPGQGECVKRLAALCSHHRCVEIDWQLSEIPAYDSLGIDSYLFLSDQGLY

TSK

>SG-AL_contig_4478_1_-3_2463_2819

MSSCSVATTSWCVVIGSSLLSVGPPLPTITSVSCYSEIHCFGVFFWKVFGQGCLRSRLGF

LAKHKRIRFLPHCSTHTCVHLPLNKLYSQSLVWTAYCLAPRQLVYSSYFHLPFLWTGAL

>SG-AL_contig_4479_1_+3_3_206

NNTTTTIQQQQQQAWKKGHPSTKDTESTKICTAMEQMKHIHTKLSLVRAQTTNNEPRLSI

DLILPTNT

>SG-AL_contig_4480_1_+1_34_222

MVSRRGLGDSQQEGFCLDGSQHDVQALLVTSSLSVGVEGGGGQVQWERAQGRHSNTTSLG

HSS

>SG-AL_contig_4481_1_-2_329_598

MLFLCWFFFVTPSFEKGDYFILPGSHVCLLIKCVCKIFSAVSGWIFTKFGSRVWTIEELL

MREFGRERAKVKVTLAQKRCLKVTLLKEGV

>SG-AL_contig_4482_1_+1_373_537

MANQMANSSALTSVNYWFKSLSFSPVVTHPYELSRNLNKNMKKAHKVQVIHKRSF

>SG-AL_contig_4483_1_+3_84_197

MFKSDFAQSEFDSTFPTKGAKSTNNNLAIAAKRCFGAS

>SG-AL_contig_4484_1_-2_1127_1399

MNMAWSKVKLKDQYVMEAGVCMHFLILAVALICARYRERKRVKDCSIMLFICGLGPILCN

RPTISNPVRVNVGTFICLKRPLSYETCIRTV

>SG-AL_contig_4485_1_+3_894_1139

MFLSYVDYLFLTRFKIVLVLPALFDQSITMGLLRLKLKTSPAGWESQELSSFVPVFVFIL

FFFFFPWIFLSSPFCLSFFFFL

>SG-AL_contig_4486_1_+3_1803_2066

MIQLMFLPPLTLICSLSGSKLKYISFKREERKKGKTRRKKKAATYFTTTTVVTAKKEFPV

FHHSLCVCVRESVCVFNDYFLRFYTCFK

>SG-AL_contig_4487_1_+2_980_1279

MMNKIKNSRNVHVRSPTCIHCGSTQCTRILTKDKQKEQKQQVTNNDKDTERPNRISTDTE

QQTNGLPQTYGTTDIFMKHGCEPVAQTQPFCAYNNRVGLI

>SG-AL_contig_4488_1_+3_63_257

MQYIFFVFDSKAVSIIRKQTVNVTLYFRDVCVCVCVCACVRALCCVCMSWVGWPGKGKSA

CIHII

>SG-AL_contig_4489_1_+3_495_710

MILLGWWSNPSIPVSPPTANNLSNTSMYKHCPCNSQHLSYPVRTISHSFQLVVLGLKCAG

REKCYHVRSKFG

>SG-AL_contig_4490_1_+2_854_1243

MHLVRIKARAPYSLSLCYLHRNPAVKYELNDSVTMLDHVNKCKHDAGTLLLGDFNIALLK

SQNSRCSTCSLFRLSQLIREPNRTITASETLFDHIYTNKPETIRNASVHLNMPSATTVLL

IEQNFSNSPK

>SG-AL_contig_4491_1_-1_13_720

MAAPCESTGGKLLAGIFLILLAGMHAEDEFEGSRTLQVEHSFDQGASPEFSKRGTVVIHS

LKSNKAQFSQAASLSPQEKDQLETLSKQNGIYRLRIPLAKGGSSEATSYVSTFTTACAVY

ESGLRDRLMVNFDTTGEVLGVSILPAVGTCTGASVNSANLTSWKTTVDVVQTVAGPVPDT

QTYIDKMKRDEQERAKGQQGDNRSFFGKYWMYIVPFIIMMIIMSSADPQAQGGGGR

>SG-AL_contig_4492_1_+1_1_216

VCVRTCARAIQIGTERRESRRCSHTHTHKVTALLIVYTAQNIPEISILMYICYKISWSHL

QQTKLPVFIQQK

>SG-AL_contig_4493_1_-2_29_2797

MRTLMDWIEDMEVKIVSSREVHVEDLLNKLETEYADAMAVKEREVEEVMAQGQQLMKNSS

EVRASDVEQRLQRLHDKWCHLQAVADFRKRKLQETVLAVKQLESSMRNLSRWLATMEQEL

SAPVVYHDCDMQEIHSKLQHTQDIQHDIEHHSAGVSSVLNLCEVLLHDTDACPTETEFDA

LQHAMKNLDRRWKNIFNLCPERRARIKETWDLWEAFGTDCRRFSEWLGNMEVEIGDAEAD

MPPSVTKEDIRNYESLQRKVHDHLTDLESINKQYRRLAKEGRIDNKGVLRSRMQEANDRW

DALQQRVTQLMQGLRHSASIREDFVTTRLSLMQWLTEVDMQLTNIEHLSQLDVPTKMREI

QRIEEEIDDRRHRMDYLNQAAVLLIQQGSGEDAVRVQQELDEFRQFSRQVLDRVNTGRSA

LHRISAAQVEDIAKVEERERYAQTEMSTDILVGQRPPSTEWDTRELEAYLESSPPDSPPY

KRRAGAGGAVLPKASVSPIRRSVSPVRSASPLQRSSSPARRSLSPFRSLSPTRRSASPRR

PLSPTRRSSSPFRWSKDVTDSACSAKIDVLVEQLADAVDAASSHLDAAEQQMDSYSSADT

TPGNKEVQVVMECEKHVDTVQRLDRLLKIEAGSATIPSIDAQVRGVTERWEKIQSRAAEH

NIRISQQQRDLTKFVSDVDSLLMWLDEAEALQASHNTMPTDIAHLNLVIRQHKDFMMQLE

GKRARFLSIQLLSKNYINLKTSEGRQLQSRVRELERRWEAVTSRAADTQHTLQGALLQCQ

EFHHTVHDYLLWLEGLESRVRRCEPVRLSAETSLLWDKYNILKDLRSELEGNQSAVMSLQ

ETADQLLVSGELPEMSAARDKTHIISNRLRSLLHLTNAYLQSLESKLNIKSSGVDTREGR

LPTVIHPTLRTRTRSPFALLRKL

>SG-AL_contig_4494_1_-1_1_159

NCCCFDTDSSLLYSLLDSTMNKYRIILYKNIKESTMNKIRIIIYTLQKLKERN

>SG-AL_contig_4495_1_+1_1_270

RTCARESSFVFVFLYGKAVTQQQAHSLSLRNQDPVVRSHQDWRLLWLSTAVHHNKQSLCS

LTGRTSPWGGQMRTKGKLQVGLQQVAHNSR

>SG-AL_contig_4496_1_-1_100_333

MKHQQAFISFPFFCVIHPLKKKKKVEGDSDSVVYVVLDSLCLLLLYGSLPYLNQNSAPVG

TVITQFGDHSEWTTACQQ

>SG-AL_contig_4497_1_-1_166_834

MALGSCYSCIKYLMFAFNFLFWLLGCAILGVGIWLRVDSNVAKYVNHAEEINIFYTLAYV

LIAIGIIIMIIGFLGCCGAIRESQCMLAAFFVLLFIIFVILLVFGIWAVVEKDVLKDEVA

EMMQNAVDNYEKREDSQKFMDTIQTAFDCCGANQGAKDYATPMNVPDSCKAEFRDRPCDK

EFFDWIAEHLIIVAGVAIAIAIVLILGMIFSMVLCCALRDTVA

>SG-AL_contig_4498_1_-1_127_435

MNPSSQVTSQTEPDADSIKMFVGQIPRNMDEGDLRKMFEDYGPVHSLNVLRDKNTGQSKG

CCFVTFYTRKAALDAQNALHNIKTMPMMHHPIQMKPADSEKRN

>SG-AL_contig_4499_1_+1_76_165

MSTYYLQLIFTPNLPHLFLFNIAIRAAIHY

>SG-AL_contig_4500_1_-1_1_111

VAYLLNLGHILLNLGHIFVYIWVIYLLNLGHIFAKFG

>SG-AL_contig_4501_1_-1_1_123

VAYLLNLGHILLNLGHIFVYIWVIYLLNLGHIFAKFGSYIR

>SG-AL_contig_4502_1_+2_2_112

HPCVCVCVCVCVCVCVCVCVNQNQNQNQKRFIAIKVT

>SG-AL_contig_4503_1_+1_1_252

HSIVKGPTDRCHSIVKGPTDRCHSIVKGPTDRCHSTVNTDKDGFSMRLSQVNTLTCCVTL

WPKWRIPVPTCNSVAKMENPCSYM

>SG-AL_contig_4504_1_-1_196_525

MKKKKTGNDIKFTGWIEKGVYCIAHFSNGNGSFMTYDEFNRKYNTNVNFLVYNGCKVSIK

KIHTGNRTNMPNGCAHGIPTALKIIYTVPKGVRRYISTTDHTFPNSCSKW

>SG-AL_contig_4505_1_+3_3_62

LKYHSIAVLLSVWGKLFSVP

>SG-AL_contig_4506_1_-2_2_172

KHKWKNICSELYPFLNDIEKYGSSYGLHKINNNLFWKHVLKAYNSFCRNVKSTSPMY

>SG-AL_contig_4507_1_+2_2_145

FFLQYQPTNTFNHTFCYRLNVLTTFVHNHAPQQIANILDGLQTKIFHD

>SG-AL_contig_4508_1_-1_1_216

RVLFRFFFFKKSTRRSVYLLIINYNHACVCGFCSCFTHKTCSWLICFSLSSDFENVPIKL

NLQRNELYITIV

>SG-AL_contig_4509_1_-1_214_510

MGVSRLTLQHTERGCPFIRESKLCHVHQETSCALCTEKLSFLLCLVHDNKFIASRFMSRI

DSLQRGRTAKLKTSPCLILPQWHSYGDGALCIVNKPLSA

>SG-AL_contig_4510_1_+1_295_570

MDGEAAIGQKIAELPVCLSMSMDGPSKPVVEKQKENARENEGRMFISSRPLLVQAGGKSK

ISKLSSDHWEKEVYCFLESRLSKGRKTLIFSI

>SG-AL_contig_4511_1_-2_176_442

MTMMGFPTFCEFEIFMFIPLNKKPQSKRFSLVKCCHCWPSFSMMLLLVFCFVFISSVAIM

FMLILGNIGFSWLMYLISALAHSTSTEVS

>SG-AL_contig_4512_1_+2_233_484

MSETNLEQATAQTFEILGEGRPDCAPMMEYILEFITGKFISSGKYVHTFLIIFVLLETQN

TIYLSEHLVKMGRQSRAIIVTDSN

>SG-AL_contig_4513_1_+2_2_220

GGEDWQSHHSHTHKTQAQIACSYLCMYTGATCSACQATNLHHLFLFINYNYPVNTFHKSY

TQYSFQKVEIKFQ

>SG-AL_contig_4514_1_-3_3_176

FSKLIWTHCALRSDRTQSVIHAEALISHTHRHIIRIHTLIDVHRSIRTYRVNNTYTYL

>SG-AL_contig_4515_1_-1_1045_1224

MNCLYFICRLQSYVLGRMPDSSKITEVKMLKEIIIQWWLTQWRHSFLAVAIPGNVQILAI

>SG-AL_contig_4516_1_-1_1780_2028

MIVHMCYVQSLSESRSILPAGSWLHSLSRPQNALSLHWSFVYFIVHHVEYKLLEHQPNQC

TNQPVLNSELWNYVSSNDFTFWN

>SG-AL_contig_4517_1_+3_3_275

KNSSYICQRVHYYPAHPLSYFGAWVTLKSFTHHRGEVWCLSTSLLQQCIIWPQQFFYQQR

FYIWYDSAHILYRHSISESRHTQRDEQKKIS

>SG-AL_contig_4518_1_+1_136_333

MSKHSCIPLIVHSFIKYYYLQSFGECLRPVIKFKMMDQRWTSTRRPVFKCCRALEGLEEV

LTDLDG

>SG-AL_contig_4519_1_-2_38_220

MRAYGIPPKVTTAIENIYNNSRCCVRTKDGLSDWSELLTGVCQGCLLSPLLFVLAIDWVL

K

>SG-AL_contig_4520_1_-1_31_219

MRQFISFVMSFELLIELPRWVKSWMTLISFCPMYTADSNGCGPTSMFFVFLARVPSAPGR

QFH

>SG-AL_contig_4521_1_-1_13_2163

MGDEDEPSGGSVVPAQPAGQNVPKPDPSHQDVLRVESVNFNVATNVQRHHTDQYDITEGH

DPQLVVRRGQEFTIQIDFSRPYNNKEDDPRLVFEIGDNPKVSKGTHVDFILSDKDEPGKW

GAKLAKPGKSATITIFTPPDCLVGKWDFKVDVVKKKEKKTSIFRYNHKNPIYMLFNPWCK

EDSVYMEKEADREAYVLNDNGKIYSGSFKRISPKPWVFGQFSGKVLDCCMYLLDLSEMRA

TARGDPICVVRKLTAMVNSQDEGGVLTGNWSGDYSGGKSPLSWTGSTAILEQYYSQKSPV

LFGQCWVFSGVLTTVCRALGLPARSVTNFASAHDSDTSITIDLHFDSEGNSLERENEDSV

WNFHVWNEAWMSRPDLPAGYGGWQTCDATPQETSGGVYCCGPCSVRAIKEGEVNLQYDGP

FVFAEVNADKVYWMKQEDGSCQNVYVEKNTVGRKISTLSQTGEREDLTLQYKYPEDSPEE

RVAVRRANQVGSSRKDVYTEGPKDVEFEICFDSENTFVGDEFEVKLCCSNKSKENRTIFG

QTSASTMYYTGVVADQVAKQKIDGIMLKPGETKNISFKVNAETYQDKLKDCCMLSLSAMA

RVVETKQIFTKKDDTRLRKPHLNIKAPATGKVGQEFEVEVSFTNPFAKTLTDCSLEVEGP

GLSTAKTYPQSDVAGKGTFHTTLKLKPAKKGKREITIIFSSRLLDDINGSHVITVQA

>SG-AL_contig_4522_1_+3_435_596

MDQKAYKSIHHGLHSQILILNFSPPASHKIYTVCMSTAHVFVLELIVVQLFPSY

>SG-AL_contig_4523_1_-2_1418_3637

MERVGAQRRCLLVLFTLTLTASVRCWQTEWQPSATLSLHEDKSVQKFRGKNNQRDDFRML

LVEDDTMLVAARNKVYNISMTTLKKNAEIQWSPEKPHIDVCQKRQKSFVECQNYIRVLVK

KSPKTVYMCGTNAFKPTCRTYRQQDDGTYSRTLKDQTGLALCPFDPSHNTTAIYADGKMY

SATSADFNSRDPLILESTKMVRTEQFDSKWLNEPNFVSSFEKDDKVYFFFRETAVENINC

GKAVFSRVARVCKQDDGGNILLYNTFTSFFKARLNCSVPGDFPFYFDEIQSTTEMGRGNY

RPTYDSGDRSDMVYGIFNTPANSIHGSAVCAFRYSDITRTFEGRFKGQHTVWHNWLTVPW

DQTPQPHPQQCTNQSRTLPDTTLNFIKTHPLMNNAVPASGGAPLLIHTSFKAQFTKIAVD

WMVHAADDRYYDVMFVGTDDGRVIKAVNKGSSSKIETVVIEDIRVFGEHDPVTDLKVFRN

KAKGIEKLIVVSKENIVSIPLHRCGVRKTCRTCVELRDPYCAWEGTRCVNAERGIQNVMD

GWRSGWERDCEEVTIYEDSKEEETQVTTPETSQKCSCPPYETEKKQPEWVKDDKDDTDTK

VVIGGENSVGGKKGNRRKGGPRVGQSSEQTPVPPPAPSPSVETLAIAIVVSIVLSMLIGF

FIGYKVAGCRGANNSDASYMERTCSLQRSRNRLSSGEHPYYNPEHAMMPKQMNYVVNVKG

KLNTGSVETKPVTKSNKVYL

>SG-AL_contig_4524_1_+2_170_358

MGDSTYKLKCTFYFIHWERKTQANTRIREKMMNSDTCLHYDRQILTEEINRKFSRAHTRE

RKY

>SG-AL_contig_4525_1_+1_847_1140

MKHTKKNNLCRLQQHILSITEDEIHSQHYQENYSNRAFTQHRSLLFYKTYVNTKRTLYQE

HKSYTRITTTKHTQTHERAHTQLCNTVYIRSNIYTLIQ

>SG-AL_contig_4526_1_-1_7_687

MALNYYYDLMSQPCRALYMFLRLNEIPYVDKTVALRKGEHRTEEFTKIHPFNLVPAVEDN

GFRLIESIAIVEYITRTRNVAEHWYPTDAKKRAKVNEYLHWQHVNTRFQCALLFQYLLII

PRATGQPVDQDKVEKQRGGVQQVVEKLESYFLKDTPFISSEVITVADLFAACELMQLFSV

GEEGLYESSPVVKRWMERVQREIGPLFEEAHKVVYLVRKMYKGQSKL

>SG-AL_contig_4527_1_+1_118_321

MRDYDGDDESDDTDEENQRENASLINDNNNNNNDNTIWLTDHTLVGFAHAKFSVWSTVHS

IFYYLSCK

>SG-AL_contig_4528_1_+1_115_234

MTTTWSTARNIHKRSVTQKSIHPSTQPAILWVTMKVTARP

>SG-AL_contig_4529_1_+1_31_222

MVLKILILILVVRSVDPQTFFSWWKAVLALPIRVRMSASVPPCLSTVLPRSGKLNQILIV

QNPT

>SG-AL_contig_4530_1_-1_640_1281

MNVNGGDLENATFLDYILHFCTFFWKILFAFIPPAQYLGGWPAFIVALAVIGFLTAIISD

LASIFGCLIGLKDAVTAITLVALGTSMPDTFASKQAAVGEKTADSSVGNINGSNSVNVFL

GLGLPWLIACIYWKVKTGEDFEVPAGSLGFSVVLFTSCAVIAIILLVVRRFTVGAELGGP

PVFKYISAIILVLLWGLYILLSSLQSYKIINVQL

>SG-AL_contig_4531_1_-2_236_3643

MSFICIRRKKKKRGHKSSHDDLWVDPTPRHVPAISLTIPPPVCSPLPPSLSCDSLLLLST

PASCTDGVQPQSNPEPEGEGEEDGGEPQTPKPRISSFLIRKKQSNRSLRETNSFSLRKMN

TQVATGVSRSASTLNPVPPVRLTPTSTLCTDDFSTSVRDKYYKALFAYKAAYRGELSLRE

GEIIQGRERDQNGWILGKKLHSKEEGWFPAVYVDQMTGDLSSVDKLGVPEGAGDRRSLSQ

LDSDVYGLLAVNRNKSPDQSWVGIEHRVSKNYSTVSEPDDNTQMPLREGDTVTVVEALDS

GWWLGYHGDQVGWFPGDHVELMEEKLEVDLDAIPIPLKTCSALHISNNTLDVSPASKRSS

LASSVDSRDLHDKKMTHTLSRESVLSDGGDRPRPVRKAPAPPLPAAVQRLHASASISGTS

TLSRNPRPMRPAPAPPMHKPRCESVGSTDRWVNAGKLSVSGNSMETEVRVITPRKQRKPK

VVRVPKERLGLDKISSCPAPSCSPPSATASPSVSPSSDGSSSVGHHPVSDTSEDVDSVVS

KGDASEPCSETSSQKGSEKMDSNDVPQTNASVTSSSHSSVEQIQLQTGPKELSANDKSNS

QNSSEETLVTEDNVPTTTDNTEETSLSGEIFQQLSAELTIAIPELTTGSARPEEKDDNSP

TLNDSSARSSRILDDASVGNSTTLDDTSVSDCRTLDDTSFSGPMTLDDSSVNDSRTLDDS

SERSLDSTAYVEASPTLHAPPPVFPVVQTESCDDSEKAVSNDREKSPHKDSEEISLETES

VQDSVVTKEESESVCNKAEDNETKITDTGSSVRNSLTDSFLSPMMVGVRTSSPITIPKYI

TPPSILLEDADESSVDGLQPSGQSSPASSETSSRSSSSSKPTGYWKFHSSTNGHNPKPKP

TKLTKVAPPVVPRTSHPARDSEEGVVEVPPAGFSGRVHKIVHQINRMASVDEQPGLLRKP

SLQHLSATAHSAGPAEELKTDSQLQSSDNVKSMNPQTSGSPLPHRSASPSLSHHSAEDAD

STLPAPIPETLTNGGLMSELSESSSTEKRIVSDLPDVILSSEQADHENTQQRMSDCDEQR

SQSQSREGTPESDGGGQGQRQSSRKVRAPLPPTSPPPSVLRKMHTLTVAEEQSTEC

>SG-AL_contig_4532_1_+3_3_179

SGLSPRLVHQNQDKRVEHKTVKPQNNVTLNPLCSLRQIRLGCAEDTSHSAEFITTRLQN

>SG-AL_contig_4533_1_-1_208_1266

MADRNDSEAGTETRPDVFKWHGTSLIFIPLMMAAGIVGNFLVLYITHFRWKNSIFSFFIK

VLAWLDVSNLVVAMPLLLIITLNDHTPHFMAFCHSTTFVALFTAMTSAVVLVVIAANRFR

KLCQPQRSQVTLQLATKLTIGCFLLGAVIATPSVWLFSKDTVSYQADVSPVNISYCFIKS

DASPPLMIAWAVVMGGTFMSITVSLVVLYGFTILALRAHDKRIATMMRRPSAVSNNSKKI

SKKHTLVFIAVTVVFFLTYFPYFITVILLLADRTIESHMGPVAKAFFDLAKLFPLLSNMS

NPIIYSFTSERFRSECQKVFKLRPCKKLLNLKQSILQWRPIVESCCNLQTHKK

>SG-AL_contig_4534_1_-1_289_414

MNVTFRFISAQLFTALKLYLINYITIKPHLCAGTAGYVFEIM

>SG-AL_contig_4535_1_-3_33_284

MFVCSLYANFSRLCMEHVLFQIALKKMFLNILFPGLYPKHSVFTALNFQCCDWQALSFII

VFSLVLTVCLPHLSRNVVSGELTG

>SG-AL_contig_4536_1_-3_12_1568

MAALIRSRLLSAKNLLQLNLRCMSAAAVPQPTTNPDIKYTKCFINNQFVDAVSGKTFPTI

DPSNESTIIEVAEGDKADVDIAVKAAREAFRLGSPWRTMDASMRGRLMNNLADLIERDRQ

YLASLETLDSGKVYTQAFLADLALTIKCYRYYAGWADKNHGKTLHIDGDHFAYTRHEPVG

VCGQIIPWNFPLLMQAWKFGPALAMGNTVVMKLAEQTPLTGLYIAKLTEEAGFPPGVVNV

IPGFGPTAGAAIATHMNVDKVAFTGSTEIGQLIPQMAAQSNLKRVTLELGGKSPLVVLSD

ADMDHAIEQAHFALFFNQGQCCCAGSRIFVEEKIYDEFVERSSVRAKNRKVGDPFSSVEQ

GPQVDKDQFTKILSLIESGKKEGAKLVAGGDRAADKGYYIQPTVFADVQDNMRIAQEEIF

GPVMQIMKFKGLDEFIDRAHNTIYGLAASVFTKDLEKAIYLSNSLRAGTVWVNCFDVFDA

SAPFGGYQMSGNGRELGEYGLEAYTEVKTVTIKIPQKNS

>SG-AL_contig_4537_1_+1_292_543

MCVCALMKCVSKIFSAVSGQIFTKIWTKGVNIEESLMHEFGRERAKVKVTRAKKVPKINT

FQGRCVNFNFSATSVSIQILFSLL

>SG-AL_contig_4538_1_+2_182_343

MHHYFFSNSTTTYYYYILFIAQPTIKAISGLSPRKVHQSQEDNRVDHKTTLNPK

>SG-AL_contig_4539_1_+1_100_216

MTDLQNRLYPLNSLRIPKPTKIVWGAILMNATPVASILH

>SG-AL_contig_4540_1_-1_403_756

MITPDCILDVVRGLYPNPKGIPCLWHEWWKRSVADFIFHVFMYNLIIGMFVMVLSCICFL

LPALVFQSCALLFLSSSHTHKTHARRHRKKTFRPPPFKSASIGNVAMGLSSHFPLVQL

>SG-AL_contig_4541_1_-2_95_1978

MSGDGYHCPDAKHQQALRDMANLLRIHSIQSTNASKSGHPTSCASIAEVMSVLFFHTMKY

STKEPRGASNDRFILSKGHAAPILYAAWAEAGLFPVADLMNLRKLTSDLEGHPTPRLNFV

DVATGSLGQGLSCAAGMAYTGKYFDKVDYRVYCVVGDGETAEGSVWEAMAFASLYKLDNL

VCVFDVNRLGQSEATSLGHDMEIYKARAKAFGWNTYVVDGHDVDALCRAFYDATTVKSKP

TCILAKTFKGRGIVDIEDKDNWHGKALGDKADGAVKVLKGIISNKGSLGLKPCQPEVKAP

VVSVKDIKLSENPTYSEGEQVATRAAYGTALVKLGKTNKHVIALDGDVKNSTFSINFKKA

YPDRYIECFIAEQNLVGVAVGCETRRRTVPFVSTFAAFFSRAYDQIRMAAISQSNVNFCG

SHAGVSIGEDGPSQMALEDLAMFRSIPECTVFYPSDAVSTERAVELAANTHGMCFIRTSR

PNVPILYSATETFKIGQGKVVKKSASDKVTVIGACVTLAEAMKASEELAKSGINIRIIDP

FTIKPIDADLIIKNARETGGKIITVEDHYVEGGIGEAVCSAVCECRDITVKKLAVREIPR

SGKPNELLEKYGISSSCIVKAVNQVLSA

>SG-AL_contig_4542_1_-1_304_3786

MDNLEPGLPLRDHGSSRDFREYDIDCDADSHRATFMQLRLPKRPRRPHKGGHSRKRTTEK

SSVDTVSPASLTPSEQIQLLLGDGDEGHTARKIFCQMDVLHKLADSMQWRETARWVKYEE

DVEEGGERWSKPHVASLALHSLFELRSGLTSGAVMLDMDAHNVTQAADLLLDHLVNKKLL

EEGMRGEVKEALQAPHTFQHNKYRPNRRPSTAHDGMDLFGRRLSVMRNLSMAVKRDSQAS

LQRMQGNPHSTPNLEVLNGDLRESPSSAKLNVDFMRKIPEGAEAANIWVGELDNLRYQVV

AFIRLCEARTLGDLTEVPLPTRFIFFLLGPKGSHSKNMEIGRSISTIMVDEVFREVAYRA

RNKQDILAGLDEFLDQVTALPPGEWDPKIRIEPPQSVPSQEQRKAPHPPPGEEKEEEEEE

SHADPTLVRSGRLFGGLVEDIKRKLPWYGSDFKDGLHIQCVASIIFLYLATLTPNVTFGG

LLGQATDQYMGTMECIFTAAVVGVLFALFAGQPLNILGSTGPMLVLEMILYRFCADNELD

FLPFRCWIGIWTTFLLLVIVAFDLSALVRYITRFTEESFASLIAIIFIAEAFKKLVGIYE

LAPFNSDPDAPLDYNCTCLPPNDTADSNLTLADMDTDNVTSLMTTLMMTPMTTTMPGYNV

TGNMTSNDSMVEWMTMSKKMCTDLGGELFGSGCGTPHYKADVFFLSVILFFVTYIIASSL

SSFKNSAFFPTFVRQTLSDFAVLIAILVAVGIDVAIGIPTPKLKVPEQFKPTRDDRGWLV

NPWSDRNPWWLTLCASVPALLAVILIFMDQQITAVIVNRKENKLHKGNGYHLDLFVVAIA

ILICSFLGLPWYVAATVSALAHIMSLKKESECTAPGERPTFLGVREQRLTGLMVGLLSGL

SVLFTSVLKFIPMAVLYGVFLYMGVAALRGMQFIDRILLMFKPAKYQPDTRYLRHVPIKR

VHIFTTIQVVCLVVLWVIKSVKSVSIIFPIMVLGTCFVRKALDYIFSQRELKWLDDIMPD

ATRKEKEDAKILHDPNSKDNDTDRKEKESLLGEGGYDRLVEIKVHHDKLSEGDSKYGQRF

THHPSIDSDRVNISEELSRTAMWNQLRSNSAIPLDADNTEKKPEKKKRNKDTSNGVDLKD

SPKKSAAFYLHDDGEDHDTKA

>SG-AL_contig_4543_1_+1_1177_1434

MYDFNIINIDVCSSINPCHIYKKNSKKLSIKIIHKLNTQLKFSNNKEKQSKQTKNFKTAE

KETMLTHLLTKGQTDRETDTYHRLSV

>SG-AL_contig_4544_1_-3_48_1388

MDRKVPPPPIKLPSEKLREKEKNKNLMPIPIVPFAEIRAMFEKKGKGQKAKKEQEENKTK

EEAKPKSQTTSVPTSAVPSISISTEEEFRREESKPVSVKRSLFDKPPVHPSRTSPPPPVE

ETVQVAASSPTSAQQPTIPRPGHRATNGVSDSVDGNFVSSPRTEEAFPPSTSPGRSPVTE

KPPVSPSTSFSVEPKPGRDLKDSSKKHSPKDSEKDAQQQQPKSPPKESKQQFSPSRSLRQ

FFGRSKDKSSEKKSEGGGSSLDSSFSSGDGVYEKEPRRLGGSEGSRRYDSKTQPTTSFDR

GSASGKDLDSSFSSAEGQPSGADTSSRSLEGPPSRISGTPQSSAFSFPASVSSRPAHSGN

SDRYQRPPSRPEDVQPPPRIGKPEVREAPKFQRRLPGAGTSFGHVSSSASSENGDYLPGS

PPASPSGAPESPTSPSGEKKSVKETTV

>SG-AL_contig_4545_1_+1_1009_1224

MNPCEKMTWMVPNHLTQSVVQTASDQIYCKSSNFNVVYTETKKLNQNNFPKRNRSKNFFL

QKSVVQNSSNTE

>SG-AL_contig_4546_1_-2_80_1060

MKPILLHGHERSITQIKYNREGDLIFSCAKDAQPNVWYALNGERLGTFNGHNGAVWCIDV

DWNTTQVLTGAADNTCKLWDAKTGVCISTLETNTAVRSCGFSFSSNYVMLTTDKTMGFPC

EVLVYDIRDGSQMLGGEPLQRITLPMTEAKITSAIWASLDDLILTGHENGECKQFEIKTG

DMVNSCREHTKQINDIQLSKDGNWFITASKDFTAKLFDLQTMDSLKTYKTERPVNSASIS

PLFDHVVLGGGQEAMSVTTTSTKIGKFDARFFHLIFEEEFGSVKGHFGPINSLSFHPDGK

SYSSGGEDGYVRVHYFDQTYLDFRFEF

>SG-AL_contig_4547_1_-3_609_1049

MYLLTSFPYYLLPFLILHCPVLEVAILGTSFVYSTTVSSPLLCASHPLPLPLNHLYSQSH

FKELLPLFLLSRTLKIKYSVLMKSTTNTLTYWIDIWSQQTRIPHHTTHTHTHTQGLVSCH

FCVGHYLLTVLVCSFSTCFRHIRCTFC

>SG-AL_contig_4548_1_-3_1203_1490

MCEAVSSGIWSQKAQILFSELTSSVQVSHLKRRMETRSEFKFHTCQGGWRLGVNSSFTPV

EGDGDLGVTCTVCTLYFVKLMMLLFQILFIWPLLQS

>SG-AL_contig_4549_1_+3_1005_1220

MTVSASIPTCFVRYMPLYFDIMLRQGLLLGNFFFFYSIYVAIVCSYLYITDQNGSFFSSF

FDFLIQSWAFLL

>SG-AL_contig_4550_1_-1_151_408

MSGNAALNKQAADLSFVSPKYRIPISFAATILLLRSSLSLLQLLLLLPLLNYYKHYYSSA

STTATSIASLVIVLSDAFEMISVAVC

>SG-AL_contig_4551_1_-1_1_189

SVLAISSVLLLSLSLPFFFPLLSSAFFCLPGPFHFLFSSKTSHNFLLNRFMMYYLCCFAL

ATR

>SG-AL_contig_4552_1_-3_3_113

IHSTFFFTSKPSHDFLSPPLPRQFMMYCLCCFILVIR

>SG-AL_contig_4553_1_+3_90_251

MKTNSCLALLPLNISTRSLPQKLWLSHSCTSSLVVITPYLYIDIYIYIYIYIYI

>SG-AL_contig_4554_1_+2_1823_2077

MTTKLVHQNFDHFLIQPSNNGADNFLKLEFFSRKIHPTQTVPCYQLQTMGQSSLFSTTIK

TACQAAGKHTDSSFRRTARFSHIFT

>SG-AL_contig_4555_1_-1_25_270

MNMNIALQKLFSVAKGTKLYYDVLNADDVKPHCCDKWERKLSRKIDWSSCFYKIKKIQDV

NLKWFQIRIVHRIIGTNVILQE

>SG-AL_contig_4556_1_+3_3_272

TPSIVPPRIRDTLCVTHFCFNTFGVQLQDVLPALFFFTPVWSKSIATPPKYEVCSVSATY

VRSFVRLERSSPLLPHLTFRSHYSLPTAVP

>SG-AL_contig_4557_1_-2_41_223

MFLPVLKTGHRLGPIGGQSFSQIRVHFNNLIPLYIRCDVCSYIFVMYYCDVSLFLLTCVI

K

>SG-AL_contig_4558_1_-3_3_113

YGPLAFKKLMTTENQGNVDSPGLICQEKYRFMNRAKT

>SG-AL_contig_4559_1_-3_9_6392

MAPVGPGDQDLGLLTLTTSLQSTMPQASAVSDVRVSFVSNSSHSARAAESVSVNSSVPLS

VCTADVTLTSVTGRDTACTSVTKKDVTLTSVPGKDTPSDVTGVDSVDSSLLGAAGGDAGS

NPCVSDGPPLTEQTKDWASATGKREDKPWCGRLHQEASLSVSGESGNGSTKLGLPSSSLN

SSQVSVMHSMPTTTSIPTSSSLAITTSITTTTITTSCVSTPVVTNTSSSSAVTPPSLGKG

HSDRPSKVKGAHSRLVEQSVKPDPLFAPDIKPEAPSTKSEPKSSASRDRVKVGEAGDRHD

CTTPRSDPDPKDKKAHNKPSQADKKLGKSASKDASLDRSDKKEGDRQEHSSSQKTADRVC

HETGSRAKSKDDLHGKAKESDSNSKSKEKESGSKSKVDPSNNKDVESQSRVRDPQTATKT

KEAEVRPKGKERKSESHKTKVKESGAHSGKGDVKDSDSRNKEGDTHTKGKDKEHHSRSKS

SEKDSDSAASGKSKDKTSDSHSKSGAGKSKESSSEGHSKSRLKHKEDKDEKGKKESRDPS

TESEPRSKSKDVGHGDMYDKVKSKDKHHESHKKEKKSSSQEKEEKKTPEKTETVKEEREK

TPKEEKKSHGKSLEVKKEEKKTADSHEKTKEKSKVKEKSEKHEGKAEAAKPDTKHKVKAT

ELENPQDKKKTTSKIPDSSLEEGEQKMHAAKDSQKPADGGAKSSSSKSGKDSSKNSNKSN

RGRETDKNKPAKRCKNSENDKSKDKQKEEEDRQREEEKRREEEKKKEEEKIKEEERRKEE

ERRREEERRKEEEKRKEEEKRREEERRKEEEKKKEEERRKEEEKKKEEERKKEEEKRKEE

EKRKEEQKKLEEEKKREEEKKRKEEKRKEKEKQRKEKEKEKQRKEKEKQKQKEKEKEKEK

EKQQKERAKEKEKAKEKKKEKEAKEKEKEREAESKRAADDDFPYHLLEEEQVFTSMYDTI

KRRSCTKKNPISEVSNDTRKKLSQLQSRRDSTKKTKSKVPLDDSSSDESSLSSLEFDTDK

SDSDSHFSAKKKVTKKKTIQPTVKKKRAVISSSSDDEEEAVAALVRKGVSTPKPKTSHIF

SSSSESEGAGRDNTPERPAPKSKSSPAKQSDKKKMPKKQAKKRKSAEDSNDSDIPKQQPV

AKASKKSSSHLTTVSELSLSDDDDDDDLEDIDLEPPKKEKKKVEKSEENLAKNKKAHTEG

PPVKKAKLSSSKESVSETRDKKEKKEKLADKKPAPKEVKKMPVVDNEPMDVNTESEVEKD

HDKDKRREEPAKESKKKKESSSKPKKTPAEQKEKSTNEPSMQTEAPSVQKKKDKEEHGEK

KKKKKGKKDDDGESQVGPAQDKKGAATKTEVTSTSSVPVKLVEEAVTSDPPVMEEKVKAV

VKEEEQSTPTPSVRTAEEKANKPKVEQKEKKKRPKKSETDSSAQKSLKTSAPEPEKTSAV

ADKQSPIKVKEKESVVEEGKSELPATNANLEVKKEPGAKLGKEKEKKERKSKGKTAEKTD

QMSESGALLGKEGQSCDPNMQTLADDMGKVKDKVEVKEESKDSSVGDDPGKAKPKKKKKK

KEMAEKSPQSFAISSLVAETVKKQPEPEPPAPVAETTEELDPFVSFWGHPAPLIHKKRDS

IPRPDDGKDKEEKDKKGEKIPSLDAAASPDKMQTSALPAEEDRDTTSVSLQCKPESKTST

SSEDKGKPAWVSLFGMPSTMDHDNKPLINIPEEAFKPKAEELGEKSDSEAPVKAKKKKIK

KDKKDPRKGSKEIDPLAAVEMCLEATKNSTEEDTTVLLQDVVDKKASFPDRRFSYEDIAI

SKDSSPLFSESRDLGSPMDKVDKGSPKLQDSKEFADLSQPVGEDMGLFTPFTKPERVDKV

AFTATEPASSLAEPEEPVEPEEDGFVQLERKEREEQAASEEAALAVQAIMGEEDTSQFTF

DTGDTHPMTAMHQEPTGVMDPTATPTSPSTALMDPIALQGQPETAGPIIPEASEAPQTSA

QSVEHEQSLSVMEQLQEQQADKVQPPEDVVAEPGKKTPRRGKKQRSNSKGPKDLQAKEEL

PPPPSQEVVPPTPTLKDYFPGLPYSDTNMPFMPSEEPKHVPELIKSLPELSKVDDTESKK

KNLDTYDFEFEDDDPGIELIVESAMSTL

>SG-AL_contig_4560_1_-2_2_181

SSVFTIRTSRPPLTDQIFHKMKCPVISVFLILSLSVCLSACLPACLSFCLSVCLSVFLLR

>SG-AL_contig_4561_1_-3_87_341

MFSRIFVRRLTTPASGQSAGIVPSNARYQKIQELQNIFCRDDGLLVWQKMGSKDKMLYYA

TVLITVVGFIPSLGTIGKMSFPKKA

>SG-AL_contig_4562_1_+3_129_341

MMQSPQRPVSSTTLGPKCRHQINICLSDCHRHFISCTTLLLQSTVNTSFKPVTCISFINK

RYLQNMLTKTP

>SG-AL_contig_4563_1_+1_439_699

MTLIWTTNTWTAIVFPNLSEYELRQIKKKIQKNPKNPPQQDCMQTLRNITAKYAMNKECS

FRIIWHKLHTMSIGYKWHIIRKKMTVK

>SG-AL_contig_4564_1_+1_58_351

MQKLYTLNNNSLELFIKLFDSQVQPIVQYGSELWGLDKSVNHCEFVHLFALKKFLGVDMR

TPNDFVYGETHRYPIYVNSAVNCIRYWLKLLHMEEFRI

>SG-AL_contig_4565_1_-1_103_1377

MAEVIATRTVLADSGEKTRPHFRTYLMTDANAGGHSVAQIEAKRKIRHMKFPSLKGRHDV

DSFQTDVQEKAFKKWNDDGDYHAEAPYRSYDNIIDPVSGFVSAGGDVDRNTGHDRIRSLV

QLNRTPQSAPPSSHDSVRAKEPAAPPELRRENTWSPGAPTAWNSRKTSDIWIRTQLGGWT

SDFDPRKPAPELKRAKSMYIPKPPSEASKTSRDHLALQYMYSPSTQRSYAEVPWDTILAP

KMWAPVNTAENKPDMISHRWSNKKYDPAAQEWQNTGRAWDWFQRRHGYYKTQPINFASHC

PRVQQIPLYGGCIGGENLEEIDNPAEPFHPFTVKRVPIPRPSETAHRPNIPGYKGCTLWQ

GHYAPANTHPPPEPSIQPVTSLIHRPFPMPQDSGAKREAEMSRMVTLVPPCNPFNTIEKE

EVVAA

>SG-AL_contig_4566_1_-2_2_139

TEMDSDPPFTSVKQVPLQYSCGSVCGTEMFLIPALFSYINQMLTCR

>SG-AL_contig_4567_1_-1_412_1293

MRMKDYRVICAACLSFFSVLAVLDSGQGQHVVDKEADFTVVDTNGQPCLVASINITVYIK

YHTDQSALTRQANTTLTRHSVKDKSSHYRGHCGKHLSKLSISWNNHAFTVNLTFLLQDEL

EGSRDERRVDMDDIPPGTHSSTTWSLYQVSLTYNTTHNNVFPSALNPSVTSVSAQNLNLF

RTPLGLSYICRTPGVVEIRFKNDIDLIFNYVHIQPFGVGNNLWANGTQVMCSQDINYAVE

DKYPILFIVSVCFAIIILVGTFTYAGARYVTWHCGHSGYRPLAREGPKDEMGFE

>SG-AL_contig_4568_1_+2_92_325

MKCVARFSQLFLDGSLPNLDQRCGLLRSRLCMSLEQRGPRSRSQRPKKCLKISTFGGSCV

NSIFSATSETIQILFSLL

>SG-AL_contig_4569_1_+2_125_1366

MDLLQSCALVFVFGFSVVPTVSREVNVLQDFRSWNEARQACQSQVGLGSCDLITKSRLNE

EWSNLGLSDDDSYWVGGQYFYRWLWDDDKGAPNLPLFQRHGCYKLAADRLDKSRLNTLRY

NGLHQCYTACENQGFLYLQGDSCYCASTQEGEATTSDHCNVNCTGSGDTCGAKTDYFSVY

SIPPEIHFVDEYSTVNCAVGRISNDMLRVQAALCNRFRHFFCESDLQADEIDARSESFKQ

AVYRCETRHGSALPTLSAEKSVGQLLSDLQTLSDKLTDPVNGALFWLPLRKHVRPVWVDG

EEGEDGGNFGDCLAAIPRTASYRLSNLPCSQTHAAICACTSGTDPGDSGNKDEEETLALS

GTVKAVIFLVMALIILAVFIPTFICLRRHQRAKHQLARAMEERFATGENPIYKG

>SG-AL_contig_4570_1_-3_1224_1496

MVTFESWAYSLEDRCIHLNKQFRFTGSQFVVLNANCCEGEVNTDFPACSSCAALLLYTCT

VPGHTPIASITTIALFKMCFLALQVQPLVLV

>SG-AL_contig_4571_1_-2_2_262

VTVVFVELGDTKIELLHPLGDKSPIKNFLDKNANGGMHHICLEVSDIKSAMEEMKAKKIR

TLSEKPSTGAHGKPVVFLHPKDCDGVL

>SG-AL_contig_4572_1_+3_777_983

MKFFSLFPRNSYFFPKETWSVPVENNGSEENSHACKMKAATQYPSSAYKYQEINLIMSKY

FPHAQGTQL

>SG-AL_contig_4573_1_-1_1_285

ELEAVRRALLALEAHTSHKVVKLLTDYTLHYSGLLPKQGRGWGTHSSSLSLQSETLFHWC

HNRKTTLLARHIAGKTNILADALSRTHTVVRQNGP

>SG-AL_contig_4574_1_-2_5_358

METPLLSSILSLRAESASLVSRQEAHAPSQAHSGQSEHPGRRSQQVSHGSPDGVDPELAS

TRSSLESLVLPDDRSLCDKIQQETAHLHLPSPRPRGLRDRRLHSGLARSPGLCLPSLP

>SG-AL_contig_4575_1_+3_3_239

GCPTHRNEPRTLKHVFKTKWTTEKTIMCPAFLSVALHKPAQQENHNLPVSDTASLTKEAD

HSPHMKLSFSVLVWPITHL

>SG-AL_contig_4576_1_-1_67_486

MAFRAMATIVRKVVSSPKAPKAVGPYSQAIIVDNTMYMSGMIGFFPETMAIVPGGAGPEA

EQALKNMGEILEEAGASYNNIVKTTVLLADINDYATVNEVYAKYFSENKPARAAYQAAAL

PKGAKVEIEAIAVLGDIKDE

>SG-AL_contig_4577_1_-2_152_250

MFYMFTCFICYDDVPPTLLYVNSFILHCIYIIN

>SG-AL_contig_4578_1_-2_2_220

SQFFSASLFGSVDRAVDPRSHVFSSIPARSGHLTTQHFITKDSLDCQYLACNSGLSFHSA

ALRSAVQRRYHPL

>SG-AL_contig_4579_1_-3_480_797

MNLVTRPLDNPAILCLTSAFGSKCSNHHAIPSPLFFFFLLLYFKKTTKTKNNNKKHPQYI

AVKSFSDFEMEIRTGAQLLYFTVTSFKKKRRPLAVIFVEILCYCCE

>SG-AL_contig_4580_1_+3_3_158

FSFPPPLTPSSLLYLMPQRPEIHTFIHTYIELAVIIILTGGVHASAFRRLKI

>SG-AL_contig_4581_1_-2_2_199

LSLSLSLSLSLSLSLSLSLSLSHTHTHTHTQISIVSFSSLKFWINSLQTHALYGTCIVWF

FKRQKC

>SG-AL_contig_4582_1_+3_354_533

MACTRKGFNSCQKDKPRRHKKHFLFLLFQYETNALWAKLCDCSRTIFARALQFGRSVIKI

>SG-AL_contig_4583_1_-3_393_638

MNSTFHIKIPIFNSSTDVFYILKKIIVWPACGDSCVVVCIQFIVLLVCKILVLMCVKVER

NTRREHGSRESSERDTPFKQVF

>SG-AL_contig_4584_1_-1_22_888

MYRLRICCRIVLRQRRNMAACTELPTGGFVFENCQRNKFIEAQGYQVPKAYKTGTTICGV

IFKDGVVLGADTRATEDTVVADKNCAKIHYIASNIYCCGAGTAADTEMTTQMISSELELH

RLNTGRLPRVCTANRLLKQLLFRYHGYIGAALVLGGVDVTGPHLYSIWPHGSTDKLPFVT

MGSGSLAAMAVFEDRFKQDMSRDEAMKLVRDAIAAGIFNDLGSGSNVDLCIITKDKVDYI

RPYDEANKKGVRQGKYRYSRGTSAVLSTEVKKIDLEVITTEVRPVPMET

>SG-AL_contig_4585_1_-1_58_444

MLTSGNAHCAVNRARTLTNGVITNGVSNGVSDKDSPLSDSPSSNCENQGRGDGQDSSPDY

VMQYSKIENCDQRNRYKGDFNREYAEYRFLHGKVQHISERFEHLKERINNTVEGSQDYEA

LRNKVVEEY

>SG-AL_contig_4586_1_-2_14_262

MYLGKNAPFCAEEENFFSPSEKGKQDLFWWWFCIEEGKHFFPPVTEEGNNCFQRKDSRFF

PKFQMSKIVTKKIIWVCGRCVNC

>SG-AL_contig_4587_1_-1_1_225

YYPSRSLRLYSYLLNIEPFSTAFRCSTLPRHFGTNFQTRGSVPHAMTSQRFLAGERDFIA

ASETRADCVAFSCKI

>SG-AL_contig_4588_1_-3_1065_1568

MRQAGEVTYADAHKRHRNEGIIEFATYSDMKNALDKLDNTEINGRKIKLVEDRPRKHSRS

RSRSRRRKSRSRSHSRRSRSRSRSRSDSRSRSASKSRSRSASKERKSKSEDKDKSDTKKR

SKSRSRSRSPSHSKSRSRSRSRSPDDHKGDDSPHHNGDAQRKEDEEDD

>SG-AL_contig_4589_1_+2_617_886

MLKDLQLQKLQDRRFHLHLIFMYKVVKGLVPAIPAHTSFTPTEKTKEEFNQNRFPTVRQK

IIAKHAIHNNRAFKIPDSHNEQFKNSFFVK

>SG-AL_contig_4590_1_+3_3_212

AGSAGQSVPPCCTRAEHVQSLRRTYFATFPFFFEVPPRYLTIMNLGSLERMNLLLSAENI

SDVNILTMLA

>SG-AL_contig_4591_1_-2_62_2011

MEMNTLLKKLGFKSTDHMQEEFCTNNYYEMLAVVHVFMSGIVKESYHDVFLEQQFMCPSL

GFFEFLKKKYLDQILSWQLPSGCFGEESAIQDQHLHKRPPKLDSSSLQLSQKLKGVPDVN

RLDFRKQQISKQLEDLKSNNEISKLSVHQKNSKNDQQDVLHKQPWKINVKRSVLPDNEYH

PHAKLPVDKEKNVFTNQGKDQQHPQKVNDMHMKQGELPVIHANVADQKNQAPPSLVKETQ

PQNQNAAARMNNRNIVNKPRYPKLKGIVPAELKGGPQVETHNSQEQKQEVPVAQNFKYVR

DGASAVRLQDKSQNAPLPVLVPDMPQHDGQSGRKLLGSYVASGGSRRKLLVEKEMPGGCL

AHKTGVAGGALVMYLRYLIDPGPLDLFAPHDFFPDDFQALLGSKRGPKEMNFLRNQMHNN

NGDKHNQVEFDTVVGLNQQNEDEDDEEEEEEEEEEEDDEDGEYDDREELYPDAREGAGQQ

QERKVVHFGANDNPQLKHQDDGYDDNKEEENDEYNDENEDEEEEEEEGDYIKRVKGDDDG

VANPLEKDNIDDDTEYTYYDDDDDTDRGIIKRHKPQAPPQKGVRIRTHNRHKPHPPSIDQ

VPMGLPDSPPTKTVVVLLSATSLLLIVFMYRFITRRRFHFRHNSRGFFKL

>SG-AL_contig_4592_1_+3_852_1130

MLQAAIELEMLNPKTMALSTEPASLLTSVFGANAVSAVSCSLVIQSLQYDTQFSVRRGKS

DSGRQGEVSMPVIIWRSVIGCDWLCRLLVQFAN

>SG-AL_contig_4593_1_-1_88_402

MNLGVVFRSQNEQHGINATEAAHKMEQQKEKKKIIFQKTALGLILISYSVISDCARFFLL

KCIEYACSFCLPSACLNLPSLEECLSLHKSLQNGREMGPRFYIRH

>SG-AL_contig_4594_1_+2_143_1411

MTTTATTTTTTAELEMGGEGEVVMTENQLQPFQHQVAGHSLMFRHGADTICKALTPRELE

VYQTMPEILKPHVPKFKGVVAVNINDLENEGEDCGDDPEAKKRKAKLPSILRRLTKITHY

TGNTYQFILMENIVEGYQRPCLLDLKLGVKPYVSKRPNKTSDTLGLALCGMKIYQSRTGQ

YVNLDKEAGRMMDENDFRQTVRHFLFNGHHIQRDLIPPFLRQLEKLKTAISQLPTFRFYS

SSLLIAYDADDEHVLNGLQLPTTRTDKLRSPLSSLNSNEFPTVREQSSPCSEHRETQHLR

TSVRRRSQQQELASFKTGNNANNSIKRAIGTHERKRSFSDEITGRSLHRCCSCDSDIAST

GTTVPSLKRPMESADSCQRKVDVRLVDFGHTTYAGIGEETVHEGVDGNSLLGLQTLIDIF

AQT

>SG-AL_contig_4595_1_-3_48_326

MVLLKFLDQHHRCLYLSGLGSGYFDRNCKALSRSLNPQPCHAVSIQKVTPQHHHHHPHSH

GSSQHCEKLSQILRPFFLCSHGDLHLAGLKVCW

>SG-AL_contig_4596_1_-3_39_260

MIWLLTLASNSGLELTSVQTLAHISSGIVAFRRLMLLLVHCVLCSNIFLDASMHAVWPTL

CRCYVSAQPANCLR

>SG-AL_contig_4597_1_-3_3_221

RFSYQHLLCLYIVGLFDTRIYNQYKRSVEYSPTMQSQSTVELHSIIIIPISVSHHSVVKN

VSDPVSFQALFSW

>SG-AL_contig_4598_1_-3_24_221

MCLYIVGLFDTRIYNQYKRSVEYSPAMQSQSTVELHSIIIIPISVSSHSVVKNVSDPVSF

QALFSW

>SG-AL_contig_4599_1_-2_8_583

MDWLTFSYSVKKQSFVSLAEEDDDDFYAGKMAKVVTLECKQLTPSKQLYRTRVNIETISS

NVSSTLMTFLSKISDKLNWTLPAILIGSIVTASITNTPTPLQVALGVLVREKYLLQTMYD

YGVMCSYDEVIRFKTPAAAHTEIVRDNKTTLDASKGLVQAVGDNLMQTSLVKMASNLHMP

LLCCLHKASRTQ

>SG-AL_contig_4600_1_-3_99_2039

MAHLQMKEGEYTSTIYGLIKDQRYSEAVQILNHQLQSHPRSRAALSVLAYTYYQMQDFVN

ASDCYEQLSTLHPEVEEYRLYYAQSLYKACLFQEAMKVACQIDQPQYQGQITKLQAAIKY

GEEDLPGAKSLVEQCPSDDPDTEINFGCLLYKEGRYEEACQKFSAAMQVVGYKADLSYNI

ALCHYMMKQYAPALKHIADIIERGIREHPELSVGMTTEGIDVRSVGNSMTLHETALVEAF

NLKAAIEYQLKNFDAACEALTDMPPRSEEELDPVTLHNQALMNMEAEPSRGFQKLQFLLQ

QNPFPPETFGNLLVLYVKYEYYDLAADVLAENAHLTYKYLTPYLYDFLEAVITRQTSPEE

AYRKLDEMATKQTETLRKLTKQVQEARQNHDDEAVKRAVNEYDEALERYIPVLMQQAKIY

WDMENYQQVEKIFRKSVEFCNEHDAWKLNVAHVLFMQENKYKEAAGFYDPIVKKNYDQLL

SVSAIVLANLCVSYIMTSQNEEAEELMRKIEKEEEQVAYEDPDKKIYHLCIVNLVIGTLY

CAKGNYEFGISRVIKSLEPYNKKLGTDTWFYAKRCFLSLIENMAKHMIMIRDSVIAECIQ

FLECCELYGRDVKAVIEQPLEEEPMHPGRNTVSYESRLLKSLLLQLN

>SG-AL_contig_4601_1_+3_3_176

KPLNKQKHWWWRGAQAMLIVKLYPYIPSTELLCLHFSDHYDTKTGKCTVEIYTHIYPT

>SG-AL_contig_4602_1_+1_2464_2676

MPKTPYASWLKKLNKGISKYTLERKDMQSILMSLFLCLHHKPLLGVFINLFCFQSPVNRV

YLEFDLCNKWH

>SG-AL_contig_4603_1_+2_1175_1666

MVQSVKNRPSGKQFCRNRVFILEGSRQGFRNTEEGGLFACAHQHNHPSAIHHKAEVEKKL

LEQIQLGHYVVANSAPAILSVLEAIPKDDGSVRLAHDGSRPVGTAMNDNSISDSVKFQSL

QDAYKMAKPHYFMAKVDLQAAYHSVPIHFDDYTATGLQWKFENM

>SG-AL_contig_4604_1_-1_169_5298

MAGSNPNSLKWVTFFDENKGNDQAVAPPAKKSKKAAAAAPPPLVPMMMEKDSGDHGKSSK

AADKRLSVERIYQKKTQLEHILLRPDTYIGSVESVSQQMWVMNDETNLMELREISFVPGL

YKIFDEIIVNAADNKVRDPGMDTIKVDIDPENNKIKIWNNGKGIPVVEHQTEKMYVPTMI

FGHLLTSSNYDDSEKKVTGGRNGYGAKLCNIFSTKFIVETSCNEYRKAFKQTWTDNMLKT

KEPTITESKDKDFTSVTFCPDLTKFGMDGLDRDIVALFTRRAYDVAASTRGVRVFLNGKK

LPVKNFRDYMDLYLKDKTDEAGQPVKVVYEQVNPRWEVGITLSDRGFQQVSFVNSIATTK

GGRHVDYVSDQCVTKLIEIIKKKNKEGIKIMPFQVRNHLWVFVNCQIENPTFDSQTKENM

TKQAKSFGSKCQLSEKFVTQVNKCGIVESVLSWMKFKAQAQLNKKCHSAKHSKLKGIPKL

DDANDAGTRNSEKCTLILTEGDSAKSLAVAGLGVIGRDSYGVFPLRGKLLNVREATHKQI

MDNAEINNIIKILGLHFKEKYDSSDKLRTLRYGRMMIMTDQDQDGSHIKGLLINFIHHFW

PNLLQQNFVEEFITPIVKVTKGKAEHSFFSLPEFEEWKSHTDNWHTWKVKYYKGLGTSTS

KEAKEYFSDMPRHRIPFRYGGTDDDASINLAFSKKKIEERKEWLTNYMEERKRRTEMGLP

ELYLYTKDTRNITYNEFINRELILFSNADNVRSIPSLMDGLKPGQRKVIFTCVKRNLTQK

EIKVAQLAGSVAEQSAYHHGENSLMSTIINLAQNFVGSNNLNLLQPIGQFGTRLHGGKDA

ASPRYIFTMLSPLARHIINLKDDSLLQHLYDDNQRIEPEWYCPILPMVLVNGADGIGTGW

STRIPNYDVREVVANIRRLLSGLDPLPMIPSFKGFRGTIEELGENKFVCSGEVSVLDDVT

VEITELPVRTWTQNYKEEVLENMLQGTDKTPPMITDYKEYHTDTTVKFVVKMSPEKLAQA

EQTGLHKVFKLQTNLSANMVLFDNKGCIKKYNSVEDILREFFDVRLDFYERRKHYLEGML

GAESSKLDNIARFILEKIEGKIVIENKPKREIIQLLVRRGYDSDPVKAWKEAQAKDKDAE

DDDDSASTASDTTGGPDFNYILNLPLWSLTKERKDELIKQGESKREELMELRHKTPSNLW

EDDLDQFVLMLDEVEEQERSDVTGEALTIKMGKLGKVGKVKSKKAVVDTMPSPMGRRIEP

RIDHAMKIKAEKAEAAKKTKAARGKKEKLILIEDGLDDLEGESGSIKDNGDSTVQEPLSL

RLNLNADGTATKVKKPRKLAGTSAGTGTGKGRGKGKPPGKGKKAGKKRNPWSDSEEDDGE

ISDVSDMDADNMAAAVAAAARDRGPRRAAASRAKYTFDDDMEEEERVSSGDDLANSSYSF

KPSAPPTRKPPSPALPLSDGDEDDDIPMKTTPKKTFGGISDDDDDDYSAKPASQKMFGGI

SDDDNDYPMKAAPKKTFGQDNDYSAKAASKKTFGDISDDDESFTVETDSDTAAKSKAMPA

ARPTTTAPAPKPIMPVEFSDSDDDGFSLDSKSASKEKDGMDDDLIVPKSSKPAAKKPAAK

KPRKDAGESKPKKTKTKRPKNSDSEASDEFVPKKKKAPAKKKAASKMTEDGDSDMGGGDI

PPASRRVSTGRSKAPVKYTFDDSEDDFDDY

>SG-AL_contig_4605_1_-2_8_1906

MAAFGSRRFASCLQRGLLSASLKQKPSPSTNRFSSSSKVNSLKLLAATSLPRVVRCARSL

PLLGTTATVGVGVCLYGGIALCASSSELNERLLTAARLGVHKEIAKLIEQGANVNCHHDL

GWTPLQVAAMNAHPHVVKLLLEAGADPNAGDLYTTPGQMAREKHLHSLQVLITREEEFSH

RLSNRANFRDCTALHYAVLAEDVETTKIILDYGGDPTKENTLGHKPIDYCRTEEMKKVIE

DSARSFEEKRAREEAEERRRFPLELRLKEAIIGQESAIRTVAAAVRRKQNGWYDEDHPLV

FLFLGSSGIGKTELAKQVAKYLHKDLKKGFVRIDMSEYQGKHEVSKFIGSPPGYVGHEDG

GQLTKQLTEFPKAVVLFDEVEKAHPDVLTAMLQLFDEGRLTDGKGKTIECKDAIFIMTSN

LASEEIADYGMQLREEAKRLSKLRHNKIDELDIDDITVSRKFKDLVVRPILKKHFRRDEF

LGRINEIVYFLPFSRAELGKLVTKELNFWAQRAQSKHQIQLLWDHRVVDVLADGYDVHYG

ARSIKYEVERRVVTQLAMAHERQLIKEGNVIRVTVTHPDDLLTKLDDDVDEKEEEEEKGL

HGLKIQVLEEGSKDKYNDISFDTAENPFTIEFK

>SG-AL_contig_4606_1_-3_177_3758

MTEIESYSNNTLRWDPGQDDDFDAGNNSSKLFERSRIKALADERELVQKKTFQKWVNSHL

IRVNSKINDLYVDLRDGKMLMKLLEVLSGERLPRPTKGKMRIHCLENVDKALTFLHEQRV

HLENMGAHDIVDGSARLTLGLIWTIILRFQIQDIMIEEVDNSETKSAKDALLLWCQMKTA

GYPNVNVRNFTTSWRDGLAFNAIIHKHRPDLIQYDKMHKSSAIQNLSNAFTVAEDKLGLT

ALLEPEDVNVEYPDEKSIITYVVTYYHYFSKMKADSVQGKRIGKVIDNAINTENSIEEYE

TLTSDLLEWIEQTIVILRDRQFANSLTGVQQQLAAFNTYRIVEKPPKFDEKGNLEVLLFT

IQSKMRANNQRVYLPREGRLISDINKAWERLEKAEHERELALREELIRQEKLEQLAARFD

RKAGMRETWLSENQRLVSQDNFGADLAAVEAATKKHEAIETDINAYEERVSAVVAVASEL

EQENYHDIDRINARKDNVLRLWNYLLELLRARRMRLELTHNLHKIFQEMLYILGWMEEIK

ARLMSEDYGKHLMGVEDLLQKHSLLEADVHVVGERVKTVNTQAQRFIQDDFADVGGYRPC

DPEIIQDRMSTLRQAYEELCELAESRRKRLEESRTLWQFYWDMADEEGWIREKEQLMSSP

DLGHDLTSVNLLLSKHKAMEDEMQARHSHLQSVIQASQDLIQAGNFGAEKIQERIDEINQ

QWESLIDLATYRKKRLNEAVDFYQFFADADDVDAWLLDTLRIVSSKDVGKDEASVQSLLK

KHKEVTEELKNYQSAIEALHEQAASLGEQDRESPDVQSRLSSIDKRYQDLLDLAKLRKQR

LIDALSLYKLFNESDGVETWIDEKEKFLTTILVTDDVEELAIMKHRFDSFDHEMNATASK

VAVVNQLARQLLQVEHPNAAEVVQRQNQLNSTWNNLREMVDQKRDEINVAHGFQNFNIEC

NETIGWIHEKAKLIESTDELGNDLAGVMTLQRRLSTMERDLAAIQAKLESLQNEAERLEG

EKPEEAQAIREKISEINNVWMDLKDMLKERDEKLGEAGELQRFLGSLDHFQQWLARTQTT

IASEDIPNSLADAERLLNQHQQLRDEIDTYAPEYAKIKEFGDKVTEGQDDPQYMFLRQRL

QALDDGWHELLQMWENRQQLLSQSLSQQMLLRDAKQAEVLLNQQENFLSKEEVP

>SG-AL_contig_4607_1_+1_1_153

NMYIMTPEHVHNDTRTCTSLYTWMPSENAASQTTNIPIMRSIMHDRFSNSK

>SG-AL_contig_4608_1_+3_3_200

ETVLKSAHRRGLNYACLVGTEVAHLPSSPTTQVLMGDNLVRAKLCACSLQSTEWSMGTQP

CSGLMK

>SG-AL_contig_4609_1_+1_406_612

MRQICPNSVKIQSQTSKKKKKFLPTHFSYFPRHTHTEIIRIPWVILNHSSSDSIFPSSIF

WGENRERVV

>SG-AL_contig_4610_1_-2_2_124

GRVALESTVKTEHHKAKKSVEGNINDSDFLCVCVYFQFNIY

>SG-AL_contig_4611_1_+2_275_529

MDTFLHDYLILNKLVHNTHTHASAHTYAQGDRLRQTERQTLRLSHTHTHGTLSPPLSLSL

SLIVTHTHTHTHTHTHTGILLMYLA

>SG-AL_contig_4612_1_+2_2_154

KAGDPGLWMHDCHHSDMDFPDKVGDTELRMYDCHSDMDFPFTVILQLYDTS

>SG-AL_contig_4613_1_+1_106_258

MVNLKKNTYAYITYTSLHTSFKPFFIRYTHIDHMALNQHKGVHIFYFNLQF

>SG-AL_contig_4614_1_-2_2_208

FFFFPPPLPHGDTGGSFSINSIPTFWKFCARSFLFSIALFVSAPLPFFTVVCFVFALPVH

STFFFPARL

>SG-AL_contig_4615_1_-1_1_168

LLLLLLLFQVYIIIIIVVCVCVVLLLFLFSGWFMFLCKIFIVTVYHMIRPLAEMAL

>SG-AL_contig_4616_1_-1_1_177

HHHHYYCCCCCSCVCFVVFVVKSFYVICYVKQGFILQPTNPPECVFCDCLLLHLVILCT

>SG-AL_contig_4617_1_+2_2_133

KKKDHPGFKQSNKQRYSVTAPSCKRTTTLLLFDKDYKYGVQFSG

>SG-AL_contig_4618_1_+1_1_138

KKNKQRVGSGTGNVSSLAEVTGRHKFRKLISLLLCLSTSVMKENNE

>SG-AL_contig_4619_1_+3_3_200

QHNYVLSAIPFFPQNSTSTTMFSQRYLFSHKSGPPQLSQNLYFKLTGRMYNKLLQYLSFY

NSQTTP

>SG-AL_contig_4620_1_-3_3_245

GLGASKTHHRDTLDSDSAVVSRSSKNFLILGLGLLCRVFCVRLRLQGFALHTLVAQLNLY

SGETCIFLFFHSRSWNSKFFV

>SG-AL_contig_4621_1_+2_2_193

LSLSYTHTHTHTSQNLKFHDKYALHGSTTSINSIHISSCENHAPVNRTNLLQKSFQILTE

HQLI

>SG-AL_contig_4622_1_-1_1_465

CSSDLAAVLRNARSVGNFVASIRSTLPRLCTAALVHHNGSAKYLHSQNQVFHPSILKNHS

LTQSRFYSEKPPLTIDMLQQRVMLVLRLYDKIDPEKLTLDSHFMNDLGLDSLDQVEIVMA

MEDEFGFEIPDTDSERLMRPRDIVQYVADKEDVFD

>SG-AL_contig_4623_1_-1_1_138

FALVCVLESLQWHTFHSPLGLKMPIIKVNKVQTSPETCVFCFRLSF

>SG-AL_contig_4624_1_-3_3_260

SNFLFSVALFVSAVFSFLYCRLLFSAFPVHSTFFFPASLHTIFNFIHISSSSSLKYLTVT

FSISQHISSSLKHPTATLSFHHHSNT

>SG-AL_contig_4625_1_-1_4_255

MNDWPPSVPKDYGICAAGETPGHKPGQRVKENLLLPMQQAYPVATLMKFKEVICIVRSLL

MCWYLSLLCIYIYLSVYLCICLAS

>SG-AL_contig_4626_1_-3_123_725

MSQLNLSEVQMCGEQLLAVLAGDDMEAVTLQRLHDITQQVAALRHKQLQQLKDDVQYLTS

ELNETCDVGEDCGDNTLGDLANMETALKNRVDTVQSQSATVVREYQQLKEQLTTLQKEQQ

TLREERQSSKDAKTAVLPKSRCDANLYSNITNLRWQYNCEPHEVKGFITNKHDVKTFNLN

SHELSRFFITNYLWDLIEEDW

>SG-AL_contig_4627_1_+3_18_239

MLFVQLSHRKKAAQKPVQEKVFKVEEPLHCMRHFHSLGLGSRVPVVQRVLTIGDFFSYLL

NYRLSIIRGYGLCE

>SG-AL_contig_4628_1_-2_38_496

MYYVNDGVYGSFNCLLYDHAEVEAKLVNAPVNSMEFLTSVWGPTCDGLDQILKNAKLPEL

NVGEFLNFPDMGAYTLCAGSTFNGMPRPDVFYHCAEHVWNSVIPADLKVLEPCSPNSKSL

PMLKAGHRPEKDKDILSISPKTMFVMEEEECPN

>SG-AL_contig_4629_1_+3_258_410

MSNIPDKFSNTVCNSRSKIITYHPILNREKSIFHGSLKDIAYTVLKSTACL

>SG-AL_contig_4630_1_-2_146_1579

MVVLTRRSARLAHTRSAATGMDDPMAVMDMQISTITSTMKPRAAKKRPSVSARQFSCQRN

RCPGSPTSGSVARATRRPIRPVDRQKMVDWLVERLNGEQLTNNLWWLEKGETFAVKWSHA

SRHGFKMDERQLVKAWAKHSGIYEEGDEEDHKKWKANFRCALHSLREVEEITPKEGRKGQ

NAARIYRILDKPREKKASKSKNSKSESSDDDQKGDDSYSGLSADRGSPASLPLPVAETLT

PVAVSESSGSVNLPNFQRLGMSTSGMTYVQQNHLSGGCMTIKGEPPQPNPDAIPFQILHF

PGASSSSSSTSSTTSTTSSPLGCPSLSFSFSPSYPIPTCTTLPTDLLEKKEMEGMIPVGI

MTRLKPRERTPSGDSGHPQSPPEKMEEVEEILEEKTLCISELGDVVEEISADEGIEMAQE

FIMEIVEGQNCQPTLFWTELPSLPPSNPPEAVVTVGVISSGEESHTYTSLEALFMDTQ

>SG-AL_contig_4631_1_+1_124_309

MLTTVYITFSMLLTLTINKNKTNQKGGRVEARTETSFLILTEILSLLRFPLMRTLICYLF

HD

>SG-AL_contig_4632_1_-1_1_192

KKIDHTLSPQPAVLESSSTSLHSVVNTVSRHFTSGCCQVTFQFVKSLTSPNSDAKIVSRH

QAMM

>SG-AL_contig_4633_1_-1_967_1242

MILHGCESHPGELYMCVQPPVYPAVNGYLTFFKVRESSEGEEMGTALIMLALRKVEVSTT

SLPYSLRVTGKPYLISVAVVVIIIVVVVVVEV

>SG-AL_contig_4634_1_-3_3_62

LLEKKCITGKITNFPHFSRE

>SG-AL_contig_4635_1_-1_346_594

MYISTFIVARYTFLFVLNLYYMSLPANEIRFYRCFLYLTASVYHTLCALHFCYFKIIRKM

GCFLFELFWGSFCFSVNMWCCGK

>SG-AL_contig_4636_1_-3_3_374

YCHFSVSSVEAWPSAISAVLSSAISAVLSLFCVKCGGLTKCYLSCIVTFLLCVEAWPSAI

SAVLTLFCVKCGGLAKSLSQLYCHFSVSGVEAWPSVISAVLSLFCVKCGGLAKCYLGCIV

QVCR

>SG-AL_contig_4637_1_-3_333_566

MQTPAVLVSHIRSILVYIYAFMIEWRRFGCDDICQPYITTLACSNLDKAWLEFKMPAKSL

GYGYVLPPVQVFICIPCS

>SG-AL_contig_4638_1_+3_630_779

MALNLEIFKSKCTFLGVLGYFFLFSACWVYSCFLEPPNSNMAYRIFKVHI

>SG-AL_contig_4639_1_-3_246_2000

MSTSRCGNKDNEEEAGTPAATTTTASHPSPSPPSSQNVIPDNVHISSSSSSGSSQGNHHQ

YHYTSDDSNKSNGVGASQRLWKRAVNRIRADDPSQLLRILKGATSHKDTAQARHRRHLQN

YINKLHRRDSTLVKPWTEEEHVRAKRSIQMTAAKDEEDLMNMMGLQISENRGAGLDVEED

PNSEIFNKDVIRWRLLTTGFSTRIPVEDQRATLDLAFRMWSEVIPLRFVEDTDSNINDVD

IEVAFGRGSHQNCEHDFDGNGGEIAHSQHAGNMHFDDEENFKSIRSYSPDGIFLLRVAVH

EIGHVLGLAHTNKSQSIMYAIYHGADLSPEFELSREDRTEVQKIYGVCKGEFSTVFDWVR

RRPDNRFIYNTYFFRENHYWMYENHANRTRYGDPLYIAREWDGVPNKLDGYLHKWYFNGN

DIVNEAYFFKGEHYHKYDSDNDRVYEGWPRLIRDDFGPKPGQTEGIPDNLDTVFFDMRDK

NIYFFKDDMVYVYDPKQPQDSQGCCERKRRIIEEFPPASDNDVPLPDQLDAVYYSYGDKT

QYFFKGENLWRNRVFDPRQRRTVNSVEFLGKWYDKWLDICDVQAH

>SG-AL_contig_4640_1_-2_293_436

MSSSKTCICCFDTSSVKTKIFHPFFSSHTISKSYFSDWKNFSEESFFL

>SG-AL_contig_4641_1_+2_53_202

MTQTRHSVTHKTSTLMPPFVCVQANSLKLLVSHSQKAVQYIFSSLQEKKL

>SG-AL_contig_4642_1_-3_162_884

MEEMHPEDLIECPYDESHMIRARRMQCHLQACRKHHASGAEIACPFNAAHVINVEERRYH

MTRCPDRHKVHSRMVNEVLDKTDNRGCVEKPQYRPIRTFGETWDMQDDGSNRQAHHHWEM

PAANLQVKIREEEFEGLNPRQRKQLYLQRKAELAEVSKKQAQSMNIPSLRTPHHDTSKSR

SLAISQQQQQQQKKNQPVVPVDIYQGALKGPQGAGVAMGRGRGMGQGVNAPRRPPGRGRG

R

>SG-AL_contig_4643_1_-1_652_837

MRSGFMYTWHGLVCTCLKTSFVALTKKKLMYKWKLKPKVVLESVHNLCLWHYFKPKSYEE

CI

>SG-AL_contig_4644_1_-2_38_238

MLQSFWWCQCFIDPVPLPISSLCQHIGFAQDQGVCQINCGQIKKKKKSNKQNKQTNKKPG

ARFGWRP

>SG-AL_contig_4645_1_-3_117_482

MRCLYSRSDVSSLIPAPSIKLARFLPMLMIQSFWWCQCFIDPFQSQFQARWVLHRIKELD

NSWQVWVEHIIIGFCFCFVAAPSSSPLQRHWSHTAHSCYFTHHQPQVRSVKIPVPGSPWD

TR

>SG-AL_contig_4646_1_+1_1_159

ISPLKNKKERKRGGLETANHQEAEYMYMHFLSPLDSAIYTHELSLSLTLKHLK

>SG-AL_contig_4647_1_-2_2_142

KNACKCLCFISDIDCLNYVSCIVSIICNMIIYMSTMFLSQLFELCVL

>SG-AL_contig_4648_1_+3_360_503

MYSYNVFIFIACICLQSFFCVIHELFCSTNLNFLLLGLFKVRGGWTFQ

>SG-AL_contig_4649_1_+1_223_390

MTIWVLFWYVFLQYIHGMYSYNIFIFTACICLQRFFFAIHELFCNKNVNFLFRGLF

>SG-AL_contig_4650_1_-3_3_107

LFCSLLKTHLFSSNTCKKSMLMCAYISYAFYIYLF

>SG-AL_contig_4651_1_-2_182_1276

MRCCSFVLNLAVLVLIVQDGWGQTDQTDLRLRRREKQRYRLAELARASTSFGVNLYRELA

ARDNSNIIFSPFSVFTALSMTLLGTDGITKSQLRAVLVQRPRQGIHQALHAVFASFPSSP

DANVTLRVANAVYYDAQQVSITDSFNRNVRRLYGAYPRMFERPNPEIPINNWIYSQTGGK

IQDFFNPGDITSDLVLILLNAVYFQGKWKDKFDERSTQDGDFTTASGQVVRVPMMSRTGR

YPVKMLGGELGALVLELPYGNDGRFSMFVLLPTQVNGLSDVEARLTSRLLEEAVSNMPQA

TRVRVQMPKFTLRVKTSMKSVLQAMGLTDLFSGSAQLSRMATSRRPLAVNDVKHEAVIEV

DEKGT

>SG-AL_contig_4652_1_+3_465_734

MYMKISFKNTSPPDYSRRFYDKMQDQPFVNRTLSNSQYHSIENCVCTYNVLLSLEIMLYL

GEVSLFQATDNKNNKGVGTIVQQTGLSVLV

>SG-AL_contig_4653_1_-2_68_496

MLQKERQEGHPNDKQGPPPPLPPSLSLSRLHVCIYVACHSHHHYHVMAFFLVSSLGFCGN

MEHTCSHHCGILMSYPISSLGLCGNVSYTCSRSRQYHVMTILRVSGLGLCWNIKDTYSIF

TLWCARLLIDFNQTPCGVEEAAT

>SG-AL_contig_4654_1_-3_3_206

NANNRQHGLIIQKDRGLQKPSESVLKICDLMEQMLQRMLCITEQKLPQGPGVPGYLHMQC

YKRLGWCV

>SG-AL_contig_4655_1_-3_3_197

SPVQSPAPTPKFACTTWQGSVIFRSQEIHSQNNDEARPLKHQQKVHNPVLMAMRRFATVI

SLFCG

>SG-AL_contig_4656_1_+2_95_649

MPSPDEVADMYLAAVELLASHRYEQYEVSNFAKEGNYCWHNIGYWTGQQYIGIGPGGHGR

VWMPVEGTDLRHACIQTLEPENWMWEVEKFGHATRKSILQTKKDILEELVSVGLRTKWGL

SDKNWSALLPFVPLEELLGSSGTSHWIKNGFLVHTQQGLQASPQGLMILDTILPDILLDL

EALLS

>SG-AL_contig_4657_1_-1_22_2523

MQEVMQRQRNIRNMAVIAHVDHGKTTLTDSLLAKAGMIASSQAGDKRATDTRKDEQEKGI

TIKSTVVSMCFDVDKEVLPLIEEERVKSTEFMVNLVDSPGHVDFSPEVTAALRVSDGAMV

VVDAISGVCVQTETVLRQALAERIKPVLVLNKVDRAVFEKQLAPEELYSSLRTVINSVNA

VVSIYAEEDSPMGNLVLDPCVGNVVFASGLHGWAFSLRQFARLYAAKFKVPEEKMMKRLW

GDHFFDPITRKWSNSRTESNERGFNKFILEPLMKMLRSSKEGDVETTCQLLDKVGLKLSA

EEQEMGGKGLMRTAMKRWLPAADAMVEMIVTHLPSPVKAQAYRTACLYEGPLDDEAAMAM

KTCDPKGPVMMYISKMVPAADKGRFYAVGRVFSGTVHSGLPVRIMGPDYKPDCRKGKDLY

EKKLSGVKLMMGGSVGSLEEAPCGNIVGLVGLDNYLLKTGTISTFERAHNIKVLKFSVSP

VVRVAVDVVRPSDLRHLIEGMRRLSKSDPMVQCTSDDGQYVVAGAGELHLEICLKDLETV

HAGVPLKKSDPVVRYCETVSALSDQVCLSKSNNKHNRLFMTAEPMPSGLAEAIDEGTVSS

TQNVKERARFLNSSFNFDINQAHKIWCFGPEGSGPNMLVDTSFAVQHLQDIRDPVVAGFQ

WASSEGALCDETLRGVRFNLVDAKIHSDPAHRGGGQVIPTTRRVMKAAMLTAKPRLLEPV

FIVEVQCTDDVIGGVMGVLAHRRGQIVEEIHSSDSPLCVIKAHMPVNEGFGFTEELRGST

GGKAFPQCVFDHWQVLPGDPFDNVSLAGNIVAQVRQRKGLPVTIPPLDTYLDKL

>SG-AL_contig_4658_1_-3_3_311

CAHDPHCSLLAGLTVVPGPEVPVSCAASGADPKPGPSEATALRDQALKPRDASPARLASL

RLRQPSLTDPSLSTGGRTRAHPRSRAVSSALEASGALPETRSL

>SG-AL_contig_4659_1_+2_2_307

LRFNNLNLICAHTYTYTHIRAHIHTHNHSLRFNNLNPSHTLTHTHTHTISLRFNHLNFTL

SHTHTHTISLRFNNLNFTHTHTHTHTHTHTHTHTHTNTLRFN

>SG-AL_contig_4660_1_+3_3_140

HTHTQHSTHSPARVDTLTLTHTHTYTYTHTHNSSICMSIYKYNISK

>SG-AL_contig_4661_1_-1_256_2412

MSRKHCRPPDQAETVSSSFETQTARMEAEESSVKAAVATSPDESARKDDSVHIKSEDISV

GPQPDAVNINSDGMQVEAEVKSQIDSSGLQPNMENEVKSEEIHHSEKVNIKDGADSLIEV

EEGAKPLDAAAADDADDNGTNDDDMRIDGINDGEEERTEVKTSSPVAGTEVHPRDSGFTS

ENFKIEITELPRKIGFVQLKKRLSTTLGLQPHKAKMFPNKPFAFVCFSCEEDRDQALQVI

NGHVWKGRTLSAKKARPSADPMKRKHDGSGAGSDNAKTAKPDNDLPATTRLRSSVEPLWE

VPYEEQLQHKADKAKEALRSVASHPEVRCQFTKARKENEGLCCPLLPIIPSPVLEAYRNK

AEFSVGHSEDGNESVVGFRFGLYRDGTDCVGSPAEISIISADSRKVVECFQKYVRQSKYP

LFDHRTHGGHWRQLTVRTSRDGGGTMAIVEFVVNNTRQDQMEEEKESIHKYFTEGEGKEA

NVSTFFFRPCTPKTSNSTSEEPYELIYGDGYIHETLMGMKFRISANAFFQVNTAAAEVLY

KMIAEWCQATPSTTILDICCGTGTIGLSMAKNVSKIIGIEMCKEAVEDAKVNAQLNGITH

VKYHCAKVENVISDLTHNIGTNCVAVLDPPRGGVHSSVIKSLRSCSHLMRIVYAACNVAG

AKQNFIDLCRPTSRKTKGDPFKFVRAVPIDMFPSTNHFEVLVLLERDIRGANSASTGTA

>SG-AL_contig_4662_1_+1_19_1059

MRKYYINSNLISVIENLYNKATSAVFCNNNIGDWFRTTVGVRQGCLLSPTLFNIFLERIM

TDALEDHSGTVSIGGRPITNLRFADDIDGLAGNECELASLVEQLDKASSNFGMEISAEKT

KIMTNSKESSKKEIKVNGQILESVTKFKYLGSIISDEGSKPEILSRIAQTTAALTKLKPI

WNNKNIAISSKIRLLRSLVMSIFLYACESWTLNADTERRIRAMEMRCYRRLLGISYKDHI

TNEEVRRRIENAIGPHVDLLTIVRQRKLKWYGHTTRSSGLAKTIMQGTVNGGRRRGRQKK

RWEDNIREWTGLELRNTLRKAEDREEWKAVVRRSSAAPRRIPNLRDR

>SG-AL_contig_4663_1_+1_1_393

ACEVAIGPEPSPKSDTFCVWVTPGPYCGAAVAWCVAGVAWCPCGVTSGGAPSGPPSAATA

PGPCCNCSSCLVGGRSRHSVPRTGQAGTTGAGCPPGRWPVPACRGGVVPRALRPAGVGGR

RSAAKESPSGE

>SG-AL_contig_4664_1_+3_174_431

MMCACACASVLCMCKHTHTRTCVCVDLCVSMCAHIPYSTCLHVNVCPRLSLCVHVFVCMY

VCVLQYMYIYMCMHMSVRVCTHVWCV

>SG-AL_contig_4665_1_+2_233_565

MMSVLPSDRKNAAQKPVQEKDFFFLEWKSHCTASTALSQPQKPGSSGTKGHHDWRLLWLK

PLVVWGFLLLLLLLLFLTRHWVGGRGARWLFSCFHKPPNTDMVYRIFNVRI

>SG-AL_contig_4666_1_-1_49_402

MGILFESVKKYKDQDYQALQKECKRKGTLFIDPEFPPDDRSLSSTPGKFGNIVWKRPKQI

CDDPHLFVEGASAGDVTQGRLGNCWFVAASSCLASVKEIWHKVIPEYKSQEWDAQKPD

>SG-AL_contig_4667_1_+1_1054_1224

MHTNIPLQCSHPASREREKTEQTVQLIKPYQPSPFSFPFQGNLKMQGSAIITPSPLY

>SG-AL_contig_4668_1_-2_1412_1777

MPLKNLFQAHRSYCKCLEYQMMVYIHTYYAIAEGEWWRYQVKMVAVFSQKGGGIQSKGCW

YQAVLGDLLTFLELDSENRVIYPDIHLNTNLHLSQVHFILNPFTPSPYHQLPCRKLVKRV

RE

>SG-AL_contig_4669_1_-3_1305_1448

MSTCTYSVSVLSLLTPLTTHCCANHPHLLEYASAINITHALLCIKKKK

>SG-AL_contig_4670_1_+2_650_799

MFKKITLYNLLKLRLHTLMYIQYVLDCLYTELLLYGLLLSCLHLEEKITF

>SG-AL_contig_4671_1_+2_2_103

FTCLWCDIASCLSIFTYLWCDRASCLSIYMYFVV

>SG-AL_contig_4672_1_-2_2_259

CWGFCLTFFFKLSLSLSLSLSLSLSLSLWIDFVRLKLYCYFFSVLFFALQYTLFSRCVFL

NILSSSYLLCKSINYYFVASFYRIGY

>SG-AL_contig_4673_1_+3_3_263

GYRAASRFTSNTLRLGIAWCPDALHLFQHTLLTGSTFHSITRGLGWDQSLWRLTRGGPTG

VLAALVLSLAVAVITAGVGADTHLVLS

>SG-AL_contig_4674_1_-1_1_162

CFCCRYCFVTHIVDLTFCILVAAFVADTVSLLTSRSDCSFSHSGGFIVLLLLFC

>SG-AL_contig_4675_1_-1_1_162

CFCCRYCFVTHIVDLTFCILVAAFVADTVSLLASRSDCSFSHSGGFIVLLLLFC

>SG-AL_contig_4676_1_+1_46_231

MRTKIIIQSDQNNTKRNISHKSLSFTSSSILGNSLNNTGNTHVCAETFHATESQRQILKP

KQ

>SG-AL_contig_4677_1_-2_2_160

LWDNLDLALTQSDEDCSMLVFCFSVFLYEHALTFVETLNMTSWRGMFIYSFIH

>SG-AL_contig_4678_1_+1_1_591

SFSAPGVKETGTSGRLAGACVTRSGRNSLKRSATEESLSGVLSERLLRECLLRERLPWEL

RLRDLLPWDPLLWERLLREGTLRERLLRRWREEEEWASSDEGEQDEECLRRCFWAGALSL

LWSFLRLGLSLPCAGPSVETCREVWAGAVPGCVWSGRDVGACAAGGAGAGCMECSMEAAQ

GLGQPQKDGKSRHVPCD

>SG-AL_contig_4679_1_+1_1_186

EREREMKQSTKQTTKKQPKMNTQATTLHPCYHNTSHGNFIYNSHQFYKQPYIQFHNIARS

SL

>SG-AL_contig_4680_1_+3_135_407

MYKFNQKSQKLITTSDSICTMNKAYHQAHSEANCIKHFPHMINTTPFVFAMRKECKLASV

LCMQNCHGRNKCAVTFTIRVLRGTVILTKYL

>SG-AL_contig_4681_1_-1_1_162

VSSREVFRKKTTKKPQKTELTVRTQLKHVHTVMLFEIFVIRGNNCFYYWYWYIV

>SG-AL_contig_4682_1_+2_149_304

MTKISNSITVCTCLSCVLTVNSVFFYIFFPEHFSTGNIPFYFFFLLSIKKKP

>SG-AL_contig_4683_1_-1_1705_6108

MEIEEALKEIQGSLDISAMDLSSNLMDSSANDSSTDTCTKTSDNESTSPDDFDLPTGPKR

RRMNTDTLAAVSSGSGSPLLLSGDSSMADTPTSSPAGTLPRDFRQRYKKDELWKAIESNY

QYLMDKEIIETCQSTKNAQEEDVVPNVSFTEFLKQYQELIDWLNQTQRNTQRTVTSLSEK

YLNQAYHEEMLEKCPRREFLTKYSQQLLVRYPNLGDQVTMRMQKFNTQWALVEAAITPQQ

GEANMQSMMQDLECDLAALRRWLNATESRLLPLTIKADWTDEELEERLRHHQVLQQDIES

HSRIVSAVLKVSERVEREASCSSGSDCQSLQLLALNLERRWHCIWLQSLEWQCRLEEAIN

RRKGLYGSGFNFSGFTLPTLDESCISGSSDLDLKSNGNITTSSNGSLIELSDDSLLFIGT

RTEHHVGDYSSSGPASPHSLTLPLHSPTLPSPKLTNFLDRSSGDSANISECESKMGDSPR

GVPLSQDGVNLSLSMSSDGEADIRGQWMKARQEAKDIGYSSESQSNDETESMQQQLVLEC

RYPGIIEVCSDSPLGEKMTLPSPGSVRQDYYCMTHVDLDSTDKTTTDDGSATDDRESQAG

RHVGSRIQEGPGNLGLVSQGAVVTKTGFVNQVDTTRNFSPRGSHEDITVKSYLESSDEVD

GAPLTELSAAQRGNLSESDSAAESHDKIRYLIDHAEELVKTSPRSTVPGSPQRKMSPSKQ

TQTVITETSSVESSCDASSENSEESGAEEFSTATDDADETLFDSVINLDSTVDSLTSRED

MSLLIPNSITVDSARLRKQTGPRGGKKDRPWSVVGVQDLKKMDFQPLSTSESAIDHMRPH

TDTDSSSSQFVPPRHASTFPRRARHRGYQRAFSACEQSSPRATKRKLKYSSSSSATDSQA

SRSSATQVAASTEDEDDRTFVYDSAEQLRSSQGIYTCLVESESETTDEYVTASMDEVVTS

ADEDPHNSTGSFSETAWDNYQMMYPTASEDATEEVLHWEPVDDMEFDDDFNFSFPKQSSL

LSEAISKRANKMRLRGTQSGEDSDSDMEDFRSVLEDSEICLKVVDHSLKKKRNNPMGTGL

ASPGKYVEMLATCETHMNCVKDVCKHLTQEDIGLGDVQRVQDLLCQWEKLHSVATDRHQQ

SQQLTVVSEALTSMKMTLNSVTQCLNFDLFTSKEELEATIATLRVKQEELGQQSQVLGEH

RQVIQSFVQQHPSVNVTKYLEELHTLYKATTHTQHRVDSQLQSLQKNWAVWCEYLEGQRE

LDTLMAADRDRLYALVCQREVGHRVTRIEVLRELETLQSNLCVYESKLSVLQAMRQRLTR

VSDDDTQRSFLASLADLRNQLHVVSQRCRQMYRDMEEEKACLHADQSHIRSLAMSPSTLL

ESHTAAHTRSQLDGYDAERAVEEGGGHRQGGQGRAWSWLRSLPVQVVALMMVAGLVYILD

PDLLADLAGFSGLTLTPELRHEKGPSGL

>SG-AL_contig_4684_1_+3_1965_2276

MMFLMEGCFTKLVTHEHSSELQEISCSYRKPGCLILGIEPVKVDCCGLPLISKLKSISMS

VILAKHMFHVHRHFPPLKNKTKKPTTEGIKEEEQEKERTEQKKK

>SG-AL_contig_4685_1_+1_865_1014

MAPYQPGSSNLPCITQNILIRLTAHSTLQMPMGDRRWLSVSYTETRSINK

>SG-AL_contig_4686_1_+1_37_330

MVICLDWNELDWILFFFITTEFFRVKYGLLSAGKARRQNTAPPTFFAALFFLYACIFVHL

SGGIFYGILPGTTPLLPGVLFRALSACCTRELGSHPKD

>SG-AL_contig_4687_1_-3_57_266

MILIAPFWQAHPWFPDLRSLSHVPPRELTPSPDVLRDHPCESRDATPARLASVRQLLPTL

YDSTSRPRVP

>SG-AL_contig_4688_1_-2_716_967

MSRACNHCAVQNFNLKKKVHLYIYFCVHFKVDFFLKQNLAGMITLWPWALRVCVKCVLHS

GHPSSSHLKNKTRTRTITQDLVEG

>SG-AL_contig_4689_1_+2_278_457

MFPKYLVDKNITCSLQSVTDGMYLPNTWENRTKTLFLTQCYTTSKSSRHVNLCITRAVTL

>SG-AL_contig_4690_1_-2_164_517

MVAKVEADRLLNRNQNCCVCEDVAAISLCLDCRDMLCSSCVKVHGKLSSLRHHKVKSLVS

LTAEELVANPAPSPCANHPDKSCELYCTTHNVSVCHVCATSRHRGCPEVLDLEEKAGE

>SG-AL_contig_4691_1_-3_231_371

MNKETKIIQKQVDDSFVLRSLQEFLEGDNLKVKSETCHRILDIICFY

>SG-AL_contig_4692_1_+2_179_415

MFCMCITHEVGSDTCRSAHMWIWEMGKISTLYPSAKVSEEVRTQTFQTPQFGSQVSAPFD

HHACQIPTSRYLLPCQIMY

>SG-AL_contig_4693_1_-3_18_1679

MARFDPPQIQDNPEGWGPCVMPEMYKDLPYQPFSKEVRLGKVSDWTGATYQDKRYLGKYS

SAFGGSQYAYYHEEDESSFQLVDTARVQRPIYQRGRRMFQQTKQRRERERRQQQAQANMQ

VLTKTQKNRERDRQRMLKKWQKQFGKQMQENRNKAPMKHRDASVQIKDDWPVVEEMDFPR

LTKLNLPDTKDAEDLMRCGAMEVYDKTYDRVTTKNEKPLKRINRIFHKVTTTDDPIIRLL

AKTQGNVFATDAILATIMCCTRSSYSWDIIIQRVGKKLFFDKRDDSDFDLLTVSETATEP

PQDEGNSINSPRNLALEATFINHNFSQQVLKPGEDKYQFENKNPFVQAEEETEVASVGYR

YRKWDLGNDIVLVARCEHDAVMYGPNNEVQFINVKSLNEWDSRYSGGVDWRSRLDSQRGA

VLATELKNNSCKLAKWTVSAILAGSDQLKFGYVSRQHVKDTAKHVILGTQQFKPNEFAAQ

INLNMDNAWGILRCIVDTCMKLEEGKYLMMKDPNKPVVRIYDIPDHTFESDDEDESSSDD

DEESESEEETETAK

>SG-AL_contig_4694_1_+2_470_568

MSPNTTILRVKLRLKILRNGLYLIFAFCFHYSF

>SG-AL_contig_4695_1_-3_18_1865

MAFRLSRVSAPLPPDKTMELGHHILKAHIYKSVGRQRGYSSRDMQAFWMCLSSHSLSQAL

VTHRNVFSPNVKVSRLILLAGGNPNVRTEFMNNAPLLCVASKEGFSDMVTLLLEFGADVN

MTSDTGMSPLCYAASSGHREVMRLLCVRNSRPSLQDKQGQCAAVHAAMSGQLDALAFLLQ

LEWRPLQGEPGRHDATLQCMASAAAMGNRHIVEYLHHATRGEALDVVDTLLGETAMTAAC

LHGRKEVVQYVLEEGADIGAANSKSFPPLLCAVKAGKWEIADTLLLAGADMEQTDKYGRT

PLMIAASEGHLGVLEMLLSKGAQLERTDKEGLTALCWACLKGHVTLVQSLTQRGADIHHS

DHSGRTPLHLAAFYGDAQVVQFLMDKGAQIEHVDMHGMRPLDRAIGCRNTAVIVCFLRKG

AKLGPETWAMASGKTDVLLLLLNKLMEDGNILYKKNRIKEASQRYQYALKKFPREGLGEE

GRTFHDLKLNFLLNLSRCKRKQNDYSGAIDLATRALELKPKCFEAYYARARAKRDDQQYS

SAQTDLMEALRLAPNNREVRRLLTRIKEECTQQVSRYESGLPSHEMDRISEDEDDSSLAL

EVPSQNTQHRPEETAL

>SG-AL_contig_4696_1_+3_1131_1598

MTGTTAKLVTKSDFQWKGQRSTLPGIAQQVKLGDFSSLQELPMEKSEVNPAWWCPVGKGW

RFFQSPKSIYVYTHAHVKYPISYVQWLMEIRKYHRVIRHRESVPQNRCSEQKLRRRGHLE

PQSDSQPVRESTARCSLFLVHTVCVSLDKEIQQQQG

>SG-AL_contig_4697_1_-1_169_615

MRTTFLLSLATFACLAAVQGGMVQEACSTMTCAQCYQQNSGLLGQFKPKCDANGNYAARQ

CHGAECSCVDSSGVQIQNYRAEIGDATNCLCARDKQAYHLIGKEFLCADNGSYEPVQCLG

SVCFCVDEKGEMKPGSATVSVGNRNGLSC

>SG-AL_contig_4698_1_-3_597_854

MLLKHIKESLEQKSCPWLHSVQKSTFKDKQKGLCVFWGLGLLLFLSWNKRAVLGYIMYKN

PLSKVNGNVSVFWGFGVLLLLFCFVT

>SG-AL_contig_4699_1_-3_75_1469

MMAAFAQLGLDDWLVQQCKSMGLKQPTPIQEKCVRPILEGQDCVGCAKTGSGKTAAFALP

ILQKLSEDPFGIFALVLTPTRELAFQIADQFKVLGKSINVRVTVITGGMDMMKQGQDLAV

KPHIVISTPGRLADHIESCDTFSLKRVKFLVMDEADRLLDDGFGAQLKPILSRLPAKRQT

LLFSATLSPTVITLKDTVLKKPFEWIQKSDRATVEELKEEYVLVPASLKDAYLVHILDQW

MQKNPKSSVIVFAATCKYAQVLGMMLDKLQFSCAVLHSMIPQRFRLSSLSRFKSNQTRIL

ISTDVASRGLDIPTVDLIINHNIPNRPRDYVHRVGRTARAGRGGQSISLVTEFDVKLIKT

IESHINCTMTEHPTKEKEVLEIFAEVAVRRREAEIKLDGEDFGEQKRINKRKQLILQGKD

PEEEEEERKKRKKTSGKSREEVKTAKEQRKEKYKAIFAKKKKLKR

>SG-AL_contig_4700_1_+2_242_343

MPRYRQLLVYPKLQVSWMTGAAIISCRSRTCPLA

>SG-AL_contig_4701_1_+3_3_86

VCQTGAVRSGCDKFPLGKLYSTTNIAIN

>SG-AL_contig_4702_1_+2_638_904

MPISLCSSSSLLSADVQDIVSSDESISIFIFQLSIDILFCLFQCNVHIAIQACQHTSIIN

TGVEFHHDRSANNQFQEVSWCLLLAHVDS

>SG-AL_contig_4703_1_+1_1_201

RRRRQRRSRRHRRSRQSRGEAGRGEAGRGAEEKVKKKKRNEERQVRKYTYHLKNVLKARH

KNLKLTT

>SG-AL_contig_4704_1_+1_973_1254

MFLGASIMRAVLIAFPLAALPSPTLSRVRFPLTAGWIEGCMQFFSTYTLTRAPYYKTHTR

QNGEKKDKRKNKEEDEKKKKKEEKLGVLFSSQVG

>SG-AL_contig_4705_1_+3_3_128

NFVVTLHVLNLVLFIFRLLSLFALKCLTQAVNENRFEPALFG

>SG-AL_contig_4706_1_-3_3_140

SVVIIVSFYRYIMVILQGRALTSSLWFEQLSLELIAFFVLELNCLQ

>SG-AL_contig_4707_1_+1_1_165

FFLSKKSLFKVSLELLSSIFIKTTEDDPNNLRYAKGKHGFILFFYNRSVSVNFMV

>SG-AL_contig_4708_1_+1_394_672

MCMYTCACMFVKCTYIWGLQFALFNSQSFTFFSALMTLTHTYTHSLPKHTISLFLITHIT

VISYTTTVNSFYALDKLSRLITYEKNECIIYNR

>SG-AL_contig_4709_1_+3_3_266

THTHTLSLTHIHIHTHTHTHTHTHTHTRAHAHTHTHTRAHTHTHTALNDTKLKLSLNMNT

ITNEGKAKNISDKQTTLHWTVIIIYIFK

>SG-AL_contig_4710_1_-3_141_542

MKDIEAFENPYSTGQTRLMNILMQLRKCVNHPYLFDGVEPEPFELGEHLVEASAKLVLID

RLLKWLYISDHKVLLFSQMTHMLDVVQDYLGYRGFTYERLDGSVRGEERFLAVQNFNQND

ETFVFLLSTKAGGQ

>SG-AL_contig_4711_1_-1_136_735

MTGFYDYSKFNFLLRVSSYSRQFEHIVDAVQVVLTLDLAFEEKPTVRNFSFKELFPVLVF

RSLLLSGVSAVARLTIFNCQNGIHNATDPRTHKIIACAYGFSWSKESRKDIPDHHFAFWS

SGLCVGLCLPAVLTDHVHSPGRCPPLLRSNTAAMEHVPKKPTAVAEGTATVWHWRQSFYP

SCPCPIYPAKIKEKEVDTSF

>SG-AL_contig_4712_1_-3_72_173

MLPSSVSRTLNQPVSMGRRYKELSSKLLTLGHCR

>SG-AL_contig_4713_1_-2_2_175

HASDVRLGHVDVKNFAIVIICRIWVLTKSPLVVLDFAEYGILLICACDNTYTCVIIMK

>SG-AL_contig_4714_1_-2_20_559

MGGLLSALKGLIGKTEMRILMVGLDAAGKTTILYKLKLGEIVTTIPTIGFNVETVEYKNI

SFTVWDVGGQDKIRPLWRHYFQNTQGLIFVVDSNDRERMGEAREELNRMLNEDELRDAKL

LVFANKQDLPNAMKTAEVTEKLGLQSLRNRDWYIQAACATSGDGLYEGLDWLSVQLKRKP

>SG-AL_contig_4715_1_-3_3_287

VFVSLSLLCVFVGVFVSFLLLCDNMYLAVLKVILGVGGGILVWLYCTHFPFILFLLSIFA

CVWLLKKKKKNFFFFFFFFLFYFCVFFFFLIIFFL

>SG-AL_contig_4716_1_-3_87_2711

MTEEKKERGTFDDSAFEALEKDFQETLAELMGDRSLEKFRVEYEKLHKALKKSHESEKRL

MQKCRELNAEIVANSAKVATALKLSQEDQATIASLKKEIEKAWKMVDAAHEKETRARETI

QSLKQEITNLSKLVEQGAGLTMGQEHSVNELLKMKEDLTKERDEQLQEISRLREDLSEAN

TKQTDLEQKKSDAEEKIQELNQDIQVRSNEAQRESRKKDKMERELKQSKADLDAKNSEIK

SLTSQIEKHKSENQKLEQQLKEQKVLNERVLKDVDISSTRLAKLQTDFENQVLAADAMAS

ENQTRVAELKQKEDEVNGLKTETQKLNKMREMIQRKLRQVEDQKGDIERERETLRSQITG

LERELDAAKKQAEADKKAIEDLVRERDILNKNLLKAAGATQKQLHLVKLHEQSKKNLEQE

IQNYKEEAQKQRKIIYQLEKERDRYINEASDLTQKVLQHMEDVKVREMQIFDYKKKIAEA

ETKLKQQQNLYEAVRSDRNLYSKNLIESQDEITENKRKLKIMNHQIDQLKEEIQTKEAAL

VKEQLEHQRVEKEKETLKAELQKMKQQAAESKAYIEAQEAEERKLLKIIAEADAERVRQK

KELDQVISERDILGTQLVRRNDELALLYEKIKIQQSVLNKGEVQYNQRLEDIRVLKLEIK

KLRREKAILQKSVANVEDLRREVYHIQRELLRERTRCKALEEELENPMNIHRWRKLEGSD

PSTYEMIQKIHTLQKRLIQKTEEVVEKELLIQEKEKLYLELKHILARQPGPEVAEQLQIY

QQTLKEKTKQMKAMASELNMYESQVSEYKYEIERIARELQEVKKKYFMQKKKEQQVKERD

RALAQAGAPPIQSQRADQPRFTGGGFNLKTTKTAA

>SG-AL_contig_4717_1_-2_2_931

RQNLELKEEISRLTSAKEECCVENTRLSKQLEESEHLCISLQEQLKEFLEPSGPINICSP

SILGSSEQNKTDLEKAGEDLDIKKTVICNSKGTQTDKVSETKKRDKSSRSRSRDSERAEI

QSLTQKVKTLQEQLKMLEAENLELAEKSARSHDSSSDEDDPLQPSIKHKPRQKVKTLERQ

LQVLHSENIRLADKLGGYSSGTRLDPEEVIERIVELEDFCTDLQDRLREAEGHERQMRDK

LRVAEQTINELENSEALYRDRCDELSQLERETKKQLHSLQTTGRELREIIMDKDILEQAL

KEKVEFLEKV

>SG-AL_contig_4718_1_-3_3012_3356

MCLAHQKRRRNEQQDRNMHKIDVSFNVHAPRFPRLMEDTSHPLHCGCLFHFFDCSYCDYV

VIFIFPSFVICSWTCRRIVNVNYSLYFASFVAKAYIYIYIYIYIYIYIYSLLGTE

>SG-AL_contig_4719_1_-1_523_3474

MNTVSMMVGMVATAAWWYGWLWPLIYVSVVVLTAYYTAQWNLVRGKFYQPAPPPSHITVA

SVMLQKMMEKDAAGSGARHSVPRKTIISRSLDNALQEILELICRDFILNWYRGISSDSAT

LINSVQCEMWQLIDNISSRLADIDVVTVATQDVVETLYYHFKSIRLAQKRDGDQGEGTQN

PFLLHPWLRDEEREEECLRQVTEALLLILLPPAYARCDGHRHLLREIISTSVFQVTINML

CDPDYINEKILAYIKQQEKLMADTKRTYTYAASYEDFVKMINVSDSLDELKQMRYKIISE

IMQATTINNLKKAQGINTDKERSPKSTAKGELLKARNLKRYINQLTVAKQLVEKRMQGLG

GNGATQQSPSSDQDVTFPGQKVLSFAVIMEKPKAREFFMRFLELEGKDFMLAFCIDVEAL

RSCNKVEQHQVGSEVYQHFVAKSTRPLPIDKAIIKGMETFLRGDGGPEAFYEAERQVKEE

LENQHYHSFFVSDLYHQYVSTLEEEEDEEEEEEELDNLSIVSNELFLDDKGEEVEEEEEL

LADQSYHARQLLRQLDTKITNKTQAMQALQSSHKLEKDKVKKLYEDLEQEVEKMRSERRE

LEVHIERTKLWTENIGHWSVTVVSASVEVEEERKVPQFVLLVSLDSRPQSGSGEEGSKKD

LRCLSIPDTDKTSTMQKSLDSPQGWAVQRTLDDFYALHDRLAQIGSWLQKKDLPSQSRGP

FRSMDAAYLEKAKTTLNEYLMAVMQDEKMIHSETLYAFFAPSPQFLHQPTTQRRNKFSVS

LLKRSLPSLRTETSDEDDFLFFSEDSGKEDRTHDSIAKPLYGLLGEVFELRGMFKWVRRS

FMTFVELTFGSNINRQLREMVKWTYSEQMLLFYMRTFKESMWPGGKLADNPPAKTEAQKF

ETRMAAKNKLLQNIPDALKNLVGEDNARRGTIKVFEVLQNKTLNKHLIYNLLEIFLLELC

PELKKTRQTLRESVQQPPADTVSS

>SG-AL_contig_4720_1_-1_217_1902

MKQEVELIPETDEHFKEELTLDISREEPELEEVQFQIDTSVVDTDVSFKETIEVETEQVE

LADETFKQEISLQPQVQPSEIQVELEVKEEEAEMETTPPSIETLMEERPQVETTPSVATM

PEKEEEKPAEVEQLISEQLPEDVVPEEDVEVQRPREETTEEIRETITVAEETTAEVTQLQ

VDITETEEAGIEISEIERPQEEILEEVRESFDVEEKVPSEVLQLQVILDEQEEDMQPIHE

VAQPLEEITEEVRETIQLETEKPSEILVEIIPKEGSPKELTAAEVERPDEEVTEEVREVI

VVEEERPLKTEQVEIVSEDLEAREITTETEVTEVSVTDMELDTTEITSVQKESVFQVELV

QEDIETPHTEEVSIQISPEQPQEDVTQQISDLVVVQEEKPTDITALETPLEEIEESAPEL

ETLKPREEVTEEIRETIELDIPISEVSEMQMEVTPEEVGEVSLEIQFEQPQKDTVVDTSS

LVTVESEKPSEVEQAEETVVEDIPQEEVTTEVTRPSEDITEEVRETISVSTQPFQVEETQ

AEVDKADTEQVPEDTPVQEVTE

>SG-AL_contig_4721_1_+1_61_300

MFRTKKLMFGMFIMKMRMNHQKSLGIRMSGVGKVPETKLCDKALCSPSLLSLVSLSFRFL

SPSPYTQKPLTTHNGQPFML

>SG-AL_contig_4722_1_+1_1531_1776

MNKASNAGPTGYFSSAGFNDFGTFPRVVMFFECFSLAIRILCLLGILFQSKVLQQLSDMT

ISPRNLVLYCHDTLSASDLCKE

>SG-AL_contig_4723_1_-3_45_1388

MARVRHIVLTSRLRVLDYFQDFDPLRSGSISKGRFRRCLATLGLSKLAQHDLNDKQFDLL

CAAYQDPKKEDQVLYTKFVADVESVFTQAGLEHSPTTTVPPQEVFRVPKPGTVDWTEADD

GHSDLYEATMTRIRSRVEQRRILLKPVFQDFDRHNNGHITRVQFRQGLTMLEIPISTPEM

AALEARFFNDVGANYLAFLKELEPQEPPEFMYVKRLEEVRQTNAKGALPELHAESDLEKI

LLKIKTKVSKERIRVEDFMRDYDKLRSGRMMKTSFRRAVKLARLDLYESELAMIEDKYQS

TVDPEYIDYKRFSDEIESIFTTKHLEKMPLEEVDPFKPPVEWELNAMSPENNEKFMRCMA

KIKEKVRKHRMQLFPLFEDYDRVHNGSVSRPQFRRVLSELQLESMVSHEELELLWQQFQV

KVGGKDDVNYIAFCEMVYHMAGFEWRKP

>SG-AL_contig_4724_1_-2_2_100

NNVFGCPALYCFCFSLLLFSFLNSYFSFCLDIN

>SG-AL_contig_4725_1_-2_17_1411

MASHFDTMAILLVAFLAVLTPVFGDTPANCTYEDIKGTWVFSVGPGGNDRSVDCSNFTGP

AHKQVEITLYYPDIAQDQYGNLGFWTIIYNQGFEVVVTGRKYFAFSKYTASKQKTVSYCT

EILPGWSHDVLGHDWACYSGAKKGSEAQSGIKVSAGLPSKVLTFEPRIFHNDLDLIKRIN

QAQTSWKAVAYPHLEGVKTEYLLKMAGGAASRTVQRPPVAPLTREVFEAAAKLPADFDWR

DVDGVNYVSPVRNQGGCGSCYAFASMAMNEARVRIMTNNTHTPIFSPQDIVECSHYSQGC

EGGFPYLIAGKYAEDYGLVLESENPYKGVDGKCSTPASVSRYYSTKYDYVGGYYGACNEA

LMRLALYENGPLAVGFEVYPDFQGYSGGIYHHTGLDSGFNPFQLTNHAVLVVGYGVEKST

GEKFWITKNSWGEGWGENGYFRIRRGNNECSFESMAMQSTPIISL

>SG-AL_contig_4726_1_-1_22_291

MGSVPPNSHQSTSPSSPTSSSSSSWGVGGMVPRLAWLLLAQTVLSMAVTDSGARDWAHTH

TRPAQPGVHLLHTLHCIDDDTGLTELCDHQ

>SG-AL_contig_4727_1_+3_561_758

MANKHSTSKPVTQINVSSCDYSLKMSLTELLFTACSSYITIFVAKCTVNLKVSLTVLHIT

YNNLCN

>SG-AL_contig_4728_1_+2_2_151

GMDKHLARKFRGLDKHLARKFRGLDKHFAREFCQQDNCLGRISLTGHMFS

>SG-AL_contig_4729_1_-2_8_808

MFETMTSAFIDEFPHLLRNKKVLFTAVMCCIEFLLGIPCVFQGGIYVLQIMDWYCATFSL

MLLSFTECVVIAWVYGVNRFYKDIELMIGYQPGMYWKVLWCFITPATILFIWFFSVSQLA

PVTYGDYQYPTWAIVFGWMLGLCSLLPLPIVMLISIIQEKGTLWDRVKKLSRPADNWGPA

LPENREAYLASLEPHRRPKAAFEIPSPIYSPKKVVTSSGASVNVLMPEERKLLSDSVTTV

SDRSVVGQSTEFTSMLSDGQTPPVSKC

>SG-AL_contig_4730_1_+3_3_182

FFSRLFLFIYYFFCNQSILTVYSVNRWWGVGGEQEGMIRTMKKGTVNEKQTLHHHRVHIT

>SG-AL_contig_4731_1_-1_40_327

MKKENTFVSVACSSPQCQEVLDPVQGWEGELAKERRWISPHLRKVEVTTTLSTTLSTMLS

TTTSGSWNSLQLFMTTYPAALSERTSGKNPTSVFMN

>SG-AL_contig_4732_1_+3_132_248

MQQKGPRVSQPAQDEKLGTPEGRTRDAWIISVRSSQLNY

>SG-AL_contig_4733_1_+2_494_778

MAERKHFGSFILTVSFKKKKNRKLSLFSLPIHLSINPSTYLSIFSSYTHACMHPSNVICL

LMYVCIYLIYSHTHTHRLKQNSEESTAWVKSGREL

>SG-AL_contig_4734_1_-2_653_778

MASQDYWKMIVQRSMRVLVKECLEIWMGASVCVCVCVRVRVC

>SG-AL_contig_4735_1_-2_59_1807

MASNVQHVLLLFVGVLLALGSSQTVHKMSVRSDIRFRFATTEMMCVMSNDEYVAKELKFD

VTLPKDGFIANFSMEIEGKEYPGNVKEKETAKKQYEHARRSGHSGGLVEQSARHSNKFDV

SVNIAAGSNVTFTLIYQELLRRRFGVYEHSVYIDGKTLDNIRDYSIEVYIQDRNEIRNVR

TPVMRKEITNNAVEEPQNTITQITSPAGNIVYVVYRPSVQELQKQGPGWFVVQYDVESDS

AGELLVVDGYFVHFFMPPDNLPPLPLDIVFVLDISGSMAGRKMDQLKDAMRIILDDLRET

DRFNILLFDGNVQHWKRSLQEASKSSVIHAQDYVKAINYRGSTNLHLALMKGLELLQGSR

EAARVPMIFFLTDGEATAGVQNSKTIVSEVSQKNEGVVNIYSLAFGRGADYKLLRQISAK

NKGFARKIYEASDASEQVSGLYREISTIVIRGLTISYHSTSNNDGGDDANEIRVDNSTIT

ICELPTVFGKSDFVVAGKLENADALIRTPGKAGGLRLAVSGLGAEQKINLVVEENDLSHL

ILEGDNPFWTVPRDLSKMAERAWAFLMIKQLLEEKVAVQEEAS

>SG-AL_contig_4736_1_+3_57_263

MKQYTDPGIVHRIQEHNIMTNTHLQKTFTNPTLSLKHDMIRTSDAWHDILWLPSIKGSER

KKNYRTQTL

>SG-AL_contig_4737_1_-3_183_395

MMGSFNKTCLVSNAVYLMCLLLEKKNNPKHNTPFICIRIDTDFIILYLAPYTVPCQTSCQ

LVCSAGPEFRG

>SG-AL_contig_4738_1_+2_365_598

MAGHGSEGNTPSQALKGGKSADWLADDGAPVSVLNLLLATKFYSQWSKPAGLGLHKFNMI

SILAKQSSEKLPIFEKGG

>SG-AL_contig_4739_1_-3_75_221

MCVFIFSPRIIKKSRMIHGLGSSMFVIHENDRISQVVMRIRSFKPFIVF

>SG-AL_contig_4740_1_+3_3_80

KIFKKKIKKKKKILKKKKKKHAKYIL

>SG-AL_contig_4741_1_+2_179_316

MKPALFFYKMVHTHTHTHKDKLYTLYKIIRIRFRSNSRDTSLSTDM

>SG-AL_contig_4742_1_-1_100_324

MRIIIIIIIIIIIIIIIIIIIIIVIIINYHREDFLTILSWGRALGFLLHHIITEKVLVHD

VSGNVFGVSGWLSQM

>SG-AL_contig_4743_1_-3_1734_1997

MSLNGEIAVERLCKILKTCYQTHQHCRRILNLIHSNSVMYLFCFTVICCLLIFKNSYFRR

KKKTGFRLTQWFLSAVCCYINNIWMECT

>SG-AL_contig_4744_1_+3_3_263

QINGTIHIRIFYYFHHSPRHQPQCLTQLLLHNYYYCILFTAQPTTKAISGLSPCKVHQSQ

KDNCVEHKTSNSKTKILQKNTVHDMLS

>SG-AL_contig_4745_1_-1_76_318

MAFMTCGYIVFRGAPRSECQALDPMRTCLSCSVELMCNARAATFLTDFLSHLVHPAIEHM

CGSQGRSTRGILDMFPIDWRK

>SG-AL_contig_4746_1_-3_3_182

CLFFFVFREWRGGLAFNLSHSHLSCSNCCFVLVGEGVCLCENKHFVKPHSVTFHCLKMWS

>SG-AL_contig_4747_1_+2_497_700

MKSSGNKSKPHTFKPVKPAREQNKKRKEWITPDTWQAIENRRALQKKNSWKSNQRDFKRD

IKYNTRKL

>SG-AL_contig_4748_1_+3_2466_2636

MTDQMDSTFTVLKHKALSGTHQHKTYFFHAASYLLRQPPAKRHHHIVHIVIKGLTKL

>SG-AL_contig_4749_1_-2_2_118

MESCLLYRNMSGNAKQFVFRALMENGIDQKSLLWERGSW

>SG-AL_contig_4750_1_+3_3_275

LIYFYPLLRYTASFLHFVQYHVNHLYRVSTIMTTNLCTYHSNVGNNRLHGSSTSIFHQTI

NQNYFHPVATWDFLHKVTKNPVPCWISVSER

>SG-AL_contig_4751_1_-1_1_345

QKHREERIKKKERKREAQQQRRAEAGDDEDDEEDEEAEVLLDGVDSDDDPFQYIPDPDKL

AARSESDEDDSGGDAAEDNSEDSEDKSDDDDDDGSDGGDDDGDDDNDDDSSEEDD

>SG-AL_contig_4752_1_+2_2_289

LYCIASSCIALYCIVSSCIVLYCIVSYRIVLYCLHRLELYCITLYCLYLHCVVMCFTVLS

CIILYRIVLHCIALDRLVPHCFALYCTAASCIVWYC

>SG-AL_contig_4753_1_-3_75_530

MMPGSTNSGTIPSRVLKRPASSNSEHRQTQHAGVRSRGSNSPMSSQAKYRQQSGGPTATN

QSVQQDNINIGDVKLEQISTDELLSLASQVNEGASSSHVPPVVGQQNQTGQGPHSLNSTA

GQPVWIKQEMPDDSQSLGGHDTGWPRSRDGQS

>SG-AL_contig_4754_1_-1_55_927

MRKIEKLEADTFSKQQTLEQLRREKVELENALEQEQESLVNRLWKRMDKLEAEKRLLQEK

LDQPVSAPPSPRDINNGDTARNLVQHIDNLRGEVTGLKSQLRAAQIEHAEKMAQYAQEER

HIREENLRLQRRLQLEMERRENLCRHLSESESSLEMDDERHFNEMTKQHEGGMVRPRTVS

SPIPYGSSNQPQPMSPAGTYVAAKSPPSPMRRSSGSHPGQPFHAPMQPPHSSVSHPPGQG

TVSSAAFPFMPGTSPPMFSSVQMSSGVQRPKVIDGKFAKPTGIPSDKNEKT

>SG-AL_contig_4755_1_+1_139_330

MNQNKAVTVLMNPSDTQLKKHCFKILKQTLCVHLSLLYVSVMTLINLVFAFRYSLYTSSR

LNLL

>SG-AL_contig_4756_1_-3_24_440

MASFFSFSEWLDMHELEQPVIDVLKEAGLCEEKALLLLSESDIRELKLAKGDTLLLLQAV

KDLQTKKGCGPLCASVTVAGQSTSAHQAPRSVDELLTQFSTLNLNTTNLAGGPGTSGVIG

GRYYLKIMNYVLAQWRIGV

>SG-AL_contig_4757_1_-2_131_295

MKQILNLKKSLSLIYRSKPRLSAVHKPLENAPDHLQVLEMTSWKKRALLRERRDI

>SG-AL_contig_4758_1_-1_91_480

MVNDAEKYKEEDDKQKNRIAAKNSLESYAFHMKSTVEDDKLKDKIGEADKKTIVDKCNEV

ISWLDNNQLAEKDEFDDKQKEIEKICNPIITKLYQASGGGGMPNFDPAGAGAGGAAPGSG

SAGPTIEEVD

>SG-AL_contig_4759_1_+1_55_267

MCLSKKMYENEKHKKQPKKPKKTHAIIKFPLAKFGPKLTRRRSRWNTQGQRSMLNEAHDT

LKLRVSGNGQC

>SG-AL_contig_4760_1_+2_2_103

FPDGDVFPGFCRTVSILSLRTVLNLPATPGCSQL

>SG-AL_contig_4761_1_-2_563_823

MCITELTWIRHCLLVGKFSAHIRRLGHCLDQTCVNIHSLSKIHLIQSLTHSFFSTSEPLK

LYSLLLTTDKVAALGESTRLFTEILLS

>SG-AL_contig_4762_1_-1_94_345

MFACRANGGASGPTKAAHTKRRKQPKHRHKRGGSSLDCCGLGWDATKVEENTPNTTTKEK

VTGKTERNNLRPPLQMAGHGSEGK

>SG-AL_contig_4763_1_+2_206_505

MTGVRVEVMLSPCGLYTHMGPTRVDVSQPASTVPWDGCGVRRGARCKKDSSLFRDNMTVA

CYVNKGWVWAHFSILSLQAEDLLRWCHAKGILLSARHIAG

>SG-AL_contig_4764_1_+3_666_1004

MSLHQYFTQSITLEKLEKSAHCIDDSKNTSPLSTLHEKHGHPAFPYFTENVTHTQTTAEC

TPSAYCVFPSSPTLDKCISHVVRTLVFQLEGLGSKLLGYCLMGKGWRFFHFPD

>SG-AL_contig_4765_1_-1_1_183

QDNNDFSALLVIRRATPSECLSSFLPELIINFFPSARYSLSLCVHRVSLMPSPACSCPFH

S

>SG-AL_contig_4766_1_-1_118_1227

MEDRMLTCLTCRVAFPDLNLGRDHYKTEWHRYNTKRKVAQMEPLSADTFKLQMAAKRDLA

SKEKESQGVFCKICNKHFGSQNAFDSHTRSKKHKEMEAIAQKVGIDRSQMMTHSTRKPEQ

EEMEVQSGEAGDDDDTVSDEESWDENEEVLGLEECLFCSHISSSLEVNLRHMTTKHSFFL

TDAEFICDLEGLVIYLGELISQGHMCLWCYDRSKMFRSIRAVQKHMVDKGHCKMKHDETN

CEIYADFYDYRSSYPDADEAAGEEETVDPEMLISSGFELALPSGSTIGHRSLASYYKQNH

GRRQVVKAKTPLSKVLANYKALGWTGCIDAAVSQRARDVHYLQRMKKRFQMKIGTQANKM

QFHYREQNPK

>SG-AL_contig_4767_1_-3_156_257

MRFIGQDFLSVLFVFCYIYISSHSVVKNAILIKV

>SG-AL_contig_4768_1_-2_83_1579

MDKAKMTNYYGRALLIAVFLLALASPVVCNPAGPAERQMSYEGFQVHRLTSDDPLTLSET

LEHLEQQSMVDVWSRGSRSADVIVAPKHISYVRKRSADRGIRSDVIVEDLQSELQKDFVQ

RQRRRKRSVLDVTSRYLAYDEMQAYLYQVAREATNASVTVDSIGLSYENRNVSVIGIRER

AYNLKPKQAILIDAGIHAREWIAPAMALNIVNKLAFGRDQEARRMLQLFDWFIVPCVNPD

GYVFSMSSATNRMWRKTRTKQYNARCYGVDGNRNFGYEWNPAIGGSTDYCSDVYSGPHAF

SEPETRNIRDWLESHRNQAVAYVTMHSYGEYILYPYGSSRNKPVADKALLETLGTSFKQE

LQKAGHNYKVGNSATLLYPAAGGSDDYAKGTVGIKVSYTLELSPASSTGGGFQLSKNKIR

KVVEDTWPGFTAMANKLYTELNPQSSSGAYLGTGDNSVNGRGRIYFNSFVDGYLKRCCEQ

YSSFQYKCCTALGRSVGSG

>SG-AL_contig_4769_1_-2_962_1249

MYFVHIDHTFDILCPIISVGALFSAFVNQCTRIHFIGMSVFLLSIYICKLCVFSLYTCIR

ISFTKQMRGGSMYIFILYISSHRCPTFIFWHVFYSC

>SG-AL_contig_4770_1_+1_1_264

LNAEKTSAIWLGSRKDSSLSYKQYLRIVWNPPKFRILGVWLTNDVKNCESLNYSEKLYEI

KMLFKLWMKRQNTPLGRVAVLKSFILSN

>SG-AL_contig_4771_1_+1_46_285

MGNTHSNISPTFTWSETYNPSSGKIHQQSAGKPKPMHLVQTHTHTHTHTHTHTHTCLIFV

QIYSFFFKRRLKKKQKTEWP

>SG-AL_contig_4772_1_+2_2_157

THTHIHRANIIINVYIINTVNTFKTIVGTNVNVKQWVNKNTNKAYFTHTSLY

>SG-AL_contig_4773_1_-3_114_395

MRLTVDSEPQPTGPTDASKLIQPRDHKAKIVVWADLTVDDVDRLNESLNVDHNKQVLADI

FHLDGWREDPKQAIVMDLYYYTLQFARENNFNRE

>SG-AL_contig_4774_1_+3_693_1112

MLITSPFSGLGMGSIVTPRVMEMTFTFSVMEVLLSMHFEHVFCKPLMALSSNVSLAGLSV

SQLVQYQEDQRVQQQSVEENEGPGNLVAHDHTTHAQDDLLIRLQSSVALLSLLFVEGAEG

VEAFLILRGVSADCRIIGTT

>SG-AL_contig_4775_1_-3_153_1550

MPPMQTTSSAEAEANPSPDGADLNKMETKKSTTRKNEDNEERKGYFVVEIPYWLCGCGTL

LSIVVGALAILTMVFMTWAPKADIEDWILEVQDRVGKGGVDLFRTYDLNRDGYLSVYEFE

PLIPHLKNLTGSIEEVMPLETYLEYTPEDGEEVLILQANFTPLKLDTMTKEDILNMDING

QNTLFGLRNWQEPYRKGSAFGASEFQSLLPEDMENVPLGKPYHVLPYTGSTEQKVGSYAS

SAIHSSSRYYPPVPKGKEHLLFRLLGMMHHRPFVDMRFPPRGTVACVQAFNDKYVHVVFR

MHAEFQLNEPPLHPFWFTPGQFVGDLVISRNGSHVQYFHMRVPNEKRLNVDMEWMTGVAE

GEEGMEVDIGYMPLMEWTLNRQSYLPRSLEEALSTEPESSPPQPPDQIEWTSEVPRADIL

RLIEIEFYPFKTVMYHNLTRAAQLSKEENKPLHAVLLWGALDDQSC

>SG-AL_contig_4776_1_+3_3_170

KNNNKKNQCHVHKLINNNKTTYVTVQHLLDCTQSNVQGITNNKRVRYSHCTGHRQS

>SG-AL_contig_4777_1_+2_2_175

QKKADDRKGKKKQQETNRVIENKKCLAQNFQKVWTLLLLKEPTGGDDHITCTAAHKSD

>SG-AL_contig_4778_1_-2_764_970

MNSMDICYLTLFFVCGRTVVAWYNLVLMSFWLCHRSFFRCTLTSLFWSLMCKSIKRSLCT

CVGYLSAKH

>SG-AL_contig_4779_1_+1_1_150

FFFHDHHSRSLGFIPVFVPQSQTSKRVHMPTFVISPVRNSSPDGSKQHTN

>SG-AL_contig_4780_1_+1_796_1053

MYRNSTLVQTHKKHTSKCKAHHFHATVIVLNIARACALARTHTHTQTHTHSLTHSLNLGY

QYKLCYTNNVSVLRMRTSTAIYLQNN

>SG-AL_contig_4781_1_-1_4612_4902

MWPLTPPNCRCDHHMLVFTFDFIVQVFLMKWIALTYISRRSDTYATHFPMLSSLCLSFSL

SISPHLSFLCCVVREPLLVCHYSADVRLNHERMGLNL

>SG-AL_contig_4782_1_-3_3_230

HTSPNPSSFTRNPNTITRQHMGAIDVEPPWGTHQRRPCTAVGSLWWSTTVPVLIQGHCLL

NPLLTTVIAQSWNQTE

>SG-AL_contig_4783_1_-1_1_219

GCPIFCTTSVALISHCSLRLFHTSPNSSSVSQNLSARKPVGASDVELPLGHPPEETLHCC

RVTLVKSNSACSD

>SG-AL_contig_4784_1_+2_2_226

YISKRRTSLSRYLFLRELSTKGKNCLLQIKTKSKRNSSANSSKSHSSTLAFSIGLKSFAQ

IFWQCLLIFCTIVII

>SG-AL_contig_4785_1_-1_1_192

EGEETGTTLIMLAPRKALVSTTSRLYDVGAMEQSLPFLDFTQSEMCYGLTQMAAILQPSL

IHCT

>SG-AL_contig_4786_1_+1_1_177

LSLSTSFLRVKGLTARYKHPCLYWEETQNTHNNDNSIHSAFILNESTLQQNNHQYIYNA

>SG-AL_contig_4787_1_+1_1_165

LTLFPYTTLFRSVQHESTFLLHTLNWCALVFKLQRAQSRNIPKTMSHQQLLMKKI

>SG-AL_contig_4788_1_-2_131_790

MKFPLLVLCLCFLAFEKGENWVSTACEGYSQTLWCEIGSTILVSSANYGRTSTTVCDPYI

LFPTSQLNTNCVSTTATAKVATQCNRRTQCQLHANNKAFGDTCPGTYKYLQISYFCLQQQ

TPLYYACEGSTLILECPFRNQSIWIQHANFGRSSRSICDPNVLGIRILGNDLNCRAPSST

AIVSALCNGQQRCEVKTNNGAFGDPCFGTYKYLEVSFTCD

>SG-AL_contig_4789_1_-2_2_376

RALLLPKVSAPCSVTPQSFCTVLCYSPEFPHHALLLPKVSAPCSVTPQSFHTGLCYSPKF

PHRALLLPKVSAPSSYSPVFIKSFISIGQVWPKQSKTGTVLHKLDMVECMMLKEEFSIAI

LPPKC

>SG-AL_contig_4790_1_+2_191_421

MCTTPHTYKPYALRTLRTGLDNDKTMVNNIPHRQQQDGPLGMIRIHKAPQNKRGAEYVKP

LRLWSLTLAPRYNVYNH

>SG-AL_contig_4791_1_+1_1_132

KKINKKKSSNVPHIDLYELTLLLSMDTFCQVIPDLSVNRNPIYL

>SG-AL_contig_4792_1_-1_931_1089

MFTILHKKLTILSVVMIRKLSTRLFFQCAPPVTLTSSVVLDHRELLLKLPNSV

>SG-AL_contig_4793_1_-3_3_1007

MAKLQEVCSLPAHDERAWCVSWNPTGTLLASCGSDKLIKIWAKEGEHWVCKSTLADAHTR

TVRSLGWSPCGNYLASASFDATTAVWSRKGGEFECLATLEGHENEVKAVTWARSGGLMAT

CSRDKSVWIWEVLDDEEFECASVLSVHTQDVKDVKWHPEKEILASCSYDNTIKLFKEDSD

DWCCFATLKSHNSTVWSLAFDKTGSRIASCSDDKTVKIWQEYPPGNPEGVATKDNESTWK

CVCTLGGYSLRTIYDIGWSHLTGDIVTAGGDDAIRVFREDGNSDKNQPSFSLVASVKKAH

SQDVNSVAWNPAVEGLLASCSDDGSVKIWSLVPEL

>SG-AL_contig_4794_1_-3_3_212

AESSWRWVRAHAKRRRLRRNSRLLDMAIAKNRTMAGNTKCTVCKRPRKTRAQASLKYSTI

QIHSIYHILI

>SG-AL_contig_4795_1_+2_275_613

MTPEDAGNYNIEGFLPDWCTHLLYSCGDVPPWQVTLEFCKRCINTKLFNLYGPFCQILQK

FPLLQTILSATVVSKYKQIIQHTHRKTIKVIKLELSSFYHWNKNKTKTKNKNK

>SG-AL_contig_4796_1_-3_3_500

IWVPGHVGIRGNEVVDQQAKNATKQEQAKTRVPFTDFKPQVAAYIQSLWQEEWTCQQDNK

LFQIRPDLKVFLPSVSGYRKEETVLCRLRTGQTFLTHSHLLKGDVPPWCFPCHAPVTVKH

LLIDCRDLHDVRHKHFTADSLRTLFRDVPPWAIFDFLKEVNVFNLI

>SG-AL_contig_4797_1_-3_3_83

GSLYPTSLIRGFSFSTSRASPIFLHGE

>SG-AL_contig_4798_1_+1_1_165

RCWIQTTSHLHMCIPHNHTPKQPMTMELAFKQKNPLINVQMKPKASGSMNRTAYL

>SG-AL_contig_4799_1_-2_113_316

MYIQICIRVCVSNILTVVVKHGYSYYLFMHTKSIIAFYRYLYNHVFKSFVSFFFFHFNND

VPRMSYLY

>SG-AL_contig_4800_1_+2_194_322

MLTQSNQYHFQSNFNLYYKTKFKLIITANDCILHRITCESQLF

>SG-AL_contig_4801_1_-3_117_1961

MADNGAVPETREKMPIDDPEAAFAENELDDSKAKFINGGMDDTKVVISGSDSDASFVGLG

KEELKRYAEDPFWIRLRWILFILFWVGWLAMLVSAIVIIVLAPRCPTRPDLKWYDKDVAY

NIYSKSFLDADKDQDGYGDLEGIQKKESYLADLNAKILWLSSVLPTEGDNDRAILDYKTL

DAKFGTIDAFRAFCKKLLKAGKQVIMDLVPNQTSKNHTWFKQSQKKDGKFANYYVWSDKD

PGWKRADGSPMWLMDSERQQFFLSRFDGTADLNLTNEDVIAEFKEILLYWASVGVNGFHI

NDLEYLTENSTLPADDAEMSQTYNFADNADVVDSLRTITDALDNKPGREKLLFGTVTSTS

AEDVDLYIGGDGKKGLHMVSVQLGQLTRTISAQDLEQELAPYVAINTTRLGWRLTTSRPG

TDRVISHVGEDRMTIAHALQALLPGSSLPYYGDEIAMDSGTEVGKKTVTPMQWSKEINAG

FTTGTPWTSVSNGFETKNVDSLTAQLGDNTMMNSFQALNELRGNESLSFGKTKFCSKEDL

LIFSRAAPGFPSFVVVANMGGSTAHKFIGDECVGERPEAELVFTSHKTEHLDKVLKLDEA

ISIDPNQVLVFKFPA

>SG-AL_contig_4802_1_+3_33_731

MREDRTADITQQSEMSTIVEEPSNAVDTSANISRASRRSERSMEKSFVPKSPRHSVQPEP

IAHPEPMAHLEPEPVPAPQDTFAQIPDGIVDNPTDPTTIEPTINANLDLEGPPSVAPFSV

PPPSNAPASVGPPAAPTFDDNDEHLEAMPENEEQDEKRLNKRTQQMQYTLKTAFTYSQQL

SFKDMTRNLNRKQVTSRFYTLLVLKKHQVVKVEQREPFGDILMSEGPHFGRDC

>SG-AL_contig_4803_1_+3_3_185

ILFIAQPTTKTISGLSPRKVHQSQEDNRTDHKTSNLKTKILQENTSQDIILTIKFLNSMP

S

>SG-AL_contig_4804_1_-2_176_421

MITSVHFEKVMIDLSFSFVVEIFKDPSLVLRTRLLQRIKMVASRHDAKLKIFVLRAILTS

NVINLTSNIKLTWFCTHMYLDY

>SG-AL_contig_4805_1_+3_1065_1208

MIKICYRSAYSHARNSCYAGRVVTCLLSYTQSVNQSGIKLKELLDRAA

>SG-AL_contig_4806_1_+3_3_164

TLQVIVVVRQIFKGVQLCFDIPTRGCTSLLCYLPSSFPKFRFRKYLMVDTITGV

>SG-AL_contig_4807_1_+1_1_294

AGWSVFPSNYCCLVSLLLAGQFFDQFIAVWSVFPSIYCCLVSFSLNLLLAGQFFPQIIAV

WSVYCWLVSFSINLFLVVSFSLNLLLAGQFFPQFIAVW

>SG-AL_contig_4808_1_-3_504_929

MYRYINFHLLHNYCIPSVLCCDNCSRFIIEAVQNKIESNNMFCSFLFLTLYLSILSVQSQ

RFICLYQLLIHVSTVKSGVGRHSLVTAKQTACSRFPTYGSIFRNKSGLTSSWKDLVTIDE

GVAPDRLYSRSGCVSDFGWSER

>SG-AL_contig_4809_1_+2_134_454

MTCVAECVSPMCQKCVNDYLIASPVFIHTPHLSNIKYSHRHHFHSHSPSVPSSITTNPNI

FLSLSFNNHSFSSKRDRPHILSSLTMHHQQIQTLTFTGYSYLKTFSV

>SG-AL_contig_4810_1_-3_12_1391

MASALRKVAPALLQRAVWKSSSPLFFARGAGKVWMPDKEYYDKFNLPVMYPDESTSSWKL

PPFNEHAVVREKQVENITLNFGPQHPAAHGVLRLVLTLDGETVVRADPHIGLLHRGTEKL

IEYKTYTQALPYFDRLDYVSMMCNEQCYSLAVEKLLNIDIPPRAKYIRTLFGEITRLLNH

IMAVGTHALDIGAMTPFFWLFEEREKLMEYYERVSGARLHAAYVRPGGVALDMPVGLMDD

IYEFIEKFGQRLDEVDDLLTENRIWKARTVDIGVISAEDAMNYGFSGVMLRGSGIQWDIR

KVQPYDAYDQVDFDVPIGRNGDCYDRYLIRMEEMRQSLRIVLQCLNKMPPGEVKVDDNKI

TPPARKEMKDSMEALIHHFKLFTEGYQVPPGSTYTAIEAPKGEFGVYLVSDGTNKPYRCK

IKAPGFAHLAGLDKVSRKHMLADVVAIIGTLDIVFGEVDR

>SG-AL_contig_4811_1_+1_1_267

PTRRSSDLLHVFRLCSKALDCLLPLGKVLDLLAAHQQKAIPRSFGFFSHVLHGCFRLSTP

SATSKAIYWDIFSPLAISCRTFSPARNLP

>SG-AL_contig_4812_1_-3_105_551

MKTALIAFVAIGTAFILLDTGIAIKCFVCNSFHQADCADWFDNVTQHLVKCPDEYSKCRK

VVQEVWLGDHWDVRYIRQCALDGQIGMYEGRICQERYGSYDVRMRYCHCDNQDGCNSAPS

LIPTTGFGQFLPFVVTLIAAGMPYILVQR

>SG-AL_contig_4813_1_+2_1019_1252

MLLCEMEGMAAQHKLSTECNHSMKTSPSVHDLLHITTVTVEGTSELAGSGGHDGHLSSQP

TLLYGTVITTLHLAGRHR

>SG-AL_contig_4814_1_-3_174_470

MESSGNSKQDSALNAHAQNKSLHCTALLKAAPSSKCPWPAVSFIDFTKVFDSIHRPSLWK

IMRVYGIPPKITTARENTYSNSRCCVRTEDGLSDWSEVL

>SG-AL_contig_4815_1_+3_42_221

MDREGRKKQTTGKERKTAETKRAIEKRKLLAQNFQKEGILFILKEPPASPWGYSYNSEQF

>SG-AL_contig_4816_1_-2_221_394

MKPSIHCVNVAVHIFFCDFFSEVKRQEKSLLHLKCLQSVLSCSFLKITNHGSFVVCCL

>SG-AL_contig_4817_1_-2_2_274

AISLRISPTCWQGLEVLPSLWILYIIVYACLILLLNKTRLSAMSHAALHWRRPPRSHFGC

RTSPSPSSLFPSEVSTDVCNYIYHSNPHWIR

>SG-AL_contig_4818_1_-2_437_616

MPWSISCIPIHHSRNISTSLPVYHYLFTHHLHSLPIFHPHPLSLSSPPLPSPNASVISES

>SG-AL_contig_4819_1_-1_334_420

MCHIATHRSLSTYLIDSTYTCHIATHRSL

>SG-AL_contig_4820_1_+2_2_337

ERGSCRIHCWEKRWVFKLDLKAERVGVWRRESGSLFQADGPETAKERWPTVDCLNFLIQL

RLGVSPLKMHRLCYSINTTGYSCSLCKLEAETKVFCIHMFETHSNPKTVYSW

>SG-AL_contig_4821_1_-3_498_752

MGKPLTYYYYESLTGFICFLAVIWKCNGVQLGHPHMCGACVNVCGCVCVRASMCRYVCAC

FCVCVCVCVCVCPSVCTHLCTKSIF

>SG-AL_contig_4822_1_+2_2_214

HNPTHLHVNNPTHLHDTVTTPHISVTLSHISMTTTPHISMTSTPHIYIIIINNNNNNNKD

LFTATSWEASS

>SG-AL_contig_4823_1_-1_1_225

KISTLYSPGKVPEVRTQAFQTLQFGSQASSPFDHGAVMLLLLELLIIHNCMYVFVCCLFI

RSCSVCFIKAIALKL

>SG-AL_contig_4824_1_-3_1680_1982

MEKSSSCGMTSRLNENCQRLTIGKVKVCSTGDCFCHHQILCPVWLFGGLIGCSSRQDPLS

LGCGSKQFRHTEVGPVFDICHPALPLSLGCSNKQFRHTEVG

>SG-AL_contig_4825_1_-3_693_995

MAIFLSHHEGRHTLFSGLCMLGVFVSHQTLAWLTGSLTCIFDIVHAGRLEESPPLYPAGG

TQVSLNPDLLVGEYPNHLTVWQGLVKVCLCGSGRLCTCFQS

>SG-AL_contig_4826_1_-2_41_220

MKSTPTFTISLPAKTASHTHIRYVFINVACVVFTHTYGWSKREELRNRKTETKNGRDSKH

>SG-AL_contig_4827_1_-1_322_426

MFKSVHECPAYIGPEQLLLNTQQQYIYVISSGGWP

>SG-AL_contig_4828_1_+2_8_346

MWRSPRTALTPSSDCAFHPTQRLRMLKDDRKRPYHAVSLPRKKPQKNAVNLLARHHAQST

APCPMQSREYGHHHDERWIGHIIQIEDNITLAPEEDAREEDPRTLSVKEQKKN

>SG-AL_contig_4829_1_+3_81_341

MLKDDRKRPYHAVSLPRKKPLKNAVNLLARHHAQSTAPCPMQSREYGHHHHERWIGHIIQ

IEDNITLAPEEDAREEDPRTPSVKQQN

>SG-AL_contig_4830_1_-2_2_166

FFLIIIPLLHQVVYIQSSLFFAPFRNYVYISGFGMEAECFVGQNLIISDILTLDI

>SG-AL_contig_4831_1_-2_23_736

MADTESSDTSKASSSGTSSGSAVTKMSRLNRLRELELRMNEARKLNNREASAEEERNKRP

ENWEAKRKRSEWEEQQDQLKKRCEETGEDFEIAKLRDLGADEVERWERKKKKKNPDTGFA

DYEQATHRQYQRLTKQMKPDVHQYERQREKMGDEFYASANTLGLHQRKDPEENVDRMVQD

LEKQIEKREKYSRRRRFDEEEDVSYINERNMKFNKKLERFYGQYTAEIKQNLERGTAV

>SG-AL_contig_4832_1_-1_82_1473

MASKAKVLLAASDFISFINKSPSPFHAVDELKKKLLAQGFQELLERDSWKTKPLGKYFVT

KNQSTIIAFAVGGQYRPGNGFSIVGAHTDSPCLKVKPVSGKVRSGFCQVGVECYGGGIWN

TWFDRDLTVAGRVIVKESGSLTHRLVNLARPILRIPHLAIHLQRDINDSFGPNKENHLCP

VLSTCVQAKLEGQAVSSEESDTTASKHSPILVKMLCEELGVTAEQLMDFELCLADTQPAA

IGGALEEFIFSPRLDNLLNAYCACQGLLESLSDGSLDQDPNVRMISLFDNEEVGSQSAQG

AGSTFQELVMRRISASLENQAAFEESIGRSYMISADQAHALHPNYSEKHESNHQPAFHKG

IVIKCNANQRYATTAITTAILRQIASQVKVPLQDFVVRNDSPCGSTIGPIMSSKLGMQTI

DVGAPQLSMHSIREMGDTTSIVHCTDLFRGFFQHFPSVLDSAKF

>SG-AL_contig_4833_1_-1_16_513

MHGKILEQRTRYKAEPFLSEAQMGFRKGRGCTDAIFTLRQLSERTIEYDREMNLAFIDKE

KAFDRVNRDILWRVLELHNVRGQLLDNIRAMYANSMSAVRTQEGLTDWFEITSGVRQGCV

LSPLLFIVYMDSITKEANPDPEALNELLFAEDQSLAHEKEEQLQEH

>SG-AL_contig_4834_1_-2_38_295

MKQIYLTWGQVCCIFCCIRNEFAKIWVKICYFMMSRGWVIAHLDIPKKPKFSWTILTKYM

KYESIFLFIKTCIACPNKRDLSSPKI

>SG-AL_contig_4835_1_+3_204_443

MQVLMNRNIDSSLYAILCQSRPQKLYFLEDIKMCFYPPSGHCEMTRYFDPHFCKFIANTT

KIQQTWPHVRYICFMNYLFL

>SG-AL_contig_4836_1_-3_315_521

MILCVELFMLLQYHLFFLMVSLCDVILLSFICYFFLFYRFLCVVLYFHSNITHFCFVSSF

VVFYISIQI

>SG-AL_contig_4837_1_+3_261_479

MQSKFKACLCTANTCLLFVHTCSVVDNQQAPFPASESSKRTAKSTEPMFVFTMYKPTNTL

GRGLFAVGQGNIF

>SG-AL_contig_4838_1_-1_139_435

MLGQDVTLCIVGNKTDLEKDRHVPVQEAESYAASVNARHFHSSAKLNRGIDELFLELSKG

MIEKYDGTMGRGRTGGNNRRNVVVVDDDQAPAPKSGCCG

>SG-AL_contig_4839_1_+3_3_161

HTRTHTCVRAHTHTHTSHIHTPTLHTHTHTHTHTGNTITSVLFYICSPDEWLG

>SG-AL_contig_4840_1_-2_608_715

MKVPTIATAHYSLPPQQYTSSYYTNGTLLPDTETVH

>SG-AL_contig_4841_1_-2_2_73

LAQRQYTSSYLRDSTLVFTTETVH

>SG-AL_contig_4842_1_-2_2_88

KQYTSPYHRDYTSPYHRDSTLVLTTEMAH

>SG-AL_contig_4843_1_+2_2_331

TKTKQNKKLVQQIYSQISSSSLFSLIHFLPTILVPFSGGKQNTVNLRRVGICFKIVLGLF

IYFSSIYDVYAVFIFHVFTSLLLYLLSFFIYLLIYIQVVHTCSLLCSIKT

>SG-AL_contig_4844_1_+1_568_873

MRAVYHTPYACTLFWSKMAEADDAGLSDKVQHESDENRARRLSHVFIFENQVRKLSHICI

FIRITLVCDTCLMMESRESTSSPPFRLSLQYMALFTISVLVM

>SG-AL_contig_4845_1_+2_173_382

MNDCISDTQVSNRVNSLDYTTEHTVFTIHSQCYITHLYTSLKKNGTAHYKRIHHLLQGKD

YYMSKRGKNN

>SG-AL_contig_4846_1_-1_196_2007

MPVVEIVPKFLEKLTDEELKKESKTEAKNDALSSIIKALKQLVSRLPNQEETIKNLEIFR

LKMILRLLQISSFNGKMNALNEVNKVITNVSFHANRHQQLEEDEWLTAEKMAEWIQEHGV

LSIVLRDSLHQPQYVEKLEKILRFMIKEKALTMEDLDRLWNAQSGKHDAIVKNVHDLLAK

LAWDFTPDQLDHLFECFQGSWSHATRKQREKLLELIRRLAEDDKEGVMAHKVLTLLWNLA

HSDDVPTEIMDQALTAHIKILDYSCSQDRDAQKMQWINRFVEELKSDKWVLPALKQIREI

CQLFPEAPQNFPHTQRAPHMYYRNNVISQLQNQHSIVMLVSQNLSNYMVRAHQYAVEHPT

EEPSSVLPDGRFHHMLQVQERLRFLRFLLKDGQLWLCEPQARQIWHALAENAIYISDREA

CFKWFAKLMGEEPDLDPDITRDFFERNILQLDASLLTENGITCFERFFKQVNLKENKLQP

KRRGGCLMDDMELIGLDYLWRVVLLAPDDVADKAVNLLKEIFTNLGPRLQQNQVDIHEDF

IGSCIDRLKALFDTVTVLEKDKDCSNRLIQETIRMVRVLNVLKEYVAECDEAYGEERVIL

PLSR

>SG-AL_contig_4847_1_-2_2_199

PREWNCRHAMARLLNAFSCTVSVRPSSYQRAAQTSPSNSRDYRSLNIIRKQFAWNEMYVR

LCCLDK

>SG-AL_contig_4848_1_+1_1_105

ISPFKQQHSLLVPVIRVINTARCQHKKQLAVSTSF

>SG-AL_contig_4849_1_+3_261_488

MCRYTNMYTTKHKSTEKKYKKHSSFIKYITRRNRLRQMNTLTFFRKCQCQMVYYVRFYTH

SQRELGNGVGVVGFAT

>SG-AL_contig_4850_1_+1_1_273

SLRISHSGTLFLTHSLWISFSGSSFPTVGNFQISHSASPFPTVSRLWTSYLVCSFPAVRS

LRISCLGSSFLIRSLQISYSGSSFPTVHNLR

>SG-AL_contig_4851_1_-3_507_737

MDSSCTLNYTFFFFLLLEGRVPNFCFLVHSWFLLLVFFLLHLGILRFLLLSDVFLSSSIP

SVASYFIWSSIFIFLKF

>SG-AL_contig_4852_1_+2_86_199

MFVKLYCVYQSLHLHPFLIFSVHQAHPPTLLYYYFCCA

>SG-AL_contig_4853_1_+3_1689_1895

MNTCAAIPAACYVHERTFWILFHHNALAALVHMEFIQCQDTVLRKLKPKLNTYTFTCNCC

NQNNLYQKD

>SG-AL_contig_4854_1_-1_52_318

MEGKWLHHRIVHVLLLKSLCCTQPSPYNENSVYRVNGSSALKDRHFVTYPFFFHVMAFFQ

ILRNLSILRVMFNIFIILKAVVESTVNLP

>SG-AL_contig_4855_1_-3_333_662

MHVIIHVDYQGAKSESDFFVIVSAHYITMWRAVPRLGTQQALLRLNESLLTVLRTHQGCG

DAGLLSKVPTDSTSHGCGQVPILQQSSSWSIQQCCQSSLPLSALSSLSLL

>SG-AL_contig_4856_1_-1_124_4155

MERLGLMSFVLLLAVSFPLLVANPLVTSSSSTPTQPFVTNDNDNDTEIINNNINSSISNS

SNSIDGDITSINDTITRSSNYSSSTTPSLRPSRCQYQYLEVVRNELFDVHGENLPFFVKR

FFIANSETWCYLSRQDLENKLNNSSDAEVIAVFAFGCLNPEGTDIVFMEDEEAEELEASF

SDNQTSSGQGGDDGDGRRLPQGSSSYRYYEHVISYFQVTRCRMENLKGVDMVANMSDLRV

LSVYAHRGWDLEQLVTQPEMCRAFRNVVALSILADWTTQNYLHHLAVCADHLHNVVEMVF

TNDSLTSFPPVLYSAVPNLRALEISNNNLTFPVDFPWPSGFATLPRNLSRTYQFNVHYNF

ANSFDMPPNLFRRIYLLDGNRITNLTHFQFDSDFQYISIQRNGLKQIAAGVFDSVPSLQF

LSLAYNELEVLPEKLFARLVNLKRLELQGNRLVTLPTNIFRKQRRLENLNVEQNRLEVIQ

EGVFTKLEELTVLKMKNNSIREVKKEAFTTLSIKLKHLELQHNPLKTFPVIVLLLRGLLN

TNLQYTDIEVLDFVEISRQTSLSVLTEALKNPTTGEYVDIKDNPAFQKMISLKYSQLRSV

VVYNATRETLGIFLLAIKHFTFDMEGSRLSCDCHILNVTHQLQKWKTDGILTGEEACLKG

WVCAWPSELSGTPITVLKDNDTYCPLLEVATPGDGGSRNYSSCPEGCACYVRTKVNTVIV

DCQNGGLTALPEWVPNNTRELWLQNNTIQTLESHDYLARLHSLRLTSNNVQELSIDVLQR

MPELSELHVDDNWLTGLAPDFARLTQLRVLRLAGNPFRCDCTTLWLKDWLLGHQTITEDW

AAIKCTTEDDDGQVFTAVPNDQFICKVSASLGQLAIPIAIPLSLAFLLLTASVIAFLQRR

RLKIFLYIHTGYHPFDRDPPDDHALYDVTVCCDVTARAWVLRNVIETLEEGGRYKVFFYN

RDAFVGLTTSENVRHCVKNSRRIVVVLGKDWQQDDLLKTATKHSLVKCHKDMPHFLTVLL

HQFSVKDINDKDLQHYVHGGRYIPTDDKHFTKKLLYEMPHVKAAKAAQQQPKKGHHLQGP

EPIALYDIIAAGENEQKDRLNRLENRLSGNGVLARQVSHNEVDVDLRHSGVAQYNIQIVD

RQDSQQSDDDNPEDDSDEDNDNNDGGADGDINRQVAGNVNGNAEHHPRALQNQMSVDSHV

SHQLKTIFVWYADTDLAFTLKNIVNPLEKRGHTCILQDRDFPLGAPIQENIVHAVENSTR

SIFVLSNDTADNDWFNFAFHVAFDRHLQARDRLVAVVTREEVEIIRFLEEIRQVIKTSSV

LSEDDPWFWKRLKTFVHLDHAELG

>SG-AL_contig_4857_1_-1_226_387

MKGGNYILFKHLAQQRGNLFPAFPVLVMGSFSRVHTPVTVSTTRYFGQRNFGKL

>SG-AL_contig_4858_1_-2_113_2236

MKTASGSWGPQWLLRMSPDKQDTLLKMMVLAIAAVLSFSTRLFSVLRFESVIHEFDPYFN

YRTTRFLAEEGFYSFHNWFDDRAWYPLGRIIGGTIYPGLMITSATIYHVLNYINITIDVR

NVCVFLAPLFSSFTTIVTFFLTKELKDAGAGLVAASMLAIVPGYISRSVAGSYDNEGIAI

FCMLLTYYMWIKAVKTGSIYWSALCSLAYFYMVSSWGGYVFLINLIPLHVLALMVTGRFS

HKIYLAYSTVYCLGTLLSMQISFVGFQPVQSSEHMAALGVFGLCQIHAFVDYLRSRLSRE

QFTLLFRSLVLLSATLAFGAAAVATALGKISPWTGRFYSLLDPSYAKNNIPIIASVSEHQ

PTTWSSFYFDLQLLVFMFPVGLYFCFTKLTDANIFIVMYGVTSIYFAGVMVRLMLVLAPV

MCILSGIGISSILTAYMKNLDSSRRDKKPKKPDSTYPLKNEIANGVVLIVTAFLIMYTFH

CTWVTSEAYSSPSIVLSARGGDGSRIIFDDFREAYYWLRHNTPEDSKVMSWWDYGYQITA

MANRTILVDNNTWNNTHISRVGQAMASTEEKAYEIMRELDVSYVLVIFGGLTGYSSDDIN

KFLWMVRIGGSTDRGKHIHEMDYYTPQGEFRVDREGSPVLLNCLMYKMCYYRFGAVYTES

GKPTGYDRVRNAEIGNKDFELDVLEEAYTTEHWLVRIYKVKDLENRGV

>SG-AL_contig_4859_1_-2_503_613

MNACASVVENVGNCTVHTSSKSVHVIQILSDWFIRPI

>SG-AL_contig_4860_1_+3_3_104

KHRKQHVEILIYYNSQLQLTQSMAEVHKSLLSTD

>SG-AL_contig_4861_1_-3_456_677

MQSTNFCSCESYHAVFVDEARPDRHQNPIVGSLSILVSGFTGLFLTSAYNTFQKIIKSPN

KIQHCTVQLAATVI

>SG-AL_contig_4862_1_+1_1450_1689

MDYSIRVFRALLRIFMSVSFKYYRAGELTLNVKVILKMSASYCLTFGSSGKVLSTFIVAH

CYTRNKCMHSSGKMVSTFIV

>SG-AL_contig_4863_1_+3_3909_4478

MKCTESICCILSSIVADDADPPWVSASPGGDVVHLAFYHQPFICVLRVVFHFTPTICLQL

FPLRILDTLTTQTHFERTTFYPTAAVQLNHRVYLVNSLQLAQSKVAALSEEKHSLSPLCF

YRRSSLSKPAKFPRVPQSVCCVFRNAVCMAPMTTHGSANDSVLFQRDYGLLQTLTFGYFN

HCDSALGLVF

>SG-AL_contig_4864_1_+1_73_1230

MISGCSSSGFSFSGASSFGTLTSTVTSSVSDGFSGEMVISSLNCSCSMISGCSSSGISSS

GSSSLETGTSKSTSSVSVGFSGEMLISSLKVSTSSGFSTTIVSLITVSVVSSGLAVSWGT

SSTLSSVTSFTASDTSSVIPISKTTSSDSVGFSGEMVISSLNFSCSTISGCSSSGFSSSG

SSSSGTTTSTRTSSGSVGFSGEMVISSLKRSTSTISGCSSGFSASGSSSSGTATSKVVSS

VSLGFSGEMMSSSLKLSTSSGFSATIVSLITVSVVSSGLAVSWGISSTTSSVTVSDTSSG

TDSGTSTSVSKVTVSSPSFSGEILISSLKCSTSTISGCSSSGISSSETFSFSVITSTVTS

VSVSFSGEIVISSLNCSYSTISGCSS

>SG-AL_contig_4865_1_-2_107_205

MLIYFLFSSVLFSLFDSVVIYISTLCYRDIRNV

>SG-AL_contig_4866_1_+1_133_324

MIPRFYIKRFLENLRIESIDSIITHILPSYYHSHICYQIQTFFIVIDIILTNLHQGCYKK

KFRY

>SG-AL_contig_4867_1_+3_696_872

MVNKMSLSKLGRKISVFIENMVEKNARKSDHHNNRTVPFLWLVPGWHDRGGAMFPSYKF

>SG-AL_contig_4868_1_-3_3_122

VVDFVVVYISDGLLVIHLSSQRRIDFFFNVFMFSCRILIS

>SG-AL_contig_4869_1_-3_45_272

MHGYIIICLIKRIVFSLSVLSLIGVDVFMHERVIIQFVKGIIFSLINQPCVWAIIGVRLL

CPGVREIVESRGLYFL

>SG-AL_contig_4870_1_+2_884_1375

MYYLPAFTLMGVVEKMNRCLAGSWKTPRGVTLVSPENSVLILLLLFVEQLYNPLTACLAR

CSCLVLIYIFFLAHAWVFSCFHNPLNTDMAYRIFDVHIYLMFLDLFCMLIHTKGVRALAG

LQYIDLGDQKNPHPLPTRRGARGGFEHRPLEPSNLEIQCLHYST

>SG-AL_contig_4871_1_-1_382_639

MYVCVCVCVCVCVCIHAYTCTSLYFLVFFLDLYFNFVVRSTASCIIIYFVQLFEFRACSR

GINRTQAIPYVLHPILPVIYFMWQTP

>SG-AL_contig_4872_1_+3_3_152

PSFSVCQHSSFFFSFFCFNGSKARKKHAQQYHNMPISEMFLFNTLARKQE

>SG-AL_contig_4873_1_+3_267_485

MFIQRRKSAKRRTILSPNAYNSGPYKIIQYPLPFCLTFRCFFPFLFLLPSSFALLQLKSQ

RKGARKLIISTKP

>SG-AL_contig_4874_1_-1_64_2868

MAQILDGKAVSSDIRQKLKQEVENLKGTFPGFIAGLAIVQVGNRSDSSVYIRQKLKASQE

IGVTARHVQLPSSSTQAEIIQTVQHLNNDPDVHGIIVQLPLETDVEVDAHLVTNTIAPEK

DVDGLHQSNAGRLSRGDLDECILPCTPRGCLELILRTGIEVKGKKAVVLGRSKIVGAPMS

DLLTWNHATVTVCHSRTADLKAEVGQAEILVVGIGKPQMVKGDWIKPGAVVIDCGINSLP

DATKKNGYRLVGDVDFESAKQVASWITPVPGGVGPMTVAMLLKNTVDQAHRAAVVAGGAD

QWRLDLRPLVLKTPVPSDLAIAQSQRPKDISILAQEIRLLPEEVDLFGKKKAKVSLKVLD

RLADNKDGRYVVVTGITPTPLGEGKSTTTVGLAQALGSQVKKNVFACVRQPSQGPTFGIK

GGAAGGGYSQVIPMEEFNLHLTGDIHAITAANNLVAAAIDARVFHEATQSDEALFKRLVP

TVKGKKRAFCKIQLGRLQRLGITKTDPAKLTPEEVKQFVRLDIDKETITWQRVMDTNDRF

LRKITIGQGPQEKNMTRETQFDITVASEIMAILALTSDLRNMRERLGKMVVASSKEGNPV

SADDLGVSGALTVLMRDAIRPNLMQTLEGTPVFVHAGPFANIAHGNSSILADKLALKIVG

ENGFVVTEAGFGADIGMEKFFNIKCRYSGLVPNAVVLVATVRALKMHGGGPTVTAGVPLP

KAYTEENLDLVEKGCSNLIKQIENANFFGIPVVVAVNAFVSDTQAELELVQKLSCENGAF

DAVISTHWAHGGQGAKDLAVAVDRATQETSNFQFLYDVKLPIEEKIEIIAKKIYGADGVE

LEPLAKEQVARYKRQGFNDLPICMAKTHLSLSHMPESKGAPKGFVLPIREVRASIGAGFI

YPLIGTMSTMPGLPTRPCFYDIDIDPDTEEIIGLS

>SG-AL_contig_4875_1_-2_1976_2242

MGINSEDFIDFCLHSHVLFMESFVMRQILLADNFTHFQKVFLRCQVLYLLSLYKVSGGGR

AWISTILDILHMCAQRYTNFSLEAHSHLN

>SG-AL_contig_4876_1_+3_1173_1340

MCKYDIQRTQQQSVYILQRNTVYNAVLIFMLTWTLSLIPNLRFAVKINQPSLNPIA

>SG-AL_contig_4877_1_+3_1101_1412

MRNRKGYFCFVRVMTVVVSAANNLLVVGRLPKDGRTALVNRVRAGVAVQGRVAGKVISEL

CHCQECPDQCTVSDGWVCLCVSLCVWSSQNELSSLADQTINYDR

>SG-AL_contig_4878_1_-1_61_459

MSTLSDPRESEHDPSDLCVHHSGQIGQGATVRLQCSQPITGQYVSLVLPTDSEPLNFCEM

EVLGKPALGNEEKWTSETETVTSIPSTSPAYSSQSSTDGYKYKSEEDVGGTADTVASYYY

YFDSDDSFDWDLH

>SG-AL_contig_4879_1_-1_76_468

MSTLSDPRESEHDPSDLCVHHSGQIGQGATVRLQCSQPITGQYVSLVLPTNSEPLNFCEM

EVLGKPALGNEEKWTSETVTSIPSTSPAYSSQSSTDGYKYESEEDVGGTADTVTDYYYHF

DSDYSFDWDLY

>SG-AL_contig_4880_1_-2_671_859

MSTLYYSIRLAFNRRWQMFSHLYFGNICMVCQGEYQCDLAILCQPNKQLHFQKLKIVLFN

TSV

>SG-AL_contig_4881_1_-3_6_233

MFTKIYTGLNKSSLIVVWNYVAAVTSRTHRATVLVVLTQSMQQIQHNNETIHTITTTTVT

FIQTIFFVCTVLIHQK

>SG-AL_contig_4882_1_-2_1091_1405

MKGQAREPMKGQVIELMKGQVIEPMNGQVIELIKRQVIEPMKGQATEPMKRQATEPMKGQ

ATEPMKGQATEPMKEQATEPMKGQIIEPMKWQAIEPMKRQATNRL

>SG-AL_contig_4883_1_+1_292_528

MTVSYFGHACREFGLTISLKKTNIMGQDVSSIPSISISNYVLEVVEDFTYLGSIKSSNFT

LDTKLNTRIGKTATAMACL

>SG-AL_contig_4884_1_+1_1396_2049

MANLVKRVWDNPILTTNTKMKVYQACVLSTLLYGSKVWTLYSRQECKLNAFHLRCLRRIL

GITWQDPVPNKNVLAQVGIPSMFALLTQSRLRWLGHASHMQNGRIPKDTLYGELAAGSRP

AGRPALRYKDVCKRDLRAGNIKPTDWEAVAADRSSWRLVKAGIQTSEKRREDRQEGRRER

RQQRAETAPTEPGTDYTCRNCNRSGLYSHSRRCNSTAN

>SG-AL_contig_4885_1_+1_85_480

MFGTICEHLAQYWSTLEGSAVFSTLTYTKSFCPALPCPPCIDVVKFPSQQKLEYCLQYYA

PCTLPFTVCTFCTNLSPRCVGLFNQSTARVLVAATMMSCVSLYSPLFHFGFLNLFSYSWM

LYDGVCVPQLES

>SG-AL_contig_4886_1_-2_158_388

MLQGHSVYYTVCKGSEKEHCSCGLSIAFFFFFFFFFFTQRFLHELRDFPMKRLSIRWCFG

FFCFFLFKLHGVCLINL

>SG-AL_contig_4887_1_-1_67_357

MALCPTNLPMSWWRTGMREMDAMCDLGLWCSLFDRTVICFKICVCECDRSVCAYLIMFGP

CLSLNKQLFLCLVSCLSAYVILFGLFYSSYNLSCSCN

>SG-AL_contig_4888_1_-3_69_305

MLVALKFAKMIQTCASKYPSIQVSFRAIHFYGEDSVMQQRSCWLILGLMEGMHTHFSHFL

TFLAQCMRLTILDQVCKHN

>SG-AL_contig_4889_1_+2_1079_1684

MVQVGDGLSHGPSCGHFSTVQVGDRLSHGPSRRQTFPWSKSETDFPTVQVVDTFPRSKPE

TDFSTVQVEDRLSHSPSRRQTFPRSKSETVFPMVQDRLSHSPSRRQTFPRSKSETDFPTV

QVGDRLSHSPNHGHFLHGPSRRQTFPWSKTETDFPTVQVRDRLSHGPSQRQTFPRSKSET

GLWMFYCNKGHKLQKTKTNPAA

>SG-AL_contig_4890_1_-2_62_337

MTSCHVSVDTIYIESCIFVMPCTFPLFLFLWSLKCLYIAALAENTASKIMQLVWLWHVSV

SAVHQPCHLWERDRGWREGKRGCAWTCRGEDF

>SG-AL_contig_4891_1_+2_2_79

TCTAKAKEPEQRAYTHLRKRCSEHKH

>SG-AL_contig_4892_1_-1_1_207

SVSMQPSYLFSPCAEPYVSAWVAAKPSTGLSLWQRPHVGLLPGAQTSPAMEAYSAAQSTA

SVRLPEGLP

>SG-AL_contig_4893_1_-2_74_172

MVNSARKETFDYELQFTSSYDDRKETLKQLASK

>SG-AL_contig_4894_1_+2_701_1273

MLHESFADDTQLHQLTSIAEIDAPTSWTCECIVDLKDWMNLNKLQLNEDKTELMLACSKI

FLQHPSLPNSILINDTPVSCSSSVHSLGVTLDQPPSFQQHISNVCKVAYLELGRISSIHH

YFSTDVTKTLVCAFVLLRLDCCNSLLTGLPKYLLDFSESRITLPDSFAELLNLTTFLLFF

TFSTGCLFLIK

>SG-AL_contig_4896_1_+1_34_351

MVECMTLNQRFRQRLLDKFAIRKYIAENRQHANNKNKSYHINTPVQVLGRLILNTDKTVK

YYHCSSTDQTAIKRITLYLQHTTETCNMSWFTITIIQAEACLLIFF

>SG-AL_contig_4897_1_-3_42_308

MISMKPKDGPALTARKKLQPLKVPMETEEEHANGNDLMTRVRSVVSCNWKDMRREFKNVD

KDGQGTVNELEFRRILRQFCANLSEEEFD

>SG-AL_contig_4898_1_+1_1663_2004

MKKKSIHSLSFSLITPNMHFIESVRAHAHTHTHTHSLSHSLSFSNCFYSQSETTNTDLSI

HQQLSIRKKPQQKQTVLKQKHNRLHAASTTSYKTGSSMQNSTLHSNTKLQLVTN

>SG-AL_contig_4899_1_-2_2_157

GMEALIHFILIFFRFIFNVSVFVLIFLVIFFKTHHKSCNFWFSSSAERLRLH

>SG-AL_contig_4900_1_-1_1_111

MRPYSTSVLFKKEGSFHQILRCFALCWYGYGSCYAVA

>SG-AL_contig_4901_1_-3_48_263

MRVEIPDPPAPALLSQCCLPLRRARIYEHDVPEIRFFIVRSGKIGKVLSFFSVRDFRAQV

HSSLNCVQSFPT

>SG-AL_contig_4902_1_+1_1_147

GKMGGAALWQCTLPGRAAWISDGKSILTNKIQYNTTHINTPLHHYQKVH

>SG-AL_contig_4903_1_+1_1558_1908

MLMNREKKEKRKKENAKKKLHSKCALSAVKQLNQQCKVLRGWGGGGDTVYTHTCKNIYLR

AHTHTHTQLPQTQTAVLVEMESLHSPLQNYEIIIYKQEAASHISTCTHTHTYINTVI

>SG-AL_contig_4904_1_-3_171_371

MENSFLLYIYQILLEITMVLRIIAITQMLCLLICAHIFKFAAISTLNGFAHVVCPFCCFA

ECYFPDL

>SG-AL_contig_4905_1_-1_274_327

MLNFSALIFVIYVQWESH

>SG-AL_contig_4906_1_-3_765_917

MDTPSTFKPFSIQGGQNRLVITCPLVFNLIRTGHMYLEHIHVVLTLSALHM

>SG-AL_contig_4907_1_-2_77_1231

MAASQIENVEQILDKYIPPEELAAVKRLLYGKELRKLVLPEKAVSLAQEKNFELQAFGFD

AQREDTRSARIVRVGAIQNQIVAPTSAPIVQQREALFRRIGEMVEVAALCGVNILCFQEA

WTMPFAFCTREKFPWTQFAESAEDGPSTKFLQEMARKHNMVIVSPILERDELHGDILANT

AVVISNTGAVLGKSRKNHIPRTGDFNESTYYLEGDTGHRVFQTQFGKIAINICFGRHHPQ

NWMMYGVNGAEIVFNPSATINTLSETVWSLEARTAAVANSYFTVAINRVGTEYFESEFTS

GDGKPAHKDFGHFYGSSYITGPDGSRTPALSRTRDGLLVAEMDLNLLRQVNDIWNFRMTQ

RLDMYAKELTAAVKPDYQPPLVKEQ

>SG-AL_contig_4908_1_-1_385_684

MLKSALRLHSTGNKNIRACVVHGVVEGVGTDFRSKQDIRCQQQGSVTFGRFHIRSDSECA

RLFASTLRPNIQEKLCLFLYCKPQWISFKLKKTTTSFEVD

>SG-AL_contig_4909_1_-2_347_433

MCYYCKICLHSTSFIVSFFQHSPHLKKKK

>SG-AL_contig_4910_1_-3_375_689

MVYHKRSERSRYTVLAGSCRMLTIKMNVADVIGLMTGRTDQTDTKHLVFQPWSLPYMLTK

QTEAKQYVFRPSRDLLQANKQKGVITLNVSAPKVFYRQTKRTNRK

>SG-AL_contig_4911_1_+1_2116_2451

MFLSRKKGLLVGLFNQDKNVFEYKLPLNAKRSVLAAVGCSPVDWSTTCPCRHHTNPHFHS

GHRCQPPLPPPISVRAWPACHFDYCTLLHMTTYHTASTFPPNWYVRIGCRTF

>SG-AL_contig_4912_1_-1_1_123

GTNFDNSSMKNRWKWDWMEAVMKEERLGKHLRKITTSGHAV

>SG-AL_contig_4913_1_-1_232_384

MLWAHIEFGHHILSTLLFRLSPYISTFHLYFFLSLSLPTADLAAVQGSSYY

>SG-AL_contig_4914_1_+3_2619_3002

MSTLLFSPSSTIIYTHTHMQSQNACNLLDKQCIPMFIHLSTRSLSTCSRADSSFTVLVQS

QKSCVNHKTHLFCTKVNTNCHFPHSAMHEFNNVLSCCIWTKNSFRSSEVILTRGTANRLG

RWTSNQKV

>SG-AL_contig_4915_1_-2_80_808

MGSHRAQLEKKVTELTNKTKLNKQVVQSLVEHYHLKCKDKGLDRDSLRDDLMNQFEILDD

FLLDRIFRTFDISANTQHLKLDDFVMGMAVFLTGSLEDKMKFCFHVYDLNGDGYITREEM

FQFLKNTMVTKAHDADQDDADEGIKELTELALKKLDHDDHDGRVSFADFRAAVLGDKLLL

ESLGQCLPDEKCRCRYLKMLGDPDMNTPPTTISSKSRRRTTRAARSAKKEREQAKCATQQ

KAH

>SG-AL_contig_4916_1_+1_469_696

MPIPKNNIQCNIVCAMYNKLFPCTNKRQYVIQYVPSTIPLSFPHKILMSHTMCICKAQYL

VSFPTKHIHVCNSFLF

>SG-AL_contig_4917_1_-1_1_249

HRHVRLLLIRRGSVCLVFVLIYAMADSDSPLTRSAKLEESSENGPKKSDFRFTEGLTLEQ

IREKQTDFIEERNWDQFHAPRNV

>SG-AL_contig_4918_1_+3_810_1235

MELTGMELPCQNSLLCGMSLQTEDSLSLKDNSANVYTYLSSSKRNTTLNSSTRQTAYHDI

IIYTSGRNRMRKESNNRIHYWLIVFHGNLQLLFPLTGNRWRESKNKTKNHFGIIAMPAGE

IDCFMLYCKSTAETNKKRNFAW

>SG-AL_contig_4919_1_+2_20_391

MSKFLEEMQLFVIIFYNYTPNTQHGFLNWKCTPHFIILMPTLHTTYTAKMSGLNESTKSK

QCSLTTRHNFSISRITHQRYRMHFSNVECHTSVHVIIAHPIDHCTQLTAERTRQQQINQS

HNEI

>SG-AL_contig_4920_1_+2_158_253

MQKLKPYVLGQLFPLRRAIPPPSSHQGCWWCI

>SG-AL_contig_4921_1_-1_1_267

LPICASSALVSWKQFLSLDRHGNFTHSLGKYYHILNPSFNNAFRTFNTLMVEKVLAMLCN

ILCTKKEQKCIESLHHCAREMICYFLSST

>SG-AL_contig_4922_1_-2_65_376

MTAALHTGAYPVKYGLKTAKKNTPFRPVNGTKNTPNQWHSSDLGHSLHIQPSPNCMSSGT

GLCSDLPCDWTLQLMNNSCRYSSLSHWFVLEYPSLKMQLGKYQL

>SG-AL_contig_4923_1_-2_80_238

MIQTRSHNTLGPSWFGVWLNDRNTHTVQEADKATGEVSYAALDSAQGVLKRSD

>SG-AL_contig_4924_1_-1_19_3075

MASSVPVVAVVCCLFAVLTSGKQLPFRNDDTASCGYSSCNPVKDDMINVHLVPHTHDDVG

WLVTVDQYYYERVQYILDSVIPELLKDPSKRFIYVEIAFFERWFREQSDSMRHIVKRLVN

EGRLEFILGGWCMNDEAATHYNAIIDQHMLGFEYLRENFGKCGKPRVGWQIDPFGHSRET

ASLFARFGFDSLFFGRLDYQDKINRELHKTMEMIWRGSPDNLGTAADLFTGALPNGYNPP

IGFCFDIFCKDKPIVDDPRLHDVNVETKVDAFLAEMSMQALEYATNHLIVTMGSDFQYQA

AHNWYLNLDKLIKYVNARQANSSKINLLYSTPSCYTYHVNRANKTWTTKEDDFFPYAHRP

HSFWTGYFTSRPTIKGYVRQTNNFLQAVKQLDALALLEDTDNSTFHLRILAKAMGVLQHH

DAITGTEKQVVTYDYAERLNNGVLEGQKVYNDAIKKLGGGMKVDQAFCTLLNISVCSMTE

NGNQFQVTVYNPIGRPGLSYVRIPVSSTRNVGFSVVGPDGKPVSSELIPVSPDTMRIPER

HGDSAKYELVFPVSLVPLGFSVFSVVRSKGLQADVQDRKVTAVYDEDLVVRNEYIALAFD

KKTGLLKNMTNLEKKISIDLTQSFDYYMGMAGNNSDSKLQASGAYVFRPNGTSPYGMKPG

HAKLTSTTYFLKGQLVQEVYQEFTPWMTQTVKLYNQQRFAEFQWTVGPIPVKDKQGKEVI

SRFQSSLKNSKIFYTDANGREILKRELNHRDTWKFNNTEPVSGNYFPINSRIYIRDEAKK

VQLTVLTDRSQGGTSLRDGEIEIMVHRRLLYDDGLGVDEALNETGCDGKGLIARGSHYLM

LEPIASAAAGHRDLAERLFMGPQISFSQTKSKQVKAVWSGVRQALPDNVHLLTLEQFKSY

GPVPSATQPFLLRLEHFYEKGEDPVLSKPVTVNLQKMFSTFDIVKATELTLGADMALADL

KRLKWNYPHKDDHFADKKDSTLTFKSERNGDTAESDSLTVTLNPMEIRTFQISIRPVSH

>SG-AL_contig_4925_1_-3_1965_2273

MIISVDMEESWIKCEIVEHRIQYKICCLCFKIITGTAPLYLSELVRIYSPPRNLRSSSDN

RTFCKPPFFKRKQHGRRTLCALANKFWNPLPLSLRHCPFSLYL

>SG-AL_contig_4926_1_-3_321_533

MVDYSQKNYYLVFFTCFRMFGCPHRNSKVKTPATGVASPYSLVAHWCDAHLCGDAGGSFS

INSTPTFVRVA

>SG-AL_contig_4927_1_-3_765_1028

MHCGTSRAAYRYGEPVFVTDSHQNEHGVPEIRFFIVRSGKIGKVLSPFFFSVRDFRAQVH

SSLNCVQSFPTTAEVTSQEWRQLSDVGR

>SG-AL_contig_4928_1_-2_263_538

MEFFCRILPETTAFLLCVIYFVYVVCALGNVLVGSEQRVVSVVGLLLCDFRTADIPADGD

LCVGLCISHGLRMSHGLCISHGLCISHGLLNT

>SG-AL_contig_4929_1_+1_130_204

MRSLTAHNNCTLPVLRESFLTYSLQ

>SG-AL_contig_4930_1_+2_2_106

FLTYSLQRLQSGCFVFQQRCSSFLDCTANSQISCW

>SG-AL_contig_4931_1_+1_1_201

EQIKQPHCHIRSLLYRGNDVRVSHHPICFVQLLEVIGSVLVKMQECTGENAFSSCPSCSS

CLLQAAL

>SG-AL_contig_4932_1_-3_3_242

VYRTFKCRLWAQRTVKTSCFRTLIIGKSLWHQEQRKPTIYCAHLFLSLTHTHTHILTHTS

LTHTKLSLVFRNCSNCSRLI

>SG-AL_contig_4933_1_+1_124_417

MQILLQLHLLVYSHSLNIKKNNKKNVFSDHFHSFSHIQVMVVFITQVLFAHQTDYIDNKS

FLYSAICKLIWTHCAYEYVAETANGIYVTFFVHAWCFH

>SG-AL_contig_4934_1_-2_2_274

SPASGRPIHSPRESPLTILQRTLNKNNYLRSPYSHIMGVSNKPIQFVKSLYTDPFRWSLV

KSAAFFIAAVFISREMASAEKAAQLAPTPAP

>SG-AL_contig_4935_1_+3_27_410

MFALLTQRHLRWLGHVSRMQDGRIPKETLYGELATGTRPAGRPALRYKDVCKRDLKAGNI

SPAGLEAVAAHRSSWSIIVKAGIQMSEKRREERAQTAEGRNSTHRARRRLHLQKLQQGLP

LENRAVQS

>SG-AL_contig_4936_1_-2_2_202

FSTLCHGLSPGVSPVAQIPLSFGTEGGQSLISKAKYLWLFCNSNKIIVFYFFPFFDLTIC

WYNESEK

>SG-AL_contig_4937_1_-2_2_202

FSTLCHGSSPGVSPVAQIPLSFVTEGGQSLISKAKYLWLFCNSNKIIVFYFFPFFDLTIC

WYNESEK

>SG-AL_contig_4938_1_-1_613_798

MLDGKRQLDNSDVNLCKTKGLGSDGPRQCSGNRCWINLFSPHPLPSYFLIQSKTNTKMVL

LF

>SG-AL_contig_4939_1_-2_2_217

CDTETAFCESRTDLFFLSLVLVSLASFQSTLSELTKSMLIFFSLCPNISSNRLLDRKVSF

TYYYENTVYFEC

>SG-AL_contig_4940_1_+1_187_354

MPIFNRKHYLLSTFYSTRFFIWTSFDLFASCLTIGLLRCVGILKIRSNQETHDKTI

>SG-AL_contig_4941_1_-3_3_245

VFFNSLGEVAQSPCTSLNGLRGKRGVILTTSPPIILYQLRSLFLAHLFSRCRSEIHTLSG

SPTDLASSQMVLSVSSFCQSS

>SG-AL_contig_4942_1_-1_1_249

LGIPLRKLTSAPCPLSWTGQTQLETCAPSSSFSTTYSEPRSTALLTPVSSPSTVKWSMTS

PQSRSPAESLRLYFRPAVRHGKT

>SG-AL_contig_4943_1_-2_2_208

PRFFVFFSTECCLGILSLSWEMCMSHLQQYQSDHQRLGKLEPETRTDNNGMCSIDKTRPA

QVFGQLAFV

>SG-AL_contig_4944_1_-2_539_2392

MADTDVTNEGATLFTPVSTTPVEEVSMRETDTGVMKGRAMPSTLTPITSLTKRDISMTDT

NVWNEAATPFTPIHSESVGKLISSGRDELNKGTISFTLTPLIPVAVREVTVTRATEVNYN

DTTSITISPIMPVAVREVSMAGTDVMEEDATPFIPVPGGPFSALTPSMWPPDVSRLHLSP

GGPTAQSDHKYDEFGFTIQETEARQQQRVPFVEDPWHRLQWTPYLNIPTNNHHHHHKPDV

TPHTWDIDNMCWNCSRAFHRKTQHHQAECQPLRSEKLREMVREGIPHSLRPHLWMRLSGA

WQKKEKCHVPYHTIIAVSSNDQLATSKQIEKDLLRTMPSNACFSQQSSPGVSRLRRILRG

LAWRYPDIGYCQGTGVIVACLLLFLEEEDTFWMMCAIIEDLLPRSYFTSSLRDIEVDQKV

LRQLLVSFLPDIDQVLRQHDIELSLIGMHWFLTLFASVVDIRVLLRVWDLFFYSGSVVLF

HTTVGLLQLKEDELRRLDNSAQIFNALSDIPGSVENVEQLIELAFRVSGSLTDEVVNILR

QKHRTYLMADQTVFSDSSTSDSLPEQRKDSTSHSWWWAAWGWSLWSRRLKGGEEGDWGPA

AKNVRQTELMLDLREAIL

>SG-AL_contig_4945_1_-2_185_3043

MVSGDNVTVTLTGGSPWGFRLTGGGSMPLQIAKIRKKSQAHAQGLQEGDAVVSINGTPVH

DRSHDEAVDLIDSAGDTLTLQIYRGDADDLGQLLASSKHHAPLLPPQAEGQGDVVVTSST

STSSVTEAEGRRREVQQNVFVSKDGNKESTTFVRREHTITTSSELGDVNGNDVGVGGRDR

NLTAQIEVSTSLESAPLVSLSPQPALSPKPSSPKPALSPKPVSPKPVSPKPVSPVPFAPS

PQVLAPGPVSSQSQSHIELQIQSKPKADGPMWVPKGPTFKAIPPKVAPGVAPKPALKQQF

TVKPTFNAKAVATATTPTFAPQKFQVSATAMYSQQAQSPGGQQVQAKFQPGQQFSPGIKQ

QHLSQSSQPYSPGSQPFSPMNLSSPVSPVSQPFSPNSPGGGSTPMFNVRNNTGEGTTTWQ

PNMWLPGQEPEKQKMQQQQQPQQHYQPPPKENHTQNENIEIPKRMSIKERQQMLLNHAAT

TKHSLGARQADPNFDAPPEEVQRMAQQKLKIFGPPVQMSLQGPMEPWDDLGLVRTDSVES

DGSSPTSSLHRKKKMYADSAFYEDPTHKYPTIEEQMKLCRLISHSLTSAANKRARGAKMF

AKRKRKSQRWVHEGHSEWSSSAGDVANIEELESELGVDEGGSKQLFMFRIPKLKQRITSP

ERNTKMSMKKDEFERMRLQSQKCDHRTVSPGTCFDIAADLKAHKGRGGRLFERRKNRADK

FIIDESNAKMAGPKTTKLDNILGTAPLKSSQSPWEAAQSNQAGKVDSAFDHLNDIERMQK

MNQIMKVYSPPKPAQPTREVLQPFNPPPVKGPKTDLRGSGIALLQGRNFNRFARGWCAAG

GDTQDKAGSMASHHVGPQQHHQHQHPNQTHLAAQDYPVESAPRGQPRVHFGDYNPRPRAW

QSAAQPGDSYSCPASMSASWASSDFDSYHQSPSGAAGGGGGLYSPNELPASDL

>SG-AL_contig_4946_1_+2_110_367

MNCSIERLVPLSLVISQSQQNNLPEGFNFGLKPAETSETHTVQGNLFICNQFRNPSLNVL

LSLACQVLFFSHMKSNSVLIALAPLF

>SG-AL_contig_4947_1_+1_463_669

MSSSQAHLTQYPATVTTDHTGKQGNCYHHFTFLCITYKSSKVTITITLPKGNYYHHFTFH

CVSHTSPTR

>SG-AL_contig_4948_1_+2_353_538

MTFCIFQTQTVDYLAPIFFCNKIRACNLFKDSLHWKLHVCSLQVCLVSSRRLLTPRGKNR

TE

>SG-AL_contig_4949_1_-2_434_964

MSMRPSFFRGLWDDPWEGMRQPSRLYDQHFGHILPDDLCPAAAVLPFPFTSRPMFTPPPV

SLTRVQPPQQTGISEVLNSEKEFRVSMDVQHFKPEEISIKTKNNRVIITACHEERPDEHG

FIMRQFTRQYVLPNDVDPASVSSSLSRDGILILKAPKIVLEPPKEHIIPITQEAEDK

>SG-AL_contig_4950_1_+1_565_867

MVGVEAYVIFGTWISLWWQQVAKARRKAVLYESSEKGTTSRDSKGGDLRALRTRVRSHLG

SLLGALASRAVRPLETSRRRKSSERVPPPPPPPPPRGGGGG

>SG-AL_contig_4951_1_-3_489_716

MVLPEFVTKVQQPGCRPRSFRIPSLVSCLDPRDARSKEHLLCPVRCVKYYLKRSVPRRSS

QRRLLISLNENYSRDI

>SG-AL_contig_4952_1_+3_165_599

MESMRSQASLCKQGRPVSSSNLTDAARIDAPPRNAVGSAWSVGPELPHMGASRASILAAG

PQLDETDPQFCDGRVDSRPTDVLDRYPRLLHLVLKADLLATGSTCGAPLDLFVATQRQTV

SLPRPWLRTGRPAVVRRASRPLVAY

>SG-AL_contig_4953_1_-3_51_227

MGHKVLIIEWGGVVVSWFHGSCHCMIWGWMCVVVLYCMHYALCAVERHFSIFICAVKVV

>SG-AL_contig_4954_1_-2_2_2446

SDGNAGFLRAARDGNLQEVLEYLKGSTDINTSNPNGLNALHLASKEGHLNIVTELLRRGA

NVEAATKKGNTALHIASLAGHEEIVKILVDNGAHVNLQAQAGFTPLYMAAQEGHSEVVRF

LLNSGGNQSLATEDGFTPLAVALQQGHDRVVSVLLEHDTKGKVRLPALHIAAKKDDVKSA

ALLLQNEQNNVDMQTKSGFTPLHIASHYGNTNVSNLLIQRGADVNFKAKNNITPLHVASR

WGKSNMVQLLLDNKATIDEKTRDGLTPLHCAGRSGHEAVVDLLLEKEAPHSSKTKNGLTP

LHMAAQGDHVDCARLLLYHKAYVDEVTVDYLTPLHVAAHCGNVKTAKLLLDRKCEPNARA

LNGFTPLHIACKKNRIKVVELLLKYGASIDATTESGLTPLHVASFMGHMNIVIYLIQHNA

NPDTPTVRGETSLHLAARANQTDIIRILLRNGATVDAKAREQQTPLHIASRLGNVDNVVL

LLQHGASGDATTKDKYTPLHIAAKEGHDEVAQVLLENGASHDVTTKKGFTPLHIAAKYGN

IKVARLLLQKDANPDVQGKNGLTPLHVATHYNHVNVAQLLLENKANPHATAKNGYTPLHI

AAKKNQMDIATALLEYGAKPNAESKNGFTPVHLASQEGHTDIVSLLLEKDANVNARAHNG

LTPMHLAAQEDKVPVAEILVKYGSETDSQTKAGYTPLHTACHFGQINMVRFLLEHGASVS

ATTKVGYTPLHQAAQQGHVQVVNLLLKNGASPNTVTNNGQTPLAIAQKLGYISVVEILKP

VTEVQVTVPASEDKYKVVSPETMQETFISDSEDEG

>SG-AL_contig_4955_1_-2_128_505

MASETEKAQLATKGGDTLFGKIIRREIPCKFIYEDDHCVAFNDIAAQAPVHFLVVPRKAI

QQLSVAEDRDEKLLGHLLLVAKKLAKEQGLDKGFRVVVNDDVEGGQSVYHLHLHVLGKRQ

LGWPPG

>SG-AL_contig_4956_1_+3_81_254

MQYSGELYLCAILNISGCGSDGMSTEKQATTLMSTVTHRPSVADTRLTFQGRIAGQRS

>SG-AL_contig_4957_1_+2_2_151

HTQPHPHLKLITFQFLKATTFGTTVTDTRDNQCKPVQITLPRVSDAKRLL

>SG-AL_contig_4958_1_+2_35_217

MKDMQFHIKKTHKNTKVNQTFRKQNLTIAPKFFSIQTFINQHTIKQVTFCHKSQSRSTTV

C

>SG-AL_contig_4959_1_-1_1129_1473

MPCFFFIHAICFVYMYIHTYIHTYIHLYIHTYIHPSIHTYIRTYIHTYLLTYLYTYIHVH

CRLWMGIYILTYVHTYCAYMSIVNFGYIHTYIHVSVEFGQLWVYTYIHTCQCRIW

>SG-AL_contig_4960_1_+2_209_355

MKKVREIKMYPINIFLKKMTTPFNKNVIANLSTVVFVIIIIIFFNLQKK

>SG-AL_contig_4961_1_-2_2_124

LLCFKSLNDQAPLFIFELLHLYVPSRLFAPLLTPEYTNFLM

>SG-AL_contig_4962_1_-2_608_847

MFRSSHSSRISCLLHSLHWLPVKQRTSYKLSLLCFKSLNDQAPLYISELLHLYAPSRHLR

SSADTRIYKLPHVRTKTYSQ

>SG-AL_contig_4963_1_-3_357_683

MDVHALLTALLLLLFFKDNYSCGVSGYCTGLIFIQRQCNCRLFGVLHTPVYTVSSFTHYQ

CFPVCIVINIYAQLYSVWSFFISRRKAQVSVYVWFLFLYDFCWFLFTQL

>SG-AL_contig_4964_1_-3_3_233

NLQNLTVILNSYKTTIVHPETPTQTETHANQPPANQPTNNRAQTSPNQLINQEPTNRTRS

ILKNPYCNPCISLILIN

>SG-AL_contig_4965_1_-3_60_263

MFVQLYLHLICLMIDYCSKMFSLFLSLSVSACLSLSHTYTHTHTHTHTFTHSCMHAHMFT

YTLAYTHT

>SG-AL_contig_4966_1_+3_126_224

MHQCYMLRDGRIQVQCRQHVHKPKRQQGLGSST

>SG-AL_contig_4967_1_-3_228_1295

MDIKMGGPESDSRDTFVVTVPPSTTDISRREKSSGGEMTEPKEYPLRQRLATVFCYAVSA

SLIVIVNKLVLTSYRFPSFLALGLGQMVVTVAILLVAKTARLVQFPAPHKDIFKQICPLP

LLFFGSIVFSLGATQRLSLPMVVALRKFCNLFIMIGEYFVLGVVATYFVQFTVFLMILGS

LIAASHDLAFDMTGYAFIMATNLCSSMTGVFTKQKLNTKTLGKYGLMFYNCLFMAGPTFL

LALVTGDLHKAAAYEHWHDPLFVAQFLMACVMGFIVNFCTMLSTQYNSALTTSIVGVLKN

LFIIYAGMFIGGDYVYSLVNFVGINVSVVGGVMYSYATFRSKQRTNTPSDDSSSKV

>SG-AL_contig_4968_1_-1_106_1044

MMAQQKRTAAEAESLLVGVRAIVFDIEGTTTPISFVKERLFPYVSKNLEKFLTDKFEDAE

TIKDVSALRELAQKDKTDGMEGVVEIPDTTSDKEEIIKAVAANVRWQMEKDRKSTELKQL

QGHIWRQAYESGQVKGEVFEDVPPMLRMLNEEGFKLYVYSSGSVESQKLLFANTTEGNMI

EVFTDFFDTAIGPKTDCESYKKIAQEIKLESSCILFLTDIPEEAEAAVAAGLRSALVIRP

GNADLTDEHLQNFACIEQFDELYGDEDEEDIKRLHQDDNGEAEDDDEVDEDEDEEGEEEE

GEGEEEEGEGEDA

>SG-AL_contig_4969_1_-3_3_173

RGLTLVRQSAYILGDPQSVKLGNANNKQQQHWAFLLLCECDFTGRGDCYHGLTILYI

>SG-AL_contig_4970_1_+2_122_241

MMYLDDLAIKVSEKFRKTHLSLRQLLLQLHLFTISYLQFS

>SG-AL_contig_4971_1_-3_75_329

MAKSQIRCLFLVMVEPHVLLLLMRPLGWFDVLSKHHRGLINSSRKMLQRNMTPIFMEKHK

HSDLKVIIHEEKLKKLWKSWRVDHN

>SG-AL_contig_4972_1_-2_53_2209

MWEETFKSYTDSKPFGPTSVGVDISFPGVEHVYGIPEHADSLALKTTKSTDPYRLFNLDV

FEYELYNPMALYGSVPVMLAHNEKRTVGIFWLNAAETWVDISSNVADKSIFSNLADFVTG

NNEIPQTDTHWFSESGVIDVFLMLGPGPKDVFRQYGVLTGTTPLPPLFSISYHQCRWNYN

DQDDVRTVDENFDKFDIPYDVIWLDIEHTDGKRYFTWDATKFPASVEMLRNISSKGRKMV

TIVDPHLKRDDNYAVFKDARDKGFLVKNKDGGDFDGWCWPGSSGWPDFLEKHVRDWWADK

FALTEYVGSDLNLFVWNDMNEPSVFNAPEITMHRDIKHLAGWENREVHNVYGMHVHAATM

EGLLKRTNYNDRAFVLTRAFFAGSQRTSAVWTGDNMGEWGHLKISNPMIMSLNLAGITFS

GADVGGFFRNPDNELLTRWYQAGAFQPFFRAHAHLDTKRREPYLLPEENVNIIRAAIRAR

YSYLPYWYTLFASSEEDGAAVMRPLWVEFPKERATFGIDDNFMVGSSLLVRPVTDQGATS

ANVYFPGADQIWYDVETYETYKGQQEVNIAAPLSKIPVYQRGGSIIPRKMRPRRSSSLMF

DDPFTLVVALDSKQEATGYLYVDDYHSFRYKKGAYGLIQYNFASHKLHSIVHHEGDQFTT

KEWLERVIIVGLPHEPRKVTLHKSGQTSDLIFTFDNSKKALTIRKPGTNILQTWTISLH

>SG-AL_contig_4973_1_+3_2112_2438

MHESADYSDLLESFVLDTFFKNHFELHCVACVYIPKKQTVKKLSKEPWLSSQLIIFLIYF

ILHEKPKSKERNLLTINSLSLSLFISLYLSLCISIIFSCLLCRSGLARL

>SG-AL_contig_4974_1_+2_1241_1930

MLFKIWMKRHLTPLGRVAVLKPLIHSKLLHLLILLPNPRDDSSLICRKCFLFVWNNKQDR

ITRKTETQSINKGGLNVPDLKNVLLALKLIWLRKLQKTNHKWRNLALINYKFLGELDTFG

PNITYKFTHDNKFWTDVFKAYREFYYKVEPKDVRDILSEPILFNERIKVDNRVIPYLKRA

RKGIHFIADFMKEDGSFLSQTEFNRKFNMNIDFITYLGCKSAIKKYKKTA

>SG-AL_contig_4975_1_-2_2_1000

WSPKVGPSPEACFSPALSEDVVDLESVNSESVRSSPKALSPSGEWATAGRALSPPGLHQE

DSSHPALSSLIHVDNSLSDSSLDDDCPQTKQPDFEDRIRKLDDMMMNKPNSSAMTSSSAH

QTTPSSSTSYSSKFRIVRKKDSESGGLAAEKKEEKPSEIVQTILKRSSILDQDYRRLGQQ

QNDTNLAALAQYRTTGVKQGGFLPSRPMPYVDGVGVEVACGGAAPVTTAWASMQQPTTTT

TTTMAGLPGLMVTTPSLIQPAFRSPFPASSPSPSTTSPRPYPLSAALPAFPVSVTGACSP

RPCTGPTLSVWPTPTLTALSPSPHTAESAAALE

>SG-AL_contig_4976_1_-1_271_1482

MKTVPNAKVGFSKAMSAGWIRIDKAAEGGPRVFRKVETIEDTVQLCLLRIQDLCLDGVPE

ASKAEYKKRKLIAEVKETGYRLRKGANFTLTVEKLEVDLTPAMIHSGAWKTKQFKPYNFD

ALGVPPDRGHLHPLLKVRTQYRQIFLEMGFTEMPTNNFIESSFWNFDTLFVPQKHPARDA

QDTFFISDPHTASAFPMDYLQKVAKVHGDGDYGSTGYRYEWQMEETEKNVLRTHTTAVSA

RMLYKLAQQETFKPVKYFSIDRVFRNENLDATHLAEFHQIEGLVADHGLTLGDLMGIINE

FFMKLGIKKLRFKPAYNPYTEPSMEVFSYHEGLQKWVEVGNSGVFRPEMLLPMGMPEDVS

VIAWGLSLERPTMIKYGINNIRDLIGPKVNLQMVHENPLCRLDK

>SG-AL_contig_4977_1_-1_55_315

MISDINGNLYSFGCPEFGQLGHNSDGKYFVTSNKMEYKCEMIPRKVNVFIEKTRDGHVIP

ITDVDVREIACGLNHTLALDSRKRVFS

>SG-AL_contig_4978_1_+3_540_860

MPKHVGKKFRTVHAFGKKRRKRKVTKTDEQKPAEVVTYLAVLSAPTCDSTPPLSAKRKLD

FFPPENGCCQQTLIVQMKIHRLMKAYWSQRWNFAPNDHQTSLLMCQH

>SG-AL_contig_4979_1_+1_1_258

EKTLHCCCSNSTCAKTQPAKSCPDNGNYFICEVRVSKLPSHLSFPIWSRMISIKFIHSKI

SNDLFPPSFTNHHVLFLFFFFSFFFF

>SG-AL_contig_4980_1_+2_104_304

MADLLAATKFSQLHFGRLYIHTQQAIYRINHVINSICKSKTENVSTHFRSTKAYMVTQSG

KETIIER

>SG-AL_contig_4981_1_-2_533_688

MKGHKSGTIFHNAMVMRMGLYSTFTFIIYPGINQAQETQTPVVLVMKFEQHP

>SG-AL_contig_4982_1_+1_103_312

MLILTKIHSLSMVRLDDRTSPGPAPSQPVRVPSARHTCINFLSFAGSYQNDPCENQSDMS

GDGSLVLESV

>SG-AL_contig_4983_1_-1_1237_1467

MVRFNYSDVTGPHSGSANQQPSRQTSFMPTAVFSIASSIQMTLVRTITTRAGMAGREFDV

RIGKYLAKQRFKEKSPF

>SG-AL_contig_4984_1_-3_2187_2360

MSAFKRCSPCMQKKENLGSVVGCGAGMKNLRLMSSKLCRKWGWTKIIAQFCVYINRIY

>SG-AL_contig_4985_1_-1_271_462

MFIPPNLQGLTLHLNLRGSMTSVCCWEKNQQCRICCTFGAVAEEKSYFPFVQSVVNKEVL

FSFC

>SG-AL_contig_4986_1_+3_3_269

MIQWTVAKTEGPMAVTSWAQCVPVGKTSVKYRNQVKLSHDDESKDFNNSKLRMPLNIMSK

ITTKSSEVFYQRQLPKNMLGSLLGFKACL

>SG-AL_contig_4987_1_+1_1_111

FLRVSTQVYWHLIYIKVHLLTTKSNAESNCVNIHQRK

>SG-AL_contig_4988_1_-3_3_95

ERERVESMCTCTCLHVYVSVGKRHVNVLSND

>SG-AL_contig_4989_1_+3_822_1238

MSKLYIYIYIYIYIYIYPSHQEILKHLLSLFQSAEVNECRLHSYVIPEQCGLTHQNNMQT

NVQASTNILPSEVRKGGYPLVQLVGSFKVTENMFSSWLQVTFRHHQKLYRSKNKNERKTE

KKKKPNQNQKAQHWKRLHV

>SG-AL_contig_4990_1_-3_3_242

ANEPPSERLRPDAVIDYLHRFPSAVISYLEYLIFQRKLEKETYHTHLAVLYLDAVLQLMK

KDGVKKEELDIARSKLRHML

>SG-AL_contig_4991_1_+2_311_613

MSSKSNVTFKNNVTTPPSSKQVQCYHTYTHRSNVTTHTHIRPISLILHTHTQVSNKSRVP

FKNKLTTYNTHTHTHVQQVQYYHVVQLLLRTSLQHTHTHIQ

>SG-AL_contig_4992_1_+3_3_140

AWLTPGYVMKVSVECSPVTQSQSEIELHSIITIIPILIIVALKKIV

>SG-AL_contig_4993_1_+2_2_295

LDGTPQHCHYHPHSHHHSIVKNCPRLCGPSLPCLYGDLSFGGFGGVLESQNEQRMGSGTG

AVQKMGQQQKNKTTHTQHAHQTPQTRTGSTMFECNSLK

>SG-AL_contig_4994_1_-2_14_1639

MGKEREVEEDVETETVLASEVPFWTSARLGMAILGFLGFIFVYALRVNLSVAIVCMVNRT

AIAHHENASSLAAATNASFSDKKDVDVNTECGNLAQSANASSSSLVEGHLMWDKKTQGLV

LGAFFWGYLVSQIPAGLLAQRVGGKWCFGWAMLAAALFTLLTPLVANHSFVGVMVLRILL

GAATGVTFPAMHCLWGSWAPPLERTKLMAFTYAGAQVGNVITFPLAGFLCKYGFASGWPS

IFYVLGGLTLVWVALWMFLTSNTPSQHRRISDLERRYIVASLRKVAQSEESKKNVPWKSI

ALSLPNYAIIVSNITSDWGAYTLLTNIPTYISEVLKFDIASNGLVSALPYIGMWATMNIS

GWLADIIRQRKWLGTGGTRKFMDAFGKVAAGVLLVGLGYVDCTQPAVAIALLVLAVSIAG

FQYSGFLVNHVDIAPQYAGILFGISNSIAAITGFVSPVVVGIITEERQTRAEWQIVFYVA

AAIYLFGAIFYVIFAAGVLQPWASKDASNALEVQEPVLLNVTKSSGMDGNGVGDKLVNHN

AD

>SG-AL_contig_4995_1_-3_3_182

VSSSVPACNIKIGLFTAHLYAPIILVVSLFHMSCSPPPSPFQDLPAHRALCKLKLKESAR

>SG-AL_contig_4996_1_+2_149_418

MSKAVGRSSAVNSAMPELKKKKKKEKRTHFPSYIHLPKVLHCPGECTTQTRDGSAMTKPT

RTEVKLHNLPTLTSTRDQQLQAPISSDELA

>SG-AL_contig_4997_1_+3_516_899

MAILVIILIKAVPSGNNIMHINPVTLVYIFLIFSEVFISVHFFPPVYLREVCTLQKLCFA

CFFITFNFPPSCPRLFQHCYYILFIAQPTTKAIQDCHYIKSIKVKKTPMLQHKTLSNLKP

KILQKNNS

>SG-AL_contig_4998_1_+3_3_110

NKTLLQNNTVHDILCQPLSSLRQIRLGCDEDTSPST

>SG-AL_contig_4999_1_-3_210_1331

MSAAHLESRDTTFQHMHSGLIKYLSKDEDKKDQDVMTATFHFLNPGRLLLTGLEPIGQTD

HPTCVALWWKGSHHLLQFSLLRVGKDLEHKPDVVWPFSSAITCSAVSTCTSHLAVGLANG

NISVWDRYIGLQRGVVNMNPASGVQMLRFLDPSLYPAASMEYPPYPQRTATYLLAQYQDS

SHYLYDVGKATQDQPLCIGEKPDHDDEVQTLLESVPDLPDLSLMVQKDGTLYLKDIQTGV

RICQVALPENHQLVTPWEPVVALGGRGQLLFVKGSGEECGESEETTDTSSVFIFTLRSYP

TLDPYWHKLRTPAQLQVHPTIETRLDALMKERLAQQALRKQRLQMRWGMLRSDLAVIQNA

KEVNISTAARPFHM

>SG-AL_contig_5000_1_-3_3_116

FFVVSLCVGENQCLYYIFIGCLCSGLVLCFGRYVGPKR

>SG-AL_contig_5001_1_+1_1_351

TRLYTPGQNSSYLNQTLYTWTKLFIPGPDFIHLNQTAHTWTTLLIPGPDYTLGPNQSYLD

QTAHTCISHFSQQRHFTYLFLAGRSIHRLGSHPLPEMSHSAPNYTFFSNFTTLLNFS

>SG-AL_contig_5002_1_-1_1_303

PRPYVFHTFGSYACSTLVSCAISRPGCSSLPFTCSTHLVRCIFSYLFPGLGLCSHFVFQA

RESTRHRTRLDRSLWASIGLPIPRSEDRSTPFPCWEQEGSF

>SG-AL_contig_5003_1_-1_1_336

TQSPAGARAQYHPLSFQPVDEASRTAASSAPTPDFHVFQAPRPHVFHTFGSYACSTLVSS

VISRPGCSSFPFTCSTHLVRCIFSYFFPPVLDFVAILSSKPGNRLGIGRGST

>SG-AL_contig_5004_1_-1_1_177

FFFSLSPFPCFCDSLSLTSSPSYTHICVCPLEEQEPMVCHIRLINEDAFPLFHQVMSVF

>SG-AL_contig_5005_1_+3_303_590

MINWRQQLYTTKSDNVNTYPFSHPMMDTIILLSTAPHTMTIHAQIHSGFTFMSGPVITQH

FHFLLHSHLSHTCSFICTTISLLLTSEILLSISHKA

>SG-AL_contig_5006_1_+2_461_649

MTQRYNTAVTVHERLSFCCKYVLMGSSASDLTHSQNNSPESPETERSRKLCDVMSCSSRG

NYF

>SG-AL_contig_5007_1_-2_101_1648

MTKVELCNNVTSGDYDGVVVVADTPAKLEGDPQLHPLAQAINNYAKINENIEKEVSIIQT

DAVPSGRLVYSPTGPLNRDYDDVRRFYDAGEKGIKRALDAGCKKPLLVQPQDTTFPKSGH

VALLGALHALYVPLEIREACEDRRQKAETLGVLSQTLAEGTGKKGYELVDALEKGRIVTR

DIGGSDPERMAAPRAAEYIKQAFEGTSVQVKIVGDKVEIEKEYPLMAAVNRAASVVERHQ

ARLVFLEYEGEGPIDKTLFLVGKGITYDTGGADIKAGGVMAGMHRDKCGAAAVAGFFKIL

AELKPAGLRVVGGMPFVRNSVGEECYVADEIITSRAGRRVRVGNTDAEGRMVMADVLCRF

KEMAGEAVNPELFTIATLTGHAVRAMGPHYSIVMDNGPARKIRTSYQLQACGDIYGDPFE

ISTIRREDYDFHKGKSEYEDVLQCNNQPSSVTPRGHQSPSAFLIMSSGMDKHGNDSDKPY

PYSHLDIAGSSGPFPGVPTGAPIVAMAARYILPRIN

>SG-AL_contig_5008_1_+1_1_207

FFFCSFPPFYLFKQSFKRLSGTISWRYKTTTTLFKPFEFESRQNILCTQCKLFFLLTVYE

QTYLLISSR

>SG-AL_contig_5009_1_+1_1_171

KKQMTVNEGKRAKTQRAIEKRKFLAQNFQKAGILIILKEPLASHGGNQSQCLPRSQE

>SG-AL_contig_5010_1_-2_650_787

MSASQGCSSSESGSRPDSRSKRANTCKCPSRLTSRSAWLLHFQQYR

>SG-AL_contig_5011_1_+1_2491_2658

MYPPHNFPTQNVVQSHQAGLVNIIAPFTCFKILQRKHHVHQQNIYITQTIILFIVQ

>SG-AL_contig_5012_1_+2_197_355

MTVCTISFFTTLNQMDHDKYKPNIPVSCYQLPVYFSLPPSASIVKQHKWKVKQ

>SG-AL_contig_5014_1_-3_1062_1391

MLLQSVKNLRICDDWRIDFIEQVQVTVREFAEKVEWKMMAYLFSIIIIFGDRFTNERSMS

FFVVACGNKAYRQVRVAHLCEGELPGWSKLVHGSIQVCRTMKASHVTVYM

>SG-AL_contig_5015_1_+2_2_85

PISDVQGSKTDHDRCHKCTHEPSVRVIS

>SG-AL_contig_5016_1_-2_185_331

MPDIEVIFHIREGGGNGLAFVIQFYLYTLRMTMFVRSTFLIGVNVLCPL

>SG-AL_contig_5017_1_+1_1465_1698

MCIIYHIFQTKGRTVYAIGMFLTRITKIYNLIFKIFNESNTLLIFWSRIWSSFIHYKHIQ

LWHFPSITLIKKAAVKEN

>SG-AL_contig_5018_1_+3_549_716

MSKTGFPLPIILEYQTKVISEPAFTSLKIVILVNLPYQSIKDFDSSRLTISITSQI

>SG-AL_contig_5019_1_+3_18_221

MGVRLYQHGCLLVPSLVLQGERADDLLHVRLYHHECCGEKELTIFYLFACIIVGVNCTIM

DFTGRKSR

>SG-AL_contig_5020_1_-1_1_132

VWYHAGNWWRKTHQGSVQNGDIALLSYRGRDERRRKNGVKETIP

>SG-AL_contig_5021_1_-1_274_444

MLVFPNTYDKKNIFWGEWWWAHQLSNVSVEISAQYVLLGVYLCFSSGSPPQKHLPMR

>SG-AL_contig_5022_1_-3_186_863

MASNMILYWGSGSIPCWKPMLVLEEKGLHGYQSKLLSFSKKEHKGEDVTKLNPRGQLPTF

KDGDVVICESGAICDYLECKYGDQGTRLLPTDPALRGKVQQRVHESQNLQRIVIEGVVYY

QFRTKPEEVDKEHLDGKKKEAREELQRWEQYLADQGEGSYIVGKDFTMADVFVFPFLAFG

VRSSLNLAQFPHLEKYYNRLMERPSVKATMPPHWSEEPGKDFLKGV

>SG-AL_contig_5023_1_-2_2_235

VNVFFVISIILLRAVLLSLFYHLTVILQVVSFISLELCYPSSFYIIFAIIQLLYHYYFCG

MFCFGFLGREVGGGGPTF

>SG-AL_contig_5024_1_+3_3_182

HVHNLFSFHIFIHPFLHIKFYSNLCINVFLVIFFLFKASGDFAETLKLTWFSVVMSQKAW

>SG-AL_contig_5025_1_-3_126_380

MYILHVTKKKNDISCLRCACIYNHDSHVNLKDSTSMMQFCVCVCTVTLWFLPSKDSSLSS

AMPYWTTSFCYTHLCRLLLLSCCSC

>SG-AL_contig_5026_1_+1_1_195

RQTDRNRDKEKERDRQTDKEKEREREREKERERERERERERERESTYVCVVLLKFHNCSS

YQLIS

>SG-AL_contig_5027_1_-1_1_114

LLSFCAILPCISLCDSSIFIVTVLKYRSYTLGAYMIYT

>SG-AL_contig_5028_1_+2_2_103

SSSINVISIIIICTHLAICLCLYYCSILTHVHNG

>SG-AL_contig_5029_1_+1_256_453

MERTLLHLRASWDIGCPNYTLPTHIFFTIAMLFSRERTHCLFLDTSWLVLEHGCLPPRHA

ESAWLC

>SG-AL_contig_5030_1_-3_24_1220

MTSIWPWLIGFAAMMAVVFSTPIDNTTQNSIEINVFVDRWALKRWKAMVATSSVMSEKIN

ETKAAITDYVYSVFNDVNSIFKELMRYGINLDVRVHSLNFIEENEIAPDAKLGFRHVDPL

PVVQNLFRYLWYRNYSYIEHVMLFTGLDVNKSDEHFYSETCGNYPLSVLEATPSGSAALS

MAYQQALYMYVELDGQKNLCGNNYIMTAVDGPSSPTNFRFSNCSIASIKDFLETMIDRGN

CMSAARTNTPPVSAPALGERYTPDDICKLTFGNTSYFCRSLYGYGGKYTYDSMCLEIYCK

RKSGRCAKVQAPDGFVCGKDKQCSLGECKPSLSSVSGSSPASVAADKCPQGDSPVANDEG

QTCSQAVSASRGKCYNGKFQRTCCDSCHRVATGPTENVN

>SG-AL_contig_5031_1_-1_49_1437

MAGVWLLLIGSAAMMAVVYSTPIDNTTQNCIEIRVFVDRHAFKSWKDKVATSSTWLQRVN

ETKAAITDYVYSVFNDVNSIFKELMRYGINLDVRVHSIHFVKQKLLARGINKTTGQLSPG

WALYLLSEWVNPNYPAIDHNMLFTGLTLKKKPTQNYPESAYYGSTCFSDPFSVVEATTSG

AAALFMAHQLGLSMAVEAEGSHIFCDNGYIMSAFKTHSSPTNFRFSECSIKRIKRYLEVV

RDRENCLTAANTNTPPVSAPALGERYTPDDICKMTFGNSSYFCRSLYGYGGHYSYDSMCG

VIYCKQDSGLCTQVRASDGFVCGKDKQCYLGECKSSLSSDSGSSPGSAPAETCPQGDSPV

ANDEGQTCSQAVLASPRKCYNGEFQRACCDSCHRAATNLTGCQYGDMASECSDFSRPQEC

NRDHDVLCCEFCRPYYTGIAGCEYGDKDSDCPHYIATGTYSCE

>SG-AL_contig_5032_1_+2_146_265

MAINKEDLWKTYIFFLFMRSIHQLQINYKAFFFNLYIVDF

>SG-AL_contig_5033_1_+1_178_447

MCRKMELPHCTEATCSSQGQHKFHMENLLQQDIHTVQQNTTQCNTHTLCLPVEHICVIFF

PEAKIKYTHEFMLNANGLQDSYFSLVKTTN

>SG-AL_contig_5034_1_+1_1_168

LEFKILMKMNKITTLTTQLKENRHHKHKTFKAKKIRSKVSSDVMQMLLCSTRKMHM

>SG-AL_contig_5035_1_-2_128_1726

MQAEAMSRKSRKASNKIQIKNVPSSVTCDDVRNAVESLIPGVAQKFEEAGAEGAVYVTFD

TVESAQEAITSLEGFQLEGTSLQVDFANNKSPRRPRSNGNSSSGMNRQDLPLRIVVGSEY

VGAIIGKSGQTIKNITQQSKARVDIHREDNVFPETVVTIRGSSEACTKACQEIMQVMQAE

AQSLSRGECPLRILCPNSLCGRIIGKKGNVIKGFMDKSKTNIVVSSFEASPYPSGMGDMN

NYYVDRVITVTGKLEDSIKAEEMISEKMKQCYDQDLNTYQQQQMMFGGLPTLPMMPGGMS

SYPSARPPYPYMHGGSMQGGSSSYYGRSMFGGGLIPPPPPQQLEVIFLYIPEQSVGAVIG

SRGQNIKNIMQMSGARIKIMGADVKNGSGSSKSGQQGQQSPDDRKVIITGSPEAQWKAQF

YIFEKVKTEMMLTAQDEVHLRSEISVPKSSIGRIIGKGGQNVKEMQRVSGAIVQVPEDQG

EEEEVPVTIIGHFYACQSAQRRVRALMNMKAQGLLPLSHRPARPQHALQMNGN

>SG-AL_contig_5036_1_+3_30_248

MVSVLAKREFTWQNSSFSVIHSTISNLCRIQRLEGRDLLPWSARPVEASITALAVTKEMR

DRPSALETRRSRR

>SG-AL_contig_5037_1_+1_562_699

MTLRSPQSEELQISLLLYCIVLYCIVLYCIVLFCIVLYYFCHNGFT

>SG-AL_contig_5038_1_+1_202_300

MVSILAKHVFRKKYTFFASYTVPCQIVSRIQRL

>SG-AL_contig_5040_1_-3_2388_2714

MFQPTSALQLVPLLFKPVSVNKVSKSFPVFVSRCKDFSSLTCLLICLCEQGVKASLSCAL

FFLTLFVMNMMMMMMQTLLYDAFFSETFHLCVFLASIFATVNICSGVKW

>SG-AL_contig_5041_1_-3_93_1085

MATEVRPTNRFQPPDWFTSNYTISTNAERQRSASHQTRQEARFLRNETDSQTKWDQYSNN

VRLADRVDDIRKWKEILEKTLADLDKEIADLSEAKELTENALEAKNIPTDTAIECLTIRE

GRHSIDVVQDEVENQLHKEVEVIDGIKKALQQRIGEAFEQLCLLQEARQQVQADLQDKNI

ALGIDVDQYNLTDRSSQISYKPDSLRVPKGSTTPQQWEDFSRYNKERADAEMQASNRLRE

AIHHTLHQTANDLEAQKIATEYAFRKRIHEFERAKDELEWQKKNTEDEIAEMENDIRGLE

ESIRAKINPTKLAQTRLENRTYRPNVELCRD

>SG-AL_contig_5042_1_-2_2504_2689

MLLIFYYSCCLCVIVPVACIILFLLLILLVFYCSCCLSCIDPVANVACLLLFLLLVFYCS

WC

>SG-AL_contig_5043_1_+1_1_129

ISRGERADGQQRAGRPEEEKYHVKSRYGLLHAILLPSFVTSPV

>SG-AL_contig_5044_1_+3_186_332

MSLDYGLLVSIDTGHNREKSYNSSTNNLKTIISYRCICGYRASNYNGHR

>SG-AL_contig_5045_1_+2_128_562

MRPGGSAPGAERSESPGSGREEEPLVEDAASSYSCCSMGEDLSLKGVLSPLSFLAQEVKG

TGSSGTPGQAGTPSGGQRRRCPCLGTRRGSACSGSACSGSACSGGASSRRRGRRPTRGSR

TRSACATTVCWEPYLGSISPSMVGC

>SG-AL_contig_5046_1_+1_1_285

IKPRTQSKPKTKKIYFHKTYSSCTILLLSRSGKTYAHIHIYILIIVHIICTSNGQHTIIT

NIKAATSNLTVLDYSQTFFSKSLFLMNLLSDLQYI

>SG-AL_contig_5047_1_+3_3_278

FRNGGGKSARQRRWADLSRRRGLFNTSVRILLQIMGMSCLPFCVHYKCDEDVLTKALSTF

GHGGQMLKILDFHTEGSGRPGSKSSSSTSPSG

>SG-AL_contig_5048_1_+3_3_197

PISQVNICADLLVPEPPLCVYAYRTSNTHVRDPVSHVCVRWLMETHRYPACTLYSPFTGS

KLHWL

>SG-AL_contig_5049_1_+2_323_502

MKKREVRWLYQMIEYMKCVCVCVCVCVCTRACVRVCVCAIGRSEFDSAWYLLMGKGWRFF

>SG-AL_contig_5050_1_-3_249_659

MPRRGRSSPMFSRRPAPAPAPHSRPMTTAPAHPAPAAAAPRQPGLFGQMAATATGVAVGS

AVGHTIGAAMTGSGGGYQQEVAPAPAPVQQQDQVYNNPCQRELQQFLDCAQGQADIALCA

GFNEALRQCKLQYGVRP

>SG-AL_contig_5051_1_-1_118_1380

MAQQESVASLTVRHRFSTSSSAFLTQLGATVGGGCVGEQEMDVIMLDHCYAKPWSAHPDA

SSARPVRMLFMPKQIRTSTLEMLQRNVDPPIDVVDAPPAPSMPFDINKARNLMTECERHA

SYGRTDSQEDWEEKISKSGWTIQQTRLFNKVTKILQSDRLARLAMESRRYECEPLQSSSK

AYINRYGYEEMEKALADAANMELKELMAKTSFYGLMVDESVDISVHKNLIIYVKLMVDGE

IKIVFGDNISVPDGRAETIFNAINKFMDDYGIERHRLVGFASDGANVMLGRISGVATRLR

GINPNIVTIHCTAHRLALAAFHGAKACEQLLDLQRTLTQVYTYFEYSAVRYERMRDLQDV

MNVHVKKFKKPTSVRWLSLEDAVEAVHKSWEVLVAVLQNEWEEKKITEAKFLARAAMLLM

F

>SG-AL_contig_5052_1_-1_7_1644

MAIRWQVHFLVFFVFSFVLAASNGRHVSSESVAPTRGEPWPLPSSYNTTGNVNTLDPVTF

RFVAGGVDCDILREAFVRLHRAIFGHLAVKSYTDRLRGGFVQEMQQHMQTGLQQVWVMVG

TPCQGAYPSLQSQESYILSISGGKAVVSSNEVWGALWGMETFSQLVYRSNTGEFLVNDTT

ILDKPRFAHRGLLLDTSRHYLSKVNILRNLDAMSQNKLNVFHWHIVDDQSFPYQSRVFPE

LSDKGAYNPETHIYTPSDVKDIVEFARLRGIRVVSEFDTPGHSQSWGNGQSDLLTKCYSG

KVPDGQFGPINPIFNTTYDFLQKFFKEVASVFPDHYVHLGGDEVSFTCWQSNPAIQQFMK

KMSFGTDYSKLEEYYIQKLLDIVSSLNRGYIVWQEVFDNGAKLRSDTVVEVWRAGWQNEM

ANVTKQGFKTLLASCWYLNYISYGEDWTKYYTCDPYDFNGTAAQKALVMGGEVTMWGEFV

DDSNVLSRLWPRASAVAERLWSPKDYNMPLLAEARLVEQRCRMIRRGIPAEPISGPGFCE

EEYRGY

>SG-AL_contig_5053_1_+3_3_179

SQTSNRKWAAFVLFTVVTLLLPTRKGSAPTPFRKNFSIFVRHGLTLSELCEKTLFSQRP

>SG-AL_contig_5054_1_+1_2227_2442

MLPSTCNCKQLCSPQCVTVNISIQSEKKNCKLQSSLQCVNEANFPSTMTICDWKLQSSPQ

YATGNCEVLLNM

>SG-AL_contig_5055_1_-1_1_183

LDPRDARSEERLLCPVRCVKYYLKRSAPRRSSQRRLLISINENYTKDISATTIPAGCPLP

S

>SG-AL_contig_5056_1_-2_29_1372

MAGTVSTVTKFVSRFGSNIQNFLAPSVVGASQQRHYAVGKRIKVDNPVVELDGDEMTRII

WEKIKDRLIFPYIDVDCKYYDLGLPYRDETNDQVTIDCAEAIKKYNVGIKCATITPDEER

VEEFKLKKMWLSPNGTIRNILGGTVFREPIICKTIPRLVPGWTQAIVIGRHAHGDQYKAT

DQVVGGPGKLELVFTPQGGKPESLTVFDFKTGGGVGMAMYNTDESITSFAHASFQYALMK

NWPLYMSTKNTILKRYDGRFKDIFQEIYDKQYKAQFDKQGIWYEHRLIDDMVAQALKSSG

GFVWACKNYDGDVQSDVVAQGYGSLGLMTSVLVCPDGKTIEAEAAHGTVTRHYREHQKGN

PTSTNPIASIFAWTRGLEHRGKLDGNQELQRFAQTMEKACVDCVDSGKMTKDLAGCIHGL

KNVKPEHYLYTMDFLDAIGEEFERQWKA

>SG-AL_contig_5057_1_+2_350_547

MPPCTGPMKQNSGSNSSPFRSCHGQHSHPISAQLKMSAQFRKTRKKVTSIMLPLWLPSSM

NSQWCG

>SG-AL_contig_5058_1_+3_327_524

MPPRTGPMKQNSGSNSSPFRSCHGQHSHPISAQLKMSAQFRKTRKKVTSIMLPLWLPSSM

NSQWCG

>SG-AL_contig_5059_1_-1_133_573

MTSFGWKRKAGAGVIRKASEAFEEEAKDEELDNEVTRGEVDWLTLAPKKRLIGLEDAKAK

SRRLCDEGATLAEAERYWEAIKKWDEALLMTPGDSRMLEMKAQALMSVGEAFPAVHTAQQ

AVDADPTWWVARQTLGRAELNLGELRL

>SG-AL_contig_5060_1_+1_313_534

MLPLFSSLVSQSYLFVVCCYMLPLFSSLVSQSYLFFVCYLFFVCWMKMHMPKNLHKCYFF

VITSYCGHYLHSHQ

>SG-AL_contig_5061_1_-1_133_375

MTTVLQGKLEAKRNPGRPPTTYMNIITKINGLKIHKVAHRSRDRGEWRRIAVSSTAAANM

NPMMPTGDRLVFEQARNNILH

>SG-AL_contig_5062_1_-1_1_204

MSTVSHSKPELYNCSLEQLLLTSIFVTDELPPSPSYKYIRSHINPFRCPQFLIPSQIYIT

TPLNSSLL

>SG-AL_contig_5063_1_+2_533_727

MKVVVRSGDIPRYIALKKKIISWVKLTININTLKRQQLLVFSTSVKVMTGSLWAQVYCSL

QKNPT

>SG-AL_contig_5064_1_-2_1487_1729

MFLRWAYDKNCMINKRSSSVAFGESITDKSAGSGAALEAVELVCNNFVCSDCSESALMQL

LTHVQAWAVLVYMATFNPVTP

>SG-AL_contig_5065_1_+2_2_136

FFFAFKIHFNQIKLTISGHVKRKQKPKIMNYMRGRERNRGDIMNL

>SG-AL_contig_5066_1_+3_510_725

MEPKSLLNYQFLNGTTIFAELPILRHPASIDLTYGQVDHRSVSCCQSGMLLLSCNAQTHT

EFIRRIPMRQSL

>SG-AL_contig_5067_1_-3_324_1343

MTSLRAVVCFAVCVLVFVWSAQECQSRRGGGSRSSSRSGSGYRSSRSRSQRYRSRSRLVR

VRLGRVRIGSSGFTSSTWKAAALSGNIYGGSRYLQSDFYRTHPDRTPPICTNNEAKAPNG

RVYGYFICPRETEPANYTYCCGPTNREFCCPRDYYGSSTSASTDKRSPVPAVIGVIIAAI

VVIVAVVAYKKRKKIRKFFRKKAHPDKSPLEDTKREETLGDEAPPDDGYQGNDGAFSNHS

NNNNSSYPATEAETAPPEELFLATETKTAPPSAGPFPAAAPPSAPPVPANFYHPPPAPYC

PNYSPDCLPPARPPTPPPLDPNRPFVMPPDPIPYPPKEEI

>SG-AL_contig_5068_1_-3_882_1163

MDLYLLYICDGNRFVPCWNRCRLVIFNIPWEKGLRGTGGDCFTSTWRLYVLCVSLCCLCV

NRSKAGERTRMCVCVCVQIVSFCPLFMHGSLLIL

>SG-AL_contig_5069_1_+3_825_1373

MCPNPSPASVPHLWGSIFYVYRLPILRVRIKGWARARVRVKLALVLSDCWARARVRAKLA

SVLSDCWARARVRVKLALVLSDCWARARVRVKLASVLSDCWARARVRVKLASVLSDGAKC

CQMEQSFVRWSEVLSDAAKSKINMTAVIVQQPIRGMYMGLCLDKSLFCTPSMSVSWPKQS

KAY

>SG-AL_contig_5070_1_-2_92_571

MGIYLAKFLFFMYFSPCVSGTHSLSVPMCQALSLSFCVCVHWHFSHSWARTYIHRHTHNL

FLHSLVFAHTYILMAQYGTVLSFSQALSHTHTHTHTHTHTHTHTMVFSHSFSCNGKFEKW

LLANNFYPWKIKYIVIVIVIVSLSHTRTRTHTHTHTMYKT

>SG-AL_contig_5071_1_+1_1_261

KKILKKIKKKKKKKKKNSLLAIIKKRKKKKIQIKFSHLTGSKPNSRIMQLMATCKNNDNN

NFIWYSANIPRRQHVCRSLHSNGLLKK

>SG-AL_contig_5072_1_+3_1353_2288

MESHKSLLPDNRPTEPHVVIKVEKGKADDRPTHSETPPVNKPISTNNRLLLLFLHHDDDQ

HHHLLLRHYEYHHLLETSSLFQQLHLQEIAVVDAVVLVGVHGADLTSHLVIDVNAHLLQP

LHHVLTGGAPASVCVELREKLAPHAGLGVSTRIRHKIDVLVHCHSAHFGLLLLLPQNFSS

PIDLGQPQQGHESRELLLVQESIPQHVSHGLKLRPVVQSLGRLVHLVVGPGLWCLFTYAL

PHLNAEITCRLGEQIISELQTLLVPTLFCHSTLTEQLLVQHTSRLVGPTWVRAASVGLHY

AKYFSTVSYQIF

>SG-AL_contig_5073_1_-1_31_1035

MGALCVVAGVLLFIPAVSYSFSVLFKVTLLTLLMSIFLSVFFTPMILSFVGCEKSREEMD

KDKSLNEDLLRQNKESPISENGHAVRAEDENERNAIATLPHTQQGYGDSNTDKNWSDYVI

SPTEANMRVIGNGTAGERYTKMLNGRKPLTDFPKADYENVSIGGTCPHGLSSNSYKNARK

SLPSNGYYNTFNQYENWQRRNTASRDLYDLPISFPSERREGLRTYGIMDTDFRNPPQPVR

PLRRKDVAPLIIRKRPTSREMKEPKSLRQTRRKEEIQADIVASLNNMDDDFHESPYYEPV

ASLEDVHHVACTLSTNGGLGATQAYVTENFHDIPS

>SG-AL_contig_5074_1_+3_675_821

MCRFFLMWNIIKCQDCANLNETGQLLQSQPYRNLSTGAWNRPHIKCQGT

>SG-AL_contig_5075_1_-3_30_581

MGLLTILKKMKQKEKEVRLLMLGLDNAGKTTILKKFNGEDIDTISPTLGFNIKTLEHRGF

KLNVWDVGGQKSLRSYWRNYFESTDGLVWVVDSADRMRLADCKRELQVLLVEERLAGATL

LVFANKQDLPGALTSQEIQQAMDLDSIKTHHWLIQDCSAVTGDNLLTGIDWIIDDIASRI

FTMD

>SG-AL_contig_5076_1_-2_71_376

MQLKCKPTTQKSIMYAYHKIKNMLLYECVHTHTHTHTHTHTNINTHTHTYTQPHIYTDMH

AYTHLHMHAHRDTYIQPHTNARRQTYVHQRKACKQICETYTE

>SG-AL_contig_5077_1_-1_166_498

MDLEKAEDRADTSERRVRELEDQSHALTNNLRSLELCEEKASQREDSYEETIRDLTQRLK

DHECRSETSEREVARLQKQIERIEDELESQKETNQKLREEMDQCIGDLQNL

>SG-AL_contig_5078_1_-1_1_225

KKKKKNPTCASGLYLALRRYLTFVRVGEGKTARERIGYCSHKAGSGIMRSVPVSSLSNNL

TIIRQPVGGVRESEG

>SG-AL_contig_5079_1_-1_913_1116

MLFWKHYSLQPYIYNDHLFVQLGKAATLVVLHVYSCYRLVTLLNTYFVYCLQQVCRNCVL

LCKLSCIG

>SG-AL_contig_5080_1_+1_205_387

MPKTDADVIIIIIIIIIIIIITIIIIITILSTTATTALTTTTTTATATAPLPRQHHHHPK

Q

>SG-AL_contig_5081_1_-2_2_142

CVCVYVLCVCFVCVYVCVCVCVLACAHAHIICCMFYFVEVLFMRRFF

>SG-AL_contig_5082_1_-3_3_194

LKEANTIQTCGISGCPIIQGCEQATPGKGKKRTYVSCHACRFILCFHCQRKIALINAWII

LVTL

>SG-AL_contig_5083_1_-2_2_130

FFFFFHYRNKTGNSPKASLLLLKIYALICNEVFNLFPRIFFFL

>SG-AL_contig_5084_1_-3_1650_1832

MLTWEMGEISTLYQAASNPGIPVGRPVPCPLDYHARSTYNHGEKKKCVGYEYLITGKLNL

G

>SG-AL_contig_5085_1_-3_153_578

MLEVTNLSPFSFSLEVKKMGKCVFSQAWLTRSQYKNWIAPASDGSKHVLSEIFHKSSNAP

FCREILIQFGFEFGMENFQSGLEKVWEKYGILLGHICILASTRLESYRHLWCWSGTLNTT

LNDASVKWMRLDNVVPVFPTNP

>SG-AL_contig_5086_1_-3_3_206

HAAHSGSSTHHKPQVHSVEYPMPGSLWESTMSSRCRATHQRRLCTAGGSLWWSSTVPFLI

QLTKDSAF

>SG-AL_contig_5087_1_+3_3_200

GKTGTTPSSLIHFMDASLRVLLKFPDQHCRCLYLSGLVDTRIYYEKKRRVQPCHAVSIQK

GTPQHH

>SG-AL_contig_5088_1_+2_674_898

MSEHNQSTWQSGYAADNWLEGRGFESPSVHKKSLWDFSALTGSYPELSVLWARWEDWDHT

AKSYPLYRCIIEGVA

>SG-AL_contig_5089_1_-2_59_1306

MNDSLAEATSLEQMLLAVYQPYRPYLVKYKVFEEVALGKQLDNIRLDHEEIFDTVQLLAE

SVSKIFSAANTSSERCRHLTNGCCYLGLLEAFKTFFCSYTREFRRVVVNIREKCRAGESE

ESEDWSNFQHSLRIIQTCGFLVMHTEEMDKQLITSILQTLAHLTPSHVEHSKKVNSGLHT

LSFQASLFLSNKQDVEHLESFVIQLEEGDTPSVLTEVMADLGRLSEDVHKLAFEIVFAQL

KNYLANVSTMEMWTSQSAGGALTSDLPTFSLAPQEYITKIGQYLMTLPQHLDPFTMQDSP

ALTITLKHSKLPYTEEQEPPEHLADLWLESVARGTMHVFAEEMLKIPELTPHGTKQLITD

IEYLRNVLDDLGLQTSETLSQIENLLKVSPHEFADTAEVMPQRISHSIAAMRGIEV

>SG-AL_contig_5090_1_-1_331_675

MKSYRQYVTMAAGELGLNLARVFEPPKVIKRYTVLKSVHIYSKHMVQYEARTHFTVFELK

HLTGSTADTFLEYIQRNLPEGMAMKVTKQRMEKIPDHLTPPAQPTSHTASTSPAS

>SG-AL_contig_5091_1_+2_101_199

MHEFSVLASQWPVKRQTILGFHMAFRQAQREGC

>SG-AL_contig_5092_1_+3_3_86

VSHGFPAGAKRRLPLPIFTLFCNLGFIN

>SG-AL_contig_5093_1_+3_3_188

TKQKKTKVTKKSVRMIVTLQLLKANSKDTLNYILHYFWQFSRDENKAIHFSIGAEYKNYF

FM

>SG-AL_contig_5094_1_-1_16_753

MEHSEELAPREPTPIPAMSIFMQAVDWLLEVKAIPFTEQALAHELLTTPHGKTPAYHIAT

ARLKLQKEEYEEAEESLNQALQVEHQNPDGWALMGHVKYLSGDNDSARQCYERTLSFVAD

ASETHSIYLRLASIYLQDGEFQHAKQTFLMACKKSPSCVSWLGVGISCYRLGELGEAEDA

LMEANILNNSDAEVWGYLSLVCLRTGRKVEAEQSYKHAVKLHLQDTALLTEIQAEQKKVG

FGSPEF

>SG-AL_contig_5095_1_-3_111_1127

MTEQGGSPIDTDCITLTRFVLQEQRKYPKATGDLTQLLNAILTAVKAVSSAVRKAGIHRL

YGISGSANVSGDEVKKLDVLANDLFINMLKASFTCCLLVSEENEHAVEVELENQGKYIVC

FDPLDGSSNIDCLVSIGSIFGVYRKTTGMPACEKDALQPGHQMVCGGYALYGSATMVVLS

VGNGVQGFTLDPAIGEFLLTERDITCKPRGKIYSLNEGYEKFWDQPTKEYLKSKKFPESG

KPYAGRYIGSMVADVHRTLVYGGIFMYPATSEAPKGKLRLLYECNPMAYIMEKAGGKASD

GKRRILDIQPVNIHERSPIFMGSKQDVEDVEAMYKKYGN

>SG-AL_contig_5096_1_-3_63_437

MDLSYLKSHRTASRFLEFVDFWALSLIQLVKDDPTFYLLLSLQLFETCDIYNINKYGHSL

ISLSNKDNMIVLVSSVFTHFLAVWFFSAFLVCFLLRQIYFSLLVWSWQRSRDLFAGTIEL

SMCCE

>SG-AL_contig_5097_1_-1_94_348

MIMYRNGLPWGVAFEDVFEGIYHPAISLYKNAQVTVNFGPDFKFPPTDQPDYRPMSEAAD

QAMVEGALADVIYHIDNEGKHPEFQ

>SG-AL_contig_5098_1_+1_253_369

MQVDNSRQSKSASMTNGFLSSHVYFFLVDKKGRNRKAQK

>SG-AL_contig_5099_1_+2_98_247

MLLVLISQLTTKAISNMSNNYETVKSIVFTLVIIHLITWTILLFRIVQTL

>SG-AL_contig_5100_1_+2_173_337

MLLMCPECRQFEEKNTNTQFVPVRTQNVTCPDYEGLFPDVLLVSSLCELKVVLHS

>SG-AL_contig_5101_1_+2_20_271

MVTDLFQSQDFSLLEIFFTFISEEHTHTKAHTFLHMSNVVSDVYMSILLVVWPTHVYMSI

LLVVWPTLFMLLQTLRTPFSMENE

>SG-AL_contig_5102_1_-2_215_886

MAMYPNATHGYMPFVKPAHKRQAAKRSKADKDSLKTKAAEGTKTYKVTMRDRRGFTYTYI

IQADASAKNVPVLCESRVTAPDSSSSPLLSNSLPNMRRSARFPVAAPETRSCASLAEGKE

DLTKVGSEKKGLFNSLLQFIKKRTTSKAPTPKTLTPGPKSKRKLKPLPQRKRQRTVSAMS

HLGTIQESPEEDNLELEDTDSYSHDSVTSGNESESGDGQGSCPP

>SG-AL_contig_5103_1_-1_97_426

MTRGRIELGDVTQHNIKQLKRLNQVVFPVTYNDKFYKDVLEVGELAKLAYYNDIVIGAVC

CRVDTSENQRRLYIMTLGCLAPYRRLGIGTAMLEHVLKICQEDGNLDNVF

>SG-AL_contig_5104_1_+3_1392_1721

MGGTITGNVPEADITKVNKITTSFFHCPKFQHKFNTWQQFLEHRKVTLFQMNTSLHIHPE

VMSCKSEEMVTRKPCGNTGEADTIIPCSIHFWSTGYSHPLSTAHYQLLII

>SG-AL_contig_5105_1_-2_182_406

MCVWPPVYPAVNEQLSSKLGKVNQQGRGDRHCPHNPGKEKLRSLPPDLRVRGQPLSFFFF

FYLVHGYVDMRAIQK

>SG-AL_contig_5106_1_-3_9_218

MSRQFVLMDDVLLKIECKYQMLHVAFVLGIFAFLLVCFVILYYDDDYYFCIVSSIPDAYH

FHSLIVILFF

>SG-AL_contig_5107_1_-2_38_553

MAARRIAKSAVDWTAFKERVPLRQQEFYRAFKAKNDNYVNKVHQFPEELPKIDFAFYKAR

LPNPAVADQFQKAYEAVSVPYPTDKSNMLKDIDADEKAAGELSKAYIAERQEEINDAKLL

LSKIDSLPKPEEMTMEMYSYYFPHLALDPVKRPSFWPHLPHMQPGHKDSHLI

>SG-AL_contig_5108_1_-3_3_272

QFNSSSSSSSSSSSVFCLFSSSSSASSSSPPRPLSSPPSCSSSFSSLSFSFFILLLLLLH

LLLHHHHPLPSPSPVLLPFRLAELHHYTWP

>SG-AL_contig_5109_1_-1_757_1023

MTFLHSIETLFRLIQHILVNTRQLPSHLHPTVLPTCPLSATVLASSLAFFRLHRVRGVFH

YCKLYLFSYIIVTAGKNGTQNFHMRMRTK

>SG-AL_contig_5110_1_-3_3_134

KSRCQYKLQGLHIVILVDLYQPTSRIASILITQPELCNPPHTSF

>SG-AL_contig_5111_1_+2_128_271

MLLHSLHTLSSVGVHASSFYKLLNMTWLTGSLTFVFDVLHAYTHEGRV

>SG-AL_contig_5112_1_+2_80_271

MGCDLHVIITQLVLVWSISGLQKHSKAMQTRSSVIKTLHHSGIKQYSIQSMKYSTIPLNY

TKEK

>SG-AL_contig_5113_1_-2_518_1429

MSKQVTECLSPPCGVNMALSQSPASRARRMNARYTVHEVLEQLQTCGSDVDDLSSDSEGE

ENIGDSDFSADRSDEQESSSEQETDDVDQQQFEGAESDISATSDQQEEVEADPDSDIECF

LSAVLKPWSQDFSFFPTVQDFNGGDDRGLHVDHMESHAPLDFFWEMFDDVILALIVDQTN

KYARETIVRPLTKGSIFHHFPADGITKNDLLGFLAIIVHMSLDHKPEIADYWTKSPILQS

SFASNIMPRDKFIMLLAILHASANDSFVPRGRPGHDPLAKIRPVYEHLRTKYRSLYTPGQ

HLCR

>SG-AL_contig_5114_1_-3_339_2096

MSKTTQRFEEDSISSYQPRTAQKNVIISRSSIGPRSRTSGGMFGGSFSSQRMSMSFNPAV

YSQMAGTGIADFRGTREKEKKEMQDCNERLASYIEKVRWLEAQNKKLEAEVEALRNRKQE

DWKPIRDMYEGELEQARKVIAELSTQKGVGEGKLAGLQDEIQSLKELIAQYDSNAKDYAK

KIDQLNSQIGEYEGEVTTLRLRCGSLEDEVAKLRALLAKYKDENARLRADLDAETAAHIE

QEVLAQTKAEECEFLQDVLDKLQLMQQEPVTIKGLNLEDYFKGELRRSVQEMQQTFDERM

DQATLEMDAKYAAQLNQLRSGNVRDNMEAEHAKGEIKRLRDQLAERQSRIAELESLLASI

KAERDNYHLQLNETQQQLEALQIESSRKINELQMQIEALMQQLSELIDAKMSLELEIACY

KKLLEGEENRTGLRQLVEQTIGVQSSGAANLADIIGQSNLSSSTTARTTVQRSSKGAIGF

QSVDPAGSCVTLENTSSGSRAKTQSMKGWKVIKEVNGKQLCAADLGERELRPGQALVLFA

KGAKSIATSDNEIIVDQFSWGTGTGQFKLIDSDGQEKANLTVRTQQ

>SG-AL_contig_5115_1_-2_521_835

MRHGFPSKVSPANVETERGWAKMIRGTKFASKDFRAEKGCTVYFSTTKFLLLSIFCLLTA

LSVADTTKKPYCPKSLRRSAASIQALEPTMSFAAMTMSVPTKQGP

>SG-AL_contig_5116_1_-1_430_720

MTFSFGKLAAKWVPHKLTEEQKQRRVHAARDLVNCFEQNGPKRATGVVTGDETWVSFYGI

ASKCKNKAWLGSNDQSTKLASKDFRAEKGFSLYLSTT

>SG-AL_contig_5117_1_+2_2_286

QPCTHFSYADVQVFLHKFGHTAALLSFEALFRSHFGLSVLFPLFPVLNSCFHRRIVEYEA

TLIPATLFTSVAIFITPSPSCSLANMYNVSECLLK

>SG-AL_contig_5118_1_-2_380_532

MNWVQKACIKSSVRFFFFFYFFVLNLFFFFFFSLQQNKKKTRIFNFCYFRR

>SG-AL_contig_5119_1_+3_3_203

LLLFVEGRRVRGGGGGCIITFFASLLVCEFTILWEVTLFLKRDESVCTYWHTSKSNFYSL

KLFCYYL

>SG-AL_contig_5120_1_-2_20_418

MAAPYKLRCNVVGHKKDVRALTPALFPEGGVISGSRDCTTCIWLPNEHDASFTEGQVLKG

HSNFVASVCVMLPDDNHSQGLIATGSNDSTILLFAPGQVEPVAKLTGHSNTVCSLAAGKF

GFLISGSWDKTAR

>SG-AL_contig_5121_1_+2_383_622

MRDALHAIRLLYMNCPSLPVHRQEQKRDRRPMDIKFKIILMFLHWWQIQMTLAYADCTKI

SWEVKTVVYDGCQREKGCLL

>SG-AL_contig_5122_1_-3_81_1067

MSHFVTDRAYDYFDLGDESDQESAESSEDEIDIILHGTPEQRRRLRHNHAKERQAGQSNS

KSSQHGRDQYQSSSEDEFEKEMNAELDKTVSSLEKSRGSKQASGPSAASSTASKKEAGKQ

DSSASAGKNKMPYYNDIYFDSDDDDNYEGEGDSRVHKHHTKPSNDDLLYDPDMDDEDQRW

VDQQRRRQHGDNSQARGNKRAHGKKQGVPKSDAVLDCPACMTTLCLDCQRHDIYANQYRA

MFVMNCKINRSELLSVPEQANKKKFKKSKKSKGHSRESTDTGNNVGDSSSSSSSKDMFHP

VLCKECDTVVGVVDVEEVYHFFNVLVSQP

>SG-AL_contig_5123_1_-3_150_1382

MMAWKALLYAVTLVTVMIHVAVAKPVDKRGPPHPDPVEAGSKQDDDMGTGLEYDNYLRQV

VEVLESDDTFRKKLETANVSDIKSGAIALHLELVNHTIRTKLDEIKRLEVQRLQELALIK

MKSMSGVGVNRMDIPGHLDLRNPHSFEMKDLENLIKKTTHDLEEVDKRRRADFKEYEMEK

EYEYRESLKELSEEDKKKTEEQHEEMKKRHMQHEKVHHPGSRDQFEEVWEKDDHLDDQDF

DPRTFFNMHDLNSDGMWDIQEVEALLQREVDKMYDARNSPEEDDPNDRYEEMSRMREHIF

TEIDTNKDFMITLDEFITYTGKHGENAKFKEDDGWETLGEKPEFTDDELEEYIRQHHPDS

MTPHPDPSLQFQPEDKVREQMMQQQMHQQGMQHPQQGMPQQQFQQGQGMQH

>SG-AL_contig_5124_1_-3_75_1325

MSFVNVGPDSHFPIQNLPYGVFSTADNAKPRIGVAIGDQILDLSVVKTLFDGPVLSKQQN

VFDESTLNAFMGLGRPAWQEARATLQRILSASEPVLRDNADLRDRAFVPQAKATMHLPAK

IGDYTDFYSSKNHAFNVGTMFRGPENALMPNWTHLPVGYHGRASSVVVSGTPLRRPVGQM

RPDETQPPVFGACKLLDMELEMAFFTGPATQVGERIPIDRAHDYIFGMVLMNDWSARDIQ

KWEYVPLGPFLGKNFGTTISPWVVTMDALMPFAIANPKQEPTPLPYLQHSDNYSFDIKLE

VALNCEGLPKPATVARSNFKYMYWTMKQQLAHHTVSGCNVNPGDLLASGTISGETEDSYG

SLLELCWKGTKPLDLGHGVTRKFLADGDEVILTGMCEGDGYRVGFGTCTGKILPASV

>SG-AL_contig_5125_1_+3_153_392

MKLKIPLDTTHQQLIISNALFKTETENTQQLHFFALVCFVSCLDSENPKSIPFQCLTPGS

LKLKRNINNYLRNKILNKKI

>SG-AL_contig_5126_1_-3_93_353

MSGRMFLRRLATLPKGQPAGIVPSTARYKKIQELQNTFCRDDGLLVWQKLGRDKIFYYIT

MLFMVCGFVPSLTTIWKMSFPKQTPEE

>SG-AL_contig_5127_1_-3_2046_2339

MERNCDGEKKLTVIYGGHLLLHDVCIIYFVSFIEINSHFIEKCHMEVGPRDFFSQRVSRK

FLPNFLSNCASFWLRSNILTAFIKHSLVNFCWKNPQNS

>SG-AL_contig_5128_1_+3_135_308

MYNSHSHTNTQTIFHTYLLSCTIATHTQTHNFSHLSLFLKNSLELSEKGRFLEWLDHL

>SG-AL_contig_5129_1_+1_535_744

MTTQLPKLALRGRVSTTQKYCMIDLSDVSLFEAEGLKNWHSPPYPSFIPSKIGYTQDTVV

YYYSERMYTI

>SG-AL_contig_5130_1_+2_2_217

GEKEGNRGIGEVCVYTHTHTHTHTHTHTHAHSFINFTCRHRNPHFSLPKLYTQYNISNIF

IVIKDIVCIYDK

>SG-AL_contig_5131_1_-2_803_1036

MDHVVILSNKFISPTPPHPVWSSLSTKFVIPPLHCMSQYMVLSICIILFVNVLMEPFRSA

GGKGRMKKGENVERKKIA

>SG-AL_contig_5132_1_-2_23_262

MKVIASFEEFIMDGLQCLLIILVFVIFVLALPTLSSTSSLNRSFTALLENAICKMGVSSV

PSVDWLEDASHLFRIDQSVF

>SG-AL_contig_5133_1_-3_30_431

MFFPVFFITFYFTKLVSNHSKIYWQIFILLMSVWDWKGKLLINFYRVLYVLQFCIYLFCH

NAVFVRNSGSCDRVERVNTVQRRFVLMCFCVSIFDHWHVFRISLRTTFCCCGFLTHVLSA

HSTQDLAVSSHPKD

>SG-AL_contig_5134_1_-2_305_784

MGNCWCPDHFVCANPQCGVKLIDIGFVEDGGNLYCERDYERYLAPHCKKCNKTIIGECVN

ALQSTWHPDCFLCAHCKLPIGGNQFHVEEGKPYCEKDWAAMFQTMCAGCDFPIEPGDRWV

EAMGKNFHAECFNCATCQVSLEGQPFVAKAGKAFCKKHAR

>SG-AL_contig_5135_1_-2_2_274

VTLFYKDNSIRSCWTFLTASPWSACWVFSCFRKPATDMKWCKEHNVPPTKPSSLQLANFL

AYLHAQAGLSASSVRGYRSAICTSILQLGGP

>SG-AL_contig_5136_1_-2_2_343

QASAQATPQPTPSRQPVRGGEPGSSRPAVEATAPQRLGGADHRGGLQASLARNEAPPPAV

TSSPTSGASRAGRSPGRGGGRPAQQGGCGSPTPPPWTRLLWQTLCRAQDLGWLA

>SG-AL_contig_5137_1_-2_2_901

DTVNGDVDTYFERLNSLFKANNIHNEAKVDTFISVAGLKTYKLLKALAAPAKPTEKTLDD

LKKLLKDHTQPQESIVCRRAKFYCRRQGPSENVSDFAAALQSLAADCSFDNFLNQALRDI

FVIGLRDLETQRKLRAVTDLTFKKAVETALTRETLSRELTGIHAAVVNQVSVAGAKGGQQ

RQRQQGNSRPYQGHRSVVWWSSGGGGSVAARGSSTGARKNCYRCGKAHDPSTCRFKLYEC

RLCGKKGHLQAVCRSGSSRQASSHYIEREHAEREEEVEEVEEMIDGISGLGLFHHRDNTT

>SG-AL_contig_5138_1_-2_56_214

MHLDFLKDFPASENMSHKQLRKNKKNKKKREKDVTLYFIYPFSVEGSKLPFMY

>SG-AL_contig_5139_1_+2_77_181

MQGCPSWYSIKKKRGKLCLRTHVSNRAERSSRSKR

>SG-AL_contig_5140_1_-1_220_363

MITEDKLWGSPLMYRNYNRNQGLFWCSHSHQNFIIYPEMSCIGQLMKE

>SG-AL_contig_5141_1_-3_57_203

MSSEQINEPTEESKILPWHIAIPRCYYTVAGKGLIKQSLVNFMDFVQEK

>SG-AL_contig_5142_1_+1_1_480

VSSAASSSQETDHPDPSSSSFSSSLLPPSVSPPPLSASSPVQLVQLPSFLLLLVSGGLLP

LASCVLPPVCVALPAAVFSVLPAACVAPPLPPPGACALPLPLHVQQSRPLLLPLVQVPLH

LHLSADENECWWSPQLVRRRLRPCYDDFCEKKCPLLVPRL

>SG-AL_contig_5143_1_-2_2_166

LPPLPWDVISVGADVKPNKFVSPCPSVIPFPPFLHYFLSPSLCPLFQHNVSMHFV

>SG-AL_contig_5144_1_-3_102_1034

MAVEDRAAAKELDQWIEQLNDCKQLTENQVKTLCEKAKDLLSKESNVQEVKCPVTVCGDV

HGQFHDLMELFRIGGRSPDTNYLFMGDYVDRGYYSVETVSLLVALKVRFKDRITILRGNH

ESRQITQVYGFYDECLRKYGNANVWKYFTDLFDYLPLTALVDGQIFCLHGGLSPSIDTLD

HIRALDRIQEVPHEGPMCDLLWSDPDDRGGWGISPRGAGYTFGQDISETFNHSNGLTLIS

RAHQLVMEGYNWCHDRNVVTIFSAPNYCYRCGNQAAIMELDDSLKYSFLQFDPAPRRGEP

HVTRRTPDYFL

>SG-AL_contig_5145_1_-1_37_369

MPSDKTMGGGDDSFNTFFNETGSGKHVPRAIFVDLEPTVVDEVRTGQYRQLFHPEQLITG

KEDAANNYARGHYTIGKEIVDLVLDRIRKLADQCTGLQGFLIFHSFGGGTG

>SG-AL_contig_5146_1_-1_1918_2547

MVRQQSYTVYSDWVSDETVTVTDISISREAVCEMGFPEYLVQVAIDIQRLDHGTDFADAA

HLFTAVEDLCQNPQKQADALMRLYQTEQAQKEEANKKHTEVTNQTKENNACYTNECALYP

SAEVMRVVEPKQVPETKAQLRERVKKLAQENRKLRQRKLCRGCRKVELASSGVTFLPCGH

FITCEICSEKFDDCPACGKSIMGTVRTFLS

>SG-AL_contig_5147_1_+2_2_145

THTHQHKYSSSTRTHTQSMPLNFDNLMESNLARCTCCFFVRFSCQSDR

>SG-AL_contig_5148_1_-1_1045_1203

MLHIFHSTIPFRLALVSHHFLICEQNKETHNTLLLRSTLNPSCLKWSVDPWSG

>SG-AL_contig_5149_1_+3_3_155

EGESSTSEPKRPLPEETITFPKQISTQTVLYKLQQTILFHKKFKVANSNVM

>SG-AL_contig_5151_1_+2_173_352

MKYSLWLDSRTSLTGQFTSAISKQVLIINYPCPSLTPSNRDIQLLCILKVLSKCHLHTDA

>SG-AL_contig_5152_1_-2_362_1507

MLSVPWFLGWALRPYIQINRTGTPTITTDTRSGAAADDGMTSSVAAKAQVAQLSSPSAAR

HVMARTPSPTSPTANTPTKPYPVTIQPITAKDNSQTKSAPLSQPSTTIAKTTAPRTAAAR

PAGSPKPATPTSTKATVGKPGTPEDIAGNLVSTKTITLKMSGEWVQQQLEQFRFSFDEAD

RDKSGFLSLDEVFSVISKHGFKGSKDQAKQLFAQLDANNDQKLTREEFTAALDKLPRNTI

KEFVLRKTFQQMDKDGSGKLSRTEIEDATKSQAGPDVPAEKIAQLLIVLSKDTKDDVVDY

EEFLQVFGIQTTGDVMETIFRELDKDNSGYLTKEEIMAAIHAESELKLRAAKISDLLIAW

HKDSDKKINYQEFVQVWTKYKN

>SG-AL_contig_5153_1_+2_695_988

MHFTLCNSNSPVSWRAGWTKDFLRCSYMPHKAGSCLSARAQCFFAALSSALSLFTCMVCW

RAPLRVRKQSCVGVVAQGQTDFRASPSRPEARKCLRSL

>SG-AL_contig_5154_1_-2_116_316

MQKAHKQQTSLKQQQTKIKKGARGLGKNKQTNKQKTEVFWKQITGASNKQGKITTQCEFL

IYNKWDK

>SG-AL_contig_5155_1_+1_22_216

MHTISCCQIQHERNIIHGFLLSSQTPQQYGFLNLEYTPHQFGMHFSFHYEYYCPFGMLLE

QSSRY

>SG-AL_contig_5156_1_+2_1055_1351

MDRNIAVVEKYKTRLYGQQTLGLGSLSQLSFSPSPLFFFFLSAFSFLAFFPPAPPFILLY

SMKSKQLHTQYMASKMPPILCLSKAIVSLSVEQKNSVTT

>SG-AL_contig_5157_1_+1_1378_1587

MCVYTHILYHLSVLLLIPNRVQEDISILSEYLDLHHTRGDICNVSISCSKRMKSTPTVPS

ALHPNLTNHL

>SG-AL_contig_5158_1_+3_3_203

FFSNLFYIFIIIIKDLYKKEIAFSRLHIYFIMNREINDLRFQGNLHIFQVTNLLTVNIRN

VQTLVNS

>SG-AL_contig_5159_1_+1_172_477

MATMDITNRRSHQFSLFQPNTTYTIKCSNCSDAHCNVSQSFSPKWQYIHSVSLACPFTCH

IITVIHEVCSPSCTSGSISTVRRYLRVWGQMAKRERERERER

>SG-AL_contig_5160_1_-2_80_748

MGIESMSKEPTKVVYAPVAIIYWTLFAFAVFFALLIGLYYLLTGQGARFDIGWFLMSTSP

YMWGLIGTGLAIALSVVGAAWGIYTTGASIAGGGVKAPRIKTKNLISIIFCEAVAIYGII

MAIVISTNLASFDITNMENKVDWRKNYASGFKMFGAGLTVGISNLACGVCVGIVGSGAAL

ADAANSSLFVKILIIEIFGSAIGLFGLIIAILQTTGAGMVATK

>SG-AL_contig_5161_1_+3_831_1034

MKSRITTTTKLCTYKTIRWEVAENGATVLVLASSTVIHIHVIFDLLTAQFPQPDQVLSVQ

VLSEMNTF

>SG-AL_contig_5162_1_-3_78_263

MRIRTHTCKIQYKIVYRPVLSTMEICHEVTSSLTNTRTHRHAHTDIVVEFRKTDKLNFDG

DK

>SG-AL_contig_5163_1_+2_245_514

MVNKMNILYTEKKVNKTQNLCPKSAIDDLNDLHLYAQLYFDDDELKMMMILVIMAIIMMK

MMIVIAITVTVIITIIIIMIYGMHKYGMHK

>SG-AL_contig_5164_1_-3_1038_1415

MKCRACTDTIVESMQKIWKVEFMGSLYSNLLLGVYLHCLVPDSVFLSVLDLTTSLSTHLL

FVVPSASFIARTLSSGKSQVKRVVIFTVVSIFVEIGSIDKPVIPPVDVILQRRSAFSLLE

IDGCEF

>SG-AL_contig_5165_1_+3_255_431

MTFFLLCKTNRIIKPHLHYIVFQFHHGTFGYFLFKSAVRQLRHCWSQAQLRKEGGWASG

>SG-AL_contig_5166_1_+2_119_319

MIGISRWELLYCELGLNPRHEILESTALWTGLASQSFNHFYKYVTVSISITLKLTIHVNT

FPAAILS

>SG-AL_contig_5167_1_-2_2_124

PVIASASIPICFVRLTPLYLEITLRQVKDYSLKNDIVFMGT

>SG-AL_contig_5168_1_-3_1551_1760

MMDSTAVSNLIANDESGLKYLITEYTCLDNGNKNWFDSDMSWEDEDEHHKCEACVLAMHI

FFAESCMSFV

>SG-AL_contig_5169_1_+2_2_220

KSSASTVAFSTDKSSHDSRSAPVDSISQTDGNGLNLALFIGWCLLIRPQDLAGYAAPAPL

KPIMNIHEHSHEH

>SG-AL_contig_5170_1_-2_275_433

MSSVLLLYDATITIIPQDSPTNYASIYKFYLYFVNIHLALECGSFLRFYWQPY

>SG-AL_contig_5171_1_-2_2_445

DSLFKVVVQVNSDLVFKVVVQVNSDSLFKVVVQVNSDSVFKVAVQVNSDSLFKVAVEVSS

DSLFKNTELPTVVLFLVPLQWPCCHTTHSSSSTHCQRQIHSVKVPVSGSLWEPAISSCHK

GHPPEETLHYCCRVILVEFNSACSDPTY

>SG-AL_contig_5172_1_+3_96_281

MYYVLSISFVCKTCTSSMFYFFLFNICIMFCQYYWYVKHPQVVCYTFFDLIFVSCFVNNV

CV

>SG-AL_contig_5173_1_-3_21_455

MLLHLDERGKHNRVSDVRQTLFKFGFGYVWDDQGVESVNMFIRCFKQRLMDCQWQDWDDN

INTSKRFGFYRTFKSNHFQEEYISVDMNKYVKAALVKFRFGISDIAVHSLRYKIHTDTDL

VCPFCKAFLENELHFSLCCPDLKEL

>SG-AL_contig_5174_1_-3_87_320

MLYTLWYKTHTDTDVVCPFCRVFVENELHFSLCYPDLKELQEKFIPAKFHRNPNAFKLAL

LLSSKNQKHIKCFAEYLY

>SG-AL_contig_5175_1_-3_21_239

MLLHLDEQGKHDWVSDVRQTLFKFGFGYVLDNQGVESINMFVRCFKQRLIDCQWQDWDDN

INTSKNSNFTEHS

>SG-AL_contig_5176_1_-3_63_1463

MAGEQRYTLQDDIEVDDPEMYDLIRAEKLRQRKGLEMIASENFASKSTLQALGSCLNNKY

SEGQPGQRYYGGNEIIDKVERLCQKRALEAFRLNPEEWGVNVQPLSGCPANFATYTAVVE

PHGRIMGLYLPDGGHLSHGFMTQKKKVSATSVFFESFPYKLDPATGLIDYDKLAENAKLF

LPKMIIAGISCYSRSLDYKRFRKIADDVEAYLMADMAHISGLVAAGVVPSPFEYCDIVTT

TTHKTLRGPRSGMIFYRKGVQKVLKSGEKVKYNFERKINEAVFPGLQGGPHNHQIAAVAV

ALKQAKTPEFKVYQQQVINNAKVLAKGLTELGYTIVSGGTDNHLVLVDLRPQKLDGARGE

KILEDIGIAVNKNTCPGDKSALRPSGLRLGTPALTSRNLKEADMEIVVQLIHEAMTLAKE

VNASCGPQFDEFKKKMEEADVAAKVKALKQKVENFAVVFPMPGFDDW

>SG-AL_contig_5177_1_-2_209_496

MIVSHHHTMDHPDTGVISNFGDGRYHILSKECQLLLPVSCLNVIKDYQVNGCRLYSVISV

IVKCQLDRSLDCLFHQRVLVVKLLYTPLVQLYSLIL

>SG-AL_contig_5178_1_-1_1_198

EVRKAIKCLKNNKSPGIDGITAEMLKSGGECVVHWMCTLCNQVWNSGEVPEDWKNGAIVC

IPKKAT

>SG-AL_contig_5179_1_-3_126_269

MLLPLKNNSEFSLCLKFSKVVLIRFTYSNSCMCHYQKNGCVLVYVRFF

>SG-AL_contig_5180_1_+2_101_1096

MEGERQARLGNLQDMTLPQLQELLDRQTKLFSRQKFLRNLPDGGQKVKDLADKLQQMISQ

RQKCLPSQAHTDSLHSTTSSQHTLDDDRSIVRNGSPVTQINDALSRSETVPKTSQRDGGE

IIDATATLSDCMNSLVISELSASRMTDKSDKNSGPPRDGGNLEMRFPHSYENIMKRSQVT

GNKRAPFKPNSSLKIGDIHQLPDEFKLKSKSTASPASSSTTASHPSDGQTQVSRKAVVKP

HEDIAVTGPVYKFKGAKEISVQESILLQRQQQEKLEKMQTLHAAERLAERLNIKTVDYNP

SAPDMEYRSLDPGRDDDICDSDTAADEDDTLD

>SG-AL_contig_5181_1_+2_200_295

MQASIWNQMSNHFIKKSSDQYYTGLPATSSLC

>SG-AL_contig_5182_1_+2_35_2431

MMPPQMPQQPPPNVPPPPQFAPDEMSLIYERIVRDLEQHLQVVSIMPQTPMTLQLHALLE

AAIQTRNMREIRTTTVLLHKAVEGLLEHYVPSSITSDQQELLLRFRECHLMVLKGLADAR

ALGHAWAPKEITKYLILMGREESKYNLEAVETLMRSGLMNTNQFDFYMSSILENASHPAI

NFVMQVMQRICSDDKNSPQNFTETDFPQTLEGLNTLANRPRQSPEGLENLMTNLRMPTSE

NVALTLTSGGPGNASTMMQSGITQAREFDDPPGLHEKTEFLLREWVNMYHSPAAGRDSSK

AFTCFVQQMHQQGILKTDDLITRFFRLCTEMCVDLCYRALSEQSHSPTLVRAKCFHTLDA

FVRLIALLVKHSGDNSNTVTKINLLNKVLGIVAGVLLQDHEVRYTEFQQLPYHRIFIMLF

IELNAPEPILEGINYQVLAAFCQVFHILRPSKAPAFSYAWMELISHRVFIGRLLALTPGQ

RGWGMYAQLMMGLFKFLAPFLRNAELSKSTQLLYKGTLRVLLVLLHDFPEFLCDYHYTFC

DVIPSNCIQMRNLILSAFPRNMRLPDPFTPNLKVDSLPDIAQAPRILSNYVNIIQPTSFR

KDLDSYLKTRSPITFLSDLKSNLQSSEPGSHYNIPLVNALVLYVGTQAIQHVGSKGQTPS

MNTIAPSPHMDIFQNLAVNLDTEGRYLFLTAISNQLRYPNSHTHYFSCCLLYLFAQAHTE

AVQEQITRVLLERLIVNRPHPWGLLITFIELIKNPSFKFWQHEFVHCAPEIEKLFESVAR

SCTMKTPATIREPETTEVH

>SG-AL_contig_5183_1_-1_139_264

MCAQSKTVFRKPVQCDYFLLVLLFNPGTVLVLVANIIKHILS

>SG-AL_contig_5184_1_+2_131_232

MHKTPHKMLYSLSTVMQHMWNNTPVMCTTYTSKY

>SG-AL_contig_5185_1_-1_1_165

GDAGGSVSINSIAPFWNFCARNFLFSIALFVSAFYLPLLMSAFFLSFPSIPLSFF

>SG-AL_contig_5186_1_-2_2_337

CVCVCLPVSLNCFYGILPGITSLLPGLLSHALILCCIQDPQFIITSKGLAPSHHSRTKGE

GDPCPTLDLWLPKWVLYPLDHTPPVLGPLHLPGDHRPSYTVTDYDTQCCKIC

>SG-AL_contig_5187_1_-2_2_271

VLYCIVLYCIVLYLFLSQWSYHLKLGLLSWGGSALPQSSAAHFFFSACWMFLCFHKPPNT

DMAYRIFNMHSFDVLHACTLEGGSGTSSSA

>SG-AL_contig_5188_1_-1_1_573

PPFEPLDVVPFSLLSYKTAFLLALATCSRRSKIHALSGAKDNISFHSNGDLALAVLSEFV

AKSQQPGSHPRSFRIPSLVSSLDPRDARSEERLLCPVHCVKYYLKHSAPRRSSQRRLLIS

LSENYSRDISATTISRWLLAMVIRAYKELGSFFLLDSSRTQSPGVLCRLPPLPSYQGRLG

SSILAAGIHVH

>SG-AL_contig_5189_1_-1_289_432

MLTWEIGKISTLYPPGEVPEEDSNPGLPDHPVWKSSVFTFEHRAHRHK

>SG-AL_contig_5190_1_+2_746_988

MVKTLDFQTGGFGGPGFDSSSVTSPDGQRVEIFPISQANICADLLVPESPSCVYARRTSN

MHVKDPVNHVSVQWLLETRIH

>SG-AL_contig_5191_1_+1_31_246

MYPEIRASPIRPIQNRTRTEGGKFYFTTIPQCSFSRQTNLNYRLNRQKRQKVFKIPPLEQ

VHVSIYTWKKKK

>SG-AL_contig_5192_1_-1_1_288

PDLQSSTPVKRSKTAEIQTSPVTPKSTVRNVVDVSTSISPNAFMKEKAASHQRLTACDTG

SKLSKEEEVLFTDLCRRKLKDSDDKVTVRCKTGGQP

>SG-AL_contig_5193_1_-1_226_2463

MRSLIKEGQSTGNWHRLIRTIGLVFSNPDSLMRSFRISPVHSKMEPDPDKDVDDTETEAM

HIDHLPIQECGEQVCTPSTSADNLSKGDVSATEDSQCTVDIESLHRAYDQLLSLPELPFQ

GALIYALISLSRDLQMDFKYHHVVEKNPNLINIFVIIMEYPHLHSPEYLEAAFPSFCKAL

GLMPLPAQAHLVKVWASFGAQRLTEMLHMLQQLITVRIVNNEGRWGRSLNLNDDEAVTGS

CSVMKIVYFASLYGGVHDSEELLREEKLSNEADVQNIALEGAIGMEPKEQSQPKEDPLAQ

ELKLSPINVRKPLIPPEEFVNEPLNEHIEISTDYGYYRAEPESKFSFMLHSFVLNTASKH

MQMYFDNRIHMLQERRSSLLQTLMHGGPPMPYLRLRVRRDHVVDDALVNLEMMAMDNPAD

LRKQLFVEFDGEQGLDEGGVSKEFFQLIVEELFNPDIGMFTFKEETRQFWFNPLSFETES

QFTLIGIVLGLAIYNSCILDVQLPMVVYRKLLGKKGVFQDLFDVDLTLASSLQQLLDHEG

DDLEDVYMQTFCIGFKDIFGNDVQHNLKEGGGDIPVTQSNKQEFVDLYADFILNKSIDTQ

FRAFKKGFLMVTNESPLKYLFRPEEVETLVCGCKEYDFCALEQSTEYDGGFSAGSETIKN

FWQVVHEFSAEDKKRLLQFTTGCDRVPVGGLSKLKFIIARNGPDSEKLPTSHTCFNVLLL

PDYSTKEKLKERLLKAIMYAKGFGML

>SG-AL_contig_5194_1_+2_203_397

MKRKRGATQSLHAALEKHVQGGGRGVLKTRHKLSAPLFIIFKKAMLLATRSVSHLFLKGS

FEMIT

>SG-AL_contig_5195_1_+1_124_729

MLTETWLYSTGDEAYITTMTPGGYDFYSFSRSGSRGGSKGFMIRRGFSNYISLIQLNCTS

FEAVELRLSKNQVSSSLVCLFRPRPTKRNKLNSSNFFFQEFPDTLSGCTNSSCHVSFLGD

FNFHFDVSTDRQIIQFKTVLSDHNLIQVVNVPTHRLGHTLDGVIIQSENCTLSFDHAQYY

PGLSDHSASVCTLAVKRPPPRR

>SG-AL_contig_5196_1_-3_54_233

MASLNCFETVPVEREALIMAVISRTKSVRHFVRDVGIRSSIQLFVGDLTIILCMALSLSG

>SG-AL_contig_5197_1_+3_78_380

MLELYNFPIFSQTCSVLCSQLPIKTDLQPHELIDTYNKGLRQILDQHAPSVTRNIRDHPS

VPWINDDIRVARRKGRQAEKKWRKTGLTVHRQIYDKREGCC

>SG-AL_contig_5198_1_+3_3_149

PTHRLGHTLDRVVIQSENSIFQLCTVLPWSFRPQCFILSLCQGPHIADE

>SG-AL_contig_5199_1_-1_475_2172

MIGVMATVILQSSSTSTSIVITMVSSGILDVRPAIPIVMGANIGTTVTNTMVSLAHSMDP

VEFRRAFSGATVHDVFNWLTVLILLPLEHFTGYLYYLTLKIIQSLNMEDLRTKKQDLLKQ

LTKPLTKRIVQVDKKVITAIAEGKGDPDARLLKVFCVFNKTETVVMVNKTLEDGNVTLVN

ETVMVKTPVEECDSLLSKLDLGDSTSGLFLFVFSLLLLSGCLYLMVKLLHSLLGSQVARM

AKKTINANFPGPFACFTDYFALVVGAGMTILVQSSSVFTSALTPLVGVGCLKLERMYPLT

LGSNIGTTGTGILAALASSSATIQYALQLALCHLFFNITGILLFFPIPIMRRFPLRVARF

LGRTTAHYRWFAVFYLLMMFLVFPGFFLALSFAGNIVFITTLCLVSALVVAVVIINIMQR

NPRLHPCLPPVLRDWNWVPLPLRSLQPLDKLFRAMCTPCQHICCDCCLRKCQCLGEGSET

NIDSDDEDSDNEDNTDSVMCCCRSSQGMQRIQQSESKVAMEEGHMAVSSAWSKRTMRPIY

KPSFKSQPPHHSQSTVPLIIIPESVV

>SG-AL_contig_5200_1_+2_2_130

PCAATALTRPQWSAGPQTIYNNTWGIFPLDLHYLLFSSDFKVL

>SG-AL_contig_5201_1_+3_150_347

MCFCLFGIIKRPNYQKKKNKKSIHNEGLNIPDIKKKYIQASKLFWPRKFQNTKHKWTFIS

SGLYPL

>SG-AL_contig_5202_1_-3_171_1778

MDQVADNRMQQAFQRYMPLSLPGLSHYPDFFAFSLVLLATVIVALGVKNSSLVNNVLTVT

NIVIIALIIICGFTKADIHNWQLSGDELPPSGAGSGGFAPFGVSGIMAGAAVCFFAYVGF

DFIATMGEEAENCRQTIPVSIAVTLFTNLVAYCSVAAVVTLMAPYFLLDVNTPIAFVFER

AGWSAANYVITVGAVCGMTSSMMASVFPMARVVYAMASDGLIFRCFATVSSRFKTPLIGT

LASGGLAGVMALMFDLSELIDMMSIGTLLAYGLVAACVLILRYETDEKLITGPSGGTVVE

APLSTTHGFQLKDLIKPGSSTPTSVSARIAKSSIAVMCVCLIVEAVFVTVCADQLEALSA

WSVVLATLLGAVILLSVLIIGCQPQNPLELRFKVPLVPVLPAVSCLANIYLMLKLSSATW

IRFAVWLIIGLCQYFLYGLQHSTLNGGPPDERTPLVQSDEKSCASTIPSKDQSPHGDHSP

HTDLSSHKDQSPPTDLSPHNDYSPHSGPICQHVPQHSNACHLSSSYNAQHARDTRQ

>SG-AL_contig_5203_1_-1_121_510

MAGCGQWVKKLLRKKVFSEDDLHRSELRRCLTTLDLIGLGTGSTLGAGVYILSGQVGRLS

AGPAVVLSFLFAGLAAGLAGFCYAEFAARVPRAGSAYVYSYVTVGEFMAFVIGWNMLLEY

IISVASVARA

>SG-AL_contig_5204_1_-1_7_273

MHSHTHIHTCTHALTHTYTCACMHSHTHTHVHGCTHTEQLHIILCKHTCIIFLDFYSQVT

STSTIIIIIMGISNLLNLNSPKQSQTKNI

>SG-AL_contig_5205_1_-1_448_738

MLPWLLLLMLLSYNFARHVRVLHVCTGLLRVVAGLFGVVCHQQTADAVSDLGPFANIILA

FQCSLGLTVKYPPPPFFCALFCEDFYTHVCTHTHTRA

>SG-AL_contig_5206_1_-2_86_631

MLSDCILIVFISICTALLGEGLTWLMVYRTEKYQKLKAEVEKQSKKLEKQKEAGENADRN

TKKKLERQEERLKNHSRDLSMVKMKSMFAIGFAFTALLSMFNTIFDGRVVANLPFTPISL

IQGISHRNLPGEDYTHCSFIFLYILCTMSIRQNIQKLLGLAPSRAASKQGAGFFTPPSPL

TK

>SG-AL_contig_5207_1_+3_1737_1943

MIPFLSVQLCEGVPKPFPYCNTFSNPQNGLISGDFRLFLTTSQAGAHANEQDSGHFSPCT

ILQPIFINM

>SG-AL_contig_5208_1_-1_1_255

VFPCGDAGGSFSINSILAFWKFCARSFFFSIALFVSALFPSLTVIHFFCLSSPFHFLFFQ

QAFTQFSLLLAVTVTSLNQFRNQSV

>SG-AL_contig_5209_1_-3_42_377

MKVWVVNKHQLTSTVVKQDTGLRTGRSGRPWLETPWVPHLVDKGGGFSDLPGLNRCVDLL

VPEPSLCVYAHRVSNAHAKDPVAMSAFDGLWKRKHTQNVRKLDFSRHTMPSP

>SG-AL_contig_5210_1_-2_5_1213

MLFGFNAMACDRIIVQSDWAESLREQCGTAWVTFKTAVPPSVHSELKNHGMSENGLPYVV

AEDGFSASFTTRKTMSVTYTGEAATITRKFVAPTITFSTIHKAKKGVHCVDVTSGGLGVS

CDNVGNLLIWQTDNGEVRRELRGHLGDVYRCKFFPSGVVALSVGADMQIKIWSAETGKEA

ANIVGHCAAILDCAIVERGRNIVTCSRDGTARLWDVSQQAELFAWTDLGGDINCCDLAAT

DNSVQLGVPDEAPSDKEILTEGKMLVLGSEAGSLLSFGLHSRKKIFDVSCGSPVNCCMFL

SDTQVACGTQDGQIFVIDARNPSQPLRQWKESRSAVLSVLPHKQGLFISTGDGSLFYVNE

EYETSVEMTGSDCDPVYSIAMDGTHIYSSCRDGTISKYSLNHV

>SG-AL_contig_5211_1_+3_324_470

MLNHCREKEKWKIPRHMAEKSLFQNHVIFCKAINHIKVNGKHLHHVPGF

>SG-AL_contig_5212_1_+1_178_324

MPLSQVACLANRSHSILFCCLTQAICFKLSSFIPSVLHNMPILHFDNPV

>SG-AL_contig_5213_1_+1_244_534

MGWERIQNDSGIIVYLRISPMCWQGLEVLPSLWIPYTSVHACPTSLLNKTGSSAMSHAVP

HQLRPRSSHPGCRPPPSPPAPLPSEGPIDTCTGLSLV

>SG-AL_contig_5214_1_-2_728_4312

MVKKYSSADKQPQQAASQAPAPQPQRQDGKRNKKNKKDLNKKTKEGSDMDAFYDTQAQTS

QESGSVAAPTEQPAEATPQASTSSASTPAEAACSVPQAAAASLPPEAAAEPTGEAAENTE

VVEESTPPAPSPEASSPVPEVVEPAPQPEPVVEEPDAQPPPPTPEVEVEEAPKEEPAEKP

APELAPEQNGVEGEDEGEPEQLSMSTRLPNGLEITYETGQWSPANPEGKKQYGREFLLKL

QANPESRKAPNLPDMPEIVIDPRMVKEDIQKAVLVAGRPLADFTPGFFKSSYGRVGQLPT

VKRNSQQRGRGGPGSQTGDQQPRVITSIRQDVQLHKSDNAYKPLFQKDVSTLEGDKETEA

ILRLFKGILNKLTPQKFEKLVAQVLELKINTEARLTGIGELLFEKAIAEQKFSSLYASLC

YHLLKLPKVRVEKENEKKKGEMIFVDLTFRAVLLTKCQKEFEKDNKTQEALDVKKAAIAT

ADEDKKAQLKEEYARDLKEARHRSIGNIRFIGELFRLKMLTENIMQDCIHKLIRLHDEES

IECLTELLETVGPFLETNDKSKVRMAHYYKDLAVMAAEVMPEDVGKLYGVQRTSHRDPDD

QLSRRVKFGIINVLERRQEKWRQRNAKTQNKPTTIEEIHKKARQEQQEAQFLNSQPRPEP

AAARGNRGKGRGMGQGDLDGWNTVPTRQVKQQIDATRMKLTKNYVDESTLTLGGGNKFQN

WSRGSQGGGGGSKSQEQEKMGNRYTALNNLTDGEGRRGYGRSPARNENRMGGARSMGGDR

DRRDRDRDRDYRKTVGRSSMEERDRAVANTRSMLGASSPSNPSSRNASRDNSRTRDLPPQ

AAGGVPSGIDVSQMTPDELARRLNTIAEELHSNGNIKETCESIVELIGLDYTVMVTTMLE

KLLEKPSGSRRLLGQFLFEMVKERHLSIHCLNKGIVEFLQFADDLEIDIPRIWDNLAEIF

AYLVMDNKVLPLCFLLDCMPPLDTQRKKFVPKIFNTAAGYKSREAIQKQWAASGLKLSDF

KTKDSIENELQWLKGNVVYDAVRNLMQKNASNEEVFKWIDQHVSKQEQGEDRFVRQLIMA

ICEGYCNIPNADWKKENPLARLKTRTHLMLKFVDSQHKETQALVGIQILMDKLEHPSGLA

NALFNMCYDEDIVSEDSFFEWKKEGGPGDAIPGKRVMVMALVPFWQWFLNEEQKS

>SG-AL_contig_5215_1_+1_394_591

MLKGRPYISIVKLIMLENLHQFSQPSSKCSKKGKCFCHFMNEQSQVLTSVWQTCIKTVLI

NLLSLD

>SG-AL_contig_5216_1_-3_3_290

CCNTFLSKLKLKHSLTHTHTHTHTSVCKHTCLVDLKLFFRFFYRKREILSVLKRVFIIDG

GRKKVIVELVRSVLWTLCKFFFQEINRIFVSKKKKI

>SG-AL_contig_5217_1_-2_41_514

MATQQRWIPLESNPKVLNKYIANLGAEEKWQFVDIYGLDTELLQMVSRPVAAVMLLYPLT

DKAKDESQKIGTLDTCAPDQLYFMRQSIGNACGTVALVHALANNEDKIAFADDKHFRKFL

DESKALSPKERAEYLENDEDMGTAHEESASEGDTQAPS

>SG-AL_contig_5218_1_-3_45_338

MVRLHAGLLCGSARQHRASQRLQRSEWLAWYGPSQANSRSPSPVELAKLPGLPCHLGGPS

REHRQGLQVRCLHYPTSPQTWFPPRCSSTGRPSSWPGP

>SG-AL_contig_5219_1_-1_1168_1422

MLLSVRVFCPFFFFIFIIIIILDWLIDLCLYHWKIKINQSVIIVFFWFSSDFSTQLPISL

SLSLSLSLSLSLSLSLSLSLSLFIT

>SG-AL_contig_5220_1_+3_1065_1394

MLGWFCQLFSLLLCLSLSNTHKSKIHFALESFRKPLSVQIFCHSKNILMMIFSPHKILCE

QSFSQNFLYDQIFSTNLSCMTNYFSFSFFLFFPPKLSNTFLPVVRMIMLE

>SG-AL_contig_5221_1_+2_572_871

MVLLGTGMLEASLLLTWLKPDEGTPHSSPSLLYLPIPQPGKGSGTHSQLGRLEYTRTSFL

NKVQTRAVRLQSGSSCSAPTQTSPKMICTFMTNIIQELST

>SG-AL_contig_5222_1_+2_395_607

MNHFSVALIITAETTIGINVKVFAHAWRFHVSISYQTPIWLTGSLTCIFDVLHAYTHEGG

SGTSRSAHMLT

>SG-AL_contig_5223_1_+3_45_206

MVCPMGSGLAGSCPVCYHYDQTALYCSVLSTFVHHCAQDHAICRPVEWCLTHTG

>SG-AL_contig_5224_1_-1_376_600

MQCEMEELKLDKALALFRDVYMHEVLGWFSYMEKMLRCKVSFACFSCIGYFSLCYSAVVE

NWLASRKQYMMDFAC

>SG-AL_contig_5225_1_-3_78_500

MNLQLEILLFLGIITSAFGLECYVCNGQFDNRDKCIKSSVQCRQNQDTCLTHIRWQAPEF

WTPRSEKIHIIGKQCATLSECNHLRRSVGLECMRDWYRNWQCYECCQGDRCNYYVTLGAS

GVTSSITLLLGALLFVWLLRR

>SG-AL_contig_5226_1_-2_2_163

VFVLQQFLISNISITECYSLCNVPIFHIDCVACVFKFCPDFIFLKMPVLLGDCH

>SG-AL_contig_5227_1_+2_2_103

ITDVFVRLTISLTTTSIIIDSFCQTKNLPNNNQT

>SG-AL_contig_5228_1_-1_1_207

LLLLLMMMMFWFFVCFLLLLVVGFVVVVVLVFWRGGYFFLSPLLLLVFCAFVLIYLFNCL

FLFLWKFYC

>SG-AL_contig_5229_1_-2_2672_3031

MRGGRLWSVTWYIDYKIARPDHKGEVGFLLLCVQLERHRYVEVTGVFLMICCYPCDDELQ

SANLFLITNHGIKKACFVTLPIRIIRRSHLQRELALYDLFRDFANQNNQKVSLAKRVGSL

>SG-AL_contig_5230_1_-2_2_202

ESVHDPHSTLLAGMAQPWFPDLRSLSHIPPLELTPSLDLFRQLSSGILHLNPNCSCRSEI

HALSGAK

>SG-AL_contig_5231_1_-2_197_274

MLSSCNSIGTVFYFVIVLCLLMSILF

>SG-AL_contig_5232_1_-1_301_414

MCFCLVLPDLFLPKLPDVRGHQSLSLSFFFFLTLISFC

>SG-AL_contig_5233_1_+2_8_220

MIPRLHTLDTIMIPRLHSLDTIMIPRLHTLDTIMILKLHTEHHNDPKITHFGHHNDPKIT

HLGHPSIPPVT

>SG-AL_contig_5234_1_-2_62_1120

MKLKKEITDSPQCFVLWQITAKNEFNAVCIDKVLSPSGLNLILGGTDGIIRVYDADGLPA

SSEPEARVDTKGGPIQCMATHNVTRLGQIDMLSADSYGTLIVVCGQQILSRQNLASHALT

CLQVQEDGRGYIEIVSSTERGLITACQASTQLWTINLNDYVKVSRRSFVAVKCLLSVELP

DCHGHNLQYLLAADDTCHLHLILHGQIVMSFITPTVVTSMAQGMFVPPSQLDIPGNPVGP

AGASCKQVALGTATGAIYIFYNNAITIEEIARAESPITHLAALQLADNPLDLLMCAGHFK

ALSVYQLGKLIYRHPTSDWINAMVTADVDRDGTKEVIIGCMDRTVTALKFSSV

>SG-AL_contig_5235_1_-1_34_1020

MSEGSSDEGVNSDTWVPYKDRPEWKDVTPVPQDDGPQPVVQIAYSEKFKDVYDYFRAVVA

MEEKSDRVLQLTKDAADLNPANYSVWHYRRVVLGDLDKDLTEEMRYITAVIEDHPKNYQV

WHHRKVLVEWLEDPSQELDFTQRILRDDAKNYHAWQHRQWVLQKFSLWDKELDYVDSLLK

EDLRNNSAWNQRHFVISNTTGFTDDVVKREVKYAQDYIKKAPNNESSWNYLKGVLMDRKL

SKYPGLKEFCQKLYDDKNRSPHLLACMVDTYEEMLEQGCDNSENILQKALQLCSSLGEEQ

DTIRREYWQYMSRSLASKYGKEMPSEMSS

>SG-AL_contig_5236_1_+3_3_269

WKHEHTQHAHFILPSLAPSCIGFSLSLGFYHVVEKVGLCMGNSRFSSTLCPNLCPGVSPA

AQIPLSFGTEGDQSGWREFIVVRALAPKS

>SG-AL_contig_5237_1_+3_3_257

ASCNSKQAHCAYLSESSAPADTTNTNRIRMPAEAAAALLVAERVAERSLTMASQKKLTAF

LRPSTSSASCSNSEPAAEVKEPESK

>SG-AL_contig_5238_1_+2_2_115

TPIKNKQKKKKKKIKKKKHPYKKKKKKKKKKKKKKKKL

>SG-AL_contig_5239_1_-3_15_509

MVAVWRALARQGRTLNQLYVLRQRLAVQPACFISTSKKNKDVAVPVQPMPKSEELKKLEE

HFADTNPDADKNWVSYGFEPTDPEFDNFVHHMTMFFTITLCICWGAFILAYQPDHKNLNW

CSREAYLELERREREGLPLVSPDYIDPSKLELPSDEELGNTEIIV

>SG-AL_contig_5240_1_-2_593_844

MYISILDPQLLSQAASLTRSTQDKSIVACFGSILDLPCRGRRTCEGPFFFFYCPIFCIAP

VSLPPCRSFQFSNTPPNPLKVRSP

>SG-AL_contig_5241_1_-3_18_1559

MADDWGSLVQQQEQKLAERVDKLDLQQTPATTAATNSVTPAVAAPVPRAVVSSGDHVAQA

PQQTQGAVSGAAAPNTSQGTEEEEDKEVKKEDAALLQKLLHSKLVNSKTDIEVLRKDPNS

PLYSVKSFEALSLKPQLLKGVYDMGFNAPSKIQESALPILLADPPANMIAQSQSGTGKTA

AFVLTMLSRVDVSQNYPQCLCLAPTYELALQIGEVIEKMSKHMTGLQIRYATRGERVVRG

EQLSEHIVIGTPGTVMDWSHRYRALHLEKLRVFVLDEADVMISMQGHQDQSTRIQKRLGS

QCQMLLFSATYDKEVMQFAQRIIPNDPVVISLKREEESLDNIKQYYIECFNQEEKYTALC

NIYGSISIGQSMIFCHKRITAAWLAEKMTADHHAVGLLTGELTIEQRAAIIKRFRDGKEK

VLITTNVSARGIDVEMVTVVVNFDLPVTQDGRPDCETYLHRIGRTGRFGKNGIAINMVDN

SRSMSIMRSIEKHFNRKIEKLDTDDLDEIEKIGS

>SG-AL_contig_5242_1_-3_33_236

MDSKLKGKSLFLMSQLRKKKKLCVKVDGCGSNLTSFSFYLPSLSMRQSILIHLSSCGISL

HTSEDNFV

>SG-AL_contig_5243_1_-3_24_1175

MQQEVAFKEECVKFYLCELALVLDYLRSQKIIHRDIKPDNILLDEQGHCHVTDFNIATQL

EDDELATSTSGTRPYMAPEVYGVAINERRGYSFPVDWWSLGVSLFELLKKRRPFSMHSNT

TMHDMWASMMASRGQLIHLHNVSDGMKDILRLLLEFEPQDRMCSLAQLKEHSYVADVDFE

ALLARHFTPPFIPSKEQLNCDPTYELEEMIIETKPLHKKKKRLAKQNSRQSSEDKEDLEL

QNLTKRFHYYNREENKAEKFASSPLVQPGSPTNIKSSMPSLEAPCSSQQVTAQQQHNYSQ

HQRLQEGPIVTLPSPSSPEEDIDGKVDGPVINVLSSPVPTDRAHSSQQAASTADQSSLPR

TVAFQTHSDVDSPRKAAVTRVLDT

>SG-AL_contig_5244_1_-3_57_647

MASLRNAVAVCAILFVCCLGGQSKADESEEELVKEVLFKPEKCGRLSKTHDMLSMHYTGT

LTATGAKFDSSHDRKEPFEFQLGVGQVIQGWEKGLVDMCVGEKRKLIIPPHLGYGDQGAG

EMIPPKSSLTFEVELLDIKDGTPPQNIFKQIDADEDQRLSQEEVAGFLKEQAAKAGPESG

IADSEHETIVSQIFEHE

>SG-AL_contig_5245_1_-2_2_106

SHVFCSSYNYGNSWTKLATSIPRTLFPVLIIPCSS

>SG-AL_contig_5246_1_-3_3_239

AEPEHLQGVSSSDLQRQQQHVLQEQDQGLEALSKVIGRQKQMAIDIGSEVDLHNEIIDDI

ADHVDSTDNRIRRETRHIS

>SG-AL_contig_5247_1_-3_15_404

MKFSVGFLLALMAVALVLVTAKEPDAEEKLCQQRYLLIDITIPCDVTEAQLLDSLPGLDV

LYVFNVPLTKRYIINIRQNGNILPLQRLKIPSVATKVYRAERSDHFYSRFGADVSYCNNL

KPLTGGDITL

>SG-AL_contig_5248_1_-2_50_265

MWSKEPITVLNHTLSLLNGLFCSVPIAPLESLVSYNKRRKCRRMPQLRKTCYSLREVVLS

GCDLTYEAFRVP

>SG-AL_contig_5249_1_-1_1033_1272

MGRHGVSLSGHNQLYSKVTAAENKKFLSVFHKGFLGHLLAGLGKWFILTGSQLFGQTRSH

ALQVSLKFKNSKIGLPVTVI

>SG-AL_contig_5250_1_+1_1_225

PPSSHTSNLTVYSNGLAEHGLFKMNILHVMNRWKCLHTMEHCDLKCSPSHYPLYPLPQIY

PQALEFPLKVFFFFF

>SG-AL_contig_5251_1_+3_3_155

LCTVKGYNCALTSLQEAAPVYSIISLLISEILLPKIPLGRHGYRRMKMTLW

>SG-AL_contig_5252_1_-1_7_351

MALDLQGGPHWVKNFVDSPVIVNASSAQFYKQPMFYALGHFSKFFLPGSKHVELSSNQKT

DIMSAAVLRPDGTLALVLLNKGSSDVKLSVHDPKVGYLNVAVAAKSVNTVLWRSK

>SG-AL_contig_5253_1_-2_2_130

LDQIESTPGPSGHQLQMPLSTKPVDLACWGSVCVVVVFLSENM

>SG-AL_contig_5254_1_-3_99_293

MESIMDFFSTSLKLSSCTQHKYTLCVGIVKKMGKGWVLLRLEERSLSLLLYAQLLIKLPL

FSYLC

>SG-AL_contig_5255_1_-2_92_451

MATHQQQRQQQQQQHQQQMTTTATTSTAMTITATLTAMTTATTLTTTATTAAAMATTSTA

LTTIAATSTVTTSTATITTATTTTMTTTTFYMHITHPDKHAPLLQTHACIHTHAHTHSYT

>SG-AL_contig_5256_1_+1_184_450

MHFILPSPAPTYIGLSLSLGFYWSCGKGGPAALGNSRFSLTLCPACVLESLQQHRFHSPL

GLKEANHIAVAVLGVWCLDDHWWSKSNPV

>SG-AL_contig_5257_1_+1_472_657

MFKPYLVIGSCSAGGFFSIYSILTWKLCQKISSFLLLSLFLPFFSSFPVVCFFLPYWSIP

LS

>SG-AL_contig_5258_1_+2_2_223

ARSCPPRASSTRGPCLTQGSEAPDRHYSGLLPERGGGALAQPLPQGRVSASLVSRQEAHA

PSQAHSGQSEHPGR

>SG-AL_contig_5259_1_+3_189_329

MLPSPLWPWSKALSVNDTSKMLRPTLNCCYALQCYVGIVFIHGKLLA

>SG-AL_contig_5260_1_-3_375_2819

MSVKKLNSPQWKKFQPPPTAQDPAILSFHQSPTNTLHSPTSGVAPTMPPALREKGIIDKR

QHSYGFIQCWDRDARLFFHYSEYNGNIDTMKVGDPVEFSTSTDRRTGKPIAVQVTCIDVT

QAVPTTLSDEEFVGTIIQEAKPVKNKNGTVPSNQDGLGRVSYEHSGECFFIPFAQEDILD

PTTKLKTSDQVIFHLGSDPLTGIVCARSLQLVIPPSQRYQGVICSMKESFGFIERADKVR

EIFFHYSEFRGNIEDLRLGDDVEFSIQMRNNKDVAVEIEKLPDGTVIFEDLTMEKVVGRV

LKTLKVQNGRRPSDPLGGRIFYEKDKGMHEIQFGDKDQYGDCTLQQGDVVSFNIATDRRD

GLQRACQIQLLEETFANGEPRETGVIATLKDGYGFIQCADREARMFFHFSEFMDPEYIPE

DKAEVQFTVMPDPSSASRMVATRIKVLPKGSVTFMTVYPSMYVGTVDKEPATHKNQNKNK

EAETGSIKYDLNSKIQTITYSIKDLKETNQVPRYGDKVEFQIGEQKANNRKVAVNIRVLF

RSPTGRCQGYVATLKDNYGFIENDKHDQEVFFHFSAFEGNASNDLDLGDEVEYTLARKSS

KMSAENIRKLTKGTVGPGEDIQYSKGTVQGKIVRPMRIVDPEQDEYCGLVQVGIEVEEGE

KMETYPYGITSLADKRDFLQKGDIVSFQVAKVKGTDVLRATNLAAQRKFIRAKVDSVKGQ

FGFLNYEAEEGKKLFFHMTEVHDSAQISPGDEVEFVVVQNQRNGKYSACSLRKISEKQRP

ERLISRLKSFTDDNAPRVVVERPPRGPDGSKGFNM

>SG-AL_contig_5261_1_+3_75_416

MKQCTLYYTLVHNYTFCTNAPEISLQRQVVIKTRGFHFTFHNTVMINNITSVQQYISTSQ

YHLQIYTENVENIRSVNGFFLHDLLIQIPTDLPFYNIQNFVKAQTHYHGKHDLT

>SG-AL_contig_5262_1_+1_253_483

MKPLRCFDALPLECLMIWPSLPHIIYHNHHISWVNPVYFLVNFCLILGCATCDLRVLKAV

INSGVDLWNTFSHSDRR

>SG-AL_contig_5263_1_-3_1506_1688

MYVIFILFHVTFNIVMCMFLLSHFCPLLLFVNVLVLELDPGKPYTYMGIMSRVQCSIKFR

E

>SG-AL_contig_5264_1_-2_2_274

DPSSPEGGKYQRIKKLYIILQSHKRPYRPRQTTPQNGHSVHNSVTTASPPSRQSKTAQTA

HHKMGERERERERERERERERYIDIKKKRER

>SG-AL_contig_5265_1_-3_198_593

METPTLIRASIREGDWASSIDLMDACLHGLIHPSCRKHLRRVWKAQALQFRALPFGLSLA

PLRLTRVSRELASLARGEAIHLSCYLDDWLILAESSTLCCLRTQRSWSSAPPGGTRPTPG

SATSCLHSLSTS

>SG-AL_contig_5266_1_-1_1_105

ERRKRKRDDSCLQINPRYMADYPSSTDSGMNFQTA

>SG-AL_contig_5267_1_+1_1_129

THTHINTAHNYTQKAQIYTSTAAHPCSDTFFKGTGKGKQNLDK

>SG-AL_contig_5268_1_-1_94_270

MYTSNFKYVKSDLFQGFLNFTSRAILAVWILYLALPLHQVLLECKIDATPLLSHHLIIH

>SG-AL_contig_5269_1_-1_1_159

AYNPVAVTCGDHLGFFFSLHICMVLSQEWKNGQVTGSLMMELRSCGYSFKDYT

>SG-AL_contig_5270_1_-2_2_118

IYACMHNNIVFLIILMTKVILLLRLLHIYSDTSGLVVLT

>SG-AL_contig_5271_1_-1_1_495

KCELSLKQAFHFLRISFNTNRWQLLLHWRGSEVQSSVPSPPAYNYQSTRFSPWHLGVSSP

LLPLGRVLTHSLLRVFRTCWAQYFYSWDAMFQLGPWFRESATVWLDLTALQASFPVVSPK

PDLYTDASNEEWGYHMDPHHASYTWSEDEKNLHNLLKLEALATPC

>SG-AL_contig_5272_1_-1_250_450

MDSLVAALRKSSTCPEPGPHCRTDPIDLSNACRAFYQSRLESARAIRERWGPSSANGVAS

SCSRSQS

>SG-AL_contig_5273_1_+1_1123_1365

MNKDSAHVCFASNEQKPCLELSFETKLGHAFLEGYTHTVRKDWITKQGMADNSKDLSCQK

PTVLLKQAVFSSKSFNNFTHQ

>SG-AL_contig_5274_1_+2_23_307

MHTHTHTHTHTHTHTCTCTRAHMHIAHKASNDRYEMISLFKTRREKKKGVYVHLSLVTNQ

IEASYVYSKNNNKKTSFGSTWKTRKKNKQQTINRT

>SG-AL_contig_5275_1_-3_546_839

MSICMRESKTCAYFQGDVCVAACCTRGQCFSACLLGAQEQLLEYENVGWYLVLSLWMSKG

HWQELTSVSSWYIFGLFPGPSRTEHLLCRYEGETNNLS

>SG-AL_contig_5276_1_+2_2_190

IHQLPARMLVDNVQEWKGLKPSQTRLTPKQRHKLHTQINIFFHTKPISEAKSRRKACLLV

LKN

>SG-AL_contig_5277_1_+3_3_119

PILLIQQVINILIMRSEFTLSSLWLHLQTDKDCKMAVSK

>SG-AL_contig_5278_1_+3_138_320

MQLLARPEYTPHTLINELRPMHFYTARHFLLSSNINLKKKFTHGHTENIYLFKWQARCSF

H

>SG-AL_contig_5279_1_+2_2_217

TTLRQGKQAEEFSCGSYIDKVYLLKDKDHEVYDDNAICYQNQQKIRDPYRVNKTIDITIN

TTTNAFCSCKTG

>SG-AL_contig_5280_1_-3_237_1478

MAKTVILLVIVAFGGFTSAQRGDIQRSPTVAECQGKAVDIFFLLDSSTSIWIRHYLQVLQ

FLSDVISRLDVRADDTRIGALTFSDNFQVGFDLDRFRSKEELLGAIKEETLPYRTGVTNT

HLAIRHVRQHAHFRPNVTKVMVVVTDGGSRFPTATREEARLAKEDDGIYMVVVGVGLYQQ

EEEWKAIASDPDTQFIFNVTDYDLLQALSDRLPRVLCPLPPVVFRGECPVTENADLFFLA

SRNAVPQAHDVITHLTPSFRPRDRLHVRYVMEVCDDTVDRGFQGLDKHCNRSHDASEQDG

VSLMAEVRRAARDMKFARGANQVAVVFVDPRSVINNRIEILEEARRAREHDGITIIVVDV

GVGDDDSSYLRALTSDKKNVIKYPGSDNMESLQQLLHRMCESANFSFGPEPEPV

>SG-AL_contig_5281_1_+3_3_182

EELRARARASLPDSALVDKAAWESCRRRRNPEREEHRAVRSSWAAGKRPKGQLFEPAEVG

>SG-AL_contig_5282_1_+2_59_178

MCVSCNSKLCLFVSVCFLYSVTGLPINFILKSISTSSCWF

>SG-AL_contig_5283_1_+2_2_292

RVRQPNKQPTVLRKASRVKQPAKQPTVLRKASCEIARQTAHSPEEGLSCETAKHTAHSPE

EASHVRQPYTQPTVLRKASQCETAIHTAHSPEEGLSM

>SG-AL_contig_5284_1_-1_1_96

FFCTLVFLHYLYVVGICVCMFECVCVCVNIGS

>SG-AL_contig_5285_1_+1_76_219

MIPSQRQGGGCAGCCVREDQNAFFTAVRALEHRGSSCPSPPYSSSRIS

>SG-AL_contig_5286_1_-1_64_579

MPAYDEETQKLIEVADSVRNEYNEADTKVRNAENEINGLKKYLEVDYGPDEEYSALRGQC

FEFTDREYTYKLCPFDKAVQKPKSGGIETSLGSWGHWYGPEEDKYDAMKYENGQSCWNGP

NRSCHVNLHCGIENKLTGASEPSRCEYQFEFTSPARCKQSKDTLSVHEHEEL

>SG-AL_contig_5287_1_+3_291_692

MPLQTYPFQTFPWTQYKTQIWLKITHKHKHIRPHSWKSKYINPNITFSFLRKLEKKTKQK

NYNKNAILDQQKKTSHFSTYENTRFALWKRNAVFTFGFKNLVTVMVITGVASVDPDCKFT

NKQVSNFTSPKTSE

>SG-AL_contig_5288_1_+3_270_416

MCWPHGINSLGCPGFSSWTGQVLFSFRYSLCGCLGASLQDEIPSLCTPS

>SG-AL_contig_5289_1_-2_299_631

MRACLSFQILLNFTNILFCFLFISHVINQSSKFVLFVSSLISSDLLRKCAHWTVLYIFLT

FILKKLCLIPVCCLLVKTLVCTSSKHGISLSRRKDFMYLFLLSSWSLNGVS

>SG-AL_contig_5290_1_-2_50_220

MEHQLNTVNEESLKVSLKIHRGKTKFMTNIDTTDQIQIEGTEIEKVTNYKYLGQTIA

>SG-AL_contig_5291_1_-3_15_227

MEHQLNTVNEESLKVSLKIHRGKTKFMKSIETTNQIQIEGTEIEKVTNYKYLGQTIAMKN

ETRDEVLIRIK

>SG-AL_contig_5292_1_-1_1_339

DCEKAFDSVEHEAIFKALRTIGVNETYVTILEDIYTGATARGHLDNQDSEEIPVLRGVRQ

GDPISPKLFTATIQEVFKNAQLEEKGINIDGERLSDLRFTNDVALTTESIEDM

>SG-AL_contig_5293_1_-3_39_236

MQVPKKVLHNHRCMARKTLRRLQGSVPSDWPLTPYLRSFPAGQPEAWPIAFTCDTRWAST

NGSYRC

>SG-AL_contig_5294_1_+1_67_252

MMQEAGREGKDVKGLKLITHSYTLSQPASKTQAGLFSHAKSSRNNSEYQYFSLPNTIFNC

QA

>SG-AL_contig_5295_1_+1_43_219

MDNHLTMPQKDIFLFIQPSINTSLNNFLPQSETGLNTNTAKHLLLQHKINTLHNMHIYI

>SG-AL_contig_5296_1_+2_233_469

MLTSLHMLARLAYYNPNLNPNLNPKQGRPHNTAGKKPNWNSNISHIRYGPSLDLCTASAL

DSNQALSQRPCHHAFADDI

>SG-AL_contig_5297_1_+2_86_235

MQNIKVSTLIILKVMSVAGLRKHAKTPSMPSARTSCKKDPMGPNATILEK

>SG-AL_contig_5298_1_+1_1_195

LRRATVFMGRLVLLIDPWTRRSLVSSSIPAFYIKTGLFAAHLNPNHSGGVSLVSHRFSPL

PHFKT

>SG-AL_contig_5299_1_-2_2_163

KNLFVCLFFCVCVPITMLDFSPVCLFSFQTFTNQLKKRKKKRSQQTNSGTSKIY

>SG-AL_contig_5300_1_+2_2_67

TNQKKKKRDYIFYYIFDAVKQI

>SG-AL_contig_5301_1_+3_3_134

FFWHSAIHFICKKSLCNSNDVGNVCCTKQNKKEASSVFFFLTFQ

>SG-AL_contig_5302_1_+3_696_839

MLFYQLFNICCKRKQELCYSVNCSIFVLKGNRNYVVLSTVQYLYQKET

>SG-AL_contig_5303_1_-1_40_396

MASSAVLRLSGTFTSSLKQVFSRQVASVTVPSRVAFTQHKEVEPCSLVPVREQSTEAAKG

VVKRKPYSPFQTNNNAEYALARVDDLLNWARKSSIWPLTFGLACCAVEMMHFAAPRYDM

>SG-AL_contig_5304_1_+1_1081_1254

MKCITAKVITQHNSKGDNAAEQKGNNADTNHHDPYNLGKKAKPNKSTFPVRFKTFTLT

>SG-AL_contig_5305_1_-1_1_306

FLSFNVVCFFLPLLSSAFFLPLRSSAFCFLYCHLLFLSFTVVCFFLPLLSSAFSCFYCHL

LFPAFTVICFFLPLLSSAFFLPLLSSAFFLPLLSSAFSFLYC

>SG-AL_contig_5306_1_+1_58_285

MKEKGQKQSNRKEEISSTEFPEGKDTIYTETAPWHPHGQAVVQRRHPRLIHREHKYVSIK

IKYISIGKELWSNGDI

>SG-AL_contig_5307_1_+3_18_176

MKRKRQNVGALSLCPPDLINYDIAFRENTHTHAQKNQKQNNNTTNKIWQTETV

>SG-AL_contig_5308_1_+2_2_112

SIVLTDLFSINKGLSTRKGIPNLLIVYYTSYTNYCSL

>SG-AL_contig_5309_1_+3_297_473

MTITEFNLLLCCQCKCLHIQQFSSPFSKACIFNNKPYWFMFSKKYFNSRAPNGVHLCVI

>SG-AL_contig_5310_1_+3_2424_2723

MGGKACFNNQRKGLMQGTSIQTSWSIFPCKTASVRHMSFTLSFNICYQNHCTVRKKMTEK

STCHDTILFFFFFPIKKEWILIFIFFFLFFHFFLFLNKNK

>SG-AL_contig_5311_1_-2_77_934

MVKALADHDAIKHNFNSKGISLLGVKNRMMMQYLQDLTVIMTTKLSGESIKDHPAVWRLI

ENRTYMEKTLTIEQKLKYEISKLVKKHRFGDSGEPDGLKFKPQMDNFNVQSEDDDDEDEE

EGEDKDDSEDDDDDNKKQSKGVYRPPKVAPMSYTGDETVEETEKRVKKRALSNALLADLR

AELLDEPEEIVENPDLHRIRADKQRKEREEYEEKYFVRTSVSKKEKNKRRQMATMSSLEG

LVKFNDYFGDEATTDGPAAKKRKTSSKGFKGKGKARKGKGKKKWHH

>SG-AL_contig_5312_1_+1_145_321

MFIQWRKSAKRRTILSPKCNTTTASDPSITNHAIRRTHAFCIYVTGIYFWLAINSVKGC

>SG-AL_contig_5313_1_+1_187_357

MYTPVNSHCLIHASMKSAIFDFMYLYIICINHNNLFIRLELHWKEPRLIGGSKVKRS

>SG-AL_contig_5314_1_-2_2_178

EWYSALENSSKSLVYKNFKPKFGMEKYIAQLLDVYVSPLIKFQSSCRKLKIELGRHRGI

>SG-AL_contig_5315_1_+3_696_1028

MELVGENPVHHHPSLPLPKPFHRSLVNWPKWCQCFDRYCIASGLALRSCAEPVNTYLYAM

GDCADDIPETTDVDEETVEYDDLKQVFATPQKCCGRTCKIQQTCSATRGIN

>SG-AL_contig_5316_1_-1_1_90

VFFFIFLKKKKKKKFKEFTGLHSHFFSLPS

>SG-AL_contig_5317_1_-3_3_206

LSVAITTTRRQLVISFIVTNNGMCHTLEIRDAISHILYLVQLCTHVYYRHESFPFSCLDE

DVSIQIYT

>SG-AL_contig_5318_1_-3_3_152

YYYYLLVLYRLCIIYLICDYYMLVLYSLCIYASIRMTQDVLATGDKMSPC

>SG-AL_contig_5319_1_+3_63_218

MSSCSANGKMSGQNNDIHTLLQNTFFYMLYHVIWFIRFSAPWCGLQIPPIRA

>SG-AL_contig_5320_1_+3_3_161

GEAEQMKDLGKTDQYIKENISNFMLRKENISKFVLRMFSFNQKTLLKTTTTTL

>SG-AL_contig_5321_1_-2_230_2704

MKQHNLLVTIGEDEQGVNPVIKVWNLDKMDKGIPSCTRLTRAIPANKPTPVTCFTVHENL

NYMAVGFENGAVLLFKGDVTRDRHSKPRLLHEGGRPISGLAFKSSGKDVFLFAVTESAIV

SINITNKDTKIPLEEFGASRGNIVMSDTSQDNQLVMGRQDAVYFYQPDGRGPCLAFEGEK

LQLHWFRGYLIVVGKESKSMPRAPPMGSHSLEMNLVTVYDINNKFIAYTAPYPEVIDIMC

EAGAIYILSGDRKLYQLQEKDTSTKLDILFKKNNYLLAISLAKSQQLDQDGLMDIFTQYG

DHLYAKGDHDGAIQQYIRTIGKLEASYVIRKFLDAQKIHNLTKYLQALHKSQLATEEHTT

LLLNCYTKLKDVSKLDEFIMTKDREVDFDVETAIKVCRQAGYHRHALSLAEKHQKHEWYL

KIQLEDIRDYQAALTYIARLTFEAAESNLKQYGKVLMSEAPQQTTDLLKRLCTDYKPTNQ

PLVDQTSLEGAVPRIQHANAEEFIHIFVNNAEQLTEFLEHMVEVQPNSSSLVYNTLLELY

LHDMVHQQSISTRVDMERKTLELLKNPDAGYGMEQALVLCQVNSFKAGILYLYEKAHLYQ

QILQYHMEHNEFVHVMETCKKFGQQDPQLWVHALSYFAQKEAQCKPQLMEVLSHIDKRNL

MSPLLVIQTLAHNSTATLSVVKDYIIRRLQMEQDQMSEDKRLIRQYQEDTAKKRSQIQEL

RTSAKIFQASKCNICNHTLELPSVHFLCEPHSYHQHCFESYSESDMECPACAPENRKFMD

IIHAQEMTRDSHESFHNQLERAPDGFAVVADYFGRGLFNAPKPGK

>SG-AL_contig_5322_1_+2_8_280

MGRGPERERERECVCVCVCVCVKACNKSVLLLRRKAIFLRGWEFVFPDQLLSLLSCLALP

VIMQADYLSTEQDQTAPMQMRKLNCMLTWLS

>SG-AL_contig_5323_1_-3_54_227

MVIRTYKESGSSSLPYSPRAHEFGALASSAVFYRSLPVRGVLEAAYWWRESMFTNYYL

>SG-AL_contig_5324_1_-2_143_406

MSLLYRTRRHWQWTPSPWTGLPSLVSSLDPRDARSEEHHLCPVRCVKYYLKRSAPRRSSQ

RRLLISLNENYSRDISATTVSRWLSATV

>SG-AL_contig_5325_1_-1_28_966

MVSASPTCVCVIKCRDSHFMLVWFGLFCVSVVEINSSCVLCVKCRDAHFMFVLCFVLFCV

SVVEIHTSCFVLCQLSRFTLHVFCVSYVKILSSCFCLVLCRMSRFTLYVRHVTFVKTKYT

PRPLQCSFVLFIMMYQFTVFSHVHSVMFIQLFLFIQLFLFIQLFLCPFNCSCSFSCSYVH

STVPIHSVVLMSIQLFLLIHLFLCPFNCSYSFSCSYVHSTVPVHSVLMSIQLFLFIQFLC

SFNCSYSLLCSFNCSYSFSSYVHSAVPIHSVMFIQLFLFIQLLCLFNCSYSFSSYVHSAV

PIHSVVPMFTVHA

>SG-AL_contig_5326_1_+3_1152_1493

MLFNMATQRRNRLLRNTSLQEAVQLSHQPGSGEEDGVVLSLCSVWKVEYVMEVKYSVLHK

FWFLAYAYLFGLLLTRKLSRIFTSSKLSSKCMSCIFQRHSFMSAPFVVTFFIIF

>SG-AL_contig_5327_1_-2_107_226

MALPQIVFELQVKHNRVHDFCLHIASKIRGVSTNLFSCFG

>SG-AL_contig_5328_1_+1_1_213

KAEKKADDSKRRKRSRNKESNRKEEISSTEFPEDGDTIYTERAPGIPTGINLKNKQLKMM

HHTYEDCEAVT

>SG-AL_contig_5329_1_+1_52_222

MKTNCQHTTKISLTATTQPKIHFQTMHRKCERENVFTVIDMFMGYISTDMYVMTNVN

>SG-AL_contig_5330_1_+2_383_601

MQKEIFHRLHKIVSKSTAETPFRFLARALNLHSRLLLSLLTAGSKPTNHNTIKGKKQKSR

MAKLLPSCMIHLP

>SG-AL_contig_5331_1_-1_40_540

MSLSLRAEFALDKNKKKKKRELADEQKQEIKEAFDLFDTDKDRYIDYHELKVAMRALGFD

VKKADVLKILRDYDREGTGKISFDDFNETMTDMMLERDPQEEILKAFKLFDDDASGKISL

RNLRRVARELGENMTDEELRAMIDEFDRDADGEINEEEFIAIMTGDT

>SG-AL_contig_5332_1_-1_1_99

VIIIIMGIITINITLTSSVSPTSSLADDKKLMS

>SG-AL_contig_5333_1_-1_958_1275

MLPFWKASKTMCEYHSFISCVLCGYLYSCVGKELYNCHLFVLIISCFWAFPFLPPSVRSG

DLVIHDTFISVLALHVVFTIVQNRYCFTFCEIFCFCLPHNCFILHV

>SG-AL_contig_5334_1_-1_1_210

KVKKRGRGDGHRPHNAGPRTVEVSTTSLFSPWTSGLCHYEKNSNFCIFMLVFLCVFFIFI

LSLAAVVVFV

>SG-AL_contig_5335_1_-1_139_279

MGLGWWRLSCSLPARVRIKPGELYMCVRPPVYPAVNGCLTFFTVKER

>SG-AL_contig_5336_1_-2_50_934

MLEDPFPDLQITVSSFLNSTESRYKCERCTKSRKFFCYTCCTPNPQLKDKLPKVKLPVKI

DIIKHQSESDGKSTCPHAVILAPDDVTVYTFPCIPDYDKDKVVLVFPCKEASTLKDLVAH

SSVRSTKTPPSSASNGASTSPSSTSQPDSSADDKTSSGKRTADPSDVVDSCPDKKRLRTT

SCPFERAVFIDCTWNQTRNIIQDERLKDLCRVELRDHETQFWRCHEGLSTSCLSTIEAVY

YFMREYHEEVVDTTYRNEYDNLLFFFTYFYHTIRERQQGGKELKAYQRKLKDQCS

>SG-AL_contig_5337_1_+2_203_478

MTKSISKVKENPDLLRVVILHDSAFEWSAWEKLGHSYGLCVGTQKTPMINTTVPLHLSSF

HVGQKESGYVCRCCLLRQEARGHAFSFGMLIG

>SG-AL_contig_5338_1_+3_255_482

MFTSAQNGKTKATMSTIQTTIFIQSKLFLLNTLTCNFTYYFSLQQPSLEEEKIVKEYLEQ

TSKQTFMELHDYMKDE

>SG-AL_contig_5339_1_-1_10_990

MAEAELNVDSLINRLLEARGCRPGKTVQMTESEVRGLCLKSREIFLSQPILLELEAPLKI

CGDIHGQYTDLLRLFEYGGFPPESNYLFLGDYVDRGKQSLETICLLLAYKIKYPENFFLL

RGNHECASINRIYGFYDECKRRFNIKLWKTFTDCFNCLPIAAIIDEKIFCCHGGLSPDLQ

SMEQIRRIMRPTDVPDTGLLCDLLWSDPDKDVQGWGENDRGVSFTFGADVVSKFLNRHDL

DLITRAHQVVEDGYEFFAKRQLVTLFSAPNYCGEFDNAGGMMSVDETLMCSFQILKPSEK

KAKYQYGGLNSGRPITPPRGGGPPKKK

>SG-AL_contig_5340_1_-2_110_235

MFKLWIDITMYFVLILYCISVACAIKIHQNINSIPTFRKFCA

>SG-AL_contig_5341_1_+1_139_333

MVSMRIHNERGSGASRSAHMLTWEIGKISTLYPPGEVPVVRTQAFQTLQFGSQVSSPFDH

RARLV

>SG-AL_contig_5342_1_+3_135_278

MLTLFTLSENKSFILLIPITTIVISLCHTHTHTHTHTQTQHTHLSVQM

>SG-AL_contig_5343_1_-2_2_112

NPNQWQNNSTLKKNLFYLCSSLFQEAKELRLCAVYFF

>SG-AL_contig_5344_1_-1_2167_2544

MCQLRSTRHCFLCCSFFLFQSKQLVDCSDCQHSIIYLSYLSSVENGISCVRCCKCSSRIE

LHVEKLDGRVPRVLYFMLYSSSTRIICGGKNLALCACSVSECMICSGPSFHLLCLCSLDG

DVCICI

>SG-AL_contig_5345_1_-2_116_919

MEQIAHLEAEQEARHKQELEGLDGQNDQEVDKATEAVSTLSVSGDAEVPEGAAANDVTPQ

EHQQSKKTSRAQKRRDKKSAKERERDVRLKEAAISNRQGPRHQENLKLQSLLAQRGLKVF

QIPSDGNCMYSAVKHQLSQRGIQETNESLRQKTAQVMRSKPDDFLPFLTHPETGDPYTPE

KFEEYCNELVSTHAWGGHLELQAMSMALSIPMEVVQAEGSKVMVGEDYSASPLILVYHRH

ALGLGEHYNSVVERGPDDDEDEEEETAR

>SG-AL_contig_5346_1_-2_59_241

MYLNSLCVLDSVGFSSRSLAETLRTCTEVFLYNCGVFLTFIQVAHATSGNVSLVVGNSTV

D

>SG-AL_contig_5347_1_-2_1022_3514

MATTRWKYVLTVNVLTTLLTVIHSTRPSLAMGFRGMEENCILSFVVPRDKVKSSCDMEER

VTRRLNAMETKVTLYKQHVQELQEQLTKEREFHRQRLHELNERVVHNERNPADGLLEKRI

QGLEEEMRKLRMKAAQSPGLGEKPHARGAGLGMMPTGRSREEEEKAITNLVKNVVHSEVQ

RLFDNYTHYVGRYVRDQALAFNQIMNMVPRNDQSFHHHNLPPNWPDKLARNDIEPKSTSQ

PVHRDMVLLNELESIRSRLNNTGKRLADHMHNVFNNVNVTSPRPMSPTAASKVTDSSGSD

GVPSHHDSVKGNGLLDINDTSSGDTTDVASDRNITLAFTQLANNTIHQDAGSQKNLHVTS

SADFSSGSRDSASAKSRMSYNADLNKVPDPSGQRAGGRQNPDLPSSNGSGWEESMVHDQS

AGQWIQVLLRTEMQRILNQQVDEVLSLVSKHSSMVDSKLSDQERKFSQLEQRLAADIKHL

EQVVNGLKLQMTSVSQGLQTLYSRTDKAEELQDTVMELKKNMSMEGGTRLLATLLDEQGK

KVRKMERLTDIYKQSLEHYRNETKLEYRDLKERLNKETTVLRSLHTDLFHNVTEHAKQLT

EQLNTSANKQMEEIKQRVQVMEDNSLLLQIDLSSFERQLKSKTSKSERDMNKLNDDVGEL

KQKTRNLQKNFNSAMTVQKRLSQAVNTSTTKVQQLQMEVKLNVLDDWLPYNFDYMKTRTD

CFGEQYIRKSGFKTGRLVGVVLCTNKRYKIFLGQSPTDTFKNIGDDSGMGEDHCQFVNAR

GMSKVKVSPFRTSTETEPGYVRTHWGQEPRPELLNIIRPSPAWYECGVSIP

>SG-AL_contig_5348_1_+1_871_1107

MITQNISNSTLSLTCSQMRGITLSHTHTYTQCRVTEKAHSHCIILSSLFLFFSIIFPRKI

SLNLYYLCNLEKPKPNHPD

>SG-AL_contig_5349_1_-1_109_333

MLCSKLLRLVTLDYSERGWENLSIYYLFLICMVVCLEMASFTLLVFEKNVKIVGFVAVII

KRKFVVTADYSNTYT

>SG-AL_contig_5350_1_-2_98_1087

MSPPDPNKITKVVVHPLVLLSVVDHFNRMGKVGNVKRVVGVLLGANRGGVLDVSNSFAVP

FDEDDRDRNVWFLDHDYLENMYGMFKKVNAREKVVGWYHTGPKLHHNDIAINEMIRKYCP

NSVLVVIDAKPKDLGLPTEGYIAVEEVHDDGSPTTKTFDHVPSDIGAEEAEEVGVEHLLR

DIKDTTVGTLSQRITSQLMGLKGLHSHIRDIDTYVGKVVSGKLPINHQVVYQLQDIFNLL

PDVNLMDFVRSMHIKTNDQMLVVYLAALIRSIIALHNLIDNKIQNRDAEKSEGGKDIKKE

KKEDKGDGDSKKDDKSKESADKKSATKSKK

>SG-AL_contig_5351_1_-3_237_1124

MVAVFRGIALGLLVVAITVHIVGFVTPNWVSAKLDMRGTQGIMSANSMGSPLLNQIGMNI

NMGLWKYCMKSNIGQVMQNAMAGAGQGMGGQGMPAQDSDGTANQDSDSSWQGADLDQGSN

LGPSAGEMTDDSSSSIGDGMDGLSDMCRSVNASETPGWMKAVQALGIMSLIVAIVGLASI

LYSCFGESKGDRARLLPFIIAGICVTAGVQLLIGVAVYGTEFEKMMQDQMSMMGSASPAL

HDLAENIKKNTNLGYSFALDTVAGVLMLLTAVVVAIPAMKGWDSSVQFGGPRNMAV

>SG-AL_contig_5352_1_+3_168_512

MGWELQPQSAFLLLLLLLLLLLLIIIIANVCVWFLFIYLYIWRWIFLRNLAMDNLFAAMG

SLTCAKCMLHTGPRFIISSQRLAPRLQLKVKWRGGGGGKIPVRPEIESRTCRILG

>SG-AL_contig_5353_1_-2_98_1114

MQYMVWTLLLVSVMTPHLLVRAQSTNEIFGSDGRLIMDPFELLLFQMIVCSLPPEAGPCE

TIVPMVFYNRTAGQCQNFTYGGCKGNANRFKTEEECEIACTPSWCSLPAETGPCKAFIPS

FFYNITAGKCQFFVYGGCEGNDNRFETMGECESACSLPNTTSVAVLAAAAAAAAAAAAGE

TVVTEINLDQTQPAISSTGPLQPPAAEAVITEIALGGPPQSVDSSTAAAAAAALPPPPPA

TAAETGVTEPSVDQPAASVVSSTPGPETSSDTGNASSDHTQSHQEACSLPPVTGPCRSYI

RSFFYNAIEGQCQLFIYGGCGGNNNRFRTKDGCENFCNP

>SG-AL_contig_5354_1_-1_82_267

MHFKVLLHLKTVFTGSLRINFMLDQCYNLKTKLLTCDEICSLTFIVNSISTNIQTGSIYC

HF

>SG-AL_contig_5355_1_-2_476_751

MKTFSSDYTSKQKTASKQKTTHAYTHIYTLQACTCMCMHIPHGDSRGSFSINIPAFWKFC

ARNFLFSVALFVSAVFLSFPVICFFLPSRSIP

>SG-AL_contig_5356_1_-1_1_93

NNNKPDSWRHILHLFSLVCADVDYMYCRKIV

>SG-AL_contig_5357_1_-1_22_633

MTGQLKSGDDMVDFWVELMSRYPAIMVIIDPLRKQEKDQWMRLCDRITERCFVVGQKIYP

RPGLLKDATLPDPIMSGATVLAYENLTSVSDVCACVKKMEEASNQIILSACSGETQDDFI

ADLAVAMNIRFMKMGAPNRGERISKFNRLLQIESELEANNKLAPQVDHEFKHMALPPDNE

EAEGAPVSPTSPKKEDSKKDGKKK

>SG-AL_contig_5358_1_-2_728_952

MQEIKWVCFLFVLFIIFISAKCIVKSYVTFAHWHKHLHTHTHTQNAHTHTECGNKSNGEK

QHTHTHTHTHTHIKN

>SG-AL_contig_5359_1_-2_482_817

MLRISFLKCGHAKLVWPTVGPKFGKRSIRPIYRPVLRCFKIIHLQKFCIDLWSRSKTSRY

RIYCKALSDSTLHYGPFHPLLDSYTMVAGQSCKSIVSLTAKPVTLNLIGPND

>SG-AL_contig_5360_1_+1_1_204

KICTLTCKQNSETPEGYPTRINAITPTFKKYTTNNINNKICTLNNLQTEHQNISMILSKD

QYQCLDFL

>SG-AL_contig_5361_1_+3_369_575

MKTVFFSLVDLNYSRNQYYEGRAHLLLNKVGHPFTAGWTGLRSHKAPLPKSLGRARAGRP

ESGSSTSTP

>SG-AL_contig_5362_1_+3_3_137

HTHTQTNMYACLRIDAPQTDRYAIFPGMINLLMAQHIQSFSTSFH

>SG-AL_contig_5363_1_+2_2_199

TTEQVLARLGLLSGLDPDSDDVMQTSLGKVILSLTVTQKVLNLCQVSIFSSKNQNLKKHF

LITIVK

>SG-AL_contig_5364_1_+3_3_152

IGSIRHYLTLDATKTLVSVYILSRLDYCNSLFIGCPQTLLRPLQQVQNCS

>SG-AL_contig_5365_1_-1_1_207

HVINYDFPNFVSDYIHRAGRVGRVGSQHSGLVTSFITHQWDVEKLWQIEIAARKSAEIHN

VNANIRRKL

>SG-AL_contig_5366_1_-1_1_207

HVINYDFPNFVSDYIHRAGRVGRVGSQHSGLVTSFITHQWDVEKLWQIEIAARKSAEIHN

VNANIRRKL

>SG-AL_contig_5367_1_-2_53_1504

MNGSGQCSGLLLRAFQQTASCYFHQQLCRTLATYPSTDLPRITTPHKVYAHLQKLKKRAS

SGSIIKKPSFKSPLVIACKRSNFNHHRGQTYSDLGPQTLASYGWKHRKSAGDHFTLLCHD

KNPALLSQEANRSFHDLRLDDDIVRAVEALGFSIPTSIQNAAVPAILRGESTLCAAETGS

GKTLAYLLPALNMLLQRKGYRADAEESINTPSIIILTPARELADQVTRVAESLQEHLDFS

VYSVCGGRGTKGRLAWPVRRPMDILIATPGVLRKLLIAGHIKSSGLQQVILDEADTLLDD

SFMDLVDRVLHRLQIPTLEKMGVPGGEGNSHGGYVAGVQFVLVSATMPRGLEKSIGAYIP

VDSLKKVTTSGLHRLMPHVPQKFIRLLPSQKVEELVKLVKMESRQPQGSMMIFCNTSKTS

FFMNHVLIEHGFKPAIINGEMPEKVRRAAFERFQSGRHDILVGTDIVSRGVDTVSVRHVI

NYDF

>SG-AL_contig_5368_1_+1_127_303

MTHYTYCGPVRHTLSVPLRVMCKLSPPDLSPIAITTLLSNPDTAVTGPHSQSVWWINDT

>SG-AL_contig_5369_1_+1_1_282

KKKLEKKKKTRQEEYDEAEGRDCRRTSRTRKKDQRKKQERKDRSRIRKRRRMRQKNKNYK

NNKNERLERKRRKIRRDRKEETEEDMAVWPNTLS

>SG-AL_contig_5370_1_+2_65_337

MMRLVPIASPSLLYLGLSQVPIHSWVDWRPHSHIKFPGRDSDPCILMRADWKSTTLPLHQ

PGLIRIIPAGYTGSTMIVGYADSMIQAYSII

>SG-AL_contig_5371_1_-3_291_1685

MKMQPASLTSLWLCVFSCMLTSAFAIRYEPNWKSLDSRPLPAWYDESKIGIFLHWGVFSV

PSYSDEWFWWHWKGQHDASDVKFMKENYKPDFTYADFAPQFTAELYDPGKWAEIFQAAGA

KYLVLVTKHHEGFTNWPSKYSWNWNAMDVGPNRDLVDELANAIRKKTDIHFGIYHSLFEW

FNPLYLEDKANNYTTQKFVETKTMPELYELVNNYKPDVVWSDGDWMVNDTYWNATHFLAW

LYNDSPVKDTVVTNDRWGKNIRCHHGGFWNCEDRYVPGKLMKHKWENCMTLDKGSWGYRR

TADLSSIYSMDDLIALLAKTISLGGNLLVNAGPTEYGSIIPIFEERLRQMGQWLGVNGEA

IYATRPWTYQNDTTNPDVWYTSKKEGSGTVVYAILLKWLRGTTLQLGAPIVTDQTSVTLL

GYSGGPFKFTGQVTQGVTITVPTISFIDMPCQWAWVFKLENLMNQ

>SG-AL_contig_5372_1_-2_2750_3175

MVGHIHEYFSPSPSTLFVAYLTPFTILCNDDLLFHLCHACLRTEETLTLYFLNDCKLFFQ

ARHDNNCHDQIKCVLPDTIYAVCSVQSSQTSGLLKSKLAISVSTSYGNIYVCISAQFFAS

QFHKSWYSEFSFNSNILCNLLE

>SG-AL_contig_5373_1_-3_99_1586

MADQGTVADGDYVVFKTDDKCKVFQLSNSRNIFFEKAKLKSKDIIGQKYGTVFEVQQGKL

MVVQSSDNGASAQPLSATSVAAAAESGKDNRDLVASGENQKLTMEEIMKMKEEGVTGQDI

VEKLVEQSETFKTKTEFSQEKYIKKKKKKHMLVFRILHPCSRVLLDTFRHAPSKICNLRA

DSLSQLLCYSNVMSGCTVAVVESCHGLILGSVMERLGGTGRVVHLYPGNDMNRHVVSQFN

FTADQTKTLHEFPLSKLASLEDGTLLPQTDTSDKTTASVRKSETTGENLTGENSNEVKRE

QDSKNTVDSKDTVPDSKDSPMLVDKDSESDDDDDDDDEKGEENGSADGSKEHAETKTGRN

GKGCKRQRTKNTRNNAAEQEAKRHLKAQRLLQAATILKDKSLDSLIIATKYDPVAILLRL

LPYVAPSRPVVVFCQHKELLVECYTKVKENGCGVQLKLSDSWYREYQVLPSRTHPLMSMS

GTGGYLLSFIRVTSQQ

>SG-AL_contig_5374_1_-2_2_217

QLPLFYTYFILSTCFILNILKQTCSLCCFTFTVFSVSVKTVRRAVEAVLYLLYHYVLVNA

TRCSRTICQQLL

>SG-AL_contig_5375_1_-1_130_2481

MGERVSLQVLVGTVVCAVVVVITVTASASSNDNDNDVFAAHYGRASVSSYPFGDPRFCQD

MARKDGDKLQRFEVLPGYGWDNLQNEAEGQVVVFNYSKCRTTEDARFLLPDSVSAVPIKA

SRVQTFAELITHWKSWTSITSKSVNLEAGLSLNKFSISGKFSADFEDIKSKQVGDKSVTT

RTQLRYIRYGVQLQPDTPLHPVFKSRVLGIAAAEELGEKERARYLAQLLVRDFGTHVITK

ASAGAALLQVDQLREEWVNSNIEHKSDILAAASGSLFSIFQFSSRFESKTDQRFSDQYKK

SRTSSSILTLGGPVFRPANFTPDMWADDVDRDLVAMDRSGDPIYYVLTAATLPELPTSTL

NEVYNRVLEAVETYYEFNRLPGCLDPTSPNFSPSANTDDGSCKPPPTNLTFGGVFQTCSL

KPGSNNGNLCEGLTQVNPLTSDYLCPDKYQAIKLHSGTTSSSRTEHVCKRYLIFWHKCHD

DFYHSQATYNMYWCVATSGVSRDSGYLFGGLYTSTVVNPVTRAATCPPTFFPLSFGAAAD

LHICISDDYEFGSEYHLPFAGFISCQAGNPLAVAGDGSSNKGPNQVKSAGAKSKSESKSS

KVHGNLKMFFKDQADDWPKRCPDGYSHHLATVDQGCEISYCVRTGSMNGPVLPPIKRPPF

MPAPALPAPASENTVIFDPATLTWKRNKEAGQVPPSAAPNQASADESMSPGTAAGISIGA

TLGCVVIATVAILAVRARRRRIMPGYRRLQNPLLDPQEDYGSVNTTERRPASTMVNVAPV

ESTP

>SG-AL_contig_5376_1_-2_545_850

MRDKERLLCLVHCVKYYLKCSAPRRSSQWRFLVSLIGRYLRDISVTTISHWLSETVIRAY

KESDTSSPLESPRVHELSSLDLSCLPSLPPYQGCPGGSLLAE

>SG-AL_contig_5377_1_-2_1787_2140

MVSATTENSYSNMIVSTIHTVIGVCQICLHFFLCVFICCRYISFSVNILVDACYWFVCCC

SHCFLLPFPTDTPTGSHIISLFCVGTNSAPLEQFMTCQHIRFTSFRLLLFLSIFRLLA

>SG-AL_contig_5378_1_+2_2_118

FFFSKFNNLLMGICTSHSFGVTNTNTAVSSTNISCSDFS

>SG-AL_contig_5379_1_-3_3_212

GQCGCLLDFDMAIVVGWAINNNNNNNNNNPTDIKHMQELLREIKCRNSWSYSPSQLHGGT

KLVYYVWYLF

>SG-AL_contig_5380_1_-1_61_264

MYFLEFIVSSTKIRSPTLKYETQHHTMSFRPHAGGPHTISMHYGDLFSEDFHHSLTLPVF

FRSVIIYH

>SG-AL_contig_5381_1_-1_31_240

MVKTLDFQTGGFGRPGFEPRAHRLMSGEFSDLYKVNIRADLLVPEPPSCVYSRRTSNTHV

KDPVSHVSVW

>SG-AL_contig_5382_1_-1_1_510

PDEDETDDNPDYKPPAPKSLDEILTQDKEDESLRKYKEQLLAEAGAGEEIVVDPDNPLNV

IVKKLAISVEERNDMEIDLTLPKEKIKESKLVLKDGCKYRVKIFFNVQRDIVAGLRYEQK

VYRKGIKVDKGNHMVGSYGPRKELHVYSTPVDDAPSGMLARGDYRVESRF

>SG-AL_contig_5383_1_-3_417_713

MLDTFRLLKGLFYNVSIATTERTVSYSKRHKSCIFKECLRHPIYCVSITNKHKKMSLRHP

IRSASQRTHLRHKDRKKEIKDNCHLMFHASERSLCYRIV

>SG-AL_contig_5384_1_-3_432_1142

MAKAPAIGIDLGTTYSCVGVFQHGKVEIIANDQGNRTTPSYVAFTDTERLIGDAAKNQVA

MNPENTIFDAKRLIGRRSEDPTVVSDKKHWPFNVISEAGKPKLSVEYKGEKKTFFPEEIS

SMVLVKMKDTAEAYLGKTVTDAVVTVPAYFNDSQRQATKDAGTISGLNVLRIINEPTAAA

IAYGLDKKVGGERNVLIFDLGGGTFDVSILTIEDGIFEVKSTAGDTHLGGEDFDNRM

>SG-AL_contig_5385_1_-2_2_196

QVSHFFSTMKSNSRVFLDYHCLLQSYFNILRKILIEKFHKIILHPNKELIILKHLAFGWS

PNLIK

>SG-AL_contig_5386_1_+1_1_207

ACFRLIRKTGTTQSNLIHFMDVSMSVWLRFLDQHCRCLYLSGLVDTRIYYERKCRVQPYH

AVSIRSGTQ

>SG-AL_contig_5387_1_-2_401_2296

MPAGLNLEMSGQRYKRTNFDEDELTSNGGGTPPPTDYSTNVVFKGGWLSWHRRSTQERVL

IVVLALMTLTLVVLASLLAVKDSTIKDLRKQDKKDGVCLTPECVTIASSLLNTMDRSIHP

CNDFYQYACGGWIKTHPIPSGHARWGTFGTMWQENQLVMKNAIEQPESSFSSEAERKAKR

YYLSCMDTNKTVEKLGAQPLLDLMQQFKANASQSGMPDEGWVFQEMLEKVQSFDISALFS

VWVGEDDKNSSQNILQIDEDGLGLPQRDYYLNKTIEEDKVLSAYLKYMVKINELLAPGRH

PNITQHMTDVIKFEMDLAEITTPAEERRDEEKIYHKMTVGDLQRQFPLIDWVQFLNKFLD

QTGAKVNETEEVVVYAPEYLEKLCGILNTTLATLEGRRAINLYLEWHVVKQLISYLSKPF

REAKREFAEVLSGETGKEDEWRNCITDTDSVLGFALGALFVKNAFHGESKEKAEKMIDEV

KTAFKNNLPYLKWMDPKTRKAAIEKANAVVDMIGFPKYILNVTQLDEEYHLLHINASEYF

QNNIRNLKFMLHKNAEKLRKAPKENVWGMTPPTVNAYYTPTKNEIVFPAGILQAPFYDRN

FPKSLNFGAMGVVMGHELTHGFDDQGREFDKN

>SG-AL_contig_5388_1_-1_34_399

MENQVIVSDYTQMDRVLREERNYILEIVKKIKKAGCNVLLIQKSILRDAVSDLALHFLAK

MKIMVVKDIDREDVEFVCKTLGCRPVASLDHFLPENLGSAELIEEVQTGSSKIVKATGIA

NP

>SG-AL_contig_5389_1_-1_160_1395

MTAAGREWRVEASKFAMNTFNPIRSIVDGMKLTPNPDKPMIALSIGDPTVFGNLPVPEAA

EDAVMASLKNRKYNGYGPSAGYEHARQAVAQYVSTPTSTVEAKDIILCSGCSGALDLCVM

GLANTGHNILMPRPGFSIYKTLAESMGVSVRLYDLIPEKCWEIDLKHLAELIDDQTVAIV

VNNPSNPCGSVYSKQHLQDILQVANTHKVPIIADEIYEHFVFEGNSYHSMASLTTEVPIL

SCGGLTKRFLVPGWRLGWIVIYDRNNVFSEVRKGLMNLSQRILGPNTLVQAALPDIFKNT

PDHFYSDTLKYIQKNAEKFYERFCNIPGLKPIMPQGAMYMMVGIGMANFPDFQSDLQFTE

AMVTEQSVFCLPAICFQYPGYFRVVLTVPAEKVDEACDRIEQFCHDHYQGPE

>SG-AL_contig_5390_1_-3_297_488

MTYKNIRCFCLMWKCFKTYFPASCTFGFQNEGTILKLLKGITVFCFVAFCSLIFVPFAHL

SVSF

>SG-AL_contig_5391_1_-3_3_1007

FLFSVDINKEDGEGEVYTEKADSDDSGNEEPIAEADFYSSSDADAANRVAKQVVHKTDRL

AERIGNLTLPRERDQVPEGLVDSLPPEVLLVVFRNLDDISKWTAGQVCQRWRQILDAEMS

QSKWKHFISLRWPLFQPQYKVHCWRAIYSQLLKSSPCRYCLESMMLQSTPPIEENSWRHR

RLRSELKTLKSDPPEGIQATPLDRHCCHWQASITGPQGSPYEGGLFLLYLQIPHSYPMRP

PKVRFITKIFHPNISRHGDVGLDSIHHNWSLALTISKVLISIQSLLTDPYCHVCMEPKIG

ALYERAKPEFERIARRWTWKYAMHDYVMPTDVDLT

>SG-AL_contig_5392_1_-1_133_402

MKTTSSMDPNDMMKEIRKVLDSNNCDYEQRERFLLLCVHGDPNTDSLVQWEMEVCKLPRL

SLNGVRFKRISGTSIGFKNIASKIANELKL

>SG-AL_contig_5393_1_-2_272_1288

MCSTTESEKCATPTTSEKKLQWMYSVIKEAEENDLNVSCDKNSDCDEEAVVDQSVPTGNR

IIDVSLLNDSISFHLQCKHCGHSVSLMETGRQGLDSVLVFHCSNRKCNEQKPFNTSKKIQ

VGNLAISSVNRRSVLAMRSVGCDRSELALFCGIMDMPPPVEKRSYTKINKTIENAALEVQ

TEATKCAADLEYKLAEKEEDTNLRDIDVSSDGTWMTVGHSSNIGVATTIGCVTGKVLGVG

TRSKVCKSCEVWEKRDKNSRLHRLWKARHVCTLTHIGSSGSMEGHIVADIFSQSTENNLR

FTRFIGDGDTNTFKIVSESKPYGDEVDIEKIECVGHVQK

>SG-AL_contig_5394_1_-2_131_544

MVNNSKCNVAHKKPCTSISCKRYQKPTTSLQDVLNISKRSSTDSSRLPQLRLKTRFTCSL

STPRTSRPFYCALNELKNDEHLCYHLMKRMVVPILLYGCKVASKLQICFYRIIFRVNKST

PTSMVYGKLGKFPLGVQT

>SG-AL_contig_5395_1_-1_94_3738

MQDMTGLRWLRLSSTDLQTVPAALGKLNKLEDLTMVHNSLQSLGKELPQLPSLHMVNCRY

NKLTDTGIPQQLFTLGDLSIVDLSHNNLREVPPELENARSVLVLNLSHNEIEMIPNQLFI

NLTDVVFVDFSNNNLEMLPPQMRRLTSLQTLILSNNPLIHAQLRQLPVLTALQTLHMRNT

QRTLSNFPTGLDTLTNLQDVDLSDNALPRVPEALYKLGSLKRLNLSDNEITELSLMIDVW

VNMETLNLSRNKLTALPVSIHKLASLRKLYINCNQLDFEGIPANIGKLHNLEIFSAAKNN

LEMIPEGLCRCGKLKKLILNNNRLITLPDILHLLPELETVDVRENPDLVMPPKPTELRKN

MEFYNIDFSLNTQLRLAGAAPVPSSGNTPPAKSDPVARKLRLRRRRDGQESSEKVLKGMR

DVAKDKAEGKAKGKDEDKEPVKPKRWDEHLERPHLDYSEIFEEDVGQIPGITCWEIENFL

PNIVEDALIGKFYEGDCYIILKTFIDDSNSLSWSIWYWIGEKASLDKKACSAIHAVNLRN

LLGAQCRTIRQEMNDEDEEFLNLFDNGVSYIEGGRTSSGFYTVEDHDYELKLYRATGTQS

VHMERVPVVVDALDKNHVFLLDCGMKLIIWVGKKSNLVTRTKSRLIAEKINKNERKSTAE

ITVMPQGQETAEFWKLVGGPPINLEIKESTFTNLDLPKPKVYRVGLGKGYLELPQVEVPN

NRLVSSILETKCVYILDCISDVFVWIGKKSTRLVRAAALKLSQELHAMIQRPKYATISRC

LEGTEPQIFKTKFIGWDDVIPVDYTRTAESVIRRGADMKVIMERDKMQTDLSALFIQRQP

TMPPDEAEQLSADWNEDLDGMESFVLEGKKFVRLPDEELGMFFSEDCYVFLCRYWVPVEL

PEGEEEDMDEEDLPEDEYKCIVYFWQGRHASNMGWLTFTFSLQKKFEALFGDKLEVVRQQ

QQQENLKFLSHFKRKFIIRNGKRPYRAGSPGQVPVKPSTEFYQLRANGSPIATRCVQLQK

PDSSLLNSCFNYILMVPFDSEDLKGIVYVWVGSKADHSEAQIAEEIAYKMYKDSYTIQMI

NEGEEPENFFWVGIGGKKPFDTDCNFMNYARLFRCSNEKGYFAVSEKCADFCQDDLADDD

VMILDNGEQVYLWVGKKTSDVEIKLAFKSAQVYIQHLRNKQPDRPRKLLLAMKYKEHRKF

TKCFHGWGKYKEVPK

>SG-AL_contig_5396_1_+2_206_493

MVFTVEWYNATYSRAPRNSIFSRLICVSHIRLACLKQQLRLLTNITFTPLRSVWVSRIQH

CESAISSSLLLFSFTYTFSFYSLLHVFSFLYTYTYI

>SG-AL_contig_5397_1_-2_2_3496

ESYQKVQELATALVSRADSNAAIEVNVTLGQLQSKWVQLQRWAERRSEKLQDIPEQWRGC

EESIEELLSWLKTIRQSLSADLPETYDDLHSDLTRCKDISATLHSSEDKLDDLKKQEQML

KDLIQEEDMNLLHQRIQLLTKLWDQLKSQVELREQRLKDAISRWSSLEHRIEELRQWIED

TMGKIGSLDQEEKVRPLDLQGAAESSNAEGKAGSSDMVGSSDACGKIGSSPEVHVEDQLN

KLLTEYAEPMTSKGKEVEEVTAEGHQLMKLSSDVRASDIQQRLERLQERWQCLQKALESR

KHKLQGTVQSLSQLDSKLQDLKVWLTTVEQKLASPVVYTQFSRQEIQTQLDHAQDLQCDI

DNHSTQVQSVLVLCSTLLEDGDACTTDTEVAALRDTRNTLQARWRHIADLSAERRARIKE

TGKLWEAFLSDTESFNEWLSKMETEVDEYKVSMPAAATKGDVRKYEALQEKLHDRLRDLE

SINKRYHRFFREGRTDSQGTLKQTNQEINDRWDAVQQKVTDMLQDLRRSAEAYENFMSLR

HSVMKWLKTVDIKITDVEHLSHQDIDDRLKEIQRIEQELHAERYRIDNVKEGAAVMVQQM

SAEGADSIQQAADQVNDSFTQLLDHVDSCRQHLHSLSVGEEQNIKSTEASVSETAAVPPD

SPPYMRRAGSGGTVLPQATVPPIPRPPSEVAADVSESGAADSEAGSRECSVKIDVLLEQL

AQAVSEASVCLDDADRQLQAVDSSDGTLGQAQSQSVLECKKKMDRVQQVHQLLKREAGSA

TFPAMEQRVKEVTDRWEMLQSRATELDMRMSRRQQSVNKFSSDVDAQLAWLEEAEALQAS

LDIMPTDLTQLSIVIRQHKDFMAQLEEKRPRFQSISLLSHNHIDNSTPEGRDLQARVDEL

EDRWRRVSDRAAAIQKALQGALLQCQDFHHSIHDHLLWLEGIEAQVQACEPVRLSDELSA

LRDKYCKFKKIHSELEEKQPAIMILRESVDQLLGSSDSTEIATARDRTYLIASRLQSLSH

KTASYIQSLEAKLGLRPSVALKEARRLPVHQPPVHTRRRSPFTYFRKLLFGEPTISLPAD

ETDSANLLTVPETAPTTADVSVATRPSLLRRVLKTAVPIQLLLLLLLGIACLLPVCEEEN

RCLLINNFRNSFYPMLQYNDGTPPM

>SG-AL_contig_5398_1_-2_1097_1375

MNVQLWYTHPPSNLHSHMSTNTEDCFDDDTPKPKCLVGKIYFWGQHKHLHLPWLSIKIKM

GVGPTSLSATVSSASVQKEINCESHSDWPMSSD

>SG-AL_contig_5399_1_-1_1_258

NCLWTDLPGSKTGKCHPDDLHPRNMAFRLPAALRLAGVRYMASGSGVGEFGSGAGKGGGS

GGAVREAGGAFGKMEAAHEEMYFKKL

>SG-AL_contig_5400_1_+3_69_227

MSLHVHVHMYIFIYVSMYVLYACVIMLVYLCIYVDSFRMETYKKKKVSCFNKL

>SG-AL_contig_5401_1_-3_81_938

MADSEDKDVPMEEGGEMDEDELLGTEGKNGEDEQEDTMKEDAAADGDKNEEKKEEKEEEE

DLDDGGAASDVEFTTEDDRVPQEETPWKWHIELYPLAPEDVTMDNFVKMVSTASSSHVVF

KDNERGEKRGEAHFYTQDTEAAQTLMRTICCRKFTFFRPFIYLYHLKEGTEDEYESAYLR

LDLIESAKMRWDKQKKNKGEATSRIAKVSDVPLDTSKDFLKVLFARAYLVKRESDQSEEE

AKKAKEAAEKDEAKAESSTADKSEENDEEEDKKEEEEEELVGEFNG

>SG-AL_contig_5402_1_+1_58_171

MLTHAGSVTELLLSLVGNLELLEVSVLPIAMLEQNRHC

>SG-AL_contig_5403_1_+3_51_272

MLTHSGSVTELLLSLVGSLELIEVSVLPIAYVGSEQTLLISSEVTQQQWTVTSGKWPYGR

FRSLASVEFLALGL

>SG-AL_contig_5404_1_+1_1_231

KKTTKKQERERERERDLGHRWSRDGKTNQRVKNKKEKDENLLEITSRLKLFLYMYIQHLT

LLNHRYKSLTVSEYTPK

>SG-AL_contig_5405_1_-3_39_1070

MVSPGGLSFIGRSKEGLGDIPAALAIKRLHLDEAIAMVAKRVGADLREEAPVKDAVFNKQ

DGLWTVSLEDDDRTFRSRVLVCADGAPSRLATQLGLVTRPPDSNCSRAFVEGGTHCFQAD

GVVFYNKHMLPGYSALFRHPNDELNFCCYIIPGNPKVKSEDLAYWHKFLLEKDPSIVKAL

GNNYKIERMKAASLRLGGEKVSTSDHVLVVGDAAGMIDPLTGEGIHHAMEGGKIAADFLG

ETLSVGNYDANVMQIYHQRWMKQFGFDFKWSMLFCQLLYRFPILIDAATAAVQRKGETFL

LKWADIMTGRVPKLTMLHPEFSVVITFELMVLIFKRLFRITRNK

>SG-AL_contig_5406_1_+3_3_221

SWLVRPLETSGRRESSERAGPPSCCRVEQIADLWPLTALEARTCEVARKPGRFASSTGEE

AWGSTWKGPRQAS

>SG-AL_contig_5407_1_-3_3_278

DVGDLSPAEASWDIADSLDLFSNRVRQAQTIIYRIHKLGRTSRPCQSVELMRRRMAYAII

ILDSFPSHGICSVGVYGIYSEHGDAILRKLKT

>SG-AL_contig_5408_1_+2_80_172

MTLSPDFQISLSFLLKKSASIFKNGSQNSYQ

>SG-AL_contig_5409_1_-2_2438_3310

MLTTASVTRLMSDHPNMTSSNAPRGSVGKNRTGMILRIPGPLNPDNSNDAQGCDSPQLIR

RMMMRDYGPQAPFDKSKETDMIDGAVWQRKLRENNSNTHVNDANIINNNFPEGTTSQDCG

ADKTHSGLPLLDDMTSCDSEEGKVRSLSCESEQCTEKGTENSSQNSLGPETGPVLKRKSS

LKDKSRNQSNSNNKSVSVPSLGESGPPSDLSLRDCRVSSTSSGSSDNAADSVSPDITPVN

LKRRISFPADSVLTAVIQDGDTAELIRILTGRHGPGLVHVRGEGGVMRGVD

>SG-AL_contig_5410_1_-3_3_188

TTGQLIKSHGVVQDISHSMRLLTNDLFHLDDRIDIIQSCKLLPDINIPVPTPTPEAASTQ

PA

>SG-AL_contig_5411_1_-2_1178_1591

MKNKLTLLQFAHIYTNTRTHRRTDTDTETHRHTETHTDTDACTHMHARTHTHSLSLSCGI

TSKQERLLNKKTLNYNMNISAKIFIFTKTQVTLNYYYYYKSILYSGILQTVWTHCALHII

RLFRGAWTTQAVRRADHI

>SG-AL_contig_5412_1_+1_1_252

MLFFFCLIAKQHNDGDTLAQIIHHHHHHHHHHHRRHRRRHRHPPHLHPPPPPLHLEGQHL

HHHYPPPHHHHHLHHHRHPHPPLL

>SG-AL_contig_5413_1_-3_45_239

MLRERFCYLMDSSQCTGSMRRLLAYAMIVWDLFPSHGICCACDAEFIQARRFSHEKNLIV

FLSFA

>SG-AL_contig_5414_1_-2_308_502

MCSCFKTLPVRRAYAQAYGLCHDRLGSSPSYGVCGIAAYGIYSECEEAILRKMKLEVLTK

CAAYK

>SG-AL_contig_5415_1_+1_1_282

SLLSPLQATAHSQLLINDSSFKHPFILRPLSRHPAAFTNSNCSLKQPTAVKLLTSSIQWH

SLYCSLQAPCCFQTAHSNFIQSLLNCSLQVSNSH

>SG-AL_contig_5416_1_-1_115_1644

MQIKAPRDWRPEDAKARQVNAPPVHSRYRKFDLYRNVQQFFHIDNHAIQVAQTDHDNFTD

LIYHLVYSANLQSDLEKTRVIFRWMTSKNMYTISFKDGAKAGTPEEVLLSFKNKQGTYAR

IFETLCRFAGVTAVVLTGFAKGLDYRPGDKFKGNEYNHSWNAVLIDSNWYLVDSHWATRY

LVSEKNMPENLVYEYDDFYFLTDPEQLIYSHWAHRKEWQLLVQPITLLDFENLPLVKSYF

FKCAMFFVSHHNGVVETKKGRIALTLGYCKPTNFTYKITFADTGEEMYQGQKLKNFALQH

HGQNQTGTFVFRAPRPGQYYFTIFAQLLTGELGVKNIFTASCEYKVIADAASTDATPLPA

CSDSSWGPGVAVDQLGLVANPPDGIVTTDDGRVNLEFKKTKPAYVLCKLRRNGMKDDDLE

KCINEKDTGDAVQVTANLPSKGEYGIEVYGNDPAKDGDTYTHICQYYVHYATPNEQQQAF

YQESPERRAAMGEPGQTVMAPGQYPGGVIG

>SG-AL_contig_5417_1_-3_6_719

MAVSRATQLLRLGVGSAALASIPAMTYNFKVYATDDGPDESRMVKITELSVYENPEDRVQ

YRFVPEEISPFRQIISDARQAMWEYLETVQETTDKVKEKFEIGKAHTQGLIDYLQNDPGT

LPRAAVITVAGLGGIVAGYRGGIMRKLFFSASAVAAATSLCYPNEAVALTTDAYTVTLES

VKSLWSSEDSNAEGSKVIQGSNPTTEDNQMTTVKGKSSVPGDPGQSKEEDKDMYTTRS

>SG-AL_contig_5418_1_-3_648_842

MLVSWCSPRSPWSCFCSFLYCELGWTFSWHGWLILVLRQVEMGNFSLILILPSFLWPFSR

HATFV

>SG-AL_contig_5419_1_+2_2_253

RLWFTISPSAILVRNASPNGSLFISGIFVHGQESHEIRTHTSGDKFSAYFFGGNRFFFQR

KMFIQWRKSVKWWTILSPDEYLYK

>SG-AL_contig_5420_1_+3_918_1226

MSSTTYYVNILGVKVAIYIIVQTKDKRKGRFVAQGPASSTRPSLSSTVTPLQSNLAESAT

RRTASSLAMSSASSWASFTASLASSRTLSILSWWRRMNGRMKR

>SG-AL_contig_5421_1_-1_394_648

MLEKMGKCFFHFIWEKVHDYIYIYIYGIECKNKDSPLSLSFRKLLTSFHLSLLFQSGLCS

VVLQVAVFLFCFVFLFTMLVLPFFF

>SG-AL_contig_5422_1_-1_658_843

MLSGPLNLRSIVIILLLLKYYLYRQLHVCTHTNTLAYTLTCMHTHRQLHVCTLTSTLAYT

LT

>SG-AL_contig_5423_1_-2_164_457

MYVCMYVCANVGKCVCGYVFCVFFLHIDRWLRSMCVSIYTYSLLGAFRCVIDAMCDVEYY

VYCIHLLFVSYHLFCKAPKAVFWIVCYISIHYYYYYYY

>SG-AL_contig_5424_1_-3_3_176

GCAFNPQAGHRFSSRLAGSVDRAVDSRSDISSSIPACNIKTGLFAAHLNAPIILVVSA

>SG-AL_contig_5425_1_+2_1169_1513

MPNIAHTVIETDSMIQNAQHRSYSHRDRLIDKPHYFELFDFITQTSGRKSLGETRYKLKL

TNTSIRLKVCKCPLDLSPVTYQPKTFLLQELVSLHCQFHFPQTFASNLQPIMSLA

>SG-AL_contig_5426_1_-2_14_1063

MASNGGASPVDSEEKPKSRKPKDTKFKQQKLPAWQPILTANTVLPAFFAIGLAFIPLGVA

LLVTSNNIKELEYDYTYCTSQSNPNQTCAQFFEETNQTFQTCTCVITLTLNEDFSGTVYM

YYGLTNFYQNHRRYVRSRDDDQLHGKTMAPGDLQKDCDPYRTGSNNSQTVGYAPCGAIAN

SLFNDSFAIVYKGSPDVDVGLLKTGIAWVSDKNVKFGNPSDWQRSEYVQPPNWNVPVWNL

SSDSDNNGYKNEDLIVWMRTAALPTFRKLYRRINHTEATFRDKLPAGNYEVTITYQYPVT

AFEGTKSIILTTTSWLGGKNPFLGIAYLVVGSLCIVLGVVFLIIHLKWGK

>SG-AL_contig_5427_1_-2_2_1315

MAACLRHVRSFAGSQLKKQSLFVQYQGQLKQAHANHRCLSNCLRTYTSCSRRHCLVTLTT

VTPVRWSSSRAVEQKVAAVIEAATDKPAATNYSDYIPPPPPIPSEQDVDVPEILNALGEP

TLSSVGLGGWSPSGIVQNALDLLHVSCGLPWWEAIIVGTICVRLMMFPLVIKSQRNVAHM

HNHMPTMTRLQEKFTLARQSGNAVEASRAGHELMEFMKRNEIKPFRNFLVPLAQLPVFVS

VFVGLRQMANLPVESMKTGGLFWFTDLTLPDPFYAMPLITMATFLVTIELGVDGVKAGTM

NHTMKWVMRGMPIIMLPLISNFPAAMLCYWFTSNAFSLVQVLFLKIPAVRTFFKIERMVQ

HPPSALPKKKGFLEGFRDSMGNAKLAQQMEERQRVDALNFKKAGQGPIQKTYSYDPTKQK

VQTSTSKSKPTSSSKTKS

>SG-AL_contig_5428_1_+3_1740_1898

MMYIFSVLAQVTCLHTHSFTLHVCDFVPVSFTHGSLHSLFLYSHVMFMFLFEP

>SG-AL_contig_5429_1_-2_158_682

MSCLKCNKGLIKQISQLVFDMAFTKILAKFGHNSWLYVNVIMLKVQRLIIGYRQCRMILG

KGDSVWSDHTVIVNSDLLLKMMKIVLPFLFVIFFPRFILTVPFNIVICNISKKVLIVYFA

SNYLLLMSVPSYLGAVSHQMYGPWLPEIYVFNGLYFSNCAQYTMFVKIQITFLVF

>SG-AL_contig_5430_1_-1_211_1287

MSQAVADEHEGQLDTAAEENGREAVTAPEDAANESTLPLTSSPPSSKARGSRGPSQACLK

SSRMATSLEDITSFRLWRGIVAEFLGVMLLVLVGCGSCISLEEGKSSSVVQIALAFGLSV

GTIVWIIAHISGGHINPAITLALLATRKISLVKAAFFVLFQLVGAVVGAALLMGLTPKRY

RATLGGTNLDASINAGQGVGVEIFITFVLVFTVFASIDSRRRDLNGSTPLTIGLSVTMCH

LFAVQYTGSSMNTARSFGPALVTGNWKNHWVYWVGPITGGVAAGLLYEYMFAVNASLTKV

RAFMLSSDYDDEKFHAQKTKIRIIEEDPEAAMECVPLSDAAEDKPPVDEVKSLDEPVKV

>SG-AL_contig_5431_1_+3_3_134

SSLYARFSFTLYNIDFSIGDIFSCCYFFIPLMYRFMYYMVCGNR

>SG-AL_contig_5432_1_-1_1_147

WEILGWKILFFFFSFFFFPPFFLFFFFSFSFWSAFIFAYLVLGFSSFFN

>SG-AL_contig_5433_1_+1_316_549

MLAAATHTHTHKLSTYFYPFTLGPLDTHTHSQAYPFQLHTSFSKSFSSTGIKQISTLPQF

MSNIREIDRKGQREREAV

>SG-AL_contig_5434_1_-1_1_177

IYIFLGCFKGRPKIKLLVACTSCSKISMTWSKKSSSTSQLAVPKFICSNQSVKSVAREK

>SG-AL_contig_5435_1_+2_41_259

MDMQNTFKKSDTLCLSFTQQEIIFAPINFHPFKVMRSQMNGPELLKRCFNKAHAHMTVKF

LQHRPITTNRIVK

>SG-AL_contig_5436_1_+3_69_371

MNTIILNKMLESKSKSNQDASKCNHNVIISKIILAQVFLFVITQNVQMARSFYTHFFCST

LIITNEIFLKQTFVNLLNILKQTYLKVLIEHFKMAIPQRYR

>SG-AL_contig_5437_1_-1_925_1230

MYYMYFLFITNVTCCRDRNSVLPRVEIQLFQPLGRAVRGQPRRMQVLFLALRGSRIMTGY

LDQSCTFLRGKNTLLCFMLCGTRLREREGGGGVSFFFANYSL

>SG-AL_contig_5438_1_+3_36_284

MYLNTNHIYTYGESGFIIDLHTFDSHMQQRTSIIITFFPKISSFILFSKLFFIFNICCAN

NIDPRPYGMHYQPMGKNSAIQTC

>SG-AL_contig_5439_1_-2_1226_1489

MKVCIYCPVLYKRTIWLFFPVYYHLMYERRNFFLISLVERVDNDLHHLEKFWSNLYVCVR

EREGRGREREREREREYPNESVAFFFFF

>SG-AL_contig_5440_1_-1_199_414

MWCVMVSCAVYGDVFMARCLCSKSALLVMSFLSVTMDQWCILIIINGVCVCLSQEHFPGS

VYWMYLFVLTIN

>SG-AL_contig_5441_1_-1_61_1227

MPSIDVVNPPQQTEEPKTEESPALNKPAIGIIYPPPEVRNIVDKTASFVARNGPEFEHRI

RQNEVNNPKFNFLNPTDAYHAYYQHKVKEFKEGRGQEPMAPKAQMLFQRPQQEPVKLPEV

FVPKDPPAEFEFIVDPPSISAYELDVVKLTAQFVARNGRQFLTTVMQREQRNYLFDFLRP

QHSLFNYFTKLVEQYTKILIPPKNLKEKLAIESLGYKKVLHDINYRVEWLKFQDREKKKE

EDAAERERVAYAQIDWHDFVVVETVDFQQNETGTLPPPTTPDEVGARILNQERYERGEIA

IDDAVEMEVESDDEEPEEEEEEEEEEESEEEKDEGPVVPPLPPPPKPAQPSDDTEPQDMD

EESDEEDRPPIPPPPPRPKIAPPPAPPPP

>SG-AL_contig_5442_1_+1_49_243

MPMGPSSSTHPAEGSCDAGGEPRPSSPGALLSASPWPPTSSRGSPGSWLPRPVRRPSASP

ASGLA

>SG-AL_contig_5443_1_-2_1211_1366

MKENYVEGNGEWNLLKPKMDHPFTDDTWIILSQMILLQIWFIYVAQHRLWIN

>SG-AL_contig_5444_1_+2_1814_2065

MMDLTMQNQYKQVFRPMIFSVCKNIYSVPRQITLHAIPDHSQFTYNVPDSRIFQLLTFSH

NPPRQLIYHAQKCTYAMYAMYVYT

>SG-AL_contig_5445_1_+2_89_256

MIPMISQLHKGTIQKQTNKHSKSGNVFTNKIHPAILKARNKPKCAEDKNLKAILYD

>SG-AL_contig_5446_1_+2_1679_1924

MLFSQKHKHCLFPDTSQLVIEHGCRLDTQKLHGTVDRYKALDWLVSVEVGKTCEGLIVIC

DPFSHKYAEKYPSVTSFYTWHS

>SG-AL_contig_5447_1_+2_965_1279

MCVCSLQSTQLLMGICTFFKFRERLSSEGEEMGTALIMLTPGNVEVSTTSLPYDLRVTGK

PSPFIYLSITESYCYTYRGTGLMSEPINPSHHSAYLDACASAMES

>SG-AL_contig_5448_1_-3_450_728

MPVILVSPFFFGLFVQDSITDARASKEVERCYGSNKTQSQHKSTQGNPRHKQQLRQSQIS

QHKAITNKQQLRQSQHKSTQGNRRHKQQLRQST

>SG-AL_contig_5449_1_-3_3_302

EVRFFTVCTEAECSVSHSQKLVANGQVERAFPLVRLKTQLSRTIGPVGISSPGVCVCVCV

CVCVCVCVCVKSVILVLEWSWCHTHSYCKHIMACFFKKKI

>SG-AL_contig_5450_1_-2_11_265

MKRGGGHSTSYHELGGGRRRIPVIMKGERAKIPPSLMTASATTRTSTVTATTTTTTTTTT

TTPPPPTTRMPGRPPLSKRWRKKSK

>SG-AL_contig_5451_1_+1_190_432

MMDHFPNMASNASGVSLTLITQARLILPRITQHISPQVHRTYSFYKLVKSLTRNSPTPTS

VHTLCSNDRNIDAIPMGSGKD

>SG-AL_contig_5452_1_+1_562_1029

MRTHTDMNIYRHLNTTINFCQVPKYSNSRSLPLNTVKQWLEDHFHIHIPNKSSECIPTFQ

LNQAHTYIFLPNQAYVYKQVINYINIYFYIQAYVNAQWNDNRQACPITLRFLRSEEVLTS

TLGQHRKGSAHLSTASLSPVWYPFSQLGGLGSYSNF

>SG-AL_contig_5453_1_-3_3_263

VSEAVLSTVKQLLILFTVPACVTKRPGESWYLSYWNVLSVSKLVMLVYVCFRHALCHFCE

CAQNIVLTVILYCPELSDSRHSKRMMM

>SG-AL_contig_5454_1_-3_462_1580

MPSREFFNINKMSCDTHDCCSPERLRDEIRTSGNVVIVDCRAQADYVRAHIRQAINISLP

SLMQKRLKRGSLNICNVIQNNEAKERFSRCCKSHQMVLYDECTTDLASNPSSVLNLLVKK

LRQDGGKVSFLIGGFSTFQERFPEYCQTQEITDNTILGLCNLRISDDSSYSSCESSGDLE

NTPHMSPFPVQVLPYLYLGNAKNSADLSQLKQNGIHYILNVTPNVPNMFEDDGDFKYLQI

PISDHWSQNLSSFFPDAIAFIDEARHNKQGVLVHCLAGISRSVTVTVAYLMSRQAMCLNE

AYDFVKKCKPNISPNFTFMGQLLDFEKALVDQTSCQCSAHHRTSSSADDEDDGIPASDSD

SDKTSPPFEISPV

>SG-AL_contig_5455_1_+2_2_121

QHTMSALTTKGLQQGFDRYNMTTTTTHHVSTNDQGITTRL

>SG-AL_contig_5456_1_+3_1296_1592

MSIDRKSTKITTIRKWKGNQIRGKGSNLKTGHEGWGKIPVSLREVNGSTWKTQEGNMVGK

RREKYSHSLHYKPTKAKVNIGILTETTFTVSNWKPTLTI

>SG-AL_contig_5457_1_-3_3_1136

DHLHIRQKLEATQKWRDSVSLSKVLPKTLAYSTPAASIDRFSSVPAETSSKRKHETSDSE

VQTFVPDACDGKEGNGDDQSWKRPVRLKRKPRLFIQDDIPQTKIRRKPAWFRQGNILPQI

KNCQKTLELTAKTTEMVQAITDLKATSEDGGKGAKVAENPAKRNRQLAALLRRAGRLMKE

WKEAYSDLAERYDALVYKINTQLAQKGARVKASTCVPVSSGAPVPEDISVTSKPEIEIDI

AEMGENLTQGNLGGHINTLNMVSSSAVNSVAAENLSLSVTNTVSLVNIKMEPVDCEDIEQ

KWKQEPMESDLGHAQREPIESDMGHARTEREWKQEPIGSDMGHTRTEPIERDMGHARTEP

IERDMGHARTEPIERDMG

>SG-AL_contig_5458_1_+1_1459_1779

MKRVTSTLICQTSEENLLHIPSPIIHICNQSGHTLTIPFAPTHSHILQLCTNTSLNSHSA

ESLKVHNAPSVSVLLNVVPNKTISFFTLPMISHEESLQTRLQRVPPG

>SG-AL_contig_5459_1_-2_1532_1783

MSPHAKDIFVDISYVFTHIILDLKQLTSFQLTNLHAAMSGINMVNLKILSACRAYAQAYG

LCHYHFGLFSILWHSQRWCIWNLF

>SG-AL_contig_5460_1_-1_1_156

LVVIFFPQRCIIFLVYFVLNASSFHLNIWDFIILLTLNWWFSLLNSKFVILL

>SG-AL_contig_5461_1_-1_1_99

DKSYSCGQGDHYFVPRTVATAVVKVIITLSLGL

>SG-AL_contig_5462_1_-2_17_520

MAATTAASRMQPPNITVETSMGSFVLELYWKHAPNTCRNFAELCRRGYYNGIKFHRIIKD

FMIQGGDPTGTGRGGSSIYGKNFEDEIADDLKHTGAGILSMANSGPNTNGSQFFITLAPT

QWLDGKHSIFGRVSSGMTVVQRMGMVETDGNDRPLDEVKIVKAFPTPV

>SG-AL_contig_5463_1_-2_194_490

MESRKWTKDRARKSLKLGLIRICESSCIAVWGMFAWYTFLRNAQLFWQEYINSKFKVNRS

AFISLFFVFFRSLSPSLFVSVSFSLALSFPPRNLLMCHN

>SG-AL_contig_5464_1_-3_3_110

IKMGCEQDLRNTDTTRMTGALRWAANRTYETLTPPE

>SG-AL_contig_5465_1_+2_467_640

MHCQAYLDFMLLSVFVANVCFVFFQDQGAGLKPNPKHLLERLHGGRLKVKDCRPIIHH

>SG-AL_contig_5466_1_+2_935_1159

MFTYKTCSGESQNISRGRDHKSTVFVIQQYKWTEKISPENVWTESYGQINHNKYLMCYAT

IRYMLLQIQTIGKCV

>SG-AL_contig_5467_1_-2_1067_1291

MASLSSLAHYRVTLCLVIAMSGFCQWVCAHKCVYVSVCARTQYACVYASMNVCVCRCMHV

RGEDCLLVAFYTAVM

>SG-AL_contig_5468_1_-3_3_164

RERERVPFPKFSTVNGYEFLLCDNLCKMYIRLYFDCQDGPKVTLNCAKKCTTQN

>SG-AL_contig_5469_1_+3_135_350

MFLLLLGQCKAFVSFDHVLMQVLSVPKEFSTIDTLELNVMAMLFVAVVAQIGKRVESLFA

LNADIQLVNTPH

>SG-AL_contig_5470_1_+1_208_309

MHIQLTMGDCVIVMMCVRVCVCVCVRVCVQMYLC

>SG-AL_contig_5471_1_+1_1_108

GGASSGSARRSLVVGGSPASGWAARRTLVLRLKHSG

>SG-AL_contig_5472_1_+2_2_163

FFLECSATSSNIYFMLSLLGIMVTENKLVKGAYTISHQSPPRGKNTMPYRQIKQ

>SG-AL_contig_5473_1_-2_20_1168

MADGTPRLSWHQNQVMIKLYKDHEALWNKSCPVYSVKSARRSSLQSVQEAFKAATGLTLP

IHIIKTKIKSFRTQFTKEHNLVLQSKRKGAGPDEEYRPRYELYDKLSFLRPVVTCHKSRQ

PLEDSSNAAPDLQDPTDCSQHTPTLQDYEELQDEGGQEFHVYNSPPVDDNNDNDDNNDND

DEDSMSCDLSCDSIPDDKHMMRELIEEQNRRLGQGVGSSNMGSTHMGSSHMVAQDQDDFS

AVHNSEQTSWPKTNRVYQSGRRNSDQGSRKRRDSTSVDAEESGLNSAKRQRVEEEEEKSG

AALINTMVSYLLERMKRREDRKRAKQARRRQEMSVGQSSDRLWVKHVLQQISEIGCEQKR

KMLKLKISQMIIDCQMEDLQQKQ

>SG-AL_contig_5474_1_+3_522_773

MLYTYRQHDTDTAVECIIIYTHRMAVGYPTKFFLKNFQQNHATDMEVVEQLYKMLKVSSS

AKLYRIYSNGTAIALAHANHTPLH

>SG-AL_contig_5475_1_-2_509_703

MLDKRTTTETNKKVGVGRQEKIKSNNFLQFANRRSQNYLDNLNKIIFLLERISVTSFIML

KDPDC

>SG-AL_contig_5476_1_+3_2652_2867

MIVEVRSVRQARNVVYPKTIITGFSQCGFTQQYCSPIFTESKANRNLSSYVMISTGSLKQ

SIMHVYKNYKRK

>SG-AL_contig_5477_1_-3_273_1604

MALSLWGIVGLLALSAYLSFTTASTTSRSNHIPTALPPTTTTLTTRHTTNHPTTTTTTMT

PVDFKRPRRRSRRCKCGKRLTKEMNKLVEDRLHDFETRYLRHLQEDRDTALSRKTTNGRL

DTLERRLYKMGDDVTNTREALSVMQNSLQQLKQDMQHATSEVTALNVSFRQLDVALQNVS

RFVRNMENLLPAGATVCPPEGCEVTVPPTVHTYPRHCDEVYQQGGMQFKGDYYIMIKPDG

VSHPFKVLCKIYNNSGWTVIQKREDGSVDFFRNWGEYKKGFGSLEGEFWLGNDYIHYLTV

QEPTMLRIELQDWEDRDYEANYGSFRIDDEEGLYRLHIAHYQGDAGDSLRSHWENHDGMP

FSTKDRDNDGRYYDNCAEHFHGAWWFNNCFDSHLNGKYYHKGFHKNYFQRDGLQWNTIHM

YSSLRAVHMMLKPAHNTHPPPSRP

>SG-AL_contig_5478_1_-2_167_1933

MSELALLSVSDKGGLIPFAEKLSALGFHLVASGGTAKAMRDADIPVSDVAEVTGAPEMLG

GRVKTLHPAVHGGILARLTDSDQADLKKQGHEMIRVVVCNLYPFVKTVEKPDVKVEEAVE

QIDIGGVTLLRAAAKNHARVTVVCDPSDYDKVIEEMKKSPVKDTTLDTRKQLALKAFTHT

AEYDAAISDYFRKEYGAGEAQLSLRYGMNPHQKPAQLFCNQPKLPVTVKNGSPGFINLCD

ALNAWQLVTDLKQVLGLPAATSFKHVSPAGAAVGVPLSPEEAKLCQVDDLTNLSPLAVAY

ARARGADRMSSFGDFVALSDVCDAMTAKIISREVSDGIIAPGYDREAFEILRKKKNGGYC

ILQMDGDYKPPLMETRTLFGLQMQQRRNDAVIDRSLLQNVVTQNKEIPTGAERDLVLASI

TLKYTQSNSVCYAKNGQVIGIGAGQQSRIHCTRLAGDKADNWWLRQHPKVLGMKFKKGVK

RAQISNAIDIYVLGTVGQDMDTAVWKDLFEDAPEMLSQEEREKWKKELKGVSLGSDAFFP

FRDNIDRAYQSGVEYIASPAGSTNDQAIVDACNERGIILAHHNRRLFHH

>SG-AL_contig_5479_1_+1_1_147

FFFFRFRNFTSSNNVQHTFFKNKLLYKFMNQTLETKHPASLYVFYVYTF

>SG-AL_contig_5480_1_+2_86_346

MDEFGSCTDSEQKKDISYTLISSSNAKGTELGTQHKPAMQQCFAPKTQLCSIKENWVNTL

SALLANISNEDCQFQRQTDEKHATAKW

>SG-AL_contig_5481_1_-2_2_127

NSNTFIIGAVRAIIIIIIIIIIIIIITLIIISKSEQYIYTQD

>SG-AL_contig_5482_1_-1_1_147

KKFSVMRSVTWSQQRCTLYSSITWKAAERQTWLSTRVSYRAERSSRSKR

>SG-AL_contig_5483_1_-3_153_287

MTRIQSASLCTCKHLPVQMASNPLIVYVKKNLIILEPCEHFSKLT

>SG-AL_contig_5484_1_-2_407_574

MISKQMYISVKFVDVAFMQKLFITVWSRCQKSCTTHLQNICCKLSCLLLLFDILFD

>SG-AL_contig_5485_1_+3_27_248

MNKIFEKPQTHSNSRFKTDPISPLRSGTCKRLHQQTPAPPTPPHLAKNSKILQTCKDKQD

KRPTQIHHHKRRQR

>SG-AL_contig_5486_1_-3_24_230

MSTLPRTYPLMIHSMASLRSIISVIFYPVLGIIISHCILSLYAATNLLGHSIIEVTQRSV

CLCECVCAR

>SG-AL_contig_5487_1_-3_12_1607

MSAIKTLNPKAEVARASQALAVNIAAAKGLQDVLRSNLGPKGTMKMLVSGAGDIKLTKDG

NVLLHEMQIQHPTASLIARVATAQDDVVGDGTTSNVLIIGELLKQADLYIAEGLHPRLIT

EGFEMGRNKALEVLEAVKVKKEVDRTTLIQVARTSLRTKVHTDLADLLTEHVVDAVLAIQ

RPGEAIDLHMVELMEMQHKTDMDSTLVRGLVMDHGARHPDMKKHTTDAFILTCNVSLEYE

KTEVNSGFYYKSAQEREKLVEAERAFIDERVHKIIALKKQVCEGNKRGFVVINQKGIDPF

SLDMLAKEGIVALRRAKRRNMERLSLACGGMAVNSVDDLTPDNLGEAGLVFETVLGENKY

TFVEECKNPRSVTILIKGPNSHTITQIKDAIRDGLRAVKNAIEDGCVLPGAGAFEIAAYQ

ELMKYKDQVKGRARLGVQAFAEALLIIVKVLAQNSGLDPQEAIVKLQQEYAGTHQPVGLN

IKTGEPVQPLEEGIVDNYRVKRQLLHSSSVIATNLLLVDEIMRAGMSSLKGD

>SG-AL_contig_5488_1_+3_3_188

GFVFVFFKSVRFFVFCFFLSSFTVFFVSKFYLFMHDRNKSFCLPLPSSNLISFIKLCLKG

KM

>SG-AL_contig_5489_1_-2_650_1039

MAIRRRFASVPPMYMSTKRSKNYAGMPSLYPVTGMSRAARMAYDTRAEDYLTDRVTNASF

FSNYALDEGYDTAKKSRGRDERLLRDATHAIVSSESYATPQASVAARRVVRGRSMAPRAT

SAPPRPLYPE

>SG-AL_contig_5490_1_-3_3_149

KERRKQRKKRRKKGQETLKNIDFRLSSLLSFSFRVCVCVKLHVTYYKSS

>SG-AL_contig_5491_1_+2_221_508

MQTRKKERINPLPNITSFTRYLAAKYHIEENNAKLTWSVHTFKEKCCHTCQWVQMKINII

RCLFMTCCLLFAFPSLTLWGSPNIYALCLTRVRPRV

>SG-AL_contig_5492_1_-1_1_180

LVELPIYVFRSVVLNELAFRRFVFSFVCETNVLPECHFDEEIFCCCLTCIRLQSFVFAKQ

>SG-AL_contig_5493_1_-3_39_209

MLHACRPVHFCISHHEVKTTIILTSSPYFIFQHALSLICHVFKHERSHQYYHYSCQA

>SG-AL_contig_5494_1_-1_175_825

MSREAKINELKLKDRIIELEAEVASLKRRLDELRKAKNTTILKREREVLEVGAPFGRRES

QQSDMTKNSELEKKLQEAKESREAEMEALRKKFASEMADIKNSTKDCGHEEELQFLRQRN

ETLEIENVVLMEESRELRARVDALVMDLSIKEAKWCETEEEFKMKLKTSWAEKYKDWMAA

TEAKIADLQRTNELLRTYLKNSRSDGKDPTMPDSDKL

>SG-AL_contig_5495_1_+2_164_346

MVTADTTKICLSPPQKNLVHIHHWKRLFFSSLKSAIIVMLPTQSQSLIQESHCSGSDGGG

R

>SG-AL_contig_5496_1_+3_3_290

TLGVHQIELVVQTGPGLGDSGGVAQHTHSTLNLRQVTSRYHCWGLVVYAHLESCGTPVDE

LDGSLGLDGGDGSVDILGHHITTVQHAAGHVLAVTG

>SG-AL_contig_5497_1_-3_183_302

MSCVIIYSSVWCTLVRFVAMHSLVFLIYWCGSECHLSQVG

>SG-AL_contig_5498_1_-3_1839_2207

MNARIVNDLICKQYNNRISWLFIDGLFLSNVDPQASPRRKSFLQVSVGLTRHLGNSDRKC

EYDLVRILQLFYSKFGSSILADMSRKYKCYEVGYFSCLWFHNMRRLRDSVSMAWLCGHLL

LSI

>SG-AL_contig_5499_1_-2_2_145

VVAVVVFSVCMCTHALAGMLVCLRAHIVAFISVVAMGVSNCFDVGGGL

>SG-AL_contig_5500_1_-2_152_1600

MGDVVDGPVLSPASLQSYSYEGAPGTSTTADYDLVTGIHPGYPSNTLASGAGSVSGGSTM

GGGRGYSTNTMQSSYNYTFRNIRMSGHQQMMDFGAESNMVGVPPGALGEEIRVDCCLITN

DGKSVVTGSCIGPPQVWDMQSGELLRTMQGQTMGSTDLHLACNDRLLVGAINAELEANQD

SVPKGVNNYVLQIWDFITGRALDMGADVEVCSALTMMSNNDKVVFGRTDKFRGGTTVVVW

DLLGNEAIKTMRYDSPVGHNDFVNFLALSQNENFAIAGFTNSFDNFAEFIVFDMTLNHYN

VTDPCILRLDANPDCTAILPHDEAVTGMRNGELIIWSIRTGAPSRQLMAGSGYHAHTREV

KAVAMSEDNKYLVSGSADGTVKLWDMQSERHIDTFNGHTDEVWCCTISPDNEMVVSGSRD

RTIRLWKLKNGSEICCFNAGVDIFHVTMPRDKSTIVALGDKLGARKLIMLEVCRSKIRRQ

VSN

>SG-AL_contig_5501_1_+1_1_270

NNNSNNTTTNRQKIKHQQKKMHTQKKCIHMRIQKNTYEFTQPESHTDLYTHSENRLTRIE

QEEAQYFSAVQTAGIIFDGAFHTAGVARTS

>SG-AL_contig_5502_1_-2_2_220

FHVSVSQRTLTWHTGFLTCLFDVLRAFTYEASSGTSRSAHMLTWEIGKISALNPPGGIQA

GSNPDLPDGSPLS

>SG-AL_contig_5503_1_+1_427_699

MNVKSYLCLFVGNLLLLENVLTDDNNVRFVLTGHVSSVQKFKFLYYGTTQRIPGISFSFT

FIVATHHMTFYFCKSGVSYIFVSLVFLTFNC

>SG-AL_contig_5504_1_-2_491_1555

MASSEQQPVATQEECSKFFKEFLAQAKEEFQKKWDHPPPANACLDDFDRIKTLGTGSFGR

VMLVQHKGSNKEYYAMKILDKQKVVKLKQVEHTLNEKKILQAISFPFLVSLEFAFKDNSN

LYMVLEFVSGGEMFSHLRRIGRFSEPHSRFYAAQIVLVLEYLHHLDIMYRDLKPENLLID

SQGYLKVTDFGFAKRVKGRTWTLCGTPEYLAPEIILSKGYNKAVDWWALGVLIYEMAAGY

PPFFADQPIQIYEKIVSGKVRFPSHFSSDLKDLLRNLLQVDLTKRFGNLKNAVNDIKNHK

WFSTTDWIAIYQRKVEAPFTPRTRGGGDTCNFDDYEEEPLRISSTEKCAKEFADF

>SG-AL_contig_5505_1_-3_87_3377

MALKLDKEAFFRRVKKVYSSWAKATGEDSLAKMDALVTSVGVDEEVVYSKSTSLQTWLFG

YELTDTVMVLSEKVILILASKKKIDFLKQLEGNKENDQAVPPVKLLIRDKGDKDKANFQK

LISAIKESKKGKTMGEFSRDKFPGEFMESWRSVLKSGGFEKVDVSNAFAYIMAPKEESEV

KTIQRACQVTSDVFNKYLKDQIMEVIDADKKVKHSKLADGVEQALQNKKYVTNVDNSQLD

MCYPAIIQSGGDYSLKFSTVSSDNPLKFDAIVCCMGVRYKSYCSNIVRTMLVDPSEDVQK

AYEFLVKVEEEILAKLQHGTRLSEVYQAGMSMVKSERPELEAKMTKTFGFAMGIEFREGS

LLIGPKTNAPAKKGMVFNVNVGFAGISTGKEGKDCALFLGDTAMVNEGAAATVLTNLKKR

VKHIGIFLKDEDDAEEEEEEVAMPKETDVLLGRGRRNAVLDNRTRTDAPSEEKRQQHQKE

LAQKLNEEARERLKGMKSDGGDKKVRKSNVSYKGFGQFPQESEVKDLKLYVDRKYETVVL

PIFGVPTPFHISTIKNISQSVEGDYTYLRVNFFHPGATLGKADGNTYPNADLHFLKECTY

RSSNTKEPGEISTPSSNLNTAFRLIKEVQKKFKTREAEEREKEGIVKQDTLVVNPNRGNP

KLKDLYIRPNIVSKRISGTLEAHTNGFRFTSVRGDKVDILYNNIKHAFFQPCDGEMIILL

HFNLKNAILFGKKKHIDVQFYTEVGEITTDLGKHQHMHDRDDLHAEQAERELRQKLKSAF

KSFCEKVESITKNEVEFDLPFRDLGFHGAPFRSTVLLQPTSGCLVNLNEWPPFVVSLEEV

ELVHFERVQFQLKNFDMVFIFKDYSRRVAMINSVPMTMLDHVKEWLNSCDIRYTEGVQSL

NWAKIMKTITDDPEGFFDNGGWTFLEPESDNEEDDEDDEDEDSYAPTDAESGDDESSEED

SEESNWEDEDDSESEEEDLGSSEESGKDWDELEEEARKADKQKDRDEEEVRRHGGQNPHG

KHRSSHHKGGHGHKGGSSHGHKGGSSHGHKGGSGHGHKGGSSHGRKGGSSHGQKDSHGHK

RKHSHGGDKSPAKKKRH

>SG-AL_contig_5506_1_+1_1_159

TLTLTQEQRRGWGWPYLFHIFIFNNNQKFINNSNHIPQKYIYLKYNTIEYIFA

>SG-AL_contig_5507_1_-1_1_63

ERADPCFSIPVQAMTNMRPPS

>SG-AL_contig_5508_1_-3_3_194

EIRIEQKPFKFFIPYENYEKQNFVNLPFHVFFNPSPCFESSILKFDRLTPNNIYTSLISV

NLMH

>SG-AL_contig_5509_1_+3_267_383

MFSRHCHMKPKNLTSGQRAEIVHIPVENILVLDAVSQDG

>SG-AL_contig_5510_1_-1_853_1062

MILYLFYLFCLVVSNLLCFFSFFFSETSATLDFCFAKYHLPLTGEHLCTYINGNQCSGYP

KLRPTASFQR

>SG-AL_contig_5511_1_+1_796_927

MCSRFFIQLFPVAALSSLPLTSLKASTVRSPLPCCVIFPVPNFS

>SG-AL_contig_5512_1_-3_69_338

MHFCDFKERLLSYFKAILYDIKRFKIAFTGPLKITITSTRNQTWLVYHGLLWKKYVFLKS

IHFCIFIFVIIFIIYMGNTRMSSSNVILTF

>SG-AL_contig_5513_1_+1_25_132

MHSQIQPSNINHSSSCKTDENCLNAILKKKLLLKCY

>SG-AL_contig_5514_1_-3_3_179

SYSSSRQSHFHIAHPRIDMHKCSLVFSGASLWNSLPQNIINTRSLPSSKENLTKYLMSI

>SG-AL_contig_5515_1_-3_1425_1625

MGSFGKNGTTHLHVVHFTDMSLRVLLNFLTNTTGICISMAWLMPGYIMKESLRVQSCHIV

SIQNGTL

>SG-AL_contig_5516_1_+2_2_229

PVSQVSVQINENTQLTHPQKRTMTAHMARFKNKHTNRTQKAHLFYINPHSSCIPGTQKRE

EKSGWLAAQNKDKSHR

>SG-AL_contig_5517_1_+2_110_244

MLNIMNLDHVMHDTNDSETSSYDTKTLKPLAKMTVQHNYQLYPKF

>SG-AL_contig_5518_1_-3_3_275

PRGRPLAPVDRRARGSTPPPTGLGLTPWLPLFHRHPRNRPRRILPPRPQPDQPRPDQPPA

PVDPRDPFPGPFQGSGSRLRSVRRSPVEAAT

>SG-AL_contig_5519_1_-3_12_584

MAGSRLEKFGTIYKRVQGLLRSGAIKHEDRPMWCQVYEAFPPRDPPVYERPVPDKVVPQI

LYPEDIIRAQFYKIYGNPDVMDLSNEKVKTTCQRFVDKYIELVQTNTTGQQVFEDTAAAL

KAQGLRLRTVTELQDSSTQAMETGSAPRHVAPTQPSTLSVASIFGQDTITQAQAHDTDFI

VDEDGDTDPKL

>SG-AL_contig_5520_1_-3_1098_1343

MIDICLADHISSRRQAHCCIICMCHTSAIEWIEGMCILQLNYSMVTYVSYLRGSFCDRYA

YIILRTFGIGKVHFCVVCYVHR

>SG-AL_contig_5521_1_-2_62_568

MATAAARPLISVYSEKGETTDSNVVLPAVFRAPIRPDIVNFVHFEMKKNGRQPYAVSEKA

GHQTSAESWGTGRAVARIPRVRGGGTHRSGQGAFGNMCRGGRMFAPTKTWRHWHRKINVN

QRRYAICSAIAATGVPALVMSKGHRIEGLPEVPLVVSDKMEELKKTKEA

>SG-AL_contig_5522_1_-2_41_208

MRVQNSRELTEVNPELYILVFLRCYKPDQVCTMVSGSIFIFCVLCYILLKTPRKKI

>SG-AL_contig_5523_1_+1_667_900

MTDSESCPHIVILHPQYFFFFLKGIILIQFNDSNKSTFSPSAAKNLSPTHWVLTDIDIIC

KSQLGIPSSQYCGKSIHR

>SG-AL_contig_5524_1_-3_144_1118

MAPEMYEEHYDEAVDVYAFGMCMLEMASSQYPYSECKNAAQIYKKVTMGVWPEAMDKVAI

PEIRDIIERCICIRKNERYTVKNLLQLDFFLEEYGFKVELVNKEDEVNNRVDIEMRLRVV

DAKKRKDKHKDNEAIQFSFNINTDQPESVAQELVTSGFLYEDDMRIIVRQIRDRVVQVKR

ERERRAEAGNAQNIQSVNCDPSTAQQTGTNTTTVSQQSPSQQQGTGDSKASQGQVSTQQS

SHSTTGTSAVAVQKQTTPPGPSPGPIVQQTQYATGEVDVRPAGDGRGGKDVTDSAVPQSE

PVPTSINQQETSASNLSHLDMAAAQ

>SG-AL_contig_5525_1_-3_18_407

MFPPIALLHGDLGRLIAGTRLRTLDTAMLNANRTFYKGARYPWESAFTGLPTSPSYSCET

FEVHVTGDASWMMRQYLQLTNDTDFLAHKGYEAVAGIADFWVSRSTYNKTKQMYEILDVT

GPDETHRDRK

>SG-AL_contig_5526_1_-1_1_180

KLLHSLHSPSSVGVHAGYFHDSISHRTLACLTRSLACVFDVLHVYTHIGGSSTCWSAHIC

>SG-AL_contig_5527_1_-2_50_295

MYLCAKHIVATVIREKNFLSHKNVGYEQSPFDTSSYTYREGSDTSRSAYMLTWEIRKIST

LYPPGSTQAGSNPDLSIGSLTS

>SG-AL_contig_5528_1_-1_1_189

FFLPQSIQCCPMHSEETDPCFSSRVCDSTPVSILPQEFFISMLYGSNICRTLVTLPDNCS

CVS

>SG-AL_contig_5529_1_-2_44_1249

MEHMHQVMALHPTYMEAFYTAEDSLLKDMLGPLTFQYRQYIAIMAAARHQCLYLMKLHAS

EFLMNNGDPQWLEGLDKVPQKLRNLAEVNKILAHRPWLLKKDHLEKLLRGENNWSLSELM

QALIIMIHFHSLSSFVYGCGISTDHDHVTSLRLSSSGEEESDYSSDSSGELNSNLIDEPS

LELLMQKMEQLRVESHQDDGPTQEELLERFKSVEKQSVEISVPTKEKSSRIEEAQRFVTD

IDFAYSDFAKRAHDDIQTFRVQDYSWKDHGYPLVNRLYNDIGNIVDKRFDVAFNLTYNTM

GNNHEVDTTAFRQAIWYYIHCMYGIRHDDYDYAQVNQMLERGLKTYIKTITCYPERVSRR

DRDGVMKALKHSEKVHVNLMIMEARMQAELLYALRAVNHYMT

>SG-AL_contig_5530_1_-1_496_651

MEVLDSDILLKKELVSSIVHSFHRVFEPQNHIYIYIYIYIYIYIYIYLSIYL

>SG-AL_contig_5531_1_+2_287_334

MEFHLGLRLRGRAVLY

>SG-AL_contig_5532_1_+2_2294_2605

MFLNVVMIHDNANDGDDGIEFYCGLRVRDRAVLYVSFHNMSWHQPGPKDTDICGAGQETG

ATPTLDDASMKWMIPRLCSPSLPSEPIAPSALIGAGQGRKKKDS

>SG-AL_contig_5533_1_+3_510_710

MGLVTSNIWLLLPLYIVSAVYGFITNFKKKKRFFPAHLLQCFCVTAPNPLILAFSNTPPN

PSKLRSP

>SG-AL_contig_5534_1_-3_123_1040

MPKVRPSKKSRQHQEIQKQGINFNTGIGQHILKNPMIITNMIDKASVYSTDTVLEVGPGT

GNMTVKLLEKARKVIACEVDPRMAAEVHKRVQGTPMASKLHMMVGDVLKTDLPFFNVCVA

NLPYQISSPFVFKLLLHRPFFRAAILMFQQEFADRLVARPGDKLYCRLSVNTQMLAKVHH

LMKVGKNNFRPPPKVESSVVRIEPKNPPPPINFQEWDGLIRICFSRKNKTIGASFRFTKV

LELLEKNYRIHCSVNNIPVPDDFDMKTKVNAVLEKNDFEKKRARSMDQDDFLLLLHDFNV

EGIHFT

>SG-AL_contig_5535_1_-3_738_1037

MWSEYCQQIFLYNKPVHIMYNLTLFKSWNSDLKLVGHRMQVIHKSVILSTMLVPSLLFIL

IYLLLFFSFFFIFLGGGWYVTMLLSYAFLFLCRMSSYVTV

>SG-AL_contig_5536_1_+3_654_848

MLIKFEAVIIITPKKPLHLKHRSFKPCTTILIYEGKDFASCNTFYAQVTRHCYLHAKKFS

KFFLF

>SG-AL_contig_5537_1_-2_5_514

MQRYNRRGRHFISKGRTRATCAERCAKPTDDGMYALSRGPSKIVASTRRGTPRPIEVVPE

NSRQEQSERVDSDATMSAEPRPIFNHNGRTRNHPRPSGNNGVMSSTHQELYSFLKASWER

AKLDYNKRASTEGGPEWYREKEANPAYANFKKVDWEEFFVKKTMRRYTES

>SG-AL_contig_5538_1_+2_110_328

MRAMPIFSPSLFPNPAQGQAHFYSWMDWSLFAQGFLLGSGLNSFSQIGKRQLYLYANPSL

LLVYSKCDLRRRR

>SG-AL_contig_5539_1_+3_33_218

MMTLLKLFFCFSVMVQNNTQFTVYIFPVQYQDKESIKLIMDSVSVYRVCPECVCTCVSVH

KL

>SG-AL_contig_5540_1_-1_349_897

MQSGDGKSEQGAESMDQQKEAAAGAEAGPSGSGQTGAEGEELLGACGGSWRPNTVADVLK

MQGVEQLFAIQPRSSCPHLSGVQPLPPEGLDTSLPCQDCRDRSENWVCLTCYKVFCSRYV

KEHMLFHSVAEEHMMVLSYSDLSVWCYACNDYIDNPVTFPMKNAAHCHKFGESLPTATLL

SNE

>SG-AL_contig_5541_1_-3_120_242

MVLFSCYHRIKPSKIFQVKFFFVSFFLLFCFTLAGWLVVSC

>SG-AL_contig_5542_1_+2_260_457

MYFCISFFSQGTLSNKTFQQDSLQQNSSTRLFATKLFNKTLCDKTFFNKTTTAYVIVLQT

MAFPEN

>SG-AL_contig_5543_1_-3_3_125

EILSDAFGDVEPTKIVYSVIFDHMQGLQKSDVAQYMSFLSH

>SG-AL_contig_5544_1_+3_546_833

MKTEFEYCQKLLRKCCSHRFLKKSKKVLILGTFLAHVTLTLALSIPNSCISDSSTVHTFG

PNLVKLHPETVEKILLTHFIKKHTHGHTSLQNFPFF

>SG-AL_contig_5545_1_+1_118_300

MDLKKANTKRKKANVRKWMCLVGIKIGTQDGQAILIFFLLCHVDDTKIYTDLENDSRSSK

F

>SG-AL_contig_5546_1_+2_149_304

MVDCHNSENRILIFSQIAEKVLPSSKSANSRHFWGPCDLDLGRWPSLFQAHA

>SG-AL_contig_5547_1_-2_509_736

MRVSHHISLLLQDAVKQPDQTKHDQAGCSSLFCHKMAPMEQMKAATMTTVEAVSFNKGHM

LHRQPAANKTEMIKKI

>SG-AL_contig_5548_1_-1_367_552

MPKIETEKERDLRKSSHLDQQYTNRSKLSPQNYQATVFNLSQRCFEVQINKDVTVYIYGD

SK

>SG-AL_contig_5549_1_+2_530_784

MSLFQYFTKHFHLFQLSHTPLVNISVYLSQKFFISKFISVYSNISYTLGYHFGLFPSHKP

LVNVGQFIPFSFSQTTGYHFNLTNS

>SG-AL_contig_5550_1_+3_729_992

MCIHASKKTLLEQNSKTKRRTTKVMICRRGQTSSLRLSPLLTVFKLDGTSLSNYMYSNTI

NTSERAAHQTTTLMLCKRGQTLTDCYSL

>SG-AL_contig_5551_1_+3_81_251

MSVLSGLWNSKTPSMHTNQQGLAVRQIQRDLMLLYLKNSLSLSLSLSLSLCFLVFYL

>SG-AL_contig_5552_1_-1_1_138

RVHFVSLALKRAARSIQACSTSTSSDIGTVHVHDVTFQEQSFNTCG

>SG-AL_contig_5553_1_+2_2_118

TVLVCIIISIISFNICVCEFILIQTSTNTGHRDSKHTSY

>SG-AL_contig_5554_1_-1_1_174

EPSNQETTTPPHSLPPTNESSHSLLLISHPPHCNDSSIQLGSLKAQVLMLSSFCPANS

>SG-AL_contig_5555_1_-1_31_2070

MASERESEEFSVPLPDAEHASLQATWGLGNHLLVYATGKAPPRLAGDGVSGVENGNIMEV

RWGQDMHGATTRKLINESHSVFVMLQGQVTSLTGTALYNQLKEASRQYRSIVKACSLELQ

LLLSDSTDEAEKEYYSIQIELLEFMQIVWSLCEILFIDITPGEMVLVQLLNWVRWHFSAG

REMAQDVLQEEVPEEHPAYWEAVFRLLLQGEIERVRKLLSLHSCSQTQPFNSVEEMLKKM

PQWGNLRSVSLAEFDLKWRHWREECMRRYDEGQFTAFSQLERVVQVLCGDVVVFSELKDL

CGTWYHLLISQLLYQNPTVRPTDLSFHVQACMEEFQRGGHRMGSIDNILQAALEFDLHQV

IKDSSICLGDRSWWFGAHLTDILHHCGQIDSQKLPFGSNLREYLLLEYVAGLMSHNSLWQ

VGIDYLDHCPVFGKDHMKLYIENIPLDSERKANKLIHICEERDLSEQVQSICKIMGVRCS

RDGRLGSALSWFLRSKDVSAATRLTEKFLEEYKENGSFSHLDLLDHLGPTMLLTQQLTFL

GKYREFHRLYQDKDVKGAASLLLNMLTARAAPRHFWVTLLMDALPLLETPQVVLGSSQTY

ELMHCLEELTQELSTTAKKSPTPHSVAAAQATETTSSLPIVAVQSQTLSQGDLDKLKYLR

LALCRNLSRSIVHEGTIVPS

>SG-AL_contig_5556_1_+2_2_181

RNTHACRQTGYSLSLSLSLSLSLSLSHIYTKTDKCHGTSDVTNTCLCNKHFISSETFVFV

>SG-AL_contig_5557_1_+1_109_465

MVFRYSFSFWIAEWLRRSSVFSLRSCLFSSWSCATFSSSSCIKAFFRSRAVCAATRFFSF

LLNIFSSADKPVSRRRFLDPPPSLKKQPSSATSAIWIELGRFPSAVMTIAFWKDIFGES

>SG-AL_contig_5558_1_-3_315_548

MLGTLTIEIVSPPSGNSVLAILSFLLLSLSLLFFSFLYCRLRLSAFPVHSTFFFPASLHT

FLSPQFMMYNLCCFALAT

>SG-AL_contig_5559_1_+3_36_197

MSVSPEPHNSNMAEDRYKRRMKSAFQLLNRQTLIDTQTHIRSRNSPTDILHLFM

>SG-AL_contig_5560_1_-3_3_989

GGNRAINEYTFRARATDNGVAYRCEVTNLVTTQPLTSTFTLTVHFPPRGVTISGGGGTLR

AGRTITLTCVSTNSNPRSTITWFARGRQIPNSDVTNTYSSSSLGGQVTRSRVRVTLGQED

HNVMYTCQATNDVGQTVADTVTLSVLYPPSPPTISGYTEGASIRANELERLTCIAVGGNP

EANLRWYKNGQLLRDSQYKVIGNIAQSEIGIVARPDDNGATYKCTATNDATSSPLEDSVT

LTVLFPPTSVNITTSTNEPRAGQGMNLTCVSSSSNPAAEIIWLKGGKRIQGVNRGVVDAD

HGGKNTTNVLHLIPTSSDHNAVYGCRATN

>SG-AL_contig_5561_1_+2_272_547

MSVNFLVLLFLSCFSPNLHLKMERVIYSGPRHYVSMGEREFDGYFHLRVIIIVLHSTDFA

CYLLPEGKAFTKIRSTCFLYSFYLSQLYCQML

>SG-AL_contig_5562_1_-2_443_706

MHSPMFFPQQRMNWVLLLLPVKQASTLSVVVLGLVFLWFFLFFVWNTGCMQTILFTNFKA

GKLVGEEKGVGSELEEHFLYVPQTLGTY

>SG-AL_contig_5563_1_-3_72_488

MAEPVVKVPGQKLAYKDDTTKCGPGTYAREGIIHSSLAGCLHKETLPDGITLVSILSDQL

QNIVPSVGSVVTGKITSVTPRFCKCSIFSVGKVQLKEPFRGMIRREDIRATEKDRVETYK

CFRPGDVIVARVMSLGDAQ

>SG-AL_contig_5564_1_-1_268_1488

MVLSQRQKEELNKAIADYLSANGFHTALEHFQKEASMSGEIEKKYSGLLEKKWTSVIRLQ

KRVMDLEAKLAEAEKEVNSGAPTRDRRSPSEWIPRPPERYVLSGHRSPITKVVLHPIFAL

MVSASEDATIKIWDYETGDYERTLKGHTDSVQDVAFDSTGKFLASCSADMTIKLWDFQGF

ECLKTMYGHDHNVSSVTFMPNGDFIVSASRDKTIKMWEVATGYCVKTYAGHREWVRMVRV

NQDGSLLASCSNDQTLRVWVVSTKECKMELREHEHVVECLAWASEASAPAICEAANIDTK

NKHPGPFLASGSRDKTIKFWDVGTGLCLFTLIGHDNWVRGLVFHAGGKYLLSASDDKTLR

IWDLPNKRNHKTLQAHSHFVTSLDMHKTSPYVVTGSVDQTVKVWECR

>SG-AL_contig_5565_1_-2_893_1273

MYMAVNDSVKRFEILLNVSLLVHDVNFPKGLFFSASTDTLVHNIWIFTVANQTSLLYCIP

YDSHCISPLYKIHIDYLHCIYIPMDHTCLLVPFLILVTRTRLNTSAGILLHMLCNMPLLK

CICKYLY

>SG-AL_contig_5566_1_+3_3_86

PSPYHSPGLGLTASMGPKLIRLHTAGQD

>SG-AL_contig_5567_1_+1_508_666

MLVYLHALIHLHGATAYKNASSHTRSQITIGHDNNINHFKVFLLKRAHSTLQN

>SG-AL_contig_5568_1_-2_74_1429

MVTAGQEELVVVLETLPDEDTVPKDIFCHFSTVYEEAGKGNTVSNMGHSIFNQPFLDSGS

HGGFLYIRSTFQCTNKLLMPATPFLFAILLQKWETPWAKVFPIRLMLRLGAEHRYYPCPL

VSIRNRKPVFYEIGHTIMNLLADFRNFQYMLQQIRGVSIHMEDKKTFINFPRNRYEDVMK

VVSSSNEHVMALGASFSLEADSHLVCIQNEDGNYQTQAINIQNKPRQVTGASFVVFNGAL

KSSSGLRAKSSIVEDGLMVQIMPEMMAALKQSLKDMTDFTIGCGSVTIPQPEEVVTVRWV

DDDKNVNVGVKSPVDGMSLEGVESVYVHNATDYVGERYAIRWTQVFFLQTESGTSRWEPV

DLSRVAETLATAVCIALTPHLQKLNEAGLTKIGLRVTLQPDSVGYEVGSNGERLPDFYIN

DLDNALIPVIHGAISTNQDGPIILELVLHLIH

>SG-AL_contig_5569_1_-1_151_2748

MDSDVMDKCCKMVGAIFSRHTSPSRRRDASPQAVDVIRSVPQNGVLAKEATGAYKAGVWV

WDVSHARTSPRPQRHKHGDGDVSEKDQRKCQSMERSGGQFGIYPQLPPPPPETKTKTPSM

TSVLDNDEKREKLSKSPPPPPSTQTEKLDIREKYMTLPATNANASSSSKQGKRHSLATLS

RNPHSEDPGSTQPSRNSRRSKDEEVRRLAKTMVNSIENLIGNLHGIVGDLRQLVQQIDAV

TESIDTTCPPARPQRAIKAGPERKMIVTTNQIFTQTDLDQQCRDSLGHSEWVQKYGYMQA

SSPHRYSYPQYYSPVSSVSSPSGYDTDRRMYRRSRSSRKSGDSGHPSQKSSSSQHQSHKF

GAETRDTVLQLRQIEPARTGQQRQVPWGFGGAPFEETQVHSAIPSKSSYLPDPSIPFLPV

ESPPNPAPTLFGAYADEEKTIHVKDSDSLPDRGLKTWKEKMERHSGSADSLTPQMDWSYL

VLTDHNLTTSVEGFNLSPGSRHMDPLSPQKDWFKLQLSSHKSESRRSQKDRSRLSQTGVK

MKHHVGAIPGIPEQREPCDSSYDSNCDVFLPADTCSSSSTGSVPPKRTGASVPQFLVNED

HQKQNTDQDPQTVCSVSQETGRLGVFPQVQQSIGGASLGWSTRSKALPNWPSEELSSIGA

SDNEIDDLSSPFLQQQFQYPRRSSAKESNPETKNGPEVNSGMNRTISVSDTIASGGFEVR

NDIETSPESDTLGPYGGQYESAMESRLETLTNEVEGSDFSDADADVGSDVLEQSLTSLED

SSSVKYDRELNTWTSYTMTHIDSDDVVSECTSDVLNENYFDTAMSNPGFDDVSLNFDDIG

FLPLPLQTSKFDGVSPRIEKVSPSLE

>SG-AL_contig_5570_1_-3_186_350

MHACMHVCVSACVCVCVCMCECVHVYVYVCVCVCVCVCVVAAIVVHVVVSQKKIT

>SG-AL_contig_5571_1_-3_147_2006

MSEGAQLNGPASQSGRFIDTVDPEDPEYIKNLQRPAEVKEDMKQMEGRSRVSVVLNSQAF

KDELEAVIQEQIRQGGPASIFALQQISDLLLPHSRGGPSFSRSSPVIPINDIRGVETFGY

SKQEKVLRCKLAACYRLIDLFGWTHGIFNHISARVSQDQEQFLVNPFGMLYSEITASTLV

KVDVRGEVVDTGSTGLGASKAGFTLHSAIHQARPDIRCIIHMHTSAAVAVSATKCGLLPI

SQEALICGKVSYHDYRGILVDQEERDLLGRSLGPFNKVMFLRNHGVVTCGETIEEAFHYA

FNVMAAVETQVKVMPMGLDNLILVDEETQEKTFKVGSMGGGGADTSGRKWKCGELEWDAL

MRTLDNSGYRTGHIYKQPLMKQEKKERTNSDVELPPASSSFTYVFDGDYEHSKYISPIKT

ALERQKQAYKAGWLASPNTYKKQEIEEIGTTTPKKITKWVPGETSQQKSTAIKVENPNQF

APQGSNPKEFKEKQKSIRKDYYEEKVSAGPTSRILEGISWDEAAKMKEGGDQTIIVGAAS

KGIIQRDHQHNAVVYRSYYSANPFDSMNEEEVKAYKQQVERQEQGEEPEDVPGPEGRLIS

TEERMQNIQLSREDVQEDTE

>SG-AL_contig_5572_1_-1_106_2253

MSETQFYQPGQEVKFQCQATGSPPPTYTWQKNGKNVNLTDSETRGNELRINGTLLFPGDK

VDADTEGVYQCLAENEYGTSLSSLVHLTMARLEMAPMEQPDRMVTPRIGSSLIIPCTSPH

SVPEGHVHWIVEYPIGDMDLLEDTTYSDVKLSDRVTMDYNYSLHITNVQQSDEQGGGGYM

CVTRNQVLRAMAMGPKTFIRPQSGRFRPPPMPARLMWSSPSYVVALLGTDLKMKCIFSGN

PTPRVRWAKGGVRALPSSARLESAGQELVLTDVKYSDAGDYECWATNSLIHIMNELRSFH

VEVQSAPQWVDAPQNVEVEAGQTAEVNCSASGLPQPSTAWFVNGMNLSDVVASEERFQSE

RFDNTSDRLVFHNVTTDDRMVIQCNVSNRHGNIWADVSLNVLATGVENQATTTIQSTSTN

DQETTTNIQTSPTNDTETATTTQTTTTSDDQNTTTNAQTTTTIYDNETTTSTQNTTNDNQ

TTTSTQTTTTTYDNEATTSTQNTTNDNQTTTSTQTTTTTYDNETTTNTQNTTNDNQTTNT

SQTGTSDNPYSTTPTQTTITTTSDGSQVTSANQSSATENPYSNATTQSTTTGIPKPRSST

ETGREGATVILTCTNASDLHLSVTTFKDGEEINLTNDRFALQLNGAFAMKKVTRHEGGIY

TCHVTNATWDTIPIVVVIMPFVSSNGAQSPPLHMFSFALCMMSILCTICCLCKGIP

>SG-AL_contig_5573_1_+3_1383_1619

MYTHTHTHTHTHLWDYCDRKNESSPTLQLSTVRPQAPRIQPSKKQQKPGNTSPRTLSSSS

FFFFFFTPCSGFFFFFLFF

>SG-AL_contig_5574_1_-3_3_179

FHSLTSLFLILLTSRHVLFKKSSVLKKNKFYSPSSSRRYPVERRIRNAFLFNYLFEKFV

>SG-AL_contig_5575_1_+2_2_124

VISNQYIVILSCAFHHWVNQLQGCICFKCIASLICMLLFFT

>SG-AL_contig_5576_1_+2_2_487

RVCASLSLSLSLSLSLSLSLSLSLLHEPHYLFHVTIISSIRSLTDFSLSLSRLHDPHYLF

HVTIISSIRSLTDVFHVVIISSIRSLTDVFHVTIISSIRSLIDVFHVITISSIRSLTDFY

YSLSLSTSRSSLPLLSVSTDFYALFCNISAIFNHSSDAEGCT

>SG-AL_contig_5577_1_-3_258_506

MPDLFVYSDFECEKLTYFPSEVIVCLAPPLSLSLSLSYAIDQTIVCVFRGGVVMGRWKQS

AQTQASKSEMLEKREACRRHLAT

>SG-AL_contig_5578_1_+2_1271_1795

MLRLLHSSVKRPVYPKAMAAKGIEQRHLFHLYQAVILGVIAYGLGLTTLSQTNLLDTIQN

EAMRVILRNTKDTLNETMWYPFDLSLMQARHKVEQVKVYFRTLENPQNPLHEIFKTTKSN

QLHRRKSWLEQAEESIQEACRLTKLNETKEWEKYPNHFKDLYTTIIPPNLGRHCR

>SG-AL_contig_5579_1_+1_1_171

FLVSSTTKTAWLRRVVVCNLCGCPHDNIPRRLRTHIEIGFIHRTLFCFWLIQTFFFT

>SG-AL_contig_5580_1_+1_151_267

MPTTVCITFTEVCQWHLLKYRPLPSTYQPASCINSKAKT

>SG-AL_contig_5581_1_-2_110_241

MHARTHAYQVQIIRVNSWFAFVAFHVTFERLNNTLFLCKNILTD

>SG-AL_contig_5582_1_+2_2_157

NLSLRPSSMFVLEISQVPSHHCHCYQLSLVMLVPSSLSTSSYTPSIKSKDVA

>SG-AL_contig_5583_1_+3_1932_2327

MKRVVFCSASTGPYNFQLLIMWHCVNIHVCIIMSVTIGMCMYCWSYEYILICVHLRVPVF

MSMHVRYRCFCMYHCLLTKGKLKVWRLTLYNISWLWPKPLPANDKRNAEAYINCYALQRY

MWIVFIRGKLLA

>SG-AL_contig_5584_1_-1_301_555

MKACRIFMHTCFSHHCKITHIYTKHTHTGSLSLSLSLSLSHTHTQSYIHKRFNDTLHLNL

SGEGTHYFSIIHSHTFINMEDCKKI

>SG-AL_contig_5585_1_+3_576_767

MLVRWSLRAIDLHTIDRLFLFIHLVHHVLRNQTRSFCLSSSSSRSPSHLEFSLEDEMGRL

LQRI

>SG-AL_contig_5586_1_-3_3_200

QTDRRVHHDIIVDCESVELFSCGLTMTSCTQWFHSSRFCLSLTVICIISKCLCAKLDKIP

IKPFFK

>SG-AL_contig_5587_1_-3_201_968

MMTWLWRGEPLCVVLAVCLWKLGVAAPSLQAGDIDTDTQLWLADEAFYDDVDVYNKPQGD

QAAKQPLELCPPVPATLPDGSCQEHKCTSDRECGDSGARKCCYNGCVYTCLPELAPAAFL

DWLREPRRRLSSGLSWLVTGPDEANEEEPCSTSPVDSDADPLLCPEGYFCSLRDNGDLTR

GIPNQGICLKLADEDTDDADSELLTTDRREGDQPVKHPHACDLGGRMLLEGHVLTQDGKQ

CICKRRQLVCEEGEKD

>SG-AL_contig_5588_1_+3_582_794

MHQTGHKCPSGVRPKIVDSRRMLISGEGSPGSWFLARNSSRNSRETEHLSGSYVGKIAAK

GVNFTSMSARG

>SG-AL_contig_5589_1_+1_94_315

MGDIHDTENSPYNKINCWTKNGRPFQCPPYLAGHWSRMGVMVGEGAEQTKPTLHLLYHGW

EGAGTDQTNSPPFI

>SG-AL_contig_5590_1_-3_237_500

MKLVIYKRIIVNTDIMDSLFSQWNDLTMKVLYNTLAVFKGCCAIYCCVLVCEGYYICTLC

VVVDLASDILYIGPILGSGFFSPLFCWW

>SG-AL_contig_5591_1_-1_454_759

MRNLAVLLLLVAVTGLSSAYWIDGLYNNREFYDDEDGVFSENKRRGLGQSKDLKDLCIPW

RAPCPSDASLSQQYAFLRCCGGLSCKCTFWGNNCKCESRLGR

>SG-AL_contig_5592_1_+1_28_462

MTNNKLKLNDDKTEAILIKSKRASSSTFAQASSVCVGSTNIVFTPSARDMGFMICDDMSL

ESHISNICHSAYLEIRCISVICPYLTTHAAKTLVCAFVQSKLNSCNSLLSGCPQYILSKL

QKVQNSAARLVLKARKCDHAQPLLY

>SG-AL_contig_5593_1_+2_194_490

MICDDMSLESHISNICRSAYLEICHIRVICPYLTTLATKTLVYAFVLSKLDYCHSLFWLP

TIRLWQATKGHNSAARLVLRTRKYDHAQLPLYNLHWLPV

>SG-AL_contig_5594_1_-3_3_155

LEANEACKDEAEHDCICKPSKLVLQQNFESLVACQRCIVGSQKGETCNILI

>SG-AL_contig_5595_1_+2_59_1471

MLDFVIFAVTAVVVLLIAVIYMYPSSRRISTIPGLDSTSAQDGNLVDIQRAGSLHQFLLQ

QHSERGSITSFWIGDEFVVSIASPGLFKQQTNVFDRPGELYSIMLPVFGEKSICFLNGAE

GRARRQLYDKCLQHEKLHEYSQLLNKISTDLATKWGTVAKGEHISLQQYMSAFAIKAVLQ

CVMGSCFTDDKEILSFMRHFDQVWVELEYRIRDPEIPADDSPRGKMFESALKNMRALISH

ALEHRIKNKRGNKEELIVDHIIAAHKNDTDGQVGDCLTYLVTGIPRLASMLTWCMYFLAS

HPEVQDKLNAELQHEFTGDSSPAAVFKLRYLRQVLEETMRCAVLIPFAARCQDFSTEIGG

HKIPKNTAVIQALGVVMQDEKLFPVPNKFDPDRFSVENSKERDTLAFCPFGFASRRHCPA

KDLTYMMASALVAILIRKFSVSLVEGQVVTPFYGLVTKPEDDVWVTVQKRK

>SG-AL_contig_5596_1_-1_1_189

STKASSGPVKAKDGTLLSKGEEKLARWAQHFSEVLNRPEPASPADFKDPQDLLPITTGDF

TER

>SG-AL_contig_5597_1_+1_1_228

MSVSPWHCKAYVTVQCWAATGTLSMSVSPWHCKALCLLFGYTICIVLYKPLSLQKKAILI

GQSSSVKSPVMIGNNS

>SG-AL_contig_5598_1_+1_85_351

MGGSWESDIVSILAKAMRIKVAFSSLAFIYCTMSDCEQNSKIGGGVAPRVRVGLTGKNRS

ISRDEGDTFGTVRSRSPEIPEVKVNEGIL

>SG-AL_contig_5599_1_+2_80_235

MNTRKPDTQSIIHNNKKPATYSIIQKIHILILPRTQSYTLIYRFNSKQKVHR

>SG-AL_contig_5600_1_+1_112_255

MTGTTLWGRIELETRDLEPKALSKEPASLCYKNYVPGWTQTTNLSVNS

>SG-AL_contig_5601_1_-3_3_209

AAAPPLFCFHFRYPSLPCEDLSPKRTLHSCAVTPSLTHSLYHIVIYHPPVLLVLRERSTV

RHRYTLWHA

>SG-AL_contig_5602_1_-1_1_810

MQTPLQSALRICLASNCRRLQFQFSSLSCPVLQGKPAHSQSSGLNSIAEKIHSPSRFARL

PLWTTPGDSVLLNTCFHNDTHGAGVPARSFSTSACNSAKELSEKGDVVYTGTISGMIKGV

KIFSLSTSAIGICLQPMLLFSNQELPTALKFALGGFINFFVFMNPLIIHYIAKKYVTELR

WNRRTGIFTAVTFSFLVQRKELQFTAEDVKVPDIPGMFTFLEAKGTPLFLDPQAFENSEA

YIHLMGFDKPLDWEIPQHRESSAKGDTQKR

>SG-AL_contig_5603_1_+3_168_377

MKQIQWECITSLYRYTSMQSFKRRVIKATTIQELNTTQKAIYIMYALPEIVIILCKTKSG

PTRVSIDQTF

>SG-AL_contig_5604_1_-1_34_1056

MVNSSSLASLQQSDTDSMANSASTNSLQNLTSTAVIASILGSPTDTQAQGDCIGDGGVKT

NGVTASSPLAELKPSDLESKAAPSPTVDLTTDVNNATTTIITIGQENSNLNAKNVKSEVN

GDTKMPKEEMENGEKQHNGHMDDAKDTNSPDTLIMDISRLSLEDGIPCEKVPSSTTHIMC

NGFGGQRSSPDSIQQNEAEKGCVSNCVEEEADVGDVLEMNSKLQSEEGIALEVEDTPLPN

PVTLTDTAVMQQSERLEAGFEEDCTQQDDSRLGASSPTYGLVRMESQDDQNDSSNPNVPK

NKPEVDPVNEICTDFPVKIADLGNACWTYHHFTEDIQTRQY

>SG-AL_contig_5605_1_-2_155_1927

MDMDSDAEPIEESDEDDLDDNGMGTDEEEPDSEESDEEEEPGVQSNVREDIYGRLVDSKG

KLLSDKEQSGAYIPPAKRAAMLAGSGQESVELERLRKQVKGLLNRANESNMQSIANAVED

MYMQHSRASVNQTLFTFIQEAVVSSILTPERLVAELAMLVAILHGNVGSEVGAYFLQRTC

TKYEELRKEEQYGGEHTMDNVVLLLCYLYNFKVAHHTLLFDVISQFVESFSEKDIELLLL

TMKSAGFYLRRDDPEMLGNQISSIQQKARELEESGTAVPARVRFMLEVLVAVRNNNVRRV

PGADVERTDRLRQLAGKFVRGGSLADNQLNIGMKDLLQAEHKGRWWIVGSAWEGRLDANT

SSGKASANAAPASSVVGAVSQKLVHLAKKLRINSDLRRSIFFVLMSSEDYEDAFHKVLQL

GLKGQQQQEIVVVATLCCKQETTYNPFYSFFLQKLAAHERKFMIAIQFHMWDMFKTMEDL

SSSSRNNLAAMLAHLLASRSLSLSVLKVLEFGILSKAVIKFLKHIFTTLFTTVPMAELKD

IFRAVGQLQKFKALADGLRTFLVKYVKMEGEQKKKLVEALNELSLNRSMMM

>SG-AL_contig_5606_1_+2_2_205

HQHLEVQPSCTQPTTQILLFHVLAFGSNQWYRSSNAYIHQNILHQKLIKKKNKNKKKFES

FSKDLLII

>SG-AL_contig_5607_1_-1_19_276

MLLARWGPAHQFTMQTAAALCCLQGGGQLTMQTAAALCLQGVGQLTSAPCRHMCESRGAC

LRWICWPGCMLECRRKALPVWVEMTV

>SG-AL_contig_5608_1_+2_2_214

GAGSWAFVRDVAENPISHGCPSGGPAGEEPSAGGQASAAEAACGASWLGGWGSSAKRRVP

PCPCVLRPWRR

>SG-AL_contig_5609_1_+3_477_626

MVKPRTTNISSPWVKLKGLAALYKVPGFSVCKSFQTFCWSEQHLSPTFVK

>SG-AL_contig_5610_1_-2_497_655

MILKCVFNIPVNILLERVLSSPCLLTEYSMLSPWRTCIIGHFLPLHFYNCAHL

>SG-AL_contig_5611_1_-2_143_331

MSVLEATTANKLPHLQEALQNIRCTRQVLQWIPSHCGISGNEEADRMAKLGAEHEQKDNS

VSL

>SG-AL_contig_5612_1_-1_526_759

MAAKKSNIPVAEYRQKLHLTLPPHISINHVAFCMGCDNLIFTAYFTFTYIYIYILNVNIL

QQKDGQATPHWHFMYYDS

>SG-AL_contig_5613_1_-2_104_1891

MKPVWFPVILVLPMCVARGGLSSAVVPSHADNSTEADTSSNADGTKLVCYYTPTSYFPAG

EVPANLCTHLVYAFGSISTSPPNIAAPSAGVASIWATLTSLKQKNPQLKVLLSLQNGFPE

LVGPDVSKMQQFAGNSLAYLRRYQFDGVDMDWEYPKSSQKTAFAAFFKLMRQAIEKEAAA

TRKPALLLSLALPNNQFVASRSYDLPGIAAAVDFATAMTYDFHLYNKGDTKTGYNSPLFT

PPGEYKYFSAAAMAEYYLGQGFPGSKLLLGIPTYGRSWKLANPSSHGLHAPAVSKGDPGP

VHHLRGIYLYADACVAIKRGASRVRDPGLGAVYLYKGSNWVAYDDGTTVAAKCNWVRKNG

LGGVGVWALHLDDVGDVCNEGSLPLITAMKYSVEYPHLSRKSSNNHRLETPKQESGNRFR

LNWEEAKRMLSNQTGNWFQSDQQGGNQIPLNQGNENRIRLSNVKTQEEPSTRDGMTHPEL

GQRWYDPYDTEINRVDLEKPQNRKSTNIRKVQQRTGVSEPILERRRHVFNDVVVPMESAD

EEQGKPRATDGDHENHDINSQDSTENALVGMLKSVFQRLVRAFLNHFQSGPKTSAE

>SG-AL_contig_5614_1_-3_33_1151

MATSPNTSASGTLTPKKKDESFLEKIGTLARKKKIKEVNELQEEGKNAIESPTSPTLPDI

GPEGYQLGESEERSMIEPNSRESAKLKDLIKILLDWINDELADHRIIVKDIEEDLFDGQI

LQKLIEKLANVKVEAPEVTQSEIGQKQKLRVVLDYINRMLQLPPVWNQQKWSVDSVHTKN

LVAILHLLVALARHFRAPVRLPENVCVTMLIVQKIDGVLQTRRFIEEITSTNDDIGARFE

RDAFDTLFDHAPDKLNVVKKTLVTFVNKQLVKVNLEVNDLDTQFHDGVYLVMLMGLLEGY

FVPLYNFHLTPTTFEQKVHNVSFAFELMQDAGLQKPKARPEDVVNLDLKSTLRVLYNIFT

KYKSSTSTSGAIH

>SG-AL_contig_5615_1_+2_2_205

KLNDDKTEALLIKSKRASPFEFAQTSSIHVDSPTLHSQPSARDLNHTSPTFVIQYILTFA

PSVFSART

>SG-AL_contig_5616_1_-3_639_932

MESNEFFFVLSIKKLVYDMICKVQSRDVISSSRLQKLMSAPHIISFMQYCFFNYLFLCIT

VVSNLCSSCVFLVQIPHTFYYVHISKAWPWVFRIIHVQ

>SG-AL_contig_5617_1_+1_679_903

MQGMSHQKITTQILYSFSFSLLKKMMQDSICAKPSPQTLCEGDPSPQTLCEGDPSPQTQC

EGDPLPQTLCDGDPK

>SG-AL_contig_5618_1_-2_1373_1651

MSLLLVLFYSLYLHTRTHTRMHAHRQTDGDTHTHTHTHTFTHSLTYTHTHSLPVLECSAM

LSSLENLSFENVKCMLSFRMRFIRMVVGLCAKV

>SG-AL_contig_5619_1_-3_3_353

VPVSRAAPGAVPKPGPPQATPLRDQARKPRDATPARLASLRPRLPTLTDPPLSAGSRAHA

HARPREVAAALAASGARPETRSLAARSRRKGTESVYNSKWSKWLAWCSSSQVDPRSP

>SG-AL_contig_5621_1_+2_275_1012

MLDYQPMQFKLDPRLARLLGIHTQTRPVIINALWQYIKTHKLQDPYEREYINCDKYLEQI

FDTKKMKFADIPSKLHPLLSPPDPIVINHVITWRDKGVPWNKSLTSAVRLDGPDAKKTAC

YDIDVEVDDTLKQQMNSFLLSAQSQQEIGGLDNKIHETVETINQLKTNREFFLSFARDPQ

EFLNNWLISQTRDLKTMTDVVGNPEEERRADFFYQPWSQEAVCRYFYGKVQQRRLELEQA

LGLRNT

>SG-AL_contig_5622_1_+3_54_230

MRRFEVLSFDFLRNSLTQKFNLYFCIVQKLGTDEQLQLIRKSSSVCLEKTRRSRAENSK

>SG-AL_contig_5623_1_+3_348_620

MKRECRVEHIATELKLGAQTFCWTHDHHFGEQLKDFSASMTFSQPLSEFSQTPVTFQTFK

DLKMALHFSKAFKDCANPDRKLPLHTCSLFS

>SG-AL_contig_5624_1_+1_19_339

MSKFTNFQRPFIPFFKTLRGQFPGILGLIWTKTISHTGRHIDRSTRMLSRDATLLFKDLC

SMTQECHIEHIAAELKSGAQTFCWTHEHHFGGQLKDLSASMTFSQPL

>SG-AL_contig_5625_1_-1_1_180

VREVHAAHGTSVYQLICKMYTQTTIEGLVEGAKTSLSDRELNLGFSHPRWVVCSLGHTLH

>SG-AL_contig_5626_1_+3_282_563

MDKVTNTKWKIRCKRTKCVRLTGTNGLYLDAPHNHPYIKSVNIPYSPHPFPKLIHRYHHL

TYLGPSSIYQYIIPEMHQPPPSIPLSLHSPLTLS

>SG-AL_contig_5627_1_+3_3_281

KCTNLLPVYHYLFTHHLHSLPVFHPHPLPLPHHSLPVCHPHPLPLPHHFLFTFITCITAN

SCSAKTRKTIHLRCQHFFLKYMDTSTRYDLKMV

>SG-AL_contig_5628_1_-2_23_625

MESAMDISGKRLPLPVSAYGMGSIAPMDYMAAYRGLTTAPLSCGLESSIYDKNAELLRKR

LYPDLVTNVPSGSKRIEYPYVASVPPEQYQAFYCCRCMENQPGDIRTWSVEEVVKLLSSL

DGCGVYSEIFREQRVDGKILVLLTRDHLMQNLGMKELPAVTLINRVAKLLLDSTRSSGCG

MCQRLASMVPAVQSAVRAGYS

>SG-AL_contig_5629_1_+2_248_466

MTNTKYTENVHVNILGNMLTSAFSVQYLSLCMKYEMIIINCFGKFSRGMKWMHEAMIVMI

NFPRQLSIFLPKW

>SG-AL_contig_5630_1_+1_1_270

VYFTPLTGVLFAFPSRYWFTIGQSGVFSLGGWSPHIQTGYHVSRPTHRAHSMCIFVYGAV

TLYRAPFQTLPLTHTLIQALGSSPFARRYW

>SG-AL_contig_5631_1_+1_130_243

MRSTQLCQQLSGRLLEPTQNPMQWQGLEMGRGMGTMKH

>SG-AL_contig_5632_1_+1_232_411

MKQNAVKTKHDIDTRDMHTFPSLSSCLFFVCVESGEFVQERFIALRQFHIPSTQIRQQFL

>SG-AL_contig_5633_1_+1_400_666

MDFSWNILGVFQVCFGSFPRSALGVFEKCFRGISGIFQGPFTCPGVSWEIVVKELWKCLM

DVLGDDSVSVLFRVCCGDMHSLGGATGVF

>SG-AL_contig_5634_1_+3_3_179

KTTTTKHNHFLIQQEHYTHIIYITMYNCAQNKCFYFTCLVKIHQYIYTSLYIQSTNKNC

>SG-AL_contig_5635_1_-2_308_574

MLPFCALFSCVLVLLVPAKKCNSVLLATPKSLVWICGVLQCGTASQPGRDGELRTGWLEF

TALSYVTSEMHSNILRANRAVYRQQNQCI

>SG-AL_contig_5636_1_-2_2_205

QCFSTKCLFVLVIGLTSRYCWKLCGRSVACDHCPKSRGLVRCGCGCTSYLMSLVVHLLDV

RHGGLLFC

>SG-AL_contig_5637_1_-3_18_293

MEEEQGPYLGEYEGGRNERDERHGEGKATLPNGDTYEGLYENGKRHGQGVYRFKNGARYI

GEYMKNKKHGQGTFIYPDGSKYEGGWVEDQRC

>SG-AL_contig_5638_1_+3_3_98

GGLCVCVCVGVCVCVRERIDKKKNVSPITSAF

>SG-AL_contig_5639_1_+3_3_74

QTLTGQKSTLWSLLIIPLNRYKKI

>SG-AL_contig_5640_1_-3_3_80

LHCSYCDCFIFQSLKMYTNNFTLTIL

>SG-AL_contig_5641_1_-2_83_2194

MFPVSHKTVFVLDHSSYFAQPCGEKMNYDMYSRNKAPPVGIIPAAHINKPLWTCSVECLF

EYMRIVFDIYPDYKLIRVVVGNKSLNSWAHKEMNMNQLAMGLANVGPPFVDRGGGNDDEE

YSAVQGLSQAVEALCQISPTQEVYMASDAQEDTPLVNNGRIICFTHMKTEAHVSSMEECV

VEAVNQHNKLAASSDTLIPINKVEVIFIHITPVKEPTYISDRPKREISPEVTRELHSTQS

GTHLYYKMIGLVQKHYDLASTTVTGIPMKEEQNAQSSANYDVELLHPAAVHSDLIRQLQE

QPENTVVIPSREGLPPAITLKWCTPKTNIVELHHCMGSFRITPVDVNSRPSACLTNFLLS

GRAVMLEQPRKTGVKVISHMLASHGGEIFIHILPTCRSILEDPPSISEGCGGRVTDYRIT

DFGDFMKEHKLVPAPPGLLGDTGVKPCQKALDHLERSSRFWPIVITETILGNLQMSVSPL

RTLLAKEELTDDEVLECKKVVYNIVGMESRNEALPVSAVGSRGKGPKREEQYRQLWGELE

SLVRAYTDTSEQHAKVLECILDCKKPPPPGEEAYSSTSRVTDTKPVAEDRSESAQVEQGW

KDLDRYQKMTEREKQELRKTDKVIAPPPFKKAKTEELLMKPGGQNLLSMWTSRLKAVNSK

RHVEFAGCAENPDSLKVELYPHLTEDNTEFNKLVDVKPQRGGKS

>SG-AL_contig_5642_1_+3_1434_1682

MVINLSFFSFSFFFPPPLFGSQLPEIQKWVNILHTPVICLHKQFQSQLYKMCPKKFKGEK

GRWYSLSTFVLKTATKNQKERYL

>SG-AL_contig_5643_1_-2_74_181

MDARSHGLSSVPARNFKVILFAAHQCFNRSGGVSVS

>SG-AL_contig_5644_1_+1_358_540

MSQSKDGTSFNGIWNLFKRDSSHSDSSICAPPKSARFLQPVQNPMQWQGLEMGSGTRPIK

Q

>SG-AL_contig_5645_1_-2_2_499

IPLDLLTVKTVFLLSLASSRRRSEIHALSGNPSDIAFLADGSVSLKFLPEFLAKNQDPGD

PSPDVLIRPLSTILDRSDPDRHLCPVRCLKFYLDRTKAHRASKRRLLISINPGLEKDISA

GSVSRWIAQAVVKAYGQARLDLGSLNPRAHEVRAIATSAAFTRKPP

>SG-AL_contig_5646_1_-3_3_143

YGLSGWLCYKQYDWVQVMATEKNCCFYVLSDYVCSTILIIALTVFVN

>SG-AL_contig_5647_1_-2_29_271

MHGVVERVATDFRSTQDIRHQQQGSVKLGRFHIQSDSECARLFASTLRPNIQKKLCLLLY

CKQQWISFTFKKTTTKNPQKF

>SG-AL_contig_5648_1_+3_210_500

MLSVKSRVTVTKLSPILSNCIRYNTISSSSSKKMQRTNTKRNNPVVWLGQPVHVLAPFPL

RIWSQIQATELAAMWFAAKRMAHQIMGHYLFLYLHSL

>SG-AL_contig_5649_1_+3_678_815

MRTFSQRWVTPVKTIMACMVQNKRKLKQNPCLPIRLPKSRNPSHSI

>SG-AL_contig_5650_1_+2_749_1129

MESLLMNQSFFLFKHKSVLYNSNRAFFLHSPPQISLQNSSQTLRLESILCQITGIGNDVH

TDTYTYIKKLRTASKTYNQPSTVDFKYINYNHFYPALNHKQRKNRSKSPVFSYEQHTRKE

NQMIEEQ

>SG-AL_contig_5651_1_+2_116_463

MSFEVAQITLTIQEQRVTFFLIYRPPPSKKNKLSVTLFFDELPDFLDFCHQCQNSIVIAG

DFNFHVDDPDNVDASRLCKQLEMFNLTQTVTEPTHKCGHILDLVVHRTDDSVVHST

>SG-AL_contig_5652_1_+2_2_301

RHSKTAQNLHHALREGGGGENCVKACWKNESGMDQEGRKMQTTVKDGKRTETKTAIKKRK

LRAQNQFLEGGDTIYTERAPGIPTGEEKKQIPGERKQINR

>SG-AL_contig_5653_1_-1_958_1911

MASSVTHISTASSALYQSTVEGSHIYRETFEDRIKQWRTWLLGQDYEGRLVCMGRTLMDW

MLLIACYFAFFTALVAMFVVFMALFYWAINRNFPTIQGPFAIVQTPGIGFRPQPNIHNTI

IRFVKGDGATYVHHLDHIEAYLKYYENQNQQGENYIDCNEIRERRTEDLNKVCRFDLIDL

GPDCVKQQNFGFDDGQPCVLLKLNRVFDWMPEEYTDDTVPDSIRDHWQPWWITVTCEGET

AADRENMGEITYWPANGFHFKYFPFRNQQGYRSPLVFVRFENPHPGILLMMTCKAWARNI

VQDSNEMVGMVHFELMVD

>SG-AL_contig_5654_1_+1_106_357

MFADILCACGNMWVCMCLCVYVMDQSMHYFDVSMDSGVWVGAWSLYLPSALHGFWCVGGR

RGCVCILASLCVCVKFIFWILLYI

>SG-AL_contig_5655_1_-3_3_194

KQKKKKKKKKKKKKKKEKKKKKTKKKKVWKKKKKKKKKKYMCVCLASSLSSHEWVPDFLQ

SWGR

>SG-AL_contig_5656_1_+1_385_966

MQIEFVRKEAYLELQQASFLFSHNENTAFTSFCLFFSPQESPQRRERAPDLSEEEYKIVL

AALNSHRQFRQQNREEKLLYRKVYKHNLKSDLVADPYRGQDVHRLIKNGRILLPPNEIDP

CIDFFYKLSLGDGAAKLAKKISEHYFGISKNHVISFLNAHPVHSKSNPSFKNKAPLQPII

TQQIMERHQIDLVS

>SG-AL_contig_5657_1_-3_450_713

MILSSRLILSRNIQSVMPDLPLLCACENNDMMYKKILKLVIIFKLPGGVQILRYVRSLIV

DLLSPRITSNLSYCPIWVLIINKSVCES

>SG-AL_contig_5658_1_+3_3_272

EEEGERRKGRQRQRQRERERERDRDRERERERERQRETDRRKHKDQENPQRYDAKGQIII

GKYNRLYSLYISLLDTQNSFGTKTCPNRFS

>SG-AL_contig_5659_1_+1_22_303

MHPHSHWERGSNRQDFPSSNLQPHPSKHKGENRPPPLCSILNQHQNFSNSKKRIESKPKQ

RNNFHLCGRPGILRSRPNNWAKVQVRVRVRVRVS

>SG-AL_contig_5660_1_-2_1499_1789

MYSCSPMKVDFSTKFCQGQLLCCCGIFNVCWMCAAHGTSVYQIHLIRKTSFLNAQKERYI

AYILYLNKGNIFISLFNTQTERITIVSFSLCYRHCSS

>SG-AL_contig_5661_1_+2_515_862

MNITHTHTHTHTHTHTHTLSITTPSAFDKRGSEFSKKCIRRCCQSLCMKMFQIHQQQMQK

HNQLSSITVPLHYTYQNVCKITKGKHKTQSTTIDHWFYILLTHFNLSANFLQESGG

>SG-AL_contig_5662_1_-1_133_288

MIRFTRDDYKDCFITFLCYLDTIYVDIVIVLLLTFISKFLRVICVITCMHGI

>SG-AL_contig_5663_1_+2_1232_1408

MSVFKAQSLTITLGEMSVFKAQSLTVTLGEMSVFKAQSLTVTLGEMSVFKAQSLTVITG

>SG-AL_contig_5664_1_-2_155_508

MATGLILAGLGMAVVGFAGRYALRYGRQAQHTIKQQLDALPASGAFSKYYKGGFEPKMNK

REAGLILGVSPSASKAKIKEAHKRIMLVNHPDRGGSPYLAAKINEAKDYLDNTKPGGN

>SG-AL_contig_5665_1_-1_253_3762

MARLGQALSEVDRKVAALKQRFETLTKEAAELKIKLDKENETIIAAETLVSKLDGEHQRW

NEQVGELSKELQELPMRALLGAAFITYLPSAPEDLRRERMNAWMEAIGITHFDLRRFLST

ESEQLTWKGEGLPSDELSMENALVILQSSQRPFLVDPSSRATEWLKGHMKNNRLEVINQQ

DANFSTALELAVRFGKTLIIQEMDRVEPVLFPLLRGDLTAQGPRYVCQIGEKTIDYNEEF

RLFMTTRNPNPEIPPDAAAIITEVNFTTTRAGLTGQLLGITIQHEKPELEVRKTDLLKQE

EDLKIQLAQLEDSLLEELASAKGNILENKELLDSLNKTKASSITISESLTESMRLQASLD

AERNSYLPLAMSGSTLYFVISDLAKINNMYRFSLAAFLRLFERALETKHDGSGTDLRIRN

LSHQLQQLVYEYVCRSLFKADRLMFAMHLAHGIKLDLFGDNEWEAFTGMLVADVKGEARA

PSWVDEERASSVAALKANFPKLYQQLDMDDGGLWSNFARSGQCEQEFPSAVEKRCTPFQQ

VLLVQAARPDRLQSCMGLFACRALGMKELSPPTIGLKKLYQETIATEPILIIISTGADPS

QELQELANETVGHGQFHQVAMGQGQAELAMQLLHQCSSSGEWLCLKNLHLVTAWLPSLEK

VINTMKPHPDFRLWMTAESHPKFPTILLQSSIKITYEAPPGVKRNLSRTYETWTQEYISR

GGSVIRSQALFALAWLHAVLQERRMFIPQGWTKFYEFSLADLRAGADIIDRLCVPGKDLQ

WNFIHGLYEFAIYGGRVDNPFDMRVLVSYLRQFFESGVINTQSKKRLGPLRMPNSTNIKD

YQEVIASLPEFDKPSYFGLPENIERSAQRIISAQVISQLRILQRGDAKASRFDKEVWATQ

LGPILNLWKKLNQGTNLIQMKVAMPTDKSGKQSPTLAFLLLERYNGVKLVQSVHASLAAL

SKVIRGTQLLTSEVQMLAGALMHQETPAGWLSHWDGPEDPIQYLKGLVTRASAIQVWVEK

AESGSLFRESLDLSELFRPDTFLNAVRQQTARELGCSMDNLKLVSSWRGGIQGSRLPFKL

GGIQLEGCSFDGTRLSENQHDSPSVSSIPPCVVAWVTKDSPDPYGSEDTISLPIYFDITR

ERIVSRMEVPCGGNMNQWLQCGAALFLRSQ

>SG-AL_contig_5666_1_-3_3_167

LSLPLSFLYCRLLFSAFPVHSTFFFPASLHKIFSPPFSPPQFMMCYLCCIALATR

>SG-AL_contig_5667_1_-3_312_488

MCIRGNVHCFALICLHIARIGCTKILANSKDMKKSTLFYTCVLANRTGWCNDVEKSLNY

>SG-AL_contig_5668_1_+1_1_162

EEYRLYILVFLSSDISRRIQTLHSFPKCRLLQGQFVSSLTQIAETVGYPVLFGL

>SG-AL_contig_5669_1_-2_593_739

MKRPECCMKVTLSVVSRSTEGFGMLHKGNIEVEYSINCKGKFECPVYVK

>SG-AL_contig_5670_1_-1_1_159

LGSWEEAKRATLRAGRSWLAAWPLEGKGARQEATEPGVGLHGRISSTDLLRPL

>SG-AL_contig_5671_1_+3_3_86

KKYTIHRRERLTMSLHQKSVNVMNCHLL

>SG-AL_contig_5672_1_-2_5_1924

MATSKLKARGKPIAAKHPKFAGYEPASKPSVKIMKCIGFQKFRYFGENRPGKKYQDKDFL

QLALCHMSGKNVRVQIVCFDTERQHAIHPNGLVGRDCESGVYNKVFPISKSTQIIRIPYL

RTERILTKKTKKEKDNKEEGNNMKTVFEDRAKILPSPYKEQCLAQAAMDQKYDTTRVCFG

VIITIEDSSMEPLFCLSNVVQNASEKTTLRILKLSTQFVSAQEGGYIDIFTSEDGPPITA

GKCTVKVIVYDGEGDVHWESPEHKPANDQILYKRVVKYRVPAYCPNPATDCPVRGVVSLS

CMQSGQNCSCDFCYEPGADSARKLSTIHSNKPLRKRPYVSEPTGEEKYNDHIKWRVKEKV

RSRQNQAESVPDLDLTTRAELSHNTSPIAVSNGVTNLMPVSTIEQDNVPLHQQDDIVLAK

DLVTGEVVYIRTPQTHNPSSDVVVYVHDAPQALNSSDNITILSDTPRAVAVPVSEWSNGA

STSMATPATTTIRTLESVGQARRPSSDQAAGPSSRKRKYVSPWIVGEPESTPLINGDISF

TQPKTQVLDISYPATSVEEEFKREPAESGPPILTPGQSVQITSDTVPGMSNNLPSGTLIS

SANLPSLGTLLANGVLEGVKFDETGNLLSSSVFEMVRSYM

>SG-AL_contig_5673_1_-2_62_316

MSASVMSEKAQYQMKTAPFDARFPNQNQTRNCWQNYVDFHRCQKIKGEDYEPCTYFKRVY

KSLCPNSWIEKWDEQVESGTFAGKI

>SG-AL_contig_5674_1_-2_2_118

EEVRTQVFQTLQFGSQCLHHSTTAFVFSNNYYNHYALME

>SG-AL_contig_5675_1_-2_215_4555

MATPAESAAAEGAASVNGAAAAEVDNGERSENFQGPHKWRQHLRHVNYVPKFECSEKLSD

TTDQREISDRKLSVVSEEDQTQDKNNSKKTDPEIPPWRQEYLEKKKTRQAGKVANSGCSQ

IPVSTLMRRRSQHLTSAESAFTRQVVNSGTNARKKTSPSNSFRQANQSASCTSAGRRTPS

QGDAPSTYLTQKRIERVQRARLYLLQQTGPNSFLIGGDSPDHKFRVIIGPQTCSCGRGPH

CVHVLFVMLRVFQVGESDPCLWSKTLKNYEVENLFRIYHDKRSLRLTRRRQEKAKENSTP

EEGKETVAAMLEEASTPSENDTGSTREEEDTCPICLLEMLEGESLIKCENSCHNRLHHHC

IAIWFEECRRQNDPLSCPLCRTPWTSSTTEIKTSAHSENSDVPEAPPNTRSSSPQPAREE

SDGRLPHAEPVAQEFQTVAAPWIEALGEDLVNCLFSRNWSFRETGLKHLSKEIIATLLRS

AGEGRSGAIVSPTRQAATHAMLETSSKILAHMCGDPVYRVFVACLRSVRTLLSYTPCRDQ

QQRQRLQKLLRPVVNAIFHKCTDGNRRTSQLSLSTLLELAKGQAGELAVGREIVNPGTEG

LDGLDFVVRCATEDYELNSIPWQWLLGRLYVIDKLMEEFPNDFLPRQEVPDSAGAAEEAA

EAPEEVGAAAGESPGRKPQAPIVNYERLMSVAHFSVKAVCSSHMRIARMSRRVFLMSGKL

GSHIDTVIDQLEELLSNISASSSVLSLKRKLRRIVADFQLSEKIVHELQHGCEKLAGGRF

ESSTSTPLDSPVSTPRCNSPNTIPSEELSESMRVHSNTEKVAPVAPPNTPTHRKCRKLRR

HSARRSFRDAPVVKAPPTSPDETQEETGDKDSDVKNEILKVEEDRLRVEEDRLRVEEDRP

KILKVEEDRLRVEDISIEIKVPHTPPNPPRTLACLTPVIKVRPPSTSEELMNIVRHTENL

SLNLHIDPEVPSVSQASVAEEHMILDEGEIKTPPSTDLPPERTTRKKCCCSCHDRESGLY

DDAASQTSGTVAVPNTPGSFRNRFHTAEQESMSSDDFLEFSTPSSSTNEKPVSFKSEVAE

SPKSSPSHTLSEASGNGDCACKEEVEKEEAEALAKAMEVSEEQSPIPVVPGLTPTTREEV

ITIRIQPDEVESKNSIKSLSSCDRQGPQIYYEGIHWVKGPLLGTGAFSTCYQARDAQTGV

IMAVKQISFCRNSPVEQESVVEAITAEIHMMSRLSHPNVVRILGATKQGCHFNMFVEWMS

GGSLSYLLGQYGAFTEAVTIRYTRQMLRGLAYLHDNHILHRDLKGANLLVDSTGQQLRIG

DFGASARLASQATGAGEFQGQLLGTIAFMAPEVLRGESYGRACDVWSVGCVMIEMSTCRP

PWNSHDISNHLALIFKIASSVDPPPIPDNLSPPVRDLMLRCLEQNSEFRPSAKDLLMHPL

FTKYSNY

>SG-AL_contig_5676_1_+3_420_581

MCLMGSLTSFSTLVMAAVFPSHGTSGVSAYEIYSQRGDAVLRKLKMNLNVQIRM

>SG-AL_contig_5677_1_-2_62_3478

MMMMQILISSLKKGRAWYGKFIKSDPLLYGFDISTPMAYRPSYNGSPLAYALLFPELMGA

LGIRVFYFFKEIAAAYKCHERACQNFSMKCYNDGYVTRVKGKCQCRCVGRLDPDTGCQTM

LMKYKIPLEFPGGKYALIASKNGCPESNTNDTDSPLQFHTGNLTHYFSAAWTYGTFGLAG

QYRADGLVEQHFCVRDAPDNGVQWPPGRYCILRFGGQCPAGFDESFIQFDNRPPNSTWNH

WFGTLPDGIYGDDIRLEFCCRQDGWTTLEPLVLPNTKPFVLFSPLWWGCQDVQGMLYTHT

FVVMKHENGSTVTERGKGPYTYYFPNRGSLYLYYCYYEPLNTTTQQQGIATDCGDHRGYC

PFWARIGECDINPNYMRYHCKKSCGLCQPDGCGGVLELHENLRTVTLTSPNHPDHYTNYL

ECQWFIKGPENSTFVLVFNDFDIEGSDGNQNCVDKLMINHARVGQEPLTYCGTGPRGSIR

SITNLMLVTLTTSRTGTRRGFNATVTVALAEDHCYNVDDKGVNYRGRVNWTRDSEPCVPW

TEVGHCEHSALYGGDFYDGLDENYCRNPGNGLRPWCYTQAEHCVRNYCDVCFLEKPFDKF

VDCQALREEGLCRNQYLATKGCAHTCQDQLIRPGTPVIHTQVTCPAPVTPADAYPLTHNT

SLYPAGTKLTFSCKFFAGNVTTRCLLDGTWSPVGYACGGCPDGWAPYGQMCYRALNIRKT

FYDALGLCQAYGYDSATLVTPADPKLINFLFTLRGDQNTFWIGLKKESYDDWQWLNGSAP

LDNRWFQGKLPNATKKGCGYMNIDQQWSQATCSATWCQHTVVCQMKPKEHVLCVDKRQDC

ERLVRQTPTLCQDQQYTALAWMMCPRTCGFCHNKSDVQNCTDGWEKYGDGCFKFPTTSRK

TYSKATSICRSLGGVLATATDEAEQQLIQHIVSNKWEVKPVWLGADDLASKGNWTWQDGT

RVSSGRWARGKPDNDPDDLTHCMVLKNGKWKDDYCQKKRCFFCKRLSAYCLLPAPTANAT

ITAHSNVQILPVGKHVIYSCKDGYVPASGVAVRACLPTLELTGSDLICTVADETLTESSS

VPLIERHHVGEQYRAYTGPSGNLKITRAGHVHAWTFYSRTTSTVTFQVYRPLANLSSHQ

>SG-AL_contig_5678_1_-1_73_225

MYVRHFEEGKAMLSNVYRIRDKNFLSIICVQKKVFYILQVIPYIHKNIYFN

>SG-AL_contig_5679_1_-2_1865_3415

MFTCCVRMLRSPEDEDNMDNDFRWKKGQTNRAYSQGGNYDLRPPIFYIESGDSTDVDSKR

YDISDVNGVMTSKQKLDPNSQLQRKSKLHNEKLPSFSALDRTKRPRIRNEETVMEEDNSS

LGYEPLTFETFRSASPPLAVIAPLSNPKIHPHTNLAPGQKITNPHSSQKTRRQKNGKVDV

KSNTSFDQERGKSSNVHRDSQQPRLPKLTHQISISKEGAVTFQCKDMTNEGLTNKGYCSD

DEVSDLPRSSPSSVDDLPTPNPRHTSLSRSVQRPNTLLLLPQSGHHSPSPSRNLASTVLD

ESSSAFDTDTFSQTTRSRQLEDPISRTRGCINGGFNVHDSSLHDNTSVGSDKNYALGGMK

EGRNDAVSDTKYVPSSEAKNENRIHEVISPSAVKKIPPKKPKRKNPPSNVSTAASRDALA

KCLTSNGVTSSESARHSDADPQVDTSTASSHDVITCVSSKGVMSSDHEDHIEADLRHDDD

DDVFEADDVVLLMPPQCFMDHDFAGFDGFDEVNGSLV

>SG-AL_contig_5680_1_+2_59_244

MQEFCLYSLRGRSIFTSLKWVLNVYFKPVCFLFIAIVSVDTSAKMGLLWHCINAHHSRQS

LY

>SG-AL_contig_5681_1_-3_171_953

MFEARLVQGALWKKLLEAIKDLLNEAIFDCSSTGIALQAMDSSHVSLVTLNLRSDGFDTF

RCDRNLAMGIHLPSMSKIMKCASNDDIITIKAGDSADNVTFVFESPNQEKVSDYEIKLID

LDTEHLGIPDTEYSAVVKLPSAEFQRICRDLSQIGDSVVISCTKEGVKFSASGDLGTGNV

KLAQTTSTDKEEEAVVVEMNEAVCLTFALRYLNFFTKATALSPQVTLSMSQDVPLVVEYK

MADMGFLRFYLAPKIEDQEAE

>SG-AL_contig_5682_1_-1_148_381

MSALEATTASKLPHLQEALQNIRCTRQVLQWMPSKCGISGNQEQGSQWCAFVRNTRTETN

KNAMKLRRGGRQMQRNSQ

>SG-AL_contig_5683_1_-3_3_203

TLLSMDSVIQTTLLSMDSVIQTTLLSMDSVDQTTLLSVDSVIQTTLLSVDSVDQTTLLLL

IVSFRLP

>SG-AL_contig_5684_1_+2_2147_2554

MEDLENNTTATKVAAIETIVMAQYYLSLSLITFVLITKSTVMVVVMVPTCYNNSLSPSLS

LNATDTSILHYFNTFIVFKLHLTLLPCGIIISILLVGTIYSELFLMPIESLNFVIIRSSC

SSSSTSSNSVIIKSYY

>SG-AL_contig_5685_1_+2_281_430

MRGGWRWDNKNSKNQNHQAIQTDWQTNDLPFSVSWPRIPTLQNRLFSFKS

>SG-AL_contig_5686_1_+3_48_278

MPRDVAKLDDLSGDGKSFTSVPSVSIIVVVVCCFRSPAQHSGGHPICMHSVLPQSDNEYI

WMRRLRVWQFFSTSPCR

>SG-AL_contig_5687_1_-1_1_144

TIQVNWTCVYADGSVQKMVQVVTHTPLVVKIHWLCMDVYCKPCYRESK

>SG-AL_contig_5688_1_-1_73_1344

MQSSLIFRTGVRRVAPRLCAWHRHTTSLASTRNILEGELSVIKEAGTWKSERVIVSAQGP

TIQVKGQESGILNFCANNYLGLSNHPAVVEAGKKALDTHGAGLSSVRFICGTQDIHKELE

EKIARFHGREDAILYISCFDANAGIFEVLLSPEDAIISDELNHASIIDGIRLCKAHRLRY

KHRDMDDLETHLKNSQSQRQRLIATDGVFSMDGNVAPLKDICSLAEKYNALVFIDECHAT

GFFGKTGRGTEEYCGLDGKVDVINSTLGKALGGAAGGYTTGPKQLIDLLRQRSRPYLFSN

TLPPPVVACASKAFDLVMENPDLPQKVLSNTTLFRSRMAEAGFTVSGDPEHPICPVMLGD

ARLASQFADKMLEQGIYVIGFSYPVVPKGKARIRVQISGSHSRQDIDRAVDAFVKIGKDL

RVIG

>SG-AL_contig_5689_1_-1_244_1239

MSSDASVPREYCDEGPSKWRSTRFLVSCMACGGMCVLYFQRVSLSLAIVCMVNHTALHRQ

QAAHQRMLNFSGDQEREANVSAISIDNRVDNLAYQTYANTSGEYLSTSQRDLDRCSSEND

AASGVPNSEEDGPFIWDKQTQGLLLAAIYVGYTVMQIPASYVVHRFGVRLTSGVSILVMS

VCHILCHPAALLSPWAVLVLRIIMGVDTAFAFISMYAVWTKWAPRTEHGQLVGITFCGEM

IACIAVFPSSALLCRYGFWGGWPSIFYTFGSLGIVWSVAWFILVRDSPETHPRISASERQ

FIVANRVDVSPYTGGKLAVPWGSILRSQCFWA

>SG-AL_contig_5690_1_-2_854_1153

MAADDDNRSLMKLDKDGAYMLVTQKMSMNRHKCLRWQNQAQSLQTDPAHTLTTQSTCIHT

PASKNRTYILLLMSTLSHGKKKLTISKAQRWMLGIFTLQC

>SG-AL_contig_5691_1_-1_256_441

MRPPLYLAVNLYLNLFMDGKVSSDGEEVGTAVIMLAPRKVAVSTKLPCDLKVMGQPLPLP

YT

>SG-AL_contig_5692_1_-3_639_932

MEIQNAHAKKILHTHFNMRTLTNMESHTRTNIHTRTHTHTQSHVHTHPQTLTCTLTYTRT

THTETQTHFLFMCTHIYTQHSISLSHVHTNTNFPSTPT

>SG-AL_contig_5693_1_-2_212_430

MSSSYSHQWLFLPLCVPLYVECIINLSVAIVHQLHLPHSSCPHVLHSLQGRESCISSTFV

LYSRPGQWSHMLR

>SG-AL_contig_5694_1_-3_99_4991

MWLIQVVLGAWLLSMATSQDSSVAQSCLLDNSVGCALSRPPQYIVLVPRRVRPNQVIQAF

VTILKMEYDSEYVNVRVSIVKDNVEYTGSSLRFDRPSSRLMQLQMPPNAQFGSYRLRVEG

SLDREATGNIFVNETDIDFSSKQASLFIQMSQPIYRQGQTVQFRIVPIKPDLMPNYGSLT

IYVDDPTGIPVRRWLGLQTNAGGLVSQSFELSDQPNFGTWNIRVDAFGHTYRQAFQVEEF

WEPRFDVNVTVPPYLLENSTTIIRGIMVANHTSGRPCKGNATITVFFRPREEVWNATRGW

ERPYYNNMMPNRNPNLGPPEYKAIADIPVMDYYMYLHYDYWFVDYYDGGVTFDYPREKLQ

ELAKRSGEYGTLVDSGVYFWLNVTDWYTGMNRTGWAGTIIASSRIILRWIGEPVRTFKPR

STMKIQVAVMHADGTPVRDPQASVTLSESISNTNSVGTTSNPQTRSPVNGIVEFETYLSS

QAQLLKLTARYNNDPLTKQIEMRATRYYSPSNSYISIMTSTDHPKVNEYMIFHVKTSQFV

PRIFYQVVSQGNIIIGDELEMTSKQKTFAVALSREMVPIARIVVYYIRQPEEIVTDVLNF

FVNGTRQNRVSLGINLAKDFSRDSIEFNAYADPGSYVAFSGMLLDLYNRGLNDGITENKL

IDELATYDKPANGTYRQLWRVSDTDYEYKFFTSSDYGIDANTSFSTAGLLILTDAELTRI

RNGELCKEEENQYPCFSGDGQCYSIEQRCNGRFDACTDGADEYGCDYDDSSVKHMNAMDR

VSRIMRFYDNSSWAWQEIFVKPDGRVDFRVTVPKYPLTWVINGLSVSRDLGLGIMTRPVM

YDATRFMYIQVEYPQTIVRGEQVGVRVTVFNNWYEDDYIEVLITMHGSDDYEFVLVEALG

WVSSYGPKTHKGDHQTIVFLEPGESKDIYLPIVPAADLIRGMLTFHVSATCFMERDEYTG

NITVIPDGVQNYYHTPYLVDLIRYASIEVPYLDVVVPEQFVVPEVREHLYVPGSGRADPS

LFGDVVTPGFFQDYLNAENILLRPYGAGEMVAFNFAYNIQTLKFMKSSQQLSDKRLMTTL

KMANVVLQRVLSYMNGTDGSFHMFRDHPVPSVWLTAFVAKTMHEARFGEWEQDLFIPLEL

INKMVVFLCSKQNKTTGAWDPDPRHTIYDRKMASYGAIKEDRMYADRIPLTAYVLIALYT

TKDVSEDALGCLDTARVNGANFLSIQAPNIPKNEVFHLAITAYALSLSLEKSAIAFNRLW

TLVRNDSEGLYFADQKVPENPSETLNNERFLYPRIELMNDGYSVQSTAYALMALINNNGR

KEHRDSLMRWLNSMRNSIGGFASTQDTLVAMDALSKFTQVDPNRNVFDLMVHLTSTSTPT

WSRRHFLRKHDYTTLYLDSIPIVWGMVKVLAQGTGRALMQLTTTVNVEYDHLIKPTQLTD

MSDRDSDPIQFFDLMVDYIQWHGRNFSIMEMKPCARWVYTDRSLTSGLAVMEIDIPTGFL

IMNDTLRDYVRSRMVPNLKRAEFYGRKVVFYFNSLDESYTCVTFQAARWYPVANATIQHR

IRVYDYYEPGMHNTTMYTTFNLFTLNICYVCGSYQCPYCPYFNVATAIKATITMLCLVSG

FLIQRYFLLRS

>SG-AL_contig_5695_1_+1_2308_2562

MYSQHSLTLSLNAEFKRKIHNCYAKVLSINPKHFTFLSDSAMKQKHVVKQSHFTSMISIF

LPQTYQHHHNELTPTLNTKDSQFSI

>SG-AL_contig_5696_1_+1_394_606

MKKWTQEMNPRHVNIDEQSQLQFYREGVVHVWTTSQWKTYKYDQITNSPTCFSRQALRKG

TAEGKGREREH

>SG-AL_contig_5697_1_+3_66_434

MVKDTHSRPIQHHLKSCTPSLPTHLTVRQHGDKIVVKMHFIHMLNKKQTNKQKGGSSLLT

TLSQNLSTLSLHNLSSPSLLLLLLLLMMLLLLLLIMLLLSITTLIYTLSDTHKHTSSLCT

LIP

>SG-AL_contig_5698_1_-2_401_604

MLVPIFFLIYILCMLPCSCLCILNKCIDSFLFRNMHRSSKHEVLFLGSAIYNISCFHLLA

FDLTMGSL

>SG-AL_contig_5699_1_-3_102_278

MNLCESDFSRVLFDSVGELVKTVIDVICFQGIECAFVLHGHRKRDCLNGMTILFKKKFI

>SG-AL_contig_5700_1_+1_19_324

MEWSMVSKAADRSRSVRREILPASEARRSSLTMFRFLGTGDFEHPAWISDYPLLFRAVRM

CLCSAFLVLASDVVCVPQLNTLGVTQYICTLSYHSETTSTFG

>SG-AL_contig_5701_1_+1_1_405

VGVVVVVVGCVVLTVGGGGAAVVVVVGVVVVVGVVVVIVGVVVVVGVVAVAGVVAVVNVV

VVGAVVIVDVAVVVGVVVVGAVVVVGAVVVVGIVVVVGVVAVVGVAVVVGVVVVVVGCVV

LTVGGGGIAVVVVVG

>SG-AL_contig_5702_1_-3_3_3146

PTTATTPTSTTTPTSTTTPATAITPTSTTTPATATLLTTTTTATTATTPTTTTSVPTTTT

VAPPPPTVSTTQPITTTIPTTATPPTTATPPTTTTTTLTTATTPTTATTPTTATTPTTAI

TPTITTTPTTATTPPTTATTPTTTATTTTTANTPTTATTPTTTTIPTVTTTTAAPPPPAT

PTTTTTPTTATTPTTATTPTTATTPTTSTAPTTTTTLTTTTTRTTATTPTTANTPTTATT

ATTATTPTTASTPISTTTSTTTTTPTTKTTPTTATTPTTTAVPTTTTTTAAPPPPTVSTT

QPTTTATSTTATIPISTTTPTTTTTPTTATTPAITTLTTAITPTSTANTPTTATTPTTAA

TPTTTTIPTTTITVTTATTTTAAPPPPTVSTTQPTTTITTTTTTTPTTATKPTTASTPTT

ATTPTTAITPTSTTTPTTATLPTTTTTPTTATTPITTTTPTTVTSPTTTTTVPTTTITTA

APPPPTVSTAQPTTTATPTTTTTPTTATTPTTATTPTTATTPISTTIPTTTTTPTTTTPT

TTTTPTTATTPTITTLTTATTPTATANTPTTATTPTTTTTIPTTTITVTTATTTTAAPPP

PTVSTTQPATTITTTTSTPTTATKPTTTTKPSTASTPTTPTTPTTATSPTTTTTAAPPPP

TAQPTTTTTPTTTTTTKLTTATTPTTATTPTTATTPTTATTPTTATTLTTATTLTTATTP

TTATTPTTTTTVPATTTTTAAPPPPTVSTTQPTTTTTPTTTTTPTTATTPTTATTPTATT

TPTTTITPTTTATTPTTTTTLTTTTTRTTTTTPTTTTTPTTTTAPTTTTIVPTTATTTAA

PPPPTVSTTQPTTTTTPTTTTTPTTTAPITTTTPTTTTTPTTTTTPTTTTIPTTTTTPTT

TTTPTTTTPTTATTPTTATTPTTTTTPTSTATTPTATATTPTTTTTPTTTTTRTTTTTPT

TTTTPTTTTTPATTTTPTTTTTPTTTTTPTATTTTPTTTTIVLTTATTTAAPPPPTVSTT

QPTTTSTPTTTTTPTTTTTPTTTSTPTT

>SG-AL_contig_5703_1_+1_1_252

CTYSRRWRSSSCGCSRCSGSGRCSFGWCSGGCRCSGANKCSGSSRCSGSSRCSSSGRCRG

SGRCSGGGRCSGGCGLCCTYSRRR

>SG-AL_contig_5704_1_-2_131_373

MLVFSCFHKPPNTDVTYLYLYALRVYTHEGGSDTSRSAHRPMLTWDIGKFSILYPSAEVT

EEDSNPGFPNPPVWKPSVFTI

>SG-AL_contig_5705_1_-2_2_160

FLKNHEFALFYMKTIDGITSSQIFSRSIFGLNPDCWHNDFGICWIPFNRSPRF

>SG-AL_contig_5706_1_+3_297_425

MSQPFVQPFHTSACINVQHFPYPIGSQIKINANYIQVIQQAVL

>SG-AL_contig_5707_1_+1_1_189

INLELIGTLFSISKSMKMQQNRSKQTDIYKIKVQSRLRTQHECTPELYRGKNGGFDPNVR

PPR

>SG-AL_contig_5708_1_-1_370_564

MLCVIVVRSLYDSIIGCGSCPACYVQDCRISPLSFAADSLPSSLHVFCLLFPKYADMSSE

FDCEM

>SG-AL_contig_5709_1_+1_235_474

MCPWMNCVVHVLSMRVRVCVCVCVQSNRLLHRRRTHSECMCVCVCVCVCVCYTYVQKFSS

ISQTCVSHFYFTVLSTLCQK

>SG-AL_contig_5710_1_-3_1689_1952

MIGALVKVGQRAGPFLCRRLGIELETSGLLFVVRCYCHLTISFSGCSFVCFFVYMLLFFL

LVCVCECFSVVVFNPKGIFLGFFFNLGM

>SG-AL_contig_5711_1_+3_927_1112

MCHFPFVFLSWCKGCHRFFLMLPLPPPGRFSPFSLQWPCFGVIPECSTKKRNATSVMPHK

HS

>SG-AL_contig_5712_1_-1_82_1383

MRPYSVCGRTTYSMYTQIRRKLCESFKGSISLSCCGRHLSTYHNRSVDAADSDESKRRRL

VLGIETSCDDTGAAVVDDRGNVVGDALNSQTKTHVQHGGIIPPIARDLHKAYIDSVVTDA

LHRADVTLKDLDAVAVTVKPGLALSLLVGLQHAKKLVHQSGLPLIPIHHMEAHALTARMI

HRVDFPFLVLLASGGHCLLAVAQGVSDFLLLGTCLDDSPGEAFDKTARQLKLKNLPECSS

LSGGAAIELVAKEGSPQAFQFPQVMTRLPDCHFSFAGLKFAAKKVIQAEETKLNTSVSSV

VSSAPDICASFQFSVLHHIARRVQRAFLFCDMKQLLPEERILVLSGGVASNSYLRRGLQQ

VCEMNNCQLVCPPPPLCTDNGIMIAWNGMEKLLQGTGQVEDPDTVDIQPKCPLGKDISRD

VIAAAIKLPRLKLR

>SG-AL_contig_5713_1_-1_250_1008

MPGVSFDASQYKTPHVSRQDYGFAWVPSANSTFPPGYDPRLSQGEGERKEGVDSHTPPLN

AEGKGGFHNRSHNTDKTKIDDRLQLVPSMSSASSSQQHVSNMPSNKSNNLGLKSQTLGVN

KLVIKQYKPKGPPPKFVPRQIIERKDFQPTKTKLSAAKVKEEEVNREIRREAFRLGEDQG

PSLPSDPETREQLLGLRPLVEPKVEASVMASVKAIRKRMSKFICETVTKKLKPMEAPETQ

SASISDKGSSKTN

>SG-AL_contig_5714_1_+3_3_275

TEQATSQHTHTYNTFTVSLREILTTDHSQLKRHRRKHEFIISTGKDMIMRSPGDINGHLT

TPENDETKSGRKHRVRDIFYPCSNCNTWTDG

>SG-AL_contig_5715_1_-3_3_194

FFFFLFFSLSSPPPFFFFFCFCPFPPKKKRKKRKESHSVQSLMFVHRYLGNEIVSTGMSI

HYPF

>SG-AL_contig_5716_1_+1_1_159

FDNLAVVTLKLCQGHPTWAIVSLVQGITNAMKPIMVTLVCNGDVLSTQINNRH

>SG-AL_contig_5717_1_+1_1_141

QGITNAMKPIMVTLICNGDVAVHTNDIISIKIKGGLFIWLDHFFNNG

>SG-AL_contig_5718_1_-1_184_507

MSTEPVRLYGSDPLLSTRSLHMRNTWICFTAMPSFSEISSVHWQKRKQYVLCTAVLWWTH

SLWGKQCKNRLPMYCCFMADPKPLGKSRVLVTKQKPHLINCFFFFFLL

>SG-AL_contig_5719_1_+1_1_189

STKFYQLIVNCHTTAQPKYKYIRLINFGVMMRYVYFDNLAVVTLKLCQGHPNLGNRESDS

RHY

>SG-AL_contig_5720_1_-1_1_297

SALFQTELNINWIPCATNLFISLLHPTFVTTSIFILQLAACAQLQTLFPFRFPDLNYQLS

ASVLLLFQDHLPRIDFPLSLRQTPTLSALKSNLKTHLFS

>SG-AL_contig_5721_1_+1_37_219

MTELLITSCFNNILNKTSSNSCQLFTDIANNSYFSQPRLKIQTVNPSYKQSVLKKVESAL

L

>SG-AL_contig_5722_1_+2_2_223

AAAEIENPNVDEAGLRLNITVTKQCHQTWTIISPVHDWATCMLILMHSLSLLLNLNPRGS

QYSSKLGTRSTRKD

>SG-AL_contig_5723_1_-1_31_729

MAPITIIKTLLMPNISSGQLQRALTHCARTSLQQDLLRCLDSADVTQAVGKCLCADHMEQ

PDTWWNNTVLSQSADPGMSDDVYERLVARFCGPATTVEIATPSPPRQAVEVRSMGPAVAE

TGLRFSSLALTPRQVALVNELSPEPRYVYLQGPPGCGKTVVLALKALQWLNQGRTVCIVS

TSDAGRAVSALLEHQVRSSLGAAAAGAQFQRLDYDFRNRGEVSRAVSDLERLA

>SG-AL_contig_5724_1_-1_1_150

FVQEGFVTPRQFHMCSTQNCQQLSSRFLAPAQNPMRWLGLEMRKMGSMKN

>SG-AL_contig_5725_1_-3_1005_1241

MLFSWLLSLYSFSCLLIRNLRFVYLSVFQSCKAYKSTFTKFCIAFKHFFITFLIQFHKCT

NGLLLKRTNTNSKALRWFT

>SG-AL_contig_5726_1_-2_317_637

MVTLCIYLCELEMSSVRTTNLSILCTSGLLGGDVLGICGIVSFVLTMHCISSTTSQIHFQ

HQNLGWNYWCAFVFIFTYFLIVTMCSVVSRLIHLSEPVMLNHLRQMF

>SG-AL_contig_5727_1_+3_3_164

HNANSKTHSISRQLKNTAQDRQTAKELSTDSSRQQKNTAQGKETAKRHSTASRR

>SG-AL_contig_5728_1_+2_44_247

MPCDVAKPRQLPPLDGGEERFLGAHKVLNLAPHIVICSVLQEGDAEQPAQAFVFPRGGNF

AFFQDTAG

>SG-AL_contig_5729_1_-3_321_587

MCCLLCDELCYILYAVVGICVVTCLISIKCKDHSCVPLNPAKQSLLHRYDTILKILTFST

LHCRSIFYVSIVNSITVCDDVCDCVLLHF

>SG-AL_contig_5730_1_-2_41_400

MQCDAGDTSCLNNKTYTVAWQFLDIPNIEFVTSPMTLLNIKTVAYSFYPNLHLFIQSGNE

QGLFDTIVQGDKAALRLLKPLVGPAEFDVTLVLENREFSKQVIVSRHITYVKIYVSEYHI

>SG-AL_contig_5731_1_-1_784_966

MIFCRSASNVSVYLLFDVQVILRTFTQRTISQEPLWNSSHLLHHTSGAGKATVFTAAPIT

V

>SG-AL_contig_5732_1_-3_3_134

RRGQRQNRHAVIPFSFFSASLCMSLGHWPLTHCVSTLGLSLAVS

>SG-AL_contig_5733_1_+1_685_933

MTRHKPQQQQNSRNWFHNTQLQLVFIPYLYACIYSTTRVDTVMHETIRHRMHELLKHMYR

SRLVTETKYQTKIQQNPRELNVV

>SG-AL_contig_5734_1_-2_893_1135

MTSDKDILLCILFVFTVFYACHWVCHFIMVFHVYVQLLSPSHFLFLLQICFRFSWVFFLY

VIIMTPWCFFCFRVKVFLLTI

>SG-AL_contig_5735_1_-2_32_136

MYKQDFSAVCRCVFTQFQPGLLASVGSHRYKFQRA

>SG-AL_contig_5736_1_-3_126_233

MYNTHSTDKVINSMNPYVSICTIAPSLNTSYHFVLI

>SG-AL_contig_5737_1_-2_2_187

PTCLLRLWSALLEQTPTLSPPAAHFLLLQNFPKNPPVFFSLMSVSTVPTTPTACLCPLGL

RP

>SG-AL_contig_5738_1_-2_248_1261

MASASLSGFSMTGWNANSFRPQLPLPPVVYPRCCGKGPTFSYFLQSKLLALAFLILGIAF

LVTFICAFLPFWFILRFNIFDSTFGSEGLRQLSKDTGIFFYTVDDYVSLLMLEKISNRVV

VPMLFKVAEVMEVMSVAAVACSFAGAGILAFRKYASVTGVLFLAALATFAALGQIFMVVF

SMVSLLSSSNDVCEPNQANCAYTDNLVWRNLPFSDQFRRINQHTTPKMEANWALYIACIG

AALSIAGCVLLWLEGLMVCRSVGEIRYQQLRQKRDVHENERDPTDFIFSPPSRSRPVMYS

EHPNVSYAPPRQTYRPPPSFSGASSQSGSAYVSREVDL

>SG-AL_contig_5739_1_-1_352_489

MPWDLVSFSLVFFSCFQGGLCDVLMVFGARCSGGFSSCVHCCTWVM

>SG-AL_contig_5740_1_+3_2754_3083

MYAAIKKKLEDKMTNEAVIKKLNVKRELLSKIKSTKLSYFSHITRHDSLQNNILTGSTEG

RRARGRPRCMWTDDIKEWIGMGLSGCMRTMADRELWKDLLNRSLDTGKVL

>SG-AL_contig_5741_1_-2_2_208

FGIHLVFYALSLSLSLSLSLSLSRARARARARFIFKYYVMLGVLEVEVEEGILGIEELCV

FSVLVSPLL

>SG-AL_contig_5742_1_-2_116_1627

MALVPLEKKGKKLTWNIKDTNDPRSKLPAINKAPIVDYDALSWLCSIQGLASRDTPDTSE

DAHRMFMENYHKMFVPEPKKTGFPVHAEKRAIAGKSSTQSESVDATAGRTTQVGKKQATG

SSQIEEFTHNIKDYLPLLHKERRSEDPEQIRTDNIKTLRRLLRKLPFERTATDNDRIFSI

LKTFDFFADNISNNVLKELCVVAQLDTWKEQDFTVFGNTGLHMVLRGKVMPLTDPHVYTG

LEIDELELRSPTPLLENCSTMLEVGDCFGTLQAIKSKQPSTRLLSVKTVDANCEFLKIAT

SDYSKVIVQIQQREQTEKLNLLMSCSQYKLWPRQPLQQVASLIEWISYPPNTVIVSEGYK

APFIGFIKSGECHVLRQVDVLHTLRSGKKEKKTKQVVMGKLGQSDSFAEISLLLEEPITC

SIVTATDMQLGVVRPERLHELDEVTVQLFKQSNTRTFGNLTKEDIQNEYMQQEQKRGWNE

FKHSMVVDVINANGIRPGYGKWAK

>SG-AL_contig_5743_1_+1_1_309

ANTPLWPGLLLDRRSEWPTLINRLVVHCPSIARRLRLGSNGSGGTVASFPTRGALVLYSA

GLPQVGIPLFGTRWWVGTKGTKITVYNYGNQINQTSQTYGFGF

>SG-AL_contig_5744_1_-3_120_1163

MPRMKHNLCVKTQEQKEEHENLLELEAKVRNEIKCSIMHTRLDFTSETELGKGNFGKVVK

AKLKPDCETKEEEERCEKKDVAVKMIHDTITNNDEVSRFLKEALCIYHLKHPNVVELIGI

VMKGLKNSYMLVMELADMGSLDKFVHKKSHSLGMQTFKLTLKKLAIDIAKGLEYFCQQKN

RLHGDLAARNILLFGSDGPSTRNITAKLGDFGLSHEMNGGYYDLRKSKTKSHRLPVYSLP

PETLKGYSTPELRFTIKTDVWSFGIVLWEMFSGKRPDDFLNPSNPETPSPSSLLKRYLEE

KYRLPELDNFPRKMYEIMQCCWQESPGQRPDFTELRQQLEDLTDNDMA

>SG-AL_contig_5745_1_-3_3_146

HLDPLRSLSHCKNCLGIFGPFSSSLRIISSFDFCSRCIPGVSSRQGLP

>SG-AL_contig_5746_1_-3_1017_1301

MVGGHLSVSISGRDSLSRSNMHYVTGTLSLVMKMIFPYMTNFLKTTTLRNITSDSFHFIA

SLLHVETTMSPGPQVGPDESIAMLQVAIMPRTKPL

>SG-AL_contig_5747_1_-3_351_2912

MLEDSPQVMEKKGASRQPNLTKPNSTFERKNDPNNTVRYHGRYSKRRDNGVGRGTPPEFS

SRKPYPQKTRSAVDKRPRQRGSSGGRQREEIAETGLLETESAIPHGLSKKGNLNYLLNFT

FARREVVGSNQRRWVKRLGSSFSKECYLQANCQFVVKDSGDYAVQACDPDALVSWDLVEQ

VWTFSFGQTTCPICLQPPAAGKITKCGHVYCWACILHCLSEGNNNSCRCPICFETVQGTD

LRSVKVIEKQEHKTGNVITLQLMKRARDSTYAVPCVHWEDREGRPQHILDPLNTEFAKVL

VASAAEIQKSVIEEERQALLQHLAAAEDSEVCFIETALVQLSERESILQGKTQAAQENAA

LLEQLGTGSGSESGNKERGDKGSNSMTAATPPPPVVTTILPRNVSFDGSKIRKQYADAFE

ELEEEEEESFVSGEFSDREDHEDSESTDSLMSMSSPTPDYSSSLPLFIPGAHGAAASSEK

EPLSVPSDDSLGLVPAEVADEDTMTTEEAVEQLSMPAESENVTVPAEAENGAAASNNTPG

RGARNQSKRYFYFYQASDGQHIYLHSINARCICQEYGSLELGPQTITAKIVDREITFMKE

DIRKRVRYLSHVPKACEFQVVEVDLQPPTVSQRTLRHFAGELKRRKQYRQKVKRDDRRNA

RKQNIEDKKNLFGIYPEVKLALDNPDHFPAPVAEGDERRRLSSVSDTASLSSLDISRPEL

NVNAVEFLPSPKTESSSSNTPCAFSFAAALKKAKQPVPSVWGASSTSTVTQAMGAAAAAA

GGPPLSRRPDSEESDNDDRVPVPLFKNSFSDAISMKLETLCAKGDGGEQANSKGGKKKKK

KEKKLLFSTDMMRR

>SG-AL_contig_5748_1_-2_50_865

MAQRLFSSLGKLGVGIAVVGGVVQSALYNVDGGHRAVIFDRFRGVQEDVSGEGTHFLIPW

VQRPIIFDCRSRPRNVPVVTGSKDLQNVNITLRILFRPQVPMLPKIFTNLGEDYDERVLP

SITNEVLKAVVAQFDASELITQREIVSQRVSQELTDRALQFGMILDDISLTHLTFGREFT

EAVEMKQVAQQEAERARFVVEKAEQLKKSAVITAEGDSIAAEQLAKAFGEAGQGLVELRK

LEAAEDIAYQLSASRGVVYLPQGQQTLLALPQ

>SG-AL_contig_5749_1_+3_3_278

SPDEKGGGLGEGQATPLRKKTRTTKTSTSISPNISVLRAQARGGWRVFVDGLCPRRGNRR

RWFYLILKCNNCQASECSKVQSAERGRGPGAA

>SG-AL_contig_5750_1_-1_169_1326

MHVQPMPMPAPLEREMMPRPAPAFMGQQQPDYRIHELNKRLQQRNEESDNLWWDAFATEF

FEDDASLTLTFCLEDGPKRYTIGRTLIPRYFRSIFEGGVTDLHYVLKHSKESFHNTTISL

DCEQASMVTQHGKPMFPYVITEGRLLLEFTFDDLMRIRSWHFAIRQHRELIPRTVVGMQQ

PVDPTLMEQMSKNITRQGLTNYTLNFLRLCVILEPMQELMSRHKAYGLNPRDCLKTTLFQ

KWQRMVAPVGKESARPSNKRRKRKGSASNNSGVGNNSRDVPPSGKQKRSPGPHGFNIGSS

VPGDVMVVGEPTLMGGEFGDEDERLITRLENHQFDGTRPGPEEDGTEQKFSTSPAWAQGD

KKTPPSSGAGGPPPPSINPEPEIKTE

>SG-AL_contig_5751_1_-3_3_167

LVTALTCNCTDVLTCNYTDGSYLYQLCLVLGRASRNLGGHVDFPLVAPRTAKNDL

>SG-AL_contig_5752_1_-2_560_907

MVDMLYGKNITWFGMWNYCLRKKKNCVIDKAYSVEIHSWLCHLCMEEKWLLGTDCKVCVL

IGFWLHGRIFFMVTLCRTLVVQHIVFKVSRYADKVQLISCRFFQRYIHLHDFCGLL

>SG-AL_contig_5753_1_-1_1_801

MVSAQHGALHELLTEIVFWREVYKNFMASNASGSSKSRTSSHGSAGSRGSKTPPNSAGKK

GQGDSAGAGAKPKVSKPSDRLNPKTVDHFSGGGKSQSAFASLYSNGGVPCRLVHGSVKHK

LAWDTPPEQLPFDPVLPTLAEGLRETVHPYTQVSHMGFKEMLESQGAGDKAAPLLPKVVP

PLRAALIHSNSEVFERGLDGLNYLSDAVGPALNPHLKNLLMALSKRMMDKKYRDKVTDTL

QLLEQNGGKEVLPVIKAKVPTYSSIFG

>SG-AL_contig_5754_1_-1_76_561

MGGMRTPMMGMGMGRPGPYDRNDRFGNAPMGMGAPMGPFGGRGRGRGNVKGFYDDYDSYP

MNYSNMANKGRGGARLPPGPPGCHYMSKTGHSVHMRGMPYLAIEQDVLDFFAPLKPVGIQ

FEYGGDGRRTGGADVDFATHQEAMEAMKKHRSNMQHRYIELF

>SG-AL_contig_5755_1_-1_43_1101

MASELLSKAKDEYISDNYQRAVELYSEALSEDPDNEDALVNRAQAYLKLNQNEEAVKDCD

KALVLNSKNVRAYLRKGTALFDLKDYRQALDSFKEGAKQSEPGSQFDIWIEKCETKINDG

KTCHKESSAANTEPTQVAAAAAAAPAPVKPKYDWYQTATHVVVTLLQKNTKKEDLVLDTR

ESELKIHTKLANGEDLNLELHLRHPVVPEQTVAKVMTSKVEIKLKKAEGVQWTKLEGDGQ

KFALKLPNATNATANGGSAATKYPSSSHYTRDWNRLEQEVKEEEKNEKLEGDAAVNQLFQ

KIYADGSDEVKKAMMKSFSESGGTVLSTNWSEVGQNKVEVKPPDGMEYKQYEK

>SG-AL_contig_5756_1_-3_267_449

MLVLRQDRISTGICRKNRRLIDKSQVSRKCLLILLLSAVYSVSGFINSVIDKCGIHRIIY

R

>SG-AL_contig_5757_1_+3_3_107

FCSVICLYGLFMYFLLLRSISYNFFMLFCNLGMIN

>SG-AL_contig_5758_1_+3_2229_2477

MPRALSVGMASLPSGNSVLEISSFLLLSLYCFCLFFFLLLSSAFFCLPRPVPLSFFQQAF

TQFPPPPSTVYDVPTLLFALVTR

>SG-AL_contig_5759_1_-1_31_138

MSQGVWGTTYTVENEGKAYVVEFAQRLFWELRGVCS

>SG-AL_contig_5760_1_-3_138_983

MSYQYPVSYGHGQNFRRGPGGRFDDSSPVNQDQNRKLFIGGLSYETDDQSLRNHFEKYGE

IKDCVVIKDPTTKKSRGFGFITFKETKGLDEAQKERPHSLDGKEVETKRAMPRDDPQSHQ

TVTKMFVGGLKDTTTEEMIREAFSGNITKIELIKDKATGKLRGFGFVTFDDSDTVDKHVL

TKNFTLNGRRVEVKKALDKNEMGYGPSGGYGRGGDYGYAPGYGYSPYGPAPSRGGYGGGN

YGPHGGYGNQGWNQGGNYGDYGYGHQGAGYGSGGYGGNYNRR

>SG-AL_contig_5761_1_-1_103_2967

MASMVDQPCYTLINTTTDSEPPTEIQLRQDLEKGDTRTKIDALKKVIQMILNGEKMSSLL

MTVIRFVMPMQDHTIKKLLLVFWEVVPKYHPDGKLMQEMILVCDAYRKDLQHPNEFIRGS

TLRFLCKLKESELVEPLMPSIRQCLEHRHSYVRRNAVLAIYTIYRNFDFLIPDAPELIAN

FLEGEQDMSCKRNAFMMLIHADQERALNYLSSCIDQVTSFGDILQLVIVELIYKVCHANP

GERARFIRCIYNLLNSSSPAVRYEAAGTLVTLSSAPTAIRAAASCYIELIVKESDNNVKL

IVLDRLVALKEVPAHEKVLQELVMDILRVLAAPDLEVRKKTLNLALELVNSRTIEEMVLV

LKKEVVKTNNVEHEDTGKYRQLLVRTLHQSSVKFPDVASTIIPVLMEFLGDTNELAAADV

LVFVREAVQRFDHLRPLVIGRLLDVFPSIKAMKIHRAALWILGEYCTTTDNIQSVMTLIR

QALGDIPIVDDEIKKAAGEVKEDEMQSGGNIQRLVTADGTYATQSAFSTVSVKKQEERPP

LRKDMMEGDFFIGAALATTLTKLALKYIKLTTDKKRQNAFVAEAMLIMATVIHLGKSGLS

EKPITDDDVDRIATCLRVLAERSDLMQDIFNEACRGSLSVMLAAKIEEDKAAQEAAEKKV

VKVQADDPINFTQLISRADMGPGENLFELTLSQALGMSSKKDADPIGSSKLSKVTQLTGF

SDPVYSEAYVNVNQYDIVLDVLIVNQTADTLQNLTLELATLGDLKLVEKPQPLLLAPHDF

CNIKANIKVASTENGIIFGNIVYDVSGAASDRNCVVLNDIHIDIMDYIVPASCTDTEFRQ

MWAEFEWENKVTVNTNITNLSDYLHHVMNCTNMKCLTPEKALSGECGFMAANMYARSIFG

EDVLANLSIEKALHLGKDAPVQGHVRIRAKSQGMALSMGDKINLSQKKSAKTLAS

>SG-AL_contig_5762_1_-2_2_118

HKGDLSIYRPHLFSISCHANYIQLMHIKTGVEAECLCHL

>SG-AL_contig_5763_1_+2_110_313

MFPVGCPQRSSDLFIFSLLDFFFLLFFKLCQVIFDWRVIGYITCSCFPLLFSCSVVYWST

VEDCISYW

>SG-AL_contig_5764_1_-1_166_660

MADTRARKAGIGAEIDKKLEHRYDQEEQAGTTQAVQTWLNAALLNDHPAIESHSQRDLHH

ALRDGVMLCKMINLLLEREGKPKVHLQKKVSTMFVAYTNIDNYNKGCLDYGLAKEFTVQS

ADLWEGRKGPFLNVINNLHSLGFLANSKGFKPEYTGEIRKTLDCE

>SG-AL_contig_5765_1_+3_3_203

YIYWVEMNKDYVGKEKTTRVLVSTFWGASKWQYLSCQHHQMSHDCIIPSTCVWPVQECIT

DQDTCIW

>SG-AL_contig_5766_1_-3_153_959

MISAMGGKREVRRCVSLVEDRRHTPDNIQSLVDTTRNHMTTFCPISIQIEGCSDDEILDN

PTSQQDSQHMTVTRLQVNHGRTPPCHIAPHNKSRNSTSSPSFRSSPNFLEVPENSDLSTV

KATAVRLNLRTRTSSLLAWQATWLDKSRAPPEFPVPVEAEGSMDKLTEERKKRINHALGW

LKTELREMQSMDQHLARQLLALRHDIHQLRLSRSCQQHQDLLDDVQSEIEEQEELSDVLD

LPNASLNDTPLQHIGVTRMNICARRFSTC

>SG-AL_contig_5767_1_-3_372_650

MKSVALFLTSICKTKNGVCVAMVKTVCSNTEHINPFPRSSVSRVFVIESMIKTIITSTYV

NKTIITSAYVNGTSVIIINSSFDHIFYHVAHQM

>SG-AL_contig_5768_1_-2_959_1129

MYGNFCVRRCRMDPTNVRCFRACCPNGYMDVFSHCAKERVNDSKNSNLSKKNNSKYC

>SG-AL_contig_5769_1_-3_12_755

MKEEEGAHGQQPAPTAVPALVQDSSASEQKQHADSFKNYLQLAHDNAEDGAETVVAESQS

QERSVAQPVLEEAEPPSRIETCQSAEPESQHSATPTLSETPSAQIDSSTEEVVQPSPTPS

TDTKSKTVEPPVASVPVSMESISVVEEEKVDVEERSKVREALRVAVEEEEEECVLKTTEK

GDLVVVGSGHGSPISNTSESKELSDVDDEWEQDLDIELTEEDMREAEALARKLDDNINLE

EDDWESWD

>SG-AL_contig_5770_1_-2_35_709

MEAAREESAEVMAMFTDVHVRQTIADLFAAGVDTSRITLDWTLLFMAGHPEIQKKAQAEI

DLATGPGGRMPGMGDRGKLQYTEAVLYESMRMGCAVPTALPHKTLRDTTVGGYDVPKDTM

VLVNIWAVSNDPKLWDQPEQFNPERFLDDQGKLKTRPEGWIPFSAGRRVCLGESVAKPEL

LLLLACFLKRFTISLPEGAKFKLDHQMAGFSAHSPKPYKIVVTPR

>SG-AL_contig_5771_1_+2_1817_2044

MTLNANARAHTRTHIHTELENTICPLVCNSNSQLRRQDKFAKQQNVTVHDYKYYTLYLEF

TGYKHLTAPCHRITMY

>SG-AL_contig_5772_1_-3_216_314

MVKFIYHELCSIAVFLFSGLTRFCVQCGCLVDF

>SG-AL_contig_5773_1_-2_803_952

MEFFRVKCGPLSPGKARHHSTAPPFFSLFFFCMRVFLFTCDSGFFYRILP

>SG-AL_contig_5774_1_-3_9_3068

MLKMADTFGDELFDVFEGKSDSTESAKQGTSEADSKRADELFKKIASKRLADNEGSEGPF

KKQRNDESSHVEVMPRIQVHKVETVEACQHEVAVPPDQEFVPLHQGGSEPAKEYPFILDP

FQKEAILCLENNQSVLVSAHTSAGKTVVAVYAIAMSLRDKQRVIYTSPIKALSNQKYREL

YEEFQDVGLMTGDVTINPTASCLVMTTEILRSMLYRGSEIMREVAWVIFDEIHYMRDKER

GVVWEETIILLPDNVHYVFLSATIPNATQFAEWVCHLHHQPCHVVYTDYRPIPLQHYIFP

AGGDGLHLVVDENGDFREENFNTAMTVLREAGDAGKEQRGRKGGSKAHESSIFKIVKMIM

ERNFAPVITFSFSRKDCEAFGMQMARLDFNSEDEKKLVEEVFNNAIDSLSDDDKKLPQVE

NLLPLLKKGIGIHHSGLLPLLKETIEILFSEGLIKALFATETFAMGLNMPARTVLFTSSR

KFDGKDFRWVTSGEYIQMSGRAGRRGLDERGIVILMVDDLMTPTVGKAIVKGSPDPLNSA

FHLTYNMVLNLLRVEEINPEYMLERSFYQFQNYAAIPALLEKLKKSEDKFHNMSIAEEEQ

VSSYFRIRQQLDNLGRDLHNYILKPIYLLPFLQPGRLIRIKNKGDDFGWGVVVNFQKKVD

QSAATEETGPLYVVEALLHLSRDSARSRSVSNLKPCAKGEKGEMQVVPLLTKLIDSLSAI

RLYIPNDLRSAEHRLSVLKSLQEVEKRFPDGVPLLDPIEDMGIKEKGLHEVIKKTEAFEH

RLHTHPLHKDPRLEDLYNSFDKKTQVGNEIKALRLELKKKKSLLQMDELKCRKRVLRRLG

YATAADVIETKGRVACEISSGDELVITELLFNNVFNDLSPAQCCALLSCFVFQERASEMP

KLIEELSGPLRLMQDTAKRIARVSKEAKLEIEEDEYVESFRPHMMDIVYEWCNGCSFSKI

CEMTDIFEGSVIRCMRRLEETLRQMVQASKAIGNTELENKFAEGIRLIKRDIVFAASLYL

>SG-AL_contig_5775_1_-2_104_1030

MTIYKTQPGEKKSDKEPEVLTEPLTGVKDESEFEEAVGTAPKLVRVTYFPRRHRTWVNLC

LILTALLVLGTGTIAAILLYRHLSTKVVRGRCGVTYYDGFYHQQPEPQIMADPPPADYNS

EPAGVTELDFFEEDIEVSEVDLYERLTIPKFDEVDETVVWHDFDRNLTAIVDPNHRTCYI

MPLNRTRISPPRDLIDLIVKLKTRYYMPRASVVREQYRVVLPPMNDLTVLGSIIMRECFH

YYTFRLERYVSGVFKRSTDEVSHPMVLTAHLGYFSWKKSDEENMIYKMDVFGEQVVETKD

LQTQPRIAQ

>SG-AL_contig_5776_1_-2_959_1132

MCCAVDRFVLCSGYVCFKCLFLICTDPKGWAEVSECNHKCNPMIVYVARTYLTCNSLL

>SG-AL_contig_5777_1_+3_201_425

MVSVYSKPRFDIYVRHVLHRKSSPSFVRQHSIVTKNAIKTQQSTIHYTTLHYNTIQYNTI

QYNTIQYNTIHFSTI

>SG-AL_contig_5778_1_-3_3_4826

MERLHEITSQAPETPHEAWFQTAYGEMIQNALDRLKNPSNPSHPHASWHLFKQVYISLQQ

RAAKRGSLHLKIDEISPRLAALSKSLIPMPGIGLPGQMVTIEKVCNTVQILPTKTKPKKM

VFIGSDGKRHPYLFKGLEDLHLDERIMQFLSIVNNMFVSASKGQGKRMEYRARHYSVTPL

GPRSGLIQWVEGATPLFSLYKKWQQREAYAQTLKPGAAANPGGSNNTPVPVPRPSEVYYS

KLTPALKEKGMETTDNRKEWPVSTLRKVLEDLMDDTPDDLLAKELWCASGGAGEWWQMVQ

TYSRSTAVMSMTGYIIGLGDRHLDNVLVDLATGEVVHIDYNVCFEKGKNLRVPERVPFRM

TQNIETALGVTGVEGIFRLSSEHVLRTMRKGRETLLTLLEAFVYDPLVDWTTGNEGGYTG

AFYGGEMVSVGGAGEVGKSKKEMEQEITLSMFAIRCAEMAFAWNKNKEETLVAVPELQKS

VELWVSVLDHLEASQEAMDSLLELRGLLSEAASDPSHSIFSLSDRYSDYSMLQETREEVV

KAVEEKMAEFSRWHHLHKVAVESVQGQSFQKLRAEIAKPHDMGPASFTAAKDFLQGAGQS

KHVAQCEQLEGEMSGLLKQRQTLLTSTLDILHSYSAIVSQFGTAFANNNRAFQFLGYLQE

LMSNFSSTKCEEITSRFQETYGPQVSQNSRSQLLLVAESRLETIITDTNAKLVKLVERGG

REDLSTPSITHEMALQEAEASIHTFLQESGPAAEKSLMGFVVSALSSLSKRYLLMESASS

AAGERLMDLTSRDGDWFLEELSSISGNVNHFLSTLERNTTVGTIDQFKELHTALCATHTV

YVAIQDLHMNFRSIILPEALKLMQSQEPSILSALSQLESLVGESAQPLESLVAQLEVMHR

NVVMGIQGDNNDVLTKVQQMQTLFSHLLQGLHQPGPDLVQDGSHKDLSPGQMLLMGFDGL

FTRLDTEFNQLLSCVSNLHIPDAWGKVDAVREARALQLSPWSSETRSLLSLLFFLKRLQA

MQEFFHMVTQLAAALQGLNGGAVYDDEQMAKPIKKFIAEFVRKQMIGFPSQLLGCMMCVL

VDALGLSVESEVALKDVGALAKVPLEDMCKKAVENNLRAGHFLHTHLTQATTLTSAQDLA

WRKLDLAKRMEVGVSLLKGSYQRWQALLTCFQWLHEDTVTNTTHNSSSLALQSRSALMAH

IKKILSSLSAQEVLITACKERYAGLESHVTQRLRWAAGANPSLNTTLQNFQDASAARAHL

LEEESQLVSDVMSICQGVLHFEAWRTRTQEAVTTDTTFMSLLNKCYESCLMLESTSTSVT

AEELQILNIKLLQPGERVDSVWLEAVTDQVKVSVADAEKNVCAQYVELSSCRSKMVEAVA

QLRNLLTAHHKLMSDIRSILKTMAKLEEQDYGDEINPDSIRHYLSSYKTFSENITTALKS

VVSEEASRDTMVTVQGALQDIMVQIPSIYDRLMQFAPPLLQQDQEEGDSPRTEVTRVMKR

GDETSGSPAHRLLMLKEGSSSVLGSPQHREKAGAKKDKVSRDPRTGKAIQERNSYAVGVW

RRVKMKLDGRDPDSSKRLSVPEQVDYVIKEARDPDNLAVLYEGWTPWV

>SG-AL_contig_5779_1_+2_656_820

MSIHNNDNNRNTHTHTHTHTLSLSFSNTHTFCMRNIHKVCFVSQLRHYPVSITFP

>SG-AL_contig_5780_1_+2_2_97

RFQIQPETCLKYKTEGPELSKLHRFGRTLHIL

>SG-AL_contig_5781_1_-1_34_360

MLLENEAFLLELTKLFQKAKSSGSVTLTMKRFDGHNKPRPRTGALPEPNEYKCLIRAALG

NRKISTVINHKDVTKFQMAYGNLLKANIDGLKKKDKKSARGKNKSKATQ

>SG-AL_contig_5782_1_-1_1_438

AADESERGRKVLESRSQTADENIDTLESQLKEAKYIAEDAERKYDEAARKLATTEVDLER

AEARLEAAEAKCYELDEQLHVVGNNVKTLAIQNDQASQREDSYEETIRDLTQRLKDAENR

AHEAERTVSKLQKEVDRLEDELLVEK

>SG-AL_contig_5783_1_-1_745_1134

MLWMWGVPATPVYPELLWPSSGSEARASGVVDSKGWLVDWHPGQVAKPLDPLLCDVMADR

CCPNLLLDLFVSNHVSFGLACCFSEALISQVVIFLSRVLSPGLALVSQHGYKDCTDKTGF

CFHYGCLGVL

>SG-AL_contig_5784_1_-1_1183_1548

MQNASEGAEEREIICTVLEICFSATTPTNTMFLKLSMKQRWHLPSVVGPWPKLSCTHTSR

YGQISVASRSTLHSFLARVFFVHVHQTLFRRDGSLSLKRCLNVPCFAAMFSQLFVEDFGD

QF

>SG-AL_contig_5785_1_+2_203_316

MGCICFTHLFPKYLLLHPPAKKKNSFNYFTTRSLFVML

>SG-AL_contig_5786_1_+2_2_142

DLQHLSITLTYPDNILKSLFSNATKSNRCDSLTKCEIQGYNLKTREV

>SG-AL_contig_5787_1_-3_51_1049

MLSTSRRTFLRTLSLLRRYNSTLVIVEHDNKTLTPITLNTITAATKIGGDVSCLVAGSQC

AKVAEEVSKVAGVNKVLLADSDVFQGFLPEAVTPLILATQNHFNFTHILAGATANGKSLI

PRVAAKLDMCPISEIIGIKDANTFVRTIYAGNAVQTLTSQDKIKLITVRGTAFEAADTGG

SAEMGQAPAEGVSNDQSSFLGQELSKSDRPDLSSASVVISGGRGMKNGENFQLLYTLADK

IGAAVGASRAAVDAGFVPNDMQVGQTGKIVAPDLYVAVGISGAIQHLAGMKDSKVIVAIN

KDPEAPIFQVADYGLVKDLFQVIPEMTELVEKK

>SG-AL_contig_5788_1_+2_275_520

MLSDFGSICGIQCLVHDIVITEHGQGNEGRAQNAQWADSAAAVSNMHIPIVVEKSLPSPP

NLWCLIQMQPLNLHDKIGRAHV

>SG-AL_contig_5789_1_+3_3_206

SDQESNPGTLMRALYPLGHRSTYHGLVGSFDRTMDSRSDVSSSVPTRNIKTGLFTANLNA

SVILVVSA

>SG-AL_contig_5790_1_+2_158_313

MQEKDFKVEELLHSDNSTLLFSEAYIYIPVYIYTHYLYSFTTLWGPAVLHTS

>SG-AL_contig_5791_1_+2_140_307

MAQGISSEAKILADLATQLQALATQPSQVNTVSVKLPEFWTKSPEVWFARVEAQFG

>SG-AL_contig_5792_1_+2_2_337

SSELGVIPLRWFWVNRFVFVNISSEKRKLRRCTSVCDFRLEWYTLLLHHNTFKLIRFLTI

YYCKTKLQIISFAFSLPNVEIKSHHFLFITNRTVSNQTFLELANPLPLSNSN

>SG-AL_contig_5793_1_+1_1_201

CMTSISNCFYKFSERHGLTVEWQYPLVFMNIYNCFSPSVCMCDLSRIRFTNESNKYEHGM

IYMCLQV

>SG-AL_contig_5794_1_-3_69_716

MSDSEDDVPQLSAHALAALQEFYQEQSNVEENMTEALSGNIDKFSPQENWQLSQFWYDEA

TADRLAREALSAAGDRGRIVCVSSPTAYKRIRELKGEGVQAYCLEYDDRFKVYGEDFVFY

DYNEPLKLPADLQNSFDLVLVDPPFLSEECLCKTAMTVRYLMKDKVLLCTGAVMTELADR

LLGVKPTAFFPQHKNGLQNEFRCFSNYHSDLLSETH

>SG-AL_contig_5795_1_-3_366_497

MFPKQVMIVLVCGDLSLIVSSASLSTSSPPTRLLFYTSLMKLAN

>SG-AL_contig_5796_1_-3_642_830

MILLINTYLHASTVLPLGQLMCHSRCSCNFSFHGARSFVPFPPFYSCTVNSTYKKLGCKK

TGL

>SG-AL_contig_5797_1_+1_100_336

MSEDRDHIAETIFNLNYNNNKNERKKDKRRKNVHIYTHSKFIRLEQKKNKNKKKTSKGQS

RKSKSLRKQGTCPVQVSAT

>SG-AL_contig_5798_1_-2_281_1066

MAKDSQSDDCFQVLLARIQQETNTPQSKLQILYSSRGYFNAQQASAVLHALSRPEDKLKA

AKMLEPRLCRMSCQEARDILTAVNIQNDRLIVLDSIKRVLTDYQTVVGEEYVLSAFPFET

DKHMALNILRTVRSDVVDKIGAGGHQGYAAMGGLYTQARPLHPHLYGSLPEQAKQIPGQG

KIEVPLTAQPGVVPSIYTGHPSYAYPADKSYAEDRGYPGTIGFPATVEGPSSYPGGAPPL

GNHSGAPAPTGFPRLDSRGFVY

>SG-AL_contig_5799_1_-2_2_250

SSERRPSHPRPQPSSGVRERREGPPLLSAAPHKPDHLLAGEHWVCSRAGFWLPLSDQLTG

WFWDPSVGHWGFLAGPSPDGESR

>SG-AL_contig_5800_1_-3_474_620

MITTLDSFETLVLPADLKTTDHLAEQLFNSKSISADSFKMVLMRSPLCF

>SG-AL_contig_5801_1_+2_2_220

ETDRQRQRLIYVSVHAVCVCVCACMCECMHVWAHRCIFVIATFQNQYFKPQRAQYKRQNN

HFMHKCNTHIKYI

>SG-AL_contig_5802_1_-2_101_2920

MGPKNQPLVTGVSPKEGPPGTRITIRGENLGLDVRDFIGLNICGMDCTYFAEWKSPSKIV

ARTGPGKGKGDIMVITRSGGKGSCTVGFRGYFLQSGPLQESAVWIDESQTVRANLDHGRP

STTTTLKEEEDPLGISDEGDRPRMTEEELLEIFPEASGNMSLENFSPGWYLLENHHNASF

VDLKAGLDYMKRRASQRSEGPITVVRSNLTAILDCLDSLTTTYEKFAADDIRGNCMDSYA

VLLMQAKSCADGLFQEVLGRKDKADSTRNALSVLTKFRFLFHLPLNMQRNIQKGDYNIVI

NDFEKAKSLFANTDVKLFQKVYKEVERRIANFRDMLHKRLLDPNNTLDEQKKLISYLTSL

ECQGDPAWECLVSQKNHLIELLEDCKDDHLETERTALLADAASSTQGTPASASKRKPASA

SPGAIFAAKGKSDLAAGGWRDRTPQKVMYVEDLTEIMTENFPDFWKLGQAYFSGEFNRTR

EIPEKGFKSDTSKHSKLKQMIAELVGLFANFVRAAFLPESLENLPDIEHEKFGLWPTVAK

SMVAGTWLPHCVRTIRSCVATLAGLDLTGDSMIILQDLAFDLRTNCMFTLLKQAISDVKS

LCNKQTWAVETDDESGGTTQLPALFENIVSETVQHLHEVVVLNKPGEPEIFSQRPIQKEA

TVLCTQLLQAFAHCLEQLAFSPPLHRQRHDNRSAHLPKILAEREAEMDDDEEHLPSLDKR

LIIMLSDCNHTLEVVIPRIIDNLNKHGYVEMHKADEAAKNTYKQLDQRLFDSYIEQKSDP

IIGAMEPCMYRGGFSWKACPAPTGVRNYLKENLMSMIEVHAEVFSISPKFVSRVMEKVTE

AVVEEVTRLIHCVDSFGAHGALQARLELMALRETVALYSTKNTDSCFNDAEKCIPALSKE

GNQMLKELLAKFKSRMKFQLMCFGAVALETKTNKGVHSTC

>SG-AL_contig_5803_1_+3_300_542

MVPFFFCTPNYFSRRHYFFFVENRIFCCENLRFGRQFPCLIKLCTTRLATLEQENEEREV

MIDFNSSNILRARINEWFIEI

>SG-AL_contig_5804_1_+3_3_119

YIYIYIYIYMQMMLQASSHSQDITLNTPVLINFSYCCPN

>SG-AL_contig_5805_1_-1_13_291

MHTASPPPQHTHAHTHTKSSKFSKSFNPNGSFRKTATKRTHTHTHTHTHTHTHTSSKQTM

SALYSLLILLRVVSASDNDLGHIWISTNSFFHL

>SG-AL_contig_5806_1_+1_1_315

SGRLLRKCPSPARLLGEPPLRERLSGAPAPGPSIQGAVCRGGGKASSDAGKLKKESLHYH

TWTRAVSLFPSLLRLESSPPCGDSVVARCGGAKPNQGLGCRWSRR

>SG-AL_contig_5807_1_-3_45_320

MCVCVCALTRPSVLEKCKCTLALTLKEFDDHGLQTCHPFVHGVFFQLALIIVFMVKCHRS

AVHLDHDQKKSSYVFTSVSRFCSIKKFQSKKK

>SG-AL_contig_5808_1_-1_289_690

MLVLDTASSFRNRHSFCCWRCFFILFHFFCHNRFTVRNLDCSSWGERLRQCRFCLFQICH

VNLEMGSVCYLFVCCLLRFWIYCVKTGLLSFSRKSVYPQCNTNLFFLFFLNCVQLSNLLK

TGFFRRFLFFQSSF

>SG-AL_contig_5809_1_-3_3_224

RSNVSSSVPACNIKIGLFIAHLNAPIILVVSLSQRRPPSLPPSHFKILRIGFYAGLRSLP

DYYWQIGMEHMWNC

>SG-AL_contig_5810_1_-1_58_2022

MTVMLNTSDGCILDHDQALTSAYSLCIMPASFSLSQLYTNEEKECRNHHTRDESHQCSVD

VLSSHTQYSESLTSKPQCKLNPYALSAEKYINLEPPCKSNQIIACTYELTNQENLTDQEK

VSEAGCNLSDANSGDTAWHHRGHECVRELTIDNFTVISRRKRENLSPECIVNETKGRHQV

LIPDSKKQKNSIVMCDSLTDEQNNVTDCDSSRNEQKSGTAMYDTSTDDQKSKITSNRSTF

SNSGPEFDQWQSVPLEACKLKRLSLRLKHGFFTVKVPFMMDSQFEKVKTHFSQEEVINGE

CRKHIDVADIQDMIHSKQRKRRLEKKARSSEEERLLTVHSSLADLGRKQEIFSKHIPPPL

DNNLLSRQQAQLDLVGDIFAPLLMSADGSPLLHMGDCLQPLVINTYGSGSFCGSELIHRR

VSHENTEACVIDIDGAGYALPPKSQFLISNLQDFCDHLYLELEEGGYDLIVLDPPWENKS

VKRKKSYDMMNEDGLQQLPMARLVRPGCLMALWVTNNERLEDYSLHTLFPLWGFTAVARW

HWLKVTQTGDLISSMEGHKKPYEVLLLGRASRGGSSDVTKERKMEPPESRVVMNVPCSLH

SKKPPLSEVLQPYLPANPRCLELFARNLWPRWTSWGREVLKHQNLMFYEVMDHPC

>SG-AL_contig_5811_1_+3_183_404

MKSTTTEPLCESNILMAFLSVSVNLAAPEIPRLENDNTYKSGFRPISYTFEVTNGSEWNP

RFEIKLNLAMEDWI

>SG-AL_contig_5812_1_-3_69_476

MASTLLRTSAGVVRRTIAYSTVRAASGGGPPIDHQKGKVASDQVTMPDTLGHAVGIERYE

LLAKLAGNEDPFEMNVKKRAKGTVDEPTLIPSLYEKRLVGCICEEDAVSINWMYLHKGEP

RRCECGYWFKLSDLPH

>SG-AL_contig_5813_1_+2_8_376

MERERGNLLCGLCLPETWREREVTYTATHGRHLSQCSDQQPVTHRAERGSGQYKPQFLSI

YWPPSVLKDYGICAAGETAGHKPGQSVKENLLLPMQQAHPFHNTGKIQVSGKSQYSLELV

KGE

>SG-AL_contig_5814_1_+3_3_173

QFTFLQICVFFKLVKCHTGFVILELVLTILPHHHFICGGLQPFLLIQEGMLSPFWSL

>SG-AL_contig_5815_1_+1_616_864

MQLQSTVYSNNRLVLCSILIHIGSKQQEAEASLSGLKTEIPQKFERTEDRNPIMDLSLSL

LPLSIPAYYSIPSSLPIPSPHFK

>SG-AL_contig_5816_1_+1_1222_1590

MTDAHRKAEHHSSKQLQQSICCLLLAPLASFSQSLNSSAFFFSNRYCPSTMYAYSKPASF

NLPPKTSPMPASSVTGLSLMGSETKFLCFCDVEDMFLYLAMAKSSARHVTKLEFCVYNNL

QQT

>SG-AL_contig_5817_1_-2_2_226

ETSTVEELLLLRSGTRCLSLFDTVCHCLHSNPISKCFFSSSPFSSAHFNSPALLMYFCRM

CCVTGLCFGCVCVRL

>SG-AL_contig_5818_1_+1_40_255

MGHNSEYTSPFFPPSLPPPLSFPHKCLHKHVDIAHNIGSYVFNTYTMTTQNIRCTQHAHL

DIQQARCLQHIQ

>SG-AL_contig_5819_1_-3_3_161

GGGGGVVCLYFCIAPLSFSTSPHRMCSLIWLHIYYAALVLCLNCCLTSERGCK

>SG-AL_contig_5820_1_-2_44_190

MTQSFHSQPKWPSSDSGKLSCGPRAHTGTQTGAVVKYGLNRKQPSMQSE

>SG-AL_contig_5821_1_+3_3_269

KRRRRRKKKKKRKKKGRGGGGGGGGAGPHSTVAMHFPQGWQLKRDTANLFGRERYHGHII

LIRQSLIVFSVLTRKIPWTSRPDTVMIVL

>SG-AL_contig_5822_1_-3_18_209

MTQSFHTQPKWPSSDSGKLSCGPRAHTGTQTGAVVKYGLRNVEDINYNGKFSETVHLYKA

WIED

>SG-AL_contig_5823_1_+1_1_237

SSLYPPGEVTKVDSNPGLPNPSSLEVECLHHSATVPVVVDTTLHGEIISLVSGTVFGGRG

GCVFTVNISALLEDSACFK

>SG-AL_contig_5824_1_-3_654_896

MLFTLNLCDISCFKTSACCNVFRSMVHSQCPFSAVGILSDKQFQVSTVPKQLVFQSLYCV

CCKVSHFSASSQNKFSQKWAG

>SG-AL_contig_5825_1_-2_146_346

MCFYGIFFKIGVLEIGVFISASSVIHCLLGCSSDVELSWMDSNFCSPSEKYGLCVLSFNS

LHAHFFF

>SG-AL_contig_5826_1_+2_788_922

MVQSKYNYICLINIGVLTRHLYFNNLVVVNLELHQGYPNIGILRV

>SG-AL_contig_5827_1_-2_104_235

MGTGCPFCSVICLYGLFYVCVCPFCSVICLYSLFYVCVCCFKVF

>SG-AL_contig_5828_1_+3_3_206

FFFTLNTIYFDHILTQAHTISFMIYTDIFHQSVSELRKLTSKHKRPSAEEASVVSCYHAA

TATHHNRS

>SG-AL_contig_5829_1_-3_117_3980

MRLKEELANPESADIVDLRLHHQQIGRALQKRCVALRKVKQDLERGSQSSVAVSLTGENV

VELCKENRTLLDTLVSGNSMLEEIEKQYYAGFTHNDTVLTQSSASNLHTAPSQESDLNSS

ADVAALKAEIQQMLDVIRVKDSQIVDLKQSLADLQHQYAASRQSSGSLSESLLQLETAIS

EKDSELKILHTENDQLMKVCEESKSKLAESDMKMVSLKSQLQETESALQQVQEDLDTARE

TLEHLTEDNALHLEQKKELDSAKHKIQQLETELSQAIETLSSHKAGIEELNSKYSDQRKC

LEKEQAKVTKQEDVIADLRHTSVSLDEELGTVKWQLSQAMKYLSEEQRAMIMEESADFAD

TKSPLPALEYVDKPAIELTLNSKIEQSGMQNGEFVAQDLDEKSDLSEKHADLVRQLEEKE

EIIADLQQNNTSLMQMLEAKSLTSQSGDSTQLEVHRLQSEVKALKVEKEQMLAVMNEKSR

EASSLKAEVMRLMSVVSAEKSALDKLQKDNQELMSRSSPPRDDSLEDMTREAMHNLSRLV

RDREVEIEALKQKNETLMAVLQEMNQGEGATKGAEGANSSGSTLASQLVDKENLEKQLSS

LQAEREQMVAFVNQKHSESVAYHAEIQRLAALRNEEIQRFEQLQADYDALCPQFEDKQQT

LLKAQNDLINYRQKYAELEVRHGELLQRSNSSETVDAASFHEVKEEKERLLSKHAELQQD

IQTRGEKLLATTDKLKEVENALLARDSEVSALKKQVDSLHFQLQGLRNETADLKQEKQAA

EQHFTEWQVQGQTLKDLNSQLTLSVREKEFEVASLQERIQKLTALVSEQQGEKTQVAQLL

RDQEEASGLRASLQQEVDQLTLAMKQKQQQLSDMHAENLRLKEKEQKLSRELERLRSHLL

QIEEGYTREALEAEEREKVLRNRLAHAEEQLASTASQVHSASQQASQQVESLQQQLHSIA

SQRDAAYMQVASLQDQCQQYAASLSNLQLVLEQFQRDKESQIAAETERYQEEARQLKEQT

ARLENELQQAKVELEEASDGLAAASRLSEQLDRKEEALQAMREEVHHRETALRNAEEEIQ

KLTSSKEAKVDRELMKNLLLGYFHTPDRNRMDAIHAIGSVLGFTQSDFHKIEESEGGGWM

SSFFRRSRSPGSPSTTPTQQTRGAGANQSFSQLFVKFLEVESTPPPAPVRLPAEEMAREA

QQRHHVKEWDARDKPAFNPFTAPRHVSMPIPVGAAATAVASSGSHLLMAPPPASPTATFP

VFTPFSTSDAATTPQNSSAILKDVLGSR

>SG-AL_contig_5830_1_+2_1031_1285

MSPEKVPCSLDLALQYPVSSVLMSYDRIPLLSSAHDSMTCLSVGIGAHYEQGLFDVFYPI

LLCFLISCLNVYQIPLQPHSLNLSR

>SG-AL_contig_5831_1_-3_18_479

METAASFTFLTVLLLAVFQYGESIMCYRCYSAMGGCSDYVKWPMYVWRECGAEFCVKVIE

KVPGEEPRYLRECEHNLQKSARHRLRMPILRRHGYCLPARQNDPSNPLELTDSRFTYCFC

NDWNGCNTAAPRHGSLPLLGLLSGFVSLLLWRLL

>SG-AL_contig_5832_1_-2_2_112

EEIKGLNLCIYLLVCLVRHLVVCFLFYVCISHNANLY

>SG-AL_contig_5833_1_-3_84_1136

MDGNLWDLAIHQAKLSKEEAKSSEDSAVFLLGTKNAGKTSIILRFLDRDEAPKPTVALEY

TFGRRAKGHNVDVGHMWELGGGTWLSKLMDIPLNPDTITHTTLFLVLDLSQPQELWFTLE

TLLRMAKSRVEAVIADMKSTDPDLKQKLQKAAWERVGEENPDKSMMEPFLIPMVIVGSKY

DLFQDFDSEKRKIICKTLRFVAHIYGATLQFFSTKQEQLVSRMRALISHHLFGTTPSKSM

QTEHSKPLFVPAGLDSIQQIGSPPISDKDIGRVQAKTPFELWKVAFIGHFPQEEKQKMSD

PSMVEDPSKDPQYKEHAIDSLRAQKDEELERYRRLAERRAKEAENNQYAAY

>SG-AL_contig_5834_1_-3_519_728

MQSQSNSHAQTLKHTHEYTQTHTFFLESHNYSSKCLQCTLQIWTNFCIILYKILKFFCQH

MYHCQHSICT

>SG-AL_contig_5835_1_+1_1_285

TLTGKTAANAFAKAYAQESNISVPRKMEKEIRQEEKERRNHEDTHDAMKKDLTIAELKKA

IRQLKKKKSPGPDNITNEMLQHVGNIAQQKLLDIF

>SG-AL_contig_5836_1_-2_2_265

ERYEYAPQNVKFEKINIGDPVYLTKYQRANKLEDKWQPYYHVVEKLTPVTFKIRNQLDNA

IVKVCADHLYVAKLDDWPLISVEEEKDQ

>SG-AL_contig_5837_1_+1_1_126

NFHNTVVTWKENKKNNEHVYCVPSLAYKRSHVCTHKCMLTEF

>SG-AL_contig_5838_1_-2_107_754

MEKPLNCTIKSPVSLTVGEIKVDMLCGCKVIDISFPNIMSPEVGEIRFKNYYVAILTVKA

KFRSDQTPRESGEQPKWRTCLKRVKLMPDPHSETGSHDFFSFTNRQFRCELNNVMSLRLI

LQQPSPVWREFRLEDLRLFRSPGGPKTSPLPAWLMDEPKDKFQKKIVEGVPDLEGLSANL

QQLWALAEEVSTSQTSQSLGRFEVDGCYDINLLAYT

>SG-AL_contig_5839_1_-3_9_1160

MAVPRKLICFRFPGEQAVRLQKNAVALFKGGERHYASLSKEQKDKLADGPGLEDFIRGDV

IPMYEGQDSIKRVKGQKLRLPQWLKREIPMGENYSQLKSSLRNLNLHTVCEEAKCPNIGE

CWGGQSGTATATIMVLGDTCTRGCRFCSVKTAKNPPPPDPSEPVHTAEAIAQWGLDYVVL

TSVDRDDMPDGGAAHFAQTVREIKSRKPEVLVECLTPDFRGDLTGVDAVADSGLDVYAHN

VETVAELQWLVRDPRANYQQSLKVLKHAKQGRPHMVTKTSLMLGLGETDDQIIEVMEDLR

KIDVDCLTLGQYMQPTKRHLKVKEYLHPDKFQHWQEVGDKMGFAYTASGPLVRSSYKAGE

FFIKNLLKKKCGDASETDTESHSA

>SG-AL_contig_5840_1_-1_478_1101

MSLAGFFMNEVLDYKDLPQKVTAVSRCFRAETSDIAEEKGIYRVHQFTKVEMFGVTADES

GGESEQLLQELVDIEQELFVSLGLHFRILDMPTEELGAPAYRKYDIETWMPGRGFWGEIS

SASNCTDFQSRRLNIQYRDQEGNLKFAHTVNGTACAVPRMVMVLLETNQQQDGSVVLPQV

LEPWLGTTVLTPPRHKDPFIRLHDRRKR

>SG-AL_contig_5841_1_-3_1248_1481

MSVNYFTHHKMQKLSYLLYLIVCEKKNYAFYYQHIDLKNYAYTNTISSDLFITTGFTDTS

VHAYKHVYKHGPYSSQAR

>SG-AL_contig_5842_1_-1_28_228

MACPLCKDTVENEIHFVLCCPELYDLRKELIPFKYHGMENIFWLTLLLSSKNENILRDPS

LYLSLSI

>SG-AL_contig_5843_1_-2_2_331

EILLCFLSIVLSGCLHVCVCVCVSHTHTHTNINRENDQLSLSLSLSLSHVHTSRYKDNKD

EMACPLCKDTVENEIHFVLCCSELYDLRKEFIPFKYHGKENIFWLTLLLF

>SG-AL_contig_5844_1_-1_28_258

MAYPMCKDTVENEIHFVLCCSELYDLRKEFIPFKYHGKENIFWLTLPLSSKNENILRNLS

LYPYKAFKRRQSIMSMN

>SG-AL_contig_5845_1_-3_513_824

MNDTLSRSQFWKKCLNNSEYRLLVWLDHLCFVCVLLICFCGVCVCVCVCVAVIVGCKAHG

GCKETVVVVMKHPPPVSVYVCFALAHTHTHTHTHLCIFTCIHVH

>SG-AL_contig_5846_1_+1_1381_1671

MDVSLMSCATPSYNVSLHNLWQQLGLCRSRQSDLLSFHNILQRFGLDGLGLKVSLVLMTS

SKIALIPFCVSEEHSKYFWAPILCANALPWNRQKVHC

>SG-AL_contig_5847_1_+3_1422_1685

MVLKYLSYMFDLAYHQGKLPDFHLLDTPRTFPLLLTHAYNVTPSTRTVNAISFSIPHPSP

LFLFVMFFKAPHSQNDSKRKQYVHYNDE

>SG-AL_contig_5848_1_-1_1297_1767

MWWAGQLTLRFPCQQLTPGFVSQNYVVLSLSKVGKLCCVLCVCSLCYKAGPHFAFDLKKS

ASCLLVGCTGCFVLPELCIVYLVCKAVCYGAEREVSWRLPTAVLLGHGTGPVGHVGWFVS

GVLWSHRMSWLVEYCGHHCANEISTTTLFGAGSQSCR

>SG-AL_contig_5849_1_+1_1084_1410

MQMYMSCYKLVCVCLFGFFLQVCKIFSVLSCNCSHALLLLSCICICFLLKCILKKFERKI

IFLNPLFFLSLPHPLALRKCLFASYFFSKSLITHSISQNGYQLSNNRNQ

>SG-AL_contig_5850_1_+3_90_275

MATDKTCTDCAVGFFFAKYDCINISVCDFMHFYYLKKKERLLSYFRAILYDFKRFKIVLQ

DL

>SG-AL_contig_5851_1_+1_1_237

SIDHIFWKCLYTKRFWERIEELLKSECVNCMNVKLTENMVLFGIDENMHMDSVFDLIILL

GKMYIYKCKCEKTAKHNLF

>SG-AL_contig_5852_1_+3_162_269

MKIHKIQEIKLKWLQIRIVHRILGFFAVTSVWKRQY

>SG-AL_contig_5853_1_-1_1_261

DTYIHTGGGHHVQLDTYIHTYIHTYIHTYIHTWHHVQIDTYIHTGHHVQLDTYIHTGHHV

QLDTYIQGIMYNLIHTLGTSCTTQYIH

>SG-AL_contig_5855_1_+2_44_349

MLYFLYSNKKQIKIQTNKTKTKIRIKTNKNSNKPENKSSQLLITCICCASSTYIMFHNII

MRINQTSGTIIIQTCCISFFTSMESQSKGPTFSSNRTILSKV

>SG-AL_contig_5856_1_-2_977_3373

MASLGQCAVKGNVGHGGRGGGVWWLLLVELCRRWWWWVWVVSFAAVWCFSGVSATAAPTA

ITKVTKLTVSAGENKVLQLPDSSITLSAFVLPKAKEDEEYHYEWSPVGQPEGSEERATMQ

GKNTNTLKLSNLIAGLYQMKVQVTADNKFGQAFVNVTVLPPKRKNKPPVAIITPASQQVK

SENSFILDGSDSTDDDGITAYHWEEVSGPLQEHQIDDDTQTLKLKDMAPGVYVFRLTVTD

TDGATDSTSANVTVIKETDYPPKANAGSDVIIHLPKKSVVLCGNASTDDKGIASYEWIKK

SDALTADMTGVRTVCLQLNNLELGDYTFTLKVTDTGGNTRTADVHVYVKPETNRPPVAHT

AGEITVMLPSDSIVLDGQNSTDDKEVTGYKWTQTGGTNSLTISNLNQAVAVATGDIAVGD

YEFKLTVSDEEEQTSSDKLKVHVKMNKNRPPEAKTGGDRVVLLPVSMVSLDGSASTDDRG

IASYLWERDMESLAAGDVVNQSDHQAVLQLVNLVAGRYVFTLTVTDEQGLKSQDTASLLV

KNGETYKDLVELVLDTDIHHFTQDAKTNIVKQLELLIHQSSQHSGTVVEVESTQLELTKG

QLRLTFFVKKKDGSTVSGVDMVRLLKDKLDDDVILSYSIVSLDTVLCQNNCSGHGQCDQR

TKLCHCEAFWMPNVIKAAYRHDSNCEWSILYVVIVCFIIIVTAIAGTWGVICCCRSKRCR

IKTRKRHRYSLLREVEDEDDKTKLDLMPKGKIQNSSVMISESDFSSEEETLFINSKPANG

HSKPPNGIVSRQHRNKLKT

>SG-AL_contig_5857_1_+1_1_210

QRTTTQYNTIRYSTTQHNTIQHKTILNTIQNDNTTQHNTIQYNNHSHIKKKEEKHRSLKK

TDEDLYALLL

>SG-AL_contig_5858_1_+3_393_596

MLESTYMHFFILSKAHTLAAIFTPCLSVCLSCTNAHAHSSTHSGPHICSIHISPDQYNKC

CIQCLAPF

>SG-AL_contig_5859_1_-2_2_259

AVRCPGPAHARARSFFLFPSGHVSAASRTPTSSPSTPDFVSFQAPLCYIHLPLGSHACST

LVFCVISLPGCALPSLIAQRPATTIW

>SG-AL_contig_5860_1_+1_1_156

FPTRRSSDVFNFTIKQIIQNTMNHDLSVPRLIRLYSSFLCVTLYAKKHKGNI

>SG-AL_contig_5861_1_-1_1_645

QREIDRFVNWCNENHLELNVGKTKELVIDFRKKKPGSSKIVIKDGVVEQVDTYKYLGVII

DNKLSWNENFTALTKKINSRMYCLRKMRSFNVCKQMLQMFYTSVVCSVLTFGAVCWGGNL

TKHDKDRFEKIIRKAGGVVGKRQDTFSDMYSRKMKDRFMIILADETHPLHDDISSRRSDR

SVRFRAPRARTSRYMNSFIPTAIRAFNQDVQYSQR

>SG-AL_contig_5862_1_+1_1_93

RCVEHLEQMYYCTKVSEPLNELMLLNKDSSV

>SG-AL_contig_5863_1_+2_44_271

MVQRKSTNLYQEYKHFMLDVLSQKHLKCTAYYHSTYRPDSHTNLLVLNLLAYIKLNYLSL

LEQIRIIQKLSNGKGH

>SG-AL_contig_5864_1_-1_1_114

IGRWKLYLYNQASFSVTCCSKSHSCLFSISQTLIYDLH

>SG-AL_contig_5865_1_+2_2_70

GNIKTDILTALKCALENILMSIL

>SG-AL_contig_5866_1_-1_19_240

MGQPLPLSVYVGQNSIATYDRTVWYWMRLGESKLIRNMGTNLFVKNCNHPSPRHLIFVHD

SKHSGFGNLYFTAW

>SG-AL_contig_5867_1_-1_247_465

MCVWAPVYPAVNGYLTFFKVREGEAGMERRWALPSYRWPLEKLRPLPPHSPTASDNLDLF

YHASVVCVCLQVC

>SG-AL_contig_5868_1_-3_174_374

MILPQLMKKGYSQNLHVTCLVSIESLHGTFVKPQTENGLWFFICGHTNGLLCNWLLPSHW

RPLAGVT

>SG-AL_contig_5869_1_-2_122_1012

MSVLIGGGSGFVGRHLTKLLTDNGMKVTILSRAAGPNRITWSDLQRSGLPDGCTAVVSMS

GENILNPMKRWNESFKQDVWASRVDCTQTLAHAICQAATPPKVFVSMSGVGYYKPDTSRE

YTEESPGGDFDYLSELCTAWEAAAKLPSTVPVRQVTVRSGVVLGRDGGMIQQIYYPFFFG

LGGPISSGQQWFPWIHVKDIVGIVFHAITNDSVSGVLNGVAPEPATNAEFTKAFASAMCR

PAFFPVPGFVMNWVYGPERGKVILEGQKVIPKRTLESGYKFSFPDLTSACKDLSSGS

>SG-AL_contig_5870_1_-3_3_368

LFVDDFALYATGKIFAGVQRQLQLCVDKIQKWAEENGFKFSATKTECIHFHNLREFFPDP

EVCLQSSKIKVVTEAKFLGVIFDQKLSFKPHLQYLKTSCQKALNVLKVVAHTDWGADRRT

LL

>SG-AL_contig_5871_1_-3_3_224

HLNLPLLLRTSASQSSTKHRLCLRQKEKSHRPRVSIRHQPDTTKQIGTGRWGSQQINSDT

AARYWVSVLDRQRA

>SG-AL_contig_5872_1_+2_2_136

CGMVCRSDCLILCHSHVCGMVCRPVCLILYRSHICGMVYCVRMPE

>SG-AL_contig_5873_1_+1_1285_1551

MYLQMKFLQLWLCFQPYIAMLLLLIPAAYLLFTADVSEQFANFRHCLKPHQTPPLWAICG

SCNEQHGNSFYVQQVLRKFPSTTGRWVFC

>SG-AL_contig_5874_1_-1_121_849

MRLTVVAFSYFKKYVQREIAKATGVSVNKPLQGWREIRKKKWFYDENRPWTDAAKAANAP

NKRHRVPLLEPIADIDWTVFKGDRVEVLSGPDKGKLGIVNCVIKERNWVFVEGLNCKYKW

VNRSATSTGSMIKTEKPLLVTTEVALVDPSDGKGAQVQWRYTEEGQRVRVSLRTGRVIPM

SRIAEDETEDMVARSGYAEQDCDTKETDVQKVTFKPTLSTFDEDILKQMNIKDDRKRAPT

YWY

>SG-AL_contig_5875_1_-3_363_617

MIQYNAMQLNTITFVNTMHTSLLIHNRNLQDLSVCNRQTIKHTPKHRNESCYILTFLFNI

CEPLVTFNLCDIYDRILHIRSLKIL

>SG-AL_contig_5876_1_-2_281_562

MESAKSVSCFMLPIVNLLTEACHVGEARINSLMQLCYSLTKSGWLINCLVCVLNLKEVLH

EEHLLHILVISSAVIAWIKFFNGDYEKILYCLYV

>SG-AL_contig_5877_1_-3_111_566

MHREGIVFSMLPGESSPQGSPPPNLSFMEVLCEFTNKLMKQDKKTVLNYLDKHLTGGLPA

NRSEEWQPLLTYRNSLVQGEEQVNPITLKQASAKRYGKRSKADVDVSAGSFREVATPGPM

TTPQMTSTAVKQRRIEGDDGSEVGSEADYDDA

>SG-AL_contig_5878_1_-3_600_995

MPALLVAASERQKWRILSAEASHESSPTPTLVMGLEEEEADHDEIICHLPHQNIVSFSRQ

VSWHETKSSQSIFSLLDLQKSLNNGHCFRQPARVAEGCCANERCSSHLHLSRLRVSVWVV

AMFSLISKAREK

>SG-AL_contig_5879_1_+2_2_163

FFFKKMCESFINRVDTMTRSKTEQIECQQHHHSTLTKKKDLKRCSHQLNVSVTT

>SG-AL_contig_5880_1_-2_47_322

MKYVKLLGDACVIQCSYFYIYTGACMHACVYVCLVEVILQLSSYCFFLYWFVTLGCHRCR

TPPHLVQLYGARLANLCACGLQFPCCEWVPNP

>SG-AL_contig_5881_1_+2_146_670

MAEVHVIGQIVGASGFPDHSLFCKWGINTGGAWKILAGLREGQTQVDNPQNHEAAFFGHP

IDIHFATKGIQGWPRIHFQVWHQDQFGRNELYGYGFCHIPTSPGTHEIDCPTWRPTGSFM

ETVSQHFLGGGAQLKNPDLIYSGADRYRLHTIAMGKVHLRLGVILRNFDKFGIEC

>SG-AL_contig_5882_1_-2_53_262

MDFNDGHNQKEKVQNGRFLEWLDILLFLIQRPVQRYTWISNCEMIISLFGAMLLHTLTQS

NMNIKQCFCL

>SG-AL_contig_5883_1_-2_161_2104

MVSPTPAGDEDGGSVTPQMRSTPSVMEKSTGDISAEEYKARLAEKRRQAREKAEREAEEE

RKRQEELERQEEERMRQEEEEQRRHDEEAMRLAEEARKCEEERLQKAIEAEEARKAEEAA

RLEQERLAKEEAERKAKEEAERLEQEKQEKARREEEERLERKKRLEMIMKRVKTESPAEM

TKDSSKSVTSSPSKSINSTASSDTSPTDPENKTVLAMAAHSAAQDTSAQPSTPAVSQEEV

DVQDGAVRGEDESQAPVAAPCPPSPVMTSDTQPEPDQVPDAMEKSGVVEKSEAESVAAEP

VPAVAISPAPHAEGGGDRPRFKSPLLQKLVEGKDDAGSSPGGGPKFKSPLLQNLLGKSRA

RTMVQDHSASAPNLSQMDTSSDQQADSDGPDVMSSSMVDVGAGDSSHKEVLNRNEEKDVD

TSEDSSSSSHSSPSHVSGKTVVSNGAEEGASIAVSEPSPEDAAFIVQPEPMEISTPMESS

AVAPIMATSTASFWSQQDSGVSFGSEDLRTSAAPSGSLQVSFNGHGPPRAAQGQALEDSS

ISMRSMESVTSSVTDSAAGSVFSSVSSLPQGQGLSGGDKDFEELIDLSVNNKSATSTTTT

SARTVLDYSEDLANFNKLEEGSQETTTGPPPPIIAFEDRRQDVSDLLS

>SG-AL_contig_5884_1_+1_64_177

MKPHALTLMQSHFAKKRNTVRASFVCCYEISFTSEKAL

>SG-AL_contig_5885_1_+2_209_403

MLETLFNPCTLLKVWDQETELKCRRSCGYQVLREEGVACPFYVSNLCSDDKTFLYIWIVY

FQSCQ

>SG-AL_contig_5886_1_+1_142_273

MSSICYLMCMRKDTLKINYPYSVTIDLMLSPVSYTPSQHTTHTH

>SG-AL_contig_5887_1_+3_69_326

MIRPSLISLRMFWRELAFAISFVSLGSNQTLFLPHLSTAAESRFCRRRVLMVAYPHVSQQ

RKFDVCDRIRCMLGVASDSTAEKRFC

>SG-AL_contig_5888_1_-1_40_1764

MASGSGNKPPSGGESTPLFLPIRYRMSTTDSASGATAIPATASADEASASGGAGGDAGSQ

TNRSIVVDATFFEEQFLRCQVCQEKFHLTERPPKSLPCNHTFCVPCLTQIFNHAQPQSRR

NLLWADESLDGPLKCPTCRVEIFVSKNKIKDLPNDHRVVQMMDFLSQAVSTTQNVCNKHE

RQPLNFFCKKCLKPVCRDCTVLDHKEQQGHAIVDVKEALKDRSGEFNQIEEQCRQTLERM

KNRSDALANASKRLDIQERQLRNEIKETFIEYRLLLEKRQEALTHALQQRTRDQKALINT

RFVHVCEHGTQLQKLFDAFSKAKGSNDVEQLFNLHKEMKDHEEQFNSVAAAEDHELFKSC

QFESTSEGEFLSGISSLGEVSDKDDHTLKEPVSAHQLLYLDNEDRRERERLMQYEMTGAD

QELEDPEPDVMDDVIEYRPGRHRPDRERDEDVEMHRLLAAYRQLAIADQSGHRTSREEME

QEPSGPTISRPVHPRRRPRTTQLPTSSVAAQLGLFTPMGLPFLPESEDSNGHSRSGSRSR

RSASSREGGSGSSGSGLRGYRVERHAHYPATDDSN

>SG-AL_contig_5889_1_-3_3_173

LIYTHSPLTQTEPKNTNVFMIKPYHIFFFTYLFMSKLKHSLRSHVLLEVHLSMLFFW

>SG-AL_contig_5890_1_-3_645_821

MGTALIMLTLRKVEVSSTSLLMILGIWDKITCCCCCFFFWGGGGGGGGLKGGGGKEKTH

>SG-AL_contig_5891_1_+3_1605_1913

MLGRQCWVDNRHKSFLRRFRYKNNDYTFHSCQYRFQKVTISTTIKSNIHLNITIKIIRNK

NNYESGCLYFLKIFSVTRRMVTSKSTTTTTIIHSRQHQNQQQQ

>SG-AL_contig_5892_1_+3_204_305

MKLRYNAKPACWGVGCLSQSVKEKFLKSHRNYGK

>SG-AL_contig_5893_1_-3_882_1202

MVSKNTCRHQKKGGAALWRRALPRKLVQNAHSKVVVKRNKQNTTQYNTKQCNKYNTTQQN

TIQWNTLHYNTIQYNAMQYNAIQCNAIQCTTIQCGAIQYNTIQHNTM

>SG-AL_contig_5894_1_-3_63_1541

MEILRSPWVFVTSLVLLLHIVLLHTSTASRPHIVVIMADDLGWNDVGYKNPEVHTPNIDK

LAEEGVKLNYSYMQQVCTPSRVAFLTGIYPFRMGLQNLVLRALENNSMPVEHRVLPERLK

TLGYATHYIGKWHLGFCHYNMTPTRRGFDTFYGMYNGKGGYFNHTSKWSGYDLQDNKGTD

PQNFRVAWEDYGKYSTHLFTNRAVKIIDEHDLSTPLFLFLSYQAVHGPLEVPEGYLQLCP

NVVDETRKRHCAVTAAMDEGIGVVMQALRARGLSDNLITLFTTDNGGPVRLGSSNWPLRG

SKITLWEGGTRAVSILHSKTHLPYAPYEWKGLIHAVDWYPTLLQAAGDKSKIADIDGVDS

WKKIVRNSAKSARQGFVYNINDVKNNAAIRYNRFKLIYNKGGNPDGWYNPPGNGASEEPN

GSYSEYMLFDVEKDSEERNDLSGVNKEHIVKTMAKLKKQLATFRQGLKPTTRAAVIAEGK

PRFHGGAWVSGWC

>SG-AL_contig_5895_1_+1_1_288

ADDRKGKENGRNKESNRKKQMTGKERKTAETKRAIEKRKLLAQNFQKAGVLFILKEPPAS

PRGMHMRNLYSKSQFNTEYQHLSLSLFRTLFTSGKC

>SG-AL_contig_5896_1_+3_3_143

DKLGRARLTKQLLQWNASTRILLCRAYNSQPTSTCNPCQLAQYKHAS

>SG-AL_contig_5897_1_-1_1069_1305

MRVHVRAHVRTHTHTPPSLIPSHHHHQVNIIVLTLTHTHTHTHTHTHTSPSKLSLFCFLF

VYLFMDNLKTEKKRLLANI

>SG-AL_contig_5898_1_-3_411_653

MVEIFPISQVNICADLLVPEPPSSENAFKSSNMHVKDPVSHVRVRWLIETRINTACTKKE

KKGWRCTAAKRSPRESSPNFT

>SG-AL_contig_5899_1_-1_166_342

MSPPSQASAISRGRCCIPMTTGNPPNTQTSELSSSASGTLEATWPLSSADLGRFTSALD

>SG-AL_contig_5900_1_-3_549_845

MLVHFGLIFFTKCIKLNLVPIFVRYCIFPVHGWRIVLYCFIFWSQWIYHVKFRLLSRGER

IAAVQRHPFLFFFSACCVYLCFYKPLNSYVLHCKITCDM

>SG-AL_contig_5901_1_+2_611_715

MAMNPKLGQAHSIMHSKVSFCLCLCQFGTSPTLQG

>SG-AL_contig_5902_1_+2_116_265

MFQMWTPHFQPPSAPKGRCPFTLTHNDILYASSLDQVGQTVVTSFFVFVF

>SG-AL_contig_5903_1_-3_3_185

IYLLNKLREVGLCPPSVKLMCNNHNFDCVYIVNLFHWCYCREVCGVHIGRVGCIHTWHVF

V

>SG-AL_contig_5904_1_+1_241_522

MSLSSLSHIKGSCSQSYTHTHTHTHTFSECDCECEGSLFENKRSELHYLAVQVFRYVRLC

NTVKKLCLLKRCLHVYTEKMYQRIFNKFCVIYIP

>SG-AL_contig_5905_1_-1_1_180

CLTERKINVVKTLVLHCDSFINWNTKGVTITMQTILRNILCLPWSLCFSCHAAVTFEVAC

>SG-AL_contig_5906_1_+2_194_379

MLVIFQERFCCRSYNFHFPICLHGFAWVKSSARKSLRARPPVHPTVNGYLVLFKGEEGQV

MR

>SG-AL_contig_5907_1_-2_386_628

MLEISFFFSIIRIVSAFLFSIASSLLLVCLTSPFTCIFSRNPCFIFSSHSPFHPSWSIPT

VPTSFCLCTVSQKQCAYTHPS

>SG-AL_contig_5908_1_-2_35_199

MKCVSKIFSAVSGLIFTKFGPKVWTVEELLMHEFGMERAKVKVTRAKKVPKISTF

>SG-AL_contig_5909_1_-2_83_436

MKGHKIWDNFSQCCDVENGDNRDDAMVFHPGLRLHDRAALYAFFQYISWCQPDLRVTASC

GVGQEILSNNFNDASVKWIRLDMCGPSLHNERTACSALGRHRSGLKNPTMILYGLMGI

>SG-AL_contig_5910_1_+1_325_459

MDQKSRTHILSTSNRGELCCKKHTHDYHYQSHNTYKLLLQNKKYR

>SG-AL_contig_5911_1_-3_147_479

MYCAHAFFEVAIMCCAHAFLEVANSSLQRKVSVRKSHWKTVIFFKIQGERAEELAFFNNA

CTHFGSETLVLCDQTRCIFFSDMHEQIFTCTYKCLHTPRKRDSNAEMLKKK

>SG-AL_contig_5912_1_-1_1_78

RFGGGREECGFMCLGNISCNMSMKIC

>SG-AL_contig_5913_1_+1_100_324

MLPATPDSSQKKQNKKQKKKRLIRDPGAASKWLLYIRSTTFTFALNLPTMQYCFCFLTCS

TFFHFWYSIYCFQIF

>SG-AL_contig_5914_1_+2_161_325

MYIFNHKRMKEQSGGFHTTWKTVQVHSKKSKDITNFIYIMFLKFCMFWQNLLQSV

>SG-AL_contig_5915_1_-2_2069_2284

MLVFNSNSVHSDGVQGKWVLFCLRTCLNPFSLLPPPWGKHWMKCKKLHFCVSLQLNTECM

REGWGVCWFWLV

>SG-AL_contig_5916_1_-3_3_194

VLVVFFIQVLLQIELSFAVIVMCKTCGEKGFTLLSTVSYCRMSVSILEVLHHRNSDEYSS

VSAT

>SG-AL_contig_5917_1_+3_933_1118

MRQLMNTDTGKKTNFCLSKRMSSLSLSSFHFSYIHIIQAKLKVSTSYLVQPPFKPIQRVA

VI

>SG-AL_contig_5918_1_+3_54_1256

MAAYSACMCVIRRCSHLRNSKLITRALIRNEICKIRLPTNAGKSLFLRSAMLYSTQTPPG

GSSKELVDDILKEKLAASRTADDKTSSSEDTSSDKKGSGKGSEKKSGRWTGKNAWKLGLI

FLGGWSVMCGGMLVYVWGSPPLDPEGKEIEDEFSELPRFTAMFKRAFKEMNLFTKKIQEP

SRERLLPDPLKYPYLQPPYTLVLEMTDVLVHPDWTYSTGWRFKKRPGIDYFLQQVGPPLF

EVVIYSQEQGLTADPLVTSLDPQGFIMYRLYRDATRYMNGHHIKDLGCLNRDLSKVIMVD

WDINATQLQPENSLLIRKWSGSDEDRTLIDLAHFLKTIAASEVDDVRKVLEYYSQFPDPI

AAFKENQRKLQEEQERLAVEMKQQEEKKASGWSISSFIKRR

>SG-AL_contig_5919_1_-3_42_227

MLVKQIHGKTTVLLLEFFHSKGPVTYSCHLKNQQPASQNIQEFSLNLGENYNHLSWSVSK

QI

>SG-AL_contig_5920_1_+1_184_336

MNNQKKVKTNSELRINKQINIINYLIIIIDGTNLLGMVMKKTVNRFSLNKL

>SG-AL_contig_5921_1_-3_393_974

MHFRCLSRIFLEILRRINPTICIFLPSLHMWRTFFQMLVRSLFRLYDCDCLVLKGISSRS

YISYGLLFFNISCWFVTFEYFLLVCYFWNTRCAFFFLGMQDVLFPLSEYKMSFFPLGMQY

VNFFLGAQDVHLFSLEYKTYIFFPLNARWSFFPWDTRCALFVWDSRSTFFPWVTRCTLSP

HGMQDLHFCPEFCI

>SG-AL_contig_5922_1_+3_840_1043

MASCRHLSNQTPFITELHHSLWHTDYTLAPLKCGNVPPLQNTILQSNTILTNRTLFTKHD

YLQNQHHL

>SG-AL_contig_5923_1_+3_597_830

MFGVGKTFYSLFSLFNGLSGEALKYSFTCNFSSSSGGIGPLNPSMGLCPCTPPGPGQSPN

PCLFRRIGLQTILMPVSS

>SG-AL_contig_5924_1_-2_782_1114

MQSTVQCFVVLSCVLFKTLGSTVSCFVVLSCVLFKTLGSTVSLSLSLSLSLSLEIFPIFL

VNMCAADLPVPKRPLCVYTCRISNMHVKDPVNHVSGWLMENENIQHAVSKD

>SG-AL_contig_5925_1_+2_2081_2335

MQRLKHHKNRQKKSKTGKRNIFICKQLNSTTWISTLSLHCRHAAHLSVLASSWCFMDVQR

STKTNAVSLNFSPLFYALDTFSGWI

>SG-AL_contig_5926_1_-3_1296_1565

MMSVQPSDWKAAQKPGKRKIFEKWKSHCTATIALFWPQKPGSSGMKKSHPDWRRFLHQPN

NQPTKLKITQKANNPIHKVMFASVRVVRFV

>SG-AL_contig_5927_1_+2_692_1228

MRQVWSELNLGCDNANVMSGQHKGVFSFIKEEQPQVFFAGCTLHAIYYAAKKATTHMPPF

DTVLADIFFYFDKSSECKARLKEMQDIFEVEYRKIIKHVPTRCLSISKCLQRLKEHSDAL

KVFFSEEKESVSRTSNSKKASSYDVTSVDNIFQFVKSPTNHLYLLFLLYTVKVFFTNFF

>SG-AL_contig_5928_1_-1_10_1269

MAELASGGRPLATNAVADDCEPPATRHAAHTAGTATTTTTTTFTLTHGAGGSPIAGDGAV

LDKSSGETLSAEKDSASTKEEASAIFAASGSPATSSTLTSQSVRDGGAFTSPQTTSGDQS

SRQSSVAVSSPGKTGIQVINSAVAVSSSTKVGTPNSHHHIVTDSWTTKIGTQTCQHDTGN

ISSTTKTGTPAAHYTLNASSTTKAGTQSTQPTVNTSFSTKTGTSTTHHLQVVTASSANST

NASLPRTGSRTLHNGHDPSFSFRCSLLPVRPLLSKVGEVPEDVENDSEASVASSGSTTTT

FLLPLPPVPSDMAEAIKRQQKGAGSVRYWKTKHLQSGRSQTMSLKPNGGFQSAMFPVLKP

SPLNTSSGGGGGMVVNSNASPGAESRLSKAKERLLRQRGREAMLGDLRRTINNNSNSSSS

>SG-AL_contig_5929_1_+1_1_237

TGKAEKRDDSQERKRGRNKENNGKEEIASTEFPEGGDTICKCVFRLYNIISDVLSLSAST

PDTSDGHKMILQEDSKTIT

>SG-AL_contig_5930_1_+3_288_545

MAFCPQKCNVLSCTRNPINFNYILKSHINAYYTLARPHLEYCCTVWNPHTEDQTKKTEAA

QRRATRYVSSQYETTHSVTSRPEIRH

>SG-AL_contig_5931_1_-3_705_887

MVDNNSYQFLHISQNGSTFFPLKKLKIARLFTYCISPLWERRKHFVFQLKLTDQSVDFTV

C

>SG-AL_contig_5932_1_+2_23_262

MDKKILQTRKVRPALTKTDKWELNSDKHLSVTSNSIKSEIQRNEMSTKTKHANSKITIAR

KIMYKRGRQRQTEVENHSKR

>SG-AL_contig_5933_1_+3_261_506

MRQSEPQSREVERIQIHRSITIYFQQKSCPTLFFVYLFVFFVCFVLFSPTNCPKAKQREY

LISFTVKHFFHKFPLYGREVVS

>SG-AL_contig_5934_1_-2_209_322

MPSWSVSVSFSQQAFSFLSPLPPTVYDVYLCCLALATR

>SG-AL_contig_5935_1_-3_138_434

MITTVWDIAFSSCFVSEQFLTRFKNYVGDCKALGLSYINLLDCSLYSIFYHYWIQTLFMY

HSYYYAKVTAQNCDGCFGCIDGTFFFSKKVIFISKIVKD

>SG-AL_contig_5936_1_+1_1_198

KSYIIICLLSQCKCVLTWQCYVTTKKQKFNLLTISQIPLCIQIQYQEHRKCHGIPRSTSV

TVQLLA

>SG-AL_contig_5937_1_-1_484_699

MCRDVYILCLSVLCLVIVSFAHSFVRHSFCRFFAFNSHHFAASIFFSPFRSCISCLFTAM

LLLFFYCIFFSF

>SG-AL_contig_5938_1_-1_379_1677

MAEDQKPHVQVKFFTKQSQYAIPDTPHTVPADVDREQLSALINGILFEDTEGSPSEDQRV

QFEFLLDGEFVHTTLSKFLESRENLAETLLEVEYVERQTAPKPEESLEHDDWVSAVQGTR

GFILSGSYDHTTRLWSTKGKAIMTIPGHSAAVKCVAWIKHDESDPVSSFVTGSHDQTALV

WQWRRDNNEVDCRLSCRGHAASVDCVAVSPTGEQMCTGSWDKMLKLWSTDCSIGDDEESN

DTERPSKKRSKTSSMQTRVPVLTLSGHKEAISAVQWLDSQRVVTSSWDHTLQIWDLQAAR

QTSLLPGSKVFLDVSYSPLSQMLIAGSADRHVRLFDPRSSGVVVKCAFTSHSRWVSGVDW

SPTREHLFLSGSHDGILKLWDTRSPKAPLYDMNRHEDRIMAVDWSVPELMLSGGVDNKLH

IFSYSDANLATSD

>SG-AL_contig_5939_1_+2_179_649

MDGAELWRRAVLGESNPHFTRRKNSIVIKHESNSIQSNPIQSNPVQSSPVQTHKSTFSQT

YTQYRLMSQTHKDHKGFAPDIQTAKSVTQRSQWFCPRHTSSTVSCLRHTKIAISRSHNDH

KSFALGIQHSQSHKDHKSFAPDIHTAQSVTQRSHVDV

>SG-AL_contig_5940_1_+1_730_1335

MNFMRDFSLPLYFHCSSLFLLLSTTCCPLRSLYCLFYCSHLSLYHLFYCPLCFLYHLSWC

PSVLSIICPSALCFLYHLSWCPLRSLYHLSWCPLFSLSSVLVSPLFFICSIFPFVFSSFC

SAVPSVLSIIYPSALWFLYHMSSCPLCSTIPSVLSIIYPAVPSVLSSVLFSPLLSSSSVL

LSPLFSSSSVLLSPLFSSSSVL

>SG-AL_contig_5941_1_-1_1_234

LGKILDSTSQKTLFKTVLGGRMWRSLPQRHCYRQPLLSTLPVLRCLESGCPIHHFFLFLE

QTVVRKKIYIQNLCQHFE

>SG-AL_contig_5942_1_+1_313_579

MYVYTFFFFRQQISVCTVSWAFVHKHARIHTHTHTHTFTHSRTYTHIRTHACTHAHAHTH

THTHTLAPSLTFSSLNDMNIDHSEYWHLR

>SG-AL_contig_5943_1_+3_99_1187

MTLNPLAFPKGVKLVCELCQKPAFVQCTQCRVTYYCCVEHQRADWLGIHEKICDSLVTLR

QPTPFLPSEEERQKRESLMLVKKRQMIDLTRTTGQKLLFEGKHEQAVPAAMQSLRFAIDV

HGIASIELVPSYLILGEASIGLGRLSQAEEYLAQAEWTVLKTPDVSSAMKSRLHRNLGLL

YAAKGDFPESLRHLADDIYHASEEYGPDDIRTSGGYFHMANVFFRQGKRDVADSLYRQVT

DIWHSHLDRIVKSRTRTPATPRGVGPGAIEKEEEEEEGLDEAQEAEAIQVLNSIYDMREH

QSSRRSQDLVSVCHALAMLYFLLHDIKKAKEFGRKAVVASENNPDDNFSRNIVEFMKVCE

RVR

>SG-AL_contig_5944_1_+1_55_204

MDKINRTILLYECSRHSSIRFKSKRLINLHEMCCHSLNKINDAVEIQNIS

>SG-AL_contig_5945_1_+2_2_406

TAALIIDGSVLSQGASSRDSQTAALIIDGSVLSQGASVGLTYSLWRVSQDHSGFSFSIPW

HLQRWCTWNNSARGNAVWRKLNLKQTVLCFTLCERKVRPVHPWRFSVHCLESSFFSASFR

VPEGRCFDQVVDPII

>SG-AL_contig_5946_1_-1_64_384

MPAHSGPLHCPALPISENQSAEEAYKISTVLGWWPHGGLISLAPMSFLALGLGLVVCLGA

RSSMRSNLNATEVMQKMRKKRKKTYQQSACVILFTCCITRWWYGYGG

>SG-AL_contig_5947_1_-2_179_595

MLEIALLQIDAYCRSKGCVIAGYYQANENFDDNQLSFVAKTVGKKIQEYFPDACLFQIEN

GRVNPDSVQEVYRVYTVKDNNWKEVDKSKSTAEEDTKSTAAELLRSGAQRKVVDFDNHLD

NVTNDWRNPELSELIATYI

>SG-AL_contig_5948_1_-3_738_1049

MLHQLPVQARVDYELSTLCYNFFSKTTPAYISDLLTVYTPRHLRSSLDEHILCIPHVKTK

TFGEHSFSFCAPKQWNSLPSDICHLQSLSSFKRALKTHLFRQYY

>SG-AL_contig_5949_1_+2_77_355

MVKSINTRITRKCQDIFPTANYVYFCHRLRSWAAILTSTGVYTRVDIYMYGRLGCFLFCF

VFSPCHEGRHTLLSGYVQAGYFRVSTSHRILTC

>SG-AL_contig_5950_1_+2_2_130

VCVCVHACVCVDVFKDKCMSLCVSACVHNSMCVYVTLRVNLFK

>SG-AL_contig_5951_1_+1_28_252

MPQCEGIHCSICLVHLMFCSASCRCGSLLAVVASMTDMASVRKNTRMIGALRCCWVGLNS

RQILSPQLYQKSQPA

>SG-AL_contig_5952_1_-2_101_1696

MTSQNGDDAVDQTSEEQLLTEDDLQDEDLENFNKLTECGIDKKVCLELIKIYRTGKLCHS

ELDKRALDALKEYSATDALEVLKQFCEHSLEHVGNKSAYLCTQMKNLKKTKASGGAAQAK

KPDESKVQEILKRTGYDFDVTAGQRKYGGPPPGWTGSQPGAGHEVFCGKIPKDIFEDELI

PLFEECGKIWDLRLMMDPASGLSRGYCFVTFNTKEAASEAVKKYDGHEIRKGKKMKVNIS

IANLRLFVGNIPKNKSKTDIFEEFTKRAEGLQEVIVYGSADNPKLKNRGFAFLEYDTHKN

ASAAKRKLTTGRVRVWQCDIIVDWADPQEEPDEDTMSKVKVLYVRNLTGDVTEDQLKEKF

GAYGKLERVKKIKDYGFIHFEEREHALKAMEELNGEKIGNLEIDVSLAKPPTENKKKEQR

KREQEIRMMARESFGWDDYDYYLPPRSQGPMRGGMRRLPPPEMYYDDYYPYDDDYYYGSY

APPPLPPHMHRGRGRGGPYAPPAAYAGRQGWWPSSAWGQQRPRRNGNTRGGR

>SG-AL_contig_5953_1_-1_37_546

MLDQKAAAAAAAQAPPAAPMQILPSQPNQEKEEEKKGEEELQQSLAQQENMKISGSNARH

MVMQKLMRPAECTVLVLRNMVGVEDLDDDLKDEVTDECEKFGKVNRVIIYQEKQSEEENA

EVIVKIFVEFTKQEEVEKAIASLNGRFFGGRMLTAEKYDQEMYNANDLSG

>SG-AL_contig_5954_1_-1_142_1761

MMKASSILASTARATSYNMVLQVMIRLMTFVFSAVVLRYISRDLLGVLNVRLTLLYSTIL

FLTKEAFERACLSHRDSQHWRHVINLMWCTVPLGVVISVALSAVWLYLLERPDPMAVAHY

GVGVVAFAVSAWLEVLVEPLYITGQAFFFIRLKVFTLGLSQVVKCVTALLLVLVFPHWGI

YSFSLAQLLSSASYVAMYYAFFAYHTSSRQKKEDDDGFPFASVADFLPQAVHGETFFDAH

LVHLTWSFFKQSFLKQILTEGERYVMTFLNVLSFADQGVYDVINNLGSLVARFVFLPIEE

NGYLFFSQTLNRGKPAKEQAEEGVSLSSSVLGMLLKIVTLIGCTVVVFGYANSYLALHIY

GGDRISAGSGPTLLRWYCVYVLLLAVNGITEGFVFATMSKEQVDRYNQKMLVFSVLFMTT

AWFLTTQIGSVGFILANCLNMLLRIAHSLLFIRGYFDSTPYKPLSHLLPSSPVMASLAVS

LLITALSEGQLVAQGSVMNNLMHIAVSAVCLLMVLAAIFFTEKTMVRVIQEHVLHKQHSE

>SG-AL_contig_5955_1_-3_147_278

MLHALSFHTCLNYCDLENWPLLFKPKHMTLTNNKTWHLFVCVCV

>SG-AL_contig_5956_1_-3_507_884

MCKYTSPFLQSHMRAHTHIDCHSLICMPIHIYFLTYACSMHRQVNMFMHPYSNAHDIMMT

NSHFYSCSHSTNIHVHPTHMHTQTTHHPITPPQERICLYPQEKAPKPNPPTPIPTPKTSK

QEKKKK

>SG-AL_contig_5957_1_-1_1_162

KKKDILKIIILLKNLIYLSKQGVKSFTDFYARTIQCGCLLDFDGLYVVTALIWP

>SG-AL_contig_5958_1_+2_869_1078

MPEFSSLHTVVFTKYILLAVTLISNYLQWHFAKIAYNFLGLTKPAHSIQSVIYTGNLDVI

IILQSPQPIL

>SG-AL_contig_5959_1_-3_1215_1415

MSYAQLNFNICCLSLLYFVKSCLFIFCCCEYCIMLPGLLICCVLLVTAILLMLLHVLQLL

PVLLKFC

>SG-AL_contig_5960_1_-2_77_244

MSNVCYWVSISVSSQFLFLSSGCHSLQIVWLKVEMICVTVNRVCVNHCTCLYHSFA

>SG-AL_contig_5961_1_-1_307_1638

MQRDQENRFFTSAHDRDSPARRDREREKDSPAWREREKDSPARREREKEAAKLEKASDSS

KGDSDKADGSRKRGRSPPRSQGRGSDSGDRSRKRWKGDGSWSETEGGDVADKDAHRQKMG

GMEQPRAAEPVREAGDSDRPDTAKALSSASGTPTDMAKGGRPGRGDIFGGRGNEDRIPPD

ARANRGDDSFHRGLGRPGFGMDGDSSLHQDSADDSKDASLGNMLYEDDYEKISSDESDFE

ADDGEKPSQNIVSVLDIDWASLAKKPTPKQTAGSLLKRFHPANVFRQIGISRALAGDALF

EKVKAICKEGVTVAGTNEEKDKENVDQSSETTQTKGTKREAPTIDTSLQSDVPVLHMAAV

RQKRERANLLRNLGPFRRALCARRDLEIRRQLCKVDKVYEQPAVFPIHVMDTDLCKLSMQ

LFRQGRDFVERKDSECDGKIELSI

>SG-AL_contig_5962_1_+2_2_79

PKALKRTVGSELDNKLKCPQGCKTQN

>SG-AL_contig_5963_1_-1_556_807

MKPFLRCKRPSVCSCKNRFTWVSKNNISTNVCVGVHSAVENLHEIWRLRFEPNVDEIPVS

DSGFVVKFVTRSPMCLNPYPSTKL

>SG-AL_contig_5964_1_+2_2_130

PIYTRKFSQQICTNPKNDPWGLQKVGFPPKVGRFSSLNTTWSR

>SG-AL_contig_5965_1_-2_11_382

MAADDLCQHPERQSRIVMKLFEAEEARKEKENAVIKLEMQNRPVVDSKPSKTELQEKVKK

LAKENYKLKQRKMCRACRKVDLASSGVTFLPCGHFITCETCSENFDDCPACGKMIMGTVR

TFLS

>SG-AL_contig_5966_1_-2_71_928

MEAKRKKEEEARKKQEEADRKAAEALKIELAKQAKEQAQRQAQLEQEHRDRELALRLAME

DQNAVEDISLPPPLPRSDAVKAMQQAAANKKYDLQKWKYAELRDAINTSCDLELLDACRE

EFHRRLKVYHSWKSKNKKRTDGGEHRAPKAVEQAANQAVAQRPAQQQQQNAVQRYFKIPF

ARPADEFRDSNKKHGMWYAHFDGKWIARQMEVYPDKPAVLLLAGVDDLQMCELSLEETGL

SRKRGAEILANEFEEDWQKFKGADYLKKNATKVSSKFLQTRLGIAK

>SG-AL_contig_5967_1_-3_135_1202

MNHYMIVDADEKFSQLVKFLKTHHQDKLLLFFSTCACVDYFSKCLQQIVQNIQVLSIHSK

MKQKRNKVFAKFRSMASGLLVCTDVMARGIDIPDVDWVIQYDPPSSASAFVHRCGRTARI

GNTGNALVMLLPAEDAFIKFIKINQKVPLTEMEKSADAPSMVNKLRQLASKDRALYEKGM

RAFVSFIQSYIKHECNMIFRLKDLDFGQLATGFGLLKIPKMPELRGKTVSFTPTDVDINS

IRYKDKAREKLRQQKIESGEAKLKQARPQKTEAWSKQKEKKSKKQIRKEKKEKKMKSQQE

ATMSSKRKREDDEDDDLEDLEEDMRLIKKRKKGKISEDQFIQKFMGSEGTTEDLSD

>SG-AL_contig_5968_1_-2_62_835

MMMMQILISSLKKADIKMTTQYTTIQKISLAGMVIGSLMCVVGAFAPYWIVSDPKGFSGL

ADLVGKVVKGNMGLFIYCIRVNVLGLAKSGCEVLPMETDSGWFHSARVSACGCVLLGGLC

GLISLCLACCNCCKRFIVLGILSFLAAAAGAFCAIVFCQNMDAFTSMVLGFKLTSYGWAF

YIFCTGTGILGVVSFTACFAAPENPIHGMVLSAPGNQLQVVNSTVTNHPYMHLQEQGGPM

GHPPPVDPGVTVVTANGY

>SG-AL_contig_5969_1_+1_19_312

MKKKKSDQETNYLFQDFNRFLRNSLPRSQNVTFSQTTNFPIHLFDFYLLSPSVSFAKQKP

DIQETSYASFPIAQFSNSIINGVSFMPFKILYFQTISP

>SG-AL_contig_5970_1_-3_90_3179

MSDKMSDSEPDSPEEQMELQPPIAGPSVLLKSLTKPETGIEKEDGDFEAKIQENRSNRFN

FLLQQTELFAHFMSTGAQAGGGGPTSPLKMKPGRPRMTKKMDEKSKLVAAGDHRHRRTEQ

EEDEELLTESRKSHNVLLRFEKSPSYVKGGEMRDYQVRGLNWMISLYENGINGILADEMG

LGKTLQTISLLGYMKHYRNIPSPHLVICPKSTLANWMAEFKRWVPTLRAVCLIGDAEERS

TFIREVMMPGEWDVCVTSYEMCIKEKIVFKKFNWRYICIDEAHRIKNEKSKLSEIVREFK

STNRLLLTGTPLQNNLHELWALLNFLLPDVFNNAEDFDAWFNTENCLDNNQMVERLHNVL

RPFLLRRIKADVEKKLLPKKETKIFVGLSKMQRELYTKILMKDIDVVNGAGKSDKMRLLN

ILMQLRKCTNHPYLFDGMEPGPPYTTDYHLVDNCGKMAILDKLLPRLKENDSRVLIFSQM

TRMLDILEDYCFWRAFDYCRLDGSTPHEERQKSILEFNAPDSSKFIFMLSTRAGGLGINL

ATADIVILYDSDWNPQVDLQAMDRAHRIGQKKQVRVFRFITDNTVEDRIVERAEMKLRLD

NVVIQQGRLVDNTTNKLGKDEVLNMIRHGASHVFASKDSEITEEDIDSIIAKGEQKTDEL

KKKLDSLGESSLRTFTMDTQDSVYQFEGEDYREKQKLGGIGGWIEPPKRERKANYAVDAY

FREALRVSEPKAPKAPRPPKQPNIQDFQFFPPRLFELLDKEIYFYRKSIGYRVPKNPDLG

SDADRVRKEEQLRIDEAEPLTEEELTEKEDLLREGFTNWTKRDFQQFIKANEKFGRDDID

AIAREVEGKTPDEVIQYSTVFWDRCNELQDIERIMTQIERGETRIQRRIGIKKALDAKMA

RYRAPFYQLRIQYGTNKGKNYTEEEDRFLVCMLHKLGFDKENVYDELRQAVRQAPQFRFD

WFIKSRTAMELQRRCNTLITLIERENMELEEKEKAERKKRGPKSLTPKSAQKRKADGTPD

NRGRPKKKKV

>SG-AL_contig_5971_1_+3_48_281

MRRLRVWLFFSTSPCRQPYSDFRGSRYVDLRDWKESPPFTHIGKSHHPLPTRYYLSKIKL

DVLLEVQYHNHLTLLACS

>SG-AL_contig_5972_1_-1_130_1407

MPKVKQMRGDKVRRGGPLAEEIISEHEVKSSGRKKVRKRKDEDDEYVESDLTKKILSQAC

IQQLELEEEHGACPPERPQSSYTKRGKGKQVLLGRSTEDDECEITTKEDTEAADADGMLG

LVTLDQDAEKAFETFMTKKGHRSLADIIQSKLTEKKTEIETVMSDDQGASIHNISPEVEG

WCIQLRSILAKYRSGKLPKFFKALPLMEKWEECLYLMRPEQWSAAAIHAATRIYSSNLPG

CLAQRFYAAILLPRIRDDIAEYKRLNFHLMQALKRSLFKTGAFFKAIVLSLCESGDCTLR

EATIISSVMRECSIPINHAGAAICQISSMNYTGASSIFLRTLLDKKYALPYMTIDAVFEH

FYRFLHDDRQMPVLWHQALLTFAQRYKGDISQDQKEGLYEVIRKHFHFEISPEIRRELES

QVSDRS

>SG-AL_contig_5973_1_-1_361_540

MFGMGIKPQTLRIEDRGIDHLMSYALIRRACPWLLIWCDGHVLSLCRVSNELCLLSSFQH

>SG-AL_contig_5974_1_-3_459_782

MIKSTLCLHSHHRHKLWMSFVADVKKTVDKTIISLSLRISIKVQTSLSLVSLPKRKSTET

INFVVSRCLLKSVSRFKPNLAIVSQGSVCPRVKNCAFEHCSVFLCVCT

>SG-AL_contig_5975_1_-3_144_833

MYPPFVDTSKPLYLFSSDLCRSLEIDYSHGYGLKGIDVDRFAVPPIAFANVSVNPENEGF

CTPSTNCLPAGLLNASVCRDGAPVIFSLPHFLYGDPDIRNRVVGMNPNVEEHQSVIDIEP

MTGLVMNAAKRLQINAYLTNISDIKETLNFREYVVLPVLWLNESATIDDKSVSDFKNKVY

LAVKVTEAVQYGLIALGAFLLACALILVLKTVITHARKKVMESAPILVTD

>SG-AL_contig_5976_1_-1_1_216

LSGCPQYILGKLQKVQNSAAMLVLKTHKYDHAHPLLYKLHWLPVQAGADYKLSTLCYNFF

SEITPAYISDLL

>SG-AL_contig_5977_1_+2_2_298

NLKLRFYNSNVLSTLLYGCETWHLKTSQEKKLDSFDLKCLRKILGIKWTDFVTNEEVRSR

SKQNPVTSIICKHRLSWLGHTARLPSSRPANQVLNWNPG

>SG-AL_contig_5978_1_+2_131_361

MSVFGGLWKHEYTLLCGDVGRAARISHGKSIVTKTIQYNSFSAIYGLIFASFGSWSESLR

FCKYINLNHFCAFLMSP

>SG-AL_contig_5979_1_+3_114_284

MYILSFVLVCLTVAAIHSPHLTSAISETSATGISRNNPHHHQHKTQRTKCSQQIKDT

>SG-AL_contig_5980_1_+3_813_1103

MMFSGIANAKMTTTNIIGRRHCSPSIMNSSELAMTLTSHPFQKYRVTLCPFHTGRSDRTG

SNGSPQPHRRNGLCISCFMVLLEQRGWSERVSERDSL

>SG-AL_contig_5981_1_-2_713_1036

MFLHFSLCVLSSYLGVSKILICTLSEVVFPFSTSLSDTVLSFSTSLSLVSSLSSPRVSDI

VLSFSTSLCLVSSLSPPLCLILFSFSTSLCLVSSLSPPLCVILFSLSP

>SG-AL_contig_5982_1_+1_1_180

KNKNYDEKTKMQKPLIVYGLCLLVDFPSVVEVPMNFQKRHLEKNENLVPDRCTMQSQGQL

>SG-AL_contig_5983_1_-1_1_195

RPLRPFFFPPPPPPPPPPPPPPHFPCIKLKIVSAPPPRALHPDPPKNILVPSHKNELIRP

CNLSG

>SG-AL_contig_5984_1_+3_663_866

MAMSNLYRTISMSKVVKAVNNLKTNKAPGKHGITSEMLKASILSIGTAICKLFNAILQSG

TYPNQWKE

>SG-AL_contig_5985_1_-3_177_284

MYSFHTTSSFCMLFSSFSICQNFIFTSLQQSRFVQK

>SG-AL_contig_5986_1_+2_98_325

MSLLVTDCDLETTVHLSKVLLKPTCFQIILVVIHWTSKPEGLECLGSNSPRAPRLVGKWC

RFFHLPGQHMCRPASA

>SG-AL_contig_5987_1_-2_128_313

MGKKNKTKKPKKNRSKSDDFVAISTARLTDWMTRETTILNNQYEYYELHPFITCSPRIQR

HL

>SG-AL_contig_5988_1_-1_43_255

MLFVIIKVMRTTHHYHGCFTPKSCLMSMFAFAFMRRQARKKMLVWHIEFMSFGRKLLDML

VYIILFVCFGG

>SG-AL_contig_5989_1_-1_571_963

MEEVEKIKQHNEFLFSTPFVQLEKYTNSQHEMRRDFFFSNHYVTRDTCSPLKNLNFVLPN

AVGSVYIILRKIQKVQMCQLTVITVISWVSLCFLFKLEDSQTIFRVSVQSTKVQYEMEIF

QAHMTGSGQFE

>SG-AL_contig_5990_1_-3_402_611

MFFTVPNGNPIQSSLRCCLFFSFRFPLFWILSLFLSGLTKKSFSLVVIWTLDLSGFLPLL

LRARKSYKCP

>SG-AL_contig_5991_1_+2_740_934

MGVTQISWRVFNSNFFFDKNHHQQTPLASISKLFWVDELYESHRISNSVLMNPFTPSPYP

PTTKT

>SG-AL_contig_5992_1_-2_11_292

MDGDSPHMALQGVATVWCGLRHFACVIGWQSFWPKPRHTVDCYPHSPTVMFIAYMDFCTV

GEPEPESVMFCGKYLAEYSLCFLFGSHFVFNIFV

>SG-AL_contig_5993_1_-1_1_1062

SSSFLFCSSHSSSSSSFLFCFSHSSSSSSSFLFCSSHSFSSSSSSSSLFCSSYSSSSSSS

FLFCSSHSYSSSSSSSLFCSSHSSSSSSSFLFCFSHSSSSSSSFLFCSSHSSSSLFCSSH

FSSFSSSFLCCSFHSSSSSSSSFLFCSSHSSFSSSSSFLFCSSHSSSSSSSSFLFCSSHS

FSSSSFSILFFFFFFFFSFFSLFYLLLLLLLLLLLLLFLLLLLLLLPPAFTCSHPPLSSS

FSPPPPCFSARLYSFFYSPSSPLLLFLLHLFFFLLPLLLFFLLLLLLLLYHRLLLLLFTS

PFLLLSSLLLLLFLFFFLLLLLLLFLLLLFLLLLLFLLLFFPLPPFHRLLLLLL

>SG-AL_contig_5994_1_-1_1_129

PYLCSAFLMVVPIFVLGGSQAVLHRLGKFPLLILVSIKSSIKQ

>SG-AL_contig_5995_1_-3_21_221

MGSIYFLYKSHINSICLSYRYCHVPFTRSAFLMVVPIFVLGGSQAVLHRLDKFPLLILVC

IKSSIKQ

>SG-AL_contig_5996_1_+3_387_506

MIKRNDCQELFAIPDINSRKKCAEYFCMSSVIRNIQTISV

>SG-AL_contig_5997_1_+1_286_495

MFTFFSCTSHGQSQRSLVFPANVHHLSLLPSRGWDGGKGVTNLYNQHGTENYFFLPLKLF

RQEAKINLSM

>SG-AL_contig_5998_1_+2_167_340

MGDPATCQGHRGGEKGRCENKVLITESNLFYTKNNIFSCNIHNTDSFSSHFHTQGTMN

>SG-AL_contig_5999_1_+1_4_255

MYRNSEYCLFFLRERERERERERERENVLCRVRLPNNPKQKCKNIVNKITSLLKKIHFHC

VFVNRPVSNVLIATLAGPTDDQFG

>SG-AL_contig_6000_1_-3_192_374

MLFKLTYRYCLFLSLWSPYVTANKAMCWSASIIYMHIVDISVDSVNMVRSFFPVDIVNNT

F

>SG-AL_contig_6001_1_-2_83_2746

MGKKVKKDEKPPPDDVFDSLLVESRQAATVVLMLSSPEEDVQSKACEAIYKFVDKCDENK

KLLLDLQVIDPLLHLMQGEDATVRRHATMALGVMCQNPDVRRYLRKKEDSIPALINLLQP

EIGDLVVNEFAALAVSFMAIEYSSKVAIFDNGGIEPLVMLLSSCDPDVQKNSVEALAQLA

LDYQARGSVREYDGIPPVLELLKSEYAVIQKLALLTLDRLMQDVENRSIMRELEGIAKLL

DFMAKPEWADLHVMVIMVIASMLEDVESLEQMKESGGLKKLVALITDQPPPEEESKKGDK

KGGSRAGKKSAGKDSKGKKGSADDDEKETAPGGESIIPALPDVKMSAAKAIARCARNSES

RKLLHEQEAEKMLILLLGHESVEVQAAAAQALGIMCENLTCRDSIRDWDGLPPLVKLLGT

DNGEVKEAVTMALANLTTSNINNCQEIVNLNGLDPVISALSDSREEVVANVACVLTNMAQ

EESLRAEAQNKSVISALIDPLRSSNTNVQSKAALAISAFACDADSRAELRESGGLDSLVM

LLHSGNDEVRKNAAWAITVAGVDETLASEICKLGGLEILQQIQLSSTRQSPFINSAVERL

LDSNLSAKYALTGHLSSMNLIEDGFYDPGQLKPDTKFRTLEEYLGDPLHDKRPVFYVNAK

QEASIAVEKSDDADSKADTKSISGKTTKVSKQDSKGKSKAQREKEEKQKEEELAAQMQKE

AEAMAEKDQKPLEPPTDPNFVKYLEEVNERILPLPSAKEQIVALAQFVSDKMGGAIEKGQ

LPCFGWELPLSQIRFDCKTNVVPIGKIKMGIHNHRALLFKALADRLSVGCSLTRGQYNRA

WNEVTIAEEDESSGAAKYPPRSFIVDLIHEPGKLLSQDSADYVAYQKI

>SG-AL_contig_6002_1_+3_1227_1637

MFTFCSPLLSLKKTTLAHNITKQSFYTRQLTFTILSPQRMQNVQPMTANQASFHTSMLQA

ATHQTCQERRRVLKELKAHEWSGINSNHILGVDQSQSSGLNSITSLLYMGLIQSHLSSRP

NSMTFEKWTKISVVENI

>SG-AL_contig_6003_1_+1_40_327

MMMMKPKATHAHLNTHAHTRSCTVVSRCERKPPKKQEETREEGTQFTLQAQKEPRLEKGF

SFIQFRVSLLSAFSSRARVPYFPSDRGVRISSSLSG

>SG-AL_contig_6004_1_-2_380_553

MGLNPHQSIKEKSLWNFSAPTGSYPELSMLWVHWEDWDHTVEPHPLYGHKFEGVAQIT

>SG-AL_contig_6005_1_-1_79_918

MFRNQYDNDVTIWSPQGRIHQLEYAMEAVKQGSAAVGIKSRTHAILVALKRAPSELSAHQ

KKILPIDDHVAVAIAGLTADARLLCNFMRSECLDSRWAYEQPLPISRLVAVVGNKSQIPT

QRYGRRPFGVGLLIAGYDSAGPHIYQTCPSANYYDCKAMAIGARSQSARTYLEKYLDQFP

DCSLEELVKHGLRALRDTLPSEVNLNTKNCSLGIVSKDMKFTIYDDDRVASYLALIEGEE

RSTAAAVAEEGGEAASGAGEDMPAPVPVSDAAEGEEAMES

>SG-AL_contig_6006_1_+2_434_649

MNHSAMIIQYMMKFTKNNRAFCLHHILYQYINIQINCSALSLQNTDCIKHYTCQSFLKTI

ICRFSVLFVHNL

>SG-AL_contig_6007_1_+1_1_186

FFFFPSLPSLLLQPADHLRLFLNHKIVESTIIENHRTTLNHTSSALIRMQQPIHNISQIL

NN

>SG-AL_contig_6008_1_-2_11_238

MSSELISEPTKGSKILPWHTAIPKCYYTDTGKGLRTLIYLGALADSDCLVLEGFFLFFFV

LFLANISINVIKPVFI

>SG-AL_contig_6009_1_+2_2_187

IVTKKERTTNTKKNIHRSVDLLREVILNGAGKLCIRMYIVTELTRSRRVILLDIYEHFHD

RF

>SG-AL_contig_6010_1_-1_10_1245

MEGGSRDKEQLLALKVMRLTKPTLMAYQSLVSDSRDLCGDIFQRLNQSDLSKPRDLDHFP

LGDLLTLPQNFGNIFLGETFSSYISVHNDSNQLCKDITLKADLQTGSQRINLSSVGDQPV

PELLPDHSIDDVIHHEVKELGTHILVCAVSYAHPSGERMQFRKFFKFQVLKPLDVKTKFY

NAEDFVTDEVYLEAQIQNITPGPIYMERVSLEPSPQYLARELNTPEGKEGRLYGGVDYLS

ANDTRQYLYCLSPRPELYADNKVLKGVTNIGKLDIVWKTNMGEKGRLQTSQLQRVAPGFG

DIRLTVESVPDTVTMETNFNIVCRITNCCERTMDLTLVLHNIAGQGVMWCGVSGRQLGKL

PHNESLDIPLTLLAVTPGLQTISGLRLTDNFLKRTYEHDEIAQVFVIDEDTF

>SG-AL_contig_6011_1_-3_66_1142

MSDDSDSEIPDLVPADIKKVPITIISGFLGAGKTTLLNYVLTEQHGKKIAVILNEFGEGD

SVEKSMSIGQQGELYEEWLELRNGCLCCSVKDNGVKAIENLMSKKGKFDYILLETTGLAD

PGPIASIFWLDEELCSDIYLDGIVVMVDAKYCEKHIHEVKEDGSINECQRQIALADVIII

NKTDLVTSEELHKLQQEVRSINSFAKLHETTKAKVDLGSILDINAYGGHGPASMEDLFGQ

RGFSTADVHVIDKSVTTTTIEMDGSVERERLDGVVQHLLWEKNINNSKGNPMEILRLKGM

MAVSGEDQRMVVQAVHELYDVQFTTPWGSEQRKSTVVFIGRNLDHKILKEQLESCLISR

>SG-AL_contig_6012_1_-2_2_268

NLVKFCPETAEKILLTHTHTHTHTHTHRSWVIWKTSLFIENKGRSKRAPSIKQFSFTGRL

AATLEAFARRIKKFQRLFFLRKRTAVVMQ

>SG-AL_contig_6013_1_+1_25_240

MKPVSSFLLLFVSALSCFCPFFFLYCHLLLSAFLVHSTLFSPASLYTIPSTPAPCNLCSF

LAVLLWRLGSEH

>SG-AL_contig_6014_1_+3_84_209

MVKLEGLEGRSSNLLRYLAWWVKGRDFSDLPRQHKCRPASAY

>SG-AL_contig_6015_1_-1_31_123

MACVHSMQNALFRFSKADTGAVIVNLHVKFN

>SG-AL_contig_6016_1_-2_506_709

MILFNCLFVTVLGKKKKVSRLLQISQLCMDESLQFAVYILERGLATCTFDYNFSQVSAGT

SAVRVTII

>SG-AL_contig_6017_1_+2_2_187

KKKKKWPQITELNNEQKPRSAASRKFMLLLSCANTFRNGPLGEICKTDSGWMNTMLNDSL

PH

>SG-AL_contig_6018_1_-3_525_716

MMFKFILHCVRHTSMTNVDALINRLKKQMNTQTRTHTVTPPHLKKDQASFSKLRTGFLNI

FVKF

>SG-AL_contig_6019_1_+3_129_260

MLDRNCLLWYMESCYLSLIFVHSNTLTVKHMHKLTFQIKMGSLS

>SG-AL_contig_6020_1_-1_154_558

MSDEDSSTPVAGRAVLQPGQSPNGSDSQIETGSVTSPSASSNPPSSASTPDHLHGSGLTS

GPRLHPSHGDRPSSAPPHHLEKLSGVILRTMGDHYQSSTYGQTLRMMGDQMTFENEIRRA

LSYSDLDRLQSNDNR

>SG-AL_contig_6021_1_-2_26_391

MAVASMKGKLISVIGDEDTCTGFIVGGIGELNKHRVPNYMVVNKDTPAMEIEETFKSYLR

RDDIAIILINQSIAELIRHVIDQHTSPIPAVLEIPSKDCPYDASKDSILRRAKGMFSAED

FR

>SG-AL_contig_6022_1_-3_3_278

PSPPFPPPFPSPPPPPPPLPFSPPPPPLPPSLRSPFISHSLSLLLPSLCLPVYPIMSSQV

CIAVIGIKEKIAYILLTSGGKVTKISLSLKLF

>SG-AL_contig_6023_1_-1_349_624

MRSIIINVPIYFDFKKKTIACYANLRLQKKRYKYLSHIKIMKLSERQRYRSHCLQDSSAF

EHGHCLKWSVWWAACFSKIFRIYLNRLVIRNV

>SG-AL_contig_6024_1_-2_26_475

MAAATESATIVEDLKDKRIPNPMYWSTAQVADWIEELGFPHYKSCFETNLIDGRRLICIE

ASAFPRIGITDFEHIKIIAKSIRDLLTIEEPDWTRSISLPPRSHLGMYLEQKSAHGKQID

TTTLIQFEERHQDPKWRPPLANHCLILPHD

>SG-AL_contig_6025_1_+2_2_154

SFYCTTPMHTRQYMRDNFKAFIQCWLPDKIHTSSKAPRIETMCHKDGRGRT

>SG-AL_contig_6026_1_+1_94_447

MDDMCQIHYHKRTVPMRATRKPYCISLSAYPLPHPFPKLTHRYHHLTSLSPSSIYQYIIP

EIHHLLPVYHYLSSHHLHSLPVFHPYSLSLSSPPFPFPHASVISESGKAKNPHFHSTL

>SG-AL_contig_6027_1_+3_3_86

HLLYCQLRMYSYRDLLCVRNFQGEVVAL

>SG-AL_contig_6028_1_-1_7_384

MSLAWTTPESPNRCCMGQLSEGTRSVGRLKLRFKDQCKNSLTEFSIGVGNWDEIARDQVR

WRSAVFTGAKTFEENRIQHAVEARQRRKCPTVADASTIFFYCGRCQKPCRSRIGLFSHER

RCPINN

>SG-AL_contig_6029_1_+1_274_732

MNRSKMNSNLTCCVFCSPAMNRSRVNSSLTCCAFCSPTMNRSKVNSNLTCCAFCSPAMNR

SKVNSSLTCCPFCSPAINRSKVNSSLTCCPFYSPAINRSEVNSNSTCCPFCSPAMNRSKV

NSKLTCCPFCSPTMNRSEVNSNSTCCPFCSPAM

>SG-AL_contig_6030_1_-3_9_326

MISLHTHARTSCRHTYTYTSKYCSACTHTHEKFICTCTCIHLMQTNLDKKNHYITDCWIN

GLSIRRVDGGLCRYLNTVYTDKRFDCLLTVLYVRENDPQGQVGEGW

>SG-AL_contig_6031_1_+3_246_485

MTMIINRMTTTLSTCAVNTGRHGHQNVFSNVFYLNSSITSTPRKTRISQPLKHHKYRYSR

KRRKRRNLHTHSPLPHNDHR

>SG-AL_contig_6032_1_+1_1_303

LPSCQSKTAQTVHHKLREGGKRKVCESLLEKKKWNGPGKQKKADGSKGKKKGRNKESNRK

EEIASTKCPEGGDTIYTDRASGIPNEESTPTIPFKLEKQKH

>SG-AL_contig_6033_1_-1_175_372

MFFSYRSKVASSTVVPGPSLSVSLPPLELPPRPDLLLLTRSGLRHKPSLLLLLTWSLCGA

RCPHEV

>SG-AL_contig_6034_1_-2_362_589

MSQTYRHLEPRRLPAGHILTSSRRRSEISALSGNPADLAFLTDSSVSLKFLPEFLVLNQA

LENPSPVFLFCPLPLS

>SG-AL_contig_6035_1_+2_2_148

HRLGMWIMAWSALPVHTQTTQGTLSPVTNPNLMHRCKQYSVIHVLINPE

>SG-AL_contig_6036_1_-3_1143_1346

MLGIYIFMNGELLYVTHLYLLLFFGFCFFLVLFFFALHFLSVIMKPFRYFRVDRHLFFTD

IKHTARVV

>SG-AL_contig_6037_1_+3_1191_1487

MQTILTITQYAGANVPATRQTLCEHTHWIGLINDYVLLSQQIFLHEKWFALPRESMSPQY

DTTLFIIPLYACIFIYEGGFFYRILTGTTLLLLCGLYHS

>SG-AL_contig_6038_1_+1_187_363

MTRKWVAPSCGDVFFSKWSGPHSTRKKFGFNKQTKKTTTTNTHTHTHTHTHTKKIKSYL

>SG-AL_contig_6039_1_-2_572_853

MKISPQSQTESTSRFLSASLLFLNNPIEITLRVLTNPLTNKKLKHTLPQTQCITHTHTHT

HTHTHTFTRIEEIQNERDTDRDRQRQEKDSMIIQ

>SG-AL_contig_6040_1_-1_1_222

SSHTNSLPFLASSVFLHARNAALLQYTCYSDVLTNVPDLFCVLGTTCMSRSAHWLRLPCK

KRVQTLNELALAVL

>SG-AL_contig_6041_1_+1_79_294

MLDIQIKQFKNECNAYVKRKKRDMCGLMPVYAGIFLKEMCPYRLLVKFWTPEIMLCRLIV

LSKSYYSRARSY

>SG-AL_contig_6042_1_+1_2386_2739

MDCFKLSNVDLASSISFVASFDRRSLSRNASPTACHWRSLSRISDVSSMACFNSCVCFFV

ASTSLCCTATAASAVSPARFPKFSRITSNAAFSAASSASFSLSLCSANRFCSSFVFLA

>SG-AL_contig_6043_1_-3_1587_3644

MSEPSTASSTTQMQNAPETIARESNTGSIQDPEAGNWESDVQKYFASIVAGGVAGGCLLL

FLLVVVVVMLWRRRSRRRSAGDIEDGKEKGGRIYDEIAEGKKQKQKDKKKERKTKGKQWD

KDNNNVSAIELVAIDTAPDIITAHDNESIYETLDGRRAARAKAGHVTVEGQSESCNVRGP

MHGSYVMTQDATQDSTYDHLHRFHKPRPPHQPPAIPEKRFTDPEVTTRPKVPPKPRLVHS

ADEEARAGGGLGNRDGGGGEEMGTGCGAVDWFGTGPAESLTDSVRSSLTELARQSVSGGK

RIVCQLTMVGVVENGKPVPLKPPAASNAYFILEPSRDSVEQGEEPSQRSPAPKFRFRSEA

SRDDERQGGDVGAGASDYFVLEPLKQQNDLEEFIDTQHPQCFVDCRKRESQDSCSEGGQH

GSQCSLHVRYSVDSFLSLKGDSSSQSFARFHSCPGECPSKENADSPPPLPPKHLTYRRSF

SSPQSSQYRRTRKDSTPAQKKMDAETLKARIQQEMMALGFLGDTDTYNDVDDTVSNRDSM

NSDIVPCSERPATNDYYVLEPQLSSEEEDQAKTDPEVQFPSTRGTDSGRNKPRCHQRQQA

PTPPPRQPGRNGHNRSKSEAPLMNSDKDQSGEQGPVIVSLKKNRPSDKYSGGATAGKASR

DDDHREPFRPRFKSESGVLPERLKRH

>SG-AL_contig_6044_1_-3_1113_1295

MIDVKYNFKVSNLFTKSVERIMIIINHSCVALFSNLIWTQCALHSYKTECDTYRSTGITH

T

>SG-AL_contig_6045_1_+3_813_1148

MWIATLKYVAIWQHVSFETRLPNFPRTTIILLYSSRKVSILCYYLSIARLFFKRAFHLAF

SFTHSTMIFDEKLQEMLQHRETGQINSVLSFIKQTAYCDAEDNSHEFAMLKM

>SG-AL_contig_6046_1_-1_25_381

MTLLHHLVAEAEKEAQDVLDFAKQLLPVLTEASRLNVENLSSERRQLEANVRKLETQVTS

AGEDVLHQFEQFIEGAKEELEETVNKMNTVDELTHKLCAHFVENEKSFKLEECLNIFKT

>SG-AL_contig_6047_1_+1_448_624

MINCRANIVLFDHDFRGTSFKTTNALWSQWIFSLQLINCRMCLGNVRLQRNFVQITNPL

>SG-AL_contig_6048_1_-1_1312_1500

MMPCRCTFFWFDFFPLLFNNDYCMIFLIMYRLFDTPRSEILRSLHILSYLYLCKLLLDCL

DTP

>SG-AL_contig_6049_1_+2_410_571

MELLTVHTYLCYAGESMLTLNLLVIHYHRINACSTCTYLCYFGKSMLTLNLLVI

>SG-AL_contig_6050_1_+2_380_478

MSIYFKYYWAGELTLRVKVILKMPSASYSLSFG

>SG-AL_contig_6051_1_+1_1_168

GGGGGRRVLFFFQFYNCEYKLNRKLNSRISKIKTAADFSYMYKMYGWIFFQIYNQK

>SG-AL_contig_6052_1_-1_1_267

QSGLEKVWEKYRILLGHICTNPVKFLHFDPDSYQKVDPDSCHGSLKCGKSCSFMTSTITT

APSMWNTVLLSITYVIRQVSLWYWYVSTI

>SG-AL_contig_6053_1_+2_68_331

MPYVATKTDFTFQHLSFELAQLSFAFQHQLFHLLCLYHPPPSTQNRVKDSLFIDEFSELQ

TDCKMLKGSYVICGDFNFQYDRVHTDVT

>SG-AL_contig_6054_1_+3_12_239

MQAVPLRRPRTSRPTAVPRQTQAPRYPPANQRSSLPSVPGLAHLLHFHGSKTTPGEPQQL

KRGWTGGPESLTWQVY

>SG-AL_contig_6055_1_-3_72_3323

MSDSEESNFSDAESEGGDLNEPLNEGSDAAPEGSDVEEEEKKGGSDDDEEKDDDADEGEK

KDGDEDDEEEEEEELEEEDYYDEEEEEDGQRPKKRKRKGALGFIIDEADVDEEDEEDDEE

WEEGAAELIDTRSTYEGPSAKEIESHQRLKQIWENQDEVEIEAYYKQRFGDTSTTDRFGE

GEEMSDEITQQGRLPGVKDPNLWVVKCRMGEEKAVVVHLMRKVIAYQLTEEPLQIKSVVA

KEGLKGYIYIEAYKQTHVKQAIEGIGNLRIGYYQQQMVPIREMTDVLKVVKETANLKPKM

WVRLKRGVFKDDLAQVDYVEPAQNLVSLKMIPRIDYSQPRGAVRSASNQEQVKKRKRKPP

QKLLDVDAVRAIGGEVSTDGDFLIFESNRYNRKGFMFKSFAMSAIVSEGIKPTLAELERF

EEQAEGMDIELAPEKSSTGEATHSLSTGDMVEVAEGELVNLQGKVIKVDGNKITLLPKHE

DLKDPLEFPASELRKCFKMGDHVKVIRGRFEGDTGLIVRVEESMVVLFSDLTMHELKVLP

KDIQLCTDMATGVDSLGQFQFGDLVQLDPQTVGVIVRLEKENFQVLNMHNRVISVKPQSI

TRKRDTRHAVALDSENNNIQVKDMVRAMDGPHSGRQGQIMHLYRSFAFLNSRMMTENGGY

FVCRTRHLASASSTRTTSLGSMGGMAPMSPRLSSPAHPSSGGGDKLAGGRGRGTTRRDRE

LIGQTVRIRQGPFKNYIGIVKDATEDTARVELHSQCKTVSVDKARLLTLTGPKAGAPAGG

QTSTYGGRTPTYGGQTPLYGSRTPMYGSQTPLHDGSRTPHYGSQTPLHEGSRTPSGAGGS

AWDPSNANTPSRMNDFDYSYDDTASPADFAATPNPATPGYQTDSPSPVGPYTPQTPGSAY

SPYHHTAPSPGAFQAPSPGGYSTPSPAGFQPSPSPGTYAGTPSPSGMGFSPMTPGAPFTP

QTPGTAMDPSMSDWYTTDIVVKIRESHDNMDIIHQIGVIRSISGGMCSVYIPSEDKMVNV

SGDHLEPVQPEVQERAKMILGEEREVVGSVISIDNTESVFKSDEGNIYMVPVRNLCKLMS

DMDS

>SG-AL_contig_6056_1_-3_117_368

MQTCITASSSTTASVQFTSNTSTQPIHMTGCLSVCPYVVVKDFLAFLELSPGLEIPTGPI

VRDNQVFRQTSGNALSTCPLAINF

>SG-AL_contig_6057_1_-3_3_167

GVGWVYILLSVIQTVQSLSGRLYSKESEYSISLKLYGHMLTLTEYHSPSFYSNST

>SG-AL_contig_6058_1_+1_439_762

MNFRKPPRMWIEHDDCDVTDNKIAKPSICEISGSGFNPSRCWTLPSVRWMSRHTGLQRLV

WVFMWDRRPILEAHVLPQALHGKESCSPGFCFRRSLFCRTAAVLHCSN

>SG-AL_contig_6059_1_-3_96_581

MLKKITCLVLFFVLFTESSSFFMPLHASCKIEWDWPGQQCPDIQTKIVAQIGKWNNTDCG

TSEKCRYGLVCDKPGYLTARHVTPEKHYVDDLSFTFESKGKGCHSEGKSRSRIWYAVLDY

GTNYCNLENLITGSGLDKIPGYTETTSDAVCTQFTSANCEKY

>SG-AL_contig_6060_1_+1_1_198

PIENLWDELDRRVRSNHSPPRDAQHLYQMLQAEWHALPQRIFTNLVNSMRTRCVDCQNSH

GGYTHY

>SG-AL_contig_6061_1_+1_1_198

PIENLWDELDRRVRSNHPPPRDAQHLYQMLQAEWQALPQRIFTNLVNSMRTRCVECQNSH

GGYTHY

>SG-AL_contig_6062_1_+2_161_502

MELHSILTIIPILINIIALGKIVPNSVALHRPALMVTSVSVGLEVCWKAEISSMGAVTQE

RCRRWGSPPQKKEKRKKKGNVFLLPSSSYTCNVKRSTVVAGHCKGLGFCVSWKH

>SG-AL_contig_6063_1_-2_35_2134

MAAGREVEGRKSRKSESQNEEEEEMEFEEEPDFNDPPDFVDDITDDELLADLIKKKPKTT

DGIDNVIVVDNIPQVGPERLEKLKNVIRKLYSKFGKVVNDFYPTEGDKTKGYMFLEFSSP

QHAQEAVKMTNGYKLDKQHTFAVNLFSDFDRYRNIPDEWEEPEIRPYEDKGNLRSWLLNK

DCYDQFSVIYDAGEKTAIFLNSADPSKVEERNRWTETYVRWSPQGTYLATFHLKGIALWG

GEKFVQIQRFSHPGVQLIDFSPCERYLVTFSPLADNREDPQAIIIWDVRTGQKKRGFHCE

SQTTWPIFKWNPDGSYFARMGVDTISVYETPSFGLLDKKSLKITSIKDFSWSPTDSIIAY

WVPEESNIPARVTLIEIPSRQELCVRNLFNVADCKMHWQKNGDHLCVKVDRYSKAKKVEE

KDQLKYSGIYYNFELFRIREKQIPVDKVEIKEQVLAFAWEPVNNKFAFIHGESPRIGVSF

YAIQPKGKVEHLKTFEKRQANHLFWSPNGQFIVLAGLRSMNGVLEFVDTQDMTVMAQTEH

FMATDVEWDPTGRYVTSAVSWWGHKVDNAFWIWNFQGNLLVKQPMERFCQLMWRPRAPSL

LADKQIKEIKRNFKTWAQKFETEDAMRKMTASKELIEKRQSLMEEFSRMRAEKEELYKEM

KSKRLELRGGVDTDELDSHVDNFEEETIEFLVKVEETIEE

>SG-AL_contig_6064_1_+3_3_203

THDFERVYINFFGLNFNTFVRLRYFVLMTFGKEVIGDKVHSYGLNSWLQDTQHISIHVCV

YYDCLPA

>SG-AL_contig_6065_1_+1_37_261

MLKSGGECVVHWMRTLCNQVWNSGEVPEDWKNGTIVCIPKKGNLTECDNWRGVTLLSIPG

KVYCQLISNRKRDVV

>SG-AL_contig_6066_1_+2_44_289

MTETLKSDDECFVHWMCTLCNQAWNSGEVPEDWKRTLVKKCLKAGRAEPLFAFPRSATLP

NVITRRGLLSCPSQARFTASSS

>SG-AL_contig_6067_1_+1_1525_1764

MNTNYTSCQCSLIPCDASHHQCNKLIKNGWITITWLLKKPNKTLNCQRDFSLFHFLNRYS

TRFKFLFLKQSKTISFYYVS

>SG-AL_contig_6068_1_+1_1_111

HTHTYTYIIHTSLFSISHSQSLYGQNNEMTWCTTENK

>SG-AL_contig_6069_1_-3_6_1244

MGTRVMTEYSYPEGQFSSSSRQREIWGDQENVSAGYQVPKKDSSQNDHIQKIAGVSIGFC

SIFAEQLISHPCIVLRRQCQVHHNGKGYHLTPFTLIQTLLNLQRTQGGWTLWKGIGGVFV

VRGINLVSENVISEVTNFPRDVSRHSSLKKYGEHILLKTMGFIICTPFYAASLIETVQSD

IASEKPGILDILREGVSRVAGWGAPHSGRLIPVWHLVGPTIALRLLHFFISAVAQYTVLS

SKRSEQQERRDLPGQRGLPLTPQEQHFPDLLANFTGQLLADFVLFPLETVVHRLYIQGTR

TIIDNTDTGLGVVPISTRYEGFVDCFRAIFAEEGFQGFYRGFGALVLQYMAHALILRTAK

MLFERLSQEFGPPPPHMIRMVNLSGQRSPPSPSSGAYSDLSAPSFDRSEPRHL

>SG-AL_contig_6070_1_+1_1_177

HFAKVDTFSENYWHFAKVNTLSQENYWHFAKAYTLSWKTTGKQLWQLRKICLGYLFMHK

>SG-AL_contig_6071_1_-3_129_1310

MPSSVGKFANQAVVLCLVSGLVSVLFRCAHGHGGHGDEESAKLIIVSFDGFRWDYLSRTN

TPNFDRVIRGGVQARYGIKDAFITKTLPNHFTIATGLYEESHGIVGNMMYDPVLKETFNL

KNRSQTASSRWFDVGAEPIWVTNQIQKKNGRSGCMMWVGCEAPIKGVVPTRHIPYDPTMP

NNTRIDQTIEWFTGPYPVNLGLIYFQEPDHTGHLYGPDSQEITDMIGGLDKVVGHLLDKL

EKSGLLQHTNLIITSDHGFAATDPDRIINLDDYIDPTAYRVFTVSPVAAILPDAGLENEI

FANLSSGAQKNSHFTVYKNSTVPVEYHYTHNRRIMPIIVVAKESYSIVFNHSTFVTKGNH

GYNNSLQDMHPFFIAMGPGFREGAEVPTFNNVDI

>SG-AL_contig_6072_1_-1_313_477

MKRYSVFLYCFVDQTFSLMAKIKLFLYTVFHNTYYFDIYDRKRNVLQYIPPPKKK

>SG-AL_contig_6073_1_-2_8_1882

MLTVAGLMVLMCAGVSAFYVPGVAPVEFHAGDPVEVKAVKLTSTKTQLPYEYYSLPFCHP

EGGIVYKTENLGEVLRGDRIVNTPYKVKMARDVKCQLLCQANKESMSVKLTQEQAGIFMD

KIKHDYYVHLIVDNLPCATKFEMESKEPQVEQGFKLGFVVGNDVYLNNHLKLILSYHTEN

NEDHRVVGFEVEPYSVSLDQLNKDANGVCSFKAADKKPVPQKLNEKGETEVMFSYEVEWR

KSTVRWASRWDIYLAMTDVQIHWFSIINSVVVVFFLSGILTMIMVRTLRRDIAQYNKDDD

LDETVEETGWKLVHGDVFRPPRHTLLLTALVGAGIQVFFMAFITIFFAMLGMLSPASRGA

LMTASIFLFMFMGLFAGYYAARLYKTMKGTLWKRAAFQTATVYPGIVFGACFLLNFFIWG

KHSSGAVPFTTMLALFFMWFGISVPLVFLGFYFGFRKTPYEHPVRTNQIPRQVPEQTWYM

NHFLGTLMAGILPFGAMFIELFFIFTAIWENQFYYLFGFLFLVFIILIVSVSQISVVMVY

FQLCGEDYHWWWRSFIVSGGSAIYVFFYSIFYFHTKLEITEFVSTLLYFGYTFIIVFTFW

ILTGTIGFFAAYWFIRKIYGAIKID

>SG-AL_contig_6074_1_+1_1_243

PPPKKKKLQHTTLTGSTWDHSTNNKRHRSISNDDEKSKHHPLQKSRADFFEGKHQQKSKK

QLAHVTTKWRTAEHNCLAPLD

>SG-AL_contig_6075_1_+2_2_247

IIYILFLYLGTLNVHGLTNKTKRKNLYTYLKKHKTDTTCLQETYITKETVREREWRGKLH

YTEGTQNSHCLLIIIHKQRNTL

>SG-AL_contig_6076_1_+3_159_380

MEIKKEKCTDATDNKQQNININKTLSSHRLLAQKVCWNSALDHPQQMKGSTTEIIQVQIQ

SSAQLKQGNSQKPT

>SG-AL_contig_6077_1_-1_1_522

VSSLFPSPCHSLCLVSSLFPLPCRSSCLVLLLFPLPCHSSCLVSLLFPLPCHSERNWSSH

FFLLPCHSSCLVSSLFPLPCHSLYVVLSLFPFPCHSLCVVLSLFPYPVTVCVWSYHCFHF

PVTVCVWSYHCFHYPVTVRVWSYHCFHYPVTVCVWSYHCFRYPVTVCVWSHHCF

>SG-AL_contig_6078_1_-1_1_237

WSHHCFRYPVTVNVIGLITVSITLSQFVSGLIIISISLSQFVFGLIIVFPLPCHSLCLVS

SLFPLPCHSLCLVSSLFPS

>SG-AL_contig_6079_1_+3_195_464

MFSQVDSNDGSFHGNGWRVILGGTTDGELSYISMLTLFQRHLTVTAGNAIDTKHNSTRNL

VRTSRPCRHAGFMCRLMAFHYFAHYITWNL

>SG-AL_contig_6080_1_+1_181_330

MSKHNLKSPREHAFSFIAPSIWNSLPADLRDVPTLSHFKSQLKTYLFTQA

>SG-AL_contig_6081_1_+1_73_375

MKKRKHDHLTPLLKELHWLPVKSHCQYKIASLAYRHFDRSLPPYLLDRLCTYQTSRTLRS

STQKFLMVPKRNLKSAGKHAFSFIAPSVWNSLPVDLRDVPT

>SG-AL_contig_6082_1_-3_165_926

MFCLKTAPLVRSAVHSRILSNGLHSTSALNAKVAVVLSGCGVYDGTEVHEASATLVHLSR

DGAEVAMFAPDRDQLHVIDHTKGEPMEPKRNVLVESARIARGKIEDLSNLEASNFDAVIF

PGGFGAAKNLCTFAVDGTAMEVDTNIKRVIKEFHQAKKPIGLCCIAPVLAAKLLPGCEVT

VGSDEEEGGKWPYAGTAAAIGSMDCTHVKKDVTECHVDHSNKIVTTPAFMCETAIHKIFD

GIGLMVKGVLNLAK

>SG-AL_contig_6083_1_+2_326_529

MKSVYRKEEGTEAEGRGGQKVVSTEQFLEGQSLFSHSEMCNIIPNYLKQIKFIPLLLDVS

CSPNSIRK

>SG-AL_contig_6084_1_-3_456_1049

MATDAHDASFQSQDAAQMSRKRRQSNAHQDMAGPVIRRMSRLEARPSIQYGGSRRMSYAS

RSSISGHSWGMGQIKPMVKLANTYRLAPKPEEKFNSATARKLMTSVLQSYLEGERYDKTL

SISLSKNLADVIKERMKDQGFSQRYKYVCVVTMGEKKDQGMAVCSRCVWNTDTDNYASAS

YTKGDLFAVATLYALYFE

>SG-AL_contig_6085_1_-1_643_876

MIEFREMKMYDENKTCPPFPFPQRPGGHRGIVPAFQSDNTDLSDLRKNLCVRPQIHPAMN

GYLALFRTEKRIVCLAGE

>SG-AL_contig_6086_1_+1_1_264

VVLLVLGHDRVILRFLDLKKSIYYGGTILALECQKKKLDRLIDRIFDGSRIRRRQSLFTI

LPIKHKQENRSNKTRNKLTSLKRGRCHT

>SG-AL_contig_6087_1_-2_14_781

MAAERRKVASTSDDKALDTDKQFLERTQVNAVIRDVIGKMIANRPDDPVTFLADFFEALV

EEQSSLVQRALRIITMTHHSRPVFEINVRDAYSLLNKHKVSKKLKGVNGAVYTELLQALC

KTLPSTVSVKLVKKLECFDYEAVHFDVFRSSIFTCCVLREYAAVAEQLFASLDIQKNGKA

DKALCEAVLDQLKTALGSSRNDVKRIIESSYNLAPEGLHQALERALSREQAQGVFTAEQF

VTEACDSFLAKVKKLK

>SG-AL_contig_6088_1_-2_2_823

LASSVRYLPRCLRRFSACRAQLENVKDFPHVRAQISTGSACRLQGCHKESTLARLSCSSV

VTSFQPCRTFHCSSALYEEIRIVTTPAFPESVAEGDVRWEKAVGDFVAEDESVGEIETDK

TSLALFAPAAGVIEELLVPDGGKVEAGQQLYKLKITGEAPPKKVEPAAEKPAAAAPEAAA

PPPPPPPPTSQPSTAGPPPSTPPPVPPMPSQPMASVPTSAVKPTPATSTTPSSVTGTRTE

QRVKMNRMRQRIAQRLKDAQNTCAMLTTFNEIDM

>SG-AL_contig_6089_1_-3_3_371

QVQTGQEQLCCRLHPGDFTLHTSINDGQEFAGSIYQRVNPDLETAVNLSWTSGTNATRFA

LGAKYILDKDASISAKVNNSSQIGLGYTQKLRDGVKLSLSALIEGKSINQGGHKVGLGLD

LEA

>SG-AL_contig_6090_1_+3_3_269

EGVKGFRTIKCIPASSALSRNIYNSMMNHSFVLTSKITRFCIKVTSINSITHVIFPFSIF

SYTINMHIVNTNHTDCAFHSLPHVRSLLF

>SG-AL_contig_6091_1_-1_1_351

MVPHMEPMVPHMEPMVPLQEPMVPLQEPMGPQEDIQLVMSRLHRLHTLLLPLHMVPLVSN

HTMGLTLGKGLPLQLRMVVTLLLAMDPVDSLSLDNILVSTLRRCSPEEENQLLQTSA

>SG-AL_contig_6092_1_-3_3_236

CAAGETPGHKPGQSVKENLLLPMQQAHPFHDTGKIQGRGQSQYSLELVKGSVRTQQIISG

NYHPGVWCQCVSQCSERQ

>SG-AL_contig_6093_1_+2_335_484

MYFYTCLKECIANDTKWGVHLGLRKTAGITASAFLLLSNKETMTQIQIHD

>SG-AL_contig_6094_1_-2_2150_2446

MHLCMQHLSDYFSPCVCIAPDVLLFSCSSEWRMLLVEVQSASFCVCTLGFNLCQLLQIIQ

SIHEVQNMYILSPLLAVATKIRLFWQNCFTCYVFAVIIF

>SG-AL_contig_6095_1_-3_219_383

MIAQPVTQLVACLPKYVYDIIICVCMLSYCIFPFTPSPPYPPAFCRTSARVNQQP

>SG-AL_contig_6096_1_+2_2_256

WSNGEDSWLPNWRVWKAWLRILLWHLAWWVKGGDLGLEGLGSNPPWAPHLVGRDFSDLSG

KIFADLLVPVAPFVCICTQKIKYTC

>SG-AL_contig_6097_1_-2_329_586

MFPSFHFQYLFTPFTLAPQHTTKPRFILLEATHWRGPCTTAWSGGTSFSQVLEISISVVR

QNNVIYKTVFNNKNKDKNNDITKKLI

>SG-AL_contig_6098_1_-1_388_651

MGRVKGTQMKSNIGIALLYCTVLYCIVLYFLLQWIHHVKFGLLSRGERIGTLFFFFFNAC

WCFCAFTSHRTQTRLLGTLMCTSDVLHA

>SG-AL_contig_6099_1_+2_155_445

MHAKYPVSDASVQLLTETRKYLVYTPPRKWSMTANMVSNSPKTEYADYAATQCLCSVGQA

EECAYKLLRKHCLEWGEKLMHYFFCSFDCNLFIIAFL

>SG-AL_contig_6100_1_-2_74_760

MDKKQPKHHIRQEVCTTRPSYRDGRKLTSVKVYTIHQESKYLLIQNVPAVGAKDQLAKLL

SAYGTIDDLHPLDEYPADKFTDVYLVKFQRIQSARFAKKKLDNHSFFGGNLHICYVPEYE

TVQETREKLQERRKVIAAKVRKLEKEAESDSSHQASGSKPANTPNGSQLSLPTSQQTLAL

PPPPPLSQPPPPPPLSQPPPPPPPAMPSVSVVNQPNPSPAAGFHQNFHS

>SG-AL_contig_6101_1_-2_2_199

ATCGFCDVIIKIKKSEFFSIDSDSFSPQIVTLTYEKCFSVFQEHRDISSDHHTCFFNSVF

PECDLM

>SG-AL_contig_6102_1_+2_143_358

MMNPLEKIHLIPTQIQLGVGQPCPVSKNCSPPYHMFTVTAGTKVSCKWNQEYFLLSDLFF

SHFKGPVNTILN

>SG-AL_contig_6103_1_+2_2_199

LIFVMPFLKFCLFFLIISTLSLHFPNLYYSAQHFSCPTSCFPLTSRFVCNKYWIFTALCC

SLWTPE

>SG-AL_contig_6104_1_+1_955_1275

MQKVVMTSNLTKICIKKVADTELLLTSQNDQRKVSQIYNTTFYLPKVQNGDSVLLISLHF

NLTESKRNPAKVMDFREVYHTTREVMKHSHRQDFTHVCMCSRFHCLE

>SG-AL_contig_6106_1_-1_157_312

MSYFNMVEFGSDSVSMLKTMIIVLVIILNFYVQSSRSIFGNLFMYYMCSYLI

>SG-AL_contig_6107_1_-1_1_159

KKINLRENISRSELPCGQPTGNIRFPSGLNACFKPYGSSHKGDLSMRLLKITF

>SG-AL_contig_6108_1_-1_241_390

MHTLFHVCFGSHFKQVKSASALRSEFLVTLLCFSAVQDFLHYKSGVYPAK

>SG-AL_contig_6109_1_-2_23_1021

MLNYTLVYWRRNDTLTSLALDSSFLTSGAVTCVLLHKRNSREPVLEGLLPSTRYTICVEN

GFIDSCVLSSFTQSSSPSSSLPSSVSQWPSNCVTIYTPKTSPPRHVITKSSTVTVVAVVS

VFILTLLVVVFVVFLLRRHNVAFRLLMDSVCGKCCCLSCTCCGHSGGHTSQDRTRLSHSS

SQISRTTFIDDDSPRDNGTCKRPLLKWLRPCQKKPKSSTYTSANMLLAPPPSSNPADGFS

HSESLPLGFDLIDDDCVFDNSPSEQPAAVAALPALRGGRNKPRHRLSRKRRGYVAYSTRK

DKNIRLTTVLESPIGDDDDDFCESFLGCGHSSI

>SG-AL_contig_6110_1_-1_1_171

FVCLGFFPSQFNCFAMCILIPSFVITRKIIPIGRSIVLVKRQYQTIYLLSALILILR

>SG-AL_contig_6111_1_+2_548_787

MLQALSKPTEVTINVSRCWINFKSQPVSTNIANTPSGMVTCKIIIENNSGSTLHKDEGEE

GKQHKSASISNPLTLHPIHQ

>SG-AL_contig_6112_1_+2_2_97

SKTSAVSLNSGPFVLYLRHIFRLDLKSVFFSL

>SG-AL_contig_6113_1_+1_193_468

MSDQSCSMLQGHGELTARSRSCLRGFEGRCLRWILKIRWDQRRDGQKIWHMQPLAVISFS

YQQYCQQGERRDGDGWATFSGWTKTKHHMMAR

>SG-AL_contig_6114_1_-1_1_153

FSFLYCQLFSAFPVQSTFPPCKPSHSFWPPLLPTPAQFMMYYLCCFTLATR

>SG-AL_contig_6115_1_+2_968_1228

MLFQAKQRANRMGEPNLNARHGLQASGLQSRSCCNREERNAHAPIVKPTSGLLCVCSFKL

KRTLFSLNPRTNKSNNYAFFSFKHKRS

>SG-AL_contig_6116_1_+3_3_122

QILNFSMYADFSYVPSGNMICFICEHALFLNFSKLCLLIM

>SG-AL_contig_6117_1_-3_3_281

CLSLCLPLSLFLTLSLSVSVSLKSAVSPSLHSLSLFLPLPPPFSLPSLSLFHLAPSHLVI

FPPHSFLSFFLLFVSSLSVWLFLFEFCFVLFLR

>SG-AL_contig_6118_1_-3_3_122

VHVDDDDDDDDDDDDDVVLMIVMTILTKTMNMMMRIVFFF

>SG-AL_contig_6119_1_+1_1_384

EPGLLEETIEGGQIQDRSPTPRGLGNHKHPRVEPRTWVQQLRDRPLQKELDDHLPQDPLL

LLHAGHVRGRSRSQGWPGIQPQQPVPRFQHRNDPLVEPQLAPTRSMPREAASLEGLNEWR

CWVGAKSL

>SG-AL_contig_6120_1_-2_35_532

MGSSTVGQPDPSPGVDGGARRGARSKKDFRTPEFMDLVLDWPAFEARGARVIAPPGRDSV

SVLGALVVGKFLKDSRGGVWGWYPENHPAFKVAYPSPQHRVLVPQLLSFPPLASLSFKTL

GEWDRMEWRAVLEASTAHTSLWAMAWRLRVSKILLRISASVCSALG

>SG-AL_contig_6121_1_+3_105_248

MEKSRHQYLVFLNPLTPSAYPPVPAERVKVDGVPAGRPHRYPAGRVTV

>SG-AL_contig_6122_1_+1_1_273

FLRAYKEAGSSSLPDSPRAHELRALSSSAASQHSLHVRDVLDTAYWRSESTFHKALPPSL

QCRRSVLHLLHSGQCRYRRHQGRRSLPSSSL

>SG-AL_contig_6123_1_-2_2_175

GSLTCIFDVLHAYTHEGGSGTSRSAHMLTWEIGKISTLCPPGEVTEEESNPGLLNAPV

>SG-AL_contig_6124_1_-3_3_155

KQIISDDLPQTNKLSVMIYPKQTNYQFSSDTLFFKDSSIRSHWIFLTARSC

>SG-AL_contig_6125_1_+2_116_694

MSSQKGNVHRTRAQKHKNSTAFKNTKYDTSQRTKNMLNVTQDGLCERCKEKIEWKIKYKK

YKPLTQPATCTRCHQKTVKKAYYTVCQPCAMSLGICAKCAQKKDVVSDFAPNEQTQAAEQ

SEMEQQIKFLPERERRTLFRLKAQGKLTEGLKGEDIDESDDDVDTAKNSDADDCPSEEED

EDEEKVDAHTLFE

>SG-AL_contig_6126_1_+2_2249_2707

MDTICHLQLQGVCFHKGISACHSMYNEHLSHWHANQRTAVLCIHREVAVLLFVEPPTVPA

FLQRQAISLQRAEAHFQHQGMDHNPESICGAAAGGSGEHISELSRHGGRGRLATQHGLLQ

AAVHHRVLQRTGGFALSVFSEGCSPNNVLSMSC

>SG-AL_contig_6127_1_+1_1855_2100

MVVSQIHMKSTVMSITDCTSCTRQPAFKADIPFSQIKQCTEKKNLSGTKSLSLSLSPSLS

LLHTHTNTHTHTHTHTHTDSYH

>SG-AL_contig_6128_1_-2_611_850

MKEMLQCSFSLSVHLLLKSFHEYTFHSLHSFADRPTVCVCACVCVCVCVCVCVFKVTVTI

SLTNPPPQFIALFFCCWFFF

>SG-AL_contig_6129_1_+3_246_623

MCCVTLETWIGLIALMYYVQKAYSVLNSFFQFLNLSALQLYFHVLRHTRDMDRVDRTYVL

CTKSLLSSKLLLSVSSLSPDCFTAIKTSRFSLTALFSNILAKPYKHLSRTVKTIRTVNYF

LLVCFC

>SG-AL_contig_6130_1_+2_2_199

FDVLHAYTHEGGSGSSRSACMLTWEIRKISTLYPPGEVPKEDSNPGLPNPPVWKSSVFTI

QPPCSC

>SG-AL_contig_6131_1_+1_196_330

MMVKTKQLLYSHKLHAHHTQVSSTLRLIKLLSMDFKIKKIHILKL

>SG-AL_contig_6132_1_+2_521_796

MRYPKFAFSGSRFQTTCANELSPLFSGPTLCRCVHLLSLAALPFLTLQRVEFLSMVGCTE

GHTHQVFLKLAICGVRLEGRRSISMLTRPLVK

>SG-AL_contig_6133_1_+1_271_531

MYVCVQTCLEPPEPSSCIYASRTSNTLVKDPVSHVSVRQLVETGKHPACIPTKDVVCRLC

SNITPAQCGHNNVHTCIQENTAQNEKK

>SG-AL_contig_6134_1_+1_22_240

MMDFVNPVDGIHRRTRYTHNTSNRDDARALINALHRKMQLTEKKVSPTKTTHANVSSLQN

NVQNLTLQLIELC

>SG-AL_contig_6135_1_-1_187_1275

MSQVVAATNKQLLEKYLQLDQPDDGVMAEYIWIDGTGEGIRSKCRTVTKEPKKPNDCPVW

NFDGSSTYQAEGSNSDMYLYPVALFRDPFRGGKNKLVLCEVFKYNKKPAETNRRKTCNEV

MERAKDSKPWFGIEQEYTLLDYDGHPFGWPKNGFPGPQGPYYCGVGANKVYGRDVVEAHY

RACLYAGVKIAGCNAEVMPAQWEFQVGPCEGIQMGDHLWIARYIMHRVAEEFGVVVTLDP

KPMEGDWNGAGAHCNYSTLAMREPGGVKEIEKAIEKLSKHHVRHIKAYDPREGKDNERRL

TGFHETSSIHDFSAGVANRGSSIRIPRQVAEEEMGYLEDRRPSSNCDPYSVTEVLVRTTV

LDE

>SG-AL_contig_6136_1_+1_55_180

MERNSRVKKQKKQITQELLSMEAKYGFYCRMQGSVQLPKTAW

>SG-AL_contig_6137_1_-2_62_658

MDKPESNTLGISWHDSHWIPMLNQNNVLDYFSERSNPFYDRTCNNEVIKMQHLSPDHLVN

MTGLEFTLLHAQEPILYIIRKQNRLSPTQVTPLANYYIIAGVVYQGPDLASVINSRILNT

LTHLQSAFEETHSFSRYQPSKGYWWEFDKKKEEKPEEEQKKDEDKKKKEEPSSMFQRQRV

DLLLGEIAKKFPPQFVPRP

>SG-AL_contig_6138_1_-3_3_419

SVSSATKSKLSLFSAPDSGPSAGEGSQSDASTFLHNKLEFLQPDKIRDASKRSPSDPDYD

PRTLYVPESFLSKQTPAMRQWWGMKSQHYDTILFFKMGKFYELFHMDSTTGVEELGLMYM

KGEHAHSGFPEIAYSRYAD

>SG-AL_contig_6139_1_+2_104_325

MMDLLPSVVGTSHMDSTVISQHARRELVDLIINLGIISRKIAQDPISYLYFHEATSKMNG

RLDTVPVYAYHYLK

>SG-AL_contig_6140_1_+3_3_200

GEGYRRLTVRSFTALREVWASKARSARRQLLALEVEGKPCSDKSWRSGGDPRGATRLTDE

AKKKGL

>SG-AL_contig_6141_1_+2_551_778

MMVMEHKHSFCFRPLRIIFPLYHHCDLQNYMIQTRFLQKLAHKAMKLLMTYFYILTFCEC

MALKFSHANLKLSIFT

>SG-AL_contig_6142_1_-3_3_425

AKQEATYPAAAATNGAPSAPNRVHTSKAAKKNAKRKEKKKQEQSNEDAPCTAQLTESLEQ

VSLSSSSAPAPVKQDSGDGKAESAKKLKNLKKKLKQILDLENKISSGELKNPEKDQLEKV

AKKSMIMDEIEELELELNDDS

>SG-AL_contig_6143_1_+2_122_448

MISNRFFLSVNLFAVLFIPACIKGLVSHSNYTYMSIQVVVTFLKSFNGNMQLKWKGKVAD

AEDEIYQQSSGAVRHVIRWNTPSIGRWKIFDVAPQASVWSVRGLVGSAV

>SG-AL_contig_6144_1_+2_95_217

MYITVVTNLTKPSWSSHTKNFPFTFFTIFHAIFAMKINEYI

>SG-AL_contig_6145_1_-2_560_940

MYPTLTDVNPLSVFHKHQSPHLFLLKHKPAFDLSSANKVHIFFQYGKKHISIHSLFNTPS

PQLLQFIHPFIQPFHNKSPVTTTLYTTGNWLLRPREWKCCCVMIVPLKNKLSLAQVSIQP

SSYQIVA

>SG-AL_contig_6146_1_+3_306_641

MEYFMTPFFDTRQKIKTLTMEYFKTPFFDTRQKINTLTMEHLKTPFFDTRQEINTLTMEH

LKTPFFDTRQKINTLTKEHLKTPFFDTRQEINTLTMEYFKTHFLTLGKRSTR

>SG-AL_contig_6147_1_-2_2_190

TGSTGKQVDHLAVTRKLMLELVPTSTSSLEVDMDVAVQVAPLVLLLPPKSKSSLSESGVY

DSV

>SG-AL_contig_6148_1_+1_1_534

DHRPVLLSISGSPPTDPPQYPRWNYKKAKWGLFSIRTNELTRDITVDGRNINNVVRDFNA

SIIKAAKETIPRGVRKNHTPYWTSTLQDAHDDLTRAREEAETNPSQENNIKLQRCKALYL

KTKVECTRRSWRDKTSKLNLEKDTTKLWRLARALNDEGNREQKITLEEDGKTLTGKTA

>SG-AL_contig_6149_1_+3_3_245

SISGSPPADYPQHPRWNYKKKQNWGVQHLYQLTDTRHYSGGKEHQQRGLRLQCQHHQGCQ

GNHTHRKQSHLILDVNTPGCT

>SG-AL_contig_6150_1_+2_329_601

MLFIFHRLQVQAHAHTHTHIHMHKPVQLQDLKCAVTPSKHFVSLRATHEYNCKSGFYHGV

CTSMAHGLAYISYIWYFMQRTLFNKPTGWGT

>SG-AL_contig_6151_1_-1_310_888

MKTVSKTDIYDPLGIRHLLPPICLLNQIRQKTTTTKKQQKTPTKSMSCVVNQINIPIQFS

HVVCSNFCEMLGITFITFTYYFHTACRACSKNKCFTYTTMSLRLKLLFPPSKLSLGLKLL

FPPSNLSLGLQQPSPPSKLSLGLQQPFPASKLSLGLKQLFPASKLSLGLKQPFPASKLSL

GLKQLFPPLKLTL

>SG-AL_contig_6152_1_-2_257_340

MIFLFSFSGWILTDIYHNYISVYLFLFC

>SG-AL_contig_6153_1_+2_2_439

QFTLKKKSNNNKKQNKNTNLYTYRHCLLNIDLLANDKYYITVFVINCHKQSVFFPSSSSF

IILFIYFSKISIFCPCFGATLLRYVKMLMFNIFNQVIILCLWTGHDRMNHHIFFFFSFAI

TPGTQPRGRNGSSPAAHPQAHTAPPP

>SG-AL_contig_6154_1_-2_2_115

RQRIMTWLKLLNINIFTYLSTVAPKHRYKIDILEKVNE

>SG-AL_contig_6155_1_-3_435_524

MIIVLSAKCSDEDKLRDLRSMCVEHGYIHV

>SG-AL_contig_6156_1_+3_3_248

VCVCVCVGVCVCVCVCGCVWGCVCVCVCVCVCVGVCACVCFTSVLVVLLFKVQFSGLYFM

FSPDVFFSTLKCCRYYDSVFNA

>SG-AL_contig_6157_1_+2_224_418

MMLLLLLLLLLLFCFVFCCPIFCATSVVFILQRSFCLLHTTPNPSSFSGNPSARKPMGAS

NVEPL

>SG-AL_contig_6158_1_-2_2_220

RSWKIHHPYEGGICTVLLLWLFVPTVTGIIQCRSMYHCMWYVAFYSSECLYLHVAFPVLC

FFVRSFLCCLFLL

>SG-AL_contig_6159_1_+1_1_252

CCCGIFYISKVHAAYWAWIYRFVQKTGTHTTTEDLMEKGEKLIQQGLEPWTYVLLAFQAD

LLSTGLPLYFTTDLLLVLRDLVGE

>SG-AL_contig_6160_1_+2_53_238

MKLRYCYFISCLQVTEFTRRLSVFCFAKKRTLKCAVAAVYSMIGRPCVCKCTPPYTPPYD

KQ

>SG-AL_contig_6161_1_+3_213_503

MAVLYVVEQCFVFVWGLKQTLKEKNRSSDLSSTLGDNGIMGRLGFPLRIMKNNDQNQSVD

KGQSHSLLVVRKERRVETQTGPIRQTAVEGQMEFKAY

>SG-AL_contig_6162_1_+1_310_603

MHIHGKVYIVVTAHNCHSTNLHGQSMYSGNIYLLLTLCVTVTPVTNTKRVKFIQRLRTCD

TYFSPFCSMHIFPPTPFFLRIYSLYTLSLSLTHPTYIK

>SG-AL_contig_6163_1_+3_570_818

MMTQMHQTQSFPCATITLTLSLLHSIHQLPEDGARELRVSDITNAHLLASNSAPSYRMLQ

PPFSLKGFSLGLAKPRIRKSSSM

>SG-AL_contig_6164_1_+1_328_762

MLTYPSSFRNLHLLMNTRCTKSVLLFILLLFTVYVQTHLMKCPLFSVIPAHSLKHLCACA

HTHTHTQCTHTHNAHTHTHTHTHTHTHTFSLSSYKLSFSLQLYTLFDIFQLKACLHQKQF

FSNVHLHQSKTICCVIHWTIPQCCS

>SG-AL_contig_6165_1_+2_227_454

MTEASVISHWSHSVFTVYSSVEDRGHHHYLGSLTVLSIHISNCLLLPSSVRFVLIVLFIM

FNYKAVNCLEVKSPKL

>SG-AL_contig_6166_1_-3_198_377

MQQHYLSFVYENLAMIIYNETRHECNRLLYNSCNLDLLERTLYSHKDILYYISLCIKVTL

>SG-AL_contig_6167_1_+3_3_173

SDFFFFHQYACFYFPLSPKPAKRTVSELKQNQECTRETNISCNNIKCTHPNILSLMN

>SG-AL_contig_6168_1_-3_864_2648

MIRKEPERGGGRRPLLAAFVGNNRQMLNDIRDSLSHLRKSQLEGDGEGGMQQGKTELSQS

TPNLADKAAATPTGTKKGLENHTKTLAEIRETLRPFQNASGGRDRPQGSGDGDVTRSKIQ

QIMALGVDEDIAVRALKLCNNVDGAVQLLCQIIESRNGSSKISAGLPSYPVNRRPSFESK

YGPSSPSPGASETNSVRSDSPSLSNVPPTARHNAQVNGHQTPPPLPPRAPINTPQPPARG

HTPPPLMQGEATAATPNGSAPQTSSADIPAGHVQQMIKRMSPGQFRTQVVHNQTQPAGQS

GMTNLGLQQRGLGPMTGMATRQALAAYSQGQGQGVALSQQLQQGLVLAQGSLPSSTASST

SSSASSTPALSGPRLTINLSHPPPPPYSQAQVQSQAGGTPTLLHKAVEQSHLLGAQLSDY

HLALAPPDSPLNMSTTSSSVTSGFSDNSGGLMQGVGPARGPALPVVADRRPMAPHAPVIM

QSVKSKEVHKPLPQTATAPMSPPPNLPPTPQHNYISQVQAQVNMSVGSGPVPSTPAALVT

ATPTTTTTTTHTRNLQIHITGQAPHHVVHSHPHTGPQYATEPLMPPPHTALPARP

>SG-AL_contig_6169_1_-1_76_1455

MSSFAPTSGAQASSGNYAAFDAADAEFFKGIGQIPYKPDAGPEESLVYKHYNPKEVVMGK

TMEEWLRFSVCFWHTFRGTGADPFGFPTLVRPWDDGSNTMANAKRRLRAGFEFFSKLGVK

HWTFHDRDIAPEGDSIKQSGDNLDVMTDLALELQKKTGVRLLWATCNLFAHPRYMNGAST

NPDAHVVAYAAAQVKKGLDIAKKLGAENFVFWGGREGFHSLLNSNVRAELDHMAAFFRMA

IAYKKKIGFNGVFLIEPKPKEPSKHQYDYDAMTVIGFLRTYGLEKEIKLNIEPNHTTLAG

HSYEHDVVMASAYELLGSIDANTGSPDLGWDTDQFPMDIKNCTMVMKVVLEQGGIAPGGL

NFDCKVRRESTGLKDMFISHIGAMDSMARGLKVAAKILQDGVIPRYVKERYSSFESGIGE

KIAKGEATLEDCEKYVQEKGEPPRLSGQQEHYENMLNYYV

>SG-AL_contig_6170_1_-2_2_205

CCCLFVCLNKCLFMNSVIKQQFITHFNIFCTTMAIKQTDLVQIYSLARQKRHFRNYFCPQ

FCNYFQHL

>SG-AL_contig_6171_1_+2_2_139

NAVVKYHTVMSHHLSSLRLYLNLWCSMTSCKTDPMGPNATILEKSV

>SG-AL_contig_6172_1_+1_175_339

MYLTMQFVSTILKVGQKFYDLDWQYGLVRSNPFFSSKVLPFSVCLDKHVLFLCKL

>SG-AL_contig_6173_1_-2_50_2308

MSNGTQVFVGRYCGNTAPPVISSSSKRLIVKFRSDSSNSASGFSANWTTGCGGVFTDTSG

VIMSPGYPTQYEDNLRCNYTILGDPQHYVLLSFDSSVYELEGGRTNCPFDFIQVFGGRNA

SASQLGKFCGTSVPGPLHSLGAMHLRFWTDHSIVKVGFKARYRIAECGGVFTEPYGVIRT

PTFPNPYAHNSNCTWTITVNASNVVALKFTSFSLEAGSRCQYDYVAIYDGDTLSSPLIGR

YCGTTPPGLIKSTTNKLLVNFVTDSSVSSDGVSAGYWSTYGPQQGCGGVINQTSGVVRSF

TLGSTGRYVNNLDCRWTIFVTPNKVISLNVTDLDIENGGPGCSFDYVAIYDGLSNNDAQL

AKLCNNNAASQTSFMTASNVAYIVFISDHSIVNRGFNISFQQQDPVCGGTLTATQAAQNI

TSPNYPNAYNQAVRCRWTIDSGSSSRQVSLTLVDLNLLNDTTCSGEYIEFRDNPMGVYGR

SLHYCGTRLPQTFDSIGRVVQVHYNLLGGSSSRGFRLTYAWADCNKNYVGTNGRITSPGY

PNVYPRNIDCGSTVTAPAGYTLSFYFTRFYIESHSQCRFDYLRIRNGTNSTSPEVVKLCG

ADLPDPVFLSSNTAYLDFHTDYSAQHLGYDITYLATTSGVGCGGVLYSGINGSFTSPNFP

GNFSDATTCTWSIMVPARRYVSLNFTYLATPGEEDCQNNYVEVFNGPTTLSPTFGRFCSS

LPGTLRAVANSATVKLTSTGSSPAPSFRIIYYS

>SG-AL_contig_6175_1_-1_73_1035

MMEGGQLQARQAPIAVWEEETQQWFIHLTCGHFTFWGTPPIHVVFTMPSGERHVSKDYQN

GDFYLRVKPSLLQDGAYTCEVPAEVLPNMCFIATPFNLGKAMANVTVDDMKTRLSVVESS

QKILLEGNRQLKERLTKIERKYDDQLGNDSSFVTGPFQAQLDSLQRAQQTMQDQIEDLKN

ATQKQDEVALDTFPCTPSSYTTEDSPKRSVERGDGGMCDMPGRGLSEGWYRFMMNGQDAE

IPTSCTKAHRCSTDATGWLDLQGKLVPKPDHTVEVRGCASWDGDCCNWETPIKIHNCGPF

YVYHITPFTTCSLSFCLKEKS

>SG-AL_contig_6176_1_-2_29_628

MSGNVGMEQLIPVVNRLQDVFSSLGIPLSLDLPQIAVVGSQSAGKSSVLENFVGRDFLPR

GSGIVTRRPLVLQLFNSRSEYAEFVHTKGKKFTDFNMVRKEIEDETDRITGQNKGISSVP

IGLRVYSPHVLNLTLIDLPGMTKVPVGDQPQDIEMQIRSMLFEFISKDNCLILAVTPANS

DLANSDALKIAKEVDPQGLR

>SG-AL_contig_6178_1_+3_69_230

MHATFCLAVKEFLTSVCTGQRDLLWAIGCQRSDNVPHTSCQRYYLYAKTFMSVC

>SG-AL_contig_6179_1_+2_293_520

MRQIHRKYALVRFLVSPTSTDIKHVDLSWSPTSETGIHKETEGEENDSINLYRVFYQSLG

PPQGRFNIYVVFWDVA

>SG-AL_contig_6180_1_-2_2_166

PCSHYRDALLHSLSCYLLFTYCLIYLMMLLRLFYPLLETAYESQWISCFSTDICP

>SG-AL_contig_6181_1_-3_336_539

MSTRLLQEAGGLGEGVKGLTVKAGSKASKDFVGLLMAKIFTLSLPHPIHKLQKPNPFTPP

PYPLASCR

>SG-AL_contig_6182_1_+3_24_149

MPSRSAADTGKKCHCIKYKQNQWNVLWGISVNNGLFIHFSNI

>SG-AL_contig_6183_1_-1_10_1365

MRKLHFFCCIIMGQKQSGGSGGQGGEKSDDKDKKRKYEPPIPTRVGKKKKKTRGPDAATK

LPLVTPHTRCRLKLLKLERIKDYLLLEEEFIKNQERLKPQEEKHEEERSKVDELRGTPMS

VGNLEEIIDDNHAIVSTSVGSEHYVSILSFVDKDQLEPGCSVLLNHKVHAVVGVLGDDTD

PMVTVMKLEKAPQETYADIGGLDNQIQEIKESVELPLTHPEYYEEMGIKPPKGVILYGPP

GTGKTLLAKAVANQTSATFLRVVGSELIQKYLGDGPKLVRELFRVAEEHAPSIVFIDEID

AVGTKRYESNSGGEREIQRTMLELLNQLDGFDSRGDVKVVMATNRIETLDPALIRPGRID

RKIEFPLPDEKTKRRIFTIHTSRMTLADDVNIDDYVMSKDDLSGADIKAICTEAGLLALR

ERRMKVRNEDFAKAKENVLYRKNEGTPEGLYL

>SG-AL_contig_6184_1_-3_66_872

MPATESKSPEKKSADAETDPKKGSEKKKDKEEKKERGRDKQKRGGSSSSGSNSSSSGSSS

RSRSSSSGSSSSSSGSSSSRSGSSSSSRSSSSSSDSSHERDHRRRDTDKDKNKKRKRSPT

PRPNKVHIGRLTRNVNKEHIQEIFSLYGTIKFVDLPNDRIHPNTSRGYAYVEYENADDAE

KAVKYMDGGQIDGQEITAVPVLMPRAPARPPPRRSPPRRPPPRWRNSPPRFGGRRPSPPR

RRSPPRRRSRSRSPARRRRYSRSSSSSSR

>SG-AL_contig_6185_1_-1_802_990

MQANIHTHIYTKCFKSSRLVQKLFGIMFAASTVSVKIKSVFCSSQKQEKMLNPQSLIVVF

QSA

>SG-AL_contig_6186_1_+3_126_269

MHLSGLELVCLVEDGVGDAQCMFSTFAQQEQAIAVHVSHSMGAKYSFP

>SG-AL_contig_6187_1_-3_933_1160

MCRTNTVHHHHLPPSDKFKTTWMHPRSKQWHLTDYIIVRQRDIQDVPITQAMRGVECWTD

HRLIRAVLKLRIAPTQ

>SG-AL_contig_6188_1_-2_188_1924

MGKLKANLFKHLSKEDFRVLVSVEMGMKNHEMVPGPLVASIAKLQGGGCHKVLVNLCRHK

LLSYESTGNRRYQGYRLTNSGYDYLALRVLCGRDAVTSFGRQIGVGKESDVYIVADEGMQ

EYALKLHRLGRTSFRQLKNKRDYHKHRKSASWLYLSRLSAVKEFAYMKALHEHGFPVPKP

VEQNRHAVVMELIAGHPLCQVMKVRDPAALYSECMELIVKLAEKGVIHSDFNEFNLMIDS

DDHIIMIDFPQMVSTSHTNAQMYFDRDVQCIRQFFSKRFSYESELFPVFEDIKRTGSLDR

EVQASGFTKKMATEFDNITEQMAREDSETDTADECEDESSELDESDTTEDEEDDEPARHG

KKRHVPVMDYKDEQLRFRLALLNVDEGEKPEVDPEVEFSDIERERCQEGEDTVAVAEETE

PGADVCKNKVISTEDGAVVGKVGLETCVKEADISAEGAEAGAGVQYCEESDSDHGFVPLK

AKASTCPQTSEAEQVSEGSDSESVGMHHCHRHHVDAASSVQSSIHPELIKRRATAQFKKK

LEKQKAQRTRKRGEAAIITKQRRENAAEIKTSMSAFWYQ

>SG-AL_contig_6189_1_-3_1773_1970

MFTPHSHHACSHPPHVCLLDFCLFLLLIDWSFLFVNFDQNFTFGVVKTHVTVDMLSHYWW

NIHRLR

>SG-AL_contig_6190_1_+2_2_178

PEQRTLREMGRGRVHKKNCTPSNVSHDYSNTDAILVPANTCNLLFPGGSGFYGIGLLRY

>SG-AL_contig_6191_1_-3_72_356

MAKLGAEHEQKDNSVSLSEMKTIIKSLFRAPQLHNSYHQLSRQEQVIILCLRTGHNRLNY

HMNKRFRLVPSPCALVEKLTRQQNTSSRTAGSTGR

>SG-AL_contig_6192_1_-1_121_12366

MRVGWASVSADPCAEVGASADSYGFDGYLARKWHHGSHDAYGKPLQKGDTIGCMLDLHDK

TISFSLNGEMMMDCLGQEIAFRNVEVDNNAGFVPAFTLGAWQRIKFNFGQDVNTLKYFTC

CGLQEGYEPFCVNMVRAMTFWYGKEQPLFETVDMSHPELLISRIQGGNNVGPCLKVSSKT

FGTLEKVHLEYLRLSLPVKCKDSFTSSKDRHLAQAVLEKRLNLENVRRREMDVEEEVSAD

EEDYSVSTEMVNGGPDLTLRKTTHHTFLDDDAMSAAERRKSYQKNMKAGKGHSLDDSSVA

SHSTGGMKAAASESQLNESSDSFLSPPKDKRTSSKLSLLADKMLDTAKGITEKKDKKSKS

SFPTIFRKAKSRDPSPNNARSTRSTEYSSLDRKNVSARTGHLDRKKKSPTKTSVPPVRVT

GLDGEDSTDLDLDKELIPFEFQDAMEQRHAFSQFSEDSDFGQSEAELNELNAIAEQIEEY

SYSVRIFPGQDPSQVWVGWVTPSFHFMEKSFEMKKVRHVVISQLDMDYKLKSSVSRKNCY

MVSAGDLQQRYADTNQEMSSKRASPGLVIGCSIDTSTGTLSFTVNGKEVANKFQVEPGFK

LFPAVICEPTSKEMFQFELGSTRTSLPLSAAVFRGPKSLVPTCPPRLDVQVMVRSSWSRV

PNISVKTSSLKLSEIRGWSMICEEPVSMLAVHIPDEDRCLDVLELIEYNHLLDFHTKTLE

LYQAVCSHGNHKVANVLTQHVDERQLMYTIRSEYMPGKLRMGFHDLLISMHLDSHTRART

MTENEYIVPLMPSTHSLSLYRKLSPTSDTYGIKHQIPNMEESVSIRPQLAITEKEIEDRI

QKNGKDSTAPYFPMDKLKSFVMQSLTTAMEKGAAHIRDPIGGSNANLFVPLVKVCNNLLV

MGLLNDDDLHHLLSLLAPAVFDQEHCHNPSGFGLLSMQLDEPVKLEVCRVLQHLCDMQLR

HRVESIIAFSEGFVANIQNDQLRRYNEIKNADLPSAVTAKKTKEFRCPPHEQMRKMLRFR

EQDDTEECPCREDFRENLRHFHSNLLDHCKMPELNVEEETLLESVASPKPTLRQRLMALV

WKAQELEASTQSAETPPDNMQKLITGTMVRWAQEDFISMQDLVREMFSLMHRQYNGTGEL

MIALEKTYVISVKNRLDINQLLKALGIIRSLLMVQAGHDEEELMKDSLKDLMDNKVFFQH

PDLMRQLSVHETVMQLMVNTLNKAQLQQQASAGAAPQDPSSSQGQKAPHSSMDALTEQSK

DAAAEMVVMCCRFLCYFCRTSRQNQRAMFEHLSYLLDNSSMLLARPSLRGSCPLDVACSS

LMDNNELALALRESHLEKVAIYLSRCGLQSNAELIEKGYPDLGWDPVEGERFLDFLRFCV

WVNGENVEENANLVVRLLIRRPECLGPALRGEGGGLLKAMKDGVRMSEQIAASRDVTSSS

FLTAMNDDDDSGGNYLVQSKYNFHTLPPEDDEDYIDMGASILDFYSSLVDLLGKCAPDAE

TIKAGRSDSLRARAILRSLVSMEDLEGVLALQFILPVHIANEEEGEGGLGGGGMPPGLLP

NHKASIVMFLDRVYGIEDQSTFYRLIDEAFLPDLRAATTLDMAAASESDMALALNRYLCM

SVLPLLMKHSYFFRESEHMSALLETMLQTVYRMSKCKTLTKGQLEMVSDFLVAFTQQLRP

PMMTNLLRKLTVDVPALSEHTIIPLRVLTSHYERCGRYYGSGGGYQGTASEEEKRLTMML

FSGIFDSLAQRAYDPELFSKALPCLSAIGCALSPDYSLSHHDDSWLRQTSMDLDGTFNPR

PVDTARVNLNQSLESATNKFGEHYHDCWAMKKIEEGWMYASHYDDDRKCHSLLKPYHLLD

EKSKHRYTEPVRDSVKAMLAWGWTLEQDQARFSSNRDSIKRRASKANVHDVGYSPRPYDL

RNITLTREMLTMAERLAENAHDLWAKRKREELDSIGGGVHPQLVPYDILTDRERKKTKEH

AQELLRFLQFLGFRISSGDMGLERRRESASRGSRESEADSGVGVSATERRFAYSLLEKLL

EYLDNAALNMRQMRPSSRFSRRESYSTATEDVKFFGKVVLPFVEKYFHAHRGYFIAPPNS

PQSSSGMASIKEKEMAASLFCKLAQTLRMKITAFGHDVNISVRCLRMLVQAVDFRSVVKN

GPEMVRSSLLPFFNNAADDLTQVVENLMKERFSHVKGTISRGATSLNYVHMVLLPVLSSL

YDHFGLNHFGTDLIVNDIQLACYRILNALYTLGTSSSAFSHRDTISAELARHRPALGECV

GSFASCFPVAFLEPDLNKYNRNSILFGLEGKISEHSLEAQEVMDQLKENLPGLDKIVAEI

EELAHSGGKYQEAPHVIEVTLPMVCSYLPFWLAHGPDGTIREGSNLVTNVTAQQMNSVLG

NVLKLILNNIGSEDAPWMNRIATRTQPIIANSTPDMIKDHFLPIARKLKENAEATEQLEK

RLAHEKRISSEGSEDLEQYVQDKFTILVRDIYAFYPLLIKYVDLHRSHWLKNPAAEAEEL

FSCVADIFTMWTKSLFFKREEQNFVSHNEIDNMALIMPSQATRLSMTKVDSQDKDTANKR

PGGKRREKKSRLEAHNSLNVACLKRLVPVGLGFFSGREQELLQQAKQKLLQKDPEGDIED

FLLHSLTTEEGPIDDKVRWQKVLYNKIGDSKTSGTTQLSQLRTIERIISMAKVMHGLHMV

EHPSVLLKNAWKKVISSQRKRAVMACFRMIPLHGTPRHRAINLFLKSYKLQWLESEDVDK

NILITDLTESQGAAEEPAEKKEVEEEAKPDPLSQLITALSRGAQEQQNSLPEDALYISYA

EIMSLSCSGEDEDDDEGDDEGPGPTFEEQEMEKQQLLFEQSRLADRGAAEMVLLYISASK

GEPGDLVTKTIDLGISLLRGGNTDVQKKMLEHLKEKKDVGFFASVSGLMQQCSVLDLDAF

ERMKKAEGLGMTSEPGSGAGEKNMHDGEFTCKLFRFLQLLCEGHNLEFQNYLRTQAGNTT

TVNIIICTVDYLLRLQESIMDFYWHYSGKDIIDPSGKENFCRAIMVAKQVFCTLTEYIQG

PCSLNQLALAHSRLWDAVGGFLYIFAHMQDKLSKDPDQLDLLREFMKLQKEMMIMLLSML

EGNVMNGPIGKQMVDTLMESSANVEMILKFFDIFLKMKDLTSSEAFLEFDTNEDGWISHK

EFRKAMESQKIYSEEEIEYIMMCVDANHDGKVDFTEFTDRFHNPAKDIGFTMAVLLTNLS

EHMPNEPRLERLLDKAKSVLDYFGPYLGRIEIMGSANRIERVYFEIKQSHIDQWEKPQIK

ESKRSFLHSVVNEGGDKEKLESFVNFCEDTIFEMQHATSISAEEQRIAAMRSGAGMHSEG

GMLEPVFLIFRFLRDGIAGLFSLFTWSNLKKCYHAYRSMTYPQLLLTLIKLFFRLLLFLV

VVFFRVVWTLLKFFVNMMMGDKSLEEPKPEKLTKGPIALPVGPIALAQAQAQAQAQAQAQ

AGMAVNGAAIVPPAELETGPSPVIGSIPPADTQDASKSREAGPTQNGIQPGSSQDLPSTS

TAPPLATRQASELEDDVFMSEAAATTAAAPAKSPPPPAYAPEQMSASATEEPATSLEDEA

PLDVQGFDYGKYVLSLFARNFYNFKYLALSLAFCINVILLFYKVSRVAGEADDDGVEDSL

LGNATAAAEDDDDDGSSEFVVIEDDYYYLEPVMRMLAFCHMLTAFCMLVAYYCLKVPLVI

FKREKEIARQLEFEGLYIVEQPSDDIRANWDKLVLSTVSFPESYWDKFVKKKVRTRYAEQ

YDYDAITSLLGMEKTSGSTEDNHTGSRFIPAFLSNVDYQYQLWKWGVIFTDNSFLYIAWY

FLFSVLGNFNFFFFAAHLLDVAIGIKTLRTILQSVTHNGKQLVLTVMLTCVVVYIYTVVA

FNFFRKFYIKEEDGQVDYKCHDMATCFVYHLHTGVRAGGGIGDEIEPADGDSYETYRILF

DITFFFFVIVILLAIIQGLIIDAFGELRDQLEQVKEDMESKCFICGIGKEYFDKVPHGFE

KHVMNEHNFANYMFFIMHLINKPDTEYTGQETYVWELYQQRCWDFFPVGDCFIKQHESEP

SG

>SG-AL_contig_6193_1_-3_219_6329

MDGWDITNAIEGFVIEAGNTIAVKARDDIADTNKALYWVAPPEYLGNRMYSYGGELIFTV

LFTLPQGQDSEGIIRPDVILEGNNMTVVHYHPQQPQNSVALDMEVSLFEYNFRHAQSGAE

VTREQFMMILYNLQALHIRASYFNLVDEVRLSDASMEYATETGPGEQASTVEECQCSPSY

QGTSCEECAPGHYRSRTSPYLGICVKCNCNGHSEECDPVTGECFNCQGNTTGPYCEQCLT

GYYGDPTREDCHICSCPLPLPSNNFATECRLAEDGIRTYCTCIPGYYGPTCESCAPGYYG

NPREVGSYCQPCSCNNNIDVTDPRACDRFTGVCLLCQNNTAGDDCSECQDWYWGDAVVRK

DCTACTCDRCGSERCDKAVGVCDCKPNIIGSDCSQCAPNTWGFEYCDGCRECDCGMGSKS

PQCDLRTGRCDCQPGVEGEKCDRCMHGHWNLGPQGCQVCNCLSDGATGCDQDTGRCNCLP

GVTGPSCDRCLDRWVLVPGQGCQECDYCIHLLLDDLDVLDRNVSTVRRQLADVSVGVAAF

NRLSQYNMTVNQLRPQVADLYTLDKDELQAMLDPLKQRLATVESDARETLDKANQGVAQA

DQVQESVDTLNTDAADMESLVREQVLGAENAVQYVKQILQRILASIKVTNIERYIGEVER

SMEDIYSRNFTMEDNDTVTELNNAKELIMKIDALEQNVEAQLNRSLDTENNLNDILERLL

DLGENTETSTALKALDDLRRLRAQNMEAIDGVTGIIEALEEDTRDLLDKATLLAVDAEEA

LDTTSVALEELGLDSSRMEQALQDLIGMVDGLNDGITGMEPLVNRSFFHSEELRTQAIEL

DNLYNNTRDLSANAVTAAQAYQNIVKFISAAHNNSMEAVLDAERALNETNGNGEKAVESL

RRSRELKQEADDLDSLTRLTLTNSLMNAKDATEALENQKVTIVTDLNDIRTQLDQLDGRS

SFARAQDVYDRALVGLDKTNATEERIDRIIALLPGDQTKLESIRPDRTSASLDIKNTASN

LETVRTSQPEIMDLLKPLQVNATRLIGVGEKLDLSVEALREKIELAREQANRIRVGLKFL

GNTTVTARNPPKLEQAGSYSSLSMYFKTTESDALLAYIGGQQRSENIQNDFLSLELIDGR

VVFKFNLGSGPAVIASNRLVNDGQWHQAVAERIGKSGTLTVRTSKEADDVTEGNSPSTFT

VLELNPATTNFYLGGVPDDMTIADVVTRRPFIGSMEDIKFDDIPVGLWNFEDAENNRVGE

NARDVMKEIVTNGMRFNGMGYAVLSRSKLGLRPNKTDIQLQFQTFSESGLLVYMSDKKRD

FMSIELRDGRVVFQYDLGGGALTLEGTDRLNNGEWHSITISRSKQRGLIRVDNQIVGEGS

SPGSLSELSTTEEIFVGGFDQILVPVGNVGNYGFDGCVRNMQFGTIAWDLNENQKAKGVV

RGCPAKVARIATYRKARSDGFIAVDLIEGIGSKFDMTFKMKTNQSKAMMVYIADDTQENT

FSVGVIQGKIVLKSMQDGGAVEMSSRVNTYSDNNWHYVSIMKDGLLFEMSIDDYEMLQTM

ADTGTASLDTTTPIYFGGVPPEFRIADGTMPSKNHFVGCIGDITVQRKFVNLASIPESKR

MGVQLVECPLHTEDGEVVAPGETTTQPTVDGGTVAVQTTMAPDQCALPLQPRIPDEAEVE

AGIRFGSRLHSRVEFVRLPHGMRLKSQFSLSFKTSGQNGLLFYVADRRHIDFLGLYMVDG

ELHFGFNCGSGAAKIMSGDTYNDGKWHKVEFGRVQRSGQLMVDGQLKGRAKSRGGTRSIN

VNAPYFFGGLSEAAKKAALGNVGDGWVSFPGCIRNLTLKNVELKQPSQKVDIQTCSSAYE

LGSFFPASGGYLQLVDRFKVGRDLEINMEIRPRSQSGVLLAVHGRMDYLVLQIVEGQVIF

ITDNGAGAISTSFPLTARNSLCDGKWHSIRANKNKNVVTLLVDGVQSEPALGSARVSSAD

TNDPLYIGGVPDTSAKGVQTQENYVGCIRNLQLNDKPQPLSSGQPTGDIQLDSCPVS

>SG-AL_contig_6194_1_+2_14_223

MKSFIQQLLSKRTTHKKNSTENWLLESWIVVVLHAMNNIPLLSQVTPITNSMTEMGRVCM

SRFSGYVMWQ

>SG-AL_contig_6195_1_+2_125_376

MKITSKPGKKSQASLSKVCPHHVVKLSGRWLNLFTASLLKSVLSPAKSHIISFSQPVSGL

VAAQSSDSILVQCVVNKASECLTE

>SG-AL_contig_6196_1_+1_1_156

PPPPPPNILVWEYNTLQCCACNTEVWTLYFLQLYNRAVKTTDTSNFGTKMHE

>SG-AL_contig_6197_1_+1_7_369

MKAIKICALDQTDEAWLDKRGELPLSNLTAQVCVGGNLAQEKLGACSLQSPSLSGYLTLS

RVRKAEAARERELGTALMLFQREDEVSTTSPPYNLRVYQPLVITRLTVLQAEPRQERSAV

K

>SG-AL_contig_6198_1_+2_2_202

FFFFDVKNFISIKISFLCFTKKQKITRFANLELQHRTSPCPSVQNGLSVKFFSFPLSENI

KDPEEHT

>SG-AL_contig_6199_1_+3_3_113

GEVPIVRTQAFHSLQFGSQMSSPFDHRALKIIQLAYA

>SG-AL_contig_6200_1_-2_146_937

MAVNVYSTSASSDNLSRHDILAWLNDSLQTSYGKIEELCTGAAYCQFMDMLFPGTIPLKK

VKFNTKLEHEYIQNFKLLQNNFSKMGVDKVIPVEKLVKGRFQDNFEFVQWFKRFFDANNQ

GFEYDPLAARGGEPVGSANNKGALSKAPSNRSVAPKSRVPPSRPAATRQPANNVRSPMSK

PSSNHAGGDSQLVEDLRAELRQLTLEKEGLEKERDFYFGKLRDIEVICQQNEDQEMMKGI

MDILYATEEGFAPPEDDFEEQEEY

>SG-AL_contig_6201_1_-3_3_188

SFPPPPPTHPCKASHKKRVVTGVYPVGFSYVTIRSKVVIIWWGKLVLGDYVLLKLLTTSS

PN

>SG-AL_contig_6202_1_-3_192_380

MLPAIFMDNSEDLGLRYAGKTSNFEIFPNRNSTTLFSKFIKFCSFILHFFHRKWNRCVNI

AKV

>SG-AL_contig_6203_1_-3_231_2675

MGLGVDEAFEGAGASTGMEIWRIEKLKVVKQDPETHGSFYSGDSYIVLVTKQVPGSSRLE

WDIHFWLGKDTSQDEKGVAAYKTVELDEGLGGGPVQHREVQDHESKKFLSYFKSGIRYLE

GGVESGFKKVERDKYETRLMQIKGKRNVRVRQVECHCSSLNQGDVFILDCGLVIYVWNGP

FSSKMERIKGAEVAKRINDEERGARAAIKIIENGWDSDPGFYKALGSQEEIKPADDGGDD

TEFERTLAEAVKLYRVSDESGALEITEIAEKPLKREHLDSSDCFILDSGPSGIYAWIGKK

CTKNEKQSAWQNATEFLTLRGYADWTPVTQMVEGGETPLFKQYFSQWTDKDAQTGLGTVY

HVEKVAKVRNEKFNTSLLHDHKQVEKKDEMMVDDGSGKVKVWRVEYGDLVAQPEETYGIF

YSGDCYLVLYTYTKGNSRMERYILYFWQGAKSTTDEKGASAIFAQRMDDNELNGAAVQVR

VVQGREPEHFLLLFKGNKLMIIFLGGINNKEPDSSTRLFQVRGTKSVNTKAVQVQPRAGS

LNSNDVFIAETEKVVFLWIGRGASEDEKNVANNAVQHICPDRNADLVTVEEGLEPQEFWD

ILGGPEPYATGASLVHHREDMPPRLFQCSNASGRFLVEEIVDFSQEDLCEDDVMLLDTFT

EVFVWVGSGANETEKKEALATALEYIRTDPAGRNEDNTLILHIKQGFEPTSFTGHFHGWD

PEKWSEGKTYEDLKREMGQENVAVTTVKEELANYYKTHPIDVLKKKTLPKGVDPSCKERY

LSDADFETVFGMSRCDYLELRPWKQLQLKKKVGLF

>SG-AL_contig_6204_1_-1_1855_2088

MVLSHCCFGMRNGSAFFCYDLKYRVALKTHSCFFLCLFCISNCLPHPHPKYLHPDFNFSP

LIPDSPSPSPDHLMHFGK

>SG-AL_contig_6205_1_+2_2021_2236

MRTVPNLFSPAALPFPSQKEGPFYNWLDCDSILKPMQESGHSSIQVPCSFVLDSAFRNEC

TNHYTTCPLALL

>SG-AL_contig_6206_1_+3_627_836

MIVKVNSRLKCTTRANKVLYNAYVHFALNCIICGSTQNIIMYILYKKLIYTHSLFTCSLQ

VNIIISSLIS

>SG-AL_contig_6207_1_-3_87_335

MYVHCKQKYKESIKKSRLILLLGTGCRLCASCGLLCSSPCVRIILGMEIVSVKCSIRMTL

TFANFVFSFNLVAYVLLIVRSVI

>SG-AL_contig_6208_1_-3_84_338

MSKTAEKKKIWKDHENAWQTHHQVMEPPAKQRKVCAVTDTHHSPTTQLSAYYNQQSACYN

QQSHHYNQLSHHYNQLSHHYNQLSA

>SG-AL_contig_6209_1_+1_112_309

MQDVVLSIPIEDDFTCQQHMMKGASLVAPRNCVPQSDLCHTKTKVVHIGDFSSVVGTLST

PYSPRR

>SG-AL_contig_6210_1_-1_103_594

MQEKLREGEENVEKFLASMASLETDKASIVTEQDSLRQQLTEMTSQLHDAVTDKQQEHLH

NEELQQEFSQLSQQHSALKEDLAKKEAEIMSLQESLETNAQKHSEVTKQLTEQVESVLAD

LSQTKQQLSETSDNLSEVTSLKESVEGEKNSVVQKLEQTSSELK

>SG-AL_contig_6211_1_-1_820_1158

MLCLIFSVKTLECPLCFLCLVTISMLNVNFLHATLFLQYKLGRFSTVIPLFCPSTWLRHT

MKNQVLFLASPLQIIQLLPQILGSLFFFFKYLQVQFEMCPSFLAHCQSFCPLY

>SG-AL_contig_6212_1_-1_145_342

MNTHTHTTSHYTRVCMHTHTHTLTLTFTHSHTHTHTHTHTHTLTYFYLCSSFNMVVVNFL

CAMKSF

>SG-AL_contig_6213_1_+3_3_200

YCSSHSGGVNLVFHRFPEGFNSRVFCRLEFTRLSLADLGESHMKLSRYGKSRLNKSEFNL

IKFAQV

>SG-AL_contig_6214_1_-1_1_3846

LLFTVLQNTAKDHAKQSHDYFNLLCRLLNYAANNDVSLPTAETLLNNEIQWLKRVRDQTL

KAGEVQVDEALLEGHLGITRELLSFTSAERKFTIGSENGGANLIRELVEDFIFPASKIVL

QCRKSHGEFPAAQMVPVCASPQPVIAAFDLLVALCTGCIHNLKFLSSMLIEMYYTNEMPL

QEWEYVPPVGPRPHRGFVGLKNAGATCYMNSVLQQLFMIEPLRRGILSVDGAADNIEEEL

LNEEKPESETNMDLGGEEERKDEEKGSREDERKDYNMGVLRQVQVIFGHLAASKLQYFVP

RGFWKHFKLWGEPVNLREQHDALEFFNSLVDSLDEALKSLSQSALLEQHLGGSFADQKIC

KDCPHRYQREEAFTTLNVDIRNHQNLFESLEQYVKGDLLEGANAYRCEKCDKKVDTVKRL

VIRKLPKILAIQLKRFDYDWERECAIKFNDYFEFPREFDMEPYTVQGLAKIEGEIIDDDL

GAATVTSTRYRLIGVVVHSGQASGGHYYSYIFHRGQHDTHAKWYKFDDGDVMECKMDDDE

EMKNQCFGGEYLGEVYDHMLKRSAFRRQKRWWNAYILFYERIDSEDPSADLAKEMQKLTV

SSNNEPSTEGQVIPAGESSPNQIKMPLPIERCVRRQNVLFMHEKNQFCAEYFQFMKRLLL

CNNHYINKLLQEKMMQEVDQVCMTSVELGCKFIFNVALHTKKSFRGPALEWYESIHMLVR

SSAAVRSWMAHNVFFAHPSRFAEYLLECPTSEVRSAFGKIVVLLAHASQKDGPCPPPLLQ

GLYVSQPADPNATLSDHILSAVLTLLRKEVSEHGRHLQQYFHLFLMYCNLGIEEKEQLLK

LNVPQLLMLVALDEGPGPPIKYQYAELSKLYSTVSHLVRCCDISHRCNSSVSNQPPLANN

PYADPHCPRPLMTLQPQVEDILLARTIYVKKLIEECNNSEDTAKLLKFCSWENPHFSSTV

LSELLWQVAYSYTYELRPYLDLLLQMLMIEDSWQHHRIHNALKGIPDDRDGLFDTIQRSK

NHYQKRAYQCIKMMVLLFTNCAPAANMLQTNGDLKRKWTWAVYWLNDELERVNRPYAANT

QYAYNNWSPPAQSNETSNGYFLERSQSAKQTLNKAFELCPEEEQEEPELGEDYDGQQEES

GNVPTYLNQQQQQQHAHQQPTSNQQQQQQQQHVPQNSPSTSTQQHPSQQQQTQQQQQQQQ

QQQPANQNQNQGAPVSSTPVQNNQVHVQSSSQPAQPPSNQINNPLNQGQSQGQGQGKQPA

ENLETGEEEDSNSSKNKDSSAE

>SG-AL_contig_6215_1_-3_60_4379

MENKEVTLTCEVNKPDKKATWQCNGKDLAPSDHIQISADGRTHTLTIPAVQLEDEAVYKC

IVGDRKTSGRLSVKEEPLAFITPLTDQKLMEYETLTLECTVSKPDRAATWYKDGTKMTPS

DRVTLTSKDKTHTLTVSRVDVDDEAEYSIKVDNLTSKCTVLVEEEEVEFVRHLEDIDVTE

IPGTATFECEVSRVNTVAQWFCNNQPITASDKYVMESKGVIHRLTINEVEGKDEGDYKVV

VKGKTSEAGLFVEVAPQIFVDKKYDETIYLYAGQSTAFEVPFTGNPQPKVTWTFNDGDLP

DKKRMEAETIYNMTTIRMSHVQRQDTGNYTVTLLNDNGKATITIKLVVLDKPSPVKEVKV

TDITAETATITWQAPDDDGGKPITQYVVERREVNRRSWNKVGDTTTLDLSVSDLVENNQY

IFRIFAQNDVGLSEPTESETITAKNPFGVPDAPAAPEVTEVYKDNALVSWQPPANDGGSP

VTGYTVERQTSFSPRWTVVNKSPVSELSLKVDDLIEDNSYQFRVIAHNKAGPSEPSQPSP

KIVAKDPWTVPGKPGTPDITGTDKTSVALKWASPDSDGGSPITNYLVQFRPRGTTQWLLA

NDTVTVTDTTFSVTGLKEGKEYEFRVAAQNRAGTGEYSAPTKPVKVIKPIVGEPPMLLEP

LQDTTVVAPGDAILECDIDVGEPEAEIKWYRGKKEVRKSAKYEMSYEEEVASLVIHKTEP

DDADVYRCEATNPLGRVQTEGMLSIHTHPKLEYDNKLKSAQTVRAGTTLTLTVNISGIPS

PTVAWFLDAEPLQKSDRISIDTTENYSTLTVKNTLADDTGLYTITAENVVGKAQADFEIS

VKDKPSKPDNLHVTEVLKDQIVLSWEPPTSLGNCPLKGYVIEKRDAKRNTWMAVQNVDST

TTTYAVQKLLEGNEYFFRVAAENEIGVGEAAELFDGIVAKSPYDVPDAPKKLQPSEVTRS

SVTLTWEPPESDGGSVITGYIVERKSPSTSRWLRVNKSPIRDTVYTVPDLTEGSEYEFRV

IAENAAGMSKPSQPTGPIKAVDPYKKPEAPSKPEAIDITKEYITLKWLPPDNDGGNAIFN

YVVEMRVCGSTRWLPASQNIKVPETSFTVKELVEGTTYEFRVSAENKAGVGPPSPSSGPI

TAKEPIMGEAPTVLEGLPDLAVMEGDTTIFECKISGEPLPSIKWLKEQRVVVEDKRHSMV

YDEHNVASLTIRDTTQKDAGSYTCEASNDLGTVTTSGVLEIQAAPTLEFDNKFRDLISLH

VGATLKIPVKVSGIPTPRITWAKEDGVMKSGGRITIHTQDDSTTLTVKKVTKEEDGLITL

TADNEVGEAKAKFDVEVLDVPTAPQGPLEVSDITADSAILHWKPPKDDGGIDLTAYIVER

RDTKRAMWTKVGSVDGVTLDFKVANLQEGMEYLFRVSAENEVGISEPLEGDSTVVPKSPF

>SG-AL_contig_6216_1_+2_77_187

MQTELKSTNINIFMICTIGMMPKLFGRFHNDRNSNNA

>SG-AL_contig_6217_1_-2_2_553

VVTYLGTTLVNYSSCCPPCSDCRVAYWGVWEGFGPWPYVTNHTSQQRRVSPGCLTNNCTG

PRPFQTRRCFEDGTCDVNDYIRISTYEEEFNCFAVIQCGTMAMCDSHIRSVKLYHKRVNQ

VGKWELLAQAQHGVSEGRFVRVTDDLNANTANPRPNIPSLCAGLERCYDYFTYDIYPVLE

ANQG

>SG-AL_contig_6218_1_-2_77_493

MALPHLGWYKLNIFATSEGTKGSLPCVFTYLFELTRIRKNAHPFPKQFADWTSGCHLYEP

KYLDVDKDLSEVHFKVKVPHAKKVAVKARDEWTHLHKCGDMWEGDVHLEQYRKKDVSVNL

NACTGSDETSFATLLQYTI

>SG-AL_contig_6219_1_-3_15_251

MGTALIMLAPRKVEVSTTSLPYDLRVTGKPLLFLMWACNHTELGNVAVIQIASVHYCCKF

LLEKKKFSNQLENYSEWKI

>SG-AL_contig_6220_1_-3_477_608

MHTDNFLKATNIQHKNFHHCRHMYITRKIAVISKMRFKNTLKQQ

>SG-AL_contig_6221_1_-2_188_958

MLLCGSRWRMPEQISYQKFADDRGESSGSPSGTNHGVNARIGVNSIRNIRGASAHPPCSG

PAMYISNNHPNTMDVESSRAIAEDYIRYCLERNGLSWPNGQRDLTPNQHQRAMRALGDEF

ESRFSQSFDDMINQLHITTETAHHTFTTVVSEIFGDGVNWGRIVALFGFGGKLAVRCVQQ

SMPQLVSSIVDWVSTYVDTRLKSWIAEHNGWAGFLEFYEGDPEKRSKDSWPSFKKICSVA

AAGLGILTLGALLTQKS

>SG-AL_contig_6222_1_-3_147_470

MQTSFLVQGLHMRLIMPSDHVSTSPLSPANITNATLVQNQTVIKQHPQLVAAWFTLFDII

MLIMLLPFFDRVIYPRIEQRTGSPVSISWRIVVGMGFATAAMVMAGVL

>SG-AL_contig_6223_1_-3_3_188

VFVFVILVTTYIPLCLCFCHIGDDIHSSSLRLCHIGDDIHSSSLPLCETIKEVQAVAVNG

KL

>SG-AL_contig_6224_1_+1_499_597

MTKLDTVNFSLCFGFFPFFIWTLLKWYYTTRFK

>SG-AL_contig_6225_1_+3_540_707

MPSNIILNEPESSNIMLNEPEPSIIMLNDPEPSNIKLNEPEPSNIMLNVHETDLSR

>SG-AL_contig_6226_1_+2_2_301

MHMKTHTHTHSHTHTHTRMHVQAQIHTHSQIEDTHHTQQVFSELEKIIQCAKTVSALYTH

IKPLNIPFIFKCTCVDFFACRIIGTAKLHSDNCYFTVLSF

>SG-AL_contig_6227_1_+1_1_186

ATFPFGLGQERHILVHHTLTHHQSGGYNDSNIPFHFLSVTKLTQAAFSLALQRWLSRLGQ

TY

>SG-AL_contig_6228_1_+1_103_270

MEYSRTKISARITVMSYLEIELAMLHPSNMPAPTSCLWDKTIKHAINGREKTRKVT

>SG-AL_contig_6229_1_+3_3_182

HTHTHTKKKTNRSFHFSITPTVHDSLLLFLFIFLSIFNRINSRLCKNLKQSKNAIVLWNH

>SG-AL_contig_6230_1_-1_1_180

YGHSGRLGYKQYIIIIIIIILHLIFYEFKETLESFSSYKYSVGLDGISYQFLIHLPRNWK

>SG-AL_contig_6231_1_-3_711_938

MMRYIYTDNLAAVTLKFCQSHPSLAITSLTRSVTKAMKTIMVTLICNGNVAVHTNDIITL

MIKGLFIWLEHFFNRS

>SG-AL_contig_6232_1_-2_107_211

MRNTYQDTMEVLDSILTILTFKLLSKSCSVTAVCF

>SG-AL_contig_6233_1_+1_52_228

MHELETEKAKVKVTWTKKVLAHFLGRCVNSTFSVTSDNIQIVFIVVAVYHFLRFTSSFI

>SG-AL_contig_6234_1_-1_34_996

MEGSVDGTATSGLQETRTTSPSFLGSDPAVGTEEHGAQEGTVGVQNSSSSKGVESGLRDN

TSTHPISHDMGGPDPASQPLSANLSLEESNLRTRHLTRKRKSTNSESDSSSEPAPKLSCV

ECSVGNRSSVESSKASDSSDIPPKLYKEGRKTNVVEKMEEDKGGKKPEDKLTKEEEKGKK

EEEKAGLKNMSHHDRNILDATPEEVAKKIAAAGSKRRVHYTYVLEKVTPGQSYFDTPVLP

GRTRSQGSKGSKESSAPTGVTLTVSAAPAGSQMIGPQSVGAGKEDTGDPAAAGSKRSTRS

LRTKDGGESSQAKRKRSKEHR

>SG-AL_contig_6235_1_+1_181_462

MEFCQEHPICSCGLFYVCHRHAEPQFVISSGRPEPRLDSSGWGEQNLSTWQSKLELLFPP

GYFNHWATAPLSHIKHKQEKHGAVNQNKCTDGVR

>SG-AL_contig_6236_1_-2_11_802

MAGDVTASPLVVDAFQAEIGATEPWPEDVCLYQQYQVEQITFPDYANCLSVKTFLYMSGL

DFRVEVRTNAEEMSPSGKVPFIHVGAFLVSELDPIIAFVNARGFSLTAEMSDTQRSEMRA

YMAMIENILVNAELYLAWLDDTVSTEVTKPRYGCPYPWPLSWMLPLRKQREVCARLGSNG

WKEKTLPQVCEEVRTCCQALSERLDQQKFFFGNNPTELDAMVFGHLFTLLTTSLPEGHFA

EIIQQFPNLTTFCQKIDERYFKQN

>SG-AL_contig_6237_1_-3_243_347

MLKKSIRLRLYFVYCIFYTPPPPPPPSNPPTTYLL

>SG-AL_contig_6238_1_-3_3_92

FVCLFLSWNRNNMSTSDITPQWFPIKIRII

>SG-AL_contig_6239_1_+1_1_126

NKQTKTNGEKLTNNQRRHQFFLYFGKHHPFNDCKFSMLTSHL

>SG-AL_contig_6240_1_-1_304_1395

MSSLAAKSRLPAGFYKTEIVATKWEVPQKYQRLSPLGSGAYGQVCSAEDNTGTNKPKVAL

KKMARPFQNEIFAKRCYREIRMLRHFDHENIINLIDLFTPNTSLETFADVYLVTGLMDSD

LHDILKYQTLNDDQIQFLLYQILRGLKYIHSAGIIHRDLKPNNIGVNQNVELKILDFGLA

RSMAEEMTGYVVCRWYRAPEIILNWMHYNSNVDMWSVGCILGEMIIRKPLFKGVDHIDQL

NKILSLVGKPSEDFIQAIASVDAQNYIRQLPRMPRKNFKEFFHGANPLAVDMLEKMLDLD

PARRINVEDAICHPYLADYHDPTDEPTAEPFDHSFEGQTLDVHGWKSRVYSEVTSFVPKP

VPVE

>SG-AL_contig_6241_1_+1_3373_3684

MSENTESLVAVTFCTQLLQNSRIYTKFVTVKFCISPVALKTGYSFLCKAYRTWPKGSTCC

TNTPTFISQLGFFRTDTSDVPKKFKSRLQVVKKNASDFLLTRLD

>SG-AL_contig_6242_1_+3_1149_1391

MMCRQQLLIHNYFKAASIQHFTISDRPILSYLKLLVRNENQITLALNLKYSYSHMHIYVT

LLLTNFHLSFILSADTSNNTK

>SG-AL_contig_6243_1_+1_13_264

MGPRQASYFDHWSCRLTLNPFPGRGRRGSESLGELLRQAGQKTPLWAGGGVHGTRGREGE

SYRVGRSCRTEARRAGVASRDSHG

>SG-AL_contig_6244_1_-3_333_665

MKWLAWCEPNQVDLHAPSSVELANFLAFLATLEGLVKGYRSAICTILQQLGGPALSDDFL

LRKVSRGLTAREAKAPRRLPKWDLALVLGTFRSPPFEPLDVVPFLLLSYKM

>SG-AL_contig_6245_1_+2_2_268

TLFRSVHCTNAGEIIGHHLNHVHESKQTSKRGRHLGNEKDINNLNTTSPATVHPHNINNY

NIVAFVQLQISNKTAWNMSKNNAPKSQEQ

>SG-AL_contig_6246_1_-2_65_1795

MLGGRLRVSQLQGLLQAAATQWQGRALPAVTMATASDVKPFHYQDILELESKVNTPWKKL

TGDYVSTTEVKGKKILQVAPEALTMIAEHAMVDIAHLLRPGHLEQLAKILKDPQASSNDR

FVALELLKNANVAAGMVLPGCQDTGTAICMGKKGQYVWTEGRDAEAISRGIYNTYTNRNL

RYSQVAPLDMYKEVNTKTNLPAQIEIYATDTSHYDFLFIAKGGGSANKTFLFQQTKALLN

PEKLLSFVNEKVKTLGTAACPPYHLAVVIGGTSAEFNLKTVKLASCRYLDGLPGEGNEHG

QGFRDRSLEEEIHKLTQNIGIGAQFGGRYFCHDVRVIRLPRHGASCPVGIGVSCSADRQI

LGQIREDGVFLEQLETNPVKYLPEVEAGDLGGEVVKVSLNQPMEKVLHQLSQYPIKTRLS

LSGTLVVARDIAHAKLKERLDSGEGMPQYMKDHPVYYAGPAKTPEGYASGSFGPTTAGRM

DSYVDQFQAAGGSMVMLAKGNRSKQVTSACKKHGGFYLGSIGGPAAILAQNCIKKVDVLE

YPELGMEAIWRVEVEDFPAFIVVDDKGNDFFKEWQVE

>SG-AL_contig_6247_1_-3_54_263

MCTYMLCVCEKLGKYLPGIPVLTDCQALYPVKILAGCQVYLSCRHHNQEHGFCFFFMEDV

CCGVLHRSAC

>SG-AL_contig_6248_1_-2_2_280

PRASRIPPLVSCQDPRDARCEEHLPCPVRCVKVYLFRSAPRRHTQRRLISHLRVLPEGHL

GDHHLLLAADHLWRAFNSSGFLPPTGQPQNPRI

>SG-AL_contig_6249_1_-3_3_263

LGFHSNGNLVLTALPEFLDETQQPGSRPRIFRVPSLVSCLDPRDARSEERLLARSAASST

TRNARHLGVPPAESPYLPRRDLLEGLI

>SG-AL_contig_6250_1_-3_3_227

IQACYILFINCYNVRLFVHKHWHLIGRKSLFRGVHRHLIEGSRFFVHSHPIERNRFVSDA

RWCMKAKSSFFTECT

>SG-AL_contig_6251_1_+3_294_476

MDQEGRKKQLTVKEGKRVETTAIEERKFLAQNFQKVILKEPLASPRGFLYTKICSVTVLK

S

>SG-AL_contig_6252_1_-2_2_1801

RPLLFKLATEQWILHNFSQEVDHVAAIEIQRFWRGYRVRLKLRVLLKTLLTEVGGKPNEC

VCSVSICQPSSGTARNIHPSSRARPEKRGKLQPQAVKQVKTKKSTKTIKPGARSSWEKGA

SSSVEKLGERLQPLSVRSPKRTITKDSRSREERSNTDLGLVQEKGSSSKQQQEGDLQRGS

GLQDKEEVPAKHLKMTKKRASRLALEASYDRYVQQALKQGQTSTDMLQFTEYCAHRIQIW

WLLKHHVFLRERFIQRLIELQKKAAMESAAVMEAMIQEASRKVPKKKREGPLTEREAAVS

IQRAWRRHIDIQVYRYYRDLINFKTRGNPAVMLRCINPNEAKLLDPASGTSVRFRLAGDR

FPPNIYYKIFTHRPVQDMCANSPKDYTVSQAKQLNVKHRHNKVASLPPQQDDKNGWYKRI

ENNGWRLVSDRLIHHIMNDPITWESSRKTYQFDHSKLKRKQDVEQRKKTRKIEWMKKMYK

QGMLHARADDHETVQLIEGAAAGMMATVDNQGPEALEDWEVDELLDWTTSLNFEEYFSTW

KDLATSAAADTQLEKSMSIGTSKSDPYELTVSTAPSRFQSTRQSRHTPATLTSGSIHIKT

>SG-AL_contig_6253_1_+1_253_714

MNKLGHASGTSHSLNLSSLDFIKTFVNQYKFHVVQKKKLLKGHKLADNTAADWAASKYQS

QLLQSHELLRHMWISGAQQTVPRFTTRRVIALESTERASETKGCPQVPETKQLHMSGTVR

AEESSRQRKDRAGKVSGGNCRPLNQQAEKGDDET

>SG-AL_contig_6254_1_-2_308_484

MTMVGLCSYLFLIWWIERLSVRCGETSCWSVLQDCQHCHVQHCVIPLLFFPVERPDSRH

>SG-AL_contig_6255_1_-2_137_487

MPRSFLTMTAMPAPMLHDWDDVRSGWRKPPAGPTAGRRMAVTLSGLDRVIHGNLQYGPYY

PQLVWCHNLAKLPCTQGSYYKLQHIYIYMYIFNRIKGKLVPQTTNINKIHLKYSSTT

>SG-AL_contig_6256_1_-1_100_231

MFFPFCFFKKIVFIVCWDEGGAGSPFSSGALFLVALLAMVLRYT

>SG-AL_contig_6257_1_-3_3_203

LYPPGEVPKEVRTQAFHTLQFGSQVSSLFDHRARGCFCFQKPLTQTWLTGSFTCVFDALR

AYTHKEV

>SG-AL_contig_6258_1_-3_1158_1424

MHTRAHTQLESLFFFFFFFFKYLKLLFGLTKTCAKIFLLISLKYSLILCAFGGFVFPVVC

LFPCFSFQLGHFFLSPLPFFVSFLSCKSC

>SG-AL_contig_6259_1_+3_51_278

MHFYDFKECLLSYFKAILHDSTRFKIVFTGALYFMTVEIGNLKSPFYHSCAEHSFRLILA

ALRQYTRNHAKFTHNV

>SG-AL_contig_6260_1_+3_114_269

MNKHLTANYTHTHTHQHNMHQSYSRNRYYYYYHHVLFIAQPTFKAISGLSPR

>SG-AL_contig_6261_1_-1_85_177

MLKKIDFSGLVNTQQFHLNLAIKVLFKQEHI

>SG-AL_contig_6262_1_+2_542_841

MQNHLLGPQFQSPCHAPSYVAHGLFFLTLFTSHSRQQGWLKMTLHMIYTPLYKSHHRHHH

FKPFTVRLTTFEVIQTKSSKYIHICTRFIFTTKKFTIFWN

>SG-AL_contig_6263_1_-3_6_1079

MAAYSELGLIGTIEENEDVPDLDVDSGSDLEGDGVTVKQQKKKKQKAVIFNSSFKFSTQY

EESTPATVPFLKKKASSTLDEKIQEMRRRMKRKKVDVDLEKDVEDSDSSEEETMATDVVK

IKEKKTKKKKGKKGAQTEEGQEKQEFAESVDSYDAKLTFSDLDIARPLMKGLSTMQFEKP

TPIQAACIPIALRGRDICACAVTGSGKTVAFMLPILERLLYRPVERSVTRVLVLVPTREL

AVQVYQVGRQISQYTKITMCLAAGGLDIKGQEAALRLGPDIVVATPGRLIDHLHNAPNFS

ISCVEILVLDEADRMLDEYFAEQMKEIIRMCNFQRQTMLFSATMSEAVQDLAAVSLKD

>SG-AL_contig_6264_1_-1_130_255

MMSMNMCASLIVHLVSECTVLLSRCLNYNSVFTFTVFVQNLV

>SG-AL_contig_6265_1_-2_2_172

SSDLQHLLLTESAVGHKYEQFLNQRNTVSCAQCSLITGHSVYNITFRLYSWLRCHDQ

>SG-AL_contig_6266_1_-1_22_1650

MAAPETAKAPVVPDMHVVGDDDDDAWLYGDEGKDKEPSGDGPDKSESRKKGDEIPKGVVI

KKEDREEGEMSGEEGESQEKDDDDDSDDDNVQVTIGDIKAWSTSDQQPRNLFKSGTNYQK

PATGTPVKPAAATKGLDIEAQGTINGVPVYEFDINSIENEEKPWRKPGADITDYFNYGFT

EETWQQYCDKQRHLLAANESTPKTIAMNASVSTLNRTDKDLGVSTRPPPPRPRPQPPVVS

KPQVGTIDVIGGTAKDSRRPDEGDPIPVAGVASHLGRKFMPVPGLPPTSIPLPHLPPVDY

SVPPPGLPPPPHAVPPPGMNVPPPGFPPVPDPFDPFYGVPPPDRSMPPPVFEDRPPYSYG

GPTHPTYNSNFSDASRPSGPQQWESGSTLSEQDSRDRDWERERDRREGSRRDRERSPPSH

LQRVEEDFGSREAKHRSHERERERERDRERDRERERDRDRKRDRDGRRRSRSADRDRDAD

GRRDRDEDKHRSRRSDARRHKGDDSDTESRSSSSKHKKSRRRRDKDDDAGESLAGDDTAS

KGE

>SG-AL_contig_6267_1_+3_267_443

MLIVITYQKAEDIHHIHKMKYAVCSLFKADSKHSDNSTHICQYLADSLILHKTLRLAKF

>SG-AL_contig_6268_1_-2_77_2191

MQQQQQQQQQQQYRDPPPYPGHSKQIFSNQPGLRQSFSGSETSTDVSLSSTENLATWQRQ

EPQGEETQTPLFQPLDDGGYSILARLGMPPSAIEAKGSADLVSSSPSPFYPAGLQSYYSY

STQHSQSIYATSSSSRRDDLTNVYSSSSAGGATRQSLTVPHHHWQSHGLPPPQSGHYSRA

RSLTPTYHHPHPYVNQHWTPGEEDISGQIAHNPQMLPYVSALPPPPQYPGSKALELDKTD

IRRSSEAIGGKSEHGHSQPDLTGLSDSSDSRTSSQKSSVLGQLGSHTSSEPAFSILRSGS

SSPGSQERIEQAVELLTEENRKLREELSHCLKKVSKLQKFELEIQKVHEAYESLVKSSQK

RERLEAAIKLKLEEEVKKLQSQNEHLMAQLQQCGPSKKQDSDDSTSSVGSVQRKSQREAL

DAKLAATQSNLSRLEEEARKKDAQMGRIEKLQKAFSSLQVTSERREEMEKQLRTRLEREL

ETYRSEGRGSSSGSTTPKEETKSVTALKQILNEKETKILQLETEIVRWEQRYVELQAVQH

MEDSGPQHAHLAEKEKLIDEAKTEKLRHMEELYQSNRRIAELEARVKWLQTQLTEKEAMV

KVYQRAPPSLPRSSSVHAICCSPHHSPRPSLIASTAYTRPSAPTSTSGGATGSYCDFVTI

RHVKTGSTSAIETGRKMSLDDTLTSQDHFPTSKSDSEDETRLWQV

>SG-AL_contig_6269_1_-3_915_1142

MQCNTLLKVSPDFLSLRSCILWWLDFHMAPFLFCLSNYLPHSVCLSVCLSVSLCVSLSLS

LPLFIPQSGFNLNKPV

>SG-AL_contig_6270_1_+3_3_242

TSDGDFRDVNSDLLDDSLWSNDADCCDISTDLSGEEEARFSNKSLFLLNPPVLPELELDT

DDTEDAADEPVVEVPLGAVL

>SG-AL_contig_6271_1_+3_372_665

MQIPLTLTKSCIGKNYYYKFIIMHYFLLFLHCSFSFSIFTKRLKTDLCTCVSTQKCVCMD

FSVRPFVDLSFTSSNSDHTDYDMYKCANFLAVHEHTEF

>SG-AL_contig_6272_1_-3_45_479

MSRSAAPLSVLYRWFRGPFANLTRTVSILSNRTRMSSTSTNEVTPPVDQELHVQHDDKQQ

EFVISLGGDHRALLQYTRTPQGAVDLWHTEVPPVFRGKGIAKLLAQAALDHFSRHETSMI

VSCTYLQQYIQHFPQPEHLKHIISE

>SG-AL_contig_6273_1_-3_3_311

IHNTKVPKSFQTLFGFCRAHSLELLCRCLVLFFRRHRTRTSSFVTWSTISWSVSTADVCA

SLSMFINSSGVHNCAGGNRAVKCSNRLSLSATLSGTGTGTLSG

>SG-AL_contig_6274_1_-1_37_282

MVRPDDRPLCDQVQQEASDLRLPGAGPRGDGGGRPLPRLGSPDQLRLPPFPIVPRVIRTA

RVENARMILIAPFWQAHVPPWS

>SG-AL_contig_6275_1_-3_189_299

MISCTSPIPAFTFSDFSLCICKIYHKKCQNKFVLICH

>SG-AL_contig_6276_1_+3_864_1103

MQSCPQFLLLFRFYFSSSVSIQRIPCYRVLNPFTSHSKDSMVPSFKSFHVIHILATRGKP

TPTARGGQSIRQIPQEIICC

>SG-AL_contig_6277_1_-1_502_678

MPRYVRLPSQLLSLLVYSLFFPPPTLWVFLVRFLPILPLDTRFMFIFLSGGFGFFPYEY

>SG-AL_contig_6278_1_-3_81_899

MTEQQLDCALDLMRRLPPQQIEKNLSDLIDLVPSLCEDLLSSVDQPLKIARDKTVGRDYL

LCDYNRDGDSYRSPWSNTYDPPLDDGTMPSERLRRLEVEANGAFDQYREMYFEGGVSSVY

LWDLDHGFAGVILIKKAGDGSRKIKGCWDSIHVIEVQEKSSGKSAHYKLTSTVMLWLQTS

REGSGTMNLGGSLTRQVAHDASVNDATPHIVNIGRMVEEMENKIRNTLNEIYFGKTKDIV

NGLRSMTPLESRVKQEALRNDLVAALSRRPQAQ

>SG-AL_contig_6279_1_-3_33_245

MSYRTGSSCLQTAAKKHLKMLRAVLPLLCLVVISHVTEAKLTIGAFNIQRLSDGKTEDAA

LVEKIAQILIR

>SG-AL_contig_6280_1_+3_33_1103

MVCMSIQMSTTDEIQDIMDAVGLDDSEYDSSGDEYIPSKEELREREEEEMDDDGPCTSKR

TAKKRKRETEWDEEDNIPLATLQDDIPLARIRQNDKNGDWEEEDDNPLASFVLEDVDGSD

QDLQDAIQNPVWTEGPFEPPKAEFTGDLEHPPEGELKTPYQYYKYLITNKMLKKVEDQTN

LYSVQQHGTSINTTQKEIEIFIGIYLRMGLIKAHCVRAYWDSKTRYGPVADAMAREKFKK

LASHLNSVDYSETKEEDKVADKLWKIRPWIDDLQDSFCKIPAEEHNSVNEIMVPFKGHSS

LKMFMKSKPHKWGFKMWGRCGSSGILYQFDVYQGSCAKNQQKRSPYGVGGGVVLKMT

>SG-AL_contig_6281_1_-2_2_112

LGVMMRYVYFNNLAVVTLKLCQGHPNLGNRESDSRHY

>SG-AL_contig_6282_1_+2_911_1252

MGVACTIMGVACTIMSLVPTWVSLVPPWMSLVSSCMSLVPSWVLRGEKVDDQQRAGRPEE

EKCRVGLLHAVSPGLPWSASASQSPTPGPRPGRWKQDKVVDCWVQAKYGVALCH

>SG-AL_contig_6283_1_+3_318_518

MKKWRTLIMGVSDCRLKGNGTKQIHGNYVLTWSGVDPGLKATHGVGFIMHPDIAKNVLET

NFISERI

>SG-AL_contig_6284_1_-1_151_600

MAEDAIKAKHDEPKRAQRATSNVFAMFRQAQVQEFKEAFTMIDQNRDGIIDIEDLKDMYS

NLGRIPPDDELKEMLKEAPGPLNFTMFLNLFGEKLSGTDPEDAIRNAFAMFDDEGKGFLP

EEYLKDLLSNMGDNFTADEIKQPSPMEGLH

>SG-AL_contig_6285_1_-1_115_717

MSKSKDDLIHENDKLVAEVSRLKVGLDEVHEMFVNLEKTFVESKASFSLSRYGELKDMIK

NITSDEMMKKVHSLTENQADEKKGLLQNARALAEGERGAAGGDQSPPHKSGGLSSFFKKI

KDVTGFSDDPLDIRCLDKDDIQKDNERKRRDIHRLNTRGLRAYNRLEQLQRRYQASKSYA

PPTRYQELKDMIKDVISDQTL

>SG-AL_contig_6286_1_+1_85_279

MLVYCGREEANCGREEVNCGRLNTVELLLPKIETHEAPVFVRNLEKTKIPICPTLRATAE

SAFWV

>SG-AL_contig_6287_1_+1_469_771

MANLVTPHMLALLQIYLSLSHTHTHTHTHTHTHTHTHYCKHHTFPCMHCRCVPYIPTHTC

THVHTRTHYTVTFVQHLGQHIQESSSRHNLDEKCQIYTTLK

>SG-AL_contig_6288_1_+2_194_373

MHIIAFLFNYKEVHTCSLSFLSLDKKSFHPLAMNVKIKKKLKLKKPQFVLTINTVHYVLC

>SG-AL_contig_6289_1_+2_2_193

FFFISYRFYSSTSKQTFGWEGVGWGRVRRSISKQNIFTPSEKLQIMQQYSFSVNSSVFKN

NNAG

>SG-AL_contig_6290_1_+1_13_261

MDQTSNTPSWQSGSSKASILSKIIYLWILPPNPPDNLIVSLQKSASEYGTRNKDKIHGKT

SVKQVQNGVGIPDLKKKTTKSQC

>SG-AL_contig_6291_1_+1_34_321

MIICIPKGDKGKDLIKTNCRPVTLLNVVYKTMSACIGNRIKPVLDSLINEDQSGFLSGRC

ISDNIRLIDDLISYMNNENLSGLLVILDFEKKRLIQ

>SG-AL_contig_6292_1_-1_1_183

VIKFLKSDDCSHREMCKINVKLFVESLPSVKFQIVLWKSLFCHLKFLISISLKQDVSPTV

L

>SG-AL_contig_6293_1_-1_61_1362

MSDILAALFHFETVCKELGVEAHPYIAKMLDREKESSALAKERGEFKSSMHLYLAGNNHL

LCDARMTDADCPLLAKFLDKNSFVTSVDLRYNNITDEGATIIANMLAENNTLHELNLMCN

DIGPAGAEALARALHTNETLKMLRLNGNKLETKEQKGGMYFAQALQINTTLEHLDLGDTD

LRTESLIALATVLLHNRSLKSINVNRPILFTHMEEPTVHFAKMLKVNRSLEEIHLQKYDQ

RDFGAFRLAENLMHNFTLTYLNLSCNRITRDGVKELAKVLKQNTALQTLDLGYNRLEDDG

AMHVAEALATMNNKLKCLVVVHNNISSAGLCAIADAMKTNTTLTNVYIWGNNLEEPACIA

FDNLIKSDRLELYNTDVQPYIVDGVTYLCELNNGIRRHYYYAGVYGDDVPEWQPRGENPR

SGKKMESMSFSMFD

>SG-AL_contig_6294_1_-2_2_271

CIAPFGIRLLPHLQKASIDASLISDISKFPESPLWMLKCPNVHFDLAAYKKDTTSLLAYT

AYFSDLCQEFPNFQQISDMPGISELPTDFY

>SG-AL_contig_6295_1_-2_2_208

HLQYLKTSCQRASSVLKVVAHTDWGADRRTLLRLYRALVRSKLDYGCIIYGAARPSYLKK

MDPIHHQGL

>SG-AL_contig_6296_1_+1_109_342

MGVTHAYTICNHCTGNHITEIIAKTQIVHLKFCFVFSCFQFFCLILFRIYKLVHNHLFLL

LPFFSAVFSCNISSPSPP

>SG-AL_contig_6297_1_+1_268_1632

MSSDEESDNDMNANKSTEDEERRKSMAKKVKREDGDEEKSQDRTEKEKNGEETNVDDVKK

ESDDDKKIPDNASPSQSPKEIKAKSESKEVKVKSEDESQEGDKKGKARDSNGSKSEQDRN

SPSLSGKEKEREKKRDRGEDKKRDKEQDRDRKRDKDKEEEKKRDREEERKRKEEKDRKQQ

EREAEKERKKREKFEQKRREKIQKIHFEQTEQKLIQMDDEIKNSLVKDNLDVDKCVAVLE

DLDKLPVNLVMLKKNPTITKTIKQCRKFKDSDRIKNKAEQILNRWKVILSTDSHTAKAIE

KLEQKKKEKVRTKSESNKENSIGSHGDAQLTDQTHKDQPDGVPVPPSEPSAQNIANSEAS

AITPTTTTTPAASESDGAVVPPPSTEGESATAQPGTSLVSSGSVLENGEIDSAGDGNAFV

DTNAAGNMDVTGEGDAEGKSESAVPLTNSVPVAPV

>SG-AL_contig_6298_1_+3_3_128

CYCLSQVFIVSHLLYFCSFYLVCCINICHEFGFSSMYLETDL

>SG-AL_contig_6299_1_-1_1_168

SPKLFTATIQEVFKNAQLEEKGINIDGERLSDLRFADDVALTENIEDMERQLKHCE

>SG-AL_contig_6300_1_-3_63_341

MDKDVWAKFEQVTIIAMYLGVFVLGLFKSCYSILGCDSENECVKISFICLLRVVSYYLFI

YHVRFLFVLFICNEAKAASYMMSVNPNNFLMLS

>SG-AL_contig_6301_1_-3_189_1172

MFASGLSYERAKQTLEAALGKEQEHWKPIQNRPASSTQEITDMERDKEAFWLIQLSQSFD

FAPETYALAVSVMDRVSTLVKIRPKYLRCVAISCLYIAAKTLEDDEAIPVTLDLVRKSQC

GCSVSEVLRMELVILSKLDWNAKTVTAIDFLHLIHFLMMRSVPNLLIHLEDMTPSRLLAF

LTRKLLKVLCHADITRYRPSLVALALISLELEQVIPDCWLKLHLMMQTVIQAENGEMISC

RERMASLLSQYGMLITGYRKKSTHSVKSKKRKVEDTESDNIYDGIKRLYEETGNPSEKLI

RPKRGSCASEMHLDAEDIHPFVQTVAAV

>SG-AL_contig_6302_1_+1_427_708

MKEERTFKVAKDLSSNTTLRLLQSRTVCTDFFRGQTGYDSKECKGQTGKTLQSMHEPEAG

GGGDERLPSLINIFSNYRPPAPQSALSRVRCRAV

>SG-AL_contig_6303_1_+1_568_843

MAPKHCIFQIKLVHVYCDHNHYDCTFNDVTRFGLCRDKVAKATILVRLITSLLTLFSKHS

FIPKQHIPPISQLIQIFYSNSILHPVSLEKNF

>SG-AL_contig_6304_1_-1_1606_1935

MPFVLMTNSPWALVLWASVLIFCFVFCSPTPVLYIDMCFFIHADDVCVVAWLGYRTVCMQ

GIGWLINVYDQLYSSSDVIRYYQSIRVRYRLGSERPNKTILVFAKVHDIR

>SG-AL_contig_6305_1_-1_10_423

MIRKGVPGAEAADQATEMTTVADPFLGQTGSNTDHSRQESADSGLGGMGTSYNLPRTPED

FLSNMEEMDIHDGGPKVQGQGDFGSMDIASVGDASDSHNMDSEDLVPSLHEDVISSELLK

DVENVLNSNKVDNLLTWL

>SG-AL_contig_6306_1_-1_430_771

MLEAVSGTFYINSYFATTLFCSPFLSVGSVLTAKLKTEGCKSFKKNGSHLSVLFHFPNIV

IQFKAIFVPRFLIAVRIAGCKLFVFMFILYFAILSFCQPFLLVVSSWQSSKLKL

>SG-AL_contig_6307_1_+1_1111_1323

MEISVFGPREETVVQMNPYSGYKVLNKISLKGFDMLQINHTVGKNKTTTTKNKKRKGKDG

TASKPHMHGGR

>SG-AL_contig_6308_1_+3_54_257

MQLRYATKPELRCKVGQDLPINDREIHAPDVQTSIGRRTEAMDRSESIPLTFYKERTLNI

NPPPDKKR

>SG-AL_contig_6309_1_+1_1_171

EKRKLRAQNFQKVGILFILKEPPASPRGKTQTWRNKADNVSIAAYVHKGHICDWLST

>SG-AL_contig_6310_1_-1_967_1179

MLPVQDVQLSVLGQTAMHCKIEVMEKEMQNRHSKCQKPAYKPHALTRFEILLNLLLHTIV

YRSFTSLSWNR

>SG-AL_contig_6311_1_+3_225_542

MPRSTSSCQIWPTFSPFGRAHNVSNGRAHNGQKRLYTQCQERTCSYMSEMVIYYMSATAK

HRKSATAMYYISATAEHITSTKAMYYMSAPAEHGTSAKTVERSTTC

>SG-AL_contig_6312_1_-3_93_2198

MSKRRKALIDSDSDDSDSGEDIEESIRALTKRRRTSSASASDSKSESQSEAQNKTAVPSD

SDSDTSNSDDDWTANGKKGSPKKKRVISKKVARRSSTVASSDSEKASASEPEEGEVSDSG

SGGDEPEAKFSDGFDENLIGDEEDKARLEQMTEKEREQELFNRMERRQVMKTRFEIEQKL

KRAKREERKKKPPVQQLPQERTRPQSTPDAVSTATISERSKERRKNIEDRKDSKKTSAFQ

DLKARREEKLKKDEEKKQQQQKKLKASDIYSDDEDSEGDNESDNEDRKSVREDRKRSEEK

ENQSDQSDAESEASRRSSSPSEDEDDDDTTTRSRRRVQYVTMKEELDKIRLSRSKLERWC

HMPFFNRTVEGCFVKIGIGNHENRAVYRVAEIVGVVETAKIYQLGNTRTNKGLRLRHGTA

ERVYRLEFVSNQDFTESEFQKWVTTMSMQGVDLPTIPDIEKKVADIKKTYQYSLKESDIE

QIVAEKQKFKKNPHNYAMKKTHLLKRKEEAENAGDHESLSQVIRELEELEQRATELDRKR

TNNISSISYINQRNRIQNILDAESAFAVEVKEMKSAVADPFTRRHCRPTLVTKTREPDDL

SKLQKQEEEAAMAADQAKEASASKTMGDQLSSERKSSDKTDGGEHRVSEDLFEVHDFEIK

IDLDVPSAPSVVVPSASSGGPREGAPRRSLNLEDYKKRRGLI

>SG-AL_contig_6313_1_-2_8_568

MASMGKQKSSNLDKSLSKGKPEVNVATFALLFSEIVQYCQNRVYTVPELQTKLAEMGQHV

GSHLLDVLFVREKGFKREIKLLSMLIFIKSNFWKTVFGREAEKLEQANDDERTYYIIEKE

PLVNRFISVPKDKGSLNCAAFTSGMLEAVLNGANFPAKVTVHWHKGTCFMIKFDESVIAR

DKTIDSR

>SG-AL_contig_6314_1_+3_213_506

MTGLSMQNISQLGYTTVRKGLEPFVVTWEEKKKGFKSFIYLHSEHRDGRKLKKQRFQPKH

SDKSKKCTLVSLCELTEADIQTISYGDKCKINNNNNNK

>SG-AL_contig_6315_1_+2_20_442

MGNGFVLVGDFNFHYEDLSKANTRRLRDLVNMFSLVQPVNEPTYRSGHTLDLVIYRDTDN

LVLSTRVCHDLASDHNSILCSLCICKPVQNVKFQTIRSLNKINMNDFSADVSDGLTSNMS

ITDFNSHLSHVLDRHAPLYKL

>SG-AL_contig_6316_1_-1_1_162

VSGRQVWIRSHCFLPPLSGWLNRSTRTFSPCATFPPLELPPHPNLLPLSRSGFD

>SG-AL_contig_6317_1_+2_2_151

VLNIMEHHLVTSSSINQFQFHHTVGAIPNSVVCFPRILCTLVKKFQQKQN

>SG-AL_contig_6318_1_-1_106_354

MKGIIMGLFYLFSGVGSFLGTTIIITLSTSNTWFHAHDYGNINCRLPCTPAQPYVTQSCH

LDNYFYLLAGIEVLGALLFLIVA

>SG-AL_contig_6319_1_-1_133_1236

MLLLTCIVSYLVATVVADDPIVTIIETNPVVNPIEGKIISEKNKDVQLTCVVENKPKDKE

VQWMGTVSRNGTVTTVPISTGTRSYDSFKYSIDLPSPTSWRLRIQNGQVTDEGIYTCQVQ

VSNQNYAKAFHDLVIVETPQIADLETSSDVSKSEGESLVLKCQASGRPPPSLKWTRMAGE

LLPSGGREHLESDLLIPVLKPENAGIYKCTASNSAGSDYREVRVTVNFSPILSAANSVVQ

QAQGYVKELVCDVKGYPTPKAKDISWTKRGSPLGFDNHIFIRNIPGASSRITSILTFWGV

REDDYGEYTCAAKNEKGSGSLKFILQESATPTEDRTGQIRAGASILTVNIGTIIMLLSVC

LLHRLPFH

>SG-AL_contig_6320_1_-3_3_272

RCQFARDNVYHRHAGKISSPHASISKQLLFTPSICHQNTNILIYHFSQPGICFHTNSQTF

LRKMPAIGQHSTLIAIDLLEQAFPNPSSPI

>SG-AL_contig_6321_1_-1_1_513

HSHPLLPDHDIEVAFDVDIDDEDIHLINRVRHAINLALGSKTNTAEWGPDAVAAKIQTPA

RERLLELLYKTREYKEPEYSSKDYVWSLLNPQEIVTHEDDFDSDFLTLLPPHATVRLVAQ

NVKAMDHQRNHIDNLHRIASTHTRGPITCNLCKVTVQTSQLLAIHLETERH

>SG-AL_contig_6322_1_-1_163_1968

MFLKPFRVKTQTTVKGSDRKKLRADIERQYPSLTSAAVADLIPNKGDMTQVKIEAHSDEH

FMVYCFQKNPVFFESFKKLYPTVYTLWKFPDMLPVFTTYPPVFGKLLKGADLMLPGIEVK

GEHHPKMFGNLQKGDLCLVRLKGNRAPVLLGEALLSGEDMYMSAMKGKGVRTIHIFQDHL

WEMGDKSVPPQIPDSGSEDESDDDEDEGKDDNECPAQDAEGVCQSLEQLTVEGKREDDDA

CTNGIGHASNDLDGDVSGDAADGAAGDDEDGGATGGEPSESSSPDMDELLLSCFKWAIKG

RVSKSDLPLLTSNLLKGYMQPFCAPGVHLDVKKSSYKKFSKFLHHMQSEGFIKLKEISKG

VDAITQIDKSHRELRGLEIPEITSEEADEDSTDIKFNPPEIKEVLCVTAAMLPLLSPKGY

TKGSSLTPQELRQLVTEYVRTENLQEEGSKEQVKLDPILAELALTRNEGDKSYLTWNTLF

TRLCDKMQPGCVIQLAGKAPILKKGKLPAITLKVEQRGSKKKVTLIQNLETYGIDPRQFA

QLIQRKMACSASATHSERQNGGMDVQVQGNQAEFVVQQLLGTYQIPRKYIEGMENVKKSK

RR

>SG-AL_contig_6323_1_+3_3_137

WGVDGWHQDLLCAQPLSACCLLSCSCFKKTPHVSFSQAVVILLKQ

>SG-AL_contig_6324_1_-3_852_1469

MGARSVSTVYPGPQLSEMDPSGMRRPIDPPSADVLEWCTHLLLQHYQLGLTPMAYPLMTG

GTCGVPQGQCVIVPPAQKFLHSSSTDADEGTCSRQARESLSCGIPDPPYILALEDVTSSR

KDTSRPQAKEDTSARDQPTGGQSQSAPSLLLRRRLLRGPATQLGRGAPLSLPLWWYHLGI

QREARDQPKDWRPLTDQTTYQRVSTG

>SG-AL_contig_6325_1_+2_251_406

MLPITGQEIKTDIATCIKTAEILFSLFFLSLYKLMTGCNQSWAQMERPRSHR

>SG-AL_contig_6326_1_+3_3_173

LIFMFSQIYHHQLVQLLKLRIQLEYFTQLSLFLNEGKQVELFLYNQKLLPSHFSFII

>SG-AL_contig_6327_1_-1_61_1365

MWKIISLLVLMLVSASNAASTPRERYRLMNTACPEECVNASLEISFVVDGSSSIGPLNFT

MGLQFIEDFVRMFHIRPRAVRVSMVTFGFTEHHEFDFDTYDNNHDLQKAIAQIHYPFERY

TNTSAGIRHMLTQQKPKARPGVRHVAIVLTDGNSQEWQETKNAAAEAKMANLEVFAVGIG

INVSDEELHNIASDKRHVFKVSTYAALEEITDRLTHQTCNIPTYPECEMDPVDLSFVIDS

SRSIERDNFTLGMKFVTDFVDSFLINRASVRVSAVTFDVDVNTGDAFGFTAYPYKKDLLD

ALNRIPYRDGPETNTGAGIRFMREEQMSHGRRDSAHVCVVITDGNSQNWTSTEHQAKLAR

DAGIVMYGVGVGKTVSFKELLRIAGDESRVLTSDNYAELNLIKRNLTQVTCTGVLSALEH

KSEHFKSQPVPHSSF

>SG-AL_contig_6328_1_+1_487_723

MIHTLQKLVQADWKVSALPVRNPGLNNTNNNNNNNYNNYNNWRFACYLYNGVLALWLKLN

LHGKQEVEVRFQVGSFSAG

>SG-AL_contig_6329_1_-3_171_1328

MVGFNHGGRAILEAAYGPGNAGQPILLDELNCTGTEATILDCGHLPLGSHNCRHQEDASV

FCLVPSSTEDPGIGGRDTTIASTTTGQPLTSILGNSQCGLRPLERSVRQKRQAGDGVNMT

DLTAPREVEPLRFEKIVGGDLAQHGMFPWQVGIQKIYNIYPNGEMIVSHWCGGTILNKFW

ILSAAHCFTNLPKSRVLLRVGDHGLKMTDKGEQKFLVEELIMHRGYDRDSHDNDIALLKV

KSANGQGIKFNDYVQPACLPSSVSDYTPGTQCYISGWGETGTADKAPNNLRAARVPLLAF

NVCQYLYKGSLTRRMLCAGFLEGGIDTCQGDSGGPLVCNIHGVYTILGVTSWGFGCAKPN

APGIYARTHEFMTWIEDNIRSHSSTK

>SG-AL_contig_6330_1_-1_16_378

MEQQLNTVNEESLKVGLKIHGRKTTFMTNIDTTDQIQIERTETEKVTIDNYLGQTIAMED

RTRNEVLIRIKAQRSIFGKYREIFQDKHLPIRLKRKLFNQCILPTMTYGCQTWPLTKAIV

Q

>SG-AL_contig_6331_1_+1_190_321

MSDCEQNLKTGGGIASKVQVGLLGRKVVLATMKKNLWNHQVWSL

>SG-AL_contig_6332_1_-1_1_120

YIGGSADSAAGHLLSTSQFLVVLCLLYLVCNCNFCVIYVQ

>SG-AL_contig_6333_1_-3_3_284

CREPSLAHRRLKLALHYYLKLQSEPENPAYNCVIKPQFRDKFENSPRCIPPFGIRLLPHL

QKASIDASLISDISKFPESPPWMLKCPSVRLDLA

>SG-AL_contig_6334_1_+3_1002_1193

MRLDNLYECSVSFFPLLLTFDPSRWSDNFADRTRHSMDNHNVLIYCLSQCSSVHCLSNQP

FKLI

>SG-AL_contig_6335_1_+1_1_186

TVAGFIQLNCSCQKSRHMKHRSLQEIWRRQKYPFVPHCERLPSQHSGCNCNFSVLKLYCA

PS

>SG-AL_contig_6336_1_+3_3_158

LSLHDALPIHFIDLQSLFYATLQFNRDTNFQQHCNTLTDELKHITISTTSNV

>SG-AL_contig_6337_1_-2_47_1699

MQLLSPTMRMVNSVSCLLLVVAGLTFVGAASIFKREKRQTGQYRTRGEWASDNGFEPGTT

YASDTHSNYTITTGARQDQLCDVTIDYETDIEVSYQCAYDKLVLNAGRPDSITVCNQATG

QTYTTTVPKGETVELRFASDDEVELTGFNLTYRVLCHDYENDGGTEIPFVRKIDRQGSGE

FKSPGFPTYTYPPNFDRTYLAYTTGPRIHIKYFVDIEEEAQCLFDYLVIYAAERSLTLCG

DKQSGEMDITVKNNTLKLRFHTDRSANYGGFSLNFTVTGAGPSNQSPMMRNKASWQGWRP

GRSQTSFHNGIHQLISRDPNLGPLAHSPPVSYGHNSRPGHRYFWPGSAAISHPPEQIKKI

DVSIPSDENSETNNVDQIKNIKVFLPQDKQFPIDQIEKINVYLPSDETRKPEADHFPFLY

NWNQGIADNRFPYGQINDNHFFLPSDETQNPQASYFRFPYGWNQGFGDNPFFHDQIKNIK

VSVPRGKKLPAKPVPFPIDQIEKINVYLPPDENQNPGANYFPFPYDWNQGTADYRFPSGQ

NDRYDQNSSDY

>SG-AL_contig_6338_1_-2_65_736

MAVEWKYSTPVQCSEDVQREPPSFQTDRKVSVPDIVPDAARADQPSPAGRPDRQGNLPGT

SPTGNVQPTVTRDKIILTPDHSEGRIEAEGLASVLQPGGIFRMGVENHTQVFHTSQNATG

KRRPSNIKNKHKLTESSAEPVIKHLDVISAGLADDYLRLGRHLGLADSMLSQIHLDYRAD

GLHEVSYRVLARWKQQFAPVTVRMLAKTLLDIGRGDLADELRHG

>SG-AL_contig_6339_1_-2_2_169

HLKELFLMTEFDSLEHHDTKKVTDSHIRTHKTTIKKRNKNNEYNKNIRNNVRKKNR

>SG-AL_contig_6340_1_-1_10_270

MKNRGVDHKEILRLAGEEWRNMSAEDKKPYEKRSVEAGKKYEREMAEYRKNEGKASASAQ

RQEVADDDDDEEDDEEEEDDDEEEDDA

>SG-AL_contig_6341_1_+3_699_1166

MRWQNIATIMDESQQCNVIYNDCSFSLCHTHKWQACVLIKQHNQTLSRLFRIHEPETYKI

LHIFYFCFKSATSPMNYTVKMENSCTVLSFNSTVAVQKTMEEGNAAATNKSDRWFFSCLL

NNSLETDAESEARKDISFKTHAGTQNSTVETSLLSG

>SG-AL_contig_6342_1_+2_254_430

MGGAVLSPRAFSGKRARISCGKNSPVTKNESNPIQSCPQLLSSVSQLAGLSRELHQLVN

>SG-AL_contig_6343_1_+2_2_199

LATYSLVTCNGADTLFPQVKVFVLAYPSVMYTSLLEVSSLVNTVVPHHSILYHMFSLVSC

LYCLFV

>SG-AL_contig_6344_1_-2_485_1678

MMSFEIPPGLTDLLQEFTVAVLRERPSDLVTFAANYFNKLNDKKNGDSNEKRGGGVRFIE

NAEPEKVEVSDEPMQTDSEDDDFEPPPVSVRYRRKSVSAEAYNPETDEDSGESKVIHPKS

DEQRKRLADVVKNILLFRSLDPEQMQEVLDAMFEKLVEEGEHIIDQGDDGDNFYVIDSGT

YDIYVEIEGQPKLVGNYDNSGFFGELALMYNMPRAATIVASSAGTVWALDRTTFRRIVLK

TAFQKRKKYEGLLESVPLLKSLDLYERMNVADALQSRTFEDGDQIIKQGDTADVMYFVES

GQVRITMVNKDSNDPSLGEKELTVIEKGGYFGELALVTHQPRAASAYAQGTVKCAVLDVG

AFERLMGPCMDVLKRNINEYEEQLSAIFGKSVTMKDIR

>SG-AL_contig_6345_1_-2_380_1081

MEDIMTMMMQDNSLWVFAYGSLLWKPCFHYQRKVVGHIKGFKRRFFQGNTNLRGSVSQPG

RVAILVPDEEEVAWGVAYEIHGQKDVQHAVEHLYVRENSKGGYYCMVNTFHPRPPPPQDD

DSSDSGSEEAVTLEPFSVLSFTATSSSHLYMGPAELSTMAREVVAARGQAGTNSEYVLRT

AEYVRDHIPEDRDPHLFGLEKMVRELVAEIQHRRKSAGHDSAALPALQAVPVPV

>SG-AL_contig_6346_1_+1_1_408

LSRSQPTLHHVRLVLVTSLVVCCEVEELLPLGLQHLHAVLVGVNNILKQVQFLAIQGSVL

VSITQFIGRAHNLVRQAHLGVDAKARACQLQFIQEFFRTGHVNSLLTHHGSGKVGAAFAD

TCQHPDSILCHQTRNL

>SG-AL_contig_6347_1_-3_888_1088

MPSEETTSGENMGTGRRDWGECSYTCQEGEYETSCSVKKVEGVKNLTKSFGSCYSASWQC

PWNQERD

>SG-AL_contig_6348_1_-2_38_1402

MDSVKQETREQTLQLQHRLVQAEQCLQERESSMEEMEKEQQRQTEELRRQVEDLRLQLDN

TERQLKANKLFLDEQMLEREQEREEFLKEVDKLKDHLYNKEKQTDSEGRLQHEIQDLTEQ

LQARIESQALMRQQTRELQQTLQERDLSAQDLRVWINTLERELDQRGDTEEQLKGRIQKL

ERRLTIGEADNQSDTSASPVLTPRPDSPTTIPLSPAQPGSQRRESRISLELELRKSRQAE

EELVHEKDALQKRVQEQLLQISALRNQLDEMRHWTGTPDPNASSTSAADLREQLDSEREK

LEKIEEELSTVRGNQEAMQAELKDKTQEVEQLRQQLNESVASTSRIKELEEERSSLQSHI

AELSSAGPFPPELLDEKNAEIEELRQQLIKTETEFGSLQAEMKEKEELVSDLQDTLHSLQ

RGEACLDVSLSFNEPLPLNASFTERFQEEERPSSV

>SG-AL_contig_6349_1_-3_93_188

MTSKLSTPPVTICEVYNALITSHKLELMVLMA

>SG-AL_contig_6350_1_+2_1361_1663

MLLTPKTGLKGSVMGETQAVLQYSAARSLRHTRLSKLSTVEVISHHHFIIPSSVHRQQIP

FYCLAPVQFKNYYYILFIAQPTIKAISGLSPHKVHQSQEDN

>SG-AL_contig_6351_1_-1_1_285

DEPKYRENVLELVTWCDQNNLELNVSKTKEMIPDFRQTKPIPPPLVIKDKQVETVSEFKF

LGLITSDDLKWEKNTGKIVCTFFGASKSLVLRKTF

>SG-AL_contig_6352_1_-2_86_313

MIPDFRQTKPIPPPLVIKDKQVEIVSEFKFLGLIISDDLKWEKNTGKIVKKAQQRLYFLR

RLKKFGVKKDILIQFY

>SG-AL_contig_6353_1_-1_1009_1182

MIFQTMSTHHMNRDSCSHSVMKCKYAYNLSQNFSVGDITLIFVSIIYARWHYTYFCFH

>SG-AL_contig_6354_1_-2_176_1762

MEGQGEANGPDGQQISKKALKKQQKEAEKAAKKAAHKQQNAQQQQENGPEKEADVSEGRY

GNLPLNQSQEKIQRKIYKLCELTPALANQKVWVRARLHTSRAKGKQCFFVLRQQKWTVQA

LSSVGEKVSKQMVKFIAGVNKESIMDVEGIVRTVSQKVESCTQQDVELHAEQVFVVSVAA

PRLPLQIDDASRPETGDELATVSQDTRLENRILDLRTVTSQSIFRLEAGVCRLFRDYLTR

QNFVEIHAPKIISAASEGGANVFTVSYFKTSAYLAQSPQLYKQMAICAEFDRVFSVGAVF

RAEDSNTHRHLTEFVGLDLEMAFNFHYHEVVDLIGDMFVHIFKGLQTEFAEEIALVNKQF

PAEPFKFLEPSLRLDYVEAVSLLREAGVEMEDEEDLSTPNEKLLGRLIKAKYDTDFFILD

KFPLAVRPFYTMPDPNSTKWSNSYDMFMRGEEILSGAQRIHDPDFLSKQALKHGCDLEKI

KGYIDSFRYGAPPHAGGGIGLERVTMLYLGLDNIRKTSLFPRDPKRLTP

>SG-AL_contig_6355_1_+1_1_213

LPTRQRCLGGELNTGPSVWKSGVYPHDYRARVIHVSTIHVYIIKEQEKPTQTQTTPTAMV

LKGGVENVNTK

>SG-AL_contig_6356_1_-1_22_513

MVTCMDEGIGNLTAALTTSGLMNNTVFVFSTDNGGQTRLGGNNYPLRGAKDSLWEGGIRG

LGFVYSPLLTNRGYENKELIHVSDWYPTFMELAGASRDGAPPLDGYSQWSTISERTDSRR

RELLHNIDPLRESHGVPQFPDTFDTSVHAAIRVGKYKLITGNPG

>SG-AL_contig_6357_1_-1_1_150

KRVGYCPDNAGPRKVEVSTTSLPYDLRSWDKFYVYLLHKCISIRTVPVNA

>SG-AL_contig_6358_1_-1_283_480

MPTLLSTGNYGSITWPLIIRLWGFNYPTLLSTGNYGSITWAEIIRLWGFNYPTLLSTGNY

GSITWA

>SG-AL_contig_6359_1_+2_74_373

MHTFGTERAKVKVTKAKNVPKNSTFGGRCVNSTFSQLLTIFKFCFHCCDSLPLSSLHVKF

YFIRSFYKVMVTFTVRKSGFAFEQLKFLFEFSHLFVFWSF

>SG-AL_contig_6360_1_-3_3_185

YVMQIILKNMGDVENDSGSCKFYLLSRSCHATAQPKHKSIRLINLGVRMSMCILTISQLR

P

>SG-AL_contig_6361_1_-2_233_430

MKPIMVTLICNGDVAVHTNDIRSGKIKGFYLYGLIVSLTTVAVGYVHILDCQKAKLPEYK

RRRKFK

>SG-AL_contig_6362_1_+1_1_201

GTEGGQSTTVVKKKSNHINKPFITRVIKSFVWTATSPLQMKVTIIAFIALVMPRIRLAIA

KVWMTLA

>SG-AL_contig_6363_1_-2_119_571

MLQQHTVLQSQQLIHPQSLELTASSEVILLDNLDIFRKNGFDFVIDQTAPPTQRVKLKST

PVSKNWSFGKDDIEELIFMLTDSPGVMCRPSRVRMMFASRACRKSIMIGTHLNKTEMKKL

LCHMGEIEQPWNCPHGRPTMRHLINLNMLPK

>SG-AL_contig_6364_1_+3_660_1079

MFCHAQKIIVKSKSHHILVYSQSTMNVCSSQVFLRSTSCMRKSRSHVLCPGMHAKAGYSG

SSSLEWSRFTLSFPEPVSVMPCFTKQKECFSPSPNPVACSTPANTQSMQHREVKANTKSM

QHPEVMRSDGHTMGAGRTCR

>SG-AL_contig_6365_1_+2_2243_2602

MCVCVCVCVRARVCVVVVFLPEFLFDPCQVSNSKTFVFLRHNQSEKPDLVFMYIYIYFFI

YLFTSAYLFICSVVFLYENVWLLLEVCFFPVILIIVIILLLLLLLLTQVLSLEGPQKKEH

>SG-AL_contig_6366_1_-2_1151_1609

MNHEQVVTLTDEQVAEFKEAFSLFDKDGDGTITTKELGTVMRSLGQNPTEAELQDMINEV

DADGNGTIDFEEFLHMMARKMKDTDGEEELREAFRVFDKDGNGFISAAELRHVMTNLGEK

LTDEEVDEMIKEADLDGDGMVNYEEFVTMMTAN

>SG-AL_contig_6367_1_-2_2_208

VREVGRTNVSPSGCNPLLCGNSQTLRQQQPPPGAPVLRQSRGTRSSLLGRPPCHTTHQRC

PRRSAATSS

>SG-AL_contig_6368_1_+1_307_696

MFALLLQSSKAPRKHSKAEFSALQASLISFSLSPLILLKSGEHWVMFWHLTASLLPSHAA

NTAVKSRTHFMFDGLSVREKTKQRWIQHQLDQALCVPQMKPSCTTLQPKPTFWPLCLSLP

LNLGRVARTD

>SG-AL_contig_6369_1_-1_1399_1599

MQSLFFLNNYTGNYFIINYHHHHPFFVLFCCSCCYCCCFNAYILGFLFFLLFLCNMFGIF

FMIWVFF

>SG-AL_contig_6370_1_+3_12_308

MSHRTLSWLTGSLTCIFDVLHAYTHEGGSGTSRSPLYMLTWEIRKISALYPPGKVPEADS

NSGLPNPPVWKSSVFTIQPQAHLIIVSKNHHQRRKTVKL

>SG-AL_contig_6371_1_-3_69_356

MNKQNKTGINIFFFFFTIIITSEYLCFHPLCPCFCLSICLLIYLFIYSLSTLILQFDFKL

ILVSAKKHTNVHRFIDSYYYSQLFKTTHTPGYILCR

>SG-AL_contig_6372_1_+2_74_304

MNKLDLSSLNKYCKVLVMISLLQSHRAMEFVLLERLQDRSLSKVLKRTCCCPCSRPTLST

TPVKSKGEDKANTTWSR

>SG-AL_contig_6373_1_-2_83_394

MAKFFTQEGQTEFHVETFCCDEKIQTKPKQNKHLKKQKPFHSLYCLVVDKCKLCSIYLHV

CLIFKILISFVNLSFGLHAFPYMLTVMIFPSMCMLFELLRTIII

>SG-AL_contig_6374_1_+2_2_196

LHHSTTALYIDIYLSRLLALLGACKYVRIHAVWFDVNACTPMHIPVLKKMHTQIHYTIFP

HAYFV

>SG-AL_contig_6375_1_-1_133_387

MSICTTFCCLFFFVLSIMFFYACMHIVRPMLCMRYVAAESSYSVFGGCAFWVFLCFHKLP

PPVWLSGSLPYLLNGLCAYTHEGGS

>SG-AL_contig_6376_1_-1_1_177

LVNCSPVTTQQEPSDGWRGAGFHLKIFFGSPLELCYVYIFHFVVSVISCSNCCCCFPLP

>SG-AL_contig_6377_1_-1_1165_1437

MNQFGTVWGILINIISSTDALPLTTTQPYPDPTSATHIHTHTHTHTHTHTHTLVDVNISY

TFCFSQISVKLFLTPVSPGFCVCVFVCLFVF

>SG-AL_contig_6378_1_-3_276_461

MTVYCSILLCNVDNTHTHTHTPGVAVLFAILRSGTQSLQKNAFQTTTTKKMRSVSKHKSI

NQ

>SG-AL_contig_6379_1_-1_106_294

MQRLCSCFELMIFSMDHLCYLSAPYLLFAFPCYQCYMFISNVALLLCSVICMPFYCFCTL

WYV

>SG-AL_contig_6380_1_+3_393_551

MRLPYTAVPCSLLTHPPYSAGMKTKKFLHFSLYGLGEIISITSFRDALQSCKP

>SG-AL_contig_6381_1_+3_84_278

MCRLLLAGMKTKKNLHFPLYGLGEITSIMSFGDALPSCKPWFGCFRLPTDLISLKTITPV

FLTTC

>SG-AL_contig_6382_1_+1_721_2058

MMVTLCVVQELPQELPQVDRLLAGAGAAVRIGQMNWGGMGHHGLTDLTSSVGSSNSSSSS

TGTRAVDVIFRTVVGHTVACRQMNTAASSPHRRRRRQRTAGAVGAGGGGDAGGHMGAARP

VVAQMVGGSVMVWVRADVVGMMVVTLVLVLVLVVLVLVLVLVLVLVLVLFSEADWGEAAA

IPLPSATNTSLRVGAFNIRTFGKTKMSDDIVAGRIKQIVERYDVLLIQEIRDKSVDSVRK

LLGMLQRPFNFVISEPIGRSSYKEQYAYFYRSDRILLLDTYQYEDPEDVFEREPYVVKIA

PMFANHDTVSLIGLHAKPSDATAEIGHLRDVINKTLEEMGHDSEPIILGDLNADCSYADL

DARLEAHDYLTVNTNTYTWLIGDDTDTTTKTTTDCAYDRIIVRSPLQTHVVSNSSIAFDY

VHWLGLTEDEALDVSDHYPVEMILSF

>SG-AL_contig_6383_1_-2_2_223

SSKPVLSLSLPLSLSFCLSDFPPNLFSLSLSLSALALTLWALMAELVERQIHIPELVSLN

LGCGTLNWPSFCPS

>SG-AL_contig_6384_1_-1_157_6264

MSSKRGNEDDEEMEMEEGEEEEEEPADEVRVEKRGRKRKNKDGSEKKKKSKKKKKRYSDD

EGSDFGGGSDEDDRSFEITPKDIKKRAPVRPQPKTSADICNEFGLKNTEINFSEDDYKNI

TNYKLFSQYVRPLIAKDNPKIAQAKLVVLLGAKWREFAANNPFKGQAQGGGGVAGGGGGN

SSSERTEAVSAVVVPSSSAAASPATPQRDDDDDGDATEEESGGGGKSSKGKKNKGKKAVA

PLKIKITKKRKKKKASSDEDEGGYNTSDEEFERQLEEAAILKETEKAEAAAAPARKRTKR

GRGQGHRKKKTKTTNKFPNDPDAEGYETEHQDYCEVCQQGGEIILCDTCPRAYHLVCLEP

ELEEAPEGKWSCPHCEGEGIQEQEEDDHMEFCRVCKDGGELLCCDTCPSAYHTHCLNPPL

KTIPDGDWHCPRCDCPPLKGRVHKILTWRFASKPPEQEDELDHTHHEKSKKTRPPLREFF

VKWHEMSYWHCEWITELALDVYHPAMYRNYIRRVDMDEPPPLEDGSSYKVKDEDPHNLEE

RFYRYGVRPEWLEIHRIINHTRQRDGTMWYLCKWRDLPYDQATWEKEDNGIADMKQFIEN

YENLRLIMCGSNDTQTEKVSKGKKGKKEKRDKKKEKEREEEVAGKIPPSHPTTDLRKKLE

RQPAYLDETGCSLHPYQLEGLNWLRFSWANGTDTILADEMGLGKTIQTITFLYSLYKEGH

CKGPFLVSAPLSTIINWEREFELWAPELYVVTYVGDKDSRIVIREHELSFDEGVVRAGPK

AFKMKQGCNIKFHVLLTSYELVSIDAAMLGSVDWAVLVVDEAHRLKNNQSKFFRVLFNYK

IGYKLLLTGTPLQNNLEELFHLLNFLTPENFHDLQGFLDEFADISKEDQVKKLHDLLGPH

LLRRLKTDVLKNMPSKSEFIVRVELSPMQKKYYKYILTRNFDALNTKGGNQVSLLNIMMD

LKKCCNHPYLFPVAAMEAPRTQNNAFEGSALIKACGKLELLSKMLKHLFGSEHRVLIFSQ

MTKMLDILEDFMEAEGYKYERIDGGVTGSLRQDAIDRFNSKESQAFCFLLSTRAGGLGIN

LATADTVIIYDSDWNPHNDIQAFSRAHRIGQANKVMIFRFVTRNSVEERITQVAKKKMML

THLVVRPGLGNKGGTMSKKEMDDILKFGTEELFKEETAEEDRIVYDDKAVAQLLDRTQVG

QEEKEMAMNEYLESFKVASYQTKGGDAEDEPETEILKQEAEHADPAYWEKLLRHHYEQQQ

EDLARTLGKGKRIRKQVNYNDAMNGHDEEAWKENLSDFDSDFSNSAHEDDDDDFEENRDG

DQTRTRTRRRANEKDRPLPPLLARVNGQIEVLGFNARQRKAFLNAVMRWGMPPQDAFNSQ

WLVRDLKGKTEKVFKAYVSLFMRHLCEPGADNAETFADGVPREGLSRQHVLTRIGIMSLV

RKKVQEFEAINGTHSMPYIKALDAIEKLKKESIKAPSPKTESKADKDAKSEKSDSKDKDS

KDSKDPKSEPAEKAAAGKDGEVKEEPKEGVEVKKEPEDEEKKEGEDQKEEEEKDEEKKEE

EEEEKKEKGDEEEKKEDEKKEEEKEASRPETEKEEEGEKVEKMEVDVKIEEEKVEEKKEE

PMETDAEQKTEKDAEEEEKTEEAAKEETKDKETKENEDDKPTKDEENTEKGEEEEKKEGE

EKKTEGEAADKPEDPDKEAVKEEKAEDKESKDKDKTTDAKKDDEKKSDEKKEGKEEEKKD

GIKKEGDKKEGEKKDGDKKEDETSQKFMFNIADGGFTELHTLWANEQRALQIGREHEVWH

RRHDYWLLTGIVTHGYGRWQDIQNDLRFQIINEPFISEQGKGNFLEIKNKFLARRFKLLE

QALVIEEQLRRAAYLNLTQDPAHPAMALNARFAELECLAESHQHLSKESLGGNKPANAVL

HKVLNQLEELLSDMKQDVARLPATLARVPPVTQRLQMSERNILNRLVNPNQLPGAAAAAA

AVMAAGAGVASGSSQQLPLKTGLPGQPSPSSSHRSSPAPTPGTSGQSDKKETIVLD

>SG-AL_contig_6385_1_-1_94_216

MNMIHTHAQTFTYEHIWRTKSLCMLDGKYICTVVRLCDVMF

>SG-AL_contig_6386_1_-3_15_221

MLVIITPSPNFRPTGSCQQIGIAIGLSVDLRLPQDSSFPRHFGPSLLHNGVRVADSIPET

ETIQWCCPL

>SG-AL_contig_6387_1_+1_208_339

MLPLLFVRFVFRFLCICIITSFDVCYSGKKNLSTLHLFFKLSFF

>SG-AL_contig_6388_1_+1_151_318

MSSTASNDLKDSEPKQIRYRRDFQISGNGTYLILKPYQQVTDLTCTWGFAKTQGVF

>SG-AL_contig_6389_1_-3_45_626

MGRFKKTSSSMDVANEESSIVNEDTFVDVDGYSSSDSSDDDESEDIDVEDLESDDYDTDS

GSKPRIVKKGHHNELERRRRMELSSNHGDLKSLVKKLFPRAKVSGRDRTLSLASLALQKV

SDRSRAIKKSGKALIYAYKCIRATRVPPEKLAELNADIKELEELFGRDGTSPDKEYLEEI

KVKDAQRKKSKRRQ

>SG-AL_contig_6390_1_+1_247_408

MIKKNVRSLLHKVYEIQLEGIQSDIMQVPEVCHHRIFKLTTIKTTITLQKHSNY

>SG-AL_contig_6391_1_-3_225_329

MYISQLRIQGCQFIKHNEHVIVQETLYLDLLYYVL

>SG-AL_contig_6392_1_-3_255_485

MCVCWKFLIVNETSQLYAHEKWVSNSFRFPDNFLDYFLVERDGSMLTVTVSKQFIFLFLL

VFTVFYLSLSNRSSNVS

>SG-AL_contig_6393_1_+2_395_736

MRRGPILLGLQCHVNNNRVLHGEYSMTNLTKREHDREAKVWWEFNAKPTTIRVSHGKCHR

QDKLMKSVYQKELIKNSIRAGKQTSKKTNTHQKNTPLMLLMLKYLCTVTKKIEC

>SG-AL_contig_6394_1_+3_129_347

MNSKKVQVNSEECFHRKQNDRTEQAITNNNKTQGNVTHVLNQCKSPKYKIPLPPINKLPS

MVTGKNNITLKKK

>SG-AL_contig_6395_1_+1_685_942

MKLLTPLPLLSSHQRWEYADCAAMYADCTVWAKLSKHAYTLSRSSYVEENLYFFPFKDNT

EHPMYKVQLFKQTKEKFNESSHLLLI

>SG-AL_contig_6396_1_-3_12_1067

MADGGFSVEGSENIQEILSKQLYLCQFLVAISILREGGHFVCKLFDLFTPFSVGLVYLMR

LIFDQVNICKPVTSRPANSERYIVCKGLRANSHPVQLYMDSINRDLCQYMSAVSTIDLYH

IVPLEKLLDDEDFFTYIYNSNVRIGKKQIVNLKKIQTFAQNVDLYEARQSDVRKSCLEKW

KVPDEVRTAPARQDPRTVFENMVTTEKSDYETFTPTELYKSNLHKIDSIYDYMCVVSGDQ

RRSFLLSCGRSQVFFWDGQGNGKWRKPDNLRVELPTQTLLEVEFVVELKGEGKGQRRLTT

IHIMDALYLCGKDIRQKHFRERQDLIQKFVKAVHKETRFDLTRLVMADVFRM

>SG-AL_contig_6397_1_+3_3_173

ERERAFTLLTNRNHRRPFFKKMFLINTYNFSQSVYHPALHSQIKTIETRVNFWLTGE

>SG-AL_contig_6398_1_-3_69_980

MFSFGPMFPEMQAGGRSFSTTYRCYSVTMLRDREDVERGGKIIMPPSALDQLTRLNIQYP

MLFKLTNRQTNRETHCGVLEFVADEGRIYIPYWMMRNLLLDEGELVQVENVSLSVATFAK

FQPQSPDFLDITNPKAVLENALRNFACLSQGDVVAIKYNDKVYELCVLETKPGKAVSIIE

CDMNVDFAAPVGYQEPQRQSREEGASETDGAPMDTHNVDEAEFRPFIGQGNRLDGKKKGT

ESNPQASAGGQQRRGIPNYNYKKGTITFIRNSRPVANGNSETNGDKNFEAFSGQGQSLRR

KTRK

>SG-AL_contig_6399_1_+1_736_915

MHVLQVGTKLEISDLESTALTSYSDSSEQSLQSSFVHEPSQTAQNRALQSSSAHEPFEKS

>SG-AL_contig_6400_1_-2_2_238

QGFRLYSVKNKSCVASLQLLKSLNYSQQAASVTSANVRIQVASELLPTLYTPAVPLNILN

HTSCSIQQFKSLSLVHSAI

>SG-AL_contig_6401_1_+3_168_311

MYAFTGGWTGGRIFHQIGLNLCRQIGELYLSTIQSSSIKNGEKIRKSF

>SG-AL_contig_6402_1_+3_717_881

MEASRHPYKETCSYGPVLPSLTILWNKESVAHTLLHHRLYLYSTVSLVLSLYMKT

>SG-AL_contig_6403_1_-3_1314_2267

MVSMSEEKEKTAATPFSLKNAGSGFEGRSSDIFGGLDVLERKHISHEQNRTDLDDFADLR

HDDDNDDLPNRSSFVTTIRPSATESLWQRKTDGNQQGNRHRFTSPDDVQGTTEVGIHSDK

KRDSDDAFERGGRPKSEGRSTLFRRPSGRPPGRCQNQRKRVPDHGKGCQSWTHYSLEDVK

GSDMSEKSNTQAALAFLDERRKLREKQEEEMSLGEEEAFDVRSAACSKGLFSFTKRSKTD

KSSSISDKDKSAASKDKFDRDRSSAAEADRPDTEETEGAAEEESVELRFSGMKDDATKAS

FKSRKGIKRNIRTRDNDD

>SG-AL_contig_6404_1_+3_567_821

MQIYIYMCVCVCVHTHTHANKNFYINKYKEKSVCTHTHTHTYSSTNIRRNMYVHACTHTH

IYMHTLTHTHTHYLSYYHSLSFMYI

>SG-AL_contig_6405_1_-1_91_1176

MAVSNGDGLDALEKKIIRQVEYYFGDINLSRDKFLREKTREEDGWVSMDVMLNFNRLKSI

SEDKEAICKALQKSTSGLMEVSEDLTKIRRNPDKPLPDNTQERKDDMAARSVYAKGFPKD

STLDTLMEFFEEHGKIDTILMRRDRPDRTFKGSVFVTFSDKSDGDTFLATAGIKYQDTEL

IKMSKEDYFKMKTEEEREKKKNKPGNKKEEKKKEEDKRMEDQITKGAVAYMKGLPKDTTL

EDIKTFFGDFGDVAWVEFATGDEKALVRFREGEAESALKKIKDQNDGKVIMKDSELETKV

LEGEEEKEYWRKMFKDIADRKQKSRSKGFQNRKRKFGNRSNQKGNKQDDDGAPAAKAKKT

DG

>SG-AL_contig_6406_1_-1_295_648

MFAFSFMQHVTSLVENMQVFFSLKKHTRRRNFRTGFLLDNMLEPNSFLHSKLVDSRFMLW

HCFLLNNVLEMNAFLTFKLVDKIFQDFASKQHAGNENNLINLVMQPSLWSAFSFSECV

>SG-AL_contig_6407_1_+2_2_100

IVTHTHKRYLYTHTNCIHTQTVQNVKTESDRIF

>SG-AL_contig_6408_1_+1_1891_2523

MIDNINHNPSSTTASDSFHGTSISLVQQPSRSDSKPQINLDVQGKRSVQPLPSTYTFVPQ

VERRKEAAIPAVSGPCKEDICKEVANNPTQQPWLENVYCDISSESTVSNVIQKDEHFPQD

LITKGAMHSTDNSAFTECSSTSMQDYDEPANPVSISWRVFHASNQKHQQHGVAHIALLPL

LEDNAHSQSLIKHTMHIIKAVLNASTLDNFQ

>SG-AL_contig_6409_1_+2_233_535

MQCTAMTILHPQSVQAPQCRIMRSTLILFVSWGAFHASIQKHQQHGEAHTALLSLLRDNA

HSPSLIKHTMHIIKANVECLNPGQFPVIAIDQPLYTLAKQI

>SG-AL_contig_6410_1_+2_2_121

LCVCVCVCVCVCVCVCLYTYTHIYIYSHHLNNNPNPPTPT

>SG-AL_contig_6411_1_+3_162_311

MTTFSNTDHSHNPTFLVKQNTSNKDWTRVTKILQVGKTLNKTSQCGEVVS

>SG-AL_contig_6412_1_+2_2_190

HEAAKAFQRARSAESLRASLQCLSTSCRSLSTVWCQFILGLPLLRCPLGSSSGLGWQVWK

TEV

>SG-AL_contig_6413_1_+3_3_179

QNKKHKKQTNKNKNNREKKEHPDVRAMKAIATSDLTFLDKNSSTSMRKCILKKISSTRG

>SG-AL_contig_6414_1_-2_284_439

MDLISLILCAYKYRMEMCSFMVGKMHVFGGCGETVCVCVCVCVCVCIIIIMS

>SG-AL_contig_6415_1_-1_772_1014

MCCYYVPDFMVLRMFVWVCDSDCWRMFVFCHADRDLVSWGILKTPVRPFMLGTYLMVNYL

PQNKFFQSSFTGSWRFIFVWY

>SG-AL_contig_6416_1_-1_652_957

MHIQDRHTFRFFRRTYGPLLLDAYSRFSLLFTHNANSLVCVIANCICGYLACFMCTFPHF

AAVFSQSFYSPLEEVDNKKKNLFTLLLYILLTVLILQWVYVC

>SG-AL_contig_6417_1_+3_48_227

MIIVIGTFHCSLPISHFHQKCSHSPGHRYDTFIKFWYLPSSYHVKKLSADLYFMRYMMVI

>SG-AL_contig_6418_1_-2_113_508

MALPTGVSCMVSITLAVVLFAVQQIFRQQIGGTEYMTIAGGFLGSVIFILLLTFTSNLEQ

IIFFKGYQARLFPEVLICLFFAMFASGLVHRVCVTTCLIFSLVALYYINLLSNKKYAPVV

SATPTASSKKRK

>SG-AL_contig_6419_1_+2_2_70

FITTGFNNATLYTSIEIDVSHYQ

>SG-AL_contig_6420_1_+3_234_311

MYHKLGLNFLLTTSFKGLSFYGLVFF

>SG-AL_contig_6421_1_-2_110_382

MITVYISSVTLKKELHAQQNHIETVLRGKQVPFQMIDIATDPTFKDEMRQRCGDEKALAP

QIFSDDFYCGDYKSFQQAVEEERILEYLRLS

>SG-AL_contig_6422_1_+2_404_643

MAPEIKENNYNTHLKTKIAADASSIQMALSKCNSLVLINRGKSKLQILHDMFCYICHLVF

LNLSVSGECGSVVLRATLWF

>SG-AL_contig_6423_1_+1_1_168

EVLPKQSLLYLVLRIKVHLRKSAPRMRYVGHDCYCFDKDHVKTIAILSVMVDVLTK

>SG-AL_contig_6424_1_+3_1386_1583

MLRFHPFNVHINSCLKQNKKKQRNHVTEDSSGHRNKGTKRGGGEPTKIKRNKSRHDYSNK

KNETDK

>SG-AL_contig_6425_1_-2_218_418

MSIPSLIQFSVFPRQCFHARNQFHMHQRRKCHGMEKKIKKIMASAIRQRRSPTLFEIRHS

PLPDHLL

>SG-AL_contig_6426_1_-2_239_394

MSYSNGDHDSTSSENDKNANAITTLCHRQSINARHLERRNQVHSQLVLPPLQ

>SG-AL_contig_6427_1_-2_119_2194

MGLTIRTQSMTEAELKGGSITRDQRQAVLDDFFRLACLQAMSEEASGGVVDPSGMEHVVH

VRLTRTEFAEALGVQPSSIFVRNIFLLADTDNDGFVSFHEFIGLFAVFLKGSAEEKARLM

FNVYDMRRRGVLTRDDFSKMVRSLLELSENAGDLHDDNTVTQLVDVIFREAGIEQEDEMT

FEDFRKVFASKEYASTLQAATLANAGGVVPAAAKTTRNVQQKKSRMINSYRKRRIARDAR

APPGSNSNSDATVERQGGRNTTQSQRIRNRRSQIRISVREPKSEPLTDKGKYLYRLTKFV

EVYRLQIFWVTLYTLVTCGIFIERAYFYAVGREHAGLRRLSGAWTTAMIRGSASVIMFTY

SSLLLTMCRNLITRLRETFLHRFIPFDSAVDFHKYISVIAMIATIVHIVGHGTNLYCMCT

QTTINVNCFFREYFTASNGFATFHYWAYQTITGLAGVLVTVLIFVMYVFAHQRARREVFT

AFWFTHSLYPLVYILTFLHGVGRLVQDPLFPWYVMGPLIIFVLDRLASSSRKHIEIPVVK

AELLPSDVIQLVFKRPANFAYRSGQWVRIACLKLGKREYHPFTLSSAPHERHLSLHIRAV

GPWTTNLRQRYDKDVLGGEPYPKLYLDGPFGEGHQDWYSCDVAVLVGGGIGVTPFASILK

DIAFKSRTGALINCHKVYFLWVTRTQKSFEWM

>SG-AL_contig_6428_1_-2_2_145

EIFLISQVSICADLQVPEPPLCVYAHRTSNQHIKNPVSCVCVGWLVET

>SG-AL_contig_6429_1_+3_3_236

LRHSDSAWHKLLSISTQITSSSAVFWVSHTPISSTDQDTYFFKSLNEYLRTSRYAHASAH

AHKLQGTDFLKVHTHTHS

>SG-AL_contig_6430_1_-1_1_237

RLNSKCDGEQEEEEEDNYKKSMSVHEQIVKQVLLLYYFYFAVLDLPCGIWAVLPGRVHRC

SAAPLIFSLFLVHAWCFRV

>SG-AL_contig_6431_1_+2_2_142

QPITFAQIGPNTQQMHCLQQNMFILIPHSPPAFFKFVKTCLICLDTV

>SG-AL_contig_6432_1_+2_2_130

QPITFAQRGPNTQQMHCLQQNMFILIPHFPPFFLICKNLFNMF

>SG-AL_contig_6433_1_+3_606_830

MDKDQHIIMYWNGQRPPHNYIDGMDKHHHIIKYRNGQRPSPKYVDLMDKDHHIIMYWNGQ

RPPHSYVLEWTKITT

>SG-AL_contig_6434_1_-2_53_937

MKDLPKGCTKMEDGGSTWGSIVNFFKYQFTFQKDACVTYYEHLIVDPIVRVSPMEALTVA

VVQTLLKPMRTVGTEISEFIRALVKDLPVQWQMISVTLVVILLVFILFLTCGYRVRIPFL

LALEPSHGDNVEVKALKETIHQLRETMQNMEALNRPPAAQPQPFETVGPLPATENPPAIG

SPPAVAIPPASANPTAIANSPSAATKPVEQGGGVPTGAARSRQHSQPPFTTPPRFPVPIQ

EENTSRRETEQSDRFEEREADSGCAYRETETLGAVGRVDQSGDCIATDSNPLLKD

>SG-AL_contig_6435_1_-2_332_517

MRMYHTCMQTCMLACMRARARTHTHTHTHTHSHSLNHAHTLRHVSTFCGRSLALQFCFKK

FC

>SG-AL_contig_6436_1_+2_398_607

MEMIGSGRKIFFLCPAFPCLPLYSFFNKNNLFLTFGKLNLALSTSCKNETLSRYTLASWK

VLDKVREKKI

>SG-AL_contig_6437_1_+2_2_166

RRQYSKEKTAETKRAIDKRKFLAQNFQKVGILFILKEPLESPRGTLSFKKPSPFA

>SG-AL_contig_6438_1_+3_3_167

VLPDVCHIISVVNTEDKAHSSADHVITALSVQIRVSTVCQNDTIDVCTVLKCVWS

>SG-AL_contig_6439_1_-2_2_190

KRYKALKDHSFFPLFLSFLIATNGKLRAIFIPHKVLIDDSAYRGQSYQRTASLCVTHLLY

LSL

>SG-AL_contig_6440_1_-2_2_163

KPEFTCGIGIKNPHILAYYSETPVCEVRSNCGFSPLQNTGSVMYHQYQLNLCSL

>SG-AL_contig_6441_1_-1_1_168

KAGVHLWKWHKKIHTFWLIFQRPLSVKSVQIVGFPPSKYRQCNVSPIPVEPVQFVI

>SG-AL_contig_6442_1_+1_1_351

IFQAGNDVEQNLQYCAQLGTHRQDTFRAAAIRNGNTGFEVSSQSFDVGTSLPNDGTCVFA

LQQNSKFHTGTFPTTTFREIIRTAHGFIIIQRTLHSSFNTIHVIMISHLESRGARAD

>SG-AL_contig_6444_1_-1_541_705

MKGLTLLVSLLSSIACSFTNFKYILFLFFVVFFLNFFCCCFFFQFSKEDGTCVYV

>SG-AL_contig_6445_1_+2_2_127

ITKPLIPLFKNTRINSLLFLPKQFLFSDIQYPTKLGESYNQL

>SG-AL_contig_6446_1_-3_87_176

MFVHIEMSTPSIQFLTFDVVMEAIQVCISM

>SG-AL_contig_6447_1_-1_1_147

KPNKNKQSNCSVLFLFFLFVHLKGKSEHHFILTRKRVKTYLGWGIHCLH

>SG-AL_contig_6448_1_-1_73_480

MNLLTKGVVLRGHSVQTKDKNPFIVRDSNGEDRETPSVRLTIGNIPLSFSNEEILHSIEK

MPGIKIRSRLMDERARDEHGKLSHFKTGRRFVYIDTPSEPLPKTVSMGIFQASLYHPGQK

TPVCSKCLGKGHHVSA

>SG-AL_contig_6449_1_-2_1025_2356

MDASPVGIARILIQVQENTPRVISYASRSLTDVERRYSQTEKEALAIVWACEKFHPHIYG

LEFDLLADHKPLQVIYGPRSKPSARIERWVLRLQAYKFKVVYIPGKTNIADSLSRLLTPT

PTESNRLRRKFLERGDDLDLANALKIAATFEAVEDQAQEMKTKEGESVSKARSAKHESSQ

RLTQCCRCGGNHYGRDPNCPARGKTCSRCGGANHFQAMCQSKNFERSKERNRKEKFQKPK

THFYGRRRNAVHQLDAEDSTDSEEDKTNLFMTNSVNNENIAARKEEETRHSEDEEETYVF

ATNSSVKNNKITVKIGGMPVTMIVDSGSDTNIIDKNLWKELKKQKIKCTSRKCVKKLFPY

TSDKPLHIIGCFDTEIQAGEISVMAEFCVIEEKGEALLSRDTATPLGVLKIGINIQNNIR

PENSFCQAWTATNSTLRQWPAIHI

>SG-AL_contig_6450_1_-3_90_1640

MAWMVQFVATIIAVIYGDGIQGSPLLAGEPYKEILDNPNFRPKPENPPNKTIDELITDAY

GGLPIYMSDNFLDKGGLITAELDMMLSNDQYKAMYTKSNGRYKRKATRPLMYRWTDKTIP

YEFITSHFNADEVYYIKKAMARWEETTCLKFRPFRHTDKNRIRFQNGNGCNSLLGMNGGV

QPINLQSPGCRYSGLYLHEVGHAIGLVHEHQLPDRDQYIYIIYDNVAPSMRVWFNKYSSK

DVNQYGVPFEYSSVMHYGVTAFSKDGSQTIRARDRSREKEIGRVYLKGLSFSDIKVVNLM

YRCNEHCAADPNCKEPGYLDKNCKCVRPDNGGGTTTTTKPGVCSNIWDGSECEAWARDGE

CDLNPSWMKENCKKACDECKDTGGGKPDPKECSNTWGDIKCVGWANSGECEANPIWMKEN

CKKACKECGGTGTGTGTGTTNSLCKDNNQYCSSWAESGYCQSNWYTQENCKKSCHTCDAS

TDVSDVNTEEDIGNNATRMMSQLMMIPVFISIVRSYL

>SG-AL_contig_6451_1_-3_246_1013

MSDREDCVYQAKLAEQAERYDEMVDAMKQVAGLDIELTVEERNLLSVAYKNVIGARRASW

RIISSLEQKEDKCEEKKCINKGYRTLIEKELKDICQDVLDILDRHLIANANQGESKVFYY

KMKGDYHRYLAEFATGNDRKEAAENSLVAYKAASDIAMTELPPTHPIRLGLALNFSVFYY

EILNSPDRACRLAKAAFDDAIAELDTLSEESYKDSTLIMQLLRDNLTLWTSDMQGEGGEE

QTAQQVQDIEQDQDGS

>SG-AL_contig_6452_1_+1_1_105

NNKQTNETHHNHTAKQSHKKHLSDLLTLCTVSMIC

>SG-AL_contig_6453_1_-3_741_1058

MLCRLGVALQSDIHRIDLVRQLVDFHHFYLFYLNIYIFVFLEKKKGERKGLFYVDWKEVC

VCVCVCVCVCVYEQIVSVDFSAVIEWIFFFFAYNMTIFEQGHFGCF

>SG-AL_contig_6454_1_-3_1545_1718

MWFYVCLLIFGILFISSCLLFAFPSLTLWGSPNIYALCLTRVRPRVHLDEKIACMAVF

>SG-AL_contig_6455_1_-3_27_4976

MNLVLEVFQITLRKGYAIPDLKADLAALYRKAGLKNVGMVFLMTDAQVANEQFLVLINDM

LASGEIPDLFPDDEIEEIIGSVRNEVKGLGMEDTRENCWKFFIDKVRRLLKVVLCFSPVG

STLRVRSRKFPAVTNCTSIDWFHEWPEEALISVSARFLEEVELLSPEMRESISKFMSHVH

KSVNEVSHQFFVNEKRYNYTTPKSFLEQINLYQNLLKKKNSELQAKIIRLENGLEKLRST

ASQVDDLKAKLASQEVELAQKNEDANKLIQVVGAEQEKVGKEKAIADEEEVKVSKITVEV

EAKQADCARDLAKAEPALKAAVEALNTLNKNNLTELKSFGAPPPAVTNVTAAVMVLLAPG

GKVPKDRSWKSAKATIMNKIDQFLDNLVNYDKDNIHENCRKAIQPYLADPEFEPDLIRAK

SFAASGLCAWVINIMSYYEVYCDVEPKRIALAQANADLSSAREKLRVIKAKVSDLEATLA

KCQSEFEQATAEKLRCQQEAETTAKTIELANRLVGGLAAENVRWAESINEYRENEKTLPG

DVLMITAFVSYVGSFTKRYRTDLFDNHLMPFLKGLKYPVPVSEDLDPLTMLVDDADIAGW

NNEGLPSDRMSTENATILTSCERWPLMIDPQLQGIKWIKTRYGADLKVVRLGQKGYLDII

ERAVSNGDVVLLENLGETMDPVLDPLLGRNTIKKGRAIKMGDKEVEYNPSFKFILQTKLA

NPHYQPEMQAQTTLINFTVTRDGLEDQLLGAVVVKERPDLEKLKSDLTRQQNEFKITLKE

LEDNLLARLSAAEGNFLGDYELVENLETTKRTAAEIEVKAKEAKNTEVEINLARESYRPA

AARASLLYFIMNDLHKINPMYQFSLKAFSVVFDKAIDRAEPSEEVKQRVINLIDCITYSV

FVYTTRGLFEKDKLIFITQMTLQVLIMNQEVNAVELDFLLRFPATPNVTSPVDFMNNNSW

GGIKALSNLEEFRNLDRDIEGSAKRWKKFVESECPEKEKFPQEWKNKTSLQKLCMMRALR

PDRMSYAVSGFVEEKLGSKYTEGRAMEFAKSYEESGPNTPVFFILSPGVDPLKDVEALGK

KLGFTEDNRNFHNISLGQGQEIVAEQAMELAAKEGHWVVLQNIHLVAKWLAKLEKKIEEY

SEEGHENYRLFVSAEPAGSPDAHIIPQGILESAIKITNEPPTGMFANLHKALDNFNQETL

EKCARETEYKSILFALCYFHAVVSERRKFGPQGWNRVYPYNTGDLVISSDVLYNYLESNT

KVPWEDLRYLFGEIMYGGHITDDWDRNLCRTYLEVYMHPDMLDGELYLAPGFPIPPNSDY

KGYHAYIDECLPPESPYLYGLHPNAEIEFLTTLSENLFRTVFEMQPRDTGAGSGGGVSRE

EKVKSLLDEIVEKLPEEFNMTEIMAKVPVEERTPYVVVAFQECERMNMLLSEIRRTLKEL

DLGLKGELTITADMEALSNSLFLDMVPENWAKRAYPSLFTLGLWYADLLQRVKELETWTS

DFQLPAAVWLAGFFNPQSFLTAIMQQMARKNEWPLDRMVLQCDVTKKTREDMAGPPREGA

YVHGLYMEGARWDTQTGMMAEARLKELAPTMPVIFIKAIPVDRQDLKNMYACPVYKTKTR

GPTFVWTFNLKTREKPSKWVLGGVAILLQV

>SG-AL_contig_6456_1_+2_317_454

MTSEPSLQARPLHAVITLQQLLASLEGLCYDQTGKARLSPHTYTFS

>SG-AL_contig_6457_1_+2_2_124

CSSDLETSTFLGASIMSVVPISSPSLFTFPNPKEGQVPINS

>SG-AL_contig_6458_1_+3_378_560

MFACYFHISVLNCVRAIGPTVCMRACVRVCVCVCVCEGVCVFVCACKYLYSTFFFSFVFI

C

>SG-AL_contig_6459_1_+1_898_1116

MFSYLSITTVFLTKCLCMMSKSSSTMATPSQNYHQHHTCCTGSQSECKNRQPIDMSVRQI

LTDKRITRLRDDF

>SG-AL_contig_6460_1_-1_7_348

MLYGSRRSLYSTYTVSGMSYILPVMTKSPNPYRRPLDNWPIAFCSLFFNPIFGIFAILLA

EQSKIYYAKCEYLRASQYGVYAKGVAAGGIFCTVTLLTLIIFIVIWSHIRYMGY

>SG-AL_contig_6461_1_+3_162_317

MKIVCIVSYKLLGVLDVIFLTHTEPYMTVAGVCVCLSLCVCVCVCVCVCARV

>SG-AL_contig_6462_1_-1_1_210

GGIYNPFPGVNVTVVLSERKGECLHGLTILFLCPKRNTETGVEAHDLKGIMEHSQWLLTQ

PYVTFSTHFS

>SG-AL_contig_6463_1_+3_249_725

MVLLFLEQRCSSFIHISKHISDSSTPTHTSGISLLSINFASSFHRKKCCHCVYKCKILKN

MHPNAQSVRDKATESTAVSAASNDTVSSSLHLHQQTDIPTFKCIPQNRSAICSPAQTFIY

FKLVILKTLPTCWIYVQAYGICHYYFLSPSHSICDARRI

>SG-AL_contig_6464_1_-2_2_358

WNSKKISNLKLRFYNSNVLSTLLYGCETWHLKTSQEKKLDLKCLRKILGIKWTDFVTNEE

VRSRSKQNPVTSIICKRLSWLGHTARLPSSRPANQVLNWNPGGQRRRGRPKMNWRQTVD

>SG-AL_contig_6465_1_+1_220_1086

MVYHKFFDGNKAPITLYEKDGRIFSSHRTLTYWHQVASMSSRDEDVLLCGYPKSGLHWLF

EIVTMLKRGNPQLSTDRMENNGFLMISSLADLDNLPCPRVLTTHRRFHELPADFIMKRRK

IVCILREPKDVCVSYYHMLRSVKAVDYDGNFDGFVPLFLEGHVPFNSWAGWVLSFEKGLR

VHPELSVHVIHFERLKEDPVKEITRLAHYLGAPMALVEDIASQTVFSSMKSFKEDQPNPF

GSYYKNNTDAIFRSGETEQWRQYFSAEEAQQFKAQLQKQLGRPSLKARL

>SG-AL_contig_6466_1_-1_1_219

MEADKMKQSTDSNFTDSGRKPLNTDMDSSIDEHISRLVEERDTLLRTGVYTTQDKIIIEL

DRQIREAIAQRNR

>SG-AL_contig_6467_1_-2_185_244

MSNYNKYDLHQGKTVSGFKY

>SG-AL_contig_6468_1_-1_19_213

MAAQMSFSNSRDYHSISRPVTAVHKLLCTSVHKLLCTSVHMLLCTSFMTDSLWFCEKSYI

PYTSH

>SG-AL_contig_6469_1_+3_57_215

MYRRQTHNILLNRGGLAVTRTYQVLLKRDSNLCTPEPSDWKAGALPLRHPDSH

>SG-AL_contig_6470_1_+1_55_183

MEMTKVKITKAKKVPKISTFGGRCVNSTFSATSDNIQILFSLL

>SG-AL_contig_6471_1_+3_1020_1190

MTRCSIESVIVIVQSFQIYKNWFDLQQQVLIKLLNQIFVLTVLYFFRLLEPGGVMGR

>SG-AL_contig_6472_1_-3_15_326

MVRTTPCQPLHCTTADQVAWPPDQDASQPTSPSSLQHQMLGLQSQRKTTAALDRWCGRHT

PHPQHDSVPGFPSSCRPPTSSSHDSSWYKRTDKVKVSIQFFNTQ

>SG-AL_contig_6473_1_-1_298_474

MWFVNMWALCYVDRVYCYLMFLCVWEVFCGFISLLFSYVVLPIYLSIYLSICLSICLYL

>SG-AL_contig_6474_1_-3_159_311

MMNNRTFDASYKIAGFRFTKKLVEFKKMKAYIRKLLHCASDTVRFSSFFLR

>SG-AL_contig_6475_1_-3_150_395

MRVSITYSIFMWYWYCRTKLKLNLFSSSSPPSFPSPSFPSHSPSSSFPLSFFLSFFLFCL

FLLHLPRQSFFRYCDCDFSDFV

>SG-AL_contig_6476_1_+2_1079_1387

MKAVWYMVFPQIPTQSQLHNRTYITTEIKLAEKSQTFKVCCLLVFFFLCFVLFLFLFLLL

FSPYMAAVFKVSKQDLTAWTPDYQSACISRFACICFTEQCRFR

>SG-AL_contig_6477_1_-2_242_370

MVIMFLCFNKINLFFYTVMCVLGVFFLGFFERWFLSYYLFFDN

>SG-AL_contig_6478_1_-1_1_207

IRKAMKQAKESWIDEQCENINSNLARNNTKQDYQTVRDLTSTRQVWKKTEEKEILARSTP

SPLLRSLQP

>SG-AL_contig_6479_1_-1_1_144

RFFLLENRCAGRSTVTVLCALVYLLASSTSHVSCSFSFLKNPSASFRV

>SG-AL_contig_6480_1_-3_129_842

MATHGSRQLPRGQSGWPRDKGPSASSSYNKGYNSSNSSPLSLTLERTVNLYPLSNYTFGT

KEPLYERDSSVTARFQRMREEFDRMGMRRSVEGVLIVHEHGLPHVLLLQLGTTFFKLPGG

ELSSGEDQTAGLKRLLTETLGRQDGAPMNWVVEDTIGNWWRPNFEPPQYPYIPAHITKPK

EHKRLFLVQLPEKALFAVPRNYKLVAAPLFELYDNSAGYGPIISSLPQALGRFNFTYK

>SG-AL_contig_6481_1_+2_224_493

MTSTLVSLLTFLTSSNCLLSGCQLSDSPGDLHQAVSSRPVVSSCCNLCPRPYDLSTTCYI

FGSSIPPILPFSVQRSSNSAVLCSKMDSTA

>SG-AL_contig_6482_1_-2_2_100

IVIIIIRALPFDTTRGLSQLWPHGSLIPFLCSP

>SG-AL_contig_6483_1_-3_171_389

MIRTIGVLVLLLSHEAVNLLRKENLPTVNFCMGMLRETLHIFMVDTWTYQMCSMFPVKGK

TDCNNGDHKNCCF

>SG-AL_contig_6484_1_-3_3_110

WTVEESFMHEFGTERAKVKVTLAKKGLKLALFEEGV

>SG-AL_contig_6485_1_-3_297_464

MRLNFACSSVFLSLQRVSCFQTNNGLLSYKSFFMSHKYIQVNISPQMKCVDVRNRP

>SG-AL_contig_6486_1_+1_1210_1395

MNIKLKSKFFIFVIANILVYNSQVNKQKNRILVSYISFCTIIQSTDDCYSQNTAGEKILC

MS

>SG-AL_contig_6487_1_+1_94_243

MLMIYGLIKMMMVSAVVMYECNLMVLRTNMTLCSQSLTKFNDSVLCRSFV

>SG-AL_contig_6488_1_+2_23_256

MYRWKLDAFKTKYTRNIMCLCGQKITTSQILSCIKIKRFIPILSQKTVDEIFESTSLTYD

FFLSAYQKVQSLVIYNSL

>SG-AL_contig_6489_1_-1_331_2271

MGPERESRGVCVAVVFAVLLAVTALASAQSEAKPRIYSLKVFSDIKFHFATTLVTSRVLN

PAETAQEVVFDVTLPNQAFISNFTLEIGGKVYPGTVKEKEAAKKQYDKAKQRGQSAGHVS

QKPRETNRFKVEINVGAQEKVTFNLTYQEVLVRRLGVYEHVIYIDPRQTVEDLSVEVAIE

ENREITYLHVPPLRKDVLTDVDLSASHSDAVVVRPSLNRAVIVYEPSAQAQSAVSASGLS

GLFTVRYDVDRELDGGDVLVVNGYFVHFFAPVVDVPVPKDVLFILDTSSSMSGEKLKQLK

EAMRSVLNEMTEGDRFAIMEFNSGASFWRSSLVPVTSQNIENGVKYVQRRSARGWTNMNK

ALSDGISFLNDQGSDLGSAMVIFLTDGVPTSGETNKDKILSHALRRNKKEMPIYGLSFGE

DADWTLVKKLTAQNNGVARRIYEDSDSALQIQGFYKEVSTMLLTKVQAKYLDDVVDPESL

TKSAFKNYFNGSELIIAGKLLDASANSISVNMEALGASGEIVLVSSSKPLDVTDMTASGL

MDLEDLAEITEKMWAYLTLQQILKQQVGEEDAEKKVELKEKAVGLSLKYGFVTPVTSMIV

TKPEEEGLGDLEEDDSDLQDDSNVPSRSSSSGRYSSSSRRRGGGGGD

>SG-AL_contig_6490_1_-3_3_212

FSFICYYYYEYFYDDSYDDYEDDGDSVHDDCRVECNNDVGERKVRRREGGYLLHLFPFLS

FLMFLLLSLT

>SG-AL_contig_6491_1_-3_3_83

LFVCVFVIFLDKGMTVREIRILMKNVL

>SG-AL_contig_6492_1_-3_3_1052

RPAASKPTPTPSKPSTTTITAADTDLLPSQKHQSAFNALTGYESDSDEEDAGRRQSDFFS

ISSEADGASSSSSGLFVVKNSAPSLNQRDEDRLRTSDSISKDSSNVPSSSSQTKQTPSVS

VISESPVSSSSSSVLASTSADSGNVAPHEINDSGDGNGDDDDGDNENEVSSTVGVPLVNQ

DAPLNFSSTMGGVSWSTTSLTSPMLPEMYAAGSTSGGASYWQHEHYAQGYGEDIEQEASS

SSGSSNIQQYMEDEQFLRISGKRKRGQEDIQIIDANVDDCVDPSELVKGISEESSYQPHR

KKDNMPSMQQRRKKQITYLAFQAKERELELKNAWSQNRMTKNQSRSKYGF

>SG-AL_contig_6493_1_-1_115_336

MTTAPPTTTTAATTTTAPTTTTAAATTTTAPTTTTAATTTAAPTTTTAATTTTAATTTAA

PTTTTAASTTAAPT

>SG-AL_contig_6494_1_-3_27_1004

MANLRQTPLTCSGHTRPVVDLAFSDNTPYGYFLISACKDGKPMIRQGDTGDWIGTFEGHK

GAVWGATLNRDATRAATGAADFSAKLWDAEKGEELQTFAHKHIVKSVDFNKDGTLLLTGS

NEKLLRVFDLGKPDAEPKSFTGHTSNIKGAVFLGDSKKIISASDDKTVRLWNVDTCEEMQ

QLTFDSAPTSLNLSPNGNTVIVTYGQNVAFYTVDTLREIKKFEADAPINSASLHQSERFF

VAGGEDFKMYKFDYETGKEIESFKGHFGPVHCVRFSPDGELYASGSEDGTLRLWQTTIGK

TYGLWKCMVPDETEVQNSTPDVKVEA

>SG-AL_contig_6495_1_+3_1011_1400

MMQVSVITMLMAPQCSGNSLLPHTQSVATVPTVVLPAPRGGCGCREHTAVHHMRQKRWLK

VKLSKCSLMIQVQMPAECPRVLSMFKFTTTCRIHLHFIYVKDDALRSQDGTSKDSNFNQV

DDNEHSTQQW

>SG-AL_contig_6496_1_-2_701_868

MCDTHISSSMRMVSELVSSLDMQCYIASDVQKSVRITYYHSSVNIQHTTHTFTLTE

>SG-AL_contig_6497_1_+2_629_826

MQRILHALTTSLKPRWHTSRVLAFHSKYMGSCLVQGNLCMQPPIDPVVNGYLTLLRIGRQ

SSKGEE

>SG-AL_contig_6498_1_-2_2_280

SPTTSTTFATLRPHPIIIISVQSVLLSLNMKMVWPVTCFHLVFSVSSVGAVPCILLKEHF

FPLVQQIKCVCKGLHLFNEYCEIYSNNEGTLLI

>SG-AL_contig_6499_1_-3_732_953

MADKIDSPPPPVCGQQLLCELKYMSVLLYVWPLLLFFYPTIEAILLCFRRYKIDLTERFA

HVFNAFNPWSEYCI

>SG-AL_contig_6500_1_+3_96_281

MLNVFYSYTNTTLRGEISLAQLLSTHCSASKFIMHVFSAMIFYKLIYQGSPQTFTTRNKF

QI

>SG-AL_contig_6501_1_-3_3_281

WGSPNIYALCLTRVRPRVHLDEKIACMAVFLVPRHVGNHTPTSEESECDESPLNKLSWPH

KSHVLVKCLPFMLSAVAVLATTGSLSSADSDDS

>SG-AL_contig_6502_1_+3_3_179

FFFFIVNHHLIQTVKRKKHNVFLVNQHNDAYKTKRYGGWCGKRGRRKSKLVTGTILTMF

>SG-AL_contig_6503_1_-2_38_397

MRSETYISVEDASLLIFMLNVKYIFCVCCPWLEYAVIVKQSGPYHCSFLMSHLLLVSFSF

VLVVLTPPVLTTLPMCYLYSCLFIYLVKVGQRREDLTGDKHKMYTIAYIALQIPSSTLLK

>SG-AL_contig_6504_1_+3_213_413

MGNPCLNQFTFQVSMVQIIGHTHSSLHHDFLLLHIYSDDSIHRHLGRKVSINATSLNKNC

LLATGKV

>SG-AL_contig_6505_1_+3_207_305

MECGSCTSMPPGLYINTGNVFNIVRRTRSWCLK

>SG-AL_contig_6506_1_+2_2_238

EGGAHLLCLTALPTPTLNRVRYPFTAGWTGGQAQFFLGQIQTGAIRMECGSCTSMPPGLY

INTGNVFNIVRGTRSWCLK

>SG-AL_contig_6507_1_+2_1010_1252

MKHLKHANMSVTCTTMYRNHWGKFETTDKEVKINTYISKTTMNIDTEFWMARSRNNFYAN

TNREKLVNNQQNYRGNNSSMM

>SG-AL_contig_6508_1_-2_92_310

MKPFHHTSFILDGFNPQLFLESSRDITLKKQSLDPASSTNGRTEDRNHEVTSKHKFAQKL

FPDVYRFSFISLI

>SG-AL_contig_6509_1_-2_146_262

MGTFSQEAGITREALLFGVPSLCQCVRMLFVQMDSQRSS

>SG-AL_contig_6510_1_-1_238_402

MKRGRTVSSSMRSGEFVLPLFYTSHYHSTLLRLLIMSVWYYSVIFICHSERGKEE

>SG-AL_contig_6511_1_-1_1_279

SRSHTRTHTHTHTHTHTHTHTHTHTHTHTLFLLDCMFASVFISLLLCQPAPPTSNLPECR

HSVSTLHYCCYYQLSVWRTVLVFTLPAKSLCCS

>SG-AL_contig_6512_1_+3_159_317

MNTISSQQKPRHSLPERVERFLSVASIPAHNIKTGLFTALLKNILGCQLSVSQ

>SG-AL_contig_6513_1_-2_362_469

MLPVLINRYNTVRVDIDTILVYMVSFKCTTILKVCF

>SG-AL_contig_6514_1_-3_69_230

MSPPTSLVVAAGAGLCFLGYCIYFDRKRRSDPDFKKKLRERRMQAKQQKTKPRT

>SG-AL_contig_6515_1_-1_88_3198

MATVQITSAAGVLSLLDEPEPQLKMFALQKLDRIVGVFWAEISESIDKIEVLFEDQQFKS

KQLAALVASKVYYHLGAYEESLTYALGAGDLFNVNDNSEFVQTIISKCIDHYTRLRVENA

ELSEDEQKPVDLRLEAVVNRMFQRCLDDKQFKQALGIALETRRIDIFEKAVKMADDIPTM

LTYAFKVCMSLIQHRQFRNTILNVLTNLYMGLHVPDYINVCQCYIFLDQPSAVADILEKL

LKGDEDSVLMAYQIGFDLYESATQQFLQCVMAALRAVAPIPALLPDQESKPIPSTPVQES

SGADDKKEKEMPMDTAEEVAVETPPAAEAATPKPAGTAVPATVAKDLSSLSDTDKALQER

LSRLLTILGGETTIFIHLQFLIKSNKTDMLILKNTKDAVRNSVCHSATVIANAFMHYGTT

CDQFLRDNLEWLSRATNWAKFTATASLGCIHNGHEKEALNLMSSYLPKDTTAGSAYSEGG

GLYALGLIHANHGGEITEYLLGQLKDASTDMVRHGGCLGLGLAAMGTARQDIYEQLKFNL

YQDDAVTGEAAGLAMGLVMLGTKSAAAIEDMVAYAQDTQHEKILRGLAVGISLTMYGRME

EADALVESLTRDKDPILRWAGMYTLAMAYCGSGNNQAIRRLLHVAVSDVNDDVRRVAVTS

LGFLLFRTPEQCPSVVSLLSESYNPHVRFGAAMALGIACAGTGLKEAIALLEPMTNDPVN

YVRQGALIASALILVQHNEVSCPKVTHFRQLYAKVISDKHEDVMAKFGAILAQGIIDAGG

RNCTVSLQSRTGHTNMDAVVGLLVFCQFWYWYPLTHFLSLAFSPSCLVGLNADLKMPKVE

FKSAGRPSLFAYPPPLEEKKNLTKEKVETAVLSITAKHRRKEAEKKKEEKMEVDEEKKDE

GQEKDKKATAVTTPTTTTTATPTTSTSAAATPATSTTVEEGSKKEGEEEKEKKEPEPSFE

LLNNPARVMKNQLRVLSPREGSKYRPVKELSHGGIIMLKNVNPDEEEDLVEFVQAGGPKT

EEEEEPEPPEPFEWTED

>SG-AL_contig_6517_1_-1_1_246

FRSLLQASLYQSEGRLTRLVPVASCSYRMSFGSLPSSMRRNMSQSSKAALGQQEIHAGNF

GTLQGVTVWVVVLSVPGDRYIF

>SG-AL_contig_6518_1_-3_3_1070

MASTLSKSLLAVNLLLLFVQVLGGRDFYKILGVPKNADLKQIKRAYRKLAKEHHPDKNRD

DSEANERFHDLAAAYEVLSDADKRKVYDKHGEEGLKQGDVGGDPFSSFFGDFGFNFGFGG

GGGQRDRDIPRGGDVVMDLDVTLEELYTGDFVEVLRYKPVSKPAKGKRKCNCRMETVTQQ

IGPGRFQMSQQQVCDECPNVKFVPEEKTLEVEVEPGMRDGQEYPFVAEGEPHMDGEPGDL

RFIIRQVKHSRFERKGDDLYTNVTVSLQDALTGFEMDIVHLDGHKVHVIRDKITWPGARM

RKSNEGMPNYENNNVKGSLYITFDIDFPKGTLSEEEKEALKGILKQNTKQTVYNGL

>SG-AL_contig_6519_1_+1_124_372

MAYRIFSTHIQCSAGIFYFHRPLNTDMAYRIFSTHIQCSAHVYTQEGSGTSKVCSYFDLG

GWENLHPLPTRQYPGRVNFRPSE

>SG-AL_contig_6520_1_-2_2_133

YFLGINLSQRSFKPEHRQKCTSFFFRTSYKKDTINLVLQIVYIE

>SG-AL_contig_6521_1_+2_752_946

MFRIGCPQRFFFLSNFVNSYLTGECGLYDLFLFSSANKLFKYIYIYIYIYIYIYIYIYIT

IYYFI

>SG-AL_contig_6522_1_+3_3_185

GIFSPGDGIICPKLTVIGGSCTLITEPACGYLGFMSCRLASRWPASHQPTSSVNHTWQDF

Y

>SG-AL_contig_6523_1_+3_3_185

GIFSPGDTITCMKLTGGSCTLLRLQACFTPTHQLCLPYMARFLLTFLDTSTADKVLCMVP

E

>SG-AL_contig_6524_1_-2_2_163

LNQRHPSYRGEIVTMVQPILLHVVFVFFFVRFLICNAGVKVCFMNSLPKVDNIC

>SG-AL_contig_6525_1_-1_346_624

MGVKMKGVYLYGLTVSLTTVAIAYARILAVPKIKITRIQTAEKIQIEILAAERRIPIFNF

SRSMSPWKRVRQNKTYCEVMTMTKSNLNSFRSC

>SG-AL_contig_6526_1_-3_3_179

EGAKGLNDTLCPHICNGGQVYSCQTLTYRMTHFVHTSVCSSGHVQLSDTNLHKGDQYCL

>SG-AL_contig_6527_1_-3_3_68

QRVNSNRKNQRVNSNRLRESKG

>SG-AL_contig_6528_1_-1_16_993

MGRTKGKNQDGGGGPGISSISQSSNRASSLSPAPNTSGSSASTPAQTDLRIKDELKELFN

LIREIQDERSRGEHNLINISKTHERMQQESRITPYYKNKLKGLYTTAMQDAEAEAEILRK

ALEKITEIKSLRESKKQDSRQPKPIMRRGQLMTLLQSTATSLPLWVSKPGEKPPPLCGAV

PAESSYVCNKLDKVAARVKSAEGEENWILATVVSYNPQTNRYEVDDIDAEEGKERHSISR

RRIVPLPIYRANPETDPEALFPKGTLVLALYPQTTCFYRGLVEEPPKNHEDDYSVLFEDT

SYTDGYSPPLMVAQRYVIACREDKKK

>SG-AL_contig_6529_1_-2_467_811

MCLFFSLNKKDFVCFCLCDYEHFHVNMDCDFLVDSDILCLQHELIARKSESVFNRMIRIQ

SCGSRAHVLRINFTMSHECYQARFLWPIPCTKYTHRASPRMEEVRKIKSGLKSCL

>SG-AL_contig_6530_1_-1_466_696

MSVEVTNASLNEYDHCHNDGTEKVLNCSMKDKSVDGFGSGVGHRCSLTCVQDGNSAQPIF

KVRQCGNDLNMQFMKEC

>SG-AL_contig_6531_1_+3_78_194

MPNLTMKMKYALYLVENCLACSVPRHVNGGTVHCSSADS

>SG-AL_contig_6532_1_-2_1757_1972

MVRSISSVSMMCVCHVRMGSLLYRTLVTVSPSCVAWIRDISSDALVVTSAPPSLLCSVTF

DLVTPRFLSRTC

>SG-AL_contig_6533_1_+2_2_73

DDIFSSFLKTVICFGYNQKPLHKS

>SG-AL_contig_6534_1_+3_471_695

MKSVPVVTFSNETVSSEVDGCFVSLVCACDKVKIAGSKRGQAYGYFLSVILACIQSFWWY

QCFIGPVPLPISRTC

>SG-AL_contig_6535_1_-3_540_638

MSIVTEICETLNVTEYYVCRKRFMWTNNVELFI

>SG-AL_contig_6536_1_-2_2_250

LFSGLLSSVVSLSHLSSLSLVYSAMLSVYSLVYSAMLSVYLICLLFPWSVQQCCQSILWS

IEQCCQSISSVFSFPGLFSNVVS

>SG-AL_contig_6537_1_+2_122_400

MGEQKHGIHIETLVTQEVASMIVFLSKPGITVQDIFHNITKDVRVYFPSSVPTVLQCHSQ

HSFLTQDPSHFQERGIPSHTFMPQIFIIIIANK

>SG-AL_contig_6538_1_-1_13_789

MARRYDSRTTIFSPEGRLYQVEYAMEAIGHAGTCLGILAKDGIVLAAERRNTNKLLDEVS

YSEKIYRLSDDMACSVAGITSDANVLTNELRLIAQRYLLQYQEPIPCEQVVATLCDIKQA

YTQFGGKRPFGVSILYMGWDKHYGFQLYQSDPSGNYGGWKATCIGNNSANAVSLLKQEYK

EGETSLDEALALSIKVLSKTLDVTKLTPDKMEMATLTRKDGKTVMTILPDSRVEALIKIH

EAEEAKAEAEKKKEQEKKK

>SG-AL_contig_6539_1_+2_2_121

FFLLCKPTLFSYNKKKYNFPVSIGFYSAAKWDHNILLIWA

>SG-AL_contig_6540_1_-1_1_189

THTHTQKGDYFMSASACKRNKKTSCPWQNNSDKSTLLAKQKYSCTERKNDGTAVIHIGRR

EAL

>SG-AL_contig_6541_1_-3_789_1010

MWSVSLTAVAYSPANSVLRRAQYMKKAGYIPHIFVPPRHNVHTQTDSWRQSGQIAITSKI

AYMLVSLFCVSAIT

>SG-AL_contig_6542_1_-2_2_145

PPFFSFPHLSFCSFFYPKHRFIFFVSSLPLFPMHLFRVHSNVLKHSMS

>SG-AL_contig_6543_1_+3_3_68

AAHEHTYFQLGKQNCSLSHTQT

>SG-AL_contig_6544_1_-1_10_213

MFLIGWRVQAPPGGRKNLGLPCVGVKVIRRAEALFESGTGVPLSGSSLTLLSPLPFGTSR

HCIAGQEG

>SG-AL_contig_6545_1_+2_236_487

MMYQRPGEPSPYRVNMVMRRQDAGIQTSADFPLMQKNETLEQSQHALISTPQHMPWWLVW

FRGISSFIMARSRASVLGLGKFFG

>SG-AL_contig_6546_1_+3_105_458

MSRDRSRENLILGHILLTSPALTVPQHHAPSRSCPSYTTVKQEISTQNVYKFSVFICSTR

QTMYTDSLQSSVLTWKAKAHVPSIPLYIQLRVCCAQTSIYQCKMTQLFCSVLKLKPSS

>SG-AL_contig_6547_1_-3_3_83

CLPSIVVVNYNDKRLLYWPFHISAVFQ

>SG-AL_contig_6548_1_-3_120_1769

MATVLHGRRKGQARVTPEMPEQGSMVLSGGPDLRQVTVLPKSEWDRIQRELNRRQIEEER

VRRIRAEHEEQKQKSKELVKSWGNTLAGQRQRKLEARKQREEKEEAERLQIDIEEAKYQA

GQRRAAIDKAKTQQYYQTDRVKNFHSALQLTEVLKERDAQVELNILKAKASEGQDHELLE

RAKKDYEDSIRCDQEAAMKRMQAARDNANFVLKQVDEHLKEHDKVKVEDAAEGEELKKLA

VAYEREKERLENIRREERLQMMRDNRQQIHDVQKMKQLREVQEEEENEECRIFAAAKRKM

MRLRAEKEKEIYTQKQQQLDRIRQKLAAQMKQAVDNEDERIEKALQEKEEKLMKEEKEKE

AKLKKMIHEQAEHRMRQMHESEEKRKLARREELEQVQLRKAADEVFRRNEMEKEGRRCED

SQRRTEFLFAQMDEQRTMEENAKKQQLALDKANEELVKKEEQQFQEYATKVIDHCEQNGR

NVYPLRKAAREGAGGGLGPVFPGKGGVRPSYMTQDKSGVQLPHYQRVTTENIKEQIYGKA

DSKARLGFVW

>SG-AL_contig_6549_1_-2_908_1138

MPQTSQNAPLKKKQNKKKKRIAGSFQCQKSHKILSVSQRREKTANCCPVTLEEFHSAGSV

VVIINVIVVVIIVVIVV

>SG-AL_contig_6550_1_-1_472_618

MTANICHEFTELPVCYYLYLWSDQMRANICHEFTELPVYFTIYIYGQTK

>SG-AL_contig_6551_1_-1_46_237

MEKSSPVGLNQKLFCFRIIISCDIQDLIRCTETHKLRTDFYKHFYVAPTLGLSEIQVGFV

HMEM

>SG-AL_contig_6552_1_+3_354_668

MLGTSWQYRSHCHANNITLKNNKFSCHRLEKLIKSITGTERLLRTKQLWQTVKNLPQKFT

CTQTTQQSLPIQVTYNLIKWNDKSNNLTTSTSAPPNLSPHFLSVA

>SG-AL_contig_6553_1_+3_429_647

METWQKRLRRMMITLTMMTGRDDYVLIITRNVFIKITTCLQCYTPKTKSAMVICVNVYFT

FFQDFCLFVCLWF

>SG-AL_contig_6554_1_-1_370_1698

MMASGRDLLTVLVVVSVLSATGAQNDLLGSPEEGGFSVPGCPNQKMDLILVLDSSENEPV

FQALKDVANNFVQLANPDNGLVRVGVVAYSSEVYRRIKLSEFSRTVDLVSAVSSLHYQSG

RRDTAGGLESMRHLFKTSFGDREDAPNVAILLTSGNSVLRADEVSKEARLAHEEGIHVFV

VGVGVSNEDEINDIVSSPSDETKFLVDNIADLSFLPDIVFAQSCYRLQSQVVDELRASNR

LDIVFLLHFSNQLTKASLARVQSFIKDVVNKADVGSGSVRVGAAIYRRRAFPIFRLNQYQ

TKEGVQSGIDGIGLNYRSASASAAAGLEIVRDTMFTADNGDRPGVPNVVIVLTDANSDVD

VADTVGAAQQLRDSGATVFSVGVNVDYKDELEAIASSPSKTYIYPSFDQLPTARDDIVRQ

VSALEGMMTDGQLDEGPGSVTVD

>SG-AL_contig_6555_1_+2_113_355

MQNSIHDTTFYHQFSSLIAKKNHLQSLAINCNMCETSHLHILLVDVTAINAITKLITIIT

TLITIQSTKEIRTAFNQVTNT

>SG-AL_contig_6556_1_+1_136_306

MHMELSKCNEPLLKLNKFLISIIVFLPCEIQAALLGEYVLLLCSSTHFLYACNFVYL

>SG-AL_contig_6557_1_-1_109_858

MNEPRRKRHSASTKSRSDYADHQQRSGPHSSGAQRPKNLHANTQRMNSMDHVRKIVQEEG

RTARNLVQYSVPLEDEEYAGRPSRQWAKSRPRPERTGSQSGSGKTPPPEQRQRHRSMSEL

IKSPSSEHGPKKNGVDLRARYWKFLFDNFQRAVDAIYQTCEQDESVVECKEVIMMLEQST

KDFKSLIERLHVMRAFEDAAKDGDRPTSIAWEVRKMSPGKHASASPGPVPHLAFSVDITH

GQVKPTNSWA

>SG-AL_contig_6558_1_+3_3_266

WNVILHVRVLQNVILHFWDFVEFHFWGFAECYTSYISGVLQVIPVTFVGFCRSYQLHFCG

FAGHTSHISGVLQKVIPVTFLGFCRRSY

>SG-AL_contig_6559_1_-2_500_820

MVTLDPFLTPPPFFPSSVCLNVRFLCTVTDQTKSLFLFAYLSLLLLLLLFCFYHYQGRQP

CFPSPGAEDTIIKAGNSAFPLLELRTTTCSYSTCKCSPWQSFLFHNY

>SG-AL_contig_6560_1_+3_363_686

MHRHWHTKQTKVHSHFTFTFIQTFHTDLTKIHAHFVLIAPDPLILFSSFFLFETFLSFHF

VFFQCPYICFSHSIIIHIVSPSVPFTFILFSFLIFLPCHFHFTPHCLV

>SG-AL_contig_6561_1_-2_161_352

MLKMYEQTSMHHPQTLNLPSKQTHAPNERAKPRTSTEHKNKQTNNKTHRSFKLITLYMTL

EHTQ

>SG-AL_contig_6562_1_-2_2_616

VPGPPNAGAFKARTVPPTSFRKFYERGDFPIALEHDTKGNKIAWKVEIEKLDYHHYLPLF

YDGLCETSHPYEFFARQGVHDMLEHGGSKILPVIPQLIIPIKNALGMRNHKVLCTTLKVL

QHLVISADLVGEALVPYYRQILPVMNIFKNKNLNSGDGIDYSQQKRENVGDLIQETLEVF

ERNGGDDAFINIKYMIPTYESCMLN

>SG-AL_contig_6563_1_-2_224_481

MSDKVTHTFKRLCAIYGLTTAVYCMRGYDSSHTAGAMVSTALAAVPVNRNGLMHGQICTH

YIALYSTYEYQSHPHQADRVYSKVPI

>SG-AL_contig_6564_1_-1_166_1380

MHLPLTACLIVVVLLVGLEARRGEHHHKKRGRKHGHGHHKNSWEKRGRKLAATSDKQEIH

TRKIGWLNVPEVTGCKCRHPGEICIQGPDKQPTCVQKHHLKESTRLFKHYHEMKEKAEED

LNEVDPPPHNMLHDAASDPLHNIHIHVVSHHKAPYNREGSHNDDKVQHQENGHHFLGLKP

KPGKHHHKTSHVPMKAEDNYVINPQMTSKMCDSDALDEMRRRLTGWFHLLRGKEHHHHGH

RSHRHLSVKKELREDDGQCRCLRSVMWEFHQIDTDKSHSLSKAEVKVIDNNDMEPCLHPY

LLSCDKNSDGLLSHKEWCCCFPQKESESPCYAKLDELEATKNTEKYIPSCDREGYYHKEQ

CMGEAPSTQTCWCVTPEGSEIPGTRTIGRAHCGKLDNMGYPKSMS

>SG-AL_contig_6565_1_+3_3_125

PPPPHPLCDHQSLSLKQREQDSVQLPKISVDLSLSFPFLFI

>SG-AL_contig_6566_1_+3_393_584

MQLIPAPPHTTSPKFIQLKFQCQEAHGSNHHGATHQRRSCTAAGSLWWSSAVPVLIQLTK

DTAY

>SG-AL_contig_6567_1_+2_413_634

MLWLLFYHDMIMLVVFLPGPFFPVSSEVFSDTCIHVVPLVVLPTLCCCIVCKTYFLWVTP

PRKQSYFFFGRECA

>SG-AL_contig_6568_1_+2_47_193

MHIWKKITEYKKRVPTHCFFSNFAQLLLTGQVSEGIAYFSSLLHHLPQP

>SG-AL_contig_6569_1_-1_28_597

MATLRGAAALSKAFRPLLARRTLSTTAVLANWNKDWKPGPFPTTPQERAAAAKRYGLRVE

DYDVYSNDGMGYGDYPKLPMVSGDARDPYEDWDIPELKRNYNEPLHALADIIGEDRWDAS

ANPRYSYLNMFLTLAAFLGTLGIVYYLTLPYPHFLPVMPKQYPFNELYLEKGGKPEEERV

VVHYTFKVED

>SG-AL_contig_6570_1_+1_154_306

MYKVNIIKRKSHLHFSTQKVNSIILFSNSKHVKFHLVTFVTFKLIQQEEES

>SG-AL_contig_6571_1_+1_172_384

MKAGPTLACMSTAEWQPMAYCWTATANQSLISQARNKSKIKLLEMEEQQGTKHHQRIGQM

WRSDTHEIQLS

>SG-AL_contig_6572_1_-1_382_576

MAQWIKHPRTLSWTGCFLTPLLDLKWCSACRLLDEMINLKLYVQHALSACQRTYCNIELS

LGQIL

>SG-AL_contig_6573_1_-2_74_2443

MSRRRRESYSDDEEDERSRKRRRTEVVDIEDRLESLITRVGEKSTSSLESNLEGLASVLE

ADLPNYKVKIMRILCQCVVMLPEKLTIYTTLVGLLNAKNYNCGGEFVEMLVRNLKESLKM

GEWENARMMVRFISDLVNCHVLVAASVLQMFDNFVEVTLEDNIPQVRSDVYIYMVLSSLP

WVGRELFEKKETELNKLLDVVDAYISKRQKIHFPALRVWSVDEPHPQEEYLDCLWAQIRN

LRSNKWLEKQINRPYLAFDSVLCDALQHTLPQIIPQSHNDEMVYPLPRVIFRLFDYTDVP

EGTVLPGSHAIERYLIEEQIDRIIHTNYMERKDCAAALLSYPVKNLPLNYMIVEVMFAQL

FQLPCAPYLELFYGSALIELCKLQPSSMPQVLAQASEMLFERIDHMHTICIERLVNWFSY

HLSNFQFHWTWEDWSEVVDVDLALPKAMFVHEVLLKCMRFSYHQRISESVPETFAKFVPE

KPHPEYKYEQEGAGSLPGTMAAHQLMNSIKAKCTPDEAAQVLRDLPNKFPSGEDNDNSAY

HPLKIDVFVSTLLFLGSKSFSHSFAALAKFYPNLKSLAESEEGQICLLKTLYSTWRTHQQ

MMVVLVDKLLRTEVVQCQAVANWVFSDQMQKDFTSFFVWEILHGTIKKMTKQVNQLQSEV

DDARDRLDAAKRKEADGMEMFSDEEVPTEEMIERMEERLETATSQQKNLFLVIFQRFIMV

LTEHLAKCESRGIDYNTPWYKWVIERLQHIFLQHHELVFKYINTLENLLFTSEIDIHILE

VFQQFCALRS

>SG-AL_contig_6574_1_+2_35_217

MSVQKYDIPNRLYSFEMIFCLTNQTAQLSAWHQDNRKTRQDKKETRNAERGIGEERGRGT

N

>SG-AL_contig_6575_1_-2_56_364

MVPFPRLHFFMPGFAPLTSRGSQQYRALTVPELTQQMFDSKNMMAACDPRHGRYLTVAAI

FRGRMSMKEVDEQMLNVQNKNSSYFVEWIPNNVKTAVCDIPPR

>SG-AL_contig_6576_1_-2_128_703

MQAIKCVVVGDGAVGKTCLLISYTTNAFPGEYIPTVFDNYSANVMVDGKPINLGLWDTAG

QEDYDRLRPLSYPQTDVFLICFSLISPASFENVRAKWFPEVSHHCPNTPIILVGTKLDLR

EDKETIEKLKEKKLAPITYPQGLSMTKEINAVKYLECSALTQKGLKNVFDEAIRAVLCPK

PKPKKKRTCEIL

>SG-AL_contig_6577_1_-1_316_747

MTVYDFYLFDRNGNCLYYAEWNRRNQPGISKEEEFKLMYGMIFSIKSFINRISPKDVQDG

FLNFKTSKYKLHFYETPSGLKFIMNTDLNVGNIRDVLQQMFSSIYVEYVVKNPLTELGEP

VTSELFRTKMDDYVRGLPFFASKA

>SG-AL_contig_6578_1_+2_200_574

MSHKQFVESDQAKVKQSKLLTFFTSGKNKVGHDQKKEFERSVMSRSNDLICRIIVDSNLP

ITMFDTMAKGMKAVFPDSKIAERIQCCRSKATALIKEMAAQTSNDLRSRMKSGPYTVPTD

SSNDT

>SG-AL_contig_6579_1_-3_81_1685

MPPKRKAAAAKGGPAAKKGKKAAAAEPDAEEAPETMKDKITALKAADKGKVKKFKPDSYC

RVSGATVHEDYDAMLNQTNIGHNNNKYYVIQVLKDSSGRFYAWNRWGRVGEPGQNALKGP

FGVATNAIKDFEKKFKDKTRNNWADRASFKAMPGKYTLLEMAGEDDEDDVDAGPVVSVRS

DQKVAKCTLDATTQTLLKLIFNEDMFKDAMKKFDIDVKKMPLGKLSKTQIAKGFEILEEL

EEAVSKKSPKNKLQMLSSQFYTAIPHDFGRRIPPVIDSAEGVQMKYDMLAVLGDIEIAQS

MQKEKGKEMEDKEEVPHPLDVDYGLLKTQLQLIDPKSKDFKVLEKYFMETKPTPSSWRKL

KLNHIWSVNRDGEDSRFKVHDGIKERKLLWHGTNVAVVAAILKTGLRIMPHSGGRVGKGI

YFASENDKSAGYVGTAGKKGIMFLNEVALGKEKVILRDDSSLTKAPAGFDSVLAKGTQEP

DPAFDTKISINGKDVVVPQAKPKPTGLSSSFYQSEYLIYKESQNRIRYLLLFDFN

>SG-AL_contig_6580_1_-3_81_221

MLTFSRVLLLFAAVITFILLTMIIQISRSVPSVGFLLKFPGLLEQSI

>SG-AL_contig_6581_1_+2_2_217

SKSTQNTHTHARTHTCACTHTHTHTHKTKRKTDNCPLCSLIWVIFFFSVWLGLPHYCLFS

LGILHMCSNKPN

>SG-AL_contig_6582_1_-3_90_215

MLPPFWLAMNRDLALLKVREGKAVMERRWALPHTGFAVFEGI

>SG-AL_contig_6583_1_-3_96_1487

MAENQVGPIDITSEKDGGIILETLRPGEGEESPCTGDRVTVHYVGTLEDGTVFDSSRDRQ

EPFQFNIGKGDVIKAWDCGVAKMRKNELARLTCAPAYAYGERGMPPKIPPNATLIFEVEL

ISWMGQDLTKDKDGGVIKQMVEQGEKYATPNDGSTVTIQFTGQHEGRVFESREVTFTQGE

GDEEGIIPGLELATLKMKKGEKNRLIISPKYAYGDKGCPELNIPPDATLQYEVEMVNFEK

TKEPWEMDVGEKIEQSRIIKEKGTASFKKGNYQRALANYQKIVTFLEHETTGLKEGESQD

REQLLLAAHLNIAMCHLKLQNFCQAEDACNQVLEVDANNVKAFFRRGQSRLERCEYGTAA

EDFGKVLELEPDNKAAKKQQIICRQREKSAYAKEKLLYTNMFKKMAAKSDGGFNLYQTMP

EVGEWNNDMAKNMMPLQDERDAFEDGADGYKKDLSPRPGETLED

>SG-AL_contig_6584_1_-1_1741_1995

MKAFRLARRKQSCSRQRSVLLIKVHQNLFCILNKRCTILLVEFEVGVHTCFIFPCVFVCS

PVCADYTKPELLYLLGKYCTIKLSV

>SG-AL_contig_6585_1_-2_2_208

EELKIKFTAAKSSKQSSKCSQTDIQLRNAVSETLCTGSVPHSWTISYHARSRTVLNARTS

VLILSHAIR

>SG-AL_contig_6586_1_-2_2_64

VNSVVFVMFHFDAWLWVRFSL

>SG-AL_contig_6587_1_-3_120_1496

MKGKGKAAQQQQEKDCPLQFPDLTIVNHEEECKRYTAILGRQPTETISIEELEPVQTDLE

VLLASVGRRLKLLKTEIQILQNWQDKKDAKTPIAKGKVQSETPSAKRGKGASSTPEERPS

KKFKDSGGKAALPPSSVASPIARSKSKLPQVKGVDGESVHSPAELPRIVKNECVDRFWQA

MESYCRPITDEDIKYLDEQIHKVEDEESTYFKIPPLGRHYRDVWAEEDVLEEMTEGNKIN

DKKRHSNHTNHAFSNATTNGANGTTEASLLLKKAESSLEESPYGPITQRIVAALLEENIM

TPMDDIITDLGENAEEAPAISPRALAKQLNITNSVALERRLQRELEEQGILEPEEEVEDD

PSDEILTEIKQAQVNLKPLLQWKSTHLKDLMKKAKEQQSRQQIWKKFEECDAKLHEEYRK

LNVARQKKKPSLKRERESVKKMLKEWWSVYQMLDDTYNQ

>SG-AL_contig_6588_1_+2_38_229

MATHSASTWHLHSLSAFQRQHCLQPIRQPHTKWRPVCSPQEPSPAETTLPFPVRPMRLLL

PQCW

>SG-AL_contig_6589_1_+2_2_205

VSLILSVSLPGCKKQTNKKTNKQTNKQTKTTHTHTKKNTDTNKNHTHTYTHTHKKKTTHR

SISVTRTI

>SG-AL_contig_6590_1_-1_1_324

QHTHTHTQLYTHIHTHTHTHTHSIHTHTHSTHTHTHTHTHTLTHTHTHTHSLSPSLSLSV

TCICLCTHAHTHTHPHACICLCTHTPSHTERVRAIIITITSDSATFLS

>SG-AL_contig_6591_1_-3_3_236

ESSLNFTRPIRCDKKERNTNISTRAVCIQIKICGHAGNHEKNMFSFSFFLFLSLVVFVRL

FSCVLFVNLRNWHWENIN

>SG-AL_contig_6593_1_-2_182_388

MLYALQYNLLYILCCMPYNITCYILCCDYPAPLHSLFIECIYYILWINVYETFIQAAISS

LVTAPSSLL

>SG-AL_contig_6594_1_-3_852_1079

MQLSEWTLYCTICVLHVLMFFVWCFSVSSLKLHQKLCNDNECVSAHIVEVCMFKRRYGRG

FPRKSIVCLLINCVCE

>SG-AL_contig_6595_1_-3_3_176

SDIVSPFLCLFFVVVCLFVCLFVVVFLFPFQCAQSHKQVNGEKDTKTKIFFRNIFFGF

>SG-AL_contig_6596_1_-2_2_337

FTHQARCPRRIRTQAFQTLQFGSQASSPFDHHAHLFTAHLNAPIILVVSASCCIGPVLPP

PHFKILLAPKVLCRLKESARNYWQIWVEYISNWLIAMNPSGINSKLADVVIA

>SG-AL_contig_6597_1_-2_458_583

MDIASSFFSFIFIFAAFALLQRAVLFQQPPLENEWCVCLKGD

>SG-AL_contig_6598_1_-2_155_316

MGIRRLHDWMMCVVFRCRCCCGICRFFSFFFFFFFSSSFFHRFFFNSFFASIFM

>SG-AL_contig_6599_1_-2_20_457

MKIEELQAQNDKMKAHLMKVDKDFAMMMASQGSLTSELALAKGAMGKVSDDELESLRKHM

SGLESQIEAERTKGRMTQEVANKDLASTKHELLKIREMLDMAEKELEKKVSMTTPFKNLK

SMLQKKNEQIKDLRKRLSKYESLGDE

>SG-AL_contig_6600_1_+1_1384_1614

MTVHQEFPVFVCVFNDTWWCSLLFAVTVFETFHSIKWLVCCSHWGNDSVLVAGYLHILSM

RRYVQFKSLPVITLPPP

>SG-AL_contig_6601_1_-1_184_1758

MAATTDDIQQQITTTIPQFFHGKNVFITGVTGFMGKVLLEKLLRSCPDIGQCYVLIRPKK

GVQPVDRIKQILDCKLFEKLKREQPRVAEKVVAVCGDILEPHFGMSADDQQRVCQNTSIV

FHSAATVKFDEELKLAVQMNVIGVQYLSNLCRHMPHLQSLVHVSTAYANCDREEVAEQVY

DPPIHPHKLIDAMEWMDKDTVEVLTTRLIGPRPNTYTYTKAMAEALLLEETKGLPVAIVR

PSIVGASWEEPLPGWVDNLNGPTGILVAIGKGLLRCMHGNSHCTADIIPVDIATNAMIAA

AWYTGVEKPITTLVYNCTTGQINQITWGQVERMSHENFMKNPCNTVARVPNPRFTLSNWR

KMCSMTFDHLLPAYLMDAYLRLVGRRPMFVRIQDRLWKAVGTLEFFTSRQWNFCNDNIFM

LLNQLSTEDKKTFPFDPRAINWPQYMENYCLGAKQFILKEEISELPQARVKLLRLQRIQF

VLKIVGVVVAWRMFVKRVPVAHSLWNLLLSWVLWFFQKVPMLARS

>SG-AL_contig_6602_1_-1_115_372

MFSSLALSNVGAHSVWEKTSPTQTVRTCAVFREKADVKMIVTVSTCILVISVVKLFREKA

NMKIIIIFLACKLVIGVVKLYRGKQT

>SG-AL_contig_6603_1_-2_32_334

METTMYTNILSWKQQCTQTFCHGNNTLRAFIRTRSRHLAIVTSLLSAIKIRQFNTPSLVF

VCLASTKLISPVLSRPTRTLTSDCSSAHVTSFKIFSPVFTY

>SG-AL_contig_6604_1_-2_2_556

MAAPNKSSVKIAPGAVVCDECELIGDITIGARTVIHPKARIIAEAGPVIIGEFNIIEELV

HITNRFPEGEEQKKNSQVMIIGNNNVFEVGAQIDAVKVGDHNVIESKAYVGPQVELSSGC

IIGAACQVTTGERLPENTVIYGAKCHRRVQRERPPAQNLQIDFLTKILPNYHYLKKVSKA

QSPQK

>SG-AL_contig_6605_1_-3_30_1778

MVGCGAIGCEMLKNYALLGIGASQKGQITITDNDLIEKSNLNRQFLFRSHHIQKPKSTTA

AQAVLEINSALHIEAQQHKVCPQTESTNYPDAFFEAQDVMVNALDNLEARRYMDSRCVTN

QRPLLESGTMGTKGHVQVIVPHMTETYTSQQDPADEDFPYCTVKSFPATIEHCIQWARDK

FEELYVHQPNLFNKFLACNADMTAVVHKLKAGDIVDGGLRVSKIAASRPHSWDDCVRLAR

LRFEKYFNHKAQQLLHSFPLDTKLSDNSPFWQSPKRPPTPLQFSLDDPQHLSFITSTARL

LCDIYGITLPQEMSIDSVNTLLQSVVVPPFVPSNKKIETDETKTQEDIETDTSGDEVVAA

GERLAQVVMSSKAESALKPMVSVEFEKDDDSNEHINFIAACSNLRAQMYSIENVDRLKIK

RIAGRIVPAIATTTAAVSGLVTIELIKVLQQRPLEAFKNAFLNLALPLLLLSEPGACARK

VLKEGLSVSVWDKWTIKGNKGFTLQQFLNSCKENFGFEATAVVNGVKIIYMPIMPPHKKR

LPQTMMKLIKPERGTAYVDLVVCFEDDAGEEIEGPPVRYFFGI

>SG-AL_contig_6606_1_+3_675_875

MPINLGIHEFPLDWTCSLKLGKNSSINSWKNLCRQLVLQQTQRSLLVDMTPPPPPPPFLF

LTERKLY

>SG-AL_contig_6607_1_+2_17_259

MIYANTDYQKHVQWTIININKRVHLHFYSEWLNSLFVCRNHAKILRLYNRVRKHRREIYL

NIITSVIVSINKRSVISRLVK

>SG-AL_contig_6608_1_+3_219_329

MIFQFTFFSPAIEYSNFIIKQNSKVGIHNFMVSVVKV

>SG-AL_contig_6609_1_-1_1360_1665

MSFGQFVEASSPILALPRSFQKSCLQMRLTFLFNLTDEVDLPFQPVHGNRSFCHFILYSV

YVHSKNLVKVLYGVGRYLIHFKDLRSIFRVLHVRERVVPFCV

>SG-AL_contig_6610_1_-2_74_3070

METQQQALVARETRLKYLQQQEARHRQLASETVRLQRIREKVEAQELKLKKLRAVHGQVE

QYKTSNGNLTDSELEAVKALFNEKEKELAVASSRVDVLNQQLKEAKAMVLITTNGSTVKG

DPHALALELDRLRQELVHRNKLNEHQNSHLAAQRQLLKQRKDEAAKMDARIHELQQRLKK

RRSQQQLQPNAKVGDKNVEQNLPGSRLQSQGRKPGPPNVAAVEPYIQYAPKDVVKDDLYS

KTGFQKQDPKYQTLPHNTKFIPPGDDSSKPGGVAGNQEVNNNDKNRGNGVSGVKPVEFKI

PALISSHFSAHRGQSGSAANRTPTDSPVSGAGVADSSKSSSQPSAQGSGSSSTPQQLVNK

LPKGPRPFVNTFGKSGLPKWPPEPKKDEDTAPKPASHQLPPHHPQINIFDEERQAGSGQS

SPASSEGSGPLVKQGSGVFVHPLAASSQNPPATSTNQTGSGPPQENKFLGQPGSPLDFRG

VAGLQEPKPPPNTPQGSSLVNPVDQGSNRAANVKPGARGGDDPDSSRLSGNDSVNVGMPQ

ATSTPQGAEGKTGSTVHLNQRPAPTYRYASKNVIANTYMGRLGSSALERYQQLNKQLYST

IDSTQGNNAQTQQPAQKPNQKKTLANELRAAFNTQQQEPSENLAGDSQGGMTQFGLVGAP

NYPDIASDKGSFKSNTPKHIRRRHSDSDSEDAGKAFHRGDKSRQQNSQSEDGSNNNNVTQ

NSSTTDAETKTSNAAQRRRTNSESGGKSKNTSSNQESGTTDSKTKVVAKQKKTILKGDNR

RKSNNRVSFDPLALLLDASLEGELDLVTRTAKEVADVSAPNDEGITALHNAICAGHYDIV

KFLIEFGADVNSPDSDGWTPLHCAASCNNLPMVQFLVEHGACIFATTISDNETAAEKCEE

DEDGYDGCSDYLYSTQEKLGIANDGTVYAVFDYYACNQDEVSFRINTKMTVLRKGDDVEK

EWWWARLTPNKEGYIPRNLLGLHPRVIPSDYQPHETSDC

>SG-AL_contig_6611_1_-3_3_194

KRDSHLCKLVQSDWKVCVLSLHQPRLSISIMNCSPSKLRMMSVQLSDGRRLHRNLSKRKL

FFFY

>SG-AL_contig_6612_1_-2_1229_1630

MLQSCAKEFLELQSNPVFGRLHRQYQDLHMITKTQVLVVCQRRPHFADMNQSILANPYVY

KSSKVGHIGHSPSENTAWFQVCDRGHPFLEQRLLEICTEVKTIMLVYIKKLGLYREIYFS

NLNWSVLGAARYSS

>SG-AL_contig_6613_1_-2_197_1780

MGPGLTPEGKDIDECSRMPNACENGRCLNTMGSYRCVCNKGYKTDQSGKRCVDVDECQQR

PKVCDFTCSNTLGSFVCGCPSGYVLNMDQVTCRDLDECTMMRHNCQFQCINTPGSFECDC

APGFRKGLRGQCTDINECVETPGLCGPVGTCHNTHGSFECVCPRGYKRDKTGTKCIDQDE

CEDGKCEGECENTLGSFKCICLPGFSEHFGKCVDDNECSDGYVCGLAVCVNTPGSYDCEC

SGGASFDLNLLSCVDQNACGGSPCLFGCSPSSGASGFTCDCPTGYVPVGQGHCMASNPSI

SQYPPGVQLPHVPGPSGGELPAGEGCYNCNHDFGDLPLSKPYTGRTKRSVTETSAMLEKD

EDEDLNWVVSQFHEQLVKEKVMSVGDIVRSRRSVDPYYNWRHLQQKSAQNASQTAPARQR

ELTYENQTIILRLRRKQTRPKVRIIKVLPSMVALKHNTRYTIASGNEGDVFSMHERRGIS

SLHFTRKLTEPAEFHLQIQCEPIVSDVDVDGKPMHLDSYTIRLVILVQ

>SG-AL_contig_6614_1_+1_754_1050

MCFGLCWHYALFVSESELVYVGRGLFLLSAIAWTFSDHGVMFSFTPWCGFSASGELPPRA

GVFSIGSTVPGLSVVVVVVVRIDTLWWCWCGDGGQSDHG

>SG-AL_contig_6615_1_+3_3_206

HGSDATFDPRHMESTRPQKALVVKGLRLHLTERRTEERSLVIRFLLLLRSFLMFNFLWTQ

RSCSHYIY

>SG-AL_contig_6616_1_+1_1_219

FFFQSLKKPNNNVSCVLLYDIYRYNKTCSRHNEIPPRCSNIICLLSCLVLSCRVLSLIFQ

KMSLKSDQSVYVH

>SG-AL_contig_6617_1_+1_256_450

MNIKWFQIRILYRILGTNINTGVQILIDIAFVILKKKALRPSFGTVILANDFGIGLKVCL

TLNVY

>SG-AL_contig_6618_1_+3_3_167

QLNQLILHRIIQICTTSFLSQYSKHKQFLRLITYDWTKRSQHMLINIKSDHSRSV

>SG-AL_contig_6619_1_-2_143_328

MGTALITLAPRKVEVSTTSLSYNLRIVGQPLSFFFNKLVLLVVRLNVGFKLTNTCLSGHK

HG

>SG-AL_contig_6620_1_+2_416_604

MAFNILGVFSETSECQAVASVSSWIHLHLFFSPACLTATTVVTSPSSQNSMITIVVSEEK

MAS

>SG-AL_contig_6621_1_-1_1870_2064

MRKSQSSTTLQCKRRGNRERKKGINSPPPLRTYCRSYFPYILRGFGVGLLWALCVCVCVC

ACVHI

>SG-AL_contig_6622_1_-1_142_267

MPFHTESRSAGLSMGHDYRRSTEYLDHNLTGVILKQTLTLQS

>SG-AL_contig_6623_1_-1_157_1113

MMNNTKMDVDRLNSCVQRYKFLSPRFSSNYSQRTDCDLSSRSHAVAQEKVHQTGSDSRSK

PSSSFFRLVSSWKASCESNHVSVPTATDRMEQAKESLKKMDASRPSSIISEVEIQNYLAK

ESGKDRLLKMFTRDHIGQSSPEFKFMYQVTLQSAAATFLVMTTLAGRQAKQEFIDRNRAT

VFNTRFQAFRRLQDAVFLFSMKEGAKWAVRVSLFTISFLGISQSIAVYRNKSSAWEYAIA

AGTTIGLLKANMGLRACIVGTGFGAVMGTMMGFVALGAMKAFNETQEQRHYWQIQKELEK

EKLLELKGKLPDSSAVETA

>SG-AL_contig_6624_1_-2_2_199

PVTGVQTCALPISGRLCSFSSPLSTGAVLISTSTQTTGGLPIINWANRRKDFAITAEEIF

TVTSLD

>SG-AL_contig_6625_1_+3_69_278

MIPLFVFIPVKLCINLFYLFSGRFGVCFFPFPHLQSLIFFTSMHRCSMTFLFKMYKCLIC

LGLKKQKGKK

>SG-AL_contig_6626_1_+1_202_426

MDLLQSFHLHNSHIFQQLIEQSEQVCQNLSATIVTINKYNVSDTLTRNKYFKSQHSNSKL

WKNKIFQIGKMSINI

>SG-AL_contig_6627_1_-2_98_448

MSLQTVPKDLRNLRACLFCSLVKTIEQFEFDGCDNCEEYLHMKGNRDVVYECTSSNFDGL

VAIMSPEDSWVAKWQRTERFVRGCYAVSVTGRLPQNVVRELRGRRIEYRVRDNSQKS

>SG-AL_contig_6628_1_-3_87_710

MDGTNQLEVAADVLSEFVEVAIHSVLYCRCVYPPGVFVRKRKYNIPVQVCLHPDVKQYVS

SVCTSVRTLLLQGVVHRVDIVITTPLSQPLERFVLDIGQVSSNIGSHLKDDQYLLRLEQS

LRALLLKLNVSDSLLQPLPSDCTWTVHVHTKESAAADLEDQLTNRDFPWVEAEESEQQLK

DAQLVPLKSLNSHLFKIQLYAEESGRKT

>SG-AL_contig_6629_1_-2_11_286

MWLKVLTSHSTLKFPGSQVVLEPPIAWGTFWRLRAWHHRHLALVSEGNSMLTNPYRACSG

HLQVCMPIVLVVWPPLQVSYFCTVRIHHLRWV

>SG-AL_contig_6630_1_-2_329_481

MSKQYKDAFFGVCFVFSLFFSTNCEVLIFFCTVCGYVAWHCVQRRLQLMQM

>SG-AL_contig_6631_1_+1_796_963

MELLLPFTLKGLYIYDIFCAVFVHTRTTFIYLSVFVYKRRHILSAFKEDMIQILWQ

>SG-AL_contig_6632_1_+1_40_264

MVSLRRKLSEKEGPPNRRMVAVADWGARAVTGIDSPAGWTLAEGEPLAWDPRVSSRREGA

PPDVRIWERSRTPQE

>SG-AL_contig_6633_1_-1_958_1281

MYAAHGTLVHHLFSKISTHSTNHGLLEGERKVPSDWELNPGPLVSHVCTFNTFTTELSMV

GWCRGTALTFQCACMNLHGCKSCRGNFSAACSLPSSEWVHDFLRVRER

>SG-AL_contig_6634_1_-3_243_488

MVTSVSVSSTTSMPEVNSGGGLWCQWTLVHGGFCLRAVVLVSLLGEWGSPLTRSDSATER

SLSLVADLVGSVLNKLDHNRHC

>SG-AL_contig_6635_1_+2_371_535

MANQMANSSALASVNYWFKSLSFSLVVTHSYELSRNLNKTMKKAHKVHVIHKRSF

>SG-AL_contig_6636_1_+3_984_1196

MYACFFLIFLIQLLQPWNLNSTLSPKVTRSFQFKDNVMFCLAQSIYMQILLQTASCVSSS

THLPTKILTNT

>SG-AL_contig_6637_1_-2_119_2908

MSEEKPPPPKGRGRARGRIKEHSGPDKPGRSEQEHPGRAQLVQQPAVLTQEAGPSQVASS

RAAHRGASQTGQRQSEEGVAGRGGGGKPPVEGMERMHIGQGSEGGAVGGEPEVPTFPRPR

FSRYVDGTFRPEGRSKKGNSGDPLMLVGNFFKVKFPETWGMYQYHVDFQPPCESRKLKWA

LLKNHEELIGKVKAFDGTCLYIPHRLPNDETTVTSLRIWDDQQVRVTIKFACELRDFSGS

TLHVMNIIWRRVLAMMGMTQISRHYFDEKKSVELIKHGLKVTPGFQTSILQYESDVLLEI

DITHKIMRRETVVDQMYRIHGYMNQPRLDSEAFRKAVGKEIIGTIVMTRYNKKTYTVDDI

DWDVKVTHTFQKSNGSRISYVDYYKEQYQATITDYAQPMLVTRAKRRDIRRLESQGITDR

NIHLVPELCVTTGLTDDNLANYNLMSDLATHTRINPEGRVKRLLTFRDALKENENAVQEL

RDWGMEVEDKLELIEARQLKPEKIHMRIDPKSDKEVEFEYDQQEADWSRQMRGKLMLCPV

AMEHWVIIYQRKAADKARDLSSMLQRVGPPMGMYIHEPERVVLENDRNDTYIQAIKARVV

QQRFKMVVCLLPNNKKDRYDTIKKYLCIQWPVPSQMVTEKTLQKSQRLMSVATKIAIQMN

CKLGGVIWKTPIPLRNVMIVGIDTYHDSSARGKSIGAVVATINSDHTRYYSTQTPQIARQ

ELHNHLRLSITACLRSYEEENPGETPEKLIVYRDGVGDGQLQLVYDHEVPQIRAAIGDRK

LTFIVVKKRGNARFFQRGSTQSARGRGGGGGGRGGGGGGGPMSGFVNPGPGTVIDDVATK

VTWMDFFLISQSVRQGTVGPTHYNLIVDDMLIKPEYYQRITYKLTHMYYNWQGTIRVPAP

CMYAHKLAFLVGQSLHSDPHAKLATNLFYL

>SG-AL_contig_6638_1_+2_2_157

TNNKNKTRNTPHTRKQQQQNNKKQQSPPTILFQISHTTMDNEMKEDLIHKLI

>SG-AL_contig_6639_1_-3_63_2450

MATTTQSEEDRVLPEAEKNVREHEMGEKLSPETTSDISATPSQLPSASAPGFQTTHDNLT

AEERERRAQGYGAADFCFVSENRSGSDEATLPNEGSQLANGDNINTVCAQPLVPTSEPVL

VQTSQSHLSSHPPASEPSQTPQALDAKPQDSTPADSNSSPAKQTTADCQQQGDATLKDHV

PPPSPTAMDTLAEEEEEEEEEEEVDHLVNAHQDSDISSKISQEMQLFGERVVRRLSSLLE

SQASESDSCIEEGRTDLLRNPARQRKPAGDESTSHQREHQFSEHSALLEQEQTDSSSRVD

GGVVEDSAHECLDSSSAPHLVDIEGLSQEVGATPPKDIKEDIEPILHEELNTFSTADLPG

ERLVASDTTETLSTDHETSASSKRESTTSTATDETLTSFKTRSTDSAEFDEAFILPESLV

FVRSVNEGVEGEGAVVEPGRIVKRPAIPAQLTRHGATSETTTPHKPRKKRRRQVGSYDVS

TRPDVNEPEPKRGSKQIQEVIDAYCDDMSDGSDISSSTDDILSDEVVHKTTYSRSEQQPR

APTSIDLEHHTKDIVRKPKPPADRSTDSSDESGELEGKRQSSSSHDSSFRIPEIEETFGE

LMGVKTFLEDLEDKTPTSEPLPVLAGAEKLPSKDFVLATQHRRRSKTSPARTDRTESDLD

VSEHRDDASTDSMYADDEDEGTELLFQRSYSEEHGGEVVLSCTIYNSSHDNDGDSDEFWA

DAEAAAASDQNYKTLETAADAAGSAFQNARIQMHDIHQHLQDLRRQMELLQDDITSTSLT

LTPDYSLESEQRPVTE

>SG-AL_contig_6640_1_-1_1042_1311

MSMVYQQFSISDLYCSLTNVKMTILIQTQCLLGHVQLLLPMLVTIFSLSLFFLTAFIVII

QIELSWNFTFLFTKIEQSHNLITCHFFNLL

>SG-AL_contig_6641_1_-2_86_1081

MCAKEDVAVTNFRKYLRINTAQPSPRYGEADEFYKLMAKEIGLKFKSVEVVPKTPVAILT

WEGEQPDWPSVLLYSHTDVVPVFPEHWKYPPFDAHKDEQGNIYARGAQDMKCVGMQYLEA

IRRMKAKNIKLKRTVHVVFGPEEEVGGEDGMKKFVHRDEFKQLNVGVTLDEGLASPTEEY

RLFYGERSCWWFTVTFSGNPGHGSAFIKNTAVSKLNKFISRCLEYRDQEEKRLSSDPSLR

FGDVTTLNVNMLKGGVQPNVVPSEVSATIDVRIAIDEDLEKFEDMIHGWCKEASPDENYT

ITFVQKCNTTGKSPIDCLSPYWAAIENATYSL

>SG-AL_contig_6642_1_+1_712_1014

MGNMYSCKLSHIHTHYYAHTHIHIPERGILHHIYLHYQPSVEGTVATLLTMNLTVLLTSI

FRNRVSTYFSFKFWTTSISACTDNYPTSICLHSWYQTADHG

>SG-AL_contig_6643_1_-2_2_211

FFFPSLYLHLVLTLRFNFLCFLASCVPSPLSTFRCLLPQFSYLRATWQPSPFPLYAQQTP

EHLCFPLDRC

>SG-AL_contig_6644_1_-3_3_197

DDFLTIFIHLTVFCSNLGVTKIQIHSLIFWLVERAACHLTWVFSGLRSWVLIGYQSFGGQ

ASTSL

>SG-AL_contig_6645_1_-2_1631_1927

MKKRGQQTTRITTTITTTTITTIITTNFKCTTALPINSTYLWRWISWIHFMVTVRNADLD

KYDMYTGTFKKAHKFNANENKWINKEASLQTSQGIDQPN

>SG-AL_contig_6646_1_+1_328_594

MLQRASMAQLVEHRVHMPMVVSLNLGCATGLLNSPSYHIVVMVVSVQNTQSPQPPPPPPP

PPPPPTPPPPPPPPPPPPKKKNKTKKNKK

>SG-AL_contig_6647_1_-2_2_205

LNPRRRLPKRQPRSRQRRRERRRQPRSNCPCVLPCFCRDLGLCSGRPVDIRVNILVCMGT

VLVTELGL

>SG-AL_contig_6648_1_-3_819_1109

MIDSWQSMHLGIMCRLMAYAMIILDPFPSHGICGIDAHGIYSERGIAVLRKLELKLKPVS

LFQVLIHILLIMLLCDRIMGTFCRIQGNKENIQIKCA

>SG-AL_contig_6649_1_-2_2_355

LASVRQLLPTLNNTSLSASGPTHAHPWPRALSSALSASGVLPATRSLISLSRRKGTESVY

NSKWTKWLAWCEPNQVDPYAPSAVELVNFLAFLATSEGLASNTVKGYQGSQWCAFVHN

>SG-AL_contig_6650_1_-1_109_303

MVILFDSLVLYRLATLENTHSVIRSKLKLCFFHELTAICALLKMLKIRVCVTLIVGNTAG

CRYLV

>SG-AL_contig_6651_1_+1_208_357

MPLILALQHTTNPKLFSQSQCKEKEAHGSLQCQVEMLSPLVPGQSVFDCV

>SG-AL_contig_6652_1_+1_1_63

AKRLAKANLLVTGFYFKLFKS

>SG-AL_contig_6653_1_+1_1336_1602

MFPWAGGRWSVNVDGCQYVGSIRQLWMKVCTVSSVVGKHFLTLYSTVQSAGTIFLMSVCC

LLFAFPSLTLWGSPNLYALCLTRVRPRVH

>SG-AL_contig_6654_1_-3_111_1184

MRLSVESDTERMEIEDLDENEDQVYSKFMRAHKCYDLIPTSAKLVIFDTQLNVKKAFFAL

VYNGVRAAPLWDTVKQDYVGMLTITDFINILKRYYKEPNSSSGDSSPPPTARMDELEDHK

IATWREVLKAELKPFVFIDPDASLFEAVKALVDNHVHRLPVVDPSTGNAIYILTHKRILR

FLHIYLRDLPKPSFMGKTLKELCIGTYTNVITASKDMPLIQALHLFVENRISALPVLDDE

GMVCDIYAKFDVINLAAEKTYNNLDISIEEALKHRSAADSYEGVATCLDTDRLEDVMENI

VKAEVHRLVVVDENRQVKGIVSLSDLLNYIILRPMGVEGSAAKGAAAAASEPVVFTSS

>SG-AL_contig_6655_1_+3_348_461

MYMCYAVRCFIKTDDPFFLLSFFVCRFLQKKLYKTVIS

>SG-AL_contig_6656_1_+2_176_412

MKRKSKKIEERLNTTKKQLKPELHSTHAASVSPIPNPRSDTQGNLPKRPQGNKKNHRKQH

RRRERERERIMHASSLSTS

>SG-AL_contig_6657_1_-2_98_373

MSTRVATAPNVAVNRVATYRELDNDLLRQAAFQVLDNEVDLKLLTKSLSPMEDLIEEDRP

WDWDRLFTEVSSELTTQWEKTKEEAEPEQAET

>SG-AL_contig_6658_1_-3_99_317

MDEDDLDFGMIAKSRNVRQGRRAAAAANPAADMDHDDDLDATMQHDAGGMASETSERPNK

AVSGWGEEAPRKP

>SG-AL_contig_6659_1_-1_1759_1965

MANRDLVNSLYLVNPFTPSPYPPPSCGKLCGKGEKVRGLTIKELGFHWLPTVINVHVRFC

ELFLPSAFS

>SG-AL_contig_6660_1_+3_93_290

MRRSSQTKKENSVLSLTPLCKFGASDPETGTYLLQLIQQLRLADAPNIQHKFCTDPSKYG

YEAWQS

>SG-AL_contig_6661_1_+1_709_831

MTMLVGVSCMQPRNGCPRFYDTAVQVSWELVHTFRYFKKKK

>SG-AL_contig_6662_1_+2_815_1030

MNHHNHTTTSSLTNNINDISTLIAHYKSDNIDQQSVKQKIKDEDKIKACIFCLTGFQNFS

NDEPFNLDDANK

>SG-AL_contig_6663_1_+2_272_559

MTHKLHSGPPLSPGKGDPGGPAPKRVPSDPQPKPDRVQDPRNTTEPHIDSLFPLYSCFHR

RAAKTTTQECTPIGSNRVVLWQICVFMCSLRGVYLA

>SG-AL_contig_6664_1_+3_108_245

MFTLLPRCFTFSNPEHGQVPSHSWVDGLEERSETRTECLAVNYESV

>SG-AL_contig_6665_1_-1_43_450

MAGKQVPPQKEPKNMVHQNAILCETVTKENRYQKLYTNYSINPFKEFYVIAGKPNQTVED

GEEDDHFQQVIKRANQEPVKKYKRPQTEAQEVGWITKPLIQSDRGDTRLNFFRQNCPITK

FMDKAWLEKEQQTMGN

>SG-AL_contig_6666_1_+1_577_840

MIRFEPTKLCEGLLEKRSGMDWEGRKKQTTEKEGKWAERKRATEKRKFVAQNFQKVEILS

ILKEPPASPRGKCILIHDFVIMQIILQN

>SG-AL_contig_6667_1_+1_1_159

CVCVGVCDMTPDSCVVFHTLNDAQDTNMYKNQKAQSMSVTMKQDIQSMHEDKM

>SG-AL_contig_6668_1_+1_148_306

MQVHSVHSSAVCYLLVLPQSQCEIFIWIIHISVYHLLYCCRDPSISADQHLTG

>SG-AL_contig_6669_1_+3_3_194

WCASAGCTCSRLTTGCSRRTADSLIVGCQLLQTLFCAWQVAPRLHGKSARHFHDLSKFSF

SLQF

>SG-AL_contig_6670_1_-3_720_1034

MGRARESNLSQCLHIFVRCVMQRLGNHGTCNTVFGCGRGSGGSSKRSIASMARVSSMIYP

VDCPAFSLTPTLRGSVRHGCLTRSTRLQIDIALRVVRVHKLSRHL

>SG-AL_contig_6671_1_+1_109_324

MFHVTFFSCQKTKQTLSRKKINTDVLKNLSSRGLTAYITWGSRCVACMCATPKTRREKER

GRDRQLPVRYAV

>SG-AL_contig_6672_1_-1_100_897

MASGSAILNKTSAQASKDNEVFLQRTGCADASCLRNLSSTDVMMASPWNVFPYWSMADLT

GMPIKGRFDGALLVVDGYVLKEAPLRAWQNGSGNDVPTIFTNTAQEIDFLPLHHDITSWT

WEKYINTVTMELTPFSTFIADKALELYPPRLALTPEYQYTSLVSDARATCPIEVLADTAS

RKFHSPVFEAVITSAPSSPINVFGLFVSRYAFHTWDLLAFLGDFEAVKFHPMPKDLRFQR

QLREQIMAFARNGLPKASAWKTAQNC

>SG-AL_contig_6673_1_-1_1_897

PNKVTVFGQSSGGTAIHALLASPRASGLFHRAWLASASAILNKTSAQASKDNEVFLQRTG

CADASCLRNLSSTDVMRASPWTVFPYWSMDDLTGMPIKGRFDGALLVVDGYMLKDAPLRV

WRNGSGNDVPTIFTTTAQEIDFLPLHRDIASWTWEQYTNTVTMELTPFSTVISNKALELY

PPQPALTPEYQYTSLVSDARVTCPIEVLADTASRMFHSPVFEAVIISAPSSPINVFGLFE

SRYAFHIWDLLAFFGDFKTLKFHPMPKDLGFQQQLRGQIMAFARDGQPEASMWKTAQNC

>SG-AL_contig_6674_1_-1_28_486

MPCHPNDRHTCGGNVEPPLDSTPPTWAPLGSTSVGQLDPSLETDGVGRRAQCKQDSSAPL

PQKHHFGTWVLGGSRPVRSPPPPVVATGSIKVEALHLRLHAQAGVESGGLPTISVPLVES

PSLPQGMRNDLRPRRVPRSHQRPRPPACLRSPP

>SG-AL_contig_6675_1_-3_9_185

MNIFIIQLISTFWTYFEHLVYEHEYFVTFITSMNTAHFCFVFFYFFTYFAVSYKAYNVR

>SG-AL_contig_6676_1_+2_2_160

TKVCCIHTCYKGYKIFMFIDQMFKISPKGRYQLNNEDIHLLTHMHTQHTKPPT

>SG-AL_contig_6677_1_+3_1704_2003

MFIVVTHSPCPLPEIIFYQQLAQYWYPGLPLHSRSLRSTDQNLDWHMNQMFKILRELVFL

LHESGLTHLVNILIMLGHLTHRKFIPATNTHFSILSEQIQ

>SG-AL_contig_6678_1_-1_394_570

MYSLNTWLCVCVRACLWGVELIAYVYNVGLSACARVRGSLGTCSICIQCINIRLIKGGL

>SG-AL_contig_6679_1_+3_3_206

RRETSGSEPRSTALLPMQGPLTSEQRTAAIQQKTDCRKRTQVHRTLSHCRAHSHLNSVQQ

CFSTEDRL

>SG-AL_contig_6680_1_-3_9_215

MYGAAYGDALSHQGQQPQFRTANLLGRERLKNTSTKCKGQHHSQYSTSLFPRTSHFLSLL

SLQLLHHVL

>SG-AL_contig_6681_1_+2_119_487

MPLFTPSPHHGGSKKISVKGKERERDRDREREREREREKERISDFFKKLITQSALFISFA

LYCIKSIGEEKGSFNLFPVEIKLLRRRAILYNSVCSKIKLSNQDFSLMFIEIKAEDFFSR

LRI

>SG-AL_contig_6682_1_-2_317_664

MTLKLLGISKIPFCSVIGSGIGSICANLYVSVLSIVVHIPPPIPQNKEIEKSAFFLSLCT

VGVFLLSLSLAIISESSRNICWTSLSLYLSLRLVCQPYFFLDIFTALAIVLANTVS

>SG-AL_contig_6683_1_-1_1837_2154

MKFFLSLCIMGVFLLSLSLAITSESSKNICWTSLSLYLLSSYLFLCQPYFFLASFCIFTA

LAIVLDLPSLKLSCPTVKPCATMKLWLAVDSILNSPAAFKSTQHRS

>SG-AL_contig_6684_1_+1_1267_1509

MTGLKQRFPGCLHNGPGIRHSTTSILTTSPQNISQILYLLFLQQWWDWVSVYILKNIYWI

TEVVRYSEINIITKYNKNKED

>SG-AL_contig_6685_1_-2_2_223

VCVCACVSFSVCVTLSFFVKAQMYKVHQRIDEVKVLLTLATFGGFLLSKALNLSWILQQR

GSWPGSMFLPLPAS

>SG-AL_contig_6686_1_+3_855_1166

MHSVINRYDTLHIEYPFEESVIFLSVTILSASKLSAFTGSWVFFFPSKPTHTHVKINVDI

RHRINIEMPWEVTLADQSQSFSDVQPSFPFKHLLLHEHADHVAQ

>SG-AL_contig_6687_1_+3_3_179

CDNANAKGLTYWLLQSQQTRCYKQLDILGADPSSPKTDTHLLLFYFHSTTAKYITNFSY

>SG-AL_contig_6688_1_-1_172_915

MSCGTSCARMLLISFNFIFWLSGVAILGVGIWILVDPDLQDYIDVIHTTDEEYFKKAAYI

LLAFGAFIFLVGFCGCCGAIRNSKCLLGLYIFFLVVVFAGELAAGILAAVYKDEIIDEFD

KGLTKSIQNDYKDNAGAWDAVQKELKCCGGLNPTDYDSSTWQNTQTNQNIQIPLSCCELQ

NDQPVDQTNCQSRTSNYHTTGCKAELEKWVKDHSVILIGTGCGIAALQIFGLVFAICLFR

SINEEKYH

>SG-AL_contig_6689_1_+3_1293_1541

MMLNFFLRIYFSTNVCSGLDLAWKGTTDEALCVDTCLVPLTDGVIYLFICLFIYFEVCRD

LDLALGRTINEDVYIDATHAEFL

>SG-AL_contig_6690_1_-3_3_182

RSFWLPLAREDMFHTSSSLFGFTLCLVLSFLNLSAAWNSHASVHLIVLTCSVSPAVVLCL

>SG-AL_contig_6691_1_+3_3_191

RIACISCAIGAMPVPAASISIRLYSLRVYCRLPKGPRISSQSPTCQRFTTCDMRPCLYTL

IRS

>SG-AL_contig_6692_1_+2_3188_3457

MITQHYNDECLHTSTSDHLQWNVWDKPLHPKVHMPVTSIKPKRKRGREKKGRSVVGERMK

EKDRKEKRETEKEALLHPMIDQSKQGQIKC

>SG-AL_contig_6693_1_+3_732_953

MERYVGKDIPLWTDGMKYSDTVSSARYILVHSCFSQIQTILLSLLVYKNRAFCSRSCFLL

QIQQVTSSPFKVHY

>SG-AL_contig_6694_1_-2_2_634

MMRKRRILILRATKMKRMSWARVKRRRKERMARRTMRKKMRKRMTMRKRRMLALPGGRRG

STRMKRKTHNHVYSSNVISSSSSTSSSSSSTITIMTFTTTIHPHSRRNIQHQEIREKVGS

TRERENGFFFCFLVPLHVLDPSSCWTLKWVVPTKFCVVLAVDAVWGQPLSRTNRHARHRG

LGQRTRGLAVYIHPPPPLLFVCFFIFCFFFF

>SG-AL_contig_6695_1_-2_1055_1258

MHTVNCLFLINGSECVCVRVCACMLWSSCLRFTKYVPDLHMNTLSYRAIKLEVGVCVCVC

KLAFLVIH

>SG-AL_contig_6696_1_-2_170_1474

MWKSISFVALFLVLSSKAMQVDAGRQRRQSADCPRDCTGTPLEISFVVDGSASIWPANFT

FGLNFIEDFVNTFQISPSIVRVSLITFGERAYEEDKIDFDEYTDNTSLRQAIANIPYRSG

IRTNTSGGIMFMLSKQKPKAREGVRHVVIVMTDGNSQERKETKTAAQAARADGLEVFAIG

VGHSVSPEELHNIASDDRHVFAVASYNMLEGIKKRLVYQACDVPSYPECEMDPVDLSFVI

DSSTSIGQDNFTVGMTFVKEFVDSFQVNPSAVRVSAVTFGEIVYEEDAFNFNTYPDKKEL

LDALSAITWRHGTATNTGAGIRYMREHQMIHSRPTAAHVCIVITDGQSQETEETAEQARL

AQQAGIVTYAVGVGIVGKQLDEQELLNIAGDPARVITADNYAELNLIKEELTDLTCRGIL

SSLSIKATKFQTSEN

>SG-AL_contig_6697_1_-3_120_284

MIYTAQKCLSFPSSLPLFPSLIKVICLFPIQQWGLSNSAPSKESFERIQLSAFKV

>SG-AL_contig_6698_1_-1_172_243

MVYVSVCASYIAVLIKFCFTDRKS

>SG-AL_contig_6699_1_-2_110_268

MFRCTVHHKKKKREMQYGLIILFVHLYNSQNVIVWFHGPRGCKIIINIDIRQH

>SG-AL_contig_6700_1_+3_3_134

QLFNRDMQLSLKLKYHFLVIFLIAIIHFGLFQGCIHFPLSKQKS

>SG-AL_contig_6701_1_+3_3_272

TWTAWKTWTSGDSEALTVVSPKAPPSFSGPFLPSFSPWRPFRIRSDVRRPVQVSPRDSFP

PGTVSSSTQCFGKYRIAGSVQHISLPRNNL

>SG-AL_contig_6702_1_-1_115_1788

MSATVKQQLQQELFLPSDERLIAMVPVFKVPRKRKGSYLCAVLNTSEAQVTAHLYQVKKG

DRGDGYKKKLSWLLRDLRQVDGKSSEKETTDFDLQFEKVYKWTAMKIEEKENFIANLWRL

SQRFLIQKPDFVNVPNRLLEDVTDPREISQKSQSVDDLSMAGEDYQALSAKEESDLEMLM

AECQVAMSNAEVFAEQLSKHLSVLDGANIHSIMGSEDQVLNLMRLLDEGIQEAERIEDKL

NSYDNILANVKEQMEVMQDKDTLVTLRNTNHQRLLQHLQNLVVQLDLDLKHVTALCDGDL

TSPAGIMECTAAAQALHRCMNADIHPSMLKLAAVEEQQKQFKKFSVSFARRLAYFLNNLF

IHQGNEIGETLSRFVGDLKMPQHGSTHKGLVIYADLMLWLKNTDPGVFEKLSTYYVECLS

RLYIREVSEFLEFARQRLMSKEGKGKHSSNTLKPSGSSTSLSKMVEARGRSSSMQSVDSA

HTYGSESDLAARSVFDQVLEKVLVELERVCIAEQDFCFRFFHLIDKTVQPNPESANKDGE

QEGAEDIWQPQSSGTGTE

>SG-AL_contig_6703_1_-3_129_896

MSMQPNHTIYINNLNEKIKKDELKKSLYAIFSQFGQILDIVALKTLKMRGQAFVIFKEIN

SAANALRSMQGFPFYDKPMRIQFSKKDSDIIAKMKGTYIEGERRRRDQDDDHGRKKKKKA

PPTAGPTKTSVPPAVQPMMTQPPVARGPPPTVPAPVPPMAASAAPPVAAAAAPALPEQPP

NQILFITNLPEETNEMMLSMLFNQFPGFKEVRLVPGRHDIAFVEFETDTQAGAAKDALQG

FKITPTNAMKITFAKK

>SG-AL_contig_6704_1_-2_2_187

SLSLPFSHSVIDSLFINFLCLFMFLILLVSLFIPSLFMNSYIYYFILAWFWCMVASFHLS

SC

>SG-AL_contig_6705_1_-3_3_290

FFSCLSSCSSSFSLSLSLSLSLSLSLSLSLFSHLHSTRSRPRNCSSPNKQGYFSFSSFFC

FSSSSLSSYFCARFLHHFLFACLCVGSTCFIPFAVY

>SG-AL_contig_6706_1_+1_49_231

MTRNNTMKKNCPTGKKINIKIKERKLAPKSMLIVISNALKPAKLKKTKPSHTIHTKPKQN

N

>SG-AL_contig_6707_1_+2_2_208

KQNNTNKTRENLQTTKAHKQQQFSVPFSSSYKDKSSINRSPKTPRLTKHTFNCMVVNNYR

FKIYPQYLP

>SG-AL_contig_6708_1_-1_259_1755

MEVTEQLDISEVPEERPEEAGIIEIDVEQVEAAKEQPAAFLTETVEEVTEQPEVLPVEED

ATEKTLIVEIPAELEKTAGEQPSESQIDVVLEEMVHPDISVMEKESPEAAVETEVPSDIA

IVAPQQPAEYQTEAIEEQAELPVVAEVPQEQQAELVPSEIPTEVEDTVTGVTEQFKTVPV

EESIAEREVQPITEDESRDAGVLEIPVELVETAEAQPAEYETRPVEEEVPEVTTTEGVEV

VTEVLPEAEELPTDVEEAITKQPEEFQTKETEEVIVVSSIEEQPEEAVSRAVQEEIEQFA

TEFVEKIITDVQEKQAQEKPIIPEEAPREEQQPTEEEKPEKEGEEILETYVTGLVHDILE

QAETTAVSTVTTTVQQQIQIEIPTAEEVVKEEIQIDFPAKPEEEELEAPRFTVMLSDLTV

MEGSKMVLEVVVTGKPMPELTWHLDDQPLTESQEITVVTEQGRSVLTVLQVLPDDEGEYR

VEAVNKVGRCTSVAYITVL

>SG-AL_contig_6709_1_-3_3_80

AVCLSLKSTGAVCLSLKRERAIDVFS

>SG-AL_contig_6710_1_-2_878_1066

MIWLQVQFTGHKYISLNGNGSHLGHSCNRYLTQFTVGKGKTAREKSKSHPQKARPTLKKL

TQE

>SG-AL_contig_6711_1_-3_111_464

MSVIELFEETDTITKIKISWNLHWQTTHNAARLNFIVLFFFHKTEILTDIKQIMPEWLVF

SVKLFCLVCFHSVGWVLFFDYHNRTDISDNSCTRVINRQFKCMHEFQQYPKNMSLSVL

>SG-AL_contig_6712_1_+3_3_200

THTLTHYINTDVSTPYKNIFHLGNTLFREINQQKVSKLVKRSIFPFCYHMKCITRRSRQA

VIGQNR

>SG-AL_contig_6713_1_-1_1_507

DCLNYCSSDGSRPDVVTEAPPVDSSDPNVVCQLPEQVGNCRASLPRFYYSPERGECVRFY

YGGCGGNSNNFETEQACNDYCRSDDICQQPKVIGPCRGGFTRYYYDSLSAACRQFIYGGC

QGNLNNFGTMEACQKKCVHHGSPDMGPDSRTREVCTLNPDAGTCRGYDI

>SG-AL_contig_6714_1_-2_86_2377

MACVRLSWAARNACKTGIFSRRFECLSRQISTTSAMAGKYLKLDTSHGDVAVVRFDDPDS

KVNTLSLGMQTEFREVLKETQSNPAYKAIVLISGKPGNFIAGADIKMIEACKTKEEITAL

SQNGQDAFSKLEHSPKPVVAAVMGPCLGGGFEVALACHYRIAVADKKTGFGLPEVMLGLL

PGAGGTQRLPKLLPIPTALDMMLTGRTIKADRAKKMGLVDQLVQPLGPGLGNPEENTLKY

LEGVAVDFARGLASGTLKRTPKKKGLQDKVMDLVLSYDMGKNFVFKKARDQVMKMSKGLY

PAPLKILEVVRTGVDKGQAVGYKAESEGFGELGMTKESKALIGLYHGQTACKKNPFGKPQ

KPVQTLGVLGAGLMGAGIAQVSIDKGMNVLLKDMNLQGVARGQEQVQKGLDAQVKRKKIS

SFQRDSIISNLDPTITYDNFKSCDMVIEAVFEDINIKHKVIKEVETHIPEHCIFASNTSA

LPITQIAEGSKRPEKVIGMHYFSPVDKMQLLEIITTKKTSKDTAASAVDVGLRQGKVVIT

VGDGPGFYTTRILAPMLAEAVRLLQEGVTPKQLDKVTTSGGLPVGVATLADEVGVDVAAH

VAEDLGKAFGERFGGGDVNLLKEMVSSSYLGRKAGKGFYTYEKNSKNRSENDGALKLLQK

YHMEPKTELSDEEVWYRLFSRFINEAVYCLQDGILSNPIDGDIGAVFGLGFPPFHGGPFR

YLDLHGAGPIVSRMERYQQLYGIQFQPCQLLQDHAKDSSKKFHS

>SG-AL_contig_6715_1_-2_449_616

MYEDVGVHIIIKVSSVWMLYCRYVCEPPTPTFQSECISSALTWEKSCVSSLQSTQL

>SG-AL_contig_6716_1_-3_126_254

MCMVVLYFTSCCWWNSLPSKVPHRSLELTLVEKFSEAFGFSQS

>SG-AL_contig_6717_1_+3_2640_2909

MSKPFRVYPNPFMSIRSHYVHCNLFTHSYLSKPTDANLNPFMSTQIHSCCCPFKFMHLGL

CTGLIYVPSSTNNKPMIWECRMIPVCHSKL

>SG-AL_contig_6718_1_-1_1_258

PVRLQCVEIECAHRTSALPRDSTASTSETECCVCFVWQDELHSWATGKKCVLETSVWCVG

SVNSPCWCTMKRLKCLTLDGMEKSLE

>SG-AL_contig_6719_1_-2_119_2656

MAGVSRLQMMEHVHLAGEEEEDIYSGFNDYNAALDTEDLAGDEQFQKAVLRTSHGRRPPP

TAARGTMGMAMGAGANARLGTAARMGTAARGRMGLPSSMGRPMTGMMEADSGPRPMTAIK

GAGFSSTGNRGMFDPMNQTTKGPAPPLESKPEDSPEEKIRQLEKKVNELIEESCFANSRG

ELPLALEKAKEAGRKERVLVRQREQQSMGEQINLDLTYSVLFNLANQYAANEMFAEALNT

YQVIVKNRMFSNAGRLKVNMGNIYFRQKNFPKAIKFYRMALDQVPNTHKEMRTKIMQNIG

IVFVKMGQYNDAITSFEHIMSESPNFKTGFNLILCYFALGDRNKMRAAFQKLLTVDLKTD

DEDKYLAQGDDKQASLLLEVIKNDSLRQIERERQYEAERCIKYAAKIISPAIEGSFSDGY

DWCIDQVKNSQYLELSHDLEIDKAIMFLKLKDFQQAIETLKSFEKKDSKVASTAATNLSF

LYFLEADLAQADKYAEVAMAADRYNPSALVNKGNVLFARHDFEKAREFFKEALSNDSSCV

EALYNLGLTNKRLGRLEDSLDAFYKLHAILRNSPQVIYQVADLHDKLGDTAQATEWFMQL

IGVVPSDPSVLARLGELYDNEGDKSQAFQYYYDSYRYLPSNIPVIEWLGAYYIDSQFCEK

AIHYFERAAIVQPNQVKWHLMIASCYRRSGNYHKALETYQHIHKKFPDNVECLKFLVRLC

TDLGLKDAQEYANKLKKAEKAKELREQRVTSAGRKGSGRGRRGGSADSDREGSAEAVRKP

HGRERRKPNFDGDDSRPYQGSQAEVDASYSDPLGPQMERPKTAAKRRDPNEDEFGDEELG

DDLLPE

>SG-AL_contig_6720_1_+2_2_307

IQNVLVVPLSQDRAHIYWVTPQSVKLGNAKQQTTTGCHLVDYSFIKWLKVQIPASLAPFL

VLFPCVYVWCAFCLCKIKKKKNSQNTLMYIVYFRHKIFSSLK

>SG-AL_contig_6721_1_+1_1_123

FFFVISVNDLLTVIHCDTRCMTRKIQFTVFTHSQLSTSYTQ

>SG-AL_contig_6722_1_+3_633_941

MVVSVTYGLLVMNLAYPNSSQACSMGAVSWDSEGHNVKAVFACCRQSKARCVRPSIIMLE

GMEFLVCTEDRLQHFCLLKTITPNPTSTVMHHTSNYLENIWEI

>SG-AL_contig_6723_1_+3_3_371

KTSEDQGQDLNHSAKDIHAVGSDEEFAQALDARRQKLNLPRANTTDDWEKLDEKNIRRLD

ALIGKSTLEHKLATYGDIVYATCTDAYGVKQLKEETPPMKSRCQGSFWSRRNKRELHMAY

NFP

>SG-AL_contig_6724_1_-3_417_671

MQLVPHSTKANLYCLNCSKCVPLSVIIFFSLLLVFTDVFHFRCGFLLLSSFIPVSNLVTT

VSGKKKLLALSYQHLLSPGQLTKYH

>SG-AL_contig_6725_1_-3_6_1226

MMDGIDDSPDLLAEDQEHRQGRSDTVDLTDTSLLVDISDSLSERDKVKFTVHTKTTLQQF

KKPEMSVVRQHEEFIWLHDRFVENEDYAGIVIPPAPPRPDFDASREKLQRLGEGEGTMTK

EEFMKMKQELEAEYLATFKKTVAMHEVFLQRLAAHPTLRQDMNFEVFLEYEGDLSVRGKN

KKEKLGGFFSRITKSVDETLLSGQKDVDEFFENEKVFLMEYHAKIKDATQKSDKMTKVHK

NVADSYIQISTGLVQLATIENTELAKVFTRVAEALEKARKLEGRVASDEDLKLSDTLRYY

MRDSAAAKDLLYRRTRSLADYEAANKALEKARTKNKDVPQAEKHQDQCCKKFEKISDVAK

KEIQEFKTRRIAYFKKHMVELVELELKHARAQAQLLKNCITALEEES

>SG-AL_contig_6726_1_-3_504_1130

MIGCHNGLTTVLQKLNLQLIGKWCCAHRLSLLAHWASSAVPVLKKIQEMLVVIYHFFRYS

AVRYNHLKELKKIMEEVVRKLKKPTIVGWLSLHDAVKAAEESWGCLVLILENATEKDATA

DSLLKELNTYNFTSTLCLLRDVLNPLVKCSLSFQRDQIDIEETTNALSLHGPRVTKLCTG

LDSDGQYQIIAFQASASRKDSQLLPAESL

>SG-AL_contig_6727_1_+1_79_225

MMFTYPSMAYKINNPSESESLSLSHTHTHTHTLISIYMRIKTSIIERRL

>SG-AL_contig_6728_1_-1_439_678

MNGDNQAFLQVLCIFFPGCVGVSFHLDVMSVNCRSISQLNSLVLGLFFSISKITGWRKEL

VRLVPSFLCQGHLLFKGELN

>SG-AL_contig_6729_1_-3_6_1511

MAAANTAAASATASSDGEGVRSQASQAMSGNQSSQTVAAFLQTMGDNHSILPGEGFVMDA

FLCVAEQLTFSDMITIFMGQHQPIAQLRRPMRQFVSERVLNGSAATDENLQRGTDTVVEE

FLPDVMTMVSGAAVVDNVDLRATLRKFFVHHLLTLFKLIVNYNVSDEQFALQFYNGFRRT

LGEFVILMPACLQDGQPAFNAMIENRLTGQLTGLNPMLQQWMTSMTLQHVASFQQIQSIS

QSDVEGYMERHPTAPAAPRVVAAAASPQAGMGEGERKTEGRSGASASRESPMEVDSPPHP

TPTLPSAPAAVARSLASQSAQVPRTLDLRRLGERMETRSRTQEGRIVNGNLGSAASTPAA

TITSPGSVQDNWQEVVPSEWVPVMEADVQRQKTQRSQGPFSDAYLQGMPPKRRRLMSYEQ

AGNLSNMSDYLPAAVKQAAGRAGVEPVSSSDNLVAEATDAQELQGMLEAEVMRSLSDRLE

QDDDYSAERFPNAQQYFKPKPS

>SG-AL_contig_6730_1_-1_277_1326

MKVEDVMRCSFSAWYETFEDVTIKSIILPIPQEFLDYLHADSVVLPEGSLQGAYSSAWSD

DSEEEEETVDWSSKGDCKEATMPDCREFLQEVDSTISQLGGKVFPKLNWSSPKDASWVSL

NNTLQCSHASDVCLLLKSSNFVTHDLTQPFIHCEDSEVASKDVVLQYELVLRQWADISTG

HEFRCFVRHGKLIGICQRNHTKFFQHIVDSKKEIQEDIEDFFEEVELNFGEEAGCYTFDV

WRKGKNKILLIDFNPYGPVTHALLFDWDELAAYDESDGLMFRCVESEHGVQPNGLSQFCM

PLDFVDLATGQDPFKLMDLMKLGQHQDNSSSDDDEEVEEDGLSVDNAADR

>SG-AL_contig_6731_1_+2_2_163

IFNGKVRTQSSCCEVTVVTLIFALYGPVKGSRLNTSTQLCRSQSTLILSPHIPI

>SG-AL_contig_6732_1_-2_68_214

MVGIERAPPENPSPTASCAIGLNGATETLVRNSHVHSFVSSLYRRAFSN

>SG-AL_contig_6733_1_+2_77_256

MPVADCPRCERNGVQWLRCAKRGGEGFAGCGFAVQCLSQTSWSLVTAADFAVPWLTQNRY

>SG-AL_contig_6734_1_-3_999_1304

MTVQQRSPGLLVCFVLFLYFVGGFLFLYFVGCFFVLFCLIYTATVLLFLLQFYIYIKILK

VLLYNLIISYSQFQISCEKAGCKGRRISCRFQTYRTFFFYCY

>SG-AL_contig_6735_1_-1_544_840

MKKQPLSTCCVLTYCTYFTQSVQCLIFSFFYLLPEIFHDQQTQCQRLRINYKILFSCGFM

LLVRFLKLYLFGFSLVNWLFYASYQTDPIKRFQIQKKKK

>SG-AL_contig_6736_1_+2_2_139

LFLYCDKWGKKSEAVKPHSHAHTLSLSLSLIHTHTHTKLHYKAKRP

>SG-AL_contig_6737_1_+2_2_79

NEPVKSRECSSMNGHIIKLFCISDQK

>SG-AL_contig_6738_1_+2_2_181

LQTLNMFANNHTRNTIWSTKMFSFFLRVLMLAIVTSSRANRMNKQHHLQTLNMFANIPYI

>SG-AL_contig_6739_1_+1_196_336

MGIYLAKFLLASYTLSYTLPYPTCAEFEDWRREEISSCGQSAEWRRL

>SG-AL_contig_6740_1_-1_589_720

MCSRNYMVVLTFSKLRTFFIQRQNFVSNAATSGKCISHHNFDNG

>SG-AL_contig_6741_1_+3_237_509

MGHFPAKAIFFYVICKKKTKLSEHKIVVSLQISLPSNANAATNFAHFTDCTVEQGNRLPP

ILLVCVCVCLCACVCEYGGEILQKLNDKYLS

>SG-AL_contig_6742_1_+3_3_164

YCLVPIIHLLIYYVEGCITSYVHGLILCSCWKKSNETQVTQFRNFARTKSECPK

>SG-AL_contig_6743_1_-2_182_589

MSYSVLLGLETAKNTKTRCCRCHFMVTFPYSVLCIYHFMYVLRCKCTFCCLWGIYACVCV

CVRERERFHSLLNIDPFGIRGICQRERLHCLLNNDPFGWGYVCVRETERIILFYISTRVV

MDPCKCGKKMVWIGRD

>SG-AL_contig_6744_1_+2_116_265

MSSPTHKPQALDIMHNYPILLDTVLLVKHTMVSRMFFCLLSSNTSFVNST

>SG-AL_contig_6745_1_-1_1_177

YSEAPVMSASCLMRWQGSPSQFVSLTHHVTIMRGFEMTCIKIQQRCVPLLVCLSFQSCF

>SG-AL_contig_6746_1_-3_135_458

MSKTAKEKKIWKDHENAWQSHHQVLEPPAKHKTVSAVADIHHSPAVNCQQSTCYSQQSVC

HNQYWHPTTTSSHITTTSHQSATTSSWPTTTSSQPTPTLPVHMKKSSK

>SG-AL_contig_6747_1_-1_148_4854

MTSYTSEVADDNDSVNSADTSSTQEGEFINPKGVRFMQTQQQQQTKEGYPLIPYGLPCVR

ELFRFLISLTNPIDRHNTDVMIHMGLSLLTVALETGADHISCYNSLLYLVKDELCRSLSV

LLQSERLSLFAAALRVSFLLFEAMRSHLKFQLEMYLTRLSEIVVSESPKITYDQREIALE

SIVQLWRIPGLVTELYLNYDCDLYCSNLFEDLTKLLSKNAFPVSGLFTTHLLSLDALLTV

VDSIDQHCHHRVLSATKSSASAEGDSQQSEGSASGVEGEAKPDPSGAAEPPLSSFRGPPS

TGFTMAQFLMQKQDPDFGRSAKKEQRLGPQIKPNRMAKLTAKIPSREDLAEFKQKKRAYF

QGTEYFNQKPAKGITFLQEQGLLATPLDPGQVVIFLKENPKLDKKMIGEYIGAKKNKVVL

EAFVRSFNFEDMRIDEALRLYLETFRLPGEAPVISWIMEHFAEHWHKSNGEPFFNTDAAF

TLTYAIIMLNVDQHNHNVKKQSIPMTMDQFKHNVSHVNGGKDFDEDMLEDIFNAIRNEEI

VMPAEHTGLVKENYIWKVLLRRGATKEGVFLHAPTGALDSELFTLIWGPTVAALSFVFDK

STDETIIQKAVSGFRKCAMISAHNNMSDVFDNLVISLCKFTTLLSAAESPENIPVTFGSN

YKAKVSARNVFGLAHRHCDILREGWKNILDCMLQLFRAKLLPKSMIEVEDFADPTGKVSI

VREETAAVTRSESGVLSSFVSYFSLSESAATKGPTQEELEAARQAQNCVKDCHLESLIND

TKFLRHESLSELVKSLIAACQGAEEQGSLGGQYDEDAGVFFLELLVKVVLQNRDRVTGIW

SAVRDHLYWVFVRSREPGLLVERAAVGLLRMSIRLLRREEMAGQVLGALRLLLVLPGPVL

HSLARQLTYGLHELLRTNAANIHAANDWSTLFTLLEVVGAGANPPPVMQVDSDVDVAQVL

HDAGAQSDSEVAEGGGASSANDRGYTSDSELYESASRHQHSHRGSPELALRPVPDNGSWM

LVNREDAASPSFPPAPLRVNQYSIELREELVRIDRRSLMKAAETLAFLVRDAAHVTPHNF

QSCVHAIRIFVEASVNGGHHRSPLRSSSMRERRGGGHPHNPPRGRYHRDSKMKKSRSNPT

QLTQISTSDDEGEEEAALTSLHSLSIQLLDLMHTLHTRAASIFAAWAQESSENSEQAGSG

EGEKPIDAATGTLWATCWCPLLQGIARLCCDTRRQVRSQALTYLQRALLVHDLQTLSAME

WEACFNKVLFPLLTKLLENINAQDPSGMEETRMRASTLLGKVFLQHLSPLLCLSTFTALW

LTILDFMEKYMKADSSDLLSEAIPENLKNMLLVMETAGIFQTDAGTESKLWRLTWDKIDT

FLPHLRDEIFKPFEPEPKKMDRSETGEESSAHIASQTTGDREGIQQQHQHQVTEAPVMDG

QQMSGVPDVPLEGITVIPTGHVAVAQQQDVIQRPESPLLSQPEDSTPSTSYILHPPLPVV

TSSASPAPPPAAATAPVSGLPGVHIPLLLNPDIFQSTTIPYISPQSTVEMMEAQKTQNTD

TPQNMDTPR

>SG-AL_contig_6748_1_-1_232_891

MKISTSCLRIVILGASQVGKSAVTVRFLTRRFIGEYSSGADCTYQTMFQYEDAAVKVEIV

DCASPWETSKRQIMEADAFVVVYSITDSSSLKQAHSIMDTLTAMPDLTVPVLLLGNKTDL

QHLRQVSRHDVTRLTYQYQCRHCEISAAESYAAVDLPMQEVLREGLENKRLRLSQVKRRR

GLLENVSKRLGSVFRRKSMEGIPQKRKLSRVAENPNRRSV

>SG-AL_contig_6749_1_-2_2_151

FFLHSMSKTSYVDLLIAALNSLHLSGVGGLRRDPHPIILIPFIYLEVCTC

>SG-AL_contig_6750_1_-3_156_908

MAKHTLILVRHGESEWNQKNQFCGWHDADLSDTGIHEAKYAAELISNKGFSYDVAFTSVL

KRAIKTLYLIQEGTDRHWVPVYRSWRLNERHYGGLQGLNKAETAAKHGEEQVKIWRRSYD

VRPPPLTKDDKEWSGTDPKYAKLDPKCIPACECLKDTVARVLPYWSDAIVPELKEGRTVL

IAAHGNSLRALVKYLDEMTDEAIMGLNIPTGIPLVYELDENLKPIKHYYLASEEEVKAAE

AKVANQGKVKK

>SG-AL_contig_6751_1_+2_2_142

KKKDWVACFVNSLNPMKSKLVTKFNTILSSYYNLYYSDQTSSKVFVC

>SG-AL_contig_6752_1_-2_2_118

VICTWSPGYFMLSVVYWSTLICVCVFFHIRAHLCSYMIG

>SG-AL_contig_6753_1_-1_130_462

MIDSVCEREGEIVCVKEREKEYTYMLFFGCVFLLLLFCSCTETSCALKCMREYVCDLFPL

FFTLLLRQSQSDFGGACWVFSCVHKPLNADMAYRILTCIFDVLHVYIPIGS

>SG-AL_contig_6754_1_-1_199_363

MLMPGTSNLFYRRLDLCVVFVSGRHVIALGSHMSGGLLMVRVSSTCGLCFCGGVP

>SG-AL_contig_6755_1_+2_2_112

STKRLKEHQKRVDPVPCEHLRGENHTTDLSDISMDTP

>SG-AL_contig_6756_1_+2_2_160

LLFHRHDLMFSHLTIIFIDIVSLNKTRHVHFASHNPKMKSICYFTDPHTQTLE

>SG-AL_contig_6757_1_-3_15_245

MWIQYSNQNLDISFVYTYLSTSVFFVVVVVFKQLTINVHASFEVRGLLLFLAVSVLLMSH

SVPTLHHLFSKKNFSDI

>SG-AL_contig_6758_1_+2_2_88

NVTEIFFTKQMMQCRNRMTHQQNGNCQKG

>SG-AL_contig_6759_1_-2_2_100

THTHRGLLEYFSVPVFDHEPVLVIFYSLNLPLS

>SG-AL_contig_6760_1_+1_1_87

LFDSKTLMTVTLNNSNNNNMHKITPPLTT

>SG-AL_contig_6761_1_+2_962_1264

MDGPSTFTAYTINLPQKASTRFIFSIKMNDPSTFTAYRTKISPCKNSYRMTLQHFECEQV

PHHPFSHFHQKHPELDEPSTFIAYAINLSHIISLHRPNFTT

>SG-AL_contig_6762_1_-2_137_232

MFYSCLICACKLQNLVFALANKDADVKKKKIG

>SG-AL_contig_6763_1_-1_46_246

MCLTFQLLCLHGVLKTLDTLRTMAAVVVTNTLKTGKNHDEIIWISVSVFSLDCNLNRFTI

MNVMNMT

>SG-AL_contig_6764_1_-3_51_1307

MMVTSRTQEIAGLLQTVPANLTALTSQCQTFQAALNECEREAQRVCPASDSIQSTLQSLL

GAYDSLCGTEIVCTLRVQTCSQDLSKITDITNALAQTPPNVQVIKEKCSRLTGKVRPCLQ

KLQSDCSNVTSVAASIPALVSSLDSLCGQGALCQLQLGRCHLSFANLSASGDSQDADKLQ

QVCRAQKAAKMCLATEVDAGCRELPELAPMINTVNSTIMSIVCEGGDSGSGDDKEGCPQV

FQCLSQMRELTGNNCSSMSQMMECLSGLKGVCNVKETTVTFKQLESVAIQTCRDVMSNSR

ALENCTQLGKCITESDVVRNLYTAGNQAPNAKQLKRMFQLILDANTWCQYLEVVSSCYVS

SADTCDLDTNLVSSMSQRSTKLKDTCPASSSDGVRGSALNYLLVAGLAIFTAWLQFLGF

>SG-AL_contig_6765_1_+1_106_264

MATQLRSSLFSHSNCRWFITSVIFKCCIHIKCIPQDMWSNIHKIKGYTLTRFS

>SG-AL_contig_6766_1_-2_224_1435

MLRISNAGDSKQGESGGETSESDNNNSTDTIKSNPHAVTSNGHAYVNVHDRDNNNDDEDG

EGEKPRPAEHGIYDSQRVDSGSETDEFVDTSDQIIQPASRTSSIQGEVSYSASPSVDGRR

ETRVRFAPYLNTAVSPNSCMVDGVTSLHNTALHDLSTSSSQMLKDRLALGTPIGMTFLPQ

SGEYIPRNLSESLHVNESFSSLASLADDSVAGDDLTEERDTALDLPEDTESMMDVIHDKV

LLTSTSDLDFMTSRGGEEEGPSQATGGWNPVTHGGASSAPRGARDSGRSKEGRTRHRPLP

PGSGGGGRGGRGQGRGPEISMDKQILLAMVRMQQDMAAISNRLNTLELYVRLHREKQDKK

AVKKSSWWPFSGMPKTLVFLLIVWPFLAPIIVNLICQRRHRAPR

>SG-AL_contig_6767_1_-1_1_507

NAATGVVRGQCLSLVPVSGNERACARQGGQPLLVDRGNGDLSEANCGRSPSRTACPSGYS

CNIAPNDAYAVCCPGGAVPVEKPGTCPAPRGDVLGICASTCSQDSECSGTDKCCKNPCGA

FTCQAPESPRPSCDTVTCGPAERCVLRQVLCVRAPCYPVPVCVAVSKPG

>SG-AL_contig_6768_1_-3_153_1946

MNYNSVPRQQASVPPLEERSQLDELLSDLLSDQVIVTSPRSPSGPWSPQSPSSPQQNTMK

GAKTVTTTTHTYHTYSGTDSHAGRSPADKVLIQRAEVTYKVPGVGEEKQHYTIAGEPPEL

EKPRSPENAYSYSHSSSSQIDQSRRFQSLERDMNRSEQIMGMQQNPPTRTQTLPYRTDYE

NRYAESQRYNTLDSDQPSWLQEQQQKLQRKRDGRDASGRTEQEKQLVSELRFAQNRYYNQ

RTASEAEEKAAMDPYRQPPMHNGPLTSSTLPANYAPPPQGFGGEVGHFSTYSQSQYQEYR

SGGDRSNNKPPPSPTFQRSAAPSSSMPVQPPARSSSRDYMKSRSHSSTSNLWQSSPPPTS

HSLERQYSESVLDRDHSVGVPAPRRVASSQSTPPHSPGTRSPPHMGPTATTTYTTYRTIH

KEVRDDPLGPAPVQPAMAAAPVQAQAAPPQTQPPVNPQSQHNYITEVFVHRVGGEKQGHD

KQDGSEDTRLDELTRSLQAATESIMEDRMSISKQQKQQQSFQQSSSWQEHYQSQQQQQSQ

VQKAQQEGPPPKPVNVIEEDRITLRPIGPGMQTLEPENTHSTSTLSRAATPSFPTSPV

>SG-AL_contig_6769_1_+1_298_504

MVNVQKMCTVLSTKNPVQTHVYNKCVLQFNTPCIRDSSKKTHSFIHKKHDHTHVLKLGVD

WLSLILNNQ

>SG-AL_contig_6770_1_-3_108_1574

MANLCGPLTHFCVLIFLLVAVSLTFAGDEDARAADDVALVRSMRSVSRRGGRRGNEGIAV

DSGGRGGRRGNRGNRRNHGPGRRGGRGRGGRGGRGGRGGRGRGRQGRPGRRVFSRREQRL

IGRLTQATAIFSLQMYNILKQDRGELIFSPHSIHTALTMTYLGAREKTAKQMMKTLGLKR

LRKNKAHLAYAALVNSLTNSGNVTLNVANAAYVKPNLPIEETFRTGLQNMYKAEFDHFDY

QAVGGPEAPINAWVELKTENKIQNLLRPGTISISTALIIINAIYFKGNWKDKFNVEDTTQ

QIFYQDGGSTTSVQMMNRVGRYNYTRSNELAAHIVELPYQDGRFSMYVILPVTRNRLADV

EQRLTLERLNDALQAMWTRKLDLYLPKFKTESEFSLRRVLRSMRMIIPFSPRANFTGICS

VGGVAISDVIHKAVVEVSEEGTEAAAATAVILERMSMPMQVRADHPFIYAIRDNFSKAWL

FMGKFAAQQ

>SG-AL_contig_6771_1_+3_3_278

MSFIIYIPIQKNGLSCLHFRGVNSHIIIFMSFIIYVPLQKNGPSCLHFKGVNNGLSCLHF

KGVNNGLSCLHFKGVNNYIHVIYHLYTSPKER

>SG-AL_contig_6772_1_-2_2_178

SVRTRVSEGGGDWLVLFCYKCLVQVTVLSHSLSDSSPAVCFAAVSDPLSYLCWLVGIDR

>SG-AL_contig_6773_1_-2_71_202

MHRKTSAMFKDLLIDASLLIRSKLSKFLRLPTRRHPRSMEDILS

>SG-AL_contig_6774_1_-3_987_1640

MRGQRSREKDSLPEIVLDYQYQTPPIPRKTGTLGPSVQGDCPMAGHLAHHFEAWRKVTDS

PFMLQAVHGYLLHFNAQPPIVSPSSQEDGNNFKRNKAAHMDRAIQSLLTKGAIEETGALP

VLQQDVSGSKERGRVEADHQPQAPEPLYQSPMLQNDNYNRCLPAHSRGRLSYHLRPEGCL

LHCQSTPGIVDSFSLYGRGRLRGIVQLSLLVCQKAPYA

>SG-AL_contig_6775_1_-3_471_644

MSISFFASHTNYKTFMDIHTHASLSLSLSLSLSLSLSLSNRQGKHFSPSAQSLMFSAR

>SG-AL_contig_6776_1_-3_3_224

NFRGRKFYFPLKRNSVRRKNSGVQKKNSTGSGCPQLALCSRATTARFGYLNSECSFVHQL

ANSRRQATSTEHNC

>SG-AL_contig_6777_1_+2_2_235

CVQFDSIFLVLSLVHKADTPRRKILQKASTHFPVDQASSQATALAERGFVVHEAELSCGE

RESHQSVSLVEKKEKRKE

>SG-AL_contig_6778_1_+1_1234_1473

MEFVPLERSLFHWRDFRAQARAKILWRTRCCLCNRPTFPQHQCKPKERLKPLQLGVSEGK

ITVAVTYQWNIQTCPLLTHV

>SG-AL_contig_6779_1_+3_54_1196

MAPRRSKSSKNPPIFSHEFLIQNHADIVSCVAMVFVIGLMFQATSPVASMFVTMQHNVSM

NDSAGEEITMYTCGRKDVFSVFFYFLICVVIHAVIQEYILDKMNRKMHLSKVKHSKFNES

GQLLLFYFASVVWGADIILREGYVTGLSQLWEGYPQDHATLPFIVKFFFIIQIAYWLHCF

PELYFQKVKKEEMLGRVQYISLYLVFIVAAYILNFSRVALCMLVLHYLVEFVFHAARLSY

FSEKSDLASHAFMAFNVLFVLVRLGTITLAVLTFWYGLEQTSQESVDPATGNYNTQLVRI

NCLAAVCLMQAWLMWNFITFQLRKLRERSSAGRKARSPSKKKPIKAAGSGDDDYNSLPEV

DQNSATENGSIRSRAKAKKSN

>SG-AL_contig_6780_1_+3_453_665

MVFKNPSLSLSLSLSLSLSLSLVSCTYTHTHTHNQYNSPFPSILSISTVIKIINVCNYNL

HGMWTSTQIWY

>SG-AL_contig_6781_1_-2_2_133

IFSASSSSYAWGKSCMFCCLNIYTFTQYCIKMCVLSGQVVIGLH

>SG-AL_contig_6782_1_-2_23_223

MVDWVTADGTSRQRSTWLQVPYQSQPRPSETFFAKKTILEPSVEETTCSETLMVELIAEL

NLPITPV

>SG-AL_contig_6783_1_-3_3_110

RQRLFIKKGGVTDSTAFPWKIHRKVTVLQKSENGLH

>SG-AL_contig_6784_1_-1_139_588

MTSLATMLHVELPHINLLSKVDLIEKHGKLSFGVDFYTEVLDLSYLLDHLQVDPVFAKYR

RLNEALVGLVEDYSLVSFTAVSVHNKESMLRVKNAADKANGYVFGKKEERSMESLMSCAV

GAEFEYQKIKDIREKYVETESSVTMDDEDS

>SG-AL_contig_6785_1_-3_168_260

MFCSEAQFTLAGRVGLLSRDESACVYMGGSS

>SG-AL_contig_6786_1_-2_308_481

MCTSACAIILLMWHYYFKLDFFLFLFFFKCCSRILRSKLWMLHKLYKSDRALVESHKN

>SG-AL_contig_6787_1_-2_323_472

MTRSDSNPAYHYYYILFIAQPTTMAISGLYHVKSIKVKKMTALNTKLCQT

>SG-AL_contig_6788_1_+2_188_295

MMIIIHQNGDVQGLQFYIGSEISLVASGVYSANGVT

>SG-AL_contig_6789_1_+2_980_1192

MSHHQVTVRARLFLKRERNKYVTYNPHTHLVFTETFILLVFQAWSPQCSLVIMIYDYSFY

SNRFALLCCYC

>SG-AL_contig_6790_1_-2_278_460

MDNVHSNLQINSEIHSIPVGCSVIHICYYRHRSVYTHLANPYRHFLFKHGHRLLQIHLQS

V

>SG-AL_contig_6791_1_-2_2_142

LGSANKQNLSHLGSTTLLTEPSRPCFKKTTKKNVAPVSLCWSVFLCC

>SG-AL_contig_6792_1_-2_389_547

MFYDYDYNVIVLVKSGFTRSDVNTFGFVLSIFFFCCVVGSSGETLYYYYAFVK

>SG-AL_contig_6793_1_-2_140_1144

MGDTDFKEVNHGKFRQDWNQTSTGVGHHHRPGYYFPDTEFRALLREPLPPPLARPDEVTR

DQHFKTTTGTFHDPKHPSLLYGERIHKKAPGHWKVNYVKDLAEKLGNGGWRKPLTMGNQE

SEMKGEYKGLPGLRARYHFDDVYYPQDFTLQDHHKEGPTKYGEGSTQNPKLQGQPFYVRD

KGILTNLDPYLSTTHKDHRSFKPAELKAYPKKDVPTYWQCEEYPKAWGHGLKHNPLPKHT

VPRERLPMRDTMVFPTATKIPRPPKAMVPVPHGGLKSEYTDHFLKQDHVRQKENYYCAVD

TPFTLPKAGTKSICTAPKMYNTEYQHVGSEKPITV

>SG-AL_contig_6794_1_-3_3_164

EVLKNCHTRNLLIQMYSWSHCGKTECIYWVTPRVLSWGTQTTSVAQCNSFLDKL

>SG-AL_contig_6795_1_-1_73_441

MVRSAFSGGTVTVRQTLSNPVIEEAFLYIYATNETDVKEALKSFDEVAKDKFTRKEINDD

MISTLDDEDVRRIQQVGEMYKVELQIQQMASRIICDGLYNQVCDCINEISTIIREVDHKR

QTK

>SG-AL_contig_6796_1_+1_145_258

MFSVKRNVVRGSKTDSQCLSEKCSFLFLTQQCSLSREL

>SG-AL_contig_6797_1_-2_539_793

MASCLIMMMMMMVWPLCMLPALADDLLVTCFLHQPFQRLFHISRFPKHWSSRVYWNPRVA

HLPANILKEQSSPSVPIVTGWPWGD

>SG-AL_contig_6798_1_-1_142_375

MTDNDQLGCHIVGYFSKEKNSFLNYNVSCLLTLPQYMRQGFGKMLIDFSYLLSKKESKVG

SPERPLSDLGLLSYRSYW

>SG-AL_contig_6799_1_+3_570_746

MRISSVVTRQKQTNKQTKNESNPIQSNPIQSSLEVHQGMVTAEKNTNKQANKNRTVTVK

>SG-AL_contig_6800_1_-2_3317_4240

MWLWASVCTFAFIMGGGKDPHQQHRGQPLLQEPRFGGNGPADMRGGPPGGFGGPGGGFGG

GFYDEFDSYGGGRGPFGGPPDRFGGPRSQYDQGMGPQSGGQGCVVMVYGLNMQKMNCEKL

FNLFCLYGNVVRIKFLKSKEGAAMVQLGDGISVERAIQNLHNCFFFGSELQLSPSKQAFL

QDVMNPHELPDGTPSFKDFMGSRNNRFTNPEAASKNRIQSPAKILHFFNAPPNITEEELG

EIFETCYGKRPIRTKQFPSKTERSSTGLLEFENKTEAIEALIMTNHHPIPSTKSPFVFKL

CFSAAPML

>SG-AL_contig_6801_1_-1_1_201

AARIPHGKSSVTKKINTNTIQYNTIQYNTIQICCTLHTGGAFMKISLEEAYGSVSFVHWG

LSVSCQG

>SG-AL_contig_6802_1_-2_314_472

MCYRRLVPVVCVCVCVCVCARARAPVHYCEKSDYCLTIEMVTVICWSESSGPN

>SG-AL_contig_6803_1_-3_18_203

MLLHVCVLFLYLSQILVRLFLKVEMVWEIFRIIHQKSQYVRKVLCKDRFVFCNWLSCMLV

WG

>SG-AL_contig_6804_1_-3_57_221

MHFVRKFGMHSSCDSKNVCFLGVPVTFCASWNQLIAHSHRIIVNVPRICDRTNSF

>SG-AL_contig_6805_1_+2_2330_2566

MLLKNISIIHPHLLVNIDMFFIRMTRNIRYTSAESKTAISLLFTAFNNPMLHHGPHLHHG

LTDPQFVKNKNMNNLMPTF

>SG-AL_contig_6806_1_-3_3117_3728

MVCSAFYQNTLSINTQYFDWSILPLRKSNQQCYQVEYTILCESECVYSLIMHIKLLKWDG

REQMRQKNHCPFCHQNEGHYWSSGCYLTNQWTVQMNCTAKVFVSSYWSTKVKNCSDTTQI

LYNKELFEDMFTCLLFIELVVEIKAIDLYWQQLSGISCCAASSLCIHLEILLMSCIRSLI

HSCQNNLILWNQAMLAHISMHRFV

>SG-AL_contig_6807_1_+3_708_980

MIEIIGKIVGYSSLKPLSCHTSEDVRNSTVQPENSFSRQPFHKTFAAGISCIQQKLHHVN

RPLQKRKKTVPLRNQRMSVCHIRNKYLYVKH

>SG-AL_contig_6808_1_-1_76_228

MSIHLHKISMLDTSRGETFLALQLDIIPYQSELPHPRSQKIAQRGLVGRCG

>SG-AL_contig_6809_1_-1_136_1116

MDMGSNIQFESLCLDVTAVKQEVLSDIPDNQSSSGPLNSGQEMESTDFGGVVIERLGHQI

EGTQLIVVREGMQQEQQLPFPIMLHAHSDLSAPISVSPDIETPVSVLPLSASTPSQSSSS

TDLHLISDGAHKRYSKKKQGSHAGFQPGNKCWQLQARWKKAKGNRRKLHQGTSADKTISS

RDEVKVNKKGPSRGLTSPRTVPSKATKTVKKGKSLHGSKTVKKVSSSKAQAPNKKGRSPN

ASIVSQSSKPKGPIHTLKKVPWTGQQKRQPRKCCRVCKEKGLFPMSCYFCPRCPRQPGYC

RIRNCFSSYHSKMGLRYCVSSASGKTS

>SG-AL_contig_6810_1_-3_99_1820

MSDPWNDILTVKDRKNTLRAKMMQRKKEREGLVAELIGEASTGSVSSVASNSSVSATTAE

SQSGLSSQGNTGPGGTKTGDGNWGSGLTTSSSSIASVSSPTLEYKGDPEVEKKLLLVLCD

ISLDIPSDSSVIAEHTSRTLGRKVELSAVETLLRKFASQSLISIIAKEVDGKTQLTVISL

DITKLEAVSGEKSEKRARESQDGDEEGQPSKSSRMGGVDDIESLLSMQSTKEREEKKANE

EILQLLSKPTAKEMYLVEKFKTPGGAQLKEFCPYGVKEECRKFSQGIPCNKLHFKKIIQK

HTDESLGDCSFLNTCFHMDTCKYVHYEVETVASDSELVLLKKEATLAKQASEASHGNQVR

MFPPQYVQCDLRSFDMSVLGKCAVVMADPPWDIHMELPYGTMGDDEMRRLDIPSLQDDGF

IFLWVTGRAMELGRECLELWGYKRCDEIIWVKTNQLQRIIRTGRTGHWLNHGKEHCLVGV

KGNPKGVNKGLDCDVIVAEVRATSHKPDEIYGIIERLSPGTRKVELFGRPHNIQPNWITL

GNQLEGVCLKDPDIVRMFREKYPDGNCMAPPKGK

>SG-AL_contig_6811_1_+2_176_319

MNYYFVTMDSPCEIRAGLPVRARCRSAALPFFFFFLKLFYLVHAGSCF

>SG-AL_contig_6812_1_-2_1304_1642

MRTLSLFILLTLVFFFFVILVRCWWSVKSTYRPMEAPVTMPANSGVAAAVGTVTRMLPLL

MTKVMTLSAVASAGPTVDDTLLNTAAVAAAMFVCHSMFIRSRATPSYTSATHK

>SG-AL_contig_6813_1_+3_27_1277

MTLMLVSVSPLDKAYQSTQSALQYGQFAKAVKNHARMNLDDTQDIIAELRQDISKLRDKI

AAASEPNRDDVAKMEDLVSDLQLAKKQTWEEKERLSAQFEEQRKINLANRGILEWVMDSM

KKGNREMQEKILLLQKERDQLSMQYKEKRQTVDALKDDLQQKIKDYTKFTESGKKSESET

KKLVTAIHDLKERQKRETETLKKIKEQLRDVQHRQQRERENASTQITAIKGNAEMRQKVE

MEERQRLEKEHKMMVEEEMEKAKLDMENEVAEIQMREAEGYQYTTKEGAQLEKALAELKA

DKPVVTMKLQTLHQEKDHLSGELEEVYRMHHEQLELQQLQHYQTFRSYREMFEEQKAAID

QRYRKLLEDCIQDAVFLSSRNSDLTAENQSLKQQIGELKDVVTKLGGKIPSSVDSSI

>SG-AL_contig_6814_1_-3_192_1196

MSVHQIPQNETSVTSQCNQQTSIHHHTATASNLPAAKTNSSPRELNPEQNKPASKTKHKI

PAISSKHEASRSQPMSCKPAVPSSGLFSKFSSTPQMSDDLDEFEETSYQRPTVSTKLNPW

RPARGKTLAQKRPSETPDASYVSKKIKTSAGCSSDTKNSPVTKLLFAEQTKPLFSASKTN

PGEKSQPLISSFSVKAPSNKTSMNKTTAANFLGNTSVTSSQNSQQSKFLNTKLDKNPPSN

LAAGQTRDSTSIDVSRLPLSNTVTAPVCKQPQTQKELKGKGTSSRERQNVGGGKEKMWEK

ETRKTGAAASLDERVTSCPLCQVAFSTAMSQIDVD

>SG-AL_contig_6815_1_+3_303_479

MSGLTTARHFFFFNNMSRECSRIIIQTGRLWLYTQLLNTYEFVMCMCLCMHACLCVHAC

>SG-AL_contig_6816_1_-1_1_237

ASCYRVPEFKLPFCLLSLYVSLSVCCLLYYMQGKCETGESYISFLFIFIILPKAEFRPVA

FVCSAVFLCSVLHYTIHFV

>SG-AL_contig_6817_1_-1_1_234

ASCYRVPDIKLPFCLLSLYVSLSVCCLLYYMQGKCETRESYISFIFIFIILPKAEFRPVA

FVCSAVFLCSVLHYTIHV

>SG-AL_contig_6818_1_-3_3_386

TSGAFPSSDDALTLWHTHFLWRWRSQSGPFGSRQSQARTPLEPELPLPASPSWSSFGRLL

EQESRSRRLDDPWAGTPPSPPACRSGVPSSFAVRKVGWTEDFPSGYNPLHRGNSQTMRLQ

QTPGAPVP

>SG-AL_contig_6819_1_+2_2_172

QCNELVKESSGNDLAIVFNACSLPARVSQLVNKATLTVPVIKMGDSSTRTKCRLRAS

>SG-AL_contig_6820_1_-1_1_111

LCSHVTTARFSYLNSERSFVHRLANSRRQATSIKRSC

>SG-AL_contig_6821_1_-3_192_545

MALLVGWAINNIIIIIIMIIIIIDLAVFYHRLLLILIWVCFCPLAFVDSILAVISLLLIL

CLPLAFVGFCCVHIFILKSGYVGTLVVQHCYMLFVHGIRRWDIMGVFCYRQKRDCHSS

>SG-AL_contig_6822_1_-2_98_472

MAFLCIFCCFEVFLIIFYAFFQPGDDKLNGMAGVLQHSLILPALIKELNNNNKSFLYSAV

LQTYLDSLSYLTQLHDTECDTCRSTDNTHTHTHTHTHTHIYNQNTHMYTDQYIHTHKVNS

TYRYL

>SG-AL_contig_6823_1_+3_3_146

PFCSNKPNRKYSKNQLTFIIHNLYFIKYLNKQITKKSTTLLLTSKCEH

>SG-AL_contig_6824_1_-1_1_201

CSLASIIGLTLRRKIENSFPEKYKVDIFIKEGTHQTVDEINKQINDKERISAAMENPNLR

DLVDRCV

>SG-AL_contig_6825_1_-1_19_237

MHKDCRGLHEAAATGQRTDRFIPSKHVAPEEKSSIQAEANFCELIIKMNLPLSTADIMSK

TVRSAFPDSATAV

>SG-AL_contig_6826_1_+3_60_209

MTKNVFTATMNMYIPLPSQVFLHIREHFSTLFGFYCFLRENLYMWTILIK

>SG-AL_contig_6827_1_+1_157_507

MFCKETATHPEFAGLLGEEEGISVSTMSRIDRNITSPSNLQTPGEYPSFIMIKIVQLSSC

SPDLISGCGCTNLLCAGSETVSSHCCFRLWSDHLQCTWGAQCRHRLTVLQYPTSTET

>SG-AL_contig_6828_1_-1_640_783

MLDSNLVKLAKSQMKQCHVSSQYDPNNGFCLLKLAYKYRYEIIDYRTR

>SG-AL_contig_6829_1_+1_1_228

RSDVSSSIPACKIKIGLFAANLNAPIILVMSCSHFHFKNLPAHNVLRRLNLRSLPDYHRQ

ILVKCIRSCLSAVNPP

>SG-AL_contig_6830_1_+1_208_294

MNSRKAWVGEHCGKRLQDVQAILTIIFLF

>SG-AL_contig_6831_1_+1_1930_2148

MTASNVCVSIFVSTLTGHEFVEDERKTHDKEAEHKWQYALKISMTRVLLLLVCSFVKTVP

WKLALQMFYTEMT

>SG-AL_contig_6832_1_+2_2_217

PTHVHQHPPTPTPPHPNRSPYLHPPSPPPPLHHQQNNRSCNVVPSSKTVTLYSHKPTTKN

RRHIHLTVTQTS

>SG-AL_contig_6833_1_+3_147_404

MECKPLQKLIFYSLYMEVTLVDLVPRDRIKSCNKHFQFQFSPDSVSMLGTNSICTNAANA

MGWERIQNDNGISHTPAHKPYTLARS

>SG-AL_contig_6834_1_+3_3_293

GGGAPPPPGGVFGGGGGGGGAGPPPPPPNKIELEYFFLKMICKLPCSMQRKQNKNLLFST

FLYELQCLVIIVLSHTLKFQNFLEEIRNCVNSHHITR

>SG-AL_contig_6835_1_+2_2_178

FTFLQKKKKKKKKKKKIPFFFFFKLWTYLLLCRSTQWQSHKDMDIFCCVDIRSSDHTMT

>SG-AL_contig_6836_1_-1_1372_1695

MKVGVFVQQFLPLNRMQHALQRQEQPLFNGTDDMQISLIQNGVKENKNKFCVSLCSGAIV

CKKWQLPVILCLLSCEISDFKHPWQGKYVLDFIKNGVKKKLSPTWMPI

>SG-AL_contig_6837_1_+1_856_1173

MLSKKWAVICSIKNYWNIIIQLTKFQSDSITRLPTRRSQRHSKMHNVHKQKLFQTNNEHR

YVDIQKLPSNTHKRGKPCISTVIWLVMIVTTRIHSKRGKPCINADI

>SG-AL_contig_6838_1_-1_10_1659

MNNAGAETDAQNVQTTSLSLAESPDVPQVQANVPSTVAVPLQAQVSTTLSSLQTHPPAMV

AALAAPPPPPPTPKSSRRAPLTDPRNSDTPRRRTQRFIKRRKFDDEIVESSLVKSERGRL

KGPATSIPTLTTPAIPHTSIATPEPALPLIEKPGKPVGGKPIEPLIKSEPRLPAPPPVKK

KVVSQKPAPKVSKTTKSSSKRVKKPKAPPPASTKDLGRWKPQDDVALITSVQQTNDLQAV

YTGVKFSCHFTVKEIQERWYALLYDPTISKLSMQAMKQLHPETITAIQGKSLFSQNEENL

LAKLQSTSSPQLETFQELLTKHPEVFHPLRTTKSLMTHWQLMKQYHLLPDQSVQPMPRGD

HVLNFSDVEDMLKDEDLKDPSNELVEHELTLADRHNKRDIRHLEQELPKWQVLVETVTGI

SPPDFDSQTLAVLRGRLVRYLMRSKEITLGRTTREQSIDVDLSLEGPAWKISRRQGVIKL

RNNGDFFIANEGKRPINVDGKPVLAGNKQKLFDNSVVEISCLRFIFLINQDLINSIRAEN

LKQGATAAVS

>SG-AL_contig_6839_1_-1_1_144

LTVRILAMECPPAVEERQRERRLQDHSYENEEGFCCCLFCGGLYNCRF

>SG-AL_contig_6840_1_+1_1_105

YVLSACFYPEGCHRENELMLAASWKTPRGAILVLP

>SG-AL_contig_6841_1_-3_105_437

MPMSKILYVMLAFAAFRNTKIPSMRKQNKFQNKTTTMGLASRWCITNCSTNNGEGSRRNF

KAKPGSPLSGSSSLLSTVSLFSPATPIRVNARRYYKHTYTHTQTFCCQIDR

>SG-AL_contig_6842_1_+1_28_261

MFKSLKNKHDNFFNFSKTNKITLVLKKKSKHDILSSDHGVLGYGNISMACSHENEDTSGH

DKAGHTSQERELCVSQPY

>SG-AL_contig_6843_1_-1_1588_1944

MYPYTQVYTPVCVTGAECCMMVWQFSYRSPPVPRGYAARWAVLFCNACIDQLTVFFLYLC

LEFFCNSAVFAFWGVCRKHLSLLNLLVSRKVRGVFLRLVTLACVLFLTTSVEVTPVFQI

>SG-AL_contig_6844_1_-2_65_208

MIFENLLQNRMVSHWCHVRLSCVYQNHLSFVLFSVYSHRFRHFCFVFV

>SG-AL_contig_6845_1_+3_87_203

MHTIKTTACYHIHCHIIFISPQLNSINALLFMTKVSLSL

>SG-AL_contig_6846_1_-3_570_899

MRVSVTVWSLHTEHVSSWECEHLVALNSERMRRRVLQRLQMFVWIEQNEFSEPCSSGQVE

IGASIVLSNEWKQSMIYILSTVILFFSAVYLIYVSSALSNFLPHQSPAYQ

>SG-AL_contig_6847_1_+3_294_428

MDTVYQLSLKNNHLMPALPIFWQGSYRFVTPKFTSFSQTFKTIRT

>SG-AL_contig_6848_1_-2_83_859

MTDKLDLSLDDIIKQNKKTRGRGGGRGRGGRGMNRGRGGGGQRRGGGGGGVFRGGVSSRS

RRPPPYQRPKELPDVWQHDMFDGSNGGYRRGAAPVRTGGVSTSGKLLVSNLDFGVNDSDI

QELFAEFGQLNKAAVHYDRSGRSLGTAEVIYVRRTDAVKAMKQYNNVPLDGRAMNIQLVG

ATEGGIQSRRPSTGGSYGSGQRGSGRGRGGGGGFSTRGRGGLRGRGRGRGRGGRSDKTTP

SLEELNAQLDAYNSKMETD

>SG-AL_contig_6849_1_-1_394_588

MPKYPPSIADDAVSGAVKTFKSSDRTIHETSINKCLNMADIFLCIGHKKGASPNDEELHV

DAYRF

>SG-AL_contig_6850_1_-3_222_374

MFYLSKTHSSVVIFCPIFVYNIVTCTKCCWCGVVMYLNSQLLHFGREREIE

>SG-AL_contig_6851_1_-2_8_724

MAAPTSRMIATCRLLERLVLSGSASTSSRLHTAQKHVGQVNRARRCAGGVGVPSLSSQYP

TVCQPVASPLSVPCRHTTWYTDKRISGNLGAVGVGKVDPDFTPITDQTEGLELAQRVNAS

LEQEHGRLFAVVYINGVQRKVTVEDILVIENEFPPTVGDRIRLEKVLLVGGTDFTLLGQP

ILSREQVRVEATVIEKTLSHNKVDSTYKRRKRFRKMKLFRTPHTMMVINSIELGSVPEA

>SG-AL_contig_6852_1_+2_380_781

MTDKSKPVKSDHLLTFSGEKNGNRDTNKQRGKSRFFVLQKHRLERKTNLFIGTICVDHTN

HHQHITGLLTLLSFVHIITRERKKSNKAETAPEHLKANTNCSTDQTGDNTHDINAEKGKT

PGNKYCLLQANPFS

>SG-AL_contig_6853_1_-3_69_590

MGTLKYVHVRCLQKWIKSSEKLSCELCHFDYLITTKTKPFREWEKLDMTTAERRKIICSV

AFHIIAITCVIWSLYVLIDRTTEEVSNGNLDWPFWTKLIVVAIGFTGGLVFMYVQCKMYV

QLLLRWRTFNRVIQVENVPETEEFRINALAKIKVKMEESGGVTNSVALREVESL

>SG-AL_contig_6854_1_-3_102_422

MGLGEYPPEFNPRIHGPYHPGRYYGKADVPLGQVKLGEIGGWLSRRSYTPQAMVACINRG

YWRWATKYLLAKKTSIAPVVQFTACLAIAYYFLQYRSHTNHRHAKYH

>SG-AL_contig_6855_1_+1_1654_1938

MYTLRKWLYDSSTATRTSQLITGGFTNHKDDKDDDRHKRRGSGRRQANGLVGRTEDRKTI

LQTRQNKTKQTKQNKKPKEHTEKARAKPHSVSAQI

>SG-AL_contig_6856_1_+3_30_236

MSAAYGCIHTYTHTHTHTHTPVVGPSYYALNSCKLEHVQHCLIYQATMGVSNVLPMVSLT

QAYSISSLT

>SG-AL_contig_6857_1_+3_1023_1589

MKMDPVLDQADRAIGIANDIAVHAKSKTEHNQVLHNLMKVAAQNGLVFNSGKCHIKTESI

TFFGMTYTTKSVKPDPAKIKDRQVMPQPTSKKEVQEFLGFIMYLGLFIPNLAERTAPLQD

LLKKELAFLGEDHHRVLPATEGHHVKEINITVLRHHSASNVASGCKYQRTWNQLDPGWTP

HRMCIQASL

>SG-AL_contig_6858_1_-2_2_232

LFISTRFGATDPVLFISTRFGATDPVRQVTACIHTGLYEPGQFQNFFVPKDDRMVMFWIN

ICHVYCRQLKDSGALCT

>SG-AL_contig_6859_1_+3_72_503

MKNRKIFGMLVMGVLLHSLALVEFFSDIWSAVGELGWYVASSLSACEEFHEAGKGLANWG

CVSCVPLWRRGFVSHRCHHVIYQDHLLRVSHKHIKTGGDIVAYTTFIEAGYRVHIHRVHK

QTENEQLRGGCVTKWAYIQCGLQL

>SG-AL_contig_6860_1_-1_1_153

SLHLCLDVILSSVFDFHFYVPCLIGSDIWPGWEIRNEYNVVNQVIVIMTSA

>SG-AL_contig_6861_1_-3_57_338

MDFSSPPPGGKMGAADKEHLMDQVKQQIALANAQELLQKMSEKCYKKCISKPGSALDNSE

QKCIAMCMDRYMDSWNLVSRVYSSRLQRERNRLQ

>SG-AL_contig_6862_1_-2_122_1465

MANSNALQKAIDLVTKATEEDKNKNYEEALRLYEHSVEYFLHAIKYEASSDKAKESIRAK

CVQYLDRAEKLKTYLKKKDKKKPVADGEKPNSSSGGGGKSGDKDSDSDDDSKEDPDKKKF

ADQLSGAIVVETPNVRWDDVAGLNGAKEALKEAVILPVKFPHLFTGKRKPWRGILLYGPP

GTGKSYLAKAVATEANNSTFFSVSSADLVSKWLGESEKLVKTLFQLARENQPTIIFIDEV

DALCGSRSENESESARRIKTEFLVQMQGVGNDNNGVLVLGATNIPWTLDAAIRRRFEKRI

YIPLPEAPARTEMFKLHLGSTPHSLTEHDLRELGQRTDGYSGADIGVVVRDALMQPVRKV

QTATHFRRVRGPSRADPSVIVDDLLTPCSPGAPGAVEMSWMDVPGEKLCEPVVTQSDMLM

ALSTQKASVNEEDLQKLVKFTEDFGQEG

>SG-AL_contig_6863_1_+1_1_219

KRLLQRWCVPEGEGVIRRGKCSGCALRGGPCSGIACSGSSGSRDVCPGSSGFGNARTGGG

VGRRWNERQPTRG

>SG-AL_contig_6864_1_+2_2_193

TTRGAGSSLSSTLSASPLVGGLVSEGPVPLTSLPVPRASLGTRERRTYALRAGSWQRGNP

SPAI

>SG-AL_contig_6865_1_-1_1_138

HFSQKLISFSRSEKPIPVLRFEVPIRQTVRADDNRRHADFVMSLQE

>SG-AL_contig_6866_1_-3_288_548

MIKVLSKAMSVSICHVSKSSLIYCLLFVYLACLLIYFFVEYAGGILLHLIAGYPELLVFI

MCQNQLCACMCTSVFPCKCIERKVFEI

>SG-AL_contig_6867_1_-1_784_3477

MSTTETQPSANPLYDLMDDDMNQQEVDSSANPLYDLSTDNTTGDLTFETNVTTTTTTTTE

ESSANPLYNLMDQDQESVEVTKTETTASSDLLGMDFGADVSMEKSTPSEDIVVQANNNYD

DQVVPDIAAQQEAGPRDVAEEEEEAGEAFDQNVELFIREEGEEQAVSLEPEGAVEVDVGQ

QQQQQQQEEVMEEVEEEQKEQLERPRSRSRSPSRHSISSEGEVLNEEEDMRQDERAVFLS

QQEEQRKYDEEKRSEEQAPQVSDPTDDYGADDGNVAREQQDKEDFHIQETSSVKDQFEQA

PQETAQQQQQPIKEPAISEPQWEDEPEEGVYENEPEQRPDVVRETDRNIGAELPSVGTAK

SMRDKFVAGQVENTSKDMKREVTPPAVVDGGVFENEPQYNADVVHAGDQNENEVLPEEGT

ARNLAARFKEMEKQGAVPVPSPGKREITPDRTGKVEYVSEPRGHVDKYEPEVQAGVFESQ

PAERDDVVKSDQLAEEVLPEKGSAKNIAARFKQLSTDTTSSTPRGKRELTPDTSGRVEYV

SEPTGFVEKYEGCTESGVFESQPADREDVVKSDDTNEDVLPEQGTAKNIAARFREMEKTT

KSPPSPSKVKEFTPPRENGASPPSAGVYESTPKVAADVVHSDEQQEEVLPEKGTAKSLAN

RFRQLSSSDSTPVKSPRSKKEFTPPPGDAGVYENTPKQFIPDYNQPAESGILESKPESRD

DVVRSEDPAQSEQELPERGYTKNLLSAWRQKESESSKVSPPSSGKPKEFTPPREEPRVAQ

QRPAPRTPKSPLAGTDMNDGSVHPTDLPGQYQPQAEASVFESQPERREDVVREDDTDWNQ

GMPKADTTKKMLAKFKHIQQQANSNSSTPAKPKPTARKEGDDRNSSETTPDQVGRSKS

>SG-AL_contig_6868_1_-1_331_474

MYHSSFLLSLTYGCFVRSFVILLCRCAVVLCSQDCFFWFFLILFICLF

>SG-AL_contig_6869_1_-1_2164_2595

MYEMLVSNLKRKGLSSLMIYFFFLYMCKNIMKQWILCVLLFTCHLIFFSIDENLFHVGVG

FSVYKADCYMCKVFSMQRFLLWNQQVRLFRTWVFTQIAYGAIAAHQCQRWLKKKKFYNYI

VSRSLYSLCMPHITKKLYIFCAGT

>SG-AL_contig_6870_1_+2_821_943

MEMNLKRHKACVHVEYTVLLKHHLQTVVSQISSSPISDLLT

>SG-AL_contig_6871_1_-2_938_1078

MLCVCICYYFLCMCVMSLLHFLLLYVICLLYMSAYRVVVVCCLRSPA

>SG-AL_contig_6872_1_-3_33_221

MLCNYYTTGAVSWMVRCWILLIEIFFKKILNSYSFNLLSGPKSRTAPCSYSIQDCHLTFY

NNF

>SG-AL_contig_6873_1_+1_1_294

IFSNAIFTQTFPIREPFLLFNHQIHLLHTSQTINQIAHNQIITQCYAVTSVNQGKPSVIL

NHTTTVGKKVTKTSLSNFRQAASFFYTFLFSISFLFKV

>SG-AL_contig_6874_1_-2_182_472

MPPVTLWCITSASACINQISAMVGASVGERDFLGHFLWLCCVREDLRSHDVMLVIHRKLW

RDVFRKAQLSKSGDGSGLKSLQKWAIPISEFGLAFEK

>SG-AL_contig_6875_1_-3_4620_4994

MGTVKGTKGGMVVRGRAGWLEKQPEQWLISTSVNKISHLSMGGGNVRPGPQGLHLGEGSD

IAYGPNKAKVQSKTRDQQEIIHLCIQHIQQQTYCYCIPMCTDNHNVVRASIIYILANSST

LHMVI

>SG-AL_contig_6876_1_-1_991_1251

MISNLKIMYFVCVCVCMCALTNLHRYCTQKVNSYFSWCAVATVYIMRLLLVFFRLITCTT

VFTGKLFFQHIFALSESCPFFHFKVVC

>SG-AL_contig_6877_1_-2_350_538

MYLQFLSGSRICIFTVTAAFILLSLSTAPQPFLSYARSCVCYFIHLNVWSVKWFSFFQPD

LSV

>SG-AL_contig_6878_1_-2_2612_2944

MFKVGGRGTGYLINNSTSVVQNINDFAISIMEKKKRRRKRGNRHASATMHDNGNAREFTF

EIHVLSRVKGPFPTPIYPEREQLLQKQVWFHFFNTVNCRFPCTTSKTMLEV

>SG-AL_contig_6879_1_-1_121_339

MCCLGGARHVSYIPDCDVTVLQSDLLLRSSAFPLFVCLFVLLLFFPFSFKSKRKAVKQIG

NTLQIIRCENKVG

>SG-AL_contig_6880_1_+2_2_478

HQGITKCRERAKTTVWWPRISTEIKDLVSSCASCLQKQPSQRREPLKPATPPERPFQRVG

TDILLHCGTHYLVLVDCHSRYIEIAQLANLTSCHVISKLKTLFARHGIPETVISDNGTQF

TSSEFQNFAQAWNFTHVTSSPHYPQSNGQAESAVKIAKS

>SG-AL_contig_6881_1_-1_1_159

CGSHVIAYQRDSYVIAYQCGSRVITVGQQLEVRKTIELKAATVGERYNSSRAC

>SG-AL_contig_6882_1_-3_3_167

FCYPFSLSIVNLCIRYIGIFCNFGGCLCSCFIFYSSLSLFVFLNTVTLYIMALSI

>SG-AL_contig_6883_1_+3_465_611

MFYQNYQCVLTCIIKLGLKMSDCLICRHSLIHIWLLHRQAVPFKFSQSN

>SG-AL_contig_6884_1_+2_2_118

VLSIMSYSGSIITFGRHPGRQDTEHDDTDSDQKETSFIQ

>SG-AL_contig_6885_1_+1_193_423

MPIFLFLKKKVNESHDFCLFSSMQKVFCTKCIQSVRQFNAHPQPPRAFYPQCEPDFECSI

LDICLCNSSINLTGQHL

>SG-AL_contig_6886_1_-3_615_950

MGFFGPDQLFPRVEHTMGSLGKLGPHSQGSSTLRLNMSLKVLLKLPDQHHKCLYLSGLVY

ARIYYERKHSVQPCHRVLVQKGIPQQHYHHPSSHHSIVTNIFPDSVALHALL

>SG-AL_contig_6887_1_+1_19_213

MPFLRSPLSCPYSHCMLAISIFWGNIWTNKKSTTQVLTKSGPFCFSFGFLTKLWSKVNCM

VNAPK

>SG-AL_contig_6888_1_-1_1_432

SSSSSSTSSWLPGIIASICIGVVASAAIVGLIIHYRITHKSTNYVRAPGNEVTERRGDAT

TGSGDIQSGSTVINIELQHALPIQVSSEQVTAEDISVKQQTTSVEAVVDIEDTQPKTVSM

AIAECDPVAPSSAAAWHVHRQQSS

>SG-AL_contig_6889_1_-2_224_460

MDSIYYPCITSHHNNCIYLYRSVLAFSIMNYFLKMIRKKEEKKNEARERERERERERERE

NDNDNDNDNDTLFSNGRSY

>SG-AL_contig_6890_1_+1_1_294

SLCLCVSLSLSLSLCLSVPFSVSLSLSLSLSFVIFFSLSVAQSLSVSVSVCLSVALSLFV

SVSLSVSLSASLSLSLSLSLSLSLSLSLSLSLSLSSLF

>SG-AL_contig_6891_1_-1_7_4284

MAASGIEGMMREVVVAQSVTPPWKEIVVNTGASASTLQDIKVPDSCGGFTYRESSCPGSP

VRNRFIYWRTWGDVLELEESSLDMMLTGGKIRYRLQDTPIVGGVSISETNSHVIVLVATV

ASIHRLVFPHPCRRQQTEAFSLSEHISTSVFFDASLTTATDSKNLHLLNPGGSITAHLVS

AASCVSGDGEALFALGTNAGTILLLRMPPVGITGVVIQHELSHSSVMQKIWSGLVPGAIR

GHQQASDTPISVEIQPLRGETYIFALCRDLRLRVWATRTRECVLTVNLQDMLGGASRLQP

SSAGHVIKQVQSSTMAALTLCVYLSFPDKSQFVLLEPVVCDGRLSMLQHALLTQPAEDLI

DFCVTADQLITLWTKSDGETIARAAVLTEGGPTGEWRDVLLEHPDTSEPLIPPNRDPRDF

FLNLIFRPNHFSHHDIIRALTVYRRSHDSTQNADALLMSINIREEVINAVSLEIRNCASE

YEMQEEAYDNLQREQWGRFYSCCCQYREVGAKLQGVFTDTATGLVCLIRKATLTYLRPCD

QLERLYLCEQERITPKDLDDLSYNVEGMSQYALCEDLRKLCDIVQLVRSEITEEVVTQFD

LELQMMESPEELAERLVDTVFFEDVDVPSEVGAQIDVRLQTMHHLTACLELMQLLMDVLE

MQQQQQSMMDDSVGDAARQLSVSHLFTSPEACAILGQAFEQMCVTRMSFLRDLIVVQILA

LRLGQKRSVSPEMCQTIQEELLPNFSQLLRSYMLLYWSSQALTSPSPTSSLDSNMRRLAS

LELSDSGGFTATRQTTSDTTVLESFIQAVGGTQARQQIAMTTEQDFQATWSHDLMAFVHS

LASLIWPLSDETVFPEFLAGSCQYSALQEYVRLLHPWCVWNSSATMFFLGLSYLYFDEPH

KAQQCFVSASDGVATETFLAQKLLQGHSGNHRQLTLDYYLKVIVQFQEYGMPDMVIDLAN

RALCLADLEDPNVATLQSKVFKYQLELGHHQEAYTAMMDNPDPARRHDCLRQFVMVLCEK

GELEDLVHFPYRDLEDEVVAVLERRARSVDLATYNYYNVLYSFHIYRNDFRKAASVMYEH

GVRLGREMGGVRALQRQAQSYLVAMNVLRLVQPEYAWIVHPQLQSLEVSVTDKESGGDSP

SKHTVDGVEKQPAKGKKKVQIVELRQLEKDYLLLDARLRLIRKNPDPALASGPTPAADEM

VGILVNNGLYDMAVIVCQAFGLQLFPVFTSLALRCVTLARCSSYFMLGDNKETSEAWDWL

RENDIRSTTQSTDNSATDHVWKLLQRYLESFEECDARYHRCVCEKLLSQAFPLPAWLVNS

YKVWSTHQLLRVYLDYDLLQPAAMLLMEYLDALMGVLQGQDAPVFGLKGILDHRPMSVWV

PYTCADQLLGALHGHQEDPMFTQLHGQLTEKISTYHHKVSQLSDES

>SG-AL_contig_6892_1_-2_32_1237

MGVEDTAKKSFFSSKPQLRNRSTVFTLGNRGNVLTSDLEAPVIVPHAAAKNETRYSFETL

FRSLHFALMDNCCREYLFICDFFIVSGTGAQSLFDAIFSKTLSMFLKQMEEYTSDCYDSI

AVFLSVHIVFRYRAIMAKRTIPALDRYWEALLNMMWPRFEYILHLNVQSVHDCEPQKLGH

IDCRPHYITRRYAEFSAAIVGVDQTFPSERVGRVLGQLQAEVENFVLKMAAQFPNRREQL

IFLINNYDMMLNVLVERTSEDSKEAEVFRDLLTARTQEFVEEVLVPHFGGMITFVKDCEN

LMDKGQLDSVTGQEARVQQIVRGFNNDWKRALEVINQDVMRAFTNFKNGTQILQGALTQL

IQYYHRFQKVLSQGPFRAMQIRNELINIHHVMVEVKRYKPTF

>SG-AL_contig_6893_1_+1_673_972

MSYQILLCRFYISCSTVKTTYLQGQVTSNCDVSSSIPYALAKAFLYLVTKGWLNSNSTIF

KCRITSSNSCVSSKLYRRSTSQISDIRRLATLQYNFPTVH

>SG-AL_contig_6894_1_+2_734_862

MVLCGSERVIRPFITIPDDSQIKKCILPHVTGLTTHSAHYESL

>SG-AL_contig_6895_1_+3_96_188

MANHIQDQPSSSLINTHTHTHKLHQKHMKIE

>SG-AL_contig_6896_1_-2_62_247

MARKICSGLHQSLCHSVCAYPSRKKLPPFGYLFGHLIICKYVGYQEPGKIIFKDLILFFL

YQ

>SG-AL_contig_6897_1_+1_403_708

MKSRTYFPCHGCLKSLISHVRKQQHPLLHLNRPALLFQALWVSSTFALFSLTPFWISKIC

TSSVALNKSYSCLCKACCTWSKGSNCCTNTPTFISQLPFFRN

>SG-AL_contig_6898_1_-3_6_896

MADDLGDEWWQDSYDSVGDSAGEESGEEKPQTQKRGMKRPAKDEVEADCSDKSDQKVPAK

KSKSAPTKQRKHGKKKKKKQFVRKKITDLDEDTLKKGGIPADIQALLRPSIVVAKHDSSP

SGYDDNDSDDATLSADSDFFPVHEEQPVGDYLTDVLPGWQLAVKKAKLPPGSPLMLILCS

SAIRAVQLNRELKSFLGKDCRTARLFAKHMKLEEQKSFLQKKVCHASIGTPNRVQALLEA

GCLKLSQLHSIVLDWNWRDIKLKRMVDIPDVRRDLTVLLKEHAMDHIKHSPCRIAIL

>SG-AL_contig_6899_1_-3_1074_1259

MDNDSAMSAFFGDFFFTPSFLFKRENDSDLFKACVYLLTKLCPVAFVPIVEFENADWFLI

LP

>SG-AL_contig_6900_1_+2_2_148

IQRLEVGRPLKCERGFMERNVLAVKKKGLRKCQIKKLQNPGGKINEGIL

>SG-AL_contig_6901_1_-1_1_102

LVPPDVRSLCNLIQQDTFHLCLPSPTPRRFHSGL

>SG-AL_contig_6902_1_-3_3_206

RLERSSGLRLPPFPILQRVIRKARLEKATLILIAPKWPSQPWYPDLSLSVPCSTSGTFPR

PDLLLQPR

>SG-AL_contig_6903_1_-2_212_358

MYAFACIQSFLNLQVYTHAYVHICIHTHTHTLTHTHARTRRLTFNRATD

>SG-AL_contig_6904_1_+3_30_320

MAFHKQRQIPMRRAAKWEYTCPFQSLSVRMSVTDPQSLEKVFMNSECLLANVSPTKMGNI

TEASPYTLTGVADILILPQEMASSGRAPESQIGRAHV

>SG-AL_contig_6905_1_-3_231_362

MLYLFHYLLSLSIHSEGLGLNIMLCQCQLFKNLFHFDALGLPNS

>SG-AL_contig_6906_1_-1_1_213

CPLPWQHVSLSQRSNHNPGNEAVDQQAKNATRKERAKTRSVQILNLRLLPISNPFGKKNG

LVNRTNCSKSV

>SG-AL_contig_6907_1_-3_132_398

MGPVARPLSLRGSVQHLGCPHATLAGSPDISAGPATPKTLVGFRSSAEGHDGVTAGRRRL

QTEERREQRYSPGPGMVTQALFFQLPRVR

>SG-AL_contig_6908_1_-1_97_1092

MSEPVKVCITGAAGQIAYSLLFSVAKGDVFGENQPIIVTLLDIPPMEKVLNGVVMELMDC

AMPLLKKVIPTTDEKVAFQDIDAAFLVGAMPRTAGMERKDLLEANVKIFKSQGAALDSYA

KKTVKVVVVGNPANTNALICSKYAPTIPTENFSCLTRLDQNRAQAQIATRLGVANSDVCN

LIIWGNHSNTQFPDVRHATAIVNGKTMTVSEAVKDDSYLKNEFVKTVQTRGAEVIKARKL

SSAMSAAKATADHMRDWWFGTKEDQWVSMGVISKGLYGIPEGIIYSYPVCIKNKQWEVVE

GLDINDFAREKMEITAKELCMERDTAIEFCSH

>SG-AL_contig_6909_1_-2_2_1168

VTEEQQATEEPAAEQDTTEVQKDTTEQAATEEKPESTETQAATEEKPESTETQAATEEQP

ESTEAQAATEEQLESTEAQAATEEQLESTETQAATEEQPESTEEPSAPVEQSQHEDESAP

AEEKTTSADTSSEQPEPPLSTGEPSPNEETDAAETPASVEETSPPAEDQSAEEKHACQES

QSPHGEQQVPVEEQPLTEPQAIADEEKTPTAEDTSLTETPVPTEEVASAVDQSSAQEKPE

TAEQPTSEEKAETEQPTHTGEESAPAVAEPTTADEQELGEQSEDQTTPAEQAVADEQSES

PQQTETPKEQTSTEQAVPTEEQASTEELPVPAKEHPTEEPESASEPEQTTVLSPTVMVEA

TADETAVTADSADTSAAADEPSEPASPSQ

>SG-AL_contig_6910_1_-3_117_329

MIVLDRLVCRVLCPNHFSLRLFTVVRLEASIRFQRRSNVPTSLSTKELRSVCILSLGLIF

FSLLQTLSGHF

>SG-AL_contig_6911_1_+3_486_1022

MVKELFAEANIPRASISACPCNSTNHTKYFGFNSNINRTHKHHPMNFHKSYKTFGSDLNI

NRRIGSCTFNFQQTYTQQQLSLDNGNKIFCSKSNPNINRPILQAAVPLKLPQTVHSHSSG

YLAIRSIHCTVMWHEQEGGGEEHRKEQGFRSTQHREKGVRCRRGGEKCQKRKQKYKKIL

>SG-AL_contig_6912_1_-2_197_364

MHLQNLYLLLQHELYIHSHVVYVDFLVLHCVASCVQLCAHTFVFARERARERAWVT

>SG-AL_contig_6913_1_-2_2_202

FFFFYLFAVIIIEPAYSMDVVYIMIGLEQKLLLLFLCLVMYVFKSLTVPMIQVRFLLMSA

FRECLQV

>SG-AL_contig_6914_1_+2_233_391

MIGSHPLTMSDHTQQWSDSKLFTMLGPNWLKQQFDSDPQTSIHVDCSTCRSAT

>SG-AL_contig_6915_1_-1_169_312

MFLLVWVFHLQPFVFFLKPCGHYDCFKNNFDQCISVSSLVSGLELKVI

>SG-AL_contig_6916_1_-1_1_87

NVNLCQNSYIINFSPFLMGKAQVCECCQN

>SG-AL_contig_6917_1_+1_145_297

MKMASSVTYQAWDSKILKKEESKGRGLGAAKGPGEGLGAKHFCFLTYKNLP

>SG-AL_contig_6918_1_+3_189_320

MLQAGIKLKTIVLKSKALSKEPTKPHKIGGPYGVRTHVLREHRV

>SG-AL_contig_6919_1_-3_558_683

MAWVIIVDIITFLFVSVVFILFISQSCLFYFICFSVFRIHSL

>SG-AL_contig_6920_1_+1_856_1185

MCVVKTCTHLYFAHLYSFRLVVPIVPMQRLQKNMCLQDWSSIPHEELHTALKTSIPIRPS

DGQERVHQWFQILIFISCLLLSVVTCLEFNNVLVLQSATSMLLIWTICTI

>SG-AL_contig_6922_1_-2_131_952

MKGFGKVSPPVSPTSSMASSSFLDSRNGDSKKNDARLTANAKRQKYFRRLFKFRQMDFEY

AFWQMMYLFYSPQKVYRNFQYRKQTKDQWARDDPAFLVLLSFWLCVSSLGFSLVLGLGVV

EFFKFLLWVVFVDCIGVGLMIATLLWFVTNKYLLVNPPRGQDVEWAYAFDVHLNAFFPLL

MILHFFQLPLLNHFIRPDYVMGCMFGNTLWLIAIGYYIYITFLGYSALPFLKNTRTMLFP

MMGVLLIYVLSLVFRLNFTSHLCNFYAYRVIRHI

>SG-AL_contig_6923_1_-1_1483_1743

MLNTRCTVRDHEIVHNLMKHSSFVLLFQCLGSVHVCVCVCVYVKMTYPPDVEECLQSIIM

NCIENIEVLEFFLGGGLFFCLFLLSKL

>SG-AL_contig_6924_1_+1_658_1017

MAAIGEHVSRVRALYKGILKLHRGLPLQMQALGDQYVKEEFRRHKEANKEEVDTFMLEWT

KYYVTLAKQLGPRRKFRTLGENLSGDLLNNFNSEQIGQLKELWEATTIPPSQASSSKEKR

>SG-AL_contig_6925_1_-3_300_2273

MCTNTAGSYVCTCFSGFLPGSDGICVDFDECADPEVCRGTAVCDNLPGSYRCFCPTGYHY

VIDDCQDENECLTGVCGENTYCNNTAGSYACLCLEGYHFTNGTCVDIDECAADPCPGVLR

CMNGEGTYFCSCPQGYRRQGDTCVDVDECSDGDNACDSNADCSNVNGTYFCQCHDGYTGN

GFVCFDINECRISNGQCNQECVNLPGSFQCQCETGYQLLPDNVTCVDTDECSQDNGGCQH

ECHNDPGSFRCQCATGFQLHADSHTCVDVNECQENDGDQCEYSCTNLEGSFECSCPSHLV

LAPDGLHCEAISSCQQENGGCDHVCVNLPGGGFRCDCYSGYQLTSDSVHCEDFDECQELN

GGCEQECHNTDGSYRCACREGYILRRDRNSQDVCEAVCDPPCRNNGRCVSPNQCVCPPGY

PGPQCSPICQPPCSHGGTCMRHNYCNCVQGWSGRGCKEAVCEPQCRNGGRCFAPNTCQCT

GGYAGPICQTATCYPPCQNGGFCVRPNVCACRNHHTGPHCERRLPSHPEYSLCDPPCLNG

GQCLSYNRCRCPPGYRGRRCGRSFRPDCRPPCSNGGTCLHGNRCQCPRGTSGYRCQNNVC

RLVTYTQSIRRGMRRPVAESYTVPCGRFGWSSCTAYRMLQRFVYVTSFKVGYRRTCDP

>SG-AL_contig_6926_1_-3_117_944

MATSVLSLSLWLACVALCIDVASPFPSLFGLLTNPFASQDNKVYLFAVSGRRKPFQFVND

AGEIEGFNVDLIKEVCGVARKRCEMVLTPFTECTFTERNINYPGRGLMAGWFDVCPGYAI

SPDRKSAFDFTLPFLSSSATFAVSPHNPSGFDPDAEDFSAFTITHLTGAYTNAACLNRLQ

KKFGNIIIASNLPEAKALLLNGTAQVLFSPRSSIPGLKVLPQKVSCAEGGTGMMVKKGST

LPYWWNRAFRAYHASGRFQTLCEQEGLKYNRTINCV

>SG-AL_contig_6927_1_-1_331_516

MQLVKCVDCTNNWVYIKKVYKVHLVVCVCLKYSHLGVFKYFCSFCSKSYSELYSDSHIFL

LI

>SG-AL_contig_6928_1_-1_49_477

MYLYLSLASVLSRCRLLLEVDVPFLHCPLSCALCMQPAKLSPVQSLMSSVHFFLGRPRVL

FPSAIPTRIALGSAWCLIIWPKYERFFYLILRRSSCCTLSISRTATFVTLSDHLILPNCL

NIHISNASILSSMSLCTVQVSAP

>SG-AL_contig_6929_1_+1_136_252

MTKFISRCLNSLFICTTIQEKIEMICMNNIYNLQGKDKK

>SG-AL_contig_6930_1_+2_377_748

MTKEDKHYGIKFCGIKHTGIPDTELPFPDISFKSAKGLMHTETVCVRTYVYMYVHGCVYK

IILILIGEAYFGFVLQERAGAGMGSITGEDVSARDSVVRMNRISPHEIPHSLYGFGIKVS

GILN

>SG-AL_contig_6931_1_+2_2_166

CRGLGTAQNTTLHTSSCRCIAHKQTDQWHRSSETVITSKIMCCWSLYLCLVGVSN

>SG-AL_contig_6932_1_-1_229_390

MYHLQTAHLARRGTTHNMKKVVRSTGTVCDLVFIVSVHFLYVGHIACFIVSISE

>SG-AL_contig_6933_1_-2_368_622

MILSFCSDSKPREKRKTSNRNVYCYKIPSELPFSQCDLSFGVFGGVLESRNEWCSGSETQ

VVQKMRQQKTHNRQIKKDTQGKKAF

>SG-AL_contig_6934_1_+3_1572_1793

MCSWLFVSENNSNIIQNLQTAQKQLLCHTLNFLIGKEKLFYNTENGHVHMNFYDLWFLCY

SVLGSPLGMPEALS

>SG-AL_contig_6935_1_-1_1_117

YKCPVIIIVSLACNVDQRNSKYCYFQRYTKYEHSHSFGD

>SG-AL_contig_6936_1_+1_1513_1713

MSMECLFKLDVKHLVINGRTGVLINMQIYYVQEKEKQHFKCTLFHLQLFNTELVMYSSNN

NFSFTHR

>SG-AL_contig_6937_1_-3_42_254

MMRTRMKTTRMKWWATTSMSAACFPARCALICVLTCPRVSAVTVRLASTSRMTNAPAPSW

TIERTRWTAAA

>SG-AL_contig_6938_1_-3_42_185

MKTRILCRADLRVTVHIKFAIFCHSRILRRLDFCVCIKFLFCHKPVAM

>SG-AL_contig_6939_1_-1_130_303

MISFCSFVYFVWGFLFSLSLTNTQALAHTCTHSLTHSLTHSLTHSLTHTSHIHTHSLT

>SG-AL_contig_6940_1_-2_917_1135

MDRWVFCPSVDGYLLEYGITNKKNNIQMKLHLSCKPPLLNFFPQVGTFISLDEITVNYEN

SLWILTLNSKHEL

>SG-AL_contig_6941_1_-2_314_2347

MPLYCIVCVDTAGGFVTAVAQRPQASHCCLLRLRLKKERHSETSTSPTPQRDIVLHSCQA

DHRTIASGCSDQYIAAVQGHELSVYSCKKKTCRKLTTDAKNPFTCVACHPTDCCIVTGHA

NGKITFWWDFSGKEKTVISSNHWHSLPVQCLAFSPEGSFLLSGGHECVLVRWKMDSEYKD

FLPRLGAPIYSIASCPLGTLFITSHTDNVLQVISSNMDVMHAVRGLARAHLGMNTSGQVP

VGLLCDPRSRALVTNGRPGHLQFYCMDTDTQLYNLDIVGHNYISPENLKKPAVVTEVEQA

AFDSTGEWLATFQKWEDGDLIPMYCLKFWNYNKKQQNYHLNTTVELPHYKVVRCLQFRPQ

GTPETSESYKPAMVSAGDDGFFKLWSLTTENLPGKTVKRWTCDAVGHYRQKPARIARFSH

DGTSLAVAFDSLLTFWDPDTNVMAERLSFGDQPILCMEFGGKTSTNCLVLTTSSHLTVLD

LASYSVSMQISLPSAILTTDGDLMCAVSAQQDLYVFRPSEEKPVFYRKKVMENPVVSAIF

LPQSTMEEEEEVEVSWQSQSHLYLMDNRQRLFTPRLQAALETQTPSSRAQILLQQVGPPK

TALQVLLSSGHSQHESVSTTPTAADRNYTPQAILDVMNTPLNTMSCFHFVSQHFMDSLLT

KRKGLEEPMEVDQDDEGE

>SG-AL_contig_6942_1_-2_209_1501

MATIAGCLSPSGFKRSFLFCMIVLIVIYSASQTVETSGETNRKGHLQPFGESGTRKEIEV

RSEFPDPYDFLVNYMQQLKPLKLSGVAQHSRAVREWTDDYLLQLNVPQDSVVQLETKKKE

DRNQETTEMHFHEFLKTYNHTEHYMVDDVPPYLRPDVMVPCSLQCPELMEEGLAFAMMWF

SSGGTKSVIHTDSFDNINCLLRGEKTFVMMDPARDREKIKIKTEGAYSDIDVDSVDLSVY

PELADVEFYHVHVRAGDCLYIPYQWIHQVRSFNSNVAVNFWWNHYASMGLLEEKQGSACQ

GQCDPDLTLDKVTLHHEDPTFDDHNSVRDWLRNVILNDLFTPEAALKQIRMEFGVEDLPP

PAERLTIEMFEKLDLNRDQQVTKEEHAQISEETWKEVQRDIATLAVILDDGEDAERREYA

GYVDAERKDEL

>SG-AL_contig_6943_1_-2_2_94

LFVCFFGAVISKYEDNFYLLNFWLLCLLLLT

>SG-AL_contig_6944_1_-3_324_758

MLSFGLSFVSLPVFLCRLLNYCMFVLWWCFFVFCFVLFVFFRGRGLFVVLLLSVDMCVCL

CGCFRLSFGLSSCVVVSGSLFGLVVFVDAVFGAVILFVFMQSFGLLVPLLLHFIMGSGVG

SNSSRESTSLLFQIMVKKTQRTWQG

>SG-AL_contig_6945_1_+1_40_153

MILTRVCQFANVLTSPEVFIRTSGGLCGWPCLRGDHMM

>SG-AL_contig_6946_1_-1_103_591

MYFAKARKQMDEQDMREVQEKLEKYQRSDTARVQLQQLKISRAAEEGSGSRRSSRGDFRP

DELPDGAFVSPKHTPSQVSPAKIDSVTVSRSAEYIGSFSVTGADEETRTRQVETQLESLR

SVTKSKKVVLIISLDGIKVCSEDGQSVYMFHELKRISYATCDP

>SG-AL_contig_6947_1_-3_186_3953

MASPVILIIVCSLARFVLSQKPEFLSTKSQVTAVKGTTAVIPCSIRNIGNHKVLWSFERV

LLTFDTRRITDDSRITVNRPYQSEWNLHIVDVTKEDAGTYLCQVNTVPMMSYNVRLTVLD

EAPSPVMPEEVVALAGSDVEIKCKLPDLNDIGVWGYKADDRSDRRIISENNRFTFRVGQG

QDNHVHTLVISDVRAGDAGQYFCRARSGTEGKTSLRVGVPPPPPSLEAKAESPNRLMVRW

RKPTSRNTDVAGYILYVQNHETGKEHVFEVPPNQKLSGEESFRVGDLSTKTTYTVRLATR

FQLGDQVTLGKRSPEVTITTPLFVPEAPGWVKVRRISDGEAEVQWSPPSPSKTSGIVRGY

KVYYTPLTSRKKPIVNQQKIVTVEKPEGNRWKVRLTGLRPKTRYHVQVGGFTHKGDGRRS

KTTTYLHKALPPELSLNGRVLSDNSVLLTVTSLGSGRMANVKMFYTEATQPSWKRLDFSP

QWKFTVVGGLDPRTSYFFKVKAKIGGKLKQCVTHITTPPHDLQAPQGVQVSATASTSIEV

TWDYVIANNKTSVPLGYIVLLEETSGKDDEEKRHRQSVVFNRTSVEVKDLTADMRYNIQV

TAFNGFGEGPRTDGLLFHIAESDVKPDTVSEPFPPRFTHELPKRLTVQSGDAVKITCVAD

GLPVPEVQWYSNGEPIGVSAQGSNQLFLANVFTSTILTCRAKSQWGEIEANTMVDVARVQ

GGQGKLIIKLMSDNVKMSTFTLAWNLQSGDPKSIDRFEVVISDKTDRVLLTQTLPGPTRS

FHLRGLQPGTEYRAVLRAFGAQGNLPLAEARATATTLRTDGSAAVATTPSAPVTPLESRY

RGQIALSIMDVKPSSAVLEWDFSGLDPRHVQLFELKVKSRSNFTLLTESLKPNLNVMDLS

LQPGQIYYVQVRAFDRRGRVLALNTTRVETPTQRAPPATTPSVINTDNPVLKRASLALTS

HAVTGDSARLDWKLSGAQQADIGQFILKIKDSDDRTLLRQEMTREARSLTLSNLTPRTAY

FVELQALDDKGAVVAATTGSLTTSSQTAEESDPDPSKLRVFTEELDKNSVKVAWSTPAKL

LAELSGVTVKMTDIANVTLLTQQMSPLTRSLTVRNLQPGERYKAYVELKGARGDTVRSGY

LEFDTEPTHGMPGRPQVTNIEVVNSTAVHLNWRNTPSPSPAARSEDITGYVVNFVRVDQN

NKPTAGTYQLPVFGSTQFVVIPNLRGGQRYMFQVAARNQYSFGPLSVPVTADLPRQ

>SG-AL_contig_6948_1_-2_29_1204

MTVAMEVEMSSCSRCSRCHRLVFDEETMSGWSADDSNLNTTCPFCHAKFVPNMQVYVKED

VEGKLSPEMISSMHLDDLPLSLQEAAAAVRRRCTSECLTNTTDHSALGGGYYNCYPGSLD

VSRSPLSLSIDEDVELPTSPAAVPEMKKDFMTRSSCTAEPLVFPYLSPLVLRKELEYVLD

HEGDLCLASKSFLDQHPIIFWNLVWYFRRLEIPSHLSSFLLTAATFNSQEASAKGAGQFD

SRNILIRPMWDNVHIHDEMGLPMYMAWDAGNKSTVVDALVTESESFSRPCMFQIISSIQR

NDVLTAIKLVAQGRRRLMGRRRRFRSLYREVLFLSFVACGRENIDHDAFDREFTLAFDEK

LSPAEVRRLQADDKPRSANIQWCRRVFCELEI

>SG-AL_contig_6949_1_+3_3_137

HNQANLTFSLSKELMYISFITATLKSYIIAKTYIVLCCLKHTAKI

>SG-AL_contig_6950_1_+1_259_612

MWYIYSDLHVMSRVCVCACVCVWYIYTMSRLCVCGISTCDVTSMCVCGISTCDVTSVCVC

GISTCEVTSVCVCVCAHVRVHVCACVVYLHVMSCVCVCLHACVCVVYLHAMSSVCVCM

>SG-AL_contig_6951_1_-3_51_683

MSEAYDFLFKFLVIGSAGAGKSCILHQFIENKFKQDSNHTIGVEFGSKVVNVGGKAVKLQ

IWDTAGQERFRSVTRSYYRGAAGALLVYDSTSRETYNALTNWLTDARTLASPNIVIILVG

NKKDLEAEREVTFLEASRFAQENDLMFLETSALTGENVEETFLKCARSILTKIESGELDP

ERMGSGIQYGDASLRRLQRQQQTLKRPDCAC

>SG-AL_contig_6952_1_-2_23_1150

MADDSTSEWLRECSVCYSRGLEKNFITCEGCGVMVYCGKACQQKDWNSENTATSSSSHQV

LCGRMKRYADREQELCQLPFSFAKETTDRTFCEQRLVEFLQRHIVYGLGLWRRESPQLHK

TLPFRKCGYFSHKNEDKWVLAEESSLLTLCPDKTVPSPSAPLLDWQQYYSFRGLPFHSPV

AAVLNYPLTLYFIMTTCLRNDEPELFRRLVSGEMFIIHILGVEKEVEMKEAFLECARLLP

GCQLEMVLIGKLLSKSVDGYHWQKDNLRFSVHRALYHKTQLPPPHLAVGFNAGLPAYPTW

PPTLSKLMDQRTPAYFTDQSEYSCDCVSSAISGIGIPGVSPVLVNPFRSPLRIVTREQNM

PWFPNAFLYRLKYEAT

>SG-AL_contig_6953_1_-1_49_1647

MTFQPAPYPDDVEGDFGPYSCDEIVVARTSLLHTVQRENNSLWQSIKYKLGILPNGYRLS

TFDPDHVWLPHHVPDIWSAPVKLHDPHRDRANDCNPEGSQGMEYKVKSRLGYGGADRKGD

QVPWGSGALATDAESSWPSDQGDGDSQDSGRSPNKRRRKDSKDDGTPSHFQGDHMQNQPS

DLVAKKSQGSASSSTSVASASHSHTSVDGSSTGQGSSADVKGAGGGDHLYEVVYQCDFCP

HDSQDETDMQHHLSQNGHYSASRFLAQRVGAKGKLRHVYLSVAGLMTDPAYQFQGQVAAC

PTCHSTFRDLRSCLDHSLSNHKRYGQYSFRHVDSSHTFQMQAHSDLCTECHQTFEAKGEG

QGLHAHMGKTGHMALYDGQKTEDPMLVFLCPCCPLTHANYFEYKEHVFSKHAAQTETQGV

VTAACIKLGRSQPARTMPPRDPGIPMENILYDFSKAPLPGDPGFRGVMTKSKLRRLTRKL

RRKTEQLATHTSGQQQSRKKKKKKKDKDNRKERLTAFKEKMHATGTSKKKKNK

>SG-AL_contig_6954_1_-1_76_1905

MGRRSINTTKSGKYMNPTDQFRKEARKRELKKNKKQRMMVRQAVLKGKDPMKLLEEMEAI

DRMEYNPLEAPSLNEKVLRDKRKKLRDTFDRVFKLYEKEKPEYAIELKKAEAEYERTRQK

LVQFFEQVRNAERVQLDQIPLPDAPVDQTLPAMIPLPNEIPLPMLGVYPEQMPPSILKKR

SAYDQELIGPVLPENLTKKRKPPGPPPGPPPPLSDSEDEYDPAKDIADPLEVDFDAIQHP

DDLAVLLAQTAEPPTKPRKIRFEDDDELEKDAPEKDTRLKLGKIKKKKGSSASSSSSGGG

GVSALQARMLQMAGQEIPDASSESSSSDSESDEEREEKVRKAAELKQRMDEIARLDDRPP

GTEKDEDEDDEDEDEEEEMKRRQPSTRPVPPGPPPGAPPGLPPGLPPGPPPGAPPMFMRP

PMMPGAPPGAMPRLLPPGPPPGRPQGLPPGPPPGLPPNLRAAPPRVPPGPPGMPPPRLMR

PPGAPPNIPPPSVIPSMIPMPASNPNVLSAPPSIMKPPLRALGDDETKSSATIEAKPKIK

SGMGDVTRFTPTAVKIKRNAQQKGRLKPTGKADEMSALNVSNKPTPTPATQTKTKDDAYD

EFMKEMAGLI

>SG-AL_contig_6955_1_-3_3_593

QDGDRDRWGPGAGGGRDGRPRSGEEGRYYAPGEEPITADRDRWAPGVVVPLGAGRDGEDP

NKKDRKDSDKDDDKDPKKKKDRKDSDKDDKDGKKGKKGKKDKDDDKKRKGSDKDDDKDKK

DKKKDKDADKPEKEKKGGLFSKLKKPKDSKSKLPEEEEDHSRPEDHSYPDDRFRPGYGPG

DDKYGPGEKYGPGAEDR

>SG-AL_contig_6956_1_+3_1245_1373

MQYKRCMHVQVLTEWPSISLNSILLDVPNLEYTAALSHYVNLT

>SG-AL_contig_6957_1_+2_1445_1690

MLNQFSMDHYLHNYGTDQSVNTTPPHLKTLGTGGKIEQRQTANKHPTPTSQQQKKKNGCG

FFFPEFLSASALYHSHHCEHAH

>SG-AL_contig_6958_1_+3_2865_3281

MGTGPNTHSEAISIFISWSYTKQSEIHNTTRLSSSLQSTSHIKNDSPFTFLSMLTKLFLM

KALHGISSPGVRAVFWTQRLYYQFSFTIQLTNHHLSLHHLQVVVCFLDQLLNLCRVADSL

VAMETILCPAHGVLPKVVV

>SG-AL_contig_6959_1_-1_1_204

LSVFVANGFFLGSGCWPEAQPQTFIRETAWGSSQGEGLQTDYPPYTVARFRSRTLGCGFS

CRDPRPGR

>SG-AL_contig_6960_1_+3_123_248

MMSMASRSRFSTIESMTGSEPLRMVVNWSAVLHLETTPMQSL

>SG-AL_contig_6961_1_+2_2_202

QPMHQLPEAVANWWLGRGSQMVNRYIPQYIPGTMPAQLLMILFYNTGQTGFRQWELQTSC

CCCLPHI

>SG-AL_contig_6962_1_+3_606_899

MFIIKVVQDRNFLNKHLLKINVNQWSNRLQHIQIPTPNFVHNQKVYNLKTPISSPSSMTS

ETGNNNNNEDFYHANLVYNHTTKSYSFSFSFLKTAFLR

>SG-AL_contig_6963_1_+3_1248_1550

MQGMSCRSNMCMVLYQATSLTKMVRLQAFLKCVKCLVLNVTVLYCFNSVMLGFFSYRVAL

HITGVAMASTFFFFLSHIFVDVLCVSGMGEAQSLTHLSTVC

>SG-AL_contig_6964_1_+3_303_410

MLRLTKSPHFNSHVQFTMTQLFTRAIPVIQRIVKTK

>SG-AL_contig_6965_1_-1_496_732

MLVKDQTMVLSSCLKEKRLMQLGHLFFLGGNTCCFIVICSIKVLGFFFPVLFLHLCLRFG

QFFVCVLAILLWSSVCVVF

>SG-AL_contig_6966_1_-1_475_4323

MEAEDMADESKTDEVKSNEETKTDEEAKKEQEAEAKGKIPIKLTGKSGIKPAASVPPWKM

FTRPIPKSSAQKRLLGPRRQAATVASSSGIGPAIPNEDPAPLADFLTVGVKKTVPVVPDE

VIEQPPDPKTLRKENKVALSAEKLEEMKILGIDPSSLVPDVKPKPPPPPSQNLSLPLARN

TGGAELAKTESGTSGPANTNVAGSASDQSQKQGQDLHVNSNIITSSTGVEGRAGLFSMPS

APNVTPFPPPPPLGPVVRPAGPPRGFGRRPPLLSNPNTPMPLPGYMPSMASFAPRYMSRP

APMNMPPPFVMQATPPILPIPPMPVCVALPPAPPPPPPPPLPEGPAPIPPPPPSPPKPED

SSVSAGDAGLPGIESASADEVIKSSDRQPSETPPTSNEAEKPDVSDSAPATDKTLTEEAP

QTTASNISLPEQVTSATTLTLASQVTTTPSLPQEVTPITIPSPEQVTSATALTLTSQVTT

SSSLPQEVAPVTIPSPEQVTVTTTQVTATTALPDQVTAASQLPEEVTNTTTTQVTATTAL

PDQVTAASPLPEEVTNATTTQVTATTALPDQVTTTSSLPEEVANTTAPQLSELVTATLAT

SPLPLQSTATATPPFPEQVTVTIATPPLPEQVIATTPSLSDDVTATTPPLSQQVSATKVT

TPLPEHVTDTAPLPEPVTAITTPSLSQQVSATTVAAPLPEQVTNTALLPEPVTATTTASL

SQQVSATTVAKPLPEQVMDTAPLPEPVTATTTPSLSQQVSATTVAKPLPEQVTDTAPLPE

PVTATATPPLTQLVTTTTSFPEQVTAPSLPKQVMGTTMLQVTAATTPSLPLQTAAPASPS

LQSEVDVEGSQVEFQSSSAVALTVQITDVGVAEESSDIVAKPSTSSAEETPASTVSESAP

QVTLEVEPQPSTSAPEVAASDESTEPTSTLSVTFPVKRNVARRGRGARRAVPVQSMPAEE

SVEPEGSDAPAAASRRPRTRSQMRPVPQRQTRSRTRQQKIEEEEDSVTASDETVVHKCEG

EVGSSTAREAESPDGSKASSTSACVLDLTNPPADSSGGSEANTEVTSSSTAVKSVTVVEP

PSVDSALNLTIPPEIRGARQGRLEQTMPEDLSCAEPQDLSTSSSGKGKGRRPPEMPIDLT

MRLVPTQSVDLSLPEEPQPMETQPSTSCPSSSSESVLNADCLDPQTMKDTLLSSTSAMNP

STSNLSELEVGEEGDMVVDASSQMADSAGLGVDPEGELVDFSLAPVDFEELVDMEVEDSN

AGQPEAGDAEDSGGAADSTFSQN

>SG-AL_contig_6967_1_+1_1_222

KTTEQFFFQQNSWKGLSRKNKITGPCKTTENFQIVLKQSTYQTSGIKPRKAICPTPRWQE

YFLSQQTNRDFKLI

>SG-AL_contig_6968_1_-1_502_1260

MALSSIFITGASRGLGLELVRLILSQSARPRHLLAGCRDPAKAEELQSLAKQHSCLKLIT

LDVTKDSDIEAAYTQATACLGEEGLNLLINNAGIFDKPGGTLPQLTREKMQSHFNVNATA

PALVTQKFLPLLKTAADKNSSQPVSYQRAAVINISTVMASIESLLSKGHCGGYHYRASKA

ALNMITALMSVEFKESGILVAAIHPGWIQTDMGGPSADFDSKTSARNVWSSIAKITEKSS

GLLHNYDGAVLPW

>SG-AL_contig_6969_1_+2_2_160

EEAINTVNNFERKSRLFLNTENPLPYDLATSVIHQSSTWIIYTWTGTNLGLKY

>SG-AL_contig_6970_1_+1_553_1029

MFCKLIDFGKALQPKTSEEFLAEPLFYSENMKIGNAAFFCKNWAEKHLYVVKNLVNSDGA

FMSFEQFKGKYTVNTDFITYTGVLQSLKSYVRKEGLRIENDVSVICSKILKCIPSINKRA

QLCYKILTQDGTRPNCCNKWEAKFSEEIKWSVACTEVKK

>SG-AL_contig_6971_1_-3_3_293

SIFLVHTSRKKKKKKKNQNNQKKKENREQSVFLFVICGIVCIHIHVYVFTFVFELMDQCC

VYAWYFYEHRRTQCSTFCKMCKAAEMNIFVCIVSSCL

>SG-AL_contig_6972_1_+3_321_503

MPAKSIYSYMLSFQQPLQNHRAFQRKYKTSSSVIICESHVLYMIRNMTRSYIYVTQNMKP

S

>SG-AL_contig_6973_1_+1_1366_1671

MATRLTVTWTTKRRRISSIRVQATTSYGGPIQPGTLHISNNTTKQELERTDYFIPHWTDD

LTTFTNDVVCCLFAFPSLTLWGSPNIYICTLSYESETTSTSG

>SG-AL_contig_6974_1_-2_707_1042

MHAHTLKYVCSSTGTHMHTHTHACARTHTYTHAPTHSCMHPHTHARTHTHLYACTHTHTH

THTHTHTHMHTLHFQQPFIVILTNVVLTLSLRHTHTHAHAHTHTHAYTNIQI

>SG-AL_contig_6975_1_+1_253_402

MSNEISPNIHIIWKTNVSLSILVICFTLKVSINDHYLMNRQSLFKTKFLQ

>SG-AL_contig_6976_1_+3_3_113

RKKKTTPFGFIYCTMSDCKQNLKTGGGIAFMVRVELL

>SG-AL_contig_6977_1_-2_344_496

MPWAITGDTKYVYVCICVHMCKHTHTHTHTHTHRRSAPCKSCCLEFVHTHT

>SG-AL_contig_6978_1_-1_1_240

SVRDGSRQAVLHSQNKTSVVTGDLDLRCSFVYHLHFSHMKRNIGRHVYTHRLEGKRGRVQ

VFGVGQGERVLLIISGRLSL

>SG-AL_contig_6979_1_+3_291_380

MTHYTLQALLCWFWLLKCCFHDSIRITVTL

>SG-AL_contig_6980_1_-2_515_895

MHTRTCARSHTHTRMQCTHCRAVCLLRCNKLSLNVYCFGRQILNPYCDQCDGHQIEDLCH

FLFVCLKYASLHIKCVPLYLYRFPTSFKVKLLCLNLNNKLAAKVGMYISESLRIRDGSLS

IIKKKLV

>SG-AL_contig_6981_1_+1_1_183

DREGRKKQTTVKERKTAETKRAIEKRKLLAQNFQKVGILFILKEPPVSPRRNKKPQVGEN

K

>SG-AL_contig_6982_1_-3_3_134

EETINRKWQGVKDAVSSTCQEVLGPKKHSVKEWISTETLKLKSE

>SG-AL_contig_6983_1_+2_2_118

TDLRTVRPFTSQDTQCNMYRGTDLTHTWIRIHTLSDIHK

>SG-AL_contig_6984_1_-3_39_2723

MSNNAVLKIKWDPKNLEIRTKSVEKTLEPLVTQVTTLVNQSGPSNKKKGRSKKAHVLSAA

VQKATENFIVQGEQIAEENPEVRADMMAAIDEVKTTGETMNDASIEFAADPCSSVKRGAM

VRAARALLSAVTRLLILADMVDVFMLLNKGHSVEDDLAGVMNATSQQGLMDSFKTLGKSI

AELEQLAAKRQADLKDPRRRDELAAARAMLKKNTMMLLTTSKAYVRHPELAAAKANRDYA

YKQLCDAINIISGAVQAAGASDAHPYEGPGELASALDELDQKIIMDPLSYNEIRTRPSLE

ERLESIISGAALMADSSCTRDDRRERIVQECNAVRQALQDLLTEYEKNAGRREPSENLEK

AIDNMNKKTRDLRRQLRKAVVDHVSDTFLETNVPLLVLIEAAKAGDERGVEEYAQVFTEH

ANKLVEVANLACSMSNNEDGVKLVRMAAAEIDALCPQVVNAARVLAARPRSKAAQENMDI

FKDAWEKQVRLLTESVDDITTIHDFLAVSENHILEDVNKCVIALQESDADTLDRTAGAIR

GRSARVCNVVTAEMDNYEPGRYTERVMEAVTMLRDQIMPNFAEKVEVAVDALNSNPPQDV

DENEFIEASRLVYDGVRDIRQAVLLNGIDVDDSDSEVELEDEYEAYTSVADADEYGKSDR

AMMRQLPEEEKEKIQAQVEMFRMEKEKLDMEVAKWDDSGNDIIVMAKQMCMIMMEMTDFT

RGRGPLKNTMDVINAAKKISEVGSDLDKLANSIADECPDSQSKKDLEAYLQRIALYCHQL

NITSKVKADVQSVSGELIVSGLDSATSLIHSAKNLMNAVVLTVKACYVASTKYKKGNAKK

PVVLWKMKAPAKKPLVRREDQNELNAKVRKGAMRSHIEPLKALSEFQEITSDSFC

>SG-AL_contig_6985_1_-3_117_992

MSLTKCLRMTGVLRAMQRQSTCAAVVVPRRHEWNYSSREGIPREVLTMLTQDYDFSWKGI

KAYMEGSRWMALKEDHKFIAARHRTLGPDLATAHFLVKRGAKVKFVGSNTWFQRDKKGYI

YLPNQYVSGMYLEAIDCTGMQMSYVAFDNMVHLEQLRYLNVSRCMNLDDWCLARLYQFQD

TLEFLDLTDCRQITDNGLACLHVLKKLKGLKLTGLPNVRDMGLMVLLLEEALPDVTIFGL

EPERLELPSVGDQDTPMLSGRLQSITAGESTQDVHQEQSMTLLSGGQQNVKS

>SG-AL_contig_6986_1_-2_2_106

SFLQEDDGLGKGVKELVFICMALLIVLFGYIYVFL

>SG-AL_contig_6987_1_-1_1_114

LRVTGKLFLYTSPVFVFSVASLHPCLLVLPSATSLTYF

>SG-AL_contig_6988_1_+2_1046_1507

MRDLMICFVKPSAPYGEAVDFKNKLCQKDAQILFSEAVKDKIEKKEECKLRDVRITEFYK

VAKQFYFSACDFLKDKFPLDSELLEHTCVADTSLRIEGVKDSDLSYFRTRFSCLIPQEYS

KNVTIEEFADYRSHGSIPLQRGNELMQPGLLLEN

>SG-AL_contig_6989_1_-2_47_1018

MFTQTLSPAEVTEGEKLTLSCAFTGQPDPNIEWFLNGQLLTSDDIVSITTRSSSSRLEIG

EVVLDDEGDYVCKATNTAGVASTKANVVVRAAKKSVVEQERETMEEAPPPPSVSVDPPRF

LEHLQGQSVMDGDQVTLQCRISGKPDVEWLANGNPVTDSDDFKHQNAGDIYKLIIGEIYP

EDSGIYSCKASNAGGSAISSATVFVSVPDEEPSDPVFTTWPLSQNIEEGNPVTFSCALDS

PAALTVSWSKDGRSIDDSGRFKLSQDGNAYSLTIPAALSTDSGTYTVTATSEEGSTSWSC

SLAVAIGDSGDEARVQELLQQAVE

>SG-AL_contig_6990_1_-3_165_290

MMYEFVHCKVQCVGLYYVCWMCDNLFKMRTPCSVTLCVLQEV

>SG-AL_contig_6991_1_-1_223_1092

MYWWKETTELESKGRVTILPNNSLRIVATQLNDSGLYRCFASNPLGKTFVETVLNVVVHG

RYSDWGEWGQCSASCGQGVRHRTRSCDNPRPMNGGRDCVGERQESASCQLTACPKDGQWG

NWQTWSSCSASCGAGQRQRQRQCDNPTPQRGGRPCEGDAVQLDRCNDGPCPIDGEWGMWE

PWGPCSLSCGVGSQQKHRQCNNPRPQFNGAPCEGSEVEQQPCRNQQCAVDGQWGLWLTWS

PCSLSCGGGLRSRQRRCDNPQPQFSGADCVGSDMQRDYCNNDPCPVHGNW

>SG-AL_contig_6992_1_-3_3_236

FFFVFIFYNNFVGFFWGFFFFFLRIVKMFLSLFSRSDLIIFKASVVFCYPCHRPFATFMM

WTVVHIHIHLIKYEIGSG

>SG-AL_contig_6993_1_-2_128_946

MGPGGGFMGTGYGRGRGGRNVKGFFEDEYDDYSSGGYGYGGRGRGGMRGRYSDALDDRRG

PGSQYMSKTGHSVHMRGLPFQAQEQDIFDFFSPLQPVRVSFDFSSNGRPTGEADVDFATH

QEAMDAMKKHKSNMQHRYIELFLNSTASAQHNSDMSGYGGSDMGSGFGGGNMGSSMGGGG

YSNSGGGFGSGSNMGGGYNTGGGSMGGVGNQTGQYGVSQQYGSSSMGGGYMSGNSQTSSY

GGQGNYMGSSGSGYNSMGSNAMSSLANTNFTAF

>SG-AL_contig_6994_1_+3_3_179

LKKKKLLFFIFDTKDSQTNHSLQPMALKYIMRTKKPNFKFRFKCKLQTICQSWPNFPLF

>SG-AL_contig_6995_1_-3_54_242

MNRLNTTTQFLSPTLSLFLFESACTRAHTISCSLYKYMYKYMLLVASTFLLSLHECIKTV

ISL

>SG-AL_contig_6996_1_+3_18_236

MPGEQLCISRQSVNSIRMLLMLEHMHTVYTHLYIHINKGKQQMIIPPPPTQIFGLLYRLP

QGGHKSLLALSSQ

>SG-AL_contig_6997_1_-2_284_397

MGGRSGHNSTTVYLLTHTHTHTHTHTHTHTHNARAREK

>SG-AL_contig_6998_1_-3_789_950

MYIHILQFLCRFLVKKPTPLSDSIVLILIQALSKTFKTHTNFQELNTGLKRTIL

>SG-AL_contig_6999_1_-2_44_304

MKIGLKVKKQQTKLVSQCEMHSLAKFCGDPMHIFQVMLHESYIYMKNELSRPQVWLFLCF

ADCISLLQTVETCQKKWSCSRHVLVWE

>SG-AL_contig_7000_1_+3_3_230

LVDRVKGLLYCLKPSSPLRSYQGCHHIKSIKVKKTMLNTKPHHTWKKNSAKEHNSLHIIS

TIIMFIFLQANTTRRC

>SG-AL_contig_7001_1_-1_370_1281

MKTTNFGKTCNVLLHSNKTPHMELVQERVAQLEAQHREDAFFLADLGNIVKKYEKWTELL

PRVEPFYAVKCNQDAAVLRLLAHLGTGFDCASKDEISRVLKLGVKPTRIVYANPCKQTGF

IRYAAKENVDLMAFDNEAELGKVKAAFPNARLVLRILPPANFKVQCELGNKFGCHPRDAR

TLLCKAREMGLNVVGISFHVGSGVAEAAAFAAAVEVAAEVFDIGQTLGFDFDLLDIGGGF

PGHAAAPITFEEIAKVLNESLNKYFPPKRNVRIIAEPGRYFVASAFSLTVNIIAKRSVNQ

YADK

>SG-AL_contig_7002_1_+2_419_583

MQTCYCLNSHVCIRRQFIGFCTTGTQLLMPRVELSLRNGSSTLKTFSCTGAFFFQ

>SG-AL_contig_7003_1_-3_3_164

FVCFVLFNLALVWVCLVGFVHRRSSPCTLVITNLLTYCFLYMYIYSNRTFFLKI

>SG-AL_contig_7004_1_+3_114_1556

MEFMPVVLAAGVGKGLDLLTSRYPKALLPIGNLPAVYYPLAMLERHGFGEAIVVIPRSYE

KEIRTTLLEDCNIKMKLIFESIVDTQLEDHGTMHSLRLVRSKIPENTDVLVVGCDLITDL

NLNHLFNLFHKTDASFTMLLSSLKESRDLPVPAHDKAKLEKERRANADIFVYNEDNGRLI

IMDKKSNLNGEVELDSGLMKMFPQLTITAATDCHLYLLKRWIIDYLTIHQDCEMMKSLKQ

DLLPFLVKKQFPSEDRLEVSRFWTKSPVEGEAPARLLKLGKEHDVYAFAEIPAALDVSKN

TLDLAETMSAGITDVDFTAVDKNSGALHVTRDDDVIRCYTYVQNSGFCIRSNTFSRYCDA

NALIAKAFPTLVPHNPPMVHPSVKAPRSAQVSDCFIGESSTVGEKVLMTKTVVGAKSVVH

NKAKISNSIIMDQVEIGEDCMVMNTVVCSGTVVGGKCEIKDSVLRPSSNVQSASKHSFQL

I

>SG-AL_contig_7005_1_-3_255_584

MYNGILFVLSGRYSFLHFFKSFHDKGGNIEEGEWSCKSELLGGIGQGVLLSRFVIHVWCV

HILYQTPKILFDQQWSPAMYQWVLETLKCLICFVCLFPVCFLLLLLVLSV

>SG-AL_contig_7006_1_+3_3_62

SSKKGYVSRGEKTKNLISKY

>SG-AL_contig_7007_1_+1_1_87

VTFYFPRVMNNNNCQCRRSNLHYKKGTPT

>SG-AL_contig_7008_1_+3_195_290

MTLHDLQTNSDILSNKEIKFTKKAELTNSPQH

>SG-AL_contig_7009_1_-3_336_623

MYFNSSHELLEHFEVTLLDLNSITRTSNMSTYVCPLLLLIFYILIIVTIITIYYYYYCGC

CLVVIAVVLLLLLSFDDDFNIAYGLREGGDRWKEVL

>SG-AL_contig_7010_1_-3_3_227

PHAPSSVELANFLAFLATSEGLASSTVKGYRSAICTTLHQLGGPCLSDDFLLCEVSTWTW

FPSLLQNSFPPGLGH

>SG-AL_contig_7011_1_-3_3_2414

PETIEDVEPKTVHVELQAEEVAAQELAPVEKAPEEETTAAALDIPDSEESATPLVAKEEL

AITVEGLEAPTTETVAPETTEVFSVETIAEKTTTETEEVSHEFIVRETAEAVSHEEILEE

TPVPESEQAAPERTTAESFQTLITEIEEAFAEEFSQEASITLEEVEHPKLTVEGVPSQET

ETPLEEEFELLELPATEEVEQEVVELRPQGQAPTFSQPLHNVEAQEGQPIRFETVVTGSP

QPEVSWFLDGEPIKESPIYHIITEASGTCILELPQSFPEDEGEYECRAINEFGTASTKAD

LYIQGPAEEQEVHVTEEVLSEKEKLVTAEEEKDAVSDEERKETSPEVFEAQFVEIKEERF

APEIRLALEDTVTQDGGQVRLTANIVGVPQPEVTWYREDEILSKTEEYEISQDDQNNYTL

FIHDVLPEDAGKFVVVAKNELGTATSTAQLTVEVEEIEEQVRPEDVSGLAPEFLEKPRFQ

TVQEGDTVTFKGTVEATPVPTILWTRDNMPLPEDQRFTVAATQDETTSTVHHVSLAISEV

TPQDAGTYRVQASNDLGEESVSVSLIVNRVSEEKTDFRDQLQRSEMDIPDKPTTVEAEQV

DFRGQLTTEVKFATDVSVTETTATVEEVVEEQPQQADVQEVTVEIGRPEESQPEFLVPVQ

DLTIKEGEAARFDCQVKCTPEASIVWYHQDKPLKSDDIYQILPGEDGQSSLLIAEAFPED

AGEYTVRAINPLGEVTCSAVLTVEELVSEVSSVGSRQDEEQEEPVPETVVHEAAVKIQAA

FRGYKAREQVKILKETKTTEEEFF

>SG-AL_contig_7012_1_+1_625_849

MFCQTHISVHHVLPNSHIAITFSLYPLICHILLTIPQPLSKYSFKMIGPIKVLCNTPPTS

FQEHLQIDQTHRGFI

>SG-AL_contig_7013_1_-1_193_768

MELLKIQMQDAGRSARIDLMTTGEAAASGVKSTKPVIKMSATKIAMQLIRERGIMGLYQG

CGATAMRDITFSAIYFPLFANLNKLGKRREGSDQAVFYHSFISGCIAGCTASFLVNPIDV

VKTRLQTLTKGVGEQHYNGILHCFKTVMVQEGPKAFLKGALCRILVIAPLFGIAQTVYFL

GVAELLLGLKKP

>SG-AL_contig_7014_1_-2_467_727

MHVNDHIRVCCNFPSFTPLQHTCGVKENLNTATLVLVCIKTNMLHLSSSPSWSILVMSKQ

TSFIIIIDSWFLCIETNTTFIIIIIDG

>SG-AL_contig_7015_1_-1_187_423

MYLQYIFDHSGIFSVICRLLSLWKSMSPFYNTTSFSVCLSLSVLLYFSSCIKRCFALNIN

TYKTVPIKKLIGKINIFKK

>SG-AL_contig_7016_1_-1_1_120

RQSAYILGDPQSVKLGNTNNKQQLINNCFSVLAVLMMTLC

>SG-AL_contig_7017_1_+3_933_1229

MIFQTTNLIRPTLIETTVTCFGNVLDLPCGGSLTPVKSQVCLFSHDCVLYREIYNYKITY

FALLHEYFKRQEIWARKWRMQFNAKTCYILCTKEKLSHL

>SG-AL_contig_7018_1_+3_192_374

MIFSLNNTPYITAAFMYTIQLPLLFRSPFQHMASTVLVNKVFSGFFYILWRLPRIFGVFN

T

>SG-AL_contig_7019_1_-1_73_1029

MFSMFKNLVGSKTLTPEDLAPVLDKMKDHLIAKNVAADIALKLCESVATKLEGKVLGTFG

SITTTVKNALQESCIQILSPKRRVDILRDAMECKQQRRPYVITFCGVNGVGKSTNLAKVC

FWLVENGFRVLIAACDTFRAGAVEQLRTHVRKLNALHPPERHGGQEAVQLYEKGYGKDAA

GIAMEAINYARDCHFNVCLVDTAGRMQDNEPLMRALSKLIKVNQPDLVLFVGEALVGNEA

VDQLVKFNQALADHSNMTNPRLIDGIILTKFDTIDDKVGAAISMTYTTGQPIVFVGTGQT

YTDIRNLNARAVVNALLKA

>SG-AL_contig_7020_1_-3_12_155

MFEVVARMAVSVCISKTCSVNCPPQFCRMNKCWYSLHPSASKEHLLPM

>SG-AL_contig_7021_1_-1_1_192

FFFFLVNKQQILVVEMYAATAVWVCCVRYNSSRIKKGAIYNSYKLSNSKVVDLKWVLFTA

AFVS

>SG-AL_contig_7022_1_+3_258_410

MKFMKFVDFSSHLQIILWKRKGNNISVIVSKRYECINIADNKQMGEVIMKY

>SG-AL_contig_7023_1_-3_36_1097

MYGRLLLLCLAAVAYAKPGSVAWFKVERASQIEDASFRLRYDPYDTTWAQFKQEHNKVYE

NEEKENKRREVFMKNIKFIEQHNWQYYNKQKNYYLGVNQFADLTAEEYQQYNKLKRNKTD

GKDSIQCTQYLPPLNWVLPTSVDWRTKGYVTPVKNQGQCGSCWAFSTTGSLEGQHFRKTG

DLISLSEQQLVDCSSRFGNDGCEGGLMDRAFEYINAVGGIESEQEYPYKAEDEKCVFVKS

EEVADLHACKDIPSGSEKSLQQAVASEGPISVAIDASHSSFQLYKGGVYDEPDCSSERLD

HGVLAAGYGSLNGQEYWLVKNSWGEQWGMKGYILMSRNKNNQCGISTAASFPVV

>SG-AL_contig_7024_1_-2_170_1105

MEPSMFDSNEPESAVKRLKRNMTLDFNGSGAKAKKTAALLQSPDLHMLKLASPELEKMII

QANGMVTTTPTPTQFIFPKFVTEEQEAYARGFVDALAQLQSSNDPKETALPFPPSQAKAI

ITSTASNSIGQSFPPNSTVTESAALPVVTSLQQVSTTTTLPNGVNLSTAGAAALPVSSAV

GVVSHPKPPLPSHQSAPVVDIQLRQPKLEEEPQTVPCLSSSPPLSPINMESQERIKLERK

RARNRVAARKCRTRKLERIARLEERVKDLKGQNNDLLTTATSLREQVMKLKQQIVEHVNS

GCQIMVTQNMAL

>SG-AL_contig_7025_1_+1_457_606

MSEINRNQILLPDVKYGLRFTEPKKRKQEYSLSLLHTHTHTHTYIHTHSY

>SG-AL_contig_7026_1_-1_1_108

RCPRRLKPRPSKPSSLEVKCLHHSTTVPVMWHAVNS

>SG-AL_contig_7027_1_+2_2_133

FFFNNSKVNKYISENYRILHKAKCLEVYKPHKAPRENVIYMIII

>SG-AL_contig_7028_1_-2_2_91

AYTVLQFLTVGGIYCFTVPDCWRHILFYSS

>SG-AL_contig_7029_1_-3_3_131

AFPVDNLTFFYPAYRTHCYTYTQMHVVIFAVFLFIVFASFVVD

>SG-AL_contig_7030_1_-2_80_4294

MVDAHLASGQPSLGRHVLEVTLQKEGDKGIGITIVGGETTSSLDLGIFVKSVMKDGPAAR

TGQIHAGDRLIAINGISLEGVQHHEAVQLIRDSVDSVTLLLSQMRPPVTVRRKFDGIKTR

FDIDDDDAECHSDIEDQETSYQGPSYHPANMLNSAAACQSHHHYHHSPIGHSHHPTHNSS

DIVDNDNMESFKVSDALSERLQFSDNDSVNADARDEVLLSEEMLAAAELESQSYSSGDIN

RMLNTQSQLNQEMEELEILGDIANMTVDNSDTDSDFESAVQATVEGRVTRSSSSKSTTGR

DPNPLRKAISMSSPDVVIYEVVLEKHHDSLGIRTSGGERREGSGRLYIQSLEPGGPADTG

GILQPGDCLLELNSQSLQGATATDLQKALQSAPAICLLKVARTISLDQLALGQRLEELNK

RSSMHPAQSAPAVSGDTSLDELAGQEIPISPPSGFASAAPTPVKEVPSIPIVPPPPPLSL

DLLQVEDERSNNTQSDVDSDVLDSSRSELEQICVSAEKYLEDDPGDFDLDAAVTAAGLNA

SIHEFHRNMQSLGDKHSGSGGHAGDSDTNGDVSPVETAKNKSPSTVRSADSGKQLAGKTR

SKSLVRQHHVVEAAEKGNCVAEDEQQSNADQVQDFTVDLMKSAGGGLGFTVVGGVSTTGG

CCVKAVVQDPALSDGRLQPGDRLIQVNDQDMTGLSHFEAVTFLRQTSKKVSVKVRRYSHT

PQAFVISPQQPRNKGPSNQVQHVAGAITRKERHVQKSCRNTEPDENTSESVLDQKQGLRA

SAEGNKEQPQSVKDLAKCLDTPKAIIRPYTYGQQDNSETENKVTMEICQARAESNAGVLQ

IVLDKSQLVDSLGFSLVQRHVDCNQSGIFIKTLTPGGIAHTHGQLRMGDRLLQVNGVSLI

GCSHTKAVRLLREAAGSVVLTISRFPEGQVDSSEPEISSGGTSSQNIDANMGSVPSDSTY

SSDDVTDDDINDILDEAMNFLESEHRDKLQTPTPDSRDQETPTPSDSRQQVTPPHREHDL

PTLSTKNESHPLPKLRSFSVSTVSMSSQLPTYKINSRANTPRNSDNTLRNMDNTFRNSDK

AKPVFTGYVRKVSSLELPEDLTDKSMSSLPILRMKKGAEERTMQLLQYLADAVEQEEPLL

EFQKLRQMKATDNMEVAKLVENKARNRYRNVLPYDCNRVKLQTDSPVGSDYINASDVQLS

VNGEEGNLCYIACQGPLPATQGHFWQMVWETDTSVIAMLTQEMESGKVKCHKYWPQSCEE

ALDLCDGKYIVRLVKEEKFEDYDVRHLKLCNTRRKEERVVVHLHYTTWPDHGVPDTAYPM

LQFLRLMHQFHKSGPFVAHCSAGIGRSGVLMAVDTALARFETGDELDLFDIVSELRHQRY

GMVQTQEQYLFCYTACMEALLSMER

>SG-AL_contig_7031_1_-1_1_222

AALPFPTLYRVRYPFITGRIGGCMHNSFFSFLFLVEPDWNGAVRLEGGKSISVPTGLVLA

SYSYTVSLPQSYSP

>SG-AL_contig_7032_1_-2_230_856

MTGMRQMTLTLACALTLTCALGAVPAQGVSLRDESARWRLELEPYRSPQHRSRRHVFPGT

GMPCLTVAFLTEMKGRFRERIDPNDDGKATMPEVRQYLSNFNPEVTDDQVNRFITRRDLN

GNGIIEFIPEYILDIATPDMTPEAASEWFELEDSDDDGYVTRAELLRIAQRLGMTPQQAD

ASVSSYYMTVDADKDDKLNFDEYKVLYSE

>SG-AL_contig_7033_1_+2_29_250

MTTSVVHTHTHTHINQTQSSRVVQNGNMLGVKGRLLCIGAIHFKLFIHNNILTPSTATDV

YRFKSSCKVLKCWF

>SG-AL_contig_7034_1_-1_388_657

MLFSLLSSPQKMNFEYFNDMVVMLRRTLCGLSMAFIMCPCSCTTPPPLPQPLFPVNGLML

NTVNHSYSLSPCIPPPEKYVVKGMGIQDEI

>SG-AL_contig_7035_1_+2_2_262

LFFILTKVNTHRHSHRYRYTHTHTHIHTYTDRHKPHTSCGKVWSWKIDSDKILITVLGKR

CAGTPAAIVLHISHLLTSTGYQFFCSH

>SG-AL_contig_7036_1_-3_264_518

MCDVQVIFINKPLVRKMRKNVRSISHTRTSRGHTDINPEDCPYQTKLQHVRSEQDRVDDL

GIIVVIEFVMAKFHCWGFCSERNGI

>SG-AL_contig_7037_1_+3_132_464

MPMEKKIETTRYIFKSKFTASFNRLLLYYLQLQHRLHVAITALLTSFNDLILCLVKALQD

CIMCGFCLDDTLRKTLLELFIQKYRFLEYYSCSCHHFGGVWVYMLHSVGSF

>SG-AL_contig_7038_1_+1_1_141

FFFFFPLALLFQQTGKIISTDRQHKMKETKYMPNIIFTNTWYNRMES

>SG-AL_contig_7039_1_+3_195_590

MSIQHVGSIILVLNSTTTDKSHMKTKSCMLACHISKFNSSKNTAIKEQFVLTLLIKSKTF

KEDNRLEYWSHISNEANGDNKIKPRSRKCQSITYLYSTNCQTNAYLILHRAFHNTQTSRW

PCSWIQSSSQWI

>SG-AL_contig_7040_1_+3_837_1001

MKVVQTSLRSDRLSVMAGLAPFPRCFTFLNSEQGQVPIYSWVNWRPHTRWSSSQT

>SG-AL_contig_7041_1_-3_36_305

MSSDESDCEIQDQDLPSAAECEERCQRFAEVTGTDTALAMFFLQDRQWNLDQALNAFFEQ

NGGGDSVIAVAAAAPAAAAASIASSTSSVS

>SG-AL_contig_7042_1_+1_247_429

MCSEKIKCSKTSGKWTMFCHESHFTFKDLNATVDSLHYTAAIQATEKVHHSHMKYFIYRP

H

>SG-AL_contig_7043_1_-2_293_1933

MDYEPGFSSIAIREAKTGNQHNPPIDKPGQLSQYSVGNVRSEPDVRQDGDGADPRDLYKR

VQSGGDIPTKGLQKPAPEKPRAFKHQTTDERRPRPEPVNPEKERRRKEEEEAYRKKRLEE

LYEEERLKKMKKDEADAEARRHHDYYTNAQKSPISPNRFDDAATSLSPSTSGSLGTLERK

DPQFSVPPERRRGFQIQGKARALFNFTAQNPRELPFRKNDIIYLMRQVDKNWFEGERHGR

VGIFPTNYVEVVTSIEAAQAAAMQAEGQGRAKYTFTAQSSVELSLRKGEFVVLLRRVDEN

WYEGRIGNKQGIFPVNYVEVTREPSTPLITPAPSVITTPMTGTPEMLSPVGYDAAPTPPP

QPSPSAFTTQRSPYSFNQPQGYGPASTSLLTPASSVSRQQINLNRHEPDFSGPRSPVSNP

KSPISNPKSPISAGGRLGSPAGKRASPGPSPGSGPTSRLQVDIHSSAKPFANGQPPSSTA

TKVTDDDLAVTRYRALYAYRPQNEDELELREGDEVFVMEKCDDGWFVGTSARTGCFGTFP

GNYVQKI

>SG-AL_contig_7044_1_-2_2_181

GGGGGCFSPSGSPDMLTWESGRVSTIYTSVQGGSNPSLQAPTVWKSSVFTIRPPSPSIQV

>SG-AL_contig_7045_1_-1_451_765

MPADYVDVICVMVIRIGAILSYLAICVCCGLFNDVKSCAELATVAEHALLFPFSPDFLQQ

MGTVRVTEYCFFLMPEMTVIIAESLWIVMIVFAVIYYICSFHFSN

>SG-AL_contig_7046_1_-1_217_423

MGDHLGTSGAVGTKKTTKIIGMSIHGPFCGANCQSGLFVFMYFVIYAILRYFTLFESPLF

VCVSAIWRC

>SG-AL_contig_7047_1_+3_165_473

MNTNYYTKSQHLQMIKHNRNKSEVSNFVKVYVKTKAYIFFFLMKGKKGKTKELEERNIMF

FVKATGTTVGPFPRTFSGGISLMQQVEQNFSQRLYIYKQNRTN

>SG-AL_contig_7048_1_+2_2_115

VIGEVTKQVPTAVRKHCRDSEWPHNFGGDNVEILGQRK

>SG-AL_contig_7049_1_-1_1546_2247

MVSFHSEFALTDGMSGLVSFRVCANGWNEGLASFRVCVGGYKCRLCMPCQQYIFSSKCVV

NSIMIGWGKKSTGPIVLPYIIIFYRYIMVRAGVTSGGKKYLDLVRVGVTSGENTALIWSV

RAGVTSGANTALIWSVHAGVISGGNTALIWSVHAGVTSGGKIPWSGPCWCDIRGKKITLA

AKEPAVTHKYVMFLSDRHTRARACAHTHTHTHTHTHYIYIYIYIYIYIYIYIEP

>SG-AL_contig_7050_1_+3_396_497

MQSHTHTHTYAESHTSFIHHKVHRHTQVIIYPPQ

>SG-AL_contig_7051_1_-2_590_745

MASQTRGNSKAIKAVTVTLICNDHVAVHTNAIITLLIIGGLFIWLDRFFNNS

>SG-AL_contig_7052_1_+2_104_247

MKGVCEQYIVQYISSVGTDRERERERERERERGLNSDQFIHFVGYIVL

>SG-AL_contig_7053_1_-2_2_220

HTHTHEKTLRKIPFEWRKEKINGNIQTSSTFFLRHGILLRKALELRKNYTFTDVTANGNS

LTQISLAEYVYDC

>SG-AL_contig_7054_1_-1_142_417

MNFEQATYLSIDMSRFTMNTLIRLRFGISSITIHSAQYRQYTEQDLFCPFCRLFEENEVH

ICLCCPGLNDLHERFIPRKYCSCPSRQKLSCC

>SG-AL_contig_7055_1_+1_154_279

MYVYHICTSNIKISWGKNKETERERERERERERERERACARE

>SG-AL_contig_7056_1_+3_1059_1289

MYRAGHQLVTNVHNKNIILQHFKVTQLCTHTPKLGVKINNRLLSQKHTSLSELNKCFTYI

TLTNCFVQRQTLAICTG

>SG-AL_contig_7057_1_+1_49_282

MVENGLALSPEKSHMMLFNSGYDPEPLPSFKIEGTELEYKQVVKFLGVFLTSKLTWNSHI

ENVLTKARKNLKSFKDFE

>SG-AL_contig_7058_1_-1_1_117

KARYQYHNIIVHGCYQYHNIIVHACYQYHNIVQGMLPIP

>SG-AL_contig_7059_1_-3_126_347

MENKYVRVLNNNFTWNLTDDEGNSDTPVASYDCKEIDDEINGAYSPLLDAFFYGTVVGKM

FENWYGMSPLGDVM

>SG-AL_contig_7060_1_+1_43_279

MPILQTHPHKYTLKITLIYILTYTSWAELNECHPHNTLFFSLKGSLQKKNHIDHMPRLPP

STHPHTHTHTHTHTRAHAH

>SG-AL_contig_7061_1_+2_2_196

PLLYLRQIHVRDPGAGNSWGLSMEGHKSDLIIKETLTQAGSVTERFLLSAGGSVGSALDQ

LDRCS

>SG-AL_contig_7062_1_+3_33_758

MNICIDERMVRNRGRFTFRQFIKDKPTRWGMKLWVLADSQNGYTYDFEVYTGKGIPVSKN

GLAYDVVMRLCKSLDKQGYHVYFDNFYTGVQLVKDLLSKKTVCCGTLLTNRKGVPVEFKD

TKTFAKGPRGYMRWKREGNLLFLQWLDNKPVSFLSTIHKKANKYKMVKRRNKVNGVYRPV

YVRQPVVVQDYNINMGGVDKSDQFTGKYETLRKTHRWWKTLFFHFLDVARVNSFILFNDF

RS

>SG-AL_contig_7063_1_-3_93_206

MGTRCPFFSVICLYGLFYVFLLLRSISYDFLRFFVIWG

>SG-AL_contig_7064_1_-2_179_283

MVSVLAKREFTWQNSSFYVFFCGFKVFFIIIYAFL

>SG-AL_contig_7065_1_-1_199_486

MSQVTVPIMMTFLWQQWRKQPTVHHMLQFYHIYRKKERKPIHFSAFFKHSMYSTVRFTIN

IEAAEPIPLSAAVHNLRATETWIHPFLCLISTLSKI

>SG-AL_contig_7066_1_+3_219_461

MVDTILPRVILLVSSSNPTEHHYCIQLANIILRLCYKNQTVADIYKTHHYVHRYTDCKNS

YSYLPQYSKYTCNARFHLWHI

>SG-AL_contig_7067_1_+1_61_351

MKISDLAIQTEEEKFSHLQGFLFCCCCLFILPPHLLCHFSGVDPTPLIPAPPHTTNPKFI

HLKSQCQEAHGSQQCQAAIGPPTRGDPVLLPGYSGGV

>SG-AL_contig_7068_1_+3_12_293

MAVHTFNSPLKLPCLQKSIKQSTPPPPLFRVSLSHIQNIVHYMAVHAFYNFPLSTNEITL

PSKTNKTANTSLSLPLSLSLSLSPSPPLSLPLWA

>SG-AL_contig_7069_1_+3_315_569

MISFGCVPEKNTSSPHSLFKTRYTLYSYTISLTVQEYVRLQMNHSGIDNVLTKQKLCSLH

SVWTGKDATKQPYNHKDISEYQGKM

>SG-AL_contig_7070_1_+3_3_383

QKYVEILNQNLWPAVAKYFSDKPWVFQDDNCPVHSSRYTLQWKRENSIPSLSWPSQSPDL

NIIENVWSKIKSALNRRPQEVNCCADLVCVVQEIWVSLSPAYIQSLYNSIPIRIPHVYRA

NDCISRW

>SG-AL_contig_7071_1_-3_96_581

MPPMKLQSSDGEMFEVETEIAKQSVTIKTMLEDLGMDDDEEEAVPLPNVNAAILKKVIQW

CTYHKDDPPPPEDDENKEKRTDDICSWDAEFLKVDQGTLFELILAANYLDIKGLLDVTCK

TVANMIKGKSPEEIRKTFNIKNDFTPAEEEQVRKENEWCEEK

>SG-AL_contig_7072_1_+3_3_251

NASKWGGTRIVRNYTPFAIRPTYPIALADFSFLWFLTRSTPLATQLDQRGSQLPATPVKS

CPTTVINLDDATFHVALFCMRPE

>SG-AL_contig_7073_1_+1_1_444

CFVWFGLVWLFGRFVFLIFVFVLFSVFVLFLFLCCSSVCSSCISFASSYFPSCSCLCCSS

FFSSFFFFFFLFFLPLFIFPSSFFLFFLFLFIFFFYYYYYYYYYYYYFVVIVFFFFFFFF

LLLLLLLLLLFVLVVLRLLLLFLLLPFL

>SG-AL_contig_7074_1_+1_367_618

MNILTMNKTNNTKLIHPLIVSNATPFKFITVSNHSQMKAKPERIKQRLLDLVIRLYVNKF

NIQQCEYIQAISLHCQTQNLKHRP

>SG-AL_contig_7075_1_-1_1_411

WPAQPWFPDLQSLSHEAPLELPLCWDLLRQPRSGLTHSNPQLLCLHALVLCCKACPHNNH

GHLRTWKYDCTAFILATLLKAGTSWKPCESLWLPDAKELNSYTTTNGKRGRLGAQGISGP

NPPLEQPNSQLLGSPLF

>SG-AL_contig_7076_1_-1_67_675

MMDETYDQTFKKCMELIAEDTEKLNCQIVVVPSWRDAHHDPVYPQGPFIWTHKPQHLHFV

SDPCTLVINNVVFGITSTDILFHLGAEEVAQCPPGTTDRLGRLAQHMLDQHSYYPLYPPA

DDVNADYEHFEEYVKMSVIPHVLIVPSDLRYFIKDCNGCCCVNPGRLAKGQVGGTYAHLQ

LQPSTSNMPASVVSTTAGQILKI

>SG-AL_contig_7077_1_-1_148_309

MQICYSNYVDTGYFFRRSKSSSSLSIPFTTGLFIQYQNCSFMLNILQNSFTELG

>SG-AL_contig_7078_1_-2_470_676

MTYGIFNVHVYTHERGLGTGKSAHMLIWEIRKNLLPLPARREVSRWTEPRTFRLEVHCLP

ARQIMMSPI

>SG-AL_contig_7079_1_+2_2_184

SVCVVCVCECVCVSVCVRARARASERREVEGQLYSYVGVFMQPVVMCTLVFLIVFCVLLS

Y

>SG-AL_contig_7080_1_-2_14_787

MISKKCVFHLFGMVNVIELTERRLKKALKMDVFSLLDIKKYIEALKLSWLRKFKTTKHKW

KNICSELYPFLHDIEKYGSSYGLHKINKILFWKHIFKAHNSFCRNIKSTSPIDLLSELIL

YNELIKIGNDMIKLTSWIDKGVYCIAHFLNGNGSFMTYDEFNRKYNIDVNFLVYNGCKMS

IRKYIQETELNLLNGCAYDVPTTLKIIYSALKGVQRYYDILTTDDTLPNSCSKWLEKLGT

NIPWKRVFMKIHKIQEVK

>SG-AL_contig_7081_1_+2_47_367

MLHHPRTPNHSHSQPADHSPDTQNKHTAVVCVYMYVNLDPVPAAGQYNIHHMCGISRCSC

EVCTTPLPVSPDTQPMNWSRDQGVKQNMLMIQPLAITTPPTKWRHCC

>SG-AL_contig_7082_1_-2_239_388

MLVPSLFPLFIVFIYHFVVLLGTNILYSHLLRLGKCIFIFIYLVSVILPI

>SG-AL_contig_7083_1_-3_3_215

HSQVCTYADLEDWKNLHPLPTRQGAPGGFKPRPSKQCKTVTTVKTKFKYCQKLLRKCCSH

TFLKMCTTHRI

>SG-AL_contig_7084_1_+3_3_161

LARFSQLFLDIRIFTKFGPKIWTIEESLIHEFGMVRAKVKVARATKVLKFSTF

>SG-AL_contig_7085_1_+1_1_99

CVCVHIHVYTRTCEANMTGMTPYPPNILQRCSQ

>SG-AL_contig_7086_1_+1_1_180

MTAYSTDQTQTVTAYSTDQTQTIVNILEILRDHFWPSLKWNYTVVSWCAYTDSDNDHMRQ

>SG-AL_contig_7087_1_-2_152_331

MFTHTRTRTLKYRTEGKQFTAQLRERETMTVILTFQWQELLVKCHFSNLLIANLCVCLCI

>SG-AL_contig_7088_1_+3_3_170

VCVCVCVCVCVRERERERDERDTQQRETHTHSLSLLLLLLLLLYSRCSNSRCSSLI

>SG-AL_contig_7089_1_+3_12_614

MATTFHFSRETPPDSMFSDFQALNKFQNEQFHELTQYVFLFLTEPGKSSRLMTQLEEFAQ

QNGVGVGPLKNVIKSLLVLPNYALKKSLSPSQLQEDLGNLGLSDDKATFFTEQFQANQAV

LSKGALDLTLMVNQLVDMEWKFGVTASSSELDRVGNTFLQLKLVINTGNGTKNVYMELSL

PQFYSFLHEMEKAKASLEYLS

>SG-AL_contig_7090_1_+1_1_297

LVDTAKTAFYSSLVSGSNTCKQLFHNMSSLLGKSKAVTLPSVSDVKLLPNMFADFFHNKI

LAIRTSLHSAPQLQNSSQPFSGTPLSTFSPVSEDFVKKV

>SG-AL_contig_7091_1_-3_288_536

MFFLMKVRCSSGIPGVTPQGRPLHGILRGGHWTHSRAFLGPGTGMLVTPSPNGGMRLGAP

QLCRPSRSVHSGGGGGWPPGVLG

>SG-AL_contig_7092_1_-2_2_388

LCLSLSVCLSASLSLSLSLSDSVSHTHTHTHTHAHAHTHTHTHTYTLSHTHTHTHTHTHS

HSHTHTLTLTLALARTHTHLHSHSQTHTHTHTHPEQGNGRVVMKGTFIKTTGQLFTLSVS

LSTTTLNNI

>SG-AL_contig_7093_1_+3_3_182

PSPYPSGEGVKWLEQQELLTAVRMNERTICTSECMTIYKYPQLPQDRPTAYTTNQSLTSG

>SG-AL_contig_7094_1_-3_213_956

MDFLYGHDRNFNVFEDPDMTDMMEPDLFTTSSMGMMDDSILPSLMAEETDNNWFINSIFE

EQDLVYNDKYLGEAATQPCIKSEHSYSSNNLSPSSPLGLGKGDATDGLQNQSSSLELNQN

AQFIHAASTATQPPSIKPESPSHDPLLTAMTSSDTMSTFTHPATIILATTTSSSTASAYS

HNGQSVFPTTTVHIKTEPMETFHTLQERTVNLDTVIMPPTPPSSNGSDSDGSTSPQRSAP

NSPQRQSP

>SG-AL_contig_7095_1_-1_475_597

MPTLFSPLCCKVTISCVNQSAPTMKITNTLFRIIFKPSSFR

>SG-AL_contig_7096_1_-3_105_1091

MAKRLNVIFGHLHPQDGSIQFRSTSAVDIGSGISSQKFLYTVDDVPSILTQEQRKFYEDN

GFLVIRGLVPQEKLDLYRNRFKEICCGDVKVPGATIMKDVAIAKSEFAQGERAITKLQDF

QNDEVLFSYCCLPEVVKYVESIVGHNVLAMHTMLINKPPDAGKKTSRHPMHQDLHYFPFR

PAERIVCSWTAMQQVNRENGCLVVLPGTHKGELQAHEYPKWEGGVNKMYHGIQDYDPSQP

RVHLDMEPGDTVFFHPLLIHGSGMNRTDDFRKSISCHYASDACHYIDVTQSVQSNIAAEV

MEIAKKRIGDADVQFDFVDIWKLKSRPVN

>SG-AL_contig_7097_1_-3_3_932

GRGNQGGSGFGSGNQGGSGFGSGNQGGSGFGSGNQQGGSGFGSGNQQGGSGFGSGNQDGS

GFGSGHQQGGSGFGSGNQQGGSGFGSGNQQVGSNFGGSGNQMAGGQNFGGGYNQAGSGSS

FGGVGMNSGSNFGGGGMNTGSNFGGSNFGGGNSLSNTSSASWGTGANTNTAANSYGSGNF

GTNYSTGNVAGNFNNANNPNANTGSFGNYNTGSVMGTGTNYGTGSTGNNYNMGTSTYGNT

GVGTGSTNYGSTNFGATQSYGTNTMATTGQQQQGWNYGQNQMGDTTSNNTWSTGMQGGFS

NTTYNTGNRF

>SG-AL_contig_7098_1_-1_1624_1917

MTIGNGYLTKSCSILLLSLNEFYLTYYQICPLCEYNVCRCLVNYSYFIYLLVCLSLYSLM

STDACLSVYSLMSADACISLFIGVCRCLFISLFIDVCR

>SG-AL_contig_7099_1_-3_6_299

MSILQYYLPVWPFLCIFCCFKVFLIITRRGDAGGSFSINSIPTFWKICARNFLFFITLFV

SALFLSFTVVCCFLPSWSIPLSFFQQPFTQFPPSPHSL

>SG-AL_contig_7100_1_-3_99_308

MHFTGLFICLVGISVCLYVHMFMRLHVCVCLCRPSFLWLRVFITRCLLSTTADGIKREIW

RRTLKQFKKK

>SG-AL_contig_7101_1_+2_1091_1237

MELGEGKIIKLTFVGKKSRKTVSYYSKSWCVSTNAQIYTVDEPNNQCLD

>SG-AL_contig_7102_1_-1_1_177

IFNLLLALKKKKRKKKRPLDLLIINLHVHLEFSLEDEMGVYILKLNNCCVLMCTKLEVS

>SG-AL_contig_7103_1_+3_189_572

MLLLRTRLQYCYKFNSLHPNYYDHHNHDYKYLIRTCLFCSIETSSPVFLKKQNTKINCSN

SLGLKAQSVLLILKIKCHLILTVTINFEHQLTSNVVHINTQQLFHFRIYTPISSSRLNSK

CDGEREEE

>SG-AL_contig_7104_1_+2_140_265

MTMYMCVCVCVCVCVCACMYARACMCVCKFINRSKGGFLLFF

>SG-AL_contig_7105_1_+1_829_1089

MQHHKDQEETEQLEGENSDPDFECEASDSNTFFFLHLFHTQQLHISIYIYNIDCICLLFN

FFFNRPIELHKKQHKHKACSFIFQCAV

>SG-AL_contig_7106_1_+2_2_283

FHWVSYKIENVKYCVCVCVCVCVCARACARRAHVCIIVCMLGNERPLLLFVSFYSVNHEN

HFITTEIGSVLRSKQFKTKTPPFFKTLFLIQLRP

>SG-AL_contig_7107_1_+2_2_151

ITTKKKRRKKRKMKKHRRGGGRKTGSIPNHRVLTGPVMKVQVDYRKKHEK

>SG-AL_contig_7108_1_+1_1_942

RSVRDPLSGVCVSLTASLSSSTLSTTGAPTSVCVEKDLNQNAKNIDGGLLVEHRQGDDVC

DSTNEKDETDSGYRSGTIPEEKLPKAPSQATLNRKERRRKIQTFNHFVPKAALEETEDGE

SFQGFLRVTLNLIRPITMELGARPPSIYELLTREHIVEQSTQHIAFYMPRDTVKSIHISS

KTTTKEVITSLLKKFHILDHPRKFAMYEQEFNEKNKLVKLRRLTDKDFPLFALLSWEPEQ

VKHYRLVLQENETGEIVWDAFSEPELKNFMIVLDREEGEAIAQLQYKYRVMKRIILQRMK

ELRQEKHALKESTA

>SG-AL_contig_7109_1_+3_1041_1370

MSVPLYPCMIWNPGGYLGNKVKCKMQMQGKMQWPRCRINVLTKGLLHWTVTRSRTGSTVG

KGGDTTNGLGWTFDVERYMEGHHWSWRHTVTNVTHQSALPSVKEQRIIND

>SG-AL_contig_7110_1_-3_3_161

RGDFVGGRGGGITTKTQYLYVYKICRGSNVSLKAKKGRGIHHRGRETSLCSQA

>SG-AL_contig_7111_1_+2_479_556

MCWLMVQAMMIWNLFPSHVLCGFSAC

>SG-AL_contig_7112_1_-3_3_299

CIPHVKTKTSGECSFSFCAPKQWNSLPSDIHHLQFLSSYKRTLKTHLFQRVFTDGSKSDE

GVACAAFNSTTGKSFSEHLPKESSVYTAELTALLLALKM

>SG-AL_contig_7113_1_-3_3_197

FTTGWTGSCSQKVDQPGSNSSSPKKTNIYTNKKNLVPFWVCCYLKINELRRKRSGVVVWC

LKASY

>SG-AL_contig_7114_1_-2_170_352

MGTNEIQSTFTGAFVDNGEMNRHMFLNGTCLVFMVKTVIIQCLLSLHKIYFNKPWLVFYQ

T

>SG-AL_contig_7115_1_-2_317_538

MKCYAEENFGPVLSAWRCTPWRTPSTPILMATVTAIFTTNGATARKYTVECDVGQMGVNV

PIPMPLPMFSFTDS

>SG-AL_contig_7116_1_-2_2_646

SDHVDYQNWNPSNARVQCFKDVLPLIEKFLPIEKSDSPKQDVSRSDRLIQLVLKGVLYES

CVEYCQQAATGSDSAAIAYVSLLQDTGFSEADLSLLAWLQSIPYDTFGCPFEQKSVNVDV

RPLVKPSLEASWSEQILVTPIKPKMFPHSAIPAARPRSAEIMTRSLNPQFDGLSSGLRRD

PMVTSGDLAALSRSLAPAPSLSTLPPPPMPQGPRN

>SG-AL_contig_7117_1_-1_46_792

MDDFLGSILKSMEMPPRLGGSDRKKEEAQRKMLEKQKEAEKKRLSDFREKIQKRIHEFIK

DGKQEKYKFEPMEKVFRSIIHEVAEVAGLTSFSFGHEEEDRYVMVWKKEFAPSDEELLAY

RRGQEWDPEKAKEMAKLKEEERLSAEAEAAQRKKMTPASNYQDKYRHLIGDNKVCKDDAR

STAINRNYGFVSADNKRDKRTIEEVLAESRAKKRMRTDGGPSSQTADTGDNADVAGATAS

ESSNPPSSS

>SG-AL_contig_7118_1_+1_70_390

MRLVVEFIDKFNLVTMRLVAEFIDKLASRMRLVAEFIDKLVSRMLLAEVLYKAVFRMPIV

AEFIDKFVSRVPLVVLLDPFVSRQILADVFDKVTSTSHCGELNIARD

>SG-AL_contig_7119_1_-3_201_362

MEKNSSSFCECTVMQSLFFPSPFSASIMNVECLQHCDRVLFIMPISSGQFRGGI

>SG-AL_contig_7120_1_+3_390_638

MASQLFNMDCRIFGDNFFLFCGPYTSLFLQMINKLAIFKPFHGLPGSFDTVCNTFLKYNT

GKVHMKRENRNVLTKWENIVFFH

>SG-AL_contig_7121_1_-1_424_606

MWSPWFICIVFVGYIYNLLLHCLREKKIPHCCCVPPYTFICCAHDEITYCVQSLFLGIGK

L

>SG-AL_contig_7122_1_+1_850_1095

MCALTLANICTHTLLYTLRLQLPLSGFVSDVSVYEIVVYIQDSTTSFWFSFGLFQVCYEI

VVVYRQGFYTLLRQYLLLFEMN

>SG-AL_contig_7123_1_+1_1_183

INTSTLLLKQNTEHLNNDNRTTGNDFFRVFNGQSSSATSDLSMFESQIEKCGKAFNLSLF

L

>SG-AL_contig_7124_1_-3_147_293

MRVISLFQLSTSKGLYDEQIFKHSTFVLEFSTSFTEFLKLPGVTFLVIA

>SG-AL_contig_7125_1_-3_249_398

MLLPSAFHLVWQLSEQSLGYRPKAVGKSFFRLCVKVLKCIIWIRHSSLIY

>SG-AL_contig_7126_1_+2_23_490

MQQAHMLQNGQMHKKKHWFDFIKMILHEIGMNHIGNKQPTLNENKLKYAMKKKQLEYKHI

FFWNQQKNRNTSKLCLYGKATTSYRLEPYLINITKSNHRKALCRLRVSVHGLQTERGRYT

KTPREDRKSSVCGVIKDELYFMDNCTRHKETRLKLI

>SG-AL_contig_7127_1_+1_202_510

MSSASVGAREISASVSSGVGFSGISKTASSSSCSEPSASSKTSSTSLGPRDISASVSSGA

GLSVISNTASSSAGFVISVSSVISSASLGPTDISASTSSGVGF

>SG-AL_contig_7128_1_+3_3_200

FGNVFSFRGSKRDLCFSVFRRRFLWYFKDSFFLCRFHYFSFFGDVFSFCWSERYFCFSVF

RCRFRR

>SG-AL_contig_7129_1_+2_272_460

MHWMGHKCLSGFNLSKIVDSGQMFLSGEGSPGSWILARNSGRNLKVIEPSVRKVMSTGLP

LRV

>SG-AL_contig_7130_1_+2_983_1276

MLYVLLRYTLSSQSCLVSYVLFIRYSLHSTIYVYNIYIYIFNKKDESAAVPWLILVKENT

RFKFHYTMHHAINSSEANASKYFTNFSLQKLSWKQYTD

>SG-AL_contig_7131_1_+3_855_1217

MSRQRTFKVQTINTQTMNTLKYRQRTIKTNKEVQRHSNKEVTEHSKYRQRTFRQRTDNEH

SYNKEVQRRSNKEVTEHSKYRQRTFRQRTHNEHSYNEEAQRHSNKAAQRHSNKEVTEPLK

V

>SG-AL_contig_7132_1_-1_280_438

MLNWEIRKISTIYPPGEGLPNPPFDHCIRQHRERERERERERERGVQTSDSLF

>SG-AL_contig_7133_1_+3_117_437

MRVCCRLQLLPQIFHITHYVRKLYKFEKDVKNKRTISHPVLSVVRNQPAEYDQFPRRSIC

LTQTTRALQPIPSQACTAIDSVVRHFGNVREDNPWPGSKPGAPSAMS

>SG-AL_contig_7134_1_+1_1072_1488

MRQHITQVCHVAYLELKCISSIGHLLIDSAAKTLVNSCVLSRLDYCNSLLIGAPLSVIQP

LQKVQNCAARLILELLAIESSTSLLSMQAACICYNVVSCTAPAYIAKLLHAYSPSRTLRS

SSDAQLFKIPRYRRKTHAF

>SG-AL_contig_7135_1_-1_91_870

MVTQHGKPMFPYVITEGRLLLEFTFDDLMRIRSWHFAIRQHRELIPRTVIGMQPVDPTMM

EQMSKNITRQGLTNYTLNFLRLCVILEPMQELMSRHKAYGLNPRDCLKTTLFQKWQRMVA

PVGKESARPPSKRRKRKGSASNNSGAGNNNSRDVPPSGKQKRSPGPHGFNLGSSVPGDVM

VVGEPTLMGGEFGDEDERLITRLENSQFEGTGVGQEGEGVEQKFSNSPAWGQGDKKTPPV

SAAAGPPPPTGNPETEIKTE

>SG-AL_contig_7136_1_-1_1_159

RLVVCEESIYDGSLERTFLGTCCRILFYFFNLKYVSCDTNSFNGNVYCKISFY

>SG-AL_contig_7137_1_+2_1553_1828

MNVFARTEQERHCYTILHVCTRIHTTPSFYMREHTHAHTHTHTHTHTHTHTIFLWLNKKL

SSFDIINPNNQLHPFQIPNIVTVTGPYGFFFK

>SG-AL_contig_7138_1_-1_247_360

MLTIRLCSRCINDQSFVIQMNFAGMCSVIIVTHEATDK

>SG-AL_contig_7139_1_+1_1_201

VFYGNGSLVLVIASFSRHFPLPAAKKTLENRCIESKDTNFFPTKKFFPMSASSDEEFLSA

FISGSDN

>SG-AL_contig_7140_1_-2_2_229

DSLSVCGRIRSRDVAGPRSGSAGPRPFCWVCLASASVHMLTTIYAIQMCTGFLFCLGHLS

HTRCVQISSSASAVSV

>SG-AL_contig_7141_1_-3_3_74

FGSCIDRGVCLILFCFIYYSCDCS

>SG-AL_contig_7142_1_-1_4_600

MAQIGQLALTKPAPEFSVQAVVNGEFKTVKLSQYKGKYVVLFFYPLDFTFVCPTEIIAFS

DRAEEFRSINCEVLAASTDSVFSHLAWVNTPRKQGGLGNMKIPLLADKTLELSRQYGCLK

EDEGITFRALYIIDDKGNLRQITMNDLPVGRSVDETLRLVQAFQFTDKHGEVCPAGWKPG

ADTIKPSVNDSKAYFSKAN

>SG-AL_contig_7143_1_+3_495_716

MIVYPHECMCRILSVDFQEDFQLNILSRQLRMFPEAAVSHTSNCQVFDFLSTPSHVNQSK

SNQHSQRLLHNKTR

>SG-AL_contig_7144_1_-1_484_858

MCTVTPVFVTVPELQEFTSCCRTALWRIDVFHSCKAQYAHTHTQTHRHTHTHTGYILFLK

KKISLIVKYKYCGGVFLFPHLFFVSFVLFPFLSFVIFHGFLCNILNVLLSSIHVCVFIVS

VRIVS

>SG-AL_contig_7145_1_-3_3_155

IWPRQGDLSAPQCSQSDMVSILAKHVFRKKYCLHNMHQIENIEQLLNQIVN

>SG-AL_contig_7146_1_-1_730_951

MGLKSHYSGVSGFESKSHSSSSLLYLASSSMTRVRYPFTTGQTGSSLEINAQFSTPDLNL

CSLIGKQEIYLYTN

>SG-AL_contig_7147_1_-1_1_207

DVLFTASNLCLCSLPLKSLWTTAGVVNWRPPCGLFSSEIICFCFQIAQLSSFVCPCFIYC

VWVVTNEIC

>SG-AL_contig_7148_1_-2_173_745

MGHHYRLVVSVITPDGTCTLLWSVSVWARHHRWRFVSLPVVAETVHKILISVERKEWSLS

SVVAIDSISYRRKWRCTVAPHDDEVFYPPSSTPCVTSPPPRATTLDSSDLPRGVPSIYIS

DENDDDESSSTAQFTMRIVVISVVLVVLLGVFICWGCQRRHAKKRQYDVRRIEHAQNLQN

DVIIQPVTYQW

>SG-AL_contig_7149_1_-2_74_319

MRALGARLQQEWGSSSSNQQDVPSDLVSLGREACGAIVESAGAAVKLSYNLALLRRDSIL

AASNLPAEDFPALRTVPMQPSS

>SG-AL_contig_7150_1_+1_115_366

MNTSQDVCINLCSPCLGKEKISHQASEPKRVTDLRHRERLVICSASCMLYVSRCSSLWPG

AYNIAEGVELHTWQIFVTLRHTVH

>SG-AL_contig_7151_1_-2_2_118

YEGIASTFLGASVASAVPIFSPFSQSQHNECSAHLLTTT

>SG-AL_contig_7153_1_-3_141_266

MTETLRWAANRPVFMLQAGFKLKTSDLESTALLAEPASLPKI

>SG-AL_contig_7154_1_+1_1_123

FIYLFIFGYHADFHNVLGLTGRQEDKFFCLSLIPSPTKQNC

>SG-AL_contig_7155_1_-2_2_256

LPITTTSLTTRSFACSSPAGRKNGNSAQESSSSIMKSYVLLIVLILIFALVWTVGDACTI

SPECKNGWALDKSGCKCICRNYLRC

>SG-AL_contig_7156_1_-3_870_1058

MLFAHEKRKAECCSFQLTVNTNNLRTDFLYAVCLLSMISFVNLLLFFYFFFKIPFHYTTV

TLN

>SG-AL_contig_7157_1_-1_544_819

MLLSLSNKLISSFSSLSLSLSLSLLSLKGKTACVTGLSDPLELPLFRTVFNQTPPNGKCV

STKWKVCFLYTFACFFVVTKRNDCQGLYFQVE

>SG-AL_contig_7158_1_-3_459_800

MHSPVVICQSTRTIEQDRYHSLTICCHFTTVPDQCCLAEPRGVSPTQPSPCHMLHFVFLH

FKLSIYQTVGMNILKTFLGTVQGLISNGGGLSFLSLIQIAPFLFVSLDNYLRTA

>SG-AL_contig_7159_1_-2_218_805

MYLFMLVLISCCPAFTPKNWHNSALCPLVTTLTGTGCVHHWSLTESMNDTFIHGSIFAFK

GFGRGCPRLLLQAGKPHTGQANPAPATADESWSESDAEGSQFFCLFLFSSNLTIHFSSKV

EVAWWIVPLSRSLSCPLAVFSGTGGEGLFQGVLISLSWCPSVTVFNKQFTIQSDLWLSIV

FRPGDVSCLRHLVVQH

>SG-AL_contig_7160_1_+3_441_674

MIHFKTRHTDFVTMNLNQFSMFYFDLLTLYNCSHLTTRFHIIFILCYAVYNTTKLETNVL

NLRFEILFSFSFSSSFFF

>SG-AL_contig_7161_1_+1_1_243

SIYVSLSTRHTHTHTHTHTHTHTHTETHTHTHAVTHTHTHHFTRLRDGLFQLQMHKHHFI

GLRYDPMLLPHPHTISLLIFS

>SG-AL_contig_7162_1_-2_89_415

MAADVTKPCELASFRSCQKRFLRASRREACSVRTTLVGLMLEVGCAKPFPQTLGFKGLKP

HLVSVLRQCSSGMCQAFSSGTVLQRLEVSSGECPQAVFKMYTHRGECSG

>SG-AL_contig_7163_1_-2_23_607

MEVGTSVQENLSLAGDTAHIPDRCFDQLVRRACQGVLSDVDRNTIEDDPSFKSVDKAVLK

AAYSGMVALALEASKHDMDADGITSFLEECKFSPDRIQGFNNIFLAQKSAIQILLGSIGN

CRPHIVDVDWRLDYYIKSNHMERINEAVYLISLKTEMPGQEELQEVQFTCSLEQLQDLVG

KLKDATKCLEKLAQM

>SG-AL_contig_7164_1_-3_123_311

MLYFGLVIYFLKLNSYLGMKIYYLGHDFSFGAEPLVYLMKHLFFWIGGCPPFKDVIIFLC

AFC

>SG-AL_contig_7165_1_-3_417_623

MLRLDHSMTPFHDICHSSFVRDILPEYPEKCSGQCVMADDVTKQVCSNSLLPEVLAALQG

CQHMTVQKR

>SG-AL_contig_7166_1_-3_3_83

FCTPNTGRNKDLLYSLILVKTKIICTA

>SG-AL_contig_7167_1_-1_16_1278

MAATDVLLQQAVDLRKTNTIHASEILTKIVKQEVRTLDDEAIRVKEQAILEIGNLFAETK

QATELAGLIKFVRPFLGMVSKAKAAKLVRSLVDLFLDMEAGTGKEIELCKECIGWAKDEN

RTFLRQALEARLISLYFDTQNYQEALALGSILLRELKKLDDKALLVEVQLLESKVYHALG

NLPKARAALTSGRTTANGIYCPPKLQASLDMQSGVLHAADERDFKTAYSYFYEAFEGYDS

IDSPKALTALKYMLLCKIMLNVADEVQAIVSGKLALKYTGPEVEAMKSIAQASHKRSLAD

FQKTLGTYKSQLAEDPIVNAHLKTLYDNLLEQNLCRIIEPFSKVQVQHVANLIKLPVDTV

EKKLSQMILDKKFHGILDQGAGVLIIFEETVVDKTYANALETIHSMGKVVDALYAKAKKL

T

>SG-AL_contig_7168_1_-1_1_135

GRFAQMAIRTFSDVVPGKGLKGSVCKRMDQELSWANLPKIHRLLG

>SG-AL_contig_7169_1_-2_290_451

MQCILSYMDILCQVVFAHRGLESFVWIHFFFTSHFVVFISDQFFVDSSLYCRNA

>SG-AL_contig_7170_1_+2_2_325

PQKKKTNNKQPHPKQTTKTTTTKQQQQKPPQQKQQQNKTKTNTPPPHTQTTTTTKQKPQT

KQHQQLTTKQTNKQTNNLPATTKPDMPRKKAVSRENPVIRWGQRWRKW

>SG-AL_contig_7171_1_-2_2_244

FCLVGCLVLFLLPHLLCRFSGVDPMPLIPALPHTTKPKFIQNPSARKPMGANNVELPRGH

PPGDSYAARSLQWNSTVPVLI

>SG-AL_contig_7172_1_-1_1_114

PDMAVVVGWAKQYILKMIILYASGRLIYFNHIFICIRL

>SG-AL_contig_7173_1_+1_205_432

MINKKSVLEMLVVRLIYRLHIKRKVKNHFQDIIFYTVQHVYYMLQQIIHKSQIRLITLQN

GLRMTIPMFTSHTINV

>SG-AL_contig_7174_1_+2_2_82

RKLHIKWLYTRSEILLIDHIHVHDLLY

>SG-AL_contig_7175_1_+3_18_209

MFPHNIYFKTLLANGIRHGLDPSLILKQSQPLRAKPQQELIKDWYEKEGKNIQLICYTYS

SIYV

>SG-AL_contig_7176_1_-3_33_254

MQTVIRRMLQQVIFSKHVIWSGSILDLEQPIVEGICFSSIFFYQFQSCFIGFSTVVLLDL

VQLSRKLSLSSMYV

>SG-AL_contig_7177_1_-1_118_303

MESRSDIWSSVPAHNITTVRCPFQWVIHSHGVIQVFHRSRPPFPLQDFAQVQGACQITFG

TF

>SG-AL_contig_7178_1_-3_3_299

GGDPHGRGQDRHVTSLDQQILLALMQLQQNVASINDRLAALEMSINLQREEEAKNAQRES

MWWPFQSLSKKWVAFLIVWPFLTPLIVNFICRRRLRSSR

>SG-AL_contig_7179_1_-1_52_363

MMNGAAVPTAQTTVAPAMPASIATSRQPVLGVAALSTQLAVTSPPSVSAVKSPSAPSATL

INFDNPTVQKALDNLIQSGPNLLKNISQPSLYPSTTNAPPRLSY

>SG-AL_contig_7180_1_+1_1_243

NGTDIQLSEVVCNLGVTLDDTLSFQQQITSISHICYLELQRISSIRHYLSEEAPKTLICF

VLSRIDYCNSPLCRCPKHLFS

>SG-AL_contig_7181_1_-3_1842_2141

MLTHEQTYRCMCEREQEQELATSLLVAYFFFFNSSCILKQESLYDCLKLGRHDLRGQSSL

MVEIAVVAPIIFFYVCFLFWFGFVLFCFFLFFFKFRSPFR

>SG-AL_contig_7182_1_+1_226_417

MNCPRARIALSNIMENYYLVDVWRELNPDKRPYMCQRFNSTQQGWLNYFLISEQLMLDIK

NAAA

>SG-AL_contig_7183_1_-2_269_457

MLIWEIGKIASFYPPGEIPEQVRTQTFQLEWKSGVLTIMAIDTMIVFIVTRQTQFSANVN

IIL

>SG-AL_contig_7184_1_+3_636_893

MSELTNREIVSMVSSFQFLIPFTIFQPASKCYDLLSGLSEILLNLLLSLFIQFIQFRLLH

MRLIGFPTAIVFEHLTFYNDSLMQTD

>SG-AL_contig_7185_1_-3_318_653

MIVVCKNVSHPELRKLVTSCFTMIEPQERGSVEGEKEEYCATIVAEPTVSPTPPSSCQDE

CDLLGKGGFSHEDAPSLLNTWGLVTFTHTVVELSLASVRESEAGSFLSLKKW

>SG-AL_contig_7186_1_+2_743_958

MRPSTSMHTCLFCSPWSVKTKHMNRHKKKKWIWQLFLNVTTTTCYLNLSPIARSHVCTAC

CSAAYVMHARVR

>SG-AL_contig_7187_1_-1_1_420

HWAGRPVAPSWNRRAWRSSCPTTWPCCSGTPTSLLRTFRPYGLSPCSLPLFSAPSRSPSS

PPGPLPDGIRRPSPHGRGPPAVPPPRRANHLSPGPPPRASRQPGCQKDTCSRQRFCLVPP

PGPGLGWPPGHDFRLPAHFG

>SG-AL_contig_7188_1_-2_95_343

MARGSIPMSLLSALYLFLLSVLDWWPCYFSCGLYICLVWGSLFCLLFTYCLLRYWHVGQQ

RRSASPVCSGPASVWYSRYSSRP

>SG-AL_contig_7189_1_+3_3_197

RTLINHGHTHTRTHTVEADFIEVCCCWFAFPSFTLWGSLSIYALCLTRVRPRVHLDEKIA

CMAVF

>SG-AL_contig_7190_1_-2_59_316

MPAQCGTNSVHSRTPETRQHCSERWHKSTNQQTKQGRRKKRINEQMSKQKERKNKIFKKI

IAIYHLFLHNTYNACFVANSSFKPKK

>SG-AL_contig_7191_1_-3_189_611

MKNIWQQLRVVTSTLPHCSQTQREQHRLLVWGQTWKPVFSTEACSRCKIPTHKVFRTNHI

MIFQHPQFACETLSVHCQGPFSPAVWILGKLTAGQWRFHGCFFTASSCSTLPRHFRMKFQ

THDSACLDVTSQCFLTQRASE

>SG-AL_contig_7192_1_+3_63_248

MDWAWYGMKSPFFCACWVFSCFHKPATDMPYTTGSLTCIFDILHVYTHEGGLGTGRSAHM

LT

>SG-AL_contig_7193_1_+3_324_563

MAKCKNFGQKGFGNHARRPITDQIGNHAKRLIAEQNGNHAKRLIAGHNGNYAKRPTSEQI

GNYAKRPSLNRLEIMQKDHH

>SG-AL_contig_7194_1_-2_116_328

MTPSDLMYQPSSFGRIRHQVREAQDRMDLHRQMLDRYLPEEGAESSDVAARVLRKMDMEK

RETIGASSGKT

>SG-AL_contig_7195_1_-1_1_225

RGHTPQSTTLRVFTQLCTLTECVQLLQYGCRVTQGHMVRNKYYTASLKVWVTLSFTFEGS

GYVFVYVLFTCFFTN

>SG-AL_contig_7196_1_+3_219_446

MIIILSVFTKLENVLPINIFQMKTLVSRFYWEQNLFPPKIKKRKKKKYQQTSFQFETAYD

NYFKCENSLGKDFPVS

>SG-AL_contig_7197_1_+1_475_735

MKCRPLSVGKARRHSMALPIFLVVFLFPLYVCILVYLQKWIFLRDFARNNSSVASGSFTC

TRCVLHSGPQFIVSSKGLAPRPPIQVW

>SG-AL_contig_7198_1_-1_1_249

RLKLPSSIHCLKFYFIQRNSMIIAIAFFLLNVDNCSKLNVSMTLRIAAAFEVMIVSFSCY

VLTHQMQYSSFGCLYSSMIWVFS

>SG-AL_contig_7199_1_-1_52_639

MPQRTVQSQTSLMNSEPTYQSSQVLSDQQNNDVKPDRIHTKSSEAKVDKCSRPNRRKRRA

SGKTIMLEMDTGDLDRWLNVGLTHHLDTSTRIAVFLLAHYEKNMGQAVQGKNCQNCGHSL

HLFCPACKKLSPTPSSPPPPSTTTRVSSLPTATTSMATAEQQQERLSCSSHSCLFPTTLS

TELTSSLITQPPPPPS

>SG-AL_contig_7200_1_-1_256_915

MRFDTTSKFVVPTPAHMEKLASLLSRLQRSRIATARPITSLLGMMEFLDPVISLDQAQKH

PLQRTFQERWSQSHHARDANLILGQWFLDVTASWTSPAPLLAGVQISTPPHKVALYMDAS

TGGRGAHMGSQHVAGSWTEEGASWHINTLELGAVCGALVTFKPEIRGKVVRLFTDNTTVA

FCVNKQERRHTWWTFLSEPKPSCGATSEKFLFVRDTLWGN

>SG-AL_contig_7201_1_-2_128_964

MLHVQGVDPGEVRRGAEEEHEWLLSRVLEVPPSHSHSMKTSVSWHEKMHHHSPALPAEGI

PAPSHLTDSGDKPSASKLSEDSGDAASQGAQSKGKGGQAIAKDRRRLFKKSASLDSEVGA

CIAHAGMTTLVPPAVPSAFSPSDFLATIKGKLAPAFGRKSKEEVRAFEPRPPSPLFIPTT

VPDIPLRPPASSTSTTSSSNVDPTTSRQREGGDALDADVLEDSRGRGFSRRQDAQRESRG

DARDIRWRSMSADRATRPLMVHTDVIVPAQVWQFSETAV

>SG-AL_contig_7202_1_+2_2_130

KNERERERERERERERERERERERAFHFYVSKFNHFTTFSSTI

>SG-AL_contig_7203_1_-1_100_807

MLGHHVPGMVWPGGRLHLHSGVNSQGVMQFTFRQLLDAHYDRKPIFGCIGGQEHEPSWES

SYYLRPFGICMQFIKKGSSSSIKLAEWLKLSRQFANNWSHPVPLSEYTWEDVASGEMWKA

KTVAGLYLLESVDSLKSTKEKASRLRQAYNIYKEAIESHLHFPGYWHKNFALVCEKLRHV

ADSSETIPLIKTTIAHFKAYLHSGTSDPQTGTIKSAIKKLQEYVDYMTQSRDDGNR

>SG-AL_contig_7204_1_+3_3_212

LPGNLHTECRLPGNLHTNCLITWQPMHWILSLFATPHEKAFQALNKRKTHTHTHTHTHTL

TLTHTLHTYI

>SG-AL_contig_7205_1_+3_708_938

MSFILFIDIIIFLNKNSFFSLAHFDNEIKLIHKLSTNNNYGKTSKTVVVCFASATLDFWN

TKLGEKEKLFWISIILH

>SG-AL_contig_7206_1_+2_77_343

MNCGGGWGQRKLCEDLLEKRKWNGPRRQKKAEDSEGRKKGRNKESNRKEEFLVQNFRKVG

ILFILKEPPASPRFFFFNCRYCYVPIKLL

>SG-AL_contig_7207_1_-1_391_720

MDTVCITSSPSLPQAPCLPLKPLYSERMIFTRYDQLHVLEISLPSLTGPHKPHATPPFIA

AGSKGQRHARASRVVAEGLLVGHGFRSRGRMGGGAIVRFVFLKKNYFKGH

>SG-AL_contig_7208_1_-1_1_612

GLIYQHDHNASRPEVEKTFSFPLELVLKVFDDKIVVAFGERDGIKVGHTVLSINGVPSEG

RFLKDGRDILETLAMEENYPINIKFGRTKLTTNEKIMLASMFHSLFAIGCQLSPEPRSSG

IEMLETENFKLHCHQTMTGIKFVVITDPKQTAVDGLLKKLYEIYADFALKNPFYSLDMPI

RCELFDESLKLAIDQLERMGAASV

>SG-AL_contig_7209_1_+2_83_409

MSATFICTKRYHPTLLLQTFIQSIHTYICVYICVHICINTHTYIYMYTYIYIYFLLLLYW

PPSVPKDYGICATGETPGHKPGQSVKESLLLLMQQAHPFRDTGKIQGRD

>SG-AL_contig_7210_1_+3_312_728

MGRIIDWNISFGEGELRTKTTLYDRVHFIDINGCSHLSARRSFTQPPALHTSDADVHTSI

QNWLGKNINTFLVNARSLFSQLKNHSFYCVQAAPIPLSLPVLMFSNKMLFHQAALSLMAR

YLSVPIMTIGCTGRGLATR

>SG-AL_contig_7211_1_-1_271_1569

MMNLASLLMVVLVSGGSLLLQQTMAAPAAAAPAAAAPRVGGAAPAKDVLLGCKDNPADIY

FLLDCSSSVWIVDFEKQLEFVVSLINYLDIRPNATRVGVGVFSDDFYHELDIGNYPDKNT

LIRVVRNTPYLSGNTYTGKAIHGMRTFGFRPEVVRPNVTKIGVVVTDGRSRHRTNTVSEA

TLTREEGIWLFAIGVGNSVDEVELSAIASVPQKDFSFHVASFDILSTLARTLAISTCSLE

QPQADGAVCGHKKQADVMFLYNAGAISAQELEMVKEFVEYVVGKFSMSSGNIRAGVISRG

TQGGDIELSQYIRREDFLSALLSQPKPKLSPLLKKLRTDGFDPTNGGRYNAQKMVIAFVD

DVFDDARDVRYEALLLKYDEAVIHVITLGSKVDPGQVKQMATSQDTITDFGSYAGLKDDK

KRQEFLDKFCVDL

>SG-AL_contig_7212_1_+3_981_1133

MSPPQTYVQIRHVICQHYVQCLTLSLPHPHPYLSACCRRLMDNARGERVKN

>SG-AL_contig_7213_1_+2_2156_2377

MSHTQNENKPVSETFLKHHRELTLSRVTNYKHTATVTNHHLRNLVSVCFNTRDLSLINEK

LFPKPILSPSCKYI

>SG-AL_contig_7214_1_+3_384_584

MIKTNKQTEKQTTKRTKKTFWTELTVYNNTNETETKRTKKTFWTELTVYNNTNETETSEA

DTAEKYC

>SG-AL_contig_7215_1_+1_1_171

SIKIYIQIQHNEHSNHLWLICTQSSSPPPPFIGKTRETLKSHTEISKYLDIMNTDVT

>SG-AL_contig_7216_1_+2_446_544

MHANFCSLRRTSPATSPSLENKLHHFKQSITSI

>SG-AL_contig_7217_1_+1_1813_2148

MHVKDPVSHVSVQGLMETRKYMHTPRNGVGLSTDDRVCRLCSNINPAQCGPNSQNNMHKH

IPNNTAQNTEETGPETRIERPATVQFGWRSLYRLTNVNDQPAPHNRPHNWVL

>SG-AL_contig_7218_1_+3_90_296

MKDFWRLCSIFLHNACLLENCSQSHMQSKPLVSGYLSCQQAILKKKEKCQINVRENDTFH

RNIYHVHLL

>SG-AL_contig_7219_1_-1_250_426

MLFQTSHIDLNSWSPWILLWLVSDKKTPQKTKKPPQKTKAPLYALNCLLYIIVISYRFS

>SG-AL_contig_7220_1_-1_55_1788

MSPQIAHAFDTEVWLRRQQRELAAKNRELATVRQELEAWLRRKDRKTSNTTNFSASIMTM

TLRLQEKDILIASLKRELTESRELLTATKKATAEASSSSESLSLSLQTQQQIAHLRSELQ

RAKAALAAMQEQSDSHSDVSEDKEDREHIEKLEAEIRLLKSSVDMSAEDFLQKVEELRKE

LSEEHQRHVRDIQELARQDLELKLRELQSHHDHEMEQLQQLHQRKLEQALAQQQQELEKR

HKTELNHLRVQHQQEIDRLRNEELASLMETSVAQQLHADVLATQQMDEHMIGRLTTEDDA

GTDLSETCPPHIQALLDRLREAGEHILSVSELEQLRRHLGGHRDLAGSDGLQEAWEGEKL

SLVKTIHALKELLSQADHVNAGLEESGSDWRADLIHALVEVYKQERLGLEAELHTLRLQH

PQQEHANTIKNFDNRLHEQEQLQQSGLDAMMRAERQSLLLELEQLQTRLSQQRQEQQDLQ

THMTESLAQQEENASNKTWKLQREVQVLEYKLQQEKVIEEDLKKSLESERRHVAELSSEL

SRDKGTLISVQSELQSAQLHLSRTKDALEREQNRFTSV

>SG-AL_contig_7221_1_+2_104_373

MHNLGGKTFIAFCMHKVGSKRHSQLYNQCTFRSHYQDNTIIQQHSRIIIIHIFTSGCSIY

AFIYTFQHRYCGSEQNTNFTLHRQSLNKSL

>SG-AL_contig_7222_1_-3_3_242

KKKKSKQVIKNVWICFLDDLYKHWSFVPLLPSFTCASKYFVHFFFSWYASLLKRVDKNGY

WFGLIFSVSIIHHILVLETK

>SG-AL_contig_7223_1_+1_1_117

SNIFQMHCHSDLRNKTACFMKLKNDIQLKTKQTKLNIVL

>SG-AL_contig_7224_1_-1_385_1848

MNLKNLKPGGEKRFIMDNVRLFAACCFFACVQEFLVVNALTMDELISNAAETTEEFSFFQ

GSNDKPQIKVELDMIFTPQQWEEIQRASGTYGDESGRHRRKAIRSSTYRWPNGQIPYEIA

PRVFSSRSINEIQRALNEWQKYTCLTFRRARSSDRSKIRFDNGGGCYSNVGKIGGSQTVG

LAQGCRYKGVIVHEVGHAVGFQHEQTRPDRDDYVQIIRQNIPPHLYYNFQKYSRASVNDF

LVPYDYKSIMHYGPKAFSVNGGLTIKTIDPKYQNEIGNRQGMSFRDIKLANLMYQCNAGC

SNKNLRCPGEGFVDKNCRCMCPGNPIRECSNTGTETTTAGTGTGSKITTTTGTGPNECVD

RQKSCSAWAARGECHRYRFFMSTYCKKSCEICTEENQRCKDMAEHCKYWESQGYCRKKYV

TYMRSNCAKTCNSCDSVNTEVGDSNTGGTSGSNDNNDNDADDENAAGTSLPTLLSVIVMV

AFSVVLHY

>SG-AL_contig_7225_1_+1_1_300

FPSLTLWGVTQYICTLSCESGTTSAFGWEGCVYGCFLVPRHVGSHTPTSEELFYYDGPSL

CTTVISVCANFSWHFSELSIKAWSHLFTVMRVQMYAQTVS

>SG-AL_contig_7226_1_+3_120_314

MYKKSFQKSTLYNVLISKIQRQAGEPDEADDTKRNSTNTSQGCSSSTDGEETQDKYTVLS

TTLKP

>SG-AL_contig_7227_1_+2_293_400

MSEMAVPDWGTSQTTSVHLAMAIYKNKYDFYRMGKS

>SG-AL_contig_7228_1_+2_2_229

SFRLVLVDDLGRFRCLGRISLIFSFAASSVPCCWKVSSPCLLFFAHLLSEMQSSRQEGHP

IIRQPVCHLSHLQKSV

>SG-AL_contig_7229_1_-2_110_1066

MKKLIFLGAIGLLFFAVAVKAEEEAGTVDVDLGKSMEASRTDDETMQKEEEAIKLDGLNV

AQMKQMREQAEKFAFQAEVNRMMKLIINSLYKNKEIFLRELISNASDALDKIRFLSLTHK

DALKATDELGIKIKADTENHVLHVTDTGIGMTHDDLVKNLGTIAKSGTSDFLAKLGEASP

TELNDLIGQFGVGFYSSFLVADRVVVTSKHNDDEQYVWESDAESFSVVKDPRGNTLGRGT

TVSLHLKEEAHDFLEPSTLTNLVKKYSQFINFNIYLWSSKTVEEEEPADEDTTEEADTEK

KEADEDEEGKVEEEEEEKE

>SG-AL_contig_7230_1_-1_196_2205

MKAVKEKERDRRSYPPRNRLEMTVNMTGTERIQYEDWKAERQKVDQERMDRQKTSSGEWR

RAWDQDKQYKDYDESSGRTEPVKRPVGRIGSGRFDRGDREDRRAEGSNQNPKAANTMQAV

QPVPENDRKPPAGRGRGRGRGRGRAQQPGGAGEKPPRPRTWSGGDDARTVECSNDMLVVK

IDKSRGCDDHSVEVEYDGPDEEIEKRKSSPPKRKPGRRSPTKQKQQQQSPQFQDAEVPRG

KGGPSPPAFISTSSQDSSIYEGRPASLSQSISVGSTDEWEDITGTDSDMYTTDEGESYRR

GSGRLNPEAPVFLPTSPDIQSSAPHSGFSLAPREQRRCRTSTSSRASSNSSQASNCDVGG

CSKQIQSPDPKRSQPLALREDRQTTVDDKEGQQAEEPDDKDDQQVEEEDVNEDLLEEKEV

QDAAAYEDAEQDYEEEDEEEFYNCEKDEEVAHLSAYESPREVPSSTEELSVNVHSRNARH

SDVNSQQGTGASLADELSAAGAPLHSSSSSFATHTSVQEDFTSPELDEIEEDDVVEEEET

VPAVVDDMVPEGEALPNTATALDTMMEEEEAASLDADSSCQAAPDDSANSSPEDGGKQMA

TTEEDGSSKDELQGPSSSQLIFSPGKALQESAESTVMEEPDAQRINGHTADAASDDGVTM

ESLSSTTLSA

>SG-AL_contig_7231_1_-1_751_1074

MFTNIVYASIASSCFCFGLFHHSWFGCTFVVNIYRKISIINGYYNNGYSIAIGIESNISV

GFVLSNQTESFTQVCLSHDCCCYFTYLFHMSYHIVFECLSLLISKSLC

>SG-AL_contig_7232_1_+1_82_192

MHVCMDHISRITQLITNQCKCMTEQSSAKDNQHKQQL

>SG-AL_contig_7233_1_-2_119_2662

MSAYPVTDHDKRKQISIRGIAPVENVANVKKSFNRHLHYTLVKDRNVATPRDYYFALAHT

VRDHVVGRWMRTQQYYYEVDPKRVYYLSLEFYMGRALANTMVNVGIQSSCDEAMYQLGLD

IEELEEIEEDAGLGNGGLGRLAACFLDSMATLGLAAYGYGIRYDYGIFSQKIENGWQLEE

PDEWLRYGNPWEKARPEYMLPVNFYGRCETDCNGRNKWVDTQVVLAMPYDTPIPGYGNNT

VNTLRLWSARAPNSFNLHFFNSGEYITAVCDRNLAENISRVLYPNDNFFEGKELRLKQEY

FLVAATLQDIIRRFKSSRFGCRDPVRTSFDTFPDKVAIQLNDTHPALAIPELMRILVDIE

GLAWDKAWEITVRTCAYTNHTVLPEALERWPTSMLERILPRHLSIMYEINFRFLKLVEQK

WPGDLDRVRRMSLVEEHGEKRINMAFLAIVGSHAVNGVAAIHSDIIKKDTFRDFAEMFPD

RFQNKTNGITPRRWLLLCNSGLADVIADKIGESWVTNLAELRRLEPMSKEEELLRSIMKV

KQENKMKLAAYMEQNYGVKANPASMFDMQVKRIHEYKRQLLNCMHVITLYNRLKKNPNMP

FVPRTVMIGGKAAPGYHMAKLIVKLINNVGRTINNDPIVGDRLKLVYLENYRVSLAEKII

PAADLSEQISLAGTEASGTGNMKFQLNGSLTIGTLDGANVEMREEMGPDNIFIFGMTVEE

VEALHKRGYSAMDYYNSDQELKQCLDQINSGGYSPEEPGLFCDIFNSLVHHDRFCLLKDY

RSYIECQEQVSALFTDPMKWAHKCLMNIAASGKFSSDRTITEYARDIWGVEPTDIKLPAP

HEGPSNRK

>SG-AL_contig_7234_1_-2_2_187

VGVCAHAYADMVVVVCVLELVSLMYMCTGCTLHTNAGVSRIKNQNHMKILLNGAKKYRFK

VK

>SG-AL_contig_7235_1_-1_172_2499

MGGSHPHNAHSDPNLGTPGYSSSSLKSPHRVPHHHHGHRDPQPHNTTPSPTATGLTLTSY

QRQSPAPAATAVSQNPAAQRPKLRGMGLQRRVSLEDAQRELTSSLSEMRLQEGDHVLFDY

VANSIVGKDTIFNGPFGQRKVIYLDYIASGRPLTFIEEYINKEVLPNYANTHTTTSYTAR

QTTVFRNEARAIIHSTTNATDEDVVIFTGNGVTGAIHKLIHALSFTEPPVVFIGPYEHHS

NILPWREMNAEIVRIQQTENGLVDLEHLEEKLQEWSPKGLRMVGSFSAASNVTGILSNVD

TISILLHKYGAIALWDYATAAPYVRLDMNPVVAGDDKGLAYKDAIFVSPHKFVGGVGSPG

LLIAKKKLFLNNIPHDCGGGTVLLVRQTGHRYLQNLEEREEGGTPAIVGAIRAGLTFQLK

DAITPELIMHREQQLFRAARESWGKVSNLVILGNLEVERLPIFSFLVHHPETGYYLHHNY

VACVLNDLFGIQARGGCACAGPYAMDLLGISEEQAKDLEELLVTPAIPDKAKKRAPQQHF

GDSVREIFKPGFTRLNLPYFMSEEQFHFVLQAVTMVAEQGWKLLPQYKFNPRTGEWKHEG

SQESKQQMHLDQLSYSKGYLEVQLPKPPGQDTLPHSPQDCLNQARRMFAEAKQNITTIPD

QSHLFEDFARSSRWFLLPSEANEVLHAYSQIRSLGPTKPFICPKKTQSKQPALYPEVPGS

NISSSNASLSSSPRTIATSLASSGDTPGHGKEKKSPLHVRKIKVSAYQNGGPKWKL

>SG-AL_contig_7236_1_+1_214_546

MRNNCQQSHHLTNHCQQSHHLTNHRQQNHPLTNHCQQSHPLTNHCQQRETIVNKRNHCQQ

SHSLTYHQQNKEADIDWPPSVPKDYGICAAGETPGHKPGQSVEQNLLLPMQ

>SG-AL_contig_7237_1_-3_30_899

MTKWLQDVFDVPLVIQLLDDEKYLWKDLTKDEAHALAYENAKAIIACGFEEDKTFIFSNS

DYNGGMFYKNIIRIQKRVTSDQADGMFGFNGETNIGRIFFLAIQAAPSFSSSFPDIFNKK

TNVPCLIPCTFDQDPYFRMTRDVAPVLGYPRPAMIHSKFFPALQGSQAKISSSEPTSSIF

LTDKPKQIKDKINKHAFSGGRDTAEEHRKYGGNCDVDVSYQYLCFFLEDDEKLEQIRQTY

TSGELLTGFLKKELITILQKLVAEHQERRAKVTDEVVKRYMTLRKLKYDF

>SG-AL_contig_7238_1_-3_1371_1589

MVSNQSFSNFFLFVLFSCFYVCVSVFVFVQMCVFWFLAGGSLSLLACCMCMRVFNFIKPG

ILLPQEIRSLNSA

>SG-AL_contig_7239_1_-2_341_1789

MDVETELSLWLEALEPDNVTSDGSSEPAIKKKQRLNGQKTCRVCGDRAVAHNFDVLSCES

CKAFFRRNALKPKIKPCAFDGDCEITPKTRRFCSHCRLKKCFRSGMKAEMILDADGKKAR

QDKLIENRRKRQEAQAQQQQQEQQQQPTTPSLQEAVTSTTENLLARSGSGEEPCTTDTEE

WYIPPLSSATQVNRSLRGSSALSRLLERAKKQATLLDFSVLTPQQSTAPVSELDIDVSFQ

HVSRDQLPSDPLKYWCLKDEERNLLRELTVAYQETVFKLPDRMDTVKPSNVQNILDRCQR

FARDIVNFLKRLSDFKALQMQDQIAVLKGASWRTFLVKTSANFIVEMDAWIKDDGYVSVK

DFTDVSPEHKDRILLLARYSAALKHIMRNDPTTFAVLQVMVLFDPRDPNLNNRQLVSELA

DKYALLLKHLLQSQWSFDYADYYLGAVRGKMQEMPWVAENIQVMFMDWLHLMEPLMREIL

NLS

>SG-AL_contig_7240_1_-1_160_408

MALPLKKNTPNNNQKTPFLFRTCLSVWLSSYLIAAQTSSSNLKDCQTYSLWMVSGVNTDG

ERKVGLFTGKSQEYASFFAPTLW

>SG-AL_contig_7241_1_-3_174_608

MAEADWEEVTYIGHRAKANQLRSKQAINSAMRTGAEIDTSKKFTAGQNKQSSASRNTAKL

DRETEELHHEHVSLDVSKLIQQGRQAKGMTQKDLAQKINEKQQVINEYESGRAIPNNQVM

GKLERALGMKLRGKDKGQPFGGPKK

>SG-AL_contig_7242_1_+2_2_214

PLLSLVVPEEVVSGPDGRGTLDLDVEDVIIRGKLTRLVTQQFQLQVHELTFHPNPFRHCW

SARHVSRSICP

>SG-AL_contig_7243_1_+1_1_144

FIAQSTTNAISGLSPHKVHQSQEDNRVEHRTSSNTKARSQFMTYYLNH

>SG-AL_contig_7244_1_-1_49_177

MYYLVMCSMRKRIFIFECVLTHRLSCTSVKYMSVICILLIEQV

>SG-AL_contig_7245_1_+3_90_371

MCKKCNLMSSLCRLISALYSKQEEIYVNKGIIHKKSNLMFSLYLCRFNTMEENICAFFSF

QLEIRQVKFTLTTRVIYHAIIRQNKLSAKTNNNN

>SG-AL_contig_7246_1_-3_6_305

MLFIDVFSVWFGLFCFVLFCFVLFFNIVNLDAVPPPTSPTSCFLPIYTFSCSRFNTSFGL

NVDLYKMHIIINVYHECIITCTFLLLVFIEVVLKIYLLGC

>SG-AL_contig_7247_1_+1_1_150

FFFFIITTIIFFFRIWIFFPLNTCQQCMKSLTQKCTNIAILKKVNLYNKL

>SG-AL_contig_7248_1_+1_1_156

MNSQWCGMRYPFGIFRNCLRSESEGPPVLALRHQNFKQGKGKYFNFSTGIPQ

>SG-AL_contig_7249_1_-1_1_1128

TEPALSQEEPVTAEQQFAASEETSPADHKSAAVGEQPSPAAEHTAHAEVPSPAPIEQSSA

EQQSSADKSNVGHSDQLAREDEQSASEEEQPSEHSSLEEGKCLEDSALAKQQSAEASTPI

DKSAEDSVPVEQPADSMPVEQPAEDSVPVEQPAEDSVPVEQPAEDSVPIELPAEDSVPIE

QPAEDSVPVEQSEDSVPFEQSPEQLLTSEKESTKQLAVVEQTAEKTASLDEHSSEDPASV

ENQPTGQSAPMEGQSVENTLPAKERSAEESAPVKHCADEPTREDEQHLGQPAPTKEQPSE

QSSQYASHVVEQSDKELPQAEEQYTEHSVYIEDQAAEQSDQSAPVEEQAAEESPSAKQTP

TEEQPTQTQELQTPAE

>SG-AL_contig_7250_1_+3_342_512

MFYTQSSLRQLNDIFQRHQSQLCHHQNQLKNDITKSGGKHENQCGTCPMDFRPSVGQ

>SG-AL_contig_7251_1_-1_1_294

ESSKGLHLQKHVLQHTHCILASLLKWKLPTTWKPQRRVDITTCADATYIIWTHPETLKMP

DYQLRSRSYLDQGGIQSMPCHRSKRFVGIRFSLWNILT

>SG-AL_contig_7252_1_-1_1_294

ESSKGLHLQKHVLQHTHCILASLLKWKLPTTWKSQRRVDITTCADATYIIWTHPETLKMP

DYQLRSRSYLDHGGIQFMPCHRSKRFVGIRFSLWNILT

>SG-AL_contig_7253_1_+3_318_554

MTCRVSQYSHFHQEHRNAVSVTDNASVCMQNNICLSVDLQILVTGVSNEENKYFVKYMNL

KPVYHDGWNLIKFRNLKKV

>SG-AL_contig_7254_1_-3_24_335

MHICMYTCAYLHVSVARRLEISIQGYGGRGDSACVSMYVHAHVRYVYMYSCHVCIHVRIP

TRSCNRYVSFLCRLLAFATMIFSVIYKIAFMVLCMWNHLCGTLS

>SG-AL_contig_7255_1_+1_1_234

THIYIYTYTYIYFFFFYYYYTHTHRIPASPFITEPQDGSCTYKSKTPSIISRFHSGQAFF

VYTHTFFLHGTKKITCNK

>SG-AL_contig_7256_1_+2_104_256

MFLQFLLHSASLMNEFYYNSSPPLLDLPLPSAPTSTYQHFVLRQSILSPFI

>SG-AL_contig_7257_1_-3_51_383

MHALGRAKDDIGFHSDGDLALTVLPEFVFKTRQPGSRPRSFQIPSLVSCPDPRDARSEER

LLCLVRCIKYYLKRSVPRRSSQWRLLSSPSTRTTRGTYQQPPSPTGSWLRS

>SG-AL_contig_7258_1_-1_58_219

MRTHKEAGSSSLPDSPRTHELRALESSPAFPHSLLIRDVLEAAYWRGASTFTNH

>SG-AL_contig_7259_1_-1_88_3912

MSGPPQMPNDFLTFQDVVTETAHPIRLYSRYIDRIHIFFRFSAEEARDLIQRYLTEHPDP

NNENIVGYNNKKCWPRDSRMRLMKHDVNLGRAVFWDIQNRLPRSVTTIQWESSFVSVYSK

DNPNLLFNMCGFECRVLPKCRTTHEEFTHRDGVWNLQNEVTKERTAQCFLRVDEESMNRF

HNRVRQILMASGSTTFTKIVNKWNTALIGLMTYFRESVVNTQELLDLLVKCENKIQTRIK

IGLNSKMPSRFPPVVFYTPKELGGLGMLSMGHVLIPQSDLRWSKQTDVGITHFRSGMSHD

EDQLIPNLYRYILPWESEFIDSQRVWAEYALKRQEANAQNRRLTLEDLEDSWDRGIPRIN

TLFQKDRHTLAYDKGWRVRTDFKQYQVLKQNPFWWTHQRHDGKLWNLNNYRTDMIQALGG

VEGILEHTLFKGTYFPTWEGLFWERASGFEESMKYKKLTNAQRSGLNQIPNRRFTLWWSP

TINRANVYVGFQVQLDLTGIFMHGKIPTLKISLIQIFRAHLWQKIHESVVMDLCQVFDQE

LDALEIETVQKETIHPRKSYKMNSSCADVLLFAAYKWNVSRASLLADSKDTMDGATTQKY

WIDVQLRWGDYDSHDVERYARAKFLDYTTDNMSIYPSPTGVLIAIDLAYNLHSAYGNWFP

GAKPLIQQSMAKIMKANPALYVLRERIRKGLQLYSSEPTEPYLSSQNYGELFSNQIIWFV

DDTNVYRVTIHKTFEGNLTTKPINGAIFIFNPRTGQLFLKIIHTSVWAGQKRLGQLAKWK

TAEEVAALIRSLPVEEQPKQIIVTRKGMLDPLEVHLLDFPNIVIKGSELQLPFQACLKVE

KFGDLILKATEPQMVLFNLYDDWLKTISSYTAFSRLILILRALHVNNDKTKILLKPDKTT

VTEAHHIWPTLTDEEWMKIEVQLKDLILADYGKKNNVNVASLTQSEIRDIILGMEISAPS

AQRQQIAEIEKQTKEQSQLTATTTITVNKHGDEIITSTTSNYETQTFSSKTEWRVRAISA

TNLHLRTNHIYVSSDDIKETGYTYILPKNVLKKFIIISDLRAQIAGYLYGVSPPDNPQVK

EIRCIVMPPQWGTHQTVHLPNILPDHEYLKEMEPLGWIHTQPNELPQLSPQDISTHARVM

ADNPTWDGEKTVVITCSFTPGSCSLTAYKLTPSGYEWGRQNKDTGNNPKGYLPSHYERVQ

MLLSDRFLGFFMVPNQGSWNYNFMGVRHDPTMRYELVLSNPKEFYHEVHRPTHFQIFSNT

EESLQIGADREDLFA

>SG-AL_contig_7260_1_-1_205_876

MFNAMFFDLQHPVVITDAIGYWPALTTHKWSLEYLQKIAGARTVPVEIGSRYTEEDWTQK

LMTINEFIEEFITNPKSDTKGYLAQHNLFDQITELKDDISIPVYCCLGQEEEVDINAWFG

PEGTVSPLHQDPKHNFLCQVMGRKYVRLYSPDFTPHLYPHSSKLLENTSQVDCEHPDLHK

FPKFAEAVYLECVLKPGDVLYIPPKHWHYVKSLSLSFSVSFWWQ

>SG-AL_contig_7261_1_+1_1_246

HTDTHTHTHVCILGREKSGQKNGRRKVKSCLTMLVSHGNELAENPNPFGSSSTRAAPIFS

FHHRSMPVHFHYNLGVTLQVND

>SG-AL_contig_7262_1_-2_107_259

MVQIICHELCFLAGFLFSCLMRFCVQRGRLLDFDGLYMVTALIWPWWSAGL

>SG-AL_contig_7263_1_-3_180_452

MGTGYPFCSVICLHSLFYLFIYLFIYLFIYLFMVTSKCFFLRCFYNLGMVDCAEWLASSA

QLSLVCLNIRSLMDGMICHELCSFAGFLFSV

>SG-AL_contig_7264_1_+3_396_587

MQLKVGLLKTCRRIYKGFQCNFRGSSWRTGLSGLRNFQAMCCMGEAERFGPSRNCCFRCQ

KEIL

>SG-AL_contig_7265_1_-2_17_2035

MERMENYKLLERLGKGGQGSVYLAENKEENKNYVLKKVECFDESEANKAFHEALALQKLD

HPYVCGYKEFFVMWDKEESSMFVCIVMDYYKNRDLRLIIGDYQEKKIPIPEEKLKKYLGE

ILEAVIYIHSQNIIHRDLKPSNIFVKDDDTLCVGDFGVATVMGDRRTCPRNTVGTTVYMA

PEVMTQPYDERSDVWSLGCMLLEMTTTALYNQQQLASKLAEIKKDPYLLEEVFEEISKHY

SDDWIKTIRTMLKVKHQTRPTADALLKMSFVLECVEASNSSLLDKRKRQQQTVNRIRPLT

EGQDNPEHIIQYIADTIDHEQCVQEGLEALRQLLDTANATPTQPQEGEGTPRPPILLDQA

SKNLISLAMWDNVANKDLQLAGCAVLTALVIHADANDVLFTPEVLAVLGKIMKEHKGSSD

VQQAAAGTILALSANNNASDALCQLGAIQDLLAAMRADPNNTALCAACCNALWGLTIKEV

NAKIASDENAVREVCLALQNHIDCAEVVEAAAAAMVSLCLDDNCFETVNSLDGVGVLVQA

LDTHVRNAKVVKNVCMVLSTLVEPNEECAYRVLTSDKPDDQKPAGIPVIIKAYELHRDNA

EVVEAIVNLIVELCEYGEICQDLISYDISVPLREVRSKYHKNRDIMGPCEQALDKLGAGK

AMAPRKSSNQAHK

>SG-AL_contig_7266_1_-1_40_1755

MYTSARCLFSFSLLLPVLLCTCSPVKHEDEGHHRERRAAPTLSDRAACQPGSEWPSTRVN

TTQRLKDMRDAMVNLSIDAFIVLSDDAHGSEYPADSDLRRGFISGFMGSAGSAVVTLTKA

ALWTDGRYFLEAEDALDCNWLFMRSGETDVPSKSEWLTSELNAGDVVGADPFLVSNSLWM

SFKSALEKANITMKLVYNNPVDYAWKDQPSPPETTINALEMKYAGRSWQDKVSDIYKEME

KKDIHGAYIVTSLDELAWLFNLRASDIAYNPFFISYAILEYYGANNESNRVRLYIYDKDS

RLTKDPTDSETENKLYIHLNTGQDGSCAAQTSMCVEVHEYIPTDVVQDIENLAAASAQNK

FWVTYICNYAIFSAIPEAQRVQARSYIALAKAQKNPVERRGMETCYNRDSAVLVEFLAMI

EKEVKEGRHWTEVSAAKELKTRRQAAEKNRGLSFASISGSGPNGAVIHYRPAPETDRNLT

TSEMYLLDSGGQYLDCTTDVTRTMHYGTPTNYMIECYTRVLMGSIDLADVIWPEGLYGRE

IDALARAPLWAVGLEYRHGTGHGIGAYLSVHE

>SG-AL_contig_7267_1_-3_567_869

MTRLSKVSILYFSCELNVWIHGIKMSEAVSLYLFCLASMSTNLHLTLGGRGYVQCLFLKF

FHVGVSYDRGQRIHYCTLYMLVKTFIVAKLCGFQYCFCLVI

>SG-AL_contig_7268_1_-3_3_80

FSVLHYFVLFPQLCKCCLTSGTVAYS

>SG-AL_contig_7269_1_+2_530_934

MPTNTHTDAIMCIHRQTILHHTHTHFPYMYQQQQTVETFLYHTYTYMHKLVCTHTRTIQM

LSCVCTLTQKYLSVLTHTCAHKEDRHRTAIYVVKDVKTRHSEMYIKCSKTYKPTQDNTTF

KVYKIIFNNTYEGLP

>SG-AL_contig_7270_1_-3_369_581

MCQCVIFFHVPLSAIVCGTAPAQLISSVPDTTKPTETEVTMGAENERPQNLIQFFSVAMV

LKNGDDGDMIL

>SG-AL_contig_7271_1_-3_42_1421

MASHIFRTGRAAADRLLSLHGARAASAGAPALLAASSHQQQKRHLSIHEHMSMGLLRDAG

VSVPRFKVASTSAEALQQAQEIGKLSGAPDVVVKAQVLAGGRGKGTWDSGLKGGVKLVFS

AEEAQDIAEKMLDHKLFTKQTGKDGRICQKVMIAERLYSRREFYFAIAMERSFGGPVLVG

SSQGGVNIEEVAIENPSAIIKEPVDIITGITKEQALYVAKEMGFSEGSIDKAAEDIIKLY

NNVFLKHDATMVEINPMSEDNTGEVYCMDAKISFDDNSAYRQKDIFALRDWSQEDQRDIK

AAEADLNYIALDGSIGCLVNGAGLAMATMDIIKLHGGSPANFLDVGGGATAQQVTEAFRL

ITSDSKVNSILVNIFGGIMRCDIIAQGIVAAAAELDMKIPIVVRLQGTRVDDAKAIIATS

SMKILACDNLDEAAQMVVKLSTIVDLAREASVDVKFELPL

>SG-AL_contig_7272_1_-1_841_1149

MCVWLKQQNEINDQVINCCYISLWFVPTFHSVFSCFVVNCVKHVRYVKKPTVILCTGSVL

CRTVINGHVHKSVGNLYPSSSPSHPLLRLCPYVVLCWMSCGDS

>SG-AL_contig_7273_1_+3_1098_1286

MKSISQQKICSIKEHELFVLITKIHAVIMLHVFSFTTTTCLLAKYTHQLRLTFFHTALIM

QGL

>SG-AL_contig_7274_1_-3_18_1979

MEDLSVEVCGNNGAYYKAFIKSISTTEATVEFENNWQPERHVQLLSVRLPPKESTAKPDY

RENEKVEVLSKAQEDDAYGWWQAKIRMLKGEFAVVEYAGYDSFISDIVPIDRVRRINSNP

PISPDSFQQFVLEVPPDLREGCQGEGALAELRKHLGGGAIISYVVADNSIHVLSTSAAVT

KRASMIGDMFLRNVRQKLLLKQRTEEAVKKLQSTKIRSGYMEEFAVREDLMGLAIGTHGA

NIQQARKIEHITGIELDEATCTFKVYGETTEAVKQARGMLEFSEETFPVPRDLVAKVIGK

NGRNIQDIVDKSGVVRVKIEGDNEPDSQRDQLDQVPFIFVGTQESISNARLLLEYHLEHL

KEVEQLRQAKQEIDMQLKSLAGPQAGPYFAPPRDRWNDGGFVDERNRRGRGGMRGMGGRG

RGRWNPDRPDYGGNRYDRRPNRGGYEDGYHDNNFPYRGQGPRPYNNYDEESRDTRSRRRM

TDDDDTVLDNADNASVNSQDDDRRSDRRRRRRRTRPRGGSNSAPSGTETDTNVSSYRQDR

GQRPPAGPKGGPHRPLNQVKSEDSASMNGGGGAHSPHPVANGGGGEDGSATASKSDSQQS

VPKEQRSARRPPPQTSSSSQNKTANSQPNGGDSSDGKAKTASGPRPKEQVVNGE

>SG-AL_contig_7275_1_-2_203_1510

MMKVIFPMANGIIMRSADDPNMRYTFIETSNPNNAQGFLMSYPGLIVNDQELKLSMKEKD

LEILPKLLFENRRKDGNANKEEKEDGEKDETKKETEEGDEKDDKTESEETKDDKTESKGA

EPEKRELTEEERQRRTQTNKQKKKRKREFMEEVQREWSAKRRREDPEMMMPPLARGRGGP

PFGRATFEEAQELLEIQDKVSSLLSLQKQLEVTISTQMDFIHHSEATVRRRAEEMRLQDE

AYSRGYAFEAPRGGFSRGGRGAMQRGFSPRGGGASSPRGIMGRTFGGGGKARDFERSDSR

EDEFQVPGNKRFRRVSAPRYETKAPAASHEDASKGSKTSEGGGQGNKAQEPKKFGQGAQS

SGEGGNKAGAGTTINTGANKLGGADKSSPGNQFAGSNTGSGSNVGNKSRFSAGNNQTGSG

KFGGGGVQTGSGNQGG

>SG-AL_contig_7276_1_-3_657_1115

MCKMDFNRTVPSLPFDRATTCFTAYSTMQIGACALWIPGNGADNGLKKQRTVSPASNSKK

RTSSSKSWQKERVEDDYQVILLRLRTGFNKLKNNMAPTEVGPSPSTSCVHMCSSYSGLRT

IWSEGIALHVKLHWTQQYLERTADSILRIGLTI

>SG-AL_contig_7277_1_-3_330_479

MRPLVHPVVIGYLTLFRVGEDKAGEDWKLHSQSCQLEVGWSHHHHHQSHD

>SG-AL_contig_7278_1_-3_510_689

MHKNEHITSTMDRKWREGERSWIKGDNLFPMTIRKIFSDVLFIGMFLMVITNDDSGDGVI

>SG-AL_contig_7279_1_+3_177_521

MFVFLLWLCVWLLRLFCRDTNAAESLSEGRDWKSVPPPPPPLFEKATVVLFIRGFTTLSK

AWASNTIRCGGQLPALKCLSGGFVHTPIKCQRCGGDLRGLPILRSTRLGHLAGAL

>SG-AL_contig_7280_1_-1_436_1080

MATATRLHLVRLSLSPSPETHSHSPIVAEDQATNFPRSDKETASYPDTTLQAYREARLHL

DSLLKSMRSQASLTTERQLVSLPDLTQVAHIDTSPRDAVRSAWLMGPGELHMGASSVSIL

DPGPQRDEMNPSQVHERCIATQSADMVDWCAKLLHLYQLSSTPKADLLMTRSTLGAPLRH

SAIMTPPQEFLHPTSKNVDEGPVVGRRGSCSLWRA

>SG-AL_contig_7281_1_+1_1_171

RSHNMNFTTLSSSLTAFWFLNEYSFTTTNQKPAVSSHTRHGSRSSQRNFQHALKSPD

>SG-AL_contig_7282_1_+1_103_267

MNYALAVLREFKKACGTQARTPVSTETRKIVWFAIKPAHSHLPSGTHGISNTLFT

>SG-AL_contig_7283_1_-3_57_227

MQVYKTLLFDFEKMLNKKCQIRHNTKLTENLVLFGMDTEIHTDPAFDYCDYACKIQM

>SG-AL_contig_7284_1_+1_319_864

MLIQLFQRKKDKHTLIANLQAHNYRKYYVKTERYTPLIFLMPNFILIKQIQNRTKMVTLY

NVLVSHLSHITDTFCIAVTLKMSHFWACLFGKHNLNYDSNLIKAHLYIAGKSTPTFFWLL

LTLKYWARPIQTCYFGFFNRPELRPTVRKEPSNNIPETSADTFCFLSIIQTHTQHFPTQP

HA

>SG-AL_contig_7285_1_+1_193_306

MFLADDPSLAASQQAHRSLSLSSYLVMSLTLSISLWSP

>SG-AL_contig_7286_1_+2_2_181

RNATTLAHTSSYIPVPRTVKTAIARCTRIGSDEAVRRQICVCLRGIDVVYLLVRIISYII

>SG-AL_contig_7287_1_-1_40_540

MVHSQAHTTFSASPNFRSIPMKNSQSFGYPSLRPQPEGSKTIKVPEGPYGVPDVMLHGFN

KTHAAQNLVSSHPLEFSEKNWLTHQQKMDMVMLRNSQGIHAPMRLHMEQHFAQKVQRLPC

LPSSNVLLDTLTGRDEMIGFEDILNDPSTAEVVGHPHVLMEKRLGIL

>SG-AL_contig_7288_1_+2_152_355

MQVYRAAVLSTLLYGSEAWVLYRKQIKRLEQFHQRCLRSIMGIKWKDYVTNIEVLERAQL

QSPRKSST

>SG-AL_contig_7289_1_+1_112_327

MIIHYGQTIKSTVYTKSVEHTRPHTLHLTKKLIDEIIGYISTQPPSPHTHTHTHTHTHTQ

THTHYYELNEIL

>SG-AL_contig_7290_1_-3_3_452

FILLAAIDQRGRVSCFSSLWFFALSRSRLFDLLAQPGIVVLLSSIRPIFCCLDHISPRNP

QPVEVANFLAHLSSVKGLAASTIQGYRSAISTTLLQLGGPSLSDSFLLRDTIRGAKLKDA

KSPRRLPAWDLFLVLNSLSKGPFEPLGASL

>SG-AL_contig_7291_1_-1_406_816

MVSSQLPRPPHYKPQPVETANFLAYLSTNRGLAASTIQGFRSSISATLLQLEGPSLSDNF

FLRDTVRGAKLKDTKNPRRLPAWDIFLVLNSLRREPFHPLGSILLDLLTFKTAFLPQWGC

QGLFQNKQYPHLLEILC

>SG-AL_contig_7292_1_+3_186_680

MYHHYSPHGDAWGSFPPFVHTGPDDIGLEVSSFYHSCSSLFYHLFFRLLTYQSTCVNFLL

KEILGTFFVFLIRTNKFLPHMDTDCSRHDHARTQKRSHHRYHQLGCDGITEAHHHYTRYF

PSDYVRVFSSFFPPHPTSIKYPIPSKEDKCTMKIQPASSTEQSNY

>SG-AL_contig_7293_1_-2_113_538

MAGGNPTAADSSPPAEDHLYDVVDPPLSSDRKNTAAHVLPINTNNDGHSRERQKPHQMTA

RKGTRVKGKQKRNITTPNSAETDLSVIFEVCGDAAVVYENAGSSGVGACAVGTEGRRTDL

YEPLGYRDQQPVYTALTTGRKK

>SG-AL_contig_7294_1_+2_365_940

MISIKALLSVLRKPHTNYQGKMYMLTVTLLFLSILTKHHPGFQYTHDLEPQAAWNEHYSI

KIKLVCHTSWHPHTLLNYPEQSSCHSLSSLTLYGHTRQSEWGCSFTCNCHSLSSKGNMLA

KPKGKQSTDPQFHHHNIVLPHPGCVKRALQRTTSPMTNTHLHKLLSWQLFNKYHQKLEQP

FTVLKDRFYISP

>SG-AL_contig_7295_1_-2_2_271

RRYTAEEVARIVLELPYDGDNSDDENMIGNNSDVVLDAVDKSDNESRDSDYPDKDYTLPS

TSTGRGRGRVRSGRNRGLIVSYLILSTLNI

>SG-AL_contig_7296_1_-3_309_443

MDFHFVVSLKRICAGIVAWMCVFGGHLDNCLKLFYPAFPFKVLFM

>SG-AL_contig_7297_1_-3_150_332

MGPVLQCVIHGQRWFHHSLMHNAAIHWIRWFHHSLMRNAAIHWIRWFHHSLMRSAAVHWI

R

>SG-AL_contig_7298_1_-2_56_271

MYNTWNSLFFHWLKVKKINTFCIVERMSPQRKCRDSYPTSWQKFSTIAAVKMSLRDFQWK

ILKLRTLLHYIK

>SG-AL_contig_7299_1_-3_189_449

MPYILREKIGKTSVLPILRTVYFPSLFRVQSLFRVQTGVCSSSEFRVCSSSKFRVYSCSE

FRVPSLFKRNSPHSDNSICAPPKSASN

>SG-AL_contig_7300_1_-3_3_137

MAKQIKIETSFLTTYTSFSNQKKGMQRHERLKWSNQKRECKDKKD

>SG-AL_contig_7301_1_+3_393_596

MSNVNQTFVHKEKQTVINSSKEMNIHLVFISNKSLKNVQIRGKQIQTQELDGKPALIPRG

KYLVNCGR

>SG-AL_contig_7302_1_-3_3_143

ALPILKLLHKGNAFDTVQKVEQHRGKNTERKFTWKIMYQAHTCDSGF

>SG-AL_contig_7303_1_-2_2_109

DETRLCGPSLPNEPIACSTLGRSQSGPKNPTVIFLD

>SG-AL_contig_7304_1_-1_1_171

HTHTHFDLNIMSALSSSLNHISQGHNFVQLKLSKHAKQDTAKKSILQTNKRRNKVQS

>SG-AL_contig_7305_1_-2_80_304

MYVMCFAYQGCASGLAAQGQRVTFYIDFDKCYTCITTAGAELASFVLISKQLHQILISIK

TAEEKVYVFNQYQNS

>SG-AL_contig_7306_1_-2_53_250

MLLQMLYYSVVFQFMFLMFISKRLVHDVTYQSYIIIYYHLVVASIYDYMICGFKRNYCNK

RVFSFK

>SG-AL_contig_7307_1_-2_254_646

MLQDLKSTVPQQVLGSRTWDANSKHASASMAITLWDAHFDFNDLPTVPIHTFMEEAIGDA

EACQAARAAAGKVAGLKVSDLVLNSLPAKVKKQGVKSAVYQKEQGFFDEVADHTFRTLGE

QALSQRIIQPL

>SG-AL_contig_7308_1_+1_268_507

MWTQIKADSHAYCAVLYCVTEMWTQIKADNQAYCAVLYCVTEMWTQIKADNQAYCAVLYC

VTEMWTQIKTDSHAYCAVLV

>SG-AL_contig_7309_1_-1_1_210

FDVSILTIEDGIFEVKSTAGDTHLGGEDFDNRMVNHFVQEFKRKHKKDISDNKRAIRRLR

TACERAKRTL

>SG-AL_contig_7310_1_-3_3_164

LKMASCSLFSNSSFRPRLQRGFLLLLNKKLVNLSDNFNTRNGKKKAFLDCSDFD

>SG-AL_contig_7311_1_-1_76_846

MIPPNASLVKYDNPVLVSRNTDKKTPRAKALKTSPTPPSGTGPVPNPPSKQKLSPLDAKA

AQQTDEILNSILPPREWTENGQLWVQQVSSTPATRLDVVNLQEELDRRLQQRQARETGIC

PVRRELYSQCFDELIRQVTINCAERGLLLLRVRDEIRMTIAAYQTLYESSVAFGMRKALQ

AEQGKADMENKITTLEDEKRDLERQVNELKAKCEQIEKRAAEQRQVEEKKHTEEIQFLKR

TNTQLKTQLEGIIAPKK

>SG-AL_contig_7312_1_-3_33_1010

MASYNRHTALHLAAERGKQECCDLLLEAGAFIDAQNGAGHTPLHVAILKGQMKVMQFLMS

SGADVTRRPYNGNTLMHLAASGGSEQCCHLLLTRGLDINQQNGDGNTPLFCAVQTKQVKT

AQFLLNAGCEVDVRGLHGMTALNEASFLGHTALLKTLLLHGADPAIADDAGTLPLWFAVD

QASLENMRLLLAANSPFLSRSSLSPSIGPCNPLELAASKQRDVIVIWMVAACGQHAATCL

HSYLPQLEQSAGSRDLTEKWREMVREPQSLLRQCRCVVRRSLSSQVALTLADSLKDMTLP

QVLKNFLCLADLSDLKPAEGTSSPSS

>SG-AL_contig_7313_1_+1_2737_3057

MRKAKPHTKYPSLGQRLAKRQAPRGHHPTWLLLTLCDVSKDRGRPITRGPSREGGRVLVV

RHGKYCLLKGNFGINLLFNCTYFNHVLQVQSGTTRMVGRNVLVIGWM

>SG-AL_contig_7314_1_-2_179_439

MYGQIFLSPGSYLESASTFSLSCYHSSFFQICSKYISVSAALRLITIFFNNKCTGVPAST

ALFSLGIKFLPAIGLSVMTLSLSTSSL

>SG-AL_contig_7315_1_-3_120_1205

MSATMEQREISAPRVGRFKVYIQGDLKTTQFIILTVHDLGCNHSMWGHFLGHSAMEEIMR

RAAFIHVDVPGQENEAKDLPSDYTFPSMQSLGEDLVCVLDQLDVRSVICLGEGAGANVVA

RFAMAQPDRVLGVCLIHCVGTTAGFMESMKDKLIEWKLEQIGMNPTAETYLCLHRFGSFE

KAQNKEQLQKVIENFQHSLRSRVNPHNLRLYVHSYMKRTNITDNIGKVRCPILMVTGSKS

SFNDSVHGLFNRMRACLDKKNVDYLEVDGVANVLEERPERFAESFLYFLQGLGLVGGVPM

PRVQRSASIEGGQGTPPGRTRSMSMEEADLPRGIYSMSPPKFGGSKLSSSPPSSFASSPT

KG

>SG-AL_contig_7316_1_+1_226_318

METDIVLALLGAWISQQWLHVAKAKWKVAEN

>SG-AL_contig_7317_1_-1_1_255

LCGDAGGSFSIDSIPTFWKFCSRNFLFSIALFVSAPFPSFTVVCFFLPCRSIPLSFFQQD

SHNFLSPTPLPAVYDVLSVLLCFGD

>SG-AL_contig_7318_1_+2_2_199

PYRGMFKKKPPPPRFVLIQKYTLLTDVYGMIKNQISVHIHKYKSLRGCLKYRISMVDTHS

NSFCIN

>SG-AL_contig_7319_1_+3_459_797

MHVIDPVSHVCVRWLMETRKQPACTKKKKGGAALRRRILPRKKSLNFTRQIHYDKKLINT

IITPTYKFTLLLLFFVLIRQSNTSTSVHHVYIHPIVLFSPCSYSNKTLFTPSS

>SG-AL_contig_7320_1_+1_46_453

MNLGLVACGGTEISGVESTTLTQHRRQGSKTKQVTTRIPQSPLSSQHTAVQHREDNGHQR

RSMQVGKLALFHYTTPALWTAVPQIQDTKRTFHLLLLDKPFNFINWNSVHISICFHYNMS

FSFIDRNSVYFHLLVL

>SG-AL_contig_7321_1_-2_2_130

ILNHVCVLIRSQNCMNFNGLGISLPITASKLKHRSVFVSYYSC

>SG-AL_contig_7322_1_+2_32_214

MGKENFPAEKPCCSSKNRTWLLSYHMLLLFCLFFSPSTMTKHSHSVQFLFCITFSLINQI

C

>SG-AL_contig_7323_1_+3_3_242

FFLYIYSSLFPLLGFQCRSRYRMLNIQGSLQTDTVLCTVEQTKNQQRGCISMQNTLLSRE

DKIKPNGHVCISDLTSMTNR

>SG-AL_contig_7324_1_-2_2_184

KKKNPSPSSAFSTFSFNNSSLSFCTPFSYTHIFQRLICECQKDLCRNLFRTLPTCFKFAG

T

>SG-AL_contig_7325_1_-1_184_1665

MNFPEPIRDPEIKFTQLFISNEFVNSVSGKTFPTINPCTGKTIVELQEADKADVDKAVAA

ARDAFKLGSPWRRMDASNRGNLLYKLADLMEKNMVYLASLETLDNGKPFRAAIGDIYFSI

QVMRYYAGWADKIQGKTIPVNGDFMCYTRHEPVGVCAQIIPWNYPVAMFTWKIAPALAAG

NVVIIKPAEQTPLTALYMASLVKEVGIPTGVVAVLPGYGPTAGAALTAHPDVDKVAFTGS

TEVGQIIMKASADSNLKRTTLELGGKSPAIFLDDVDVDSAVAEAQEACMTNMGQCCVAGT

RTFVHEKIYDEFVKKSGELAQKRKVGDPYAPDTINGPQIDDEQFKKILDLIEKGKSEGAK

VESGGGRHGDSGYFIQPTVFSNVTDNMCIAREEIFGPVQQLLKFTSVDEAIERANSTTYG

LGAAVFTKNIDKAMMFVNGVRAGTVWVNCYNAVCPQSPFGGFKMSGIGRELGEYGLQQYV

EVKNVVMKIPMKNS

>SG-AL_contig_7326_1_-3_132_854

MFLTRSEYDRGVNTFSPEGRLFQVEYAIEAIKLGSTAIGIQTNEGVVLAVEKRVTSPLIE

PTSIEKIMEVDKHIGCAMSGLIADSRTLIDRARVEAQNHWFTYNENMSVESVTQAVSNLA

LAFGDDDPDPGAMSRPFGVALLIAGVDNNGPRLFHMDPSGTFIQYDAKAIGSGSEGAQQA

LQEQYHKSITLKDACKSALTILKQVMEEKLNATNVEMATVTPDRLFQMFSKEELEAVIED

L

>SG-AL_contig_7327_1_-2_179_628

MSEFKISEKQYTDANQTFHLFDKKGNGTVSTKELANVFKSLGLQVQDDQLKEWADAVDEE

AAGFITWEQFKPVFEIKLQEDEDEREMREAFRVLDKGNKGVIPVDDLRWLLKSLGDDLTE

DEIEDMINETDTDGSGTVDYEEFRTLMNSN

>SG-AL_contig_7328_1_-2_92_232

MHMKLLAHNIVCEIVRTHIVSLRVFHPFTPFTYPAGNCMMETYMVRE

>SG-AL_contig_7329_1_-3_39_2123

MYRAQDLWDEAYRVAKAHGGATAAKQVAYLWAKSLGGDSAVKLLNKFGLLDAAIDYAAEN

CAFDFAFDIARIAMKDKLPDIHLKYAMFLEDEGKFAEAEAEFIKASRPKEAVLMYVHNQD

WDSAQRVAENHDADSVADVLVGQARYAFEEKEYAKAESFLLRAQRPELAIKYYRDSGMWQ

DALRVCKEYLPHKLQQLQDEYDREMTSSKSGNGGGGAGAEGMVQQAREWEASGEYARAVE

CYMKITPSVISDQNIMEKCWMKAGDLAIKFLGPQKAQVVVEIVGPRLAEIHRHGSAAELY

LSLDMVKEAIDMFIAAGDWNKAKKVAKELEPKYEPYVDDRYKEFCRNQGKAEDLANVDVI

GALDMFADKGEWDKCLQMAEQQNNFKVLHKYVALNASHLIKGGNSDAALELYVKYGVPAQ

QQNFNIYKRIFTDIINKRDLNKPEAYRVWADLRDMLFDLHENFTKSSDANSPQHEEFENM

LIIAHYYCTRSAALSQKSLESIAGKLSVSLLRHTDVIPADKAFYEAGIICRNIGWENMAF

VFLNRYLDISEAIEEGTLDMLDHSDFQETDIPFEIPLPEKAYLTAQQHEDIKEWVLAISM

DQKVEQSLPKDERGTYEASLVATDTGIRSLPCVISGYPVLRNKVEFKNTNKVANKDDWNK

FLMATKVSHSPELQDVLKFVGKWCGATPNPSYSFQ

>SG-AL_contig_7330_1_-1_55_1500

MGTAPNGRHFFIDHNDRRTTWDDPRKPRERSGSTHRHDSPTPIFRSTSTDDLLSNLGALP

PGWEERTHADGRVFYIDHNTRTTQWEDPRLQKLGGPAVPYSRDYKRKYEYFRSKLRKPNN

LPNKIDIKVTRRNVMEDSFRVITGIKNTDYLKTRLWIEFDGEVGLDYGGVAREWFYLLSK

EMFNPYYGLFEYSAVDNYTLQINNLSGLLNEDHLTYFEFIGRVAGMAIFHGKLLDAFFIR

PFYKMMLKKPIVLMDMESVDSEYFNSMMWIKENDPSDLELHFSVEEDHFGEITERDLIPN

GRNIPVNDENKLQYIEQVIKWRFVSRVEPQMKAFMKGLNALIPQNLLQIFDENEVELLMC

GLQDIDVNDWKQHTAYKGEYNPNHPVIVHFWKAVYSFNNEMRSRLLQFVTGTSRVPMNGF

AELYGSNGPQLFTIEKWGNPTQLPRAHTCFNRLDLPPYESYSSLRSKLVTAVENAQGFEG

VD

>SG-AL_contig_7331_1_+2_968_1180

MYTNQYYVTGQLILLTQNNNMTEYEISTYDVRTTKGYTFKTCRTCCEQPFMKATPLPMIF

SNPHPVSFFFF

>SG-AL_contig_7332_1_-1_1_714

MVLETLTEALQAYLSEIGRCARAYAELAGRTESMVSDVILTLVDMGADLRGIPAHAKREN

KTVLVPPLLSSQPATQKTLQVGERRPHPPHVPDHLPPFPDPHTYIKTDAIRQPFSEYQLM

REKVATQKLSVERALTRYIARTGMTQSLFKDDFRIYPLIAVKPCPLPYLDALLSKHPKDQ

DLDSQDQETHSASVAIQRSSGLDEIGDSQIDEQDSAETDAIDNPYLRPVLMPKRKRYS

>SG-AL_contig_7333_1_-1_487_828

MYSFVKSCRLKIYSPPISCSFCLQPETRRAISELIPLPNLMHGMFTLSWLFVTKAFKFSF

HLLSQRASSLLSIYKQNIILLSIQSSVCVQAKYYSALWVGVAFLMNIMNILLCL

>SG-AL_contig_7334_1_+2_2_211

FFFLNIKPFIESCLRTVLVVGELHQERKKLNQGIVDRSSRRRSSLVVVVVVVIYVSCSRG

FVNSAKGKGK

>SG-AL_contig_7335_1_-2_2_190

NFSFIECYYYYYYYYYYYLLLLLRGHVSIIIIIIIIIVVVDMIAIIVVAVMWIQFCNNLF

IVE

>SG-AL_contig_7336_1_-3_3_131

THQTYTCKQCRLNTIYKKTCTNRGKEVVVQFIVGRRGGFLNWI

>SG-AL_contig_7337_1_-2_2_106

RISLCMGMRSNIFLFSNGGVGKLNLSVIIQHRDHH

>SG-AL_contig_7338_1_-1_1015_1269

MHFYGGELLVWCLMQILSSIDSVQLYHYHCYCCCCCCDDDVVVVVTHVMLSIINIFIIAV

VVNEYYFLSVFLMPIIILCVHHYFI

>SG-AL_contig_7339_1_-1_58_585

MGGRRPLSRLEQSDRLRLPALSDPLQGGEEGQDRAPSTHPDRTHVAGTNMVSGPLGTNTH

SSSVTPSKRGPAPATTVGHDAPEPPTVLPTRLAPVQAQVCASELKTPASRSVAIQAVLSA

KGASEDLKALVADAQRPGMESLYGFRWRKWLDWRREHKVPHTKPSSLQLANFLAYL

>SG-AL_contig_7340_1_+1_472_666

MFSFITNSQLGELDAACTTFFPPSQTLKQSGCKAGVLSLHQAGFIYAVFFLLKCPWHLYY

LNDEF

>SG-AL_contig_7341_1_-2_134_232

MFDLHSLRDEIKIGVQISSNVSLLQLFQMLKKK

>SG-AL_contig_7342_1_-1_1_297

WVTPQSVKLGNANNKQQQTPQHSRLRQKTEETSLNFHFGQYYTNRSTLSPHHTLSFAMQQ

VGKAPALGSERSENSEVRYPTSRIFSLMVNSGCHKWQRN

>SG-AL_contig_7343_1_-1_349_837

MTGSNVMTLHLKAQNWFQICYMLCICVCVKGFVVRKILSEKLVPLLSYDVRPPPLPCFLS

AMHHVISFIQLVIHYPQILCPSSFSADPSNLCAVKYVDDAYHLCGTRTCHVQVNFGHCPL

TCLSHQVTVHQSPHRMCHMISPSNRLHKVSHNVTVQSPHRMMC

>SG-AL_contig_7344_1_-2_899_2311

MSTGVLLHTLTTTLTSTLTSTLAPDNEVDTLADGFALSTQPDAGMTKTTLLTPVARPPGV

RPAAPDYLLHTQFVCDRVLIPLVVSFGIVGNLLSLVVLTRKEMASPTNCFLTALSISDIS

LLLLQIPMFFGLNAQVASTDSYRLFVRYYTVILYVMTNVFLTCTCWLTVAVTVERFLSLR

FMMHPRLICSMVRAKRVILAIFFASFLFHFSKFFEYMPNTDLKSVRPLLPTELSLNKAYD

TAMHITNITLAALLPIGALIIFNSFLIYFLATHRRRMLKYKSKSGAGLGLGGNKGNTGGN

GGGNNNSASVDMLHVSTVVVAVVLVFVTCHSLGVFVALNIAVHGRLAIFSNHLFVAFKHI

NGLLVTVNSSVNFLVYCSISRKFRTMFTSIFLKRWLAVSGGKTRPWCLSLPPPDNSLATG

VILRAGGNNANGYTRSSNDHALAPTGPKEATFARPSPTNSSGIRSLDQLDL

>SG-AL_contig_7345_1_-1_1_216

FLLFLFLADSALLRALCFAFRSFQSFSKPCFSAGSAVFICFLRFFFWTLILSISSWRLDF

FGGVFSLSCFTP

>SG-AL_contig_7346_1_+1_1_147

ARRQKLNLPRANATDDWEKLDEKIVQRLDALIRKSTLEHKLAKAHWNTN

>SG-AL_contig_7347_1_+2_2_223

PSSGNPDSRRQRVSTLPWLESLQQHKFHSPMGLKEANIFYKPKTFCYKFNILLSGPESSL

VSLGLLNTFFAIEL

>SG-AL_contig_7348_1_-3_177_260

MKLPIIHRCLFIHPFAGKTKLFKIFILL

>SG-AL_contig_7349_1_-2_158_1399

MAVDESIMPWRGRVAWRQFVANKPCRYGMKLYCLCESTSGYILKMKMYTGKEGDKREVDH

GPNVVKTLTEDYLNKGHTIFTDSFFTSAELVTHMQANGTSYCGTVLKHRRGNPPDISNKS

LKVAKGKTVVRQKGDVVAVRFNNRKDVMLLSSKYTAQPVNTNKKQRLSRQQRQRGEVARN

VVKPQIVHEYNHNMNGVDHFDQHLSYYSFNRKTVKWWKRAATHLLHMAKIEAMILYNKFE

EKQKTQADFTLDLIVQLTDSGVTPPEAATENQAAGAEDHGAEAQAENRGEEAEQHPHNEE

GPNGGEETEHQEGPPRKKKRVQPLDFERLSHSDNPHKPVPNPPTAKRERPPKDCVCCTIS

GGGRNQQYIRRAQSSFMCQKCQVTLCLYPCFHVYHSVKDYKREMRRLHNINDSD

>SG-AL_contig_7350_1_-1_94_2859

MPTLTEGSRAGSGPLAVSVSLDDMEEERRSPTNLLIDTMELVEARKPMHVPNHLLETKTF

TTIGQNSMVHVKPETIHFDGFEVNEKHVKKLVILNASSDVLRMHLIPPQSKYFSIKYKKG

DRMVPGLTLECTIEFQPDEWRYYYDCVRINCPGDENLVIPIHGYPIMSTKEFPRNFTFPV

TAVGKSASEVFPLRCMAPVDFEYELIYIQPHPAFMIHPMSGIVPANSEVEVTVTFAPFEF

QTASMTVQLNISQFNAQPIVCKFIGISKPGMLKEKTLTQLALETSEVMDPRCVSPLDRAR

YNRKLKIKVKKVPQPPSAKSIEYKGIQFPPTIDTPYAVGQVLNQEPGKLKVKDLRDTVLG

KQETTETSTRQMKEARFEHAVRQNVYEERQNQLRWQVKVGDEQISNKQRSLILDARAQAY

SYYKFNKREDPVPEDEYGRHKTKINFRRTIRDVSKLGEQGIMFDPYMNDVWATRNAALDT

FIQAVRKVIIRNRGEARLNSLRAVVLDWGRRQPADEEEEDGEEQEASHAADIDKDKPEQN

FHIAAPRIQRTRFPTYVPPNVKDDMAADALGEVELVPTHVIVKEKVPYFNLKVPQMYRLS

GYSQHSVHNACSGYVPPRLVKPLRSGAQDEVILLPQTPTPVSVRTSMSCDLGITEGNQTL

AEALPEEKEKESPREIVQPMVIATLRPPEALFRPIDYPPLHIMNPAPGLQVFQAPMPYAE

VDSEFHLCPLPRYFRPDYDINPHASTQRKYLDREDTIRGMMSWKKFPSQGLTSLANIPTL

TNVWVPRWDDNFSPELMPTTVPALFDGLPSDDAEHLVEESQWDEESMVVHLTPDMVNAQF

ALVDASLPAVDDSKPASDTFPFGNKMPSTNIPVGANGPVPREKREEELEYFMTKKYNRLG

SKVTQKVSTLNSLLNQAHLKLQ

>SG-AL_contig_7351_1_-2_11_244

MLRFSVENGFGMVWFSQRISDEKPLLAEYEDRLIRLFRQIWHSQIEDSGIYDHYFFLLTV

FSKPRNRYQWLLINGIEI

>SG-AL_contig_7352_1_+2_2_154

KILTVTPVNLRVKKKTKTSVKLSCHYSLYSGILTQFTECQDERNQKSAKQS

>SG-AL_contig_7353_1_+3_3_206

QPVHQLPVGRVSMDRVPGGSWWVGPRESKGLRIPGLFNYNRGFCNVLDPSFVPTVINMAV

HGKIMDIS

>SG-AL_contig_7354_1_+3_3_65

STVAEKVKKPIPYPNINCIGT

>SG-AL_contig_7355_1_-3_702_923

MKLSALGWTLTAHSAAVLPACLCSPLYYSFTFVMILSVVFSFSYFNLDVITLVESLQGIQ

HSLFCVYKESSAVV

>SG-AL_contig_7356_1_-3_3_242

YKVYCFTLSTPHSVFALYKVYCFTSIQQFHILIHCYPHGQARGSFSINSILTFWKFCARN

LFFCCSLCFCPFSFLYRRLL

>SG-AL_contig_7357_1_+3_1164_1388

MKTLFIYLVIGIIHCKNRINSNIMTWRKYQKRGVGRENSWEGGEKKIEDSREKKHTKKIR

RGLINIMVLIRLAMA

>SG-AL_contig_7358_1_-1_49_1656

MSIPSAVLAAGWIPFVVVLVLTLLFSAGYIRYYRSKYDNELSSTLTAIFALCVTLLTSDL

VPVDIFLVSYMKNSDGSFKDWAINSTARNDLANSVLYAYYALYGIICFCFFILLPFMYFF

YEEKDENSTCKTRCCSALKYLIVFVIIAAILMLIGAFVPTKSLPNVNSTDWQKIEALFEG

LGSNGGEDAISFVISVFSLVGLLGFVTYTAYGMSALPMELMSGKKSAKKERMAVQSKRQD

AESRRQAIRDKYGITSRPSARGYQREQSLEEEERQLVRQERHLEAKEKSWVQKCLVLLRP

FEIVIGILFFLLGLLVFISLLLTNIDKAMHSLGYKIGYALPTRHLPNPIDIVLVFCQKVF

PLDYILMTGVIVYFVLCSMTGVRNIGIWFLWIKMYKVRPRRTVPQGLLLFCMILMFLVMA

INIVLYELTPQYSSFGSQHYLKNVTGAGNVTKTSLEPCTTEVSSDECTITRMALLLTRFF

YKMWFFGAAYYFLTWAFLVLVLIGFIVTLVQGRRSAIHGEVDSDDFEESDDELIHA

>SG-AL_contig_7359_1_-2_2_208

SWILVLSPPWLVQSCCMMLNLLMVGEMWLTFSEKPLLKTFLPHIQRQAVLLQLQWACEHL

SVRCFRRRA

>SG-AL_contig_7360_1_-3_987_1271

MGLTYEIHYSRTDYVQSAINVSIGHYSVSHLLSEQRLCKLLNTQFAEMLRNSKFVSVIMF

LRTVLVNVLPNFSCESHTVHSVFVEASKPLVPRIN

>SG-AL_contig_7361_1_+3_3_122

LCLSLSPPRNDLINKQHIHTADRGMPTSTLSHHSKLLRNK

>SG-AL_contig_7362_1_+2_284_460

MEKGDDISRRGASRFWLEDLRQRTGQKCLQGLTYPRSGKGDRGGKGEGSTGYRFQVGKS

>SG-AL_contig_7363_1_-2_287_499

MSYIIKSFMKVYKAAEKPSISFLCGIISDFFCKYENMICGSGLFSKSCFIFYRRIICFKI

SYDTFIKDFTK

>SG-AL_contig_7364_1_+2_101_310

MLTWKIGKISTLYPPGKNAQGGFEPGLPNWSSVFTNPPPRHIAFEKKKTTKKQQQHIFGR

HLSVLHLKYV

>SG-AL_contig_7365_1_+3_3_194

WNDPLFVLMSVNDLSLSLSLSLSLSLSLSCATTITQETIPIPPQITRAPTTTAQNPLRCY

THKL

>SG-AL_contig_7366_1_-1_157_1638

MTTADGSLPASEPVAPVSVTTQAEITPINNNITVSSTHVRPVIVPPSPTPNAQRHSPSPI

PTQHSPIPVPQPSGPTLRIEEESRAPPPAPTEVQRRGVSVSSCDDEEELNTSTGSITQVP

RRQVTISDATPVQIMQPEPAVVVNDEQDEEELPHVIYPDDSSAGRPYPPYEAIPACEPDL

SRRPNKSALKGGSGQGGPLSPPPPFPASSSLSRPLETSPSHTHASSPGPPDVSFNLSMNR

SGSPNLGASASPMSGRHVGIVIPRPQLLSYQMHGAGDSDKENMAPPLPPPPALPPYPHQS

QPDYDNLPDDSDSDDGEIHYKDDYEESSLAAKVARQDSLARFLSNRPTKQDLEDKNIIPA

HSEREKADLREQIGSKLTRRLSLRPTLEELENRNILHSQSLDDYQKEKEAKRRFLIRKLS

FRPSVEELRERKIIKFNDYIEVTDAQEYDRRADKPWTKLTPKDKAVIRKELNEFKSKEMD

VHEESRHLTRFHRP

>SG-AL_contig_7367_1_-1_151_831

MMTTTRLSAVVSLSIVVSLIIPPASSYQCNNEVKARYTDVLEARLELLETKQKTLELKNR

ELEDEFTSLHTKNYDLETQVNELKNKNIALDARLKSLKERSKVVEDQMDSMGINMTVLNT

AVDSLTQHAERQQGLMQSLDISFQARSTEPRINLPASGSTLVLNQAVTNQGNGYNTTTGV

FTAPLNGTYFFLATAWDYWAAHSSTLSLVVDGHQIGSGETSKELFYQ

>SG-AL_contig_7368_1_-1_4_1650

MWRNPNDSLLTVNEKRVINDVRFSVEQPYPQFWNLHIDDVASSDAGEYLCQVDTVPPQVK

RVVLHVNVPARFTRDEPSHQSLRVREGESLELDCNVTGTPTPLVDWLRRLNDSYVWESMF

RQVEVLTLSDVIRYEDYEYKCYAENGVPPGVTRHFKVDVVYPPVVTVLTPRVRQVQGKNA

VLDCEALGHGDGMSLQWKRNKRVIYENSKYEIEVSSEGGRKMSSRLTLKSLEEEDYGEYE

CFSENSEGRDSRLAVLSESSVVTIAMSNDRTPEVSEGNSLRLLCDVSTASEEGAADTVVW

YRGPLGSSNLQGLGMKQLNETERELLVKNMTHGEDFMCLAGNDTESPVFFVTSVHVLYPP

EVTVLTPRVIQSPGSNATLQCSVTGQDVLSSIDWLHNGHILYGNEKYRTEVVKDRERQVT

VGTLTIVTVSEADYGQYQCMSVNSEGTTTQTAAMSEESPIRVDMSEQTTQRVSEGRTLQL

VCKVTTGQPSSSVFVWYRRHIGTGDVESLTGEHTLEVPNVTRGDDYVYLCVVDSGSPPAV

SHSLSIHVD

>SG-AL_contig_7369_1_-2_2_217

HLGSPPSDSLPRRPLPEKGHRVSLRLQWSDWLAWCGSNQVDPRSPSPVELAKLPGFPCHL

GGLSHEHHQGLQ

>SG-AL_contig_7370_1_+1_1_114

TPLSRSPPPHTHTHTHTHTHTHTHTVQRTMWMKPTQYR

>SG-AL_contig_7371_1_+3_3_155

KRVIPTIWYQLSYEITTTKIQNTKNDGRNCTQKIPDKQKMSRNTTFLFLIK

>SG-AL_contig_7372_1_+3_117_224

MKHCSEVILRFLDEQKSVNAERPRNVCIKDADTQIC

>SG-AL_contig_7373_1_-3_165_1496

MTGNGGEIQWCFSQVKGSVEEEVADADIISCVEFNSTGELLATGDKGGRVVIFQRDKASK

NAVPSRGEYSVYSTFQSHEPEFDYLKSLEIEEKINKIRWLRQTNRNHFLLSTNDKTIKLW

KVAERDKRPDGYNLKDETGISLDPNNITSLRVPVFQPMELMVEASPKRVFANAHTYHINS

ISVNSDQETYLSADDLRINLWNLEITNQSFNIVDIKPANMEELTEVITAAEFHPQQCNTF

VYSSSKGTIRLCDMRDQALCDKHAKMFEEPEDPTNRSFFSEIISSISDVKFSRNGRYMMT

RDYLSVKVWDLAMESHPVETFQVHEYLRSKLCSLYENDCIFDKFECSWSGDDRHIMTGSY

NNFFRMFDRESKRDFTLEACREITKPRTVLKPRKVCTGGKRKKDEISVDCLDFNKKILHT

GWHPLDNIIAIAATNNLYIFCGKD

>SG-AL_contig_7374_1_-2_2_376

SGALPATRSLVSLSRRKSTESVYNYKWTKWLAWCKSNQVEPHAPTSEGLASSTVKGYRSA

ICTTLQHLRGPTLSDDFLLREVLRGLAARESKEPRRLPKWDLALVLGALRTPPFEPLDVV

PFSLL

>SG-AL_contig_7375_1_-2_2_265

PGGRAQSRFTTPNGRSGCHGVSPTRSPHTPPPQSSWPTSWLSSPPQRDWRQGLQICTTLH

RPGGACLSDDLLLCEVSRGLVARDSKQP

>SG-AL_contig_7376_1_-2_2_286

CLPQELSRRKGTESVYNSKWTKWLAWCGPNQVDPHAPSPVELANFLAFLATSEGLASSTV

KGYRSAICTTLQQLGGGGAFSPTTSSSRSPQKAPQ

>SG-AL_contig_7377_1_-3_993_1265

MLGKIKVLSIQPENSFSLRIARLHAFKYHFTHTHTHTHTHTHTHSVCDFSVTLCVCKCVC

VCVCVCVCVCVCVCVYFILTGRRLLNLVLVK

>SG-AL_contig_7378_1_-3_273_872

MGETHETDTLPQEMTTEKTRKERRNGMWNIFEGVDNAVLEQLGLPEEVVRQLVTEADDTV

SEHLAIHQADSPPLGDCRCQRARILRSFSATVGEEMRAALQNRHRRNAVGNLFEVLTPSQ

KLLLVEHLQEQLERLDCSCGAKDAEASTAAATSHRGSDSNSSAIILALTELSSSSSSASS

PQPGSSSVSSPRAKEEVVCR

>SG-AL_contig_7379_1_-3_279_485

MEIRAECPVTVGTFYVKAASSLVKIQAQCPIAIETFSIVTFRKCNKVKRRDFCGALLCVL

LFFELCLHS

>SG-AL_contig_7380_1_-1_1_264

MPKNSGMPGKLNLRVFLCAILFTDYTRCYRYECVEVGSSLMEIRAQCPITVDTFYVKVAR

SLVEIQAQCPIAIETFSLIFRKCNKVKR

>SG-AL_contig_7381_1_+1_124_324

MTCETILFIPQLCIKQNNNPHNSAHCNISHTHTHTCLMGKTPTQTHINTYTFTFLALIFG

TEHIKIT

>SG-AL_contig_7382_1_-2_65_4468

MSSQPGTSRATRSSKDKNMKSTAVTNQASQSPSFKTEKPAPKNLATVEQMRMAQMIDSGK

DITNAHRVKEICEMTGRSEDDVIVALHDAQDDVEAAVILLLEGEQGQGEWEVQDKKKKRS

STLKSEKEMSSSPAQENDRGDPRSDRSRDRQDFDRHDGPTRRGRDRRNGVPPPRLANRGR

GRDRNNYRGDRDRDRGGFGNRGNFGDQNDRSSYNEVETVNGEEDWEVDITASNDLKEQRS

SKDYRDQRRGDKDRGFGGDRGFGGDRDRSFGGDRDRGFGGDRERGFGGERDRGFGGDRDR

SFGDRERGRGFGGNRGRGRGRGGGFGRPSSRGGRPRRFDRNDSSEGPHIDTWTNETAENA

EKENSGWGETWAESTEDWCEDQWTGSLEDTKVFTSSQTKTPVEADLTTNVSDSTNAIGQR

LDVGSLFSKSAEFAKAPDFTKTGEPTSGDSFFASYNQQAAESIKNSIGIGSSSARQNLSN

MPASAPQSLGTLPAMPQREMSHAQTQPPRSSVPSILSQATSSAPSSMHSSSTSNAPPSHL

PPVPATAPSASVSTPPMAQPAQPQQRRQQRSKLPPPSKIPASAVEMPGHAIPQQLDVQFG

VDFGAESSQFGFGSSTPNTNTPFSSSSPASNSSVTSHLGESGKSQEASRQVHMVTSPPAG

SQTSQSSMPSMEASPARPSVFPDSVYASPSKSESPAKSPAPEHKSGNLMNSSRSSQSSST

MSQGSLSTSKQERDTSNMVPLSMANGYAASSYPTHSAHSQSHKSSSSSSQTYRQAGPVSQ

TSVASYPNQFSSQTQYSGAGSNQQNQYPSAGQAQFPGSQGQFPSAGSAGSSAPTPSQYMQ

SQAQYPSGSQSSFSQAPVASQGQYQAGGSQYHGNYQSSSVSFQSQPSSQSGQNPLYPSTT

QQSGGSYPSGSSSFHVRDSQSTGNMQPSVVNLQQAAGSLQPSVVNLQQPSGNLQQSVGTH

ASPAVSQPSNSYQNQQPAPGLTKATTAQNTNAYNTQTYSGPHSGQHASPTTTQTPSAYNS

QTYSTQHASLHNQATTTQSPSSYNTQQTYSTQHSSQHTQATTTQSPSAYNTQTFSAQHAP

QQNLQTSPLGNKLGESLSKMTIKESASIESRTSQYDHVSSSSTTVSLTSSSSTANSSSIS

TSTSTVSATSTPVVTTSTLASRVTQSIPLTTKAPPNLPPGVPLMNPHLFMGQAAAAGGLP

PYFAGLQSLQQPMYGYEDLQLMQPARLPLQTGNFYDLAAYSGVPSTIAAGRDQAAALGTA

PFTGAATDNKQMNRVEAQSPSQSTQQQSTHNAPQQTLPFNIHYSYYYPGAMLPGAAGFQY

PTMFPMPPVTNTAAAHVPGTTTAAQLPKTYGSHQGFPSKGTGTTADMNTGTYANSRTQGF

DKQGFPAGTPPPFSLPLANASQAGPLGAPTAPFGTPFLPMMPPQAHNQTLLHHPMAQDSG

TGSNRSLQQPTNQGKPAAAKNNYYPSWN

>SG-AL_contig_7383_1_-2_2_163

KKRNLQRTTLASVCTGAPNPPESSHACFFNITGCSRFLLTATATADCEITDPCF

>SG-AL_contig_7384_1_+2_2_181

KKKKTKKKKTKLKKKKKKEKKKKTLSCINSGSSGLASFDIFPFRCLFMLLRIHHSYANQD

>SG-AL_contig_7385_1_-1_118_249

MITLTIRPDSFLVLFRLLQSRLIYVGCDHFVSCMLYDGQVEPEL

>SG-AL_contig_7386_1_+3_588_848

MNNTMDSAAEKFSILSDCGFRRGCHDLMNCLSRLFLPKCYQSNYRVASKPAGNVVHPHCD

KTALSVALSELVSTKRDIQILPPPPPP

>SG-AL_contig_7387_1_-1_154_276

MFSYVSCLYFLINTFASIINKRVHIVLYFLLNSSNVINDPL

>SG-AL_contig_7388_1_+2_179_403

MVIGASSYCVCCCANTLLFRASSYKLVLFFSFCPLYSNDGTTSCAKLMSPKKITMESSII

IFLTSIHTVQEHKLM

>SG-AL_contig_7389_1_+1_355_483

MTEEDPFLYKGNGRNKIMLFYIQAREKRRGRRWKTKRQLFLFQ

>SG-AL_contig_7390_1_-2_983_1297

MLRVYDACACVECSFNVFLFLVGYIFYFSLSGLKCYLLTYSLELVGKITNVETTTVSLTL

NVDNLKQILKNDNSAGKKGRRNTIVVENMTLHPSWHVQLVELFSN

>SG-AL_contig_7391_1_-1_1_210

PRASRGKPFSLCSATVQTATRLLPLRLDVSSNNHCKRASFLFLLGKSTVRGHSKHHHAWS

QRSTQQIQQD

>SG-AL_contig_7392_1_+2_1109_1339

MMAVVSNNKTCIMIVVVSNLLIMQHQKVMNIQTIIHFMFNLCADTFLFYSLLWQNCHYIY

LLSYSISRQKKIKNAFV

>SG-AL_contig_7393_1_+2_2243_2473

MSVLNIMLVHCPFPSIPVVLAFHRSCFPPHFKTLSVHRVLYRTKEFARSLLADLGRTHMK

LSECDESLLNKLLTPNC

>SG-AL_contig_7394_1_-2_8_3385

MASGSIEIPLRDTDEVIELDLDQLPEGDEVLSILRQEAAPLHIWVTLALEYYKQGFIDDF

VKILEVSRTDGSLDYPNSERDQMRSLDTLAAHYVQQAHREKNKDKKREMFTKATLLYTTA

DRIIMYDQNHLLGRAYFCLLEGDKMDQADAQFNFVLNQSSNNIPSLLGKACIAFNKKDYR

GALAYYKKALRTNPKCPASVRLGMGHCFVKLNRLPKARMAFERALQLDSQCVGALVGLAV

LELNSKNQDAIKSGVQLLSRAYAIDPANPMVLNHLANHFFYKKDYQKVQHLALHAFHNTE

NEAMRAESCYQLARAFHVQEDYDQAFQYYYQATQFAPGSFVLPFFGLGQMYIYRGDNENA

AQCFEKVLKAQPGNYETMKILGSLYANTFDMEKREIAKQHLKKVTEQFADDVEAWIELAQ

ILEQTDVQGALSAYGTATRILKEKVEADVPPEILNNVAALHFRLGNFQEARKYYEASLER

SKTDAQHDETYYSAISVTTTYNLARLYEALHEYDKAAKFYRNILRDHPNYVDCYLRLGCM

ARDRGQIYEASDWFKEALQINQDHPDAWSLIGSLHLAKQEWGPGQKKFERILQRPQTKDD

AYSLIALGNVWLQTLHQPMRDKEKEKRHQDRALAMYKQVLRNDSKNIWAANGIGCVLAHK

GCINESRDVFAQVREATADFCDVWLNIAHIYVEQRQYVAAVQMYENALKKFFKNHNVEVM

VYLARAYYKCGKLHDCKTTLLKARHVAPHDTVLLYNIALVQQKLATSVLKDEKSNLKTVL

TAVQDLELAHRYFSYLSQNGDRMKFDLNQAAAEARQCSDLLSQAQYHVARARKIDEEEKE

IRKRQEEERDAIRQKHLEEQMEKRRAKEEMDKKLLEQRAVFIEKAKTINIQAVSEEKGRK

SGGRKKRADDGEILSEGSDDDMPKKKKRKKHAGSGSDDDEGDSKSRKKKRRSRKNLDDSD

DEGEGSSRKERRKKPRKQREPKEPKKKKPMDDDDGLTAKQRRKIVSKAIISSSEGSDSDG

EKLKIADESGSENEGGTGKRRRIMSSGSDSDEGGKRERRSRSGSGSPGSRSGSGSRSGSG

SRRSGSGSRSRSRSGSRSASGSRSRSASPRKSGSESDGGGKKSGDE

>SG-AL_contig_7395_1_-2_2_172

HKVLFLWNVLLGVYMRSVIVCHSEFCFLMLDDHPHPSPCECKLTFRTSLTSILSSLR

>SG-AL_contig_7396_1_-3_369_524

MGYDFAVNLCSIQSSQGLDVRLVHMAFMLLGMSRVKIIVWCYFWLPGIFMCV

>SG-AL_contig_7397_1_+1_319_780

MAEGREADQATGLMTARNQSREDVFYATVDLLTSKQHIRVGCWNVQTLFQAGRLSQAVKE

MNSYNLTMLGVNETRCTGTGKQRLNSGEVIIWSGRQDNHHQKGVALIISKKHVNTLLQWK

PINKRLLYVRLNSKYTKLSVIVAYAPIDNADEEE

>SG-AL_contig_7398_1_-2_152_724

MFKNTFQSGFLSILYSIGSKPLQIWDKKVRNGHIKRITDNDIQSLVLEIVGTNVSTTFIT

CPADPKKTLGIKLPFLVMIIKNLKKYFTFEVQVLDDKNVRRRFRASNYQSTTRVKPFICT

MPMRLDDGWNQIQFNLSDFTRRAYGTNYIETLRVQIHANCRIRRVYFSDRLYSEDELPAE

FKLYLPVQNKK

>SG-AL_contig_7399_1_-1_430_561

MYINIVPSQVSHFVVEIQDFCWQFTHKYTVFTSSVLSLFFFFFE

>SG-AL_contig_7400_1_-1_64_363

MIRQEVQHECAKKMHLFICNFSSNSAKNKEPEALWSRFLQKIQYSGTTAFSLCYSLPSHP

LRLQEIMKPHMTVQTIYPGTVKQNINANIVEQTADPNIVQ

>SG-AL_contig_7401_1_+1_397_573

MKFMNMLYNVNFYLMSALCNACNVRHYSGQNVNNKMMHSPFTFLYHPTLALHYAEKITK

>SG-AL_contig_7402_1_-3_315_710

MQYGSRFALFWPELTRLPRNRYKDASLRSPTHEAQLVRARSLFLFPSGHVSAAGRTSTSS

RLDSLLWLFPGVLVPRHLPFGFLRLCLGLLCDSAARVRSSLADSSVVVRRIFFLLFSRSG

VWPLWLPSPGLE

>SG-AL_contig_7403_1_+3_3_239

FFFTHDHFQRVKIFTHDQFQRVNKFFTHDQFQRVIFFFFFFKLMTSFKDLNGLLTTSLQN

TSYTFLLNSLPGLFIMSMI

>SG-AL_contig_7404_1_+1_382_651

MHINRMLNITLARLTAAQQYKQAQKKGGGEGGGREQIAANLETKVCTRLEGMLPLHKTPQ

HRLRDALQAKYRALTLDNCPGLAIFDGIWT

>SG-AL_contig_7405_1_-1_277_552

MHTQNLKYALKDTVSHVSVQWFMKTQKHPVCMLTKDGVCRLCSNIMHSVSQTMCLHASEK

KHCSEWGLKRKHFLLGRFSNALTASLMGAVVE

>SG-AL_contig_7406_1_+2_2_265

YYILFIAQPTIKAISGLSPRKVHQSQEDNRVDHKTSVNLKSKILQNTVHDILSKPSSSLR

QPRPGCAEDASHSAQLIIPRLKKKRKKI

>SG-AL_contig_7407_1_+2_848_1117

MQPISSQLHVKTSSPLTLPIFPSLAQFNNMYNRSGFSSLITFSIFQFISKIQHLKEFTQT

NAVVIINSYNAFFKSFLFFKSTTMIVLSSI

>SG-AL_contig_7408_1_+3_189_461

MGEGEERAEREAEETIKNRSPQTETIVACFGSILDLPCGGPKDWLSTLKGSYKTFLFSAI

FPPSAVSVSNDQIVNSVQTAFFVCLFVCFSV

>SG-AL_contig_7409_1_+1_1_228

VSQITLSSGQWVPSCFPSSSETVVCWTRLLFGPLFSGFNSLHTSRSVQSIPPIRVLDRRA

VSRVRRVWGGDKEELV

>SG-AL_contig_7410_1_-3_849_992

MCLFIHKQCLYRLWLPEDIPSNIKINILTFRSRLLSSHKFSVSFLTRK

>SG-AL_contig_7411_1_+1_649_894

MYCLIITKMMVDHKVYVQNSTRLGEHQISNDKSTNAASALKPQNRIQALILSLSTSFQYD

QPRKLVDRVRVEFLQLVDGFQE

>SG-AL_contig_7412_1_+1_1_72

RQTDRERSITHFINSDRSNKTLMM

>SG-AL_contig_7413_1_+1_40_204

MYFILFIQTEYICDVVLQNKFDFGNKMLGGIQFHNFHKMIMFFTCLVQQGFLNLF

>SG-AL_contig_7414_1_-3_3_149

GSYMCAHRHIHTHTLNYTCSFIFSSLDVNFIALDLHIPPAFPLLSPNLQ

>SG-AL_contig_7415_1_+1_442_570

MATVMDSVIKSFAKFCLLQKFLYSDVCFLSRGKSVGFLGNSQI

>SG-AL_contig_7416_1_-2_1067_1249

MYVVLFSFCAIHYVCLNNSYSLLHDFFNLFNFLLLLLFGSFCHSGFLCQICITANVKCIE

T

>SG-AL_contig_7417_1_+3_3_197

FFFGFCYFGVVYFFSLLFLSSPRLACFYLINCIVVLATLTGINMYTISFYIGFFLGGCLR

KKKKE

>SG-AL_contig_7418_1_-2_533_772

MYRVYQVIMQSPIGKVVSVYLCFNVQTLLMKNCLCVADGKLLALEGFCSFVGLYFVQNIL

HFYSKLSLYIGVLTALAAFY

>SG-AL_contig_7419_1_+3_3_221

VVGVEGVGRYTRVVTHVIACYCGGKQPHKIRAWNILAIFLPGDRTDRGVGLHLTGDVGWP

PQSYCIGTEWLEG

>SG-AL_contig_7420_1_-1_1_153

NYSRFRKIFKKKKKKKKKKKKKKKKMVINSIGVRTFCQRIAHKYYSSPLKT

>SG-AL_contig_7421_1_+1_400_678

MYQKALTAMAVMKTGLPIYKAVSVSTTFHWDYVHHYSFLNSCSFADTFIQFVNNPKRHCP

MFNVNQSLKNNFIFNACYSYLLLSTCINSVHTF

>SG-AL_contig_7422_1_+3_3_149

VKFSYLPLQVWRKAWWGPSGSPLPFFFFFFYKTKFPPLKSKDYIPTLEK

>SG-AL_contig_7423_1_-1_1_372

DGIWRPSPHGRGLPAVPPPRRADRLSPGGLHPCPRLNASQAARRTPEAGRVFGLVPVPSP

GRSLASRSRPQVLTLPALPPPAKGQSSVGSLACHASAWRQLRLSDWVVEVIEEGYKLPWE

GAKP

>SG-AL_contig_7424_1_-1_136_834

MMESTEPQTQKCAYCSSDTSSDGLPLRRCAGCKMVSYCSKDCQKRHWKNHRAPCRKAKSE

REAQASSLRHQVAEKVELATLCSLLSIVRQPPKPWNWPDAILEAQKLFPFKDIITVFDEI

DRKKAVFARNVGMMRNIAMVAPLQMVALGKIATSPSTTSRQKRMELQDAAGQTRHFILET

FLRKRSAGFRHEFAEGHFVCIERPEDFIYHSIIFGRLSWHVYDCQNVHILKPE

>SG-AL_contig_7425_1_+3_3_227

INHSCTALFSKLIWTHCTLHSYRTQSVIHAEAPISRTHIDTEYTHSDVHKSIHIQGVNNT

YTCPLSQTYTHEMTF

>SG-AL_contig_7426_1_-2_269_5065

MNNILSSGEVSGLFARDEMDEILQELIASMKKDFPRRPPSNENLQEYYMSRVRKNLHVVL

CFSPVGEKFRNRSLKFPGLISGCTMDWFQRWPKDALVAVSSHFLSAFDIDCQPAVKQAVI

STMGMFQDMVAEVCVQYFQRYRRQAHVTPKSYLSFIGGYKQIYKEKREEIGNLANRMNTG

LEKLIEASESVNELSKELAVKEKELAVASKKADLVLAEVTVKAQAAEKVKAQVQTVKDKA

QAIVDAISVDKAQAEEKLEAARPALEEAESALQTIKPAHISTVRKLAKPPHLIMRIMDSV

LLLFQRRLNPVEPDPERPCVKPSWNEALKMMNASNFLGSLLSFQKDTINAETVELLEPYL

TMDDYSLENAKKVCGDVAGLCAWTSAMSFFYGINREVLPLKANLAVQEARLQVADSDLST

AQATLDAKQKELDEVQAQFDAAMAEKQTLMDDAEACRRKMNNAEALINGLSGEKIRWTEA

SKSFDAQIRRLVGDVLLATGFLSYTGPFNQEFRNLILRSWKKEMVSAKIPFTDDLNIIQM

LVHNTVISEWNLQGLPNDELSIQNGLIVTKAARFPLLIDPQGQGKAWIKNREGDKDMQLT

SLNHKYFRAHLEDALSLGRPLLIEDVGEELDPALDNVLEKNFIKSGSTFKVKVGDKECDV

MSGFRLYITTKLGNPAYTPEVSARTSIIDFTVTMKGLEDQLLGIVILTEKQELEAERTNL

LEEVTSNKRKMKELEDNLLYRLTSTQGSLVEDESLIEVLKVTKATAEEVSEKLVIAAETE

LKINAAREEFRPVALRGSILYFVIVEMSMVNVMYQTSLKQFLELFDLSMARSHKSPITTK

RINNIIEYLTLSVFQYTARGLYEMDKFLFTILMTLKIEMTAGRVRGEEFSVFIKGGAALD

LNAVEPKPKKWITDMTWLNLVELGKLPQFTQILSQVGRNDKAWKAWFDEDAPEECPIPDG

YATTLDTFRKLLLIRSWCPDRTVPMAKTYVSEAMGPAFAEGVILNLESMWGESNVRTPLI

GFLSMGSDPTENIERLAKQKGYIIGAISMGQGQDVHARRLMNVSMLEGRWVLLQNCHLGL

DFMDELLDTVLTTENVNEGFRLWLTTEPHPKFPINLLQTSIKFTNEPPMGVKAGLKRTYG

GVTQEQLEYSNMPMWKPMLYSVAFLHTTVQERRKFGPIGWNIPYEFNQGDFNATVQFIQN

HLDDVDPKRGVNWVTVRYMIGEVQYGGRVTDDYDKRLLNTYTRVWFTEHMFSDNFAFYTG

YKIPKCKTVEEYRNYIEGLPLVDTPECFGLHPNADITYQTNTANSMLATIVNIQPKDAGS

GGGETRETIVYRLADDMLDKLPEDYIPFEVKERLKKMGLLQPVNIFLRQEIDRMQKVITE

VRITLMDLKLAIDGTIIMSENLRDALDNMFDARVPNTWRKISWESTTLGFWFTDLLDRNA

QFSSWLFEGRPMSFWMTGFFNPQGFLTAMRQEITRAHKGWALDTVILDNDVTRMMKEDVH

SPPGEGVYVHGLFLDGAGWERRNARLIEPTAKVLYTQLPVVHVSATNQEKQRSMNLYECP

VYKKPQRTDLTYIFPLMLKTNKNPDYWTLRGVGLLCDIK

>SG-AL_contig_7427_1_-3_3_239

PPQEFLHALSRDADKGTCLPPGRFCLVAYLIPPPPYPCAERGWLFVQGYFRPQVGEAPSA

QDSPQRAFQQKVLCGLFWR

>SG-AL_contig_7428_1_-1_1_279

VGSTWSTGPGQLHTGASSAGILDPGPQLDEMSPSQECRWPIVPPSADVLVWCAHLHHYQL

GSTLIADPLKTCGTRGVPLSLCAIVAPPQEFLH

>SG-AL_contig_7429_1_+3_681_863

MCKKITHVQSVSVPTCSAVTLSTCIKICKTGDACPTSCNSSSFISYPNLVKNMLSGAVSH

R

>SG-AL_contig_7430_1_-3_3_197

HVYTHTCTHTHTRTHTHTHTPTPTHPHSHTHTHTAYCVKGCFDWTVTHTHTHTHTHTHTH

TLITV

>SG-AL_contig_7431_1_+1_487_666

MRRITPTDITTDTASKARLYFSSIANTKLTSVFERRYGRDCQINFYQENFPFMIHKHAPD

>SG-AL_contig_7432_1_-1_139_351

MDMIMVSLKAISFYRSVMPCVTLLLNILASLLRAKSTWVQMRDDLTSWLQRLSEWDRLFD

KLKWASYWLLY

>SG-AL_contig_7433_1_+3_3_215

KIDFLTAVGFLVYKIKTNCSFLVKKLNFFKICSLLSIFGFFACFWSNVDEYLYIFTTASA

HDNNRCINFSM

>SG-AL_contig_7434_1_-3_237_2243

MSALRSTCMRAMSPHRKTLLTSISHQDVSPSHCQDPCRAATFSSSCRCDTSDSQPTYMQA

KNAFGKETVRQKLGMERGGGPKQQSVSKVLSQVHLSSWAKGNLGSIFNGLGAFATNVPNR

LALEIGRLVGRSGQKSFQNRDSQNIDMTSAGVAEDSTHRLDINSKMEQLTDMETKREQHT

FEKSVEDTNQGILSYHHAYTTPQYVPAKVKKAVKSDEEVGWNVFRKIPIPFKDSSNAEKK

KPVIRKDFVSRSAIEARTRALVASIKTATSSSSKLSRTQDLSRHLKQYPSGRHEATESKV

VPLLLRMIESGEEELKVEAREALALLGYTEPVRGRGIRILCLDGGGTRGLMTIQCLKKLE

RQSGCTVSELFDYVCGVSTGALIAAMVCLYRLPLEKCEQLYLEFSRQMFSRGRVVGTGGL

VWNHAFYNSDQWEEILQAEIGHKTMIEFSRDPLVPKMSAVSTLMNTPRMKKFVFRNYNLP

PSTYSQYPGSCIHELWQVIRASSAAPGYYEDFPLGDYVHSDGGISANNPTALAVHESRLL

WPNEEIQCVVSLGTGRYEPILELAGSNKVSLKEKVAKIVDSATDTEGVHVMMTDLMPPGT

YFRFNPYLSEDLQLDEIRQDRLDIMQRDTSMYLRKNDLKMKKVVSTLKQKRMPHQYAMDW

LKLKADTYK

>SG-AL_contig_7435_1_-1_61_288

MCCKGLEVLPSLWILQVIVCAFLTLLLKKLDYQRYPMKPFTGVGYPDLNLVAELPQAHPL

YSPSEGPTDICNYMSQ

>SG-AL_contig_7436_1_+1_1_120

TMLQVYVCCTGHYLYMFQIQPLPAELASTHKTASNLIFAC

>SG-AL_contig_7437_1_+1_196_426

MAMLRKKLIFQLAGLRVGSSIFGFSCSCSSHTVLWPHEVPCSLHPGCLSESLSSFPDNKL

EMFLAGIRCTHPNQSRQ

>SG-AL_contig_7438_1_+1_1360_1626

MPKRLKTNLINMMRFYSLVSLIMLYITLKKTPDKLILSYIKKRGVGDETERLFRQNAHPS

ASTSTFIQFMLFLTQPNTKAIQKVSSYKN

>SG-AL_contig_7439_1_-1_1354_1665

MFMELDNLFPCSVLKGLIIHHSLCFHFDCCGLGKGWGWGGFIHTKENILNFLDSGLQGHT

KVDLCQYLPVLWLRIYFNFAAQRPSLHEFESDSGKQNVCDLLST

>SG-AL_contig_7440_1_+1_1_246

CVKACWKTESGIDREGRKKADDSKGMKKGKSKDSNRTEEISSTEFPEGRDTIYTERAKVH

DHTVNSNSIPIITKNNNRKKQQ

>SG-AL_contig_7441_1_-1_178_564

MLWTFTITESLCSYLSGLNCWCVCVCVCVCVCVCVLFSYFYFIYFIEFRHCSVDIMFPSD

FHVYAVCLYMNGMCACICMVYMYVCLYMYGMCVCMLTDIYLRILVTLSILLFKYVCRCIS

YILFIYSFI

>SG-AL_contig_7442_1_-2_20_244

MLRERERERERERERERDLYFSNGKDMTQYNTMLLGQFSMPAICLPKERGRSMFGEYVLS

LVIDLSVKYINHDSP

>SG-AL_contig_7443_1_+2_170_265

MIKLYQPSVLRKIQFRNFGFHVTFVAKWCNQK

>SG-AL_contig_7444_1_-3_27_1781

MESQVDVEGGGKHLSLRRYLSELKDEPSVSTGALRCKQLDCVRAYYYLSVLHYLQQQYHN

SSQDACRPESSPAHQTSAAIGVSSEEKGTGSVSVVRTDGSEHHADTVNDMVKSEIQSETA

AAVGGDEDGGAIASLVKLKDFVPKDGDGGSSVDLPQLAKQSGDTGNSGPYLVAGGKKKGK

TDSGDSCVDTTATTDSASYCADSRKGEESDSHAGKDPVPKDGVKVQSDVCGSSSWQLSLT

RLLQLSKGLLWDVQAKRLALTDTLGYIHHAISQHLLTDRSREVAKTLSRHPHAGNNHNHA

HHHHELDNTPSFAPVLADTVASHLPANPLLDIDDVGVPSVELEGGGCSLGSGSEGAESVV

SPSVNNSAVLSSLPSPSSPSSSASTAVSSPPNSTKDLGRKERNNPPDPSLDALPDSNFDL

DLVTLGQQWDGSRRVLWQDDPMSGSGGRGSESRRGAVDSSVHKPTSLLSPGKYINVPDEW

YSLPVDEKYGRSYVTLAILKDEQDFVMGELKRMSDSSQVPFPNPFHSVETLLSVCLISDS

QLLSAVDKHNYVKSLQSWAMHFAVTAQQRSNTDALFASAEKDIGL

>SG-AL_contig_7445_1_-3_3_143

VEGLGFCFVIMADNAGSWWIGWGVKGLMCHIWRKILGRKCRIIICHI

>SG-AL_contig_7446_1_+1_478_636

MLRTINPLTPHPIHQFPAASAMTAKQQRKPFTPSPCAPEGAKWLIMSNASFFV

>SG-AL_contig_7447_1_+1_334_765

MRACFCMIYSVLQKDVHVLFWFLIPDFFFFYFCILNVLNTLTTKIRKPVLFRQCYVDSSV

KIILSDPRVKIILILMSIIIIICVRLVLLCVTKHDVMCVCVAVSLLYVPSSPKMRNRNNL

LQLLLFLILFSFLILSTSFLHYQP

>SG-AL_contig_7448_1_+2_389_655

MSQTHICAVRRQGGSSTSMLSRSAALSYNLKNIFINTHTDLSLFPLNHCPFIKGRITTNK

EAIKKKKKYSITQYHFRIVAHHRSDLYFL

>SG-AL_contig_7449_1_-2_182_385

MVHGSICNCFLCHGIRIVPHNYTNPTKREISRQSICNCFPCHGIRIRFVIFISGKGRWLI

WLDSWFFF

>SG-AL_contig_7450_1_-2_2_271

IPYLNVCECVFVCACVHVCVCVCACMCVYVSERVRERERENVCVCVCVCVCAACTRIYLQ

KRYSFMLYVLEIVAKCNMCCCYVLSPSIFL

>SG-AL_contig_7451_1_+2_191_352

MSVFGGLWKHKHTQHAHFILPLPAPSCIGFSLSLGFHQCQRKGEPAAWATAGSP

>SG-AL_contig_7452_1_-1_370_681

MCCTGVGAGIHCDQLYFFVPPVFKQVLYSHCWYLFFDVENTHTPLHSHCANAGVLTFCIC

YKLQVDKQLFSPIASRYTGFFHTVFVQHSSLKVTGCQSTHKTKC

>SG-AL_contig_7453_1_-2_50_217

MFTALPSTCSVLLRVVFGLLFFAPSASKIALPLVVFFIVHSQYMTITFPSFLSDDV

>SG-AL_contig_7454_1_-3_132_1397

MGLAEMGRKAIEIAQNEMPGLMMMRKVYGPQKPLKGARIAGCLHMTTQTAVLIETLLELG

AQVQWSSCNIFSTQDFAAAAIAKTGVPVYAWKGETDEEYIWCIEQTLVFADGQPLNMILD

DGGDLTNLVHEKYPQYLEDIKGLSEETTTGVHNLYRMMAENKLKVPAINVNDSVTKSKFD

NLYGCRESLVDGIKRATDVMLAGKVAVVAGYGDVGKGCAHALRAFGARVIITEIDPINAL

QAAMEGYEVATIDDALGEARIYVTATGCKGILRPHHFEKMLEDTIVCNIGHFDCEIDIAW

LNANCEKKESVKPQVDRYTLKNGRHLIVLAEGRLVNLGCAHGHPSFVMSNSFTNQVLAQI

ELWTKTSEYKLGVHMLPKKLDEEVAAAHLSHLGVRLTKLDDDQAKYLGVPSTGPYKPEHY

RY

>SG-AL_contig_7455_1_-2_122_1657

MSTVNGVNGLDKSESGWKVGLINFANKYLTAETFGHKINVSGSALKKKQTFTLEQDAREE

MLQAVYIKSHLGCFVSTDKYGNVSCEAKEPGETEKFIVEYDKTPSGRWAFKHNVHGNYLG

GTDDNLKCFSKNFGDEELWTVQLSIHPQVHLRNVNRKRYAHVWDDQLQVTQTIPWGEKSL

LFLDFVEGKYALKTVDNRYLHTSGELLPQRTDDAMFTLEIRSGQYRGLAFRDRHGCYLMG

VGATATMKGRNKTVGKDELFTLEDTHPQVVLYAYNEKKVSIKQGVDVSANQDEEEGDKEI

FQMEYEPYSGKWAFRTVDNMYWSLEPLGGIQAVGKAICPETLFDVEWLEDGKVAIRAKNN

SYVYNKPTGSLIAGSESPSKKEKFRLKIVNRPVLVLKSDFGFVGKPLVGKSPGAGKAEIV

CNRSLYDIIQVEHASKTGTYFLKADNGNYWSVAEDGSVIADGTGKSAFELQVRDNRKLTI

RAVDNGCFIKGEQNGLFRALGQAVDSSVLWEY

>SG-AL_contig_7456_1_-1_1_276

SLYCSVPVIQIIVSFCPSHSGHCCRSAPVIQVIVLFCPIEAIVSYCPSYSGNCIVLSQSF

RLLYRSVPVIQIIVSFCPSRSDNCIVTALIWP

>SG-AL_contig_7457_1_-2_1289_1570

MDLVTVKLVWATVFGTQLPVFFHCSFVILPVSLVGVALFSLSCLLCVYILYTIFSALCCL

LILVVNFILLVCASLYSSDYTLCCFLSIPRQGST

>SG-AL_contig_7458_1_-1_19_219

METSAKTAANVEEAFINTAKEIYQKIQDGVFDINNEANGIKIGPQHSPANQNMPSGGGGA

NQGSNCC

>SG-AL_contig_7459_1_+3_345_578

MLSVFYSSLAFLHHEVYFVSKHTCVCVCDLNFYQFSLLWMRKLCQVLTVIDYDRAAIYEH

GLYSFVLLMINPFYWDWK

>SG-AL_contig_7460_1_-2_2_238

SIPLSKRPTRSLRRGPRFARLEPLSGLSGRAASGPRPGGESRRVPACCGPGGDERFRKGA

KEGRGLHRNGPKGRKVLSR

>SG-AL_contig_7461_1_-3_423_545

MNLLRLHHLLLMSLGLMTYSLLMCVDKETTKVKEHTHACFY

>SG-AL_contig_7462_1_-2_845_1126

MSSFLATSIFFSRFSRQERQRRQKTVIRNFVAFGDALFDYSFFSTSLVFEYVCDFSCIHQ

YAYTRLTIPGVYMIILPLFCRLSFPLVNRPMICC

>SG-AL_contig_7463_1_-3_189_683

MADLRAKKAGIGAEIDKKLEMRYDQEEAAGTTKAVQLWINAALEGEHEPIPSHSQRDLHA

SLRDGVMLCKLINKLLGAKGLAKVHTQKKVASMFVAYTNIENYNNGCLQYGLAKEFTVQS

ADLWEGRKGPFLNVINNLHSLGFLANNNGFNPTYTGEVQKTIDFE

>SG-AL_contig_7464_1_-3_255_464

MDIYNNRACFMITVNISKNDATTCFHQNATLAYHSTTILLISTECSRVFLQWLDNMSIFT

GGVFVHFISN

>SG-AL_contig_7465_1_-3_348_662

MTPTTYFHQKATPAYHSTTILLINAECSLVFLQWLDNMNGIHRGCFFVHFISCTQIIMQL

CVYNITNSCKDQLIGGLQAVSLCNEAEKFVACCAHYKCKRGVVDQ

>SG-AL_contig_7466_1_-1_1_177

KYTHVIYQIEALFHALFHCTTHGIIRDIGNNVCLILTLKKIHICMLSKCDQTKKEIKFS

>SG-AL_contig_7467_1_-1_1_147

FALRNLMVKIICHELCSFAGLLFSGLTRFCIQRVLDFDGLNMVTSLIWP

>SG-AL_contig_7468_1_-3_159_371

MERSNLWHLLNCFLPQYILISVEAKNLTFSTRPLRLVIPPLFSKFQMIALFVFTGVQSLP

STYRFLFCFSF

>SG-AL_contig_7469_1_+2_2_226

FRSINVVHTPQPAASRKTGDRMFQHQSGRKGSNHLTVHVEAQRHTENIQGTDDHRSQNIF

NNGEAEVIVLRKISP

>SG-AL_contig_7470_1_+3_3_200

FIPISLIFNYPENFNHIQYDVLSQLNSQRLTLSLTNASILHSLSDKNQLHGIFNCANFPL

SFVIHI

>SG-AL_contig_7471_1_-1_1_198

SVTVDKPAIRSPMSVGKPAPVSACVDDNSPSVSLSLSSVSDSCMHQTVSKYWGQGGDGNS

DGQCPC

>SG-AL_contig_7472_1_-3_39_1709

MLGSRLIMKILTWNINGIRASKRCLRDVLDSLNADVICLQETKITRDMLDEPSAIVDGYN

AYFSFSRKRSGYSGTANFCRDAVMPTAAEEGLSGQLSSQSEGSVGCLGNNGEFAWEELQS

LDGEGRAIITQHQIELENGTVKDLAIINVYVPRAADRDDRWTYKLRFLALLQSRAESLLR

HGRSVIVLGDVNTSHKRIDHCDPSCQEFLREGSRRWLDQFLRAEDRDPELAAADDDTSSC

EAILPDAEGGHFIDAFRFFYPDQKEAYTNWSTVTSARQTNYGKRLDYILVDMGLQEFMED

CQILADFEGSDHCPVVLQLRCKSVAARKCPPLCTKYMPEFQGKQQTLLTFFSKRKTVDSE

RKEAQNVASCDKPRSDSEKKGAESMVSCDKQCSDSEKKGAESMASCDKRRSDSEKKGAES

MASCDKQCSDSEKKGAESMASCDNLCSDSEKKGAESMASCDKQRSDSEKKGAESMVSCDM

QCQKGHLKRTSSDKTWNSSGRLKKKKIAGKGDNYVLTSTVGGGKQASLLSFFHKPASKSS

VTVDKPASRSSVTVDKP

>SG-AL_contig_7473_1_-3_6981_7433

MIVNDKKQDHYLFYFSIINHTDYKPLPSHPSPNSYTKNYTLFDRLLCDSLERNQHKTKNA

AATDAELNITFLEIMIQVAEHNPPSCLPCPNKYLPIFSFTQPPWLKNASPAKSQCLQSEL

HMINFLHKSITQYCNCNQHDSQKSESAKQPN

>SG-AL_contig_7474_1_-1_436_1530

MAGRVVAGETNKTVLKKFLELPQSPDKVMVEYIWIDGTGEGIRSKCKTLDFEPQKPEDCP

EWNFDGSSTYQSEGSNSDMYLLPVALFKDPFRLGPNKLLLCEVLKYTKLPADTNNRHTCN

QVMQEAKTARPWFGIEQEYTLLDVDGYPFGWPKNGYPGPQGPYYCGVGSNKVYGRDVVEA

HYRACLYAGVKICGCNAEVMPAQWEYQVGPCEGIEMGDHLWVSRYLLHRVAEEFGVVVTL

DPKPMVGDWNGAGAHTNYSTEPMRNAGGLGVIEEAISRLSKKHAEHIKAYDPNEGADNRR

RLTGHHETSSINDFSAGVANRGASIRIPRQVAEDGYGYLEDRRPSSNCDPYSVTEIIVRT

TVLDK

>SG-AL_contig_7475_1_+3_282_476

MSSCRSPFAPSPASCIVSHDSRAKTEPCHSLTLSTSFLQEAVRQGEGVKRVKFLQLVGWV

REWKG

>SG-AL_contig_7476_1_+3_3_197

SLPHPIHQLPALSAMTAKQKLNPFTPSPCPPASCIISMTAKKKLTLSLPHPIQQLPTLSA

MIAKT

>SG-AL_contig_7477_1_-1_1063_1269

MLCMVCKLSLFATVLANRLFSRQDFFWFMFCFVYCFIKMAEIVNECFFTSIAFLCFIFHL

KIELGGDSL

>SG-AL_contig_7478_1_-1_448_741

MTSFFFVASCQNCLLYHCRCQRGLVFVCWFGVVFFLNIIVRVKFTSSFLGCCVSFKSSPV

QCRDIVMEQKMDNTFYLIKPFLNGSIDTYSLVAIASVT

>SG-AL_contig_7479_1_+3_3_164

SNACFDIDYTDLKKNGGPVLSAIVKLHIHKIIMTIWKFLQTALFLKTIKQKLPL

>SG-AL_contig_7480_1_+1_1_285

TDLKKNDGPVLSAIVKLHIHENYNDHLKVFTDSSVLENDQTEAAFVIATLKVEKSYHIGK

GFSIFTAELAAILMALNYLLSLPISLFRVLFCIDS

>SG-AL_contig_7481_1_-2_35_235

MCALHHTDYSTHRSYVCVTSYLSLFPQVCGHYFTLLPLGIFRTSFITIFITQLLTVRNLQ

AGLFLPI

>SG-AL_contig_7482_1_+3_3_179

SIALFVSAPFPSFTVVCFFLPSRSIPLSFFQQAFTQFSLPPPLPHFMMYYLCCFALATR

>SG-AL_contig_7483_1_-3_78_1718

MWKAAAGTNVKITTQASEDDDWETDPDFVNDVSEKEQRWGAKSVEGSGHQESLDLKKLRE

GVRSDDTAYKKEQFDAGPKASEGYGGKFGVQKDRMDKSAVGHEYQTELSKHSSQTDTAKG

FGGKYGVQSDRVDKSSVGYDYAGKTEKHASQKDYSTGFGGKFGVQKDRVDKSAVGWSHHE

KVEKHESQKDYSQGFGGKFGVQKDRQDKAAMGWDYEGKTDKHESQTDHSKGFGGKFGVQK

DRVDKSAVGWDHQESLQKHESQTDYSKGFGGKFGVQKDRQDKSAAGYGDVTKTELHPSQT

DMKKGFGGKFGVEERKDQSAGSFDDMEGVSSSYQRTKAEGSSEGARNLKSRFENLAKSSE

EESRKRADEERQRRQAKEQREKEEQKKQQEERLKQERWEAEQNKANEPEPEPEEPRKPAR

QMVPEPEPEPEPEPEMSYNEAYQSSDNPYEETESEAAPAPAVQHEPEESPYEMDDNVYNQ

PEDNSAQSGGGHTAIALYDYQAADDDELTFDPDDIITDIEMIDEGWWRGACHGRSGLFPA

NYVELQQ

>SG-AL_contig_7484_1_+2_2_262

EIKDRWLVNLSDRALTQTEQSLLEKGLNCAVSPEKLSMVWYQNSVMAHLSQLRRGSTSML

RMCRYSEADETPPSRTSPPRKDKHSLC

>SG-AL_contig_7485_1_+2_584_844

MFEHFVSDSEEAKNDPEEVLDKLARYCHPRSNEVTESHRLWSTAYHEPSDVFMTGLRSFL

TGLRSEANLKETRYCLVYQTKYITFVV

>SG-AL_contig_7486_1_-1_157_2649

MSSYQMNASGGPASTMGPGSQNYMDLSDMPVDNKQQQTMMWQQNQYMADSGIHSGATTQA

PSISSKGHGDELEDGDMDPAKMMFDWGLTDQYPPQPEQVDDINQQLNQTRSQRVRAAMFP

ESLPEGVQIPSTQVHPDQPTAVQRLAEPSQMLKHAVVNLINYQDDADLATRAIPELIKLL

NDEDQVVVYQAAMMVHQLSKKEASRHAIMNSPQMIAALIRAMANTEDIETTRCAAGTLHN

LSHHRQGLLAIFKSGGIPALVKLLSSPMESVLFYAITTLHNLLLHQEGSKMAVRLAGGLQ

KMVALLQRNNNVKFLAITTDCLQILAYGNQESKLIILASGGPGELVRIMKSYTYEKLLWT

TSRVLKVLSVCPSNKPAIVEAGGMQALAMHLGHQSQRLVQNCLWSLRNLSDAATKMDNME

GLLHMLVQLLASNDINVVTCAAGILSNLTCNNQRNKVIVCQVGGIEALVRTVMQAGDRED

ITEPAVCALRHLTSRHPEAEMAQNAVRLHYGLPVLVKLLHPPSRWPLIKAVVGLVRNLAL

CPANHAPLREHGALPRIVQLLIRAHQDTQRRASISSNGPPMTGYVDGVRMEEIVEGTVGA

LHILAREAHNRAVIRGLNCIPLFVQLLYSPIENIERVAAGVLCELAADKEGAEMIEQEGA

TAPLTELLHSRNEGVATYAAAVLFRMSEDKPQDYKKRLSMELTSSLYRGDNMNWSEPPGF

DDVGLMPEESRDQMYTQQPPPHHGSQGSMYSGSHPDMQQGRPPTGYDQVPVNSMQGLDLG

GPGAGGGYGSQMDNLPDLGPTSADLNFDSIDPNMGAPPGDGGPSMWFDTDL

>SG-AL_contig_7487_1_+2_245_382

MAVDRSNMSVNHMLLNLILSLLHPIHQLQKLNPFTPSLQKLVDRMR

>SG-AL_contig_7488_1_-2_86_1741

MSFKDRMFNNSGRRGGYGGGSSGRDSGFRGGGFQNSMKGMQPGANLRKPKWDVSRLTPFQ

KNFYVESPVTAGRSAVQLQEFRRSHQITVSGKYIPNPILSFAEANFPDYVMGQINKNSWT

NPTPIQSQAWPVAMSGKDLVGIAQTGSGKTLGFMLPAIVHINHQPYLERGDGPIVLVLVP

TRELAQQVQEVAVEFGSTSRIRSVCVYGGAPKGPQLRDLERGAEICIATPGRLIDFLETN

RTNLRRCTYLVLDEADRMLDMGFEPQIRKIIEQIRPDRQTLMWSATWPKEVRKLAEDFFS

DYVQVNIGAMELTANHNILQIIDVCMEHEKEDKLVKLLNEIMQEKENKTLIFVETKRKCD

DINKRMKRDGWPVMCIHGDKSQQERDWVLNGFKNNRCPLLIATDVASRGLDVDDIKFVVN

FDYPNSCEDYIHRIGRTGRATKTGTAYTFFTPTNIKQSGDLINVLREAKQQVNPKLIQMA

ESSRGMFNRGRARWGGGGNRFSGGRGGYGGRGGGGRGGGGRGGGNRFSNSYSSANGIGGS

YQKSGGYQKNGY

>SG-AL_contig_7489_1_+3_3_146

FFLQPVLQNFICIQNSRNKCFKQNNKTEHNFCLLNNCIQLINKQETAN

>SG-AL_contig_7490_1_-1_2269_2604

MSLSTQKRRQFETCLGFFRYACLSILRGIRAQSFGLFISLPYYVQRYKFVLWFIISAMLC

SVRVWSLFKNNSSHSDKSICAPPKSALNYLAGSLSLPKTLCIAKVLKWEGEG

>SG-AL_contig_7491_1_-1_2056_2427

MYKPDIRMVSCKPYMQGKYNPTKTKSMVITTRPKHQLSKLCLQLSLDGVNIEQVSEHHVL

GVFINEKLTWETHVDHLSKTLRIYFFLVGFHTLSTLKQKKYFTTPISNPILTMHQQYGMA

AVKF

>SG-AL_contig_7492_1_+2_251_454

MSVKMYQCLIYYKYRLFSFIFLPVSVYEKHQKLFKSTFCQISKTHNKCIWLFCPMFRPLN

LAFLRVKS

>SG-AL_contig_7493_1_-3_3_170

GGVSALCPPGEVPVIRTQAFHTLQFESQASSPFDHRASSGNVCGFLALCCKNKDQL

>SG-AL_contig_7494_1_-1_310_642

MLHQLPPSFMVVGECLGCGKVVCGPLCHVICPFLLFCPFSFPPVLFPAGWSLEFSGSCHM

TIPSQLSILYCGQQVFQASHMLGDFSSHFIVCVWSVKEMSRSLLKHLISTA

>SG-AL_contig_7495_1_-1_1_258

DWKASALPLSQPRFILLQTANNTEDYQTQITSKINENKNKNYAFEHDIKGFCFVLFLFFY

TSLLTLSVSIQFISLPLQCTQKQSIV

>SG-AL_contig_7496_1_+3_3_65

DTIDTERTPVIPRRSLNSFKC

>SG-AL_contig_7497_1_+1_1_120

CVCVCVLYVFVTAARDGFADWDLIFNTNKAEKFTAKLTLN

>SG-AL_contig_7498_1_-2_14_1444

MSAPSKRAPDSPGEGPRPPKISSSGASDVKLESFLRWCNDNKFNISPKVCVERERACAQY

GMVAGQDIAAGEVLFSIPRPSLLSPDTSSIATLLHKAQDELGEDSGWVPLLLALVYEYNN

PQSSWRSYLDLVPDFAQLDLPMFWSREEREELLAGTGVEEAVSRDLSLLEKDFNTKALPF

MQRHPDVFSPACQNLDLYKKMVAFVMAYSFTEPASKGAGADGKGKNVEDEDSDGDEEEVE

GCPPPMMVPLADILNHVAKNNASLNFDVDALRMISARDIKQGEEVFNTYGELANKHLLHM

YGFAECFPHNHYDTVDLPVSVLSEVVQSQTADAGLMAAKLLFLREQDLHQEGESYVVGRE

GVLTDEELQAVLKVLVMSADEFKEHAEKDGWSDAESDDANQDASPLSFAKMPDLPASWKE

ILSRCAEVCLSKLGGTEEEDKAAMKTEGRGMASRARYAMYMRYGQRQLLRQLHNACR

>SG-AL_contig_7499_1_+2_2_163

KGKGVEAKLTLFIFHSHETYLTRLRSLAKMSGLNSRLVATSILILDLLLGHISD

>SG-AL_contig_7500_1_-3_12_1070

MGRKAIEIAQNEMPGLMMMRKVYGPKKPLKGARIAGCLHMTTQTAVLIETLTELGAEVQW

SSCNIFSTQDFAAAAIAKTGVPVYAWKGETDEEYLWCIEQTLVFANGQPLNMILDDGGDL

TNLVHEKYPQYLTDIKGLSEETTTGVHNLYRMMAEKKLKVPAINVNDSVTKSKFDNLYGC

RESLVDGIKRATDVMLAGKVAVVAGYGDVGKGCAQALRAFGARVIVTEIDPINALQAAME

GYEVSTIDDSLCEARIYVTATGCKGILRPKHFENMLDDTIVCNIGHFDCEIDIAWLNNNC

VKKESIKPQVDRYTLKNGRHLIVLAEGRLVNLGCAHGHPSFVMSNSFTNQVLA

>SG-AL_contig_7501_1_+2_17_1807

MPQAHHLAFIQNCCAGTSCYLVCTTQTRTQVTMPNIEKRESWASRIDFLLSCIGFCVGFG

NVWRFSYLCYKNGGGAFVLPYLCCIVIIGFPILFLEVAIGQFTRRGAVGIWDICPLMKGI

GYSTLVFGCMLCTYYNIILAWVGYYFVVSFSYELPWTQCHNDWNTDNCTLQQAVNISGSQ

GQLEVLHNVSNSSLGQHLPDVNITNDLSGIQAMRKGVSPAAEYWWRGVLQLSEGIEKPGE

VCGHLVGSLAICWVVIYFCVFKGVRWSGKVVYFTATFPYVLIIILMVQAARQEGASTGVA

YYVKPQFDRLGDIKVWFDAASQVLFSYAVSIGVLPAFGSYCPYNGNCLRDSILVAIANSG

TSFLVGFVVFATLGHMAHVQNETIEDVASAGPGLAFIVYPQALSLLPVPQLWSVLFFFMM

ILVGLDSQFANVEGVMAALWDVFPRWYRHRMLVLLIFSIGAFVLGISMITQGGMYVFQLI

DYYVTGAYTWQILSLLEVVTVGWIYGANRFYDDIESMIGYRMGVWLKICWKFLTPASLTV

IVVFNLVTYQPLTYNKTYIFPLWANGLGFLLTLLVLILIPLYLVLTLVRTPADTLRD

>SG-AL_contig_7502_1_+2_1490_1705

MLPSKNSKKILQTWLPHLFMNYKSLFFLPSWCFHLSAPFLLIFTNLFVKFTFLLFRSSSS

GLYFQKHRYHIN

>SG-AL_contig_7503_1_+2_617_751

MQAFHIIIHNFMHCGKINTQQSKLLLNLFWGVLFKSVLSSGSLGN

>SG-AL_contig_7504_1_-1_397_645

MMAVAISSPSLLYLSPTLNRIIYSFTAVWTGGRTHKDFSQASIERVHICAVRLKGESSTL

RQPRRPHSSVKYPGYRPRIEQHF

>SG-AL_contig_7505_1_-1_1_102

THTQKNCKIRPKVVWCLLSDHVLRLLQCVIQKAH

>SG-AL_contig_7506_1_+1_1_243

EDRKKADDSKGKKNGRDKESNRKEETASTEFPEGEDTIYSERVPGIPTGEFANKEGSNKL

FTLDAKTVMTLHCKKKNYLIS

>SG-AL_contig_7507_1_+1_1_129

PSLCSAGAWVSYCSHLAYTKEGPAAQESNDIFVLTVVVVANAS

>SG-AL_contig_7508_1_+2_2_136

FFFFICWISDICSTWEKRTSLSSKRARNRRSPLLRYPFKLIISSS

>SG-AL_contig_7509_1_+3_3_233

NRHLVPIAVFTISRPSRQSKAAQILHHKLQGGKGGKLCEGFAGKKKVDWTRKAEKSDDSK

GKEKAQKQREQQERVNF

>SG-AL_contig_7510_1_+1_121_384

MAVGLIQVQTTLIQNGCKPDSGSDHTGPKWHVATVWGHVGRTGQKGKSKGCLITYKSFES

EVAEASTFLGASIMRVVPISSPSLLYLP

>SG-AL_contig_7511_1_-3_384_578

MNSVTCIQKLANRASISAFTIIILLLFVSIPSSRQHAHVNNNIFKNGASTDLDMLTVKCN

KIHMI

>SG-AL_contig_7512_1_+2_71_730

MFLSDFDQLLDHYSLLPGNLLILGDINVHYDNHDSSDTKHIRSSLSHHNMTQHISEPTHQ

AGHTLDWIISRNHANPATGNSVVLSTSLTAGLPSDHSAILCFLDISPPTRTRHTVTRRNL

KAINLATFSADATRLLSEKSHNLAEHFDTTLHHLLDTHAPATTRLLPNRPPAPWLTPEIA

HAKRARRRAERRWRKSKLTIDKQLFHTAKTRVSTLIASAK

>SG-AL_contig_7513_1_+1_1_345

VVGAVVVVVGAIVVVVVGTVVVVEAVVVVVGAVVVVVTAGGGGATVVACAVVVGAVVVVA

HSCGSSCSGGSSCSCGSWCSCGSWCSCGSWCSCGSWCSRGSCCSCGSWCRRGGCC

>SG-AL_contig_7514_1_+2_836_1096

MYVNVYILTQNCMHFNSELWDNSKLRHVYFLCYCCHLVAVCVPDTDLLTPNFHLEYFSAN

LGVFFKNLVYSDVAYCRKPIMVKCTSF

>SG-AL_contig_7515_1_-1_217_507

MRRSWVQPLCGPRDFFHWLACVFLVSWTQDLNPVGMPGALSVYIVSPSNGNSVLAISSFL

CSVSAVFFFPLLSSAFFCLPGPFHFLFSSKPSHNFLS

>SG-AL_contig_7516_1_+1_1_96

LLLLHIVYSQADHIKSIKVKKTTALNTKIHQT

>SG-AL_contig_7517_1_-2_2_163

FCASNFLFSVALFVSAVFLSFTVVCFFSAFLIHSTFFFPASLHTVSASPLHPSL

>SG-AL_contig_7518_1_-1_16_1104

MPTHQVRVHHLNKGENKESVLYRLEKGWVLRFLLGPSLHAALVRLFCNHPPNPQTPFDRH

TYHELEWRCPTGMRSDLTDVYADVKIICSGSFNYFFTIDESVDISKADGQGYFLVDPTMM

VGRDEEITMDCLQVQTVITKLLGPFSGWENKLAVARETGYNMIHFTPVQELGISNSAYSI

RNQHVLSPVYSQGGSTQYSMDDLEALVKKMHSEWRVLSLTDLVFNHTSKDSPWVLQNPDC

AYNLKNSPHLRPAYIVDCILRYFSDEVAEGKWESKGVPVEVSTEEHLLAMRQILHNDVFP

KHKLSEFFTCNVDQVMREFRRGVEENRRAVPGDTEVTIKQDPNYRRNQSSVDIDLALHHF

NTD

>SG-AL_contig_7519_1_-1_16_261

MYFTEKLSAITKHCSLCILFICMFIRLLVYILYISTMLINEDVDIFIVLLYVRVVYVLYI

RCDLYSLLIGSYLISTNIFLLF

>SG-AL_contig_7520_1_-3_756_971

MRRRAYYYYYILFIAQPTTMAISGLSPRKVHQSQEDDRVEHKTLSNLKTKTLQKITNWSD

NVKDWTDMTIPD

>SG-AL_contig_7521_1_-3_63_326

MLFSILSPTDYRTFQTTVIFSRISDTSMSVESLLKNKRHLLFPMCSPTDYHTFQTAVVSP

FLPLIFFLFFFFFFFSFFFFFFFGSFFL

>SG-AL_contig_7522_1_+1_337_687

MYYSQYVSNHFRKFHFLTLCFRFFTIFFLTFKNGGTQMGRKLAVPSELMECIYTCTSLKG

PVNTILNLLKSYKIASKYESKRSLKSQKCMKSHTFTLMQSYFAKNATQSMRVSSAAM

>SG-AL_contig_7523_1_-2_1076_1408

MIFSCTVPSVDTTVTCLCCSVCDACLYHRGIVLPLFIHILTLKNIVSFWWLEYKCTSGIY

FAWALPFNNNNNYSIYMALNLDNSNRSALTHQTYTCKQCRLNTIYKKTCTN

>SG-AL_contig_7524_1_-3_348_542

MCCGFFFAKYDCISVNVCNFMDCYDFKEHLLSYFEVIFHNSTRFKVVFTGPLNSSYWKPI

HTILI

>SG-AL_contig_7525_1_-1_1_126

SVDFGKKKKYIIEFCFYGLHIKKKKKKTVNKIFLQKFTYKHC

>SG-AL_contig_7526_1_+1_670_936

MRTEQSIHMLTHTYKLTDVIKIFGVNKRERETSYNNLYIHYIYTHTYSISLYIHYIHIYT

HRQYIIYTHTDTVSAYTYIILYTHRYSIK

>SG-AL_contig_7527_1_-1_1_201

LSLLGRGVYKKKTLFFFCHYPRKKKKKKNLRCCESFSVNEVVQQITHTKGKIPETSKHTL

SLHIIMK

>SG-AL_contig_7528_1_-3_123_278

MRKGLENEDVLVCTSGRGTSLDMNVCVCVCVCVCVCVLFEIIWYCFYCWHHR

>SG-AL_contig_7529_1_-2_44_379

MGGGPLGLGDPDDKSLRSVEKEVMIPLKMKEKAKAEKCTLEVREFGECAKEQGLLLPFMC

RKVAKKLEVCLTSWYQNPEFVAQCTQEYLDERSQYRRTGVKQKMKKKDAVIG

>SG-AL_contig_7530_1_-3_2247_2450

MYITPYVCIHLSAYNIDFFPCAVFSQSGTSNCQLSVDYCLFIMSSFYSKAILSSSVYFYL

DSIFICRQ

>SG-AL_contig_7531_1_-1_277_486

MFMYASMFLHAHYTIFKSECFPPGDARGSSSINSIPTFWKFCARNFLFSVALFVSALFLS

FTIVCCFLPS

>SG-AL_contig_7532_1_-3_693_884

MSGPMTVMLIMKVLTVLMRARLSISATKPHMKHTHSLFLSHTHTHTGSIIHIILDCVVHS

TACA

>SG-AL_contig_7533_1_-1_349_798

MQTRSVLLSRSLDKIASASTLANTETAERLRNQHVYSLQRVIAAVTTAGMNTEEEEDESL

DTDEWSHDSDVSENLPTVLMRARLGIRATKPYMNLSLSLSHVHTHTRGHTHRHTQRLNNP

LACVAHCTVYVSVCVCEFICGLVTLILIIQ

>SG-AL_contig_7534_1_+2_38_247

MYPTCMHACVFFYCSVYPPSVRINILMIYIIGGNCDNKIFVNINYPQCNKFYCLTSIHNY

CILHKMSGIL

>SG-AL_contig_7535_1_+3_3_371

RCSYKLFRKTNRFKAIHKLFCKTNRFKAIYKLFCKTNRFKASLTLSRSQQEKTKGNFSAA

VFHGGSTHAVLFHHTLTTSNHSKKRPTGKENPKNGPTKCWHYFGILALLLLTVVVVCCLR

SPV

>SG-AL_contig_7536_1_-2_62_1471

MSHTVMLRTLSQQLLRSNKNLLNGVGLRHATKASSSTNLKDVLESLIPEKQKEIVEFRKA

KGTTKVGEVTVDMMYGGMRGIKGLVTETSVLDPDEGIRFRGYSIPECQKILPVAKGGEEP

LPEGLFWLLVTGEVPTEEQANSITKEWNERAALPNHVITMLNNFPCNLHPMSQFSAAITA

LNSESKFAKAYAEGVHKSKYWELVYEDAMDMIAKLPTVASIIYCNLYKEGVTPCAIDMAK

DWSWNFTSMLDFENPNFTELMRLYLTIHSDHEGGNVSAHTCHLVGSALSDPYLSFAAGMN

GLAGPLHGLANQEVLVWTTKLMEALGGEVSDDQLREFIWNTLKSGQVVPGYGHAVLRKTD

PRYMCQREFALKHLPDDPMFRLVSQLFKVVPDILMEQGKAKNPWPNVDAHSGVLLQYYKM

TEMNYYTVLFGVSRALGCMASLIWDRAFGLPIERPKSFDTPHLRKLVGHS

>SG-AL_contig_7537_1_-2_2_64

QMGVNHVIKLKIMARNFTERV

>SG-AL_contig_7538_1_-3_279_1490

MRTAVCGMVIKGMLDKIEREEKQEQKRQKKQESAEERQKRLNSVSQQRALFKHKEALKKD

ILKKRSAMEKSLQQEIFAEISDTMKRQKKAGMKVGASPSGAAAASQPSGPTPTMLVKKRE

GEPPKITSYIPGMDSSRTSKTSASRKRKAGGQSGPLNSSKQRLITIGAGSKGRTEKLYCV

CRTPYDETKFYIGCDLCSNWFHGTCVNITEEQARFIDSYICEDCRKQQENTSEELYCLCR

TPYDENQFYIGCDRCQDWFHGRCVGVTKSEADSIDTYICPNCQQKEQANPIAQKVLTSTD

YVTLLKIVRSLKAHKMAWPFQAAVDPSEVPDYYQVIKDPMDLAIVENRVISKTYARLSDF

VRDVTKIFDNCRLYNPTDSPFFQCAEVLETYFVQILKSSRQQFT

>SG-AL_contig_7539_1_-1_154_5217

MSQILPIRFQEHLQLQNVGINAANIGFSTLTMESDKFICVREKVGETAQVVIIDMNDSAN

PIRRPISADSAIMNPTSKVIALKAGKTLQIFNIEMKSKMKAHTMTEEVIFWKWISVNIIG

LVTDQAVYHWAMEGDSQPQKVFERHTNLAGCQIINYRTDAKQQWLLLVGISAQQNRVVGA

MQLYSVERKVSQPIEGHAAAFATYTVEGNPNPSTLFMFAVRGPQGGKLHVIEVGQPPQGN

QPFTKKAVDVFFPVEAQNDFPVAMQTSPKHGVVFLITKYGYIHLYDIETGTCIYMNRISG

DTIFVTAPHEATSGIIGVNRKGQVLSVSVEDENIVSYITNTLQNPDLALKMSSRCNLPGA

EDLFVRKFNNLFQNGQYQEAAKVAASAPKGILRTPQTIQRFQQVPSAAGATSPLLQYFGI

LLDKGQLNKYESLELCRPVLQQGRKQLLEKWLKEDKLECCEELGDLVKAADPTLALSVYL

RANVPQKVIQCFAETGQFQKIILYSKKVGFTPDYIFLLRNIMRINPEQGLQFSQMMVADD

EPLADLNQIVDVFMEFNLIQQCTSFLLDALKNNRPAEGPLQTRLLEMNLMSAPQVADAIL

GNQMFTHYDRAHIAQLCEKAGLLQRALEHYTDLYDIKRAVVHTHLLNPEWLVTYFGSLSV

EDSVECLKAMLTANIRQNLQVCVQIASKYHDQLTTDALIELFESFKSYEGLFYFLGSIVN

FSQEQEVHFKYIQAACKTGQIKEVERICRESNCYDPERVKNFLKEAKLTDQLPLIIVCDR

FDFVHDLVLYLFRNSLQKYIEIYVQKVNPARLPVVVGGLLDVDCAEDVIKQLILVVKGQF

NTDELVEEVEKRNRLKLLLPWLETRVHEGVNEPATHNALAKIYIDSNNNPERFLRENQFY

DSRVVGKYCEKRDPHLACVAYERGQCDDELIKVCNENSLFKSEARYLVRRRDPDLWATVL

DVENEYRRQLIDQVVQTALSETQDPEEISVSVKAFMTADLPNELIELLEKIVLESSVFSE

HRNLQNLLILTAIKADRTRVMEYINRLDNYDAPDIANIAITNQLFEEAFAIFKKFEVNTS

AIQVLIDNVNNLDRAYEFAERCNDPAVWSQLARAQLNQGMVKEAIDSYIKADDPSQYMEV

VQVASTNNNWEDLVKFLQMARKKARETFVETELVFAFAKTNRLADMEEFVSGPNHANITQ

VADRCFDNQMYEAAKLLYNNVSNYARLAITLVHLGEYQGAVDGARKANSTRTWKEVCFAC

VDNEEFRLAQMCGLHIVVHADELEELINYYQDRGYFEELIALLEAALGLERAHMGMFTEL

AILYSKYKPEKMREHLELFWSRVNIPKVLRAAESAHLWPELVFLYDKYEEYDNAILTIMT

HPTEAWRESQFKDIITKVANIELYYKAIQFYLDFRPLLLNDLLMVLTPRLDHTRAVNFFV

KTNEAPLVKPYLRSVQKNNNKAINEALNNLFIEEEDYQSLRTSIDAYDNFDNISLALRLE

KHELIEFRRIAAYLYKGNNRWKQSVDLCKKDKLFKDAMMYAGESRQVEIAEDLIEWFLDS

NNHECFAACLFQCYDLLRPDVILELAWRHNIIDFAMPYMIQVMREYITKVDKLEQSEAVR

SEEEQKAEDRPIVLGEPQLMLTAGPASMGMGVPPAQPGYGAAPGYAGVPAGMPPGGMPAG

MPAYGYSM

>SG-AL_contig_7540_1_-2_2_322

LSLLCLSWYTSHCVVSLLEALLNQNHSYCDNDPIVLHSHTQRLGHSISILLCLHCHCQEP

LMVSLSCVFAPIFLPVYAKCNALAQMCMREYFVLLFLYCLRLSIVWL

>SG-AL_contig_7541_1_+3_3_182

FFFCIFIYLLQRRTKNCVHGETSETTKYRLNPTELHPTATRTHAQHYKALPSSRERNLNK

>SG-AL_contig_7542_1_+3_1212_1544

MENKHNNNDDKDNDNDDENKNTRINGIQGGGPTQMVELTIFYSFSMTHTHTHTHTHTHSK

TPEKGKPYQEYSFLLWYRYHHHHHHHHHHHYYYCIVYSPADHSDHIKAVTT

>SG-AL_contig_7543_1_+2_1496_2008

MVEQCPRFFLISQHCSLIQFSFAFFMPCFILLLTSLYHLAPSASLLSFLSSLLSHRLRMS

AVTQGFFIGPCLPRVSLAALVTAVLKVVISVSMFVSSSASRRVCGANFPPIIAWRASVTS

GAFTFFFRSNLILECPCLLMFLRQVKSHQQQVVIASNVTSRKASCLGDVHT

>SG-AL_contig_7544_1_+3_51_233

MKGHRIWDFSQCCTGLLQSQRTMEFVPLEGLQDTSLGKVLRRACCCHNVMIKRMGMTVMM

L

>SG-AL_contig_7545_1_+3_594_839

MNTKTKYTLCIHRDEDRLFTKWKTGQFFSFIHLSTYLFIFIFVCNFYKSACAESILKGPV

NTVFECKNTLKCIRAFLQDLCW

>SG-AL_contig_7546_1_-2_209_376

MKCMNNLRIVLCKLCLNSAIHVWGKKISVLMHHPCCIFVMLICHVISLNKTKKETV

>SG-AL_contig_7547_1_+1_178_300

MIRTTLRINKRLARLKTIKKQKPFNINTQKLKDMKETFEIN

>SG-AL_contig_7548_1_+1_43_222

MPPTVANLKQLKTYTCTYMYTLHTYLVKSPAGITTNSQKYWKRCKLLWKKHAVTIIMQFL

>SG-AL_contig_7549_1_-3_48_179

MMLKIRAAYYVKVTLANVTASLTSSQRYCWTFLLSLSSTGIDYF

>SG-AL_contig_7550_1_+2_176_271

MAWMMASNEKAWSSITHSFEILYLSEYVTIMI

>SG-AL_contig_7551_1_-2_863_1096

MIDQKVSCVLLFYVCIFLYVCMCLSVFVNTFLQGFVKCRLLDLYLFEPYSSKPLRNHKYI

SLNGSGWFKIYTEHNTFY

>SG-AL_contig_7552_1_+1_451_693

MALLCVVKLCLVQMLIKFDEQSFFIYLFLLLLCLLSHLLYHFSGDDPMPLILALQRTTKP

KFIHQSPSARKPTGAMDVKLP

>SG-AL_contig_7553_1_+2_1160_1612

MQVPRKQNSNTCAGLSETMGIQGKCSRSPVQRNNPSQAEISVGIPYVKGLSEKLSRVFQQ

HGARVFHKPSGSLRDRLAHVKDRTDKSKVCGTMYHTQCSNCPKDYTGEIEWSFVNSMASY

RIIRSDTHNKRWTRIGSYDLIDCENFLSKDR

>SG-AL_contig_7554_1_+2_62_283

MFSVQRHHILHVMQCFLFNVVASFMSCHVSVQCSHTPYVMQCFLFNIVTSFLSCNVFCSI

LVTSFMSCNVFCST

>SG-AL_contig_7555_1_+1_1_228

SLFHRSCSPPHFKTLPAHGVLHRIKIIAGRFRWSACGIVGVQWLPLEQPSNSMTLRSHES

EVVRIFSPSLLHLPQL

>SG-AL_contig_7556_1_-3_162_305

MIRLKMLCFLYCIRPKNKRCWFMLVLSYITPLFYLYYFMVVNYKDVRT

>SG-AL_contig_7557_1_+3_444_758

MHPYSTALLQVHQEIRYTHQGSAHLLLHFMSKKFNEYTHTPLATVYTNQIIFSSAVNQLF

KANIQLSLSSHTTDTQSKTNVAAYFLPAWQLAILTESMYRIYHLY

>SG-AL_contig_7558_1_+1_1_195

IKQHTRQRSTTKVHTNFSFSFSKYNHSNVTSLTPHFLNPRAESLVIFHMASYNWEHGLTL

HRTDE

>SG-AL_contig_7559_1_-2_221_322

MHAYEYCVLYACINACMHACVRMYRLTRGAYISV

>SG-AL_contig_7560_1_-1_64_2028

MVTMFLWLFSTTLIVASLAAPTPKCPARCISCMNGVAECSKRSLVTPPKDFPSGIRKIIM

DGNHLRVLGARSFQSMKALEVLRMKGNKVRVVKKAAFKKLPNLMTLDLSNNQLVKIYSRG

FEGLKQLQTLSLQYNKLRSMARIFDATPKLYQLNLAFNRLRSIGKNDLKTPTRVHYLDLR

NNRITKIHPEAFVHLRLLRYLFLNKNPLVTVPDMTFGSQVLQLVDFSNCRLTHVPKTMPQ

SVSDFRISDNKIMQINDTDFTNMTNLRLLALNNNELHFVANGAFTKLNTLDEIWLRNNKL

VYIPRGLPDNLRKLYMDSNKIREIEDGLFTNASRLDYLTVENNRINRINNKTFMGLKFLR

SLNFQGNRLRVIEGGTFDSLGNLSTLSMSNNPLEKVEDNAFVNLENLTFLHMDMCGERMT

LEQNFLNEMPKLKQLSMMNSPGLGKAFVDMVATTPISPLKQLTEIDLTYNDLYTLSPNIR

EVFPALNALALDGNSWICDRRLVWLREWMMHSDVSFSKYEEVICEHPYSLKGRTIRDVNE

EDFVEVAPTTEAPSMDDYNKMMADKQAGNSTSSNALPSPAAMHDNSVMIGAKLPPDASML

SSKEDKKDKKLRSKKTRKQRKIKGKGRKKRGKKGKGRKRRNRRTKKSTAADPTSG

>SG-AL_contig_7561_1_-3_219_344

MEGVCFVVVGFFFIFVCFSQNFKETPELKQNCICKCYEYRQV

>SG-AL_contig_7562_1_-1_166_348

MEGQPSLNRDTGFELPAIYRQVLTSRDRHLSNKDSQTTQPTSVQQKHSQHLSNKDSQTHS

Q

>SG-AL_contig_7563_1_-1_1_87

NTGMRTSCPFCSVTYLYGLFVIFFFVVWG

>SG-AL_contig_7564_1_+3_45_218

MVSMSMIVHKPHTVTSLMSRHQRPSHTSEYHKRRYKEWGRQGRLWGAKMELNQDYTPL

>SG-AL_contig_7565_1_-2_485_823

MFSQNCMCVCVCVCVCVCVCVIQKYFIIYFMYTHLFIFKYNFYLFINLFCMISADAAVLC

FFSKIWSFSLSLSLSLSLSHTHTHTHTHCFQLSSSQYETIFHKKGPEYFSPCK

>SG-AL_contig_7566_1_-3_222_554

MAARKVVAEVPAWKRALQRWAYYKSYFPQLGLMRDDCLNETADVKEAIRRLPTAQYDSRQ

FRISRAMYLSNRKEILPKEEWTKMEEDVRYLTPLVDEVVKERLEKLEWQKK

>SG-AL_contig_7567_1_+2_101_382

MHTYKFPAVEFIKYSQLLQEPNPDHNKIPILKSYKCNYKQTNNKTKRQAQFLRVKHLLNV

LLYTRARMHARMHTHTHTHTHTHTHTHTHTHTIT

>SG-AL_contig_7568_1_-3_30_215

MWDVIHTHHISSSPSPHHVGCHPHPHLIFTIIPITGDDIHTHHISSSPPPPSRGMSSTPT

HL

>SG-AL_contig_7569_1_-1_76_1089

MSSTPTTPSPPPHHVGYHPYPSHLHHHPHPVGCHPHPPHLHHHPHPVGCHSHPPHIHHHP

HPVGYHPHPSHLHHHPHHVGCHPHQPHLHHHPHHVGCHPHPPHLHHLPITWDVIHTPHIF

TIIPIMWDVIYTHHISSSPSPPSCGMSSTPTTSHRHHHPITRDDIHTHHISSPPSPSVGC

HPHPPHLHHHPHQWDVIHTHSISSSPSPPSHRMSSIPTTPSPPSPSRAMSSTPTSSPPPP

SRGMSSTPTPSLPSPSRGMLSTPTTFSPSPHHVGCHPHPPHLHLPITWDVIHTHHIFTIT

LSRGCHPHPHLHHHPHQVGCHPHPPRLHHHPSHGMSST

>SG-AL_contig_7570_1_-2_23_673

MSCTPTTSSPSSPSRGMSSTPTSSPSPHHVGCHPHPPHLHHQPITWDVIHTHHISSSPSS

PSRGMSSTPTTSSPSSPSRGMSSTPTSSPSSPSRGMSSTPRTQLQCYYHHLGCNTRPQSS

LHHHYHITRNEFQRHTSPPYHMEWNPMPHIFTITTTSRGMESNATHLQTCFHCMGCSPPQ

FIIRVITITRDGIPHYNRFSLTAPTSHGMEPTPTTHR

>SG-AL_contig_7571_1_-2_59_3166

MKKHLNEVPAVVVIFFDLDWDEVMWKERQMECATRVEIVRNSLQGRGTKIAVVLLQKSAP

LPPGEDVGAAERAASFCSACDISAKSLYVLPQTDHLLGFTIRLENAFYELAQSYYHTEAR

KVKSHKDFLNKTTHQLLFVRHQFKIAFFNELKQDTHTAIKHYKQAYGHIVEQRMHDTNML

EIKTIAGFVNYKICRLSFQHNVPLDAIAQFRKHIDFFKKRTGIPELAFEHSAWMCRQFQL

FGDLFDEAIKLGLTAIQTQHPGFYYQQAANHAIFRKQLCHGLCVDQDKQTQQGLLDSLDH

LDFYGQRPWRQGHQSIEPPDAGREKEGIISLQAKERKVDHCWIIIPLLSSAVAQFRKYKS

PRMKRYLMVQMGEEYFHAKDYNKALMLLNKVTFDYRMEQWWPVLTSILETSLKCAYLTAR

VQEYTSNCMELIGHNSQCSVDKKTRIQMNMLQVLAHESPSSEPGLDQSCVEQASKAWSEF

KSKAQVFTIDMQGLIPFIDCKARFVGDTVAADGQVTVEVCYRVSCPLPVRFSKLSLQMSN

PRYQHNCELTDVRGMNAASASREEKEGNDLLLEPGKPRLYRFSFLPLKDDVGHQIEISSI

TLETGTDTGCCALLRWAGAGGESISGATTDPFPARKPSSSSAGQAFDWAMFKVNPVIRVT

PRQSLLQLSVDHQSPCLINEFYCITVTTTNTEHTSIHNAILSLGLQKDQDSYDSTTHICS

DSPEFDGTVMAQPAQVDLGTLKKGEKVSHQFFVKCLQSGTRCLSVKVEYNVEVKQGSHSI

TCQCLKEEALSVPAVPPFEVSLLLHSLKFEPVEAVHAEEPFLLMTDIRCTSPWPVDIKTS

SVDLSEFIKTASDLFESQVEQVQLKKTECASECLWLVAASCIQPSVAMGTYTIHWRRQGQ

EWPYVSTSFPLPTVNIEHIPISVHLQLPAFGRVKTLLPLTYLVRNRTAYVQELEINMEST

DNFMFSGNKQTRFRVLPGEEYRLMYNFFPLVAGQVALPRLHVNMLRYPGTMDDLIYSMLP

SHIFIRPVGKSLQIPT

>SG-AL_contig_7572_1_+3_456_887

MFRLVFSFFIFYLGCGIFTLYFNLIFKSPAKKSSSYKCISCGWVQVNGWLRVRNGEYGMC

CCPPSFGPSFCLVGISSPSQISNIMTMFVFICIVLLLAFCLTSILLFLFFQLFYACMFRL

FWVFFCVSNFMGETYLPKLLELRK

>SG-AL_contig_7573_1_-3_78_326

MDTDSQADLQANRHGHRLAGRFTGKEIWTHTHRQIYRQVDRHRHRLTDRFAGWQIDTCSQ

ADSQAGRKTDIDTGSQADLQAGR

>SG-AL_contig_7574_1_+1_1_651

VVPAVVVVAAVVVVPAAVVVAAAVVVVPAIVVVGAVEGGGGGGAVVVVAPVVVGAVVVVG

AVVLVPAAVVVAVVVVVGTVVEVGAVVIVPAVVVVGAVGGGGGAVVVVAPVVVGAVVVVA

AVVVVAAVVVVGAVVVVGAVVVVVAVVAAVVVDAVVVVDAVVVVGAVVVVPPMVVVPAVE

VVPTVVVVGAVGGGGGAVVVLAPVVVAAVVDVGTVVD

>SG-AL_contig_7575_1_-2_2_310

KAASLSVVTRWVLERGTDIEASVTANYALSRLCTGTCSSFAFRSSLLQVDSRSLHLSTSR

LFIMQSPQSSQSSKRKCLFFEAKLKIIEEVEKEYYELHPFITC

>SG-AL_contig_7576_1_+3_177_533

MNAYVHICVYHISAFLFVYVYYVRNCAWAHAHVSLCACVCVSVCRSMHVCVHTFVLYAIT

HKKRNETPMQKRAKGGSGRGWEVGSTVYQIKTRATRNTDWRKNYREAPSYSKAQVWAGR

>SG-AL_contig_7577_1_-2_74_280

MWVVFIFVFANACGLCSFLCLPVHVSCFLGQYCDTEPTIGNHLTSILFPVFWKRGLGGGI

SQLSVCSVH

>SG-AL_contig_7578_1_-3_3_107

GVVYFFFSELRESSKQESSHFAPLLGFTVFNHFCC

>SG-AL_contig_7579_1_-1_265_1695

MATESKVSRLETADPGSKVEHQVRDTEDLVQRYKDLFMQDRFSDIVLRVGDQRYPAHRFV

LITASGVFEAMLSQSRWKEARQPEICLTEEEECVAEFQHFLGYLYSGEAMLKTTSVLPLL

LLADKYEVSCLRRSCLNFMMQHIVQSPNTNRALTWYQYAQMTGQSDLQDKCLGFILSNFD

TVMQAPDWPFMVLPELLGFLASSDLVVDSEALLWSHVERWLLHDNNKDMLEDNLREVLPL

IRFMMMPPKCLLRVEESSVCVDHKHLFADKLNLAYRKHSLAMDDGDLSSGDAPTPQAPEP

YRNYLSEGYCVNHKFSLPGYATINKIDSRISLDCKARHRFVSPSRERQDDMTIFSVYFFP

RGYFTTLTIYGSYMGRQTSDTTLKVIRRRPDLVPMKVEVTLMLLGKQNDLKYAAFTHSWS

VTFTKDNHTLSVDKFLEVEKLMEENSPYLVEGMLEGSVFLKIQDMGDHLLDKPKDKK

>SG-AL_contig_7580_1_-3_18_1499

MEKKRRARINASLAELKSLLLEVIKKEGARHSKMEKADILEMAVKHLRQVQRQQYTSGGA

DPTLSDKYRLGFNECAQEVSRYLGAADDDDAELRARLLNHLANCITSSDSPSPLSTPSSS

PALGGHVSSPGSVVGSSGGVVTPVTPIPVRPVSSKPGSVSALSLIPQGDPGGSPPSAAAA

AVLVPTTEINNNVVTLANNNSSNIVSSTGDGGVMGGVELRGVSHQLTLPTAAPSAPAGRR

VMGEVQVVGPKAGGGGEVALVLPANVMTGGHLPSYIIPLYASPHPLPTASLSLTPNNAPQ

PPTAALPLPLNVMATTATPQVSAPGNAPSLVTLTPGAALSPAVLPPSSVGHVIAAGLNAP

LAWQTATPVYSASFSSATSLSSVSSTVQASSTSLPGLSPSLATSSVTSEMRPSEDRVSSS

VSFRPEKTPQVMVPQSAVASVICAPPAGRSGVSPESGERLSLLHSQLMRPQADAAAPVAG

ALPAADGSVWRPWW

>SG-AL_contig_7581_1_-2_29_2311

MSSLAQLHQHLLHSLPALWQDLSTKIHHLHSDLASHYQQAGQQQGQGQAEKEGGGGERKD

DGSGGGEDADEPEDTPRMRWSEGVKKLLNKTRGTLSNVSRHLQQTWHQVKDASRRFWPAK

DSILSHMAAQVKDSMTRLSHKVQGKASFWFRKRSKWGKPGDGKATFPQDREGQDQGRERS

KRKRDNSKEDRPDHQENDHPHKKKSDPEKGWNKHDKGGIDKHHHKHSPHRKRKDDSEQLE

DSHHKRDDKSRKHDLKDSHDDDDDDENRQRNKLGPHPPDERNHKDQRESKKTEPDRHHHH

QSHSKDGKKYNAHSKNHREDSHGKKDKKEKYHQEKSRDKDDDDDDDDDKRSNKSQADKVG

QFSTKAKDHWSHWDKAAHKARYKVQKRFHKLFTKVGAMSKTSYNNMDRGDLREMFEDVEK

VWGLYQDNKHLDLPESGHKWLQCQFTFWMKASPRCVRYNPQEECLQLLAPWQMGFVQELR

ARLQTRECLRVRYISCIDGGGFEPDPEDYYKYVCAEDDDFHRYNKDFGDVDDADDDIDND

DDEYERKKRKSQASPKGQREGEGDKTEHDSHGLWKAENGKESMSPTPSQQVNQSDDNLHT

WYFNWVKGRNNMRSLEHKADWLFERASERGEAREQEHSADWLFERAAERHASREDEVDTA

EHKEGDDHHKRHGDRMAEQEGHHGKRGHQGERGHDGERGHHRERGHHNSKKGRHRKGHGG

KEGHHKRQGQGQGKHHKHKDKGHHFKRYKKDYRQKYGRTTF

>SG-AL_contig_7582_1_-3_90_2543

MSAKTEIPGVNNAVEKLQELEDRMRSDDFGFRNNDVDNMDKVIDALHELETERRKMHEEL

EKETIQSSILRHQLKILPGQIRDEVMEAVMSARLSNANVLLELQEKLKRMNINITTLDKQ

ARELEQENASLQPERELLKQQHEEIISQLNQRMAEKASLQIALNETRDKVRQANQDIVDL

EDGILQLKEDLIQERTEARQEKKRLKKAVTDTTQKTREQKDQNVTKKKELDVAQEKLTDS

EGNLDAVRKSIRRYETSRAKLEGEERALAAQLSKQLKMNDQMRIRGAEIMNSRIQEERDF

KTKQQELLDTIQQFEDDILTEGARSSELEKRKVELLLDLREKEEVREEDSEKVADLDSTL

QASKEALNTKAEEVGAMQTENIEMSEQIEALAESHKAVLAQLNKQIEEYREQLSKERKER

LEVQAMKDTVSKDLDDLKTENQFFMSEMTNVVQDGKKKHQDLSNEGMTLQRTLKADEEEI

GGLEDTLDNAQKKYQDMFDDYQKRTDMMEAEVLSLETGLEEKARLKEERTPIFQQLEVYF

NQRTEEYDKMKKEIVAMKNKRQSLEDEIKRMKKDRGSLRQPQEQMRKELKVKRQQLMEQL

QSQGKSTNDMEQQILTAGCKVRAVIEENHKFDESSKKLEAELTELKKQMDENERIKMKLQ

EELNKQKQELVKKWETDNMMQEFFASRDEATAEAFGRLLSRTEKRENKIDMITERLKEEL

QFLAGFLDNISTRRPNGSKRKVRIQGGRRKYQSRQSRRSRSRSQSKERHRSRESRHSSHS

DDDRVRQQSKSPQRTPTALSKECSQLSARHSTQSTVNE

>SG-AL_contig_7583_1_+1_7_273

MADVADVFLHRQILVNLRSKVFHRGLERNINTTNICILTTNQLKLRSSAERHHVCFVCVS

SILVCCCSSRTTHHQYIHIPIITDIIIHT

>SG-AL_contig_7584_1_+1_1_135

GTTSQLQLVGSEFCQCSVLFVAVHPGQHIISACLNATLDRRKITR

>SG-AL_contig_7585_1_-2_632_820

MDTTMVYFMDTTMVYFICFLKPGCECEIQHFPLSTPIVSYALWWQRQQLNKTMIMTMILM

TIT

>SG-AL_contig_7586_1_-2_2_79

TVEESFMLEFGIERAKVKVTRTKKSA

>SG-AL_contig_7587_1_+3_135_254

MHVLALPKVKTTRTQRAEKIQIEILAAQRKTQIFKVSRSP

>SG-AL_contig_7588_1_+1_1510_1809

MDSCHRHTDTYTTVHSPSLLAQIRLDQVDVHSSVVGCSQPGSVDQSWLYSLYIDCRICPV

YSAVAMFGKLSQTRRHVHSCFHCQLRMQADMHAHLLWAVL

>SG-AL_contig_7589_1_-2_890_1078

MHISSQSTDQAEGFSLFDFFIFVFLSWFDVVVVIVIPISMYLRNLCSPSVHVFISLKKLW

PFF

>SG-AL_contig_7590_1_-1_25_1074

MKSIMAVFRSLSSIEKTVEKCKISHNAKEARLVFELHCRHGIVKKHNLAYTECESPQAVF

SKDLCPNKLTAPSKLLCEVVVNFQTNQEEITLTVQPEILSLKNYVDDEPDPNKVVHTELS

LAPEEFDLYQVGVDTNITFCLKELRAILAFGETTGLPLNVNFETAGRPVTFCIDSDQTFE

TNFVLATLADAEQTASQMPSSQRVQQAANSSQSHRKNTAPAAAATKQGGNRLPSRKSSSL

NGHLPPREGVREDDDLGDISTEELAAAMEVEQEWEEAERDQPPQNHETGNDRPSWIRKGE

KNGPSQNHGNKRKEPSHSLESERDHLSRNRGGERDRPSHSGASHSSRGKE

>SG-AL_contig_7591_1_+3_3_224

AHAHAHTTHAQSLGADKDIRCAQSQVKSHKPHHNKTHTDFGQVNQDQLNLNKEKIGGGTN

MCNSQMCHTYKKTV

>SG-AL_contig_7592_1_-1_31_1017

MSALKEISPLQVSSKENTPKKVKSPSEKKENKENNEVKLKIERHAELQYLEAVQRIISEG

TLRSNRTDIDTMTIFGMQMRFSLRESFPLLTTKRVFWRGVVEELLWFIRGSTNGKELAEK

SVRIWDANGSRDFLDKNGFHEREEGDLGPVYGFQWRHSGAEYQDMHTDYTGQGVDQLNEV

IKLVQNNPESRRIIMCAWNPADIPKMVLAPCHCLAQFCVINGELSCQLYQRSADMGLGVP

FNIASYALLTLMIAHITGLKPGDFVHTMGDAHVYVNHIEPLQEQLKRKPRPFPTLTIKRK

VDNIEDFQFDDFKLNGYKPYPKIEMQMAV

>SG-AL_contig_7593_1_-1_1_285

AADLTRSLGQVVRPYDRPLCDQIQQEASDLCLSHTGPRGTGSGRPLPGLGSPDQLHFPHL

PDPPQGHQEGERRESAHDSHSTLLAGTALVPGPEI

>SG-AL_contig_7594_1_+2_602_868

MKTKPPSLDWYAKGEKSQYWKTTLQNEHQVSIPVLQSQFLVVRVQQFRKHLNDFIFWMKG

RPMNQYAAEEKKKKTDEDKKKTGKKKWCL

>SG-AL_contig_7595_1_-2_2_250

FIASDVVDYTQSCWLTRYDKCINYKQHCLKMFDVYSPQPVWVVSCIPPPLCFVIPCEITR

YLLEWWWSEMRCEVRWSDQSGYR

>SG-AL_contig_7596_1_-2_38_1711

MANVQRSLLSDEPAWKALQEYYDQNGSKLNMAKMFKEDGDRFKKYSVELKTGNGDLLLDY

SKNLINDEVMQKLFSLARAQWVEEMRNSMFHGEKINFTEDRAVLHIALRNRSNTPILVNG

KDVMPDVNAVLKHMREFTNEVISGVWKGYTGKPITDVVNIGIGGSDLGPLMVTESLQFYQ

VGPNVHFISNVDGTHLAKVLKPLNPETTLFIIASKTFTTQETITNANSAKQWFLSSAKDA

SAVSKHFVALSTNAPKVKDFGIDEKNMFEFWDWVGGRYSLWSAIGLSIALHIGMDNFEKL

LTGAHYMDQHFKTAPLEENLPVILALLGVWYHNFYKAESIALLPYDQYMHRFAAYFQQGD

MESNGKYITRSGQRVTYSTGPIVWGEPGTNGQHAFYQLIHQGTRLIPCDFLMPVETHNKV

QDGLHHEILQANFLAQTEALMLGKSCEVAENELKKSGMSPDQINHILPHKVFEGNRPSNS

IVFQKLTPFMLGVLIAMYEHKIHVQGVIWDINSYDQWGVELGKQLAKVIQPELKGPNPVS

SHDSSTNGLINFFKAHRK

>SG-AL_contig_7597_1_-1_337_573

MYILRVNLCTVRMRTVMSILFDHIIPDEHLSHVYVGFVKTMTEHCSVLCFVPCSFRLLCC

CGSLFSLLLIMRVVCKFLL

>SG-AL_contig_7598_1_-1_1384_1563

MKRKKNSHLTCLNAFLVRIQFCGPLEHREHVEIGTWLFDHTYWVLWPYLIVKFDFRLLST

>SG-AL_contig_7599_1_-3_105_317

MAYRIFNVCICCSAYIYIHMKRVQACTYVDWEIRKISRCDPGRIEARPSDWESTSNHLII

VPVFVCFSHRL

>SG-AL_contig_7600_1_+3_48_230

MIKKNFITRIMIYVQAFFACLSVCFWSSLNREVGYNSNSGITRYHTKALLPDAAETTHFT

Q

>SG-AL_contig_7601_1_-1_1_192

HWVDFDHSRAYGCLCICDLEYNFLCSAVCSSTSGIRYGMEMHVMNVAYSQRWGWCHVDTI

SING

>SG-AL_contig_7602_1_+3_102_308

MESVSYYICIILLLFLLAKALFHLIFGDVFWILTPSHHPFGHKMRQLMFMILFFMHRWIV

LCCSPALLR

>SG-AL_contig_7603_1_+2_848_1180

MLEDLQLQKLQDRRDSTYRLFLAHTFFYTDKNKIRIQPNIFPNNKADNITAKHAIHNNRA

FKIPDSHTEQFKNSFFVKTVIDWNHLDEETVTAKSVNCFRTRVSALTRQYT

>SG-AL_contig_7604_1_+1_517_756

MCVHACNSLYSTDFKGKKTPSITFHKSIIIYNPNTTEQQITPTELPRLHQQQHWPNKRKK

KTKTNKIMKRDRLQLQDYLS

>SG-AL_contig_7605_1_+3_3_176

ILSFTANLQTTNPVTYVFKKKSEQIFANYQTRSHMHSRKSLNKSISAVKYPQNKTIWQ

>SG-AL_contig_7606_1_+1_388_660

MPDQAHCEGKCAPFFSEFFSFILFYLINIVLCCNHLQPKYSVFITGQTVHVVETVCRTPF

SNQPHFKSAVYAKLPSKTLKVSKCAKGRTFY

>SG-AL_contig_7607_1_-1_1171_1434

MPFTCGRNMVISQTLSCCFVTELSALQQVSDQHQVHGKKSLWKSQRLTRTTSTDSEMCFL

SSINVKTILIFITHDFRDPELVRCGMTK

>SG-AL_contig_7608_1_+2_272_526

MCFIFIVPFCVDSGMDRGRKRPDHCYRWGFIQNAGTGLCELVSVDGLLGNTHLVWSTCHI

LIHVCRRDLYKGHGYVSCAFSKSSM

>SG-AL_contig_7609_1_-1_1_111

KFSHHVTTTHTRDRAKHTANTRPEEGLTYRSKCRRQL

>SG-AL_contig_7610_1_+3_789_1052

MCTLFYNGNGLVGTHLVHTLNKISPYQTEGLQVPMAVNKVQQHLAIEHGTRAHTTNFEIT

VLQEDTFVRSLLQQFHRLWSLNQYSCQK

>SG-AL_contig_7611_1_-1_1_231

PAVDLLGPRGGPPQLLGHSFQAKLPSTASYFHPGPLRPRPSGHRPTSRRLFPRPKSLWTS

PPPMPCARPSSSSLRRL

>SG-AL_contig_7612_1_+2_302_397

MQATSQETCVDRLHSDRRDLYGHILTHNTYMN

>SG-AL_contig_7613_1_-2_257_469

MDTWGNDSVGNKLSFLDRGQVMSFVKQCHCLAVLVPAYPLAAMIASKNLTLISLPYPPAS

CIISHDSKAKL

>SG-AL_contig_7614_1_-2_2_367

VAAGLSSVAENFTKLRFNSLLARRDAVLQHSSLPPEEVAALRYFPAQDVSLFGPYAPQFV

SLRAEANREVASYAASAGRVAGVVPTHPPSPGSTPNECSPLQHLHSMLRVGASWGSTSGT

FW

>SG-AL_contig_7615_1_-2_1607_1822

MRCLVFELRYQTVFCILVYAAYVMYFTVFNKTITSVFFQRHCTNTCLHISMHAWTKAKVS

FRCIWYYEWILF

>SG-AL_contig_7616_1_-2_2_184

LKELNGKIKCHELFLLNGKIKCHELFSFVGLSFSGLKRLCFQRGCLSDFDGLNVVTALIW

P

>SG-AL_contig_7617_1_+1_1_195

PGRKPTPPPKNSVQRRLQGEPPQAQPARTRAVSSSFIPRPSGRPLGDPRGEGRAGLRVGY

GKCTS

>SG-AL_contig_7618_1_+3_123_272

MIISEGTSEHCKKISNGYVHPHKLFIHDCLSKETPTIQRVPFIHKDTELH

>SG-AL_contig_7619_1_+1_82_324

MVSILAKHVFWIKYSFWLHILYHVGLEQNLKIGGGVASRERVGLIGEDSSISRDERDTFG

TVRPRSPEIPKVEVNEGVLGA

>SG-AL_contig_7620_1_+1_1507_1893

MFRLVGDVTTKVPAHNAVPGGVVLFIKLFLDVSGDVFFDVEFFQGLSGAVHRILLHVLSH

VGILDHCFAFRHLSSCRLSQAVQNHAKPASVLTQIYKIADAVVCTTLSLSNTAEDVNLKT

PGSPSQTPA

>SG-AL_contig_7621_1_+2_5678_6271

MINGKISKLLITNKITGKYNQLNPLTPSAYSPASTSFPHRPKEPFEAFTLMTQHSQTQMF

YKNLIPHQPLIYNVKISSLAHYFPITCTQHNPMKNLVIEMMNIHVNYIQLEAQHFVVSLV

KSPKGKSVQNTKVMSACNYWVCHIYLLLLLITFRFTQQILFFPIIFNLKAQRNFIYIYIR

QIINYAGNISFFFFFIIK

>SG-AL_contig_7622_1_+3_240_404

MKRIQLTFPSVTCGYTDVTFAIQCCLLYSPCLSICYVHCFTMILVCFIIPSFCSN

>SG-AL_contig_7623_1_-3_3_554

RRSEIHALSGAKDIGFHSNRDLALTLLPEFVAKSQQPGSHPRSFRIPSLVSSLDPRDARS

EERLLCPVRCVKYYLKRSAPRRSSQRRLLISLNESYSRDISATTISRWLSATVIRAYKES

GSSSLPDSPRAHKLRALASSAAFHHSLSGTSWQQRTGGGNPRSLTTTFETFGSLMQMVCL

ASLS

>SG-AL_contig_7624_1_-2_707_1024

MTWISHCLGLKIVVKMTWTSHCLGLKTVVKMTWISHCLGLKTVVKMTWTSHYLGLKTVVK

ITWTSHCLGLKTVVKMAWTSHCLGLKTVVKVTLGQGVQDCKVGGRP

>SG-AL_contig_7625_1_+1_265_459

MMVCTVRNHTCVSLNGDFSGHNSNNNNNMISIFKSQKEKNIKYFCNDSWPVNELAFTIMV

IRCMA

>SG-AL_contig_7626_1_-3_447_614

MAYFCPKGRKNLHSGHSSLVRCRTGLNQLHQRTLKILRIYCLSSQETSPDEVRKAI

>SG-AL_contig_7627_1_-2_2_262

DLHQKRHTDTSDDNAVYQDYIEKDREVKRSAQKDKRIWLEDQANDAQNAASRNDMRAVYQ

ITKKITGSTKASSGPVKAKDGTLLSRG

>SG-AL_contig_7628_1_+3_189_302

MFSACFLTVNGNILNKLTAYLTSFVKIHLICCSQFLKI

>SG-AL_contig_7629_1_-2_299_1807

MGRKLNLSHLSEEECERILHVLQKDFEVRQIEKERIGAIQNTLKSEENKTYVLSQQSKFN

KNCCIRCCQVFGLIFNRRRQCFHCGYNVCKDCCSYQPDSKHYVCNACEKQKDLSKQSCSW

FYTSVGQKFRRFGSAKVVRSLYKTTSYCKLRHAISTQGGLNNNKLIIITNKDNNNNNDNK

DRRANSSDTELDRGYTGSGQSRSVQVTSRRTSRPRLPAAFRDDTGDAGDEDKEEGDDSDE

LPPPSPNATSRPTSTITRSLGSHPVARAGSVDTLNLLSSASPSSKRHHRTRTPDPSKPLR

ASDLGKRRTPDPGSGKGRARWDDGGGGSKAKSQNETKENIYKHAFESARQAEEHKFKAKF

DLLLNDLHRTLDTQSPKGLTDSFASTSYGEVMLSFRGRVKDLLVGFTQRLQLAYESFDSS

DSPEDHSQQVKQNVSRFLEDLLGESLDLTSDEAVSDLSSLSDDSGNDRHAFEDQIAQAVV

SKILDSHRREVHHTLYQDRRSRH

>SG-AL_contig_7630_1_+1_1_204

RLEESKLFLGLLDIAGYFILGNGGEAQIPFSGGQIYHLLLREQLVCFFIRDRWEHHAFST

LLPVLWCG

>SG-AL_contig_7631_1_+3_54_236

MVNQCLSNHFLLCYYVKSQQMLCQNTEARQTGGNKKSGHHEICYKDVNLLFLQALDKYVT

D

>SG-AL_contig_7632_1_-3_3_338

PFATAGALLQTHILLARRDAFWPPRSRPTGLCSLAASGPHPLRRFCSGSLVFQSGVRQGC

PSLNLSNLLARWRELVCNDWILKVLESGFRLPWVGSRPPLLPSPPPSQRSSR

>SG-AL_contig_7633_1_+3_3_155

RNLLIQMYSWSLSRKTECILGDPQSVKLENTNNKQQHQYSMCVLKKTHEKE

>SG-AL_contig_7634_1_-1_94_1791

MKGIVISPKGTVNSVMAKKNSIHWFRKGLRLHDNPALLEALKDSASYRCVYILDPWFAGS

SQVGINKWRFLLESLEDLDSSLRKLNSRLFVIRGQPADVFSRKFKEWDITHLTFEEDPEP

YGKERDHAICALSAELGVQVVIRTSHTLYNPQHILEANNHQPPLTYRRFQSILSGLSPPP

TPLEPVTASTVRHTCTPISDDHDEKYGVPTLEELEFDTDCLGPVRFCGGESEALARIYHH

LERKAWVASFERPKMSPQSLFPSQTGLSPYLCFGCLSPRLFYWKLTDLYKKVKKGQEPPL

ALHGQLLWREFFYSVAANNPNFDQMVGNSICVQIPWDHNPEALAKWAEGQTGFPWIDAIM

IQLKKEGWIHHLARHAITCFLTRGDLWISWEEGMKVFDELLLDVDWSMNAGTWMWLSCSA

FFQQFFHCYCPVNFGKRVDPTGDFVRNYLPVLKYFPTQYIYEPWTAPESVQKGARCIIGK

DYPLPMVDHPHVSHINMERMRQVYKTLLGTTSVAGYKRKLDLPKIPKMDMFYLKANMFGS

NTSSDEERKQVSVIRRCPQDMISHSK

>SG-AL_contig_7635_1_-1_1_162

LFRILRLTWKTMFCTLWVIMSDVHVYTVCVALLLSREFVRQFEYCEYVPRCMHH

>SG-AL_contig_7636_1_-1_40_453

MVLPEFVAKSQQPGSRPWSFWISSQVSLGPRDTRSKERLLFSVRCVKYYLKCLAPRHSSQ

QRLLISFDENCSRDISVTIISHWLSTTIIRAYKESSSSSLPDSPRSTQSPGVLCRHLPLP

YYQGRLGSSLLAAGIHVH

>SG-AL_contig_7637_1_+1_175_483

MVSVLAEREFTWQNSSFCDWYWILSDPADWILTSCIPIWFEWHYLPCLIPRYRGSLSTEN

VQHRWTNCLNLKPDLCNLGMPRDPFLIGHCDRNRKTMRVCFAN

>SG-AL_contig_7638_1_+3_177_386

MNEATLTVQVAKTGSSSMRTKRQLVARILNDAFLFFVPQTIFSADTISFFAENRIFSRGN

LHFRGQIPCL

>SG-AL_contig_7639_1_-2_2_337

FKKKKNGGGGGLTIARRWLCEIKYLNKTWVKCQINPLGRTAVLKSLKLSKLFHLWIILPN

PQDHTLDKLQKKIYIYIYLNLFGTKKQGRINQKTAIKKNIQMGGLGIPSLKQ

>SG-AL_contig_7640_1_-2_2_253

SHAALHWHRPPRSHFGHRTSSSPSSLFPAKGSTDVCNCIYSSALVGCCGLCMHINGNIDY

LRHGNCPQKRKFPQQKIQFSAKKK

>SG-AL_contig_7641_1_-1_1075_1266

MWQKMGFNLICPVLSSLRKRCISLKWYKFPSHFQFSLLMASKHEIYKFVSLSLHSIQTEI

SVIM

>SG-AL_contig_7642_1_-2_638_841

MAYNRGRSTAGQSYISMLSDRYREPDNAQSAGRPAIVYGSGGPGGGKKQTQRSEHTTYSF

SQEEQLTQ

>SG-AL_contig_7643_1_-2_149_340

MKKVVYNTMVLNYLTGQCSVAIHISWYASFRPGNTYTCCVGQMAQVSTKWMIINCIVSGF

PISP

>SG-AL_contig_7644_1_+1_3664_4071

MWNAASILTAGILNITAWVSSGNFCAVAAVLSVHREGYVVCVDSIKVTAFSITTQGSISA

IKITTFSLKDSTSAKMTAFSTFVKDCMCVIKNTTSITALKTQPSLSLNQKHNPFYHCTKN

AAFSITDLQQPSLSLY

>SG-AL_contig_7645_1_-3_3_215

FSAVSGWLLTKFGPKVWAVEESLMLEIGTERAKIKVTRAKKVPKISTFGGRCVNSTFSAT

SENIQILFSLL

>SG-AL_contig_7646_1_-1_1_267

FIDVTTNRHITDIVVEFRKTDKLNFNGDKSMALEPQLSNFKIAILRNGHLKCLESAKNYN

CKASTGKFKQSKGKVVLFEYNGVYLTKTV

>SG-AL_contig_7647_1_+1_1_156

DSTQAQLTSVQTIIDSCGPWKFKQNPGKAWVRTSHYSLGLLWHEEIQSFPLW

>SG-AL_contig_7648_1_-2_71_625

MLWRCLNPTCHDVTLCPLCYVMDKHDVKHPFERVDYEGAAGEPVKKRSVSLRIRTMGIFP

GAKVKRGRDWIWGNQDGGAGCLGDVIGFENVAPTSSRNLVRVKWNSGTSNSYRLGLQGCV

DIECVDEMPGTYYYRDHLPLVDTTSTRTRSTAQAPSTSAKTSSPASLPGSEPQSSPASSS

KGVDR

>SG-AL_contig_7649_1_-2_8_259

MDQPFAGLLILVVMYIYTPPPYPSLRPPQKRTSPCLSAILFRPYLCGGCVLAGCLLPGLF

FLLLLSLSVCPSCPCVRPQSGHSI

>SG-AL_contig_7650_1_-3_3_251

PSFFFFLNTFRKKKEICLDQSLCLKRPKFINRDSVWPFVPMHKVWNKFTHQQLEESLCIF

WITVIPNTGGSQLYLCIIEYQLI

>SG-AL_contig_7651_1_-1_1_141

IIIIIIMGLVLHFDQLISTTVVALHQMHRHRVCCLCWLYNTTLERPS

>SG-AL_contig_7652_1_-2_5_265

MGVPACGRVCVRIYLGARVLGRVCVCVCVCARARVHVWVRECVCVCVCGLFGVACVCVCV

RMDGSLSQVLFVCSVQTEPCLTFKCKC

>SG-AL_contig_7653_1_-2_467_652

MQVTPFWPVIMEELLAQQDDLLSGRQGCCLWRWSLFLGPPPLFVSSPLQYRKLRFFLVKL

FK

>SG-AL_contig_7654_1_-1_160_1473

MSRALSRALRVTHKLEKANHVRSIKPLPCFRLLLSSANARFVPAVAMSSAPAISAPECLF

HPLDFPHKTLMGPGPSNAPPRVLGASALPLLGHLHPEFTQIMDQVKQGIQYAFQTQNEWT

FAISGTGHAAMEAVVANLLEVGDVALVCQNGIWGQRFADMVERNGSVAQKLVRPMGEVFT

LQEVEERLKTHKPVLMFVTHGESSGGTAQPLEGLGPLCHKYNCLLVVDSVAAMGGVPLLM

DKWEIDCMYSGSQKVLGAPPGTAPISFSDRAKKRVEARSSKVRSYYFDANELANYWGCDT

GPRRYHHTGPISSVYALREGLARLAEIGLEASWANHKNCAQQLHVGLEKMGLTLHVKEPA

VRNPCVTTIQVPEGIAWKDVTDYAMKNYRVEISGGLGELAGKVWRIGIMGYNCTPDKVRL

VLRAFSEGLVSCGYKLPK

>SG-AL_contig_7655_1_-3_3_191

LCSYLIGRRLYRNLCKRKCCCLVSGRATVQQQLHSLGPRSQVLRYICVILHKQIVVYLIL

GID

>SG-AL_contig_7656_1_-1_1_243

RTNKFCKELKKLIPNSVLLYRRGLDLKKVIPQAVAQDFTDLIVINENMKTPNGMLICHLP

DGPTAHFKITNVKLTTEIKGA

>SG-AL_contig_7657_1_-2_275_1843

MMSTTMTSMTTAMTNRETNRLHRWAMWRMTVNEGIISCIRDNITTRVIYPDSNTSDSQSL

ATSDMADFHEFPFITPEQMSLARKLNLREKYGSNHGLSDDAYFIFGLPRINVGKDIPEVT

STGTRSVYESFSELGETVNNNLQGREVTDNNTTLNMTYSELGPTPDDQSLASHDMEIQRI

NGYREEGWKSPPPPVPRLNLYSIQEEFNNNAGDSTTRYLSDRYQDKDTTQRFSSDRFSEE

ETTNHGSSNYLQRGNMSRDIDRTTNHYMSNDYAEREAAARFSSKLYREEEMSNPFSPNHF

HERETTNRYSSNCYGEEETTNRYSSNRYGEGETTNRYSPNQHQDEMIPTYSVREVIDRYE

SANRYASTSGPINNTPRETSNRFSFDHFNMNARSPPESPRRDIYPPFSSSRQNSHSPTRR

SYSPLPSSPHHNYSPLPTAQSYPALPTMHSRQSYSPLPPTSPGGFPSGGGQGVGSPGYGN

KMRPTRLLSDAEAERMKRQAYYDWSTPTTERYITEFASHNRYS

>SG-AL_contig_7658_1_+2_1946_2257

MPCRQRQAGGWFSGQQLMPCRQSCRRYAAERRGNRTPGDPWSGRGTLGQSDPTECRQTLD

SSQPLSHFLIPDAADWKMPKPRIPADVSLCLQARIERTEPRPER

>SG-AL_contig_7659_1_-3_282_977

MISSLAPNFLRSVVAKAAKRKMSSANKRGAFIVFEGCDRSGKSTQCGLLVDRLNKNGVPA

QLMKFPDRTTAIGQVINSYLQQSCQVEDHTIHLLFAANRWEAMPKMVDLLKSGVTLVVDR

YSFSGVAFSSAKPNLSMEWCRQPECGLLRPDTVFYLSVSPQVAAQRGDYGTERYEKQDFQ

AIVAKQYQLLKDDSWTEINAEKLVEPLSDEISAHAMKVIQEAQSQPLQHLWI

>SG-AL_contig_7660_1_-3_3501_3710

MFSLFDMVCIDLFSFWHLFFNMSAKVDMKVCIDLFSSGICRFVHFRHLFFSMSVKVDMKV

CIDLFSSGIC

>SG-AL_contig_7661_1_+1_484_849

MCPRCLITVVNMYSIHYTQCAGYMERSARQLTGYTLLVQKAHNTHRYSATDSHLTHTDMQ

ITQSSFGYFSTLKHYFISCSQSFPPFYGSVHTTLNETMWQQQSWLVYLVFTCTANFLEKH

SC

>SG-AL_contig_7662_1_+1_151_393

MCLTFCRDYSFYYFETSFITLLIVISHTLLSVLQIVLNPIRDIERETGNMLCGMLFAVAW

GLMHRAPQRTQDSTSCRFLKL

>SG-AL_contig_7663_1_+3_3_92

GSSVAGMAFQAIVTASHLRLMDRMNHLRNP

>SG-AL_contig_7664_1_+2_128_403

MKEELSYQGKNAEYSDTTRMTAHQNEQCKSQFCCQTKLLLFYISDTAPLSVGSNCSLLSI

KGQFPCQILLTVFLDGFLLLGVKGFKNHIQNS

>SG-AL_contig_7665_1_+3_3_143

KKKLCHPSLNRHRPTMVSERNSSNYRTVPSIMKKQYSVKSGMLTLLS

>SG-AL_contig_7666_1_-1_157_294

MVTLYMTSESSQDQFLSAFSGSSDNFWSALSGCNIITSHYFYLYTF

>SG-AL_contig_7667_1_+2_2_118

ERDRNREDRRNHVDDLKTLIKEYIKSYNKFPFMIFANFS

>SG-AL_contig_7668_1_-2_62_1519

MRHTFINRSEGRRHSSITSSAAQFRMATSRSPLTVISVFLLCFVAVSLALTGDEEARAVD

DVALARSMRSARWLPFTLIGNERAVVHRRSRRQRGAGSLPIFSRRARFLLSRLATASATF

SMQMYNFLRQDHPELVFSPYSIHSALTMTYMGARDSTAKQMNKVLRLRRLRRNRAHQAYA

ALINSLADSGHVTLRVANGVYVKPNRTIQQSFRAGVHDMYSAEVGQLDLEAEGGPEAPIN

AFVAFKTVNKIQDLLSPGLINNMTSLVLVNAIYFKGDWQDPFNENSTTEQPFYRGDGSTT

QVHMMARTADYQYTEARDLSAHIVQLPYRDGRFSMYVVLPTSRNGLATTERSLTWDRLNR

HLSNMTPTRVALHLPRFKTESKFSLKETLQTMRMILPFTRSRARFTGISYIGDLYISEII

HQAVIEVSEEGTEAAAATDVIIGLRSSRRNAVEVRADHPFFYAIRDNESNSLLFMGKYSA

QEQESE

>SG-AL_contig_7669_1_-3_30_224

MRFKAGYGYIEEEHVKRVCKKKSTCVLKLIMATMKKSMVKLSKTSFPLSLIFFVSFSVCL

SCSLC

>SG-AL_contig_7670_1_-2_2_154

VKPGCKKINIGYGYIEEEHVKRRKKIDKLVMVTLKKSMLNVVVKKKNRHAI

>SG-AL_contig_7671_1_-3_12_1736

MAALLSLKDYDSGSEGSEDEQDQEEEQTDGFNAHLQPLDRSKSIASNMMLVSAPAVESNI

DPSGIRHIDPLTKEVKYNPKYEELYAPQVGPSNPNLTQQAQAERNTLAGFAEPSHFNDFQ

FENQRRTFTSYGYALDPSVNSGTVDEKVVGDTGTAEELKGATVFEKVPQKAEKRKREKNT

DPSDIDGYLGPWGKFVDEKTVMKPSEDEQKELDEILAKRSKAGRSTDDKPLEEKTTLHIK

DPFDYQGRSFLHPPQDVGVNLRSEEPPEKCFLPKKQIFTWTGHTKGVSAIRWFPCFAHLL

LSCSMDTKIKIWEVYNNRRCVRTYLGHKQAVRDIQFNNDGKEFLSCAYDRYCKLWDTETG

QCKSRFTSRKVPYCVRFHPDDDKQHLFVAGTSDKKIVCWDIRTEEIVQEYDRHLGAVNTI

TFVDQNRRFVSTSDDKSLRVWEWDIPVDFKYIADPSMHSMPAVTLSPNGKWLACQSMDNK

VCVFDVLNRFKFMRKKTFKGHMVAGYACGIDFSPEMSYISCGDADGKVYVWDWKSTKLYS

KFKAHDDVCISVLWHPHETSKMATAGWDGVIKYWD

>SG-AL_contig_7672_1_-1_73_444

MLFEDYPYMQPSFNAFKGMTSAQLRKDKTMYSHALTVMYSIGSLVENIDDPEQLVLLVKK

IAYNHLARDVGIKYFQDLADMFPKFLEVRAGPEATPFIKESWTKFLGVMNSVVQEMEDEQ

KSKS

>SG-AL_contig_7673_1_+1_58_315

MLLSFFRIVIVAVLLPHCYCCCPSSTLLLLLSFFRVGIVAILPHCYCHCPPFALLLLLSF

FHVVIVAVLLPRCYCCCPSALLLLLS

>SG-AL_contig_7674_1_-3_3_227

PSVSLPRRPLPEKGHRASLHLQRSDWLAWCGPSQVDPCSPSPVELAKLPGLSCHLGGPSR

EHRQGLQVLHYPSSP

>SG-AL_contig_7675_1_-1_166_291

MSKTAEKKKIWKDHENAWQTRHQGMEPPAKHRTVSAVADTRH

>SG-AL_contig_7676_1_-1_355_576

MCAFVNTWYKWLLFCLFVVTFFFLLLLNTWLTECVYLLAFMCVCVCVCAQVLLMFINFVF

LWFSLFLCVLEFCV

>SG-AL_contig_7677_1_-2_2_139

SVQICCKFLTFILILCRRNHKPHKCDVKKMMLNNQLTSYSAWRSLQ

>SG-AL_contig_7678_1_+2_158_280

MNIHTHPFLFLPSLCHKLIRVIPALIPFAFLCVPRVHTGFS

>SG-AL_contig_7679_1_-2_2_142

FSMFFLNAEPFLLTDSLPCCRVVSNWEKNHSALLTPLSCPWILFIYL

>SG-AL_contig_7680_1_-1_208_2025

MVDLQHLMLLALLKDFNFWTTRWCCIKRPRKLSSVVIVVLKDVSISDYEEHPDSFPFLKN

SFLSCVEIVSPAQYNSTVEKDVCTVPLFISQLKCYTKREDVHRKILKLISKPTDSNASET

EFQQDFQTLQEVGQKESCVEKAKPKEPGFEDNFPRTSLLLSTSELMEQRYPMPVDSKNKL

YGDFAFTKDCYSPATESSPLFAVDCEMCHTTENQFELTRVSIVNEQKEVVYDELVKPYNK

ITNYLTKYSGMTEELLSPVTKRIEDVHRDLRNLLPSDAILCGQSLWNDLNALKMFHPYVI

DTSIIFNQSGDKNIKCGLRKLAFNFLGQTIQDSKKGHCSIEDAKATMDLVKLKLSKDIEF

GDAMLGGVYFPNIRTYFENPSKAQNVGETFSAHPSSVLAPSDTNADSFASRSSQSSSKDT

DSSSGLCVQRISSGSVTDRTNGVIPDVSSKRKELEGVNHRTDGDTHMSEVGMDSVNEGND

IGQVPRQQCQEHVGQREAFFQQEGSTVMMSLFDLLADRKKSAGLVGNKDTISRFQSHTQL

ERVEVRDDSEACSKAVKMGSDKDFTWVELSAFANSLAADFHCSNNDADKRKNLFTQLDDL

VQEMVT

>SG-AL_contig_7681_1_-1_1_264

QPTTRAISGRSPHKVHQSQKDDRVEHKTLSNLKTKTLQKITNWSDNVKDWTDMTIPDLLS

AATNRPACRRMSAASALRSSRRLQRSWD

>SG-AL_contig_7682_1_-1_532_858

MRIHSPLFIHCLSHALSCGYLKSILLIVLSTVLYFHVDKILAAGGWVYFSHSFFNVRMSR

QWCCVCWLGFICLFQLRNSSSNNCVLHELNMFSIGFCVFEMFFTANVAR

>SG-AL_contig_7683_1_+1_370_651

MSVLCLMLRGIVDTFIVGDICHSNMNRCMLSFLRRHRHLFNNASLLRTQCLITAYTVKQY

MIQKMIQTLQKEYVKKMKFFNFPFGEEKNDEKVD

>SG-AL_contig_7684_1_-1_229_4170

MEAHELKKNNNNGEDIQLDKEELQQAINQANLEASAEYKKAQAVKSVNAAIDSNDPEQLL

KAFQNSLAQFPQVYDFAAPLYLEELASIKAEKQGELDYDEIAAALIVLNAVAAINQAVDA

GDALTTYTCLTDPQAHLPEVEEHIADTYQSALADRKASKAESQSANPLLTHADIVECVET

VHQKVQEEQERIEAIKSINEAIDKGDEHETLAALQLPSAKLSSIDPLQAVHYQVLLAKEK

DKKAEKTQDDGAVLWLEEIQSTVDHANSNAEEGLKMSRGIQAANEALTEGNVEGLVAALT

GDAGLQNVKTECGPTYAEKLKELQQEKMGSGETGSGWMMNRVRDGSKFFYNIHTQQYAWV

RTDDVIKDYSLLTKDEIQGVILEVTAEHNREMLFRANEDFIVLVQAHTRGYLTRKAFHDR

LAFLEQQLPAITTIQAWFRGSKQRQAYQDRRQYQKDNTPAVVKLQSYVRMWRDRKRYRDR

KQFFKDNEDSIVKIQAFFRSNLARQDYKALMYDENPSLAVVRKFVHLLDASDNDYAEELE

LQKLRQQVVAEIRSNQQLEQDLDQMDIKIGLLIKNRITLQDVVTHNRKLTKRSDDTSSVR

GLKALSRESHERMEAYQHLFYLLQTNPTYLAKLIFEMPQSKTTKFMEAVIFSLFNYGANQ

REEYLLLKLFRTALEEEINSKVDKMTDIVTGNPLVVKMIVGFNRNQRGQNALRDMLNPLI

TRVIEDRDLRINTNPVEVYKAWVNQLESTTGKTSELPYDVSTEQALKHPEVQEQIEESIK

RLQALADQFLHKILTSLDNIPYGMRYMARVMRAALVKKFPDAPEKEVLKIVGNLLYYRYI

NSAIVAPDAFDIVNIGAEKALSPDQRRNLGSIAKILQFAASNKGFGGESAYLSSMNDYIR

QAHEKFKKYCAQACDVEEPEVRFNVDQYSDIIMIAKPVIYISVAEIVDTHSLLLQHQETI

APEHEDPLHELLEDLGEVPTIDELLGTELTGDEATNETLRAQLAKTEISMTLSARVEVHT

DDKADMKSLLIRTKRMIVDVIACQPGDRLTDILETASSATQEEKHQALVKKRDILDKKLT

QSEKTGAKIVRHASVVGDTSLPLETMKTKIRNNLQVLELAGMVSHRDHYQDLINAIVQDI

RNQRRYRNNRKQELLRVRNTLNSLNEKRTFYESQIDYYNQYVKTCLDNLQHKSKKPKKTG

FFRRQGNQNARKSVHYNAAQLHEKGVVLEIESLPQNQFKNVLFEIAATENPGVFDVSAKF

MGVAMDKVELVFQDLLQLQYEGVAVMKMFNRAKVNVNLLIFLLNKKFYGKELGK

>SG-AL_contig_7685_1_+1_1_246

GRAECQLYSKVFVNTHTHTHTHTHTHSLSLSLSYTHTHTHTHAHTKTKIGKVSVKNNVFL

FCFVFFFFFFLFFFVWVFFFLT

>SG-AL_contig_7686_1_-2_812_943

MLILCLACIYIKLKQAVTDSSHKGKLGSNFCHESIYEETNVAKA

>SG-AL_contig_7687_1_+2_950_1216

MTVWIYLLCCCWCFFLLWKTPNFGYHAIKKFDKQITRFHCTCTDLSIINLPSKFQINIFY

FVFLTFFFPSILKRLVYRDSPGKNTLGME

>SG-AL_contig_7688_1_+2_2_139

PPPDNFQSSLHISCHYFSQLLPLRLSHSMSLALQVSSSVFVSTREF

>SG-AL_contig_7689_1_-1_4_1257

MTTCVPQVSNDLSNEALNKTQSDRSVAQSSQGSRGVMSSVRDMLNRSQDAAEDLLLDLTY

VTERIISMSFPSEGHDTTYAFNLKDVVRMLKTKHGDNYLVLNLSEKRQDLVKANPQVKDY

GWPDHLAPPLERLCSVCKAIDAWLNADPRNVAVLHCKGGRARIAIIIAAYMHYSNICASA

DQALDRFAMKRFYDDKLGGLPLPSQRRYVHYFAGLLSGAIKINSNPLYLHHILIHGVPNF

DTRGGCRPFIKVYQGMQPIFTSGVYNVTDNMQKVCISISPGIPLRGDILIKCYHKKGGGR

QHTAGGGGSREIIWQCQFHTCAIQDGNVVFSKQELDDGIHDSRFPDNGKVEFVFGPTSDV

PVSVSGFKSDVTVPVDDNEDSLTRSDSYENFHKALAAQGLDRYVGPRDTSYGSHQHQT

>SG-AL_contig_7690_1_-2_2_2731

EEQLTQDGRTVYKTKEEKSFNEARNDGQPAHITSFSKLENGSVGTGQPKRQQDYHIQKTV

TTKTSERATIPQQQIYLQQQPQPQQQPVYLQKQQTTYVQQQQPAFSQQQQQQPQIRYAQQ

QQPTYVQQQQTTVTSQQTKQQQPSFVQHQQSAFTQQPKQQQQPSFVQHQQSTFTQQQTKQ

QQPSFVQHQQSAFTQQQTKQQQQPAHQTQQQPRYGQQPQQQQQPQQQQQKQPQQQKQLQQ

PSYQTQQQPRYGQQQQPQQQQATYKTQTQTQQQVTYQQQKQQQQPRYGQQPSYQQQQQPS

YQQQQQPSYGQQQQQPQPSYQQVSYQQQVSYGQQQQQPQQQPVTVQKISTIKTGSYGTGN

QPSSATITTITTTSTSTTNYEGAAPWEEEEEEGVYENQPLERPDLVRESDKNIEAELPTM

GTARSIREKFVTGQVENRQAGFKREITPPTMAAGGVFENQPQYNPNVVHYGEEVQNEALP

QYGTARNMAARFKQIGKQGYQSGPTPAKREITPDRTTRIEYVSEPRGYVEKYEPEVQAGV

FESQPAEIPGVVRAHQLPEDILPERGAAQSMAMRFRQMSIERTPDMPRGKREITPDYTTK

GEYVSEPQGYVEKYEGRPESGVFENVPVNLEGVVKADEYEDDRLPNRGLARNIAAHFRDV

GRWRPPPSPSRMKEFTPPREEGSALHGYGVYENNPQYADDRVHATDQQQDILPQRGTTRT

ILTRFRKMSSGENTPKDPHFKKEFTPPLDGGGVYENNPTQFIPDYNRRPESGILENQPIV

RHDVVKGNDVPLYEVELPERGLARNLVHAWRQKESESTKITPTGSGRPKEFTPPREEPRV

MQQRSGPMTPGAQGQGQVFIMLSGGGVHPSELPGQYQPLAEASVFESEPVHRDDVAREEE

TDWMMGMPRG

>SG-AL_contig_7691_1_-2_113_217

MMKLCGVDVDSVECYLTILCHSFSAGSLTMVHLTF

>SG-AL_contig_7692_1_-1_28_174

MFHGSRPHLFHLPPPHLISKRIGFCAGSRSLLCNCWQMECSEFFLNKFL

>SG-AL_contig_7693_1_-2_44_319

MFSWLRVCTLFSTHCAGAMLPHSLHTPSLVGVHAGYFCASISHQTLAWLTRPLICLFDVL

HVYTYGGVQALAGLGVQKSLHHLSTRHSTQQS

>SG-AL_contig_7694_1_+1_1_207

CILLWVCLSMSFFSTMCDEKIVLAVWQWANEHPAVDSIIPGSYFVFRKEHLLSLLPSFRI

LRARFSFHL

>SG-AL_contig_7695_1_-2_2_184

ILPFPLPLKKSSLTFCIQGIGISGKKNNCRFFEFLSSVSSAQMIYLVSVKYGRGLLSGYI

L

>SG-AL_contig_7696_1_-2_1040_1204

MFMVWCIWNYWHCDIFLEQTEKLNLRFFRKHFFTYLSAYGCDFSIQFDTLPFTVH

>SG-AL_contig_7697_1_+1_1_279

RVKRAVPISSPTGGFTFPNPEQGQVLIHSWGHKHKVFNQVSVNLGSQTGRCHSSNWAVRL

EGISVELRQSDWKVEALSHSGLMKSIKKMLNHR

>SG-AL_contig_7698_1_-3_3_137

KCLTIVAVRTRKHGKWTRTEKKTTDRKKLSTEQTESLFQLSTPHV

>SG-AL_contig_7699_1_-2_2_94

QTCALPIFLSHYKVCNITPCLVDSVCWAAVG

>SG-AL_contig_7700_1_-2_56_235

MCVLCVYVRNCVGVCVCVHVRICACARVCVCACVCLCVCACLGDFLCTRVCVCEREKERF

>SG-AL_contig_7701_1_-3_3_296

LPRRATHEAARAFQRVRLAESLRASLRHLSTSCRPLVNSLEPVHFWSSSLSLSLWVPVQD

LVGRSGRWQSSSVAQPTQRLQRVNQMAAQTSSWNLRDY

>SG-AL_contig_7702_1_+1_91_348

MPHAAADCRFQDDPQLNDPRMDLSSSLATYQLDPKPVHPYDFAVFCTPVCKKIRPICHTA

KKICQRNRQATILPRHDRNQFATLTF

>SG-AL_contig_7703_1_+2_5_241

MDQQGRKKQTTVKKRKRAETKKESNRKEEISSTEFPEGGDTIYTERAPSIPAGKVSSLGS

EVVETLTSLKDQHYEGGAH

>SG-AL_contig_7704_1_-1_202_459

MFSDVHKPQNTPISRRGDTGGSFSINSIPTLEIVCWKFHVCCCICFCPFSFLYCHLLFFC

LPGPFPFLFSSKPSHNSPSLPPVYDV

>SG-AL_contig_7705_1_-3_9_1019

MADDGTKPAEAAELPKDVLAPAEGPRENLVATAVKFLQNPKVRPSPLSQKKAFLERKGLT

SEEIELAVQRAGVADDTSSSQTVQLPPQPQYPAAYANTALVPAHQPLPLPPPVSPWTKAR

ELVTTAAIVAAVSYAIYNVFKTYIQPWLMGKSEMETRLERLETNIAQLHASVTDTNAQMV

DTLKVIQESLMVQNDRSAQNSEVTLRQESRALGEIKSEVSSLKALLLSRRQFPPPPSTSP

IIPTWQKTVSRDDETQQSLTGQTEGATSQGADPATATQHNTAPSSDSSVEKANPSSEDSA

HSLQSGKGDDAISQSASSNEFEMVKPSSDSDTPPDLD

>SG-AL_contig_7706_1_+2_755_895

MINFETYQRWLDKFVQNCIKSQPFEKKNSKGMHQGILMTEPCRNTWI

>SG-AL_contig_7707_1_+2_2_190

SAHKIMLIWGNPKNLHPLATRRHPDRFEPRPSNWKTSVLTTRLSRPSVPVSIIAWFCCCY

FLR

>SG-AL_contig_7708_1_-2_113_556

MWIKACGRPHDQLNPDVITKNYYVCSKHFVDGRPTEKYPYPTPYDGQPVRRGRKRKNTER

PHNCPPPPSLKSTATKSASVSTPPPSGSPEAVTPSPSSRPPSQRSQRDRDPASSSPSSPA

APQDKDNPDWIPTVAMGPHRSAKSADFS

>SG-AL_contig_7709_1_+1_412_735

MIIRLQNIQTQHGNHFSCMCKWQNQPPCKWQNQPPLSIEFNSILLNIYRSTLRAMVMDSN

ILAYFKVVQVMIIFFLVSILKLLAARLAERQIHMPVVVGSKLAFLLPI

>SG-AL_contig_7710_1_-1_1_117

SSHINKPPIILMLMSFVWTATSPLQIKVTITGVIALVMP

>SG-AL_contig_7711_1_+1_133_339

MQSVQGRQADMMGVTPGETNKHDGCYSRGDKQIGWVLLQGRQTCDGCYSRGDKLMDGCYS

RGDKQDCCY

>SG-AL_contig_7712_1_+1_1_207

SKATHTHRQMHAHTRIYTVHVQAHTHTHTHSHTNTMTDTQTCRHTGKYYYYILFIAQLNT

KAISGLSPH

>SG-AL_contig_7713_1_+1_130_291

MGSGRVSQLSWLKYSLDWTSYIFLASNMSQIMGTFLIKYRQVVISKGQTDGHTH

>SG-AL_contig_7714_1_-2_65_196

MSILFLFFFHPFSVVQIRLILTSVLKERVNTFTMNTAGGGGGGG

>SG-AL_contig_7715_1_+1_1_201

KMNNNSNQTKKKKIIHPLYMIVWSLLKRDSSHSDNSICTPPISASNYLADSLSLHKTLCT

GKVLKWG

>SG-AL_contig_7716_1_+3_3_218

RQTDRQDRQRDRQTDRQTDRETDRQTDRQTDRQTDRQTERQTDRQSERERERERESSDNC

GLSNSKRLVTTT

>SG-AL_contig_7717_1_-1_238_375

MHAFLSFMVCERWKLQTFLIHESHNYYDASNFSWYCLYISVLKRPG

>SG-AL_contig_7718_1_-1_181_648

MPGGHFAPHFDGHFVKNCQERSLQTFMLYLNGDFEGGSTNFVDEKQTLFMDIDGKYCAED

KNIIYRIRPEPGLAIIFNHHRLHEGQKLKSGVKYILRTDIMFLNTSQNIRSADDEKALQL

VQEAERKEAAGDCLEAAALYRRAFKQSEEVASAYGN

>SG-AL_contig_7719_1_-1_61_984

MSFRYISAARRGERLLQRMICRNISPHEMQRTIHLLSRHSAPETLSSIVRGWSVLRQKMP

KGFEKFFPKQGKQPAEGSARPTPKEAPKAEPPPPPSRSAPRQQSAQQGEKKPDFSFTMGK

GKNPFSDPNDPNTKVLIGLIAGVGIVGLLGLYQNQYREITYKDFINSYLSRGIVDKLEVV

NKKWVRVKLVPGTQVDGSSVLWFNIGSVEAFERSLENAQLEMNMEPANFVSVIYKTETEM

ANILYSALNILLPLAVIIWLFRRSANMFQGGGGRSGSRGGAGGIFGFGQSTANHQGGHWC

QVQGCGRL

>SG-AL_contig_7720_1_-1_43_222

MVLAKEKADHKNGSSKRKGRFHPLPFSKRRKIVTPEYLSQFSSHYWKTIIFTWESAIVNV

>SG-AL_contig_7721_1_+3_111_230

MRTVSVSFSLFVCGFFCFFGGAGVLSFFLCVCVCVLLFFF

>SG-AL_contig_7722_1_-3_1560_2009

MAKRWLSLDHSAHPLHRLCIQPMKISTSLSTHTWLPQSHIPSYKHSHSGSGPFLVPLILE

FMLSRNNRRCSALLALPLVGKATVSQCFSAVFFFFSPHIFLHFVKKNFFFDFIFSPNSSF

CSQLLFFSASADSSFLLFLPFFLIHFFSFF

>SG-AL_contig_7723_1_-3_516_755

MYKILVKLITLPATRLCRQAYEILLLQGQSGKMNWVSQIKKVLIENGSGIVWLNQGVGVQ

KHFTAEFAEFKQSWRSEYEK

>SG-AL_contig_7724_1_-3_357_764

MTTIMLVVIIIIVRRKRRRSSLGFFGCCCCFAIPSSALLQRHRFHAAHSNSSTHHQPQVR

SKSKLRSPWEQAMSCHRKATHQRVLCTAAGSLVGVHQCLFSSSLLTTLPTKPPTLTTIAQ

LQSGARLRKQTPNPQQ

>SG-AL_contig_7725_1_-1_1_183

KTREQTESIEFVAYVQHRTSVLFYLFKTKSYSAPITPHPLTPQAPCDGGSRIVAAKALVC

E

>SG-AL_contig_7726_1_+2_2_109

SKPLKKKKKVIPNPAIPFTNIKHVLHTLQTFRVVKI

>SG-AL_contig_7727_1_-2_284_517

MYCLFACFVCLFVRVPFGCVCYFTFLCVCVCVCVCVCVSYMLTVYVRVLYVDSVCVCVCV

MLILCMCVCCMLTLCVCV

>SG-AL_contig_7728_1_-2_557_949

MQLFIQAQQLYTIEVEGSETVEDVKALIEERERVAAEELAVYCGGKPLEDDARLAVLEDQ

TSLEVEVRMLGGKVHGSLARAGKVRGQTPKVEPQEKKKKKTGRAKRRIQYNRRFVNVVAT

FGRKKGPNARS

>SG-AL_contig_7729_1_-2_2_175

SFRQTRPHVLISKFLCLTSVFSKQSVQGVLLLNCDNSGTISPILILFSPLERASKIHL

>SG-AL_contig_7730_1_+1_223_360

MSYLAAHWCSGNPFFNFYLWNVRATRPDGSKGISSWRLIPPSEQPI

>SG-AL_contig_7731_1_+3_126_248

MVLQMHIIFETIMHVNKCNENVSAMIILLSAMFHHLSFSDS

>SG-AL_contig_7732_1_-3_3_230

CPHLLEILCQQFSSFLLLSLFLPFYLSFTVVFLFFCLPGPFHFLFYSKASHNFLSPRPSL

CQFMMYYLCCFALATR

>SG-AL_contig_7733_1_-3_3_131

IIRHRIRNYFALGCVIVDYTRPIEKNGQIIKMCLDKRSLLTHL

>SG-AL_contig_7734_1_+3_3_233

SINSNAYIINITICKSCSRTWHLLRHTEAGKFMIRINKHALRRIFIYIFCLHSSTLKETI

HVSIMTPPTNPGEYELR

>SG-AL_contig_7735_1_-1_583_1440

MAFSLPATTYPPFNCQSEGRAVRWTKWVARLKNVFVGYAIDDDVRQKALLLTFAGDELND

IVDTFPVDSITPKEEGETHFQKLVDAITYHFNSPVMPKVKVETDMAEGEVPQEKMDDAHD

PPKASDLKIEVDVAVEEKPQKCCKTEEDRTESKDPDPHEEEDPDPHEEEEDSDGQGEEEE

PPAKKKRASKYTLPLDINRLSHSDSPHQLVSNPPTEKQLRPSRNCVCCCVPAGQRGKFIR

RVTTKYMCKACRVPLCVEPCFYVYHTVKDYKREMRRYCDSNGHVGD

>SG-AL_contig_7736_1_-2_2_109

VLLNTLPWLCPIHNVNLKCQCFLCLKTFIFVDYSGT

>SG-AL_contig_7737_1_-3_396_824

MKDVKEGGNYVAENTDKKVDYHLDEGELKKEEDNNGDKNFDKQMCHHGDDKEMKYVKEEG

NYVDENTDEKVDVYDEEREVSFKEEGNDGDEKFDKKICYHKDKSITELGKYGKGKVVGRG

KDRADHNIIQKDDKKVNNTLTKM

>SG-AL_contig_7738_1_+3_3_194

CVWICLCVCMCVCTWAFVCVWICLCACVCACVCMCVYMLYARMRVLARTCVCVCVCVCKV

IYKQ

>SG-AL_contig_7739_1_-2_2_163

HILMRFVCACVCVCVCVAQFYLPVALILESPYFTCIEQFMCEKNLTVSPDAQKV

>SG-AL_contig_7740_1_-1_220_330

MFILNFFCNIYILRMTYRFLFICYSASSILYICYPLF

>SG-AL_contig_7741_1_-1_3157_3414

MVLCGVTAVIFSFPPSQRTVIIVRVTWCSMHTLFCVVLDPSFVMVFSHFLSFHLRCTCIQ

SSHFFFVICMFWVTCFSFFIVANAHF

>SG-AL_contig_7742_1_+2_1850_2155

MCEAFTAVFILRKTLFSLSLTLKYMVHPPIIFYKITLSYKQNNNNSRYKKRWEGGAFKSS

SLFHSRYHFRILYHSTSSSKRKTHIHVKYKGRYVHYNTLLYI

>SG-AL_contig_7743_1_-1_1441_1650

MAIIELKSVINYSYQSLHAHLSLICIIVIRFFFSDRSHSFMKLPIKDSHSSATLPFEDSA

EFLAFVLGGF

>SG-AL_contig_7744_1_+1_1_291

VFCFGNKHFAYLEVLHFVLATSTCVHSWRFCVLFWQQTPVCISGGFVFCFGNKQFPYLEV

LCFVLATSSFHIWRFCVLFWQQAVSILSGRFVFSFGN

>SG-AL_contig_7745_1_-2_1061_1291

MVLEYSSVNLSLPPSLFRLHDICVLCSSYISVTLSFVCNAFLICCVKICWFFSFKICFSW

RFKNSEYVTMTIIVLFI

>SG-AL_contig_7746_1_+1_271_513

MTYKLKEAATQTLQEKRENAQFVQLWKLTFISLTFSTRYKETRLNPVFFCFFFVCFVCFF

LRKGFRGKKYFQQFIALNRVF

>SG-AL_contig_7747_1_-2_149_1195

MADVNLTAILYKKDDLRLEDKPIPEPGPGQVQLCMQQVGICGSDVHYWTHGEIGDFVVKQ

PMVLGHEASAVVSKLGQGVTSLKVGDRVAIEPGVPCRQCTYCKTGRYNLCLDMKFCATPP

YDGNLARFYVHDADFCFKLPDHVSFEEGALLEPLSVGVHACNRANVGLGNKVFICGAGPI

GIVNLLVAKARGAAKVVMTDLDQSRLEFATKMGADQVIQVKSKDPKQTAVLVEEALGEPA

DITIECSGAPPSIRTAIYATRSGGCVVLVGLGPDLIELPVVNASVREVDIRGIFRYVNCY

PTALAMVASGKVNVKPLITHRYTLEKSIEAFEAAKRGEGVKIMISCVRK

>SG-AL_contig_7748_1_-1_577_2121

MTSYGSFSLAALLYRLVQGVAERWTLGSSVLMVFAVLTLFTYLWVTHRWTSSFERMRDIT

CPLPLPPGSMGCPFFGETLEFIRKGGEFYASRLRALGPVYKTHVIGSPIVRVVGGEYMRH

VLMGEHVTVSSSWPRCVRRLMGPHSLLGMSSDEHRKIKPLMGALFTPTVLSSFVPRIQMV

VSQHLDQWCQTSAEEGQVLGFPSCQRLSLDLSLEVTLGQNMDRSQDHSLQRAMKQFAENI

FSLPVAIPGCGMWKGLKARERIVEEIRHKLDMAEGRSEADFLSIVELMQTEPTLGAQRQL

IEDNALELFFAGYSTTSSALCSSLLCMGRNPEALQKVEKELEEEGLLEDRTKELTYDVLE

RLHYVYSCNSEVLRFYPPAGAVFRTALRTLQIGGFQVPKDWVVAPSIRETQQTSALFPDA

ERFSPDRWMHMSEEQRERSRFEYLPFGVGARMCVGRRLAILIQAVFLVEVVRRCRFQLKN

PHPEMTFIPLSKPKDDLPVVFHSRLDDCSNSHSSC

>SG-AL_contig_7749_1_-3_147_1871

MVENRATNSMFVFPMFRWVRPFYHYFIQHLDTSLPQHDEYREQRQRDLEEKRKTYQFTQK

APGMPVQIRTLPPDEQFSFEYKWDIMKTKMQLIATSKLKQIVAGTWKTLTDLMNVYTDKV

FYKPRGVDRWRDDLAFGRQRVCCQNNLLIELCTAIPDKLPVTDSMLEPVLQGLSIQQTME

QKRLFICDLHILEGLPVKPNLTLCVPICLFFLDTEGQLRPVAIQLFQTPGPDNPIFLPTD

DPLLWTLVKMWYNNADSAYHQALTHLGFTHLLMEGVVVSTHRQLSQSHPVFKLLAPHFLF

LIAINSRGMAVLVSENGWVDKTMNYGLKGMFELIRRGFKMWRLDLHGTLPEDLQRRGLDD

PEILPCYYFRDDAILLYDAIHKYVSSYVDLYYDSLDKLTGDVEIQSWATELVTPRDNDKG

GCGLLGVPGNGSLTSTDQLATILTSFIYTCSVSHAATNFPQYEEYGFPPNYPGLLRGQPP

RTKDACTEDDVLKSLPDLPTTLDIMTVTKILSQRGTKSLGDFEVQYIFDPRAMDILEEFR

RDLSRISQEIKERNERRPFQYLDLDPACIPNSISI

>SG-AL_contig_7750_1_+3_3_263

SFESAAIKSCLAHIFHGLKSCHYPNVMSSIEPFFTASRCSSLPWQTRDSVPHDMTSKQFL

AGERDFIVADKTRAMCCVFLQNMTASA

>SG-AL_contig_7751_1_-3_27_143

MRSHTFALMQSYFETNKQTNKTKQNKTNKQNPREFCLLP

>SG-AL_contig_7752_1_-3_36_221

MKNGKSPGTDGSGLEFYKFFWRKFGPFVEQALHQPLRDRELSTTQQEGLITCIPKLITQE

NT

>SG-AL_contig_7753_1_-1_220_621

MKNGKNPGTDGFGAEFYKVFWEKLGPFVVRALNQSFRDGELSATQQEGLITCIPKPDKPR

EYIKNWRPISLLNILYKIGAACIANRIKTILPNLICEDQTGFVANRYLGDNVRLIYDLIS

YLDIKKVTWTLTLP

>SG-AL_contig_7754_1_+2_1529_1762

MHAIPHTHMNAIPPSTTHTQSCHPSFDNTRACHPSLDHTPACQPSLYHTHTHTIPPSTTQ

THECHPSLYHTHACHPSL

>SG-AL_contig_7755_1_+2_140_403

MNIQSPFYVLFPTNHSKTFFCYCPMIEVNLYLFEIKMKRQEKAGFLLLLSSLLLLILVAK

VFCVSVCVCVCVCVCVCVCVCGCECVCV

>SG-AL_contig_7756_1_+1_1_270

MEQDMNKLNTNEDNLFSLSADAKKKTDFDSGFVNTVVTKFTFNPHTLSNTKGNNINKNCE

HKIPACMLSVVIKPNECSNMLDSTEKLGQR

>SG-AL_contig_7757_1_-3_114_560

MRVTIFTMAGKKLAQFSLSKELQFPNSIAVHNNEIFISDNRGHNVKVFDYEGHMLRTIGQ

EGVTNYPIAVAINQRGQVVVADNHNNFNITIFEKDGTLVGGFESKTKHAQCFDLAVTDDG

LAVLTSKDYKVYCYPYQEDSHHYIQNQRR

>SG-AL_contig_7758_1_-3_33_578

MGSVTDDDVIRRRLLIDGEGGNDDKRLNNLLKTFIRWYSSPEDEDSHNTHQRMLSMLAQC

EFTMEKSVQVHAMNISEQENYEELTKDIEKKIQEATEKITECKAELQEAKRIRKNRQEYD

ALAKVIQQHPDRQETTHQLQSLDKELKSFADTKEKLEEKLELRRKQFLVLITSIHELQRI

LE

>SG-AL_contig_7759_1_-2_2_178

FFLSLFSQIVFDIISASLQIICMKSTLCMYSSVALHPIYILKSCKSHCLWQQNVGLIQY

>SG-AL_contig_7760_1_+3_12_266

MSDCEQNSKIGGGFAPSVRVGLTGGNVVLAAKETPGTVEFRSPEVLEVSKGKKNERQEAA

SRIALKGVKEKAAEVAVALTLWALG

>SG-AL_contig_7761_1_+2_338_568

MRKKRFYLLIEQLNRLGIYIIIFHLVTESSKVHDMNECWLRAHPSGQLETACSQRLDTFK

NSVSSASKGAITELALG

>SG-AL_contig_7762_1_+1_415_654

MYICHTVHGRIHGKDVSIPEKLTLLSERTPENNVRISIMNGICQHQYENNTKYMCAKNVR

IKAFLDSPPSPCCTFPNHTG

>SG-AL_contig_7763_1_-2_299_3745

MPTLRRSKSLNDKISRQKNRVGDVVRDPRSPDVEGRTRTCVDNFQALVHLRKQTEDKARQ

LELAYSYATNGHTYLRRGPSGGVELPYASSVTSATKSSRHPDSSESKLPLNRRQHSAEGR

TERGVVRRQDSFSGTQQSQQHHQSRREQKRCARPMSMVEPRVGGLFPSQDELNRKDQVIQ

NLTESQRSLNVHNQKLLSKIDELEFENSQLRDILNAHHIQVEAFTQSRCASTRPHRSISL

HGDAMRIPSSSVPSSAVVSPHSETSVSGYGSLADSPSGDGFTAGSDVRVVPRTVGVGDYD

DDTLPLSGVSEVSLECKNDTLAKTSSSSSAASRGSRALTLNLDSDRSGHDSNYEEKVDGL

GYMDDAGGGEEEGCPRIEELAKQVAELSSQRDTVILRYKQLVEDNLKMVKRDKRIQEELG

GLLERHGETVEQVLRSELQIVEEQLKDLTEDVQVQVQRALIALDSPGYSSSESSVDAMDS

AVRDMLNRLVDLIGPDYQRLGRRLTVPQQELDKIEKMTQDASTQKNQDACTSKTRDTSTQ

KTKDTCTQKSPQNTCTRKTWDVLRAWLKRRGGADTDTVPPAVRHLSQALRDVDKDMVHCQ

HTPVSGPERQALRNCLQFALDHVADPRDVLDHLIALNELTPASISFVQEVMEKRERAVCR

LWHVSEYRGTGVVSRLALALARSHYPQVSHRLRDELEIQNSRVHSQCHPASEEPVPAPIP

VSVPISVTAASASRPSFAQARSRPMSRVPRFRSKTVDSGSQPVKSPPRPDSSVSAAAKAK

PPLLRQAPLARKERELSAPQKERPKSGVQERTVPAPEEKSTPSSKKAPEEKSTPSAKKES

PQAPQQQERRILPIPRPFSPSLREKPASPAHKDRPALHLPKERPFSPLGKDGDKTDRASQ

KDRPSPQTQKDRPFSPFEKDKSTRPFSPFDKDKQSRPFSPFEKDKQVRPFSSSEKDELPT

RPFSPVEKDKQSRPFSPVEKDKQSRPFSPVEKDKQSRPFSPVEKDKQSRLFSPLEKDTPT

RPFSPFEKNKSAAPAQSERAASQSQKERPFSPFQKERSSMKAVTSVQKERGVASAQKERA

LSRERVHSLSQKEKQSGAPVKERSNPQSQKERGTVWDKRSSVGSSAGAAPASGARNSVVL

EQGGRISRV

>SG-AL_contig_7764_1_-1_1360_1560

MVVDMYDLFVYQPRNSTTRFRVLFKDALRRCMDSFPLMCKFCWIFFRYRSDMLLSVQRMI

LMIVRFC

>SG-AL_contig_7765_1_+3_399_776

MDMLPFFYSFLVFQHNSKTAKAQSWKLQLHFSVFLFVCCATSGYCDLKSKTPAGLQNYMI

CSAGVFCSINSFFFCLGLVAYSFCQNYFCLLVNSLGLFIFPLKIRNVLKEGMEVQYYWTI

SVMWFL

>SG-AL_contig_7766_1_+3_39_215

MHSNSSWPISIMVCIYRSSNIPSLITRDYINRRRRQLITFIKTFIEVTYHSNISAKVCL

>SG-AL_contig_7767_1_-1_1_219

PPLRQSPPPPPPVPHAQDAALAAEVTDLLDKGAVEVLPHPPGPGFYGRLFVVPKTSGGWR

PVLDLSSLNRFLV

>SG-AL_contig_7768_1_-2_2_223

SALHDSQCSSLGWVDLCQLTCHMTFCIIWVRQISANSHTLYDGQCSSLANPCRTFCVVLL

VLGRAEISSNSLVM

>SG-AL_contig_7769_1_+1_112_426

MDVPMKDKDIEMKTTVHKVTITMTTEETSPTHTKNDRNEDITLPPGTMCPLSETMSIIHL

IIEIPICTLRKTVICTATVPPCQATGRDPLRPFMPDEQQKKTLIV

>SG-AL_contig_7770_1_-2_68_1168

MGELEKLPIWADILIVAGALLVLSCCVYCVCKRCCRKRKKKEGKKGLKGTVDLKSVQLLG

NAYKEKVQPDLDELPVNMEDNEDAESTKSEVKLGKLQFSLDYDFQKGELSVTVIQAADLP

GMDMSGTSDPYVKVYLAPDKKKKYETKVHRKTLNPVFNETFTFKAPYAEIGSKTLTFAIY

DFDRFSKHDQIGLVQVPLNSVDLGRVIEEWRDLASPDLESEKENKLGDICFSLRYVPTAG

KLTVVILEAKNLKKMDVGGLSDPYVKIGLYQGSKRLKKKKTTIKKCTLNPYFNESFTFEV

PFEQIQKVTLIITCVDYDRIGTSEPIGRIMLGCNSTGTELRHWSDMLANPRRPIAQWHTL

QEVPEKN

>SG-AL_contig_7771_1_+1_85_183

MFANSDTFIVVVVVIIMPKLYKVYPLISTRSLG

>SG-AL_contig_7772_1_-2_2_187

FSFFCFVYVFIYLFVMGLFSTMLPLFSVSELRKHLLTHTCTLAFSLDRLQQVWDLTHILI

IK

>SG-AL_contig_7773_1_-2_539_1240

MSTLSAFQKEKLTYFFKFFEPNESNELDDKSLEHFMKRVLEYTGWDLQSEHSRTMREVHQ

AFFEILFDKMSDGTRASCTVSLDDWLSVWSNLLPGCKGIANFPIWLRLLPKQLFRIIDRD

NDGKITTEELYNFYMHMVKLPVDEARANSHLAFAQMTDNGHYPLNLPNFEQIFSNFLLGQ

TPYGPGRHIFGCFEHQVPKTPFRLIQPAPDPDDFDMDVKVYLEKRPKRMSLTGI

>SG-AL_contig_7774_1_+3_3_164

KKKKIFGFSDLYGRSTVDWPHTFKINGKKNRNASVHSKMKEVRAHFAKHTVFEP

>SG-AL_contig_7775_1_-2_116_1063

MSTMNQKPNEAGWFYHAPAKKENREPVGAPPASQIPGLGEITVLPPEEPREMVFRDTDSK

YIQLAKMGGRKDLLTIRPNSGTKREAKGYPRSDWFYLEDNAQQDAARQDKDKEPWKFLLP

DYMVYQSMGETTLPVENPDAPREGIPMRRAPYAVDEQLPSKGKSTSMKPPHTRRPGCIPK

TERGRPHSSQDDSPRLKYQPLPKEKEEPTNMTKLLSYSYEREWHDKHQAWEEKQAKNRDK

SQLVKSPELNKSSRKSPTAPQKSGRKQKYDTNNNHMPTARKPEEEKKEPFKLSKFKNVSA

RSDTHQRPEVLAAMGK

>SG-AL_contig_7776_1_-1_253_603

MSDRKTCFSVGTGVSAGLCLWYYLLWMMTLSQVDHHEAPLASCQERQNLSTCIPGLRKME

LDGWPEAMGMTAVVTDDTASHCAVVLGMNSLCLGAAKDTPIQGSSAGFTKLIHCQCS

>SG-AL_contig_7777_1_-1_337_540

MIQLSSFAERRERESDKGCPITIWGAMWKKPCYSWLSFESLQSDGKIRSLPLPTMPRRQR

DGGSKLDQ

>SG-AL_contig_7778_1_-1_1_174

SKLPFRDADTKEWTISNKVNLYYVEKLFLSFSSCQPFLKILNQTYFYSFITNYKHKYY

>SG-AL_contig_7779_1_+3_3_86

QNFHRPQSFFKDLKLLLLLYIVYSPADR

>SG-AL_contig_7780_1_+2_314_697

MDRGCLSRLTAGCSRPTAGCSRPTAGCSDVTAGYSKLAAGCSGVTAGCSKYTAGCSYLTA

VCSGGCSKLTAGCSDVTADCSKLTVGCWTIVGVSYCKTLFFAWQVAPRLGESAEHFHGLS

KFSFSLQF

>SG-AL_contig_7781_1_-1_73_339

MPDNLDRLHRKKNYFSLIGCDMRSEYSQHEGGDDDDSSDESELSMDYHREKSLSTRASRV

TLATLDQMIEEENLTYAEALWDHVTMDPD

>SG-AL_contig_7782_1_+3_297_560

MKTKLKCVNKSTVKFKNDLKKKKREGGTKLFLIKLHRRQEESKQERKKERKKESKKARKK

KKEKKTNIKMKQRMKDQTKSKKHVSPPL

>SG-AL_contig_7783_1_+3_3_179

SLSLSLSLSLFNTHTHTHTHTHTHTPGVDLNISDFFRGIYNEVNSKHSYTHTTLKPVCE

>SG-AL_contig_7784_1_-1_58_723

MAHVQNETIEDVASAGPGLAFIVYPQALSLLPAPQVWSVLFFFMMILVGLDSQFGNVEGV

MAALWDVFPRWYRHRMLVLLIFSVGAFVLGITMITQGGMYVFQLIDYYVSGAYTLQILSL

LEVVTVGWIYGANRFYDDIESMIGYRMSVWLKICWKLLTPASLTVIVVFNLVTYRPLTYN

KTYIFPPWANGLGFLLTIIVLILIPLYLVLTLVRTPADTLRD

>SG-AL_contig_7785_1_-1_100_288

MYMYWIGSILIPVIQKSGRFFGATLHPLPFYLVCIVTSSFWSLSGKGVRVCVCVCVCVCC

KSV

>SG-AL_contig_7786_1_+2_521_718

MTTQLTDTSPQNRKFQHSHTHTLRESESISLRTMNCVQILNRTIRALFRLAPPTLIVHVF

DVEGLN

>SG-AL_contig_7787_1_+3_21_263

MNKKANVPCVSNTVFIIQSLLRDKITSKHHRRIHEKAMKTDWAHHPKRKGQHHRDCPSRA

TIRKMQEKTQEHMATNCRRRT

>SG-AL_contig_7788_1_-2_134_1036

MNILPKKSWHVRTKKNIERVRRDEENAAEEEKERQRRVALAEQEARTEVLRERTKKRHHH

QIEASASCPSLTTEPASEPDRKMVRSSADITLNQGGHINFFRDIEAGLCREGSNKEHQAE

RAAEKEKWEKNVGLLTYLGQSAVESQNDKPWYLQKSEPKKTSPYEQKEKKREDHDRKLKQ

SMDPLREMKRHMDKWHKHKHHSAQKTGHHSKRKQTPKADSHVPSCSSKSKTSKTVEQLRA

ERLRRELSERQKTAAVMARARGEVAPEAAHNHVSDADRDRSRGYNSRYNPDFVRPVSRHR

R

>SG-AL_contig_7789_1_+2_1865_2035

MISLSTCLPATISNMHVKDPVSHVSGWLTETRKHPACTPRTSCKKGPTGPNAIILVK

>SG-AL_contig_7790_1_-2_809_2704

MAPRRICVTWLLASTMVSLIHGLSISSHEVPTSAHEGPILSYKGPTPSQEGPISSTSRTD

QREQRHKVSTTTSQLPSTSTLLPGDNDDVNDAEADAKLAADVLHKFGYFRKGKPLHLQLE

SPEASGHSRTERDAQNEAIRMFQSFFGLEQTGVLDKATITLMKKDRCGVADIPEEKVEQT

HHLTRDIQSPSDYSLLGTKWDRTALTWRLEVASNQLHPNQQRHTMEDAFATWGQVAPLKF

VEKKNRGKVDIKIKFGRRHHGDEYPFDGKGKILAHAFGPGETEVAGDMHLDNDEKWSVTQ

DHGSSDLFLVATHELGHSLGLSHSRDPSALMFPYYGLTDTLGQDDIKAIQSLYGIRIGPP

EVTPKPRRTTTTTTPKPVTRFPWAKSATYTDRKRPINKPRPRCGKFDAVIQDNPMNKDIY

TGIRGDSVYRFNQKGVMAGYPIALDALYPGAPSKADAVFTIAEKSATYFIKDNKVWRYQG

YRLDAAYPKTLNPSHFRETIHFVLPVVDTSGIQRIFVFGSTYWLEYNFDALPSYTPHSHV

ISQYWPGVGYDVIYAVTGYDGHIYFISPNSHVVLDSQFQRRAMYGGKQEGHPHWLQPACH

MTGGSSGLSNRRSFRSTVVVTIFAILMCALRK

>SG-AL_contig_7791_1_-3_723_989

MLTFIGHGLFILWYVIYFILPLLLGFYIKKNIIVIIYFVVVVCLFVLLHFACVLNNVFSD

AFITLFWLYHGIFSKCLQVPVGMSGALSV

>SG-AL_contig_7792_1_+2_1508_1807

MTLVSSCILSRLDYCNALLLGVPAPLIQTLQREQNSTACLFYKSSELLKQLHWLKIKQRI

QYKSACLCYKITTHEAPTHLSELFEMYTTALFPVPSFLLR

>SG-AL_contig_7793_1_+2_2_271

PPPPPKKPPTPPFKVGGGEGGGGEKKKREREREREGEGGGGWGGRERERERERERERGER

FQAGQLSLTKISIRYTHTVGINTTFKRFVK

>SG-AL_contig_7794_1_-1_1558_1851

MQLIALKLPLRENSKYTDVCMPFCCFLFSICVSLFGIYFLCIGKDFRRLVEENKMCLIFL

ILCLSPVPYHAEFQSPKVLWVVMFVCVCVCVCTCTCLC

>SG-AL_contig_7795_1_-3_933_1145

MSIQFLDDHCVQACVPPPLASSIHSTSLAILLTPGKNQQAATFPSLHVQSGGVPSPLTLF

SKSSFLFLKGK

>SG-AL_contig_7796_1_+3_3_341

WTYTACPKTLWAGHVHIALGAGQSGRPQHVHIALGAGQSGRPQHVHIALGAGQSGRTQHV

HIALWAGQSGRTQHVHITLWAGQSGPSLCVYSTSQGLNSLQLLSDETSSVLTV

>SG-AL_contig_7797_1_+3_627_839

MQQAKEHQSEARIQLTMPQVWNKNLIQFHSHPSPKSKSRKCSSIVTILQIKQTAEQNKCK

RTTPFPFTQTV

>SG-AL_contig_7798_1_-3_264_428

MFSLLLKPSLFCQNLNSADIQSFISVDYFCKMYVNFKLNIAKPVNVKQQLKMAWQ

>SG-AL_contig_7799_1_-2_1079_1273

MTLLHEIWIGWLFLHSFSHYVVLVFSLCLFEVGSFAQSYTTSFFELNRNENLSCFEICYL

FVYLN

>SG-AL_contig_7800_1_-1_88_396

MYIKQVIIQGFRSYRDQTVVEPFSPKHNVIVGRNGSGKSNFFYAIQFVLSDEFSHMRPEQ

RQALLHEGTGPRVITAFVEIIFDNSDNRIPIEKDEVVLRRVIG

>SG-AL_contig_7801_1_+2_2132_2530

MVNRMQFSLLAQNCVSSRFKNTVLTHRLDSAAIPFFFLASGRRRTKGTLWVFGAASPCLS

FHSTHSSLSFLRAHGRLLCVCCILLCPCLILRKWDKKKSLLHYKTPVRDIFLYSFVNYFR

LFVFFLLVFPFFF

>SG-AL_contig_7802_1_+3_3_203

KPSSSKYSVLTIRPPRLSMAERFETVVPWWSNSQTECQQENQLQACSVSFSRHLAENRIN

HSRRSHL

>SG-AL_contig_7803_1_+2_146_295

MAYAYGAYGIYSERRNVILRKLKLKLKLIHQNDKAEKRIASVREYEKSRP

>SG-AL_contig_7804_1_-2_2_247

GTPPLPGPLRQVLPETLGSEAFFPAEAPHLHQRELLEGHLSDHHLSLAVGHHFEGIQRSG

FLPPTGQPQSPRTQGLPPLPPH

>SG-AL_contig_7805_1_+3_3_179

FFFSSFKFNKTKTLSHNRFIKLKILQDIYGYISSLSPSLCKECSLFPHQISTWRATFLK

>SG-AL_contig_7806_1_+1_1_168

KKTHPQQTHSPNIDLDDKNYLAHTLQDSIQDMLYIPVKDICPPGDKNHSIHTCVKI

>SG-AL_contig_7807_1_-1_106_582

MSVDRHQFGSFLFMLLCVFVCGGRGRGVGLELEIAFSTCCFSCLLKPTENYVTCAHLYFP

SYGTSQIHFHMHLSHVPPGFIVVRVHSSQFCVPFIYSWYECSLRERRKRKREDKGKVTSV

PFFLFCFVDASSSHICGTGYAFFFFLKKRSIAFFVHLGP

>SG-AL_contig_7808_1_+2_170_313

MATIDLLVITYSEDHPTSMTSLSLSLSLFLSLSHTHTHLAWKTLRKTS

>SG-AL_contig_7809_1_-3_585_911

MFAVCIYRDRSILMTVWGLFSFFSFFFVLSSVFSYTLLALTVQVLCCYTGCILHLQWVNP

SMQANAFFVGVHAWVFSCFHQPPNADMAYRIFNMCIWCSACIYTGRFRH

>SG-AL_contig_7810_1_+3_42_182

MKSPVTDKFRNLKFDRHLCTRQLQCSFSLSLSLSFSPRVYDKLCSMR

>SG-AL_contig_7811_1_-3_1482_1766

MLYRPLQQEVAKNCCCLSVDCVNVSDIHQTYGLTSPSPQLTPPSPQLTPPSPQSMVPIGV

REGKSTFDFSSFRGFSLSRSPRTSQFFLSTGLVCQ

>SG-AL_contig_7812_1_+1_1_210

SPPPPPPPPPPNMKTEITAVVQQENSETPHEMYTPHLTVCMGSVGVLYTAQHLEVLLQRH

DTMLKLTKSI

>SG-AL_contig_7813_1_-2_2_181

HHVQCILAVFSVCVILWFSIISVVSEVFCCLMTGHCTDMVVLEPWISTLALLTRYAVHLL

>SG-AL_contig_7814_1_-2_74_310

MLDVSLGCRLLVFCPLGFHIICASLILSRHSGLYDLSGASLPDSVLPPSLRVHGIFLLRR

CVLFCYVFKCLGSLWCIFA

>SG-AL_contig_7815_1_-2_23_757

MDSTIHCWNIPSSNIDPYDSFDGSVLQDTLLAHTDAVWGLSIHSSKMQLLSCSSDGTIRL

WNPGTKSPLLNTFVVETERGVPTSVDFVRCDPTQLVSSYTSSNAYIFDLETAKQVMSLDT

LLHCDTSGGNQINSVLSHPTMPVTITAHEDRHIRFFDNNTGKLIHAMVAHLDSVTSLAVD

PNGLYLLSGSHDCSIRLWNLDSKTCVQEITSHRKKFDESIHDVAFHPQKPYIASAGADAL

AKVFV

>SG-AL_contig_7816_1_-3_381_737

MVLVHCSRILAYILPTKLRRHIPLYVLVFLVPFLFPYGQYNCALPVFWNLHFFPDLLEYD

GKPLDSASPQATTVSVVMPSLPAALFFGLVFNALLISFSRMFGSLCSGYFGVNWSLMLL

>SG-AL_contig_7817_1_-1_103_390

MRISIGKTEVMKVCRRSGDLNIYIEEAKLKQVAEFKYLGSIFTEDGRLNREIETRVQKAN

NISYQLAPLLKHPDIPMETKAKLINSIFTPTLTYQC

>SG-AL_contig_7818_1_-2_29_256

MRISIGKTEVMKLCRRPGDLNIYIEEAKLKQVAEFKYLGSIFTEDGRLNREIETRVQKAN

NMSYQLAPLLKHPDIP

>SG-AL_contig_7819_1_+3_543_812

MAASTSIIYLHITAASTSVIHLHITAASTSMTMSLWTYNRCLVNSFLLSSTHTHTHTHTH

TLCCNPAQSESFSQILSVFFFEDGRLSYLN

>SG-AL_contig_7820_1_+3_561_851

MKPGPTISVAFNISNINHFVEAQIQTSITLCSNSNINQLVFSQLDDHVYHFCFAAFLGNL

QLPIFISDFFLDQLSSHQDKASYYFLILDFVELPPRP

>SG-AL_contig_7821_1_-3_81_1373

MEAANSSFLWSEENAPTEESVLGEGEAFRQADLDPRHEKTGLTQNTEGLLVQTPTLPKQT

KKGGKARKVPPSPFTPMPPYDNMTTPALKNEVQKYGLKPVGKKRMKLVLKDIYHKTHQYE

TDSDYEEESHLPDLGPPILSREEDQTLAAKQPPVLHKQSKLSKPRGAACKAGPPPLLHKQ

QKQGKLHAAGPSRKPPVLAKEPVVGVEKEPPVLRREGDMNKPGKRPKTKRGLAQKPKEPV

KALSTKRGQKKLQEAVTASQPADYPVHPLYHSPSKGMQEDMGKQKRVHLASPAKSTEDCS

SSPEKGFFSSQDSDASERPDVSVFEEERLGSDDDEEITPSQQVAAKDLPERLMVFFQNHP

LLYPKVLTYEPLDLGVVKEDLKAAGIKCSHDKLLDFLDEKCITFTTKNMIPPKRRNRGRK

PQSRKLAASQK

>SG-AL_contig_7822_1_+3_69_320

MKRRKSYLSINCSSLKVVEEYCRDTGHKISRKNTEGMARQSKSSKRTIRGALEIMQHREV

GYKLHESLVVPTRWLAGERFLCCF

>SG-AL_contig_7823_1_-3_3_524

KWFKWCKEHNVPHIKPSSLQLANFLAYLHAQAGLSASSVRGYRSAICTSILQLGGPSFES

DPLLRDTVRGAALRCARSPRRVPQWDLLLVLDCLRSPPFEPLSGISFDLLTLKTTFLLAL

ASSKCRNEIHALNGTLPFTGTVRSPSGSSRNSLLRPRFRETPCLLSLSGPCLTL

>SG-AL_contig_7824_1_+3_1530_1931

MYQYLEHIQNIKISRRLMMNTNRRWTLLKLCICVYHIILTCASNQTIPNWTYMNGSLVSL

QNSQQKKIVSKNHHSEPQTKLWSFIHGLSSNTIKEWLQAWTLPLVRFQYTWMAFDMGSAL

DLMFLSHLRSSLCL

>SG-AL_contig_7825_1_+2_161_2140

MERDGLRHATLPKTRNMRMKTTQRIIPARSSPGPRTASFVGRIHQDGGGNYATGGSNKTI

RARNKIVIGTWNVRTLREAGKIEQLEYEMSRYRWNILGLCEVRMKHFGETLTSDGHKLYH

SGREDKHEHGVGFIVHKDTADAVLGCRPISSRIMTIRLKAAPFNITIIQAYAPTTDHEDE

EVEDFYNEVQKTLNEVPKKDIIVVQGDWNAKIGEDAQEDWEGTCGQYCNQATNDRGLRLL

EFAKYNDLKIMNTFGPHKPSRRWTWHSPGGEYHNQIDYIMIKRRFQSSVNIAQTRSFPGA

DVGSDHELVTMTFALRLKKNKKRGNIRIKFDVDKLKDPNILTTFQANIGGRFAPLLVLDN

DQDLTPDDLVETFNETLSEEASKLLGKPRVKKKWMTDEILALCDQRRSLKKRKKDPEGGK

LYRETNLKVKRSIKEATEKWIEDQCEDIENSLKHNNSKKAYKIVKDLTGTKQARATTIES

KEGKCLTEEKEILERWTEYCSELYTHVATNKDPNVLNVPPSSNNARHSILRAEIIEAVSS

LKPGKSAGIDNITGEMVQAGGEATIDMLLLICNKILQTGVWPKPWTQSLVITLPKKGNLN

LCQNYRTISLISHPSKVMLKVILNRLKPEAEKIIADLHHVFIDFKKAFDRVWHEALWSTM

>SG-AL_contig_7826_1_-2_296_1606

MSTPKRESYEKAEEQQRRKTSARSMHSSSRQSGSSTNTGVLMVGPNFRVGKKIGCGNFGE

LRLGKNLYNNEHVAIKLEPMKSRAPQLHLEYRFYKQLGQAEGVPQVYYFGPCGRYNALVL

ELLGPSLEDLFDLCERKFSLKTVLMIAVQLLTRMEYVHSKHLIYRDVKPENFLIGRQSAK

KPHVIHVIDFGLAKEYIDPETHKHIPYREHKSLTGTARYMSINTHLGKEQSRRDDLEALG

HMFMYFLRGSLPWQGLKADTLKERYQKIGDTKRATPIEVLCENFPEELATYLRYVRRLDF

FETPDYDYLRKLFTDLMDKMNYECDWQFDWVGRQPSTPISVAPDAVASQQRESGAQTRER

HPSQPRAPTNVQRGHHAWGEQPRHNHYLGANIAADRHGGSVQVVSSTNGELPTDDPAVHS

NTPITPQPDVEVVDETK

>SG-AL_contig_7827_1_-2_2234_2548

MFRGLFPEIYYIYIIIIKLFHLTQQGSNVFMIFFIFLSITWLLLSIFVYSFALKSQVCQK

LQSIICFCIEQGLQFLFVFICFGSCQCALATTVIMFSLFVILPKF

>SG-AL_contig_7828_1_-3_162_362

MRACVHVCVCVFMRAGECMCMCLCVHVCARAHVCVCVCVCVCVYRSSFLLIVNFKGRLFR

WDRKMGV

>SG-AL_contig_7829_1_+2_2_172

FETHTHTHTHTHFSIYKTMFTSHVSRSIPRPPDPRLFFMIFHDFPVPSLLIIWLFIP

>SG-AL_contig_7830_1_+1_1_195

EGDQENGFSVQVLQRRVLSGFEGGIPKGVYSLLLAPTHPFFPMRRVDLECTRSMRKYNEP

RHVWR

>SG-AL_contig_7831_1_+1_1_78

LLWTPHRGTRTLAHTLSFMPVSFIKE

>SG-AL_contig_7832_1_+3_186_338

MMSSDSTRSQNPCPSLTLSTSFLPEEQEKQASLTLSGRLFAFLDACMYVVW

>SG-AL_contig_7833_1_-3_309_485

MEWRWGRLLSYCQVQCTEQTFCKFVCNITIRFGFFVMFLSCHKALNCGEYRYLNAKKKK

>SG-AL_contig_7834_1_-2_110_262

MVWYASICNNRQVWAFQKTGMRKMFHNCKPITVYCGKLSSTVAATRMVHAP

>SG-AL_contig_7835_1_-2_2_430

VCSVGVCVCACLCGCVRACVCACVRASVCVCVCVCVHTRERVCVCVCVCVCARARACGVF

SENEFQCVCVLCVCVRVRVCMYAYMRVGEYACVSMHKCGLSGVNFLFGWGFFYACVRICV

TCVGARECLQKIIFNLIHPHVIV

>SG-AL_contig_7836_1_+2_563_799

MRRIKKEREKNKKKTTKQTSIKTDRQKSVSTREYTERSKRNIPVQTTMAKIIMINTIITI

KVTVTTTTAAMTVVIMVPI

>SG-AL_contig_7837_1_+3_207_401

MLLHRLLFTFLAAPSTLFQCTTSRRSSRRSDTPSTLTSFRWHLAIRDPDDLSIGSCHETT

HQKRL

>SG-AL_contig_7838_1_-3_42_239

MLGVFLALDAVGICISWYCVPSLEQPRYTGREVLRKLRSYWLAFRQPKLWLLVPLFGARG

IIFSLH

>SG-AL_contig_7839_1_-2_11_556

MATETAENTSGIPQADFVEAVDEFMKKPENESAEAVLRRFDELHSKYKFMEYNLNTKKTR

LKSQIPDIRTSLDIVKHLQSKKDSEDPLESRFLLSDHLFAKAKIPPTDKVCLWLGANVML

EYTLDDADDLLEKNLTAAVKTLAQVEEDLGFLRDQMTTLEVNMARVYNWDVKRRQAEKAV

KS

>SG-AL_contig_7840_1_-3_3_1199

EKKEEEEEKKEDPASAFATRFKKFREEDEEEQLDFGETAEEETKEEEQKEEEKKEEKKEE

DPAAAFAKFRKSRIEDEEEGFDFGESKEEETKEEETKEEEKKEEKKEEDPAAAFAKFRKS

KIEDEEEGFDFGESKEEETKEEIKEEEKKEEKKEDPAASFARFRKSRGEDEEEELDLGES

KEEKTEEEKSVETKAESTLQEEKEEEKEEDPADLFAKFRKSRMDKEEELDLGGLKEEEET

VSEKEAEETKEEEKEEEKKKASSIRTPVLRRRYIYDIEEEDEEEEKPSVEEKKEEESPAE

RLRRLRAERQMEEAAEEEPVPTRRRRQVVEEKTEEEEKPAEDEREARRRRRREEAAAAEP

EDDDREARRRRRREEREKQEQPEDEDREARRRRRRARDE

>SG-AL_contig_7841_1_-3_3_161

ITVCTCTVVCVCVITVCTCCTIVCVCVCDYCVYMLYSSVCVCDYCVYMLYNSV

>SG-AL_contig_7842_1_-3_3_206

PPPPPPDEVDKCIEDTSLVRTCKLKFGSSYHQTTKKSSQSHFVYTLVGLNGLKSWTSPPP

TFMADKIG

>SG-AL_contig_7843_1_+1_10_279

MCLYIYIYIYIYIYIYIYTHTHTHGQHVMIYSFYNEQMMVPFLMLVLQNMIFNIFKCTDC

DIQHFLNYLQCVIDPFLSLMGSFYVQECTV

>SG-AL_contig_7844_1_-3_102_737

MISPLRTRPPTPCPLLPLTPAVRRACGVISRPSGVGPTPGLPPCCTPCQPRRLLLLRHLC

RSLPSRTRGQRRPPPLFKSPRRFPRGAGAALLGGTAAGTGPRLVGGVVSTPLLAPSRRRT

PTPPPPPTAARAGVPLGTGALAARGPAARGPAAGAPMAGVPGAGAPTAGVPGAGAPMAGA

PGAGAQSTPQIGTPPLLTSCDSSCQILCRLRQ

>SG-AL_contig_7845_1_+2_191_307

MKQNHWCTPSHSKEYEECHVFDVSIPKTDDDKLQNVLKT

>SG-AL_contig_7846_1_+1_1_153

ESEKEKEIMSMRERKRERERERERERERERARFLYVFLLIYTIVYILHVHI

>SG-AL_contig_7847_1_-1_40_741

MVEISTLPYPVCFDVDGQTGEAFRLLRDPEIGLTVNIEMGQGQRKRDHPLKHGSVYRVYI

FRVAVILQDIVMEVTRERIHVHGKTFHWNVTHSVRFPGVWIMMTESGVLRFVLDSGVEVD

VKKFIHPASNLLGTDFLDVEIENEDRLSSATDGLLGQFVHKKITMTKLVNKGNKMVAHLR

SRSGKKRRHGSAYLKERKDPFFNRTTPCLNLRSRGTRLLDHPTKAYKVRNLFYV

>SG-AL_contig_7848_1_+1_919_1170

MTIILPGPMTGRTDIHFHKTDDSVCKVLEPQEKNLKKKSSRFSGRRLKGKEQKQEGGRKK

KKEGKTVKEGEKHTSCKWVGVGIS

>SG-AL_contig_7849_1_+2_1523_2173

MADECDDGTLKEELIRDRIAVAVADDDLSNDLQAKAKLTLPEAVQISRQAEKRKQSQPIR

KRKQSQPIIRASERVDFVHKRRDSQLHSTNSKSPQNSKSRQFGHCKYCGREPQNATSSSC

HKQGYYKIVCRSTKDRQVKKLVEEEDSYSSSDGFLGYINSIQDDDDSWSAQTLLNGKPHT

FKLDKDAYVSVIGESWAKDKKLVKTKKSLKGPGGTSL

>SG-AL_contig_7850_1_-1_94_504

MRVTDFVSEVPQHVKLMDILHLCGLCSKSHQEVIGRYLTQRLGATTSVGRVEIVKQLCNS

RETKLAVLGCLLHRHSAFISPSSTFQVTNSVHFRASFDGARLSPDVVEELLDCVLSFLNE

GEMAEALNACWKYSYWP

>SG-AL_contig_7851_1_-3_2166_2636

MILYTLNNVFAMLRPQKLGKYSNMIITNVMTMMTILYSNMIITNVMTMMTILIMIIIDKG

TRQKHSVIHDHTHTHELLHVALPILFGCCSAVPCFLFICTFIELQFTLTSNKEGCLIVWN

VRTHIFSIGNVNFHVLQVFHYLLPGIPCGLVFWMASV

>SG-AL_contig_7852_1_+3_438_710

MTFQSVVRCTNHFTTLTGSIAVPIFSPGQCTQIHNEPFIWFWIKHQPTSLWLDWSMAFCS

CISSVHPSTPSAYSPASCRNSVTMDHLYNST

>SG-AL_contig_7853_1_-1_4_546

MSTTESQVPQASQAPAVPTVYGGVASVDGNFTSVTLGDGDGTVEKKKKVPRRILHFSDGT

LEEYSTDEEEEDVPDQPLVDPKTLKWMPWVWYYVSTAAYKTWSVADFCGEKLAWWFGITS

PKYQSAIDEYYRRQEEEEREKEEEEKNLSRTTLNTELSVSSVNVQVVEDEKVVVGDSLQK

Y

>SG-AL_contig_7854_1_-3_3_65

DYRARLELVKTLDSLCTAAKT

>SG-AL_contig_7855_1_-3_705_2057

MSENSSDTSNYTPLECDADTSGEAKLVQETTSSSSELSSEDVAECASQNAAKAERDQHTA

SISEDVPGDDDDDDSDAVPNNDICVGNREGESDLVQAVRQNHLDRIRQLIRSGRDCNAVC

KSEGVPCTVHVHREGFRKDLQMMGYAPLHVAAIHSSLRTIKCLLTMGAHINVRDKNERTA

LQLAAAVNRLQVAQLLIESGAEINTRSISGRSPLIEAVVNVNLDLTRILLGAGANADLTD

IKGMTPLCAIISNMAGPSRNLPTGQAIVQSLIDAGCEVDYTNPAGASALMLASGLKITSV

IPMLISAGANINLADRAGRTPFSMAMEGQRNLTTIKTLMEYGAATLQPDCKGWTPLSKVL

KYGAVSILRLLLSVDAVPHTYDILTAPRIIQLRQYLPEFDSWLQEEFYQPRSLRRLCREV

VRREMSPHNLPKVKCLGLPRTLNDYVLGATD

>SG-AL_contig_7856_1_-1_1_165

LDFLFHHMAFAGSVHMKIFRDRRHYLENTETETVLYCIVLYCIVLYCIVLYCIVN

>SG-AL_contig_7857_1_+2_239_412

MDEYLQARGLFIRITYHPFFCFLYPLIVAHTMCVCVCVCVCVCVRVRAQERERERERE

>SG-AL_contig_7858_1_+1_988_1224

MNPICSQLTVNFSLKKTTTYFICISCNNKHLNNLIIIIIITGFHQFRRTMKFMPLERLQD

TSLGKVLRRTCCWPRSRPT

>SG-AL_contig_7859_1_-2_1214_1519

MLIWKLVCLVWVRFSILYVSAFHCFVLHSYDITVSAILVRRFCHPLALISMIRTVNYLHW

SFLFCFVFCLFVCAAQSFALLLYRYPHATHFGSATLLQTYRN

>SG-AL_contig_7860_1_+3_369_668

MMMMKAYLQIMTLLLCMMHNYHKDLLLKTTISYIIIIVVSHTDLPRVPVTVYFKVGSGTI

YTDTTQYLHSTYVHCKRHNQVLENNRSPVHYCKLRINLYV

>SG-AL_contig_7861_1_-2_68_205

MTDFNLLYIHRSDSCINMQHYFQCALKLLNVACFWLYYFVYLSTLK

>SG-AL_contig_7862_1_+2_236_424

MVNQDSRVIFLDTAIKWTSNWALFLSALSPFSFINGKDCFSPIPKFMKISTYRNYILKIH

AQV

>SG-AL_contig_7863_1_+1_1_144

SLLTHSVVRHTQTVCQIQCVPHYLVCKKKSHKCANKIFYKYAEPKEMK

>SG-AL_contig_7864_1_+3_90_272

MQYSTLAHWMIVPHLEAWQNCFLYPFQYLSKMSLDKSLNTQPNEGSANTRKTVTNQRGYI

N

>SG-AL_contig_7865_1_-2_2_247

PLSLQLCNTSIPISDFAKNLGVHLDSTLSMQNFASQTAKSCYYHLRRISLIRKHLSTEAT

VRLVLCFIMSRIDYCNSLLSGV

>SG-AL_contig_7866_1_+2_179_340

MGNTIISNSLLVNFQFHILFNHIYVIKCDISLHVLFIQLGSLDSGSKYTSFKIK

>SG-AL_contig_7867_1_-3_126_299

MSLFAWHWLSGQVHNPQVSYCDCWITVILIRIENVLYHLLILSVFSCTIKKFWTSKKK

>SG-AL_contig_7868_1_-3_291_422

MFGTHAFSHDILLMFKIYRHAFVKFQNLWVGGQKDINCLVCKSL

>SG-AL_contig_7869_1_-2_2_529

CSSDLAARRIARSAVDWAAFKERVPLGQQEFYRAFKAKNDNFINKVHQFPEELPKIDFAF

YKARLPNPALADQFQKAYEAVNVPYPTDKSNTIKTIDDEEKAAAECTKAYVAERQEEIND

AKLLLSKIDSLPKPEEMTMEMYAHYFPHLALDPVNRPSFWPHLPNMQPGHKDSHNI

>SG-AL_contig_7870_1_+2_122_262

MCPGIQTAGVFLSVCLEYGLSDPMRDRTCSSVLAVALIHGDEKSPLG

>SG-AL_contig_7871_1_-1_1_237

ETFSSRFENLRICLKVYSVVCRLEVSYGSGSVRSLKDHIIGKRHKERVKTAAKNPVLPDM

ILPLPSLSLKEFCHCLCLI

>SG-AL_contig_7872_1_-2_311_2617

MYKRAIEIQPNFPDAFCNLANALKEKGQVDQAEGCYNQALTLQPSHADSLNNLANIKREQ

GHTEEAIGLYSKALEVNPEFAVAHSNLASILHLQGKLLDALGHYKLAISISPNFADAYSN

MGNTLKEMQDLQGALQCYSRAIQINPAFADAHSNLASIHKDSGNIPEAIASYKTALKLKH

DFPDAYCNLAHCLQIVCDWKDYRDRMIRLTEIVQDQVDKNRLPSVHPHHSMLYPFTAAQR

KAIAASHANLCTDKVAALHKTTYTYSTDLRASGGRLRIGYVSSDFCNHPTSHLMQSVPGM

HDRSRVEIFCYALSADDSTNFRGKIEKEAEHFVDLSKIPCNGQAADRINEDGIHILLNMN

GYTKGARNEIFSLRPAPIQAMWLGYPGTSGASYMDYIVTDHVTSPLELCDQYSEKLAYMR

DTFFVGDHQQMFPHMLRRIVLRFVDQHSGVVVNWIFNGVDLDALQQLAVSVKEEVQASLH

GLMTDAENGGAGVLRTTVIELPLNVIQALQNMITTNQSQCNIGNVLVQNGLTTNQTNTKS

ATGEEVPRCVVLSARASYGLPENAVVFCNFNQLYKIDPDTLKMWVDILKQVPNGVLWLLR

FPAVGEANVLQTASDHGLPAGQIIFSNVAPKEEHVRRGQLADVCLDTPLCNGHTTGMDVL

WAGIPMVTLPAETLASRVASSQLHCLGCPELVAHSRQDYVSIATRLGTNIAYLRAMQAKV

SLARTTSPLFNTQVYTENLEDLYYRMWNQKQLHGQADHLANDNDLRSFS

>SG-AL_contig_7873_1_-2_38_871

MGRTRNFDPNEKPPDLHWEDDTVCKYFLCGFCPHELFTNTRADLGNCSKIHDDALRKRYQ

ESSRFEKMGYEEEFLLCLQTLISDVDRRIRRGHQRLALNNSQNTLTGGALAEKEEKINVL

TEKINTLVNQAEELGCEGKVEEAQGVLKLCDQLREECIQVQNHSVKDDLSQFKQMEVCMV

CGAFLVIDDAPQRQDEHIMGKQHAGYARVRSEIEKRKMQKKMKLDEREQKRRQDLEEQEK

ALEKECEGSCRRERQDRDYERGRYQDKKQRSRSRSHGR

>SG-AL_contig_7874_1_+2_2_115

FFFKATVTGLYFHNNVMRLYSNLLTTAQLLNFNKSRPL

>SG-AL_contig_7875_1_-2_2_106

GRSPGEESKTKVIPSNFFGVDDDVHHYQPDTVIAY

>SG-AL_contig_7876_1_+1_1_210

LYRLELYCITLYCLYLHCVVMYFTVSSCIILYHIVSSYIVLHWTVLYLIVLSCIVLHHLV

SSGIVLSCIV

>SG-AL_contig_7877_1_-1_118_1851

MASLPCPDFSVPLLLLLVLGVTCLCHVTHGWRVLLVPLPDTSQCRQAVSIGRELQTRGHQ

VTLFVPDYFRARRCTEPSPPGVMDIEVRAFDTDEEAAEVIEDKLERLGDEVMRGGTGLLG

DSSSAHEVMRDVCNSYVMEARDDLDQLRREGKVDMVVVDGSPGGRCLTLLPAYLGAPFVA

LSSFIHPFDSSLPLPTNFYPAPLSALPGNMAFLQRLSNFFYYVSYTGGDSLFGSFHQGNA

LGDRVSRLEHGKLIKKASLYLENSDYIIDYPKAVFPNFVQVGGLTASPARPLPGPLAKFF

DEGADTGVIVVSLGPSVFTPNKNLEDKLLTVFRRLDSRVLLRLNASRSLNRLKNVQTVSW

FPQNDALGHPNTRLFVSHCGKNSFFEALFHGVPMLCCHFAGSDVLGTAARVAEYGVGLSL

DLLTASSDSLATAITTLLMEKAYIRRMKAASKLFHSRPQTPAKRAASAIEHVLKYGGGYL

RPRSLEMTFFQYAGLDVVLTIFTAILGVMALVSFVIFKHFLPVLQKKEVKAEVKDDLAST

SSESEPTTKKVAEHENNPHASIVMYIFFVLVILFYIFV

>SG-AL_contig_7878_1_+3_57_224

MVTYFYKNNLCNVIKISHMYKQYNHYTVIYNSSCLGPLNSSCEMYPSLSLSSCSKI

>SG-AL_contig_7879_1_-3_3_290

VSLLVHRSVLCSPVDLQTDLQAVAARVSFSKTITICSLYLPLSSVVTKLDLLDLIDQLPR

PFLLLGDFNGHSPLWGSDHSSPRGLLLEDVFSELDL

>SG-AL_contig_7880_1_-3_27_332

MSARPTRDGVTGGVSLLVHRSVLCSPVDLQTDLQAVAARVSLSKTITICSLYLPLSSIVT

KLDLLGLIDQLPRPFLLLGDFNGHSPLWGSDHSSSRGLLLED

>SG-AL_contig_7881_1_-2_2_82

IIIQYNTDICSSLLCIQICIHHCSALK

>SG-AL_contig_7882_1_+3_282_407

MVLHLHWYSLQILLHSRQYIELLETLHFQRSIVFQRICRKCF

>SG-AL_contig_7883_1_-2_92_343

MIYIPMSYCSFHHNPSPPSSNPNPCHIGPREQNFLLPTFFLCKSITHYKPWLDLFYLWEC

TCLFCFFEQFFFPAFCDVLLFLSQ

>SG-AL_contig_7884_1_-1_547_1812

MLLSQSPLLHGLWARHFMSKLRFKAIQSFLKVCNEKTENNIVDKLCKVRFLHDCIRRKCM

KLYQPYKNMSVDERMVRNRGRFTFRQFIKDKPTRWGMKIWVLADSLNGYTYDFEVYTGKG

TPVSKNGLAYDVVMRLCQSIEKQGYHVYFDNFYTSVQLLKDLVAKRILCCGTIIPNRKGV

PAEFKDTKTFAKGPRGYMRWKREGNLLFLQWLDNKPVTFLSTVHKKATSYTHVKRRTKVN

GQYHPIHVRQPSLVKNYNINMSGVDKSDQLIGKCETLRKTHRWWKTLFFHFLDIVHVNSY

ILFNDLRSKNKDVPALERPESYNQLDFTLELLKEMAGITADTEVPLFKKKKAEPPPPPPP

SSHHIVPVNSDKQRNCVLCYKVEKKERKTIVKCRTCDKHFCFQSARNCLLAAHEETCTAF

SK

>SG-AL_contig_7885_1_+1_553_813

MSPLSGNSLLELHLFYQSFCLFLFCQCHLCLSIFLPHWSILLFSFQEILVKFWGVSLTFT

AQQRRNMQTAQHHVACTVWAKPPEQHA

>SG-AL_contig_7886_1_-1_595_897

MPEAVVCHALSYTFLVCTTFSLVFYFLMNGLFCVVLISNCINVIVSVRPAMNGWSWDQVC

WSCNILLLYVNVTFFPEISLHLHKPTLNLGKNCDNYVGLGN

>SG-AL_contig_7887_1_+1_256_360

MVKIKCHELCCFAGFLFSGLRWFCDQRGCLLDFDG

>SG-AL_contig_7888_1_-3_381_1784

MAKQVTQQTFDDVVKENMWEFEMAVEEAIEDAVQQFESQGVNLANIIKDASLYCGGDGED

TTVHPVVSALQKLSLSFDSDSPDTVSVMSDLDIVCDECNIDLSHRCLAGTNQAYPTLLKL

LKKFAADERVIKAILRCLCSLVNGQPDLLDEEGSVLFIHYLSDFRESELLLVVVRLVRFF

CLKHESNRQNFIGKGLIKLLSGLLQSQQSNPELVKEICSALRVLTFDDDIRVPFGKAHEH

AKMVVTEGDALRAILQICEAYGDNAGVLGELFLTLGSLAVRNEFCEEVLNLGGVQLILQA

FEKNIADKAIVRQALVVLRALAGSDKVKVAVVKQGGVELVVAAMVKHASNTTIAEGACGV

LAALTLRNPAHCNKVMECHGHDAVVQAMKIHIKESAVQKQACMALRNLVARTREHSAAIL

ELGAEMLLNLAGSNHKECEDEAKAALRDLGCAVQLKELWTGQKGALHY

>SG-AL_contig_7889_1_-1_1_165

SHHHLHQNESDETRTVCMKEYLSIIYVCECVCREHMCAYEFVCLCVPTHIICILT

>SG-AL_contig_7890_1_-1_100_1980

MPSDAKKKRDAKKKEQMKKRDQKKVTKEEVNGEAEEAGTVQNGHAETNGGTGEDGGGASI

AAVTKKLDEMDIAAQHRSVTGVLASHPDSRDLHIHNMSVLFHGVELLMDSKLELNVGKRY

GLIGLNGCGKSSMLAALAGREVPIPDHIDSFLLRREMPPSDKTALESVIEVDEERLRLEK

EAEDLSHKGDVESHDRLMAVYDRLEELDATKATAKAGFILHGLGFTKEMQHNKVKNFSGG

WRMRIALARALYLRPSLLLLDEPTNHLDLNACVWLEEELKNYPRILVVISHSQDFLNGVC

NSIIHMHQKKLQYYGGNYDSYVQTRLELLENQAKRYKWEQDQIAHMKNYIARFGHGSAKL

ARQAQSKEKTLKKMVDGGLTEKVTSDKTLSFYFPSCGTIPPPVIMVQHVSFRYNETKPYI

YQNLDFGIDLDTRVALVGPNGAGKSTLLKLISGELIPSDGLIRRHSHLKIGRYHQHLGEQ

LDLNLSALEYMMKCYPEIKEREEMRKIVGRYGLTGQQQVCPIRNLSDGQRCRVTFAWLAW

QCPHLLLLDEPTNHLDIETIDALAEAINEFDGGMILVSHDFRLISQVTEEIWVCEKQTIS

KWEGTIFDYKEKLTKQSRKERAKFIPQ

>SG-AL_contig_7891_1_-1_820_1098

MLSRNFRMVRWIYLQYITLMKLFGYFADQFVGCCRSCFSLSLDLSVLYMPVLFFWCSDGS

LIFILACFVYVRVYEVFVALQTNSFIKNYAMFR

>SG-AL_contig_7892_1_-2_284_505

MMLELEPVLLLYQVMIYTVFECHNMKCNGFVCNRIWLGSDVCVCVCDRYEYTILEQICDE

LFFEQLSAHNCWYY

>SG-AL_contig_7893_1_+1_64_336

MDAAKESLKIREITGHTQTNRQGLGLTKTEWWSKAKGKAKRDMVIQEMRKEEDQKRLQKA

VQQSQQGQWTTWESALQRSLTWNDIWHMAPL

>SG-AL_contig_7894_1_+3_171_413

MVIQEMRKEDQKRLQKAVQQSQQGQWTTWESALQRSLTWNDIWHMAPLRISFILRSAYDL

LPSNANLVKWGKTDDPACPLC

>SG-AL_contig_7895_1_-1_316_441

MLNPLREKTSLGPFYLFIYLFIYLFIYLSIFPKLRPGWCRGG

>SG-AL_contig_7896_1_+1_64_201

MHKNLSFLVILAFLLLSIKCTIQINILVSYFCIYQFCGPAKGRNLK

>SG-AL_contig_7897_1_-2_44_298

MLLSDGTFLLHVQVKQSDFVPWSLEFVVRYLTKRVPYTDLSIVNKASAQTAAIQDVQCLH

CTGFDVSVCLIVPSVLCVNMFMSLP

>SG-AL_contig_7898_1_-2_104_814

MVFVAEFPSDSSRVLVYASLLSLYSLTDYLPIYEMAELQKLVQNCHGFNSDLMKMFVAKH

RSDSIKPDLPPAPVLKGTGGASIFSTTSTTTTGTNTTDNANIIMQKNMNDNQQNNTTTTT

TTTTINSDNKAILQNQNNMDNRNLNANVNLNANRKMAKAKPASPAASPDQPDPNANVSEV

SAVLEEVRGAMSESRQQLVDQVTEVTVDAHLATCINTQKAVADATARFLAMKASGQL

>SG-AL_contig_7899_1_+1_79_195

MIWTLSQDNDLQFLTINHRLLFAKSNTHLKNERAVCNIQ

>SG-AL_contig_7900_1_+2_2_154

FFIAQPTIKAISRLSPRKVHQSQEDNRVDHKTSSNLKTKTLQKITIHDILS

>SG-AL_contig_7901_1_-2_38_625

MQEVLKEGSLGQINFMQASFCVPAATVSRLTKKDLGGGGLMDLGCYLVQYANLVFREPPE

RTVVVGKLNEDGVDMSGSIVLKYKGGAMAVLLYSIDNAYGNNSLTLYGSKGNLQLEEPFH

CPTHMKVAGGEVQEFPLPPSPYTYTFVNAQGFVYEARCVRQCLLDGLLECPRMPHEDSIS

IMTILEEGLRQLGVQF

>SG-AL_contig_7902_1_+3_51_314

MLDLICLYKLIKSVCKAVEWIISIQSRLLTRCPIQWKQAPPCVALLTRSQQSQEPLHSRA

KSHGRKKCPSLLPLTKEHQRLIGVFVFF

>SG-AL_contig_7903_1_+2_149_388

MNIFISKVFESYNYTTENKASKQNAGKRREWGGSVIQDLFRAHFTAYLQSKEVNTSVEPT

IYCNDLSQKSKTEKQQSGEK

>SG-AL_contig_7904_1_-3_1326_1589

MDHAVLLSHLQLKFGIFGNVLSWFNSYLSDRKQTIFVNGHYSSPSPLPYGVPQGSVLGLI

LFVLYTQPLSEIVSSHSLFLRSTQSCIS

>SG-AL_contig_7905_1_-1_544_759

MILFHSNNKLLLFAAPVTLSFGEYVLSVIIFLKKELKPFPFVPARIYYCNSLLGVCHKCL

LSRLQKVQNYTA

>SG-AL_contig_7906_1_-1_1_186

FLKKFLFYFIVLYGALFCLVRRSNCFFSSIDLRNRKMFDHVPLFVTLLRLLLLYAVITGD

NW

>SG-AL_contig_7907_1_+2_305_643

MCEHPAILFSLFLYFYDCSMRKKRQEKNPTHIFACVPSFSLISHTFYTHTHTHTHTHTHT

HTHVSPMLARTHTHTRVCARRLASTHTHTHKHRWACTYTHTRCACACLHTQSP

>SG-AL_contig_7908_1_-3_93_449

MEDYVGQNGGGYPYGLELTPSQIIISWGRWDWEGWASELTAYSLPELQEGNSDQRELFPN

TKPCGLTNVALNKPAIQSTYHELYGNPERVVDGFKGTNIAQCTHTADEDNNPNRWWRVD

>SG-AL_contig_7909_1_-3_93_443

MEDYVGQNGGGYPYGLELTPSQIIISWGRWDWAGWASELTAYSLPEPQEGNPDQTELLPD

SKPCGLTNVALNKPAIQSTYHGLYGNPERVVDGFKGTNIAQCTHTADEDNNPNRWWR

>SG-AL_contig_7910_1_-3_120_314

MSTFWRQVGLNYINYSEICAKVVRRCLKPELRVAAAGREETIIKTAKWEAGKIVKQAESG

ERLSQ

>SG-AL_contig_7911_1_-3_3_164

THMRACTHRKKVGVVEVLNHTSIFIIFTHLCICDALSGVRVKEVDEQMKRYIIK

>SG-AL_contig_7912_1_-1_1_105

GIRTSVGASLQMEISVIALLFSLKLASSFSVKLFA

>SG-AL_contig_7913_1_+1_307_666

MKNIILTVLQHLLSHNQNVNKCTLKLLCPLQVNSFTAYILIPTSTACKIQIIPLGWSLVL

VNNVSKYLFAFMLNIFICIHAFILIIHVYLFLHYFSSILQNDLKKKQQKTPTSIQSKCCN

>SG-AL_contig_7914_1_-2_2_190

ATFTPFFFFCLCDFDLGPLYPSMVHTVGPNLVKIRLETAEKILLTHFINTHTHTHTTHTH

TLI

>SG-AL_contig_7915_1_-2_2_232

AVVFCFVFVFCLWCFFRFAKRSFFWLKILYFVLRFVDSLIFYKLSKFAELTKLSIFADLL

SVKLSPISCAMYMNAFH

>SG-AL_contig_7916_1_-1_499_636

MVQQLAMPALLLSVHDSFIMFMCCNFTIQIEIFHAFSVSCDHVSVL

>SG-AL_contig_7917_1_+3_138_434

MIPTARTQVGIVGFGYALQEPVQRLQLVSGQDIVGVTDITRIQSQPAVLWVPQRTGHFFR

KPVVGQGLELLALAKFKGQGNQGQDTQILHVCVDKEWVY

>SG-AL_contig_7918_1_-2_65_1093

MASCNEEQVRFATDTFAELCHKFTSQLTERKQAQRGIGILCQAIRKIQTHPALLTSIHPD

LCQLCLLAKNMKPALGFLAEDITEISRESGHYDAKDFLCYFYYGGMIYLALKDYNRALYF

FEMAVTTPSMAVSHITMEAYKKFILVALVVQGKIPTLLKFTSQVVPRYIKPLCQAYHDLA

SAYTTNNPAEVRAAITKHMDTFTRDNNVGLVKQCETSMYKKNIQRLTKTFLTLSLTDMAN

RVQLPNPREAEKYVLRMIEDGEIFATINQKDGMVRFHDNPEKYNSAAMLMELDKELQKCI

KMDEKVKEMDREIAVNPQYVQKMSGMEDVVDLHPGSTSKGQSF

>SG-AL_contig_7919_1_-2_59_499

MGEGGPSENLRLREVSRIWWSPRTGLPDVFRDGTQRGSWVPTGLPPGDLGRGAVSAPAFK

TAFLLALATCSCRSEFLSLESAKDVIGSHANGDSALPSLLEFRAKTQPPGPCPRFLHISS

LSPAWFPETPGVRIAAFASPLRQVLLR

>SG-AL_contig_7920_1_-1_10_1146

MADTEGKVITCKAAVAWEAKKPLTIETIEVSPPKAGEVRIRILATGVCHTDAYTLDGHDP

EGAFPVVLGHEGAGIVESIGEGVTSVKKGDLVIPCYTPQCYDCKFCKSPKTNLCQKIRAT

QGKGVMPDGTKRFKCKGKELFHFMGTSTFSEYTVCAEISVAKIDPKARADRVCLLGCGIS

TGYGAALNTAKVEKGSICAVWGLGAVGLAVIMGCKDAGASRIIGVDINIDKFEIAKQFGA

TEFINPKDYDRPIQEVIVEKTDGGCDYTFECIGSVHTMRAALESCHKGWGESVIIGVAAA

GQEISTRPFQLVTGRVWKGTAFGGWKSRDSVPKLVDSYMKGKLEVDKFVSFEVPLDKINT

AFEYMHTGRSIRSVVVFGD

>SG-AL_contig_7921_1_-3_120_359

MMIIVNVARNDIFSDYLLCIRGITLLWCGDESAKSFHGKRKVRKLLFSLSCFNCACCKHC

SVNFCVCIWLGHIHSHKLLK

>SG-AL_contig_7922_1_-1_124_720

MTYSRIIYCAFVEYLCCGVLMKVQNLSVESERLPRVWRPYLHKRVLYSEILDRHMVVTVT

LRTMDLIDSHNGLDNYILKTHEVDLCSKLGMDLKREMLVTLARESCYPDDPAKRQEILNK

YKDFVIPLEEAEWIGLSVEEAVEKVEKEQPPPRPLKDIFMEELAQELSETSVTDTSSESF

LGRFNPFSSTPADQRDRKS

>SG-AL_contig_7923_1_-3_78_407

MLSFLIYAQYSLVISWPQLVSQHCCHLHPLLSCSLPSLIPVFGLKLTFVKLILCYVHPSK

VAEKLTWLNQGTGRNIAMVCDCRWWMKSVVLASNKHYRRVSLNISSEYIV

>SG-AL_contig_7924_1_-2_1334_1573

MNIQDHLIYCVGVVLCIYMKVQFIAFGEFGNNRICLYVTAISLCVLTANLWSVWMLSFIG

YKIYLLSPAVGFFFKRPPLA

>SG-AL_contig_7925_1_+1_184_420

MAYHSVSACINTSMQLWVHPKVNTIFYGCASCKRFQDITMSRIINHGETFEEGSVTNKWT

EKPTKVGNSDQQVLTSFGK

>SG-AL_contig_7926_1_-1_31_282

MKEIRDRGSQFRHFKVPALQRGWTGAKRAGTSIGPPDPVGDYTFEGFDTRILEFKLVTNM

TGNLGRKQRFSSFVITGNKNGLAG

>SG-AL_contig_7927_1_-2_23_328

MQLRTSHSLSLSAQTPRMADICRPIQLNITRNVSFFGKVTADELWGSVYGVSNAGRKRGR

GKRGRKKMNLNRGQIIGVGKNNMVWPGLNAPVVKGREVINIK

>SG-AL_contig_7928_1_-1_181_297

MLFTSSSFLVCPLWSVHVKVTLSVKACKASIKRSWFGDN

>SG-AL_contig_7929_1_-2_2_268

KIFSAVSGRTFTKFGPKVWTVEELLIHTVGAERTKVKVTKAQKVPKNSTFGGRCVNSTFS

ATSNDIQIVFTVVTTYYYPILTSRQVLFH

>SG-AL_contig_7930_1_-3_3_200

PPPPPFFPLPPPPFLLFFFLFSFFFFLKKGRFFMLPGSDVCLLMKCDSKIFSGVCGRIFI

KFGLKV

>SG-AL_contig_7931_1_+2_2_148

KKKNKNKKKNPTRQTPRKLRSLAPHSSSTLGVMGKDFFFFKRDLEYRSM

>SG-AL_contig_7932_1_-2_38_235

MCLSVWSLWFLYLKLPLCGVWKVLCVVSEEITETYVCFCFDLLWSKCHNHRSNIETALMF

EREKCT

>SG-AL_contig_7933_1_-1_175_10536

MCKLEWRGDEQCGSCSSGYTGEDCSVRESEISKPVVLAYCLIQSGGDLFQFAGKSCTIHN

VGSYYLVGLHQFHIQVSQFPCPRPRQKEPCIKSVSVSLHNQHVTVSFLRANTVEVLVNGI

ATDVSQLYTIGVRGCDVSVVSSLLATTITIPRLKLTLFVSVGRVLGINLAVSKQVCCSQN

TTGLCGACHCPSTPGPLPCGLSSPVVGGVPLPPPPLPLVIDAQDNGRKIRKKVKALRVPE

DKDLSGTVLMEGAGPGNMMQFHDSSAITDRLTHIGSLQSIQFLFKSCSSVDPACWGVILS

YAKKKSLTVSLNDTLIVASDTGLFSTSLAVTDVTWYMLTLTYDPEVEQLSVYLVKSRDDI

KKKVIPLANSVLEGGGQLSLGKWVPATGSDSNQPKQGFTGSIDELRIYSRYIDNMEVGAH

WNKNYVGNEPGLEYLWKFDEMYSSASESSSAVHDIVRNHTLHIPQPPFSAARLVFSDAPI

PFLPSSQSPAVLLPDDIELVRRKRQAVVDISMSQYRTCENIFVNSQFGRVCVNASQSVQT

QYLFRCYSALYLTGDLSASQDSIVNYATFCQEVLSLSQWPAQSLCTDQPQVAAVTVVQSL

CGTPCHFGTKPTSTPDLCLCHHGYWGPACDQVCPGGADNACSGFGTCNQSSGKCSCPIHR

QGASCSSCSLGWLGDSCEISETPIPSGNGSSTHKAWLLPLGHVLSADGLGFYVSSPGVYS

LLAISDRIMIQGKFIRCFENFTCATFVSVLAGDMNAGYARITIQYPSVHDGKPRVYVSNS

SSSVDSEVFFHGVTVSRMSLTRVKVAVSGGVDIISSSCGLYLTVAVQIPTTYLTATSGLL

SGSGLVSASQKLQHLNSLHNSSQLSFCQGGVKEQQTLSARSSTPVLNGVLPVTLTPLTNV

TLNFVSFLIADCEVFVHFPTDQHRRQLSSGFSLDLGGSAVFRNFSLPDTSDSVMVELMVR

QLAVTDQRQVLLSFTHTDILVLYLLNNTLYIEASTPSNVSSYNMGVGLDRGQWNKLALTY

NGLTGTLIFYHFSSNAWLERRDFLIQTALFSTPGILAVGSWQPPHDGDPHPRVYPFQGEI

ENLLVWDNIIEPNLVPDIWLMDPAVARPALTLAWTFDEGHGFTTLDFVNDQSLMLPTPPL

TSPQWLASDVDYILTRSGVSGVDLVLAHTNSVSGTDVCSAFITGFNSSGCQAVSNATKQD

LQAQCHYALTVINYTQAALSTVLTYSTLCVDSLTFPPSRLHTVCDADQTWNDLAICSPDC

VFGASMLSSIVTATNSSLCSCCAGYYGSQCQFVCPGGSQSPCSHHGVCESDGTCTCQPNW

AGSGDCSICSSGYLGVDCSIFTTSPLTTGSQTVAMVTMTGDVITFSGLHFGLSGLCGVYT

VFKDTTSTPHVEVQVLLVWCSYGSCTAGVAFTSDSYNITVLPSPQSTLPSVYVNGSQFEL

PLNHTFSPTLTLTMTSVHSLTFSVGVGMEVVVMAKGGYVEVMMKVQSSACSGSMGVLDVC

SPGTSAYTPLNSHTLNSLHSDIDTNFRVTDGDLLFQQFNENSAFSTTASSYSLSFNGTSA

WSGPITVGSSTGLQDFTISLHVKPHSYGGTILSFGKNVTLSITSDNPLRVKCGTVDLDSG

LRLMLDKWNQLLVTFMGSENVMHVFVYSQDTTIQHQVLPDQCGLTVVDGAVMSLGEWVPS

PHDQVRQLRNTFSGEVDELVVWSRPIPVAIVYQVYNMDVDVTSFRAILASLLKLNEGLGV

IAHDNLLSTNNLLLPPSPWPTPLWVLSDLQLYSSSPLHSSSLSWTVDPAWVSVCDSFFTS

SAVLSTCSSSSAVWFRHRCLHLASTSGSSEGAVVAMATSISVCSAMTVVDVTTLYRTVCD

LTSTPPPWLQQSCAHCAFGSVTTTTTTNLTCSCLPGFWGTGCYDYCPGGAHNPCHHHGRC

AEDGTCWCDRHWMGKECDSCAQGWKGDDCILSSNLTAVPFSSSLHVVGQVSVLARVITFD

AVTFDVVKYGVFNLIHRADQSLYLAVKVTPCPAGVFSSHCITSAIVGSQSGSMEINHRSF

SQNTITLFGQTTELVVRGKRTVGHLQFVYSSFSTLVISTTDLSVNITITVINNNLLVTVS

ARKDAWFTGNSTVSGLLTSCNTQLSIQFSACLNQSHQVCTQSQALSLPPSCDQRLSLTAL

LNFLTFHLYIAPSNISPNGSIITEACLRLNNSGTIVRGVSLPAQHFSLEFNVKMFSLSGV

LVSYMVDSSNYFILGCSGSQLAVFTAEGTVLTGLHMTVNVWTQVLISWHSALSSVEIYVT

GVDGLVAFKSVRLHMHVFVSAGVLTFGQAPARVNIATNIGVFLGLLDEVRVWSRPTNPSV

VSQTWKMAVQDTTPDLHLHWPLNDGSGITASERRRRSTMYPMDVQNPPTWEISDLTLISK

EMLGVGDFTVNGADLDDYLFPVQPSTNATNMASAQATCGTLLGNLTSSPECSRIFPDTSS

LLQQCSAMVYSTDSGLSSELFTLALAQLCQHSQQLQASPLQSQCGQLHYLGSVLPFYGMD

CTDTCLFGWTPTLSSCLCYHGYWGSTCSLLCPFTDHGVCNAYGVCANVTGICSCSPHWLH

SVTTTLAYWTGNMTAMVSTYACTDCTAEWTGGDCSVTVTVVKVSVQWRAAIAFSNYLTTW

DGASLALVTPGTYHLIQVQGVQLTALFIPCTQRRLCRLISAVSLTMETFHLKVSASGAKF

DNFTATVKIEGEIEDMTFPFSKSWGRASLIWWRANYLRVTMDSKLVVLIGTSPVGLTVST

KLHQDWFSQTTGLFGNPDGNHLSDLLPSSSIPQADLTAIKISEHQRKNFLLTDKDLSALL

GESMAGLNLSTCGHMLHLTHHSLTFSTLDLIVSTQQLTLTLWLKVEGLLSAPTVVMELTT

SVGMISVSVHQHLILLTWFGTETTILHPTSIHTWTYLAVTWEGSAGSLSAFAIQESGFDY

STISVGRLSGQFTITDLSLVGPQTGQSAVALDLFRLWQSAKPLKSIAQDLRVYAPDSDLD

TKTSLPVLLMWAGFDEGEGDSTAMSVQTMTAPMNVSGVISVNAQPSPAAPSVWTPSTIPV

LDVALPDDVFHTPDTNQLAQACLKVLQESSLLTQCGDLHAVFSIYLEACVREYQRTGRIN

VTDTVANLLAFYCHTTNDVGECQLDQYVDFCRPIPDEELPVWIIIVICVGVLILASCCCC

FVVFIVTKKKKKQRKREQDLAGSVSHLSWEAETSFTRESSADLDSVQPDLGFVNPAFGHV

AASSRPVGAKDKHGVHHKGKGRFHSKNTKIAGTDGSSGQRPDSGSSDQSGFEEKPHLVAW

SSDKNRPISGRRDKWAQEFLKHSQAPLGFYTSQEEPDIFIYDPEQNEREGEMTGARPPQA

TAMHSSRRGRDTGMTSQASASGETPQTLASHNIYRLQSVPDQEKVILDELEVEEPTPIVF

CHHDDRDEPYHASSRRRSSQHGTVDHINLAFDEN

>SG-AL_contig_7934_1_-1_82_441

MADDTAEENILDYEDEENETAVDGAAGDGQVKKDVKGTYVSIHSSGFRDFLLKPELLRAI

IDCGFEHPSEVQHECIPQAILSMDVLCQAKSGMGKTAVFVLATLQQLEPQDGQVSVLVMA

>SG-AL_contig_7935_1_+3_903_1184

MCISHSLLTSVKFMPASIPITTANKSLSRTSLVTTANKSLILPDTDFFPQLTSHMLLVYT

SIYAILLAAVHTHQIPVGIKDNPHREHISLSQPI

>SG-AL_contig_7936_1_-3_1341_1484

MWIIPTVQQGDTSAQANVKVRSEWSVSAGVFISLLFRICSPLSLSSYV

>SG-AL_contig_7937_1_-1_85_465

MAAQISSLNSRDYQSVTVQIAVQVGVFTTNIGSGAMLVGVEVKLPIFILTVWVQVWLEKT

MLAASGPPSCQWESDTVQHCGKSSSKIEEMGTSMILGLRKVEVSTTLHPCTYDLRVMFSL

IVVKVSP

>SG-AL_contig_7938_1_-2_2_277

TFRTTGTRLPRSREWNCRCATAPTLKTFSCTGYCAVFFQSNSCTDVILELKGLPISMLTA

NSVFFLLMNSYVNNHRCLQLIVCAFVTNVTNV

>SG-AL_contig_7939_1_+2_611_961

MKRRSVITSKVYSRMYRNKCQINTPSILSSPIQFQGQVNYQLLTGGLMVHVHSRGEFRTD

TRVAGNVLANRSKKWQKSLTRGHQNNSCDSSGRKSLSFHHLQHPIVISHTAMESHTQ

>SG-AL_contig_7940_1_+3_3_218

EKVLFAHLPPKVLILGTLLARVALTMALSDPNSCIGYSSMVHAFGPKLVKIHLEITEKIV

LTHFSNRHKDTR

>SG-AL_contig_7941_1_-1_322_2640

MENVCFLLLLVCGTCSAVNGQYAAVRESNTSVLSTDDDHDASTFRDSGGGPENTADSTET

LPITFSTKTEHSSTNDATIKSSPNAGARVVDENVTGFADSPEDFNTTTTRNSADTRKATT

ITEMAASERNATQDNTTLTLTDATAKENRHPTRTHTVIMTSTQYGLNLTNDTQTKKDEYQ

TSSRLDVTMMNSTPDNLTLKDIEKKKSEVPSRPHVVMLNSTQDDPTPKDTQTTTNDAPSR

PHIVMIVADDLGWNDVDWRDKTMHTPVLTRLATQGVVLNHSYVQPVCSPSRAAFMSGFYP

YHVGLQHDSLRATQKAYLPDNITTLPQHLQQLGYRTHMAGKWHLGFCNWRYTPTYRGFDS

FVGFYNAVQDYYTHTGHRHGYDFRYNKDVFYSANGTYSAFVFSQYIQHVISQHDVTTPLF

VYLPFQSVHGPLQVPQYYIDRYCSHIQNATRRIHCGMVAALDEAVGNVTSTLEERGMLDN

TLLFFTTDNGGPVHKGANNWPLRGSKATLWEGGTRAMAFLHGPNILKHSGTTYDGVIHAV

DWLPTLVEAAGGSAVTGLDGVSQWQSVQGNKVPSAREEFVYNIDELKKNAALRQGKYKLL

QGDPGHLSGWYPPPQLESVFQPPAYRDTQSAAVDGISNQGNVHLMHILSDRYVSGETSRQ

YDIFFDTQLLNSSSKHPSTLRDGEGTAGSDATGNPVLAMVSENEVSDKGYQLYDLQADPN

EHYDVKKKYPDVFKKMRALLDEYRKSLVPANFPPHDPDSYPSNFNGVWSPGWC

>SG-AL_contig_7942_1_+1_1_99

LWPVTSPEMSSLLSDLLFYPRRHLSNSRIFIND

>SG-AL_contig_7943_1_-1_91_201

MPVKKNCNYVNKLVAFWGLSFFLQGSIFTHNHVPTPM

>SG-AL_contig_7944_1_-1_1_132

TDLLTRTFRAYVFPLFFFFLFFNYNFFKKSKLTRKIKKWMSLNS

>SG-AL_contig_7945_1_+3_3_164

YYILFTAQPTTKAISGQSSHYVHQSQEDNCVEHKTSLKLKIIILQKNTTHDILS

>SG-AL_contig_7946_1_-1_4_1425

MANATRKHHKHRTSRRRLAAVSFLSNISLDGTHKDTKLAIYSRKHKKHVAKEEESDAKID

LSAAEDQLGREVTQEGSGQDLSLKQKQKHSEEPVHPLSEHRRSVAEGVQPFSRGDKNADG

FQASVSTPGSHKRWRTASLSSERDRQAVKKKLMHMLSDLGSRKENAEPYGERSRRSSSMS

DNSSESAPKEVRFLSADNQRCMSTGRVFFVSKGRSPLAVCSVLPFKKKPFFRQEATLDEP

RPRVRHTSGSRSISSSEGLLHMGLTVSARVEEGQDVSYSRFLIPSKNRLLYRANSDNVTS

PAEGTSHTVAGHEVLRSMSADACPSGLRPGHALDRVPEDVMENYDPHILDDPELNSGSYR

TLMTFPSYVVSVTDYVKPSVLKKELNEKFRERFPSIQLTLSKLRSLKRELKKIAYTKCHR

DLWTVAQAYVYFEKLILKNLLNKQNRKLCAGACLLLACKLNDVKGADLTKLIEV

>SG-AL_contig_7947_1_+3_363_560

MGGRDLSVSVSGQLSEDGANYSPLLLLSPTLDRIRYSFCRFTELETICTRFSWHEPGLKQ

CSWVRM

>SG-AL_contig_7948_1_-2_2_232

QSKTAEIVHHKLRRVERRENCVKACWKKESGMDQEDRKKKQTPVKEREMTRTKRAVEKRK

FLAQNFQRVGTDCPSVV

>SG-AL_contig_7949_1_+2_2_286

PTNQPTNQSTNQPTNQPTNQPTNQPTIKQTTKQSHDNSFCHNSFNKTNSVGSWHENEICV

MCKFSKKKLLCSSRRFSAGMSSLPTLIYFVIHYIP

>SG-AL_contig_7950_1_-3_42_146

MENTQVKKEIIFLFYYCFLGERTFFCLFVFSLPLL

>SG-AL_contig_7951_1_-3_42_146

MENTQVKKKIIFLFYCCFLGERTFFCLFVFSLPLL

>SG-AL_contig_7952_1_-2_2_211

FFFFFTLGKIILADVKRHLYKGITDLLKLTIVSAHDSVSDLVVRYRVKSETARMFVDFST

VWAHLSSDTL

>SG-AL_contig_7953_1_-1_223_933

MEAKDRQKFKKFTVKASGDMDDTLTTDQKADSESYATTDTSRVKSQTLPEHNRSKEHLMH

LGDYLLEEADTLSPPSLNLNPSTQRQDASTLHNGTSSAKLPSPREGGGFIASALNRDPPQ

TSFQFQADFRILRNDLQAFYTYFQRIDPALYPKLFGKSLEAPMLLKIVESIRTCGILAQD

DCFHQLKGLSEVNRFSTTLMFCSSKDKQGIKEIFQHLKNMGNHPECDIDNLMKKYEV

>SG-AL_contig_7954_1_-3_3_308

SSFFFFFSPPPPPLSVSLSLIFFVSFSYLLKLSFFNLKKTCLLYFSLFIFSFFSSHFFLY

MCSLFFLNKYGPKYEIRKIASDCYNRAPVNKIFAIPLFSIAR

>SG-AL_contig_7955_1_+2_14_391

MARLSKRVWENKALTEHTKIQVYQACVLSTLLYGSETWSTYMRQERRLNAFHMRCLKRIL

RISWQDHNPHSDILQRAKIPSMYSLLSQCHLRWLGHIRHMEDGRLPKDILYGQLATGTRR

VGCLSL

>SG-AL_contig_7956_1_-3_3_233

NVNGPHFEIYHSERSGGKGKCTARIFPPAIYESVDHFILFTAVGCNVFSFVNGCDCDGLT

GTACFKFLLLKNTHMHA

>SG-AL_contig_7957_1_-3_21_812

MADAPKKFTFRVIEGKDFPSLSNKDNQDYLMKWCMKGRLKAQLYSFDQMFQAYEKDQFVL

DFFKDENVTTTLQTMSDSGKWSPVGLTAESVTATQIPATTMSMAFFDKLYEDDQIVRSSS

GHIRKCMDEFFEDFTISDELRQMLLNEDSDSYCTFNESDREQFLFHLFEHICLGGQICQY

EDTIDPYLDVTKQLYKDLISVQKNPDTKDLTITSSVFKVTAADKNGTYYPSVKEHRQNFS

YLIVDPLKRHVTVLYHQFGAIAFT

>SG-AL_contig_7958_1_-1_493_2019

MDLSLFALIPWLALLSDCGLANGSSYGGLFNNNNLTWVGARQACRNTQTGCDLSVQSTQQ

LDFWKSVSLTVGTSYWVGARLKERPQWKKSKKPLIEQLGCYEKPQGLKSTVRALRYNGPE

RCLTQCDGNYVALQDTTCYCVKKDMMGSFTQELSQEECHINCNKEAASCGGTDSVSLYKI

PDQTLYEKSGKSTAFFWQRTFTNEAEQILFTNKPGKQKSGFCASPSNDYKIVALGKTCPA

GTELMSFEGVNIMQFLEDTSSNSDWYTTSFSLPLVFSGKRLWLNGEEATVASGYGPCVTI

TYDGQNYSLVSKTCKERLPSVCSCFGHTFTTDASTEQTTITTTVPVTTSPEPETTTSPEP

ETSPFAPTMTEDSGPEETSGRMGMTTMASESTASAGDGLSSNVTDQAAEQETDKDGNLLF

PFQDMQENDGPSRGAPPASPGQSSGSDDSPASAAASIEVIVGTAVGIVALVLIIVIVAIV

MRRRRRQNSATLLKSKAKPAPGRVTNYPI

>SG-AL_contig_7959_1_-2_119_259

MCILKEAVILQSCMFLGNTSFWLYHRASENKKDHRAFSKHMKIKRQH

>SG-AL_contig_7960_1_-2_2_169

SVSEMQKQFRHTTTFIVSFAGEAKVHEMTKPPITYFILAETYIDIDKHTDSRMDMT

>SG-AL_contig_7961_1_-3_3_266

FVCFHPKPQQSSSIPPFSVFCSCFFVVSVCCLGWLGCHVIQTTDVHAGAMLSPLWTQHPH

MGPTRVDVGRPTRSIPWDGWRSWEQGLM

>SG-AL_contig_7962_1_+3_1041_1247

MPCVPKYGTNEVDLPAFWMILETGRGVQDCVVQVSPFCWHVCRKVNPYCWHVCRKVSPYC

WHVCRKVSP

>SG-AL_contig_7963_1_-1_1_117

LLLLVFLKEILIMCNCHHYEIYYYEIIFITYYECNINVI

>SG-AL_contig_7964_1_-3_213_323

MRDEHTAFLRKVFAVETNACDSTVIADFLCCHFLLNV

>SG-AL_contig_7965_1_-2_134_394

MCADVPVPKPPAYVYTCGISNMHVKDPVSHVSGWLMETRKHPACRISNMHVKDPVSHVSG

WLMETRKHPACNQQGLAVRKIQWDLML

>SG-AL_contig_7966_1_-1_1_282

MMVSHLILVFSADHPSLGCDAIHTCRLVLCAGLQCSSDFFSKVFGSLCSGYFGGELWFDV

ALISKTDKIFTKRQQVKNTLIRFLAYCESDCHKL

>SG-AL_contig_7967_1_+2_56_280

MCADLPVPKPPSCVYACRISNMHVKDPVSHVSGWLMETRTYSACTLYSPFTGSKLYWLWP

LPWILPVSWKGWACC

>SG-AL_contig_7968_1_+3_87_449

MCVCVCVCCFLFCLFLFCFFKGPVGFFFCCFLFFCLFVFFVRFFLHKGLKIRGMVYFRTH

TRVFFPNPNQVSYMVYFSAEQERLGLVKSCTVQACFAIFNHIPQFLFCVCVCVHVSVFSV

L

>SG-AL_contig_7969_1_+3_216_320

MILSRQWLVYNALFKYSIAIPGIVKQTMACLQCTH

>SG-AL_contig_7970_1_+3_147_338

MIINSLIRNKAGSVKNTITIMEQRSFSTTMIARSKEYYYYYYILFIAQPTTMAISGLSPR

KSIK

>SG-AL_contig_7971_1_-2_14_238

MMAEEINRLRAQEEVLEARLRGRAALHARSGGSGGSVFVRWGSQTCPHSAECVYSGVMAG

SHYSTSGGAANTLCL

>SG-AL_contig_7972_1_-2_2_202

KICTLYPLGAGQAGTNSDLPLQVQHPNYLGTMPIVSVFILFHFVCLSFFSMGMGYYGIVN

FMLRMGL

>SG-AL_contig_7973_1_-1_7_441

MAGANCGQVEVPRNFRLLEELENGQKGSGDCTISWGLARDDDMKLREWNGTIIGPQRTPF

ESRIYTLKMVCGEKYPHVLPTVQFQTKINLKGVGDNGVVNLKEISGLNPWCPKYTLQNVL

CAIRDSMSVKNNKQLQQPAEGSTYF

>SG-AL_contig_7974_1_+1_1_144

KTEPFHSLTLSTSFLLTLSLPHPIHQLQNLTLSLPTLIQLPAGSWWIG

>SG-AL_contig_7975_1_+3_3_191

RVDGRQGHEGVPPPVPFRSENPAVEETFFSPSAVEESTFFSPPPATGYFCAELGHLLLQL

GLA

>SG-AL_contig_7976_1_+1_436_807

MKLNKTLNWNSCFLKLHKIQYVNRKWFQIRIVSRIIATNVILKGVNIVNNGKCTFWDITK

DSIEHRFWKFDKVQRFWKHLENYAHNLKFSENLILFGVECCIKTHVIDFIILFAKPRLHT

RYTK

>SG-AL_contig_7977_1_-2_275_364

MLQSVEKSQRRKKKKKKKKKKKKKKKKKKC

>SG-AL_contig_7978_1_+2_2_241

HVALQSHATFQPVESRSRSRSNLFYLCHAGTSHTLHVPTLLIFLKCFPNLSSFTTASIQS

HFQRFFTLESPKFIPLFMQG

>SG-AL_contig_7979_1_-1_208_345

MKNYVGIKSCKVLKIHYYFDTLMKTILFCLYYFSDLYWIAFISILW

>SG-AL_contig_7980_1_+3_3_209

LLTFSVIYLSSWIPRKQDNSTLKNINIKLIYKCFLNFFVWDSNVDKACTKDSFHFRFKHT

ELLSAIMNP

>SG-AL_contig_7981_1_-1_34_843

MAELPHLGKHCLVESCKQLDFLPFDCDGCGNTFCLQHKAKDDHQCDKRLLQTANADYSGP

KGFACSMTECTHRELTPVTCEHCHLNFCLSHRHQQDHQCTELKPSPVPNAQTAQHVANIL

ANKQPPAAKKPLKSLKARQTAARVALMKLKGRATGDKGIPDTERIYFTVALPVASKLPPK

ALFFSNKWTVGRVIDAIADSAGLHNPNNTGAAQKLRLFGGDSGELLAVESRMEEVMSEAS

GVFSGSPLIMELVSSDVTCLPDLNNYQTTL

>SG-AL_contig_7982_1_-2_809_1003

MLHNTISSLVCSHSNIKRMAQRFSIAIFCIYLDHHFCKNKHSSVRLKYMARHSEIVSVLL

GHQET

>SG-AL_contig_7983_1_-3_3_161

DTFLCFKKGCFQLSGVCDFIKLQDLFSCIYKVHLQLAEADLCTPPVSIIIPLS

>SG-AL_contig_7984_1_-3_549_779

MRVYWLSWEQHCAVNTFLSVIQHSESFKAISPACVRACQPPKHSRQRSCSVPGTRQKVML

AYFHSALSDIIYSRMFS

>SG-AL_contig_7985_1_+2_2_175

VPSQANTCFKPPFSFPPPRPFLPTPHTTAPEINTFLHTYIESAVIIILSGGVHQLFAD

>SG-AL_contig_7986_1_-1_415_534

MLPCRHVEAFSCQCFLSLMSSATSCPLACTLLSNGTLVWD

>SG-AL_contig_7987_1_-3_162_440

MLLTKLRAVHSCTLWVFFGSKVETDLGFEAIFNGCIFGWLQQKAQYVLQIDSTMYFLLCM

LPLWSYIYIYIYIYIYIYYMFNCFLVTVLCSIF

>SG-AL_contig_7988_1_-3_3_530

TEYKFRLSAKNDEGPSKWSDTVTFKTLPDRPSPPSKPQIKGKVFSHAFRITWEPPNDDGG

ADISKYIVELDDGQGYETVYEGCDREHQFEQLTPGHQYRVRVACCSIGGRSEFSDCCLAT

TQPVAPGQCQAPKLQGKPKATSLHLRWSYPDYDGGSQVTQFAAQMISPDNSMREVY

>SG-AL_contig_7989_1_-3_141_371

MWKAQALQFRALPFGLSLAPYVFTRVFRELASLARGEAIRLSCYLLFAVFFGCCLLYLLD

CDELVHQKGHFQVNRCC

>SG-AL_contig_7990_1_+1_1_396

LVELLRQAEQKTPLWAGGGVHGTRGREGESYRVGRSCRTKPGVAGVIPERGCLRRWDLRS

GNHGCGCPFYSRLLGKRGPLSGVGLEPPGGTELQNPLECGDGRGCWTPKDGPNAQVAGEA

DSFVSGQRHQFP

>SG-AL_contig_7991_1_+1_34_219

MLVSLNMTHVSQNIVFPTDLTLKSLVQSSQNAYVSPFLPGFMTGHFPVYSPLNVLKHLTA

FF

>SG-AL_contig_7992_1_+3_411_638

MLSTNFVHVLSDINKFCNCLKCYQQILYMLPLVAYFNCGFYCFCFACLERQRKCVCVCVC

VCVCVVTATRSQIITV

>SG-AL_contig_7993_1_-1_16_480

MAASKRLGKELSDIRNSSMTTFFQDIRVDESNILNWNALLVPENPPYNKGAFRIEVNFPA

EYPFKPPKVIFKTKIYHPNIDEKGQVCLPIISAEHWKPAIKTGQVVQCLMDLINAPEPEH

PLRAELAEEYTKDRKRFMKNAEEYTKKHSEKRPPA

>SG-AL_contig_7994_1_-3_3_164

ISNSHACLGKITIFEFRCVRLLLQEIFEFLCVRLLLQEIFEFLCVKLLLQEIFV

>SG-AL_contig_7995_1_-1_208_477

MDISVYLMLRLLMSNFMCPSSPVPLDMGRRPCSLDLNHFVSLSLSLSLSHTHTHTHTHTH

CFPIFPPYPLPPLVSLLNTCFFLSSLCSLN

>SG-AL_contig_7996_1_+2_131_304

MINIDSTDQIQTEGAETEKVTNYKYLGQTIAMENRTRNKVLIKIKAGWSVFGKYREIF

>SG-AL_contig_7997_1_-1_202_1089

MPFGIKPVCSSCRVTTSTLWRKGAQGEILCNSCGLKQNSNGGKDGQNGSATNSKNDNGGG

NGNQNNSSQGGGPVLRKSARIKPGKSKVQAATKALATKGKGRRNVFKKSQPIRAPRSVAT

VVTGSSIFYGGLYYQVGDIVSLVDHDGGVFYAQLRGFMSDQYGEKSAIITWLLPTQNSPQ

DRFDPSTFVLGPEEDIPRKMEYMDFVCHAPSDYFLDKNTPYPVISKEPNLCFIWTSLGPE

IRTTPSRDEIFGVKDEEDGMATSAPEIPSTSSGKTSKDKEVKDKDKNKDDRKMTKM

>SG-AL_contig_7998_1_+2_188_340

MSVFGGLWKHEYTQHATLFSLHWLQAVLALASPLDFIGVVERKGRACCMGN

>SG-AL_contig_7999_1_+1_1_192

HQLCLHKKRETYTCVSQMSNCASSELRWIWVCGILYPSQTGDRMQHKKSQHLIFKNFIFI

QKIL

>SG-AL_contig_8000_1_-1_106_525

MPRRGRSSPMPSRPAAPARPMSTAPAHPPPAAAAPPAAAAPKQPGLFAQMASTAAGVAVG

SAVGHTIGAAMTGSGGGQQQQQEVAPPPAQPVQQQQEVYNPCQRELRQFLDCAQSQSDVG

LCAGFNEALRQCKLQYGVQA

>SG-AL_contig_8001_1_+1_1_234

PTAPTGYGAKERKGPKIHKNTPLHSKVSSIGNIIYKASKRLVSVLSSHMSLTEHNEEKKI

AKIGPPRNKYPMMLQHCS

>SG-AL_contig_8002_1_-1_163_426

MGKEEFDQAAKDVKNLKSKPTDPEMLEIYGLYKQATIGSCNTDKPGVMDFKGKAKWEAWD

ALKGMSQEDAQTKYIAKAGELKAKYGSA

>SG-AL_contig_8003_1_+1_1_135

YKDWPDSPWTPLTQIRRETHACVDIPYRRETRPCVCSLYRRETHS

>SG-AL_contig_8004_1_+1_1_243

KNPVIVLFCNPRSRIFCAGFYACQSHHDSCVTEHRLSHRLDTEVIGAKFTECICIAFFAR

LHERPFSCIFPSERLETPHCI

>SG-AL_contig_8005_1_+1_1_282

VIVLFCNPRSRIFCAGFYACQSHHDSCVTEHRLSHRLDIEVTGAKFTECIRIAFFTRLDE

RPFSSIFPSECLETPHCIFLWRRTGQCLNIRRCP

>SG-AL_contig_8006_1_-2_2_205

LTLHVPISGGPAIPSRSTSYASCLRHVDLSYCNSVAEPNMRGVVTVCRGGLLVMNYYKQR

VQPYNDLI

>SG-AL_contig_8007_1_+2_602_739

MTCTKARKHLSYRYHQSHDISTVKSKSTELAQVSSVQLIQNTTLVH

>SG-AL_contig_8008_1_-2_107_469

MQSFSFPFLNMREVELEQPVFGANYIKGRITAEPNGNWQGEAKFKMHFTHGGAIEFGQAM

LKAAALASRNRMQQPPPYTPAMDGNIYAAPPPAYMPPQDPYYAWVPYQTFPTAPPDNSVY

M

>SG-AL_contig_8009_1_+1_1_144

HRATVLVILTQSMQQIQHNNKTIHTTTTTTITFVQTIFFVCTVLIHQK

>SG-AL_contig_8010_1_+2_2_235

VIIHHKRIVQFQHFTCSENTTCRSHTSHTKRTQSHQRHIDPLKNIGKHRSDSVQAKVSKL

KVPSENTDSIQAKVSKLK

>SG-AL_contig_8011_1_+2_191_316

MIFLVKYKKGRSNNDSLNATSLNKRDRAFYNIMRINDNNNNK

>SG-AL_contig_8012_1_+3_12_323

MKIKSDKSSQLLQPQAKLSATLSETVRDYSKYCKRMCNKKNVNMTRMFTVNKICTKNVFY

QECLQSRYIKNVYITHKKCVQECVQSKMCKIRKCTIMNVYIKNT

>SG-AL_contig_8013_1_-2_1112_1348

MKLLTEMFATAVHLKIIYDALFWEWPMRVGSGNTVLCPYVINLCSSFPVHAHCAWPYKHC

LCSAFGDFRSLFSVIHSGV

>SG-AL_contig_8014_1_-2_2_238

YGRVPVSYANYPYPGSYYVQRRRRSLAHRRFPHIHRRRRSGSSKQLARSKRTKRDLFDDE

LYYNPEEQYQLQQAEEEAE

>SG-AL_contig_8015_1_+3_3_221

SLSLSLSLSLSLSLSLSHSLSHSHTHSHSLTHTLSLTHTHTHTHTHIFFFSFPYMQLETR

TQSFNITVFISTE

>SG-AL_contig_8016_1_+3_3_68

SSPATDFPHNLNAIWLLTNEKE

>SG-AL_contig_8017_1_-2_2_286

VCVCVCVCVCVCVWVWELCVCVCVCVCVCKMVCSAILVSENCHYCVAYNARSLKNVKNGL

FPFRFVHLETGQIMETNKQKPTKILNQTSPVFCLV

>SG-AL_contig_8018_1_-3_798_1580

MMYCSDNYGMQTCLSVRFLPILYTVFGTRTPVCKKIFQVQSQQQQKNSPNKQKLASLPKE

KLSCWFWLVKRLSYICAKMFWFHNPRSLGSGGKTVHGNVLTAKSARKMFRFQFCPQPQSW

CQMSGSDRDLSITGSFDSAVDSRYVSSSIPARNIKVSLFAAHLNAPIILVVSVFRRSSFS

PHFKTLPVSSCSHVETFLYHIFLDKYVAKTISDKNMTVKDIALGVLMGQGTDLHTEAISV

LLPFPHPDGSSLLLSSQKMNF

>SG-AL_contig_8019_1_+2_2_139

LKVCLLKTTQLKVCWILLGFIVLDSYFCCENFYKFCCGNFTIRNFP

>SG-AL_contig_8020_1_-2_2_538

MVESQRVMSVFEETIRRVEIVTLLPYIMQNMDRFRISLGSELVELLKHHSVVISSYDEIR

QELDIQLERRAAMAMRKSPAPSEDADKELMEGKENMDEEAAEEEAAKQEDEGANIMRQES

TLSERSAAAQSTDSQLEATMRNLSLVAQQMSHSTRNILRAFTLNPAIMTVVTDHMSERG

>SG-AL_contig_8021_1_+1_1_135

FRSGYVHILAVPKMKMTRIQTVEKIQIEILAAQRRIQIFYFSRSP

>SG-AL_contig_8022_1_-2_44_274

MSVLSDYDIYIYSVYVGLGAFTIFMHMCMRVCMCLCVLVSDYCVRVCMSCVCMHLRVCAL

LTCACTDTMLEDFICSF

>SG-AL_contig_8023_1_+2_2_112

NLDHWMLTNLDHSYNRKTKMHEYPGSWLIICVDATKM

>SG-AL_contig_8024_1_-1_205_339

MTQPFIITHNTLKYEAKKSVLSRKPQCSYQQKIPRGNKISQDDNM

>SG-AL_contig_8025_1_+1_1_243

FFFFLLHCFILFLSLIHFHPFYIHTLSKCKQHWELAASVTTENRQNFSKNLQNNNNPHTT

QIYYTLLGTGTYILLTQLHFS

>SG-AL_contig_8026_1_+2_2_97

SLTGKTTFSATLTFKESFHEINIKILFFSPLK

>SG-AL_contig_8027_1_+2_80_235

MSGANVALVTHFSVEVYKTVVVFLISQIKSNHVAYSPADHIKAISGLSPHLK

>SG-AL_contig_8028_1_+2_62_394

MQSDWLSQKEWPIRMEHTSYLFWALSLYTIIIQIIGTQQSGPLQLKLKLTQLKVCYALHS

ADGSLLRAKPLLGAVMGDPILSRSVVLLTLQFCVCVCVFCDSFKAEVQKCN

>SG-AL_contig_8029_1_-1_187_375

MTHNTILFGQFSMSAIAKIYLIKLEQNVRAINKNNNKHTPKDKKYCLCKQSTGWPQVATL

KLS

>SG-AL_contig_8030_1_-1_514_3228

MAAERKRLKEDRLRTKNWKRWGPYLSERQWATVREDYSADGDCWRYFPHEHARSRAYRWG

EDGLLGITDRQCRVCLALALWNEKDVILKERLFGLTGHQGNHGEDVKELYYYLDSTPTHS

YMRALYKYPQVAYPYEELVEENAQRTVFDDEYELLDTGVFNNDCYWDVTAEYAKGTPNDI

LAVYTLSNRGPNAARLHVLPTLWLRNVWSWGSECDAYLEEKPQLSQMGPGKVLISHSVLG

TMVMEVDVGPEGDLPDLLFTENDTNHKLLYKGVNATPYVKDAFHKYVVDGDQNSVNPDRV

GTKCAAHYVVNVESEQEVTLRIRLYHHLEKPKATFAKAFDDLMELRREEADCFYDEVVGY

KSHSEQVVARQAYAGLLWSKQFYHYVVQEWLQGDSQQIPPPSERLSGRNVCWQHLFNRDV

ISMPDKWEYPWYASWDLAFHMIPFANIDLHFAKEQLMLFLREWYMHPNGQIPAYEFAFSD

VNPPVHAYAVLKVYKASGAKDHRDILFLAKCFHKLVLNFTWWVNRKDPDGKNIFEGGFLG

LDNIGVFDRSKPLPIGGRLEQADATAWMACFCVIMLEMSLILARRDPIYEDMASKFFEHF

VFILDAMNNVAGTGLWNDVDGFYYDHIRGNNFSMPLAIRSMVGMVPLFSSLVLHETELKR

HPGFFKRTKWFLEHKKSLASKILQRTADDNGNGEDTLLLTVVTKEQLVRILHHVLDEKEF

LSPYGVRSLSKHHAKNPFKFEVGQETYCVDYQPAESRSKVFGGNSNWRGPIWLPMNYLLI

ENLQRFDFFYQDTLKVECPTGSGKFMRLRDVASNLSQRLTRLFLPDPFGSRACHGKEDRY

SKDPNFSDLLLFYEYFDGETGRGCGASHQTGWTALIINLMKSAHHRKSLSGDSTSSLCGG

SHGLR

>SG-AL_contig_8031_1_-1_343_1716

MEFSAFSWATCSLLLLALGADLSWAASWTYEGEKGPLYWHELFPQKCSGKHQSPIDIQTE

VTMYNSDLRDFAIWYDPPRPGSTMTVLNNGHTVQVVTTGDFYVTNAGLPYVYKTAQFHFH

WGHAKHHGSEHLIDGVAYPLELHIVNYNSDLYDNIGAAVQATQGLAVLGVMFEESDEDNE

DIEPVIRAMAQVQDPEEGNQVEIEPLSLRRLLPKDITQYFRYNGSLTTPGCFETVVWTVF

AQPQRISKRQMATFRAQLQMRHKSGGHHKRSVRDPMPALRSERRKERRALQVLDELALTP

EQKERFRRELVETKENAEIMAAKMDEENAGLTHRRTLEAVHKSMDHENHADDDDDDDDDD

DDISEVEIEYIQEKLVNNFRPVQPLNGRIVYRSFKLRESSHSNCHHHHHSDSPKHSDLPD

EETLRKRLKLYASTTGAAGSNIISLLLLLVSCLVALFL

>SG-AL_contig_8032_1_-2_2_238

LRKIFCVFVSVRSDVCVHASVWVCCTQMCVHVFMRNIMGVHACSHEKRAYEQEQACSHER

ERMNKNKLSNLKIIFFNVS

>SG-AL_contig_8033_1_+3_3_341

MVLSTEPASPRYVCIHICTNIFSFIVDRDLSLHSHMHKHIFLYILLTDLSLHSHMHKHFL

IYIVDRPKFAFTYAQTHFLIFFADRDLSLHSHMHKHVFLYILLTETKFAFTYA

>SG-AL_contig_8034_1_-1_34_258

MHACCLFLRMRTWSELGGFPVKSRMYGLSCWGFLFVCLFFGLDAWAWLSESTFSLREKSQ

NCNLIAGPVLSLGSY

>SG-AL_contig_8035_1_-1_1_153

LQAVLALDSPLDFTCVTERVGLLHGQQQVLLNTLPWLVSQSLSSSTNSIVL

>SG-AL_contig_8036_1_+1_1_342

SGGSRCSGGSGCSGCSGGSRCSGGGSRCGRCSGGGSRCSGGGSRCSRCSGGGSRCSGGGR

CSGCGGCSGSGRCSGGRCSGGSRCSGGSRCSGGSRCSGGGCSGGCRCSGGGCGL

>SG-AL_contig_8037_1_+1_1_561

LCCTYSRRWRSSSCGGSRCSGGSRCSGGSRCSGGSRCSGSGRCGGGGRCGGSGRCSDGGR

CSGGSRCGGRGRCGNGGRCSGRGSRCSGSGSSCNGGGCGLCCTCTRKWRSSSCSGSTCSG

GSTCSGGSRGSGGSRCSGSGSRCSGDCRHNGGSRSRGGGRRSRSGRCSGRCSGGGCGFGC

TYSRRWR

>SG-AL_contig_8038_1_+1_1_108

EETTDKIKYTTKLIQSYIHKKHLFIIHTINLFCIMQ

>SG-AL_contig_8039_1_-2_221_538

MDNVPLSSDSDGNQSSVEQENVKNMTFSTGSLSLDADSNVDDDEETQRLISQVLELQNTL

NDLSSRVDSVKVENLKLKSENQVLSQYIENLMAASSVFQPATPKKK

>SG-AL_contig_8040_1_+2_110_313

MSLEESFRIIFARCHWTFHCKSFDAAALVLAITLPYLTLQCEAWHIGQGPGLAIFLYRLP

SVQLLSGR

>SG-AL_contig_8041_1_-2_635_931

MFIIFYTFLVHELHFYLSVLSMDCISDKLYSPELEVPIFFMFIDSVLFYVICFCNVYMFI

IFYTFLMYEMLKAAEQTAADRERWRVLVPAMCAMLDTGG

>SG-AL_contig_8042_1_+2_2_469

RGRERERGRGGGGESDNVFIVKANNPYTVYTHNHTPTYLCAYIYIYTYIHTYIHAYIHPY

IHTSIHTYIHTQTDDQPASHIHAHTTTTTTTNDDDDDDNVDDNDDVNDVDNDNNNNNESV

CVCVCVCVCVRERERERERERERERERDLARNCFFL

>SG-AL_contig_8043_1_-1_85_711

MDRRLIKPSAKRCLFGRPNDNDGFFERQKQLESAELQHFKRKWSLHSDEGEYEWTKASLD

IPEVYTKPYYHKCRRRAVRPCVGKCLSFVDSDEENICGVRPPSPEAVQYCDSNTVEVDLG

VFMGIVPPQPPEEQVTPKKSELGGSGDTPMSNSTNDTDHVAVSDRVCSQLLKRRRLTQKR

LTDMWRIVKRRQQSLTSTKSHQRVRINRL

>SG-AL_contig_8044_1_-1_160_336

MFSAIKCIDRKSEMFSPHTKKCTYRNSLNCSVSITNELSFVCERKRSCKTIRTTTTTKC

>SG-AL_contig_8045_1_+2_2_265

LYYSRTVALGPTGSVLQLVFVILHTYKHANRERERERERERERAKKCLSLPKRLSSVYHC

QRDCLHGRSLYLVAIFVLGRSARQAYDY

>SG-AL_contig_8046_1_+3_3_206

GSEVVEISIIFEVSITRVVPNSSPYLFYLPQLLTGSGTHLQLCGLEATCTVFLKPDSNPC

SQTGRREL

>SG-AL_contig_8047_1_-3_3_242

CLTAQVGVEVELPPSSLTAQVGVEVELPPSSLTAQVRVWLEKPLYLQPSVHIAEKGYLTL

FKVRESKTVKERRWPPSSHW

>SG-AL_contig_8048_1_-2_152_364

MHTCTDTNTWSFLESFTQTVFQFFSLQSHLFSTFLWNGIYLLLLLLGNVNKCINQYIDGQ

VVSACGIQFKK

>SG-AL_contig_8049_1_-2_2_406

GGGRGRGGGGRRGGEKKKKLLKKGLHRPKRSSSALGSILDLPFGGPKDGLSTQWRKFGLS

LFPRSSFLEAFVAKICLKPRQFNTHSTVCSFRAGSGYVFPLMSMVNRLTVTDIFCACWVF

SCSINHRGRHGLEDL

>SG-AL_contig_8050_1_+3_3_164

FQSPKSTYVQTYKCINPLCVYACRPSNMHVKDPVNRVCVQCLIKTQKHPACTLS

>SG-AL_contig_8051_1_-3_282_1499

MLGSEKREAGQTSSASEHTTPVSLVHGGGGINRTTPVSTTPRPNSFLCAGNSRGSHQLGS

NSSSESGQNGSQMSNMFGVSQSSHLSPEVKDVLPTSAQNCSGWWCSMCSKSFRSGDLLSM

HMQFVHRKPVPSVSASGGHQTAGMSTTPHTAVLPTAASTSSETFFIEPSIVSQSFLSDIV

DADLFQSDNKMSSSFSSTSQASKPQFTDNRMKEFPSNVQSSWMQDTQTTTKGEQNRTFES

QLSYSSQLLVSGGQNTQTMSNPFYVYPSFSPSSQGGDVMNTDYSSASCSLGTPGTLTSRM

LSRSSFTRRKPFKTGSRCKHCHKIFHFSYAQFVDHVQNCTKEKVGLECGQCGRSFASKVA

LKDHEDSMHSRKGFQCRACGLVFKWRTYVYQHRYKCAAYRIKKELT

>SG-AL_contig_8052_1_+3_117_215

MHRQSETALARIDLIYAMCKLCHVGFTNCSLLT

>SG-AL_contig_8053_1_-2_20_3052

MYQDVCGRYESAKQMESDLRQQILNLKKELLDRDDEVSKLQEQTKVMHEQLAQFDRSSPF

LDADLAAADRSTLEARKDLAALRQLEKTIEKLDQSMDQDRDATLEFDLSHSQRSARHSTP

SREERSQSGQPATRRARSLSPQRYRREVMERGGGGEQRLTDMQQLLELKEKELEAMRRAH

ANRLERLRSVQRSEKLLQEQIKTYEEHQPSRKKKKHQRSDPRQLQRENSDAVWNELSYFK

AQHRKLEVERVTLQEEADELRVQASVDAATVHELRVRLQAQREEFQYQLKKAEVATVNKT

DMEGQLVMLQSRVQNKDIHINKLEKDLQEMAVDRDNLLEEKKALKNQTNRLQQELSQCRI

QVADLSHDLQRKQRALEESLHSQGSHQEGETTTGQGQAGGSTVVPVRRKPTHRFRKPGRN

NPSVKQYQQVLNKSIEKMSGLFSNFREDGWEEISDSGETEEWGECEEEEEDTEATVDSLG

GAIVRQARGATATCHRSTPSPPLHMARSTVGKTSRRGRGQDRQRRVATSTRTKGSQSGLS

PPSKSVRVQIPSPLARSHSPTRERVVLREVATSPIPWGEEPTTSAAAGSRMHGVMKTDSP

QIAVLKRRIAYLTRQLALFRATRTIGLKKFAELKASNQQLTSDLNQANQRLRTTKLTCQR

LSGDVERVQREKQSLEQQVSAHDRTGEMDKHTESDKRLLEARLKMSTSEVSRQAGTIRSL

KTEVDTLQDQNRGLQDRVNHLERDNNQKRNLVESQRIKLKATQDATKTHDDAMEELQTKL

KLSAESTEKMRIQLDSLKKRMRVVTQEKRDYEDKYLQASLSLEKKAKELSATREQRSQLE

AALSKLESLSQQQLQRVASHSEAAVDAAQAQVSATHSTLSQYQQFVKLLCGELLDRVTRT

RGQLRDAAAAAAREEQARAAQDSDTASLKRAQDTARDILNISQSDLDDIMSADGDHAAQE

SSYVHEGKKDRRWLRKCEKAIGSKDDFVRPLVSLILQKVDERAELLMRLTS

>SG-AL_contig_8054_1_-1_529_741

MFVCVGMFVCVCVGMFVCVCVCVNVCMCVCECLCVSVKQLHFLMKVVVFYIVYIDDKLKK

MLYFVSRYFVN

>SG-AL_contig_8055_1_+2_263_406

MHVKDPVLSHVSGLLMETQKHPACSQQGLAVRKIQWDLILSLKNKVTN

>SG-AL_contig_8056_1_-3_33_641

MPGVENWTEKPLEVVQNIINRLPAGAKETAAVKEYFAKQLKCVDALAWVPSINSQALHQV

AADSLVRYRCMVQDMFDPELYLAKYKVTNRKTGKVTQCSGRYRDVASCATGEQIHVDDPS

NVTCERQTLYCVPVPGESPWVKSLYSKQCYKPSTPSTSSSPINRKRVRDEEGGDDGGEKT

EEEAMNGGGDMDNGDHSADHSLA

>SG-AL_contig_8057_1_-2_83_280

MFRNASSHLGHRIFTCYAHTLQIVSNLQTGRKPYHIIGVLKPAQPYQVFSHDLRLLDDHC

VKGMQC

>SG-AL_contig_8058_1_-3_12_1571

MASLLSRVNALQICSFLCKRPAVCLPLFKVTNHRYSVHPDNKFCKTAEEAIQDIPDGAKL

LVGGFGLCGIPENLIAALLKTQVKGLTVVSNNAGVDDFGLGLLLRQRQIKRMISSYVGEN

AEFERQYLSGELEVELTPQGTLAERIRAGGAGIPAFYTPTGFGTLIHEGGAPIKYNEEGK

IEISSKTKENRQFNGHDYIMEEAITGDFALVKAWKADKAGNLTFRKTARNFNPPMCKAGK

ITIAEVEEIVEVGEIAAEDVHVPHIYVQRIIKGPKYEKRIERLRLTKEKTDSKPPSDADL

LRERIIRRAAAEFKDGMYVNLGIGMPMLASNFIRPGINVHLQSENGILGLGPFPRPGEED

PDLINAGKETVTAIPESSYFSSDESFAMIRGGHVSLTILGAMQVSQYGDLANWMIPGKMV

KGMGGAMDLVSSFGTKVIITMEHNAKGGKHKILEECSLPLTGKNCVDMIITEKGVFEIDK

EKGMMLVEMADGENVEDIVEATGCEFAVSPDLKPMQQVEV

>SG-AL_contig_8059_1_-3_486_647

MTQAKAEAYTKRWLSFAMLSGDCPYPRQAPSNNLPSPSFVFVVFFSFILFFWHV

>SG-AL_contig_8060_1_+1_1_147

EKKNHTKILNDLKQRSEINCTCSNKVYIVIGIILKLPAFGFVHQKNGSI

>SG-AL_contig_8061_1_-3_189_368

MTSRLTPYSEIFSPDLGASVENAMNGQARQRRRRFVATLMKKWPACWKRANSALPAMKGF

>SG-AL_contig_8062_1_+2_2_196

EHKPTHVQSNQQLINTISMILMMTSVSDIITHILISFLTQEGMELTSASLQSETHGRLSV

IIPNS

>SG-AL_contig_8063_1_-3_3_284

NTIQYNTTQYNTTQYNTIQYNTIQYNTIQHNTNPGQGVPVGCFNDQAAFLSHFICPNFKT

PSVALPPCCSFQSLTHHHTVAHSVAASVPEVHWR

>SG-AL_contig_8064_1_+2_116_454

MLTWEIGKISTLYLSGEVSEEVQTQAFQTRQFGSRVSSPFDHCAHHTSTNPCSQIGRWEL

YLYARQTLTIYRVIFCPGKFNSSTYLAKKHNGDSNLISVKNQSSTGDTLYSLL

>SG-AL_contig_8065_1_+2_725_886

MWMVFNDLCVCACACVSSSSSMFVLAERRLWYRVSSFYSIQHNVLSILLSSVPC

>SG-AL_contig_8066_1_-1_1_207

FITVGQNLHSIQPANIFQNNNNDVNYLFSGKYIFSLDEAWNVIRNSKEFKLIKREKVHEI

LKLMDGTDT

>SG-AL_contig_8067_1_-2_2_127

SINEFYSKDISVTTYLSLAADHLLRAFNSSRFLPPTGQPQSP

>SG-AL_contig_8068_1_+2_65_361

MTALMTSPIYLRFTESTDLKNYYCTAFHTTEKDKWRKAKAKYRQVSGRYSFGQNTAQHFI

YIFIFQSAEKGGRCLDGERSARLKESTVQKSFCQLKLNM

>SG-AL_contig_8069_1_-2_98_283

MMPFLELTYSFTFLGIYNFQGYKSLGACTEQDLFESASETILILLIIQRTGSEKDKNTPN

RC

>SG-AL_contig_8070_1_+1_88_279

MTFSFILKCELKMVECSYEHMKIVSMDKMNCVAKQKHRSPHYKRTKMYDKILCTGVGFTQ

QHFI

>SG-AL_contig_8071_1_-3_3_203

PVRKFLGFPVVVVLLRLTFSCTCLNNMEEDDADQFSDDDFYNDLDKSDLSELNEEEGAEF

NAITSKA

>SG-AL_contig_8072_1_-3_3_122

FFGLLFPISFCVCYLNSFHGHTSFIVLVTVHICSHFNLLF

>SG-AL_contig_8073_1_+2_2_259

AFTYAQTRFLIYIVDRPKFAFTYAQTHFLIYIVDRPKFAFTYAQTHFLILLTDLSLHSHM

HRTFSYIVDRDLSLHSHMHKHVFLYC

>SG-AL_contig_8074_1_-1_2134_2403

MCIYIFMTVHTYIHTHELVWTQCCAALDVRVLFGFVLLFVDFFVVPCLPPPSQFLEGCLC

IRATMWSSCDAVCPPEPTSHHPDILSLDSP

>SG-AL_contig_8075_1_-1_217_1239

MSAKPTWMSGGSGSGVYDPVTTADNVRVQYSDQDDGWLRFKGRKYNFGTYRNSGLKGLCD

DIRNRSRPKCLLMGFIATVVLILLILIVVIASSRHSQLQTPGFEKWVNKSTGLNFQPIGH

EGTVFIPFTLQSGKEDSYLDYTKQLEAALEPYETIHIHNGPNIVCNDSYAPADAICQQPL

TVFGTGCVHAHRYGYSAGQPCILLSFTLPQNYTVRALAPKDGQVYVDAEAVLKERFSPKH

VGISCEPTTPEDKKYVGNTSALGDAIEFSPKMGFPTYFFRQRTVKRFVPPAVMVQFRTIR

DSVREPHMVRLTCTAWGKAKDPQGNLVNDKMFTTSFLFRIH

>SG-AL_contig_8076_1_+2_53_874

MEGVAQLSWPTLQVSACISGTWLTPGCIVRRSTENSPVTQPQSTMERHNIIPILIYHYCA

VKNCLRFCGPSSSPAFMVTSFPLGLGLVENENEQNGGSDTEATQKQHKTKITSFSQELRV

LFLEQTHLPPGSPDFDGVVIADGDERLLVARIEGHAVHYVVVRETLEMHAVVTLPQIAPP

VLRTACNEMVADIQEEEETLVIGRVGFVGPHDCADLGQGHQVDDMDVDFLGNGRGFQHSL

DLVVLRPLPTVHVQISVVRRESHAHNMSLQVEGV

>SG-AL_contig_8077_1_+1_718_876

MVCHGKQIFNVNEMLPSCKTVTKLTHFLKIRANVKRTTNSSFETPRRNKTYLE

>SG-AL_contig_8078_1_+1_190_339

MNINISDLFPMVTILFHHCACDKDYGAFTPTPASCWHLDGSQSFKILIIL

>SG-AL_contig_8079_1_+1_457_636

MHLEQQQHVSQLQHLLGFTNFLYQTTDLSVTENSDTAAPWQSNVQVGNRRPITRAMAVLI

>SG-AL_contig_8080_1_+1_844_1071

MTCAKRKNGIKQVDGKCPKQNVTKQVDGECWFKLHHFGDYIQLTAELVADAEVQLTVRTF

VDTQALSDPPYILSNF

>SG-AL_contig_8081_1_+1_607_720

MCDNHEAHYGKIIYSVMFLCILETFMSVFTFLQPPIPE

>SG-AL_contig_8082_1_-2_101_706

MKVLAILCFLGLVYSVWSGASKRNENCSCKHGYLFIDDNYCRCGVGECRRYLWTGKEAKA

GKGFKVPKTPGVGWSLCIKAKPTKFERITVDIINKGGSRIVSHFDIRMDALGMKDAVAIN

DGDVSHGNIYWNTQQNIYPRPYPFAVGREFDLKIVAEKENEFEYFVDGKSVGRRTLHVPV

REAEAVYVYHQADVLMVNTWCS

>SG-AL_contig_8083_1_-2_2_91

KKKKSSTKNNEFLLLKLHTNKNLVTSINVN

>SG-AL_contig_8084_1_+3_45_245

MSMFCSASKTRQNHWTITNTGTGTLSIPPTYTRTLSQHLMFSLPALLQSSHLLSHPILSC

CVQCKNI

>SG-AL_contig_8085_1_+1_1_198

FFFFLLLFPLSCLYFVCLLFFCFLFVCKYFDSIGFFCIILSETVPLLPCDLSACCTRNLT

LSSHPK

>SG-AL_contig_8086_1_+1_139_240

MCALPSQNSLLHNLKVRMLTVHPFCSTFLLYYYY

>SG-AL_contig_8087_1_+2_899_1186

MLWSGKKYQQERNDHNHSKTQLSMDSETALPMVHTHTHTHLFSLLFFCGVHNSFDGLYDN

KTSLSLSHTHAHTHFYHLVYNLPCPICGGLQKPCPI

>SG-AL_contig_8088_1_-1_163_267

MCGELIKFIKTPVPMSDINSMTRSMTVVLFLFSFQ

>SG-AL_contig_8089_1_-3_3_173

RPPFFPGPFFPPPAAPPPPPPTHTPPHPRVKHLASLTFTDVHIRPKLLSWPLLRQTC

>SG-AL_contig_8090_1_-1_226_453

MLIRQDHPCVHAPSDSFAVYSPTRSISKCHPPIPSPFVCVCACMLLFGFFLFVLFGVGFF

GGRGGCLVGWFSKLRK

>SG-AL_contig_8091_1_+2_38_181

MSASGSEESAGPEQRSAIHASWCLLRRMSQQVCLATGLSLKTGHRTKR

>SG-AL_contig_8092_1_+2_227_523

MTITNTKPTKLVPMIFLTFTPTTFSPSSKTTSTRRIYCIHHKCIPRLHFRGVLTDLQKNM

SSRRSTIFTTSIFLTFIPETLSLTYKNISSRHRYCINEK

>SG-AL_contig_8093_1_-3_162_320

MRLHFPLLLCFHFMLSLDVTKEALFKLCYLKFVGAEDYMSWAYFFLKLPFNFF

>SG-AL_contig_8094_1_+2_287_517

MNRQTSSWTFFIHIHTALPWVNMKSFPPYFPQRGKNHHDKTVSPGGLLIIYQSKNLRSIG

KHDRKHYTCTTIYNQSL

>SG-AL_contig_8095_1_+2_2_187

ICIKNASKRKDYKQNGTQKPTACHKRNRLLIYCTSVVSTQRQLQILVCLTKSPSLKQRIL

DP

>SG-AL_contig_8096_1_-3_270_413

MRESALVQLVNYHIQLRRLFFYYYYYYYYYYYYYGQLPYSITQTFLLL

>SG-AL_contig_8097_1_+3_3_116

VVVKFQEGKSPVLQLGIVSRAVDDNVIQAFCKCVTRGL

>SG-AL_contig_8098_1_-1_106_1029

MMACRDQTRPAPVDEQPVHIERQTAPAEDESVPAIEPSSQQQLLLTERQSNSELQSTTET

EQSVSEEVKEQSVFTEEQSLHDEPMQFEDQQESRKEPTPAQEQKTPVKEQAVPREESSVQ

PKELSKPASEEEQYAATEEATLSNKQLTPTEASTPASTPADEQQTPTEETTHADELQTPT

EEYTAEQQTPPVEQISAEERAPVEEKVSTEEQQSSTLEETPAEEQTPEQQTPPVEQTSAE

EKASADEQQSSEKTPAEEQARSEEKEPAKEKQAATEEQAPAEEQAPTKEIQEQTAPAEEQ

TAPAQEQA

>SG-AL_contig_8099_1_-2_2_205

PSGLSHPSLGLLPGRQGRGTPPLPGPLRQGLCSAPRRHSQRRLLISINEFYSRDISATTI

SRWRPTTC

>SG-AL_contig_8100_1_-1_79_1452

MYISKMSNKKSSGLDEISPKILKLSLPYISESLTYIYNISIECNIFPTALKEAKVIPLPK

TKDLSQVSNFRPISLLSVISKPLEKHVHKHLLDYLERRNLLHSLQSGFRQKHSCHTALTR

LADTWLSTMNECKLTGTVFLDFKKAFDLVDHDTLLTKLHAYLVNHAAISFFRSYLNKRKQ

KVLLNGSYSSEGHVNCGVPQGSILGPILFSIFINDLPLHVSNPTVCCDLFADDGTIHTQE

KSIMTINLRLQESLNEVSEWCTKNAMILNPSKTECMVICTRQKRQLGPLSLNLSMGGSAI

KQVSDHRLLGVIIDHNLTWQDHINYICKIISRNMYMLSKLKYLTDMNARQMFYNAHIRSH

IDYASTVWDGSSDIHLKRLNSLHRRAMKLIIPDTTLTTDEKLKKLGILPLSSHFMFNKSI

MMFKVANKSVPQYLLNLFNQRVLSYSSSRQGHFQIAHP

>SG-AL_contig_8101_1_-1_226_690

MPRLLTAPLKAVLSDPLVMVPTTPSLALTTKTWPPTTMLYMSRKRPKLRRSSRPCHVLTG

STRPKRARCRMCRPPPVSPTLPGAKHTNCGSLRPPSFAHNTPLMMSGGKGKGVQTETSRQ

EHPAKNGHNWGNNNSWAARTATGILVRRCSELENR

>SG-AL_contig_8102_1_-1_1_117

KKFLAVKGLQLCFWKLYSQSFSPVSFRVRKFVIVRTCWK

>SG-AL_contig_8103_1_-2_203_406

MDKLSPASVCHHGAELFVLCVEMKKNLTTSHSSLKPWSLLCNSHIQEYLGGGGWGFLVSF

ACLTLLCH

>SG-AL_contig_8104_1_+1_961_1224

MLTHLFIEPTCLCETASNATTFSDATLSAGFCSEEKGDSGGGRCESEKERKEVRFLYQSA

LRLTQSVSDTVKNWLPTDKWREHFHLSA

>SG-AL_contig_8105_1_+2_2_73

PNFNLPSQSKDIVNKEQSQVSKQN

>SG-AL_contig_8106_1_+2_1271_1558

MARTPFNNVHPVASVTHSSDLTKTLQNYITHNNPCVLQFLSIYKSDYVCWTGSEINSMAA

ACKNTICHKMTHSGVMSVQSHVVDLAVHPSTIQVTV

>SG-AL_contig_8107_1_-3_258_371

MFLCLFCFLVVVFFLYLSLPVKVDFSEEFLPGITYLLP

>SG-AL_contig_8108_1_-1_58_348

MGVDDDDDDDDDDDSPSLFDPTFHLFNDRVVISGLPDPRQHRRRWQRRQDRRQQGPGAGR

LGLRDHRRQQRRQQRRQQRRQQRRQGRRGERVQPQAL

>SG-AL_contig_8109_1_-1_1_261

GSFFAKVRLTLIDAAPFGTTISYKGLSIAAGNSNASQAVGQAMANNPFSLVVPCHRVIKS

GGAVGNYAHGRRNSVKQWLLKFERGEV

>SG-AL_contig_8110_1_-2_3068_3475

MFLGVVAIGLFSDFYGCFVLIFRTHCSNFYEHWVLSFMNASSHFDYRLWNAVATVLHSTD

GIISGFLYDTQKVQLILSSFPIFYIFHQVSLSLQLNAKTVILCRHNVMTCEAYLPPPSVV

LYFSIRAEPPCICKCV

>SG-AL_contig_8111_1_-2_62_607

MLVLLLGDLHIPHRCNSLPARFKKLLVPGKIQHILCTGNLCTKESFDYLKTLASDVHVVR

GDFDENMNYPEQKVVTVGQFRIGLCHGHQVVPWGEIESLAMVQRQLDVDILISGHTHKFE

AYEHEGKFYINPGSATGAYSALDSAIIPSFVILDIQQSTVVAYVYKLFNDEVKVERIEYR

KS

>SG-AL_contig_8112_1_+1_1_75

LYSPWLEWKKCNYLFRISIKVPDKN

>SG-AL_contig_8113_1_-1_706_1083

MAGLFFYWLQMFHIISVLKDMVTMHVVKIYYEKKCIHYFLIQCLIKAQSKSPVILSAVGG

RGVGACLLMELCSKLVYLRLRSWFVLSFIKINNLKYVYFDSHTHTHTHTHICKSWKLAKY

SFKRFS

>SG-AL_contig_8114_1_-1_55_591

MASGTCSEASALKKDFCPCHPSPQRTLQQSSSLEQSIGRVLPDKRWCQTGVPTLSSTLQP

FLERIVQESLHNYYTFISFGGKPVCSLHFADDIDLMRGSNNEFQDLSDRLVVRASAYGIE

ISMEKSKIMVNSTSDISVDNNINSEQLEVPSFKYLGATLLRTAPAQQKSTLRLPQSQQR

>SG-AL_contig_8115_1_-2_71_952

MHLFTVALVFSFCSHSVLASNVVDMKIVDEPSSYGFGNSNLQHWGAGLETEEKLKMRVKP

ANVSGPPHLSHLRKRCFSKTFENYKYNFCPFSNVTQHEQGYHWNPYNGVLGVWQEWEVEN

NTFVSMEMQDGDDCGQIHRSVKVKLECGNNSEIMSVSEPSTCHYVMIFSTPLVCHPQSML

VYPTLSADLRAKWDRLEGQRYRKELTEKGYKKRLQLILEEAGLRLTAKKRRQLSAAVVAH

EQTQEHAVQFHSLPHCSQMYRRLQQEVEQLRAELATYKNSSETRSAVNSSLTQT

>SG-AL_contig_8116_1_-1_385_528

MLSQMIASSKGSMGRGRGKGGIQNECMIWLCLCFCFKEQFVFIMNSPL

>SG-AL_contig_8117_1_-1_118_318

MLLDNVQMYGYKTNKYFIYYCTHSHAVGCCRFLTQPSQTTEKTLCGIMSHFFFKHRQHTP

KATHRPA

>SG-AL_contig_8118_1_-2_47_352

MIRKEAYVHKSVMEELKRLIEESEVMQEDDALWPTPDRVGRQELEIVIGDEHISFTTSKI

GSLVDVNGSKDPEGLRTFYYLVQDLKCLVFSLIGLHFKIKPI

>SG-AL_contig_8119_1_+1_1_159

NAMLQIITQRNVTNYNTKLELDTDWVCRPSYAPVVIISIHQPNHCHFSCHPKQ

>SG-AL_contig_8120_1_-2_59_181

MTKFYRHVCVCVCVCVCVCGGRAFISYAYVIDFIIDLFFFA

>SG-AL_contig_8121_1_-1_235_546

MASFHYSYSLVPGSFVPKRDVWICLSSCCRGNVLTNKEKHKHKTCVAMALVLCCSLINQE

ARGFFVIVGGYLFRVRSLLLTQWAQCVCVCFGMHTSDGGFMFGL

>SG-AL_contig_8122_1_+1_1_294

GAHLLSLTTLPSLTEESQVPIYSWVDWRLHAHIKFPGHDSHSHKLVQADRKASTLPLHNQ

ASYNIRNMTNYFSSPYRFQKHIIHVKRPADYFQNRNLH

>SG-AL_contig_8123_1_-2_71_199

MIFDLCIYFYDLEFVMFLLVCIFVSVCSILYIELLIFKRVQQC

>SG-AL_contig_8124_1_+1_112_372

MFNARSVGPSIKRSAIYDFVCDNDVDILLLTETWLCQSGDEAKHRDLTPPGYTLFSFPRS

CDSNLKRGGGIAIIIKAPLAEHCTITT

>SG-AL_contig_8125_1_+3_33_290

MFNARSVGPSIKRYAVYDFVCDNNVDKLLLTETWLHQSGDEAKYRDLTPPGYTLFSFPRS

YDSNLKRGGGIAIIIKAPLAEHCTIT

>SG-AL_contig_8126_1_-1_100_1197

MSLFGTSSFGQSNVFGSTATAVNTNPMKDFEVTSPPDDSVSCVKFSPGVLQSTFLIAGSW

DNKVSCWEVQQSGQTIPKAQQTHTGPVLDVDWSDDGTKVFTASCDKTAKLWDLNSNQCIQ

IAQHDAPVKTVHWIKAPNYSCVMTGSWDKTLKFWDTRSSNPILTVNLPERCYCADVKYPM

AVVGTAGRRLIIYQLENQPQEFKGMESPLKYQHRCVSIFLDKKTNAPTGFALGSIEGRVA

IQYVNATNPKDNFTFKCHRSNGASNNVQDIYAVNDISFHPAHGTLSTVGSDGRFSFWDKD

ARTKLKTSEQFDQPITTCNFNAQGNIFAYASSYDWSKGHEGYDPQKMKPHIFLRSCFDEL

KPSKRS

>SG-AL_contig_8127_1_+1_1_210

LPCCMKQRSKKDSPHKILHLLNNLPCAQTTARLPGGPTNSQSKNARVGHNRWLGGDAESH

SLTRKASCVN

>SG-AL_contig_8128_1_+3_3_254

ETLAQQASRKAQSNAEITYGSHHLHLSLCVVEGFLDGVDVGVEGVDGAGNDHAQTSCHQG

NAHQNLPTSLHDDFNVDLATLKIF

>SG-AL_contig_8129_1_+1_58_387

MAADATHTDCLVLFCCCCCFVVVVVGFFLGGVCAKYDCIKGSVCDFMHLYDFKDLLLSYF

KAILHDATRFKIMFTGPVSFIDVYFRCLFIIMMLHYSASSLSLPKTAGFR

>SG-AL_contig_8130_1_-3_423_596

MSVKNMQSATQGALGALKNKMTTIKAENDNLVEENEALRVSLDQKQQEVEKLETDMAG

>SG-AL_contig_8131_1_-3_30_944

MMELPENLPSPSKTETERSPSPSKTPTKTPPTLAITPTPTPTTPTTSAVTTAIPTTPTPP

TTPIKPTTPIKPTPPTTPIKSTTPTTPMPTTPNPGFSSPPGSSSKSEADETRSRCSRHSS

EDSSTAEDKVESLQQDPESEDGGDVEDGAGSSSSPEKTPVKQPPDMLDKKRKFDQVMEEE

TREEILRKRERQEEERLKMQILVSNLSEEQLNRYEMYRRSAFPKAAVRRLMQSITGSSVS

QNVVIAMSGIAKVFVGEIVEEALDVMEEQEETGPLQPKHLREAFRRQKMKGMVPSTKFKK

YAFRR

>SG-AL_contig_8132_1_-3_252_617

MSYNSVIPSQLMGMQRKVILVPNLMYGVCVYIFILICHCHHYYYYFHHSTSLLLPLLLLL

LLLYYYCQCLVGVGTPSFRSDSVSSIPCWGKLCVWPPVHPAVNGYRVREDKAVRERRCTW

PC

>SG-AL_contig_8133_1_-1_16_1032

MGEEKYGPEITIMGAPAPSTPAEKGPEYPMCQRVLTALFYAVCATLIVIVNKLVLTTYKF

PSFLALGLGQMLITVVLLAILRMAGFVHFPMPHRDILKQVWPLPLLFLGNIVFSLGGTQR

LSLPMYIVLRRFSNLFIMLGEYIVLGQVATRFIQFTVYLMILGAFIAAVDDLAFDFVGYM

FILATNICSSLSGVFTKQKLNNKTLGKYGLMFYNSLIMVFPTFVLAYTTGDIHKTMEFDQ

WGDPLFSVQFLMGCIMGFLVTFSTMLCTQYNSALTTSIVGVLKNLIIIYSGMIIGGDYIY

SFLNFVGINVSVIGGLMYSYATFVAKSRPSSANGEVSKV

>SG-AL_contig_8134_1_+1_31_258

MKESIHTADRQFHVVIMHSCSPGFTQQLASTAHRTVSFNILFIHLSHLSTECVADTVVNT

TPINSYYFQINLSLPH

>SG-AL_contig_8135_1_-1_559_882

MMYISIRNISPSLYYAWRLFSTGELLTIFPSPMLLHFSIISCSFIHGLLVGSVLRKILYH

VYVLLFLKKVPSYVYFSSYHTLTSHSTRFWNCSCLYFKFGMLAKGVQQ

>SG-AL_contig_8136_1_-3_3_161

KKKLFFFFFPFFFFFFPPPPPLQWLYYDCAFFLTCPVQCEAARPCGLVQASHP

>SG-AL_contig_8137_1_+2_2_106

KHSHSQQNLQTTTTTGQLEDYSIISLTLVFSSDTK

>SG-AL_contig_8138_1_-2_65_343

MNRKPVQKLKKRGNMVKFGRIPCSYFYTNQTHPFSCILSFFFSCLPFSSPLHFQFICRPV

HYFPYRSTPIYNSHSHTHRHTLFHTYLFLKKSH

>SG-AL_contig_8139_1_+2_140_280

MVHIFGPNLVKIRPEIAEKILLTHFFNRHTHTHRQKNTQTHIASQVI

>SG-AL_contig_8140_1_+1_265_429

MGCTHFSTYRSSHIHPNGKPDEVRTLQHLAWTERFSSYIVLASIFWDPGSLQKSR

>SG-AL_contig_8141_1_+2_2_73

GAVLRQRASSHRENIPNLHAVNPL

>SG-AL_contig_8142_1_-2_215_385

MILCAVNYVPTTVLPVACAQYTVTIINLVCHIACAQYIVTIISLVCHMSLFGFSDTL

>SG-AL_contig_8143_1_-1_118_1227

MNMANRFSQEGASPSPPLAYHVSLASQGMNQMSLSSGSGAVGPPPVQSSSSPMMGMFGMS

PILMPKAPRLQHQPSTGSSTSRSNSMDSEHSGTHHSSDLVTEPPFQPHSTPPLHPHRGGM

GVMGMVIPGTSPVSTSSQSSISSESSMRDRELASLPRRRSGSIQEEAAYTQALLVHQRQR

MDKLTEDIEDKRQILSQLRREVSDAEKSKIVENSLRTTFPTADDISRLCESNRRLQTDIQ

MYLNEIDMYKNGQTPFSIIDPMGQQNFFNNMPTGPNDLFTRPPIHRPRTPPPSPPPRPPP

PRVAPAPVQRVNSNDLSTTVPAPAPGHTQGGGSADSEEGEKWACSACTFENHPALKKCEI

CEMPRLVPGS

>SG-AL_contig_8144_1_-3_3_431

RLRLPSPGPGGLRDRRLLPGLERPPGLRLPSLPDPSEGDQEGEAGTGHLDSRCSEVASTT

VVSGSPLPLPRSPAGASSSPRPSASAKVRHPPRKPAPAPPSRLETLRDSVPARALARTRS

RPDTVDRSLVLGGASTDLRSLVQ

>SG-AL_contig_8145_1_-2_224_658

MMMMVVVMMMVMMMMIVGHWFDFQFVLICFDSPYIFVSLLSCDKGQLAANLRLLVSSVKF

AFSFSFRAFLVHVFCFNESFTLPETESTTKRNTRKRLFLKKNGVYMFIPIPPPIPSTHTH

TLIFYFATCSHTHLHTFYTTVVEYP

>SG-AL_contig_8146_1_+1_1_162

PNPQDLYMTKYSRKDLEFRLIVKSQIQFKQTPPTCPIYIHTKSSLSYPHTPEEV

>SG-AL_contig_8147_1_-1_1_132

FFFFIVLVKGDCQLGLTILFPVFQWTHKNVNNCGSQKIFNLIRL

>SG-AL_contig_8148_1_+2_2_220

LFFFAVLQNTLLVFVLLLRCPDIASKLRPALCVNSKSVNKDGQSTSRFRFIYTCYQYHCA

LSVGKHAAMNSPS

>SG-AL_contig_8149_1_-3_3_281

VCLSLCLNLRVGENIGVCTCVFMHVCMYLCACLMKIVFFSVLVYVLPYYIACLVGSCHES

VYVHLCVCTCVRTSMCLLNESCFLFCFVLCLTL

>SG-AL_contig_8150_1_-2_41_139

MCIFWYHLHDMKGRISVWLDHPMSLYQLNTSIF

>SG-AL_contig_8151_1_-1_1_288

LTIPCPYLYTNQTHPFSNILLFLFLYLPFSSPLHFSVSICRSIFPLIDLHPCTIATHSQT

EKQFFTFIRSLFPSGDARGSFSINSIPTFWKFCANN

>SG-AL_contig_8152_1_-3_78_263

MAYLCFIHFIHLRVRRAGHRFYLKIDHFLCSGSSAMSGLISIEGGDRVVIVSSLYAYFFF

FF

>SG-AL_contig_8153_1_+3_3_131

FFYKFIGAIYLYQYVYKPPEEKYAVVCVWFITYDNFGIGIQSQ

>SG-AL_contig_8154_1_+1_769_966

MTKTSSSPSLILPSVYDFLHRQRQYVQTQTQQKPGHKFSFILIKRDLIFVVGSCNKQDLL

TLSISF

>SG-AL_contig_8155_1_+1_1_204

PPTPPPPPPPPPNPHPPPPPPHHTHHRNHHHTHTHTQPPSQPSSHTHTHTPSHTHTHHHN

HHHTHTHH

>SG-AL_contig_8156_1_-1_91_309

MTTHALAFMCLFYLCIFCLFVYFFKNLVGCSTVNRTTVYYNLEYCYTWRVAVCSRKVTVC

VYTTFTLLALSYN

>SG-AL_contig_8157_1_-1_220_342

MCIYWLCQKSKLPKYKRLRKIQIEILAAQRRIQIFNFSRSL

>SG-AL_contig_8158_1_-2_2_229

LFFFFFFFFCHSALIYCVHQEMNENDQCDTDNRHLNFTVQRKQHIWIQPVSSGIGGEWHK

LSIYHDRVDFVDRWQC

>SG-AL_contig_8159_1_-2_263_916

MFQPSLPSEKLHAITGLGYGVVDKVFLEFDQPIVDSDVFRLNILWDDELPHDVDIRHSWT

RKIYSFEVVHEHILVGWLSGKEALYMESLTEEQVGEDCATVLKKLLQKDVPAPIRVLRTR

WGSDPLTRGSYSFVKVGSSQQDILELGKPLKSNGHAKPSLLFGGEATHPKFYSTTHGALL

TGYREANRLIKLYCPNPCEDHTDEESDDNEDNGLKMNF

>SG-AL_contig_8160_1_+2_95_256

MNMRTILNALAYIENSALLTLQDRRSPRDRITTTLHNTHGLGNTTTCKQCYFIS

>SG-AL_contig_8161_1_+3_141_209

MILTAACQLPSCHGLSLVMIISF

>SG-AL_contig_8162_1_+2_2_211

FHPIPFPTAFHAGIVKIRSSVHARPVNLTALVHNQSVRHFGQKCFPKWVVICFWYFCMWS

RITRNKNTKW

>SG-AL_contig_8163_1_-2_593_853

MVIVTGFNDCYSCLSTTCNYWLWEGVGEREFVLIVVLSSLSFQATIMMILIMVVVVTVVV

IMMMMMMMMMMMMMMMMMMMMTKSVVL

>SG-AL_contig_8164_1_-3_3_236

PGSPLELLVSSHSQDKTALVLDYFGSAYSAPVYSGSCRIRLSIHTESASPWSPSCPSSDD

PLRLLKQRPQHEFTLRGK

>SG-AL_contig_8165_1_+3_3_221

ALPIFFFKKSQLYCRKTLAKTICCILCTQLVLMNSLHFNELFTKKKTVTVTLVKHSPNNH

NKQDQHFTTNTTG

>SG-AL_contig_8166_1_+3_3_116

YTALTAKLKILFIIILLVTSLDPSVSQLIVPRGFLHLS

>SG-AL_contig_8167_1_-3_966_3308

MHSGRRKMAGRGASSLSMASSHAVTVVVVLVLMAVIRPSAQFTLIHRTPRAQHGDAISVH

GKLVANDNTYQPDPLYLHHHQQPDLLPLPHHQQASPLSFHQGPHSASLATSDSDTLSILP

PQAHTQPATNFRKTNHHGGEENFQTRHRFGTTPTQSRGSTGEPDAEAALNLKRDGLGKVG

ESTRKQTVVVGRSRSKHQQIATSSKTPDKQRGGSPVDPDIRLTKVVQVELNGGEFAQTSG

NFQREPAEPLPGGADEKLPGGGSSVESAGSNSEVLLKGSGQWSDKAVVVEEEGESQSLQL

PVAHGEGDTHVSKMVLTVIGSLTAILGVCVMAAIFQCCCRRKRSSSTEEKDQDQKSSRSS

RSSKSPSPQKVLVKDVEDEETEKGQSEQDKLLAEEDEDLGDFSVVQRETDILRSYRKPPP

PKNRARASRVPRALKTVDPKASPNVFQELLESEEGITTATNQTPQKATNGLTSPETMSPE

MHSSMLDDIDSTIAAMHVKDDKSTPSPSKSVDRSREPSAEKLHVAKNSGDSSQLKSATPK

NGSDNSQLKPRAKESPPKSLPTSPSREELAKALVQELSRSVSKEMLSKPQSKASTPKTSK

ETTPKSQSAGASPKPVHNKNPSASPSKDSVGSSSNGGSAKQGLSPEEEEDKKEAGGNIFV

LIDPTTKDKANDAGGKSKIPVRAASSPTQPTAPLRFSAGKDKRFPVPSVHGDQMDQLLKS

LKTRLKEDDGANPKQPSNKVSSEEESAHDDEDLDPQGRDRSHSDPTRQQAEKGEKPHQTH

L

>SG-AL_contig_8168_1_+1_1_138

AVQNKMHLIGQIQCHICVNKLPLVLDIHVICNRSEPDLRHGHFFFF

>SG-AL_contig_8169_1_-1_1_123

IPLEQTPYSKLETRQTQQYALVRILGTEFIFDASFRQTFCF

>SG-AL_contig_8170_1_-1_31_1020

MSAKNDESTAKPTSSKETDKDNGDVDGGTVKTSSAAKTDEKPGPQVTSDQRAMVTFRGML

MPQLSEPVALQVEKVKVMELRDSDVLLCSYPKSGTHWMWRILDMLLSGSAEYSSRSLSNT

MLDFENIDKINNMDVSLPRLLATHYPFDLLPQQVEVNKKVKVVHVYRSPKAMAMSNLLHM

KRLYSPDLTVERLVDMVLADEAPYGSMFSYWDKIKEFEKQNPDINVFHVAYEDMKQEPVE

CIKRLAAYLDVKVTHHLCSQIARAVNFDTQKQEEAKQSQSPNLATFYRKGEADDWKNHMT

VAQSERIDDVIRKRADTCLFSRHYLSSDAV

>SG-AL_contig_8171_1_-3_27_902

MASRVSRRVVQVVNLGRMGFLEAQDLQLRYARPHLEEMAGRPNAVGRDTLLLVEHNPVYT

IGIRTKDYDQIEEARLKKLGAEFYRTNRGGLITFHGPGQLVAYPILNLHHFQPSIRKYVA

TLELTLINTCRRFGLQARTTENTGVWVDDSKIAAIGVHATRFVTTHGVSLNCNTDLTWFR

HIVPCGLKGKNVTSLTQMLGKDIPIKKTVSPFLSAFEEEFGCELEYGLFDSEDASLLKED

EEGTGHGVTDGSIGHPPPIQTAQQGIRLLSTSASASASAGVVPQPRRKVCMW

>SG-AL_contig_8172_1_-1_613_816

MAEVNEFIFLLFKGLGPTLPSCLNFIGVCSRGIHRTRAIPYALYPNLSSYLVHSLTLQKP

YVLASLEM

>SG-AL_contig_8173_1_+2_2_220

AWPPDQDASQPTSPARLQHQMLRLQIRRKTTTALDSWCGRHTPHPQHDSTPGCRLSTSSP

RDTSWYKRKEKVK

>SG-AL_contig_8174_1_-3_3_149

KKKTEGKKIWGKKKKKKKKKKSTDLGCPRTVSALFACYYRPFQSLELRA

>SG-AL_contig_8175_1_-2_38_190

MRRASIQSLITLLCHSATFKPNLRQFSVLRKAGGKQSAYTYFKRKSLWFKN

>SG-AL_contig_8176_1_-2_2_274

SPDGVDSQLSSARPSVESLVPTHDRSVCDEVQQETSRLRLPSPGPRGLRDRRLLPGLERP

PGLRLPSLPDPSEGDQEGEAGAGHLDSRCSE

>SG-AL_contig_8177_1_-3_2217_2495

MSTRCKLRQLCPLLDTMLETLISSLFFKPSHFPTPLIFLGWSMTELFVVLRKFMVMVEDS

YGDRQTRWQTQSRGKTWIVNVIDLHLSFSFLFS

>SG-AL_contig_8178_1_+1_1426_1803

MFSITSSKICWLHMQRVSMYEQCICQPHSTYNIMYVPSKSLVSKPAPELCVSHNSTKEQL

AFFWSFNTTATTLLLLLRSWAATPTCTRICKWAVTCMAFSPCHVGSHASFSGVCHYKTEL

HKQHRE

>SG-AL_contig_8179_1_-1_205_426

MLECFSLSFRKLIYVLLLMKLKCMLCVFAVIQRSVISTVERKREKDMTINIFLGIVCNRK

LGSFFIKIFISHQQ

>SG-AL_contig_8180_1_-3_291_530

MYIQFHTHHEALKDPACCIQVPQTLEEFQQQFYILILLHVWCFSWALKFLLVFSWQIAHS

DQPVASRIQDDWKGSGVLIR

>SG-AL_contig_8181_1_+2_41_406

MYTETDRQTDRQSDRETETERQRDRERDRQTDRQKDRDRQRDRERDRERERESERQRETD

RERQREKDRDGYRDRDTATQTDRQRGQEVINRSRLSYNPEDEGQWVAETSNGSGTISLLY

LP

>SG-AL_contig_8183_1_-2_224_586

MMLMWLQLYVTNHAAFNIDEVVMLDKICPCRNFPLSYPPKILFFKLYKPTDNSPVRLYTQ

GPDPALSSYDNNGTADNAEHTHTHTHTHTHTHTHLLPISRPCQRIGFCAGSRTLPDTYWQ

I

>SG-AL_contig_8184_1_-1_148_288

MFYTTRQPACTTATTTTTTKKRKPSKQHGNKKQQELQNVVCCLRSPA

>SG-AL_contig_8185_1_-3_180_5501

MKPSETAALITILGVVISVFVDTVSSAACEGCTSTICICAGDKGERGAAGLPGLRGPQGL

PGFLGPEGPVGALGMKGGPGEPGARGAKGYRGETGLPGFTGVAGIPGQLGLEGPVGPPGY

PGCNGTKGLAGLAGQPGIPGEPGQPGRDGFPGPKGEPGSFVTDGLTIGEKGGQGDRGLNG

IDGLAGIEGSAGEHGPDGEDGIPGLDGIPGLKGMKGDKGADGIGIPGPKGQKGMKGELGV

AGITGTPPSNSTRLTGEKGEKGERGDKGEEGDRGLPGPDGPIGEAGVGGMKGDKGMAGPP

GRRGKRGKNGPPGPFGQKGMVGEKGFPGVPGVDGLKGITGDAGYDGQRGVQGPPGVDGEF

LESGGSGSQGPRGRDGQPGPKGPRGPDGIDGEIGREGLPGLPGLPGPPGFSGEYGIPGDS

MKGDRGLAGLPGQDGVDGLAGFPGLPGEAGEKGQPGEDVTGPTGDRGLPGLDGLPGLPGL

PGDPGLDGQKGEPGIGVRFPGPAGFPGLPGEIGEPGFPGTDGLPGQPGLQGFPGDDCGVC

PNGVPGAAGDPGDPGFAGADGFAGAVGFLGQKGEPGELGRQGEQGREGQPGIPGRPGPDG

LFGSKGFKGDQLPPPDIELYRGAKGMKGNIGYPGMPGMDGPQGDTGTRGLPGLYGPQGIK

GEPGQPGDDGLDGLPGLPGESGRKGQPGQSLVGPMGRKGQGGLPGLEGIPGPRGDKGLQG

DPFIVQLTQGDPGEPGLPGLDGLPGMDGIPGEPGQLGDDGFNGLKGMRGEPGLPGLPGEQ

GSNGPKGYPGLMGDPGFPGLDGRKGEVGPDGLPGLPGRPGLTGRKGETPRFAPEGEPGEP

GGPGEFGFPGNDGIPGEAGRPGADGLPGQKGEPGLAGLPGYPGEAGRLGDDGLPGLTGER

GRNGEPGAPGLSGLRGVPGDDGLPGIPGEQGGIGEDGAPGLPGLPGQQGLDGLPGNRGLD

GLPGPSGPKGETGLTGRPGSPGVPGLEGLPGRPGEAGIEGEPGEAGEPGIPGEVGSPGFD

GVSGLPGLKGREGVPGRPGADGIRGERGQDGLPGLAGIPGEDGRPGLPGFKGDRGQSIAG

EPGRPGSMGFDGLPGLDGVTGEKGQPGPMGIPGLAGLPGDQGDSGIPGEDGRMGLPGVKG

MSGQDGFPGLPGEPGGPGVQGPPGLSGRPGERGSVGLDGLPGRDGPPGENGEDGRPGLPA

LPGLPGLQGDDGPPGQDGIPGEPGQLGQDGLPGLPGERGEQGEPGFDGRPGFDGEIGRTG

EPGPVGRPGENGAPAPPGRPGPPGEEGIPGLPGLPGQRGFMGEDGFNGRSGPKGQKGEPV

PTTRGPQGFPGEPGVPGLSGRQGLKGMDGLSGLPGLNGMKGETGRPGPSGLPGRPGEDGI

RGEDGVPGLGGLPGLPGIDGREGLPGLPGIKGFKGEPGFPGYQGGGGAKGSRGQQGPPGR

PLPIQDIPRIKGEPGLPGPVGFDAPPGPRGESGNDGAPGLSGLFGQDGEPGLSGLPGRPG

ESGVPGRQGTSGLPGLPGEPGRDGEVGYPGVAGTGPGIFFTRHSQTSAPPECPIGTVRMW

EGYSLLYVMGNGRASGQDLGEAGSCLPRFSTMPFMFCNINNVCSVASRSDYSYWLSTPEP

MTPMMQPVEGRQIQPYISRCSVCETPSPIIAVHSQTMTIPDCPLGWSGVWIGYSFVVGTG

AGHQGSGQGLNSPGSCLEDFRSSPFIECHGRGSCNYYATSSSYWMATIERSDQFRRPLQE

TLKAGDLRRRISRCQVCIKDMSTGPAFFTYASTK

>SG-AL_contig_8186_1_-2_2_631

KPASSSSRAADRPSIDKGNGQYEETQYSDGKVERVYSNGSREVLFANGTRKEISSDGHYF

IVSFFNGDIKQVFPDQRVVYYYAESQTTHTTYPDRLEVLQFPNNQTEKHYPDGTHEITFP

NQTVKYLFPNGTEESIFPDGTILRIEKNGEKVLQFPNGQREIHTQQHKRREYPDGTVKTL

HNDGRQETRYATGRIRLKDKDGNVILDRQY

>SG-AL_contig_8187_1_+1_1_192

STCTNTANAMGWKRIQNNHGISHQPAHKPYAPVSSATKQVKTRLLYQQLKTPVDDAHTHL

YIGG

>SG-AL_contig_8188_1_+2_2822_3118

MVELDISLHSQAITNIIHYKMVKLHTAFIVIKPQMIHTCTATNNKVSYKLQIIHISHAFF

LTLTHKSAKKTVTHQEGTCVPTRNTEDNTAHQGFTGCKS

>SG-AL_contig_8189_1_-1_91_276

MHIYIYMWNQMCAVKSSGVNLFLFKGIEDVRKADESCQSLFLHVWCKSHPKVCCILSLFF

FF

>SG-AL_contig_8190_1_+3_3_164

RERERERERERVCVCVCVCVCVCVCVFFITDKTDSDLEYIFSSQLYNCSSLFWL

>SG-AL_contig_8191_1_-2_1286_1432

MGRVEIFLISQVNICADPLVHEPPLCVYAHRSSQTHVKNHDSVRWLIET

>SG-AL_contig_8192_1_-3_222_575

MQVPTHARAHIHTLTHTNTHTHIHSCTHARTHSRKRAHTHLNPPTPVYSHTHTCAHAHSE

LNTHTHTHTHTHTNAHFSLFCFISSPFSEVMHFYFFLSKIKSGTWFYFFLLKINTSIQ

>SG-AL_contig_8193_1_+3_3_146

EREREIYHFHIKSVLYFQLSTVYITSFSTHGVSQYTRTIFRHITSRQH

>SG-AL_contig_8194_1_+3_3_152

CTRGLTLVRQSAYILGDPQSVKLGNANNKQQETTRYKKNEVTYNINIRKR

>SG-AL_contig_8195_1_-2_17_904

MQKYEKLEKIGEGTYGTVFKAKNRETHEIVALKRVRLDDDDEGVPSSALREICLLKELKH

RNIVRLHDVLHSERKLTLVFEYCDQDLKKYFDTCNGEIDPDTVKSFLYQLLRGLGFCHSN

NVLHRDLKPQNLLINKNGELKLADFGLARAFGIPVRFYSAEVVTLWYRPPDVLFGAKMYS

TSIDMWSAGCIFAELANAGRPLFPGNDDDDQLKRIFKLLGTPTEDQWPGMTQLPNYRMFP

MYHVTTTWQQVVPKLNSKGRDLLQCLLVCCPSDRMSADEALQHPYFADLPPSARQP

>SG-AL_contig_8196_1_-1_169_1497

MALSTTCHLLCVLIFVSYVPCAVSAAKSRRLLMILLDGFRFDYLNQTSQPLPGFDKLAKM

GVKAEALVPDFPSLSYPNYYCLTTGLHTESHGMTGNYMFDITREESFLIGTNPEQYHAHW

WDGGDPIWITAARQGKKSHMYYWPGCGVEIRGLRPTFCQHYAGVPSMEDFRSALNQSVTL

LQSGQTDLAGIYFEKTDKMGHYYGPFSAELNSVIRELDPVISDLVDQLSQPEMSEVNLMV

FSDHGMTGLSQDRVIDMTEALDRSEYITILEGSSLASIYTMPGKEDQVFSKLDNFHPNMT

VYRKSDLPERWHYKHGPYVAPLTAVADLGWHIYTAVMKEQNMSKVIKGMHGYDNAYRDMQ

GIFLAMGPAFKAGQKVERLRTVDLYQAMCDILHIQPSPNNGSRSHFSPLLSSDVTISEEE

NSASAMAFVLLALVFSFFLFFIV

>SG-AL_contig_8197_1_-3_897_1103

MCIECFQKVGSVCVCVCVCVCVCVPSSGCFSLLYNIPPNPESFHTVPVTPPGAVSVKSPS

GFPPEETLH

>SG-AL_contig_8199_1_-3_120_1373

MSFNISPIIYLFVCCVAANASAPDSLRLVNVLYRHGDRSPVDIYPNDPFKADIWPQGLGW

LTQIGMRQQFALGQFLRSEYDNFLNSTYKNTEIVVQASDENRCLMSAYCNLAGLYPPVKS

QQWDENLLWQPIPVHTVPEIEDNKLALEKPCKRYDELYYKEIHGPVVQEEEKVNAAFYQL

LDHATGVKRENISVVWEVADTLFVENAHNLTLPEWTQGKWKNGTVYSKLMELRDFSFELL

FNGTQLSRLKGGPLLKEFIYNFMNATSGTGEEYHKLYMYSAHDTTVAAVLDAMNVYNQRA

PPYASAVLVELHEVEGHYCVKMRYRNSTAVPPHTLQHPKCQQDCCPLNKFVEVTRSRVPV

DWEAECDNPVTSSSHDDELLSGTILALTVLFIVLVILFILLCTSYLRQKRVVKTFENI

>SG-AL_contig_8200_1_-3_258_386

MFTCIFKKVCQSDLFSLDASSTSSSFIVHKVIFNLLGFFLHLL

>SG-AL_contig_8201_1_-1_184_1116

MGLVKRENEGLKAQLDELRQHMGATLSERYTSQQKGTAKIMNDYEQMRKELAKERENNEK

LRIQVRNLELRGEQNDRELMDTKTKLEIEAAKRPSSTSIANDQQGWKSAVMTRMYEEKIK

GLEEDVDKKTKQMSDMKTLLRDAAEREQTLLQEKEELQQKLAILERFPAGSQVTDTNIMR

EFQQARLQIERLENEKKELVNDLRLAQQQVGGGEQVTSDILLKARKYEEMAKENVEARLE

VKSLTLDNQKLQLEVDRLKKELESFGPDFFEEIEDLKYNYKQTAQRCTLLEDKLRQLSRQ

FGVSVGVPGIN

>SG-AL_contig_8202_1_+3_693_1040

MCITHVGLIFLFSFLHPFLTVHPPFTPTSDSRFPHLVTFTPSLLFAVCACQSSWRARPIF

SHPYQLDSPCLTASGKVIGCRGANPSEHFLGTIWNFGGEHSEIFFYEHSSTEHVCI

>SG-AL_contig_8203_1_+1_1_153

SWWTNAGVMLSDSLPTAPVGMLLLPHASHDAGAPKSKSKSVIIQSNYSVLP

>SG-AL_contig_8204_1_+3_114_275

MLKWKLHLFIVLLVLLGLVIGNPLCVRACVRACVRACVCVCVCVCVCVCVCTVC

>SG-AL_contig_8205_1_-2_2_118

TTLTCTHKIYRNSTCPRIGSIHVSWNYDTIYTPWNDSNW

>SG-AL_contig_8206_1_-3_1365_1664

MKKIVFTIFTNSFSLSDVSLCVCVCVSMCVYMHVCAHVCAAQNLDNCGTYMHSIRLLWNL

HSLGCSCGWEQKLTTIVNKPKLCLLQNPDGVQRQDTTWEG

>SG-AL_contig_8207_1_-1_154_1083

MGVLCDLASLSRPSLMTGGRVLCRRTNIPAKLYQAAAKLTGGNSMHFARLSSLCRNCCSL

PVFGMHLQHAPFQLQKSVLLDGRSGLQRTMRGALRGRGSVWTRNVDVRLMSDQAKRSDSY

YRRTGLIYVCAAGIFMLGMSYAGVPLYRMFCQATGLGGTVSRGHDTSKIETMQSVPERVI

KVRFNADVASSMRWNFKPQQTEVKVVPGETALAFYTAKNPTDKPIIGISTYNVVPFEAGQ

YFNKIQCFCFEEQRLNPHEQVDMPVFFYLDPEMAEDPKLQGVETITLSYTFFEAKEGFQV

PLPGHMTQAQ

>SG-AL_contig_8208_1_-1_70_897

MASALSRRAVRTLQARLYSAAARQEAKESNAPFPQQGPKLTKLPNGVTVASIENYSPVSR

IALVANAGSRFEDGDNLGVTHCLRVASGLSTQNSSAFNITRTVEQAGAEFTCSSSREYMF

YKIDCLRDNLDLVTKTLSQVTSAPAFKPWELSEVQNYLAVDLATLKTQPNAYVFDLLHQA

AFRDTLGRSLYAPDFMLGKYSSEQLLHYMKTYFSAGRLALVGVGVDHDQLVAQAKQFTPF

TPAGIVNNKAKYSGGEIRMDTGGDFTYAAVAVEGPS

>SG-AL_contig_8209_1_+1_1_150

PGGGSPLAGYRGSTLLGGLGGNAPLKLLIFSFYKLFWWPLLDLITEFHQS

>SG-AL_contig_8210_1_-2_296_664

MLVVNIFTSLSYLHLPSSPFHTLYFPPSPIDLHFPPSPIFTSLPLPSSLPSPLIFTSLPS

PIFTSLPLPSSPPSLSHLHLPLPSSPPSLSHLHFPPSPIFTSLPLPSSLPSLSHLHLPPP

SSS

>SG-AL_contig_8211_1_-2_80_271

MKCMVPKKYYAAKFNNYRLGLISVTVVEIIIGCMFLFSVPKMIDQWIFLWFCCCCCCFFL

GGWG

>SG-AL_contig_8212_1_-3_3_398

FFWPQNCKKKGGGGGGGRRGIKTLFSLTVLQTTHHKHWYTVVLLHLQFYPLLHKHRNRIY

TLAHALACARARAHTHTHTHTHNVCFHSSHLNVSDVLKRHTNLYCGRYHFAHICLNNNLS

LSLKQKYRHCNL

>SG-AL_contig_8213_1_-3_81_1277

MALIVSRAISAVQPHARQFLFVHAGRAQAISVPCMTQVANLTTTHGEPAKRKYPAWPYKT

RPYNSLYQFFDHTRKRFDDNTKIILIEGNIAVGKSWFGQQVAKEFDMKFVPDVKDEKIFL

KGNNFDLRSLNEQLPARSGFCDIETFYSQDGPAALLKTFGRTQLTMYYYHFHYYVSALEH

LLNTGQGIVMERGVFSHMVFHNVLRRMGHMSASALKYLNFVYDNTVCEMWRPHLVIYLDA

PLDFVRTMISKRNVGWEAKSPIITDEFLTTMDKVYKEKYLPYMSKYSEVITYDMTDLPDW

EIIVEELESLDLDNQPFDNEDKFKDWQSEFEDDYNLMRMNIAKKWQIDNLFSMAKPWEAP

ELLIHPDDADVVCQVVREHPEVKYRAGYNPETTNVLFKW

>SG-AL_contig_8214_1_-2_2_265

ERKYDQGQQLCLCIHCLETGEKHEFSTLHQKNHFSCEQAVSDVQHTQLKSRTVTDVLNMI

IMCFQESIIMDKIFCLKMKVDSRGCDVQ

>SG-AL_contig_8215_1_-3_90_308

MHSFKHFISSLGPTMQGVLLKWAFESNFFRFFFFKQWVRFVCVCVRERENGFSIIVCFHC

KKGCDLCFALNNT

>SG-AL_contig_8216_1_+3_3_452

PPTHTHTHTHTHTHTRARTHTHTHTHTHHTHTYTHTHAHTHTHTTHNTHTRTRTHTHTHA

HTHIHTHTKTHTQTDCDRHTCPIPHILSHHTHPPGSNLTTTNKQANKQQTNKQTNKHTYK

QKANVLPYHTHTHTHTHTHTHTLQILSKTD

>SG-AL_contig_8217_1_-2_158_1270

MAEKHNFGVKPVTCHAVSGDKNGLALSLNDHEVKIYKKNGSKWQETDTLDEHGQRVTGID

WAPRSNRIVTCGADRNAYVWTHQDGKWKPHLVILRINRAATCVKWSPEENKFAVGSSARL

ISVSYYEDEHTWWVSKHIKKPIRSTVTCLDWHPNNILIAAGSTDFKARVFSGYIKEIESK

PGPNSWGSKMTFATAMAEFSNGGGGWVHAVSFSPSGEKIAWVGHDSSVSVGDSTQGNQLV

VIKDTFLPFMGITWVTENSMVAAGYDCNPVLLTYNGGQGSLSVVSKLDIPQEKAAGTISA

MNRFKDLDKKASSGDSSAELKTQHQNTITQVSVFDGTKDHCKRISTSGVDGNFIIWDLNS

LERSIQALKIS

>SG-AL_contig_8218_1_+1_220_624

MKTSKTVIYAHVQRNTGCSTLYQQSSFSNSFIHVIIILEVTKIGFHTLTNSLLFPNHLYM

FYIIVKVVVIELPDRQAGYMTKIWLQSVYTIIEKSQLNVLTSWACVFQITGLTHLNLRIC

EIPTALSKDVVRMHL

>SG-AL_contig_8219_1_+3_69_398

MEPWTWVLQLRNRPLQKELGDLLPQDPLLLLHAGMEGGGVGHRGRRASSQPRQPAPRFQH

QNSEVVEPQLALMRSMPRQAACLEGLNDWRCWVRAKSLGCCLDGCAPPWP

>SG-AL_contig_8220_1_-1_202_804

MRMEKQEQALGEEIAQLLLKGAISKLQNTGRGFHVRMFVSQRPLGVGDRSWIRLPSITTS

SQQIQNGDPGSYPGHSAWRLGYLHQSERHLLSDPFPSSTPPVSEVHVEGQDVAVRCPPFR

TVSGPQGGEGTGSLGQSGGHSAPGVPGQLAPGSVPDLGRWFRTVVDREHLLTAYVPGGAT

GTCEVRLYIIKGTWKFILRGT

>SG-AL_contig_8221_1_-2_101_586

MAQVADGGDSFSTGIRKLAKRCNSFFEEGSSRKTCVGFFIDGTNVGFIRPNVAAKFQKFP

DVFDVVESDSKDGRRAGVHLSQNLKTAEERTVAVNTVVEKMREDSKDLIALRGWYNEKYK

VSTSFSSEALMYVERAASPLFGIVTYGTHINGYTYNDSGEML

>SG-AL_contig_8222_1_+3_930_1199

MCCVRCCVRVQFSQKHVLRNSWYWLAWRSQLFSGLIILARPRPTANQPNQRRWPRGECTH

HKKWHHMRHQDFDTLSHILSYLGLMHCTLG

>SG-AL_contig_8223_1_+2_2_238

GGVKQLLPKFQLLHGILHRKAMIIKQFKLNIWTCTTASGGVKQLPKFQLLYGTLHRKKKN

IQFLHPTLVYFIQKREAIR

>SG-AL_contig_8224_1_+3_3_392

KKKRRRRRRRRRGGGGGGRRRRRRRRETEEEEKKKRRRRRRRRRRKRGGGGGRRRRRRKR

RKKKEEKKKKKRRRRRRRRRRNPKQQQQQKTGGEGEDSDHTKKYNQIQVIIFVRIFGKIV

NKSHFTYTYK

>SG-AL_contig_8225_1_+2_2_187

CFNDGWRAVTIIFMKKNGACRFGGSFEYCRRMDDLFSWTVTRAVDIHNEFRGDGSEFAFG

GC

>SG-AL_contig_8226_1_-1_1_165

LFLKRTHISKSFKSVKLVSRGIKYFIYIPVRSVCSVKPRKDCWSPFPLHPLSPSI

>SG-AL_contig_8227_1_+2_140_244

MNKYDLKLQDLLGQWVEDFKGQSAYFREPISSAEC

>SG-AL_contig_8228_1_-2_116_223

MQILFRVSKNHMRNWNFQALVHQGKKGCMIDSNKLI

>SG-AL_contig_8229_1_-3_240_509

MHNPCMSGGTSRNEWSTGGFDWRATVCWTRLVIVSCPLCGFLKYLCLIVHFLSLFFSLVM

DVHSEWPYGGMNFDWYPTVNFDWYPCRCEL

>SG-AL_contig_8230_1_+3_213_749

MCGAQAFWEVIQASFTAHSVCSYANTCWIFTVLCKPDQVSHQKDTKRERARIIPETEPCL

NAVSFPLSLSHTHTHACKIYLKIKIYASRIHATVEVEPEVFSFKEYNILYTLFHISPTIT

YHFTSQIYFFGRRRKSGSSRGGKGAGGTCSSGRGGSRNSSGSGNSTTCFYWNVFWFVSV

>SG-AL_contig_8231_1_+1_1_111

VPHISNHTPYLCCMFYLKGILLNTLEFPPESEHTVLL

>SG-AL_contig_8232_1_-1_412_1284

MWGPWTRCCTASLQRAKKLWTVKMPATIMTLEAIDYKPKGFKAVAVALSNRQVQLYKEKF

LVDVIHTEDVITGLRFGRFAREEGTLVMSTKSGSLFVKILKRSAKFEEKDLSAGPPAAQS

VKLNVPKKTKLFVDQTLRERENAVAMHRTFQRDLYLLRLNVAREYAKALDNSMNPISSDP

TEPLKLSAQVQGIGPTFKLTVNLQNTSLTQASSNLIIAFEYDDRLYSFRKKFIQVPMLVP

GLNYVFDTFVDCQNDKGVSDNVKVFVLKQGKSVPLITGVISMPVAEVVVMS

>SG-AL_contig_8233_1_+2_1631_1861

MPHKPTPIHPPTPLNSHYSINSQAILNKSRNYFPSNSLRPHYNSVNMLHFTCSLQNYISH

QILYDCDYNPINMLPFA

>SG-AL_contig_8234_1_+1_1_444

FLRLGLLGLGRGLRFGCLRFLYLFRLGFGCLLCLVPQPEGTRSPGTFGLLQGAVLYASTQ

RQLQVIVDRLLVLPHVEIGHYVFEQRLTRGPTALFQGCDGGTNHLAVFRVVGRFFRLGCF

FLWRLHRRGWGACCIVRHDDVQILLSVH

>SG-AL_contig_8235_1_-1_1_117

HTHTHVKQYSDRMQQERKETSFLSLSDLAKFKNNKCRKK

>SG-AL_contig_8236_1_-1_115_2232

MPGDDGLGPRVRDNNDGTICVEYQPKCEGRHEVQMMHNGNAVKGSPFSCVVDSTGGGFVT

AFGVGLVGGQSGEEETFTVVAKQGTAKDIDIKIDGPAKVDLKRKDNPDGSTNISYLPMTP

GAYNVSIKYKGTPIKGSPFNAKVSGEGRKRSQLSLGNSAEYSLKVIEADVVDLQGTIQTP

AGNIEPCILKKMGDGEIGIASFNPRTKGEYKVNVMRHEKHINHSPFKVIVGDNEMGHAAG

CKVTGTVDKGKANTPNEITVDTTNAGYGGLSVAIEGPHRSDIECYEVHDRKYVLHYSPHE

PGIYILNVRFADDHVPGSPFLVNVTGNPSGRVRETKSKQMEAAEASVPNKNCEFILHIPG

TNPFDMEAAVADPDGTSELCDVMDLEDFHYKIMFMPKMNGVHTLSVRHKGMHVSGSPFQY

SVGQLSLGGSHRVQIGGPGIEKGEVGVKSACNIYTREAGPGLLTIAVEGPAKAEISIEDR

PNGFLGVLYKVSKPGMYGLHVKFDDQHIPGSPFMVNIAPDSGAARQVTVHSLKDRNLAVD

KPNTFTVNFNGATGKLHASVRTPSGGHEDCFIQEMDKGVYGVRFIPKENGVHYVDVKLND

AHIPDSPFAMMVGAASADPAMVSASGETLEHAKCGVKNKFVVHTAGAGSGLLAVFVDGPS

KAALSCKEVDDGYEFTFNPFCPGDYLITIKYGNIPIAGSPYKTNVS

>SG-AL_contig_8237_1_-3_3_287

CGSLDLSFSLYWAVALTFSLGDMGVLELLLQLFPLLYCFPCVCGYPFSLDAFPVAGLFCC

FSHGFVKVVNSHHCFILEDAELIPQCDVEFFDLVL

>SG-AL_contig_8238_1_-3_3_248

LLSFFFFSLSLSLSLSLSLSLSLSLSLSQNRNGNVIVLFQLVKFCCWFFCFFLILTSVDS

RVMMTGGDIILRSQHTQKSLLY

>SG-AL_contig_8239_1_-3_12_1538

MASSGTKSYGLFIPKKANKGALSKPASANVFGDDSDEESGTSAVSQTVNRDSTKAKVKKQ

TQLEIDKALQEDPNVYEYDTIYDNIQSQKVSTDVKEAHKVDRKPKYIAGLLKMAEVRKKE

EERRIERKVQQEREEEGNQFADKEAFVTSAYKKKMLEQQEEAERERQQAAMEEMLDVTKQ

KDLSGFYRYLYRETTGAAPADSQDADNTEDMDKVKVKEEPHSDNENDKSSSGKETKHHKE

KERRHSSDKHQDRKGHTDKVRRSRSRSPAHKSKSDGSSRRRSRSREREHRHHRDRRSRSR

SPTHKKSHKHRHRSRSRDHKRDHSSSHDRTKRRSGSAERSRKRDEDDKPRKETRKRSHST

KEESSPSADKKDESVKNEASAKSPSPSPERSSSPVMLPARPPDEDLDRDDPDEDLDRDDP

VSESESSADEDQGGKKKSKERTEGKEQTAESGDSGRKREAPPESSGSKKEEPAAKYGRKT

TEGEVLDARQRYLARKLARERAKPVVDPE

>SG-AL_contig_8240_1_-1_1_225

FFCFVIYFYSTHHDGITFLSEACLRHVLVGQFLILFLVHPVPFSVVSFLNCDMLTRFYGG

IFYIWGKVSLSSVCA

>SG-AL_contig_8241_1_-2_5_1288

MAGRSPKAQRTFVVGVGMTKFERPKSKDWDYPDMAKEAGVAALKDAGIPFTDVQAAVASY

CYGEPTSGQRAVYELGLTGVPVFNVNNNCSSGSTALMMARRLIQSGLEDCVMAIGFEKME

GGLSERYTDRISPVGRHMNHMVALGAEPGIIQPRMNQMTSDVIKLFAYAAREYMDMYPQV

TVEELARIAQKNRKQGENNPRASFQKAPDINTIATKAMLCHPITFGMTAATADGSAAAIM

CSEAYVKRHGLENKAVEILAQHMVTDLPSSFGTSFRDLCGYTMAKTAAQQCYSDSGLTPA

DVQVFEVHDCFSCNELFMYEALQCVPEGQGLQMMQQSSWRSNKQGGQLLYLNNKWVFNPS

GGLESKGHPIGATGLGQCAELVWQLRGDAGKRQVEGAKVAMQHNFGIGGAAVVTMYRKYQ

PSVPQARL

>SG-AL_contig_8242_1_+2_611_727

MKIITDKNKEQWSSQEHCKNSHHRNVQQVSIKLGWWGKK

>SG-AL_contig_8243_1_+1_304_546

MPVCATEINTHTHTHTHTHTHVFKHTHCVNLPERIKLTVSGKCIVTYCPLWHWLLGQQQR

SSISVCSGPASEWYPKCVSGP

>SG-AL_contig_8244_1_-2_992_1261

MSHQTLTCFTRSLTCVFDVLRTYTHEGGSGTSRSAHILTWEIGEISRQKKKLYQYFSNSH

VSQIWQRKIVNYKTRMCLKIDTHCMIYYLK

>SG-AL_contig_8245_1_+3_3_308

ASLRVLLKFPDQHRRCLFLPVVDARIYYERKRRVQPCHAVSVQKEFRHTIIIIRILVHYH

NIVKSCPNTLRPLQWCGRWERKTVRMLIELYYMHPNQLHFFC

>SG-AL_contig_8246_1_-1_7_567

MATSGSLLLRSLGRRLAVCHWSFKDKTFTARWTEWNRLPAACSLARFQHSDSFGSSTTTG

AVATAAEAEKTPVVIDNDLVVKLLKEMEDNKPVSGMPDPYQKEHKKCILCQHNLDVNYKN

VRLLSQFVSPYTGRIYGRQITGLCIPMQKQVATAIKQSRIAGYMPYMHKNSEYMKDPRLF

DPFRPAK

>SG-AL_contig_8247_1_+1_1_123

RWSGRHTPQPHHDCTRLPVCLQAVSFTPRDTSVYRQTEKVK

>SG-AL_contig_8248_1_-3_177_1727

MLRPTFPGMLRHVAKNIFHMVFVVNPADDGSRELLKMAEAFYVHSAPIRLGIILAVNGDP

KVTGREDAGVAFACTFDFIKADQSSAKALSFITDVYEKVGSGEISAQSIIAEFSAQYPGE

DMDMVFGSNSDYDDTRRAGVDFVSRSGLSGFPQVLINGVPMEKKYLSHDAFEEGVVTEIL

KQTPKIQQAIFNGELSDRMEVLDWLMEEDNVMPRLNSRILSPATTHLDLTENMDGSSLDE

PDTFTLLGSREMSAVVADRMKYITKRDEESVRAVSLWVVCDLETAEGRDLMYSAIKQAKS

SNDLRLGIVFNSASPSGTNLVSRAVQVALQTLPNAMAKSLVTKLVKEENYADLMAGTKTL

RDLEVHGMDMDSYLRDVEKEKGHFLQSHAVFVSRVLHFTPSQRGLVINGKVYGPLEPEES

FTPDDFSLLEKLVSVQSGLKLKHQVQGVGIPGHKGSDMVMKVSALLSTQSSSETRHKVSF

KGDKHSVVKIPAAADAPAFTVEAIVDPASREAQKMAP

>SG-AL_contig_8250_1_+1_7_219

MLTWEIGKMSTFDPPPGRRCLGVCVCVCVFCTYICVCCAFCVTLCMRVCMSVCVCVCETV

CVHVFLFCLFC

>SG-AL_contig_8251_1_-1_1_135

HPHTHPHPFSEINVDFDVKEGSKLRQRTDPVKKESTPSPNNVDFD

>SG-AL_contig_8252_1_+2_602_832

MHLFPHMIYSHFKLGTVWEGGGGFQSWENSTNIPSNKLQTPFQEPTFDSHARYSLVSHSG

SKAYLVTEVLSFAAQQK

>SG-AL_contig_8253_1_+2_2_409

CCDQNIHCIVLYCIVLYCIIFFWSHWIYHVKFGLLSRGEHVTTVQRHPFFSFLVHAVFIR

VSIGHRTRTWLAGSLTCVFDVLYAYTHKEIPIRSDLLPQTALSLSLSLTRSLCLLLADSH

THTHTNWRVSLMQSLI

>SG-AL_contig_8254_1_-2_89_337

MAQGEGHSFISDQQSSESVDKILAVAGALYWVQKRNCARDKNRLKILSVGLLLLLYIVYS

PADHYGHIRAVTTQSPSKSRRQP

>SG-AL_contig_8255_1_-2_2_250

TLLGDGDRSRKLLGCGCCAVGRAPRGDGDRLRALPKGHATLFDPVIDSIRSVYSVLSNRH

VVQHAMLHCTISQHWSTRVTDKI

>SG-AL_contig_8256_1_-1_154_333

MIRSDQFHSVLSSRHVVQNAMLHCMISQHWPTKSLTRPNQLRHTPGPNRFHVTDVIFKQS

>SG-AL_contig_8257_1_-2_2_346

ARGFKAHRRDVVSGRSPPPYQCPRDGGCVTLPPRLRRPAQGQGGQAVHGQHDCRLLREQA

GGHALLSPLFQGGVASEAVPEHRNFSECRSYSRERQRPRRLVEQGPLHHPHGVDP

>SG-AL_contig_8258_1_-1_211_963

MTELLEMLSNNRNTLQARDDQRRKDAKWARYMRCDGSPDPTNQGEINTYINLKIENHERD

DADNVLKDSQLDLALIDELEFLLTDTPLEDLPHVERQRYRETIGELQHLIQAKLDAASLQ

VLCDASKLQDQETSNLQYTLKNDTISLCTWGNLSKNPRIKMFEFTESGFAFDIPRVLTLS

SCAMRYLHTKFDHFSPYSKAYYPRRKKKEEPVAPEPEVPPEEEKKPEEAVEEEEKKEGED

EAENVDKEDTM

>SG-AL_contig_8259_1_-2_191_319

MSVVMFCTQLSCLVDMFVVMFCTQLPCLVDMSGYVLYTTVMFS

>SG-AL_contig_8260_1_-2_140_283

MSSYHCVRSHHSDMQYSMTCFLDNSLGNILALLCFSPEGNTEPFDFCI

>SG-AL_contig_8261_1_-3_444_1484

MMDYTYQHQYNMYHDYYTPPYAQQYPPYSMVNFLTSGTGTTPTLTPTTLHNLEQTFMELQ

SVSHNGSGGASSGSGATGSCDPHTQSGFVPPIVDPSSREQSQDSFESSDTDWEPAVKRVK

GEYGSILSSSSSSSSISGAPQRKYTRRSRHEKLPPEEEHRRAVRRERNKVAAAKCRQRRV

DHTNSLVEETEKLESEKSDLENEIQALQQQKDQLEFLLHAHVPICKVEPRNFKIKSEPTD

FTTLTTSACTSLHPSMSSSSSSRPKSLALDRHRHGDLPVVVSCAGPGVPLSTPSSGLFTY

TSLDSLVDGSTGLTPLTTGPISCSSQVHRSESESGSDPVNSPTLISL

>SG-AL_contig_8262_1_+1_1_255

VISVFHRSHSPPHFKTLLVLRVLCRIKEFTRQLLADLGGAHMKLSKCNESLLNENKPTPK

ILMYYIYICHAAGMYCPPIISVDSV

>SG-AL_contig_8263_1_-2_218_409

MLGEILPILDLDFKQIEKRSEGYRTEDKTPNFPQPKQTLEFYIVNEDLSVSCGACGMNIL

LTWR

>SG-AL_contig_8264_1_-2_122_1795

MAGVADGVEGLTLAEIYTSESTGCDKTGDGSKDKPFKTVIQAMRTAGKEPFPPIFVDSKT

EGERYDTLSKTQKKKVTSLWQTEQKKLNKKAGQETEKAERREKNLEEAKKIVIEQDPSLP

APKQIKIRDSVANRDVRVKVFGWVHECRRQGKNLIFVLLRDGTGFLQCVMTDKLCHTYDA

LTLSLESAIAVYGVISKLPEGKQAPDDHEMKVDFWEVIGAAPPGGAENLLNEDAHPDVQL

DNRHMMIRGKNTSKVLKMRSVIIQCFREHFFDRGYFEVHPPTLVQTQVEGGSTLFKLDYF

GQEAYLTQSSQLYLETAMPALGDVFCMANSYRAEHSRTRRHLAEYTHCEAECPFISFDDL

LNKIEDLVCDVVDRVLKSPYASYVKDLHPEFQPPKRPFRRMNYTDAIVWLKENSVTKDDG

TFYEFGDDIPEAPERKMTDAINEPILLCRFPAEIKSFYMPRCAEDKRLTESVDLLMPNVG

EIVGGSMRIWDVEQLESGFKREGIDPTPYYWYIDQRKYGTCPHGGYGLGVERFMCWILNR

YHIREACLYPRFVDRCKP

>SG-AL_contig_8265_1_+1_1033_1290

MLRFHLTRLHFHYKAPKVSLSSLVSLVLVFNTRVLNLVSTRLLCHFSRQPRHVRQDISKM

KIWLQESERSEEESKRSFLNIQVNQE

>SG-AL_contig_8266_1_+1_799_1503

MILIPRLTVNPQLSNLSIHVVQSPVAHTGYLMAVKRKQPEKSGSIPPQTYKKQCPETETA

TTLHPTTTQAISGLSKKQNKQKQKTAMSPVFNHFTPHKQDYAMSVTSKGFILMMMLRLLR

CFILQEYSIFYKPTVNTGAINLDKSNNQKLMSSAKLHLFHSKTRHLNNETSWSNINCSPK

SKYSQSQIDVLCNATHLLHAKTRYLNSVTSWSDIQPLTQFKVFSMIIFTSVMEIQ

>SG-AL_contig_8267_1_+3_255_485

MTLKPATALYFRKLTLGSEDNYSSYNSSVVNLQPQLSLCEQYTVYSIVIKKMSSSILSIM

LSQLHFYTHRHAYGRER

>SG-AL_contig_8268_1_+3_1428_1661

MSKVNKCADQLVPEPPSCAYACRTSNMHIKDPVSHASVQWLRETRKHPACTKKQKKKMGG

AALRRRALPRRAARISHG

>SG-AL_contig_8269_1_+3_207_1703

MKRYHWNILGLCEIRWKNFGEMNTPEGHKLYYSGREDRHEHGVGFLVNKDTVNTVMACRP

VSSRIMTIRLRASPFNITILQAYAPTSDYDDEEVEEFYGQIQDVLDEIPKKDILVVQGDW

NAKIGEDACKDWKETCGPYCNTHTNDRGLRLLEFASSNNLKIMNTFGPHKISRRMTWHSP

GGTYQNQIDYIMVKHRFQSSVNIARTRSFPGADIGSDHELVLMTFALRLRYNKRRGNIRL

KFNVDKLKDPKIMEAFKANIGGKFAPLLVLANEGIDLEDFTDSFNKAITETATEVLGKHR

PKKKPWVTDDILELCDKRRELKNKKNEPEGRKQYNDVNRKVQTELKTAMETWIDNRCKEI

ENSLERNNSREAYQIVKDLTDTTQAKTTTIQDKSGKCLNDEEQVRKRWTEYCSELYTHNA

KGNTTVLVAPQPTNNSKDRIMREEIVKALTSLKPGKSPGVDNIPAEMIKAGGDPMADMLL

VICNKIWETGQWPTPWTQS

>SG-AL_contig_8270_1_-3_3_215

VSVLAKREFTWQNSSFNLILMFLSDDHISACGTVCLVSRTSPFPSDIILHRTSNMACKLF

SNRIVGESADM

>SG-AL_contig_8271_1_+1_553_837

MCLSENSSMFKNICSCLQLYTVIHNKTIQSICILVIYSIRRLSLPQYQNKKTSLSGMFTG

PNTIYPDISFWTCLLSPFNIKKNSMPKHITQAWLV

>SG-AL_contig_8272_1_-3_15_608

MLDSIRTQLGMGEEEAQTGAAIACYLAKQGASLIACNQQGKSPLDVTTDPRTVEILQQFA

AAFSATKTKLSPGLEEEEETGVCEVCCNQPATVTFEPCTHCLVCEDCCIKMKKCFKCKVV

ITAKKRPDGSMVGSNVNTMDLKRRLQEMEDSLLCAVCMERKKDLVFKCGHAVCKTCSENL

QVCPMCRKTITLRIPLYT

>SG-AL_contig_8273_1_+3_861_1037

MPTDTNLIIITVIIIIIIIIIIIIIVYGQPTTKIISGLSPREVHQSRDDNALNTKPRQI

>SG-AL_contig_8274_1_-2_2_226

VPHPPPPPLPSGEDRERRGRLPSQRQSSASETREEEGSTSSSVTSSSQDCDVAVVSQQTQ

RRAEEDGSDSTNQLV

>SG-AL_contig_8275_1_-1_118_1074

MQHSQAYHHYCIFYLATLSCCMGGINWVLGGKSQMHVALQFLKLMMFLVLSHFYWVLASR

ALRCEAAVKRIMYPTVGVVMFYYIITAVLAMFNMNSEESQCMEPYWLLMSAMEVVLVQFF

ALAAVHITRRLNEISTLDSVRWAQKRDLWCIVFVLEFSAVVTLLYDSTIQIVGDKEEGCG

AIFNYKQSLYSPIFGTFMVIKLLIPIWAMLYAFQPNVVSGDQEQTYPAYSEDGTCGSAYS

DDSQYRQLYHPTDMYHSVVNSCLPDISPSPNSTLEPRLGLSNPALCRHSTATLEPICEEG

TLGGRKGSQGMLAAARIGR

>SG-AL_contig_8276_1_-3_312_1202

MDRQPLLGEGQVTLEVSNPREIKREARYKRSEVTSLHHSYPLRLLVPEHASASGCRWIFS

LAFGGGLVGGDIINIDYHVGDHAAVLVTSQESTKIYHCREGKVSEQNLNYRVCDEAFLAV

LGDPVVCFADACFKQTQRVRMTSSSSLILLDWVTAGRIALEEIWQFSSYQSLMEVYVDED

LVFRDNQHLSDTPYQTVRQAMKGYQVLGTCVVLGKDVQQLVAHLKQQYSRPHSIGQQLTT

ELICSYSPLEAETKETTLRGCYVRFLASTTALAFGVVREITSAVADILGGDPYSRKY

>SG-AL_contig_8277_1_+2_4853_5377

MGSCSHKANQPQEENNEKSMHKPFSLWCRGRRESQQLTIACNTVARSLPNTHGHALSELC

SHADDNTKDPDLQSTTASDVLSHHQLLCWKPTERKKVIQFPLPLCQLAPVSHCFVFQVTN

PNHASSRAKEKGEKTHSYQLATSSVLPQLQTLLLLAPAQHTIQRLAEWLKIQNRK

>SG-AL_contig_8278_1_+2_530_709

MRLQQKKKRKKGGQICTRSPIITGYAFHKNNLFGMIMQDSLPSVALKVTLDNYVKYLAQS

>SG-AL_contig_8279_1_-3_168_1334

MSEVWLISAPGESTPQKTWDTLNRATSAENLSSNYKFNIPELRVGTLDTLVGLGDDLAKL

DIYAESVTRKIAQYLGEVLEDTTPTRDKLIDNLKINDATPAHYLHKFAWDGAKFPIKQQL

KALAEMVGKQVSQAESDLKIKANTYNTLKSNLQSLERKATGSLLTRNLVDLVKKEDFVLG

SEYLITLLVVVPRGGSADWFGKYEMLTDMVVPRSSRVLHEDQDALLVNVTLFQRVADSFK

HKCRENKFVVRDFEYDEAKILAEKKEAEDADTKKKKQFPPLVRWLKVNFGECFIAWMHVK

ALRVFVESVLRYGLPVNFQAMILVPNRRSQKRLREVLKQLYQHLDNSALMGMESESQVVD

IPGMNMGSADYYPYIYYRINLDLLEQQRL

>SG-AL_contig_8280_1_-2_200_2008

MLESGGPFYLSSELLNLLQQGQTGGDSSRVMNINIHGQQGSSGQGTSGLQQQEQLLYKQQ

RKKASSKLVKSSDSDNKDMSSSSNSGVVVIGDMSRAKSSGTLSEQEMNDKKKYLFEKQAY

EAAKLENDLHSQEVQTLEEVIKDYENKKKESEKDVAKDFAAKLAQAKTEQEKEKIMTDYA

NNLSKLNNALEVQKQKQMAELREKLLDRRRQSKKNLHHQHIAEAQKEGVGADEVPDITLQ

SRDDMVHDLHNKQQTLERQMAEIKSASIHPVEQVFDEVDDDDSDFYNFMEGLGLRREKTA

DILKRAAERNQLMKTSSEEIIRRLKERKGRKVSPKLSAEEMKNMSQDEIDGMKEAREMQN

EADRLSEESLLLKAWKQHEKEQLRLEQEAALRELLKSATDEERDRILADFHDQSCRMDNK

RDEEKQRQQDKLMAKLASRKRMNEELDRDKAVNKELDHITKNHAAKGDNASAEVIANISS

QLNGDKLTAEQEALVQDQLTRQEALEMEHLKQQRRTDQQLDEELASQQRAVSDQINQQKR

LVLDSHKAQLDRELLLKGDKLSDEEHKRLLAKFKKEQEAIERNLESERQRQNKALADKIA

ERR

>SG-AL_contig_8281_1_-3_3_170

EYALACCIFLPYNLEGMIQTDPAREGLWRGNRGRKTQRDRPTKTNRRTDIQTDRDR

>SG-AL_contig_8282_1_-1_94_354

MLLSRCIILSVSSASVKVPNSISHQVLLSRCPILSVSGSPCVSILSVSNLSKYEPAVCPV

ASQGGSISVPRSISVPRSISGPCNPKI

>SG-AL_contig_8283_1_+2_2_106

VTMYFNLCLIDVPFSGLALVTNYHHILTSSGVFPQ

>SG-AL_contig_8284_1_-3_30_386

MLCHVIQRVMVSVSVSCHVIFHVYGILLLTRNVSCHIMLSIMSCDLSCHVIDHAVHHLSW

YASCSPSLYHNIYHVIVSFVLIASLCHDLYSLSYVMSLYHCSIEIHSCEFMCTYTHVSS

>SG-AL_contig_8285_1_-2_2_286

PWRSFLKAWYPLILKGFPSCRCVSCMQQMLTLFSCKKCSSSVFLLEIPSAFQCMMVIPGV

SFSLVVIFLSASHFSSSSLAPQADGGTSSHKGGLL

>SG-AL_contig_8286_1_+1_184_756

MHWNAEGISNKKAELEHFLHENRVDICCVQETHLQEGKPFKIRGYQALRNDRQGRKKGGV

MTLIRNNLNASETKTHTGEAEYIQVKVTTSNSTLDLVNYYCPNNKMLSLDTIQVPDSGFL

IAGDFNSQSQSWGYETLDKRGEEIEAWQDENHLILANDPTDQPTFYSRRWHSTTTPDLAL

CTEDLHKRLSR

>SG-AL_contig_8287_1_-2_239_403

MRACISVMHKEGLIMNHFMLKKAIHFSRFISVFCSEMSSTVKQGSVKVPPLPKAP

>SG-AL_contig_8288_1_+2_278_784

MKATCPFLSGSWGSTKLSAQNTTHAYQYLNFQSDHHKYSEVRTPFSTELTPPIQKHSNTT

CGPKTRSSISLFQNYICDSVKRKFLPVHPLYPRPVRRTGTPSTNSTPEPTKPLKTPRPQP

VPPKTKGKLTKNVQQSTTRPVKGVGVNTWENSPSAGHRIPGNHTSTEAS

>SG-AL_contig_8289_1_-1_43_2427

METIWLQRSNADIPWGFRLQGGRDYGVPLSVQRVTPGSVAAAGLCQGDLILKIGTISAND

LSHMEGQELIKRAGNILQLTIKKSPVPPGSYGSQVTAQLHDSSPMSPTGRGINIHQQNHY

SPTSHPATHTPLPDGSTRIQIQRSGPAPSSGPPLPPTPKAYEDNTPFAFSDSNRLPDYTI

PYRQADPYRDSSGGQQNAYGVTGGSMSLPRGVGYGERRQEPRSLQINLTNQPSSPASSQS

QPAKFNPTQAYESRIGSSRHPDPLDEISAGYPSSMGSSAFSTPMSPQQQPLSPRGGSGHP

GFSKIQVSSSPYSEPSRPTVNSYSSSPYSPQQQQQQPSYSNRNQQGPMSPTSSYTQQPSL

QRGYSQEQPYGQDQQRGYYDRQYDRPASPPQIYQQRPQYQQPQQPQQYNSYGTQNSYSEP

QPDPYSMPRPEAPPSYSQTFPRRQASYERQQSHDRNQDRGQYNDGYVSNTVFNQQAGVSD

FGYDESAAAPQHSQPYRPPEPQLYKPSAPQPYRPAEPQPYRPPEPTRYEPMDNAYDARSG

YRRTSSQHDSQPFSPGVRSVQPDFSRPSFSSQYDGRGGDWGAAPPPPPAPSASALASGSS

WTPTRVGDVGEDLSRLGLTEQPPPVPPAPVAPPPPPPPPPPPPPPPPPPPPSNFDWGSIS

RPAVQSTEGEGPKDEVLSTVLSKSKGPGPKPFSYGVDLSELKKKIGPPTAPKTFKKKQSS

PAGGYDPSDSFAMYSPKPSAGYVQSDYRRKPDEGPRAEIDESPININMGTNPKKQSMSFK

VLQWMTDTENTPDEP

>SG-AL_contig_8290_1_-2_368_613

MWRMRQLKVKQYLQLRYFWQYLKFWLLRTKIFCLKSQTFCSIGILCNIDVFCCISVYMHV

LILCCYILFIFMAISAVSVVHF

>SG-AL_contig_8291_1_+1_724_1047

MHLAVPSTVNFSKPCSNTFLLHGLAHFNFCLHRYPFPLPSVFTAGIKQLITVDNACDCSR

HGCRLNLQSLKKQPVENAFSLCLPGNVMARENLVSYLGGRLCAHGNRC

>SG-AL_contig_8292_1_+3_288_413

MKVHSLTNFHTHTHTHTHAHTLGKKNISHHIYLKGQLLLSFL

>SG-AL_contig_8293_1_-2_2_358

MPDRNLKWSVQWCLTIPPSLHFISPPGTPAILVKNKMIFMLQFTRIGLNVEAQSLCVPLL

YEISQNKVQQVDNQGSPALTAIGAMLSRFSELHHNPNECAIFPAVRELMTNLVIPVTSV

>SG-AL_contig_8294_1_+2_2_82

SSFISPEAFTRSRRPWECENLNSPLSS

>SG-AL_contig_8296_1_-3_27_1421

MASWITLIALLALTSAVCFAETEGDDAVFLSPLIAAGRISEAKSLSRVRWDFVKEVNHTE

LESYAGYITVNETSDSNLFFWFFPTKKNVTGVPVLLWLNGGPGMSSMVGALIENGPLKVT

EEGRVVESKWSWTTEFSMLYVDNPVGVGYSFTASSSLIPGTVEEMTPHLYSLLVQFFTVF

PEYLQSDLYIGGQSFACQYITALGKHIHDHNHASPPPSLFLPLKGIYVGSGFCNAAVMYT

EFYEYMYYAGLISDYTRRTYSAVVNRVIDNVLATGRTTVYNVLDILGGTVNLTGSYNIDN

SFGGQRSLDTLKKKLGILLTSATARQALHVGQANGNVSFEAYSFYVWNYTKTFSFFRSAN

EEYVFLLENYRVLHYSGNLDLKMNVPATEKFLHQLQWTGADRYYNSTRYPWYRDDNAFLG

WVTQIGNFTRVVVRNAGHQVQYDQPEAAFLLVKNFILDKPFLTEK

>SG-AL_contig_8297_1_-2_131_850

MYPELYENMYYAGLISEYTRRTFQTQMKRFIDKVACSGRVDEEDIWEINGIFLNITDGFD

IDSQLGREHSIYLPTKKLETLLTTPTARQALHVRQAYVNETFKSLNYSVWNHVKSLSWFR

SADEEYVFLLENYKMLHYSGNLDLIVSVAATEAFLHQLQWTGADCYYKSIRYPWFWDDNS

LRGWFTHIGNFTRVVVRNAGFRVPYDQPDSAFRMMKNFVFDIPFVTTENTTLETEYMEGL

>SG-AL_contig_8298_1_-1_4_1005

MLYVDNPVGTGYSFTGNASGMPNTIGEITPHLYSVLVQFFTVFNEYSQNDLYIGGQSFAC

QYVSALGKYIHDRNHETPPPSLLLRLKGVYVGGGFCNAAVTYSEFYNNMYYAGLMSDYTR

RVHNALAKSTIDNALSTGRVDGTDVYNIISGIITVTDAFDIENQLGKKYSIHSLKAALNT

LLSSPTARQALHVGHANGNVSFLAANNDVWAHIKTLNFLRSANEESIFLLENYKVLHYSG

NLDLLVSVAATEKFLQQLEWTGADRYYNSTRDPWPRGDALLGWVTQIGNFTRVVVRNAGH

QVPHDQPEAAFLMMKNFVLDMPFFPPAESTTDEN

>SG-AL_contig_8299_1_+2_260_388

MMHLCCVQSTITPKHIAKQYFIVQPAKSNKLYIPQLLEYIQQK

>SG-AL_contig_8300_1_+3_198_395

MIRFCCVYNCIQDDTFLLCIQLHTTVQKFHTISLPSKNKPNQLRILTYLILDVTYCIQAD

IHDTVV

>SG-AL_contig_8301_1_-1_7_2298

MKIHPSTVGFGASPKSQKKKSLKGGWPVMRSAPPVNISRGEAERLVNSATKLLAATTEWD

LIHKLDPAVTISEIDNSKQPRARSANKRRPLTAKYTWDINGHRVPSAKIKAYDDSYLDKV

PRRPFLKYRRSPSPASSTATEDSAQPPARPLPPPPKPPTPPPAVERPSSSKQRQRTVINL

PSEKIRDEVKVTRRSPRKYEMSSQTDRHLLDEYDHMEEEVKEGPWGEYQVYVRTGDRIGA

STSADVNITIYGEKGRSPAINLATSKHNKVKFQRGKEDLFIIAVHHVGKMRKIKIGHDRT

QLSFAWYLESVTVYDMNDKQIYDFPCERWLSGQDDDHRTYRFLPVDRQRTFVDALEKHET

TPSRSRKSKSDSDTSETESESTPHRNDRTSMRAKRSQRSKKSSTDSSTTSDSETDSEGEA

VVRMSASTPKEGKKEPAKEDDSEQKAPGTVIQFKKKGSQKVEEEVVLNERGRSHKPVEHE

YLAGYKAGVEAVEASDRKHKEEERTRDRQLLSGASIHDACRSGDLSRVKELLDKFPEMKD

FKDESGWTPLHLAAASGHVDIIKWLTSSGRDDLDVETPTGYTPTHVAAMNGHINAVMVLH

AMGADIGCKTVDKQTPLHLASRAGHLECVKWLVANRAELSAEDSFGRSPLYLADEYGQTT

IVDFLRVCERDLADPNSTFAQMHRAQQRGGSVALPTIKEDDKTSTGSDRPQIGDTSTSEG

SRTKAKEERLLQEKREVYQQQRSHMHAQGVSFLDSVRQDWETEA

>SG-AL_contig_8302_1_+2_1013_1210

MRLFQSLSLMTEFVYAFVFYPPSLNMALSLQKLCSITFICFSKVGACLFHAIFISMQMML

KACTIP

>SG-AL_contig_8303_1_-2_131_250

MRLQNVLYVYTVLCRYIPKPSDYTLIRTIKQIHRNIWTYN

>SG-AL_contig_8304_1_-2_2_226

RISGITDLRDKFSCFRSLHYILPCTHPNQTEPDSDYLVMWSDADNITAGQCCGVSNFPWP

GVGTEHRALLWLPAG

>SG-AL_contig_8306_1_-3_213_956

MSCGASCLRMLLIAFNFVFWLSGVAILGVGIWILVDPNIQDKIDVIHTSDEEYFKNAAYI

LIAFGAFIFLVGFSGCCGAIRNSKCLLGFYIFFLVVVFAGELAAGILAAIYKDEITAELD

KELTASIRDDYLTNQDSWNAVQKEMKCCGGLGPGDYINSTWIQKQNDSDILIPESCCKLE

NDKIVDKVECQKKTTGYFNDGGCKGKLEKWVKDHSIILIGVGCGIAALQILGLVFAICLC

RSLNEEKY

>SG-AL_contig_8307_1_-1_778_1059

MKLVLFAYCQRQHIFTLFWTLFCTGCGQLAVYMICIMSDMIQNVLTVMIWYVIYNIYVIV

TDGCSLNQNYVCYSSKWNPLFSFCLFVCFVYLLY

>SG-AL_contig_8308_1_+3_3_299

VKDPFDLFYSFVLAHVVFPWKQDLGFALACEEDGLWLGFPLEGVAEGSNSNQGKKQQCHR

ETSPSDVTIHPDVLPSLRHQTAITIFHNHRNQQRTAIQN

>SG-AL_contig_8309_1_-2_539_1903

MDWLCMSVPSILGMVSCLIFSLPPAQGLALRSRYFGTVGKSTLGLKRTPGGTSRGGSSGS

RDEIRNRLRSYRRSVLREFGGIFDPGSQGMLTTVEPQRCSLQDPDDIQRLLPGRFLSEED

LMHTVPGTKTWVSNSDMASTALQRRLVPPLSASLLDTARLQQEEIQQRQQQQQQQQRHAA

FDIKTDGAQFQDTVCNNCMTEIGYEIMEQVVDFMGQSHTVINFKNAFQFIPSGRCVNEHS

PCGPSGWQCRQVTRAHWTLVWTAGMGVNLVAHEVPSHCQCLNLGGPAATSHRSAAADTLS

DVNPAQVEGNVRVFGWAGLKMDHSNSSSGPTYTTSNRPHPNPTNSDLNPVNPSLHPTGLD

PNPVSTDPFAASPGSYSVSPGRNPVSPNPYPYPVSSVVSDKSLFPSFKNSNAWGWSLTSM

VPSTSSSSSSSSSVTSSPAKNPSTDVAEDYRITFS

>SG-AL_contig_8310_1_+2_2_415

FFENNKRVFITNLEISITVYKHISSKILPSSISISHAQNNPQIRRTDIKTSTPYFHSKIN

HHTDILYSSSLISRQYALKKRSTTNPRSLSEHFHFSAKNFDCLTTASRQSGTNHPVGCCN

AGADKLTNDWQKKSIFTT

>SG-AL_contig_8311_1_+3_81_230

MFYQFLNSSRIYKISLFHNKHLQLLIITHSPQHNILQHNLVLQLYISFGD

>SG-AL_contig_8312_1_-1_49_1344

MMKHFVLLFFAMVLVADAASFKDERRRKDRPDCQEKCRSRPMEISFVLDGSASIWQVNFT

LGLNFIEDFVDLFDISQSAVRVSMVTYGDVVYTQDAFGLSAYDNKENLKAAISQIPYRSG

IKTNTSGGIWYMLRQQMPEGRPKVRHVAIVLTDGNSQEADLTKQAAKDAREAGLEVFAIG

VGHTVSLQELHNIASDNRHVFVVSSYNMLEDIKERLAYQACANEPHPVCKMEPVDLSFVI

DSSASIGKGNFTIGMTFVHDFVDNFEINPNTVRVSAVTFGDGVYQRDAFDFDTYPDKKSL

METLEGIKWRHGQTTQTGAGIQFMREYQMAKARPSAAHVCIVITDGESQETEITAEEARM

AQAQGIVMYAVGVGEVGKQLNEEELNNIAGDPDHVLTVNNYAQLNLLKDKLAKKTCEGVL

ASLSLKSEQFRQ

>SG-AL_contig_8313_1_-3_210_395

MLYKQQKVPKTFSDVRSLSLILTCVCKHHCLEFLLNSMKDVSTADNLSSAEHICSVLLPK

KL

>SG-AL_contig_8314_1_+2_263_535

MMLLIFKLNKAGDMVSHQTLLEKLSAYHFNEHSIAFFQSYLKNRIQTVFIHSKYSEKWSV

RFDVPQGSVFGPVLCCLVIKLLTFHYTLNPL

>SG-AL_contig_8315_1_-2_764_1282

MYIYFVVSILSQCMALLISFVHLSLSLSLSHTHTYAHTHTHTHTHTHTLSLSLSLTYTHS

HTHTHTLVSISFFFLCLSSNVDMCHIMNKNFPLPPIHTHTPTITLSKTQSTHYILNAHML

MKTSAMHVIFEHWSFLFVLNTHTHTHTHTHTHMQACVDKRKHTHIYIYIYCKS

>SG-AL_contig_8316_1_-2_86_2497

MGLHRSNSHLNAEEKKNQLSGENKEKAKLEMVKVDVDNNMTDGVHIGLVPLEQQKRHVAD

IMRRIPAGSEVSTVLVGQVDYMIRPALAFVRLSEGQFLENLTEVPLPVRFVFVLLGPEKA

GMDYHEVGRSISTLMSNQAFHDVAYQAENRDELLQAINLFLDDSIVLPPGDWDQKTLLPI

MDMARKRAQIRRRHKQKEREALLEKGAPDIPADPLKRTRHFFGGLFNDIKRRYPHYISDF

RDALSFKCLMATIFIFFACISPCIAFGGLLDEKTKGLMGVTETILCTSIAGMVFSLFCGQ

PLLLIGATGPVLVFEQSLYNFAESYHVEFLPMRCWIGLYVCLISIITVALEGSFLVRFVT

RFAEEIFAILISIIFIYEVVKKLVHTFEEHPLVTSYNCETNMTDDPFGLTFNSSDGLNFT

EAYNSSYYSMAPPTGNTTTTELVNLTDTLVHNYHKEPENQPNTALLSLILTIGTFLIAYF

LRIFRNSRFLGRSVRRALGDFGVCIAILAMTLLDYIMKDTYTQKLQVQDTMSPTRPDKRG

WFINPMGMKHHMPVWVIFASIIPAFLIFILLFMEVQITEMILNKKERRLKKGCGFHLDQF

IMGVLAFVCALFGLPWMCAATVRTMAHVSALSSYSKTHAPGEKPHLLGVKEQRLTNFAVH

LCIGLSLSLGPVLRAIPVMSLFGVFLYLGVSAMSGVQMFERIKLLLMPVKYHPSVGYVRR

VRVLRMHLFTVIQLVLLLILLAIKSTAAALIFPFALILLMPIRHRIVNRIFSPQELQELD

KEEEDSEEEDEGDPDFYQMAHMPI

>SG-AL_contig_8317_1_-1_1_303

THTHRDTHTHTERHTHTHTHTHRVTHTHTHTHTHALLYLSPTLKRNRYASTVGWTGGAST

VFLRSVSNLCKTRAVRLEGGNSVGVRYKRLKVYRDPEVIGE

>SG-AL_contig_8318_1_-1_301_684

MCIVNQSEMMTNKLRKNMFSGPTLLGRSPSALLPCFTFPNPEQGQLPIQSWGYWRPHPQS

VSSQFRLEPCSQTGTHAVRLEPVQSGWNPHSQTGRRDSASAPVRHLVRGRIQTTGGARGQ

MSKVKVIP

>SG-AL_contig_8319_1_-3_636_881

MDGLPLLSPVVWQKLSLRARYLFHLRIILFGRVSFQFTFSNVFWLCMCLCVLTSHLFEIY

CLILYKSCAVSLIEITAFLILL

>SG-AL_contig_8320_1_-3_6_293

MFTSILSVLNLCNVFLLDVYFNLNVYTFCQYSIYPIFIDVYFNLNVYLCSQPFSCVSVQV

CVCTYCYPNKLYVPCCFPLPFSFLLCIDHIGSRSYN

>SG-AL_contig_8321_1_-1_820_1023

MERTMLVFSFLPSFFCAFISFISLYKMKDFNLFQQEMEIYVISVSTLPFHLSCIYNNTVH

VCKCARCM

>SG-AL_contig_8322_1_-3_264_608

MLECALLVLGWVFFQGRVEYLHGVVGANAVCVHVVVVIPHGSAENWCDIVKPCPNEIVSR

LVLQQLPSARRRQWKASGTKHSVPKRPMNVLILGTSLYSPASSRSCVFCQMHWQK

>SG-AL_contig_8323_1_-2_2_208

SPLSSTPEEETDCVTTTVCQSFLCRLLLAATFRCELDIQCASSARPSPVSVLVFCRLLPL

LAEKHVWPT

>SG-AL_contig_8324_1_-1_37_363

MLFKNGSKVCVKFRNRQKYMTSSSLQKMHDIQQPWGPKTWNLFQNNSSHSNNSVYMCSTQ

ICQRDLKYRNNTDLQNMLIFSPPLSIAVQGLYFSFLHLLLVYYLFLCLF

>SG-AL_contig_8325_1_+3_345_713

MGRGSALRLATVFVVVVVVGGGCGLARSVDKTMDLGSDVSSSVPACDIKTTLFIAHLNAA

TILVVSVFHRSHSLPHFKTLPSRRVFHRIKFARYQLADLGGVYIIVRVYMELSECNESLL

NKL

>SG-AL_contig_8326_1_+3_48_221

MSLILHAYAHGGSGTSRSAHVDPGDRKNLHPLPTRRGVRGSNPGLPNPLEVKCLHHST

>SG-AL_contig_8327_1_+2_2_193

HAGYVYVSISHRTLTWLIGSLTCIFDVLHAYTHEGGSGTSRSMLTREIGKISTLYLPDGG

SDEV

>SG-AL_contig_8328_1_+1_1_144

WSGPVFDEGPLFGLGGPIFDLGQSNIWSRVVQNVKSLLEHIWAVCGRG

>SG-AL_contig_8329_1_-3_45_539

MMGMGPYIKYSAGNATSRLSQAVSQATSQPFAVSCVNANYTDSGLFGFFAIAQAREIEKV

LRAAFSQFAAITKTGVEEKDVTRAKQQLKAQISMYREGGDAVLCDLGEQALGSEGILSTA

DLLSLVDGINATDVSNVAKKLVNGKPSMAAVGNLSRTPYIDSLLQ

>SG-AL_contig_8330_1_+1_409_741

MISLLYEGQLKRNIAKVLQRSESAVSKVLKRIRQSASVESQRQDSGCGLRSKIDNRAARS

LSRLVVDCRKTLRGTTGEFITVTGLNIFHGDCAKETSFDGFQKRNCKKERL

>SG-AL_contig_8331_1_+3_3_335

TTIKTSHHTNNPLLSRDDITNSITNIKTSYHTNNSLLSRNEITSSITTIKTSHHTNNLLL

SRNEITSSITTIKTSHHTNNSLLSRNEITSSITTIKTSRHTNNLLLSRNEI

>SG-AL_contig_8332_1_-2_281_655

MCIRTQNMKYTSNKDPVSYVCSVACGNTKTPSMHIMTNHQSISKQSVKQIQWDLMLLSLK

KIVSENTGNTKTPSMHIMTNHQSISKQSVKQIQWDLMLLSLKKIVSENTATLTMGLAKFT

SFKSI

>SG-AL_contig_8333_1_+3_87_182

MLNSVMLTCTQLSKTWAIKLCSFFRGAVTSLA

>SG-AL_contig_8334_1_+3_756_932

MQDSACHCPVGTRNNWIYLVFLSFFGGTDSRLPFELLMANEGAMEQAKEKILWLTKGKT

>SG-AL_contig_8335_1_+3_3_98

FFSFFSSDSLFRAMQLNAYIYHILHSIHVLLI

>SG-AL_contig_8336_1_+3_273_1070

MNKTRIRTVKSVNQTYKSTIKTSSQTDKQINKEIRTFQSLRLHQNGDHKCSYFPGCTKII

NKNTIIFARLHRKPCIIQRTNNRSTLHTHLLRCWSFVQFKPKQQIGELAQINHSIVSGCG

LDEELDLLIHQSASCGRFNGLQPFPGLVGIQFAATVCVQFFELVHDLLGMGAHAHVELFK

LPDAHDSIMVLVEGFEHDSRLVLLQAKLRHQHVDSVWLAQVAYIVLHVTVKQLQNVLWEL

FSVERHAVVLCLVMTSVDSKSCCQWR

>SG-AL_contig_8337_1_-3_324_515

MPELLLPVQLFYYQLVTISKTIFRGTFDFREIHTVQHTTLYDACEKRILVTFHVNKVLFL

LQKK

>SG-AL_contig_8338_1_-3_282_941

MPSYCVPGTPVSFDIDASQAGSGNIEIRVNEGRVACSVQNRGNHCFTASFVPEHSPHLVD

MTFNDQEVDGSPWKVPVIDMDSIHIATSSKEIVPVDVPASFTIHYGHCPLDQITVNIQGL

QGSSLPCEKMTESDGDMQVTYIPCQVGNHLISVQCLAQDVNGSPITAKAYNPAAITVTSL

EDGFVGQPVSFTIDVHAAGEGQLQIMVNNGNLPNEVEVRD

>SG-AL_contig_8339_1_-2_875_1042

MFRLERLLCIKEVCLHTPLPVLYAILSEYSCVIYLCPPFQCTLLFSVPLKSGVDSK

>SG-AL_contig_8340_1_+3_759_1220

MLILPFTHGRKVISYRSGFWKNSLGLHGCLHFHLSDLDRWSHGARTRPQMANFHSNDMSL

LSWPSMISRAAPTFLPEVCFLVVRILMLFCVTWCLFLYNGIPQLFVVFWCTLTPVPSTLV

VHLCIHCHAHPRLCSEPLLIFCVCGQFVFVFWVN

>SG-AL_contig_8341_1_-1_1600_2040

MGTDVIAFASCISLSVSPSLLFSAFPSVCLPSSFSLSLSLSLSLSLSLTHTLTLALSLSL

ALSLSLALSLSLTLSHFLTLSHSLFLSLFHSFSLFHSLSLSLSHSLSQIELHLPLHPPPP

PLLSLSLSLPPSPSPPLSLFFFFFFFF

>SG-AL_contig_8342_1_+3_408_707

MYYQIFPYNSFKIFHEDLCFSKGLAMCTCFFLKNEQTRNKQITCPQQSKQTNLQIWYQQT

NRMNHYYYILFTAQPTTKAISGLSPRKVHQSQQNNCAEHK

>SG-AL_contig_8343_1_-2_29_433

MDDYLESSIRRYDDPLHILEAQKARLEAQMSQGSRQSLRLVAEERKLHEAGSRRPAQFEC

NPDDTQGSQPMSLVSERQYPKVDRLELPHSSSEDELPDLAEEMTQAFLQQENQHTRELER

RLDAQINNFTQGLDN

>SG-AL_contig_8344_1_-1_10_819

MLLAVTRQAQSRLSCLSKLINFPAVQACSRGMTSTGGVRYLGQEEAQAIDEELFSTYAFS

VDQLMELAGYSCAVAIAKCFPAHTLSKHGAAVLVCCGPGNNGGDGLVCARHLKLFGFSPS

IFYPKQSSKQLFLNLKTQCEKMNMAFVGSLPDDTATLTGSYDLIVDALFGFSFKGPPRPQ

FAEILDKLKQVEKEVPICSIDVPSGWDVEQGCEDGLQPELLISLTAPKKCATLFRGQHHI

LGGRFVPDSLAEKYDLRLPAYPDTDPIVEL

>SG-AL_contig_8345_1_+3_870_1295

MLHDGCDSQVIWTLVFQLEGLGLNLLGYCLAGVKDVDFFRSPRSTYADLLVPEPPSCVYT

RRTYTHVKDPVNPIGINFGGLWKHENIQHAHPPKWSMTALMVGLKKRAIHEKPAPTSIFE

YTWELQPTNKQKPRMLLTRQMQ

>SG-AL_contig_8346_1_+2_599_844

MFFKSTLTYTIRHYLALTLLTISLFKSRLTFSHLTKFSYFSMRSNEQQNTTKENKIQSLT

IHISQAVTRTRDYTLISVFRAP

>SG-AL_contig_8347_1_+1_436_696

MSVTYTLYRHPYSHHREKNVYYEIEKCDQTHSTNIIINFKTNSFIHKMNSMNHHIVLSDA

EVSKEENYHHSGTVYKKPQTPRQTDMQ

>SG-AL_contig_8348_1_+1_103_270

MQYGYGLRSTIKMERARDVDNKLTHSLTGCRFVFITGFPTPLDKKVKEFFHSKHIP

>SG-AL_contig_8349_1_-1_34_1470

MRAEMRYHRAVEDLKTKQLQIQDHQKKFQEMQIKLKDFAKLYDVIKNERNKCVNLIQTST

QKAAEMKEKIKILQNEIEILRTAVMQKDRLLQKQRLKHMNSIVIRDGLRNEMAKQQRLEE

EMREKREQQKMDMAKLNLMINQGEEQMVKLRKRYEKNVQHRNDRGIKLIERNEEVCVFYE

KVNIQDQMIRNGEVELKAREEEIRFLQMQLQEEKRSRELLQRSMPSKRHLESELVTLQIQ

LQQCQDRMLELEKELENPYDEKRVRFLGGNDPSPAEIQTKVEDLETRLAEKEEQLLEKDL

IFEQVQRLVERIRNKAQVGKDDTLTLAKRVNETQGKIKEATRKMMALVSEVSMTQAQAIQ

LQQQVKDGEGELEQCYLRMEKGEPPSVTLEKEWMRLIRDEHRRMEDREEKRLEEEEEEQY

KLAGGVYTTAEPRPNAYIPDDDSELPIPRPYGSHAPFKPTETGSTMRHIRKPIPKPIEI

>SG-AL_contig_8350_1_-3_105_1763

MKNCFLLCLLPLSGALGTLLGGYKPVDNKDPVIQSKLRPILESNGELGSLPIRRVEQQQL

YKVVNYKVYQGIPNNTGRETCVKITCYASGVCDGAKQCDSHHVHEAPVKRRVRPHLVGGQ

EAGSREDVEGALQYFVDRMNLMSNNMFRMVAEDVTNVTKQVVAGVMYTFTVTLATTECRN

SVENKGKQLGQCGLTNNTERQQCRVGVWCKLNGEYSVTSQKCGQMMSRHPAQALLFGGDG

HDSCHVHLKAFHKFKVDFDRQYLSDEEEASRFQTFCTNMKRIKVLRDTENGSAVYGINKF

ADLSESEFRKMYLTPVWDQTTPRPWMRPAGPANPHSEAIPDRFDWREHGSVTPVKNQGQC

GSCWAFSTTGNVEGQWAIKSGKLLSLSEQELVDCDKLDQGCEGGLPSNAYEAIMKLGGLE

TEKDYSYKGEDEKCHFNRTDVAVKINGGLNISSDENEMKAWLFKNGPISIGINAFAMQFY

FGGISHPWKIFCNPDELDHGVLIVGYDVSSSGEPYWIIKNSWGASWGEKGYYLVYRGSGV

CGLNRMCTSAVIN

>SG-AL_contig_8351_1_-1_43_1038

MLYVDNPVGVGYSFTGNVLGMPLTIEEITPHLYSLLVQFFTMFHEYSQNDLYIGGQSFAC

QYIGALGKYIHDHNHASPPPSVLLPLKGVYLGGGFCNAAVMEAELYENMYYAGLISDYTR

RTYGAIVNDVIDKVVSTGRLDAESASKITAGMANITDTFDIDNQLGGEHTIQFLRDRLDW

LLSSPTARQALHVGQVNGNVWFRSLNFAVWDDLKTLNNFRSANEENIFLLENYKVLHYSG

NLDLSVSVAATEKFLQQLEWTGADRYYNSIRYPWFLDGISLLGWATQIGNFTRVVIRNAG

HQVPHDQPEAAFLMMKNFVLDMPFLTVKKRDP

>SG-AL_contig_8352_1_-1_43_1035

MLYVDNPVGAGYSFTGNVSGMPLTIEEIMPHLYSTLVQFFTVFPEYSQNDLYIGGQSFAC

QYISALGKYIHDHNHASPPPLLLLPLKGIYLGGGSCNGAVIFTELYENMYYAGLISDYTR

RTYSAIVNNVIDKAVSAGRLDGEDLYKMLAAVENVTENPDIDNQLGGEHTVHLLKDRLET

LLNSPTARQALHVGQANGNVKFTSQSDEVFEFIKTRNYFRSANEDNIFLLENYKVLHYSG

NLDVIVSVAATEKFLQQLEWTGADRYYNSTRDPWFLDDALLGWVTQIGNFTRVVVRNAGH

QLPHDQPRAAFLMMKNFVLDMPFLTVKERDA

>SG-AL_contig_8353_1_-2_422_610

MSMTAEDGELILLHCDYLTVLHSSYMHPSVSQDWEQDMLFDGMGDIHLCTVGNMFSMQSN

PFF

>SG-AL_contig_8354_1_-2_17_367

MRARTHTYTYTHTHTHTHTHTHTHTWRTQASFKWHRTITRDERMESTLDVQCLNLVQREV

LAVQPRGSGILLWVYKPTFLPSLRSLLDLLAVTPAWFLSLRPIAGLLTRNFKSWDST

>SG-AL_contig_8355_1_-1_421_585

MVLFFQICWMTIFHELCFLSNWCNDMVQRNRMISMFVSCYYDQDVCQLLLLLLLL

>SG-AL_contig_8356_1_-3_45_527

MGGNYGAQSSGGTGMGRANTNTESYQTSTGQGYMTGNNSMAGSGNMGSYSGYGSNDMVEG

YNTSGSYDDGSGTGGYSGYNMGDYSNYNQSGYGNTGNMASYSTSGGNTTGMMNTSNQLTP

AGSKRSYGHLLPKPDANPQVDYVGQHAQGIGGLYEKRPRLF

>SG-AL_contig_8357_1_+2_695_877

MSCPRQNSVGKSAFASEQKYFAHLLERKGWCCTVVTHYPGESSPNFTHRKYFVTTNNYSI

C

>SG-AL_contig_8358_1_-2_44_310

MVPGEFPLSRLTSPSLWQGAPSRTTRAVPSFFSKFCTSLAWYSIRSQNWEFSPTPCLAST

LTISNSQAHLSVPHFYCCGSPGVFLEPWK

>SG-AL_contig_8359_1_-1_7_915

MADVDKWIEIAKQCKYLPENDLKRLCDMVCELLLEESNVQPVSTPVTVCGDIHGQFYDLM

ELFKTGGEMPDTNYIFMGDFVDRGYYSLETFTLLLTLKAKWPDKIVLLRGNHESRQITQI

YGFYDECQTKYGNANAWRYCCRVFDLLTIAAIIDECILCVHGGLSPDIKTIDQIRTIERN

QEIPHKGAFCDLVWSDPEDVDTWAMSPRGAGWLFGAKVTAEFVATNNLKLICRAHQLVHE

GYKYMFDEKLVTVWSAPNYCYRCGNIAAVLSFTDAEHHEAKLFKAVPDSERVIPPRTQTP

YFL

>SG-AL_contig_8360_1_-3_591_761

MHRHGHSHSCTCAHTHTHTHSLTHTHACTHIRTHTHTHVKYFKIQCRYYCPSFSLKW

>SG-AL_contig_8361_1_+2_1490_1744

MCIPTPLRCFKCQKFGHMRDQCSETEVCGTYSKQCTRESATAQPSVLTAEVDILHLPRTA

LIGFKRGKFNVSRLKGGFLSLKPKK

>SG-AL_contig_8362_1_-2_2_193

EFTRAMGMEVECKKMLGTNRSQKYSLVVDDGIIQSINVDPDHTGLACLLCIRTMKSWSSP

GTKT

>SG-AL_contig_8363_1_-2_2_178

LSMSVSFVCSSVTLSALRYLHGLLGCKLVYRNVSFAQDKINNFWFWKQIILICSLLMKS

>SG-AL_contig_8364_1_-3_987_1286

MFKSPFCMWFFVVVFCLFVFCSFCFFLLILSCLPFQTNGKHISGDYSQIHPEDLGEKSIG

EVVWIRSKILYLISQLFLGVLHFFSPNFEGKKAPVSDFSE

>SG-AL_contig_8365_1_+3_243_374

MLYRKKNDCFIEAKITVGHSHCKSFMDPYSASMIIIKINLKNIP

>SG-AL_contig_8366_1_+3_1665_1919

MRVEVYTKSVSQSTTLRLWQYQYLLPCTQVQGPCWLCPAQSGSPDSRMESPFTTPRIAAV

ISITDRPFLSYCIGSVSVSQVKRGS

>SG-AL_contig_8367_1_+1_130_339

MLLPSGLSTCSSLHGQCVLSISVNRLFLRTISLHFKSVIFLRESSHHSQQKATAVITNAQ

AFPITKPLIP

>SG-AL_contig_8368_1_-2_2_187

VHPAVNEYLTLFRVNQQGKGDGHCPHNASLEKGCLYLFTPQQPQGYGKTFTLLYAEIEHF

DA

>SG-AL_contig_8369_1_-2_155_421

MLTTLSWSVYMVYTVEYNKMVIEHSKHLYVVHSDKNTDMFTKLSRPVCMVHLNLHVLMFA

KLCKPVYIMVRKSEHTKNITLHRPNPWTS

>SG-AL_contig_8370_1_+2_26_283

MHQNTMSVIFMITMQHRKKLISNKTRNKNTFLQSQSKPVSTNWPSLHSAHQLTTITLHNA

LCKTLSTLINAHVSSICMIAIQTFCT

>SG-AL_contig_8371_1_-2_356_577

MKHVQKTGLHTLHKLIITLCILVTRFPRGKFSHLVCMYASCPGHSRCTPDFPLQTSSNCH

TRNLHLTPALACYW

>SG-AL_contig_8372_1_+2_2582_2809

MSLSETEMVVVCFDNHTYWTVTGRTSRLKRIDLSTEVITIDNLVEVFFSSLCSCEYLYLF

GLWPRQTNVDCPFQCS

>SG-AL_contig_8373_1_-1_256_465

MLFCKFCLICFLFIQIAQYTFYCMYIDLLFISVCFVVAVFIYFCLFRAYSYVFILYIVMK

SSLVILRPSG

>SG-AL_contig_8374_1_-2_170_487

MVEIFLISQVNICADLLVPEPSPHVYAQRTSNMDIKDPASHVSVWWLIPLLKHGNTQHAY

PPKKSAQTVQQHNTCAVWARQCAYTQLRKHCSKQKLIKPQQIHTNK

>SG-AL_contig_8375_1_-3_96_1061

MDMMAQQQCAQQQQKRTAAEAESLLVGVKAIVFDVEGTMIPITFVKDTFVSYCRDNVEKH

LTETFEDAETIKDVAALRDLSQKEKESGVEGVVQIPAAADGKEEVIKAVVENVRWQIEKE

RDSAELRQFQNHVWRHGCEFGGIRGEPFDDVPPMLRMLSEEGFTLFIFSCECAELQKLLF

ATTNEGDITDVFTNFFDTTFGSKTECETYKKLASEMKFPAEEILYLTDTPEEAAAAVAAG

WRSALVIRPGNADLTDEHLQNFACIEQFDELYGEDDDEDYKRMHGDNGEAEDDEDDEENE

DEVDEDDEGEDDDGEGEEGEDA

>SG-AL_contig_8376_1_+3_84_368

MPNMKKVPSRHLSNGTTPKFWGTEEDQLNPWVVGSTLRWATSFFLGHVNSDGLSTTPHRS

RRFDPQVGHGFRTEDSRSDAPSSVPARNFKNWPAY

>SG-AL_contig_8377_1_-2_20_400

MPFLQIASFTHNKINSTEGFSHPMLEHLNLSNNNITNVSGLDANKLSRLHTLELRGNKLE

TTDGIYLPNLKNLFLASNMIKKIEGLSRCTGLTTLHLRDNQLEALDGFPEELKELQYINL

RGNLVTA

>SG-AL_contig_8378_1_-3_102_1460

MAMAAGDPWAYTPPLSLWQLVEQNVSPEEQDEIKSMLGTSLIETTLDLHNEVKMLLEIWQ

DYKEETESESHKTLPLAEPPLVRDRLIQEICFLAASVRGKSASKGVNPSVLLSRPNSQIL

DYAEQSMRSGSSLGSTRPQSARSSDGRQTPISTSEHASSRIALDSTISEEVSSANNKLNY

LQFDEVCNRLREILTKEMEQLEADVAFLHNCLNDELDGHNHGGTPALSREPTLTDLREER

SLLEKDLLSAKSFPNTPPVNKAPFLPGSAFYGVNKMQTLGPVNPKLKTVHTDSTAGTGKN

TPLKASHHVAQILPQDSTNCISSSDTDTVSDRMSFNNNTSYKTSQILTQNVISQSGQMPD

QSAGKSNERSYHKHREKDDSRKKCEGSGFHRHMRSPTRVKVVPVGSVHEPRVDSAGSRTF

IPTPPPAEEPPHLSRPNSAQRFREMVMSHRDST

>SG-AL_contig_8379_1_-1_76_6315

MSLVHYKFKSSLDYQHVKFDGLHISVGDLKKEIVAQKRLRLGDFELEITNAQTNAAYKNN

STLIPRNTSVIVQRVPARPCAASSTQKSWEAYKLEAAKARQQKQLSLEQLKQTTDLANAN

ASEIDKVMAAVAQSNKGFEPSNYSGKHHSGPIPEGYLCRRCHQPGHHISNCPRQQENFGP

HIKRTTGIPNSFLTFVDDPLAPGALLTQHGQFAVPTIDAQAYKEGKKERPPFLPQPKGME

PEKPPIPQELKCRLCHDLMTDAALVPCCGNSYCDECIRDALLKTDDHQCPTCRQFGVSPN

EIIPNNSLRTAVSAFTNDSGYSKARRKSLDGKPLPSQQRQPPPQSPQPAPHHQPPQSPQH

LQPSGHHQPPPGQHQAPPSHHQPQAGQHQPPQSQQHLQPPGHHQQSSGHHQVPPGHHQPQ

AGQHQPPPGHHQPQSGQHQPPPGHHQPPQSPQHLQPSGHHQTPSNQHQATPSHHQPPPGH

QPPPGHLQPPPGQHQPPPGHHQPSRPQHHEGHHQGLPPRQQAPAKDPGGPRSHREPHLRL

PHHQPGPGHIKPLLDTPQIPTPFNQLPPSHPQQHPARQPPPELKASPRIPSLMDTALDMS

RPPPGHVPAAQSSSGHSLPPGPVSQPSTVPVASHQHHPHHAFPGDARRVQAPAPEAPPVY

VSSTLTSTESSTRFSPRSHSPRSAYEDENNEQVIAVVGSRPPHREQRPPPAGSAFPPVPA

TMPPQGAAPVPLGYPQVYRAAPVIPNPAGYSYPGAVPTPSYPPHVYAGYPHTMAPAPVPA

SKRDVFEDFARDLLERKPSDKLKGSRSPSRSRARSYTPRRSRTRSRTRSRSRSFKGRSRS

RSPRHSRQRSFSRGRSPTPHRSVSHARVSPGQANKSPGRDRSQTPVDGKPRSPGRSHSRS

PGRRSRSPGRRSRSLVRRSRSPGGKYRSPGRGSVRSQESNKYRSPGRGRSRSFSRGRSRS

YSRGRSRSPARRRSRSPRSRSFTPRRRSRSFTPKRRRGSRSRSRSRGSPRRSRTRSRSLS

STRRRSYSISPARITQPLRPVGSISQRRSVSPFRRRSASPNFRRKGRTPPPGSFQPFRGG

RGRGRGRGRGFRGDWVPYQQRQFNDGRRPVANQFGQSSEEYLRHTAQQYDAYYRDYYAKY

GPNAPAPPPPTPTPTPAPWNQNQTFPDAQGGHHFPARQMGQMHMGWQQQQQLQQQQQQQM

QQQQQMQQQQPHPPSLLPQRHSPPQQRIPDGRGDMGERERRSAEPHPPGVGPSSDGFPGN

RRDYQRDGQNYNRDFYNKDQNRDFNREQDRDKSRDQSRDFPRNQSRDFDVRRNSPPTDQG

RSFPRDQNKDFARDQNRDSRRDQNRDFSRDQDRNQGRDQGRDYPRDQGQDFTSRDQNRDY

PRDQGRDYLRDQNRDFSRDQGRDYPRDQRRDYSRDYRDNRDHRDNRWRDHRSRDNYKDAN

RDGQRETSYDRDRHSRTDRDSPDKSRNSSEKHGTRSSDKPIKEKKKKPEKDNVVDKEKVV

SKKKAMKRSSSGDEKVLPIKKSKKAKTDDATGEKPLVKKKVLTKKKKKPVSAENKHNSDS

ETPLSSNLIQVTITKDSSKSEKIAKTEVAGKTKKVVRKVKKVNATSSGTEDEKAPKSAKP

SVDSASVKEVPEEATSVIDKVPDTTSGSPVVGVKTEQADSTSKPEGKVGSPDKQGPVIEV

SAEGLEQTPEGGEDAEERAAGSSDDELMLHPPELSKWERDDFDWDTSNSPHLSKKRKLVP

EKKITMLPRAVLNRVETYMAKKPVKPTLASASTTSAMKPQRRVYLEDKFEQAAEEKEERR

VKTTRTSSLDMQITVTKSEKHSPDQHDRSRQDRRSHEDTRSVKKSKDADEDRPSQRKGYS

DKHRHSIDNSRSRRDSSSPRRDRDRRYEREEGRKRSKDRDEHRERERHRERDYQHRDRED

RHRDREVQHKDRDFHKDREEQKLERRTSTSEKGSSLSAPKDLRHKLEQARTKDVPKKTQR

SLDRKESVIDEAKFEPDYEELEESSESGSEEAIADQHSTVSTEETSDVEGSPAKKMRTNS

GDEGSKKKHKKHKKNKKRKKNKKQKREKKHKKDKESKEKE

>SG-AL_contig_8380_1_-3_12_1520

MESLLEQQRRYHEERERLTDAMTKESLLQKKTPREQINSDTRMKLLLDRFLDVTTELKEL

YEDKDGVRKEEIQALAGPNEYIEFRKRLTAVEKFYAKHPNEISIPMSIEFDEMAKQRDSQ

ADETQNLVEFSDEEGYGKYLDLHECFVRYLNIKGVERLDYITYLASFDHLFKIPKDRKNT

AYKEYVLFLLDYLSQYLSKVKPLLDQQEVMEVVGSQFESDWADGIVPGWPKEATGALTHA

GAHLDLSAFSSWEELASLGLDRLKSALMALGLKCGGTLEERAQRLFGTKGKKLDELEPTL

FAKAKPGKAGKSGRESEKQKEIAMLEAQVYRLGELLADQRAATRENVQRKQAQTGEEREE

DEEENYPESDSSEDEDEIPYNPKNLPLGWDGKPIPYWLYKLHGLNISYTCEICGNFTYRG

PKTFQRHFAESRHAHGMRCLGIPNTAHFANVTSIEDAMALWNKIKNGKEQERWKADVEEE

YEDSQGNILNRKTYDLLKKQGVL

>SG-AL_contig_8381_1_+1_34_249

MRVCVHVLVFLFCFVLGFLGGSCLFVCLCVTDQTVKSVLIVFCHTVDRWFCHKGHFVCWF

VFCLFNLFCQGR

>SG-AL_contig_8382_1_+3_105_227

MCVRGYRKSKRFSCISEKLTITICHTVWLPLVPGELANPDT

>SG-AL_contig_8383_1_-3_108_314

MRVERYLPPHSPVFLWLSENLKPPFSQCHHFLLCLLVAVLSITQYRGYLNYVVQLLVSCN

HSIGGIFSW

>SG-AL_contig_8384_1_+1_157_231

MMKYTKCKSFTAMAFLAHPHVHIFP

>SG-AL_contig_8385_1_+3_3_242

NHSCFNPDEFQLYNHVTNNDTGQRLINIILTYLPVNMCGTHKNAMGNTTECNILLIYNFY

IRKRNSVCDLKKAISQSANQ

>SG-AL_contig_8386_1_-3_6_677

MPMPGLGMPSPGPFGAAARLGMPLQAQAATSVILVSNLDEEKVTPEALFTLFGVYGDVHR

VKILFNKKDNALIQMADPHQAGLAITFLDKVRVWGKNIRVAQSRHAIVQMPKEGQPDAGL

TKDFVNSPLHRFKRPGSKNCQNIFAPSAVLHLSNIPPSISEEQLKSWFADYGNVKGFKFF

QKDRKMALIQMNSVDEAILALIGMHNTQLGENSHLRVSFSKSTI

>SG-AL_contig_8387_1_-2_5_103

MFPSLPDFHACYSIVEASDSLVDFHASCESVKL

>SG-AL_contig_8388_1_+3_2061_2465

MTENLTLHRRLLVLHQGMHHSWTVCAEILSELQAASPSSTPAKATVFWWVKHFQSGQSSA

SDRRRKVKNNKKCNEKLIAHVKAVINEDARISLQKIASTLSVSLASASNILRKRPGYHKV

CTVNLPYFDAPAEEG

>SG-AL_contig_8389_1_-2_23_844

MAMLLLSLVSVIVLIIFTASSSLVVAEKSDPLIRVTDDNWKDVLEGEWMVEVMAPWCPSC

QSLTPTWQSLADRSRDLDIKVGVVDVTENPGLGGRFLVKSLPTIYHVKDGEFRIYTGPRE

KNGLLSFVEDKKWQDIETVSSWRHPNSFQMGAVGIFFKFSMVVRNFYHLMSSEYGVPEWL

CYIIFAVFTLIVGLLLAVIFVCCCDCLFPPKYVPAHQIAREDVEDDRDDESDVIEDISHA

EGEGNEDGGDAGLRHRQVGGQGPSEGKEDEASSS

>SG-AL_contig_8390_1_-1_1819_2034

MRICSDQPTTSSCATASPQDTIYIHHDISSLHPRSHPPCFFSLILVRYGQYGNPDTFYPV

SCFVHCVMFLTN

>SG-AL_contig_8391_1_+2_2_133

THTIIIINHLLSGTHTHTHIHNIEMINWTVFKLIPSLNVGHGSV

>SG-AL_contig_8392_1_+2_323_622

MQILTILRAKTINHQAQNSAFLFLKFSIQKLKRRKIQHGKIKTILVLSMEELHQSVTSFS

LNISAENKISFKRFLQNKCCINQTIVTSLLCRQKRFHTYM

>SG-AL_contig_8393_1_+2_2_151

KNFKKKKKKKKKKKKTPPPLPPHSRLVNNTAEHIATAHPSFYNITTQASE

>SG-AL_contig_8394_1_+3_3_62

NVGMCIKMKGKFSFMQEGHD

>SG-AL_contig_8395_1_+2_209_367

MIANYLILYSNIAKNNPAFSLYCATVTDNHIIIIIYCLQPSRPLYAISGLPPR

>SG-AL_contig_8396_1_+1_58_222

MTTTSSSPSRQKSQQHAWKVGENAVNKSWVTASAMNFLFSEIHMCMTPPKLIGET

>SG-AL_contig_8397_1_+2_2_193

EGFTLHPYLPIRPREAPVTGCSEGFTLHPSYPPRLTQGGPSDRVLRGIHTPPFLSPYTDP

GRPQ

>SG-AL_contig_8398_1_+2_635_814

MLKLENWGPFKHKKEIINQTLLLLLLLLLLLLLLLLLLLLLLYIVYSPADHYGHIRAVTM

>SG-AL_contig_8399_1_+3_3_206

NTLHRLVADFPAVCSEVMLVTAHWRETEEMYTSLVFLADAVRRNCFKETDDEFVEITDLT

DEIVQFSI

>SG-AL_contig_8400_1_+3_195_518

MQTKKSTNSHYTILNPISASATKSFNRFATQFTCFCLLTHSKYTCPFSKGMYNAQICKLL

WMFSDLFSLHTSYTDKIHKVGQINSQSVIDLPSLTKTGILSGDLPVAI

>SG-AL_contig_8401_1_-2_110_1711

MMAAVTKSCLPRVKEEEDINQEVPQNYVVPVPPVVSPSRSSATSTTSTTTLQPSFLDSSS

SPISWQPAGSLPDCAISTTHFSSSLQLSTAQSIQPPPTSLPTAYVSEEHNWQASQACVRD

RNAVMFNNPLMSDVTFRVGAGGSEKSFPAHKYVLATGSSVFFAMFYGGLADEMKEVEIPD

VEPVAFHNLLRYLYTDEIHLEADNVLATLYAAKKYMVSHLAQACVNYLETSLSARNACVL

LCQGRLFEELHLMQRCWQVIDAQAEEALKSEGFADIDHQTLDTILSRETLNAREASVFAA

VMRWAEAECRRQDLDVTPENKRQVLGSSLNLVRIPAMNLEEFANGPAQSGMLSAQETTDI

FLHYTANKKPDLQFNHAPRAGLMLYWVHRFQSSAYRSNQWRYRGRCDSIQFSVDKRIFIV

GLGLYGSSNGSAEYLVRIELKKNGLLLSQNVTTFKSDGSSNTFPVLFENPVQIEADTFYT

ASAVLDGTELSYFGQEGLAEVQCGKMTFQFQCSSDSTNGTGVQGGQIPEILFFC

>SG-AL_contig_8402_1_-2_59_550

MAESGEGEESASPLSLLKRFYALQEERVEGYRFLESGFSAYLESAPHYDFDLYRSLVHEI

TQSFKTISEEVMSIEQQFKTLHGMSRIADNLHRIQMSEKEKLEHTVQLQLSRQGATDNPD

DGCLKQQVAGLREKLQEIVQQINDHLEDLKFESEDLYNSEGADR

>SG-AL_contig_8403_1_-2_53_292

MLDIDSMNRYVSPINPAVFPHLTVVLLGIGIFFMAWFFVYEVTSTKFTRDLYKELSVSLV

ASVFMGFGVLFLLLWVGIFV

>SG-AL_contig_8404_1_-1_1_237

PKPSFLVYAGCFHVSISHQTQTWLTGSLTCVFDVLRAYTHEGGSGTSRSAHMLTWEIGKI

STLYPPLRRYPRRIEPRPS

>SG-AL_contig_8405_1_-3_57_242

MVGVDMYLLYVCLPLPLMLIKRLGLHEMRRCTNSYYYYYYYYYYYYYCTLLIKEKKSHQE

IT

>SG-AL_contig_8406_1_-3_60_653

MGKKSKKEDDDVITADQVDDEGDEGEEEEEEYTVEKVVDSRTRNGKKEYLLKWKGYPDSE

NTWEPSENLDCPDLIAEFEDRKKKRDMEKKRKSQNGVEDTSSAKRKKKVTDSKSTEDDNK

PRGFDRGLQPERIIGATDSSGELMFLMKWKDSDEADLVPARQANVKCPQIVIAFYEERLT

WHTHNDADDDDKGKDDKS

>SG-AL_contig_8407_1_+1_124_393

MDALYDTLYYNIMLYIARVFTCDIKTRLHYSEVQNLNNKHKTKKTKKAIVKLKAAPFNKD

TDAFRNNIKARNNRTTTNNINKQKSFKKSN

>SG-AL_contig_8408_1_-2_194_427

MGLFISSQQDGGIPESNQQFMSSQERSIPQHTEDLQPSVEEGFTDQCSVSTVCPSPQKLN

RQDRTYVNVDGVKETAHL

>SG-AL_contig_8409_1_-2_2_166

GGEKSLSSHELNPEPPTFCADNLTTGPPFHSVYIFIIDLQLENVYPMKIRVCQYQ

>SG-AL_contig_8410_1_-1_118_957

MLNEPEVWRTMLPDMPLTALIRNLGKMSAIKLLDDPEMVKLVKSKLTNETALKKARVHPY

TILLAQYVYSHGRGEKGSLTWEPNSEIVATLDSIFYQSFGLVEPTNKRILIALDISASMG

SRLQDGFITCREAASVMAMVTIRAEKNCDVVGFHTNLIPLDQFKDTSMTLKQVIHSTSQL

GFGATDCSKPMTWARHHKKEYDAFIIYTDSETNSHRVPPYMALDKYREKMNIPTAKLIVV

AMTSFGFSIARPDDLHMLDLVGFDSDTPGIISDFLTGGLD

>SG-AL_contig_8411_1_-3_3_293

LFVTKFNKRLLIYVSPVQDLEALAVDVFSLDWAPLISYAFPPFPILGRVIRKAREEKARM

ILIVPFWQAQPWFPDLRSRSHVPPLELTPSPELLRQP

>SG-AL_contig_8412_1_+1_1147_1449

MCSSLRCIYFLPQPIFLLLRPGPSFKRDKTLPFSMHVFVPKIGIDPPLPLRITSALFLNT

FSSVLHFFYHFLKNLLYCHNNLDFVLSYCLPFSIYSLIICW

>SG-AL_contig_8413_1_-3_174_527

MKSVCEKLGADSSERLRVSCEENRGCTVVGGKGRSEVFSLVWCQCSGVLFPPLPPLLFCS

CVHTVELKHQVVLCTCYCAFRLSPETWLRVCVMCCDRVVRFAALLQAMTGLAPSAREQ

>SG-AL_contig_8414_1_-3_87_683

MANIAEQRIRREFKEVVKSDEVAACNIRVELMQDTYTHLRGEIMGPPDTPYEGGRFVLEI

KIPETYPFNPPIVKFLTKIWHPNISSVTGAICLDILKDQWAAAMTLRTVLLSLQALLSAA

EPDDPQDAVVAQQYKEHHDIFTRTALHWTSVFANGPKKDPDCEEKVATLQNMGFEENQAR

VALSTNDWDLVKAAEYCLS

>SG-AL_contig_8415_1_-1_1_204

THTHSLSLQTIYIYYSDVHTQSYCVHGSGFCRAKVMFERFLTCYYFCFFCCSDGNSFCFR

FDYYCYFL

>SG-AL_contig_8416_1_-2_2_103

LALLIHAVMTRFMQFALCLATVRQHWIFCAQLNN

>SG-AL_contig_8417_1_-1_1_111

CVWGGGGVFFLLFCFNFYLHLAFFFYNNYQLQKHIDE

>SG-AL_contig_8418_1_-1_43_756

MHEDNYTSDAESEAVVRKMGLGSQTESIGLSQDASDELAEEVAIKRQRIFKVVFVGDSGV

GKSSFIHRFCSNTFRAAFSATIGVDFQVKPLTVGDNIVVLQLWDTAGQERYRSVTKQYFR

KADGVIVMYDVTSEQSYLNVRQWMDSVKEGVEDGTVITIIGNKTDVVEADPDHVTKVKDG

ARMAVEYNALFYETSAKTGSYITESMLGMASLLKDKEDKALEAALKLTDPIKKKGCCK

>SG-AL_contig_8419_1_-2_29_547

MAAAMGSYEDWLTDKLKSLSPEFDTDVFVSYIISMLDTDSSAEEVTESIEEILSQVLGEE

ERESACQEIVDQWKEFNSDALAAAAAAAVKKDAGDVLSEIMEQQSLATVKENKLTAEEEA

HKRAILAQYAHISDGEETDQEVEETAAFNGLKGKGESPNALLERNMNAASVIE

>SG-AL_contig_8420_1_-2_2_181

PRRRRTAKKALFECQVLVKTRGRTVFEKDSCFVHFLFSSLKFIYIFGSLAGCIFMKGLLC

>SG-AL_contig_8421_1_+3_3_203

SFVLLQSRQCILSKRSVCSLVCLFFLFVCSFLPLHGRQPCLPSPGAEDYHLQLPYLTKQP

LAVISCS

>SG-AL_contig_8422_1_-2_2_259

SSALFVSDRSHQTADDNHQLPPDEGSGPHDKDLSDLFADVSSGDVTYRCFKCAGSSEQPG

CNVTLLRQNPEDYLTDCNGHCLNITS

>SG-AL_contig_8423_1_+3_3_86

TDACMHIIWPIDACIIIIIIIIIMNIDA

>SG-AL_contig_8424_1_-1_742_1293

MSLASCLLAWMALVVTDEYTTCRLWSLGCCVPGVAPSTSPSEGSNSRPERQTLYGKVRGS

VNTMSFLDNRQVERYLGVPYASPPVGDLRFELPEPPKPWRHVRDTTTLPKACPQTNWELS

YVKIHVPSFKTTDMDEDCLYLNIYVPVTTSSRAGEEGGGLPVLIHVHGGSNEVGMGALFH

GDIL

>SG-AL_contig_8425_1_+2_2_265

RASCLSPYPCLWCDRASCLSPYPCLWCLPIHVYGVRERIACLPIHVYGVTELVACLPVHV

YGVSLSMYMLVACLPIHVYGICLSMPLV

>SG-AL_contig_8426_1_-1_1_192

KALTTCRLWLSMHHTKIKVQFQRIAVKNILQRFSIFGVTNAISDVIMTGVAFTGKACLCI

RQVK

>SG-AL_contig_8427_1_-1_16_252

MRDGSKFLENYVPEFDATVVTRILDAGGRITGKAAVEDMCMSSSSITNTDGPVRNPHDER

CVAGGSSSGSGALVASGQV

>SG-AL_contig_8428_1_-1_1_168

DGVQSVKHSQVWWSSRWVARFVPWLEHTAPVHPVVNGYLSVFRGTVGKAAKDKRWE

>SG-AL_contig_8429_1_+1_1_165

KKQTKTNPKTNSFHKLEVNFQSPSTTENPQQKHLTETVSLSQAKSMQKLSYFFLT

>SG-AL_contig_8430_1_-3_3_155

VGVIFFVRRESLFFFEDVFFFFLFEVLFLLIVTHHVHQYLCFCSLIWFIFK

>SG-AL_contig_8431_1_-3_360_911

MHSYTGAGATKCCFPVPIYDPASFSSHVLFFQLVVGDFGLSTYQQKAQFGMWAMFAAPLF

MSVDLRTISEEALAILKNKEVIAINQDPLGVQAVRLFEMPNSVSIWLKPLSQNCTALAFF

NKWNQGRPTPLSVTLGDYGLKNPNGYNVSDVFDGQQSGIFKPATEMNLLVDPTGILLLKA

VPLK

>SG-AL_contig_8432_1_+3_45_368

MPVHRTRKEWQQKDYIEYNKDGQAIQAVSIFTIRQYSGRGMGDQISYAGALAILAVSLFS

RNNSCMDFSHLLDSVGKTLHIFHDSCWWFNYQKLQATSVFLLPSFQRN

>SG-AL_contig_8433_1_+2_2_367

PKEETKKTQKNSQTLHVHISVPESFHAVSTYRRLIREIFLTLFRVGESKAARERTGLAFI

KLALLPNVSCCHTALSAIILRQAFYNDHSQHLKMKPFQSEAAQGTLSCKLARPRSQAPYH

TN

>SG-AL_contig_8434_1_-2_407_748

MRFLFCFLFFSSFKAKYCQACVNMAARRYTAEEVARILLELPLNSDNEIVIGNKGDVVLD

SVDQSDNESSDSDYPDEDYILPSISPGRGHGRVRSGCSGRLIVSYLILSTLNIS

>SG-AL_contig_8435_1_-2_602_877

MLLSTEYVLARRFPLDSYSCSLRKLTQFTTLPVLGSLVEITVELKKNYPCALLSSFRLCK

MWDVAIQKLMPAFLGQTFLYLLPPAQVSNHSL

>SG-AL_contig_8436_1_-3_342_677

MIAFCTSGNLLGQMVDSHTSFIDNITSNATFKKKKKNRVHVIFTTPTHPPTCTHTHTHRV

VKTRLPLLPPPSLFHSLSFSLWCACARAHTHTLLLTHTHSHMLPHHRHLCFC

>SG-AL_contig_8437_1_-3_27_332

MARTQHPSLHTSPDLQCTGDSHHTNISTMWKMMIVLLVVAAVAEGYILDTATMAKIAEAL

KKFAAGTGYPGKRSILTEEAYTEPRRAEAYGAASGDFVFDRK

>SG-AL_contig_8438_1_-3_330_623

MLPCIHSDTNACMHVHAYVYTHMHRHIECDWLLKSYASYVCTSLTKCAYAHTCKCTYTHI

YVYNIYVDIYTQTRTYTCTHMYIHTHIIIIIIIIIYCL

>SG-AL_contig_8439_1_+3_144_260

MHQNKIICQNNHPSKQTVTSACNASCFILITQQKKWKVQ

>SG-AL_contig_8440_1_-1_1_492

QNLSLSPGGSSDASSSSSVVSAIPASSSSSAPPPPPPSNAQRSEPAYLRSAAEKYAEPED

PVKPSQHQSRSFKYLQDLMDSGKEPPKALKTVNPPALHARSVSPAEDRSEQSATAPVQLL

GQNKPGRVKAIMSQRYNTPIGLYSENEVMTQFEGQSKFLITDED

>SG-AL_contig_8441_1_+1_1_198

SVCKTVFVNKFGIILSQFIIVLQTIIVTKHFLSLNYCGKKTYLQIQKDQMLLSVLCCDFC

FKVKKK

>SG-AL_contig_8442_1_-2_32_1585

MADSETNRSQIEERIKAQGTVVRQLKAEKAPNAKIEEEVAKLLALKAQLGDNGPKKFVLK

TPKGTRDYNPQQMAIREGVFAKIITCFKRHGAETIDTPVFELKETLTGKYGEDSKLIYDL

ADQGGEITSLRYDLTVPFARYLAMNKVTNIKRYHIAKVYRRDNPAMTRGRYREFYQCDFD

IAGQYDLMLPDAECIRIVAEILSSLDIGDFVVKVNHRRLLDGMFAACGVPDDMFRPICSA

VDKLDKTFWEDVRMEMVDEKGLDPEAADRIGEYVLYQGGLDLVDDLLNDPGLQKQKNAVE

ALEEMRVLLKYCELYGIADKVLFDMSLARGLDYYTGVICEGVLKGYTHAKGAGGSEEQIG

VGSVAGGGRYDGLVGMFDPDGKKVPCVGISIGIERLFSIMETKARTTNAKIRTTETEVMV

VSAQKNLVEERMKLCAELWKADIKAEQSYKKNPKALTQFQYCEENGIPLAVVLGQSEIEA

GIVKLRIVATREEREVKREAMVSEIRKVLQDLSLASAS

>SG-AL_contig_8443_1_+2_191_343

MIKKINLTDESFNPYAGCRNISRAIIRYTVSWLSRQKLLGRSVWLVTVTFL

>SG-AL_contig_8444_1_+2_2_115

KLSGHFEVLKFILSEMSKRRNDYSCYMTFGRKYHMFYQ

>SG-AL_contig_8445_1_+1_1_276

ISLVSLTDKAFDQGQNGNDESDYQVVMTFGFPNRKSLSSNLPGSHLVSKGGDFSGLPGQR

ADLLLPEPPVCVHTCGTSNTLIRYPVSRVIVR

>SG-AL_contig_8446_1_+1_1507_1905

MQTFLSISVNMYRKQDDGLGVLKVPTSNCKIHYFGNQYTVSIHKHHHPPNLICLNGLLCL

CVCIFYVQLLFCMFFILFCYISLYQCNKTVILLAIMYPISISQSLHCILFKISSQVKNLP

LQYMHDITSLSLS

>SG-AL_contig_8447_1_-3_723_2177

MAKNGNKVLTLDTLNPCVKAMEYAVRGPIVARAGEIDNELKQKGANKPFRKVVKANIGDC

HATGQKPITFIRQVLALVTYPDILMKSPDFPEDAKERARRILGGCRGNSIGSYTDSPGLL

VIREDIAKYIQERDGFPSCPSDIVMSTGASDGIKSVLTLLLTGQAGKGKAGIMIPVPQYP

LYSAAISEFNAYPIRYYLDEEKHWSLDVSELNRAINEAKPNCTPRAIVVINPGNPTGQVL

TKENIQNVIKFAHENRLFILADEVYQHNIYAEGSQFFSFKKVLMEMGAPYNTMELASFMS

ASKGFMGECGYRGGYAEVINLDPDVKAMYMKSISAKLCPPVAGQAVMDVIVNPPKKGEPS

YDEFIKEKENVLKQLNEKAKLVTRLFNSIEGMKCNEVQGAMYAFPSFELPPKAIEAAKAA

GQTPDSFYCFALLEATGICTVPGSGFGEKPGTYHFRTTILPQVEELKEVLDHFKDFHQQF

LAKYK

>SG-AL_contig_8448_1_+3_3_113

PCLCSVAYGNMKTPSMHTLKWQVTAKEVSSCDNSSYH

>SG-AL_contig_8449_1_-1_1006_1200

MLYLSTKSDGEKNLRRGRKRKEREHKNKNGYYMGTSKEGVKKKSTQSELSEEEEKETSVM

IYVKQ

>SG-AL_contig_8450_1_+1_409_540

MGLVNNFYCDFWLEMKDSIDHIFLKCLQKILGKDRRIVEVKIHH

>SG-AL_contig_8451_1_-3_222_452

MLFWKMCMFSNCINNVIMFYNDIHVYVCLYVCMSKYVCVHVYVCVYVCVYVCMYIYIYIY

IYIEDELKKKKKKPILL

>SG-AL_contig_8452_1_+1_349_513

MELSHNSEQYCLLEREKKNISQRSYLQKSTPTKFVDFLKMFREKKSVSALQVAYG

>SG-AL_contig_8453_1_-1_1_432

SLSPPPPTLPPLSLSPLSLSLSLCRSYVTWPSSFVFCLGLFFLFLSDFFFCPSPFFVFYL

PSFLISIPSPFTLYPLLSASFPFFLAPSFFYIVNVCWWACPLSRITHIVTSLWSVPHTHP

HSTLYLTCPWPGFVGEVCFCCKYI

>SG-AL_contig_8454_1_+2_2_196

AHVDLGDWKIFTLYPPGEVPEEVQSQTHVLQFRSQVSSPFNHCTFKIFSLSVFGQYHLFW

ALYLT

>SG-AL_contig_8455_1_-3_3_1286

YRDFLSRSGYKPYYLRQQELNQQKDQQERDTRGSSRITRPVSSISAFSSPSSPSSSTSSS

SSSTSSSASSSSTNPLTSTATGTTTVVTTTTNSVFRRSYEPPKRDEETETQRKARAKHAR

QTRRSTQGVSLEDIEKAEEVLRQGDNKKNTTEDTSSRASIRDSQQETTTATTTVTTATTT

PSSLSSSSSSLHNDRTSGLSSRDSLRDDTSSYRRRNFEDRLDGDSTRTSFRRPREDRDST

TNNGTSSTDITSSYVPRSQRNALSTSDITSSLSSSTGLSRSASLRSKLRNGETETTPEDD

RKKDETRETKKAESPGTEKEEKKETAAIKARRKRQERRSTGINYVPGEEEKGKENKPEEE

EKEESSENSSRFGRYSSSDVPSDSSSRNRPVSYSEYSRSTGSLDEYKKLYEEEKEENGRL

KKELDQCK

>SG-AL_contig_8456_1_-2_2_196

IRQLEGVVDGISVNSSRLRSVFRHANCSEFVVFCSDQSKVVPMTSKLFGKVHIRLAWVTR

THPRH

>SG-AL_contig_8457_1_-3_3_203

RSRQPASSHFRQRPFKTLADWDQTSRHLLLSCARADAFLSGLSARLKFLGSLNPGSLPPQ

GPTLLAV

>SG-AL_contig_8458_1_-3_75_203

MVTNARRAAYQCFCNVLTYYCKIVFEFDYIVKMNLPSAFFKTM

>SG-AL_contig_8459_1_-1_1_120

VSSERLVPSSTATQNPEEGGKLSQCNLKIESILLVSSAGS

>SG-AL_contig_8460_1_+2_2_211

SLSDETISRGPLCSICLAYVKEPMATKKLSLVTFCKRYTFQDKQSVHAHMNATTIIKRML

MVTIHYPPKT

>SG-AL_contig_8461_1_+1_1_66

FFFFFLKHAFIRFHCYNMNSCD

>SG-AL_contig_8462_1_-2_26_226

MIIPPACFGFKYGHPSRMLWIQIWSSLQHVLNSNMIIPPACFGFKYGHPSRMLWIHIWSS

LPHALDS

>SG-AL_contig_8463_1_-2_89_199

MHVPCMLLLDCVPDPVISYNVTILRFSVSQVSLPVTC

>SG-AL_contig_8464_1_-3_114_719

MAARKSTVIMLVCVCFAALALSLTSGAPTSSSAATSSGTSPAASISSGTAITKSSTAHTT

SHQSTTHVSQTSASSQTSETTAHLSTTTAPENGTVQSTDHTTVSNTTHFSESTTIFNGTS

ESPGNTTVNPNVTTSGNATSTEMTSVSTPSPSSTTPASGDSGRSFDVASFFGGIALAVGL

TIIILVAVRCYTSNKKSSYSAM

>SG-AL_contig_8465_1_+2_2_232

VQEGVLQNWDPSRSGNSQKVQEGVHQNWDPSHLGDYQWVQEGDLQNWDPSRLGDYQWVQE

GDLQNWDPSRLGDCQWV

>SG-AL_contig_8466_1_+3_3_206

IWNFLGTFINRLYVLHTHKFDKVKKSVFMVKKTKIFPKPFFFLFFFFLSTNINFSSRTYI

YSHVLTCK

>SG-AL_contig_8467_1_-1_1_180

FFFFLLFYFLINNFFFFLFFFFFVFCFFFSKIIFCLTVVLLNFQFMAFPVVMACIICRCV

>SG-AL_contig_8468_1_+3_3_158

TDCAYIRIIPKKNYPLKQSDTREIYPLKKYSGVVFTIRIKSLSLYVIQLCDF

>SG-AL_contig_8469_1_+1_1_138

PSNNANYLYPCISLTGGFIFSKSQFSLLPHCNMAVKMVKFGFTVST

>SG-AL_contig_8470_1_+3_195_365

MCYQCGELCDSQPSNNANHLYPCSSLAGGFLFSKSQFSLLPHCNMAKLVKFGFTVST

>SG-AL_contig_8471_1_-3_60_269

MEFNYPYFPLVLHTDFVFKKQWIGIFVLIQYHDLLCVLSSKYVLSFLFTSFHLVSLVPRS

GSASPSEVSH

>SG-AL_contig_8472_1_+3_471_620

MTMQEVLFSMCYNCCNSAKLSGQSNGSFKHPNLVQIHSKNVAGDRAVDSR

>SG-AL_contig_8473_1_-1_34_333

MAEDQVAAATVSTNEVQDQIETGPEVADNIQKLVTSEKESSESQAKKAKVELQSLPTRAY

LDQTVVPILLQGMSVLAKERPPNPVEFLAAFLLKNKNQHE

>SG-AL_contig_8474_1_-3_3_254

YPGGVIGLDNATANLNLDNPDSLPPPPPELLQQQYLYGTFPDSRQQAVMSNYAPTRGQGY

PTYAPGSGQLNMGQPQQAVVSSPV

>SG-AL_contig_8475_1_-3_891_1121

MPFLQVFSSNPPSSKFTWNCLSKDPLSHGFTQLSQEDSLLLDHKLAPSYASVSSRRWQIG

TVSPNTFLVVLKPTPFF

>SG-AL_contig_8476_1_-1_76_231

MELSEWRESLLIKLQTSCTKNYIVLRTWDGRVKSSSVISKKVILHIIYLYLV

>SG-AL_contig_8477_1_+3_3_248

NVRILCSPVCVYTQKGFRHEQVCAYADPRDRKNLHPLPTRGGDLGGFEPRSSKPSSLEVK

RLHHSTTTPVRWQRPHSLRLAV

>SG-AL_contig_8478_1_-1_37_261

MYFFFSFFFSFILRRFFVVEKVVLQSTLGILNFFKVTVSFSSKGIFLGIANFVCLNCDGL

NSHAFYEMNFCCHLT

>SG-AL_contig_8479_1_-2_1937_2188

MNCLITQAFGSITEPPSFGFRDISSLKHCVPHSGETPEESFDRLENVCHQVKNRPCKVMT

FLQYYVPVKQLTNTSGQFFIQIMI

>SG-AL_contig_8480_1_+2_2_181

VHAGCIRVSISHRPRTWLTGSLTCIFDVLYAYTHFTTLCVARLTASKEALITNVLTCSMV

>SG-AL_contig_8481_1_+2_1754_1954

MLALGRGTSILHGNQNDRKQLYRQIKSFLKIIKGPRILVTVTQTLDDASNLGIPNLLIFR

PGLRSKH

>SG-AL_contig_8482_1_-3_3_191

SPAEQYAQATSVQRCVYFRYHICFLNLPLPHPVHLLSFSRLAGSVDRAVFSGSDVSSSIL

PAT

>SG-AL_contig_8483_1_-2_77_1429

MQKALSVQCSPQSQSSTPTASPSSVDTEHTGSHLSGQSGRLHSGHSGQLVPSGQLSGQMA

GQLSGQMLGQQTEQSRRQLLVRQPNVNQTEQQFPAISKGSRSFEESSGSIASSSMYRSGS

VPNLTVASTQQGAFPDPVTSTTLVYRDFDQDSTSLSSSGFIPELSLLTKVVSDSDTSSNI

PTVVSHHSPPTAASKSSSAWPWGPNQEFSGASTSSDASQSRTYPFTCASSVTRTPQSAQL

TTSQAFRGGVFSSGSTVTPGPNFLVSSGSSISAGTVLSSAVPYYQRPRSSSDLARKVTST

VSSGSVSSSLSQQNLPGLFDAAVTTYSPRDVYPSFRDSVTTCVTHSSALQETAPVCVGSE

LSASISEPIPHWYSFLDDLESAASLSARQGAFSDAGTPGSSRTRSDAGTPSSVDSPYSQQ

LLQQQQQQQESGGDGNIPTIISDMLGQAPPS

>SG-AL_contig_8484_1_+2_101_322

MIIIEKKTTVQSKYKSSQTCPFQFMFRVQLTTGPIPGLSNQTYQNQTRRIRTKYNCSTRK

SPHSSQTTVREAGH

>SG-AL_contig_8485_1_+3_258_614

MVNKQNNPSVRQSPDTYPSILFSLSLSSFHLSVIHSFSLCLPSFSFGNLYTCLFLIAICK

INMQTAGPGNNMKHPSHFTAFETLCKTVIRNAAGMSVLSVYLYVCIAPAVAHTKFIQQH

>SG-AL_contig_8486_1_-3_114_1217

MPFYAVKSGRIPGVYNDWASCQEQVKQYQKPVFKKFPTEAEAWDFVNGVKPNASASTEPS

ATTANCTETSYGDSMNSFFQGDQRHKSSASASNSATRTPDEVADQSAAFEAIGMLRATAK

ALRATAEQLMTGVDSLTSQIDHLAGSLTSAGQRPMGTAFSTSSVRDNLKRTYSDFTTLGE

ASTGRKNVRDAAAKKHKKMKSDPAGCTDCGFTGVPFPDHEGKVVYTDGASFDNGYATARA

GIGVFWGHNDPHNRSERLPGRQTNNRAEIHAAETAVRQAKERGIKNLILYTDSQFLINGI

TAWIKKWKKNGWKLTTGGDVINREDFEKLDQTISGMNIKWMHVRGHYGVEGNEAADRLAN

EGAKKPLV

>SG-AL_contig_8487_1_-1_1_234

PRPPLKVCWRDWKIPVQLGIEPGISFFLGRYFNQLDHNSMIIQIIMLVRLSCEAEVHTLC

CLGSFNMQSLSFVSLFNN

>SG-AL_contig_8488_1_+1_382_678

MVTGGINRFSLNNGYNNIMVVKCCLHSLVHHDNFVATDINHIQKKNNHTHTIQLSSTETR

ICTETLSVISISAFYLAAKMYKQIPSLSFFITPPASHQQ

>SG-AL_contig_8489_1_+2_35_214

MGEPNLNARNCLNASGLQSRNCRKRNRKVCLLNGKVCLFEKLKRVEYACTNCKTHKWSIP

>SG-AL_contig_8490_1_-1_553_1035

MADPLMTGGTCGVPLRPCAIVTPPIPRLRLRIRELIVDRLGRLYLVAYLIFPYPCAESCS

FFVRGYYTPQAREVPSAWDKPARGSIPVNALAPPATMVAERPSHRWYWHSSRPSPLIVPP

LDPERGEGPAESLAAPHRPDHLPAGKHWVCSRAGMWLPISD

>SG-AL_contig_8491_1_+2_2_85

FFFLTAVNWKSLFDNTSQINYCVIHNQH

>SG-AL_contig_8492_1_+3_273_713

MDACCSILSECQTFYLINVKLTERDGAKDDVLIRFWGGLLFSELCMLGSESGNLFFIFCV

FILLIYFLKRGNLVTAVKESTKLKNLSFLRALALSENPVADEDDYRMEVLIALRTLERLD

KDEFTDEERHEAEEIYETRKAEAEAAE

>SG-AL_contig_8493_1_-3_177_260

MSMKHVVFILLSFILTLPVNRSHLQKKK

>SG-AL_contig_8494_1_-1_1_252

NSALVGSYSRKLFLTNPKYSKRVRHLAPGGLTLRGVKVNDAGNYSIEVTVENKQKSCRKT

HTQTLTLQVGEGVMVEGGQLKARQ

>SG-AL_contig_8495_1_+1_583_819

MPNNLVVDATHFQTAIISMGWQDYSLPNHFYIYLLHLYTIHSQHPHFLNCLLVWLTSTPL

ITTQFFCKTEFSCEGRKKH

>SG-AL_contig_8496_1_+1_2698_2985

MVSQSQVDSTLHMFKTYDFYSGQSSDTVIHVMNIIAFNCPTRMAESARLLLADLGGTHTE

LCEYEVQTPRTQTGTQYISSFLLLLVVLLPGCIHKG

>SG-AL_contig_8497_1_-3_300_464

MSKVNCIGTVVFEVNSGTRGVPPLTEFMNYQQSDCVIDLPNCYLASMIFSHVARL

>SG-AL_contig_8498_1_+3_84_239

MPNVCWGRHSLSFRSRSCVLQDPNLRSHTHDYMGCPNAETDSDNVIGNMTAT

>SG-AL_contig_8499_1_+3_423_722

MSILVKLDQPYLTVYEYTHNAHCSHLACSHSVYSHAYFPLMVQACLQWFANLPCGTLASN

SAFFENYRFFFFNMTKHTQCIGAEEAISSEFMRGDELQQR

>SG-AL_contig_8500_1_-1_136_321

MHCKNVWSQRDSTYLLTVILHWYFFVVKDSRVAMQHATLVSVVISIVHLGFTSHSIGNLS

RY

>SG-AL_contig_8501_1_-3_483_656

MTKSVHPKNIWYCIMSVLLDQSDEEWLSWYCIISVFWGIYFIKRGGTGTICTNTTGKF

>SG-AL_contig_8502_1_+2_2825_3238

MCSYVPPEGSPFYDHFDLDNGMNLLEECLTENKIPGDLNNRTCDISKEFSTNNIFDMQHT

SQSNNRLSQDSTLNSDGKSLLNMCTSLGLCILNGTCKGNLKVAIYIFLILEIVLLIMLYF

RMICFHSFMNHLSYMLMK

>SG-AL_contig_8503_1_-3_417_557

MCSNSDFSFLLNFFSYAFRIEDHVFVMDSIITVHSLFYIRFVIKCYL

>SG-AL_contig_8504_1_+3_258_383

MTMKQTPPHADNSVTMFSCSNPMNITRTLLPGVLAPTLMNFK

>SG-AL_contig_8505_1_-1_319_633

MGLASLEGRSPNQVLAWGLLLVLDALRKNPYEPPNNAPLSMLTSKMVFLYTLATGRCRSK

LHGLSGAKGDIAFYQDSSVSLTLLREFRAQNQPLEEPSPQLSSIL

>SG-AL_contig_8506_1_-2_743_1336

MGTSIPGSSEPQSSPSLGPFRKNPYELPKNAPLSILTAKMVYLLTLATGRCRSKLYGLSG

AKNDIAFYQDGAVSPMFLLEFRAKNQPPEEPSPRLAISLLTDIMDKSDEERYLCPVCYFK

YYWDRTKSMHANCRRLFLSINENYWKDITAGTLSGRISQMIIWTYQSARKDTSVLNPRAH

KIRALVMSLAFSQSVRST

>SG-AL_contig_8507_1_+3_3_140

YTSQLCSIGGTIRPYMLANYGLLGGIIRPYYTLANCLRLGAQFVLI

>SG-AL_contig_8508_1_+2_89_256

MVLGAIIRPYTLANYAFGGHNSSLYISQLCWVFLDIIHPYTLANYALLGAQFVLIH

>SG-AL_contig_8509_1_+2_89_256

MVFGAIIRPYTLANYAFGGHNSSLFISQLCWGFLDIIHPYTLANYALLGAQFVPIH

>SG-AL_contig_8510_1_-1_1_366

FETLSPKTLLSVETHHLPQRKLLEGHIATTISCWLSATVIRAYKESGSSSLPDSPRAHEL

RALASSAAFHHSLPIRDVLEAAYWQRESTFTNHYLQDLRVSNADGLSRISFVAANAVIDA

SM

>SG-AL_contig_8511_1_-1_316_600

MLMWLPYHQQCYQLFSHLLDWVLCTLLMPTTSTAQHMRSIQWVTVSQIWILMFSHPGFLD

SVMSLLLVIIFLFCFWLTGGVLTTFLCTDFDSINC

>SG-AL_contig_8512_1_+2_245_439

MPSPYPAASCIISHDMHCKTLRLSCPHPMQQLPVLSAMMTTNQKLNPFTPYPIHQLPAGG

CCWIG

>SG-AL_contig_8513_1_-1_16_294

MRACFSEKYFSKIFSELCVICGSLPDLGQRYKSSKNRKYISLDRSVSKSQRPANDPHRKE

TGTANIMYNLFFVFKFTFYMYILFIHLLQFTIL

>SG-AL_contig_8514_1_-2_176_271

MKSGFELKIIPVKFYKLWNQERILTCYFYLFG

>SG-AL_contig_8515_1_-2_110_3781

MLTKFETKSARVKGLSFHPKRPWVLASLHNGVIQLWDYRMCTLIDKFDEHDGPVRGICFH

QQQPLFVSGGDDYKIKVWNYKQRRCLFTLLGHLDYIRTTFFHHEYPWVLSASDDQTIRIW

NWQSRTCISVLTGHSHYVMCAQFHPSEDMVVSASLDQTVRVWDISGLRKKNVAPGPGGIE

DRLRNPTQTDLFGTSDAIVKHVLEGHDRGVNWAVFHPTLPLIVSGADDRQVKLWRMNESK

AWEVDTCRGHYNNVSCCIFHPRQELIMSNSEDKSIRVWDMSKRTGVQTFRREHDRFWVIA

AHPTLNLFAAGHDNGMIIFKLEKERPAMAVHQNILYYVKDRFLRRLDFTTSKDVAVMQLR

GGSRTPVSTLSYNPAENAVLLCTTASNVENSTYDLYLVPKDSDSQNPDSPEGKRSAGLTA

VWVARNRFAVLDRTHTIIIKNLKNEITKKIQTPNCEGIFYAGTGCLLLKEADCVTLFDVQ

QKRTLASVKISKLKGAIWSADMSHVALYSKHVISICNRKLENLCTIHENIRVKSGAWDES

GIFVYTTSNHIKYALTNGDHGIIRTLDLPIYITRIKGTNVYCLDRECRPRVLNIDPTEYR

FKLALINRKYEEVLHMVRNAKLVGQSIIAYLQKKGYPEVALHFVKDEKTRFGLALECGNI

EIALEAARALDDAACWERLGEAALLQGNHQIVEMAYQKTKNFDKLSFLYLITGNLEKLKK

MMKIAEIRKDTSGHYTNALLMGDVPERVKILKNCGQKSLAYLTAATHGLQEDAEELRESF

GEDEKVPDLYPGARLLQPPPPICLQENNWPLLTVSKGFFEGAMASRGNPSALAAAVDDEG

AGEGWGADAELVLDDGGFGAGEGFEEEGISAGDGEGGAWDVEDEDLELPSDLDMGAAATG

IGEEGYFVPPTKGTSQSQVWCNNSQLPVDHVLAGSFETAMRLLHDQVGVVQFDQYKPLFL

QTFSRARTCYVGLPSLPPLFGYPHRNWKEAGGRNGVPAVGLKLNMLVQQLQAAYQLTTAG

KFGDAVNRFRSILLSVPLLVVESRQEIAEAQQLLEICREYILGLQMETKRKELPKDTQEE

KKRQCEMAAYFTHCKLQPVHMILTLRTALNLIFKLDNFKTAASFARRLLELGPKPEVAQQ

TRKILSLCEKNPTDKHDLKYDQHNPFDICAASYIPIYRGKPVVKCPLSGACYLPDYKGEV

CRISKVTEIGKDCIGLRISPLQFR

>SG-AL_contig_8516_1_-3_3_92

QIYCHLLTKVPCVCLYNALYNIYHLVLFEK

>SG-AL_contig_8517_1_+2_1472_1873

MKSLQGCMRLVEREGKMHCMGDSCHAQHSLCMKTAIAILFQHSYTKRISTMMRLQKMTTS

RHLQETEPCNKMSNGSNSNELCRSFLHQPMQTNAPPVTGIVNYAGQVLPLSLFIFSLFFS

LFPVSTPISLPPLF

>SG-AL_contig_8518_1_+1_1207_1368

MRTLYFVSENHCQRMLSQSKQLLRLYSGTCSRKLTGNGLCGFAPETNSRWERTE

>SG-AL_contig_8519_1_+2_152_292

MMYQICHQMKSINRGDLYLQPDIYTYMQSTQGEQAQSRLITHGPMAP

>SG-AL_contig_8520_1_-1_364_621

MRQYTFPSGCDDLFSDAICLCRSLELPLYSCVCVSKHVRVCIYVRANVRTREGVGVTHPN

LENCKIFDNKNMFIKKKKNGRATRRE

>SG-AL_contig_8521_1_-1_49_237

MHFPHAEQLWQCIFPMQSNFGSVFSLRRSTLAMHFPCGEQLWSCTFPYGEQLWQCVFPRE

SNF

>SG-AL_contig_8522_1_+1_22_270

MGKTHCQSCSPWGKCIAKVALHRESALPKLLSMGKVHCQSCSPWGKYIAKVALHRESTLL

KLLSTGEAHCQSYSPQGKCIAKV

>SG-AL_contig_8523_1_-2_2_172

CMCVYARACSFVRSCVRECMCLYSLESLLSSYQYLSTETSICLRVCIIHGWGYSPYS

>SG-AL_contig_8524_1_-3_96_4268

MTLEYGSAEMTDVTYRFGPPGPPGGRGIPGPPGSKGETGPLGLNGVPGTNGLPGPPGHVF

VIPLGSNEGKQSNYAEHFRTMLDQHMLAMRGAAGPMGMTGLPGRVGPAGPPGVKGDLGNT

GEQGPMGPFGLPGPPGPMGKRGRPGNDGDRGVQGPPGDKGVPGFPGMPGMPGEKGHRGVA

GTPGETGNMGPDGEPGDSGEPGPPGAPGEIGPRGFLGPRGRPGPGGPPGQPGNEGPVGPK

GNMGSVGSPGAPGQTGPSGQTGPPGPVGPLGPPGIPGSTGKPGLPGLPGSLGPSGLPGPP

GEHGAKGDQGPMGLQGVLGYPGPRGIKGDSGSRGSQGTKGDKGASGHDGMQGDMGLKGDK

GIRGPRGPMGVEGIEGAKGDDGSAGEAGLQGAFGEKGRVGAPGFPGYPGAPGLKGMRGRV

GRRGRRGARGKRGLPGPLGETGEPGIRGQRGERGKQGIAGPFGPKGDPGPPGPPGFTGEQ

GIQGPPGPVGIIGPPGAPGVPGKDGQMGMPGDRGESGLPGSPGQPGPVGVMGPTGPTGEL

GSDGERGAPGNPGPPGDQGVAGQSGKAGERGPAGPQGNPGPSGPPGLGGFPGGRGFPGSS

GKPGPRGEPGPPGPPGPPGPKGSNGLHGPAGSAGRPGTPGVRGGIGPVGPKGAPGSAGPA

GQQGTEGGPGPRGYRGPTGPVGPPGMDGDKGDRGGPGVMGLKGDYGPQGPPGPQGPLGIQ

GPPGPTGPPGEPGTRGPQGPSGPKGSEGPRGLLGPTGLNGLQGMPGPPGPKGETGDEGKK

GPTGPPGSQGAAGPAGPSGPPGLPGTPGGEGPRGMKGEPGPPGSAGIPGADADRGPPGPR

GPKGEDGRRGSPGAPGPQGVRGIDGEKGDAGGFGPPGPQGERGEQGTKGETGDPGIDGEP

GPPGLDGPLGPAGEIGLPGIPGKQGEMGIPGLPGSAGEPGSKGDRGVRGPSGQRGPPGPP

GGSGPTGPTGPAGQPGPVGEAGVSGPPGTPGAQGPAGPQGPLGKKGSRGIKGIKGHRGEH

GNPGPEGQQGMKGEQGVPGPPGPKGESGPHGPPGPRGTPGNDGAPGIPGAMGAPGPKGEP

GQAGAKGEQGNPGPVGSPGQPGDKVEVGVGDLAAPRQLKKRKRRRRRRRSPDATDIDTDI

QSVYARLIERQQDGGPLQLQEVIQEVSSMMLQHIDQLKAQVEKIKFPQGTRENPAMSCRE

IVLGHPQYQTGWYWIDPSQGSGDDAIQVWCNVTVDYIETCIYPTPKTKMVAEKAWTKPNG

KTRWFSRLKGGSTIKYGSSVQLGLLRLLSEEATQRFTYHCSGSVAWLDETFGSYDKAIVL

AGDNDFEFETGNFPAEFIISDTCRDRRQSGSTVFEIRTNKVDRLPITNFQPLDYGHPGQK

FGYEAGPVCFH

>SG-AL_contig_8525_1_-3_279_509

MYRQERALTANVYIRQCCARVYCDVSAGVCCCVDWQMFRSLLDVVIIFSGPEAVGFKLRN

LLLNFIRCIRRADSRAK

>SG-AL_contig_8526_1_+3_3_128

CTSGPHLLHNLQKVTTIHIIQGKFHNFLDDNARQIIDEEITQ

>SG-AL_contig_8527_1_+3_534_755

MCTHTCTKYALFNIHWLMLLIPIAHVGHYSCVSSSRCFCWVRTLCPTDFLNPRHFVLLLL

SPFYHGCKTECGMK

>SG-AL_contig_8528_1_-2_116_562

MVLLFAFCTFISCSVQMSSGLSSMPFSSFLFSGHFLSFLLSHLSVSLRVCSCPRLSPHTS

ECIHLLLVSPTQPTLSFPVSSFPETQLCQILCGLSITLFSFHTKLSSPCDAQSDLFVILK

RVFSAMLEMSFTFDIRVCFHHVSRDVSVR

>SG-AL_contig_8529_1_-2_131_520

MFVFMSPRPPSLVYLATPFPPPSGGWARVTPWGRGILGPAEVEPWVSGGPPTINPLEGGA

PPWAKRTTGSNGDLSPPPPRVQSVASASGFGSPVGFPRRPTASATVTNASQAELNSSLQS

GFPRGPPQGG

>SG-AL_contig_8530_1_-3_15_317

MFLHSLLTPSSVGVHYFCVSTTHQTLTWLTGSLTCVFAVLCAYTPTGVQVCRYVDLAENK

PSDWKWLLDCHACSFVFYFASVILVYEKCFIVFHLACVDVT

>SG-AL_contig_8531_1_+2_362_556

MDETHVSHLLSIKEVSSSGINAWKRQQLHNFLFKDEEGLAGKHVVTGLFLHKAKNYTPFL

HLVNL

>SG-AL_contig_8532_1_+1_1_159

EQLFCCHGIFNICLVHAAHWTLLYRLIKKTQGLVERGEKSLFDQELNSGVLAS

>SG-AL_contig_8533_1_-1_28_1059

MAAEGMPPPLEDMTVTLEQARKLRDLRLGRQEQSPGARSSTEAKTKKKSTERSPPAATVP

TSHSHTEPSTTSTQEKTKTVKSTPDSKSGSGDFGGMKKGFLFGGSKPSHASGAQQTKAES

QKSKADSGKADPIPFIKPKTQDKDSGLKLDEVQKAMESTKGFLQDKDWVTGDLLEKVQKN

ETLNKRLGDPRFMQAVTEFQTNPQAAMVKYQGNPEMQQFLKEFCGILGEHFSSLGEKEES

QNNPQAQNPSNTVHTGPQITELTDEDTSQSVRPQESTVPVDPHVQQILVDPANREILMDP

KIQQLIQNLRSNPEKAQRMLREGDTAFKQKVDRLISLGLLKFQG

>SG-AL_contig_8534_1_+1_1_78

FTLVIIIIIIIIIIIYFYIVYSPADH

>SG-AL_contig_8535_1_-3_858_1028

MFDRTKVIIGFLLISPMPSPFKKVKGGKMSDGPNVLVAECSILFTFLFDLSDCLVNF

>SG-AL_contig_8536_1_-2_2_133

LRSVCVCVCVCVCVRERERERERERDALDSFSNSYVQNRKQRSG

>SG-AL_contig_8537_1_-1_1_798

DAAEYLDKETEYTPWSKAFDELESLGNKLSNTDVFPAFKAFVAKKTSPLYAKLAKKTPAD

YFETELRKRIVQLACMFDGPECVSDALTGLQEWKMNPDRNETSRAMPYMMYCTGLRQGNT

DDWDFIFERFKAESDPSEKRTLMNSLSCTNNSKDLNRLLDWALNSGDINSENFIDVLHST

IFNPVGANTAWTFINSTWDSHPEKYDTNDFWVQSTIEQFGFAFHTQDQLRKLQDFVDRHG

DLGEAGQAFLAAMESITNNIAWMEKN

>SG-AL_contig_8538_1_+2_2_214

RILLLHDTEQRILLLHLLLSFICLLVPSWVSRGERADSQQHAGRPEEEKCRVTSRSVPSW

VLRGERADGQQ

>SG-AL_contig_8539_1_+1_733_1131

MVNKRQFLLIQKTVSTVLWTKTPSPLSHYSIQPPTHSTNHLPTHNTTYPHTIPPTHTQYQ

PPTHNTNHKCTHGKTLMTTQYCQIHLAKAPIVFLSFFLFPFPFLLYEEKRGCKERQEEKE

RKQRGEEEREGGE

>SG-AL_contig_8540_1_+1_184_384

MKKIKVLVCKKKTPPPKTEFSLVLQLLPFNRDQLRSPSDSNQRTHDLLICVSIILCYYIV

WHYSVPP

>SG-AL_contig_8541_1_-2_65_1819

MSFQLNMPPSEIGRLARSEQSAGGGERQKQSREDWKKMKELEEARKAGTAPAMQDEEGKD

INPHIPQYIMQAPWYFGANTATLRHQRPQAERYTTYSQLEEYVQKGIKAGPVATKYRKGA

CENCGAMTHKKKDCLERPRRIGARFTGDKIAPDEILPNELVQDFEGKRDIWTGFDPKEHQ

RKLFEEFGKLEEAKQTLKEEALEKNLMEKADEAKRTEDDEDKYAEDMDMPGQKFETKNRI

TVRNLRIREDTAKYLYNLDLNSAYYDPKTRSMRQNPLAGTGKESKLSQYTGDNFVRFSGD

SEEFAKKQLFAWEAYEKGTDVHLQADPTKLELLSKEFQNRSDQFKKSAKDNILDQYGGQE

HLETLPKQLLLAQTEDYVEYSRHGTVIKGQEKARVKSRYEEDVHPNNHTSVWGSYWKDGR

WGFHCCHSFIKESYCTGEAGREAHEESSLILAPPGSAGAASTSIVVRQTSSADEAEVPKS

KEKKKKKKSKKRKRKHKKKRDSSDSDSSGSDADSDSEERKRKKLKKALDAEAAHQEKMSE

LMAVDERKRPYNSMYDVKAPTEEELEAFRMKQIRDEDPMAAFFSK

>SG-AL_contig_8542_1_+1_19_108

MRSRLPVLQNEVIILYDSLVRFEIVRIQSF

>SG-AL_contig_8543_1_+1_274_573

MHAMDIIVGFFFRSPHLTFSNFGLRGVNDMPFVFFSHFTLCLFASLHFASTSSHSYPPPL

YPPSTMVWSMVWEREREREREREREREREREREREIYIIK

>SG-AL_contig_8544_1_+1_694_1035

MTRKPTKHKPIAKLSLIIHSDADSDLVRKRIAWLVYMCVCVCMERGVEPLCIIIHQLLKH

LCEHDPDCGSQNQLPCWMRRGMDGVGEESVYVLVVVVGRRVEIKPLRLFCCCFF

>SG-AL_contig_8545_1_-3_3_182

VSVTVSFSPNCNIVSFLCQCRCPSLSDTCTYTAVPYNYLLWMISMPCTYYTLVCTFSVLR

>SG-AL_contig_8546_1_-1_166_441

MPVSVFVLVSYESLFLLSCQCSFWCHMSRCFFCHVSVIVRLGCHVSHCFFRHVSVIVLGV

IRVTVSSVMSLFWLSCESLFLLSVSLFILGVM

>SG-AL_contig_8547_1_-2_347_565

MSVSLFWVSYESLFLLSCQCHCFGCHVITVSSVMSLFWVSCESLFLLSCQCHCFGCHVSH

CFFCHVIVFVLGV

>SG-AL_contig_8548_1_-3_138_2390

MELERTTRSGLRAAASSSAHGTSSGTASGSGVAMSIASSIHSDAGSEASQSQTEPTTITS

WKKRVRSEYMRICHMRKFKRADEVKNVFSANRQHIDDQLQKQEDWLKKQTLPKLIAPQVL

PNSPITRKCEVTSGNNFPTQTVALRVMNAVKPVPTMYSWAPLQQNFMVEDETVLHNIPYM

GDDILDQDTAFIEELLRNYDGKVHGGRNTNFISDDIFVTLTQSLTQLYPYTGSSVPEESE

SGEESQSSIQSTSDGKKVDVPQVIFEAISATFPEKGNASELRDKYRELLESQASDPNTLP

PECTPNIDGPNAKSVPREQTLHSFHTLFCRRCYKYDCFLHPYHPTPSMMSRKMAQKKQES

EPCGPDCFLHITGVKRQTEEEADSESASSSPPRLSGRKHKLAGSSHSSNSSDDSDRSNDA

TDVEISFPERRQPPTAEWTGAEESLFRVVSDMYRNNYCALSKLIGTKSCKQVYEFAIKEE

AHIPDLKEEHTQTPPRKKKKKHKLWSVHCRKVQQKKDGSSNPVYNYQPCDHPGQRCDDSC

PCIMAQNFCEKFCQCSSDCQNRFPGCRCKAQCNTKQCPCFLAVRECDPDLCQTCGADQFD

TVKISCKNVSVQRGLRKHLLLAPSDVAGWGIFLKTPAEKNEFISEYCGEIISQDEADRRG

KVYDKYMCSFLFNLNNDFVVDATRKGNKIRFANHSINPNCYAKVMMVNGDHRIGIFAKRP

IQAGEELFFDYRYGPTEQLRFVGIERDEAAS

>SG-AL_contig_8549_1_+3_3_146

FFFSLSSFFLAIQFHSFPNNNDFTLSRGIYNAMQCSIYVNMLNNAIFL

>SG-AL_contig_8550_1_+2_23_358

MSQSSRWQERRMATPRAQDAISLETLISRRGARERPNGRAWNWRAWAFHMRRGCFLQDGW

MRPWRQASMGTIEEAQLPSRIEALIGTGSPSRSGADQELVEGRQVQHRAPAT

>SG-AL_contig_8551_1_-3_3_260

GEGGRELWDVVSSLICVCVCVCARASVCVCVGGGGGGGGVVLFLFCFFNETELIALVTPH

KIQSATTLPSKQFSITHCLPCGQNTD

>SG-AL_contig_8552_1_-2_1052_1471

MKACVITVLVVVTTLCRHCVAAPSAYFCGSECRQPQSCYLLLLLQDSMRVYVCMCMCTFC

FPIRMEFSQFCQDNLFVTMGSFMRANYCLLHIRPQFIVPSERLSPTSPLNIQQGGEKSVQ

LQFTQPTSGFFCFSDRCFIH

>SG-AL_contig_8553_1_+1_679_903

MPYAVVLFSLSLIIFVFLTKSTVMVVVPTRYNNFLPPSLLMLLIFQFCIISVPLLFSSFN

SCLAGLYAVFCWLEL

>SG-AL_contig_8554_1_-3_786_950

MFTSQYVHYVCACVCVCERERESISSMCVKIFQFIPSVSLVPVLHREMYNRPNKF

>SG-AL_contig_8555_1_-1_1_222

AEIFLISQVNICADLLVPEPPSCVYACRTSNRHVKESVSHVSVWRFMKHENTQHVQKMSV

MVNLLTVDINGKTR

>SG-AL_contig_8556_1_+1_370_813

MIYTQRNEKKKLFNFNTLNKSYNRNRKEKLLTSMHKIKYITSTHNHTTSTHKEKELHDFH

TQSYNFRTPGKKPTNCTTSTQSCNFHIPREKKCTTSTNKSYKCHTLEKQNKTANFHTESY

NFHTPRGEKTTQLPHTIIQLPYIQRQQK

>SG-AL_contig_8557_1_-1_1_180

QSVVDFDLFAVFERKGEKTVMGTIVTKYKIRILPPSPTPTPLSPILFFDICMYSGVTGVF

>SG-AL_contig_8558_1_+1_97_231

MTVEAGLGVATLNPSQQHLFTSSDSNLFQTDPQLNGKHLDIWLFC

>SG-AL_contig_8559_1_-1_1_243

SLSLSVFASLSLSLSLSLSLSLSHILSLFLSLFLLSFSFSSSLLSHLSCSSDRFFTLSFV

SRYTRNVCTFVCVCVCVCVCV

>SG-AL_contig_8560_1_-1_100_228

MPTGAVRFSNCVFYQHVNKSCCVLCNVRCFVVHIDFPPPLLPT

>SG-AL_contig_8561_1_+2_2_142

CNRNIKKIHFNQKWKVITNRHFKKKKPRGVFLFKISLLIFKVFKKYF

>SG-AL_contig_8562_1_-3_93_278

MDRHPSLLLQPSLGWMLHSCVNLWTTSIIITSAVLATSPSFMSGNMDNSHHHYINCASFT

CG

>SG-AL_contig_8563_1_+3_426_1451

MAMYLFSGNPIGVQEVYGASPAVQQSEPLKVQENTPLTPFKDRVRNNEAMLVGFLAENSL

PFSMAPKLIDLSQALARVTKVLENMSMDRTAASYKLKYGLAQTFEESLLEELRCTPFALN

LDEAMNANKQKVVTILVSHFSKSLQRIVINHLTSFVVTRLTSETLFLKLEEIFEHFRLPW

KNLLAMLMDSCRIMRGSKSGLEKLIRDRKAPHLLDVDGDTCHHAHSAAKTFCAAFERHVE

QLMNDLHTDLEWSTYQRDLLFKLCAPLGSKPTKPEQFIQHWWLSVLQVGQDTLRQFDGLT

LYYFSFLKESHRVLYKDIVTDILNKRKVEETARTAFPTIKKK

>SG-AL_contig_8564_1_+3_3_200

VTQLMQLHEKHEFGRPGDSTLPFGLAHSIDVGQAQNSFLGNFPCMQRNLIIFFRLCSKAK

CSQSLT

>SG-AL_contig_8565_1_-2_32_259

MHKCVCVCVCVCVCVCVCVNNVYSDCFILKSSLYQCHSFKLIMQIHFLMSVLYCSFLISC

CLYSHLAQYFIAVPSL

>SG-AL_contig_8566_1_+1_1_225

FFFFRFSDILLLSDYGFQTLYCYARSAVTKHLFAFWAFLDYETIFDAYSPFPSPPQTRSP

SLMIVFTVSTERIEF

>SG-AL_contig_8567_1_-1_928_1092

MHRKMCLVTLAAVGFVLRLILMTVSLVCWLLCCLHYVQQVIDPNMWKTSVNSAGT

>SG-AL_contig_8568_1_-2_2_154

GCKKHFHVYSTTFVHKFVTFWFLFSLSPPSRKFSGIPNVHLIPIKNSSAIN

>SG-AL_contig_8569_1_+3_309_443

MISGLKKKSNHTYYCDGHVAVACGIFPFVWVRLKLGFSCPGSVLN

>SG-AL_contig_8570_1_+2_608_808

MFCSKQYKQHRDIKHTCSDPLVQRFTFLAKQCLFIHKLSQVHHSNKRQHITSLSTLFICP

TAFQRFH

>SG-AL_contig_8571_1_-3_3_149

CLIHINWTLFLFFITLSLSLSLSLSHSLSLFYHFLNLLLSVGKIGAGIG

>SG-AL_contig_8572_1_-3_3_143

FFLYKYCNTAHKVDYSSVVGYVCPLVLWKSTKFVLEENPRNYLNSIL

>SG-AL_contig_8573_1_-3_513_1112

MAQGRLSPHCATQTHFVPLVFLSSATVLPPHLFPWLPDFVDVDDDNNVDDDVDASCLCGI

SFNQTACSGREQMSFLVFRLLFFWPLLLCSCFTLASFNASFAQAGEEGRVGGLVEVGFES

LTLGLTGWEWGVCLWVVSVLVSCCSSPNIIACSKQRTNLSISFFTVTKAVQPTHSPTPLF

HLVHHHPYHRWGEREGRGGG

>SG-AL_contig_8574_1_+3_3_134

LYTIVHFLAPKKLNCVMCGSVKSERHRYRNGKTGKWVELGGGNP

>SG-AL_contig_8575_1_-3_84_374

MPAQIAVPDCCSSSSRPECCRRAYSAQARAVCCDAALYPQHDTCCLVASAIGLSQCCTSA

GFLSPCCSPILTLPPPTTTTTTTLAPPPPPTTTTAAP

>SG-AL_contig_8576_1_+2_122_379

MTLSITSAHLKKRLPSKQMYLNNFFFFKSRHLPHIPMHAFTETEPTIIRNTPEIFSACLC

KSPLQFSHPKFLYKLSDLLTGICYFV

>SG-AL_contig_8577_1_-3_351_755

MKNTLTHFVMCVENFVHCTHKNASEYGRVMLMCNLVFFPFFPVIKTLVINNSNFKIPVRK

VCDERGVVGCILRCAHAHQHTCTQALACNVNYMPSLFYKNLSLSLSLSLSLSGDDGSSGI

AGVLSTAWPCLLWGA

>SG-AL_contig_8578_1_-2_470_661

MAISNLQLHCFCTGAYNSVAHIFKAHIVIHCLWGKVLPLLHASCAHTCSCQHNFILLPGL

SAIP

>SG-AL_contig_8579_1_+3_3_437

LIQEQKSVNPSHTHTHTHTHTHTHTHTLCLSVSVFVSVSVCVCLFSSLHLSLCLCLCLSV

SVFFLPSSVSLSVSVSLCLCLCLSVSDCLSVSVCVCLCLTVCLSLSVSVSVCLSVCLSVC

VCLCLSVCLSLSVSEYHLSICVCVC

>SG-AL_contig_8580_1_-3_132_569

MAAKGKCGPNVDVYKKLFKEADANGDGFLSIVELRDLLKRGGSKLSDSQVADTFVFFDGP

RGDKRITFEEFCAGLDQILMFVQKIGELFKELDTSGDGMLDKNELKVLLQRTGKNFTDAE

VNAILKEADKNCDNQISFQEFLDACT

>SG-AL_contig_8581_1_-1_1_210

LAEALKIAFVEKYLPYVLLSSNPKSTFTYLILQKLLYIDLYLKEMPFYFVYIFYLNIKCE

ISVVLACQLQ

>SG-AL_contig_8582_1_+1_100_285

MLLIRSSFHKKKTLYLICCFFVTRTDPMYFMSFYFITFINCSFCSVWFGLVVYYFFTCYL

FF

>SG-AL_contig_8583_1_-2_761_1030

MQLKVACHACICFIYIIYTSNWVTCFAQYCVSVAVYYDSRFFLRRGSCTSLERIHCDEKI

LYKKYTKYTVFIMLNKMYCMFLSIYFLKKY

>SG-AL_contig_8584_1_+2_353_547

MVIIIHQLVLANNLYIQAHSCTPSSLSPHHHQHTKCKSYIYILYIYIWSWLQNGIERTVR

TTNQV

>SG-AL_contig_8585_1_-1_73_366

MLPSTPMATHPCIASPKSPDSEGSSELRIVLEPKEDEVKEEMKDDEEEMMEMETERPVMA

VEALPPVTGPAPPLTLNADLTPVALTIPPSEKQRAIVK

>SG-AL_contig_8586_1_+2_110_514

MTLILLASQAYLPLRTKHTTAIYQTLQSRHVYAQLHTTHTIAIYVTHHSPIKLYTHKAEH

WHKASTAATQLHCQREIHLTFHSCFSNCGHPRSTLCSPVGVIPIRPFSSCPFPFCPRSFR

PVLFHPASVLGAVSS

>SG-AL_contig_8587_1_-2_53_256

MIQLCCVQSTITPKHIAKQYFIVQPAKNNKLYIPQLLKYIQQKARPIFFQGNPGFWHTLN

KILVCRIF

>SG-AL_contig_8588_1_-1_1120_1341

MMIQTGDKLIGLPEDKPECSRGKIHGTEQHLTNYTVSGEPNTVFKVCLFIADCNKYGRND

LKFSSSSNGILNTL

>SG-AL_contig_8589_1_+2_686_934

MVQCNVKSGIVVATYNTPFSIQALTFTGRRQRVVRRHPDRAASGRLIFIHEMAQWLADDR

RAQAKFAIPLTACREGRLTHGNV

>SG-AL_contig_8590_1_-2_5_568

MADDAKSFLALMNGLTRKSYYGQAEITNEFLKEQIYPDLTQEEFDHVLTRSRGLLKSMVS

ADMDMTQLEAFLTAQCKKRESPLTEEQATAMRKFWKSNKTKIHESIISQTMWGNTLQKVS

WRIDLKSQSRHANEINAPSAIMELQIADSLQKDKAAEVVRFEMNEEKLASVLSSMQEIEI

QINQYVQK

>SG-AL_contig_8591_1_-1_1_93

RHRKKYCLCLFRTCSVYRHKKKLFFMLVGNL

>SG-AL_contig_8592_1_-2_164_289

MYSLYKYKANIVYISLEPIVYDGTSENNAVYVSLEPIEYTGT

>SG-AL_contig_8593_1_+3_3_104

LHYGFLTHVTLFFLVLTHTISSKLASTDCFFPCT

>SG-AL_contig_8594_1_+2_185_826

MEDRSKTYCWSAGDVAEDHTVTYLVRNALCPALMAVFTHGFKKPKLTGGHCHPWRFIQEV

AEREAEKDIQSVYSSLVLCQTYRLDEENCKVLTPEEVLYRAVQSINTTHDLCDAHMDLRF

RSLLCCGLNEQVLHLWLETLCSCVEVVDKWYHPWSFLRSPAWVQIKCELRLLSSFPFHLS

CGQELPKTHASHPDTVRQGICDMLLTHQLFSWDL

>SG-AL_contig_8595_1_+3_3_143

HSFPTRRSSDLHTKHHKRIDNFRVHINPTINVPLCIHISRPLKVGSH

>SG-AL_contig_8596_1_+2_194_265

MQVPTIKKMTYASQSEMHGTENTK

>SG-AL_contig_8597_1_-1_1_183

QLHHAIKSKQSEHYGAFTSSEFKCLRVMKATALHIGNRTFFSNDFSLVHSAQLNHEHGLH

T

>SG-AL_contig_8598_1_-2_1187_1573

MHACMYFMDACAYIHHYDLFIGRHLHARARTHTHILKYVYKAREAMTAPHEIFVYKSGNT

VSRDTCICYVLLLLSLKSCKGLFYENLCYNSVSLWSLNIFKDLCHYICAIFCNECGIHDT

CVYLLKRWR

>SG-AL_contig_8599_1_-1_403_3564

MEDTLSRLQGKDLALSIPLESEREKVEEGPETAPGAPPESVVESAKEVAAPGGPDGKTSR

PGSKTGSRSGSAKRSASRTSSKEGKDSKKDSKRSKSGSPKREGSAKKSEGKKSSRGSSPK

SPKRKGSGKKAKTPEPEPEPEPEEPTGPPPPQPGDDDWEFVDLEVELALADVLARQWDGV

EKSYVNNCKHVFRQVRLERENVYRYFFQIRKDFLAYLRRPDHKQEFVSQWQKDYNAVPDD

MRDDEETRAELHQRVDDLRERLWNICDERKEQAERELETIQTDGWLDDRLGILSNHYITT

MQAELDRFQDTVRLLRDYYKGMDGQIPDELNVNYDRIPLIELPVERPETPESTHSEASVT

PTSDEPPGTAKSDRSKSPRAKSPKDRSKSPKDRSKSPKDRAKSPKEGGRKSRTPSAKGKR

SRSKEKDVVPEASTPSDGAGLRIKIPLVPRRPVSPDPDAKPGTTPGKDKKDKKGGKKADE

VPGAESPLPPQDPDEKLLFDAQCFAISAIGQIISVEMAAKEAEEEAEKVRELEKEKEKEK

AKVTKGKPAKKGGKSRSPSPKKGKKDTDSAAPTPPLSEDLSEEDKQKKQTRERMRQEYYF

AVKEEEMATRARIELIKAISMAVLQDLKMKADGAFKDMNDWLGARFLKEMESIETMSEVM

RHAVENSQRMKEQVILHQDDFLVDTDHHVLKSPSPPPVPEPSESVMAEHFTVDQLFHFYQ

QFSAIAPSGTMSSKSFVETLENTVAVTHGMEQLPDSWMHITPSQIQEISNTLATETDYVD

WRRLLLALTWPILTPTQAQLLETLQRFRDMDQKNTGFVTREQYDRMELWFEVGPSIPGAY

DRITHLKKILFDIFADHTGSLSKLDYISMLLYFSAVPNSHEGFLRALSVASNCHMPRLQK

PLPVSTAAIHIHPERATSVADSIIPESAEDEREDKGPVEPPMTADEIPPEAVDACVPLDA

LFRVFHHSEISSGDSHRFSVSVDPEDVTSKDKLAAVYVELGDESLRPVPYKVLIEHPLIQ

DIVVACKIFKALDLKGILSNTSDNTELNSLKTMD

>SG-AL_contig_8600_1_+2_2_184

WEFDKSVKLDLMKNSKSKTFSALDHHNKKRNTGMKSVFFQNTHKKIPQILFFKSILVQKL

S

>SG-AL_contig_8601_1_-3_222_323

MGLSLAMRLLASSSVHLAVNGYLNLISTGKGKTL

>SG-AL_contig_8602_1_-3_375_1892

MDTEAAKNYRKKKIEEIGKMTALSQDEIVSSTKTVIQGLDTLKNEHHQILNSLLSSLKTI

KRESGDTNLVEEKTNILKKSLETIELGISEAQVMISLTQHFQNIEAEKQKLRAQVRRLCQ

ENGWLRDELANTQQKLQFSEQKVATLDEEKKHLEFMNEIKKFDSGDSNVAAEEKEAETPS

QPLDLGFPDDDDDVHSPEGLLSPSQPGAMASAASGGYEIPARLRTLHNLVIQYASQGRYE

VAVPLCKQALEDLEKTSGHDHPDVATMLNILALVYRDQGKYKEAGNLLHDALVIREKTLG

KSHPAVAATLNNLAVLYGKRGKYKEAEPLCKRALEIREKVLGKDHPDVAKQLNNLALLCQ

NQGKYEEVEKYYLRALEIYKKRLGPDDSNVVKTQNNLASAYLKQGKYKQAETLYKEVLTR

AHEKEFGKVEGENKPIWMRAEEREENKGKNKDGPPLPDYGSWHKASKVDSSTVTTTLKNL

GALYRRQGKYEAAETLEECAMRTKKM

>SG-AL_contig_8603_1_+2_2_142

KTNKKTGIDLNKPYQSKLNWQLKGQACLSPCILESDHDFQQRKVWSY

>SG-AL_contig_8604_1_+2_1151_1489

MSKGQLHKLGFCISSRNICSLFINNNNLIDYILLQYYTVSWQVTSHVEQSVNQLADTDTL

ELPLQIICPQPCLNATHVCHSFLFVQEQALSRSRKFQPEVKKTFHFKGVTEPF

>SG-AL_contig_8605_1_-2_44_298

MYTIENLFFGFHVRIWTCTRTVPARIYRQLRTKIFQFRLPRLIQWAAKESMIFKRYDSWF

QSQQGRFLHCLSIDCNHILVKCFVY

>SG-AL_contig_8606_1_-1_1_303

CCYYYYCYCCCCYCDCCCYYYICCCYYYICCCYYYICYFCCYYYDYYYYRLLPLLLRLLT

TTPIIMRRRKRRTATTITTTVVAESVIFKVVTCPSSVFLSH

>SG-AL_contig_8607_1_-2_74_1213

MESVADNNVHLTTLLASDSKAVDSSSSVQPTSLLQAIAATSSSATTTPVTPVTPTSTAVK

MEVDPSAIKEEIKTEIKQEPGEEDTFPSTSSQFKEEPVASEGTCSPKTESVGENSQSGNL

DIKPSSVEASASQPSKPCNKKEFKPDELRQALMPTLEQLFRQEPESLPFREPVDPVVLAI

PDYYDIIKKPMDLGTIKRKLDTGQYTNPWEYVNDVWLMFDNAWLYNKKTSRVYKYCTKLS

EVFEAEIDSVMQSLGYCCGRKFVFNFQVLCCFGKQLCTIARDATYYSYQSRYTYCEKCFN

EIEGDEVELTDDPTQPGTKIKKDQFEKLKNDQLDHEPFIDCQECGRAWHQICALYLEQLS

QPFICDNCLKATGKKKKENK

>SG-AL_contig_8608_1_+2_290_697

MSLEVLFCELLVIWSSFPKVNFEQLLRNCVEIISNNLWKRMGWNGMMHFQSQKYMHLEPC

LGNLSLYSIRHLGFVGIHFVWKVPPNLSSIYVTVEVSSFFHPNPVCQWTSHLPNYSCIFP

PTWDLNNKNLATKRFL

>SG-AL_contig_8609_1_-2_17_931

MVQPPYPGLVMYNDFTQPPPAHMGIPPVHTDPKTGLPRPPMYAAYPPHSQFSHPLYGPDF

PQVPWPRPAGYPITTGAFSGSYPPSLSISRYGHPSLLPPGIPHPGMPHPGMLPGHKTDMM

GAAGMQDGHRLDGQEERSASKSSAPEKRKNYIKKPLNAFMLFMKEMRPKVIAECTLRESS

AINQILGRKWHALDRSEQAKYYEMARKEKDLHMQLYPGWSARDSYAVHQKKKKRKKSEPS

LTQENKTIAAECPNAKKCRARYGMDQQSQWCKPCRSSLHENCDQSTAELDVTQNANPPDI

KYISL

>SG-AL_contig_8610_1_-3_1467_1631

MLLAMWYWMFILSVQLSVCLCIYMFTCMYVCVCVEIPDSAIGVDCRSVVTERIQA

>SG-AL_contig_8611_1_+1_721_1014

MKTNNHLIFLPLNISTRSLPQKLWLSHSCTSSLVVITPSASFHLSHFSFSPFSLCTCRRT

WNLQFVQKGYIALGQFHTRSTHICKQLSGRLLEPAQNP

>SG-AL_contig_8612_1_+2_2_178

WVNFSSVFDVCPFVEVSQRIKNVVPKSAPKRTGRMYDYLVLFMNACCLKYSAWQSFRDF

>SG-AL_contig_8613_1_-2_128_2506

MSSPKEDAFSIHGPVKVQPTKEQLTPHKFALLVLIWDQFHLHKLQTSMTDIDFDDDYPWR

PYTEREKRQVMTTLLDLLQSPDLALSELKARLKLSLKQLLMDRFNERLDNFQDQGFLSVV

SFIEGMGGILSHSDQNDVTSMFIDRTSVVGLFIRRMLLAYSQMSFSQLCSFQAHLKSYLS

GKPVTTYSDMVSAQYQQNNCVEEMLSRSLAETGMGTSAQLSASDLHPADLSVASPIRDDK

EKVSFSHKQAEFFISQQTSFLSVNENRALPPKELQKKLSSMLEQHPDIAEAHFLSYLNNL

RVKEYCTAVHNLYHYFDRNAQLTGDAASNSKRSEDEVSRRYAALSLAGLHFNFGHKKEAK

AALLEAIRMAQESNDHVCLQHALSWLNLLADQEGGKAGSQIERSVGKSDELNLSYLTSLG

VQALARHNAFATATPSSVFDYMMKSNVINCQNSLYGMMSISGAQRAGLWSYYGNRECAAM

CAQMVLCLDTQDKGVFYNGESICISLCLLARLHADRGDHASTKEILHIASRWFHPHSRHA

HLWQSCEQSLAFEHALLQGRWNEAEKAAHNLRSINDTEGRLHLARLLRERGEMTASLGLL

QKLKDEACDRKDLTPDFLCRVLLLMSEALMATDNHTTALPHLTHCLTHATSHHLHYLASL

TTLHLAIVQLRMKLAGQALQLIETAMLGILSHGGPYDCARLLYAYARCKVAAAQKASTEE

KKTALLGAVSLMTIVVNKFQSLEVTARVKDALYYQARLYHQLGYTAERNRCAHRFKQLDQ

QSPTLSKSSVHAV

>SG-AL_contig_8614_1_-1_118_576

MLAVVVLLFALSWLPLYVVNLIIFFFNPGDDSEEMQIIHDSVIPVAQWLGTSNSCMNPLV

YCLFSKRIRERIRLMVTCASRSEARRALSRYSSTRHMTVDYCNGQVKLAFRSNVSPNRLT

SLTSYQNKPMLLASSADNSEDNVLNSDTAVTMT

>SG-AL_contig_8615_1_-3_3_230

VFDVLCAYTHKGGSGTSRSAHMLPWETGRISTLCPPGEPNKDRTNAFRCEVRHSNHWLSG

LPILVSKRQFTDDTSF

>SG-AL_contig_8616_1_+3_3_227

FSSFASSRFFCRFFPFFLFVHLLSFIRFSLCEWIDEVTAKGHHSVNVRTDGCLPWFAMQL

IVSVEFVFISFVYLL

>SG-AL_contig_8617_1_-2_2_175

VKGLTFIGLCSKLGADIGLCDVIQSCEMLEVNLGALAVKICGHFVNTVLLFEISPSPF

>SG-AL_contig_8618_1_-1_40_2382

MSPDLDTGEEQDVTDTKQSDPWEHIVSAVTNTQRMKWLRQRLTGARNDSVLVNNIIDFVL

FPQPIDIEKLRRSLYHQVGRAELRLKGIQSMLSLIHKDFLIPSVKHSILCGWQGLVTVGT

RPRKHHPRGSPPLPHCLSDVQLIPPCDRILLEMTFSELYRWAITQLRTCVIEADMAFKAR

GINPAQPSLDNSKEQLSLGSLPPSRFVLATLGVLVAEHHAHSLSLLLNSGLLALTQSILR

LAGIDPEVQVPETGSMLCTVVEEQKIRKQHQQMPMTGPELAAMMKIGTLVVRGVDWKWGD

QDGPPPGLGRVIGELGEDGWIRVQWENGGSTNSYRMGKEGKYDLKLAQPPEVPENEDEED

EESESEKAKPESKHPTSLVRKSTVGLLKVLSLCAGIHARRAQPEAIGALSGLMHSIIEAG

CNYGLNTNSAAIQIASQQHLRWCTLGFVRSLASDGVLCRALSTRRWITLLMHVLEEDQGS

KYAKNINRQILTLKLLRAVLPHWDPSSDVSRMNEVIHSLFHLLGSVLTSCATDPTLLTAD

IPGGPIKPPYPRVSVTATYTSTVAEEMVAVIRRLHSLDVWNPHINCYITSRLSTIISVLD

TKLLEMESPSSELTGTILAVLAVVGGVDSRPRLGGQVQHETFELGTVARITPKGKIHIQF

SGNKHRICRLSELTPVPVVEFHVEKMAMSEEDLCNWARLISLSMNILRSADRDKEVMRAP

LATSTGSIEPVPPPTPTSQDLIRQQILLGVMRATACLYRHQNNLRSILCQTVPTDPTPSP

T

>SG-AL_contig_8619_1_-3_9_332

MHLAHAKACTAMKDSCLAKFCIKETTSIGKTKMHAARERKKRKRGGIALQRNALHRESSL

NFTQELNTSLLTHFVHTHIFYANAHTHPCTHTHKHTHTHTHTHTHTHN

>SG-AL_contig_8620_1_-1_106_405

MAHCLPSERSQIKIPLGTDFCLLSTWYRFLSPLYLVQIFVSSPLGTDFVLLSTWYRFFFP

SLLGTDFVSSLLDTDIFAFSTWYRYFFPSLLGTDFCLLST

>SG-AL_contig_8621_1_+3_3_227

AAFDTIDHGILLYKLENLYGISGSVLACFKSYLGDRKQSVFVKGYSSKCADVQFGIPQGS

VLGPILFILYTTPLS

>SG-AL_contig_8622_1_-1_334_567

MSVTDTFAGQECSSNFLLNIFATLPSISWRLPFGLVSLLGGELQLSGCMSRSNRSGKLLE

KMSLTCKSETLLLVVPCG

>SG-AL_contig_8623_1_-1_460_738

MATCFPQGERPHLTSKKIRCDKNKNRKEKQKQNKTTTATKQTKRKQTTTTTNNKIQSNPL

TIYNSNNSNNNNNNNNNNNHIAWFMLQVNNTKQ

>SG-AL_contig_8624_1_+3_3_149

MANEKKTDFSLQVFAKTVNMLIAPNIFSTVSLSLSPTESTFVISCYIHT

>SG-AL_contig_8625_1_+1_1_150

NLSVSDKNITSFCEESFQKHNYCWKCTRLGTAQHPALETQIKDGRFFNMW

>SG-AL_contig_8626_1_-1_112_2559

MESSTDFQKEVERLNTELAETAREKVQAAEYGLVVLEEKQQLQAQYDELESQLEVLKTEL

ECAREALTKHHIDLRRHHQTGVHHEELFMEQTAEREEHFQSSISDLEAELKAVNSSLERL

SLDNERLAANVSELSHQVEVLEAQRNQLKHENREYKIRENRNYADYAELEEENITLQKSV

SQLKTSQVEFEAMKHENKRLHEEMEDLHLQVEEMTNLRRVVEKNLEEALSSLQSEREQKH

SLKKELDQRLTRESMFNLSSLAHFGGLTEGLSVSSYSTNESSQDSGAEEDGEGQDHAHPA

LKRIEADFSFFTKDGEAVPMPRPGVVGDILSEIQVTEVGKLEQLLQQSEEEKQEMQKALD

EARKLMEETSQDLIEQKKRADQLKSYIASLSAVRDLSTSKLPNDLEVSFDETMLREMEEE

DDPERRALLQLRNNLQLKDKKYRTALNEIGSLQAEIMMLQSAAALGPGAEFNDALAELKS

KCMLYEDKIKEQARDIKSQTDMANRAQDSISTTQAELVRITEDMAQLYHLVCEVSGETPS

RVMLDHAKSSTQTIQSSDSSERESSEEPATPTGENKLLPSAEEKTSPSKPASNKQKSKSR

REKKAAVSEAQGDPTSCEKLSETVVDQVKFLKQAVEHLMEISQNWNRDGTDGSGDHAEDL

MEMQDQIVKLKAMLSTKREQIATLRSVLKSNKSTAEVALTTLKQKYEKEKVIVRETMLKL

RNELKSLKEDAATFASLRAMFAQRCDEYVTQLDESQQKLAAAEEEKQTLNSLLRMAIQQK

LVLTQRLEDLEFDRERRNMTTRRAGGRGTGKIPNSK

>SG-AL_contig_8627_1_-3_93_449

MPKAVSRLEILQVCKLLQCVRAKDKHQVEKLTTSGVPHLINYNDSNEGNTALSIAAIAND

DDMIEFLLELGAHPDVMDLKGRTAAMRAAEYGHVNCLEKLLTAGADMTLSDLEGKGVIF

>SG-AL_contig_8628_1_-3_105_251

MLFVPCMVRLVYFMSFLCLFILSSLPLCSYDELRALKEATTSEVLFHLD

>SG-AL_contig_8629_1_-3_45_590

MSGLFNLDFLDDVRRMNKRQLYYQVLNFGMIVSSALMIWKGLMVVTGSESPIVVVLSGSM

EPAFHRGDLLFLTNYKEEPIRVGEIVVFKVEGRDIPIVHRVLKVHEKEDGTVKFLTKGDN

NSVDDRGLYAPGQLWLDKKDVVGRARGFVPYVGIVTILMNDYPKFKYAILVCLGIFVLVH

RE

>SG-AL_contig_8630_1_-2_2_94

RLKCTIRIACEQNIAVNVTFISVHLIRVFYC

>SG-AL_contig_8631_1_-1_43_285

MATGHDQAVGWAMVSLSSFIFVYYSIWVIVLPFVEPGVFLHKLFPPRIFAIAGPLLAGVC

ALALIGMFVCYIALKSKKKKS

>SG-AL_contig_8632_1_-1_82_570

MSSPFTVLSSRPEHAFPHIHPQGHQVFAYSSLSPVNAISGGISPTTLSLIASPRTTPRTT

PIPQRWGSQFMEENVDYNSMLANIMPTVNPDEPLLPEERYFPAMQSGEAMDSNGSGGHPS

HSSNSSQTAPPPSSHPPHPSSAPPAMPPGGNSPKPSTQSQGST

>SG-AL_contig_8633_1_+2_35_160

MHENTMHSLLTIFNKKSWKLLTLIQLNPTWIPKQISPWCGEK

>SG-AL_contig_8634_1_-2_2_172

HFKTNKQTKNKNNKQTNKLSLAWVSMQPSNQIAAQKSSLNSRDYQSINQSCAWSFHA

>SG-AL_contig_8635_1_-1_1_507

PLCNQVQHEASTLCLPSAGPRGDGSGRPLPRLGSSDQLRLSPLPDPPQSHQEGQGRECAH

DPHCALLAGSTVVPGPSEATALRDQALKPRDASPARLASLRPRLPSLTDPSLSTGCRARA

HPRSRAVSSALAALGALAETRSLAALSCRKGTESVYNSKWSWWLDWCSS

>SG-AL_contig_8636_1_-2_653_997

MCVCVYTCVHVSVCVCKHVSMFVCIIMIIMHTYIHAYVHTHPSLCLCIHNTSVCLSLSVH

VYTHTHTHTHTHSLTHMCIGVNMYACLYVYMCIYVYTCVCKHTCIFECIHMYMWW

>SG-AL_contig_8637_1_+1_172_417

MISRCWSPACAGTTTLTYYILHVLSYKHMSVITAVLSIWTTERERENTHTHTHTHTHTHK

ERETTHTHAHTLNSVDKTVIIY

>SG-AL_contig_8638_1_-1_1_303

KITIIIIIIILIIIIIIIIIIIIKARKLVTGTDYERIPLLVDTHEIGLKLGWGKKQQRNW

SLDSQRIPPLAAESPLLCVQFAHAGESILTLFTGMIYSIQE

>SG-AL_contig_8639_1_-2_2_220

HLSDNGLLESYQSAYRNDQSTETTVLSVLDGLLTKADERQISLVALLDLSAVFDTLDHSI

LLKRLEVTFGIQG

>SG-AL_contig_8640_1_-2_2_136

DPNELKNFRPVSNLPFISKILEQVVLSQLLKHLSDNGLLEIYQSA

>SG-AL_contig_8641_1_-3_120_218

MQIYSQYLIFISFSYFHILSFQKYLRRLYCHNY

>SG-AL_contig_8642_1_+3_48_161

MAGKREDKHGLITDYARLWLNVIHLFSPVNPFTLTPNR

>SG-AL_contig_8643_1_-1_1_117

VGFFFFILISNYSVFGVSVLTVAVMSQLYLVIGHQQKSP

>SG-AL_contig_8644_1_-2_47_2668

MAVSLAISMSSLACLLLNVFILPQNEATNVQIETADGSRHVFEMKTMEKSGSYLLCANCK

SFSTGPIPTPSALRFNVRFTPTGEIVSACETHRKELPVGLCSAQGADNNGSCMNDNVCLG

NTAQEEVADEIVKRNLLPFPSGDQEIDHSVAHAIVEMNAGLWDRAISMLNSILDKYPENV

AAHYARGVAYARKGQESQHLALQSIADFNVCVEKEPNYPEGFERRAEVYITMNRFKEALS

DLKSALALRPNNRIRFSSGIVNLFLENFVEAEAEFKKNLEEGAGDKGPVYIFSYFHLGLA

QYYRGRLRNSIEVFKEVLKLQPNHVEACVSLAQAFRELGNYKAALGRFDQALSLSPNHTL

SMQLKGSLLYHSGDPASARSVLQQCLRVDPENTSCQYLLALSTVALGEFYQGIKLATRVM

IKSLPELKISPERVRAQYLREYGRFLHASLETPLNHLHPEAALDEKMRVPWVQGMPLTLR

GQGLYREQPGLQPEISDVKVDTQDWSQSSARTLLCHADKVGLSMQVSADGFLPNRRLNLA

MGLAAIHIAQLAEAKWKNMKGPGKSTERPFSWKELFSIASRYRQLVDPEQPVLWVDSLPD

YQTEEGHREDITLIRGPVASVRVSQYFDLAFKLAKTMLEHYTGMGAVSYSGLSTDIEKAK

TCEELLQIARKRQINPNGFLVSTQVPATVPLPGGSSSGGGSEDRLDGTVLVLTEDPNGRI

VFALSMASTPARTAAYASQMDHFLMQLHQEVQRTGVGKLSDMDPVLSAILSLVYYFYNLT

PLTRGSSVVAYAVALGLIMSLGRQVSGKIPAGKLLDMEAMLSGAPDAFIAVMRQWMNIKK

TAMPLSSLPKVSEAFPTIRSILEVLAVNTTAVCG

>SG-AL_contig_8645_1_-2_1142_1372

MNDYRGICLSVISSKGFCAVIDRRLQDYAEENNVIGDHQAGLDLKEDIQQLAICSCYWLS

YRNSFHVTVKFVVCYIL

>SG-AL_contig_8646_1_+3_3_167

FFHWSQFIFHESNSYSVNLKSVKSYISHAQTPSTSIPHQTTKTMKEKPIIMDGLF

>SG-AL_contig_8647_1_-1_433_657

MYLFRPCSSLERQINMSWQNNCGCLHSSLLKLYESPRLRYFSIYLNKYEADLKTYVQKIC

VQLFVDGHLPKLSQA

>SG-AL_contig_8648_1_+2_158_301

MTAILRKQPVKFLCTLLHIFLSNGISKLTKLPEFNIDNKTYTLAKTQT

>SG-AL_contig_8649_1_+1_1_87

YSKKCRSYVCLKSLSCMTAILRKWPVKFL

>SG-AL_contig_8650_1_-3_201_1583

MNRRATVPNPTSRPRHSSSTQTRSGGPQRLSFNEYSPLRGIGRLDTRNLHRQNGNTDFRR

PTLRHLSPMLRSSDTMLVGPLESPTGDVLPPISSASLRPSISPSLHGIISSSEDSDSESD

SPMSPPESSPLQSPASLDHLAMPQANGSRPVHAARDAVLARELLQSEMDLMMQRDEEMAR

QLQDEIDDDDEPSPLSRPFSMFMPPGFNSLQARGRHYPSIPRRQEGGPGGQTRDRRGPSA

LRFMHSPPERSGVESMGMGRAMRLLMGVDPPRGIAGPGLRRSSDTDSPDSLRRHRFGPEL

DGLDTPTDEYDPDERLLAGGLDPPDDFLSMMNDPSLMLLMLLLRAPDTLYQNEAGVDLDD

YEALWELAEQLGEVRRRGITEDEISRLPTHKFKSQNGEASEACATVSSSVQCQICLVDFE

SGDCLRGIPCKHDFHKDCIDEWLRRNATCPICRQDIRPDDK

>SG-AL_contig_8651_1_-2_83_466

MKYLPNFFRKGQLSKMFFLFPDPHFKKTKHKWRIISSTLLAEYAYVTHIGGLVYTITDVK

DLHDWMVQHFTDFPLFQRVPDEELETDIVVKKLYDSTEEGQKVTRNAGEKWLAVFRRTED

PVVSMEMS

>SG-AL_contig_8652_1_-3_3_203

GSFIINSIPTFWKFCARIYSFLVLSLFLPIFFPLLPSAFFYFPGSFHSFFFQQAFKQFSL

PPPSCRL

>SG-AL_contig_8653_1_-3_1047_1277

MLSLSLSLLKAGLCLFLLSCFFLHTHTTGYVCIIFLSITLEMQYIINVSLRSFTFGVFNN

CVCVCVLFWRGGLFCFV

>SG-AL_contig_8654_1_-2_59_259

MDQGGLILDQNCPNCESIYLIIFLLFGNGYSIKVKVTTTQIILFACIGKDFFGGLLTDSG

QSWTTYS

>SG-AL_contig_8655_1_-3_309_563

MNIFIHIIVKSIICSRYVYPFVYFNYLKHLTKVCYHNHKWDNNNCSVIIYRYNLFKDHSK

LSVCENVQLLCYVFMLSFFLFHIPP

>SG-AL_contig_8656_1_-3_150_3476

MDLTRLILFVISVFCDVHRTNSFNIDVRTAKVFQGNSGTGFGYTAEFIRQSTGAIRLLVG

APKETAASRMQSGIHYYCDPATRSTSGSCLSHPSPPQPGVTVSSGRFGEDQELFGLALTL

ANETTAVACGPLWSDMQWWRIGYIFNIGRCLTLPPSDNHAGETPYKQSKKYIGKVELEGK

ATRQMIYGRAEYGFSADGDGVGRVVIGAPGFSVSRGGVVVLDTRFGTFNLMETILDPEPA

ILLGYAVAVGNFCGSNRFCFAASNRQVKGKVHLLEHVSDSVFRPLFMKKGEQEYSSFGSA

LAAVDVNGDGWADLLVGAPTFTMYTNESRSYDQGRVYIYISQGEVLAGQDNFREGRTLSG

DETSFSRFGSAISNIGDINRDSINDIAVGAPVEEEGAGALYIYLGTPNGPTNKYSQKIRG

QEFTPPLSSFGTHISKPTSDLSSYNYPDFAVGAPESDAVVFLKTRLIVNAQVSLKASPNP

VDKTNQLCPRMFSGRESGCLNVTLCISFTYSTTGLAGSATSSFRSLDFVVDMDVDKLISS

DSARRVRLYEPMDGSTKTGFRKALTVSSASPLCQKYVGILKQDQINRNPFVPVQLEAAFS

LNTTSFPDIAPMLNLSVPTTATEEVKFKNKCGEDDRCQTDLSLTAHVHYVPDSDEYSFNV

INSTKQVELVLDVLNKQETAFGMKLTVNITGQIDFWTNTGNASLICDDVENSMLNCRGWK

PLGQDEVVHIRLRFLADSVPLEPQNNRITFEVKVMPTDRTENPEVNAFDNVRTLETRTAI

VSDLRLDGSSTPSLTHVADSDRGDVSLTHKLLVSNQGPSFLPPTVLNVTLPVRDSTATDI

LRSANVTIKQEGGVVVECPQLLTTGSVVGTTVPGGTATTTTPSSFTTTSGGGGGEGGGGG

GGTGGKTTQTPGSSTTDDPFGGGPDIIPSKRRRRQSGDAPNAPSKDKNDGTTYNMDCHRY

QCRTYQCPLNALQPRQRAEVNVTVTLYRAAVVFPGSTDTLLYKTTTSVGQPDHPLFFQWT

EPRIREVVTRIKRLSSGDEINIWYIIGGVIGGLFLLFLIGLLAYFCGFFNRKDKDKLEKL

KRQSGFYGGNRSMKKSQRASSSGSASDKE

>SG-AL_contig_8657_1_-1_37_366

MQRLRNIARTTKSKVPGVGLTPTSTQTKDQLATALAVTHRSTASLGQFMETLPKEKLKGL

GKKRKFDSNDMGDEEKSRQKKILKRISEATPILNVNRAVNASQHNEEKQR

>SG-AL_contig_8658_1_+3_360_644

MVIRERGSRSDTAHGSAMDEPHSSPSFLYRSQPQLDKMEVASTKFPASLKLYRQTGRQGL

YIYGIQRRMSSCMPSKNSCHKGSKWQTKNHAQINS

>SG-AL_contig_8659_1_-2_140_1096

MFAHRGVFANHANRHHTRNDRQTAGRENQGLLEDHYTVDEVLGKGGFGTVYSGHRRRDNL

PVAIKHIMKHKVTLWGRIGNHEVPLEVSLLQKTQNISGVIQLLEYFEKTDCFYLVMERPE

SVMDLFDYITEKGALKENKARQFFRQIVTTVQEVSCRGVLHRDIKDENILVDLETSQLKL

IDFGSGDELRDGYYTDFDGTRVYSPPEWISLQRYRGMPATVWSLGVLLYDMLCGDIPFER

DSQILRAQVTFKSTVTPQAQDLVRRCLSLRPSDRPSLEEIMDHPWMQADLNARLHADNRQ

EDIVNANLDAISLSSQESI

>SG-AL_contig_8660_1_-1_1_462

EEEEEEEEEEKEKKTKSASEEEEEEEEEEPPKKKAKTEKKTPAKKEAPKKELPKAKKQNG

KQEKAKTEKKEPKKKKKVADSDESDSDDEPLAPKKNEPPSNSDLKEVIKKILDGANLEEV

TMKTVVKQVYEKYPTFDLTDRKDFIKTTVREIIS

>SG-AL_contig_8661_1_+3_195_386

MCFVVLLFCFTTEKKFSEYLIQNPLKLVNSGTISLPDKETITKSLINHLRKNKQENMPSS

EHYV

>SG-AL_contig_8662_1_+2_83_211

MTNSTLQLGDDKTEMVLITPKTVPFSCSLPHSVLLKGTDIQLS

>SG-AL_contig_8663_1_+1_388_495

MHKFSTLPNKQRKLMNTSSDIHIHNKICSGSRHFVI

>SG-AL_contig_8664_1_-1_241_630

MIIVTLLLRPSTRCLCACTTRVYDESRLAGCLIRKLVCTSSTDGTEEMTHRHLYSVDGLL

PQRDAPHATPLNPVSACVSWGLTLGVENNRLANPIPERASVCRKQDNDLSVKELTYQHGT

TNWRQTCCSR

>SG-AL_contig_8665_1_-1_2053_2334

MGVFILLIVRVLICTNAKLNMLIFQARTRTQQSTDCTLNWKIFFFIICFCGNIAILMKVL

AVENHFCHYSRQLNTSKFQQEDTSGLQICPFSTV

>SG-AL_contig_8666_1_-1_712_2241

MSTLGIPLLSRSRRPSALIEETDVKAVKEIKYNLAPNRPRIHSRSVSQSSTDSYSSASYT

GSSSDEDETNPRDKQQKNSKGSGDFCVKNIEHNAFGRREIEIAEQEMPGLMALRKRAEGD

RPLQGAKIIGCTHITAQTAVLVETLVALGASVRWAACNIYSTQNEVAAALAETGICIFAW

KGETEEDFWWCIDRCINAEGWQPNMILDDGGDATHLMLKKYPAMFNMIKGIVEESVTGVH

RLYQLSKSGKLTVPAMNVNDSVTKTKFDNLYSCRESILDALKRTTDVMFGGKQVLICGYG

EVGKGCAQALKGLGAIVMVTEIDPICALQACMDGFRVVRLDEVVRNIDVLITCTGNKNVV

NRQALDRLKTGCIVCNMGHSNTEIDIASLRTPELTWEKVRSQVDHIIWPDGKRIILLAEG

RLVNLSCSSVPSFVVSITATTQALALIELFNAPQGRYKQDVYLLPKKMDEYVASLHLPTF

DAHLTELSDDQAKYLGLNKAGPFKPNYYRY

>SG-AL_contig_8667_1_+1_1_144

RSCKSWGLRCLCRNDEQFCSSINNGMFFHSLKLLCSLPPHSFASVTQT

>SG-AL_contig_8668_1_+1_1_237

NTNVFHRYIQRTPTRHTISQSHTKNTPRECWTRHLISVTHKECQATHMIFHISQVLTEKV

HHHTFKVSSKSSASHYFFF

>SG-AL_contig_8669_1_+3_534_683

MNYVRRIYSRHILSGMTKTSGVLEWEKTNSPSEPLCPPHNHKTTPLQVKF

>SG-AL_contig_8670_1_-2_2_130

DRCVSVERRCYPAVLAGTLNQPVSMGRRYKELSSKLLTLGHCR

>SG-AL_contig_8671_1_-3_549_716

MGMTQFWGHHHQTSGKLLFKTKQKTNQPTNQPTNHNNKKPKTTPKQTYKKNPNNRK

>SG-AL_contig_8672_1_-3_120_1391

MEADGAEQSSEEQGEDDSAEVATCYCGAPRKLGTLELMCGTCGGWFHARCTSASVGKSLP

FMVCYQFYCKNCGQGDVESFTKKQATFSQVCHQAMANLTFTAQQEGEERPEFSKDMDIIP

FIEKNWDNMTTMTRRTKQTWHKTVLNTMMKEPDLYVVSPEDGNEAYFTLINQDLTKIAPV

YDSTKAGPSGSQSAADSGTPGKGRTKRRGNFDSLPVISNKKKNDQGVGPKLPPHGYPLEH

PFNKDGYRYYLAEADPHAPNRQAFDESTEMAGKPIPGYLYRVFQGTQVLLSMHDRAIQLK

LTDDRLTVTGEKGYSMIRASHGVITGQWFYEVTIKDMPEPSHCRIGWSQPLGNLQAPCGF

DKFSYSWRSRKGTVFHQSKGKHYCDTAYKQGDTIGFFISLPQPQDLSKRLPDTFKDRPLV

KFKS

>SG-AL_contig_8673_1_-2_2_157

FEFLAAQNRIFHTLVICNSSSVAMLKECRKKDGVTSFKLQLPQSYLLWKLLF

>SG-AL_contig_8674_1_-1_178_1746

MSADNKKDTIQWEVATFLPTEMSAREQRLHEAASSDDVDSVRKLLSEGVPLNCRNSLDRT

ALHCAAAQGHLGVIKVLVDRGANLELQDKFGMRAVMWAAWFGHVDIVQHLVNSGCSKTCT

SKQGLGLVHCAAANGHVDILNFLSEFLAPDKVNVVDKNKGQTALHLAVEGSHLDTVDTLL

HLGAHLSIKDKTGETAVHMAAVKGDGQVMRRLLLVGVEVDDTNMEGRTAVHLAAEHGHQE

ALDALLEYNANPNSLTTKEFTPLHVAASEGQSDICESLVKAGAYINAVNCQRNTALHLAA

AGNHPEVALILVENDVDMDLQNSRLQTALHVAVESGHLHVVHVLLAGGANLTLREKGGKS

ALQLAARGNHVAIVDMLIRAHRYYIMMSERETRQEEEVEEVLNLRQPAHPGAGSMRDILW

TLATRQLRPGEWKMLATHWNFLPEHLQAIEHEYTGANSYKEHSYRTLNIWLHGVRADENI

IKGLFEGLVAIGRKRLAEQVRRRANRAAADKGKTSSAKRCVVA

>SG-AL_contig_8675_1_-1_1_252

DLFCEFEHLILIRSPSAIFYNIFRIFYLHKICQVQNGPVRNKYFNVCLGCTYVSFIFLSV

YYFIVILYLFCYSGHLLLHKCADC

>SG-AL_contig_8676_1_-3_2400_2723

MHIRTHTYARTHTHMQACTHTHTHLHTHTHTLSHTHTHAHTHARTHAHTHTHTHIQHTHN

THTHTHTHTQVSWISCKRLDSVQPYSGKIKCVQCQIISTRKCRKQIPC

>SG-AL_contig_8677_1_+3_3_185

PDGIPWKSRAAFLLVHIMKRLGIKKQKENCLYLMCQTFLTAGHCTSEALSCAKQNAHRVI

S

>SG-AL_contig_8678_1_+2_491_766

MASRVYWLWCNPFLSGLHTPNTPYTHDIYRLSRKLEKNPIYMTKTSKSCKNESSLSKRKV

SHGIIDNRISCSMILDCTINVVTRSMIVLYCI

>SG-AL_contig_8679_1_-2_50_259

MLLQQELKLHIIYMLIYVYVLCSLATYVVAMFCLPFCLFCLFVFSMYPHGVSLWNKKNIL

ESLKYTAVQH

>SG-AL_contig_8680_1_-2_1121_1339

MTIILGVRCGWSGCQCVGFTTPNHQFPVHNHCLFFSFFFFSWCEVSYLYFFHVTESTLSS

AVHCHLWIKGKKK

>SG-AL_contig_8681_1_-1_358_555

MRSVSFSGHRIHTQNVSEEVEKGRLYQFCFWRLWWCVSFYFLMKTCILILDKSHITIKFI

FIIKQF

>SG-AL_contig_8682_1_-3_3_143

RQIVDVYIILQCSHILSSSNENSTFTPVGLQCFHSGLIIDTMHKWQI

>SG-AL_contig_8683_1_+3_3_179

KSGMDRDGRKKQTTVKERKGAEIKRATEKRKLLAQNSQKMGILFILKEPPASPRGDKAG

>SG-AL_contig_8684_1_-3_30_2057

MAPPKKGRSDDLKQEEILQAVVIADSFDVRFGPVTDKKPRALLPVVSVPLLDYTLEFLAT

QGVQEAFIFCSHLSQQIREHIKKTRWREESKDMTVKVILAEGSSSMGDAMREMHSRSLIR

NDFFLLNCDTIANVNLKELMRDHKARRQKMKMSVMTMTCMKTLPSDPIRCAEDSITMTLR

TDTNQVLDYQKVPDCRKFKIPLNMDDRITAHQDLMDGQLYVCSPDVLTLFTDNFDYCTMD

NFIRGVIVNEDVLGNTVHVNVLKNQYCARITNPYMYGIVSQHLVALRTLPLNLNRFVLHD

EDQMVCRGKNVYIGKGASIHIDGYKQIYNTALCKGSTVGANCIIRNSTIGPDCHIGDNVK

MYNCHLWTGVRIEDGCELNNAILCDGVTVLANTTVQPHTILSWNVTVGPDITLPPGIRLQ

DTPQKDEFDDEEEEQPIPEVTPEYGSRSKAFLFKADSDSDDEDEDDADWGEALADALRRG

HEDNDDVCRNFRLETSEEEEVDEYGNFCSELTATLTRAKEEKINLENLILEVNSLKHAYN

IVIKEVHHAVVKVLVEMPLNNNPNAQGKELITAVLRHLETHKGLLKHYIRDKEAQLDCLY

CLEDFAVDNDRMLSVLSKVVQYLYNKDILEEYVITKWYHKDAVEDEEEEHEKIRKQMEEF

VNWLEEAEEESGNSED

>SG-AL_contig_8685_1_-1_136_1587

MFRNPRDCTTYFWCVHEQQLVRHCPEGLAFNAATTNCDWPDNVDCSQSSVISTKATAATT

TAATTSTQMPITTFVQSTTTIVTTARLTTPSATTQPPATTAMPQITGFPGCSRRVCYHTN

WSQYRPGSGKFLPENVDPFLCTHLAYAFAKLNGNRLAAFEWNDESTEWQKGMYERFNDLK

KTNPNLKTLLAVGGWNMGSAPFTSMVATAQSRQEFADHSVTFLRQHGFDGLDLDWEYPAN

RGSPPEDRDRFTLLVKQLRETYKRDAAKTGKQPLLLTAAVAAGKNTIDTAYNIPQIAEHL

DFISLMSYDFHGSWEGVTGHHGALYTHPQETGDDRLLNLDWAARYWVEKGLPKNKLVIGI

PTYARTFTLTSADTGRGSPASGGAPAGAFSREKGFLSYYEVCQMNSTADVRQRIEPQKVP

YLVKGTQWVGYEDVDSVREKVRYVKTNGYGGVMTWSLALDDFRGSFCGQGKYPLWSAVNT

ECLR

>SG-AL_contig_8686_1_+1_91_294

MRWVVCHHNTTNNECPCSLGTFKWGRSLFMFFPPLNLISYVCETIRTHLCRCLRTWVGDI

IIEKISSC

>SG-AL_contig_8687_1_-2_824_1192

MLLLALFHTHSSYFNLHLCTNSISSSLDFLCTHIAGIVYARVCVCVCVCWLALLHVCVMS

LLNLLSNSVYYFFLNNCKMLLWYMCVVQILFYSYVCVCVCVCVCVCVWHDTLQSMCFIVL

NNI

>SG-AL_contig_8688_1_-1_97_1977

MSSHSSRSSTPTSILSKLPPIITNEADREFLYELHGFIDRELSKINPQSEEQRYLVYKSV

FNKVIEHVSAYKALLTAIKKEYEDTIETIRTGQREAIFLQGKLKAMASEPSTLRNYKKRA

DELEERISIVKKDNDRLQKELQELKAEHAEREKKPEMKEPPKRELKKDSRRIPGMTLEEA

TDLPTLHKKNERLERTLKELNISLKTRFLPKTQKLQLKDTLDNKVNYRDQLLYQSQVYKA

RGQRLKIALEAAQAYNRVKPPHQTVGDAVMLAFQHAMGAAEKEQAAAAAAGEQGDGAASE

ATQSSTFEDDDPNKEKEAEMMLEYIEKFNELFEDNKFEEAAIHAANSPKGILRTQATLAK

FRDVKAGYQGRSPLLAFCDAMMSTVKTATNRPNEGLSLDCAKAGLNENRLDLIYHWLTQD

KLTTSVEMGQLIRDHCSCKVPCMCGCQGLAQYVFQRQRAHSDAMLCLLQQGKVQMAIQYA

QAKGTLEHDVLLHLLKKCPSVQLVETLLKDEDLEDGRGGPLLPMGVVLTTLCDIDHWEMG

KSWLQKLVYTASSKTDKMPGGEMLHKLVSTDDMTSHSQWLELVRRLQTDGHEELSVQLMA

VVTVAMSMKLAFQVLRHESDMQCDALD

>SG-AL_contig_8689_1_-1_91_270

MENPDVTVCCLLRFQNILQSGDDGRDVNFADVPQMAWRFCINASYNDVLFTHLLYFFCLN

>SG-AL_contig_8690_1_+1_1066_1404

MPEHFFLHRPFSKPFYNTRPHSHLYWFVTAHLHRHTDNSITIQDLVSALFFGVFFFAQDQ

GVCQIIAGRFGWSARGTKCDKSLLNKTVNEEQGVKRPHYSQGQQYYEGSAHFL

>SG-AL_contig_8691_1_+2_161_337

MTTQKYFNTHTHDAASAETSCSCNLQSIFINHNFLSLIIFTSLSHTHTLPHHLYLSLSL

>SG-AL_contig_8692_1_-3_3_224

AFKNCENRKDRRADRRGSHLDKTSAFESGGPGFISYRHLCLVGKGFAWWIKGGDFLVGKG

WRFSDLPGQHVCRS

>SG-AL_contig_8693_1_-2_521_829

MSSVARIVNRTDAVDEEGVANKWEWLSKIAKIGKTEEQTGVVVTWVKHQTSSRKVLGSIS

LDTFAWWVKGGDFPIFEVNMCADLPILNPLRVYAQAEYQNMHI

>SG-AL_contig_8694_1_-1_55_183

MSLVARIVNRMDAVNEGVLPASGSGSGFQKLQKLERLKSRRAW

>SG-AL_contig_8695_1_-3_3_347

EAWIWAYTDGSTTDAVKRESAGVYIPKWTRAGRSYTNWTSLHKLHRTKVEALVHATNINN

TADCDSQIVFLTDAMSVMEAITTSKLPHLQEALQNIRCTRQVLQWIPSHCGIPGN

>SG-AL_contig_8696_1_-1_16_1572

MADKAHFQLKVHELIPDNGQRDQLFDSLRRYQSSQKLHQLLVELRRLLDEPDKLELFEFI

RPLILLRHQMEYSYLAPPVPGVRLRTIRLHRASNQSLGFAVRGGYEFNVGVFVSKVESGT

QAEKQGLKVGDEIVRVNGFTIAEAIHEDVLSLIKSRDEIVLKVTHIGMLPVKEHASDSVT

WQYVEDMDSKRALEEVLAEKQVHRGKGIELKIFIDCTGYDSIGCGILSGPHYYPGIFVEK

VRPGSLAQEAGLEVGDQIVEVNDTSFQNIGHNEAVVVLKGSKQLNVIVRKHAGLPLFQRR

QTSSIGSSVAGVSNRPPSTPSPPSSQRSAPSSRPWPDNSRLDESQLIAEQLSRSSLYNKR

TSTSSTAAESSFYQQSSEAHTISTQAEVHSDSDSYHGSGGDGPPIATEEFNPLEIDGREL

KAFSFPKGQTLGIEMEGGMGTPLAGKIVVAAVFNSGAAVEQGLQTGDQLMMINGRKLTDI

SVAEAEQILEEAYNGPKEMIQLVYAQSSFLNDEECVTYF

>SG-AL_contig_8697_1_-2_980_1309

MYNITTKMVGCAVDSATKKMVDNVLDIISLLNSTTTTTTTTTITTNTATTTTTTTTATTP

TTTTTTTTTTTTTTTTTTTSKATTHHGKGKLSSPVSVQNYKIMRVLFEQK

>SG-AL_contig_8698_1_+2_2_181

LSLSLPNTHTHTHTHTHTHTPHHYAKAIPGDVNVTSHTTKIKMIIQFSQKNRHTSQHHYY

>SG-AL_contig_8699_1_-3_3_413

CHVLFTCSSPCVIVCQILSIFGCHVLFTCSSPRVIVCQILNIFGCPVVSFEVLRHLTDSP

VIFCHIPVLALFSFLLWYLLQLCCKQLLTVFIVYKGSRTLCICELGIEICICSLHLIYSL

LYVSIYHADLHKVSPMK

>SG-AL_contig_8700_1_-1_187_327

MCQYFHCRFFLIPLGRFCSFKQNSWKMSFQYRSLWNMCCLCHFYSSD

>SG-AL_contig_8701_1_-3_285_443

MKDSIDHIFWKCLHVKRFWERIEELLKSKCVTCANIRLTENLILFCVDENMRT

>SG-AL_contig_8702_1_+3_510_701

MKSYMKNMALGTTLFSKRSFLYTRQFSFTTERWTHKNGDKKKKTLLTSQLQDLSATIRNY

PWKL

>SG-AL_contig_8703_1_+2_2_394

QPQESESRPVRPTTAEANKQTDKRANKKDLLDRVNCPVSVSLVLLQTGQLILSKSFFVVV

CLLVFFCSFTRLHRCTADKPALPPPGQRTTTCSCSTLICNENHQSNIHITTKRSIERATR

IDRHERIRQHI

>SG-AL_contig_8704_1_+2_2_115

TQGTYTRARTHTHTRARARARTRPCVRACVVYKSGLSE

>SG-AL_contig_8705_1_-3_90_461

MAGRVVQQGVKQVKPILSTNPGEAKRRVLNLYKAWYRQVPYVVKDYDVPINVKQGRDKVR

ELFLKNKHITDLRTIDLLVMKGQMDLVETAQIWKQRPHIMKYFEDTVNERPKDFLGKFYD

GHDP

>SG-AL_contig_8706_1_+3_3_380

KQTTIKERKRAETKRAIEKKKFLAQNFQKLGILFILKEPPASPWGHSTDYSTDYYHHHST

DYSTDYCYHHSTDYSTDCCYYHSTDCCYYHSTDYSTDYCYHHSTDYRTDYCYYHSTDLAL

VKSFVS

>SG-AL_contig_8707_1_+2_398_625

MGKKRAGYTYCSKTKDVSHIFNSTINSRWEMWKKKILHFQFNNRMVRKVFSIQEWETKML

MLKSSFHWYVMYTMRE

>SG-AL_contig_8708_1_-2_71_313

MAKCRVVFNATQVAELISQTGSDDDIAVDHYESSNKSVEDEDFVADIPENDDDPAYSTDE

DGSSRRSKSEWHRQCSFVNVQ

>SG-AL_contig_8709_1_-2_65_430

MDPAVVLARENKRKKKIEKQMKRLEKFGRRLKPIEEIEGDPKIRREVESGLRKRVLPELS

FEEKERRALLHKEWARYKMRRHKKELKVWARVQKAQKRALDELRFESEELYQQAIQLDHT

LI

>SG-AL_contig_8710_1_-2_497_775

MTYNVQVVMAAHKHSKTLSLKQLMPGEFSCVKVEILDSMKCSKTAAIAHCLIFNFHIYIK

LCSLVFLMISHCHFLNSFYYQQKLNFSQWLPAL

>SG-AL_contig_8711_1_+2_1256_1414

MMYNINTGNINSTTGVCYQTRHILAGRIFQYIYFTTTGTIYRMLGHVTFSLVI

>SG-AL_contig_8712_1_-3_171_542

MRFDTPSMRVFPTPARLQTLRLKLASLRAWSAAPAREISLLGLMESLASVLPLGRTRKCP

LQRALRTRWRQSASSWDQVIPLGGWFLEATQEWVSASDLAEGVPIALPRPQATLYTDASC

IGWG

>SG-AL_contig_8713_1_+1_1_462

LFRSLYSPSSSISTSSVTSTEADVEALNLDNEGDPLTLPFLLFLAALGVTSTICSSVSAS

SFSCSLCPCLNLFSKRLRFLLDKLTLFFMFFSLFFSMSLLKSCTSSRFSCGWCFCLATAL

CPLRHFIVFLCFSVFFLFFNWPKTPMVEFLQNTN

>SG-AL_contig_8714_1_+1_175_405

MEEWRVKKGEVLVCRKVVKKLKSANAMHSMGLTESGGKKWMRGRCRLMAQYMDTPNANRK

FNLVSSWIFQKLYTHTT

>SG-AL_contig_8715_1_+3_3_221

LSTWNRTRREVFLQTRVKGDKETTLRGAVGLGPVQVKPETANRAQSLSGSDLSKIVERGW

MRTSGEGSPGSWY

>SG-AL_contig_8716_1_+2_299_739

MATCFPREGQPAFHAEKFRCDKKMNPIQSNPIQSNPNQSNSVQSSPVKSRPTQPNPTQPT

PIQPNPTQSNPVQSNPVQSSPIQSSPIQSNPVQSNPIQSSPVQSNSVQSSEVQPNPTQPN

PIQPNPIQPNPTQSNSTQSSPTQSNPT

>SG-AL_contig_8717_1_-2_191_382

MVPSRNERLESTPWNKIGRKCTEIVQISEDRHNQYLLHFNISNSFTPSQYPPVSCRKLMD

RVKK

>SG-AL_contig_8718_1_-1_331_522

MCILKCRVLLLLLSTDFLNKIATNHIWLATSSHSNNCFCWASYKGEKCLQILRKKKAKTE

SAQN

>SG-AL_contig_8719_1_-1_1_225

SFPITWHLQCWYVWNLFQAVLTKLKLKLIVQGVPCFWDQTVQVWEKIHLLFMKSVTIFGF

RMRLDKEEGINVNFK

>SG-AL_contig_8720_1_+3_1059_1316

MLVLKVLLGEPWPQIIHGEDFLAHAGEVLSFYMAYGCHANFHYYSFLIVFHCVVHIQGAM

HTSAYTLISHKLKQNGLFKTTTHTHV

>SG-AL_contig_8721_1_-3_246_809

MSKLSITWNNNTFTLNLTFLLEGEGQSNQEGQLDMDVLPPGINSSSPSWVLHQVSLSYDT

ANEDTFPRAYDGKVESASADNLQLFKTPLGLAYMCKTPKAVTMEFKEVELAFIYMHLMPF

GADSQYFTNETVECSGDLSSVDAEDDLNTVPLIVAGVLACIVILIIVVYATVRCVTRRAR

QSQYKQME

>SG-AL_contig_8722_1_+1_520_717

MFLNFTFYKTFLGGKAIVIIDIVCNFSVQYKLLFICHGFLLFYFFNCAQLNCQFQVTDTM

ARKKTS

>SG-AL_contig_8723_1_+2_1046_1213

MTHLRDKQISYAYIKTDTTRATKSNQIRTKPFQTVQNEDTKLEPNHVRQYRTKTPN

>SG-AL_contig_8724_1_-1_157_393

MVIGIHFGHRCLGHFHLMLTLMFFILSQKIVWESSGQNEMLKAWRRAQYMKKNLNGLHFS

LFSTQCKRERAHIKSAFKC

>SG-AL_contig_8725_1_-3_108_794

MAASQNCSPLKVLCIHGYRQNAQSFREKTGAFRKIIKKKAELVFITAPNVVPPLETAEDS

TEASGGDDSMGWWFSRQDDYFHAQDPSDCCKGYDLSLQLVLQTVKEQGPFDGVLGFSQGA

AMVSLLLGQQEQQSEKWFQFAILVAGFKSRSSPHDALYIKKVSIPTLHVFGDTDKVIPKD

MSEDLLQFFEDPVTLQHPGGHFIPAGGPQKKVYIEFLDKMTEQKKALSS

>SG-AL_contig_8726_1_+1_262_546

MHKYHISMTTFPTLHNIVDEVSHSNSFTLYLSKNNLLMYFSIKFPSSIFTNFVNYSHFSN

VTAFKRIPLKNQIMQKMQLNNTVQSETSIQPWMAN

>SG-AL_contig_8727_1_+2_17_169

MNIIQYSCLPVFCACVFVSFCVHMCVHVQWPVLTNLLNRRQQSIFNTKINF

>SG-AL_contig_8728_1_+2_701_1042

MFEARTKGYQELQGCVKQELKGLIRTAKMFETRTKGHYKNCKASSSQAVSVVYKTAAMYH

HHQIAHSPSTPSILCKKNFHHHIVFTFERKILFDSGMSYFPSAFKTEGNFRQCL

>SG-AL_contig_8729_1_+2_11_385

MYLYGLSRLYCVVLCCVVLCCVVLCCGVLCCVVLCCVVLCCVVLCCIVLYCIVLYCIVLY

CIVLYCIVLYCIALHCIVLYCIVLYCIVLYCIAVIFVTMDLPCEIQAALSGECVAAVQSL

FFLFF

>SG-AL_contig_8730_1_+1_1_207

LNQNSLFDILSYMSVVDKLQSEKFDSRFVLYMFVVDILESAQYTLIHVLFYMSAECEHSE

VWLMVVTYV

>SG-AL_contig_8731_1_-3_594_2474

MPSESSRQSYHHSSSHSDQSKGQPPPLISPHKSSSKGSGENTIFRPFEQNEASTSSVTVN

NGSDKTHSAVSLLPRQFNVSSDVRYNHMNNTDKETSSSSSSSTVPNNMRLFGNGPESKLL

TSNFLINHRIEKFDFKSLARECSSKDGASNTAPELASKPAAVVVIDDDKVTPSLAPPPPL

SSIPPVVAVPALAEPVPTSRLDARERLLGKARAEGRYQSESEEECELEEEEERRRDRLTV

VGSMVRVETAVTASKRAMMEGLGLVTQQRKKDMQQKLFRKRRKKLREPSVSPIELEDDWV

LPNGAMETEPLRTLPAVALEGEKNPKEKALFLHSLLLMEVPPEQKKAKEEIRQVCEQVRR

RRAEKAKAKIRRKLEKEQQELLKALNRGSRNNKRKFDEMAGDSTTPADGGTSNIEPLHIS

LTQIHEQHMTAARMDLEKHQQLQQQQHHRVNHTYSSVGGSSSGATTEGTRPVIFPEGRDR

QLSREFAEQFHESVLQTTRQKEHLQKLGQMGHHHQDMTVTKPSSYSNHLLPSTSSPSGDT

TPPFVWPGIERVLESYMHYRREKQAEKKMLVEKCCQLHAQNVEHTKTAEKLCSRLEVLRE

EERRKTAQRQQLEEAITNLKKCLHLPR

>SG-AL_contig_8732_1_+1_1_132

FFLKVLHTLMFIPFILNGNVRMDKTANVKQANKSVYEENHVVVG

>SG-AL_contig_8733_1_-1_433_669

MVIALFVHIYVCMCDCIACMFTCVNANDMDICVYVRARTRTMCVCACICLCVCVLPCFML

LRSQVQSLLTLHRQICICI

>SG-AL_contig_8734_1_+2_68_187

MPSPVRINSCLGCRTFFVGTDGNCQWPSAVSMNAPLKWCG

>SG-AL_contig_8735_1_+1_1_114

AFCIASENKQLFALQDFFVGSDGNRRWSSMVNMNAPLQ

>SG-AL_contig_8736_1_+3_3_242

EKFSPLNGRTEKVLHARTKTFHRWTAVTCRGQYERLMPSPVRINSRLPCRTFFVGTDGNR

RWPSVVSMNAPLNCASFLQH

>SG-AL_contig_8737_1_+2_44_226

MNQSSSTVWIWHTKCVCVRMCACVYVMLFSFIFESDGGQTKTDTLSWPTKTTSICFSHFI

T

>SG-AL_contig_8738_1_+2_71_847

MNWGLKQEVPYMRLRVKVSQFADDTLLTLEGDRASFVQLFEILNESEKCSGLKLNFEKTC

NVWFGQKRNSNKVYMAHKRMEWNLNKYKISGLWLNNDLSNLTAINMVDKIMEVQSLFKIW

SRRIWTPPAGVAVLKPLSKRVYLWILLPNPPDSQMNQLQNMCFELAWDKKTNRIKRPNSV

HNIENGEIGILNIQAFAHALKLSWNRKFALGTPKWKIILQTLDPEIEYLSCYGTKKFIKC

KRNKFWKDDFLTYDNLNNH

>SG-AL_contig_8739_1_+2_2_286

IFNLSLQSCVVPHCFKKSTIIPVPKKAQPTCLNDYRPIALTSVVMKTSERLVLQFLKACI

PPSFDPFQFAYKPNRSVEDAVCTALYHVLSHLENP

>SG-AL_contig_8740_1_-1_109_261

MTQNMIKGSAHSVFQQICWAYRQTEMGQRKEVCKPSKIAKPNVQRFSLQLK

>SG-AL_contig_8741_1_+1_28_285

MVKKKKKFTNVRAQRQPDGAIRLSQTSTSSALIRLSPVSDCIKINLFLFTHAPTCTFYIT

LYIRTHSSLNLHTYIHTVNVHTLLDV

>SG-AL_contig_8742_1_-3_423_713

MGLVLTSYLTGSDLITPALDKGRQLFFTQHVNMLILLRARLLQCFRLANCLRHCITLKRV

SMAIDRLQINSKKKKKMRVTFCAYLFLKLGKLAPELS

>SG-AL_contig_8743_1_+1_1_222

FQTTCWWITNNIYAFWSPLFRGLSPYCYSAGVLQISHHFITEQDVFPVIIPSPVQSCPTP

VSPEFSVQSMNNSI

>SG-AL_contig_8744_1_-1_1_234

DRRKTLRDITGEFNIVTGLNCSWRTMQRKLHLMDFKRRTVKKKVVIREVKESSGETGVGQ

DCTGLCMFIGNTSCSVMK

>SG-AL_contig_8745_1_-3_210_311

MCNSNQWLYERFHFKCTRVYQFNIQTNETEIVCS

>SG-AL_contig_8746_1_-3_528_725

MIIVQSVLTWFVSHKIQFFAQSSSSCVLNPFLSGSKDILFSASHLHMIHNSSILVLQTRF

FLLFEL

>SG-AL_contig_8747_1_-3_399_539

MPCGANKQKEPWLWPNALPANDTSKMLRPTLNSCYALQCYVGIVFIH

>SG-AL_contig_8748_1_-1_1357_1581

MKTCMDAMVTRLLCWLLSMLIGTGYLHVSIWMYGKHIAYHMDIVTMLLFCTVVSSIGYGC

VLAHVMVYMSTVVCS

>SG-AL_contig_8749_1_+2_2_238

FFLTAVNNFIIPLFITNSNFSLESFTPMHMSIKLDLHTHFQIIQNNLKISTTFKLKQMIC

NRQRESIWTKITEDKWKLA

>SG-AL_contig_8750_1_-2_2_103

SARRMIMRDHVLLVYAGMHRQTQPLSYSPTCWYS

>SG-AL_contig_8751_1_-2_47_1093

MAKAVSEGRVDVPDLVHCLRSERCLMVQAFDQYSFLHKALLEAYTARGTSVEMNDFDSIF

RETISSTKPYPRVDKEFQTLMQLQKLTPEHHFNTAKQEENVAKNRNTDILPADDHLVYLT

EHVPDRNQYINAVFMPTFRDARGSLLTQLPLPSTVVDFWRLAYGNDVKTIVSLSSPNEES

ELADYCTYWPRDEARSLQSGPYRITLKKKHERSGFDITTYSLSVAKKSHKGVLETKEVEL

LHYRGWTGEVGGNTSDILHLLDTLTSSQTMSKTNCMILQCSDGVTKCGVFAALCDVVNRM

TFDREVDVFMAVRRIQSVTPKALTSLVQYRYCYEVVQSRVREMSIYANT

>SG-AL_contig_8752_1_-3_645_827

MVLVVHVKLVCLFLVYSLMHFCSLKKRCRKGRMLIQFPLQRILRLMLFAVYLYSNTYFSV

I

>SG-AL_contig_8753_1_-2_164_517

METGELGKKFPHLPILFRYNSLKLDMITKTGKNKGFPITRVIIEQQGVGDLNFSQGQHNK

ENVHLFSHAALPSPSQWWGKTQLQWSGTVVFKPGPKSGYPSICQRCLIVSTSTFGTRV

>SG-AL_contig_8754_1_-3_345_2393

MDYIAKRKRLDEREVKKFIRQIVSAVDYLHRLGIIHRDLKIENLLLDQNRDIKLIDFGLS

NTIKVSTAPEGTAHAQEYLVTQCGSPAYAAPELLNHRKYGLQVDVWSIGVNMYAMLTGSL

PFTVDPFNIKTLYNKMISGQMNPIPESLSREGKDILRKFLTADPEKRLTITEALRHPWLS

EGRNKPVERHPFPNKLKTSDLDTDILKHMSDNLGYRMSEIIRFVVGNVPSSASATYHMYY

RKLQRHRAEQRSQGKAPADTKSVEATRISLVLQDMTPRVTNPGAPTRALRARGDSFDTSR

DLHVMHGPRKSMEKTRTATATPSTDMESLLGKERTVLSSLEHSLEDVRADAKENKEKIKG

HQASSSITPSSTPHPDTFLTHSPKHTLSPGSKVMVKPSHAHHPTGHTHRTDQGPGSITQE

PVGGTRHLKITPKATARSGTAETLASVATGQHSHVSSDTPRKGRILIPLSRTFVPLRRGI

KGLGGTGTSQPDTVTSNSHPERGTPHPRVEGGRVIRATLPTNPAYIHGSSTSPVSSVNTS

HCTRHAAGVAKRSPNAQFRGAPHRTDGSVNHGGVSRLMPHEAKDAHSKRPVSPQALPLVR

RRLAKRSRQDGQGTHHDRPSDGDKDHDNSAEGDNESVTVERLVTSPDCRASAKTPISQSA

ASSVARDLHMLPAITALGTPHHS

>SG-AL_contig_8755_1_-2_2_4930

MADGSRTVVCLESLLSCVQVRSRTIMNVRTVILCLCLFALVASAQEDPPRGRSGCYDDAG

RAQRCMPEFVNAAFGLPVDASNTCGVSKETEYCLQTGASGARKPCYICDARREGLNHPPE

YMTDFNKQDNWTWWQSETMLEGVQFPTTVNLTLNLRKAFDITYVRIRFMSPRPESFAIYK

RTKEDGPWIPYQFYSASCESTYSLPRRGIITRSNEAVAICTDEFSDISPLTGGSVAFSTL

EGRPSAFEFSESPELQEWVSATDIKIVLTRMNTFGDEVFGDPKVLKSYFYAISDLAVGAR

CKCNGHGSSCQIIRDQNLEDRLVCKCEHFTAGPDCGECLPFYNDRPWSRATERDANECQP

CDCNGLSDRCYFDRELYEQTGHGGHCESCRDNTGGPHCEVCLPNHYRRMPENRCLACGCN

EMGSETLQCDDQGQCRCKPGVGGQRCDRCLPNYYDFSDTGCQPCACVVAGSLNNEPRCDS

ITGECDCKENVEGRRCDRPKPGYFGLSEANPFGALACFCYGHSSVCTSATGYYGRNISTN

FATGNERWTAVSRSGQDLDTQYNGITEKLGVSAPSMEVVYFNAPDRYLGDQRFSYNQFLT

FELQIGEETARPSVVDIIIESNDQRIATHIFAQSNPVPDVASQNYSFRMHEDPQFQWSPR

LKPQEFISILANVTALKIRATYNPDGVGFIDNIHLGTARQAFNDGPEAGWVEQCTCPEGY

IGQFCESCAPGYRRDVMNGGPFARCVPCECNSHSDICDVNTGRCICEHNTEGNNCERCAR

GFFGDPTQGTPNDCQPCPCPNNGPCVQLPNGDVVCTECEEGYGGNLCDICLDGYSGDPAG

HHGSRRPCERCTCNGNIDSNAVGNCDTTTGECLKCIYNTGGFYCEKCLPSFYGDALALPK

GQCQACNCNPTGTIIQPGVLGCDPVSGKCACLPNVGGRQCNRCDPGYWNLDSGTGCEACD

CDRTGSTNYTCNENGGQCQCRSGVTGRRCDTCQPFFFGFSQSGCQACNCDPIGSLDLQCD

QYGYCPCRPNVDGRRCDRCQENKYNMSAGCLDCPQCYDLVQEQVTIHRGKLRDLTSLINN

IGNNPSLFNDSEFLSVLGQVNKSINVLLDEARGASTGDGTIGQQLQELKDALREVLEKCG

QITRSIAAAASESKDSIRDIEVAEAAIRRAEMSLREADDYIGREGRQALAQALEALQQFG

KQSQQMTEIAQRATDESIKQMKDANMIDNVAKNAVNTSQEALRLAEETLRMPDTNRAQIE

RLTREFSDASELYDRTEELAKVALERANEAHKEALDLLREAQSPLPTVDVRQLSEDATKI

KEDAARIKLRAEELMQNNKDLLAEVEQQTNLSRSTLTEGNHLQQRVDELLAEVDAARDVA

RKAVESGERTLREANETLQTLLGFDRLVSENKDAADDALKKVPEIENMIAEAMRTTQQAR

EALRGAESEAKEGLRIAEMAEFTATEASKEAEKIRDEARDTKNKAVDLKDQTETLAQEVQ

EANKSVTAYEQQADADEGMIDEARRKAREAQITADAAADKVNKALDQVTSIKNILTYLED

VDTDRLAELEAELDRLETELNQANIDSEIETLTTQNDRIKCWRNKYETELSQFKKDVDNI

AAIRDSLPDDCFKTIDIESPLNG

>SG-AL_contig_8756_1_-3_39_872

MYTTPLTQLINNHYVHHEIFADDRQLEHSDTPENCDSLISKLQNCVSDVKQWMSNNKLKL

NDDKTEAIRFVKHTRISDVLRPSTVALGSTEIPFSQAVRDLGLYIDYDFTLKHHIAKTCR

ASYLELQRLSFIRPFLTKQATQTLVSSCILSRSDYCNALLLGLPSTSLQPLQWVQNSAAH

LIFMAPKRQQISYNNYTGFPLNVGYNTSPPACATKFLPVMHLSVRNFSSLCSSTLSSLFL

RQPYFQNPTFLSKATWTQGLFALCCEVLELSLRYASSF

>SG-AL_contig_8757_1_-2_80_931

MGEAGKSSQDDPELNELLNSALEDFDKVKLSEESETASTNNTATPQSDAGAAGGKPQGAE

ADPLSEDFSEMFSEQFASQFEETMASLMSQNPQVMQQFEKLAQAAETAGDSQEAQQNFME

TLTQTLVDMSENADGIQEETLEDQDQGQGQAEGQGDDFMPMMQELMQTLLSKNVLYPSLK

EISSKYPTYLSENEGKIEASDLDLYRRQYEVMTAICSVYEEEKADDSAEVKSQRFEKLLD

LMQQMQELGQPPKDIVGDMAPGLEFDENGMPRLPGMSGSQCRLM

>SG-AL_contig_8758_1_-1_832_2157

MDEDDLVPCAALPRPPKNYNVLERDSQRCVTPDQMFRGHLGPLPFLLIIDVGHMEGLDPF

MDDFAGEEILFAFKEGSQTRVLMHDSCNKSLSLPLSSSLTFHLAEHEEELTLSQIVARQG

QWPVRVKFARADRLEFVMNDTAKDIFTDVTFFGTLTLVCVYEEPFYWCLSLKGSGEVQDM

RPRVIPRNLHDVSFAPVKGILGVNDSDSTLKDMLAELSLLAQQVPMDDDPFFQELALVSN

GPTLVKEVKPYIAAGTFIRTRHKPPNQKLARSDHPPPPLPGRSSTPRKADKADRSDEVPY

SALEQVADDNMHYYSVCAQFPSKRPPASIPSKVSSALGSVSSLPVPERPRGARTGSDDTS

KMMQSSRSDIRATLDSLSIDDLSQKLKELGLGKFAKKFRKHRIDGVLLQKLTEKTLKSDF

KLSHLEFLRLNTFVTEGHIPKA

>SG-AL_contig_8759_1_-1_211_1698

MMDEVPEAEPMDDTSLKKMLLQFEKRVNKNQEMRIKFPDMPEKFMESELELNDILQQMHI

IATVPELYHVLVELNTVRSLLQLLSHENTDIAIAVVDLMQELTDVDTLNESEEDATALVD

AMLEGQITAMLVQNMERLDEGVKEEADGIHNTLAIIENLTEFRTETCSEAAQQGLLMWLL

KRVKLKMPFDANKLYASEIMAILLQNSDEVRQLLGEHDGIDILLQQLAMYKRHDPTGKEE

MEYMENVFNCLCSALMYSTNRARFLRGEGLQLMNLMLREKKLSRNCAIKVLNHAMTGVDG

VDNSQKFVDILGLRTVFPLFMKTPKPARAGPTFDEMEEHICSIIASLLRNGHGTQRQRVL

SKFTENDHEKVERLMELHFKYLDKVRQCDDTIEKEKLRLKHMGEDVDDDMEDEFYLKRLD

SGLFTLQLIDYIMLETCASGSSSIKSRVMQILNMRGGSIKVIRGIMREYAGNIGDAKDPA

AREAEQSRILQLVDKF

>SG-AL_contig_8760_1_-1_1108_1260

MFTEYCTPISMLCYSSTLFDVIINMLCAILLDHLTPRPFSDFLLHSSFKNI

>SG-AL_contig_8761_1_-1_148_1422

MEKEKERDRKYGAEMRKELEGYVGFANLPNQVYRKSVKKGFEFTFMVVGESGLGKSTLIN

SLFLTDLYSAEYPGPSNRIKKTVNVDTNQTVLKENGVQLTLTVVDTPGFGDAVDNSNCWQ

PITDFIDCKYEEYLNAESRVNRSIMPDTRVHCCLYFIAPTGHGLKPLDVEFMKRLHDKVN

IIPLIAKADTMTPDECREFKKTILNEIAQHKIKIYEFPDCDDDEEMKIQKKLRDRVPFAV

VGSNRVIEMGGKRLRGRQYPWGLVEVENLEHNDFIALRNMLIRTHMQDLKDVTNNVHYEN

FRYNKLAPSSSDSKIKPGSLTKDPLTQMAEERQEHNLKMKKMEAEMEQVFEMKVKEKKQK

LQDSEADLQKRSEQMKKSLEQQQKELEEKWKAFEKEKGAWEEQWGERRRSLENAKDTKKD

KKRLF

>SG-AL_contig_8762_1_-1_1_99

QTDFSGQFLTQLCHFQCSHTPNRRINLKGQPNI

>SG-AL_contig_8763_1_-2_2_175

VIAGDWSSDVCSSDLITDRCWLIFMPHPPTLFTPGCSTKRVRSNCRDLHVVLGNSGQV

>SG-AL_contig_8764_1_-2_2_376

RPHPSSKSHFSEQELLLLGLFQASLCFDHLPFGSSPALPWSSVRFRFQGALFPCRSPSIV

VRRIFFLLFFPVWYFGHYGFQVRGSTRFEAQLDQPLWGSLGLLIASFNPFIPSPSVHQVP

IISWL

>SG-AL_contig_8765_1_+3_57_185

MAGYDANFGDRLKKNCCSFFQDGSTADSDDVKTVWGVCTDDML

>SG-AL_contig_8766_1_+2_401_598

MYIIIWKYSVHLITGTLFYLCLFITLCVTDTFSTITKLLVTCILKQCVLPKLSSGNTLLL

KKKKIH

>SG-AL_contig_8767_1_+1_97_264

MCSQVSNHSRTKHIQVCASTTRTLRICSQVKHLYFLETNNHLINAPKFEKLEFDAQ

>SG-AL_contig_8768_1_-1_394_642

MCARWCLHAMEFTRHGIYTRPECVLASEVTASTFRKTAFLFCIIHWIPFDRFSFNLIILL

SCTILNCHLFKGFSTLYFGRFPL

>SG-AL_contig_8769_1_-1_37_1134

MDICQRSQSGIYDINRNSGSGVHNVVPPTSGGFTLTFNNNQINNQINALHSQMAATQTLG

SSQGVSEIPYLQYSIQSGTYLSYQPQQQLGPTVPSQRQSAQFPCSDPFRQPLCVPSLVVP

ALPSLNSPSQPNAVSMVTQAPQTLQLQPQLISQTVPSQHLTPVTMIDNGRPVLMANAHVN

SWPASRQVLMGPWAPVPALTSQRTVPQTLVPPDTLTASAESWQPSLMNSGSQQWNMGLVP

SSYPLSSHYAAAASHSSSKRNTKQSRPNKEGTSTQLSPVKKRVKESTPPQTELLGAEELL

RVRGDTWGVAHNLMLETKPVDTQQTIVIRDSPSPSHSVITISSDSEDESDRRGLPSHHHH

HHHHRH

>SG-AL_contig_8770_1_-1_676_900

MLLMDRLHNFGQNTFCIISSSKSRLFRQNIDCCRVLGAEENGEKSFLALKTRSQYNWSFV

FNARPHFIGPFVGMP

>SG-AL_contig_8771_1_-2_140_253

MYVASNIRSREWVPCLFRVREARAAARERSGHDLHRPS

>SG-AL_contig_8772_1_-2_257_442

MFTNIKFHFPVSLFSFVQLTDVISGIGYHPCCNDYFTMTWLPLTGVIFSHMNGTFVCYFF

FK

>SG-AL_contig_8773_1_+2_113_292

MMVIPILGNFCAHQNKSEVPVVGHSRGYTRTRGEFPLWGTLEVTPEVISEQERSSRCGAL

>SG-AL_contig_8774_1_+2_2_217

KVLFINIGENSPNLTHTHTRTHTHTHTRAHTPRTQTNYTNTSDCRKVAVFAGAFQRKQEA

CGHALSFGAPIG

>SG-AL_contig_8775_1_-3_1497_1844

MRVVPICSFAALPSPTLNRVRYPFIAGWTGGCLHKVAPQLGSNSCGQTGVLSLCHPGLLF

NKFTYNQVRAGGIRELFLSIQAINALPFITHYTFNEQRFHKMRKKKIIEPFSSYST

>SG-AL_contig_8776_1_-2_245_493

MDVMLKRHTSPTLFFFFVGQETKVSKNPGFISCLWRNVYEFPLHIELSGDDNSYVKMYFY

AEVVTAQVKGDRDHGLTIFYIHS

>SG-AL_contig_8777_1_+1_190_492

MQNSKIGGGEGSPPLVCPPRGGVYNGVSRYEGDARQTFCAGDPEVAQVVVSEGGFAPPVG

CFQDVPDREGVFEGSRRRQGPEFVGSGTVGEGGGTRFLVGP

>SG-AL_contig_8778_1_+3_612_758

MRAVSIARVLTTLTDLRYVVWVCTTTRSSGSYISVCPCARRMSQVPRGV

>SG-AL_contig_8779_1_-1_19_270

MTSVQLSDRKKAAQKPMQEKVFKVEEPLHSDSSTFLASEAVFGWYKELSLLLTTLAVVFI

SFFFVVFFLTRHLEVCMLGVFIFP

>SG-AL_contig_8780_1_-1_142_2988

MAKDMKKMMRQHVKLQAKMAEIDPDLPRLLQMVAVLVQSIHGEHENAGRAVETRLAAAKE

AVKPLNEKEAAAKKALSSWDKKYKKAHGAAPEDTDRDEEGQALAEAAEDSKKALDIKQLE

IRALTLLTTGIYKPEEQQRPKSASRATPEADVDQQLSALDNKVIDLEGEKDRLQEENNKL

QASVGDLEAEIADLKEQLATQSAMAAVAVTADTSQDTSELEREIDSLNAALQQEKAAHDA

TRDELESLQKQLQTLKDEMSALQGQSQADVHKATAVLEANLASKEEQLAEAKKKNEEMEK

ERLASVPMDTAKEIKNLQNKIAMLQKEKEEVKATAASQGAAMVEVKAQLDVANKALEAQR

GTNRELEGRVKAARAEKEKAVKELNNQVDKKEQQRAALESKLKQLQAAGAVAVTATAAAR

GGAARGGKGAAQGEDTAALKREIRELNARIKQLETEARRPVAVAAAVESAAPDRNTQKRH

EKILKELEKRLEIEHSKATKLAEAQKVKDEELKTLNKDVKDKDAEIAKLTAELATLGLAA

KEGVAAAGKVKSLEVENKKLAEENKVLAENFNSERVLRKKYYNMVEDMKGKIRVYARARP

LSNTEKERGNFAVLKSPDEYSLQVTSQRGVKEFQYDQVFMEDSTQEKVFEDTNNLIQSAV

DGYNVCIFAYGQTGSGKTFTMIGDRDQRFPGIAPRAFERIFEMAHENKSKFDMQVTAYML

ELYNDRLIDLFAKPNTYDEDRLDIKKDKKGMVFVQGAAVKEATNAKELNALFEEGSKNRH

TASTKMNAESSRSHLVIGIVMESTNKSTGNVVKGKLSLVDLAGSERVGKTGASAEQLKEA

MSINKSLSALGDVISALSSEQSFIPYRNNKLTMLMQDSLGGNAKTLMFVNISPADYNADE

TVISLTYASRVKLITNDASKNADNKEIARLKAVIAKMKAGEKVDEDELP

>SG-AL_contig_8781_1_+1_1_333

DRRKLCQLIPSILYYKERLSFYTFETYSIYRVIWQQINEDNLRVTDINILFLCVCVCVCV

CVINILTFLRFQYYHNTKYSPSLLAHTNVIHFNNTSISPSETNMLYFVPRK

>SG-AL_contig_8782_1_-2_890_1144

MFDLYPLYLVTDDKMFIMTFVFKFCFNAVFFHEITTDTECAYYRVQVASESFLFNNRHQV

HTHTHTHTHTHTHARTHVRTYFASD

>SG-AL_contig_8783_1_+3_1200_1313

MPITLQWTVAHCDIWGNEQADSGKSRKQTKTAIVPNLF

>SG-AL_contig_8784_1_+1_1_195

PDSSFCPKGLCVCVVTDQTVHSVPKVFVFAYLSVFLFVCLDHTKADNSALPPLLGLRTTT

CSYST

>SG-AL_contig_8785_1_+1_316_492

MSVREDREGVWGGRERKEEHFRTIIHEDRQVAHRSQDRGEWRRTAVSSTAAANIEPDDA

>SG-AL_contig_8786_1_+1_49_252

MTTQESICNETAIQVAETHVKEEDRKNIHQAHCTNTSNEVIVLSTGRSVVNFDTGQNLVD

GLTLRLPH

>SG-AL_contig_8787_1_-2_2_559

KSQQPGSHLRSFQIPFLVSSLDPRDVKSKEHLLCLVHCVKYYLKCSAPRHSAQWRLLISL

NENYLRDISAMTISRWLLATVIRAYKESGSSSLLENPRAHELRAFASSAAFHPYLPIRDI

LEAVYWRWESTFTNHYLQDLRVSSADSLSCISFVAASAVIDASIQRADHRRRSLPSSFKF

CLSWIL

>SG-AL_contig_8788_1_-3_21_410

MAPSIKTLLALLSFVTGAFGWKCAKYRHYFSQQPDYIDCFWDCCGNSFDRHCCAPIGIIV

GCAICGAVVLAAVIVFVCCFWQRRRRYGIQPHIFQNRKPVSAYPAAQSTVTNPAPYVSYG

QKPPPDQFFH

>SG-AL_contig_8789_1_-2_32_391

MLEIARQKNIYEKDFCCYLDDNPLPMEKSTYDCAVSSGGMCEGHIPTSGLRQLARVIKPG

GAIIIVMREDFLQQVADYSGKLEKEMKDMEKEGVWRLATRKTIPRYFYHYSGVVFTYVVL

>SG-AL_contig_8790_1_+3_3_227

YFLLFFFISSRHTHTHTHTHTHTHTHTMLWRIMLWSSIFRLRLRGRAALHAPFYELSWHE

PGLRDTDTWSVGQVI

>SG-AL_contig_8791_1_-2_2_199

FFFPHCYRTFSDCILMYPSSSVCSLTVSMLVALLTVHLLCMCLECGLFLCKKTRNVFSKC

VVIFHF

>SG-AL_contig_8792_1_+1_79_219

MRPRWMMVFLYTILTTQGISTKNSWVVHLDTIHTLFHASTFCSERSV

>SG-AL_contig_8793_1_-3_1050_1361

MQVFLEPIQLTGPVRWTASQEEVIDKTPTDTRRTVEKKELVTEFLQDVDTVWAEKTPRHP

QGVWMCVYQLVLNLQCLGFCLFVCFFVSIHELQLPHALNHTSGL

>SG-AL_contig_8794_1_+3_894_1121

MLAIKSGRCTFQEQALSPFGFGTNEALVLSGRVSFFKMCRVSDKYEGMWPNNEQTSFNHE

DKIISTVHNLVYSTEY

>SG-AL_contig_8795_1_+2_2_100

YVEVIQVRIQVKIGIMLSHQKCFNFLFTQLKID

>SG-AL_contig_8796_1_-3_72_1313

MRNKKRGKAAKKARAAMLSDAREEAFAKAPHNFVFTRGKVGKSIRQLAMDCRKMFEPYTA

ANLKVRKSNSLRDLVEAASPLHVTHFLCFTKTEVAPYLRFMCVPRGPTLTFKIQEYCLKR

DVLSALSRPNIEQSVFQAPPLLIMNNLSGESMDMKLMGTVFQNMVPSIDVNKVKLNSIRR

ALLLNFDKESKSLELRHYLIRVAPTGINKATKKLLRPKLPDLGKFTKMEEFVEKGGNMSE

SEGEMDGPHNEVVLPQEVRGRGNVKATQSAIRLKEAGPRMTLQLVKIEEGLGDGAVLHHE

FVSKTPEEQQNLKAMIERKRQLRERRKKEQLENIRKKTEKKEKHKQKSLEGMKKKAVQQA

SEAADASSDDDAAYYKEEVGKSPDKDLFPRKRKRQAAKKGKPEEPALKKKKRRP

>SG-AL_contig_8797_1_-2_170_328

MLAVVLIFFLIIFQCLCLFFLLFFQQSSSLVGTVTFLCLLTCSTWINSLESCQ

>SG-AL_contig_8798_1_+2_71_160

MNWGLKQEVPYMRLRVNVPQIVQFEGLECS

>SG-AL_contig_8799_1_-1_1_225

RFVIFNAQTFEIEQQNFISSLYSFILKVLILVKNSCQPLCLGRGCVRVCDDSTAAAATGF

PCVGSMEDSLSLCVC

>SG-AL_contig_8800_1_-1_73_768

MMQTVGAALLLCLIFTGGETKICARKYSRIHREHTMCLTKNSDAVKVYLTEREKDVIVST

HNEIRAAANATNMQKVKWDNELAKLAQKWAMQCPSGSEDNPDKRKTPDLPGIIGQNFASG

RADIFEAIFHWWNEHSNFQSGGVARHSPDSRNYEQLVWAPADRIGCAASTCTNTHYYICN

YHTGPVVGEQVFEAGPPCQRCPNHCEEKLCDCTGSGICMNGNTRDVATCECN

>SG-AL_contig_8801_1_+1_1_279

GQPASQRNRAKLGSQQFNSSAECLKKEHYLPLLTTFKLSKWKIISHPNKHNKQKNKLGGK

AVSGREREQQTERKTTHNTATQSKSKNTNRQVT

>SG-AL_contig_8802_1_+2_2261_2689

MKFNAKKCYILNVCHKSSTTISWTNIFCSRSTPYPIFKSKSTKPSPGLTTSTGLQRKPMP

SSVCSNIISTFAPSLAGKQRTSLSLDPSWSTAPQCGAPPSERHRQAGRHTMQSCKIHHTR

LQIPRPRLHHGHAEGPRPPNTPR

>SG-AL_contig_8803_1_+3_165_257

MSTKKQDHKLCCYTVILNKFECKGIHALVSI

>SG-AL_contig_8804_1_-2_113_1510

MHSTWRTVEEWKAMNVPIATPSNEACKMYDAALTQYVTWKDQDSVGGIEGALEKMMAADP

DFVMGQVIKNGLDLMGSGRSVKLDKEFQADIDNMVKLAESQSGVSASEKKHTKALKLFSE

GHIVPATQIWEDILVDHPLDTLALKFAHDSYFYLGHSAQIRDSIARVIPHYKTSNPYYGY

ILGMYSFGLEETNLYNQAEKTARKGLEINASDAWSTHTVCHVMEMTGRQDDGITFLDKTV

TDWEPCGMLACHNFWHWALYHIEKGEYQAAMDILDSQVKVRACKSGAMLDIVDIASLLFR

LQMEGVKVGDRWQEVFEVIRPHLDDHILVFNDVHLLMACIGAGKDEAVNHMMDSIENFVK

TAEGDNHEVMKEVGQVLCMALVAYKEGDFATAVDLIQPVRYRIITIGGSHAQRDVFNLFL

IQAAIKSPRVEHHRLARTLLAERKALKDSAPMTDRLMARAMALHAD

>SG-AL_contig_8805_1_-2_152_268

MQGSNNDPPLLSVKHNIYMDFAVILCCQRLLGFCSGITF

>SG-AL_contig_8806_1_+1_550_747

MLGLTDDPNMVKIHPQTVNTSCRHFFMQIYCTLIPSCQPHPPPTHIPQNDSDAHTQAGIR

KGQGNE

>SG-AL_contig_8807_1_+3_75_209

MSKYIHNMSCHTLITLKPTLQLFAIPNAFIRIKMNTFSRLSLSLS

>SG-AL_contig_8808_1_-1_58_270

MFIHTRTLSKHTHTHTHTHTHTHTWQVIHRVISVFTARGQFFNKKIQFECTEFPLCMSTH

KLLTNSKVLIE

>SG-AL_contig_8809_1_-3_312_569

MVVTVGSSIYHFPGGGWGPPHKRERSGCSQCRHYMKGLPERLIKHLETIKPKSTTNRSTL

REDPSLSTPKSEPLMRKNVSCLIYLL

>SG-AL_contig_8810_1_-2_185_358

MAGDFIPESELKLTGIQKYYNSFTKRGRFNWVVTTYGTLFMAYLLFGRGGKKNTAPKK

>SG-AL_contig_8811_1_+1_790_978

MLLHNKITALEWQKSIVCCCFKIDRCHKPSELERGEGKAGVGGRFLIVAFFSRNYHPILL

FGS

>SG-AL_contig_8812_1_+2_2_223

KKEEKENTLKQYGHTPRPSHLAKTVLQGTVPGGRKRGRPKERWEDIRERVDFHFSDSQVA

VQNIQRDEENSSGK

>SG-AL_contig_8813_1_-2_92_403

MDLYARLKLIARNIDEKKKILSLKGKWEILMKRKKKNSFLQREVGNVEKKKILSYKGKWE

ILMKRKKILSYKGKWEILMKRKKILAYKGKWETLMKRKKGSGKY

>SG-AL_contig_8814_1_-3_87_749

MPTNPDEPYYCSQQINIPPELMDILKQFTKAAIRTQPKDVLAWSAAYFRSMAKGELPPVK

ERLEMPMSTQKTDTGLTAGLLKVLNKQLGPKRTVALQFIEEKWLDLALPKEQFDDLVRVG

SFSGQVEWNKFFALAASALGDNITDAMKVICEILTADPEGGPARIPFPLFRELYQYLAKI

DGEISPQQVSDVNNHLQYEVDKQEGYVMPRNFMSPDCPKLF

>SG-AL_contig_8815_1_-2_11_808

MASAEPEKASDASNSNTEEGSTENQPASDPGTSERPDAFSNFYQEVKAIEQRDSVLTSKQ

QIDRLTRPGATYFNLNPFDVLCIDPDTPLNDVKKKYRQMSILVHPDKNKDDEERAQKAFE

AVNKAYKTLENEEGLKRCAEIVEEARSRTNEMIKQKKKQLKKEGRPQDIPEDDSERYKHA

VYVQTCKLFADLERMRQERETREMHERKRKAEEEVKEAERQEMEKEWKKNYEESRTDRVD

SWRNWKKGGKKAKGSFRPPKVKTEKL

>SG-AL_contig_8816_1_+2_44_274

MLQARQSRMKRNHFYYRKYGRVEYQFAFTNRRKNSNNTDSGYSFAGAPVINVHRTQASIC

KISHTLYMKHKCIQIPA

>SG-AL_contig_8817_1_-3_1365_1649

MLRIIAEYYSGICIHDVQLSALNSHDFPLKMSVDLFSLKFSNSLFFESFSIWKIALHFPQ

TFLNHGNHGNTRYIRNSCRCYSCNVLPAASDLMFS

>SG-AL_contig_8818_1_-3_546_869

MTNVIITFMKINHTLKDLKTEASTFFCCCIGLMALRLFFFLFFFFALNPYFIFLKKKKNF

FCMCFCVFFFFSFLCENFDFSCSKKKGKRGGVVFFFFFFCLGGEQKKK

>SG-AL_contig_8819_1_-2_38_208

MIKKNRLQFFLGEFLLSSQLLLARCAYEYIYLIKGIQYFNIPHLRNMKNTFWSIHEL

>SG-AL_contig_8820_1_-1_7_1998

MNVEKARGRLLILKDQIHEYKYEFNVRLLALRDKKIRIIEEIRQLVGQLLDVQKDLRPSK

HRPAPSIPEMHLDEMPEKRLEYTRDTLIKFKQEMIEQEAAVAKKKGADESGFGGFGGSSG

TAVDKQATPAAKQMSFMNTKAPSRQEETKEDADLELEDEVEETLSPLEKELQETEQIRLE

YEQSRLLERMREMVDKFDAELRLLRHEKFKLDIVMKNADLRQVTLFEEFVLLKEYEKSED

VLAEKVSSKQQEKLDMHNKILEVQTRLETKRKDIEKLQEKERSVHQQFLSTIGDNKFADY

LTKVFRKRIKRTKKKATEGEGSDEESEDDSDDDSDWEESDEESESEMGGYDLDICPPGCD

QMLYDAATTMREKRLDCEEMLAEEKKNSEGLKKELEALQKKARIIDSSLKTAQNDLEAFQ

LEKQQKLNELNMVTTLHLHQIQHIINSTLPSDLTNTLVFDSPGVVRLQHRIKELEHEKTL

QKKHMKESRRKHVQLIKDRKHFDAKICEMEAKCDSMMVAKFGCTVDMEKLEAVTVNRTIE

ELKEKLRQTELMCAEELQTYDSQIGERRSQITLLIRNNTQRLNQLGMLLEENKAYQRSLD

KRQKNLGDEYVAVRRADVRERQRLVQLVQLQAQEIDALKDEILLLSRKGGHILPPAQPPM

SHSP

>SG-AL_contig_8821_1_+1_457_636

MFRICCVYYIFCPPSLTHIHTVLHVLLNNLTTSAKFHSSLFNTVIDISSFCFDNLLPYFC

>SG-AL_contig_8822_1_+1_1_273

QNTKYARQRSRKSRQWLAHGNTKTPSMHAHQRKSMQTVQQHNACTCGPNNMRNASEKVKK

KKEKAVFTKDTPLMYIKLIVLHIYTRYSRVH

>SG-AL_contig_8823_1_-2_2_697

VLGGGSLEVIQEGVVAINVSVCLVSRPEVCGPPTKAEISQPDNSPALTVRDVIIIACVCA

VIIIVLLIILICFICRRRANAKEYENSGIPPSASNGGVPGKKMQQGQGQPQAYDNADGRY

PTFASSSEYPPNYWDTHPQTYDHANNLSFDSQLDRQMYELELNRRNDMALYGRADGYRGQ

QESIGMQGRPMSPKDKYIDLEDPNDPSLQSQESGYSTGDPNKPKKVIYEVVV

>SG-AL_contig_8824_1_-1_262_438

MFLYKEKYSLGNKESFHNCFKCDAMIQYHFDEKLCFIYSAFAAFFSFKIKREYENDKKK

>SG-AL_contig_8825_1_+2_2_187

RTLTWLTGSLTCIFDVLHVYTHEGGSGTSRSAHMLTWEIGKISTLYPPGEVPEGRTQAFQ

TL

>SG-AL_contig_8826_1_-3_3_476

DDEETSQDDKKGHDVTEHNIAEENDGATENSYDKGEDESNENKNNVGGEDEQANEECDPL

AFLRWRRRPAAFIFLDPNCIVQKDGQTLIQQAPSFKLEPLKKKTEEAAPLSMEPVVLNET

IISPKKPAVVASLAEKANKNGKGGRSRYSYGRLKKGGF

>SG-AL_contig_8827_1_-3_525_806

MYVHFNIWVHSGIFVPRDVYCSFVGHQFDLFHFIFVSGFAVFAVLFWIGCYMLLGFAVWD

LILKVKKIYKVQRPELRDQQLDDLAGDFILSGVM

>SG-AL_contig_8828_1_-3_3_518

HQVLQAVYDGDTGWHLYAIHFTASRPFQILIVGEKYEETFAGESEIFLDFVKFNHSSCGK

KGAVVITPPTMAPDREMSSDSRVAGTLTPPTLLTPSSPDHSLTTDWTAEVDTTDRDATPQ

GTHPTDIESDGVRRTTEEGSEVSGAESEKRGDDDDGDGDVGGVVVLVVCVCG

>SG-AL_contig_8829_1_-2_23_532

MAALVRIFGRQSQCLLRQQGFFMQKRVAQASSTAYEEMRQYWDKNRQLNRPLSPFLTVYK

PHLAMMTSLTHRVTGVVMQCSVALVSITLLALPGDFTHYLELVKSLSLGPVIIYGAKIIL

AFPVTYHFLNGIRHLAWDAGVGFGLKTLYKSGYFVMGLTAVVVSYFVFGL

>SG-AL_contig_8830_1_+1_961_1233

MDVLHKARWCKNDQHTVSNKDVFAGCVSENHQWKTTQVYTKKCLRPVPTQILLLPRGRHK

MERQTKYIKIQDTYLVRKTRKAYAIQTTQTY

>SG-AL_contig_8831_1_+1_1_162

FIYLFIILHCRTGLFVFVGGGLLFVVCVPQLNTLGIAQYMCTLSYESETTSTFG

>SG-AL_contig_8832_1_-3_3_86

RVKCLSVIFHHHAVWGHQWKCKSICDLS

>SG-AL_contig_8833_1_+1_451_627

MIFSLPPFTNGQTILTVSFFHWEKNLSSRRESRFSVYECHYDLEKASDKIKLDAKRGQW

>SG-AL_contig_8834_1_+2_2_202

CLCVCVCVCVCVCVRARVRVHVCVCTRVYVCACVSACVCVCVCVHACSYVCMDVPCFIFS

ISSYAYD

>SG-AL_contig_8835_1_-2_2_163

LSHMLGTHGHPLQPDLCPLQSKPMLYFDYTYIIVCHHFLFVLLSSVCFRFVVCC

>SG-AL_contig_8836_1_+2_83_1552

MCIYVSASVSVCVFLSLYLCLSISLSVCVCLFLYRVSISICVSLSICVSLSLYLCLSLYL

CLSISLSVSLYLSISVYLSICVSLSLYMCVSLYLSLYLSVCVSLCLCLSVCLSVCLSISL

SLCVCVSLYLSLYLSMCVCLSVCLSLYVCLSLCLPLYVCVSLSVSLSLFMCVCVSLSICL

SISLCVCVSLSLSVYLSMCVCVCVCVCLYLTLYLSLSLCLSICLSISGYVCVSLSVSLSL

SMCVCLSISLSISLCVCVSLSMCVSLSICLSISLYVCVCLSICLSISLCVCMSLYLSICV

CFSICLSLSLSVRLSLYLSLYLSMCVCVSLSLYVCVSLYLFLYLCVYVCVYLFMCVCVSL

FLSLCVCVSLCLSISLCVCVSLSLSMCVSLSIGLSLYVCVSLYRSISLCVCFSLSRSLYL

SLCVCVCLSLCASLSLRVCVSLYLSLYLSLCVCVSLCLSISLCVCVSLSLSLCVCVSLYR

SLYLCVCVSL

>SG-AL_contig_8837_1_-1_556_798

MLCAVHLVQCTVHLVQCGVHLVQCTVHIVQCFAYCAVYFRLQTLYFSRIVALGPIGSVLQ

LVLAILHIYRLKACNKQSKMH

>SG-AL_contig_8838_1_-2_314_1279

MAYGCQSSAVPAVSLLLVLVVVGGECDAGRLITTQQVLPSACLEWFTQRQQDCPIKADWD

PHSWDNRWGVLNETCCSNLNGTVLHCVRNGTSNFPACLPDQKVPAGHAGRLVRDKEGNPQ

FEVVPCNSSLAYQSEDRVSSSVTFPYCLGHRSVCHKEKGQQLLCRGGPTADDWCTCSPGY

KPNSPHCVGIFTNDGDCWCQELSCAAGYTAVAIPTPTPTRLCASLTNGSLTTHFGCLKDS

SVTTLPPTPTGSDTDRFQPSKRGNTSSSDSPGGPSIADTTEADTTLRVAHVYMVIVPQSM

FLLLLGSMVVLANCRSDRVTDW

>SG-AL_contig_8839_1_+1_1_189

KLVEMSVCLSVCLSVCLSLSLSLSLSLSLSVIPGGFLLCFVLISLVLTNAYLPCLLHAYI

CVE

>SG-AL_contig_8840_1_-1_403_564

MCMMHSCFNVSTLVYSFVYYMLSCSLPSQCCYCEYSSWNTITNTIKPKQVSCLL

>SG-AL_contig_8841_1_-1_1_189

THTQSTAHNCTYQLSHTPGRFRYFLLHSLMNETINKPNKLKTKTPATKNLNHAKHFAVVH

EKR

>SG-AL_contig_8842_1_-1_1060_1389

MENVLYFCVWINKLITMYVNAGMHAHPHTHAQTRTCAHALTHTHTEHSTAQHSTAQHSTA

QHSTAQHNTTQHNTTQILYDQLCMIFSFVCNKMFWSNDNGVHAIVFFVHE

>SG-AL_contig_8843_1_+1_1_300

GGGGQICAKLQQHEDVNAKEMPHFQMPLSGGGKKNSTVKMEKSASAVTEPDVNKCLDSVR

HMPSIYLYVYFQHFCEWTFPKLDSKLVGYGNLGSLHVSQA

>SG-AL_contig_8845_1_+2_2_352

GSVDRAVDSRSDVLSSIPACNIKTGLFTAHLHAPIILVVSVFRNPVLSPRPSPPFKTLPG

HAVLRRLQGICQIITGWSVYGIVLSAMNPPLNKLNSKLCTTRNPQLSSADGKFLLCV

>SG-AL_contig_8846_1_+3_291_809

MLISRPTLIEISNFFFFRHLFCQGLYTLTSFLSWYINISSMSLTQNTRFLTHTIVCLAKM

LFIRFPSLNFWTCRAVRDILSRGARQVLCVFGQPAVLWKVRFCGWFVRKVAQPSVPFSRN

LTAIIVHARSVGIHYPATASHKIHGIMVVLVSIIIFSNQGSCLQFISPVKLFY

>SG-AL_contig_8847_1_-3_1167_1382

MLRRISSLHSSDLGRHGDRMTKIVSSNSSCKCRYALDGISCAVFIGTYCHFSLSVWFVMI

VAFAVNDESWHS

>SG-AL_contig_8848_1_-3_174_434

MVKSVSEVLGNMGACYVFLLIFINVQGMDNLSVIQSCQVKFIYHVSCFTLWTLLIVKFDH

HCFKLVCFYCQVWSSLFQTLWRFLYQV

>SG-AL_contig_8849_1_+1_3037_3315

MCYMCCILPSIICLSRRDINKQFRQFLKIHIFLKIIHMLNRRDINKQLRQFQKVSIFLKQ

LIKIPCHTCKIQVDLHHCLHKSIYKAKPTTPSG

>SG-AL_contig_8850_1_+2_119_226

MAFTPSHTYEQCPSSFHVNLNHCSFKSQRLFMQNPM

>SG-AL_contig_8851_1_-2_179_313

MVWFEILASQKVASLYGFIHGVSDVHVDLGKVNSICRKYQLKKGH

>SG-AL_contig_8852_1_+2_149_202

MHNNKNYILLINKTPTSS

>SG-AL_contig_8853_1_+3_27_242

MFQTHSSWFFNFTPNQLVNVKINMEPAVHTAYQSHIHFILKIHDMRPHRAEKWLHRGELG

SPLCSKDYELIV

>SG-AL_contig_8854_1_+1_751_1422

MLFTMHKPELEMSRGRFENKNKPKAFIKYIENMAGVDKSDQMISYFPMHRKSSKWWKKPF

FHLLTLCTIQTMILLNKHRRSRDLKQMPLEKVVKSLLLDLPSVRLVLPSSGADSPSLPMS

RLEAQHFLSDIPPSKNDTKPRKGCHVCCAKMKASGSSREDLKNKRLRTHLQSSQCKISMH

NVALCATPCFELYHTKKNYKLQIHFTVCVNMICSIHVLGVGVSM

>SG-AL_contig_8855_1_+3_234_383

MALLISLTSDIFMVPRHVTCKTVNGGHWFQCDNHDTQSEQNKSINEIIIR

>SG-AL_contig_8856_1_-2_2_160

NGRVGSDLGIGACTSSGCSLVNCVNGSHSWLQSISDFHNNDGYLYNTLSRKLL

>SG-AL_contig_8857_1_+2_464_922

MYHWNVNSFVFDVYYLINQFNQEIEVGIFRKNHVLLFHWQHLPDLLHFCTRVCVCARRER

ERKKEIYICCLPVCCSSECGSWSVCSYLIVVMNSYSYMHTTCDFMESAVCHCSPEIKKSS

GMVIRHVILCLQLKVVNSNTLIYSDSLFLIIKP

>SG-AL_contig_8858_1_-2_272_565

MLLDLQSVLMDVMLIASANRSPRVCTRLDWHSPYLYCTVTVQLYLHCTVMVLMYLYCTVT

ILMYCTPSTSVLLQYYCTVLIVQAYCTYSTSVLYCTVL

>SG-AL_contig_8859_1_-1_139_393

MRCPAVAAMLLVFVIVVMMSPMTSATRHYELTRDDDDIRTTLEDEDVPNQDGGPSPAERL

TLYILRQKCFHWLSRGVSPLPRFCF

>SG-AL_contig_8860_1_-3_93_1970

MQDFAVVEVVFFPSEGSNLTPAHHCSDFRFKTYAPVAFRYFRDLFGIQPEDFMVSLCADP

LQELSNPGASGSIFYCTHDDEFIIKTVQHKEADFLQKLLPGYFLNISQNKRTLLPKFYGL

YCYQCGGKNIRFVVMNNLLPSTVHLHEKYDLKGSTYKRKASKHERAKSSPTLKDLDFLDL

HHEGLLLERDTYEALIKTLQRDCRVLESFKIMDYSLLVGVYNLDQAAREKAAQGPQSGTD

NTTIPEPAGGTGENRPARTQSLSRGKSFKTRIGQYSTPMESIQANAVDKTEDEDVPPGGI

PARNSKGERLLLYLGIIDILQSFRLKKRLEHTMKAMVIDGDTISVHRPSFYAQRFLNFMA

SRVFKKVPTALKHSPSKKKGLQGRLRTQTLNEEMTSQQSKMTEKAARAISFSDEQAVATG

YGGKPDLVADTQGLAASPVAGRLSGERQYQSLSSINTNGTTDSRRSEMMTLATYQSEEAM

GAGLRYPSTTLRYSPPLSLSESTPTHTDHTEGTPSFTASSPSCSSDVLDINPTGESTLTT

ATLHPHHRSSSGHSPADTPTASPLLTSVSQSQSRSSSGQSPSEAPTIAPQSTAAVAPLPV

LKTDSSITAETELSETSDDKDEVTYL

>SG-AL_contig_8861_1_-2_179_295

MAHTCQYYIGYTLIALAVERNFEIILSFCIIVRIVTVPN

>SG-AL_contig_8862_1_-1_193_1539

MVETEETVGEVIEVITDMPAMQEEKPSEVEVALETAIEETPEDLQVQITPEQPIEEVIEF

LMEETVSKDIQPAVVQVAADEIPEETPKDFEVAETEETTEEVIEVITDMPAMEEEKPSEV

EVAFETVEETPEDITIQVESQQPTQDTVQETKETLTVGEETPFEVDLVLVEAPTQDTEAL

ISPQTEEAAEQPSDELSEFVVVEKEELAEVDIEVMPEDTKPEDITIEVSPEQAPGEIIEE

VRERITIDDKQVSRTETEESVSETTLEGLPDQIDLHKTEETVTETMAEVISIEEEKPTEV

STFEMEMDVPTDLPTEVQLESLLETETTTITDVAEVIEVEVKQKEEEMVMPNFITSLKDL

TVTEGDNVTLHVSFVAGPAPTVTWFLDDEPIESSEDFQVTTEHNFSKLYIPEILSDDEGT

YKVQVENQVGSCVSSACIFVKVPEKEIEK

>SG-AL_contig_8863_1_+2_1127_1654

MNGACTLTMTVPFHNINRANHPTLLHHALEDLLCEVFHLQLFSVLGHGHSISPMSQLLSL

LGHGHSVSPMSHNAMTTWYPQCLQLLSLLGHGHSVSPMSQQRHGHTVSLLPQNYHGHLVS

PMLQQTWSLGIPDVTTTPWSLGIPMSQHYGHSVSPMFPILVFAWPWLLVISNIATT

>SG-AL_contig_8864_1_-2_2_1414

PNAPSAGQPTYNQPGFGAPPAGGYPAQPQPGGYPQQPAAPGGYGQPPAGYGSAPGGYPSG

PAPNQYGAPQGGQYPASTQPGYGAPPQGGQYGAPGQYGAPPGGQYGTGGAQPQAGGYGAP

ATSGAPGYGQGPGYSAAPGGQFQGSVPSAASYKIKEEGTLKPYPRFNAEDDVQKLRKAMK

GLGTDEKAIVDILGYRTSDQRQNLKMMYKTCFGRDLMQDLKSELGGRFEDAVVALMYRSD

EYDAYELRRAMRGMGTDEAALIEILCTRTNAQIHAFTNAYKMMYGRKVEDDIRSETSGHF

RKLLISMSVGGRMEQQSVDLQKAQTDAQRVYQAGEKRWGTDENVFNMVLASQSFEQLRAV

FDAYGKVSGKDIEHAIKSEMSGDLSSGMLTIVRMIRNKHAYFAERLYHSMKGMGTDDRTL

IRVVVSRCEVDMKQIKQAFQNQYGQSLEAFVRDDISGDYRRLMMALIGGMP

>SG-AL_contig_8865_1_-3_3_314

TDLPSALACISWGGSLSDDFLLREVLRGLAARESKQPRRIPKWDLALVLGALRLLPFEPL

DMVPFSLLTCKTAFLPLWPPAAIVARSRLLAEPKTTSASTPTAT

>SG-AL_contig_8866_1_+2_2690_2899

MTSRLEVCGCDILCGFGGTRSGAIAILLFIGRGFRGLIPNGFLEVLEDLGGDVSSLVAAV

CHLLGAQRLW

>SG-AL_contig_8867_1_-2_365_427

MTNYIGTQHVAPWLECDKYIF

>SG-AL_contig_8868_1_-2_2_217

PSLPCSHGDLGFRGFGGVSDLESQNKQCEDSNTGAVQNMEQLTTTKNGGGGDGVCMCVWW

RVALIFIKRSSS

>SG-AL_contig_8869_1_-3_3_293

VRCLAHTVQALFCCTPSLVSGHAGYFSVSISHRTLTWLIGSLTCLFDGVQALAYMLTKKI

GKISTLYPPGATRFQSRPSDWKSNILTSRLSHSSCHG

>SG-AL_contig_8870_1_+3_3_353

GRTPTFQTDCTSLSLTLGKNIAHVVSNPCSCESIHDPVQGLQKVKQRGRGDGHPAHNAGP

ERNGSLPPHSPTASGIWDNFLEGTRSGRSEKKKSPTDEPLFLSFKVNEHRVLDLCKC

>SG-AL_contig_8871_1_+3_255_398

MCTHTHTHTHVRAHTHTAFLRSEPTMFKNTHQNNTTDKVIELQPQTDF

>SG-AL_contig_8872_1_+1_1804_2022

MTATAIKSNHRQSLNDRNPNHRQSFNDKILKTNLCLSLTHTHTHTHTHKLPIGNSHSLGV

FEHLQAESFIIYK

>SG-AL_contig_8873_1_+2_104_310

MHQPTHASYPVPCCSDRLNILCVRKKKPSINIFSHICMIIMKTDASPIFMRKQDKKEKKR

QKLKRKEKE

>SG-AL_contig_8874_1_-1_28_678

MQTIKIAGRFGRNGLATRLFRNFSTSEDKVYELRLYQMKPERYRESLDLLSKYWTVRTAH

SKDVGSWMSEIGGICETVHLWEYDSLSDRMKRRQNVANDTVWRQEFLPKFLEKSLKLTCL

LLKPHPGTQVITTFPPSETAVYELVNSKPDVLPQMTEDDEIVVGQFETIYGQAEGEFTLF

RYTDPDVACERAYQRRKAQNLKGLSRFLVPCHWSPLK

>SG-AL_contig_8875_1_-2_2_136

KLRICRHCVSPHSSRVFSCSQELSLLFCDVIHRAFYEYSCRSLSL

>SG-AL_contig_8876_1_+1_517_795

MAEISRLPAPPLREVSRLSTPSLKKVTHLPECTFSLKEVSRHPAPSLKKVSRLPAPSLKE

VSQLPVIIIIIIIIIIRTHTHTHTHTHTPEQQQ

>SG-AL_contig_8877_1_-3_99_353

MDLPCEIRAALSRGELVAAVQHHPFLYCMLGVLCFHKPPNTDMAYRIFNTHVLHVYSLKR

GSGTSRSAHIMTWEKWRFLLKSLFS

>SG-AL_contig_8878_1_-2_338_529

MLLNTGLFVAYLCTCHPNSVSTVVLNRIYEFLPFEDLTCPGLNGKVGIILATSHLWETTG

FRRG

>SG-AL_contig_8879_1_+1_943_1221

MHRPVIRMPIKPKFSHKPLTADLAFSLILSLPHPVQKSGVPACEALILCSVYMWHTVLSL

CPVGLCCDPGFNDRGFVWILIAYFFFLGALCGF

>SG-AL_contig_8880_1_-1_46_1038

MKKLACVLHTFETLPRGWMGGARNLLVGHSMCCTSVRYEKPGENCRTFSSQKLSNPQYCI

NLVKNHDYENYLCCLLLPKQLHRAAFAIRAFNVEVAQVRDVVSEKMIGAMRMQFWKDAVE

NLMEGTPPQTPVAVELAGAARYFKLSKRRLKNIVEARAEQLDTDEFRTIEDVEKYAENTA

SSLHYLMLECLGVKNVHADHAASHLGKAQGLTTVLRAIPYHAQRRRVYLPLDLIIKHKIS

QEDVIRGKSEQNLLDAVYDVASVAHGHLQTARSFQKDVPKDAFVAFLNTLPCDQYLQKLE

SVNFNVFDPKLQGKNSTLALQLWLQKQRRKY

>SG-AL_contig_8881_1_+1_1_225

ERVFVYNYLRTNRKIIIKKEEQTNLVVKLYQQSTFYGTVRYVLCNNNLNMGHAHIDSAIF

TGLNRLDKADSFDSI

>SG-AL_contig_8882_1_+3_12_227

MSTEAASYFGNFLSDRSVRLLFHYFIYMDTLTIILPHKQTYGARAIILTSVTIHLTDFNG

NKQTKKVVNKTL

>SG-AL_contig_8883_1_+1_1081_1347

MSQKPQASNCTTLDLFPVLTYRWVFFSRSEHCIPRYMYTHCLAHTMQPLLCTPSSVGVHA

RCFCVSISHLCFHKPPSADMVYTILYLHI

>SG-AL_contig_8884_1_-3_3_236

LNSQPDTQLDRLLYIPTGWLAGQPPNSLSPCSALDFHRLSLAISLMGYLERPRPGFKVVG

SYCGHLSAFGECCDSGTK

>SG-AL_contig_8885_1_+3_3_131

TSSLIKTQSLRNTCQTLNNSIQSVRYICTTTPLNSLTGLRQIN

>SG-AL_contig_8886_1_-1_1168_1299

MVLCEPLLDYFHSCLSLNVTEQSYMSKHPYLCLGESVLLFHLIN

>SG-AL_contig_8887_1_-1_85_195

MVVDSVELIHSPFTGFSLAASLDFTLATKRVGFGLAR

>SG-AL_contig_8888_1_+1_1_66

HLVHKGEAYVKTSNVLSCSFCS

>SG-AL_contig_8889_1_+2_179_382

MSPVSTRLSQQIYGTICLEMGQLVLEMYRTICPGIHETICPVNMGKFVLKHIEMCRTICP

RKILNQLS

>SG-AL_contig_8890_1_-1_31_1026

MSPMMNPHLQMGLMIMLVLHIHAEKKEPFTGVLAKQLNSLSESPSSTPSTDPSKRPSILG

DPVTAQANIVLSNLKQQLNQVPVDEEAKKYLSTSSNPAVKTIMAKFMQNARYLSLSLLVN

LGQMVMSMQANSHQEGPMAQAAAAAASSIARHSGGSLLKTPGPGKGLLGDVPRPNPNPAT

MRFMKTLEAVASQKLGGQNPAFISALMTNINASPAIGGLEKSSALGGEKRSLLGEPPQHL

KQQKEGGSGGGMGGPGGRGMGNMGNMGGMGNNMGGMGNMGGMGNMDMGSGMGNMGGMGSG

MGNMGGMDGMGNMGMGGGGMGNMGGGMGNMGG

>SG-AL_contig_8891_1_-2_2_61

GLSKPLCTLAVVMLSFEEKK

>SG-AL_contig_8892_1_+3_162_293

MAYRIFNMVSDVLHACTHGHWQVCPYVDLGDQKNIHPLTTRQGV

>SG-AL_contig_8893_1_+3_3_179

VCVCVQHVCVCVCNTCAVCERETCLTAQILFHKFRPSHVNGFQLVLSNLLSCKCLSMCM

>SG-AL_contig_8894_1_-2_2_103

NKDGRVFMFKLVKLLCFFWLQMLNRSYMYILSPF

>SG-AL_contig_8895_1_-3_609_1205

MPSSSSHRVALGAHAHAGASSSRLRPAPANLNLSRGVGAAGGVGQLSPVQASVQLGQPVL

LNAPSQLEVRSDYRHGAAATLHGSPIHHYHLVPAHQQHQMAALTAATAATVGQYGPFSPS

VPPPPAHQSPRHVQYTAHPLPAHMHPVLHTSSIPSFPSAQVHPHYTGGASYLNSAPTSAG

VYTTYQIGPIKARPYQYFA

>SG-AL_contig_8896_1_+1_46_255

MRTCVCVCVCVHARARARVCCVPTKAFVSSRKQNFINKTTQQPFTVRLTNVELTRFIFIS

KTRKEKSTNH

>SG-AL_contig_8897_1_+1_1_114

SIPLSFFLAIQQTFTHPPSPLHFMMYYLSVCYFALVTR

>SG-AL_contig_8898_1_+1_1_159

IKWKGWRPQHSLILLALICHELFFCRFFVFNVVVSDGLNVVTALIWPRWLAGL

>SG-AL_contig_8899_1_+1_1_216

RSKVKKKKRKKSTTIQQIFQQWQCCSCILKQIKTRQPTTLFKSFKQKVTNFHQDEEDSVI

IPILQFLLSPQA

>SG-AL_contig_8900_1_+1_31_234

MITEHLSNIVCSSRCSHFGRSVFNMKTLPWAVGCARKEDIHFSSILKNKQTISQLVNVNM

VSGEIPAF

>SG-AL_contig_8901_1_-3_3_161

TRECLSLVAVPFAVYILDKTGGSNGLTERVLVRLKTFFRTKTLGFCCLNKVSE

>SG-AL_contig_8902_1_+2_137_388

MCVQKSHRHICYKEREEGYGAHRHLYKLQCLMLTHYLQLSFRLLVLLTGLRCVELKCTEY

CYSWRTHSFHLSSKHEHVFPLHHK

>SG-AL_contig_8903_1_-1_58_174

MYRPKRLAHSKALANQNGSYKVSIFDHDSIHRILMTGTQ

>SG-AL_contig_8904_1_+2_128_217

MILKNTVDLLFINCCMCVQISVSNVCLLFV

>SG-AL_contig_8905_1_-2_2_133

FVVSSEGQDSIQNITQGLGEEEWEKIPSFSRNQTWALSLPRQVC

>SG-AL_contig_8906_1_-1_1_297

HPIIHSERTPKKRSLQGACDSWFAPSIDLFVTKFNKRLPICISPAQDPEATAVDTLSLDW

APHSKKARIEWARMILRASFWQAHLWFPDLRSLSHVLPL

>SG-AL_contig_8907_1_-1_1_501

LSRGHPIIHTERTPKKRSLQGACDSWFVPLSDLFATKFNKRLPICVSPAQDPEATAVDAL

SLDWAPLSKKARIERARMILRAPFWQAHPWFPDLRSLSHVPPLELTPSPDLLRQPRSGIT

PANPGMLRRHARLQCGSSCQLCTTPPLGPGSHEHHPRPRAASSALPA

>SG-AL_contig_8908_1_-3_3_98

PTWQTNRGYKSQNKRRLRLFFFFNFRLKTFYH

>SG-AL_contig_8909_1_-1_1_129

EFVEEGFIALQIWLTDRSIDYSHAASSQSGTSQCLCDHPFPQL

>SG-AL_contig_8910_1_-1_13_813

MPGGLFAISRKWFIELGTYDPELRYWGGENLELSFKTWMCGGSVVIAQCSHIGHIFRTVN

PIKWTSSLGTRNYVRVAMVWLDEYKNHYFERTGYRLGDYGDISGRMAIRERLQCKPFSWY

VDNVYPELKEQVVFDATYAGEIKHELTGLCIDKMDKTRHGPALFSCHRQGGNQFWYLTED

RYLQIEDQYICETAEMSGTVKLCQKEGREEWSYNPVSRQLIHVKSGQCLQKNTTKEHSQE

LVLKPCQENSSLQTWKLERRLELNEIR

>SG-AL_contig_8911_1_-3_42_611

MKLWVAALLLAFACYIPCGAEEETEARLLVSKNILNLYLVEEKDLTVEYRIFNVGGSSAL

NVQLKDDSFPEGDFAVVRGNLQVSWERIAPNSNVTHAVILRPLKSGYFNFTSAEVSYLAT

EGDSDRQVGYSSAPGEGGIVPLKDFDRRFSPHVLDWLVFAIMTLPSLGIPFLLWYGSKSK

YDTPKAKKGN

>SG-AL_contig_8912_1_-3_3_149

YLCFVNISICVIMCIYLLICYTLECYAVCHFGNSKGDKWIRYLYEMLRF

>SG-AL_contig_8913_1_-1_106_1590

MHRPKTAAAILGRTAECGRVHQLEFEPTRCHNLESHLPPKRSKPPPWQKNIDQPLVPFVV

GPGYILSRSKTKYAVTIKDEFFDVTQEVDHKKKQVDTERDSLIVQLQQQISDLTLYLEEE

RLNHRQTKQRAEEFLRDRLEQANAQHQEHIRDLEGEHRDEIIRQKAMHDSDFTQYQSSTE

NQIHRLTKEVEFLQGAFESYKSTLHQEMHDKWKVKEEDLRRECEQNQHSAMHDIKTQLIK

ERNSERSSAAKEHHKAIEALRKEHKKELDALTRRFSNAAADIQRLKKTTADLEEAKIELQ

QIKQSYDETCQQLANTTRSLTDTKVRLMDFEQRFQEKVMEVDDKYRLKLQDMMTQNTELK

RLYVQKCGELYDEKTITELRTMEEVRSAKAAMESLIRSKHKANVSIAMTPDVESPMRSAK

VRPSSAPGTKNEKERAHWSAGETDHLNKQQELIKPDHVIPPEDPETERIRQELLATEVRE

VTKEDLLSSFEVSPL

>SG-AL_contig_8914_1_+2_53_196

MEVGSGGGGLQSQSSKFVLNTINTKIIQKSLQPQNFYFEYKITLSILH

>SG-AL_contig_8915_1_-1_496_765

MRSSLCRLSLYSELLSAICRDLLIIVRWNAKLFIFHSSFYGRYWLTSYCRMKFGDRDFSV

HPVLTASIACLMTVYLDYSQQKRCLHFHFC

>SG-AL_contig_8916_1_+1_394_588

MHLLHSAIHRLKNEIRFLQLSLGEHFPDKSSRILPYDHHILTTHISKLTISIYVYASKLL

FLREV

>SG-AL_contig_8917_1_+3_3_164

NNNKNITDKVESSDSDVISLVTTSRVSHRKKSILTINRLFLFLGFAPVCTFSVR

>SG-AL_contig_8918_1_+1_199_423

MLFTTALPGVSLFCSCIGCCEAEPLELAGVWSGRDTDNPGKDTIPSLLGSFHSQHQATAH

LVVCGKNVSWIVCCH

>SG-AL_contig_8919_1_+3_18_224

MHESIRDACPCIHVKNKNHNHKTLQVKLFCRGKTVFSNMKTINWTNEERPLKNRKLITSN

IPRSQRGSS

>SG-AL_contig_8920_1_+3_3_119

NAHTHTHSYTHTVTRNPCIQISLKTINVSKQDRPIQYCA

>SG-AL_contig_8921_1_+2_152_376

MFHNRRIDVTFQRNDKSTYNSFLFLADLLPKFKYQIITLQSKLNMVITTHSEKHQFRHFF

LTLYFHLSKDFFSSI

>SG-AL_contig_8922_1_-1_109_294

MLCILRQAMLMTLGHLQHDYHNCLEKRHMIKFNIGITQSTLLVTGDKDSFRIQITAMKTC

LQ

>SG-AL_contig_8923_1_-3_3_662

LSPASLSLSLSLSLSLSLSLSLSLSLSFISLIRPYNLAFFVPTFFSCLFSLSPSFLSLCA

RLFSLFLSLYLFFFYFSHLSVFQIHTLFICLIFFPFSIFLLAGHLSFYFSCSFSFSFSIR

FLSFPIFISLSLRSFFFSSLPSLLLRPFSLFLLPFFPLFVFLCHFSTCPSLVTFSLFLRL

SVSLYSHILPYFFFIFSVLSPCPFKVFCVDLSIFLYKDVC

>SG-AL_contig_8924_1_+1_2350_2538

MCVCVCICAHAHECMHVSDFDLDFHFKTYYSRITHPISKYLGNNSNTTYQISNCVGDNVY

PLL

>SG-AL_contig_8925_1_-2_746_952

MFIERNLGKNSCLCQCCAESFILIFPLLCIMQSFQLLAVRLNKQTSNNIIIFTEQNLNEH

DQEVCQSET

>SG-AL_contig_8926_1_-3_105_776

MIQYIKAVADDGDEPLELPVEEDGTILLTTLSAQFPGSCGLKYRNPDTGTMRGIRLSDGR

LYPPDKDWGKQVFVCVFPKVEGKSSPSASEHPVVKPVKSSSRQKCSDLIVLGLPWKSTED

DLRNYFSQFGDLLMVQVKKDPKTGQSKGFGFVRFAEYESQLKCMAQRHMIDGRWCDVRIP

NSKEGAQQMMNRKVFVGRCTEDMTAEDLRQYFSKFGEVVDVFIP

>SG-AL_contig_8927_1_+3_2448_2741

MLCAYLSLRGRRKKKNKGNALYTHTFTEGPFFFKEPKVKFLGLRTWLIITFTVINMSSTG

CNDSVCFLTRLHVDMNKAEINVRMCICVLHVDMNKAEI

>SG-AL_contig_8928_1_-3_3_233

FIYLFVYYLSFDGKLFSISCKILFSTTLNYGQVVRYFFKRIQPGTTPVLLWIFCIVCEVQ

AAHSTLIYCIISSRRVA

>SG-AL_contig_8929_1_+3_2604_2933

MYFYLKSCDKLHVTYRPCRFIALLNVSLGSCLSLCSIQLLSHKCYHYCVGVNLFFTFMTD

DVLNSVSVSFCSVGYCLFVASWGNTSGDMEIASLSCLVSCLCEREIFSHT

>SG-AL_contig_8930_1_-2_2_298

STSCKYNTTHNHHHYTYHNHHHYTYHHHYTYYYHDTYHHHYTYHCHCTNHHHHYSYYHRY

SYYHHYTYNHHYTYYHCHYIYYHHNCCSSTSCKYNTTHN

>SG-AL_contig_8931_1_-1_28_624

MAYLTEISKRERQNAVIIEKLELELKGKNNDKDEEIQKKNDVIKRLQAELHQIEKFSEEH

IRRVRADAEKQEAADEKNSDGKRQKLQSEVGQLRSQLQNATLEHRETEQELRRKKFKIET

EVENWIQRYDQELGERQDEFEEIDAIYTEEKKQLRELEERFNTLEKEYVGIMEERRLARE

KREKAERELAMLVKAATTI

>SG-AL_contig_8932_1_+2_119_325

MGLSSRNSSSAHGANVQHSLPEYISWPVAEQTLGSILRSLNQDNLRKISLVIFFIFPTMA

LFCGSSQEI

>SG-AL_contig_8933_1_-3_3_275

LRLSPLPDPSQGHQEGERRKGTHDPHSALLAGSTMVSGPEVPISRPTPGADPEPGPPQAT

SLRDHPCESGDATPARLASVRQLLPTLNDPP

>SG-AL_contig_8934_1_-1_10_219

MRKVICYSHKPVYGEIVVTACLLLKSFSVWQIDENVRIVLFQREAGGGVESEYSLVLFDC

FKDTGSLVTK

>SG-AL_contig_8935_1_-3_63_242

MNLVMCLTDSSFDWSQSASHCIKVSWWFHSFIQRDHVCTSSHQRVSVSANKVVLQTSKKK

>SG-AL_contig_8936_1_-2_83_1978

MTRTVQKKKGKTEEEIKVVIAQHDHKKEKAAVDKVFVARLARIFKIIVPGWISMESWYLF

LVAATMIARTYADVWMIQNGTAIESAIIGRDMALFKVHLFKFMYAMPAISVVNNMLKYGL

NELKLRFRTQLSRYLYSRYLRGFTFYKMSNLDNRIANADQLLTQDVEKFCDSVAELYSNL

SKPILDVVLYTVKLTGAIGAQGPAYMLLYLAVSGLVLTRLRRPIGRLTVAEQKLEGEYRY

VNSRLITNSEEIAFYQGQRREQHIIIATFNKLVKHLRNFIFFRLQMGFLDNIIAKYVATV

VGYLVVSRPFLDLTHPRHMTSSHSELLEDYYKSGRMLVKMAEAIGRIVLAGREMTRLAGF

TSRVTDLMRVLEDLNNGRYERTMVTSNGPREGAESSMKLKPGNGQLIFKDHVIKFEKVPL

VTPNGDVLIPELSFEVKAGMNVLVCGPNGCGKSSLFRILGELWPLFGGSLTKPEKGKLFY

IPQRPYMTVGTLRDQVTYPDSHEEQMRKGTKDEELEDILNKVQLGYILQREGGWESVQDW

MDVLSGGEKQRIAMARLFYHKPQFAILDECTSAVSVDVEGFMYQYSREVGITLFTVSHRK

SLWTHHEYYLKMDGRGHYEFRAITEDTSEFGS

>SG-AL_contig_8937_1_+3_552_764

MKLTVLLKLSDQHCGCLYCSGQVDAWIACKREHGGQPCHAVSVLKWNSIALYVVCVCACV

CVCVWGANKKN

>SG-AL_contig_8938_1_-3_117_467

MSVHHNYICHVMFLVHHTHSHTPHVMRYFLFYIVTSFMSSNVFCSTRSHPSCFLFVWFFF

VFFCFLFGFVSFYPVTASMTLFVVAFAVAIFLFCLYFFLTHTQTYNTMEFHFQTETV

>SG-AL_contig_8939_1_+2_170_319

MSQEMSLPYCNVIHGAFYKYHPSQSPSSYNYFLNTETTSHHGVVVNAMDN

>SG-AL_contig_8940_1_+3_3_260

FLSFFFLSLSLSLSLSLSLSLSLSLSLSSFSPVVSVFHWKTVLKYVEMTVLQYNIQAHKG

QNIWKNNVCVLMKIKLYSECIRIHGC

>SG-AL_contig_8941_1_+1_154_300

MRQKIQYKLDHNSSILVFKIIFLTKKKSYDHNWYTTETNMVKADSILVF

>SG-AL_contig_8942_1_+2_254_400

MDLPCEIRAAVSGENVSLQCNTTQFLFFFLCLRAILFTKELEVIIAYYK

>SG-AL_contig_8943_1_+1_1_207

HAVCIHVSISHRPRTWLTGSLTCIFDVTMFCNCCVAVLLYEGLCLYMNKSSPPSLSLSLS

LSLSLSLCI

>SG-AL_contig_8944_1_+1_325_495

MALNLEIFKSKRTSVHAGCIRVSISHRPRTWLTGSLTCIFDVLYAYTHEGGSGTSRS

>SG-AL_contig_8945_1_+3_3_137

MTGRSGSQTSAQNYYYYYYILFIAQPIIKAISGLSPHKVHQSQPR

>SG-AL_contig_8946_1_-2_2_232

ICCLCWFLWVRSLVQLIYSLPQHTQHTASTTFQKCSHPSLAFFFPLPATKIVNTTGLYGI

GYIICLSTFHVKCYYQL

>SG-AL_contig_8947_1_-3_264_449

MFIEFELCIFSPIKASSGPFLMVLESDNSWYEPSFSSLFNISLIQPFAHLSDFAHLSDFA

HL

>SG-AL_contig_8948_1_-2_89_220

MFELLFDILAFYFLCACLFSFLSSFLYFFSFFPLLTFDFCNSVN

>SG-AL_contig_8949_1_+1_97_189

MTTQINRHWRKRTDACSSSTATIALDKLRHK

>SG-AL_contig_8950_1_-1_19_393

MGGMRFTTFDLGGHRQARRVWKDYFPAVDGIVFLVDAHDRERFVEAKAELDSLLTDEQVA

NAPILVLGNKIDKPGAASEDEIRHWFGLHNLTTGKGTVSRSELPGRPLELFMCSVLKRQG

YGEGF

>SG-AL_contig_8951_1_-2_2_274

CSVGAYGIYSECRNAILKKTSETETEKIACLFTPATFYRSKYVSTIYGKILSTHLYHTEV

TVFRQKGVMNWFEWSVPIFEVTLIENVCCLI

>SG-AL_contig_8952_1_-1_382_792

MLCCMFHNTAGYGMVLDLTTDAGQGNMSVIGNVVLIVLFEDCRHVGSFPVIWEASFGQSF

PEDAHSFRILTGILSAPAALWGLFLLSSFQTLSSDISISVMVGKGSPGGDGILLVSSLVK

TDSYCLFSILALSLGAE

>SG-AL_contig_8953_1_+3_3_134

LPIYNIIQDIELSHYLKKIKSLSTCNTRNKCMGHETNANTAQHQ

>SG-AL_contig_8954_1_+2_227_466

MDCKKNTYNNIVLSLTEASDILNKCKRSLYIYTDIQHVPTVAEDVHYTMQKHQQKVNTFH

NKQRCENYIFVCATLSHKKL

>SG-AL_contig_8955_1_+3_141_287

MYNLRAITREDSPISLGLEKVWSGVSSAVYCVTHLVGRDYSPHASGILG

>SG-AL_contig_8956_1_+1_13_255

MDRQGRKKQTTVKDGKAAETKRKFLAQNFQKVGILFILKEPLASPRGAICLQILLKMFYS

LPTPWANQTSPNPFSVKIFLT

>SG-AL_contig_8957_1_+3_147_395

MQILQNMFVLFKQYISNNNKIYMHYAVTKNRIFKNCCHVLFLQIYPFCSTTQSENEATIY

NRRSYPWQPNGGTRLLSVLILAH

>SG-AL_contig_8958_1_-1_142_1038

MSFFIGDLCNEKDVLAALERVACVFHCASPSPLSNNRALFHKVNVEGTKLLISLCKKSGV

KRLVLTSSASVVYEGKDIQAGKEEELPYAAKPVDAYTETKILQEQTVLAANSEDFHTVAV

RPHGIFGPRDPHLVPTTVGLARQGKFKFIIGDGKNLVDFTYVDNVVHGHILASEKLTKDS

PVCGKAYHITNDEPVFFWTFMSRVVEGLGYEAPYIKLPYLLVYVIAVLLMVISKLISPFF

SFTPTFTPMKVALAGTHHFYLCDRAKKDMGYKPRVSLDEGIQCTVQSFDHLKNGGDGDK

>SG-AL_contig_8959_1_+1_1_294

FFFKGLDVSLLFFSHSSLLKIKTVQANKMHKNTQIKQIKCVKILSVKHQVTFFSGEFLHD

KWAGYSISPEHTITFIFPIQMYSPTIRMLVLQPMGFLW

>SG-AL_contig_8960_1_-3_3_161

PPLQRRPVLVVLPSSRRSLGKPAPPLDIEKRRRDWISKINVCRPVACLLLKCM

>SG-AL_contig_8961_1_+3_12_440

MHTHLSMSLQRHCQSKSCRTVCAGSNGTCYFPVVSVHFVELFPQFLLRKRGVTGSFSSMQ

PGIRFQQCAEVPIESGNVVLCLVHASQHFFFLFLPPDLCFQLFLVKFFFRYCPPLVISEP

FRRCTQWSSFAVFVNVDLKRHSG

>SG-AL_contig_8962_1_-2_2_82

IINYYWNFFIQHLYVRSFKHQSCTHTV

>SG-AL_contig_8963_1_-2_2_130

SAFQLFLKLNQIFLIMMEAFLRCCRSVNFLKIRKTDFLLLMYH

>SG-AL_contig_8964_1_-3_171_296

MYIGLIKDNDTDDRVKPFQSMLCKITSPLEICSNSCSSVTCF

>SG-AL_contig_8965_1_-1_391_666

MIHGHDTYIYIQSCMYVSPDMLMYYSLTVKENHLCKGKRFMSVHVVLLWVFTDAWMCMCT

ESGVCDSQLLCLFRYGYCVVLLSSECICPCVC

>SG-AL_contig_8966_1_-2_47_259

MVRRLTHRSLPVRVMTSPLSDTDASELGLAIHDHVIVADYNAVLGLERRVVIALWCGWYD

DTVHAMSRCTG

>SG-AL_contig_8967_1_-1_139_1569

MEKCLQCGKRGGADVIRRCSSCKMAFYCSRDCQKRHWSHHKVACRKTRDKSLHNMDLNSG

HQTAETGADEKVARKTTAPSVPDSGKCSDGPLETCNKCGMQRSGMKKCGVCLEVTYCSRD

CQRADWLTHKVTCQAVDDVITTEGYRLTEQREQAAERAKATKEDQQKEALHSLLSDMFLT

PQPTFTLTWEEARSRAQRLYPRRRIIDKFRDLQNDCPWMGILRRESVVAVVKIGALYPHI

FRHGRCLEDAEGNASFVLFHTPPDPGPYFRYDQLREGNFLCYEHAHMHFFMDGTVGFRID

EASSVHIMEPSKETPKKSPSTKTSQPSPPPPAQLWKEARSRAQAAFSRERLVDSFLDLDP

NDLTSRTGVMPVNPEVWVNKGETHHTLKEGVVTATLLLRLESPISHSLHLRWAVQDRTGK

NGHLTFRFLKSPSPHFRYEDLHKGHFLCVSRPHILVQTSDFYFINVEDASTVKIIEM

>SG-AL_contig_8968_1_+2_869_1135

MISNQLPNHSEQSFRSKLPKIESENKRLEKKLPFLSIARKKEHYYYILLIAQLTIKAISR

LSPCKKYPPSPNKTQQLKLKTQSILQVSK

>SG-AL_contig_8969_1_-1_1513_2016

MLISNNGWFFYVFFLFFCGVCSKGIHCTLTIIYKANNSCLADFWHLQKSQCAGKTWKWGK

QGCACWVFSSFNKLRNSDMTYGIFNVGCVFDVLMCIHMKEVEVLQGLYMLTWKMGKNLHP

LQSCFFVSCTVIKEMKFVFNCTRNCTFSTFSCSVVYLPLSVCRSYALT

>SG-AL_contig_8970_1_-3_210_1670

MDDTHQIPNPDSSASSSRSDVGGKEDRGIVQKPLQRPVRTCAVLCGAFFFVYTANLAIQN

LQSSLHQAAGLGVTSLAVLYGSIIFFGTLSPMVIRFLNAKRTLVLAFICHLLYTLSNFYS

TFATLLTSSLLLGIMAGPMWTAQGLYCTATGMTYAGIKQMESHGALSLFNGVFFAFYEAT

QISGNLIASLVLMRGSYNETHTSTNRVCGAAVCPGTEMNSTQIEQPEKYIVYILMSIYLA

CNIVGLLLTVFALPPLPRDQEIQAGESSLKKSAMSCLSMLLVPKMVLLLPFFMAQAMNSG

ILLAEYNQKFVSCAAGIQWVGYVMTVFGVLTAAQAVGLNYAAKVLGRRVLFTAAAMADLS

VNMTMLLWDPTGTSVGLLFILPCVSGFAEGIFQAQFNSLVAMVFPDQVTGAFATYHTSKA

TAFTITFGLARFLCLNQRLYVSLSLSALGLLGYFIVEVMLHRNKQRETPTDNAPTLVSHI

SLDDKQS

>SG-AL_contig_8971_1_-2_2_145

KINLRLKNMHIFQKCHPVLSSQSGIHATHCWNDSDKHQNQIKMINLIS

>SG-AL_contig_8972_1_-3_1077_1253

MCSAVWSASPKGHKGESTSQKRLFMWRFSLLCTVRRWRIMTCSCRDSWWYFPCSWGALD

>SG-AL_contig_8973_1_+1_1_126

KQSKWLRLVNSKQSNQLQMIYKCKSKPKHEQMYKSNYPILLT

>SG-AL_contig_8974_1_+3_3_302

IWYELFLPTLNKHAPLRRKRVKHPTMPPWLSQEMINAMALRDKLQKEICFPEYKTTRNKV

KTMVRQAKKSLFDRLVKDNKDVSSLWKAMNLITKGKGKRS

>SG-AL_contig_8975_1_+2_2_211

KDNKDVSSLWKAMNLITKGKGKRSLDTPPPNLTANKFNDHFLSVSNLLAHRCKNTSQYAP

SENLINFCKQ

>SG-AL_contig_8976_1_+3_3_203

PPALLSPLLKTEEGKEEEERRRGKRADYAHLKLGICSTHWNSGSSNLSVVNDFALREQTQ

DTHHMSI

>SG-AL_contig_8977_1_-1_127_516

MDGGRPPSSSFTTTAPATTKLKTWREHKPRGMHPPSAMFFTALVPLSTDRPTGSAVCCFS

DRNAVTVYAHYDEGTHTHTQDGLTHTHIACACAHVRVHTHASVSDKESVLGFFVVFFCSR

VFFFFNVLSC

>SG-AL_contig_8978_1_-3_111_227

MMIFTDELCTDYFPNPLFPFQCQKITKMTPQNPKFCPKI

>SG-AL_contig_8979_1_-2_23_2053

MLKLERPAIVESIKFGKYEKTHVCNLKKFKVYGGMTEDHLIELLDNGLKNDHIPETFLVR

NKIDGNHFPCRYIKIMPIQAWGPSFNFSIWYVELYGVDNWQQVKDCMNWYNTYREKEAIR

LCLKHFRQRHYMEAFQELSKKTKVSLEHPLLTELHELLVLKGEYEKCEDVVRQACQEGLF

DQYISQQDYKPVWTPIQHLPHTNKEPMSESRPGMRGGHQMCIDVNSETIYMFGGWDGNID

LADMWAYHVPSQEWTCISKNTEEDGGPSARSCHKMCLDPERKQIFALGRYLDPSMRTASV

LKCDFYMYDIPSDRWTLITDDTYSMGGPQLIFDHQMAMDTDQQTIYVFGGRVLASPTEAE

RTQDQTFSGLYSFHVPSNTWSKLLDDCTQLRSRIGHSMLFHPVKRVLYIFAGQRSKEYLN

DFLGYHVDTGTIEVVSDGTNKESCQAGFTQRATLDPELNEIHVLSGLSRDKDKRDNVKNS

FWVYEIGRDKWSCIYKNENTGQQYWIKMQHIEPVPRFAHQLVYDHKRKKHYLFGGNPGKD

SLPKMRLDDFWSLQLSRPSHKFLLRKCCFLIRKFHFLEMASRDSCAAMSYLQTQVYETVN

KDDPKECEEFQLLTTNLFETYQAGDSPLMEGDTPEYFSQRTELFDRLVSFFPENMTQPKG

NLIDMIPTSIAHTGGIH

>SG-AL_contig_8980_1_-3_3_275

QQCRWIPLPPSGAICFLGPSPIQYPRRTDVAWWSCALLLVMTSCGALRFSFSGLSLFHPH

KFLPDHSLHSPSCRSPPIDGACWFCLWGQQV

>SG-AL_contig_8981_1_-1_88_219

MCAPTGDDCLWCSLILFLRFHSLSPPTEFFLITVCAVCPVDHPD

>SG-AL_contig_8982_1_+1_46_273

MNLECQVQLWMLSLSQLSSPLDSVQDVFNCTHEVRILNIRHRFNLTLTPIRLRETNSHPL

FTDTLLILYKLLKDKK

>SG-AL_contig_8983_1_-2_68_1114

MSLKSRITRCRRHLRSILLLIYFSLLGFALFNYKSHCVYTMPDALLQMSQIGHSSEGKCH

VNLDELDEVKVDIFSEPTEEQIKERNPEVQKGGVWSPRNCQAWQKVAIIIPYRDRYPFLM

ILLNRILPMMQRQQLSFHVFVIEQAGDKQFNRGKLMNVGYMEALKFDKFDCFVFQDADLL

PETDKNLYMCDPHARHLASAINEMRYHVMYYNYAGGVIALNRENYHRINGYSNSYWGWGN

EDDDFSARILESGLLLTRPPEHIGRYKMVRHKKQTRSESGNEMFLGWRSRWMTDGLSDPV

GMNYTRVGLDERPLYTHIKADIGDPPAKMRLGNPDELVSVWWFLKFYFP

>SG-AL_contig_8984_1_-1_145_1680

MVKNNPSDVEMKDATSPEGSSVDGKPTEKEEEKKTEEAKKHPELLTLEDIREQIRHIEKS

VTTKETRFMSRVMRSLVNTRRRLTSRILRRLIAGYFTVPEAAQRKESLLQFLTEDADQAV

SSLTFRPRSAKQAGQPLPVEIEVYLHLLVLIYLLDSKQLDKALACSNLLMATLLPLNRRT

LDMLAAKCYFYHSRVYELMDQLDTIRGFLHARLRTATLRSDFEGQSVLLNLLLRNYLSGN

LFEQADKLVSKSSFPDTATNNEWARFLYYLGRIKAAQLEYSEAHKHLVQAMRKAPQQSAI

GFRQTIQKLAIVVELLLGDIPERSVFRQPMMRKPLAPYFQLTQAVRTGNLAKFNETLAKF

GPKFQSENTYTLILRLRHNVIKTGVRMISLSYSRISLTDIAQKLALDSPEDAEFIVAKAI

RDGVIEATIDHQKGYVQSKETSDIYSTGEPMAAFHQRISFCLDIHNQSVKAMRFPPKSYN

KDLESAEERREREQQELESAKEIADEDFDGFP

>SG-AL_contig_8985_1_-3_3_200

VSFNLCEVFFIQWSSLPEYIYILLKIVTLYEYAFITSYCLFKLYILIKTLAIYKAAFIAS

CHLCSQ

>SG-AL_contig_8986_1_+1_466_684

MGEVYGFIDCRQSWRCWNWILYICVYKGSSIKLSWVIGEQGCRDQLLLGSSIMRAMPIPL

TCCCTFPNPEQGQ

>SG-AL_contig_8987_1_-1_1_258

FLFILTRVSDVLYVYIHRGGSGSCKFAHMLTWEIGIDFHKSTVYGVRFLFILTRVSDVLY

VYIHEGGSGSCRFAHMLTWEIGIDFH

>SG-AL_contig_8988_1_-3_3_317

DPTGNILEHTHTHTHTHTRAHTHTHTRTYTQPQTESLLYINLNQLQVIKEHCKFGGQSSR

HTNLCYIIPSKLEGQFLIAEKLPFQKPSSPLEKYKYYIYGKHHRQ

>SG-AL_contig_8989_1_+3_201_380

MEHIPQSQELHTLYLKIGLALSTRALYDGYGINLIQSITKLQHLACKDRQNRTELNCNNF

>SG-AL_contig_8990_1_-3_156_455

MSRQLRGSGYMSFTSISQNTVHYPWFNGIAFRTRAEEGTLMQIILSTGQVRIQLLGGFIQ

YSFEDNSVALDAVQVSDGQWHYIEARWYQGRMELLLDYGQ

>SG-AL_contig_8991_1_-3_3_74

KFLCVCCFRGSRFCLIFFPLEDLL

>SG-AL_contig_8992_1_+2_1208_1372

MWNYHFSISSLLNCPFQRTFLILHQNMIQNAVPLFQTAHKTVKSPNIFSLLSVFI

>SG-AL_contig_8993_1_-2_50_190

MNWGLKQEVPYIVRVWVWFLFVFESTRFSLLSNVMFSFHCEHFGGHF

>SG-AL_contig_8994_1_+1_541_900

MYTHTHIHTHMYTHTYIHTCMQTHTHTHTCTQTNTHTCTSVTSLLPTDKQQYQQYKHTKS

FCIQYEVSSIHICTRIHLFFIIQSQQCVYHRFVGIQEAWGHRGYLWLQYIHKCIYIHIYI

>SG-AL_contig_8995_1_-3_135_1085

MSEELKLDLTCFHDDNEGKAIFLKRPGAPPVDTHMPLSMNPIPGGDDHPDGAEEDAEPQP

ADRSQTEPSKEVVHSLSGDKTGPSLPPIHPQDALPIVADNLDRPPVRRSVTPSDFSNVTR

PAVLRRAATPSDYLTSSDSVNNSKAGGRRRKDSKNASSEALHRSVDDVSLSASKKKPFRT

SLKNLFQRKKGCVILDELEREYTKELVRRCLINNNTHTNNTTTTTLNNNHHHRLHNHTQQ

AEDRERKGEEEGEGKNNNSVSTNNIINSTNSQQQHINNNNNNQHDGNGSSLWSKMRKSPL

FSLAKSPDSATAEPCVV

>SG-AL_contig_8996_1_+2_677_1003

MKTLMSVLGDRHILLFSFEKNVPVLLNSLLNEYVMVSGGNIIHRHNYQFILIIQSLQGCN

SCKILFLHCQCVVYLFFLCVFFYLFVYLFVCFQISMDKAQTIFLFFVGF

>SG-AL_contig_8997_1_+2_2_190

GKHVAIVWHHLFFCVCMCVFLFACNWIFLPKFARDNSFVAMGCLMCANCMLHTEALSIFL

PSD

>SG-AL_contig_8998_1_+3_402_650

MTNPPTTHTYERTGYTFFFFFLSIETIAAARVKQEGQILISGEEYQIWTMNDKTTQEGNC

FFLKISIDITAAARVRQKGPIII

>SG-AL_contig_8999_1_-3_171_881

MMLGGGDDDVDEDDEPMMDLEGMKKLNLNKADSTKPDEIMESTTDAADWRLEVERVTPQL

KVTIRTDNKDWRVHLEQMHQHRDGIDSSLTETKVHLDRLHDEISHTLEKISSREKYLNHQ

LEHLLSDFRLAQDQLAETKEKYRQASGGVTERSRHLAEVTEELEKVKQEMEERGSSMTDG

APLVKIKQAIQQLKKEITQMDIRSGVVEHVLLQAKLKDKNTQNKQVHSSTTSDGFNI

>SG-AL_contig_9000_1_-1_1_141

FGQRSAKFVSIIDEDPVVNITCKCLNIFFFNSSPERPWEQRSTENIL

>SG-AL_contig_9001_1_+1_1_114

HTLYLMKLSIYILKKNKLKGDNKQVAMLNYSKSSENGS

>SG-AL_contig_9002_1_-3_435_641

MMWEFLALGLPPEERLHCCWATLLGHLDGVNSTCFNPTYKGHCLLTSLLTTVVAEPDRVS

NPAHHSLNH

>SG-AL_contig_9003_1_-1_613_837

MFWNFLEYVFSQHCKCVSNMKISRNTVCVCVCVCVCVKYKNTQNIRSHYYICIKSILMNA

RLTSVFQIYPFFKGS

>SG-AL_contig_9004_1_-1_1_132

SGILEVSKCSGILEVSKCLGILEVSKCSGIPLTCSQPSKRQVTS

>SG-AL_contig_9005_1_+2_2_61

TSVPRNFAVLRVIIKKKILA

>SG-AL_contig_9006_1_-1_610_1512

MYDNLQSHAVIPTPLRIGFLGLGIMGQGMVMNLLRSGHEVTVWNRTVSKTKEFSKAGAFR

GISPADVVRVCDITFSCVSDSVAVRDVVFGKEGVLEGIAEGKCYVEMSTVDEETVQDVAD

AVMGRGGTFLEAPVCGSRVPALEGQLIILTAGDRKLHDDCFSCFSAMGKKAKYLSNEVGT

AIRMKLIYNMLLGSITASLAEGMALAEKVGLDQEDVQEILNLGNLKCPHISIKGHAILNQ

RFDPHFPLQHQQKDLRLVLSMGDSVEQPLYIAAAANELYKKARQLGVGEDDVAAVYKATS

S

>SG-AL_contig_9007_1_-2_449_775

MDNFYGNDMELYYVLGVARGVASCVYKTKIKITRLFLQVQLIPFKAGISFAVAAECELGD

LTKKGHTMIKRGGIYHMEGVGVLTPKTTFYDDNEFSVFLSRLPRSEEHK

>SG-AL_contig_9008_1_-1_274_1008

MTLQWTFVAACMYAEIGIITLLLLPFISPSRWQTIFRSRILAGMKSYANIYFNVFIAVLL

LLFFESVREVRKYSEPISVEDLKHHPEAETLYHMKLFRAQRNLYIAGFALFLWFVLRRLV

TLIASEATLIAESTAAKRQAESATAAAQQLLDEKDNSKNVNKEDVKDKGDIPENRDAIAL

EKARAQLEQTKEDLYHAKLEFESMKKQAESVTKEYDRLLEEHSKLQKKLSMLEGAAEGSE

AKKDN

>SG-AL_contig_9009_1_-1_1_189

YKTEKDGIQETRVEHRVVLSSNDDDFDYDAALADAIRAVTEFNPDMSVERIECVQQIEEV

KDK

>SG-AL_contig_9010_1_+3_36_233

MALRDKLKKEKCFTEYKTMRNKVKSMVRQAKKSLFDRLVIDNKDVSLLWKAMNLITKGKG

KRSLDT

>SG-AL_contig_9011_1_+1_115_279

MHIKMIFINIHRKITNIKTDLQQGIKQEMGGERNRLKMVIKKKRKKKRVLIINYA

>SG-AL_contig_9012_1_+3_774_959

MGLIEMEKPVQWICGNIKETLFTLQITFTSLYRQVTSACLPFTVVCCCCARNNQFTFTGQ

FG

>SG-AL_contig_9013_1_-3_3_122

VSTWMGDHIGAPGSYWHIKQQQQQSFIVPGVHENDELWCA

>SG-AL_contig_9014_1_-3_285_443

MTGPVSTWMGDCLGIPEAVGTKKKKERERKKREREGEKEREFIFVFFHFFWLK

>SG-AL_contig_9015_1_-1_112_813

MRISLAKNLLKACVSSIPHAHHFHASAKQLYPLIPIVIEQTGRGERSYDIFSRLLKERII

CIMGPITDDLSSLVVAQLLFLQSESSKKAIHMYINSPGGSVTAGLGIYDTMQYVQPPINT

WCVGQACSMASLLLCAGDKGMRYSLPNSRVMVHQPSGSAVGQATDIQIQAEEIVKLKKQI

NQLYVKHTGQPLDIIENSMERDRFMDPHEAKDFGLIDHVLEHPPTLEEEQGSSQ

>SG-AL_contig_9016_1_-3_99_242

MNVQTAILHLLLQLVQSSGRKCLIEKLTSVTFCVFLISLDSWIPFVST

>SG-AL_contig_9017_1_-2_203_802

MTEPCMDTTAPQAGQDGNETVMKTSNQASLILHRNTGVTTGTETNRQTEGGRESMSKPSL

RILGDTGTKSEVREEMNDQNIDIETRTERGIETGIMTRTEIGTDITIETNEEITAETETT

MIVDIDEHAGYNVFTETEMTMIVDIDEHAGYSVVFTETEMTMIIDIDEHAGYSVVFTETE

TTMIVDIDEHAGDIRLPNWR

>SG-AL_contig_9018_1_+3_585_740

MLPASTKVTRSLTHTLSHNPLTLCFIIDYILHTLLPNKKENIFKYRIISLTA

>SG-AL_contig_9019_1_+2_845_1102

MNLPLNLSPISCKCTSFSCYKCTSLMRWHGYISWHKDPEYIEKENILLVFCSPLPPLFSL

STDLNSVENDNSDNLNGHIILDHNNK

>SG-AL_contig_9020_1_-2_242_2098

MDYVPSPDPFCTPDFLKSTFSPSPSSLYMYDQEHPDMHTPLHTARTPLGQVCTSTELTTL

TPVEPEFDLHPQYENSNNLEGFFRGALGSSVMPAGACEASVGGQAGGKQEMGLVTISQHD

LNKLKPGPTLTELNMDPYLFQDVDAIVIHEGGKLPRGASVLPQKQVDVKTENFAQVQPES

DPQKTFTPLKSVRTNQSPATVVRMLHQSQSSPSPSHTSTTSLTQLLTRNRTQNVAVVSPI

PVTTPAATHTTIKTEPSDMDKTTDSPSLHKMVTSPPQQNSRVSPPTIAQSPSPPGGRQVM

PVVGQSRPPTLGQQGKSGSVEEKWKEIEKFIHDPNGEQAEASAKDRKRKRHASGASTMSD

EESVKSQRVGSISDDDFDSDDDSDMSDTPFEESLTELAKKSKQYFWQYNIQAKGPKGTRL

PLNISDTDPHHPSNFEDPVFDATNTTLVGIRHGGKARKGDGNEITPNPRKLYQIGQRLCR

LNREINNVQLASHLPASVRSKSRKEKNKLSSRACRLKKKAQHEANKIKLFGLNCEQGELL

EVIRLLWPCLKDRAKQQMKENIPSSLSSDTLTHQINNIITSQYKTAIAGNTADYVNQVIA

RVEDGDPVGGLAIKLKVKK

>SG-AL_contig_9021_1_-1_757_930

MERREVCICYVNMTQSFLVFFSDPSGKQSPDYNFPTIMILVSVYFLPPDHVNVNVVQF

>SG-AL_contig_9022_1_-3_3_230

AKLRLTGWKQRYLFAKSHLSKFKKKTTTPTTTTTTKKPTTTNSWFVFSASGVSVSYTRDL

FMSGCPTHTQTLVVIF

>SG-AL_contig_9023_1_+1_91_312

MKPQTSRKTSEKPIHSRTCQKQKTSISAKSRFSPTGAVKTRHSTAYRSEHVLFRYFQLWI

ASSYKPLPSQASEC

>SG-AL_contig_9024_1_-3_618_833

MPAGWLASFSIENDTASSFVRFDVFPNYIYGLNLVVLFFIQSKPILIILKIALKIIQAHK

CTYDMEFVCNID

>SG-AL_contig_9025_1_+2_524_724

MFRFSLIYFTSVVNILLHPNFQTGTTKWFSTELKRITCKLTTYMKLNATVMAHVYISEYW

IVKYHLE

>SG-AL_contig_9026_1_+1_67_222

MHYVVRLNFVKTNWLLCASSLRLLHFCVFFIPSFADFASVPYIEMCSMLHVK

>SG-AL_contig_9027_1_-2_137_271

MKTCNKTCIHPYLRSQYAGTDSWRGMPGTTASPPPNPQNEKKIRI

>SG-AL_contig_9028_1_-3_3_191

EVQCLHQLTTMPIKTCQAGFWALFVLILKMPLVGFKGKTVSILHVELSLFLFKCLQLASI

YIK

>SG-AL_contig_9029_1_-1_778_930

MRIHSNVQIRQYIYSVDVHDHNSLSLSLSLSLSLSLSLSVSVALCLCLSLY

>SG-AL_contig_9030_1_+3_3_77

FFSFKRLLQQTENINSVTTTKTGTK

>SG-AL_contig_9031_1_-1_400_567

MIISSENVPLPVLFIRNVRLWELLFFLVCCSIVFIHSETNASLSENCPLQLLCTFV

>SG-AL_contig_9032_1_+1_1_348

PKNRAHQYKKRNDEPARQLLPLRQCNHNDPPNYSTSSKLTRLKCLVAQVTLHLIFSKSLR

NREETSAQIFSVLAHIHAPKSILPSRNQPLAAFHLTGLWSDCYCSCCCCKDPSKNN

>SG-AL_contig_9033_1_+3_57_209

MINLMKVNISAENLPHPLLNLPSFLKQANRHINMHQSNAICERFRAFQYYV

>SG-AL_contig_9034_1_-1_49_1764

MYRLPSLLRPALAKKLAPGLVRCYAKDLKFGADARAVMLQGVDMLADAVAVTMGPKGRNV

ILEQSWGSPKITKDGVTVAKGIELKDKYQNIGAKLVQDVANNTNEEAGDGTTAATVLARA

IAKEGFEKISKGANPIEIRKGVMLAVESVVNQLRSMSKPVTTPEEIAQVATISANGDKTV

GDLISSAMKKIGRDGVMTVKDGKTLKDELEIIEGMKFDRGYISPYFINTAKGAKCEFQDA

LVLLSEKKISSIQSVIPALELASAARKPLLIVAEDVDGEALSTLVLNRIKVGLQVVAVKA

PGFGDNRKNTLQDMAAATGAVVFGDEGNLYKMEDIQMQDFGQVGEVTVTKDDTLLMKGKG

DKADIEKRVQQIKDDIELSNSEYEKEKMGERLAKLSNGVAVLKVGGTSEVEVNEKKDRIN

DALNATRAAVEEGIVAGGGTALLRCISILDSMKTDNEDQKTGVDIVRKALRMPAQTIAKN

AGVDAYVVVEKVMNETGDIGYDALNNQYVNLIERGIIDPTKVVRTALVDAAGVASLLSTA

EVVVTEIPTGDKPDMGGMGGMGGMGGMGGMGF

>SG-AL_contig_9035_1_+2_35_181

MITTLKTNQNLYFFMITPVNSDQSHTKEKKYKDMKHEVLKQTTFKIISI

>SG-AL_contig_9036_1_+3_27_134

MLYTSTEIAKYNQSCHITSVLKYIKVYRKVTTHTFA

>SG-AL_contig_9037_1_-3_60_272

MARFFVTYQIIVSVTHCGTFFNQLKLCAHYTFPCVMFFQGMLEGKLFSFVVFYFIQKRKI

GNVALLFLLLV

>SG-AL_contig_9038_1_+3_3_203

CMYVRACVRECVRARARARARACVCVLCVRAYVSLIKLPNNQIIDEYIMIWLRQKKRPNH

PNVGYSR

>SG-AL_contig_9039_1_-2_2_262

SLFSFLFSFVVVFVSFLLHDLLSCFSGIASMPLCSVLHHHTCLRLFTTRPIDFHSFKVPV

TGSPSKPSDVKPPWGHCHGCYCVPCSP

>SG-AL_contig_9040_1_-2_2_127

GDNFTADEIKQTWKEAPLEKGKFNYSEFVSILKGKQEDETAA

>SG-AL_contig_9041_1_-1_1_195

KKKSFCEVCGRYYQILPINFPVTCHLDVYVNLGGNKSVLLRICDLFSILVSRSFGIFYVY

ISTFI

>SG-AL_contig_9042_1_+2_2_94

RSREKVSRGGGGGGGGGLTDIYLIINSTETT

>SG-AL_contig_9043_1_-2_32_2500

MVLDHLFAKLSEERYHPQTRITKQDPDVTMQMMEVYNELIKDLLQVQVGSSTGLLDLTET

ADRGVHAKKATTVPVTDASSANSYFRLGWGRRTEATTDHGPARKNAALVLHIDLSVKVGD

NPQPYKSRFTIVELPGLEKLSTDPTHVQLREGPKLSQSLVALNHVTSTLADHPFPDRLIQ

YGDSKLTQLLQEELGGNFKTRALICLKPRTDTDISNAILKFCTNLSHIRNFPIVNDALAQ

GLLTQKGARIIELKQQSGTGPVHMTSTAPVYSDSSEELRRLQTENLVLKDQTERLQLRLD

RLQNKFGDVANTKTDLSQQLLLSEEEKLKVSQSLVDLQIENNKIREEAEAAKFELTNKII

LLENQLLENEAERDKFKKSSTHAKERLTEMETDRKDLADEYVVLKTNYLALTKEHQKELS

RNEELAIELLNLVNNKAALMRQVIILTNGDPGEVGDPNAEIARVKAIVTKNSSGKVQEEA

ADKILGTQSDREAVEQTLFASKKRYDSEMDRLKKELGDDNKRMETRMTGIMKELADARNL

ARERQQKISELNAKLITIRGEKQATDTQLNRLQHKVKDLGEDFRARLVKYVEDIADYVDK

GSQGSQSDARLRDYCDSMLKDIRRSHKEREQQLSEAAQQFRARLQKTIHDYESVLVAYRN

LRQTCEARGFELEDLGPDEYQLKLSDTDLQTSHLRELDRVKGDLGRAKNELDTLKMRYGL

FGEDKETMSEITVAGRSDLWGAMRKQLREFTLNTQQQLEQERARLLSENHVLKEQLKESQ

QYIDSHLVRYKQEVVRLRKLLGMNEDGVVLTDRGVGKIHIRRR

>SG-AL_contig_9044_1_-3_675_1013

MFLLTVSKAFSKSIKATNNLRFRENFLYKCHQCKHVVYCRVILFKLGLVFTGNILLLHPI

LQSPINNNSTIQFITSIAQRNSSIVVRIIDVTFLTQWENNRLCPLFKKNASIE

>SG-AL_contig_9045_1_-3_3_209

YYIALYRIVMYCFGLRRIVLFHIVLHHIVLYYVALYCIALDCITLNCIVWHSIFSQLVLI

DTNQYDKFI

>SG-AL_contig_9046_1_+1_1_216

QSCNYDLFDFTILFAKHYLYSCKMKSCEPNMNVLKKLHSVFQIKEFNAKLNLDLTHFSAK

QHYYNKPMFTEN

>SG-AL_contig_9047_1_-3_54_1181

MDTYDVIANQPVVIDNGSGVVKAGFAGDQVPKYIFPNFIGRPKHVRVMAGALEGDTFIGP

EAEEHRGLMSIRYPMEHGIVKDWNDMERIWQYIYSKDQLQTFSEEHPVLLTEAPLNPRRN

REKAAEIFFETFNVPALFISMQAVLSLYATGRTTGVVLDAGDGVTHAVPVYEGFAMPHSI

MRIDVAGRDVSRYLRLLLRKEGMDLHTTAEFEVVRQIKERTCYLSINPLKEESVDTEKVQ

YTLPDGNTVEIGPARFRAPELLFRPDLIGEECEGIHEVLSYSIQKSDVDLRKTLYSNIVL

SGGSTLFKGFGDRLLSELKKLAPKDIKIRISAPQERLYSTWIGGSILASLDTFRKMWVSK

REYHEEGVKAIHRKTF

>SG-AL_contig_9048_1_-3_3_110

VGVTFNCTELISLMTKGDVALGDNKRGTIDLLIVRI

>SG-AL_contig_9049_1_+3_75_392

MITMHELVMLQVTSLNKSLTADITHKWFMSSMNQLVSLQVISPSKSLTAHITHKRFMTSM

NQLVSLQVTSLNESLTADTTYKRFMTRMTQLVSLQVSTLSKSLTTH

>SG-AL_contig_9050_1_+2_257_442

MAFSTRKTHTHTHIRKHYTHIQSDTQTHNCTYIQTDTHPSLSSLLLFSLNTLGKSLQRLP

VF

>SG-AL_contig_9051_1_-3_3_203

GEEMDTALVMLALEKGLRHLTPRQPGGLWEQPLHFCIMLKMICHYAGFGILTVNREGGGR

CYNTFCY

>SG-AL_contig_9052_1_-2_386_688

MSETFVYNRLFYLFIFIILFITFFFLLTIMNQFQNMYMRYIDHGFRLVAIRFDLSSLIFR

HLLMLTDLSPLDQARTMCSDFMKMKPQKPDCTFPHEPIVRY

>SG-AL_contig_9053_1_+2_158_322

MASFIHPTHPKTDADSCTPHPSIPTILNRTYSSISLSIHRQKGRERAIKENKTKK

>SG-AL_contig_9054_1_-1_1540_1788

MERLHQMYNSIRSLVLYFTGKGFHIFGFCCRFMAYAVTTLDLFPSHGICGNGTHGIYSKC

RDTVLRKLKLKLLLLLVCFVLFC

>SG-AL_contig_9055_1_+3_168_323

MGFKRSRQSRTLNMSQVLKTAFWHSLRFQSCRTICMYMFNYDLILSKNCKNT

>SG-AL_contig_9056_1_-2_170_445

MRPASPCTKDTLFDGFTKEGKGRYQYLRRRYNTIPEKKYAFPIISSWEYGWRLEDVIEKE

DIKKPPHGRTRIVNDTFYTRNGVGISPQLNTA

>SG-AL_contig_9057_1_+3_96_227

MPYTSFVINGVCVSNADTSFVINGVCVSNAVFYFLTWCLCMQYI

>SG-AL_contig_9058_1_-3_3_71

KRYMLKLTMFIEWQTHFSSTKHA

>SG-AL_contig_9059_1_+2_77_247

MQSMASAYKNKPSSPLNMHQVMPSNNPNHSGELLNSPPHPTQSRPLQTRSSSHLVIG

>SG-AL_contig_9060_1_+1_706_987

MESVHAMLKRSQLKWAGHVRRMDDQRLPKRLLYCELDKGKRSQGGQRKRYKDTLKASLKC

CGINANLWEEAAQDRTIWHSLVNSNSAIAGPKPG

>SG-AL_contig_9061_1_+3_792_1040

MHRNGSVSADGQRPDHTPRVCDGEVLKYLIHIFRQKWRIFNSRLYTSTHTTTTIHSYHYI

QWKVSGRFNGPTRPFHHTHIFFL

>SG-AL_contig_9062_1_-3_348_674

MASSILTDAVSKSMKRKWMMTKSITVVYRQNYGKNENGKSVCCGVSFESVQQLFSPFIFV

TINQSSIMFMKYCMMLEFYICYFDGIVFNFSVCFGFFFVLFCFCLDLSI

>SG-AL_contig_9063_1_-2_2_268

INDLVPELPKGIHAALYADDLVLWCTEEYATTATYRMQLALDRLSAWTNTWCVTINKEKS

SATLFSLSTKAQPGNLTLEDATLQCKDQQ

>SG-AL_contig_9064_1_+2_143_991

MSATEIDDTQTSPVDTEDSEIDSSLSELRAECEKIWKQVKETQAQLEETRHPPSSALSGS

QPPLVTILREKERHLRAEVEIARELDLQTLPNDPKVLETALWEELQREVCQLRETLSLVE

SQRQQLETSIQQEQQYEQQLLAVSDKLQEKNDAVVGQDLPEDAVETVAQELETKINQARQ

TEHSIMRRMAEFVGKHFPLPEPDQVAKISKELRSRKKQNNKEVVPLKDILLELMTKCVET

PSDPYVVLTDRHWPAYIELLLRCNIVLQHPDDDRRVKLVPFHL

>SG-AL_contig_9065_1_-1_475_621

MTNHLFPRQVQTKTLQKYSIKRTHILCKNNPKHPYINTNKAATIFIRCF

>SG-AL_contig_9066_1_-1_127_591

MFHSLNLSGGDRSTCAHDTSAPRPLTLAVRSKESNVKVFTVDVWVSGRHHMVERRYSEFE

DLHKQIKKLMKTPEFPPKKVLKWNNKVLEHRRLGLEAYLQGVVMYEAIPKAVFEFLEIIM

EDDGMEFENTSTTYAPKHQPVVTFNKDAYLQDVNR

>SG-AL_contig_9067_1_+1_1_153

KKKIKKKNQQSKQASNSTNKINRLKYQQICHNKDHNNRKSMICFHNKVLLS

>SG-AL_contig_9068_1_+2_2_151

VLLSLFLLLGTFIQLPQDIYREGQDEEWNTTGHLIVNVKHTIVYCISVYL

>SG-AL_contig_9069_1_-3_246_1100

MMTKRTAILYTLLFLLFSLYQTSESFSFPDFSLGDLLKGSWVPEFIRNRRLTGSSTTTAC

NYPGVVAITLFGKTMLYTGIYSNGAIRIPDDALSLLKLANTNNPLTIQTDKGSSTAEVAK

GAVNAVSNKDGTSTIKLTKDFSFFTCPANNAILYDKATMRVDISTCKIVSYGRQVKDLGA

YDGVLRTSSLLENPVQSCCSYVLPNLLGKSPNLGHPNVASSPSSFSCAKSKEGICNGDIG

APIYCTDSLSSSEVVVGMVDSGTCSAGETFVSIDLTGGSLNGFSS

>SG-AL_contig_9070_1_+1_1_132

KKKEEKSHMMENQSNHPEAINPCINFTQSKGPQEMLNFDYPDVC

>SG-AL_contig_9071_1_-3_489_659

MLYSKWAVQNQYNTKSIMYYYYSIIYISDPHSSGQMRTIIYIILCTRNTLSPKFLIV

>SG-AL_contig_9072_1_+3_9_383

MVGVSNCKHCSLLGRWLHDLVVSLPSIFMVFPNFPFLCSFRHGVLMAGTKICSALSFFLP

VVFFSVLALKTPRLIFSSSRNSLKTMTISSYIYISITIKTNLYDMLLAVYCSPPIIFFLH

PFKTI

>SG-AL_contig_9073_1_-2_2_202

VFFFCVCVCVCVCVCVCVCVCVCVLSELSKMQLFLIIQVYILIVNRCIFSKKQMFSIKFV

LFYFTSI

>SG-AL_contig_9074_1_+3_3_86

FFSPASCLLAVYSFTKITTADLTAKVIT

>SG-AL_contig_9075_1_-3_234_380

MTWSSDLTDHLSEKNCFKKKSHYFPTFATGGKKIVGFKGDQRSLALFYF

>SG-AL_contig_9076_1_+2_80_304

MKSGTKSLVIRETIIHRDKFCTRCLILPNNSTLSINVVTLNADTSQSNVSIINIRCHIHH

QPSVLRGWVEKRERE

>SG-AL_contig_9077_1_-1_13_477

MAPIGKEDRLGTPVADGNDGDDSQNLWASILGEVQSGSASKLSTTRSLLVLGDNECGKTT

LIAKLQGVEDPKKGAGLEYYYIDVRDEYRDEQARLNVWVLDGDQSHTGLLKYALTEENFE

DTMVMLVASMAQPWSVADTLNKWVNVLSEHIDKLR

>SG-AL_contig_9078_1_-1_154_756

MPGNEGCEEPRVTVASHIHHLFPEARIIFILRNPVDRLYSSYLYNHAKHHAVSSRTFDMY

VRDAISLYTDCFARYSLRVCANNATLFRQDKVMLIISLYVVFVRDWMELFPREQLLVVST

EHYHHNMDDHLNKIFTFLGLRTLNSTELQVITNHTIYNKGQHYSGAGPMMTETRQLLEDF

FRPYNQKLASLLNDPQFLWME

>SG-AL_contig_9079_1_+2_146_262

MLSIVFIFQPRFSSAQPMVTSLFPTWMRLDQMYFFLFFF

>SG-AL_contig_9080_1_+2_26_259

MLQMCDSSDISQIIKPKSLAEGVNIMPARMELVCANADEDVLLDLRTASVLSSFSLSSLL

VIQFLFLFSFFSFFLTFF

>SG-AL_contig_9081_1_-1_1_198

CDDMSLESHIANICHSAYLELHHISVIHPCLTTRVAKPLVCAFVLFKLDYCNSLLSGCPQ

YTIGKL

>SG-AL_contig_9082_1_+3_90_191

MLTQMYLYTRRKNKVIHCVHASPEDHQNWLIFNL

>SG-AL_contig_9083_1_-1_1606_1878

MEEMLQENMDLTVYFTRGTVNCPAYIHLYLSSKFSAVLEIQTAVVVVFLYCKRLISEHVM

NGVAQIVDRPAGRIIHSAFRQDSNFSVFKLH

>SG-AL_contig_9084_1_+2_530_775

MCRFELVIAGFCWCNVLSSVLCSVLRVVFVSAIYCCCCDSVYCVFCKLVCPFVHICQIYS

EYQRVGFLLGRCSVNCVNGMNG

>SG-AL_contig_9085_1_+1_589_798

MITNLQNTVWKTDSTKHIEIPTSNFEHQIYRPTFMCGCGVVMMVLVLCVCVCVCVCVCGP

FLLFVLFYFF

>SG-AL_contig_9086_1_-3_18_668

MADDSTLWVGGLTDQVTEDHLYELFLQAGPLEKVVRPKEKDGSSRKFAFVEFSHAESVPY

AIQVMDGIKLFNSHLRLKGRTGSQNDSGRSDSPASRSYDSRDSSRGSSDYHRSRSYQGHP

HQKPGLSRGNSAEDRRGTNPLSQPFSSSAAYNSANMQPQSFEDRRNRLLNKQNMSLGAHA

QMRGGQQQYGGQNNYAQSFSGYSQQSGPQQQWYLDNY

>SG-AL_contig_9087_1_-1_247_492

MFEVSLIATFTRSYLIKKSAWLLEDTNENCNNVNFFVTHCFIEQLVVTEILCITAALSKN

IDEKNEAIIVKCHIITALYLCL

>SG-AL_contig_9088_1_-3_132_539

MACNICEGLIRDVLTKHLLSGHLLSTHQCGFTSGRSCITQLLNTRNNWMSMLNENKPIDV

VYLDLQKAFDKVPHKRLVFKLKGYDIQGKLPKLIEDFLTNRTQYVSIGEISSENISVTNG

VPQGSVLEPIFYVFYQ

>SG-AL_contig_9089_1_+1_517_723

MFVLHCSHFISSGCHNVNILIHKKNTCPCIVYISYLKFSTLSIKLKNMGKPFRCTGQGKS

IRFLTTSHA

>SG-AL_contig_9090_1_-2_626_778

MTCLRRLLSMWDSEGPFLFSCPECKSLSKLDLVLGSLLPYMPVVQQVVCKF

>SG-AL_contig_9091_1_+2_743_1015

MITPPFTIPVKQKWKQQQSIPVLLWPAQSPNLSPNENVGAVLKNKAKSCMHHTATPTDLK

YELKVAWNKVSVWYLQKLVYSLPRRVQFVAS

>SG-AL_contig_9092_1_+3_54_149

MLFTHLPPKVLILGTFGGLCDLDLGSLCTKHA

>SG-AL_contig_9093_1_+1_1_129

SACFLLEILSVYSAILPHKPDSLTQFFISFFYFVYLKLHTNHI

>SG-AL_contig_9094_1_-1_1_216

SAICTTLQQLGGPSLSDNFLLREVSRGLAAAEDRTPRRLPRWDLALVLGALRAPPFEPLD

VVPFSVLSYKTA

>SG-AL_contig_9095_1_+2_335_472

MFNLKFWSDLIRTGVGNLLCCPSLHIHYFSSIQTSQIKTPRTKSLQ

>SG-AL_contig_9096_1_+1_109_219

MLMVHFFLFRAVLSHMCVYTLFWLFSLHSADVMLLHS

>SG-AL_contig_9097_1_-2_56_283

MSMRLHIGALRGNILCPDLYTEQAEQDAANSDTDAHDIPDSEEVSLRPPQSYPGCFGLPY

QDHNYGAPPPLSPPPR

>SG-AL_contig_9098_1_-3_39_287

MHIFIQYERSHCSLRLLFVCCFFLFVCFFSLYFFQCMGWWLVGWFLFFSLYCENKLISFQ

LTITNPCYDDCFSKNKMCLFFKD

>SG-AL_contig_9099_1_+1_115_219

MDWNGQIFGLSGPKYEDIYFKIFGLFGNGSNLNVG

>SG-AL_contig_9100_1_-2_290_445

MMYGGTIIHIGDLHVCLLSFAVEVVLPHLLTSTLLQISHTQIGRASCRERVL

>SG-AL_contig_9101_1_+3_3_62

FPNLEESDSLIDFQMCAGCA

>SG-AL_contig_9102_1_-1_1_258

SPHSPFCIFRSCRRDLLEYSIDSISGSKQKLTKKCAVVYYMTYDGEYLAGTARMLSTGVT

EHHNTTQASWTVGVKEHHNTTQASWT

>SG-AL_contig_9103_1_-1_166_345

MDAVYLATDEICDGGIILEADAYAVVQKVAVRNDDLPLGEQTIAQVLQSAKEQIKWSLLK

>SG-AL_contig_9104_1_-2_131_439

MSDFDPCECVWNHESAMQRLINLLRSSQNSCTDSGCLTDDLSGPGSPYGNSNMMMMMMIG

WLVVATALFLLRPRSMRDLGDGKPSSSGGGGSNNPPPPGPSVH

>SG-AL_contig_9105_1_+1_1_225

TEHIAHSLQHLFSKYVALLAQTHRELISLSPIFLSTPTSTYSSRNCRHNFLHDRRCVLPT

FFIAKSDQASWQLTN

>SG-AL_contig_9106_1_+1_115_333

MTFPTASNQTTKQSANQSVKLSAKISHTGASNRLSNQPSNTNLTSRFMKENDIKHVHEMV

FTRTHDHYRTIIR

>SG-AL_contig_9107_1_-3_675_1052

MCHMSGVLEGGLVQESLVDLYTVTECIVTKQCPSVSLPVPPVIRKGEVKWGRRLSHFMSI

TVTLFVASSAVFPSCGSFSLFLLLRHMSLLQHFSTLLHKLILTCAVSLSFIFLSYYSPPP

LFPVCC

>SG-AL_contig_9108_1_-1_913_1146

MSITVVHVVCEILLDFWFVCLRFVWIFVLSTMLTFLSSKMVAVNRKGGLMKKKWNKVIRQ

FMEAAAFGKYTLHYSHIV

>SG-AL_contig_9109_1_+3_1242_2069

MLLKDILLEKKGIRIKLLCMYVSFSRTQIFMFASLKDCYNCWEWVKFVFLIFRHKIIGTR

TCAPGGTLHTHRMRTQRHATHTSHAHTGTLHTHMCSQRHATHICAHRGILHTHVHTEAHC

THIVHAYRGMLHTHTCAQRHATHTSYVHTEARYTHIIRAHRGTLHIHHTCTQRHHTCTQR

HATHTSYVHIEACYTHIIRAHRCMPHTHHMGTYAHSTFQLKFCPFFVLTSGDEIVTSSGL

LWLSFLSLFNLLISYSFIFIFAWFLFVPCLRTDLIM

>SG-AL_contig_9110_1_+3_3_227

ILSKTKHTRCTLKAKHPQTTIRRNTWYFFLVIRLCKECKKDMNKQYGTKHPNDSWYIYTQ

KSNLQLLSFFKKKFK

>SG-AL_contig_9111_1_+2_242_604

MEEGQNTGTKTGADAASMLAQVKAQAYQYSIGTNTNYGQQFANSIQTEALYKKMWFLCPL

ERLERTKNVYTQHLQKPKQNKTKPADKPASLKMKMSNKKTGPPSLPILFKTKQNQAKDNT

L

>SG-AL_contig_9112_1_-1_1_153

VLIRRVCGLQCMKVKTAVDIFKTRDFRSFLCLVNFISSDSILLIIFLFVSD

>SG-AL_contig_9113_1_+2_1241_1465

MKLVDRVREGKGYEDQLLINCTLKTNMSTVNLLSLKLHFPSCFLNNTSFLSSINGQETRT

HARTHTYRAYHFTLI

>SG-AL_contig_9114_1_+1_1321_1620

MSYKAYINRKVTKNKQTKKNTRRLAYKKKTTTHTRRLAIIKIMASFVQRVTCKVILHNIY

YIISNYTVRYLNSNANLRVNLFKLISRAKSDHSSLTFKGS

>SG-AL_contig_9115_1_-3_3_179

MRIPILCAGFLASFLLKLSTSPPSYSSPFYINFFFILLKNIYMYPTCTVFFWSTCGLGS

>SG-AL_contig_9116_1_-3_135_617

MTKMGFPKHLISLIVTLYNDQKATIKWNSQNCEYFSIKKGVCQGCILSPHLFSINTEQVM

READIEDMGIKVGSRNITNLCYADDTALMSDNITSMKRVLYRVDAVGKKAGLNLNFKKTK

VMHPEGEESQEYQDIQINNATLENVSDFKYLGSVKANDGTC

>SG-AL_contig_9117_1_+1_1_234

LNSLSLLVALPIFFFFQSSYKFFIYSFMCPYLIRKTSTTKKYSHTASQMNTTAATTANII

LYIKSTCIYTIIRPPSLV

>SG-AL_contig_9118_1_-3_198_1466

MLTNVLMVLIFAGGLPVSPVSGLRWSPRLMSNKVKVCSGQNIQLFWNFTLDRAERLKGMV

WDCQSEGKKEEILATYVDDHFVPMSTFSGRLAHLEEGGIELSCATILESGKYSIAVTTEN

AFNRMTVHRKSVNVNVVDTPKTQSGNLHVSKDEEAVRDVTSTWHVQLSCGNFTDRGHPPV

GVTWTTPDGLRLDSSSFQDGIFHLLVPNPVVEGNYTCSIPPSQAAAQCLSVDSPLLDTVS

VYVEENAARFSLLEARQQELLDKLTAENQRLLNVFEGISNRTDDLEERLDKVQQLSMPVS

FHARLTGNTQLSSGSTLKLPKVISNQGQGYSRTTGYFTAPVNGTYFFIGTTGSYSSGNWA

NMYMMKGGTEVARTYLLLYSNYYSMGSCHVTLYLKAGERVWLQSSTSNSYFYGSVTSFSG

FRI

>SG-AL_contig_9119_1_-3_45_2483

MAALTVLVQESPAHNISALDMLVNMAKKKGKREAMLASDTLKNLFTSETSPLPDDRRLTP

FDARPFSTLWQTCSGNRDNVDRRLVMWHAEDLLRQKYAAFVKALDVMSYDTLVATKEKAL

AIMHSLLTGRPEQENVLLPMLVNKLGDPNHRIASKASYLLIKLVEEHPNMKCVIVGEVEQ

LVYRPNVTERAQYYAMCFLNQIKLHKSDHALATQLISIYFTFFKVCVKKGEADSKMMAAL

LTGVNRAYPYAKVKRDFMSEQMATLYKIVHIINFNTSIQALMLLYHVIDSSNSESDRFFM

ALYRKLLDPGLRSSAKQPQFLNLLYKSMKADVVEKRVKAFVKRLLQAGSVLGPNFLCGAL

IVISEVLRERPNLLGIRQVGKQSSGTDLDMDDEEEERFVDLPLPPEFQESSTAESREEKA

EPVEGVKPETQTGGSWLHRHNLTEQQDYNRYQRNPLHSHAQHECLWELQSLTHHFHPSVS

LFAQTVLRGEAIEYSSDPLQDFTLIRFLDRFVYKNPKKKEAEQKRSETVGTASGKRAKVA

DSVPVNSAEFLQGGEEAVPEHERYLYRYFSQKAEKKKEEEEASDVDSVSDTEFDAFLDRH

ERQVDHDDLDDMDFGLDFAGKLGGKKKGDKRKSKADSDEDMSDDDDDNEDDDDDDFGGLS

DEEIDFGDDDDDGLAAEFRQEMEGLGDMDEEEGDIEDKDLSGDELKQMMSSGKKRGSDDE

FDDRPAKKKSRKNDLASMFAAADDFADMLEGNADAALTSAGTWDAMSNNDKASAKQIAWE

AKRERQSCGKNWRDKKHRGGKFFKSGHKKKRKM

>SG-AL_contig_9120_1_+1_118_222

MIHTNKPSKQKQQMELSMSQHNSTFNDAVHIFVNE

>SG-AL_contig_9121_1_-3_96_254

MFLSAAVKAADYPEIRKRTRTQNRKMLIRAHRTHALTHTHTQTRTRTHAHTHT

>SG-AL_contig_9122_1_+3_3_188

FCFCHLPRLQCFTILLANSHSENELTNVKQSVDTEKKAGIIFITTYALQNGILYFTALRV

LL

>SG-AL_contig_9123_1_-1_1_240

TEALSPSPGSETAAPPLADHEGPSVSPQSPAGALQPSNSPRPTPPPESESSPNESPLISG

GASHTFRYEPTSSPTHGSTG

>SG-AL_contig_9124_1_-2_2_292

HQGPSVSPQTPTGALQPPNTSCPVPPPVAVESSPNEAPLDTGGASHTPIAGPSRPAPVHM

AVPRAGQAAPPVHRRPLSAGGWGSSFLPGCGPPLASP

>SG-AL_contig_9125_1_-1_97_609

MANLQSEVIELEFMEFSRGLSTISEVDFAKILLRYTSLDRGQIEECLERVRSRIPQEKGI

SLEDFRQFCQFLNSLDDFSLTMKMYTYSQRSVSQEDFQRAVKVCTGHTLGPHIVNTVFQI

FDSDDDGHLSHQEFISIMKDRLHRGARSHLMHTKNSWDSFRACVRNEMRSF

>SG-AL_contig_9126_1_+1_955_1188

MQQGQDFLQVHTYDHFFFSFSKYVHTYNLGKTLLSHMDSVHKFFVFQKMYIHTILPKFTF

KHLYALNGKAVSLFTVIT

>SG-AL_contig_9127_1_-1_79_192

MYKGINPTKQVENGAEAKIGRKPIYKMSLIILCIACSC

>SG-AL_contig_9128_1_-2_1259_1426

MCHEDLYTLRDLSDCRARRLTQSLLIGIVASIAVMAYCEISLKTTFRRSKHNRGCH

>SG-AL_contig_9129_1_+2_2_211

SAFFCLPGPFHFLFSSKPSHFFSPQPRPLPQFMMYYLCCFALATRLDTVNTSCSFCSVIC

LHVLFYIYIL

>SG-AL_contig_9130_1_-2_2_145

SEYLENLSETYSPWVWEVILFQIIHTLSSLLNFVETRGTILDFSFTSI

>SG-AL_contig_9131_1_+1_346_513

MITCHQQACSHADTNSQFYSSPNFFFQNEIGSFPPLQLFSQGLNTKRKTNRCVTLQ

>SG-AL_contig_9132_1_-2_2_208

KCVIMICFSFSYWFHNNNGKIWKIIYTHKNMQYVRLFPACEIQDIFDWIAKCVVSYSLVT

NVDVKLQSN

>SG-AL_contig_9133_1_-3_186_374

MLANQHFVLFILILVTLTLFFRQMVYTGVQNCRNEEAVLKSAHFSRIKKKKKNWGTLHNN

TLL

>SG-AL_contig_9134_1_-2_86_313

MQHPAVLRSRRCIRAYALRRESGRPVCLHDPCPRSIPPTGSEEGPSTSEGATFSSAPPAP

GALAIPPLGDRESAHG

>SG-AL_contig_9135_1_+3_1944_2252

MYTGCQQYMYVNCGYVQELIVPSNDKHTKYRQQFKQMCNHAPVHYDDKTKHVTQVESVNK

DCLHSFFLFYLFFCCIILSCINLSYLLFNRLFMLRLLSKLGHQ

>SG-AL_contig_9136_1_+2_413_649

MRVCVCVCVCVCSYFLSMRYFHSILISCIFVYFSFLFMDVMYWFASFDLMTRLTIPKHNC

RNCMIWISLFLAVFLIFII

>SG-AL_contig_9137_1_-2_119_304

MTYLYGKIVSDICSLEVYLHVQDISLLMSHPNLLQFCVIFFFSFPNHVPKRINLCSCCHS

QA

>SG-AL_contig_9138_1_+3_516_746

MYIFPRRIIKSNTEPVCIFSSTPNNTMFSVILTYAQLTHSDFNNSSVLSQNHLVCRHLSF

PTKSHNRKYLQVPFLLR

>SG-AL_contig_9139_1_-2_23_1597

MMLVDLPPRGQKSGERKSWLWGYRQVEGAPLHPLGFALQVDHVSTDVSDWKVTRVVYNGQ

MFYEIEDLMDRYNEESLRKVRLAMDAYDRGFSSYERRGPHRFDSTGRGPRLVEPDGRRFT

VDGQFVQYFGWSFNFRSRCSTGVQLFDVNFQDGRIAYEIALQELAVFYSGHSPEMSTLTF

YGSSWFIGASSHELVPGVDCPATAHFVDSFHFVDSPSPRRYKHSICIFELNSGIPLRRHY

TASRDDDGFQYYGGVVADSLVLRTIAVIWNKDYIFDYVFHLDGTLEIKVSVTGYLQTSFS

LPRERPFGHQVHQDLIGNTHQELFHFKMDLDVAGSSNRYETIKMEAQDVPNFWFPSQNTT

KMRYRGTVVKTERESMWKQESSPLLHHVIYNHKLSNRYRTRRGYRLVHRSPTSFVLKGAK

VARAAGWAATPMLVTKYKDVEDASSSLYAQGDPWDPILDFSMYLEDNNTIVDEDLVVWAT

LGAVVLPCAEDVPSPTTTLNSYRFLLSPYNYFNIGLSTVVSDAVH

>SG-AL_contig_9140_1_-3_78_308

MIVEHVRSFKHVSVTCHCNLTFECDFYAVLSKQKKEVKKNCVSESGLYVNIAEYTDNISN

KKHAFTLKIVRVLLDKL

>SG-AL_contig_9141_1_-1_1_156

LQSFGGQPILLRSSNRPFLLARSVLMIINIIAYSFGCMFPSASLFNDPNLRC

>SG-AL_contig_9142_1_-3_177_293

MALAFMLLGRECVNLVLFTGDYVLECNPLVWVTFIFFFF

>SG-AL_contig_9143_1_-2_56_226

MCHAKQVRIFQIGFFHNVGQDLFPGYPSETYEKNWAWSIYTSKSNCQVSGNTLPYCM

>SG-AL_contig_9144_1_-3_1173_1844

MPSQALIMRSRSRSPCTSPSHSPSPSPSRSSIHSPSRSPSNCPTPDRRRREDEEEGEEGG

RGGGYERQSRRYRGTRVHVGSLARATSVRELRTQFRKFGDIVDVWMARTQPCFAFIVFRH

REDALQAVKRMDGETLHSRRMRVTMARPRTKGTRSGAFNPYLRCYQCGKRGHFSRDCRAH

QWSPSPSDIRAQRREERYRYDSRYNDPRHARRSSTSGSATYRRQ

>SG-AL_contig_9145_1_+3_150_368

MLPFMINVQFASHCILYSYNPSFPIGKEGIISVLNHVQLLVPALTADSTNLKNCELFWHK

PKDHISIFKYFNV

>SG-AL_contig_9146_1_-1_325_522

MEDNNNTGYIMCLAQGSRCLQTTLQTNKQLTANYTHTHTHTEAHTHACAHTHVSTSTYIS

HNGRNQ

>SG-AL_contig_9147_1_+3_549_1091

MVRQLKYNLWFSGFADQFRLLKRERRQAERHWLKSGLTVHKEIYETVKHKVVELVEGAKT

AFFSARILASKTCKELFQNFNSLMGKEKTSPLPSGPESLPGIFSSFFSEKILTIRNSFLQ

TDQTSPISERINYKVACTCFNIITGSAPTFSQNLLLSTYNLAVSTVLRLPSSPRTLQTEM

S

>SG-AL_contig_9148_1_+2_2_64

FFKNLYLTFVSFYLQRKYLDC

>SG-AL_contig_9149_1_+2_2153_2470

MVYSFLSSRTETKRWYRLEQIQKSHVSFFFKLPDSWLVCTNRSSIIYIYSNFHSALCVCV

CVVVVVCLFDLLLSVWNMVCCCCQYGIWFVVKMEYGFVVNMEYGLL

>SG-AL_contig_9150_1_-1_88_2550

MPTPKRLRDLIREIRSARTAADERSVVNKECATIRDSFRDQDNTYRCRNVAKLLYIHMLG

YPAHFGQLECLKLIASPRFTDKRIGYLGAMLLLDERQDVHLLITNSLKNDLGHQTQYIQS

LALCTMGAICSTEMSRDLAGEIEKMIKSSNAYIKKKAILCAFRVIRKVPDLMEMFIPSTR

SLLNEKNHGVLLTAVCLITEMCEKSPDTLHHFRKLVPQLVRILKNLIMAGYSPEHDVSGV

SDPFLQVKILRLLRILGKNDNEASETMNDILAQVATNTDTSKNVGHAILYEIVLTIMGIQ

SEAGLRVLAVNILGRFLLNNDKNIRYVALNTLLRVVQADYNAVQRHRTTIVDCLKDPDVS

IRRRAMELSFALINSNNIRGMMKELLHFLEICDPEFKADCCSNIVIAAAKYSPNKRWHID

TVMKLLTTAGNYIRSDVVTVTIQLIAETAALHAYSVQQLFRAMQASYAQQPLVQVASWCI

GEYGDQLFSTNVEDEEPLQVTEDEVVDMLERIVGSANSAVVTKEYAITALMKLSSRFTVT

VPRIRQIMTLYGSNTNMELQQRSVEYTSLFSSFDHLRPALLEHMPLIETHHHTLENGLEN

EEDDLLGGDPVSRTNQQNLQQPQQQQTGNILEILENGQTGTQSESSKLGGAADLLVDLLG

DLGPPQPLPNNTVPAVPVGPLDGLLSPSSPVSPSNNMINGSAGIPLTAFEKNGLFIKFMC

ERDQSKPSSTLINMAATNSTPFPMTEFVFQAAVPKSFQLTLESPSGNLIPAMNTGNLTQV

ISVNNPHKQAIRMRLKITYNHNGTAVSEMGEVNNFPASLWQ

>SG-AL_contig_9151_1_+3_69_230

MAYATIFLDSFPTHGICSVRAYRVCSKHGDAVWRKVKLKLKPDRNWYLSCRFMN

>SG-AL_contig_9152_1_+1_28_315

MDHWMMSVSERTTGYCLYQNGPLDDVCIRTDHWLMSVSEWATLCCLYQNGPLVVFYIKTD

HWMMSVSEWTTGCCLYQNGLLVVVYIKTDHCIRTDH

>SG-AL_contig_9153_1_+2_2_163

MKNRKPSPIKQPKKELSLFRLDTRLNPPLLFRSSITVCIRMDHWLLSVSEWTTG

>SG-AL_contig_9154_1_-2_95_424

MEIINRLLITFFETMCLRRGIGMVGGATPGKRLMGLRVLSCTDLQDLGNNRILVAPASDI

GWFSAFVRSAIKNFTIAFFFPACLTVFMFKHNRAAYDVLANTIVVETEDL

>SG-AL_contig_9155_1_-1_88_537

MASQHFPCVVVIVFCGYCYQQDTHTHTHARTHTHTHTHTYACSHMDTYNHVHTHDFMRTT

CVAVSIYMQELEKGYSDVLFSFGYSVFIFCFFSLFSNLICSHTQLYFLYDIIIPNWRESR

SYVFIFYLQHLTTLGHSTPFFPPLCIFPRL

>SG-AL_contig_9156_1_+2_2_184

PPQMVSFRVGRFNSLMDRSPFWRLPWGLRKTFGPEGNLMYAVGCPQRSSDFFSPLSLPSH

I

>SG-AL_contig_9157_1_+3_594_806

MPELLDAAPRETEMENPVSCCNPRAPPSNNHAGYGIGRNKPESSCNCIHAHTRKRDIIMT

DSFPVGNAQFV

>SG-AL_contig_9158_1_-2_137_1210

MNMVCRCQAWMSSRLFRCTPQIMDRMNECNIWSSGAESSAMTDYTDKVKRKESKGLPIIH

HYKYVSPLPLNHRFAMRKFHGVLRMLRKDGIISMNQVVEPEKIAQETTSLVHDEDYVQRF

FSGKTTPHEQQISGFPWSEGLVDRCRYETGGTLLAAQMALECGLACSTGGGTHHAFPEYA

SGYCLINDLAITAAALLHLHTVGRVLIVDLDVHQGDGTAFMFEEDNRVFTFSMHCQGNFP

AKKQNSDLDIGLSVGTEDKEYMQLLEGHLPWIVDKFRPDLVLYDAGVDAHIKDELGKLCL

TDEGLFDRDHYVLKLLAAQGVPCATVIGGGYSRDLDALSQRHSIVHRAASKIWEDSIL

>SG-AL_contig_9159_1_+1_94_279

MDTALIMLTLKKVRSLPPHSPMTSGLWNNLYLLYLASKISKHQVYCFSENCFHGQCMDSE

IK

>SG-AL_contig_9160_1_-1_10_1173

MSEVWLISAPGENTPQKTWDALNRATTAENLSTNYKFNIPELRVGTLDTLVGLGDDLAKL

DVFTESVTRKIAQYLGEVLEDTTPTKDKLIENLKINDASPASYLHKFAWDGAKFPIKQQL

KALAEMIGKQVSQAESDLKIKANAYNTLKSNLQSFERKTTGSLLTRNLVDLVKKEDFVLD

SEYLVTLLVVVPRGGSAEWFAKYEKLTDMVVPRSSRVVFEDQEAQLVNVTLFERVADEFK

HKCRENKFSVRDFVYNEAQILAGKKEAEDADTKKKKQFPPLVRWLKVNFGECFIAWMHVK

ALRVFVESVLRYGLPVNFQAMVLVPNKRSQKRLREVLKQLYQHLDSSALKKDTESQVVDI

PGMNVGSSEYYPYIYYRINLDLLDQQRL

>SG-AL_contig_9161_1_+1_1_201

SLCLSTSFYQLPLFIQLPLPIHQLPLFIQLPLPIHQFPLFIQLPLPIHQFPLFIQIPLPI

HQLPESW

>SG-AL_contig_9162_1_+3_54_242

MKFLSVINKIIIPIRFILFLYLLQNVTLSDTCCPCSFLLFLYFPFLSFLFFLFHLLSLYC

SFF

>SG-AL_contig_9163_1_+3_267_467

MNNPRRSDKSIRSQDSRSRRGCRILVMHEQYPPKSPSLLICLGFTPLVFLSTMRPQGKVY

CQLDHLK

>SG-AL_contig_9164_1_-2_293_580

MLLSAEMTLSRYLKFPTSSSSSPLTVVFFVLLVFSFMILVFPGWIWRHSDQTDSIIYRQT

ILLLVIYLAFFPCYVITVGSLPCIFPCYIITVGNLP

>SG-AL_contig_9165_1_-1_319_999

MARPEMPKGYLGAQTKWKEWGLYTVSHNSPEITTNTGSVKIDIKTGCAMKVTANLIECGG

KEQEYKELVFTQTQCRENSKERIVTLLVTLPSAGFYKLQIYALLACDDSNSLPNVYNYLI

HSTADHPGPVVPFPKQFAQWKDGCYLFEPLHLKDLDRALFKVTIPDANAVALTVEKEWFP

LKKGSDGVWAGEVQGLHKFKGKVQKAALNANFVPEDTKYTSLLEYSL

>SG-AL_contig_9166_1_+1_592_849

MTEVPEVLWEEAAQDRTIWHSLVNSGVAGYERRCTEDQKQRYQQQKSRACSNPQPGLHPT

ISCPHCSRHFKARIGLISHLRTHTSQ

>SG-AL_contig_9167_1_-1_724_975

MQYIDGYDIFDGLYLKKKCISSSVMIVYYFENTEFTQAISFSAWDIRAPTFWGKGEFFCL

FVCLLFFARLVSSAMYFFPIDSIV

>SG-AL_contig_9168_1_+1_109_279

MLLNPIDITSSFHCKKIEIQYISHMIMSINFIHKISSSSMLYSQKLLDCQICNRILL

>SG-AL_contig_9169_1_-3_63_335

MAYVSGGQVLESRSPWRFSVVPDFFWGIINFIVLFFKTMINPDLNSRGSSHTSDYRPPGS

GPGGPPRPPRRRYGGFGGGGVAPSPPPMGGG

>SG-AL_contig_9170_1_-2_2_115

DSVFCEHLRGENHTIDLSGISILDTELHPVKRKIKETV

>SG-AL_contig_9171_1_-3_615_827

MTKRREINPPHNQHTHTQTNPFTSAGYLSLAFTCVLVPDSSDIRYYWIFQPLLVAFFSRW

FPTCAETDSHL

>SG-AL_contig_9172_1_+2_26_229

MTRLEHWGGQRRNRTVFMLQAGIELETSDLKSMAVSTVPTSPHKKKTKKLARLRVEPTTP

KLSAWRSN

>SG-AL_contig_9173_1_-2_2_127

NSPFIFHKKKPLITLSLINSKLEFCLNNYYAKFLGTEVYLDW

>SG-AL_contig_9174_1_-3_102_1142

MSQNADPTRFDGMLMNMAQQCEGGIQELLGSFFGFLERKTDFYTGSGSAQAEELVMSSFR

KHSAAAQKLKKEQMEKEKKKAEKKAKEREEAERKKKEAEEKAKEEPKIKELTDAEAEQLE

KELSQKDGEEMTVPASGDAKTVSANGDAGVSEKKEETKSADGDDDEEDEKDKGKLKPNSG

NGADLPNYKWTQTLSEVEIRIPFNVSFPLKGKDLVVDIGKKRIKAGLKGHPPVLEGEMYN

SVKMEESTWCIEDKKFLLINLEKVNQMEWWSRIVTSDPEINTKKVQPENSKLGDLDGETR

AMVEKMMYDQRQKEMGLPTSDEQKKQDVLQKFMKQHPEMDFSKCKFS

>SG-AL_contig_9175_1_-1_202_1524

MPGPWAFFGASRFSPPNWFCQEDCDDSCATSSNFQRSGTATAASSSHDTFQEMGPHLAPD

QESRHPQNDDQSQAASNSEQSANFNPLSFASDFCNWAGKWKEEKKYAKVMWKKVFWSMYK

SCQQGGQFLFCPDCLQTFTKESELDKHSQENHPTPREHNCFICQQPYSEAVSYNKHIRQH

CETFHMNTRRGRGRGRGHGRMGFGRGMHHIFKWLCKAAKPYDTSNSAQFGQGHTQDTSTD

NSPGSSVSEDSKECPVCHKKYSANSRECYFERHVNSHFGVDMYSYKCEECGKKFLEKGKF

TAHQAKHKASLFVCEVCGNSYQSKTSLKRHEKIHTGERPHKCPDCGEGFIELRELERHRM

KHTGQDPFQCTVCNKGFTLKDSLLNHVRRQHPDKSPVLCIKRAMPLSEMDRKAGSETQGG

SVTVKTEKTSEPQEAAYMELD

>SG-AL_contig_9176_1_-1_406_594

MYWSHLVQFLCFVLKIVPSPEEKKTCSDLINIVSSFFPPLCLVSQLVPATLFPRKIIPNY

ITP

>SG-AL_contig_9177_1_+3_393_644

MFISHARSTASSYVHSTALCYAHVIIARLCSHSSLCVRTHTHTHTHTHTHRPTVTAAKEA

QKVSRQVKALYTLHIIFVCTTRIL

>SG-AL_contig_9178_1_-2_2_163

SDWLAWCGSSQVDPRSPSPVELAKLPGLPCHLGGPSREHRQGLQVRYLHYPSSP

>SG-AL_contig_9179_1_-3_3_149

FFFFFSFIYIVVRVLRTLFLLRDIYRIVIFMLRIYRCKLGNGSTVVNHH

>SG-AL_contig_9180_1_-2_2_112

LEKTETGVKISKMMVNMLECIHECIYWTAVLTNSVCE

>SG-AL_contig_9181_1_-2_203_427

MNNWLTVCLGLPLPAHHPSTSSYIGAVSVIQGACPCLCECGEKKGYLVLMNCGMREMYDN

GKEFVVNFRPRISYF

>SG-AL_contig_9182_1_-1_61_441

MMVMIALCTVSKSVMIVMIALCTVSKSVMIVMIALCTVSKSVMIVMIAALCTVSKSVVIV

MIALCTMSESIIVMIVLCTVSKSIMMVIVHCYWCKKKKKFPVVMTLCATGTLRLREWNCR

CATVLPL

>SG-AL_contig_9183_1_-1_1_195

GGGSRQNCSVVSVSSSVMQKKLFESLVFISFLGFLLFVVVGVFFSDSDAFLCHDNPSNAQ

NDHVK

>SG-AL_contig_9184_1_-2_2_202

VCVWSFTVILISTGKMCHADLSSRVSYALLALTCVCCDICDLIHCTADIVIHVHVANVVG

TVILVRQ

>SG-AL_contig_9185_1_-3_201_338

MNLFKSYGTIMYVRKYELTFVLLCLCSWINSFKHIFKLLTKNLAEI

>SG-AL_contig_9186_1_-1_1_105

CSSDLRYSSKSFGLNRANNPLVLHNETIPVSLTLR

>SG-AL_contig_9187_1_-3_36_674

MASNFAVTVTVVTRLVCACVLFHFSLATCPQDRSPVLDDKVMGSDVVVKGRVRGQVKVNA

LDSSYTAVVDVHCVFKGGPVPHLINVTEAGKEEGKCVQTELLEGGIYLIYLRHTAGGAYT

PTFTQDPADRKYLDELQYVCGLHVQLPTGIDSSKTTASCPPDSFAADYEVDCPDTSPPSP

APSSAAETLKAVWTMSCFVSAALASFPDLQLLL

>SG-AL_contig_9188_1_+3_3_335

GEDREGGKGVADQRGPVEGEGIHRHRLGVLHRGDVDRKPLVELGRKEVDHRDEPCVPDSL

EGLLLEGPLRVDDGVTCAQSVGQDVGFPDYVSCRQGDPAILAPPQKNLGPR

>SG-AL_contig_9189_1_-2_2_193

KKKKSVSLWLNKNIYRGGKMKTEWMRCIIETCALPEENSLNFISTIPHGKVQIPFLLHGW

LGKR

>SG-AL_contig_9190_1_-2_2_253

GTPPSPPVCRSGVPSSFAVRKVGRTKDFLSGCNPLHRGNSQTMRQQQSPGAPVLRQTRGT

RASLTMRPPCHATHQVPTQNCRHL

>SG-AL_contig_9191_1_+1_10_303

MLSHCIVLYCIVLYCIVLCCIVLYCVVLCCIALHCVALHCIALHCIALHCIVLYCIALCC

VVLYCIVLYCIVLYCVVLYCIVLYCIVLYCVVLYCIVL

>SG-AL_contig_9192_1_-1_124_1104

MADSDKLNIDSIIARLLEVRGSRPGKNVQLTEAEIRGLCLKTREIFLSQPILLELEAPLK

ICGDIHGQYYDLLRLFEYGGFPPESNYLFLGDYVDRGKQSLETICLLLAYKIKYPENFFL

LRGNHECASINRIYGFYDECKRRYNIKLWKTFTDCFNCLPIAAIIDEKIFCCHGGLSPDL

QSMEQIRRIMRPTDVPDQGLLCDLLWSDPDKETMGWGENDRGVSFTFGAEVVAKFLHKHD

LDLICRAHQVVEDGYEFFAKRQLVTLFSAPNYCGEFDNAGAMMSVDETLMCSFQILKPAD

KKKFPYGGLNQGRPVTPPRGNTKAKGK

>SG-AL_contig_9193_1_-1_799_1122

MTGELPFNQTPSGITNILAVASSRALTVVQTEWTFSARSSVESPVSSVERSLCNQIQQDF

PFYASLVQDPEAFTIPFYASPVQDPEAFTIDVFTLDWSGLLACAFPAF

>SG-AL_contig_9194_1_+1_1_162

FTLLSFQVHSCQNCTEHFHKNCSQQRQSHSSLLGLVHKLYRIMNSTCRLSEMTD

>SG-AL_contig_9195_1_+2_965_1375

MRMCSSRMFMSVMPAILRTGGSDSVFSEGMGPLGPTTYSAQGLLSLKAWVRSDPRRTALK

VCSLKVWVRLDPRRTGTFVVCLLFCFLTKTNKCYIDKVIKFSEDSFKTFFLLLIKNYPCG

SMGTHGVRERLKIMGYS

>SG-AL_contig_9196_1_+1_1_201

CVLPYQETPGRTSAELRSLPAVVPQSSPLTLRPGTLDESTCTCGPDSLVADCRPTFNPRD

ASFQIVA

>SG-AL_contig_9197_1_+1_211_399

MNKKIPHHGDQKQTRIMTTHNISCTVDYGDKPLKYKSHITEDNRISCNINWGTNNFFKNP

ALW

>SG-AL_contig_9198_1_+2_284_529

MHTAKQYKLCNQYVNVSKQIARKTNHKMLARHSALHVHYAVITVLLNDITISWIRANAEL

ANNSTFRFHFHILRTNFIVKAD

>SG-AL_contig_9199_1_+3_3_188

KQMTVKEGKGAETKRATEKRKLLAQNFQKVEILFILKDKYTQISFVNNIYEKRNQAEIKL

QH

>SG-AL_contig_9200_1_+1_1_204

RVLIPGLPDDSWEDREGGKGVADQGSPVEGEGVHRHRLGVLRRGAVDRKPLVGLGRGEVD

REDEPSIP

>SG-AL_contig_9201_1_-1_193_2868

MPCIPDRDVSLTTLSPCALPLVSSQAQQVKLTWQSRIYDTSAAKGNRFPSRQHSGVELCA

DRFVSHIPPFQWQQLLAAVREDDEEKQVTTVHSVQGHVSEPLSDGHFALDVSSEGGGGGV

MQHFTEYSMSLHTNMMLQVQVTAGMSRYLLTPQIQLISLTPSLDVCMEHRSNPSSCFAKA

GGCLAISNTYLDENSYQQHFMPLLALEAAESAVCNKESTTIHNVTIRWNESSSTYTGGEA

DVFGQFSLTHSFCKERNLFTGSQTHEDLELFGLEKSMPVPMDLLCVRYKSLEVAGNRKLD

ERVSALMQMGSPGSWVAHCVVLQVSCEDHRMVNVRLRLLSSVKMPAQLLDSLQASQLTCT

VECIPKTNLFRQMEFSVACLKSASRLARDVAVGRKPASNIDQSSVAFLMGRVEGLLNSEQ

KEAVQMALTQPFTVIEGPPGSGKTLMAGHLACLFAQRNRMLAQPASTEMARTQVLVCAAS

EVVLDVLSACMRSQAQVCPRVVRIYDQQTEDQQYPVPRSTIPPSKLQVASISNPELQGSG

LHHIIRSPSSRYYQQIKDYDSLFGLSPQSITDSQVEEYRNLVFQAELEELRQAEVILATC

STAASPKMAKAANVIQVIVDSCESCMEPEALLPVILNRSIQQLVLLGDRQQLTPTVMNHT

ARSLGLGRSLLERYANTAIPLTKQYRMTQGITEVPSVCFYNDYLRPSELGPGSAAKSLVF

SHIMGREKVQMAATDTGVMESLCNVKEAESVVHFVEDLVQKGVTDSRILVLTQYAAQVFL

IKQHLTLRHLNNVTVNTVNQCQGCEYDFVIFSTCRSIPRSEIHKMPSDAWLHSHLGVSGD

AHQMNVAFTRAKEVLIVTGNKYLLQCHPLWKKLLDHCRKERSVITGIDAADM

>SG-AL_contig_9202_1_-3_15_218

MCLAQGSRCLQTTIQTNTVKLCYSESGRPRHIFSLLPSSRYSQMTNLPRETKGNNLGTGA

VDVFIINR

>SG-AL_contig_9203_1_+2_320_589

MLEKSMYISHVSSCNEILSCDRKIASLIYRLQLNSWRTKYVHNACICSNVMSVYHLFFYC

SILISEYNNNNMDFKTKFSNVKEILYNKRH

>SG-AL_contig_9204_1_-3_15_266

MFYDWLDVVYFPSYTCAVVSFVVVSVIVKRGCTVNVVTIGGVDFHICRYRCLKIKHQSCC

LSSGRRNFRLLTRSFWCFRGSYGI

>SG-AL_contig_9205_1_+3_102_317

MSNCLPSRSTLVDTTMLVAAPNIVDLEDNSSLSFHFYLLCANNITAMKTTIRHFHRVQQN

NRMLNSNRCILQ

>SG-AL_contig_9206_1_+1_1_171

FFFKDDFSLFLLLFQLAWTDDNSHLLCSKEIFNNKILALSTTIHPILKTAYKMKKKQ

>SG-AL_contig_9207_1_-2_116_1894

MDFFPDPMESGNLKIKGQRRPLAQPQGDTDRGFMAGYQCSTPYPAATVSLAVEDPVFSLG

IQDRTHRKGVEFDTGLGNFTLAGNDAVMGSVSEMEDGSSLVYDMGHGTSEQQPAFLESMV

TSVRDAGLSEQDCNIVWRKNFKSKLVIDSTDLQVVEHRCEVTEKKEGYVIRGGDQDLCQL

VCKRSDSPHQTLNEGVICNSQVLCSENGDYNEGKQYVVFCDTKSEGAFGEAVMCEDLKTH

KTYIRKKVHGQLSRNEVTVPVVMKGTAGIPAVYGVISRGKTTEIYQEHAGLSLRRLCKSE

YAPRIRDPGMLLGLAFQGFATLDKLHSKGIQHLDIKPENICVDMGQKVPSLKLIDFGTSQ

MPGEVVTAGLTYEYLSPESCLNILKVATGQPPPSEGRLGPGADVWAFAQSLLYCLCGYDV

MVYMCTEKTAYPTDLRPEELERKRVECLLKTQRMTDKDITSRLIHPEWPQVLQVLLVNTL

RVNPEQRWSAGQAAAFILEYLKTVAQKSAAPPQVVAPVSGFTLAPSNLSLRVHTSQNGQE

NLPVQFTSIAAPSAQLPAFGELLPHSLNKMEAKSGKRRCQEESSRRGYKVMKM

>SG-AL_contig_9208_1_-2_179_334

MFQLARMRYATVSEFRGKVLVSIREYYDKDGEMRPGKKGISLSLEQWNALKE

>SG-AL_contig_9209_1_-3_162_914

MAAKETPNSKKVSDFIENFKKQTEALVTDIFPQKVLDIEEIYQKMIAEKLSDVHTSVNIP

IPEPALVLNDDEEPSSKKRKLDANGSDSANSVPAGTPVLVFPNGKVPYNTYIHRWAEDVK

PLITDLMEHANMVKMWISFLIPKIEDGNNFGVSIQEDTMAEARQVESESATYLDQLSRYY

ISRAKIISRIAKYPHVEDYRQSIRELDEKQIVCLRLTLCEMRNHYASLHDIIHKNMEKIK

KPRSGNAENLY

>SG-AL_contig_9210_1_+3_126_248

MHISMQIHTQRTDVPLPYTCMKTVNHHSLSPHSCTSPPPPP

>SG-AL_contig_9211_1_-2_413_3982

MAAVGDDCYFYYYSSCAKGSLCPYRHSEAARGCEITCTSWQAGTCFRPNCRFRHMEIQMD

RSQIACYWESQPVGCIKPHCAFKHFKPRPAGALIPQTTTVPGHGMLPPGGLPPSEHEEVP

RSSSGHSSPTVPPVIIPVGDDSDMESTGTHSNVGTPSKSELNPAHKSSPRKPDPEVAHKR

SGLKAPPSRLDFSSTSEPQMSAPKPQKSSEHSRPRNSPPLRPHVSSPRRFQASPPSRSQV

SPPSRSQTSSPPQHEVTSPPRRKPVSERLGTRSTSSHAPGTKASVITLKPSSTEKPAVQV

LSIEEIYRQRALESLKRARARAASASREPESLVKSEDPESDCRRVIVDSEDPQQTEEAER

PSSPKKARISVRDRLGAKVSPKKVTKIKKRKRPVPVPEPDPDPEPEPELESEESEPESEP

ELELEPELELDEYSEDDDIVDVDDREPVKVTITQDARPLKKLVNRAGILPSGSRHLKKKL

SNSVVSTPKRVKKLIPSAPEVVESRKLKKIVSGDSIIIERKPLKKVIAADPAVKRKQERK

MLLKQSIEERKSPKKAVIYDTVLEHSVVERKRSKKAIIHDPVAERKRLKKLMVDHLSKSA

VEKPRRKITLVDETERKTASVTGLSLSEIRQRAARKCVPDSEDQVSSDGSRSPEMSTLET

RRRQLLKKLSSPKPETSEDEARSDRSPSPEVLSLEEIRRRKALKERKQKKSEAYKPVSGS

EKKPIVKPAVSVQSLTEIRQRKQQKDVRTQSLTEIQQRKQQKNVRTQPVYDISLFTDRRE

ERSKVEEVEPSVLSLDEIRKRKLKTDSVKDRPALAKRTKTEQQIYVPPPRKNAVKMEMVP

LRKEGPAIKKRLGDISVSRTQTEEKPAVTVRPESVEVKTFSEIMAEKRRKLQLEKERQKK

AQGGQEENGQANKRPFRFRPIVFGEADSDKSECSIPLVTKISSDDDKSNSPKRSAFVRLG

QTERAGTTFSSSMNSRLTTEASASQSSSVSSQLPSKFNISTEDVSKDSRVGIIKSSSSHS

LSSVSADAFHCAPSSPRPKSTKSNPTGEGPSHFSSTPFVNPCTQSQPTPTISAFPSVPQQ

STVCHQESATTPNPHMMAIDTLLTSKNKVQIQPSPASASTSWAYPLPSSHQATSGDTAQA

AESTTSTDLSTSRLLSDSGDDLLKLDADIVTVDDNDQLDDLLMDIDSLLA

>SG-AL_contig_9212_1_-1_19_279

MAAARIIWRDHKMFENFFEDFFADANTEHIGENRHCKECRPDYSFSYPRKKTTIIPYLFQ

ICQLQIATNPLKLCLCCTLFISVQLSF

>SG-AL_contig_9213_1_+1_1_207

KKVFKKFSNTLRSLQMIRAAAILDSARKLRPVTCIFAICLLKCAWKLRSAGSWAQLINIL

MNAELTIAF

>SG-AL_contig_9214_1_-2_203_379

MFAFLCIKAGDCVSEIPLHSCPFFFSFKKGVNCKHLHSLHIGYMLYRHGFFCRLLVTLF

>SG-AL_contig_9215_1_+2_134_298

MKQNITTAQYWPISLANYLLPSYSDRSKSGSVMYSKNKAKHKNHQLFKSVQAQFS

>SG-AL_contig_9216_1_-1_1_327

MSKSNIIVFRKGGYLGMRERWLYDGTAMPVVNVYKYLGIFFTTHLSFVVACKDQGSRAKN

ALFYIIQKLYTVNNNSLQLFVKLFDSQVNQLCNMAQNCGDWVNLCIIVN

>SG-AL_contig_9217_1_+2_47_223

MERIIQQTHHVARAESTCTLGHVTARAWGGQGMYVSIGHKRLKTNGTRGRGGNGQSSTG

>SG-AL_contig_9218_1_-3_3_227

HALSGAKDDIGFHSNSDLTLTVLPEFLAKTQQPGFRPRAFRTPSLVSCLDPRGARGEERL

LCPVRCVKYYLKCSA

>SG-AL_contig_9219_1_+2_47_214

MHSVRLSSVTMKKPTDFYLSHSSLKVAQIVEGTDVPYPTQVLKQQRCSDVGSTKHC

>SG-AL_contig_9220_1_-2_164_646

MLTEKLGAQYSPRHYVVASTDSISLKKIIKSEKTASGAANSKSEYTVYSVPRSREVKQSW

FTTFFTTLYATLYSLRLVVQLKPQIILCNGPGTCVPICYAGLLLKWLGLADTRIVYVESI

CRVETLSLSAKLLYPFADSLVVQWPTLVEKYPQCKYIGRFF

>SG-AL_contig_9221_1_-3_9_743

MATISVEDLKKMKVNDLKKELKDRGLPTSGTKDVLLERLQAALDGTEPISSVTAENNAVE

DNDSVEKSIDQLVGDTIPTAPEAPVESTPVPVVTSKENIPEKTEEPASKPEKKVVSLTVM

PEADRMKARAEKFGGAVSDSAKKLMRAERFGLSASSSSGSTNTVGISAPKSEDMEKMKKR

AERFGTVVSNKLSKVDEDERKRKRLERFGAASTSPTEIKKVKLSSTDSDMEEKKKKRAER

FGLTS

>SG-AL_contig_9222_1_-3_123_347

MEDLSTRLQTLALQQKVAVSLEKTEPQYVLDLAAQYMGGADSLLAASSSNYSVRLFSRCR

LAQTNVLTGHEQAIT

>SG-AL_contig_9223_1_-2_32_490

MPVPGAGVPVASPSGIPMGLPPSGWFPLLFAPWVSVSAHHPAVGFLIQGYALHPVGSPWR

QWSSHGLLLPPHGRGGPMDASGPRYVARPLCLLVMGTPHGLCQQLGGRLAPCQGDSCYSP

DYPGPVCPACPGPQGPVTPVPRLIPGLWWPTQE

>SG-AL_contig_9224_1_-1_1186_1341

MFSLFANSEKWRVGPQYYIHLYKICVFPVLLSSREIWGYEDVHVIEQLQTEF

>SG-AL_contig_9225_1_+2_2_313

CVCVSFFSHRSKSGNSNRRTQTIGRQTPTTGLRNSKQQNDKVCGFSPAAADTAELSTAQH

LSYLLATPDDLYLFAAVAAQPSERTVQALSFRHPSQTPSVSGCR

>SG-AL_contig_9226_1_-2_692_937

MEVLSASIYKWCSHSSILGEKSLTVLQNRFYMAYSFQFIYILTMCAIIKVMNKKSSACQN

LPLLPLHFVRWVISWLLCNLFM

>SG-AL_contig_9227_1_-2_107_2644

MSHSYYNKQWQEAQATLVDLLQQELPPEPPKPERDRLAAFQLLATMYIKYIQIMRRLEQC

YDQVVHPQKRRVLRHCLDGVMGRVLEIKHEMVNLEFSEYHYFDDVLSDLKLTPNDVEIPI

PHYFVMERAKALKEREKVLGQILAKLGPQDVVQDKEEVKMPWDQAIRIIQVHERARQGRL

RARFMREIRQQEEREKQAQTRGAPTLDPDVAAMRIQKIWKGFAQRRKTRKEREEEMIFIG

MIPPPLPNSKNMPQAFAYRTEDERRKTQDVHEQEFQQALVTIREKIMETEGPDMKETMMD

QIRQWFIECRDATGKFPEFPDADDGGSAAIFKEKDPAELERELKEKEEEKGKGKKDKKDK

KKKKDKKKDKKGKKGKKKGDDEEEDEGWKMAPSNFVPSINEASGNYRDIWQTRSEDENFS

QKHDSELIKEEKRKEVETEIRTQVDELMRTELKNLKAAVDREKAKKGKKSGKKKKKKKGK

KSGKKKKGKKEKDLTPDRTIEDLYEELVTQGIIVRPSAVRLADFEGEYSYLGTTLRQANI

EPMPSLSDVRRLVTEFGILPLGSPAVHEQAPLTKSILLAGPRGSGKKMLVRAICNEVGAN

MFDISPANLAGKYPGKDGLKMLMHLLFKVGRALQPSVYFIGDCEKTFKKKVPKSDPTDPK

RLKKELPKALKTIKADDRLLFIGTSRCPFEAEMKPLLSCYQRVILIPRPDYASRNLIWRK

LILKHNGVITSALDVSSLSKVTDGYTPGHMVTAVQQVLTDRRVQMLSKKPLQSVEFVAPL

ARIDPIYKEEEESFKNWYAKTPLGKKRAKAAQADEDDGGKDKKGKKGGKKGGKKGGKKGK

KGKKKK

>SG-AL_contig_9228_1_-2_2_370

SIRAPFRSISLFILLLIKCLFLNNRSFPLQPPAVVSILIYWLEFVFPLIGPVLGCFCCCS

CCCCFLFVVVVVVVVVVVVFQLFCICFSPIFLSFQLYSFSSQSVFFLSCFLFSHCRITSV

KNL

>SG-AL_contig_9229_1_+2_314_511

MYIFSYINACILCKVSQQYFVKHEQREEATQDSISRPLVCNNKIEQRDKNLGQSSQWCLI

FNTDRK

>SG-AL_contig_9230_1_-2_257_709

MKNVYLVCIVEIRFTCDLLRKTQVPGSYFGVCLMYIIYIVPKMHMLYYLGLDNDWSVINP

NSNLNSDIRYASCAEMDRLCLSVKQRNQISEQVMVQKMPLVKHKMIYRTHTERYTSTEQC

TVFQTQNTHYACMTRNSPHLQHYITSYHRDR

>SG-AL_contig_9231_1_-3_153_452

MISKTTCLPFTIHETVAGKVRCLYLLCKYQEGTCAVDLACGEACCESCPMLNLKVRLKNK

NSSLTVKEIKGQRLYFRLYFAYMLNDILCMLLFIEQTFLS

>SG-AL_contig_9232_1_-2_2_190

PVKGYFHTCTSSYSCLGCREEKLIFRFSGLLAGWYSVCLLPCGDTIIFQLDQYSLKLALS

VSE

>SG-AL_contig_9233_1_-3_3_263

RNSLQIELHTTCAGCRVWITPTQQFLEMDTPKEDNQPSTKAQIMIYICGDCHKENEIKAR

DPIRCRECGYRIMYKKRTKRMVVFDAR

>SG-AL_contig_9234_1_-2_860_1144

MQSSIFICMITQVPHKSTEKKILKILQGVRCFLRFPRVTRPGISKRYKLYVMCSISEKQA

LPTRHIYTSSESNHKCSHDVNKPHSNNDLLNESSK

>SG-AL_contig_9235_1_-2_170_286

MPNVFHYYSSGAKSAKDSSGLRTPLVYCASCVSPHGMNV

>SG-AL_contig_9236_1_-1_139_513

MAASGINEHKEGCRKQRVLDRAANARRVIMGGLTDKDVLAAQEMARDNPEAPGYMRKMGS

PPGIVGTHTGPCDMGCQAADCDVAGCHSPTAIDKLRREGVRAQQLVAYSAKIPNRLSSVN

YTQQW

>SG-AL_contig_9237_1_+2_2_253

STLTISNLYNTAHVCSIFGQNPPNHREIQCKDASRWRPTHDAQVARAHATSSSFFRSLVE

GQFGRFKRGDGRAGLRVRYGKCTS

>SG-AL_contig_9238_1_-2_266_505

MCCFHHIQQWKFKSRDTWCHVVRYMLGRSYPSSQSSHPAVEVYVQRHMLSVFFGRCWAGH

VHPPNQTDPSVSSLQVFKGC

>SG-AL_contig_9239_1_-2_2_163

QHYSDCLVVTPFSSFSLVFPALSISVILSSQFEAFSFNVRILICNSLYSISFDI

>SG-AL_contig_9240_1_-1_73_237

MLLSGKWNLNFLSTQQGYYAKLQAASPPQSPLPNSSHLSFLNFPLLSKLNIILTV

>SG-AL_contig_9241_1_-3_3_212

TQICFNALCYYVYNFASPESFLNSEKGNKKHLNLCWIGGRIGSHGFNHWATTPLTSVLLE

TKQLVLLVLG

>SG-AL_contig_9242_1_+2_68_277

MRERVCEYITICVLAQVSVCVCVCVETACLNFVLVYCFFYCLVMCLSPVRCVITICSSTS

QLPRFTYYFF

>SG-AL_contig_9243_1_-1_19_654

MVSEIHIDDPELVEQLLKENQTNNAQQSSSTGTDGKGKSEGHLTPNGVAPLPAAGPVRRM

VAMYDYDPQELSPNVDSELELSFKQGDIIYITGEMDEDGFFAGELSGVRGLVPSNFLQEA

PLSDEDEVLDSASMVSPSRSQGSISAASRRSDSLSTLNNTGRKDRLSLSPPVERPIVLDA

PTDPSPAVEEEKRKKKGGLLNKGRSILKKFSR

>SG-AL_contig_9244_1_-2_185_1759

MLRFLSRMVVIAVSTSVVVPYTVAALCHLFYSHEPQKGRALARSLKPKRVYALSVAIMQS

MVLRARYVRLYLQWHRYYITADPSITLKNLAYGRNNCCLDLHLPVSHRLTQPTTKRVSKP

VVMFVHGGAWSSGDKTMYGALCAQLVKSLDVLVCCPNHSLYPQGCVDDMIQDIVDCIQWI

HNNIHDYGGDGNKVMLIGHSSGAHLCSMALLELLHDQQLHIGPFPPEVTEAASMLFHESH

YGRLISPNSGHNGKDHLEDSSGSSESFAVVSENGNSNKEGEASSLGSSMASSTLTSMIEV

SHADLDPQLSSMLTSTPADAASVEGGKLDEGSSQQGEPEKGSGDVSNMAKPSSQEGGDGE

EESGDDDDDNDSVVTVRPKDIERHATFLDLAQSIKAFVGLAGVYHIGDHYQHEMWRGIEH

LSCMTPAMYGPEHFERFSPSTILLSLDAPISLPHMVLVHGTEDYVVPVTSSQMFTERLAR

VSADVSLRIIPECDHFDICFDLMKPSRRFHSSLMTLLLETANSVF

>SG-AL_contig_9245_1_-1_286_1779

MRNFDEVVLTRSAARPNSLDLDNDLHTDMESVFPDMDNNNEVQPASPTRNNRKKKRRADA

PAKKFLDLYDATGEFLGNGSYASVQTYRNKSNGKEYAVKMIEKCNSLSRSKVFKEIEIFH

ICHGNENILNLVEYYEEEDRFYLVFDKMYGGTLLANIERRGHLCEREASLVVRDIAKALD

FLHSKGIAHRDLKPENILCEHLDDVVPLKICDFDLASGINCDGDGTKTPELLTPVGSAEY

MAPEVVDAWVGESFSYDKRCDMWSLGIILYIMLCGYPPFYGQCGEDCGWNRGEACQDCQE

MLFNRIQDGDYYFPSVEWGGISSVAKDLIRHLLVREPRKRYCAADVLKHPWVCNPPAAPL

ATPHVLTRNNSTKDLESFAETAISINRMMQQHMHITMSAAHSFFHSHSSSAASRFRADST

SIAAPDSASSNGSNGSTSGDEHDDFLTAGFLDGFENLHLTRPGSSKLAKRRATLHDCAFQ

SSSGSGDSLSLSSGSWRV

>SG-AL_contig_9246_1_+2_2_241

FKWAWPIFWITLIHTECVEQRCRRVLVWTDQLGYCFLNNYPAKHCIPRPPDDVDGSSVST

TIRRKFKARGKNKANRFPFS

>SG-AL_contig_9247_1_+3_273_413

MMNQQVYITRNFHGNCFTTITEQILLYEIQFFSVSMVHVEQEYKCDV

>SG-AL_contig_9248_1_-3_117_728

MSGQAGSVTVVKCDVSKEEDVGAMFHMIRHDTSLGHVDVCINNAGLAHDAPLLTGDPAQW

KHMFDVNVLGLLMVTKEAFALMQEKNIDDGHIILVNSMSGIRIKKNKGGHCYTATKFAVT

AIREGIRNELREMKSKIRISQVCPGFVETEFVQRQFNEELAKAVYSSMKCLQADDIRDSI

LYILDCPPHVEVNDIHVRPTEQEA

>SG-AL_contig_9249_1_-3_15_752

MLKVGDQDITVLFVQAPPLSWPLSRYGYLVAADSGVIGFTLLLVLPLLTHVVHMPDTHLV

LMAIGFRLVRSMWAGFCTHTWMVFTSVVLGAIGGLTSPGLRSELSKTSNPGEVGKLFSIQ

ASLETLAKLVGSSVFTTIYALSVHFLPATAYLCEVVVFLLMGALVIWLIRLLKDDEAHGL

VMGSSRTYQSVNKYDENLNRQLVPSKETSLDIPQSDDDDNNCDDDDDDDDDGPRKEKRGI

VGAFTP

>SG-AL_contig_9250_1_+1_1393_1602

MDYWMHYIIQSQFTRRGGFKSSICDVCHILNISWRLKAPKVRRLRERGYRPRPLSAGSVI

RVAWRTAESP

>SG-AL_contig_9251_1_-1_10_1395

MQSHSVNMDYDFKHKTAAERSRVEDLFDYEGCKVGRGTYGHVYKAKKKEGVDLHGSFGHR

GGHEYALKQIEGTGISMSACREIALLRELKHQNVISLQRVFLSHTDRKVWLLFDYAEHDL

WHIIKFHRSAKANKKQVNVPKNMVKSLLYQILDGIHYLHANWVLHRDLKPANILVMGEGS

ERGRVKIADMGFARLFNSPLKPLADLDPVVVTFWYRAPELLLGARHYTKAIDIWAIGCIF

AELLTSEPIFHCRQEDIKTSNPYHHDQLDRIFNVMGFPLEKDWEDIRKMPEHQQLMKDFK

RSSYVNCNLMKYMEKHKVKADSKAFHLLQKLLTMDPVKRITSEQAMKDPYFLEEPLPSSD

VFDGLPIPYPKREFISDEDNEDKTDTASKAQQGGDQSSSHNQPPAKRVRVMAPTTTSSNS

MVSTDYQHVHNMQAGNQQQNLPFSSSSTSHTSSNAAFGQRII

>SG-AL_contig_9252_1_-3_492_1016

MSPKCASVGNMADTPEAATQREFPNKRKDSRLPEAVEVEEIESEEQAADGVYDSRSTSLS

SDRPSSCDHSRTSSAHSQLGSEDTMDRGDRPVDGDGREKNKGPSVWQSFKKAGNEFNQDT

RRMFRYLGRKLHSRRGQTKVKEIHILPGEDDDRMWARRMAGSGTSRSSSSSDPST

>SG-AL_contig_9253_1_-1_316_3861

MKTDIVTYGMAAKLYMERHDARVIKTWQDGEITWIAWAHSHTMTVTRDVLLKMYDHTLEL

RVWDTKDKCSTRARFDRPKAFRLPQPKPGESPEDVGGVKAMVMKQMGGFSKMQSPKADAV

RPLPKRCAFPRADTKKRAATATVGTPSQTDQESGVGGKALVAVPNKAGSRGASGRTFSRL

GKLAGTERPVQSPGGSAVKDMILPEDSATVRRRQRKAEAAAAAAAERAKKFGVCMVPFRM

ALLFSGMQSITSRLHQPVSGVEDLFITVSLDQPLLSEDQKRALNPMIITVRSVTDMPSSP

MTFDELRSKCQPVYCKYQFFRQPSHTTVAKEHGKTIYWDDKNVVLLGPQEPSELREYLSG

PAMSVEIHDRDRKTEDVKLKATLFGDDLEDEKISNVGTVASRRTLHNPFEGRNKPWDPYG

VCKVDLSELLLGHRFLELRLPIHNCPLPDILGVEGTGASKLMGVAGAVDGPVDRPLSSGD

YMRSGAALKIQVELAHPLITPAKLAAKEELESTPQCPFGRIVFVFAYKNTTLLYQLQQMV

TAINARALELDDMPQHVIDAALSTYKLSVEQQLSSSLDIITGFHVLDGVRHIFVLEGLKE

QGIKQLWEQLPTPESSDVRVLYNSDLSFSQRLYGPLDVDLCRVKLHEPLDLVVQQSLLYV

RDMVPKPCFHALVRLEEMARMHLMKDVVKNDMFLTSDMVVSMSKEFGVPFTAEDFDELRP

DWEKSPVLVRAETTTETPGVMIQTSRDWTPIDNFNRTFVDRMAERKEKGSSHDFINENKD

VIKRTSDLNLLIREKTRVPVVSADVAKAHNYSTQTLNSTELANEALRNVLKEKPDSRFTR

SQDFHHSSTVVPVNVPALAKEDARLSKELWRTPTGWIYPGKRTMIESNVHPKRPDTARVD

ELEEKWRENILHVSQLQPPLERDKYPWDYRNHDMELYCKPPPMFKEEPLSIHLPGVKQQV

EKMEAKQKEANEWQSRIVVDDTAQYFHRVLPHTEMTSSGFYSSNQIDRLTCLLKDKPDKL

SLKRTHVRDIPPLSVVHYPAVDTLARAQGLPLQPAIPGEGQEKVPGYKPGPYDLSSWSME

DNRVPAYVYDHRKHAEQHGTDFNALHKERQQLWKRPIIPLNEEERDNHLFRTPLATLNFY

QPLPAVGASAMEPGDYAPTMDAATTTQDLPQSVMHERGMVYV

>SG-AL_contig_9255_1_+3_3_245

SCVENQNNTSLYGQKWNLVRSECKFFFFFFFLIFVFTFLYEYFCIFSISADKICLACTGK

QVCVYLVYVHLTNNMYVLTIL

>SG-AL_contig_9256_1_+3_3_164

PPQGISEILDSLLFSSRLSTPGQALGVYSAMLFASLAIYLCFLVVLLVLRVLFN

>SG-AL_contig_9257_1_+1_127_333

MERIQLTAEGTGHGFPDCRQHFPVQHAHHQQGPDEAVYDSQHRHFLNVDALVDGGDGGER

RFDRVPGPL

>SG-AL_contig_9258_1_-3_177_404

MLNVLFLTMVLCCSAKCGLPFAQHYKFTSTCRTHENKALKDRAILVGNFGVYLMSSVCIL

CIESRFRAPPFLHIDF

>SG-AL_contig_9259_1_-2_134_763

MDDEQETYKLWRIRKTIMQLCHDRGYLVTQDELDQSLDTFKATYGDKPSEGHPKRKDLIV

LVNHNDNPTDQMFVFFPDEPKVGIKTIKAYCQQMQSDNIMRAIVVVQTGMTPSAKQALVE

MAPKYILEQFIETELLVNITEHMLVPEHVVMMPEEKTELLARYKLKESQLPRIQQGDPVA

RYFGLKRGQVVKIIRSSETAGRYISYRLVV

>SG-AL_contig_9260_1_+1_1_273

IHRRTHWWTHTWPHTWHHRTWAHGAWPHRYRWASWSHHGTPWGSYLWCGARCIHVGRGRN

HGTGARGCSTLERICGDLSCLVRCRCVIHQF

>SG-AL_contig_9261_1_-3_3_1637

MAAVARLCSRALQQHTISRRILPLSAMQWYSNAAGTAENTLHVKHKIDDARERALLGGGQ

RRIDAQHKKGKLTARERLHVLLDEGTFIEYDMFLEHNCADFGMEKEKFPGDSVVTGRGLI

NGRVVFVFSQDFTVFGGSLSGAHAKKICKVMDQAMLVGAPVIGLNDSGGARIQEGVESLA

GYADIFQRNVMASGVIPQISLIMGPCAGGAVYSPALTDFTFMVKNTSYLFITGPDVVKSV

TNEDVTQEELGGAKTHTTTSGVAHRAFEDDVDALLQMRDFFNYLPLSNQDASPIRSCDDP

WDRIVPGLDTVVPLETTAAYDIKDVILGVVDNFEFFEVMPDYARNIVVGFARMQGRTVGV

VGNQPKVAAGCLDINSSVKGARFVRFCDAFNIPLITFVDVPGFLPGTSQEYGGIIRHGAK

LLYAYAEATVPKITVITRKAYGGAYDVMSSKHLRGDINYAWPTAEVAVMGAKGAVSIIFR

GSDNQVEQEAEYVEKFANPFPAAVRGFVDDIIEPHTTRKRINRDLSMLVCKKQSNPQKKH

ANMPL

>SG-AL_contig_9262_1_+3_558_734

MINTHRPQVRGVYRHISFHFTFHFFKPFFSPMYSRSDVSSSIPARNIKTGLFAAHLNKH

>SG-AL_contig_9263_1_+2_2_265

VLTVGGGGAAVVIVVVGTVVVVVGEVAVVGVVVVVGVVAVVGVVVVVGVVVLIGVVAVVG

VVAVVGVVVVVGVAVVVGCAVLTVGGGG

>SG-AL_contig_9264_1_-1_61_918

MLDKAEFLSKIGDKAAALTQFRQTYDKTVALGYRLDLVFHLIRLGLFYMDHDLINKNLEK

AQTLIDEGGDWDRRNRLKVYRGVYCMSIRQFKEAASLFLDTIATFTSYELMDYPQFVTYT

VLCSVIALERPKLREKVTKGSEILEVLHGLPKIKHYLFSLYDCHYSDFFLSLAVVEELMQ

TDRLMAAHYRYYVREMRIMAYAQLLESYRSLTLDYMADAFGVTVDFIDQELARFIAAGRL

HCKIDKVGGIVETNRPDSKNWQYQSTIKHGDILLNRVQKLSRVINI

>SG-AL_contig_9265_1_-2_110_250

MIGYQLHTEMQMVSALLPREIVLDGRKELHDVVFLCLCFSGFRFTHK

>SG-AL_contig_9266_1_+3_48_323

MFLCLNTDGWLINRKKEKQYSVTTKRHMNPVIDVSINQTQNEAGKEEGAGGAEQKESVDT

QNGEIHLAHRAMLAFVSHYQPFNINYIQCSTN

>SG-AL_contig_9267_1_+1_763_900

MCSVLFEFVINPEKSCVCVCVCVRARLCVCVFVSCVMYVFVRCDVY

>SG-AL_contig_9268_1_-3_3_149

FFFIHFVVVHCNISVITCGEVLVCFEQIKTTNKKFLYCPDQIKNKYIYI

>SG-AL_contig_9269_1_-1_1_396

SRDEPDRVRLVSLTDGHDEYRGCWEPGTGQDRKGRDIYGPGTHGDKDRLSGGRVGEDGGR

VGDCVRGDKVGQQVGQDRERYAAGEDGQDLYPGRDRDKDRWGAGKHREDRGRFATGSDGD

KNHYESGKDRDW

>SG-AL_contig_9270_1_+3_3_194

FFLLYHVILFVQVTLYRYSDSAFNIIQSNLSSDTDTNASAIQLQTAKTQNQSQIPHAIKH

LSYT

>SG-AL_contig_9271_1_-1_730_906

MVERKKECIVDIIRYSSAVRVSQFTPTWVLSVCFLFIWFIFLLYIIIMCLLLFLFCFLI

>SG-AL_contig_9272_1_+2_2_244

SLAALPSSTDQVQVYISARFTEGRTHKVYIYYIYIYIILGGRTVVKGPSGKWEKSEDWGS

SPGWIGIFLPPPLDLEQWSGC

>SG-AL_contig_9273_1_-2_179_598

MQAPANNTHFSRQNPLQIYAMLDKEDTQGGAAVGSLFWDDGVSVDTFQKGEYFLANYNVT

GRILQVSVVSSNPTAVPDVNTLRYADIIVYGVTGANTAQVTDSSGAAVPNTFVFDETLQK

LNVTLTSGITMNSNFTMSWA

>SG-AL_contig_9274_1_-2_92_349

MAATEVERVVENNMRNLTLSGHVGFDSLPDQLVNKSVSQGFCFNILCVGETGIGKSTLMD

TLFNTNFDSTPSSHDLPGVKLKAHTY

>SG-AL_contig_9275_1_+2_2_208

ARRRSAAPPIFFSFLMHAGCIRVFISHRTRTGLTGSLTCIFDVLHACTHELKGFRHYQVR

TYVGLGDRN

>SG-AL_contig_9276_1_+1_1_312

NNDNNNNDFFLHSAILLTNLDSLTKVGINVTTLAHSWCFHDSISHRILRDLQDLQRAFCV

RTHTKRVQALSCRPAHMLTWEIGKISTLCLPTRQGARSSNPGLP

>SG-AL_contig_9277_1_-1_694_1113

MSLDAFIGKWVEDKKEGFEQLAQSIGLAADKVEFYKHARTEISYARDGDQWTINVGIVGI

PNGSRVFHFVLGQPYDSVNIDGTPLKSIITFDGCAFVEQHKEVGPKESEMSILRKVDGDK

MEVQTTMGGSTMKSIYSRQA

>SG-AL_contig_9278_1_+2_2_136

SCDAVAIGVEGVRGLYDRQKNITMSSFRRNCGDKKALLANGINYQ

>SG-AL_contig_9279_1_-2_1145_2008

MQHNTIQHSSVQYDTTQHNTTQHNSTQLNSLQHNSLQHNSIQHNTTQHNTTQHNTTPYNT

TPYNTTQNNKIPQRNTTPLRTTQDNTTPYNTMQHNSVQHNATQRNTRQYNSIQYNSIQYN

SIQLHTIQLHTIQLHTIQLHTIQLHTIQHNTIQLHTTPYNTTPYNSTQYNSIQYNHTIQL

HTIQPHNTTPHNTTTQYNSTQYNFTQYNHTIQLHTIQLHTIQLHTIQLYTIQLHTIQHNT

IQLHTIELHTIQLHTIELHTIQQHNTTPHSTTPYNTTPYNTTTQLHTI

>SG-AL_contig_9280_1_-1_25_762

MVAGFQDDLDSDDDLRTPATSKASAPSSSSPVIKADIDISSEEEDERDLSGRKPAVMPDA

DLSSDEDLHQANPAVSVATVDITDSEDEASQAQKHMVQKNSHKTSDQSSEDSKEKDKVKK

EKVKVMQKKSVTASESSKKKSVIVDSDEESSSQGGDNAAAEGENSVAVADNQAFHIDVPQ

EDFGNWLDQFETKPSKPSSTKKSKPVAVTPVVLSDSEEEGPAIKPVSSDIEEAPAKPSTK

KKKKKT

>SG-AL_contig_9281_1_+2_2_106

NKKTKQSDKLTKKLSQHIVFTSKRCSLKKVWVPVF

>SG-AL_contig_9282_1_-3_150_353

MCLRATLTLCMLHCMMDWKLNHYGMVTVTLFLSVEVKSDIKKWAGILFMFVVKSSETAIK

VSKMLKKK

>SG-AL_contig_9283_1_+2_113_223

MLYHQLLSEFTLIVCNVFRVRRLRALVYCYMIEWAFT

>SG-AL_contig_9284_1_+2_2_286

SRSAILVKNASPNGSLFVSGIFACGQESREIRIQSGDKFSADFFCGNQYFFFQWKMFIQW

RKSTKRQTILSPVRCWINTKKVDMVAVSLPGRRIF

>SG-AL_contig_9285_1_-2_224_430

MPFHYYVVFGNVVASIFHTTSMALSLHCVFQFFNNSPPLLKSGSSIRSPKKSQGSYRVME

VWKSLGKMR

>SG-AL_contig_9286_1_+1_109_393

MTLYEPCYDQPTKTYTAKLIDLFESFGLVQCVCVCPYTQAGHTLDWIVHRADQTVVRLWS

ATKRSIRSLLSSLSAPFLLSPFSLSLRSGQKSHCR

>SG-AL_contig_9287_1_+3_3_143

FFFWLSETLFSLTNTISWHSITKALHFYYSELWHINSKINLNGLNNA

>SG-AL_contig_9288_1_+2_2_190

INFEYQLTSNFVHINTQLFSFRIYTPISSSRLNSSVTANKKNVCLCVCVCVCVCVRVCKF

INR

>SG-AL_contig_9289_1_-1_1_165

KLVPTPFFVQTSSECQSSTDNSCEFLTETFVIAVMQIKGRACSNLLNAYTFVTSL

>SG-AL_contig_9290_1_+1_286_489

MHTEKHTAQTSACICRTCIASQSHFSEYQHLSLSLFLSFIIITHTHTHTHTHTHTHTHAY

THRMCPYQ

>SG-AL_contig_9291_1_+3_780_1049

MKVKGEKGRDRAECVSFIGIAPWLPFQLDMKQAPETYFQFNYETCDQNMHTKHTHLHNQN

SVCCQQQKKIDKKMRQKWISGYYVTLHNSS

>SG-AL_contig_9292_1_-2_41_310

MLHKHKSAASRLRQSRFACIWRLLSLRFVGRVYKRMCVPEWLRSSFGLVKKRLNKPRKAS

ALPSLVFSCLAAVYTLLSWPFCTLTTAPSH

>SG-AL_contig_9293_1_+1_10_225

MCMCMCTCVCVCVCVCMCVCVCVRVRACEYVSTTVCACVCFLHMHQILFHRKEKVTNSKS

NSISIYTMINRK

>SG-AL_contig_9294_1_+1_154_276

MVKTPAWDARGHGFNSQSGFFSPFHYNLSDGLCDSFSEMKN

>SG-AL_contig_9295_1_-3_279_1418

MSSVRSVQSLQGFRGIVFLFTICAFCANVGGVNTHKAIMGSFLNNLWQKIAGALSGQGSP

KYTFPDTLEGFGYHFNHKGELVGKNGGRFQFVVRKDDQNYNQQHYAALGELVTEHLYNIM

ETELKLERVFVPLDAKKEEAQSFIFRSKDAAENKKLMVVIHGSGAVRAGQWTRRLIINED

IDTGSQLPYIRRAVADGFGVIVMNTNLNEATVNGRPQKIRGSEKPEAHGVYVWQHFVQPS

KAQNIAIVAHSYGGIVTYTIAEKFLRDFQKRVFSVVFTDSVHADGRAFWSKAVKDFMKER

AVNFVSADVPLNYRIASSSSDDCLLVSAGTDEHERTSHSCIESAFRFIRENYASWTRNGS

MAPHHFFMSLPDKGKFKSEL

>SG-AL_contig_9296_1_+1_592_795

MLDLCRNVLDIFQILPMCYCDLVHCRCHNFQKQRFLHLLVFFPKTISKSYLFFSRTMSKP

VRFVKTIF

>SG-AL_contig_9297_1_-2_2_172

THTHTTTTTTTTTTTTTNINNSKYIHTSVPVTRGILTVRTLPLKSNARDQTVAYQRT

>SG-AL_contig_9299_1_+2_95_208

MSEIISRHLSDSIFHANDVSFSVETFIRTCFSLTISLL

>SG-AL_contig_9300_1_-2_302_631

MGKKNVMIVPVIKMIKYNSYLFVYCCITRQNDTRCDIIITWQIVYCLCLFLLAVKPCAYY

SLQHKINLQIVLFFLTVPHAFLSLCCASRSFSADRFSDVVDFLGILQKQF

>SG-AL_contig_9301_1_-1_112_606

MVLAVLFCCCIACQQTYRFSLTVVWQTYRFSLTVVRQTCRSPSLLYGRHTSPPSLLYGRH

TNPPSLLYGRHTNAPLLLNVRHADPPSLLYGRHTNPPSLLNGRHADPPSLLYGRHTDAPS

LLYGRHADPPSLLYGRHTDPPSLFSSWDNLCDVVLELFQLSQSFA

>SG-AL_contig_9302_1_-1_1_288

STLICVYKKKSNIKKGKEKKRKNLGKNRRLERISLGHSRNILHVCNATNNALNVIFFFIA

QAELSICYNFHLLQDYICCQYPVDRTHLKPFCFIFF

>SG-AL_contig_9303_1_-3_3_143

LFSQKRQHMHLCSLHKHFKYKTLKMYNKRNTTKLCDCVSQHAVPWLN

>SG-AL_contig_9304_1_-2_2_127

VPQVCFRTLSSAFTAHVPLIGQLPDRSFHGYQIFDFVCVCLN

>SG-AL_contig_9305_1_+3_561_806

MTILTIFCVLPIWVNVNMVDESKESGKQCTTFTTQQVLERLYLLSSNKDIIIENMKRFSA

FCLSMCVCVVHTEIIDFLSIVD

>SG-AL_contig_9306_1_-1_1_294

KEGKRAEAKRAIEKRKFLAQNFQKAGILFILKEPPASPWGLSAGQTWCCFDVFLCMHATG

VSERVCVCVCMCVCKQHNLYNPCLVYVYIMILYRLVIA

>SG-AL_contig_9307_1_-3_1203_3407

MMATVTGKLVLVIVALVVTVVCFVTGLMIGWFLLDAPDPDSMAGSSNTTSTTSSGRRLAV

GAGVSAGHSGHYGRLAEANVTDADPTISTRLMQEIQAGNIEQHLRFLSERPHLAGTEGDQ

RTVDYVKQEFEKMGYGVTLAPYDVMLSYPAEEYFNRVVMLDGRRRTAIFTSDMGAMTGSE

WDSELTWPYCIYSPSGKVQGKLVYANYGRPQDFRTLDKLNVDVNKAIIIMRYGKISRGKK

VREVAKRGGKAVVLFNDPADVNINMTYNVFPATWWLPDDDVEHGNIRTSWGDPATPGYAA

IDGAYRIPKKQMDLPTVPCHPVSYKDARILLQNLEGERAPDDWQGQLGFIYHVGANTPGA

LPVEVDVKNTLETRRVYNVLATLPGAIEPDRLVLVGNHRDAVAYGALDPGSGTAVMLEMA

RAFMALYNQGWRPRRTVMFCSWAAEEFGLIGSTEWVEEKRRWLTDHLVAYLNMDTVLQGN

YTFDMSATPSLKAAGIRAASKIPYPHSPNRTVLDVWKDVVTDERSPGQPWIRRPVSFTDF

QPFVHLLGVPVTHSRFSFVEQDGELLYPIRNFPLYHTAYDTMQLATMMDPQFAVSNAVAA

IVAEMTRDLIDSLFLPLVPEDYVMDIASHLETPMDNAMDKFNSSVSHLRAALDEFFEAAQ

SLRSEIAKLEMDTVDPFEVRRINDKLMLFEREFVDHQSGQHMFLSHGSNSKPTLDKQTMS

QLTQALKAAAYTLAT

>SG-AL_contig_9308_1_-1_613_867

MRFMVSVSLGSLSFSLSGHVCTGFTHPLSNIRFTVSVSLGSLSFPLSGHTCTGFIHPLSN

VRFKHKHVLFILTVHFSVIHSTISN

>SG-AL_contig_9309_1_-3_402_632

MMNVKNIVDVFHLKWCSSVKYRNVPCAVSHLSFSIFSQSFHKKPFFFIMNFLKDHESINF

HCDHNTDYWRKMLAFFL

>SG-AL_contig_9310_1_-2_428_1111

MNVPKIKGTHNAMKSGILAAESVFETISNEDAVSNSPTAGLNPEVYEKKIRDSYVWKELY

QVRNVRPSFHSSLGLYGGLAYTGVIWYLFQGREPWTFKHGGLDHNKLKPAAECQPIEYPK

PDNKVSFDLLNSVALTGTNHDHDQPAHLTLLDDSVPVNRNLAIFDGPEQRFCPAGVYEYV

EREDGNGRRLQINAQNCIHCKTCDIKDPSQNINWVVPQGGEGPAYNGM

>SG-AL_contig_9311_1_-1_49_249

MKDHRTWDNFSQCYYDKNGDDNDDAKEFMHLGLRLRDRAVLYISFHNFSWRQPGLRVTNI

CGIGQKI

>SG-AL_contig_9312_1_-1_97_279

MVLQHSEDSICLSDICHALEIENIIIVIALSIHKQAKSVYILITVISCHMKISKEELKPK

L

>SG-AL_contig_9313_1_+3_117_272

MTNQCPKLCHKTGARSLTPRSMKPSSPRSTEFTSSKKKGQPMLQLSPVSSHV

>SG-AL_contig_9314_1_+2_89_235

MNKWQSTFSVHIDSKIKQHLKTKLRPSSHLSTLLLLLLLLLKRKKPNRW

>SG-AL_contig_9315_1_-1_1_162

YLKEYLYLQLLVLKGNPTSRPPSFPEGKIRRTKINHLFSHTTHSYMHTQCSTYK

>SG-AL_contig_9316_1_-1_1_486

SGQPPEVPPLAFTVFRLPGHEVRHSHSTSVSDSGPAPGTSAQAVFPLGLILSPRQGYCLS

SGPHGVSSASAPSGQSSQASIAEGSPVPLEPVSDQLGSGGPFGRLVSGGDAGMGLCLRPG

RGCPHCSPSSSGGPLHGRLQLGLGRARGFRPHRRDLVSGRGP

>SG-AL_contig_9317_1_+3_126_422

MCSVCVLLLACTPFPLFLFYCLFRCGWKFVFIALNPWIFFSDLSVFIKVYPKPVPILPLV

VQIHYNNNSSSSQSLKKKKKLVRGRWGEDANSKAIHSRC

>SG-AL_contig_9318_1_+3_3_146

SFMSTFDEPINQTGNKSISQTAHKSCHHKNSGTTFQQINTFTNNVNSL

>SG-AL_contig_9319_1_-3_3_512

KPRDDVGQPGAKHDELSNAPRYDGNDPRHDGKPGDAARYDGWAWQHDGKPGHDRDGESES

GHDRHESAESHWHHEHDGTTTHDGTTDGLWYGHAARHDGHHEHGSHGHAECWRDAECRCH

AVRFPATHRPGILLLWQCQAVSCVRWPSCRLLTLSFSLPHFISCLEESKG

>SG-AL_contig_9320_1_-3_3_305

SIRNGTPKDHDYYPHSHHHSIAKNCPRFCGPSCSTVVVTSVSVGLEVYWTAKMSSMGVMT

PEWCRSLGSKTKENKTKQNKTKPNNPKKVEWTCVLASCWLV

>SG-AL_contig_9321_1_+3_282_554

MNGASKLTKNRQKEKTKIVIFTKTLPNTSSIHMRLSDSNPQINTNISGLSKEKRQFYTCT

RTSRKTSKQSNSYTEIYHRAETETVCNDALI

>SG-AL_contig_9322_1_+3_78_314

MHSTCIHTMPIHIYIHIHNYIHAQILQKKLDKLHNYCNEWGLKINQKQTRTEKKEKTKTV

IFTKTAPRYLVYSHEALRL

>SG-AL_contig_9323_1_-2_278_496

MNSSCTKFHTDWLLYSAMFDSSAYFINVINVHFLSNLRSTYRLMISESFLYRTTSKLPCI

VHSAAHSHLLPGV

>SG-AL_contig_9324_1_-2_473_772

MNQMLLPTPLLSQNISPSPSLNVCKRRDNTCSGRQSDLRYNESGRGRRWENAGVEMMGVD

HFSIVSVSVFSKQRLHARNKLHMHQRCKCHGMEKIQNDNG

>SG-AL_contig_9325_1_-1_1_288

FFFFLYIKKKKVGGDLVSCIVIVGKDNFSVLSFSEVWFLFIYAVIFIYLYLIFLPFLFWK

LFLCSIILPFLFWKLFQCSIICTVSTIVNWSEHVKI

>SG-AL_contig_9326_1_+3_27_242

MPCNIKVSFTPSIQVFLGLPLALNLNYLKLTLKTRINSCTMNALYCLAYNNSWNNIFFHY

GKKSFRHKRKLA

>SG-AL_contig_9327_1_+1_1363_1692

MTDANQPRVLQLSTLPCGFRVLQRQPFSKTSWLMSRNKQCVRSRENNIAMVKNYAWGVCN

SDTRYPERLAGGVKFFPFPKWRRSQPNNHEKMHVLNQGLQSTTRSAKHHW

>SG-AL_contig_9328_1_-2_584_844

MSLSVCLSVFCLSVCLSVHACMQSLRLSLTAACLLSLSPPSTSLCFSRVCVCVCVCVCDV

ILHTCVYGCVQVCACMRVICIVCSRRS

>SG-AL_contig_9329_1_-1_55_2799

MQVNIPLLRSQLRGFEILDALRLHRQGFPEFMNFREFVQKFHALVPPASRPTANTEEKQA

VNQVLDHLDVDKLNYRVGLSKVFFRPGVLSQLEAGRDEKLTGIVTQFQALCRGYLGRKKT

EKLRVQHLAVRCIQRNVQKYMQIREWPWWKLYTKVKPILNVHRTEEELLEAQAELEAMKT

KTEKLEKERNEYKAACEKLESRLVDVTADLMEENTTSNQAGEMLEAESAERMRLEKELKD

LQAKYSPLKRQNEKLHMEVMQARVWQAQSLEEELEDDTDGGSSVYKERCERLMRELQMTK

KQMQQQHEEDLEQELSSRKMVEKRLHEAVEEAEEQRRQVQVARKKTQRLTAEMQDIKLHL

EEQMARNNELERKQRRFDSELGIAHEDVRDERHMKEKLQQERDQLLSEKYSLEQTIQNLR

MDQQSAVEKAERLDKELNDILVSGKDNSEVVSLKRGKHELELKLREQEEELDDQAGQIQQ

LEQTKLRLEMNMEKMKKQHQADIEEKEEELEDMRTKTQKKLRSMENQLEEEYEEKRRVME

EKAGLERQLQEYSARAPARDRESEKRLRRNLRKTKALLADAGTMLAKQKHVDGAKQQIAQ

LRNQLDDAEFTSSAAVKAKKRMELEIQDLNQQIEDLSRAKQEVESKNMALLREKSDLQSN

LEDNDEEMSELMKKYKAAVQQQSVHQITMTDQLHQIEELIKERDLLRQQVADLSARVQGY

EEGSVDKHSVSRLETKLKDFESRVELEQSQRHRYESQVARLKEQVEKITSEKEDMSANRQ

AAEEVNKRAQKQLRDLREELTEAQKKEMEACQKFKERENEVEMMEVEVAQNRADLKLAFK

RITDLQAALEDELDSDDDSLQLDDSDLDSEDDDDEDLDTYLGNHHATGHRSQSTYSSPRA

DRYTFASTENGVTDA

>SG-AL_contig_9330_1_+3_18_281

MAEDSSHILIKNRILTRINNNGLQPRQASRYDYCYKCYFIYFLNFIFNFRYILLKLIPYL

NKKSFFFPKINIKVFKIYTKSIKSTSNL

>SG-AL_contig_9331_1_-2_386_583

MNVCIGTFYHTTLGSIRVITKLIIIHYYYYCYNYCLSLFSFGYLFWKVTHFYKTCIKVSP

STHVSV

>SG-AL_contig_9332_1_-2_644_898

MYLSVCLFIYSILIRIKTKRSCMFCEGNISARFLSCLWIDLHPVWDTDVTNGKFQLCDFN

KHCLHIILVMNKNISMFQQGFLGLL

>SG-AL_contig_9333_1_-2_32_2782

MDDCNLEKLVIKPGKLSPAFDRNVTEYSTVLASSVDKLNVDPLTSDTGASYSISGSGGSK

TVPLKEGEVTDIKIEVTAEDGTVKNYFIHAKRLSAKDAALSDLRVDEGILEPDFNADVTE

YSCILPCQVTSVKVSPVVPDPKAEALLSGKKPGEAIPLCVGETRVEIQVTSPDGSTKKVY

ILDLIRKPLPRYVKFIDPKKALEFECPISLSPLYRPVTVKDSNPKHTYSAPFMDSVTRTS

KVDLLNGQTLPSDWRIVDRELEDKMSSESAVIPLTYGGATEPVKFGELAAQLEKCNVAPK

VEDPKDRFAKSAFSATHTVESRKWEKQLQQVAAETDPAMLVFAANGELDKYYASLPTTAQ

GFHLFNEGESPMDYIQNAIHFYATAIKFKAKDASLHFQLAMALEEKYFAEDMFGLKKTEA

SSDIPSFNIQAKESSKQEEVLAICKLRKVEVDAPVAHQLKALDEEYHHLLDNGQGDKADH

VQGLYVWYSKKISQEGAAAHKAEDAGNPLGQAYLKYLDALVLDESKAIYNFHVGRLLITQ

GNYTEAVKRLEAALCWNPQHQLSRIYMGLALSLLKEGPGSRLKECVGYLLEGMEVLLTHL

SKQAATPEENTRKNTLYAETLLRPTNVHLLRGLIQLGRLLSANPDIKDAMAAQDVFHTAA

LLATQAMPDVHRGDVYRQMEWVLLDAHAILLEMLSAAGKDEELIAQRCQRLSALIDNATI

PQNQQLLDLQEKTSQNLVYIQPCNSHALYLLGSSQFNKYENSSPGDAATKVLHDARTSFE

ACIELQGKAAEGAVPEIVAGQQWYQDRQKQEEEKAKKAAEDKAKAAAPAAKSTTTPARGG

AAAARGGAAAGRGTAPAARGRGAAAAPARGGAAARGAPSAKRGAAAPAAAGRGGAAAKAP

AAGKAPAGTDKAAAPAA

>SG-AL_contig_9334_1_-2_2_406

EETPYLRLASENPEAIAFDAFTLAWSGLLASAFLSFPNPPEIAQEGKTRDSDYDSDRANF

ASSTLVPRPDLGTLSLRAHKVRTITTLAAFQQKAPLRAILEAAYWRSENPFINFYLRDVR

TTRADGSKGYFIHSS

>SG-AL_contig_9335_1_-1_1_213

WPRVSPLFPLVPRWPSTRTPPAWAGARTWVQRTSPGRGLRTKPASTSTSSRWRRCYAPSS

PSPINSGARRS

>SG-AL_contig_9336_1_-2_2252_2521

MCLSCLGNKYVLSCLFHPFSICCYSLFVCFSDLNGDNIAHSGACCSWVSQLGSWNQVSPQ

SKKNMAIYPASVHADTAYSFQSITKLETNA

>SG-AL_contig_9337_1_+2_56_283

MDTNPSYPCRGFVVRISHFGFVVPFCSISFLFLISNLWLVSLPCTMESVNKIVAAQFSCY

LASHTVCSKLSLGGVK

>SG-AL_contig_9338_1_-2_2_217

DLKSKTRRRKPSQERLSDKTEVCDHFMTHSGPSGGIKTNQYQPSLPVSFARDCCYVIYAF

WMSGTECRHGAF

>SG-AL_contig_9339_1_+2_2_160

FFFFSMHCFYLTPDNSKYVFSFTPSDLSYWLIVIKTSISCIHTQKSSHNLTMN

>SG-AL_contig_9340_1_-3_3_824

YVLNVPKEISDKLWQVYRQLPYKIIFRSNLTSPDPERVYASPWIPQNDILGHPKIKAFVS

HCGKNGQYEALFHAVPVVAVPIFGDQPYNAERMRVKGFAEVLDLRTSTAEEMVATIMQVA

GDPKYKAAISAASRLFQQEYHVPMDEAAFWLDHVMEYGGAYMRSSGHEMPLYQFMLIDVI

AFLVACFLTCLAVIVGTLYLFFRLFCTKERRDTVLLLVMDDKALFDNKKKVRKVCRKAIR

AGKKSFLAFSKLLMLHRCSEEQGRRSYTRKLECA

>SG-AL_contig_9341_1_+3_108_353

MSTRYVYIIFYFVYIFSTLSQRVYSQRAERVATLQCHLFFIFVCGKVFVWVFLLFCFVLL

TYEGGCFYRMSPGTTHSLPCSL

>SG-AL_contig_9342_1_+2_2_274

VIIHHKRKVQFQHFTCSENTTCRSHTSHTKRTQSHQTHIDPLKNICEHCSDSIQAKVSKL

KAPSENTDSIQAEVSKLKTPSENTNGIQAEV

>SG-AL_contig_9343_1_+1_157_309

MICLALFDFHTSGNVLHLQPQRIFCLFCFCFFFGCFLFCFVLLPHLLSHFS

>SG-AL_contig_9344_1_+3_54_347

MRTPLIHSIHCKTHETVKCFFYLINSLRKQSLIHTQLNNSSKKKAKQTQSDGMLQAPIQG

TWYNQLFIFVKHSASERLEDINMKVLCVGMIWFVCIIY

>SG-AL_contig_9345_1_-1_1_285

WSHHCFRYPVTVNVIGLITVSITLSQFVSGLIIISITLSQFVFGLITVFPLPCHSLCLVS

SLFPLPCHSLCLVSSLFPSPCHSLCLVSSLFPLPC

>SG-AL_contig_9346_1_+3_3_209

SRFQLCQKIWLCHKLDFAHHKAQSITQDTQLSTREKKCYFFFLMSKMECVLFVTCTVSSS

LFFITSDYC

>SG-AL_contig_9347_1_+1_628_774

MFLGFMLFQKRKSTHWLQEHLYILYIVMHSETHEIKEKSHTDTGFFFFF

>SG-AL_contig_9348_1_-2_83_316

MHYSLNCIFVYIYVYIVQNCNYSQWLLRLSLGFFHFFFLSHSFISLLSVYLSVCLSYLSL

FCYASSVFFMYIILHRIA

>SG-AL_contig_9349_1_+1_136_360

MYNFSNTFILILYRKYSQYYASNITFFFLHENNLRPFFWFHKTKCSYYQFVQLLLHSLNL

ENKNDVPFLYLKCSL

>SG-AL_contig_9350_1_-1_463_699

MYGLSSYEIKIHGEYLDSTSHDVEQFNIIIVLLTCDTVEILYSDRRMTDHIGKEFTHAVF

ENHVIVLCFIIKVSFCSFV

>SG-AL_contig_9351_1_-3_12_161

MFTSFKGPVNTVFECTNALKCVGAFLRRTYVGNRYNFYFYHLPFKSQSLR

>SG-AL_contig_9352_1_-2_2_259

HTHTQSCTCTDSHTHTQTHTVTHTHQMNQRQKRTLSIETNTCTIHTKSIHFFFLCLLTTH

TLHTHIFPFPKTHIYTYNFSTRKILQ

>SG-AL_contig_9353_1_-2_1223_1402

MKLTERDATFIYVYIQSSQLVIQISVFYVIDSKQYRISSVFIVLELVRIIVMLGVCSGGM

>SG-AL_contig_9354_1_-3_702_5015

MPEPIVVAAMSESTVSVLPSAVPPGVGGVSSMESSTVNSMHASAIVDHGDITVSLTDSLD

NAPLSIEHPFPSMHTLAAKVSPATSFMAETFISPDEVTQTTDAESDFPPGLDEFTSHLSA

DDARVLVDLLKLAVADRVGSDSRDTLSNILTSLGKANSQVSEMLLELCVTELEDVASDSE

YARSTAHPVTQESPHPYTDDTSISGHVKIPGAEALRVEFDRRCSTERRHDPLTIMDFSGR

VLGIKSGREWSDWSQELRVPGEELRWKFTSDGSVNGWGWLFTVYPIMPAAAPLDLLSDRT

ILSRPSIDLVTCLLDFKLENMMDTGIVPRMAASLAACAQLSCLSANQRMWALHKLRKLMS

ACCGPLINVNALLSSPTSESPEPEISRPFHMLSGSALAALVKGLPEALQRQYEYEDPLVR

SGKHLMHSPFFKVLVALACDLGLDTLPCCAEAHKWAWFRRYCSAARVAASIVTRSALPSV

FTEEVGKKILDIASDGESGNSERMEHDVFKLEQDEQLLQWLNRKPDDWTLSWGGSGQIWV

WGHNHRGQLGGVEGAKVKLPIGCESLASLRPVQLIGGEQTLFAVTADGKVYGTGYGAGGR

LGIGGTESVATPTLLESIQHVFIKKVAVNSGGKHCLALSAEGEVYSWGEGEDGKLGHGNR

SPCDRPRVIESLRGREVVDVAAGGAHSACITASGELYTWGKGRYGRLGHGDSEDQPRPKL

VEALKNHRVTDIACGSGDAQTLCITDDDTVWSWGDGDYGKLGRGGSDGCKIPMKIDFLQG

LGVSKVECGSQFSVALTKSGAVYTWGKGDYHRLGHGTDDHVRRPRRVSALQGKKVIDVAC

GSLHCVACTDSGEVFTWGDNDEGQLGDGTTNAIQRPRMVAALQGKKINRVACGSAHSLAW

STHKPVSAGRLPSQVPMEFNHLHALPMPSLRNRLVLLHHFSDLFCPSLPMFHLQDTPRLK

GVGAGVTTVSMGAGGGLMEGQEEATTGLDALRGVLVSSAKEAAFRKVVQATMVRDKQHGP

VVELNRIQVKRSRSKGGYAGPDGIKSVFGQMAQKMSVLGPDSLMLPHRVWKVKFVGESVD

DCGGGYSESIAEMCDELQNGSLPLLIVTPNGRDESGANRDCFLLNPSLSSTMHQNMFRFL

GILMGIAIRSGSPLSLSVAEPTWKQLAGMPISISDLTEMDKDFVPGLICIKEMEEEALRA

ADMPFSVPSATGLETQLSHKHSRINPDNRSDYIKMAINYRLHEFDQQVRWVREGMSRVIP

VPLLSLFTGLELETMVCGSPDIPLTLLKSVATYKGIEATAPLVQWFWEVMDEFSTAERSL

FLRFVWGRTRLPRTIADFRGRDFVLQVLDKYNPPDHFLPESYTCFFLLKMPRYSDKLVLK

EKLKYAIHFCKSIDTDDYARVALTTDVLDDDTPEASEDSDMDMGFMESEGEIVDSDGS

>SG-AL_contig_9355_1_-1_157_1656

METRKRILVGIALVIGCLDMIIAGSIRSFNTYINAIMKTFNYTQTEMEVMGAIINFGVTL

GFPAGLLYERFGGRVTSLVAMLLTGGAFSLAFSTTFSKTFYSQDNRAGLQDFYFFLGGFG

TVFTYMAAMMTTVMNFSLAHRGKVIGILEACFSGGAALMTLIYGEFFIQGHINDEQKQDL

RGFYLTSAIAFIVTNFLGVVFLGVYREPDVITLQKWSSRSALVNNNETDNEADDKQTVGI

DLADISPDVGEEDVTGWKLLKEFDFHFLTWSYIFCTSLQFTFLLNITTFLQSNHLESQNT

LLTTLSFASTSLSKFLIGLLSDLIVHRVPHVAITLVTTAVQTALLAVCIFKGDRFIVLLI

ATLGVSISTGATWCLTPVMTSEYFGIRCFGRNWGSMMLGSAFMGLGLQKMFGALYEMEIT

VPVSTDCYGLRCFRWSFVVLAGLSLCSCILYVGLLQRRLKLRKWRRPEKETKKEQDCQLQ

RLIPPPVPDANSNKVVNKDE

>SG-AL_contig_9356_1_-1_136_321

MTRHHSANYTFLPVRHHSTKCSYALAGTVRHSFSKDAFLLVRHNFTSLRLSPAWTMRHYF

TT

>SG-AL_contig_9357_1_-1_136_321

MTRHHSANYTFLPVRHHSTKYSYALAGTARHSFSKDAFLLARHNFTSLRLSPAWTMRHYF

TT

>SG-AL_contig_9358_1_-3_3_320

TRHHSTKCSYAPAGTIRHHSTKCSYALAGPARHHAAKCSYAPAGRARHHSANYTFLPVRH

HSAKCSYAPAGTARHSFAKDAFLLARHNFTSLRLSPAWWMRHYFTT

>SG-AL_contig_9359_1_+3_858_1112

MNNSNGMQRYEKNADPLLFSMGTLRGTYQLYLVTYYSLFRAKNNNKNLTISITKQKRNAY

ILIFMLIQIYAFISFMTSNWAGPAI

>SG-AL_contig_9360_1_+2_2_160

QEAAKTCRKFPLPLELGTMKDVNASNLHYRNLDSITQTRVVSFCAHQFTHLSR

>SG-AL_contig_9361_1_-1_418_615

MADRPDKCLLAPVHVVAALGIFGHCHITMYSASCSISRGTEENGQIQHRSKRKRAAGYKL

SWNTFR

>SG-AL_contig_9362_1_+1_58_249

MCKVYSACPLLSLVFVLVSVSPYVKQRLWGSGSGELGLILTTGAVVKWVNTGLPNGRSRV

QFPA

>SG-AL_contig_9363_1_+1_187_378

MTAEQNVCSNSYECTQSSLMEVTTTSSRVWKCTVSTTLLNCVFVIFHVYFVACVFLSLFF

FFFF

>SG-AL_contig_9364_1_-3_66_407

MAQADPRIKKIKIQTGVVKRITKEKQMYEKEATEIEGKIEKLKAGGADEYVLRKQQEVLQ

ESKQMVPDTQKRLKNAYEELKVLVEAETDLTETEEYKAAQMALDDAKVVVEACE

>SG-AL_contig_9365_1_-1_166_417

MPFSVQTSYVKVCPQPERLLGVEMLQPQRFFLVPVSSPCFPFPVHKGQITVSSRKVSSTR

FETCKSEMVLSVCSTLVDYSTPAQ

>SG-AL_contig_9366_1_+3_3_296

KKSTQAINVYASPLHLSNNIQHLFQIYSITVYTQSHCHIHCILHASSISFLFTIQSDTKK

RVIEFLKYHGIHILHQSAVQNALSVLLQYTATSRRQTF

>SG-AL_contig_9367_1_+2_1628_1918

MHPHTTESSHQQYSPQTHKNLPHRSLTINNSNQNNNQDQVSLKKKYFFGSCFQMPCMFQT

TVRSQLLSHFCNGLQLRSCKENRAMYSIQLWNMAQIF

>SG-AL_contig_9368_1_-2_524_940

MVAPVPVRLLRLRESIFSPLYPLLTTARALASLLDTLESLAPLLPLGRGLTTPFYWRPEH

AGPNTPTLGMLRSSWAPGSKTQFQLGSISLHSNQASWSTFWGQTLPPHGCIDLRMGSPLG

SPPTSGTWSEDMLASTLTF

>SG-AL_contig_9369_1_+2_1847_2272

MNRIELMSNLQRLPNKHPHQISCLLQNDGQSIHHGKTLLLKLLILSVCRGVNQTVTDMEF

KFRLSISNGSKQNQVNLSELSIDIIASVERGKRCTEWKLASKQKYAQAYSFDPYTFHLQN

TFHTHGEFFLTPCLASILTMSN

>SG-AL_contig_9370_1_-3_3_170

VDEKGTIAAAVTSVEIMLLSGPLSVLVNHPFLLILRDNEARLNLFMGRVNDPSLQA

>SG-AL_contig_9371_1_-2_218_2446

MKDKVDSDSEEDASDDDDDYQSDGGYKECTKAELNKFKRKLLEVHCKRFMEPNCELEVKE

ERDYLTNLMFSYLDSINNDYQLSYEELKLRQNTEHIRNISDICELTDLIWYDDKLNADGH

LSLDEFEQAFNNLDGAETSEESKPVDKKPSLTPVIHIIPTLATVGNGLELKCGLAAKGSD

VVWRRHGMNIHDLGMVELLVLDDGSLFFSKMGVHHMGNYSCEDSTEPSVLQVHSLKVQMP

PIVKVSPHSQIHPSKLNAKMKCHAEGVPKPTISWQMNNVPIASQAGHYALESNNELLVVF

KTDYQKDTGAYKCVARNQAGTSEDVASVFIIGDKERTLGMDFTKIKSGKFAVFHHDGFTV

YDPDDCLKDRSVHRDFGYFKFIPDNLDGPLKLCNNGKECTWGTVVNVKDGFLYASQPDMN

RIVVIETTKNWIPVQVIATDKQPVQLHYVEHLDQVWVLCWNSGEDDSTKTIVVIRDASKL

VQHRSVHTQPVGNHFDQIQALFIAPSNDLRHSFDYGYVTHNGQQGLTKLDLDNMRYVKAV

DLTAYGCVPRSVAFVPIGGHVVVECVSPLNHQTLQLVMDYITDAVVSTAVLSGRPHVSPD

SRHVVTVDDLTGKVSVAGVNNEGALEAAYEVTVSASISDVAFYPASGFGYLLVLTSADDD

DIITINLSTGNVAKMKGTDRKTTSSDWRPSPVRRSIISGDIFSNFLLTPSKSSLAIMDMK

FHHIACEYPQSPRSQAAIFVGHH

>SG-AL_contig_9372_1_-3_3_209

SYFLKARLLVLVKCAVVVLVMSGSSSSFCFCFFHTCHSDILIIHSFSGYRHIRHSNLAHI

LSCSRLCYS

>SG-AL_contig_9373_1_-1_1195_1491

MLVYVGLPNWRQFLGTTEQIGESSKLCGELPPLTILQIQSCQVFKTSCLPVRCTRPSYDI

HIAAACMMPEWFVNYYRPAPKFPGKWNSAELIISWQSRM

>SG-AL_contig_9374_1_-2_53_484

MATAEWYLVVLAFLSFCSLGAAIQCYQCDSNEDVTCPSDEPFDTSKNAVVDCNSFEANVP

GQFCMKIQQESPGWGGWKKTTRRCGSRTDFGVAWGCRWTWDYTGIFRETCYCEDRDGCNG

ASPLSFSVLSAVLVLLAFFYNMLL

>SG-AL_contig_9375_1_+1_7_261

MYLWSHCGKTQCIQGDPLECQAGKHKHAYLQSPLSTKTWTEMLVRSKIKQLSPLLTWLYT

TFYITGIFQHSHTHLTWNSFGSFTG

>SG-AL_contig_9376_1_-3_3_203

CVYVYGCVWVGVMYVWWVYFAPGHSLSPGGSSVIIAYIILCICLRILLLYIKILDCICCR

SIIFFES

>SG-AL_contig_9377_1_-1_1_291

GGHPAPVVPAWPALADATPAPATHQATAAVATRPWCSRHGRGHGWGPPRTSRCRDTTPSL

PHTWRPLRCNTGQVLSTRRRYRSWGRALAQRRLHRHP

>SG-AL_contig_9378_1_-3_57_956

MWNLRKAASRLAVLSAVLKQGSVSRSPLVGRCHSSQTVTKHTDMIVKLLPALEDNYMYLL

IDESTKQCAVVDPVEPQKVMKAVKEEGVILRSVLTTHHHWDHAGGNEELIKLGGKVPVYG

GDDRIGALTNKVSHGNEFKIGELNVRCLSTPCHTSGHICYFVTGPSGGDPAVFTGDTLFV

GGCGKFFEGTGQQMYTALVEKLGALPAHTKVYCGHEYTVNNLKFAAHVEPNSDAVQKKLA

WAMTQRSSNQPTIPSTIQEEKDYNPFMRVSESMVQTHAGKTDAVQTMDFLRSEKDNFRAK

>SG-AL_contig_9379_1_+3_3_110

VLMQIQIKNLKDQSIIPKKISHDQCRHSENRLMYSV

>SG-AL_contig_9380_1_-2_2_151

IHSCCSLDIKLPPAIGLSHDSEPLHHIQFVMLSLSLVSNLLLKKVSFSSS

>SG-AL_contig_9381_1_-3_498_761

MIRLHCTSLNFSTSMHIRVIFAPLLTPESTNFLTENKNIWSTFFFLSDSSLESASMSSSS

CCHSSFFHNYSKIISLSAALRPITICQL

>SG-AL_contig_9382_1_+3_3_155

KKKKTQIQKQCDPQVEMLSTENKTSQSQVVILVRNEVSSNGCGSIITIMFS

>SG-AL_contig_9383_1_-1_73_1488

MESSDNFIDPENEVKKLQELVKKLEKQNELLRGKQKLSLDGYVPNGDVDKSAVTRNSNSY

RQVQAESSSDSWKHKTAEGGLEDVDVLDVDNLSLKDEEDSWLYSSPKAPTPQQSNISLYK

WVRQDFDHPSPELESARRSLLSKLDEVARMNRSCSTPALGTTVSPTKSLSLLSRSTEDSH

KNYSSSREDSHKHHSRPHQRHSMLAMPGSRIENGIFARPKKSQESSPVVGEREKVEESSY

HQHEATDVEKLARQQEESLRQSLATYTSPKRVLPNKSMSIASDTDNSGSPAGSNRSSPAR

YDFGGTFLSRHANSFGSDSGTPPDSPRTIQHSTPYSALTYTEQGPGGRNLTNMSRLQYPP

PSSHSSDSSLEHQSAGSTDDLHLAPETRSNSRLPTPGFRSASPAMGSLRPGLANNQGLST

QRSGLPTPRRAIPRPAASTVRSSLPMARRSGIPSHRPSSAQGKDESWRDGCF

>SG-AL_contig_9384_1_-1_145_393

MRLFKKLLSHSQCLEELDLEDNLIGEVGGREILDGLSYRKEQKMGGVKVRTTHRMTADTF

NSIVKVGSGLKKKKGKKGKKKKK

>SG-AL_contig_9385_1_-3_2529_2909

MLPVVRGKSFGRGEKNRVGCDCFTLSLCLKKALQPVFIFKRNLLHEQLSARYIDISVHGV

QHTCSCVHTHTHTHTSMFTTYPPTHPLKMHTLTEACKYSKKGSVYKYGNTTTSNCLFYRF

MKRDSMV

>SG-AL_contig_9386_1_-2_20_310

MNCPWGHSVETSTLIGQQNKTKQQKPPNKNLTADKNEKKMALQCGDALCPLRVDPLRQSR

NNINKIENNVNIPCWHGTRLRILCVSVIIVHYNIITL

>SG-AL_contig_9387_1_+2_95_274

MRILKYYRYLVFKVRKAHLYHYLMHNIYFSNRREREREREREREREGEEGGGSPSPFCSE

>SG-AL_contig_9388_1_+2_1403_1783

MLSGNAYHAEFLTNERKYKGAIYCPVTHSWAGSHWAEMWVNERETCGRRTSHLQDQTWLQ

DLFTRSTTTTTDGGMDFGLLHFRCHRLRFTNHHKVPSKKAYHSSSITHELTFQIITKLLC

SLQAHHL

>SG-AL_contig_9389_1_+3_3_233

KKRRKNKTQTTTKTGHCCKVNNRHTIFLFFRILIWLLMLINIMQSFLIWHHKLFYQKEKN

CSYSLITLISTLDFVKI

>SG-AL_contig_9390_1_-1_133_510

MHTCVFFFSRTQPEIFLHFFFIAVKIIVGISCIWGRRLVPRNFCRIMPRNVFLLLKGVDG

WKLHPVINLSHCEHVYNFNLHVCNPYYLPGRFLFLVAQKNCKYQWLRAALINTTFLCVLE

VASACS

>SG-AL_contig_9391_1_-2_2_205

EEEEEEEKVPPNPLTDEMMAESLSLLCKTGDGLSHAYVKVDIHERGVTDINLMKKFIHLR

YVDVSKNA

>SG-AL_contig_9392_1_+3_63_254

MMYIHSHIHSGYTIRYWCSSRLNIFVRIVSLTAYSVLTVGHFHIFIRLRPCLPCGIFSSE

ISEN

>SG-AL_contig_9393_1_+1_106_222

MADQCRTSTHRPVFKCYSPLEVLKEVLNDLDQWTTWNRF

>SG-AL_contig_9394_1_-2_1100_1354

MILFPVISEFLKSIKYFLRYGWLKLPNVPSLVLRNLSFQDFHWILHIKSLATIEQALVIK

VEQFSLAQILHTLSTLSTHNLKAIV

>SG-AL_contig_9395_1_+2_770_997

MKRRDQAKSVSTHQTALNMAWFVFASSDRLSRSKNTWHPTVQGELKALEHTWGTIRKLVE

KDRSGGPSLLPYMPVT

>SG-AL_contig_9396_1_-1_1_237

GTPRPAPPRRLTPTPPPAHATTGAGVPGAGAPMAGVPGAGTPTADVPGAGAPTAGASGAS

PSSDDAPTPKKCNLTSSVF

>SG-AL_contig_9397_1_+2_578_868

MMWLVLCLYRMDVNFFPHSKQCFLRYMNARCLAHTVQALCFCTVCTLCLWSMRSRTHPPG

LWDLQGAEGGDLAGTCEPAGEAVRACGCAAEDDPFHP

>SG-AL_contig_9398_1_-2_2_112

YFRTLINALHFICQTMELKVITAVTTNFICWQGHKEL

>SG-AL_contig_9399_1_-3_138_347

MLILMVLVSGNNMFLPYIEAFLFEIEHDITEIKNRFSTYQFIASSSPLFYKHTHNQYTNS

IPVQHNGHQC

>SG-AL_contig_9400_1_+2_113_343

MCWRVWETSARSSAKSRSSSVEKRFHLMPLGWFSVVHLITQSMTMLKNSADITHPCLTPV

FTLKLCSPFPTVQVKLI

>SG-AL_contig_9401_1_-1_613_906

MQEKTSRLNTFTQQVGLKINQKKTGDDTEHPNLSPIQVDGTDLPTTEEFIYLGSTVRHDE

GTGSDIKSRLSKARNTFRMLNSLCRSPQNSTCVLSTLL

>SG-AL_contig_9402_1_-2_2_160

LILLSGLTCSEDIFIDMSKIDKRCIVICVYMFSVLFPLFTLFRNKEKEKKIVY

>SG-AL_contig_9403_1_-1_34_651

MDKEMYSHMSLVSDCSLIIDRGIALHKMIRLITLALGGEGYLNFIGNEFGHPEWLDFPRI

GNSESYHYCRRQWNLVDDQMLKYKFLNNFDRDMIHLETRYSWLNNRQNYISMKHNDDKVI

IFDRADILVFVFNFHPHKSFTDYKIGVRTPGVYRIVLDSDAEHYGGHKRLDHNTVYHTFN

EPWNDRPCHTYVYIPNRTAFVLAKCD

>SG-AL_contig_9404_1_+1_448_702

MMRHFLNWVLKLLVKGVFVSIIMVIGWVVALDKGDLSMGGDKPCCSFTGVHSSSAWHACL

SRMRAMPSGGCCPDGLRTSWFCLLM

>SG-AL_contig_9405_1_-3_81_281

MGASILSLLYDITFTKWVVLAFYRSSQQFGTEQRLSFAPPLYFIDLFVYSIHESKPLGLG

QDSDCNL

>SG-AL_contig_9406_1_+1_745_900

MEKNITANVHQDFHIRMSFPLGSHHTTMSLVIMQLVTSVQHFDNAKRKNSIE

>SG-AL_contig_9407_1_+2_2_202

YIYIHIWKTDMFNYKQLECFYFQSHYKVFQSSVSTGFLVLGTGKVHLFHSALSAITSSTG

TMTLLFV

>SG-AL_contig_9408_1_-3_3_1919

FTYLWDIREPKKPVHSFQTVAGAYQVKWNKVTNNLFATTHEGDIRIWDPRKGNTPQQYIA

AHLSKIHGLDWSPDNQYQFVTASQDGYVKLWDYTNPKKCCGSTDSGAPVRRARYTPFGAG

LVTVVVPQLKRGENSLYLWKIVQQTQMQPIHTFVGHKDAVLEFQWKKQPDGVRDQQLVTW

SRDQSLRIWRIDPNLQRLCGHDSGDPGEVVDLTGTSYNSSESTLKDLRDLTLGDSTVDEE

LSITLSTGSMTHQSLQLEFNAIRNMPNVSAEQLDVMNRLCRVSAICGGHSMHLECLFPAA

YPNNVAPSFQIIQTTLDAAIQSRLMKILNETAMKCVKHNQNCMEMCLRQMVSVLSTYDDR

RSPESEVPYSHTQQPIVQASGFLPMYSLGSFQDSSVPFPRTSGARFCSNGLLVAFGVPAE

MKKVSGGSEMTPKALSDLAAFTQTWTGPSAAFSLPFFSRSPPTPSSDTMTISDFFEFKGR

RSSRHSHSSRRRRHERDGDKSTKKQQSVGKVKIYNVEALIGVSRHLAQHYIMDLNDIEGT

CRHNRSAAAAIGRKDLVQIWSMVATMSSKKLQPDLVPQQVVPWALSPFGRPLLKHILDQY

TAQFDVQTLAMLCCLFWTPDLKPRTPVQRSTSKTSLEFT

>SG-AL_contig_9409_1_+2_755_925

MKAGSRNILPAPKMGAGLNELRTICSKVSTCYQNNCCFLLLSKCYSLIPKVLYVQPF

>SG-AL_contig_9410_1_+1_382_564

MLFPDFYKNYILKVSNVFRTTVFLLFYPKVVHRCLQVLFVKKKKRKCFHTIMKLKSKWLK

S

>SG-AL_contig_9411_1_-3_72_365

MNQTLETSPELVSLRHQLQEKDQYINHIEKDHEKMRAVHDEEERLIVTAWYNLGMQLHRQ

AAEERLANNNMGQSFLARQRQVHSTTRRAQTINTLHTS

>SG-AL_contig_9412_1_+1_1102_1245

MKICCQLFYLKRECIAQLHHLLLPPLKETISSENEHAFPCIRIRFPQL

>SG-AL_contig_9413_1_+1_448_618

MLTLRNLSTQIDVHTRSQAHKKTNVNVENIQMQQKHKEKKCKCYRKLINVTSIEQQQ

>SG-AL_contig_9414_1_-1_562_906

MRSALNRHKSQNTQNIHIHLFKTKSGGNVTDHLSDVPFIIDHLSDVPFIIDHLSDVPFII

KSEILHTNEGCDINVKKASKGIPLVSMSVQLNKKLKIITTFACSKKNVNSVILCR

>SG-AL_contig_9415_1_+3_396_641

MGKKMATVKRYCNESSILLHSLNSFYPSILIPKHYSSFVFFHNVKFSLSIELFTDTGSMI

MQLVCTVKLASVHRCSHIFFFF

>SG-AL_contig_9416_1_+2_38_787

MKVEGKVALVTGGAMGLGKGFCNMLLENGAKVCFTDINEETAKTTLNEFQKQFGDSKVIF

VKANVTSDSDMQDAFKQAKSWGGSLDIVINNAGVTNEQQWVETININLTSVVRCTLLAMD

YMRQDKGGEGGVIIQVASAAGLVPVSFSQVYCATKAGVIMYTRSVAADSQIEANGVRINA

LCPGFVNTSLVAGLQTPEGLKFIQENGGMMQVEHVAGAMYELITDDSRHGAVLMMTELKG

KNYITFPEYT

>SG-AL_contig_9417_1_+3_3_221

CQFLPQVSHDVPQFSSTDEAVAVLVEDPEGLPDLLFTVRVLHLAGHHCQELGEIDGAVAV

SIHLINHVLQFSL

>SG-AL_contig_9418_1_+2_2_79

RSSDLYTDIDSLTATVVIYFISHILR

>SG-AL_contig_9419_1_-1_76_243

MAHPIPPSPLPQEYLKTIQTKPVKHLTFTLSETFLRFSLSPRHTHTHNTHSLKSEY

>SG-AL_contig_9420_1_+1_1_168

FLTHIHIYIYIYIYIYIYIYLYLHIHTICRLHILYTGIAGYGVSLRHGGPTDLQKD

>SG-AL_contig_9421_1_-3_177_305

MSIHLSDFRQDCKVHVGLCIAIRMQVLTCRLLLVKWNLEWKTY

>SG-AL_contig_9422_1_+2_2_148

QTQANPCIYVCVYVCGVCVCLCVHASVSVCVCVSVSVCIYVCKHECMYV

>SG-AL_contig_9423_1_-1_4_213

MLCVKEDPDPRKCLSEGKEVTRCGFEFFGKVKQSCFAEFTKYFECIDNSDRQMSLKNCRS

TQATFDKCIK

>SG-AL_contig_9424_1_+1_274_411

MLVLLNVSSVFVLLSLWQCLLHLLLLIIIRLTELKRLTISKFCVKI

>SG-AL_contig_9425_1_+3_96_323

MHVCTHTIIVQLFMKAYLSACYLLVLRYPVWWMWMCVYMCVCVCVCVYACICLCASIVSV

CVCVCMCGCMYHCMGV

>SG-AL_contig_9426_1_-3_3_293

KKKKKLSRGNNLLTKLSFIKTTHPQIRASLALLFRQSYPSCRKGNPPSSSDKNLGTQAPH

RRTRFQFQFKHTCCDLGHQQQTSPCTDSTNTLQKLHN

>SG-AL_contig_9427_1_-2_368_2119

MLLANTGSHWRWRRTSLGVVPQVWWWWLLLLLVLLSSMCAGNDNAKRLYDDLLRNSGYNK

LIRPVGNSTDKLIVKLGIRLSQLIDIDEKNQIMTTNVWLRQEWKDLDLLWDPVEYGGIKT

FEIPSEDIWLPETVLFNNADGNYQVTLMTKATLHPDGLVVWEPPAIYKSSCIIDVEFFPF

DVQSCKMKFGSWTYDGDQVDLVHICEPFAKTGETIRKGIDLRGFYPSVEWDILEVEAQKN

TRSYPCCPEKYPDITFTVKMRRKTLFHTVNLIIPCVAISFLTVLVFYLPSDSGEKVTLCI

SILLSLTVFFLLLAEIIPPTSIVVPLIGKYLLFTMILVTLSIIVTVVVLNVHFRSPATHS

MSPWVRRVFLSILPRLLIMPRPYPEGESGNSEKAKKFVVRTCNGIEMRQPLEGHERDDLT

TRPPVGYSYSQPCYQPHPHYSHEASPESRLLRGRSEDILNPLDRQSLSSGTLRRTRGGGC

GTGSSSYSREVLDAIDSVNFIAEHLKKEDQDSSLPSLWRRRYPVIIEDWKYVAMVLDRLF

LWIFTTACFVGTFGIILQAPTLYDDRQPLTPVESSESCYASYSS

>SG-AL_contig_9428_1_+3_3_236

VDSRGGCVGGGGGGNWFLFRHVFPYSIFLRNCVGKVNVKHHLTGRSEKSVCVIYNDLTSS

ELMFCVVVGYHVQITVLS

>SG-AL_contig_9429_1_-1_1_186

SWHFLTHRRGHWELITWHAYTHHLSHSTTLLLLQNTPPSWLTNILALCHKRGKGQSLIEI

TG

>SG-AL_contig_9430_1_-1_109_258

MHFKAFLHSKTVFTGPLRALCVFTQSCTLTESVQLLLYGCPSHPGLYGEK

>SG-AL_contig_9431_1_-1_769_948

MFLDIISIIMTLNICRPISFKIYTVVTPSKVNNFHSYDLNISRPVSFKIYTVVTLPKLNF

>SG-AL_contig_9432_1_+1_1_297

FFFFFLTIQNTFLLYNDAPKNNSTCVLQSYAIVMKEKKKEEKISKTTMYSLPHSRKTNGC

NIYLFTTSENFHNKEFLQIKGFFFFSIYVRRNTREKNSG

>SG-AL_contig_9433_1_+3_789_1181

MRGTPRRHGSKQSDLLQLFHADNVVGITTLALGLYSHNRCCQQDWHLFSDTNPTQIHSSI

PFSVLVLSPSEVKIIFKTGYASSNLFRLMHVTKYVRLYSPFGVQSIIQIWLRSVHEQQHL

TVLHAHIHTHT

>SG-AL_contig_9434_1_+3_117_215

MDESRFRESDFCAHSTAMGIEIYTHNLWQRLKP

>SG-AL_contig_9435_1_-3_9_272

MFLEWVKKHCKATTKDVFLMIRRAKTTIFTDAKETTPLIDVKRIVEGILKIAPENQRLYR

EDLAMTDDNKSLGDYGLNTNNAKAQAPA

>SG-AL_contig_9436_1_+2_149_409

MNIQDGCSRSSVPRNQNHRKRKFQWASHMSKASQRSCQGPSSNMVPELFTSHQAPSDTTD

KRSVVPYITSSDKTTLGKQNGPLLRDS

>SG-AL_contig_9437_1_-2_2_100

CTSRFREILVCLCLCTISYTCACYLRFEIYLSI

>SG-AL_contig_9438_1_-1_436_738

MPIINVCLFNTSKVFFFNNSDLINNELIILNFLLKTKTCLHKLTEVSSAKSKCFLCNVPC

CLPGMMKQCLLQMELDQPFLMHVESIHVHFGISYRGEYITG

>SG-AL_contig_9439_1_-3_3_416

TCPKRVESVYPSLLKHRHTLVKIYVSILLTYARKCVKIHPCASQHVHPSLAKFSFPPLKS

RGKQQEWQTASSRQKSCFRYPRVLYFILLFYFVPLFFFRIFYFILNNLVSSSHTSFYDCN

ELTSMDIVACASASLLSI

>SG-AL_contig_9440_1_-1_34_321

MLISHCDAHPSLSLFEYMANNCTLSMLGLECMVIVNCIVPVWFSNEIEGITLGNDLIFKG

NIYWVHDCKCVNICVHVFVRVYIYAYGCRCICAYMC

>SG-AL_contig_9441_1_-1_127_303

MDAPLKGVVSNFLINTSALGLVDARMYHDRICSKSPVMKSLKQNSTVLSSPFLIFFGLF

>SG-AL_contig_9442_1_+3_15_233

MTANKLQLNDGKTEAMLVASNRVSSSQVLPLSIDICGSHIQFSSSVKNLGVYLDSTLSMN

LHVTNTCRSAYFE

>SG-AL_contig_9443_1_+1_100_375

MTVTTPDKYMSFFNIISYKKCSFQPDISAVYFINIYHTYMLGNIEVSYFICFSERCTRIT

LMNTADIIRKMTFFLLSRVIIPHCNISELIVT

>SG-AL_contig_9444_1_-2_77_340

MLLTYCFIASLFFKMYACCTCDLNGKAPDYSFGLGINAFTFIDLIFVVIHLFVCLLAYLF

VRLFVSLCIFFSLYTRAAFSVSYPAVNG

>SG-AL_contig_9445_1_-1_133_2316

MDSTTAMAETTLPDFISNLQSTTARLSMTSASPVHYDVSSTSEAQGMRMTSVSVMMMMVT

TTANGWSFANTSTSWSSMEDVEQFLQHKNDEFTMLVLPAMIFLAILFAVGVSGNCMVLYV

YNRKLQKGTIRWFVQALAIFDLLSCLVAIPGEIIDMRNNYTFGSSPMCKVLRSVSMFCTV

ASGMTLMVVAVERYKRICTPLRKQITPKGAQVIITVSTVAASICAAPACIIYGPQTIPTD

NPAINGTDCSTADVFMGTVIPLAFSSFQFLLFVLGALGLIVLYSLIGRKVWSHAHFRKMG

FRAGINRRMSFFFNGSECSSPTTDDTVFAFPVKRNIPAVVVVAEEDNSNTSHGNVDPNDN

NNVVHTKAEKVATVEGFPSEVEKMEKTPAYSEPCGDDIADRASMDTEKHYPEDEHSEPDT

LTPHDLSLVNDLDGREQENVTSLHQTDLPSGVAGQAREKLSLHNIGLANRLRPKETEMLP

PPHLGLSNEIDRQSPESLSLDHKGLANGLDQQQTSEPHQAALPNGVEKKVPEVLTPHPTG

LPNGLVRKVPETLTPHHPSGTVRPVSMEQPPTPTGNFPHTLDTFRRTKSTSRPISRDRVK

SGDSAVVRGRYRRRSTHHTQSDMRRFLHRSESVNSGTRRTTLMLFLITLVYLLSFLPYLI

LMIVKVLDEESLSGRGGGWELAHNILLRSYFINSMANPIIYSFCSRTFRKECSKVLHCRC

CRSPLFVY

>SG-AL_contig_9446_1_+3_3_236

TAQILLINWGERKLCEGLLEKRKWNGPGRQKKADDSKGKKNGRDKESNRKEEIGSTEFPE

GGDTIYSERAPGIPRGTR

>SG-AL_contig_9447_1_-1_346_450

MSHFNSDMFPWLTFILNLVKLGSSASFRAKMRCGN

>SG-AL_contig_9448_1_-1_217_471

MERTLLHLRASWDIGCPNYTLPTHICFTLAMLFSWERMHCLFLDTSRLVLEQGCLLGMRK

PHGNVDNCKARNWLASIAVGESARD

>SG-AL_contig_9449_1_-2_203_1300

MSEGSYKEGPSHVPEPTLPEDFMEEEELNREYELRAEMGDEEFVDRKEETVAVIPEKLSP

EEVPERDISFKITLEDYSPSGSPRSPRSPRKDQDQEVSPSSVSRGRGAKKNQGEEKKVTF

SQATLSPRQQKMENITVVGPEREEVDVKEESEVLPDGTVHSTQRVSHHKVKHVRHTHAAQ

GGQEVVYEADEEVPGSGKEDVYEMFEHPAKLMQESNEIEQTLDDGRKVKKHVVMNRMVHL

VKMHHESFDEEHHKLEEDDYEIEEVVPGTESAFVAGLDSDYEEEAEDNDKKKRDKQKMSG

SGSGMSSGPDESNLNGGAASPKLVTYIHGSDVDDINQFRHHGRGETTLEVVEDMLAKGQL

TLDDPQ

>SG-AL_contig_9450_1_-1_46_594

MAGGNKNKLSREEELLLQDFSRNVSTKSSVLFYGMALIVSVIPIWLFNRIHQMDLYQSAV

IFAVGTVVSAYLVAVAYRNVKFVLKHKVAVKREDAVAQAVMKQFADDKKMTKKEKDERIL

WKKNEVADYEATTFSIFYNNALYLVLLVILSFFVLKNATPALNYAGSVGAAAGLLALFST

SQK

>SG-AL_contig_9451_1_+3_177_452

MYYLIMFPFPTSLSPLTHRNLTIAVLQVYPCLYPMPIAHNPLPLSLSSLTWEHTTQQTPE

NTKQKTFDFKQVLTSGNCFDLGYQFIPYLVTD

>SG-AL_contig_9452_1_+3_42_353

MQNVPFLLQPLEFGARCCENSIMYNPPCVLKQLSKLICSNSTTAKLPCQDVLAVCVLLMS

ECYEKNLMSCSNALIFIFIFMNFGCYLCLVIFSVCVCVCVCVCA

>SG-AL_contig_9453_1_+2_1034_1327

MNKTNCCKYVLYFTHMRVVYYLYSIFFSLDLSVCPVLFCVFLYFVIVWLVVCSVLYDCFC

FKKRNTFVECGRCEIVYRHVHNTMEESSTEPVNCQTKN

>SG-AL_contig_9454_1_-1_250_1902

MTDLETKEEANLTKDTAVEEQVSRRLSALLSDLSLKKAIIRLIHHLHKKSGDGFTEETAA

SYLQQLTQISVQQGTLQTQQHKEDKTKKLFIVDNREGNPRQSVICLNAEACRDIESNWDR

FLQVLVSAIHFVLGVNLDSLLLYRLLKSPRQADEALDEAGIKECGFQTEKDHSISSKPGT

FVPVRNHHQLDNHFHELNEGEHVAYEVYDPLMVCGDTDSKDIVPVYIYATIIREVCDSDV

ESIPLFAKRYYIDLGPDRGEADVSVTRLYRIISETTVQRSPTSSLKQQTAPHKEKDDQIQ

DLSAILKDIRQVLTKVWKVSRKEECQCVMKRLLLEWHPDKNSGKESLCTHVIQAVLHYSE

LLNQGESLPEDDSTEGASDSVDGWSVKSTSNLFARSYTQRQIYRARYMADHNMENPQPGE

GWRWFHTAQCDMAAARASRGICEHGRNWVCFLCHQAVEKALKGVLYFRNADDDGLQSHSL

PQLARRVSDAPLVQLAQQMESRMGPHTRMRYPDVLVYPNVPADVYTEDDVDMACDVAWHA

LERAQYLMSVM

>SG-AL_contig_9455_1_-2_356_571

MSVFGDLWKQKQLACTSPKAEYDCLNSGVKKTKNRHTCKSPLVYSSAGGGYFQEQFIAVC

VQISGDLTASCT

>SG-AL_contig_9456_1_-2_2_160

EVQLPPSSLAAQVGVEVELLPSSLAAQAGVGVELPPSNLTAWVPVWVEKKKTV

>SG-AL_contig_9457_1_-1_1_120

LWTCDSLKQYDCLNLFTPDPIHQLPPASCKLVNRVRESKG

>SG-AL_contig_9458_1_-3_15_1016

MVLTCRFYKNRFPEIDDVVMVNVRSIAEMGAYVHLLEYNNIEGMILLSELSRRRIRSINK

LIRVGRNECVVVIRVDRDKGYIDLSKRRVSAEEVLKCEEKFAKAKAVNSILRHVAELLDY

GSNGKLEELYEKTAWYFDEKYKKPGSSYEAFKHAVSNPEVLDECDIDEKTKEVLLENICR

KLKPQSVKIRADVEVACTKYEGVDAVKRALKKGLELTTEAMPIKINLIAPPLYVITTQTM

ERTEGLELITKAVDAIMEDITAAGGVFNIQEAARVVSDIDEIELEKQLAKLEEENREVAG

DDDEGDEYDEDGEKKGKKGSDDEEDDDEIDEKDG

>SG-AL_contig_9459_1_+1_391_561

MINKGGSWWIGRESQRVNHTSEHWPIPMQQLHAATRIMKQLNLGYVVWHPAPPYFMK

>SG-AL_contig_9460_1_+1_1_168

FFFQYESKVCSFIVHCHSILMQQCSNIQQHISQHILRLALNQQLLGTTKQLWTGDI

>SG-AL_contig_9461_1_-2_95_232

MFSNYITYIFTYIFLMIGISVTLSEGRSTNKSLLQIVGAFYEILST

>SG-AL_contig_9462_1_-1_106_249

MHILKPFMIMILFCNVPCCCKSFVNHVCVHMFTGKGNKGPYCSSIQKK

>SG-AL_contig_9463_1_+1_1_243

FAQACILESLQRHKFHSPSGLKEANTIRVEAHLHCQSSQVFSHSPQTLLFIKGRWKQKKA

RWRIEGVFNIIAKWSIKYRQN

>SG-AL_contig_9464_1_+1_625_828

MPTPGRHHTTSLQHRDTQKDKGNFHPKAIQKLLQHIPTTMQPDPTNSGQPPRSRNQQVRR

PYSNAWSA

>SG-AL_contig_9465_1_-1_73_1620

MSNMETQSELKKLSDENLAKQPDEVFDIICKIGRGSYGSVFKSLHKETGQVLAIKQVPVD

TDLQEIIKEISIMQQCDSKYIVKYYGSYFKNTDLWIVMEYCGAGSVSDIMRLRNKTLIER

EIATILYYTLKGLEYLHSRRKIHRDIKAGNILLNNEGHAKLADFGVAGQLTDTMAKRNTV

IGTPYWMAPEVIQEIGYDCVADIWSLGITALEMAEGKPPYGDIHPMRAIFMIPTKPPPSF

RQPDKWSTEFIDFVSKCLVKNPELRATAVDLLEHDFIKKAESCEILQEMIREAKQIQEMQ

NQVLFNGDESGEDEDKDGTMVKKTDEPDGTMKAQSEGTLVPEAGGTLVVNSAASTLQSEL

GTMVINQDSEEEEEEEEDDGTMKRHSTATGGKEKYRPAYLEHFEKKEQEEKARMSNKTQP

VSSNPAQQAPPQQQQQQQQQQPPPKQVTPPHHFQRSFIDGDFEFLRTLAYEELEQRLSDL

DPEMEREIEELRARYQAKRQPIQDAIDAKRKRQQNF

>SG-AL_contig_9466_1_+3_3_116

HIAIYMSNAPQHTTPRYNNLTHSYLHVQRTLTHHPTLQ

>SG-AL_contig_9467_1_+1_1_117

CKSTFYCMQMFSYNMLRIIIGKQFITAREVMHADAVHQM

>SG-AL_contig_9468_1_-3_51_221

MHPAGQGSQAEAREVCMELGHASHVVQLCQDREGDRGRCWGWPCIGGGGPALTPLEG

>SG-AL_contig_9469_1_-3_2145_2483

MEYELYGLFIVNILRTPKWKIVVRHDRNLSFSSETADSLLEQIFFLCTKKKPTPQRDHKF

IILICCFCFCVCVCVCVCVCVCVWSFYLDMRKHLKLLKDMSGPINRHKGVTVV

>SG-AL_contig_9470_1_+3_234_512

MQNSKNSAIYQSIKICSEHSNCIHNVYSNTFHVDEINLQIMLNVNTFSKKLRSFHFHFKV

HNQRTVHTSNSIHLSKRIRTDGHIQIVASTSPF

>SG-AL_contig_9471_1_-2_872_1060

MCLPVWILSTVCKLSLIFECMFHRYSVELPYLNFLVFAHSCHVYFIVEIPVIIQQDVIIL

FVE

>SG-AL_contig_9472_1_+2_2_208

FFFFLCAKNRTFYSGNNLNGVNKIKVQFLKSNTSFRAKLPLSTQHNHSLVSGLLILLDNF

KLFNDTRER

>SG-AL_contig_9473_1_-1_43_381

MYWQGLEMGREMGQVYQALTPPEWFGHWWAADKPIFMLRAGMELETSDVESMALSREPVS

PLNCGPPEGQTYNPHIISIALKLTGHCAKKPNLRAVRGSNPQLLGYQHSSVTN

>SG-AL_contig_9474_1_-3_69_1382

MIGGLFMYNHKGEVLISRVFRDDIGRNAVDAFRVNVIHARQQVRSPVTNIARTSFFHIKR

SNIWVAAVTKQNVNAAMVFEFLHKMVEVMQAYFGKISEDNIKNNFVLIYEILDEILDYGY

PQNTDTGILKTFITQQGVKSQSKEETAQITSQVTGQIGWRREGIKYRRNELFLDVLESVN

LLMSPQGQVLSAHVAGRIVMKSYLSGMPECKFGINDKVLMESKGKSSLDDSSRVSGGGGG

KTSIAIDDCQFHQCVKLSKFETEHSISFIPPDGEFELMRYRTTKDISLPFRVIPLVREVG

RQKMEVKVVVKSNFKPSLLAQKVEVRIPTPLNTSGVQVICMKGKAKYKASENAIVWKIKR

MGGMKECQLSAEIELLATNDKKKWTRPPISMNFEVPFAPSGFKVRYLKVFESKMNYSDHD

VIKWVRYIGRSGLYETRC

>SG-AL_contig_9475_1_-1_286_786

MARSKKANAKKGGAQKGGVKKGGVKSPAKAKMTKSSTMMATAKEAVAYLYPSKNKGRQWA

YLQDDAKEALASKGIKVPEETSPGKKAAGKASKPSMKRDSTMAKTVAEAKQLLDGKVTVA

KGRKARTPKKTSPTKPALKRAGTMQNTAKEGQSYLNRGKGKKGKGRK

>SG-AL_contig_9476_1_-2_113_2413

MMQDAPRENVKDNSEVRTVEINEGNLTPLQAVEQKAHVIPTQSNFGPDMSGSDDDEASEV

DSFILDENDMDGNEEVLETSKFLLAAETEEGQQQLQTVDPETHARLEALLEAAGISKLSS

KDGKALTDPEVLRTLTSSVSSALDEATQAINCMRSEQQGQAQGAEGAQALSERDPGTVRK

LYESGSVPDTTEEGESLLSLACSAGYYELAQVLLAMNANVEDRGIKGDCTPLMEAASGGF

VDIVRLLILHGADVNAQSSAGNTPLHSAACAGHTNVVKELITNGALVEVHNENGHTPLME

AASAGHVEIAQQLLAAGAGINTHSNEFKESALTLACYKGHLEMVKYLLDAGADQEHKTDE

MHTALMEASMDGHVEVARLLLDSGAQVNMPVDSFESPLTLAACGGHVELANLLIDRGANI

EEVNDEGYTPLMEAAREGHGEMVALLLAQGADINAQTEETQETALTLACCGGFKEVAEFL

IKAGADMELGCSTPLMEAAQEGHLDLVDYLLLESANVHAQTGTNDTALTYACENGHTDVA

MRLLSHGAFLEHESEGGRTPLMKAARAGHLCTVKYLIKQGANVNRCTSTNDHTVLSLACA

GGHHQVVEELLNNGADVNHKLKDGSTMIIEASKGGHTTVVKMLLEWPNRIMLNSSMDLAQ

PDMPHMNMAQGALEEPRVPVHGLANIVPPNEPDSQLPTCLNFSTSPGKVPERQNSGKAMG

KRAVPASMGVEGKLAGKTNNTVITDGQQMNHEGVVLADLTDEKATEK

>SG-AL_contig_9477_1_-1_412_582

MKRVHIIIGLCVFLHNNTNYNQPLHFISCWFLCSSGKIPLSVILILTPLDCSGYLFN

>SG-AL_contig_9478_1_+2_38_274

MTTKWCTKITAPWMARPLTFNNPFQPHSTTHRHTLTQAETSDTIYTHSVCVSLSHKLAQA

ETSDNIHTHNVCVSLSHTN

>SG-AL_contig_9479_1_-1_121_663

MGNVFAGLFKSMFGKKEMRILMVGLDAAGKTTILYKLKLGEIVTTIPTIGFNVETVEYKN

ISFTVWDVGGQDKIRPLWRHYFQNTQGLIFVVDSNDRERVNEAREELTRMLNEDELRDAI

LLVFANKQDLPNAMNAAEITDKLALHNLRSRQWYIQATCATSGDGLYEGLDWLSQTLKKK

V

>SG-AL_contig_9480_1_+2_110_334

MDGQKHDKLITEKSFMLMYCTMHLHRTNTPPLLLQTLSFTVSKFYLLSTLNGSRKIRHII

NNCFNKKHYQKWSLI

>SG-AL_contig_9481_1_+3_3_158

TLHDILSQPLSSPRQTRPGCAEDTSHSAQFTIPRPQKSVKYYKKYFEATKNT

>SG-AL_contig_9482_1_+2_749_901

MLMAQWAESKSASPFPNCEGHTTAGYFSADDSLDKLDGWPVSLLSYVSWFH

>SG-AL_contig_9483_1_-2_125_1636

MRTYNEEMKFLEKETPFCWKIKKGFVDNMKVDGYFYVNDRLEKLMFDELKQSCNSKGFGG

FLPGMKQIANVAALPGIVARSVGLPDVHSGYGFAIGNLAAFDMSDPKAVVSPGGVGFDIN

CGVRLLRTNLHERDVLPIKEQLAQCLFDHIPVGVGSKGIIPMTAQNLEEALEMGVDWSLR

EGYAWAEDKEHCEEYGRMLNADPSKVSSRAKKRGLPQLGTLGAGNHYAEIQVVDDIYDEF

AAKKMGVEFPGQVCVMIHCGSRGLGHQVATDALVAMEKAMKRDKIDVNDRQLACAHISSA

EGQDYLKGMASAANYAWVNRSSMTFLARQAFAKMFKSTPDDLDMHVIYDVSHNIAKVEEH

YVDGRQKTLLVHRKGSTRAFPPHHPLIPVDYQLTGQPVLIGGTMGTCSYVLTGTEQGMSE

TFGTTCHGAGRALSRAKSRRNLDYTTVLAALEEKGISIRVASPKLVMEEAPESYKNVTDV

VDTCHAAGISKKCVKLRPIAVIKG

>SG-AL_contig_9484_1_+2_143_322

MKVWQYCNVKFLQKYYNIGTSLKVILVVCCLRSPAQHSGGHPIHMHSVPRERDHEHIWMR

>SG-AL_contig_9485_1_+1_154_309

MCTQLQQHDITNNHCYMLSSEHLSYSLNLNWLAYENSGFSACLLINFHLILS

>SG-AL_contig_9486_1_-3_3_191

DFEESNELWITEEFLLKTFRVHAHMHVTYTHVAPVYVTLIFIQEYHICSSIKYVAYFCSC

LMS

>SG-AL_contig_9487_1_+1_166_360

MIIVKEKYFLSNILSKLTYQVNLSIVCSRKPIKHSVQENLSNTKRKIFPIKHSIQVNLSI

VCSRK

>SG-AL_contig_9488_1_+2_2_253

NSLSKSTYQHSVQENLSNSLSKKTCPTLKKNISYQTFYPSKPINILFNKPTKHSVQDNLS

RKSIPQKTIKHCPRKPINILMILF

>SG-AL_contig_9489_1_+3_216_353

MVVTAYMHTLTLPKIKITRIQTAEKIQCEILAAQRRIQIFKFSRSL

>SG-AL_contig_9490_1_+2_110_427

MFKKRSYRGQGWEKRVKACCKKESGMDRKGRNKKQTTVKEGKKTERKRAIEKRAFLPQNF

QNADHIRTFLSKQINNPSSLKTFMFCGDSDAVESDAVRNTTVQSNC

>SG-AL_contig_9491_1_+2_59_196

MFPYHNHDEVCSNIHPFTFYCTQLPQEMTIMSFEKINETCLCTPCK

>SG-AL_contig_9492_1_+1_223_348

MIWIINECVFMEQKIITPPLDTHLVYCLPTSAGNTENNVGWL

>SG-AL_contig_9493_1_+1_190_417

MPVDHYGPYTLTLVISTHTQSSLVHTHIFTHSRHQYTHTHTVVNTTHTRYQYTHIHTHTQ

LSSVHTHTHTHTHSCH

>SG-AL_contig_9494_1_+3_384_656

MYLFLFFWSSKCRLRSSAHIIQQYFLDASYPSLLFILNSNQSSSFFSPVTETTFFIQAAI

FFIQSLNIFKAILILCLPGEQEGRERGILPS

>SG-AL_contig_9495_1_+3_381_563

MWIMEWPLLSASARMLAFRLCELQTGRSRCPCTTSGDPQPSLRGRRSGPPPPPPCLLAKQ

M

>SG-AL_contig_9496_1_-3_114_302

MHILIFRMRVTKSPILLKGSSAGEQKRVNARPAMHPLHCTVLDVWVPKSPTSSRSGCTAK

RHG

>SG-AL_contig_9497_1_+2_83_373

MNGWKLNLVGAPRTTEAPSKKKEKECLRRRPDLGRSRRSGQGGSFRGGTKNCGHIPCVIS

MALLPTELTWGLARREKVQVPRLSWPPWSDKKKRGLF

>SG-AL_contig_9498_1_-2_2234_2653

MTAKFFRSRQSFFLLLLDLSAAFDPLDHKILLSHLDQNFGIISAAFDWFQSYLLERKQTV

LVGGNRSSPESVPDFGVPQGSVFGPVLFKLCTTPLTPLTDMYSVHHEMYADDTQLNHSES

QSNYNHMIQTLQDCVTEVKT

>SG-AL_contig_9499_1_+2_2_259

TNEEVRSRSKQNPVTSIICKRRLSWLGHTARLPSSRPANQVLNWNPGGQRKRGRPRMNWR

QTVDRDLQLVDKRWSDAVRLSADRAC

>SG-AL_contig_9500_1_-2_137_610

MRHFFLNDKKIYFDQICAVWVFCFGFNHKKNKQNKQKNNQGCCCCVAVFLQLFVHFLKIQ

RILSTNCLSLLPLFLSLSLFLNFVLILLYFKGSGKGWILIQIFLLWVGSCQIRLEFFFLL

FPLSETTATEVFSVDRLFHRGSTILEGRNFLFNVCSQL

>SG-AL_contig_9501_1_-2_152_409

MSWNEIVPISNIAIFSYVCGNAITVQQYQSPNQTFVVCAYGNSITMPYTDIVPILTSMLC

RGIKHQSLNHKVFKCACRNSIIVPST

>SG-AL_contig_9502_1_+3_282_437

MKSENFVSERSIQSKRGMSTKNSRTHHYSIDFQNDYLFTCLGYLRIVCMIVY

>SG-AL_contig_9503_1_+3_3_245

LNFSRGQHYEGSAHLLSLAALPFPTLNRVRYPFTTGWTEGHAHTVPRGRFEFVQSEGWIE

FYRYASHASELVVVCCLRSPA

>SG-AL_contig_9504_1_-3_84_221

MSHFSSFFVLCFSLSPLVRSHFSSFFVLCFTLSPLVSFSWFLEQLI

>SG-AL_contig_9505_1_-3_117_338

MANCKASHVSLAFQSGCLGLSKAHRIRKFELAASNSPSCEVLPCSAGEDKAAQERSERCP

HKAGPRALKPLLPH

>SG-AL_contig_9506_1_-1_1_522

DLLTLSSAATSQHLAEIERQQPPTPIASSDVSNNSNNNNNNGISSSSSSAHHHHHHHHHN

SSGAPHFLAHPPHTSPGPVVPSTPSPVSHHHQQHHQHQLPHHHHHQQQQQQQQQQLQGAY

HQPVLSPSLSVDEVSQALSDDSCVFMPEYEQLTSAGGAGGSSMSGGGQWGGQRD

>SG-AL_contig_9507_1_-1_1_111

TFTMIRQHMHTHRAHTHTHTHTHTEPCFNLSESLGPF

>SG-AL_contig_9508_1_+3_3_257

FFKYTLTILIIPFTAIPHERCMDIQSKHGILPLKRERQTERDKEREREREREREKKNGPQ

ADEAHFREKRGTEKERGRQIKKREE

>SG-AL_contig_9509_1_+1_88_357

MHTKQLQSNHPTSTAGIKTWKHICLHNAESRRAPLHMFLLCRYYCRHDCCRLPAGPCYCH

ILISLSKSPSLYHEDPQVPPPLLPRLDPML

>SG-AL_contig_9510_1_-3_717_950

MLTEVSQEMFRLLLHLLTEQIPQRICLVVNEISFLNFSIFVYIDMNQFTVSSRQFLVSSF

FVYLYTIYMNHCFKYVEK

>SG-AL_contig_9511_1_+3_234_479

MSEHACLCMCICMCVHVRVCVFISITVLSRIFLYHTFFTCVYSVQACTCMYCIFFFFFFC

VGCFFIFFFFFFFFFFFFFYIP

>SG-AL_contig_9512_1_+2_743_1036

MYSKLRKLLLSHSLFLSHSFTHTHTRNCLFFFPSTANRIHTSPNSIPNSIKNTFYDHVSN

YEKICKRILPIENKVCKLPKLTVNLKKNNSQHETFCYK

>SG-AL_contig_9513_1_-1_310_507

MPVLFITCELGSCFAIVLCNLHHFLNGKNVVQKKPSYKNSEFVYWSLTNYKCLSLFFFFL

FMLMKC

>SG-AL_contig_9514_1_+1_559_804

MQSFIYQKPELKTKEKKRNLLSQSTKTTPLYHIYMTNHCPYKASLKANTRFIFLVHIKKT

LACSHLQPRNRSSIFEAICSLA

>SG-AL_contig_9515_1_+2_80_301

MVSENMLCFHLNKYCNFNFKTHQINAPTVFQQLLRVRADIQNISHPLHSTPKPSHIHLIL

HLKGVFCFLNKCLA

>SG-AL_contig_9516_1_+1_1_189

GNPANIAFLRDGSVFLKFLPEFLAKNQDPGDPSPNILIRPLSTVLDRSDLDRHLCSVRCL

KFI

>SG-AL_contig_9517_1_+1_202_462

MHRNQTNYMCSLVDDNFLHSIHIATEKQELNLPIHKHYKTNTELDTSNQANQNVRGKCAT

NQYKKQAIYTHNEELSFLRCFGFHCLF

>SG-AL_contig_9518_1_-3_3_203

CLFLFCLFWGCVCVCVCVCARARVLENSYIGVIYGAIWIYALGVVGCMLLCNGCFDFCAC

VFLFSLP

>SG-AL_contig_9519_1_-1_61_216

MICYCIYLLNFSFSLLLHTFLSLDPLLPPMSFVDFWCKLSSVPVSYCRGPND

>SG-AL_contig_9520_1_-2_557_832

MCKCVCIHTFKLSCTDFCILLFDTIFFICIGLFVFYYSYLRPGILWLLCMEFSGFCVQND

VHFVCSGICERYLDPPSCPMSSFIFVSHILGP

>SG-AL_contig_9521_1_+2_1013_1246

MKEGYSLLVSRFDGEATFRFPHVLLCQAEWLAVWQSPSLSAKQCSFGVSTCLCMLGSSNP

VSLFNFTMTCPPGFPMLL

>SG-AL_contig_9522_1_+2_1046_1267

MYKHTIILLTIPLLQPGQQVKVECRVETSRHQLHQVTCLQNASVLSNVQNPTSPLTTSPT

HTSSPIPSLPSPSL

>SG-AL_contig_9523_1_-2_8_2461

MADRAMEPISDSPQLEGQYFHTDGDPERFVPTTDILNLDNIGQASDAGDVDMALGGGPVT

KVDDDDTVELSPGGILDEETVGRGLSNLGRSADGMHQVYLSAAMPGFSLVDISILAQFVH

LQKVEIPYNSIADLSPLGKLPHLLTLDASHNCISVMLDFTPPSNLQEVDLSYNQLSAMAD

LSAHHALKKLVLDNNTISEISGLEKCRRLHHLSLAHNQIETIQGLNGLPLRHLNLCHNKL

KKIENLDTLKHLQRLNLAGNRVRSLQGLADHDLLETLDLEDNEVIDVSEISHIQRLRMLR

EMNLLRNPIQELPDYRLAILFRIPSLTELDRHRVEVEEKVSAKNMFDPSLEVVAARDHIM

HVVYNFLQPSRVWDSTLPNVETPYPMLVLVGPQGSGKKDLAMKLVEEFSDYFGFGVSHTT

RPPRSDEEHGKDYFFVTMEKFEMDIKMGQFIQTNKHNDHWYGLQMEAIESVAREGLACVV

HMELEGVLTLKNTYFEPRYVLVMPLDKEAHEHRLRDRGLYTEEQMERTLQRADTYCKYNQ

DHPGFFDMMISSEDIADAYRRLRRLVMDYLGISPSTPDASSQAYPVTNNTGEAETYQAMT

GAASTNAMGARTWSKPSIPDSMSQARGQMGSAAGRGIVEEESYKRRHSAAKEVVAGYVPP

IYEQILTQFPRTAPQTVEEQQSLGLDDAADVRVASAPNNNAIANPEIPGSPDSTSSNASS

SSNLSDLDSATELQRPGSPKTHPGAPATHTQPAANDNLPTEKVNPLTLIAEKGYNSASKS

RLPEARPPSATRQMLGSGQGNRPGSDRHKVLPPITAAQ

>SG-AL_contig_9524_1_+2_635_880

MAMFRVSILAPFILMRSMLPADINDMKKHMYFSFTSMFSFQIYTNRPWEKKQVMHMCVCV

CVCVCACVTVRVCVTARLCVCV

>SG-AL_contig_9525_1_-3_189_350

MMKFLDKSKPAFFIFCRHERLPTASDFSWVWNIFQLLMICYQQTLCIGHRTFVT

>SG-AL_contig_9526_1_-2_53_250

MLTAAAIGRKVLGCVVCVIHLVLIMVLFKPSLGGAGDQEKRNLTDVNVTVPQSLPDVPLP

TDPFFC

>SG-AL_contig_9527_1_-2_548_802

MFKFKELSTCIKKQNRMLCAVHPRKQDTDMISRCKRLLIPLDESFMSRQKICGGTKESTL

WVRFLYHFGWQDQETMFMPVKQNNI

>SG-AL_contig_9528_1_+1_568_807

MFYLLTMTESGTSLLHSQTYVVNPWKALTHLAMYIMTKHSHLSGDVSSSMYKFSTVIINS

FSSLRVFCLIRNQTQQQTDL

>SG-AL_contig_9529_1_+2_2_265

LLLLFSIIVVGQLIVTTVSVFLFQALSGKGLHPSKCCFTSDMLSARPPIVTILARKLHTR

SNTMPSTQSLYATSFFQLQRLFSLADQR

>SG-AL_contig_9530_1_+3_273_398

MLNLTNSYFAKNKESFLKQNTTKQSIQNYFSCCQVCSQKLKT

>SG-AL_contig_9531_1_+3_234_347

MNATGLWRLKLTAGTTQTLQQYITHSMKTNATGSFIAY

>SG-AL_contig_9532_1_+1_82_189

MTVIAQLLSHTEHFFPFRSSFPFFVLSLSELKTPMF

>SG-AL_contig_9533_1_-3_165_599

MDSNGNLCGYGNYRGRDKLFFFDLIQCGRLGPGVFVNGCPTPQVCVSSCPSENYVWLESV

DPIKGDDKSKLICKEGVDKSAKSLADLVGDNDCAAYYLKSTSIINRCVPIDNLFGAVKGA

VSIANKTVVDNYNNTVTGDGLTTAQ

>SG-AL_contig_9534_1_-2_1298_1555

MWIWKCNVDVSHCFIHLAQLSLSYVSEKCEQDSIDAHSFCIALHTSSQQNLSLKGRLSLP

GEYINFCCCCCCFVLCIHMFLFCYQS

>SG-AL_contig_9535_1_-1_19_2130

MDNPCTGWLSDGAWDNITELDKLTNFHGIITSFEQYPRDWNIWYTSATPETTSLPGEWDN

ACNELQRMLIVRSLRPDRVSFCATSFIVNNLGSRFVEPPVLDMQQVVEDSTTKTPLIFVL

SPGVDPTSALLQLAENSGMASRFHALSLGQGQAPIATRMIKEGVKEGKWVFLANCHLSLS

WMPQLDKLVEQLQVEEPHPEFRLWLSSSPHPQFPIAILQQGIKMTTEPPKGLKANMKRLY

NLVTEQQFQRCHKQDKYRKLLFCLCFFHSILLERRKFLMLGWNIAYEFNDSDFEVSENLL

SLYLDGYDETPWDALKYLIAGINYGGHVTDDWDRRLLMTYVNDLFTDSVLSQPFFKVSTL

PTYYIPRDGPLSSYKEYVSMLPNIDHPEAFGQHPNADITSQIQETRMMFDTLLSLQPQVS

SSTGESREDKVLDLAANIFKQIPENIDYEGTAKILSTDPSALNVVLLQEIQRYNLLLDEI

RVSLTDLEKGIQGLVVMTLELESIFICIYDGRVPPAWEKAYSSMKPLAAWTRDLVQRVEQ

LAKWAQTAHPPTIFWMSGFTFPTGFLTAVLQTSARQNSVSVDSLSWEFTVLTVDDSNITG

PPKDGVYIKGLYLQGAGWDKKNSVLIEAEPMQLVCAMPSIHFKPTETKKKSSKGIYTAPC

YYYPNRAGTSGRPSFIVAVDLKSGEKNADHWVKRGTAILMSLDY

>SG-AL_contig_9536_1_+1_181_372

MLEERYPQEAWIRAYTDGSATDAVKRGGAGVYIQFPNGQRQAEAIPTGLHCTNYRAEVEA

LVHA

>SG-AL_contig_9537_1_+3_3_137

FISYHDFIFAIAPLSGNSNTILATSSAVYLLAAMLACLTTFRFKK

>SG-AL_contig_9538_1_+1_883_1074

MCDLVVAKSNTVVCQPKLPHTTEIDFACLLTSNGQSMATGQLYRPQSRMWKIRERREQIH

ITDT

>SG-AL_contig_9539_1_-2_2_424

DLALVLGALRSPPFEPLDVVPFSLLSYKTAFLLALATCCHRSEIRALSGAKDDLGFHSNS

DLALTALLEFLVETQQPGSLPRTVRIPCLVSCPDPRDARSEERLLARSAASSTTCNARHL

GVPPSGAYLSPSTRSTRGTHQ

>SG-AL_contig_9540_1_-3_3_242

CLVGADFSLNVTSLNATPTVLLPPFLYLGVSDHVTSGLVEQGISQVVILCSRGLVRVEVW

HWYVKVEMNGSQWQIMSHVQ

>SG-AL_contig_9541_1_-1_112_1536

MWKQVFLLVLAAFLFGCGSEAAVASQLERKESSNSSPPGTIKCEKPGFKLFVKSAEFSPA

SEKFCSRLCRRVPAENSIEECIPTQYNFEKGCKRRFPAKDVWMVYKKCQNLISEGKPCYL

DQMEGLSQPKGADIQYYCVNEKSTKDPCSVKTTGETRDLYHDLSSQTQHRRCLCTGKVSF

RETEWDVKLVPVRYLHFLDPTIYSDRPMEMSSAILTSNAGEEAMDVSARTNLVMASVNRT

SPTFAITVQIPKSHHLSGFLWIHFRDDNVNINCRTYSGSIEVSVRPDGRTTVSTSTATDV

EWLPLYVVGAAVFIIAVIVAVVVLVCVMKKSRSSPTINTPTPSSSQPTTPTEEHSSTHLL

NSVYTEVNPHRHNHQHPNNSFTSNHHDYHPPTHTHRSSPPNYEEPLLHTASSSSATPGAD

YATLQRHQGDRVVVHDSGNADTYDHTLMCADPYQALERGARRQPVIDNVYDSTER

>SG-AL_contig_9542_1_+2_200_646

MFIYNAISYRLTCGSLTGIHIFSLIVWVLFCFLFLLPIFCIASVLLPARRSFQLSNTSSK

PIKTEVTTRAGQRRATESGTIFHNATMMRIWDDDDGAIVFHLRLRLSDRDVLCVSFHNIC

VFHLRLRLSDRAVLCVSFHMCVPSKIATV

>SG-AL_contig_9543_1_-3_3_116

MEREEERKREREREREEGNVLCAHQVLSSISTDINELD

>SG-AL_contig_9544_1_-1_379_618

MPGYKNIMKGSAEYSLVTQYPSTMELDDIMTFIQHHSHSHKSSQHCEKLPHVLWTCSHGD

LSLDRRGGALGSQNEQQWGQ

>SG-AL_contig_9545_1_-3_3_230

QGHVKVKWKLHCGKRQAWSSASEVTEENTAEHKNLSKNRYLFSGNSIDRATCQPHRQAGT

EWVRAPAVLTKSSWAS

>SG-AL_contig_9546_1_-1_25_888

MLSRQMTKYGSAVCCRCDFVVNFMSTKAAIRQGLVSSSSTFAKPKGLRPTETRATHLNAS

HKVPLRLEGIQGPISTPLTLRESTRLLASAEKQVNDGFFQQGVQDFHLFTDLHHQKRAEI

SPLEGKLEEGACPQGVQGYEKTDADDEDGCIYFFTWLDTCSQYGQVNCDQQLEGIQSGRP

TLRQILAEQEARITEIADAYRKFSQEYPSAQGESFQGAQDEFHLPDINMPCPQGVQGDDC

VGFKLWLENCHHFGLHECLEQLQGFQSGRVTLAEIFEEQDAIIREVVR

>SG-AL_contig_9547_1_+1_58_201

MIIMVMIIIIIIKTKNDDPCKYFNRSQSVEREKSNQATKATNTRDMLL

>SG-AL_contig_9548_1_+1_1_192

STHIPSLSHLPKGLRHSFPTLPFYLPNICASHYTYFSSCAIASFNQTGNSTKCNVVRPLC

RRSK

>SG-AL_contig_9549_1_-1_85_11430

MLKGGVSPSVTAQLDEFVDPKSGEKLSMIQAESRGLVSVKKSDDQIDISEALQSGLVDKD

TGLFHDPVSGDAIPLDEALLCGFVSFKDSTNGHQEEENRPVIEHIQKVGPSRLSMSTAVA

SGLIDPEHGTYTDPNSGQTISVIQALQHGIIRPTLSVAESTPPSSDHKPTTPSHQYVQIT

EGSPFTPKHGTVVDKDGHLISKTFTEVTTTSGSATYTSKPGFSIDSSGQVINTATGDRMS

IAQAVAAGIVDSEAEQAMSDIQVMGGGDLPSPDRSSSASPSVGRLADSFDNMLQELAAEQ

DQLADFERVLKKDSQMGDEAYKVRAQLESHKGVHDEIMSKQQPILSLIYKAEQLTENYQE

ELTPEQVTELTTQSTLLKAALEKITRSSDRRLSHLNKAAEELGKLEDEMSNFQKWMTSAN

SELARQEECLQRFEDLKPLAEKQKELSSDVVSHQADLRFMSMAVQKYMEEAKLYKLEIDS

FRADRVRPARHSLISMECVAADDVKDRLKDVTEEYHDLSSRCHSLGDRLADLSGKHRQFN

DAAFKLLDWLTETEGQLASLKQEAGSPEPAQLHAQLDRLKSLSMDAVSQRGQLDEMQRRG

QDLTNSLSGQATDKQEVAKLQGVMNDISTRYTALTNDIGARTTGLQAAITQSQDVTQAMD

GLLNWLDSAEQAITSQTPVSLHRPVLNSQLQGFSVLQADITNHQSALEAVRASANELIKT

CDLEIAKAVEQRLATVEKKFSDVQAHTEERDHDLEDIDQGLREFKDKLETCTTWVQGGIQ

RLDSKDLAKLPSEDMTEQLELLTAEKKSQEIHVADLRAVADRLINDPRTGDASAVQETVV

ELERNLAAFDSLLAARHQEADVKEQQGTAFESAKTLAVLWLSQMEARLDEFDAVAIEVDI

VEQQIVQLQPMLQEYEDYAPKIDEVNDLGNTYEAMINPNERPMSPIRRLGRSRRLPGILS

PRLRTPSPTFPASPTAHRQSPLSSESSGVSSRKSSADNLLLDDLSESQQQLLDINQRYEI

VGERLADRQQELQLMLTSIRTFLQDLQDVLQWLDDRDKQTLTYQPLPTNEKEAKKRLKEH

EAFHRELLSKEGLVEDIRKKAQELLKTRRGVPGEESLQQQLQELDDKWHGLRASSEQRRK

SLEDMVSDLRDLREHEEQLMRWLGQKDRMLDVLGPVAMEPAMLASQLEQVKVLKEEFAAQ

EPIYDQFLNCGHAILDRCDPASQDSKAISRKLDTVSKAWNRLQSRLDERTKSLSSVEGIS

TEFAALTRGLADWLSDFTDRLDGLSHISSQPDKQEQQLAEVMELESDLMEQQPRLKRARD

LCRQLCDKAKDPSTKADLRSKLAALEKDLGDTTKKLEARKAAVEAASRDAAQFKADCHDL

LAWVNSATDRLQEAEAISSDPNTLRQQTRQSRDLQQELSIKELDIKELLGKGEKLLQDSS

PSAEVRAMADTLGELQGEWEHLKQEAAEREARLTTADSHAQHFSEHLDKLVLWLTMTEEK

LDKLKPDDLDRRDIEQTLKDLQGVETDLQKKSHALDKLRHDGQSLMDSVDSGKDDIDGQL

KAFNDRWDKVNNAVTDRSAHLEDLSQRLAEVQDTLAEANSALTKWEDKLAAHSSLGSTAK

DPKYIDKIKALLEDSGWLDTQLGTADSLMDGVEADGGNASDLRAEKGKLADRHQTLQDEL

GELVADMETGSQIVEQFQGLLKIVGGQLSELESELDTKSPISRDDAELDKQNSDMKEFLA

RLDDKIENMDDLQHQAGDLGSAGYVSDPDALRSQVDTLCKQHATLKAKAGQRQKDIDSTQ

HALEQLTEALNAARQGITKAAADVDAMGPTAGEVSAIKALQDELKGFVKSTVDPLQKQLD

VVISQGQGLIKSAPPGSNTSGLETDLESLTDRWADLSEKVSEREKNLDSALLHAGKFQDA

MASLLTWMTETEEMVANQKPPSPEQRVVKAQLQEQKLVQKMVSDRAPNVQAVKESGSQLL

KGKDPKERKKIEGELKQLEERWDALNKKVFDRAAMLEEVQSLANQFTEVLDPLSTWLDAT

DKEFAALEPQSADAEGIEKLIGELQKLQDETGERDPSVKQLAATGKDLQDFCKGEDVILV

QMKIDDAQKRFGELRSRVEDSLEQMEEALPLAQHFQEAHQGFLAWANKFEPELRSMEHSL

PESEEAVEELLNQLTEVQPWLDVINSEGAELSEVTPGDASLRVEDLIHKDNKRFDNLREE

IEKRAEKAKLVRQKSMEVTGELSELLDWFVETDEKLKNPSPVTANPDALHKQLAQQKAMN

EDIAAQKSKTRDALSSGKKLLNDHSLDDESVLRTKMDDLKQTSDSVSATANERLSTLEQA

LPLARHFREAHTDLVTWLDEVEPAVAQLDVPSVDADQVKKQQEEAKVLKQEVQDRKPAVD

RLNKTGAALASLCDPEGANEVNAMLDDDNRRMDEIRTRVRDRSNSIDLAMQQSAEFSDKL

ENMLDNLTVTAEQVKHAEPISAHPDKLREQLEETKAIEEDMEMRQAALDSVKSAAEELLK

QAGNDQDEAVRDVRQKLDELTQLFKDIQDMGRGRQRALDDTLGVAEKFWDDLNTLNSTLK

DLQDNLNSAEKPALEPEAIREQQEELEALKEDIEASQADLEEVQQTGDMLMGMVGETEQP

EVQKTMEDMGANLAAISDQYNKRSNELESALVRAVHFQEELMKLLVWLQGAEEKMADFQP

VASEFDTIKKQWEELKLFKAGVDPKHMEVESLNQQATELTKDSSPEQAVVLKEPLTLLNT

RWGVLLNNIGDRQRDLQMALLSAGQFDHAFKVLRNWMDTVATTLEEVEPVYGDPKVVEIE

LAKLRIVQNDITAHQESVDSIGEEAQRLMTVEGASEAQGLKAKMEDMNATWDDIHNKSRQ

KQDMLEDALREAQGFTGELQDVLMKINDLESQLITSKPVGGLPETAKEQLDRFMDVYGAL

EKLEPDVESLTLMGEKLSGKSSGPALANIKQNLTNLHQRWDHVRNRANDRKKKLEDAVNK

AGNFHEEHNKFIAWLTETEKILNNLHPVSRLVDKVTTQIEDHKVLQKDISSHREAMVALD

KLGTHLKYFSQKQDVILIKNLLSSVQHRWEKIVSRSAERTRHLERGYKEARQFHDTWKDL

IMWLVEAEQALEAETAIGNDPDKIKTQILKHKEFQRRLGSKQPVYDGVNKAGRTLKDRCP

NDDMPTIQAMLSELKTRWNSVCSKSVDRQRKLEEGLLLSGQFNEAMDALLEWLAKVEPAL

AEDIPVHGDLDTVSSFLDTHKTFQQELGARATTVAFVQKSAKEIISKAEGDTSNLQSDLI

ELNSAWDRACKLSVSKQERLEQAHKLADEFHKKAHQLLDWLSDAERQLRYKGPIPDEEPL

ILQQIEEHKTFEESLLRQEANLRETLNIGQDIMKRCHPDAVPVMKQWLSVLRARWEELTS

LGRQRNQRLVNGLTDLRHSNHLLEELLAWLNGAEVTLTDLDRQPIPEDLAIIQQLLKEHQ

DFQNEMSSRQPDVDRLIKADKRRTSVSQEPISHIPVLRASTPRTPTRRIPVRVGGSGRNT

PDRFGRHTPEHYGRRTPDVYSGRQTPDPTGRRTPEPQFRNPRVGALFNKWRNVWLMAMER

QRRLQDALDHLNEIERLKNFEFEDWRKRYLRWMHHNKARIMDFFRRQDRDRDGRVTRKEF

IEGILSSKFPTSKLEMEKVADIFDKDRDGFINYKEFVAALRPDRDPKPATTEEMIQDEVR

RQVCKCTCVKQFKIHKIGEGKYRFGDSQKLRLVRILRSTVMVRVGGGWVALDEFLVKNDP

CR

>SG-AL_contig_9550_1_+2_467_781

MPKPYLERAHVRAHTHTNTHHMQTRNEDQSMLKVSSLYTFKEDFSPCTPPRRTHNPCTPM

RKISILYTYEPSRKISVPVYLQRFQSLYTFKEDFQSLYTYEEDFN

>SG-AL_contig_9551_1_+1_28_213

MGGTFCPPPPPTPHHFSVNNGSMSQCLQCGTRQLNTNENDGHSYVQGLVQYRPTSEREWY

IG

>SG-AL_contig_9552_1_+1_766_1032

MTKKVFIMLYLNQDKMSITLNLNQIHHQQTLPHPIPRQTFQPSHCQTLLLFPLRLMRLVN

SLLLPLQTPLSFPFSSKAYQSQSWIHQMK

>SG-AL_contig_9553_1_+2_50_244

MTGREKFLFFAFYIQSKLLVNEKILNCKNILTYQQNPNHPRPFSSTVHTARSASNRWGKK

IVIMP

>SG-AL_contig_9554_1_-3_156_296

MVSFSSVWIFYYVLTFFSSGLVVSILRCHSLYTKQISITHIPCLDLY

>SG-AL_contig_9555_1_+1_76_978

MFEFKHIKPFDRSYIDNPGPMVVFATPGMLHAGLSLQIFRKWAPNEKNMVIMPGYCVAGT

VGHKILNGARRLEFENKQMLDVRLMVQYMSFSAHADAKGIMQLISQCSPANVLLVHGEAA

KMKFLRDKIRKEFDVNCYMPANGESAIIPVSHNLPITASVSLLKRRSAVANDESSDPIFT

KLLHGALVMRNNKMTLMEPSAAFRELGLEEHQLRFTCNIMLDEEGTVTDTAERMYKLIKV

HMKQCPVQYSSDGSISVGDSVLLNVTGDDESKTILVSWSHQDEDLGSYLIKLIKSNVTVA

C

>SG-AL_contig_9556_1_-3_414_2432

MLSSTGFSGILICLLAVFGVVTGPVVTSASRSSSANSTITSSASSIFGITSRGNQRRGSQ

PRTTNFRSVPYDLKSPANPHWNSFLEEVAQQTSRDLADLRANGVNDPSIATMWNIYIQTL

SDGAALPSIRESRWTGGGEGGSAAPGAPGALGGSMQMGAGSSHSSFQDSWFRSFFIDPAD

KAAGKDMFSSFTSFDSAPPQGLGTNGHSSKSNGAPSSTAGTTAKPSTAGPAAKPSQGSAS

SPNSQQARSFSSDPQQLVKMFSQFGRPSSTQQKQQPRQHFQLQSRQQQTSQQKQQPGPIF

QQQRQQQTSQRKQQPRPIFQLQSRQRQTSQQNHQPGRIFQLQSRQQQTSPRKQQPQQSFQ

QQTRQHQSAQSQKQPDKQHPLQQSYQQQQRYQQQQWYQQQWQSQNIPDPFQHHQPYQKPV

PPQQHYHQQQQRRYQQQLPYRQQQQQRQQKPQNRLDPFRQYQHHQKPVTPQHNYHQQQQQ

RYQQQQQYRQQQRHNQQYSFQQHRQTQQHTFQRYHHSQHPVSPQQNYPQQQRQRYQQQQR

HQQQGYQHPRQQYQQPPYQQYQKQAPQQQQSSVSTDIAHTLNDLLSPTVLRSLVSQTSPT

SQTSHTSQTFRNNRRQPSPWTSSNYRSRSAVAGGISPWQSYSPYSLRQRREASPTLPASH

LHSSHSSVKTPSD

>SG-AL_contig_9557_1_+2_2_136

TSDLPPKSSVTFSTFLKHCHFIGPRRRHKYKCTFAVPVFLSLCVV

>SG-AL_contig_9558_1_+3_3_110

HTHTLFIPHSTHMLYDLPHLHCIQNKLDLTRCHTHK

>SG-AL_contig_9559_1_-3_3_767

DDAVLKKQPQPVLTTELYPTQASKFGAVERSAPLWRDSAASIDSATGAHPEPVTAASYTD

SATVDPQQKYMARLNDQPQARRPEYIMAESYSDSKKAFHPEPITIEAYSGSGKPFHSEPG

AEESYSGLGKHPHPNDSYDGSGKRFRPEPGAEDSYSGSGKPSHLEPVTAEVYSHSGKAIR

PEPGAEKSYNGSGKPFHSEPGVDDSYDRSGKPFHPEPITAEIYSGSGKPFRTEPGAEEAY

TGSRKPFHTEPREEG

>SG-AL_contig_9560_1_-1_1_273

THTHRGTCADTLRVTDLLFRIRSKCAILLCLQTQISYLGSAFVFRWALIFRTKFPLWLRG

RLNSFICSCRNGWAIRIMIFFLSALSAIIFI

>SG-AL_contig_9561_1_-2_200_493

MILYRHCHTLLLCFSLFRIVLHLFSHSCRAQQSNYQRFLTTVYLSSRLGCKSSYFHDVII

IVCYVSDMDATPPTPILSHSLSFACVLLSYTVFLNTLY

>SG-AL_contig_9562_1_-3_93_1997

MSSKRDRRSRSGSPDPSTSRPKRVLDLVSTRRMSPPGRYQQRYFRGGSGRGRSSRRPFVY

RNSRGVTVYRGTSQRYYPRRSRYSSRDDSHSRRRRSHSRGHSRSRSRGHSRGRSGPRGHH

HRSKSESSSSRSGSRHRHKRSRSHEKRRPSSGRDDSRTHIRDSPSSDDKVPVKKKPRHHA

SASSDRDSLHRSRSDSNEKQELYDRSQHHQRAKHEEDQTKLCVDRRPSQPDEDDRGMVEK

YKKHSRSMLPEVHHESKKSAAALKGKELIPQNRLHKIFSLISQDADFELDEDISIAIQRN

PYAEPSEDSTVTVVFDEELFAMIYPNEGRKHKPIFDREEIKVFGHDKNLADDPDFERRVI

RVKPGKSSQQADSGPSSSYQYRSSLEITRTVKRNQSVSRSRSRSATPTKHRNPQFRLRVS

TDPRFEARYAELQRREGESSRRPGLEPNDLRHELSTNRKSSSDSRSRMDRDKDRHGDRRM

SLEQRSDKTDLKKEREELPDFSRRKDKYQYTPWLDQPELIPKDPKYFLHDDRESLDPRDR

GRGRGRFFRGRTNFRRPYRPFRSRGNFSGPYRGRGNYFNDTRSRSPRRSYSGHHNDDHRS

VEREWKHDKFSELETDSEKKSHRDDDQHPHSTSER

>SG-AL_contig_9563_1_-1_1_210

MCYCCVCVHVTMCMCYCCVCVRVTMCMLLCVRVHVLLLCMRACYCVCATVVYACMLLYVC

MLLCVCVHVT

>SG-AL_contig_9564_1_-1_7_348

MCSSVPFEVSTQTILSKANLLATGCMRGAALVHGVSATPSQNHLPLSKDADKGTHSSLCL

VAYLIASLSFVERYSLSTQGFPLSPGEGVPLCQGPARKGGDPSQCPRSSFDDSH

>SG-AL_contig_9565_1_+3_327_599

MHSSMLVVFDAGSEKTNKTDPLRQKCRQNGLHSFWCTCVYTPITCFSSHILSDQSSIHHN

LHQYMFVKMFNCNNIKRMKHEHICTEYLSPL

>SG-AL_contig_9566_1_-1_1_174

RRSKLIMAEAVSLRINPTRWQGLEIGRRTGPMKHRHHLNGWSMKMGSEQANFYAAGWD

>SG-AL_contig_9567_1_-1_1690_2037

MFIGNCLLFCRIVKKSIKITCFYSDIFIGNCLLFCRLVHQDRRKEDSVSNHKFHRIYIDN

CIPQLSLVAYVIYNNKNKSFLYSAILQTRSDSLRITYYLPPFLYCLIHQWSLCERR

>SG-AL_contig_9568_1_-3_57_209

MGNLVLGLYCDMLLGRGVGLVLGQFHDGIKKTLFSCPYYLKVIYCPFLYVM

>SG-AL_contig_9569_1_-1_1156_1332

MHVHLVLCTWLLVNFLKLMAKRITSNVMCIGQILQESKSQNCITWTWLLYCTMEKVLIC

>SG-AL_contig_9570_1_+1_1_132

NKTKQQNHVILPMIKMFKKDNKKRFHCGRTFYSETVLLLLVLFL

>SG-AL_contig_9571_1_-1_199_642

MKHRKHWHSFCGTKDGIRVPENVHVNHDDRLIRVAPEHDDSHAMDMLHEYNRGLIAFKNN

TAKTCYLDRLDETFEDGYARWQGYEEVVHKQNHTLRVFPEKINIQVVRHIADVHIFEHCG

GEEVEYHWVMEVTEEEIRMHPENSYISV

>SG-AL_contig_9572_1_+3_162_266

MSTVSTFCVSPMLNHQCSYVISYKSCVNPICPNCH

>SG-AL_contig_9573_1_-1_1_174

KCKSCFDVSLFCPLSLFPHSQYFSFSPLLSTGLSTSFQSPSLCVSDYICSEREVLGNK

>SG-AL_contig_9574_1_+1_847_1152

MNASGFLKNKLVLHLKHCTHSHTQIYLTGLFSSSERQEQLFQMTDFERQYSLSCSLHIHN

VNVISSHSHSKEKINTSVNKQSIKQQKGHMLQLVASSINYKT

>SG-AL_contig_9575_1_+3_3_149

VSQIHKFFYSSFLPANLLIFLTYHRYWSTHYSVSSHLMFLLIVNKSIII

>SG-AL_contig_9576_1_-2_29_217

MSVVSPILKRWQSLCSDGITSTCNGATDSTLVDIVDFNSCMTPYGHWFAHCRRIEDCCAI

FRS

>SG-AL_contig_9577_1_+3_684_1070

MDSTFSILLVNCQCLGDFRKRRDVMYYLRNKKCSAFFLQDTHFETKMESYISAEGAIRVI

LPHIVQIQGEWPFLFNNNFEFNVKRVYKRGNGNCIIMIVQIKEMTVVWGNIYGLNRDNPN

VYNTLKNIY

>SG-AL_contig_9578_1_-1_124_978

MDPALLKEREAFLKRAKATPTVEKRKIKPSSDENASKKSKPNKPPQPKAPKPLDYKTAQG

SSQYRFGVLARIVKHLKGKHQEGDTEALLLDEILDETNQLDVTTSVRHWLQTEALPNNPK

VALKESSEGRKFTFNPKLDIRDRKGLMRLLKNYDVVGKGGVLMEDVEESLPNAQKAVKSL

GDHIVIVTRPVDKKKVIFYNDKSLKFDIDEDYRKLWRSVAVDGLDEAKIEEYLTRNGISY

MQDAGIRKVSAVPRRKKGVGRKNNKFKRHNDHLVDMLKDYSDVNK

>SG-AL_contig_9579_1_-2_185_376

MYHPHVYKHSHQNDHLSNIFFKVLITFREYFCFSWKCLCFSLIFCTLFSLCRCQNFYKTG

MHEN

>SG-AL_contig_9580_1_-3_147_272

MSVLKMSSSFSSHCDYYTNCMVSTLTCTKHPLFCVQVLHKKG

>SG-AL_contig_9581_1_+3_27_290

MPQRMTVTRRTRRTSTADYKLLYRIIIVMGDFNAKIGSNNKGYEEVMGQQGLGEMNDNGE

RFADLCATSNLVIGGSFFHHRRIHKATW

>SG-AL_contig_9582_1_-1_4_1332

MASKTMLWAVVLTFFTLSAADVSKRTIETMLYPDCIKYPSCELMIGQNASAPNASFPNVA

YVRASVGDDILHFVMTTVKLPSILVVHTVGDANERLNFDWDRLLLANNETSMAGAVYTNG

SLYHGSIKYSFALTFSRLIEYDDLHDSASLTEAEKDNNTLWNIRNFTEFLWMNVTEGQGG

KSLIFNTTQPQPAPYANDSAGSMYFEFEINSDSGRDSDLPRLIYNANNTQMNFALDKYVP

SYNKSRFALEMTLTANHSSEAMSVEETRSIDDEFSPGVFQVVNWYSAGKSDHTGYIQWKP

VCYLAAKRGRSVGTEVTKYPLQGRGSITKNTTDLLGESLAFAFFGDAMKDSSKVVQRSSN

ISFGLSQDGYYLKKNYTVWTASVGYGKAPVESISTTVIIIIIAGLGLPALIIILGGIYVC

VKKRRGGYEEIDHEVSRNNPRLN

>SG-AL_contig_9583_1_+2_890_1051

MCFPHPDQVIFPHESHFTLKHILIHKSQISQAMRFKDCRRMSAELSAVHWFEVK

>SG-AL_contig_9584_1_+1_316_498

MGPKSERKRSDGQSDGIPKNQDVICSKHCVWGFYASNTPQWKRWVSTVCLRTNRRCCSLC

V

>SG-AL_contig_9585_1_+1_1_117

FFFASHIVHIFIKLVLNSLFWATEFFIQIMFVNSFSLFN

>SG-AL_contig_9586_1_+2_917_1126

MKKKQYSVKHRLIPNFTIFYMAKRILLKFDRPADISTSPMVFIRLSGWLCGWPCLCWMSG

DHLVRVPVYR

>SG-AL_contig_9587_1_-1_115_1086

MSVSQQAIKIFGSPWSAPAWMKTNKNMTGNGTLIGQPGGPYYKTWANYFVRFLQEYQKHN

ITFWGLTAQNEPTDGYIYKHFFQAMGFTPEQQRDFIKLDLGPALHNNSFGDIKLMILDDN

RLLLPYWAEKVLSDPEVAKYVSGIGVHWYIDEFSPLVALDYTHQEYPDKFILATEATVKV

VKIGVWEHAQAYASDILDDLNHWVTGWTDWNIALSTIGGPNWVHNLDNSPIFVNATSGEF

YKQPMFYAMGHFSKFLPPGSVYLEMSSPSPSPNIKAAAFLSPDSNVAVVILNMHQNPVDL

SIKDGTAYINIKMPAFSLKTLVWK

>SG-AL_contig_9588_1_+2_2_178

FSFLLYMLFSLIKFSQTEKSKQHTHSILSVMFYYSDFSAQKMQFTPSSNLGLLCPRQMF

>SG-AL_contig_9589_1_-3_36_2666

MTTADMEATGKSRMFCKLLVCTVCVFALLQTTTASNIVSFHQRVEKVSADVKSDIGSHSR

SKRSSLTESERNDNKCDSQKEKFANAVRDGKRLETTHVFKDETKFSLAMAWAGKNDDGVL

VVLTSELVSFQFAVSTLWRSKDFGRTWQNISSAVHNSAFRKSDGLQRNPHDPKWIYLISY

EHFFYGSEDGGETWTKFGLVDTVKVELVFHQDEKYKDHVMIITTSNKLIMTTNNFKTMET

VRDGGVHSAHWGSESSNTAKDMFVTHGSVTHQSPLPFFEKPVLYYKLLRRSTDGDEWVAK

REYVVDFGLQGDFLYASVYKSKPTLDSERLLVVSNDGGDHWNEAQLPTLAGDRFFSVLDM

SEHLIFMHVDNPGDTGHGTLYTSWSRGIFFSESLQRHLYPNYNSITDFYKVSSMRGTYIA

SQLSKDNSIHSMITFDLGGEWQPLPKPQDVSCKTEGEECYLQIHNAYSIHRGVNAHPPLS

TDDAVGLIIVNAHPAKNLQTTDPDIFVSSDGGYSWRKALDGPHSYIIADSGGLIVAVSLK

DPFPKEVKFSTDEGRCWHMYQFTNETVHHTGLLTEPGGKSMTVAVWGYTNKDKKWTVSVI

DFADVITDHCTRADYETWMPHETMKKEDPKRQGCLLGKRTTFQRLKHDSWCHNGYQYKPE

SSEKVCPCSIADFECDYGYHRPEGQEKCVKTLNFQGPEIDVCFRGHEEKVISEGYRKLPA

DVCNEQGGFQPTSKYINLTQECSGRFKVPTPSPEELEKIRKEKEEKTEQSHGTLVVALVL

VALLIVAAGLAYFGYRMVLLRRHKVVYRYSMLNQTDDKDFDEEIERTLTGGSHPHPMFGE

TSDEEELPAEPRQNGHIQKGRPKTRGYHDDSDDDMLD

>SG-AL_contig_9590_1_-1_448_5346

MSLGLMDPDIIADDTNLLEGIIPERVAASTSESDVSLSGNSSTLEEASVATVNEAVGAAI

NSTPLQGVDFKKFLDENGEHLSDTLSDAESVGSKSTTSVKQSLAQTFVTVAHSEELAQRK

DSSSSISGVSAVKADLLQEEGSNGVSPEVSQVLEWDYDHDAFDGKLLLEKCQDGMSTSTS

DSCSEASADHGGILAEKGVPDVDMPAPEGEEAHPFEESEDGFENIDNIADFPLGMSKETQ

HMMTDSGISEKGFLDPIADAASKRKSVEFDNKVSVVDLPGESAFDNKVSMVDLPGEVVVE

RRVTVDENLQKDLMDGHTRQERPLSLISTTSADTGYVPDTDSETGTLTTNSPTEWTEKQL

NANLAAAVNADPRQRVHIPGKSKQALPIGKGLPGEIVKETETDSDMYSDADTAAVETAPE

RTIETDDRKKTACSDGCEENEGEEEEGETSEEETGSDEEEEEEEEEEVVDDDRPEDDRVE

ADRVGPSGDTHPIIQQAPGPVAGTIQVPKETGEVVKDMTESASLEIPGAEVVTDTTNTSE

PPASDLSIQEQEQEAPVQKSEPDASEGTDESSKTISPDGGESSNSRHTSKKKISPVSTPA

DKKILAEHKKAVKSAHSRLADYIKTPPPAKPKEEKMEKAELKSKKLNAGDGKTSKTGGKK

QDNGSSSVVDKAKANLGEEKMEKPAPVKEEKAKVKRTAPKSKWGNIMSQIEASKDTTKLK

PKTEVKSSLAVYLSTPAPPANPKKEGEKDNPNEDNPKPKSKLKLVKHSKPDFSNVKSKLN

VSSPPKQAGNPRDASSPSRSSTPKHFDSPKDSGNIRNNPKDSGNPKGNSKDFGNPKGSPR

SLGNPKDSPDTGKLKGNPKDSANLKGNLKDNPGHRKGSVNVKEKNPKTIAVKKTSISKVK

TGKLLVEHTETLDNCSTMSQATSSHTDLSVFDETEGTPSAPAAASIVSHLERKRNALSCA

GLRQKNERSSSVASTKSDASVQRSVKSMNLRNAQKFEPSSSNVSPIKKSTVKSSIPPPNQ

NRFRNKAGSGLKSGQPPDVVSTLARGSRAQEVARLESLCEARTKELNYTKMQLRASLQAF

DAMTCLLNYCTNQLNAFSCPVITAKLKTAEGKLTEQQETIESLSQQKTQLDQQVQEVTGA

KEVALQEVARLKQEMVQAEEAYSAAQQQLKEEHTAALKAKEEELTSEHEKTVQQMQKCQD

KQAECTRQSHKRQIMELTAEHQMFTNQVKLAHLEEIQELRNKHDLQMEELHAQHRNKLED

ITRRFESIKLSLSEKVQTLCVECDELRVRAKTSEEALQRDADVKVQLALAPYLHLPKEIE

SLKMVIDMRTEEIQKLRRHNMDLEKQMEELPIAQEKVASLQQKVENLQAIINIKTDHEKQ

LHEKCQVLMRKYDRESRANKRLSMDYEQVVWRMSQSADLTGSVESLGARQLSSSSPSRSE

DHHSRASPSPVRRSQSSKGESSPVGVTRRQRKISGGDEDFDRKIRCRSATFSLDKSKSPR

SKAEAASRVNGTSSSPGSPNKHILSKSADYTDMIVAAINNEHEESFSSGRDSADISSMES

SMILETAQGVSYNINVVENGSADTGDKPCTTDDVFSEHPLQMDPSIPTDFFVQSADMELE

SEVTVVVNSPTSD

>SG-AL_contig_9591_1_-1_1_180

RTPPHTHTQCVHRSKCTHTHTLMPCVLEEWTILLLLRSPFDPIYTHNDRPEPKHFSDSEL

>SG-AL_contig_9592_1_-3_129_707

MATNRKMGDDEEMLLPGGDNVHVQQPYFAQPQVQQPYPGAASAPPAYASPHPHAHQAYPP

GHAVPMGQMGASGTYSHNTTVVINQPAPVVRAGPRVWSSTMCSCCDDMGSCLLGTFCPLV

LACQVSMDMEESMCVPCCVPGWLNVLRTKLRAENNIEGTVMDDCCMSMCCHQCVLCQMSR

ELKFIRRSYQPSQ

>SG-AL_contig_9593_1_+2_518_841

MLVGKPTEVAASAPTASLEPSQWLVASLGWEDVLVSFGGVDRYDRRFGVPAPQAALGIVG

GEAQAALVVGKREDAFGAEVVAPKLSGEDGSVVRDQSRGGVRLGEHQH

>SG-AL_contig_9594_1_-3_3_110

GSQRVNRKLVDRFKEWKGFKRETEWRGGPYKVYLYA

>SG-AL_contig_9595_1_-1_1_225

RRSYCLKILSVGGQKFHFIAVHCSIVLRILRARHVGAQSRFVQLEVVTVLDLVICLLGTL

FKSCIGRWGESIFSM

>SG-AL_contig_9596_1_+3_270_530

MTNILSLSLTHTHTHTHTHTHTHNPPIIHKYISFDVTSRDLQTINLLISRQIIWTLCVYV

CPCSTGITKDQNMDNYSLPNRLQKLID

>SG-AL_contig_9597_1_-1_4_423

MCYCCVCMHVTMCMCACYYVYVCMCYCCVCVHVTVCMCACATVVYVCVLLYVCMCYCCVC

MHVTVCMYACYYVYVCMYYCCVCVCACYCVYVLLLCMCAYYYMCYCCVCVLLCVCVHITM

CMCYCCVCVRVTVCMCAYYY

>SG-AL_contig_9598_1_+2_2_142

LVSPQRVNGMSSKSQWHEPVYPQRASGMNRYILKEPVACAGMSSKSQ

>SG-AL_contig_9599_1_+2_1145_1432

MQGVRQQTYHQIMEFMVIRFITTTNTFHYLPADKLVPFCRMDLPQCIPAHFIDILSLKSF

SHSIFYSLHPSPIFMMCASVRVAVSSFFQFFHLRNH

>SG-AL_contig_9600_1_-2_68_283

MKLWELLVCTFIFIDEVKGWTSDEEFVPGGNAKSSENETEGEKKKTSKTKQKQKTPNIRV

YKAKSSSVCRPV

>SG-AL_contig_9601_1_-1_25_1329

MFRSSFLTNTKRVYSAVCARRLVSSSKTTLGQTSQIVSTDDKPIAEDILYKEDHIQLRES

LRKLIEQDINPHVDQWEAEGFFPAHKVFKKLGDAGFLGVTKPTEYGGLGLDYSYGIAVAE

ELGSINCGGIPMAIGVQTDMATPALARFGSEKLKKEFLAPSIAGDVVACLGVSEVGSGSD

VASIQTKAETKGDDLVINGDKMWTTNGAQADWMCLLANTNSGLPHQSKSLICLPMNLPGI

HISERIDKLGMRSSDTVQVFFEDVRVPRSHIIGEEGMGFTYQMLQFQEERLWATAAGLLA

LQKLIQETIQYTRERKAFGQSILYNQTVHFTLAELETELELLRALLYRAALLYIQGNDVT

KLASMGKLKGGRLAREITDKCLQFWGGMGFTSQVLVSRFYRDFRLFSIGAGADEVMLSII

CKYMDTLPRPPKKVK

>SG-AL_contig_9602_1_-3_3_164

VIESWSRIPGQTEGWKYYYVRLPLCKAYGIQWLRLRKCSCQKGLSVEVSFQKIC

>SG-AL_contig_9603_1_+2_2_229

FFKRFPPPPPFFCCYFSLNFFCRLTLDIVLSFIHKTQLPTIYTHTPRRLQYSMITEWVKV

YNFLELTSTHKGRALS

>SG-AL_contig_9604_1_-2_2_199

TPKANLRLAGLPVTPILATLCMEEFEKRVLDTYSHTFLESGTEMLMTPSPNNMNTKLKVS

PNTSVQ

>SG-AL_contig_9605_1_-2_23_268

MQCYRETYMTEYPCIDKPSTASANHVFCSVYRLDFKVSHGKSTDVSQHVKSARHMEIAAV

GGATLKCILLCSDNKMLLPRPT

>SG-AL_contig_9606_1_-3_30_254

MTEHPCIKRSTVSANRVFCSVCRLDFKVSYGELTLQCESICEICQARGDRRCWWCYSQMP

FCYAQIIKMLFPRPT

>SG-AL_contig_9607_1_-1_511_846

MILIAPYWQAQPWFPDLRTLSHVPPLELTPSPDLLRQPRSGILHANLAMLCLHAWLLCTT

LSVAGPPLSSHPRTRVVSPALSAAGALPATHSLTSLSRSKGIESLYNSKWKK

>SG-AL_contig_9608_1_+1_265_543

MKKRKHDHITPLLKELHWLPVKSRCQYKIASLAYRHFDGSLPPYLSDRLCTYQTSRTLRS

STQKFLTVPKRNLVSRTTCFQFHCTIYLELTAS

>SG-AL_contig_9609_1_-1_730_1002

MLDSSGADISPSEQTECRAGVTGLTSWSECQTFNRKVRGSTLPGSSCQLCVQGGDLFRSP

GSTYVQTWPELSSFVCISTHVKDPVSHISVR

>SG-AL_contig_9610_1_-1_133_405

MFDMYRDNLKGRVLKIHQNDDMQVFQFYTGSNISLVTRGVLSAIVLHDHAFFSSRLDVLH

CCEPSVIKKFVLLAIQLVVMQNYSSLDGKLL

>SG-AL_contig_9611_1_+2_2_220

KKFYSLLFCGSGVDSPLSTSLANLVVHTNFTDLEAPKKLRTMQMVMKMTCMIQKKVRRKQ

MRTFLDLDIRHRD

>SG-AL_contig_9612_1_-3_39_161

MYMYVCMFITIYNQTYVLKKFDVIKITKIILTKIFDITGSY

>SG-AL_contig_9613_1_-3_576_755

MVHTCGLNLVKIRSETDEKILLTYSIHTHTHARTHARTHAHTHTHRDTSTQLHTHTHTHT

>SG-AL_contig_9614_1_+3_84_188

MNNCSVKDKPHLSRHIKRYHYFTGNTVYQDTVHNL

>SG-AL_contig_9615_1_-1_259_423

MQYNSIYICVCVCVYINLNLTDVYIILIKMYKYTYPYECACLYILLNLIKMYKYT

>SG-AL_contig_9616_1_+3_327_482

MNLIQLSEDSDTDDSKKNYKFLGIQCISDLQLYCFTNMICSHFKLKTTGATN

>SG-AL_contig_9617_1_+1_163_300

MLSMNEDGSAHIFNHTVYTHVTTLMKGSISLIRCYGILTSMKTTVS

>SG-AL_contig_9618_1_+1_1_144

HDALPISLFKKKSFKSDVFFGLSKIPCLVSPTFLTISNTSWGTLKQGH

>SG-AL_contig_9619_1_+3_240_347

MFQTPKNPLSLSSALNFHTRSNTNEIGHNIQPLKVL

>SG-AL_contig_9620_1_-3_918_1256

MVCLKLTFQMEQGFVVWIGITCTHLSFNGGDHAMWHGIKGSVEWMLLQPLSALYITFKKK

NSIRLNLSGYALVIVGLYISVSPVYLVDDVYVTVCTCCSVVLQYNWLPEKKKK

>SG-AL_contig_9621_1_-3_3_335

PQLLSAKCKRRQAGRKWLKTGLATYKQILQALTNAVNTLLLQAKSTIYNAKIFACATSKQ

LFNITITLLGKEKSSLLPSSIAPCQVPQKFAISQKDCLYPKQSRFSDTDFT

>SG-AL_contig_9622_1_-2_50_220

MQLYQQLFVISHSHSHTHTHTNAHTHTHTHTHTFTFTYACICIKLFMHEYSSLKSEV

>SG-AL_contig_9623_1_+3_90_209

MFISPIVVLQQMKHYTHPSLEFTPEQAFMLRWAIYNMTDS

>SG-AL_contig_9624_1_-1_91_1215

MGKNKRGGRGGSDRGKGRGWKQDRQAKLDSKLKKHVFKEEDEIENEGPCEGAEAAQLSQV

PLAMWDLEHCDPNKCSGRKLTRFGLVRPLRLNQRFPGIVLSPMGSRPVAPNDRQIVEQGG

LAVIDCSWARLDDTPFSRMKCSNPRLLPYFVATNPINYGRPWKLSCVEAFAAALWITGYR

EHGEALLSKFRWGGSFVQQNGDLLEAYAACTTAEEVQAVEDKHLKQLQYDHDHRQDKDLL

DIDTSVDVCNPNKPVRANYLPPSSDESEEDEEEEDSDEEEEEDENKEGEEETADSENKDA

EDKDSERTLLDGNTSGQNPGCPSVSKSDAESEEQCGAEMNSDTNSVDEDHSDEDCDSSTG

VSSKFHHLNLAPKNG

>SG-AL_contig_9625_1_-1_847_1047

MSFVLAVTILSFISSLLSYPFMFVPSDVCRQIFASSNQHHEHASSREDIVRLLSSSHIVL

FFLSFFF

>SG-AL_contig_9626_1_+3_27_269

MRVALPGKSSSPWYGTTLVFFYFSYMCVFLFPYESELVGFIPTELCQGQLLCYLGSLTYA

KCIPHTGLRSKRLAPRAPLKV

>SG-AL_contig_9627_1_+1_1_204

WSLQPQMQSLWSHLLESLRQWLMTGFSERQYAASKILFSGTDCENTEDSDQGPDLVGSGV

QYSGVLSG

>SG-AL_contig_9628_1_-2_47_166

MQIGTPVLLLVALTVNRHFSSFSIFKNHSFSSSSSMHLTD

>SG-AL_contig_9629_1_+2_161_310

MNESHTIKKALLMNLVHTEAAIHTQTEKNNYSNETLSQYFLFPIFCKRNA

>SG-AL_contig_9630_1_-1_184_378

MLIYKQSQPLLKKRSSHINKPFIIRVNISFVWIATSSLQIKVTIIASIALVMPRVIITKV

WITLA

>SG-AL_contig_9631_1_+3_2040_2294

MNTNYIFYQYSYHKNTFKSQIKSGSSLQQNTKSADYCVKKKFGQMKTISEYIHSKTRLEK

KLVKNRLSNKIILPTEHQNSYACTN

>SG-AL_contig_9632_1_-1_2356_2652

MEFLYMDKSFCLLLLLHIQLILNLLIICTSSFLLSCTQNVILPLQLFSCIHYLHVYYFVV

IALLQFKAVAFGFCVEVSFLSSLSFWNSTWEIESDCCKF

>SG-AL_contig_9633_1_+3_3_200

KKQKKTLHKKTTNWIHFEHTIMLKNAHNVHETCDRVVSILSIMTLVQFLAIMNKLVMNNN

PLSNKK

>SG-AL_contig_9634_1_-1_1_213

VCVCVCLLNLIFFPILLSSLFPHPTIIFLSLSVNVCIMPLAFLCLYLFVFVSVSVCLSLS

LCFCFCMSVSV

>SG-AL_contig_9635_1_+1_61_231

MAYGLSQHPVSFKVNKTRLCWGCQPFRLFYHPHIRKQRKNIILKNTLKQQKIHKKAI

>SG-AL_contig_9636_1_-3_27_224

MPNLKKCIRKLSNTNHKWKNITIALYKKVASLDLPTHQLSKTNVFWKNVYKLVPLCLLNF

LTGNTE

>SG-AL_contig_9637_1_+1_364_555

MKSLPNFKLDDVVSLIFFSFFKYQSSSKSNGRKGLTQMIKETMNIKQNFKIRNYLNPVLN

KKHK

>SG-AL_contig_9638_1_+3_294_458

MPTSFTRGEGKLPWKKFRQVSRKTQFCHSGIKIPQPAVLSLQPNNPLQTFELRKR

>SG-AL_contig_9639_1_-1_169_2046

MSRAQAESVIKNIIREISQECASKGQAVSETLVAFMVKAVVLDPANEFNVDRTLTKDDVQ

KLIRLCVERLMDNRSPALDTVKMQVYFDMNYTTRSDFLEEHRRVLESRLQPVVREITDSR

ARTREELESLYRKIVSAVLLRSGLGSPTDIAVVREATAALQSVFPQTELGSFMNLTKRDK

ERQLLELTMIVTGIRLFNKECGKGGEGIDDLPAILNEAVPATTQSVDSEIQSTLKSAYKY

TALIEEMHKRQPQEGPSIQLLKEALINTRQHEAFLRIILNDVISCAQQVEMLENQLASRM

ETLQATVQSKTAVPTAQVYPQFIHLAQLWSGFQDEMVLLSVLSNILASLEPFTRGHKELF

SDDVLTPYLEGLSVITDEERIQSTAVPEQRVNPKDYQVENRDWLFPETTKNFSKLPLQYK

GYCAYTLGKYDRLLIPANPEIGVMRYRDHYYAFSTREAAYEFAENPQDFIHLVAEGAKRS

PELIQLLELHTQFSTITPYSQSRDAGKMVEKPVTKCDSGTQTDTHFMESNIVKSYEWNEW

ELRRKAIKLANLRTKITSSMQTNLSNLRRDNVTQVYPLKDTDTQTKTDGYSNVPKPQVFL

AGLRGGGMSVPTNMTKVDLTIDVDQT

>SG-AL_contig_9640_1_+2_2_142

KYTHTEKRKIHGWCHMTVTHCPQEGQPTSHMVKLHYDPPKINPVKSC

>SG-AL_contig_9641_1_+3_51_239

MNDNRNIHLIQLQLACCTLPHDGGSNKYTLNSVTTYMLHFTSQLVSDSVSLDSRQHHGVR

MIP

>SG-AL_contig_9642_1_+2_836_973

MKSRHISSSFQAVYIQKISCRLFTITMISHHFLNIIKKKKIKKKKQ

>SG-AL_contig_9643_1_-1_361_1341

MAFLSSWTTRCLFVIFLPLLSISATNAVSVKCGECQKSLCPELNYYSCEGKLVKDRCGCC

TVCSSSKNQPHPLISNDDDHGESEGSACSKVKCPKFKVCVENMQGLPLCTCPGPFICKRR

SRQKPVCGTDGVTYESRCYLRIAACNSAARIKVARRGPCNGAEVPRPRTCDSFSSCRRRK

NQNPVCGTDSKTYMSRCHMKIMACQKGVKIKLKHHGTCEAPSSSGRAPTHAQRMPGVLIS

KLKDSDKNTPVNDVRGRKERRRRKKKGGNEVSGKEEGKKKRRKQRRDKRKQRQDRRRKRR

KTRGNSRQRRYQADRQYLRNRGHIQWK

>SG-AL_contig_9644_1_+2_11_298

MYCSTFTQGDKIYTCTQASTEKAQHMFEIIIRHKIFTRDECRLGQRLQLPFCCHVIMHSP

FLFPACLCQVCDLELFCLKTILDPYKIHVKKIEKYT

>SG-AL_contig_9645_1_+1_1_210

NTQLVSHTHTHVHQNISYSKPFSDPTPFVTDLNQALWKQFHTQTRLSFTSRQLVDPCFLR

RWNIQTTNSK

>SG-AL_contig_9646_1_-1_1_354

MTDYYHYTNTAGIQGIQSSLCILESSRPDDGDDARFGRGVYLTTIDPSKGKRMIIEKCWG

PNQFPAFVNNGRVDYAIRVRLSPNQVKRTPKNQYVYLHKGPLHLSGLTWEIIQVPNKY

>SG-AL_contig_9647_1_+3_153_353

MVQIQEEWLICLIIILNLKLETSIKGENVITAGQVKELTVLLVNIYGPNRDNPAFYNTLK

NYNHSKP

>SG-AL_contig_9648_1_+3_84_224

MYFFRMGISAVFLLHISQIQEEWLFCLIIILNLKLERFIKEKMETLL

>SG-AL_contig_9649_1_+3_318_512

MGLGYRIHHKLSDTQYSYTSPTVGYISLPACVIKVPPMKWLSLFCTLYVYIMDKGKQHKK

RHVKH

>SG-AL_contig_9650_1_+2_56_253

MCVCVCVCVCARTCVRVCIKEEKAIKFSEWKNISPNQSLSYFSSSSYLSTLWQQTRFCVG

NVKEIS

>SG-AL_contig_9651_1_-2_356_568

MSIVCDIGFVSQLILVIAGSKLLCITVAFSSCVSSVLPSVQCLHVSPHRFCFSLMMYTHT

PYIYNLQSSDM

>SG-AL_contig_9652_1_-2_749_952

MGKFKLQTLLKKGYYSIINRKRMFTSCKIVIRYLFPFSCLVPRLYSNQQFKSTLKWPPAL

TVVFCLFV

>SG-AL_contig_9653_1_-1_79_4824

MAVSVSISQQKPSVAAALTAELLKNSGHAVNWSLGQDNALRVDRDTVFSSSASISRYLAR

SYPASRLQGETILEKTEVDHWLNFASGQLSCASEFSTALEYLDNVLKPATFLVGHALTLA

DFAVWETLQGSIQWHRVLQKSAPQNVERYYQFLCTQVPFSTLLPQLPKVEVKDTKKGDVS

VKKEEGKFVELPGAEVGKVVTRFPPEASGYLHIGHAKAALLNAYYRDEFKGTLIMRFDDT

NPAKEDAEFEKVILEDVAMLGIKYDKFSHTSDHFDTLLALCEQLLRQGKLYCDDTDPETM

KKEREERKESKNRNNSVEKNLSVWAEMKKGTEVGQRYCVRAKIDMDSNIGCMRDPTFYRC

KPEPHVRTGLKYKVYPTYDFACPIVDSIEGVTHALRTTEYHDRDVQYFWILDALNMRKPH

IYEYSRLNLQNTVLSKRKLTWFVSEGLVDGWDDARFPTVRGVLRRGMTVEGLKQFIVTQG

SSRSVVMMEWDKIWAVNRKVIDPIVPRYTALLKEEVVPVNISAAKVHAKDVPKHPKNEDV

GVKPVWYGPKVYIEGADAACVKEGEVVTFINWGNIIIKKVHKDGAGKITSVSAELNLENT

DYRKTQKITWLTEVEGAEDSSAGGLVPTVCVHYDHIITKGVLDKDEDFKAYINRDSKKDQ

EMLGDPCLASLKKGDIIQLQRRGYFICDEPYLPASPYTGRASPCVLFSVPDGHQKEMPTA

GSKHKEEGTKKNTSAKEKAQSKKGKAAEKSPQEEQPPQKVGSASVNDLDNQIRAQGDKVR

QLKADKAAKGDIDSAVQVLLSLKKQYKEATGKDWTPPKPGAAPAAAASSSGAGDDLDAKI

REQGDKVRKLKADKASKGDIDSAVQVLLSLKKQYKEATGKDWTPPKPGAASAAAASPSSG

AGGDLDAAVQAQGDKVRQLKADKAPKATIDAEVQVLLSLKKQYKEATGKDWTPPKPGSAA

TKAAETKEMPSIQGKAAVDSAEALGLKAEADAQGDKVRQLKSGAGDKAEVDVAVQKLLEL

KARYKDLTGVDLAGGARRDKKSGADNKGKGGGKGKGVGGDADKGAKAAKDTEAGREVKKV

TRLGLEAKKEENLAEWYSQVITKAEMIEYYDISGCYILRPWSFSIWEQIQKFFDAEIKKL

GVENSYFPIFVSHSALEREKTHIADFAPEVAWVTKSGQSELAEPIAVRPTSETVMYPAYA

KWIKSHRDLPLRLNQWCNVVRWEFKHPQPFLRTREFLWQEGHTAWADCEGAKEEVYTILE

LYADIYENLLAIPVVRGHKTEKEKFAGGDFTTTVEAYISVNGRAIQGATSHHLGQNFSKM

FEIVIEDPDTQEKRYVYQNSWGITTRTIGVMTMVHGDNTGLVLPPRVARIQVVVVPCGIT

TCLSEADTKALYDTCEKMAADLKAKDIRAHADTRDNYSPGWKFNHWELKGVPVRLELGPR

DIQARQVELVRRDTGERMTRPNTNLSTQITELLDTIHNNMYTKAEADLKNNLAISHDWND

FCNALDQQKLIQAPFCGEIPCEDNIKTDSARDVVVEEGAPAMGAKGLCIPFKQPQELAAN

TRCIHPACSKTAKYYTLFGRSY

>SG-AL_contig_9654_1_+3_135_296

MYTMCFFLASVYVKTGLLWSFGNPWTGNIRGQNSLSQEVFHKITSPASGWLHSF

>SG-AL_contig_9655_1_-2_833_1366

MAGRGVMEVITLCFFSAHNYVFLSLLFVLANQNVRDYVCGQKNHRRCRKTSTFAHTWLQP

ISPYPLLTWNESCLKYAASPFCHCLGRCGHTDFEGVGPSSLHSNSSIPSSSSFHRVTCSH

LSLPSPTQIPSAVFCYPFHHPNLPCHRFEFFALDPRNCDIHGLENHFRNLEGKKKSGL

>SG-AL_contig_9656_1_-2_140_331

MFKWAWFDLGVTFFHICENRFLIIEGEMLVASWYVYTHLSVYRRTECAKNFSDIHGCIFT

IFRS

>SG-AL_contig_9657_1_-3_3_305

FFFFLLDFHFCSSFPTSHSVAFIHEHRYHLVNFLSVSIKDAALKIFSAVTAFSLRPKMMN

HTSVKSVIKISTLTTLSLALFVASLRAREKNAEESFFNFLP

>SG-AL_contig_9658_1_-1_280_438

MYQLIVHYWKRIGESTLCFLCFCCCWFFYSLAYSSSLSCSLRHNEVGNIHCLQ

>SG-AL_contig_9659_1_+1_2440_2664

MLMKAVDFGGCTSLTKTLAIKCKESRDREKYSFHDRKHTPTHSLSLSFSRSLSLSHVHSH

THTHTHTHTHACAHR

>SG-AL_contig_9660_1_-1_31_246

MCWVFVRVDQFWRVILFVPWWPACWVFVPFGQLVLGDIVCSCWPTCAVWYCLFLLVSMCW

VYFSCWLTLCVG

>SG-AL_contig_9661_1_+2_20_202

MNRKLVYVMLFFKSYCRATQNHMSRADPQTANFRQTSGPDTIVQCFVEKTAACCCYFSPT

K

>SG-AL_contig_9662_1_-2_299_436

MFCIVFIFVDVLICFSNIQRLPLLTVSNAFIIVSKSYIQFPRSSMF

>SG-AL_contig_9663_1_+1_310_522

MGNDREFNEEICSRKCISPQQRKELLMYGCLQLSYFTLARLESNSTAPNWSTDNHNKNTF

SYTLHKSKAKY

>SG-AL_contig_9664_1_+3_261_419

MAKLNSIHSMDESVLPSLAILFLAYDRLASESKTFFHNLNLYRPFQNYHKVKR

>SG-AL_contig_9665_1_-3_66_428

MSSRDYTNPTAQWNPASLTSPKPENGMVAFEGQPSGNTSLSERQGEHYNPQSLTFAKDDD

TLDQLMGDQFVAVGSQEKIVSAQNQSCAQTVHNPCQAEAPWTETDLRYTQSSATISSVSS

A

>SG-AL_contig_9666_1_-3_582_1418

MIREFEGARLAVKTPFFPGLLEALIMPVLLYDLIIGNIEGARGPDEPDLSILNKKEQEGA

PEETAAIQTRSQAKERMMKPLQTAIGEPGVEATPAEFKEKQESDPSLAGLWALAKKNSKS

EIRGGTMTFSVEKGLLYRIYVSTKMGRVTSQLVVPAQFRQRVMSVANESIMSGHLGKGKT

GDRVMSSFYWPGIMTDIHRFCALCDVCQRTAPKGRIKIASLGTIPFIETTNSPYVAPVAI

VNKKNGEQSCSEVGNVTTAVLLPRLAYSWSRQHRGRPPE

>SG-AL_contig_9667_1_-3_81_557

MSDEDKDVDIESDEEDDEGGAASQPHTPADKRAHHNALERKRRDHIKDSFHSLRDSVPEL

QGEKVSRAQILKKAAEYIQAMRDKNCTHQKDIDDMKRANQQIQSQIKHLEKYADQSRIPE

RPKERSHPRYDDGRPVSESSDDDDINHSSRKKPKLAPDS

>SG-AL_contig_9668_1_-3_45_2051

MVPTFDDTGHIVEQSIARGRLTSVWLGGLVNPQALLTAMQQEKAVLVNKNIDQITWKCVV

LNSQDVAEFDVDEGGLFVNDIYLQGADWNYDQDKLTESKSSLFHIPCLYICPIVKPNPEQ

RSAEDRDYEGADKEDTPTPTPTTAEQPPPPRAEDPPADNKEAGEQEEGAKEEEGKEEATP

KEETTTPKEETATPKEETATPKEERPPSEKKEAVAVHSPKPASQKSTSVTGDRDERDEGT

AGMYDCPVYMNKSRQIQVSTLPVNCSTPEEKWTLANAAFILDRGLPEGASKKSRSYLLLQ

RLPTTMRGEGEEEEQVEEEEEGEQEEEEDVEEPQAETGSQISARTQQSQLSYKPMTPKAS

PLPQGLGLTRESAMIQGSQEQIADSVEPPLVSSRPVSGQQLQQGSRPGSASRAPRPPSQV

SMHSRGKESETGKGSIQEEPEPAEGEEEAVKEEGGEQKGKEEEGESNVEVTPRGDEPEIE

KLDRQGEHERGDDRVLASPKEDQMEEKEKAETEESEKAQKAASPPAADAGAGLQSRTVTP

HPGSISRGPSAASRRQREEERATEATQETTRSAEDVPGVPPVKEDETGRGGQAEAESEAE

PGPSPKPAADARSTSFTERAEDSQHVETDIDAGGGETDEAEAAPTQPASKPTSGKSVGRT

SLTSKGKNK

>SG-AL_contig_9669_1_-1_997_1539

MEVYVDDDSKLTLHGLQQHYCKLKDNEKNRKLFELLDVLEFNQVIIFVKSVPRCMALAQL

LLEQNFPAIAIHRHMTQEERLSRYQQFKDFQKRILVATNLFGRGMDIERVNIVFNYDMPE

DSDTYLHRVARAGRFGTKGLAITFVSDENDAKILNDVQERFEVNITELPDEIDISSYIEG

R

>SG-AL_contig_9670_1_+2_71_244

MQTSSSGDEVKDSIASEKLCVYLGIQYWFLKVKHLLNEFYSYQGGELSADFRGRPLDF

>SG-AL_contig_9671_1_+2_2_289

FFFYQDSAEFISQKASNTIAVACVSYKRKDKYTISMILINRHRELKISNTSHIFKTEESL

LESQFFSLFIAKADCFEMKLLGGKKARKTTSTACVS

>SG-AL_contig_9672_1_+1_1051_1263

MASKLTKKKREVQVSSLIYSMGPEAEMIFSQLGLTATRAKSFDTVSGKFDTHFYCFYPKR

NITHERATCHR

>SG-AL_contig_9673_1_-2_263_826

MERKLRVLVTILVVVLVQTIDSVAAKKDPRDCEVCIDVLTKFSNTLTDADRKDPMKVEKK

FSQFCKGLKGRSHKFCWYLGAVDNAPTRMVSAMSKPLTSFIPVDKTCEKLKKVDTEICNL

RYEKQIDLANTNLKKLRVNELKKILSVWGEDNACKGCMEKADFIREIERLMPKYDPEAHK

KRSEKTEL

>SG-AL_contig_9674_1_-3_777_2228

MKSWLCWVVVVVCVLLVMEVDCRRRDRRRPGHGRMRQRHRDRAHHAHHRRRGHSLDGWRA

LMRRLEFGYGRQRNWWDGPNVCHSEESDHEESEVNDITHHHKAALQVCEESQTAYRCKTT

VQSPEGQLSKTELYECCHGYARERSDYGCPERLRMRDLITTARKLDLNDLLKAVNTVGLD

EELTRGNFTLFAPVDGAFPLPTGPGLMLQDQHGALMVSRPMLDKMEDDLQAVLLGHLVPE

ARRASSFADEETLETGSPYDSTIRINFYSRPEKLTTANCVKVTSTDNMATNGVIHTVEKV

LPTVTSSLMDIIQSNEDFSFLKTALARANLVSEFQGEGKVTLFAPTNAAFDRLPPRLLDR

LLRGDPKCLEKVIRQHILPHVICSTVITGRAKSRNVLRNYLNLTRTDDDKIFVNDAQVVR

ADVMATNGVLHVIDAVLVPDDALDLVSVANKQGLTELVKLVGEAKLASTLQKAQNVTVFA

PSNE

>SG-AL_contig_9675_1_-2_221_340

MALGGLIVKSSDPKYEVSSSVPAHNIENWPVCRLNASVIL

>SG-AL_contig_9676_1_+1_1_207

LTWEIGKISTLYPPGEVSMVRTQAFHTLQFGSQWLHHLTTAPRLRGIRFTQTIPYALQLN

LSIINRQTP

>SG-AL_contig_9677_1_-1_97_438

MSYSSLYGAEYAIPNHQNQDVPCCLCRAPQASTVMIAATLTCPLGWTTQYSGHLAAGYWG

GEASTEFVCLDGSEQDMPNGGRDDSSSLFYYTVSMCGSLPCPPFIQNKVVTCAV

>SG-AL_contig_9678_1_+2_2_214

KSSKLLQANNLTARSRHTHKHSSNLIQSECQLSQKASRPTAKLKTNTCLINQMNETRILC

AVVSAKCMPTP

>SG-AL_contig_9679_1_-2_26_181

MKITEMLWYSWSSLVTILHMATCAYICGYTFAQMHAYACPPSESEEGDVAEL

>SG-AL_contig_9680_1_+1_1_117

PDKAAHVKVRMSNCTDNISQTGIPMNKITVTIERDKCNN

>SG-AL_contig_9681_1_-3_33_197

MGTCPSLGMGKVRKRGRRDGDCHSNACPRAVLVSTIRLLNDHSGGKNTYFLTTST

>SG-AL_contig_9682_1_+1_1_942

LPSFYLLLFLPSVFFLPFLLSSSFPSFCLLLFLPSFCLLFLPSFCLLLFLPSVCFFFFLP

SVLFLRFLPPFPSFLLSSSFPSFCLLFLPSFCPLLSLSSFCPLPPSLPSVLFLPPFLLSS

SFPPFLMSSSFPPFLLSSASFSSFLLSSASFSSFLLSASFFPLPSVLCFFFFLLSSAPFF

FLPSVLCFLFFLLSSSCSSFCLVLLVLPSVLCFLFFRPSVLCFLFFLLSSCSSFCLVLLV

LPSVLCFLFFLPSVLCFLFFLLSSSCSSFCLLLLFLPSFCPLLFLSLSSSALSLNVGCKK

YHCDIHDLPSSSTK

>SG-AL_contig_9683_1_-3_390_500

MIKVSPKHEIIGEIPVFTWRENLMLMLVGYPHLPCHH

>SG-AL_contig_9684_1_+3_258_389

MLIYLSFMYIYHLFGCVFTGLSDCRYPKPHVLVKQSTLVPAASF

>SG-AL_contig_9685_1_+3_3_260

IVLCCIVLYCIVLYCIVLYCIVLYHFFVKMDLPCESPAAFPGRACRRSAAPPFFPFFFLV

SAGWMFSCFHEPPNTDMAYRIFNVRI

>SG-AL_contig_9686_1_-1_25_333

MFYVLLFVVIACILIQLNYLNRALDTFNTAVVTPIYYVFFTSLVIVASLILFKEWGGLKI

EDILGDLCGFLTIVIGIFLMNAFKEFNISFRSLPFWKKDTDTP

>SG-AL_contig_9687_1_-1_790_978

MNDGGLVCWLLLMQAEYSCHLLAHIPTSGYQLVSYHNITHSNRHFLFSLQEKDIFYESLH

HTF

>SG-AL_contig_9688_1_-1_22_243

MDADHNCTGAESTSFSTTCTQFEKGTTLIIQILRKVEVSYHFTPRHPRGYGMTLTFLTRL

NMYICRPHGTETAC

>SG-AL_contig_9689_1_+3_3_218

HTTIHVTIIHDSPYIRATFNCNTRQLSIQLSSTSHYTSRLMSPNHKTHHIYRKHTGLNII

NYRSHECFVLFS

>SG-AL_contig_9690_1_+3_3_149

KNKMVNYTYSFPYSHLKLKRHYFLLSMTFKTDHPPVKLIHNTVVNLYHT

>SG-AL_contig_9691_1_-2_38_1105

MERNAAMSALMGKKAEDIPDVDLENLDDLLEALTAEELEELNGDFDPDNSLLPPSQRCRN

QTEKEPTGKFNRQKLLDFLEKSAKTEKDWDQNKPYVKEQKGKKFVAKEEAKTVVNDDDED

VETEWDEILTVASEEELVDLAAVLGFHSMLTQTQFYASLRDEKIHEGGFTGAARAQELKM

VPDEAPNTTDVEESIKQLKANDAKVKTLNLNNIKNISIERLLEICEALKDNTSCVHFHLA

GVAANDKVGKKLAESLSVNKTLRTVNVESNFLSGEVLVELVKAINVNQNILELRVANQKP

EVLGNKIEMQLSRLLQENKTLLRFGITFEYADARIRTNERLKQNFDDLRKQRVAKA

>SG-AL_contig_9692_1_+1_4_420

MLQYTHTPPTSPMLQNTHTHIKNNVTLHTHTPNKSNVTTHTRTHRSNKSNVTTHTRPISV

MLPHTHTTHTCDGVPTSPESLLRTSLPHTQSNKFSVTLRTNNTPTHPGKSNVTFKNKVTM

STHTCPMSLILLLRTRLQH

>SG-AL_contig_9693_1_-1_142_372

MKQVIVASDFHTVYTVAACAATGRSSLLTHNTFRFQPKAYILLLTTWLAFLQSDEKSNSV

LLYSTCIYSPILRFSTT

>SG-AL_contig_9694_1_-3_93_428

MPVVCWWPPTRSDTAMSVQSFTLSDQFFHCRPYLHPPSTVPCMMVLQRLSCFVTWPNHTS

FRRLMVVRRGSWGAHKVLDLTPHSHLFCASSRRCGATFSNMDKQLKQSHTNG

>SG-AL_contig_9695_1_+3_3_164

IIYIFGAVHHLVAESNMSTPGHSYFFLQTELKMEACLLCTTLIFKPIVGRLVFL

>SG-AL_contig_9696_1_-3_3_200

TVSDLLFGLVLHIYKARFTRNIFGQAASLQFMHCLYVGAFLCVVLKDRICCKRGPGLCGH

FLFPLQ

>SG-AL_contig_9697_1_-2_2_181

FLRFVCAFVFMCMQMLCTLCMSMYAREGVSVSVISVRSPNIPGGLPDWVAHPWANAQDVI

>SG-AL_contig_9698_1_-1_1_201

LTFTVYGSKPYDLCTVGEILTHAFLKGKLLFILIWIWVSYSPRVTRPDPLTAALVCTVTL

FSSQCVA

>SG-AL_contig_9699_1_+2_332_673

MSITINTILVNVHNSISLICTFVPSWVSWGHNSTIPVNVHNNRYDPCKCALQSAAKSKSY

RQPQQRSLYLNFQQPQSKHYKGLILNTTNSVCVEHLKINLVQLCAIISNKSNNA

>SG-AL_contig_9700_1_-2_41_202

MFVVWWVGWWLLCLLIGGWSGWLVFRRTILRKLYNLDELKRMDQLCLTVGGVWS

>SG-AL_contig_9701_1_-2_2_199

FFFFFISAYIDIFSYCIPASTFPFRFYFQIGHSMGVCHSFILCFVTWLSQFSAVSVTMIS

KEAWLH

>SG-AL_contig_9702_1_+2_2_169

HEYETKCTQEISLSRDDAQSAQSVNVSITREKICWPMLCKGVWVSFAYIVVEGPRQ

>SG-AL_contig_9703_1_-1_85_408

MSYQLYRNTTLGHTLQESLDELIQMGQISPQLALKVLLQFDKAINTALSQRVKSKVSFKG

KLNTYRFCDNVWTFVLNNVDFREAPATELATVEKVKIVACDGKSENKS

>SG-AL_contig_9704_1_+1_1_216

DNKVLPHLKWTREGIRYTADFMKEDCTVISEVEFYRTLSININFITQSGCKSPIKKCIQE

NMTIISNNQRHP

>SG-AL_contig_9705_1_+2_2_211

GFAERHIIFLGFCRTSYYFSGVLKNVILYFWGFEERHITFWGFCRTSYYFSGVLWNVILH

FWGFVERHIT

>SG-AL_contig_9706_1_+2_1877_2044

MTTRKDVHNFHFDDERILENVKIKKKVVKIKLDKYVMVHFLVINKRKKNEKVAQMN

>SG-AL_contig_9707_1_+2_140_388

MKSTGTTLIGAESPRPWWVRDWVVTTSIPCLYKENDDAYEGTGCPITPRSQGSGVVETPT

PPGASTMRAVPTSSPSLLYFPQL

>SG-AL_contig_9708_1_+2_2_82

IFNFLLVVGSAGKVLSNSGIFSPYLLC

>SG-AL_contig_9709_1_-3_15_452

MYYLYYTFANMVVLNNFRRQRGLKTFVLRPHCGEAGSATHLVTAFMLSESIAHGLMLRKV

PVLQYLYYLAQIGIAMSPLSNNSLFLNYHRNPLLEYFNRGLNVSLSTDDPLQFHFTKEPL

MEEYSIAAQVWKLSTCDMCEISRNSV

>SG-AL_contig_9710_1_+2_182_370

MCQPVRESAVHSIVTSLTKSCIHDPIPTSLTKQCLTDLVPLITTIINTSLSQIQFQSNLR

KQL

>SG-AL_contig_9711_1_-1_160_2460

MIAAEEEAKVSKPQSLKLEGANGKESVEAAVEESDGPVSPSHYIQPPTPDCPPPSPSTAL

QGIQQKINPQDKRKSKDMETITETTLLHSVPGASGTAQSEPQNKESEGVKMSKEETATSA

TTWSSEGSPRLSTREQRLSTGEQRWSRGSQPMEEEKSRNATKSNTSTNSATTTTTTTTSS

NGSSGSSVMTMSDVGMLQNKVEVEGEVVEMRRSVSSGDHPIDWDSNVFAGLLKGSTPGGP

RNVVSQICENVVVKTVRSKRRSTPSTIVTFDPLMEREEDRDVFDAGRVLDEIDEMTGSGA

TTACRQQSCDSARKSDDIFDDSEEWAKIAATLKTFGVGMARESVFVREYEASFTKSLAAG

PHKIQSVGEWLDSQGLGQYENTLVANGFDDTDFLGGSIMEDQDLATIGISSEDHRMAIVT

AAQRLPPVQPIDQRVLPESVEKWLETLRLSQYLDTFVSHKYNTMLRVIKLWELELNTVLD

ISSLGHRKRILASLGDRRQLERDTSMSPARVRRRGSEEDEPRSPFDHIDLYRDYTGVKAQ

GTDSSEQRSPKHNPLMTFDKSEENGPGSTQGIRDSQIHIRPPHLMHTTGSIKQWRHRPEV

LIKGCCNYTAHYLGSTVVKELKGATSTQEGIAKLKKSTEVINKIPTIMLSISYKGVKFID

AKSKKVICDHEIANIFCACQDGDHMNFFAYVTKDKETSRHYCHVFSVRSRELAGEIILTL

GEAFEIAYQMALKEKAEEDAVQFERQLSHSDQDDATSISSKASINTV

>SG-AL_contig_9712_1_-1_1_117

FCSRVYPYAVFKSAFGKKGEGPVRIQLCTGKHEHRSSFL

>SG-AL_contig_9713_1_+3_174_464

MTQNQPSVHHDDHLSHTKMRHGWVLVGIGEGEGNRKEDGLRPIEQKLTTKTGQANQTKLG

TNYNNNIQSHIFFASNKSVVMLSRRKITRKSTPIKPI

>SG-AL_contig_9714_1_-1_205_864

MKGGADSGFRHVRPEEYEQRLLHFSGTRKYIEVKEVPLCKSRLKSDDVFILDQGMVIYQW

NGSGSNKDERFKAMQYVQDLKSERNGRPTTEVLEESSTDDAHDFFKALTQDDDDDDDKEF

EAADQTTELFRLSDASGSMTFKEVKKGGVDKQDLDTKDVFIIDTKKALYVWIGKGTSSGE

KKNAMQYAHKYLMKTDHPLLSVSCLPEGREFRGFFSAIAA

>SG-AL_contig_9715_1_-3_216_362

MPIDTFCVRFNDNINCMGLDPRPVVIFGENLCCIYSWITDHDRYKISAS

>SG-AL_contig_9716_1_+1_1_171

FFFFIHKIFFITLLIFTNSNTPFLSLSLFKQLPLFTYMLKTFSSVHLQDQSIQNCKA

>SG-AL_contig_9717_1_+3_3_140

PKNDKNSHVIAAPFPRPGINTHHLAQGKSSLVTTQTSCSPFLLSLL

>SG-AL_contig_9718_1_-2_107_442

MHFYDFKERLLSYFKAILYDFTRFKIVFTESLERISTRSLPSLSMQHLERISTRSLMLSM

QHLSMQHLKRISTRSLLSLSMQHLKRISTRSLTLSIQHMASYFFPFLIAHIS

>SG-AL_contig_9719_1_+3_270_350

MQNIGQWCVKLLNNIKYPPPPPKKNPQ

>SG-AL_contig_9720_1_-3_51_281

MYYRAGTRNLHFARHEYYYDLQPVRFDENYVEVTGFMNNIHQAKAHVTVRPTKDHPQDLS

TFILIKLEALVTANSTR

>SG-AL_contig_9721_1_-3_51_281

MYYGAGTRYLHFARHEYYYDLRPVRFDENYVEVAGFMNNIHQARVHVIVRPTIDHPQDLS

TFIKARLEALFPTNTTR

>SG-AL_contig_9722_1_+1_451_570

MQNMSLVLTVHTENPSVNTIPANKHSMVPFWQLEAHVLIK

>SG-AL_contig_9723_1_-1_1_120

HTHRHTHQNETDDFLLYSCSKFKKYFNTEHHLNTVEGLES

>SG-AL_contig_9724_1_+1_1_243

SSTPSPVRNIIHSTNVDVCVCVCVCARARASEMATSWQISTIHTVLVSCRILETANKRNR

RPPLACPSSVATGQMFHPSVL

>SG-AL_contig_9725_1_-2_8_2188

MPMWEGEEPTPSEGGPTPKQRLLNWIQSKVPDLPIKNFTTDWNDGRAIGALVDACGPGLC

PDWEDWNKKKADDNAKEAMDAAEKWLDVPQLIKPNEMTNPKVDDLSMMTYLSQFPNAKLK

PGAPLRPRVNPARVRAYGPGLEPSGNQVGAPARFTVETFSAGKGEVEVTILNPKGVKESC

EVVFNNDRNLTYSCVYVPTMEGQYKVSITFASKVIAKSPFTVTVEGAAGDASKVSAKGPG

IEKTGVVATKKTYFEVFVKGAGNGKVDVVILDPHGRKDTVRPSVQPVSGKDSTYLVEYVT

ISHGLHSVNVFFAGSQIPNSPFGVQAAPPSNAKACYATGRGIQPRGVRVKDRAVFYVHTK

GAGDGEVAVQIIGPGGMEEKCQMKKLPDQPDVWECVYTPSKQGNYIINVTFGEQHISKSP

FKIEVGPTWNSKIRAYGPGLEGGVVNQKAIFTVETNGETGALGFSIEGPSQAKIDCKDNG

DGSADVWYLPTVPGEYAVHILCNDQDIPNSPFMAEIKPGADFDASKVIAKGPGLEKNGAA

PNKWAEFTVDTSAAGEAPLLITAMDIDYKPLEVQVKDNHDGTYSCRYMPTRPVKHTITIT

FGGVAIPNSPFRVYAAEPSDPSKVKVYGPGVTDGIQTMLATYFVVDCKLAGPGDVAVGLT

DSKGVDVPIKTTNNEDGTFTVDFEPKVPGTYTIVVYFADTEVPQSPLKVKVEPSIDVSGI

KVEGLDS

>SG-AL_contig_9726_1_-2_20_160

MTVGREKEHVEWGKGYLYEVHSIFLLLVFKENCHLCRGDVMGCNIHV

>SG-AL_contig_9727_1_+1_28_285

MGRMRSGRIPLVPTRIAPTMEYIHTSYFPKFSSATNFTAASPASPLAGISLSCATSTDES

RALLARFVPLYTLDLARCISPFNTCV

>SG-AL_contig_9728_1_-1_61_1023

MASLATMLDAYFGKSAEIFTVIGALATAFFAFKATSSILNGLFSYFLSGMMGLSLNLKNA

GSWAVVTGSTDGIGKAYAEQLAKKGLNIVLISRTKSKLEELAKDIESRFKVQTKVIVADF

THTDIYANIQQQLEGLDVGTLVNNVGMSYGFPEFFHQLENRDQLVMNIVNCNVTSVAMMT

SVVLPTMVAKKSGVIINIASAAGSIPTPLLSLYSGTKSFVDFFSRSLHQEYGAKGITVQV

VLPYFVATKMSKIRKGGLFAPYPDFFVRSALNTLGLQSRTFGCWSHAVQDKLTNLLPASM

HMQFMLKARARALKRKASKSE

>SG-AL_contig_9729_1_+3_2025_2273

MTDYSSKMFSLMFIQLHLHLTCLMIDYCSKMFFSLSLTHTHTHTHTHKHTLTHSITHTHQ

TISFSLLYSNCSSNCRLIYQYFH

>SG-AL_contig_9730_1_-3_216_527

MISGNLFSSVGWSAMLITWWSWARPPCGLFFLPGLTGSFDRAVGSRSHVSSSIPPRNIKV

GLFAAHLNAPIILVVSVFHRSRSPPHFKTLPAHRVLRGLKESAR

>SG-AL_contig_9731_1_-1_1_126

LKLKKKYMKRCFPLLGARVYFKIKTNSTQTNNIYSIYLTQCY

>SG-AL_contig_9732_1_-1_58_600

MLPYIGLFVVMSGSGFAFDAIIKRRLLSKLVVRKLATFLAFVVPACLLVFMSHLQCQQTV

LVLVLLVVAVALTGLAITGGFFLSPYDVAPRYAVNICTVTNTIAAIAGVINPYMVAALTV

DQTREQWMMVFYVTGGLYVSGCVVFCLLAKAHVQPWADPVLRDTECGKALNSTPLQLQNS

V

>SG-AL_contig_9733_1_-3_63_1550

MLTGTENERQTFAETYKEKNKEVKKSTRRDKRQFADNLAAEAQTAAEKGDSRTVYKITKT

LTGGFTSKTTVVKDKTGKVLTKDIDQLNRWAEHFRETLNRPDPDEEATIEDMGFKIEMRR

GCITQQEIVEAIRQTKGNRAPGEDRVTADMLKADPKMSAKALTKLFNKVWEEEKVPDTWK

KGIIVKLPKKGDLSECGNWRGINLLSVPGKIFCRVLLQRMRQAIERTLREEQAGFRSGRS

CTDQIFVLRTIVEQSIEWNSSLYINYIDFQKAFDSIHHPSLWKILKAYGFPLKVINILQD

MYADNQCCVRHDGQQSEWFHVQTGVRQGCVISPTIFLVVIDWVMRKATSDRARGLVWGLT

ARLEDCDFADDIALLSHAQKDIQEKTSRVDQIASSVGLQIHPDKTKIMKVKTKSAEKTSV

RGSALEEVEHFKYLGSYISADSNIEKEISTRIGLAARAFSGLKNIWKSTVLQTKTKLKIY

KSNVRSILLYASETWR

>SG-AL_contig_9734_1_-3_1848_2027

MNRSFLSSFFEVTEGIIPQVTQNSIQINCTNAISLSWEGAKFRFLPVALFAENGFEENLS

>SG-AL_contig_9735_1_+2_2_175

DRKKAAQKPVPEKVFKVEETMHTDLLVILEEYNQINSIKTIHSSIITANSIITDKWCS

>SG-AL_contig_9736_1_+1_1327_1680

MKTQLNITSDNTNANLIPTLTLALTLTLGMNKLFKEKTDPKGEFWGRARVMETAGSCPQN

GQSQLCHGRHLGKETEADQQAPGNAQFSKRRKPGSIKTIVAGGDWLMPYAPPGAKRTE

>SG-AL_contig_9737_1_+1_154_327

MDPVLYVMFLMFSLSTDDWFPVSLSLDSGILSMSPAFSWDVMLADCSGLSQITTTQKS

>SG-AL_contig_9738_1_-2_143_286

MTHMCDPFNTSNCSSFLFILPVCFHVLQVCLQNLTLLYSHTDCSPCID

>SG-AL_contig_9739_1_-1_22_210

MKPILASNTVSCRTVSIVGPLGRPVSAKFSGRTDQIKVIKKNRCVAIPGHTKIGKDKARQ

SVN

>SG-AL_contig_9740_1_-3_93_407

MAAPLMVSTDFLLSLGMRNIGRSFRGSPRSNKNLKVFQLVSAKSSKAVCYCDRIWERNLP

FLHGGVGCGLLWAVYQHAHAVFAPRDGEWFKVDNIPVCESKGKLD

>SG-AL_contig_9741_1_+1_1_204

HTHTHAHRLTAVKTIPRHHQAISHPHRQSMSQNLQSHKPTVTETISKQQIEGTRPSTQFI

PDSRNLSH

>SG-AL_contig_9742_1_-1_1_729

MASPGQKVSSAGAEKPRGEPKMGESVIMKAYLVAYNVAQMLGWTVLMVKMLDHLIRERQS

TKLYGEVERILQIFQTAAILEILHSAMGIVKSNPVLTGFQVFSRVFLLWGVVWSVPQVQT

SQFIVYFLMAWTVTEIIRYAFYFFSLLGAVPYALKWCRYTFFIILYPIGVMGEVMTIYQA

LPHVQQSKMYSLSMPNQFNIAFSYHYYLWFIIASYLPVFPQLYGHMLKQRKKIISSPPRP

KKE

>SG-AL_contig_9743_1_+2_83_313

MKTIIKPLFRAPQLHDSYHHLSRQEQVIVHHLRTGHNRLNHHMNKCFWLVSYSSCAPVEK

LTRQQNTSSRTAGSTSR

>SG-AL_contig_9744_1_+1_25_339

MFIVSLAISALGNGSGSVGRGDLGGEGHGKRLGMGNEDGEEGHLLSLPVSRPHLHKRQTK

TMVNHHQHHHASFSFPSPSLLLFPFHFSFPNSLSNLCFNPSIHSS

>SG-AL_contig_9745_1_-1_52_240

MGENQNVGTFWLENYERVLEKSLVIIFLILCDIYFVDPSFCFELNLLSESMHLLSFDDSC

LKS

>SG-AL_contig_9746_1_-2_680_799

MHVSLIFFCDRFLLHNYTTNQLLTAVLIVIWFSNCCLISS

>SG-AL_contig_9747_1_-3_3_2384

STSTNPHETEDETQTSSSFLDLSEQQSATVSTGSLFGPASSKPLGFFNRYRPSKFLKESS

FRLHSSALGSALCRNGDDSSDDDRDGSEGRKKKKRKKLLYFKSKHKNIIDPAFVAELGSL

ESSMDKLTISEQAFIRVKPGEVLLPSIFRLTIIDVKKKKKDKQVLEPPPVPEKSKKTKVR

KDSKEREPPMPELVKEKAKNVRKKSMPEDNHPPSLEIQVTRDQCLPPKKRHRMMYEAEST

EESVVSPKETTVPEKRKVGRPRKNPVPDSSGASEKAACIDSKSDCSKPVQDRSLTSVTSP

TRLSLKKRHNSSGGTSTEKTEDSLLVSSQGSCPSPVLACPRHAHQKSALDISCAECTLLV

QSQPQTTMSKSHTRQQQTAQDSSSHTGRASKSKLSLIRKQNISVNQSRRKGSSNRSEARG

GSSLISPSPSPRSRTKMIAAALPSAEVVINPLPESTVSAVVSSQPTRKSLHMQCNTSNLS

DSGAEDDSVIRSVCSEFEPLPLEHCLKRKRPETDTPNRNKKRFRESSSGCEDNIQKSPFS

DTGLKRSTDDSQPPRKRYQRVGLYSDFYKDDEPKKRNEVMVRNPAKLSYSKGDSTPSLLP

PPIHFGKHFRETVIPFQLPYDIWWLYTHDMLSKKEAPRPKFKKIRNNVYVDVKPNTKKFE

PHPCNCKPPANSKEKGCLEDCLNRVVFTECTPETCPCGEHCSNQRIQRQEWAPNVEKFLT

PDRGFGVRTRTAIQPDQIILEYLGEVVSEQEFRRRMTEEYSQDSHHYCLNMDGRTVIDGY

RMGNLTRFVNHSCE

>SG-AL_contig_9748_1_+2_2_226

PTRSIQNTNPYLTPFCHFQTHMKTLAGIHPHNHRTDYDAHHSIILFRLMLYCSVSILLPR

FYQILYFTFKRRRRR

>SG-AL_contig_9749_1_-2_128_421

MGARAKILSILLYIQGLKWSRSQIVIPCFSCRVNFFFIVTLNGADSKSDGCQTENLGHFV

LILYLKIQCDHHGFGFIPVHCHILADFSLRLKYFETFE

>SG-AL_contig_9750_1_+1_352_726

MIPLNKSSVNDHQSQLTQTINHLIQLAQGVSSLSTVCRLWNGIRTDSNVHSIIVSSFLLV

HITGSHILKDQSNQQNVCSMSKHYTPDSCWLKLGYAWAFGHSFTYIGQKLTVKWIHTTNH

AMLQA

>SG-AL_contig_9751_1_+1_1_159

RSSHHTTCLFLKKPQYSLTIQLSSQLTPHHCVLFTLFKRIPLKEQVHQHSQTG

>SG-AL_contig_9752_1_-1_13_999

MLGVKNRFAALKVDDDEDESTRSKGASTNQNQSNTAAKKKNKKKKKEADEDLRSMAFGTG

RPSGKQQKQPGSAGHRSQNNKPVRTEQDWADWQRRDIEATEELYQQDLQEALLVSKMEAE

QKKETEKLMSTEVQAGAMLADGRNGGKKKKKGKPMTLSEFNQLPPETVPGSDDENEDPSP

PPVHTRVPVECQDTRYFEDIASDANKIMHKERVQENFQKHYVQEIAMKSRYQEEIEKLQK

KIKKLETRLEEQGAELKNVKGRNARLCKLVADGEMKEKSDILSQVEELTTDKEDLTEQLA

ALTAELEKERSKNHALKLENDKLKGKHGK

>SG-AL_contig_9753_1_-2_2_199

IPLEQTAISLLIIHTHLHFCMIAILTLLAAVSLYIMYRFLNYYLWCVCFFFSFPLFTFLV

PYMVVV

>SG-AL_contig_9754_1_-3_3_401

FHTAFLQSQREGCHYLLLLYFDVSTCQADAYYTSNIESNKSDNNLIKVITLCKWLCRIIT

ASWQPEEHSYCWLVFILTEYYTTMMFLHVKQMHLIVLVILNLVKVTRQFASGFASSSQQA

FLEKITCLIQKHR

>SG-AL_contig_9755_1_+2_260_418

MIEFAVFSCRNLYFLPLFCCFNFFPFFFCFYKILLSYVFSFLLLGSGSLLFAP

>SG-AL_contig_9756_1_-3_36_8696

MHIPYDEFEKVLASCFDVFSAWDDEYDKLQGMLRDLMKKKREEHLRMVWRVNPAHKRLQG

RLDHMRKFRRQHEQLRQVIIRVLRPTTKGRTGTPGAEEQDMKTEPVVDEAADANAIEEVN

LAYENVKEVDVLDVSKEGADAWEAAIKRYDERIDRVETRITARLRDQLGTARNANEMFRI

FSRFNALFVRPHIRGAIREYQTQLIQRVKDDIESLHEKFKVQYPQSKSCRMSRGHDFPPV

SGSIIWARQIERQLNAYMRRVEDVLGKGWESHVEGQKLKADGDSFRMKLNTQELFDDWTR

KVQNKQLSVTGKIFAITSQRGATKNILKLGVNFQTEIIQLAKEVRNLRWLGFRVPLANVN

KAHQANQLYPFAISLSESVRTYDQTLKKIDGKDSIKLLIAGMRKDVQNLVAEGAGLTWES

YKLDPYVQRLADTVFAFQEKVDDLLSMEEEIDLEIRGLETCGYNATTFREILTKIQKAID

NLSLHSFSNLPQWVARVDEEVEKRLALRLEAGVKAWIQCLQGVKEDPTDNSMDTDEPKQQ

AHKPGGEPHIKPLVHELRITNQVMYVNPPVEDAQYQLLQELFAWEAIVTMLPRIQHSRYQ

VGPDTESEMDRTYRKVLTQLPEGSQVLDDAYGAVQKIVGDFKKYVNVWLRYQSLWDLNPD

VLYGRLGCNLKLWMATLEEIKSSRKTFDTSETQTEIGPLVVQFGKVQSKVTLKYDSWHKE

VLSKFGSMLGNEMAEFHSQVSKSRSDLEQQSLDTANTSEAVGLITHVQALKRKMKSWEKQ

VDMYKEGQRILERQRFQFPSSWLYIDNVEGEWGAFSDIMKRKDSSIQTQVASLQMKISSE

DTLVENKTSTMLQEWEKEKPVAGEMRPDIALKNLTIFEGKFNRLKEERDNVSRAKEALEL

AEPGQISPSHERMQVALEELADLKGVWSELARIWEQIDELKEKPWLSVAPRKLRQSLDAL

LNQMKEMPARLRTYSSFEHVKKTLQSYAKVNVLVVELKSEALKERHWKQLMRRLNVNWVL

PELTLGQVWDIDLQRHESVVKDIILVAQGEMALEEFLKQVSEVWRSYELDLINYQNKCKL

IRGWDDLFTKVKEHINSVCAMKLSPYYRQFEEDAQAWEDKLNRINALFDVWIDVQRRWVY

LEGIFTGSADIKHLLPVETSRFQSVSTEFLGLMKKVAKSPLVMDVLNVPNVQRQLERLAD

LLGKIQKALGEYLERERASFPRFYFVGDEDLLEIIGNSKNIPRLQKHFKKMFAGVASIVL

NEDDSHVIGLTSKEGEEVVFATPVSIAAHPKINEWLTLVEKEMRVTLAKQLAAGVQDVAA

FRSGQIDQAKYIEWADKYQAQLVVLAAQISWSESMEQALQTVSTGGDKEAVNNILKGVEN

TLNVLADSVLYEQPPVRRKKLESLITELVHQRGITRTLIRNKVNSPKSFEWLMQMRMYFD

PKNPDVLQQLSIQMANAKFNYGFEYLGVQDKLVQTPLTDRCYLTMTQALEARLGGSPFGP

AGTGKTESVKALGNQLGRFVLVFNCDETFDFQAMGRIFVGLCQVGAWGCFDEFNRLEERM

LSAVSQQIQTIQEALKELASPDRKDKSSPMTVELINRQVKVNPDMAIFITMNPGYAGRSN

LPDNLKKLFRSLAMTKPDRQLIAQVMLYSQGFRQAEKLASKIVPFFQLCDEQLSPQSHYD

FGLRALKSVLISAGNVKRDRIQSVKQGLMDRGEPVNETTIAENVPEPEILIQSVMETMVP

KLVAEDIPLLHSLLSDVFPGVAYTAGQMVELRRELRKVCAEMHLTYGENDEQGTQWVEKV

LQLYQITVLHHGLMMVGPSGSGKSTAWRVLLKALERVEGTEGVAHVIDPKSISKETLYGN

LDPNTREWTDGLFTHILRKIIDNVRGELTKRQWIIFDGDVDPEWVENLNSVLDDNKLLTL

PNGERLGIPPNVRIMFEVQDLRFATLATVSRCGMVWFSEDVLSTDMIFEHYIQTLQNVPL

EESEEDARYMSRAAGSAEGKEDQVSPTLQVQRDAASVFMPFFSLDGIIIKCLEFGKDLDH

IMDFTRFRALNSLFSMLNQGVRNVLAYNRTHDFPMQTDHLERYLSKYLIYSLLWSITGDS

RLRVRQELGDFLRGITTIPLPPSNALIIDYEVTPAGEWASWQAKVPKVEVETHKVAAADL

VIPTIDTVRHEALLYTWLAEHKPLVLCGPPGSGKTMTLFSALRALPDMEVVGLNFSSATT

PELLLKTFDHYCEFRRTPNGIVLSPVQLNKWLVLFCDEINLPDMDKYGTQRVISFLRQMM

EHGGFYRTSDQAWVHFERIQFVGACNPPTDPGRKPLSHRFLRHVPVVYVDYPGEMSLKQI

YGTFNRAMLRLVPSLKPYADPLTNAMVEFYMMSQERFTQDMQPHYIYSPREMTRWVRGIC

EAIRPMETLDLAGLVRIWAHEALRLFQDRLIEDEERQWTDQNVDMIALKHFPNIDRETAL

CRPILFSKWLSKDYVPVDREVLREYTKARLKVFYEEELDVPLVLFDEVLDHVLRIDRIFR

QPQGHLLLIGVSGSGKTTLSRFVAWMNGLTVYQIKVHNKYTAADFDEDLRTVLRRSGCKD

EKICFIMDESNVLDSGFLERMNTLLANGEVPGLFEGDEYTTLMTQCKEGAVREGLMLDSA

EELYKWFTQQVIKNLHVVFTMNPSSEGLKDRAATSPALFNRCVLNWFGDWSKGALYQVGK

EFTSKMDLEKGGYVAPSNFPFAYENLPATPGHREAVVNAFVYVHLSLHQANSRITRRGGR

STAVTPRHYLDFINHYVKLYTEKRSDLEEQQLHLNVGLQKIRDTVQQVEELQKSLSIKRI

ELEEKNNAANLKLKQMVKDQQEAERKKVTSQEIQVALIKQTEVINEKQSSVMEDLAQVEP

AVIEAQN

>SG-AL_contig_9757_1_-2_2_136

QFFLIGCQNYAKALVAMSKCEIDLVEKHMDSFLRPKFFSAKKLEK

>SG-AL_contig_9758_1_+2_431_580

MMLYHLADETNVFNVISVSYMRFIHFLHVYADLKKIIHPFFTCVHRLKRN

>SG-AL_contig_9759_1_+2_2_238

DDVCDFLRHTIVSRQLPEGLSVYAVECLHIVNEVDIKGRVPFQRLFQDDDVCVPQLNTLG

VTQYICTLFYASETTSTFG

>SG-AL_contig_9760_1_+2_167_325

MMNDQDLNDIWRDMNPDCYRYTWPHTNPFQQSRLYFFLISDSVLHYAEDSDIL

>SG-AL_contig_9761_1_-1_136_1683

MCSVGDLKNNLRILQSEARQMKYGGEFDLDGLTKGNPHALLPLYNYAFTTFNSQFSAEVI

DILDDNLYMKSDVRFMESVYRILRDVFHFKPSMTKEQFFTVNNFAERKIIMCVEVLRLVQ

QRCRFLGPKNPTNPHIKVSSVVDNRRCSKKSSTDLVTAEPSGDVVLTRKVSDKGRVKPLS

AYMAINEKDNWQLKVGYFQPRGDENMENGAKEDGKERTGRTEREDHGQRVEKSGMVASVV

REFSPASVRNRGVEVVHMPHDKHENGHLRVADMLPKSQSQPQNDWDPTGAVECILAKVRD

LPAQLTNFMKAVEGRLQKIEERLDNVESVTARITQAEGKTELPSDIKHEIDTLQARLLLL

ENRVTLTENKTANSVSTECIRSVSATEDKKMDFAPSPFVSLERGEDLFSGSLMDNFSPIR

QDMGDYNRSSNNTLNSIADGDNRRSSTPNQSSSARPPPCNSSKENSEKRPAVSTDTTLTW

ENLDASTQQQVERINGLLKATQVLLPNIAGQGPVDT

>SG-AL_contig_9762_1_-1_385_483

MNLIMYACIRFKYRVYFYFRPSFSTSDPCNEIN

>SG-AL_contig_9763_1_-1_625_888

MSRLSLNSQFCLSQLKSYLEIFLLVTFSRIFLKLLIVNLSDLMLSRNIPCFLFYSKNIGF

ISWLIRSKEYACVFSTISTVFTYMVVTF

>SG-AL_contig_9764_1_-2_119_259

MILEFGNYILSHDCECYCQNSEVGAWYMNFTVLVSSQFFPLSVCFFF

>SG-AL_contig_9765_1_+1_496_792

MCGSLLLLHAKHASGAFPSIIIKTPNTDVFLLCPAHQLELSADLYVATGTGTVSCHPVCS

LFSQFQRNVDLLFSSVLLSLHAFTGCDSISTVVLRANGK

>SG-AL_contig_9766_1_+3_618_869

MCVCVLYYRILYIYIYIKCSLTKQCNAIYTCALMTLYDHISENWGFMSEPHKESSLTINK

YLYLQLHFIHRHRFVFSLNHSPNL

>SG-AL_contig_9767_1_-2_2_133

QILFSHAIHNILINYLLSLQMSDAPGTLKKEEEERGERKKKEEE

>SG-AL_contig_9768_1_+2_110_265

MVEYIGTQYIEEVNSLRDSSTVGSKWSETPDQSGAYLWHNVQVSTFFVWLSV

>SG-AL_contig_9769_1_-1_49_369

MVDNPDYEPDDKLYLYRDIGAIGFDLWQVKSGTIFDNILITDDEQFAKEYVENTWGKTKD

PEKKMKSDQDQEEARKKQEEEDKKRKQEEDDRYAKMQEEEEEEEEEF

>SG-AL_contig_9770_1_+3_126_338

MNTKKTNTQKKTSKTKHSNLKFIFFYSLHVSFIFTSIQPHITIVMFTHSHKHTPWNNTKC

TVTYCTHKRGE

>SG-AL_contig_9771_1_+3_126_362

MYLHHIFTMITKFSYKFCVKKFSYKFSVYKSIIYSTRHLFFYRNELQVHYLQTQDISVST

LTSTLFQFSDLSHQMDNEF

>SG-AL_contig_9772_1_-3_126_1619

MSAIDNTELDEPRRKSLKVHRAAHMDARFRCSEFISHFADAFLSKSHRKEDVTNTEVFAT

PFRHASLPNLIPDSDYLEQLITELRSLDMQDKNNDLYKFKQSIDLKHVSSPCISMFRDIL

QDEVLPWLRDVTRFPLNDKVDMFCSNYDYTDVLLCHDDELEERYFAYIYYLVPDSWGKED

GGTLDLFDTDDKGQPGNIVKSIVPAKNLFLFFEVTPGSFHQVSEVLAEDKTRLAVSGWFH

GPPIRRPAPYVEPPMPITGCGGIEENVFFDWINPVYISQEIQNDIKERFESESELELSDF

LKDEKYEELVAAMASVENVWSHRGPPNKRSYDVCSPDSLPPAVQTFIQILQSDAMFLLLS

SMTGLHLHDNPAAEGEGVPNGSDAKNDMAKCRVEVQRFKHGSYTLLHDTDAHVGEMALDV

VFFIGGMEWSIESGGYMSYVAKGEDEELLSVCPSPNSLALVYKDKETLRFIKHLNHRVTK

AKESRYYTVSATYYENAD

>SG-AL_contig_9773_1_+3_135_224

MTEITSGHFYTDQSPLDSYLQESCLQDSYL

>SG-AL_contig_9774_1_-2_125_769

MVVWASCNWCCLSTWCLTQVEMSLLLLLLWFPFSFLILPPTQVAQELEFVVLNTSVHREQ

KKTNPYARNPYSLVGHNMRMLCTYTVNIGGDDYCAQKENTNLTVQPSQSMTPASKRLFEN

RHVVISQTKCNLTVEFQNMTEEMEGMYKCRDLTTSESAPRRVKVGKPPVKPRNISIVYSP

NHSIALNLDPVKTGRDLNSEGTTWTVQYKWRRKGE

>SG-AL_contig_9775_1_-2_77_1948

MAAPDKLKALFEDLGKPTLRGVRKCPKCGTYNGTRGISCKNKACDAVFKEKESKKGHSAE

AVKILTGSATQVYSVRLRDRGPDYRGFVQLPIVQDQDGNPATGVDVSVLAQVAQCLVSSC

SRSTNNSSGDLTSDLTKWSLCPHIKAAIECNQEAQPLNIKNSVLNSLQVASEIKQVIWAL

AADTPGPIVQRVTKNIMVVKCKSSTTQPLGFLHFAFFETTRNRQQPDYKFQCSCKAFRAM

KGGPRDDAKKRCVHFYACICAFASDDKLAEEFSYYINLSNQAFPGIVAQPAVVEAEQQQE

QRQVLTVYQPVDSLAEIADGTATLEVIVTESLLDSPPEKKMKKDDPLAGVVAQASSALLT

LQEQKSPGMLQTAAVRASPVKQVLQVGKPQPTPKKTDRGLSVPTPLDENSVSLPFQQWLA

SVTERINQTMHYQFDGSPEPLVFHAHQMMFDCLQQRISAGSRKKRLPNCTSSFTRRDALP

LGSFTKHTWQIVNILHVKQIFDTPEMPLDITRSFVENRDGTYDLYEVPRVAVENMDDAFK

TKNQTPIRPFELKTFLKVGTTMNDQKEPTPFIIEWIPDILPVSRIGELRIKFEYGHQRNG

QIEHRVWVPPQDLPMQTIQVQEIG

>SG-AL_contig_9776_1_-1_4222_4512

MTNTKFTESPFLSSTLYLYLCLLLLILFVYFSDEILFGGLPITSSIYGNNCISWDQARIM

CAIFLNYSASQAVTLSIQKILCLCPGTVVVISQAHHG

>SG-AL_contig_9777_1_-2_2_160

LPSLCLLRYTVLAGNTREYTQRQKGQFTVTVQFIPAKGKHSDLWMKVNCWVSD

>SG-AL_contig_9778_1_+3_1668_1976

MGENTETVLYSVDYRNTPSKTWLHVARLTWQSAPSQHSQKTSVSLFFPPGAASANDSFLL

LNSFVYIEMTDEKPKKSEFCLELRFKKWGIFKFLFKPSSYFNH

>SG-AL_contig_9779_1_+1_85_264

MMCTYGTALPTISDIYKYNVFNCDYLQKERNGTNLYADCKVNDLQCANGDNLFTDSLKKK

>SG-AL_contig_9780_1_-3_126_266

MVRTDRSTCILPSFGANFFGYCFVYGRFYFLILSSFRKKKKKKKRKI

>SG-AL_contig_9781_1_-2_209_823

MADIKLYYFDVRGRAELARLVLAAAGQKFEDIRWDREDWPKYKPEAPFGQAPFMEYKGKK

YGQSVAIANFFAREFGMYGKTNLDALRIDEVMCLTQDIWQVLVKVMFEQDEAKKAEMEKK

NESEEFPKFFGFFNKMLGENKPNGVIVGKDLTLADICVYDIMYSLCHAKKYDAGAFFPDL

KALKDKVEAQPKIKAYLAQRKVTEF

>SG-AL_contig_9782_1_-3_87_719

MEEGVLLEFDSMLPDTLPDTTEDPQKSLANLRAQFFGLNTANHSTSSNNNTNLLDLDDLG

GHTLTTNRNEDSDSDVSSDERGMEGTFSPGDSGVILSSLPTSPDDGDGRIQLISYTSKNI

PTTDTTTTIVTTTTTQRPQSLPMKESLPTSTGDTLSPSSANLVSAQDMAALSPRSREIFE

KARHFAEMVQKPNPAAKRGEEVGEGEDVPKQ

>SG-AL_contig_9783_1_+1_133_384

MDEGRVGQRGLGHQCQSGGGGVHFLQAQSFSHLQSCGPVQPLPQSHLPAEQLHLQDGQPQ

SLVHSHLSAHPHGVWRHASVHPAP

>SG-AL_contig_9784_1_-3_3_143

SLSSPILPEKMIKLEKCSHKIKTTITTTKRNHCGYLNGKRYTRSGRM

>SG-AL_contig_9785_1_-3_135_281

MSLISLNICQVLCRWYNKMYGCSVVVFWFAFIWFLFVFEKFKSSSMLLI

>SG-AL_contig_9786_1_+2_2_181

HTLSFSLSHTQPDVILTTITLSLKSKPTQVHNNLSQESTTSTSDGCLYKSVNSLSNGITV

>SG-AL_contig_9787_1_-3_108_281

MNMLKQSHVVCVRGISSGLLVYLSLCAKVVTSIVELILFLVSTHLVFMDDFLCFVSRG

>SG-AL_contig_9788_1_-3_3_218

SPAGSWAPARSPPSATGRSRSPGGCVLLSGQLLHRGTGGMVQVGSTCSFGVVLVVLSFLF

ILFCDSITCCLQ

>SG-AL_contig_9789_1_+3_12_230

MKAARELKGQQPNLHSSFFRVSTSYRYHRFFSAPPFMLIYTAHFDSPIRYQPHAHSITVP

EPDIVQVNQYHLP

>SG-AL_contig_9790_1_-1_97_1956

MTALSAGSIELMIQGDSPNLQPVLQVINLKKLASNATSDRYRLLMSDGQTTYSHVMLATQ

LNTMIDNNELDALSVIKVNKYLRNTIQDDKKILILLDISVLRKGSEVKEKLGNPGQLKPD

GTGSTNGMHHGDTAPAVRDNTSRPAPAPARPPLNPSRTVGGNSVTSPAMKTSKSFGGAGA

PTGPGTPSSRVHKIATLTPYQNRWRIRARVSQKSSVRTWSNSRGEGRLFSVNFLDDSDEI

RATGFNDAVDKFYELLEVNKVYYVSRCTLKTANKQYSNLKNDYEMTFNNDSVIERCEDDA

DLPCMTFDFVAINEMEKHPPNAIVDVVGVVKVCHDLSTVIARQSQKEITKRDLQIVDQSG

VAISLTLWGSDAQNFDGSSNPVIAVKGARLSDFGGRSMSVLGSSQLIINPDIREAHVLRG

WYDREGASMDFSTFRSEGPGAGGNSNWKTFGQVASENLGHGEKADYFTTKATVMVMKKDN

CMYQACPTANCNKKVVDQGNGLYRCEKCSREFPNYKWRMILQTTLSDYSGHQWVSCFQES

AESLLGVKADDLGDMRQNNEAAFDQVFQRGIFKEYIFRLRTKVETYNEESRLKTVCVNAT

PVDYVQYGQHLLQEIKQLGL

>SG-AL_contig_9791_1_+2_35_280

MNWGLKQEVPYMRLRVKASKSNLIHEQRLNECTCNNGMSRQAGNFLVAKNLSFSLICVMY

ACIGLSVSLQWFLWHIRILNNH

>SG-AL_contig_9792_1_-2_2_208

DFQPGDNTQLCGDHFREECFNHSGKLICLKEVAVPTVFDFPDHLKKVHYLKIILKKHAYT

HRCTHTHTH

>SG-AL_contig_9793_1_+3_117_314

MELNKEFVFIIIMYKANHMHVHKCTTNVRQNTLTDIKQLDYCLGNTALFVYYFSCIRCSS

LYGLFQ

>SG-AL_contig_9794_1_-1_1_306

TILLTRLQHTFGISDTALSWFSSYLLDRKQTVLINGIYSQPAHLTCGVPQGSVLGPVLFT

LYTSPLSTIIKSHSLKHHFYADDTQLQDSDSPNNIQTLLTRT

>SG-AL_contig_9795_1_+3_114_308

MLHLTWFPCTGVAKYYHLVTNNTMTMKQIILVFFFCTYHHIHTTFASGQSVWVIIKSRND

CQGTV

>SG-AL_contig_9796_1_-3_93_875

MPEYADVNIEVTDDMIDQASDKRNEAMDAMAEGRFEDSIQAFTDAIKLNPTSALLYAKRA

SAYLKMKKPKKAIHDCSKAIELNPDSAQGYKWRGKAHKLLGHWEESYHDLTLACKLDYDD

DANMALHDVTPNAKKIMEHKRKYERQQEERELKKRKERIRKAREEYEKQKKEQSQEGPTG

GFPGFPGAFPGSMGGMPGMPDMTTLLQDPELLTALQDPEISAAFQDVATNPANISKYQNN

PRVQQVINKLAAKFGTGAAGA

>SG-AL_contig_9797_1_-3_3_92

NSYRWKIKYHCHCHCHCQLFSVEKTTRKVT

>SG-AL_contig_9798_1_-2_764_1051

MYNLQQLNQGYQQVFSVACRLCLILPCAILSHFHRQQSCCFTYKLPASSQGHLQACKAIT

VLFGIFHYNFHLSLLLGQSCAFYRPHHCNVYVYDVY

>SG-AL_contig_9799_1_-1_202_2688

MKFSVTPPPGQSAFDQWKDAMKAVNRLPMGLPPEFRKKVWLSLADHYIHKLKIDWHKTVR

LAFNERSNPDDDKLGMQIVKDLHRTGCSSFSGQDNEEDRAVLKRVLLAYARWNKRIGYCQ

GFNVIAALLLNVMERKEDDALKVMIYLIDHVLPESYFDNNLRALSVDMAVFRDLLRLTLP

TLSRHLDRLQHAAQDINTGACYEPPLTNVFTMQWFLTLFATCLPKQVVLRVWDAILLEGS

EVLLRTALCLWGKLARRIMKVDSADEFYSLMGELSSDMMEGTIFNSDDMIKAIYAIAPFP

FPQLTELREKYTYNIRPFSSNPAPSRKASRMAKAVLYSDEDELDEEEMRAITSFPGMSPV

QSGKGKGTDISLVGPGAYGAESEMGMGSRDSVYMERMSTDITALKMQYDKLRQRQRQAHV

LIAAAASAKKAVEVKQKSQAPVLVPKIESPLAMNHLFVGKVQGGRNRLVTHGPRIAASTA

PRNNVLTKQLQAVAAAQAASRSKHSPTKHFPTTKNNNPAPRPDQREGEEEKQPQDCLPEE

TLTGTENANRACDRAPTAPAEKESVSEIHSPEPDTGCTVMEDGKSVVEDDRSAAEDGRSF

VEGRSVVEDGRSFVEDEKCKEEADGQEQSHGSYLELATDQETFPPKADDDDSSDSSGDDT

SCHAFTLRQLGEEIDCKAFSLDADVDNGIELCPVNSSNAEIKEEGDSGVHLSSPSLDGSG

KSSPEKKSPRPISIQLWSESSPCLNGNMTYDCQENTGQGDSNRNSHKSQITIGRCNSAPN

GSSHMTIMSMLTPATTPTHTQPTSPSTPSAPTAVSSQLLYSPSHPPPLS

>SG-AL_contig_9800_1_+3_3_293

INTVIIIVVVVVIIIIIINNNIIIIIIIIIIITVCTLINKKICTYNCNSRYKPTWKLITL

FVFQWMEWLSTWMYTRYIHTFAMPTCFMKGFCLHKLA

>SG-AL_contig_9801_1_-3_3_251

GCCSNFLSNTAGICISQAWLTPGYIIKVSTEYSPVMQSQSTMELHSIITIHSHSYHHSIV

KNCPRFCDPSCRALMVTSVSVKK

>SG-AL_contig_9802_1_-2_1115_1369

MTNRTPPRHPGPQQGKNKQYTNTLTRQTGEYTITHKTNYTSTHKTKYTSTHKIKYTSTHK

TKYICTHKTDKVHRYSQDKVHKYSQ

>SG-AL_contig_9803_1_-1_1_165

EGVKGLTPCFCFDKVTRRTDSLSMEVGGMYAVVGCPFVCLNVYIISLSLLMHLQF

>SG-AL_contig_9804_1_+1_334_630

MCTSQSCKTGFYRPEPSAALCTLGCSSETNIACHRASFLSDSSCLWGCLQAQRLGTDETA

RMVGEKKAETIESCGFCYLSVFHHVNKRGIKCTAKEKSV

>SG-AL_contig_9805_1_+3_3_131

LPVRNDPESELDGYCSREEKLSTGNWLENHPCLSKITKERRES

>SG-AL_contig_9806_1_+2_203_385

MFLVATVSNLVVMIQLLNSSIQNVIKYISPPTLGTIWICSNLIIKSKEVPRYTNIPSRPT

L

>SG-AL_contig_9807_1_+2_362_505

MVFHMGGHLSAHGKWQYGNVEVRVTNSYKYLGMVFTTKLSLNVGIQKR

>SG-AL_contig_9808_1_-1_61_246

MLSLCLPFSLPLPLSTFFCLPGPFQYVHSAVLLQSIFKKNFYTFLYSGDDKLSRMAGILS

TV

>SG-AL_contig_9809_1_+2_2_151

CVTCVCVLVLTQIYYIKSDIHCHTHDNAARDFYKLHSSTFPQFGFSFSML

>SG-AL_contig_9810_1_-3_18_461

MFQVFLASVCRAEYVVTLSLVKVHFVTPSGHVSSTALLLTGVNIIFSIHLATLLFETFMS

CIFLNTSDFTTIKDLLSYHYTPLFKSLHAHRIKILLQPKFVPESLNSSTIIFRLYCQTQL

ISTTTLAVPVISLSRRTKHNTSSIITAI

>SG-AL_contig_9811_1_-2_1160_1513

MLLIKVQDKTSFAFLCVNFCWWSGLCLPYLVACTVKPGEVVFALFLAVCALPGCKKKYMV

GVCKHLSVCVYGEGSRRVVCLSEHVCDWVTVGVLCVCVCVSIFCVSPMSAFFSSVCVC

>SG-AL_contig_9812_1_-1_28_846

MGTDTRSAGQALQSLFGQSPSEDARTRVVEDLLDMKVLPEVDAYGRQFSADRVQERLSKY

GNFSTMNKLQGMLGSLEDKVRQAGSQGSAREVSEQLSQLSHRYSTAGTTVLTRQVPHSFS

MANLSGHALWQTFANRQGSEAEECVPEPSTSAQDNYDDLCSDFAQVCQQQISAKDAIRTH

EQEITSEMELTERSKGPERELHAQKQQEATQQLQAAKTQLEKLNAERSRIEKLLEKSAVA

GSNPAQSEESGTSTASDTKKTDKPGRKGGKSKK

>SG-AL_contig_9813_1_-1_130_243

MTNAEKILHLVMRKSDIKIKTFWRSENVVMRLKLFDNI

>SG-AL_contig_9814_1_-2_2_778

LGDSTPAAATNGLDSNGLTPGSEDNLKKFICKHNGVLYENDLVQIGVKSEYRQNLARIGI

FYGNKTTFQLTNFTADISCPGELATLLSVQPKPVESTVDGGAQVQQLFNIECVSEFSDSP

VLSVSFVYSGSQQRLSLKIPVMVNKFMEAADMNAESFFQRWKALSQPNQECQRIFNAQFP

QDTEQSKQKLLGFGVSILEGIDPNPDNFVSSGILHTRNQQIGCLVRLEPNKQAQMYRLTV

RSTKGSVAQTVCNLLESQF

>SG-AL_contig_9815_1_-3_129_458

MHDKLRMNNFRVVLCFALHGTFFLSKIQTVCVPCVRIDVGHVSYLWHCQCLSVSFSLISL

TCFGFDADDLAINATPPFLFSLCRLQCNTALRSESARHAFCRKQRDVVGV

>SG-AL_contig_9816_1_-2_2_1072

MKTLHWKKHHKMVDMEEKKVEADQPKIVFKKRGKFRGQRRQRDSSDEEGQKSSSSDDDGN

TAVKIDRKAHKKGIMQSTKRFKVETKQAASSSSEGEQEKTQDGGVSVSYKTSGTAKRVGP

DDMGATATLEIDTELDKDAQAVYERSLQLQKELKGKEDDKKYRGMNNYAQYYEKKDTAQG

NAASGHVRKGPIRAPAHLRATVRWDYQPDICKDYKETGFCGFGDSCKFLHDRSDYKHGWQ

LEREMDEGTYGQNDDENYEISSDEDDLPFKCFICRKSFTNPVITKCKHYFCEKCALGQYK

KSQRCFVCNQQTNGVFNPAKELINKLSKRKGENDNASDNDDSNKEHDSEPPSEGEET

>SG-AL_contig_9817_1_-3_228_812

MACAATLKRSLEFDPVHSPSHSSSKRRRCMPIATSSPPVKFNRSYTSPFSGATEKLTTDK

IAASIAHEVKRMQRRRQLQFPSTATQTCTFTPTSTPEKDDVPSCSSSLDHSPQVSQSLNN

LFSSFPHTKKDVPLFTFRQVSLICERLVRDREEEVKEQYDKVLNCKLAEQYESFLKFNHD

QLHRRFGKSEMSYVS

>SG-AL_contig_9818_1_+1_28_246

MGTLLILKEPLASPGEEIDHTHLQICFIVGILQTITNYFNHLAILQLHHKRIILPSCYNP

TLHEVYCYFTTIK

>SG-AL_contig_9819_1_+1_478_825

MRQPVNVAKWLVQWTTDWKVMGSNQHQSKKLLWDFSAMTTSTQSLACYELIGKTGTTQSS

LGHFTDASLRELSKFPDQHCRCLYLLGQFEARIYYGRVYSTALSCSLSPRWNSTAS

>SG-AL_contig_9820_1_+1_367_585

MVISGLSWLSTSSNTHSYMGREREREREREREQLLYVTHGIMKTDIALHDFMLHFNPHPY

LPQQRYAPQRQKF

>SG-AL_contig_9821_1_+2_44_142

MLLAQVKLKILNHQSTAVVLERVGRLKWGGCDS

>SG-AL_contig_9822_1_-2_1187_1453

MADITFSLLLTYTCLYQPPLLAFFPTGKMRRQQKKQGKRMESVVWKYFLSYIRSQIFPQL

PSDQPTASSCLWTKWASKNLFMCLWMTFY

>SG-AL_contig_9823_1_+3_336_425

MAISQYCVTLRSDILIIGNKIPVLFREGSF

>SG-AL_contig_9824_1_+3_207_350

MEHIHHHYTQVTPSFSHPIHQSWWIGERVKRVNNIVHQHENFNIYRHM

>SG-AL_contig_9825_1_+3_279_431

MKWKQDKPLMLLHSLLTDQILHKYSFTTLKLSERKGKVCNDKNKQRSVYKH

>SG-AL_contig_9826_1_-1_250_492

MALFVTLYEIEPDYQRISERERWPLSLYFHAICCLFMFVTIPCTFITSSKSLRPNFEAGV

YHHHFPLSVICRFEFSTYGSA

>SG-AL_contig_9827_1_-3_3_167

NRTGEYTVIWYRSGLSTPSPSVLFHFSFFLSFFFVVVVINKIVSLSHTQIYVSIC

>SG-AL_contig_9828_1_-3_2181_2354

MYIFVLADHLVHCLKPMCVASSPLTSCFFFRLSGTPPTPRSFSHSPCARVHKELSVPH

>SG-AL_contig_9829_1_+2_878_1114

MKRSLLTGLDFFLPFPVLGTSVHISFTFSSTMLQCLSNALTLARSFLLLRQLMSTWVLFF

TDWVSTDSGPVLNSSCSRA

>SG-AL_contig_9830_1_+3_3_158

HWPIQGDKIYIYIYIYIYIYVCVCVCVCVCVNFNSVWHAEFRRKIKLHLSMV

>SG-AL_contig_9831_1_-3_183_641

MPPTRLPERPKPPTGQQMLADIAGADDNDVVFLTGQSIDLSDLTPTTPDGGAEGGDKMLT

QASLAEADRRESAQRAYHKALELLEKVQTLEESPSYLEAQFNHLNNLGEEVSKMIKDLQE

SSNHVTPKKSVQKDVEDSKDAKVSKPKSNKKKK

>SG-AL_contig_9832_1_-3_3_209

LSSLSLSLSLSLSLSLSLSLSLSNTIDFFLSFSLPLLLDKKNCSWSRISIKDVKSLHHDS

NSPCHQPLH

>SG-AL_contig_9833_1_+3_2451_2738

MPGYIMKGNVEYSPVLQSQSKMVPHSIITIIPILICHLSTVKICPRFSGPSCPPFMVTSV

SVGLEVCWRADMSGMGAVTKGQCRRWGSQKTPTKTQ

>SG-AL_contig_9834_1_-3_3_170

FFSCFYLLSKLGHCPQIVRIRVLDKCANMKSCHYCFSGLCFSLSQYCTNRLCEINP

>SG-AL_contig_9835_1_+3_36_131

MPSKFPYICMRTKSEVVTQPHGFFNQLATKIT

>SG-AL_contig_9836_1_-2_1487_1771

MDTSGMVVDAGIRNILPSSEKEKESVFISLTLYAHFTSYDPGNLVHKWLQKLLGSLTVVG

AFNGNIMPVSETSFQLNGSGYLARSNVLYYVFESD

>SG-AL_contig_9837_1_-1_859_2175

MPGEKEAAATETAPAAEAEEAVKAEEATAAKPEGKEKEKEKEKEKEKEKKGKTEEPPKPQ

MKSVVLTGFGGMKMIKVQQKPQPTPADGEVLIRVKACGMSFQDLMLRQGVVDNTPKTPLI

MGLECSGIVEALGQNTTGFAVGDRVMALTDYKAWAELATAPATHVYKLPDNISFQDGACL

LMHYITAYILLFDLANLRKGQSLLIHSVGGGVGQAVIQLSAMVGDVTIFGTASPHKHDSL

KDKVNHIFPHSVDYVQEIRKVTSEGVDIVLDCLCGEDTNRGISLLKPLGKYLLYGSANMV

TGETKSFFSFAKSWWQVDKVNPIKMFDDNRNIGGFHLRRLLFRQGQHGYVRGVVDKLIEL

YGQGKINPVIDSVWAFEDVGEGMQKLHDRKNIGKILLDPSKEPAPKSTPKAAAEGQAENA

EGEGAQKAESAEPAAANGE

>SG-AL_contig_9838_1_-1_184_525

MFLVDFSILVFDVCFFCCVCLCLFYVSYANSCSPSVKKKGIVYNAKYINYVCICFIYSVI

CLFYVSCENPFSPSVKKKRKNRHHVHINPFTAPARKFSRPNVHSVCQEPGNFPG

>SG-AL_contig_9839_1_+1_241_399

MSRINPLPHNVLTRNYFFNDSVISTYQELCCCLGLLKSLFCKSQHASLSCKDY

>SG-AL_contig_9840_1_-1_928_1119

MPCHLGSHTPPSENTSLFLESPGKSLSCLKMMHKYALHDNGYCVFGAISCLCTTDRTLRQ

AIYT

>SG-AL_contig_9841_1_-3_3_425

QTEDVPLVDLNRKNKRQQSDSQSAHRGLKSVLALKNRRVEREDNRSTNSLAHELTATEIE

RVELEKNKLKLEIEKNKIEMEKARMEMELLRQNMALTKFLTTNAQLEQANLFYALRKAER

EMGLQPAVTELDISLPFEQSI

>SG-AL_contig_9842_1_-2_23_889

MNVGVQTLETIKEWYNNTMAQADPRVLGFPLMQGPERLAMVISLYLLAVTQGPRVMAPFK

PLELTRLLVVYNFAMVALASYIFGLCVTIIIGDGFDFVCDTVDRTLSAKYGSTFIYEAGW

WFMFMKIIEFADTMFFILRKKFSHVTFLHVYHHSTMAIISWIGFKFVPGGQTIMYPLVNS

FIHIIMYTYYGMAAMGPHMQKYLWWKRYLTMLQISQFVLLIIQTAVNAVSPCEFPKIFSY

TVVFYTFSILLLFLNFYHKAYLRRHAKSKTTTTTTTTISSSGDNDKKKE

>SG-AL_contig_9843_1_+1_943_1371

MPTGPTSVGQQVLFNRYTPISKACFGSIQKVFSYLAGEVDPWHPSAQCSGPAAAPVGICY

SSYTCLSTVIALLFVTQVEKWLPDLQDVWLHNSVFFDIFSSVSSVCSLFLHCRHLLFVLR

FFVIDTSHHCMPSSFLHIDFNVW

>SG-AL_contig_9844_1_-1_211_309

MLGFRQLTKKSSIVCVFAGVIISVDFNFHLFIL

>SG-AL_contig_9845_1_-1_466_732

MNYDSDENDYSADANINRSDQSSNKDDVIPSTLTNRSKIVTTSQSGHGTSQQTFINKLSV

KQRQQKLAKDAIYTFANNILKYEWNLLSS

>SG-AL_contig_9846_1_-3_717_920

MDVKKIINKKKTHNQCQILISVFLCSCWGRNAFCVGMLSLRLWILTAVWLQSSFLTDLQI

ILIIHYFC

>SG-AL_contig_9847_1_-2_2_139

GVKGLRTRAKDLPDSGRPFARVLCRRCYCSFLQVQLIIYLHCYVNG

>SG-AL_contig_9848_1_-1_1_156

QSSDTVIHIIAFGCPERVNHLNYSSNEKDEYMGLDDLSPICFAKDFPEIFLW

>SG-AL_contig_9849_1_-3_3_179

LTACWSSRECLEHQMGGLVLGKGDNQSGSVKTYVDSDFNFSSSLSFSLYVLLPLITVLP

>SG-AL_contig_9850_1_-3_3_272

IHYPRNAPTSSQYTTLSPLTTYTLCLSFILTLCLFPPHYFSSYFCHFWIKRKSPPPKKIN

NKNKKTHQTPFHVVGAFAALSWRWYQCLSD

>SG-AL_contig_9851_1_-1_352_1998

MQSLKRRLSFRKKKDHLPECSKPHQWQEDEKKVREGTCSFQVRYLGCTEVFDSRGMQVCE

DAVKALKAGKGKYQRAVLYVSGDALRVVDEISKSMIVDQTIEKVSFCAPDRNHEKGFAYI

CRDGTTRRWMCHGFMAVKESVSGERLSHAVGCAFAICLEKKQKRDKETGVTVTYSQDRTS

FTRTGSFRQTTLTERIVDPQSAIVAIEPVPVRKIENPFAVQRPHASATALQRQGSFRGFE

KLQEVSSPFKRTLSLRLNDLPSTLQRQNAVLESPSQNGNGVGETIQEVSPEHEDSITMMC

QQLTQGLSAMSSEDPFANYGCGGVQGHVSSSSTGAHLTHQASMPVSSGISQQHVLTRQHT

SPASHTIMPASHSSPQMMATNPWANSSADSRVPDPTMTSVPASSPNPPAQASPSNPFVAS

AHNGSPAATYASASPAQQHHQRLQGLQHNRSHSIDTGELSMPGRGWTQHQAAQQAQGQKP

TLLSMARQGGANFQQQNGAAASPWPQPVSSDTTGGSQGVDPFDVAWAAKAVNKPNTFSGK

QVKQFEVQL

>SG-AL_contig_9852_1_-2_2_130

SPAEETCLEQRMICVCVCVCVCVCESVCESVSVCVCMYVCVCV

>SG-AL_contig_9853_1_-1_1_123

ETPHECNRLLYNICNLDLLERTLYSRKDIVHYISLCIKVTL

>SG-AL_contig_9854_1_+2_23_259

MKEDDAARVRAGWALGGSPWRRLCTEFLGNRVGFRPKQSKTGTVLHKLEMVECMTLNVRT

NEGLLDDLCIIHLMTKSQK

>SG-AL_contig_9855_1_-1_157_312

MRSFQESSSSLLQSRLNGWLAFSACHHERSSQHMTERGKSRWQARCVSPCPC

>SG-AL_contig_9856_1_-3_120_1910

MNEGVQTDMDKGVAVSSQEAPADYSLKYKAGIAPARVTKKASEYQKEYSWKKGVPASPLL

SAEQIVYNSNAQISPLKKAVVPKRTEYQMQFKTYKMPEVVSLAPKPSQEPPVQRGRSKTQ

MKRSKSVGAFVSPDRMQPAGSAPVTSADDPRLLRESRNSSPVPKVPVSHGKLKHVASEYR

SNFRSPAVFSYESGAWKNAYPPQLLQTKNGSEKKAEPSGEEDAPLSSWFAEVLELRRRAN

EYKRRAQGTHFSREHLVQLLAQQTGYWDADTARSSTVASLDALALEPAKPAGLPSFKSKT

EGGQTSVAMDKMSMMAIRGDSTENGDDSASTVCNSQQPTQKGEKMGKRGRKGKRSRMAWQ

AETASERESAVEEQEAQDTNQNAACDAQSTEDVDEMGDAEEQGRLPTPILRQQREKVQRH

HLDRTTPSVGGVLLSVPEQLQFSARSGDSEETVTESQPSPKAKFSTEKLHTSPLLGKPTP

DTHALRDDGVESDQALDTQYIASPQVTLAKSGRNLKALQRRLNAKVPATIDERMGETYNK

DLGNFHVEDLPLGKLGDKDEDVLSLSAMSIASSGSLASEVYERARRRRDQFWGKPKQ

>SG-AL_contig_9857_1_-3_1239_1463

MKRVYAHCQSEERSLLCKSCSITWVPSVQTLYDVSYSSSQFMPEGLYIVVFVFGGLQCKL

VFTVLFVPVHSSTYT

>SG-AL_contig_9858_1_-1_568_855

MSILNPQTREVRALATFSVFSHSAPLKDTLAEAYGRSEDFINHNLRDIHMIGADSSLGVS

IVAANITISHLWLCSSNQRGDTPFHHYLNSAPVFIW

>SG-AL_contig_9859_1_-3_3_605

SDRLRLSALPNPLQGGEEGQDRAPATHPGSSLLASSDLVSGPTGALTRASSGSASQEGPT

PTATVRGGAPEPSPVSPPCVAAVRTHLCPPDRTGPAPRSAAIQAALSAKGASRDLKALVA

DARRPGTENLYDFRWRKWLEWCKEHNVPHIKLSSLQLANFLAYLHAQKGLSASSVQGYRS

AICTSILQLRGLSFESDPLLR

>SG-AL_contig_9860_1_-3_129_602

MLQEFMGGKELNKSIHPDEAVAYGAAVQAAVLTGDQSEAIRDVLLVDVAPLSLGIETAGG

VMTKLVERNTTIPTKTSQTFTTYSDNQPAVTIQVYEGERAMTKDNNLLGRFELTGIPPAP

RGVPQIDVSFDIDANGIMTVTAQDKSTGKSSNVTIKND

>SG-AL_contig_9861_1_-3_93_761

MTRLVERNTTIPTKTSQTFTTYADNQPAVTIQVYEGERAMTKDNNLLGRFELTGIPPAPR

GVPQIDVSFDIDANGIMKVTAQDKSTGKSSNVTIKNDRGRLSAEDIERMVNEAEKFKEAD

EKQRQRVESRNKLESYVFSVRQALGDAGDKLTTEDREVVSKACEDSLTWLDSNSLAEKDE

YEHKLQELQKLCSPLMAKIHSAGQNCNSSSSCGSNGPTVEEMD

>SG-AL_contig_9862_1_+3_87_272

MRMTSNTENKQNSQMKKIIKTKNARTNKWLLNELKIKQTTPKKNTTNKKYYIPAPNNLAD

KK

>SG-AL_contig_9863_1_-2_2_202

LFFFSGYFRSDLETQFMLLPLLFQFYVLCGIDFVLVCVSGSWCKCNFCLNDKSKGIKSGG

VLYIKKK

>SG-AL_contig_9864_1_-3_108_311

MKSPLCGHHCLLFRDESFCTVSHCIIFCGLFVTLFIVYLMSFNYVLCVTLALSLWPKALP

PNGTSEML

>SG-AL_contig_9865_1_+2_2_280

PIFFFNQVVPLFCHIHRVTRRVHHIIPLLPPNHTDLNDTDILTTKGQNNVNSDYSVDFLV

WLPITTQHEEKACILTHTRYSVLQQEYSKRVLQ

>SG-AL_contig_9866_1_+1_1315_1560

MQVIEHLPRYTQLVRCHSVIDATTFYELIFIQIPWVFMHTSHPGLSFLARSPTQTISNLY

KQNFFAIRNFLHSCISHSALSH

>SG-AL_contig_9867_1_-3_3_176

FFSLLNNEQFIRCHHKRRGVETKTTKNNQSLQSVHLAQGKCYQELKYIYTIVFTLLPQ

>SG-AL_contig_9868_1_-3_564_788

MGWVECKLRESWRALLGRYGWRALYFITVSVLNVGFPQKKNPLYCSGRDMLTREVQQRLY

YACVSMFRSRATLFR

>SG-AL_contig_9869_1_-3_78_485

MGMALTMLARREIGILTISLHYDLRILRQALPFSFTFIFFWCIPVQCLFLYACCVCWLLV

LIPECFEADMVTTKSGTSVGVQSIFEPNIPCQRSSVWSVGCRKTVLTSHWHHHSSLWCWT

IFLYSISLQSFFFFCC

>SG-AL_contig_9870_1_+1_553_795

MVATWLDFAKKHATICLEALSFLLNFTGGLLCPRRKETLHQIFLAHGCTSSPYPASVLHS

GYGTPNGHNISSRQGSWEECK

>SG-AL_contig_9871_1_-1_1522_1953

MRKRYADSVPHNLSEEQKQGRVNWCTHMLRKFDGGRSPHVWDMVTGDEMWIYQYDPETKQ

QLAVWVFPGENPPVKLKRNKTASKQMEACFFAKSGHVATIPLENRKTLTVHWYINHCYYY

YHYYFRSFLYSAVLQTVRLTAHYT

>SG-AL_contig_9872_1_+1_1_231

HGGDTLIQKLEELFTSCWNEGVVPQDMRDAVIVSLYKNKGSKSDCSNYRGVTLLSIAGKI

LARVLLDRLIPSIAEVN

>SG-AL_contig_9873_1_+1_121_516

MDSRSHVLSSILACNIKLACSLPILMLQSFWWCQCFIGPVPVPFSGPCQCKGFWAVSRSL

SDSCWQIWMECIWNCLSVMNHLLNKLQTHGFFLVCFSTASSSAPLLCHYPHTAHFGSPTH

LTPPNPPKLRSP

>SG-AL_contig_9874_1_-1_91_378

MHACIVVMFIQRIFSWSCFGIQSKWIFFFLIVEFFVSIDITHQVPAVNLHSSSILRKETL

IQIGLYQQFVCLLTDRMVFTAGCLPHFVHVQWPTTF

>SG-AL_contig_9875_1_+3_3_137

VQDKKKKRRKSSWHGMMKWRSHKHSRHVPLGGSRGMPPEIYKKSD

>SG-AL_contig_9876_1_+3_171_512

MTFQPVDLHLIYFIYVMLAQHTQLMSQRCNLPQMLPQPFLLHNCFHPIPFPTTFHAGIVK

IRSSVEARPVNVTVLVHDQSIRHFGQESFLKRVVVIHFQYFCVWSRITQNKNTH

>SG-AL_contig_9877_1_-3_186_356

MQSCEHLNSLEQTELKVTLKIDQCVHTTLVASIFSCYRVAEQLPLCTRFHCARMFVR

>SG-AL_contig_9878_1_+3_819_1097

MYHPSLCPTLSYSCVHTHICTDKNMHAHIHTHTTCTSMLIHMNTHVCTNTARNLCQHIFF

IHTHHTHTRTRSSQNSLMEINIGQIKGEKMVIL

>SG-AL_contig_9879_1_-1_151_1926

MVRSGLCVVVRHIVKVADAALPGQSLKQLLGFRQGSLRMCAEVSGWTKLCEVELPHSLSC

LVHQIHRLSVQSTTPPKDRATELPADLIKLEAHFLKPPKVHNDDRRRRTELSMLQADVAD

PEKRDSVLQNGRLDGTCFELERVTKLPHGWKKFKVQKDVVEVNGGGGEGLMTGVVDQSEG

LDRCGKGEADTEKLCQVIRSLNYLDIQFVHLYSEGVEMTIADLCLFSYFYFLLESVQFSV

CGLSEHLPRVVSWLRHMITLPRLELASTQCGFNFAGLKQALKDNMAEGGERNGLHFFTPA

MEDVTEDDMELSRRCRTKHKAIKPEVLQALDNMKDRGIEPTVGAHPRGSEVGLAWDTFPA

FVHPKDGEVPAKRVQRKCQQLENLVTAVMEVARPGDIIVDFCSGGGHLGIAIAYLLPQCT

VYLVENKEESLAKAQSRMAAMNSDNIILYQCNLDYFVGKFDVGVCLHACGSATDMVLHQC

LSNHAAFVICPCCYGSIQRTHLLTYPRSRLYQDRQVTYMEFLTLGHAADQTEFNIALEQQ

GRQCMNLVDSDRAQLARECGYTVTLTSLQPLACSPKNNLLIGQKRLTAAVTS

>SG-AL_contig_9880_1_-3_177_1463

MSSLRSAWRLTHVTDFPQNVGVFVACHNWQNRCRQINRRCSTWQCVKLFQNYGCQRLPLV

HSNVSNQTQRRLFSASKQCSVNRSFFLCSSKSSRKSASVIATSATEARELLNTENTQLRP

RYIYVHTSVNRWEDVPGGLLFDTAYLDSMPGEKINPSVPLVVSLHNSPGSFYDMQPVLAA

FIKNGCRVLAPAFPGSGYTEGILASYSDVFSHSTSEKAEFVRDFLYTLGVKKVDLLIGHG

AGCYPAMRLTAGKDTKDMFKSTAFLSPWPHRHFRGTKQKNLIPLLHDMWERPYYRPMARA

LSWFLANTANTKSQDRITTVYTLYNLDFSEVGGLNVALGMQNVPRVMFFAEDDPFVEAEL

LYELAEMMGIDSDHIRHFTGQLEREQMSQFPSCLVFDKGGQNVHLKQPGVISAVLFNLLK

SVRPAFLAV

>SG-AL_contig_9881_1_-2_593_4570

MEKRLPKRAGSKKKVESQRGYRYVTPAVRLERKQISMLNPPPGPSQPPAPDSHTDSTSDA

GTRTTIVGKKGPLEEEGSSLVLRAPPNGQSPKHTTEEPKTAPSKKEVPPAEEQALPGKYQ

KEEGEETSRTKRTEEETRTQELSPVTKEETQVTYQEQTKETQELSSRTTTREPEVRTTST

TEREPSTKDDGSRSPTTTKTTSAVGRPNVQFADQPTRLETADLVEFFEEQAPVRKDSEVQ

IAEQSVRSETTTVMEFFEEQPVRQESVHVARQTTKQETAEVIEFFEEQDHSLEDVNRSTL

LVVPSPQHSSLQRGQRSPSPGSFHASVGLHHLDNLIRLMEQLSNLKDENAKLRRKCDYLE

TTKNLLKARSELMSTSDSSSASGYQSLPAARHKGHHHHHHHHRHHHRDGSGTRDAEGGRP

RLFSAEDVQCIEISESASDSRPKRPKGAMHKRSFSTGSLEVEILDETSTTDGRITKSKSG

KSVFSSKSPKQKSKGSKWARVKKVLTGQFWYEDLGTTLKSLKEFGRSAQRYSTTSAEGQD

FSPPSQQRSLDTSLDTDLHGSRLPRVPSSSAEASSASRTPNIPGPGDSGGVEELGTEIWM

GPPGWWEQYEASKRQSEASNSSDVSSVIEVKTMYLGSNQKDTERLLKVKSLTRRQSSPSL

TTKDAEGEEEEADEEAHQVHRSASYKDEELDLPKELSVSSPSSKSDSKKLHRKAWGRVKD

IIHVRKDSVKKRSRRAAEREKESSEWSQGEEISEVDMEGLMEEQLSGVFAEGIVSRSTPK

TSPMVMPRTSSSSKSFSESPPQGADPSASAFAAYAKMATGAGGPEMAALLAGSLSEDFAQ

KLQAWQELQLRKSSSFKFGGGGEAEGESQAGVAGEGGGGGGTTTAADTQELLLSSSSPAI

TTGESTDRARAKGASASQPNLEGQVEGALLPAEPSPTEPQAPTVNVEELQRRISESFSRK

LAEWERRKYRRPTTPEMEPKSARSRKEKEDRSRSKKSREEKERERLEKAREREMQKVEKE

QVKLEKEKVRIEKERLRAMERQAKLERMKGRLSQADSEPPSSPAPISGSPPMSTALTSPA

LDSAAQYKVTADFKKKLHEWEQMKSSLTLPDQGASTPEGATPSPQPTTSPPTFTLGPSPP

GAGGEGGKPPPLSLQPYYPGDTPEDRSPVDKTSDASYGDDSTSVTEESYTRSNITSLERA

NSELLQEVARKEEEYGNLQEEVNKLNSKLDKVRQEHAAEMARFRMELEAGTVTGPVHLEV

GELESTVRELEEKIALMENAGDQLAESMETAAIGKWQSVEGEEAVSQQLVDMVEQMKGML

VQAAHT

>SG-AL_contig_9882_1_-1_16_1536

MAAYGVHLGTCSACLAVYHDGKTNVAANDVGDRATPCVVGFTACDMEVGASAKQGIVSNA

RNTVCHVKRILGLSFEDPVTQDYIASSSVKVVNQKNQPMFEVEFKEKKQQFSAESITEMI

YKKVLETAQSHGGKGIQDAVLAVPGDFSAVQRKAASAAASRAGFKVLRLINECSAAALAY

DLGQLDNTETFKVLVYRLGGTSHDATVLQVQNGMYRVLGSCTDHSFGSNNFTHVLQNYLA

AEFYKMYKLDPKESKRSMARLLLAAERCKHALSIVDNAKCDAESVMDGIDFHANISRVRF

ENLCPRTVEQCTQLIQSALTKASCSKEDISKVVMCGGGTNMPLVQRTVARFLPSSEVFSS

VPGDQLIAIGAAKEAANLANMDKSELEAAEENNTFQCLAKDIGIETADGVEIVLSAGTIF

PFRARHTTTLAADQTSFRMVICETEQESSAEAKKLAKLVMKDLPAGASIKTTFHLKREGG

LHVTAHEASSGQQESVHIPSMGIANHS

>SG-AL_contig_9883_1_+1_1_183

VPIYSPSLLCLPEPCTGSEVPIHSWVNWRLPHTVFHKSGSNSCSQTARQELCLCTDQGLC

G

>SG-AL_contig_9884_1_+2_2_136

HDALPIFFFVFFLQNCIISLMILLPHKETDHTNSAKQANNPPINP

>SG-AL_contig_9885_1_+1_1_294

PGLPTAPSSPTLPVLEERSAWEGKEGREAGYQNPILSGGARHPKSGVVVRVPPPNPSPGV

LEIFPRLQGTWSGHRVPARRGNNSGGPGLKGWPIQHTA

>SG-AL_contig_9886_1_+2_2_184

FFFFSMSGCMVALVYCSIDRINLKEEKKPVSIEQTVRLLMNIRMMKAILTIPSPFSPTGI

Q

>SG-AL_contig_9887_1_-1_46_885

MLDFFTIFSKGGIVLWCFQGTSQLFTPSVNDLVKNVILQQRTGTDTYNHGSLTLKYKLDN

EFELVFVVGYQKILQLSYIDKFLSDIQLEFRNKYKDELLHGSVSGNFDFTSSFHSVLRQR

EEESKQEARAPKKMRAFEDSQKSKKTVASMIENKKEEKPKGGNKGKENKTPKAPSAAAPK

VGATVAKPAVASPAATNGELDEETLRKNREAFFARGKKKTEAPKSPESASNKKKGKSARV

WANEGRDVKNLDFSGAAGDTEGAQNGSMEDSKSMPTDAER

>SG-AL_contig_9888_1_+1_1_222

SSAHTWACTGAKHVGGTVGGSSASYLCLPEQVLLYWEGQRRHVCECHEIRTPRLCRPHGC

DQDELRVLDPGLPH

>SG-AL_contig_9889_1_-2_1460_1687

MIKKQDFYRQFRRKPGIIFRSRSILPCHNFCIQAQILSVLISTFTLLFFLINLSFGYAIY

KHDAPVFPHFPQSKQG

>SG-AL_contig_9890_1_-2_5_322

MWLWCLLCVCVFSGESMIGHWVKPRQQNQEYTNSGKLKRWSKEVGTDPEIAGSQKRNVIG

YYSRVSDACVCHVVSTWDMLEQIRHTAMLYKGHNFCQIVGCAASVA

>SG-AL_contig_9891_1_-1_97_846

MSKYTLILVRHGESEWNQKNLFCGWHDADLSDTGVNEAKYAAELIKKKGFSFDVAFTSVL

KRAIKTLYLIQDGTDLHWVPVFRSWRLNERHYGGLQGLNKAETAAKHGEEQVKIWRRSYD

TPPPVLTPKDKEWSGNDPKYAKLDPKVIPASECLKDTVERVLPYWTDAIVPQLKEGKKVM

IAAHGNSLRALVKYLDEMSDADIMGLNIPTGIPLVYELDANLKPIKHYYLATEEEVKAAE

AKVANQGKAK

>SG-AL_contig_9892_1_+2_119_409

MCSMHLAYVKESMATKRLSLAKFYRKSGPIGEQKHKLQKRKKKGSGAVQWRHAFPGVRNL

HYYYYILFTAQPTIKAISGLSPHKVHQSQEDNSIDYK

>SG-AL_contig_9893_1_+3_3_191

YSKLYNKRGLDFSKLYNKTGLDFSKVYNKRGLNYSNLYNKRGLDFSKLYKVERTTKYHII

NVD

>SG-AL_contig_9894_1_+1_346_630

MQWLYHYKNSPAQVFLCNVVYLSAHDSASPDLDFRATPMCIDSYTATSSLRPNHHYYYLH

LQNFACYKIKFNVLSGLKFLSYPKSLIFSVNVTEV

>SG-AL_contig_9895_1_-1_1372_1605

MQVVQTHMVSISIVWIRSNWTVLFFCPFLFVRFYVSLNWTVFFFFFFVFFYVFVVSLVIT

EFFCMGGMLLFLGKAHLS

>SG-AL_contig_9896_1_-2_113_292

MCMYEYFFFCVRVLYCYLDLTCVIWTGSKQRNKQMSVCVCVNPLTADLTKAGQYSDSELN

>SG-AL_contig_9897_1_-3_3_827

MDRSRREVDESMVMLEEHQKEASRLQQTSEIELEKVKARLHQRLQELEPLPELLRTTELR

LHEANEKLLQYERRNTDNTKLIAELTAKVETRGESLDKLRDKFSDSQEESRMMQSKVDAM

DHRIKELDTANKELAAQVARKEEALHQVNLRLEEKGRESGVLTRQLENALSDLRQQQDNA

RDRFASKERTYQSRVVDLESQLSQCRAEIARIKREKEENERKFNSRLFDLKDRLEQCHST

NRSMQNYVQFLKNSYANVFGETAPYGPTPLKPTLP

>SG-AL_contig_9898_1_-3_120_716

MAQIGQLAIQKPAPEFSAQAVVDGEFKTIKLSQYKGKYVVLFFYPLDFTFVCPTEIIAFS

DRVEEFRSINCEVLAASTDSVFSHLAWVNTPRKQGGLGSMNIPLLADKTLEISRQYGCLK

EDEGIAFRALYIIDSKGNLRQITMNDLPVGRSVDETLRLVQAFQFTDEHGEVCPAGWKPG

ADTIKPGVKESKSYFSKAN

>SG-AL_contig_9899_1_+2_260_550

MTKDIRKIKNHTYISNSHERTNSQVTWQSPPNDYIFRNFYSVWKFKSLQIFSFFLNKRVT

RKFNSFHVIFLCLTPSLPQYNSILVLLCARAKFCFLR

>SG-AL_contig_9900_1_+3_129_236

MLKCILCECETELEGACMVILCTCDHESEWKWVRSD

>SG-AL_contig_9901_1_-3_69_221

MLRHINVINLAHKSLNPPTSTQTHTITCAQDNHARAVKLHFTHKVHLNTHV

>SG-AL_contig_9902_1_+1_88_417

MKISSEMTVLAGLGPGGSRSAPPNFFHVFLCAIDLDTFRVFVWYVLTVLTIFPSSLYLYS

VIFFSPFFSSNSSVFGGSTPSSPSSTCSAQLFPADMILSGMVQSGCGIMN

>SG-AL_contig_9903_1_+3_3_140

IQQSYNTLSKNFTEPLTCDLIKQKLYFPSFKYTYSVIYYAPRPRWW

>SG-AL_contig_9904_1_-1_115_729

MDFLQILFALCVLSLTGIDAIWFHLGQNQKKCLREEIHKDVLVTGDYEAHENGHRVDLSV

TDSKGHILYSKENAVKGKFAFTTEEYDMFEVCFEGKTMSGVQAPDREVYLNLKHGVEAKS

YDELAKAEKLKPLEVELRRLEDLSKSIVDDFAYMRAREEEMRDTNESTHSRVMYFSIFSM

ACLLGLATWQVLYLRRYFKAKKLIE

>SG-AL_contig_9905_1_+3_141_278

MKKYCCNSLQSFLRFTVGISLMNWLVMVMNKVPPINRCKKMRQNQR

>SG-AL_contig_9906_1_+3_885_1271

MNEASCLGVNRTGVQDEVCMLGVVTSNGCRASVWRGVCTQLRWFQFHTRQFCFQGENFTG

PLGLMLEKSVDCPLTSVNWRWVPALLISWWACMWVSCWAFVLPIPRIASPGQSAPALGVP

GCTFLTDNC

>SG-AL_contig_9907_1_-3_123_842

MAEENGEEVMLCASDDIPQGIEEQRVGQQDASEDPELEAIKARVREMEAEAEKLKEMQNE

VEKQMNLSSPTSAATPSPFPSQEEKIESDARSVYVGNVDYGATAEELEQHFHGCGSVNRV

TILCDKFSGHPKGFAYVEFADKDSVVTAMALDDSLFRGRQIKVNPKRTNRPGISTTNRGR

GRGGFRGGRGRGSGFGGGFTGGYNGGYAGGYAGGYGYGGGFTPRARRGRFRGRGNFYSPY

>SG-AL_contig_9908_1_+3_138_326

MFNIPHTHVRESVKMLSHTLSSPHNPSPVHRDKHTDITKNPPTIGSCFEPKAPLTFTICI

TRS

>SG-AL_contig_9909_1_-1_28_3771

MNPLSTDPYNKETKKYMASASPLIGDKSAVDDTLREVSNLIKSTSSLMPVASQVALAAHK

DASVSLPLPAKINNLPHQQLVIKHKAAANSQVLEVHQNGPTSLQGQHLPSSTHATVHHST

GITISHHQGQGGAHHPPSSALNNQASHQPPVSVPNSQGYGGWHHQPSSVINSLGQGGHLP

SAVHSQVVRTSLHPGSVPSGLPPGASPHLTSSVANEAVSASSLSAGHNSQSAAGLPFHNA

VHVIGNNTALAKNKGHKIVGKDLPSPVSQGLHPLVNSGKPSASQAPRTASQGHPSHTSSA

TAPYRNVALHHMAVTFSHSQHTASPGSLGPHSSTVSPGTHNQGSGIQGAPSQVPVSHGST

SLVPVSQDSASQPPTSHTAGDKLAASLPSLLSSSSTSAVHKTTATHTLASTGPQPVSSLG

SQPVSSSHKMIVNNALHSLIDLALDNSTREAQTTTAPGLRTSEGDQVKHVSANPSSVNTS

VNITTGLDLASVASDLVADTSLGDFALLGDSNSHNDDVFGAEDLALITEPRLSAYLDMGV

RHGTTNGNMGVSTETRAGVNITSMYTKRSSPINVNMLNHIYAAGLPQPRRLTESVQRLVK

PLPNPENSLLPGRTCKSPSASSRHSSNGSASPGRASQGGSRSPGASITGASPARNLTFDK

LTPNQSSAVHNSFVSAGSNSLASHMTHPTFGGSLNCVNTSVIPAAPHPLATLQSSASSRH

KTGNGSAVPANVSVALSTVGVASAVAVSSLPAASSSSSALSSSQSVMPHTVTDLAVSSHN

HGNGPTISQDSPQSRLQLNHPRYSVSGPVSKVPQPPAVSVHHPHSHPSMSINVAQPDLRQ

PESTAEDSIVSRVSSPAVSSLPSSVSAVCNRVAIRPSHQAVPPDPHASVSVCNTSRVENV

TGKTSIHPPSNVPHPLLTSLSSVHHLSPSSLSSPVMCSSSSVVAQLHLPAACVSPTHQNS

TVTSVSSSFDLSTHTSVVTAGHVHPASAATSSVCVSNMLAASTSAEFPKTPRLVSVGHSV

TSQSQHGLAVTGRVTSAQGIASLNMTSSRGTSTEMRSEPTGQTFSSACEHSTVPSSLSDS

LAKITHSGRILPQSSPPVTVDQRGTGVINTYTVGAHLSAKPSSKGSTSASSDSCVSKVPS

MAENLSHTGRLKESDGEEKVSGPSATVPVSECSIQDSTPNSQCGQTENLHSKPPLLQPAP

PLTLPTLIPSATPPPPPLTPVLLQDTGKKEVISNPCQAPLQTNMEGSV

>SG-AL_contig_9910_1_+3_3_152

GQNSTPNLRSSRQCKVNINGFKQQLHAIIQPMHRVWSQRLVEITVVLGNN

>SG-AL_contig_9911_1_-3_3_74

EMIALFSVSLHCMLHFISCCLADL

>SG-AL_contig_9912_1_-3_435_716

MNNFLYILKAVIMTPMLNCCDDFYVPITVWICVCISCSYEDDAINYIYSIVNSVMVKIGC

CCDKCTRQICIFENVNCGITAFGFIQEPCASTLY

>SG-AL_contig_9913_1_+1_1_201

EGEKGGGGGGGEETDRKRQTETCSMKYISYTSLIKMDSLVTNATDHFTHFEFSRKLIQIP

MIRAINT

>SG-AL_contig_9914_1_-1_157_420

MAGEALKDPHMQRFVEMETQRQRFQQLVHSLTEKCWDTCMGNPGHKLDSRTESCITNCVE

RFIDTTNYVMNRLESTPAPSGGGESSGW

>SG-AL_contig_9915_1_-3_270_515

MAYVGVFFSFRKGDIQLHSLCSPHSLKLRVLPTVMPDLFLTCRLVVSLAFPCLKMVVRVV

YQKHQTVYPCIDHTFILICLHS

>SG-AL_contig_9916_1_-1_1_150

SLSLNLSLCGRVGGEVLVGGKSVAVIILNFHCCKFSRLEMQLVCFSSKSE

>SG-AL_contig_9917_1_-3_63_251

MKKHRREGVEKTGSILDHRVDLLAGPLPFNLLSLPKFNFIIIYLNKKKEKRTVFEAQCIS

FLM

>SG-AL_contig_9918_1_+3_3_203

LLLSLFINTHTHRGMCMPTHAYTLISASRFKSCWIFSLIYILLSYIYLENSLRCETLVSK

HIQIVLQ

>SG-AL_contig_9919_1_-2_83_1531

MSQSKMQAYMERHKLGVLFEDLMNRILRDMPEEPMIYLLRTIYKKAGMEIPQDIRYGGLR

KSVDLVRSRSPERANRSGQHAVDPKSTSPSREYERPWLTHQRRMKPRRSADDHDASGAKT

GKKRPEWQGDTKILATSFDELWEDREGQPRDSAAPPGKKPKSKNDPRSSWAAVGLESGDT

FTSGDYHGPIHQTSGSEEDLLAAEMMRSSGRDTDSPQDSAELQSVSSSSVRGQRADSERH

RRELEGMLHSGESQKPSASVTRDDSMADVEDEALELLEDAEDLQREGVRNIPATGYKLSK

IMRERTNENVVKLNINLSPRGYGGMRVSDSMQSFGGYESDRENASPAGGRWESEDEFESV

SQVTGPRRPVWNVPDSDGETAVPRSNRKHHRKHQQVRQDLTASAPNPLRPVTLTAGDLDL

AANQSWSPGLKVQTEDSSPELERKTDITSARSGGWVVPDDSDAASVQDWNQRVKGRKRDP

RTY

>SG-AL_contig_9920_1_-2_149_415

MEQLIQTDSCSNSLHTVRLWTKGRHIHVCIISIVLKEDWFKSTLGVSLYIYLSVYGQQFF

SRNLLLSLCMGDLYRMFPRGKVGNENFSL

>SG-AL_contig_9921_1_+1_1_162

FFFFKDNSFINTRLNNLHTSPIVNSWQEIQCFDSFHIKKSSQNKILEKKWVFQF

>SG-AL_contig_9922_1_-2_131_274

MLFINSHLLQIIILQMLFWHTHKQEYYVIHVVNPTLICFHNLEYKGQN

>SG-AL_contig_9923_1_-2_2030_2170

MLCLLFCEFTVRTSSPNKRVVEPVVFFSNSCMLYKIMTPGYTQCLIL

>SG-AL_contig_9924_1_-3_3_242

FFPFPAPPRTHTPRKRGPFLGFWGPPGGGPPPPGGGGPVGPSPPPPPPPKKKTFVIFFPE

IKTDQQCTNLKTLILKILTV

>SG-AL_contig_9925_1_-1_25_3765

MQVQDFMVTELEILNSQRLDLIESLSDLKPPASTKSAVYKWRQQMQKITQEIDAVSQMHM

SKLHEEMEKVCQFCLEKIESIKQSLMDAGVLTESKAKQVIEEQMLPLVGEQQRIFENNLE

TMEKAQEEHSRKMEKEMHSLFKFAQGVAHLWDVHEIGLAKQERALQEKLEDCRHDHDYAN

QEREANLDIVMDRMRQDATEDALRDSLGKALEMLTKISEYYMEFHKDQTAIVHTYPSMVQ

SELDNFDNAVCTFFSVDRTHPDEKVRQEAETAATEAEAAATEQENASRSPSVLLSSRGSA

RDGKKSPKKPRTEDEGSTESVKPVTMAVGEILSTEKGTTFYVLTVAGEHGVPDDALAAEK

KDDPLSVGGAFVTELPALAEDKEVDQEEEGKPDYISCVDLPPSHILTSKKKMRMNFLNHL

ETWCEQAKERAASVVVAKCEELNSELDLRLHLHQPRSRRAELDVHNVRAAELVMHAERVT

RHCKGISQSLTELRSRFSAMSSQHNKLAQKFRQDIEALEVIFINATKSSRLVALQNQLGV

ELEKFMSVIRTSLRQFRQHLDETLQMLRESNARFIKSFKLFSDGGNFCPEEIEEYRKRLE

KMSNKIDSAEGSIMSELEGMESKRLDVATKVSNEFEDRFKSHMFDLLFMEKVARWLTNTQ

VKIKAEVAASNSQAQALATHLTDLERRIDACEKPNLDKEQITASQLNEFLKVMMEAFHRR

SEYLNCLRTGGPNRPPSNQPPTGQMSDSFDNLQGLSPNASKVGFTGDGVTPVSKPGKQPS

EDPSVNVIKSILKTQKAKFRFGSDAEMEGDFGTTGGAAPVATPASVMPGSEGVREKTKSA

LSQASTRLNGSEKTAGSRRPTLPSIPASDMSTGQRRVPSGQSQRRSTKLAKTDKKFLLFG

EKETEEGVSASIAEETHFVASIQRTLREALDGLLATADAYYRQKGVRVVTRPQVLQETYE

QCQEVVTQKLQSYYQQCDDYHNQCLQEFRQQLTKMEQLSARVPALVINDLVKVHVGRAQE

AHRTLQQQFQASLDDINKKQKDNENSLRPSMGHPHLAPELEALCQQESVRHDDYLMAVAK

QTKSLQACAVDHARTVLDALTRLSEQQLTQFDGLLVVDDVEKGRVEPTRYPTSELIRRKN

AGEALEDDEDKDALPRGKGNWLGIPNNQLVAENRPTKLQLTSTVQTAKTTLGHSATIKAR

DSAYEDCKVQFEKTLANIEKDKERLARDEQHWTASWLTSVQRVKDLY

>SG-AL_contig_9926_1_-2_125_751

MTSSGEFGNPLRKFKLVFLGEQSVGKTSLITRFMYDSFDNTYQATIGIDFLSKTMYLEDR

TIRLQLWDTAGQERFRSLIPSYIRDSSVAVVVYDITNANSFHQTSKWIDDVRTERGSDVI

IMLVGNKTDLSDKRQVTTEEGERKAKELNVMFIETSAKAGYNVKQLFRRVAAALPGMEQP

ERRPGNENIELKPKEPMMDSLEQDASCSC

>SG-AL_contig_9927_1_-2_119_370

MSAVIDAVVFCSSSSSSSSSKSGYIIRSCCCWHCCHCLVVFLVHHHHLKVDYSLNNKMFR

GKWMVFLSISLFPFLFCSLAFFFA

>SG-AL_contig_9928_1_-1_1093_1500

MTTSFGHIKLKLKIKEEPRGKKPERKQKTSEPYQIICLFPGETTHIRTRGAIMRQADGVT

SLVVIYNLYESDLQHCSMSLKLIRLQFFNKLKIIPKSGGRERERERGEGGGWNGRGGMSL

ISSRKVQAQRFSNVWS

>SG-AL_contig_9929_1_-1_91_618

MIGGSGIRAWGGSDRDYTYDELLSRVFDIMREKNPEMIAGDKKRFVMRPPQVLRVGTRKT

SFANFADICRLLHRQPKHVLAFLLAELGTSGSVDGNNQLLIRGKYTQKQIENVLRRYIKE

YVTCHTCRSRDTILQKDTRLFFLQCETCGSRCSVASIKTGFQAVTGKRAAIRAKTA

>SG-AL_contig_9930_1_+2_1793_2134

MVMPHILSTHTSEACSSWLGCMYALSWWVGPYCAGNISEFCGLEEETKDKQEFLKKMQVN

GPIRLKEKKRKGRIKEVTEKRAFLAQNYVQMRKLLLFAFPSLTLWGSPNTYALC

>SG-AL_contig_9931_1_-3_270_443

MSRLLLSCMYMHNQYRFLFQFWCVVMLMCASMWEKKGIIYMCVCVCVCACFEIPILCQ

>SG-AL_contig_9932_1_+3_267_422

MDKRQILFFNLISQQQQKKLLNLTFLAITMIAGCEQSKLSQILENIFFPYKK

>SG-AL_contig_9933_1_-1_1_117

RDGHTLIMLAPDKIGLSTTSPHSPTTSGYLGGNPVLFII

>SG-AL_contig_9934_1_+2_488_664

MQEDVEVLPSEFHGQLVEGSEINQQVNASHELVTLLLDNWTWDRYFLLFFFYQISQQCS

>SG-AL_contig_9935_1_+2_2_121

IHLYPQNTVESALFRALNLPPLSLASHEFFTASRVVTTLM

>SG-AL_contig_9936_1_+1_19_318

MVCRPGQQIHPLLSKLPVTRSNPHCSSHHGHITTTPTTCFSSLHSLCIVSSSVQVNKMCY

CLSDTPVRILTNNVVKGFNIMLLSWSLASLGLGLGVLDRY

>SG-AL_contig_9937_1_+2_2_97

AVMVCFHMLVSEAWLKHHQALRTHLVFLWNSS

>SG-AL_contig_9938_1_+2_2_187

MYIYIYTHTPTHKQTCRYICSTSTTLTPNTHTHAYTSATRHKRESRRKVKPCHAVSILDG

TP

>SG-AL_contig_9939_1_-2_2_223

SHATHSGWLLNIPPTPISFSDNPSAWKCMGVGDVKPPCGHPPEETALFAGSLWWSSPVPV

LIQLTKVTAYQAPY

>SG-AL_contig_9940_1_-3_108_1847

MAYYSVAHLLQGGIVDGSKPGPANIRPEQMTLEVWDYIFMQTSTYPSKCNIPKSTLDCLR

AEFQYWYPVDVRMSGKDLIQNHLTYFIYNHTAMWPQDNSKWPKGIRANGHILLNSEKMSK

STGNFLTLSDAIDKFSADGMRFSLADAGDTVEDANFVEKMADAGLLRLYTLLEWVRDMAA

QRDSLRSGPPETYTFSDRVFLSEINQTIRETQNHYDNYMFKEALRTGVFEFQGKRDKYRE

VESKGMHRDLVFRFIEVQALLMAPICPHMAEHIWGELQKMQTGSSGESIMHASWPVAGPE

DEVLVRASEYLMDAAHEFRLRLKTVLHPGKNKKASPPTHGTIWVARTYPPWQHSVLTTLK

ELFTVNQGFPDNKVISTELKKKTQLQKYMKKVMPFVQVAKENFSKHGQRALDLTSEFDEK

EVLETNLVYLCNTLELEGLDIQFSEEADEKVRDDCCPGKPFISYRTEPSVSIRLVNQQQC

SGLFEVSLPVFEGDVTTKIAARLMKGDRSKIKDVSAVKLLRYKDPESGWRSIPDLNGAGD

NKVTVSTSALFHINTSTNTVTVEENGRKVDLGRQIVYLVA

>SG-AL_contig_9941_1_-3_3_77

FFFFGGGCWFLLLLLLLLLLLFLGV

>SG-AL_contig_9942_1_-3_3_188

FFFHFFSPSQETVRIIVISLFVSSVFKVGQVCIHVGGVFFCDCLFPCMHLKREKLCFVSS

IL

>SG-AL_contig_9943_1_-2_2_829

KIVRTTERSGLIRARIAGAKAATGDVLIFLDSHCEVGPGWIEPLLARIKEDRRAVLCPEI

DLIDKDTLQYGGTGSFSVGGFWWSLHFSWRPIPPHEQKRRAELPDSSVSPIRSPTMAGGL

FAADRKYFFEIGAYDPGMDVWGGENLEISFRVWMCGGSLEFIPCSRVGHIFRSSHPYTFP

GNKDTHGINSMRLAEVWMDEYKRLFYTHRRDLLGQDYGDISERLELRKRLKCHSFKWYLD

NVYPEKFIPDENVHAWGMMRNPASNLCLDTLQKDEK

>SG-AL_contig_9944_1_+2_146_349

MTLTKGMLWHRARKLLSQLQCNLSIVFIFTDLIKMSCIGPVQAQFFPFVKSACYSVLTDT

PPPPKTTT

>SG-AL_contig_9945_1_-3_3_413

EYLWPFWLLLRSVYDSFKYQGLAFSVFFVCIAITSDVICYLFIPVQWLFFAASTYVWVQY

VWHTERGICLPTVSLWLLFVYIEASVRLRELKNLPYYLDLCRPFAAHCIGYPVVTLGFGF

KSYISYRLRLRRQKVVQ

>SG-AL_contig_9946_1_-1_61_606

MVGHHFTRRRALANGISVSGSAVGSFVLPNLMRYLLNQYGLRGALATIGAIMFHVCACAA

LFRPLTNYRHHRPAKREGSASAYSLNEEENVSASLPQDESVRSFQPKENDLTCHSLDEED

CNFSPEEDDITDLITPKPDESGELSGNMEESNVSSLQLNKDVEHLTGNSHTTPTSPTDDP

KP

>SG-AL_contig_9947_1_-1_1093_1311

MNAGADLKDRNMEYKLKWEWVFFFCVPVCCLTVKKSSRHFVYRSQITFIHFSSKITHTHH

EYWLERMGRAMFL

>SG-AL_contig_9948_1_-1_1_369

PSLSLSPPPSLSPSPQHTPLSLSLSLSLSLSLSLSLSYFPCSLPLSFCLSSYLFYSLPPP

SSLSHLLLFLPLPSPPSFSLPSRLQIQSLGKKGGRGSLMHSTEFCVVMLLLLLLISSSWK

IFC

>SG-AL_contig_9949_1_-2_83_436

MQNKISQQEETERQESHHVKRRMERHTFVSDDSFLEGLPKSDLFYITQLQDQLIKEGTLR

SQTDFDTFWERIQQPEIFYMYFRVPKSDDVTKRDDFNDNASSRAASTWKGSASTLQSS

>SG-AL_contig_9950_1_-1_139_312

MPKFLYTFIHNTCIYCVLNMNAGTQKLYICVHTSVVCVHVCLSAHDTCIDSYACAHTH

>SG-AL_contig_9951_1_-2_200_487

MDCSSMGSTLLVLQRAGSVQSEKDCVVSETVWEYLSIFNYVSVVLRIKLTLNVLVGVYLR

INSNRFRFMLCCVRGRLVTVILYCRYWCRYRRCCCC

>SG-AL_contig_9952_1_+3_3_194

FFFFSIPHFIGKRSLLQLVSKFCLFTSAMLTIQMNLLKQTNRQSHIYHPIQHQIIRYTPN

KSNM

>SG-AL_contig_9953_1_+1_1_219

KINTAIYIQEKLDYSQILPPFQKDTPNINACAARVKLDNSLILNVVSIYLSKGPDNLNTE

WLRILQDQNENWL

>SG-AL_contig_9954_1_-2_164_352

MYEDARYVTKCWVLLRIKVACQSCLSGFRTFLKPWFQTTLSGCWQGCHKCKGERHEQQMF

VQV

>SG-AL_contig_9955_1_+1_382_534

MTILFSTLTMQTLIPTLPCPIHQFPVLSAMIAKQKLNHFTPAPYSGSWRIG

>SG-AL_contig_9956_1_+2_152_307

MTGNIKHVHLLIRQVHMMTFTVLLTFILFSDQVLYATPVFKLVKSIFHMRKK

>SG-AL_contig_9957_1_+2_2_229

NKETPPPQQFYSQRFQIKQPTKQRHTKKLYMHVGCLDWSKCHGFSLKRPKQQKCINHDIN

KTNLNKTNTKASFVLL

>SG-AL_contig_9958_1_-2_11_391

MRDGIFVSQVHSRGPAIESGKISVGDRILNVTIGYENIVYEDALTILSYASPYPVQVTVQ

KEQTVPTNTQSGTSKQLNHPLYRSQSLDALQNIGRDPLFGPSRTHGRMKSDTQIKRKEVA

HKAKQIS

>SG-AL_contig_9959_1_+1_1_102

GKGCPLEPSQVKVPPQFLSGIRKDLKQSTSVTEI

>SG-AL_contig_9960_1_-1_181_300

MEDGIECFHITGTYDDKSSDSSSMNYPKFFLASATIELDY

>SG-AL_contig_9961_1_+1_322_606

MISTCVSTQLSSIVCHLVTKVARKQDHGRPLLPGQLLLSLPCLPLPLAILCCLERMQCGP

LAQTLIINQVFSLSLSFKENRPFHVGNDKVSGSLL

>SG-AL_contig_9962_1_+3_3_242

SSLQPGSPRGPPQGGPGGQGPPHRPASAGDWVFLVDRCTWVSSSSPQLEGELAWHPYSGC

WWDFRVSRCQDSGVSSPAAP

>SG-AL_contig_9963_1_+1_1_111

PIHQLPAEGVKGLNLSEPNQIIQIAVQAFAHHHQLLQ

>SG-AL_contig_9964_1_-2_2_217

LTIKNNNCHQYCHLCVTKRTAGHPNTHQKLCHATAAPSDIWFNIPVLTLLQSVSDKTPTL

YFLVNSSRNPGM

>SG-AL_contig_9965_1_-1_76_408

MATTLRTFTVRLLGLTRQGLGQTAVAVRHHSEDSSTTKSSKPKRRKGKTPAGKLDEEWEG

EEEERQPQGEQEPLERFPNDINPITGEIGGPRGPEPTRYGDWERKGRVTDF

>SG-AL_contig_9966_1_-3_3_143

TSEIHIPPVWGKNDIVHLFEKKTHLDQQKCIRVCLLIKSVNREILAL

>SG-AL_contig_9967_1_+3_3_329

LPVACLDSGGVGTGLDGLPILVFSTISFPSIYFPHTNTDRHCSEILNSLAGRVSRFQACY

FSAISRNSAKPVLNCRETQKFSEKKKIAQHNCATLRGTLTTRASSRLAA

>SG-AL_contig_9968_1_+2_194_412

MSTSLKNKLEMPDYYLLTTTPAGLQHVSPCWKVLEHVNLLCKVTQEYTPIRMCEMEKCVY

KQSQTLNQSESQE

>SG-AL_contig_9969_1_+2_2_253

PSCVYAHRTSNTHVKDPVSHASVPSFMETGQHRTCAPKMSVQDNYHGLSKLEVDYRIAQQ

IKKKNQDEILRRDQDRPSGSSSLL

>SG-AL_contig_9970_1_+1_1_117

LPYPIHQFPALSAMLAKQKLNPFTLSPCPIRKLVDKVRK

>SG-AL_contig_9971_1_+2_2_193

CSFRHGVLVAGTKIQFALRSASFCLLSSFLFLFTSHAFLFTQPLSSNVSSPLTKRVRGDM

LTMQ

>SG-AL_contig_9972_1_-3_165_341

MHTCIHIIYMTLQRRISNFHYICFQSINNHFTSLSIYHSTNQSFSKSADDPISSPSIQI

>SG-AL_contig_9973_1_+1_778_1050

MHPALSGYLTLYRVGDGKAAKERSGTHPQTPAPRVVRSLPPHSLSTQFRFMGLALPFHYY

HYFLNCRETRSEAPSYFQSVGLTESAMRKVT

>SG-AL_contig_9974_1_-1_154_324

MLCILLFDVFSLRNLVKCEGNLKLLYHTVTLWYTVFLHHSLVKLSPPWGCQGLFQYK

>SG-AL_contig_9975_1_-2_1670_1897

MTKTWQMSTMKVETVSGSVEDTYYNNIFSFSEFLDVLNFVNNRLVSFVLDVCNFPSVTSV

IAVLDVLRRRFVLTFA

>SG-AL_contig_9976_1_+1_3970_4260

MLLLLLLHLFIYFTVNLFIVMLSTLFICNVPNLVFHLRVVFKNTHCIILIASIFDKTQER

YNVSLRILYIYPAVFSFPGHTTLNPLTLSTSFLHYQP

>SG-AL_contig_9977_1_-2_2_211

CRDQTTHSAKDLQPQRRSVIKNKSGWGFTAQQNENTTHEDSAAYIITTSSLTIEVKAITY

ALKCLVHLIR

>SG-AL_contig_9978_1_-3_33_236

MSYITRSVSVVINFRGKNSENKSSSLQIRSVFFLFCDNFCAKHCSPYIFMIILLLFSHIV

VSEKYALK

>SG-AL_contig_9979_1_+2_2_142

FPLPLPCLRQVHVRFFGVGDLWRPSLERCVESLLTRELLTQFGSTTE

>SG-AL_contig_9980_1_-1_1_114

PTVHLALHIFFMIDLWPLLKCSCSNVLQSVFNVVLFKL

>SG-AL_contig_9981_1_+2_278_445

MQQRANTDSNKTTQRHTHAHESDRQKTNEKTTTKKRKHNNKEKMNTKSPTEREKSV

>SG-AL_contig_9982_1_+2_116_262

MERKKEMKIGNMREREREREVQVTAGNTITSVILARVTLPRVTLPHVYH

>SG-AL_contig_9983_1_+3_33_320

MIVDAIDERGTLTDDAIDETGTLIDDAIDETGTLTDDAIDETETLIDDATDQRETLLIVD

AIDEKETMMIVDDIDERETLIDDAIDETGTLMSLVR

>SG-AL_contig_9984_1_+3_180_314

MKRQDHTCLGKYGVGVLLQAQLSYMLIPFKYNWLTHKCCITKSII

>SG-AL_contig_9985_1_+1_67_234

MHSLHFRKIKTGCTSTNTIQILLFQKNENKTLNTYPPCDLSNSHALPVTGHRSILI

>SG-AL_contig_9986_1_-2_26_445

MAGGNKNKLSREEELLLQDFSRNVSTKSSALFYGNALIVSAIPIWLFWRIHQMDLYQSAI

FFVLGTITSTYMVAMAYRNVKFILKHKVAVKREDAVAQEVMRKLADDKKMTKKEKDERIL

WKKNEVADYEATTFSIFYNN

>SG-AL_contig_9987_1_+2_2_196

SGFAHPKDVAQPSPSRVSGFVHPTDVTQPSPSPLLNLTDRPADATSCGHLLTGDVLLPSN

FQNQL

>SG-AL_contig_9988_1_-1_1_60

QIDKSRSLRDNRSVKILKRQ

>SG-AL_contig_9989_1_-1_1_396

MAKVYKDKSQQPIVSLNDGGYNVTQSGLYLIYSSVMFHDIKPRQSQAVVVNGEKRFKCMD

SVDYVDKDLPPDQQHKALAKYKTCAMTGVTHLSVNDRLEVQNLYAQTEIDLSTDATYFGA

VLLSAGEINKSP

>SG-AL_contig_9990_1_-3_6_266

MQVKSVVLVAVISVILLTNESHTWVFGSGCRGRCKGAGSSGGRRYRSRATGIRYCLCDGP

VRFLARHRRHANTLLQQQQALQYHVAQ

>SG-AL_contig_9991_1_+3_3_338

WNDTVKAAIKEKKRLYKVWVKSCLDEGYVKYRTARRHSKRVVKAAKHESWKTYGEKPSKL

CKHSPMEFCKFESHDSEMKLLTLPQSSITKMETPCMMKTKSQKDGKNISKIF

>SG-AL_contig_9992_1_-1_1_234

LPSALGLAYFPSLPWPAVCNGGRRLYRSASPVLFSLSWCCLVCFPPLSCVFTPNHSSLSS

IPPFSVFSCFLVVSVRCL

>SG-AL_contig_9993_1_+2_26_202

MLCSSISVETCMSGLYSSAFLASTGPGSQVITACIASREPALLQDCRGCVFSFYNTAST

>SG-AL_contig_9994_1_-3_204_278

MFTNENTLHFLCVCVFVHLLACSFV

>SG-AL_contig_9995_1_-3_243_1556

MLSRLRQPISQAARQWFNVVANKSTQGAPAVGPIKIGAVSQAIFEREDTYGAHNYHPLPV

ALAKGEGVFVYDVEGNRYYDFLSAYSACNQGHCHPKIVAALKKQADIFTLSSRAFYNDVL

GEYEEHITRLFGYDKVLPMNTGVEGGETACKLARKWGYKVKKIPENQAKIVFVAGNFWGR

TLAAISSSTDPDSFGGYGPYMPGFSIIPYDDVSALEKELQDPNVCAFMFEPIQGEAGVVV

PQAGYLSKVRELCTKNKVLMIADEVQTGLCRTGMMLACDHESVRPDLVILGKALSGGVLP

VSAVLADDEVMLTILPGEHGSTFGGNPLACRVALASLDVLQEEKLAENADRMGCLLREEL

SKLPKQIVSVVRGKGLLNAIVIHTKHDAWDVCLKLRDNGLLAKPTHGDIIRFAPPLIITQ

PQLEECLDIITRTILHMK

>SG-AL_contig_9996_1_+3_3_149

LVQHSPSKNSKIHNEHYPTSSVLVVLVRRHFHMFIAQLTTEDISGLSPC

>SG-AL_contig_9997_1_+3_708_881

MQVLGRGRVGGYFYLFYEFTDFNLKPPNLPKTENRTSDQEDFFFYFYMTIFTVFILKH

>SG-AL_contig_9998_1_-3_141_1433

MNSLTRPLPHHCLWRCLPSSISGSSSLSVTQEVKQFHRLCQKRNLHVLLYSDNFRHDGFI

VASVGSRQQLHTAQKVCVEFKVQTGLQDSFSLLGVDPDCSDQELKAAYLDKAKAFHPDSS

ADTADPYKFSQVKDAYKAVLEHRKGRALEEEEEKEADIIFDIKHTAPQHRQYLEFEGVGY

GTPSQRQRQYQQHRVQRVTENVFQHRIQKLASETEDALVVRDKQQAKKAIISNALHRIVE

DLIQESMKKGDFNNLPGQGQPLEHSAHNPFVDVTTHNLNKILVNNGFAPEWIMLQSEIRK

EIRQAREILALTHSRLVQPRSAKDTKEWHAAVVKFKHQINEVNFKIHKFNLIVPFLNKQK

VVYEAEKEVKKVVDNVQDYLPEDSDIHLVWDVPLPEYSSSQSAGQQAIDWKQVWRDIKNV

FRSSQDKEINS

>SG-AL_contig_9999_1_-2_2_172

FCLFVSLSFCFCLSVCAVVFGFSFVQGQMYTDWPLVVSAIIDVMGNVTVVLLFNLRW

>SG-AL_contig_10000_1_+3_3_191

TTPGVRLTGQKCQCEKGVRLTSPMCHGQWRPVVNPEGVWLGRCWLGPLGRQRTTHDISQG

VFV

>SG-AL_contig_10001_1_-3_3_248

STTVIERAKHTSERRSSLPISLARLRDMERSEAPSKVRQPLTDQTNYQQVSTGCALLQAF

GYLCPIGSQVGSGTLHLRSGAT

>SG-AL_contig_10002_1_-3_426_1484

MPRDTPHSNLLTVPEDKEEKEEETPDWELEYTEEPHASRRREILRKHPEIKHLMGYDPRI

AFIVTAQLLAQVVLCWLLQDASWRIILLLGYVLSPIFNQSLGVAMHEMGHNLAFGHGRNA

WMNRVLGIWANLPLVVPMAITFKKYHADHHRYLGHHVDDVDVPCKLETYFFHHPFTKLLW

MVFHPLFYAVRPFYKNPKPLCGWEVINLIVQLVFDYLIFTHFGIKSLVYLLCGLAFGLSL

HPLAAHYISEHYFLRGGDPTHSYYGPLNWVLYNAGFHNEHHDFPYIPYSRLPEVKRIAPE

FYENLPQHSSWLRVLWDFIFDETVAPHAKGEAGVKKEEGCVQSEVIRYRPKVV

>SG-AL_contig_10003_1_+1_295_456

MSTRERRSHDYYNYLLKYIYYKYLLKSHVQITQIPPEEGLFSMCAYGRSTDIPV

>SG-AL_contig_10004_1_-2_2_178

YISMCQCAGLMRTLTAYAIIVLDSFPSHDVCCVGAYGIYFECGDAVLRQVKLKLVKIET

>SG-AL_contig_10005_1_-2_2_178

YISMCQCVGLMRTLTAYAIIVLDSFPSHDVCCVGAYGIYFQCGDAVLRQVKLKLVKIET

>SG-AL_contig_10006_1_+1_1_75

LRNSSNAGITLCLIILNADSKTAKE

>SG-AL_contig_10007_1_-2_191_451

MPEVTYLYCDVNTTNIVIQYLKSLRYVTSFQLFRDIMQQCRHALDPYITGHCDVKVTSLR

HRPIRNHLPGRWFCLFVCFLAGFFFFF

>SG-AL_contig_10008_1_-1_1_294

VALGECYEKLERLQEAKKCYRKAHSIGDVEGGALYKLAKLYARLSENDQAASAYTEYILE

AERRGVHSHDEISQAYRYLANYHWSRRQLDDAYAAAQK

>SG-AL_contig_10009_1_-2_374_1237

MMDAFTLLLILGLSLGSVVRAVEAKCPADYRKRVETCIMEAQLAPQAGGGVTLVLKKDTI

VALCDRGTLRKTIDCLEEIHSRCIYNTTVIQELDRLYSVPNWEQGEQLLCQDTHLFENNF

DCLFRSASSVTSCILMRTHTFRMGLAFTDPSNHVRVRDVTCSFAESIVKCICSPLESNCP

MDMTKRVAGTMRFFLPPYCLNDNANVIKTTPQPKTTTPDSLAKKVDPSLPDDTAKPAEWV

LLDETALPPEAIPPDDSTKQRESSLRNDITKLPGFVLPKDTSEPEVAE

>SG-AL_contig_10010_1_+3_102_275

MCTNINHILTNFPQFFYFINSNDTKRHRTFKAEGQFQSYHVFIWKHTHLQSNNTHTHT

>SG-AL_contig_10011_1_-1_1_327

GQQNLLGEHWSERRPQTQESQVPATHALGKQQKSNQKHVVFFDDQEQIPAKQEQIPAKQA

RLATSTASLTRDSGAPRQEMVTPVGSQVESTQVSQQHHSAIVQPVALPA

>SG-AL_contig_10012_1_+2_419_676

MERSESIINPRFLALSEKETVLDPICVCVFIYVCQHIGIMCRLMAYTIIISDPFPSRHLR

RWCMWNLFQRGDAVLTKVKLKLCVCV

>SG-AL_contig_10013_1_-2_125_298

MLLYCGREGVTTHDFLTSLHQIPTPQFKTINHTTNLAQSHVFMIVFLGYVLGGCVLLC

>SG-AL_contig_10014_1_-1_1786_1989

MKKRKDCEIHYFQDQSTCVCFKICFCWILSLSTDQRTKRGCLCVCENNCKRASMDIVLVK

GEIMGLFC

>SG-AL_contig_10015_1_-2_53_259

MTKTSKSHVCQVYETSDCDILWPLSTFLKNLFECCVDTEKGSIKQFCMNVEQILPKHSVV

SLDVLEPDW

>SG-AL_contig_10016_1_+1_148_243

MTCSTAHSSVMASASCTCQCVLYTQSYTQKAL

>SG-AL_contig_10017_1_+3_75_173

MADNTGSWLIGCRSGRINVHTLMSCVRAHTTYG

>SG-AL_contig_10018_1_-3_183_545

MQAHSQMCINMHIHAHHKYMNAHTHTHTMYTNVLKQVSIHCGCIQNIHFEKRWYLSLPQF

IHFLSIFICTIIHTYFLAQLLEFIPFYIYLLIRNNCFHFLCCCFVPCCVAKNFYTLKKFK

Y

>SG-AL_contig_10019_1_+3_78_233

MAESENTTVVCSVCNTPTTDANTKHNRNKTNAAEQLKMFVNELKIESIAIYF

>SG-AL_contig_10020_1_+1_376_579

MISCSYVCLPASMDQVNEIVDVAWSAYINIHHGFLQPCLKAFGAVDQEKRAKPESALQLT

ALLSSQHS

>SG-AL_contig_10021_1_-3_132_440

MYINENMNAMDLKTPAKVRRFAHACSSYATRVERVTLSNVRNVVMKARQSAEVQIQVLHA

FPDPLHQTLSDRVAECHDECCISGPGHIYNFIDLIHTGRQANV

>SG-AL_contig_10022_1_+1_1096_1620

MSVELQVKNICRSAYSELRRISTIRHLLSADTTKILICSFVLSKLDYCNSLLSGCSKHHL

DKLQKVQNSTARLVLKASKYDHVQPLLKSLHWLPIQARIDYKLATLCHAFFADTASFYLS

NLLSVYTPSRQLRSFSDSKTLHIPLLRTKKIWRAFLFFCSPYSLELVAARTPLYT

>SG-AL_contig_10023_1_-1_199_3690

MKVEWFRDGEPLVHGTRYMPAYDFGFVSLDIHWVNSEDSGVYTCKATNDYGEDTTSATVI

CKALRAIIMDTQLPGEGATRLQEMEEHWRSQMVLDERYVEEPQTRSPPVFDLKPEPVETG

EGEPAKFLVKVGGYPRPRVSWWINGSLVVSGTRFKLTYDGMMHHLEIPRVREYDAGQIRV

VAKNPQGEAECCTSLLVMPREDWRARLRQAPKAEMEIELERRRKIELRSVELEKAFKKPK

ATEFELHQLERMAEDKVKFQKDEEVITTERQYISVHSQLRHTEPSLAAQPVYVKQVTMEM

TQPHEDVTKMAEQQITLERGQPPVFTKPLRPVRVPEGSGVTLEVNYTGLPPPIISWYRDN

FEIHPSRDFQISTTATTSTLHIPEVFLEDSGMFSVKAYNKFGQVQCKAKLTVVEGEEPER

EKSPEFRSLMRDSRSTQGEPATFDCQVTGIPPPIVHWEKDGIPLGESPRWKFIDEAENHT

LVIYEVRPEDAGHYACVAINSVGKSTCTARLDVEIPTQPLEFLPSEPEVVSSTHAPRVEE

EPRTMEVEEGEPVRFTCRITGKPAPVVTWYHNNQLVKPSKYFQMSSTTEGLHTLSIMEAF

PEDTGTYKCVARNKMGEVACITFLKVHGVESEPEPSHPMALAQPPSFIRPVANTMVPEGT

PARLDAVFSGHPTPEITWIRNGIQTLHDSRDHKIETQDSMTTLTIFSALPEDTGLITCRA

RNQAGTAECSAELYVQRSEEVEKLAQQLHVTVAQRAPPEFVKPLQPVLDVEPGQPVRLEA

EMSSVPVNRVTWYVNGIEIPPSDRTRIVQEGRTIVLIIINVGVQDTGEYTLKVQNDLGEV

TCKTTLTVKGREKFVEIPDTETTETITVTRTVTTIEREEQVVTQMVKPQVTTPLEPEIFV

RERGIARLECQIQSHPPPLVTWFVNGMEIKPSPHYEIRYEEGKSTLLIIEVGPEDTGEYT

CRAVSELGEAVSSTTLYVQEPLQEEMPQLQDTIQMVEPSPMMEAMVAPRFLEPLPSLQAV

DGQEIKLSCRVSGVPMPRISFFHDSKNIDEDEEFVITYDSETGEINLIIVEVFPEDQGQY

ICVAENPAGQATTSSYLAIMESEETVMELEPQAPDQEEVFTEDQEVTITLDAPITLDAPI

TTEPMDMEEKPVQEEVIVVSEVAE

>SG-AL_contig_10024_1_+1_97_444

MSFERAATSQNKMNVTTKYYYYHYTTTTAAAATADATVAVAAAAAAATTTTAAAATAAAA

LCILFYSVFCILYCYCYLYNFCSPFYFSSPSSSILPLLALKGTTGIYKAGSSSEAA

>SG-AL_contig_10025_1_+1_64_207

MIVLCRSLKWFEYFPHSFVSFNFVSWPTCLLPVPSMIFHVAVDCLLLF

>SG-AL_contig_10026_1_-1_496_6078

MGVGDDVAPRFTQKPALKQEDGGKKLVFQCVLEASPQPEIAWFRGTTPLTVSDRIGMRVE

SAGGAAYNVIMEILGVTQGDAGTYKVTAKNRLGEVSASINLNFSAGAQKQQDGIAPNFIQ

KPITRQENNGQKLLFECVLTADPAPAITWFKDNAAIAPGGRYSVRAEPRDNKTYFLVLEI

NGVNAQDAGNYKVTANNALGESNATIRLNFDTSEVSQVTGARPVFLQKPAIRQVGDKIVL

ECKLTADPKPQVSWIFNNQPISFGPGSHLTPKLISEGSTHTLTLEIAQVSMKDAGEYRAY

AKNDVGEATATITLSFEGAKKPELPAGKAPHFTQKPTIKQQQNLLILTCLLEANPTPQIR

WFRDSTEVGAGGRYSITLQRDASGADLYTAVLQITGPKPEDGGTYKCTAANDFGESNANI

TLNFAGGEKAAKPPGGIAPTFTEKPKVSQDASGKNIIITCQCTASPKPEIMWYKGTTLLQ

SSARIVPSVVEDGNKYSLKLEVLNFTKEDGGQYKVTAKNEMGEGSANITINLEPPKEPPP

QPQGKPSIRLEDNGKMIVIQQAISSASKPTATWYFGNQPVGAGDRLVPEVTQEGNLFYLT

LKIPNFTDKDSGTYKLVMKNNGGEATTTASVNVDALRPKPKGEGPKVVQKLNPQSVNDGD

AVEFLVKVTGTEPLDIQWAKDGKAITSSDIYSVTYDKGTCRLYIPEVFPEDSGNYSVTIK

NQWGSASSVASLQVKEPKEEPKQMGKSEMEISVESKSVEMQMTKPEQDQMEIQVQSKQKE

PEPAKKEPAKPVEEEAKKQAAPAPKEEPAPPAKKPEEPKPEPKKPEEPKPEPKKPEEPKK

PEPKKPEEPKPEPKKPEEPKKPEPKKPEEPKPEPKKPEEPKKPEPKKPEEPKPEPKKPEE

PKKPEPKKPEEPKPEPKKPEEPKKPEPKKPEEPKKPEPKKPEEPKKPEPKKPEEPKKPEP

KKPEEPKKPEPQKKEEPKPDEGKVSVSTEKGAAPAAQTGEGPFFKKKPTNQVIVEGENLK

MNIEVELEGKPMPNVRYLRAGREIREDSRVTISTNKGKGRSTLEIRKVKLTDEAKYTVNL

EQDGVVTDSAVFSVFVKDPKDSNLDFRSLLKHRDVKKTKEEEEDPDWGNLKPVEKGRRES

EVDSLKVNLKKVEKPELELPEKVPDTFTKELQDAKAKEGDKVEFVAEFCKPNATLRWFKN

KLEVFHGHKYHFVNDGNEYRLVIPCAKPEDGGKVTIECNGVQSSAWLYVDAAAPDYHFTQ

KLPEQYEIKRKKTGMLECFVSDPRAFVKWFRNGKPLEYTPGKYEIQRRENRCILRIVDAQ

PGDEAEYSCTCGDATTTCKLRVGEPEWEFMKPLDDVEAVEREKALMECDVSDPEADVSWW

RGEKELKSGGKYEFLKDGLKRRLQIKNCSSKDDGKYTCKVLDQTTTAELYVSPDVKFIKK

LVDKKCKEKEQILLDVKATNPHKHPVKWLKDGKPIPDTIRFDTTQKGEIYKLTIKDTDFG

DSGEYALQIGERVTRGNLNVEPLPRPPKIDASKVKEVLVKKGENIAVEIPYDGAPVPRAK

WSKNGEPLNEANLDTETVPRLTKLRIPGAQTSDNGQYELTLTNDAGSDKVPVKITVMGPP

SPPKGPLDVVDVYCDRCALIWDKPTEDGGSPITHYVVEKCDQETGDWEKLCETGDLEVDV

PDLVLGHKYQFRVSAVNAEGASDFLNTDGAVLAKDPWDPTTPPGNPDILDYDKDYVELGF

EPPEKENGAPVQGYLIEFRQKGDKDWKKGVETKSPDNKGTVEGLTEGKEYEFRVMARNKA

GLSEPSTVSPSVLTRSRKVKPRIDQPSLPKNVRIKVGQKFSLPVKYIGEPAPQVVWTAKT

L

>SG-AL_contig_10027_1_+3_3_179

FTHHSFSLEHSRGGFCNLAVGESVRCKLIIYLEDIHSKVAMFKSQLMHLKLHVIFFNCI

>SG-AL_contig_10028_1_+1_1_171

LCLPIHLSKQKPKKPPLSVSSQFWSLKQVFFFPATNYINLNLFLELMTNLICLNSPF

>SG-AL_contig_10029_1_+3_9_233

MPNEHQILKIYQVYITIIFIYLFSVCIGKKSLQKVFKHITISPNDSRSSHFGQHEKASPS

DLYFWHMLTQMCLET

>SG-AL_contig_10030_1_+3_3_161

AVDLRSAVSSSVPALNIKNWPVCCPFECFIHSGGVSLAFHRFSRSMPDNYWQI

>SG-AL_contig_10031_1_-2_2_304

QSLWQEEWTCQQDNKLFQIRPDLKVLYLQYLVAEKRKLYFCRLHTGHTFLTHSHLLKGDV

PPWCFPCHAPVTVKHLLIDCRDLHDVRHKHFTADSLRTLFR

>SG-AL_contig_10032_1_-3_384_527

MAGDHVFSLYSRQVGETTPLFSTKSNRLRKSIQPVVSTKSNRLRKSIQ

>SG-AL_contig_10033_1_-2_2_145

LDNVLAFIYLFIHLFIYLFIYLFIYVEMAEERGGIKLCMSFPFWELLF

>SG-AL_contig_10034_1_-1_1_129

FIGWFVLFISFIDISFFHLLLIHILHYYVFLCFILVFICKLCL

>SG-AL_contig_10035_1_-3_117_1331

MILICAAVLAFTFVARAGAALDLASPEVFSANFYVNTNRHVLPHGTLGAAAAKTHWLQHG

IDMGLQATGDFHSAQYLARYPDVVSAFGAHNYRQAVIHYLTNGLTENRLGYLEGGYGGRW

TVSDNEHRLFISASRRMGAAIDSLVWDHHEFINAWDHGRELQMAMNFVPNGECFNPTEAG

GRDDSQRNTTHTQIQHVSASGNTLTTQVLPAFWLQAGRTITLTSGTNCQVGMAALNTQNT

YPHPFSKNVTLKCPHTSQPCLHFKSTFTIAGSIPSYTTLQMEAPTGYLTGEFTKIQRLDP

VTGRLGGHSNRKPVIFSTPDDRYAMGVFAPDNQDTDLYMYYGAAQFPSGTAPAATNKWNV

VFRKNSINDGTTHVLVYDVYICVGRVSDVTACLMSLTHEGGNLIG

>SG-AL_contig_10036_1_+2_2_73

LLQEEGQGEGVNRLMLTNIPLLSQ

>SG-AL_contig_10037_1_-2_236_508

MKLLKEKKSAVLSVDGQELIVIKCCTIIKTATKIQQQTNTHSCKYLHTQTHTHTHTHTHS

RTRPHIHTHNTQIPYEFNPINQTAFGVDRTS

>SG-AL_contig_10038_1_-2_326_706

MEQQHCGIQHDLAAQNAVLIHMEQELVLWHSASTMAFSITLSHRMQCWCIWNNSTVAFSR

PCGTECGVGAHGPTASTVAFSRPCGTECGVGAHGTTASTVAFSLTLPHRMQCWCKWNNST

VAINGPC

>SG-AL_contig_10039_1_+3_801_1022

MLYLLTKMFCFFSRKECTFFKNKKCAVYSPEIQADKATESSFTESTHKQQCQHFAQYFIK

SNIWYKILYIFCVH

>SG-AL_contig_10040_1_+1_190_417

MRTYSHEYKYALYGQAHKLLYTHTYKERFSFTRKSVHFIQRPFSFSCAIKKKKIKKGNCM

TEVRKGQDFHEGTTLS

>SG-AL_contig_10041_1_-3_6_1187

MATMSYHEVLLVTESSGQKCSVSAWDLHTGTGVRTFKGQAVKAGCLCLQAGHLLIGAAAG

KPALQLWPLCKKDEPQQRIVCPGPVSALAVSPDGLFCVASILDKTYIWEMSAGDLLAVVN

DIGEVKTIQFTDEGSHFVAGTSSGLLMISDFAEVVSTTRHSTRRVMARKTITAHSQPITA

VFVGCGGLAARVVTSSKDNTCKVWDVLSGSLIRTLIYETMPCSVIMDKQENFLIVGMCNG

DIMCSPLYAQTSQVEVYMSRNATKAGEFVLQGNTGAVTCLALSTDSRHLASGSADGKVNI

WDMEQGRISRNIDVSGAVGNLLIIPPIVQSAAPTHHGPPISFKQEIASKKEGRGRVFNIF

TSDTSELTVPDYCADIAKMLKLSSTLDQGKEDGE

>SG-AL_contig_10042_1_+1_289_588

MTLASVLLLLVTDNVIIVKTFMYVYVHDQRYVCVLLDSASMLSFVQVCECVYFDLSIYLC

KHTHTHTHTHICVCVCVCVCVCMGYQRFCMYHTLCLVNAQ

>SG-AL_contig_10043_1_+2_1373_1675

MTNNEDWKSKRSTSSHRFPVAGFIHFQHCWMRGTDAAQPGDTGNRGVMVKGANILPGKRE

KSEVWGWTLIGPGFFSPPPPPMTSVRPRAAVWVLVFLFFFK

>SG-AL_contig_10044_1_+3_3_161

LETNIKMSQITITCVQYDAGYIFSHKHKKHAVLLKSRKTFWDINPPHYIRMII

>SG-AL_contig_10045_1_-2_191_484

MSTSAILVKQEVHEGIVPRGKKSGKILSPKGKISKSPSPKKTKHLASVTRTRKRNVAELE

KEDSPEKPATKRRKQAAYESIIESPKEGGKTSQKSITK

>SG-AL_contig_10046_1_-2_2_169

ESIQDTRINFRSLRSVFNFVPIPSAYSKRIGFSFFLVNLEFLNQDAMSQVLSQPAT

>SG-AL_contig_10047_1_-1_148_282

MAPEEGRFGKNLDQCTDATVHRNVTGGGESAPKHCSFRISGTITS

>SG-AL_contig_10048_1_+3_981_1178

MSQTDKKIKPSTATHNSNSDAPKYRAPEQTTKQKVFKCGQSAVLVLYKACCNAKLKDWDR

YIGPDQ

>SG-AL_contig_10049_1_-3_123_302

MQPLMRRMTWRNCYTLHTECAFLFLALLVWFIVGTLVRNPFTASAYPPTSCKKLMGRVRE

>SG-AL_contig_10050_1_-3_3_188

VTGCDQRARAHTHTHTHTHTHTRTHARTHAPTSKQHCVQYTQPLLREQLRNKKNKTFSEQ

RS

>SG-AL_contig_10051_1_+2_2093_2389

MLHMCDSPNDFITGLCLSQQSKLQFVNIRLQFCATCILSHPLIHTYIHTYIKRVEMCQLV

LFTPFKTLPFQSWSKGGSVCAYTQICAVSHSRPYSKDQF

>SG-AL_contig_10052_1_-1_1_696

CSPFEELCPEGLFTCDDGQCISNVELCNGINDCRDGTDERGCVTCSDDEFTCESGQCIRL

SQRCDGVRDCPDATDEVNCPPRPRCQPGDFECADGSCIDGRLQCDGVPDCRDLSDESNCP

ARRTCSPQEFTCDDGQCIDRRGRCNQTPECLDRSDEENCPCRDNQFTCESTGSCIDLARR

CDRRRDCEDGSDEENCACADDEFRCGDGRCIPESRKCDRRRDCSDGSDERNC

>SG-AL_contig_10053_1_-2_2_160

AGGWWTGLGIKRVTASLGRHIPYRWFSTNGTVFSTKTVLELIILLPLYYHTGC

>SG-AL_contig_10054_1_-1_1_165

FHSLTYPHSSCRRLVDRVREFFGDHYMETPVARKNSSSHPKGLRVPIGDGVKGLQ

>SG-AL_contig_10055_1_+2_113_220

MLLPKSWFLSFDQAVNTLILSFHFISKHIFTWHPDL

>SG-AL_contig_10056_1_-1_19_609

MLSYSCQQSCNKNLKCGNHVCEDLCHPGDCGVCSLLPEQVNYCCCGQTRLADLNATPRTS

CIDPVPTCDLTCNKPMACGPENSPHMCQQKCHDGPCGQCDGVTTLTCVCGANQKDFLCRE

LDKFTAEEPFKCERRCNKKRMCGRHKCNELCCTREVHICEIVCGRPLNCGLHKCEELCHR

GNCRPCLQASLEELTCR

>SG-AL_contig_10057_1_-3_318_461

MRLATGSWVCAFFAYLLEGGLLSLGAEKGFHSLGKLKGHFPGWESLGK

>SG-AL_contig_10058_1_+3_570_863

MPWKIIISNKTCKVVLSFNFSKRCLSHILSSTSCHQTLQRNHLHVSIQHFIHSFHILSTH

THTHTDFGQSNSCTSFKKKENQKQKSRRKGIPKYLPTR

>SG-AL_contig_10059_1_+2_2_217

NKVLNHHDVTLTTKLKVYNAVVLPSLLYSCETWTVYRRHLKQLERFHHRALRSILGIWWQ

DKVTNTEVLDRA

>SG-AL_contig_10060_1_+3_477_611

MTKCLFWHGNSMKTEAVLQQTHHRHFQPNKRKKKKRKVRECSSKT

>SG-AL_contig_10061_1_-1_1_186

FFFYGTRYCTLSGASYLSIFLFPASINIVAFCCFCSSSIYIYIPKKMCLQVILEYMLFLF

LL

>SG-AL_contig_10062_1_+3_1158_1337

MAMPQIVWKKDVDYNVSFCNDPSLNYEYGTSHRVFTASGTDLRVQSLTSETSGNESWAQT

>SG-AL_contig_10063_1_-2_176_373

MAVCSRFLFTVEFDPQTGKSPSQGRGHCDGLEYCQTVYQIYSFSCGWMCGLLKVDIDVVN

NKQTSF

>SG-AL_contig_10064_1_-1_850_1068

MWRKNQINVHHTHQQSHVQSVCKRFCKEQIDELGIVCKLRFKLRTILVNVNMFYLHLLFL

LIFFFFLLLLLTL

>SG-AL_contig_10065_1_-3_132_341

MCPSCLAEGALPHHRQTSLNGVPTTYIYQSGSTLMANPLMTGGTRGVPLRPCAIVTPPQG

FLHPSSTVAD

>SG-AL_contig_10066_1_-2_38_790

MKDHMSENVRRMRQIQKKCKEKETEAVKPVKAMWKSDRYTDIPSKVMQDLENPPPSRPSS

ATFLRAHSRAGPPVKLESRPGMPDTTDRMTVPLASTANDVKMEHHNYDFIKLNGINAKRS

KTQRAPSASALDDLKKKNESAMADYNMGEIPSYLKSRKKQWKKEEEERIANIPDPAMPPG

HRSLPEHERLETLTLLQNKETELVRELGALPIGKDTMRVHQKRRELESKLAELEEAVRIF

SRPKVFVKVDS

>SG-AL_contig_10067_1_-1_1534_1644

MLRKILIHCREHLLTYLTKHAEIDVNTLPRTSTYMLN

>SG-AL_contig_10068_1_-2_2_97

GCRTYTFRSKGSEPVRVKVEEYCFMKVRDSKA

>SG-AL_contig_10069_1_-1_1_240

RVFTHTHAYLRACLCTSTSAVNFQISSQAHICNHMNTMFAEKVFVPLIVDQMLLLLPKHS

VCISIICHCHCKCMHMDVSV

>SG-AL_contig_10070_1_+3_330_566

MSNLENHVCVCVCVCVHVYECAHVCVGVRLFCGKILSVNNTRDLGQLICIKMRRSLHRVT

FEGKMIMHPKKLKSMKTIL

>SG-AL_contig_10071_1_-3_3_128

GRERERGGGGVRTRVHCHGGADYMGFQSLFNSEQHEESLWLF

>SG-AL_contig_10072_1_-3_504_692

MHFGFCSHFVICWHICLWFAPVAWQAGVWNGPWILIWYKDLIPGQSGWSIFTTIVHLCMF

FRC

>SG-AL_contig_10073_1_+1_1_138

FGFFFFSLFFFFFCTGSRSLQDNYSFIYWQSWVECIWNCLSMMNLS

>SG-AL_contig_10074_1_+1_46_369

MLTSQNYTSASCISVAIYRYLSCRPPLKHTHNQNKAKTKPPKPHTHKTCKSKTSFILSIN

TNRKRHEEQSQSDKKMNGLMTKRRRKERLQHCTHSIVHLPILNHEGIY

>SG-AL_contig_10075_1_+2_167_310

MYNGAIYVATTSVRVKCAMVSVGRLAPHSQDSSTSLMHHWESCSNFLT

>SG-AL_contig_10076_1_-3_3_209

STRTKCRLVAGILNCFFFFFLVPQTIFPADTISFSRKIEFSPTEICVFADNSHAWLLLAG

CCDLSTPLG

>SG-AL_contig_10077_1_-3_15_311

MADGPLLQCKIITNKHTTGQTVNPYTGCRDISRAIIHILSAGCRDKSRAVGSVCLVSYSC

LLWIRKAIVSSAFIRILISWAQEPAFLSFQAHLSKHMAK

>SG-AL_contig_10078_1_+3_36_191

MCGKSSYRELSSPQSTTIYVIHTYFQTSGWVQCKLLIRATAHILSDYSDYEA

>SG-AL_contig_10079_1_-2_11_556

MPDAAPPIELPPCELSKLEEISELFSSVLPSPIRREKLSVAIENDGYIKKLVDLFHMCED

LENMEGLHHLFDIFKSVFMLNKNALFEIMFADDVIFDIVGVLEYDPSLPQPAKHRDYLRN

KAKFKEVVPMTNSELENKIHQTYRVQYIQEVILPTPTVFEENMLSSLSSFLFFYKVGIFS

II

>SG-AL_contig_10080_1_-2_509_907

MANGSMLELAQSSFHDEEDDDELDETLAERLWGLLEMFPQPVQNVLQTAFSKSFIALKVG

YSMSRSVMWIAATSASILVLPVAFESERSLQQQQELQQQRQILLGPNAAVSGSAGHGLLP

GMAAMPPGMVPPQ

>SG-AL_contig_10081_1_+2_26_193

MYLHSNIISTHCHKLNHFCYPKTIPENCHSSNILLLFYNLEKCLPTFLSQHSKTMF

>SG-AL_contig_10082_1_-2_104_718

MSILAYNGAAIIAMKGKDCVAIAADRRFGVQAQTVSMDFDKIFEMGPHLFIGLPGLATDV

QTVSQRLKFRLNLYELRENRRIKPQTFMSMVSNLLYERRFGPYFVEPVIAGLEPKTFKPY

IASMDLIGCPMITDDFVVSGTCSEQMYGMCESLWEPDLEPADLFETISQALMNAFDRDAV

SGWGGVVYIIEKDKVTKKLLKTRMD

>SG-AL_contig_10083_1_-3_174_2813

MKTMDKVVDMLYKRIHDLDTLETHFQQQLKEIERDRDELHELCRQDKALIHDQCVKLQEL

RLQRTQLRAQLSAVEISERECHVRCGHLEDTVCRLQDRVDELQVHEQRLLELFRHLKVTE

KLWPKQPSDQDDVPPSQSQFSTQELCSQSKAREVEKEKDKLIINVHDLEKFDKYSEQHTQ

ASNQDGVNGDMASQQQQSVEENLRLWHHVSHQHAQLEVLHSQLSASGEVALELRQRVDML

QDKKKCLLEQVRAYQSRDVDLQSRVEELERSEEALKVKVSELQHRENCLLARLMEYDTIG

CQVLDTLPLAQKQQLPQTLEACQRRVLVLTRLLEDAQRQLLDSERQHTQTDRQPSDSIHQ

QKQVQKQLSGSHHVDTRKSEDVQWQVCDDVPQQEHIQQQLYENHLQTEQIQEQLSDNDYQ

QEQAQRQLHEKDFQDNKRIKATQSQTQMSDIDLQQEQAQRQFCENHHKTEQAERQLPDND

HRQELDKREKDPHDDKRTKATQQQLCENHHHQEQALDKNHQEDKTKTAPQQLSDNDHKDD

RIEDHGSQLSDNDPQEEQAQRQHEEDHQDDHWTEAVEQMCENCCQQVSEKSHNQDLAQSH

RDENDHHPSKDQEYTEKIASITKATGDTKCEDGTEARDDETNRMTQDIKQKAGQRQENDK

QSKQNIDSSKDIVELFEGLQDPQLLLSSEAHKQLSQRQAPEAPTRSDTRMWRPTPRGDRQ

CGERGAVPEPPSHLRVERVEGDSVVLAWQPPVLDQQGCSNGARVVGYRIYLNGRVCQQPH

NPALASTTIKGVRRSRDPARAQQRLAIETLTASGQVSPRLHLVMALQRDGHSTVTDLSSS

ETISSATGVGYNAASRSGDLQSSGEISGATDVGDSEVTRL

>SG-AL_contig_10084_1_+1_1_168

PLSNILFGAESIFHSLLSFFPKAIKTRVTIKKEHHNFLHNSNANAQHIILFKMIQT

>SG-AL_contig_10085_1_-3_870_1172

MFTLVLFRLVLFMSNSRAVFLMLPFFSFNKTSGGLFRLITDETLLCVCVCACMREIKGSV

QECFSCLSSDCYEIGARHRFDLLDSGRCVEGRHNIYRVSTA

>SG-AL_contig_10086_1_-2_695_4015

MDSGYIKEGLTMSQWTRGNGFNLFSSSSINSNEQAAVTLTGYTWKSESNTDSNIMASTAS

YQNIPGVPRRRPPPPPPRTPSPEPRTPPALPPRSHGQRKEDGAAKPAGEGEGKVDETKVD

FRSVFRQWENNSPREDAYTAQLRKLDVKFSGMRGSPSTPPTSMSVMHSRPPMAISTSYKR

QPTVAQEATSPSLASVPTPVLTSVASSPSLSSLSSTVAGSSSSRHHSGSDISSVTDSQPS

VPLPSSQTKDPSCESPVKGLVDRDTKPTMASVMSSAKPPQLPERSLPPRDQKPGVSRAEP

LPPRGDTPLSKDRSDFPGKGSERQFLSPPEVLRDRKVLDNRLKGEGGGSMSPVKDSSRSP

AGRRRNVPLPPPPLSPDQQDGDLDPDRLPPPPPELLGEGGGSFVAGDQENVDQFADDSYG

AYRNRGSRQVGTYKRSSSSTVIRPDSQLLKSAADAHAVFSDRRLGNQSWNSDSQLQANRK

VEVQQGPPGSTSSNEPPGRPPDRAGSNVPAFQADSGLVRSVSRVSGVAQPLTSPGSSPGR

GRDDGKVFTARPFLVEQSRAVSDKDSTNSGDTSPGSSRLIRDKPEQTTAPSSSSSYSPNS

SSQRWANATTFPSPLRVAHGDTSSVNNSHVSSTGPVVLRASPQSRDGKSAGSPTTPSTQN

TPTSRSRLLSSQSHDGMPPSADNTKHTQRYAEVAPFPSDSRGLPGHVRTFSQSSDHSRVS

STPYSRRESVPVPPEAIAMVSPRRKSEDQLSDTEKEEEECVIVSHGRQPSQEELECDQRA

KELAKEVAGSEKKLSDVLNTDATKKRMKYMDGLFSGPMDSEVKTPTGVRRHQSQGARDTT

LSKPPLQNSLSQTAADEDQLNKRNSLPDKYFVSAPKAVIEMEMRKKEETSTDLTKNIHDN

DTLIRHKEEVMKKLHKKLDQLMEERKGLQQEIDDNDALGKRVCETVDAKCTNPSECDKFH

TYISDLEKIVRLLLNLSGQLARAENAVQALDDSADSEQKKRTLEKREGLRTKHEEARQLK

EHIDKRSDNVSAFLRQSLTEEELNDYSYFVKMKSKLTIDLQELEDKITLGREQIQELKKS

IPERSTPPDPHSSTSAASPPPSPVPVP

>SG-AL_contig_10087_1_-2_233_454

MPVICRAATFVAVSVTSLFFRDWVVNPVPNPNLEDQWITLCQATTLQPVQLGWPTSSRRY

CLHSSRGPCSMQAP

>SG-AL_contig_10088_1_-2_2_232

IWEFLALPSQFCFLDLDLHGLLVGSCPLYTICCKCRYIVSFNQVSTKKIKIIWRPFPHRN

PVSENCINVLVSAISYH

>SG-AL_contig_10089_1_-2_515_697

MSPIASPRKKHKTAGFGATFVSKIFRLVQLICVTVVVYYCWLWWLTLEIEEDLRQLSRQQ

L

>SG-AL_contig_10090_1_+2_665_982

MLSHLRVSLVIYQTTQPWTSDTITPRAICLTNAPQSHKGGLLKLGYLSHSTIRTLDNCAT

VTQRWTFEARLSQSLDNQNCCCPAVLALTSLPPYNQAILASLDKDT

>SG-AL_contig_10091_1_+2_2_265

KTKNKQNQQKKKRGEGGGKKVINFLFQGTIQHLRFDLNRFVSAMLEVFSFYLHFAQVFLN

LSAIIKVFFRSSKFVNSSQCLSTILLFF

>SG-AL_contig_10092_1_+1_142_264

MEKSNPVNSKERKTENCHLFFERLSACVSFSKQNKMQKLQT

>SG-AL_contig_10093_1_-1_256_480

MVRFHSYDSLSVATLGTCSVLLCIHMQLRTSCLRPVHGGHSLAGGVVVKGLMGLAQNNIT

GSESMYIYDGVEDVH

>SG-AL_contig_10094_1_-2_218_412

MLLAGIKLGTSDVESSRLYELYCTALYTFFFFSQQIFLVKLWLCACVCMYSKYFMKGTCL

FMNGF

>SG-AL_contig_10095_1_+3_69_182

MKTAINLGKFLFSLQNVALSSIQRNIQTRHISTNSSFC

>SG-AL_contig_10096_1_-3_3_143

IFKFSLAKWLKIVTWNSVEYICLKTVTAKFCGFTLEDITKKDESAIH

>SG-AL_contig_10097_1_+1_667_1182

MTLRLPKCYSISTFESLFLIHKHKVELSLVFACFLQSWQSLKTASMHPLPFLNPNWASLK

NGSTMFLVRCSRILAYILPTWLRGNIPLYVLVFLAPFLFPYGPYNCSLPVIWNLHFLPTL

LESDSKPLDSNLPAGHYSFSCDAIHICGLVLWAGLQCTSYFFFSRMFGSLCS

>SG-AL_contig_10098_1_-2_1010_1213

MVFVGSFTHSYCVLQYKILTCCLNKNIGIGGAFFFFLFFFSLSLYPIEIGGTVVSLLLFF

TLSLMGSE

>SG-AL_contig_10099_1_-1_1_96

GFLFSGFKRFCDQRGCLLDFDELYMVTVLIWP

>SG-AL_contig_10100_1_-1_391_609

MLQHEYKQRTHTYMHTLCTQEYTHTHTHTHTHTQACMCMHTQNHIMCRLNKVILRDETIR

FFFLFVFFLPVGE

>SG-AL_contig_10101_1_-1_643_1014

MCKLADQYKSAHPNKSFSWNSDIQVFFIQTSVTRHLLSPAFSLMTAFGTTSVLSVLEAHS

PSTLVILKALASHTVVQIHKNFQTSPGTKSKRRKSISWVFSTQGSSLWCTKSVLLDMWLQ

VSHA

>SG-AL_contig_10102_1_-1_1_186

LFVFFVLLLLLLLLLLLLCVCVCVCVCVCEVESVSISICHNINIHHKSEEKKGNRVFWLK

AF

>SG-AL_contig_10103_1_+3_180_311

MFSYLLRTFSCMVTNVLIVFDFILLDLKFLSITCLLNGFSLQIF

>SG-AL_contig_10104_1_+3_180_329

MCRQQSCMHCYLQTTLHDSDSTANVQPSAISIHKLTHDSRMLSILIRQIR

>SG-AL_contig_10105_1_-2_566_973

MIYIPTDIIVFLSALDVWLSDEIYCYECVLSETFFSLSNSFHFLKVLHLFVSFLSPLPPP

LLPLFLCDKRLCFFCPFFFSCRLKKIVLLWSTILYVEYFILFVTLLNSKLKQSIDMNYIR

HLWNNAIFFQGHTLCE

>SG-AL_contig_10106_1_+1_1_216

TFHVKKEQQTTVYGCIQSEKALQNGVTHLTNRETRYLYSIHIELVVSTGSFAIRPTYPIQ

NKTHMNWGKEVV

>SG-AL_contig_10107_1_+2_2861_3235

MWWWWGEGARTSPLINKPTSNPNRTQQHCRTEQLRAQKTFTRTRLSDIHSPDRRPYSPDT

HLTKDIHQKQETQQTSTRYLRHYIMYRRHSNISTHQEVQRQDFFQTHNCHSLTEDIQTQD

IHHIY

>SG-AL_contig_10108_1_+3_3_227

EEVARTVLELPHDSDNSDDENVIGNKGDVVPDAVDQSDNESSDSDYPDEDYTLPLPSAST

GRGRGCVGSGCSRGL

>SG-AL_contig_10109_1_+2_119_1246

MITVVRFFCVFATVFAAAPDTLQLVNVVYRHGDRSPLKVYPNDPYKNVWPQGLGWLTPVG

MRQQYELGQFLRSEYDNFLSPTYKNSEIVVASSNANRCLMSAYSNLAGLYPPQGDQIWNP

NLLWQPIPVHTRPEDEDHVLALEENCPLYDALLWKVRHSPMVQHVDRISAALYRFVEEKT

GIQNVSIVDALDVADILFCEKTHDYALPAWTQARWGDVAVYDKLQEINDLLFGLLYNYTE

SSRLKGGPLLNIFIQSMQKAAAQKEHYKMYMYSAHDSTVAALVNAMKVFNQQPPPYATAL

LVELHHVDSDYCVKIRYRNETTHAPYTLVHPDCRLECCPLDEFVAVTRSHVPVDWKAECW

NGSVITSTPRNLLVLF

>SG-AL_contig_10110_1_-3_3_188

VGCNLHQDNQNHLIHCQIDQQHLEQHHLYFLSCFHRRYCRRCAVIPTQFSPPLLQSTSWQ

PY

>SG-AL_contig_10111_1_-3_903_1178

MKGLNKIKFTICNHLGGASLYKVMFMNFKVVTGVFRIRTILHLTERFSDGYLRHAVSVSV

SNNNYYVCKVHFIHLFKSPLPFHVHFPKASFQ

>SG-AL_contig_10112_1_-2_248_517

MINFDFTILFKSCNCITSNRRNTLFKGKQNLFLFLSVLLDSRSALKLLFKKVDGGGMSLS

QMLSKRALFILQSDSSYVSSNRSNTLYKGK

>SG-AL_contig_10113_1_-3_3_254

VPNVIPSETSLERTLPRCPLPLLALLDCFLESLLPTPYHRENILTCRWHIHLLLFVSLFV

YVFIHLFINKTGIHQATQSIIRED

>SG-AL_contig_10114_1_+1_34_261

MACRSASLGSAVVAAVAAAVSVSGSRALLEFRCDEVWSSLSCSCDASCSLMTSSNEQGTI

TLVFPHGLDTCDAMMM

>SG-AL_contig_10115_1_+3_3_269

HTHAHTEGDIGTDVKHHAQITLVHAGTERRTDIRVHQLILMRRQREEKQTNLSSHCSVLK

KERQRQKDTSHISNHLDVILIACNTQQTE

>SG-AL_contig_10116_1_-2_905_1027

MPMAFQPLLVTAHKCKVFLFYYLSWADVQTISLNLSWADVQ

>SG-AL_contig_10117_1_-2_128_889

MDVDCSLGKTVLRPLACLLPDADKTSVLVILNRPLEKHLLKHLWQCSLFKAVTDGGINRL

HTCLEGEQDNYVPDIITGDFDSATPDVVDFYRKKLVPIISTPDQDETDFTKCLRITIEKT

KSMKVDQYVVIGFSGGRLDHEFANMATLFKATSFTPKPVYLVSEGNLICLLPSGETVIQA

DSGLEDDWCGLIPVGCRCDHVTTTGLKWNLDDHPMAFDGLISTSNTYSEKTVTVKTDQPL

LWTMGYRFPHTVQQ

>SG-AL_contig_10118_1_+1_1_102

PKMWSIANELNKHAQIKSIYPGLHIHFCFDFHYC

>SG-AL_contig_10120_1_+1_1_213

TKTKKKKKPPGEGKKKKKKKPPKTRINQSINQEVKGTDNEKKKCLNKLSHFKISCYHRAG

IFFFIASMYFQ

>SG-AL_contig_10121_1_+2_476_754

MRIHTKGDSGTSSYTHMLTWEIGKISTLYPPGEVLEEDSNPGLPDPPVCKSSVKKVIEVL

KKNMYSKQALCADVSPCTSIFINSVVLFRVHVS

>SG-AL_contig_10122_1_+2_539_721

MFSLALKKLNEFIGNRTLNTFCLTACFACLYFNSSRLFISKQQLNFPQHYKQTLRANRKL

P

>SG-AL_contig_10123_1_-2_236_466

MKVVFLTLPLKTMRKKSLPQGRRWLAPPKMFSLLARPSSGGDKGLCHVSHDASAARYDAG

WFVYPTSCIQLLRSNVN

>SG-AL_contig_10124_1_-2_632_1114

MCSLFQQIPEPDASIQSISIDPMGTMMAAVNNKGNCYIWTMTGGQRDSPTQLHPKKKFLA

HSKYALKCLFSPDSTLLATTSADMTCCIWRTADLSLMTKLKTENQRWVWDCAFSGDSQYI

ITASSDNNARLWSVDAGDVKREYSGHQKALTCLAFRDEVVS

>SG-AL_contig_10125_1_+2_1109_1591

MMDAVSFDATEMLLLGDFNSDLLRPQQSWIDTFSLYNLSQIIDRPTRETPTSKTLLDHIY

ITDEQNMNKLCVLVSGCRPTCETPTSKTLLDHIYITDEQNMNKLCVPVSGCSDHFPTCLT

WDYKRNKNSQSWSQTNHIYHLQNSTKATFLLTSKIPPFYLL

>SG-AL_contig_10126_1_+2_47_232

MRWKRTLNSQHSSPRSDPLHTPSMIYLPLSWQRLPSITKRRTSWKRPISSRISSLLGRAP

FL

>SG-AL_contig_10127_1_+1_361_648

MVPKREARDLVVDICAKRRLSKVSSSLRSLSGVGATIMRSVSKLIPRKWKVSVGLSSHLL

WFNTKPSWLQRLITLHVWVRQRSELSAKKSQSSRQQ

>SG-AL_contig_10128_1_-2_80_1573

MKFSYKFSNLLGAVYRGGNIQFLPDSSVASPVGNRVNIFNIKQSKSETLPIEGKYNFTAV

AVSPDGCLLILINEEGEALLCSMSSRSIIGTYHFHDKVNHVSFSPDGRKFAVTRDTVVLM

FHAPGKSRVFNPFLLYRTFYGAFDETMCVDWSSDSKAIVVGSKDMNTRVFAVQPCENLVV

HSMGGHTDAVVGAFFEHNSMNVYSASKRGHLCVWECNQNTGDLVPKKTKAQKRLEKGDDD

SDDDENRRAVEEDPEPDEESGVLRVRYKKVKRHVFRGAKGTDDTVAPNLMSCDYHKSSHI

LVSGFSNGTFMLHEMPDFNLIHSLTIAEHDTGNRSITSVTFNPLGDLIALGCADLGQLLV

WEWQSQQQALKQQAHFSRTSCVKYSPSGQHLATGGVDGKVKVWDTTSGFCFVTFDQHAGG

VTDVTFIQNGKAIVTSSLDGTVRAFDLTRYRNFRTFVATRSVQFSCVAVDPDGEVVCAGC

QDVFEIFVWSMKTNRILE

>SG-AL_contig_10129_1_+2_2_199

SHQTPTCLHDFLMCIFDVLHVYTHEEGSGICRSAHVLTWEIGKISSWKNLHLLPTLYLPG

KVPMEV

>SG-AL_contig_10130_1_+2_77_301

MWRAREHSISGQTELAVLSSYYTLFIAQPTIKVISGLSPRKVHQSQEDNHVDHKTSLNPK

SKILQNNTVHDILSN

>SG-AL_contig_10131_1_-2_89_319

MIITIFSVHIIQTSLLAAHLSAPVIRVVSVFYIHPRCITCSPFQHFINTQGFSQAEEVCQ

IIAGSFGWSTYGIVWVQ

>SG-AL_contig_10132_1_+1_1_384

AKYILRSSTILNSNSKELAIESSTHFPKRAKSITSLTTINTYTSDLFEISRIQPKNIAIR

PSFSTIPRWELSNACFDIDYTELKKNDGPVLSAIVKFHIQENYNDYLKVFTDGSVLENDQ

TGATFVIP

>SG-AL_contig_10133_1_-2_2_193

KKKKKKKKKKKKKKKKIKKKKKKKKKKKKNWIRNDIIYDTVLILLIMNFIHLWEFSLRYN

FTVL

>SG-AL_contig_10134_1_+2_38_247

MDQSTLCHSCQCSDTSSHHKSCLTSPLSCHKGCVCLHRGQCLRHPWSREGSGCGGAVLSK

SWAVCVGPVI

>SG-AL_contig_10135_1_+3_1929_2234

MSSQDIVKSTFCTSHCKNKDISYLPCLRQDHFFNMKYCSFLLLYQLRKLLDFHTFTIVFL

KILIFLYLWARSASSECKECHNFYLKLTAVQLQCRQTLLEAF

>SG-AL_contig_10136_1_-3_249_1556

MIQESKYLGGDMEHTHLVKGLDYALLDKVRAEINSKEKEDEEQMETVFTQSKEEEDPEEK

IQFKTRLGRNIYRTLFKSKLPQRNELFLPKRMAYAVDLEDEYGDFDIPNTVIRSKADCPS

LETPTTLTTNDIVINKLTQILSYLRQGKRELKKLKRKEKGKLKEEEKPRDKGAPLEDSIY

GEIGEYMPSFAKPKDKRDRKERDRERDRDKDKDRDRRDRDRSDRDRRDHDRDRDRDRRER

GRDRDEDRERPQDRQRKSYFEKPAEEDEEKDKPSQTAKDLARSINDKFKGYAELEEEQKK

LTAKVEEDRIKKLKAKTEIDSYAECYPGMMESADANEDSDDEADYTKMDQGNKKGPVGRW

DFDTQEEYSSYMSSREALPKAAFQYGVKMSDGRKTRRAAPKDEKAKLDRELQQINRIIEK

RKGPIIGDSSSKKTKF

>SG-AL_contig_10137_1_+1_1_117

SYTFLHFMFKPFLQCAGNGTLGKCRHKTLNNSKTDGTSR

>SG-AL_contig_10138_1_-3_87_2969

MVEEEESLRSNMCALAILSCRPNSHPFQERHISLHEPVKIGRSVARTRPTASNGVFDCKV

LSRHHAILWYEAEKFYIQDTKSSNGTFINNQRLCKSGEESPVREILSGDLIQLGVEVIEN

NRRGEKITHGCIIATITLYHPDGREAKPIPFLDAPNQPGIKIQTQDLYQLAQYLQEALHR

EKMLEQKLGNLQQLVLTTQEAAEGGWQALIDEDKLLSRLEMLETQLHVYSKNHAEDTLRQ

ELMALQEDKINYETTAKESLRRVLQEKLEAVTKLSDLERSLTSAEDECGHVKEMCESSQE

ELRQLAIKYQDQLKEIQELQDKLQEAEESHMEEIQRTEKERQELQEKLEDMLQQESSLTN

KIDSLQADNDFTREQLTAMKAKLESMKETNQKEDKALELGLEGFSSEDIIPVVDKDSAPE

NLEEKLKELQTQIEVYKVKLENSDKKLKESSDRIEQLQKELEKAQTESAQYIVKMTSLEE

KVSLSHIHMNQMMENALDGLKLQLKESQKQSTSSEDLISSLEAHIRQMEKQLMQQVVVDR

LTSLAWDKNQNSDNLDDTMCESDQDSTVTVTIFTNRHDAEPSSLPVQDSNLLASVSSTTS

SLSDLTDESENVIEAREAQKKAEAEVQKYRDEVKESSLQLQKSTIELDELKAQYFEAQQL

AKERGDLVSDLQEQMRKAEAAAKEVKQQILDLRGDLENERTKHQQTSADLEETRSQLQEA

QQAAKQSHNEAEQLRNRTKLLQEEVEMEKKECHASQRLSHGDSAHSSRSNRSKAEFSALK

EECSSLRKRIQAIEAEMKMSRKENLQLSSEYNKLQESYKQLEALKEKLTNGELTARQNLT

DAQKDAQKSKQDVRESGRTSSTSTSAVKVTMSGRKLHLSGKQLQSKNTGDELAQLRSQCR

HYSQEVSSLKSQIETMETDYQQIAIRSKKLSFVSSIPLLMLLFAILLAVYPTLEQLTATT

S

>SG-AL_contig_10139_1_+3_534_917

MHSTTHSHTCTSKLIKHDNSFTIIPVTNYASKQFSCMFSKVHTFSIQILTVPTHFETCLS

KQMPSFLHEQTRHNIHTHAFKLIKMNIFCTLNKLCIQTIYTHVFKLPIFFSIPIPCIQTF

HMHTFKLI

>SG-AL_contig_10140_1_+1_1_321

PPPPPQEIFVLSLKYADQTFLQPCSTCLRTVQSHAHAIPYPQPILPSACLANERCLFQLQ

QDGPHATDILGVFGVLGVELTQAGDLLPRLARHFLVTVLDECFFCRS

>SG-AL_contig_10141_1_+1_451_795

MYLGHQDRKTEHQHSHNNKKPEKVNTQHKRKSDRQKNIFAQKETLCRETHKHICTTRKGK

YAGSVQQRTWFYIQIKQRKQAFQDGRPRMTCWYCLPLELSRNLKDTLYFQNHLFF

>SG-AL_contig_10142_1_+2_2_379

KIGGREREREREREREREREREREREGERGAQQLSAKQHQEAPGRTTTLPTPFSPPLTPY

TIPILRKSLIIITEGKLLFTGYITTRDNALPFNKHFSITLFFFSNEMFFSHEIFINKKDY

KSFTWV

>SG-AL_contig_10143_1_+1_1063_1476

MKWYYDALFLTGGGGWDSIMTRELFPAPRFTLSRVVAKCMDDQACAPVHPAIYGYLTLFT

DREGKAMRGRGDGHYHTKAGHMLRSHSPIMLRKTITFYLFLTRITNMGSQSTHKHNLVTQ

KEIGTKDCGLPKSPMSIH

>SG-AL_contig_10144_1_-3_3_1436

MSAPWRATLKVAHQCLQKKCFIDIIPGRHSKLWLQRFHSTSSSKVLSLHDRGILQTVFPE

GSVVELSHHLNRGQTVYCGFDPTSDSLHVGNLLALIALLHCQAAGHTSLALIGGATARIG

DPSGKTTERVAMTAQEVENNVLKISENVQRVMHNYSTHIHKDTKQPLPPFKIINNMEWYQ

KKNVVQFLSTVGRHFRMGPMLAKHSVQSRMSSAEGMSFTEFTYQIFQAYDWLHLLQQHKC

SIQIGGNDQLGNIVAGYDLISKVSKQPVFGLTVPLVKSTTGDKLGKTAGNAVWLDPNKTS

PFHLYQYFFNMADADVEKYLKLFTFLSDQEINTIMVKQQASPEKRVAQKKLAEQVTLLVH

GDTGVESALRWTQALYGSSIEAVASLQPEELGRLFGQVPVQEMFLDPGMTVMDVCMKAGC

FDRIVDAERIIKAGGVYINHCRVQQPDFVLIPGQHILSNNTTLIRIGKKNYYIISWCQ

>SG-AL_contig_10145_1_-1_4_1533

MAKVVIAGRSDCPYLARVELLGDRLVRNLPSFQLHKIVKTPDEWQHWLNQTCQERGWQHK

QSPLVWRELIDRGGKGVLIGGASDFQEYVKGYYGLEASLTSDDMRKIGSENSTTKQEIDA

EDAQYRALSKPLHVCITAAQSPTCYSLISSISSGEVFGPNTEINLRLYDICATDDSIIRG

LAMETQDLCDGLVRGIEVVKNTTSAFKDCSIIVILDELLQNDEETYEDWLKRNCNFFSAY

AKVINEVAKKNVRVLVAGNGPVNFNAMMMIENAPKLPRQNIVALSRGIEHRAKAVIGQRL

NVNTAGVVDLIIWGNVNGKHFIDNSKSRVHGYDGAVWGPPSYSVPVVEMVHDNKWLEKEY

LEQVQQRREAVESALGHPSSGSAARAVSSMLTHWWTGSPLGQMFSLAVCSEGWYGVPKGL

VFSYPVTLSTQGYWSVIQDIELTDETKQKLQETVKDLKKEREVIYPSSRPPTPEPEKPAE

TKEDKAETADVKESESSEAAVAEGEGQAES

>SG-AL_contig_10146_1_+1_25_189

MTSQSKLATTERTRANHKTCGDIINTHLVCKNSQFTKIDIPLNVYSFLGLLFTQN

>SG-AL_contig_10147_1_-1_211_1035

MSKELPNMNAPPGVYEMFNENRKYYFEADISPLPLPPSSHPAPVPPGFPRLRKSRSQPHL

ATTVKLLAGTHLSTAPPPPPAENLQLPTAVMAPVHRQVSSSDTSSTYNSLETRYQTEPTY

KSSRILKSFHSEFRLFGHNRDNVNSNANSQVGRKSASTPNSPRRASSPELDLSAVQAYTD

SDPWKLSLVKEETAEQVGVSGSVAPVAHSLSDDTETKSQSHMASLMTSIPKDTMDGELKG

AKIPLTRKMMTWLTTSQTQVPVNKTEFNAITPTSF

>SG-AL_contig_10148_1_-3_1032_1154

MSRLILQYSRYISYMSDHFYPAALVAAFHQYSVLLEPWRFS

>SG-AL_contig_10149_1_-3_135_383

MNKLRQTILLIKRSNMQLFCECVCYVILHLVSTFLFCFLVGWLLLPHLPSYYKLFMPAPQ

YTMTSKFFQVKSQYQEVHRSQPI

>SG-AL_contig_10150_1_+1_451_720

MSSTFQLESEAVLTVNLTFSLPNGSVQHRTEDFSPIATIARLKEDMANKLGMSVEQQVWT

YNGVKLIDNQTLDDVEFDSDDTIEVSCEQA

>SG-AL_contig_10151_1_-2_50_211

MSAEVGGSTFIHATECVKKMVPFVSDLPDYQIFSSNIHLPCAGEDLLMYVLHSL

>SG-AL_contig_10152_1_-2_2_265

RNRLQPVIDLQEEWKHGYGTFVYTDLRCCLIFRHDCACAIFTRDMKSWVWHVCVVCVCVC

VCVCARAQYLQEKRNHGYGAFVHCVCVC

>SG-AL_contig_10153_1_-3_255_1085

MKEHTGASRQKKGTDKSAEAKKDKSSESKDEESKISPDVLWDLYQKMCDVLLEEDRLDEL

AEVALLGLTCPAFIADPIKNKDAEFICLTACMMTKNGQFAYNFIKNICLKEPESTMAWNL

FNQSLTYSTEIRHHKFCLRRLLRWPDHLALGILNGHNSVQNGSYRCALGEYMAVFRRIPT

DPMISLCIGLCYFHICCQRFTTRKDWLALEGIAFLNMYLKLRGECQEAYYNLGRAMQQLD

LGYAAVYYYKKGLALPPVLEDKDGTFDLKREIAFNLS

>SG-AL_contig_10154_1_-3_861_1058

MNNNYALIFLGQIGGYGYEYKLCPDPFCLLATVPLFANLFQFIFFFFPGLFFFFFFFFSP

TVNSHT

>SG-AL_contig_10155_1_-1_1_660

KKNIDLDDVKLAVQLKADQSLTKPPPKDLLQDLARQKNSQPLPPIKPYGGPRSLPDRYCL

TNPNYRLKTSKKPQPQPRIQFGLPAHQRISLPTQGKGLAVKSIGGSGGQSVTVLPKANPS

GTVGAGQQLTIIGKPTVTPKPTTRINTGPVAPTGIGASGLAQLVTQTTPISSSSSSTSLL

PTVTTSSTTVVPNISSLGSSSTTITINPLKRKAEDDDYDG

>SG-AL_contig_10156_1_-3_3_215

AEAYVPQGLSPYAHGPSVIIFDTKTTKPAHVPQVSKPRPVAGAQGAAQASSAALGEGLEL

GVVPSNPRYQK

>SG-AL_contig_10157_1_+3_3_206

GVGVGWLLVAADCCSAGCSGLTGCSSSSVFPIFAIFQSHSVATPSLTASVQLTILANVKN

YHTLDQNN

>SG-AL_contig_10158_1_-3_36_296

MVTVQRHPLFPQSLVLGLNHKVESWLFLNSSVWEQTEQHYLRSVHASLECVCTLVDPVGW

LTPCGQRHIVAPSADSGLSGYQEGEKN

>SG-AL_contig_10159_1_+3_321_518

MTMLLKFQYVVAFKTLQFFHAKRIHEISSLHITLPTHQLPAEIKTSQLRMHTACLHTLQE

REGRKT

>SG-AL_contig_10160_1_-3_3_680

MIMQKKAERRRRRREEARSAASQQPAEVERDEQAMEQEPKENAALPPNRPSPKMAAARHP

PELHSQQPQGQQHQSSFASAFGQTSHEAPPAYGHYQPKPQKNLMRPRSFENIQASSQADA

SNRQTSAPAAARGPGRPISGIGRSQHPTVTRDEKRGLVVSNSRMVPGQRPISASNPRPKP

VYGRKQFNNAVNNPSAGAPKAVFGSYAQTYGTISSSQLASIQGRKS

>SG-AL_contig_10161_1_+1_19_288

MHARTPTVQTCTCTCTSTDAHTTSTISISLTGEGTAMTRSTMYVYTFSLYPHYQSRVHSA

DAHTSFMFITILGNTDDLSLARSICQYVKN

>SG-AL_contig_10162_1_+1_58_330

MVGYHLFADNTQLQQPCKVTDIPTLTLSLQTCVADIKTWTDNMLKLNDDKTAAFLFCGSD

CPPPSSISIGSRTVSFSKPETLDILWIQIFP

>SG-AL_contig_10163_1_-2_2060_2293

MYLYIVALSLTLCTLIFLVVSHSHSDDNYFIYGRVKETGEKQLEKGLLAFKHCCIVFHIF

EISGVPISLFKNALKVNL

>SG-AL_contig_10164_1_+1_1_393

WVCGGERERERERERERERERERERCEAITNGTDCVCVCVCVCVCNILCRIFLVILSVCL

WYVCNFVDLDNYATETTNGINATVLVHAGCFRVSISHRTLTWLTGSLTCIFDVLHAYTHK

GAQALADLHIC

>SG-AL_contig_10165_1_+2_170_535

MYINKRKKHSKIKQRKSDIKNNINNDNNNNNDTNKYNNTIESQTFTPSHTPLSHTFISGL

PLSKSYRHSSQRLYHYDLSPDISKRSNMSVYKLSRLLRKTCKHKICILSKIFQNLILCLW

WK

>SG-AL_contig_10166_1_-1_1_132

RERESSTKLKIALIRWPKHIEPLSSLIRSFYISLGPSLTCDWDC

>SG-AL_contig_10167_1_-1_82_996

MRCSSSLCTSNLHSHSDAWLNTVRVMECIAMLAYVLCALVDILQNCFLRGIHPPYNTTVE

ILAVLAGITGISGAVAFGIRWSTNKTMFSTAVHDPSLADEYSSNLQWGFGLVIAASAIIL

LSAVAIGYSRHRQAQSMFTATKMSVVKTGDREPFDEQVSFRSYENPDSSSMMLVPVSHFD

TNKTSDESSDPPPYALPPPYPYPSACNFFVPIANSSLVPQIVDTSSTGAHPQVTNNTNLS

LSLVGTDRLFTGPPHTFVSPASTICVQNKRPVVRPIFIKTDGGSEIEITPSAQNSSGLPS

PFNHF

>SG-AL_contig_10168_1_+1_907_1305

MEIQKYPACTRSTPFTSCRLYWLSPLPWPPWFCRKEEPAAWTTAEPGSLYHRARVCALDS

SVWCPSPQSLGTEGGQSLTGGKSRTFMHRMTGTLNWAASRPTFLLETGIELMTLDLKSIG

FIHCANHPNVKCG

>SG-AL_contig_10169_1_+2_2_205

HTHTHNILTAERKESENNKYLHFQFAQNCEQQHETDRNLKITEKSPQQQNMYKIISIASS

SRKTIRTQ

>SG-AL_contig_10170_1_-3_2076_2402

MKEVTRLLGIKHLTTTPFRPMCKGLVENFNGTLKVMLKRLCSEQPRQWHRSITPCRLPAE

RHHTGLQASPSVRQNYQDTYAHSKGAEDEGNGDARGEKQLLVRVRLLAL

>SG-AL_contig_10171_1_+1_121_261

MYTFPTSVSVRIKRQCNSVITCIQYSTLQYLFSRFCTENYLSVYIQS

>SG-AL_contig_10172_1_-3_33_200

MMCICYSCIIDRTSWECGYFMHGLCDYSHTNSNVILLYNSVLKKNVWYSHEIFICV

>SG-AL_contig_10173_1_-2_197_796

MSSGSVISKLKRLFATHGIPEKLMTDNGTQFTSREFELFTKQWNFTHLTSSPHYPRSNGL

AENAVKQAKQLLEKSKTGNSDIMLGLLNLRNTPRDGMGSPAQRLLSRRTRTTLPTSSKLL

KPKTVNAAKVSQQLKKARHQQKKSHDKSARDLRPLQPGEVIRIQTDKGFQRLATVKAQRG

APRSYVSPRTKWTIYETDDI

>SG-AL_contig_10174_1_-2_26_868

MASRDRPEHKAPPEIFYNEEEARKYTSNSRMIEIQEKLSERAIELLALPDDTPCYILDIG

CGSGLSGECLTENGHVWVGMDISPHMLEVAREREVEGDIMESDMGQGMPFRPGTFDGAIS

ISALQWLCNADKKSHKPHKRLYTFFCTLYASLSRGARAVFQFYPENSSQVELITSQAMKA

GFTGGLVVDYPNSSKAKKMFLCLFAGGASQHLPQGLGTESASGMKKEAVSSAREQWKNKK

SAKLNRDWILEKKEKRRKQGKDVPLDSKYTGRKRRTLASRR

>SG-AL_contig_10175_1_-2_74_397

MSDGEDEKASDLTIVDDVDDEDDDLPLSDDGDADIDSAGEELSEVVAHMDKPQASPVLNN

NSEVKDSDVTLPAAVNGVGEKESPENSSQENKEGFELDVSHKPIACST

>SG-AL_contig_10176_1_-2_8_301

MAREQLELQLQEAVQEITTEKMLAEENAKIKYEHHILKLEEELRKTQDELDSQKELHHKD

KRALDHLRNHFASLPLRDVMPPGVVDKDQVSYVDYIGF

>SG-AL_contig_10177_1_+3_123_245

MKKQLSYTGRDLQSYKSQYFGQVAMLQTGRYRHEREKRGTL

>SG-AL_contig_10178_1_-1_985_1893

MALQHESSVNKSDGVAQARGILRVVNICKFIATACMLPDVLKLLCDHSKVFQRDVLDIEQ

VSDVLHVTLQSISDLETQPGPVYRDFLQIIRDSGEYQGVALQLTQDVEHSVSTIMAQFAR

NIRTEVDKRFPEDDMQVQKDLTTVLDPRKIPMHADGLHTYGLEALDRLLKTYDGLIADSV

TLTAACARELYPQLKGMMARHRNDWSLADICRTLILQYKDLFPNFYLMAHISVSLPVSSV

PCERGFSAPNRIHNTQRNALSIDSVNNKVLIMHEASQQDFDESAVLTRACEIFLEMKTRM

RSV

>SG-AL_contig_10179_1_-1_1_186

EFIAGDWSSDVCSSDLGTRCPFCSVICLYGLFCVFFLASKYCLQFFTLFWLTDFYAFFVI

WG

>SG-AL_contig_10180_1_+2_116_307

MRNKATKWSKIVHATSNNIFTEKKTKQKQQQHQSRKAALQVLKVMNSPYQLFDGTRTCSQ

VYSV

>SG-AL_contig_10181_1_-2_197_382

MVSGGKIEWWVAGCYLLSFYLFLVMSVIKYIFLLTFIEFCMAIFAVLSCLTKQLSCSELQ

LQ

>SG-AL_contig_10182_1_+2_710_1066

MKCIYALNTSRTHDIHLTSSNHPESVMQSSNNSKSTKGWEGGNAVAAHSSIPLDCNDVKG

MMYTKLSSLPETKPTITYNRKFPTQQKIATTTATTKESHIQTKIHISNTELATFLYTMK

>SG-AL_contig_10183_1_-2_449_634

MLNELKCVNFILLYRCLCNVILPFKCLSCPPHNFDSQGMGGTWENCAFFRFAIKLRVVLD

PN

>SG-AL_contig_10184_1_+2_1676_1849

MHLKLPTRRIYDQYLLINPSLPSPQILLETFCLLTSHTHTHTHTHTHTHTQIHYINQN

>SG-AL_contig_10185_1_-3_3_215

FFFFFPLVSLSVFSDSVLKNFYIYFCSLCFTVFFCVVFGFRPFYLIIFLEKREEFKEVHV

GVGWFVLCVCV

>SG-AL_contig_10186_1_+2_227_397

MASLLTPLTLTPCSTHVMFVGCYKYSDNGLRKTGDSCTCMQLRSIRKKKGFIIIIID

>SG-AL_contig_10187_1_+3_261_431

MAQSTVMGNNRLNTTLQQKEESGQNTPKQPGSKDRKILYLFTCYRDWKAIPPLTQNS

>SG-AL_contig_10188_1_-3_834_1034

MLVDMCRFFWESGEECVCGDSVYKFVLISWDIYRDKASSTHPLVGIVHDANSNHQQNQHE

MIQSKYL

>SG-AL_contig_10189_1_+3_327_656

MHTSMYVYVCVCVHMCKKNIMSMIMNLLQYQCIKLKFYHISVSFVSFRLLKMISTNNIKC

MDADAVWSLFKRDSLHSDNSICAPPKSASNYLADSLSLHKILWVMKPKKH

>SG-AL_contig_10190_1_-3_279_473

MARCSVPFRMFGSYAEIGLFVYKRCSILCFFQLFSMGLEVYQITCVKCLIKISMTWKYTK

HNRHF

>SG-AL_contig_10191_1_-3_636_821

MTSVKQDICIYLCLGASSALKDLPLVILHYLPCAHAYICKCVNCLPVLMGKLILIKDSIA

RY

>SG-AL_contig_10192_1_-3_15_443

MAALFGNSAGRSQSKNLVEFRAGKMVMKGKMVHPDKRKGQVYVHQSDDSLMHFCWKDRTS

GNVEEDLIIFPDDVEFKHVPQCTTGRVYVLKFKSSQRRLFFWMQEPKTDKDTEYCKKVND

SLNNPPTPGSSRGGGLPSELSGL

>SG-AL_contig_10193_1_-3_858_1148

MESVCLEVYSNEWKVCLENLQQWMERECLVSVPQFAFALYDDWNKMKLLFANYTCELCLL

CFFSFLLALIGNICAKFMKISLVISYLLVTFTLSCMY

>SG-AL_contig_10194_1_+3_3_92

KIKKCHPTWQISVKIHCRTKFYNLAFVFLS

>SG-AL_contig_10195_1_-1_31_408

MSTLSDPWENKLDPSDLCVHHNGHIGQGATVRLQCSQPVTGQYVSLVLPTDSEPLNFCEM

EVLGKPASGDVPQPADTVDGCSDSSVLTNVALNKPAIQSTYHELYGNPEKAVDGISDTLI

SECTHT

>SG-AL_contig_10196_1_-3_168_446

MGQFEVHAVVITNRGDCCGFRLANFRIYVMSTLSDPMENEHDPSDLCVHRSGQVGQGATV

RLQCSQPITGQYVSLVLFTDSEPLNFCEMEVLG

>SG-AL_contig_10197_1_-2_8_571

MEVLGTPASGNVSQPTNITVEYTGSSVLTNVALNKPAIQSTYHGLYGNPEKAVDGVPDTV

IGECTHTADEDSNPNRWWRVDLMGQFEVHAVVITNRGDCCGFRLANFSIYVMPALSDPSE

SEHDPSDLCVHHIGQIGQGATVRLQCCQPIIGQYVSLVLPTDSEPLNFCEIEVLGLAVSG

DEDESDGN

>SG-AL_contig_10198_1_-2_2_412

GNRLSDFGVYVTSTLSDPWESEHDPSDLCVHHSGQIGQGATVRLQCSQPITGQYVSLVLP

TDSEPLNFCEMEVLGKPASEDVPQPADTVDGCSDSSVLTNVALNKPAIQSTYHELYGNPE

KAVDGVSDTSISECTHT

>SG-AL_contig_10199_1_-3_66_257

MEHIPCTQRFHERLNAAFSSAWNEISSKREQDPVLWREMPVPIRNPFLKIVACRDNGKDF

PCRQ

>SG-AL_contig_10200_1_+2_2_160

FFFFKHESFDSDVMIPYTYREEKETKQTGPLSTLSISDCLMGQVQEMKTIAKK

>SG-AL_contig_10201_1_-1_1_354

LPSQEVSAGAAPGETSCFQEGKGEGLQTEWVTPSQAVSAKTLPRPSTSNHSAPPSRQPLE

ACCTLAPAGTQRVDRFGAGSGVQAAVAGRPAAPVTYSRRPPYVPSTEKQEQVLGEEIT

>SG-AL_contig_10202_1_-3_3_263

NGELWGVLHICHFTSFHSYIGSCNIEYSYIALYHRHMYMTDLCISYFRQCVQLKCRLNTS

AIILDLKSRDVCGLVHNMGSAAPCDTN

>SG-AL_contig_10203_1_-2_128_1591

MAIVPLSCALLASLNVVCGFYLPGLAPVTYCEIAQDSCKNEVPVYVNRLNSIETVIPYEY

HSFDFCKKENDYDGSPVENLGQVVFGERIRPSMYTLKFNQNETCRSVCQRHYKEGNKEDL

DKLNFIKKGVSLNYQHHWIVDNMPMTWCYHVEGGEQFCATGFPMGCYVNKRGRPKDFCVT

DRQFSQPDTYYLFNHVDIIIEYHPGSVEDWGASLNGNRGRIVSAKIIPRSIDHKANKESP

CSASSPVLGMKGSTLTGEIKVNYTYSVIWTRNTQVKWSSRWDYILESMPHTNIQWFSILN

SLVIVVFLSGMVAMIMLRTLHKDIARYNQVDNAEDAQEEFGWKLVHGDVFRPPRKGMMLA

VLAGTGTQIFFMTLITLVFACLGFLSPANRGALMTCALVLYVCLGTPSGYVSSRLYKMFG

GEKWKSNVILTAFVCPGVVFGVFFVLNLMLWIKQSSAAIPFSTLIALLALWFGISVPLTF

VGAYFGFR

>SG-AL_contig_10204_1_-3_3_338

YEGSAHLSLAALPFPNFEESQVPIHNWVDWRLHAYVSCQVSRARRLEGKCSTGANCHHIK

VLFTKQQSLPCLNVISRYKGINPTNVRIDAETTVSQSLLRCQSIYRYVPTLL

>SG-AL_contig_10205_1_+1_1141_1641

MHGHDKSGLLVDMVAHAHLHKMYIHCIRYIYRYIYSSMITLLYTVGTYQYASCHSSSRSR

TTWRPTMHTQTQTPARTGTHRNTCTLTSVKTVAHAHTHIHTPICKCTHKEHSSFSYRRNT

PHCFHIHTDVVCNMYTSMNRKTNSLRSVQLMKCQGHSILHTGTAQST

>SG-AL_contig_10206_1_-3_324_623

MEREAVDTSLPWGRFVRMDPSPLHPCFTFSNPEEDQIPIPSWVDWRSHAYIKFLRRDCYP

SKFLQADQRQADLFWSPVSSFIKSYSHARLLPLCCAAAAV

>SG-AL_contig_10207_1_+1_1_174

FSTCSKQNFFPTTIAALVTSVYDQENSGHEVSNSTPEEISPLTMQTILDMSRVYIFHT

>SG-AL_contig_10208_1_-1_46_258

MKGGEYFMVVFNMIFSISTAFMRRVICFAFSVTSRTAASELAGWFLIKFITDSIIVILFS

DLGRTVTRTDL

>SG-AL_contig_10209_1_+3_90_188

MPRFVTRFICCKFASTQRNKFCYDKVFNKHHQV

>SG-AL_contig_10210_1_-1_94_291

MKNRRAWRLLLQYCCNSGLLYGVGLSQYRSLVGCRLWFLYSANFRLKTTCMDEEFVLPHY

RCFRNW

>SG-AL_contig_10211_1_-2_113_220

MCIICTIISLLPVYSMFSLSIYNVIYYCCKIWPWSK

>SG-AL_contig_10212_1_+3_3_158

KHTRAHTHTHTHTQTHTHSLTHTHTHTHTHTPLQRLYHRRQTVDLFGPPNPA

>SG-AL_contig_10213_1_-1_1_105

GVSNIYPQPLSKTVRTRTSGSTECCTQLRFHCYYV

>SG-AL_contig_10214_1_-3_993_1223

MSSDILFGTELIVGLSPTVDTVSNTIGKTRTTHRLLACTICHYWDCSDHTASFFPIGSCT

AVFIQSCVHQKSGQGNP

>SG-AL_contig_10215_1_+2_2_229

FILLLSDNALDKNLFIFSSSMFSPGFSPRFTLCSASCEVMKPSSDLSSLEALLIPLEGTG

SSPEDAICKQYQNEAL

>SG-AL_contig_10216_1_+1_1_84

DEMKLSAQIQYNTRGLVTELDIKQSTKN

>SG-AL_contig_10217_1_-3_3_137

MTFIEMYRVTLSDHRHINVTADIEKRNCSFIVFPKVRGCRTKFAV

>SG-AL_contig_10218_1_-3_2037_2306

MVVHARSVKRLQISGAPFQCWFVSLHFLFLSCNLQPHLSTVSISSTILIFLLDCQMIFVS

MCISLFLCYAYIFTVFICQPFYSNNASLLF

>SG-AL_contig_10219_1_-1_343_456

MKLLSACMRHFVFLETLSIICCFIFGVFDMGMVLPPNL

>SG-AL_contig_10220_1_+3_99_284

MKMKSCSVINGKEKQIKTNNNANSLPYNNKDQITATIINSQKSPSPIKILLLIMKYNLKS

SC

>SG-AL_contig_10221_1_-3_3_158

ILFIAQPTIKAISGLSPRKVHQSQEDNRIDHKTSLNLKTKILQNNTVHDILS

>SG-AL_contig_10222_1_+3_156_281

MLFNKQINYSEEKLINKRARQNTTDRVVQAWELSVKSRISEK

>SG-AL_contig_10223_1_-2_524_1144

MLKELGACMDPKNLDPSFSHPAGNDQNVYVLLDVCHMLKLLRNTFAMCGPLKDKDGNIIK

WKFLEELHDIQEREGLHLANRLKSSHLCWQSQKMKVKLAAQTISSSVADALEFCRDGCML

EEFRNCQRTVNFIRLVDAVFDVLNSRNKFCQRHESSHETRDGGANYIFLGLCLQLLCLHD

RGGSSSEDSLPPRSPQEFLSTDTWVNH

>SG-AL_contig_10224_1_-3_3_218

DLLQFHCILFLHALLHIEANIAAMSNLQLHVVAINFFFCCNIPGKHVPRLLGSAHLNFFY

GHFFCGFDIQLK

>SG-AL_contig_10225_1_-2_98_400

MPYKVYEKHPDLLFVNRHLQVICAKGLCHPVWNPNFKDASVHHLSSAPFDWHTDTLAVHW

TYPDPEEFASEQTLSDSITMFSSIAKYVLRKAEHHLEKELV

>SG-AL_contig_10226_1_+3_612_902

MMMSGILKKKEKKYMTIKTDATFKHFICNDDQYKYCFLNITETAQNTEEKLSSFFFPISI

YCMVFKYSIFLLASIMDKNSCKTGQFFMNVTLLATGQ

>SG-AL_contig_10227_1_-3_1494_1670

MKSWPWPHLWPFRQSVPIKEILKVWPFRQSVPIKEILKAAYWRSENPFINHYLRGLHLA

>SG-AL_contig_10228_1_+3_78_206

MKTPSMHTQKMWHKNLCVDCGHAQQKRRIKILKGACTNMEIST

>SG-AL_contig_10229_1_-2_59_442

MKGRGALFLFVWICHSTYVSNSQRQGDGGLGSNLEGMINSVETGAESANNPLFSLAPSTP

SPDEVNCYVEIPAIRVGGRCVRLGRIRWRGRGSRRRSSVAWGCQSGIHLDIGSPDCHNNN

RRSRRIGG

>SG-AL_contig_10230_1_-2_2_172

HVLSSVPTRNVKVGLFAARLNALIILVVSVFHRLCSCLHVETLPAHRVVHRLKECAR

>SG-AL_contig_10231_1_+3_57_503

MGSISKKGTAKLAQLVKLDLAICDASLQQYNCAACEQMPLLVMTASPTWKTKETQTNPGV

VSSSATSQNRKAEFACCANISYGNKCIMKCTRSMGPDQIYAYGNKHTVAMKGSYFAKGLK

ASSMKLARVTSLCERPATSCELKVTCTLL

>SG-AL_contig_10232_1_-1_37_225

MKKKFKGFVDHVQQRTLQRVMINFIICARECCSKAEPKVRHFLYLACFSSVQSSHLHHSI

IHY

>SG-AL_contig_10233_1_-1_442_585

MRFIPSLFQRYVEVCFLYDCVRVVLKFIVLKFISCLEPFSCTMPADSC

>SG-AL_contig_10234_1_-1_1_231

SCAPGTCSGLNLGIFRCGPGSSEFTSFSLSGLLQGYVESADDSWHLIQLSYCHDQKCEKN

HHLSKIAQALALMNSCL

>SG-AL_contig_10235_1_-2_260_523

MESATDFFFPPPPVPVGSSTFGSFRQGYQLAKFLVRTPIFSTVPTLPCVCIGVCFFCWFF

NPPFKGVHIGRFRKPPNTDMTYRISNVR

>SG-AL_contig_10236_1_+3_252_353

MFYSLVYKHNMQPTQSQQQKTPPPQSCNITQALF

>SG-AL_contig_10237_1_-1_1_162

VIMGQPLFLYDTKPDCLVSLTSFIEMRSVKDRLDECRVLICWCKCFAAFIADCF

>SG-AL_contig_10238_1_+2_2_163

ISDTSHTHTHTHTHTHTYTLALYDSKHNSAHTYIHTYITYIHLYKYTDHTRINK

>SG-AL_contig_10239_1_+2_107_373

MPYNTTQHYTIQHNTTRYHTTQHNTIRYNTTQYDAIQHNTTQYDAIQHNTMPYNTTTQCD

AIQHNTMRCHTTQPNTIRYNTVPYNTTQH

>SG-AL_contig_10240_1_-1_925_1437

MYMFIGCVTILCHGNVLFYFLSPPPHVCLNHWCRIVLYLLSSNSYKINAWGKGRGGASIE

AYSGYCVLVQFNNVCIMTSVSLCFSLCLSLCLSVAAPPPSPFSLSLHPSPSPFSLPLPPP

PSLSLSLSLHPPPPLSLSFPLLSLSLPLLSLSLPLPLSPPPPPPPPLPLSP

>SG-AL_contig_10241_1_-2_113_3640

MYNQDEEMFVGDDESDDVTTELWQEACWIVISSYFDEKGLVRQQLDSFDEFIQMSVQRIV

EDAPPIDLQAEAQHTSGQVETPPRYMLKFEQIYLSKPTHWEKDGVPSPLMPNEARLRNLT

YSAPLYVDIKKTVIKEGEDPVETMHQKTYIGKIPIMLRSTYCLLNGLTDRDLTELNECPL

DPGGYFIINGSEKVLIAQEKMAINTVYVFQQKDSKYAFKTEIRSCLEHSSRPTSTMWVNM

MARGGGGAKKSAIGQRIIAILPYIKQEIPIIIVFRALGFVSDRDILEHIIYDFDDPEMME

MVKPSLDEAFVIQEQNVALNFIGARGAKPGVTREKRTKYAREILQKEMLPHVGVSEFCET

KKAYFLGYMVHRLLLAALGRREVDDRDHYGNKRLDLAGPLLAFLFRGLFRNLMKEVRMYA

QKFIDRGKDFNMELAIKTRIVTDGLKYSLATGNWGDQKKAHQARAGVSQVLNRLTFASTL

SHLRRLNSPIGRDGKLARPRQLHNTLWGMICPAETPEGAAVGLVKNLALMAYISVGSQPS

PILEFLEEWSMENLEEVTPSAISDTTKIFVNGCWVGIHRDPDQLMTTLRKLRREMDIIVS

EVSMIRDFQDREIRIYTDAGRICRPLLIVENQKLLLKKSHIQQLKEREYNSYSWQDLVSS

SVVEYIDPLEEETIMLAMTPDDLVEDKEAAYCSTYTHCEIHPAMILGVCASIIPFPDHNQ

SPRNTYQSAMGKQAMGVYITNYHVRMDTLAHGLFYPQKPLVTTRSMEYLRFRELPAGINS

IVAIASYTGYNQEDSVILNYSAIDRGFFRSFFFRSYKEAESKKGLDQEEIIEKPNRETVQ

GMRPALYDKLDEDGIIAPGTRVSGDDVLIGKTLTLPANEDELEVTAKRFAKRDSSTFMRR

SETGIVDQVMVTINHEGYKFCKIRVRTVKTPQIGDKFASRHGQKGTCGIMYRQEDMPFTC

EGLTPDIIINPHAIPSRMTIGHLIECLQGKVAANKGEIGDATPFNDTVNVQKISKLLQQY

GYNLRGNEVLYNGFTGRKLNAQVFLGPTYYQRLKHMVDDKIHSRARGPVQILNRQPMEGR

SRDGGLRFGEMERDCQIAHGAAQFLRERLFEASDPYRVHICNLCGLIAIANLRNQTFECR

SCKNKTQISQIRIPYACKLLFQELMAMSIVPRMMVT

>SG-AL_contig_10242_1_+1_19_144

MCMLRKGWVCICQHQACIVCVACVRVFMCMSTSASKCGTIWA

>SG-AL_contig_10243_1_+2_2_205

PSHRPTIITQTYNHHTDLQSSHRDGHGGRMVTSLNFQTGGLGRPGVEPPWAPSLLGKGWK

FFQSPRST

>SG-AL_contig_10244_1_+2_119_268

MQAYVNSRLAPDEALFNLLNKRKRKKLKLQVLRLTTKSSCHLFQQKDATR

>SG-AL_contig_10245_1_+1_1_108

HYFIHLQMTNIFIDYTSDKYPLDQLLVPLFHTFTNH

>SG-AL_contig_10246_1_-2_1073_1189

MVSQCYRTSYSNLEQQFYLEQYFLESRPIHDFAIMYICN

>SG-AL_contig_10247_1_+3_3_206

SVHGSDAACYPRHMEYTRPQKVSAVKGLKLMHLQLAVWPYLLFKSYPDPSDKSLLVHFLK

HTHKHMCV

>SG-AL_contig_10248_1_+2_2_115

HHQQQQPNTKYIYIIYELIKLLNAAPTSSWCGSLLVMK

>SG-AL_contig_10249_1_-3_108_524

MENQYGTKPKPHKLSKKEYEMLYIEVLYTVKHKIGVTSGSHSPYQQDLFQYAKEAFSVSP

EDHATLLARATEEKPPIIILNVTVIEAAGLEAKDADGFSDPYCMLGIMPGQQDNDSGGVY

NSSDEDSSPGCSPKLPKDR

>SG-AL_contig_10250_1_-2_1445_1615

MICQPVHAVSIIAMGLSKNNRTGTFHSILRNFCAGLQGCQCTVRSGSVLGYRVVGAQ

>SG-AL_contig_10251_1_-1_91_267

MSLLSLHEFVTLTGGLFFFRIMHLVLFLESYVHVNRGKPAKLSTCTHTCARAHTHTFYC

>SG-AL_contig_10252_1_+1_1657_1968

MMLQCLMLQFSSCFCIFLRLFPLHYSSELIVTTKLLHICLFKHLSFVILHSEMSLTATVG

HFILGCLYHCKECLIPFLIIQIFQVVPKICVVEVTKVSTPVLIN

>SG-AL_contig_10253_1_-2_23_2179

MTKETPVKPQTPKDTPVRPQTPKETVVKIQPPMPKEKPAKSQQQTTTTPAAPSLDTVAPV

ASSTPRAISPRSASPSPRAASPREASPASRTPVRVLSPTPGADEDGGSTTPQSRSTPTPM

EKSSGDITAEEYKAKLAEKRRQAREKAEREAEEERKRKEEEERLEEERRLKEEEEQRLRD

EEAMRLAEEARKAEEERLQKAIEAEDARRAEEAARLEQEKLAKEEADRKAKEEAERLEQE

RQEKARREDEERLERKKRLEMIMKRVKTDNAGEGAKDSPKSVNSSPTKSATSTASSDNSP

LDPDSKTILTAPAQSAVVAESEERKRSMDSNAEESRSPANGTMSPASAPSPVPFAAQEAA

TPMKTASSQENILETKESTPTSSRSQSPIVSAMLSSRGETPTSDSGSDKPKFKSPLLQKL

VEGKDDASPKFKSPLLQNLLSKTRVGARMGLTQDYSRSSPDLSQVDPGTNGPASRASPTV

MAMSSSMIDMDTVNSASKEDLNKTEEKDVDTSESSSSHSSPSRTSRKTPMSNGEEGSSTV

EVSVSSPMETSTPIESTAPGVMTPSSTSVWNEQDSGVVVGSDEMRSSTASTSLQVSLNGH

TLTGISQGQEGLVDSSISMQSVDSVTSSHTDTAAGNVPSCQSPKLEHSSDDKNFEELIDL

SISNTPTSGVTLLDNSGDLVSFNKLEDDESEGSPTLPPPIIAFEESNMMRHQDVPDLMS

>SG-AL_contig_10254_1_-2_2_268

MSFRIVVRNRKCARGCNITLHCVVILMFFLPVLCFWIWSSCQSVNWARESVVVWFHTHLL

DHALYHRHQMCIMCTKNKNQKTVLKVYTW

>SG-AL_contig_10255_1_-3_624_899
[truncated: 11,544,935 more chars]
