# Supplementary material for: Multiomics analysis of the giant triton snail salivary gland, a crown-of-thorns starfish predator
Source: Sci Rep. 2017 Jul 20;7:6000. doi: 10.1038/s41598-017-05974-x (PMC5519703; doi:10.1038/s41598-017-05974-x)
Supplement: Supplementary file 5 — Dataset 3 [file 41598_2017_5974_MOESM5_ESM.doc]

**Supplementary dataset file S3:** Lists of genes identified from *C. tritonis* SG-PL gland.

>SG-PL_contig_1_1_+3_3_890

HVLAVTGVTLHHLVGRLEAGVGDLSHRQLLVVGLLGGDDRGVGGQREVDTGVGHQVGLEL

GQIHVQGTIKAQGGSDGGHDLADQTVEVGVGGTLNVQVAAADVVDGLVVHHEGAVRVLQG

GVGGQDGVVGLHHSGGHLGGGVDGELQLRLLAIVHRQTLHQQGGEARAGATAEGVEDEEA

LQTGALVSQLTDSVEDEVDDLLADGVVTTGIVVGGIFLACHQLLGVEELAVGSGADLIDD

SGLQVDEDGTGHVFAGAGLAEEGVERVVTTADGLVRGHLAVRLDAVLQAVQLPAGI

>SG-PL_contig_2_1_+2_2_160

IADLDSGLADVDGDTLTHVVGWRLAYGQMQEKNVENERTVLTTTQCVLVRRAE

>SG-PL_contig_3_1_-1_100_609

MQKDSDKNILIFDLGGGTFDVSILTIAEGSLFEVKATAGDTHLGGEDFDNRLVQHFSQEF

QRKHKKDISNNARALRRLRTACERAKRTLSSSAEANLEIDSMYEGIDFYTRITRARFEEL

CADLFRNTLQPVEQALRDAKIDKSQIHEVVLVGGSTRIPRIQKMLQEFMG

>SG-PL_contig_4_1_-2_146_2149

MKWFLLPYCFAALLLSTAWIVQADDDEEEGETKKKDKEGVGTVIGIDLGTTYSCVGVFKN

GRVEIIANDQGNRITPSYVAFTSDERLIGDAAKNQLTSNPENTIFDVKRLIGRMWDDRSV

QKDISFYPFKVINKNNKPHIQAAVSGETKTFAPEEISAMVLGKMRDIAEAYLGKKIVNAV

VTVPAYFNDAQRQATKDAGTIAGLNVMRIINEPTAAAIAYGLDKRDGEKNILVFDLGGGT

FDVSLLTIDNGVFEVVATNGDTHLGGEDFDQRVMDHFIKLYKKKKGKDLRKDNRAVQKLR

REVEKAKRALSSQHQVRVEVESIFDGEDFSEQLTRAKFEELNMDLFRSTVKPVKQVLDDS

DLKKEEVDEIVLVGGSTRIPKVQQLVKEFFNGKEPNRGINPDEAVAYGAAVQAGVLSGEE

DTGDLVLLDVNPLTMGIETVGGVMTKLIPRNTVVPTKKSQIFSTAADNQPTVTIQVYEGE

RSMTKDNHLLGKFDLTGIPPAPRGVPQIEVTFEIDVNGILRVSAEDKGTGNKNDIVIQND

HNRLSPEDIERMINDAEKYADEDKKLRERVDAKNELESYAYSLKNQVNDKEKLGAKLSDE

DKEKITETLDDAISWLEGHQDADTEEFKDKKKEVEEVIQPIISKLYEGAGGAPPPTGDEG

DEQERDEL

>SG-PL_contig_5_1_+3_105_488

MATPFVADDFPDYSDEELQAIVRELEKYEEEALVESDIEVGEGEDDENSELGEESDPEDN

VPLATFQQAWHPAVVPPAVKKFDLLVGPNHTLPTDARPSCVYVCSLLVNCSMNIECSLHL

VLLWKEKW

>SG-PL_contig_6_1_-3_2697_3254

MNLIILFKDTVSSQIFRSFGLNIGGFINVITALFLFLIFINLRGLIPYVFSPTRHLAVSL

SLGLPLWLSLIISAVFFNPSSVIAGLLPMGAPAPLNPFLVIIETVRIIVRPITLSVRLTA

NISAGHIVLTLIGNYLTASFFISSIFSIIILLSIQVLYTIFEFGISLIQAYIFCLLITLY

SDEHPH

>SG-PL_contig_7_1_+1_1570_1821

MVLWRFNSCNATHTFLLKKLQKSFNTSRTFSFLNALLCGVSTAAAEPFKFHFLQQILLKS

QNTGYTFSLLNGLFCGVSAMQYNL

>SG-PL_contig_8_1_+1_385_558

MWVRVRVREWVDTYMAVWDFYPSLKAVILRFRVVYAGYFPVSTSRRTQTWLTGYLTCV

>SG-PL_contig_9_1_+1_1_210

CKEFIEDEIHFVLCCSFLENFRKEYIAPKYFRTPNAFRFNLLMSSKNNETMKKLCVFLYK

ACKLRETALS

>SG-PL_contig_10_1_-3_33_2447

MVTELSFRLDQVQDSLDEQGSSTSAQMELNKKRELEISKLRKDLDMANAQFEASEASLRK

RHTEAVNNLSDQVDYLTKHKNRVEKEKQTLVVELDGLSGELDSVSKAKIALEGRVDGAEQ

SAARLKGQVDDLTRQLNELTMLKARLTQENFDLQHQVQELDSSNAALAKAKNSLQYSNDD

LKKQLDDESRQRQNLQVSLSGLRSDYDNLNARLEEETETSSTLRNTLSRVSADYAALKTR

FDKEIAAKTEELEETRRRLNLRITELEDSCEQLRIRNSGLEKAKAKLANELKEVTIELEN

TQIIVQDLTKRNRQLENENGALLRRVDELTAENGQLRSEKAALEQDNQRLRVANAELTER

NDNLQRENKTLSDQLREANQALKDANRELADLRQLRAQLEAERDSLAAALKDTEDALRDA

EAKLANAQAALNQLRIDMENRLREKDEEIENIRRSSARAIEELQRTLVEVETRYKTEISR

LKKKYETDIRELEGALDNANRANAEYLKQIKSLQMRVKELEALLDDERRIADDLRNQLAV

SERRRVQLGQELEDARSMLEAAERGRKNAENELGETTVRLSEVTIQVTSLTNDKRRLEGD

MAAMQSDLDDAFNRMRAAEERAERLAAENARLADELRQEQENYKNAEGLRKQLDIEIREI

TIRLEEAEAFAVKEGKRLVAKLQGRVRDLEGELENEARRGREAMAGMRKAERLYKDLIAQ

TEEERRQLAELSSLNDQLSLRIKTLKRQIEESEDVVNVTMNKYRKAQALIEEADQRADNA

EKNLQSVRRARSMSVTREITRVINV

>SG-PL_contig_11_1_-1_298_3897

MPGDSGPSGAIGPSGTNGPPGMPGMPGMKGHRGFPGRPGRDGDTGRRGEKGPAGPSGPVG

PPGPNGARGAQGERGRDGPPGAPGIRGNDGSPGSSGPPGPIGPPGPGGFPGSPGTKGDKG

ESGPSGSMGPAGPTGSDGRPGSPGPTGQPGVRGEDGIPGPKGGRGAIGSAGGQGFPGPQG

PPGAAGPPGSAGLKGDNGRDGTPGKSGDPGPQGHPGAPGERGQPGLPGNEGSDGPMGPQG

APGPAGHQGESGSPGAAGLPGRPGMPGNPGKDGARGMAGERGVPGNPGLQGQAGPPGPPG

QIGSPGYSGSDGAPGARGPPGNSGLDGTPGEAGAQGQQGAPGQQGPAGSRGEPGEQGREG

AVGNVGSPGARGDRGPPGPAGSPGGAGGAGPEGEMGPAGNPGAPGFQGLPGPAGPPGENG

NVGQNGPTGPAGDAGSPGARGDRGFPGEQGLSGTRGAPGERGPAGPVGEVGLSGPPGPPG

SRGDPGSPGNIGLGGERGQPGRAGPKGPPGPTGESGRDGEMGPSGEQGPPGNLGFPGPSG

VVAAKGDRGSMGPPGEQGLPGTTGRPGPPGNAGPQGLPGVPGPTGTQGVAGEEGAIGEKG

DTGPQGYPGEQGPAGEPGLPGADGEKGDTGEPGPSGPSGQPGHPGAAGPPGQTGPAGGPG

MAGPVGAPGERGSPGEVGPRGVSGEAGFPGTPGLDGSPGERGQPGHQGPAGPAGEAGPQG

ERGSPGFPGAAGEPGPPGPAGNPGEAGGRGENGKDGSPGPSGPVGPPGAVGYPGDEGVMG

ERGQDGLPGLPGRPGSKGNRGNPGPPGPSGPVGQTGSPGATGNAGAQGERGERGERGTAG

PRGPSGAMGSLGATGQKGPGGKAGATGDRGDKGWPGLPGPIGPPGPPGSNGEAGQAGPPG

PPGNMGNSGTRGLPGRPGEPGPQGRPGNTGARGSMGDDGPKGPNGPRGPPGSPGSPGYAP

VWPAERSQQKGPDPYMQYDQPLETIDPYEGLNFAREALIRARNPVGKESNPGRTCREIKE

NNPDFKSGSYFIDPNLGKASDAIEVRCNMESMETCIRPSMADYGKNRWTQSEHEGQYFMD

DINGGKEFIYKTEFSQLKFLQLHSTGARQQLTYHCLNSQAYGTRLLMYNGEEIDTVESVY

KRVTRLSTHDECAHDNQWHSAVFDVRTNRTDILPITDVLLFDVGRENQQFGIELGEVCFS

>SG-PL_contig_12_1_-3_48_929

MEKIWHHTFYNELRVAPEEHPVLLTEAPLNPKANREKMTQIMFETFNSPAMYVAIQAVLS

LYASGRTTGIVLDSGDGVTHTVPIYEGYALPHAIMRLDLAGRDLTDYLMKILTERGYSFT

TTAEREIVRDIKEKLCYVALDFEQEMQTAASSSSLEKSYELPDGQVITIGNERFRCPEAM

LQPSFLGMESAGVHETTYNSIMKCDVDIRKDLYANTVLSGGSTMYPGIADRMQKEITALA

PPTMKIKIIAPPERKYSVWIGGSILASLSTFQQMWISKQEYDESGPSIVHRKCF

>SG-PL_contig_13_1_+2_2_1078

ALPILREDVLSSWDGSVWTVRCKLKEELLSKDRRFWTETDELREEVLSSLEALAVDDGGA

RFIILLLRDPHLLEGGQRGQDGATDPYRVLALRGSDDFDLHGAGSQGSDLLLHPVGDTRE

HGGASRQHSVGIQILTNVHVALHDGVVGGLMDTSRFHTQEGRLEERLRATESLIADGDDL

TIRQLIALLQGGGGGSSGHFLLKVQGNVAELLLDVTHDLTLSSGGEGVATLSEDLHQVVS

EITTSQVQTEDGMGQSVTFINGDGVGDTITRVQHDTGGTTRSVQGQHGLDGHVHGGSVEG

LKHDLGHLLTVGLGVEGSLCQQDGVFLRGHTQLVVEGVVPDLLHVIPVGDNAVLDGVLE

>SG-PL_contig_14_1_-1_148_375

MDRPFLVHCQQQDCISSSSASLLILHSCLLSSSCILLVMSSSVCPVIPLSVCSVKVNLEL

FCNPFQKKKKCCPLGL

>SG-PL_contig_15_1_+2_1823_2167

MSKSYRNRGYLPRTQINKKAKNNTFNINLKSLSNKNIDLPPKKSKAENNLITRMLAYSAK

KIKANPAAPYSILNPETNSDSPSAKSKGARLVSATQVTNHIKETGNIIKANQINS

>SG-PL_contig_16_1_+1_94_201

MKESKPDNQPTNKINLTSIKHHHHSNGVNNLQITAQ

>SG-PL_contig_17_1_-2_224_838

MSEIKLVYFDLRARGEPIRLVLEAAGKKYEDVRLSFDQWPDEKPNSPYGQLPYVVYNGNK

YGQSAALASFFAKEYGLYGKSNLDALRVDEVVALCQDFIDAMYKAKFENDETRKEELTQK

LHDEAVPKYLGFFQTLLKDNGDSGFFVGSTVSLADLFTYSTLDSLIAEKGDAARTFPAEV

KKLRSTVEAHPFLKTYLAKRKSVPF

>SG-PL_contig_18_1_-1_925_1158

MKGTGIDLEIDSVSVRPSLSPVPIWELRRARFDIHHTDLKKEDNTNLLASLVKCHIASSY

SDHLKRYTDQKKRQVQRI

>SG-PL_contig_19_1_-3_3702_3995

MFFLQVFKALKFACNCTVEKNVMSLYWVGIGEYWGGMRSPIFFLQKATHVQGTYPLVAFS

QVHLYVSSDGTCCPPQISFMFHSASGISYIHSRPSQVD

>SG-PL_contig_20_1_-3_3_194

KSGDAAMVDLIPSKPMCVEAFSDYPPLGRFAVRDMKQTVAVGVIKSVEKVEVTGKVTKSA

AKKK

>SG-PL_contig_21_1_-1_1_177

FGPPPPPRLPIGCDLYIIALLLLVHTFSCECLKNKNKKRKKKTTTVNKTKKRIKKSREW

>SG-PL_contig_22_1_-2_1595_1852

MNILVPCKLVYQSVMLFEAQLTIQAMAALLRTESCQYVREEIKLYNYIRTERCQYVREEI

KLYNYIRTESCQYVREEVNLYNYIRT

>SG-PL_contig_23_1_-3_117_1463

MTSKTAKVPILSTAGAVPIKNDKGETTMEKVKVTRYVTGKRPDYAPDSSSDEEDEFGFQA

RRKAQPRLDEEEEERDDEEKEEQEMLDRRLKRLQQRERVADDDSDEEVDRVTRHRRQVIE

PEIMAEESDDEGKGGRDDKGSDDDEEEEEELDEEEIERRRAFRRQRALQRRDEEEVMDVE

EEKSEGETEEESSEYEECTDSEEETGPRLKPVFVRKKERVTIQEREREEQRQKDLEVEAK

RLAEDRRRQTLRMAEQEMRREMEEEKQVEDMLGEVNTDDENDEEEYEGWKVRELKRIKRN

REEREALEKEKMELDRVRNMTEEERRQEFRANPKQVTNKAPKGKYKFLQKYYHRGAFFIS

HDEDQTYKQDFSQPTLDDHFDKTILPKVMQVKNFGRSGRTKYTHLLDQDTTQFDSPWIAD

SMQNLKFHTSKGGGTKQVFERPATRTGQK

>SG-PL_contig_24_1_-3_1833_1979

MRRGGAKSFVDDLVLGRGVVLSRAATSLRSIETQPWTRRSEERRVGKEC

>SG-PL_contig_25_1_-3_315_1292

MFDEMASGLDQLRTMTTVVADTGDFEAMKKYKPTDATTNPSLIFAASGMPSYQKLIDDAV

SWAKEQGGSEDQQLTNAMDKVCINFGCKILDIIPGRVSTEVDARLSFDKDAMVSKAKRLI

ELYKAAGISKERILIKLSSTWEGVQAARELESKYGIHCNMTLIFNFHQAVACGDAGATLI

SPFVGRIFDWFVKNTDQKSFEPLDDPGVRSVTKIYNYYKKFGFKTVVMGASFRNTGQILG

LAGCDLLTISPGLLEKLSQSTENIVRHLKKDKAACSDQEKVTVDEKMFRWEMNEDEMASD

KLSEGIRKFAVDAVKLENVLRQRLLK

>SG-PL_contig_26_1_-3_135_320

MKVFAACLLICLVLAGCCQGFSLGQVGICVEACNQERNNCPQGQTCRSNGCGHSCQDDVA

GL

>SG-PL_contig_27_1_-1_142_1623

MKAKKLSSAGYVFRKDRENDKIQKTTWRCTRRGCPGRAYTQWCDGDQRMAVHHTQAHIHG

PDPDIPDSVEWVENAEVRRTAPISLSLTQDVPREVPWVRPSRPRVDAATQLSVHSSQGHR

YSSGECSATTSAAATPKRKSHDEPEVDIDFDVDLDVHFDSVGRQHSSSQFLNLPGTSSGS

VQQTTKVPRIMAEGQVPQAAVEAVLVRSQGMPAGSQTVRGYDFNNGIDYHELFRSYTRTG

FQATSVGKAIEEINKMITCKMQPVPEEHRTEMNLNPCGREKSNCTIFLSYTSNLISAGTR

EVIRYLVQHNMVDCLVTTAGGIEEDFIKCLGPTYLGDFHLSGRELRNKGINRIGNLLVPN

DNYCMFEDWLTPILDRMLHEQREQGLMWTPSKMIHRLGKEINNPDSVYYWAYRNNIPVFC

PALTDGSLGDIMFFHSYKNPGLIVDIVEDLRRMNSQAMYSVHSGMIILGGGVCKHHTCNA

NLMRNGADFSVFVN

>SG-PL_contig_28_1_-3_135_1091

MFAHRGVFANHANRHHTRNDRQTAGRENQGLLEDHYTVDEVLGKGGFGTVYSGHRRRDNL

PVAIKHIMKHKVTLWGRIGNHEVPLEVSLLQKTQNISGVIQLLEYFEKTDCFYLVMERPE

SVMDLFDYITEKGALKENKARQFFRQIVTTVQEVSCRGVLHRDIKDENILVDLETSQLKL

IDFGSGDELRDGYYTDFDGTRVYSPPEWISLQRYRGMPATVWSLGVLLYDMLCGDIPFER

DSQILRAQVTFKSTVTPQAQDLVRRCLSLRPSDRPSLEEIMDHPWMQADLNARLHADNRQ

EDIVNANLDAISLSSQESI

>SG-PL_contig_29_1_+2_1103_1369

MLDSLVRVTRRVGWRADRFATDPVPAGPPQSPSREAVGRHGCPVSASRDIPGGRGGLRRR

RLLGPRADRDRRGYTSARRGAHLPSPAAF

>SG-PL_contig_30_1_+1_76_258

MNSTVNFDCTLNTELLSSMYIFIHNSVAHHISKIHASHFNSFSHTNSATHQTSKAHAKHS

E

>SG-PL_contig_31_1_-1_1_1110

SAEGTDSGSVSCDDALRSLCPDFKPDALLHLLEEYWGESGVFSFQPETQTDQTSTSAFGQ

LGVIDAVGGELSPETSLVGSAATNVDSINELIQSDHQYHKEEPKPPSVHTSLSNSSVSTN

LPVGRQVFTTSSSNILPDLGTQLIFPSGQNMVSTSTVKCSPSRATLTRGNTSSVNTSTSA

SASDKLSLATMVDPNMVYSPRPRTVQPSPVDTKDSVPDMDNSSFLSPDMLESLDGLLSFD

DLTDFSLLDQKPQQQQQTVITSSSSPSGATTEGKVSQRRPSPTSTTVKHPVSTFDSSNKI

QALKPLPSKTEYQDGHLSPDSGFTSDGTSYLSDSSASPVSLTSPDMEDLSWQEEFVRVNM

ELFPDVGLGV

>SG-PL_contig_32_1_-3_123_713

MQLCLVLVCCGLMVGPIWAGTVESVAVAKHNQLRAAEVETNSASNIYALKWNSALAAQAA

TWTRKCRVEHTPGLDRNIGQNLYSAGYIGSEKPKATKVLKDAIDLWMEEKSLNDGTFACC

TRLTMTCCHYTQVVSSRSKEVGCAVEFCPGNQIIVICNYTPMGNFNDQAAYRTGPPCSGC

KKKDKCNADKLCVSRKG

>SG-PL_contig_33_1_-2_275_1597

MASNGIDGESSADRNVEIWKIKKLIKSLEAARGNGTSMISLIIPPKDQIARVNKMLADEF

GTASNIKSRVNRLSVLGAITSVQQRLKLYNKVPPNGLVIYCGTIITEEGKEKKVNIDFEP

FRPINTSLYLCDNKFHTEALTALLADDNKFGFIVMDGNGALFGTLSGNTREVLHKFTVDL

PKKHGRGGQSALRFARLRMEKRHNYVRKVAEVAVQLFITNDKPNISGLVLAGSADFKTEL

SQSDMFDPRLQGKVIKIVDVSYGGENGFNQAIELASDALANVKFIQEKKLIGRYFDEISQ

DTGKYCFGVDDTLRALEMGAVDILIVWENIDIQRYVLKNHTTDEERILFLRPEQEKDKTH

FTDKDTGVEMELMEQMPLLEWFANNYKNFGATLEIITDKSQEGAQFVKGFGGIGGILRYQ

VDFQLMEPMINDFDDFDLDDY

>SG-PL_contig_34_1_-2_59_1015

MTSIWPWLIGFAAMMAVVFSTPIDNTTQNSIEINVFVDRWALKRWKAMVATSSVMSEKIN

ETKAAITDYVYSVFNDVNSIFKELMRYGINLDVRVHSLNFIEENEIAPDAKLGFRHVDPL

PVVQNLFRYLWYRNYSYIEHVMLFTGLDVNKSDEHFYSETCGNYPLSVLEATPSGSAALS

MAYQQALYMYVELDGQKNLCGNNYIMTAVDGPSSPTNFRFSNCSIASIKDFLESMIDRGN

CMSAARTNTPPVSAPALGERYTPDDICKLTFGNTSYFCRSLYGYGGKYTYDSMCLEIYCK

RKSGRCAKVQAPDGFVCGK

>SG-PL_contig_35_1_+2_269_409

MMNQQVYITRNFHGNCFTTITEQILLYEIQFFSVSMVHVEQEYKCDV

>SG-PL_contig_36_1_+2_2036_2302

MATDNEIITVEKHCVLSHLETAAHEKVHRRQVLLYLFYPLMQILGFYHQYTMYWQARCAL

YTTVYMDMYRIYTYMKQSARAHREARVNV

>SG-PL_contig_37_1_-3_3_584

PLEPELPLPASPSWSSFGRLLEQGSRSRRLDDPRAGTPPSPPACRSGVPSSFAGRKVGWT

EDFPSGCNHLHRGNSQTMRLQQTPGAPVPRQTWATRTSFTTRPPCHATHQRCPHRATATS

PCRPLLGGAGQAGAGRSAFGGLRQPPQTGPGHLAAAGARRSLALLRPTKVVREPESRTQG

SPFALLLHGRPAHP

>SG-PL_contig_38_1_-3_117_1007

MPCRLLLKSLILLMTVTSPGAGDVYTSTSTILTTALSEGKLLEGLNKFIGFEYDNMNKMS

QFLWERSKANHLVSKEDLQWLVEHPNGAYFIVKLFTQHWVPLANTYPVLARMMEPVLSEM

PTDRDFEGALSSLIRLQRVYLLSVDHMYGGNYTGHMGPPLQPEDTYLLGQRAFEDGYLSE

SVQWLELALRLMYTYDAFPATSSVFGGNHAAAPLVTLAQVASLLGRVYFFMKNQTKAQEM

YELSSALDPQAYDVKNLKTDLDGNGLEYRHAESEEWEHNMTTLCLRPRHHTVAETTP

>SG-PL_contig_39_1_-1_142_765

MGISRDRWHKRRKTGGRMAQLRKKRKFELGRPAAMTKLGPKRIHTVRTMGGNKKYRALRL

DHGNFSWGSESVTRKTRIIDVIYNASNNELVRTKTLVKSCIVQIDSTPFRQWYEAHYATP

LGRKKGVKLSAQEEAVINKVRSKKVMKKYEARKKVAKVEQALEEQFSAGRVLAKIASRPG

KHGRADGYVLEGKELDFYLRKVKSKKGK

>SG-PL_contig_40_1_-1_151_630

MPASKRDMSYYDILGVERNASERDIKKAYRRLALKWHPDKNPNNKDEAEKKFKEISEAYD

VLSDKKKREVYDMYGKEGVHSNTGFSDDDFAHSGGHFHFHFRSPEEIFRDFFGTDDPFSR

IFGGGFQSGNMFEQSFTGFPGGNDGFFSSSFFSSDPFSSS

>SG-PL_contig_41_1_-1_97_291

MMKNLRPALIAILLLMALTMITLTAGEEKAEDSMRLGSMMKRSAHLGQPDSAPYCLCTSP

CPCIG

>SG-PL_contig_43_1_-3_3_272

FFLFPPPPFPPPPPPTLLSHNTRSIFNKVLFLLIRVRILVGNALLILGSFTSSSFLQLTP

PPLTRNFIPVRKKVSQYDTMLCEHNIKRYM

>SG-PL_contig_44_1_-1_1_99

VTGVQTCALPIKKNPFMLCLFLSFFKDLWYVCS

>SG-PL_contig_45_1_+2_41_283

MRCDFFFCAILSHKRYTVNDPSAGSPTETLLRLLLPLNDQVRASSRPTAPPGEGRAETNP

RPSLNRSIGSSDGRCVQRAGT

>SG-PL_contig_46_1_-1_70_231

MTWSLSTSSTRTPLQRRKESLMRRKGRKNWLKNELQFLFCFFPSCSSPIQVLKT

>SG-PL_contig_47_1_-1_373_636

MGAVDGDGDQGFEGSSKEAKGSLEMHLTLGASLDVEDGVHMFMGGSQTPGEEGEEFNDAE

DESHFWMGTSGGDGDAGVEGRCKYLTQP

>SG-PL_contig_48_1_-2_89_1216

MCDDEVAALVVDNGSGMCKAGFAGDDAPRAVFPSIVGRPRHQGVMVGMGQKDSYVGDEAQ

SKRGILTLKYPIEHGIVTNWDDMEKIWHHTFYNELRVAPEEHPVLLTEAPLNPKANREKM

TQIMFETFNAPAMYVAIQAVLSLYASGRTTGIVLDSGDGVTHTVPIYEGYALPHAILRLD

LAGRDLTDYLMKILTERGYSFTTTAEREIVRDIKEKLCYVALDFEQEMATAASSSSLEKS

YELPDGQVITIGNERFRCPESLFQPSFLGMESAGIHETTYNSIMKCDVDIRKDLYANTVL

SGGTTMFPGIADRMQKEITALAPSTMKIKIIAPPERKYSVWIGGSILASLSTFQQMWISK

QEYDESGPSIVHRKCF

>SG-PL_contig_49_1_+1_1_264

FFFFWVFLHNLFLKSHNYTYVSVTLLRKKNSALASMPGDFYYCISSNECNVDEMSLPDRC

SLTGVGQLQDLLSTPTCTLSYNDVIKGY

>SG-PL_contig_50_1_+3_270_443

MTAGEEDFLTYKKRHPTNGLHTHTHTHTHTHTLAHHTSPKWLTEHSVKGKQLIWKRNI

>SG-PL_contig_51_1_-3_165_458

MRTFYLTLLSILGFFMALLALSLQYKHFLRVLLSLEAATINLFIIMFALSNNTTYSGQTS

LILITLGACEARLGLAILVAIIRAQGNDYVSRFSLQKC

>SG-PL_contig_52_1_-2_146_466

MGIVEITTADQFEEMVTKSTSGKLVVVDFFAHWCRPCKMMAPKLEAFADEHKDVEFYKVN

VDELPEVAESCEISAMPTLHFYKSGQKVGDVIGFNEQKIKEAIVKWM

>SG-PL_contig_53_1_-2_368_808

MSSGVTVADETKSAYQAIKMHQKGLKKRRCSVFKLSDDLKEIVVDSVLTDLFSEDSDEKH

FETLISQLPKDDGRYLIYDFNYTGSFGPSSKMIMVLWCPSSIPVKKKMVYASSKDSLKKA

CPECVEFQADCCEDLCEKEIQLKLCKV

>SG-PL_contig_54_1_+1_1_1284

HAHGLAGDQVHHGSITGLDHLGVVLQLLAGTPVALLLQLGKLAGNVSSVAIQHGRVASLD

LTWVVQDDHLSVELLALLGGIVFAVAGHIATTDVLNGDALNVEANIVTRQSLRQSLVVHL

HRFHLSGDGVGGEGDNHARLQDTSLHTAHRHSADTADLVDVLQGQTQGLVSGAGGGQDGV

KGLQQSLAAGAALFALDGPTLVPAHLLTGLQHVVPVPARDGHKGHGLGVVTNLLDVVGHL

LLDFVETSLTVGRSGAVHLVDADDELFDTQGVGQQGVLTSLTLLGDASLKLSSTCSHDQH

STVSLGSASDHVLDEITMARGIDDGDVVLAGLELPQSNVDGDTTFTLGLQLVQHPGVFEG

TLAHLLGFFLELFNCSLVNTAALVDQVTSGSGLARVHMADHHNVDVNLFLTHIAEFRSST

TCSSGAQA

>SG-PL_contig_55_1_+1_1_252

FFFFFGESVYFPAPKCTQKHFQKRKYRSNQINKQTNENVDTDIGDNSYVVKRNFKFNLKV

LSEKNIIHRSQIHTDHKMITVETT

>SG-PL_contig_56_1_-1_1_1080

MCKAGFAGDDAPRAVFPSIVGRPRHQGVMVGMGQKDSYVGDEAQSKRGILTLKYPIEHGI

VTNWDDMEKIWHHTFYNELRVAPEEHPVLLTEAPLNPKANREKMTQIMFETFNSPAMYVA

IQAVLSLYASGRTTGIVLDSGDGVTHTVPIYEGYALPHAIMRLDLAGRDLTDYLMKILTE

RGYSFTTTAEREIVRDIKEKLCYVALDFEQEMQTAASSSSLEKSYELPDGQVITIGNERF

RCPEAMFQPSFLGMESAGVHETTYNSIMKCDVDIRKDLYANTVLSGGSTMYPGIADRMQK

EITALAPPTMKIKIIAPPERKYSVWIGGSILASLSTFQQMWISKQEYDESGPSIVHRKCF

>SG-PL_contig_57_1_-1_148_954

MATPAKAAKTDGKNNHDAADEQADKEQQEAIEQIDEVQNQIDRLNENASEEILKVEQKFN

KLRQPYFKKRSDLIGKIPNFWVTSFVNHPQVSALLTEDDEEALQFLSKVDVQEFDDIKSG

YTISFTFESNPYFENDVISKEFHLNEAGEPSSKSTAIKWKEGKDLTKKTGTPQKGKKRTH

DEQDSFFCWFSDHGDAGADELGEVIKDDIWPNPLQYYLASEIDEETGAGDEDDLDEENLD

GEGEEEEEEDFEGEEEDGAEEEEGDEEEG

>SG-PL_contig_58_1_+3_123_398

MGISRCHFFPDFFMPPPPIGPFPAALAAASFNAFCSSFCFCLKATSSSSISDFCFFGALR

GFFFPPSLPDMMKIFSPRQMPVRLARAWEKAD

>SG-PL_contig_59_1_-1_4564_5031

MNCIWKAVFRIFFSFRRRLRHLSILRDDGSALVSLHQSCLEQRLEWDSSPILAWQSLPQL

LQAKLDGGSRACFCCLGFLRCLLSSSCWLHLSSPLLTPSWTAWCQPLQSSASCSQLSGSM

PVSFRSRLHTSLKRSAGRPTGRCPVASLPYMTSLGI

>SG-PL_contig_60_1_-1_1_180

GYIQYNNNNNNNNNNNNNNNNIIGRSLIVSCLKGTLKSVLTNQDIFHMLDAVLQKQGDIG

>SG-PL_contig_61_1_-1_1561_1749

MSYFLCLAFQLVGRTVSVFGFSSSSLFPSHHPALCLPFSSPSHLSCVYMFCFAHVVTIKQ

NLG

>SG-PL_contig_62_1_-1_259_2172

MNNAGPSYPMASLYVGDLHPDVTEAMLFEKFSTAGPVLSIRVCRDMITRRSLGYAYVNFQ

QPADAERALDTMNFDTVKGRPIRIMWSQRDPSVRKSGVGNVFIKNLDKTIDNKALYDTFS

AFGNILSCKIACDDQGSKGYGFVHFETEEAAKQAIEKVNGMLLNGKKVYVGRFIPRKERM

MIMGDKMKRFNNVYVKNFAEELDDDKLRELFAQYGKIISAKVMVSEDGKSRGFGFVSFEE

PEGAEKAVEHINGTEVGGRIVYCGRAQKRAERQAELKERFEKIKLERMNRYQGVNLYVKN

LDDTIDDERLRKEFSAFGTITSAKIMMEGGRSKGFGFVCFSSPEEATKAVTEMNGRIIVA

KPLYVALAQRKEDRRAQLASRYMQHMANLRQQQSAQFSQMFQAAPGNFFIPAMAQTQNRF

YTPHGGQMPMRANPRWNTPVRPAQQAAGFQGGMPPAQMRQRAPTQSAAARAAMNARPITG

QAAVPPSAGGQPGPRPMGPGVSRPPTAVQAAPAQQNRYKFTNNMRNAPAAVQPGVMPQAQ

QQSVIIPGQDPLTATMLAAAPPQEQKQMLGERLFPLIQRMYPELAGKITGMLLEIDNSEL

LHMLESQESLKAKVEEAVAVLQAHQAKEAAAAAAAKDK

>SG-PL_contig_63_1_-3_87_404

MLECPSVRLDLAAYKKDATSSLAYTAYFSALCQDFANFQRVFTDGSKSDEGVACAAFNST

TGKSFSEHLPKESSVYTAELTALFLALKMVYQSHHKDFLICSDSLS

>SG-PL_contig_64_1_+3_3_263

AFKLLTQTNCFQAALTIQLLSNCSLKPSNCFQTAHSNHPTAFKLFSPTIQLLSSCSPKPS

NCFQAALSNHPTALKLFRLPLPIIQLN

>SG-PL_contig_65_1_-3_30_1352

MGQKQSGGSGGQGGEKSDDKDKKRKYEPPIPTRVGKKKKKTRGPDAATKLPLVTPHTRCR

LKLLKLERIKDYLLLEEEFIKNQERLKPQEEKHEEERSKVDELRGTPMSVGNLEEIIDDN

HAIVSTSVGSEHYVSILSFVDKDQLEPGCSVLLNHKVHAVVGVLGDDTDPMVTVMKLEKA

PQETYADIGGLDNQIQEIKESVELPLTHPEYYEEMGIKPPKGVILYGPPGTGKTLLAKAV

ANQTSATFLRVVGSELIQKYLGDGPKLVRELFRVAEEHAPSIVFIDEIDAVGTKRYESNS

GGEREIQRTMLELLNQLDGFDSRGDVKVVMATNRIETLDPALIRPGRIDRKIEFPLPDEK

TKRRIFTIHTSRMTLADDVNIDDYVMSKDDLSGADIKAICTEAGLLALRERRMKVRNEDF

AKAKENVLYRKNEGTPEGLYL

>SG-PL_contig_66_1_-1_4_1284

MATGTLYTFPKNFRAYKALIAARYSGADLKVDPSFKFGETNKSDSFLKKFPLGKVPAFSS

NNGLNLTESNAIAQYVGNKQLQGESAEAAAQVQQWINFADSEILPAACTWVFPCLGITQF

NKQETERAKAQVTKALSVVNNHLLTRTYLVGECITQADISMACNLLMLYEQVMDPEFRKP

FQNVNRWFTTLVNQPNFKAVTGEIALATKMAQFDAKKYAELHGGQGDNKKKEKKEKAPKQ

QEPKKEKAKPKAKEPEDEEEDIPKPKESKDPFMAFPKGTFDMDEWKRTYSNMETETEALP

YFWSKFDKEHYSVWLGEYTEDLSHSLTFQTCNLVAGMFQRLDKMRKHAFGSVVIFGEDKK

NQIGGIWIWRSHELAFTLSPDWQIDYESYKWTKLNPDDAATKKMIDEYLLWEGDFGGKKV

NQGKVFK

>SG-PL_contig_67_1_-1_187_411

MRRQWQQLQVSEGVPAAVQSSERRKRLDRVSEEYDYILTQSLSLSLSLCLCLCLSLSLSP

PLSLSFNRRKMNSNT

>SG-PL_contig_68_1_-2_2_493

VTRYIERYSTGPVAKTPCGLAWFSIYKPNSLAAGAAWIALAAANAGIDADDSRKFAVEQI

NFLLGDNNRTADGCFSYQIGYGNKYPLHPHHRGISMCDGCTLYGALVGGPDVGGFHRDTQ

ADHVMNGVSLLHNAAFQSALAAIVQLQKEDAVPGTQSLCTCPSQ

>SG-PL_contig_69_1_-2_20_580

MEVSRLFVRSFACFRKERAVRLISTTHALNMPLKAGDKLPSVDLFEGNPSNKVNTSELKG

KVVIFGVPGAYTPTCNNDHAPGFIKNADALKAKGVSSVVCLSVNDPFVMDAWGKSLGASD

KVRFLADTCAEFVKQADLDLDLTAVLGNIRSKRFALVAEDGVVKRINVEPDGAGFTCSKA

EDVMKFL

>SG-PL_contig_70_1_-2_824_1192

MLLLALFHTHSSYFNLHLCTNSISSSLDFLCTHIAGIVYARMCVCVCVCWLALLHVCVMS

LLNLLSNSVYYFFLNNCKMLLWYMCVVQILFYSYVCVCVCVCVCVCVWHDTLQSMCFIVL

NNI

>SG-PL_contig_71_1_+3_1059_1763

MVEVTSPLRDQSGATSLGDVASACASRSTHAESSSSLPETVAAGPKVLSVAVLAVDLLFV

HGHGGRVQGLVAALAVEAALMPRGVDADSPLGIVHSLAAAWALGSGAAAASSCSYGRILG

LGVHLALLVAEALSLVHAEGASAAAKAISLGSVLAAMTLLAVQLQLVVSHGGAVQHLVAH

PTLEAELVVLGPSGHSLLGGVHRLAALGALGLVWRLEGHLDCWSLYCSRSDMQPG

>SG-PL_contig_72_1_+1_157_315

MQTKGNSYILYTLHILYNIIIIIHPQFLAHSFHHNTSKCITIWKLLCTHWEKS

>SG-PL_contig_73_1_+1_1_270

LNRHILFYRSIGHHAEAFLRGQLRGSYPKGLALGTVTGRPEAHTARRPLYSLKFRRSLQK

FCNIHCFIFTSGQGHSGQGQQEQDGDQGRA

>SG-PL_contig_74_1_+3_4236_4478

MNTQLGNRLFSTFGLQFFDCTHPSKHRCTRLLVWFDHVWLVPPYFPFFHFHFFRVYTFLR

KSCFINKGVRVCACTVRERKS

>SG-PL_contig_75_1_-1_70_612

MIIFKDAETGDELFTDACCPKLSDCQTYYSFQSKLTEQKEGEIDDKLIGGNASAEEQQEQ

MEACVKRGLDFALRSKLEPFDFGDKKLFMKFLKEFFVCYKKHHLEKHPELTDDDHKDELT

AFESRLNAFGKLLVSKFKDAEFFFGENDMEKKHSFCAAVWNEDGMSATVYVFRGCLAEEK

Y

>SG-PL_contig_76_1_-2_110_1417

MLARAVVLAAVVAVCAGDLTYKIISKKLFSFEILLAFTSPRDISGWRMRVTMPSRTKISL

VRTWDGDWTRERKNTGYLQNMDSTGPMKAGETITNIMTVTCDGYCRFPFELQVVEPQEGG

SISWDGVPASGLGPPIGAGQGTKDYADALKKSIMFYNTQRSGRLPDNNPISWRGDSFLDD

CVPGGWFDNGEDFAKYGLPFGFATHALMWGLYQFKDGYERAGQLDEMYDMIKWATDYMLN

AYDDSTKELVAQIGANDNHHSPGEWDRPEGVVMDRRCVKMSESRPAPELYGEWAAALAAG

YLVWTDKGDTDYANEMLAEAVSMYAVGKASNRTEYNDIADVDYFYHSDGYEDELCLAAVW

LYRATGDSQYLKDAKNYHPSGSFARYSWHSKQVACQLLLYEETNNELYRKEVTRYIERYS

TGPVAKTPCGLAWLSI

>SG-PL_contig_77_1_+2_299_541

MKHTQLRTVRSLGATAEKSILHHCSEAGKRRGLHVSAPEPRGVFHADKDKRMTQPFKIIP

NDEKLFLTLFHLCLNLPNNSK

>SG-PL_contig_78_1_-3_126_545

MDSGSSDLDKLSASVGLAVTSTHDHDYSSKRPRMSTVTEELSFEEIFLSPAPSPAPSPAP

TPSTSYVITEPVEDKSRNRREKNNVASKRSREIRKQKFVGMEEEAKRLIVENARLEKRVV

ELERMAKQMREILVAKMARK

>SG-PL_contig_79_1_-3_984_1166

MCLREIGTEQKQYTRIYQSIMQNFKENVCHTANYLYLGIHRLRECRYTKNKEAVCHTANC

F

>SG-PL_contig_80_1_-1_79_585

MFKRVCVLFALAILMANCSVAVNWTSISGALKSVSIGPSGIWGANNADNIYYRASTYDLN

NVQGTKWITVPGKLKQVDVGTNIVWGVNSDDNIYYRHGISEDNPTGSAWVHVCGKLKHVT

VSNEGHVWGVNNVDQIFIRVGANNCNPYGVSWKRIPGSLKQISAGFSGV

>SG-PL_contig_81_1_-3_858_1445

MAAKSVKCVLVGDGAVGKTCLLISYTTDTFPEDYVPTVFDNYSDDITLDGQTISLQLWDT

AGQEDYDRLRPLAYPHTDVFMLCFSLMSEASMKNVKVKWYPEIRHHDPNVPVVLVGTKLD

LRQDKDAVRQLRSNKQEPISTSQGNELAKAIKAKRYFECSSKTGENLKTAFEETVRAALH

LDGPNKVKPKPKCVLL

>SG-PL_contig_82_1_-1_1147_1395

MKAKAILLNQNETNSVSYAFCLLFAVYPTLPGRDYTIQGKVWVYHIVQGGKRVMLREKVV

GVLTISKRSKLKKDYIIQSLHNV

>SG-PL_contig_83_1_+2_4142_4825

MTRFSSIPRHSHLSVFSRHTRSTRGPNDAHRTRWTLDSLFASETRWSRGTRVSFWTKRTR

DTLLASLTTLAPDTRFTSLTRRTWQTPLSSCSTTTSATTSPSGTFQTSRSWVAWETWGSN

TTLFTKGSLGSNFPWRSIVSLCPLNALNAHGARRSTHSFVSFGSMTASIAICTRATFVTF

LSLTTSRSHITLTTTGTRVTNDSLQAHGTLISLSTIVTWRSRKTSRPQ

>SG-PL_contig_84_1_-3_3_272

VHGCAVHCTLQGIKLSQHHHHQPFSLLASPCIAVFSRFRCAQLTSSPTPVVELCTHSVVF

HPPCGLVSSDWPHCPSVHLPVPLTCHHVQP

>SG-PL_contig_85_1_-3_51_260

MSVFGGIWKLEHTQHAHFILPSLALSCIGFYHCRRKGGPAAWATAGSPEHFALALCPGLS

SSTDSIILGD

>SG-PL_contig_86_1_+2_131_310

MNIRSKYKKQLIFCRASYLSPKVVECFIINLGRKLMILAIRITGRPGFKRPWVLCLVGKR

>SG-PL_contig_87_1_-2_2_253

GRQSFLDTTDPGLSWSASGLVSSHLHVVCTMGRVGDQHSGEASKPFYSLVFYVMEDWCCP

HHFSDWYMLRSPDLQTFITSVCQY

>SG-PL_contig_88_1_-1_235_1254

MYDTSPSSPFKGEIRQLWPENFSTKAFAESLLELDPALEETLDLSEFETNTNPVGSPSGF

PDRASGSPPVTTRDLLTLTPAVTTQYLAQLEGQSPIFTADHGVAVNHPQQHPAPSPPHLA

LAADDVVQTFPDDVLMPEFQSLVAMTTAPSTSTCQQGGAEWAQTPFYSTVKTEDLTNLTA

CSVEASLPGFTSTTTTSSICKSEPFGQDDLSCSQFTDLTDLSPSPRSTRSMSESSTATSV

MSDTPRRIGKGGSRRRQVPKGSEEYKEKRARNNVAVRKSRAKAKEKQRMTEARVKELADN

NDALQKRVEMLTKELNVLKGLFINVGAKLPDDLSKLLEQS

>SG-PL_contig_89_1_+3_45_278

MVSILAKQGVRKNSPFWHHILYHVKLVQNSEEKEEGIALVVRLGAPCQRDWLVSRDKGNS

GTISSGKSEIVKVMGNEG

>SG-PL_contig_90_1_+1_1459_2256

MKMSLALPSKPVHFVCIYRPPSSKKNKLRDSMFFEHFPSLLEHCNTLSGNMIILGDFNFY

FDRPSDSNTSKLTDLMTVFGLSQSVDGPTHQHGHILDWIMHRPDDNIHRSTHVSHALESD

HFCVVAYLDVMVPPNPPVFTQTRNIKAIDRLAFKADLHDHLKSCPDPSAEQLNASLTSVL

DHHAPSSRRKVTVRKSTPWFNAISDQLLAAKRERRRAERQWLSSRLTIHKQIFTAAKNFV

NRLVHAAKTAFYSSRVVESSTTKQLF

>SG-PL_contig_91_1_-3_3_272

QFALNPFPFFFSFLHIHEKKSWHILASMYTNVCTQTLTLSLTHTHTLSLSLTRTHTLPLS

HTHTHSPSLSHAHTLSLSLTRTHTLPLSHT

>SG-PL_contig_92_1_-2_110_1321

MTLLVAALFALTCLTVPATSVRQMILGVTPFTEEQMAILLSAIFKTEVEFTPPPEINTQI

QALIPTDNSQALREEWEKAGMDVTTIVSDLRELAQVEEQHRRRRDAENVNPDTTLNHNYY

RSYDQIIEDIKKLVQKFGPTLIRFDESGKNPARTLTAEGRIIHIVELGTQTEKQKPVILL

EVNIHAREWITNAANLFMMDWILTNYRLSGGAAKMLLDNYTMVVVPVANPDGYEFSRTHP

DNRMWRKNRHVFEFCTGVDLNRNFDMMWRGDLEGYLWFSRSDLNISPDEERQCPLLYPGP

SANSEPETQALISIFHHYQENVVLHFFVHAYSEVVLMPWSYNSIAANKIHQEMGDIVARA

MDYGEAKTVAEFARYNASGTSLDWALNEKLTESPDSKFIALGME

>SG-PL_contig_93_1_-1_70_2283

MDESALKATFTIHVIRRSFNLAVSNMPVETTRNLTGDKMADDFETSVPMGTDQVAFIVCE

FARKTKMTNRNVKVEVWSRQEVVDQMNFALDFGVKTIEFLEQYFQILYPLPKLSMVGLTD

FAAYSTEVWGLVMDADEIFSFNDQTITIIDKMITATNVANRICRMWQGNLVTPVSLGDFW

LSRALASFMENKVFDHVYPDRRMMDYLLSDVQKAMIEDGFESSAPLSDGYDELNGYPKGA

FVLRMLHSVIGDDKFRKGMKKYLSTQRYGSAHDTDLWNALEDDELDVSIADFMSTWTQQM

NYPVVTVTTEGQTVTLTQERFLQDPSAEDPGDYILPFGYKWDIPFTFTSSDQQNFDITAS

DVIWFTKDDEQKTITIPTLSEDSWVIGNIGLNGFYRVNYDDNNWRKLLAQLHSDPTAIGP

LNRVQIIGDMFALSRAGLVNKTRALEALEYLDKETEYAPWSAATDELYELSKILSNTDIF

PAFKAFMVEKTRPLFVNLRMTPVENTTETQIRRLIASTACKYDGPECIEAAVTHLHDWKN

NPDNRIPEVMHYAVYCGSMHKGNTDDWDFVYERFLAESDPLEKQVLMTSLSCTNNSNDLD

RLLDRALNSQDFTTENFMTVLQYTILNPVGSKRAWTFISNTWDSQHEKYRADGFPYTISY

FGDVFSTQDQLQMLQDFVDEQDSLGEDTEAFSAVVDDITINVAWMERNAGIIRNWLQKQG

FSASPGSVALNINSLTDQ

>SG-PL_contig_94_1_-2_26_1465

MLHFAIGDDQFRKGMKKYLSTWSHSYAHNTDLWNTLEDDQLDVSIADFMNTWTQQMNYPV

VTVTTEGQTVTLTQERFLQDPRAEDPGDYNSPFDYKWDIPFTFTSSDQQNFDITASDVIW

FTKNEEQKTITVPSLSEDSWVLGNIRLKGYYRVNYNDNNWRKLMAQLRADPTVIDPLNRV

QIITDTFAMTSAGLVNDTRALEVLEYLDKETEYAPWIKATYKLTQMSKIFSNTDIFPAFK

AFMVEKTRPLFVNLRMTPVDDSMETELRTLIAKTACEYDGPECIEDAVRYLHNWKNNPDN

NRIPKAMHATVYCGSLHKGNTGDWDFVYERFLAERDPLEKRILMASLSCTNNSNDLDRLL

DRALNSQDFTTYDFMTVLQYTILNPVGSKRAWTFISNTWDSQNEKYRADGFPFTISHFGD

VFSTQDQLQKLQDFVDEQEHLGEDTEAFSAVVDDITKNVAWMERNAGTMRNWLQKQGFSA

>SG-PL_contig_95_1_-1_232_339

MSSWYIIFLTMVYELSTVFCLCTCVCVFVTLCMCVC

>SG-PL_contig_96_1_-2_152_550

MESLTEEQIGEDCVGVLKKLLKKDIPAPVRVMRTRWGTNPLTRGSYSYIKVGSCQQDIVE

LSKPLKSKGSAKPTVLFGGEATHPGFYSTTHGALLTGYREANRLMKLYNPSSCEDDSDED

SDDNEDGGIKISF

>SG-PL_contig_97_1_-3_69_977

MSKLLRKYLLVLLLLLPTTILFVGQSKVSSNVAFADDSMEGEDDEAVVETDDGPASSDED

VTEDADTGVTDQEQEEEEGEDRALKPSPDADTVIHFVKPTGNGQELPAGKVVRVLVGFTN

KGYGEFTVESMDASFRYPQDYSYYLQNYTTQTLNRLVESKRQATFEYSFIPNEAFSGRPF

GLTVNVNYKDGQDNQFQTAVFNETIQVVESDEGLDGETFFLYVFLAALVILALLGIHQLV

TTFGKKRLSKPRPAVEMGTQNADIDYDWIPKETIKEMNRSPRRSPKQSPRQRRNQRSSGS

AEE

>SG-PL_contig_98_1_-1_67_1335

MREIVHLQAGQCGNQIGAKFWEVISDEHGIDPTGTYHGDSDLQLERINVYYNEATGGKYV

PRAILVDLEPGTMDSVRSGPFGQIFRPDNFVFGQSGAGNNWAKGHYTEGAELVDSVLDVV

RKESESCDCLQGFQLTHSLGGGTGSGMGTLLISKIREEYPDRIMNTFSVVPSPKVSDTVV

EPYNATLSVHQLVENTDETYCIDNEALYDICFRTLKLTTPTYGDLNHLVSATMSGVTTCL

RFPGQLNADLRKLAVNMVPFPRLHFFMPGFAPLTSRGSQQYRALTVPELTQQMFDAKNMM

AACDPRHGRYLTVAAIFRGRMSMKEVDEQMLNVQNKNSSYFVEWIPNNVKTAVCDIPPRG

LKMSATFIGNSTAIQELFKRISEQFTAMFRRKAFLHWYTGEGMDEMEFTEAESNMNDLVS

EYQ

>SG-PL_contig_99_1_-3_153_1187

MDSVRSGPFGQVFRPDNFVFGQSGAGNNWAKGHYTEGAELVDSVLDVVRKEAESCDCLQG

FQLTHSLGGGTGSGMGTLLISKIREEYPDRIMNTFSVVPSPKVSDTVVEPYNATLSVHQL

VENTDETYCIDNEALYDICFRTLKLTTPTYGDLNHLVSATMSGVTTCLRFPGQLNADLRK

LAVNMVPFPRLHFFMPGFAPLTSRGSQQYRALTVPELTQQMFDAKNMMAACDPRHGRYLT

VAAMFRGRMSMKEVDEQMLNVQNKNSSYFVEWIPNNVKTAVCDIPPRGLKMSSTFIGNST

AIQELFKRISEQFTAMFRRKAFLHWYTGEGMDEMEFTEAESNMND

>SG-PL_contig_100_1_-3_93_623

MVHTTNPLYRKLGQKATNDLNEAKLQTKILSLSCSFDGSECTQDANAFMEDWKENPSENP

FPYHPRELWYPAVLRQGNRDNWDFMFDLYKDGDPSDEFALERGLCSTNNSEDMNRVFDWV

LRNNRTDDFVRLVFCASLNPSSANIAWNFISNTWDIHHEKYTASRWDFPATIRILGK

>SG-PL_contig_101_1_-2_2_463

QILGDIWDLARAGLANQSRALEALEYLDAETEYLPWTRVDDVLEELRNILFFTDLFPAFE

AFIVHTTHLLYRKLGQKEPNDLNEEKLQEKIIFFSCLFDGSECTQDANAFMEAWKENPSE

NPFPYHARELWYTAVLRQGNRDNWDFMFDLYKDG

>SG-PL_contig_102_1_-1_115_663

MYYAGTISDYTRRIYGARVNDMIDKVFSTGMVDTDDLLKMLAAAGNVTDNPDIDNQLGGE

HTIHHLQENLDTLLNSPTARQALHVGQANGNVQFTALSYVVWEFIKTLNFFRSANEDNIF

LLENYKVLHYSGNLDLVVSVAATEKFLQQLEWTGADRYYNSTRDPWFLDDALLGWVTQIG

NFT

>SG-PL_contig_103_1_-3_198_893

MEAELYENMYYAGLISDYTRRTYGAIVNDVIDKVVSTGRLDAESASKITAGMANITDTFD

IDNQLGGEHTIQFLRDRLDWLLSSPTARQALHVGQVNGNVWFRSLNFAVWDDLKTLNNFR

SANEENIFLLENYKVLHYSGNLDLSVSVAATEKFLQQLEWTGADRYYNSIRYPWFLDGIS

LLGWATQIGNFTRVVIRNAGHQVPHDQPEAAFLMMKNFVLDMPFLTVKKRDP

>SG-PL_contig_104_1_+1_205_627

MRDASSSSPSNGGMSSVAVASSLAASGTPSSSSIPKPSLTMRWIRLAWDVGSSKVKPEDS

RAVSKSSMHKSFTDLSFLSASHLTLRVLMMGWSALISKCFLAAIYPMVLVSRRAWAFMIR

SMLADQPYCDVTMQHGEDTRR

>SG-PL_contig_105_1_-2_2_238

GSEGGAWKQVAGSLKYISSGNGAVIGVNSNDDIYIRNGVNFKNPTGTSWTKLNGRLDVVD

GVDDSLYVGVNSADNIYHS

>SG-PL_contig_106_1_-3_159_455

METEAYKSLYYFGLISDYTRRTYSAVVANVVNRSLSTGRFEWIDVAEMLTGTDGVTDNAD

MSNMLGGEHTTFRQEFYLNVLLSSPTVREALHVGQANGN

>SG-PL_contig_107_1_-1_115_756

MASWITLIAFLAMTSVVCLAQTENDGAVFLSPLIAAGQIDTAKSLSRVSWDFVNKVDETQ

LESYAGYITVDETYGSNMFFWFFPAKENAATAPVLLWLNGGPGESSVFGALVENGPLKIT

KTSRVVESEWSWTNAFSMLYVDNPVGVGYSYAANPAGLTNTVEEMMPHLYSVLVQFFTLF

TEYSQNDLYIGGQSFACQYISALGKYIHDHNHAS

>SG-PL_contig_108_1_+3_186_500

MIFIKKHPVVLYVFGTVIVVKKHPVKKKMQHLLHLSLSEVCSSFKERSPVILAMVLRPWQ

RVSLMPLSSWILFFLSHGICGIGAYGIYSRRGDAVLRKLTTENEN

>SG-PL_contig_109_1_+2_2_118

RDLKVQFQPEASKNQAQDSVLNFYESKPKKRQRRNKPQK

>SG-PL_contig_110_1_-1_133_270

MGPVDHATATLRVLSWSPSPPRVRQTAQHPHTPRQHLHRRSCNTTF

>SG-PL_contig_111_1_-1_28_474

MGISVKDVNQHEFTKALAAFLKKSGKLKVPEWADIVKLGRFNELSPYDEDWFYTRAASTC

RHLYIRSPAGIGAFTKIYGAKKRNGTAPSHSCRANRSVARRVLQSLETLKLVEKDPNSGG

RRLTAQGRRDLDRIAAQVKSKKAEAPKSV

>SG-PL_contig_112_1_-1_1_177

NQSMNQSVNVFKVCISHFHFENIDQFQILPLGKISKTTNFENMILFQIYKDICLSLSGG

>SG-PL_contig_113_1_-3_3_242

NGSEGGAWKQVAGSLKYISSGNGAVIGVNSNDDIYIRNGVNFKNPTGTSWTKLNGRLDVV

DGVDDSLYVGVNSADNIYHS

>SG-PL_contig_114_1_+1_1_225

SIILCTLPITSLSKLVVIYVCFIYMVFLFPPTNLICLCFICSFMLLFNLAVVVDSSLIVP

DHCFLFVNKTISFCQ

>SG-PL_contig_115_1_-1_154_483

MLPQDKNIPSSIISDDTASPQKLSAEQKSDNAYAGRRKGALADIKINNLLSQFHRTQETP

GKGSSSPHRPVNPVVVQSKRRRDRLEKATNKALHDIHRLLQTYRHLVLKD

>SG-PL_contig_116_1_+1_2602_2964

MVNTTKFIRFSLITITKIIYIKKFQIKAIDKNQNSNILARLESGCVEFRHQDNKLVLVYK

LYQAARRRKLSMVSWQYLTVSTRKSNNHDLEQIITSRTKHPKQISMYAGHRNCSNAKKKK

F

>SG-PL_contig_117_1_-2_215_1651

MPGAHDQLSKYNKIEAFVKGFKVSTSSYKRVMHLLNQEMNHGLHKHSHPVADIKMFPTFV

RSLPDGSERGDFLALDLGGTNFRVLLVRLQGHDIDIQSKTYLIPQRIMLGTGTQLFDHIA

DCIGKFVRDHNLSGQTLPLGFTFSFPCRQEGLAKARLSKWTKGFRCEGVVGEDICNLLHE

ALKRRTNCDVEIVAIVNDAVGTLMSAAHVDRNCEIGLILGTGCNACYMENLGNVGTWDDE

KNHPDQVIINTEWGAFGDNGCLDFLRTEYDRELDTFSINPGKQIMEKMISGMYMGEIVRL

VLERLTMEGLLFRSVDTECDLFERGRFFTKYVSEIESDTDEFFRNTKQVLEEIGIENYST

EDCKIVKYVCTLISARAAFLAASGLATLINRMNKSEMTIAVDGSLYRFHPRFHNLMCLKI

QELVKPGLKFKLSLSHDGSGKGAALTAAVSLRLRNMKYTKENVASKNETPVRISIPQEA

>SG-PL_contig_118_1_+1_190_462

MDQEGRKKQTAEERKKGRNKENNRKEEIASTEFPQGGDTMYTERDAGIPTWGGAILRHIV

WDIKKTQTNKKNPTNIIYIQNLHPKLKYTWS

>SG-PL_contig_119_1_+1_451_648

MFKKRNVLHCNIIQEYIRLQIYMYVECHLYFSHQRSTQLNISPAAESSLPIISTKNSTFP

RLQRAH

>SG-PL_contig_120_1_-3_231_3989

MASNMEEELVPSVDDQAFDPEPTPTYDAAFPALTSEHRELNPALVNTGAWQPKFQPRTSK

CTQVFTVPMVERRFKDPNEHKFGDETEQAKICKDIMSKYGVAIEISLARDQSMTVVINGR

SDNVMKARREVVSKLQTQAMYQVKIPREHHRFILGQKGKKLAELELATATKITVPRAEEN

LDLIKITGTKEGIEKARHEIECISNEQAKLAFERLPVQKAFHPFVCGPFNQNIKQLIDET

GARINVPPASVDKDEIVVSGEKEGVAKCKHVIMGIFEEKKRKCQTVSVEVKKSQHRYVIG

PRGNGIQDILQNTEVAVDVPPLDSPSETITLRGEQDKLGPALTMVYSKANSVATSEVTAP

AWLHRFIIGRKGANIRKITEEHPKVHIEFTEGQDKITVEGSPEEVQEAIMVLEGIITDLK

NRMDYAEIEVDPKFHKHIIGKNGVNITRIKNETGVSVRIPSDGDNSNLIRIEGDPAGVQK

AKQELLEMSQRMENEKTRDIIIDQRFHGTIIGARGENIREIREKFNQVQITFPDQGKKSD

IVTLRGPKQDVDKCFKHLQVLHKEMLESNYQAKVKIFKDFHKNIIGKGGANIRKIRDETN

TRIDLPGEHAESDVITITGKKEDVEMAKNKIEVIQKELANVREVTLEIPHRLHNSIIGSK

GRLIRAIMEDNGGVIIRFPQEGNTSDKVVIRGPKDDVENAKKQLLELVNEKKESSFTAEV

KAKPQYHKFLIGRGGTNIKKVRESTGARIIFPGSGDPDQEIITLMGKEEEVKMAKQELEN

SIKELDNIVESEMNVDMKHHRYFVARRGEVLRNIAEEYGGVTVSFPRSGVKSERVVIKGA

KDCVEGAKKKIAGIVADLEAQVTVECIIPQQYHRTVMGAQGMHVREITRMHEVGIKFPDK

PMPNGRRAEGEEEPVANGDVSSEDGSEKGPRKSDIIIITGKPEKCEAAKKALLDLVPITE

EVPVPFDYHRFIIGQKGRDVRRMMQDFDVNISIPPPSENSDLVRVTGPPDNVQRARKALE

ERVFQLDTEKEDRALKSFQLQVEVDVSHHPKIIGRKGAVISKIRSDHDVNVQFPDRNEVN

QSVIIITGYKHNAQSACDEILSLVKDLEEMKSVKVFIDHRVHPRIIGSKGRAVRRIMDDY

NVDIRFPRSDSAEPDIVCITGSDDAVQDCTDHLLNLQAEYLQDLMDQEYLQGFKHPPSKS

DDEPSHPADSHGFVVRNAPWHAAPDTASTEEFPSFGSVVRPKSGHAWGPMKKN

>SG-PL_contig_121_1_-1_193_912

MIVSTRVRVGRSHDSFGFPPVLTKEDRVNMEKMTVEALNMLEGDLQGTYYPLTGMSKETQ

KQLTEDHFLFNDSDRFLRAAGGYDDWPTGRGIFHTPDKKFLAWVNEEDHMRFISMQMGGD

LKEVYLRLVKAIKQLEKKLSFARREGLGYLTFCPSNLGTTLRASVHIKIPKLSATPEFKE

FCDKYNVQARGIHGEHTESVGGVYDISNKRRLGLTEYQAILEMKTGVEAVIQKEKQLAGK

>SG-PL_contig_122_1_+2_677_931

MVFKYQIPIQEHPNTHHITEITFITNHIPHEKTKHGQKRKPKQTKKQNGQPPIHTPTRVG

QAIIQSVYLFCVHSTCVMQYLPCIS

>SG-PL_contig_123_1_+2_1217_1414

MAQLNKKQPDSLLNWHFTINTNIAFYLSVKALTMQVSLCQGRLYCRIYCRIYYRKTWGVC

PNSLFG

>SG-PL_contig_124_1_-1_208_1932

MNKLAHPKLLMLWDAFEEPRTMVLVMEFVGAGELFERVIREDFVLTERDCVHFVRQICSG

VGYMHSQSILHLDLKPENVLCVAENSNQIKIIDFGLARFYQPGDSLQVLFGTPEFIAPEV

VNYDAINFATDMWSIGVICYILLSGLSPFQGDNDAETLSNVTSGEYDFDDDSFDDISDNA

KEFISGLLVKRKDKRNTIAQCEKHPWLAQQDQRIRCKRLNTDRLRCFMARRKWQKTGNAI

RALGRMASLQKLLAGGSSSHSSSSSLSSIPDSSAVQTASGANTPVFSPPVKQSASSHPLA

FAPVKEDDKVSTPGADTATDTETGTGRVEADREKKRAEDTNDGSLKTVTWRTTTTTTTES

SPAAVDTQSSPSSRRKEHQDNTLVNTSSSALSGRSKLSKEDAVTVSEVKVTNNSAPEDRA

DTSSPTPPSHLPEACNETTTSNNNNNKSISTKTNTFSPGPSSEEAPSTKCPSGQAECPPS

FLKEMVDCTAFKGDVVRFDVTVTGMPPPRLVWMVEEEDLEEDERHVVELGENGHCSLIVR

HVTEDDEGEYSCRAINSQGEATCMADLNVYRMGSV

>SG-PL_contig_125_1_+3_525_761

MNFIINSCIKIKLEEKIHKISFVLFFIIIFFPDNKFILQFHISPKPSAPSQVAPIYTTFH

TRKHPSNKIFRRKAGTCAN

>SG-PL_contig_126_1_+1_583_720

MKKMILFIVGNILKLLYFQAPTVVFSHFLHLSPVGIIGFDVSRCSR

>SG-PL_contig_127_1_+2_395_655

MIKDCYPIPRFQDFLSNLRDKKWFSILDQRKPRHQEFLKMLFTIHSFGNALRYVKMDQIP

YKIGAPGQYQRVMEDTLAEVMFPSLYG

>SG-PL_contig_128_1_-3_1533_1919

MFVRLWLRDSGMIKSLDNLRTSNFAQLNIILILLSLGGLPPMLGFISKWLVVTSRVPGPF

ILFLFFLILGSLIRLFYYLSLFFSVTLRGIKKFSVFESNINYRGFLIIIVLLNMLGGLLI

LLINSLSLA

>SG-PL_contig_129_1_+3_153_311

MESAFAVPVESQTTNARLMRWAMQLQSFLFLMRVIPGKDNLGDYPSRFTTEAS

>SG-PL_contig_130_1_-3_45_260

MILFHSNNKLLLFAAPVTLSFGEYVLSVIIFLKKELKPFPFVPARIYYCNSLLGVCHKCL

LSRLQKVQNYTA

>SG-PL_contig_131_1_+1_1_543

KEEKEEKKEEKEEEKEEKKEKKEEKEEKKEEKEEKKKEEEEKKEEKEEKKKEEEEEEEEE

EKKKKKKKKKKTKRRQRNQRRSRRKQRQKTEAKENKEEEEEYTKRRRRKRKHTRRRRKER

KKERRRRRRRRRKRGRRRRRRRRRRRRRGRRRRRRRRRRRRRRRRRRRRRRRRRRRRRKR

R

>SG-PL_contig_132_1_-1_1147_1326

MFTHAFLEEAGREFQERDVLKDRWPNVYWNEKVELRDERKIQITENDDDCRLCCGTLIFH

>SG-PL_contig_133_1_+2_965_1204

MIAHTYPSISICPGICVHSTTRQNHSYLHPLPNHVCILCVNIIFKLRATRHHNYLRQYHL

SNYIPDILITFKIFTNFSAI

>SG-PL_contig_134_1_-1_1_186

LPTSKRSHNMNFMTLSSSSTAFWFLNEYSFTTTNQKPAVSSHTRHGSRSSQRNFQHALKS

PD

>SG-PL_contig_135_1_-1_1_816

DTSSLVTVESEKPSEVEQAEETVVEDIPQEEVTTEVTRPSEDITEEVRETISVSTQPFQV

EETQAEVDKADTEQVPEDTPVQEVTEEVITLVTEMAEAEEEKSFEVEIVQETVDEREVKE

LGPEVETERPSEDHVEEVKETIVVDTEVPYEVQQTVETVDTVDTGEVAEDVHPEQPEEEM

VETYQETVVVQKEQPFETEEDETTVEQSMVEILVEKPEEAQPEEEEVDVTTETITVEGEI

TFEVKETQEEVEETETDKYISEVEVEVPGETE

>SG-PL_contig_136_1_-3_801_1028

MGTEDCLITERSLGCEVSLLWHLFELLRVMSTLLSITVLPSPTMNRLRWAGGHTHKMLIL

ALRAQIEQSHPSLLYL

>SG-PL_contig_137_1_-3_183_815

MFRSAVATLCRFGRHLPQLQQWTDSTIGVADRQLHLTGCMRVLDKDHPRKKALDKMREEE

EQALREEGSDQQMQGLKFDSMFPSIETHSLLISNTRFDELPVIHIKATHNNTIITVCDGK

GVPQARESGGTVGFRNARKGTNIAAQAAAIALAQKATKKGINLVRVSVKGIGPGRMPAIK

GLQMSGVIIVSITDSTPLPHNGLRPKKQRRL

>SG-PL_contig_138_1_+1_1_294

AVVVAAAVVVAAAVVVVPAVVVVGAAVVVAAVVAVGAVGGAGAAVVLGAVVVVAAVVVVG

AAVVVAAVVVVGAAVVVAAVVVVCAAVVVAAVVAVAAV

>SG-PL_contig_139_1_-3_72_938

MKLLFLIAVLASISQVGWSQCSTISKLPVSDASCCSRLEMYQLYQLFPSVFSNVAKPNYT

SEDCCRVRYTDPCTCDPSSPRTPSTCSDFLPITAKNSEGCCGKLYQTNITTTKCYPRYIF

ENFRIEVKKFCESHFDVTYKYTPTAKEISGMCVRTNAMPYDKCVKAKPTNPGKCYCKPAV

TSCFLPTTTTTSTTTTTSPTTTTSTTTTTSPTTTTSPTTTTTTTTTTTTPAPLCPAPPPP

RASGSRVLGGWTANPCSASGVLSFSSGKDSAQMQMCNGVIAYNPTEKTN

>SG-PL_contig_140_1_+1_316_558

MKLRFCTFLNSRKFLTILLHCSLYMEKKYVPFFLYIVNWRNCWPYECKTVYSLKLTDSLI

CFGLEHNIKCDNIVDFIILRI

>SG-PL_contig_141_1_-3_984_1325

MYSGIGMVMVLFSPFQKRVKFCSFLNFLFTLNDFKRSAKVCLRNIERKLSGVGDSALAIL

KYILPFNYEFVWKCSFLWQREFSTNNMHDKSGSFRLWVALPCCAPQGSDGAAIV

>SG-PL_contig_142_1_-3_3_146

FSWCQHYEGGAYLLSLGGLEAACTKYLIRQSDWKADALPLLQTDFYYL

>SG-PL_contig_143_1_-3_3_188

FLYNGICSWFFVVVYNGLCIWFSCIHDTVYAFLRYFRVRLFLMETERGRVKEQRILKGWG

YK

>SG-PL_contig_144_1_-1_274_1122

MFTMMKAVCVVLLLCLAFTGGEGKKRKCDLRYKIISSEHTMCLDKKPEAKVVRLTPTKKQ

AIVDRHNNIRRTVQPEAANMLKMMWSFKLERLALKWALQCSSVSEDHPNKRILPNLGVDY

VAQNLAVNPDDIYQAVLTWFQTKRNWQYGNETSLDGDDAAYVNQITATAAYIGCGQARCG

DDTYFVCNYYVGPSESEAPYEQGSPCSRCPDNCNDKLCDCPEYVSCVNGGTFDYKTCQCR

CPSPYCGRICTDQYCGMCDTSNTTYCQRISDIPAKCLDVCGIC

>SG-PL_contig_145_1_+1_316_891

MCGRLFEVLVEHVLSQAGAHSLEVGDVVTQLLDGADLFLQVLALDEVRHLGVLVAVSHLM

QVQQGLVHALLQTQGHLHGVQTCAPLVTVRLLDVLQNDTSSTHVLELHQFLRVLTFLVRH

LLEELGEPWQRHVITVEVERHGLVAVRSIELHVDLAVDAGLTLGMVVLATLTQSHICKSV

VFRLLWSFLKAA

>SG-PL_contig_146_1_-2_68_889

MALNNAHAQGGVLIFAVERILIYYNGIELHFEGPSGIPHFKGKKKGRVYLTTHRVIFNSS

DAKDIMQSFSFPFLNMREVELEQPVFGANYIKGRITAEPNGNWQGEAKFKMHFTHGGAIE

FGQAMLKAAALASRNRMQQPPPYTPAMDGNIYAAPPPAYMPPQDPYYAWVPYQTFPTAPP

DNSVYMTEAPPPYPGINPDMAPYPPAANGHDAKAKEAAASAYYNPSNPHTMYASAPPPPN

GMYASAPPPNGYEAPPYQGYEPPPAYDQATKKYN

>SG-PL_contig_147_1_+3_459_698

MCVHACNSLYSTDFKRKKTPSITFHKSIIIYNPNTTEQQITPTELPRLHQQQHWPNKRKK

KTKTNKIMKRDRLQLQDYLS

>SG-PL_contig_148_1_-3_291_575

MPVHVFENFFSPQFMIVLYVKQLFAFNVFIVDEHDIFVLFYKCFSPIHIFVLFYKCFSLI

CIFVSFCKCFSLCCIFLSHFTNVFSDTQDSSYLCF

>SG-PL_contig_149_1_-1_193_621

MIYQVKCLNTVIVQYADDLCMWMQMNMKRKRFCTYTRKIIRLELDIIGQFMNENGIHLST

EKTKIVLGNSGSNRENMPTFKLGNAILRYNQSIKFLGVFLTPKLSWDLHIEYLFTKTRKS

LNFLKIVVQTTLGTRPINTRASL

>SG-PL_contig_150_1_-1_805_1266

MYVCLHKHACVCVCVCACVCVCVCMRAHVWYMHVCVCVYVCVCVCVCVFQPFVFFFVDRE

DQMKWSFFSLHLSLSLSVSVSVAVIDSEVIWYSKSMFLFLFFILVSNPFYLTHAQMVIKH

QGFYLSFPWLMMCRVSHWILFCRFCVLILTTFDN

>SG-PL_contig_151_1_+1_1054_1368

MHAIPHTHMNAIPPSTTHTQSCHPSFDHTRACHPSLDHTPACQPSLYHTHTHTIPPSTTQ

THECHPSLYHTHACHPSLYHTCMPSLPLPHHPSLYHNTHMLSLPL

>SG-PL_contig_152_1_-2_5186_5455

MVGWGLHCQVELKKKKVNFCLFIYLFILLFLCVPSNVFSDVCMHTVLSTLCMYFVAAQSA

YSVFSGHACLVFLCLPPNTDIAYRIFKVCI

>SG-PL_contig_153_1_-1_79_210

MGPCCVGPVGQINFHGRTPCWTRSLLQRFAMGASCEVQFLSYSF

>SG-PL_contig_154_1_-1_76_327

MPGPSDEEKPAEISAPANEQPKEDKTPVEEPASEEDKEISKQESATEQQSAADKEVSLPE

EQKSSEEAAPEMPEQAEAVTEEQQ

>SG-PL_contig_155_1_-2_32_388

MLCHVIQRVMVSVSVSCHVIFHVYGILLLTRNVSCHIMLSIMSCDLSCHVIDHAVHHLSW

YASCSPSLYHNIYHVIVSFVLIASLCHDLYSLSYVMSLYHCSIEIHSCEFMCTYTHVSS

>SG-PL_contig_156_1_-1_70_237

MTFFMCLRLRSCFFFLSLFLCFFCNLGMINSVEWLASSAQPSLICLKEHNGQDNMS

>SG-PL_contig_157_1_+3_285_539

MNREMKEKTEKTVQENALKQSIRAMCSDRLCCKGNEQKFGGLTCTLDQEALEIFCGNIFS

IFLIRARPRYFFKRCDRVLPPCSTD

>SG-PL_contig_158_1_+3_225_392

MLQLILQRSKRCLSFNLFLFCLCVVWVLPSYFRKSCKWFGVLFSQKISVICVCVCV

>SG-PL_contig_159_1_-3_114_347

MCNDGLSSDHSVKGSLVFVQISCSLFIADLSTNVLCVCLVKFHLLYTFFFYYTFLSVSLG

RSKINSISIWKKVFYCLV

>SG-PL_contig_160_1_-3_354_4142

MGEQQQQPHVTMKERLIHGGRSMFNFFKKQPPKEGEEREKLKKELFDFQKCADHGFPSKP

SALAYDPKLKLLAIGTKSGMVKVHGAPGVVFSGVHKEDVSVTNLFFLPEEGRLVSVCSDN

TLHLWEINLRSGNSVLEEVKEFSMENNKMKAVSTCCLTTDSQNLLLGTEGGNIHILNIPS

FTLADQIIYQDIVMQNVPDDFKVSPGAVEAIAVHPTDSDKFLIGYNRGLIVLWDNKESKT

EQTYNGSQQLESLTWNRNGEEFYSAHADGCYITWDAKDSSEPKEASVTPYGPFPCKGINK

LQRKSSKGGRYVVFSGGMPRASYGDRHTVTAMQGDDTHVVFDFTSRVVDFILLSRADETD

TPDIRAENDEPHSLVILAEEELVVIDLETVDWPAFRLPYLQSVHSSAITCTQHVLDVPDQ

FWQKLVDAGDAQSKSYSAREWPITGGKNLAKEPSSKDLLITGHEDGTVRFWDASGTSLSL

LYRVATSPIFGIVDHACETPSGDEEEWPPFRKVGTFDPYSDDPRLAIQKTWLCALSETLV

VAGTAGQVLVMQMEREPRTQELQAYTVSIVGDKDNFVWKGHEALTPTVGDTRFDAGYQPT

CIMQLYPPAACTALAFHSEWQLVAAGTAHGFGLFDFAQKKQVAYRCTLNVEGMSGNAGTA

VSRRRSFRKSLRESFRRLRKGQTPKQRTEQTKEKSETQHIEQTPAEGETAAATGSTTEPA

APAAEEATTEPAATTEATPAPEATPAAESTPATEPTPAAEATPEPAPEATPAPEATPAPE

ATPAPETSPAPESTPSPQATPAPPQTPTATESRQGERVVEAPADDSMTSMVRCLYFANTF

IINGGSNTPSLWVGTNGGHVYVYSLTVPASDKRSTEDVGCVLAKEIKLRHHAPVISIAVI

DSKNRVLPESLEVVHERAKAPEMEGQHSVIICSEEQLKAFTLPAMKPRWKTKVTALDGSR

LRRVAFVSFRSNSGDNYSEYDIVSLNNQGELGVYSVMSLRRQILSAALKKCDVNGISSFV

FTKNGQGYFLQSPSEFSHVSLSTSNSSRPVCMVELKEGMRPEPPEPVVEVTPPSPTEEEQ

QTVAAEQPQETEAHESIETGAEAAAYDTADISTGLNDSQVNDSRADTSTADTTIDSVREI

VTSAAGTTIDSVHEIVTETVSSINQTTTMVTTDTTVVTVTAPSPLPPEVSVENEEVEAES

PQTNGPATNGTDEPSSPVDEIVDKLQEAVVAAAGGEDPATIKQKLMEETAAASSAAVQNI

FSS

>SG-PL_contig_161_1_+1_403_735

MSAYIPLGIQFYIRHTAKSLETKTFATINIMIPVLPSTVYQALESERDRKGERGGGESER

KMDREREGGRETGREREGRGRKRERERQRERELEGRTEREREGGGRKRERG

>SG-PL_contig_162_1_-2_110_1384

MSTSEVFRPSDGDLLIRDNIAEGAAAGTDLFRQRPATQGGYRFGQLSHNSFFTRHNPHPH

RVRHLKGLLDVPICSVNDDGYFASPRYSLQFPPNTFNNTKLNKWKGRIPVNAINVNSQLH

PINTITGLQYFNGLNSYPWRERAMPKIGMVPITEAWRDELKRFTDQLGDVALTKDPAKDC

LPREEERPRTTMYSQETGRLIPPPSRAMSRGNSRRGQRLTNFGGFHHIAPAPDLENVVLT

MLCQILQTEDVNAVQAWLCSSGDREKTMVMDMIKAAMAGREEYWKQYPAAEMDQGGDKKS

ILPPINGAVRPPTGEMNRLVLEEGGPGAAQPSQPPLKTLMEEKEFAIDPPTNDVFKPSTA

AGKPNSQQAPHPPTVLAPQPPPNDEAGAGKAGSASPEPKLKYTKTPLKEAADTSASKQDD

SDNTF

>SG-PL_contig_163_1_-3_3_131

VADVFLHRQILVSLHPKIFQRSGMKYHYHQCLHTHCQPIEVEK

>SG-PL_contig_164_1_-2_191_364

MIEVLSWAANSPVFMLQTGFELETSDLESTALSAEPASLEVDGKGLQLICLSCGHSGM

>SG-PL_contig_165_1_+3_897_1148

MWTIQSITQRPLQGYFLVIAYTQNQTNLGKETRCLCQFPKRNSSQGSPSRTGPLHKQKFT

KHSRTSSQGPPNRIGPLHKVHQTE

>SG-PL_contig_166_1_+3_504_737

MKETDHIQPNCNVCCIVTVLRLCSLISNVCRIVTVLRLCSPNSPTKLNKIQNEIYRSLRH

QISTKTQEKATNRKCAQK

>SG-PL_contig_167_1_-3_39_3080

MDVTFFPWDWQHAPQAWYRLEGVFSSETVLAQNLEERLKHLESRETSHLSSPYAESSGLD

RTGASSNVSSVESSMASSSEDHKEEKSSVLCEDVHDGTTENVRENHRAGNTKSFYGTETL

LQTVRNSEESSTGTVNHTGQRGTETFKQKGQSLEETDIQMFKQMGQSREANITETLKQAG

QRSEHIATETRKQTEQHTQVSATGSFIQTGQIREKIDIGTLKETGHSAKESGTEVPRQNK

EESGTETLIQTRQRVGENGAMTFKQTGQSSEENVSEALKHTGHNTDTRKLVEEKGNQTVQ

TTNDDVESAVLDRLSYTGIRRECLHEHADPRVFVHKNTVSIDTISTTASRASQVEHPPAT

MDSRSTETSHWSESFARKDRGQLTTRGKDLNTDGSSSLADDIEISQSAVRSQDSRLSSKG

EFFSTHFPGRKPQHSDRSSSDGSTTVSVTDGRFGRSKDSSRESGQRLQVRMMTSSGSCHD

EASRKGMPTLTNPDGTTDATAYMHHQSTVHTYRGNSPRVKGASVSPGRDIIDGSTDSAIT

NQTTPAQSNFNEYGQSSKTTETRINAKTIQGDTLLKESYEQTSLSDSSEQCLYSTKDILE

HQPHKNMRQLVSEPPGHQTEISEILSRKAAVETLSTNEARRNVTRTETEEIISRKEGEET

VSTTDFSTSIEDNHDFSNSDLSILSTTSDYGSMSSSNDLSSVSQDSGFLSAGLASFGDTS

TWKNSLGLEDQDHEDFTEKDKYEKTSDSLFVYEQKIIQGEGKPENGILEKSSTTKTSFDF

DSSTLQNKQSTDRNSYPSHSEEHISNFLTSEHSSGVTIKQSLQESTGRYSRTAVTTEPDV

SFQKATGLDSKTLAGREVGVSFQGTTALHSEVISLHSKEPVIRELNISFQETTGLESEAT

VSSESGVNFAEAVNMPEQSAQGEQTIVKESLLKALAEQSTQSEQTMVKEEVRTVAACASS

SSEADITHDTARRDSWIYYDVDKQWTFLELLLPVYRILCYALSRSEMPYFWETD

>SG-PL_contig_168_1_-1_442_1056

MFDGPECVSEALTGLQEWKMNPDRNETSRAMPYMMYCTGLRQGNTDDWDFIFERFKAESD

PSEKRTLMNSLSCTNNSKDLNRLLDWALNSGDINSENFIDVLHSTIFNPVGANTAWTFIN

STWDSHPEKYDTNDFWVQSTIEQFGFAFHTQDQLRKLQDFVDRHGDLGEAGQAFLAAMES

ITNNIAWMEKNAGIKNWLQERGYSA

>SG-PL_contig_169_1_+2_2_313

ETIVTCFGSILDLACGEPKDWLRGSQKTFLFPSLTPLFIQQYLPQRFPFRLCCYRADSEF

CLKGLFVRLFVCFFCLSVRFYHCTADNPACPLLGLRTTTCSYLT

>SG-PL_contig_170_1_+3_3_140

GPIFWTYLYMDMTLFLWVCYSGHDLYMDMTLFFYGSAILDMICTWA

>SG-PL_contig_171_1_+1_1_153

FFFFFLTHIFFHTLKIFFFLMQLSTFLDQHEQRAFSLKHLSYSYYYRCYCK

>SG-PL_contig_172_1_+2_62_250

MSANASALSSYLEKGYFLALLDMLIFCVSLGSFLSDLQIVVQYCSLACSMILLLFEWAFF

IAI

>SG-PL_contig_173_1_+3_618_899

MYNSVCSAHTPHKLCRNVTLGIIYQNEKHSAFIQSYLKHKGNLEIAQLTTQSQVARMDVN

TSRKLHYIHYLWFAIYLNNQQQTAVATRHRRQAV

>SG-PL_contig_174_1_-2_80_571

MATQQKAGESEVRNVVIAMDESDFANFGFEYYMKNVHKPNDHVVLLHVPEYHTVIQSPMV

MTDVSVVTDLLQDEEKRIKAFLDQLGSKLKSHGIGGKVKSVGGNAGEVICKVSNDEKAGL

IVIGSRGMGTIRRTLLGSVSDYVMHHAHVPVLVCKLEHHHQHAH

>SG-PL_contig_175_1_-1_2086_2478

MPSKFYSCGVASLQCCYSLLNMHCVFFPHTLGSRLHPVSQCDCQFSLEFRAGLYLASGVL

LPDFQSLFFSSGSNCFCCRIPPICHKNFCPVLTVSSHYGYRGLFVFRCSLMGSAAATKVE

VSSTSLPYDLS

>SG-PL_contig_176_1_-2_80_634

MAGFGSRSSSYGRSRDDYEGGSSLDMFVPAGDCGKIIGRGGSKIREMQDRTGCKINVCRD

EEGNGTRRVELSGTDDAVERAKDMINQIMEGGGDDRGGRRQNYGSSYGGGGSGSKDIINI

DSSFVGRIIGKGGSRIRELQDETGCRINVSRDGGRYGQTEVELIGSWSAIQQAKDRIDDI

TSQAY

>SG-PL_contig_177_1_-1_124_390

MTMMGFPTFCEFEIFMFIPLNKKPQSKRFSLVKCCHCWPSFSMMLLLVFCFVFISSVAIM

FMLILGNIGFSWLMYLISALAHSTSTEVS

>SG-PL_contig_178_1_+3_399_698

MDKERVSSKGEDFPTCSSKQMRGYSACRNKWVLDVGYGEHHHEFHPDLWVKISTQLRPAK

KLNIPRFPLPNTKYIHFYAVSIQLCHASSFLSLSKGFPNN

>SG-PL_contig_179_1_-2_128_667

MFRGKVGQGGEAKTDSYRTSDLAWVHDKDLTTAAAVSQRIKVITGLETDQRAPEGPSSAE

AMQVVNYGIGGHYDAHVDPFDTNEIKHALLNRSGSRIATFLIYLSDVEAGGSTVFLNAGI

SVAPRKRMALFWYNFAPAMETIDVLTVHAACPVLIGEKWVANKWIWEYGNMFHRPCGPHP

>SG-PL_contig_180_1_+3_3_272

PLPCHSIIAFCYCCVPGQTSAFFGRFANLFTAHSLIGKSLTLRFLARVFLVPLAMMWYHN

NMILLVIGMVKVDVMFVLEYSDRFGWASMN

>SG-PL_contig_181_1_+1_934_1113

MWKCRCTMTKPLSWCWKPNSSNMERCHRLETSRAAVIGIAGNQPVRSCRSNGWYPVHGNG

>SG-PL_contig_182_1_+2_869_1162

MPLQHSFAMDTGLTGRTAQPVYTTLTLRSQRRRNTTTPSTISGLINLAQLATQPTQRQAL

NTNSLSIKCLYTLQRGLPLCYNTHHTMSSACSLLHHTE

>SG-PL_contig_183_1_+1_79_288

MKEKQTNKQKHGCVNESMTEWLCTMKWRHRILYTIMDTITLFVFFLNIAITIKINQPTKN

TIERSKQKTK

>SG-PL_contig_184_1_+1_604_939

MYSFKCFTHKKIYVRTWKRTLFCFSFKQTCTAIFHHTTNAYHFHSFSSITFKFTQTAAFY

LLVHQQLSVLLKAHSHTCCGDNRDEYLQMVDDATRSPTLSVPCTDRGTGHHQ

>SG-PL_contig_185_1_-1_1_867

KNHSCHTALANLIDNWLCNINENKFCGALFVDFAKAFDVIDHTLLCKKLSLYGITSGSLE

LISSFLTNRQQTVCINSTMSKCLPIKYGIPQGSVLGPLLFSIYINDLPLHIQDLCELFCD

DTTIHTSDHDINAVSKSLQSSINQLTDWCELNHMSLNPNKTKVMLITTRQKRQNLTGKTN

LPTVFIGDEPTEEVHSHKTLGITIDSNLTWNDQINNLIKHISKKVYQLNRIKHFLNFHAR

KIFLHAYILSSINYSSTLFDSASANALKPLHRIHKRAIKTVLLKTSSLE

>SG-PL_contig_186_1_-3_24_887

MADEYDEPISDQEKLKVASNFILHAPPGEFNEVFNDVRILLNDDNLLREGASGAFAQYNR

DQFTPCKVEGCDLQALVTEHGDQGYGRFLDPRTKQTFKYDHLRKEGSDYQPAEVDKRAEP

WRLEVESAMTAYVQSHYPHGVVTVYGNTADGNITLIACIESHQFQPRNFWNGRWRSQWSV

TFSESGGSAEVTGVLKVQVHYYEDGNVQLVSSKEIKANLKVTKEQQTAKAFVNIVSEAES

EYQRALSENYQTMSDTTFKALRRQLPVTRTKIDWNKILGYKIGGELKN

>SG-PL_contig_187_1_-1_220_4440

MDNGDGTCSVSYLPEVPGDYKVNVTHAEKPIPGSPFNANIKPSTAGPDLSGVKAYGPGLS

PDGVFMEAPTEFTVDAKSVTPSGEGQVQAILTTPSGRKMATTVTNKKDGTYPVLYTPMEK

GPQTLDVTYNNAPIPGSPFKVEAVPGNDASRVRAYGPGLEGGVSNRPNKFTVDMKGAGKG

GLGLGIQGPVEAKMNCKDNHDGTCEVEYFPTKPGDYDISVTFADKPIPDSPFRVRVTSEV

DASKVKCYGPGLDAKGVTACAPARFTVDTRDAGEAPLQVTYTDQTGATKPADLVPVEDGV

FQATYVPSAEGKCKVDVQYGGTEVPNSPFQMRVLPGVDVSKVKVTGPGVQGTVPASLPAQ

FTVDTRETESADLECVVKRPDGTFIRPYIQDNADGTFDVMYTPEDLGPYDVIVKYAGQDV

PGAPFAVKATPTGDASKCKITDGADKMVPVNKETVITVDAAEAGNGKVTCRIRQPNGSDI

DIDIIDKGDGTFSISFTPQFPGDYTIEIKFGGNLVPDGEYTIQVGATEEMISPDQVDKLR

AGAPGGAPFQTVDFRFQVGPIFNFVSAAVVTPSGKTAYPKIEDNQDGTVTVRYQPTETGL

HELHVKYNNESIEGSPFLFHVDAVNSGYVTAYGPGLSHGVADEPAFFTINTKNAGPGGLS

LAVEGPSKTEIQCVDNQDGTCTVSYLPTVPGEYLITVKFADKNIMGSPFTAKITPSSREP

RKRAQFSVGSSSEVSLKVTETDISDLTATVRSPSGHEEPCLLKRLANGHLGMTFTPREKG

EHLVNVFRNGKHIPNSPFKIMVGESEMGNASKVKITGKGITEGMANEVNEFFVDTREAGY

GNMSMSIEGPSKADIECHDTPDGTCRVTYKPTEPGTYVLNFKFADQHVPGSPFTVNVGGE

PSAKITERITRERVAADVSHVGSECELSLKIPGTSPLDMKASVKSPSGLTEVCDVTSLDD

SHYSVKFVPKEMGIHTVSVKHKDMHIPGSPFEFTVGPIAGGGSHKVHAAGPGLERGEVKQ

PCDFNIYTREAGAGGLSIAVEGPSKAEIDFQDRKDGSCGVTYNVTEPGEYFVSIKFNDEH

IPDSPFRVPITGPIGDARKISVSPLQEKTLLVGDTATFVVNLNGAQGTLQARIQPPTGPM

QEALVQEMENGQYAIRFIPRENGLHLVHVLFNGHHIPGSPFRIMVGKLVADAGNVTAYGD

GLHTGKTGQISKFIVNTVNAGAGALAVTVEGPSKVKLECREVEEGYEFTYCPKAPGDYLI

TIRYAGVNIAGSPFKARVEGAGQPSGVQEHASVVVETVTKTSTTTKFSGLQQFVSDATKC

VASGPGLTKGVRGQTNTFTVDTTGAGNNMMFVGMMGPECPVDELVVKFVGNKKYSVSYKV

KMQGRYILIVRWGEQDIPGSPFHVEVV

>SG-PL_contig_188_1_-3_30_374

MVYEMLVGGGGFLCSDEGKEVILGEMECWKNPQLKMAIMTCGHIVSRGDRKECQFWDTMR

ICLSCSVELMCSERAATFLTDFLFHLYHPVAEHMCGSQGRSTRGILDMFPFEWRK

>SG-PL_contig_189_1_+3_6_326

MLATFHFISDDCLNYIHTKTTMGICLLGWLYFFHHLSRHKQKISTLVHMKNKTQIFLQYF

FFVFTNVCDFNIFTVLFLFSQMPVISHLASFFCPCGRPSCVSLSPAA

>SG-PL_contig_190_1_+2_155_361

MNTKMRQNSVKHPQTHWSKLHGAIRHQHFLYAIRTTQYNLHSYSHKTVKYTLFRSIMIVV

ITATAVLSL

>SG-PL_contig_191_1_+1_1_150

DHTKTISGLSPQLKITPPVQLKHNQNLKHQQSFSSQSKKPFLPLNLFRSD

>SG-PL_contig_192_1_-2_521_694

MTFMHSSQTHLRKKKEVIIRLFGWMGVEVVHEILIYCLLSVAYQIYYCLLSVVYQIYY

>SG-PL_contig_193_1_+2_74_295

MCLANIYFMIKMGTITTVINKSVWEWAFTTFLTKTRRKQGDGLSTSYHRFLNTTTKYHSS

PFHIILRSSHINVF

>SG-PL_contig_194_1_-1_1834_2091

MLTQPVAASQLLSDRHSSTVRTEHSQLLSITHSNTLHTEHSQLLPASCCQTDTAALFVQN

TANSCQSHTATPFIQNTASCCQPVAV

>SG-PL_contig_195_1_-3_198_764

MSGYGGRGGGFGRSRGGDDYSEGGSTDDMFVPARDCGKIIGRGGSKIRELQDQSRCRIKV

FHDDEGDGTRRVELNGSDDAIDRARDLIYQILEAGDDRGGGGRQYQQQSYGGGGGGGGSK

EVIQVDSGFVGRIIGKGGSRVRELQDESGCRINVSRDGGSYGQTDVELIGTRSQIQRAKD

AIDDLVNQA

>SG-PL_contig_196_1_-2_101_277

MAIGLIVLAPRNVEVSTTSLPYSLRVMGQPLLLWDRVCTGLVISTSKTTEDFKFMSSTR

>SG-PL_contig_197_1_+1_169_273

MYTRQVRFSPFCCSLRHSGITPPKGTMSHPKHWQR

>SG-PL_contig_198_1_+1_1_174

KKKERRRRRRRRRRRRRRRSRRQTKGVSQRKQCCAVFKRIDNLFDVYRSKEWIIRQFA

>SG-PL_contig_199_1_-1_184_684

MELAGASRDGAPPLDGYSQWSTISERTDSRRTELLHNIDPLRESHGVPQFSDTFDTSVHA

AIRVGKYKLITGNPGTSKWVAPPEDPNVVTERLPAGNQEHVKNVWLFNIKTDPEEHHDLS

DSMPGKVTKLLAKLARYNSTAVPVRFPDPDPNATPRGPNAVWGPWMA

>SG-PL_contig_200_1_-3_93_593

MYRGKVSNEKEAGPYSIRTSDLTWIDDKNLTAAAAVSQRIKVITGLEIDQRAPEGPSSSE

AMQVVNYGIGGHFDAHVDAFDKAPEGDALLSRSGSRIATFLIYLSDVEVGGSTVFIFTDI

SVAPRKRMALFWYNFAPAMETVDELTVHAGCPVLIGEKWIANKWIWT

>SG-PL_contig_201_1_+2_2_139

FFFLFSDKILFQCTGNNSGRSQDRELKSEKRSVPIIMLIIATNKTE

>SG-PL_contig_202_1_-3_45_698

MGRRPARCYRYCKNKPYPKSRFCRGVPDPKIRIFDLGRKKARVDDFPLCIHLISDEYEQL

SSEALEAGRICANKYLVKHCGKDAFHLRVRLHPFHINRINKMLSCAGADRLQTGMRGAYG

KPQGTVARVHIGQPIMSVRAREQHEAAIIEALRRAKFKYPGRQKIVVSKKWGFTKWTRDD

YTRMRAEGHLIPDGVGAQYKPNKGPLDAWKHRVAVAHQ

>SG-PL_contig_203_1_+2_332_502

MCGIISLLIFNLFISHFHLVGTIICLTMQTTAQTRLINSLLASERYSFPYVSHGSRR

>SG-PL_contig_204_1_-2_479_685

MLNKAVLFSTLEIYIINSMNYFVSKLYCCVSLVACEGLYYVILVILYCFKLFVRKLFHKF

CCGGCVCVF

>SG-PL_contig_205_1_-1_130_1518

MTYTVTTLVLLVVVTLWTADVSGFSAQRKTRSTSQTQQTDQSQHYGVLLDAGSSSTKVNI

FSYIPGEPPNFVPQVQLTDTERIKPGLSSFMENLDGVSDYISAALNDAKSKIPEDLQAST

PVYLMATAGLRTLSASQAKALLQSVRDAMSSTTINPFYFEEDYVSILSGEEEGVYAWLAA

NYLHGFFSTSMTREDSVGLLEMGGGSLQVAFLPDDPIYQGEFQVYVGRRRFDLYVHSYLG

FGSNYIAGQVRRVLSQNSNYRNGNELNDPCMLIEDVASYEAGEVTLTVKGRSKPDECLEV

LEQILESSDTALCSPKPCAIRNTYQPAVGSLPFYAISVFLYAPLALGAVEDNDILNISKL

TDEALDYCTWNVSEAVAKTGTKAKYASTNCLMGLYIPQLLVDSFGFDQHTDRITVTQEID

DKDINWGLGAMIQHLSMAFMDDLESAVHCRQGDSVLPLDRARA

>SG-PL_contig_206_1_-3_3_188

LVVWSTLCRHYAAAQSAYSAFGRLPHHVGSHTPFQWCACWVFSFFHKPPNTDVAYGISSV

RI

>SG-PL_contig_207_1_+3_3_290

INEEKNRHLFAVCRWFCRCWPSALSYLFDQECLTLQQHRLHTPVVEALFASSLSDGVRNY

PLYPGVLPHHTAYCMISLSVYLPNRRWLVFRQSPNQ

>SG-PL_contig_208_1_+1_355_624

MMVIIMITYTAPHLTKALCISGNYHRSRYTTANACKPLCGNYHRSRHTTANTCQSLYDNY

HRGRHTTANTCQPLCDNYHRGRHTTANTCQ

>SG-PL_contig_209_1_+1_1252_1623

MDRPTPYPPLHCCPPHTHTAFTNSMDRPTSYPPLHCCPLHTAFTNSMDSYAISTTSLLPS

THTQPSPIPWIDLHHIHHFTVAHYTQPSPIPWIDLCHIHHFTVALYTHTAFTNLMDRPNN

NNNN

>SG-PL_contig_210_1_-1_103_588

MRGIRGADYECHRQARRSGMSGTFRAFLSTNTQNLDSIIYYSRDRQIPIVNKKGQLLFNS

WEDLVNGAGGYINDNAALYSFDGKDVMNDSMWPQKIVWHGSSTKGSKMDGKLCNQWHSRN

GDSMGLGSSLNKHMLLDQDEYSCNNRFIVLCIQNTARSNLRK

>SG-PL_contig_211_1_-2_287_682

MINAVKYIACVLLLAGMGVMRKGQMLYCDDYSDKICTLDAIMYCLTNGTIIVGRCRAKRA

VCLEGKRLDTTFSACARSDGRLFDCSSYDHRRCTKDSIPFCLSNGTIARGRCQAMQAVCV

EKQHVDDSFGAC

>SG-PL_contig_212_1_-2_2_301

TVSGRESRDTDCETSVCRSFIPAHALQVKMVFGTVSRSLVRKFSTSAVARSQGWQQYGIP

GANLPFNINNRYKLTLLFAVFFSSGFTGPFLLVRHQILKK

>SG-PL_contig_213_1_-3_273_4637

MVGVWVRGGAWLLIVATLVALSAAAPTSEEAEESEGRNCFYNNTEYVHGLSWSPPYPPCL

VCTCKDADVLCNFQPNCKTQYASAEVRTAALAETLNSLQGPSGPRGEPGYPGRQGDRGDA

GPDGTNGVPGPPGSPGPSEMTADQAYQMYYARNYGGKSAPAYGPQYLQAQVGPMGPRGTP

GPPGAGGPQGMEGMRGQPGDSGPPGPPGNRGEPGPLGPQGIEGDLGRNGEPGSSGPPGPK

GPPGSPGMPGMPGIKGHRGFTGYPGKRGDPGSSGEMGETGPPGQAGPPGPAGPRGSPGER

GQDGPPGQDGLRGVDGLSGPAGPPGPVGNTGPPGVPGVSGAKGEQGSPGPKGGPGPQGAR

GDNGMPGITGEVGMPGPPGLSGLPGEQGASGDPGIPGPPGFPGPRGQPGLAGSPGMNGPK

GSTGLPGQSGYKGIAGEKGQTGPAGVVGKVGSAGSQGRRGQRGRPGPPGPQGVPGERGLP

GGRGMPGSPGLLGPKGEDGEPGPRGPRGETGSNGEVGRSGPPGIPGEAGPPGIPGPDGNS

GPPGEPGPIGSEGRPGEMGPPGQRGPQGTQGPQGPPGERGPPGKDGTPGTTGPRGAPGDV

GSTGEMGPQGQPGAEGEPGDRGPQGTPGPAGFQGPPGLPGAPGEAGRDGEMGPPGPAGVP

GAEGYPGERGFPGERGPQGPDGEPGVPGTEGPTGPDGPAGPAGEIGIPGDIGPQGPIGAP

GDRGPPGPAGEKGEQGEPGPTGPDGLPGRQGRHGERGPLGPQGIAGPPAIKGGPGDPGFQ

GQNGPPGTPGPRGEPGIEGEQGYPGLAGQAGSPGAKGETGLSGQKGEQGEAGPPGEPGQA

GPPGLSGVPGRRGPTGDRGIQGTPGDPGQPGRPGPQGPTGPSGLLGNPGAPGPRGIKGAT

GESGPRGRPGSDGAPGVAGNPGMKGQRGEDGSPGGEGPTGPPGPQGQRGENGIPGVPGER

GSAGTVGHPGARGSPGRRGADGFPGQQGEPGSPGAPGPNGSPGGHGAPGNNGAPGQQGQA

GEPGPVGELGPPGPPGLPGLQGPAGPAGPIGAAGERGLRGEIGPEGPTGPTGPRGTPGPQ

GPMGELGELGDDGDKGDAGNPGYQGLPGLPGPEGPTGEVGSAGPRGVPGSRGSDGPRGPM

GSDGEPGPQGPRGPPGPRGPPGDDGRRGPSGEEGEMGPPGPPGQTVYANAMTSWFQGATK

GPGVQGDAPAESRNTLVALKEVANDIEKLKAPAGTRESPGMTCRDIAMVDPSLKNGYYWI

NPNGGSISDAIRVFCKMKGNKTQTCLEPSTSEYTSQKWEFTKQDQGYWLFAGSFIEKEMF

DYGSHKSQIKMLQHHTDNGRQRLTVKCRDVVAVYDGVNKDYNKAISFTSFDEELMTSRSK

GPFRYKVLKDGCKKQNGKEGEALLEVGGSKLLKRIPILDVGFVEQPQGHFGLNIGRACFW

QKGGKGRKGRKKRRK

>SG-PL_contig_214_1_-1_7_1833

MQIFVKTLTGKTITLEVEPSDTIENVKAKIQDKEGIPPDQQRLIFAGKQLEDGRTLSDYN

IQKESTLHLVLRLRGGMQIFVKTLTGKTITLEVEPSDTIENVKAKIQDKEGIPPDQQRLI

FAGKQLEDGRTLSDYNIQKESTLHLVLRLRGGMQIFVKTLTGKTITLEVEPSDTIENVKA

KIQDKEGIPPDQQRLIFAGKQLEDGRTLSDYNIQKESTLHLVLRLRGGMQIFVKTLTGKT

ITLEVEPSDTIENVKAKIQDKEGIPPDQQRLIFAGKQLEDGRTLSDYNIQKESTLHLVLR

LRGGMQIFVKTLTGKTITLEVEPSDTIENVKAKIQDKEGIPPDQQRLIFAGKQLEDGRTL

SDYNIQKESTLHLVLRLRGGMQIFVKTLTGKTITLEVEPSDTIENVKAKIQDKEGIPPDQ

QRLIFAGKQLEDGRTLSDYNIQKESTLHLVLRLRGGMQIFVKTLTGKTITLEVEPSDTIE

NVKAKIQDKEGIPPDQQRLIFAGKQLEDGRTLSDYNIQKESTLHLVLRLRGGMQIFVKTL

TGKTITLEVEPSDTIENVKAKIQDKEGIPPDQQRLIFAGKQLEDGRTLSDYNIQKESTLH

LVLRLRGGN

>SG-PL_contig_215_1_-2_521_2713

MKGQTLESRFRIRFQKTSTIFVCFPGFGDRVAELKTIVSSFAAQHTLPFSLLPHLVSLGQ

RLAEDKEALQKLTFSRTSGTYTTVYGVAEAMKHELAEKLKQSYFSLNMDEATNNAGDKIF

NVVVQYYCTEQGKVVLEHFGSREVNRATASNLFDAVDDMMREKGLEWKQVVSCMMDNCNV

MRGCRGGVEKLCRDKNPHLLDISGDTLHVINNACKALFAQVEQVMPFTDIVSSIFYDIEE

SPKGRDVLRELQTLLNYNVPKSIIRPVGSRFLQMQTVADRLHEILDSLLIYFFAYLTVEE

QRKHQSTMLEIFHANNVSEECQARIKVVLQQQRKQQTKTGANISRKDKILDTFFTAGKKS

LFLTIMNLYRGILDIFSGYVKKFQAEKPLMHKVHSDLFSVTREFFSQFIDPEHIPSYDVS

QLTKLKFFDSSLQLSDKCLGVGPHCFNTLKAYRRDREGNPWLTDFYKALRKGYEEAAKIM

LKLPLENEALIRLSALDPGAQRSKTTPPSLMKLADQLPNVVPDDQLGNLDSELRSYTVDA

AITHIEAEETDPTFRLDSSWWSRIFQMQSNGQQKYPVLAKLVKALLSIFSGPLIESTFNI

MDDIITDDRTSLRVRHYEGVVTIKSWLKANNTSATEMKVSQKMKKYVQESYQNYQCALNE

NKRYHAAEREKRLKEATEMEQAKQSTDSLHVIKPSTSPSAPVQSSKAGATTSKAASLSTT

SKTASLYYQQD

>SG-PL_contig_216_1_-2_263_550

MGVVTFVTQVSAVTGGVNGPVLEAMLRTPTAVSFVPVGIINPWKISSSKPRGLQPKYYDK

PRQILICSVHVHTTELMMPVAVLVPVSITNWKGVAS

>SG-PL_contig_217_1_-3_3_278

DSIDREEAGSGDEIQTHRVVMRKEVHKKTVIRPDGQEETIIKEDSQIHQDSEPPEELRDS

MQQIINQFMEDNPQVPEITGEVTAEIEEGTEV

>SG-PL_contig_218_1_+2_20_1027

MLHGGIYCCCVVVLIVVVWWCLLSLYSGVYCCCIVVFIVVWRCLLLLYGGVYSCCMVVFI

VVWWCLLLYGGVYCRCIVVFIVVVWWCLLLSYGGVYCRCMAVYIVVVWWCYCCCMVVFIV

VVWCCLLSLCGGVYCCCMVVFVVVVWWCLLYGGVYCCMVVFIVVVWWCLLCCMVFIVVVW

WCLLLLYGGCMVLLYGGCMVVFIVVVWWCLLLLYGGVYCCCMVVFIAVVWRCLLLLYGGV

YCCCMVVYIVAVWWCLLLLYGGVHCCCIVVFIVGVWCLLLYCGVYCCCVVVFIVVWCYLL

LLCGGVYCCMVVFIVVWWCLLLLLYGGVHCCCMVVF

>SG-PL_contig_219_1_-1_157_1101

MVKEDKATWKSNYFMKITKLFDDYPKCFIVNADNVGSKQMQQIRQGLREKAVVLMGKNTM

MRKAIRGHLENNPSLERLLPCIRNNVGFVFTREDLVEVRELLLANKVKAPARAGALAPLD

VKIPAQTTTLGPEKTSFFQALQIPTKITKGTIEILNEVNLIKEGEKVGQSESTLLNMLNI

SPFTYGLVIENVYDSGTIFSPAVLDITDDDIRVSFMAGVRNVAALSLAINYPTTASAPHS

IVNGFKRLLAVAVETDIDFAEAEQTKEYLKDPSKFAAAVAAAAPETSASAEAPKEEAKKE

ESEESDDDMGFGLFD

>SG-PL_contig_220_1_+1_880_1122

MASGSSWLKADKKRQQMECVSITEVLEMLWTKGSYVKEASDDDDNYVLSERGEVMSDFDA

NYSQWKVKVRARLSNVHRHVQ

>SG-PL_contig_221_1_-3_861_1061

MSYAQLNFNICCLSLLYFVKSCLFIFCCCEYCIMLPGLLICCVLLVTAILLMLLHVLQLL

PVLLKFC

>SG-PL_contig_222_1_-2_920_1318

MGIPHLALFHLYTHALFKALLFLCAGTIIHNRCNTQDIRHIGLISRQAPLTVACINIANL

SLCGAPFLSGFYSKDLILELSLTSPTRLIIIILIFLATGITAAYSFRLSFCSLWGYIKSP

SFHSKQEADPYIN

>SG-PL_contig_223_1_-2_170_826

MASANDSKWQKDAADQNFDYMFKLLIIGNSSVGKTSFLFRYADDSFTSAFVSTVGIDFKV

KTVFRQDKRVKLQIWDTAGQERYRTITTAYYRGAMGFILMYDVTNEESFNAVQDWCTQIK

TYSWDNAQVVLVGNKCDLEDERVVSAERGKQLADQLGLEFFETSAKENINVKAVFERLVD

IICDKMSESLDSDPTLVSNTAKATRLTENPNMQNNNCAC

>SG-PL_contig_224_1_+2_68_724

MGGSVVVDLIAELQQEEGRCFHLTFDNLFTSLRLVDYLTKKNIACTGTIRANRLQDCPLK

SVKEMEKTKRGSFDYSTDAKTGLVVVRWHDNNVVNTVSNKVGVEPVQTAKRWSRTERQRV

DIMQPYLVKHYNQTMGGVDRMDQNIDKYRISVRSKKWWWPLFAFCVDVSVQQAWHLYRAT

PASATDPKDLLTIRRSIARVYLLRATQASSAGRPRQPIP

>SG-PL_contig_225_1_+2_1994_2197

MESECAATCLHSVDQTIRTTYVHASKKTLFGTNNTTKNNQKKKWGEGVCDIQYLGNALEW

DWKTLQNM

>SG-PL_contig_226_1_+3_276_452

MGHTVLFTSHDSGEGRKTCFCLCFLLLFVVVWGLFFFKLHGLAFECYWVGNVEGYKQNF

>SG-PL_contig_227_1_-3_333_542

MYVYFIFFLMCFFFSITFRHRLLLFSSSFFLFIISHLYQSFGFSLPFLYSHRSFNLIMRW

DFAALMIITS

>SG-PL_contig_228_1_-3_417_785

MYMNLMPRNTQIAQISIFRNSFIISLCLMIHLSSSGGATVYLAQELLKTKLPYGRNYFLL

LPIAVGTVSAYAMTRHNTRVCQDMWLAMEEKHSDITPLQERLQVSVPDKIETVTTRPPAS

PSS

>SG-PL_contig_229_1_+3_3_248

VLHQAVVKVLTTQVCVSCRGLDLKQGSFSNGEDRHVESATPQVKDEDVFVTVFLHVLFQS

IGQGSCCGFVDDPQHVETGNDA

>SG-PL_contig_230_1_-2_638_790

MKTVLSSVMFQTVKWVSVNNIYSNIYQSKHKSTYSVTVFFAFMLVFVFWFF

>SG-PL_contig_231_1_+3_132_350

MLTREISTLYPPGAVPKEVQTLAFQILQFGSPVSSPFNHRAWWQHYHAQKDPISVEQWCL

VAVLPCPKGSYIS

>SG-PL_contig_232_1_-1_781_1059

MTKVHFENVTVTWNLPNVFNLAWVHLTVQSCYKAKGFPYSSTIYNMYETAELSKYRISNK

TYTMSETSQSNFKNIQSRHVFQCATSHRAVWQV

>SG-PL_contig_233_1_-3_33_881

MHNLLRSRAIRLLQRRQQDVRDQCQDYMTNKAAGQVVMATEEEQAKQRRAAEREARRSRR

RRGRESRNIASHHEGLSSDDEESQADVTKFNMERENIDKASKTLFEDALEDFTEMELIRA

RFEEWKQQFGDTYREAYISLCIPKLVNPFVRLELLNWNPLEEESGDFEDMSWYNCMVFFG

CESNEDVDPEDSDIKILPAVVEKVALPKLKYLIEHVWDPLSTTQTSRLVNLTQRLFRDYP

TVSQKSKSAQAFLQAVVSRIKKALDDDVFMPLYPMSVLDNRSS

>SG-PL_contig_234_1_+3_681_887

MRYLKPSKECELGTTGQRVTLMGMVEDEKHNFHLGVKLIFCSCKTTWLPMKSAFVMTGSA

AFTSNPQLH

>SG-PL_contig_235_1_-3_3_437

RNKTLLRYFQLQDICVLQAFTERLAEVYNQHNATHIIKSFLIGGKKTSNSTRQRIYSNFA

ISIAKARPYLTAEETFTLPDTMPSPPQYKYFWFPLHDRQVRYLDGYDNGWNQYGNQQGYD

NDWNQYDNQQGFDNDWNQYGNEQGY

>SG-PL_contig_236_1_-3_165_518

MDACSQSEHRLGKDSPSSKLLYAKDIPKYKQWVERYYQDIKMMPAISDQDMTAMLTEESR

IHCTEFNANAALLELYKYAQKYNEELMTALDEDEFARKSKLSYKLEHVHEDMEGSSIC

>SG-PL_contig_237_1_-2_206_991

MRKLEAGVHIVVGTPGRVYDMINRRALDPTNIKMFVLDEADEMLSRGFKDQIYDVFRFMP

AEIQVILLSATMPADVLEVTHRFMRDPVRILVKKEELTLEGIRQFYVQVEREDWKLDTLC

DLYETLTITQAVIFCNTRRKVDWLTEKMHARDFTVSAMHGDMDQKERDVIMREFRTGSSR

VLITTDLLARGIDVQQVSLVINYDLPANRENYIHRIGRGGRFGRKGVAINFVTTEDIRTL

HDIEQFYNTTIEEMPMNVADLL

>SG-PL_contig_238_1_+3_3_299

GGGGGATLWCDYKRDRGWGGEYLVVWLQVRLGVGGGMDYKLRQGGRDGPPQHDTAGTSPQ

DSLVLHGTILGMGVGVECAMDRRTRWKLVSVRSRKKTAG

>SG-PL_contig_239_1_-2_47_319

MPFPRGDAGGSFRINSIPTFWKFCASNFLVSIALFVSAVFPSFTVIYFFLPSRSNPLLFS

SKPSHNFWPPLLPTPAQFMMYYLCCFTLATR

>SG-PL_contig_240_1_+1_1_153

YGRFIFLIVFKNALKKNFKWEKKNYIRAHIQSISLKRTLKLICQHLLIVIA

>SG-PL_contig_241_1_+1_1_144

YGRFIFLIVFKNALKKNFKREKKNYIRAHIQSISLKRTLKLISVNTYS

>SG-PL_contig_242_1_-2_2_208

FQRLVREIAQDFKTDLRFQSAAIGALQEASEAYLVGLFEDTNLCAIHAKRVTIMPKDIQL

ARRIRGERA

>SG-PL_contig_243_1_-1_208_483

MDLTDDHLAYQSANRDTSILNPRAHEIQALATSSAFSQCVPLKKILVATYWRSENPFINC

HLRNRHLVRADGSKGIAFVAANSTIAVTGPSV

>SG-PL_contig_244_1_+1_292_534

MRKRERLWPPRPKWAKKVWGQISDQFIQVKLVPMLTVLSYWVLRITWTFYACGQSVCLDL

TRKCVNLTSDFVWPSVGEIAY

>SG-PL_contig_245_1_+2_53_247

MSHLTYIRKSVRQAQHKFKKTPAPSHAPGPATVRKTRCVKEIKKRRREYHHDAKTCITYT

NTISQ

>SG-PL_contig_246_1_+3_4626_5636

MRAPTRVLLSRFCSSGDLNLGEASSLPSTSFCSKGDLNLGLSEPESEVGVSPRDDNIAET

IGDCERDEVGVDSFVSNIFSCDEAVFMGVAASWAAKGTGDGALAGDMVPLAGDRLSSALL

SMLLFLSSDSATTADWAGAVRMVLLSGSKGELSLEAVEVALFVGELLTDLGLSFAPSPAL

SVFTLFMIISSRFFLSSLSSSSRRAFSCLSCSRRSASSLALRSASSLANFSCSSLAASSA

LRASSASMAFWSRSSSAFRASSASLMASSSLSRCSSSSFSLLSSSKRSSSSFRFLSSSAS

LSAFSLACLRFSANLALYSSAVMSPDDFSMGVGVERD

>SG-PL_contig_247_1_-1_376_1332

MPGTVAPAEDFDAGAVAEKLRNAMSGFGTDEETVLQVMGNHSNEQRLEIAEAFKVAYGKD

LIDDIKSELGGHFEDLCVAMLTPPRLYDAKQLHDCVCGAGTDETSLAEIMATRSNGEIAE

IKEKYKEEYDCELEEDLQSDTSGYFGRLMVSLCVGGRESDEWEWADEDKAAEDAQKFLDA

GEGAWGTEEAEMNAVLCLTSPGQLRKTIEKYEELTGGTTMEEAIRNECSGSLQEGYLAIV

ESVKSKPKYFARRLHDCMAGLGTSDCDLIRIIVSRSEIDMEEIMAAYEEMYEKPLVEAVD

SECSGDYRKLLIAILNPPC

>SG-PL_contig_248_1_+1_940_1125

MHSAMLIVPLFWQVSFFCAQKIVFKIFNKPNLFHCVYYLTLYFYFLIHFSYVFLHFFISW

ME

>SG-PL_contig_249_1_-2_758_1024

MHAIKLFNIVYLSKPFTLPSSYSQCTCILSQYAPFFFSLHISFCINNCATDTSLFDECVC

EKFGGWGRCQKREIGGGLGGGGGGGGGGF

>SG-PL_contig_250_1_-2_35_322

MVEDMASISSKAVVVMVTTSREVEGMVASRVATEVSKAGTEATSQVVRGPTREWAVVTNS

DRHLATLEVAVAAEVDHKTSTPTVDKSYIIAFFKMC

>SG-PL_contig_251_1_-2_1304_1483

MTPALVYHLCHLPPLYDCMIHDHKMSQHCCHHHLIFSPTTNVHLMTYWTIKGQLSASDTL

>SG-PL_contig_252_1_-3_2568_2948

MVVLLTVIEIGYVLAVSTIVMCETTSVDRCLIHSCTIWYGFHFPVFTRSSIESPEPELTV

EDSQLALKHSLFSLFNYCHSAAWGKWSSRVHIVLRQGELERCIESSQGSLEFKLLGHCTC

LTFLFSL

>SG-PL_contig_253_1_-1_682_3249

MDYDDVVVRKVSRTYNVYRGSSPATQNRLEARIRELEDALDSERDGRVRAEKMVAELNFR

VDQLQDSLDEQGSSTSAQIELNKKRESEIIKMRKDLELANAQFEATESSMRKRHTEAIND

LSDQVDYLTKNKNRVEKEKQTLVVELDGLSGQLDSVAKAKVTLEGRVDAAEQSAARLKGQ

VDDLTRQLNDLTGLKAKLTQENFDLQHQVQELDSSNAALAKAKNQMQMSNDDLKRQLDDE

SRQRQNLQVNLSALQSDYDNLNARLEEEAETSTSLRNQLSKVNADFTSLKSRFDKELAAK

TEELEEMRRRMQVRITELEDSCEQLRVRNSSLEKAKAKLANEIKEITIELENTQIIVQDL

TKRNRQLENENAGLLRRVDELTAENGTLRNEKMALEQEVQRLRVANAELTERNDNLQREN

KNLSDQLREANLALKDANRELADLRQLRAQLEAERDSLAAALKDTEDALRDAEAKLANAQ

AALNQLRSEMENRLREKDEEIENIRKSSARVIEELQRTLVEVETRYKTEISRLKKKFEAD

IRELEAALDNANRANAEYLKQIKSLQMRVKELETIVEEERRTTDDLRNQLAISERKRVAI

AQELEDARALLEGAERARKNAETELADTSARLSEITLQVTTLTNDKRRLEADLGAMQSDL

DDAINNQRSAEERAERLSAENARLADELRQEQENYKNAESLRKQLEIEIREITIRLEEAE

AFAVKEGKRLVAKLQSRVRDLEAELENEARRVRESVANQRKAERFFKELQAQTEEERRQL

AELSSLNDQLQLRIKTYKRQLEEAEDVANLTMNKYRKAQNLIEEADARADVAEKNLQTVR

RARSMSVQREVRIVRG

>SG-PL_contig_254_1_-2_179_1537

METLAGGEEYNRPLSPLDLITGPAMSDTERLLSERIPHTSFTRWGSSSSLGDPRDISASA

DLLHSGDRSSVQRPKSAEQTRRGQHTAYPLKQSAGVGSILGYGGESLCVAVLDYVPDAAS

RSGHRHLELPLKEGDHVHVRGPVDRHGYCEAEVGGRTGLVPASHLFPLSDSGATYRPTSQ

TYGRHKKSGDSAEQIVQMFDELQDARLVSGLAHTHSSYSSQPGHSTATQTRQRRSAPKSH

GAPQAPSNLHVQEVDGNSLVLAWQPPILDQHGYSNGCKVVGYRIYLNGRVCQQPHNPKLS

RTRVEGLRHDRPQQLAIQTLAASGQLSSRVELAYQGASSLAHDHRGEDGETDTDLSSILN

SIQYKTGKKRTVMGLYDYDPEKQSPGDYTACELAFHAGDIMLVYGDERQDGFFHGERNGQ

RGLVPACFVEPVAQGSKSMQKPSAANGSASPRS

>SG-PL_contig_255_1_-3_3_227

TIDHKILLSRLENIFGIRSVALSWFCSYLEDRSQYVSVNGFFGFFSACFWCTPRLKTRPS

SIYNAIVFRNQAARG

>SG-PL_contig_256_1_-2_56_1681

MGKEREVEEDVETETVLASEVPFWTSARLGMAILGFLGFIFVYALRVNLSVAIVCMVNRT

AIAHHENASSLAAATNASFSDKKDVDVNTECGNLAQSANASSSSLVEGHLMWDKKTQGLV

LGAFFWGYLVSQIPAGLLAQRVGGKWCFGWAMLAAALFTLLTPLVANHSFVGVMVLRILL

GAATGVTFPAMHCLWGSWAPPLERTKLMAFTYAGAQVGNVITFPLAGFLCKYGFASGWPS

IFYVLGGLTLVWVALWMFLTSNTPSQHRRISDLERRYIVASLRKVAQSEESKKNVPWKSI

ALSLPNYAIIVSNITSDWGAYTLLTNIPTYISEVLKFDIASNGLVSALPYIGMWATMNIS

GWLADIIRQRKWLGTGGTRKFMDAFGKVAAGVLLVGLGYVDCTQPAVAIALLVLAVSIAG

FQYSGFLVNHVDIAPQYAGILFGISNSIAAITGFVSPVVVGIITEERQTRAEWQIVFYVA

AAIYLFGAIFYVIFAAGVLQPWASKDASNALEVQEPVLLNVTKSSGMDGNGVGDKLVNHN

AD

>SG-PL_contig_257_1_-2_59_280

MYFLAYVSYALTSSQTRLRNIKLCVSFSRSQLNKKFIMTLNRYFMTLPPSQPATLKNKSK

SLIPLIQASYWYLS

>SG-PL_contig_258_1_+2_20_241

MLVYSMIPYKFTMANNDSKSKHSLIISEILNTHCHCMHPHTSRSFPRTHTCTRVDTHTHT

HYPRKHLGNFQMSI

>SG-PL_contig_259_1_-1_172_288

MGCATFRPCIIIGFLTSAHMAQLSFLGTLLCQVSATGGE

>SG-PL_contig_260_1_+3_3_476

GQGSCCGFVDDPQHVETGNDASILGGLSLGVVEVSGHSDNSVSNFLTQVGLRRLLHFPQH

HGADLFRSQRLLLPLELHLQFGFAAVIDNSEGPVLHITLHTAVGEAPADESLGIKHGVLG

VHGRLVLRRVPNQAFCVGEGHVTGRGPLTLVVGDDLHL

>SG-PL_contig_261_1_-2_20_580

MGIDICHKNDRKVHRTEPKSQDVYLRLLVKLYRFLARRTNAKFNKIILKRLFMSKTNRPP

LSLSKIIRLMKKPGREGKTVVVIGTITDDVRIHKIPKLKICALRVTAGARARILKSGGEL

MTLDQLALRSPKGQNTVLVQGPRKAREAYRHFGAPGVPNSKAAPYVRSKGRKFERARGRR

KSRGYKN

>SG-PL_contig_262_1_+2_38_757

MAGCTDIPLPKLDENTLFVLDGGSLLHRLPWPRGSTFGALCALYVDFVQRNYMPSIIVFD

GYGTNPSIKDMAHLRRSGGIVGTTVNFNANMILSSKKEEFLANPGNKQRFITMLSDVLLA

NGHPTLHSQNDADLLIVQTAITRAADHPVVVIGEDTDLLVLLCHHVSPDLRTVIFRSDLK

SKSKKQRVWDIQWLQQALGEDICHALLFVHAITGCDTTSRLFGVGKGTATLRKLKVAEGR

>SG-PL_contig_263_1_+3_174_428

MEELDVPPSVEELSKAIDSLACSKAPGNDGIPPEVIKAGKKSALLNHLHELLLQCWEEGT

VPQDMRDANIITLYKNKGDGSDCNN

>SG-PL_contig_264_1_+1_82_339

MERWAEHYRELYSRENIVTDTAVENTNLLPVMEELDVPPSVEELSKAIDSLVCGKAPGNN

GIPPGAIKAGKKSVLLNHLHELLLQC

>SG-PL_contig_265_1_-1_40_267

MVNTAAMEVTSHLYTVTQIFPPIINPAAMEIIHPITHPAAMKNIHPITHPVAKKAKPVDW

GKEERFWTVNCKQKPP

>SG-PL_contig_266_1_-3_36_647

MGAYKYMQEMWRKKQSDVMRFLLRVRCWQFRQLSSVHRAPRPTRPDKARRLGYRAKQGYV

IYRVRVRRGGRKRPVPKGCTYGKPVTHGVNQLKFQRSLRSVAEERVGRRCGGLRVLNSYW

VCEDSTYKFFEIILVDPFHKAIRCDPKANWICNSVHKHREMRGLTSAGRSSRGLGKGHLF

NKTTGGSRHANWKRRNTLSLRRKR

>SG-PL_contig_267_1_+1_421_585

MHYVSMFTAHFSMSTDITYRCARWHVSLNDLMLWVNTPPPQKKKKQPKKKNKKKN

>SG-PL_contig_268_1_-2_14_493

MADDHEEQFEQATSGASKTFPKQCSALRKNEFVMIKSRPCKIVEMSTSKTGKHGHAKVHI

VALDIFTNKKYEDICPSTHNMLCPHVTRCDYMAMSVDITDGFLSLMDENGGTRDDLKLPE

DADLRREITNRLDNDETFSVTVLSALGEEAVIAVKKTESK

>SG-PL_contig_269_1_+1_1_192

VWRIAVYKNDLLVAAALSLLYHDLLTETYKSSKRKQNPQHQGCLLALPFRIIQFFFKIIT

FIIF

>SG-PL_contig_270_1_-2_44_133

MESTPQRLKSLIRHANNFSTATVVSSISIY

>SG-PL_contig_271_1_+3_444_881

MYACNENFTLHSKKCHFHWAGWRKNKTACFWSQSLETTSTAGFTLFHTGFIIEQKKVIIF

SVLLSSEGATFCQSGLATSWLTQHCVTPNAQHHRLGMAENCGDLVTPWALHIHEVRVGTL

HKTFKLAFSLFFLLRGVQQIFRQRHG

>SG-PL_contig_272_1_-1_10_762

MAKPQSKKHKFVQDGLFKAELNEFLTRELAEDGYSGVEVRVTPTRTEIIILATRTQNVLG

EKGRRIRELTSVVQKRYNFPEGTVELYAEKVATRGLCAIAQCESLRYKLIGGLAVRRACY

GVLRFIMESGAKGCEVVVSGKLRGQRAKSMKFTDGLMIHSGEPLNDYVDTAVRHVLLRQG

VLGIKVKIMLPWDPTGKIGPRRPLPDHVSIVEPKDEVIPPHPYSEQKGAKPAEGAPAAAA

AAAPPTPVFDQ

>SG-PL_contig_273_1_+1_415_621

MFLRCVYTRCLVVWPTLCSRYLAAQSACSIFSGLPRHAGSHTLSGGVHAGYVHVSIRHRI

PIWLTGSSV

>SG-PL_contig_274_1_+2_2_253

RFQNIVERISENFTAFAYLLFRVSLRFYGFQCVYYTQTHTTGSPAQNQLSFSTVLLMIYD

LTMVIRQNPDNHTEDANTHGIHSL

>SG-PL_contig_275_1_+1_1_147

VHKNLTSKCKLISAPKQKEKDCTLYIFSVTKRTLAIVLIMSSLTQLCDQ

>SG-PL_contig_276_1_-3_261_365

MTHLLRMHNSTLFISDSLKLNRNHLALCVILFCFL

>SG-PL_contig_277_1_+3_420_845

MPELTCYTSLWSVPSGLPPACIRPRTYKSTSLWSVPSGLPPACIRQRTYKSTSLWSLPSG

LPPACIRQRTLKSIFKSVLSDTQCTDTLVSVFLSDLTTLTRVSSIEFLHSWTKFACLRPY

TTITKRIILKVFITRDPLTSSN

>SG-PL_contig_278_1_+3_3_314

LYLCANPPTHLQITQPAVLEQISIIRFRNRKKTRIITTLHYSEYHSPLPTSIFVCVKEIT

ITMVLLYPQYYSQTHLKITKPLCVEEKKKEIIITIVNCSQYNSE

>SG-PL_contig_279_1_-2_47_274

MYVTTENAAIASVKQEHVCVCVYTSTTHDGVCREGSPWLVVGTITLRAASTDGESVGGGN

GIFRKWDAGGAVSLWL

>SG-PL_contig_280_1_+1_58_216

MLFIAGVFHNKHINLAPIPANASLTSSAKSPRSTVLQHSREKHQDWESIQSKR

>SG-PL_contig_281_1_+1_1447_1635

MFTTIDRSNFVLFLLHLCSCRPPWAKAGRKKVCLLIPLPLSIKKILSLSLSLSLSLSERS

NGT

>SG-PL_contig_282_1_+1_61_267

MPPDPPRRARLLCHLITEPRTLPPFFILDHFKPCQLHDLWFGGMLKTFNSGLKMVRHLAD

FHRWMNIFC

>SG-PL_contig_283_1_-1_142_342

MFILVFCSVLLMLLAAFRVFFHGWWSDYLNCRYYTEERRKKCDQMQCGRGGREGAVNFNV

HILQNAD

>SG-PL_contig_284_1_+3_81_1043

MQTKLKDHTYKLQSLKDSQQNFASDIKKQLQTLSTNSTNKQARPSEKPIQPTDKPNNKNN

TLPTISAESTMNPQEMYNIPTTNKFTLLSDSEDTDHPPATTVHNSTWPSSTKVHKKPVND

PASPLSHLRQKNIPTNCNALLLGDSVLEGVGERNMSTDSDSVKNLSVSGLTIHHLCQWLS

SRPPSNNIKQITFHVGINDCSDGPISVVTWGKLIRLLGRSFPKATLTASSLVPPYGQHPL

KKVASLSTANLQHICRQEGILFINNTPSFLTKSGAPKKDMYRDAIHPSVKGSRSLALNIR

VHGLQRPSPVPSTLQRPPPVP

>SG-PL_contig_285_1_+2_470_745

MVSKVSVYNAVDAFAYYSCPQLEMMIFFDVFPLCDPHPSICFTTSIPSTTEPNTTCLPSN

HVVLTVQRKNWEPLVPGPAFAMDKVPQFGFLL

>SG-PL_contig_286_1_+2_41_1036

MIIRHTESSLLITPLFHFLNEGSLSLLDTCLPLGLGQFSTVPPLLLHSLLWRRLGLWVIT

DQGMAFLVHFLHPAGRDAQLDEARELFLVQGLVLSLQVVHVLGHMLAVDVVTVNLRNVLA

LLAVVPREALGAVWDVQTTINSALHGTKDASTSGGAVETHVEVATEGSRLVILILHVVLL

SVSHLLALVDGIHAKLLQQSSGQKQSSAVSSRIVSQPDLHAISGQLVSVCSTYNHVTLDP

GVGYLTDDVLVGEANNQPVLGGIVLVLVLESQPLASIVVGATLTSPAELDLKPLEVGLVL

DNFHKPHVCKFEVLHFDHSNSIGWGLTRSEEH

>SG-PL_contig_287_1_+3_3_209

FHLPTMQYCFHFLPVLHSSTSGIVFTAFRYSQWLRCYDFLKFCRKIFKFSLAKWLKIVTW

NSIEYVWRQ

>SG-PL_contig_288_1_+1_1_255

DNPHITLQSIPHDVGFSISLVSLTDKAFDQGQIYSVILIILLVNSLSDQRNHSKAFKKGF

QMGDMYWGGGETNSEISKIIMFIAA

>SG-PL_contig_289_1_+2_101_361

MKSKTEHKPNKGEQVGTRPLSLKAARKNELERQLCQRMRVTRGTTHRPRYQLIGYFWFRP

CVLAAKIGQDGATYQRHDAHSPTWYSI

>SG-PL_contig_290_1_-1_100_1854

MVGLKKQSKRMTTKRRVKIERKVKEHNRKKRKEARKNPRKGKRKDPGIPNSLPFKETVLK

ELEEKKKQAEEERAKRREMLKKQQKKNRESLHNKNRNLTDLVADAERRQADYERKQAKQK

VGDEHRSYKSSSVETSRQTFVKEFRKVVDAADVILEVLDARDPLGSRCAELESIVLASGT

NKKLVLILNKIDLIPKENVEAWVKYLRNEFPTVAFKASTQTQNDHLSQRKVQLTKLTESL

AKSSHCLGADILMKLLGSYCQNKGIKTSIRVGIVGFPNTGKSSIINSLKRSKACTVGAMP

GVTKAMQEVNLDKHIKLLDSPGVVMETSQSPVTAVLRNVVRLENIEDPVSPVEHILSRCS

KEQLMLHYNLPDFSNVDEFLMLLAKRQGRLKKGGIPDSNKAARAVLQDWTSGRITYHTQP

PEDRSIISATICTELKEAFDINDSTLSGLPSRSGQSPLLVDSLGPVTGIVDASNLPDEDM

AEEEDDEEEDGEEEIMEEEDEDTKGKQGKALSPIVSLIQSAKSKGSETNETEVTVLQANK

ARKKAFKQMKKNRKRSDAVAGQLSSALETAFNIGLGDNDSDADSL

>SG-PL_contig_291_1_-2_302_508

MVNKMNKPSESESLPLPLPLPLPLSLPHHSLSVSPPLSPSFPLPPSLPSSLPDSGIGAIQ

WKKILTINY

>SG-PL_contig_292_1_+3_3_173

FIQEQFPLPLLCYDLSHFSTRATGDVCMFQSLIQIIYVNIFTFKSAFIKIRISLFYP

>SG-PL_contig_293_1_-2_2_202

FILLKSANILLCGLDDFTSVSGHKHILIIINVLRDDNDYNIIEGCYDCNLTSRSTNICVW

FFFPPLC

>SG-PL_contig_294_1_-1_403_1179

MTSEHSNLISRGSETAERYEKYVRPILAEFLGVTLFVCMVCMSVLNSGAASPDGTVLFSA

PAIALAQGLTIVILVMVFADVSGAHLNPAVTAGILMAGGIGPLMALFYICAQLLGGVTGA

ALARGILSDETYEAIGGGAQTLGSGVTVGQGILCELVLTAVLVLTVLMTAVDPQTKTTLF

PLAVGMAVIVGVLTGVFISGASMNPARSFGPAVVISYATSNFWAYHYVYWVGPFLGAIVA

AVWYRLFLASSENRLVLKE

>SG-PL_contig_295_1_-3_228_545

MDNVPLSSDSDGNQSSVEQENVKNMTFSTGSLSLDADSNVDDDEETQRLISQVLELQNTL

NDLSSRVDSVKVENLKLKSENQVLSQYIENLMAASSVFQPATPKKK

>SG-PL_contig_296_1_-3_123_638

MKTTACLWLLVLLLVAIDDAAVAALDAEGDSSCTKFASNEDAIAKQINGKCYYGFTTNTT

WSDARKLCFKEGMWLVSLQTEAEQELVSTTFQFRKYWTSLRLVDGIWYWYASADIKTPAG

DDLGNRLQIRPGLRRYGVAKTLGKPGSYRNVLVETMQEAQHPMVCEDDPLSA

>SG-PL_contig_297_1_+3_123_275

MMITPFFSPTLLPDQANGFKECDNCFRLSSRNWCGLFNDYRYTKFENAATS

>SG-PL_contig_298_1_+1_808_1092

MTLYEPCYDQPTKTYTAKLIDLFESFGLVQCVCVCPYTQAGHTLDWIVHRADQTVVRLWS

ATKRSIRSLLSSLSAPFLLSPFSLSLRSGQKSHCR

>SG-PL_contig_299_1_-1_58_192

MIIHAHAHAHTQKEEMAHCTLSSLFIFTILRWYTAFLRIFELFKD

>SG-PL_contig_300_1_-1_1_432

MHYKQQWGRALKEVARMKQREQEAAKASLRQQQQELEHMRLRYLAAEEKDVAKSDNQQLE

EIKNELNKLKQMEADKMKQSTDSNFTDSGRKPLNTDMDSSIDEHISRLVEERDTLLRTGV

YTTQDKIIIELDRQIREAIAQRNR

>SG-PL_contig_301_1_-1_46_483

MPAKGPIKAVQVFGRKKTATAVAHCKQGNGLIKVNGRPLDQVEPIILRAKLEEPLLLLGA

ERFAGVDIRVRVKGGGHVAQVYAARQAISKSLIAYYQKFVDEQSKKEIRDILISYDRSLL

VADPRRCEPKKFGGPGARARYQKSYR

>SG-PL_contig_302_1_-2_2_178

DVSTVTNLRDVLSSVTPQASVTSNPTLAAGPVQNVANLPRGKTAKRTARTQSTARGMGP

>SG-PL_contig_303_1_-1_292_549

MCCSMGTYLKVSVLFPRGDAGDSFRINSIPLSGHSVLGISSFSTTRFVSAFFCSFYLLFL

AVCLSAGPVHSPECFEEILVHFPLPT

>SG-PL_contig_304_1_-3_285_9392

MLDLMNQDLKSKLAEAQDFVVINSAPSQGSLADELSASSKRGQARSHSDSPAPGGSDEES

GDDSPKKVVTKLKRQLSILEEEKNGKIANLQERLADMRENEIKLSETLAEMEMTERELRA

KLALYESSEVTVEKMLKYQDKIHELRTSQESLLDQLETMESQEETLQERLEETERRLKGK

IVTLEVEMKNLKQKELKGNAKIRELEKHEEELTEKVQKQAEKENGLYVRISGLMDQVKEH

KTQITEQEKKLAEKDERCRKVASLETKFLQIEGAYRDKVTSLEASLAEAQSLLAEEEKRH

EETRQTHDEALHSRDKEHNQRIFILEEMFAQKEKGLNENFAALESSIAERDMTISQQDKM

LGEKDDQIAKLEEELSGKDSQIRQLEDATSSRQSEEQELEKNLSLKDECICDLEKCLNES

NSRIGTLEKDLSEKESHVSELEKVVSDLETDKLDKQGVIDNLEKDLSEKVVCIRALEADL

GRKECRIAEMERDINENDTLKGSLAEKDSHISQLEDNLSEKVSCISQLEQDVGGKVSQIS

RLEEDMTGKDVQITKLEQELNDRVSQVSELERDSARKGEDVSRMVKDLSERDTHVKRLET

EVLHKESQVSQLQTELEYRDGQIADLEAQLEAKGNEISDRLEEAQQDITNKDDHIAQLEE

RLIDMSSTIVQLEQKVCEKDICISGLEGQLTDKDTIISEHVTQLQSKTETEQSSTTKLSL

LEVEFAGIKSKLSQSDARCEEYSRKLTLVENELDEERANARSLKLDSDALLAENGHLQSQ

MTAKVSELETQLKEQDCDFQSRMSVRESDFHQACASLDAECESLRGELLEKDSVIEKLES

TKNDLLSKIENLKDKEKELANKLSVLELDINTLHTQKENELQDVVMMWESRLKNTEHEKE

TAVERSSSLEADLLSRNQTIQQHLEEISKLESSLNKAKAEIGTLQGTLSQLQLQEEESGM

KAEVFLQQETMLKTKVSQVEAQLEASSKEQEVQKASVSELLLQKQNLEDTLRKVEVDLSD

SHVLNAQLQETVSQLNKDTKSLQEEVTDLQRLVADLEIYQMKEQEIHKLLEQEKGRMQEL

ESQISCLKDEKQSALEESGKLHKELDLLKGTVKDLETQITRLSQAEESLKSHILAEQSSK

LEADKRLNQVSEEATVSQERVDQLLKEAREKDEQIDQFRVELLSVRQELDTHQRFTVCIE

QKDQTLHSLQENEKALQEREALLQEKLTSMEIQMGRHQEDHSQMCQKLQELEKREKELLE

QITDIEQSVIVPLEAENAELKEEVSSMKQQNEALNLQVDKQEERTREDSEELQDLQKSVN

DLQMSRTEIQSKLDSVMQELNKQESENEKLQSKVVDLEADNQNLDDHLENDRVELNKLQM

ELNKVQMELNKDFCDRVAQTDSDAQVSETELRLQQKLTVLQDAESTFMSRIIDLEEHEQQ

LCEELESTKRLLQEKEQYVEQSQTKEFLSQSQVSHDTVDASVDTEGLQEPSITKVPELFA

RVQELESVNQRLSCSLEQATAGEVDRRQLTDKLRLLEQAEDRLMERVMELEENDIALRTQ

LERSRHRARVADELEEEVSCLLQQEDEWKEKLASLTSENNTLSQKIESLENEFGCEQSRN

ENLAFQIETEQRQLQDAQAEVKLVQTKLQAVRSEYEEKLSALEESEKKIENQLDSQSQRE

CDLRREIQVLREEMEDVCERYKELEQENSRLKQEHSKVLVELKDSESQCLQLAGGVSREP

TMQAVQAQRHQTVLPHGASVSSSSMSSPGQQFSSPPSVCSNVTVHMVQSSREGAKSSKTS

DSEASLLSRIAQLEKELREARSRPEGVGGPLQDKQSSVDSSEEPCDSDSSGVQSSSVQSV

KIFTLHDALEENASLKAQLQDLQFRLVSQQVSSSFASGDGSSRRVSGDRQSAETLQLQAE

ISRLETRIAELEQSLKEAEGAVQEVVTVVRTRSVHTLEQKIETIQHTIHTQSPTWSLHQE

LLELLTSQADLQKQVSHLMETEHNLRSALKQTEDELARAKNGSGTTTLGARRLEADRRLS

LQSRLSRFGGGISSSKVQSERVQVDSSQKAKEQWLERQVADLQLAKANLERRLHEAERRL

AFRPRPTAISRDELLFRIEQLEGEQSEWRKKVEDLEALNSHYLKEKLELASERDSLEQKL

QVRAQSPGAGKEVGVVFARGRREEPLGSSGSVSEGEPSIDPDSDAAMLEKPEDVLQKRIK

ELENLEGYLRQQLTEVESDREQLHEIARKDKAMIHEQNVRIRELMLQERNLRSQVSGLED

SERSLYNRCSELEDTVSRLEDRVHELEVHERRLRELVRKLKLDEEVWMSKSGDLTASMEQ

LSSSEVSLRKMVQDLQIEKGGLSDRTEYLEVRVKELEALETTLVQRLKNYENVEASLQNR

LQHTENSEAAAHSKASELDVINMDLSSRLQRAVEENAVLSQHVAQQQGQIHEMDRKLSAA

TDAQMGLNQHVESLQKNEGSLQKKVREYELRDIDAQARVRELEQTEEILKDKVNQLQRSE

NRLKARLVEFETVGSQVLENMPSAQKQQLPQKLEDCQKRVVVLQRQLEVTQAELHRQQQV

EDDATSVKVPTAALTQMRSKVALLEETEYQMSELEKLNAQLNENILQLRQGKGSCGHEVE

LEVLKRRLHSYQNTVQKLKHHLSEGQVPSLWAAGQGQGPESEDLPARHLQWLESERVLQS

VRSLTQPPGLPHTQEEIIQAHSGTLQTGRAQGVASVPSEPEESHWRTVELRRRSQLEAEQ

RGASASMPHASATTSTALPHQEELMIGLSLPSESERGSFKSQNDTDSDMSGISHPHLRMV

GSTTHRGSAPTPGVVPASRGGGRTETRTPPMPTSAWPPTSTSAPSAYQQQRSNAGFVTDD

SFYLPSSAQSESESQEEGEAPPLPSSAPPGMAVSAPPSHPQPPPSTSADKTFVSAVALSS

VGQKGGESGGVGEGVMGQKERTQTTPLQPSLQTTSAVGLSIRQRIAQIERQLKEKPSPKA

GSKAEEEDTFTWKAKATENARLLGAAERESKQLKDD

>SG-PL_contig_305_1_+1_34_228

MKFWPHSCTLLSPLQHSVLLTIHLHELKSIQKCSHIMKVATLKILYVYLCLKTVIIIRNV

RSLEA

>SG-PL_contig_306_1_+1_592_861

MFAKMVNASLVTNCKSKHRVDRPQSSSVQMVLAYKLGGINTRFCRNILNFWSHRQLFEDH

IGLSKKLTNMNACSSTGWKVTQVSSFVHTA

>SG-PL_contig_307_1_-2_131_361

MCAYPAVDRGLFFSSPPFRTTQKIMEKRILNLHCFVFLDKSGHNYADLYSSLIPTYPEKG

RHIIKSNFCCIPVIRLR

>SG-PL_contig_308_1_-1_223_1275

MNSAVLLLLCIVACSLTQALASSIIRFEQKRAPVRLRFDPYEITWNKFKTQYGRDYKTQD

EEDKRQRVFLENVKRIEQHNWQYFQMKKSYYLDINQFADMTAEEYQQYNKLLKRNSTAVT

FTKCSQFMPPLNWFLPSSVDWRTKGFVTPVKNQEACGSCWAFSTTGSLEGQHFRATGVLK

SLSEQQLVDCSESFGNEGCNGGLMDNAFEYINSVGGLETEGDYPYTAETGSCHFDKSEVV

ADLKNCVDIPSEDEHALMEAVASVGPVSVAIDAGHQSFQMYAGGVYDEPECSSTQLDHGV

LTVGYGSENGKEYWLVKNSWGATWGEEGYIKMSRNKDNQCGIATQASYPIV

>SG-PL_contig_309_1_-3_1071_1628

MEKTIERKEKGRNKDSDRKEGIFSTIFPEGRNTVYTKRSPNTHTHTHTHTHTHTHTHTQP

PAGGFMKLNMLLSATLAFDHVMQKPVFFLCCCCCCFVVAAATTTTTTIIIIIIIIIIIII

IIIIIIIIIIIIDSNREHECFIFQLFSLLLLLSFPQFTATKKISPFHFSARMSWFLKMAQ

FQSKIN

>SG-PL_contig_310_1_-3_171_1025

MADAEAAQEEKPQEVEKPKEKKTIAEKIKGTVKWFNVKSGYGFINRNDTNEDVFVHQTAI

IKNNPKKYLRSVGDNEEVEFDVVEGEKGNEAANVTGPNGTNVQGSKYAADRRRYRNGGRG

GWYRGGYRRGGRRNGEQENDDGEIEENEDQDKGQRGGRRRRGGRGYYPRRYFGGPVRRYG

YQGGPMMPPDYYMEQAPFPRRRPFQRYNYGMNRRDYYEPGYYQPRGYGRGGFRGGRGRGR

GGRGQGGNRRNNKAEDTKQAVEQKEVEKGSGDAKADATTEDADKE

>SG-PL_contig_311_1_+3_3273_3485

MPKTPYASWLKKLNKGISKYTLERKDMQSILMSLFLCLHHKPLLGVFINLFCFQSPVNRV

YLEFDLCNKWH

>SG-PL_contig_312_1_-3_3_830

DDDDDEDDYDEDDVDDYDDDDDEDEVDDDEDDYSEEEVDVAKEEEKDTENERKNPCQEKT

CRRGEICIVGKRRKARCTCIPQCGEHEAGDDGYQVCSKRNVTYQSECELDRDHCLCRRQL

EGCSTPKAGKIRLDYYGPCQQLTPCSDREFSIFPSRMREWLYVVMQQMAQRAKIPDYLDL

LEEARQEANHSHAVIWKFCDLDVDPHDRFVSRHELLYTVQSLKALEHCLLPFLDHCDTNS

DNRITLWEWGRCLGLDEDKIKDECKNIRKASGREKK

>SG-PL_contig_313_1_-1_28_1131

MCCLIGRANEYVLSAFIFDGEGRGVDSVFENQKILNMPGFRGGRGGGRRDYGGGDMDPNA

EQFRKLFIGGLSYETDEDSLRSHFETWGEIVDCVVMRDPVSKRSRGFGFITYKDAESIDQ

AQANRPHKLDNREVDTKRAMPREESGRSESQQSVKKMFVGGVKDDTTEDMIRETFGSFGE

ITGIDLITDKNTGKTRGFCFVTFDDHDSVDKAVLKKRHDLNGRQVEVKKAQSKSDMGSGP

GGGRGGGRGGGRGGRGGRQEYGSGGGGYGGGGYGSGNSYGGGSYGGGGGYGSQGGYGGGG

YDSYGGQGGYGNQGGGWNNNFGSGYGESYGGGAMRSGGYGGRSQGPYGGGYGSGGGGGGS

YGGGYGRR

>SG-PL_contig_314_1_-2_194_1885

MATDAAQPMVEVIPPPEASMDPATTPEETLFAVNKAPETDSAPVEDRLSPEPSEGSGATV

GSAKAAGGSGSESNGASAAATGGCENKSEQDGKTVAAKTKKKEDKSIEYILRALSSLNSP

EEKLAALCKKYADLHEEHRVLQSSFKQQQRKMTVGQREKDQLQAEHTKAVIAKSKLESLC

RELQKQNKAVKEESILRQKEEEERRKEISAKFQTTIAEIQTQMTENHEKNVKLREENHDL

ACRLKTFLEQQDLREQQLDKLHKHRELENQLSEAKLQQATAVMKEHEEKHLKEKELLLLQ

TAEAQKKNAMLEAQLNMYKDRYEEFQGTINKSNDMFQKLKAEMDKMGKRIKKLEKEGAQW

RAKWEASNKALLDMAEEKTRSDKEKQLLQTKIIKLESLCRALQGEMHGKKSASPQPTSSE

ESSKNTLASAPVENAAASNAPASTTTTPPNTPEENTSRASPNPDSPASLPQSLPTGQNGP

ISASASTPVLDSSTEVESESPAEACHAAEAAALVEENAATVGSESPSSCQEVSVPSAAAQ

EPDEAETSSSTADLPGSADQEEGV

>SG-PL_contig_315_1_-3_63_1562

MTGVWRLLTGSAVVMMAVIYSAGTSTQNTIELRVIVDHFAATKWGNTVGGNVDINDYVYS

VFKNVNSVFKKLKNSGIKLDVRVVGIEIVTTEEISSEMPPGSGKRNSSEVIEELKNWLAS

KNYRQTDHTVLFTGLDLLGDDLSTANAGKAYLGKMCDQDFSLSVVEAPHSSAVLTIAHEL

GHSLNSVHDVGACMNGHIMNSSLNIGQASRNNFKFSSCSIQSIKSILRELSGKDSCLLDT

SFEWTEMSPLPLGQLLPPDQTCKLAYGYNSFFSRRVYSENGDDTYDSMCGKIWCKKGQQH

SSARASDGFICGNRKLCSLGECISSGSTEVLDTCPQGDSPQANDAGEKCKQVVTADNGEC

YLEHIKQACCSSCDEVHTRRAGCEYGDKSKSCAEFRDFPGLCDQQPDACCGTCWERSNPT

ESTCTDKLDDCQERLAKNNARCSKSAFRENCCASCADVSTNPETESTCTDKWDNCQEKLA

KNSDKCSKSTFRENCCASCR

>SG-PL_contig_316_1_-3_240_704

MKTRHIYEKQNLINWASKPLSPVSVRWLLVREYTHHAQNVRIFKMMVDFKRKNKSTWSQP

CKEQTVAIMLDCLGLTETCEEVPSGRLGRGQVGYINISLKLSNSLYSDLMASAMIIWGLP

SCGICGVRVASSAHNGRHEKLTKVFYCVIVFILGK

>SG-PL_contig_317_1_-2_2_235

STVPRLPGHEVRHPGTEGVPNAGPAPGPPAQAFVSPGQDFGPFRERSPPLLGLMEPLAPE

LPLGRVRKRSLQDNPWLR

>SG-PL_contig_318_1_-1_67_339

MVILAVYYDVPRLLGVRACMQSHACDRETGRRESDRVRVCACILEMKKFWFAACVFQHQT

CWLIHRFDDSIIWTKFFGNSVWSDCLTDRSF

>SG-PL_contig_319_1_-1_16_2721

MANPSTPTTIHRGIVKQVLSGDTIVIRGQPKGGPPPEKTLCLSNIQAPKLARRANPNIEG

SVETKDEVFAWEAREFLRKKLIGKEISFYLEYKVPGSGREYGAVYLGKDNEGENMTESLI

ADGLVDVRRMGLRKDDADQQRLVQLEEAARAAGKGKHAADASTHVRDITWIIENPRHFVD

SHHSKPIEGVIEHVRDGCTVRAFLLPTFDYVTVMLSGIKCPMFKTDETGNQVPEPFAEEC

RYFTESRLLQREVKIILEGVSNQNLLGTVLHPNGNITELLLKEGFARCVDWSMGVVTQGA

DKYRAAEKVAKEGKRRIWKNYAPPTANLDIKDKSYIGKVVEVVNGDGLVVKPADNTFKKI

FLASIRPPRLPTETSENGAPAPSGKRIRPLYDIPYMYEAREFLRKKLIGKKVNVEVDYIQ

PPNQGFPEKTCCSVSIGGINIAEALVSKGLARVVRYRQDDDQRSSHYDELLAAEARAEKK

GVGLFSKKESPLHRVADIAGDVPKAKQFLPFLQRAGRSDAVVEFVASGSRLRLFLPRETC

LITFLISGIDCPKGKRQLPSGQVFEADPYGEEALLFTKEMCMQREVEVEVEAIDKGGNFI

GWLYVDNTNLSVALVEAGLAKVHFTAERSSHYKTLLSAEERAKTAKLKVWSNFEEPKEEV

VTEEPVERKLNYSKVVVTEVTDDFTFFAQKVDNGSQLERLMEQMRQELESNPPLQGAYTA

KRGDICAAKFSADGMWYRAKVLKVEPGKANILFIDYGNKESVTSSSLATLPATFQSMSAQ

ASEYSLAGVALPPDVVEDAKQDAVDALYRDILNHEMRLNVEYRSNGIDHVTLLTAESEGG

NDIGLGLISDGYVLVEERKEKRLAKLMSDYKEAQDEALKKRKNIWHYGDFREDDTREFGV

SR

>SG-PL_contig_320_1_-3_99_1514

MSTAVMSTQLDIGETLFPNLRNSSSSSACRTQQSGYETDLRQWEMYCNTTTTTTTSSFYV

SRHRNLSGPCTGSGRSTLDNRALATPPPGVPGCRAEREGHSSRDKENHHPRGEGRSGTHR

KLDRSLSDSEDRINGNGRGVNSSRYKTELCRPFDESGHCKYGDKCQFAHGAHELRNLTRH

PKYKTERCRTYHTIGFCPYGPRCHFIHNEDDNSVTQRKNVTNATNVTRLSSQKYILPPSA

PTSVLPHRPSNLSGLKSLGSSFESVSATSSPGNLSPSHFDDQFPAVGTFSPSAVNDTYTD

AIRNGESLRTSPVFNFPGPDPTSVGSVTAPLDVQTQQLYYNKMVDMAVLDSFSTVLNLSQ

NQHNNQSNYSTNLNVCQMQQQQAAFLESLSPAERSWPLTEAMGDTGAVLPPSKSSSPFSD

DSMEGSGSLEMTSKPPAISASMVDNREFLVHRPMDMGRSMRLPIFNQLSSEL

>SG-PL_contig_321_1_-2_140_334

MFCPRNFVLCSKMAVIIQSTFSADNKLIFSCLHFSVPFLCLQVVVLIIKERVNKQNYLIN

YAYYV

>SG-PL_contig_322_1_-2_32_868

MADSAGGGRGGFRGGFGGEGRGRGRGRGRGRGRGRGRGRGAKEGEKEWVPVTKLGRLVKD

GKIKSLEEIYMFSLPIKECEIIDTFLPTGTLKDEVLKIMPVQKQTRAGQRTRFKAFVAVG

DYNGHVGLGVKCSKEVATAIRGAIILAKLSVVPVRRGYWGNKIGKPHTVPCKVTGKCGSV

VVRLIPAPRGTGIVSAPVPKKLLTMAGIDDCYTSSRGATATLGNFAKATYAAISKTYSYL

TPDLWKETVFTKAPYQEFTDYLMKNHSRVAVQKQEQKVY

>SG-PL_contig_323_1_-1_220_336

MRCFCRANTPIVHFSARSLFFNAICWFSRHDFVFYSPLN

>SG-PL_contig_324_1_+3_3_179

ANIQTHTHTLPHLQHTHTHTQTLCTCVYIQSVPHHVRSETMSEIRKRMILKRCEHVETL

>SG-PL_contig_325_1_-1_94_375

MGTPAMYTPFSKAQMAWFSNQGKRCGELEMDYYRCASKVGLNRARADCDKEYSDFHECMF

RTKQFERYCAMQAERKKQGRPYPPTPHPDGVSII

>SG-PL_contig_326_1_-3_48_164

MHLTYCSHIDGNQEEFIFWTVYGNKPIIMCQCTDLASAE

>SG-PL_contig_327_1_-2_110_679

MIKAILVFNNHGKPRLMKFYHYYPEDLQQQIVRETFHLVSKRDDNVCNFLEGGTLIGGSD

YKLIYRHYATLYFVFCVDSSESELGILDLIQVFVETLDKCFENVCELDLIFHMDKVHYIL

SELVMGGMVLETNMTEIITRIEEQAKLEKQEAGISAAPARAVSAVKEMNIPQKIKDIKLP

DLPSIGNLKF

>SG-PL_contig_328_1_+2_2_685

SSDLFFSRFHSFPFIVRCPADTEHRLELHEVRVLFVLQGEHGPQVTKQHFLLRVGTDHIQ

QLFVHLSLILFALIRHSVHLLLGVEDFTLCVLGLAQFLATKVFVVKAFGQLDALDVQLGL

SGDDVRLVDTAQGAAIQVVGACDKQQSRVQLLQEHDSLALVSACKKNQHGAWCDVGTQLV

LVLGKEFLAWVQTFPFSFCWIVAWHTRRFGNFAIRTCRWSHLAQIGRA

>SG-PL_contig_329_1_-2_2_2239

DIIQNWFFPPAVPDPLEPPPPPAGSYRLPTNLEPSHYDLSLQPHIYGNNSLEFFFEGNVT

IFFRCISSTQSITLHQDRLTIKSSSITVTDVSNPATSLPIAGVTEEEIFQFYHINLENPL

EQGKEYSIEIQNFRGDMRWMEEGFYLSSYEDGDRTIYLATTQMEPTYARQAFPCFDEPAL

KATFNITLIRRTDTDRHYISLSNMPSISSYVIDNSWTADVFQKTMVMPTYVLAFIVCDFQ

YLSGRSPKYPVEIRTYARPNKIQNARLAHNHAVELFDWFNDRFEPKYDTYLSKVDNIVIP

DFIGEGMENWGLITYQKLLLYNSQLVASDRLWLAQVISHELAHMWFGNTVSPKWWTWTWL

KEGFASYWETYAVEQLHPDWKVRSVYMASDVFFIMKLDATPLAHPLNSPVETPDEIFDQF

DDFAYVKGSAMVGMMHMIMGEHFIPAVNMYLQDNKFGNADSSNLFDSLNKYISTTPDIDL

NMTKIMEPWVTQMGLPLVNVHRIGDQLHLTQEKYWNTPGSHSDTGAYGYKYEIPITFTTS

SERNFNKPRTDIEWMSVDQDSRTIQLPSANDDWVVINLRHQGYYRVNYDKDMWKAIIDQL

NTDHTAIHEINRASLLNDAWNLYKAELLPLETALQTLDYMKKETEYGPWSVLDTETRYVH

DIIKQTELFADFQSFMQEMVDEPMRILGLDFPPDNVPPVEALTKTLILTVACAYDHVACV

TKATSMVSDWMNGLGNAISVNIRSVF

>SG-PL_contig_330_1_-1_310_579

MADTLAWRPSSVSVACVLHLVCVGDKASSSFHCVTCLQLINLQLPFSPIGCGRETILPLS

IFPSAVFFFSSGIVILLLSNRRTRFLYRNT

>SG-PL_contig_331_1_-2_71_2263

MTAFEEMGVMSEIAQAVEEMDWSLPTDVQAEAIPMILGGGDVLMAAETGSGKTGAFCLPI

LQIVQETLLDQREGKGARKSMHSSGSNKWKMNAHDRGDAMAIDPEGTLCQARDAQGWHGT

RSNKAVVGGGKWYYEGTVTDEGLCRLGFSTGQARLDLGTCKFGFGFGGTGKKSWGKQFDS

YGEPFGMNDTVGCYLDLDKGEIRWSKNGVDLGKAYDVPPSVKSEQIYAAVVLKNAEMSFN

FGDTPFKFPPKNGFAGLASASGKNVTDSKIISGGAVSAKPAPNAPQAIIIEPSRELAEQT

IVQLQKFKKHLSNPPVRELLIIGGVSARDQVDALERGVDIVCGTPGRLEDLISTGKLALH

QVRFFVLDECDGLLSQGYGELINRLHQQIPKVTSDGKRLQMIVCSATLHSFDVKKMAERL

MYFPTWIDLKGADAVPETVHHCVCLVDPKVDGEWKSMNRHIITDGVHQKDRVGPDSYSAE

TYSEAVKMLKGEYTVRAIQKHNMDRALIFCRTKLDCDNMERYLNQVGQGKLTCVCLHSDR

NPGERKANLQKFKDGKVRFMICTDVAARGIDVSGLPYVINVTLPDEKQNYIHRIGRVGRA

ERMGLAVSIVSTVKEKVWYHSNCNTRGRGCYNTRLTDSGGCCIWYDEPKLLSDIEEHLDI

TIDKIDKTFDVPVNEFDGKVTYGQKRKAAGSIYKGHVEFLAPTVAELAQLEKSAQTSFID

LKFKKKFARRA

>SG-PL_contig_332_1_-3_3_293

AYTHTHTHAHTHTHTHTRARARAHTHTHARAHTHTHTHTHTHTYTHACTHTHAHTHTHTR

TRTHTRTHARAHARTDTHHTHTHYDAKHGVDETIIIY

>SG-PL_contig_333_1_-1_382_1104

MMGETFEAGAWNVEMEVNLFHAMRGHKPVGVNRHFHMVVIHDKLSSPATKKLSAKDVWDH

LSTMYDLQALNESEILPFPNRPIDFKLPEAEYRLSGDTEFPRMQNVGLASSSSEELRAKP

EGQAKGGKASRSDSKTSHLSKVDSRGNLAELKGSSSKGDGRSSVTPEGKSSHMSKVDSKS

GSSSAGRGEGKGGGKGSHSSHGLGTSSTPVSPSPKRTKRTRHNQSANSSPASDTPAAKRR

R

>SG-PL_contig_334_1_+2_875_1057

MFFSFIFCRPVITPLPPRPPRPPLKPPRPLPRSPLPPDLSSKSSSRLDTRSAAVTGFHSL

P

>SG-PL_contig_335_1_-3_66_2591

MVNFTVDQIRGIMDNKRNIRNMSVIAHVDHGKSTLTDSLVSKAGIIASAKAGETRFTDTR

KDEQERCITIKSTAISLYYELNKDDMKYIKQDYDPENNAFLINLIDSPGHVDFSSEVTAA

LRVTDGALVVVDCVSGVCVQTETVLRQAISERIRPVLLMNKMDLALLTLQLEAEDLYQTF

QRIIENVNVIVGTYSEEEGPMGNIAVDPKDGTVGFGSGLHGWAFTLKDFAKMYCTKFGME

EGKLMKRLWGDQFYNAKEKKWLKEQRDGAERGFTTYILKPIYQIFKTAMSGKSEKVLELA

EKLNIKLAADDKELHEKQLLKTIMRKWLPAGDAMLQMIVIHLPSPVTAQKYRMELLYEGP

HDDPCGVGIKTCDPKGELMMYVSKMVPTSDKGRFFAFGRVFSGTVATGQKVRIMGPNYVP

GKKEDLYDKSIQRTILMMGRYTEAIEDVPCGNICGLVGVDQYLVKTGTISTYKEAHNLKV

MKFSVSPVVRVAVECRNPSELPKLVEGLKRLAKSDPMVQCAIEESGEHIISGAGELHLEI

CLKDLEEDHAGIPIRVSDPVVSYRESVREESSQVCLSKSPNKHNRIFMKAAPLNEGIAED

IDKGNITTRQDIKERGRYLVDRYGWDATEARKIWCFGPEGTGPNLLVDCTKAVQYLNEIK

DSVVAGFQWATKEGVLCEENMRSVRFNIQDVTLHADAIHRGGGQIIPTARRVLYASVLTA

QPCLMEPIYLVEIMCPEQAQGGIYGVMNRRRGIVLENSQIGSTPQTIIKANMPVNESFGF

TADLRSATGGQAFPQCVFDHWALLQGDVFVDGSKPSQIVIDTRKRKGLKEGIPPLDNYMD

KL

>SG-PL_contig_336_1_+2_158_325

MVYRYIIVFILSRLGNVQKSRKQAKQICDQVLTGDKSISFVLHTHAEDHTCIILLS

>SG-PL_contig_337_1_-1_1_291

KKKNEKRKKKREKKREKREEKKKKIKKIWGGGGQRKKTKKKKKRCKSIMFSVCVLRPIQV

QICQLFRIHRILFRPVESCSDVPMMSTLFTKVHFRLA

>SG-PL_contig_338_1_-3_18_593

MVSDWMNGLGNAISVNIRSVFYCTAVRDGGHDVWDFIFQQHQAEQDDLERYDLFDALGCS

KDPTILYRFLDMCLNEEVVQSDLESCVTAVTRNRYGHDVAFNFVMEHFPVLLSSFSSDTV

GIIVGRATKHFHNNFHLAQIQLLMDTYDVSGITRNLGLAKLGTIKRIRWIKNNLDTVKRW

LASKSEAVHDTE

>SG-PL_contig_339_1_-2_170_892

MTTQYTTIQKISLAGMVIGSLMCVVGAFAPYWIVSDPKGFSGLADLVGKVVKGNMGLFIY

CIRVNVLGLAKSGCKVLPMETDSGWFHSARVSACGCVLLGGLCGLISLCLACCNCCKRFI

VLGILSFLAAAAGAFCAIVFCQNMDAFTSMVLGFKLTSYGWAFYIFCTGTGILGVVSFTA

CFAAPENPIHGMVLSAPGNQLQVVNSTVTNHPYMHLQEQGGPMGHPPPVDPGVTVVTANG

Y

>SG-PL_contig_340_1_-2_143_1363

MENLMAETEVTPIPTSVQPSTSPFSLGSTFTTTSTTSINNNTRTNHHHPHSKIYIAEAAT

AGCTVVDKNEQLDHRLGETIDFQDMYSNFYDDCNNPAASVVKMEEPQLSPDWSTDHSRQH

TGFGWAEDTSLTELVPAAPTEREDLEIVLECLQKDGLDSSMGFDIYPVGTASNPPPSEQQ

HHQQQQRQSFINLTSVSNLTSAADDARVMRQQPSVSPSPALIPPSVSPAPSITDSISSSQ

SSSSSSSSADVEMFYAQQSPAAEDDFTFLESLADFTKSDLPQSVTPARTAPSPSPSTTSS

TSSSSSSSSQHRRSTIRSSNKQIRLWEFLKELLDTNDPSMKWIDRATGVFKIENSTRVAR

RWGERKNHPAMNYDKLSRSIRQYYKKNIIKKTQSTKRLVYQFYSPYL

>SG-PL_contig_341_1_-2_77_700

MAPKRNNIIPNGHFHKDWQRMVKTWFNQPMRKKRRQAKRVKKALEIAPRPVAGLVRPVVR

CPTFKYNTRVRAGRGFTLDELKGAGVNKKQARSIGISVDYRRRNSSMESLQANVQRLKEY

KSKLILFPRHASKPKKGDATAEEMNLATQLKRRPVMPISDPVRREKARAITDEEKNFKAF

IVLRQARINKKLYGKREKKRREAEEKDK

>SG-PL_contig_342_1_-2_2_196

FVKNNDGVCFLWKVPTMNNRTFDVSYGIAGFRFTQKSVKFKKVKAYIRKHLHCASDAVHF

SCFLR

>SG-PL_contig_343_1_-3_3_275

CGASVRAKAGQQFGAIKILVIASMLDVAGSLSSKTGKRNDRVEKYRNHIPADCKALEKLL

PKSQRNNRSSSNVQVEVSDLMSLPNSAFSVL

>SG-PL_contig_344_1_+2_731_1375

MVDKAEPGCIPMSPSCICLLGFRWTCHDFVDEICIFEVAFQIDVNVCQLHDVVHIFVSES

LTHGCKKMTALGSRDVALSLTVERLEGFLEVPESAPFLHAVHLLEDGQELLEGVLLLSRL

GSSSHAFDGGESGIQIHTSQQVPHFDSIDQPVTTVPEIEQVEDILDLFKLFAVGHDEEKG

GGGGGEPGGKVVEQLEKQVSQPINQSSWCAARVDG

>SG-PL_contig_345_1_-1_106_270

MCILKCRVLLLLLSTDFLNKIATNHIWLATSSHSNNCFCWASYKGEKCLQILRKK

>SG-PL_contig_346_1_-2_140_283

MGEFLFFPLCQVILCRFSINSELLASVHIASVCVQAFVPGLFFFSLTD

>SG-PL_contig_347_1_-1_160_423

MAVECILVLIQLQTFSYTDLKKITMKTAFVVGSCCFEKGQDRHCTKKKTKRLGVFFFVAV

VCFFVLFLNIHECWEGERTCGLGKRLPM

>SG-PL_contig_348_1_-1_1_192

SCLLILPSDVTSTNKINRKFDQNTTSLVNCCTGCIKRRIVSPYYCCFSTHPRQDKPFSVF

FQPS

>SG-PL_contig_349_1_-1_472_1695

MLDASEKQALFDQHARRESSSVVEEFFSLDSNAKSVKVPGWIKQEPCEEKEEVDVFDISS

TIYDSVMDADLAVSGAPSSSLWDDINASINVVDNFTDSPVPSSSSSSSLPGLDVLSKVKC

EPMDTSDVSTCHYSKTLSNSRTHSGGLVMARNNSLPFTTTPSTTATTHAQLVNVNVSSLS

NTSFQQFKSPLSQQQFTPPPPPPPVSGSNSPANTSHVVPSVLLPPTPPNSQPASPNSGDF

VVRRTPPPPYPGVVSQASTPQGNIPQPVLSCSPSLALPLAPMSAPSPGRPRSTKPQNTHP

GCSTIKYNRKNNPELEKRRIHFCTFPGCRKAYTKSSHLKAHQRIHTGEKPYTCHFTTCQW

RFARSDELTRHIRKHTGAKPFRCKVCERSFARSDHLALHMKRHEPKNK

>SG-PL_contig_350_1_-1_220_1230

MGNVLQAFEGQAPCRQATLGAGLPITTPTTTINKVCASGMKSIMLAAQNLMCGHQEVMVA

GGMESMSNVPFYMKRGATPYGGVQLVDAIVHDGLTDVYDKIHMGNCAENTAKKFSIGRQE

QDEYAIRSYKLSQKAASEGIFQKEIVPVTIPKKKGDPVIVSEDEEYKRVNFDKFSSLRPV

FQKEGGTVTAANASTLNDGAAAMVLMTAQKASQLGVKPLARILGFADAAIAPIDFPTAPA

EAIPKVLKKTGVKQEDIDMWEINEAFSVVVLANVKALDLDPSKVNVNGGAVSIGHPIGMS

GARITGHMVHNLKPGQKGLAGICNGGGGASAILIEKL

>SG-PL_contig_351_1_-3_3_281

LLLVAAVGAAIAASGAEAASSCSKFAQNNKATARLIDGKCYYGINTPVTWKEAKGICDEA

ATHLVRLETAAEQELVSLAFQFEKYWIGLRLLD

>SG-PL_contig_352_1_+3_1101_1322

MILRKKNASKHTRCCPASCSCVICDSFRNICKICSERQSFLHSDRYRSVTTAVLPLHMIS

LQIMDGTCKTRYLH

>SG-PL_contig_353_1_-1_52_621

MRIKTIQSKTYTTPRRPFEKERLDQELKLIGEYGLRNKREVWRVKYTLGKIRTAARELLT

LEEKDPRRLFEGNALLRRLVRIGVLDENKMKLDYVLGLRLEDFLERRLQTQVFKLGLAKS

IHHARVLIRQRHIRVRKQVVNIPSYVVRLDSQKHIDFSLRSPYGGGRAGRVKRKNLKKAQ

GGGGGDEDED

>SG-PL_contig_354_1_-3_3_155

SNWILKLHFCKFTSNFLFSFFLSSFSFLAFISPPPPPRPPFPFPSFVCSGI

>SG-PL_contig_355_1_-3_12_1763

MRVSRQRDFTLALSYPLTAILGWLRQGKRHKMLTARLASTLARQLPRATPQICKNAFGAG

FVAARNLQTSQQSYAAAGGAEVSSILEDRILGQAPQANLEETGRVLSIGDGIARVYGLKN

IQAEEMVEFSSGLKGMALNLEPDNVGVVVFGNDKLIKEGDIVKRTGAIVDVPVGKELLGR

VVDALGNPIDGKGPVTSPDRQRVGIKAPGIIPRTSVKEPMQTGIKAVDSLVPIGRGQREL

IIGDRQTGKTAVAIDTIINQKRFNEGSNEKQKLYCIYVAIGQKRSTVAQLVKRLTNADAM

KYTIVVSATASDAAPLQYLAPYSGCAMGEYFRDNGMHALIIYDDLSKQAVAYRQMSLLLR

RPPGREAYPGDVFYLHSRLLERAAKMNDDNGGGSLTALPVIETQAGDVSAYIPTNVISIT

DGQIFLETELFYKGIRPAINVGLSVSRVGSAAQTRAMKQVAGSMKLELAQYREVAAFAQF

GSDLDQATQNLLNRGVRLTELLKQGQYVPMAIEEQVAVIYAGVRGHLDKMDPSKIVAFEA

EFLKHLQSSQQDMLKIIANEGKISETTEEKLKQVVTSFLAGFNA

>SG-PL_contig_356_1_-1_79_1137

MSTSLISAWKKNLFSNPFFATCENDEQAPALKVNEERTIAASATEEYSCEFGSAKYYGLC

AFGGLLSCGLTHTAVVPLDLVKCRIQVDAAKYGNIINGFRVTLAEEGFRALGKGWAPTFF

GYSMQGVGKFGFYEAFKILYSGLLGEENTYLWRTSLYLAASASAEFFADIALCPMEAVKV

RIQTQPGWSTTLREGFPRIMQEEGVSGFYKGLVPLWARQIPYTMMKFACFERTVEALYKY

VVPKPRSECSKPEQLVVTFAAGYIAGVFCAIVSHPADTIVSKLNQDKGSNFMQVASKLGL

MGMWKGLFPRIIMIGTLTALQWFIYDSVKVACRLPRPPPPEMPESLKKKMGLA

>SG-PL_contig_357_1_+1_166_303

MFISIDPVVSQTMTNPRLYGSPSSFFSIFFLSFFLLLRLFPLVLFS

>SG-PL_contig_358_1_+2_2_166

LLLLSLLLPPLPLLLILLLLFVLSLSLSLLSLLLFKNSPNTAHRPSLTVIHLNTH

>SG-PL_contig_359_1_-2_158_1204

MLREELQLLQEQGSYVGEVVKPMDKKKVLVKVHPEGKFVVDIDKNIDMNSVTPNCRVALR

NDSYVLHKILPNKVDPLVSLMMVEKVPDSTYEMVGGLDKQIKEIKEVIELPVKHPELFEA

LGIAQPKGVLLYGPPGTGKTLLARAVAHHTECTFIRVSGSELVQKFIGEGSRMVRELFVM

AREHAPSIIFMDEIDSIGSSRLEGGSGGDSEVQRTMLELLNQLDGFEPKQNIKVIMATNR

IDILDSALLRPGRIDRKIEFPPPNEEARLDILKIHSRKMNLTRGINLRKIAELMPGASGA

EVKGVCTESGMYALRERRVHVTQEDFELAVAKVMQKDSEKNISIKKQWK

>SG-PL_contig_360_1_-2_116_304

MRVVKYHPPHVALYVLLAFVFSYDFKYGFHFFSVLNFFIFLFRVFNLYFMKREIDYSCFD

FET

>SG-PL_contig_361_1_+1_1_126

LFVCSFLPLRGRQPCLPSPGAEDYHLQLPYLTKQPLAVISCS

>SG-PL_contig_362_1_+1_1_696

FFFFFLKLSHLFTNFSLFSSWFTENKHPLACDSLGSPDYNSLRAGVGAFPAAEGCHNVDK

ATVVLDASLGTAGLLLLLLLRLHLWCLASHFSSTSEGAVNFSSQNADLNFQRGQVLKGGQ

EGAILQRFAGAEDSKLFCRNSLPLLYQSLNIFNSFSPLDIKGVSLLGVYEQLHRGQVKHC

QINFNCRSLPRCQLSPNVAIDNVPTPNKTKYDMTCIYSLSSTSNDKRWCKSS

>SG-PL_contig_363_1_+1_1_84

TNTDFTTHFEIFSPCSLLTACIKSPVVN

>SG-PL_contig_364_1_-1_133_3492

MAEALPHWSSTSQASLPLSDEEVFETEDAIWKLLHTNRPPEKEPKEVYVDRPRVKKHENT

QNLDSFADQNQQNVKSFDETVPSFEGVGIRSGLTVSLSLPSENDQHLSGSAQSESSQRLR

SWSDHVHTDSTEMRRRPPRFHQLERQPSIVDGLLMEIYDQWHRNRTDSLDSDTFTEYSSA

SDVFHGRWETGHYDTAVDQQPAKHYHRHFLQTQTREQLQKMVQDLQRSVTQASARLVRQL

KRRDRRVAKMSRHCDVVTAILQAASLKRQIDTRMKFSLGPGPGQSAFEQWKDAMKAVNRL

PMGIPTEFRKKVWLNLADHYISKLKIDWHKTVRLAFNDRSNPDDDKLGLQIVKDLHRTGC

SSFSGQDNEQDRAVLKRVLLAYARWNKRIGYCQGFNVIAALLLNVMDRKEDDAFKVMIYL

IDHVLPESYFDNNLRALSVDMAVFRDLMRLTLPTLSTHLDSLQHAAQDANTGACYEPPLT

NVFTMQWFLTLFATCLPKEVVLRVWDAILLEGSEVLLRTALCLWGKLSKRIMTVESADEF

YSLMGELSSDMMEGTVFDSDDMIKAIYAVAPVPFPQLTELREKYTYNIRPFSTTPAPTRK

LSRLAKAVLYSDEEEPDEDELRAMAAFPGMSPVSGARGKGTDISVVGPGVFGAEPEVMSG

SRDTVYMERMSTDITALKMQYHKLRQRQRQAHVIIAAVSAKKAVELKQKSQAPVLVPKIE

TPLAMNHLFIGKVQGRNRLVTKGPCITGSATPGNSGQTQPVHTQSLLQLASCTPNCTAIA

PPRTRTSPALSVGRGDVEGEESLQGQLEESLQICERSKTLCTGSSSEDAEGNTGNAIKST

SVLPEGGSLAAEVRPQDQEEQNCIPSSEDNENHGLSSCCSEPEELDAVRESLNLTVIPDK

DRTYNLDNVDEQHLQLATQQKGSGVTVPGSQNGVVSNNQSKNLDCENSKITRNSTTAPSV

HFPNGMGESSPKKSVSANDLLDGRAGGAHTVSKTSNDVGNVCGDGVDSKHSHIAISRSLS

LPTPSTKKTRFSPTTTPTHKHPISLTGIKVRHASSPLPPSSQPSPIKTPRPFNPFPVKHI

NTNRARTGLKLGLYTPSTLEHIQGQLLGGLVLKKARNASS

>SG-PL_contig_365_1_-1_31_360

MYDPNEQVRAVSQVQVHPDYKFVPTFSTLSYTFENDIAVLTLSSPLKMSPDNPCAVPICR

DPSFTPDGKSCTVSGWGATLSGSQQASLLLQRASMPTYSGDKCMSAFSIP

>SG-PL_contig_366_1_-3_102_767

MGFGDLKTDGGLKSLNDFLADRSYIEGYTASQADSVVFAALSGAPKASFPHALRWYNHIK

SMDRSALPGVKKPLTSYGGATPAAAAAGGGDADDDDDDIDLFGSDDDDDEAAEQLKQQRL

KAYADKKAKKPALIPKSNIILEVKPWDDETDMKQLEQSVRSVSTDGLVWGASKLVPVGYG

INKLQISCVVEDDKVGTDYLEEEITAFEDYVQSVDILAFNKV

>SG-PL_contig_367_1_+1_784_1035

MFLSYVDYLFLTRFKIVLVLPALFDQSITMGLLRLKLKTSPAGWESQELSSFVPVFVFIL

FFFFFPMDLPFIAFFLFFFFFSIL

>SG-PL_contig_368_1_-3_246_362

MFVSSSFLVAAVLLLRGIFFPQDPTYIIVRVRFAETLYC

>SG-PL_contig_369_1_+3_3_89

SRFGPPVSVTQPSTCPWLDHRVSGLYPAT

>SG-PL_contig_370_1_-3_27_554

MKVKGELREYKVVGRGLPSAKVRTPTLYYMRIFAPDQATAKSRFWYFLSQLRKLKKATGE

IVSCSRVYEKRPLKIKNFGVWLRYNSRSGTHNMYREYRDLTAAKAVTACYTDMAARHRAR

ASSIHIIRVEEVAAAKCRRANILQFHNNKIKFPLPHRVNQKLHAPRFCASRPHTTF

>SG-PL_contig_371_1_-2_455_3511

MHRIGKLLTKGPSSRILPQILRTTRGYASSVEDGSGARTGKGADRAFATQASAEPFLSGS

SSTYVEEMYLAWQRDPNSVHKSWDTFFRQTSKGAPPGQAYVRPPTLTPFPSAPMPLPLHA

VMQGAPVDEKTIEDHLSVQSIIRSYQIRGHHLAELDPLQINRGDLDSSIPAELLMSHYQL

DDNDLERVFRLPATTYIGGNEAALPLKEIIRRLEDVYCRHIGVEFMFINNLDQTDWIRRK

FESPGIMQFGIEDKRLILARLIRSTRFEEFLARKWSSEKRFGLEGCEVLIPCMKTIIDSS

SALGVDSFVIGMPHRGRLNVLANVCRKPLEKIICQFDSKLEASDEGSGDVKYHLGMSHER

LNRVTNKNIRIAVVANPSHLEAVDPVVQGKVKAEQFYRGDTEGKKVMSILLHGDAAFAGQ

GIVYETMHLSDLPAFTTHGTIHVVVNNQIGFTTDPRMSRSSPYCTDVARVVNAPIFHVNA

DDPEAVTYVCKVAAEWRATFSKDVIIDLVCYRRNGHNEMDEPMFTQPLMYKKIAKEPSVL

TKYAESLIRNNFVTQQEYEEEIAKYDRICEEAYMLAKKETAVSNAEWLDSPWTDFFTTHR

TDQMKNPKTSVPEETLLDIGQKTCKEPEDFVVHGGLKRVLRARGEMVKKRMADWALGEFF

AFGSLLKEGIHVRLSGQDVERGTFSHRHHVLHHQEIDKKTIVPLNHLYEDQAMYTVCNSS

LSEYGVLGFELGYSMTNPNALVIWEAQFGDFSNTAQCIIDQFISSGQAKWVRQSGLVLLL

PHGYEGMGPEHSSCRIERFLQMSNDDPEHFPVEDENFEIQQLHEINWFVCNLTTPANFFH

AMRRQIALPFRKPLIVATPKSLLRLPEARSSFDDMIEGSRFQRIIPEAGAAKDNADTVKK

LLFCSGKVYYELVKEREKKDLASDIAITRIEQLTPFPFDLVKAELQKYPKAKVCWVQEEH

KNMGPYQYVEPRVRTIFKKMEDSREMEYAGRFPSAAAATGNKQMHLIELSSFLEAALTF

>SG-PL_contig_372_1_-2_2_313

FRSAASETFTPSINATVSSGFESKRPQLDGREKEAADIGQTDSHAEKHTKSLNSATSIAG

NSLALGPHQEVKSSSYAKPGEDIASMPDTTSTIKRQPLSCQDSV

>SG-PL_contig_373_1_-3_150_1598

MSAPQGDIALIGLAVMGQNLILNMNDHGFVVVAYNRTVSKVDDFLKKEAKGTKIVGATSL

EDMVSKLKKPRRVMLLVKAGQAVDDFIDKLVPLLEKGDIIIDGGNSEYRDSIRRCQSLKQ

KGMLFVGSGVSGGEDGARYGPSLMPGGAEEAWPAIKNIFQSISAKAGSEPCCDWVGPEGA

GHFVKMVHNGIEYGDMQLICEAYSLMKDALGMSHDEMAGVLDNWNKSELESFLIEITRDI

LKYKDTDGQPLVEKIRDSAGQKGTGKWTAISSLEYGVPVTLIGEAVFARCLSSLKDERVA

ASKQIPGPSAKFSGDKKAFVEDIRKALYASKIVSYAQGFMLMRETAKELHWKLNMGAIAL

MWRGGCIIRSVFLGNIKQAFDKNPDLSNLLLDSFFMDEMKKCQESWRRVVAQAVTLGIPT

PAFSTALAFFDGYRSASLPANLIQAQRDYFGAHTYERLDCPGKFVHTNWTGHGGNVSSTT

YQA

>SG-PL_contig_374_1_-2_2_445

LLWCVFVFFFFKQKTAYEISRSDWSSDVCSSDLVNKLTLTLCTFGTVLQTHFRHGSGDAV

CRPCQAVCQRLHPPGEKVYQARQKRVPEDCHSDSYRVCHHGVHRVLCQTDPHPHQQHHCG

ILGGQFQHIIKRTFSIQASAFFMDIPLP

>SG-PL_contig_375_1_-2_2_121

KKKKKASIYPGSFLMMQLFFQVCVHTCAAYFSEQYMQCRS

>SG-PL_contig_376_1_-1_49_261

MQTCSYHLVGFYLSALCAHLSVSLIISQNFSFNIVTSLEINYEYFLSSFCASVTALVFWI

TITLWMYMLFA

>SG-PL_contig_377_1_-3_15_521

MKSSRLPGRLPTMTTVVVVMVVIIGLSIPVEPFIGALLAAISGLTAGQIASAAGTAVSIG

SSIGEAAAAASRSNYRVTCVIEMENWTKHNLHSPIVQITNSGSLITNPKNIWPASAQAFS

VRKPAHSATGVYGTVSWMLGDTGRRVVVMWSAPYSFNHYSNVLGVGLTR

>SG-PL_contig_378_1_-1_10_903

MASLGRVVGSVCRNVLLKTGKTNVGMLPVLHQRFLSTGQFQNLIVEKRGEKKNVALIQLN

RPKALNALCDALMTEMTVALEELQQDKDIGCIVITGSEKAFAAGADIKEMQQMKTFSEAY

NSNFLTDWDVVSRCRKPIIAAVNGYALGGGCELAMMCDIIYAGDKAQFGQPEITLGTIPG

SGGTQRLVHAIGKSKAMELVLTGDRISAVDAEKAGLVSKVVPAAQLVDEAIKTAEKIAGF

SKITVALCKEAINASEEMSLSEGLHFEKRLFHTTFGTSDRMEGMTAFVEKRKPNFTDS

>SG-PL_contig_379_1_-2_74_2260

MLRLWPLRQALSSAGARFIPACRKSTAADPAPELVEVFVNDRQVFVEPGTTVLQACAAAG

IEIPRFCYHERLSIAGNCRMCLVEVERSPKPIASCAMPVMKGMKVKTDSPMTRKAREGVM

EFLLMNHPLDCPICDQGGECDLQDQSMNFGSDRSRFTDNAFSGKRAVEDKNVGPLVKTIM

TRCIHCTRCIRFASEVAGVEDLGTTGRGNEMQVGTYVEKMLQSELSGNIIDLCPVGALTS

KPYAFTARPWETRKIESIDVMDALGSNIVVSMRTNEVMRILPRMNEDINEEWISDKTRFA

YDGLKRQRLTTPFVKNAKGELVQSDWETAFFTIVDKLKGVEGSQISAVVGGLVDAEALTA

LKDLMNRLGCETLCTEEIFPMDGSGTDFRSNYLLNTTISGVEEADLVLFVGTNPRFEAPI

FNARVRKSWTHNELRVAMVGAPVDLTYEYDHLGDTPAVLEQLADGSHPFSKVLESAKRPM

VVVGSTSLQRGDGAAVHSAVSTLANRLRTTSGADGDWRVLNVLHRVASQVAALDLGYAAG

VDGVRQTKPQVLFLLGADEGTITRSDLPADAFVIYQGHHGDQGAQMADVILPGAAYTEKD

TTYVNTEGRAQLTRQAVTPPGLARNDWKILRALSELLGVTLPYEKLIEIRQRMAEVSPTL

VRVGDVEEANFFQQSADMAKSVKSALGSEPLSPPQHVLKDFYMTDTISRASPTMAKCVQA

AKEAQTAKH

>SG-PL_contig_380_1_+1_1_120

DLSLHKHVTSADTSQKRNVHCLKQCLFYTSHTELTSLFFF

>SG-PL_contig_381_1_-1_451_570

MAPGSVVTVIFTTLGHVVISVNRHLSINFSSQETIEISKC

>SG-PL_contig_382_1_-1_1_372

AKSKLAKSNFAVGYTQGDFAFHTSVNDGQEFAGSIYQRVNPDLETAVNLSWTSGTNATRF

ALGAKYILDKDASISAKVNNSSQIGLGYTQKLRDGVKLTLSALIEGKSINQGGHKVGLGL

DLEA

>SG-PL_contig_383_1_-2_179_313

MVLCPHHSSLFLQLLPSTVFFFRKLLCLRSFLFITFFCLGFLLAL

>SG-PL_contig_384_1_+1_157_288

MSKSTSYSKPGTVPYSQWSVFLIIFTCHRIKDHISTIYISCEMH

>SG-PL_contig_385_1_+3_144_305

MALSAYISCETHSACRVLVYVCRKCLGHLGVGHQEYHRCIWHEDTSRQKCHDVL

>SG-PL_contig_386_1_+2_113_214

MTQIQLKKSKCMCYHSKNVITNFLFNTSIIKSKS

>SG-PL_contig_387_1_-2_2_238

MAPVIVRNMVAAFNIEVAAGLSDVIGYANFKEMKKTNPAYAACYKKFRQGHGISVSLTML

SIIANTVLLYYIASQCVPV

>SG-PL_contig_388_1_-2_92_613

MGDTPLHKAAQSGHLQCVRLLMSHCPILSTFNHSGQTPLEVALRNGHTECAKALEEASAS

QHSSFFPTSSSPILMNGEGIYQNGVFSRRQRESDQDMEMEEEVNNDGDAVGNLQPQPGLK

RSCQDGEEAPFKRPRLFAPSMTDKLGTVTNNNILSSYQGVLAMSVEPSKPPAVN

>SG-PL_contig_389_1_+2_2_211

EPTLCILQKAQAGTPWNVSSISIAKYPPPLFLLPLDLFGKNRSIDIAVLTNELLVSMSSQ

TKLTRHGESD

>SG-PL_contig_390_1_+3_3_173

FYIFFFIYHLTRPSISSYLEEKGKKTSTQTKKLCLQITEFSLFSLVFFVIKSTGVFY

>SG-PL_contig_391_1_-3_3_536

KTVADLRNFVHKLENRIAVLEKGGKGDAPAASSAKQPAPAAAVEDDEDDDDIDLFGDDDD

DDDGAAKLNEARAKALEQYAAKKAKKPAVVAKSSVILDCKPWDDETDMKEMEKLVRKIEK

DGLTWGAAKLVDLAYGIKKLTIICVVEDEKISVEDLKEEIEGIEDLVQSVDIAAFNKI

>SG-PL_contig_392_1_-1_103_3051

MDEHKVPIEELYDRLGTDPNSGLSPERARDILLRDGPNELTPPKTTPEWVKFCKQLFGGF

SLLLWIGATLCFIAYSIQASAYENPPGDNLYLGVVLAAVVIVTGVFSYYQEAKSSRIMDS

FKNLVPQFAVVCRGDQKLSVKAEELVVGDIVECKFGDRVPADLRVISAHGFKVDNSSLTG

ESEPQTRTAEFTHENPLETRNICFFSTNAVEGTCRGIVIKTGDNTVMGRIANLASGLEVG

DTPIAKEIAHFIHIITGVAVFLGVSFFIIAFILGYFWLDAVIFLIGIIVANVPEGLLATV

TVCLTLTAKRMARKNCLVKNLEAVETLGSTSTICSDKTGTLTQNRMTVAHMWFDGRIVEA

DTSDDQSNATYSSSDPTWMALARISMLCNRAEFKGNQDNIPILKRECNGDASESALLKCV

ELSIGQVTEYRRRNKKIVEIPFNSTNKYQVSIHETEDPNDPRYLLVMKGAPERIMDRCST

ILLNGKEQPLDDNFREAFNAAYLELGGLGERVLGFCDYFLPIDQYPPGYEFDPDGPNFPL

TGLRFVGLMSMIDPPRAAVPDAVGKCRSAGIKVIMVTGDHPITAKAIAKGVGIISEGSKT

VEDIAAERGIPVEEVDPREAKAAVIHGGDLRDMTPAQIDEILRNHSEIVFARTSPQQKLI

IVEGCQRQGQIVAVTGDGVNDSPALKKADIGVAMGIAGSDVSKQAADMILLDDNFASIVT

GVEEGRLIFDNLKKSIAYTLTSNIPEISPFLVFILADIPLPLGTITILCIDLGTDMVPAI

SLAYEKAENDIMKRQPRDPIKDKLVNERLISMAYGQIGMIQASAGFFTYFVIMGENGFWY

NRLLGIRMEWDSRGVNDLEDSYGQEWTYSQRKKLEYTCHTAFFVSIVIVQWADLIICKTR

RLSLFQQGMKNHHLTFGLFFETALAAFLCYCPGLDKGLRMFPLRFTWWLVALPFSLTIFI

YDECRKLILRRNPGGFVERETYY

>SG-PL_contig_393_1_+2_116_274

MIISPQQAYFVDTSIRQHAEAFLRVKGKLVQRATLLWARAAPIITPTVGKRPV

>SG-PL_contig_394_1_+3_441_767

MKRKPVSGNSFFRLTSNLAESFNDNFDRGLRNLLWIFIFCSILFLHKFRPYSLHFNIRYQ

KCFLIDQKIETKNTLSLLKFKFQFSQDSASALRIFPYSLTLQIPPGGKS

>SG-PL_contig_395_1_-2_335_535

MLIFCYIKYMYFNNLICGASQIQSGVCPVHRRCVQPQLLGWSVSFGNQPYARASNPRNKL

KLESSIL

>SG-PL_contig_396_1_+1_19_507

MGSKFIYVMHNVFQAMLEALLAILGHRSAHSLATGPVIALPFISPLLLTITPALSSNTMN

VPSFLLMAFLCRITTAGITFLRSSGFPFLTVATNMSPEPAAGRRFRRPLMPWTAITYKFL

APVLSAQLITAPTGRPSAMRNLPPAEPPLPLFDMLYLHLAQKI

>SG-PL_contig_397_1_-2_80_706

MPIEAVEKPVEETKPDESASSDSDDDDSPPELEEGDAAAMQQQSQVAAAAGLTEELVSKA

KQSRSEKKARKAMSKLGLKSVAGVTRVTIRKSKNILFVINRPDVYKSPASDTYIVFGEAK

IEDLNQQAQMAAAEKFRDKPPAGPGDMPSTVSQPIQEESEEEEEEDDTGVEQKDIELVMS

QANVSKSKAIRALKNNNNDIVNAIMELTM

>SG-PL_contig_398_1_-2_338_397

MEGYQCISGRVIISCMLFFL

>SG-PL_contig_399_1_-1_25_315

MVLSVERTSDVLLASVNHHQYSLTFRIRALKETHQWVSTVICVARPCQLSTVTATRNLSS

ESVVAAVLVSSPITQDVSTVTNLRAVLSTVTSQASVT

>SG-PL_contig_400_1_-2_2588_2905

MYCYLEISSFLLLSLFLPLFLPLLSSAFFCHTGPFHFLFSSKPSHNFSPTVIINTGGPVR

SLPFSCEKCFLAVFLFVCLFTSLFILMGFLVEVKYLLRISFHVGIF

>SG-PL_contig_401_1_-1_25_1428

MGRSPEGSVLWMEVLYRNQPVFGAQPAAMVYPSANLAPHMFASPSQAYTCYSFEASPSAN

SPNPPSRTPTPEYPARSRSNQESMMSQGTTLKSVLRGEVPEAYSHFLSSSSSSSSSVVDS

VRHPSPLGKRKSPFVEVEASCSPAFLGEVSTTVLSPPPEDMQTEPMDLSFKHTSSTSSEA

SSPASFHHTFPPSPAEDHQGQSSEFSLLRNLLQVGKVPQPSSSPYSHPSPSTSSASDRSC

DSPCSDSGFPARHLCGSTRVTLAKKNMYPVSARVSDWLLKVVQFANSIQCFREEVNHSDQ

LTLILNSWSRLLLLFMAESNFQFAVTPLRMEPTPCEMDGGPSPDEPTMKSVETIQTFIRK

CQHMGVESEEYYYLRMLVLFNSSYVGLSEPSKVDSFNSQIQQSLQQHVQITRPNDIMHYS

RLLMCLPSLYGISAKMVERLFCKHIDNTNAVNDMEVLLKELLQTTLGR

>SG-PL_contig_402_1_-3_75_1796

MPESRLPTAALVALLLLCFLCNGVNGTGQEKRQIHHIPDPTQGQTTAPPDPLTAWMSSTS

SSKPNVLFLIADDYRPKMGAYGESNMVTPNLDFLASKSVRFDQAFAQQALCSPSRTSFLT

GRRPETTRVFDLHTYFRTVAGNFTTLPEFFKRHGYMSVGVGKIYHPGVASGNTEDYPYSW

SVQPYAGVQPFNAHMRKRSTHQPVNETETGKLQDTTIAEYAVNFLKQHSRNRDQPFFLAV

GFHKPHLPWIYPKQFLDLYPRSKIYLAAHRTKPAHIPESTWSHSDEIRSYQDIIALNITG

PDGIIPDHYQYTLRQAYSAATSYMDANVGKVLHALTQYGFAGNTIVSFLGDHGWHLGDNA

EWCKHTNYEMALRIPMMLYVPGVTSMGHNFSFIDALAPGFDPSHDTLQHFGSQHVTEELV

EAVDLFPTLIELAGLPMPHTCPENSLHVPTCTEGTSLVPLIRKVRNLLNGESFTWKGAAY

SVYHRKWNSRSPILGYTLRTKTHRYTEWVSYDRTLFRPNFNHTFGQELYRHSDDPFEFNN

LAQSAASSATVSQLAQMLRKGWRATLQDYLQSVH

>SG-PL_contig_403_1_+1_1_138

KIPPSPFLLPLDLFGKNRSIDITVLTNELLVSMSSQTKLTRHGESD

>SG-PL_contig_404_1_-2_20_1000

MAGVWQLLISSAAMLAVIDSTPLDNTIQNSIEIRVIVDSWAFNSWAEKLETSSTEVQSTI

TDYVYSVFNDVNSIFRELMSSGINLDARVVSIEFITNQEISSESPAGSGQRDSEEVLEEL

GSWLATKDYTQADLTMLFTGLDLTIDGDTQSTSRVNVGMMCSRSSSLSVVEATPNGLAIT

GTAYNLVHSISSDLSGTGCGNGYILSPVLRTDQVSPNSFRFSNCSIRTIKASLEELHNGD

NCLSAANTNAPRVSAPALGQRYTPDEICKLTVSKSSYFCRCFYGSGGYPSYDTMCGAIFC

KGVSEDCISVRALDGFVCGQDKQCSLG

>SG-PL_contig_405_1_-2_725_937

MKQQTTLGFLKIRNGACMGSKRSLHCSVLFHLGKKNVIWVCMRERERERESVCVCVCVCE

RESVCVCVCVC

>SG-PL_contig_406_1_-3_48_497

MELVYSVPFKVLELPNLKLKKPSWVRMPSPMVVFSGVLLSYFLVTGGLIYDVINEPPSIG

STTDEKGNSKPVAFMPYRVNGQYIMEGLASSFMFTLGGLGFIVLDQVNKPLIPRLNRIIM

LGVGFTAVTIAFFLCRVFMRIKLPGYLLDY

>SG-PL_contig_407_1_-1_40_2736

MMLTHLVVRPGLGNKGGTMSKKEMDDILKFGTEELFKEETAEEDRIVYDDKAVAQLLDRT

QVGQEEKEMAMNEYLESFKVASYQTKGGDAEDEPETEILKQEAEHADPAYWEKLLRHHYE

QQQEDLARTLGKGKRIRKQVNYNDAMNGHDEEAWKENLSDFDSDFSNSAHEDDDDDFEEN

RDGDQTRTRTRRRANEKDRPLPPLLARVNGQIEVLGFNARQRKAFLNAVMRWGMPPQDAF

NSQWLVRDLKGKTEKVFKAYVSLFMRHLCEPGADNAETFADGVPREGLSRQHVLTRIGIM

SLVRKKVQEFEAINGTHSMPYIKALDAIEKLKKESIKAPSPKTESKADKDAKSEKSDSKD

KDSKDSKDPKSEPAEKAAAGKDGEVKEEPKEGVEVKKEPEDEEKKEGEDQKEEEEKDEEK

KEEEEEEKKEKGDEEEKKEDEKKEEEKEASRPETEKEEEGEKVEKMEVDVKIEEEKVEEK

KEEPMETDAEQKTEKDAEEEEKTEEAAKEETKDKETKENEDDKPTKDEENTEKGEEEEKK

EGEEKKTEGEAADKPEDPDKEAVKEEKAEDKESKDKDKTTDAKKDDEKKSDEKKEGKEEE

KKDGIKKEGDKKEGEKKDGDKKEDETSQKFMFNIADGGFTELHTLWANEQRALQIGREHE

VWHRRHDYWLLTGIVTHGYGRWQDIQNDLRFQIINEPFISEQGKGNFLEIKNKFLARRFK

LLEQALVIEEQLRRAAYLNLTQDPAHPAMALNARFAELECLAESHQHLSKESLGGNKPAN

AVLHKVLNQLEELLSDMKQDVARLPATLARVPPVTQRLQMSERNILNRLVNPNQLPGAAA

AAAAVMAAGAGVASGSSQQLPLKTGLPGQPSPSSSHRSSPAPTPGTSGQSDKKETIVLD

>SG-PL_contig_408_1_+2_2_196

NKKTTVILVLTPFISHTFYFSQIHMNVTQQLVILILFTSHICLLQKNLSYFDPHTFISVF

VLGGV

>SG-PL_contig_409_1_+1_1_240

CTAAEEGTRLLVSPHEGSGQPAGMVVAEMSLGYSRWTREEPPLCIAPQGRAFQVVLDAAG

WTKEANLAPGISGNQAGDQG

>SG-PL_contig_410_1_-3_150_1271

MTTTEKVTQSSSTSITTTAASSTMLSIDDYDDEDDSDYLETIDDDGEVSPTDDWGSDDDD

GGPDQIDDTEEEIIVTTTPPPTTTTTPTTTTPPTTPTTTPTTTTTTTTSTSTTTTTTTTT

TTSTTTTTPKPLCPAPPMLRYRRMLGGTTANSCSANGVLKFNSGTDQMCNGVLAYNPETN

ANQIYITDFCKGLMIFVKNVLKLNDFGTYNGANIPNPTSLPFTAIANNISYFASPASALS

SAQCQSVACLYNGATMKDAIDFTNCYISSFGDDVKGSDGKVGNSGMLNNVAVKQGTCQTK

PITRTAKCFVAVDPNVRGCWGDNGAPVYCRLKSTRETVLYGMLDVTGSPPPKFDMNSNNI

LCADNNEFHVLPVS

>SG-PL_contig_411_1_+1_484_885

MTDKSKPVKSDHLLTFSGEKNGNRDTNKQRGKSRFFVLQKHRLERKTNLFIGTICVDHTN

HHQHITGLLTLLSFVHIITRDRKKSNKAETAPEHLKANTNCSTDQTGDNTHDINAEKGKT

PGNKYCLLQANPFS

>SG-PL_contig_412_1_-3_3_356

CLLIVIQLFVAGIVVLLLDELLQKGYGLGSGISLFIATNICETIVWKAFSPATVNTGRGT

EFEGALIALFHLLATRQDKVRGLREAFYRQNLPNLMNLLATVLVFAIVIYFQGFRVDL

>SG-PL_contig_413_1_+2_2_199

KFESRPSKPSSLQVTSFHHSTTAPTNHHAIKLIGSFDGLIYCFVLFCFSVLLYEIDILYI

CPLFSE

>SG-PL_contig_414_1_-3_3_173

EKGKLPWHMSVSGCACVYSVIRMKFLAWCFCEWKEIIISAFKPMLPWGSVEILILLR

>SG-PL_contig_415_1_+3_102_257

MYFDHTVDLHRLPVTCQQAVLMRIYLLYSFMRASKMNKEIQTSVATLIIILL

>SG-PL_contig_416_1_-3_288_485

MTLGIDRLGETGICLPKCEMMIYICDFTIIRILLIRTMHTCVCVCVCVCVCVCVLYCIVL

YYFWSQ

>SG-PL_contig_417_1_-3_3_158

SGGGWFFYLYKSSGNLGFVILKAFIIGTLKYSAYQGSSQKINIHMELTSLKV

>SG-PL_contig_418_1_-3_177_311

MVLCPHHSSLFLQLLPSTVFFFESCFVFVAFCLSRSSIWGFCWLF

>SG-PL_contig_419_1_-3_180_389

MDMLAVVMCHIHHHPFKEIVSYTVYHEQRREASQDTTVRIGKSISGHHKHHLQRKCCVHH

QCNVTAFVCL

>SG-PL_contig_420_1_-2_2_133

DTCLNTCRECVADLVKSYLLLSPSPHPSSSYTEFYVLETVSFPV

>SG-PL_contig_421_1_-2_878_1891

MSCTGSRPRLFISHGPKKKIVPDPEPNLGNLTPDVQTDTLPPLTCPESEQDCLKLGKYVL

FRQNDRLDTVYQAIDTTTHEEFTCKVFSLDRYRDCLSTYWQVDDHEHIASSTEIILGESK

AYILFPQNHGDLHSYVRQKKHLREDEARHLFRQIVSAICHCHGNGVILRDLKLRKFVFQD

AERTKLQLEGLEDAVVLSEDDQDVLKDKHGCPAYVSPEILTATETGYSGKSADVWSLGVM

LYTMLVGRYPFHDSQPLALFGKIRRGQFHIPDCLSPKARCLIRCLLRRDPAERMSAGDIL

SHPWFRSVPSLRKPANNQHSRLQVVPEFSLPIPGPFDW

>SG-PL_contig_422_1_-2_50_496

MADEQELAEKKKKRTFRKYTFRGVDLDQLLDMSSEQLMELFHCRARRRFARGLKRKPMTL

IKRLRKAKKEAGPLEKPEVVKTHLRDMIVVPEMIGSIVGVYNGKTFNQVEIKPEMIGHYL

GEFSITYKPVKHGRPGIGATHSSRFIPLK

>SG-PL_contig_423_1_-3_1131_1496

MTTSVVLRVHFLQKLFHSLLLACRVSDHWKLRLWSCLPDWLLQYATLLFYLLEYSWSSVS

LVHLFYILFLCLLGYTSHLSTHTHTHTYPFSLTHTHTHTHSLLHTHTHIPILSLSLAHTT

RS

>SG-PL_contig_424_1_+2_872_1273

MKMDTMYTESSSQTEFTCSAQGEMTVCDNTTIKKHFPHCCSVTKITKFTEGEEQRNSALS

CTNKLMLNDKYHLFRAALAKKQYLFNQSPMMKKRAQRTGWGQITEKRADDLLIRRNTAHW

SPTQHSPLLTMRTC

>SG-PL_contig_425_1_-2_188_1390

MARAFQNPYQDYNGEPQAVNTDGKLDSEFCKNVKHSIKDLLTQLEAGLQQTRPGDYSVYT

GQAGIALLYLHLYDKLVDDPDRQNYLEKAVGQLQAPLHHLGTGVYTFLCGDAGTLAIAAV

VYARAGNMKKSKEFVSRLEQMRSHVEKDSQIPDEHLYGRAGYLAAILFVQTHLGDQAIAS

ETISKVVKAILNSGQSLSKKKGWKSPLMYAWYRENYLGAAHGLAGIFYTLLLVKDPLLRP

QIHALVRPSIDYILTMQFPSGNFPPVIGDKDDRLVHWCHGAPGWASMFTMAYQVFTDTRY

LVAAEQCCEVTWHRGLLTKGYGLCHGAAGNAYTFLTVFRLTRSQKHLHRVCKFAEWCFNY

GKHGCRIPDAPFSLFEGMAGTIYFLCDLLDPLKSRFPAYEF

>SG-PL_contig_426_1_+2_1148_1363

MYLPVLPVHLKASTKCTPTLNTSHTENDQMITSLITVTAFFFKTEWQDNSILLLLMKMIK

QLPHRLQSLPFF

>SG-PL_contig_427_1_+3_432_770

MLKCTILFLTCKFKTNTHTGKAAGKGKQFEGSNKRFHIIPTYSTSQGNKGNKKSSNENNS

LTIHRAFASKARQTDHYTVLFSFCHVFVRSNENRMGVGDKPHARCRPTFIRLN

>SG-PL_contig_428_1_-1_1_168

FFHLELYTNSVPFLQEREVREQVVGSAPAADRAVDTALAQGWEEGGGPVTKRLLPI

>SG-PL_contig_429_1_-1_25_252

MQYGSRLLHPWPEPTQSPRNPAQRRPQAAPHPRCPGHACTRNLFLFFPQLSGRTIRRSKR

GDGWAGLRVGYGKCTS

>SG-PL_contig_430_1_+2_2171_2470

MKKINGNALSLQQDFTFSTYTTIASVVNIFVISNLCYSLSLFFQCRHLCIKFVHYTHIHA

TDRQNGTTVRPAEVDSLQIPADRDRNGRDVLPYVVGATWS

>SG-PL_contig_431_1_-3_27_317

MVCLGCWPETCMIIIIMFTHTHTEHSLIELSALRRGLNLQQNILFAVFQDHESEMYIIIR

YTHRFTRAAVCRCQCTGFVVDRPVNSHSWNPSTSCKN

>SG-PL_contig_432_1_-3_99_2657

MEKTRDDIHWLSKNQQLLIPEPTVNDQKAWILINVHQMAYCRVHYDEVTWLALIDQLNTD

HTVLPAINRGQIISDAWNLAKAGLLSVSVALSTLKFLPSETDLVPWRAFAHEINYVEMML

ERTDLFPQLKTFMQDTVTRPFGELGLKISEEMAPVDVFVRTLIAQYACKYGLVGCVNVAK

RLFTEWENTGVKINANFRRQFYCTAVAEGGEGQWRNVYQRFKAEDDATELNNLRYALSCS

KDQFLLKKLLNMAIDPEEIQTQDAASTIQWVAGNPQGRDLAFQFFADKFEDIKGLLDVRS

LGRTLISVTAHFSTDYNLQQLLQFPVIRDTTVIQSVVDQAIAQTRSNIAWLETNYDIIQN

WFFPPPSGPPPPPRPAPGSYRLPTNLEPSHYDLTLQPHIYGSDSFFEGSVTIVFRCTSST

NSITLHQDQLVISSSSITITDISSPAASLPIADVTEEKMFHFYHIHLENPLEQGKEYSVE

IQNFRGDLGRSKEGLYKSFYEDGDSTISLAASHMEPSYARRVFPCFDEPALKATFSITLI

RRTDTGRNYISLSNMPIVSSNVIDNSWTADVFQRSMVMPTYLLAFIVCDFQYLTTMYSEY

PVETRTYARPNQLQDAHVAHNHAVELFDWFNDHFEPTYNTYLSKVDHIAIPGAGPMENWG

LITYQEKLLYNSSFTADDRLWIAKIIAHELTHMWFGNTVSPKWWTWLWVKEGFANYFQSY

AVEQLHPDWKVISEVEMVHEVFFCMFMDGTSFSTPLNNPVETPREIRSQFSTVTYSKGRV

IIEMIHMIMGEHFFPALNVYLNDNKFGNGDDSTLFDSLNKYIDTAPDIDLNMTKIMEPWV

TQMGYPVVNVQRM

>SG-PL_contig_433_1_+1_415_615

MNPLHRCMSLQETSGCTDSNVVMGSGVVLSLYIRDPFQPCIVVWYCANSVWQVSVYSQCV

LPMVLQL

>SG-PL_contig_434_1_+1_769_1281

MTQLVKRLLGNGMVTPVVLHGSSLCRALTKVAACFPLPSPGLDWLSLEGRAATSPSQRAR

SRSSDTPSSARPSCSRTSLNGGSTKRRATTPAGPNRRGRIWAIAGSAAGQWWKWWQRSVR

TVIVVLFIVVVIIVGIMWGWQSSKCTAWWHWWSHGWWTIVVVPCRVAFSHQ

>SG-PL_contig_435_1_-2_137_274

MMFFYTLYIIVVSDFIVTSAMFCLPSTVSKLSGSCIIHLKEKKDLK

>SG-PL_contig_436_1_+2_458_658

MDNFSSKNKNRYNYSHKNSNSLPKITVKCAYFNINFSSVPVTPKLELALTNLRFGAYPGK

NLTHLAD

>SG-PL_contig_437_1_-3_630_1055

MVGHIHEYFSPSPSTLFVAYLTPFTILCNDDLLFHLCHACLRTEETLTLYFLNDCKLFFQ

ARHDNNCHDQIKCVLPDTIYAVCSVQSSQTSGLLKSKLAISVSTSYGNIYVCISAQFFSS

QFHKSWYSEFSFNSNILCNLLE

>SG-PL_contig_438_1_+1_1_213

LTLFPYTTLFRSITSFKLHSCTHTKPGKKKYRCHITSFKLHSCPHTKQRKISFYKSLTQT

TFHFKISTDQY

>SG-PL_contig_439_1_+3_3_152

ASLASDQAESFLSFLLPNSLLLVTITGEKKCHYGVIKYCVLLFIVSNHPC

>SG-PL_contig_440_1_-2_383_580

MFRCCPILCTAVVAFLSHVREIVHEMAYICIWRLKCPLWKFCWKGDNFLFFYLSACTITC

VVGRKS

>SG-PL_contig_441_1_+3_3_233

GADQYRTFTHPTFPRFKKKSKKAEDYWLYSKKKTMNLQHTERTKDVSKMSDIHSLHEENG

RRTKQTSNETLHYQNVR

>SG-PL_contig_442_1_+3_390_935

MKKAPVNVYIYITTKYLTVCYWVSLVDVYIITKYLTVCYWVLLVDVCIITKYLTVCYWVS

LVDVYITTKYLTVCYGVSLVDVYISTEYLTVCYWVSLVVAVFPAMHTDKQTNKQTNNPPQ

KTKPNQTKHNAHQQSNQTNTTHQQNNQPTNQPTKQTNKQTTHQPSHLPHKLLQTTVSKES

VI

>SG-PL_contig_443_1_-3_702_965

MLGTEPTPILQMASSSKVVKHSDSEMKYLIELYQADDVLWKSQRKNYKNQHYWEKALQSI

HNEFIGTRNDLHLTSVSKVSCSTPSVPH

>SG-PL_contig_444_1_-3_345_926

MHFRCLSRIFLEILRRINPTICIFLPSLHMWRIFFQMLVRSLFRLYDCDCLVLKGISSRS

YISYGLLFFNISCWFVTFEYFLLVCYFWNTRCAFFFLGMQDVLFPLSEYKMSFFPLGMQY

VNFFLGAQDVHLFSLEYKTYIFFPLNARWSFFPWDTRCALFVWDSRSTFFPWVTRCTLSP

HGMQDLHFCPEFCI

>SG-PL_contig_445_1_-1_52_426

MLKRMEETTEHLRALTSSMGELDRELKEAILMDGMTNDIAKRSSARNLAADLKNTREVIH

TLNDVFNKAGHILEREKKSTSCKVNMGFNCKTEEYAKTADLLHYLNSAQSPGKKRSISDT

SMNLN

>SG-PL_contig_446_1_-3_102_422

MSDTEGDAVQAQAGAGSMDVFTAVQEVLKTSLIHDGLARGANEAIKALDKRHAHLCILSN

SVDEAQYTTLIEALCAEHGINLLRVDDSKKLGEWAGLCKIDKEGKAR

>SG-PL_contig_447_1_-1_154_576

MSPDNPCAVPICLDPSFAPDGKTCTVSGWGATQSGSKQASLLLQRGNVPTYSGDKCMSTF

SIPDKYYMGDTSSELCAGISEGGVDSCQGDSGGPLVCLNPATDSYVQVGIVSYGYLCAQA

NFPGIYTNVAAYNDWINNAIA

>SG-PL_contig_448_1_+1_1_393

FFFFSSHLFDTPISEHYTLEHDHLALPLLITTQLEVLAALDGQLLSVLAGRTLHAQHNLL

GRFGLFPENGLGLSTISRLFSVVTTLTLSIQGSLASFVLGDFVQGMFFAIFTLAKGLALL

RNVNHLEQIGR

>SG-PL_contig_449_1_-3_954_1247

MNERACLIKMSHRCWQWHCSIQAFGGFVMDFVCSVVKYLKEGIMTAQALLTSFFQIKIMQ

NVSLKNWDREVGLWLDHRVYAWFIYIFLKKKADPVLCL

>SG-PL_contig_450_1_-2_83_706

MGISRDRWHKRRKTGGRMAQLRKKRKFELGRPPAMTKLGPKRIHTVRTMGGNKKYRALRL

DHGNFSWGSESVTRRTRVIDVIYNASNNELVRTKTLVKSCIVQVDSTPFRQWYEAHYATP

LGRKKGVKLTAQEEGVINKERSKKVMKKYEARKKVAKVETALEEQFSAGRVLAKIASRPG

KHGRADGYVLEGKELDFYLRKVKSKKGK

>SG-PL_contig_451_1_-1_19_1371

MRECISVHVGQAGVQMGNACWELYCLEHGIQPDGQMPSDKTIGGGDDSFNTFFSETGAGK

HVPRAVFIDLEPTVVDEVRTGTYRQLFHPEQLVTGKEDAANNYARGHYTIGKELVDLVLD

RIRKLADQCTGLQGFLIFHSFGGGTGSGFTSLLMERLSVDYGKKSKLEFSIYPAPQVSTA

VVEPYNSILTTHTTLEHSDCAFMVDNEAIYDICRRNLDIERPTYTNLNRLIGQIVSSITA

SLRFDGALNVDLTEFQTNLVPYPRIHFPLATYAPVISAEKAYHEQLTVAEVTNACFEPAN

QLVKCDPRHGKYMACCMLYRGDVVPKDVNAAIATIKTKRTIQFVDWCPTGFKVGINYQPP

TVVPGGDLAKVQRAVCMLSNTTAIAEAWARLDHKFDLMYAKRAFVHWYVGEGMEEGEFSE

AREDLAALEKDYEEVGVDSIEGEGDEEGDEY

>SG-PL_contig_452_1_+2_194_268

MKSQFSDLQLAVVSPRCHYRRVCVC

>SG-PL_contig_453_1_+2_1007_1213

MQNKIWSHACQHRPNILKEGNGVVKWGEIQHTHTHTHTHTHTHTHTPQSETIMSIGTTFT

RSTTTKVFQ

>SG-PL_contig_454_1_+2_2_166

ALFRSKKKRKKKKGKGEGVVVGWWWWWWWREENKKEKKKEERKKEKRKLEVNLQK

>SG-PL_contig_455_1_+3_72_209

MNAMLRQSNRVTEVFQRVFYPTTDTATLIFRSNPPPTTPKRYGINT

>SG-PL_contig_456_1_+1_70_378

MNNLHSCWIEDTKNNLGNCIQMCRLLHSCWIDHRKNNLYSCWIDSTLKNLHNFLMDDRKK

SLHNFLMDDRKKSQQACTRTWDSPWSRSQNRWDSTYCIRVHTL

>SG-PL_contig_457_1_-1_22_1467

MAASLLKVTSSVAKVLATAQKAPCVSAIIAQRWRSTRAATFEQTILNVPETRVSTLENGL

RVATEDSGIPTCTVGLWIDAGSRFENEKNNGVAHFLEHMAFKGTKTRSQTDLELEVENMG

AHLNAYTSREQTVYYAKCFQNDLPKAVDILSDIIQNSLLGEQEIERERGVILREMQEVET

NLQEVVFDHLHATAFQGTALGRTILGPTQNIQSINRNDLLEYIGTHYKGPRMVLAAAGGV

NHEELVQLANKHFGSLSAGYEGEIPVLPQCRFTGSEIRVRDDSMPLAHVAIAVEGCGWQN

PDNIALMVANTLIGSWDRSYGGGANHASPLAAASAEAGLCHSFQSFNTCYTDTGLWGLYF

VSDGLQIEDMLHNAQAEWMRLCSSVTEFEVTRAKNLLKTNMLLQLDGSTPIAEDVGRQML

CYGRRVPLPELEARIEAVNAKVVRDVCTKYIYDKCPAVAGVGPVEQLPDYNRIRSAMYWL

RL

>SG-PL_contig_458_1_-2_245_2194

MCIHISWQCDGDFDCEDHSDEKHCNITCNSDQFLCEENYCIHESLKCDGQADCLNGSDEI

NCPTVAPACANNTFDCFGDGTKCIPYSLVCDKWNDCGNFEDERTENHDPCSVENPCNINN

GGCQHICYRSPAGAICDCHTGFELANNSRTECVNVNECAVPGTCSQICIDTKGSYKCDCH

KGYELMHQHSCKATKGEPELLLADRKDLRRYHLLTKKYTLLIDDKTVEGAIAMDFHFVSK

YVFWTDVSKEQIMKVSTTNNEIEMVVSQNVSVPDGLAVDWVHGNLYWTDTGLDHIEVATM

DGSMRKILIKDGLDEPRAIALHPKHRLMFWTDWGQEPKIESCGMNGKHRKTIISEAITWP

NGLTIDYVDDRLYWIDAKLHQIGSSDLHGQNRRMILRGHQYLGHPFAVTVFEDFLYWSDW

PSESIRRYNKFQKGSVETIAQGLDTPMDIHIFHPYRQVAYENSCGSDNRGCEHFCLPDPV

TSYTCECMTGYKRDPADSTKCVEGEEPVTTTSSTTTTTTTAVPTHKPSSTSAQTPRPNTT

PKQPVVTTPQPTEPTTTTEGQPINKEDNEGEGQVAVIVIAVVALIAILVLAVGIFVFRRY

RNRNVKSMNFDNPVYRKTTMEDEKVFVDKSGSRQHLPSSMQPLHTDNELV

>SG-PL_contig_459_1_-2_2_154

KKKKLSNSKMIKYPTVSVENLSLAPSSPPLLSERLMGKCFLHRGQSPMCCE

>SG-PL_contig_460_1_+3_282_566

MQFINIFLSKECCVLAVKSCTTGKTTKHRTNQCDCESNIKTVLFKSKSLAQQFFIQIVYL

SSTSLSNQASSIPYPHISTEDMIQVYKVQVITTDK

>SG-PL_contig_461_1_-1_1147_1314

MGQGYCYLTWLSRSEIYQSSGNLKLSWATSTYRFSHLEFLDHDKKKNMTRYNRVRC

>SG-PL_contig_462_1_+2_149_334

MRERRENTGKERLEKEEEGEPPSLSSLLPLPCSINKFELYKNTTTTTTTTTTTTTTTTTT

NK

>SG-PL_contig_463_1_-1_103_315

MAIQAFGIDRFKKNAAKANKGYEYVLEKLFTPVLHHQVHCTENLKDKAIKVAQAAKAKAP

ASIIAARPQPS

>SG-PL_contig_464_1_+3_3_263

KQRGRGDGHRPHKAGQEKISRTPAYHTQSPATSGLWAKLDVTFSFPLLQTCHLLLLVLQF

LPVTSRDRQTHRRTGRQAGRQADRQAG

>SG-PL_contig_465_1_-3_186_404

MTLSMFYQHCTVCASTVCVCKYNCTVSVCNCTENAVLLFGCCSVVVIVWVLARPFNIMYV

FLPRGKFYSMSDF

>SG-PL_contig_466_1_+1_3286_3549

MCTQKQRVIQMNVIKIAPSHPCTISKDFSFWLTATMLDIIVLNTNISNTKYVTGVKSCRG

QRSLEIYPPGCAQVSCDRKPGTRSPVVS

>SG-PL_contig_467_1_-3_384_782

MAMYQNLLDDSEGEKSQAMAVTTMKRRQLINKFGMCNSTCACPAVVKVTCRSVGITMDIC

FAGAEDSMTSFLVHGHYLAGQTTVLASWDHEKKIIHILLPFPDGRLILVIRSESHMFSSA

SNIQFAVSSWPCE

>SG-PL_contig_468_1_+1_718_813

MEPPFSWISIMWAFFWRLRSSFIWVWASTRTT

>SG-PL_contig_469_1_-3_36_1124

MPQFAQCLTPALQQELRDIAQAIMTPGKGILAADESVGSMGKRLQGIGVENTEENRRLYR

QVLFTSDKSLGNNISGVILFHETLYQKDDTGKPFVQSLKEKGIIPGIKVDKGVVPLAGTH

NECTTQGLDGLAERCAEYKKQGAQFAKWRCVLKIAKDTPSHLAMLENANVLARYASICQQ

NGLVPIVEPEVLPDGDHDLETAQKVTEQVLAYTYKALSDHHVYLEGSLLKPNMVTAGQAC

SKKYSHEQIAVATVTALSRAVPGAVPGICFLSGGQSEEDATINLDAINKYKGKNPWALTF

SYGRALQASVLAAWKGKKENMPAAQGELLKRAKANGEACVGKYAGGVKGSAGGQSLFVAG

HAY

>SG-PL_contig_470_1_-3_93_1904

MPSLEQKRVFAALPPAERGKSVKIYGDPKGQNILYCHDKSVIIRNMEDPSQADIYTEHAC

KTTVARYSPSGFYIASGDISGKIRIWDTVNKEHILKAQYPVLGGPIKDIDWSPDSSKIVV

CGEGREKFAHVINMETGTSVGTIDGHSSSINTCSFRPVRPFRCVTASEDRCVGFYVGPPF

KFNKTQRLHSNFVNTARFSPDGSVIISGGADGMLFKYKGDDCSACEEQGEIGKTKAHKGG

IYEICFKKDDSNQVLTVSGDKTAKILDLESGKCLVEFTNNMKEWQGMQLGCLWQGNEIIT

VNGYGHLVYQDTENPSEPKRIVMGHNKPVTSLALSADKTTLFTGGQEGIICWDIATGHSI

VVGGPGHTSEVTDMVTDGDNLISVGVDDCLRFSSVSAQQFGGGEVKLASQPKAVDARNGI

IVVACVQHVTVLEGNRQLSTMPVRSEAWAVSIHPGGVAVAVGYKDKNVKIYSLQCGTLKE

EMTIDAPGEVKCLAYSPDGAFLALGADRTVMVLSTSDYKETSKGETHTARVHCLAWSPDS

SRFASAALDSKVVLWDDVCAHAFKTRPEIKAHTMSHANQLVWLDTQNIMTAGQDSNVKHF

VVNN

>SG-PL_contig_471_1_-2_191_706

MVAGCGMTCIKYIVFAVNFLFFVMGIAAVALGIWALVDKNDMDALTKIDSDGKLEEFNAV

GLLQSGAIVLIVGGACLLILGFLGCCGAVKEVKCLLATYAAIVVIIIIIQIAAAAIAIAF

RGRIEDYLQEGLIKAVKENYDGNVTSDNAFSRAFDFAQVYFECCGVNSTANF

>SG-PL_contig_472_1_+2_791_1039

MTSTSTELQDLTSRLTNRLTISAAVYGMEISNEKSEAMAKSTTTVVQNYMNGQKLDEPTR

YSDQRWQSTKEIRTRTAAAMARK

>SG-PL_contig_473_1_-1_67_201

MSLPFCGQNGLVGTFSCSCLRMDLYHSYPIYILCGSKQEVPNAGE

>SG-PL_contig_474_1_+1_316_528

MANYSVFCMYPISMSNIPGCVQLNINIFHFVLGRAYFLLSRPSSRETQKEHHCVLINTVV

FFQLCLHCEVC

>SG-PL_contig_475_1_+1_1_201

NVTISTTIYFNVNISTITFLPKFYDMNHQQRMGMFGDCDIYTVIWLTRSAPPFFPTETVT

IKMFKYK

>SG-PL_contig_476_1_-3_45_224

MSKLIHLCFHLQFIVLFDSEWWLSFEECCRGVVSDIRVSLSFFSCLKQFGRVKFLNYDCF

>SG-PL_contig_477_1_-3_78_209

MIQCSLLGGTVRKSILEMSLLLRNMGGRGVHFRHSRIVWNEVPA

>SG-PL_contig_478_1_-3_3_152

ERERERELLLKSVERERERERERERERVVTEESVQRERERERERERESCY

>SG-PL_contig_479_1_-1_181_1590

MVPNSFLTPTSATKHITSANRHVTSNNSYVTSTSNHITSSPSYVASASVGMQEKQMLYNP

HCRRESSSAVEDILNDVNDEPEMKMVQLPTGIKREPREEKEMEVFDISSTVCDLASGSMD

LLQANSPSMWEDISTSMNMVNNINDPLMPARPPMGLPVSKVKREPMEMGDKPSCQYSKSF

YRTHTAGRHMATSNLMPFSTSASCPPHMNVGGLTNSSQMVSISGVTKSYPQPYRPTISQQ

QHFTPPTPHHHGSTCSPPVSSSSSTSPSTSHVVPCVFLPPTPPNSQPASPKSGEFVVRRT

PPPPYPNGMGGASSTSLPPGNIPRPVLSCSPSPLSVPLPPMPAPSSTPSSRARISKPQNT

HPGCSTIKYNRKNNPDLEKRRIHFCNFPGCRKAYTKSSHLKAHQRIHTGEKPYRCHFTMC

QWRFARSDELTRHIRKHTGAKPFRCKVCERSFARSDHLALHMKRHEPKNK

>SG-PL_contig_480_1_-2_29_901

MKMKLQLRRLPVHVRAEEEAEMRKRPQRRQKRWIPAVLTGRQRKPSKDVGRKRRSDAVRK

RKYGESKRRRGVGRRKNAERRRDREKKRKRGWQLKEQNKTGSREKMEERRREEEEKKKKK

KRKGLGGLSPEKKKLLKKLIMQKAAEDLRNEAKAKALEKEQFINERVTPLKAEGLGQADL

QNLVKELHAKVAELEEEVYDWEIKIRKQDFEINELTLKVNDTKGKFIKPVLRKVNKTESK

LNKLQTKESRDFRGNLKSTGQNKFALKEDEDTNKPDWREALKHGEGEEAES

>SG-PL_contig_481_1_+2_2_532

SRQQILFHQKCLSLATDTWGLSLHILGGLGLGLAALGGCRLSRLLDSFLLLLLLLICPDS

LFTLGLAHFGFLVTLGDDLAEGHTSDSTLELVGATGSLLDHLLCLALLVLATVQNSPCHL

TGVTLHGMGPLTFLAQEVENSSVHFDHSFPMARVNFDTAVRTQFDLHGAGEMQQIGR

>SG-PL_contig_482_1_-1_94_399

MQDLKEELKLKKKELENLQDAEDELLMLEGEAEGIPYQIGELFINTSTDEAGTLIEKAKT

AVQEEITRLESQVDTHKNILSDLKVQLYAKFGSNINLEAEED

>SG-PL_contig_483_1_-1_73_867

MPSDKTIGGGDDSFNTFFSETGAGKHVPRAVFVDLEPTVVDEVRTGTYRQLFHPEQLVTG

KEDAANNYARGHYTIGKEIVDLVLDRIRKLADQCTGLQGFLIFHSFGGGTGSGFTSLLME

RLSVDYGKKSKLEFAIYPAPQVSTAVVEPYNSILTTHTTLEHSDCAFMVDNEAIYDICRR

NLDIERPTYTNLNRLIGQIVSSITASLRFDGALNVDLTEFQTNLVPYPRIHFPLATYAPV

ISAEKAYHEQLSVAEITNACFEPAN

>SG-PL_contig_484_1_+1_166_441

MILLHSPSKCQATLATSVGLMMTKLMHYTMTLEPKTKTITITNRKAYPCLYQTSFCSCTV

TDRLVQTTLGKIGNTCYGIDRFSKVIPKISHS

>SG-PL_contig_485_1_+2_128_403

MKEELSYQGKNAEYSDTTRMTAHQNEQCKSQFCCQTKLLLFYISDTAPLSVGSNCSLLSI

KGQFPCQILLTVFLDGFLLLGVKGFKNHIQNS

>SG-PL_contig_486_1_+1_100_240

MNMYGEQCLAQKLSQKRKKTDLIWRELDLFSEFFPLKLITTLTDLVI

>SG-PL_contig_487_1_+1_235_450

MTLIMDKSQSHLHYTVLTLVRATIGPSLVKNCTIFSYTVLYNYLVTMDFPCQICPAPLKE

SVSPQLLVFFKI

>SG-PL_contig_488_1_+3_84_305

MDGYDAGLHSFHGSTLHLGVQLSGVQQGNIVRYHLNLLASRSDSSNVRMSPSLTGPFTLR

MMERLLSSRNSTRT

>SG-PL_contig_489_1_+1_481_654

MTKDETMGGGVYSVNIQQWLLLLKFVFCCTDVEAPFIIQTTGIEISLGFQVAVVNVLI

>SG-PL_contig_490_1_-1_46_270

MSTVKKKPKRIRIASRRRSNNVDCKRDALERHQHDVAITSTIKKKRTYCNCKKQDVLEQH

QDDVATMSPVKNETY

>SG-PL_contig_491_1_+1_1_426

LPPPHYVLFSSICRKGFCFVCVVTDQTARSVYKIYFCSLVCLFFCLFFVSTITRQTTLLS

LSWGGGLPPAATLLVNAVPGSHFFFIIIKKNIKKKTKCSRRRRLLEIKEQAAAEKDIKKG

ELWCNNQPSTGIFCFIDQSVIM

>SG-PL_contig_492_1_+3_72_263

MAYSVTYTHKRSRNSSCGLLAFQGHKEPKIGHAQLGHEKKNGYFPLQITVSVLRIFTLCV

LADQ

>SG-PL_contig_493_1_-1_121_726

MTVGKSSKMLQHINYRMRCTLQDGRTFIGTFKAFDKHMNLILADCDEFRRVKPKTAKSGE

REEKRTLGLVLVRGEHLVSMTVEGPPSADEGIARVPLGGAAGGPGIGRAAGRGVGGASGS

GAAPGLQGPIRGVGGPSPQMMAPGRGATMSAPARPNMPRMPMGGPGGPRGPPMHMMGGPA

GRGGPPPRGPMPGMMRGMGPPM

>SG-PL_contig_494_1_-1_13_456

MKSFTGGMKTSCEACATSGNQETRHRHGSSRQTVKMPETLRSVVAKFYDEYMQSTPKKLK

LVDAYLFYILLTGVVQFGYCCLVGTYPFNSFLAGFISTVGSFVLGVCLRLQSNPQNKTDF

TGISPERSFADFIFAHVILHLVVINFIG

>SG-PL_contig_495_1_+2_2_511

HLGVVLQLLAGTPVALLLQLGKLAGNVSSVAIQDWGITSLDLTWVIQDDNLGIESLALLG

RIVLAVSSHVATTDVFHRDVLDVEANVVARQGLRQRLVVHLHGLHLSGDGIRSKGDHHAG

LQNASLHTPHRHSANTTDLVDILQRQTQGLVNGTGRRQDGVQGLQQSLAA

>SG-PL_contig_496_1_+3_3_227

PPPPPPPPPPPLSPFSPFSPPPPPRQCLACISKRLTYTFSSANSVVKLNIARLLVLPKHF

NLSVLICSRYLYLVK

>SG-PL_contig_497_1_-1_148_726

MMRGKVGQRGLTSDIRTGDLSWIHDADLTTAAAVSQKVKAITGLEVDQRDPDGPSSAEPI

QVVNYGLGGHYDAHTDPFGMVPPDNAMLNRSGERIATFLIYLSDVEKGGSTVFLHTGISV

PPRKGMVLFWYNFTPALDNELTLHAGCPVVIGQKWIANKWIWTYGNTFRRPCGPHANSTQ

QHVDNMLASRLLV

>SG-PL_contig_498_1_-1_253_5109

MKLTDTAFDVLGFTDEEKASMYKCTAAIMHFGEMKFKQRPREEQAEADGTAEAEKVAFLL

GINAGDLLKSLLKPKIKVGSEYVTQGRNMDQVTYSVAALSKSLYNRMFEWLVKRVNKTLD

TKNKRQFFIGVLDIAGFEIFDFNSFEQLCINYTNERLQQFFNHHMFVLEQEEYKKEGIDW

EFIDFGMDLQACIELIEKPMGLLSILEEECMFPKASDKTFLDKLMGNHMGKSPNFGKAGK

PKKAGQVQAHFELHHYAGSVPYNITGWLEKNKDPINETVVELLSHSKESLVQILFSTPDA

AEGGAPAKKKKSTAFQTISSTHKESLNKLMKNLYSTHPHFVRCIIPNEFKEPGVIDSALV

LHQLQCNGVLEGIRICRKGFPNRIIYSEFKQRYSILAPNAIPQGFADGKLVTDKVLSALQ

LDTNEYKLGTTKVFFKAGVLGMLEDMRDERLSKIIAMFQAFIRGYLMRKAYKKLQDQRVG

LSVIQRNIRKWLALRNWLWWKMYCKVKPLLNIARAEDDMKKKEEELEKTKVELEKTEKMR

KELEEQNVTLLQAKNDLYLQLQAEQDNLGDAEEKIEKLVSQKADYEQQLKEMEERLLDFE

DESGELAEKTKKMNDEIKDLKADVEDLENSLAKAEQEKQTRDNQIKTLQDEMAKQDEQVA

KLNKEKKAMEEQQKKTMADLQAEEDKVNHLNKLKTKLEQTLDELEDNLEREKKQRGDVDK

VKRKLEQELKATQENVDDLERSKRELEENVRRKDGEINNLNSRLEDEQNLVAQLQRKIKE

LQARIEELEEELEAERAARAKVEKQRQEVSRELDELGERLEEAGGATAAQVDLNKKREQE

LLKLRRDLEEQNLQHEAQVASLRKKQQDASNEMSDQIDQLQKVKARVEKEKNALRSEVDD

LQAQIQHLGKNKGMSEKMTKQVESQISELNSKVDEANRNVNDLQSQKNRLQQESADLTRQ

LEDAEHRIGTLTKEKTSLSSQLEESKRSLEDETRARQKLASEVRNLHADMDSIREQLEEE

QDGRSDLQRQLSKANSEAQQWRSKFEGEGAARAEELEEARRKLQNKLSEAEQAAEGAMAK

VAGLEKAKARLQGELEDLMVDVERSNANANNLEKKQRAFDKTIAEWHAKVNDLQSELENA

QKEARGYSAELFRVKAQLEEAADSTEALRRENKNLADEIHDLTDQLSDGGRSVHESEKAR

KRLELEKEELQAALEEAEAALEQEEAKVMRAQLEISQVRSEIDRRIQDKEEEFENTRRNH

QRALDSMQASLEAEAKGKAEAMRVKKKLESDINELEVGLDQANRGKAEMEKAVKKLQQQI

REVQSQVEEEARAREEARESYNLAERRCTALSGEVEELRNALESAERARKGAENELTDAN

DRVNELSAQVSSFSGQKRKFEADIQAMQTDLDEMNGELRGAEDRSKKAMADAARLADELR

QEQEHSMQVEKLRKGMEGQVKDMQVRLDEAEAQALKGGKKIIQKLEQRVRELEMELDGEQ

RRHAETQKNMRKADRRLKELAFQSDEDRKNHERLQDLVDKLQAKIKTYKRQVEEAEEIAA

VNLAKFRKVQNELEDAEERADNAEGTLSKLRAKNRSSVSVSRTSASPAPAFSATAATAE

>SG-PL_contig_499_1_-2_20_1378

MKNLYSTHPHFVRCIIPNEFKEPGLIDSKLVLHQLQCNGVLEGIRICRKGFPNRIIYSEF

KQRYSILAPNAIPQGFVDGKQVTDKILSALQLDTAEYRLGTTKVFFKAGVLGMLEDMRDE

RLSKIISMFQAFIRGYLMRKAYKKLQDQRVGLSVIQRNIRKWLALRNWMWWKLYCKVKPL

LNIARAEDEMKKKEEELEKTKQELEKTEKMRKELEEQNVTLLQAKNDLYLQLQAEQDNLA

DAEEKIEKLVGQKADYEQQLKELEERLLDQEDEAGEMGEKNKKLNDEVKDLKADVEDLEN

SLAKAEQEKQTKDNQIKTLQDEMAQQDEQIAKLNKDKKSMEELQKKTLEDLQAEEDKVNH

LSKLKTKLEQTLDELEDNLEREKKQRGDVEKAKRKLEQDLKATQENVEDLERVKRELEEN

VRRKDMEINGLNSRLEDEQNLVAQLQKKIKELQ

>SG-PL_contig_500_1_+1_253_447

MKTNRVIFNHSKNRIFSAYVLGKNESIIQITTGSKTCMCVCVCVCVCVCVCVCVCVCVRA

RARVC

>SG-PL_contig_501_1_-1_355_462

MEGGKNNFTIFSLQRKGHFSVCHIWECVDKSVLGMF

>SG-PL_contig_502_1_+3_3_194

QSLGPPQRWSSMLPKQAMNVSVFVDLVRPDLSVFLFVCSFLPLPGRQPRFASPGAEDYHL

QLLS

>SG-PL_contig_503_1_+1_214_573

MNMHACTRINTHSHTVHAHTHTHTHTHTRLCVRTHTHTHTHTQSHFGFAPCAVLNDSGVG

NWLVIDTVPVFDALNWSLFEANRFHHRSSCFPPFPFHTPSSPHRLPRIILAFSPHRNPTA

>SG-PL_contig_504_1_-3_3_170

CRCTCGLAHCSVHVSVCSSVTQKFCELNPSSLTHQRGLTQSVAGIHIAVSNCEDFK

>SG-PL_contig_505_1_+3_3_299

LSLSPHFSNRYVYQFLSLSLSLSLSLFSYPPSLFLIISCSHISCCISSSFMITIIIIADG

LSLITSCVQDLASAHGLCPGIQLMPLSFWILFHPMAFAA

>SG-PL_contig_506_1_-2_149_817

MALGSCYSCIKYLMFAFNFLFWLLGCAILGVGIWLRVDSNVAKYVNHAEEINIFYTLAYV

LIAIGIIIMIIGFLGCCGAIRESQCMLAAFFVLLFIIFVILLVFGIWAVVEKDVLKDEVA

EMMQNAVDNYEKREDSQKFMDTIQTAFDCCGANQGAKDYATPMNVPDSCKAEFRDRPCDK

EFFDWIAEHLIIVAGVAIAIAIVLILGMIFSMVLCCALRDTVA

>SG-PL_contig_507_1_-1_541_684

MGACCKICHSYTFHIINYYYYYLSCMHTCIKSYCCGFLSVLLCTLHFL

>SG-PL_contig_508_1_+2_47_202

MKNSNMLSIYQKSKGTDSRNGPKLTRVEWKTLQLNNINNVPPFFYTLTTELD

>SG-PL_contig_509_1_-2_257_574

MKFFLSLCIMGVFLLSLSLAITSESSRNICWTSLSLYLLSSYLFLCQPYFFLASFCIFTA

LAIVLDLPSLKLSCPTVKPCATMKLWLAVDSILNSPAAFKSTQHRS

>SG-PL_contig_510_1_-2_41_955

MLNFRTSGSTETDSGKRNDMALIYIRSTECDDYILATLDSGGNILIETNQGVGIYRMKVS

GNFANGELHELVYKREGTNMYLTLDGVREASIVYPDYDLEKIDTIIIGGLLMDDDNTFGN

TANFTGCISNTVFIPEANLDLKIRSLKEFHLHKPQVTVHGPDVVSCSEAMLTGKVVTEAP

ESVTVTAQTFEILTMPSWEVGPARLVTLSPPRVFTEAPGTTSSPATTTSNKTKVLMARDQ

EAGVDDLTIIMAVSVIAAILVIALLVALLLCYKRRQRYEAKKDPDIELKQPLNYQSVAAV

QSSPA

>SG-PL_contig_511_1_-3_1818_2072

MFSAVNFSTRFSCVVVSQKTYGSNLMIADFPHHSHYYCNMYRQVDTVLLRMTIQCLSYKF

VGCTVYSFCTLKNDPSVWLTNLSPK

>SG-PL_contig_512_1_-1_40_609

MNKDVSFLSQPKRKCCFPKLASMNMIDLNSVNSQEISILATQIDFMKQRQVMLNSTYNPK

SGTSKGVNKVVIDYAKSMLYSVPADYPDRCFKMSYPISFSRCIPDDAEYLGPSYLGPLGI

GMLPYDGWRFRSNNGSLMITIAVTKNCVPLVEGVTPSVDPRLNGQLYMISHFQLGLKDPS

AFDLPPSCFQ

>SG-PL_contig_513_1_-3_3_182

EQNINIQIVNKKFLSCLPVHFIRFYSLPSINPEEEIIKFRKLGKKVGVECYLRMNLKSFS

>SG-PL_contig_514_1_-3_153_320

MCRHTQVKKEKTPHIHRVSLFHSLPQTCTCIYKYQINKCGICSSCSAIATIFYPVL

>SG-PL_contig_515_1_+3_3_257

FPSQIITPYPPRFAEAKSIKKNLTIICKTYVFLAFTPTPSHKPQEQDDPTMIRDTQTNLR

LTILKRMIMKTGHQYVHEAENQHKQ

>SG-PL_contig_516_1_+2_2_208

FFFLLFPHFNSFNSSRQVCIHPSQSTTVEIECTVDGNVPSRQCYPNNTKLHDTCTYVRTG

VQRLETVLM

>SG-PL_contig_517_1_-3_66_1142

MAGVWRLLIGSAAMLAVIYAALFDNTIQNSIEIRVIVDSWAFNSWMSKVATSSRELQKVE

ETKTAITNYVHSVFNDLNSIFKELMSSGINLDARILSIEFVTDEVVFSESEPGSGQRDSR

EVLGELTSWLATKNYAQADHTMLLTGLNLTQKGSDSTNKVNGGNMCDRINSLSVVEATPS

GAAVISIAAALGFSMSANHNVNDPDCKKNYIINSGEGSEPISPKNFRFSDCSIRAIKTSL

KKLHKSGDSCLSVANTNTSRVPAPALGQLYTPDEMCKLAVNKSFFFKRSLFGNGAFPYDV

ICRVIMCTNSEETTRVLASDGFVCGKDKRCSLGECKPSSVLSDVPDTCPQGDTPDAAAR

>SG-PL_contig_518_1_+3_375_782

MDLEGRKNADDIKGRKKGRNRESSRKEEISSTEFPEVRDEEMLGENFLHNNLLVINRLIF

IQPSCKGECQEKYRMTDLTEKRHERDVITIYVQIALFFSSNYKAESDTSKMCVCVCFSLE

GEIETASPEIAMPSKQ

>SG-PL_contig_519_1_-3_1011_1190

MTKRLGMTRRAIAWKFARLGSRSRGHGSSWKFPSRAANVGFPLTCREVTQRVGESCLLIG

>SG-PL_contig_520_1_+2_485_787

MKSVSRSSENLRTRRHFRCLTQEFLPKHRVLHQIDVDNAQDFLRSPPPSSILRHFRFQCR

YHRSLRRRTPPFLQLRMTEKKTLQTALSDWFLGLKRHQYLD

>SG-PL_contig_521_1_-2_14_598

MAMLAVRFTCLFLVFGLGSVFGQTVPCDTTRDVCMRNGFSSLSIFNNQIRCCLPGYSMAM

VNNNCQCTKTADAVLCSKGPDVCLNAPMRSWDGQGNMLCCPSNYPSMNSQSMMLNGMMFS

SCTCSRSGGASNVQVISGNGQGTVVFGGNRNFNNYFQKWNQRFRQTMQNRMQALQDQLRA

MRNRISVASNSWLQG

>SG-PL_contig_522_1_+1_109_414

MFCVHKHMKAVQALAGLYICSPGRQENSPAFTHWAIPKQVSNVGRLEGHCPNHSTITSFN

CILNLPLKTMKKCFFLFYYVFIYLPSRSTGWHCHGVCSEFVQ

>SG-PL_contig_523_1_-2_2399_2827

MWAALHRGQHIIDVTKAEHGYIILSLPLPIHQLPGEGEGVERLKFCKSVSLSLMFHFNRA

VCLVLAIVIHVYMYCWTIFVDGFTCPIIDDCHPCLSFVLADVYIFATYQWATCIVRPGFG

CGLFINSLVQTHTLYSASCVTDD

>SG-PL_contig_524_1_+2_2_271

GNAIYGPLLSCRPCRGKAIYGPLLSCRPCRGKAIYGPLLSRRYKGEVGASERSIALVGTT

VVATTTKTASFHMLVGVQSVVIIACMTGGL

>SG-PL_contig_525_1_-3_3_236

WKNRALLCERDQSIPEGSKWQLVAGKSVYRSLYLCFWPFSMGFFLQVFGVKMAACHSDSH

SPFIDRIGCVFACRQCAM

>SG-PL_contig_526_1_-3_255_1232

MLEEHSLGLVTSTVVSVIVYIIGLVINLSDFARQYLSVPLSMIPSILRNNGTNYMLPVSL

ADWSVSIWGVVYVCQSFWLVYAVSGICRKTAAGPAYNNPKLLPVQVHIFFSLAVLLQVGW

LVSMDKLNLGFALLLILVATGCSVATLCESYRALASTQALLVQLERSCDLWSVRILAQNG

LAVLMVWITLHATLTFALFLTFGMKSHYVAATTSSIVEVCCAGFLIIVYVITDLTVLDKY

SRLVVSPYLAFVLFHAAQLNKDWEDTHTPLFALLAFFTAFSFLALFLKVVLLLWRQLSRG

QPETPITMSHPLPTEMEEEAYLLERK

>SG-PL_contig_527_1_-2_8_1891

MTGQQKTCWKRSRRLHRHLFTMKLLVLLAFVAIGQSALLGTKKCTYGPSYWCSHIHHAKE

CHSMQHCMTTVWKNRHQASIGSTESCYDVIKILGDVRHSGAPDLREQMAKSCTQMPDEKH

SMCKDLMMNDQNEVLLLLQSDLPSINVAAALGLCGEFSDTVPHPLVEDSVKQPHVEKDQV

APLSGDYCTDCLNFFGDLQEIFNNTVLKLSEQFKEKICSKLGPFEKFCNQTTDAYAELIT

DLGVQQLNPQDMCFLLQMCDSETPADVMKKVKSIIAKVPNSVDCDLCKKLIKDVQNVLRE

PTLQNDIINIVNNDLCPLLPGDLAAQCKSYVSQYGPLLFQVLIAELDPVMFCETLGFCNA

TETKHLTVVPLGEQMSSVHVLAPEHKEQAHFGPFCYICKEVMEKLEGMLDPNATKQEIDW

ALEEVCSLLPEKLKTECDDFVKAYGSLIEEALAEMAPALVCTALGLCEGNKPVNVEHHMP

VKDDEKCLICETAVAYIKSLINNPDNVKTIEQLLSEVCGFLPSSYKLMCEEIVNVYTPDL

IKLLNKYDDPQTVCSKLGLCDSTKTAINKSDQSAHPLLFQPRVVQQAPTSSQVLCSKGPK

FFCATVETARPCKAVDFCVKYFNLKRDA

>SG-PL_contig_528_1_-3_96_311

MFKSIHLLGVKDDKQQNWYPFSGGPVASKPNDVHGIQDRVSSHSNRDTSKQRYICVIICM

VFCCCYCSHLYF

>SG-PL_contig_529_1_-1_31_1002

MAGVWLLLIGSAAMMAVVYSTPIDNTTQNCIEIRVFVDRHAFKSWKDKVATSSTWLQRVN

ETKAAITDYVYSVFNDVNSIFKELMRYGINLDVRVHSIHFVKQKLLARGINKTTGQLSPG

WALYLLSEWVNPNYPAIDHNMLFTGLTLKKKPTQNYPESAYYGSTCFSDPFSVVEATTSG

AAALFMAHQLGLSMTVEAEGSQIFCDNGYIMSAFKTHSSPTNFRFSECSIKRIKRYLEVV

RDRKNCLTAANTNTPPVSAPALGERYTPDDICKMTFGNSSYFCRSLYGYGGHYSYDSMCG

VIYCKQDSGLCTQVRASDGFVCGK

>SG-PL_contig_530_1_+1_1798_2019

MPFIACNAFWCIPSPLDESKSETSMSKKSSNFAHTHTWTFLLQPISNILIPGYFFTADVM

QIKTKTFLHSYFHT

>SG-PL_contig_531_1_+3_33_968

MANVVHSVLNMYSLQLSQVRVPQASVIISCMLYSSPGVSSSTSPLIGSRGSCLGSPVFLG

HGPVLCGSRGLWGRCSSRCGTKRLDGRGNRLAGPVAHLLHFTTGPLVLHRGSFLLLHDGS

LLGFLFLLLNLLRVPVEVQIHHDFPRVITTDGTTQTQNLASQHPPHGANSHLTLVVARDG

NVNMSQRGVSVAESNDWDVDIRGFRHGLMVNAWVGHHQQTWLTVSSLDLVGEGTRRETSS

DGGSSSSRSKLENSTLAIGTSRYDTHISWVLNSSNGTGSQHQLFPCLFDVDNVHTIRLSL

VDVLFHLIILIC

>SG-PL_contig_532_1_+1_40_156

MSAFGGLWKHENTQHALGEKKKEGVALHCGDTLSPGTAA

>SG-PL_contig_533_1_-2_38_433

MERILHGLECIWKEYYMVWNVYGKNITWFGMWNYCLRKKKNCVIDKAYSVEIHSWLCHLC

MEEKWLLGTDCKVCVLIGFWLHGRIFFMVTLCRTLVVQHIVFKVSRYADKVQLISCRFFQ

RYIHLHDFCGLL

>SG-PL_contig_534_1_-2_2_172

RLYSGNLAQPIYAHKQSQPLLKKRSSHIYKPPLFKFGFHSCDSIFLSSLIHVKCYFI

>SG-PL_contig_535_1_-2_2_322

AKENEEAFKGWLKTKQTQIKREKLLKKREEEEIKDGYYVRSRDECDQAYKEWLKRKYAEM

RRQRAAEKQRARSFRSYSRKSRKQQMMARAVRQSQAFRYVDYYGYRF

>SG-PL_contig_536_1_-2_224_469

MRKKQIRVRFIMQNSIRLLYYFLLDNKRMVLIHFCVGMIGKCPQANVHTHKRTTHTHSLH

MLIETFKTTKFKTSERHRYVYI

>SG-PL_contig_537_1_+3_747_1055

MKLQAHHQGLRTFWRLYYVMSCSIQRYRPGESNICLKKNSTHVHKHKHTHKLYTHFCQRL

FIDQCHTDCSAKRRSHDKAHAARVMCQACAVSLHINIIFLLGA

>SG-PL_contig_538_1_-1_415_1455

MMDYTYQHQYNMYHDYYTPPYAQQYPPYSMVNFLTSGTGTTPTLTPTTLHNLEQTFMELQ

SVSHNGSGGASSGSGATGSCDPHTQSGFVPPIVDPSSREQSQDSFESSDTDWEPAVKRVK

GEYGSILSSSSSSSSISGAPQRKYTRRSRHEKLPPEEEHRRAVRRERNKVAAAKCRQRRV

DHTNSLVEETEKLESEKSDLENEIQALQQQKDQLEFLLHAHVPICKVEPRNFKIKSEPTD

FTTLTTSACTSLHPSMSSSSSSRPKSLALDRHRHGDLPVVVSCAGPGVPLSTPSSGLFTY

TSLDSLVDGSTGLTPLTTGPISCSSQVHRSESESGSDPVNSPTLISL

>SG-PL_contig_539_1_+1_493_819

MTILHSQTVASQSTLRSLPHDTVKSISRFNLSPLHVIQHHTSSIYSSKEILSRHTKKRIS

KAVSLQTFTSVGPICTLTHLVAIQHNHNNNVHDNNYVKTKDRSYVIVKT

>SG-PL_contig_540_1_+2_860_1351

MLGFDSVFSHCSLCFSLMVIRLHLRAFQHLLDEIVIFDVAFQILPCVCGLDELVHLTVCQ

FLTEACQQMSQFSSRDESLPFTVESLEGLVEVGEGSSILLLADGFVDGQELVQLVSLLSQ

LEGASILVDDLLRGVETQAEAHVSHLEGIHIPVTTVPEIKQIGR

>SG-PL_contig_541_1_+2_65_154

MIFFSNLRPGIKYAEPASSSIGDPSVYIFK

>SG-PL_contig_542_1_-1_304_933

MSGLRAPKAGSNRDAQAKVEASFDAEDAKKILRWIQAATGIQFPIEESNDRYKVSENFYK

VLKDGHLLCRLVDAFLPEHMKMDFNSKTFEKRANPAFEAARERERIELFIQRIQDFGVGE

QDTFQTDCLYERTNLPKVMGCIRVFGIEVESHPSYTGQKIWPRKSEANPRNFSEEKLRAS

ENVISLQYGTNKGASQAGMNFGKKRMILKE

>SG-PL_contig_543_1_+3_3_155

THCHTHTHTGTSITVVICKKCALHVIVEGSESSCCSLKKQTKTPTPNLDYK

>SG-PL_contig_544_1_+3_87_401

MFYYCHGIVSQKKFTFYSTHQMHEKNIRTPTNKIKAYKIDPTNNQSSRTESTNYSYRTWV

VMELNVGMLSYFCFHIGWRRQLTTCVEQQICLLNIISWKTVNFHI

>SG-PL_contig_545_1_+1_1222_1512

MLLHSLHPCRFEIRPSNWKSDIPTTRLSCPPKGFNGLSDLQTPVALTSFYGRARGTTFIC

CMQCLRAFTATPEALKLSLNIKFEGCDSQVIRTPDLQ

>SG-PL_contig_546_1_-1_7_645

MLLLVAAVGAAIAASDAEDESSCTKFAENSDATAKLIDGKCYYGISTEVPWKEAKKICNQ

AGTRLVRLETAAEQELVSLAFLYRKYWIGLRLIDNTTWYWFATESDKTPATDDLGERLTE

KSRLRRFGVARLQGKEGDYQHGLTVSRQRALHPVVCEDDGVSGPSTDTVTVSVTETEATT

EGPTTATDAVSVTETETTTEGTDGDIVTVPVTE

>SG-PL_contig_547_1_+3_3_413

PPPLLSPNTHIQTNVHSVTSTRSRHRLDVTTRADAVPSAGTSWSTRQFEIKSKWKPISPQ

TKPIQFKRFAVQYSSGLQQLVFTSYEHKFDSKRMIYINMCVYVIFTLVPPSQGRGRKGLV

ILLMLQKKSLKALDLIF

>SG-PL_contig_548_1_-1_55_426

MLGDTGRRVVVMWSAPYSFNHYSNVLGVGLTRKGTHVPRDRGEWFNEMYYGAGTRYLHFA

RHEYYYDLRPVRFDENYVEVAGFMNNIHQARVHVIVRPTIDHPQDLSTFIKARLEALVTA

NSTR

>SG-PL_contig_549_1_+1_1_252

YRFQKSDNVKNARTKHNYCPSSNSVFIGRVFFREERHIKDEVFHHTKGRLRLVVWHLVAC

ITNNHPCKVANLSDPAQQSVIQEP

>SG-PL_contig_550_1_-1_1_279

QPQPLTPFLSQRLRQQLKVQMVTLSLFLSRRHQLKGLMILPLFLKWTLLLERKAFLIKKV

RQRTLVANHLQNRRHTANNCRLFSSLISFFLLF

>SG-PL_contig_551_1_-2_2186_2383

MFIIFKIVAWLLMDHYLLKNSKHAISKASLLFCWVYLWLYINLMHLVLKSFECVYAFHLV

LTAGCF

>SG-PL_contig_552_1_-3_1662_1871

MTIRLESLLQIFSLFTQNLKLWSLEFVWKLLKRDSLHSDSSICTAPRSANNYLVDSMSLR

KTQCTDMVLK

>SG-PL_contig_553_1_+2_107_310

MAYLDTKKKQSFEKGATSTDGHIGPSRMYVRSTSTIVQTRTHSFPSLLFRPSKFPPSYLR

RESNPPPC

>SG-PL_contig_554_1_-1_265_1386

MKFSVFILFTSLAFVFAEDVKTEEDIYVLTEKNFDSFVKDNEFVLVEFYAPWCGHCKALA

PEYTKAAKILMEDDSDVKLAKVDATVENELASRFGVRGYPTIKFFRGGSDNPVDYAAGRQ

AADIVNWLKKKTGPPAHELKDKDSAKTFVEKEDVVVIGFFKDQESTGALALKKAAAGIDD

IPFAITSEDDVFKEYKMDKDGIVLFKKFDEGRNDFEGDFEEAAITKHVRDNQLPLVVEFT

QESAQKIFGGEVKNHILLFLKKDGGEETINKFKAAAGDFKGKVLFIYLDTADEENGRITE

FFGLKDEEIPAVRLIQLAEDMSKFKPETTDLETATIKKFVQDFLDGKLKPHLMTEEVPDD

WDAKPVKVLVGKNF

>SG-PL_contig_555_1_+1_2368_2604

MDNDKNLLKRKKTHQKKSNKDNKDSQEYCTLSPTFSPITVLKGRSDDKDCRKRMVERRYV

SATLSTVPKETSAVSCTVI

>SG-PL_contig_556_1_-1_1327_1503

MKTKHICNVNECVNVGLVLQDKFFPLFVTLKWTCTYVSERAHLEFFPFPPVGNDNLTLY

>SG-PL_contig_557_1_-1_505_897

MAFVALKHMEFFPSTETLSLETETGYISDNVVAGTLANSFVRSDSKRCHSAVILSHIISS

ASERFNFMVTLSKIQTPVICFPFFKKKCHICASRPIVCRYDLTSLCLPISQERQDSLLVC

VYRLVKRDKTL

>SG-PL_contig_558_1_-2_29_520

MAKRGSVLLAWLLLAFVAELQVLKADAADVEITGSSVVAEGEWIALTCTVTSTDHTPDHV

SWFRDGYRLTSHRQEGMTIRTTRSTRDTYASLLTIEHAQLSDAGTYVCRSGYKVSNLRLH

VTKRGTIHRQNITMVTGIASSVTRQRIRSAILLTSVVLLFGRLL

>SG-PL_contig_559_1_-1_142_561

MSKRGRGGSAGGKFRIALGLPVGAVINCADNTGAKNLYVIAVHGIKGRLNRLPAAGSGDM

FVATVKKGKPELRKKVMPAVVIRQRKAIRRKDGTFIVFEDNAGVIVNNKGEMKGSAITGP

VAKECADLWPRIASNASSIA

>SG-PL_contig_560_1_+2_128_235

MSVNTSRWLVLQSLPSHTTTRWAVQRIDRYFMKKKK

>SG-PL_contig_561_1_-2_65_568

MVATLLTVLAAFTKMADTKGERTFIAVKPDGVHRGLIGEIMKRFEQRGYKLVAAKLCMPG

EAHLKQHYSDLSTKPFFEGLVKYMNSGPIFAMVWEGKEVVKMGRAMLGATNPLASNPGTI

RGDFCIDVGRNVCHGSDSVESAKKEIGLWFTDKDLITYEANAKPWVYE

>SG-PL_contig_562_1_+1_1_279

PSFLPSFLRFFLLSFSLSFFFLRNFYSEQSHFLLQVLLFPGLQVLIVAARHTEYFFKVSI

GQVPLFTSRIHSFRKSLLLQNINGMSLVVVGQL

>SG-PL_contig_563_1_-1_19_921

MTPHLYSLLVQFFTVFPEYLQSDLYIGGQSFACQYITALGKHIHDHNHASPPPSLFLPLK

GIYVGSGFCNAAVMYTEFYEYMYYAGLISDYTRRTYSAVVNRVIDNVLATGRTTVYNVLD

ILGGTVNLTGSYNIDNSFGGQRSLDTLKKKLGILLTSATARQALHVGQANGNVSFEAYSF

YVWNYTKTFSFFRSANEEYVFLLENYRVLHYSGNLDLKMNVPATEKFLHQLQWTGADRYY

NSTRYPWYRDDNAFLGWVTQIGNFTRVVVRNAGHQVQYDQPEAAFLLVKNFILDKPFLTE

K

>SG-PL_contig_564_1_-3_27_1439

MASWITLIALLAVTSAVCLAETEDDGAVFLSPLIAAGSLREAKSRSRVRWNFVNETAVTE

LWSYAGYITVNDAYDSNMFFWFFPARQNANTSPVLLWLNGGPGVSSMVGALIGNGPLKVT

TEGKVVKSKWSWTKAFSMLYVDNPVGAGYSFTGNVSGMPLTIEEITPHLYSILVQFFTVF

HEYSQNDLYIGGQSFACQYVSALGKYIHDRNHETPPPSLLLRLKGVYVGGGFCNAAVTYS

EFYNNMYYAGLMSDYTRRVHNALAKSTIDNALSTGRVDGTDVYNIISGIITVTDAFDIEN

QLGKKYSIHSLKAALNTLLSSPTARQALHVGHANGNVSFLAANNDVWAHIKTLNFLRSAN

EESIFLLENYKVLHYSGNLDLLVSVAATEKFLQQLEWTGADRYYNSTRDPWPRGDALLGW

VTQIGNFTRVVVRNAGHQVPHDQPEAAFLMMKNFVLDMPFFPPAESTTDEN

>SG-PL_contig_565_1_-2_137_400

MGEGGGRPPLREPPASRGVQDLVVTEDRTPRRLPGWDPTWVLGAHGSSPWRPWTWRRSVL

AYRQFSASTGRLLLLQLVLSLEGAKDDI

>SG-PL_contig_566_1_+2_2_160

SCDEHCLTCRFEKSDNVKNVKTKHNYDPSINSIFKRRVLLQSRKACQGRSFSS

>SG-PL_contig_567_1_-2_113_451

MRYPGVPDQPPEVRPHTFPVLRLPGHEVRHKDTEGVPDTSPAPDPPAQAVDSPGSVFGPR

QRDCLPLGPHGISSASDPPGQGTQAPAAESSPPTVAPVREQLGPGGSPGQLAP

>SG-PL_contig_568_1_+3_381_569

MSLEGTGARSRSHGPNTCPTQINTFLGRCVNTTFSAISDNIKFGFHCCDILLLSSLYVKF

YFI

>SG-PL_contig_569_1_-3_3_131

NAMKPIMVTLICNGDVAVHTNDIIGVKIKGGLFIWLDRFFNNG

>SG-PL_contig_570_1_-2_2_310

MVTVGELTNKLCAHFVENERTFKLEECLNVFKIFCEKVIQCKKENEQRKQQEERAERRRK

EQEAMRARSKDTWKQATTTIGRQSRAVTRLAAVAKHKEKPAQE

>SG-PL_contig_571_1_-1_19_528

MAPKKSTKATAKGEMKKVYSDPKESMLVGKAKDKAQRAKKAVLHGRHDKRSRKIRTSVHF

RRPKTMSLPRAPRYPRQSASKQNPLDAFRIVKFPLTTESTMKAIEDNNTMVFIVDKRANK

PLIKQAVKQLYNIQVARVNTLIRPDGEKKAYVRLQPDYDALDVASKIGII

>SG-PL_contig_572_1_-3_96_287

MTKSLRPAMIAILLLLALTAVTLTTGEDAAEPDSEGESFMDRVRKGDKPHCFWSRHRWVC

EFED

>SG-PL_contig_573_1_-3_321_467

MQTMTNYSRSVVGNKLKKTTKDDAFVVTCNLVFHFPNFSPVAIAELDYV

>SG-PL_contig_574_1_-1_19_1269

MGDQLHLTQEKFWNDPSSFSFDTGTYGYKYEIPITFTTSSERNFNKPRTDIKWLHLDQDS

LTIPLPSADEDWVVVNLRHQGYYRVNYDEDTWMAIIHQLNTDHTAIHEVNRAGLLHDAWN

LYKAELLPFEIALQTLDYMKKETEYGPWVVAEYETAFMQPMIERTELFADFQRFMREMVD

EPIRILGLDFPSDNLPPIEVTKKTLILTQACVYEHEACVTRATRMVSDWMNGLGNTINVN

IRAAFYCTAVREGGHNVWDFIYQQFQAEQDNKEKSRLLKALGCSKDPIILYTFLDMCLNG

EEISSSYMKSCVGSVAGSHYGIEVALNFIMDNFPVIVNSFPSRTVGRMVNSATQHFYNNF

HLAQIQFLMDTYDVSSVDWELDSVKTRILTSMQWKEKNFDTVKRWLASKNEAADEAA

>SG-PL_contig_575_1_-1_865_1179

MAFVLLGSFKQLSVNNRHIDCRTASIFPFCPSPACKLSFLYMGNRKTKSYAGCVVVVASV

MELMNHDGIWCWKNSLSLLVHVCFCFLCFSPSYEVTGVDLTVAVL

>SG-PL_contig_576_1_+1_364_687

MLGISNPWWTSLRPSSASGSHCTCQRCRGVDALSDALQGGEEGQDQAPLWPAQTWFPDLR

DLTHLPPLALPVRKDLLRQPRLGVMHPNPQLFRLHTWLLCGPKCVHRT

>SG-PL_contig_577_1_-1_829_993

MYCSADPTVKTSFKIQGVASFSVPADLHFMFCLIYLRFSCLQSSHEIRTFLFIRL

>SG-PL_contig_578_1_+2_95_445

MQMSKTQHFLSIAQLQTKKTSQCSNSFIYFFFFKLFFFKSSPFLSFIVSFRFFLSFIFFL

SISFCHFFLLSFFCLFPSLSPSLFFSLPFFLFSFSFLFFFFFFFVFVSFLIFLGFFS

>SG-PL_contig_579_1_+3_3_107

IFFFFTLNKSLFLLTHSPEQRTTTSTNNKIHTRSP

>SG-PL_contig_580_1_-1_3073_3225

MSQNVCLYALIGLLQSLRTMEFVPLERLQDTSLGKMLGTCCCPFSRPTLST

>SG-PL_contig_581_1_+3_414_617

MPQSSKIDCIMCDRFQNVMQLQHTALLPSRRFRMVAHTIMNIFYCSHRNEHFLYLVHLIT

RRLFIILT

>SG-PL_contig_582_1_+1_1555_1716

MVRPSTLASLLQPQTTACFVRQRVIVFSLLHASLPFTTRTLLSWSKTKGVHFSP

>SG-PL_contig_583_1_+2_2_184

QEDTFQSITTVSVVSNLSLYKQNITHTYKISQTSGNKQTNIHKMPQKTQTKVEPNINTDI

F

>SG-PL_contig_584_1_-3_3_272

NANRFETETACQEACLDDDEIRLSDLFWSTNENCNLPLFRSHCMAYIPSYYYNTLTGQCQ

SFVYGGCGGNANRFQSLADCQQKCSATSPR

>SG-PL_contig_585_1_+2_3419_4750

MPRKRDRDTVIVKVMRKIENANKEWQEEEQEMARGRAQTGRRTRDGKRKKTQTGRRTRNG

KRKRTQTGRTRNSKRKSTQTGRTKNGKRKRTQTGRKGTARGREHRQEEQEMAIGREQTGR

TRNGKRKRADRKNKRTQTGRTRKQTGRREHRQEEQEMARGSEQTGKTRERRQEEQEMARG

RTRNGKRKRTQDRKNKEWQRKSTDRKNKKWQEEEHRQEEEQGIARGRQHRQEEQGKARGK

EHRQEEQGTARGREHRQEEEGTERGREQTGRTRNGKRKRTDRKNKEWQEEESTDMKKRTQ

TGRTRDGKRKKTDRKNKERQEEKNKERQEEENRQEEQGMARGRAQTGRTRNSKRKRTQTG

RTRNGKRKRTQTGNGKRKRTQTGRLTTGERLETEGVRENGTITCSSIKQERNKNVGYWAS

QKLNCPKLQKGEKKPEDRVRGEKN

>SG-PL_contig_586_1_-2_305_457

MQDKFIIIIIIIIIIIIIIICLFRVDQRYDDVETYHLIISGNQNLIYHNKK

>SG-PL_contig_587_1_-3_42_521

MTNPKGYRRGTRYMFSRQFRKKGVTHLSTYMKVYKRGDIVDIKGDGAIQKGMPFKFYHGK

TGRIFNVTQHAVGVVVNKRVGNRIIPKKINVRIEHVKHSNCRLDFLNRVKLNEEKKRAAK

EQGVQVQCKREPKKPREGHFISTKYNKPQWIQPVPYEIIA

>SG-PL_contig_588_1_+3_63_296

MKGREGSQAHMDGDHRHLYCQEMSVTDISVMVIQPREEGIQWWGGGGTGRRGVNTAILYC

TSAGGFMLFQNSHMRAHA

>SG-PL_contig_589_1_-2_176_772

MACRRYMFLLVAAAFVAVALSEEVPCMTSREDCTQGIGYSTLIIINDNFHCCAAGYSMRW

DDSSDKCFCDKTSEVVACTSSACGKNDSVVSFTNKNNVTECCQAGMTMTVFQGTLNGTKM

DSCSCSSSSTWGLEVVSYSGANTSHIHFNNYLGMPHFSETSFNNWISHSLSEMREAFRRL

FDDYFYDEDDYDDDYYDDK

>SG-PL_contig_590_1_-3_3_176

FFIQWSSLPEYIYILLKIVTLYEYAFITSYCLFKLYILIKTLAIYKAAFIASCHLCSQ

>SG-PL_contig_591_1_+3_39_272

MSSCVSTTMTTSSVTQDKQSADSKQLSQRGKRNPWQLGDTSSMLHDCEPGKVSYSAFIQT

VLITTTEDDSQVNSAQWQ

>SG-PL_contig_592_1_+1_1_81

IYFIMSVQYICLIKEMFPNTCVHVQTG

>SG-PL_contig_593_1_-3_156_296

MVSFSSVWIFYYVLTFFSSGLVVSILRCHSLYTKQISITHIPCLDLY

>SG-PL_contig_594_1_-2_23_1435

MGGDEEKPETNRQPHLSKQDLNTLDINILNPLTPEVISRQATINIGTIGHVAHGKSTVVK

AISGVQTVRFKNELERNITIKLGYANAKIYKCDNDACPRPGCYRSCGSSKEDSFACDRPG

CGGKFKVLRHVSFVDCPGHDILMATMLNGAAVMDAALLLIAGNESCPQPQTSEHLAAVEI

MKLKHILILQNKIDLVKESQAKEQYEQILGFVKGTVAEGAPVIPVSAQLKYNIDVICEYI

TKRIPVPVRDFTSEPRLIVIRSFDVNKPGCEVDDLQGGVAGGSILRGVLKVGQEIEVRPG

IVTRDQEGKLSCRPIKSKIVSLYAEQNDLQYAVPGGLIGVGTKLDPTMCRADRLVGQVLG

AVGALPEIYTELEISFFLLRRLLGVKTEGDKKGAKVQKLAKNEVLMVNIGSLSTGGRVQA

VRADLAKIGLTNPVCTEIGEKIALSRRVEKHWRLIGWGQIRRGVKVTPEQG

>SG-PL_contig_595_1_+3_150_353

MVYFRTHTRVFFPNPNQVSYMVYFSAEQERLGLVKSCTVQACFAIFNHIPQFLFCVCVCV

HVSVFSVL

>SG-PL_contig_596_1_+1_1_303

FFFFFHTIQSLFHFITLCYTNFSFSFHDHEHTHEIASALHCYFLPGLKKKKRVIQTGNSL

YTVTPRQQLNESKYSKKFSSTLIQRYFHDSAMIKQITHFTL

>SG-PL_contig_597_1_-1_94_1098

MAKLQEVCSLPAHDERAWCVSWNPTGTLLASCGSDKLIKIWAKEGEHWVCKSTLADAHTR

TVRSLGWSPCGNYLASASFDATTAVWSRKGGEFECLATLEGHENEVKAVTWARSGGLMAT

CSRDKSVWIWEVLDDEEFECASVLSVHTQDVKDVKWHPEKEILASCSYDNTIKLFKEDSD

DWCCFATLKSHNSTVWSLAFDKTGSRIASCSDDKTVKIWQEYPPGNPEGVATKDNESTWK

CVCTLGGYSLRTIYDIGWSHLTGDIVTAGGDDAIRVFREDGNSDKNQPSFSLVASVKKAH

SQDVNSVAWNPAVEGLLASCSDDGSVKIWSLVPEL

>SG-PL_contig_598_1_+1_1_123

QQQQQHQNAHNIRNSSIINFNNLFSSTIKCYSGSHCLGDEA

>SG-PL_contig_599_1_-1_49_558

MAPKKATKAPAKAGAKKTESAAKESAAVGKAKDKAQRAKKAVLRGRHDKRSRKIRTSVHF

RRPKTLVLPRAPRYPRKSAPRVNRLDAFSIVKFPLTTESAMKKIEDNNTLVFIVDKRANK

PLVKQAVKKLYDIEVARVNTLIRPDGEKKAYVRLMPDYDALDVANKIGII

>SG-PL_contig_600_1_-1_1_882

SSCSSSSSSSSSSFIFSTSSFSSSSSSSFLFCSSHSSSSSSFLFCSSHSSSSSSSYSVPL

TPPPLPPSYSVPLTPPPLLLPPSYSFPLTPPSPPLLLPPYSVALTPPPPLLSSPYSVPLT

PPSLLFLLPSYSVPLTAPPPLLPPSYSVPLTPPPPSLLLPPSYSVPLTALSLLLPPSYSV

LPTPPPPLLPPPYSVPLTPPPLLPPSCSVPLTPPPPLLPPPYSVLLTPPPPLLPPPYSVP

LTPPSPPLLPPPYSVPLTPPPPPLLPPPYSVPLTPPPPPLLPPSYSVPLTPPLL

>SG-PL_contig_601_1_-2_2_1420

VPLTPPLLLLLPSYSVPLTPPLLLLLPSYSVLLTLPLLPPSYSVSLTPPLLLPPSYSVPL

TPSPPPLLLPPYSVPLTPPLLLPPSYSVPLTPTPPLLPPPYSVRLTPPPLLPPSYSVSLI

PPPLLPPSYSVRLTPPPPYSVRLTSPLFLPPFYAVPFTPPPLLLLPSYSVPLIPPSLLLP

PSYSVPLTPPPLLLPPSYSVPLTPSPLLLFPSSSSSSSSSSLSSLFFIFFFFSFFFFFFF

FFFFSFFFFFLLLLLVLILLFLLRFLLLPLAFLHVFIHFFILLLLLFFFFFCTSSFFFFL

FSSFSFFFYSSFSIIAFFSYFLLLLFFFFLPFFCSSFSSSSSSSSFFSFSSSSSSSFFSS

SSSSFLFLLFIVFFFFFFFFSSSSSSFSSFSSSSFLGRRLLSFFSSFFLLLLNFLRFFFL

VFSPLVLLFLLHFSNPPPPPPLLSLPPPPPFFEFDFFFKINFFSLKHGMKKNY

>SG-PL_contig_602_1_-3_27_263

MNGSLLPLGLALSNNYDKVAGLNDYGPFSMSQLLTVFNVTATRSVHGVKQLIGLASFSPK

GLWNLCHWGDSRTQAWAKC

>SG-PL_contig_603_1_+3_312_728

MGRIIDWNISFGEGELRTKTTLYDRVHFIDINGCSHLSARRSFTQPPALHTSDADVHTSI

QNWLGKNINTFLVNARSLFSQLKNHSFYCVQAAPIPLSLPVLMFSNKMLFHQAALSLMAR

YLSVPIMTIGCTGRGLATR

>SG-PL_contig_604_1_-1_85_741

MKIWTSEHTFEHSWDTVVTAAWRKYPNPHSPAVVGLDVVDRKVDRRGILCSHRLLSTAWN

LPGWVTMLIGMDRLCYASEHSEVDPQKKTFTLKTRNLTFSNFVTVDEVLVYSPHPEDSSK

TKLHQEAVVTVQGVPLCSRMETLMTDTISKNAGKGRMAMEWVIDTIKTEAQDLSLEARKL

SSDLSMEALKLSCEAKKLSTEAKKHIDSVLPPPTSSSTL

>SG-PL_contig_605_1_-3_489_680

MMKNLRPALIAILLLMALTTVTLTAGEDEEDSGKLGSVLKRSTDSPPRRIFLCADFVSCN

PDRF

>SG-PL_contig_606_1_+2_4469_4762

MKKKKSDQETNYLFQDFNRFLRNSLPRSQNVTFSQTTNFPIHLFDFYLLSPSVSFAKQKP

DIQETSYASFPIAQFSNSIINGVSFMPFKILYFQTISP

>SG-PL_contig_607_1_-2_116_478

MSAYPAKGDQKPAADESPIHRIRITLTSRNVKSLEKVCADLIKGAKDKQLKVKGPVRMPT

KILRITTRKTPCGEGSKTWDRYQMRIHKRIIDLHSPSEIVKQITSISIEPGVEVEVTIAD

A

>SG-PL_contig_608_1_-1_346_1794

MDSGQHLVAVDEANEHAISVWDLSRDKPHKITETKSSTEPVLAVEFHPSEKGSIISCGKG

QITFWTFEGGALAKKQGIYDKYDKPKYVLCLAFAANGDVLSGDSNGNIFVWGKGTNRITQ

AVVGAHEGGVFSLCTTKDGNLLSGGGKDRKVIEWDSAYSKTGRETEVGEANGPVRTLNQG

KGALVLIGTTKNCILQGSLDLEFSPIMQGHTDELWGLATHPSQHQFLTCGSDRNVYLWDS

QSRSVVWTKELNDGAHCCCFHPDGNIVAIGTMAARWVVLDLATHEIISVHTDGNEQIECI

AYSPDGNYIALGSRDNYIYVYQVCDGGHKYSRVGRCSGHSSFITHVDWSQDSQYLASNSG

DYELLYWTASSCKQMTTPTAVRDIQWASQSCTLGFSAAGIWPEGADGTDVNSCNRNHKET

LLATADDFGKVNLYQFPCSQPRSPGHSYKGHSSHVTSVNFLFDDSRLVSTGGRDMAILQW

QVV

>SG-PL_contig_609_1_-3_105_326

MDGPGAGAPDRCSLSGGSPSRRAGDGHFHSRRPDGFPDSDAVVADLWQTRQSALGTQASS

GRPDAPVSTSPVAE

>SG-PL_contig_610_1_-1_1_270

ARAPWQVSFAGLYDLRLSEALTTMFQAQHEGPSGGPPTCRRAANDEAAAGASGAGSPGAD

ATTTPQTGTPLPQTSGPRLQQVVGPWARPP

>SG-PL_contig_611_1_+2_266_457

MRDTTTVKTMPDRSGPGQTTTLLSTVRSQGGSEKLATGAENSSRPTEEEDGDIRTNIQTS

LKMS

>SG-PL_contig_612_1_-1_28_978

MTMSGNSADPVILATAGYDHTIRFWQAHSGICHRTVQHNDSQINALDITPDKAMIAVAGY

QHIRMFDLNSMNPNPVMNYNGMNKNITAVGFPSDMKWMFTGGEDSTARLWDLRSRNLSCQ

RIFQVSAPVNCVCLHPNQGELIVGDQSGSIHIWDLKTDNNEHLIPEPDASIQSISIDPMG

TMMAAVNNKGNCYIWTMTGGQRDSPTQLHPKKKFLAHSKYALKCLFSPDSTLLATTSADM

TCCIWRTADLSLMTKLKTENQRWVWDCAFSGDSQYIITASSDNNARLWSVDAGDVKREYS

GHQKALTCLAFRDEVVS

>SG-PL_contig_613_1_+2_515_709

MEPVSQSAITETNQPKSKNIKAFPLLSRFINVFICCHKLSSQVQCFLVGSTGVIKRTCNT

PRAKP

>SG-PL_contig_614_1_-1_421_7578

MNWLDHSKTLREQNIDPNEILLLRRKFFYSDQNVDARDPVQLNLLYVQSRDAILNGTHPV

SIEEAIGFAGLQCQVQFGDHVESKHRPGFLDLKEFLPREYVKTKGIEKKIFVEHKKYGGL

AEIEAKVKYTHLCRSLKTYGITFFLVKEKMKGKNKLVPRLLGITKESVVRVDEKTKEIMK

TWPLTTVRRWAASPNSFTLDFGDYSDSYYSVQTTEGEQISQLIAGYIDIILRKKKAKDHL

GIDGDENSMMYEDNVQPAKATIIERNTSVVSHPTSGSVAIPVVLRDGYQGDNRFSMESLN

KALFSQSSSQASGGGRASMHGQAVQQSEMTPAQRAFVITVEEGLDALHAARDELDKKAEI

PTLGSDPASKKWRENQLDLSRQKATAQLSSMNAATAQLVTLTSGDLDEVDYTAVGAAVNQ

ISSNMGQFSRDVKMLAALGEDQNTGDKLLDATRKLCGAFSDLLSAAQPGSREPRQNLLAA

AGKIGEASQEVMDEIGETAEDMDKAFQDTLLSLAKAVANATAALVLKAKTVASTTDNAAQ

QNKVISSATQCALATSQLVACTKVVAPTIGQPACQEQLIEAAKLVAKSVEGIVSAAQSAC

HDDSLLQDLGGAATAVTRALNDLLQHIKKAAAPAKAQEQHEEGVDTVINVTDRLISSTGD

AHEMVKQAKHLAQATSMLVSAIRGQAEDHGDSDMQKRLLAAAKQLADATAKLVEAAKGCA

SSPNDSQQQQMLKSAAEELRSATNTAASNALKRRLVGRLENAARQAVAAGTQLINSANQA

NKTNRNQTSQQQLVASSQALDEQIPDLVQALRTVQKNRDSATAQLNLINASQDFIQPAAR

MIASSNGAAPTVGDQASVLNLHNSVKAMSAAVAELRTAAAKAQEACGSLELESAIDQLGG

LDRELDDTKQSALSGRLYPLPGETAESAATLLGTTSKTVGSSMAQLLTAAAQGNEDYVGI

AARDTANALKVLTKSVRGVAATTTDRQVQEAVIDSAREVIEKSARLLEEARRAMQDPSHQ

GNQQRLSQVARDVSTALNNCLNCLPGQRDVDNAIRRITTSSQNLSSPQFPHTDKTYPEVQ

VDMNNTAVTLNQAASDIVSASRGAPQELATSSNRYSHAYEDFVSTGLTMAGLSKDPETQN

QIVGGLKSVSMVSSKLLMAAKSVSADPSAPNAKNLLAQAARAVTESINHLINICTVSAPG

QKECDNALRQIQMMGNVLDNPNEPVSDLSYFESLDAVMEKSKALGDGMTGISNHAKKGEL

DDFCDSVRNFANSVCGLTEASAQAAYLVGIADPASEPGRPGVVDQSQFHRANQAIQMACQ

NLTNPASSQQQVLSAATVVAKHTSALCNACRLASSKTANPVAKRHFVQSAKDVANSTAAL

VKAIKALDSDFTEENRRRCAEAARPLTDAVEELTTFASSPEFASKPAKISPQARKAQEPI

VVAGKAMMDGACHMLQAAKQLAVNPRDPATYQLYSGHSKSVSDSIKRLVSSIRDSAPGQK

ECDSAIGNLNRTIRELDQASLEAIRQSLAPRENNTLKGFQEQMINSAREILDRVEDIRQA

AKQEPENLGHLVSTVTSYFDPLAESAIGTASKTMNSKQQMTLLDLTKTVAESALQLMYAA

KEGGGNPKATQAHQSIDNAADDMKDVLQDLLQTMEEAASQAGVVSNMIDSISKSISRTDE

RITVNEHVSFVDHQTNMVRLAKQIARTAQDMIGKSTTNVGQLGVLANQMTRDYNQLANAS

CGAAAATNNSDIANRIRMTVQDLGKSCIEVVQDAGSLQANPTDSYAQRDLCDHARTINEK

VSYVLAALQAGARGTQACINAASTVSGIIGDLDTTIMFATAGTLNPEGDETFADQRENIL

KTAKALVEDTKTLVAGAASNQEQLAAAAQAAVKTITRLADVVKIGAGSLGPEQPEAQVLL

INAVKDVASALSDLISATKNASGKPVSDPAMLHLKDSAKVMVTNVTSLLKTVKTVEDEAV

RGTRALESTIEAIGQEVKAFDTTDDIPRKATAEDLIRFTKPLTMATAKAVAAGNSCRQED

IISAANLGRKCIFELFGVIKGAAATAETAEIKKRALYAGRNAALAYKELLENVHQVVLRP

TPEGRQNLALVSRKVANAVTELVQSAEVIKDGGTDWVDPDDPTVIAENELLSAASSIEAA

AKKLSQLKPRQAARKADESLNFDEQILEAAKSIAAATAALVKSASAAQRELVAQGKVSST

FTRHAVDEDGQWSQGLISAARMVAAATHSLCEAANAMVQGHASEEKLIASAKEVASSTAQ

LLMACKVKADPGSVAMQRLQAAGNAVKRASEALVKAAQQERDVQEEEESLTVNKRMVGGI

AQEIMAQEEILRKEKELQDARRRLEKIRKEKYKDRPPEDYDSSSSV

>SG-PL_contig_615_1_-2_110_745

MADREDQVYKAKLAEQAERYDEMVEAMKKVASMEVELTVEERNLLSVAYKNVIGARRASW

RIISSVEQKDEKSEDKMAINKDYRAMIESELKNICNDVLNIIDKFLIPNATVGESKVFYY

KMKGDYHRYLAEFATGADRKNAAEQSLVAYKSASDISMTDLPPTHPIRLGLALNFSVFYY

EILNSPDRACRLAKAAFDDAIAELDTLSEESY

>SG-PL_contig_616_1_-3_30_404

METLSRSMSPVMMFEMISPTVVFRYAEIFAEDAMMAPYTAAKQVEETGTDDDDVYFDALN

IWLAEALQTSDPKVAVETVEVEKEGVCVRFSPLETAQAKGTTEKDVEDFVSSLMSNLSVL

NASTR

>SG-PL_contig_617_1_-1_1366_1509

MNSKLFMNGNLGYISQEEKSQLCMHVWISTTSFYLSLQLNMFSSPITR

>SG-PL_contig_618_1_-1_469_732

MFYKNIKSTIVVNGQISNWFAVERGCSKVTSLSLFIVLCIEILAIMIRVDKDIKGIVINN

KELKISQFVDDAQLMKINCLLRNLFILY

>SG-PL_contig_619_1_-1_46_276

MTCDGINRKYNTNANVLLYNGCKVSIDKYIQETELNMPNGCAHDILTALKTIYSIPKGVR

RCYDILTADDKKSCSKW

>SG-PL_contig_620_1_-1_385_657

MCHLCRLTENVILSGVDENIRTGSLFDLIILVGKMYFYKSKLKKTLPNVTSCTRYLTTKY

QIEEYNAKLKWNLHTFREKWLPYLPLVSDEV

>SG-PL_contig_621_1_-3_81_401

MRKNIALKRVFMKIHKNQEVKLKWLQIRIVHSILGTNIILKEMGLVNNFCCDFCLETKDS

IDHIFWKCLHVKRFWERIEELLRSNCVTCTNIRLTENLILFGVNENM

>SG-PL_contig_622_1_-2_95_1003

MGSKKLGFAENFALSGAAAIISKTAAAPIERIKLLVQNQDEMIKAGRLSEPYKGVIDCTV

RTFRTEGILPFWRGNLANCLRYFPTQALNFAFKDKVKALFKTSPNESYVLKFSKNIFSGG

VAGALSLCFVYSLDYARTRLANDAKAAGKGSGERQFNGLIDVYTKTLKSDGVVGLYRGFV

ISCVGIVVYRGFYFGLYDSLKPLMLGDDANVFVSFCLGYAVTVSSGLLSYPIDTIRRRMM

MTSGQAVKYNGSMDCAMQIIKNEGFMSMMKGAGANILRGVAGAGVLAGFDSFKAAYIQWR

LKQ

>SG-PL_contig_623_1_-2_2_208

FVLCLFYGVLSENIKISITITFQNFGCHYLIKAKESEKRTDSQHHHKIYSKRKRKSRSTS

CSHSTWFCF

>SG-PL_contig_624_1_-2_371_2041

MAYRRTNVRAGDLIDIPGGLNTGSQVIVRGRVLVHETRFCINLRCAEDSDSDIALHFNPR

NDQGDVVVRNTFQGGSWQEEERDTPRFPFDNGRKFTIRIMTFPDYYRIMVNNQDFVRFDH

RIPAADVRCLQLGDGAEYYEASVQNNCRAPFMGHFCGGLKPGQSVRVRGMVKGEADRFHV

NFQCDHDGDSVAFHFNPRQNQGEVVLNAKLDGSWGDEQKSDDFPFKCEEFFDLFFIALDD

KFNVYVNEEYYTSFDHRCNFRDVCFLVIKGDVELMDVEILDPLPDDFIKEIPQGMEKNDL

VVVKGFFYSEGNRFQVNLIKGESCDDDIALHFNPRRDQDEVVINSRDGGSWEEEERHPLP

SSMSKMVPFEVEILAKKNKFKIYTNGKKVCKFRARDDIEQIRSINVCGDAYMYEVKLLRR

VDEPFTDSLPGTLEPGNWVTITGAPDDDDERFAINLQCGPDCQEDDIAFHFNPRFPEEVV

VRNTREGGGWGEEERDQPCFPFEHDDRYELAMVMLHHGIRVFVNHRHFTDYKFRMDPNRI

THIHLSGGSDYFEPEFY

>SG-PL_contig_625_1_-1_91_369

MTLPSASSTSVGGGGRGPSKSVTPRAGGGTTPRQRKTTTAAPRKAAGSSAGMWRFYTEDS

PGIKVGPVPVLVMSLLFIASVFMLHIWGKYSRS

>SG-PL_contig_626_1_+2_146_298

MLLQLDAVIKDSIYIYKKKFFFNWDHHIQPSFFFKLLLTSYFFFTHILYFT

>SG-PL_contig_627_1_+1_1759_1899

MRTYPILFSSALILCTCPTHSVSLSFFLSLALSVSEGRPTQNHKPLA

>SG-PL_contig_628_1_-2_110_343

MPSVVGCVKNKALPACRTYTMIILDLFPSDGICGVGASGIYSEHGDCLEKTETETVCPCL

FFVVFSSVCAFSKCSPEV

>SG-PL_contig_629_1_-3_150_275

MPLVGSPMLNWSSVRSRPSISVVSSSLYRPFLLSSAPQQIFF

>SG-PL_contig_630_1_-3_303_1820

MTTPATIPVFTVTTKEEEPYIMRELDAKGGARYVGFIKDLMDVLADKVGFQYNFKVVDDG

MYGQQSPAGWTGMIGEIVNKKADIAAGALTVTGPREEVVDFTKPFMTTAVNLLVKKPERM

DMGLGYLVRPFSTDFWILLLVSVVIVGFVFFLIGRFSPYEWVKVAGEKDQRGARNAFSLR

NSYLFVLSSLTWQGFREPPRSLSGRFLAAFWWIFILFTIVAYTANLTAYLLARPEQIPLM

PFKTYEDLVESNDIKAGSLAFGATQASLRKSRDPTLQSLWNKMSQENSWVNSYSEGLQRV

QKSDGHFVVFMESTTAEYLARKNCDVMLYGNNIFHKGYALATQKGSVIGSMLNKAMLELK

ETNELHMLIQRWWRFSGECSGIDGREYSQKGDSLSSLPIYPVTLRDMAVAILILFLGFIV

AVVGLILEIIYHTISTGGKIEKPKVLDSKIFKAPKFTKGKSSKPAAATSEDPEAGMSSEP

LSGEALGEGTSGEGASGEGVEEAREA

>SG-PL_contig_631_1_+3_849_1118

MQPISSQLHVKTSSPLTLPIFPSLAQFNNMYNRSGFSSLITYSIFQFISKIQHLKEFTQT

NAVVIINSYNAFFKSFLFFKSTTMIVLSSI

>SG-PL_contig_632_1_-2_2369_2644

MQIPNALTFTHCVIITVAVFVSVAQPCHSFAAFIPVSLVLHSLSVLWYLCAYLTFVITVY

THAHAHIFYHLLLIIIRFRITQNRCSDDQILF

>SG-PL_contig_633_1_-1_169_465

MSQSAPIHDMIRPLAWLLGKWRAEKGGKGQYSTIKDFEYGEEAEFFSVGQPNVQFSLYSW

DAVSKKPLHREVGFIRIQPDTNKIAIMSAHNFGVAEIQE

>SG-PL_contig_634_1_+2_2_184

NPKRWQGHISFFFLIHWSMVMPQMVKPVIWMKDVVFIKSSLLPQSCSSIVRFFFPEQWAI

P

>SG-PL_contig_635_1_-1_1_108

KSYGIPNSVNTNLICFRLFDFNIVNILVVILSSILM

>SG-PL_contig_636_1_+3_3_140

WVVQIDIVQAGTLTCKTCNCKIPLLYQCHLSLLIILVFQTVGHFHF

>SG-PL_contig_637_1_+2_749_898

MERKTPFCQPNIGHVTEKDTRNFCQLDLWSMSMPRQNTTVNMSCTQGIQS

>SG-PL_contig_638_1_-1_115_4062

MQIKAPRDWRPEDAKARQVNAPPVHSRYRKFDLYRNVQQFFHIDNHAIQVAQTDHDNFTD

LIYHLVYSANLQSDLEKTRVIFRWMTSKNMYTISFKDGAKAGTPEEVLLSFKNKQGTYAR

IFETLCRFAGVTAVVLTGFAKGLDYRPGDKFKGNEYNHSWNAVLIDSNWYLVDSHWATRY

LVSEKNMPENLVYEYDDFYFLTDPEQLIYSHWAHRKEWQLLVQPITLLDFENLPLVKSYF

FKCAMFFVSHHNGVVETKKGRIALTLGYCKPTNFTYKITFADTGEEMYQGQKLKNFALQH

HGQNQTGTFVFRAPRPGQYYFTIFAQLLTGELGVKNIFTASCEYKVIADAASTDATPLPA

CSDSSWGPGVAVDQLGLVANPPDGIVTTDDGRVNLEFKKTKPAYVLCKLRRNGMKDDDLE

KCINEKDTGDAVQVTANLPSKGEYGIEVYGNDPAKDGDTYTHICQYYVHYATPNEQQQAF

YQESPERRAAMGEPGQTVMAPGQYPGGVIGLDNATANLNLDNPDSLPPPPPELLQQQYLY

GTFPDSRQQAVMSNYAPTRGQGYPTYAPGSGQLNMGQPQQAVVSSPVIPASTKPLEVEKR

SMGQAPPAAPRDFQLSTVTQDKGEAPPPGPASTPQQKPTLEAIDRQVLQSVDDHAIQVSK

SDHNSFRDLVWDLIYSKNITNEIEKVRVIFRWLATKNLKEMNFDKVEKGSPEEVLMGLKT

GTTTYAMVFDTMCTYAGLHSKIISGYAKGADYKPGMKFTPGQNQHSWNAIYIYGTWCLID

AHWAARRIIGKQASSDEEFHYQLDEYFFLPDPHQLIYTHFPDDPRWQLLERSVTLEEFEN

MPHMKPQFFKYGLEFVTHRTAVIYSRGEIHIRLRYPAHKIAVAFNFTIQFEDGIEEFHGI

KLNRFGLQESVGGIASFSLRLPVTGSYMLFIYAKEDTPETKENVYAQVCEYKIEHESVSD

TTMEPYPPCSSLTWGPGSAFYRYGLTTYQQSSTICTREGKAELQIRQPRSMQFMAKLKHN

NISDSELEAYVIHRIVGNTAYFNITAPGRGEYGLEIYANDPASEGSTLYHIAQFLVLCQE

DVKATPMPKLPPGYLGAQPKFSEFGMTAASHQDPIIHLDCNTVTVLLKCAQSMRVTANLI

SVEDEKDYPDYVFSNTIGTSVSFIVNLPQTGFFKLQIYGIPTCDKRQELPGIYNYLINCR

QVTQAVFPFPKQYAQWKEGCFMTEPGVIPSPKGPFAGQVQVDCLPEKVNFRVTIPGAEAV

AVVANQDWTHLERSDGDEWYGQCKIGHCYNTNSKVTVNANFGGDKTSYATLLEYVL

>SG-PL_contig_639_1_+3_18_869

MRQATTGRRTGIQWTFTKQLEDLDFADDISLLSHRQQDAQEKLCRVAEEAEKTGLEINIG

KTEFMRVNNKKQDPVRLHQENIKEVDKFVYLGSIVSKDGGTDEDIKCRINKARHAFNTLR

PIWRSTALSLHNKIRIFNTNVKSVLLYGSETWRVTKTNTHKLQTFTNRCLRNILNVRWPE

VVSNEQLWDRTRHTPIESEIKKRKWGWIGHTLRKPASNVTRQALDWNPQGKRKVGRPKQT

WRRSIDAEAKAAGMTWAELKGVSRNRVRWRGVVAALCSPRNQEA

>SG-PL_contig_640_1_-3_3_266

SSIPQFPYPSGNLYSPARSFGDKKDYNYKCSECGKGFRSQQALSYHRNAKHACREDLKCP

LCGKMLGHKQHKNYHMKHVHNVDAYVNL

>SG-PL_contig_641_1_-3_144_1568

MASQAGIEDIQDLEKGARVATSVLQQRANDVRANVVNWNSYLQGQMITDEVHKFISRFDK

ASPEERANLISPKPIQFAVIFRSLIDKIAKDQTLQYILTMLDDALQEDPSRVDIFKDYAK

KCKESVWQPFFQLLERSDLFIVHQSSHIVAKIACWSKEPMEKKSLQSYLIWLRDQLRLTN

NEYHSTVARCLQMMMRVDSYRHVFVEVDGIAVISSVLVNHEKIGFQLVYQLIFCLWCLSF

NAFLAESMNRMNLVPVLADILGSTQREKVTRIILALFRNMLEKPQENSVTQDYALSMVRC

KVVKQLDLLEARKWEDPDLEEDIKWISAKLESSVHDLSSFDEYVSEVKSGILEWSPVHKS

DRFWRENAIRLNDKNYELLKILVRLLDSSRDALVLSVAAHDIGEYVRYYPHGKHIIEQLG

AKQKVMQYLTHEDPNVRYEALLAVQKLMVHNWEYLGKKITEDKTKAAGAVAEVKG

>SG-PL_contig_642_1_-2_848_1033

MVFCLKHNQVFCLKTLTCAIIVCSCSFCTWRISFTSCFVLFCFVFATGLIFCCRVSEDGM

LF

>SG-PL_contig_643_1_-2_2_121

KKKINSKMIIRNMEKEIMLCCYSDKVLMFYGSNSVSMTTV

>SG-PL_contig_644_1_-1_115_609

MRLEKCYFCSSTIYPGHGMQFVRNDCKTFRFCRSKCYKAFQKKRNPRKTRWTKAYRKATG

KDLTVDPVYEFEKRRNEPVKYTQELMQKTMKAMAAIEKIRVKRAGLHIQQRLTKGRKLRL

EHGAKILEKNISLLEPHKVAPKKKMEERLVEVIEEKDEEEEMLLN

>SG-PL_contig_645_1_+3_9_242

MLGWFCQLFSLLLCLSLSNTHKSKIHFALESFRKPLSVQIFCHSKNILMMIFSPHKILCE

QSFSQNFLYDQIFSTNLS

>SG-PL_contig_646_1_+3_510_767

MKTTNLALEHQLVHIKKKRCFTWKSTTFNHLTTFLLTNQFYSHSCFSCCAEHKRIILSLH

FLLISRAQEAEVTSSTSKVLISVKDF

>SG-PL_contig_647_1_-3_324_4544

MGVWVRDGALILVVLAAAALAAPTEETEGKSCFYNNQEYVHGVSWSPPQQPCLQCMCKHG

DIHCSILPDCEKSEQHRGDLGISATQGETVPGDISEGAPDEMMAQQGYGGYYNGGKSAPA

YGPQYMQAQVGPMGARGSSGPPGPPGPQGMEGPRGQSGDPGPPGPPGNRGEPGPPGPPGI

EGDPGRNGEPGAMGPPGSKGPAGSPGMPGMSGMKGHRGFSGFPGKRGEQGPAGEVGEQGP

AGPAGPPGPQGSRGGPGERGQDGSPGQNGLRGNDGLPGTPGPPGPVGNIGSPGIPGMAGA

KGEQGAPGPRGGDGPQGPRGENGNPGMTGEIGMPGPPGMPGSSGEDGAPGDPGPAGPPGF

AGPQGRPGLAGSPGMAGAKGAAGLNGINGLKGQAGERGSTGLPGDVGKPGPPGSAGRRGQ

RGPEGPPGPVGPQGERGLPGSRGMPGSPGGPGSKGIDGETGSPGPRGEPGASGEAGHPGP

QGAPGEVGLQGSSGPNGNSGAPGEAGPSGSDGRPGDRGAPGQPGPQGPPGPTGPPGERGA

PGKEGSPGPAGPRGAAGDVGVPGNAGPAGPRGPEGEAGTQGPAGPPGPNGFQGPPGPPGP

QGEAGRDGEQGIPGEAGPSGPAGYPGERGFPGERGPAGPAGESGLPGSEGPPGPDGPRGP

PGESGIEGESGPQGPIGPPGERGAPGPPGERGELGDPGPSGADGLAGRPGPRGPRGAAGQ

PGDVGLAAEKGQPGEQGLTGRPGAPGPMGIRGESGPEGEQGPNGFPGQPGQPGAKGETGA

TGTKGETGDVGAAGTPGRTGPSGMPGNPGRRGSPGDRGLPGVPGEVGPGGRPGPQGPTGS

AGPPGPTGVPGGPGSKGAMGETGRQGRPGSNGAAGQSGIPGNPGVRGEDGLPGGEGPPGP

AGPAGMRGESGIPGVPGETGPQGPVGQHGRRGASGRPGNDGFPGKQGEAGPPGPAGPTGS

PGAPGAPGSDGSPGQAGAAGEGGQAGEMGLRGPSGPPGPQGAAGAVGPSGSVGERGQRGE

MGPDGPRGPAGPRGPAGPQGAMGESGDTGPAGETGAKGVPGYRGMAGLPGPEGPHGEPGR

VGPRGPPGPRGVDGARGQMGPDGEPGAEGPRGPPGARGPAGEDGRRGRPGDIGPRGLPGS

PGGSAYAGAMNSWYRGSSSYSYNGGYNGYQGDAPEEARATMEALERVSEEIKKLRMPTGK

WDAPGVTCKDIAMADPTLKNGMYWIDPNGGSTSDAIRVFCKMRAEKTMTCLQSATHVYPT

RRWALTKKDMGYWSFASSFEEREEFGYNSTKSQIRMLQHHTSNGRQRLVMKCKDTVAIYD

KAINSYDKAMTLTSFDEELLTARSKKSHRYTVLRDGCQERNGREGQTVLEVSGAKMLKRL

PILDVGFAEQSEGEFGLRVGRACFWGR

>SG-PL_contig_648_1_-2_2_145

LATAQGKTLHDRVKREGDAVADPPPKAPAPDPYAAMRRVLTYLLRLQM

>SG-PL_contig_649_1_-1_1_180

LLVGAFAFLATAQGKELHNRVKRADTPVTPDPAPKTPAPDPYAAMRRVLAYLIALQRFQG

>SG-PL_contig_650_1_-2_95_361

MLSAHEKVKTMQIAVVNWRTALTPVTTDAVGSECIMNLSSISPCLPCRLSINRRDKRFPE

SGMAWFPAVLVQLTCSVCVCVCVCMTILS

>SG-PL_contig_651_1_-2_119_2395

MGLGVDEAFQGAGAAPGMEIWRIEKLKVVKQDPESFGCFYSGDSYIVLVTKQVPNSSRLD

WDIHFWLGKDTSQDEKGVAAYKTVELDEGLGGGPVQHREVQDHESKQFLSYFKAGIRYLD

GGVDSGFKKVERDKYETRLMQIKGKRNVRVRQVQCHSSSLNQGDVFILDCGLVIYVWSGP

QSSRMERIKGAEVAKRINDEERGGRAAIKIIEKTWDTDAAFFKALGSHEPIKTADDAGDD

TDFERISAEEVKLYRVSDASGSLEVSEVGEKPLKREHLDSSDCFILDSGPSGIYAWIGKG

CTKNEKKSAWQNATEFLTIRGYADWTPVTQVVEGGETPLFKQYFSKWSDPNAQTGLGRVN

KVEKVAKQSNEKFETASLHSNKTPAKKEETLADDGTGKVQVWRVENGDLVPQPEDMHGIF

YTGDCYLVLYTYTKGNSPKEYYILYFWQGAKSSIDEKGASAIFAQRMDDNELQGAAVQVR

VVQGREPEHFLLIFKGKMVIFLGGINNKEPDSSIRLFQVRGTKTVNTKAVQVQPRAGSLN

SNDVFIVDTEKEVFLWVGKGASEEEKNVTNYVVKHICPDRNSSDVVTVEESQEPPEFWDV

LGGHEPYATGARLEEQGSDMPSRLFQCSNSSGRFKVEEVVDFSQEDLCEDDVMLLDTFSE

VFVWVGKGANETEKKEALTTALEYIRTDPAGRNEDNTLILNIKQGFEPTSFTGHFHGWDP

EKWSQGKTFEDLKREMGQENVAVTSVKEVRSEERRVGKE

>SG-PL_contig_652_1_+1_1_117

GEGVKELKQCQSFPFCFSVCPSTDTSLTVHIFSLHFPQK

>SG-PL_contig_653_1_-2_497_667

MPRILHSLYVSLRGKGIKTFLLICRRMFRVTLIVMYPRKLIGETEWVHYLFKLEQAK

>SG-PL_contig_654_1_-2_11_367

MIVTIIIIIIINNNNNNNNNNNNNNNNIIIIIIIIIIIIIIIIMNHSCLTLFSILCWTPC

VFNYVAETTIGTNVTVFVDAGCVRVSISPRTLTWLTGSLTCVFNGQAALVRKEEDKTGS

>SG-PL_contig_655_1_-2_2_133

KNDGLWETKGRGGGENTLIAYFFFLVTGQTVIFSGTDDIYNIFF

>SG-PL_contig_656_1_-1_1561_1983

MVVMFKIGRTLAGSQCMRIMLVSDPCLYCTSTESSKMLTVQTATDTVMGRPKYSIKACLT

LSLPHAIHRKLVDKVRERRVNYQYCLKMLLTFQTSMNDNLRRKRGTIEVKDCRLFILLRK

WVLNYSLLILLSWAIKNCSIV

>SG-PL_contig_657_1_+2_2_295

FRVCQKRRICFYNNHNTNIPPPPPPHRRQSHDLCSRREDLRAEAADILLQAGLLVAADRR

SGMVLSVQSFTLSDQFVIFCRVFVFRFDKVLCSTRSSF

>SG-PL_contig_658_1_+2_23_295

MAMSVQSFTLSDQFFHCRPYLHPPSTVPCMMVLQRLSCFVTWPNHTSFRRLMVVRRGSWG

AHKVLDLTPHSHSFCASNRRCGATFSSMDKQ

>SG-PL_contig_659_1_-2_152_733

MPHNTTALLDCATPHYLTTTLCHTLSPHHHTVTHRGITTLYHTTELPHHTPHHRITTSCH

QITLPHCTKLPHYHIVTPNRTMPPHITHCATPHYTTTHYTLCYHTTLCHTLPHCATMPHY

ATHYHIVLPHHIMPLTTTLCYHTTVCHSLPHCATSPHFPTTHYHIVLPHHIFPPHITTLC

YLTTFSTTHYQIVP

>SG-PL_contig_660_1_+3_51_230

MQGTVEGGRRRGRQRKNWHENIKDWTGLHTYELLPAAADRDRWKREVAAAVLRFPQRLPP

>SG-PL_contig_661_1_-3_3_212

FIQARDYSSNRWGKPEDSSRNLPFPVWSPVQSLMFSCQFFRCLPLLLPPSTVPCMIVLDR

LVCQVLCPNH

>SG-PL_contig_662_1_-2_350_2404

MTSGLVIWLVFAVYHGSSSVVKGGQAPHFTNEWAAQIPGGEEHAKDVAKRNGFEIITSVD

NFDDYYILKRHDVPHRSRRSSHHHTRRLIEDGQIPWAEQQVARSRVKRLAGFRLRSDGSS

DFNDPHFRDEWYIENSIPSSRKERGQAIHLDLQVKGAWKQGYTGNNVTVSVLDDGIEHNN

TELAKNYDPYASTDLNDNDPDPMPRYDPTNENKHGTRCAGEIAMVANNGKCGVGIAFNSR

IGGVRMLDGRVTDSLEARALSFNYSHIDIYSASWGPNDDGVTVEGPGTMAQQALEKGITY

GRNGKGVIYAWASGNGGRLQDNCNCDGYTSSIYTLSVSSASEHGQVPWYGELCSSTFATT

YSSGNGGDHQVVSADLHDRCTTRHTGTSAAAPMAAGIFALLLEANPHVTWRDVQHLVAVT

SKVGPLASEDGWYQNAAGFCVNLAFGFGLLDAAELVSAAHPDNWVSVPQKSICEISADTG

SNLPQTLSSGHFVEVLIDTNGCAGQHNEVNFLEHVQFVFTLSYSKRGAISVTAVSPSGTE

TTLMMPRQWDKSTDGFRQWPLMSVHTWGEDPKGVWRFRVHDGDANESRHGVLKDLKLVLH

GTKDAPHYLRDGPVHCDVAVVSKKHTTDVTPSGGDDWGRPSLQPMGTGLMRGQSNFMTQV

IQAWMDRQKASQDDREAAIEEYLYL

>SG-PL_contig_663_1_+3_1281_1496

MFSCLFFRFRLFHVIFGYKFSLIRLSWFVQVSGGNTLDSGARARARARVCVCVCVCVLFI

YLFTYLFIYLFV

>SG-PL_contig_664_1_+3_15_269

MAEENELSTHWGFMSQSSPAWHTHDSLRTKPKATHEQAKQKGPLPCIHTTASEQSQKQPM

NRQNRKDHWTKALQQIHCVVLHTAL

>SG-PL_contig_665_1_-2_2_199

FHSRQCLIIARAFCVSLCEHVYVCCLLLKCRCDIYIYNIHRVPAVLCLGAEKHIEYIYEL

VNRKKK

>SG-PL_contig_666_1_-1_1_99

FFFITGCLVLKQSVKYSRTNYRVKIYRRVQFLL

>SG-PL_contig_667_1_-1_4123_4956

MSAATGGLADDHDVNSLLTHTLHEPGAETAKVSEEERRKFEEEFNQYYKDLERAKEDYQK

ENPNKADPYNIPEDSLYESESQRELRQIFDGQMNIQSTVREVSRKLDELLGRQELVLAKI

SSLPATGTGQVPQQVPQMQQQQGQVYMDTIKRHEVDRVLNNQNDLLQQIRDLRSVVNDVA

QRSMQQPVAVPQGGGGGGIGDQMALHEIKDRLNTVSNDVSLLVKRPMPALNTAQCPPPPS

GSCLTPVVFFVAMGAQIVILIAYMVYRQNKEASAKKFY

>SG-PL_contig_668_1_+3_513_1067

MRSKCRCSMCPAIHISSRSWLRSSSTHEPSDPPPKVVSWIQCRFASVTTFPTDPSAPNDE

RQFRVRARLVREVSSCGWNEREKEKCEGKRRKKKAGGGAARVEDGCALYTFTRQGRPARE

ARAPPGFCNQQLPTSFPCRASTDSSREPGSPYRPGTPQVTVSGGGGGRSRTPSPLPKRGV

SRSEE

>SG-PL_contig_669_1_+2_1403_1732

MILMTWFLNTHKSLLLPGLCTMFSKIRFIHKEFPFYYSGFSSHLFFFSSFFKSNYCIIIR

FFFLNNHQVYYIQFKNQSFQNATTLSTSSFVSITGKIFSVKILNTSSILP

>SG-PL_contig_670_1_-1_1_153

ICWNVLPIPFSSHSEWQCPVTLFTLSDFKAVADVILILEFTFVKYLLQQGV

>SG-PL_contig_671_1_-2_2_268

YVLTLFLIPLFNDSIKHNGSNVVIVCCQEVNEKSCVCVVIYPREYLLLTNHFCRLLCVYA

FLFILINFSFHVHIVMDIFSNERLTTLKK

>SG-PL_contig_672_1_+1_667_861

MYAKLKLLLLFAFPSLTLWGSLCPITVRPRVHLDEKTECMAGLLVPHHVGSHTPTSEIFF

SFYGE

>SG-PL_contig_673_1_-1_1_105

LTLSLPHPVTYHCHLTLSFPHPTHQLKKLVGRVRV

>SG-PL_contig_674_1_-2_65_868

MMSIPKSPLAYAVNQVVANAAKPVGKAVVSKADKIVVAPQKLAQTSYALSKLTPTGADRV

SFSLCSPAQVRFAHTDVQVPDFSAYRRDGTKDNHASSQSSAGGRRAFTYLLAGGGTVTGA

YAAKTVVNEFVSSLSASADVLALAKIEIKLGDIPEGKNMTFKWRGKPLFVRHRSAEEIEI

EQGVDISQLRHQETDAERVQDPQWLVLLGICTHLGCVPLANAGDYGGYYCPCHGSHYDAS

GRIRKGPAPLNLEVPAYTFQEEGLLVVG

>SG-PL_contig_675_1_+1_217_441

MLLSKTMHRQIRYTKFKSCYEILDVRVYIQQHRRLYCNPLQNVLYRLTVSLSVMQQFHFC

QLVLTKNTHENHLGN

>SG-PL_contig_676_1_-2_2_265

VQADYHSLIGITADLVDALEATVQGRPITPEYLQQLCSRLFSTHMRTSIDITRPGTAGEA

LRASVAPPMPSKAEVEAFPPLDYSKVKA

>SG-PL_contig_677_1_-3_99_236

MLLQRKTFLIKKVRQRTWPTSTEPTTHCKQMSIFLLVNFFLSLILS

>SG-PL_contig_678_1_-2_62_5236

MLDGTVNDRIFNWMKNFVRNFASQLDVDKGVYRVGAMTFTSRPSGQFQLNRYNFQDEVMG

ALDGLSGTGRGRVDTARAFDYVRDNMFTQPNGDRPLARNNIVMLTGNEKSLSTDRAVAAA

RRLKDLGTDIYTVGINLRNTDEIDAVSSKPLDYYQTLINSEGELEQIPGIYKYRIETATT

TTTTTTPKPTTTPRRVPVFSDTGYDLVFMLDGTVNDRIFNWMKNFVRNFASQLDVDKGVY

RVGAMTFTSRPSGQFQLNRYNFQDEVMGALDGLSGTGRGRVDTARAFDYVRDTMFTRRNG

DRPLARNNIVMLTGNEKSLSTDRTVAAARRLKDLGTDIYTVGINLRNTNEIDAVSSKPLD

YYQTLINSEGELEEIPGIYKYRIETATTTTTTTTPRPTTTPRRKPVFSETGTDLIFMLDG

TVNDQIFGWMKNFVRNFASLLDIDNGEYRVGAMTFTSRPSVQFHLNKHNFQDEVMGAIDE

FRGTRRGRVDTARAFDYVRDTMFTRRNGDRPRARNYVVMLTGNEKSTSTDRAISSARRLK

DLGTDIYTVGMNLRNTDEIDAVSSKPLEDFETLINSEPELEEVPGIYKYRMERAPPPFTR

STTTTTTTTTTTTPRPRRPTTPYTGECESTGDVTFVLDSSGSVGELNFDRLRNFTASAVR

DLEIDIGFFRIAVVTFSDSANIEFFLNTYSSKQEIIDAIRRIPYVYGYTHTAEALRRTRT

EVYNSVNGDRANVPNLVVIVTDGESNINPFETLPEANTLKLSGTSIITVAVGLSSNVELQ

GLTSPPIQDNLIFVDDYESLHSLSDQIVGPLCSDPNLCKEGPCLNEGVCLDGLRSYTCVC

QPGYYGVNCEKQCGPKTDVVFILDSSTSVGSDNFNRIKTYAEMLMRDMNPDSCDVNIGAM

KYSSAAMIQFRLGEYDTEEDLTRAINAIGYTRGRANMADALRVLRTQMFNGRNGDRPDAK

NVAYLLTDGGIEINRDITLSEADLIIDAGVRLIPLAVGLRQRNEIELIANAQGLPLLEID

SEAKLEQMSDQVLEAVMDTANQCSSAPCQNGGRCFNEALGFRCECAEGWTGDVCDKECSG

QADVVFVLDTSRYNSRKDVRSIKRFARDVVKRMAFRRGNFRVGVVDFGETARTRLTFAEG

NNKRTVKSVIGGIRPRKEDPKPANGIRRVEADIVGRLGDREAVPNYLVFVTTSMGGSSTL

QAINNLKRVGTRVIGVGINLSSSDERLLESAVSQPTRNMMFTVTDSVDLDNIAEDFVDFV

CEEKNMCAENPCRNGGYCTNGAKDYGCRCLPGFAGKNCERSCADIADVAFLLDMSGSVGF

DNFRRMKEYVYYMVENFSIGPDGTHVGLATFSTNARTQFYLNQFQDKRDLQNAISAISYE

YGNTNTAAGLKLLRSSMFSERRGNRLEVPDYAILITDGLSNVNAEQTIPEAELARKQGIH

VFAIGIGVSDNWELRAIASEPADNNAYVIQEYANLWNISDKLLDATCKDAGVCADSPCLN

GGTCIPGVGTYSCKCMQGFVGPHCEQDCQETKDIVFVLDSSTSVGPDNFELMLNYMQLLV

KDLTGAGQNHRFSLITYSTEVKTIFSFSRYNQPEQVVQAISTTRYTPGSTNTAGGLRQAR

QMFQPGFGERFTAKDVVILLTDGQSNINFYDTIPAATDLKSDGTRVIGIGIGLTNADELN

AIASGPSDVFQVATFDALKDIKTEIVAGSCRPMDGERKAPKELEG

>SG-PL_contig_679_1_+3_3_125

HISSMRWQSLEVLSSVWILYVTVFVCITLLLTEKLAINNFP

>SG-PL_contig_680_1_-2_116_328

MTPSDLMYQPSSFGRIRHQVREAQDRMDLHRQMLDRYLPEEGAESSDVAARVLRKMDMEK

RETIGASSGKT

>SG-PL_contig_681_1_-1_10_897

MADNEQKGMQLLQEADKKLKSSKGFFGSLMASFGGGSTKQEEAAELYVRAANSFKMAKKW

SMAGEAFCKAAELQLALGSRHEGATHYVDAGNCYRKGDANEAVRCMQKAIEIYTDMGRFT

IAAKHHITVAEIFESELLDREKAIANYEQAADYYKGEESNSSANKCLSKVAEYSAQLENY

EKAIEIYEQLGMYAMDNSLLKYSAKDHFFKACVCHMSVDLVNAQLSLQKYEDMFPAFSES

RECKLVKNLLKACEEENVDAFTEALREFDSISRLDKNLSAMLLKVKKQLNAEPDLC

>SG-PL_contig_682_1_+2_1448_1780

MELVGENPVHHHPSLPLPKPFHRSLVNWPKWCQCFDRYCIASGLALRSCAEPVNTYLYAM

GDCADDIPETTDVDEETVEYDDLKQVFATPQKCCGRTCKIQQTCSATRGIN

>SG-PL_contig_683_1_-1_118_966

MDEPALKATFTIHVIRRSFNLAVSNMPVETTRNLAGGKVVDDFETSVPMGTNRVAFIVCE

FTRKTKMTNRNVKMEVWSRQEVVDQMDFALDIGVKTIEFLEEYFQIDYPLPKFSMVGTTG

HASGWTWGLVMETDETFLLNDHTATLIDKMITAGNVANRIYHSWLGNLVMPASWNDYWLN

EALASFMGIMVFDHVYPDRRMMDFQVGELQKAMIDDGFVFSYPLSDDIYDQIADSKGAFV

LRMLHFAIGDDQFRKGMKKYLSTWSHSYAHNTDLWNTLEEDQP

>SG-PL_contig_684_1_-3_345_4319

MMKMKMMTVVASGVVLALSCFLLSVHAQESVNEVSRGDNAELGNLDQMQAQQAGSKAGPA

ITNGGYMAPGPPGPRGPPGPPGSPGQPGFQGQRGPNGEPGPAGHPGQRGFPGPPGPHGLD

GDEGMPGDPGPPGPVGAAGNAGPPGVPGMPGPKGHRGFSGRPGKEGEVGRPGEKGPGGPS

GPVGAVGPSGARGAPGERGRDGSSGSAGQPGNDGTPGNPGGPGPVGPPGPPGFPGAAGAK

GDRGPSGASGNTGPPGPAGSDGLPGAPGTVGQPGIRGEDGSSGNKGPDGPVGPAGPPGFP

GPQGPPGGVGPPGAPGGKGNNGRDGSNGAPGNPGPQGAPGASGERGLPGLPGPEGKRGPS

GPAGPGGAAGPTGEMGAQGLSGLPGRPGFPGPPGQDGARGESGERGSPGPDGSTGAMGPP

GPSGPRGSPGLPGEDGTSGEPGPAGNDGLDGNPGEQGPQGQPGPPGLTGAPGNRGESGSE

GRPGPQGPNGPPGARGERGPAGPEGPVGRPGNPGRQGEAGPSGPPGEAGFQGLPGPPGPA

GQNGNPGATGVPGPPGESGLEGIPGERGFPGEPGPSGQRGVPGDRGNTGPAGSPGRPGPS

GPPGARGAPGPPGDVGLGGENGVPGRVGARGPPGPSGEAGREGERGSPGEVGAPGPIGPP

GPPGTVAIKGDRGAPGLPGNPGPSGPSGRTGAPGAPGPAGNVGIDGLPGESGASGDAGAR

GARGDAGPRGYPGERGIDGEAGRPGTDGSKGERGEAGPAGPSGQPGAPGAVGPPGLNGPP

GPLGATGNQGQPGERGSPGETGQPGMPGETGNPGQPGSDGSPGERGSPGLGGPPGPPGIT

GPQGERGSSGFPGAAGEAGPPGPVGIPGEPGPRGENGKDGAPGLRGLPGQPGPAGFPGES

GRPGERGKDGPAGPTGRPGAKGSRGNPGTQGPPGLGGPVGAPGQPGPAGPTGERGERGER

GNSGPVGTVGPAGAPGAQGPQGAAGSTGSAGAKGDKGWPGMPGPGGPPGPPGPAGDNGLP

GPPGPPGSQGSPGARGPPGRNGEPGPQGRPGNHGQRGPSGENGPAGIPGPRGPPGPPGPP

GHSPVYSPAPSAGFKGPDPYMQYDQPINTAETYEGLNFADHALNRVRRVTGKATSPGMSC

RDIKERNPDFPSGEYWIDPNERSAVDAILVYCRMETMETCIFPSPSTFDRQRWTKTEGSG

QFFMEEMVGDREFFYKTDFSQLKFLQMLSEGARQRVTYHCFNSHARGSRLMLHTGEELDT

ELYKYKKSTRIATSDECAHDNQWHSAVFDIRTNKTDILPITDIRLFDVGRQNQQFGIELG

EVCFG

>SG-PL_contig_685_1_+3_5046_5624

MINGKISKLLITNKITGKYNQLNPLTPSAYSPASTSFPHRPKEPFEAFTLMTQHSQTQMF

YKNLIPHQPLIYNVKISSLAHYFPITCTQHNPMKNLVIEMMNIHVNYIQLEAQHFVVSLV

KSPKGKSVQNTKVMSACNYWVCHIYLLLLLITFRFTQQILFFPIIFNLKAQRNFIYIYKT

NYQLCGKYFFFFF

>SG-PL_contig_686_1_+2_1223_1606

MRSTDKGRQSWAFPGELMRLTRAQSCCGTALCNHLSSRRGQQDRMPPPPPHRTIQERNLF

PVQSWSCDYSARCQHEQVCSMCGSKDHAGSQHQVTPLPISAANNQHQKWPHLGHGAADNR

QQGRPQPL

>SG-PL_contig_687_1_-2_614_841

MNESWNSVSCSFACNLGMFYCVICVTYVVHGQRWKVFDHCLRNLMCYTKAPTKLESGDMC

MYLGFSFSVFLKLKTF

>SG-PL_contig_688_1_+2_755_1273

MTKHRAAIMYTNTALSKLFSVLPDDVAACDVMLDDTCDVMLGDDAKYGRLESSTVPAVDI

ANLQIGQRALNSSLGVQHESYKPVSSLRNNSLSNQFLHVLSCCTKQSAVLFACQFKFYSV

LSLDLEEYIQEKNVVVVVVIVLLLFLLLLVLFRLLHYPAIVIILILSSSTLLL

>SG-PL_contig_689_1_-2_23_280

MKFSVGVGNWDEIAQDRLRWRSAFFMGANTSEQNRIQHAGEARQRHKCPMPSGGGRFHNI

FLLWALSETVSITHWSFQPRKALSYK

>SG-PL_contig_690_1_-1_1_168

AIIQNPFNPACTNITAHQNCVKMDCNCKESNNNNKKVHPSYFPCILISQVTTPIMR

>SG-PL_contig_691_1_+1_1_324

THTHTPPLCHCPHTTHFSSKAPPHPPLLCHCPHTAHFSSNTPPHPPLLCHCPHTAHFGSN

TPPHPPKLRHHDSRAMKGHRIWDKFSQSYDDENGDDSDDAMVFHLGLR

>SG-PL_contig_692_1_+2_755_925

MKAGSRNILPAPKMGAGLNELRTICSKVSTCYQNNCCFLLLSKCYSLIPKVLYVQPF

>SG-PL_contig_693_1_-1_958_1131

MSHIQFLNCFRYIIGLETREGVFVLEAKMSHIQFLNCFRYIIGLETPSFYPLCGYIFE

>SG-PL_contig_694_1_+2_2_217

TDRHTHKLLTSRFGSKIIFTHNTQICFSDHTINLILLQPLGLPQPIHPLPPASCSAGWLA

LLVDFWTQDLMS

>SG-PL_contig_695_1_-2_158_421

MAGEALKDPHMQRFVEMETQRQRFQQLVHSLTEKCWDTCMGNPGHKLDSRTESCITNCVE

RFIDTTNYVMNRLESTPAPSGGGESSGW

>SG-PL_contig_696_1_-1_31_300

MLNLSCGLFFVSRTDNNGRVLFLGVSMCVSGVSADLFFSLSFDVVFDVFIPGRLKLLLSF

SDVGATQKSFVSYLSSISSEVSPIYIEDVI

>SG-PL_contig_697_1_-1_82_1002

MDKGQIHEVVLVGGSTRIPRIQKLLQEFMGGKELNKSIHPDEAVAYGAAVQAAVLSGDQS

ETIRDVLLVDVAPLSLGIETAGGVMTRLVERNTTIPTKTSQTFTTYADNQPAVTIQVYEG

ERVMTKDNNLLGRFELTGIPPAPRGIPQIDVSFDIDANGIMKVTAQDKSTGKSSNVTIKN

DRGRLSAEDIERMVNEAEKFKEADEKQRQRVESRNKLESYVFSVRQALGDAGDKLTTEDR

EVVSKACEDTLTWLDSNSLAEKDEYEHKLQELQKLCSPLMAKIHSAGQNCNSSSSCGSNG

PTVEEMD

>SG-PL_contig_698_1_+3_762_1148

MNDPSCTNIFVCICTVMWTEQVHLKKRTLTQFTGETKHKTHHHLHNFHSCSCFYDKYCTQ

GGRILKTTDINITLNTHTNTEIVIPRNANLLRKSLVRLKSRKKNKRCTTQQTDEMKLSAH

TIQYKRTCN

>SG-PL_contig_699_1_-1_25_225

MIGALRWAANTPIFMLRAGIELQKLQILSPQPYSLSQSTRFRNSVVHMGFEPTTCTLLSA

RRCNQLS

>SG-PL_contig_700_1_-3_279_467

MRVCIFIEFLWKGGWFGCNGCFCQDVLLKVETLGHFTYLFSICIVTFLHVFCLVIMPHPP

LSE

>SG-PL_contig_701_1_-1_172_504

MVEIFPSPICAGLLVHELPSYVYIHKTSDTHTLKILQAILSDRWLMETGKRPACAPTEDG

SMLHSVGQTTCIHTFEKIACNNTHTHTHKNKNKQQRSPLKGSPNLTRRTLL

>SG-PL_contig_702_1_-1_1765_1941

MRVCFLTLPFIIVNLFSPAAVVCLVSYKLSASSWGLPDPVLLNIVDIQSSCGHFIPRND

>SG-PL_contig_703_1_+2_2_184

KKKKKQNSQCNIYFNLNSKKLTDSLEGKKNPNKQKTNKQKTTTKNSKQAMVYESNTNLIF

F

>SG-PL_contig_704_1_-2_62_2860

MVVAGSKGSKINISQVIACVGQQNVEGKRIPFGFRYRTLPHFIKDDYGPESRGFVENSYL

AGLTPSEFYFHAMGGREGLIDTAVKTAETGYIQRRLIKAMESVMVKYDGTVRNQVEQLVQ

LRYGEDGLDGCHVEFQSIPTLKPSDHSFERQFRFDPSDERMMKRCLQEDVIKGLRGEHQF

LESLDAEWEQLCQDRQSVRQVFSTGDARIVLPCNLQRLIWNAQKVFHIDKRKPSDLDPIL

VVSGVRDLCERLTIVQGEDSISKQANENATLLMKCLIRSTLCAKRVAEDHHLSSKAFKWL

LGEIETRFQQAQVHPGEMVGALAAQSLGEPATQMTLNTFHYAGVSAKNVTLGVPRLKEII

NISKKPKTPSLTIHLVGQAARDAEKCKDVLCRLEHTTLRKVTANTAIYYDPDPMNTVIAE

DQEWVSVYYEMPDFDASRISAWLLRIELDRKRMTDKKLTMEQISEKITAGFGDDLNCIFN

DDNAEKLVLRIRIMNSDENKTQEEDEVLDKMEDDVFLRCIESNLLSDMSLQGIEAITKVY

MHLPQTDDKKRVVITEEGEFKAVGEWMLETDGSSLMKVLSERDVDPARTYTNDIVEVFAT

LGIEAVRKAIEREMVHVISFDGSYVNYRHLALLCDVMTAKGHLMAITRHGINRQETGALA

RCSFEETVDILMEASSHAEVDPMKGVSENIMLGQLAKIGTGCFDLLLDAESCKYGMEIPT

GPGIMAGAGTGMFFGTASSPTSALSPQMTPWNSGATPGYASAWSPGIISGMTPGAAGFSP

SAASESGFSPGYSPAWSPQPGSPSSPSSPYIPSPHGALSPSYSPASPSFMPSSPAVTPQS

PGYSPTSPSYSPSSPGYSPTSPKYSPTSPSYSPTSPSYSPTSPSYSPTSPSYSPTSPSYS

PTSPSYSPTSPSYSPTSPSYSPTSPSYSPTSPS

>SG-PL_contig_705_1_+3_3_209

GGVLFLLFKSQFPLTSLSSIQSDSSGRKPIKERQFHNNDTHGCEMKTRTRVVQENKFVSA

KKLTLLLHA

>SG-PL_contig_706_1_-3_48_803

MLAAVVLCMLMTSAGGYHTRQEQLNLRYISLRDLTWVDTEWVLAYRLVAGVKMSAYDYYM

DRTRNDDRLLARAFRPLPCFDPSVPSPDCHQHYHSALLNDWPSDFIDQVRVSLIADKKEV

AHILFNGTGSTSTSWFSADRVLSSSWTDLKTEPHNHFSIEGDHTPRITRRFFVFRNYGSC

PRDSGWLSVQDQQTEVCSWGTLSDSHPFPGILYSKLPTYVNFNTADVGRADFLTVTVKFL

TSTTYSGCLVQI

>SG-PL_contig_707_1_+2_2_247

CPITLRSYESEILETNFSQGQHDEGGAHLSLAALPSLTLNRVRYPFTAGWTGTTYYLTSI

SKTNPAQSISAIQIFEKISRIL

>SG-PL_contig_708_1_-3_3_83

VLFGAFVFFCFLFMWLAGVVTYHLSAE

>SG-PL_contig_709_1_+1_886_990

MYPSFLNGTIFMYWNFYLHLLLLTYITVRQPPRPP

>SG-PL_contig_710_1_+2_98_280

MTNEDGSTITEGQNSTPNLRSSRQCKVNINSFKQQLHAIIQPMHRVWSQRLVEITVVLGN

N

>SG-PL_contig_711_1_+2_164_265

MMNEDGSTITEGQNSTPNLRSSRQCKVNIMYKQF

>SG-PL_contig_712_1_+3_609_854

MDGLPLLSPVVWQKLSLRARYLFHLRIILFGRVSFQFTFSNVFWLCMCLCVLTSHLFEIY

CLILYKSCAVSLIEITAFLILL

>SG-PL_contig_713_1_-2_155_2107

MAKAPAIGIDLGTTYSCVGVFQHGKVEIIANDQGNRTTPSYVAFTDTERLIGDAAKNQVA

MNPENTIFDAKRLIGRRFDDPAVQSDKKHWPFDVNNESGKPKLKVDYKGESKTFFPEEIS

SMVLVKMKETAEAYLGKTVTDAVVTVPAYFNDSQRQATKDAGTISGLNVLRIINEPTAAA

IAYGLDKKHGVERNVLIFDLGGGTFDVSILTIEDGIFEVKSTAGDTHLGGEDFDNRMVNH

FIQEFKRKHKKDISDNKRAVRRLRTACERAKRTLSSSTQASIEIDSLFEGIDFYTSITRA

RFEELNADLFRGTLEPVEKAMRDAKLDKAAINEIVLVGGSTRIPKIQKLLQDFFNGKELC

KSINPDEAVAYGAAVQAAILHGDKSEEVQDLLLLDVAPLSMGIETAGGVMTALIKRNTTI

PTKQTQTFTTYSDNQPGVLIQVYEGERAMTRDNNLLGKFELTGIPPAPRGVPQIEVTFDI

DANGILNVSACDKSTGKENKITITNDKGRLSKEEIERMVNDAEKYKDDDDKQKNRIQAKN

SLESYAFHMKSTIEDEKIKDKIGEDDKKTIIDKCTEVIAWLDANQLAEKEEFDDKQKELE

KVCNPIITRLYQAAGGSAGMPNFDPSGAGAPGAGGAAPGGGSSGPTIEEVD

>SG-PL_contig_714_1_-3_36_488

MGRMHNPGKGISQSALPYRRSVPTWLKLSSDDVVDQIIKLAKKGLMPSQIGVILRDSHGV

AQVRHVTGNKILRVLKSKGLAPDLPEDLYCLIKKAVSIRKHLERNRKDKDAKFRLILVES

RIHRLARYYKKKRILPPVWKYESSTASALVA

>SG-PL_contig_715_1_-3_3_182

ELNSDVIRFNEVASQKFTLENILFVTFFIPFTPPLLVVLIGSVAACGRNRHPVDLPLLMY

>SG-PL_contig_716_1_-3_96_983

MSGLGDRLRSFCTFVWNGEEKTFLGRGGRSWVEIGVFYLIYYACLAGFFVGMLAVFYQTV

DEKRPKLTGDSSLLKANPGMGFQPMPDVEYTLVRFRVKPESYEPHVKSISKILKPYLEAE

LDHKQRGIDCTPDNLPQEGEACRVNVSALTQECNIDNNFGYGGSPKPCILLRLNKIFDWM

PEPYTEKNIKDSGASPELQERMLEDPNMIYVDCVGENPADRDNIGPIRYFPEQGFRHYYY

PFTNQPEYMSPLVFVQFTKPPKNIALMVECRALAPNIRYDRTEKEGGVHFELLVDP

>SG-PL_contig_717_1_+1_928_1176

MQMLLKSPNTNAYRHTHTKSIPLLSCSSTQVHHHSSLHPPISWLSQRMQNVYLQACMKYS

STFTFFEIPLEKCLKSNEGKKNT

>SG-PL_contig_718_1_+1_91_420

MFFYLTCFAMFSDALVLHWKPLKTQDYRNATFSIAATSIKLCTFLPISKFSHKHTHQFLP

LSHPPTHSHTHTRTSTYLLNVSQVKICGKRKNSIILFFLYNTFISAVSIL

>SG-PL_contig_719_1_-1_79_3447

MTQPITPDEMKEAEYSTTPSSKSGGSIADRLNQLKTSGEENWKKRVTKDKELTTPVTDVK

WREKSGLAAVRPASISDRLNKLEVSSEQWKERVEDTDAKTFTVANKITKALVSSESPLVA

KIRASSKKDLTRLDDSSNASSPVNSPSTPTKEFLPKVPLPKEIIADPEAVKNAEKKEKHK

VELRGVDSGEEEKGETVRVEVPRVDEAEIEQFFSTKEITEITDKVDMDVDDFDNIFIESN

EILQSVKKIRPTRRKKATSRNPLKTVSQFVEIQTEYIQVSTGAAEKHLRKMKTEGLKRDA

GFALEALAGLASKENFSKVELRKTEPSSLPASLAARRNPFTDLMLLHIKGRRNTQTRLVE

PSSKSMNAGDCYILVAPDKVINWVGEYSNIIEKAKAAEVASFIQQKRDLGYKSTAAVQIV

DQAKDHLGPGKHFWAALKGEKECQACGPDEEDELYESSITKTNMVYRLVDGALQPYEKYW

GAIPKHAMLDKKEVLVFDFGSEFYVWQGKEVSSSQRKQGMSLAQKLWEKGYDYSQCAVSP

MAPLRSEEDGGIPVQASQRPDWVIFGKVNQNMETILFREKFADWPDTSRLIGVKRDKTAE

VKYDFADIKAYDAKKMIPVNNLPVTLLLEGAHVGRGKKWQEDMQGFIKEQDILTLGTKVW

HVMEYEHYQMPENSYGQFHRGDTYVVRWQYMITNAGLKSLKGQAARHSLTGRERCAYFFW

QGQDSTINEKGASALMTVELDEERGPQVRVVQGKEPPCFLNLFEGSMIVHVGKREDACTN

TEGPWRLYCLRGDEAGELCLLEIPVSVANLRSCSSMVLLNTRTGITYIWHGVKSPKHKRQ

LAVTAVQNMKEKRPLELDLHEDAHIIVTEVEEGEEKAEIWLALDSKDRSLYHSLLTEPAR

YDYTPRLFHMTSVSMVFEVIEQMNPARLPGVTTPFPFFQSDLYKASQPALFLLDVGRCVY

LWQGWWPVGDEEMENVHTGSAAARYNVDRRCAMETTLNYCKERGSAGRTPEAFLVCAGVE

PLAFQNLFPFWEVDSTVHSLAILDGKREGYCEPVEDVYIKLTQTRYTLEELQERPLPDGV

DPLKLESYLADEEFEDVLEMKRDEFYALPTWRQGQIKQSVGLF

>SG-PL_contig_720_1_-1_361_666

MWCMQYLTAVYIPLSSAYHVTSPTLCMHNTTSSPVCCRQLVFFTFIPKPLSCMSCNVARK

GEWGLCLQQCCFLPFGLLHECLKGVGGSEYFLRNVKQKNGRL

>SG-PL_contig_721_1_+1_163_351

MHCKIKIDSIPCDGFCFTRLQQCPWSYLYYQTQSKCYLNTCIIRFKHKKCKVNICYHTQL

ICT

>SG-PL_contig_722_1_-1_52_567

MLEKMEEITEHLRALTSSMGELDRELKEALLMDGIKNDIAKRSSARNDDGVNCRLHLRED

ICQVWELMKDRADFIHSPSSAGKSSVDESVPNYYQLAADLKKKREVIHTLNDVFNNAGHI

LEREKKWMTCKLNLGFTCQTQEYSNIADYYDFLNSPESPGKKRSVPDMSDKS

>SG-PL_contig_723_1_-3_192_1268

MMGKDYYKILGVAKGATEEELKKAYRKMALKYHPDKNKSPGAEEKFKEIAEAYDVLSDPK

KREIFDKYGEEGLKGGVPGGGGSGGFSAGQPGSYHYTFTGDPHETFRMFFGDENPFGNFF

SMGGGGGGPRGGMFSFGGGSGGDDHMDIDDDPFAHFGRGPSTSFGMSRGQPSGRRKHQDA

AVVKDLQVSLEEVLNGTTKKLKITRKVLNADGRSTRVEDKILQIDVKPGWKSGTKITFPK

EGDQSPNNIPADVVFVIKDKPHPTFSRDGQDIRYKAKISLRDALCGTTISVPTLGGRKIP

LTLTEVIKPGSQRRIQGEGLPYAKQPTKKGDLIIEFDIRFPDKIPSGAKDILKDCIPSS

>SG-PL_contig_724_1_-2_422_640

MTIERKLHDRPGLQGPPIFWFPSLSFTSLSRSAHLVTQFPSKCDCQRGRIIQNGWTCNQP

SCEVYSGWKTRYS

>SG-PL_contig_725_1_-1_241_390

MGTVLVMLAPRKVEVSPSTLLYSFRVRGQPFPLPTVNKIFLASYLQLTKS

>SG-PL_contig_726_1_-1_307_663

MTVAVVKMKQLTAWKSGNFLPGMIFHHFRKVNGDDWWANVFQKYSSSLKLLPKVIKATLS

CFHGPLVESSFNLMGDIVDERSGRTSLETFNAFQTVKYGLKSTGKTSNTSGVRTSEEIL

>SG-PL_contig_727_1_-2_137_400

MDLSALNKFLVPVLFKMETPLLVREAIKQGDWAASVDLKDLLPHPSLSTTGTEVSPLCLE

EQGLPLFTKVVTELATLVRKSPFAFIVI

>SG-PL_contig_728_1_+1_1_234

GSIDLLDLSIDLWIYGNYGSMGSTYGSMVTTNLWIYLWIYDNYRSMNQSMDLLIFWICLS

SYGSMVTVDLWDLPIGQW

>SG-PL_contig_729_1_-3_78_1616

MSGYEGHVAKRQKTEDGRDRFDDPHKTKPSPVVHVRGLNEAAIEADLVEAVQNFGAVKDV

IMMSRRKQALIEFEDISGAKNCVEYAQSSAIYVAGQPTFFNYSTSQKIQRPGEGDDARPA

NHVLLFTILNPQYPITVDVMHTICSPYGQVQRIVIFKKNGVQAMIEFDNLESAKRAKQSL

NGADIYSGCCTLKIEFAKPTRLNVVKNDGESWDYTNPSLGGGKDPHQQHRGQPLLQEPRF

GGNGPADMRGGPPGGFGGPGGGFGGGFYDEFDSYGGGRGPFGGPPDRFGGPRSQYDQGMG

PQSGGQGCVVMVYGLNMQKMNCEKLFNLFCLYGNVVRIKFLKSKEGAAMVQLGDGISVER

AIQNLHNCFFFGSELQLSPSKQAFLQDVMNPHELPDGTPSFKDFMGSRNNRFTNPEAASK

NRIQSPAKILHFFNAPPNITEEELGEIFETCYGKRPIRTKQFPSKTERSSTGLLEFENKT

EAIEALIMTNHHPIPSTKSPFVFKLCFSAAPML

>SG-PL_contig_730_1_-3_36_251

MVSVLAKREFTWQNSSFNLILMFLSDDHISACGTVCLVSRTSPFPSDIILHRTSNMACKL

FSNRIVGESADM

>SG-PL_contig_731_1_-2_107_3592

MEAVLDAERALNETNGNGEKAVESLRRSRELKQEADDLDSLTRLTLTNSLMNAKDATEAL

ENQKVTIVTDLNDIRTQLDQLDGRSSFARAQDVYDRALVGLDKTNATEERIDRIIALLPG

DQTKLESIRPDRTSASLDIKNTASNLETVRTSQPEIMDLLKPLQVNATRLIGVGEKLDLS

VEALREKIELAREQANRIRVGLKFLGNTTVTARNPPKLEQAGSYSSLSMYFKTTESDALL

AYIGGQQRSENIQNDFLSLELIDGRVVFKFNLGSGPAVIASNRLVNDGQWHQAVAERIGK

SGTLTVRTSKEADDVTEGNSPSTFTVLELNPATTNFYLGGVPDDMTIADVVTRRPFIGSM

EDIKFDDIPVGLWNFEDAENNRVGENARDVMKEIVTNGMRFNGMGYAVLSRSKLGLRPNK

TDIQLQFQTFSESGLLVYMSDKKRDFMSIELRDGRVVFQYDLGGGALTLEGTDRLNNGEW

HSITISRSKQRGLIRVDNQIVGEGSSPGSLSELSTTEEIFVGGFDQILVPVGNVGNYGFD

GCVRNMQFGTIAWDLNENQKAKGVVRGCPAKVARIATYRKARSDGFIAVDLIEGIGSKFD

MTFKMKTNQSKAMMVYIADDTQENTFSVGVIQGKIVLKSMQDGGAVEMSSRVNTYSDNNW

HYVSIMKDGLLFEMSIDDYEMLQTMADTGTASLDTTTPIYFGGVPPEFRIADGTMPSKNH

FVGCIGDITVQRKFVNLASIPESKRMGVQLVECPLHTEDGEVVAPGETTTQPTVDGGTVA

VQTTMAPDQCALPLQPRIPDEAEVEAGIRFGSRLHSRVEFVRLPHGMRLKSQFSLSFKTS

GQNGLLFYVADRRHIDFLGLYMVDGELHFGFNCGSGAAKIMSGDTYNDGKWHKVEFGRVQ

RSGQLMVDGQLKGRAKSRGGTRSINVNAPYFFGGLSEAAKKAALGNVGDGWVSFPGCIRN

LTLKNVELKQPSQKVDIQTCSSAYELGSFFPASGGYLQLVDRFKVGRDLEINMEIRPRSQ

SGVLLAVHGRMDYLVLQIVEGQVIFITDNGAGAISTSFPLTAKNSLCDGKWHSIRANKNK

NVVTLLVDGVQSEPALGSARVSSADTNDPLYIGGVPDTSAKGVQTQENYVGCIRNLQLND

KPQPLSSGQPTGDIQLDSCPVS

>SG-PL_contig_732_1_+1_1_294

VLSTLLYGCETWHLKTSQEKKLDSFDLKCLRKILGIKWTDFVTNEEVRSRSKQNPVTSII

CKRRLSWLGHTARLPSSRPANQVLNWNPGDSEREEDQG

>SG-PL_contig_733_1_+1_562_789

MHTRTQTHCSNVLWHTHNVKSEQQRSLLLLPKHSKGWKGGLGADGEGRKGERAEALQSQK

GRLTIHKIYQKCDYII

>SG-PL_contig_734_1_-2_53_1729

MAFRRTNVRAHDMIHVPGGLNTGGQIVVRGRVLPHETQFVINLRCGEEDDSDIALHFNPR

NNDGDTVVRNSFQGGSWQNEERDIPRFPFDNGRKFTIRIMTFPDYYRILVNNWEFVRFDH

RIPPSEVRYVQLGDGAEYYEASVQNNCRAPFFGHFPGGLKIGQAVRVRGMLTDDSDGFAV

NFQCDSDGDNIGMHFNPRPNEEDVVLNSCEGGCWGDEQRGFENDFHFRQGEFFDIFFIAV

EGRFNVYVNEKYFTTFDYRCSYWDICHLVVKGDVELMDVELLDPLPDDFIKEIPQGMEKN

DLVVIKGFFFPEGNTFSVNLIKGSCCDDDIAFHFNPRRNEGEVVLNSKEGGDWEEEERHP

LPICMQELVPFELQILAKSHKFKVYSNDRKLCKFRARDDIEDVRAINVKGEAYIYEVKLL

RRLEEPFTDSVPGVLEPGNWVLMYGSPDDDVEGFAINLQCGPDKDSDDIAFHFNPRFNEG

ETVRNSREGGDWGGEERDQPYFPFEPEDRFELAFVMLSHGIRVFVNRRRYIDYNFRMNPD

RICHIYLSGGCDYFEPEFF

>SG-PL_contig_735_1_-1_97_681

MLSRSVRAETRSRAKEDIKRVITAIDRVRKWEKKWVTIGDTSMKIFKWVPVSSSEPALPR

SGVRVGPVKHTRGGKKGQGDKENVGKDFSSSSSNVYTEENTQASTGSFGSNSENLNEDSN

MSFPENSESNTMPEYSQDSNDADPEMRVALSMVREEQEALIKQEESPDSGPPVLEPETSL

DQDGPPAKKMKTGDS

>SG-PL_contig_736_1_-3_216_494

MFETGKTAQVAAEMRCYNLAILGISESRWTGSGQRRLATGELLLFSGHEEEDAPHTKGVA

LMLSKTAQGALIGWEAHGPRIVTASFRTKKRRI

>SG-PL_contig_737_1_+2_602_880

MLITLAPEKRNNQKKICTSLDFINRPSIHKQHSNTATTNNMNCTATSQKPPINYLYDNEI

AGTYYKCIKTEMAALSPNLIFYVCVCMQCLKGS

>SG-PL_contig_738_1_-1_142_375

MFCISGYNYYIYLLLLLLLLHPAIQYCMYYCQHSSCFISFLCRQYCTMNNNIYTFFSFFG

PILYLFSRQFFWSYPLSF

>SG-PL_contig_739_1_-2_185_862

MASNMILYWGSGSIPCWKPMLVLEEKGLHGYQSKLLSFSKKEHKGEDVTKLNPRGQLPTF

KDGDVVICESGAICDYLECKYGDQGTRLLPTDPALRGKVQQRVHESQNLQRIVIEGVVYY

QFRTKPEEVDKEHLDGKKKEAREELQRWEQYLADQGEGSYIVGKDFTMADVFVFPFLAFG

VRSSLNLAQFPHLEKYYNRLMERPSVKATMPPHWSEEPGKDFLKGV

>SG-PL_contig_740_1_+3_351_548

MYFCISFFSQGTLSNKTFQQDSLQQNSSTRLFATKLFNKTLCDKTFFNKTTTAYVIVLQT

MAFPEN

>SG-PL_contig_741_1_-2_335_541

MIKSNFNMQTDCSLVHPQTVKVYYKLIQIGQAVCVHLLSAFFSLQAKKCPRLQSKQLNSD

VIRFNEVAS

>SG-PL_contig_742_1_-3_507_785

MKHINVVNALFGIVYCRPTVLDLNTMTRWIAPFPGSGLLCFSVLSNLSVVLLPPTFACTF

SKTLHNKIIKIHMLFVVDSLLTLVLKLCMYVYS

>SG-PL_contig_743_1_-2_185_1696

MTHMQFPFMGTTSADDFYSGTKAALAGGTTTIMNLVCDQRGVSLLEAYDKWCGQAEEKAC

CDFGLQVALTWWSDQVYKEMEILTQEKGVNAFQMFMNYKDYFMLDDSELYAAFSRCKELG

ALPLVRAENGDIIALKSAELVSMGVTGPEGNEMCRPEEVEAEATKRAITIADQAGAPVLI

VPVMSKSSADVISAARRSGKVVFGEPIAASLGTDGTHQWNKCWRHAAGHVMSPPLRPDPS

TPTYLLDLLANNDLQLTGSDNNTYNADQKALGKDDFRLIPHGVNGIEDRMSILWEKGVCT

GKMDPCRFVAATSTNAAKIYNIYPKKGRIAIGSDADVVIWDPRQTRQISAQTHHQAVDFN

IFEGMECHGVPVFVLTNGHVVVDEGQLKVTQGLGRHVSTPCNSETVYGRVKARDLARQPV

AVDREPYTGPVVNIDEEAVMNKVEKPITTSNEPSIHSRPPTRGGGRNMQDSSFSLSGAQI

DDKMGVKASTRISNPPGGHSTALW

>SG-PL_contig_744_1_+1_589_834

MIHRTLCTFSTNTTGKLDVLGHDGHTLGMNSAEVGVLEQSHQVSFTGLLKCTNSSALETK

VSFEVLCNFTHQPLEWQLANQQ

>SG-PL_contig_745_1_-3_198_383

MPKIETEKERDLRKSSHLDQQYTNRSKLSPQNYQATVFNLSQRCFEVQINKDVTVYIYGD

SK

>SG-PL_contig_746_1_-3_3_182

LEDSSLAVWPSQLSRRLQMMLVTGFCLLLDLTSSFVTKSVHLMPKILRRHFRSNESSFFR

>SG-PL_contig_747_1_+2_635_850

MSFGAHFFHSNAAMTKYFLITTFGPDRQTRVCAGSAYAIWSIFHSNLICVAYWRKATISR

WPQCHDPTPPSK

>SG-PL_contig_748_1_+1_250_453

MKRKLETKHLFSLKHYYCVLLGLAVHIICTCANTGKKNCKHITHSALLAPVGAYGIYQSP

PVTIVLTS

>SG-PL_contig_749_1_-3_27_1457

MASWITLIALLAVTSAVCLAETEGDGNLFLSPLIAAGNINEAKSLSRVSWDFVNEVNETD

LESYSGYITVDEAYNSNMFFWFFPAQKNTSTAPVFLWLNGGPGFSSMIGALIENGPLKVT

KEGKVVESESSWTKAFSMLYVDNPVGVGFSFTANFSGIPDTIEGMIPHLHSLLVQFFTMF

PEYSQNDLYIGGVGFACQYITALGKYVHDYNHACPPPSLLLPLKGVYVGDGFCNAAVMYP

ELYENMYYAGLISEYTRRTFQTQMKRFIDKVACSGRVDEEDIWEINGIFLNITDGFDIDS

QLGREHSIYLPTKKLETLLTTPTARQALHVRQAYVNETFKSLNYSVWNHVKSLSWFRSAD

EEYVFLLENYKMLHYSGNLDLIVSVAATEAFLHQLQWTGADCYYKSIRYPWFWDDNSLRG

WFTHIGNFTRVVVRNAGFRVPYDQPDSAFRMMKNFVFDIPFVTTENTTLETEYMEGL

>SG-PL_contig_750_1_+1_190_363

MLGVISFSIPSQIYPLYQMFSVCVFILFQLCGCSCVCSKADLTCIVISLFVNNTVRKA

>SG-PL_contig_751_1_-2_182_439

MSRYFVAVLLTIAMVSSVAYGSTTAATQSAAGTATTAAVNSGTTTAAATGTTTVPSSTTG

SSAGNLVSSLPLSLVAFFLFLMTLAA

>SG-PL_contig_752_1_-1_904_1092

MEKIILYRLMHYCEKNNVLPVNQAGFGKGRSAVELLTKLTKLTTSLKQQFARRKCAVATF

FDI

>SG-PL_contig_753_1_-2_209_562

MQCCNRFSFYKTFKSSRRCGTKDVVELFLLDIDRHIKNIVENAVACPLCKDAIDDELHFV

LRYPALLDIHQKYISCIFCENPCFVRASILMANPNKGTMRNFYMYLCKALKIRSGMLN

>SG-PL_contig_754_1_-1_1_93

GSERVDETYLSSLRLPYHSWPHGFPPALESI

>SG-PL_contig_755_1_+3_99_365

MSSRHSAQNNATYWFKSTFIHKLPVKLYPPNHIPPLPPKKKQNNTKLYRNTNKTHINIAL

EKSKSMEDGKAPIVEDETKSALVLNKQNM

>SG-PL_contig_756_1_-1_295_2787

MQTVKMLQDKHRSNSSEASKSEDTIRQLRAELASIRDQHSQSVHDKSHFERRISTLEAEL

RDSKEQRRKALDEIGALQERVRESQISTHDHHEILKSELRRRDDTIQTLRRDVLNLQEKR

DAAVAESDHQLKIADTAEEQLRRSDEEVQRQETRLKTQDDEISHLRAQLEDADTEAKNSR

SKVIRLEGELQTVRNQYSEIQAQVSKLTNSLQRLQLASKDTRDLLTSEVAERTDALVRLR

TDSREAEEKLREAVANAEHQTELVRQLREELKQAKAKTQDTQLKLDEAEREKQQMQSRYT

AQAREIDRLQTDIQEHDLTTSQQETLYRSDLADREEEMSRLKADINVLHEKIAQSDGQLC

SQEETITRQQMQMRDLQDELARKETDLHKLEIDLVTTDEDRKRSEDQLSRQEISLQQLEH

DLKLIREQYKDAVEENGRLEARIQAFTINAQSEQECLNSEVKRREEAIKRLKLEQVSMQE

ANSRYEEKITQLQRETEQLKGQLRSAQSEIESKKAAAKNLESSVEEGKARLAAQLEDHRQ

LEDKMRGLNIELTSQQSQVNKLHHDLGNKDELIQQLRGDLDCLHRQQKETTAKVLVRDDN

IQKLNADMGKLRAMHQEKLHEIGKQSELIKKLQDTLTQCHYDLDLTRRRAEDDVFKRDEN

IKQLQEDLLESQTQYSACYNEVMRLEEELEKVKAENSHLAEVGSRASQTSSQLQEQLHQI

ELDLTISQEKHRTCQKEVTNRDQVILRLQTDLDTAHQKFTGCAEELNVKEKEVQRLNAKS

RDMQQEIRTQLQLLEQRDCKIAEDDKQLQRLHHEHEMTLQEPTSEDPHTHS

>SG-PL_contig_757_1_-2_20_376

MRNNRVNMIQTCEQYIFVYDALLEAIVAKDTTIAKSSFRATFEEMSQNENGVETLLEKQY

DTVQLLSPTIDRSECTAALSEENMGKNRFKNVLPANRSRPFLYTPVDDCNDYINAVFLS

>SG-PL_contig_758_1_-3_81_527

MYACARFVPAVRSVAAASGRGLLRPLGSTVVTSRQEVPQPCHSLTTFSSINFSSPSVSAL

LNQVRSFQTSTSWKDIDQAAKYIGAGAATVGVAGSGAGIGSVFGSLVIGYARNPSLKQQL

FSYAILGFALSEAMGLFCLMMAFLLLFAF

>SG-PL_contig_759_1_-2_227_1159

MMVEDRAAAKELDQWIEQLNNCTQLSENQVKTLCEKAKEILSKESNVQEVKCPVTVCGDV

HGQFHDLMELFRIGGKSPDTNYLFMGDYVDRGYYSVETVSLLVALKVRFRDRITILRGNH

ESRQITQVYGFYDECLRKYGNANVWKYFTDLFDFLPLTALVDGQIFCLHGGLSPSIDTLD

HIRALDRIQEVPHEGPMCDLLWSDPDDRGGWGISPRGAGYTFGQDISETFNHSNGLTLIS

RAHQLVMEGYNWCHDRNVVTIFSAPNYCYRCGNQAAIMELDDALKYSFLQFDPAPRRGEP

HVTRRTPDYFL

>SG-PL_contig_760_1_-2_485_709

MKFVSKSLLSCEGLTVKHVTVCRLVMKTAGLGVIFVLTFNISVPLATQSLLFLFMYYVHL

FVICRINRQQDIQCF

>SG-PL_contig_761_1_-2_95_265

MDCHGPLLFIGLFAFCFCSTCRNGEEKQTYLSLVKSSKECLYMEIRNSHNQCLHFST

>SG-PL_contig_762_1_-3_276_1115

MDGIKLTVAMLVVLSVCGLCQAQNWRWWRQQRKEQQLQQEQEHQKRLEQERLEREHQRGL

EQQWKERLERERLKQQKLEAERKRQELAQRRRERSRLKQRLERLRLEQQQLEARRQREEA

LQEEMEQLIGNYELEDLALMIGMEALGRILRPLREIPTEAPIDDCASRTTHVELEEGLMG

DFVYNVCGTGDDASIRIRFTFPGETTPVIDTTTPLQTTVATTGPPMRLCPIIPTTTPAAD

KETTTDNANVEDTTEIQDVEETTEAEELDEDTTTPFVMLF

>SG-PL_contig_763_1_+1_1_153

YYYILFIAQPTTMAISGLSPRKVHQSQEDNCTEHKTSSNVKTNSLQNNHNS

>SG-PL_contig_764_1_-1_202_450

MFYPCSAFPVLSNADWVKTETRGQSSACWQRVSCPVGFSVWANPMMYWKASVVFSYLVLI

RLLIRCCMSFACLLLIMICLFAY

>SG-PL_contig_765_1_-2_92_1207

MTATSVVGLLLFSSVLVVLVQGQAPAYRPYTRRSRGRTGGLIRVAAVQNGHSTEQNGQAE

NSTTPTPDDDPPQSWQELLGDTNIQDVRDFLPFLTRNGRPVLESDLFVPQETDDGKLVIT

GGGIMAQHDQCSPRNMSVRLDLDFSDPQVVYFPECTVMERCGGCTPSPYLSCVPQYQERI

VLKVIKGRYPYPGARNIEMERFASVEVARHRSCKLSCNLETTGCGPYKTYVPRRCACRCL

NFTQCTNNKVWNEDTCRCECPNQVPCCNPAAGPDRYCPLTFDHNHCECTLKQAIAGISNI

SQAEIEAWLASRTSQPAAGSSHFELPPDTTTPTSTTTAATTPAVICPDFFCPRQFRKVMS

ASGRCLCQSNRG

>SG-PL_contig_766_1_-3_1500_3992

MATTRWKYVLTVNVLTTLLTVIHSTRPSLAMGFRGMEENCILSFVVPRDKVKSSCDMEER

VTRRLNAMETKVTLYKQHVQELQEQLTKEREFHRQRLHELNERVVHNERNPADGLLEKRI

QGLEEEMRKLRMKAAQSPGLGEKPHARGAGLGMMPTGRSREEEEKAITNLVKNVVHSEVQ

RLFDNYTHYVGRYVRDQALAFNQIMNMVPRNDQSFHHHNLPPNWPDKLARNDIEPKSTSQ

PVHRDMVLLNELESIRSRLNNTGKRLADHMHNVFNNVNVTSPRPMSPTAASKVTDSSGSD

GVPSHHDSVKGNGLLDINDTSSGDTTDVASDRNITLAFTQLANNTIHQDAGSQKNLHVTS

SADFSSGSRDSASAKSRMSYNADLNKVPDPSGQRAGGRQNPDLPSSNGSGWEESMVHDQS

AGQWIQVLLRTEMQRILNQQVDEVLSLVSKHSSMVDSKLSDQERKFSQLEQRLAADIKHL

EQVVNGLKLQMTSVSQGLQTLYSRTDKAEELQDTVMELKKNMSMEGGTRLLATLLDEQGK

KVRKMERLTDIYKQSLEHYRNETKLEYRDLKERLNKETTVLRSLHTDLFHNVTEHAKQLT

EQLNTSANKQMEEIKQRVQVMEDNSLLLQIDLSSFERQLKSKTSKSERDMNKLNDDVGEL

KQKTRNLQKNFNSAMTVQKRLSQAVNTSTTKVQQLQMEVKLNVLDDWLPYNFDYMKTRTD

CFGEQYIRKSGFKTGRLVGVVLCTNKRYKIFLGQSPTDTFKNIGDDSGMGEDHCQFVNAR

GMSKVKVSPFRTSTETEPGYVRTHWGQEPRPELLNIIRPSPAWYECGVSIP

>SG-PL_contig_767_1_-2_110_265

MLLRFSQLFLDGSLPNLDQRLWTVEELLMHEFGMERAKVKITRTKKVPKLTF

>SG-PL_contig_768_1_-3_225_704

MTNPKGYRRGTRYMFSRQFRKKGVTHLSTYMKVYKRGDIVDIKGDGAIQKGMPFKFYHGK

TGRIFNVTQHAVGVVVNKRVGNRIIPKKINVRVEHIKHSNCRLDFLNRVKQNEEKKRQAK

EKGVQVQCKRVPKQPSEGHFVSTKYNKPQWLQPIPYEIIA

>SG-PL_contig_769_1_-1_280_1134

MMTKRTAILYTLLFLLFSLYQTSESFSFPDFSLGDLLKGSWVPEFIRNRRLTGSSTTTAC

NYPGVVAITLFGKTMLYTGIYSNGAIRIPDDALSLLKLANTNNPLTIQTDKGSSTAEVAK

GAVNAVSNKDGTSTIKLTKDFSFFTCPANNAILYDKATMRVDISTCKIVSYGRQVKDLGA

YDGVLRTSSLLENPVQSCCSYVLPNLLGKSPNLGHPNVASSPSSFSCAKSKEGICNGDIG

APIYCTDSLSSSEVVVGMVDSGTCSAGETFVSIDLTGGSLNGFSS

>SG-PL_contig_770_1_-1_1_189

RGCKQHESFAQKHDLFTQKHDLFTQKHDLFTQKHDLSTQKHDLSTQKHDLFAQKHSSVKN

KSP

>SG-PL_contig_771_1_+1_1_135

FVCSFLPLHGRQPCFPSPGAEDNHLRLLHLIIFYITCSSTETVFS

>SG-PL_contig_772_1_+1_2026_2481

MKERRYKKDPVNKISQLPLSKGECENSMLQIHELQACMQATPIHTIGSGLVQDCHCQEVF

VSHIDCASVGRISLPECVGQGFEHDTGLDEIIEGQRTPTEVVKLVQHCLHKSWGHVVSHL

LVSCLQLVAFNRTRAIVVKSLKRTPPLLNVVP

>SG-PL_contig_773_1_-2_473_694

MLICFVFVADSVDGIVCLCWLLSVKYLDFFLLQCIEVQANHIHYLFHTETCCILWPTFRD

LFIGRKQRQQKHIE

>SG-PL_contig_774_1_-3_447_728

MFLNFALEYADVLFTDASTPQYMISAHTHTCIRTHAYTHTHTNTHLGTHTRLYHLFCSLL

YFLALFIYSVQSFCLGSVDICSMCKLVAFKWDVT

>SG-PL_contig_775_1_-1_1_201

AHTHASTHTKHTHTHTHTHTHTHTHAQTHTHLHTHTHAHTHAHAHSRPYGSNTIKYTVKV

MYDYLRF

>SG-PL_contig_776_1_+3_3_164

NTSRGMFSRNGNEPKTLPRTLYIACLNNFNSKCNNTSNINLNHSIPFLHVYTCY

>SG-PL_contig_777_1_+2_20_340

MSKFTNFQRPFIPFFKTLRGQFPGILGLIWTKTISHTGRHIDRSTRMLSRDATLLFKDLC

SMTQECHIEHIAAELKSGAQTFCWTHEHHFGGQLKDLSASMTFSQPL

>SG-PL_contig_778_1_+2_179_304

MSKFTNFQRSFQRPFIPLSKTLHAKLSVLKTMAAGLIEAPGC

>SG-PL_contig_779_1_-1_301_603

MLALKSWSLPSQYPPYSLGVMVQLQVFRCMDSHLMYWTWHSVELYPRMGHVNTGLFSVDM

NLCESNTRGCPVSMSTCVGQTPGLSSVDMNLCGSTCVGQPV

>SG-PL_contig_780_1_+2_62_220

MSCGLDTSPGFVQTFHVKIHKLSKIFSRTIYPIFKDLARQLPGIWGVNLDQNN

>SG-PL_contig_781_1_+1_1_147

YRLFMSKFANFQRSFQGPFIPFSKTLSNNSQGILGVNLDQNNQSHWPPD

>SG-PL_contig_782_1_-3_6_239

MYREQEEKREISTDPALDNSDYINAPQTLQEIKDAINSVPNKKTSVRMDAISNEMLKKLP

KNVIFLHIQKMLELWQNV

>SG-PL_contig_783_1_-1_133_321

MGESFSLVKVLPFSQCKKELTIDVFTNYYRICAVGELSLCVEQMSHNHHLPGRLYILLRL

IDC

>SG-PL_contig_784_1_+1_181_459

MTTEDTNHFENTVHLHSGVSVISKKRTQKTRHFIHHLFSFSTVCNWQHLLEWRDMAEKWN

DTNQCICAYFRPIHHQFKTSMSTCICLSLSVQL

>SG-PL_contig_785_1_+3_3_188

SLHDALPICCFSWLKPVTFNLYKRFNFVLFLNLPCVDENILHTYTDNLSPPLLPLIVNTG

QA

>SG-PL_contig_786_1_+1_1_201

VYLCMIYVCCFPLLKPVTFNLYKRFNFVLLLYLPCIDDVCMRMLRGEGGEKGGGLSVYVC

RIFSSFL

>SG-PL_contig_787_1_-2_125_1456

MTGVRVVLLSLVVVGASAFYGPGDDVVELNPNNFHSKVIQSDELWVVEFYAPWCGHCQSL

TPEWKKAASALKGIIKVGAVNADEHGGLGSQYGVKGFPTIKFFGLDKFKPQDYQGQRTAS

GIVDFSISQAKSAASSRLSGKKSGGDSKSRSGGGGGGKPGDPKDVVELTDSNFDEEVLDY

DGLVLVEFFAPWCGHCKNLAPHWAQAATELKGKVKVCALDATVHTVMSNRYGVRGFPTIK

AFPAGKKDGEAEDYDGGRTASEIVAWALDKVAANIPPPDVYEITSAQVLKDNCEGHQLCI

VSVLPQIYDCQSECRNNYLKILRSLGEKFKKHQWGWLWAEAVAQPALEEALEVGGFGYPA

LAAVNTRKMKFSLLRGQFSEKGINEFLRDLSYGKGSTSPLKKAELPKIESRDPWDGKDAE

LPQEEDIDLSDVDLDDMDERKVEL

>SG-PL_contig_788_1_+3_3_152

LDVLGQVQVRWGLVHLPLGRGNCLLEVGLWPEGLGRGRACKGLGESLRRF

>SG-PL_contig_789_1_-1_16_561

MKTTACLWLLMLLLVAAVGAAIAASDAEGYSRCTKFSSNKDAIAKQIDGKCYYGFTTNTT

WSDARKLCYKEGMWLVRLETEAEQERVFTAFRFKMYWTSLRLLDKIWYWYASTLDKTPVN

IDLRDRLTPEPGKTFYGLARTQGGENEYQHSLLPARQKALHPLVCEDDPVSGEGDDTVPF

EA

>SG-PL_contig_790_1_+2_395_916

MSHLCNENTCSTNILDSLFSSLAEELCLDNDWLLWQVTFAQNFVVASSDNIDDGSLVLDF

LVFRPCLLRHQGPQAVHVDSRAVELVLGLVEVPHTHFSKISGMVFVVIDSVVVHATSITT

TTWMLPVFTDTAMTMADMASKLPGLLSFVQGHVCVFSLEKKKKHKNFYVYIFHI

>SG-PL_contig_791_1_-3_3_164

VSDQERKWFYHNLWLVTVIYSTIHFVLMCRHTLCACVRYCINRHFETVCEKSFP

>SG-PL_contig_792_1_-3_168_497

MADIPSGDPEKGKKVFVLKCAQCHTVEAGGKHKVGPNLHGLIGRKTGQASGFSYTDANKS

KGITWGKETLFTYLENPKKYIPGTKMVFAGLKKKNERADLIAYLESATKA

>SG-PL_contig_793_1_-1_1_216

DNRLVQHFSQEFQRKYKKDISNNARALRRLRTACERAKRTLSSSAEASVEIDSLYEGTDF

YTRITRARFEEL

>SG-PL_contig_794_1_-1_1_216

DNRLVQHFSQEFQRKYKKDISNNARALRRLRTACERAKRTLSSSAEASVEIDSLYEGTDF

YTRITRARFEEL

>SG-PL_contig_795_1_-3_87_599

MSSRKTKSKTTKKRAQRATSNVFAMFDQAQIQEFKEAFNMIDQNRDGFIDKEDLGDMLAS

LGKNPTETQLDEMMNAAPGPINFTMFLTMFGDKLNGTDPEDVIKNAFACFDEDGKGFIQE

DRLRELLTTMGDRFTDDEVDEMFRDAPIKRGNFDYLEFTRILKYGKKEADE

>SG-PL_contig_796_1_-3_42_1985

MLDFFTIFSKGGIVLWCFQGTSQLFTPSVNDLVKNVILQQRTGTDTYNHGSLTLKYKLDN

EFELVFVVGYQKILQLSYIDKFLSDIQLEFRNKYKDELLHGSVSGNFDFTSSFHSVLRQR

EEESKQEARAPKKMRAFEDSQKSKKTVASMIENKKEEKPKGGNKGKENKTPKAPSAAAPK

VGATVAKPAVASPAATNGELDEETLRKNREAFFARGKKKTEAPKSPESASNKKKGKSARV

WANEGRDVKNLDFSGAAGDTEGAQNGSMEDSKSMPTDAERAQVGQMSGVLKDIESESSEE

EVDEEQETPAQTASRGNTSSSGKSGRGFGMFSMFKNLVGSKTLTPEDLAPVLDKMKDHLI

AKNVAADIALKLCESVATKLEGKVLGTFGSITTTVKNALQESCIQILSPKRRVDILRDAM

ECKQQRRPYVITFCGVNGVGKSTNLAKVCFWLVENGFRVLIAACDTFRAGAVEQLRTHVR

KLNALHPPERHGGQEAVQLYEKGYGKDAAGIAMEAINYARDCHFNVCLVDTAGRMQDNEP

LMRALSKLIKVNQPDLVLFVGEALVGNEAVDQLVKFNQALADHSNMTNPRLIDGIILTKF

DTIDDKVGAAISMTYTTGQPIVFVGTGQTYTDIRNLNARAVVNALLKA

>SG-PL_contig_797_1_-2_593_955

MLVLSLQKKFSFFLFAFLFCLFFFLLFLFSFCSVFHIYFSFLFFSYFFCLFVLFLFCFVV

CFCFLSLFGYSYTLNAYVSTLFMTIYFILVSPKLGKDKCVLEVLYLGWFFVFFWFFFFHD

C

>SG-PL_contig_798_1_-3_60_362

MDTDQASGDQTNSCEENMATSSHPSSTNNSQNSATVDNEADSTEMEETSRRILQLAYVNN

YGSSDKMPEIHDDDKPLKLSNRTYISADWTPLAKRKFFDEK

>SG-PL_contig_799_1_+1_1579_1875

MDIKTLCRKRRIPILLKQFKATRDSLMLLFKNWHTQQIQDGKKNNTLNCTRPLYFEHLAM

YLQKCKTSERFKDYWLRLQTVQRKQAPVTKTCESLPYQP

>SG-PL_contig_800_1_-3_3_704

KIGGTCNTAGATATECVTGTTCSSEDPKTCKCDDPTVANADNTKCVSKIEGSCTADDGCI

DGTCTGESSKKCKCSATSIANYEKTKCLKFVMQSCEETVDCVTNGICSSDKKCECKTNYK

ATHDNGACKLAVGQDCTDDANACGVNAQCNAEKKCACKTNFVSVDSRCAKKVETACSANS

DCVMNAECKDKKCACSDGYSANNDKTCDKGGAQTVVASLLLMLAAFVTSRLISA

>SG-PL_contig_801_1_-3_180_329

MVKCKCLVSSRLRVRVDSGCSARWGEENKPDQLHRSLDKCGLRCYIHDHE

>SG-PL_contig_802_1_-1_1_420

VLFRLTQSFCFQRHVNHADDKIALNHIAATRTRNRETDLYLKSACIKKYLEIIAVILSYF

CFTEFNLFADKTCHSLGEFLRALRQTAYLYKFARKRKGRDEPCSRRDWRYLQSLDPHNQL

PSTWLTSPTLRKSPNLTRAS

>SG-PL_contig_803_1_-3_6_938

MMHSTPVIERRPEGDVFKVMSSDPLPATVTEIAEAQEGQGEKPKRKRERKPKGDKPPKIP

KEPKEKKPRTPKKEKEPKPPKPPKEPKAQKTPKTPKAQKTPKEPKEPKEKKPRGRKSKKV

KDDLNESVSSDLTVTPELGVEKVDSSDPTVPTTTTTEAGETKMDVKSDDQTTPKKKTPKE

RKPRTPKVKPLKKPSTKKRKPPASLFLKKRKRKRAGSDGSDIEMMVTPPGSPVGEDDSNK

RRSSRNTKRKKYLDDIDLNLSDDEHMLDPIEGAEGAVAMKTETEEDVLIVEKVLGGRMRK

RDAEVRLFYLT

>SG-PL_contig_804_1_+3_3_68

INCSRKKVAKQQNFQYRRCSIP

>SG-PL_contig_805_1_+2_1256_1420

MRTLYFVSENHCQRMLSQSKQLLRLYSGTCSGKLTGNGLCGFAPETNSRWERTEW

>SG-PL_contig_806_1_-2_200_472

MTVNFQKIKIVIFRKGGFLARNEVSWYGNEQIELVNRYKYLGLAFATGMSLSSMAGKLAS

GAKIRTSQILKCLGDLIALKQDFFFKSLKRR

>SG-PL_contig_807_1_+2_2_118

LSLSLLVLAISILLRHTLQIDRWQSHCLGDNGKRYIIWQ

>SG-PL_contig_808_1_+2_2_331

FFFDFTQIFYFPHFSHTAVNHFSTHSSHCIQLIFIFSFNLKILFKHCSRLKSKAEQCILF

FTTSQIYTFYTDGKNQWLPSANISTITQCNKTGPLLYDRRLVKLTIAGHF

>SG-PL_contig_809_1_+3_3_179

HLKVWTARFENPGQIARNRIFRDILIKFEKSGTNPDQPGQLAAMQTAVALTFSHHKAIQ

>SG-PL_contig_810_1_-2_686_985

MWSEYCQQIFLYNKPVHIMYNLTLFKSWNSDLKLVGHRMQVIHKSVILSTMLVPSLLFIL

IYLLLFFSFFFIFLGGGWYVTMLLSYAFLFLCRMSSYVTV

>SG-PL_contig_811_1_-2_164_700

MGLTISGLFARMFGKKQMRILMVGLDAAGKTTILYKLKLGEIVTTIPTIGFNVETVEYKN

ISFTVWDVGGQDKIRPLWRHYFQNTQGLIFVVDSNDRERIDEARDELHKMLQEDELRDAV

LLVFANKSDLPHSLHPNECTEKLGLNSLRNKKWFIQSACATQGTGLYEGLEWLSNELGQ

>SG-PL_contig_812_1_+3_1977_2222

MHTAKQYKLCNQYVNVSKQIARKTNHKMLARHSALHVHYAVITVLLNDITISWIRANAEL

ANNSTFRFHFHILRTNFIVKAD

>SG-PL_contig_813_1_-3_3_317

ITGNRVIDNIVDSSLYLLNNGNVTRTPNVPTHRPTTIDLTFISPSLAANAKWKANDGLLG

SDHIPITITLNYSIEMENAPQDRIPKFNCKQAGSANFKANLRRTR

>SG-PL_contig_814_1_-3_3_329

ITGNRVINNIVDSSLYLLNNGNVTRIPNVPTHRPTAIDLTFISPSLAANAKWKANDDLLG

SDHIPITITLNYSIEMENTPQDSTPKFNCKQAGSANFKANLRRTRTYLS

>SG-PL_contig_815_1_-1_229_6663

MPWEIAHQYKRQFSESYIHQEKKISSLHQNQSIDLGSFYRREDSTDYPSVSFDSEGQEPQ

EQQPKRTKVTFDLSYDETCEYKQYEMEEETVLEEDVSKPEKVFEPHDQIEHTHEEEVITS

IQESRQQVKEELTKDTGFESDYLDSQLTAAGATASHFESTLFQSAAEEVISSYSTAESSL

EQSLEMRPSSEAEEDRAPVKLQPTEPLMSVVSMESRLAQQELLQEMADEKDRSVSPSSEE

SPSAFTAQVELQSAIVEEPEPEPLTSAAQVPTTFPMAVHSLGKSLADNEVYESSETERDS

TEEKLSPIDEGPGVDDIEDDDAPAKSVPSEKRVTFQTDTLSTPRASEQSADEFKMSTSSS

EMSVEPTLLAASYELDTGRVCHVVSAFDISPDSVEKKLTPNAPAKSILSSPEDDVFETDA

SLLLHSEMADTPTKDDSEDLTVTKQLSSDFVPSPPTSTPRDGGIMHVHTASDLAEASSVL

EHIKTEVTEDEEQEAGHTLDESTVHEEDELSSPFEMMSPSELKETTYMAADTCFGQMVTS

AESAASGSSFADMLSSDLKEDIITNGPTEVEYVAEYDTEAVEGQMSATDAEVKEQLEEAM

VSETECEEQEEESGAVAAVWATEEEKAPSGAEATLSELQLPPGTDVNTMSTSEQTLYGLE

MPSEETVTMSTSLQRDLMSKSEVDQDSDQTQPPTEVTFKTDEQVSDQVETSAEMRASALF

ESDDMTSAEATPVGALPEQQQVVEGREDEEEEGAVEDFEEQALPHAEALIPEETEDEPYQ

LDPGSVYPGKRALEIQIPIDIDIKEDPQQYIEEEEGQETYEEEPVPERRLEDDFDLDDDN

LLGEAAGGEEEFMEQEAEEMEFMEKPEEELLRLKDSKPESEDEDEPQQETESVQATGLLG

VQGYDDLERPLSPTPDAFRQMFFGRDDDKETESPAQSGAEEDIEKTASHFVESILEDVKE

KVQSLSPEKPVEEKEYEEQMKEEDTEDAAVEEDRKADLEESEEDLPEAEQQKEDTEELDD

QVLPMAPTRSQQTKTTVLMKQVSEDIPGITITEHLSADTEETEDEEADETDFLTSLEKDV

PVQCVPEEEEEKKKKDDIDGSVFPASSEGDFHVQCIPEEEEEKKKEMDETDFPISSGKDL

PVQCVPEEEEEKQKEVDETDFPISSGKDLPVQCVPEEEEEKEVDETDFPISSGKDLPVQC

VPEEEEEKKKEVDETDFPMSSEKDFTVQCVPEEEEEKKKDDVDGSGFPMSFEKVFHVQCV

PDEEEEEKKKDDVDGREEELYFVETSVDAEMQFEMTFENEPQSKTDTKKESFLVASDPAS

AHTFASVAPLPLRDVFSSSKVEAGAGSDEEELELMEADIKMHDYAEPLSPEDHGDSSSVD

SFATVVAADAEEEDEEEDERDVEDRLAEIASMSSSMHSDILITQAEMEERFEETEDDEVK

FPQEWPKPKELAKEPDLSSSTESDRYDSKDNELSSSVESDRYAGKEELSSSVESDRYGSN

VEMSSSVESDRYEFVDRAALSVITELSEEEQFEMIEKDDLGNETGPESDPQEQFHCSPPE

GPSRSPGFGSTRYFTKPLHDKDDASVSSSLLEFERLEKQMGNSGSSSIDTSDKESIGGSY

DERKHHHYLVSSFDDKKFEYGSFDEKKLIRYKREMERDDISTTSSLAEFEKLERQIDQQG

SSSSVEEKFTVESKSSGGKSGSGSGSGSVAGSVSSLAEFEKLEAELATDSDDRRSSMDSS

LQRRSETSSLASLNEFERLERDMVANNELEAEAQKIVSYLEAGSLDSGRMFYYSSSVGYS

SSDLSEGEAAKQEHDKDSLEGRDAVEQDSLSEGEDKEELDSLDGDTSEMTEMTSSVIFAG

PAEPAASTAGTSHDLDADSLAGECIMQLSTDSLVLTQQMRTSDSSKFDTDSLQEQEDIMV

RSVDSLEQEKHSDKCDQDSLQEDIMHTSADSLELVQVEEQIPENLMLMSVESAHWSMGSS

GGTMCRSEESHTDSHDFMQVSAESIEELKSSHTEQTLKSQMSKSSEYVKEGASIEMMKIQ

MTKSSEYVKEGASIEVMHKTHATNPFLDDDGNVMESSGYSQEEEDEEEEKESPFMHWGPY

KEEKKVYTMAEWEAMKKQKLREKEERSSSPQTAEGEPGGDSIDRE

>SG-PL_contig_816_1_+1_88_405

MLSRAAQGALVGWEAHGPRIVTASFRTKKKRINMDVIQCYAPTNDSDEEDKEDFYSRLQT

IVQDRPRRNIIIVMGDFNAKIGSNNKGYEEVMGQQGLGEMNDNGKD

>SG-PL_contig_817_1_+3_561_1664

MTSPCYDIIAGLLEADLVQSDVTTDVKISGGAHGQAVTELQQVLVVDGNLEKVMLNQNQD

VKAGVGHKGEVGHFAFFSPPSPALAMVADNGVHAAVIGTETHVKVERYVTSFISIRLQVD

GEPGGIGEGHAADGDHAARAQAAWNAGQEWNRRVEDWLRTIAWLGWGYSSRGVIKGWRCE

GSPGRRPWRETVGWAAAASAMESDEVDGSFTLNHDELGSLLGQAVGEVPDVASVHFQLVH

LRLDDDADVKRSAFVELNPVLAVLIAPAVVLHVVQHHAVLVHPHVEVQGRVGLQLGPLAQ

IDAESMVRVVGDDTLHLYLHPIMYSSRDTPRVGHHHRGDCCVHVEEEPSLVLRVDFKSSR

LNAPCLAG

>SG-PL_contig_818_1_+2_1196_1810

MSAESTFGRQAAGLLGFFGSLLLGISRPTTTTSHMLDLRGRGLLCQLLEKVFTFHVGPVV

RVTLVLAAQLDLGWSTSEFVCQAVKRLIHLHCRLLLALVHLPGALCQFNHVILLSFVALF

QVVLVAHQTAHVLVQLAGLLQKLGGVHAVDVEGLDALGEDGTLLLFQLLLGLLQLLFAHH

TVQLTKPQRPPNQPFKRLALVTLHY

>SG-PL_contig_819_1_-1_82_276

MLVINNHFHAVNVYVLCFGTFLLTTHAHIQTRPLKEACSHMQRCMNERTRTHNTPTPLRE

IPTSV

>SG-PL_contig_820_1_+1_43_234

MLSRACYAQNVAVGRSAPLMGCPILALSAATITPVNCVFSHPLSSPHPPGSERFDHKVGS

NYHA

>SG-PL_contig_821_1_+3_75_209

MKNLSIIICDNLDIYKKNEYLDRKINCVVQSAQKVVMKYNIKMIK

>SG-PL_contig_822_1_+2_2_118

QPNKQRKIFRTIFGERVHEVKTPRLCDTFCISNTQILSV

>SG-PL_contig_823_1_-3_312_1226

MASSKPTVEVVLKNACRHLGEGPHWDDASQTLLFVDIQGQELHRFNPSTGQHDVKKFEDR

VTLVVPRAGGGYMIGLGRRLVEVHWDREEVKSTKEVDVGQLTNRFNDGKCDAKGRLWAGT

CGHAESGEGFIKGAGSLYKLDLDQSISKEHGGLTISNGLAWSGDSRTMYFVDSQPRNVYA

FDFDLEAGTISNQRILLTLPADTGLPPKAVPDGMTIDTDGKLWIASWDGAAVIKFDPQTG

KTLQKIDIPAGQTTSVCWGGKNLDELYVTSANMGKEELQKFPLAGSLFKVTGLGARGSPA

FVYQG

>SG-PL_contig_824_1_+3_381_632

MKFKKNRFKANNQILCNHIGHYIINLSSNFQINSSSFVFLTFLFPIILKHLVYRDSHGKK

LLAWSELTIMFLKANKTRLCQGCQ

>SG-PL_contig_825_1_-1_97_459

MPTSLYTSSLCDLLQDIPVFHGINQHELRQHMIIIKIIVLPLTKYTRKATFHDINQHQLF

IVIISIEYHNSRSDTTAEWTQCLCYKIKMFKKTKHVFPCKEKQVMKIPDLEFLMFGTRAL

L

>SG-PL_contig_826_1_+3_84_272

MPPHPTLFTRETKVFMNQNAIQYKNATRQNKKNLQQIHKLTISLRPATTNSDCELKVMVI

INL

>SG-PL_contig_827_1_-2_14_1525

MLDKSGYSHVRIVAADKKWHIAKDILKDSGLAEAVDYIGCHYPGTKSTSEAMKTGKQLWA

SEDYSTFNDEVGGGCWARILNKNYAVGYMTSTISWNLIASYYKDLPYFRDGLMTAVEPWS

GNYSVDTPIWLAAHTTQFTEIGWTYLRHGLGVGILEGGGSYVALVSPDGKDLTIIIETMS

HDHSRCIRPYLPPYSVVNQTINIKLAGSFGSIAQMNTWFSKLGFSKNPTTMFKSLSPVQF

KNGEAILSLGIDEVYTLTTLTQGQKGQYPPPPPSKPFPLPYTDNFDNYTLSAEPYNLAQQ

TGSFEIVTGDSAHGLVVRQMVLEMPTSWCQADIVDKAVNMFGNPDWTDIFVEVDTSVGNV

NNTDGVFIAARVQQGGCSSFAAAGVFFFVFPSTQKYIVTYDLARTRVLTKGTLQTQMNSW

NKLSLLVQSSKHVVTVQGAVNGNLIFNEEAALITGQGFAAIGTDSYGLADFDNLKITSSE

EGACIMNSYKNYDQWALLFRPEEH

>SG-PL_contig_828_1_-2_44_475

MKFNKMVTSDRGKNRKRHFNAPSHMRRKIMSSPLSKELRQKYNCRSMPIRKDDEVQVVRG

HFKGQQVGKVVQVYRKKFVVHIERIQREKANGATVYVGIHPSKTLIVKLKMDKDRKRILE

RKAMSRVKAMADRGKYTEETMETS

>SG-PL_contig_829_1_-1_226_1605

MEFEGVESSEAYEEDSKTDPLTTRLDLATSPSSCERHERECSLCDEPFRDRAHLFMHVQH

VHIQAASTPSSVRAQAAVSSSLSDNTFSLPKSPNQFHFYHTTSQNEETSGQSPQRQFHNQ

SGLSRSPSRGRYARLVSDFDDFASKPYACSLCFFRTSRLSELSVHVHNHLTGGSSTPPPE

TARFQPRESFYVERGSSPWKIDSEDLPSFKSVVVSDPPNSSAAVATCGNGTGFRTSCGQS

VKLQPSTVEHSKRRVLLYQETDDQTGKVVGKSSSVPIAASNSTTSSNTCHHTYTTVSADL

IVEPCPSGPSAQTVEVVEVVPNMTTTVLSDDDVIVSLTRDDLSDDDDEEVSSAGCADTAL

MDSGNNVQSDHTQGLELQNIGKTHHSSSHEKKSPPVKVGNESTHGSQKKPTSPTITGVSV

SPDSNIFENKVPHTTEDTFLEPLNPSPEKSTRKICRKARL

>SG-PL_contig_830_1_-3_645_1502

MEQPDFIGSLPSDKLFLGEDFAFTPSPPPLTTLLGFTGDPEPEVGNHGFVFADEFLKDLN

KELTLQVDTPPSSPTPTDQVAPEIVLEDTPIQIVFEDGFLQSPLSPDDVESVLSSASSQS

PPPPAVSPSTSTVDVSCLHTDSSSWSMASSELYKVVENTEKARATPYSRPHKSSKSNKSK

GRKQTASIDPNPSELELELMSKKDRKKLQNKNAAIRYRMKKKAESEVKRTEVEDLDNINM

QLREKCESLEREVKCMRDLINDIRKARGQPPLPLSQSALALKIEQL

>SG-PL_contig_831_1_+2_2_199

TLEQSKPLVLHIRSHGSNRLQPALPHAFLATPKQLTLVAVSCPVLVLGVLNLAARDVGRQ

GQGQPQ

>SG-PL_contig_832_1_-1_499_1023

MSPKCASVGNMADTPEAATQREFPNKRKDSRLPEAVEVEEIESEEQAADGVYDSRSTSLS

SDRPSSCDHSRTSSAHSQLGSEDTMDRGDRPVDGDGREKNKGPSVWQSFKKAGNEFNQDT

RRMFRYLGRKLHSRRGQTKVKKIHILPGEDDDRMWARRMAGSGTSRSSSSSDPST

>SG-PL_contig_833_1_+3_33_542

MIHLLLLGNTNSATSAACGLGVLTAHTQTPVVSQTAMGTDLLESLQILTQLVVQLVRQHL

SKTTVLDVLLTIEEPVGDLVLARIAHHGDDALHLLLTQFSSTLGDIHIGLFANNVAESST

NTLDGSDGEHNLDPAINVGVEYTQDVLKLLWNHERHDCSSPQRKRKEVSL

>SG-PL_contig_834_1_+3_159_362

MLVNKQLTHPARLLTSAPLSCLILLLAPQFTFEDKPSQVKGIVGESGRSSIPEWVGWGDR

KINSSFVG

>SG-PL_contig_835_1_+3_171_332

MLMATPDGNVESECMAVAYFANYDLEKYPFRSYIPKYFFFFYLLFSSSHTHAHS

>SG-PL_contig_836_1_-3_183_1337

MLAVFRRSASQGPRTWILGVLQGNGYATEAAFDTQPFKLHRLDQGPSTTLTVTRDQALKY

YTEMQTIRRLETTAANLYKSKAIRGFCHLYSGQEACAVGMEAAMTKDDAVITAYRAHGWT

YMRGVPLAGVLSELTGRASGCAKGKGGSMHMYTHNFYGGNGIVGAQVPLGAGIAFAMKYE

GKQNVCVTLYGDGAANQGQLFEAFNMSKLWDLPVIYVCENNGYGMGTSADRASACTDYYT

RGDFIPGIWVDSMDVISVKSATEFAKQYAISNGPILLECATYRYHGHSMSDPGTSYRTRE

EVQEVRQKRDPIGLWKERIITVGLATEEELKKIDVDIRKQVDEAAERAKMDPELPVEELY

NQIYIDPPSNMKVRGCDPTIMANSR

>SG-PL_contig_837_1_-2_26_124

MVIYVHTFVKSSLSICRFMMAIFISCIELSNMF

>SG-PL_contig_838_1_+1_289_390

MHSKTKALGTISILSLVSDTCKCKELEFTKYLHG

>SG-PL_contig_839_1_-3_60_308

MKCFAIDASMYYRLEMFGCFSYVIHVSPSSLCSGCEMCNSVAITFCDYLASRQFENQPDS

FLKELNKVQFKKNDYPGPFHFLF

>SG-PL_contig_840_1_-1_4_1503

MASSSTRGRRSPSPASKKQHNNTLREGDSFLEAHKKSTESPDLPGINERLGHLRPSRELL

EYYRKKIAEYDDEHDELVSKLEEYKVTYEEQHRLQWELRQREEEIVELQKALSDMQIYLF

QEREHVLRLYAENDRLKIRELEDKKKIQKLLSLGTATEGEVTYFIKEPPAKAIVEQSKPK

KSAGGSVRPSTAPRKKAVPVVDRRPSFEPLPEEKYKHDNEVLTLQVESLQAQLAEQTKLA

KEQLDALMEDRQVKEEEFETRRRRDEEKIRILTEKLHKTQDLLYDSTKDFLEQRYQGRAD

ERSWMGEKDKLLQQLDRCKSQLTAGSQTHRGNLNISVDAGDGRTASAEEIKSLEYQLQQS

QKMADMYREQVIQLEDELSRIREEGDVTREVFHDRSEKMTKRLQLMNSRYQDLERRRALE

VEGFQTDIKNLRQRLKDVERQLYKVTLGFAEDMDMKRPDDIDMQILRNVRSTVGRSKKIM

GELKNLKTKVYGLENDLKHL

>SG-PL_contig_841_1_-3_21_473

MPFPFPGIPCAIISSTEGNRDSMTQKRRNNGRSKKGRGHVKHVRCTNCARCVPKDKAIKK

FVIRNIVEAAAVRDISDASVYEVYALPKLYAKLLYCVSCAIHSKVVRNRSREARKDRTPP

IRFRPVRQEGTGPRAGGQGGGQGMGSGPGPR

>SG-PL_contig_842_1_-3_1737_2063

MFCSLFCAVFVLDVFALFCVLLYMCCCCFIWCFVRYHNVFSLFWSAFMFHRHPSARTWPI

LVLRSLFLTDIVANHLKLSRIFLTFLPGTILIKTNHCETGKYHPFFLQP

>SG-PL_contig_843_1_-3_3_224

SPRHLGVSSPPFSAGQSLLTPLQRVFISLDSTFLLLGRFLPVGPLVPNRSLSLTRSHSTP

AGLLRSHLPRQILT

>SG-PL_contig_844_1_-2_56_1261

MSLFEGLGYSNDKNVYIIDIGSAYTKCGLAGETGPRCIIPSTVKNSRTGIVSRIWNYSST

AELYENLKELLSVLIFRYLLMSPKDKRVVVVESLMCPTDFRECVARVLFRHFEVGCVLFA

PGHVLSLLTLGSTCGMVVDVGYTETLVVPVYEGIPILMAWQSIPVAGKAIHSRIKAQLLD

GGVVKTSQHQDQPVATVPDCLTEEILEDIKVRCCFVTEYSRGQAIQDVLLKGADASKLPT

PPPAVEYPLDGSRIVTVSGKIREHSCEALFELDDDERSVAACILDSLLQCPVDTRRELSE

NIVVVGGTAMAAGFYHRLKRELDALAAKPRYKETLGVRTFKYLKAPARENYTAWLGGAMV

GALETLPSKSLSRDAYLQRGRLPDWCCLDQETWTDDRGVQRR

>SG-PL_contig_845_1_+1_475_744

MYINEKVLAGACQLPSRQHSDLSLPSSFRYVEVGRVRYQSVFLQTLICTQRQQITLSPPR

PVHQLPAGLHWYNSFTPSPCPPASCRITLV

>SG-PL_contig_846_1_-2_41_1621

MAKYILEKTDGASYSFSEALPALSYFQSRQVRPTAWKCQMEIGPNLKIPINGYIKVKEFK

LKQSWKKVYAKDPDMIIKNERTYHMDDEEKTEVDRDDIVEGYCYGSDRVPMSAEDKESMK

YHSEKCLKVLGFTKAENVKRSHYLGDSCLSITAEKGDEAAAVALSAMINALYETNCVAIA

RRVYAARNAPRIGCLLPQIKAGYECLYWLELPFAEDIRTYTFGSLPTKEDTVANKKYTPT

EEQLAAVDNLITTMDLTTAVEDEDGEKCEAFKPKLTFNPYFQRVYQCLQHRALHPDDPIP

ELSPLIARSLKRPQEVEAACRAGAEKISKAFKLERVAKRKEEKTGEAMFKEKDAGEGSEA

KRPKMDDDLAGGIEGLTKAKVTEVGTVTPIEDFRDLISRKDKDLFDEAFKQMEERITQIV

IDSFGQQFYGKAMDCLKALRQESVKKSEPGNFNDFIQDFKDTLISKGRRDFWDKIVQEKQ

GLISKVECEESDFSKEEADTFISGEVKKEETSAPQEEETADDLLADL

>SG-PL_contig_847_1_-1_37_1311

MTGIWRLLTGSAVVMMAVIYSAPVAKAAMNTIQLKVVVDRLANNKWRRILEPEVNGDITD

YVYSVFKDVNSIFKTLRSSRIKLNVQVVDVDVTTNDITRQTDSGVVLRNLIRWLGTRQGD

NWDHTMLLTGLDLIVDDDKNKTGQTYLEDMCHETRSVSVVEALLGSTVLNAAHELAHSLG

AKHDNGTCLNGYIMTDSTNTQFPSLNNFKFSSCSIQEIKEGLKKLSKNGNCLLGGNPNAK

QESAKTVGDLLTPDQMCKLEFGSSSYFSRRFYSGDQSESYDRMCLAIKCRNGRLTSRPDT

MKSTSARASDGFICGNRKRCSLGECIPSGSTEVLDNCPQGDAPVANIEGQTCGQVKAARF

AECYAKYFQKACCGTCQGEYIGQPGCEYGDKSYSCVKYRNFAGLCDQQPETCCGTCWKRS

NPTWR

>SG-PL_contig_848_1_-3_819_1115

MMLTTRPHTHTHTHKIKLHKIICKRNDYAPKYTYLNYTEHQVKIQLEPINSGHITNAVIQ

SCHFGSGRRKREKRKTNKNKKSPQDLTNSESLSSCQNKI

>SG-PL_contig_849_1_-1_34_279

MDSRCLFLSRLKVAVVVVLSWEPVSSLVVRGGQILIQITLYFKQFWSQKHLQMPNFVCRK

MCDLLFPGHRNTYPMPNFVCRW

>SG-PL_contig_850_1_+2_1523_1735

MSSPSTIGVHNDLASCQTSIPLRSTNDEFARRLQMENSAIVQVFGGDNGRDNLLHEVFAE

SFQSDFFIVLS

>SG-PL_contig_851_1_-1_7_1002

MVLVCGEISSKANVDYQSVIRGAVQQIGYDDSSKGFDYKTCNVLIALEEQSADIAQGVHL

ERDDDNIGAGDQGLMFGYATDETEECMPLTVVLSHQLNAKIAELRRSGELPWARPDTKTQ

VTVAYKYVKGAAIPTRVHTVVISAQHDEKVTLEQLRKELMEKVIKAVIPSKYLDSRTVYH

LQPSGKFIIGGPQGDAGLTGRKIIVDTYGGWGAHGGGAFSGKDFSKVDRSAAYASRWVAK

SLVKAGLCKRVLVQVSYAIGIAEPLSITVFSYGTSKKSESELLQIVKNNFDLRPGCIVKE

LKLRQPVYQKTACYGHFGRNEFTWEIPKKLKF

>SG-PL_contig_852_1_+2_926_1261

MYVTNGKHTLTEIRRRLTASPLSNQRSTSASKPKLIKPEKPFQVTMFTKHGRLVMVTILY

TQGTEPGNILQPTAYNTHMATAHHFNLLTNLFIQLPKKEWGLEIGKEREARQ

>SG-PL_contig_853_1_-3_183_347

MQAEWKASCSTSTPYRPHMHKKTFETDVATHQHILYIHSVMYLLHFFIFIISSVF

>SG-PL_contig_854_1_-1_94_378

MGGGEKFQVPDWRSYKIEGIPELEQTQRMLAARGLKDPWLRNEVWRYTKENYGGQWKNAA

VSLGRGLKWAVAAMAITIVVDQFVSKKKDGHGDHH

>SG-PL_contig_855_1_-3_3_290

DYGEAKTIAEFSRYNASGTSLDWALNEKLTESPDSKFIALGMELRPTQRDESGYLRGFTG

FVLPPSEIIPTGREYMAGILAAASNYKNVTDKAELR

>SG-PL_contig_856_1_-3_72_941

METQKPVIFLEVNIHAREWITNAANLFMMDWILTNYRLSEGAAKMLLDNYTVVVVPVANP

DGYEYSRAHPDNRMWRKNRRDFKFCTGVDLNRNFNIMWRNDIEEYLRILKKMKSKGRKKA

RKNQKVECPQQYPGPSPNSESETQALISIFQHYQENVILYFLVHSYAKVVLVPWAHSSIT

ANNIHQKMGNIVASAMDYGKAKTVSEAVGYNASGTSLDWVLNEKLTESLDSKFIALALEL

RPTRSDESGDSRGLTGFVLPPSDIIPIGREYMAGILAAARNYKNVTDEDE

>SG-PL_contig_857_1_-1_58_2181

MMEMEQLIRQRSGPELPPDLDLIEVLWRQDVDLGVGKEVFDPNLRKELEQERELELRKEQ

EKRKEAEKQEREKQQQTVQWLSENFVIDGETGEYMPISGGGNNNFTSSGPANLYSQPQQP

VTLEEALSFVGDLQIGQPCGQQPQQQQQQGVGTVPPSNHLVTGAEGFHYMEQNLIGEAHS

SNYVQPNQTQKERIEQSWSEVYQHMFADNLTGFTNSSLVNMPPMINGGALQPELGSHIFQ

QQQQQQQQQQQYFDSQMDLLLSECKNYSDASSMNETRRVMQENTTELESDLAGLYGDSNL

QDVPLLDNLPLPSDQSLESLQSSALEGFMTYLHEPGTNNSSYLTGDLNLTELLRTDTFSN

NNTADFNLTDSLLTDLVPDDDFDLPMSQSLGDTASDSAISMGSGSPHQDINETMEMSPFE

GLEGATGGSDYDSGNYGHHRSQMHSGSNAEEENYQYSYSSYDSPASISNCSTGSNDTEHS

SGHSSIAAHSHHFHHNHTYATQPGHTPREVKKYAKKDGPRQKGPHSRDNKRAEDLKLPFS

FNRIVESPVEEFTELITKTPLTEAQISLMRDIRRRGKNKVAAQNCRKRKLEVLTTLEDEN

YETVRARDKLEQERQALNIQMRQEKERYNFVYNQVFSSLRDEEGRPYNPQEYCLQQASDG

NILLVRKSTGSATSCVSSASQPSSSSKNNHNDPEQDKDSSQQKKKRRE

>SG-PL_contig_858_1_+3_1506_1715

MLTWEIRKSPPFTHQAQYPRGFKPRSSTETQRERERERERERETEKKKESTMSAVQPGLT

TISQTTLTTV

>SG-PL_contig_859_1_+3_159_425

MPGQSVFGLEALGIFQRVINQAKSSGLATTKLGAEAEAEDDIRGGFVQLRQLISDLCLWH

SGPSWMEYIHNHLLPVEQTVGQELSSTNS

>SG-PL_contig_860_1_-2_149_433

MLCDLFFLLGFHVYIKWHLKRRLKLRKLYNFYVQLLELCAWDDVYLFKMSYSFDFILNNM

SRLLLFKLFFKVHLCFLFLRQGAHSFCTCNHFILQ

>SG-PL_contig_861_1_+3_597_842

MFFKSTLTYTIRHYLALTLLTISLFKSRLTFSHLTKFSYFSMRSNEQQNTTKENKIQSLT

IHISQAVTRTRDYTLISVFRAP

>SG-PL_contig_862_1_-3_3_218

SCCENASKWGGTRTVRNCTPFAIRPTYPSQCGHVAPICAVGILKMTVWVLSGNFCAVAAV

LSMRRERHVGVC

>SG-PL_contig_863_1_+3_3_170

LYLKATSGTSLTLIQYIQLLYKLKHRHNFCLCTQLFVQSHVRLRLFITHKKMTKTA

>SG-PL_contig_864_1_-2_788_1015

MAETKSEPAFYLLQLQVFFSLSSSVSLSHCVRLLLPSLSFLYLLSVYVVCITEVMKFSLI

SLLLHIFSVSFNRKKK

>SG-PL_contig_865_1_+1_1_168

SSYHSLALLHTHTHTHTHREREREREREREIDGCIHRYRKTKRQAHTHRQTDRLSD

>SG-PL_contig_866_1_-3_33_806

MADTKKVPKVPESILKKRKARVAAKVKAVKIAELNKKKQQEKKALIFRRAAKYAREYKAR

ESDLLRMKRQARNGRQESGQNNKRQAECCNYYVEAEPRLAFVLRIRGINGVHPRPRKVLQ

LFRLRQINNGVFVKLNKATLNMLKIAEPYITWGVPNLKTVKELVYKRGYGKVNRQRIPLT

DNAIIEKQLGSKDILCMEDLIHEVLTVGPNFKSASNFLWPFKLNTPNGGWRRKYNHFNDG

GDFGFRDDKINALLRRMI

>SG-PL_contig_867_1_-2_122_271

MYYSFFFSMLSSTSTSPVSRCELAYSLTQLHYSDYCFLCFPGNIISTNKE

>SG-PL_contig_868_1_-2_23_235

MNFTVIIVILCVCVCVCVCVCLFLCVDPFPSHSICCVRTYGIVQVPRYYFAKAKTEPEAS

ITASSTSSSVP

>SG-PL_contig_869_1_-2_734_928

MHQMVRISYEACEQKCNKHECQDSSVVHKVYELKCNIKSCIWIVFSLAGYKVISLIRKQI

VCLIY

>SG-PL_contig_870_1_+2_575_805

MHPITNKDKGDPKNHGSCFKSAHKPYNNYKKLSHTVFDVTSYMLQFFVYISVLHTLTIYL

KKQTQTNKKTNSQKISS

>SG-PL_contig_871_1_-3_33_233

MRGIISDLSWVFQPKILCSRFCNHIEFNKNRVCNAGSNVLSLASQPAVAWFVCLFFYSQC

MFLALLH

>SG-PL_contig_872_1_+2_2609_2836

MSSTTYYVNILGVKVAIYIIVQTKDKRKSRFVAQGPASSTRPSLSSTVTPLQSNLAESAT

RRTASSLAMSSASSWA

>SG-PL_contig_873_1_+2_1067_1300

MRPSGQDTPYRKAQRLLCKGSKNINKRTRLSKDEGRRRFTQCQPTTDGEDSRTVASKRLF

VRWRYLFLGHFMLTTSKS

>SG-PL_contig_874_1_+2_2_172

VCVCVYMTDCMNPLYETEKTETFKYFIFKLHCTCPYPVSVNFTVYSRKIILIISNNI

>SG-PL_contig_875_1_+1_1_210

APFFFGVGGGGWGWGGGGGGGGGEWQDLDTPHSGILIFRQFYCIKMDTCLQVKKNNLDVR

NTEYWGKNIH

>SG-PL_contig_876_1_-1_1696_2130

MFPLPLCLLSPHPPSWSKCVCDNESDDLHLCLSSDSNPGHTRDSSRPLVIKQGTDESKEI

RGRSRAGLLPVPGILSHCTVSAPVGTYVTACKTQLKCSTFNFLGFLSLYYIDQQNNLLNP

SPFLFFPLSLMTTRTFIHTHTCTSV

>SG-PL_contig_877_1_-3_165_341

MQASELYSLQALKTTINVMLNLYLFNRERRGELVVLLDTLEEHAVMMDVDYKFSQVRQN

>SG-PL_contig_878_1_+1_205_393

MRVQCKLEKHHQMRTARENIGTAIGTASYVFIPLGIYIHTYIKCQNVCIFSDLIPSRQFL

TES

>SG-PL_contig_879_1_+2_2_328

RGKRRERERERERERENYSSLCASIFGSSHRLRLTTRDPAKGLQTSDTLFFKDSSIRSHW

ICLTAGPFCSIDDLSCCACWVFSCFHKTPWYFSCHAPLTVKHLLIDCSH

>SG-PL_contig_880_1_-2_2_385

GKNACPTVQHLKLQDTLITDKQAVANLLASTIEKSSSSSNRPPAFLKVKTQKEKSPCDFS

SSHIEDYNNLFSLDELKSALQTCNDSAAGLDNIHYSLLKHLPDSCLEIVLQVYNYVWSTG

FFPPCWRK

>SG-PL_contig_881_1_+2_1100_1585

MKVSSLWRQALSPPSGLPIAHHLDKLFIVDVSLRVLHPLYKLIDLIVTQLLPKGCENVSQ

LSTSDETILLLVKDAETFNKVLQWGNTAVLADGLKDGEEGFKGDACIWILRVSTLANLLF

CGVLVESPQYVPQLTGRNLVVTLCVKQAESFLNLVQVFFCVF

>SG-PL_contig_882_1_+2_146_247

MHDAIHHRLAGKPKLDSQSKLEKPVKLKTRRITK

>SG-PL_contig_883_1_+2_2_256

LHYILFSSICHKGFCFVCVVTDQAAWSVQKVFLFACLSVFLFVCSFLPSQGRQPCFPSPG

AEGYHLQLLYLKMHPWQSLLFFYNY

>SG-PL_contig_884_1_+1_64_381

MFYSAVSVAKGSVLFVLQTRQLLSTMSFLFACLSVFLFVCSFLPWQGRQLYFPFPGAEDC

HLQLLYLKMQSLAVISFSLLLKNIKKKTKCNRRRKLLETKEQAAAG

>SG-PL_contig_885_1_-2_83_835

METSIYALLPEEEKKEEKQKRYTSKFRQTVKEEYASGRGNYRTMGQAKVPVPTTESFLKK

REKEPVLPRKEKFTYPDDDQRRPPVPDRQDQPLMGLKTNKNFITTNAVENITSVPKKPEK

KFADTRNGDTHNLIPSGLEPVYVHRKDFGETPVYLNKRKEEMKRAQEEYDAYVAEHFRRG

ALRCLTVDERRCIIDGLKANWEDIHEQYQGLSVVTDTTPKKNRKERMEAQMKQLEKDIEM

FEKFSTIYIGN

>SG-PL_contig_886_1_-2_74_1180

MWLKICILALCAVYSAQTVQIQPFRSVFTQAEGSTLQVNCTSQNSEAWVADPPGAMPTDA

VRAQADSGDNISTLMLENIQKSNEGTFRCKDKTTGDEVSFELKVFSVMTQNATFEYKVDA

MLHCGVTGDSNVYKYTWRKGSTPVADIVVEEKQAYIQHENGSLEIVNPSRSFAGLYTCMA

VFEDSTGTIRTIDIDVPYRATPKVLPFDKSKNLVQDENLVVECKVLGYPKPYITWLKGNQ

EIKVDSGSRYKLLAANTYDGMSIDNARLEIDMVDFDDAAEYKCMARSGQWPSFNSSQIIL

VRVKDKLAALWPFLGIVAEVVILCTIIFIYEKKRNKDAMLDEDDDQDGAAPEKKSENVRH

RGNTNNPRA

>SG-PL_contig_887_1_-2_113_1018

MSKKLGFAENFALSGAAAIISKTAAAPIERIKLLVQNQDEMIKAGRLSEPYKGVIDCTVR

TFRTEGILPFWRGNLANCLRYFPTQALNFAFKDKVKALFKTSPNESYVLKFSKNIFSGGV

AGALSLCFVYSLDYARTRLANDAKAAGKGVGERQFNGLIDVYTKTLKSDGIVGLYRGFVI

SCVGIVVYRGFYFGLYDSLKPLMLGEDANVFVSFCLGYAVTVSSGLLSYPIDTIRRRMMM

TSGQAVKYKGSMDCAMQIIKNEGFMSMMKGAGANILRGVAGAGVLAGFDSFKAAYIEWRI

KQ

>SG-PL_contig_888_1_-3_141_365

MPHSGVIRPRVEGGEANARRPWVVDGLPPPPSPPLPLPPQWPALASAGPLWAQVPSILWE

GPPGRIFQNRCFTKS

>SG-PL_contig_889_1_+3_567_800

MIFLIIHSFETSICENLNTNTSISIRNHCFWHPSVSIPTAFADITPKRYTPCQFWALGTL

TVITIRCSGKLQPKALRE

>SG-PL_contig_890_1_-2_65_2980

MDPVLDPLLGRNTIKKGRAIKMGDKEVEYNPSFKFILQTKLANPHYQPEMQAQTTLINFT

VTRDGLEDQLLGAVVVKERPDLEKLKSDLTRQQNEFKITLKELEDNLLARLSAAEGNFLG

DYELVENLETTKRTAAEIEVKAKEAKNTEVEINLARESYRPAAARASLLYFIMNDLHKIN

PMYQFSLKAFSVVFDKAIDRAEPSEEVKQRVINLIDCITYSVFVYTTRGLFEKDKLIFIT

QMTLQVLIMNQEVNAVELDFLLRFPATPNVTSPVDFMNNNSWGGIKALSNLEEFRNLDRD

IEGSAKRWKKFVESECPEKEKFPQEWKNKTSLQKLCMMRALRPDRMSYAVSGFVEEKLGS

KYTEGRAMEFAKSYEESGPNTPVFFILSPGVDPLKDVEALGKKLGFTEDNRNFHNISLGQ

GQEIVAEQAMELAAKEGHWVVLQNIHLVAKWLAKLEKKIEEYSEEGHENYRLFVSAEPAG

SPDAHIIPQGILESAIKITNEPPTGMFANLHKALDNFNQETLEKCARETEYKSILFALCY

FHAVVSERRKFGPQGWNRVYPYNTGDLVISSDVLYNYLESNTKVPWEDLRYLFGEIMYGG

HITDDWDRNLCRTYLEVYMHPDMLDGELYLAPGFPIPPNSDYKGYHAYIDECLPPESPYL

YGLHPNAEIEFLTTLSENLFRTVFEMQPRDTGAGSGGGVSREEKVKSLLDEIVEKLPEEF

NMTEIMAKVPVEERTPYVVVAFQECERMNMLLSEIRRTLKELDLGLKGELTITADMEALS

NSLFLDMVPENWAKRAYPSLFTLGLWYADLLQRVKELETWTSDFQLPAAVWLAGFFNPQS

FLTAIMQQMARKNEWPLDRMVLQCDVTKKTREDMAGPPREGAYVHGLYMEGARWDTQTGM

MAEARLKELAPTMPVIFIKAIPVDRQDLKNMYACPVYKTKTRGPTFVWTFNLKTREKPSK

WVLGGVAILLQV

>SG-PL_contig_891_1_-3_3_89

RGDLDWSKCISEDFGVFKTGSHQIVAQIL

>SG-PL_contig_892_1_+2_2_157

THTHKRMRICTHQTTTNQMRPILRQPTLLNPTSVHESSFVPWMHSTIHCITI

>SG-PL_contig_893_1_+2_44_205

MFLFFFLKDLKVLSHAFTHNLKYKHSGCLNHLLIQRKLSRYIKYDTVYILYNYM

>SG-PL_contig_894_1_+1_1_105

QYMKPKPNSMKPKPNSQSCSQPVQPPWHDSEPDNQ

>SG-PL_contig_895_1_-2_47_283

MVETSASLWARTMRVVSIYSPLLLYPPQPRTCSQLCGLEAAHTKLSSSVIHIHANLCSQI

GRQELYLYANQVSSVCENV

>SG-PL_contig_896_1_-2_974_1240

MYINIQHQFINTKKLKALLSREEAGEIYINYETLMQRVLLPAVTLTFISHYRTVVSDTSH

KPNSQGSDPQLDRLYSKWTFCFWLASIHT

>SG-PL_contig_897_1_-1_1_165

SSALGVSVGVTSPLAHGPSGLPCEHRKDCFSRGSHPGAKAFEAARGRLMVLSLDT

>SG-PL_contig_898_1_-3_819_1205

MAFTVQMYLSVMHVRKKLQQSDSAALRPARYQDRAVASQATWVGVWLKKTCMGPLFHPAV

NGYLILYTAVEGRAAREGGGICPGKASLRVMRCNVSVSYCPNVIYVYIHASTYIMLSESE

LNICLSRID

>SG-PL_contig_899_1_+1_817_1056

MRCVQDGMLSEGSHERHRVQRLAYVILVVKFPLLQAVSTSFLVMTYYRSTVRVPSQPFKC

MNSFAEMIKRQIFCLRVNAL

>SG-PL_contig_900_1_+3_759_1082

MTGNATSSHLSFKPATTNTDDRNITSSIPLLTVNCSILSRTLLFNNYSSFNFSPNCSFLI

FYLPNIPCSSISPNQCIVQDPLSITVHHQVFLSSKRSLLNPFPNLFTI

>SG-PL_contig_901_1_+3_3_488

LVLRDQVVHVGLSLSELHLIHTLTSVPVEEGLATEHGSELLRDPLEQLLDGGGVAHEGGG

HLQAAGRDVTDSGLDVVGDPFNEVAAVLVLHVQHLLVHLLHGHATTEHGSHGEVAAVTRI

AGSHHVLGVEHLLGQLGDGEGAVLLRAAGGKGSEARHEEVET

>SG-PL_contig_902_1_+1_34_486

MIRENENIKGININNVEHKISQYADDTEFSLAGDRVSFETCIEVLQFFGSKSGLFMNAGK

TSAVWLGSRKNSEVRYMQHLGIEWNPSKFKILGIWFTNDLRNCGEINYSEKLAEIKVLFK

IWMKRQITPLGRVAILKSLILSKLIYLWILL

>SG-PL_contig_903_1_-1_43_735

MVCGGFSCSKNALIVLNILYILVSFILIGVAAYGRAAAVVTSLTLVGSLIACGVFLFIIA

LVGLIGAAKHHQVLLFFYMIILFLLFLLQFALACACLAVNGEQKDALAEEGWRLASNATR

RDVQRQFNCCGFKDPNLGFNESLGHPDCITVAPCCDNSEAPCCKSGPSNQTEGAACPCDT

CLNKMRPVIYNAFTATGGVGLFFSFTEVIGVWVTIRYRNQKDPSAKSAAFL

>SG-PL_contig_904_1_-1_1714_1902

MKHCMFVDVAGVPDDNDHILLMVSHVSSLVCYRVLGQDFRFPPPPKNFALLLSKSLEYNQ

AMK

>SG-PL_contig_905_1_-1_736_924

MNHERHNHTHILTVRSERRWERKCNIKFARTYVLFPPPPLFSTTGLSLYKQQVYLHADQD

NVC

>SG-PL_contig_906_1_-2_2_193

VCVCVKMSSTHFQSETYDLCYIIITLVNLTVNGCIELTYVKMLKEKKNFNMVESSSSCCC

CLCL

>SG-PL_contig_907_1_-3_84_542

MAHSGVKVDDQCIDEYNQMKLDKSRQRRCIIYKISDDKSKIEIEKVIYKNLEKDQKTEYE

DIIRDLPDSEGRYIVWDMKVPAKSCGDSETVFRDDLVFMAKCFDDCKVTVKMLHASSEDA

LRRKLEGVSARLDCKDSGDMCYDHMICKCKHKK

>SG-PL_contig_908_1_+3_3_308

AHKKRLGVGGGGGLCEGLLEKRKWNGPGRQKKADNSEEKGQKQREQLLKKRKLLAQNFQK

VGILFMLKEPPASPWGIFLEKKIKKKRMGEKKGGAMHILQHL

>SG-PL_contig_909_1_-1_160_525

MGTLLISKIREEYPDRIMNTFSVVPSPKVSDTVVEPYNATLSVHQLVENTDETYCIDNEA

LYDICFRTLKLTTPTYGDLNHLVSATMSGVTTCLRFPGQLNADLRKLAVNMVPFPRLHFF

MP

>SG-PL_contig_910_1_-3_3_308

TFSVVPSPKVSDTVVEPYNATLSVHQLVENTDETYCIDNEALYDICFRTLRLTTPTYGDL

NHLVSMTMSGVTTCLRFPGQLNADLRKLAVNMVPFPRLHFFM

>SG-PL_contig_911_1_-1_130_1509

MKGKGKAAQQQQEKDCPLQFPDLTIVNHEEECKRYTAILGRQPTETISIEELEPVQTDLE

VLLASVGRRLKLLKTEIQILQNWQDKKDAKTPIAKGKVQQSETPSAKRGKGASSTPEERP

SKKFKDSGGKAALPPSSVASPIARSKSKLPQVKGVDGESVHSPAELPRIVKNECVDRFWQ

AMESYCRPITDEDIKYLDEQIHKVEDEESTYFKIPPLGRHYRDVWAEEDVLEEMTEGNKI

NDKKRHSNHTNHAFSNATTNGANGTTEASLLLKKAESSLEESPYGPITQRIVAALLEENI

MTPMDDIITDLGENAEEAPAISPRALAKQLNITNSVALERRLQRELEEQGILEPEEEVED

DPSDEILTEIKQAQVNLKPLLQWKSTHLKDLMKKAKEQQSRQQIWKKFEECDAKLHEEYR

KLNVARQKKKPSLKRERESVKKMLKEWWSVYQMLDDTYNQ

>SG-PL_contig_912_1_+1_211_459

MEDFSWGREGKQATALSTEKETTKLSAEEDRKKKPTKSTVRKKVFIAHPEKQDFHLITDK

ITLGNLLCKITNSCIILKRNPFR

>SG-PL_contig_913_1_-2_161_388

MSNHRVNTSENILDEQSSVIKQANENTIKLLLQLLPLLSLQLSQRKERNIVCVCVCVCVC

VFLSSLSLSHIHIHTR

>SG-PL_contig_914_1_+3_3_158

TTQFSHLPVDIFSPFSKMPMQLQIKAITVKLHTKCAHLYHHCVDRTGVQFRN

>SG-PL_contig_915_1_+3_3_155

TNLNLPSIFCDGKLKISHKRYQPSQNLKKDPNTVKQKRLSVLLNKKYRRLW

>SG-PL_contig_916_1_-2_2_937

EESAIQDQHLHKRPPKLDSSSLQLSQKLKGVPDVNRLDFRKQQISKQLEDLKSNNEISKL

SVHQKNSKNDQQDVLHKQPWKINVKRSVLPDNEYHPHAKLPVDKEKNVFTNQGKDQQHPQ

KVNDMHMKQGELPVIHANVADQKNQAPPSLVKETQPQNQNAAARMNNRNIVNKPRYPKLK

GIVPAELKGGPQVETHNSQEQKQEVPVAQNFKYVRDGASAVRLQDKSQNAPLPVLVPDMP

QHDGQSGRKLLGSYVASGGSRRKLLVEKEMPGGCLAHKTGVAGGALVMYLRYLIDPGPLD

LFAPHDFFPDDF

>SG-PL_contig_917_1_+3_318_509

MHNIKYASQRSCKHCQVFGGLWKHKNTQNARREIQYNYDTPTKPVVDYKIAQQIKEKDQD

GILR

>SG-PL_contig_918_1_+1_1_162

SDEIPSKGYKVIAVFRQKCRIRNFLGKCTAGKFTSVQQIQVFLTRIHSSTCFCN

>SG-PL_contig_919_1_+2_17_280

MKVKGEKGRDRAECVSFIGIAPWLPFQLDMKQAPETYFQFNYETCDQNMHTKHTHLHNQN

SVCCQQQKKINRQENETKMDIWILCYTT

>SG-PL_contig_920_1_-3_54_353

MVVQKKSKKAAENINARLALVMKSGKYFLGYKQTLKTLRQGKAKLVIIANNTPPLRKSEI

EYYAMLAKTGVHHYNGNNIELGTACGKYFRVCTLSITDPG

>SG-PL_contig_921_1_-3_171_905

MPSTSVQRGHVGSVVLLLALLGISSAEITKPNIVFVLSDDQGFHDVGYHNSWIRTPNMDS

LASQGVKLENYYVQPMCTPTRSQFMSGRYQIHTGLQHKVIFAKQPNALPLDTPTLASTLK

DAGYSTHIVGKWHLGFYKPEYLPTNRGFDSFYGFLLGSGNHYTHTSLGHTDLHDNTDDVL

EEKGHYSTHLFTEKAIDIVRSHDSSKGPLFLYLAYQSVHSPLQVPSRYKEQYQEIGDDKR

RTYAG

>SG-PL_contig_922_1_-1_82_492

MPSTSVLRGHVGSGVLLLALLGISSAEISKPNIVFVFADDQGFHDVGYHNSWIRTPNIDS

LADQGVKLENYYVQPSCTPTRSQFMSGRYQIHTGLQHRIIQPKQPNGLPLDTPTLASTLK

DAGYSTHIVGKWHLGFY

>SG-PL_contig_923_1_-3_222_494

MYTVFFISLGDTGGSFSINSIPTFWKFCASNFLFSIALFVSALFLSFTVICFFPAFLVHS

TFFFPASLHTIFSPHPLPVYDVLSVLFCSGN

>SG-PL_contig_924_1_-2_2_262

LLILLNSFPPSGNSVLEISSFLLLSLFLPFFFPLLSSAFFLPSWSILLSFFFFRQAFTQF

SLSHAPPPFPSMQFMMYYLCCFALVTR

>SG-PL_contig_925_1_-1_658_825

MLNSFAIRTVCYEASESKNHDLCCLHVLLLQSCSTQTTYFMSFFHGRNYTEIAYTS

>SG-PL_contig_926_1_-1_118_294

MFCCLCSAADYIPCCVKNSCQECCGEKHTTHTSRCVGVAGKWDSSSSGLVIPSISIINR

>SG-PL_contig_927_1_-3_333_545

MTKKQNNKKTGPVENHYGCSNPVGHDMLLCGLNRKTAHQVPSSVWNTRSCLLHESNFEYT

ASTVVCLSGPD

>SG-PL_contig_928_1_+3_753_1031

MIHTVNRNKMVFGHYTITVVSSLVSVEWCVIFCGLCVLLCLLSLSLSVLQCFLFVRFCVC

FVCLFCSLSQTLTHTHTHTHSLTYTHILTHSLA

>SG-PL_contig_929_1_-3_1050_2705

MFQNSEKSPSEMGDKTENKSPQKEQKQRTGKGFIYEVVLLDGEHVNFELDRNAEGRELFD

KVCQHLDLIEKDYFGLQFVDERISNHLKCWVSPDKKIAKQKKGGPWLFDFALKFYPPDPT

QLRESLTRWLVTLQTRRDILSGKLPATFLTQAMLGSYCVQADVGDYDPNEHRGVEYIRDT

PFAPNQTTDLLEKIVELHRQHKGQTPDEAEINFLENAKKLSMYGVDLHAAQDVNNVDIML

GVCASGLQVYREKLRISRFVWPKILKISYKRKHFYIKVRPSDNSPSENTISFKLASHRLA

KRLWKICVEHHAFFRLREAEQSQNAAMFPRLGSKFRYSGRTLYQTRQNSQILDRPDPYFE

RSQPARNTFAAPSNRSHSVDELSNRPDYWKDDMDVDRKRLPSDGKNRKDWKDDPNATERA

DLENMRPLGSEEGGALEPGANRSMDGKDRLDRMPGEDDATLNREGGEGTMGLTPAELQKK

AKEDEKERKKREKEEEKRRKKEEEDRKKAEKAAKKGKGKGDSAEDRKDLMVGGDNSEKAP

GRTPGSYGDQEG

>SG-PL_contig_930_1_+2_2_196

RERIMRERERERERERERERERVDFYIFSMILVETFNRVDFYIFSMILVETFKRVDYLHL

FNDRG

>SG-PL_contig_931_1_-2_74_415

MAQADPRIKKIKIQTGVVKRITKEKQMYEKEATEIEGKIEKLKAGGADEYVLRKQQEVLQ

ESKQMVPDTQKRLKNAYEELKVLVEAETDLTETEEYKAAQMALDDAKVVVEACE

>SG-PL_contig_932_1_+2_2_145

RVHTHTHTHTHTQRTATLFLDVWIYTLYAKPHHALKRSVDKYMNAGHC

>SG-PL_contig_933_1_-3_3048_3407

MRGGRLWSVTWYIDYKIARPDHKGEVGFLLLCVQLERHRYVEVTGVFLMICCYPCDDELQ

SANLFLITNHGIKKACFVTLPIRIIRRSHLQRELALYDLFRDFANQNNQKVSLAKRVGSL

>SG-PL_contig_934_1_+3_3_596

SSDLGVLADAVCSLVPCFVQLLFVEFLYNRLSFISGRRRHDGAADLTDGLPFPVLLLQFV

TLHHLPSIQSDRQLGLGAIDGGVLEGLSQLEGVGLCGHVGLEVHRGRVGVATHAEFHSVG

QLPVGLDPLLAEPNSGTKLFKTSGGSQIDLPFSDERFNHLDYCFYACWEERKDGILNLWL

AVCLTETVQKLLVQERPP

>SG-PL_contig_935_1_+3_609_764

MKPAPTVCHNFFLVILLLTIQSRLCVCVCVCVCVCVCVCTYFCTLDLSFNKK

>SG-PL_contig_936_1_-2_41_1396

MLRLNSRESSEKESCDQLQRSQPFGSKRKSNKDLKRRVLGGEEEFTKSLLTSKEKPKQSK

RRTAASLNAAAKKPVWEDEEDPDNLQKFEKLMGTPKWAKINEPGADDDDDSDLEELARST

GTLLATSSSLATNYINLKQCPDGNADSRNTGKLTSVRFHKTSRVLMTASTDKSIRLFQVD

GKRNAKLQNVFFDNYPVMSAEFSTWREEIITGSKHKCFYVFDMMAEKAILIPKIKALEET

NMSRLRVTPDGRFIVFLGSFGNIHLLSAKSKEWIHTLHMNGGVADVSFSSDGATMFGTGD

DGRIYVFDLTTRQCVHHFIDDGCVHGTSIALSGGYLACGSNTGVVNVYDKDTCMTSRSPQ

PLKAIPNLQTSCTTAVMNPNTEILALASSLTEKAVKLVHLPSFTVFANFPDQNDAKIRIP

WTMDFSPNGGYFSIGNQKGHALLYRVKHYGNY

>SG-PL_contig_937_1_+1_727_933

MFVDLQVGYNLSFILVCGLISKSLPVKMADRQDKDTDKKLPAWNQQKITTLGGLRKSMFA

GSYDRESLL

>SG-PL_contig_938_1_-1_52_672

MAMGAVRHIADIRADDASREGAGIIGLQAGSNKGASQAGMSMGAVRHIADIRADDASKEG

AGIIGLQAGSNKGASQSGMSMGAVRHIADIRADDASKEGAGIIGLQAGSNKGATQAGMSM

GAVRHIADIRADDASQASAGVIGLQAGSNKGATQAGMAMGAQRHIADIKVDDMSQASKAS

INLQYGSNVGANQSGMSYGGRRDIMGN

>SG-PL_contig_939_1_-1_151_285

MLFSTSTSPVSRCKLAYSLTQLHYSDFSFLCFPGNIISTNKEVHE

>SG-PL_contig_940_1_-1_37_522

MSGIALARLAEERKSWRKDHPFGFVAKPTKNPDGSLNLYNWECKIPGKKGTMWEEGLFSL

RMLFKEDYPSTPPKCKFEPPLFHPNVYPSGTVCLSLLDEEKDWRPAVTIKQILLGIQELL

NECNVKDPAQAEAYTIYCQNSAEYERRVRAQAIKFRDPSIAH

>SG-PL_contig_941_1_-1_262_5217

MLLEDYSKYKYLSHGHIPVAGVDDAQEFRSLVESMTIMGFSPDDQAALMRVISAELLMGN

MQFKQERNSDQATLPDNTVAQKACHLLGVPVTALTQAFLKPKIKVGRDFVTKAQTKAQVE

FAVEAISKALYERMFKWIVQRINKSLDRTKRQGASFIGILDIAGFEIFKMNSFEQLCINY

TNEKLQQLFNHTMFILEQEEYQREGIEWKFIDFGLDLQPTIDLLEKPMGIFALVDEECWF

PKATDKTFVDKLLAQHTSHPKFQKPDFRADAHFSVIHYADKVDYSADQWLMKNMDPLNEN

VVAILQSSTDNFVASIWKDADIVGMGAAAAVDTMFGSRTRKGMFRTVSQLYKEQLSKLMA

TLRNTNPNFVRCIIPNHEKKAGKIDSPLVLDQLRCNGVLEGIRICRQGFPNRILFQEFRQ

RYELLTPNAIPRGFMDGKKAVEKMIASLELDTNLYRIGQSKIFFRAGVLAHLEEERDLKL

TDIIVQFQALVRGLLARRNYQKRLQQINAIRVIQRNCAAYLKLRNWQWWRLFTKVKPLLS

VTGQEEKLHTKEEELKKMKETYEKQKQDVEQLESRYAQIIEEKNILAEQLQAETELCAEA

EEARSHLAKRKEELEEILHEMELRMEEEEERMVSMSDEKKKLNQTVQDLEEQLEEEEQAR

QKLHLEKVAVDSKLKKLEEDLALNEDSNGKLIKEKKSLEERLSEANAQIVEEEEKSKQLG

KLKNKYEAIIADLEERLRKEQQARQELEKIRRRLETELNDLRDQLNEKRQQVEELQAQLS

KREEEVQIALQKADEEGVSRQTTNKQMRELSTQLQELQEDLETERESRNKAEKLKRDLNE

ELEALKTELEDSLDTTAAVQDLRNKREQELQDLKRTLETNQQQHETQIQEVRSRYNQQIE

TLSDEVETTRKAKATLEKTKANLEAENQEMASDLKSVQMAKTESERKRKQAEQQLAELSI

RLQEVEQGRGESTDKASRLQQELDQANSQLEQVDTKAIQLGQKVSSLEAQLADSQDICQE

ETRQKLAVQSKLRQAEDEKESAMERLEEEEESKRALEKQVSDLNQKMVELKRKAEEDLAN

NEALEEYKKKAAREMDQLQAQVEEARTNADRMDKSRRKLQAEVEDLTIALDSQRSNVSNM

EKKQRKFDQTLAEAKAESERMAQEKDSIEKESREKETKILNLTRNLDEMQERVEQLERVR

QQQARELDDLVSSKDDVGKNVHELDKARRALEATVAEQRVQIEELEDELQAAEDAKLRLE

VNMNALKEKYKNEVAGREEQEEESKRSLLRQLREMESELEDERKQRAMAVNARNKLQGDI

QGMEQQLEMANKVKEDAVKQYKKITTQMKDFQRELDDARASREEMAGQAKDNEKKVKNLE

AELLKLQEDLASAERQRRNAEAERDELADEIGSSASSKQSLIDEKRRLEARISELEDELE

DEHTNLELASDKARKSLMQMEQLNTDLAAERSVSQKLENQRLALERQNKEMRDKLQELEG

QNRARTKATIAALEGKIVNLEEQLDQEAKDRAALARTNRKMDKRLKELQIQAEDERRSAD

QYKEQLEKMNNRVKALKRQVDEAEEEVARINAQKRKVQRELDDQMEQNDTLVRETQSLRK

YRPSAQVRTSRVTVKLGEDEGSEEGPDEQTTT

>SG-PL_contig_942_1_-1_1_219

IHFTFIIYVIKSIYFYKKNTFYFYHLCNKKYLYNYYDNYPTCYDIIMHLQASPQSLNLLN

SETNHESIYRHFH

>SG-PL_contig_943_1_+2_107_319

MHSLQHLFSEYVALLAQTHRDLISLSPIFLSTPTSTYSSRNCCHNFLRDRRCVLPTFCIA

KSDRASWQLTN

>SG-PL_contig_944_1_+2_2_100

IYLFLINRKKSQFKVTTYRAIFLQKKMCCCFFL

>SG-PL_contig_945_1_+3_3_146

RGIRERERERERERDRDRDRQTDRDRKTKRVDFYIFLLILVETFNKVD

>SG-PL_contig_946_1_-2_191_322

MYKSTHSVFQSLSLCLSSCLSVSLSLSLPPPPPTSLSYYSVSIR

>SG-PL_contig_947_1_+3_465_653

MLTKSNVVARKRSEHNMCKPERKDQISRQRKARAWKTDESCHFCGFRFTMTSTVSFDILL

FHF

>SG-PL_contig_948_1_+1_130_228

MKTKYYSQKQNKSIQTHKSLNSKLQAEWTQLPS

>SG-PL_contig_949_1_-3_147_683

MPVNCYELIARRQPQNYTLFSDLDAGISVTLSMRKRFMTALHIRRHQSYATVEVNSQGSF

TFTKPPGPSPADSIVHTRPDSQSLQVELGQYVVTVNITKKACRATIITVDMDVGANGANP

ALLRKYSGVVTKGYGSPKGKSRSKSKKGKERGSKACKANSFPADHSFVVKGKRLLRYKA

>SG-PL_contig_950_1_+2_2_172

LPIFFFHGHKMNFIAAIKFNALNTLFFNSLVLIMLVCPRQFFAVNEKLTYDNTKLKN

>SG-PL_contig_951_1_+2_1310_1474

MRECCVLMRENNKRITYLSKLVGIEEKEAYKMSRPFFIYQAYRGIIHIGTFIIRL

>SG-PL_contig_952_1_+1_1_324

DRVSSLLQQLLVAAPGSLGCVIAFVQQRVRKIVDVVDTVRQQVFLCPRIIRHTGRTGHIR

RRRGPGGHRVTPLLQHRVTFRAHIGRSISATSNGHLFARPYRARRAVH

>SG-PL_contig_953_1_+3_3_155

MFIIHLQQIEGCVGKILIKFLRENSDKKFFRMGMQKGFQGRLQHKDGLLIK

>SG-PL_contig_954_1_-1_250_414

MKTALQYPCQTENHKNFFESRQVTIFTPKVIVSRQNFLIRIKERFFSLFFCFVKV

>SG-PL_contig_955_1_-1_355_1647

MENNDPGKLAMLLYVCAVLTGASVLAAPTTQSVDLLMPEVQPTQPDTYLCRSFDVANLKP

YLTGFVPNANKDIAHHILLYGCDEPGSSEAVWNCGEMASRGSDYDSAPVCALGSRILYAW

AMDAPSLSLPEDVAFQVGGNSDIKKLVMQVHYKNVTSFLPPQNKKDSSGLTLITTDEPKR

RLAGVYLMVTGGNIPAHSIEYLEAACPFPDAQVGIHPFAFRTHTHTLGRVVAGYRIRDGE

WLEIGRQDPRKPQMFYNVTNPGLTLRKGDILAARCTYENNLDHDVAVGNTQNDEMCNFYI

MYYVDGNRLLKSNTCFQPGAPMWYLEDYKDQKALNLGAMPVTASVVPGTSKVLEATTMGP

ELPQERQVEEDQDEEEEEEEDNPVVDELLDDYLARMDPDEVEALVSQMMMEQDQRRPNEE

YDRYYFPEDPQ

>SG-PL_contig_956_1_-2_44_469

MIRFILIQNRAGKTRLAKWYMHFDDEEKQKLIEEVHAVVTVRDAKHTNFVEFRNFKIVYR

RYAGLYFCICVDVSDNNLAYLEAIHNFVEVLNEFFHNVCELDLVFNFYKVYSVVDEMFLA

GEIRETSQTKVLKQLLMLSSLE

>SG-PL_contig_957_1_-3_303_599

MFSTSSPLCGDKKYFCLLFYKNLHVLRQISAHSVAKSTMKLCVLTKHNIIITIALFPLLS

CVLYYFSGINLRDGWFINRAVDLRSDVSSSTPADDVKLA

>SG-PL_contig_958_1_-2_2_529

DGVTGGVSLLVHRSVLCSPVDLQTDLQAVAARVSLSKTITICSLYLPLSSIVTKLDLLGL

IDQLPRPFLLLGDFNGHSPLWGSDHSSSRGLLLEDVFSELDLCVLNDGSPTYFHSANGSC

SHLDLSVCDPSLVLDFKWSVHDDLHGSDHFPVVLTPSTVEREHVSERFAFHRANWD

>SG-PL_contig_959_1_-1_1_138

RVHFVSLALKRAARSIQACSTSTSSDIGTVHVHDVTFQEQSFNTCG

>SG-PL_contig_960_1_-1_1_447

SILSRLDYCNSLLIGCPRKLLRPLQQVQNSAAKLIFKAKRSQHCTPLLKELHWLPIEQRI

TYKAACLCYQIISGTAPQYLSELLQLYVPSRSLRSAADDRIFRVPKFKRDKHGGRAFTYS

AVKIWNSLPSSVRYSPSLPSFKANLKTFF

>SG-PL_contig_961_1_-3_3_179

FFASSSSLLIISHCRLINLFLYLLVVVWWKKTVKVGYCLLMFSKILIIPRREKSGHLLY

>SG-PL_contig_962_1_-1_1147_1329

MHIPQPGFKPKAFGVRMYQVEGWSLLILVGHWTMQLGKYSLSEQLSFPGRLRVCHYLGCP

H

>SG-PL_contig_963_1_+1_16_567

MVISSLKRSTSTISGCSSGFSASGSSSSGTATSKVVSSVSLGFSGEMMSSSLKLSTSSGF

SATIVSLITVSVVSSGLAVSWGISSTTSSVTVSDTSSGTDSGTSTSVSKVTVSSPSFSGE

ILISSLKCSTSTISGCSSSGISSSETFSFSVITSTVTSVSVSFSGEIVISSLNCSYSTIS

GCSS

>SG-PL_contig_964_1_-1_1_237

FKEEIRISPEKVTDTEEVTAEVVVPREEAPEEEIPVEEQPNIVELKAPKEEIGISPEKPS

DTEEVTVEVIVPKEEAPEE

>SG-PL_contig_965_1_-3_105_1100

MSEPVKVCITGAAGQIAYSLLFSVAKGDVFGENQPIIVTLLDIPPMEKVLNGVVMELMDC

AMPLLKKVIPTTDEKVAFQDIDAAFLVGAMPRTAGMERKDLLEANVKIFKSQGAALDSYA

KKTVKVVVVGNPANTNALICSKYAPTIPTENFSCLTRLDQNRAQAQIATRLGVANSDVCN

LIIWGNHSNTQFPDVRHATAIVNGKTMTVSEAVKDDSYLKNEFVKTVQTRGAEVIKARKL

SSAMSAAKATADHMRDWWFGTKEDQWVSMGVISKGLYGIPEGIIYSYPVCIKNKQWEVVE

GLDINDFAREKMEITAKELCMERDTAIEFCSH

>SG-PL_contig_966_1_-1_40_1698

MPRGQLIKMFCKLLLLIVPVCAVQALERPRGVSSGKALLYKPDKDFECLQSGETVPWHFV

NDDYCDCKDGTDEPGTSACSNGVFFCENQGYRSTYITSSRVNDGVCDCCDGSDEFEERIK

CVNRCDEMGRKEREEKELFLQMQAQGYKVKQEYMQQGKRAKDEKRARLSELERQMSDLTA

IRDDLQRKKDEVEVPEKEAKEKHEKAWEEVKAERQAEKDRAKAVVVFNELDVNSDSVIDY

VEMQRHMEFDIDSDGTVSMEEAREYLDDNESVELEAFQNRIYTNIKDIYKSPSGSEDSQQ

PDQLLPDETATPPPQQGGSNVPPPVPTHGPDSPADGADFDEDEEDDYDKEDDDEDDDDDN

EDDELGLGEERPKQEEKEEDKMPAYDEETQKLIEVADSVRNEYNEADTKVRSAENEINGL

KKYLEVDYGPDEEYSALRGQCFEFTDREYTYKLCPFDKAVQKPKSGGIETSLGSWGHWYG

PEEDKYDAMKYENGQSCWNGPNRSCHVNLHCGIENKLTGASEPSRCEYQFEFTSPARCKQ

SKDTLSVHEHEEL

>SG-PL_contig_967_1_+1_4552_5016

MAMLKDLDLPTLQDRHRQHHLIFLFKTTEGHLPAIQADKYQTPMRSKRKVRPSTKIDHIS

KKVVDQLIQNNLRCYNTDFGKSDTYKYSFFPRTETGTARTRTQLQQGQWRNSNISTLPSP

SIKILTPITIPPPPFFVCVRVCVSSNFLIYLFIVY

>SG-PL_contig_968_1_+1_724_876

MSSSEGEMGTTLVMLAPEKLRSLPPHSLRRQSWDNLFTLCSSGLYNFQWNK

>SG-PL_contig_969_1_+2_383_850

MFKKSKFTARFTFNTRQYTNSLHYNYPTKFLRIQSSATQFTTVNRVYIFSKAVQHLILRP

LWRGSTRLKHFFHDVELDTSTGFIFCHSKVVKHVIVAKIGSVGISVLVGQPFPLVGVCMT

STNVLGLQMFQLTVNIVAVTHYMLVDLRKTDQTEVV

>SG-PL_contig_970_1_+2_29_292

MKRKKTVGVGGRAINRRVSGLPQFRHQNGLKSNRLKQAWVQGPPRPLWGNRETPCWGFRG

ANPPSSERKIAKRFQGFSRKHIKQEEKR

>SG-PL_contig_971_1_-2_959_4999

MRRVLCMVIVFSTLIYKELCACQVYLQLFYESSVQGNCILTMGNRQGGKTITRHDSVYQQ

KTKKSLGKSVDGSTVDVDLLTPQFQLADFHSLSLHPAVGPTTLISSAFRPKGGGGSTYDN

DNSPSLLSKILHADEHFDSGLPADPSTVDICEGVTPANDNYVSQMTCPPSSTDTKDETVK

STCSQNHQDVSKEQFAVSLVSSNESHSFDTSQDCSLETDSDPGTRDCRDNWLAGITHTTT

HCIDRLEELKRNIEQSRTEKFVHFSNTGSAQLQSGADYDNAEYLKEPGTCSHQRNKSMSS

KTVVPADVSQKNIKTKRKAVRKLSEPWQFVHKKDTNLSKNIISGAWSRSAENSPVIVRKA

LPHPGSPVIVQRTPSQSPSVERRFPANASSSCLLSTREAESFDNPKSGTHAYHKVSSENK

GSFGATEAAHSISRQKPAEISLPLFSKSPVLFRKMPESPILLSSDHKAKSSERERSSDRT

TGDYPMFPSSSVQSSSTYHTQAPKLSCPSSSPKSQRRNLPLQEDPILPSIPLCASPTSSF

SAALSYSSPTPIQVCSQPRGTPVKDCILVSQPSTAVVLATGESQDSLQTTEEVAVQHSVN

TETNGKMVQTENCTHSQDICTTSSKHPSNTEVTEDTIKQNSESAVFGSVSPKLMRHKRLS

KNNPNSSPNEQALPNQLPATQTATSKISRNWENTGTETGGTPHADKCVHIHMENKEKTTR

VSQFLPSVSMSDNISTSGACIGIKNLGQRIRDVINESTVRTAPHGKLSSSSFGKYERLLS

KENTLLNTSAEATPTSHSFPRLSTKTNERPFYDRPLPSPTTERASSAGGRRGEKLLEELG

VKTSTSPVKDFHVTLSVGYSYHLNSFTDACSSHVSNSYQHNSVEKPGGQRQVAELTQANL

RQQSEMFSEGTESKLHEAVTARLGVPGRRLVPSDTTDTLSTDRETSGSSKRESSTSTATD

DTLTSFKTRSTADSADFEEGFVLRESLVFIRSANKEDMHSDVGAFDSQSTTVKRKLAEGL

PPNPRAGYLPPKTRKKRRKQILSQTIIARTDPPDGLLKRNRNARDIYCDDISDESDLFSS

RDDISDFPSHRDIYSDPPSSRDDIYSDLPSSRDDISDIPNSRDTISAFPSTKTNISNLSS

SIDDMSDHGTIQKRVFDRAQLLSPASHKRSFGDKSSRPMARKATIREDRSKDLAVNNEDL

VFKGHKSSSHDSSDTCVDINETSADVTDSGTIPDKPVIKSLTFEPLPKATRPDVLLPPRS

RSTGQGRRLTKASFERSGHREDGGDRDSYEQSEDTSVYCPYDDDIDKGKQLLLQRFCSPE

STKKVVLSCIIYNSSCQSFDAHGSQEP

>SG-PL_contig_972_1_-2_89_190

MYKQFLRFFVFTCTFYTNSYSLPVVSPEEEVVKF

>SG-PL_contig_974_1_+3_1539_1772

MNKASNAGPTGYFSSAGFNDFGTFPRVLMFFECFSLAIRILCLLGILFQSKVLQQLSDMT

ISPRNLVLYCHDTLSASD

>SG-PL_contig_975_1_-1_1_297

QEEWTCQQDNKLFQIRPDLKVFLPSVSGCRKEESVLCRLHTGHTFLTHSHLLKGDVPPWC

FPCRAPVTVKHLLIDCRELHDVRHKHFTADSLRTLFRDV

>SG-PL_contig_976_1_-1_16_2115

MAAGREVEGRKSRKSESQNEEEEEMEFEEEPDFNDPPDFVDDITDDELLADLIKKKPKTT

DGIDNVIVVDNIPQVGPERLEKLKNVIRKLYSKFGKVVNDFYPTEGDKTKGYMFLEFSSP

QHAQEAVKMTNGYKLDKQHTFAVNLFSDFDRYRNIPDEWEEPEIRPYEDKGNLRSWLLNK

DCYDQFSVIYDAGEKTAIFLNSADPSKVEERNRWTETYVRWSPQGTYLATFHLKGIALWG

GEKFVQIQRFSHPGVQLIDFSPCERYLVTFSPLADNREDPQAIIIWDVRTGQKKRGFHCE

SQTTWPIFKWNPDGSYFARMGVDTISVYETPSFGLLDKKSLKITSIKDFSWSPTDSIIAY

WVPEESNIPARVTLIEIPSRQELCVRNLFNVADCKMHWQKNGDHLCVKVDRYSKAKKVEE

KDQLKYSGIYYNFELFRIREKQIPVDKVEIKEQVLAFAWEPVNNKFAFIHGESPRIGVSF

YAIQPKGKVEHLKTFEKRQANHLFWSPNGQFIVLAGLRSMNGVLEFVDTQDMTVMAQTEH

FMATDVEWDPTGRYVTSAVSWWGHKVDNAFWIWNFQGNLLVKQPMERFCQLMWRPRAPSL

LADKQIKEIKRNFKTWAQKFETEDAMRKMTASKELIEKRQSLMEEFSRMRAEKEELYKEM

KSKRLELRGGVDTDELDSHVDNFEEETIEFLVKVEETIEE

>SG-PL_contig_977_1_+1_1_165

YSGLLDAHDLFIFNKQQKKKIDNIFDVNCSYRIRYTGLSSMWSNLFISFRSHSRI

>SG-PL_contig_978_1_+2_2_112

KRTPCPPLIPTNNKYNFFKKAERKKHKKRKKPFLNKL

>SG-PL_contig_979_1_+2_41_343

MCVVGNIVISNILVHKVFEWKYINLTAHIKIYVCVIYLENYTHVTFFYIHVCIFWYLGQN

KFVIPVLFDAFVCMDACACMSVQPVSSLQHSCVCGIFHDFG

>SG-PL_contig_980_1_+3_1665_2246

MTPKDTHEWKIGVSMRVEGDERGGWQQLKKPAFKSHVSTKENTLTRKPRRNKLDKMKIPF

FSSTLCKYVMITTLARSARDNIQRGGTLSHTYTQTLFSFISHIQLNAYKCHIHPDTYKVK

YTTLFCCSFVVFCWCCFLFCFFVVGVLVFFAARDATLIIKSKELFFFTAFPLCHAIHKIE

RNHHNRNPSSHPNP

>SG-PL_contig_981_1_-3_18_524

MAADGQPLHAISDYFQSPCGGISGSKKEQTPMIKTKNGKTVTGFATVARYLSQGGNIPCD

LTLEEQAAVDQWLEYHVTQVVHCGQSQHCAQVLQELNSYLSDKVYFVTHHPTVADIIIYH

GLYAVFMKLAYYEKEKYVHLSRWFDNIQQDKRIRQSLNLLPFSRTSLYS

>SG-PL_contig_982_1_-3_3_302

QYKLVHNKAGSPDGWWNWPNGTTVVLPKGRYPEYMLFDIENDPSEMIDLGGNSSFTDTIE

KLKAELKKQKDLGFKPSKPAPKPGVGKPKFHNGTWVSGWC

>SG-PL_contig_983_1_-3_339_569

MWKETVRKDCFIFLVFHVVRYLLDFLIVVLSSILTADCFILSVFFYWSVVGKTCLVNVAL

LPVLTVDMILLLKNMCS

>SG-PL_contig_984_1_+1_1_267

IGTPTTERNKLLRQAKTATYSDKRGPPEKAAKQGTKKQGRCGSCHWKSDRKTTMFCFHCS

AWLCKEHVKHICQDCTALASTSTVVTIAE

>SG-PL_contig_985_1_-3_1800_2075

MHCIYKFAVYFFFFTRIIPLCITGCEWLCKINHQTSSILLHSPTLFPLSHTLCSIHSKCN

YPSTVNEKEKAKGKKKLFDCLFYSKHRSWKVK

>SG-PL_contig_986_1_-1_1_351

MVPHMEPMVPHMEPMVPLQEPMVPLQEPMGPQEDIQLVMSRLHRLHTLLLPLHMVPLVSN

HTMGLTLGKGLPLQLRMVVTLLLAMDPVDSLSLDNILVSTLRRCSPEEENQLLQTSA

>SG-PL_contig_987_1_-3_198_341

MDPRSHVSSSVPPRNIKVGLFTAHLNAPIILVVSVFHRSCSPPHFKTL

>SG-PL_contig_988_1_-1_616_1011

MPALLVAASERQKWRILSAEASHESSPTPTLVMGLEEEGADHDEIICHLPHQNVVSFSRQ

VSWHETKSSQNIFSLLDLQKSLNNGHCFPQPARVAEGCCANERCSSHLHLSRLRVSVWVV

AMFSLISKAREK

>SG-PL_contig_989_1_+3_3_212

NRGEKYDLYVTCKVQFPLFGNQILKVKNFQIRTNLEQLVVLSFPIYVIYIITYDYTAYAL

IAIFPSIIMT

>SG-PL_contig_990_1_-3_114_605

MRHTFINRTEGRRHSSITSSAAQFRMATSRSPLTVISVFLLCFVAVSLALTGDEEARAVD

DVALARSMRSARWLPFTLIGNERAVVHRRSRRQRGAGSLPIFSRRARFLLSRLATASATF

SMQMYNFLRQDHPELVFSPYSIHSALTMTYMGARDSTAKQMNKV

>SG-PL_contig_991_1_+3_930_1271

MLKYAFLLPFHMQVLSTQQQSFYVFLVFIYFNAGSPFGSHYNNHRQRTALHGSFQKNLRK

SNFCVSFIDWSACLVSVHLCVCVHACMSAYVHVSLFECIFLYALYIPVCFVQSL

>SG-PL_contig_992_1_+2_2_133

GSNLKLNKYTIKEWIVSELNLYLAQFHEDIQMVQWSQTALWRGM

>SG-PL_contig_993_1_-2_80_280

MKMASREQPILQLGGDWVVDQSYDFSHFDDLEWPEAQGLPEVSFKPETRADTQIDELLES

YRSHNGG

>SG-PL_contig_994_1_-2_2_202

GVTCGAWVLWSDMVQYMKPKGQKKTCTNFWPLAKINIKSTGANFWPLAEISIQSVVNYFQ

ENIFVKF

>SG-PL_contig_995_1_-1_4_276

MPGFAPLTSRGSQQYRALTVPELTQQMFDAKNMMAACDPRHGRYLTVAAMFRGRMSMKEV

DEQMLNVQNKNSSYFVEWIPRSEERRVGKEC

>SG-PL_contig_996_1_-1_229_441

MSGYPGWCNQRFNKPGFFSLLLTSLYSTDQSLGLTSQQFKTGSCATKRFHEKQGIALVSC

PTVTRKETVDM

>SG-PL_contig_997_1_-2_572_958

MTTMQQGWSIKPWLILATAYKGDNNTIVYIELVKWMNIILNVITVVLNKCRVFVMIRAVL

SQFMSRLCQQIRKQAIEMLWSLQGHWVGQQHTRARTHTHTTPQHTHTHSHTHTHTHTHTH

AHHTHTTTH

>SG-PL_contig_998_1_+2_2_172

INEERNKNQKSKHARTKPELNSEHKTMTSDDATNTCTTNVQYLITINIFLKRIRYLK

>SG-PL_contig_999_1_-1_40_237

MLFDIENDPSEMIDLGGNSSFTDTIEKLKAELKKQKDLGFKPSKPAPKPGVGKPKFHNGT

WVSGWC

>SG-PL_contig_1000_1_-3_84_1196

MADSVTVDAAANATLNETAANATAKVPATIEGMSIAYLSLFFMAILPIFIGSFRSVKHHQ

EQKNSGEKPETMSSKDAAMFPLIASGTLLGIYIVFKIFSKEYINLLLAFYFFFLGVLALA

HIAGPIIEKLLPASFPNLDYHLIFTQGKGENKEEMMNYEFDRKDLVCLAVSTLIGIWYLW

KKHWIANNLFGLAFAINGVELLSLNRISTGCILLGGLFVYDIFWVFGTDVMVTVAKSFEA

PIKLVFPQDLLEKGLAANNFAMLGLGDIVIPGIFIALLLRFDISLKNNKKTYFYSGFVAY

CLGLLTTIVVMHVFKHAQPALLYLVPACIGLPVLVALVKGDIKTMFSYEDHSEEKEKADS

KDKDTSVKKEK

>SG-PL_contig_1001_1_-3_132_428

MLTVHVSLKHYHRHMLKLVSNAYCGTLMWKYFTAVFIILIGSKLVSFCCVKKKSVRVCTL

ENIWRTIVHVVSFISVCLQDGITLCGEKYLTVIAIKSCN

>SG-PL_contig_1002_1_+1_3352_3753

MGDFSGRVGPRQTPWENYLGPHSDTSTECNYNGQILALCAEFSLWITNTFYQQRLSQTQT

WYKWNDLNVSSQIDFILTRTDQKKNITDARAIPDINLDIGHRPVMITSRQQKRNPPRNKH

KRPDRISLRKLRQE

>SG-PL_contig_1003_1_-2_8_355

MVYEMLVGGGGFLCSDEGKEVFLGEIKCWKNPQLEMAFMTCGYIVFRGAPRSECQALDPM

RTCLSCSVELMCNARAATFLTDFLSHLVHPAIEHMCGSQGRSTRGILDMFPIDWRK

>SG-PL_contig_1004_1_-1_175_1023

MGASNSTPLPPPAASAATAPPSGCPMHQQNTAAPQPPSECPMHQQTMASISECPMRQGQA

TDGGFNTDNMMPPPNQRPAPDQPFPLPTERQKSTIPKAGTDETWVYPSQQMFWNAMLRKG

WRWKDDDMKPADMDHIIKIHNANNEQAWQEILKWEAMHATEGEAMPRLKKFGGKAKEYSP

RARIRHWLGYELPFDRHDWIVDRNGRDVRYIIDYYDGGKVDMSNYQFTILDVRPALDSFQ

AFSDRMKVWYWRTTASSSPDATPSNGQAVPHMPSSSDSGQRSS

>SG-PL_contig_1005_1_-3_3_479

DKARRLGYRAKQGYVIYRVRVRRGGRKRPVPKGCTYGKPVTHGVNQLKFQRSLRSVAEER

VGRRCGGLRVLNSYWVCEDSTYKFFEIILVDPFHKAIRCDPKANWICNPVHKHREMRGLT

SAGRSSRGLGKGHLFNKTTGGSRRANWKRRNTLSLRRKR

>SG-PL_contig_1006_1_-3_315_590

MRSIIINVPIYFDFKKKTIACYAKLRLQKKRYKYLSHIKIMKLSERQRYRSHCLQDSSAF

EHGHCLKWSVWWAACFSKIFRIYLNRLVIRNV

>SG-PL_contig_1007_1_-3_120_323

MRLSHTTHKHICTYARVHAILYSASGMHTLFFDLPVILMCVQKQHYITVFFLVCYSSVVI

KDLLTKKK

>SG-PL_contig_1008_1_+3_123_812

MSLPVLCSLALMLLLFASLLHLACLILPAWIQMAVFVPRVITALTGDTVAEGGGESVEVF

TNVTAVPLYSAVDYLQDRLVFVKVRLGLWEWCEQFRQVDPNCQHPGFLGGVMVVGLVVLI

TGPVSFVLLAVTSCMDMDTKKKRAITLASFILASLSGLLLLLAVMIYAILTRREVNRMLR

QTGENRELPYLGWAYGAEVMVCVLVCLAAVCLGIHLRSSPRATPEFQLVV

>SG-PL_contig_1009_1_-1_1_264

QEQDAEKTARTEVTWEGGGNKVLLTGSFVDWRETFEMVKTGEVFTATFDLPKGEHMYKFI

VDDQWMVNRNKPSKFDNAGNENNVMVVR

>SG-PL_contig_1010_1_-2_95_589

MMGMGPYIKYSAGNATSRLSQAVSQATSQPFAVSCVNANYTDSGLFGFFAIAQAREIEKV

LRAAFSQFAAITKTGVEEKDVTRAKQQLKAQISMYREGGDAVLCDLGEQALGSEGILSTA

DLLSLVDGINATDVSNVAKKLVNGKPSMAAVGNLSRTPYIDSLLQ

>SG-PL_contig_1011_1_-3_114_353

MMLCCEIVIFAGYHVAFGGGYFVSLFVCLSFSCTFHNSVFYYKAKEWSFFYCSLNNTIVP

ITQSCLYNFLRQESIYGIQL

>SG-PL_contig_1012_1_+3_1809_2102

MTSIKAAREHYAEARRQQSFMEKHEKRISRRKIRAQEIMESQPSLNKDVGFKLPPIYSRL

VAVYMTTTQMRPIGTEPAAGQTLGSDKDPMKESKAHTT

>SG-PL_contig_1013_1_+2_2_106

CIFLQALLFFTKSNVTPVSLFTVCIFTIMKRFRGA

>SG-PL_contig_1014_1_+1_943_1401

MDDTLSNELQSKADLTLAQVIQLSSKAEARKESQQLIRAALQSTRWTGNHQSTRAANLEA

SMTGPAHQATKSVETAAKNHTAGIDVLPKTRSVANATNVDAFKQFVAVLLPHNSSAALRQ

YMSCLKKKTRTTTIPWGMSFTCRMKIAGLRRFS

>SG-PL_contig_1015_1_-1_55_327

MLEGGFAGSGFITSSSKRMSDDEGDLNVDGDDTEEYGKPQFTENDLIPLSSDEPAEDRER

LALRGALLGADQVEMMKVEPSTSWEPTPGTS

>SG-PL_contig_1016_1_+1_151_303

MTRVTFQHIHEMHQQKYFHMTTCSHRLNLKSLVNMHTFSLSSGIDTKWPQT

>SG-PL_contig_1017_1_-3_3_158

HTQTHSMQKTLHVQTSVYVLYLNQYISTRRSECSDTVHHVLRVLYRHTASAR

>SG-PL_contig_1018_1_+3_3_182

RFHTKLGNELLKKNVSALRIAQKIVKLIVPSVNKEIWMQLPTQARKADLKLTQIQRCIVK

>SG-PL_contig_1019_1_-2_1361_1585

MTSWKNPALLCEGEKSFDFQKNSKWRGKCLLIILLMFLAVFHPIFCRFSESKWLPVIPTA

AHHLLFELVVFLHEK

>SG-PL_contig_1020_1_-2_224_472

MQHRHPIYPLLTFCWRAWFSPHAPPCLGFSVLSLCGIFPFLSVTLDKVPLLFCYFGKFCL

SFWQNLHCHWQIALLVTPAIFLI

>SG-PL_contig_1021_1_+3_3_1037

QQQEQQQQPTTPSLQEAVTSTTENLLARSGSGEEPCTTDTEEWYIPPLSSATQVNRSLRG

SSALSRLLERAKKQATLLDFSVLTPQQSTAPVSELDIDVSFQHVSRDQLPSDPLKYWCLK

DEERNLLRELTVAYQETVFKLPDRMDTVKPSNVQNILDRCQRFARDIVNFLKRLSDFKAL

QMQDQIAVLKGASWRTFLVKTSANFIVEMDAWIKDDGYVSVKDFTDVSPEHKDRILLLAR

YSAALKHIMRNDPTTFAVLQVMVLFDPRDPNLNNRQLVSELADKYALLLKHLLQSQWSFD

YADYYLGAVRGKMQEMPWVAENIQVMFMDWLHLMEPLMREILNLS

>SG-PL_contig_1022_1_-2_494_637

MGFIPSLFQRYVEVCFLYDCVRVVLKFIVLKFISCLEPFSCTMPADSC

>SG-PL_contig_1023_1_-2_62_556

MAPPSYSDLGKAARDIFSKGFNYGFFKLEAKTKTDSGVEFTTSGNSSNDTGKVSGNLETK

YKCSEYGVTFTEKWTTDNVLDTEITIEDQLTKGLKLAFDTSFAPQTGKKSGKIKTGYKQD

YVNVNADVDFDFAGPTVHAAAVLGYNGWLAGYQMAFDTAKSKLAK

>SG-PL_contig_1024_1_+3_132_362

MKNRPRFCGPSCLALMVTSVLVELKMCWRAEISGMGVVTLGLIAKQKQKQNKKLQKLINM

KACNLMNLLSYNPTSRI

>SG-PL_contig_1025_1_+1_1042_1227

MHKFSPKIKASNRHRKPAVSIMHKVHYKNRTSDSYSYCSDYCFISILVLFFVLLIQTLDL

AC

>SG-PL_contig_1026_1_-1_88_336

MMAGLLARSFSSAFSRSPLRATTRYLAAACLTQIVRTVHSSQPLHSDEVSKAQAAVGCSS

QPTIFSKILDKSISVDILHEDDK

>SG-PL_contig_1027_1_-1_184_336

MELAGASVDGAPALDGFSQWSTISERTDSRRRELLHNIDPLIRPHGVPQFP

>SG-PL_contig_1028_1_-2_107_526

MVTCMDEGIGNLTAALTTSGLMNNTVFVFSTDNGGQTRLGGNNYPLRGVKDSLWEGGIRG

LGFVYSPLLKNRGYENKELIHVSDWYPTFMELAGASLDGAPPLDGYSQWSTISERTDSRR

RELLHNIDPLRESHGVPQFP

>SG-PL_contig_1029_1_-1_1_291

TPRSLPPLSQSERPEAGAPWQVPFAGLYDLRLSEALTTMFQAQHEGPSGGPPTCRRAANN

EAAAGATGAGSPGADATTTPPRGLLCRRPPAPDYSRS

>SG-PL_contig_1030_1_-1_547_744

MWIRTSFEFRVCCYKCTQNSVLSDALLLRVISEFVRNGLKGKKRDGKGLDARFFFVLSFT

ITFFNC

>SG-PL_contig_1031_1_-3_9_1394

MMEEHPAGDKHGARTRESALKPRASTSAVDLDRLDIEPMEADGAEQSSEEQGEDDSAEVA

TCYCGAPRKLGTLELMCGTCGGWFHARCTSASVGKSLPFMVCYQFYCKNCGQGDVESFTK

KQATFSQVCHQAMANLTFTAQQEGEERPEFSKDMDIIPFIEKNWDNMTTMTRRTKQTWHK

TVLNTMMKEPDLYVVSPEDGNEAYFTLINQDLTKIAPVYDSTKAGPSGSQSAADSGTPGK

GRTKRRGNFDSLPVISNKKKNDQGVGPKLPPHGYPLEHPFNKDGYRYYLAEADPHAPNRQ

AFDESTEMAGKPIPGYLYRVFQGTQVLLSMHDRAIQLKLTDDRLTVTGEKGYSMIRASHG

VITGQWFYEVTIKDMPEPSHCRIGWSQPLGNLQAPCGFDKFSYSWRSRKGTVFHQSKGKH

YCDTAYKQGDTIGFFISLPQPQDLSKRLPDTFKDRPLVKFKS

>SG-PL_contig_1032_1_-2_416_1312

MIRRMTTKGSDIARQLRAAIERVNAAAQRRPEHLQGRHPRLVAVSKTKPVEMIVEAYACG

QRHFGENYVQELVDKGSDHEILQKCGDVKWHFIGHLQRNKVNKVINVPGLYMVETVDSEK

LALALSSAWSKQSCTQPLAVMVQVNTSRERNKNGVSPSDAVPLSVYIREQCPHLQLAGLM

TIGSVESSTSDEQNADFQCLIKCRKAVSEALGIGELDLELSMGMSQDFEHAVEAGSTNVR

VGSTIFGARQYPPSQTDASTPLASGLQAAGPGGDSASHKMTKNTVDVTAAQLNDLSLES

>SG-PL_contig_1033_1_+3_405_617

MLFSLSSSSFHIFIYLMIFLLPELFLLSSLVSIYLCFYLMTISRFFFCSSKLFLLSSSSL

YLLLFIYYQLY

>SG-PL_contig_1034_1_-1_151_1332

MAVAAARPLISVYNEKGETTESNVTLPAVFRAPIRPDIVNFVHFEMKRNGRQPYAVSEKA

GHQTSAESWGTGRAVARIPRVRGGGTHRSGQGAFGNMCRGGRMFAPTKTWRRWHRKINVN

QRRYAICSAIAATGVPALVMSKGHRIEELPEVPLVVADKVEELKKTKEAVALLKKLRAWK

DIEKVYSSKRFRAGKGKMRNRRRIQRRGPLVIYNQDNGICRAFRNIPGITLISVDRLNLL

KVAPGGHVGRFCIWTQSAFSRLDKIYGTWRKASEEKSGYNLPSLKMTQTDLTKLLKSEEI

RAALRAPRKEVKRRILKKNPLKNKRVMVRLNPYAKAAAKAAQSVEQNRLRKKEELLNKKR

GIKTPLPALPKGKASAKKVAQRKKAKGGKAKGKK

>SG-PL_contig_1035_1_+2_230_379

MGTTLIMLALRKVEVSTTSLPYNLRVMGQPLPFIPSLAPSCIGFGLSLGF

>SG-PL_contig_1036_1_-1_532_963

MFRLVFSFFIFYLGCGIFTLYFNLIFKSPAKKSSSYKCISCGWVQVNGWLRVRNGEYGMC

CCPPSFGPSFCLVGISSPSQISNIMTMFVFICIVLLLAFCLTSILLFLFFQLFYACMFRL

FWVFFCVSNFMGETYLPKLLELRK

>SG-PL_contig_1037_1_-2_440_649

MRHFSDSGILVVGRQTQQPGRCKITLPVQYSNLESKRHWKTSITGGDWTGLDSSTFTGKC

FDDFVHLLNV

>SG-PL_contig_1038_1_-3_57_1466

MSHIVMLRTLSQQLLRSNKNLLNGVGLRHATKASSSTNLKDVLESLIPEKQKEIVEFRKA

KGTTKVGEVTVDMMYGGMRGIKGLVTETSVLDPDEGIRFRGYSIPECQKILPVAKGGEEP

LPEGLFWLLVTGEVPTEEQANSITKEWNERAALPNHVITMLNNFPCNLHPMSQFSAAITA

LNSESKFAKAYAEGVHKSKYWELVYEDAMDMIAKLPTVASIIYCNLYKEGVTPCAIDMAK

DWSWNFTSMLDFENPNFTELMRLYLTIHSDHEGGNVSAHTCHLVGSALSDPYLSFAAGMN

GLAGPLHGLANQEVLVWTTKLMEALGGEVSDDQLREFIWNTLKSGQVVPGYGHAVLRKTD

PRYMCQREFALKHLPDDPMFRLVSQLFKVVPDILMEQGKAKNPWPNVDAHSGVLLQYYKM

TEMNYYTVLFGVSRALGCMASLIWDRAFGLPIERPKSFDTPHLRKLVGHS

>SG-PL_contig_1039_1_-1_130_2154

MIMDGLDTLSQVALADVSSFCFDTSAIGSAGRHASESFEDCLNTPVTTCSETSFFGPDTL

EPPPITATANIVASINIPDTKAVEAMLPHRPGQGSASGSSSTTSGGSGLTHTITYKGTLV

TTAMTPSSTQSEVPGPSQPNLATLFSPLNPLFSALINSPSSLTTGQFLQQQQQQQQQQQQ

QQQQPQQQQQPMPLPSDFQQQISCDFTVPVCTISDTSAPFPSTSPSPASVHSPVSQVSQA

EQDTYSQGFGSPLTPCSPTSPEAQLGLQDSSVKAEDSLGGSYSSPPPPPPYTQSLPMPVS

MDLGAVSALKQPPSYTSCTIQQAPQPQQPQPQQQQQQQQQPQETTNFLTLPAACTLPETI

LQMQISELGPNKQTLNADLRWTITPSASGQLAQLPDFSALQVNPPQFPITSTIKTEPGAE

AMEQDRFMMPTQMDFGPSSSGTSGKAASMAVLNQPYAQSPSNLKLLPVKPRKYPNRPSKT

PPHERPYACPVDNCDRRFSRSDELTRHIRIHTGQKPFHCKVCGRSFSRSDHLTTHVRTHT

GEKPFSCDVCGRKFARSDEKKRHAKVHMKQRVKKDAKLLATTASLPTTATAASSTVSLPS

STHTHTLTSSALSSSVVSQTLQDSSMISPSAHSASGTLPLVVDSTGLCSVSGLETVGVSC

SSVSPSLPLVVTTSL

>SG-PL_contig_1040_1_+3_3_293

GVGDLSHRQLLVIGLLGGDDGGVCDEGEVDAGVGHQVGLELRQIHVQSAVKAQGSRDGRH

HLSNQTVEVGVGRPLNVQVTPTDVIDGLVVHHEGAVR

>SG-PL_contig_1041_1_-1_1_201

SPGPSPGRPPRAPQAWLLPAPPPQSTASAWWRAWPITPRRGDSCTSATGLWKPSRRGTSH

PGNEGSP

>SG-PL_contig_1042_1_-3_909_1091

MLLSLLLILLLTLVGFPCMMDNPRKTVCLDKPFSYLAVIAQFSQNHPIVSTLQMSNIRSN

K

>SG-PL_contig_1043_1_+3_426_554

MKTGLFAAHLNAPIILVVSVFHRACSLPILMLQSFWWCQLSVS

>SG-PL_contig_1044_1_+2_47_232

MTRTPPRKKFFLKLTLEIKNTNTIVYAVTLYWEKNRVLASPLGHELAGILSSSVEVSSCT

LK

>SG-PL_contig_1045_1_-3_294_755

MSNRILNSGPLVTHDGNFNHWAYTPHPKSSSASTSDKKKITRKKTSSARSSEFVQEGSSH

SDSSISAPSKSSSNHLADFLSLRKVLCAGKVLKWGVKAANNHQADTTRMIGALRWAANKP

TLMLRAGTELEPGDLESTTVSKGPASPRKKLWPA

>SG-PL_contig_1046_1_-1_1_159

RKSNQFYKINGYCVELYLFIYAYYKYMHIHTYIYSYTHIFIMLMLCEVSLILN

>SG-PL_contig_1047_1_+3_3_278

HRIHFYFLTFDSPQFRPANRVFFNLSVCLSVCLSAHLTQCPDSKQNVMLAVIIFYAHILQ

FKIKETSQSISIIKMCVYEYMYVYAYTYNMHK

>SG-PL_contig_1048_1_+3_3_143

YTHTHININLSCVHKTTTANLAEQLAPTAQQHFSPPSSKAIAKTNLS

>SG-PL_contig_1049_1_-1_169_300

MIISGQLSACYLLHVHILTLLNNIEIVANQFPPTTVVIALHCRH

>SG-PL_contig_1050_1_+2_1637_1933

MAECPSSQYTPYSIADLTVTGLSNCLLGFIPKPKTKATNNIQSLSNNFERTKQQIYEQIL

NVRIPTPNITQIYTSLSLNKIRLFGKKKNCLQIHVLPRI

>SG-PL_contig_1051_1_-2_65_511

MAGVWRLLIGSAAMWAVIYAARFDNTIQNSIEIRVIVDRWALESWTEKLETSSTKVQKTI

TDYVYSVFKDVNSIFKELMSSGINLDARVVSIEFITTEEITSDSPAGSGTMDSKEVLDEL

RSWLVTKNYPQTDHTLLLTGLDLTSDRST

>SG-PL_contig_1052_1_-3_291_2495

MDEPALKATFTIHVIRRSFNLALSNMPVETSTNLDEDKVVDDFKTSVPMSTYLVAFVVCD

FARKTKMTSKNVQVEAWSRREVVDHLDYAVDFGVKIVDFLEEYFQVEYPLPKLSMVAIPD

FSSESMENWGLITCRDERLLFNDRTTSIVRKTLTARTIAHEISHMWFGNLVTPVWWDDLW

LNEGFASFMEIMVPDHFYPDRKLMDMFVAGMYRAMVEDGLVSSHPLSLPVVTLSQFNQIF

DNIAYLKGAFVVQMIHFVIGDVKFRKGMQKYLTTLKYGNAQRTDLWNALENDELDVNITD

VMNTWTQQMNYPVVTVTFEGQTVTLTQERFLQDPNTEDPGTYTSPFGYKWYIPFTFTSSD

ERNFNITASDVIWFTKDEDTKTFTVPTLSEDSWVLGNIGLKGYYRVNYDDDNWRKLTAQL

RNDHTAIDRVNRAQIISDAWILASAGLVNTTRALEAVDYLDKETEYTPWNEASFELTKVS

NMLSTTDIFPAFKAFMVEKTSPLYVKLGMNTTDEFLETQLRAMIASTACKYGEPECINDA

SNYLQDWKRNPGQNPIPKAMRAIVYCARLRQGNTADWDFVYGRFLAESDPKAKHDLFASL

CCTNNTEDLDRLLDRALNSEDIRGQDFVYVLYYTILNPVGANRAWTFINNTWDSHHEKYA

AARIALPYAIKAFGTVFSTEAQLRKMQDFVERHNDLGAVTQAFSAALESVTNNIAWMKKN

AGIIRNWLQERGFSG

>SG-PL_contig_1053_1_-2_98_448

MSLQTVPKDLRNLRACLFCSLVKTIEQFEFDGCDNCEEYLHMKGNRDVVYECTSSNFDGL

VAIMSPEDSWVAKWQRTERFVRGCYAVSVTGRLPQNVVRELRGRRIEYRVRDNSQKS

>SG-PL_contig_1054_1_-3_678_929

MFSFYQNWLLKFFLPPPTLSSTSPPHWSSSFPPITATDLLHPHSHHTRTHTYSHPHARTH

THTHTHTHTHTHTHTHTHTHAHTH

>SG-PL_contig_1055_1_-3_3_278

PCSTQVPRSQIKAITLHSIYLSSEYKHSNLSFFPTRNLFPYKFSDILKKNASNWTTQYSH

RYRSTGAGLPKPFLSHLKDEQISVTKYHSNLH

>SG-PL_contig_1056_1_-1_1_165

RETYTVVSLARSRVFETLSCLLHAMPFCTRFCPARVVCIINVHGVVFAPLGTPRL

>SG-PL_contig_1057_1_+1_499_726

MSLALDMQKIAQIILETQQIAQINITCYPSYHHSYKTYPSGLRTVLTHTHTHTHTYTHTH

TLTHIHIHTHTHTHTH

>SG-PL_contig_1058_1_-3_3_164

KVKQRERSNTCSGRDDPRLVRSLPSRSQLGIMVLPSNFRLFPCESEGAGVVGEV

>SG-PL_contig_1059_1_+1_106_1107

MRKLAGTQWGANEKILKNVYQGTVRPHLEYGSSSWMTAAQSHKQTLDRVQNQALRIITGA

MRSTPIEKMEHTTGIPPLMKRWKSKALIQYTKAKASEDHLMHKRTKELSSGRLKRSNFMR

DTRALQRSFQEQLPTTIETLRADSGPPPWEDKLKNITICTSVPHLITKDEHSDVSKRALT

LTMLEERYPQEAWIRAYTDGSATDAVKRGGAGVYIQFPNGQRQAEAIPTGLHCTNYRAEV

EALVHAANIINNVADSDSQIVFLTDAMSVLEATTANKLPHLQEALQNIRCTRQVLQWIPS

HCGISGNEEADRMAKLGAEHEQKDNSISLSEMKT

>SG-PL_contig_1060_1_-2_44_727

MATQQRWIPLESNPKVLNKYIANLGAEEKWQFVDIYGLDTELLQMVSRPVAAVMLLYPLT

DKAKDESQKIGTLDTCAPDQLYFMRQSIGNACGTVALVHALANNEDKIAFADDKHFRKFL

DESKALSPKERAEYLENDEDMGTAHEESASEGDTQAPSRDDSVKPHFITFVHVEGKLFEL

DGRKDGPVEHGKTTPDTLLEDAVNVVKMFMARDPDEINFTLMALSKAE

>SG-PL_contig_1061_1_-3_111_398

MPLDPIEGQQIHACGFAASSHHAKNFSSFFILDHFKPCLLLYLFLFIHLHSKAGESQYIF

LFALVHTLSSVLMCIYIRLHLSFGFYHAWFVIQRDY

>SG-PL_contig_1062_1_+2_770_949

MPNMTGCGSRMMKTLDFQQEGLGSNLLGYPTRWIKGGDFSDHPVNICADLLVPECFSYAR

>SG-PL_contig_1063_1_+3_540_773

MKIVQLNEKTCHWLDRFVVGVFCCLRKPLPVSFFCLFNLKLLSILFCIQSHNTRKEKTNN

TKKYLGPSISYAPPPLLK

>SG-PL_contig_1064_1_+3_147_287

MWGQTVRVCVCVCVCVRVRVWVCVCVCLLLLLFFLIYSFILKRTMCV

>SG-PL_contig_1065_1_+3_12_389

MSLHVTTLSHLKSCILHLCISDGDLHFHTWFNADGSNLLDDFGGAVQVDDTLVDAHLVAV

PGLGALTTRCFTGGDTQDLCGHAYGSLHLQLFVLCSLDQISTHLFQTLHIAASQGDTNAV

DGRLLC

>SG-PL_contig_1066_1_-2_80_2734

MSRFFQAGSDSETESESEEEVPTQRPAIPARAYQLSDDEEDTRRVVRSEKDKRYEDLRNI

IKTIRNHKKIKDMASVLTDFEGLCKSYEKAKKVVDKEGIPRFYLRCLVELLDFVNECWED

REWRDTISKNNSKGLTTLRQKLRKYCRDFETEMAKYRENPDEGDDEEEKEDRNEGAESDD

SDVAPARSEDKVMKAAKFVKDRDLDREEDEDSDDIWNASSEEESSSSDEDIPAANKLTAE

YFLKTTTEAEEKKRKERKKDKATKRSERRTKEDDEWQEVKGGAPMIMEKPKMFAKDQEIN

HEAVTKKLYEIVAARGKKGTDRAQMIEMLQELKQVSDTNNLGPAMHIKILFNITAAIFDY

NPNIATCMRPEMWEMCMDHLTTLMTMLTEMEELVVGENILEENESLEATPYRVRGCILTV

MERLDEEFTKMLQNCDAHSTEFVDRLKDEQRVCGLLETLQTYVEKNGTSEEICRAYLRRI

EHCYFKFDQTAFETTTDRSKVTSEQLMDKLCKYIYAKDSTDRLRTRAILCQIYHHALHDR

WFKARDLMLMSHLQDNIQHSDVPTQILYNRTLVQLGLCAFRQGHVRDAHNALVDIQSSNR

AKELLAQGLVPQRQHERTPEQEKIEKRRLLPYHMHINLELLECVYLVSAMLLEIPYMAAH

ELDVRRRMISKNFHHVMKMSERQTLTGPPESMREHVVAASKAMKAGDWRSCKNYIINDKM

NAKVWDLFFQRDKVRDLITSKIQEESLRTYLFCYSSVYDSLSLPLLAQMFELDRPVVHSI

ISKMIINEELMASMDEPTQILSMHRTEPTRLQSLALQLAEKVASHVENNERIMEVKQGNF

YFQKSNVQNYQQNQNWGGNKFQGRRNDRRDNQRYDRNRNYNRDRD

>SG-PL_contig_1067_1_+2_2_250

SCGRLVSLYSDRRRGICCATRLKTQASHVHLIFSNSDFPSGWTSDPNVIRIHPSVAKTSL

LTRFLHRQTDRQTDRHTAPLQSI

>SG-PL_contig_1068_1_-1_22_627

MGFSTRPILIDARGHLLGRLAAIVAKTLLQGQRVVVVRCEGINISGNFYRNKLKYLEYLK

KRCNVKPSRGPYHFRAPGKMFIRVVRGMLPHKTARGKEAMERLKTFEGIPPPYDKQKRLV

VPSALKVLRLGQTRKYCNLNRLAHEVGWKYQKVIGTLESRRKVKSKQFFERKRKLQQLRE

QAKKNVAPKVQKYQEVITSYGY

>SG-PL_contig_1069_1_+2_53_349

MFSVQHSHILHVMQCFLFNIVTSLCYVMLSVQHSHLLHVMQCFLFNSHILHVMQYFLFNT

VTSFMSCNVFCSTVTSFMSCNVFCSTVTSFMSCNVFCST

>SG-PL_contig_1070_1_+2_224_445

MKPQTSRKTSEKPIHSRTCQKQKTSISAKSRFSPTGAVKTRHSTAYRSEHVLFRYFQLWI

ASSYKPLPSQASEC

>SG-PL_contig_1071_1_+1_1_246

FFLFLVISQNDTFLFLWRFWGFQSCKDGFVKHILQAFLGESRALHVLDCTQFSCQLLPGI

LRDGFLLVFGQFLHCGSIIPQV

>SG-PL_contig_1072_1_-1_577_750

MDHILALCSLWLSSFETVIFRLDEPDIGSVWCVVNQVGTRLVTALIGQRIDLCRVNHF

>SG-PL_contig_1073_1_-3_207_1814

MVMVTTTMGGNPALQAINDLKRTGTKVIGVGISLSNSDERLMESAVSQPAQNMMFTVTDP

ADLDSIADSFVNFVCEEKNMCAEKPCQNGGRCTNGAKDYMCTCAPGFAGKNCERSCADRA

DVAFLLDTSGSVGFDNFRRMKEYVYNMVENFNIGPDATQVAVATFSTNARAQFYLNQYNN

KQQLQDAISAISYEYGNTNTAAGLKLLRSGIFSTRRGNRAEVPDYAIVITDGLSNVNAEQ

TIPEAELARKQGVHVFAIGISVSDSWELRAIASEPADNNAFLLQEYSDLLGVTDRLVDAT

CRDSGVCAEIPCRNGGTCIPGVGTFTCQCMKGYLGKFCEQDCQESKDIAFVLDSSTSVGP

DNFNMILTYVKALVQELTAGGPNHRFSVVTYSTEVRTIFSFSRYNTPQQVMDAITTTRYT

PGSTNTAGGLRQARQMFQPGFGERSNAKNVVILLTDGQSNVNYYDTIPAADDLKSEGVTI

IGIGIGLANADELNSIASGRRDVFQVASFDALDDVKAEIVAGSCRNSEEVPRKVEG

>SG-PL_contig_1074_1_+2_626_994

MSVLFHLHDTAKETAKLKKEHYCENVRHASVSRRVLSVLRHVSVSRGVLSVLRHVSVSRC

VLSVSRHVSVSRHASVSRHVFAKACVCIKACLCIKACLCQGMCLCQGMSLPRHVCVMSLP

RRV

>SG-PL_contig_1075_1_+3_3_287

PAKSLSSSLTLFSEASSLVLASMRPSLALSYSFSKLEYFFWSVLRSSSNLAFCSSVSRSF

CSICSARSSAFSLSIFMANIFFLMASMAAGFCRIR

>SG-PL_contig_1076_1_-2_98_373

MSTRVATAPNVAVNRVATYRELDNDLLRQAAFQVLDNEVDLKLLTKSLSPMEDLIEEDRP

WDWDRLFTEVSSELTTQWEKTKEEAEPEQAET

>SG-PL_contig_1077_1_-1_31_840

MAELPHLGKHCFVESCKQLDFLPFDCDGCGNTFCLQHKAKDDHHCDKHLLQTANADYSGP

KGFACSMTECTHRELTPVTCEHCHLNFCLSHRHQQDHQCTELKPSPVPNAQTAQHVANIL

ASKQPPAAKKPLKSLKARQTAARVALMKLKGRATGDKGIPDTERIYFTVALPVASKLPPK

ALFFSNKWTVGRVIDAIADSAGLHNPNNTGAAQKLRLFGGDSGELLAVESRMEEVMSEAS

GVFSGSPLIMELVSSDVTCLPDLNNYQTTL

>SG-PL_contig_1078_1_-2_26_655

MHCVSCPTVCVVFIALVHFAFLRQAAAADAQQGADRQRHSQPTRYTRSVSTRRSHRLPSD

LLPLHYDLVIHPDIYQDSYSDFSFEGRVTIKMKCISPTKAIVLNAKSLTIAQSDIVVSGP

AGNLSVVSMVLNDQVETLNISLEEELKKDENYTVQIDFSGPLRYLEKGLYFNAYKRGDQF

VFLAATHFKTVHARRAFPCMDEPALKATFT

>SG-PL_contig_1079_1_-3_45_401

MSCLYILWFMMYKMHSVSCLTVCVVFFALLYSVFLLQALAVDVEHGADMQQRRERSRYTR

SANIDTESDRLPSDLQPLHYDLVIHPDIYQDNPSNFSFEGRVTIKMKCISPTKTIVLNA

>SG-PL_contig_1080_1_-3_3_1793

MHSVSCLTVRAVFIALFYFVLLRQVLAADVQHGAGIQRRSQRSRYTRSANTDNESHRLPA

DLRPLHYDLVIHPDIYQDNPSEFRFEGRVTIKMKCIVPTKAIVLNARDLIIAQSDIVVSG

RAGNLNVVSVVLNDQVETLNITLEEELTEGENYTVRIDFSGPLKDDLRGLYFSTYTRGNQ

TVYLATTQFQPADARKAFPCMDEPALKAAFTIHVIRRSFNRALSNMPVKTSTNLDGGKVE

DEFETSVPMSTYLVAFVVCEFARKTMMTNTNVQVEVWSRREAVDQLDFALDFGVKTVDFL

EEYFRVRYPLPKLSMVGIPDFALGAMENWGLVTYREEYLLFNDETNSVIGKMTTAKYVAH

EVSHMWFGNLVTPVWWDDLWLNEGFASFMQTMVPDHFHPDRKIIDTFVTDMHEAMITDGL

VSSHPLYVPVATPSQIGEIFDSISYLKGALVVQMIHFVIGDDRFRQGMQKYLSTLKYGNA

QHTDLWRALESDELDVSITDVMNTWTQQMNYPVVTVTFEGQTVTLRQERFLQDHSAEDPG

TYTSPFGYKWYIPFTFTSSDERNFNITVSDIIWITKDEDTKTFTVPTLSEDSWVLGN

>SG-PL_contig_1081_1_+2_32_259

MFVIRDHLEWSVKEIGVESADPKDHSQHFPVNVGVVALCCVQRFGCKCHCKSSLKEHSSK

TFHGAVHFKDCGFFGV

>SG-PL_contig_1082_1_+3_45_350

MLVLKVLLEGLGIRHLGLAHRTAVHQCLGRRLLLVVYIVSLAFAIRFLLCLGLRLNLLLS

TGHADRALPGLHRSGNLNESGGYNTDFLQQLENTGKVFCTRL

>SG-PL_contig_1083_1_-3_99_191

MMFPVMSVLLLYSIMNSCAWGMVLCKTVNSN

>SG-PL_contig_1084_1_+3_1014_1373

MTSQHRQTAFVSHHKLHPVITNCTRLLLTNCFQSSQPVFTSHHRTALLSLLQYLHILSQP

HSLTNIDKFSCLNCIIHYTTNYNQTWFIIHSVMGAFITFHNSQNNLTFHLLFTSQALVSP

>SG-PL_contig_1085_1_+1_589_861

MAGTTWAPCTHFLTVPGTGVRSKSGRYVVYHSTIRVLMSNEKEILQRSKAFEWITFLRSD

CDKDTTYTRQDVLKTVLCKIRHINTHTKQDV

>SG-PL_contig_1086_1_-2_56_334

MEEAVNKLRPTNSSRSKLTHVKDVAEKAKKCGVVCHIQCTGCEDYIGETERPLDKRFKEH

QIISESSFHKHLVSQSHSTHLDETSVLSTETHY

>SG-PL_contig_1087_1_-1_175_4884

MQPSQDLPISLHEDKRQEADSTPSQLLNFTVTELHGTSLISENKMETAVESKTTSSCDET

FDLFFVPSTSQPTVEGVGEQNRSYRNSLDQDKGSSGNKQLNPSTSTDSSKVESILSPPPP

PPLHPVAVTALVHLDHPYCTFPRGAIPETDDSGESADENVSADEIERRPRSLSVDSVYLQ

YGVSGLSSQQENLSQNDKLALLSPTLSVDSISNDSSLSEHSTAAGGQCYSILTGGPSSAR

TSRPTLVSVDSQGGTDSPAAGTDGASRLHSNLLETGSNSSLLHASSLSLGPRCTKFSIGN

LGSVSNSHLEVEKESIKNKPNIGLSSDPVMKTSLSSPAIASPASPFPLLQSPGMEWDRSD

AGSECPDDLNSSSTEDKFLSSSHDSEAFGTTSQVWRHPVFHDHDYCCKEISEEYLGKPVA

PTPPTKSGKRKYVKKKDRQEEEEKLRKGKYLKRELLKQDNRFNLASNRGVDRTLAARSSL

LAESLATKPNPVGRPRKRTEKQLEEYDGEIGTKMKITGKYQDQYVYYLNKSSRNRRRKVD

EKVPSMDKIILPAPKPGDIVVPHLSDADCEAIRRGGRMALQNNNYSNPVTSQPLQPHTDH

SDMDSSIVNTILSMETEHGNLASPLDSYDGDGHGDDFADIEGNLTQEQLRLLVDCLDDYP

MEETTTTQAALDLFSSAEGVVAPSTTVDSLAATTVEEEFMVTGEEAATSHITGSSDSAAL

LAKVEPNNPSALKRDANGLEFCNYTATVTTSFTVPVPPPTSQPSVEKNLEFLKTPFTKSD

FFPTVSPATTSVSQGLFPTVPPVISSGSQGLFVPTPPTPITPSVLDVRSDDVNETPWIVT

VTLYDNDIPAIIINNMPHVRLVDIHRQILPAKDTGILKKRCQLMHIQVSNCTEMQRYFLV

QYGRAHNSKSTLVISKDEAQRLITYYAHPPPRNLRGEEGAGLRRASSYAELRSIMERPGN

SGRNSSLPFTQSRKRGGFRRRAAPTRSQGPSPEPVAQSVSPVLEPMLSSPLPVEPAASVK

GLRHKKINFREMLKGEDSSPQTSPPLEGGVDPDSTAVLEEVSVAKGRGSKTGVTAGRNKR

RASKESGSAPQSKRGRKAWRKKEEGSSHNVLKNLGEADIMVCSDSSNSAVVNSKNKKRSP

GSSVSGSLGSARSPKSKFGFKVNVKSLVSSGGLLSADGSQPRKKNLFGRKSSSSTDVTVE

VAEDIVPRSHPLRVLRISHQDVEASFPPAPQIVSPMAEVHLDLYKRASSPCVKCDTCHKF

LSVPGFLKHQHVNGQVETQDTRAEMVLRRRMLVPMNKENISLEEQRLWNEFVALQESLDH

SVCVASMGAYPLTPLSPASPVGSSLHSFGAIVSPALEESLLTMTPESPHPAQEGLDHLHN

GNLLDSETIPNSQGVTVMDERCATSPGNETGSGSDAVNVGITFPSEAAAHPWETPLSFCS

PPTLSPATTHTDNEEKYDVVNSIEPDTEIPSHPKTVVPANKKQCPYQRPRKVSPTTNVRT

SSRKRETKRLYSFEKYEYGGGKKGGRGEAEVPDFEDDFPGALLEDVNSVVRSDTLLHLAS

DAVMHEPQNA

>SG-PL_contig_1088_1_-2_800_3865

MSQQSASFDHRLSSNRSELTSRKSPSDVTSRNSPSSNVTSRKSSLDVSSRHSTSSGSRSS

PAQDSSRSTSAEDKNKSAPVQEVVLTRSSLQKSQAAPTVSRERPLERNTRDLKTCSTNSN

FSVSMDRSQLRKSELKTTDPPKTDFRSVLSKPQATSAHSKFSAQSSTDSKPSWVKTESPR

EQKKNGHDMDMKQSSSSKTDFRSVLKNRQVEDSRTSSVSRDSKPKVQSTKSETDFRSVLA

KMKHGGEDSKTTQPKASTSKVTPDRTLNKALSKDLNSIRSKFETPAEDVKSVQSKPPRPT

TTATTTTVSNVSELSSTDTNSTATASWRREWRDRSAGKQTDGGKITIKTTDNNSSIEKTT

DRWAEKNTENIRTEKTTDRWKDKTPDSGRSAALTTDKERWTDKSTDRWTDKSTNRWTEKT

TNGEKPAEKDTDNNSSTVRTTDSERWTDKGRDRWTEKTTDRDRSAEKGAEDNRPAVKATE

NDRPAAKTTDRERPEEKCDDTNRLAAKVKTTDNERWAEKTTDKWTKKTRDRWTDKTTDRW

KERYADRDGPAEKSTDDKPTLKASDNNRSTAKTTDSSRSAEKFTDINRPTVKTTDTSRSA

LRAAERERISVKTMDNTADDKVEVISSLGDTSRFSDDAKCSDSSQLSDSAISSAFSKLSE

LSDLSDSGSAVSEDVPEPHGRKRLERLKLRLGGGESGESEGAEELSEPPSIRQRLADRSV

TYGGQAVLQCTVTGVPVPDINWSVNKKTIKPSRFFQLSYRENVAQLVVAEAFSEDEGEYT

CTATNTAGTDSCSCYLTVEASSSRSSQSGSHTFEGPVSTPPHVTTLTPSTLTVRRGQPAS

FQLTFTGEPPPAVRWFLAKTPIQPDDKISIVTEGGVSTLTIGEASANNSGRYTVTVTNDL

GSDLQSATLSVEDVPECPVGTPHVYDVSKQSVSLSWCGPAYDGGSAITHYVIEVKDSNTK

EWTSLVSDCKSTSYHALTLKADTNYQFRVRAANRLGLSDPSPPTPPVVMSDPLHASDDYE

SD

>SG-PL_contig_1089_1_-3_159_1016

MRPHCSHLLRSWSSLPFIHHGALKTEVLQTLKTSRHHARGVSTISPLAAAFNSKPQRKIN

LSLRRDNAGLFLIPELSDYSGFYLLQHQVSAQVELLVEEATSRNRTRKMVIIFDELSDCL

CRVADMADFIRVAHPDRKFSEAAEDTCVKLSSLVEKLNTNTAIHSALSRVLQEGDVVETD

GIDERVAELFMFDFEQSGIHLEQKKRQRFVELNESILMLGSYFQQGAQRGTSVPKDQLPE

NLRHVFGVDGDNVMVTGLFSDHYSGRVREAAYRIFLYPDAHQEELL

>SG-PL_contig_1090_1_+1_580_807

MFHMIFSKLSLSSACETVGALCDFHIAPEEYGSWSSARCQVGCVYVCVGGGGVSPRGVGG

FSGKNSTPPFWNFCAR

>SG-PL_contig_1091_1_-1_28_345

MVRYQLAVGLQKGHKVTKVEGSRKDRPSRRKGTATKHARFVRDLVREIAGFAPYERRCQE

LLRISKDKRALKFCKKRLGCHIRAKRKREEMVQVLQKQRKAAAAAK

>SG-PL_contig_1092_1_+2_581_961

MPLIPAPQHTTNSTFIQSKSQCQEVHGSQQSQAAVKLSKHNESLLNKLKFKSYTGEYSTI

LFFYLLYTSSAVALILQIVQAVRSTCATTVNKAITSQITVNKAITSQTTVNKVIPSQTTV

NKVIPSQ

>SG-PL_contig_1093_1_-1_601_729

MGTTFIMLALRKVEVSAPLLPYDLRVMGSLYSFFLCVCVCVYV

>SG-PL_contig_1094_1_-1_97_1527

MDGLKQRSHVIVMAATNRPNSVDAALRRFGRFDREVDIGIPDATGRLEILRIHTKNMKLG

TDVDLEQVASETHGHVGADVAALCSEAALQQIREKMDLIDLEDDTIDAEVLDSLAVTMDN

FRWALGKSNPSALRETSVEVPNVTWEDVGGLENVKKELQELVQYPVEHPEKFLKFGMTPS

KGVLFYGPPGCGKTLLAKAIANECQANFISIKGPELLTMWFGESEANVRDIFDKARSAAP

CVLFFDELDSIAKSRGGNVGDGGGAADRVINQLLTEMDGMSAKKNVFIIGATNRPDIIDP

AILRPGRLDQLIYIPLPDEKSRVSILKANLRKSPVAKDVDLDYLAKVTQGFSGADLTEIC

QRACKLAIRLAIEAEIRFERERAQNPDADMEVEDYDPVPEISRAHFEEAMKFARRSVSDN

DIRKYEMFAQTLQQSRGFGGNFRFPGQTGGSGGQGSSQGGSGGGNFGGEDGDDDLYS

>SG-PL_contig_1095_1_+3_3_215

HSLSLSNTHTHTHTHTNSFSCNLGGYPEKTYNFVDLVRVCVCVCVCVTWPLVPSVLENKF

SLTHPPFVPPI

>SG-PL_contig_1096_1_+2_71_178

MMLAHFDTYRLKAMICIKHTDGSVSILTNWTPKALL

>SG-PL_contig_1097_1_+1_283_381

MECGSCTSMPPGLYINTGNVFNIVRRTRSWCLK

>SG-PL_contig_1098_1_-1_1_153

CDLNFYQFSLLWMRKLCQVLTVIDYDRAAVYGHGLYSFVLLMINPFYWDWK

>SG-PL_contig_1099_1_-3_60_575

MEKMVNSRLIWKLETAGLLATEQCGFRKHRSTQDHLVRLESTIRNAFVNKQHVVAIFFDL

EKAYDTTWQFGILSDLHELGFRGRLPIFIENFLKGRQFQIKVGSTLSEIHEQELGVPQGS

ILSPALFNLKINSIIKSVRQGCESSLFVDDFALYATGKIFAGVQRQLQLCVD

>SG-PL_contig_1100_1_+3_84_278

MKGHATESRTIFHNAMNRDDSDDAMEFHLELRLRDRAALYAYFHNISWRQPGPRDTDTCG

VGQEI

>SG-PL_contig_1101_1_+1_1_75

HTHTHTPHNGTVKCLIEWHKSRKSQ

>SG-PL_contig_1102_1_-2_224_2890

MMGDPYNDGYMEEEEEWDREGLLDPAWEKQQKKTFTAWCNSHLRKAGTGIEDIEEDFRNG

LKLMLLLEVISGEQLPKPDRGKMRFHKIANVNKALDFIASKGVKLVSIGAEEIVDGNVKM

TLGMIWTIILRFAIQDITVEEMTAKEGLLLWCQRKTAPYKNVNVQNFHLSWKDGLAFCAL

IHRHRPELIDYYKLSRDNPLENLNTAFDIAEKHLDIPRMLDPEDMVNSAKPDERSVMAYV

SSYYHAFSGAQQAETAANRICKVLKVNQENERLMEEYERLASDLLEWIRRTRPWLENRTT

DNTLPGTRRKLDEFRDYRRKQKPPKLEDKAKLENSFNTLQTRLRLSNRPAYLPTEGKMVS

DIANAWKGLEHAEKGFEEWLLSELQRLERLDHLAQKFRHKCEIHEEWAKGKEDMLQSRDF

KQCRLNELKAMKKKHEAFESDLAAHQDRVEQIAAIAQELNALDYHDVAAVNARCQHICDQ

WDRLGQLTVKRREGLEEAERILERIDQLHLEFAKRAAPFNNWMDGAKEDLMDMFIVHTTE

EVQGLVDAHEQFKATLGEADKEFSTIVSLAQEVIRLGQQYGLTPPDNPYTTLHAQDITNK

WSEVRDLVPRRDTTLQQEMMRQQNNERLRRQFAAKANVVGQWIETQLDSVASIGVQMKGS

LEDQLRKLQAFDKAIIAYKPNMDELERYNQEVQEAMIFENRYTQYTMETLRVGWEQLLTA

VARNINEVENQILTRDSKGISEDQMNEFRMSFNHFDRNRTRRLEPKEFKACLVSLGYKIR

DDRQGEADFQRIMSIVDPNGTGYVTFDAFLDFMTRETADTDTAEQVMQSFKILAGDKPYI

TAQILRQELPPDQAEYCIQRMAPYSGRDAVPGALDYMSFSTALYGESDL

>SG-PL_contig_1103_1_+1_145_435

MLSVKSRVTVTKLSPILSNCIRYNTISSSSSKKMQRTNTKRNNPVVWLGQPVHGLAPFPR

RIWSQIQAAELAAMWFAAKRMAHQIMGHYLFLYLHSL

>SG-PL_contig_1104_1_-1_1_210

IACMAVFLVPRHVGNHTPTSEESECDESPLNKLSWPHKSHVLVKCLPFMLSAVAVLATTG

SLSSADSDDS

>SG-PL_contig_1105_1_+3_3_131

QKRFEMCWSILAGPMLVTDTSFTVITYLLSQDPCGEVNIHLYP

>SG-PL_contig_1106_1_+2_749_1141

MHSYTQDRRQPNYYKCSVGVCMCVCVCVCVCARARTHTCIHMCVILCPCVYVCMHVYTCA

YVHAHSRVSACVFAFMTNAYSKHMIMFRVPLLKYTTVLLIRHTFLGCCYQCISMQNNFKN

ILTQLPYLTLS

>SG-PL_contig_1107_1_+3_327_545

MKVLSVLRLCPSLDESPCNDYFPVGFFFLLSADTKFEVKRISHLIQLSCNNNDNITKQSL

PPHLLPGPIIKTE

>SG-PL_contig_1108_1_-3_3_242

GSGGSPSVRSEVTVHYTGCLSNGKKFDSSRDRGQPFKFKLGVGQVIKGWDEGVAQMKKGE

VAKLVIPPDMGYGASGVQNV

>SG-PL_contig_1109_1_-2_71_319

MYISTFIVARYTFLFVLNLYYMSLPANEIRFYRCFLYLTASVYHTLCALHFCYFKIIRKM

GCFLFELFWGSFCFSVNMWCCGK

>SG-PL_contig_1110_1_-2_263_577

MVYYFCDRVIVTCISSECGLLVGGVCVFFLLCLTKKSFIVSLLYIKLVMYLIPRLPLLNL

TVCMLHVFFNTVAKDMNENVFHYLHVLFAFSTDVLITAIIIINTA

>SG-PL_contig_1111_1_+2_653_901

MLFVPFFLCLPFLIFPCTGGWSLRVNWILLHNHTISAFPSILWTASFHRDSHVGQFFSAL

WHLCCGQCKRCQMSLLKYLISSA

>SG-PL_contig_1112_1_-2_26_676

MSSEKFESLEDDLLSVLDEIKTKERKITGSSGEERKNAVRQVERKIEEATLIIQEMEGEA

KVAPVSYRTQMISKIRHHRSEVEQSSRNLRKEGASGTRGADNYGFDREDRLDASNRAKLM

QGNQSLQQTSESIARSHQIAAETDQIGVDIIDELGTQRETLLRTKDKLTDTDANLGRSRK

ILKTMARRMMTNKLILAVIILMELAILGGLVYWKFFS

>SG-PL_contig_1113_1_-3_180_608

MSGVVGDETWIDNKMRFEPIVPPKRETKQSLKSRKTGDIDHTMPFFSPAATSTVGRPGKV

PPPVSPKPNASFDDSFSRRDYKTEKDGIQETRVEHRVVLSSNDDDFDYDAALADAIRAVT

EFNPDMSVERIECVQQIEEVKDK

>SG-PL_contig_1114_1_+1_271_531

MRLTGRVCVCVCVCILKVILDLLNIVCVSERITGCVCVCVVSLFFKVSYIENITKISGLN

IQEVVHRSQDRATWSSITKCSGQHRTW

>SG-PL_contig_1115_1_-3_24_1688

MASTSTALTVGGDRTTGQSVRTQNVLAACSIANIVKSSLGPVGLDKMLVDDIGDVTITND

GATILKLLEVEHPAAKVLVELADLQDQEVGDGTTSVVIIAAELLKNADDLVKCKIHPTSI

ISGYRLACKEAVKYIQDHLTIPVDKLGRDCILNVAKTSMSSKIIGPDSDLFSNMVVDAAM

AIKTSDGKGGHRYPVKAINVLKAHGRSSRESTLVHGYALNCTIASQAMPRRIADARIACL

DFSLQKAKMHLGVQVLIEDPTHLDGVRQRESDITKERIQKIISAGANVILTTGGIDDLCL

KYFVEAGAMAVRRCKKADLKRIAKATGATFLMTLANLEGEESFDASMLGSAEEVVQERIC

DDELILIKGPKSRTAASIILRGANDFMCDEMERSVHDALCVVRRVLESKTVVIGGGAVEA

ALSIYLENFATSLASREQLAIAEFAKSLLVIPKQLAVNAAQDSTDLVAKLRAYHNTSQTK

PEHSELRWVGLDLVAGVVRDNKKAGVLEPTMSKVKSLKFATEAAITILRIDDMIKLVSQE

EGGKSYQDAVRAGEL

>SG-PL_contig_1116_1_-1_589_3135

MEGGRLRVGAAVAIVVGLVVMDLAVFPTSGQQTARDFFKPGVLDTCCTIGMQWSNSSGRC

SGYPAPVPGVDAGSQIACLSIVEVCCLKQVQLDMCESGKRTALDSQLCAVRDSDAGAEQF

RECCQCCQMGVAARRQSSSCQVPGLGEPCDSKFAECCTGRPSANFTIPALRDGNNDEDED

EDDEDEVVGNNIDECSVFPGQVCSHLCIDLPQGFRCDCPPGFDLSDDKRTCTFLDDREDE

MDCSSNNPCEQRCVNRNSGPECQCYEGYRLNEDARTCTDINECEERSYRCAPNQRCINTK

GGYSCLAVDCPPGTLRNSRTGQCEADNACLSGFAFNSLTRSCEDVNECALGYDDCKVGER

CENTNGGFTCRRIHHCGTGYTLDDASQSCVDIDECVLGTHNCAGGFICQNMQGSFRCVPK

ECPLGSRFDAGTGECRRIICRRGMRPNRAGTCVDVDECSEGKHRCLRHQRCINTVGSYRC

RSLVSCPPGYEATSNNGCQDINECTTNKHRCSADQECVNRQGSYFCQCPRGLRHDASGRC

VDVNECAYGAAICPATSRCVNTAGSYKCECKDGFISDENGGCLDIDECLTDNLCQHKCRN

VQGSYFCTCNRGYQLREDKRSCEDIDECTQFRGVGGRGGVCGGQCINTPGSFRCECPEGW

RSTRGGRSCQDIDECKENVAFCPQQDSICINTRGNYKCPVVRCPEGFAKTTSTGPQHSIR

CKRVETDCPECRRGLLSRSYNFLTFPSNVLVPAPLFSMTGARDPDKSYAWRLHLLSGRPL

KAGILPADLRHFRLAESLDQAVVSLVNRVDGPQDIELRLDMNITSFSSGFEGVAESRIYL

YVTDDGIGR

>SG-PL_contig_1117_1_-3_147_833

MSKPTMPCGYLGPQEKWEAMGLSTVTHNSPDIRCHTCRLEIKFKTTKPTKVTAVLLDANS

DEKYPELVFTQTVQELVYLVVAFPAAGFFKLQVHALPAEDESLSLPNIYNYLIQNTVPPS

GPVTPFPKQFAQWKDGCYLFEPFCLKGMDAAQIKVIVPGARAVALTINEEWFPLKQRPST

GKDDVKHRPWSGHLEGLSKFKGKAEKAVLTANFDEKDETKYTSLLSYNL

>SG-PL_contig_1118_1_+1_307_678

MIKLFVIGAQDSDRNAGLLQFLHPLPVMELFLVGPCLRFQIFQACGNLFKGGSVCRYLVP

AALHQVLVVGHHVCRGWGFRTSAIFHDLHHLLFITDALKWIQTHGQLPHDHSEGVDIARW

SDHL

>SG-PL_contig_1119_1_-3_3_464

QVCSLRYLCNNGGGPLREPPASRGVQDLVVAEDRTPRRLPGRDPAWVLGAHGSSPWRPWA

WRRSVLAYRQLPASTGRLLLLQLVLSLEGAKDDIGSYANGDLALSSPPEFRAENPAARPL

PSIPPHFLPGLLLGSQRYQERGSPPLSSPLRQVR

>SG-PL_contig_1120_1_+2_2_223

MCLGKNTPFGFIYCTMLDCKQNLMEEGLPLWFERGCLDSMVVLAAIKEMFLEQSALVVLT

SRRQKLMKRFSKCQ

>SG-PL_contig_1121_1_-2_107_274

MLYSVNTLFHENGQSYQLSLIIICVITCFWFLSYLEVILCFLIYPSHYIDLAFCFS

>SG-PL_contig_1122_1_+2_806_1054

MAGADRSIHSPAKSHPDEENVFTEPPGEVKDLFAWSTEDKASSFRHCFAVYCNYTTTVLM

LIMHTTNFSQQITTCQTKTKEKT

>SG-PL_contig_1123_1_-3_42_326

MESMLQSPFLLCSKSVLFMFRAPFPSVWSYFVCILFDANHSSTSSSLLLFPFTPNILNWL

FKVGTSFEFYGLSFCAILKRMTGFVGGVFVALLAF

>SG-PL_contig_1124_1_+1_880_1146

MDVNDLLMLKGKRYNFAECLSYGLCLVLYLSIAPSSLDSCRCCRKGELVSWAPADSPHDY

AQVCTLEISTQRCIFQKTEGGQSLLQWQW

>SG-PL_contig_1125_1_+3_252_401

MDDDENSTVELWLQEQQQCFLYQCKMASLNTTKSNLCSMPQEHCLIDQCL

>SG-PL_contig_1126_1_+3_507_764

MKRTIVRVWRLYSYRCFKNTYSMSTYVSGKHYLRELFITCLCSIASILHKAFLFMLLLLT

FCLAICFVFITEKQTRVLYLYYVSKI

>SG-PL_contig_1127_1_+3_2541_2759

MLMKAVDFGGCTSLTETLAIKCKESRDREKYSFHDRKHTPTHSLSLSFSRSLSLSHVHSH

THTHTHTHACAHR

>SG-PL_contig_1128_1_+1_52_519

MISFNIIVSLINTKEANNNNNNNNNNNNNNNNNNNNNEFVLHSAILLTNLDSLTKVGINV

TTLAHSWCFHDSISHRILRDLQDLQRAFCVRTHTKRLQALSCRPAHMLTWEIGKISTLCL

PTSQGAQSSNPGLPNLRVCKSPVFTIRPPHPQIITQ

>SG-PL_contig_1129_1_-3_129_653

MLVGPHPSPSAVYIGQNEIEVVQDFTYLGSSISNSSDMTKELNCRIGKAATAFNQLSKIW

NSKKISLKLKLRFYNSNVLSTLLYGCESWHLKTSQEKKLDSFDLKCLRIILGIRWMDFVT

NEEVKSRNKQNPVTSIIYKRRLSWLGHTARLPSSRPANQVLNWNPGDSEREEDQG

>SG-PL_contig_1130_1_+3_321_533

MEYILANHPCPEHFPISVSKTTRHASLRPFPLVRINISKATADTNPCWLSWTYHCLKKSL

QHILQRKSASE

>SG-PL_contig_1131_1_-2_56_259

MDSKAPMIKLARVTKVLGRTGSQGQCTQVRVEFLDDSNRSIIRNVKGPVREGDILTLLES

EREARRLR

>SG-PL_contig_1132_1_-1_652_1068

MMFEKHMVYSRLTVLSVLLCSQVCGDTLFISSPVTVSRWYGKAQRLLCRKILKIIGDLYF

LIKCLFCCCSYGTRQGVNYFSVWTLKKMLFVYLMQFYTKVQTEDLTCSYVFRYIRQMQLD

TSLLFHPAHTRFGGKENHV

>SG-PL_contig_1133_1_-1_1_144

FMMMVCVWSTVVCKLVILCTCVQCGLCKETSMRLCVCQMSSDFIIVWL

>SG-PL_contig_1134_1_-2_2_88

DGKILHPSYPPVAKQAGSNSGLLIGSLAS

>SG-PL_contig_1135_1_-3_2925_3173

MSTHDDAEITTTSSHASMCFSLLKMVSQCLDDSIYASPQMPWNGTSKIIMADAVSLHINH

LHWTGLDILKENASMSLQSDQSM

>SG-PL_contig_1136_1_-2_392_562

MCACFLLVILIMVQHYFSFLFFFLSCIFCSVCARKIFKYECQILIFCSFAVCFLGLI

>SG-PL_contig_1137_1_+3_3_206

MEKLNSSGPVIGCWGSRIQCSLASTPIFLRATRTCLGLSTQGLSPDMGTLRYSPDSCFSI

FSANTPRG

>SG-PL_contig_1138_1_-3_204_335

MSESQNSQIRLTDPFVRAGHMWYKYSSYLAHCHQVLTPLLSHLY

>SG-PL_contig_1139_1_-3_123_1721

MQAEAMSRKSRKASNKIQIKNVPSSVTCDDVRNAVESLIPGVAQKFEEAGAEGAVYVTFD

TVESAQEAITSLEGFQLEGTSLQVDFANNKSPRRPRSNGNSSSGMNRQDLPLRIVVGSEY

VGAIIGKSGQTIKNITQQSKARVDIHREDNVFPETVVTIRGSSEACTKACQEIMQVMQAE

AQSLSRGECPLRILCPNSLCGRIIGKKGNVIKGFMDKSKTNIVVSSFEASPYPSGMGDMN

NYYVDRVITVTGKLEDSIKAEEMISEKMKQCYDQDLNTYQQQQMMFGGLPTLPMMPGGMS

SYPSARPPYPYMHGGSMQGGSSSYYGRSMFGGGLIPPPPPQQLEVIFLYIPEQSVGAVIG

SRGQNIKNIMQMSGARIKIMGADVKNGSGSSKSGQQGQQSPDDRKVIITGSPEAQWKAQF

YIFEKVKTEMMLTAQDEVHLRSEISVPKSSIGRIIGKGGQNVKEMQRVSGAIVQVPEDQG

EEEEVPVTIIGHFYACQSAQRRVRALMNMKAQGLLPLSHRPARPQHALQMNGN

>SG-PL_contig_1140_1_-2_179_1099

MAKVEAFQKQLAAILLTLLLGDFVVVVVSGPVVKENMMKSLSKGSDIMAEVEKYNDMVPA

LDDWSAQVRSECEEPTLQSVQKVLDQLGQDNDILHRITSIALLQADDCPAAAISLANDLV

KVLKEKAGQEHVRLDIQGKVDHCDDVAECEQKHISFVRRTLKPYPSLVALYDVIGTAVFA

LSSDQDITHALLLDSLEDLDISTDNVDVETGGKNPTEKHSLTTRGSCINEKERNFYKKVD

WIPLLGDVYSTVSAAIYGAKGCTEEAKNRGLAQFKKITLRGASLIPVFGSIVQNTLQDAF

NFFGVKS

>SG-PL_contig_1141_1_-1_169_2211

MQGKNTNTLKLSNLIAGLYQMKVQVTADNKFGQAFVNVTVLPPKRKNKPPVAIITPASQQ

VKSENSFILDGSDSTDDDGITAYHWEEVSGPLQEHQIDDDTQTLKLKDMAPGVYVFRLTV

TDTDGATDSTSANVTVIKETDYPPKANAGSDVIIHLPKKSVVLCGNASTDDKGIASYEWI

KKSDALTADMTGVRTVCLQLNNLELGDYTFTLKVTDTGGNTRTADVHVYVKPETNRPPVA

HTAGEITVMLPSDSIVLDGQNSTDDKEVTGYKWTQTGGTNSLTISNLNQAVAVATGDIAV

GDYEFKLTVSDEEEQTSSDKLKVHVKMNKNRPPEAKTGGDRVVLLPVSMVSLDGSASTDD

RGIASYLWERDMESLAAGDVVNQSDHQAVLQLVNLVAGRYVFTLTVTDEQGLKSQDTASL

LVKNGETYKDLVELVLDTDIHHFTQDAKTNIVKQLELLIHQSSQHSGTVVEVESTQLELT

KGQLRLTFFVKKKDGSTVSGVDMVRLLKDKLDDDVILSYSIVSLDTVLCQNNCSGHGQCD

QRTKLCHCEAFWMPNVIKAAYRHDSNCEWSILYVVIVCFIIIVTAIAGTWGVICCCRSKR

CRIKTRKRHRYSLLREVEDEDDKTKLDLMPKGKIQNSSVMISESDFSSEEETLFINSKPA

NGHSKPPNGIVSRQHRNKLKT

>SG-PL_contig_1142_1_-3_822_1139

MMCQSKNRFMFMETRNICGLHLELTLFLAATHIDPNVYFPRGDAGGSFSINSIPTFWNFC

ASNFLFSVALFVAAVFSFLYCRLLFSTFPVHSTFFFPASLHTVFSR

>SG-PL_contig_1143_1_-1_1_267

HCLGEGSSGGGDYNSLEEFWAENEPLREADEISVPLLCVSSLDDPVSSSTHIQHELFRTV

PNLFLLTLPQGGHAGFRQSLQGLSWAENA

>SG-PL_contig_1144_1_+2_65_358

MTSKKESEKRTESNTTTKSIPKEKENPDLLRVVILHDFLLNGVHGNRLGHSYGLCVETQK

TPTINTMSQSLQASMTGSKNVDCFVLHAGISALKTTEP

>SG-PL_contig_1145_1_-2_98_766

MLEVNIHAREWITSAANLFMMDWILTNYRQSGGAAKMLLENYTVVVVPVANPDGYEFSRA

HPDNRMWRCNRRVFEFCTGVDLNRNFDIMWSNPFQEYLPEWRSDCNETSSYRVFCGYISR

TTNNSQDREIQCPDQYAGPSANSEPETQALISIFHHYQEHVVLHFTVHSYSKVVLLPWVY

TSVPANDIHQTMGNIVASAMGYGKAKTITDIIDNTINGTSLDW

>SG-PL_contig_1146_1_-1_118_675

MEWDSQRPRAPSTPTSELSHSTSGTSSSREKIPPYKQLNPSPVISALSKLSDDDLFRKGG

DEILRMLRRVESDYKNLLSEHSSVVKDVNRRFQIFVLEARGLKEINQKLQDDNQELRDLC

CFLDDDRQRSRKLAREWQRFGRYTASVMHSEVAAYQEKLKQLEDRQAELLSDNMELKELC

LFLDHE

>SG-PL_contig_1147_1_+3_1497_1757

MANRPVLSCACLAMLSLPCHVKPALSHHSLPCHVKPALSYYSLPCHVKPAPPCYRLPCHV

KPALPCHSLPCHVKSALSCQASLAMLS

>SG-PL_contig_1148_1_-3_972_1130

MCMPTHAYTLISASRFKSCWIFSLIYILLSYIYLENSLRCETLVSKHIQIVLQ

>SG-PL_contig_1149_1_+2_110_226

MVSVLAKREFTCKNSSFSVIHSTISNLCKSQRLEEGSDL

>SG-PL_contig_1150_1_+2_2_157

NLCNTAHVRSTPGRNPLNHRETRHKDASRRHPTHDAQVAHTRTTSSSLFSVA

>SG-PL_contig_1151_1_-1_1372_1515

MWIIPTVQQGDTSAQANVKVRSEWSVSAGVFISLLFRICSPLSLSSYV

>SG-PL_contig_1152_1_+3_171_581

MIFQKAWKERLVSNDWQTAVVAPIWKNKGIKEDCNTYRGIYLLSHVDKTYAKILEKCTMT

IVETFLSEAQFGFRKRRKRTDAIFALHKLCLKACEYQQQLHLVFVDQEKAFDCVNRIKLW

EVLESYGIKGQLLDNIR

>SG-PL_contig_1153_1_-1_103_582

MDPLQKLATDSNGAGMYVVGGILFLGIGILFLIHCGSTSHRKKRRQIEDSDVSIDPVNDI

SELNRKWREGQKKHQKKKTAVVTDFPEVEIIITPPTPCMDAGHGDNDSRGSSPSCTQTTA

SETTKQNGAKYNDVIRVNDANCNESLAPMTQTSQTALVTS

>SG-PL_contig_1154_1_-3_300_536

MPWSEASFELTKVSNMLSTTDTFPAFKAFMVEKTSPLYLRLGMTTTDDYLETKLRTHIAS

TACQYGGPECINDASNYLL

>SG-PL_contig_1155_1_-2_2_157

SIHGFIRGRILVTQQSSAADLSPCSKLTLPFEQTTKSKQFHRQLLKALWTKT

>SG-PL_contig_1156_1_-2_329_370

MLFCFVYALKYLFC

>SG-PL_contig_1157_1_+2_68_565

MIREDKDIKGIVINNKEHKISQFADDTQLSLEKSIYLVVKFGSTSGLFVNTDRTQALWLG

SKKWSQFKYMQHLNIHWNPNRFKILSMWFTNDVKGCEEINYSEKLSEARALFKVWVKRSI

TPLGRVAVLKSLILSKLIHLWILLPDPPDEFVNDLQKMCFSFVWNG

>SG-PL_contig_1158_1_+1_43_420

MNNGDKVSFEKSIRLILKFGKFSGHFLNADKMRAVWLGSEQGSKIKYMQHLNINWNPQRF

KILGVWFTHDVKGCEKNKLCRKIIRSKSFVQNLDKEKYNTSRKSRCTKIVNIIETNPSVD

PAPRSP

>SG-PL_contig_1159_1_-1_151_1221

MGLGDSMGMSGHGGGVGVSMAGVSGLGSLGPGLDNRAGYDESRRTIYKSGGSRFDSPARS

AAFDDRQGSGFDSQGRGGMDMEDRRGMDLGRGGMDMGGRGGVDMVGRGGIDIGSRGGMDM

AGNRGGMDMVGSRGGMDMAGGRGGMDMAGGRGGMDMAGGRGGMDMGRGGMDAGIAAGRAG

MDLGGQGRFDSMGGRGGLDSNNRDGYDSMRGAGLGGMDMGNRGGMDFDGGRGGYDQGMRG

STFESSGRSGGYDNKGRGGDMSSGRSDGRGMGGGARPGQARCTVTVRNIPFNLSWQDLRD

RFRDIGDVKFAEILMDQGRPSGDAVIRFGNPEDAKRAINLVDGTRLEGRTVRVVLRN

>SG-PL_contig_1160_1_-3_66_653

MAGFVSKSPVGSSHSGSSNYSGTNNSPNSRSTGSSISGPITKIREKNVYPTPWRQRVKTS

PDGEVTQIMSRTDADGLKVTKTKVTRDLRSPRSVNSPRSVGSPRNINSPASLSSSRAGST

TGSRPTSRAGSEAGSDDRGDAEVYTTMQTESRNVGGRKDTTLTYRTHVVETRTVTLPSAT

QTKISRIPKMSQKPRK

>SG-PL_contig_1161_1_-3_3_257

YAHARAQTKLTFILLAQSNCSPKRLHGDCSCFCQLFISKHTFGHLICRVVENVCTRLSIA

VTKAACFDFVSSSCVDMLKSMSAFR

>SG-PL_contig_1162_1_-1_1_171

WSSDVCSSDLFFFPFRQSVVRRRKFLLVSFPFIEWYQHGCSQDFPSENETGKEDQAE

>SG-PL_contig_1163_1_-2_2_217

TQAFETLQFGSQESSPFDHRARLRQSWSCSLQTVGIIGLVNPQIIIIILLKLLLLSWLPV

YQTTRPYIFRAI

>SG-PL_contig_1164_1_-2_104_466

MQKFISQTCQSCYCQLRRFSSIRQYLSTEAASKLITSSVLSHIDYCNSLLSGLPASSVTC

FQCIQNSAAHLVSKKHDHVTPLLQLLHWLPVTQRISYKINMFCFGFAPTYLCDHLHMYTF

V

>SG-PL_contig_1165_1_+3_738_1037

MQILTILRAKTVNHQAQNSAFLFLKFSIQKLKRRKIQHGKIKTILVLSMEELHQSVTSFS

LNISAENKISFKRFLQNKCCINQTIVTSLLCRQKRFHTYM

>SG-PL_contig_1166_1_-2_26_451

MQSSRLLSRLPTMTTVVVVMVIIIGLSIPVEPGIFKGLSLSKIIGAAESAVTVGDSVTAA

TQAAASSGYRVTCVIEMENWTKYLLSNPRLQITNSGSLVTNPKSVLPSQMQGFAVRKPAH

TATGVYGTVSWQLGNTGRRVVV

>SG-PL_contig_1167_1_-2_125_277

MLFKGKHQIIADSYIQAILDSLLSSYYCLKVLEMYSQIKNSTSCVCISLNF

>SG-PL_contig_1168_1_+1_1_462

LILTLFPYTTLFRSYNHKFISDRLFFDSIAANWVQSLFALLLHCLQTLLVWCQKAAHRPR

LLRPQVLWTVFLGAVEFPQVLLLGLVHHGHQTSDGFAHGMDFCQLGGSSTSHLGNPKGLK

FSFEFLQLLEEFILLLAPKVLCLNLTHVSCDYRK

>SG-PL_contig_1169_1_+1_1933_2232

MVFPISQVNICADLIVPKLPSCVYAYRTPHMHIKDPASHVGVRWLMEKRNVPSMHTLFSL

HRLQAVLALASPLDFTCVAERVGLLHGQQQVLLNTLPWFV

>SG-PL_contig_1170_1_-2_287_1720

MEWCPLDAEDGSRDQVLAVSAGRQMWGGWPVFKRGEGPGLIQFWTTGPLDNTQSQESPSL

TLAFTMAHNLQHVLSLAWCPRGGYLDPTSDNGDSLPRLGLLALSGYNSGVQVYSIPTPEA

VRDIVTGAERSSKQSSTPPCFKPPPSLTLKCADPDTCGACLSVAWQKGEDCAFIAAGYVR

GFIHLWHLSSASPLVKLSPDTLAPIRYFYAHVKIYSIDWSWVDPTHFVTGSWDNEIRVWD

LSDTSAPLFIYPNVMTFEKGPTRAVAYGSVSFDAIVCCDDLRDCYTGSVYAIDIANGFPS

GPRVNRANTTLAMHAACVWDMSYSPWFNMVASVDSTGLVYLSCCLPVHHLQRGQNRTARR

KGLPVFTTHCSNLKEGQDTDPAGDKDSLSAEDLHITFKDRSMEYKKDFIQTETEEDILRT

SFDKFRPADAYAVCFNPNKASSPWLAVGTKAGFVRLLRMTCVTLESHSAKVFFHQSTQ

>SG-PL_contig_1171_1_-2_497_895

MLHIATLQCVKTFQRRNWGNAGIITEGQHNSSWCFSIVERDTVSYRKRATDNLWCHHPKR

GQHFMLGRSESFRYKGASCSYSPLFHFTYLCQLWSPGVSFQVIQRYWIVQGTIILNPVTE

IVQTGVLFHPKEG

>SG-PL_contig_1172_1_-3_51_482

MRPVIKTSKPTTATTITTTTPAPAPAASCTPSQVLAFFQLLANLPLLLNGNVFTNQMTST

TTEVPLPRLMRRAVVSVDDGYCHGQLTTAQNNSLQMLLYLVGNRDINAEGPLDLYNVPQQ

VRQTLQQERNKTLLRRSEERRVGK

>SG-PL_contig_1173_1_-2_50_628

MAEKNLEGNIDASARETPSLKNKLMAISLSNASARELFSKTREKVKPWGDFINTSRFKLP

TSVAPLPKRIIKNVDHFQGNYLFVFLGLFIFCVLTSPMLLVAIAACLGACYIISIKSADK

PLVLMGREVSLAQQYAAVGVMSFPLFWLAGAGSAVFWIIGASFFVIMLHASTYVPQEEVE

GFDLEMDSIETVS

>SG-PL_contig_1174_1_-3_177_596

MQVDNIFPLKRAFQDPRPNYITLVPKDAIREDMCGFEEVTVVPEPIKTRPYRGPSFNVTY

PHTPAKELTSEERAIIAVCVTVFFLLVIIILIFVFRSRFEGGDYETEEATGAEFADDPDT

AVVYNQTGLPNMTTKYEFFM

>SG-PL_contig_1175_1_+1_934_1224

MPMEELLALSQDGNIFSLKFMHFIFTGVFKCHRTPSSLTLHLLYIMLFNRTQLPKDTSSF

IFGRTIIHLPYFSQVSKVKMMSRETARHLTPSDGIRT

>SG-PL_contig_1176_1_+2_2_262

LQLVLPLYQIQPYFAPGSTFISPTAIFCSWFYLYIKYSHILHLVLPLYHLQPCFAPGSTF

ISPTAIFCSLFYLYITYSHVLQLVLSL

>SG-PL_contig_1177_1_+2_17_214

MRLLIGLLQSQRTMELVPLERLQDRPGQSGKENLYGQWYKLQALPLWSVVQITGSTFMVS

GTNYGL

>SG-PL_contig_1178_1_+1_1_162

LRMTAKFDWPPSVPKAYVICGAGEIPGHKPGQSVKENVLYCKVYSSCFRLSTVS

>SG-PL_contig_1179_1_-1_16_900

MAAKSKVEVYGLLNDISYVQARCCAEDIYKRCPGKLEEPVTVGMLQFEWDIYIDAKKREL

RGETWQFNEKAITFVDGELIGGPEDFIKWACINHNFEEFRPEALYVTLTEEGYKCHLNSK

NHDYVYMDISIGGEEAGRLVIELFSDKVPKTCANFKALCTGEKGVSEETDYKLSYANSLF

HRIVRNGWIQGGDTFHGRGSGGESIYGTVFEDENFSVPHDRRGILGMANKGRHTNQSQFY

ISLQPTPWMDTKYVAFGQVIEGTETLCKMETEETLNERPVRDIKVTACGILTFPF

>SG-PL_contig_1180_1_-2_1628_1918

MCSGGKINSVNICTVFGNCRLVHWTLLLVPYPSPHHQNHFHSSALRETSYHFLRNTKRHD

KEYRLTWRRMAGKCFKTGLRLGFSALVRNLSLFFKTA

>SG-PL_contig_1181_1_-2_104_460

MASLSTKPKSEMTPEELQAREQEEFNTGPLSVLTQSVKNNTQVLINCRNNKKLLARVKAF

DRHCNMVLENVKEMWTEVPKTGKGKKKSKPVNKDRFISKMFLRGDSVILVLKNPLATAK

>SG-PL_contig_1182_1_-2_68_1102

MLSRIARPSQAVCILRHQFSTSSKVKKDAKVAVLGAAGGIGQPLSLLLKQMPIISHLNLY

DIAHTPGVAADLSHIETRAKVTGFLGPEELHKCLEGANVVLIPAGVPRKPGMTRDDLFNT

NAGIVRDLTDAVASVCPSAMVGVITNPVNSTVPIASEVLKKRGVYDERRVFGVTTLDVVR

SNTFISEAKGLDVSKTNVPVIGGHSGITIIPLISQCTPPVSFPMEERTKISTRIQNAGTE

VVDAKAGAGSATLSMAYAAAEFCKSLLEAISGGEGVVQCAYVRSDETEARYFATPVLLGK

KGVEKNLGIGKLIEFEVNLLKAAMPELMANIQKGEEFVEKTYSVK

>SG-PL_contig_1183_1_+2_206_529

MLNFYLNIFFPCEVLDALPRESTVHCTAPSFFLSEFCSFLCVSFWHCCLYICSCNKIRNT

PSCPQSLEIHVVNSTILRYMTFYSSCLQLLSLPCTHTHTHTHTHTHTE

>SG-PL_contig_1184_1_+1_178_396

MVATHTRYQLTTCNQSIQHVMTTVPPDLSFKMSSMSEQTALLNLLHTCICVCVCVCVCVC

VCVCVCERHTLLG

>SG-PL_contig_1185_1_+1_61_288

MHRIICTCGVLYKSVYSKANQHRCQFEVTVTVWHQYSNQIYNKRYTNSTLPNIYIYIHTH

NSSYTNFPSQCIKGFS

>SG-PL_contig_1186_1_-1_685_1788

MPSRESFNVNTMSCDTHDCFSPERLREEIRTHAKTIVVDVRSQAEFTRGHIRQAINISLP

SLMQKRLKRGSLNISSVIHNNEAKERFSRCCKSHLLVLYDECCSDLNANPSSVLCLLVKK

LRQDGSKVAFLVGGFSTFQQRFPEYCQTQEVTDNTILGLCNLRISDDFTHVSGDGSADLE

NTPHMSPFPVKVLPYLYLGNAKNSADLNQLKKNGIHYILNVTPNVPNMFEDDGDFKYLQI

PINDHWSQNLSSFFPDAIAFIDEARQSKQGVLVHCLAGISRSVTVTVAYLMSRQSMCLNE

AYDFVKKCKPNISPNFTFMGQLLDFEKALHHATSCPCTSKPSSAGDDSLPASDCDSDKAS

PPFEVSPV

>SG-PL_contig_1187_1_-1_472_1791

MAVNIDRSNMDQFYRYKMPRLLAKVEGKGNGIKTVIVNMAEIAKALSRPPTYPTKYFGCE

LGAQTQFDTKNDRYIVNGSHDADKLQNLLDGFIKKFVLCDECSNPETNLIVSVKKQMISQ

RCIACGHNGNVDMRHKLTTFILKNPPDQDLQTTTPSKGKKSKKDKDKKEENDRHSPEANA

QQQMAAQRASGGTIDVPPEQNDGDDDDWGEDFSEEARKQRMDELSGAAKGLALTDDLEKT

DKERMDIVYKLIKSKRDSGELGRCVKEVLGEAERLEVKEKVPLVLVELLLTDKILMQAKE

HRILFLGFCKDSPKAQKYLIGGLEMLIGQEYSSALMPRVPHILKLFYDLDILDEEVILDW

AKKVSKKYVSKEIAQQMREKAKPFIDWLEQASEEEEDEEDEEEEQEVVYSTTGQVGEQPI

EKPQENGDGDDDDDFDIDAI

>SG-PL_contig_1189_1_+3_684_1331

MCENFHQYEALDNITLNANMPERLRYSTAEERVAAMKNPTNLPGELEISATADTLGRQIQ

IVDKDTRIISRYGGNQAASEVPPLIVRYVTAGADVGHYDCVLPTAAAMTDDEYIRKIIET

VSPKPVTEREQRKRKCESATVTTSSPFKNSLLEKRAENTGVKKIAKGRDKAAKTVTRKSQ

KKPPEDKDASDDEEWPCYYVWRAFCEQPTSGNLGCV

>SG-PL_contig_1190_1_-1_22_5100

MQKKHGLLEADVVAHQDRMEALNSQATLFASGDHFDKDTIGKKAVEVKERYEKLKAPLVA

RKQKLTEAQSLQQFLRDVEDEEDWIREKEPIAASTNRGRDLIGVQNLMKKHQALQAELAG

HEPRIQNVCATGQTMVKEGHFAHDTIKGKINDLQDRWQQLKDKAGQRKQDLEDSLQAQQY

FADANEAETWMREKEPIAANTDYGKDEDSAEALLKKHEAFMADLEAYRSVIEGLRDQAQS

CKQQEAPVIDDLATEKVVALYDYTEKSPREISMKKGDVLTLLNSANKDWWKVEVNDRQGF

VPAAYVKKLDPSLSASRNNLMDEFTISVRQNQIETQYANLLDLANQRRENVKESCKAYQL

VREAAELAAWITEKENMMIGEEVGEDLEQVEEMQKKFDDFQKDLKANEARLQELNNIADR

LTAMGHTEAAEKIREQITQLNQRWVTLQEVSAERAQTLGSAFEVQRYHREVDETKDWINE

KDAALNNDNYGHDLASVQALQRKHEGLERDLAALGEKVRDLDSNAQRLMQTHPDQAESIY

EHQTEINEMWNSLTIKADARKGKLLDSYDYQRFLSDYRDLTSWINSMMALVSSDELAKDV

TGAEALLERHQEHRTEIDARAGTFQAFELLGQQLLQNQHYASDDVQDKLEQLAKAREELE

QAWIVRRMKLDQCLELQLFYRDCEQAESWMASREAFLAGDSVDGDNVETLIKKHEDFDRA

INSQEEKINALQQFADQLVTAKHYDSPAINDKRDSVLDRWSNLKDALIDNRSKLGEAQTL

QQFSRDADEMENWLQEKLQIAMDESYKDPSNIQSKFQKHQAFEAELAANADRLQGLLGTG

QNLIDRRQCAGSEDAVQARLESLASQWEDLVAKSTEKSDKLKDASRQQTYNAGVKDMEFW

LGEVEQMLSTEDYGKDLASVTNLLKKHQLMEADITAHDDRIKDLNDQADQFVEAQVWEAE

SINDRKRTINERYEKIKELAIHRRNRLNEANTMHQFLRDIDDEEAWIKEKKLLVGSDDYG

RDLTGVQNLRKKHKRLEAEIENHEPSIQAVHETGESLMAESEIGASDIRARLDQLTQSWE

ELKEMASTRGQKLEQSYAFQQFSANVEEEESWISEKQHLLSAGDFGDTMAAVQGLLKKHE

AFETDFQVHRDRCTEIKKEGQRLIDEGNHNADSITERIAGLEQKLSELEAVASQRRSSLA

DNSAFLQFMWKTDVVDSWIADKETQVRSEDFGRDLSSVQTLLTKQETFDAGLQAFEKEGI

QTITTLKDQLVNARHAQTTTIERRYNDVIARWQKLLADSEGRRQKLLLLQEQYRQIEDLY

LTFAKKASAFNSWFENAEEDLTDPVRCNSVEEIRALREAHDQFKASLSAAQADFNQLAAL

DKQIKAFEVGPNPYTWFTMDALEETWRNLQKIIKERDTDLEREERRQEENDQLRKQFAQA

ANAFHGWLTDTRSSLMESSGSLEEQLEATKRKATEVRSQKGQLKKIEDLGAAMEERLILD

NRYTEHSTVGLAQQWDQLDQLCMRMQHNLEQQIEARNRSGVSEDALREFSMMFKHFDKDK

SGKLDHHEFKSCLRALGYDLPMVEEGQTDPEFQAICDIVDPNRDGYVSLQEYMAFMISRE

TENVQSSSEVEQAFRALTSGEKPYITAQELYANLTKEQADYCISRMKPYVDKSGRAIQDS

YDYVDFTRQLFVN

>SG-PL_contig_1191_1_-3_3_224

LQQTTEERCEILYHLSVVCWSLSSCFLTGCLNDPGFSLHCVNPAGPLQQATEERCEILYH

LCVGLFHLVSSLTV

>SG-PL_contig_1192_1_-2_8_2461

MVGLQILGTIKLLHAKGPLYYDYDMEDYQFFDAVPHERADQDMAEKNEVFDNQPSGGTVA

AANTVTVRKDFPDTWIWKTAVTDASGQLSMVETLPDSITSWVISAFAVRDDTELALTPNP

FELSAFNPFFASLKLPYSIRRGETFQMRCTIFNYQTFSVTAEVTLASSPDYKISGNPKQK

IEVKSNSADSVTFLIVATTVKTIDIQVSAKGQKSDGSGGTFTDVVTKKLIVKPEGVKKMK

VVSFPVLLSSEMPSHTVTVYRDIAEEIVPDSLTTIVKITGDMMGNVMSNIDDLVTQPTGC

GEQNMITTVPNIYAWTYLKAINELTAEFKEVAKNFMKTGYLRETQDYRWDSGGYSAFGMI

SDEKYGASTWLTAFVVRSFAQAKQFIPETVSMDLLNGDITFLLKLQNPDGTFMEKGRVLH

DEMKGETGEGKGLSVYITMCLLEAKKYGFEFEASKLTSAVNNVKKIANGKEVKTNPFLAA

ITAYTLALHNANDQTTVNNLIGYIEAEGKAPWKKPKPGPSDVEITAYSSLALNTIGSFGA

VRDMVMWLQKQQNANGGFSSTQDTVVALQAVSEYAVSFTGTASSTVSITTLDPVMPTKTV

TIDSNNKALLQIMELPDETKSLNVTVQGQKNSLSIVKIVWYYYTIPNKSSEGSDKLKIST

NVIMLGPQRFKVRMCMNKDRTQDYKNMMVVLFNAPSGAVMENILEAKQQNSKVMRLEQTE

NENQYNFYLNEPTDECLQFRMEQSVVVEDQKPGTAILYAYYKTDFRAESEVVMPLECPPS

GCCIEDNCGTSHAAHYASLSRLLLSACVCLLLSLLIAV

>SG-PL_contig_1193_1_-1_1_222

DSSEYSYPDYSSYGQSSSYGDASSYHPSSSYEDSSSYGQSSTYGDYSSYGQSSSYGDYSS

YGQSSSYGDSSSYD

>SG-PL_contig_1194_1_-3_3_128

QNSSFNCILHVYDVVHLYLMLCLIVIYDQSSLYYDLCMYSEV

>SG-PL_contig_1195_1_-2_2_160

PSPLPLSPPPPPPPPLPFTFHFPVLSSLQLSFILKACALKIEKGKKSFLWYKC

>SG-PL_contig_1196_1_-2_71_796

MKLNIAFPATGCQKLVEIDDERKLRPFYEKRISAEVSAEPLGDEWKGYMFRITGGNDKQG

FPMKQGVLTNGRVRLLLSKGHSCYRPRRTGERRRKSVHGCIVDANLSVLSLVIIKKGEGE

IAGLTDTTVPRRLGPKRASKIRHLFNLAKEDDVRQYVVRRPLQEKEGKKARSKAPKIQRL

VTPVVLQRKRARLAVKRKRAEKKRVDAADYAKLLAMRLKEAKERKMERRRSSSRKDSMRK

SQ

>SG-PL_contig_1197_1_+1_37_375

MFTVTSTILILSQTLSLGNGAVFPCIQRSFVGLVLYTDPIWDKKTFSTHSFVVLFGPLCE

APLFGHHNLLATGVLEFGTTQSFNDGNLMLFTSTHRHDGLSNVHSGYCTLGLA

>SG-PL_contig_1198_1_+2_701_976

MTRGYRSTHTTHTKVQTHIIPSQHTNKHVRSDLISHSVYRVPCALDKNETVCLSIHHHPT

QAEILFSNDTETHKCSLIKVSQHLVWYTKLHE

>SG-PL_contig_1199_1_+3_39_230

MTQSQYKQMNQNALINNHLGFIHCSNICIPGQGRLLSNNMNVSLHLKMSQTTLYHKILPL

SLFF

>SG-PL_contig_1200_1_-3_51_1463

MAALMCRRFYLSQSRFFRDGVRTICASPIQNANAQPAKKTLTKPNVRNVVLVDGVRTPFL

QSGTSYKNLMPHDLARTALQGLLKRTGLPKDAVEYLVYGTVIQEVKTSNVAREASLGAGI

SDRVPAHTVTQACISSNQAITTGIGLITSGQCDAVVAGGVEFMSDVPIRHSRKMRRLMLS

MNKAKTLPARLQLVSQMLSPAALTPELPAVAEFSTNETMGHSGDRLAAAFNVSRLEQDEF

ALRSHTLAHEASEKGLLTDRLVYKVPGVAQPVEKDNGIRPSSMEAMGKLKPAFIKPYGTV

TAANASFLTDGASACLIMSEEKALAMGYKPLAYLRDFLYVAQDPKDQLLLGPAYATPRIL

GKAGLNLSDISVFEYHEAFAGQILANMKALDSDWFGTNMMGRQGKAGAPPMDKLNLWGGS

LSLGHPFGATGVRLVTTAANRLHKEDGQFGLVAACAAGGLGHAMIVERYPR

>SG-PL_contig_1201_1_-1_163_1173

MPVKVGINGFGRIGRLTLRAALDNKDVTVVAVNDPFIQLDYMVYMFKYDSTHGRFKGDVK

ADNGKLVVNGNAITVFQEMDPSNIKWGGAGADYVVESTGVFTTIEKASAHLKGGAKKVVI

SAPSADAPMFVMGVNEDTYKSDLTVVSNASCTTNCLAPLAKVVHDKFGILEGLMTTVHAC

TATQKTVDGPSAKDWRGGRGAAQNIIPSSTGAAKAVGKVIPALNGKLTGMAFRVPTPDVS

VVDLTVRLEKAATYDDIKAAVKEAANGPMKGILFYTEDDVVSTDFVGDAHSSIFDAKAGI

QLSPNFVKLVSWYDNEMGYSNRVVELLAFMAKKDGSK

>SG-PL_contig_1202_1_+2_2_187

PCAHLSAPRNVSASTSHACPSDKRVTSDSVGFFFFFFHLTSPIIILKLLMLCACSVLSRF

HK

>SG-PL_contig_1203_1_-2_548_664

MVTNVPWTVDYRSLLVSLKVGLFASKFILVKLLSKEYIL

>SG-PL_contig_1204_1_+3_414_692

MWSTLRQHLLISDLEIHTDTCKHVLHGYDLDLLAVVMKLYRNDFLTVTHLSVKLSYNPFS

NSRDIFKQAGFFCLFFLFLNQSKVCKAGKLSLK

>SG-PL_contig_1205_1_-2_62_403

MQTSLDEATDPWGVRVERVEVKDVRLPVQLQRAMAAEAEAAREARAKVIAAEGEQKASRA

LKEAADVIVESPAALQLRYLQTLNTISAEKNSTIIFPLPIELMQGFMQKSGSRL

>SG-PL_contig_1206_1_-2_1061_1243

MHNNTLEWQLMEYFSKFILHRSSSCIRRLFCQATSGNEYGRVLFFFPDAAICHGAWWSVE

L

>SG-PL_contig_1207_1_-2_758_1060

MGIALTTLAPREVEASTTSLLYDLRVMGRPLPYIFHIASHIQSLKCWLKYSCEPHKSKYW

TMLFLAYMLNLKWWVLLLLLLLFVFNAADSALLIVLVFVPC

>SG-PL_contig_1208_1_+2_578_988

MIRLTKKRVNALEMMSMVLQHMQTVQDDTTLSTDNTFLKMRLALTLSMTIYPKHKKKSAM

QKGKLREEACVEENKVVASVAKNMQEQFSYFTTQYPELSCSKNNNNNNKNYNNFVQSNRQ

TQVKYLSFSSEAHIFNQ

>SG-PL_contig_1209_1_-1_1_246

SGYFPVFSSWRAFVVDWVVSDEVDSVLLPLVGSPMPNWSSGRSILRVAIYSCPVFCLPAL

SSVFRSPTNLLFNILLFSVIIF

>SG-PL_contig_1210_1_-2_1067_1486

MIICFRSSWWLVVEGFWTLVGRGIITLQLEDRLHHTHALLLSKTQTSVLLSWGPLSDIRM

TELATVSFQSSQGSISALGINPISTTATNAMSWKKINPKDHGMSQKPVRKPNALVRKTVA

CLRVEPTTPALSAWHSNQLS

>SG-PL_contig_1211_1_-3_3_152

GEKTDTAYLNLFTACPQEKLKFLPRPLTTTAGLWNKLFNNLDMFYMLRSF

>SG-PL_contig_1212_1_+3_135_335

MRTPITTNTWDETMRTVNECHDETVRTVNERHDETTSMVIGDNTDAVCKLTTVGSKDWIP

WHAQCWA

>SG-PL_contig_1213_1_-3_165_1217

MSKYPIDFSTVNKNNSSETNTRGGLKSRKFSRCCVSAPLGFVLLILVILGLIVHSMYSRD

LTRKMERSGLPGAVEACRSSIENGGTTSEQICHACSGAPARTRPPTVTSTTNRDNYRLPS

SIKPSHYTLRLQPHIYDTDPNTFFFEGSVTIDVTCLQATNVITLHVDRLDIDDSDVSVMD

KASGSPINIRDILYKKARQFYRIRLNENLVVNAEYRISFDNFRGNLTTDGQGLYVSSYRD

EDEVVYLATTRFKVQNARKAFPCFDEPAMKATFDTTLVRKVNYRTLSNMNQLRSEPFGVG

WEADIYNTTVAMPTYLLAFIVCDYDHLSNNTFGIRYSTWAPPNQIQFAVTA

>SG-PL_contig_1214_1_-2_653_811

MTCFPLRFADWRRVLQPGLAVLLVVRCLISHDHYDVVDKTTAIPKQSFNQDLN

>SG-PL_contig_1215_1_-2_581_784

MRGQVLGQQPALSNMTATETSTQERSDILCSTCSDKDWVRSRWNTSKTLQTGFKVEKNQH

VKALHQKL

>SG-PL_contig_1216_1_+2_2_136

ITLAPRMLMQAKHWKASGSHLNCHNTGTSHAYASKELQVASLKLS

>SG-PL_contig_1217_1_-1_1_171

LIPTTPGLRRRLTRRIAYTPAKYKEEDEFIGFATLPEQVHRKAVRRGFDFTLMVVGQ

>SG-PL_contig_1218_1_+2_68_286

MRGNKQMHKQTIKMCASQLTEYDVFDRSLTINCIPFETAKPSLQNILTKKTKKLTNKRRS

NCTSNGPFLPKMN

>SG-PL_contig_1219_1_-3_3_104

MDASLRVNFLTNSTRICNSRAWLTPGYIMKGNID

>SG-PL_contig_1220_1_+1_1_225

IARIPKIETHTLVLHTFRWTQRYVHATRSYTRFCIYLYIQNKLCIYTSLVHTDICRFLLL

FTDFCNQQELKLPFP

>SG-PL_contig_1221_1_+3_99_404

MVGLFVTCTQAVGGITNLCSVVLDLYSTGIHADALSAMALSGGVVSISVTLGFELTQQPT

VLLVALGTRHRGTSFLHHPTLHRSQTSESCQREKKSSPHCVN

>SG-PL_contig_1222_1_+3_141_239

MLFVVCVPQLNTLGVTQYICTLSYESETTRTFG

>SG-PL_contig_1224_1_-3_960_1172

MSIQFLDDHCVQACVPPPLASSIHSTSLAILLTPGKNQQAATFPSLHVQSGGVPSPLTLF

SKSSFLFLKGK

>SG-PL_contig_1225_1_-1_325_576

MSCISIVNDNIKQDNSSKLTRARAHTHTHTCPHTHTHTYTHTHTHTHTHTRTQTDTHVHT

CSCTGFCTAFFLSHYYSDVILELE

>SG-PL_contig_1226_1_+1_1_195

KKKSQVNVTICPFLFSCLINLYTLLPSTSHVHIQSFWLPVCLSECDYSPSTDTYHTHTPR

SLAHK

>SG-PL_contig_1227_1_+2_65_232

MIVSFYHQNIFGHMIHITWHTVFQATHDFLFNYFVEKLPNPELPFQKKQPPGIEKK

>SG-PL_contig_1228_1_-1_529_639

MLLNIEHINDHKYRRNMFIDLHQLIAKYVGFCCCCPP

>SG-PL_contig_1229_1_-2_2_181

CKEWNKKGVRLSSLRVCFTSYGLSSTVNIDGRGGRTVRTLDFRLEGLEVLDSNLLEHLAW

>SG-PL_contig_1230_1_+1_2032_2748

MLPFAFALVHVATTSDVFLWEILETISSVSAEPAESSESQAPEACNLDTATDFPTGTISI

SMSETHSSAASSFSREKIDSCFSLKVDRSLGVDSYLGGTHSRFLLLVSLLFSSTGQDKSD

SAIFSESKLSDFNFFKQCVILLGSIVTIFRVLGLRKRKTGRTRLYFPITQFPLSSIGVPG

TAFFIPSISKAGLLCTVDGLPSLFFDDILASVCSFFSLLRKLSLAYCNFSWPPFHFVLL

>SG-PL_contig_1231_1_-1_1_189

VLFCSQQCFLGCMYACCLAHTAQALYYCTICILIFVGCACWIFLCFHKPLNTDMVYRIFN

VRI

>SG-PL_contig_1232_1_-1_1711_1926

MYVLLAVCSDVLLLAYYSVFPGEGRMLGKIQTSALSINLGNKELSLSLSLSLSLSLSLSL

SLSCVCVCVCVC

>SG-PL_contig_1233_1_+3_753_911

MSLVAKIIYQDCLTKCIGLGFKSIHICPVLTSTPYPLNSVELCRQYICGRLFF

>SG-PL_contig_1234_1_-1_196_324

MPSYKMCSTHTHSVLKSTPLLLTTHLAVSNRIITRVSSSSATD

>SG-PL_contig_1235_1_+2_2_139

MSLRKERNINTVKTPVEITGKLNVFHNRKFTGKGYGRHLTTFVKAS

>SG-PL_contig_1236_1_+3_3_338

ESSERLLRGCQLRKRLPQPRRRRLSCLRVGRLTVPGAAPVTERLDPPGVGGHTNQPWDAC

RGSSGSGTACRGTPCSGSACSPGRLLPRWREEEEVWASSDEGEPDEECLRRR

>SG-PL_contig_1237_1_-2_146_340

MKNLRPALIAILLLMALTMITLTAGEEKAEDSMRLGSMMKRSADFGQPHYDPLCVCFTSP

CPCDG

>SG-PL_contig_1238_1_-3_24_371

MEPLVLFPCRSVCNKIQQTSPIIRVASPQSGGHGSRHSLDGLEHPPGLRLSPDPHSVKGD

QEPQTASLRGDSFEPPTTGPPRLAALRQGMPAQHLELPSDLEANCDSFVRSTLQRA

>SG-PL_contig_1239_1_-2_2_238

EQRRPQLAVEPLVSSPCRSVCNRIQQTSPIIHVASPWSRGLGIRRSLDGLEHPPGLRHSP

NPHLAKDNQESEAREHKDD

>SG-PL_contig_1240_1_-3_15_1004

MAGPLVNNFEVPSWAGKPPAGLHLDVMKDTKMVQKLMIDEKKCYFFGRNKQLCDFPIDHS

SCSRVHSALVWHRHLNRPFIIDLGSTHGTYIGQIRLEKHKPQQVPLDSEIHFGASTRVYV

IRERPQPVGPGGLDEDGTKQTEMEGNLLGLPESETELDNLTEFNTAHNRRIASVAELVDV

PNPLKRKPKSAHVTFADEEEIINPEDIDPSIGRFRNLVQTTVIPSKRAKLDGGLGSGLEG

DHKQNTRISHFGDIPSHGPGSTLAHPLSFAAKLGIPVPNLAPDVDHEPALPPLPLVAAPF

VPEEGPKEPRKKKYAKEAWPGKKPTHNLLV

>SG-PL_contig_1241_1_+2_1892_2437

MHTFTLLMHYNPKLPSNEGDLKTKIFFFYYGLQVQILFKNVLSISFMLFPFHSPALRLIR

SRWVYRLLRQNKPTTLCTELPTTLQPSSYRLVHVQCDYTKVSRGLKYPVKNKTKTQISCF

EIGSPATTLRSNTLSFSLINSCIHSPLPSSKLNHKYTCMYSNKIKQKERQYLVFDTLFSI

PQ

>SG-PL_contig_1242_1_+1_1090_1332

MLIVTNSGPYDRKFVKDLSNITSPSSINPLASCPAATGQQEVPNVYLCPATAPAGISGLV

MNFHHALSSGLYTQTMNEASC

>SG-PL_contig_1243_1_+3_972_1139

MEFQYCAEAENSIAHRSPNVTYLVCNLASTMLALNDCNNDGFYCLSQTMLSLVDSS

>SG-PL_contig_1244_1_+1_1_159

DHEDRIQPPLFRVHKIRVTELVSWVPRSLYFYPQHSAASQSTQIWPTDTVIMD

>SG-PL_contig_1245_1_+2_2_205

ERNTKRERNKRYGLYIKPLNSARKKLTYCSRLPNASLYNMMQICTMHEIEPLTDKVNDIL

HSQITSSQ

>SG-PL_contig_1246_1_+3_927_1229

MGDSESVFPLKKKKKQKHQKTHQQQQDAIQKNPYVILIPRTTSLNQFSTLFQNHYGSFSN

ISIQQTKTSHTQNVLKMLPFSTVAGNTDNRVNCQTWQLLCH

>SG-PL_contig_1247_1_+2_1025_1339

MVLSWKRKVDVCNCWAVFCLQQSGRKCLFVRTIPTDVRSLVPSCHTILYEGVVNCYLFKV

RSRQFQSNIPFQTDLPIHSQRSLQVWFCNERKLFSASAVQKILRP

>SG-PL_contig_1248_1_-3_1260_1562

MSESVNIKQPGNISSSFPFVWNCGTFLPQWSLGTTFSYNCTHLFVFGCIHVCNNIIFICI

ICLSLPLKITRFLTPSLSCLFLFFVFLFLSVFLVSHQFSHP

>SG-PL_contig_1249_1_+2_2_100

KYRRKITHDNVSIENHRYHITQWLCQFITKQQN

>SG-PL_contig_1250_1_+3_3_233

NTHTHTHTHTHTHRTRTRTHTHTHTYTHTQPFACACTHSAFIVTFSLAASAGSPAPNSCP

FLRHQLYLQCCFTQCSD

>SG-PL_contig_1251_1_-1_91_1032

MDDQTVQGMGEESPTNLIINYLPQTLTDEEYRSMFLSIGPIKSAKIIRDKATGYSYGFGF

VDYQHPADADRAIQTLNGLQLQNKRIKVAKARMGGESIKGANLYVRNLPVPWTESELNKL

FEPYGKIIQSRVLVDLQTGVSKRVGFVLYNTRSEADAAIRSLSGSTPDGCTEPILIKFAD

DNSKKMKAPNVQYVPSPMFPHHGPGPMRNQGNRFRYNPMSGNYNMPNSGLNVNQGGYAIF

AYNIGNNATERTLWQLFSPFGTVQKVNVVMEPGKGICKGYGFVTMTNFQEAQNAINHLNG

FYFQGRPLQVSFKT

>SG-PL_contig_1252_1_+3_909_1094

MSTLFMYQFNHIHRHVEIFVSLFEIHNLQSLPLSNISLNNIYNLCLFSKLILFSIQKLLQ

YF

>SG-PL_contig_1253_1_-1_2113_2442

MPRVLIINANMTYLWQHLLDVLHHFYVFLLLPTYVTYYCTLVHRSTETVCNLEDGQPQSC

PNAGPPCLGYYVNPLRMLTYAALRNISRTKLKYISSNLAKESQYLSTISS

>SG-PL_contig_1254_1_-3_3_215

QDKDPEVWLVGRTKVWLVGRTKIQRSGWLAGQRSRGRFGWQDKEWMCTSSQQGFLSTWVT

IAVNVNDVSLA

>SG-PL_contig_1255_1_-2_2_181

KDVSVYKPWQNRCLKKKKLLISIRVGLEINITFVTVCSFRVLFLNSLPDCHDFQALFQNI

>SG-PL_contig_1256_1_+2_2_142

FFSPLYRHLVKSSRYKVFQSDQQNYETNSSRQALTKASKSNLPRLYY

>SG-PL_contig_1257_1_+1_1_195

ITEPSFIFVFAWKKVLLNFFFEYTTATPLTPNLRQTGDMALREEKSITTRTGSIIFHHSI

LMVLC

>SG-PL_contig_1258_1_+3_93_638

MAKQNDWTGNKYGTQYHRTTVIELDHEPDEGTITTFLERAKEKVKGRYLAALQNGERRKQ

PHIHIAVSTCKSPPNWARDFYRLVDEFWGNKVTCECTLKLKSTWKAFFAYLKPTTTLMVT

KDQGFRTMWNNTAEQDMQTAETSKTVKEARKLTPTNLYNLCKLNNVTQAHEWKTKMSKED

RL

>SG-PL_contig_1259_1_+2_962_1207

MVVLHNVQDDTQCYFTITQPCCNKFLVGQLTSVHIKASIYLQGEKEQHWQTVSMRTTYFC

FFQHPFFRRGLLLSVSFICLLV

>SG-PL_contig_1260_1_-1_1_387

NRGRTLGFRGAAHYKPSRGWGAPLGQADDWVQRGFEPPPPRAQSDTSASGFGSPVGFPRR

PRASATVTSASQAELNSSLQPGSPRGPPQGGPGGQGPPHRPAPAGDWVFLVDRRTWVPSS

SPLVEGELV

>SG-PL_contig_1261_1_-3_87_188

MSGVGSTPLKQCRRWDRGKKNPTTTTTSTTTKNP

>SG-PL_contig_1262_1_-1_130_354

MSGVGSSPQKRCKRWEGKGEKKERSTLFCLLDNMIVWNIISTTEVLRAILEPFNLLLFFF

LLFGFVHILKWQALL

>SG-PL_contig_1263_1_+3_705_971

MELSKCDESLLNNLQTILHWARTCSHRIYQHMCLSMEKSVQKEFTVSAHTLCAPPKAVGN

GQQRYYSVTVCMGVRAHTRTHTHTHSFSL

>SG-PL_contig_1264_1_-3_414_584

MWNILLCLHGMECEDLFVIPSPSLSEMRKYILCVHQITCASGVHIMFTSVVDSHCVH

>SG-PL_contig_1265_1_-1_37_225

MALCLRVERCLILFVDYWPKWEVFLRSLLVSASILDIFFIINFLVQNNSRVDCLSSFVVG

FCF

>SG-PL_contig_1266_1_+1_1_291

TICIITKSDLHVYYAHQNRFEIDPFTCLRPPGFNSISFCSVQPCLKVTFVLIGIPYDQFR

QVIYHDQQQLLYNCSHLTTKFHIIFITLNNASNFGSK

>SG-PL_contig_1267_1_+2_1496_1696

MDQDLIFMLAKSDAQDVGGHSGQRVDSSGDQIGVSLALGFHQGSPDLLPTSISPLSSPRP

WPLLTRR

>SG-PL_contig_1268_1_+3_3_164

CLKVTFVLIEIPYDQFRQDIYHDQQQLLYNCSHLTTKFHIIFITFNNGSKFGRK

>SG-PL_contig_1269_1_+1_1_75

VFIEIPYDQFIRIFIMTNFGTTVHT

>SG-PL_contig_1270_1_-1_625_801

MVERKKECIVDIIRYSSAVRVSQFTPTWVLSVCFLFIWFIFLLYIIIMCLLLFLFCFLI

>SG-PL_contig_1271_1_-2_2_448

VVLHLPFCCCCPSLLLLLLSSLIIVVAVLVFLFACCCCFLLFHCYCSPPTSLLLSFFVFA

SLFVAVLLCFCCYFSSTLLLSFFVVVVVFLGVLFVVAFVDFSAAVVFCCPSLLLLLFFIL

VVVVIIIVAELLLLFFILCRFCPCSLLLL

>SG-PL_contig_1272_1_-1_469_657

MGTDVIAFASCISLSVSPSLLFSAFPSVCLPSSFSLSLSLSCNGKFEKWLLANNFYRWKI

KYH

>SG-PL_contig_1273_1_-2_137_5200

MSQILPIRFQEHLQLQNVGINAANIGFSTLTMESDKFICVREKVGETAQVVIIDMNDSAN

PIRRPISADSAIMNPTSKVIALKAGKTLQIFNIEMKSKMKAHTMTEEVIFWKWISVNIIG

LVTDQAVYHWAMEGDSQPQKVFERHTNLAGCQIINYRTDAKQQWLLLVGISAQQNRVVGA

MQLYSVERKVSQPIEGHAAAFATYTVEGNPNPSTLFMFAVRGPQGGKLHVIEVGQPPQGN

QPFTKKAVDVFFPVEAQNDFPVAMQTSPKHGVVFLITKYGYIHLYDIETGTCIYMNRISG

DTIFVTAPHEATSGIIGVNRKGQVLSVSVEDENIVSYITNTLQNPDLALKMSSRCNLPGA

EDLFVRKFNNLFQNGQYQEAAKVAASAPKGILRTPQTIQRFQQVPSAAGATSPLLQYFGI

LLDKGQLNKYESLELCRPVLQQGRKQLLEKWLKEDKLECCEELGDLVKAADPTLALSVYL

RANVPQKVIQCFAETGQFQKIILYSKKVGFTPDYIFLLRNIMRINPEQGLQFSQMMVADD

EPLADLNQIVDVFMEFNLIQQCTSFLLDALKNNRPAEGPLQTRLLEMNLMSAPQVADAIL

GNQMFTHYDRAHIAQLCEKAGLLQRALEHYTDLYDIKRAVVHTHLLNPEWLVTYFGSLSV

EDSVECLKAMLTANIRQNLQVCVQIASKYHDQLTTDALIELFESFKSYEGLFYFLGSIVN

FSQEQEVHFKYIQAACKTGQIKEVERICRESNCYDPERVKNFLKEAKLTDQLPLIIVCDR

FDFVHDLVLYLFRNSLQKYIEIYVQKVNPARLPVVVGGLLDVDCAEDVIKQLILVVKGQF

NTDELVEEVEKRNRLKLLLPWLETRVHEGVNEPATHNALAKIYIDSNNNPERFLRENQFY

DSRVVGKYCEKRDPHLACVAYERGQCDDELIKVCNENSLFKSEARYLVRRRDPDLWATVL

DVENEYRRQLIDQVVQTALSETQDPEEISVSVKAFMTADLPNELIELLEKIVLESSVFSE

HRNLQNLLILTAIKADRTRVMEYINRLDNYDAPDIANIAITNQLFEEAFAIFKKFEVNTS

AIQVLIDNVNNLDRAYEFAERCNDPAVWSQLARAQLNQGMVKEAIDSYIKADDPSQYMEV

VQVASTNNNWEDLVKFLQMARKKARETFVETELVFAFAKTNRLADMEEFVSGPNHANITQ

VADRCFDNQMYEAAKLLYNNVSNYARLAITLVHLGEYQGAVDGARKANSTRTWKEVCFAC

VDNEEFRLAQMCGLHIVVHADELEELINYYQDRGYFEELIALLEAALGLERAHMGMFTEL

AILYSKYKPEKMREHLELFWSRVNIPKVLRAAESAHLWPELVFLYDKYEEYDNAILTIMT

HPTEAWRESQFKDIITKVANIELYYKAIQFYLDFRPLLLNDLLMVLTPRLDHTRAVNFFV

KTNEAPLVKPYLRSVQKNNNKAINEALNNLFIEEEDYQSLRTSIDAYDNFDNISLALRLE

KHELIEFRRIAAYLYKGNNRWKQSVDLCKKDKLFKDAMMYAGESRQVEIAEDLIEWFLDS

NNHECFAACLFQCYDLLRPDVILELAWRHNIIDFAMPYMIQVMREYITKVDKLEQSEAVR

SEEEQKAEDRPIVLGEPQLMLTAGPASMGMGVPPAQPGYGAAPGYAGVPAGMPPGGMPAG

MPAYGYSM

>SG-PL_contig_1274_1_+3_3_281

KLFKKKFIQKTQNKTKTPPPPQPNKQKQNKTKTTTKIIQFSNLLFGVSFWMKIIAYSHFK

MFVVCVHFVCTLCVLCVHFVCTLCILFYIGQFV

>SG-PL_contig_1275_1_+3_1869_2096

MAVNRPVYTLQAGTELETLLYQKNQPAREKNKQKNCTCGPTCGPPEGQTHDPDYQHGSLS

SSANGPLKYDTTECYA

>SG-PL_contig_1276_1_-3_60_2873

MKLQVQMNNLRVSQLERNKEIMLIKRQLLLQEVNNVLLQAHITRRETELYQYREARRYGP

AVRRWNTFSGQGDNEARPPLLRNISLQDGDDPLHPAAHPPASSSKHSSKHDDIPYITDED

VSIPTAGVAEGAEEGRRGLSLSPTRPLIRTKAPSHETCTDHQEQHLASPIAATTTTTTPS

SQVTAATTGHPLQSAMKSTRMVEQGKPRVVSPAVEPPGTASVSVLPAQQTPSSVPSSKEK

ETMKTSPTTATTMMTTTSEVSKVETGSPTLLSQTPTSIPSAGAPGAEGRIVSTETETPTH

PIPERSDMGLRTSSSPAQSASPDDDKYSQTQSVMECTKSVLEPEASSLHQAEPSPGVSAP

SPSGAAAGGVDITSKGQPPKAIRSPVSRQKYLRRQKEEREKRKERASSPRSSHSSDSGKP

PLPRQSTPHQSPSATHRSKSVGDMEGIESDSTQTVAPLREVIQKFEKRATVCETEERKIE

FRKTPSPTLHLPRVGLVSRVRRLKPAAELLQESQRYRSGHSIYATRIMQRYLPARTTPPG

ATGPSQDKENISGNSYVQAIVKRLSRENTPVKSPGSSSASSSLSLHRTDSPGRTHSDLVS

HIVQKLSSSSGPTDTNHKTSLSPVSSSSLPLKDLTNEGRVKKLTETSAGEEAHGPTITDQ

TSTLLDLAASDRTRATEDASLQPESVTEVAVSPPEAKPDSTISPTSHMSMPALASDQPEV

PPEDSARERAATYCVSEGDRPRQLQLQQLHGPDNGSASIEHISEEILDPALLSIRERKGE

KISPTSSTKSKNALCCQSSTCTKSKSSSSSSSQQTSGAHKIPSSPKTQERRSGREKMETI

GVLCKQSMSFDLGVSLQAQPLEAMPQRPQSAESLRTPTEGEGRTPTEGEGKERAKVRPSS

TSSEGEAPASSEEKKKSRSRLLDSSWLQKPRKFFKVSK

>SG-PL_contig_1277_1_-2_83_400

MICLAQHPGKEVLKCFALYRTYARKQDGDKTNKMNAHHTCVRALARAHIHADRHARIMYI

HCPCIANTDAEHVNRSVKPTKNKLSCTTSQYEHLPDSRVERRKVKQ

>SG-PL_contig_1278_1_+3_3_245

DQATKVQTTPPPPPPPTVVYLYFLSFRRNISSSISNRCELTYSLFQSHKNIRTLNEQGKL

KQTTDVKVSLLNERFLSSLSF

>SG-PL_contig_1279_1_-3_6_2108

MDIEELQSINYGINILKDPIVIPETHPESAILVTSRHGQQYQCNYPSHQEQKQQKEEEEK

IALETGVVELLKPLSQQPCLLKTKDWWTYEVCYGKYVRQYHVEDGHIRGNIMYLGHFQSD

FDWNNETLRESRVRSKIASGRYHSQYYTNGSKCDLTGQGRQTEVRYLCEDAEDDYIVRVD

EPETCVYVITVHSPRICSHPHLKSPTPSQPIPIVCVPLLTDQQYVQYQAHMEAVRQKEEE

ARARAKAEQEQREREKALAEAQEEDDVIEDGLADSVNSILKKSLGKEFKSVGSTTDQPTS

GIEDKPSLGWKVVKDLDDIEKLLDPQGILSNMDMSKKGQGAKTSKTTAKEDTLKNVDKKF

DEVLEQLNKKDRELQERQRTGQVEADDTVDASVASDLDEFEQEVQHLKDKQAMTQKRLQY

IRERVRKAVTSQFKDIIEEAEAEIGSEVNEDLAFKQLASSLNMLMEKLEHTEKEIKNMDQ

EIAKLGDKEASADIDEGEELEEEEDDNEEEDEEDESKEKEQESSVADGEEPKSEGVQAPP

TTKQATEKQMKDGGRWKMKVRTVTQEEIDKDGSAKLPPTKQQKHLEKSLKESLQKAGVDP

NSVKVHVKIIGGANSNSDDDTTLHVLSDEESASFQNMIVAILGGSNEAMKEQKRQKTLED

SYNFVFDQAITQDSGDDAKSSDSGDSTDSDDEALSDSVLGL

>SG-PL_contig_1280_1_-3_3_179

RVKCGLLSPRTARRHSSAPSIFFPFSCMHVFFYRILPGTSLLLLWIFFTCAECMLNTRP

>SG-PL_contig_1281_1_-3_9_221

MKVDFLQNFAWDKSFVAMGSLICDKCLLHRSSVNHLIRKTKAPRPPLRSSRGPPGKEKYT

WLGIELRFLIS

>SG-PL_contig_1282_1_-1_2566_2832

MTFLHSIETLFRLIQHILVNTRQLPSHLHPTVLPTCPLSATVLASSLAFFRLHRVRGVFH

YCKLYLFSYIIVTAGKNGTQNFHMRMRTK

>SG-PL_contig_1283_1_-2_29_238

MPAYSRDSDNIRKLSGEVERLRIHAQQPHLKLSETIREMINFCNSNMAGDHLINPEKENP

FKPKKTCQIL

>SG-PL_contig_1284_1_-3_24_476

MAAVLRNARSVGNFVASIRSTLPRLCTAALVHHNGSAKYLHSQNQVFHPSVLKNHSLTQS

RFYSEKPPLTIDMLQQRVMLVLRLYDKIDPEKLTLDSHFMNDLGLDSLDQVEIVMAMEDE

FGFEIPDTDSERLMRPRDIVQYVADKEDVFD

>SG-PL_contig_1285_1_+3_255_365

MVYISEELSRLSNGSVKSLKTASVKCNTPSLDVSVVI

>SG-PL_contig_1286_1_+2_110_277

MGQSSHLPNGFECMVLFTFKLYISCRLCCYLILPFPCLQSCLSLSVDLCMSVCVCV

>SG-PL_contig_1287_1_+1_1_108

IPFYLTSRTVHALVCQRQRSFCALIISTLYLNSFSR

>SG-PL_contig_1288_1_+2_434_736

MAMQVEAFILVHSKDSGHKTHNSNGKLYVPLGVALVVVFLPGPLWHRQVVHVCMLLNGEL

FVCLPVCWSMAAHAACPLQPGSSADGSCHWNKFSQHFVEAT

>SG-PL_contig_1289_1_+1_157_378

MLKKLFTTFNASTIQQQNATFKRFNAFENSHKTYNSLSSQQTFIQSHKHYYCFTNIYYNG

TSTSWAVALQNNYP

>SG-PL_contig_1290_1_-3_204_3116

MEMYYKTSEGEKILNKKAVYEPSSSQRCRQVVCTRRWAVAILVTMGIFILLIAIIAAFAR

PGSYLCQAVTGGQSESHPDTPTPTTPPPTKDYITTDGRRFPWKDIRLPRLVLPDSYEIFL

HPNISQSVFSGSVRIHSSVVTSTDFFVFHVKELNVTKVTVLRGEVEVEVADTMEYKTNEQ

FHVRVKQKLAADSKIIIVVDFQAALVNKLAGFYKSSYKTAAGKKREIATTHFEPTDARAA

FPCFDEPDLKANFSLSIVREPQHITLFNMPLKSTSPFQGGLLKDVYEMSVKMSTYLVAFV

VCDFKNISSHTKQNTLVRVFAPEDQIDQAHYALGVAVKVLDYYNEFFGVPFPLPKQDMIA

IPDFAAGAMENWGLITYRMTSILYDPLKTSPFSKQWITIVISHELAHQWFGDIVTMKWWD

DLWLNEGFASFVEYIGANISEPSFGMMDQFVASTLQRALYLDSFTNSHPIQAEVSDPAQI

NEIFDTISYDKGASLIAMLQGFIGEEAFKKGLHEYLRSHAYGNARTADLWQALQEASNMP

PTMTVKDVMDTWTLQMGYPVITVKRRPGTQHLTLSQNRFLLDNSGTVIDSKFKSPYNYIW

KVPLTYRTQTNPDKKLVWLKDKEMTLPVTLSDTDWIKFNLESRGFYRVNYEPDLWRRIIR

QLKDDVNTFSTRDRAGLLDDAFSLARAGLLSYDIVFELLAYFDKETEYVPISSGITGYGF

IKNRLYVTDVFPKLQKYMLRLISTRLSSVGWQNQTQHLDKLLQEVLLSFSISLNDKDNIE

KAKQLFQSWSNIPDNLRGLVYCMGVKYGDDTTWQLMYNNFTTSNVPTEKKQFLKALTYTT

DPRRIQLFLRDSMNDKIVRSQDTSTVISGIADQAGGHIFAWRFIQQHWETLYKRYGTTSF

ILGRILKSVVGPFSSQFDYDEVTAFFKDRNLGSGSMALKQSLETVKSHMDWTARNQDSIV

QWFQTNIKDTP

>SG-PL_contig_1291_1_-2_56_244

MGYGDYPKLPMVSGDARDPYEDWDIPELKRNYNEPLHALADIIGEDRWDASANPRYSYLN

MFL

>SG-PL_contig_1292_1_-2_104_742

MASRLAISLCFVVLAFVGASSLHLRRRRNTETCDEKDLDKCIGVLALMMPSSPEINVELI

DSPEKLDEMCTQVHLTLDCVANETSDCEMEQMLKISIDTIRGAAGYICSEEGKRILLKEA

KCLESPTLEHAARSCMEEIVEQASGTSDYCGVIEEIIDCARTATDELCGEEAAMFVERLS

YELVKPISNSMLGCDLQIRVARDLLRSLSIKRK

>SG-PL_contig_1293_1_-3_96_467

MVSPPLADGDIYHPPYGAHLPPYSENNPHKNFSPPDYDKLSILYDNPAFCTETSGTLRGC

LDYEEVDHFQTLRLEINDQSQPATSEADVATEPEAQSGSRYSFSAEVHSSPKRDVYRATY

VTWI

>SG-PL_contig_1294_1_+2_2_178

YRYKKIRVLQKLTNTIVKQFKKLTNVRWLSFHESVWTVVSIWGCLCPALDYEAASSTSE

>SG-PL_contig_1295_1_-2_221_874

MAMYPNATHGYMPFVKPAHKRQAAKRSKADKDSVKTKAAEGTKTYKVTMRDRRGFTYTYI

IQADASKSLPVLCESRVTAPNNSPLLSNSLPNMRRSARFPVSAPESRSCASLADGKEDLT

KVDDGEKKGLFNSLLQYIKKKTSSKSSKTANTTAAKSKRKVKPLPQRKRQRTVSVMSNLD

PIQESPEEDNLDDTDSYSHDSVTSCSESDSGDGHESCP

>SG-PL_contig_1296_1_+1_1_279

NTSLSSPRRLTSCQLAASEAIPRSSRLSARDVVDQRTQHKSKFVATTIVVTISSLRGHTI

RPPFCKRCGGFKGAQHQSRFVTTTIDVATSSLI

>SG-PL_contig_1297_1_+2_2_97

VWVGAICGVTSLIVGFTSEQSLVQYGRHWVKI

>SG-PL_contig_1298_1_+2_176_376

MGATCRVTSLAAGFTSEQSLVQYRRHWVKFHFLKFLIGCFPFLRSFQSFSCRYSWYLLSR

STTGVPW

>SG-PL_contig_1299_1_-2_158_2650

MSSYQMNASGGPASTMGPGSQNYMDLSDMPVDNKQQQTMMWQQNQYMADSGIHSGATTQA

PSISSKGHGDELEDGDMDPAKMMFDWGLTDQYPPQPEQVDDINQQLNQTRSQRVRAAMFP

ESLPEGVQIPSTQVHPDQPTAVQRLAEPSQMLKHAVVNLINYQDDADLATRAIPELIKLL

NDEDQVVVYQAAMMVHQLSKKEASRHAIMNSPQMIAALIRAMANTEDIETTRCAAGTLHN

LSHHRQGLLAIFKSGGIPALVKLLSSPMESVLFYAITTLHNLLLHQEGSKMAVRLAGGLQ

KMVALLQRNNNVKFLAITTDCLQILAYGNQESKLIILASGGPGELVRIMKSYTYEKLLWT

TSRVLKVLSVCPSNKPAIVEAGGMQALAMHLGHQSQRLVQNCLWSLRNLSDAATKMDNME

GLLHMLVQLLASNDINVVTCAAGILSNLTCNNQRNKVIVCQVGGIEALVRTVMQAGDRED

ITEPAVCALRHLTSRHPEAEMAQNAVRLHYGLPVLVKLLHPPSRWPLIKAVVGLVRNLAL

CPANHAPLREHGALPRIVQLLIRAHQDTQRRASISSNGPPMTGYVDGVRMEEIVEGTVGA

LHILAREAHNRAVIRGLNCIPLFVQLLYSPIENIERVAAGVLCELAADKEGAEMIEQEGA

TAPLTELLHSRNEGVATYAAAVLFRMSEDKPQDYKKRLSMELTSSLYRGDNMNWSEPPGF

DDVGLMPEESRDQMYTQQPPPHHGSQGSMYSGSHPDMQQGRPPTGYDQVPVNSMQGLDLG

GPGAGGGYGSQMDNLPDLGPTSADLNFDSIDPNMGAPPGDGGPSMWFDTDL

>SG-PL_contig_1300_1_-2_29_547

MTQIIVLNQNTRRESGRKVQLGNIAAGKTIADVIRTCLGPKSMLKMLMDPMGGIVMTNDG

NAILREINVQHPAAKSMIEVARTQDEETGDGTKSVIILAGEVLSVAEQFLEQQMHPTVII

AAYRQALEDILEVLRNKICVSVDTSDRTQMLNIVRSCLGTKFISKWMDLACQI

>SG-PL_contig_1301_1_+1_613_924

MSSLNFSCAQCGELWTKVYWKKIMLSRQMFSSLPSPVHVHAHVCKTLLINDGDDTFFPSF

FFMTRQELNCIFIERTNSLTKMMCKYNCVCLFLISHTYMCALIL

>SG-PL_contig_1302_1_-3_3_185

CMCACTCTFISFLFLVVCLFDQKPFTCLFALLSRFGTLYIERFLPKRSENLDRNTETEKV

N

>SG-PL_contig_1303_1_-3_363_590

MCGLKSGEPTLFRVREGKTVSKRRRDTALIMLAPKEVEVSTISFSYDLGLCDKSFERRNC

IKLVSRYRKREIVSVF

>SG-PL_contig_1304_1_-3_3_107

MLIFRKICFHILKQASFLKYNCIILIILSTLHFYF

>SG-PL_contig_1305_1_+2_2_298

ETERDREIGSHKIQINQARVTSVQCIQSQSLMTGRVEQTFFVRSIQTMQHKFKARTTINC

VILHTDCILVTSCETHKRCAPFHETIQLGFKANRIPQCL

>SG-PL_contig_1306_1_-1_115_399

MWRLATCFPQGKRPAFHVEKFRCYNNKSKKLNPILSKLKPLLKGTLDSMLGHYELNHRTF

DSMLGQSEWNHKGHLTAYLDSMNGITGHLTACWDI

>SG-PL_contig_1307_1_-2_71_1549

MRQAIVLFSCVVLAVQASNVLEFTDSNFKEKVQEHDVILVEFYAPWCGHCKRLAPEYEKA

ADRLVKSDPPVSLAKVDCTAETSICGEFGVSGYPTLKIFKGGEFSKDYQGAREADGIVKT

MQKEAGPSSKELTSVKDFEEFLGREGGSVVGFFTDTASTMAKNFQKVADSYEDLRFAHTS

TKAVLDKSKYKDEIVLFRPKAMASKFEDTAVKFSGDTTTSKIKTFLDEESFGLCPHRTAD

NAGKLKKPTFVAFYGVDYVKNPKGSNYWRNRVMKVGKKLRDEGLKAYFAISNKDDHDHEL

GECGLGEATGDKPIVCAYDERGRKFNMEKEFSMDTFEQFVRDVLDGKVEPYLKSEPIPTN

DQPVKVAVAKNFDSLVNDPEKDVLIEFYAPWCGHCKSLAPKYDELAEKLEAEPEIVIAKM

DATANDVPSPYTVTGFPTIYFAPKGSKSSPKKYEGGREVDDFIKYLAKEATNELQGYDRK

GKKKGGKKKKTEL

>SG-PL_contig_1308_1_-1_88_294

MRTATRPQLSSSRSPSPHSRTHIVAEDPTTIRSRLSEETGSNADDTLYAYRQARLHLNSL

MESMRSQAS

>SG-PL_contig_1309_1_-3_3_383

SGVRERREAPPLLSAAPHKPDHLPAGEHWVCSRAGFWLPLSDQLTGWFWDPSVGRWCFLA

GPSPDVESRRSLSPLEAQQTAQPASSSRAEGRSPSPRARSTASSSVRSRIDTSDPPVLPL

EWCPEAS

>SG-PL_contig_1310_1_-3_9_2069

MASQTTEADEVFQPPESLQAQAHVKSLQEYKQLYDKSLNDPESFWEPISEEFYWKSPRTG

AFLNYNFDVRKGPISIKWLEGAKTNICYNVLDRHVEKGLGDKVAFYWEGNDPADQGKITY

SELLEEVCKFANVLKSLGVKERDRVAIYMPMIVELVIAMLACARIGAIHSIVFAGFSSES

LAERILDAQCCTVVTSDGVWRGKKLLNLKSIVDKALDICAQREHAVSKSIVVRHLSPKDE

IIVQQDTNGEIQEEHIAKRPCRQLKLTWKDGRDVWWHELMATADGKCRPVWVDAEDPLFM

LYTSGSTGKPKGVLHTVGGYMLYSATTFKYSFDYHPDDVYWCTADVGWITGHTYVSYGPL

ANGATSVIFEGVPFYPDAGRFWSIVDKYKVTKFYTAPTAIRAVMKYGDEFVTKYKRTSLR

VLGTVGEPINPEAWLWYYHVVGNGKCAIVDTFWQTETGGHVITPLPGAIPTKPGSATLPF

FGIKPKILSEEGQEIEGTGEGYLVFQQPWPGIMRTVYGNHERFETTYFKKFPGYYCTGDG

AHRDKDGYIWITGRIDDLVNVSGHLLSTAEVESALIEHKAVSEAASVSHPHSIKGECIYC

FVTLKEGEQYTDELQVELKNLVRTKIGAFAQPDFLQNAPALPKTRSGKIMRRILRKIARG

DRDLGDTSTLADESVIDLLFQFRPSDS

>SG-PL_contig_1311_1_+2_38_253

MTDNMLKLNDDKTEALLFSGSVPSDCRLPSSISTASSTVSFSDKARNLGFIMDSDLSMKQ

HVITICLICYFE

>SG-PL_contig_1312_1_-3_3_257

SVIWQKSVKIATDSTAERERFEVGLEGSKRTRVSDLQGKRLPELWGIAGKRTVAMSFTFV

ASLVKKVGIRRRAETARWYVESNKF

>SG-PL_contig_1313_1_+3_1932_2303

MLLTPKTGLKGSVMGETQAVLQYSAARSLRHTRLSKLSTVEVISHHHFIIPSSVHRQQIP

FYCLAPVQFKKKSIDTFFFKKVCSFHGENHFKISKLFLLVSFVFCFVFISDTLEKCFKMG

PLFF

>SG-PL_contig_1314_1_+2_971_1195

MTPLRNAHLSTAKSQLSLFVCFCFVFFTVHYIFYEVQHHQAWQIQITQEWLLISYFLYQI

HGLYVSIHSFCFSNV

>SG-PL_contig_1315_1_-2_41_922

MESGGMSGQSSRPPGAQQDSQYPDAVRSNPHLPQPRGPRLTPEEVSVLEECNTESFWYRC

VPLAMAFGGTTAYLVKTGHLKPHPRYGATLKTLGAVFVAYVMGKMSYHKTCQQKILEKIP

HSHLAQSVRKSRGIKSDYDEDPIADTSLDSDYIRKEKMRDTYSSIDTSSKGLDDNLRPSL

DRDVKSKVGEESSPVDAERTLTYDELRQRNRQEYERSMATSPNSPFRQRPFPMHRPGAPS

SAPAESSSPSSASEAPSSGSRAIWDTPSPQSSGKVLDSARGQKKNEYGDVWDSN

>SG-PL_contig_1316_1_+3_3_245

IKPHKANGSKIQIAANGRQIAERRGKGWCEGIGGKRQQNPNRGNRRWREMLEGNVSKTQI

AGMGVGGKWWHNKLNSIMQQR

>SG-PL_contig_1317_1_-1_109_1263

MRKGVSPAAEYWWRGVLQLSEGIEKPGEVCGHLVGSLAVCWVVIYFCVFKGVRWSGKVVY

FTATFPYVLIIILMVQAARQEGASTGVAYYVKPQFGRLGDIKVWFDAASQVLFSYAVSIG

VLPAYGSYCPYNGNCLRDSILVAIANSGTSFLVGFVVFATLGHMAHVQNETIEDVASAGP

GLAFIVYPQALSLLPVPQLWSVLFFFMMILVGLDSQFANVEGVMAALWDVFPRWYRHRML

VLLIFSIGAFVLGISMITQGGMYVFQLIDYYVTGAYTWQILSLLEVVTVGWIYGANRFYD

DIESMIGYRMGVWLKICWKFLTPASLTVIVVFNLVTYQPLTYNKTYIFPLWANGLGFLLT

LLVLILIPLYLVLTLVRTPADTLRD

>SG-PL_contig_1318_1_-1_109_1263

MRKGVSPAAEYWWRGVLQLSEGIEKPGEVCGHLVGSLAICWVVIYFCVFKGVRWSGKVVY

FTATFPYVLIIILMVQAARQEGASTGVAYYVKPQFDRLGDIKVWFDAASQVLFSYAVSIG

VLPAFGSYCPYNGNCLRDSILVAIANSGTSFLVGFVVFATLGHMAHVQNETIEDVASAGP

GLAFIVYPQALSLLPAPQVWSVLFFFMMILVGLDSQFGNVEGVMAALWDVFPRWYRHRML

VLLIFSVGAFVLGITMITQGGMYVFQLIDYYVSGAYTLQILSLLEVVTVGWIYGANRFYD

DIESMIGYRMSVWLKICWKLLTPASLTVIVVFNLVTYRPLTYNKTYIFPPWANGLGFLLT

IIVLILIPLYLVLTLVRTPADTLRD

>SG-PL_contig_1319_1_-2_17_277

MQSMTERVNKLLKDRKVLLVNNREEVEKLEKEKAVMMAQYTSQMDQEKSAATVVGKDLER

KVEDLEDDKKRLQEELEAEREEVKRLK

>SG-PL_contig_1320_1_-2_68_568

MKKFEYKGKQWHDECFICAECDQPIGNRSFIPRDDRVICVPCYENNYAQRCSKCSGVINK

GGISYKNTPWHRECFTCTKCDKMLAGEKFTSKDEQPYCGDCFGDLFAKKCCRCVKPITGI

GGTKFISFEERHWHSDCFICYKCETSMVGRGFLMNGEEIMCPDCGRT

>SG-PL_contig_1321_1_-2_2_217

NSPSWTTTKPNTWVYLQLDPSSQTTTATRPPPCSTRMWYLNILRNHLLLQLPSWLSFAMF

LAFLCSWLLSVP

>SG-PL_contig_1322_1_-2_14_1006

MAGVKNAQDLAGFDKLLEDAERQLVVVHFWADWATQCAQMDDVMKELASDMQYVNVSFVR

VAAEDLADVSLRYNIIAVPTFIFLKGKEVIDRIDGAKAAELTQKVGKHVSFLPLKGPLPQ

EGAASKLQDLNSRLKQLINSAPVMLFMKGSPDQPRCGFSRQTVQLLNERKVKYSTFDILT

DEHVRQGLKTYSDWPTYPQLYVKGELLGGLDILKEMAESGELEEALLVQEDLDTRLKKLI

NKSPVMLFMKGCPDTPRCGFSKTMIQILNETGVRYETFDILGDEEVRQGLKKFSKWPTYP

QLYVSGELLGGLDIIKEMKENGELQEALTTS

>SG-PL_contig_1323_1_+2_1100_1330

MKRPQERPIFCYFSPGTLVVCTKEQNQREHDFHISAPSSPPLHDIIFIFLALQDITEPNF

IICSTSVEESQAIMLSF

>SG-PL_contig_1324_1_-2_863_1189

MRIHSPLFIHCLSHALSCGYLKSILLIVLSTVLYFHVDKILAAGGWVYFSHSFFNVRMSR

QWCCVCWLGFICLFQLRNSSSNNCVLHELNMFSIGFCVFEMFFTANVAR

>SG-PL_contig_1325_1_+1_1_204

ISLIMRLPISLIMRLPFISFLMYFIQYYLSPLKCHAATIIRKKKNYIDPEQIIFCQNNNR

IAIMFDSW

>SG-PL_contig_1326_1_+2_2_202

HTHMQVQTHTSLSLSPSHTHTHTHTHTPHHYAKAIPGDVNVTSHTTKIKMIIQFSQKNRH

TSQHHYY

>SG-PL_contig_1327_1_+2_38_223

MFAQLQKYMHIHVYTDSVLILVTVTYIHTHSLLSSSEKINKSYESIHTYNMPTLSSLFSE

QN

>SG-PL_contig_1328_1_+3_126_383

MRTRDMGSRKELGYIHCFPLPFPYKLKTWDCVSKAKECFQFQFAHIQKYFSYCRIYGNNS

QYLASSPTCTLFHSFLYKNRILFFLF

>SG-PL_contig_1329_1_-3_3_9689

CRQDEFTCASDGRCIPIIRKCNGVPECQDGSDEGDCGCTDDEFECTSSGECIPTNRRCDR

RSDCEDGSDELNCPCQSNEFTCESDGRCIPDNRVCDRRRDCADGSDERECACGSDEFTCE

SDGRCISSLRRCDRRRDCPDGSDEENCPCRSDQFTCETSGECVPNNRKCDRRMDCADGSD

ERDCGPVCREGELECDGRCLPPQYICNGRAECRDGRDEQNCPSCDDETEFQCVSDGRCIP

IVYRCNGRAECTDRSDEDNCVPRIEVVVTPQSLRVRTGQTVVFFCEAVGVGSTGVRVRWT

RAGNQRLPASASDDGRGRLTLRNVALEDAGDYVCSVMDVSGDFQTTARLQVDYIGPPTSI

PPDGRCRQDEATCSSGQCIPRDYLCDSEQDCSDGSDELDCDSPLPCEPNEFQCSNGRCAM

KIWRCDGDNDCGDGSDESYCPTQDPGEKCRMDQFMCQRGDQCVPASYQCDGEFDCQDRSD

EIGCSVPSIIKPPVPEITVEINGTFTIYCEAVGVPTPLIVWRLNWGNIPTGNRVTVTSVD

GRGTLTIRYARPEDAGAYTCEALNNRGSIFATPDALIIVRRTVGVCQPPRFNADALRQDE

CVRCFCFGHTETCYSSNLQISQITLGSRLELMQRATLLPVEEGFIQYVPSSGDFVIQDFD

RSFPSGSYYWSLPRQYLGNRLTSYGGDLSYRVFYDVDGFDIPTNDPDVIIEGNGITLFYR

RDSNVPPSTTTTVRVPLVETDWEKSEGLIRDDLAISQLATRQDFMLALQNISRILIRATY

DSRQNLIRIGNVLLSTAVTQSPGQGRAVYVEECSCPTGYTGLSCESCAPGFYRVQRGRYL

GECIPCTCNGHSNNCDPETGVCQSCLHNTEGIYCERCAEGYVGDARRGTPNDCEPCPCPL

TIPSNQFSRTCMRQRDGEITCTACLPGHTGRRCETCLPDYRGNPMVPGDRCTPISQADIC

DPRGSLTAVPNVNTGRCDCKANVEGQLCERCRADTFYLSSDYPFGCIPCFCMGVTQMCQS

TSWNRAQVVVSFTNDRSGISLTNMMQTREVTQGFYVDRSSRELVYRRFNNLPQDVYYWKL

PERFLGDKVTAYGGSLRFTIRYRPGADSSPISLGEPIVELGGNDIILVYRGDRQVSPNSA

ESFSIPFYETDWLRIDGEPANREHLLMTLADLDFILIRATYTETTDEASLSDVSLDIAEN

RVTGQDRAFPVEQCICPPGYRGLSCEDCDTGYTRSGGGLYLGLCMPCQCNGHSNECDPET

GICRNCRHNTQGSHCELCVPGYYGNATRGSVGDCQKCPCPLTEPPNQFSPTCFLDADGQV

TCNSCPIGHTGRRCERCESGYQGNPLQPGDFCKRIVDRCDCDRRGTVPNTQCDPVTSQCQ

CKAYVQGRRCASCQEGYFNLEESNQQGCLSCFCMGITNDCSSSSYSREVLRPQFNPDGSH

SFSLTNRRLSRTITDGFVMDASRNEIIFNNFEGIQREQESLFMQLPAKFRGDKVSAYGGY

LRFTLSYSTAFDAGQSYMDVDLEIISNDQRMYLLFNPSPKPQETEDYEILLRESSFRMLD

GSEPTREAFLTMLADIDAILIRATYNSIMSSLSLRNLEMEIAVPRVTGLRRTPEVESCRC

PEGYTGLSCQVCAPGYLRVPDPNTALGRCTRCNCNGHATSCDPISGRCMGCQHNTVGDRC

EKCAPGYYGDPTAGTPNDCRPCPCPLTSPGNQFSATCYLDRDQNVTCDQCRPGYTGRDCG

SCAPGYVGNPREPGSRCTRDDVDRRPTVVVSPITLQEPVGSRARFQCQVGGRGPFNVVWS

RLDGRPLTSRATTGIGPSYELVINRVEYGDAGRYVCTATNAYGTNRGIAELNVQRPDQPL

RVMIEEPRNIVATIGTTVRLVCVAVSYSSEANYVLSWAKEGGILPPRAIDQNGVLVIPNL

QEGDAGQYICTGSDPNSVDRATAVISIQDIDRSPTVRIEPRYLEVNEGDLIEFRCMAEGT

PRPTVRWTRGPEGALPEYASVRDGVLRIPNVRKADEAEYYCTATNQAGTASVRTIIYVTR

REPPGPAITIIIRRAQITATIGSDVQLECYAQDNQNVQLVWSRQGGLPPGSSQVGGVLSI

PNVQPSYSGNYICTGTAPTGTIGRATAVVTVISGEPREAPTARVEPDMQTVSQGTTGTLR

CIVTGQPRPTITWSRARGELTANHNVQGEILRITQASMEDRGVYICSVSNVAGRAQASGI

VDIERRQSPQIELFPQEQQTIQPGGSALFQCRVTGGMPSPTVTWTRVGGKEFTSTTKLMN

GNGVIMFERVTGEEQGGYICTATNSAGTVTLTASLIVEGPPTITISPGKRITAIVGERVD

IECVGEGEPTPTVFWRSDTRRRSDVLPESYEPAPGTAGLIFNPASESDSGRYTCVARNDK

GVTEESVDLSVMPTGLAPLVDIEGPDRMTLSEGESVELSCTARGLRNPLVRWRRPNGQPL

PPGHSVTGGTLYIPRITSEYQGKYECIVTSERGDYSASVILIVSVTPRLTVSPGQITARM

GDSVTMRCQPTGQGPFQIEWQKVGGVLPPGVREFDGVLEIRQVTAADAGQYRCIATNNAG

TSEGYATLRVAVAPSVSVTPREESLQVGSEVEFRCEVTGDPPPTIRWEKDGGSLPPQHQI

RNGLLRLYNIQEEDEGRYICIATSESGVNRDYAYLRVSNIGNIGSKIGQSVQNVDVGDRV

EFECIVTGTPRPTVQWSKLEGPIPASAIIGEGILIIPEAKPEDAGTYRCTATNVAGSVQS

QVQLFVQSKPIISQQQDLQAAPLGSPTTLSCDVYGSPQPQITWTKREGELPVEHRIEQNG

GLYIPRVREEDAGTYECRASNKQGDAVLPIVLVVGAFVPYFPQNPNSYMSFQPLNDVYID

LDILLSFRPEATDGVLLYNGQYRNDRQDSADDRGDFVCFGLNGAYPEFRFDVGSGPAIIR

GQTPLQLNRWHTVHLKRQKKNGTLLVNDEPPYYGEAPGRFVGMDIVQPMYLGGVPNYAEI

PRAAGFNQGFVGSIGQVQLKGTPLNLGASAVELYGVEQYDACRDAPCYNGGRCQVHNNAY

GFRCLCPRGFAGQRCETAGYRCYPGACGEGRCYDLSDGSGFQCICPTGYSGEGCRVGLTV

VDPAFNKTSFISYPTIENGLLSVSLKLMFKPRSRDDGIILYNAQKEDGRGDFVAVIIKDG

FVEFRFDTGKGPAILRSRQQIPINEWAMVMADRKGRDGMLIVNNEDPVN

>SG-PL_contig_1330_1_-1_31_519

MESAGASFTTAFRNTAPFQEYLLQGSILDAHCEVLLHRLRGLCDNADSTPQPFNDYEAVY

QLKNNLATQQSQYILFRVREALDDPEAPCYIRYLGQAEIGDKTRHTLVRACYDVACSNNV

RDFLNELGFKLDHEYVSRGYVFHKGRMKAMVSKIYRMQQPGKT

>SG-PL_contig_1331_1_-2_44_214

MHGYCCLTVVRYKDSPKPFCQKRFEHLHANHLFSLYSIFKNCLERENHTELFINMRA

>SG-PL_contig_1332_1_-1_931_1260

MVAPFFCMWYTMYIPFSFAVLFVCLLHFCHLFSAYLSIDWHFSRKSLSAILLMPTKPLCS

DTLVGGSLRTGWTRSAHFAVLFANKANLLCRIHICIDFLNFYLKSWGEIN

>SG-PL_contig_1333_1_-3_90_278

MCMIVLVIYRYSELHKNEVMEIFQVFVVNRVLLRRKIISDRCHSVSCVSDIFSHVWPNTG

TKK

>SG-PL_contig_1334_1_+2_794_943

MALKCLSTFFVFFGNTCIIWEILPILQNPRHLSDTPHYDRWSTVNEKYNF

>SG-PL_contig_1335_1_-2_2135_2440

MQNLMHWQALEMDGERGGERGLKKPQADTTRMIGALKWAADRQIFMLQAEIELETLDLEF

TVLSTEPTSQLLEVKSQQKETNQVLGEAAKNQTVYLILCFFK

>SG-PL_contig_1336_1_-1_1_990

RKASSSDDDSGASSSGESSGSDSSGSEHSQPETKRTPKKDSSSSRAKGGESTPGRSKRSD

RAEEDGGKERRRKGKAGTGDLSSSDGEHVPRSGPRSTVVQKVMSVSKDKGRSRDDGDKAS

PGGDGGRDRREDVESSRSKKRSRSESPRNRDSSKRLKEDRSSGRGSDRRDDRHQYRDDRD

VDKGRGWRDSDRRRMDERRRDDDRRGDRSRRDGGRDRYDRRRDRDSDRRDNHRGDRDDGE

KRRDREDRDRGSRREENKENKKDDNVAEPQKRKGGDTEGDKEADSAPPEKKAKDSDDIMT

RTGGAYIPPAKLRMMQAQITDKSSVAYQRM

>SG-PL_contig_1337_1_+3_3_1187

EFKISLSNKFQVLHELLEEETINRKWQGVKDAVSSTCQEVLGPKKHSVKEWISTETLKLI

EKRKLKKAAVNNSRTRAGKNKTQEEYSEVNRSVKRSIKVDKRNYAEMLATEAEEAAHQGN

MRDLYGTIKKLSGKFSKPERPVKDKEGRTIPDKEGQRNRWVEHFEELLNRPGPKEPPDIQ

PADRDLLIDCGVPTKEEICKAIKRLKNGKSAGPDSIPAEALKADIKTSVEMLYPLFRQIW

GEEQVPSEWKEGYLIKLPKKGDLSSCSNYRGITLLSIPGKVFNRVLLNRMKDAVDPQLRD

QQAGFRKDRSCTDQIATLRIILEQSLEWNSPLYVNFIDYEKAFDSVDRQTLWKLLRHYGV

PEKITNIIRNSYEGMTCKVVHDRQLTDAFQVKTGV

>SG-PL_contig_1338_1_+3_81_200

MLYVKQRTDIMSKPSQIWNQLMIQTTPKTMLLLHFLINCS

>SG-PL_contig_1339_1_+2_2_154

PRPTAPRNVVSLSPLTRVWRKTSLRVLFQVDSAGCGQGLQSGSTRPRVPEP

>SG-PL_contig_1340_1_+3_3_227

YLDRTKAHRASKRRLLISINPGLEKDISAGSVSRWIAQAVVKAYGQARLDLGSLNPRAHE

VRAIATSAAFIRKPP

>SG-PL_contig_1341_1_-1_31_213

MIKPFYLFKQGGGDSLRAHLQCTHTHTHTHTHTRARARARACAHTQSWGGGGGDVKKGML

Y

>SG-PL_contig_1342_1_+2_2411_2650

MLNDLDYAWISFQAEMTYLQQKYCAVHLSMDPSCLQEFSADLVTTNASVTRLRTTNPSVT

SPNKTLGYLKLLPFLTSLIR

>SG-PL_contig_1343_1_-3_72_1118

MFPELSFNIDHGYLEGLVRGFKSGILRQADYLNLVQCETLEDLKLHLQSTDYGNFLANEP

SPLTVSVIDDKLREKLVIEFQHLRNHCLEPLATFLDYMTYGYMIDNIILLITGTLHQRPI

SELISKCHPLGSFEQMEAIHIASTPAELYNAVLVDTPLAPYFIDCILEQDLDEMNIEIIR

NTLYKAYLEDFYNFCKQMGGATAEVMCEALAFEADRRAFIITINSFGTELTKEDREKLYP

RCGSLYPDGLDMLSQCEDYDQVRAVADYYAEYKELFDDAGTNPGDKTLEDKFFEHEVKLM

KNAFMHQFQFGVFYAYVKLKEQEIRNIIWIAECVAQRHRAKIDSYIPVF

>SG-PL_contig_1344_1_+1_2071_2355

MWQRPHISQGQLYGDDHSSPNCFTFPTLNSQVPLYSLVGRRSHAQICSYFLRNSYASKQI

ASVIFGDIGLQEIHLSIYFFRIVSLHNAMSTQYIM

>SG-PL_contig_1345_1_-3_129_1385

MKKVQQMTDIRSDVGYTRAWVRLALERKMLSSHLKELLSDASLLRSLYKRYAFLRCEDER

EQFLYHLLSLNAVDFFCFTNTFTSTMVTYRVLVYPSTKLGCSTTSANVWLSVAGQLGDSG

RMELQKGLLELTFDHKNMGVLTTMRIGHDNSGMMPRWLVEYILVRNELTGHTYRFNCGRW

LGKGVDDGSTERLLVAELVHSTVDTESDEVVVQSVPRTNSPKSPRKTGDSNVTLPELQEM

VGHAVNNLVKHFYKPEKERGNLTFLLCGEKGLVQCMEQVFHFGFRSSRLFRNKIFIWDYL

ERLKLYFENAMANSSVRPIPEVSKAGYWLFCNLLKKISQASEAIGKDGKFQIFICVGIRD

RCLHRWLPLMASTTVTAQMYEENSFLRNPGLTNFIVHILDSLTEFEITLESSLLRGLEI

>SG-PL_contig_1346_1_+3_12_479

MEKMGLKVSTRERVQNLNNQQQTAVATRHRRQAVQNRRRTGQDKTHSKDRAEDTRGQTAG

ANGRSNDIHKENTQQWTWVQEMGLGGERRQGGIRSSRAMTMKSGWGGSFAVFECAEDGMR

RWNLAFTYGSQQGKGMLCRFCSANKAGGLPLVPAKG

>SG-PL_contig_1347_1_-2_539_721

MSSLHCDVSHLVRMHQLLLLFSLRNEFPEKLDVNEHLLPVKNRRKVYIKAWSGKVLTRKS

R

>SG-PL_contig_1348_1_-3_57_1253

MMSGAATCFYAYVGFDCIATTGEETKNPAKIIPISIVSALLIIFVSYCSIAAVVTLMCPY

FLINVNTPLPFVFQYVGWDVARYIIAIGAMCGLSTSLLGSMFPLPRVLYAMGADGVIFRF

FAEISPRFKTPFVATVTSGVFAGVLAMIFSLKELIEMMSIGTLLAYTLVAICVLILRYET

DENMPQHATLERPPENTGEDPPIKLRYLLFPPVTDKPSSVTSAVTKTCVAAMCLSITAFN

AVVIFCEYELLAGECWAILMAAVLGILIIGFLFCITRQPRSHCKVTFLVPFVPFVPAFSC

LVNVYLMFKLDRLTWIRFGVWMVLGFLVYFLYGIENANMSIFYGHKKAEAEEGGTGKDVS

DGKGCRGRGCCGLLGSSSKKGNTEDSDADRYDSIGENTP

>SG-PL_contig_1349_1_+1_103_282

MLFLHVLLENKCVFKRLQNKPLMSQYLTSKHPHNAIIKAHLLAHHERYYLKQLNILNKRL

>SG-PL_contig_1350_1_-2_536_763

MICADYNSKYVFIVDFVDGTMLLEIKVFFSCVLSLLSVCVCLCLYVFCVYMCVCTFVCVC

VCVCVCVCGFITTDLV

>SG-PL_contig_1351_1_+1_190_525

MCKLAAWPGKIIDKCMELNRVLISAQIQSDLTTSQWPTKPWETLLRTKYLCTFHVQGSQL

QNVTGMDYPGSRVNRGFPYWNSAMQPTGLHRQVAYLSPLTPSSQALANCIET

>SG-PL_contig_1352_1_-1_46_288

MDRQGRKKQTTVKEGKAAETKRKFLAQNFQKVGILFILKELLASPRGAICLQILLKMFYS

LPTPWANQTSPNPFSVKIFLT

>SG-PL_contig_1353_1_+1_1_189

QRIQSYIYLFKKKKKKKMIVLRISFRSKNKVPDQSPGQYILDTRFSQTNVILNKNLYQKT

LKS

>SG-PL_contig_1354_1_+2_2_313

SCVYFDIRISTTETTKNSERTCQQCSVSSGEWHHGCLILITPVESIALGLRRNEPDVLGS

RNEQREDGANIVEDGKEEKIHMYSTRTVPCGLNIPSSFSVPPIL

>SG-PL_contig_1355_1_+2_2_205

GQTVIFSCLYHPPPSSTNNLTDCMFHPEFKSLLDFYSLCQGKLIVLGDFNFHFDNQTSSH

INRPFLSW

>SG-PL_contig_1356_1_-1_49_309

MSGRMFLRRLATLPKGQPAGIVPSTARYKKIQELQNTFCRDDGLLVWQKLGRDKIFYYIT

MLFMVCGFVPSLTTIWKMSFPKQTPEE

>SG-PL_contig_1357_1_+1_121_273

MNPTDLFSLFSSGVKTFNAHSLRGEYRRTNRVHWGYASSFKHCHDFQMLIF

>SG-PL_contig_1358_1_+2_356_676

MLFVFMNIVVATQAFTHNRERWKLLVQHSSVQCSYDPGCTKLTTKDLNSQSYLRIALEGI

KCSTVISIQAIFKSSNFQYQNSISENALSLSLSLQIATHSPTHVYFS

>SG-PL_contig_1359_1_+2_2_1666

PYSPCDTHSCSVQSSTYDSVFRDSPSLPQFSSSFFSKSPNFQNDNHQNVSKESSSPVFHY

VCKPNKLKICLFNARSVCKDGKADVIADFILDEHVDIAFVTESWLNAVGHEPIEKCLTPS

GYRLASFPRPSLGGGIAILYKDSLHPLLSFSQSLPFSHPSFEHVQVSYQSNPHPITFSCV

YRPPPSPTNKLTTAMFLSDFDQLLDHYSLLPGNLLILGDINVHYDNHDSSDTKHIRSSLS

HHNMTQHISEPTHQAGHTLDWIISRNHANPATGNSVVLSTSLTAGLPSDHSAILCFLDIS

PPTRTRHTVTRRNLKAINLATFSADATRLLSEKSHNLAEHFDTTLHHLLDTHAPATTRLL

PNRPPAPWLTPEIAHAKRARRRAERRWRKSKLTIDKQLFHTAKTRVSTLIASAKNIYFQN

KIVTSSSSKELFTTMNHLLGTQKTSPLPTTHSHEQLPTVLSNYFTTKIQTLREKLDATPC

RPCPPDPSFSGSPLSTFTPVSQEELLNTIRSMALKTCELDPLPGSLYSDCLPALLPFITD

IINTSLETGTVPDSF

>SG-PL_contig_1360_1_-3_312_656

MSHSIVCVTQRKLFSLKLRSVTILTMTHVLLPPDLPAGWRFPVLSVHLFWQLVIPLPRQR

KIPCMRMRLPPFGKDGTSPVASLVSLMVAWPWSKALSVNDTSEMLRPTLNCCYAL

>SG-PL_contig_1361_1_+1_1_231

GEGVEGLISKISFAKPQGTQLLYQHTTKSHLSPLLLQQNDHPDLTYPTFQHIINKYCGTK

NPTPLMPVCHSLHQFSF

>SG-PL_contig_1362_1_-1_76_1245

MSNPVFIEIAVPDQTLELRVYLKSLGAEISEENADSGILTDLKNIIEASSICWKELNSDS

DVEMVFNGIITLILVVPESDAESVVTLFCEKVGKAPHGDKRGVSRLRMLNNLFNNLDDGS

RNRAVVLTSMVRLAGQTDLLNMVPTDVEKIRAWTTKWELSTAKLQNLYRTLYDALVECKA

SEQATKVMIELLGTYTEDNASQARDDAHKCIVTCLADPNTFLMDHLLTLKPVKFLEGELI

HDLLSIFVSGRLSQYQQFYKNNTDFVQSLGLNHEHNMRKMRLLTFMQMAETKKEMDFALI

QEELVLGPQAVEEFVIDVLKTRAVNAKIDQMQKKVMVTRTTHRTFGKHHWQQIRQRLAEC

DENLSAVEGQLGTLANIKLQQQQMMVAQQT

>SG-PL_contig_1363_1_+1_745_891

MHAHIHTQPSNTNITQEHKRILQKYVSRVGGLTKWALMKGLLEKKRFEI

>SG-PL_contig_1364_1_+3_351_578

MYPEFSCLSVHLSIPASFCQWCGHVLLSAVGWGGLGFVSLLLLLLLLLLFCCFLSFFPVF

FLEAESRTRPLVNYDR

>SG-PL_contig_1365_1_-3_258_1163

MKLTDTAFDVLGFTEEEKTSMYKCTAAIMHFGEMKFKQRPREEQAEADGTAEAEKVAFLM

GVNAGDLLKGLLKPKIKVGNEYVTQGRNKDQVVYSVAALAKSLYNRMFEWLVKRVNKTLD

TKNKRQFFIGVLDIAGFEIFDFNSFEQLCINYTNERLQQFFNHHMFILEQEEYKKEGIEW

EFIDFGMDLQACIDLIEKPMGLLSILEEECMFPKASDKTFLDKLMGNHMGKSPNFGKAGK

PKKPGQVQAHFELHHYAGSVPYNITGWLEKNKDPLNETVVEILSHSKESLVSALFSAPDA

AE

>SG-PL_contig_1366_1_+1_1_138

YIYICLIITMEARFIHDNILGIQVIRIFTLSLKFQTDKLCKIVSIS

>SG-PL_contig_1367_1_-3_327_2582

MQADDFEEEEALTSERELADDAQWKIIQKNTFTRWANEHLKTVNKAIAELESDFSDGLRL

IALIEVLSSKKFKHVNRRPNFRTQKLENVTMTLKFLEEDEGIRIVNIDSTDIVDKKLKLI

LGLIWTLILHYSISMPMWEGEEATPQEGGPTPKQRLLNWVQSKVPDMPIKNFTTDWNDGR

AIGALVDAVAPGLCPDWEDWDPKKAKDNAKEAMDAAEKWLDVPQLIKPEEMTNPKVDDLS

MMTYLSQFPKAKLKPGAPIRPRVSPGINPSRVRAFGPGLEPKGNKVGAPARFTVETFSAG

KGTVEVVILNPKGAKEPCEVVFNKDKKETYSCVYVPSMEGQYRVIIKFASKEIPKSPFNV

TVEGALGDPTKVTAKGPGIEKTGVVATKKTYFEVYTKDAIKIHGAARRGAGKGNVEVVIT

DPHGRKDAVKPTIQPVVGKEGTFLVEYTPNDQGVHTVSIFFTGIQIPKSPYTVQVSPPKT

SNAKACYATGRGIQPRGIRVKENAVFYVHTEGAGEGDVAVKIIGPGGAEEKCNIRKVPDK

PGVWECIYIPSKPGNYVITVTFGGEPIPKSPFRVEVGPIWLSKIVAYGPGLEGGVVNQPA

IFTVETNGEVGALGFSIEGPSEAKIDCKDNGDGSADVTYWPTVPGEYAVHILCNDKDIPK

SPYMADIKPATNEFNPGRVIAKGPGLQKTGVQVNKWAEFTVDTKLAGKAPLQITCMDAEY

KMVEVQIKDNRDGTYSCRYMPTRNIKHTITIT

>SG-PL_contig_1368_1_+3_3_170

FSFKSRVYTLLYTLLLITYKLLLSFKSRVYTFVFVILITYKFLFLFKSRVDPLVFV

>SG-PL_contig_1369_1_-3_3_134

RERESLVQTLIGAGVDFISLSDKHLIEIHGDQVETAMIQASIRN

>SG-PL_contig_1370_1_+3_87_359

MIRGMGRNLTSSTPKTLRVSSSSLILLQPGNTQALQSNSNTTRDTHDKKSTHEGFWRWWS

WRWGNIHNHHSKTSNRQENEQSVQHNKQTSN

>SG-PL_contig_1371_1_+2_29_250

MTTSVVHTHTHTHINKTQSSRVVQNGNMLGVKGRLLCIGAIHFKLFIHNNILTPSTATDV

YRFKSSCKVLKCWF

>SG-PL_contig_1372_1_+1_1141_1656

MMTGICMILDDMNRINITHVLHKVSVTVDNSLYWPPSQSDGRHRQGTDYQHITRCNICHK

NLSQPIFIPKMFRRFSLKEMFPCMIDSQLSGVQITSNQKIHGSHSQPIQKYSRSRYIQIL

KNITSISIHNYSLHSRNNFNLFLCKQKVSQRHFLNCKQSPNLLIPNFFYIRC

>SG-PL_contig_1373_1_+2_2_226

YHTQAIPCTPPKSASNYLADSLSLRKTLCADKVLKWGVEKLFNTDTTRMTEALRWATNRP

VFMLQAGTEFEISDL

>SG-PL_contig_1374_1_+3_606_827

MDWRQLAQSSPCQGRTCLLRFAGGSFTFMPTRPLVQVRISTDKTYNNTNKITTTKHTRNK

STNKQKLITTTTTH

>SG-PL_contig_1375_1_+3_39_368

MLYVLLCKMNNPDANPNTTTPFLKMKTMPTNTDAKVQTADTNESEQKTPRVQGVFYRCKP

EAVENQCLWNMNSNNTLFHLLQPHIHRRKKISSMEIISEHLYTQTIKMVW

>SG-PL_contig_1376_1_+2_2_106

FFFKFFIIQCCKFITQHCKIALESFAWDKSKIKPH

>SG-PL_contig_1377_1_+3_453_635

MKYTLEVHHLLTKHESHERGLPGMSNNNNNNNYDIYNAHNLDKINSSLTQDTTTSEKSSN

V

>SG-PL_contig_1378_1_-1_1_819

VSEFDTPGHSQSWGNGQSDLLTKCYSGKVPDGQFGPINPIFNTTYDFLQKFFKEVASVFP

DHYVHLGGDEVSFTCWQSNPAIQQFMKKMSFGTDYSKLEEYYIQKLLDIVSSLNRGYIVW

QEVFDNGAKLRSDTVVEVWRAGWQNEMANVTKQGFKTLLASCWYLNYISYGEDWTKYYTC

DPYDFNGTAAQKALVMGGEVTMWGEFVDDSNVLSRLWPRASAVAERLWSPKDYNMPLLAE

ARLVEQRCRMIRRGIPAEPISGPGFCEEEYRGY

>SG-PL_contig_1379_1_+1_118_258

MADWCLVNGYTRSHVHCVTSCVSVCTNTANTCTAVSVTIAVDYLRAH

>SG-PL_contig_1380_1_+1_436_693

MLRMYVYYVLSACFYPKGCRRENELVVSSKLEDPERGDPGFALKFQLDPSPVFVEQFYNP

LRFARPVLFCTDIFFFFLHGLQCPDL

>SG-PL_contig_1381_1_+2_2_157

IMLNGLYSLLSGEKKSFKKCTQMRHRSDLKQAFPMQTQSIIENINNKAFQCC

>SG-PL_contig_1382_1_+1_1_96

THTYTHHTGRYLIVMNKQYKTNTGTTINGSKL

>SG-PL_contig_1383_1_+2_728_913

MKLVQGQVKRFFLSLTGSLLWFQDEKGKNERINRKTLERTPVTLVSLVSSVGRRELHDCK

TN

>SG-PL_contig_1384_1_-1_322_2496

MASDLKSVQMAKTESERKRKQAEQQLAEMSIRLQELEQGRGESTDKVNKLQQELEQANSI

LEQTDTKAIQLSQRVSSLEAQLADSQDISQEETRQKLALQSKLRQAEDEKESAMERLEEE

EEAKKALERHLADLQQKMVDLKRKAEEDLANNEALEEYKKKAAREMDQLHAQVEEARSNA

DRMDKSRRKLQAEVEDLTIALDSQRSNVSNMEKKQRKFDQTLAEAKGESERLTLEKDNLE

KESRDKETKILNLTRNLDEVQERVEQLERLRQQQARELEDLVSSKDDVGKNVHELDKAKR

ALEATVAEQRVQIEELEDELQAAEDAKLRLEVNMNALRDKYKSEVAGREEQEEESKRSLL

RQLREMEAELEDERKQRALAVNARNKLQGDIQGMEQQLEMATKVKEDAVKQYKKIFAQMK

DFQHELDDARMAREEMAAQAKDNEKKVKNLEAELVRLQEDLASAERQRRNAEAERDELAE

EIGSSTSGKQALLDDKRRLEARISELEDELEDEHTNLEMANDKARKSMIQMEQIATDLAA

ERSVSQKLENQRLALERQNKEMREKLQELEGQSRARTKATIAALEGKITNLEEQLDQEAK

ERAALARTNRKMDKRLKELQIQAEDERRSADQYKEQLDKMNNRVKALKRQVDEAEEECAR

LSAQKRKVQRDLDDQMEQNDVLMRETQSLRKYRPSAQARSRAAVSLDRLADEDGSEDGHD

AQTET

>SG-PL_contig_1385_1_+2_419_631

MFYPFSCLLMPFACTRFEEHIIHSITQALYTTQALTHNHERWRQGLQCSTIQCSNDSEGL

WDQSAHNAVLS

>SG-PL_contig_1386_1_+3_3_182

SVPSSTYIWHFWKCSFLVQILPVRPETNHLCKWALVFAIIFTLWCTTRLSPSPILFVKYT

>SG-PL_contig_1387_1_+3_2226_2441

MLPSTCNCKQLCSPQCVTVNISIQSEKKNCKLQSSLQCVNEANFPSTMTICDWKLQSSPQ

YATGNCEVLLNM

>SG-PL_contig_1388_1_+2_113_238

MAYNFETNTNSKITKYKPDITDNLTKHSCSSRKNYQHQLFHF

>SG-PL_contig_1389_1_-3_57_638

MGRFKKTSSSMDVANEESSIVNEDTFVDVDGYSSSDSSDDDESEDIDVEDLESDDYDTDS

GSKPRIVKKGHHNELERRRRMELSSNHGDLKSLVKKLFPRAKVSGRDRTLSLASLALQKV

ADRSRAIKKSGKALIYAYKCIRATRVPPEKLAELNADIKELEELFGRDGTSPDKEYLEEI

KVKDAQRKKSKRRQ

>SG-PL_contig_1390_1_+3_12_281

MAEMLKSGGECVVHWMRTLCNQVWNSGEVPEDWKNGAIVCIPKKGNLTECDNWRGVTLLS

IPGQLILNRIRDVVDREAQAAASSSKWDKS

>SG-PL_contig_1391_1_-2_350_607

MGSVLRKVASEQALLNATEVTQQWPDMAGSHGLQALGLCLKELGFGGLLRSRNEQCGISA

TETAQKMGQEKSFISSIPYDYISLFQ

>SG-PL_contig_1392_1_+3_966_1214

MVHPTHSLHTNMQNIWPTILTGNSQYALKKNKPEYPVTIYNLLCDLEFKDFTICPYPSLS

SITTFKRFKQSKHRSCLCNNVGD

>SG-PL_contig_1393_1_-1_1174_1359

MSLVLYLSSEVKLLLCIHYFKLNFKSSKVCDGYNICSCWQSERSPKSFYTKETIVISFLT

GS

>SG-PL_contig_1394_1_+1_34_294

MIRENEDIKGININNVEHKISQYADDTEFSVAGDRVSFQTCIEVLQFFGSKSGLFMNAGK

TSAVWLGSWKNSEVRSMQHLGIDWNPS

>SG-PL_contig_1395_1_-1_1_279

DTGPVPTAQETEGADLDPALTAEDEHTQDQEATPTRGKAGAEVLQAHQITQSLTERAGPL

SNVSQEETEVFGTNSFMTENSHRSFFLSWIKFT

>SG-PL_contig_1396_1_-2_437_577

MRFACIYITRCCMQIFLHIACDMILKSRVFVFRMLIELYPVYLFHYS

>SG-PL_contig_1397_1_+3_4188_4613

MYHWHELNMHAPAHTSPSHIGCPSKSFQRLDSLENKAKAVTAQAKHQPKATDQPSAKRQK

VRPETSTAVISDDEEEGQDRARSDGTTVPDITRDLLTDSNGEESDPPLEDDDDIHSEFAS

SFATTEERRPSVDQKLAKLLQE

>SG-PL_contig_1398_1_+3_168_263

MGRGRVHKTNCTPSNVSHDYSHTDVILVPANT

>SG-PL_contig_1399_1_+3_3_173

RTLCEMARGRVHKTNCTPSNLSPHDYSHTDAILVPANTCKLLFPGGSGFYGISLLRY

>SG-PL_contig_1400_1_-3_417_569

MRCIYFDNIVFVILKLCQGHRNFGSRDINNDMKTIMVSLSVMVMLLSTQMT

>SG-PL_contig_1401_1_-1_97_1239

MPQDDERYCFIVEWYEDMAARTRQFQFFYFVNSKSIEMFDIKNHRLFLRTSVKEGMLSLS

DLYLGNSINVCERLLNIVDYGDEFTRRKLTAKTERTVGLIKPDAVEKLGQVLDRIYQKGF

LLSRLRMCRMNRDQAMAFYQEHLSKPFLNALLDYVTSGPVVAFEIVGENAINAWREAIGP

TDPSEARQTAPTSIRAQYGTDKTCNAVHGSDSPASAAREIEFFFPSAGPVPPNTAKVDNC

ACCIIKPSALKAGVGGKVMSAITEAGFRVGAAQMFTLEKANAEEFLEVYKGVVREYPAMV

AELTSGPCLALEIVGCDKTQCVAAAFRELAGPADPEIARHLRPRTLRAIFGSDKVKNAVH

CTDLPDDGQLEVEYFFRILDR

>SG-PL_contig_1402_1_-1_1_156

VIIILVYRGILKFYLDSFHPACTRRCCVEMGVGGLQVDGKPDVVSEKEKPWL

>SG-PL_contig_1403_1_-1_175_1362

MTWKAHIHEAENKARRKLNIMRKLAGTNWGANAKLLKSVYQGAVRPHLEYGSNSWMTAAK

THHQTLDKVQNQALRIITGAMKSTPINKMEEITGIPPLSTRREQKVLIQSKKYECTTDHP

MKSRIQQLSSGRLKRSSFAHETRGLARKYQEKLPEQVVPPSFSLDTPPWEERRTNIKVNT

SVPHVTTKDEQSDTVKKTLTLAMLEERYPQENWIQIYTDGSATDAVKCGGAGVYIQYPSG

EWQAEATPTGLHCTNYMAEVKALIHAAHVISSRVDNNTQVVFLTDALSVLQSYNAGQLKD

LEKALFNLNALRIELQWIPSHCGIKGNEQADKMAKMGAKEEQENNPVSLKEMNTIIKSLF

RSPPKPDSYHQLSRQAQVVIFRLRTGHNRQNKHLHK

>SG-PL_contig_1404_1_-1_196_1656

MWPTTLLPVVLAFAVSGLTHGIEDKAFWMNVGQKILDEALKVKLNNRVAKNVILFMGDGM

GISTITAARIYKGQLQGNTGEETQLTFEKFPHAAFSKTYCVDRQTPGSASSGTAYHSGVK

VNFGTVGFDANVLRRNCSLINDSSVRVETMLDWSYAAGKSVGIVTTTRVTHASPAAAYAH

SADRNWERDTDIPTGCGLDIASQLIRENSNITVVMGGGRRNFIGREDGQNLVEEWKTNKQ

NEGKTARYIQNATQLRNLDASAVDNVLGLFTESHMSWELSRDNDTEPSLTEMTVKAIEIL

QKNDKGFFLFVEGGRIDHGHHDNWAKRALVETIAMDDAVAAALRVTNQDDTLIVVTADHS

HPFSLVGFPKRGNDILGLVDPVDPGQETTDGMPYTTLVYGNGAGPMRETNLTGVNATGND

FRFPVAVPMDKEWETHAGEDVGIFAIGPMAHLFHGVHEQNYIAHAMAYAACVGPNTKHCT

TKPPPDN

>SG-PL_contig_1405_1_+3_69_236

MMQTNSIIENLRWGKKPKETKHNSISFISNFTFSQQCGGKARVHDLISHHKFHLES

>SG-PL_contig_1406_1_-3_198_1199

MAKDRFTDEEKRSLDRAHNYLYQEMRQAAEAHRQTRKYMQSYIKPGMTMIEICETLEGTS

RKMINENGLDAGLAFPTGCSLNHCAAHYTPNAGDPTVLNYDDVCKIDFGTHINGRIIDCA

FTLAFNPKYDQLLAAVKDATNTGIREAGIDVRLCDVGERIQEVMESYEVELDGKTYQVKP

IRNLNGHSVGQYRIHAGKTVPIVKGGDATRMEEGEVYAIETFGSTGKGVVHDDMEVSHYM

KNFDVGHVPLRLTKSKQLLHVINNNFSTLAFCRRWLDRLGQTKYLMALKNLCDVGIIDPY

PPLCDIKGCYTAQFEHTLVLRPTCKEVLSRGDDY

>SG-PL_contig_1407_1_-2_59_2923

MDANDEKINAVLLFANRLIEENHFAADKIHKKAENINERRNTNRQRADEQLDRLKDQLTL

QQFLQECDELRDWLADKMAASQDETYRDAKNLHSKYMRHQAFESEIAANKDRLNRVQQEG

EEIMQMKPETREQIEPMLADLNRQWEDLEMTTKDKGEKLFDANRSILYEQSCDDIDGWVT

ALESQIITSEDFGKDLTTVNLQVQKQNQLENQLKMKEQQVQELESQADHLRRIEPDKVDD

IEKRRALVAERFAKIQAPLMLRRSALDKVKRIHQFMRDVEDEKLWIQEMMPRATATNYGN

SLLSVQLLIKKNHSLQVEIENHENRIMSVVQVGQDLIDEGHPQSEEFRALMEDLLRRWQE

LKDAVEARSQRLKLSEIAQQYYFDAAEAEAWMSEQELYMMAEDRAKDELGAQNMMKKHQT

LENNVEDYADVVRQLGERSRRLIEEEHPECDQMAVRQSQVDKLYAGLKDLAGERRGKLEE

VFKLYTLHREIDDLLQWIAEKSTVAGSNDPGQDYEHVSMLKEQFKEFARDTESMGQERVA

SANEICDQLIALDHSDSATIAEWKDNVNEAWADLLELIDTRTQLLHTSWELHKFLYECKD

TLERIYEKQNYIPEDLGRDAQSVAALQRKHANFEHDLITLGTQVEAIQTESVRLQAGYAG

NKAADIQAREAEVVEAWRNLHIQVETRRMTLADASDYYRFMLMAHDLQLWMDDIIRQMNT

QEKPRDVSGVELLMNNHQSLKAEIDAREENFAICINLGRDLLNRKHYRQEEVREKLIQLT

LERLDMTDLWKDRWEHLQLILEVYQFARDAAVAEAWLVAQEQYFLNQDLGDTLDAVENLI

KKHEAFEKAAATQEERFAALEKPTTFEIKDRQRKQEEEYRKQHPEEVAQAKEERRRAYIV

EFLPPEPPKPEPEPVVEEVLPVAAAVAERRPSGEEVDQTDKVDGDQEAVLQKEAE

>SG-PL_contig_1408_1_-1_79_864

MAKDSQSDDCFQVLLARIQQETNTPQSKLQILYSSRGYFNAQQASAVLHALSRPEDKLKA

AKMLEPRLCRMSCQEARDILTAVNIQNDRLIVLDSIKRVLTDYQTVVGEEYVLSAFPFET

DKHMALNILRTVRSDVVDKIGAGGHQGYAAMGGLYTQARPLHPHLYGSLPEQAKQIPGQG

KIEVPLTAQPGVVPSIYTGHPSYAYPADKSYAEDRGYPGTIGFPATVEGPSSYPGGAPPL

GNHSGAPAPTGFPRLDSRGFVY

>SG-PL_contig_1409_1_+1_328_444

MYHKLGLNFFTHDQFERVGFFYTTSFKGLKKIFSYSRPV

>SG-PL_contig_1410_1_-1_145_366

MENKYVRVLNNNFTWNLTDDEGNSDTPVASYDCKEIDDEINGAYSPLLDAFFYGTVVGKM

FENWYGMSPLGDVM

>SG-PL_contig_1411_1_-1_1_867

YLVAEEGKAQRRPTCQINAKNSSITFSWDAIDNCTDCRATGVGGNLKIGKIKYGELQRCL

DLRREDGVCYMENKYVRVLNNNFTWNMTDLEGNSDTPVASYDCKEIDDYVNGAYSPLLDA

LFYGTVVGKMFENWYGMSPLGDVMILRVHFGYNYSNAFWNGFECTFGDGNGWYFHPLVSL

DIVGHEVAHGITEKNSGLYYYQQWGGINEAFSDITGEASEAYLDEADWMMGYDITVGDIP

PIRFMDNPEKDNRSIAHADNYTEFLDPHFGSGVYNRAFYLLVHVYGIPI

>SG-PL_contig_1412_1_-1_1_195

ARTHTRTRARAHTHTHTHTHTHTHTHTHTHTAKATTNSFDLQVCPTVSENKNNSFYLGYD

CIFCA

>SG-PL_contig_1413_1_-2_35_313

MASSFAKMAGLRTSRMLLSVLKSNTSHILVPVRTKVCTETGAILEKPVRVRFGLVKVACT

VTPFLITGGLLSREFAALLEEHELFVPDDDDDD

>SG-PL_contig_1414_1_-1_16_303

MIFCIFLAVFCKAPNQSSERACMLTRARSFILWTKRSILTIILTWAKTMFYVDVIAGVGV

GAVPFCVDELRKIFVGEISVMLVSVIWIFSSTLVVG

>SG-PL_contig_1415_1_-2_692_3925

MARIVVLVSCTVFVFLTVGHYSYAEETRDLADAFSRSEVFLTGEFFTPRDTRRRFVMVRA

QNRRKGTLFEISVDFRQPQVILRLRSSDRRDYQLAFPTRALHQDDRKRFLLHFYDLDLPS

NSVRLFVDCEDIGVDKTEIPIRSILSNGVNVRQTPSFKFHVQTTLPEILMSQGCGRESYL

QPPTTPAPKLELPAWVSERVRPERPEPDPPRREGHAPSDYDSLNGRKQQIPDRLDQGPRA

VPDEFSRVINSGGDVNMLMTAALRDLTTTIRQLRADLNAQTQETRELRQTLATCAICKEG

VPAEQPGPSGPSRQRCESNPCFRGVRCVDTEEGFRCGRCPRGYYGDGVRCERYTTCADSP

CFRGVECRDTERGFRCGPCPAGMTGDGTRSGCRPLLSTCADRPCFPGVECKDTPGGATCG

ACPTGYAGNGTHCEDIDECLYSRPCDRLATCRNLSPGFTCTACPPGYTSNRVEGVGIAEA

QRQKQVCEDIDECAENNGGCVENSKCENTPGSYRCGECVEGYEGDQRQGCSPEGQLCQDG

KTRCSNHAKCVQPQGRKGYSCQCNVGYAGDGKQCFRDTDLDGIPDEELPCADRRCRKDNC

VLVPNSGQEDADADGIGNACDDDMDNDGIINNPDNCPLVANPDQSDTEYEPDKRGDACDN

CPTVPNPDQTDTDVDGMGDNCDPDIDNDGIPNREDNCIRTINPGQLDTDKDGVGDACDNC

IYIPNENQFDSDNDLVGDICDTNDDIDRDGIQNNFDNCEVNPNPEQLDTDKDGQGDACDE

DDDDDDIPDIRDNCVLIPNPDQRDTDGDGRGDICQDDYDGDGHDDLYDVCPDNGEIFATD

FRAFQTVILDPEGDSQIDPNWIVLNQGAEIVQTMNSDPGLAISYNAFSGVDFSGTFFINT

EVDDDYAGFIFSYQDSSSFYVIMWKKSTQTYWHPTPFRAVAEPGIQMKLVKSDSGPGEIL

RNALWHTGNTTHQVKLLWKDPRNVGWKDKTAYRWELIHRPAYGLLRVLFFEETELVADSG

NVYDYELKGGRLGVFCFSQEMVIWSDLVYRCNDYIPHGLLEDGSDSEDYEYDYDYEDN

>SG-PL_contig_1416_1_-2_2_361

LFRSVQHSTFALFQRRSQKDSHHGVRFNLVLSPEKLRSGLTLLPRLQQHSWTHPQVWFKH

LQKVFPAVCRFHRLQEAELKMTVGSYVWTPSGLRVTDGSRLLNRLKSTDWTWASPHETIP

>SG-PL_contig_1417_1_-2_2_232

FFSLKLQAPQQQTTAPKYKLATIYTDSHQSLKHSFKTEVFKQNEAPHRIIYYVNIHYLRS

TKLKKRHISKGGFFCVY

>SG-PL_contig_1418_1_-3_930_1217

MVFACGLSLVMCCLCVSDLPTTTVLSAFLPAHTDMTDMPMMALLTAIPVTSCLPLQNVSW

IGSIVVDLSVCIQKLAEFFFKVGVPLVCKDGRHSHR

>SG-PL_contig_1419_1_+3_660_896

MSTEKVTAQTPINFKEMTHTYANCFSLDSRKKTSVIQIKAATHQILHTWENPFFCLLQRY

KVAEDNENYLIKFSYIQFS

>SG-PL_contig_1420_1_-2_323_643

MKKQNQIKQKTNPVHASATILVKKKKSVCKGKVPSKLKIVPCVSISVITMCIRKIYSNNH

MQQRKWCCITIHVLPKGSSHNGKKNGTTRNEHDAVKYNSKQMHNSIQ

>SG-PL_contig_1421_1_+3_2166_2483

MDNPALWCQQIPHQNVRLRNSHQEAIFRLQAGQKTLNHHLFTKFHINQRDQCPCQIGSKT

KEHLLQAWLLHKSFGRQLSGRDPRDQEALQRSAGPSAFIQKTGVSI

>SG-PL_contig_1422_1_+2_29_217

MFNVCSHNRLKNKVFPQSICSTPFKLFYHKVKSSPNDSIQLMPQLIVTAHTHKQVLVLQT

CVR

>SG-PL_contig_1423_1_+2_128_427

MHVNQVHAKCQAISVPKQILFLLRSSQCTAMDRYKCRYTTLFCTSDFTYIHTHPYYIKRQ

SCHSILFPIYYKHRTDQPVLNENPAPTDAAHSFATCSLSH

>SG-PL_contig_1424_1_+2_506_679

MTLQHSEIPYAQLAARILHIRFFVTDKKYIQRRGREEKRSPTRDQPGLISGSAYNSNT

>SG-PL_contig_1425_1_-2_560_949

MAIRRRFASVPPMYMSTKRSKNYAGMPSLYPVTGMSRAARMAYDTRAEDYLTDRVTNASF

FSNYALDEGYDTAKKSRGRDERLLRDATHAIVSSESYATPQASVAARRVVRGRSMAPRAT

SAPPRPLYPE

>SG-PL_contig_1426_1_-2_596_1078

MEVKSESSVMLSNLEVLMLLRDIQAGHNNYPKPNKHQQNLGTILYETKKYLENTPCALQT

EDNVESFMMAVKYTKLTKMEKLQLVNSRPTQIVDLHLIVEESDERFSTDEEMENLLDIVA

THLPGPESEEPSDDEENEEEEEEEGVDEKEEEEVEEEYVAI

>SG-PL_contig_1427_1_-2_248_1951

MLKGEFAVVEYAGYDSFISDIVPIDRVRRINSNPPISPDSFQQFVLEVPPDLREGCQGEG

ALAELRKHLGGGAIISYVVADNSIHVLSTSAAVTKRASMIGDMFLRNVRQKLLLKQRTEE

AVKKLQSTKIRSGYMEEFAVREDLMGLAIGTHGANIQQARKIEHITGIELDEATCTFKVY

GETTEAVKQARGMLEFSEETFPVPRDLVAKVIGKNGRNIQDIVDKSGVVRVKIEGDNEPD

SQRDQLDQVPFIFVGTQESISNARLLLEYHLEHLKEVEQLRQAKQEIDMQLKSLAGPQAG

PYFAPPRDRWNDGGFVDERNRRGRGGMRGMGGRGRGRWNPDRPDYGGNRYDRRPNRGGYE

DGYHDNNFPYRGQGPRPYNNYDEESRDTRSRRRMTDDDDTVLDNADNASVNSQDDDRRSD

RRRRRRRTRPRGGSNSAPSGTETDTNVSSYRQDRGQRPPAGPKGGPHRPLNQVKSEDSAS

MNGGGGAHSPHPVANGGGGEDGSATASKSDSQQSVPKEQRSARRPPPQTSSSSQNKTANS

QPNGGDSSDGKAKTASGPRPKEQVVNGE

>SG-PL_contig_1428_1_+3_198_530

MDWEGRKKADDSKGKKKGRNKESNRIWEFASTEFPECGDTIYTERAPSIPTGVPKNIKSI

CCIIKSVKTTASTIRWYKHYNQYSQYPATSTNVFSTKNKFSDDQDSVLPKL

>SG-PL_contig_1429_1_-1_115_423

MRELVKEGKKFDFIFLDAEKSEYIDYVKLCFEDGLLAENGTVAVDNAFRYGANYLPCDSP

PSAARLFGEHVTKDKRLHKVLVPIRDGVLLIRRLSEVEPDLPH

>SG-PL_contig_1430_1_+2_62_214

MKTSESTNKVTAVNWERDGRTKQGRRMENRRKSVEPDTTISVKQIKHEGYT

>SG-PL_contig_1431_1_+1_1_267

GGLGGLQLPAVSRANRQATVFTVRECKHACNTRKPPARAPRAFYLRLCQCQRFQKYVAPT

CTSQNRRVGLSGSVTSDALGRCLILLVPE

>SG-PL_contig_1432_1_-3_96_929

MGSLRKVFVVGVGMTKFEKPGSRENFDYPDMAKEAGTKALQDAGLSFDKIEQACCSYVYG

FTTCGQRALYQLGMTGIPIYNVNNACCSGSTALFMAKQLVEGGMADCVMAIGFEKMERGP

LKAEYTNRTIPVDKHIEVMTRLRGFEPSPVNPQLFGNAAKEHMEKYGTTKDHLVKIAYKN

HKHSTNNPYSQFQKEYSIDEIKQSTTIFDPLTKLQCCPTSDGSAAVILASEDFVRRHNLQ

SQAIEIVGMEMGTDTPSTFGKSSMSLVGYDMTKNTADQ

>SG-PL_contig_1433_1_-2_32_223

MTQLSCISHLCTTIVLYLTLMYDNCLVSHTYVRLILCAPHPLLLPSVFMVFIFLSPRSFL

TQTF

>SG-PL_contig_1434_1_+1_1_183

FFFSFPSLFHICILLPSKLTIRQTIPHTYVKQEYFIYATDGKFVALVQLKDAYNENGHYL

M

>SG-PL_contig_1435_1_+1_157_471

MQLAFLEHDSTGIADCLPSCSQNGSFATSFSQMTGQTWEVAGGLFRFGGVWNRLQSPGDI

FTACDAPVPQVQVVVVETRCLPVSTAVLSIVKQLVNDKVETCRQS

>SG-PL_contig_1436_1_-2_824_1027

MGHFPLDQTSSVEAFSTRPDQMRHFPTESTARHPEEDSRGTRFVCGVALALRGRQDRTPF

LLCHSFIQ

>SG-PL_contig_1437_1_+1_202_354

MYFDFLEQYNEISVTVTDDLKQAIFANTEQNRSQKPTAVRNLLFNIFPTLE

>SG-PL_contig_1438_1_-1_964_1182

MGKHFVRNADQTHQFPHRITFCQEFRQDPQFPHRTSLEKKQEFWFRKRKKNSGPRFGIAS

LMVLDIQDGSVLC

>SG-PL_contig_1439_1_-3_21_353

MFTVMPQNLNSCMCVILSFFLVHFFLVQVVVCEYSVTIGVSLKVFHFDVYNLVLVKMICD

LDQCIFLKSTFSCVDVCILATWRRGKRCTHSNTCTHVYAHTYTQTTYTLKR

>SG-PL_contig_1440_1_-3_588_2432

MNFDHVEKGSPEEVLMGLKTGATTYAMLFDIMCNYAGLHSKIIGGYAKGADYIPGMKFNS

GQNQHTWNAIYIYGTWCLIDAHWGARRLVGKQTSSEDFHYQLDEYFFLPDPHQLIYTHFP

DDPRWQLLDRCVTLEEFENMPHMKPQFFKFGMEFVTHRNAIIRSRGEVQVRLRYPAKQVA

VAFNFTIQFENGDEEYKGTRLSRYGMQDNEDGVATFTLRLPTSASYILFIYAKEDTADKK

ENVYSQVCEYKVEHETQPGSAPEPFPPCSSLTWGPCSTFYRYGLSTTQQSPVIHTCEGKA

QLQIQQPRPMQLMARLKHNNISDSDLESYVMQRTVGNTIIFDINAPGRGEYGLEVFANDP

ATEGQTLYHNAQYLLVCQEDVKTTPLPKLPPGYLGPQPRFSEFGMSTMSHQDPVIHVDTN

VLTVVFKCQQPMRLLANLIAVEGDKDFSNFTFSNTKGSNTIFSINIPQTGFFKLQIYGTP

VSDKGQQIPGIYNYLVNCRQVTQAVYPFPKQYAQWKEGCFMAEPVVIPSRQGPFADQVQK

ERLPDTITFRVEIPGAKSVAVVVDTDWTHLEKAGVNEWSGNVSVGHCYGTNTRVTLNANY

GADKTNYATLLEYFL

>SG-PL_contig_1441_1_+1_43_420

MFMFHPNDQQKIVLQKLTECTQKTTTMKLPVLGELGRFPISLTVMCKVISFWIHTTESNK

DSYPPKAYSDMHLSESLNTAPWLNFVKNLISIGFSHVWENQGTLSVKETEIFYNNETTRK

IPSVQE

>SG-PL_contig_1442_1_-2_191_490

MLKSALRLHSTGNKNIRACVVHGVVEGVGTDFRSKQDIRCQQQGSVTFGRFHIRSDSECA

RLFASTLRPNIQEKLCLFLYCKPQWISFKLKKTTTSFEVD

>SG-PL_contig_1443_1_-1_19_291

MPDTLGHAVGIERYELLAKLAGNEDPFEMNVKKRAKGTVDEPTLIPSLYEKRLVGCICEE

DAVSINWMHLHKGEPRRCECGFWFKLVDLPH

>SG-PL_contig_1444_1_-1_115_615

MYYAGLISDYTRRTYSAIVNNVIDKAVSAGRLDGEDLYKMLAAVENVTENPDIDNQLGGE

HTVHLLKDRLETLLNSPTARQALHVGQANGNVKFTSQSDEVFEFIKTRNYFRSANEDNIF

LLENYKVLHYSGNLDVIVSVAATEKFLQQLEWPGTDRYYNSTRDPWF

>SG-PL_contig_1445_1_-2_842_2350

MLETESAYHFLDAYTPITEHALVFPLQYKRWSVDLDRGIIPDGLLKLLAQEEMQKALSVQ

CSPQSQSSTPTASPSSVDTEHTGSHLSGQSGRLHSGHSGQLVPSGQLSGQMAGQLSGQML

GQQTEQSRRQLLVRQPNVNQTEQQFPAISKGSRSFEESSGSIASSSMYRSGSVPNLTVAS

TQQGAFPDPVTSTTLVYRDFDQDSTSLSSSGFIPELSLLTKVVSDSDTSSNIPTVVSHHS

PPTAASKSSSAWPWGPNQEFSGASTSSDASQSRTYPFTCASSVTRTPQSAQLTTSQAFRG

GVFSSGSTVTPGPNFLVSSGSSISAGTVLSSAVPYYQRPRSSSDLARKVTSTVSSGSVSS

SLSQQNLPGLFDAAVTTYSPRDVYPSFRDSVTTCVTHSSALQETAPVCVGSELSASISEP

IPHWYSFLDDLESAASLSARQGAFSDAGTPGSSRTRSDAGTPSSVDSPYSQQLLQQQQQQ

QESGGDGNIPTIISDMLGQAPPS

>SG-PL_contig_1446_1_-1_1087_1281

MLLSLMSICCCWHICFQLDIILITVQIVHHNISFQSNFWTVCVLHFFHAISICLVIFKQV

KVCFV

>SG-PL_contig_1447_1_+1_274_471

MDLYKVFVTGNFIRMVTVSASVSKLLAFRCNILSVWVLRDLYKVSVSRNFICKETASASM

SEQFIT

>SG-PL_contig_1448_1_+1_1_222

KVKQQRRGVVPARSKSKPESSDLYHLTSLRPGLGLGDYLYLFFSRFSQGNVHRDKLTVMS

SCDTTLEMDARAIL

>SG-PL_contig_1449_1_-1_58_1404

MLEEVVDALTPISKFPVHLKQSIIYLFPRESAMSDPQKLTPPEIPPFPEFLNIIDVDKDS

RLALNCLARGHLIKHHTETVARQQKCLVEGQLFLRMYIEGRLRLAGEVLQQIQQQILESK

KRFDMFYTCVKSIQVLLSSMGQLPDVFFRDLLKDETLLKVHKKAEERADEIQRYRRGLHE

KLEEARPHASQWSEQCESTSCTSKVEHHLTVITNIQNRFRRDKTVKNQMTAHDENIHWFE

RQKLQEHCLRMLSILTGHCVPALDKLHKQSYQYSALLVKALARIIKVEKNNESVINCQQL

LSSRLDKIEIKCQDEIRHLHQQHEQLQQQQQEERLQQQQEQQQQPQQPQSSVDEGLGTSV

STSTITTTTTATAVAGSTVHLGELSSRASGGESLESRSIREDLQSLRQESEDLLALFSEN

MNIMRRLEEESNQMMSCGSLSRLLQERLT

>SG-PL_contig_1450_1_+1_736_1083

MYFLLPLYYCFVFYIPLISLFYLWQNLVQIDMLIDYDYPCQHVSGSLPSVVIKSGPATQK

LIFKRYNDLTFTPSSWPWSKALSVNDTSEMLRPTLNCCYALQCYVGIVFIHGKLLA

>SG-PL_contig_1451_1_+1_106_1500

MPPMQTSSAEAEANPSPDGADLNKMETKKSTTRKNEDNEERKGYFVVEIPYWLCGCGTLL

SIVVGALAILTMVFMTWAPKADIEDWILEVQDRVGKGGVDLFRTYDLNRDGYLSVYEFEP

LIPHLKNLTGSIEEVMPLETYLEYTPEDGEEVLILQANFTPLKLDTMTKEDILNMDINGQ

NTLFGLRNWQEPYRKGSAFGASEFQSLLPEDMENVPLGKPYHVLPYTGSTEQKVGSYASS

AIHSSSRYYPPVPKGKEHLLFRLLGMMHHRPFVDMRFPPRGTVACVQAFNDKYVHVVFRM

HAEFQLNEPPLHPFWFTPGQFVGDLVISRNGSHVQYFHMRVPNEKRLNVDMEWMTGVAEG

EEGMEVDIGYMPLMEWTLNRQSYLPRSLEEALSTEPESSPPQPPDQIEWTSEVPRADILR

LIEIEFYPFKTVMYHNLTRAAQLSKEENKPLHAVLLWGALDDQSC

>SG-PL_contig_1452_1_+2_56_148

MRGVNYLANSLNLCKTLCAGKVLKWGGEWDL

>SG-PL_contig_1453_1_-3_3_359

ARPGSQVSPLAGGGGTNEGNYLFQSSANSPFTQGESGTQRNATRSSPRGRGPLGSPLPYG

RTRTSTRSAPGLLVSSWEATGSPLPPRLAPARLRMGPVARPLSLRGSVQHLGCPHATLA

>SG-PL_contig_1455_1_+2_1637_2002

MQCPLPVAQCTNSDVTQKKTRSHTADSRTEVAVTGLQLQSVHHKCPGGRRTSAGRATMEE

HDYNVGYHSVTHSDIPILKPCRGFTHQPISNYLTRHFSSAFQMSLTAARQFSSACEKCHL

SK

>SG-PL_contig_1456_1_+2_197_616

MGRNVINGMQSTECNQWNAIKKNANNGMQSTECNQQNAINRMQSTECNQKNAISRMQSTQ

CNQQNAIKGMQYEMTAIKGLLKRVKRSISELHKPRGKQPAQPAKSILLKRTQTIATDQNG

LVYVWRWQDSDLKNKKKINK

>SG-PL_contig_1457_1_-3_93_308

MPTYSGDKCMSAFSIPDNYYISDTDIQLCAGLTEGGVDSCQGDSGGPLVCLNPATNSYVQ

VGIVSYGYLCAQ

>SG-PL_contig_1458_1_-1_88_312

MPTYSGDKCMSAFSIPDNYYISDADIQLCAGLTEGGVDSCQGDSGGPLVCLNPATNSYVQ

VGIVSYGYLCAQDNL

>SG-PL_contig_1459_1_-3_51_341

MICLGLSHEPRDKQRRCHANKHKSAASRLRQSRFACIWRLLSLRFVGRVYKRMCVPQWWR

SSLGLVKNRLNKARKTSALPSLVFSCLAADYHHLHSS

>SG-PL_contig_1460_1_+3_1227_1637

MFTFCSPLLSLKKTTLAHNITKQSFYTRQLTFTILSPQRMQNVQPMTANQASFHTSMLQA

ATHQTCQERRRVLKELKAHEWSGINSNHILGVDQSQSSGLNSITSLLYMGLIQSHLSSRP

NSMTFEKWTKISVVENI

>SG-PL_contig_1461_1_+1_127_303

MDAPLKGVVSNFLINTSALGLVDARMYHDRICSKSPVMKSLKQNSTVLSSPFLIFFGLF

>SG-PL_contig_1462_1_-3_75_560

MQKAAEDLRNEAKAKAEEKERYINERVTPLKADGLGQAELQSLVKELHSKVAVLEEEVYD

WEIKIRKQDFEINELTLKVNDTKGKFIKPVLRKVNKTESKLTKLQTKESRDFRGNLKSTG

QNKFALKEEDETSKQPDWRKDLKHGGEGEGEGNPGEEVPAES

>SG-PL_contig_1463_1_+1_232_339

MSRNCLPKTAKETNANTFSYSGHSLTKLGPGQKLCQ

>SG-PL_contig_1464_1_+2_80_187

MEIIRPILCFRPRNASGGDRGSKNPKIFDFYNDLVL

>SG-PL_contig_1465_1_-3_399_575

MSSLAVVFKQSHSVIICRGLTYYQTGICKGVIVTSCDMMQTFSGHCLLNFYSSSYQITC

>SG-PL_contig_1466_1_-1_259_669

MGNSRFSLTFCPGQCLRISPAAQIPQSFGTEGGQSTLMNTCMINYSYFCTPVSSQLLSLE

QNGNTLAVLMYPSFNPNHLGIQQMWAPVTKTTKFPSLHKSSAKIMIPPFLFLIHLVVKPT

HSASMSSLKSVTINLPS

>SG-PL_contig_1467_1_-2_2_265

NIISVQQNGTEFRTDDRHRDGCVGLRRNHHTDSCPVFDVEITKQGCGADVLYPHGSMLLL

VLDIHLPVPVEPTDWPTAGDTSHQDDAT

>SG-PL_contig_1468_1_-2_71_505

MLAHTAASSCSRQSGRGSACCPSWCEPQLGPPTLGCSSPQLRLPSRTPFGGSPRSPRTSL

GARRGVKVGAVTAKNGAGIPPPPHHPPQRSTATPTSDSSLGRVSRCHRRDDHSPGRSPSR

ENSSSVMELLQESLPEEVVLHYPPQ

>SG-PL_contig_1469_1_+2_2_145

SFLPSDCIQCTSNLSQTEHHSSFLQTLEAKFTVYHHSRTIRQYFFLNP

>SG-PL_contig_1470_1_+3_3_116

TTQGKAIKVYIKLKGNEQRFQIQPENVSKVLNRRTGVQ

>SG-PL_contig_1471_1_-1_217_1236

MLDIVPNSSSRTRNMPVNLAPYLLSASPPSAGFSFQSLSLTSSYTPYSTPYSFFSNTSNH

RESPVRDTVYGTAFTSSSPRKSIMSVKPNKDSESHTENTHKADAPAEEQSTSLANNTAKK

MKRVSFADHVGMPLAEVRFMLDSSADPPKFTQKKLNEITGGHAGMMLKTSMDPPNPPLIV

PFPQPLTRENFNEELKRKMVAVENVIVEGYTVTCTIFVNKHATVKRVFGRYTLDSWQSHQ

DITAFYSSSLRVNHDTYQFKIQVPLSADRSKSIEFAVCYEADGVQHWDNNEGDNYTIEWG

QEIDLEPKDSSHNDDSNGNPIAFGGMAWWCDDSDLTRPYY

>SG-PL_contig_1472_1_-1_100_1323

MKFLAVLVALVGVALCEPTIYFKEEFGDGWEDRWVDSTSKGGEQGKFKHSAGKFYGDAEK

DKGLQTSQDARFYGISAKFDTFSNEGKTLVIQFTVKHEQNIDCGGGYAKVFPSGLDQAGM

HGESPYLIMFGPDICGPGTKKVHVIFNYKGKNLLTKKDIRCKDDVYTHLYTLIVRSDNTY

EVKIDNEKVESGELEADWDFLPAKKIKDPEAKKPDDWDEREKIDDPEDKKPEDWDKPEHI

PDPDAKKPDDWDDEMDGEWEPPMIDNPEYQGEWKPKQIDNPAYKGKWVHPEIDNPDYVAD

DKLYSYEDIGAIGFDLWQVKSGTIFDNVLITDDEDYAKEFGEGTWGKTKEPEKKMKEEQD

EEDRKKREEEEKKRKEEEDAKKSEEGEDEEEEEEEEEPEDDEEVHDEL

>SG-PL_contig_1473_1_+3_75_245

MKKHPKSRQTDYTHCTGKVRWQFDFHLFFHNMLGQESLGISLPQTCLPSALGVINIF

>SG-PL_contig_1474_1_-2_2_235

HTALTLRADACRDALALFLAPQRPSPPPLKRPPPPSWGSQPRRKVPRPSSDLCPGLSRHP

PRPSGIGLQAHSRRQFPR

>SG-PL_contig_1475_1_+2_53_256

MCVVVGVLVCVCALLEMYAFSMYVHEHTHRYILKNIINTFVRWKRTIQIRMYCDVRSSQN

ICIPYVTN

>SG-PL_contig_1476_1_-2_104_325

MQRNATRSSPRGRGPLGSPLPYGRTRTSTRSAPGLLVSSPEATGSPLPPRLAPRTSTDGT

CGATSLPSGLCPTL

>SG-PL_contig_1477_1_-3_54_335

MCDRDFSLSVAEARGLVMPMAYILGISMSSEKDDRRCGNDYIMNSVHDLSKISPNSFRFS

SCSIRAIKDYLKKLHERGDSCLSRSEERRVGKEC

>SG-PL_contig_1478_1_-2_5_370

MLLTGMDLPRDRSTVTIGRGYFKSMCSYSLSVAVVSGLALTMAVNLGFSMSSTHDGGECA

NGYIMDYIHDLSKRSPNNFRFSSCSIRAIKDYLKKLHERGDSCLSATNTNAPRVSADRKS

VV

>SG-PL_contig_1479_1_-2_35_1168

MEKPFCTCKHCCLCVRCCHLLGFIRRQSTSSRPDLTMPPRRKLSELDRGRAVAWVQDCVP

KREVARRLQMSISVIVRLFQRFTATGRVHERCRSGRPKMTNPREDRLIERQALQTRTASS

SIIRRQLRVATNINISQQTIRNRLHGFNLRSRRPAVRPRLTPAHRAARRDFCRRHVRWTR

LQWSQVLFSDESRFTMNHNDGRLRVWRRPGQRFIDATVQEKVAFGGGSVMVWGAFSLHHI

TPLYHVQGNLTGFRYRDEILRPLAVPTLQLMGPTAVYQDDNARPHRARVVNDFLQQFGVN

RMEWPACSPDLNPIENLWDELDRRVRSNHSPPRDAQHLYQMLQAEWHALPQRIFTNLVNS

MRTRCVDCQNSHGGYTHY

>SG-PL_contig_1480_1_-1_1_321

PAYTDQFSSLGRESRQTHARPREAATALAASGAYPEPRPLDTRFRRKGKESAYDSKWSKR

LACAVPAKSTHDRPPPIRPGEFPDVPHHLRGLGDERSQELQVCSLRY

>SG-PL_contig_1481_1_-3_114_1031

MPKVRPSKKSRQHQEIQKQGINFNTGIGQHILKNPMIITNMIDKASVYSTDTVLEVGPGT

GNMTVKLLEKAKKVIACEVDPRMAAEVHKRVQGTPMASKLHMMVGDVLKTDLPFFNVCVA

NLPYQISSPFVFKLLLHRPFFRAAILMFQQEFADRLVARPGDKLYCRLSVNTQMLAKVHH

LMKVGKNNFRPPPKVESSVVRIEPKNPPPPINFQEWDGLIRICFSRKNKTIGASFRFTKV

LELLEKNYRIHCSVNNIPVPDDFDMKTKVNAVLEKNDFEKKRARSMDQDDFLLLLHDFNV

EGIHFT

>SG-PL_contig_1482_1_+3_1491_1685

MINYTNQLYCSTTRLHKRVHVHIWKYRTFTVDIRLSVSSHINTHYMHSHLYTFHHTHYMH

SHLYT

>SG-PL_contig_1483_1_-2_38_1036

MLSTSRRTFLRTLSLLRRYNSTLVIVEHDNKTLTPITLNTITAATKIGGDVSCLVAGSQC

AKVAEEVSKVAGVNKVLLADSDVFQGFLPEAVTPLILATQNHFNFTHILAGATANGKSLI

PRVAAKLDMCPISEIIGIKDANTFVRTIYAGNAVQTLTSQDKIKLITVRGTAFEAADTGG

SAEMGQAPAEGVSNDQSSFLGQELSKSDRPDLSSASVVISGGRGMKNGENFQLLYTLADK

IGAAVGASRAAVDAGFVPNDMQVGQTGKIVAPDLYVAVGISGAIQHLAGMKDSKVIVAIN

KDPEAPIFQVADYGLVKDLFQVIPEMTELVEKK

>SG-PL_contig_1484_1_-2_50_1084

MVKEGVLLAYGNPLLDISVNDDSCIKLLEKYDLKANNAILAEEKHIPLYDELVDQYPNKV

EFIPGGATLNAMIAVQWILQKPKVTTYFGCINKDKNGDIIKQKSEEAGVNVQFQYTDKEV

TGTCGVICTGSNRSLIANLAAANCFTEAHLDVPANWKLVEKAQYYYSAGFPLTVSPSTML

RMARHAQENKKTYCMNLSAPFLCNFFKEPMTQLLPYVNFLFCNESEADEFAKVHEYGTTD

RKEIALRMAAWPKEDKSRPRTVVITQGADPVLVATDGKITEFPVKLVTARDIVDTNGAGD

GFVGGFLAQLVQGKPIEECVRCGIYIGNIVVQQPGFTLPGLPNYS

>SG-PL_contig_1485_1_+3_3_164

RNRTSIFIIFTTPVCYAINFPVSTTGKFTIKNRDTVHTEANKLTAHRLQTPQIK

>SG-PL_contig_1486_1_+3_717_1037

MGRLGPYGKTGTLQSSIIHSIDVTMKVMFKFPDPTLQESYLSEAQLTPGYYYEGKHRVQP

VTQSQSKREIHSITTIIHILMNYHNIVKNCPRFCTGYLCSVLRSQFL

>SG-PL_contig_1487_1_-3_3_176

MTQSFHTQPKWPSSESGKLSCGPRAHTGTQTGAVVKYGLRPEFIRAFSCVVHMFYKGY

>SG-PL_contig_1488_1_+2_242_400

MALAPRKLLHDQTGKVSSIWIGHMFINAYCSVKSFVNGEKTFVSINYIQLICT

>SG-PL_contig_1489_1_-3_3_104

IDFIVDLFLCLICSVKCKVFRSLLPRMLRCTSLH

>SG-PL_contig_1490_1_-3_15_407

MERRVVLETSTAHTFLRAMACRFQELWKSTEAWELGNPFEDQRLSAFGSRVAAGLSAAVE

VTAKLRYNMLLARRDAILCHWSCSGGNLSLPGGEGVRVTAVVGRTLPSSTPNDFALTQHL

QSFSPSKQAAS

>SG-PL_contig_1491_1_-3_3_185

FFYIVFSTVIDFFFTLSLFFFTLSLVLSLTSFLPSLVNSTVIDFFFTLSLVLSLASFLHC

P

>SG-PL_contig_1492_1_+1_415_621

MNIHKEDNPSLLKHIFLVLTTPNQMSVLSSHVLYLHIPSKKKKKRSQTTPSQKCTQKSSD

LFRQEIYQI

>SG-PL_contig_1493_1_-3_63_1322

MRSKEMLGATSPYLSGVVIIVMVLFLHVSMAARPHIVIILADDLGFDDVGYRSKAMYTPN

IDKMAKEGVKLKYAYMQQVCTPSRAAFMTGIYPFRMGLQNSVLSALENRSMPMDKTVLSQ

RLKTLRYATHYVGKWHLGFCNYNMTPAYRGFDTFYGMYTGKEDYYTHISKWNGYDFHDDN

GQNFNVDWTAKGKYSTPLFTERAVKIIEGHDTRKPLFLFLAYQAVHGPEQVPDDYLKYCA

DVTNETRKTHCAMAAAMDEGIGKVNEALEKRNMTDNLITLFLSDNGGAVKKGSSNWPLRG

AKLTLWEGGTRVAGILHSKSRLPDAPYEYKGLIHAVDWYPTLMRAAGDRDGVSGIDGVDS

WKSIVKNWDTSSRDCFVYNIDDVKNTSAVRCDQYKLVHNKAGSPDGWWNWPNGTTVVLPK

>SG-PL_contig_1494_1_-1_118_276

MKEKCTVCYCVNICMVCITSINVVRCISLRHSLFPSTLFTLYSPMGMLGTLSI

>SG-PL_contig_1495_1_-1_43_210

MRFLHNCNSCVLCLELHVLCILYTTTSAPPIPSFLCHGQGIVTDLICDLALPVWLR

>SG-PL_contig_1496_1_-2_185_634

MAGVLFEDIFDVKDIDPDGKKFDRVSRLFCESESFKMDLILDVNTMLYPIKLSEKFRLSI

VTTLREDGVPDDTEYNPQAASVANRADQFEYVMYGKVYRIEGDDGSSETSQLAAYVSFGG

LLMRLKGDPNNLHGFEVDSHVYLLMKKLAF

>SG-PL_contig_1497_1_+2_2_73

FIKSLYFTIYGKFCTNTSHHDIES

>SG-PL_contig_1498_1_-2_191_361

MLFCCGHCLYYFCYSFSIVPENIDIFGVSIILLVQQCPRECCRSWGNLFWHCLCPPF

>SG-PL_contig_1499_1_-1_1144_1401

MHLFIPPNSTYQHFSLTKESFHTNTSLMRQQSPEIQPYRQALCFFMLLYFHPAPPIVERV

CTIFAEMQLRQHIHLMQAQSPLHKSL

>SG-PL_contig_1500_1_-3_81_815

MYMQEHSDQMLHKCGKCKARFFQTEILELHMNTHNQQKKYPCDQCDASYTRIDNLTKHKS

LKHHCRVMKRNRRSKAGDVETSKQTGQPTNRESSQERKDQESVPLGRISNVGNMSVKGGV

KFTCDQCGKGFSRKDHLQSHVLTHSGEKQYGCQQCGVAFAHLRSLVAHIHSHSSHRPFQC

GKCHKSFTVVTNMTKHVCKFNTPKLQCISCGKKFVTSSQLILHSRLHLAKSGTSQPSADF

DPKKP

>SG-PL_contig_1501_1_+1_223_2283

MHPGPPGWGLCSGLTTHSLKNQTVTETATSSSSNNDMVVGEPSLVEPMTRAGESLREASA

QTTLLTTKSTTRIGTWNIRTLYETGKTAQVCREMHRYNLKILGLCESRWNGTGQTRLASG

DTIIYSGQEEGQPHTHGVALLMTPEATRALLSWEPVSPRILTARFNSKGRKVTILQCYAP

TNTAELEDKENFFEQVQAIMDKTPKRDLKILMGDLNAKVGADNTNRELIMGRHGTGEQNE

NGELFTEFCTFNDLVIGGTIFPHKKIHKTTWTSPDGKTENQIDHITIGRKWRRSLHDVRV

KRGADAASDHHLVVAVLKTKLKAYIDRAGRPSHKFNVQSLKERGKAEEFKLELRNNFSVL

SQLPEETIEEQWHSLCETWKTTCQTVLGKKTRKHKEWLTTETWALITERKQLKDQINQTQ

DQEEKQALQAHYWDKNRAVKRSARKDKRNFIEELTQEAETAAGQRNMKRLYEITRTLSGK

NSKPSRPVKDKNGNTISGEEDQRARWAEHFKETLNRPAPSVPPDIPPPTELLGINTGPPT

KNEITKAIKSLKSGKAAGPDGIPPEALKADIQTSTDMLHPLLHKIWEQERVPEDWKKGHL

VKLPKKGDLSSCNNWRGIMLLSIPGKVLTRIILERLKTALDKTLRDEQAGFRQDRSCTDH

IATMRIIIEQSLEWQTPLYSVFVDFQK

>SG-PL_contig_1502_1_-1_70_1224

MVLESTFVCVDNSDYMRNGDFVPTRMQAQQDAVNLVCMSKTRSNPENNVGLLKMSSVEVL

VTLTTDVGRILARLHQVQPRGNISFLNAVRVAHLALKHRQGRNHKMRIVVFVGSPIEEDE

KEVVKTAKKLKKEKVNVDIINFGEEGLNTDKLTTFVNTLNGKDGSSSHLVTVPPGPMLSD

ALLSSPVIVGEDGAGAPMSGGFEFGVDPNEDPELALALRVSMEEQRARQEKDSREAVTAS

VAESTNLSTASAEEALLEKALAVSTTGDGASTTVTPSMDFNAMTEDEQIAYALRLSMENT

GAEDTGAAPMEVDTEGATGGGSEEKKEEEDYSEVMQDPAFLQSVLENLPGVDPNSEAIKD

AMKTLTTDSAKDGKDKDSSKKDGKK

>SG-PL_contig_1503_1_+1_1_291

HGREERRPASRRWDARARPKTAASRENSGQPSKPAGAGLHQLVQYMTPNSLFKYTLLHAI

KLTLYKCKQICPTRTSIDITEIYICSQYLWSLYCYQN

>SG-PL_contig_1504_1_-3_3_275

SLKLSKSRPECTRLHRFWSNFLKIFMGACPRTLLQQTRTCGFTTSSHHAKNFSSFFILDH

FKPCVCMCMHTTHICTHPYMNTHTHTHTHTH

>SG-PL_contig_1505_1_-3_3_290

VWVGSGREWSSRGPYCISWIWYLWEFTWKNSSIHFYKITYETVQSVQNCYRFACKLFRRM

LIKKQTIDTLILLQTDRRKQQLRCNFMAKLYYHCCQ

>SG-PL_contig_1506_1_+1_1_198

QSVSQLSSTRNFKAKPGLPFSGSSSSLLTVNSFSPRHPSRYKSQIKKQTIKQTNKKQQKN

NKKKKK

>SG-PL_contig_1507_1_+1_1_120

GEQVLCNFMCTCVHKKDLPHLSHSTIKFNIIIMVNEQTLH

>SG-PL_contig_1508_1_+3_216_407

MTNYDRKNMTKRHDDSCVFCDSTSSQKHGPNLVRFHQSITEILSIHIYLHTLKKTNHNEQ

AQVL

>SG-PL_contig_1509_1_-2_83_1579

MDKAKMTNYYGRALLIAVFLLALASPVVCNPAGPAERQMSYEGFQVHRLTSDDPLTLSET

LEHLEQQSMVDVWSRGSRSADVIVAPKHISYVRKRSADRGIRSDVIVEDLQSELQKDFVQ

RQRRRKRSVLDVTSRYLAYDEMQAYLYQVAREATNASVTVDSIGLSYENRNVSVIGIRER

AYNLKPKQAILIDAGIHAREWIAPAMALNIVNKLAFGRDQEARRMLQLFDWFIVPCVNPD

GYVFSMSSATNRMWRKTRTKQYNARCYGVDGNRNFGYEWNPAIGGSTDYCSDVYSGPHAF

SEPETRNIRDWLESHRNQAVAYVTMHSYGEYILYPYGSSRNKPVADKALLETLGTSFKQE

LQKAGHNYKVGNSATLLYPAAGGSDDYAKGTVGIKVSYTLELSPASSTGGGFQLSKNKIR

KVVEDTWPGFTAMANKLYTELNPQSSSGAYLGTGDNSVNGRGRIYFNSFVDGYLKRCCEQ

YSSFQYKCCTALGRSVGSG

>SG-PL_contig_1510_1_+3_273_527

MKYPYRFWTFLFVFFFFCLSYLFFFLVFSFFKKTSYTMTRQIWVKANKPHQRNLNVHKHK

SGSTTKTSKYVISHITDCALAPIFI

>SG-PL_contig_1511_1_+3_120_392

MPNLSKCNTSNSTCMLNTRGLKAKLFLIFSHLQMRWFICCWQIVDNVDYNNLTTLRSILL

PLKILQIKPPHLSTFPRLSGSFNFGLKAVIQ

>SG-PL_contig_1512_1_-1_16_1278

MAATDVLLQQAVDLRKTNTIHASEILTKIVKQEVRTLDDEAIRVKEQAILEIGNLFAETK

QATELAGLIKFVRPFLGMVSKAKAAKLVRSLVDLFLDMEAGTGKEIELCKECIGWAKDEN

RTFLRQALEARLISLYFDTQNYQEALALGSILLRELKKLDDKALLVEVQLLESKVYHALG

NLPKARAALTSGRTTANGIYCPPKLQASLDMQSGVLHAADERDFKTAYSYFYEAFEGYDS

IDSPKALTALKYMLLCKIMLNVADEVQAIVSGKLALKYTGPEVEAMKSIAQASHKRSLAD

FQKTLGTYKSQLAEDPIVNAHLKTLYDNLLEQNLCRIIEPFSKVQVQHVANLIKLPVDTV

EKKLSQMILDKKFHGILDQGAGVLIIFEETVVDKTYANALETIHSMGKVVDALYAKAKKL

T

>SG-PL_contig_1513_1_-2_1619_1918

MCHASTLPAHRVLRRFKEFARSSLADLGGAHMELSECDESFWHKTSNSESRDMFTSSAAA

FVTLLTGSDYFTVRGPQWLRWLSLGSTCQWLGVLTWAVLH

>SG-PL_contig_1514_1_-3_24_356

MRIRGCYACTPGFFAAAPANIDRHPTPPASGRTHAHSQPRAVSSALSASGALPATRSLAS

LSRRKGTESAYNSKWTKWLAWCGPNQVDAHAPSPVELANFLAFLTTSEGLA

>SG-PL_contig_1516_1_+2_2_190

QRGFRHLQVCTYVDLGDRKNFHPLPTRRGACGSNPGLPYLPVWKSSVFTIRPRRPSEMTC

GVL

>SG-PL_contig_1517_1_+3_60_203

MIKMYEDIYMTTALLIVVQSNMLRYKSGHNNTDKYGGSITSGEYNNIR

>SG-PL_contig_1518_1_+2_38_220

MFTKNRLRPFPMFLESLNNGAFCFLSAVLRAVRTLDVIYGTTNLAFVCSVFHMRESVSQG

A

>SG-PL_contig_1519_1_-3_759_1124

MNWNVVLWFYCAFPMTRMTAFLIVLYAVQLRIFNSDCQFLTHYSPCPRVSSLHPRSMAWG

GRGGGGRPIPIYTSVHSTSRSETDHTVNHSNLIPVSLSPQHQASEKTNDQSFGAGGLSAV

PS

>SG-PL_contig_1520_1_-1_130_303

MHIYCCSNLGVLVHDLNLADLLVPRNLIQHTVHLLCFERQTTVTKRRRGGKKIALVKS

>SG-PL_contig_1521_1_-2_686_856

MRYTVVRIHYYYIYLYWLAFVEQNVTLTVCSQSILLTLCLLKLHCLFLYNNNDDVCI

>SG-PL_contig_1522_1_+1_493_1089

MISCTKLCVLMLLSLYLYRLSSEEKKKKKETDRQTKTEERGRERDRERTGIDQSDSVNQT

GSIDIQVNLLHKPAAGAVRISSKQVLTLLIFNLQLAVCSRQIQRTPTIPYELHPSATNCL

ADSLSLRKNHIHSKLLLKVLTPWNLLPSVSETLPFPSTLFRHSTRPATCISHPLPQIPQH

SRPDDLESIRTSMKHTEDY

>SG-PL_contig_1523_1_-2_1295_1468

MRATLLGESTSPYTVPPVPPSPPFSCMRVFLLVYDSGFFFRILTGTTPSLPVGSYNIR

>SG-PL_contig_1524_1_-2_104_193

MFCTFDLGMYAAPLLIYHVETSNNRFIHKR

>SG-PL_contig_1525_1_+3_3_176

ITKFSSWYSQEVQSLQTQAVTCGLSSTNNLAFIKNVLKEIRCHKPDSLKDKQTKTTPK

>SG-PL_contig_1526_1_-1_13_1377

MASEATPDGRLQKMEVDYSTTVDEKIPKCEQLTKQGKLTEALDILLSLEKQTRTASDTHS

TGKILVAVVKNCFECKNWDALNENIVLLTKKRGQIKQAVTKMIQEACTYVEKTPTMDVKL

KLIDTLRTVTAGKIYVEIERARLTRTLAKIKEDAGKVTEAADILQELQVETFGSMEKKEK

VDFILEQMRLCLAKKDYIRTQIISKKVSTKFFDDADSKDLKLKFYQLMIELDEHEGSYLE

ICKHYRAIYDTPQIKENKDKMKEALKCVVLYLVLAPYDNEQSDLIHRVKEDKTLEQIPKY

RDLLKCFTTPELIQWKMLCQNFETELKTGSANNPPTHVFNPKQENGVKRWSDLKIRVVEH

NIRVMAKYYTRIRIKRMSELLDLADGETEEFLSNLVVNGTVEAKMDRLAGVVQFTPHKDP

SDVLNEWAAKLSQLMSLVNKTNHLINKEEMVHVLH

>SG-PL_contig_1527_1_+2_356_529

MSQKTCVYLFYTYFSCVCEKKAAVHKNSRENKKKRKQTKTISNFVAVNTNVPLRIRSF

>SG-PL_contig_1528_1_-3_138_404

MARTKQTARKSTGGKAPRKQLATKAARKSAPSTGGVKKPHRYRPGTVALREIRRYQKSTE

LLIRKLPFQRLVREIAQDFKTDLRFQSAA

>SG-PL_contig_1529_1_-3_135_365

MTQDDDDFTKVTIAAEDSSTPTISHQETVIFQHPLLSQCSNRNVDHHLESLCFVSDRYRG

KADQPMTKQVCYVRRTN

>SG-PL_contig_1530_1_+2_2_208

PAFCIITMIAKQKLNPFTPSSHSPASLQEPGGKGEGVKGLRFCRWWIGCGSERIKGCEQF

TQFKVSVNK

>SG-PL_contig_1531_1_-3_48_3866

MKIEKFAVKFKNPDKAGEFKSTFETCQKEIAEGEKSAAASASTTTTTIPKSPDKTDGNTK

SLAEMFKPEEGSWECTGCYINNKPDVLHCVACQTLKPGAKAEDVKSLPKENPFATTAPAG

TFGGFKLGGDSATPKSAFGGGAGVSSGGFKFGNATVAPAAAADSPAAAGQKDKQPSLSEM

FKPEEGSWECTGCYVNNKANVLRCVACQTLKPGAKAEDAKSPPKEDPFATTAPAGSFGGF

QFRTTPGKTDGSKQAPPAGGGFKFGGTLASSEAGGFKFGTAPKLAEAGATSATSAAPAGF

KFTFGTPSTASTSATSSTTSTFGGKSSTSTFGAPSTTSTATKPATGFVFGSKPSPLTQTG

TGFSFGASALKDSVSKESSGFSFAKPQEATKPADDSETGSELLKQLLTNDNQPETIETPA

QGSVFGSASPATSDSSKQSGFQAKLGQVTSPTTQTNKDGTQKVGFQFTFNLQKQQQSQQS

ASPKSPEVDEQGFYLNKEGEDSHIYFEPVVPLPEKVDVKTGEEDEDVLFESRAKLYRFTS

GEWKERGLGVLKILQHTDTGRIRLLMRREQVLKICCNHYITEELDLKPMPRSEGKAWIWY

AMDFSDGEGKMEQFAIRFRDPDIANNFKKVFDESKEKMKTPSKTDSGKLDPTLVNSAGKS

EVSRRLFHTEEDDDVEFLYEEMPTPDQVARARMFLLPDTFYLYENRPGCSGCIGCEDYDP

SKASASPSEKSKAATATSAAPPSQPAKQPVQEKVAPAPQGMMFGGGSGMSFSALAASSGD

IPGFGAKDPSKPFHWAGAGQKLFGAGREEDEGQEGEVAPSEDIHFEPVIPLPDLVEVKTG

EEDWTALFNQRAKVYRFDGTQWKERGIGEMKIMRHTSQLLFRVLLRREQVLKVACNHLIS

TTMELKPMATSETAWCWVATDYTESEPKMEQFAVKFKKMEVAKEFKAIFEDCQEKLRQGE

KNEQVQETLVSSSMHQPAAEGCSVRVQDIMTASSQPVTGDKGNLYYTQQLNISAQDTMSS

SSQAVTGDRGNPYYSQQLGVAAQDAMVSSSQPTMGDNGNPYFSRALDVAAQDAQISSSTF

QALGSGGNPLFSQLLSPEEVEAEGQYAEEDYDDDDEDDEDETTEEDEEEERVEFEKRVTL

FQREDDGQWKKVGMGNLRVLYDDDLNANKLMMLTDQGDKVCNHVITMELTVAVDNKKKSC

EWQPIDFATDEPIRRHFKACFSSLQSAEEFATIFRQGQKLAAESEISANLTEEIEVPEVF

SKGDRSEGAMGKK

>SG-PL_contig_1532_1_-2_74_319

MNIIYLLPCFSLFISRFRDKGYVFTGHLFNGPCEHFQYRHSHLFNGPCEHFHYSYGFAIK

ISSTNRQDLSKFRLAASCFLNL

>SG-PL_contig_1533_1_-3_60_215

MVSISLSLSLSLLRACARACSFSLSASLSDCLPLPLPMHFGVFYAKMLFHFP

>SG-PL_contig_1534_1_-1_1_147

ELSAFIDNNRNVHIQKKKGNGWRCSAAMHSPQGRTTRISHGKVIVTKSI

>SG-PL_contig_1535_1_+1_1315_1500

MVSVVVWRASHGNHPNFTMYNSTFGETLHTPLISTTTTQSTSCPDQLLHEVVTATTSLPR

WH

>SG-PL_contig_1536_1_+1_7_243

MEYDCLNGEVKTKQIKITCFKKKKCCSDHFPKEIRQENKRRFSDKVFFTKCCYKMFVTES

WISAPAIHEHIQTDFSEKL

>SG-PL_contig_1537_1_-3_51_1901

MASRNLPKVRDAEKESLYGYVHAVSGPVVVAQNMSGAAMYELVRVGHGELVGEIIRLEGD

MATIQVYEETSGVTVGDPVLRTGKPLSVQLGPGIMGSIFDGIQRPLEAISDLTQSIYIPK

GINTSALDFNKQWEFLPSKDVRVGSHITGGDIYGVVPENILIKHRIMLPPNARGTVTYIA

PNGNYSVNDVVLETEFDGKKSQYSMLQVWPVRQMRPVSEKLAANYPLLTGQRVLDSLFPC

VQGGTTAIPGAFGCGKTVISQSLSKYSNSDVIIYVGCGERGNEMSEVLRDFPELTMEVEG

KPESIMKRTALVANTSNMPVAAREASIYTGITLSEYFRDMGLNVSMMADSTSRWAEALRE

ISGRLAEMPADSGYPAYLGARLASFYERAGRVICLGNPHREGSVSIVGAVSPPGGDFSDP

VTSATLGIVQVFWGLDKKLAQRKHFPSINWLISYSKYMRALDDFYDKNHPEFVPLRTKCK

EILQEEEDLSEIVQLVGKGSLAESDKITLEVAKLIKDDYLQQNGYTPYDRFCPFYKTVGM

LRNIISFYDMSRHAVESTAQSENKITWAIIRERLSDVLYKLSSMKFKDPVKDGEAKIKAD

FDELMEEMQTKFRNLED

>SG-PL_contig_1538_1_-1_763_1089

MDVHALLTALLLLLFFKDNYSCGVSGYCTGLIFIQRQCNCRLFGVLHTPVYTVSSFTHYQ

CFPVCIVINIYAQLYSVWSFFISRRKAQVSVYVWFLFLYDFCWFLFTQL

>SG-PL_contig_1539_1_-1_64_951

MYCLFEYASNSNYSLQINPASSVNPDHLLYFRFIGRFIAMALYHGKFIDSGFTLPFYKRM

LSKKLTIKDLETIDPEYYQSLQWVKENNIEDCGLELYFSADFEILGKIEQHDLKPGGGDI

RVTEENKEEYLNLMTQWRFTRGVEDQTKAFLEGFNEVVPLQWLQYFDERELELMLCGMQE

IDVDDWERNTIYRHYQRNTKQVVWFWKFVREIDNEKRTRLLQFVTGTCRLPVGGFAELMG

SNGPQRFCIEKVGKETWLPRSHTCFNRLDLPPYRSYEQLLEKLNLAIEETEGFGQE

>SG-PL_contig_1540_1_-2_101_901

MVATPSINNKNNVSSGNNSSNNNKQPSVNNNRSINKNNTNSFRMKERSTTTTTTTTSSGP

NASGQRDYYDYDDFDDDDIGIGTMSSPRNSPSFRRHALLTTSSSITTTTTTADPTHLSNT

QVSSAVMKTNTVMKNTTAINKTSLTHTIPTISSSSHSDNSNVTDDDKVNNNNNAGGQRGT

AMTTNAVSTTSATSSSTSTSAMNTATSATVVVGGTADNGGSGGGGPIRNLRVRGQRKFLP

EKTRHKRQTLQDMTRPLKQWLLRHKDN

>SG-PL_contig_1541_1_-2_800_1603

MTATTSTATTSTAMTATTTASIAMTTIAITSTAMTTIAITSTAMTTSTAMTTIATTSTAM

TTTSTANDTNSNNISSNDDNINSNDNNSNNINSNDNNSNNDNINSDNINSDNNNSKSINS

NDNNSINSNDNNSNNSNNNSNNSNSNNINSNDSNSNNINSNFNNSNNSSSMATHQQQRQQ

QQQQHQQQMTTTATTSTAMTITATLTAMTTATTLTTTATTAAAMATTSTALTTIAATSTV

TTSTATITTATTTTMTTTTFYMHITHPD

>SG-PL_contig_1542_1_-1_94_375

MPQGKLKTKVQLPKGVKQKQKSSKKDVLRKGHRTIAPKKSRLIETAKVKKGLEKQLKASI

EQDAMSRAAHAEPKTLTILKPSTSTDCGNANKKT

>SG-PL_contig_1543_1_-2_215_2104

MRSTPSVMEKSTGDISAEEYKARLAEKRRQAREKAEREAEEERKRQEELERQEEERMRQE

EEEQRRHDEEAMRLAEEARKCEEERLQKAIEAEEARKAEEAARLEQERLAKEEAERKAKE

EAERLEQEKQEKARREEEERLERKKRLEMIMKRVKTESPAEMTKDSSKSVTSSPSKSINS

TASSDTSPTDPENKTVLAMAAHSAAQDTSAQPSTPAVSQEEVDVQDGAVRGEDESQAPVA

APCPPSPVMTSDTQPEPDQVPDAMEKSGVVEKSEAESVAAEPVPAVAISPAPHAEGGGDR

PRFKSPLLQKLVEGKDDAGSSPGGGPKFKSPLLQNLLGKSRARTMVQDHSASAPNLSQMD

TSSDQQADSDGPDVMSSSMVDVGAGDSSHKEVLNRNEEKDVDTSEDSSSSSHSSPSHVSG

KTVVSNGAEEGASIAVSEPSPEDAAFIVQPEPMEISTPMESSAVAPIMATSTASFWSQQD

SGVSFGSEDLRTSAAPSGSLQVSFNGHGPPRAAQGQALEDSSISMRSMESVTSSVTDSAA

GSVFSSVSSLPQGQGLSGGDKDFEELIDLSVNNKSATSTTTTSARTVLDYSEDLANFNKL

EEGSQETTTGPPPPIIAFEDRRQDVSDLLS

>SG-PL_contig_1544_1_-3_138_1496

MRQAKKTLKFLNLLSQSEKVAQCFTHQPLATRAASTINGFLDSLCGSKAKNFKVKDMAKY

EFNPVELLLDIVNITLRITVEDTSRTDGYLVSMVKDPDFELANLEKAQSVIISKACTSEE

TINQLAQLMEQIHVLAAVHAPVLASSGDDENNWRDAFVDQDIDQEDLEKQYTTTMQDQRF

NNARIQDSHCFISNLQGTFDPRSPKVKVIMKEMKQLTTSLPVNAGASIFVRQDKDRMDVM

RALVTGPAGTPYSLGCFCFDVYFPAAYPNVPPLVKIITTGHGTVRFNPNLYADGKVCLSL

LGTWHAGDASEKWNPMKSSLYQVLVSIQSMILTPDPVFNEPGYERIKGTEEGKIQSCQYN

WKIHLNTLRHAVLGQLRNPPPGFEKVIRHHFALQREAVLKQCAEWVKGCEDPDLLKRLRK

VADSIFEELMCLEVDFSEVEVDSEDEDDDEDED

>SG-PL_contig_1545_1_-1_511_702

MCRPASASTPCVCIYMQNIKYACYRSCKSCQWLAYGNRKTPSMQSARTSCKKDPMGPNAT

ILEK

>SG-PL_contig_1546_1_-1_115_1944

MKLCAVVVGLALFVAVNAESFQSKCIRLDVDGTVTVTATKKGTEKKEVVKLSDAHTITGN

CTNTNTTVTMTVTFDNVTNWVLVFNNKNDQVKLDQTVSFEPAILFNDTSVGNDTVGLSNS

LDLSSSNTSYLCHVQQTIKFKSETQGGYTFTVTQNISNFQVQAFGLVNNTFSPAEDCKGD

LTTVAPATTPPPIPTVDPVAAQTFLLKDAKGIVCFRLQAGLQFVVEYNTTHNTMVFSKPM

TIPSSANVTGTCATTTTNTATMTISGADYALTITEMRTKDTAVSAIQLNVSLDSTNFPNA

EKVNETVSFEMANVFALSSAKTFYMCQTEQTLHFNKTRTTLYADKVRLQAFNLKNGSFTG

DGVECSLDPTPKPITPSSTPGDNTYTVSSGNVTCIVLKGEISFSNVTYAAKGGMATADTL

NIPALNTTTHNVKFNGNCSLEGGSAQQLVITFNNNWTLALTFARHSSKDSSMLTTDYEYS

VRDIEFTYYLIPQFFPNGTQPYAKMVASQKNITSDFMKGSTGGSYKCDAEQKETLDTGAQ

FETFHLQYKAFNTDNKTDFNNNVSECSADDESNSIVPIAVGAALAGLVVIVLIAYLIGRR

RSRRQGYESV

>SG-PL_contig_1547_1_-2_2_202

QTVSVCNFVADSIFILLVQFWFYSMSFDMFTNASGYVTRLARVFKKDSYISYSCGICFIS

SVHCQHQ

>SG-PL_contig_1548_1_+2_1052_1381

MSVPLYPCMIWNPGGYLGNKVKCKMQMQGKMQWPRCRINVLTKGLLHWTVTRSRTGSTVG

KGGDTTNGLGWTFDVERYMEGHHWSWRHTVTNVTHQSALPSVKEQRIIND

>SG-PL_contig_1549_1_-3_177_3758

MTEIESYSNNTLRWDPGQDDDFDAGNNSSKLFERSRIKALADERELVQKKTFQKWVNSHL

IRVNSKINDLYVDLRDGKMLMKLLEVLSGERLPRPTKGKMRIHCLENVDKALTFLHEQRV

HLENMGAHDIVDGSARLTLGLIWTIILRFQIQDIMIEEVDNSETKSAKDALLLWCQMKTA

GYPNVNVRNFTTSWRDGLAFNAIIHKHRPDLIQYDKMHKSSAIQNLSNAFTVAEDKLGLT

ALLEPEDVNVEYPDEKSIITYVVTYYHYFSKMKADSVQGKRIGKVIDNAINTENSIEEYE

TLTSDLLEWIEQTIVILRDRQFANSLTGVQQQLAAFNTYRIVEKPPKFDEKGNLEVLLFT

IQSKMRANNQRVYLPREGRLISDINKAWERLEKAEHERELALREELIRQEKLEQLAARFD

RKAGMRETWLSENQRLVSQDNFGADLAAVEAATKKHEAIETDINAYEERVSAVVAVASEL

EQENYHDIDRINARKDNVLRLWNYLLELLRARRMRLELTHNLHKIFQEMLYILGWMEEIK

ARLMSEDYGKHLMGVEDLLQKHSLLEADVHVVGERVKTVNTQAQRFIQDDFADVGGYRPC

DPEIIQDRMSTLRQAYEELCELAESRRKRLEESRTLWQFYWDMADEEGWIREKEQLMSSP

DLGHDLTSVNLLLSKHKAMEDEMQARHSHLQSVIQASQDLIQAGNFGAEKIQERIDEINQ

QWESLIDLATYRKKRLNEAVDFYQFFADADDVDAWLLDTLRIVSSKDVGKDEASVQSLLK

KHKEVTEELKNYQSAIEALHEQAASLGEQDRESPDVQSRLSSIDKRYQDLLDLAKLRKQR

LIDALSLYKLFNESDGVETWIDEKEKFLTTILVTDDVEELAIMKHRFDSFDHEMNATASK

VAVVNQLARQLLQVEHPNAAEVVQRQNQLNSTWNNLREMVDQKRDEINVAHGFQNFNIEC

NETIGWIHEKAKLIESTDELGNDLAGVMTLQRRLSTMERDLAAIQAKLESLQNEAERLEG

EKPEEAQAIREKISEINNVWMDLKDMLKERDEKLGEAGELQRFLGSLDHFQQWLARTQTT

IASEDIPNSLADAERLLNQHQQLRDEIDTYAPEYAKIKEFGDKVTEGQDDPQYMFLRQRL

QALDDGWHELLQMWENRQQLLSQSLSQQMLLRDAKQAEVLLNQQENFLSKEEVP

>SG-PL_contig_1550_1_+3_108_242

MDNLPHLKTLSPPTSTTRNFHHYKPTNFFLFMINSLSSLNTHTRT

>SG-PL_contig_1551_1_-1_1_147

PPPPPTLLKNNNKQEKYSLIISSISLTSLLTSSTFPAPRIFTQPSSCFA

>SG-PL_contig_1552_1_-2_515_679

MCVVEVCYLFCSELQRKRKKNHDDGNYLHVCQGYQLLKTVKCFAGHARLLNLTLD

>SG-PL_contig_1553_1_+2_563_856

MANVSRCRVGENPKQLLYYNTKTQIIFFYHADCCQLTHHTKSINMQTHRSFVLLNNKDKK

NTNKQKDKKMWTFTKTKNLDSHTQKQTCIYQKTKATVH

>SG-PL_contig_1554_1_-2_2_163

GGGGGGQTLDCLNDMEVTSVFIQAAAEDKQRWNESGHIEMGSIRTYRDGMNQDR

>SG-PL_contig_1555_1_-3_1380_1664

MLRIIAEYYSGICIHDVQLSALNSHDFPLKMSVDLFSLKFSNSLFFESFSIWKIALHFPQ

TFLNHGNHGNTRYIRNSCRCYSCNVLPAASDLMFS

>SG-PL_contig_1556_1_-1_1_264

LSKCSHPFSWGFCLTFFFKLSLSLSLSLSLWIDFVRLKLYCYFFSVLFFALQYTLFSRCV

FLNILSSSYLLCKSINYYFVASFYRIGY

>SG-PL_contig_1557_1_-2_326_508

MGVSHVVQLPSLNVLPLHVCESHKILYGKCVQSAVWQYVWEVCRKHATCCSVLMKSALLL

P

>SG-PL_contig_1558_1_-2_32_397

MPPKVSTKGAKKAGKAKAVRSGDKKRKRKRKESYAIYIYKVLKQVHPDTGISSKAMSIMN

SFVNDIFERIAAEASRLAHYNKRSTITSREIQTAVRLLLPGELAKHAVSEGTKAVTKYTS

SK

>SG-PL_contig_1559_1_-1_64_468

MFDRDGNGHVSSAEVGILLRALDMNPSMGEIEDIIHKIDYNGTGQIEWEEFADFMRHLKK

RSYQDQMQEMLRAFKMFDYNNDDHIDADELRRVVTSMGEKLSLEEANQMIRVADIDQDGR

INYKEFVKFIMAPCL

>SG-PL_contig_1560_1_-1_1_126

QCLIMVIITSSTFWMCVSGTCTHKLITIGSCYKGVKISCDDQ

>SG-PL_contig_1561_1_-2_41_940

MWNLRKAASRLAVLSAVLKQGSVSRSPLVGRCHSSQTVTKHTDMIVKLLPALEDNYMYLL

IDESTKQCAVVDPVEPQKVMKAVKEEGVILRSVLTTHHHWDHAGGNEELIKLGGKVPVYG

GDDRIGALTNKVSHGNEFKIGELNVRCLSTPCHTSGHICYFVTGPSGGDPAVFTGDTLFV

GGCGKFFEGTGQQMYTALVEKLGALPAHTKVYCGHEYTVNNLKFAAHVEPNSDAVQKKLA

WAMTQRSSNQPTIPSTIQEEKDYNPFMRVSESMVQTHAGKTDAVQTMDFLRSEKDNFRAK

>SG-PL_contig_1562_1_-3_3_389

LEWSDHLRLPALSVPLQGGEEGQDERPQLILIAPLWPAQTWYPDLRALTHVPPLALPVKK

DLLRQPRSGVMHPNPQLFCLHAWLLCGPVCAPDLTTPASRSAAIQAVLSAKGASEDLKAL

VADARRPGT

>SG-PL_contig_1563_1_-1_52_642

MARISFTKDLFLWLMSIIYLFAFSSLYVQIPGLYGDHGLMPARLVMDKEARSWQDLLKEP

SLLKLTPRLGLDMQTAMDFLCLFGIVLSLYCMVSHRARDFISFTLLWMLYLSIYQVGQTF

LWFQWDILLLEAGFLTLLVAPFTLPFVRVRPSQPHDHVMLWLVRWLLFRLMFASGVVKLT

SQCPTWWGLTALSTHFE

>SG-PL_contig_1564_1_-3_42_188

MDMSRFVHNLTITEHIQQVYHLCCIGFIRCVALWGMKWLGPYYYFLYNL

>SG-PL_contig_1565_1_+3_3_155

GVFFFFGGFFLVGFFWGGGGVDIEVFLVTTIVINQRVSLIALHCTFTQSFI

>SG-PL_contig_1566_1_+3_3_71

TKAVFLRGVQSSLCYCLNSKINH

>SG-PL_contig_1567_1_+3_3_140

EINVSAVENTVTKFKMHSSTRLLTKAVFLSGVQSSLCYCLNSKINH

>SG-PL_contig_1568_1_-1_799_1176

MKPSGHREKFPTDVKLGSSKIKYAYTHPCAQTHACIHTNTYTCACTHTRTHTHSHTCTQV

SFPKVSPQYHLVYQYLKKLLPLTSCVFSQTIFSHDCSLHIGFICERTVSVIIIFYLYHMA

DVVHSS

>SG-PL_contig_1569_1_+1_790_1017

MLAIKSGRCTFQEQALSPFGFGTNEALVLSGRVSFFKMCRVSDKYEGMWPNNEQTSFNHE

DKIISTVHNLVYSTEY

>SG-PL_contig_1570_1_-2_2_5248

DLATSPERELSTLNAAIINVRKWLKKKHQCLQALKNHRSSDVLQYCVDLLGSPADKKKPQ

NADVLQRGADNKKHQTQSDIENYQALLEKLVTEAGHQQEAVDVLLDRGRRYLHQRACITL

EVDLDNLAFLWNSYTKQLFLLHDQPHTWQTHLQSGSSIPEGKENLYIHDSSVKGHLLSGT

TQPEGKENLYLNSTPVKGRKGIRVGTPDKLQSAHTPEKNHSSVSLGENGTLHSVPTSENL

SSSFAESDQIAQPASDQSDINFDVQKEAFAESQPSDQAEILFSERKSVKGKENVNQELNP

TQQASEANGKENNGTSPVKTRRVLPQIPLTPRKSSGSGDKEPGKGGEKKAEEERVADWKD

KGGEKKETGERRRSPSLSPRSKVIAASQWPAENKSGWLKVAGEGIFTTSPRPSRKILTSV

LTPHSDPNCGPNTQSHSQVPPTAVSQNLACVNTCPLPVLTTGGHLPLVESLLGKQGKEIH

NEQQQKQERTHQQDNKRDSEEQNGGADAISKEAEPRDLQLQLGLTASEENLKMEIEEALK

EIQGSLDISAMDLSSNLMDSSANDSSTDTCTKTSDNESTSPDDFDLPTGPKRRRMNTDTL

AAVSSGSGSPLLLSGDSSMADTPTSSPAGTLPRDFRQRYKKDELWKAIESNYQYLMDKEI

IETCQSTKNAQEEDVVPNVSFTEFLKQYQELIDWLNQTQRNTQRTVTSLSEKYLNQAYHE

EMLEKCPRREFLTKYSQQLLVRYPNLGDQVTMRMQKFNTQWALVEAAITPQQGEANMQSM

MQDLECDLAALRRWLNATESRLLPLTIKADWTDEELEERLRHHQVLQQDIESHSRIVSAV

LKVSERVEREASCSSGSDCQSLQLLALNLERRWHCIWLQSLEWQCRLEEAINRRKGLYGS

GFNFSGFTLPTLDESCISGSSDLDLKSNGNITTSSNGSLIELSDDSLLFIGTRTEHHVGD

YSSSGPASPHSLTLPLHSPTLPSPKLTNFLDRSSGDSANISECESKMGDSPRGVPLSQDG

VNLSLSMSSDGEADIRGQWMKARQEAKDIGYSSESQSNDETESMQQQLVLECRYPGIIEV

CSDSPLGEKMTLPSPGSVRQDYYCMTHVDLDSTDKTTTDDGSATDDRESQAGRHVGSRIQ

EGPGNLGLVSQGAVVTKTGFVNQVDTTRNLSPRGSHEDITVKSYLESSDEVDGAPLTELS

AAQRGNLSESDSAAESHDKIRYLIDHAEELVKTSPRSTVPGSPQRKMSPSKQTQTVITET

SSVESSCDASSENSEESGAEEFSTATDDADETLFDSVINLDSTVDSLTSREDMSLLIPSS

ITVDSARLRKQTGPRGGKKDRPWSVVGVQDLKKMDFQPLSTSESAIDHMRPHTDTDSSSS

QFVPPRHASTFPRRARHRGYQRAFSACEQSSPRATKRKLKYSSSSSATDSQASRSSATQV

AASTEDEDDRTFVYDSAEQLRSSQGIYTCLVESESETTDEYVTASMDEVVTSADEDPHNS

TGSFSETAWDNYQMMYPTASEDATEEVLHWEPVDDMEFDDDFNFSFPKQSSLLSEAISKR

ANKMRLRGTQSGEDSDSDMEDFRSVLEDSEICLKVVDHSLKKKRNNPMGTGLASPGKYVE

MLATCETHMNCVKDVCKHLTQEDIGLGDVQRVQDLLCQWEKLHSVATDRHQQSQQLTVVS

EALTSMKMTLNSVTQCLNFDLFTSKEELEATIATLRVKQEELGQQSQVLGEHRQVIVCGV

CPGETGGAG

>SG-PL_contig_1571_1_+2_2_553

FFFFHLFILFIQSSTHIHQFQLLTQASQIKKSGSHESFSILYWTESDSSLILLRAKASVG

VCHFNGQLLCTLHNGLAFAAGHTVRNLSREAAVLHQQHLQLLNVSDDELPEAIGTKVTRV

FGTSVTNIGHQILALETTPHSVVNTLGFAPVWLDPQVSIRLVTDKFFGPFLDNVRLDKRS

KSHD

>SG-PL_contig_1572_1_-1_1_300

WQALQKYRARPYNSANCAISELRQPLQPFAQTHVTNPDPVTRDHSVPFDVFNYEHSFHYH

YDNLQFNGMSIPQLQREVIKRQSMERVFAGFMLHGIKKSS

>SG-PL_contig_1573_1_+1_43_285

MRERERERERERERDCEMKHIRYRYVIISKTVKIDKNYEDQGVLQHSGRWAVRQQRLYHS

RIRFFESNANTSPCLFYLPRP

>SG-PL_contig_1574_1_+2_2_292

AVHPDYLGLVVQTGHNHRIPPCLEGLLPFLEGLLPYLVGLPFLEGRPFLAGRPFLAGRPS

LEDRPSLEDRPFLEGRPFLEGLPFLAGLQDLAIHCCI

>SG-PL_contig_1575_1_+2_104_427

MIKYTKVLETNQDAVYKTQERTTSTFQTSSFDITKNLGTQRNDLKKPINNMWQQFYQIRD

NFIDCQTVKTLERLSGKVEPFHSLTLSTSCKNLTLSLPHPIHQFPAKT

>SG-PL_contig_1576_1_-2_281_709

MDISDVCLCISGIMFMGMFDMRIRYIDITFVYMPGIVSWKCVHGVMKVEEISLVQPSRSC

QHIGLMRRLTAFAMITLDPFPSHGICDIGAYGIYSKIGNVRKLKVDDACIDAVETDSIML

ILYYLVFLFLCYLVLSFPNKCFI

>SG-PL_contig_1577_1_-3_63_2354

MELKIILLFCAAWLATVESRPTRWCVTNRAEAVKCDKMRIEMRNLKLRNVTGQAALPQDF

ECVLRNDVFECMEAIEIDYADLMTLDSGQGYFAGRQHNMMPIMVENYHNTPNERPTYYSV

AMVRADSNVQLDNLNSKSVCFPSIGTSAGWLYPVSKLINEGFISIFECNAIVKAVSSFFK

EICLPGALTSFYNPFGNNPTDVCKLCTGQNTEFCTTSDKRAGFDGAFRCVADGAGDLTFL

RHDTIAQMTDPVLNPGTTYNKEDYRLLCPDRTIKLVDDFLACNWGELPSHMIMTSAVRDI

PVIEGYKAFLQRAVEFFGPDGTYSDKFEIFVSNSTYDTDEFDFVFERKNQMFSDQTVMLE

DIGDKTYFQWVGDDFDQVLENMNHCPSDAVRWCVISEAEKLKCESMLLAFKAKDLKPELD

CLYGGNTTTCMEMIQEGDADVINLDSGDIYMAGRRFGLVPIAAEDYGDMSMKFKVVAVAR

KTDKATTLFNMKGKRSCQPGIGRGDGWIVPLNIFIETEQFLPDDCTIFTNIGELFVRSCI

PGSLDREYNPNQHPINLCEACASGGYRKCQRNSEEQYYGASGSFRCLVEKGGDVAFVRHL

TVRDNTDGRNHAFWARNRRSDDYELMCKDGRRLNIDRFEVCHLGEVPSNAIVTSGSKTTR

QKDIIWNLLNYGQQFFSSDIDGDFHMFDSGNWYSDLLFTDPTVRLMRIPEDRQNYKTWLG

EDFIAQIENLQKYTCVNPDSSAHIMPSLFLVLFLSLLAKIMSYL

>SG-PL_contig_1578_1_-1_58_1998

MAPVRWKVIALIGTPALLLAILGSVSGQCQIPEDYTGDWYWMDNDGSDKVEIVNLNDWGD

YNCEQIKIHDNPYANVRGVNSTMLMKKKGDDICYICLDVLYRTENILQVRRSDCYSDNDN

FARKCPGYYKIPTVDQVKTYFRQTLRTVNCITTFEGMYQFTYEVAWGGGGICDNRDSQIQ

ACQDPGSAYVDNEVFFMTYARCPEVQTSFSQTKRYQCMGSWFAMVGQTGYTFASIADTVE

MDKREKYKCLMTLKNQKSADNTIRWVMSRFAKCDTLNSMHSGPVRLVLRRIPPRSEHLAP

QCNLPRNISGEWYTQGIQFKSKVIINDTHIKYFTRKNEFEYEETYLSCQQTLGTRYLMTK

VIVGKCEVDFVCYDIVPRHHGIVRYRVGKPSKLTEDEKAQQNYMERKFREACSWMSFTFN

RNQTHWKYEVLILDPPSPVPCPVGGRYQFGQWANGELEKYATRIRGVTDRPRVQVDCRIQ

VSEFKSCTQDRSRIEVDAEYCEAVDYRGRPIGEYDEPDHILTCVGFWMEDMKSYMITYDE

EDAISKFRCWVYERISWTEMQLSRSQTARCQPGQTSDDFMMEGTGLKLYLTENERLFDDC

PQRFDPGMDPYRKATVIYVLSAAPSPFPATIIFTTAICLLVSSLLLL

>SG-PL_contig_1579_1_+1_1_246

ILRQRERERERERERERQTETDRQTDRQTEQNEASASFSLNRDKEMKRGSSTETNPYKWA

KEMKGTYQSSMNKEIKENKVFG

>SG-PL_contig_1580_1_+1_1_273

ALRPSVTLCSHFGSGGGGDAVGVELAGGKQNVGIADSGARSPVVGRADQDGTAYLAPQSL

GVTLTSCADPVLTFFKGKEAQTQHQEWKLHA

>SG-PL_contig_1581_1_-1_1_129

LLFLNNQGKQILKAFIAVPKKTQEKGEGWSYDKPMLYCCYAYV

>SG-PL_contig_1582_1_+3_126_281

MKGRFIGENIKMLYAILVKTEKENIPGLLLPVDFEKVFDSVCWSFFFFFFFF

>SG-PL_contig_1583_1_+1_454_684

MISGRDCILINSCQELKPQLLVFSCRGQTLHQLLSRYRGTHTGRHHGSDNKRHAIKWSSS

QTPSLFSRPWRMTSSPT

>SG-PL_contig_1584_1_+3_3_263

PRWQKKADDSKRKKRGRNKESNRKEEIASTEFPEDGDTIYTERSPGIPTGDISGLSPRKV

HQSQEDNHADHKTTLRLKTKILQNNNS

>SG-PL_contig_1585_1_-1_658_909

MSNTVKGRLDDLKIITSTVERNTSSLHSKESNKPSCGGTFDGMEYFLLHRSSAILIISVR

WFLICSLVVFHFKSTGFLLRAFYF

>SG-PL_contig_1586_1_-1_325_492

MKTHCVTNLTTVGSTVSRFHTCKLATELAAGFGTNQFLIEFITDLSLVVILFGLTD

>SG-PL_contig_1587_1_+2_707_862

MAMTWHSAHVLLVCGLYRHQASVMLCNNVQGRQDTLQNQKNVFFVLFLYPEN

>SG-PL_contig_1588_1_-2_2_169

ESVCVCARARRCVSVCARVCVCMYVCVCVCMNEHFYICMYACMYNYVCLFIIVRIV

>SG-PL_contig_1589_1_-2_227_946

MKLECVVAVMAALVAVSMAAKRRCQACDPDACPALNGANCLAGTTKDECGCCDVCAKLEG

QACDTDGHHDLCGDGLQCKKTANTGAICQCKFQEILCGSDGKTYSNLCQLMTTAVREQRT

NTLNVKSVGPCEPGATIVTKPEYVRNHTESNVVLQCEAMGLPTPSMAWVFTRADNQTFNL

PGDDCKMVTSARGGPGKFMITSWLQIEGLQKFHEGDYTCVAYNQHHTDRSKARIKVVERQ

>SG-PL_contig_1590_1_-3_3_158

TEVMCALHHTDYSRHRLYMCYITLTTVHTEVIYVLHHTDYSRHRLCVCATSH

>SG-PL_contig_1591_1_+3_3_80

SRSLPDNCWQIWAERIWNNLSAMNPS

>SG-PL_contig_1592_1_-3_63_299

MKTKILYGWVDLDLDLQFFQFSFLPLIRKSKLPSLLYVDLGLDVRGYVSSREVFRKKNIK

KYRIDGQNTAQTRAHCDAV

>SG-PL_contig_1593_1_+3_3_203

PCSLSLSINSSLVKSVTAMLPLLFCPSVLSSQAVLIPGLIFLMFGILLVLTGFTLYLGQV

CLTFKAV

>SG-PL_contig_1594_1_-3_51_158

MCASLYILYMSCVLISSLGNPFYLRTDVFVCVYVCV

>SG-PL_contig_1595_1_-2_119_577

MAKALCVLVDTSKKVQGTLTFTQDDGKTTKVEGQVTGLTPGKHGFHIHEFGDTTNGCVSA

GPHFNPFGKEHGSPTDENRHAGDLGNIVAEGNGIAKVSITDSQIPLTGPHSIIGRSLVVH

EKEDDLGKGGNEESKKTGNAGGRLACGIIGITK

>SG-PL_contig_1596_1_+3_1119_1361

MAFVFGLVGRIGKREVGCELFFIIAAVGHRICCCNFFCYKVGACVCGCNFMCNLLPRELV

SCCGIFMMRKKNVTVILKCVV

>SG-PL_contig_1597_1_-1_1_237

INTVIIIFIVRYKIVYCPTLNTMAICHKVASSLTRVHTHTHTHICTPKNKKEIELMYLCS

SVYNYMCFIYFHFYSVLYV

>SG-PL_contig_1598_1_-2_305_916

MFDLYRKVNSREVIVGWYSTGFEVSVHSVLIHEYYSREAKSPIHLTVDTVMRGGKMNMKA

FISTAIGVPGKTTGTMFTPVQVDIHSYDPEQTGVEVLSQGKYKQKRTVTVQSDLSKVESS

TSTLRSMLQQVNEYVDNVLAGKVPADPTIGRFLLELVSSVPKIEKDEFENMLNTNMKDLL

MVMYLSNLTRTQLLLNDKINNLPS

>SG-PL_contig_1599_1_-3_3_197

PILLRCLRKEGVGREDPLQAAVLSAAASAARRGSSSRQGLPSLPDPGDSDLPAPGPLHTG

RILRS

>SG-PL_contig_1600_1_-1_142_438

MLGQDVTLCIVGNKTDLEKDRHVPVQEAESYAASVNARHFHSSAKLNRGIDELFLELSKG

MIEKYDGTMGRGRTGGNNRRNVVVVDDDQAPAPKSGCCG

>SG-PL_contig_1601_1_-2_164_499

MKVWVVNKHQLTSTVVKQDTGLRTGRSGRPWLETPWVPHLVDKGGGFSDLPGLNRCVDLL

VPEPSLCVYAHRVSNAHAKDPVAMSAFDGLWKRKHTQNVRKLDFSRHTMPSP

>SG-PL_contig_1602_1_+2_41_277

MNFELSKINNQFNVSRIIQSLMYRWKLDAFKTKYTRNIVCLCGQKITTSHDLSCIKIKRF

NLYIYFFERQFLKYLNQHH

>SG-PL_contig_1603_1_-1_850_1191

MSYIGGSVYSYQSVTHTPNLTSTTSTSSPRGMDQRFPRGMNNAIRYKPTHGPTFCFAPFV

GHKSTNRLRWPPNHVRQLVTASARHHRRVVDGFCFQDACDLKSVEEMLCIYFCA

>SG-PL_contig_1604_1_+3_411_641

MHLNSSSYSCNKICVHENSHFNINQQEKRLSFTCFQTNTISRLCLNAHVSCDNSLKRYSN

LYQFPTITLLKLSNENR

>SG-PL_contig_1605_1_-2_284_406

MYSFHTTSSFCMLFSSFSICQNFIFTSLQQSRFVQKNACNI

>SG-PL_contig_1606_1_+3_66_284

MPDARCLAQTVQQHNTCTVFPHIESCRIPRKIQLFYEIKIQLQYSTVQVMVPFGSGLVGL

CRLFKRDSSHSDN

>SG-PL_contig_1607_1_-3_3_218

FPSFLPSLLLYFLPSFLPSFLPSFLPSFLLYFLRSLISFFSHSFLPSFFSHFSTAYAKLC

IPTYISGGYAYI

>SG-PL_contig_1608_1_+2_143_301

MFHRSSSPRSHFKTLLVHRILRWLKESARQLLADLEEVRIKLSEWNESLLTKL

>SG-PL_contig_1609_1_+3_678_935

MAARRYTADEVARIVLELPYDDINSDNENVIGHKGDVVLDAVDQSDSKSSGSDYPDEDYT

LPSISTGRGCGRGLIVSYLILSTLNI

>SG-PL_contig_1610_1_-2_125_514

MASDQQEVNSKAYPLADPSLTQTILDLVQQAASYKQLRKGANEATKTLNRGICEFIVMAA

DAEPLEIILHLPLLCEDKNVNYVFVRSKQALGRACGVTRPVIACSVTTKDGSQLVNHITD

VKDSIEKLLI

>SG-PL_contig_1611_1_-1_880_1152

MSSVRYVIHTASSQNIIESLHCFFHCDPSFLCFINNNIHLRYHITQLLFQLRRSVDVSAV

SWYCCCLVSPSVTLELSLVSRRTHLPSLISG

>SG-PL_contig_1612_1_-3_54_353

MPELAAAKLKCKNVDDPKTQEELTKMKKKLCAKLLESQAGPTCKNAAVVERLTDTSKYTG

SHQARFDESGKGKGIEGRKDIEQNTGYVASYKGQGTFDKK

>SG-PL_contig_1613_1_-1_1_189

YNVTVGGFIYFIENISLVIQLQKIHLIGSGLSCHRFGRVYVERALSSNCFSWQNSFSPAC

TVD

>SG-PL_contig_1614_1_-2_2606_2911

MSFGQFVEASSPILALPRSFQKSCLQMRLTFLFNLTDEVDLPFQPVHGNRSFCHFILYSV

YVHSKNLVKVLYGVGRYLIHFKDLRSIFRVLHVRERVVPFCV

>SG-PL_contig_1615_1_+1_1_123

SYPVAFHCHAQSCNHILWLSTAMHNHVIISCGFPLTFTIMQ

>SG-PL_contig_1616_1_-2_389_550

MLVCIYVLCVSGFFACMHLSNLTHLRPHRYRANQIMLLIAQPTTLRPYQGCHHI

>SG-PL_contig_1617_1_+2_485_664

MFCFEHYIFVSVSKIITKAGFKRGIFTEAKTVSTINDKKWLSSRIIRHNTLTYIIYLFNF

>SG-PL_contig_1618_1_-1_592_1020

MNGLMYVRPGSGFIPLFELSARSDVNGEHELPLYTFLKSKCPLPSHALFAKTETFWDPIR

PADVTWNFEKFLIGPQGKPLFRFMPDVEPFDLLPLLVQESRNFTQILANLERVVEKRVER

MKKNHKARNEIRKTHEINIQINK

>SG-PL_contig_1619_1_-2_1760_1927

MVWFCPNRSVGLSHCVGWSSVSYRVDVDRSLAHAHTVAVPYLYSRSGDSVPHRFVL

>SG-PL_contig_1620_1_-1_607_813

MILCVELFMLLQYHLFFLMVSLCDVILLSFICYFFLFYRFLCVVLYFHSNITHFCFVSSF

VVFYISIQI

>SG-PL_contig_1621_1_+1_598_1026

MPTGPTSVGQQVLFNRYTPISKACFGSIQKVFSYLAGEVDPWHPSAQCSGPAAAPVGICY

SSYTCLSTVIALLFVTQVEKWLPDLQDVWLHNSVFFDIFSSVSSVCSLFLHCRHLLFVLR

FFVIDTSHHCMPSSFLHIDFNVW

>SG-PL_contig_1622_1_-1_307_9057

MKGKWEKVADVQGNKCNVPKLQENHEYKFRVTATNKNGDSEPLEIDSPVTAKRPFDEPHP

PSQPEVVDRDRNFIEIKWEPPTKDGGAPITGYDVERKDPKTNKWVKINKSPIQTCQYKDD

RVKENQEYQYRVIANNEAGPSDPSMETKPIPARPTKETPKLNLDSLYGAKDIRVRAGEPI

NLQLGISGTPDPTVEWLKDGVPVGNRAQTQNDAKQAKLHVAKAERGDGGRYTVKVENPHG

VDTADINIVVLDKPSAPEGPIEVSDIMADQMKLRWNPPKENGGAEIMGYMLEKQEEGSNI

WEKVPGIINGTSHLVKGLKDGKAYKFRVKAENMYGASEPLEGDRVIAKNPFDRPDAPRDL

EIPKFDRRSATLTWKEPLSDGGNKIKGYLVEKKERNRDWEKVTAYPVTEKQYTVLNLKEG

SEVEFRVMAVNDGGPGEPSKHTPPHLVRDQVFPAGAPSHPNVDKITKNSVDLSWHKPSSD

GGGKLTGYVVEKKKKGDDNEWEEATVVPATATSACVHGLVEGEEYQFRIRAENAAGPGEP

SKPTNTITIADQPDKPKMDLSKVKDITVKAGQDFKIAIPFTGTPKPTAAWVLNDGDLETS

PRVAPKLTDNEVILEVSKAKRDDTGKYKVTLKNDSGVETATVGVNVLDKPQAPEGPIKAS

EMDGESMTLSWQPPKDEGGEPVANYIVEKRKEGHPKWVKVSSFCHNPSCQVRNLEPGTKY

EFRVSAENAQGISEPLETSEAILSKLPFDAPNAPSKPTCVATTEDSITLSWEAPRKDGGS

PIKGYVLEKREKGDKKWTKANVQDIADTEYTVKGLKLDKAYEFRVAALNKAGVGDFSDET

GAIKPAPPPVAPRVSRDMMGAGKDIKAKVGEEFKIAIPFSANPTPTATWAQGGITVQPSE

RFTMDTQPNQALLVCKKAEKGDTGRYTVTLHNDKGQDSATVNVTVYDSPTSPEGPLDISN

VTPDSCVLKWSPPMEDGGSPITNYVVEKQDLKSGKWEPVSKFVRNPTFEVMGLTEGHEYN

FRVMAENEFGLSEPLDSAFSIAAQYPYTAPDAPDTPKVDDVDESSVTLVWSKPKNDGGKK

IQGYVVEYKEPSSNRWKAYNDIPTADTKATVKGLKKDTEYEFRVRAKNIAGLGNPSDSTG

SVHVKPKYTKPGSPGVPEATKVGRTYADLRWDKPRNDGGSKLTGYLVEKREKGMSMWTKA

KEVGAADSQCTIDNLAENVEYEFRVAALNAAGAGEPSLACAPIKIKEKLEGHAPDFLKKL

HNVKGALGGEASFTVEFSGTPKPKVLWFKNGVEVGNAGRTRIKTDDNQCMLTINELYEKD

AGEISCELVNPLGRETCKARLDVQVPPKLDKDIRDQKVQKGDQFKIKVPFSGTGPFDFKV

KVNGKELKESDRIKISPFDDYMTLIIKDVELEDAGKFEVEVSNESGAATTNFNLKVCSPP

GVPTGPLAVADITKSSCRLAWKPPKVDGGARVTGYQVERQEVGKPYWVTIFSNCKDTSAD

VQGLYDNHQYLFRVAANNENGMGEFLEVEGGPIVAKMPFDAPEAPGMPDVAEVGGDFVSL

SWEKPRSDGGGRITSYWVDKREHGTDNWSRVNTSPCVTNIINIPNLIEDKRYEFRVFAEN

EAGMSKPSMASNSVKIKDPNAAVVPEFSSGLRKVAAVEGKSARFECEVSGQPKPEVQWFK

GSREVSDGNKYEVLTEGDKHILIVHNVYGEDADEYSCRASNKGGNRVTRADLEISSPPKI

NVPPRFRDVCTFEKGENVVLKIPFTGHPKPAVKWIRDGEELRGSRYHSDVTERHALLTIK

DASKDDDGPYRLSLENNLGNDSAVIKIQVNDRPDPPRFPVIENIRDESVVMSWKPPLNDG

GSFITAYIVEKREVPSQNWLRVASTRFAFHNITGLSPSKEYQFRVIAENFYGRSEPCETT

EPIKMEEPEDMKKKKQLEDEFGRKVRGKYDGPKINDYDKFFDDIWKKYVPQPVDIKEGSV

YDYYEILEELGSGAFGVVHRCIEKATGRVFVAKFINTPYPLDKHTVKNEINIMNNLHHPK

LINLHDAFEDKYEMVLILEFLAGGELFDRIAAEDYRMSEAEVINYMRQACEGLKHMHENS

IVHLDIKPENIMCETKKSTNVKIIDFGLASKLNPDEIVKVTTATAEFAAPEIVEREPVGF

YTDMWAIGVLAYVLLSGLSPFAGEDDLDTLQNVKRCDWEFDEEAFANVSQEGKEFIRALL

LKQPQKRMTVHEALEHAWLKGDHSNLTNRIPSSRYSKIRQKIKDKYADWPQPMPAIGRIA

NFSSLRKHRPKEFMIFDLYFDRKEAAPRFVRKPRNQVAAEGQTAKFDCKIVAASAPIVTW

MKSDMQLSQSYKHMQKYMGKDYELRISRIKMDDKGEYCVRAENSFGRKEERANLQVEPGI

EPPRPPSHEVTPRTKRVFEELPIEVPKEDTPPNFGFGLRPRFIQAGSEFKLICHVQAFPV

PKVTWTRDGRDLSDNDHYNITYSHGVCTLEVAYSKAADTGEYVCRAVNSLGEKETSCTVT

VEERAFYKPKDGSHTSSSVSRMVRRTTSGTKIGESSYESSYSSKTTRRSDRLDDSYSSSS

RTERKKRSEEEAPMFTQTLSPAEVTEGEKLTLSCAFTGQPDPNIEWFLNGQLLTSDDIVS

ITTRSSSSRLEIGEVVLDDEGDYVCKATNTAGVASTKANVVVRAAKKSVVEQERETMEEA

PPPPSVSVDPPRFLEHLQGQSVMDGDQVTLQCRISGKPDVEWLANGNPVTDSDDFKHQNA

GDIYKLIIGEIYPEDSGIYSCKASNAGGSAISSATVFVSVPDEEPSDPVFTTWPLSQNIE

EGNPVTFSCALDSPAALTVSWSKDGRSIDDSGRFKLSQDGNAYSLTIPAALSTDSGTYTV

TATSEEGSTSWSCSLAVAIGDSGDEARVQELLQQAVE

>SG-PL_contig_1623_1_+2_74_1132

MSSLLGKSKAVTLPSVSDVKLLPNMFADFFHNKILAIRTSLHSAPQLQNSSQPFSGTPLS

TFSPVSEDFVKKVIYQTTPKTCELDPMPTKFLFQYLDLLLPAITNIMNESLTSGIFPLEF

KTAIVKPLLKKSSLDPNNLKNYRPISNLPYLSKLLERLVLHQLFSHLTTHNLLSAHQSAY

RPGHSTETVLLRIVNDLLNSLDQDKVSVLLLLDLSAAFDTVDHKILLTRLSQNFGITSTA

LNWFRSYLSERKQTVLVSGNRSSESVLDFGVPQGSVLGPVLFILYTTPLTPLIDMYSVHH

EMYADDTQLNHSESQSNYNHLIQTLQDCVTEVKTWMSENRLKLNDEKTEALRL

>SG-PL_contig_1624_1_-1_295_750

MRIPLFAAFPANKPCDFFLQQIHLWSPGALRQFLALRVGSPILFAHCSDWKDMTRFVCSR

DREGRSAHAPIAPSVCYVDAKTHKWSVHIFRCKGRHLCTPSVFFCLFLFVFLDQNLHSDL

RISVHSFCCMFLLLDVILLVSLGCLVLTITVG

>SG-PL_contig_1625_1_-2_128_1639

MNAHPHVVKLLLEAGADPNAGDLYTTPGQMAREKHLHSLQVLITREEEFSHRLSNRANFR

DCTALHYAVLAEDVETTKIILDYGGDPTKENTLGHKPIDYCRTEEMKKVIEDSARSFEEK

RAREEAEERRRFPLELRLKEAIIGQESAIRTVAAAVRRKQNGWYDEDHPLVFLFLGSSGI

GKTELAKQVAKYLHKDLKKGFVRIDMSEYQGKHEVSKFIGSPPGYVGHEDGGQLTKQLTE

FPKAVVLFDEVEKAHPDVLTAMLQLFDEGRLTDGKGKTIECKDAIFIMTSNLASEEIADY

GMQLREEAKRLSKLRHNKIDELDIDDITVSRKFKDLVVRPILKKHFRRDEFLGRINEIVY

FLPFSRAELGKLVTKELNFWAQRAQSKHQIQLLWDHRVVDVLADGYDVHYGARSIKYEVE

RRVVTQLAMAHERQLIKEGNVIRVTVTHPDDLLTKLDDDVDEKEEEEEKGLHGLKIQVLE

EGSKDKYNDISFDTAENPFTIEFK

>SG-PL_contig_1626_1_-3_966_1232

MKVRVSCLCLYLSFIDLIFFSLLIFSVHHFWCRRCVFVWGGLFAGFFGLFVFYLQVQSCC

FRLFVIFQVKSCRFCLFVGHFFTQVQSCQ

>SG-PL_contig_1627_1_-1_184_666

MFVCLSASVSLSVSVCLFLSLSFAHEYTLTFALSLSLSLSPPPLLSLSPSLPLSPPHSFP

PLPSTLSPLLSPCFPSPFLPPSPSLPLPLRSPSFPFPLLSPSLPLSLSLSPSPFPSPPLP

PKTPKPTPLFFFFFIFFCVCVWGWAHGGFFFFFFFSKRKNI

>SG-PL_contig_1628_1_-3_309_560

MRFVSSFVFVGFFLFKFVCLFVCLFRFYFVFIRNLRSVRVGSCVFDVILFADCYLFLNLP

FSLKKEIISYYLGAICVFVCMCVC

>SG-PL_contig_1629_1_-3_147_359

MKCVDKIFSAVSRRTFTRFEPKIWTTEESLMFEFQMERAKVKVTWAQKCLKLAFYQEGVR

TALSQQLWRKF

>SG-PL_contig_1630_1_+2_248_361

MSHTHTQDAHLVYRQAKSTSVVYISGITCSGGSCSARV

>SG-PL_contig_1631_1_+2_176_277

MAQGSTHNIWKNSQKENFFFKYMHTIYSLPSSQF

>SG-PL_contig_1632_1_+1_805_1008

MNNFKRQVMIYLLYLQIVLFIKFSKYQFYLFIYLLYYFSVLLSLSINSIHKNTNTEKSAS

THTNQFKN

>SG-PL_contig_1633_1_+2_314_457

MFHNNLNTNDNGHNMCSNNILCICVIGLQNKARYSCIHFKHTFLTHTK

>SG-PL_contig_1634_1_-1_472_558

MLISTSMYFCVCLLLSFLKINVYNDFKKK

>SG-PL_contig_1635_1_-3_81_629

MIVPPAPPPYESETVTNASSTAQPAKLPNYVEATTLPSYEEAERSKMEEVLHHSNDNVTD

SSLESGQSREVFSDMTIGTDGMFICTFLISFLFNWLGFLLSLCLSNTVAGRCGALSGLGL

SIIKWVAIVKHKQWASEYADGESWLWWLLFFCGFLLFIRGSVQYVRVKYEWAQLNRLHRY

RLI

>SG-PL_contig_1636_1_+2_2_193

QKNMNIYSMNETRQWTMYGNCLLDKMYEKRQMNILFTCLSCFAYKGHLAENLRMACFSFI

HLLI

>SG-PL_contig_1637_1_+2_629_1111

MQQLCTNLQLEKILLKSCSVNCIYLQTFYFIFTHSHTVFTSLPFTLLHLIHKKSPTFFFF

NIISVFILTLAINMSQYYTPNHTLFTVPITPSITLYLQYTKIAACLHMSFTPFIFSLIRL

FTPVLSLTQDLQAFIKFTQDPSTDTHISTSAAFLTWTDMVW

>SG-PL_contig_1638_1_+2_1967_2461

MFLIHVAQEALCIPLVKLIQQSLATLVYKFLTVLYLGLQIFQLFMVITVCDLQILQLLNF

QELFFTDNYVTIIIVYWTLCLSFNTKSVQCLPGLVNVHFGAVDLLGQVRVSLLLCGQDFF

VFNQDPVDFTAPLLFEILKCCSSAGWSAWALHFLRPGGGTSRADR

>SG-PL_contig_1639_1_+1_1_243

ENYAKFVRVNRSGSGDRETERSPEGSVEPCLEPSRAPDLEAIMAKTPDRENKRGKRYVSP

RLWAVGKGRTHPGSEITRNSQ

>SG-PL_contig_1640_1_-1_58_282

MSNSFTNQVLAQIELWTKTSEYKLGVHMLPKKLDEEVAAAHLSHLGVKLTKLDDDQAKYL

GVPSTGPYKPEHYRY

>SG-PL_contig_1641_1_+3_876_1097

MCLYATRKCQKENTPYNNTCRHYFLTMYNRGNICLCSCTLLNTSLNWLEYKEHYMDFPYK

NTTILNLLTAEWLL

>SG-PL_contig_1642_1_-3_60_248

MNRLNTTTQFLSPTLSLFLFESACTRAHTISCSLYKYMYKYMLLVASTFLLSLHECIKTV

ISL

>SG-PL_contig_1643_1_-1_238_5550

MKVQLVFEIIKTMKNSIAILLICSSIAVTLGQRRQPQCEQGSCYPATGDLLIGREKMLSA

SSTCGMQRPERYCVVSYLEKPTKCAHCNSSEPWVEGQNEESHRVENIVSSFRGRRRRHRW

WQASNGQEKVSIQLKLEAEFHFTHLIMTFKTFRPKAMLIERSFDFGKTWAVYRYFAENCG

QSFPGISLGPVRSLDQVICEERYSGETPSSGGEVIFRVLPPFIPIADPYSEAVQDLLKLT

DLRVNFTELHTLGDTLLDSRSEIKEKYYYSLYDMTVRGSCSCYGHASRCLPVPGFETRPD

MVHGQCECTHNTKGLNCEMCEEFYNDLPWRPARQNQPNACKKCNCNNHATKCHFDAARFK

STGFVSGGVCDDCMHNTIGVNCQECKSLFFQDPMRDIRDPEICQPCDCDTFGSENQGICE

SITDDLLGTVAGRCICKKYVTGKRCDTCIANYWNLREDNPDGCEQCTCNPLGTIGDLGCD

QDTGICRCKRYVTGRNCDECYPGFYGLSEYQYGCHACDCDIGGALRSTCDQASGQCECRP

NIVGKQCKQVESGFCFAHLDHNIFEAEYGKGSGNAKVYVREPIQGSVSYWTGPGYMKIME

GDSVEISINNVPFSTFYDFVIRYDPRMPEVFQDVRVTLVRPDRVDPDGLCGDFNPQDDFK

DVALQPGARYEIVRPPSCLEEGKTYTLRIDFSQYRTGLQTPEATLLIDSVLLVPNVDFIP

IYQGPGLPEYMLNEFLRFRCQQMQYPSIKQQLPEECRRHTFSISAVLNNGALACDCDPMG

SVSLECDPSGCSCPCKVNVVGRRCDKCAPGTYGFGPEGCQPCNCHEIGARDNFCNEQSGQ

CTCIQNVGGRTCDQCREGFWGFPQCRPCQCNGNADTCDPLTGACRSCRDNTAGDFCERCE

DGYYGDPRVGVRIPCRPCMCPGGPGSANQHADTCTFDPRSQEVYCNCFPGYQPPSCDRCI

DNYYGNPLAAGGSCEACVCNNNIDPNVPGSCNSDTGECLKCLFNSEGFNCEHCVAGYYGD

ATQQSCRQCVCNPIGTNRSGGDCDRVSGQCPCLPNVVGQDCGQCAPAHWDLASGDGCQAC

NCDPTGSLSLDCNQLDGQCPCLDGRGGRTCADCEDFFFGDPNNQCFPCDCDPRGSESMQC

DKRTGQCQCVAGVTGYKCDRCARGTTGQLPNCVPCGECFDNWDRVIRDLRDQTVYLVESA

KNISVTGAIQAFDEEFKMMQNHIDEIRKILSSVNFTQVDIQEIENMLETIKRNLTDNSKK

LNSLDEELKHTTARVQKGNTEITSLREGVEKLRKLAKALQDNATDIQARDVEGAFNITKE

AEKRSREAQRIVDGTGRHLAESERIRQRTEDLINQRQADFDDQLQRNKQNLDSLDSDVSD

LSSRLSDINSMVCGAPGAPCDALCGGGGCGRCGGESCDGAVTKADTALKLAQQAEDLLTD

KEREANSLLFDVQGAKLAAEEAKDEAQMAYNAAFEAKNASENARTQLEDLLNEISKVLGT

PRAKPEDVEVVAQEVLNMRVSLDPDEIRRLADKINSTVQGLQNIDQILADTEASRELAQR

LNRSAEQASIEAREILDTAQEVRDALEGARRAQEMAENAINLAKTDIADAEADLSMIGTE

TSAARDLSDESLRLLQELKDRLEKLRTRYFKNDLDIQTAEDTINKAEIAAEAAERKATIL

ENDYEEASISLNRKYNTTADAQERAAKLAAKADRILQDTLRRKKSLQNIQQDIGENEKAL

VELSEDIDELNRRMDQYLQDIKAKHDDYRTC

>SG-PL_contig_1644_1_+2_179_358

MAHRRYTHHHCEGSVGSSFSQPKPTHEWLKQFGRLFTCFDVPLEDYKLQKHNMVNTDRDQ

>SG-PL_contig_1645_1_+1_352_531

MPKCLSTSINDNIQRSETTTTTTIFYSFVSQLAPSWESWEQQQSFKILKAFRVSGIVQFV

>SG-PL_contig_1646_1_-1_211_3030

MSPSPVEELALTDLDRSNKGASKQRRDLINAEIAVMRDLLPLPESARQRLSQLQIMSLSC

VYIRKCNVLHKLFSSLPAETDVPTMCDFSQALTGFLLITTREGKLVYISENVTDYLGHSM

VDMKTQGDSLFDIVDKRDHGSVQAHLLQGGAEPDLTGDVTFFCRMNMSRTLKRQAGFGDV

KVMHVRGHFLPLSDLSSDQQSQQQQQQQKYVFMATCSPLITPELKETLIQSNTMVFKTVH

KLDMTFLEVTKTAEFHLGWTTEEICKKSWYSMLHPEDIHEAQEKHVLLMRASHELSCMMT

VRMLTAVGDPVWVNVVMHVSQPTNSQADDSLIVCINQVISEEEAYQIRMQSHMFTLYPPR

TELWNTATLSHPQPAQPDTAFWMTAASSVVSTVPGFSYPNTSRPVLSQQHHALPTGDYIS

GQDGMGYPINPPSSSPSTAMLSSSAQTDRLKLMLKRKLQGPCHQAKIPKMAWDETPSTMG

ATGSRKSMESTVTTDRMHTSQASSQFIISGNSPRGHSWPASTQVVPPAQYQVAHVFPVTI

THQNSVKTSMLSPTRSLGSLSPPLCQPTTTCQGTEQVVPGNDVAVPESFLTPDPSPASSP

NPQTSGRSTKTKGKTAPTSAAILKELEKLADLTRAKSTSVIPNTVRCIKMEKSLEDCHHA

SRQKELPIMDAFDIESFFHNLDFDQQSVTLDWESKQQEIISEAFAAKLEKVPVSKSAAPA

MPIPQVVKQEPSDMSYCPLLAACASQQLQVQGQAKKTAPLQQPEVDPKEFEELLSYFSGD

MTATQLNLDSLLSASLSKNEAGELDSTRQQKDNQESELCGAGHLGSFLLCSTSLERAQEG

FSPSSNSDTSGDEDSSSEETLERLLRPDSSVTGQKHGGKLKRDGGEKHGCAPSFGLSPTP

VQQATQDVPGITEEDELYQLNKLLSLMASDSGMMLSEESQ

>SG-PL_contig_1647_1_-2_380_499

MQFYSRAITKLEFRREMLTKKHTRGSISSGVKKQILLKMC

>SG-PL_contig_1648_1_+1_748_1020

MEHTVHICALVLVHYNYRHDRPFLIACHYLQRILPMSRGGRLKPNILLQPRHSFSQQLVI

TASFNFHRLPVKNHHLLPHTSDFLDFRTAGA

>SG-PL_contig_1649_1_-1_31_591

MGASQSAFTEQELDDYQTLTYLTKKEILHVHKRFCSLDVGNEESSRKQKNPKLEHLKVME

LPELKVNPFKDQIMKVFSSAEDGMTFEDFLDMMSVFSDSAPKPVKVEYAFRIYDFDGDDM

ISSADLKQVINRLTGEDNALNEEQMQQLIDNILEEADLDDDDSLSFAEFEHVISKAPDFV

SSFRIRL

>SG-PL_contig_1650_1_-2_227_415

MYVFEMNVHNIHARSHFLFGYLHVEIYCLTFPPPPPTLPSIKKRSCNIFYQIAGNNHVTC

IYI

>SG-PL_contig_1651_1_-3_144_944

MECMEGGELFTRIQERADSAFTEREAAAVIHSIVKAIDHLHSMDIAHRDLKPENLLFTDK

SPNAVLKLTDFGFAKEINSYKSLQTPCYTPYYVAPEVLGPEKYDKSCDMWSLGVIMYILL

CGYPPFYSNHGAAISPGMKKRIRNGQYEFPKPEWNRVSQEAKDLIRGMLCTDPDQRLSIK

QVMASKWVGDNTMVPQTPLMSAKVLREEEDMWMDVQEEMTNALATMRVDYDHVVNIKNLG

QSNNALLKKRQRRDHDAMQDEGAMPMQ

>SG-PL_contig_1652_1_-2_338_472

MIFDKQISFINTVQMIVMIYLSTSLFSQGCLNTGRFSCCCFFLKV

>SG-PL_contig_1653_1_+3_3_149

TEGRAQMVAIFTSKNTKLMVHRGELLFERLLPLYLRFTAKKTTHDRLHN

>SG-PL_contig_1654_1_-1_91_549

MLAGKVAVVAGYGDVGKGCAQALRAFGARVIVTEIDPINALQAAMEGYEVSTIDDSLCEA

RIYVTATGCKGILRPKHFENMLDDTIVCNIGHFDCEIDIAWLNNNCVKKESIKPQVDRYT

LKNGRHLIVLAEGRLVNLGCAHGHPSFVMSNSF

>SG-PL_contig_1655_1_+3_804_1001

MRSKPNMDVDCKSDIKPCPNLPSTDTNINNPQMSETCISLSLFANTFHHIPCTPSIFLVH

KQQQHT

>SG-PL_contig_1656_1_-1_1_429

MICKMHSVSCLTVCVVLIALFHFVFFRQALAVQHGADMQRRSLRSRYTRSANSDTESQRL

PSDLQPLHYDLVIEPDIYQDDSSMFSFEGRVTITMKCISPTTTIVLNAKELSIDQSDIVV

SGPAGNLTVVNLVLNDEMETLTI

>SG-PL_contig_1657_1_-1_1_252

VGQFLGDETQTLRSLPRFLSECHLTFSAHLSRAEHQTHFIIRPPTARKSQGSGETGENPE

KQKLTWAVRERKRKEKRQPYEKWP

>SG-PL_contig_1658_1_+3_3_146

WCSRDSKANIARRYFKRCASAESAQEPPISVMCALQEVCIISETNSLL

>SG-PL_contig_1659_1_-1_1_324

HIKSITNQAFTGLASLQQLHLEDNDITTIQENSFKPLVNLRELRLNSTNLLCDCHLGWLA

GWLRSFGFQNTIIAVCSYPQALRGKSIFDVPAEVFKCNDQEISKPIIL

>SG-PL_contig_1660_1_+3_123_350

MALALTVKAETNNETCIQFQRERETERDRDRDRDRERDRERKKQGEKDRDRARDREREKT

GRERDRERDKHCFLAL

>SG-PL_contig_1661_1_+3_96_407

MCAKTVNHNLLKVPKVQHNYIQDPSNSTKLICTRNIFQREVNHICHLSQKACFLEILTVM

LGMNHLTGKIFVMYILNVMFLDSNVSSIPNFMHLTDMVKRCISK

>SG-PL_contig_1662_1_-3_255_1229

MAAVPRLGIGMDSSITPLRHGGLSLVQTTDFFYPLVDDPFMQGKIACANVLSDLYAMGVT

DCDNMLMLVAASNKMSEKERDTIMPLIMRGFKDCAEEAGTQVTGGQTVINPWLTIGGVAT

TVCQPNEFILPENAVVGDVLVLTKPLGTQVAVNAYQWLDQPERWNRIKLVVSEDDVRKAY

QRAMFSMARLNRIAARLMHKYNAHGATDVTGFGLLGHANNLAKIQKNEVGFVIHNLPIIA

KMAAISKACGNMFGLLQGHSAETSGGLLIALPREQAAAFCKDIEKQEGYQSWIIGIVEKG

TRTARIIDKPRVIEVPAKDREGELW

>SG-PL_contig_1663_1_-3_27_743

MAPNKYSVLLPTYEERENLPFIVWLLVKYFSESNINFEIIIIDDGSPDGTLEVATELQRI

YGEDKIVLRPRGKKLGLGTAYVHGIKHATGNFIIIMDADMSHHPKFIPEFIKKQMEGNYD

VVTGTRYSGDGGVFGWDLKRKLISRVANFITQVLLRPGVSDLTGSFRLYRKEVLQHLIDQ

CVSKGYVFQMEMIVRARQFNYSIGEVPISFVDRVYGESKLGGQEVVDFLKGVLYLFATT

>SG-PL_contig_1664_1_-2_536_889

MFAFSFMQHVTSLVENMQFFFSLKKHTRRRNFRTGFLLDNMLEPNSFLHSKLVDSRFMLW

HCFLLNNVLEMNAFLTFKLVDKIFQDFASKQHAGNENNLINLVMQPSLWSAFSFSECV

>SG-PL_contig_1665_1_-2_2_160

KWKKARFTFEKKKKKKKGKRKRIPQRRKNSRKQIFRKQLSFMYKTNDINRGLT

>SG-PL_contig_1666_1_-2_1175_3181

MVVDHLIAYHAVNINGKKLELGATNMSQADAFKEVEKLPEGWRFLPHSKRPRSRKHGTTT

TTKAETGTSEKSDYEEQKIKDSERNERIKKAQERSQNRQRERRGKENPKLTERGRAVKDT

RPGSLRSRGTARGRGRNLPETRVRQRRGSKQLRMQPERERQMQRDMEMFEREMHEIEKQR

QMHVQREIEMQEELERQRERERQMEREMHQERDRRNWERNQQEQWEERERRKFERIRQQE

EEDERQRYREMIRESEQMKKMHEEAIHKLKALEDQQRQEREDMERLKREIELERVRLQQE

NKEQEQRRQRQQKEEQELSRKRGRAEEGSRPGTGGWQHRGPNKRGKGDKGEWNKYDQSDS

ERLGRQGLQERHNERSAGHSSATAGPESEESVRHILEQEVQRQAEELKKSLALLDKIQKD

PSSTDADKTGYQQEKTGYNKRSYGQGYQQKLGFNKESFGQGYQQQTYGAEFKQPQTYSSL

SQNQGYDIQTQGYGQDQTQGYGQDYIQGYGEGQTQGYGKDHMKGYGQGQVQDYGHEKTQF

SYKQEGVHRQSSETGNIYSKWYEVQDSAQSGNGGWYGMQDQIQSQTLSWGVGAQEVGGWP

QKDKSSTSTRVYVRGQGRNQNAKKRERARDGGGARYGQKGRGSGGGSGGQGAGRSGDRGG

GYQSKGSYY

>SG-PL_contig_1667_1_-1_913_1101

MKMYVCVSCHTKPDNKRFVCVCVCVRVCVCVCFPWALDCIGLEYMNMNANEIVTKSSLEY

ECY

>SG-PL_contig_1668_1_-2_2_142

AKLMWNLHAFKMSLEFRVCSRRIHCPQTIPYALHPNLPAVIWGNPMR

>SG-PL_contig_1669_1_-3_1104_1316

MELAFFLFLFLCQCGGDGWVLEGVGWGKERVTGQVSMSILFPPDSFVAFLYISSCFEIVS

HPPQEKYRLSI

>SG-PL_contig_1670_1_+2_1151_1315

MFHNTNPPPQPPNPRSVIKGQAAALSRWPCQLSSPQDMDMEMVDRLSTIGTIVDH

>SG-PL_contig_1671_1_-1_1849_2151

MAWKCLSNLSIFFPPLAEVPAVMTLRVPSFKVMKPSTVVEWDTLQACTTVVAVKCETALR

GNYSCWKATHNCQRQAFKLKLLIGKPTAVDREIYNCLQGSQ

>SG-PL_contig_1672_1_+1_1543_1911

MAEVRFRLFVFIPRSQSILYHKQAQHESSCATKHTTALSSSFLPLLFLSPLIYLFLTSFS

PVFQNTSNFQCSGQSDIQSQASSRMDYIEKVKRRNWKKSGAPARRGEMDNPEEMAWLTSK

NSR

>SG-PL_contig_1673_1_-2_56_349

MFFQHNHILHVCCCLFLFFCYNFHDFFFVVVVATFFIFFYLSLSLSATLYCRFHILLPLC

FCYFYFFNVSIGVSHNDNSLLSVVTSPVDGELDLLFTC

>SG-PL_contig_1674_1_+3_297_527

MQIIPKNTGDLENNSRSCKFYQLIGSYTTAQPKYNYIRLINLGVMRYVYFDNLTVVTLKL

CQGHPNFGNRKSDSRHY

>SG-PL_contig_1675_1_-3_195_404

MPRQIQEIKDFLLTARRKDAKSVKIKKNKENVKFKVRCSRYLYTLVIQDREKAEKLRQSL

PPGLAVKELK

>SG-PL_contig_1676_1_-2_2_160

ISSTLCKGLVAYLFELIIAYLFLCWCLVVCDWKHILYVLLCIIVYFTFVCCVS

>SG-PL_contig_1677_1_+3_3_173

YTHTHTRTNIPTQKQKHTHTHTHTQTHVRTHSLASARSKSCSLEKNWMSKPLFNTNR

>SG-PL_contig_1678_1_+2_1286_1534

MLRYNEKMEKLRWDKILPDFVNSMFFRFFDKNNSFLFMLFQRFCTAFPLFKFFDENGCFL

FVCFVHDTSAFLVVIILLTVLVV

>SG-PL_contig_1679_1_+1_313_477

MPADLFFTRMTSDFFFFCYNFLYPIPPKSHESKHIVHDAVLHQWECSTLLHQFFI

>SG-PL_contig_1680_1_-2_2_229

WELSDFWRISDFFSSVFRQNWFVFFFPDQKCRNGRNRFILKRLSNLKWHKSLLRGARYRG

HFSCVNDHESFSFAQQ

>SG-PL_contig_1681_1_-3_3_164

KQHQQQQTHTHTHTHTHTHTHTHTHTHTHVDITENNVQIYVSEHVYDCLHLTKM

>SG-PL_contig_1682_1_+1_1399_1638

MASSVEVTEFVFELTMQERYDLDMAEELGLDQEVLPANDDFGNEKGEGSASDSNHSPRLG

KVFVIFCLAWILECCFHQAS

>SG-PL_contig_1683_1_-1_112_447

MREHQMIHSRPTAAHVCIVITDGQSQETEETAEQARLAQEAGIVTYAVGVGIVGKQLDEQ

ELLNIAGDPARVITADNYAELNLIKEELTDLTCRGILSSLSIKATKFQTSEN

>SG-PL_contig_1684_1_-3_57_227

MTGSNSVACPLGRARSQSLSPRGARLTMQHPHPSKFWDQSPSPSRVRQTSQHPSVSL

>SG-PL_contig_1685_1_+1_1549_2154

MLSKWMSLLSCLECRDHLSLQLDETGKINLVIHARSRLTGASSTWTITFWATKTSSSTRS

TTTTTSSTTATSSPTRVIRKCDPDVCQQLLAVTMSVIILFFLLLTFLLWVFNLFLVCNTL

LPLGVIHSFSSLAYVEGCLHTLLVLESLPFIKRHFFRFLLLLGWGYSLTTFVQVSRFWSW

FNGRRWSPFPRHIRRIGGCTWL

>SG-PL_contig_1686_1_+1_1_141

FFFTSWISLFHRRQFSNNLKQSRNTSLNSTVLHTNLTTQSHQIMKYA

>SG-PL_contig_1687_1_+2_2_61

VCVCGGGGGCDKKLDPFLKL

>SG-PL_contig_1688_1_+2_2_163

GQRWCMHSLSLSLSLSLSLARSLSLSLSLSLSLALIVTVLMDSKSEEVLLTCLD

>SG-PL_contig_1689_1_+3_18_323

MCTSCMHAFMNVYVHVGFLSVQYTPQNVECNKTYRNNKQVGFEFVAQISCAGLPSSKSFL

RVYVLSLSLSLAHTHTHTTCCPTIRKSDTEPQLTHVDNLHSL

>SG-PL_contig_1690_1_-3_81_458

MLVLCPTRELALQIQEEAEKINYKGIKSVCVYGGGSRREQVAAVRKGVEIIVATPGRLND

LIMSDIVDVTSVTYLVLDEADRMLDMGFEPDIRKVLLDIRPDRQTVMTSATWPPDVVRLA

AKYMTK

>SG-PL_contig_1691_1_+1_157_420

MAECVAFTMSQASSSVSESSSLTLHVGRHSLTGSTRRFHVPCLVMKVTRVMLSERTTGIK

EHLSMKKDSLDCNRGTLCTHWTHILMAK

>SG-PL_contig_1692_1_+2_419_583

MQTCYCLNSHVCIRRQFIGFCTTGTQLLMPRVELSLRNGSSTLKTFSCTGAFFFQ

>SG-PL_contig_1693_1_+2_2_124

SALSVLSLSTSLSWDHSSVSKDLSSSSSVSEPAVVTASFTS

>SG-PL_contig_1694_1_-2_491_730

MKIIKRSGAVCLVSFSLNQRAVTFCLTWIESHSIWCIILSQRGLKKVGSRQWAVFNLVGI

KRWLVVEFELRGSLLHYNFS

>SG-PL_contig_1695_1_-3_96_638

MGNVFAGLFKSMFGKKEMRILMVGLDAAGKTTILYKLKLGEIVTTIPTIGFNVETVEYKN

ISFTVWDVGGQDKIRPLWRHYFQNTQGLIFVVDSNDRERVNEAREELTRMLNEDELRDAI

LLVFANKQDLPNAMNAAEITDKLALHNLRSRQWYIQATCATSGDGLYEGLDWLSQTLKKK

V

>SG-PL_contig_1696_1_-1_52_330

MTLPSASSTSVGGGGRGPSKSVTPRSGGGTTARQRKTTTAAPRKAAGSSAGMWRFYTEDS

PGIKVGPVPVLVMSLLFIASVFMLHIWGKYSRS

>SG-PL_contig_1697_1_-3_33_1592

MLVLLLLVALLTMVIRPFRLTVFNVIMVMGYFCLTGDALFGAVITQFDPTQVRSPWLVEP

YSLAIMIAVNAVFLLGIVIFFLIYLLAYRFCCQGRCVLEPLWPVMTAYEYDVEGVETQKY

LAAVLRGRAVIERCRRLPSMFAPVHEMSHQICVLNAYMRESELMHDGLHPTLWAVLDEMI

DMHHRLEPKSLFGEMVQESIRKNAAHFMTLMPMFAQRLAQRDYDFMLVPPYKKRLLLKMY

IIALWLDGSRRRKTHQELTQKVLISIWEESPEVVQVTDEREYREDLYPETIQGPETEALY

MTGTLDLGRGLYDLEAVEEEEESLSTFLKRAPPTLVLKDEDFSLQDAQAGELTGADNPAF

IPDDDKDTSEYHGHSNQQLTTTEVHDTSEAPPDVVVDLEALVKGDNHSEGEATVTFVDSH

TGETNTDMPSEGELEQEDEEPDDGTTRRHNTSVSVISTKSASSAKSGASARSKTSVKSES

AGKSGDVTAASAKSAKSQASTKGSRSKGGKTFKGKQAAKQ

>SG-PL_contig_1698_1_-1_49_192

MILLMEMLLCSFFPPFPPLSCTCARAHTNDCSLPYHYHHCHCCCGCCC

>SG-PL_contig_1699_1_-2_230_529

MISKTTCLPFTIHETVAGKVRCLYLLCKYQEGTCAVDLACGEACCESCPMLNLKVRLKNK

NSSLTVKEIKGQRLYFRLYFAYMLNDILCMLLFIEQTFLS

>SG-PL_contig_1700_1_+2_2_79

KTKKKKKRRRRRQFNIETKATTLTVK

>SG-PL_contig_1701_1_-1_94_1938

MGLFACRALGMKELSPPTIGLKKLYQETIATEPILIIISTGADPSQELQELANETVGHGQ

FHQVAMGQGQAELAMQLLHQCSSSGEWLCLKNLHLVTAWLPSLEKVINTMKPHPDFRLWM

TAESHPKFPTILLQSSIKITYEAPPGVKRNLSRTYETWTQEYISRGGSVVRSQALFALAW

LHAVLQERRMFIPQGWTKFYEFSLADLRAGADIIDRLCVPGKDLQWNFIHGLYEFAIYGG

RVDNPFDMRVLVSYLRQFFESGVINTQSKKRLGPLRMPNSTNIKDYQEVIASLPEFDKPS

YFGLPENIERSAQRIISAQVISQLRILQRGDAKASRFDKEVWATQLGPILNLWKKLNQGT

NLIQMKVAMPTDKSGKQSPTLAFLLLERYNGVKLVQSVHASLAALSKVIRGTQLLTSEVQ

MLAGALMHQETPAGWLSHWDGPEDPIQYLKGLVTRASAIQVWVEKAESGSLFRESLDLSE

LFRPDTFLNAVRQQTARELGCSMDNLKLVSSWRGGIQGSRLPFKLGGIQLEGCSFDGTRL

SENQHDSPSVSSIPPCVVAWVTKDSPDPYGSEDTISLPIYFDITRERIVSRMEVPCGGNM

NQWLQCGAALFLRSQ

>SG-PL_contig_1702_1_-3_192_344

MNSFKLDPFVSLCFLLYFDILLTMWIRVLVNWIYLFILLHYSELVELFLEF

>SG-PL_contig_1703_1_+3_153_242

MNFPNFYKKKPTYVFTIILSENHNICNKNS

>SG-PL_contig_1704_1_-2_2_166

HYIRYKVSPSANHFKPFAGVSNISPQPLSNTVRTRASSSTKCCTQLRFNALHYYM

>SG-PL_contig_1705_1_-1_13_315

MSQIWPKSSLKESQQWRQQATCHKPHILTVFPTSYTTVHGNLSGCVWHRLWPITGPLVLH

GIGWKMQRLHVCDCVLIKDICDILTSIIRAEVLFDCVGLYI

>SG-PL_contig_1706_1_+2_1823_2152

MSYFKCSCTSSSQHNIQINFVQLNTIYNLILFNLKLFLLAVISLNRNALLNKHNDSCIFL

VIKKVKQKKGLKVKSKFSFCVCFPYGRLRPTVQQQTQSQQCYGFTTTIAP

>SG-PL_contig_1707_1_-3_66_16079

MSVAQAVQKGLLDSDTVESLAVSSVSSSTSSNIHIDWDRAQVTDMSSGQTHTIDSAVNQG

LIDKATAMALKAATDVNVSSPNVSQQATVKTTSILRTKTADGVSVKQTNTVVTSGSNASD

DVTSVRYITVPEDRPYTLNDAVATNQYIPQTGQIFDPNSGQPLTVGQALQRGVIDGQKSS

IVDPKTGQKVTLQQAIDKQIFDPHTGQLVHSDSMERITLQEACFEGLIPGTTDSDIQPRS

PVRQKVVKVSVSGLTLEEAVSRGLVDEEDDTFCDPATGNVLPLSTAIRLGLVTDTEASSD

LPKPFSQAHPHVQVERTLPVQYEERAVLRTKSSRQADPDHRLSFAEALDMGYINLQQQQY

TDPSSGTRMAILDAVRNQILDTSTPLDSEQTEGMNITTAVEQGCLDEQRGIFSDPNTGRQ

MTFEEAVKSGKLNPKFTMYEVKTGEIFSVEEGLEEGKIDPRTGKFVDREKNRTVTLQEAA

KLGVLAVVGAPFAAAVAAKDSLSALFDKDNKAGDETDQRQDGFTFQATTVRQTAPNVLEV

TQQQQTIPETERPEAMNLMEAVSSGCLKPKTGLFRDPVQGEDMTLKEAVQRGFIRGNSAT

VVDPKTGQKEPLEQALNDGTMGKTGRVSDPKTGKPKTLEQSIVDGLLQESATTKQSDASS

VFSKTTDKMTVNAVIDPITGNPISLSEAVQRGVVQFDDGSYIDPSCGQKMTIQEAMDSGL

IDATVVDKVTTKEQKTRAGFTADVSFAERKTLKVQSVKDPRTGSSISLSEAVSQGIVDRD

GKHYIDPVTGQMLSMDTAVQKQLIKTSALESELITEEKYRKSQQNTVTQTTSFKIQSVVD

PATSQEMSVSEAVQKGILNVAGGTLHNQQTGETVMVGEALKKGLLKGKETSPSKVEGQTS

TVKSTRNRDSVYDPQKEEYVSWTQAVNRGLVDESASTYHVSESQTMSFSEGEREGFIKTQ

QTAQPLSVKEKRKSLDILSVFDPTIGREITVTEAVSKGLLDLQKGTYIDPTTGKVTPVQQ

AYQKNLIKGQSQKESSGESVQIGEFLVKSAVDTNTGQLINVKDAIRRGIINPKEATFWNL

SKDMRMPVAEGVKQGLVILETEIASPVLTSVVQKSMTITAVVDPATGDELPMAEAISKGV

FDPNQGMVINAQTGQHITVDKAIDLGLISAEVEEGHRAQELVIINGLMITEVKDPRTGES

LTMKQAIEKGILDEKKGLYFDQPSNSYIPVREALKQGLVEGKEAVEAGLSEHKKQDDKHV

SVSQVVDRSTGQEVGFEEAVMRGLVDQHCTIYKDPITGEKMNIEDAMKQGLVAGTVKTFT

KTQTTITQRKPLGTYSITGMKDTKTGKDISVEEAVQRGILDPEGKVMDTETGRPVPLADA

IKQGMVFTEKVERSLSGISASYAADRQGAGIPFKEAMDRGFIDSSTGTFLEPSSGRRYTV

DEAVRTGLLVSASNKPFQYKGLGQQGMTHSFQSALSMGLIDPETCLFLDSNTGRKISLEA

ALSKGYLSPIAGKFGGKAVGESVVMLKGGVSPSVTAQLDEFVDPKSGEKLSMIQAESRGL

VSVKKSDDQIDISEALQSGLVDKDTGLFHDPVSGDAIPLDEALLCGFVSFKDSTNGHQEE

ENRPVIEHIQKVGPSRLSMSTAVASGLIDPEHGTYTDPNSGQTISVIQALQHGIIRPTLS

VAESTPPSSDHKPTTPSHQYVQITEGSPFTPKHGTVVDKDGHLISKTFTEVTTTSGSATY

TSKPGFSIDSSGQVINTATGDRMSIAQAVAAGIVDSEAEQAMSDIQVMGGGDLPSPDRSS

SASPSVSTAAGSGKVPTEKERPDSSVSFEPRSPAADRKGRLADSFDNMLQELAAEQDQLA

DFERVLKKDSQMGDEAYKVRAQLESHKGVHDEIMSKQQPILSLIYKAEQLTENYQEELTP

EQVTELTTQSTLLKAALEKITRSSDRRLSHLNKAAEELGKLEDEMSNFQKWMTSANSELA

RQEECLQRFEDLKPLAEKQKELSSDVVSHQADLRFMSMAVQKYMEEAKLYKLEIDSFRAD

RVRPARHSLISMECVAADDVKDRLKDVTEEYHDLSSRCHSLGDRLADLSGKHRQFNDAAF

KLLDWLTETEGQLASLKQEAGSPEPAQLHAQLDRLKSLSMDAVSQRGQLDEMQRRGQDLT

NSLSGQATDKQEVAKLQGVMNDISTRYTALTNDIGARTTGLQAAITQSQDVTQAMDGLLN

WLDSAEQAITSQTPVSLHRPVLNSQLQGFSVLQADITNHQSALEAVRASANELIKTCDLE

IAKAVEQRLATVEKKFSDVQAHTEERDHDLEDIDQGLREFKDKLETCTTWVQGGIQRLDS

KDLAKLPSEDMTEQLELLTAEKKSQEIHVADLRAVADRLINDPRTGDASAVQETVVELER

NLAAFDSLLAARHQEADVKEQQGTAFESAKTLAVLWLSQMEARLDEFDAVAIEVDIVEQQ

IVQLQPMLQEYEDYAPKIDEVNDLGNTYEAMINPNERPMSPIRRLGRSRRLPGILSPRLR

TPSPTFPASPTAHRQSPLSSESSGVSSRKSSADNLLLDDLSESQQQLLDINQRYEIVGER

LADRQQELQLMLTSIRTFLQDLQDVLQWLDDRDKQTLTYQPLPTNEKEAKKRLKEHEAFH

RELLSKEGLVEDIRKKAQELLKTRRGVPGEESLQQQLQELDDKWHGLRASSEQRRKSLED

MVSDLRDLREHEEQLMRWLGQKDRMLDVLGPVAMEPAMLASQLEQVKVLKEEFAAQEPIY

DQFLNCGHAILDRCDPASQDSKAISRKLDTVSKAWNRLQSRLDERTKSLSSVEGISTEFA

ALTRGLADWLSDFTDRLDGLSHISSQPDKQEQQLAEVMELESDLMEQQPRLKRARDLCRQ

LCDKAKDPSTKADLRSKLAALEKDLGDTTKKLEARKAAVEAASRDAAQFKADCHDLLAWV

NSATDRLQEAEAISSDPNTLRQQTRQSRDLQQELSIKELDIKELLGKGEKLLQDSSPSAE

VRAMADTLGELQGEWEHLKQEAAEREARLTTADSHAQHFSEHLDKLVLWLTMTEEKLDKL

KPDDLDRRDIEQTLKDLQGVETDLQKKSHALDKLRHDGQSLMDSVDSGKDDIDGQLKAFN

DRWDKVNNAVTDRSAHLEDLSQRLAEVQDTLAEANSALTKWEDKLAAHSSLGSTAKDPKY

IDKIKALLEDSGWLDTQLGTADSLMDGVEADGGNASDLRAEKGKLADRHQTLQDELGELV

ADMETGSQIVEQFQGLLKIVGGQLSELESELDTKSPISRDDAELDKQNSDMKEFLARLDD

KIENMDDLQHQAGDLGSAGYVSDPDALRSQVDTLCKQHATLKAKAGQRQKDIDSTQHALE

QLTEALNAARQGITKAAADVDAMGPTAGEVSAIKALQDELKGFVKSTVDPLQKQLDVVIS

QGQGLIKSAPPGSNTSGLETDLESLTDRWADLSEKVSEREKNLDSALLHAGKFQDAMASL

LTWMTETEEMVANQKPPSPEQRVVKAQLQEQKLVQKMVSDRAPNVQAVKESGSQLLKGKD

PKERKKIEGELKQLEERWDALNKKVFDRAAMLEEVQSLANQFTEVLDPLSTWLDATDKEF

AALEPQSADAEGIEKLIGELQKLQDETGERDPSVKQLAATGKDLQDFCKGEDVILVQMKI

DDAQKRFGELRSRVEDSLEQMEEALPLAQHFQEAHQGFLAWANKFEPELRSMEHSLPESE

EAVEELLNQLTEVQPWLDVINSEGAELSEVTPGDASLRVEDLIHKDNKRFDNLREEIEKR

AEKAKLVRQKSMEVTGELSELLDWFVETDEKLKNPSPVTANPDALHKQLAQQKAMNEDIA

AQKSKTRDALSSGKKLLNDHSLDDESVLRTKMDDLKQTSDSVSATANERLSTLEQALPLA

RHFREAHTDLVTWLDEVEPAVAQLDVPSVDADQVKKQQEEAKVLKQEVQDRKPAVDRLNK

TGAALASLCDPEGANEVNAMLDDDNRRMDEIRTRVRDRSNSIDLAMQQSAEFSDKLENML

DNLTVTAEQVKHAEPISAHPDKLREQLEETKAIEEDMEMRQAALDSVKSAAEELLKQAGN

DQDEAVRDVRQKLDELTQLFKDIQDMGRGRQRALDDTLGVAEKFWDDLNTLNSTLKDLQD

NLNSAEKPALEPEAIREQQEELEALKEDIEASQADLEEVQQTGDMLMGMVGETEQPEVQK

TMEDMGANLAAISDQYNKRSNELESALVRAVHFQEELMKLLVWLQGAEEKMADFQPVASE

FDTIKKQWEELKLFKAGVDPKHMEVESLNQQATELTKDSSPEQAVVLKEPLTLLNTRWGV

LLNNIGDRQRDLQMALLSAGQFDHAFKVLRNWMDTVATTLEEVEPVYGDPKVVEIELAKL

RIVQNDITAHQESVDSIGEEAQRLMTVEGASEAQGLKAKMEDMNATWDDIHNKSRQKQDM

LEDALREAQGFTGELQDVLMKINDLESQLITSKPVGGLPETAKEQLDRFMDVYGALEKLE

PDVESLTLMGEKLSGKSSGPALANIKQNLTNLHQRWDHVRNRANDRKKKLEDAVNKAGNF

HEEHNKFIAWLTETEKILNNLHPVSRLVDKVTTQIEDHKVLQKDISSHREAMVALDKLGT

HLKYFSQKQDVILIKNLLSSVQHRWEKIVSRSAERTRHLERGYKEARQFHDTWKDLIMWL

VEAEQALEAETAIGNDPDKIKTQILKHKEFQRRLGSKQPVYDGVNKAGRTLKDRCPNDDM

PTIQAMLSELKTRWNSVCSKSVDRQRKLEEGLLLSGQFNEAMDALLEWLAKVEPALAEDI

PVHGDLDTVSSFLDTHKTFQQELGARATTVAFVQKSAKEIISKAEGDTSNLQSDLIELNS

AWDRACKLSVSKQERLEQAHKLADEFHKKAHQLLDWLSDAERQLRYKGPIPDEEPLILQQ

IEEHKTFEESLLRQEANLRETLNIGQDIMKRCHPDAVPVMKQWLSVLRARWEELTSLGRQ

RNQRLVNGLTDLRHSNHLLEELLAWLNGAEVTLTDLDRQPIPEDLAIIQQLLKEHQDFQN

EMSSRQPDVDRLIKADKRRTSVSQEPISHIPVLRASTPRTPTRRIPVRVGGSGRNTPDRF

GRHTPEHYGRRTPDVYSGRQTPDPTGRRTPEPQFRNPRVGALFNKWRNVWLMAMERQRRL

QDALDHLNEIERLKNFEFEDWRKRYLRWMHHNKARIMDFFRRQDRDRDGRVTRKEFIEGI

LSSKFPTSKLEMEKVADIFDKDRDGFINYKEFVAALRPDRDPKPATTEEMIQDEVRRQVC

KCTCVKQFKIHKIGEGKYRFGDSQKLRLVRILRSTVMVRVGGGWVALDEFLVKNDPCR

>SG-PL_contig_1708_1_+2_347_526

MRNQKSLNFAWNCASRNGEFSNFCLSQAVTLITEVHMICAGGVCIMPQSEIQYLSVGYCQ

>SG-PL_contig_1709_1_+1_343_552

MLLPSSVADCSHQVSIKLHGPKPSTAPKREIIIQVEEGEKPLEPIRKEFGIGRVKRSAVQ

VLRSASEKTF

>SG-PL_contig_1710_1_+2_2_211

TENKLYKDNKLTLVIMVPWLSWYHTLIQIISSNNAAFNNHTHKTSWLMMLPITNQRGKCV

HLNQHLTTET

>SG-PL_contig_1711_1_-1_169_615

MRSLVMAIFLYACESWTLKADLEKRIKAVEMRCYRRLLGISYRDHITNEEVKRRIENEIG

PYTDLLTSVKRRKLKWYGHVTRSSGLAKTILQGTVRGGRRRGRQRKRWENNITEWTGLEL

RDVLRRAECRDEWRGLVARATEVPLRTGV

>SG-PL_contig_1712_1_+2_197_409

MDKIRVQKKNLKKQMKTALPAEKQGLEKLWKDLKAKHSALSKAESARKRKSKKKKTQERF

FREPFQFARTL

>SG-PL_contig_1713_1_-3_12_1391

MASALRKVAPALLQRAVWKSSSPLFFARGAGKVWMPDKEYYDKFNLPVMYPDESTSSWKL

PPFNEHAVVREKQVENITLNFGPQHPAAHGVLRLVLTLDGETVVRADPHIGLLHRGTEKL

IEYKTYTQALPYFDRLDYVSMMCNEQCYSLAVEKLLNIDIPPRAKYIRTLFGEITRLLNH

IMAVGTHALDIGAMTPFFWLFEEREKLMEYYERVSGARLHAAYVRPGGVALDMPVGLMDD

IYEFIEKFGQRLDEVDDLLTENRIWKARTVDIGVISAEDAMNYGFSGVMLRGSGIQWDIR

KVQPYDAYDQVDFDVPIGRNGDCYDRYLIRMEEMRQSLRIVLQCLNKMPPGEVKVDDNKI

TPPARKEMKDSMEALIHHFKLFTEGYQVPPGSTYTAIEAPKGEFGVYLVSDGTNKPYRCK

IKAPGFAHLAGLDKVSRKHMLADVVAIIGTLDIVFGEVDR

>SG-PL_contig_1714_1_+1_1042_1257

MCMCVGQEGGGRGIYMCMRHTKRMCVCVCVRARARARASTCTSVHVSHVYNSCVYSDFVV

IDKNLPSFLPLP

>SG-PL_contig_1715_1_-3_12_290

MGRYSGKTCRLLSMLLLTGSFFLVEIIVGYVTNSIALVADSFHMLSDVIALIVGFASIRI

AKWPSRKNTFGWVRAEILGALVNAVFLLALCFS

>SG-PL_contig_1716_1_+2_2_148

ERDFRLQKFYYSRTALGPNGSVLQLVFFEERESESRKIEKKAKAAFEGV

>SG-PL_contig_1717_1_-2_431_892

MLDRFGVYREEMQNVLCVTCTNWGLCRRGARLGIKLLSMVYIHFFFSFHVSNLVGRSDNH

VDMINKTTAFLLECVSSLLSVYMCLSRRLFGHFDCFWLFWRYIVQSLNLFCKHCVEFNLM

KNRSLQVCRLDFKCPVLPILFAIKPRVFYSSTSG

>SG-PL_contig_1718_1_+1_199_408

MTAIHNKNSQHTHLQQQKSKSAALAFCNCCKNTTGTNGKHRTMLKNTKSSMTDNTMACVF

LLVLELMQNI

>SG-PL_contig_1719_1_-2_980_1189

MSVLFSMCMLIRGVISYFTVIYICSIVYRFTFIMCHISTYDGSAIPIGNGKGMNTVGEYF

RLKENTKKKD

>SG-PL_contig_1720_1_-2_107_418

MGLMLLFIVSLMCGREKGKKSVSPFYLNFPPATWSLLRDRNAKHLVKLKSGRLPLSNKVY

VQGNPITWRRNQVMLSCVIIVKNHSADPGTLKFTCGGNMALAMS

>SG-PL_contig_1721_1_+3_3_77

VFHMNKCRPEKNFQSNSIKTHFRLP

>SG-PL_contig_1722_1_+3_258_500

MMDHFPNMASNASGVSLTLITQARLILPRITQHISPQVHRTYSFYKLVKSLTRNSPTPTS

VHTLCSNDRNIDAIPMGSGKD

>SG-PL_contig_1723_1_+3_66_311

MAVSQVFVKHFDRHHLSAFLLPAQVHNSSGPTPHGVRSVPGVVMGGLQQALQNWIVRHIG

LQSLTADIDLQADRHLSSSRQA

>SG-PL_contig_1724_1_+2_2_187

PSTKTVPLIDQHVLSCKIIQSKHSNSFTFSLTISVNILFFFFPCALFSAVDLNNTNTSNN

EY

>SG-PL_contig_1725_1_-1_64_4593

MGNSCIFCALKVIFTQFQYSDKSSLHPDALRKALAETFANQQRFQLGHMDDAAECFENIL

RRLHFHVANNFNEDTCSAAHCLPHQKFSMAVQEKVVCVCGASTSPLKFTEMVHYVSASAL

IAQSRQKQGREEITHPDHFGMLLRKASAMGDVRNCPANCGKQVQKQRTLMNIPDVVSVGL

VWDSDRPSVQTTTEVARVLGTTILLPDMYHHVMPDPSKLPHLQLVAVVCYYGKHYSTFVF

HSKLKTWIYFDDATVREIGPLWSNVVDKCSRGRFQPLLLLYANDNATPISVDTAPQKRVM

APGYTPAGTAEEGNSGLDRTHESIKSLPSSSRRSTTPTPKPSQQSVSRVQPHISPQDTGR

ESPARLTRDHIRQPSFLSAVSKERSKQGESGEGVGILPGGAAYKSSSRRQLLDGGANSGR

LETMLEDGGDYPDYTLSSGDQYRRPSSVSVDTRKPLPVPPNTGRNIDDQRKVTCKKDRGG

VDVVRYQVDPARGTPDFTMHQPAGQGICGKGHVRPSAHPGPERCVTEKGVGVSAGGQHVV

VKPTPTAHAHHDTADTSSHTPVQSGLATLPRKGHNHYSEPSGHHQSGSTHREMAHPTHRN

LRSCSPLDRCLPPGTPPPEYDSVLGDPHLSHSRQASTSSQASSMRSSVSMGNLHQLASGK

LSDPEQVRNIRKDAENQRSEKAMKKSESESEVLGHTSKQYIDRRMVETILKHQGVHRHGS

SCSNSSVDSDSVMGRLIANRSATTKLRVDIPADTVSLGSQKDSGCGSSDRHSSSSTGSGS

IDPFPQCFFNKSMIVPRSFNTNHAPDQQYVQLTKGPSGSHGSASSTSQMGSNHGNGPKVM

HPIKESSQEDVLKSSPNEPIGTQKFILANPSASEARPPPVPPKNFAQFQIIKSSHSLSSA

PHERESSLESLVKDESINRNFVSLCKKADALMDKCTVAETGQDMTSALYCSEEALGCLKE

AMTHKELNSKARAYLQKKHNSCLLKVRTLQKRAPASRQESHSSGIGSDVTSSVGNRSSIT

SSDSDLSDQIHRHSRQAGRPGPSPAHRPLPPHPTTSNTTTTTTMLDKARDTCGQGTVAVR

QGQTMDCKTSYHGGMAGQSAYCDSEQSRTSQTQPSGRNHLRTDSSSSRVSQSHYRTDSSG

SNLSQIQHRTDNTGGSCVPQSHSANYVSLSSISGGDSSADLYGTLPRNKSRKPGSETTAS

AEMYRSFLNRQKSLQAEVTSSQQSLDSVRSDAGSDVSNKMNKSQIRQMLQDSIVQRQWHQ

SGAGQPNSTQQSSQQSGTVQHSNHPQRQESRQAGQPSPRPGMNAGIHGHSALQQQQTLHP

RPTHLQPQQLNLSNHPSQHQQRQPPPAGYSHPQTAGQTFSMPTTSSQPVVSAPGPKASSS

NPQSAGRPRTPSQTSTSSALSISTTSHNSGFPPRSNSEMGKRSTPQNVARVTPYSHLLDA

PSSRGGGSSYLVMIPPSQPNHPSSAPRGSQAPHAAGTARPLTPASRPLETSTSQSDCHRF

RDSAGPSDGS

>SG-PL_contig_1726_1_+2_473_634

MADEHIKTFKNYLPKRQKGRKRTQSFTTECNSERVKPGQSFQSWSEQEGGLDVT

>SG-PL_contig_1727_1_-3_3_212

EYQRKVERSLTCIFDILRAYTYKWCSETCRSAHMLTQELGKISYTEAGSNPDLPIESPDY

LATAPVGILV

>SG-PL_contig_1728_1_-3_180_362

MSAVVHKQQQLMTELPKISISRLPTSYIRNHMKINLTFKLTPFVNFIYIFLKVLRQCLYF

I

>SG-PL_contig_1729_1_+3_306_2279

MISSPIYRALLLAVAMLLLTNQGQCWKCHKDETVCEIWLELEHGLTMMKGKSLITLKDGL

LFPYDTVNLTRENAIPKEQVITGDGFPEPMVVIMVNGTVPGPVIEVYENQTVRVHLHNKM

ASESTSLHFHGQVQNSTPWMDGVAFVSQCPILPGQTFIHQFKAEPAGTYWYHSHSGGQTS

MGMFGGLVVHPRSRPNEPRYHDDFTLLIMDWNHDWSDTMTFMKMQYGMYVKGKKEGAASN

LSGAHYSMFRIQSALINGRGRYTDPETSLHNGAPLEVFTVRRWNLYRFRLINSGSVFPFK

LSVDGHKLKVVATDGMELDTPVMADSIIITPGERYDFELTASDVAANYWIRAKSLERDVN

HAAEAILHYEGAPNNTDPTSRERDCTEDEPCTVINCPFHHYPASAHTKCINVEKLRASEK

MPHHIGAGATNVKEYFLNWAFPGTTWTPASVNGIQMELPHVSALTQPLEFATRCPNEGYK

CTEDQVCRCTAVLDLHGGDVVQMVLTNQGRGKGWDHPIHMHGHSFFVLKIGYPEYDNVTG

KFIRDNMDINCRGNSDRDLSYCNNATWTNSSWSGNSVPGLELEKPMLKDTVNVPTGGYVV

IRIQANNPGVWPMHCHVSLHLSDGMFLLLNESYPNHPPPPPGFRQCGDFSVPYPDVHH

>SG-PL_contig_1730_1_-3_153_1919

MNYNSVPRQQASVPPLEERSQLDELLSDLLSDQVIVTSPRSPSGPWSPQSPSSPQQNTMK

GAKTVTTTTHTYHTYSGTDSHAGRSPADKVLIQRAEVTYKVPGVGEEKQHYTIAGEPPEL

EKPRSPENAYSYSHSSSSQIDQSRRFQSLERDMNRSEQIMGMQQNPPTRTQTLPYRTDYE

NRYAESQRYNTLDSDQPSWLQEQQQKLQRKRDGRDASGRTEQEKQLVSELRFAQNRYYNQ

RTASEAEEKAAMDPYRQPPMHNGPLTSSTLPANYAPPPQGFGGEVGHFSTYSQSQYQEYR

SGGDRSNNKPPPSPTFQRSAAPSSSMPVQPPARSSSRDYMKSRSHSSTSNLWQSSPPPTS

HSLERQYSESVLDRDHSVGVPAPRRVASSQSTPPHSPGTRSPPHMGPTATTTYTTYRTIH

KEVRDDPLGPAPVQPAMAAAPVQAQAAPPQTQPPVNPQSQHNYITEVFVHRVGGEKQGHD

KQDGSEDTRLDELTRSLQAATESIMEDRMSISKQQKQQQSFQQSSSWQEHYQSQQQQSQV

QKAQQEGPPPKPVNVIEEDRITLRPIGPGMQTLEPENTHSTSTLSRAAT

>SG-PL_contig_1731_1_-3_93_365

MAARRYTAEEVARILLELPLNSDNEIVIGNKGDVVLDSVDQSDNESSDSDYPDEDYILPS

ISPGRGHGRVRSGCSGRLIVSYLILSTLNIS

>SG-PL_contig_1732_1_-2_2_331

AFPSSDDALTLWHTHFLWRWRSQSGPFGSRQSQARTPLEPELPLPASPSWSSFGRLLEQG

SRSRRLDDPWAGTPPSPPACRSGVPSSFAVRKVGWTEDFPSGYNPLHRGN

>SG-PL_contig_1733_1_-1_1_96

SSSTVLVLLLPFCIFSQVYLQIDHVLSRSYVI

>SG-PL_contig_1734_1_-3_312_1346

MDSIRLGYDSKVYHMEQGEPCGDSPPPPPQPLSLQSTPPPPQPILGTESGGKNSVEAPPA

WTSRHGDQLAECQGCIAEQRIEHTCTDANPNHNHHQHHHPSRCPDHDHQFVDVQRKTDNI

DAHQLDVQETAQDLELESEIALSEQKPRLGRNTWPRKKVRQLRHAADIFDCDEDGGEDPG

EIMVESGSNYFVSVDDSVCDTGGRLVVEYSLSRTGSVHRIPRNSKNPLPPELETVKKGTP

LYGQPSWWGEDTDICEKDTMDLPQKADSKSPRPSSLVVQSDDSQRHHHQHKDSKAIYMEI

PHTATTPERDKPLSGHHRDKVTPARSISSLSKDSLVVSPDSARER

>SG-PL_contig_1735_1_+2_500_664

MVRHSLGESMGKALSTSSKPKQAFLWPWSKALSVNDTSEMLRPTLNCCYALQCYV

>SG-PL_contig_1736_1_-3_873_1718

MTGMPLLQRTGTCASTVTWIQPHKPPVISATGAAGADFITKWKALSRIPESLLKKRKQRS

EAKEKALKAAEINKKKQKAKKGIIFRRAEKYVREYRARENDLLRLKRQARKTGNFYVEPE

PKLAFVLRIRGINGVHPRPRKVLQLFRLRQINNGVFVKLNKATLSMLKIAEPYITWGVPN

LKTVRELIYKRGYGKVERQRVPLTDNAIVEKQLGEKDILCMEDLIHEVFSVGPNFKAAAS

FLWPFKLNTPNGGWRRKYNHFNDGGDFGFRDDKINALLRRMI

>SG-PL_contig_1737_1_-3_918_1139

MHCVIHSLFICIVVWGLFQKKNSIPTFWKFCASDFLFSIALFVSAPLPSFTVICFFLPCR

SIPLSFFQQPFPNF

>SG-PL_contig_1738_1_+2_32_298

MHALTHRVSAHSFHHFTDKSIMYVCEHLQTHPVNNKHHKKSYHTLKVEKHIHQNQHNTLV

TPGQYPLKAVKENCSKQYRNKTGYGGQTG

>SG-PL_contig_1739_1_+2_2_160

YQHRSCKNAPTHFKAFLHSKTVFTGPLTFLSLSLSLCFLSLFCSTTCEGAFYV

>SG-PL_contig_1740_1_+1_661_888

MHNQLYPHNHIYIKRTKISGTRNRLYPHNHIVLLLLFAFPNSGGHPTHMHSVPRERDHEH

TRMRRLRAWQPPSTSP

>SG-PL_contig_1741_1_+1_1_192

CVSVCPSVCLSVRPSVCLLFLSFVIYSSGEVRKRGRGEVCCRLCLHACVRCVSFFCSSVQ

GDTT

>SG-PL_contig_1742_1_-1_175_546

MRDLDEVMSLGQDARLDVVRETMTVQGSRLFKMQESYKGTEIPEGIVTVETDQSGNHVMD

VTGTVFRNIHLDMKEDVHLSPDAAFDMCLQHFGDDHKRNSLSHRSVKAEVMFDNNNGAFP

GYQT

>SG-PL_contig_1743_1_+1_1_186

FFLHVIYLFTFLQTWCTLFFIQNCFHFKWNESKNTPHEHHFCPLIKHSINGNNIHTQNIK

ST

>SG-PL_contig_1744_1_-1_478_1002

MNLSKMEYIDMRLIHRYEAISVSLYRSIYIADLHGPQFCTLHTSSPFPLSFPDQWIYPQH

QHTHTHTHTHTHTHTHTHTLSLPPAHARTRARAHTHTHTHTLFLSLLHMRARARTHTHTH

THSLSLSHPHPPHLLSLSLFVCLSVCLFLTHTHTHVYVRTHARARTHARTHEHTH

>SG-PL_contig_1745_1_-2_2_199

MHLGAALNISFIEVELLIGCGGFTAYQFLTNSECYKMFFSSEGMGAKVHVREGKNPDHQL

RSPNIC

>SG-PL_contig_1746_1_+2_833_1144

MCSCQHHTSVSHHSVGHEVCCSAERFHKVHNFTQAGWLFGRAMDSRSHVSSSVPAHNIKV

GLFTVHLNAPIILVVSAYLLFVVCVPQLNTLGVNQYICTLSYES

>SG-PL_contig_1747_1_-1_1_204

FHRLHKIVSKSTAETPFRFLARALNLHSRLLLSLLTAGSKPTNHNTIKGKKQKSRMAKLL

LSCMIHLP

>SG-PL_contig_1748_1_+2_2_397

HPHLSSSSRYKRRAERRDQESPDHSLLEERYPQEGWIRAYTDGSATDAVKRGGAGVYIQF

QNGQRQAAAIPTGLHCTNYRAKVEALVHAANIINNAADCDSQIVFLTDAMSVLEATTASK

LQNISCTRQVLQ

>SG-PL_contig_1749_1_-3_939_1223

MPWMVNYILFISTPIIVIYFMYTLLFIYHHPFFILFICLLLTNIFLITVAVRPPNFRVMV

IITAHCLVVKSSKTLQICITVIDFLLFSLCLISFL

>SG-PL_contig_1750_1_+1_97_252

MDLEKDYDTLLETVIYRMSLNLIGNCENLNFSASTLRRTIYSFKCKLKCTRT

>SG-PL_contig_1751_1_-3_24_755

MLFKLTNRQTNRETHCGVLEFVADEGRIYIPYWMMRNLLLDEGELVQVENVSLSVATFAK

FQPQSPDFLDITNPKAVLENALRNFACLSQGDVVAIKYNDKVYELCVLETKPGKAVSIIE

CDMNVDFAAPVGYQEPQRQSREEGASETDGAPMDTHNVDEAEFRPFIGQGNRLDGKKKGT

ESNPQASAGGQQRRGIPNYNYKKGTITFIRNSRPVANGNSETNGDKNFEAFSGQGQSLRR

KTRK

>SG-PL_contig_1752_1_-1_1_603

TTTTPTTTTTITPTTVAAPTTTTTPTTTTTPTTTTSPTTTTTPTITTTPTTITTPTTTTT

AAPPPPTVSTTQPTTTTATATTTTTPTTATTPTTTTIPTTTTTHSTTTTPTTTTTPTTSN

TPTTTTHTTTTTAAPPPPVSTTQPTTTTTSPTTTTTPTTATTPTTTTTPTTTTTPTMTAT

PTTTPTAAPPPPTASTTQPTT

>SG-PL_contig_1753_1_-3_3_1097

ATTPTTATTPTTSTAPTTTTTLTTTTTRTTATTPTTANTPTTATTATTATTPTTASTPIS

TTTPTTTTTPTTKTTPTTATTPTTTAVPTTTTTTAAPPPPTVSTTQPTTTATSTTATIPI

STTTPTTTTTPTTATTPAITTLTTAITPTSTANTPTTATTPTTAATPTTTTIPTTTITVT

TATTTTAAPPPPTVSTTQPTTTITTTTTTTPTTATKPTTASTPTTATTPTTAITPTSTTT

PTTATLPTTTTTPTTATTPTTTTTPTTVTSPTTTTTVPTTTITTAAPPPPTVSTAQPTTT

ATPTTTTTPTTTTTPTTATTPTTATTPISTTTPTTTTAPTTATTPTTTTTSTTATSPTTT

TTVPT

>SG-PL_contig_1754_1_+1_1_225

VGVVVVVGVVVVVGCVVLTVGGGGAAVVVVVAGTVVVVVGVVAVVGVVAVVSVVAVVSVV

AVVGVVAVVGVVAVV

>SG-PL_contig_1755_1_-2_2_661

YHCHYTYCHHNTYHYHYTYYHRHYTYHHHYPYHHHNTYHYHYTYYHHYTYYHYYTYHHHY

CPYYCNHNCCSSTSYCKYNTTHHHLYTYHHHYTYYHCTYHHHYAHHHHYTNHHHYTYYHY

YTYYHHYTYNHHYTYYHYSYHCHYTYHCHYTYYHHYTYFHRHYTYCHRHYTYHHHYTYHH

HNTYHYHYTYYHHYTYYHHYTCHHYYTYHHHYTYHHHNTY

>SG-PL_contig_1756_1_-1_1_231

LFVCFVCVCLNSRIADNQNSLCLLRSCYFTRFGIFCYLLFCCVLFVCFFLELLIFRAAYV

YYSRIILNSTTIYRQVN

>SG-PL_contig_1757_1_+2_2_163

FFFNNPSLKTDLSINPSNVTYSVTFSYWPSATGFIFSRPDQLFTAQCSNNNFAV

>SG-PL_contig_1758_1_-2_5_778

MAGKQQADDLVTYVTNGLQNGDPTIIGIAVAVVVIFLTIVFFAVKSRSGNKRQGILLMGC

CESGKTLLFSKLVYKQYKQTLTSLAANTGSVTLPAVGKSLRLIDLPGHDRLRTQLLDKNK

GLARGIIFVVDSGTVQKEVKDVAEYLYTVLTDNVISHNAPPLLIACNKQDLTLSKGAGVI

QKMLEKEMNTLRVTQSAALRSTDNTGNNNTFLGKRDKDFTFADIKPVRVEFVECSARGKD

ENSEGDLKPVLDWMTRVA

>SG-PL_contig_1759_1_-2_122_940

MTEQQLDCALDLMRRLPPQQIEKNLSDLIDLVPSLCEDLLSSVDQPLKIARDKTVGRDYL

LCDYNRDGDSYRSPWSNTYDPPLDDGTMPSERLRRLEVEANGAFDQYREMYFEGGVSSVY

LWDLDHGFAGVILIKKAGDGSRKIKGCWDSIHVIEVQEKSSGKSAHYKLTSTVMLWLQTS

REGSGTMNLGGSLTRQVAHDASVNDATPHIVNIGRMVEEMENKIRNTLNEIYFGKTKDIV

NGLRSMTPLESRVKQEALRNDLVAALSRRPQAQ

>SG-PL_contig_1760_1_-2_5_511

MYMLSKLKYLTDMNARQMFYNAHIRSHIDYASTVWDGSSDIHLKRLNSLHRRAMKLIIPD

TTLTTDEKLKKLGILPLSSHFMFNKSIMMFKVANKSVPQYLLNLFNPRALSYSSSRQGHF

QIAHPRIDMYKCSLSFSGASLWNSLPQNIITTKSLPSFKENLFKYLMSI

>SG-PL_contig_1761_1_-3_6_983

MQLGILDTCIITEELAFGCTGIQTAIEANSLGQMPVIIAGNEAQQKKYLGRLTEEPMMCS

YCVTEPGAGSDVAAVRMKAVKKGDKYILNGQKMWITNGGHANWFFVLARTGSDPKVPASK

AFTGFIVDGDSPGLTREPKEWNMGQRASDTRGITFEDVEVPAENVLGQEGDGFKVAMGAF

DKTRPPVAAGAVGLAQRALEEATRYSMERKTMGKAICEHQAVAFMLADMAIGIESARLVT

MRSAWEVDQGRRNTYYASIAKALAGDVANKCATDAVQIFGGNGFNSEYPVEKLMRDAKIY

QIYEGTAQIQRMIVSREIVTKAKQRM

>SG-PL_contig_1762_1_-2_26_496

MMVVIAVIVNSILIGMGDLVHRMVPGLSDMLTVVLVVVVEHAILLIKYAVEYAIPHVPHE

VATEMAKVEFQRREALKRLETQGGSTEKSPRTRTPESPTTSSPHSPTSPVPIPRRTPQST

TATTVSSSRDSRQSLVGHRPQNFSANNSQVSYMDETS

>SG-PL_contig_1763_1_+2_428_679

MNILTMNKTNNTKLIHPLIVSNATPFKFITVSNHSQMKAKPERIKQRLLDLVIRLYVNKF

NIQQCEYIQAISLHCQTQNLKHRP

>SG-PL_contig_1764_1_+2_23_283

MDYESSLTSSPSTCEWGYLTLSWIGAGNAGPEKSWSLLLHSIMILGLQDNLCLLQLTKGC

TKGLKKEEAWGEIVQRAEWERLYRGLS

>SG-PL_contig_1765_1_-2_203_349

MIHNRYFYQRTMCSHSLESKPQTSSHNSSQKLFLEIIAQDCIIVYVCAH

>SG-PL_contig_1766_1_-2_71_394

MSYQLYRNTTLGHTLQESLDELIQMGQISPQLALKVLLQFDKAINTALSQRVKSKVSFKG

KLNTYRFCDNVWTFVLNNVDFREAPATELATVEKVKIVACDGKSENKS

>SG-PL_contig_1767_1_+3_3_101

ENCALNNASMLIDGISSIYEMYNHWNTNRFLTY

>SG-PL_contig_1768_1_+3_225_458

MIIYLYTPGNEQSACVYYHNNIYLHTLLMNNQVCLINTIHSFRFVISTMAIYGHVKLGPK

HRMTYQQTWNACGMKTVS

>SG-PL_contig_1769_1_-1_712_1068

MRLLAQTGHGPSRSTCPLLLAGRYRGLGSGLARLTAHKSRVVLVGGGAWLCLPTAQVMPQ

PCVAPPFSLLRGGAKGSTSEWCVLLGPKGPSSPPAGFCWAVVKTVVVSPPSRLGSQGGG

>SG-PL_contig_1770_1_-2_80_1276

MALIVSRAISAVQPHARQFLFVHAGRAQAISVPCMTQVANLTTTHGEPAKRKYPAWPYKT

RPYNSLYQFFDHTRKRFDDNTKIILIEGNIAVGKSWFGQQVAKEFDMKFVPDVKDEKIFL

KGNNFDLRSLNEQLPARSGFCDIETFYSQDGPAALLKTFGRTQLTMYYYHFHYYVSALEH

LLNTGQGIVMERGVFSHMVFHNVLRRMGHMSASALKYLNFVYDNTVCEMWRPHLVIYLDA

PLDFVRTMISKRNVGWEAKSPIITDEFLTTMDKVYKEKYLPYMSKYSEVITYDMTDLPDW

EIIVEELESLDLDNQPFDNEDKFKDWQSEFEDDYNLMRMNIAKKWQIDNLFSMAKPWEAP

ELLIHPDDADVVCQVVREHPEVKYRAGYNPETTNVLFKW

>SG-PL_contig_1771_1_-2_101_199

MKVSGIIKERKQKYSPPPPAMLGLLYHHEFQCT

>SG-PL_contig_1772_1_-1_115_486

MDFDGLVQAETALGTAAVIVMNQSADIVRCISRLIDFYKHESCGQCTPCREGVTWMMKIM

HRFSSGNARADEIDMLWELSKQIEGHTICALGDGAAWPVQGLIRHFRPELEKRMKEFEQK

AATN

>SG-PL_contig_1773_1_-3_135_887

MAAKETPNSKKVSDFIENFKKQTEALVTDIFPQKVLDIEEIYQKMIAEKLSDVHTSVNIP

IPEPALVLNDDEEPSSKKRKLDANGSDSANSVPAGTPVLVFPNGKVPYNTYIHRWAEDVK

PLITDLMEHANMVKMWISFLIPKIEDGNNFGVSIQEDTMAEARQVESESATYLDQLSRYY

ISRAKIISRIAKYPHVEDYRQSIRELDEKQIVCLRLTLCEMRNHYASLHDIIHKNMEKIK

KPRSGNAENLY

>SG-PL_contig_1774_1_-3_3_215

FFFLVFFRVTGLYGYKNGPEVYKLHCAICRKYRYHLGQHVTLSVQPTACLFVCLFIYLFI

SNIYYILFIDF

>SG-PL_contig_1775_1_-1_67_456

MVRMNVLADALRNISRAEQGGKRQVLIRPCSKVVIKFLTVMMRHGYIGEFEVVDDHRSGK

IVVNLTGRLNKCAVISPRFDVSVRDMETWTNNLLPSRQFGYIVLTTSGGIMDHEEARRKH

LGGKILGYFF

>SG-PL_contig_1776_1_-1_1_156

INPFRTLLPQASSTPGLRLPRKIARLATASLQPRTMRPSRSTLLRLIPPLGV

>SG-PL_contig_1777_1_-1_61_312

MAPALRKVYDQMPEPRWVISMGSCANGGGYYHYSYAVVRGCDRIIPVDIYVPGCPPTAEA

LLYGVLQLQKKIKRMRTVQMWYRK

>SG-PL_contig_1778_1_+1_163_324

MLSHLVKTGTIDSILVFTDKCQFTSNSQHQRCILHSFIALHVEWPYLGKFIAAK

>SG-PL_contig_1779_1_-2_278_406

MISVFSWGSSETRKMCMQINGWVCVSVCVFKCSLLLINAMLLA

>SG-PL_contig_1780_1_+2_2_277

IIRPLGQADGTPGDAAAIRTHHGPGPGVTAGHDGVAGDSRSPGQSPQAPSPAGAQAPVVS

GLPGLGHPDRSGNLVHGSDERVARCHSPGSGC

>SG-PL_contig_1781_1_-3_741_974

MTLFSLRCSYTISFRHRYCSRCSYTISSRYRYCLVLSLGKLLCLRNHFIMFVTMENVDHF

LCTNNTFLTSFSSPAVCM

>SG-PL_contig_1782_1_+3_3_254

KKKYSFVKSKYTQHTDDNSQNLNDKYTGKVERSTKSWKHSLKKSVSKLWLGVQSQSDEAK

VIPRVTISLKGKCSTPTFKRTSPD

>SG-PL_contig_1783_1_+1_1_303

PLKKTNKIIKKTKKKRKKQRHTHTQKQKESKLFHSQILGDQKLCQKLISGTVSAYDIFIN

NICTGTELKRPYKISKVIPLASKYCTISLSYYSIILRISLF

>SG-PL_contig_1784_1_-2_176_880

MRRSPERAKSVPPALYFREEQRRKLQAVPTFADWRRVARATSVPADLLPVSRPWYMPYFE

RRYTPYYERRGWPYYGRRGLPYYERRGLPYYERGWPYYGRRYARSLPAPVEAFVPLPSEG

LRAHKIPRRYLLRGVARAGFISPWDYYDDVPGVRRRQEEEEAEEERRSGIRPRVLASPLA

RASLARAWRHSFPRVAPSRAEQAYYRETAYGFPLLFVPCKGSKKSDRQKAAIIAS

>SG-PL_contig_1785_1_-2_89_319

MDIERVNIVFNYDMPEDSDTYLHRVARAGRFGTKGLAITFVSDENDAKILNDVQERFEVN

ITELPDEIDISSYIEGR

>SG-PL_contig_1786_1_-3_3_206

GLQQHYCKLKDNEKNRKLFELLDVLEFNQVIIFVKSVPRCMALAQLLLEQNFPAIAIHRH

MTQEERLS

>SG-PL_contig_1787_1_+3_471_623

MKSMVLSQALNKTGMFKLTGIVWDDYLIKHEKHIILTVHSHKVRERKRAIL

>SG-PL_contig_1788_1_+3_3_98

KKADSFFLMEILEAFLNDNRCTTSKSLEHKRQ

>SG-PL_contig_1789_1_-2_35_583

MALLRRFLCWYLRTATFVGFIYVMITATSALLLRALDMAAIGTDFEISRGFNVPWRSHQW

QAFLASDIIVSASHVAIIFFSLYMIYNVTQLHFVLYMKTMQWYNYFFIMYTFTEFCFSLF

EFSFYGPNTFRREYLVFIWLWWIMRTVMNGLFMLVLFSRCQEMEDEMAYELRHSDKKYIH

SYA

>SG-PL_contig_1790_1_+3_3_134

FFLLKIFFCFKKKNFCILKKKISFFPLFFFFFFPMHIEYLSFKF

>SG-PL_contig_1791_1_-1_10_2136

MDEEDLGEHGIAPRQFATSDAFSSDAQSRKRRAEVMLSTRDSILPASAALADLIVPERLP

IGIKLLRKMGWKEGQGIGPRVKTRKKKKKGNKAPGVKTYGCFLPSQSDEENEIDPGDLDL

QNVTFAPKDVTPISLTSKDNVHGLGYRGLDPRSALPTTHVNLFEAPAVRSFRGRKGIRGQ

AFGVGALEDEDADIYATDAMSNYDQTMELDDSEQFFGWTAPGARKGKGKQDQPKVPVSYV

GKLLEGFTLSAKPLPPKKVFPAPTLPKGFQPFHKFKKTAEQLVQEAIGEKPATAGHQPAQ

QKLNAVERGLMLGETPIATSVFDLIPKEDIRKLEAVKDAAKMAASRFHSAGTTSENSDDP

APSVTAATITEQTSSQQAAKPCSSGGEKVNPTPLPAGSVGVMPFHKDPPKQDRYERYLAL

VKEGVKEPYVEVAASHMTEWERHREREEFSKAAHLYRPLSNMMASRFTRGTFIDDVEKQQ

SGLVEAVTEKSEQMKAAEMKMYGKLTREEVEWHPHNLLCKRFNVPNPYPDSDIVGVPGLK

RDKFSIFSFLSVPTQHPTTTQHTDASRDAPKAKETTSSLTRELNASKRKKGAMSIFSVLE

EEEKTPRKPPPALSLSELGDSPNDTDDKAAEGGDENDQNEEENNEDADDGHPKDLFRAIF

KNSDSESSSASDKSDSEKSDEDDDMDSKEKEEMDKDDMDSKEKEEMDKD

>SG-PL_contig_1792_1_+3_111_239

MMTRFNKYQQPMSDIQFVTELTLRYISDNQLRVNRNHIRKQML

>SG-PL_contig_1793_1_-1_808_1008

MRYERNILPELCPSLVKRLFAYFCRYACSPCKVLSMFICPLPSSCVQRDQSASFSGPYSF

HFKQVNY

>SG-PL_contig_1794_1_+3_1197_1403

MKDALNTTSEDTTLFQKVYDKFPTRTVMFLAEVGPTMSAGLNNSLYILYDNTLVNFFFVM

VRTYSFHSM

>SG-PL_contig_1795_1_+2_308_883

MISIKALLSVLRKPHTNYQGKMYMLTVTLLFLSILTKHHPGFQYTHDLEPQAAWNEHYSI

KIKLVCHTSWHPHTLLNYPEQSSCHSLSSLTLYGHTRQSEWGCSFTCNCHSLSSKGNMLA

KPKGKQSTDPQFHHHNIVLPHPGCVKRALQRTTSPMTNTHLHKLLSWQLFNKYHQKLEQP

FTVLKDRFYISP

>SG-PL_contig_1796_1_-3_12_488

MAAAMGSYEDWLTDKLKSLSPEFDTDVFVSYIISMLDTDSSAEEVTESIEEILSQVLGEE

ERESACQEIVDQWKEFNSDALAAAAAAAVKKDAGDVLSEIMEQQSLATVKENKLTAEEEA

HKRAILAQYAHISDGEETDQEVEETAAFNGLKGKGESPN

>SG-PL_contig_1797_1_+3_3_140

CVCVCERERERERERESVCVCVCVCVCVKLRYACCQIAKNTEYKIN

>SG-PL_contig_1798_1_+2_1223_1606

MAVLQTCFQVTFCALLLSRCTLLTVSVVVSRDFSATPQTFYNIENPTTVDAPEDSSNWPS

LQFHLFVWLENTKHKLLKNVPLSCDRKAYQMTLLLISGLHLNPGPRRPRYQFGVCGYACK

TVCLSVML

>SG-PL_contig_1799_1_+1_76_459

MPPWLSQEIINAMALRDKLKKEKCFTEYKTMRNKVKSMVRQAKKSLFDRLVIDNKDVSLL

WKAMNLITKGKGKRSLDTPPNLTANKFNDHFLSVSNLLAHRCKNTSQYVRSENLINFCKQ

RTNNTTPF

>SG-PL_contig_1800_1_+3_2442_2702

MLYIFVYSLEKELAVLEAICSDNTLQVCSFMPNQTQVKGCQRISVCFPCKQNRLASFTPF

FQLDQCVHNNKSNVLGVVELYSLINTN

>SG-PL_contig_1801_1_-2_2_346

AIFSSKCTRGLTLVRQSAYILGDPQSVKLGNANNKQQTTTTVRSISSKTTVTQNCQKDFF

IGFVKNIFPITWQEQIRATVAAFSPWKPILCIKTVINLLYSDTTHIFANWKYTPN

>SG-PL_contig_1802_1_+3_3_209

SVFSFSMSQKVHLSLFTIFISFKIIFVAMILFTILIWSHFKLMSCIFCTFRNHTFQFIAL

LSFVSHLSQ

>SG-PL_contig_1803_1_-3_138_1112

MSRSASRSRSRSPRSRSGSSSSRTYSTESSKSCSKSQSHASSQAGGRMQSAIGLKSTIAN

MTDLKDQKNSDKLKDSDNDDRRPLGICITGLPSRSSDSSLRDGLFHEYKKHGKVTTVQIV

NENDSRHAIVSFRKPEDACKALEASQGKMFFGTKIKVTSHEGIKVDDADIRTSENDLDEY

HPKSTRTLFVGNLEKDTSVSDLISKFSAFGEVIDIDIKRQGAASAFAFVQYTDISSVVKA

LKDMEGEHIGANKIKLGFGKTMPTNCVWIDGCGDNINDKHIYRVFKKFGEVMYTFVDRKK

WRALVFFTTLEDAQYAVNEMKGRLL

>SG-PL_contig_1804_1_-3_45_308

MYKHNATQNVNILKMHIRNSVQKHSGRILRLSRRLQYEKVCSTATFETPQNSLLKRLQCD

RNGSDCDQLFSGVVTQGSSACADGFLMA

>SG-PL_contig_1805_1_-1_64_504

MQVYRAAVLSTLLYGSEAWVLYRKQIKRLEQFHQRCLRSIMGIKWKDYVTNIEVLERAQL

QSIESMLMLKQLRWAGHITRMPDTRMPKTVFYGELVQGKRNTGAPQKRFKDQLKKQLSQA

DINPKTWEQSASDRGSWRASTLRGTQH

>SG-PL_contig_1806_1_+3_3_218

LLVPEPPSCVYACRTSNTHVKDPVSHVSVRWLMETRTHPACTKKKKDGAALQRRALTGRA

ARISHGKSILTK

>SG-PL_contig_1807_1_-2_1193_1423

MNDYRGICLSVISSKVFCAVIDRRLQGYAEENNVIGDHQAGLDLKEDIQQLAICSCYWLS

YRNSFHVTVKFVVCYIL

>SG-PL_contig_1808_1_-2_1811_2164

MVSATTENSYSNMIVSTIHTVIGVCQICLHFFLCVFICCRYISFSVNILVDACYWFVCCC

SHCFLLPFPTDTPTGSHIISLFCVGTNSAPLEQFMTCQHIRFTSFRLLLFLSIFRLLA

>SG-PL_contig_1809_1_-2_167_1438

MADSRTYDLVIFGATGFTGQFVVDEVARVEDEEGGLTWAVAGRSMEKLQKVLAQSSQRTG

KNLEEKPIIIADSESHISLLEMCKQTRVVLNCVGPYRFYGEQVVKACLEGGASHVDISGE

PQYLEKMQLMYNEKAREAGIYIVGACGFDSIPADLGILFTRSKFKGDLNAVEGFLQITPG

PDGMVGNYATFESAVHGFAHQGELAPMRKVMFPNPLPRSAHRPAKRPVIFHSPEVDKWCL

PFPGSDNSVVYRTQRYNFDVNKQRPVQFIPYFCRSSRFAVYAMAFFSIIFAILAKFSLTR

MLLLKFPGIFTAGMFKKGGPTLAQIKGTTFSWTFLATGYSQKLEDPEAQHEEAPDQTIKT

KISGPEAGYVTTPICMVQSSVALIKEKARLPKEGGVYTPGVAFGNTSLIERLDKHNVKFQ

VLEE

>SG-PL_contig_1810_1_-1_1_216

SIRLSSVLKRSALHDNQPDNKVFCLLISRWKKERELCTGILLLHHEELCAPDCADPHLCV

SLDSWRRLYNQP

>SG-PL_contig_1811_1_-1_1_273

KKNFYFKGFLKKKKKKKKKNYVYSAAVTQVCYHCEVICFDACVHQLNPYTTALTVSLHCV

KLGQLRQSTAGNCLCKRLTETYRVAVFKFTN

>SG-PL_contig_1812_1_+3_195_812

MLTDMVAQTNLYASQYKAAIGQAIKPHNQLAEWKDVTIQELKVFLGLLFNMGLIDKPDIK

SYWSTSRILATPIFGDSMSRNRFEAILMAFHLNDNTQNLPRDDPGYDKLFKIRPLVTKLQ

EKFATVYSPKQNLSIDESMMPWRGRVAFRQFIKNKPVRYGIKLYLCCESESSYVCRMEIY

SGRKGDQREKDHGPNVVKRLSEDHLH

>SG-PL_contig_1813_1_-3_3_608

RWSQGSQPWDTQIALGTWFTEATSEWLDVTNLAHGVPIALRIDAVHGCVHDGLGSPHGDS

ARCGVLVPGGAAVVYQRSGAQSAAAVQDGGLRQSHSPVHRQHDSRVLCEQRGGGALCDPL

PVGRGPPTLVPRKGHPAKRQTHRRQGERPSGHAQQIQRRSSDRVDPEPRRSPSSLGCLGS

QTHGGPLCDQVQQEASRVRVAG

>SG-PL_contig_1814_1_+3_351_587

MKGKSFSHIFTQKENSSYNFHYSIFPLKLHSHNGQGQQASLTRRFLTATVVVLNIQRDQT

RPICTHFVAANYRKAQQRR

>SG-PL_contig_1815_1_+3_48_305

MVIIHSFRTDNASDYLVSRERGARETTLANIFREVKDPLIPVTLRPLPPLHRLVHTEVLS

TDSTVVRFSGHQIPPVLTGPAGRTLV

>SG-PL_contig_1816_1_-3_15_287

MNENSANVAVDEETGSAKSICTSVLTAGNLLQVTSVDEKIFKFAAKTLATGTLVLVVVKV

SDKKAKVTVNSEKMVIGAMLLKDIQKVMEKK

>SG-PL_contig_1817_1_-2_2_157

IIIIITASEAGVCYCTKVAYVYAYESIDLFIEEMFSYMTSAAVLTKTRVDLF

>SG-PL_contig_1818_1_-1_1_63

QCRLRVKNFLFVHVGCCFHFS

>SG-PL_contig_1819_1_-2_335_619

MSTLIVVPLSCALPVHYQQQRLNITRYLYLRLSSYLLHSAVSMKKSSMFHLLHDSHSTLL

FPFFFIVHFLYDSCSIFVFSFSFLFMPGWDGGRVK

>SG-PL_contig_1820_1_-1_1_711

EFPQDWVASKIGFRGPRTELANPLNYARYYLPRLLPHVEGRIIYIDDDCIVQGDIVQLYH

IKLKPGTLAAFSEDCQGMNRRITFMKNVYADFFDLKNKHFKDLKIQPSSCSFNAGLFVTD

LSDWRRYNITRQLEHWLELNSREELYGNERGGGGAQPPMMLVFYNKYTPIDQLWHVHFLG

WTARTSYSRQFVQRAKLLHWNGRFKPWGRTSSFQDIWDRYFLPDPTKQFQPVRKGIL

>SG-PL_contig_1821_1_-1_484_729

MCVCMLCARSHMCVCTCISTRVVCLREIKKYTKSSKPLRKQTTERNRICQGKTSLYGAQS

LNFVCCIALQIHGTTPSYSSPS

>SG-PL_contig_1822_1_+3_1029_1430

MCSCPFSLRGLSFFLLQMGGCCVYTVYVCLLNVYNQLLSIFWCVCVCMCSHSIANKNVKT

KQTKKTPTKKPHSAVMEIGPAVCCSCEDLNATGLILRSQWPQKPKLKQKFLVFLQMDFSR

IWSYCLSAVFFIQI

>SG-PL_contig_1823_1_+2_2_283

FVEGSFTNISTLSERGVLQTFQHFRRGEFYKHFNTFVEGELTSIQKIHHFHHTMLRTSQS

FECGNLVTHAFYVGSMLYTQICSVWVTLAPICFT

>SG-PL_contig_1824_1_-1_184_261

MRGCGLNEYLSVIHSAISSLCNTAHV

>SG-PL_contig_1825_1_-3_108_257

MPRGWFAVVVLIIFYGFYFCNGIGSCCIDIIFLLFLSWLETDSGILVSYT

>SG-PL_contig_1826_1_+1_715_1029

MLAERLEGKEKSMRLFCLFTCLFDFVLCYCFYFFMSHLLHHFGRVDPTQLISALPSTANL

KFIQSKLQCQEAYKSQRCRAATETPTRGDPALLPGHFVGVPQRLY

>SG-PL_contig_1827_1_-1_91_300

MNIFFCLFFKSSKIKTKHSGCIYTRTHTHTHTHTHTHTHIHCYTGKHTQSHMFEDQGSCP

PIFKISAASV

>SG-PL_contig_1828_1_-3_471_1058

MMAENKLKVPAINVNDSVTKSKFDNLYGCRESLVDGIKRATDVMLAGKVAVVAGYGDVGK

GCAHALRAFGARVIITEIDPINALQAAMEGYEVATIDDALGEARIYVTATGCKGILRPHH

FEKMLEDTIVCNIGHFDCEIDIAWLNANCEKKESVKPQVDRYTLKNGRHLIVLAEGRLVN

LGCAHGHPSFVMSNSF

>SG-PL_contig_1829_1_+2_2_388

FFPSQCLFFTPFLEHDHLALSLLVSTQLKVLASLDGQLFPVLARRTLHTQNNLLCGFSLL

PENGLRLATISRLFPVVTAFTLGIERSFASLVLGHLMQGVLLAIFTLAKGLALFRHIHHL

AVCVERTRC

>SG-PL_contig_1830_1_+2_2_133

RERYKKRERERERDRTERERERREERQRGERVKILETCQNQKVS

>SG-PL_contig_1831_1_+2_2_148

RYLWQSSNTAQPTTFTRLCSIHPETCISSYGAHFTPRPFPSISTPTVKL

>SG-PL_contig_1832_1_-2_8_424

MGLSFRSGGGSPDFCTARPTDPIHGHLVHRLGALTDSSHASGTWSPAKAHLHIIVLTRWR

QCPCPQCIHTPPQEQISQVVHGQHDGRLLHEQTGGGALFGCLKASREPTEVVPGQWDITI

RSPHCQEAVHHCRLPEQGP

>SG-PL_contig_1833_1_-1_40_507

MATTAAASNISYFAPPVNLLTGRCRSVACLYNAATMKDVIDFNSCYMTSYGYDVETSDGT

NGRSGSLNNVTMTKQDTCKTKPVSTTAECFIADDPDVRGCWGDNGAPVYCRLKTTKETVL

YGLLDVTGNMPATFGAPTNQKNLCSKNNEFHVLPVS

>SG-PL_contig_1834_1_-2_806_1099

MDTNQRRLLPFTLVGMLGAFSVGIVSPPSGNSVLEISSFLLLSLFLPFFFSLLSSAFFLP

SCSSLLSFFQQAFTQSPSSPSPRSPVYDVLSMLFCSGH

>SG-PL_contig_1835_1_+1_1_159

DTHTTIKTSRTAGLRQSKIHTHILTDCDTHTQLSKRQELQALDKVKYTHTYSL

>SG-PL_contig_1836_1_-1_1_198

RTHTHTYAHTFSLRLCVCIGNILVHFFSWPLQSFLPVRLLWGFFVCFFKINNSCLRDSLS

SAKICH

>SG-PL_contig_1837_1_-2_2_175

VFSELVRVGFFLLLLGLFFFIAICDTLSAPLSVSLHVSTYGLFHLLYLFISFRDFLLI

>SG-PL_contig_1838_1_-2_116_1339

MAAVLPQRPDAGTYTFASQPRALKQRKKYRDPVQQTEDPEGLVNYGNIMYDRRIVRGNTY

AQHTLPAHAQPDPIEIQRQQENRRRAIARKRAKDQLRPRSPDPVDGRKHIDVQTELYLEE

LSDRVEEADVECQTDGFLDRPPSPLFIPAKTGVDIATQILAGDLFDFDVEVKPILEVIVG

KTIEQSLLEVMEEEELANLRQQQRAFEELRNAELVEQQRLEEQERRHREEKERRMKQQRE

VVRKEKETAEKIAARAFAQSYLADLVPSVFGTLSDNGYFFDPVERDVEQGFLPWLMDQVV

DQLERNEQGRMVLDGMLREVVARRMEMYNKLDDTASSVGQASRVVSSMQREQSSAEQPAP

ATTTESQEAAAAEKPAEEQAAAPETAEEAPDSGEAAEAGGEEEAAADE

>SG-PL_contig_1839_1_-2_11_313

MDKSGEEHYLCLVCYFKYYWDKPYLCPVCYCKYYWDKRAYQKDITTGTISQWISQTIIQA

YQSANRDTSILNPRAHEIRALATSSAFSQSVPLKKILEAAY

>SG-PL_contig_1840_1_-1_235_729

MRGYYAAKHARTTTKDTSGLKVQPHGFHPSNSAESQPSSETLQISMASRRQGTESLYNHK

WKTWASWCSKESVDPIHPSSNQIANFLALLSSERNLTITTIRGYHSAICTTVKQLGGPNF

SEDFLLRDMSWGLASLEARSPSRVPAWDLLERTPMNFQRTPHFPF

>SG-PL_contig_1841_1_-3_81_908

MKARTPGFDGGSTSRDPLTCPKCKQFVVGPNAVRMKSSPKLTKHMRKLLRKRNQNKKSLT

ERQHVLLNRAMQRCNVLVLKCSPCDWREEIKLLSKEAIQKHKASLRKRIERERLAELQTG

VEESTEAQDKKALKKLRKKEKRKKSLEAKALELQHFKRLTSTPIAQGTTEKRIETPMVTP

VSSSHLVSSSLSERSSFSRTPLPETGKSASVETPSLSHTKTGNRTFVKPDRTPSGPLSGV

QKGRVNKKQLQKVLNQKKKEKLSASESLSQFLNSLK

>SG-PL_contig_1842_1_-1_265_459

MCEREGESLFCCVFDFIIMLKQFSPFSFGRFQVMRCVVWGFFSFPSVLWDETKDTSPCVI

ERLQQ

>SG-PL_contig_1843_1_-2_2_1000

MEKTRDDIHWLSKNQQLLIPEPTVNDTEAWILINVHQMAYCRVLYDEATWHALIDQLNTD

HTVIPAINRGQIINDAWNLAKAGLLDMSVALSTLDFLQFETDLVPWRAFAREINYVEMML

ERTDLFRELKTFVRDTVEQPLKRLGLEIPEGTAPVDVFVRTLIAQYACKYDMVGCVNEAK

RLFTEWRNNKVNEINVNLRRQFYCTAVAEGGEKEWHFVYAQYKTSDDATELNNLRYALSC

SKDQIVLRRVLNMAIDPAEIPAEDAATTIQWVASNPQGRDLAFEFFVDKFEEIKGLLDVL

SLGRTLISVTAHFNKDYNLQQLLQFPVIRDTTV

>SG-PL_contig_1844_1_+1_1483_1827

MFLSLLIKIAQTLNTMKLTLTLVVATQVAPLYVYGQLVVALLGRSVWSVSYLSWIRKAIV

SSAFIRIFLSWAQEPAFVSFQAHLSKHVAKIQVIGRSFLTLSKMGHGCCSNHLKR

>SG-PL_contig_1845_1_-1_1_204

KKKKEKEDFLKRKKKKKGRRKKKKKKKGGEKKGGGGIMFSFFMLFYYFHCDIFVLCDFLI

DMRVFFLV

>SG-PL_contig_1846_1_-2_143_271

MSNCFLNCTYSTMSNVISADWQCFSSLFCLSSLLPMKNSQLSV

>SG-PL_contig_1847_1_+2_2_85

TTKLTCMLSMHKITHQKQKTNYRTGLSD

>SG-PL_contig_1848_1_+1_634_813

MIQSAILTLMKTMMTIKITTSPTGLQCQTIATSVTFPRQHVSLTYHTRTNVITFTVIIII

>SG-PL_contig_1849_1_-2_2_958

PITTTTPTTTTTPTTTTTPTTTTTPTTTSTPTTTTTPTTTTTPTTTTTPTTTKPPLLCPS

STSTTPRSRRAVGGSTTTACDQPGVVAIVEDSTNYGLCTGVLTSPTTLLMAAGCSDFIAV

QAGKTYPAADGTTKTITFTVYADGGAQTVKFNTAAEFKQTSNGDSTVSVTLSTPIESSST

CNNVACSYKDATMRGRVSFNKCHLAGYGDTSTASFKYSGTLTSIPITKKVGDPCCSAFVK

DPEFNTEADVTNITDPKIKTVCLASSSGAACTGDQGGPIYCQADTGEQILVGLAGGYNCE

AGALFAAIDRTGGVYASAA

>SG-PL_contig_1850_1_+2_182_445

MYRNTLHTSVQRTEWLNSHNHNISFHLRFSADFYIVSLAIYIKGQNHTIIIYIDHKFGTL

RTHTGSLQITFQNCIPWGKITHTINTTM

>SG-PL_contig_1851_1_-1_1_156

PKRIQTQVFQTLQFESQVSSSFDHHTRQVGAIFIFPDFRHKNSESRCYIYFP

>SG-PL_contig_1852_1_+1_952_1248

MVYSITVLTMHLCYFKYMYICILYTSAGTHATTLSFTLLNTPYFENGAKPIHNIYTQWVN

PDTFLHTHTHIVKVCSHKIFIIIWESKQPKLHSSELNRE

>SG-PL_contig_1853_1_-2_47_1558

MSPPPPSNLPPPPGANLPKKKKTVKLHWRALQNVDRSHHSSLGETVWKELVPVHLDVEKL

EHLFETRTNETKQKRQDASGKKEITVLDTKRSNAINIGLTVLPPPRTIKAAILKMDTSIM

NKEGIEKILQTMIPSEEEKSKILEAQMANPDIPLGTAEQFLLTLSSIFELEARLHLWLFK

LDYETLEQEVAEPLMDLKKGVEVLHGNKTFRCILSAVLAIGNFLNGSSARAFSVAFLQRV

PEVKDTVHKHSLLYHLCHLIIEQFPDTTDLYSEIGSITRCSRVDWEELSHKLDKMEYDCK

ASWDHLRAILKHDGSSADLKGKMSLFLSDAAERIMLLQIIHRRVVTRYNKLLLYMGLPVH

EAKEMRINQFCKVVSEFALEYRTTRDKVVHMMEKKANQRERKKTRGKMILETENFARSNK

QDDELHNLLKNGYTSADERGFPGQKSRRKVDAWSLGSSRGGLTTDSEMYDTGDDEILEAC

VRTAAAPTNKPHRERRRSRNHRKS

>SG-PL_contig_1854_1_+3_3_233

CRTPWNGVAGEGWAVGYPPRPTPPPRASVPRPSLRRGEPLKSRSPNRLHRTHPTFYGPRD

AEPERALAISLLDSQAG

>SG-PL_contig_1855_1_-1_1_261

TFRTTGTRLPRSREWNCRCATAPTLKTFSCTGYCAVFFLSDSCADVILELKGLPISMLTA

NSVFFLLMNSYVNNHRMLTAHSVCFCY

>SG-PL_contig_1856_1_+1_64_243

MVVLQLFIINETHTLLPVSILWLFKKLFIRKELQNRNASKNTSFPNTMCKSAAAYTVIHQ

>SG-PL_contig_1857_1_-1_4_375

MEICHAVTSSHTHTHAHEHTHTRTHTHARARTHARTHTDTHTHTHSHSHSHTHTLTHAHR

RDHLTQAGTDMLACTSLLCSLYVLAHFESDLILQHQQVYRQNIFCRKEMHTTRFFFFDFV

TNII

>SG-PL_contig_1858_1_-2_1304_1522

MSDYWDVTISMQTFQSTSMRFRVGRKKFGSVIICIEYINTYVRLKFSNIFYPYMSDCRYV

SVSKCLSEISYHQ

>SG-PL_contig_1859_1_-2_89_439

MLRAKLDRFQWEKQQKTVLKAYENERPEADQSTVTKLSPRRDTKVAITRITTGATGLKGV

KHPQDEVVPLLLLEEKEQEVLAKNETIQILQAKIKRLEHLLHLKDIRIEDLQARLES

>SG-PL_contig_1860_1_+1_1_177

NNNSNNDDNNTNKCKVRRHAESWCKKVVFFSTQLSVVVTHTELLLASKIAQADICIGIA

>SG-PL_contig_1861_1_-3_72_1022

MGGKSSKQKKSKKENGENVAAENNAENQPAEGQATEAKAEGDNKPADGATPATEEDKAEG

AESAEKAGDGDTAKDTPAEEAKTEDGGGGTETSKEEPPAAEAAAEAAPATEETKEEPAKP

EEKESAPAEEPAVPAEETPAPAEEKPAEETPAPAEETPAPAEETPAPAEEAPAEEPAAPE

PEPEAESAPAPPAPADDATAASPSDTAETGAADSSGAAGEASEPAAPSPEQESGNNVHKA

EFKWEEGGENVLVSGSFNGWGEKIALVKNGEVFSATIDLPKGEYLYKYLVDEQWVINKNQ

PTKADDEGQENNVIMVA

>SG-PL_contig_1862_1_-2_596_1108

MFVQRTSTVTDPSVGTRLAYFSVTATQELSEVAKKGIFVYSFDHFVLVRSIPKSYRVKIC

QEKKRLLKWLDFLFLLLLLLLFVCVCVCVCVCVLLLFFLFLYSGTLVISVFTQVVINRDF

AIAHTVTFLSVDSVFHQYYLLIDTVMGTILYPVSIYTYTFYKRTLLLCVQM

>SG-PL_contig_1863_1_+3_42_149

MLYTSTEIAKYNQSCHITSVLKYIKVYRKVTTHTFA

>SG-PL_contig_1864_1_+1_271_426

MFAQGSKPSPILVWLIFICICYLLSLFCDLSLENTINLVVYVCPGLKGLIYP

>SG-PL_contig_1865_1_+1_1_402

METISHLVRVSLPDYLRSVPIPTSLGGFAKLSSEEWLSLVPFVGTVSIIVYVTVLAFKPP

PKPKDVVVNMKVQKESPKVVNMVDIEDLGNKISYCRCWRSKKFPLCDGSHDKHNEETNDN

VGPLVLRRKEAVSK

>SG-PL_contig_1866_1_-2_218_5596

MTECCQGVSLGTGSPSVSRRCVHRQATSKPGIMSLWFLLATFLVVLTAFAEIAAAQELIP

PDPRCGAGCTTVGNCRCIGEKGSRGAPGLIGPEGPQGNQGFPGPEGLPGPKGLKGEPGMP

GQQGEKGNRGKMGAPGFPGINGVPGIPGPVGPRGPPGLDGCNGTQGDPGSPGLPGLPGLE

GQPGIPGLKGAKGDAALGAPLVKGQKGEPGPAGFPGLPGAPGVDGPTGPKGEPGLPGFAG

NPGSLGMKGDKGSTGISYRGGKGFQGQKGEPGPPGLPGPYTLEPSTVIRGVPGLPGEKGE

KGDIGMKGPEGPRGYPGEPGRTGLPGEKGMLGEKGVPGIAGPRGKQGFPGNPGLPGMKGD

RGMDGLPGLPGLIGMKGERGIPGLTGPSGPQGPPGRPGGKGTVGEPGPRGFPGPVGLPGD

PGLPGLPGQDGPRGLPGSEGGRGIPGPPGPQGRDGPKGESGDSGPPGLPGLDGRPGLPGR

AGPKGEPGPYGEPGLTIPGARGPPGLPGLDGIPGQKGERGPTGLPGPPGNSSQGLPGLPG

PKGEKGQQGFSGLPGRDGQPGTPGDKGNSGGPCDRCLPGVKGEKGSQGLDGLPGLPGERG

GSGFPGPKGEPGDDGSTGQPGLPGLPGNEGLPGLPGERGLKGEPARPDVIQGPEGLKGDL

GSPGLPGEPGRPGRSGPQGPPGSPGLPGMKGERGLKGFPGLDGRPGPAGQPGIPGEAGLP

GIGIAGPPGERGDPGMNGLPGLPGLPGSKGEPGKVEGRDFKGQKGERGFDGVPGKEGLPG

LPGLSGAKGEPGLPGLPGLKGDPGEPGLSGVTGPRGLPGPRGEKGLPGLPGAPGLTGMKG

DSGLPGLPGMDGEPGLPGLPGEALQGLPGPRGNPGPPGLPGRDGEPGAPGPDGLPGLPGL

AGEVGEDGLPGLPGRDGSKGESGLPGRPGPPGEDGLPGLPGQPGDPGLKGDSGLPGLPGL

SGPKGEPGLDGRPGEPGTPGLDGRPGEPGAPGLDGLPGLPGMKGEIGVMGFPGEPGRDGQ

PGLPGLPGAQGLKGQRGFPGEPGLPGLRGPKGEDGLPGLPGQDGDSGQPGLPGLPGTKGD

SGLPGLPGGPGEPGRDGLPGLKGELGEPGIGEPGLPGLPGEPGRDGEPGRPGLPGIIGEK

GLPGFPGERGQKGESGLPGSPGLPGLDGLPGMKGNSGLPGLPGTSGEKGEPGLPGLDGLP

GLKGEPGFSGRDGLPGLPGLKGDMGRPGLDGEPCQSGVKGEPGLPGSPGLPGLPGAKGES

GLPGLPGLNGEKGGPGLPGLDGLPGAPGSKGNPGPAGPSGPPGLPGLPSVGLKGEPGRDG

GPGRDGGFGVKGEPGLPGLDGLPGMKGEPCTDSGVGPRGFKGDPGLPGIPGEDGPPGLPG

NDGLPGLPGLKGDIGEPGFSGEPGRDGLPGLPGQKGLPGLPGTPGRDGPPGEPGLPGLSG

PPGLKGDRGDDGLPGLRGDPGQKGEPGPRGLSPVINSTLLPKGEPGQPGDRGLPGQPGQP

GEKGFPGRDGLPGHDGAKGDPGLPGLPGMDGPPGPPGLDGLPGTDGGPGNPGPKGEPGPG

GLPGPTPPTGYLLVRHSQSTEIPTCPLDQVKLWDGYSLLYIEGNEKSHHQDLGWAGSCLP

RFSTMPFLFCNMNNVCNYASRNDKSYWLSTNAPIPMMPVGERDIVPYISRCVVCDVPANV

IAVHSQTLQLPPCPRGWKSLWIGYSFAMHTAAGPGGGGQSLSSPGSCLEDFRATPFIECN

GARGTCHYFANKYSFWLTTIENNEQFIRPRSQTLKAGNLRTRVSRCQVCMKDV

>SG-PL_contig_1867_1_-2_1670_1903

MGQVYQTLLHGDDNNNKCTENRFEISKPFTCMDLHNVQEVQGHFKSFQGVKLEVLIMPYL

DTFPSIASPKSQPEGFVQ

>SG-PL_contig_1868_1_-1_130_333

MKSWMLLAVLILVFASAKADEETCPELQCENGGTWVQQHCRCRCPNCLNGIRCELPYFER

FCLNRFLK

>SG-PL_contig_1869_1_+1_1_87

IDEAVMFNLDNFRSFYDAACTQYTQVMIP

>SG-PL_contig_1870_1_-2_266_376

MELMRLISMYAALHVLMMCCFSARSSSNMNPRLRTVL

>SG-PL_contig_1871_1_-1_334_426

MSDLLCAFSILDQWLLQLLLPFSEEKSLYAV

>SG-PL_contig_1873_1_-1_406_633

MPGSIEIKQYRISLSNTVFPCPLFMSALFVCVWAGSLHSEIFFLCLSHTHTHTHAHRHTH

AHTHTHTHTHTHHIEV

>SG-PL_contig_1874_1_+3_174_446

MIDLFATKFSKRLPIYTSPVQDPEAVAVDAFTLDWSGLLACAFLILLRLEKATMILIASK

WPAQPYLSLCHVPPLELPPCPDPLHQPRSGI

>SG-PL_contig_1875_1_+3_699_863

MSYFGDILQDSHDKMFSRHSILSVIWIIILLIVTSPVIRVWQKRYWQSEIYIIIS

>SG-PL_contig_1876_1_-3_45_203

MVIVICMVASEYQLATNIFTISEGQMIFSYSYSEMNQLMEFCKSRGSLRIGTL

>SG-PL_contig_1877_1_-1_373_897

MSQYLNNMEIGDYMDFRGPNGLLVYDGKGSFKVRPDKKEEPETKVVKKVGMIAGGTGITP

MLQFIRQVFKDPEDNTDLWLLFANQTENDILLRTELEDIQQQYPSRFKLWYTLDRPEAGW

KYSSGFINDTMIKEHLPPPDDDTLILMCGPPPMIQFACQPNLDKLGYAASSRFAY

>SG-PL_contig_1878_1_-1_850_1224

MWSRGPAVLCVCVCLCVSVFVSVCERERQSIIKNVCVCVHVCICTYVCITPIRACVYYGV

LPQYCWFSRRFFSSHFVSLHVMCNLSHVQSELYCVIMILGSLSLPNLSSVDDVGNGLFDF

IFIHM

>SG-PL_contig_1879_1_-3_3_167

RQFESTLTICMYARARTHTHTHTHTHTHTHTCAHARAHTELGEGGGGDVKRGMLY

>SG-PL_contig_1880_1_+1_292_471

MIPMSIFMAYNTPSNPCQFSSIFCWWVCVCVCRQMHLCKFVCVCVCVCVCVCVCVCVRVC

>SG-PL_contig_1881_1_-2_41_262

MADVGKDLGNTADPKNIQDLTTFVQNLLQQMQDKFQTMSDQIISRIDDMGGRIDDLEKNI

ADLMTQAGVDDGRV

>SG-PL_contig_1882_1_-3_105_1310

MSGDRIVRVGSRKSELALIQTRTVIELLKRKDAHLKFETITMHTTGDKILDSALSKIGEK

SLFTRELEDALLKKKVDFVVHSLKDLPTEIPPGLVIGAVLKRDNPHDAVVMHPSHHGKKL

SDLPAGSVIGTSALRRAAQLQRKYPHFKIENIRGNLNTRFRKLSEDGSTYDAIILAVSGL

ERMNWHHLISEVLSPEDCMHAVSQGALAVECRAGDEATLKLLRELNDIDTALSVVAERAF

LRTLEGGCSVPVSVYTEKTEEKLMMRGGVFSICGTQAVKEEMTCDLQEMTDKQSHEVDPK

SLQDGYITIICHEANNHLRYHNARHLGQKLASSMIKDGADVILKAAKNKTKEEILAEHAK

RKLKAEAAEQEGAKSAKVRKLEGASDVVSAAAVASTSTVSSA

>SG-PL_contig_1883_1_+3_12_281

MYKKKKNFLKWPEKKGGEDFFFFFYKTNCFIDLSLCCFYYFIDPLDSVHTSLLLGPEVFI

EDIQGLGLFSVVLDYNNTTAVDFAGFPFLI

>SG-PL_contig_1884_1_+1_289_690

MSRQICFLLIVKLYFRFLNKVSFPQRFRMRVMAVSRSRPWFCGFLYQDTPWFCCSSRSTH

HVSPASSHNHCGASDQYVQFSGSVCIPQHPCSQPTETCMPPLLPHPTPLGAPWTWVVTLL

HQPCFLQNNEQFYL

>SG-PL_contig_1885_1_-2_1241_2110

MSITYLSTLLSLSCLLLSSLFFCFLNQTAGSVQITTASTSILSTHTEKVKNTEERMGDVS

VMNPSIEQFSSYKESPAVSEKGVKHCSASCKKNAVDHPPAHFRDDCPKSPDSIESTPAQP

VTSVNNISIAKEAVCAMGIPELLVQMAIDIRRLEGESDFENASQLYMAADDLCQHPERQS

RIVMKLFEAEEARKEKENAVIKLEMQNRPVVDSKPSKTELQEKVKKLAKENYKLKQRKMC

RACRKVDLASSGVTFLPCGHFITCETCSENFDDCPACGKMIMGTVRTFLS

>SG-PL_contig_1886_1_+3_3_146

AQPTIKAISGLSPRKVHQSQEDNHVEHKTLSNLKTKTLQKITIHDILS

>SG-PL_contig_1887_1_-2_338_619

MRGNIMDVLYSRAIFSRYNYQHVNLKCLACTHLLACMYMNAYICIHVHACTLICTHKHIY

IYICIYISIYMYIYTHTHTQHITCVSDPIICSII

>SG-PL_contig_1888_1_+1_421_654

MLSASSKHTGQLDNNSTNNKDKRNVKKKKNGQKLYLKKKEKVHYNPYSAKKEWTDIRQQF

FLTVHHKQHKSFTCISDF

>SG-PL_contig_1889_1_-3_1929_2207

MIRSYVKCNKHAFDMDCLRCTNEIIIIIIIIIVVWHCHFLLCMYVFLFTCESGFYDGILP

GTSLLQLRELLCVCQRINLSKHGECFKRLFRLR

>SG-PL_contig_1890_1_-2_119_259

MILEFGNYILSHDCECYCQNSEVGAWYMNFTVLVSSQFFPLSVCFFF

>SG-PL_contig_1891_1_-2_2_154

FFFLNDKGNITNTSSHLICCSCCLELRSAEIGILLHLLFSAYTDSLMLVKN

>SG-PL_contig_1892_1_-2_68_355

MPWKEDLSGMTHIQYPRHSNPTQAPPLPAGQATHEMAIPNDLIGCIIGRGGQKINEIRQV

SGANIKISNAEDGSADRKVTITGTPDTIGLAQYLIS

>SG-PL_contig_1893_1_+1_82_510

MDPECPVLSWVWGSLVPPNDRSLCDQVQQTTPRLYLAITYPEAIAINAFLLDWSGLLAYA

FPILQIVIRKANGENNHVSDHANLAISTVVPRPSVLVSRSSSSTAASPRPPSSTTVRVLA

RKPSSAHLHVWSLCGARGSHEIP

>SG-PL_contig_1894_1_-1_364_489

MRCCWLLYVNGSSCPLIMNMYEMVHVIMNMYEMLSVLTGECR

>SG-PL_contig_1895_1_+3_3_188

GSTILHTTGTRLLRLRADAVQRLFHFKTFSCTGFCAAFFLSDSYTDVILELEGLPIKISV

TD

>SG-PL_contig_1896_1_-3_24_1481

MLLPPILCEPPSVCSSNGKSVYKCGIRLHENCTNHRDHCISSDETSFCDPKLLICRSRIN

TSCFYDNTCPHGAVCQQGFCQCRPQGHWNETKQICELPEGNTGAKCRAGSECDDPAAMCN

ADGVCECRTGFSVDPHRLTCYLKGPKCFTNKDGAVKTYFGDTVLVGLPCGFKLAKFKCGR

MVVEVFANKAYSHRIRNNYISAVNVTITGITKNGTSATNTFTIEFPRVRKYMRKNRTKGS

AWRVSPGQDRPVKDDYVHLSTSASSGVSVLLRKLMKITYTPPVLIKNTDNGVNQVQIECH

QDKFKSLEQFPVSLCGRPKDKEEGIYKQYADRVQKRLKREMPDDFFNLYVSSKAFSPVRL

ADRAILLEQQVSQRNAYCQEASAIVKGECPALQAKLYNCSHMYDSNTSPYSILDKDSYGR

CVDKVIKDPTKLFVSCLNVLCNRLEGDRKNVTCQTLKTASDLCDDIENTQPPSDLMALAN

CTTPPQ

>SG-PL_contig_1897_1_-2_77_397

MSDAEGDDVPAQAGAGSMDIYTAVQEVLKTALIHDGLARGANEAVKALDKRQAHLCILSN

SVDEAMYTKLIEALCAEHGINLLKVDDSKKLGEWAGLCKIDKEGKAR

>SG-PL_contig_1898_1_-1_1_264

GNANNNNAGPEKKNEVSTISLPCELRVTGEPYLTLPYNTYVLGTYWLLLLVYLWILKHHV

RECVRSTYVSICSTRRDMIPRKVLFFFN

>SG-PL_contig_1899_1_+3_36_251

MRMVSIPPPCCCTSPILNRVRYPFTAGWTGGRTYKVFLSQTGTHAVMLEGGSCTSTPIPT

EIGSTGQGRSAL

>SG-PL_contig_1900_1_-2_32_439

MAGKQVPPQKEPKNMVHQNAILCETVTKENRYQKLYTNYSINPFKEFYVIAGKPNQTVED

GEEDDHFQQVIKRANQEPVKKYKRPQTEAQEVGWITKPLIQSDRGDTRLNFFRQNCPITK

FMDKAWLEKEQQTMGN

>SG-PL_contig_1901_1_+2_506_655

MSYYYIKNPTGNEDGKLKFYKMPHYWNCLFASKPRFHNFIIGVHITPNNA

>SG-PL_contig_1902_1_+1_1_300

QRHKFHSPLGLKETNRVNRYFTLNKDGEINTARTRNGIRLQISDPVPKCSKVTVSSLSND

LISMGQSLSHSLNDNSFCRNSLLSPKFPKRGIEKLIIRIS

>SG-PL_contig_1903_1_-3_3_635

RVGESCTLLLPPILCEPPSVCSSNEKSVYKCGILLHGNCTNHRDHCISSDKTSFCDPKLL

ICRSRINTSCFYDNTCPHRAVCQQGFCQCLPQGHWNETNQICVPLQGKVGGNCLENFTCT

TPHSQCNNATNRCECASNYVRLDSDYTCKSPLGGSCTHERNCTPGAICDPSDNKCRLKPG

QSCAGDKANLCQSGKPCVDGYCRLRVGESCT

>SG-PL_contig_1904_1_-3_774_1088

MPRNVSFTLLILVFLGLPLVLNPGISFVEYLVSQLLGLRSGQMTKPLNFLHMNMVVDWCY

TPVSLILVFMIMSSPVDSLSQTTHLTGGNFVLQGFGQGLGLALVC

>SG-PL_contig_1905_1_+3_972_1463

MRHPQRVKTTNLLMYLQKSFSLWNLTAPGILSSHSYHHLTGHEQTMKCIYALNTSRTHDI

HLTSSNHPESVMQSSNNSKSTKGWEGGNAVAAHSSIPLDCSDVKGMMYTKLSSLPETKPT

ITYNRKFPTQQKIATTTATTKESHIQTKIDISNTELATFLYTMK

>SG-PL_contig_1906_1_-2_143_1870

MENNNASASESMPKEGSEAPATRPPPVDTGKKVLNSAGVEVSKWSIRTKVWEHLEKNDKV

NFPRPCNNRIPNFVEAAAAAEKVAEMEQFKSARTVKINPDKPQENARFLTLEADKTLLVP

TPRLRTGLFNKITPPEDANKEILRKCSTALGVKEHSKKISLKDKVTIDLVIVGSVAVSKE

GYRIGKGEGFADLEYAMMATMGAVGPDTVVVTTVHDDQIEEIPEELVEDHDLTVDFIVTP

TQVIETGCKRPKPVGIIWSKLEYEKLYRIPVLRKLRDMEREAGKDVRMKEQEGDDEEFEE

RMDQERRERGYRGRGNRGAPGRRPFRGRRGGGGRFGGRRRYRVDDSADQGDESNKENADE

SNDNGEGERGGRRRRQFRNKGFRRSKPRQSESELSDGAGGEQEGEDGKENRRPPRRRNRR

RGMGSRSANSQDNTSDGENRRSDGDENRPAPRPRRPRIETSPDGVVFVGGISRRLRVSEF

KMEMRGCDVHPVRLVWRGGRGFAFLHFQSADNANTAVKALAGLEIDGRELNVELARQDNR

PRARNNRRNPKEAASSESANQESVGSSDVAVEGGER

>SG-PL_contig_1907_1_+3_48_188

MKLTNGSSFRQNIYNFGQTDPANGIQYAIYIIQILLTYQEIRHRHLK

>SG-PL_contig_1908_1_-1_25_2208

MKSKWNIISKGFCCCSNEHFFDDENPDLERDVTAHPSNSAIGLSAHVPLYPPGKMIHVVR

SHAPSKSGCKSSEAIYQAIWASNGDFDEVLVSPTMVNDHMPDNVLDSLEKVLQRVGPQKP

VRTMTEAEHHAFMSQLSESSSATPSPSQAQGFLETSFTSGVVMRQQPTGGPERDKLARSW

DHAAEQILRSQDAGSGVASANPSTDDSTGSLTKPNFTLPLTSDPIHAPLASPETLSMASG

IVGGSMASGVIGGSVASGVVSGLSQSESIRDSLRRSFHSPASHNNLRHSLSGPPAKIDGV

HVELSPGKGEGRVQSSQYRSSVDGDRVYHQLSVHCLFAKDMLVNNGTPSHAPPLPSGMDT

QADVGQHSVYDSQSECKQTDSSVLPSQLPPEALAQPNNPHGVKKSKDDSESDSAEVKASP

GKLTKQLNSVGPTNAPSAPSPPMAASGDKVRFASPETCMRHSPVETTQGEYEHPNLHYSQ

ASTGYPHGAYHPPIRYESGDPGVNVVSKSFVSEHSPAVPSQKPSQNSSPTHVSKVPSAVS

NAPTHVCKAPTPVSKAPIHVYKTPTPSVDLPLNSAPKVVSTSPSTTPHPFVHCQSESSIF

AKHFPPKLGMKRSAEQSDICHLEDEAEVFPPTNKDLFTIKRSAEQTDITHLGDDLDVFED

ETEDTVGNLTVSRGRLAALHSTPRSSTVGSVGQRQEGGSRHASSSHSSRSDDVFESEVAL

DPTSETDV

>SG-PL_contig_1909_1_+2_2_289

MNECSFSNKRVCSICECIIAKRNARLSALCTLNMNPDYRPDLGLLLQDNQKSHATQSQWD

MPLFQAKKRIITYSFKRSCYWETRLCGFIFQFDDVF

>SG-PL_contig_1910_1_+2_104_382

MMSFKICCKLIKLLRLGSWTSIPLRIQWIIIKVDLCFSLMMSFKICCKLIKLLRLGSWTF

IPLRIQWIITKVDLYFSLMMFLSVLIKVVSRIS

>SG-PL_contig_1911_1_-2_1604_1801

MTTQVVGNFILLIFFFKYGRALARAVGTVKLLISTSKTKLMNVNAGEGTVKLNGEDLEKV

EEFTYL

>SG-PL_contig_1912_1_+2_53_238

MKLRYCYFISCLQVTEFTRRLSVFCFAKKRTLKCAVAAVYSMIGRPCVCKCTPPYTPPYD

KQ

>SG-PL_contig_1913_1_+2_338_628

MAVLYVVEQCFVFVWGLKQTLKEKNRSSDLSSTLGDNGIMGRLGFPLRIMKNNDQNQSVD

KGQSHSLLVVRKERRVETQTGPIRQTAVEGQMEFKAY

>SG-PL_contig_1914_1_+2_821_1075

MESQVAHLLDGCEGWKQKHSLLPKWCPIHMPLTGCWMITATILILDFTQSDNTNANLTPT

LALAQPLILTLGISNPYTEKTNPQR

>SG-PL_contig_1915_1_+1_1234_1551

MERDRERDMTDRKASERISHNDKQQRTKLIQNSKMRYTTRNAQYDYSVQSAHFLRRFRLA

KKQALICFFRSYFILIAKYRTHLHTEIAMFSSSKRFKKIKKKPFTK

>SG-PL_contig_1916_1_-1_103_1947

MADNGAVPETREKMPIDDPEAAFAENELDDSKAKFINGGMDDTKVVISGSDSDASFVGLG

KEELKRYAEDPFWIRLRWILFILFWVGWLAMLVSAIVIIVLAPRCPTRPDLKWYDKDVAY

NIYSKSFLDADKDQDGYGDLEGIQKKESYLADLNAKILWLSSVLPTEGDNDRAILDYKTL

DAKFGTIDAFRAFCKKLLKAGKQVIMDLVPNQTSKNHTWFKQSQKKDGKFANYYVWSDKD

PGWKRADGSPMWLMDSERQQFFLSRFDGTADLNLTNEDVIAEFKEILLYWASVGVNGFHI

NDLEYLTENSTLPADDAEMSQTYNFADNADVVDSLRTITDALDNKPGREKLLFGTVTSTS

AEDVDLYIGGDGKKGLHMVSVQLGQLTRTISAQDLEQELAPYVAINTTRLGWRLTTSRPG

TDRVISHVGEDRMTIAHALQALLPGSSLPYYGDEIAMDSGTEVGKKTVTPMQWSKEINAG

FTTGTPWTSVSNGFETKNVDSLTAQLGDNTMMNSFQALNELRGNESLSFGKTKFCSKEDL

LIFSRAAPGFPSFVVVANMGGSTAHKFIGDECVGERPEAELVFTSHKTEHLDKVLKLDEA

ISIDPNQVLVFKFPA

>SG-PL_contig_1917_1_+2_2_286

FFLFFFSACCVYSCFYKPASVVCVCVCVCVFATMLYTALCKAEAGITIRYRRDGGLFDRR

HLKAKTNLKEQEALIRDFLRLCYGHTHREDDLQKL

>SG-PL_contig_1918_1_-3_402_551

MHAIIRLMSCVHGESKYYIMSTIYFIFSVNLILTETNVTLCQFIFLNIKH

>SG-PL_contig_1919_1_+1_172_585

MLSDTTGLRQSYSCFRTYTINFQYQPTCITMYCHLKYIMKEIRNIMIKHFSSFLGNNNPQ

SQITKKFHFNMSSYVYQKTPKTFTKVLSHFNHAKSEQKRTNAIPSCHSLFRKICSKTNKN

YFIFSFITEMIFHSPLPP

>SG-PL_contig_1920_1_-3_3_434

SSLPEPGDSDLSAPGALPPGRLLKFKRGRGSAWSRYEEATLSVNLPEVSAQDRRLLHRSA

PSSVTLSFASLTDLDKLARAAQLSADYSGLCMRSLGTRLQQAWGSSSSGQNDVPTNLVAL

GREACGALVDSVDTGGTPSSHPPT

>SG-PL_contig_1921_1_-2_560_931

MCCPPVSTHDNSHQLRCLKARTKRMRGSFAHTTISFTMSPSDLCYFPSAKVFCSLRQPAL

VYPHTSVSVCVYVWGEGVCMCVHAHKCMCVFGCVSVRVHAHKHACMCICMYGHVNMCVCM

RMCV

>SG-PL_contig_1922_1_+1_1_132

QMYLWSLSHKSAYILGDPQSVKLGNANNKQLNPVQKLSNKKAGT

>SG-PL_contig_1923_1_-2_2_139

TSGHNSVELLHSAVYKVNRNGESTVPCGAPVFVKETVDKQLFNFTN

>SG-PL_contig_1924_1_-1_1_201

LSLAPYVFTKVVRELASLARGESIRLSCYLDDWLILAQSELLCRQHTQRVLDLCSTLWFQ

VNPQKCR

>SG-PL_contig_1925_1_-3_3_305

GVGFPGPGGVYSPFLLPGRLAYPGAVRTPLPSAYPEGPGSLLHPGVSGQPPEVPPLAFTV

FRLPGHEVRHSHSTSVSDSGPAPGTSAQAVFPPGLILSPRQ

>SG-PL_contig_1926_1_+3_1038_1268

MYPNGVLCHTVHTVFHFYRSTALYLQKCSSPTGLNISASKAHVSKWSFMSHHTHSISFLQ

VNSALPSEMFFTRHFKM

>SG-PL_contig_1927_1_-1_358_681

MHLAVPSTVNFSKPCSNTFLLHGLAHFNFCLHRYPFPLPSVFTAGIKQLITVDNACDCSR

HGCRLNLQSLKKQPVENAFSLCLPGNVMARENLVSYLGGRLCAHGNRC

>SG-PL_contig_1928_1_-3_3_1007

AIVHPLLKKHNLDPNELKNYRPISNLSFLSKLLEKVVLNQLNSHLSTNNLLNPFQSAYRQ

FHSTETALLHILNDLLLATDSGKVSLLTLLDLSAAFDTIDHTILLTRLQHTFGISDTALS

WFSSYLLDRKQTVLINGIYSQPAHLTCGVPQGSVLGPVLFTLYTSPLSTIIKSHSLKHHF

YADDTQLQDSDSPNNIQTLLTRTSDCYSDVKNWMTNNKLKLNDDKTEAILISTRQKLSQL

PPLSLQLCNTSIPISDFAKNLGVHLDSTLSMQNFASQTAKSCYYHLRRISLIRKHLSTEA

TVRLVLCFIMSRIDYCNSLLSGVSESSIHTLQRIQ

>SG-PL_contig_1929_1_-1_301_534

MYNFHVQCIVKVVLRLFRNILGEMASFFVGLSTGWISLSLIALYFQYFDRLCLLMFCYCY

FFASCHPALSVPLCYSCV

>SG-PL_contig_1930_1_-1_274_2484

MSDQIDQLQKVKSRVEKEKTALKSEVDDLQAQIQHIGKNKGMSEKMTKQVEAQMSELNAK

VDEANRNVNELQSQKNRLQQDSADLTRQLEEAEHRVGALTKEKSSLASQLEEAKRSLEDE

TRARQKLSSEVRNLTSDLEALREQLEEEQDSKSDLQRQLSKANSEAQQWRSKFEGEGAAR

AEELEESKRKLQNKLSEAEQAAEGALAKVAGLEKAKARLQGELEDLMVDVERANANANSL

EKKQRAFDKTIAEWQAKVNDLQSELENAQKEARGYSAELFRVKAQLEEAHDSTEALRREN

KNLADEIHDLTDQLSDGGRSVHEAEKARKRLELEKEELQAALEEAEAALEQEEAKVMRSQ

LEISQVRSEIDRRIQDKEEEFENTRRNHQRALDSMQASLEAEAKGKAEAMRVKKKLESDI

NELEIALDQANRGKAEMEKNVKKMQQQVQEEQAKVEEEQRARDEARESYNMAERRCTALS

GEAEELRNALESAERARKGAENELTDANDRVNELSAQVSSFSGQKRKLEADIQAMQTDLD

EMNGELRGSEERSKKAVADATRLADELRQEQEHSMNVEKLRKGLEGQVKDMQARLDEAEA

QALKGGKKIIQKLEQRVRELEMELDSEQRRHAETQKNMRKADRRLKELAFQSDEDRKNQE

RLQDLVDKLQAKIKTYKRQVEEAEEIAAVNLAKFRKVQNELEDAEERADTAEGTLQKLRA

KNRSSVSVARGVSASPA

>SG-PL_contig_1931_1_-3_60_362

MTKGTSSFGKRHNKTHTQCRRCGRRSYHIQKKTCASCGYPSAKKRTFNWGEKSKRRRTTG

TGRMRHLKVVFRRFRNGFREGTVPKPRRKIAAPASSTVAAK

>SG-PL_contig_1932_1_-3_633_938

MEWGLCSSSSSITIVCLLSVARSLQPASPSSAMGQGGMSLTKIDQLPSRPRKLDRSTAFQ

ALQARSLNLLIGLPSIWSEPYPTRTCSAWVALPRVQARLQLT

>SG-PL_contig_1933_1_+3_3_323

ERNIHTTNTQRKNTDTDKFVDRERKKHHQKITSVPTNIPNNHHTRLLSWYILFLPFVLFF

CPKRPTVHDKDILFHRPGQQFLTMISDTGNSFHITGNSLRSCRIEKH

>SG-PL_contig_1934_1_+3_48_236

MDREDRKKQTTVKERKTAETKRAIEKRKLLAQNFQKVGILFILKEPPASPRGCKSYKFNI

IIL

>SG-PL_contig_1935_1_+1_1177_1428

MAACVKYLRLIKIFYRVGMVPNQTAHFAIYPLEFDNGPWHLKILCWPPFQQAEWVPCSGV

GKLIGNGVKSLPTHSLQTYFYYTG

>SG-PL_contig_1936_1_+2_2_292

SSQIKTNTWAFQHPRRTLWKLRNVTDKTSINTSLLQMWQKSDSKWSNAARTIFKFVPKLG

FYNTVVSRILWFRLKLKVIVQCQLQAAVRQGGFPSLR

>SG-PL_contig_1937_1_-1_13_1104

MAALSTSRQLTRVARGSTWSALKRQFSASSSRLATQMAVRDALNTAMDEEIERDEKVFLM

GEEVAQYDGAYKVSRGLWKKYGDKRIIDTPITEMGFAGIAVGAAMAGLRPICEFMTFNFS

MQAIDQVINSAAKTFYMSAGAVPVPIVFRGPNGAALGVAAQHSQCFASWYSSCPGLKVFS

PYSSEDCRGLLKTAIRDPDPVVFLENEIMYGTPFDISDEALSPDFLIPIGKAKIEREGSH

ITLVAHSIGVGRSLEAAKELAGLGIECEVINLRSLRPIDDEAIVKSVMKTNHLVTVEGGW

PQCGIGSEITARVMESQAFNYLDAPVYRVTGADVPMPYAKSLEELALPQSFNVITAVKKS

LNVS

>SG-PL_contig_1938_1_+3_3_158

IGSHPLTMSDHTQQWSDSKLFTMLGPNWLKQQFDSDPQTSIHVDCSTCRSAT

>SG-PL_contig_1939_1_-3_1695_1871

MYINTVSSSMSMHVWVHAGCALFMEKPLSENNANFGVCDCTEMNVSIFHFHHTVLLCIS

>SG-PL_contig_1940_1_+1_1597_1998

MSNQILEIFKFIIKRLVSLDGNKLTALVSVAWQRLEYSDVDMLVTIHLNLHKVVGASHHL

GLVFYGCILGKGFFCFFLCLFPELITQILDVFLACGISGLSSTSAHHFDEFIVVLDCVCG

PCPLVPCFFLGKKL

>SG-PL_contig_1941_1_+2_119_337

MRAMPIFSPSLFPNPAQGQAHFYSWMDWSLFAQGFLLGSGLNSFSQIGKRQLYLYANPSL

LLVYSKCDLRRRR

>SG-PL_contig_1942_1_-3_387_1379

MMIAIFLAIIPVLVASEGCKLPEQLRGHWNSNRGGWNAMTITNTTIRDVRANGHNLPFTC

YMQNGSTYILQSEVQEVATVQFNYYTCVDIIPSLSNPDIVFFHVLTERNQMHQNDSVISI

LSTAEKSFDIICQKNHTQLPIGYNMFFRPDHEASLVNTTCPGILQAEFSANFTTEADYNK

CPEGFELNGCDRTHFTFNYTMCGSDTEGHVASGAPNYTCLTTVRDGNVTYTLIFMQQEVS

PEQPNHYQFACLAETMKENGELKITKSAGTCGATPSVTMLASETVSCRAPTTAAPEQTTQ

SGSSTASQITMSGLFTMLVSALLCVLVAPAL

>SG-PL_contig_1943_1_-2_83_376

MTSFFFVASCQNCLLYHCRCQRGLVFVCWFGVVFFLNIIVRVKFTSSFLGCCVSFKSSPV

QCRDIVMEQKMDNTFYLIKPFLNGSIDTYSLVAIASVT

>SG-PL_contig_1944_1_-2_1175_1417

MRKVLFMVGEGKAARKRNGHCPHIAWPEISEVSTTAYQQLYFYKVIFMGQHKRWMIDPCC

KVTVCITQYVKVLKRNIVHVA

>SG-PL_contig_1945_1_+3_540_791

MPEYILSSPKSKSGTGFTGHLLTITRLCTSTLLLTIKQPVQSFSIQKKPYNLITKPINHN

YMRQFSIISNFRTAMNPVCHSVPS

>SG-PL_contig_1946_1_+2_125_310

MRSQHLHCYVQCYHSQKHIHTIHIHLTWSSHFIYLIYLLSRPSFLLTEESACVSCISCTL

TQ

>SG-PL_contig_1947_1_-1_382_774

MQYGTRLLCFWPEPTESPRKFGAKTPPGGALPMLPSSCARAASSSCSSSRSHLSQLSVCA

HSFTPLNVLGICWSFFGLQSPSQGVFLCDSAPKVCSSLANLPNVVVRCIFSLFVFPACFF

LPLWRPIPGIV

>SG-PL_contig_1948_1_-3_1443_1622

MLHRGSETSMGILLLLLILLNQNLYLNFIILFTFSSGTSHEAAQLLAGLFSVCQHHRKQA

>SG-PL_contig_1949_1_-1_1_138

GEKKNPLKSEYGCLHGEVLKNCHTRNLLIQMYSWSHSRKTECIYFG

>SG-PL_contig_1950_1_+1_1_192

SLYKTSESKRIAIHLLDSVIWAGRRSRFRVNNIQCLPKSASLVGYTLRTQLYVVSNFSNT

RGGT

>SG-PL_contig_1951_1_+2_2_160

LVGYTLRTRLYVVSNSSAKTEDFIFINQNIYKSDLLKQASKVYVQCRTFMHFS

>SG-PL_contig_1952_1_-1_1_930

MPQSGVLILSEEEKRTLISEGYPIPSKLPLTKQEEKNLKKVRRKIKNKISAQESRRKKKE

YVEQLEKRVEAANSENSDLKKKVESLENNNRTLLGQLHKLQAMLSKVPKTAAASATQTGT

VLMVLVLCFAVFLGGSGTGPSSLNVGYSTVGQAPLVFQQPGPLRAQPNMGPGPARAADTA

DDYTTPNLKSRVLMAFSKEEPDDWCDEYRPLVPWEREESCPTGMEPEPQANDPSGPADPG

VDNVMPVVPHTLGKEDGEQQSMAVVTIAVGAKNNDSQTTDIEDYQAMMHPDIAVAPTLGR

GNFSAELETA

>SG-PL_contig_1953_1_-2_374_613

MGAAWLACSRAPATRPRERMGWVGREVFVKACATQKSQGLRGLVFCVINCVRIVDFGRHV

ALILSEIAVAADDFMRNENT

>SG-PL_contig_1954_1_-3_3_374

NISEAVNAWHSTKSSWCYHSIRRVRSKSRPYEQLIWGSVRSVGCGYAECANRRYFICNYY

GRPVLFEKPYKIGKPCTECFYRTCDDNLCVNCQYPEQQFCKTLGPEKCELFLFYMDCPHM

CGVC

>SG-PL_contig_1955_1_+2_92_310

MPTHPSDQREIPSSSSSLSVITRQLLRVTLSAVWMNSNTFCVIMTLKMTHYTLSFKTERM

LSLFCLRITYKLN

>SG-PL_contig_1956_1_-2_1412_1666

MKAFRLARRKQSCSRQRSVLLIKVHQNLFCILNKRCTILLVEFEVGVHTCFIFPCVFVCS

PVCADYTKPELLYLLGKYCTIKLSV

>SG-PL_contig_1957_1_-3_258_9461

MHANNDEREAGIEHAEMSSTERELADDAQWKIIQKNTFTRWANEHLKTVNKAIADLESDF

SDGLRLIALIEVLSGKKFKHVNKRPNFRTQKLENVTMTLKFLEDDEGIRIVNIDSTDIVD

KKLKLILGLIWTLILHYSISMPMWEGEEPTPSEGGPTPKQRLLNWIQSKVPDLPIKNFTT

DWNDGRAIGALVDACGPGLCPDWEDWNKKKADDNAKEAMDAAEKWLDVPQLIKPNEMTNP

KVDDLSMMTYLSQFPNAKLKPGAPLRPRVNPARVRAYGPGLEPSGNQVGAPARFTVETFS

AGKGEVEVTILNPKGVKESCEVVFNNDRNLTYSCVYVPTMEGQYKVSITFASKVIAKSPF

TVTVEGAAGDASKVSAKGPGIEKTGVVATKKTYFEVFVKGAGNGKVDVVILDPHGRKDTV

RPSVQPVSGKDSTYLVEYVTISHGLHSVNVFFAGSQIPNSPFGVQAAPPSNAKACYATGR

GIQPRGVRVKDRAVFYVHTKGAGDGEVAVQIIGPGGMEEKCQMKKLPDQPDVWECVYTPS

KQGNYIINVTFGEQHISKSPFKIEVGPTWNSKIRAYGPGLEGGVVNQKAIFTVETNGETG

ALGFSIEGPSQAKIDCKDNGDGSADVWYLPTVPGEYAVHILCNDQDIPNSPFMAEIKPGA

DFDASKVIAKGPGLEKNGAAPNKWAEFTVDTSAAGEAPLLITAMDIDYKPLEVQVKDNHD

GTYSCRYMPTRPVKHTITITFGGVAIPNSPFRVYAAEPSDPSKVKVYGPGVTDGIQTMLA

TYFVVDCKLAGPGDVAVGLTDSKGVDVPIKTTNNEDGTFTVDFEPKVPGTYTIVVYFADT

EVPQSPLKVKVEPSIDVSGIKVEGLDSNLPVEVEHTASIHLPRDLTKAAVKVALTSPSGQ

VTDLSVSEIMDRKVAPFTLKELGPHKLEMTCEGVPLKDSPYFVDAVVGLPKVKAYGPGLT

EGEALRPVQFTVDTHEETQPGKLDVSVEGPMKPSVDIQDNKDGTFTVTYTPEAPGDYIVN

ITHGNKPISDSPFTTKIVPAEPPMVKAYGPGLTESEVSVPACFTVDMNKEIRPGELVVSV

EGPKETDIKVKDNGDGTCEVTYIPEVVGDYKVNVTHADRPIPGSPFEAKIIPAKAPKVKA

YGPGLTESEAGIPACFTIDMSEEKQPGEIGVSVEGPKEAKVKVKDKGDRICEVSYLPEVA

GDYLVNVTHADKPIPGSPFEAKIILAKARKVKAFGPGLIESEVNVPAHFVVDMSEETQPG

ELGVSVEGPKEAKLDIREKGDGLCEVTYLPEVAGDYLVNVTHADRPIPGSPFEAKIIPAK

APEVKAYGPGLIQSEAGVPACFTVDMSEEKQPGELGVSVEGPKEAKIEVKDKGDGICEVS

YLPEVAGDYLVHVTHADRPIPDSPFETKIIPAEALQVKAFGPGLVESEVGIPACFTIDMN

EETRPGDLGVSVVGPQEAKVDIKDKGDGICEVTYLPEVAGDYKVNVTHADRPIPGSPFEA

KVIPAKAPKVKAHGPGLTESEVNVPACFTVDMSEETQPGELGITVEGPKEAKVDSKDKGD

GIFEVTYLPEVAGDYLVNVTHGDKPIPGSPFEAKIIPTRAPKVKAYGPGLTEGEALKPAK

FTVDTHEETLPGSLDVGVEGPKEAKIECVDNKDGTVSVSYIPEIPGEYKLNVTYANKPIP

GSPFMATIKPSELGPDVSGITASGYGLSPDGVFIEAPSEFLVDAKSVDPKGNGELQAILT

TPSGRKLQTLVDNKKDGTYNVLYTPMEEGPQTLDVTYEGIPIPGSPFNVNAVPGSDANRV

RAYGPGLEGGLTQEPAIFTVDMKGAGKGSLGLGIEGPVEAKMKCDDNRDGTCRVEYLPTK

PGNYDITITFGEKPIKNSPFRVKINNPVDARKVKCYGPGLDTEEVSANAPAVFTIDAREA

GEAPLDVTYTDPKTGAKKSADIVPVEEGVYQATYVPTAEGKCKVDVKYGGDHVPNSPFNM

SVVPGVDPQKVKVKGPGTDKTTLASLPAEFMVDTRNAGAGDLECQVKRPDGTFIRPFLND

NGDGTYDVIYTPEDLGIYDIIVKYDGQNVAEAPFQVKALPTGDASKCKILDGADKVIPVN

KETVITVDAAEAGKGKVTCRVRSPSDSDIDIDIIDKGDGTFAITFIPQYPGEYTIEIKFG

GNLIPNGKYKAEAVPEDEFEQYLLQRPVVDDLISADQVDRLQSAAPGSGLYQPVDFCFQV

GPIFNFVSASVIMPSGKTSYPKIEDNRDGTVTVRYQPTETGLHELHVKYNNECIEGSPFL

FHVDAVNSGYVTAYGPGLSHGIVYEPAIFTINTKEAGAGGLALAVEGPSKTEIKCVDNQD

GTCTVSYLPTVPGEYHITVKFDKENIVGSPFTAKVTPGEPRKRAQLSVGSSSEVSLKVTE

KDITDLTATIRSPSGREEPCLLKRLANGHLGITFTPRETGEHLVNVLRNGKHIANSPFKI

MVGESELGNANKVKVSGKGLTEGMANEVNEFVVDTREAGYGGMSLSIEGPSKADIECHDN

EDGTCRVTYKPTEPGTYIINIKFADQHVPGSPYSVKIGGEPSAKITERITRHKAAADITH

VGSQCELSLKIPGTSPFDMTASVKSPSGLTELCDVASLDDNHYSIKFVPKEMGVHTVSVK

HRDMHIPGSPFQFTVGPISGGGSHKVHAAGPGLERGEVNQPCDFNIYTREAGAGALSIAV

EGPSKAEIEFEDRKDGSCGVTYNISEPGEYYVSVKFNDEHIPESPFRVQVTPSIGDARKI

SISALQEKGLQVNKPAAFVVKFNGARGKLTAHVISPSGAQEEALVQEVDDGEYAVRFIPR

ENGLHLVHVMLDNYHIPDSPFRIMIGKKDAADAGKVSASGDGLQTGIAGEISKFMVNTLN

AGAGALAVTVEGPSKVKLECNEMDEGYAFTYCPKAPGDYLITIRYAGVNIAGSPFKARVE

GSGEASSSTEHASVVVETVTKTSTTTKFSGIQKFSSDASKCVAKGNGLTKAFRGKTTSFT

VDVTGSGNNILYVGMAGPKSPVDELTVKYVGNNQYTINYTVKQQGQYMLIVRWGEEDIPG

SPFAVEVI

>SG-PL_contig_1958_1_+3_3_356

MQCSLLPAPSPMALYTKRTHTHTYSLAHNHTSTRSFRHVLQSTQLQRWPPASRSPIMTPP

LSLCHSYHRRQDLFSSSSSSSSSRASRIRSSKSSSRLTSTSSSASTPAASSMGKATSS

>SG-PL_contig_1959_1_-3_168_590

MRESWDRFGCLNTTPGPLSLFHTHKHIYRPKINKPPYSVLHFLVLFRCLLKSSLYFGFYS

HYYLSYSLSLIMHQHYFCMEHFDIFAFTMASDFVVFSFILMFLSLLLSHQCVCGQNSCRC

VDSWSCDHTGHVCVALYLVPL

>SG-PL_contig_1960_1_+2_809_1012

MNSKISFFFFSILLILQSGNRLVFRLIKTIFMPMFGNKLVFSNHFNLTCIPLKIKKSKEA

GGMWTFFN

>SG-PL_contig_1961_1_+1_1027_1263

MFLLDDNFDNQLESDNSLEDAVCMNVHPSGRFILAYINVHQVFSVGSEQTWQQRSPVPSA

ASSAPPLAHRLQVYRVKMT

>SG-PL_contig_1962_1_-3_681_950

MAARRYTAEEVARTVLELPHDGDNENAIGNKGDVVLSAVDQSDNESSDSDYPDEDYILPS

ISTGRGCGHVRSGRSGRLIASYLILSTLNI

>SG-PL_contig_1963_1_-1_541_813

MMEFLLLFDLFFPETSRIKYSINFVTWVVSFIECFVDYCQGIYTSKVVFVHDCAYIVYVS

IVTSKQAREFFLECCSFLFVVCMHFALLVCI

>SG-PL_contig_1964_1_-1_76_501

MFDVNVLGLLMVTKEAFALMQEKNIDDGHIILVNSMSGIRIKKNKGGHCYTATKFAVTAI

REGIRNELREMKSKIRISQVCPGFVETEFVQRQFNEELAKAVYSSMKCLQADDIRDSILY

ILDCPPHVEVNDIHVRPTEQEA

>SG-PL_contig_1965_1_-3_375_2378

MSEWHERMLWQCSHVFATYDSSLRSVEEHVNNFIQSAAASKKFSHGEQAEADNTFLKEVF

SGCVQYSAAISVILEGFYNRDGKNTLRSEQPMYHVLCYLALFRLDELGVAHFRKFIKCLD

VSAAYKFLNFFLDEKNLRTWVKDGWGTIYDSSFVQNNLLNPLLRWLQDLQEIIEHLKKKI

DNQLQPKKVIIPTTETRPFNLTQPKPRAVSMPEEIPKVKKHNPVPRTLYKASQDQAVITK

RREENRQQAEERLMEASRRQFACANPEKTHKTREVLKNIVSVQDSKLDFERHRAKPIPSF

LTKETPIKMNTAAILREGQLYQKQEAEEMKKLANLEAGTMNSTPFEEWQASMRKKDLEAQ

LAEVERRRLMGKLSYEEAILAKQKLMEENRTRVAQMKKEAEALMQDYLEQKLAEEQMVRN

LVDQTLEGHRNARDAKKKLQESKRRIVQQVAAENRELMKQALEDAEADMRRKLELIHQIR

AAEASPAPRQKMVDLTATAGARLLSEMSIAELRERLALLKVSQKEAEEKKRDEILEAKQA

KDQQLINTLECISKHRLEQTRSAALKLEEKKKGRPRAVPTSAQLTDLQRQVEEKRQARLR

SQDQMRALSNRSGPRTASLVGQRRALEESRWQELEQTRERSARVLFGDKPYSESANRLTS

FSAVTMAS

>SG-PL_contig_1966_1_-2_2_382

KTVHTSTYIGLKTLLASAKQSVHIASFYWTLRGTDIPFHDKTADEGEDIFNTLLQLGKAN

KVSVQIAQNAVSNDTTKLAKDGHAEVRTLNFTHLLGTGVLHTKFWLVDEQHFYIGSANLD

WRSLTQV

>SG-PL_contig_1967_1_-2_20_1468

MWDEPKLKEIPHIYTVTALAWKRDGARVVCGTLCGGVELFDCSLKKTIVRNKFEITHVGL

SQVIVRNMSSNQKIVLKSHYGYEIDEVRIMGKDRYLVAHTSDTLILGDIASSKLTEVPWH

GSGNEKYYFENENVCMIFNAGELCLIEYGAAEVLGSVRTEFMNPHLISVRLNERKQRGTT

DNKKMAYLIDIKTVAIIDLLTGMNISQVSHDSKIDWIELNETGHKLLFRDNKFKLYLFDI

ASEQKTSVLNYCSYVQWVPGSDVVVAQNRGNLCIWYNIEASEKINMTPIKGDIVDVEKNS

GKTEVLVSDGFNTVSYALDEGLVEFGTAIDDGDYDRAANFLEMLESSPETEAMWKTLSKL

ALEERQLHITERCYSALGNIAKARYLKETVAIAEEVEKQVGGDGFQHYKVRARLAVFNKH

FKEAESIYLEGNHTDEAIEMYQEMHMWDEAIEVAEVKMHPELETLRRNYNQWLMETGQEE

QAG

>SG-PL_contig_1968_1_-3_291_407

MFYRDLKTVTLNTVLMPNHTCLLLCALCRPNGSNRTEGP

>SG-PL_contig_1969_1_+2_1223_1432

MKNILCITDHPKSSSDFTFYPLRPTPFSNEYLLLNILVETKFLTCVWCKTCQCWEHISHN

NYMTMHLVAV

>SG-PL_contig_1970_1_-2_77_1057

MSPPDTQKVSKVVVHPLVLLSVVDHFNRMGKVGNVKRVVGVLLGANRGGVLDVSNSFAVP

FDEDDKDRNVWFVDHDYLENMYAMFKKVNAREKVVGWYHTGPKLHHNDIAVNEMIRKYCP

NSVLVVIDAKPKDLGLPTEAYIAVEEVHDDGSPTTKTFDHVPSEIGAEEAEEVGVEHLLR

DIKDTTAGTLSQRITNQLMGLKGLHSHIRDIDTYVGKVVSGKLPINHQVVYQLQDIFNLL

PDVKLMDFVRSMHIKTNDQMLVVYLSSLIRSVIALHNLIDNKVQNRHAEKGEGRDLKKEG

KADADSKKDDKSKESAADKKSAAKSKK

>SG-PL_contig_1971_1_+3_3615_3971

MNRHINYKLWLHAKPQDKCYSGKTNSQKTIHVTVRSEKKKKSSCSTSNHLTVWVRSFFPT

PASKQVYDSYNKIKHHHTNQSFVIGGEAVIENCFCVMGTFVLNTGECIVNLLQIGQKTA

>SG-PL_contig_1972_1_-1_106_1485

MRIIKVLQLALRWTCCSQHTFPFGVSRFQFHTRYSRFCSVQQSGQLFTMKLDTPEFHQLF

TPELKQLVSLFEKYGHELRIAGGAVRDLLMQKTPHDVDFATTATPTQMKEMFEKEEIRMI

NNKGEKHGTITARINDKENFEVTTLRIDVRTDGRHADVEFTKDWQLDANRRDLTINAMFL

GFDGTLYDYFSGKEDLDARRLRFVGSAAERIREDYLRILRYFRFYGRIARGPDSHEKDVI

EAIRSNADGLAGISGERLWTELKKIVCGQYNGPIMHTMLKLGIGAHIGLSDTVNEEEYAH

VCEAATTLSPQPITFMTALLDTEDDVYTLNKRLKMSNEEVKTALFILQHRRDPKPSPPLR

YCMDLHNDTVAKEPKTKDRISELLRYQGEASLLEEFLNWTPPRFPITGYDLFNYNVPKGK

VFAKTLNDLRQMWKESCYQMSKEELMDRIEEVVKRHTSGN

>SG-PL_contig_1973_1_-3_132_1322

MPNNRHQRNHRQREEDAHNNNQQAPAIPAVHHPREVAAGAGNAMAHHAQRNDNAGALRKC

CTPGSCRSPDDTIDPEDPFDAVRVLCNNENCGEGCWMHKDCFEDFEQSVLAYLRSCGRAR

SWSEKQRLQNLWTKKGYDLAFKACDCKCGRGHLRKDLDYFPAPFNENSKKQKKHKKKNDK

PMPVVSTTHKSNSHSNQVGHQNGSGPAATAVATTTIAHPPTGTRAHQNHVTTNGNSSRTE

KKGKKTTTTMRTQDAHSNDNSHVQQLGVLDNHSKVATTTTTTNPHIITNSNNNVIHGVLP

GSGNPQLRLRTNSVSSTGSAGSHSSSSSIPSSAGSASPISSSPVSGDMFFKSRKSSDASC

DSGPTSLMGFRQRLDLSAFASLPRNKQNPYHIRMEDS

>SG-PL_contig_1974_1_+2_470_808

MDVYKSNTGYCYGQGLFACQILLMFLLWLQRNEHFGDFDLMTPATLTYVINFDKNRKVIS

CLCVQNFTAIGYQTPRYRCVRTDRQTDTHTSLSPDDHKVFPEIWKNRNYEASV

>SG-PL_contig_1975_1_-2_272_679

MASADLTWLIVRNSSSFLLKRNRQNFTLEPNNLKGRNSFRYSGLVRKRTVGVEPSKDGKG

VVLVTKKVDGCRRPGKNQNSVELKRDSRRTFRAIRKITKKGGYRKDLSMAAVRRAAAIIK

SQRPVVPRKTGRVKKD

>SG-PL_contig_1976_1_-2_854_1234

MFVHFSLDESRVCFSLDESHFHKFSLDRLVICVSFCFVLFCICVNPFLYLFVCLSVFLRC

VCVCVSPTCMCVCFSNVYVCVFFSIVCVFLQCVCVCFSNVCVCVCVCVRVCVFLQCGCER

VCYGWGW

>SG-PL_contig_1977_1_-2_2786_3055

MGRSIHPTTHTLIYAQWMYVLPFCPFVFFCVSEMSTCALVCVCVERGRKECFISCCFCWF

GFISCYFIVFISFKRCVSCDYLRTFVWYSL

>SG-PL_contig_1978_1_-1_4_1806

MWDKEESSMFVCIVMDYYKNRDLRLIIRDYQEKKIPIPEEKLKKYLGEILEAVIYIHSQN

IIHRDLKPSNIFVKDDDTLCVGDFGVATVMGDRRTCPRNTVGTTVYMAPEVMTQPYDERS

DVWSLGCMLLEMTTTALYNQQQLASKLAEIKKDPYLLEEVFEEISKHYSDDWIKTIRTML

KVKHQTRPTADALLKMSFVLECVEASNSSLLDKRKRQQQTVNRIRPLTEGQDNPEHIIQY

IADTIDHEQCVQEGLEALRQLLDTANATPTQPQEGEGTPRPPILLDQASKNLISLAMWDN

VANKDLQLAGCAVLTALVIHADANDVLFTPEVLAVLGKIMKEHKGSSDVQQAAAGTILAL

SANNNASDALCQLGAIQDLLAAMRADPNNTALCAACCNALWGLTIKEVNAKIASDENAVR

EVCLALQNHIDCAEVAEAAAAAMVSLCLDDNCFETVNSLDGVGVLVQALDTHVRNAKVVK

NVCMVLSTLVEPNEECAYRVLTSDKPDDQKPAGIPVIIKAYELHRDNAEVVEAIVNLIVE

LCEYGEICQDLISYDISVPLREVRSKYHKNRDIMGPCEQALDKLGAGKAMAPRKSSNQAH

K

>SG-PL_contig_1979_1_-2_116_832

MVLSSSEDEEGMLAGVMSLCISSVKKIRKTCLEDRHLCRLAEHFGFFSPKLCSSIGHNEL

TRFVGDCVCLAVVAGGPAHTVLQRALDCENHRFLYLFEMYYACKGLLLASSKTVITKTVV

VLPVSCLHVFAVVKEFRFFGHPLWNRVVRVRGSIDVVTRKRNDAKEHGIVCVMLLRFMKR

STLLGINSEGQLSKGFVFFDILFIYFILFLVWGKKSKWPGPVNLFEFHVVDWPAVSLAV

>SG-PL_contig_1980_1_+2_2_259

QTSQAFSRHLFRFDILHNTIGILFFSAFPVLFVFLMASFHSLLSSSIYSWGFFSRFSVFL

PFIFPPVFVQSLFFTLFLRMCVCVCV

>SG-PL_contig_1981_1_-3_48_1847

MAVNGAAIVPPAELETGPSPVIGSIPPADTQDASKSREAGPTQNGIQPGSSQDLPSTSTA

PPLATRQASELEDDVFMSEAAATTAAAPAKSPPPPAYAPEQMSASATEEPATSLEDEAPL

DVQGFDYGKYVLSLFARNFYNFKYLALSLAFCINVILLFYKVSRVAGEADDDGVEDSLLG

NATAAAEDDDDDGSSEFVVIEDDYYYLEPVMRMLAFCHMLTAFCMLVAYYCLKVPLVIFK

REKEIARQLEFEGLYIVEQPSDDIRANWDKLVLSTVSFPESYWDKFVKKKVRTRYAEQYD

YDAITSLLGMEKTSGSTEDNHTGSRFIPAFLSNVDYQYQLWKWGVIFTDNSFLYIAWYFL

FSVLGNFNFFFFAAHLLDVAIGIKTLRTILQSVTHNGKQLVLTVMLTCVVVYIYTVVAFN

FFRKFYIKEEDGQVDYKCHDMATCFVYHLHTGVRAGGGIGDEIEPADGDSYETYRILFDI

TFFFFVIVILLAIIQGLIIDAFGELRDQLEQVKEDMESKCFICGIGKEYFDKVPHGFEKH

VMNEHNFANYMFFIMHLINKPDTEYTGQETYVWELYQQRCWDFFPVGDCFIKQHESEPSG

>SG-PL_contig_1982_1_+2_116_478

MANRIIFRTVKISSVNNRAIVMISTWTCVYTPALGAQVNIKIYVQSAFFTKNNTITVPSA

VREIKAIITMFIFSGTRQQILKMIKKSSRSPFFAGFKTCNRKAFSHSISLLLLNIIYIVY

S

>SG-PL_contig_1983_1_-2_5_1228

MASGTESATETSFWLKFFTEAKIPAGDAAHYAVLFSDNRIQRGMLLDLTKEYLTDMGVSR

LGDVIAILKHAKTVHDQEAKDKALKGAKYTSPTVSPARRSTGSAKKSSVFDRLGSDSSGS

GASTKITSSGESSVFSRLGGKAGQKRAASSTSPGDSDDDDSPQSPLAYHGILKFPTSAKT

SPTVPRKKFRVTVEDEPIQITGVEKFKVTGLNKVKVTGLDRIKLGSKVDSFDSSDVQSLK

VQVSSTTTTPKAAKIKKRITTMPASSSVDDLLLSARRSLVQKQEVTTTTPLTKTQGVLSM

DAPAQPASVKTRLGVLKAPASSTTSGFGPSTQKLTSGDVRSRLGARTAQAAGSTKLTTPT

SSQVKSRLGVKKTPPGASSKLIITTSAQPKSKPTSGVFSRLGKKTSVS

>SG-PL_contig_1984_1_+2_2_199

FFFFDISIFHFIDVIRKTDTRNNPTKKRTKKSLVVTVLSSQMKGSCLKVYLPEILMFAFL

TRILRD

>SG-PL_contig_1985_1_-2_209_322

MPSWSVSVSFSQQAFSFLSPLPPTVYDVYLCCLALATR

>SG-PL_contig_1986_1_-3_3_263

APCGDAGGSFSINSILTFWKFCARNFLFSIAVFVSALFLSFTVVCCFLPSWSIPLSFFQK

IFHNFLPHLPPSSAVYDVLSVLFCSGD

>SG-PL_contig_1987_1_+3_3_113

FFFSRSIVLVYSFFSRNCRILVIKTTNNVQEVTLQIA

>SG-PL_contig_1988_1_-1_682_1023

MSKGMGWLQSASVVAMLVATQISRGQQSRCRDVCGPHSAGVMLLCVCTLFWLYGPHSVGV

MLLCVCTLFWLYGPHSVGVMLLCVCTLFWLFGLYSVGVMLLHSLHTHLWWLPAM

>SG-PL_contig_1989_1_-2_2_217

GISGGGGGGGGGGGGGGGQRWVKSRVMSVRNQIMKELNPFFMGNGCSITGLFIFQNGACS

ILLQGSDRKKCD

>SG-PL_contig_1990_1_+2_332_574

MSFPAVYNKNVVTKPELISDLTLNLYIKLSIKSKPHYCSTEQLFQHLKDQKIVYITSVSL

ANIYIPIMDMITSYKTNFYKI

>SG-PL_contig_1991_1_-3_150_644

MADLRAKKAGIGAEIDKKLEMRYDQEEAAGTTKAVQLWINAALEGEHEPIPSHSQRDLHA

SLRDGVMLCKLINKLLGAKGLAKVHTQKKVASMFVAYTNIENYNNGCLQYGLAKEFTVQS

ADLWEGRKGPFLNVINNLHSLGFLANNNGFNPTYTGEVQKTIDFE

>SG-PL_contig_1992_1_+3_1314_1490

MSNCVPEIMFIFSFMPYVFQKLCSYSLSCPTCHNFLNLPQNSVSGCNLCPVLFVTRLMH

>SG-PL_contig_1993_1_-2_968_1255

MYFVHIDHTFDILCPIISVGALFSAFVNQCTRIHFIGMSVFLLSIYICKLCVFSLYTCIR

ISFTKQMRGGSMYIFILYISSHRCPTFIFWHVFYSC

>SG-PL_contig_1994_1_-3_3_302

QDHNNNNDNNDDDLFDLRRSEALNCSRRAISSRVETCVEPLIPLGRKLAHGNYEDIETPA

GLDIACQLLGDLTACMGDLSNDCGHEENKTTMVYEMLVGG

>SG-PL_contig_1995_1_+3_1263_1592

MKPLQLTLQRNSTQKEWYDRVDINRPNENSATQINMSYLKSIHLQFSLFSLQDFTFSFTQ

KQITCFSSHHFYMCEAMPLRNVDKIPGTDMLTITDGLYLRQKFAEVRKVF

>SG-PL_contig_1996_1_-2_17_643

MFLRLNEIPYVDKTVALRKGEHRTEEFTKIHPFNLVPAVEDNGFRLIESIAIVEYITRTR

NVAEHWYPTDAKKRAKVNEYLHWQHVNTRFQCALLFQYLLIIPRATGQPVDQDKVEKQRG

GVQQVVEKLESYFLKDTPFISSEVITVADLFAACELMQLFSVGEEGLYESSPVVKRWMER

VQREIGPLFEEAHKVVYLVRKMYKGQSKL

>SG-PL_contig_1997_1_-1_16_531

MAARRIAKSAVDWTAFKERVPLRQQEFYRAFKAKNDNYVNKVHQFPEELPKIDFAFYKAR

LPNPAVADQFQKAYEAVSVPYPTDKSNMLKDIDADEKAAGELSKAYIAERQEEINDAKLL

LSKIDSLPKPEEMTMEMYSYYFPHLALDPVKRPSFWPHLPHMQPGHKDSHLI

>SG-PL_contig_1998_1_+3_3_176

SQALQQKHQGVHSQQNINTTGFLERWSVKRQDTIISEIRKREATFPNINLSSAEVHNF

>SG-PL_contig_1999_1_-2_17_517

MVNSNTNAHANITMNGQELEEVEAFKYLGATLTRDGRSNAEVKIRLATATSAMAKLDKLW

RSRSISLATKLKLYRSLVLAILLYGCESWTLSAELEKKIQAFEMKCYRKLLRISWVEHKT

NDYVRSRIETFVGKQEPLLAIIKRRKLVWFGHVTRHNTLCKTILQGT

>SG-PL_contig_2000_1_+3_39_227

MKKKGGKKRNPKMSNAKKRKPTQFFTDNFQSIHAYCYYQFLLVNFLDDSFKCKHVYKTNS

LVA

>SG-PL_contig_2001_1_+2_11_229

MNTHTYKMKPMHSDSSIQKDSYMGIILLRRTKKCSATQYNKLPKNTTHGNTIQLQLSTFA

TKYVCPVFNSCAL

>SG-PL_contig_2002_1_+1_1_273

SKTAQIVHHKLGREKTVKACWEKESGMDREGRKKQTTGKERKKQTTGKERKTAESKRAVE

KRKLLAHNFQKVGILFIMKEPPAFPRGKLHS

>SG-PL_contig_2003_1_-2_290_493

MVIVYLIRIFNFFFFFKFSVLSFWDVCMHTVLPTLCSHYVAAHLRTLSLVVYTYAGCFRV

FIGHRTLT

>SG-PL_contig_2004_1_-2_2_70

CWMFSCFHKPATDMTYRIFNVHI

>SG-PL_contig_2005_1_+3_621_752

MHGKDPLSHVSGWLMETQKHPACYQQRLVVRKIPPGRNATILEK

>SG-PL_contig_2006_1_-1_13_6690

MQTTEAYSQQLQMYGKTAEDLQAQLDILQEQKHTAEQQALEKASQITQLESKLSAALSDK

ASSDDASAAQLADMEAKVSEALEAKTQAESQLEMLDSQMSELGTRFSQLLTEKAEVEAKA

LKIKQQLEELNTMYSKVVSDKEQFESEKAGVSHQLEELTGKYSEALTEKGEMESEYSKLT

QQLEELGGKLTEVLMGKAEADNRGQKLEQELNSANEMLAHLRSVSEQSEGELSQQLQSSL

EEKSTLQGQVLDVQQQLQSVQEQLQASQGQSQALQDQLSSMTTEKDDLQTQLHSLQDHLT

SLEREKDSSQSKVDELNQEVSRHVDRVAELEQTLRDLSDQFANSQADVAAQRVEKEALQT

QLAEQSQMAVQTRCAEELETVVKDLTEKLSESQSLIFHKQEELERLQSIHESQQMEIQTL

QSQYKAIRSSQNTSETQNIVELENQIKDLAEKLIESQSAVSDKQEEVDKLQSELSQVNAS

LTSAQTQLQDRESSLLQQLTMAQTEQETTVSRLSDLENEVVELRNRLATAETASVNGSSV

GGGGDSGGGDVQQVLEAMNMQLLQKADQIVELETQLQVAEAKVHDLGDKLQMKDEKMKSL

VESMQEMEERLEQLTAESEQSTSYAADSSTTHPADKSMEDELQALKTQAAEREESLAKRE

KFVADKELTLKKALATVKKLKFQLQQSNTQLEELRKEKENLEQQLSQQPASLPLSDADVL

ESAPKEQERHQGQAAPGGDSVDSSAQSSATEELQQQLQAYIEYCARLQEQVQQLTDSVQS

GESAKAKVTALEEQVELIRQEAEKSQMETLSLRQKMAEVQQLSEKSVQELQLQASQSQDK

FREHANSLQLQLADAREECNKSQQQISSLQKDLLQTQEDNKTAQERLAEALQEAETASKE

KSSLQKVVEDLQNHVQLLSEEAKTLQPSIENMKDQLQQLTKERDSLQLAAENHQQELLHV

TEEAESSCQQNILEMESLRYTVEEKTQLVGDLQIMVENLRSAQLETIENKDSLDSQMSSM

LGMVESLQGTLSTRDSELEHMLAQVGSLKDKLALREDEVTGLQSSLASLQDVIAMTKSDL

EAVTDDYGERIQDLEAQINELEQQKEQLSTDLETEKNRSQEQMEQLARAVSQQEETTASI

EASRETFQSQIMSLSAELADAQNDIQQLNDTKAALLSELNAKKESSQSASETEDLSAQLL

AYQQEIQTLNDQLSAAKDNMQVSHSDSVEIEQIRLSQETVLREKKDLEAMLSVVQNEKAS

IEGQLQQSKIDCDQLSVEIQQQQTVLAQKDEKMSILQSNQDSTSAHLSHVLQQLDQLKQQ

KEQMEVDNDTLRGEIEGMEKIIADAQFREQESFEERQRMQQDLNKTLQDNTSIKAQLEQL

QAQSPFIKEVVQPAKDDSWDWGSQVEVEEVAITRQSLQQTSAAPPSTGMSPSGSGGEDLS

RELQEVKQRYQALQQEHEMVRSRLQVSEVKCEKMLIKLKSFKEKNEKLQLTLDEADRHAD

HRVYEARRGAEEQLHSLHTRLRDLETHNGKMIHENTTLKEAAVAASQSLREARQEVEQQR

SLADKLQREVDRLREELSGQKQIAAVEENEARHSLEAEVSSLRSQLSDLRGHHSMLQEDN

DQYQQVLDTLTSTNKDLKRQLEQLRFELSKAAVPSSSPADTTAASTKEVKILKQEMQRVE

REREEVGEELEGVREEVVTLRREKQVLSKRVEDVTQQIAQLHDQHRVEVTSLTEQIDGLN

WKLEEAAGLEEELEDLRKLSGSATDDRDKLVVEISRLSSVAGEIESLKTYCASMQKSVTD

KEQELEEIRLILNVKEDEMQKLEEALSRSKSENLTLKEELGLQKQKITELLALEQTIATL

TQELSEAQGEAQQYRRQVETFTSKLDTVRSLEADFESLSEDYSAVLRDNSALAQQIEELH

SRIGDLTKQDHQATLGGEQIQHLIREKEHLTSRVKELEQQMESLLEGNTRLTQELATTRS

SAQQLSFHNETLKAQSQLVTSGGDQMSRFTADYAKLQNQFREVVEQKNSLQEEMNLARHS

LQQRESRCQQLASQVSQLVEDRTYLNTQLATLSHSLREKESEWTTIRQQYATLHQTHIST

QAKVGELERRIALDSLQKDVDVETATEADQGAQERLQSLATEREQLIQSVLSLEGRLARE

SAALSQAQRALGEAQEQIRLQAVRALQEIKLDIEEEDDPRIPETSAMLTRHGMGYVQRLC

QLLRVK

>SG-PL_contig_2007_1_-1_1_132

HMTSQEWRSDECLRDDDIVTKPRLPSMEPCVPKSDIPNNVPEES

>SG-PL_contig_2008_1_+1_883_1530

MLGALKWTANGPCFMLWAGIELKTSDIEFHRSIKRIKQPAGHEAATCCQTWFLLFTLLSF

HTFNCPRPSFRLPWKEAQVDRGRAGWQISNTGPTSACLTCLLLRPRDEMEDVCCCVPSVP

TNDYTGYGIRRRRFFYPSIHSPSHLHISHSNMHSMQNVITTQVYHKMHGKLDWCYTCWFN

VTLIYCNLVKESIPLHYKHQHSYFTGLVEKRQQSTN

>SG-PL_contig_2009_1_-2_2_616

VPGPPNAGAFKARTVPPTSFRKFYERGDFPIALEHDTKGNKIAWKVEIEKLDYHHYLPLF

YDGLCETSHPYEFFARQGVHDMLEHGGSKILPVIPQLIIPIKNALGMRNHKVLCTTLKVL

QHLVISADLVGEALVPYYRQILPVMNIFKNKNLNSGDGIDYSQQKRENVGDLIQETLEVF

ERNGGDDAFINIKYMIPTYESCMLN

>SG-PL_contig_2010_1_+3_255_491

MYTLRKWLYDSSTATRTSQLITGGFTNHKDDKDDDRHKRRGSGRRQANGLVGRTEDRKTI

LQTRQNKTKQTKQNKKPKE

>SG-PL_contig_2011_1_+1_1_171

AGALSNRPHTSTYTYTFEHEPLTLFFVNTQTTKFIHTKIVINNSSKMLTTCKMYIYI

>SG-PL_contig_2012_1_-1_1_126

GKAVMCFFLFVVDIVSTDLLLRDEMIVLTTIFVGSGLPVFSF

>SG-PL_contig_2013_1_+1_205_309

MTQKFIFQNPLLAHHMHKKGNSLAQLFLLFVWLVG

>SG-PL_contig_2014_1_-3_3_311

KNSNNKQTKTKQNETLKEFCQRLYPVSEDVPGLYGLPGIRRNIIPLGHVVSGIGSVACRA

SKYLASNLSPYVNQHTERTIAQKQHFSLSLTISCLIQTTSACP

>SG-PL_contig_2015_1_-3_135_1040

MQAARPRMLLAPLQVGLGIQMHHQFGSQFLIDILFQLGFCTSYSEVKKFEVSAAMTQGTN

IPGITKDHFVQYVADNVDHNVCTLDGFSTFHGMGTIAVVTPGTRRQQAIRRISGTLEDVT

AVGKINIHFYNHLRQGFDSFRYTQLHDFYCEDPQSTQDLLWKLSWPLHSPRPGWSGFMQL

LSRGEHPGRSSVFFLPMIDLNPSSMSCIYSTLLFVCAEARRHRIKPVLTFDQPLWWKSQL

IVQKEPEDSDLKPIVLRLGGFHTQMSFLGCIGRIMSGSGLQQVLEVVYATNTVGHMLTGR

AM

>SG-PL_contig_2016_1_-2_116_316

MLLGSGIVFYGFCFTCAFPFSICIYNISKRSGETEYYYFYAYIFCCLLSWLVYLFVSIYR

SMNVGQV

>SG-PL_contig_2017_1_-1_10_231

MGGYYDGQGGGQYSSNNMGGGGGYMGGNSSQGSGYGNQGMTGGYNSMGAGNSGAYSSMGS

STMSNLANPSYTAF

>SG-PL_contig_2018_1_-2_2_211

VLLAETVTGLLTQLNNLQRAAASLQLKVNMNKSNISVFRKGGYLGARARWVYDGAAVPVV

NVCKYLGISF

>SG-PL_contig_2019_1_-1_88_237

MRWMRDPQIIRACAYSHRKRKGERVHIVSGHSGKDRRILQLEPIVVVVIV

>SG-PL_contig_2020_1_-3_96_479

MPKSREYLSSSEESDSGSDVPKTKKRKAEPPKKAEKAEKKAKSEDSNKAKASSSDVEKGP

NGELMFQLARMRYATVSEFRGKVLVSIREYYDKDGEMRPGKKGISLSLEQWNALKEHVDA

IDQTIRKF

>SG-PL_contig_2021_1_-3_3_167

SRRSEIHALSGAKDDIGFHSNGDLALTVLPEFVAKSQQPGSHSLPDPFPSLLLGP

>SG-PL_contig_2022_1_+3_45_311

MLSPLVRWQPVFDPVLRLTEQQTDKKNPSCSLSHLPPPPNLFSSEQKPQTCRVAFSAVSQ

RQDGSINKHVSMHRLFGLARKLANANQRG

>SG-PL_contig_2023_1_-3_48_2324

MAGEIVHLNVGGTRFSTSRQTLTWIPDSFFTSLLSGRISSLKDETGAIFIDRDPKLFSQV

LSFLRTKEVLLKDIDLAALRHEAEFYGITPLVRRLALCEDLDVASCGDVLFHGYFHPPAP

PIAPVPDKRHSAVSLEMAARSTYEGARGPSEPRAGHSRQSSVDVVRSRSHSRNSSLDLRP

GQQANLDSTKISASKQEAATCQSFHVDPLRVTMVKGHHNWICVAYPHFVCCFRMKDSTGW

HLIWTSEYLSSAVERVAINAKVVSSLQDNTQKMVAAAAAGLVRLWSIADDGDSREIGVFN

MNVSVDSLFFIGSQLVALSSTGKVGVWHSMTQHWQIQDVVAVTSYDTAGSFLLLGCSNGS

IYYIDMQKFPLRMKDNDLLVTELYKDPANDMVTALSVYLTPKTMSSSGLSGNWIEIAYGT

SSGTVRVIVQHPETVGHGPQLFQTFTVHRSPVTKVMLSEKYLVSVCCEYNHVRTWSMTRF

RGMISTQPGSTPLASFKIMSLEDMDPQVSYMAGNDFGPFGEQDDLQVFIQKVVPETDRLF

VRLSSSGQRVCVIKSVDSSTISAFCVHECEGSNRMGSRPRRYLFTGHANGSIQMWDLTTA

LDFMNKNTEKVEDGGPSGLELLRLLDQCDLSSSRCSTPCISPSPSLLIGPGLLPLTARLK

LGALQASMHSNASSSSGPGDAQAEQDAEVELEDFDTESLPAADEDREAMASPSHATTTAA

ATTTGGNNTNSSSGGASAGGEGGGGGGGSRKGEKSNYWV

>SG-PL_contig_2024_1_+1_1_117

YYYTKHQSTCCVHTVNWAVSTPDTRQQQQAKQQHKDNQY

>SG-PL_contig_2025_1_+2_2_286

YNILIIVIWGTNNLNPAVSYNILIIVIWGTNNLNPAVSPIGSLLNIKCNSRSQLTLKFKR

TQIYPRTRKDCYKERQIRPFLQENIYDKTNLPNKN

>SG-PL_contig_2026_1_+1_1_198

KERERERERERERDGPKRNRWTVIMKNLILTNKVINIEPTLPKLHNQKSCHINIYMKPVI

FNEEQN

>SG-PL_contig_2027_1_-2_119_838

MAEENGEEVMLCASDDIPQGIEEQRVGQQDASEDPELEAIKARVREMEAEAEKLKEMQNE

VEKQMNLSSPTSAATPSPFPSQEEKIESDARSVYVGNVDYGATAEELEQHFHGCGSVNRV

TILCDKFSGHPKGFAYVEFADKDSVVTAMALDDSLFRGRQIKVNPKRTNRPGISTTNRGR

GRGGFRGGRGRGSGFGGGFTGGYNGGYAGGYAGGYGYGGGFTPRARRGRFRGRGNFYSPY

>SG-PL_contig_2028_1_+2_779_1006

MVYSHWVNMLSSGIAAEISRGRSFVASHEIQYLSLLTHLRIFNRLDLWSKLASDPSITDE

KSLLMHFVYRQTNAMG

>SG-PL_contig_2029_1_+2_698_925

MQGIQEKGEASETNIVIKLLHKIYSCIHTYTYKITSDSIKTPALLKKVDTNQSKALIKET

ETKIADSPPSSNPPAF

>SG-PL_contig_2030_1_-1_286_606

MKKIHTCVYRQISQGFFFFLVWLYFSVQSFLLVYIYVIEQLDSRRKQEQVICICVCVSNN

QERLVIFSTFLFNFNIYQPIVKTNIQISEKKLNVRYLCSLPCTHTQS

>SG-PL_contig_2031_1_-2_113_2314

MMPAASNKTEPKTSPEATDPTSRSFLTILDKKVRNIEKRKSRLDVIRAKKAAGDALEKEQ

QETLNNYDGVVASLDFARDLQKTFTSNAQDLEKQQKKQLKREKQEKAANDLKRTVDLLQI

QATLDALGSENVREHYKTGKHGAVVLTEDNLNSLDEIFKLITPPPPGEANYMEQLNAAGE

HINNLLEGKERAVAGSTYKELMDLLHLIIGCGYFERAREAEEEETIAPATETEEKVEEEE

ETPSVPEVPPVNTEKFTEAPQPEVQAPSAPVQPPSPEVEEPSIPHPAQVLQAAVATHEPP

ASPFGVDVPLPGSPPLFQPPAMEPPRQPTVHKAPRPLAEIVSQVEGTFSFLQESEIDVDS

PHIDPAVVAAQPMMRPPSVPSQQPPQSNIMPAGFPAPPHVDQSSGSEAQDPLSLSQQSLT

QSNLDYSGGSQSFAQAGLSHAQNLSSGVVADAMFQPQVEETPAPPSQQQHSHPMMGQALA

SGESVAQYDLPPSIPLPPSQASQDGTQMTGQEKKFQLNASATEFQSRTMYSQMPLNLQNL

SSQPPPPQNMSSQAPSQPQPPHQPNSLPESRPIPPPTTVNTEFPSNANFAQAAGDFSQQG

GGNFQGNYQRGGRGGPGYRGGPRGGRGGNNNGNAMQNGFNNRAQNGNARPGNRGGASFQG

YPPRPDYRPDGFQGFNSNGFGGAPYQKRGGAGGPGYNRGGNNRGAGGMRGGVPRGGPPRG

AGGNRGGMNRPGNQ

>SG-PL_contig_2032_1_+2_1583_1876

MHTWHFLTRDIAFEDKNTAFQYADIAFQDKNIAFQCTDIPIQNTEIAFVQTTSKYRHHIH

TKNDTQPFKIQTYHFKIQTYHFRMQTQHLPRCSSSDGT

>SG-PL_contig_2033_1_+3_297_485

MMHMFIWPCMRLCLALVGQIKRNFTVERSLSLKWVLGAYSSELLSVERLFSQVDCGRSFH

GSD

>SG-PL_contig_2034_1_-2_2_202

LLLRYFPSETVETIVSAVTEHFDTNFHLAQIQLLMDRYDVSGITEKLQFIISRTLNNMEW

MKNNFDV

>SG-PL_contig_2035_1_-1_64_1161

MEGVTAALTDEERNAAMSALMGKKAEDIPDVDLENLDDLLEALTAEELEELNGDFDPDNS

LLPPSQRCRNQTEKEPTGKFNRQKLLDFLEKSAKTEKDWDQNKPYVKEQKGKKFVAKEEA

KTVVNDDDEDVETEWDEILTVASEEELVDLAAVLGFHSMLTQTQFYASLRDEKIHEGGFT

GAARAQELKMVPDEAPNTTDVEESIKQLKANDAKVKTLNLNNIKNISIERLLEICEALKD

NTSCVHFHLAGVAANDKVGKKLAESLSVNKTLRTVNVESNFLSGEVLVELVKAINVNQNI

LELRVANQKPEVLGNKIEMQLSRLLQENKTLLRFGITFEYADARIRTNERLKQNFDDLRK

QRVAKA

>SG-PL_contig_2036_1_-2_44_2203

MFSSSSFTPSAASSEKSSDPPLQRYKYKGHDEGQLKPKHVDLSRTVNRHAHHTSDYEIPN

LVLRRGQTFEMIITFERPFNTAEDSVVLKFVTGSRPLQSKGSVISVRKTSDALPGDWTYE

VSSVMERSVTLKVTSAADAIVGRYEMFIDSIHQPPNGGELDKHRYKHPDDIYLVFNPWNE

ADGVYLENSTEREEHVLRETGRIWLGTVGKFCVRPWNFGQFDDVCLFAALALLEKSELGD

QARNNPVQVVRAICRVINSTERDSGVLCGNWSGKYDDGVAPYAWNGSSAILEEFLKKRKG

VKYGQCWTFAAVATTLLRALGIPTRCVTCFRSSHDSDFSNPVNAHWSTDNRPRKSMDDAI

WDYHVWGESWFKRPDLPHGYDGWQAFDPTPQECNEGVFTCGPASVKAVRNGELYFGFHSK

FLFAEAHGERVHWLVDSDGNMTAFQMEKRVVGRQISTKAAGTISRLDITDLYKPPDGSSE

EKEILEKASRLCFRSMPRQIANGTEDMEFNFQGEGHKAGDVSACLKMKNDSGQPRTVDVY

LCATSAYNTGVPAADLRDHSESPIIEPHAERDVVLTLKCTDYINNKDTDSHVNIYVIAHV

RETRQRYASRDTIWIEKPHLEVKTEGRTSLSQPFQVVLKVVNTLSVPLTNGIFNVEGPGI

QRVMGVKLKKPIGPGEECRETVTLKARRGGRQEVVANFYCRQICDVASAAELDITDDAKS

>SG-PL_contig_2037_1_-1_70_306

MKNNEKYTWFYSFKDIFQPGKYLTCITNKHLKDALAKFRFRVWGFISNSQWCFSTENTEN

GFCALCKSGSEDEVHFLFK

>SG-PL_contig_2038_1_-2_47_361

MPVRVATPVEGTEVYVVGHALFGTEHNLPPSVTSGVISKVISINRIPIMIQTTCAVHPGT

SGGALVDTRGRLLGIVVCNARDNSCGASFPHVNMSIPVCTVWATI

>SG-PL_contig_2039_1_+1_160_264

MPGSHTQKMILNIPKRFQSYISMISCERGGRDLNF

>SG-PL_contig_2040_1_-3_57_506

MPLRKIPKVLSPDLLHALCSMGHGDEIVLADLHFPSSSVCKHGPKEIRADGIGAPEMLKA

VMEVFPLDQYVDMPAAVMELVPSDKAKNFPVPVWDTYQEILNTAEKKQIKLEKVERFAFY

ERAKKAYAVVHTGETAQYANIILKKGCVFD

>SG-PL_contig_2041_1_-1_1_465

MASWITLIALLAVTSAMCLAETVDDGAVFLSPLIAAGRLEEAKSLSRVRWDFVNKVNHTE

LESYAGYITVNETSDSNLFFWFFPTKKNVTGVPVLLWLNGGPGMSSMVGALIENGPLKVT

EEGRVVESKWSWTTEFSMLYVDNPVGVGYSFTASS

>SG-PL_contig_2042_1_-1_22_483

MASWITLIALLAVTSAVCLAETEDDGAVFLSPLIAAERISEAKSLSRVWWDFVNEENGTE

LESYAGYITVDETYNSNTFFWFFPATKNASTSPVLLWLNGGPGVSSMVGALIENGPLKIT

SEGKVVKSEWSWTRAFSMLYVDNPVGTGYSFTGN

>SG-PL_contig_2043_1_-1_31_420

MVNDAEKYKEEDDKQKNRIAAKNSLESYAFHMKSTVEDDKLKDKIGEADKKTIVDKCNEV

ISWLDNNQLAEKDEFDDKQKEIEKICNPIITKLYQASGGGGMPNFDPAGAGAGGAAPGSG

SAGPTIEEVD

>SG-PL_contig_2044_1_-2_2_145

PNTGIHSVKNCVCVCPCLPTNNCMHATHATMPCIYITLLLSRLKLKIE

>SG-PL_contig_2045_1_-3_3_128

VFKMCIFFLGGGGGGGGGGVLSACISVLLLSHSLPHHIVCVV

>SG-PL_contig_2046_1_-3_33_173

MGTRCTFCSAIYLYGLFCVFFLASKYCLQFFTLFWLTDFYAFFVIWG

>SG-PL_contig_2047_1_-2_119_265

MKHCGKAGFIWRYFCVYINSIMLQDIYVEWVFFGCINLLYGKLGVLEHC

>SG-PL_contig_2048_1_+1_295_513

MYSNGSFCDCIVNERVELSLCNGSSTLHRFLCRLLPINSCTDIILKCEGLPINMIKTDLA

PMLAVSLYKMCCY

>SG-PL_contig_2049_1_-1_184_603

MRLPGEGSTQSATASNPAYSPPQLRKRPGALPMSSTQAAKDPRRADMATSPLTSPHGSSA

SSRVSSSQGVQSPSQRDYMHRDSAARSSNKPYIVRPTPYYNTSTQTDGQSYAVKMSDPHA

RLCNVEIQVGEGGSQTSPKV

>SG-PL_contig_2050_1_+1_1603_1866

MSRLTTSPNKDLYTVSINVSLSKGSLSFTHKMTFCWHVFLCNYVLPFNRARNTHLSDCPG

QLSLQADKWKRSLHLSVGNQFLTVSETD

>SG-PL_contig_2051_1_-3_528_803

MSVRNMVQSCGCLHCKKVEIMYMCIISRWETFILLLLVLQGVQNYVHMGERNIHVCLFHI

LFVDVFIYIFLWGVVINGVSGDCMVCGQCVCV

>SG-PL_contig_2052_1_-3_123_1076

MSQEQMTLRGTLQGHGGWVTQIATNPQFPETILSASRDKTLILWKLTRDESNYGIPLKSL

TGHGHFVSDVVLSSDGQFALSSSWDRTLRLWDLLECKTTRRFVGHDKDVMSVAFSADNRQ

IVSGSRDKTIKLWNTLGVCKYTVQEDQHKDWVSCVRFSPNAQNPIIVSCGWDKQVKVWNL

TNCKLKTNHHGHTQPLDTVTVSPDGSLCASGGMDGQAMLWDLNEGKHLYTLDGGDSIHAM

TFSPNRYWLCAATGPSIKIWDLEGKVIVDELKPDVMTAGCGEPPLCLSLAWSSDGQTLFA

GYSDKLIRVWQVSMAQRC

>SG-PL_contig_2053_1_+1_904_1443

MSAKSVNKLFHLLNETNSSCPDFKGQNYKATFHTRSSAKGPRKKTYRAHLDGEGPRKGEH

AVVKVFRDAPGTEELCDVEIAKHVLARKFARKFNKSVTESRDKIQFTLPLKSTVEKFGMG

TYLIRHSRHLDKQEWVLIEENLTYDHEHKFELMLRKNGERTKKDPSSLDAFVHFTYMESG

>SG-PL_contig_2054_1_+3_3_212

MMETDPVCVFKSIGYDITACAFSVFMIPSTKHSGSFPLQWLLNNKQMYTLLYTIYTIYFI

YMYNNNNILS

>SG-PL_contig_2055_1_-2_32_277

MATLGSPRQVSALIRAGRYAALLSGFVYGSYHLRSLTKKEEVIQEHENKLKAARDAKLKA

DKDASSKLEMDALGKECGVVKA

>SG-PL_contig_2056_1_-3_3_116

AANRPIFMLQAKIELEMLEPEPILGSASPRDKALKVNH

>SG-PL_contig_2057_1_+2_29_244

MCQSFCWRQLDVDIISRSHPVCAPPQLASDGFFFLNGRCHKATFKTHALCVCSMCGQKRA

SFFSFVCPLVPS

>SG-PL_contig_2058_1_-3_105_251

MSDCYFSGCFTHKKISKKSVLDENLENDNFELYRFKVTLDLVYCLTLKV

>SG-PL_contig_2059_1_-2_1034_1252

MEEWPDVSQLDEMEIINKTCASVHILTHSPFPFFNMLFIELIYSHTHEYTRYEKVSESWT

TYQIVCALPSLVS

>SG-PL_contig_2060_1_+3_78_377

MREKNDGTIKTGTLFTTDHMLYDCMLILRHRVSQIHDMTESATYTNTHALWRGSVYMDSD

KQSFNGIVYTSHKTNTSSQSKETIFYFGKTSKLSQTKTNT

>SG-PL_contig_2061_1_+3_3_164

HVFRLCAYEHIFMFRQVNIFQSHTQTLSLSLSLSLSPCNPLHEHKGVVGSFRED

>SG-PL_contig_2062_1_+3_1302_1529

MLLKKRYSQTKKTEIICIVYIHYRVSILHMTENKITSFSSCVHSELQRNIFRITDKNTLM

LNLNTTSLFRSLLYLA

>SG-PL_contig_2063_1_-2_215_427

MPFSLEVKCLHHSTTAPVCLVFSELQIILSLWQACLCELFCCRESRLNILITFFFMYINR

KALVVNLLDRC

>SG-PL_contig_2064_1_+1_406_540

MPQMVKYAHIHKHKEGRVSAMMFKKKYITFSNLSLQTYRYIRTNH

>SG-PL_contig_2065_1_+3_1659_1871

MLMHPKLQQINTYTILKLEHHNILRKKQPKTSMLPLKIPNVTTNSSYPLHNMNHTKITKQ

GQTLTCLKQRD

>SG-PL_contig_2066_1_-3_3_194

LHIVILMTLYYLIFHCFRSPLYTSCTLRSSTQKFLMVPKHNLKSLSWRMCFQFHCTICLE

LTAS

>SG-PL_contig_2067_1_-2_122_736

MSTMNPEYDYLFKLLLIGDSGVGKSCLLLRFADDTYTESYISTIGVDFKIRTIELDGKTI

KLQIWDTAGQERFRTITSSYYRGAHGIIVVYDVTDQESFNNVKQWLQEIDRYASENVNKL

LVGNKCDLTTKKVVDYTTAKEYADQLGIPFLETSAKNATNVEQAFMTMAAEIKNRMGPVT

AASENKPSVKINSSTPVKQSGGGCC

>SG-PL_contig_2068_1_-1_49_180

MPLYVILILFARSIALLLLLGLNIVLIIPRLSGIFSFNFCRFHV

>SG-PL_contig_2069_1_+2_2084_2425

MSPASPHHSASHIHSLLTSYSFIADLIFRQYFHLIFIHFFFYLKKSEPDMKSMNFKDQYH

GCCLLPFRPCERFMNSMSTERAVQGISNHQSYALSLSLSHVCIICRLNRTERLW

>SG-PL_contig_2070_1_-3_117_5630

MKETPKVNLDNLFGAKEIRVRAGEPLNIQLGISGTPDPTVEWLKDGVPVGNRAQTQNDEK

EARLKVAKAERGDTGNYTIKVANVHGEDTADVKVVVLDRPSPPEGPLEASDIWAEQMKLS

WKPPKDNGGAEVSGYILEKQEEGSNIWEKVPGMISDLSQVAKSLKEGKTYKFRVKAENIY

GISDPLEGVKVTAKNPFDTPDAPRDLEIPKYDKYSASLTWREPLSDGGNKIKGYIVEKRE

RNKDWVKACPYPIPETNFTVLNLKEGNIVEFRVCAVNDGGPGKPSKATPPHTVRDQIFPA

GAPAAPSVDKITKNSVDLSWVRPSSDGGAKLTGYVVEKKKKGDDWIECAHLPATATTATV

QGLLEGEEYQFRIRADNAAGPGEPSKPTKSITAADQPAKPKLDMGRVKDVTVKAGQEFHI

TIPYTGVPTPTAKWDNNGTDLEESPRVSAKLTEDTVILAVAKAKRDDTGKYTVTLKNPSG

VETGTVNVNVLDKPMPPKGPIEADEIDGESMTLSWQPPKDDGGEPIANYVVEKRKAGSSR

WTKVSSFCHSPKCQVRNLEPGVKYEFRVMAENGQGVSEPLETEDSILAKLPFDAPRAPGQ

PKCAGTTEDSISLEWTTPKNDGGSAIYGYVLEKREKGDKRWTKACVQEIPDNEYTVKGLM

EGKEYEFRVAAVNAAGIGDFSDCTAGIKAAPPPVAPKINRDLMGSAKDIKAKVGDEFKIA

VPFSANPTPTAMWNQAGLTVHATERIKLDTQPDQALLICKRAEKSDAGKYTLTLSNDKGQ

DSTFVNVVVYDTPARPEGPLGISEVTPDSCVLKWSAPLEDGGSNITNYVVEKQDVRTGKW

EPVSKFVRNTTFEVMGLVEGHEYNFRVMAENEFGVGEPLESTMPTVAQHPYTPPDPPGNL

RVDDVDESSVSLMWTKPRNDGGKKIQGYVIEYKEPTSNRWKTYNDTLIKDITATVEGLDK

DTDYEFRIKAKNAAGLGNASESTGAVHVKPKYNKPNAPSVPEAVKVGKTYVDLRWEKPRN

DGGSRITGYLVEKREKGMSMWTKANDYTVNDCNFTIHNLPENSEYEFRVAAINAAGTGDA

SLPCAMVKVKEKLAGSAPDFTKRLQNGKGPLGGETSFNVEFTGKPKPDVIWYKNGTEVGN

AGRTRIKSDDTTSTLYIGELYEKDAGDISCELVNSFGRDSCKAHLAVQVPPKLEREIRDQ

KVDKGDQFKIKIPFSGSGPFDFKVKANGRELRESDRIKISPFEDYVTLIIKETEVKDSGK

YEVEISNDSGSVTAAFGLKVCSPPGEPTGPLNVTDITKNTCRLSWKPPKEDGGARVTSYQ

IERQEVGKPYWVTVSSHCKDTMVDVQGLYEHNQYLFRVAAINENGVGEFLTVENPIIAKM

PFDAPETPGTPEVTEVGGDFVSLTWDKPKSDGGGRITGYWIEKREHGTENWSRVNTNPCI

TNMINIPNLIEDRKYEFRIFAENEAGMSKPSMASNSVKIKDPNAAIVPEFSSGLRKTAAV

EGKTARFECEVSGQPKPDIQWYKGSREIYDNDKFEILTEGDKQILIVHDVYGEDADEYCI

RASNKGGSRVSRAQLEINSPPKINVPPRFRDVCTFEKGENVVLKIPFTGSPKPTVKWIRD

GEELRGSRYHSEVTERHAILTIKDASKEDDGPYRLSLENTLGSDSAVLKIQVNDRPDPPR

FPVIENIRDESVVMSWKPPLNDGGSFITAYIIEKREAPSNNWIRVASTRFAFHNITGLSP

SKEYQFRVIAENFYGRSDPCETTEAVKTEEPEEMKKKKQLEDEFGRKVRGKYDGPKINDY

DRFFDDIWKKFVPQPVEIKQGSVYDYYDILEELGSGAF

>SG-PL_contig_2071_1_+1_1387_1599

MSIILPFTDIKTSINARIISKTQPPSLKMVLIVSSLPTVQDLDNHVSMTQGEGLNKRKQI

WYITSTVSNLG

>SG-PL_contig_2072_1_+3_60_1445

MLQSQNMSFLVDSFLMLFSQVLFFGGGWVFFLKQLFRDYEVHHSVVLLVFSVTFSLSCTM

FELIIFEILGVMDSSSRFFHWKLGLYAILFVLIAVLPFYIAFYVIGTIRLVPQKQHVRVL

IAFGAWCAILYLFWRIGDPFPILSPKHGIFSIEQCIGRVGVIGVTLMAILSGFGAVNCPY

TYLSYFVRHVTEADIQGIEKRLLQTLEMIIVKKKRVAMAKRESLRQASLSKSSGGIWSML

KNVASGSNTENVSHLKQETGALEELSRQLFLETVDLQEAMDRIEYSKTFKGKYFNVLGYF

FSTYCVWKIFMATINIIFDRVGKVDPVTRGIEIAIKYFGIQFDVKFWSQQISFLLVGIII

VSSIRGFLITLTRFFYAIASSKFANVIVLCLAQIMGMYFVSSVLLMRMNMPPEYRMIITE

VLGDLQFNFYHRWFDVIFLVSALSSIGFLYIAHKQAPEKNMA

>SG-PL_contig_2073_1_-3_3_281

CSSDLAIGDEVVLASDPKEEEEEEEEEELEDPQDTLKASCSESSECEKYKARLDACNERI

NSSKTEETCEEELFDFVHCVDSCVGKVLFGQLK

>SG-PL_contig_2074_1_-3_135_1376

MAKTVILLVIVAFGGFTSAQRGDIQRSPTVAECQGKAVDIFFLLDSSTSIWIRHYLQVLQ

FLSDVISRLDVRADDTRIGALTFSDNFQVGFDLDRFRSKEELLGAIKEETLPYRTGVTNT

HLAIRHVRQHAHFRPNVTKVMVVVTDGGSRFPTATREEARLAKEDDGIYMVVVGVGLYQQ

EEEWKAIASDPDTQFIFNVTDYDLLQALSDRLPRVLCPLPPVVFRGECPVTENADLFFLA

SRNAVPQAHDVITHLTPSFRPRDRLHVRYVMEVCDDTVDRGFQGLDKHCNRSHDASEQDG

VSLMAEVRRAARDMKFARGANQVAVVFVDPRSVINNRIEILEEARRAREHDGITIIVVDV

GVGDDDSSYLRALTSDKKNVIKYPGSDNMESLQQLLHRMCESANFSFGPEPEPV

>SG-PL_contig_2075_1_+3_270_395

MGEDFSPSQDTPAFQFTLGKSEIFPVTYLYWFWFFFFNSYRS

>SG-PL_contig_2076_1_-2_11_274

MSYVPQICHVCAPKVCHVCAPNVLYVPQICHVCAPKVCHVCAPKVCHECTTLKGHDLFGV

ILAENVWTPEDCRGNGIQLTQLISQEMS

>SG-PL_contig_2077_1_+1_133_549

MLTWEIRKISTLYPLGEVTEEDSNPGLPNPPVWKSSVFTIRPPCPSRHTSVHTHQYRYFN

TVLQYRHFNSHNCAISMLFTEIRQHRSHGPASSNLSGCHQFQFSQDSVSMLRIFPYTQTL

QMPCDGKGHPKSPWHMPKV

>SG-PL_contig_2078_1_+2_41_217

MKNEMRFDVRKEAVFNWWFYLEKEICNFISFCFLLHGSFLLKCTARQIYTVHIAESADD

>SG-PL_contig_2079_1_+3_3_125

TNSPFWRHILYHVKLVQNSEEKEEGSPSGSTGGPLPKKWAC

>SG-PL_contig_2080_1_-1_1594_1890

MDCLHVCDNGCMCLSTHMCLTCQSLIEVKFAALCTLFTICVHGQTCCLCVINGFVAHPLM

KYSPVCAVYIQTGFSLYELVSVYMDYILLNDHSDVGSYI

>SG-PL_contig_2081_1_-2_98_1351

MQSALLLAIVALCGVVFGQRPFDRDREDRDNTIPECLRKTADVFFVLDSSSSIYIEDYKE

VLKFVSQVITRFDVSRDDTRIGALTFSNDFQLGFGLDRFRSKGEVLASVNERSLPYRTGV

TNTDLAIRYVRENNAFRPDITKAMVVITDGGSRSPGSTKREADLARDAGFHMVVVGVGQY

LDEQEWRAIASDPDNDYVFNITNFSFLDSLKDVLPRRVCLMPPIILGGECRVTENSDLLF

LAAPNGINDALDVIEELADSFQSQNRLQVRYIMEVCQDEADTNFEGPDRFCDRFGDALPQ

NDETYVSLVTQLRNVATNMREERASNQVAVLFVDDLSMRNNRFGILQEVRNAAQFDGIDI

IAVDLGVREYSNFVNGMTSSRENVINYPGQSATTSVQQILDRICYHVNFSFGEEIRPN

>SG-PL_contig_2082_1_-2_2_253

FFGFTSFHYDSVHVSVCVFLNVCLLFVVVVGGEGCLFVFVVAVVLPFSYKLQVVLYRLLF

VLFWSVSPVHRLIGDLRYSDMIMR

>SG-PL_contig_2083_1_+1_1_87

VCTIIRLHQPVTLPLAEPDPQTLKTGGLP

>SG-PL_contig_2084_1_-2_2_145

DNKLFQIRPDLKVFLPSVSGCRKEETVLCRLHTGHFSLPCTCDCEASY

>SG-PL_contig_2085_1_-2_167_355

MIIMSCARCKRSRCEIQNIRIIKFIFFIACYVSLMSIERKLFICLASVCKAVQSKLHNSQ

YRV

>SG-PL_contig_2086_1_+1_220_447

MYLYKTTHHLLHSVLSSLHLYHIIHAYHETEYTGYHSMHVNPFTPSPYLSQNEGVKGLIK

KTSILQYVSLHPLTSP

>SG-PL_contig_2087_1_-2_50_178

MCPCDFTAVCSNRILCYRPVILLFFSLYFTVFLWGIFCWIWMS

>SG-PL_contig_2088_1_-1_1_234

REHALLAYTLGVKQLIIGVNKMDNTTPPYSEARFNEIQKEVSTYIKKIGYNPKCVAFVPI

SGWNGDNMLEDGGNMNWF

>SG-PL_contig_2089_1_-1_331_564

MKLVVLFQVLLLFFGCLGGFCWLVGWVLLLLLFNCLFSGCGCGKLIIRLSAENETQNSIH

GAYLQSLLTTFKPAYAAH

>SG-PL_contig_2090_1_-1_175_282

MSLSFLVFRFHVFRFHCPFCFTSISLPLCRSFRFSN

>SG-PL_contig_2091_1_-3_9_1181

MATAAVTESPVEVRVGSQKGRGLYSTTEFKEGDVILREKPLVCAQFVWNELYKYSACEYC

LRSLETAEVMSRRLTSNPLLVLPHPECCPVDLASYTCCPQCQVVYCSEVCRQEAFDRYHR

VMCMGASHADPEHPLAKLKETWRNMHYPPETASITLLAKMVAMVKQSSKPIEMVKRFSQF

VHTTVNENENIMHKLLGEEFKVQLDILLKEATGALYCDDVQQWFTPEGFKNLFALVGTNG

QGIGSSSISTWVKNCENLVRGEEEAEQMDSFINQLYVDLDQVSGDFINCEGSGLYELQSA

CNHSCIPNAEVNFLDGDHTLTLTALRDIQPEEEIYISYLGECAIERSRHSRQKMLKENYL

FTCTCQRCLEQADDPDVTSEEEMSDGEEEEN

>SG-PL_contig_2092_1_-3_3_227

ALGPLFTNTLLLKDSSIRSTFHKHFITQVALSPTGSALQSVLLKRERERERERERNVQLD

LLLSQLHVLRYVHIL

>SG-PL_contig_2093_1_+2_740_1180

MNSKQGRAKRGEKPLNLTPSLKWGEIRKKRTLTPSPEKADTDTMEMSQSFLNLMEEKSDK

DKDKDKDSEEEDDPSLDLFAPFSAALEASEERGPPVPENMAAVLRNYGKKLEDSRLKATL

AKYRVPQNCLTLGVPRTNNSVSDQSQC

>SG-PL_contig_2094_1_-2_128_1156

MKTIMNQAKMSGKVQPMQKQQKDAKVQKQPQTLAKKEVQKQPQSAAKKEAAADKQKQQKQ

PQALAKKEAEAGKQKQQQKKEKKPQQQQPANKKQEQKGKQAAKKDAPDDSGDAERREDRD

KCTLFVKNLAAGTTVKDLKALSKDIQEVRLRITVKGKKEKRRTMGFAFLEFASEAQADKH

FKSLQGTKVKDAELVVDYVGQKSNYLADKPKKEAANPDPLRLYITGFGTTTKEEDLYKLF

PKAQEIVLPVRRRDGHPIGFAFAAFATAEQCKAAHDKVQGAKVHDRSVVVLYAKKRSHEQ

QKKDAKKAEKSKVQVTKKEEKKNQKNQPPAKKARLEVKEEDED

>SG-PL_contig_2095_1_-2_128_787

MNRLFGRGKPKEPPPNLTDCISNIDGRGESIDKKIARLDAELSKYRDQMKKMRDGPSKNM

VKQKAMRVLKQKRMYESQRDQLNQQSFNLEQQNFAIQTCKDTKTTVDAMKIGVKDFKKAY

KHINIDQIENMQDDMEDLMEQANEVQEVMGRSYGMPEIDDDELEAELDALGDEIGLDEDA

TYLDDAIGAPSAPTSEPGDSMRGDVPVDEFGLPQIPQSAK

>SG-PL_contig_2096_1_-1_73_234

MAKRRTEALSTSPALSRDIDGQSGFCETFLRHKKGKVLFGETSSPGGSNGSFNR

>SG-PL_contig_2097_1_+1_418_474

MQLFHTGIILTNLNKNKTK

>SG-PL_contig_2098_1_-2_224_412

MGKLTTSAIVIVDLHCKAPVLQRIWDHPRASIGALGHPFEQTETDMIENGLLSKVTLKQL

PDP

>SG-PL_contig_2099_1_-2_2_271

LSLPHFIHQQVKELNNFTPLTMVQYQHLCDYRQFWHYSHILLSILKTKSVLPHLSADHIS

TIYFLWSSFRLIINSIRLSFLAVNTLWVKF

>SG-PL_contig_2100_1_+3_675_986

MLSVYGAVHTLPSGVTNIQTCVCILCLEWKCILNTWILILCLPVFLNESFSFVCFICCKS

GGFVCQYTCFLTYLPACLFILLIYLFVLFGLLVCMFPFQSLEFL

>SG-PL_contig_2101_1_-1_46_237

MRKRKNNCVLRYRYICPQSKRRNCVVRINLYSGHKVLKWSHIAFLINTYLSSKHIRYCGS

LVLF

>SG-PL_contig_2102_1_+1_1354_1581

MQAKTKLRRSDPKFMYLSNEQSSLPKTCMNSSAMLSQDCIKLKCFYHFLYPSIEAKMKET

HSPSCTSDYIHHSTGQ

>SG-PL_contig_2103_1_+2_2_331

GGGGGECCYLPLWFTYNRNLLKSTSKKSLVISSYLGDRDVCGKHCDLKTEPYYYYYTHYH

FGICDCFVVWIYNPNLNNKRGLPINGRKIHGDTTYSQYVNCADVCNLLLF

>SG-PL_contig_2104_1_-2_521_1147

MIGCHNGLTTVLQKLNLQLIGKWCCAHRLSLLAHWASSAVPVLKKIQEMLVVIYHFFRYS

AVRYNHLKELKKIMEEVVRKLKKPTIVGWLSLHDAVKAAEESWGCLVLILENATEKDATA

DSLLKELNTYNFTSTLCLLRDVLNPLVKCSLSFQRDQIDIEETTNALSLHGPRVTKLCTG

LDSDGQYQIIAFQASASRKDSQLLPAESL

>SG-PL_contig_2105_1_-1_28_435

MVVGQHKGVHYWTLGQRANIPGLRRAYFIAEKKAQTNDIIVASGTDHPALFCQTFFTETP

HWIRQPPRSLIEQQMVDLGFRFQHKHPLVQCTATLSGGNSLIVSLSQPMRAITPGQYAVF

YHGDECLGSAKIRRTG

>SG-PL_contig_2106_1_+3_21_170

MRFLLHQMKTETGKGDNNKEPQLCTLYRPVTALLLNYQDNDLHWTAFNGR

>SG-PL_contig_2107_1_-1_583_795

MDGDALHLKKHINILYAFSVLRGCRKQQILHITPCSCVKICYLGMIIVLTLFLCVLCRYN

SLSLFLVAVCY

>SG-PL_contig_2108_1_+3_339_554

MPLIPAQHTTNPKLIQSKSERQEAQWGHWRIDFACSHVKTGSCFVHICTRFESEFEFVHC

TQTIAYASTQIC

>SG-PL_contig_2109_1_-1_1357_1617

MSTSFLQIGWESERVCCVGCVMFVFYSKSVFDKFLSVLNSVKFCFLSVFQQNIGNMFCVG

TLSVLNSVSFLSVLQQNISFVINFCLY

>SG-PL_contig_2110_1_-1_1_231

DIFLWLAGSWRGNYFSSFIEPLVQMQSRLLLPFCPIVIIHYLYICVCVCVCVYVSVCWLN

IFLTVFMIFKECLCENK

>SG-PL_contig_2111_1_-1_46_168

MWCEHFSSFIILSKWKLITLHNWKCFLYLMIFLNSKVFMCP

>SG-PL_contig_2112_1_+2_2_118

NVSEERSEREREREREREREREREFCLLSPWSKFHTVPW

>SG-PL_contig_2113_1_-2_86_1417

MAHDVRTGRFSVMLLFLSLTGFGTLCSCFHWNSQPDPILFACKGKPRVVFPWQMTTGNNE

TVQQVKWIFDSADTSVLVASFSHNLFLTNPDFSKRVRYEPPGILVLRAVKVSDSGNYSIE

VTVKDTLTSCLSSHRQSVTLQVGDEVMMEGGQLQAHQDQIAVWQEETQQWFIHLTCGHFT

FWGTPPIHVVFTMPSGERHVSKDYQNGDFYLRVKPSLLQDGAYTCEVPAEVLPNMCFIAT

PFNLGKAMANVTVDDMKTRLSVVESSQKILLEGNRQLKERLTKIERKYDDQLGNDSSFVT

GPFQAQLDSLQRAQQTMQDQIEDLKNATQKQDEVALDTFPCTPSSYTTEDSPKRSVERGD

GGMCDMPGRGLSEGWYRFMMNGQDAEIPTSCTKAHRCSTDATGWLDLQGKLVPKLDHTVE

VRGCASWDGDCCNWETPIKIHNCG

>SG-PL_contig_2114_1_-1_136_234

MAASDFTALLLSPLPHFQLDSLCVQTYIFLCMF

>SG-PL_contig_2115_1_+2_2_91

TNITIVWLSQNFYMLLISQSCGVGQNFIYY

>SG-PL_contig_2116_1_+2_479_619

MKLENSKRIQYGYGRGLKASCTANPHTSLSLLKLWRQVFLKECTKLD

>SG-PL_contig_2117_1_-2_98_289

MLHCMTCVVSEGLMGVSTAHLIYCLLLCNSNKICVCGCVCVCVCVCVRCIIGQVCFQGLC

ISIL

>SG-PL_contig_2118_1_-3_30_446

MSVFSSFQVLTTHLIIDQGKHCQSVWYFQCHLKKIVNFIIIFWSKETSCICSCVFQCGFQ

FFSCVCGGGLFKVYLYACFDICSLDGISPNPYLSSRPIDMCVRVFMSVCVVNRLDYRLSL

SAFQFLTFSNIPISLISMD

>SG-PL_contig_2119_1_-1_16_1689

MIYSMARFDPPQIQDNPEGWGPCVMPEMYKDLPYQPFSKEVRLGKVSDWTGATYQDKRYL

GKYSSAFGGSQYAYYHEEDESSFQLVDTARVQRPIYQRGRRMFQQTKQRRERERRQQQAQ

ANMQVLTKTQKNRERDRQRMLKKWQKQFGKQMQENRNKAPMKHRDASVQIKDDWPVVEEM

DFPRLTKLNLPDTKDAEDLMRCGAMEVYDKTYDRVTTKNEKPLKRINRIFHKVTTTDDPI

IRLLAKTQGNVFATDAILATIMCCTRSSYSWDIIIQRVGKKLFFDKRDDSDFDLLTVSET

ATEPPQDEGNSINSPRNLALEATFINHNFSQQVLKPGEDKYQFENKNPFVQAEEETEVAS

VGYRYRKWDLGNDIVLVARCEHDAVMYGPNNEVQFINVKSLNEWDSRYSGGVDWRSRLDS

QRGAVLATELKNNSCKLAKWTVSAILAGSDQLKFGYVSRQHVKDTAKHVILGTQQFKPNE

FAAQINLNMDNAWGILRCIVDTCMKLEEGKYLMMKDPNKPVVRIYDIPDHTFESDDEDES

SSDDDEESESEEETETAK

>SG-PL_contig_2120_1_+3_3_212

WVQLLKFQVSERQGSRGRPSPWRGTEATPQLGVRGATEAGEKLETEREIEGIFKQIRGNF

WRNMRKMTSL

>SG-PL_contig_2121_1_-2_317_1207

MDRQPLLGEGQVTLEVSNPREIKREARYKRSEVTSLHHSYPLRLLVPEHASASGCRWIFS

LAFGGGLVGGDIINIDYHVGDHAAVLVTSQESTKIYHCREGKVSEQNLNYRVCDEAFLAV

LGDPVVCFADACFKQTQRVRMTSSSSLILLDWVTAGRIALEEIWQFSSYQSLMEVYVDED

LVFRDNQHLSDTPYQTVRQAMKGYQVLGTCVVLGKDVQQLVAHLKQQYSRPHSIGQQLTT

ELICSYSPLEAETKETTLRGCYVRFLASTTALAFGVVREITSAVADILGGDPYSRKY

>SG-PL_contig_2122_1_+1_52_273

MEKEISNSLCNMLSSFFCFVCSDQYFLGCMHASCLVYTVQPLCCCTVCILLSSVGVHAGY

FCVFINHQTLTWLT

>SG-PL_contig_2123_1_+3_231_515

MGNPVLSIIPHNSNKYTGFAWLCLNFAVLLQERMAGCLCRQVQGERAKKMHMYVHTHNVI

ISNTIILRCQVILIFSLYQFCIIMILKRAMAIHSK

>SG-PL_contig_2124_1_-3_204_1160

MTGGPEMFFVMNADVCGDFPLQDMLDFHRTLPATAICTMLATEATRQQSLTYGCVVENKD

THEILHYVEKPETFVSTNINCGIYLFTPEIFQPLEIAFKKNLEENYNFDFQVPSKEVIRL

EQDVFLPMAATGTLFMFQTSRFWSQIKSAGAAIYANRHYLAIYRRTHPERLAVNSEGKPK

VTGDVFIHPTAQVHPTAVLGPNVTVGRNAVVGEGVRVRESIVLEGATLQDHSCILHSIIG

WHSTVGFWTRVEGTPNDPNPNKPFAKLEIPSMFTEDGRLNPSITVIGSNVQVPSEVIVLN

SIVLPHKDLSGCYKNQIIL

>SG-PL_contig_2125_1_-2_119_916

MANSQKRIMLWAVPRSLSTAMCRVMLNSGLKTQILFEAYGLSYYFGPERVSERYSDVPPV

SQYSYDNAKRQFELPYTDKELVFAKDMPYYLGQRMTADWIPDGYTHTFITRHPLSTVLSY

MRVIDKDLSAWGSFDPTECGYREQVDILRLLQQQGHRVIVLDSDDLMDKPEEMLRAYCRE

IGLEFKDSMINWQPLPDKEFMELFCGMWEPEWFSHVRTATKLNRKPKRSNGQSYDHYDQE

TCALIEKTVEENMKYYEELHSMRLIV

>SG-PL_contig_2126_1_-1_337_747

MLAFFSFSFSFFFYTTSHTHPDLNHLKPFFGKKGYKWRGGSGGEKHKHTYKNNQRNIKNK

QNSRKPKQTNHRNISVVRYMTDKIWSCHVRLKSKQIYTSTLLPLERLPLVYIGAEDSPIP

SSASQTHQSTNNCSSRM

>SG-PL_contig_2127_1_-3_3_155

ALCVHSAVLFACMVFLIYFWLLRSIFFIIFYAFFKSGDDKLSGMAGVLSTA

>SG-PL_contig_2128_1_-3_69_686

MAEQKPVEEQLAEAAGEDGENEQRAAYKPPPPKSLNEILCQDKEDESLIKYKQQLLPGAG

KEEIITDPDVKANAIVKKLAIEVEGRDDIVLDLTLPKEQIKEFRVVLKEGCQYRVKIFFN

VQREIVCGLRYEQKVYRKGVRVEKDKHMVGSYAPQKEMHTYRTPVDDAPSGMLARGEYKV

DSLFTDDDKHKYLEWQWHIVIKKDFE

>SG-PL_contig_2129_1_-3_3_197

IWQNSSITRVEDKRYGLQTEEASLIVIYKLKRLIYHKYPFLHRIHAVKRAEDNVVKIFTH

FTLCM

>SG-PL_contig_2130_1_+1_334_603

MCLCVSNIFHKHITVKISPHKPLLLETKPTQQQTDGMTSRCTLSGQPSWWYILTDPYHST

HDPQYKSTHDLNSRTQNFRSSHLGACDHAY

>SG-PL_contig_2131_1_-3_3_272

HSPHSKRYIHHSPHSKRYIHHSHTPNVIYSILHTPNVIYIILTLQTLYTSFYTLQTLYTA

FCTPQKIYTIFYTLLLFLRSFLCNLKLIVI

>SG-PL_contig_2132_1_-3_144_1259

MSKSVQTQQKQEDRPKASLEDLWAKLKKQDSKSLLKQNLTEDLYKKLKDKSTKVGGTLAD

CIRSGCEHPESGVGIYACDPEGYTVFADVLDKVIKQYHKVGDLNHPEVDFGEDKIDESFS

NLDPSGKFIVSTRVRVGRSHDGYGFPPVLNKEQRIDLEKKTVDALKTLKHELEGTYYPLQ

GMSKETEKQLVEDHFLFKDDDRFLRDAGGYDDWPVGRGIFHNAKKTFLAWVNEEDHMRFI

SMQMGGDLKEVYLRLVKALKHLEGQNLQFAKKAGLGFLTFCPSNLGTTLRASVHIKIPHF

SAAPEFKAFCDKANIQARGIHGEHTESKGGVYDISNKRRLGLTEFQAIEEMKKGVLALIE

EEKKLACGGVKS

>SG-PL_contig_2133_1_-3_156_3602

MRNLQRASDQLKGDLSLSKSSHEQEVQQLQGDLNLVRDDLKMTQEELTEAQKRIADAKSN

INSLNDDLDRFRKLQEESAMEISRQSEALKEQERILTESRQQTVVLEGHLDTARDEAERL

QASVSMYKHKFEENNHRLQNMEATVNNLQDKLQDSRNKELDKDQHIDQLRKELDNLDSAH

RNSQNQVGKCEDVIRQLTQELSNTQGDLNSANSRIKDCDKDIISLKQRLQDSQHEATQND

QRARDIDNQLLTYKSEHSHTNQEYQNKEERISRLECELDGNRADLDSKLRQISQLEVNLN

DLRAELNQTKDQKAAAVDEIKRLEQSTQQLQHNLTSAHQRHKADSARYEEQVASLKQELT

EANHRGQLLEQEVDRKEEMVKRSEAEIASCRDDIRYKVDEIERLERSLQDTRQDLNKVQA

AKQQAENTICEVHSECDHVKAELAEARTQYKDCAQELAHHEEKLMLMEKNLQTTQDQLSQ

RVTEVVRYEQQSRKLQSELKTSQDRAVNAEDESREQKMMLEKTHRDLMNIKDEFHSAVQD

VQAYKQQAQKLRVELDGSREQERMLSDQVEQQDQVLGQLQSELRGERSRHQETTKQLQTS

KKTCIALEEDVSSYNRQLRDYADKLKSRDESLVRQRADSDELHKTVRNLREDLGEKDGQL

RSTHVELQSCKKQLQQHAQDIAKYEDMVSHIKSDQERLHEQLRKSQSELMDSEHANHELR

VQLTTVQGHHKESMEQLSERSRQLACHKTDLARLQQQNATMRDEIQVYEDKVRKLQMELK

NMQELNKTTEEELRHFEERYTSLQRDLVATQEKQKQTLSDLGAKEEEVVVVRVELNTLQE

KLKSSMEDSVKLRGELEQQCHNYRSANEEIQGLRVSLDESRANGDRLHRESELVVQNVNT

WVQEQKLANEKLGNKIREQSKAIMCLTSEKELLMEQIQQLQKQNNKLRVELDDRHADEDK

LRVLQSHSAHQQVLLNQLKGRLSENEVEQDHEKKNRVATIEELHIRLKSNVENIQQLNNQ

LSSTQQDNTRLRSELEREVSLRQNLEMQLKTRDQTLASLRSQLDTRNLRTDSSPAHADPD

RLTRAALSKAMEDTGVSDPQSLEKSYWIQRVGELSIQLQQSSEYWSDKVRGLSTQLDRAR

SLTSPSRLR

>SG-PL_contig_2134_1_-1_1_210

IQYLLMFFLIHSKSTCKPRGDAGGSFSTNSIPTFWKFCACNISSFLLLSLSLPFFLSFTV

VCFFLSSWSI

>SG-PL_contig_2135_1_-2_2_154

LAKAHLIHWHTFPHVKYFLHLTCAAILKLLDEASWLLFFCKTSLIFCSLQS

>SG-PL_contig_2136_1_+3_855_1061

MIVHVINTCQCDLVSVHITEHIEVIQLCGLSRFTVDHCQSVSSCCLITSVFTVDSCLDAD

IYFSQLFKI

>SG-PL_contig_2137_1_-3_3_242

VCVFVRVGALDSGENCSCLLSSMKYTFSFFFHVFLLNELNFFLTASPVLITALIALHIPL

TPLAYLQAPSIISHDSKAKS

>SG-PL_contig_2138_1_-2_101_289

MCGVELSWSFCSSDAKIYHSVFPLQAKSLTLSLSLTLSTPASCIISHDSKAGTWPSHSIT

LST

>SG-PL_contig_2139_1_+1_25_216

MKGYSRVSQSQAKMEFHSIIITIPILMYHSIVKNCPRPLRQGERWDRKRGNKTAINPTKN

KQKK

>SG-PL_contig_2140_1_-2_167_355

MQGCPSWYSIEKKRQIVPTISLVSVHKFPIALNAALAASGNSKQAHCAYLSESSAPAETT

NTN

>SG-PL_contig_2141_1_+3_885_1133

MKLILVKKNAVQTLEGQTGETDSKTCTNVLPFTKQHENPARDTDRTTRQSAASKTLGLNS

SVTFCEMQFAFLYHYTRQAYIYI

>SG-PL_contig_2142_1_+2_806_931

MKIKSKITSLNTYQQQERSWRVVEHSKQSQKHSTQTHTLDLM

>SG-PL_contig_2143_1_-1_202_2733

MKFSVTPPPGQSAFDQWKDAMKAVNRLPMGLPPEFRKKVWLSLADHYIHKLKIDWHKTVR

LAFNERSNPDDDKLGMQIVKDLHRTGCSSFSGQDNEEDRAVLKRVLLAYARWNKRIGYCQ

GFNVIAALLLNVMERKEDDALKVMIYLIDHVLPESYFDNNLRALSVDMAVFRDLLRLTLP

TLSRHLDRLQHAAQDINTGACYEPPLTNVFTMQWFLTLFATCLPKQVVLRVWDAILLEGS

EVLLRTALCLWGKLARRIMKVDSADEFYSLMGELSSDMMEGTIFNSDDMIKAIYAIAPFP

FPQLTELREKYTYNIRPFSSNPAPSRKASRMAKAVLYSDEDELDEEEMRAITSFPGMSPV

QSGKGKGTDISLVGPGAYGAESEMGMGSRDSVYMERMSTDITALKMQYDKLRQRQRQAHV

LIAAAASAKKAVEVKQKSQAPVLVPKIESPLAMNHLFVGKVQGGRNRLVTHGPRIAASTA

PRNNVLTKQLQAVAAAQAASRSKHSPTKHFPTTKNNNPAPRPDQREGEEEKQPQDCLPEE

TLTGTENANRACDRAPTAPAEKESVSEIHSPEPDTGCTVMEDGKSVVEDDRSAAEDGRSF

VEGRSVVEDGRSFVEDEKCKEEADGQEQSHGSCLELATDQETFPPKADDDDSSDSSGDDT

SCHAFTLRQLGEEIDCKAFSLDADVDNGIELCPVNSSNAEIKEEGDSGVHLSSPSLDGSG

KSSPEKKSPRPISIQLWSESSPCLNGNMTYDCQENTGQGDSNRNSHKSQITIGRCNSAPN

GSSHMTIMSMLTPATTPTHTQPTSPSTPSAPTAVSSQLLYSPSPPPPLSSSPLKTVRPFH

PLPV

>SG-PL_contig_2144_1_-2_83_1444

MFSSTGRSCTRAWGTMRLVFRGMRTQASDNQAVKSAAAAAETGGRKDMPANDMRPLYLDA

QATTPLDPRVLDKMLPYMVSLYGNPHSRTHAYGWESETAMENAREKVAELIGAHPKEIIF

TSGATESNNIAVKGVSRFYKSKKKHIITTQTEHKCVLDSCRALEAEGFDVTYLPVQTNGL

IDLKMLEEAIRPDTALVSIMFVNNEIGVKQPIGDIGKLCRSRKVFLHTDAAQAVGKIPVD

VESMNIDLLSISGHKVYGPKGVGALYVRRRPRVRVEAPQSGGGQERGMRSGTVPTPLVVG

LGAACELAAKEMEYDAKRIEELSHRLVDRIMAECPNIVRNGDTEHTYPGCVNLSFAFVEG

ESLLMALKDVALSSGSACTSASLEPSYVLRAIGADEDLAHSSIRFGIGRFTTEEEIDYTA

DKCIKHVKRLREMSPLWEMVQEGVDIKTIKWTQH

>SG-PL_contig_2145_1_-2_20_1159

MEGMAHVRSLCFTHFRSRLLRMTVDFNLQGRSFHHGTMVLQRRRQEKAVAEASEVEEEFR

TYEVPGLKPRMGARRFQRKKRAIPPPRYRQMATDQDWTNVWPSASSFKWSVVPLPVRQGF

VETVTENEGIIPAKYANAELMKIPNFLHLTPAHVKKHCAALKDLCTEWPAGLETDEDCQK

HFPVEVESSDYVFSGPSIRDPRARTVTVKLRLSDLDLDYHARDKFIRLVGDCYRSDTDEI

VFVSEKCPLKRQNYDYAMYLLTAVYHESWKKEAWEVEKTEADMEKFFWDISKSKKTVITL

LQTIKEIDQTSSVPDEQKLQYLPEDTSEESLLAVKEVRDYRDAVMDMLNNGETQEALEKY

GQAVRTLLGIKGDVQAQPAS

>SG-PL_contig_2146_1_-2_635_850

MGISIMRWIIDAVSESGHIVPGGKVISSFLSFFVCVGLVSSVVIVIVTVCTIVFISVSSS

FMGTSMVSWVSP

>SG-PL_contig_2147_1_+3_168_455

MLSNRPNLRKQSHHIQYTPNQNKTIKTKQNNTNKTRENLQTTKAHKQQQFSVPFSSSYKD

KSSINRSPKTPRLTKHTFNCMVVNNYRFKIYPQYLP

>SG-PL_contig_2148_1_-1_49_783

MMSESHTVTDTPSKTAKGNGGIVDDFMYGSNVASAHVYIRLGFLRKVYGILSVQLLATTL

VSAAFLLSPTVADFVQKNNWMMMVSLIGTFASMFALMWKRQETPTNYILLAVFTLMEAYA

IGVIVTLYEVSSVLQAFFLTLAVTVGLTAYTFQTKRDFTVHYGCLFSLLWILILAGFMQI

IFPGDLMERGLAMGGAILFSFFIIVDTQMMLHKLSPEEYMLAAINLYLDILNLFLHILRL

LGERK

>SG-PL_contig_2149_1_+3_3_149

SDDFKLTVKISVVKFRNTTVKYNTVQNCAWKLSMYTLNSTHYAKSTEHH

>SG-PL_contig_2150_1_-1_130_297

MKGHRIWDSFSQCYDDENGDDSDHDAMEFHLGLRLRDRTVLYAFFHNISWRQPGPR

>SG-PL_contig_2151_1_-1_1_189

LFIHSSTVHFYSYLNLQIMIHTQMFRSYVKKNLFCTAGVLFVILSLFIYILFIFICIFCL

FIF

>SG-PL_contig_2152_1_+2_65_211

MSENRKSYSCVKESCITAQQMRQEGPADYSCLSLKNCHVYRLTVHGAAL

>SG-PL_contig_2153_1_+2_743_910

MSSLYNHEIFHMQFPFIYCFCRHHTGTNYSHTHTHTHTHTHTHIHTHNVLSHTLSH

>SG-PL_contig_2154_1_+2_2_172

KKKKFTLYNMKMNYSGLKYTLRQFNISLLICSLNCLIFCQYIQQLICGQYIQQLCMK

>SG-PL_contig_2155_1_-1_1_168

KYLGISFTTRSSFVTACKDLAKNELLCIMQKLHVLLEIFVKLFDSQVQPVVQYGPE

>SG-PL_contig_2156_1_-2_2_148

DGAVMPVVNVYKYLGICFTTRLSFVIACKDLASRAKRRIVVYYAEIVCP

>SG-PL_contig_2157_1_-3_3_776

QQIGALSHVVNSEEEYSGSYNWDYLLDWGPQYQPLAHVFSEIAKLKDESVTPKKTPVKTV

PQRKINGGQGLAMPQTRIVPPPIITNAPPPLGVESSETDEGDRESDIGLPPLSRSEHSQK

STPSSSSVTGSRSQRSGTGNSLPGLPRSPISYEPSYNSSALTPSFSPSLSPLGTHTPSIS

STAGGPHGDSPRSNHLHNHSNTVGLTPGHPISSRHHHHSQHPPPHYHHHRGHYNSVKDGS

RGQWTHSPSSDSDREFRI

>SG-PL_contig_2158_1_-1_1_138

SCLPGHSPAVVSLQSSPRPQGDAGHAALLLKPGVDQIDHLINWLVD

>SG-PL_contig_2159_1_-2_2_187

GAFPGTSRAGTQPSPESSSTVSEDQVQRLIEMGFSDDSVRRALRLTNNDISRATTVLLQE

AS

>SG-PL_contig_2160_1_-2_266_1684

MRRAKSLVENSSHIATLKLLEAKLGGGAKSKPDADGTRPLPPIPPSVPISKTAESVSVQT

TDTLKRTTASTSVGTESIYASAVVRDEPPPPPKQPPPQLKMKKKETTEVEEKFETVIKPD

EPQEHFKRVIVPDVEPPKAPQIDYAGFERVITELFPQNKKHFLVYNKVPWTLHIRKEVFW

PSERLENKTMLHLIYCQVAQDVYNNACVRISKDQRVKMKSMLEGYGVNKNNYLIKELNMQ

VKKVIVDTAKEWSTYFCRLFPIAGNSHYTGVKYLGISHTGLRLITREKSLVDDHLSVMED

IRFEDVVDVVLPNSTTIQINLRTKSLLFHTPRARQLKDMIDRFCQDSEKGNKFVVAVKDY

LTRESTLLSFKRGDIVKLMDPEMNLERGWLYGSLNGIVGLFPEEYVRPLARHEVETPNMK

PKLYQPPQVGRPLSREGAPGPDDRSETSQGTVVNDGKFSMMEFALLNFRESLD

>SG-PL_contig_2161_1_-3_81_185

MEVNYSVLGKFLCFGLCIPLWITYNKKAVKNIHFR

>SG-PL_contig_2162_1_-2_2_166

ICVAVLSLRLSFYHFTALFTPFELNHIDFGESHAFQHCDSKVKQVSQKYQPARIS

>SG-PL_contig_2163_1_+1_133_237

MLHEHTMSGVQSGRIWKLVLQLYVLSIIRDAQKWE

>SG-PL_contig_2164_1_-2_17_1297

MVMVSSIVCIYYNVIIAWTIYYLFMSFRATLPWSHCDNDWNSENCMDTGKMLEAESAANA

TPASTVAMNFSNVMGNTVYNATSNVTSVIADTSKHRSMSASEEFWERHVLQLSSGIDEVG

NVRWQLLITLAVSWLCVFLCLFKGVKVLGKVMHFAAPFPYLVLLVLLIRGVTLPGAIDGI

SFYIIPRWEKLLEFQVWGDAALQIFYSVGMAWGGIITMASYNKFNNNVYRDALIVPFINC

GTSIFAGFVIFSILGFMAREAGSTVDQIVTQGPGLTFVAYPEAVSKLPISPLWAVLFFLM

LFVIGLDSQFGMFETMLSGLIDEFPKQLRNKKTIVTAVTCFAEFLLGLPSITEGGIYVLQ

IMDWYCASFSLMLISLAECMAIAWVYGLDQFFKDIALMIGYEPHRMFWTIMWKFVTPSAI

LFIWLFS

>SG-PL_contig_2165_1_-3_3_173

LPWWGGGSRELAGLKPLPVCCCSFLPYTNTDFPASVSVASTTQMILTYPKCSGWRPC

>SG-PL_contig_2166_1_-2_2_73

LPIFFFQQWRLKVYMFYKFSLKSR

>SG-PL_contig_2167_1_+2_2_136

GWACCMGNSRFSLTLCPGLCPGVPPAAQIPQSFGAEGGRMPTSET

>SG-PL_contig_2168_1_+1_25_240

MRAMGARLQRERDAPSPSLEGLATDLASLGTEAVGAIVESAGASVRLSYDLTMLRRDSIL

AASILPALRTVP

>SG-PL_contig_2169_1_-3_609_2594

MTGIKMGNQLSRQQAARRWSVLREELTFPEPSWELYDGWEIYHIDIELQTLPPHVRDKIL

DFCVDEYTQEIISCEGGECTHKTHLCSKSQPCSQKQDSSDNIRLQDDVNSTEEKCLQDNG

DVCQNICVEGSESPSGLNNRGDHKNELDELLLQWNLCLRESSGSESYSKTDSVQSRSHDV

SSSRVDPDTVENREEVSASNSVLVNAELKSTVQKRESSSFCKMHEESSSSKLSSCHFKSV

HRRCLSEMESIDFTTLCPIKDPGCCSKDNNQCSKSLPQPVPTSKPNGAMNSLQSKLLACT

GNFDDISPFTSSSLPTRRQSTPAVRTRKQSSLPNSPACRKKRKFSLKDEPGWIPNPLDSD

SDQEQSEDSGVFSDYAASPKQAPNLCAELLAHKAVVRLKERRRHNSEPSGTTSTNSSLSE

NHSDSENNKSSILGTRRHSTSDSKWSLVKSPWRKNKSKLLDDSKLSVPTEVEEGDLEKEL

EWLVSDPDCETSENKDSPEEDVPVNDDSVSPTNSSNIDSSVASISPLYRQPAGALQQELE

LDWLCEPEWNNLELETFWNVLRPHLEFLLQQSETEHLPDPRVVVACMDSADQKMLDSCLL

HVQRHIWKGNYRQAVADFRAVKRHFRCCANSRLIQRGIQQDDVESLRLVFLRKRPSRYRL

WI

>SG-PL_contig_2170_1_+1_1_183

TVADSCLTACKLELTTCKLELTTLVNPYCQFRLPKVVKELIFQSIRKMISSANGTVLETG

K

>SG-PL_contig_2171_1_+3_3_314

RNESFYLNARKNVTSCYLLSTKTHNISGVFCFECIKFPPVHKGLYYSADIKLVVYFAKVK

IHVHNYCHGFSGYLGNVSKHRCPLRYNIHSRIEGAVYCSILVIS

>SG-PL_contig_2172_1_+3_99_335

MTSTSRRIERRMTAEQVAELIMNYDSNNEEIGGSFTTESEDFEPDSESESDFYTHINVHV

CLYLHICFSVIVSRPSNIM

>SG-PL_contig_2173_1_-2_2_340

LVDVNRNINSQKYAEILDQTLWPVVAKYFSDKSWIFQDDNCPVHNSRYTLQWKRENSIPI

SLWPLQIPDLNIIENVWRKIKSALNRRTQEVNCRADLVRVVQEIWVSLSPAYI

>SG-PL_contig_2174_1_+2_2_133

DKMLIKPFLGFQPKNSVACKGGNCYWTKQQLLPISSGRFQRTKL

>SG-PL_contig_2175_1_-1_73_486

MYALSRGPSKIVASTRRGTPRPIEVVPENSRQEQSERVDSDATMSAEPRPIFNHNGRTRN

HPRPSGNNGVMSSTHQELYSFLKASWERAKLDYNKRASTEGGPEWYREKEANPAYANFKK

VDWEEFFVKKTMRRYTES

>SG-PL_contig_2176_1_+2_2240_2617

MFSITSSKICWLHMQRVSMYEQCICQPHSTYNIMYVPSKSLVSKPAPELCVSHNSTKEQL

AFFWSFNTTATTLLLLLRSWAATPTCTRICKWAVTCMAFSPCHVGSHASFSGVCHYKTEL

HKQHRE

>SG-PL_contig_2177_1_-3_390_635

MCVCVCGYVVVCCSFVCIPSLLCFPACLSISLSSSLDSLLCLSVSVYLSVSLPRFSSLSV

SLSVSLPRFSSLSVCVSACLPP

>SG-PL_contig_2178_1_+2_446_676

MTHCGTSSCAKHSPTGSDRQHLYYHSHYHQSKKSINFSSFCGPSMGPKDVAGNGRRKKKN

PNNFAARPISSPPEFVW

>SG-PL_contig_2179_1_-3_132_254

MFYTVIFSYTHTCSDVSHSCILIHTHALMFYTVILSYTHMQ

>SG-PL_contig_2180_1_-1_364_654

MGWERIQNDSGIIVYLRISPMCWQGLEVLPSLWIPYTSVHDCPTSLLNKTGSSAMSHAVP

HQLRPRSSHPGCRPPPSPPAPLPSEGPIDTCTGLSLV

>SG-PL_contig_2181_1_-2_2_370

CCCCCLSWAQVGLVNLFQTSYHIFLSQVLFLGNVLCNNDVSAILLYHSFGVLKRFLLGTY

FVQDQWNLEVTTFVVLPISGLVLTSQPRKKINHFSVTMFCVSEIILTSSVFEHGIFSCRG

YRF

>SG-PL_contig_2182_1_+2_2_82

KKQQQANRPNNKIRNTVSTKPHRKWCC

>SG-PL_contig_2183_1_-2_2_430

QEPVEEVPRVKVPSPEEQFETTKAQGNKHVAKGEFEAAVECYCQCVKLFADRAAVFTNRA

LCYLKLNKAQEAEDDCTTALALEFNNSKAFYRRALARKAQKHYKDSLQDLLQLLRLEPNN

SAAKKEMEVVKGLYKEEYDQLKA

>SG-PL_contig_2184_1_-3_573_818

MLKALVGWLVYISFVFVYFLVYSLVLIFKKNRSSASGSCISDLQFVCLASISDNPVALLM

SSWLVLLHCLLVQEATLLKLVM

>SG-PL_contig_2185_1_-3_111_281

MWAYLISLLKIQSVRGLETSAGSSLMVIFPSSAVWVMIEGELWVMIEVQLRVNPFTP

>SG-PL_contig_2186_1_+2_131_238

MLTFTIKFSKNYSLLISIKYLNMCNLGHCLVQFRKG

>SG-PL_contig_2187_1_-2_110_1357

MAELQKLVQNCHGFNSDLMKMFVAKHRSDSIKPDLPPAPVLKGTGGASIFSTTSTTTTGT

NTTDNANIIMQKNMNDNQQNNTTTTTTTTTINSDNKAILQNQNNMDNRNLNANVNLNANR

KMAKAKPASPAASPDQPDPNANVSEVSAVLEEVRGAMSESRQQLVDQVTEVTVDAHLATC

INTQKAVADATARFLAMKASGQLPDMSKLSLSAGAMWEKMMDQMMPEMTKTMKFCKMLPG

FCELHADDQMMLIKSGMFEVMMTRFSMLIDHEKETMLDPSHKMVCPRQVIQEMPMGKFMD

GFFHVGAQFNPIGLTDSEIALFTAVLVFSPCRPGLADTQLVSSIQSLFQQALYCLLKKNH

SDPDDKLRKVLSLVPMFRRINEEHSKALGAMKMQSPDAFNQQFPPVHKELFSSESQ

>SG-PL_contig_2188_1_-2_182_355

MSALFTALSPSPLGYYHLSLQDNNCQLCTVSWITVVCVISRITMSAVHCLPDNGFVCD

>SG-PL_contig_2189_1_-3_261_608

MVCLFPPPLLNPPLRLHGISAVSGDKNHTTRAKLTLPLSLTYLPRSSQNHSIAHESSSPF

LLCCTFQACIPVSLASSTFAMATRQQSIVFLFCHKEMSQVVKLDICSNYQWTTCTK

>SG-PL_contig_2190_1_+3_714_944

MLKKIGLLTDCCSWVVSIIISDFFTDFGQCFFLLLFFLMVYITSHQRLQTHEWSEFCEVM

NIWYRHSSLKKKLICHV

>SG-PL_contig_2191_1_+3_894_1130

MTVQYPVKTSGCVEFITYTFFHYDLAFLFLKTNLILPQNRFLSFIFFFYLGDITYIKTLC

HKRQMRPQICFYFPLLLNL

>SG-PL_contig_2192_1_-2_11_436

MVGNSICVQIPWDHNPEALAKWAEGQTGFPWIDAIMIQLKKEGWIHHLARHAITCFLTRG

DLWISWEEGMKVFDELLLDVDWSMNAGTWMWLSCSAFFQQFFHCYCPVNFGKRVDPTGDF

VRNYLPVLKYFPTQYIYEPWTA

>SG-PL_contig_2193_1_+3_423_692

MTVSTTISHCSLQFVQEGFITLAVTAISPKLRDRIQIASGQLKHSAAFSTCFWLIPRLSV

RWTLQKGLGHKQQSCPSFQMICISLAPLFR

>SG-PL_contig_2194_1_-1_730_933

MASSSKRIKRRMTAERVAELIMNYDINNEEIRGSFTTESEDFEPDSKSESDFNTDINVHI

CLFAYSFQ

>SG-PL_contig_2195_1_-2_242_427

MPQKIHKKKPYRQVTLQNGHLVPIPVFTIPLPSHQSKMVQTVHHKLRGKGRRGWVKACWK

KS

>SG-PL_contig_2196_1_+3_54_497

MSKQQQFRKEELEEAFIMNCSPDNTIATSKLGMVIRSIGRAPTESQLKTLITEFENRGKR

TLTLQEVMAILNKYEFAPETPDNLREAFRIFDKEGNGMINAAELRHILTNLGEKLTDEEV

DEMIREADMTGDGLVNYDEFVRVMQMGL

>SG-PL_contig_2197_1_-1_37_450

MSDAGNMASELKVYRAAEVKTHKDSKSTWLIIHNHVYDVTKFLDEHPGGEEVLLEQAGTD

ATEAFEDVGHSTDARDLMKDYLIGELHQDDRSGQSYQTNTGPLGSEQTSSTNWTGWLIPL

GIAVLAAVAYRFLVAPKY

>SG-PL_contig_2198_1_-3_2028_2189

MVSVVNEQSSVCLTHLKHVLSQRFVFINAVKKKQFCWNFVLTAFPFCTYLFGRS

>SG-PL_contig_2199_1_-2_419_538

MNIHQCCIKSEIRQTKQFVPLAGLTDILSGNIKLYLTPPY

>SG-PL_contig_2200_1_-3_21_596

MDASTEGWGAHMGPHHASGTWSEHERSLHINVLELEAVRRAFLAFEAHTSHKVVKLLMDN

TTVAFYLNKGGGGRHSSSLSLQSETRCHDKSITLLSRHIAGKTKHPSRCSQQGPHCSPDG

MDPERSSARTSVGSLVPSDNRSLCDQVQQETPHLCLSSPRPGCLHWSGLLAYAFPILQRV

IRKARLEKATLI

>SG-PL_contig_2201_1_-3_102_815

MVEGGMKCVKYLLFGFNLIFVIAAIALIAVGAYVQVKLADYFDFFGNQYTGPGILLIAVG

TFIFIIAFFGCMGAVKENYCLTMSFAVLLAVVFVLMIAGGVAAYVLRNKVEDQAVTVLTE

AEKKYKINAGVTQTWDKLQRQFKCCGVKSYTEWEQLNLTTVYPQSCCVDESETECSSDAP

IPASAIKKTACEDAFITWVKDKVAIIGGVGIGLAFILVVGVLFACCLARAIRKEYEVV

>SG-PL_contig_2202_1_-1_238_426

MQFCTVNDMFIFYPMYRTDEMTTKNKKNKEVQLRIKEYNAQVGMMRTEFDFKWDNYVHIL

PQT

>SG-PL_contig_2203_1_-3_3_212

EELLRSKCVTCANIRLTENVILFGVDENMHTDSIFDLIILPGKIYIYKSKLEKNLPSVTS

FADILSQNTR

>SG-PL_contig_2204_1_-2_8_601

MPNGCAHDIPTALKIIYSVPKGVWRYCDILTTDDILPNSCSKWLEKLGTNIPWKWVFMKI

HKIQEIKLKWLQMRIVHRILGTNVILKEMGLVNNFCCDFCLETKDSTDHIFWKCLHVKRF

WESIEELLKSKCVTCANIRLTENIILFGVDENMHTDSIFDLIILLGKMYIYKSKLEKILT

KCHFLYKISYHIIPDRRI

>SG-PL_contig_2205_1_-2_506_685

MNKCVHNKLHTRCTRSIFKFFFLNCDAMLCKDQDTGASCNLKTMFVVMELHRWQLFMFGS

>SG-PL_contig_2206_1_+3_540_695

MSQETPALNIHILHPSDPKHLVMPIKLMHQFICDFLLGRACSCWVGHVTLIS

>SG-PL_contig_2207_1_-2_125_619

MTIGEDRKLFVGMLNKQQTEEDVRQLFHPYGAIEECTILRDQNGNSKGCAFVKFSTHSEA

QSAINALHGSQTMPGASSSLVVKFADTEKERQLRRMQQMAAPLGLLNPFALNQIGGTAYG

TFPQVEQPYELSRVGSFDSLSQAPLTLLSDGLVPMKLEPVYTKAS

>SG-PL_contig_2208_1_-3_39_377

MHIDISAEVLKIRTHIDISADVLKIRMHIDISANVLKIRMHIDISADLLKMCMHAGISQD

FSELIVFRDCHQQKLRSTSDERILCIPHVKTKTFGECSFSFCAPKQWNSLPSD

>SG-PL_contig_2209_1_+1_91_276

MLSQLQYTATHQEIKGCAQQRIFLKNALKKSRMSHTQSHNQGQHKTIIITIHIKQIFKKA

AG

>SG-PL_contig_2210_1_-3_39_608

MNPILRGMIRTFSEKEDEIVAPDLRSLYYGPFEYSRQDGVAMEIQRDRDHGVGDLNSVLY

SYSMPTISNWGQMGPRAQDLRDLYTNGTASVDNIDLFPGAMTTRNYVSGIPEIFYKIIQH

QFERIREGDRFWFENNENGLFTPEEVAEIRNTTFGDILKLTTSLTGQDIDKVFTCTAPRT

CVAPPALTND

>SG-PL_contig_2211_1_+3_3_338

LVVLLQGSQILTGLRELTLLHTLTHIPVDEGTLGVHQIELVIQTGPGLGDGGGVAQHAHG

TLHLSQVATRHHSGGLVVDAHLETSGTPVHELDGALGLDGGDGSIDILGHHI

>SG-PL_contig_2212_1_+1_91_243

MMLIDHIIKTKQVRTAGNVRTTSLKCLDCEMMGLMLNRMGLYRSNMLDRSL

>SG-PL_contig_2213_1_+3_3_254

PIFFFLRRQCFSLFQRVKIICVKQGTVLVSNKTNGDEKVACILNTTIKMPNLHSNDTNYQ

NTTISICGNMSILTGYNKICCCMI

>SG-PL_contig_2214_1_-2_86_241

MSEFHRTSTAGQQKFIFGVDNSDWLQVMGYSEVWDDKVETKLLHLPYRSACE

>SG-PL_contig_2215_1_-1_1_141

PDTGTVPLLLHSLHGGPAEHGEITRASSQASRVLHSSSCSVEAHILA

>SG-PL_contig_2216_1_-1_67_366

MMMMKAYLQIMTLLLCMMHNYHKDLLLKTTISYIIIIVVSHTDLPRVPVTVYFKVGSGTI

YTDTTQYLHSTYVHCKRHNQVLENNRSPVHYCKLRINLYV

>SG-PL_contig_2217_1_-1_1_291

DLRSLSHIPPLELTPSPDLLRQPRSGITPANPGMLRLHARLRCGSSCQLCTTPPLGPGSH

EHHPRPRAVSSALPASGALPATRSLASLSYSCFWAIT

>SG-PL_contig_2218_1_+2_851_1054

MTIFSRKNLRLLMTIPTIKTASKVPCLLMTPSSIKIIATTTNNNKILSINDCLLLQKLRL

LINDYLLV

>SG-PL_contig_2219_1_+3_231_566

MIYKARIYKARIMNNPSFSPQNYPHTSRYKEHIFLIPTFTTTLICVFYSCNSLVFTKITS

IVQPNCLRSTIAASPEDSGLFIRYTNQSSQSPSSLKKQKKQRNKKTTTFHSA

>SG-PL_contig_2220_1_-2_35_265

MNFEFSKINNQFNVSRIIQSRVYRWKLDAFKTKYTRNIVCLCGQKITTSHILSCIKIKRF

IPILSQKTVYEIFESTS

>SG-PL_contig_2221_1_-3_132_1991

MSEGAQLNGPASQSGRFIDTVDPEDPEYIKNLQRPAEVKEDMKQMEGRSRVSVVLNSQAF

KDELEAVIQEQIRQGGPASIFALQQISDLLLPHSRGGPSFSRSSPVIPINDIRGVETFGY

SKQEKVLRCKLAACYRLIDLFGWTHGIFNHISARVSQDQEQFLVNPFGMLYSEITASTLV

KVDVRGEVVDTGSTGLGASKAGFTLHSAIHQARPDIRCIIHMHTSAAVAVSATKCGLLPI

SQEALICGKVSYHDYRGILVDQEERDLLGRSLGPFNKVMFLRNHGVVTCGETIEEAFHYA

FNVMAAVETQVKVMPMGLDNLILVDEETQEKTFKVGSMGGGGADTSGRKWKCGELEWDAL

MRTLDNSGYRTGHIYKQPLMKQEKKERTNSDVELPPASSSFTYVFDGDYEHSKYISPIKT

ALERQKQAYKAGWLASPNTYKKQEIEEIGTTTPKKITKWVPGETSQQKSTAIKVENPNQF

APQGSNPKEFKEKQKSIRKDYYEEKVSAGPTSRILEGISWDEAAKMKEGGDQTIIVGAAS

KGIIQRDHQHNAVVYRSYYSANPFDSMNEEEVKAYKQQVERQEQGEEPEDVPGPEGRLIS

TEERMQNIQLSREDVQEDTE

>SG-PL_contig_2222_1_+2_2_355

GCVSVCVVVCVWVCVCVCVCVCVCVSLYVFVCVQYVCLQGIQLVYVFGFVVFFNILYCFF

YSCTNNAHFLHRHARTNTHAHTHTHTHTHTEVGRREKERQKNGIPHDEKVMMKHGKWQ

>SG-PL_contig_2223_1_-2_1517_1795

MSTRCKLRQLCPLLDTMLETLISSLFFKPSHFPTPLIFLGWSMTELFVVLRKFMVMVEDS

YGDRQTRWQTQSRGKTWIVNVIDLHLSFSFLFS

>SG-PL_contig_2224_1_+2_2_256

CRTKIQHLTTYPHTHTHTHTHTQKHKHTKAQTHKRYDNGSQQPVITNKVKNSCWKSNRKF

SKMSAGPSDSLCSFTFMKQVKQPLH

>SG-PL_contig_2225_1_-1_37_324

MLAGIGKRSADFPGSMVGQQRVNMARAKEFLDELELACSRFGINTKVGLSDQVMSSAYEL

SDGNKDGTLNGQELNLFENIVDAYQHCVEVLKAQGK

>SG-PL_contig_2226_1_+2_44_193

MYRFVQWLSTVKYISFCTVVITTVKYIPFCTVVITYCDICTVLYSSYHLL

>SG-PL_contig_2227_1_+2_116_247

MLIFASLFGFSYLLIYLLSKRYYISLFSVYFCECVCYSVCVGGG

>SG-PL_contig_2228_1_-3_6_386

MTGMTARDVARLARRHDVVALLDKMYIKLLRGEEMLKLERLSQDGYDSMLINFNHRDTYI

YACGNETDTVVRFVQQLPAFQSRVRQLQAAVKNQAVDVIRGLVEEYSAPHILANCKDKVK

IQSNIVS

>SG-PL_contig_2229_1_+3_381_659

MGETRVGGGGKGRVHREKGTQEAEKEDQSHMGNTHAFARAHTPVPGFGGNNRELLLFPQL

TYFTLLVIVPSTQQQNVNISLPTPSLNYHKFKL

>SG-PL_contig_2230_1_-3_3_80

DKSLFPRVIDKHCTCFFSSSPCPLGD

>SG-PL_contig_2231_1_+3_3_290

KNSRKSIPVRNNIPTKSSPQKASLRNQLCGAVSVTGVMVMTLVPVAKGRFYQFHASHSHP

VTPRGKIIQKDLQMRCKSDGRLLLCLVYHLKDDISD

>SG-PL_contig_2232_1_-2_287_1411

MKNKSEQFCLCLFHRSIKMLDNELNHNAEVQLMNQPNSAAVAHPLSEMCSSASLTVPETP

KEGVVIKVDYVGACFAEGQIRRKRIRPRFPGLEVAGTIHTIGHNLPNCNFSTGDKVVLFP

DDSLADSGYAEYLAIDDTCQLMQIPQDLSLEVATMLPGDALSAYAAVIAAKPHVEKLQQV

KSCVNVLVVGSGGLGLWTIRLAQLLMGSLCNSVRLFIADNSIDKLLTAQDHGCFDIVHWN

EEDHEEYIVERTLDACRGGVDVIIDYVSNPRTMQRSLKVLNREGLILVAGNSKTEVSISL

STLAAKQQSIAGIPRGTMNQLQDLVTFLAEGKLNPPCYAVFPIEDMKQVFDDLCECRITG

RAIFRLGGTATVTDN

>SG-PL_contig_2233_1_+1_1_210

RQHHHNLGGVVTQGAQTLQTSLRGLVILLITVLHVYWLKCGRVVSEDCTRNLLRRPIGVV

TAPWPTLTWW

>SG-PL_contig_2234_1_-2_143_343

MQRCLQCLQTLSDNTSQVMMVLSQLKFRQYLDNKTNPEKKTNPLHAAAIAQLPDVKSLPN

SIADGEC

>SG-PL_contig_2235_1_+2_944_1198

MSSRGIHCTHTAAYNVCSLPKTANSGLQVDYLNLPKTLHNRQGLQMNGGWEFVHIYISTT

VRSLFTYLCIYLCIYLYFPPHFFLF

>SG-PL_contig_2236_1_+2_2528_2791

MNVFARTEQERHRYTILHVCTRIHTTPSFYMREHTHAHTHTHTHTHTIFLWLNKKLSSFD

IINPNNQLHPFQIPNIVTVTGPYGFFFK

>SG-PL_contig_2237_1_-1_154_1170

MQYMVWTLLLVSVMTPHLLVRAQSANEIFGSDGRLIMDPFELLLFQMIVCSLPPEAGPCE

TIVPMVFYNRTAGQCQNFTYGGCKGNANRFKTEEECEIACTPSWCSLPAETGPCKAFIPS

FFYNITAGKCQFFVYGGCEGNDNRFKTMGECESACSLPNTTSVAVLAAAAAAAAAAAAGE

TVVTEINLDQTQPAISSTGPLQPPAAEAVITEIALGGPPQSVDSSTAAAAAAALPPPPPA

TAAETGVTEPSVDQPAASVVSSTPGPETSSDTGNASSDHTQSHQEACSLPPATGPCRSYI

RSFFYNAIEGQCQLFIYGGCGGNNNRFRTKDGCENFCNP

>SG-PL_contig_2238_1_+3_183_341

MILPLPSLSLKEFCHCLCLILLSLGSLSLSLSLSLSHSFTIPPSTNKYSENQL

>SG-PL_contig_2239_1_+3_168_308

MVTVFIENRVPRIKALTLIVKKPTKIQVYHASWQFQCGIDQLRDINS

>SG-PL_contig_2240_1_+1_679_963

MALARTGRHGHTHHQTWLDHVDAERKKKTFYNHMSGGHVDLCPTPLYCMKLLDPYPSRFF

VELSCIFRPSLPTHTFSYHVTATHPRQCQPHGQQT

>SG-PL_contig_2241_1_-2_59_1402

MAGTVSTVTKFVSRFGSNIQNFLAPSVVGASQQRHYAVGKRIKVDNPVVELDGDEMTRII

WEKIKDRLIFPYIDVDCKYYDLGLPYRDETNDQVTIDCAEAIKKYNVGIKCATITPDEER

VEEFKLKKMWLSPNGTIRNILGGTVFREPIICKTIPRLVPGWTQAIVIGRHAHGDQYKAT

DQVVGGPGKLELVFTPQGGKPESLTVFDFKTGGGVGMAMYNTDESITSFAHASFQYALMK

NWPLYMSTKNTILKRYDGRFKDIFQEIYDKQYKAQFDKQGIWYEHRLIDDMVAQALKSSG

GFVWACKNYDGDVQSDVVAQGYGSLGLMTSVLVCPDGKTIEAEAAHGTVTRHYREHQKGN

PTSTNPIASIFAWTRGLEHRGKLDGNQELQRFAQTMEKACVDCVDSGKMTKDLAGCIHGL

KNVKPEHYLYTMDFLDAIGEEFERQWKA

>SG-PL_contig_2242_1_+2_26_316

MNYQDYLTKICVPRPLPFNVSLHHYVSFGRNLYNFWSSSNTPDRQRQEFVACIIDELKVM

WPDLVMVHGKPKHPQSQGSTEQANGDVEDMICSELND

>SG-PL_contig_2243_1_-2_2_217

IPACSITLKTDLLAARPGASVILLVSAKSFSFIAPFPSTTTLLISRPCQCIAFCAGSRSL

PDNYWQIWVERI

>SG-PL_contig_2244_1_-2_8_280

MSPVQSHATKNWPDCYPPYHSGGISVHLIGPVHPQPNPTPHFKTSPAHRVLCRLKETVRY

LLTDLGGAHMKLSGCDKFHSFIYLFIHLFMC

>SG-PL_contig_2245_1_+3_1287_1556

MTVYSGDGPRSLSTAEASEENPHKKQRKEKKDSPPSTLYTTSNKQYQQQNETRWRRVNKM

RQCGVAPMSIHVTKTQFPPTNGNQRMCVAT

>SG-PL_contig_2246_1_-2_17_499

MNKESEASVLSPPSPTRTSGFQKVVGTVINIQSGKEPLPNNSVFLTTMGVTKAPSSGEKE

GVEEDIVPMSLHKDHLGNKHYAPSPPESLGRQSGVRQLDSAAKQDIANLLSGMASTRKIA

NSVVKQAGDDDVREEEIVATVLSREQSRLAKEAVTRDRKSV

>SG-PL_contig_2247_1_-2_1601_2023

MSTKWSRLFMTSAAMSITTTTVTDHSRDSHFNYRSDVNLAIFVFFLFFICIVLLSGVQLN

ISCQQCRHSHTYMERFYQDEYIEVYDIVAAMTQTPATDSERGSISRDNILIHRIRGVVPL

CQRFLRHDRLKTGDLQLQDYT

>SG-PL_contig_2248_1_-3_345_728

MSIMSCIPLSHAPLAFNDTSDPTRITSQSRRSRSIRGLLYDASHNDGLLESCVCSFILFS

SYPFEIKMERDRRRRNYTLHRLWLSYLLKAIDLKAVKNVLKNKKEEEKQEQNKKIKCYKT

KYKKFGFF

>SG-PL_contig_2249_1_+3_537_782

MRHSQWVRISEGPSLHASRWLAGYFLLFFVFVERPLRDTVDESTWQVSSATAKWRLRLHQ

RVLRLKMCMYNNILRLKKSLNG

>SG-PL_contig_2250_1_-2_14_1963

MDPTGQLNEYTERKEMSSDVVCMALGRVPAGEQRSRFLAVGLADDTVRIISLDPNDCLSP

LSMQALPAASESLCIIEMGGTEAKEETGEAGMQGGLYLNIGLQNGVLLRTVLDTVTGDLS

DTRTRYLGSKPVKLFRISMQGSEAVLAMSSRTWLSYTYQSRFHLTPLSYETLEYASGFAS

EQCPEGIVAISTNTLRILALEKLGAVFNQVSWPLQYTPRRFVIHPESSNLMIIETDHNAY

TDDTKQQRKQQMAEEMIEAALEEEQEVAAEMAAQFLNEERQESVYGAPKAGAGQWASIIR

ILNPINGQTLQKIALEQNEAAHSIALVKFANKPDEQYIIVGMSRDLVLAPRSLSGGLLQT

YRVIDGGAKLEHVHTTPVEDVPGAIAGFQGRVLIGVGRFLRVYDLGKKKLLRKCENKHIP

NFVTTIHTMGHRVVVGDIQDSFHFVRYRRQENQLIVFADDTNPRWITCSTLLDYNTVAGA

DKFGNITVIRLPPEVSDDVDEDPTGNKALWDRGVLSGASQKVEIVACFHVGELVSSLQKA

TLIPGGSEALVYTTLSGGIGMLVPFTSHEDHDFFQHLEMYMRSEFPSICGRDHLAYRSYY

FPLKNVIDGDLCEMFNSMDTAKQRSVAEELERTLSEVSKKLEDIRTRFAF

>SG-PL_contig_2251_1_+1_2749_3087

METPSVVLHHEFVELTKIHFPVLVLVDVIDDLVHLLVGHLHLLLDLVQQPLHLVIGDEAL

PLVVQDPEHLFVTCLFVLVCQQVLYLAVELVEKVHVVLSLHIVRHLATELEKS

>SG-PL_contig_2252_1_+1_832_1128

MMRTFLLGGKYLFRDSFSTFAISSKVMGAAAWPGKPPPMSRRSKSKPRVWPMSNTSAATS

TAAANAAASATPLPTWKLMPTTLSPISLALHSRVRASRG

>SG-PL_contig_2253_1_+3_126_527

MNLKVACWNVRTMQDSDESDRPRRRTALVARELARLEIDIAALSEVRFAEQGSLVEHGAG

YTLYWSGREKEERRLSGVGFMIKNSVARKLQYLPVGHSDRLMSLRLPLQTKGNQYLTIIS

VYAPTLQAEPIVKE

>SG-PL_contig_2254_1_-2_14_1918

MASTQNPDFRLKDKPKQEFNKIRRILLRNIPPSTEGEIQEFLGGFKAEKIEISESLDSAV

VTLRDGRQLEEAVTKLDNTKLKDNDVTVTFCASDKLICVAHLPPNYTDDEFRKMMEQHGR

MKSCFIMRSEKNGYSKSYGLLEFEEQDMEKVRKICEEIDWKEVGSHLLHADFIEPSYQTW

DRLQSRCLAVNGLPKDFTEINKLRETFSVVTSPVYCQIVMYEKESLGMGIVEFRKAEEAE

ETWELLRDTKIEDKEVSVTFCIPSKSAVFINNRIMWKFSDKMQQKCSLLPNPVTATKPVI

SNNAIVLSLVKQNPNLLEDFAKVLGELHQAYVNHMMSPGNKPGLLGPAPALPMSPMMNPH

LQMGLMIMLVLHIHAEKKEPFTGVLAKQLNSLSESPSSTPSTDPSKRPSILGDPVTAQAN

IVLSNLKQQLNQVPVDEEAKKYLSTSSNPAVKTIMAKFMQNARYLSLSLLVNLGQMVMSM

QANSHQEGPMAQAAAAAASSIARHSGGSLLKTPGPGKGLLGDVPRPNPNPATMRFMKTLE

AVASQKLGGQNPAFISALMTNINASPAIGGLEKSSALGGEKRSLLGEPPQHLKQQKEGGS

GGGMGGPGGRGMGNMGNMGGMGNNMGGMGNMGGMG

>SG-PL_contig_2255_1_+2_338_430

MTCAQVVSYTFNGLMFVSFYQVFSDMYTSML

>SG-PL_contig_2256_1_-3_3_302

VVHRTEVIKKTLNPTWKPFSLPVRTLCNGDYDRCIKVDCHDWDADGGHDFIGMFKTTMRE

LSRGRGEQNTYECINAKKKAKKGNKYQNSGVVELLSCRIE

>SG-PL_contig_2257_1_-1_10_771

MGGKELNKSIHPDEAVAYGAAVQAAVLSGDQSEAIRDVLLVDVAPLSLGIETAGGVMAKL

VERNTTIPTKTSQTFTTYADNQPAVTIQVYEGERTMTKDNNLLGRFELAGIPPAPRGVPQ

IEVSFDIDANGIMKVTAQDKSTGKSSNVTIKNDKGRLSAEDIERMLNEAEKFKEADEKQR

QRVESRNKLESYMFSVRQALGDAGDKLTTEDRQLANKACEDTLTWLDSNSLAEKDEYEHK

LQELQRLCSPLMAK

>SG-PL_contig_2258_1_-2_2_1258

KKLSDEDSNVKLAKVDATEEGALSTKYEVRGYPTLKFFRSGTASEYAGGRQAADIVNWLK

KKTGPPAKDLKTKDEAKAFVDNDQVVVLGFFKDQESAGAVTFKKVALGFDSIPFGITSED

SVFKEYKMDKDGVVLLKKFDEGRNDFEGELEEEAITKHIKNNQLPLVVEFSQDTAQKVFG

GDTKNHVILFLKKEEGDDLLKNFKAAAPDYKGKVLFIYMDTETVEHERIMEFFGLKKEET

PAVRLIKLGEEMAKYKPESKDLSTATITKFVQDFLDGKLKPHLKSEDVPDDWDAKPVKVL

VGKNFKEVALDKSKAVFVEFYAPWCGHCKQLAPIWDELGEKFKDKDDVVIAKMDSTANEV

EEVKIQSFPTLKYFPKDGGDITEYNGERTLKAMAKFIENGGKEEPAKAEETEEEEGEKK

>SG-PL_contig_2259_1_+2_2_259

FFLRLLYISNLSIDRDLFLIHEGVLLFKNYFLTVIHLYTGKKERTTIIWKYATYYSTAII

IFIYRVLLSQISEHLIIISFIHSGQV

>SG-PL_contig_2260_1_-3_165_320

MASKSGSRYAKRRGSRDISRHRSDSRSPDRRSDQATQVKRKNDRSSHAKRRS

>SG-PL_contig_2261_1_-1_31_351

MLSKKLNENDVRMLFAPFGTIEDCTVLRDANSTSRGCAFITFATRQCAMNAIKGMHHSQT

MEGCSSPLVVKMADTQKEKEQKKMQQVTSNLFMQFSNLGALGPQYLA

>SG-PL_contig_2262_1_-2_2_163

THAHTHTPIHTIHLYTRWGWGHINVMDHCLLNNGIFTFVEHVFIFQVAVHLTHF

>SG-PL_contig_2263_1_-3_3_209

PPPPLLFEGKNEKSVMYLPLSLQHEELNQGFFFPHFFRPFVFSLNIHSLNLANFGSHLSS

KTFFLAIGI

>SG-PL_contig_2264_1_+1_871_1107

MITQNISNSTLSLTCSQMRGITLSHTHTYTQCRVTEKAHSHCIILSSLFLFFSIIFPRKI

SLNLYYLCNLEKPKPNHPD

>SG-PL_contig_2265_1_+1_1_138

IYTNLNTLSLHDALPICAWCVCVCVRACKQAQNKCIHTCVHRKSCT

>SG-PL_contig_2266_1_-1_286_591

MNRKLVQKLKKRSNVVKSGRLEDKASSMVLNFLEFFSEVLWRASKQSIAIVQARKNKICA

EKGLCDIFSKEMMDGGNSTNFKVAHVTDVGNIAGGKRGTGQL

>SG-PL_contig_2267_1_-2_788_955

MLPAHICNTFWRKLPHTHTHTHTHKTKHCSIDSILFHKSCKAIVVKIKNQKPNSNK

>SG-PL_contig_2268_1_+1_169_288

MKEGPSGQRGQIPEPAKQAEWGRPGLVPTRKPQKCVNSFS

>SG-PL_contig_2269_1_-2_122_376

MDSTIHCWNIPSSNIDPYDSFDGSVLQDTLLAHTDAVWGLSIHSSKMQLLSCSSDGTIRL

WNPGTKSPLLNTFVVETERGVPTSV

>SG-PL_contig_2270_1_-1_61_312

MAPALRKVYDQMPEPRWVISMGSCANGGGYYHYSYAVVRGCDRIIPVDIYVPGCPPTAEA

LLYGVLQLQKKIKRMRTLQMWYRK

>SG-PL_contig_2271_1_-3_36_551

MAARRIARSAVDWAAFKERVPLGQQEFYRAFKAKNDNFINKVHQFPEELPKIDFAFYKAR

LPNPALADQFQKAYEAVNVPYPTDKSNTIKTIDDEEKAAAECTKAYVAERQEEINDAKLL

LSKIDSLPKPEEMTMEMYAHYFPHLALDPVNRPSFWPHLPNMQPGHKDSHNI

>SG-PL_contig_2272_1_-2_59_229

MMKFLDKSKPAFFIFCRHERLPTASDFSWVWNIFQLLMICYQQTLCIGHRTFVTRSH

>SG-PL_contig_2273_1_-3_3_206

SHHHSIAPDSVALHCSALMVTSALESLEVCPRVKMSGVAIVVQKTGHQRNKYKKIVVVVV

VADRKSVV

>SG-PL_contig_2274_1_+3_3_251

CIHCGSTQCTRILTKDKQKEQKQQVTNNDKDTERPNRISTDTEQQTNGLPQTYGTTDIFM

KHGCEPVAQTQPFCAYNNRVGLI

>SG-PL_contig_2275_1_-3_6_473

MVLKNGKWKDDYCQKKRCFFCKRLSAYCLLPAPTANATITAHSNVQILPVGKHVIYSCKD

GYVPASGVAVRACLPTLELTGSDLICTVADETLTESSSVPLIERHHVGEQYRAYTGPSGN

LKITRAGHVHAWTFYSRTTSTVTFQVYRPLANLSSH

>SG-PL_contig_2276_1_+3_18_452

MLKFTSKDHLSHFHIPCAQTQCTENTGCQTDQSTRMLSRDTTLLFKDLPAMKRECRVEHI

ATKLKLWIQTFCWTLGHHFWEQLKDFSASMTFSQPFHDISQPLSEFSLTLATFQRLENGI

PILKDLKRLCEPCKMFRHTSCHAFK

>SG-PL_contig_2277_1_-2_113_1915

MAMSEAGFRAIPSPLPPSRSASPAPSTSLASSVVAESAVSDPARLLTPRPSAQSQPEKVA

EGKKTEIKSPRVSKKPPIAPKSPPKGAKAEKKGAGGKTFVKAMDITSVQTDKNQADTTAT

PAAKPKDSPKKQKESYRPPPPPPRVKTPAEKQNLLIPDHMKEAMERSSSQAQNELENELM

KMHKAMDESLGLPTRIAEDGEGLTEEELEQAQQALKERMEEAQKKMQEAVLPGSVRSKSP

STPRKSPDLKPAAKKGKKVRVEDPIMVEEKAKKEAAKEQRQLERQKRLEEAKALQEMISK

KEAERKKKEEEAAHKAKEMEEQVELMKAEEDAIQQAEEDAREQMAALRKIQREEREARRK

ADLEKKKQDALAKREKEKKMMEDARKKEQEMLERIADAEMRRAVREEEERKRDEEERLAQ

ERYEEELREAQQQAEEEERRLQELERQAEEEAFQRIIAEREEAERQREELELQAKRMAEE

EERQRQLMLEEEKRLEEERLHHEAEEQKRKDEEKQRLKELQRLEEAAREKMRAEIERRRE

WALKRREVNLDRRKNLDGLRQKQGITEPWVFSYFIQWSREMYARPMGVSAEAKKKPMKKK

K

>SG-PL_contig_2278_1_-2_146_538

MEDRFNITSNEDILTLILWKWASAAAVYAKKDKLGWNISLRLVNVQIMRSYPSWYKPLPS

PSDGLGQRLRTFCLGTQSEKYDQVTLHTGKVSQKGILGLAYVNGVCKPKGKCAVSRSAEF

MFFVELHELGH

>SG-PL_contig_2279_1_-2_236_448

MGLLPISRPGQHVGFCAGSKSLQDNCWQILGGARVEVSNYNTNSISNFFGLCLSVSIISS

SVFVVLGRYVD

>SG-PL_contig_2280_1_-1_298_1032

MSSLSSLDTSASVYQPAQLLNWVYLTLADQQSVAGTTSQGHLNNPLNGQSPASAYSNGNN

NELWTPGGTLNNVNNLLKSLNNQQTSPLGNLTTNPFQSPGSQPLSTSPFGSMVDLADTFN

ELSLDRKPCKRPPSSYLCHLCFNKGHYIKDCPQARPKGEGLTPYQGKKRCFGEYKCPKCK

RKWMSGNSWANMGQECIKCHIMVYPHKQRPLEKPDGLDVSDQSKVHPQHLCEKCKMLGYY

CRRMN

>SG-PL_contig_2281_1_+2_1892_2146

MQNLSIGQELANPGERSRLNAKEPVHHCLSLSTPLDHRADYHAQPIYTLYTTASHFHHPN

QLMDTVQRRNKTLAHAPQIRVTDTR

>SG-PL_contig_2282_1_-3_255_425

MKCLEYSVSESPHIQVSNISVHSIVRFVNFFPFVVSQVMGTYSHTCMRACVCVCVCI

>SG-PL_contig_2283_1_-3_654_854

MLLCWHSCEIMKISTNSSYRTSLLSCPPFLSLSYFTQEQPLFPPHDAHLYRLICDSAMEL

EMKMLPC

>SG-PL_contig_2284_1_+3_288_581

MLFVHCFRVQRYRSCYYSKPLASIHSTVACCKRSYACCNSTLLLLVAAVGSDSKAVSHQL

PAYFVVGIPYIFSIMTYSRRTKTHGVGIATKISQSMCI

>SG-PL_contig_2285_1_+3_3_161

SVLAVSPLFHFCHIHHKINTKMPKSTQRRLRDRSITHQKQGIKSGKRTILLDI

>SG-PL_contig_2286_1_+2_2_460

CFCCCSCSRSCCCCYVGTCIRADKQADEWNLNHFFPFFKKTDSWHNKALPSKIKKISLSL

SLSLSLSLSLSLSLFTVQKCHCLLFIFLFFTEEHFHLFSVYCIARNLHFLSFRWFHTQFK

TCSRSHSYGMTHFSTCISSDDDMLLVLKHVLDV

>SG-PL_contig_2287_1_-1_1_252

DKLEASRNELHNLLDKPQLQGVPILVLGNKRDLDGALDEKELIDRMNLSAIQDREICCYS

ISCKEKENIDITLQWLIQHSKSGR

>SG-PL_contig_2288_1_-2_224_1564

MAAATLTSREIPPERVLQPPVNPNPQMEITYTGNTGNDMGISTMGFRAAKYNPSEWHESN

YAKYYQSFADRDQAERVRHESKRTRNETLATTNDTQADVTKKLAERCKDIDFWKAELARE

IRDVIDETDLLLAQKKRLENALRATEVPLHISTDNLNCRQRRQGLDLVQDDVEISLLKEV

EIINNVRDLLQNTIAQSEKQIRLNRDAKQNLEMDWSDKKEALEIDTGCGVLRNHHTNKQF

HDGAAKFEEIQSTPESWAQYSHDNIARGEHERMASIQLRTLIDNVLEDTSRDMREQCDTV

DVNFQKRVTEMEDAKAKLEENLKKVCTEIAEQERNIDDLKAAIRAKEDPMKVAQTRLNSR

TYRPNVELCRDPVQYALVSEVNEICQSIDALKNQLNQAENSLKDLQDNRMSLEKEISVKK

NSIFIDRDKCMTVRTRYPSTIKLQGYQ

>SG-PL_contig_2289_1_-1_13_477

MHKYFEIEDRAGLLPYLYSNFEKAKIAGFCQHLAKGAVDDKDPLCCAVFNAAGRVMAEHI

LAVAPKMDKNLLTQAGGLHVVCVGSVFKSWSVLQPGFESVMKVRGPEADIKEITCLRLKT

SAAVGATALGAREAKVKLPLDYCAFAETFYTGKFK

>SG-PL_contig_2290_1_-3_87_1241

MNDGTLHIDTSQRMLAPNQARSPSSAGSPHSPGNINTLRQELSAAHAMSGAVVHPQSVTG

QSSPSVYFGLSRRGSLSSIADSDTMHATPKFVKDTSKYWYKPNITRDDAIALLRDKAPGT

FVIRDSNTFPGAYGLALKVAQLPPNVQAKPTNDPQAELVRHFLIEPTPKGVRLRGCSNEP

VFGSLAALVYQHSITPLALPCKLTLPDIGADMVDMGAPISPEVSSPVEISMAPDVLAKGA

ACNVIFINTVDTESLTGPQAITKAIKTTIDSQPALKTTVVHFKVSNQGITLTDNNRKLFF

RRHYPVSAITYCGMDPQDRRWKRDVDNPNVQMDAKIFGFVARKQGGGSDNSCHLFAELDP

EQPASAIVTFITKVMIGQTKVKSYS

>SG-PL_contig_2291_1_-1_1_162

ASANVMSKNNFKKKTTFKTKIKLYILLLISLLQLNIRQLETKCLLLIFLYVFAK

>SG-PL_contig_2292_1_-2_5_901

MIPHPLWTMPERIMSAGKSWAEQVDEGDDGVLPPNTEEFDEKKGLKKITEYKYNEEGKIV

KTVKTYQTKTLKVSKSIAQRKTWKKFGGAERDPPGPNPANTVISEEIFMQFVNSSEQKQP

EEDDDPLGKLKNQKIVQCRICKGDHWTTKCPYKDTLAPLQEKLSEEPRKAVEEAPPSGGG

GPGPKAPGGKYVPPSLRDGASKGRGESMSAPRKDEAATIRVTNLSEDTRESDLQELFRPF

GPISRIYLAKDKVTGQSKGFAFINFHRREDAARAIAGVSGFGYDHLILNVEWAKPSGQQ

>SG-PL_contig_2293_1_+3_426_683

MTQRPLNQCRLCGSWLSTPFLSLHKMNNAHHTGWSQQHMGNLLVITLAHKGGKSHHMQSD

FQTKATTEKSTININFPLSNNHCSQF

>SG-PL_contig_2294_1_-3_81_221

MYIQICIRVCVSNILTVVVKHGYSYYLFMHTKSIIAFYRYLYNHVFK

>SG-PL_contig_2295_1_-2_893_1102

MCVCVVMYVFISVMCACVYFTDVCMCLLHWCVYVSASLVCVCLLHWYVYVSTSSLEFYLR

GIHRIWTIPY

>SG-PL_contig_2296_1_-3_3_197

QTFCFLMRIPILCAGFLASFLLKFSTSPPSYSSPFYINFFFILLKNIYMYPTCTVFFWST

CGLGS

>SG-PL_contig_2297_1_-3_3_140

KKRETERQNKISPRNPCWLKKKYGIKCHDSLLFKTETFLKCMQLQV

>SG-PL_contig_2298_1_-2_44_301

MFVSHSDTENKKRKQESSTLVNCNCLPTLPSLTSDFSSFQPPEAKIYSSVNKHHSVIVFI

CAKVCDFDVLLSKKILPFFFSFIGIC

>SG-PL_contig_2299_1_+3_69_329

MRCLVGWGWVGDQHNIAPQAMSLSTQCINNLYDLCFFTCNLSRAHAHTHTHTHTHTVTAA

KEAQKVSRQVKALYTLHIIFVCTTRIL

>SG-PL_contig_2300_1_+3_60_161

MDNHPCMSNSGHDSSIGKIHPDRKAMIKLSQEVY

>SG-PL_contig_2301_1_+3_564_914

MGGVTSRTSINPVGHQNKKWWQYFFHSLVNPSALHKLSSSWRSHLTTPHPRRTTITCGFE

LTTLTSFAQDTRPENAKLAEARQYLSPQSLSLSLTTTRLRRLKAGQRCVVSVSKWAG

>SG-PL_contig_2302_1_-1_16_1221

MAVQAFRFSGAFARLLTNNLAVASVHSRNMALWSNVEMGPPDAILGVTEAFKRDNNPSKI

NLGVGAYRDDNGKPYVLPSVLKAENKIMADNMDKEYAGITGLPDFTKAAAELAFGADSSV

IKEGLNCTTQGISGTGSLRIGAVFFAKWYTHSKEFWIPTPSWGNHTPIFKHAGLEVKSYR

YYDPETCGFDFNGALEDIAKAPEGTVIVLHACAHNPTGVDPKPEQWQKISEVMKKRKLFP

FFDMAYQGFATGDIDRDAFALRQFVKDGHQVALAQSFAKNMGLYGERVGAFTVTCGSAEE

ASRVMSQIKILIRPMYSNPPIHGARIASRILNTPDLRSQWLTEVKGMADRIISMREQLKE

GLKKEGSNKNWEHITDQIGMFCFTGLKPDQVEKLTKDFSVYL

>SG-PL_contig_2303_1_-1_13_279

MLNEAKSMYAVGKASSKSKYNKIPGIKDFYKSYGYNDELCVGAIWLYRATNEPQYLEDAK

GYYESGIAWEFSYDDKQVACQLLLYEETE

>SG-PL_contig_2304_1_-1_13_279

MLNEAKSMYAIGKGSSKSKYNKIPGIKEYYQSYGYKDELCLGAIWLYRATGEPQYLEDAK

GYYESGIAWEFSWDDKQVACQLLLYEETE

>SG-PL_contig_2305_1_-3_129_1010

MLVQHKGSNKEYYAMKILDKQKVVKLKQVEHTLNEKKILQAISFPFLVSLEFAFKDNSNL

YMVLEFVSGGEMFSHLRRIGRFSEPHSRFYAAQIVLVLEYLHHLDIMYRDLKPENLLIDS

QGYLKVTDFGFAKRVKGRTWTLCGTPEYLAPEIILSKGYNKAVDWWALGVLIYEMAAGYP

PFFADQPIQIYEKIVSGKVRFPSHFSSDLKDLLRNLLQVDLTKRFGNLKNAVNDIKNHKW

FSTTDWIAIYQRKVEAPFTPRTRGGGDTCNFDDYEEEPLRISSTEKCAKEFADF

>SG-PL_contig_2306_1_-2_986_1171

MDLPCEIQAAVPGESASPQCSATHFWFYFCVCVYFCLFMKVGLLQNFARDNSFVAVGSFT

CA

>SG-PL_contig_2307_1_+1_145_399

MHICVRKYESKYIVNHPYFSQMCKTLSVLASLHVGIHPCTHVNKHFTFNHSQYNRNDFST

VKIEYLYIIYPTLTHTHSHKSNRKC

>SG-PL_contig_2308_1_-2_893_2425

MAVQNDSTPNGSASPPVSPTVMNEVTITEEGGEKGKEEEMGKRDVFKECLAIAAGLFHRY

RKYTVVLGGIFVYLPIGVPWYFGNLATYLASYFRHKEAVVDDVVDPQWIFSAFFVAFSLA

IVLSGFLANTLGTRLTVLIAMLVHSGATFLSYFAIQHSMPALIVTFGAVGGLGAGLAYGP

PMPVVSKWLPRHIGMASGVLLTGFGGGAVFYNELITFYINPNNIQPDVTTERTSYFSQPE

VLNKIPEIFLVNGALTLGLQLVGLLLIRDPQDGDQIDEPESTELKPIVPKDHNQTGELTE

VDLQSDTVSTQSTVEMEIENKELSPLDVLKQKDFYILWMCLGLNHYGYVIKNNYYKEFGA

GRVDNDHLLTSIGTVSTIAVAVSRLFWGVITDVIGIKMTVMVLLGSTAVFASFWFFTLLA

GPGLYFLWTVIVTWILAGAFITFPLAALSVFGQQHYASNYGLISTVQIVVNLVSPPLIRA

LLDTYGWFGVFCSIAVSNLIGTVAALFLSTM

>SG-PL_contig_2309_1_+3_3_215

TVTTVKTELNIVRSCWESAVHTPSSRSANFRRSFNQCDFDLGCLRSKFKLKQFLNGPHCW

SEFGKDPSRNS

>SG-PL_contig_2310_1_-3_3_164

YGLTVSLTTVEIVYVRILAVPKIKIIRIQTAEKIQFEILAAQRRIQIFNFSRSL

>SG-PL_contig_2311_1_+1_58_279

MCVCVCVCVCACVRACVRARACVCEWNVLASLSCFWADLYQIYLNQRFGPLRSGLPMSLE

RRGPGSRSLRPKSA

>SG-PL_contig_2312_1_+1_508_675

MIFCLLLFFNRAVMKIGETCSISLKMKKKKINLHLCIHSISQFLLVDVLKIVIMKY

>SG-PL_contig_2313_1_+2_2_118

MSLERRGPRSRSQGQKVSKNSIFGERCVNSIFSATSDNI

>SG-PL_contig_2314_1_+2_344_586

MVFGGRGVISSLLLVQTQLTIQGHIRAGKIGGGISQQTSRWLQCKSQEKDMFWVFTNCIK

CVWLKRLTYKMMYRGNITRVS

>SG-PL_contig_2315_1_+1_19_216

MTKLTRKTSIKHTMVSGLSQVEALFKAGLKAYCLQVQYTFSSQGTVTDSDMPEPKVEVYL

SELEST

>SG-PL_contig_2316_1_-2_509_805

MNNTHQPLHRTTADQEAWPPDQDASQPTSPARLQHQMLRLQIRRKTTTALDRWCGRHTPH

PQHDSTPGCRLSTSSPRVQVEGKSKVSKVFRRQIHMIKL

>SG-PL_contig_2317_1_-1_1_195

IPSMHSLFSLHWLQAVLALASPLDFTGVAERVGLLHGQQQVLLNTLPRLLSWSLSSGTDS

IVLRD

>SG-PL_contig_2318_1_-1_151_468

MGQFEIHAVVITNRGDCCGFRLANFSIYVMPALSDPSESEHDPSDLCVHHIGQIGQGATV

RLQCCQPIIGQYVSLVLPTDSEPLNFCEIEVLGLAVSGDEDESDGN

>SG-PL_contig_2319_1_-3_3_200

VRSFARSFIHSSIRPSICVSVDLNHKIRELASACDGASRVCWPVFCFSEYIVNYCIKRME

MEMCLP

>SG-PL_contig_2320_1_-1_1_147

QRHKFHSPLGLKEANQSIEDIIYLSVCLSIYLYIYLYLSLSLSICLSML

>SG-PL_contig_2321_1_-3_255_1256

MLMRGLEHIAAFTPIMTTPGHNETAAGQFPGYRHRFSMPGSAWPIPEDKLWYSFNLGPVH

FVSYCTDVYLHGSKAQQTAQYQWLVKDLSEANTKRSDRPWIVAFGHHPLYCSPSLTHPDC

AKNKSLVRSGLEETLYYYGVDIILQSHPHGYERFYPLFKGVVLANNYTNPRAPVQIITGP

ASATPTATPKPTPAPSLTTAAMKTTVAPKSVNSSHGGSSGGGGGSSSGSSGSTKTPRATV

AHTAAKKDTAATTTTTSQTMTAFKFFNGTQPTYGRLRIINATHAHWELLAGSEEGGRKER

EVVDSVWLVQEQHGKFQLKDLPDDVEEKIDKNLV

>SG-PL_contig_2322_1_-3_963_1214

MVVISVSRQERSGCKLFDGRLIRFYTQFHPCVCGTCFLLLCFVDRQKCGEIFCFHPIKKL

SKTTFTFGVKLFIFGSLPLTPRLG

>SG-PL_contig_2323_1_-2_38_589

MGLLTILKKMKQKEKEVRLLMLGLDNAGKTTILKKFNGEDIDTISPTLGFNIKTLEHRGF

KLNVWDVGGQKSLRSYWRNYFESTDGLVWVVDSADRMRLADCKRELQVLLVEERLAGATL

LVFANKQDLPGALTSQEIQQAMDLDSIKTHHWLIQDCSAVTGDNLLTGIDWIIDDIASRI

FTMD

>SG-PL_contig_2324_1_-2_62_388

MNNHVVEVMDNARHPYTALSWAPPGEKKQADRPTGTWRRTIQHETKEARKTRNELTWLAQ

NCRGWRRLVDALCSTWSEEDCVNRAALISVTTKKATDVKDKLSPYLALT

>SG-PL_contig_2325_1_-2_83_589

MSNFKKTTLAGGRKKTGPKCELTEEQKQEIREAFDLFDADGSGTIDAKELKVAMRALGFE

PKKEEIKKMIAEIDKEGTGTIDFNDFLTIMTQKMSEKDAKEEILKAFRLFDDDETGKISF

RNLKRVAKELGENLTDEELQEMIDEADRDGDGEINQEEFLRIMKKTSLY

>SG-PL_contig_2326_1_+3_3_215

WTEKVLHARTETFRRWTAVTCRGQYERLMPSPVRINSCLPCRTFFVGTDGNRRWLSVVSM

NAPLCSTWSEE

>SG-PL_contig_2327_1_+3_3_197

KVLHARTKTFRRWTAVTCRGQYERLMPSPVRINSCLPCRTFFVGTDGNRRWPSVVSMNAP

LKWCG

>SG-PL_contig_2328_1_-3_39_1235

MPPKKKDEGACKPSPLIGRMGTNLKIGILGLPNVGKSTFFNVLTKNSAPAENFPFCTIDP

SESRVPVPDKRWEFLCDFYQPASKVPAYLSVVDIAGLVKGAHEGKGLGNAFLSNINACDA

MFHVVRAFSDEAIVHVEGEVDPSRDLEIIHDELRLKDMEYLRKHLEPLERSVVRGGGDKK

KKEEYDVLKKVECMLEEKKWVRLAHWDAKEIEVLNQHLFLTSKPVIYLVNLSEKDYIRKK

NKWLVKIKEWVDTNDPGALVIPLSCALELSLTDLSTDEERQNHLKELGTTSALEKIIVNG

YKALNLVYFFTTGKDEVKAWTIQMGTKAPGAAGRIHTDFEKGFIMAEVMNFEDFKEAGSE

AACKAAGKYKQKGRDYVVNDGDIIFFKFNAGAGLTGKKK

>SG-PL_contig_2329_1_-1_715_876

MCKGTFCVRLFRESSFCSLHVMDKMSLHNLDIHEHGCFDVHSFFWSPYYGQFVE

>SG-PL_contig_2330_1_-2_2_955

RRRSRSTSRTRRRSRSRSNRRRSRTPPRAYTARRSRSRSRGPRRKSRSRTPRRSRSHSRG

KGKDKKERGRDGEKKKKEKKASVSRSPSPPTLQREGSPAPHSSDDRSPARLDHSSKEETV

KQPETEAASDAALTSGAKKSRSDPAIGKSSQKRDRSESPVVRRRGKSVSRSPMRGGRRRS

GSRSPMKSARRRSISPRKRRSKSRSPRRSRTPPKKKKTRSPRRSRSGSRHRSKKDKKKER

SRSRDRKEKKEKKKSKDKREEKEESLSSKVARDYDEEEKGFNSGSDHEEMMRSPGDDYEE

DRRSIGADRQLVDMDTSD

>SG-PL_contig_2331_1_+2_185_346

MVKISTMAPLVTALKILHNGLAGQNPIVIKKTNKKKQQLRSRFVYPFSCGKKKG

>SG-PL_contig_2332_1_+1_1120_1383

MDLSPPSSIKYQIGDWKGYISSLKAYLQARREQPELNVGKQNLGLEYCFRRTGILDSIFT

LKEWTYKLCILIALLTGQRCQTLQITLE

>SG-PL_contig_2333_1_-2_1130_1426

MFILFLSLLNIYLCICLPAILCVLSVELFIALLAKSVGLLDKVADDGDFSSNTPQNQIHS

CQSCNFWDQILCRDFAKYCEGSLFSFFFLSYLSKAACII

>SG-PL_contig_2334_1_+2_2_145

LAPPPPPLNSRKYSNKLCFWLTAFHYSCILFKNTLHIIIKYIYNSSLY

>SG-PL_contig_2335_1_-1_361_3300

MRRIIALLAVASTALAMSEPNAHKPEIIASLYNERGRPEIHGIIREDETEVTLQHPLNAT

DSDHIGGASFICAFKIKESKKRPRKFEVPFEVQLKGEGSGEGVIRVKEGVHLDFEKRSHY

RFYIVAEDCGSPPRESDKAVVLIKVKDVDEFAPRFENSSYTVEVEEGKVFTSLIQLRVMD

DDASDTYGQVAGFEILTPDVPFFISKNGVLQNTEALDYSMHRNYILKVVAVDGGQRRSRP

VFVNIIVKEQCRSGWLNIPSLVRYVAGEGQKRVAESAQLRLCDKTCVPEEVKVKVTLTTK

HIGKGCDRDTYSITSQRKLCGASGDSVDLLPSPWQADWTKSLPTDDGHLMDQIFSFDGSH

NAVEVPAGHFDHHLGNHFTISTWMKHDFPGGVPVKSHNAPKEHIVCMSDGDGMNRHHYAL

WVHGEKLVFLLRQEDVAPEDMDKFKPAEFRWHIPQVNDNEWHHYAISVDFPDVRLYLDGN

LLLTDDTNYEEIDDWPLHKSNHVHSTKLVVGACWQGKQMQFKDHFRGYLAGLSMLKGKTE

SERVIKCLHNCKENLDFRGLSDMSAGTVVSFNGDMTEFTVTGGQVTEVENILRQVTYVNA

RSFPTPGRRNLRIDTTVTCNNQKLVVPAVESYVMVQEPPQAIIVINGAANLTRLVYEFQR

GLRIFHDVHIFATSQPAINSAEEEEEAYEEEETASSEDVKATQHAQKVDQVKSEQDSQKK

NSLLIDMCTVVAEPPLNLFVEHLSLPTRLLENRNLEWSETNDGVVISNADKIENYENVIR

NIHYYHNEPSKLSNRTLTLSCSSQNGRFISNKFVVRLVALHDVHDTKSFAHAQVEPAAQL

QQGLLTASGHQNAHATVASSTSNFGMAAIIVVCVGFLLFMIILGVVRIRAAHRRTQVVHV

EENQDMEWDNSALNITVNPVEQEIFEYEDNTQPSFHDDSDTDDDESSMQGDYGESSEDEA

PAPAKVPNNDLEWDHSNLGF

>SG-PL_contig_2336_1_-2_119_1153

MAKWGEGDPRWIVEDRPDATNVNNWHWKDKNANDWSKDKLKELLVGLKIENDTGSCEIKE

LTKTEGEAYVTNRKGKLAFFYDWDLSGSWQGKLTDGQKTVKGTFEIPNLGEENEIHEVHV

TFIVDKDNEEARAVKEFMRSEGDKAVRQQLEAYVTLLKNEYSQGVILPSKKADSIPKEQS

NKENIVKREMNKVVESAPSSSKTSNNIGVRIPTTTVTMKETFNCEASFLYNALITQDLLR

AYTGGESTVDAAPGGRFALMGGNVLGDFTELVPEKKVCMRWRMKSWPDEHYSNVTINLEG

KEGTTELSMKQTGVPQKELDRTREGWKVNFWERMKGTFSIGNNIA

>SG-PL_contig_2337_1_-3_3_176

HQLTTMPIKTCQAGFWALFVLILKMPLVGFKGKTVSILHVELSLFLFKCLQLASIYIK

>SG-PL_contig_2338_1_-1_1360_1593

MLVQLVSCWSPGSSRLKCLVMSKSVSSNCKTDTTEMPQLKQMHYSLLCSFRGVPFMMKTS

KLSVTFFKEKAVSRDSSG

>SG-PL_contig_2339_1_-1_193_450

MDELSEDDKLTVARARKMQRFLSQPFQVAEVFTGSEGKYVHLKDTISGFQKILNGELDHV

PEVAFFMVGGIEEVISKAERLAAEQH

>SG-PL_contig_2340_1_-3_42_1418

MTSEAKGWLSATVFLGMMVGGYGWGSLADHGGRRSVLLWSLMVNGVGALLSSLAQQFWLF

LIFRFVSGVGVGGSMPVIFTYFAEFQSKDWRGAMISALATFWMAGNILAAGLAWLVIPHY

NLGYFSSNFRYDSWRIYVAICTIPSLTSAALFLCMPESPKFLLKRGKEEQAIIVLKKVHQ

SNQCHTPFNIRRLVMWRGGEGGREERVSLQESEETQSSCCSALCSMLDLTLELFRPSLRT

VTCILVVINFTICFGYYGLFMWFPEIFNRIEKNGGTICQPYHTTTNTTNVTTISNNNDST

NTETVECEEVQSVVYVEGFLTAVSNLPGNLLTIFLMDRIGRKALLASSMVLSGLSTFFLW

FVHVRWQSVLMSCVFGAVSIIGFNALDVLQPELYPTHIRSTAFGFQTGVMRLGAIAGNLL

FGTLVDAKCMIPMLMVAALMSIGGLVSLILPNTTNIDIH

>SG-PL_contig_2341_1_-1_2497_2745

MSYFCLCKSKIILKSCIQLETDNNWQQRKLYSEDMYISLLVSVTHQDLYLQQINTQAHEV

INKMHKFATRNQKCKEVDKHELI

>SG-PL_contig_2342_1_-2_80_1552

MLHAVLGNTNQREQLLRVQLATSLGNPPVSLLQQCCNMLAQGAKHQTRVGLLMLLSTWLA

DCPVSVTHFLSNSANVPYLISQVSSSEGDELELIVQGLCAFVLGLCMVSNDDQNPAFTKA

SLKEIIQNRIGLEMFTDKLTQVAKNESYTRAAKKPHLNYKHPSEIVFDFEFTSLFKHLEN

EVLVAVSGAENMGLKAASRREHECIVENYKELIKEQDQRLNDMSQQVLELQSKHTSATAT

IEELRTQVQQLKDQNALLKAQKGGAAQGTVNGEGVESLQRDMQTLRIQLSEKEGQVEKLK

KDLTVSEARLLSQNGSGGEDGKENGPSESALLQASVSRLTADVEELRAQCAHKDNEIANL

RSQLDRAQADLLARNSQESNSGAGQTTEEMQRLLEEKTQLQERLKKSNEENLNLSNKIRA

LEEAKEAECGEKAGLREELDTLKKEQDDLLVLLADQDAKIGKFKDKLKELGQEVEEDDDE

EDFEDEEDDDA

>SG-PL_contig_2343_1_+2_290_427

MFNLKFWSDLIRTGVGNLLCCPSLHIHYFSSIQTSQIKTPRTKSLQ

>SG-PL_contig_2344_1_-3_132_353

MHTHTHTHTHTWTHTHTHSHTHTHKRGHIHTLRKNKKLSAILITTAIIIIILIIIIMNCL

YNIAKQTRHIYLLL

>SG-PL_contig_2345_1_+2_2_130

TLKEPTVTKKLSLTNSVPPKSTVIGQQKHKQTEKERKTWVALH

>SG-PL_contig_2346_1_-1_223_549

MLHESFADDTQLHQSASIAEIDALISWTCECIADLKDWMNLNKLQLNEDKTELMLACSKI

FLQHPSLPNSILINDTPVSCSSSVHSLGVTLDQSLSFQQHISNVCKVAY

>SG-PL_contig_2347_1_-1_76_330

MFSRIFVRRLTTPASGQSAGIVPSNARYQKIQELQNIFCRDDGLLVWQKMGSKDKMLYYA

TVLITVVGFIPSLGTIGKMSFPKKA

>SG-PL_contig_2348_1_-2_353_508

MANLMLFDRSVCGKESFVYMFQFNLSNDLSVSFNFIIIIISSFIVCCLRSPA

>SG-PL_contig_2349_1_-3_219_398

MNNHSCVHICQQVDDCQDVKCVEFSVAVAIQQARDEGGSYYMLVSELTYYILRCKKKKGF

>SG-PL_contig_2350_1_-3_1533_1727

MFVKFCEQEQQEFIKQSFTFQVYTEAKFCRHQFPASIKSDWWLVSFFFNFSYSSYFAMYE

DCMQR

>SG-PL_contig_2351_1_+1_400_834

MCHTMCLGRHVISFGGLIKYYCIVLYCIVLYCIVLCCIALYCVVLYCIVLCCTVLYCIAL

RCIVLCCVYCIVLYCIPLYCIAMYCIVFYCIVLHCIVLYCVVLYCIVLCCCKPHINVKVT

ASSLQHCILYKTVLHYIELHCIVLY

>SG-PL_contig_2352_1_-1_718_924

MKTCSTRYFKDEKKSWLQESEKAKKREQVFLSKCKSDPGIIESIAGSSFACCRNCKRYFY

IVHGGKKLH

>SG-PL_contig_2353_1_-3_858_1262

MTELTSQQSIPPDEGPNSGSKLRPPVKFFFSSSNYYGRSTYICKGIQLQLSRSAPSQHTP

LSMDSLRNKTKLSHLSSESYRSLRIFSHLTTSAASFCFLDIIHLAKVHGEIVMLVKVMSC

MLFRMTRLSGQTIRS

>SG-PL_contig_2354_1_-1_85_594

MLAAKFSGNFFVNLIGVWADVGGGGPARSYPIGGLCYYLSPPETLGHILEDPIHAVLYIV

FMLGSCAFFSKTWIDVSGSSAKDVAKQLKEQQMVMRGHREKSMIKELNRYIPTAAAFGGL

CIGALSVLADFLGAIGSGTGILLAVTIIYQYFEYFVKEQSEMGGMSTLLF

>SG-PL_contig_2355_1_-2_1379_1696

MTLALQFTFFSSSEVVIISSKGLCDLMANLFICDILRIRVAWNLLVAPWLPWLVFFFCCF

FFLWHYFLISNLGCVEEGKSLSYIQGMLLHIANRPRCATTHKDATC

>SG-PL_contig_2356_1_+3_3_452

GQGACEVAIGPEPSPKSDTFCVWVTPGPYCGAAVAWCVAGAAWCPCGVTSGGAPSGPPSA

ATAPGPCCNCSSCLVGGRSRHSVPRTGQAGTTGAGCPPGRWPVPACRGGVVPRALRPAGV

GGRRSAAKESPSGESWWRLLRGCQLRKHPP

>SG-PL_contig_2357_1_-1_1471_1695

MTADYAMICSVLCRNFTIDVNSLSVTSTLFLFKLLLFRCSRLCCHRTCLGKTAHFYNFNY

FVQKIFFTWFMFKIF

>SG-PL_contig_2358_1_-1_379_543

MCFCSVDKWLKMQRYFLSCLSASRSLYPFFFFFFPPCCGCFFFFIIFFFFSNKKQ

>SG-PL_contig_2359_1_+1_295_435

MELLPCLCLPNLSTPQGMADFSSHGHSSLGRSTEVDISAPYNMYVCV

>SG-PL_contig_2360_1_-3_3_215

SKITEKIVLKQLLTYLNEHQLISPSQSAYRPHHSTETALLKVTNDILLALDTGDVSVLTL

LDLSAAFDTID

>SG-PL_contig_2361_1_+3_3_137

LISHIHSINHSVTHLRLTHSYGQSIEQANIEDFADLRKMLFQTNC

>SG-PL_contig_2362_1_+1_298_531

MYANTVADDTIQCCLPTRQRLDSVLSLTPTTPQPELAGVLSQGSVQTTATLGDSVYPKVQ

FRQQLDLATVCIQRLRLV

>SG-PL_contig_2363_1_+1_802_1161

MVYKLSNNNNFSQPRNSHQDVCTTDWSFFATAHEQGPAGGTVAHRTVKHAVLIQVLQKQI

LVNYFQWKDFADVTFLNIVLHVGSAIMQEMQRELSSPWSEQQPYTLRDASTTSNQFIKRA

>SG-PL_contig_2364_1_+2_887_1150

MPYSPKNLCESSTGCSADIPNTMTLAGWYFINVFDSKLSSYVVLEGINVPLPKASMWGLH

RLMAYAVIISGPITWHLWCSAYGVNHAL

>SG-PL_contig_2365_1_+2_65_469

MKTFERLVLQFLKACIPPSFDPFQFAYKPNRSVEDAVCTALYHVLSHLENPNSYARILFV

DYSSAFNTIVPSKLYLKLKHLLFPDSICEWILDFLRHRPQVVKVSNMISSACTLNTGAPQ

RCVLSPILYSLFTHD

>SG-PL_contig_2366_1_+2_2_238

PSKLYLKHLLFPDSICEWILDFLRHRPQVVKVGNMISSTCTLNIGAPQGCVLSPILYSLF

THDCGSHYDCNIMVKFADG

>SG-PL_contig_2367_1_+1_1_117

CNEEVVPDKFCPKKIHCHRSAKTQADRERKKNMSGAARS

>SG-PL_contig_2368_1_-3_99_359

MAFTFASPTDVYYNAANVKQVDVPSFSGSFGILPSHVPSLAVLKPGVVTVFEEDGSHKKF

FVSSGSITINDDSSVQVLAEEAHPLDR

>SG-PL_contig_2369_1_-2_59_2629

MSRSQGLESIYRIAPYYFVHVLDQNSNVTKLEIGPQTYIRQDNERVVFGPEKMIVVPPRH

YCIVENPVVRDADNDVVMDSAGQAKLQHADQEIRLAQEPFPLYPGEVLKQPVSPLKVVPA

NSALRLRAVLDFEDAHSGSKRTAGDEWLFEGPGTYIPRKEVVVEETIRATVIRPNQAIRL

RARKECIDREGVAHVTGEEWLVKKTGAYLPGAYEEVVDIVNAYVLTDKKALHMRALRTYK

DDFNKTRKNGEEWLITMHDTEAHIPNVYEEVVGVVQITTLSNRQYCVILDPCDATGRPQL

GQKKQVKGEKAFFLMPGERLEKGIQNVYILGEDEGLILRANEQFKDGAVERKPGDRWMIR

GPVEYVPPVEMEVLMKRQAIPLDENEGIYVRDIKTGKVRAITGNTYMLNQDEELWEKVLP

PVVEELLMGAKDPLADRSDRSKSRDDAPKIRDKTRVINFRVPHNAAVQIYDYKEKKARVM

FGPELVMLGPDEQFTQLSLSGGKPKKPNQIKALCLLLGPDFATDIITVETADHARLSLQL

SYNWHFDTGDRSDATKAKLFSVPDFTGDSCKAIASRVRGAVAQVQFDDFHKNSAKIIRSS

VFGLDANNKVKESFRFSQNNLVITSIDIQSVEPVDQRTRDALQKSVQLAIEITTNSQEAA

ARHEAERLEQEAKGRLERQKIMDEAEAEKSRRNLLELQAQSAAVESTGQAKAEAQSGAEA

ARIEGEAAVEQAKLKAQAMKIAAESELDRLTSAREAELKYLSEQNEMELTKAKELATIET

TKFKNMVDAIGTNTLAAIANAGPEMQVKMLQALGLKSTLITDGSSPINLFNTASGLIGGL

VPTKRRRQEEEDDDEVA

>SG-PL_contig_2370_1_-3_111_467

MADYWSFQPLLLCWWMFFMLPCSTNVKKISCCSCQWHMDIFMQTCVFHARTRARARACTH

THTHTHCEAPTSCVYIVTFTCSHIVCVYCSFYMHARAHTHTHVRTHTHTVKYPPHVCIL

>SG-PL_contig_2371_1_+1_13_240

MEHDVHSNSSSNINAAPSKTDSVRKGSQLYHTILTNDNCLTKCHSKRSEKLLCGIIWKSC

FCKIHQIKDNNRNGCK

>SG-PL_contig_2372_1_+2_2_262

PRNKRTVICRNLKSIGLNVFTFEATELLSQVRGSTDSATLYTSILRHLLDKHTPPSTCVL

PDHPCAPQLHKIKRECWRAECQWRSSK

>SG-PL_contig_2373_1_-3_24_266

MHDPHSTLLAGPAMVPGPEDPISRPSTGANPEPGPSQATPLRDPPCEPSYAPSSCLASVW

QFLPALTDTTLSVAGPPPSSH

>SG-PL_contig_2374_1_-1_1_366

RLDAGCFALSRLSLFFVSWCLFGVFFPHFCGLSPQTTAVYPCFLLSSPILLFFDFSFAVL

VDRECLGNLTTGVSVGLMLGPIRVQHSLHGPPRGPSRRVNQVRPPGRTAGLGEEFDLRSN

RA

>SG-PL_contig_2375_1_+1_1684_1848

MMICRYEKGQVEKVEQKLSDTLMTCRYEKRQMSCQPCGIMGHISTSVNGYPRQQA

>SG-PL_contig_2376_1_-1_103_1584

MSDPQPKPDTKIKFTQLFINNEWVNSASGKTFPVTNPCAEQVICNVQEADKADVDKAVAA

ARAAFAFDAPWRTMDASKRGAIMHKFADLIERDLEYIANIETLDNGKPFLDSVDDVKAAV

DVFRYYAGWSDKIHGHTIPADGCLFTYTRHEPVGVCGQIIPWNYPVMMVAWKLGPALACG

NTVVLKPAEQTPLSALHCAALLKEAGLPAGVVNVLPGYGPTAGAAISEHMDVDKVCFTGS

TEVGKLIMQAAGRSNLKRVSLELGGKSPLIIFADTDLEEAVTWAHGAIMNNHGQNCCAGS

RTFVEESIYDQFVAKAKEMAENRVVGDPFDAMTMQGPLVDKDQFEKVMNYCQYGKEDGAK

LQTGGVRVGDKGYFVRPTVFSDVQDDMRIAREEIFGPVQTIIKFKDIKEVTERANRTMYG

LAAGVITNDINKALTMANTLQAGSVWVNCYDLITPQTPFGGFKQSGQGRELGEYALKEYT

EVKTVTIKLPSKTS

>SG-PL_contig_2377_1_-3_78_1814

MGKLKANLFKHLSKEDFRVLVSVEMGMKNHEMVPGPLVASIAKLQGGGCHKVLVNLCRHK

LLSYESTGNRRYQGYRLTNSGYDYLALRVLCGRDAVTSFGRQIGVGKESDVYIVADEGMQ

EYALKLHRLGRTSFRQLKNKRDYHKHRKSASWLYLSRLSAVKEFAYMKALHEHGFPVPKP

VEQNRHAVVMELIAGHPLCQVMKVRDPAALYSECMELIVKLAEKGVIHSDFNEFNLMIDS

DDHIIMIDFPQMVSTSHTNAQMYFDRDVQCIRQFFSKRFSYESELFPVFEDIKRTGSLDR

EVQASGFTKKMATEFDNITEQMAREDSETDTADECEDESSELDESDTTEDEEDDEPARHG

KKRHVPVMDYKDEQLRFRLALLNVDEGEKPEVDPEVEFSDIERERCQEGEDTVAVAEETE

PGADVCKNKVISTEDGAVVGKVGLETCVKEADISAEGAEAGAGVQYCEESDSDHGFVPLK

AKASTCPQTSEAEQVSEGSDSESVGMHHCHRHHVDAASSVQSSIHPELIKRRATAQFKKK

LEKQKAQRTRKRGEAAIITKQRRENAAEIKTSMSAFWYQ

>SG-PL_contig_2378_1_-1_148_1326

MTRNQRFNPHPGHPHPAALKKQQERAVREAYRRERKIDSYLADDENFPSFAIQLKKLGLQ

LRDIPGDGNCLFRALGDQLEGHCRNHYQHRVDTVSYMLEHRTDFEPFVEDDVPFDEHINI

LKKLGTHAGNDAIVAFAKLHNVNVMIHQLNGKPLMIQGPSIGGKVRQLHIAYHNGDHYSS

IRRDGDNTESPANICLQEDAKLSGPVYGGGSDHGISHGNTGVDHLESEVMMATGCQSVEQ

VRDALDECDYDVDAAIATLMQTMELQDDGFCEEAGPQHHIDGGLWAADGTASRIFGGGGG

VSGAYREDTSSDVSPLGSNRGGGARPKHSLAAAPSSQQHGSGRKGRETKKMEKKRRAEER

HRQRLLGSDPQRQLQREMQQEDAVVIKQNVTRI

>SG-PL_contig_2379_1_-2_200_1531

MEEMNGRAMPGQPKPLKVVVANSKRDGSTRDPREDEKLVRLFVVCPKHLKEKDLQTEFEK

YGDLDHVTIIRDRTSGDSKGFGYVKYHRAYHAALAYENCSQMFKPKFAEPQRSRDERESN

TDFYTFGQNLGFMNMKGQKRTFEEDGSFGGDNRPPPMPGPGNFSLPTGGAPGNFGGVPPN

VMGGAGPMTMLNDYPTQSSCCRIQIVAPIGMTQGYLTKLFNLIPGMEYCDLNESTGVAYA

RYATPQCAAYARDKLNGFEYPIGSQLMVQFAEENAQQQSNTGYQGGRNDELSDLRQRAAS

LLEQAGINPQAVMAAANSAAAMFGMMGAAANGMERVNYCNIALPLPQPLVSDDSVIAKRL

FIVCQPSAVSERVLRDAFCRFGSLISVYLLAGRNYGYAKYANQDCAMKAMQTLHGQNLSG

QRIKVLEAEPPKQGDDGPSKKQKI

>SG-PL_contig_2380_1_+1_481_657

MQRIIGTQQLSVNRIQKKSTTYHDQKRNRPQNPTNKILKTCKITNTNPSCCVIVYAYFV

>SG-PL_contig_2381_1_-2_2_139

VNNTKQKKVPQNNSFKWEGGDFSLYTASDICVEIVFFRLPEKNISN

>SG-PL_contig_2382_1_-1_1_99

TLCSKVTGTALLSHFPNCQVQAFLNKRIYTNDS

>SG-PL_contig_2383_1_+1_1_174

QKAGILFILKEPPASPRGSSNLKTKSLQKTTIHDILSEPLSSLRQIRPGCAEDKNHSA

>SG-PL_contig_2384_1_+1_61_363

MRVVPGLSLTAFAISNPVQGQAPIPSWVNWRSYLQSFSHSQTDTCAVRLEGRSSTFILKR

KSFVCSLQSKRVCVQPPVCPTENRYLTLFRVREDKAAKERR

>SG-PL_contig_2385_1_+1_70_246

MQACVRGSVQMHVAKNAYCSQSPVQMLVMKSEQHPQRHIHEVDDTQAPVLVIPAELVAH

>SG-PL_contig_2386_1_+1_28_339

MNSSHVVQHCCQLAFFSVVKLSFIFSTSGLYRSQHKTPFSSKKQDTDLVNSVLLVSFVFP

SVNVRHKLYMSSYLRSEDDKVHTLSGSIPARSFSSATRNMPRSL

>SG-PL_contig_2387_1_-1_1756_2004

MYTHAHIHTLTCMHPYAHRDPLCVWKSLTLCILASVSFHSVFHKSQYVILNHVYIYCCMI

DVVVSHACYKICYNCKFCSALSF

>SG-PL_contig_2388_1_+3_126_275

MKSGTYFPCHECLKLLISHVRKHRMTGNCHVLHTVALEHNSIHTVCFCSL

>SG-PL_contig_2389_1_+3_519_716

MQGATRCDRQGTKGHKHKSHVKSPSASPSIIRTLFLNIRTVLASHSAVILSIHQNCSCVP

QCRRIN

>SG-PL_contig_2390_1_+3_195_260

MFLFRWSPHTDRYGTQAYFFFL

>SG-PL_contig_2391_1_-1_136_369

MHAGYVCVSVSHPTLTWLIGSLTCIFDVLHAYTHERGSGTSRSAHMLIWEIGKISTLYPP

GEVAKEDSNPGLPGPPVW

>SG-PL_contig_2392_1_-2_137_466

MVVMEAKYEGEMCFPVFQTKLAQHKNKEKSKLLHQTKTDVQHYQDARGCTVSREGRSTLN

MMPTGHFHLLKKFAQTGTVLEKARPVLSTTHFFGPFQQANSSVHQGRGGR

>SG-PL_contig_2393_1_-1_259_651

MQLFIQAQQLYTIEVEGSETVEDVKALIEERERVAAEELAVYCGGKPLEDDARLAVLEDQ

TSLEVEVRMLGGKVHGSLARAGKVRGQTPKVEPQEKKKKKTGRAKRRIQYNRRFVNVVAT

FGRKKGPNARS

>SG-PL_contig_2394_1_-2_731_1045

MQKVFAPSQNTIVTFCHLLSFACFIILVLTKKDNCYFVTKTFIRNRMSKPLVWLKCIVVE

RCNTKIIRCLHACVDCFRKCLALVIFTIKVLFFLLSSLLLCVISQ

>SG-PL_contig_2395_1_-3_3_293

IIDLITFWLVCVCNSYVTAFSFLSCVSLFVQFCFVLDLLSAFLLLLLLSFCLYPPPLIYS

VLPTASWSIFNFVLVTMPCSMLLSPELVQVLLCSLCR

>SG-PL_contig_2396_1_-3_876_1943

MGAFNSAGGSKGAVEIPVQHESSNAAGPSHSQQYGHQNFQQPQRMAEPQRMPESQRMPEP

PLSTAQHAAPPFHLHSQKEAFLPQHAGQQGHPWAMHHNNPPQSAESDGGREIPIHHVSTH

HVPANARQQQPQQHQQPPPQSAFQPATHPQPGADPGTSTVYTIPVRHEKAQPQAPSPKKQ

YSSPQSSARQQTPPAGSTSTPPPPETRPEPSKPKTAEERAFEIIDGVMREVKALEESVNN

FKGMKRDKEYKYLEEMLTRSMLKLDSVEAEGHDNIRQARKNSVRMIEAALDLLELKAHAN

EQQVPATTASEVMDISNHATESSSETTSDHLNTKSSGRGKSPSHVKEMVLDSEVSC

>SG-PL_contig_2397_1_-1_163_339

MTVGATSLHIFAFCPQGDNVMIKGFILRCQFCACIIYKCVLCSNSRRGRSEERRVGKEC

>SG-PL_contig_2398_1_-3_234_371

MVFQRVLLMYAAHPIINHSASKKYKVSPSFVHVMYFHENTHTNMSA

>SG-PL_contig_2399_1_-3_135_1298

MSEVWLISAPGENTPQKTWDALNRATTAENLSTNYKFNIPELRVGTLDTLVGLGDDLAKL

DVFTESVTRKIAQYLGEVLEDTTPTKDKLIENLKINDASPASYLHKFAWDGAKFPIKQQL

KALAEMIGKQVSQAESDLKIKANAYNTLKSNLQSFERKTTGSLLTRNLVDLVKKEDFVLD

SEYLVTLLVVVPRGGSAEWFAKYEKLTDMVVPRSSRVVFEDQEAQLVNVTLFERVADEFK

HKCRENKFSVRDFVYNEAQILAGKKEAEDADTKKKKQFPPLVRWLKVNFGECFIAWMHVK

ALRVFVESVLRYGLPVNFQAMVLVPNKRSQKRLREVLKQLYQHLDSSALKKDTESQVVDI

PGMNVGSSEYYPYIYYRINLDLLDQQRL

>SG-PL_contig_2400_1_-1_1324_1482

MPWSVHLDKARWCISHFCGSLCILLSILFLFESVKIFAIMRCWNETNNFPKTG

>SG-PL_contig_2401_1_+1_1_81

VSRSNLVAGIIWLGKARLYKSDLTIAL

>SG-PL_contig_2402_1_-1_283_462

MKAMVKSLFYNAMVKSLLYNLAFPSHYIPAARFDLETELCYFKLLLEVMPECFGGATVNG

>SG-PL_contig_2403_1_-1_1_381

LASLGLFLGLRSPSQGVFWCDLASRVRSSLAQPAQRRGETYLCLLIFPGLVFFCHYGFQA

QGSARGILTVRPILIGIDQAPDHRSDQSSPVNRQAKRDRLHCAKRRSPDPFKGSRRHVEK

SSSCSYL

>SG-PL_contig_2404_1_-1_37_531

MVAVWRALARQGRTLNQLYVLRQRLAVQPACFISTSKKNKDVAVPVQPMPKSEELKKLEE

HFADTNPDADKNWVSYGFEPTDPEFDNFVHHMTMFFTITLCICWGAFILAYQPDHKNLNW

CSREAYLELERREREGLPLVSPDYIDPSKLELPSDEELGNTEIIV

>SG-PL_contig_2405_1_+1_1_171

FTCSTLSLIKKIEIIIEICSIKSAKTYSLKTCTLIEFYSIKFAKTYFFKTCTFEEGC

>SG-PL_contig_2406_1_+1_310_441

MVQRNTFKNKIHTRESATVPLTNVPVSVLLTNTGSLHKCYDLKV

>SG-PL_contig_2407_1_-2_14_2350

MALSGRMGNWQAELDDLLKSCRDSGHLITFKTVKSDADRCKFIFDEKLLATIGWLRDYFT

AVCQTFHQKQAERAGHCKQEGNKLFLARKDKQALQSYTQSMQFAPCRGQQEENGADLGLA

YGNRSAVLFRLHKYQECLTDIERALAAGFPDQSRHKLLVRRCQCYVRLGHRDKAVSAQQD

IVSLLHEVCDDKTTRYLEGEMDKIKAQIESLSADQSEVTEGETSPEPAVSYGVNSVVVQA

SSAVEMKTSPSQGRFLEAKQDIKAGDTLIVERPFAAVLLPPHYATHCLHCFVVLADNPVG

CHQCSSVLYCSEECRDVSWTIYHHVECRYMDLLHSVGIAHLSLRVVLTVGLSTLQDFLDQ

KKKAGVCTHDTIPGVTAEGRYERNYLSVYDLMTHTDDMVTDDLFQYSLTANLLLAILIHS

GWFLHPQPQDSAPPSEMNTANLIAFLDGDLFKTTQSTSGLNTEQVFAVGGALLRHVQQLV

CNAHAITALQATQVSQRHLVETQSQVRVATAIYPTASLMNHSCDPTIISSFQKDILIVKS

VRDVASGGEIFNCYGPHCKRMGRRKRQEELKSQYFFDCHCDACLKDLEDHTVVKQGIRCP

DCGETVIPEQEEVTCPSCRAVIRMTPILSQLEAADVHFVEGVKCLENGEHSKALGRLKKC

LDVRSKVMSANNHDLGQVHDAIAQCLACTGRYAEAADHLSHSLNVTEATFGADSIEMARE

LHKTAEVLIHAGRPKDALATATRALDAASRLLNEKDDWVQELLCLKTALTKIIADGGIQ

>SG-PL_contig_2408_1_+2_86_235

MKLLVRPIWIYVIPMISALYTFSKSQSDFFLTSATDSSLKALLLQHPFYY

>SG-PL_contig_2409_1_+1_1_207

KKKKGKKANMKRNTDQTNTTHRKNNSRSCLVRGGDKQARWLFESNAVEGTGCMSCDDLQA

TWYTRGEVS

>SG-PL_contig_2410_1_-3_1101_1547

MPCFFFIHAICFVYMYIHTYIHTYIHLYIHTYIHPSIHTYIRTYIHTYLLTYLYTYIHVH

CRLWMGIYILTYVHTYCAYMSIVNFGYIHTYIHVSVEFGQLWVYTYIHTCQCRIWVYPFI

QPASHTYTHTHTHTHTHTHTHTRYNFNCL

>SG-PL_contig_2411_1_-1_670_912

MHVRRIGANCYSKSQIYLMSYTISQHIGGTSPIVVPFLSRWVTLHVWCLDQTRRRYIHLI

HIPTLIDSSRMFVWSELVIVV

>SG-PL_contig_2412_1_+1_1_156

TIYIERAPHRGTKVAYQIHHTQLQILDLDYYSQNELNIKHSVLSNLLVTIQY

>SG-PL_contig_2413_1_-3_1056_1505

MGECIHCREQRQGCNRSTIPEKSVTRTTTTRTMKGVSMICNWMLVVVVVTTLLLSRVTPC

SSASLPQHAGCDSPTKLDARELNIRSYQANGSQPGYFRYLTVDTESGYLFVGAMNAIHAL

NLYNIESNNRKFVQEFNATPHSTYRCRIQG

>SG-PL_contig_2414_1_-1_892_1122

MLQFDRFILGFKSFTSCSLSIVDVTGIKRAGILRSVETCFLLRDVLSLVVLLMLLQLTSS

DKIVKTCFLFHVFCDKM

>SG-PL_contig_2415_1_-1_1_228

WLDHLFKKILYISIFIYFFSVAVTVLEYGLAVLFFHCILSLCLESREQYKLNFLLMVLEL

RLESRGQYRLNEGTEC

>SG-PL_contig_2416_1_-2_44_1249

MEHMHQVMALHPTYMEAFYTAEDSLLKDMLGPLTFQYRQYIAIMAAARHQCLYLMKLHAS

EFLMNNGDPQWLEGLDKVPQKLRNLAEVNKILAHRPWLLKKDHLEKLLRGENNWSLSELM

QALIIMIHFHSLSSFVYGCGISTDHDHVTSLRLSSSGEEESDYSSDSSGELNSNLIDEPS

LELLMQKMEQLRVESHQDDGPTQEELLERFKSVEKQSVEISVPTKEKSSRIEEAQRFVTD

IDFAYSDFAKRAHDDIQTFRVQDYSWKDHGYPLVNRLYNDIGNIVDKRFDVAFNLTYNTM

GNNHEVDTTAFRQAIWYYIHCMYGIRHDDYDYAQVNQMLERGLKTYIKTITCYPERVSRR

DRDGVMKALKHSEKVHVNLMIMEARMQAELLYALRAVNHYMT

>SG-PL_contig_2417_1_+2_2_178

FTLLLKSKQKNTSSYRFCNDQPKFNLITTDQLHIYFTVTILIRFFFVFLFCRFLIPVFQ

>SG-PL_contig_2418_1_-1_1558_1992

MGIYPFKSDNLLAAIVLTAVAFNAIQCDSKGAACIKTIIKDCKGCFFSTGLWQCVQSEFV

KGIVCAFLQAQCMLCCAFTTVCCVLSLCCVAPFDKSFSGRCDTTLLYLCGLVDMHSSALN

KSHHFLSYFSSWFYFMNKFHHALKK

>SG-PL_contig_2419_1_+2_26_304

MLFTLMLLRVLLRVCILKVSKQSFSLPSFKLSNSSCTQKKFFPEILFNHNLSNVICLQVI

LSLIFLLQDKDTGHIKKNVSPNTNLLTNHTVDI

>SG-PL_contig_2420_1_+3_3_146

THTHTKSLSLSLSLSLSLSLSLFFLVLVAVVVVAVVWFDCTFISLFDD

>SG-PL_contig_2421_1_-1_1003_4185

MGQKPTALLSEVRHRSRSFTEGRATSDLKDPHPSHHILLPCPHPAWKSSPTTPRDSHLDD

SDDAITPGRHPVPSPRQRDVSESGDVGKSDDVASPRESSSSSVVRHPRQRIVSDDDSCVP

GETSRDGGDVIGLNVDVNRDFTDGRDYLLSGDDFRNDDDVDEGDRGKFSGHGGGVEEKRI

QVTTEHSRTYSAAWDEYDSEEESNQFDNHLSNSSVKDTAKFSDRDSASDGTWVTSGPETG

SCTIEAAARSNTIPRHRPPTLFSAQTAIFGSKQDVKSAVVAADSRDEEQLSRFSSSETLG

RQPRGPILRYLQKTRAESSSPLASPRAFSLDSPREKKFVFPGEFPGGSSVEGEHDSKKTP

VEQTQSSSSTKAPVEQAPRSSSTKAPVEQTRSSSSTSSETSKITSQTRATQRPSHLPTAS

SKNSSTGIPKPTKKSSSIPQRARSSSQGPPQSQRKSTSGIPSKISSTSGPAQSSRGGTSS

GIPTRPRSASTGPNYSTRQPLEKPPESSRIPRVGAKSKKEKSGGRRPHPMAVMELAAKDL

QSPSLHGFLCRKSFNDQWIRYWYVLQEDSLYCYLTPDDHVTVDVTDLDGYSVTSLEDKFP

GQRFVLHLDHEDYIPVYLSASSREEMEEWNASLTSAIKVIPRVNGLHLPPQHEEGEGARA

AHVGEDRVLGIKQKLLAEVLRQTQELEKKQAARLATKHKQTSLDENELHTPPSTPPPPSV

TPTGEQICTLTRLRQRRMSTQIKMDTLQRQMQTNGHAKKSRFTFGKKKSAEETPTPQNPD

IVQQLKTLSDHLSAIDRDLIQNGGVMTLDRKTSSLTSSSSSRGRKTMSRHSQELEGSAVE

GGGKKSSSKGVDSLMTEESVINRSNSLKSSVQKLAHRTFGKTGWYRSKKSSQGDSLEHEE

SEDRNEDPDHHHHHHHNNNNTITTTNSNNNNDSPDVIDLSHRLMRTWPARLVSQDSEEDK

FGGDTGGDVFLPPPSSFFDGCGEDSPSSPVSSPRSVRSAGSAGSVDVREEGGGEGAARRS

GSSLGQESRIPVRRRRQVDPDAMAHIEAFEEMSRQILGEPT

>SG-PL_contig_2422_1_-3_3_275

RNRPKLEFRSQILNLRLLTYIQSLWQEEWTCQQDNKLFQIRPDLKVFLPSVSGCRKEETV

LCRLHTGHTFLTHSHLLKGDVPPWCFPCRAP

>SG-PL_contig_2423_1_-3_156_287

MGLAVNRSPCHVFVWLVYSACHAYPVRLCVTYNKSSSSNVKKKK

>SG-PL_contig_2424_1_+3_45_143

MLKQAGNSKYEGNDQYKECLKCYLQNNNDFKRF

>SG-PL_contig_2425_1_-3_516_752

MTQYNTERKRAPRYFTFPIPKEGQVPFTAGWTGAVCSSEGTNLVTRAFANHSRLCLTLAF

VNMSEFVLRPLTMLSLLTM

>SG-PL_contig_2426_1_+1_1018_1173

MVSFLCKIVNMASSTTHNLISFREARLTGTNEHTNKSALRNSKPKPLNRSQP

>SG-PL_contig_2427_1_-2_422_631

MKEYSLNGTISFIPLPNCRLATFLSSLALNVMPFFNFYCQTSTTNQISNMDKRNMKGIKL

LHRQWSCSVA

>SG-PL_contig_2428_1_-1_1_147

LPFFLFWVFFSSPPHHQAGKLSCCWHILEHRSFSFFAPKKKINVIELRL

>SG-PL_contig_2429_1_+2_2_151

KCLITLHLKPILHLKSPHTTKMFDHTPPMMNVNTHYLNSRQLKLLNCKHP

>SG-PL_contig_2430_1_-1_10_990

MAEAELNVDSLINRLLEARGCRPGKTVQMTESEVRGLCLKSREIFLSQPILLELEAPLKI

CGDIHGQYTDLLRLFEYGGFPPESNYLFLGDYVDRGKQSLETICLLLAYKIKYPENFFLL

RGNHECASINRIYGFYDECKRRFNIKLWKTFTDCFNCLPIAAIIDEKIFCCHGGLSPDLQ

SMEQIRRIMRPTDVPDTGLLCDLLWSDPDKDVQGWGENDRGVSFTFGADVVSKFLNRHDL

DLITRAHQVVEDGYEFFAKRQLVTLFSAPNYCGEFDNAGGMMSVDETLMCSFQILKPSEK

KAKYQYGGLNSGRPITPPRGGGPPKKK

>SG-PL_contig_2431_1_-1_1228_1521

MWRCCALIPFTFLTLSTSCLQDRVIMFLQEPGGQAERVKGLNTANYSAITASEEGWGDAF

YVFVTFLMFQALSLKSNNQMTAYGLPLHTDQFHGLRFN

>SG-PL_contig_2432_1_-3_81_431

MDQNEVFPGVTDYQSLGGACSMEDVAKVVTFLASDLSSAMTGTNIPVERGYMLTSACSLP

AVMNALSSSQWANSGDQNAKPTEESLKDLIAMKDLLGKGDGATKSSESEGPTKPSEG

>SG-PL_contig_2433_1_-1_97_714

MKKLAEVDPVEFSTEEAWQFFKNALIEVLNKACGTKKVGKGPVKRTPWWNETVKEATAEK

KKLYKTWLQSKMEEDYIKCRLASRHCKRVVREAKEQSWKKYREELSELCRHSPREFYRSI

KAMRLRDDPYGPTSVINDKGGNPLNEGNKIKERWKEYFQELLNSHQQQTTQGPFQPSHPD

ETEPTILRSEVQRALKTSPRYKAARI

>SG-PL_contig_2434_1_-1_550_870

MRVALHRDKHASHFLSLFLSFFLFFFCFVLGGFVVIIVVVLNMRLFVHQSEWFFYGNLLG

TNFFLSCVLLRAPSMLHTGPPCIIPTERLAPGHHSRFDGGEIDPVQP

>SG-PL_contig_2435_1_+3_3_125

LPPQQASKTACEYHHCLRSKPAKLRVSIILLYKIKRPLSCN

>SG-PL_contig_2436_1_+3_3_107

LPLTVTPILHSYREVPFIVKKQKVGCNVHKYYTVI

>SG-PL_contig_2437_1_-1_1_255

SGSDLALTALPEFLVETQQPGSLPRTVRIPCLVSCPDPRDARSEERLLARSDASSTTCNA

RHLGVPPSGAYLSPSTRSTRGTHQR

>SG-PL_contig_2438_1_-3_3_251

TARLPPSGLSHPSLGLLPGPQGCQGRGTPLCPVRCVKYYLKRSAPRRSSQRRLLISLNKN

YSRDISATISRWLSATILRAYKE

>SG-PL_contig_2439_1_-3_3_263

GSLAPVLPLDRVHKRPLQRALRTFHIPSLVSCLNPRDARSEERLLARSAASSTTLRSAPR

RSPQRSLLSPLTRSTRGTHQQPPSPAA

>SG-PL_contig_2440_1_+3_42_179

MFRSCSWTISSKINWVTFNYLYLKVSISYIFYLYPSKSTPTHWNPA

>SG-PL_contig_2441_1_-2_305_970

MLSVLIISLIAGVCHGQNEGLFSECDDQQTIISQCSDTHLVYLKKIDAGEVITMQRVSEI

DDPAFLLDSVKLNSICNDTNHLQLYYQCVHANLTTCLQTVNSTKAKDLADADTISQGIVN

MCKSRNELNKTCLAEKATDVKTCFATFISESDKASDQCTTYEVLIRCTEKQSVCEGGEVV

KDFVVSARPNGCRDTSTAVPRMASVSVVLLLASLGQLVAFHL

>SG-PL_contig_2442_1_+2_65_271

MGTIKINQGKAKVVNFTKTDPKIPLLFTCGSHTIQTTDQYKYLGVIFHKNGNFMYAQEHL

AKQVKQSLL

>SG-PL_contig_2443_1_+1_1_162

SLFNRDSSHSDNSVYAPTKAVGNYLAHFLSLHKTLYAGKVLRWVGVEITGSMKH

>SG-PL_contig_2444_1_-2_530_733

MVLYKRAIDFTHTYTNIFLYIHTQKENYNLVVSNSFCDASSHLLPGQRSSIIMAHRHHAA

LVVYTALK

>SG-PL_contig_2445_1_+2_119_334

MCNNISCPIYMYMSSFLRNMKIITISSKTYMVLEKSTDNSTASIHEFGLEIGKHSNHCPQ

STYMGMLFHLNC

>SG-PL_contig_2446_1_+3_27_200

MNLHVIKFYGALGTVSHTHNEHTIGSNLRQATRFYTTRLCQRVGFTRRLTAYAILNLG

>SG-PL_contig_2447_1_-1_1_312

LSRSLSLSLSRSLSLSLSLSLKDTHSFTHTQPHSLTHSLTLSCRPTHSSTYIVAQKTYNH

FHLLTVDLLISYNHFFTFFTFCILWVWFHYYLSLTIDMLSLFSI

>SG-PL_contig_2448_1_+1_553_690

MHIGIYSAKRLAQSKDNGQSEWSTQVIYFWLRRYTPYTNYKDPQSA

>SG-PL_contig_2449_1_+2_551_703

MHIGIYRAKRLAQSKGIGQSEWSTQVIYFWPRRYTPYTNYKDPAVPDPPSC

>SG-PL_contig_2450_1_-1_226_840

MADIKLYYFDVRGRAELARLVLAAAGQKFEDIRWDREDWPKYKPEAPFGQAPFMEYKGKK

YGQSVAIANFFAREFGMYGKTNLDALRIDEVMCLTQDIWQVLVKVMFEQDEAKKAEMEKK

NESEEFPKFFGFFNKMLGENKPNGVIVGKDLTLADICVYDIMYSLCHAKKYDAGAFFPDL

KALKDKVEAQPKIKAYLAQRKVTEF

>SG-PL_contig_2451_1_-2_38_244

MRRLRVWLFFSTSPCRQPHSDFRGTKVVIGKQSWELWKQGNRFKLLLCEHIPGRYVKYMI

GLPVLSRDD

>SG-PL_contig_2452_1_+1_52_531

MGIYLAKFLFFMYFSPCVSGTHSLSVPMCQALSLSFCVCVHWHFSHSWARTYIHRHTHNL

FLHSLVFAHTYILMAQYGTVLSFSQALSHTHTHTHTHTHTHTHTMVFSHSFSCNGKFEKW

LLANNFYPWKIKYIVIVIVIVSLSHTRTRTHTHTHTMYKT

>SG-PL_contig_2453_1_+1_319_426

MLETGKQKVVGQIDPHCCRCPAILTFCLITAHTSLH

>SG-PL_contig_2454_1_-1_1_207

RTWLTGSLTCIFDVLYAYTHEGGSGTSRSAHMLTWEIGKISTLYQPGEVPKEVRTQDLLC

LKVQDSIGY

>SG-PL_contig_2455_1_-2_2_151

TSNKKTLKSRNGLRGFISTAGKMYCVQKYVAQHIFVLTIPKYYCVKEKGT

>SG-PL_contig_2456_1_-2_68_193

MYCVQKYVAQPIFVLTIPKYYCVKEKALKPSLEMPSLYMYRK

>SG-PL_contig_2457_1_+1_1_261

LYHRGHRGKKEPSVCGQLESLSWCDAVGRQSFLDTTDPSTWPINNCVVFGISEKSKQTFN

CLWYVLINCFQHTDTVICMTTCLIDKK

>SG-PL_contig_2458_1_-3_3_311

IEVICCWNERVIIIIIISDSCIVLFSKLIWTHCALRSDRTQSVIHAEALISHTHRHIMRI

HTLRIADVTDQYILIGSTIHTHTYSYRHNCTYMNVYTMPHDQQ

>SG-PL_contig_2459_1_-1_160_342

MVCYIFVILENLLCDYYIVSLRPLKISLYLFLFLLLSLSLSLSLSLSHFVSLSFTFPPLF

L

>SG-PL_contig_2460_1_-1_532_771

MMFDLVNLFKQPGPCGHETSNSNHDNDTCNDDDDDDDDGDDDDDDKKKKKRITKRFYIVL

LHRIKGALDTCTSITNKMVR

>SG-PL_contig_2461_1_+3_27_287

MQPVSKPVGSYMTLTMTPVSKPIGSYMTLTMKPVSKPVGSNMMLTMKPVSKPVGSNMMLH

QQTYTYNMVMMREGNKQNLATLSNVRK

>SG-PL_contig_2462_1_+2_95_247

MTTKRKSNIIALYITMNISCQTITFSQYLQMLFFFLCKHDHQPPRYYDTLQ

>SG-PL_contig_2463_1_-2_2_634

TPTTTTTLTTATTPTTATTPTTATTPISTTIPTTTTTPTTTTPTTTTTPTTATTPTITTL

TTATTPTATANTPTTATTPTTTTTIPTTTITVTTATTTTAAPPPPTVSTTQPATTITTTT

STPTTATKPTTTTKPSTASTPTTPTTPTTAITPTSTTTPTTATSPTTTTTAAPPPPTAQP

TTTTTPTTTTTTKLTTATTPTTATTPTTALS

>SG-PL_contig_2464_1_-2_2_310

YHHCYTNYRHYTYYHHYAYNHHCTYYHHCTHHHYTYHHCYIYYHHCTHHHYIYHCHYTCN

CHYTYYHHYTYYHHHYTYYHQYTYYHHNCCSSTSYCKYNTTHN

>SG-PL_contig_2465_1_-1_85_186

MKCQFIYKFELTESSRPSRWLGGKDLNLKRQEGD

>SG-PL_contig_2466_1_-1_85_753

MGIESMSKEPTKVVYAPVAIIYWTLFAFAVFFALLIGLYYLLTGQGARFDIGWFLMSTSP

YMWGLIGTGLAIALSVVGAAWGIYTTGASIAGGGVKAPRIKTKNLISIIFCEAVAIYGII

MAIVISTNLASFDITNMENKVDWRKNYASGFKMFGAGLTVGISNLACGVCVGIVGSGAAL

ADAANSSLFVKILIIEIFGSAIGLFGLIIAILQTTGAGMVATK

>SG-PL_contig_2467_1_+3_3_116

SHETSLMIWEAHLQIHYGAKFSDHLKKKSFSRLIHSLR

>SG-PL_contig_2468_1_+2_407_688

MDFYSHILGTILWQNDSKAWNVKTDFILVYRESSCSKFKHNNHQTSQTFTSATNNKINTF

TYFTFVLSIVPINSVEMRFIHNSSQHFYYEKKLY

>SG-PL_contig_2469_1_-1_1_273

EPFPKQSPAPCNSDHYCRHQSVQGVRTDGFTSRSLYRYIRRRTFPTTNGLAQKTSAPNTP

PNSTHPLLALQNTTPRSRSEMRSRELRRGVN

>SG-PL_contig_2470_1_+3_150_293

MTKVKMNMYLIHKTRQDKTRRENTHKPKLSTMCSQCRYTIPLSNFVLR

>SG-PL_contig_2471_1_+1_397_600

MGCYSHMLWNIPGVFTCVGVFSSAINLKVVILCFQGCVAFSCFHKSPNADMAYRIFNVCI

WCSVCLYT

>SG-PL_contig_2472_1_-1_1_216

FVDNKGSTTESLIHTWKLSQELAHVTQVYRQSLCIVGMIQGKKTGNWCGQCLYKLFEICS

SDGMDELEYAQL

>SG-PL_contig_2473_1_-2_329_2740

MGLHRSNSHLNAEEKKNQLSGENKEKAKLEMVKVDVDNNMTDGVHIGLVPLEQQKRHVAD

IMRRIPAGSEVSTVLVGQVDYMIRPALAFVRLSEGQFLENLTEVPLPVRFVFVLLGPEKA

GMDYHEVGRSISTLMSNQAFHDVAYQAENRDELLQAINLFLDDSIVLPPGDWDQKTLLPI

MDMARKRAQIRRRHKQKEREALLEKGAPDIPADPLKRTGHFFGGLFNDIKRRYPHYISDF

RDALSFKCLMATIFIFFACISPCIAFGGLLDEKTKGLMGVTETILCTSIAGMVFSLFCGQ

PLLLIGATGPVLVFEQSLYNFAESYHVEFLPMRCWIGLYVCLISIITVALEGSFLVRFVT

RFAEEIFAILISIIFIYEVVKKLVHTFEEHPLVTSYNCETNMTDDPFGLTFNSSDGLNFT

EAYNSSYYSMAPPTGNTTTTELVNLTDTLVHNYHKEPENQPNTALLSLILTIGTFLIAYF

LRIFRNSRFLGRSVRRALGDFGVCIAILAMTLLDYIMKDTYTQKLQVQDTMSPTRPDKRG

WFINPMGMKHHMPVWVIFASIIPAFLIFILLFMEVQITEMILNKKERRLKKGCGFHLDQF

IMGVLAFVCALFGLPWMCAATVRTMAHVSALSSYSKTHAPGEKPHLLGVKEQRLTNFAVH

LCIGLSLSLGPVLRAIPVMSLFGVFLYLGVSAMSGVQMFERIKLLLMPVKYHPSVGYVRR

VRVLRMHLFTVIQLVLLLILLAIKSTAAALIFPFALILLMPIRHRIVNRIFSPQELQELD

KEEEDSEEEDEGDPDFYQMAHMPI

>SG-PL_contig_2474_1_+2_191_445

MLLKTHLQHHAKTRMNVPLNAAPRHLFDTAKFRKYYSVMQNNEYFILANGSITCPYVYRC

KTLYSHIYIHVFQMLEKKLPSTVMP

>SG-PL_contig_2475_1_+2_11_418

MVVWEASLSVEIINKDPMCCIHLAYIKVPTATKELSLAKFCGEKILGKQTNKQISMQEKS

LEKMALCYSDMLSHEKNSAMATKTNKMVKPFYNLPFSLKNPCKLLRRESRFSTSQRHCDL

EKASDKIQLDVKRGQW

>SG-PL_contig_2476_1_+2_2_127

WLAQRTTDRKVVGSNPYQSKKSLWDFWALTSSYPELSLLWVY

>SG-PL_contig_2477_1_-2_713_1096

MQFWDKVKIPLLNCILQGFEQEELSLSMKKGIITLVHKGNDSDKHNLAHCRLITLTKADY

NCRLITLTKADYKILTKALAIQLQNVIKTIVNEDQAGFIKGRNIADHLRFFDELTNYLNK

TARGPNSP

>SG-PL_contig_2478_1_-1_1_117

KIKAYIRKLLHCASVAALRCSFFMFLAIINLNFSNLTAS

>SG-PL_contig_2479_1_-1_1_168

ASITTKKVRQAGGKVSITTKKVGNKHCNCKERTGNCIFFFIQLRRKTVIFVGLSWM

>SG-PL_contig_2480_1_-2_2_574

PVPGPAVWSLPGPLCLHEGCQGTGVSGPRGSHPSLLLPGQLAYPGGVQHPLLSPDQPPEV

QPHAFTVVRLPRHEVQHPVIESVPDTYPASSAAAQAVGHRVRPWAPAREISSLLGLMESL

APVLPLGRVRKRPLQRALRSRWRQSASSWDQVVPLGDWFLEATQEWVSASDLADGVLIIL

PHPQSTLYTDA

>SG-PL_contig_2481_1_+1_532_762

MQEDPGLIPGLMEIFLPPPLDFVLVSIPPRNIKTGLLAAHLNAPTILVPVSAWCFISPVL

SPFPISRPCQRIGFCTS

>SG-PL_contig_2482_1_-2_272_442

MGSNLHKFIKKSLWDFSAPTSFYPELSMLWAHWEDWDHTVESHPLYGWISEDVAQIS

>SG-PL_contig_2483_1_+1_1381_1725

MLCKYVHAGHVSVCTRIVCAAFWANSLQVRREPVLTDWKVGAPPLNHPGLEIGVPILLLL

LLVTITIHAQCTDLQDEINITSFTFRNVTNYQQYNVQHTMICVLDSSEVNNQITN

>SG-PL_contig_2484_1_+1_1420_1662

MSIDFFFTTEINAHVQMPDHDTANTNVLPSITRALSKIHLIFQKKGKGLSDRGMNFYHYF

KTTFNDSGWAGENQRLVKFSK

>SG-PL_contig_2485_1_-2_71_502

MATAEWYLVVLAFLSFCSLGAAIQCYQCDSNEDVTCPSDEPFDTSKNAVVDCNSFEANVP

GQFCMKIQQESPGWGGWKKTTRRCGSRTDFGVAWGCRWTWDYTGIFRETCYCEDRDGCNG

ASPLSFSVLSAVLVLLAFFYNMLL

>SG-PL_contig_2486_1_+1_280_477

MPPRTGPMKQNSGSNSSPFRSCHGQHSHPISAQLKMSAQFRKTRKKVTSIMLPLWLPSSM

NSQWCG

>SG-PL_contig_2487_1_+1_1_174

EHPLELSDNERREEKHPQQKFYITMQSFSYAYCIAVCTHLSVLHIVEQLRRKMNEHIS

>SG-PL_contig_2488_1_-1_58_876

MLGDLPDDFLRSGRSQMQEDERIARVLQAQQQAGFVQPIPSNIIGRLSLTIVQAKLNKNY

GLTKMDPYCRVRVGHTVFETPTAVNGAKNPRWNKTVQLYFPHGVDSMYLEVFDERSFTVD

DRVAWGVINVPQSVMNGETVDEWFSLSGRLGDGLEGSINIVMSLLPVSSLPPQVSYVSAY

TTPMVMPMYFPAPVVGQQVGQPVAYPPQPGMMPQQQQQQQQQQQQQPQMTEEDLKQVKDM

FPDMEDEVIKSVFEANRGNKDATINSLLSMTAE

>SG-PL_contig_2489_1_-3_3_332

LLRIARRLSDYPGEDLNAAIHKACLARFLPRLARSALDQTLSDTGIPESRIETSLDNYDK

AVTCEVKDGLLRLGNTSVPVYNPDNRTKVPDILFYENPQHLTVMEDMVKD

>SG-PL_contig_2490_1_-3_21_704

MVEDCVRAMMMTACDVAAITKPWTIQQEIAQLVTAEFFEQGDIERHQLGEQPIPMMDRNK

KDELPKMQVGFIDAICSPVYRLFAIMTEKLEPLYTGCESNRQKWQDLAEQHQAASQHQQT

GAPSPSIQAAPAPPDPPTTEKQNPRKKSPPVTKVSPIRMSLSKASSLSKPSTQMSQLAML

RAAPVAGRPRPIVSRLTPAATRITAPATRLLPAPAAATVLATNHKRGN

>SG-PL_contig_2491_1_+1_190_330

MKKYLLILAMIQFSEVSTPIFELRQYLRYVMCFEQNFQVFDKKSFLL

>SG-PL_contig_2492_1_-1_7_741

MATISVEDLKKMKVNDLKKELKDRGLPTSGTKDVLLERLQAALDGTEPISSVTAENNAVE

DNDSVEKSIDQLVGDTIPTAPEAPVESTPVPVVTSKENIPEKTEEPASKPEKKVVSLTVM

PEADRMKARAEKFGGAVSDSAKKLMRAERFGLSASSSSGSTNTVGISAPKSEDMEKMKKR

AERFGTVVSNKLSKVDEDERKRKRLERFGAASTSPTEIKKVKLSSTDSDMEEKKKKRAER

FGLTS

>SG-PL_contig_2493_1_+2_104_385

MLTEERETVCVLYIASKHNTRFFQLACMHICQACMNLCTCMTAYVVFTSTILLCCAVTSN

ALYLLKNFFGGDRKKKSTALNSIIKRRFYFQFVS

>SG-PL_contig_2494_1_-3_375_509

MHELIIIYCLQPSQPLRPYQGCYHVTSIRVKKTTTLNTKLCQKKK

>SG-PL_contig_2495_1_+3_1191_1538

MVTSVSVDLEVCWRAGISGMGVMTQERCRRWGEGKKKKLSQGTTEIFSTHAQRVKFTTLL

LRVQTHTHTHTLTHTNISTHLFLPYDKNVYTIMNKSNMFSSIYIHMVTITVDSVFR

>SG-PL_contig_2496_1_-2_98_478

MRKKVELVSAVCLSVLTWTCFVLFCIWSQQNCSTSSVKTVLHSTAPPIFCSYVCAQFCLP

LKVDFSTEFWQDNSFVATGSFTCANTGPRFVNSSTTTNRPQLRVLWRDENNLTSQVSDLC

IVRPLAP

>SG-PL_contig_2497_1_-1_1_126

SSNQNFQEFFFILRQHRLRSKSRLNCLKKKERKSIKTNKNTI

>SG-PL_contig_2498_1_-2_35_433

MAKENVMELVRPGFEVDEDDDDDLDETLAERLWGLTEMFPEPLQKVVGNAVSLTFSALVA

GFSLGRSALWIAASSATILVLPVIFESERAQQQEQQLQQQRQILLGPNAAVSAAGSNLLP

GMGMMPHGMTPPK

>SG-PL_contig_2499_1_-2_110_889

MPQNEHIELHRKRHGRRFDYEERKRKREAREPHKQSQTAKKLRGIKAKLYNKQRHHEKIQ

MKKTLRMHEQKTKKQKAEEKCPEGAEPGYLLDREEQIRAKVLSNQIKQKRKEKAGKWDVP

LPKVKAMSEAEVFKVVKSGKTKRKAWKRMVTKVTCVGEGFTRKPAKFERFIRPMGLRFKK

AHVTHPELKATFCLPILGVKKNPTSPLYTSLGVITKGTIVEVNISELGLVTQSGKVVWGK

YAQVTNHPENDGCINAVLLV

>SG-PL_contig_2500_1_-1_7_1218

MAEAMAINSGEHIKSTQEAKNPGMETGIPSSGEQHAPPMQQKPVCVLVLGMAGSGKTTFV

QRITAHLHAKKTPPYVINLDPAVMNLPYPANIDIRDTVNYKEVMKQYALGPNGGIVTSLN

LFATRFDQVMNLIEKKATDSRHVILDTPGQIEVFTWSASGTIVTESLASSFPTVVVYVMD

TPRSVSPVTFMSNMLYACSILYKTKLPFVVVMNKVDIVSNSFAVEWMRDFESFQEALEHE

TSYVSNLTRSMSLVLDEFYTNLRCVGVSAVTGEGIEEFFQKVDEAAEEYEREYRPVYEQL

RQEKEEAARKKEEKQMGRFRIDRHAGDSTKDLLFQKPESISLRAAVEAEDDDDEDDEEGN

FRVPSSDDDEPSEVESFSRYLQQHKQRQAEKIKKNTQRSNQPSS

>SG-PL_contig_2501_1_-3_54_2051

MASLANISAKVAQAAGLKTLLGTAPKCLAAKRDFHFRIHGRNTDSKVSTSQMSRDYPVVD

HTYDAVVVGAGGAGLRAAFGLAEVGFKTACITKLFPTRSHTVAAQGGINAALGHMEEDDW

RWHFYDTVKGSDWLGDQDAIHYMCEEAPKAVIELENYGMPFSRLENGKIYQRAFGGQSLN

RGKGGQAHRCCAVADRTGHSLLHTLYGRSLKYDTSYFIEYFALDLIMEEGECRGVIALCL

EDGTIHRFHSKNTILATGGYGRAYFSCTSAHTCTGDGTAMLNRAGLANEDMEFVQFHPTG

IYGAGCLITEGSRGEGGYLVNSEGERFMERYAPNAKDLASRDVVSRSMTIEIREGRGVGP

DKDHVFLQLHHLPSEVLKTRLPGISETAMVFAGVDVTRDPIPVLPTVHYNMGGVPTNYKG

QALKYSKDSGDQVVPGLYAAGEAACASVHGANRLGANSLLDLVVFGRACANTIISENTPG

EKIGAIKQNAGEASVANLDRLRHANGAIPTADLRLNMQKTMQNHAGVFRDGPTMQAGCQK

MDEIYKSMDDIKLTDRGLIWNTDLVETLELQNLMINAMQTIYGAEAREESRGAHAREDFT

KRVDEYDYSKPVQGQTKKPEDQHWRKHTLSYVDEATGKVTLEYRPVIDGTLDEKDCPNIE

PAIRSY

>SG-PL_contig_2502_1_-2_2_118

LPGKIPLSEAALQVQSRCLKLYKKCRVAAQKKIAAQSHV

>SG-PL_contig_2503_1_-1_136_600

MAASPVISELSSSFNCLLYSAPVADMQAMCFTLALFAETPGMTPGYKMACEMALNFYGCK

ANTSLPVTAVPIPPSTTTTPPTTSTTAATTTTTPTTTTPTTTTSTTTTTTSTTTTTTSTT

TTTTTLNPNRCGVSNDLTSVTNVADRSLVFTMARI

>SG-PL_contig_2504_1_-3_186_950

MAKSGIKPTACSSNGARNKRDFGFYNGGDSNLRLDYGLPVQELCSGDDVTSKLFGDISMS

LVNAIGVQNLDLTGTCQNVQRECRSFTTYGYEEYALYCIGTTTPSTPTTTPAPASINAVL

NNVFPEVLSSLGVGESSLTSVVDNVLAAYPVISQLIPSFKAVLKCAAVPDIQAMCFTLAL

CAETPGMTPGYKTACEMALNFYKCKANTSLPVTAVPIPPPTTTVPPITTTTTTTTTTTPN

PNRCGVSSDLTSVTN

>SG-PL_contig_2505_1_-1_28_1539

MALVPLEKKGKKLTWNIKDTNDPRSKLPAINKAPIVDYDALSWLCSIQGLASRDTPDTSE

DAHRMFMENYHKMFVPEPKKTGFPVHAEKRAIAGKSSTQSESVDATAGRTTQVGKKQATG

SSQIEEFTHNIKDYLPLLHKERRSEDPEQIRTDNIKTLRRLLRKLPFERTATDNDRIFSI

LKTFDFFADNISNNVLKELCVVAQLDTWKEQDFTVFGNTGLHMVLRGKVMPLTDPHVYTG

LEIDELELRSPTPLLENCSTMLEVGDCFGTLQAIKSKQPSTRLLSVKTVDANCEFLKIAT

SDYSKVIVQIQQREQTEKLNLLMSCSQYKLWPRQPLQQVASLIEWISYPPNTVIVSEGYK

APFIGFIKSGECHVLRQVDVLHTLRSGKKEKKTKQVVMGKLGQSDSFAEISLLLEEPITC

SIVTATDMQLGVVRPERLHELDEVTVQLFKQSNTRTFGNLTKEDIQNEYMQQEQKRGWNE

FKHSMVVDVINANGIRPGYGKWAK

>SG-PL_contig_2506_1_-2_47_160

MDKIKGTWGIACILLPTPSTVKVVPEIMLIFMIFTFFV

>SG-PL_contig_2507_1_-2_65_295

MQNIKYTHERSGCKPRQCPVAYGNANIPSMHTLFSLHLLQAVLALASPLDFTGDMGRVGL

LPKQQQVFLTTLPWLVS

>SG-PL_contig_2508_1_+1_109_309

MRRTNLYTAPLIESCTKFQVPLAVSFIDFTKAFDSIHRPSLWKIMRAYGIPPKVTMAIKN

IYNNSRC

>SG-PL_contig_2509_1_+2_1064_1312

MPGYIMKGSKEYSPVMQSQSEMELNSIITSAKLSQILWPFKPCCHGDLISVGVEVCWGAK

MSGVGVVTQEWCRRWDRKGKIST

>SG-PL_contig_2510_1_+1_484_690

MHRVAYNHLSTNSFMKTQVHVHEHTQTHTHAYSHAHLYALLHTHTHTHTHTHTHTHTHVC

VCVLKNHSQ

>SG-PL_contig_2511_1_-1_166_1458

MSIKKIHARQIFDSRGNPTVEVDLTTDKGMFRAAVPSGASTGIYEALEMRDKESSYHGKG

VLKAVGNVNNVIAPALLQAGLNVADQNAVDEFMLKLDGTENKSKLGANAILGVSMAACKA

GAAEKGVPLYRHIADLAGNKNLILPVPAFNVINGGSHAGNKLAMQEFMILPTGAKTFTEA

MQMGTETYHHLKAVIKKKYGQDACNVGDEGGFAPNILENKEGLDLLKNAIEKAGYTGKIE

IAMDVAASEFCKEKKYDLDFKNPNSDASKWLLPDQLADLYKTFIKDYPIVSIEDPFDQDD

WEAYTNFTADVTCQVVGDDLLVTNPKRVQKGIDIKACNALLLKVNQIGSVTESIKAWKMS

RDAGWGVMVSHRSGETEDTFIADLVVGLCTGQIKTGAPCRSERLAKYNQILRIEEELGAS

AKYAGKNFRKP

>SG-PL_contig_2512_1_-1_1_711

VAKDINKEIKDSGSSSIVIGEGSVDYLSDVHLWEHFTGNQGCYEPRITIANHIRYVNPKA

KIIINLRDPIRRVYSTYLYTAAARERLKDPTTQKLHEQVARAVPAYKECLKRLPVRACVY

NRTLTKLHPIDIISGAYAPFVEDWMRTFTKDQIYMVKFEDYSKDMAETVKDIVKFLDLPP

FEDSDFNQVKSMPKLMQNVYYRMGPIQNRTIKILREFYTPFNIRLSKILQDDRFLWK

>SG-PL_contig_2513_1_-1_196_510

MTQTDLTKLLKSEEIRAALRAPKKETTRRILKKNPLKNKRVMIRLNPYAKAASKAAQAVE

QSRLRKKEELLNKKRGITKSLPVLPKGKASAKKVAQRKKVSKAKK

>SG-PL_contig_2514_1_-2_1379_1750

MFSSLPKMHSHNVTIFTMKIILSIHSHQNVTEMRRSLFCRSLFEHVHLMFMKQHCSHIDH

VCAWFWRQKILSVKHVDNIILFPFDFTARGEHCQLCNYLTFLMFIIIMVSLKTACAFSIP

GEYK

>SG-PL_contig_2515_1_+1_502_828

MTCSRFVSHCLFDCLSVSHSLWVCQSLIVCLPFLSVYLPVSVCLSLSLSLSLSVCLFIYL

STRIFYFVFFLCNIRIAPQMNNHISCIFLGGRVGFCCCCFFLFYLFIFF

>SG-PL_contig_2516_1_-1_1_210

GGGGGRDTISDQAIPIALLFLPDSSCEKVSKDSARLMCRCMVIFWIFHHMVYMVPFVFKR

VTILVGITGL

>SG-PL_contig_2517_1_-1_550_852

MAYFAVIFLRVCIQSCWGGFLGVFGFFFLFALVRIRSGLETLWQSMCLLSKSVSLNHCNN

IDAFRSPVFATCSALWPSSVIEQKSAYFNLHSDLFFIIMEN

>SG-PL_contig_2518_1_+3_3_230

KKGKKKLGLEEIIIFSILLVGRDMFFSSSGIMWYFLVTRTTITIQESRDLNISRHYYHIL

FIAQPTTKTISGLSPR

>SG-PL_contig_2519_1_-3_3_278

PDCSEQNGARFFGPWGVFVRADGNRKELRAQPLTCRLLDQPPLSWQPVQKYISFGSTILA

TFRPDLTRRLLLTYSFLRFRTFFSSQVRIPDL

>SG-PL_contig_2520_1_-2_2_178

FFFFNYLLCVSGRLKPFHVILVINVRITEYVIFLELCPFFFFLKETSFSCPFLCMYILA

>SG-PL_contig_2521_1_+3_75_200

MIKHYSCFLFSVPEVLYAHCFYFIWLLEQYVHKVSLLQFFFS

>SG-PL_contig_2522_1_+3_3_188

THVTLTLALSVPNSCISDSSTVHTFGPNLVKICPETAEKILLTLISHTHTHTHTHKVCKH

NL

>SG-PL_contig_2523_1_+3_459_785

MAQITGHSLNIQVCFTTKETLFFFIHNFVLFQQLMKNSWQPLIKTLPRTPNLFSLFQHLI

SRQSPFAQTFSPLFPLAHTHTYNLPLSATKKNTLSSRKKTSLNTTTHTN

>SG-PL_contig_2524_1_-3_3_290

TGREDPLHSSPRGNVGNVQSGWHHQLSSHSHQHPAGSQNAVIRHHQRRLGGSHHLASVEL

TSPQSIIISNLVDINPQRFFDILHVQMFSIPGQSSA

>SG-PL_contig_2525_1_-2_47_364

MVRYQLAVGLQKGHKVTKVEGSRKDRPSRRKGTATKHARFVRDLVREIAGFAPYERRCQE

LLRISKDKRALKFCKKRLGCHIRAKRKREEMVQVLQKQRKAAAAAK

>SG-PL_contig_2526_1_-1_1_204

TNDIISLMIKEVYLYGLTVSVTTVEIVYMRILALPKIKMTRLQTAEKIQHEILDAQKRIQ

IFYFSRSL

>SG-PL_contig_2527_1_-1_1_126

RDRKICCLLYECACDYVYRYMCACICACIHLLLWYCVFTNMI

>SG-PL_contig_2528_1_-2_17_241

MFTKIYTGLKKNSLIVVWNYVTAVISRTHRVTVLVILTQSMQQIQHNNKTIHTMTTTVTF

VQTIFFVCTVLIHQK

>SG-PL_contig_2529_1_-1_1_747

VTTKTSERATIPQQQIYLQQQPQPQQQPVYLQKQQTTYVQQQQPAFSQQQQQQPQIRYAQ

QQQPTYVQQQQTTVTSQQTKQQQPSFVQHQQSAFTQQPKQQQQPSFVQHQQSTFTQQQTK

QQQPSFVQHQQSAFTQQQTKQQQQPAHQTQQQPRYGQQPQQQQQPQQQQQKQPQQQKQLQ

QPSYQTQQQPRYGQQQQPQQQQATYKTQTQTQQQVTYQQQKQQQQPRYGQQPSYQQQQQP

SYQQQQQPS

>SG-PL_contig_2530_1_+2_200_646

MFIYNAISYRLTCGSLTGIHIFSLIVWVLFCFLFLLPIFCIASVLLPARRSFQLSNTSSK

PIKTEVTTRAGQRRATESGTIFHNATMMRIWDDDDGAIVFHLRLRLSDRDVLCVSFHNIC

VFHLRLRLSDRAVLCVSFHMCVPSKIATV

>SG-PL_contig_2531_1_+3_3_119

VVFACVSIFFLYITTLAPAHLNTFYNTFISSTLDLSHNL

>SG-PL_contig_2532_1_-1_1_81

AFKSLHEQTTTTVNNNIDVMKKILLWS

>SG-PL_contig_2533_1_-2_2_2008

FSACIPGLAGGLDPGPDLRNCKSGHFPKDDWFPLLCVQFESNALLGEFYTSADKLNTATD

IRGKLIYTYREAEVRFSDLAVVSGYTKGSNILTIYSQGSAGNLLLPQRSSSGLCLDSTPV

HFQCDSQSTCVQTLTQATCADLTPFSARVYVQSSSLYNPACPRSFSISSTGHSLQEKTQH

VDVKVNYYCTSDSSAYLKSITSTFSDLVSSQTMYSFTSGLQNVSCTDLCGADSVCLDYNQ

GLGQSGSRLPPRCSTDNSFVLPPTPTFNATTGSCDNVVLDVQYNFTWAGQTITGLVADII

LGNVALGSGEVSISQKFAASFVHDYAGASNQSDNFASVTTPYQRSGRPGYDYGKPVATGC

NTTNLTTSLPAVDQSDISQMAVWSPGINGLCQNATRRFLTFGDDMQSSCVLRLAFNSINS

CDSLKQLVLNHLNILMPANAIGRYGYNSPDINDMWIEILRDDPFAAITEANTTTPSPTST

STTADPNTMTATDSSVMENATFDPLSVRGGMCRNIISGIHLEIMYAETGHNDRYPLLEIV

GAAVRYSVSNWSMSCVGPNSVRCENNSVFTQPFLLHSSVQYTRVSPKTPQKPILFYQIHN

KENCDSDNCYRFYEDFDTSLCHYDTCWHELFFPLTQGYQGYESIYYTRGFSLVFVVFTLG

YFSVTKPWK

>SG-PL_contig_2534_1_-1_58_1701

MLLRTVCRDRDLALATLKKHDIPLDKNIKLQMEKQNEQELENLRDQNKGLRAALSSMKQM

MESMGHSVPSAVSVDPELDNEYVSSMEKEIKKLKQEKRELAEKVATAEKFGRLATQPPSD

SGPVLEEVKDTSVRSHILSLNDMLGVLRGEKVELAAAVKKQQARIQHLEEALQEAAKQPR

EQQVTIEQLRYENGAHQRRHAAESAAQRQRITLLESQLAEAQKEADMFHQASVENNAELT

GLRNQVSTLKLELAEKRPAVNFGAQELVIQQLQEELNSLKQRGAGIGFDPSGKGDPGAAG

SDSASALRLKLQHAAKHIAQLVREKQQLLELSNKLRAELKQAGLQPAMKPDHRPREVEVA

VSGSAVERDMAGRLSQLERLQYELTRQELQYAQRFAPLPNRDSHTPEKDQYKSSSFPAEA

NPMQQSMATTASSLRASTVLPAGDLTSSVGSQSVLDVLRMVDGPESPDLGFHFSASKDVG

SSMDRFPKSEVDSHGIQVSGQRVRVDQKDHPSSSRPLSSKVAGRVQKGKAPQKQRVRNYN

LRDDGDFR

>SG-PL_contig_2535_1_-2_227_427

MMNEERVSNVNKSLLIFCGLGCIVSVTSCRCRSVIVIPRICSRGTHCTWTILYALHPNLP

AIFWQTP

>SG-PL_contig_2536_1_-2_275_457

MTEAGYSFLTLSVISLNHWASCTVQPQHRQVGEKARELSVFSIFVCCSNFWLYCWFCCSV

V

>SG-PL_contig_2537_1_+2_143_256

MNDSPLTPLKSSLITLSSLLHTIHQLVVDRVQGEKVID

>SG-PL_contig_2538_1_+1_1_216

HLLCRFSGVDPTPLIPAPPHTTNPKFIQSKSQCQEAHGSQRCQATMGPPTRDSCLVTLVE

FSRTCSILIQHI

>SG-PL_contig_2539_1_+2_188_406

MKKIKVLVCKKKTPPPKTEFSLVLQLLPFNREQLRSPSDSNQRTHDLLICVSIILCYYIV

WHYSVPQIGRAHV

>SG-PL_contig_2540_1_-2_23_1222

MDEPALKATFTIHVIRRSFNLALSNMPVKASTNLDGDKVVDHFETSLPMSTYLVAFVVCD

FAQKTNMTNRNVQVEVWSQREVMDQLDFALDFGVKTIDFLEEYVQEEYPLPKLSMVGIPY

VLTKSTENWGLIMCRDNDLLFKDQATSMTDKMRKATNIAQKISHMWFGNLVTAVWWDDFW

LNEALAWLMRTVVYNHFYSDRRMMDVSVAEMEDAMKEDGLVWSRPLVTETFDTLSYYKGS

FVVHMLHFAIGDDKFRKGIQKYLSTWKYGSAKHTDLWNILEDDELEVSIVDVMDTWTLQM

NYPVVTVTVEGQNVTLTQERFLQNPESEDPGTYTSPFGYKWYIPFTFTSSDEQNFDITSS

DIIWLTKDEDKKTFAVPTLSEDSWVLGNVRLKGYYRVNYD

>SG-PL_contig_2541_1_+3_549_836

MLWSGKKYQQERNDHNHSKTQLSMDSETALPMVHTHTHTHLFSLLFFCGVHNSFDGLYDN

KTSLSLSHTHAHTHFYHLVYNLPCPICGGKQKPCPI

>SG-PL_contig_2542_1_+1_61_1581

MTDVTSILHNSGKPFHVFGFTESRLSSTVADSDINVPGFKAIRKDPTCKNSTGILIYINE

TLTFKRLDHLEHKLVEAVWIEIKLKHYKPIKIGILYRNPIEKIDWKDKFSCMLDTVSIDA

TEILLLGDFNIDLLKPKGTWTDKMSSYNFTQLVDKPTRITPTSKTLLDHIYTTEKRNIVE

ICVPTYGVSDHFPTCLTWSKKGIKIPKAGHKHVTYRSFSNFNKDLFLNDLLNSPLTSVYN

ITDPEKALDFWLSNFKRIYDKHAPLRSHRVKHSPQPKWMTKEIKIAALIRDDLLAAGDHE

SYKKQRNKVTSLKRAAKKKYFEDLVSSRNNTKSIWKAINQLTNTTPSSKSTYVSNISPDD

LNTHFATIANKTITQNRTKSNNLEKLNTFCSSKNITSNLTIPPMTVTEVYNALCHLKQTG

TRGLDGIDGKILKLSAPIISDTLTYIYNLCIDKNHFPTQLKQAKVIPLFKSGDNSQPSNY

RPISILPVLSKPLEKHINKHIYTHLDT

>SG-PL_contig_2543_1_-1_1_171

MLKSARSNISVASVDTVPTVHENLSFQSIFFNWASIKKANINRFIMFHFNFNPDCFN

>SG-PL_contig_2544_1_-2_170_3784

MLRLQAQICHLRHAASSPLAAVPTAGYRLPDSLRQMVQQQQRQQHGEATSAKVERKPIQK

LLMANRGEIAIRVFRACTEMGIRTVAIYSEQDINQMHRQKADESYLIGKGLPPVAAYLNI

PEIIDIAKDNDVDAIHPGYGFLSERPDFAQACVDAGIHFVGPTPEVVHRMGDKVEARQSA

INAGVQVVPGTPGPVKSAEEAVEFCKEYGLPVIFKAAFGGGGRGMRVVKDLGEVEENFNR

AYSEALSAFGDGSLFIEKFIERPRHIEVQLLGDSAGSVVHLFERDCSVQRRHQKVVEIAP

APQLDNEVRMKMASDAVRLAKFVGYENAGTVEFLLDKNGEYYFIEVNARLQVEHTVTEQV

TGVDLVQAQIRVAEGKTLEELDLTQDKIALNGCAIQCRMTTEDPARNFQPDTGRIEVFRS

GEGMGIRLDSASAFAGAVISPYYDSLLVKVIAHARDHPTASSKMLRALKEFRIRGVKTNI

PFLLNVLQNDKFLTGVVDTYFIDENPQLFQFTASQNRAQKLLYYLGQVLVNGPSTPLATA

IPPADIIPSVPPTPVDEHGKATKEPQGWRDVLLAEGPEAFAKKVRQHKGLLLMDTTMRDA

HQSLLATRVRTYDLNRIAPFVSHNFNPLFSLENWGGATFDVALRFLHECPWERLEQLRKR

IPNIPFQMLLRGANAVGYTNYPDNVVFKFCDLAVKHGMDVFRIFDSLNYVPNIVVGMDAV

GNAGGVIEAAISYTGDVSDPSKTKYNLDYYMDLTTQLVKAGTHIIGIKDMAGLLRPHAAT

LLVSSIRDKHPDIPIHVHTHDTSGAGVAAMLAAAEAGADVVDVAVDSMSGMTSQPSMGAV

VASCERTSLDTGLNLDNISAYSAYWEQTRRLYGPFECTATMKSGNADIYKNEIPGGQYTN

LQFQAFSLGLADQFEEVKKTYAIANILLGDLPKVTPSSKVVGDLAQFMVQNKLTPEMVKE

KASELSFPNSVVEFFQGSLGEPPGGYPEPLRTKVLKGADSIKGRPGATMEPFDFRALQDK

LEETHNVQVKEKDIMSAALYPKVMDEYLEFRSKYGPVDCLDTKTFLVGPKIATETEVSIE

KGKTLSIHTLAVGDLNKNGFREVFFELNGQLRSVMVKDVEATKELHTHPKALKGVRGSVG

APMPGEVIDIKVKEGETVEKGQAVLVLSAMKMEMVVTAPMTGTVRLLAVKPKMKLEGDDL

LMEIE

>SG-PL_contig_2545_1_-3_6_269

MKKFFFFFKFSSLKKIDLLWSIIADCEKKVSHFDKKRNLFKKKNFSSLNLLPAYPVLTMG

SVSYVHTPVTVSTTRYFWQQNFRKLEGR

>SG-PL_contig_2546_1_-1_1_282

IQHSTIQYNTTQHNSVQYNTAQFSTIQHNTVQYNTTQHNTVQYNTTQHNTVQYNATQHNT

VQYNTTQHNLVQYSTIQYSTIQQHNSVQYNTTQH

>SG-PL_contig_2547_1_+1_634_783

MQKAYNWSPLSETTQSLIFINLEHVHSKQSSPGAQPFITLIRQYQFLKKK

>SG-PL_contig_2548_1_-2_152_511

MLYVDSPVGVGYSFTNRSSGLATTMEEIMPDLYSLLLQFFTLFPQYASNDLYVGGQSYAG

KYVPSLGQYIHDRNHASPPPPLHLPLKGVFLGAGFCDPQTMLPEFPESLFYAGLISDWTR

>SG-PL_contig_2549_1_-2_44_283

MAAKGIEQRHLFQLYQAVILSVIDCGLDLTTFSQTNLSKLDRIQNEVMRVILGTTKDTPT

ETMRYLLDLPSMQARHKVEQ

>SG-PL_contig_2550_1_+3_3_266

SSACPIHNFSPCSRLPFVVLIISCSGVCGFSYALKYALTCSTLCLALIEGRSSRHRMVSV

GMSFVVPRITLFHFVFCLISRELFVIMF

>SG-PL_contig_2551_1_-2_2_205

SRVLAWDLLLVLDALRKNPYEPPNNASLSMLTAKMVFLLVLTTGRRRSELHRQTCLSKTV

FFLSQSMR

>SG-PL_contig_2552_1_-1_343_744

MPSSVCVIFYYLFLSVMFVTPSEILVKTSLVIIVLCHVRKKRMGLNRSTCYPNYILIAKF

KTKRFPPPKKTTTTKQPKQNKTTTTTTTNKPTTTTKNKQTSNNNNKQKTNQTKNKNENKT

KQKASTQTNNNLFT

>SG-PL_contig_2553_1_-3_483_698

MVTSVSVGLEVCWRAEMSDVGGVTQERCRRWGSRNKTNKKPSIFSTWKFRGWGCKGAESG

GWSRKTMKEKST

>SG-PL_contig_2554_1_+1_106_228

MVVAADDVASGRDRKQKNRKRLSTWHRNWFRCVIFTDPTAC

>SG-PL_contig_2555_1_-1_16_234

MEDLHLIEDKSHRSDSTYMRASVWVMILSYFLSLAFFCSFFSSFLLALFSFLSFVFPTRT

VTFKIMETGGIYR

>SG-PL_contig_2556_1_-1_100_255

MLNGKINQSIRNQENSIYLYSIISEICMSQKICFIVWLCSHTFTIPHEEKGK

>SG-PL_contig_2557_1_-1_4_1017

MADDGTKPAEAAELPKDVLAPAEGPRENLVATAVKFLQNPKVRPSPLSQKKAFLERKGLT

SEEIELAVQRAGVADDTSSSQTVQLPPQPQYPAAYANTALVPAHQPLPLPPPVSPWTKAR

ELVTTAAIVAAVSYAIYNVFKTYIQPWLMGKSEMETRLERLETNIAQLHASVTDTNAQMV

DTLKVIQESLMVQNDRSAQNSEVSLRQESRALGEIKSEVSSLKALLLSRRQFPPPPSTSP

IIPTWQKTVSRDDETQQSLTGQTEGATSQGADPATATQHNTAPSSDSSVEKANPSSEDSA

HSLQSGKGDDAISQSASSNNEFEMVKPSSDSDTPPDLD

>SG-PL_contig_2558_1_-1_295_5997

MRHWTGTPDPNASSTSAADLREQLDSEREKLEKIEEELSTVRGNQEAMQAELKDKTQEVE

QLRQQLNESVASTSRIKELEEERSSLQSHIAELSSAGPFPPELLDEKNAEIEELRQQLIK

TETEFGSLQAEMKEKEELVSDLQDTLHSLQRGEACLDVSLSFNEPLPLNASFTERTSLAQ

ELDQTVGEREARIDELMAEVEQLTEQLGEQQDQLEQLQQDKVALQSELQQRDEDLSQLQE

EKTQKQEALVEKESRLQQLVEQINSLQTEITSLTTFQVELQKDFDTVQSMLDEKEGEIES

LTKELLESRSQTEGSDVSEALKNELQSLKTQLAEQAAQLEQDHKSNAQLQAELAEKTEHL

QEEMKSRTQLQEEVAKKLDQLEQEKKSSLELKQEVSEKAELLEKEKKCRSELELEMSEKA

ELLENERKFHLELQQEMSEKAELLEKERKLRSELQQELEEVQRAALAGRGRAHSFPSLQV

KPLEDQLRELKAEVLHLENVLKDKEELIIYLEEEKAADGDLKEKELTDLHQELQDTHNSM

TSRITGLETTLTEQQHQHEEALQQKEEEVDSLQQELRSQAQTIQQLESEQQTLQRQAEDH

MTTITGLQREIALLTQQLEDTKRESTATDDMKALQEKLQQASQQIIELQQQVSEFQTRLE

QTSTQLTEREEQIQQLQAEVQNQNQESQRLRESQIRENSPRPKTPDSEDSETDERLKDVL

LRAQHMQSIVRDSLSSSLPLPSTTEEFSLSRWESEPGLSSNMSPQIAHAFDTEVWLRRQQ

RELAAKNRELATVRQELEAWLRRKDRKTSNTTNFSASIMTMTLRLQEKDILIASLKRELT

ESRELLTATKKATAEASSSSESLSLSLQTQQQIAHLRSELQRAKAALAAMQEQSDSHSDV

SEDKEDREHIEKLEAEIRLLKSSVDMSAEDFLQKVEELRKELSEEHQRHVRDIQELARQD

LELKLRELQSHHDHEMEQLQQLHQRKLEQALAQQQQELEKRHKTELNHLRVQHQQEIDRL

RNEELASLMETSVAQQLHADVLATQQMDEHMIGRLTTEDDAGTDLSETCPPHIQALLDRL

REAGEHILSVSELEQLRRHLGGHRDLAGSDGLQEAWEGEKLSLVKTIHALKELLSQADHV

NAGLEESGSDWRADLIHALVEVYKQERLGLEAELHTLRLQHPQQEHANTIKNFDNRLHEQ

EQLQQSGLDAMMRAERQSLLLELEQLQTRLSQQRQEQQDLQTHMTESLAQQEENASNKTW

KLQREVQVLEYKLQQEKVIEEDLKKSLESERRHVAELSSELSRDKGTLISVQSELQSAQL

HLSRTKDALEREQNRFTSVTKKQEETEKQLRDALEEEREKNNRLADMLETLSRQHSSLES

DMGRSEAIRKERESAESRFIKELQSELATEREQLHKAMEVAEEERRQRKQVEVEMEREQQ

HTNTIKQELEKLRRQTKKSLELESGKLTDTQRQLQKEQDKVEEAHRVMADKEATVLQYSH

QVQQLQAQVQQLKNKCSSLEKDMEMEHQKADNLVSLMDKERQQQLKLQQQEQSTIKQLKK

DLSQAKTEASESWRHLEMEKSRSDSLRLEMERAQSELRALSQHAGATEHQLEREHSNTWN

KLQLAQRNRDELQVKVHELEQALDRLKDRLQELELELDRSRQRELEARHDVERHKLYPSP

RSAPTTPRGEIQLQARLNTFCQQLQNVGQRLQTLTLKQRDLNISVSNNDKTNAEAGKRKS

AVETLLAELHTLQQKMASGQEAEAGSSSVNTLTQEMGELQATISKLQTEKEELKRQLMLL

EDEDNRRTPRTPRSSRNVNINNRHQETMPQYVSDVTTSDDEFIYERTVWASERLSLQMAL

DSAEHEIDHLKKELRSFRGRFNSEGFMVEVDREKMQRLYGK

>SG-PL_contig_2559_1_+3_612_758

MRAVSIARVLTTLTDLRYVVWVCTTTRSSGSYISVCPCARRMSQVPRGV

>SG-PL_contig_2560_1_-2_332_1522

MAEVRKKEEERRIERKVQQEREEEGNQFADKEAFVTSAYKKKMLEQQEEAERERQQAAME

EMLDVTKQKDLSGFYRYLYRETTGAAPADSQDADNTEDMDKVKVKEEPHSDNENDKSSSG

KETKHHKEKERRHSSDKHQDRKGHTDKVRRSRSRSPAHKSKSDGSSRRRSRSREREHRHH

RDRRSRSRSPTHKKSHKHRHRSRSRDHKRDHSSSHDRTKRRSGSAERSRKRDEDDKPRKE

TRKRSHSTKEESSPSADKKDESVKNEASAKSPSPSPERSSSPVMLPARPPDEDLDRDDPD

EDLDRDDPVSESESSADEDQGGKKKSKERTEGKEQTAESGDSGRKREVPPESSGSKKEEP

AAKYGRKTTEGEVLDARQRYLARKLARERAKPVVDPE

>SG-PL_contig_2561_1_-1_16_912

MVPRHNKILTSLVAGALAGATAKTVIAPLDRTKINFQIANKPFSFRSALRFLYHSLLTEG

FFALWRGNSATMARIVPYAAIQYCAHEQYKIILNPSKSKKHLPPAHRFLAGSLAGVTSVA

CTYPLDLVRARMAVTAKDMYHNIFEVFLKIYREEGLRTVYRGFTPTVLGAIPYSGTSFFT

YETLKKLHAEKFKGEDPSAIERLVFGACAGLLGQSASYPLDIVRRRMQTAGVTGHALDYT

SILGTARQVVAEEGFIQGLYKGLSMNWIKGPIAVGVSFMTFDLALRWLRRLEVFHQDGD

>SG-PL_contig_2562_1_-3_18_263

MWASYVQSFSCLAGQFQASLNKRMVYHQHHSFSRSHITITNLFLRTLLCKSTAHCNWIVH

VALTVFFLWPSVCLSSQPSFSF

>SG-PL_contig_2563_1_-1_1_285

QDGEEKKGGKKKNKVKAVDLAIDTEVPQLSKDALNLLTEKEGQMIMQDRLEKERADAKNS

VEEYVYDMRDKIGGEYEAYMKEEDRAVFRQKLEDT

>SG-PL_contig_2564_1_+2_17_319

MHNILMFRLNLPCSQYHQLPIHSTYIHIVCQEKCAFASIHHFTKIKNTFTCIHKCTKNAF

ICTHKYKECIRLRTQIQRAHSLANTKNAFTCTYTNTKNNNM

>SG-PL_contig_2565_1_-1_1_291

GTPCRPETTSNHLFGERDQTFTRKPTMSAPLRGALRSLQVVNKAVAHQKATVVSGPPRYR

VSMGEKVGLGLVMVAAVCAPAAYILANLDNYRGGRPE

>SG-PL_contig_2566_1_-3_45_2417

MGGSKKEKKEERKAKRARVEEEEYEQEEEDDFIDTGEVSKDVPAAALRNVDEEEASKTDD

FGAQDFRKTLELKTDHNSRPLWIAPNGHIFLESFSPVYRHAHDFLIAISEPVCRPEHIHE

YKLTAYSLYAAVSVGLQTGDIIEYLRRLSKTSLPEGIVEFIKLCTVSYGKVKLVLKHNRY

FVESQHPEVLQKLLKDPGIQESRKREEGEEENTDGFIAGELSGKSALQLKKPANGKTEGQ

PGTSSEEPQNGEETKEEVPDDIFNFYEKMDREEEDEEEEGIMKTVSFEIKQESVEMVQKR

CIELEYPLLAEYDFRNDTINPDINIDLKPSTVLRPYQEKSLRKMFGNGRARSGVIVLPCG

AGKTLVGVTAACTVRKRCLCLATSGVAVEQWRSQFKMWSTIDDSLICRFTSDAKDKPMGC

AVCISTYSMLAHSTKRSYEAEKMMEWMQNQEWGLMVLDEVQTIPAKMFRRVLTIVNAHCK

LGLTATLVREDDKIADLNFLIGPKLYEANWMELQNHGFIARVQCAEVWCPMTPEFYREYL

TTKSQKKLLLYGMNPNKFRACQFLIRYHERRNDKIIVFSDNVFALQHYAVMLNKPYLYGP

TSQGERLQILQNFQHNPKVNTIFVSKVADTSFDLPEANVLIQISAHGGSRRQEAQRLGRI

LRAKKGSAAEEYNAYFYSLVSQDTIEMAYSLKRQRFLVNQGYSYKVITRLEGLDEEDLMY

STKEDQIQLLQKVLAATDKDAEEERGVGEMGKGGIQRKSGTMSSMSGADDTIYMEYRKKA

QHPLFKKFKQK

>SG-PL_contig_2567_1_+3_516_785

MAEVQAKSVQGRGCWWSSLYPACTWHGRGKLLHQPWQRRRITRGKGSKHAEQVSRYRCDS

LPDTLEWPVWVEAKIDEVDQGLLYPKFFIL

>SG-PL_contig_2568_1_-3_12_479

MADEVLFEFLHMELVAHIHRSATKDETDNCIAKLESIGYRVGQSLIERFTRDCSRFKDEL

DMMKFICKDFWNLVYKKQIDNLRTNHQGVYVLQDNKFRFLTQMSSGKQYMEAAPKYLAFP

CGLIRGALCSLGINCIVTAEVSAMPACKFQIQIQRL

>SG-PL_contig_2569_1_-1_196_453

MSSLIYMDSMPCLVTMTVSSNCMQLHSEFRFQGSRTSHNHDSSKLVCGFYRPCVRFVTLN

VCLRNSVWGGGGLVGAEVEHLPSNLP

>SG-PL_contig_2570_1_-1_34_198

METECMCRQPNKNPEMQRLATGFIGAALRYLYTSCGFTYIHSKGSPASMWIKIY*

>SG-PL_contig_2571_1_+2_2_343

TCTHTTHTHTHTHTHTHTHTHTHTYITYVCPHKKTTGRNIGNEVSVCCSCFLLCTFKHTH

TRTHTHTHTQSSTSIHHDAYLCICLQSCKNTEHLKFMMHKGKSFFCATERCKFL

>SG-PL_contig_2572_1_-3_105_1046

MSQFIQVIDDSSDEVIEIPSENDATLLLTTLQAQFPGACGLKYKSETGAYRGIRLAEGVL

YPPEGIWGNHQYLAVFPKNQKRKGEDGYGDNPAAKRRVGEHKCSDLIVLGLPWKSTEDDI

KKYFGQFGDLVLAQVKRDPRTGQSKGFGFIRFAEPESQVKCMSQRHMIDGRWCDVTIPNS

KDSMSNSNNFYQSDSWQQSLWGAQKQNDGQNPMMNRKVFVGRCTEDISHDDLRTYFSQFG

EVVDVFIPKPFRAFAFVTFADAEVAQSLCGEDHIIRGASVHISNAAPKTGTPDKQGAGMG

RSFGGFGQGGFGSG

>SG-PL_contig_2573_1_+2_47_499

MAEEGLETIDQCGLPGKYKVWCLQFMLIPKLLWPLMIYEICTSIVEAMEAKINKFTRKWL

GVPPCLTDIALYCRQAKLKLPLKSILEEYKAGKIRLQSMLEDSGDKVVEAIKPQLKTGRK

WKVQEAMDAAKESLKIREITGHTQTNRQGLG

>SG-PL_contig_2574_1_-3_459_581

MDLFFECMLLCSTVVVLTFVLVCFCLVSRSVCVCGDVYGIC

>SG-PL_contig_2575_1_+2_59_238

MQQHYLSFVYENLAMIIYNETRHECNRLLYNSCNLDLLERTLYSHKDILYYISLCIKVTL

>SG-PL_contig_2576_1_-2_50_196

MLYANTVTEQAGNVMLWVHMRFTTPITNFVLKGVSCHLKETHYRERNPL

>SG-PL_contig_2577_1_-1_130_366

MTFCTTRLPEYLKVGYLRVKVSMFVPAPLRCFKCQKFGHVRERCKEKEVCGTCAEAVHEG

KCTGRSHCVNCGGGHPSFS

>SG-PL_contig_2578_1_+3_117_248

MCKNTRVIYLQIQEVVIISKISIGSVPFPDPWIKIYLTVLYTCA

>SG-PL_contig_2579_1_+3_264_872

MPSDKKKRGKRACKRKFQGNRYTKRPRFDGESSVDNGETDSEETECSDEVKSGECSSATP

TRSERKLLNLYTIPDDSCESSKEDSACSDDYISETDSEPNEDDVFSQECEGFRLIDVDIL

NRNIMSQLSCKFCHSDVKLYEVERKGLGSKFAFMCTNEKCDKQESFPSCPVISEGNVSIY

SVNRRSTFAMRCIGGDHAELRTF

>SG-PL_contig_2580_1_-2_224_331

MHLCLPLCYLVICDFFFISILCVCYLNVVMDDPLQS

>SG-PL_contig_2581_1_+1_133_342

MNSLPIQCRKTETDKTNQKRDITKREDLTAMPPNVCVHIINDALFTQNLKILLPKQYNCS

NSSFQELVIY

>SG-PL_contig_2582_1_+2_263_427

MLSFENFFFNTWNQKTCICKTEIGFFLQQYFRIIKQQQQQQLMIMVKILIAIIIY

>SG-PL_contig_2583_1_+2_2_316

SRRWSGSRCHLWLRSGRGSRYDGCRWPRPGGKPFCRRGGRREPRPKARREGARERREPGP

IWGVFWAVWNLGPPSLSRLHVGESRQRDRLPLADAGSCRSVTLDA

>SG-PL_contig_2584_1_-1_1_105

TCGYSGLVFLNTLGLLHHPVELLSRIQRRHVYERL

>SG-PL_contig_2585_1_-2_509_748

MKLHHIHGIMFSQYGDVAELFLPRSMHKFYWNFWIFISANVCVSLVLLQFSVCLYNFACA

VVILTCTLRHMHACGHTHTH

>SG-PL_contig_2586_1_-3_570_728

MGGFCDSILVPHVNLFPAYIVLIARSFSRVHTPVIVSTTRYFRQRNFGKLEGR

>SG-PL_contig_2587_1_+3_744_1328

MTYSHITSGTHTTEVKCNIKSAIFEACTTITMPQVHMVPTSDCYCAKVHTLHSLEARYLS

RSARHNWQAIKMALLLLYIVYSPGPLSHIRAVTMPSPSKSRRQLCRTQNLTNLKSTILQK

NTIHDIPSQLSRSLRQTRPAALRTPVSPLNLPTPDYRTVQKIILKKYFKATKKQNRPHRQ

ITCRTDIQCPSQHPQ

>SG-PL_contig_2588_1_-1_283_612

MGKKNVMIVPVIKMIKYNSYLFVYCCITRQNDTRCDIIITWQIVYCLCLFLLAVKPCAYY

SLQHKINLQIVLFFLTVPHAFLSLCCASRSFSADRFSDVVDFLGILQKQF

>SG-PL_contig_2589_1_-2_224_475

MQTCITASSSTTASVQFTSNTSTQPIHMTGCLSVCPYVVVKDFLAFLELSPGLEIPTGPI

VRDNQVFRRTSGNALSTCPLATNF

>SG-PL_contig_2590_1_-2_839_1051

MDICSYPCCVLWIIPFYRPRVCDMSMYKLLLQYRGCGLFGYCFLSVLFFPLNYTHTHTRC

FFNLLIFSKLQ

>SG-PL_contig_2591_1_-3_498_3287

MSFPVCKFFKSEQGCKFGNKCRYKHSDPLNLPAFNQSTDHQNQNDGSVRKSYSSQTSALS

QEHLTDSGEYEASVRNPRILHTDHLTDYYSYTVPGNNEDPLRFAPSFRTAETHLEKKFQE

LEATDHNEEAVISNFHPAFQQRSHRKLCHYFARSGNCCFGSKCRFEHAQPHKILHTQPCQ

ISPRFQEDRGGSRSWATENWTDGYQSSMSQDGKTEEGKGVRHSWYSQNQRSFSSYPGGNP

FPRTGPATFVSADKSAQNGSPADQPTQSGPGREGTENIDEKNQNFNQNFKSDKICQFFVR

GRCWKGRRCRFLHPRHQAPVVVGEQGETESNTKNEDKSSTSLAQNQKEETLKFSPATSAE

GTHTESVSSSKPSVMRKPRTPHVAQGTRKYCREEVDDVEGSRLRRTEIEQLVKRFPKSKM

KVNRDCAEYFQCIISFNPTDPDWPFDVKVFQLQVDIPSDYPFEMLSVTLPAEQDLPETVR

RYLEVSIEEWLEEKERQLADSGQVELVFRPFLRWLDRNIEDVVTEGLRQLKREMVAKAAG

LEFVSAKQLQKQYPGQSANSYDDGDNEDDDGSDDEDETASESQDEDTSSDYDESDEEESC

QERVDEDEDGRKKGRKSQQDKAKLMMAEPDRKGTEVTLKNLQLKESAATLFFEKMCLLIQ

CERCKATSEFMASPGRVNMVQCSQCSQTQMATYRPAMVHHFSSVIGYLDLDACLPYDVIL

QNCSAVISCLNCSKDTKAGVVAPGQPQDHWCRVCHHKLRIATDGVRFTQLEPSTTIQSLK

GSVKVVVPTRKRIPKDPAIQEGKPLPNFGTCKHYKKSYRWLRFPCCGKCYPCDICHDSKE

DHEMKFANRMICGFCCKEQMYAAERPCKGCDMNMTKVHTKHWEGGQGCRDKISMSRNDKQ

KHRNANKTVSKHSQKVKDMQSKKRTKLRHT

>SG-PL_contig_2592_1_-2_1013_1342

MFSFAIVSMILFLLCVCVCVCVCVCVFRCELLRSLLRMFVCVRVHLSLSLSLSLSLSLFL

SLSLCKSNAKGDRHGGLFSSVVSSVFMLILLLHTTSEDTYCHACCFTEHV

>SG-PL_contig_2593_1_+1_1192_1392

MSMTSEQTHNTSTQCYITKFMPFQAYIHSTLHMDKVHALTVYRHTLRTSTQDVTWTLPLH

TFKYSQH

>SG-PL_contig_2594_1_-3_996_2267

MPEKDAAPTASGDGTTAPPAPADATANAEGGGGKAAEEGAAAAPPPKQMKSVVLTGFGGM

KMIKVQQRPEPKPADGEVLIRTKICGMSFPDLMLRQGVMDNTPKPPLVMGFECAGIVEAV

GENATGFSVGDRVIALSDYKAWAELVVVPVTSAFKIPDSVSMEEGACLLMHYVTAYILLF

DLANLRKGQSLLLHSAAGGVGQALLQLIKTVEEVTIFATASAHKHDSIKDKVTHLLDHNV

DYVQEIRKVASDGVDIVLDCLCGEDTNRGISVLKPMGKYILYGSSNIVTGETKSFFSFAK

SWWQVDKVNPIKLFDDNRSVGGFQLRRLLFRQGQHAYVHGVMDKLLDLHRQGKIRPVIDS

VWAFEDVGEGMQKLHDRKNIGKILLDPSKEPAPKPQNKAPAEQQAESTENAKGDKPDATV

NGEQ

>SG-PL_contig_2595_1_-3_63_323

MRSPTAPAALANASVSSCWLSYEWQRKAKVVREVQILESCCLRPLDASAVMCAFHVCISS

LPSLGQDKQKWREDTSLLDTCVDSKSF

>SG-PL_contig_2596_1_+2_2_136

CVCVVVFKYKLTVRNSVRFLVFLQNTDLKHFRPGMFGIMINPHVS

>SG-PL_contig_2597_1_+3_171_446

MCSTTNVTWVMGDPRTVTADAHSLGINSQDGRPGAWSVTLLAMAEICEIGSETQRRQITE

VSVHAKSERHLFNAVCVMRSESKLVWRKALYK

>SG-PL_contig_2598_1_+1_1_207

SLPHPVRQLRKLNPFIPSPGPPAAKTSPSHSLTLSTSCKNLTLSLPHPVHQLQKLNPFTP

LLWPPAAKA

>SG-PL_contig_2599_1_+1_361_522

MLALGLHQGQEFYSLLASYHVSKLFSFPVLGGSDDTCMYAYAFVCIGMYTCKSY

>SG-PL_contig_2600_1_-1_28_678

MQTIKIAGRFGRNGLATRLFRNFSTSEDKVYELRLYQMKPERYRESLDLLSKYWTVRTAH

SKDVGSWMSEIGGICETVHLWEYDSLSDRMKRRQNVANDTVWRQEFLPKFLEKSLKLTCL

LLKPHPGTQVITTFPPSETAVYELVNSKPDVLPQMTEDDEIVVGQFETIYGQAEGEFTLF

RYTDPDVACERAYQRRKAQNLKGLSRFLVPCHWSPLK

>SG-PL_contig_2601_1_-1_766_1071

MTVQQRSPGLLVCFVLFLYFVGGFLFLYFVGCFFVLFCLIYTATVLLFLLQFYIYIKILK

VLLYNLIISYSQFQISCEKAGCKGRRISCRFQTYRTFFFYCY

>SG-PL_contig_2602_1_-2_2_151

LSVYLFLCLSLPLVVENFVPHLSLTCLLSYIHTIHTQQIKCSLIQQWLTR

>SG-PL_contig_2603_1_-1_109_411

MCLSEGISLIQRIYRCIYVCVCMSVYPSVIRPSIHRSVCLSVCLSISLSVCLSVCPSVSL

GVYLSNGLSVYSSVYLSVCLSIGLVFLSVYLFVCLSIHLSV

>SG-PL_contig_2604_1_-3_1050_1622

MTSQVYGFYDEVMAKYGTPTVWNLFMEVCDYMPITALVGGKVFCMHGGLSPSIDTLDHIR

VLDRIQEAPYEGPINDLLWSDPEEIHGWGRSSRGAGHVFGPDISETFLYTNNLSMVSRAH

QAVNDGYQWCHGRKVLTTFSAPNYMYRLGNRAAILQLDESMSSTFIQFDAAPRKDGQRPP

ARLPDHMYSFF

>SG-PL_contig_2605_1_+2_413_688

MHLNKHAHQYHLERLARHEKRGSQILNRALEILFHMKSTNKYFSFTLFSSLLFFYNITYS

EECKNNEILNSNNKAWHSYIIFPCLKDTVECS

>SG-PL_contig_2606_1_-2_80_1156

MWHTITLTKEDLDKFKSLRVIIRIGSGYDNVDVKAAGEMGIAVCNVPGYGVEEVADSTLC

LILNLYRRTYWLANMMRENKKINGPEQLRDAAQGSARIRGDTLGIVGLGRVGTAVAIRAK

VFGFNVIFYDPYLADGIEKSLGITRVYTLQELLFQSDCVSLHCNLNEHNHHLINDYTIKQ

MRPGAFLINTARGGLVDEHAFAAALKDGRIRAAALDVQESEPFNLATSPLKDCPNLLITP

HSSFYSEQSVSELREMAAGEVRRAILGRIPDSLRNCVNKEYFQTAANSAVRFPFSAAGLP

TEGLNGAGYATFTPAMAAAAVAMGVHPAAPDLRPVVPHSIPPANAHSDTNHVAKSEGSG

>SG-PL_contig_2607_1_-1_400_732

MKRISGSENPKEVKKREQVFLSKYKSEPGIIQSTAGGSLSIAITESMIFPSRMAEKVTLI

NMPFTVFISNCKFKVYYFNVNCFVLDDSFYLYMFVSVTKVTNNIVVGTEHQ

>SG-PL_contig_2608_1_+2_2_139

ISSTEFPEGGDAIYTERAPGIPTGGGSNLIQLNIITLMGSACQVPI

>SG-PL_contig_2609_1_+2_2_265

QINVLYQLIPINQASFKKERRTTVHHLIKLIPQIKHQFAKRKNVLATISDIKKAYDKVWH

TKRLFKLKNTGLSLARKRNILSKIHVWL

>SG-PL_contig_2610_1_-3_3_194

LFLFLLPRWFFVVVFFVWLVFLSSLFLSLFFVRLFKLLVSFVLEIFTSVWNMHSLAVLIP

NLNE

>SG-PL_contig_2611_1_+1_175_441

MTGLTPEVRLACPHQTTTTLLGTMHDFTQLWHADCLKGTLSNEHTSFKTCQHHDNCLHER

NTTLCHQRRKKTPNSKENNMKGKGKEWAR

>SG-PL_contig_2612_1_-1_664_951

MCLQDFFEFCEWILTRIEPHIFSIQKLPMCSKIILSPVIWVTASRNVRMFEQVCLCFYGV

FLYSILVKCLACWYHVCLTEIGLFSRHCYAKICLET

>SG-PL_contig_2613_1_+1_25_234

MARHSEIVSVLQKQFLYFTYLQTLIFHVDSLQNNMDQNKVKNVLIHVISVLQHCNLQTAE

LLFITKLNAL

>SG-PL_contig_2614_1_-3_3_167

SPPMTGKKKKRKKTHAPPPPPPTKDFKYWWKTEKFTVYVHLIYMFNDRGMNVPEE

>SG-PL_contig_2615_1_-3_3_389

DNPHVDIVGANLYDRFIKDTSSNNIISVDVASATSTDSSLTSLLPSSRVIPTNVDLHDQT

VEHCLSLLVGTGVYKPDTEVSKKGPDGKFIYHGHRDIAHEPKLTEPHKYVHDVNLGIEYI

FEKEAIVSA

>SG-PL_contig_2616_1_+1_1_117

LPICHVSGWPTETRKHPARTPRTSCKKGPTGPNAIILVK

>SG-PL_contig_2617_1_+2_140_331

MNQNKAVTVLMNPSDTQLKKHCFKILKQTLCVHLSLLYVSVMTLINLVFAFRYSLYTSSR

LNLL

>SG-PL_contig_2618_1_-3_3_185

YSIAIGIESNISVGFVLSNQTESFTQVCLSHDCCCYFTYLFHMSYHIVFECLSLLISKSL

C

>SG-PL_contig_2619_1_+3_471_749

MDTGVLFSSSKLVCMLFYCIVSPLSGNSVLEISSFLLLFCFCSFPFLYCRLLFSAFLVHS

PFFFPASLHTIFSFPHPPQFMMYYLCCFALVTR

>SG-PL_contig_2620_1_+3_105_251

MDPVYVKVMVVQFVGHFTFEIMNTYVCRALIDETSVSLPQTNFCALFCA

>SG-PL_contig_2621_1_-3_36_920

MPEITMSKGKSWAEQVDEGEGEGDEGVLPPNTEEFDEKKGLKKITEYKYNEEGKIVKTVK

TYQTKTLKVSKSIAQRKTWKKFGAAEMDPPGPNPANTVISEEIFMQFVNSSEQKQPEEDD

DPLGKLKNQKIVQCRICKGDHWTTKCPYKDTLAPLQEKLTEEQRKPAETPPPSSGASAGP

KPAGTKYVPPSLRDGASKGRGESMSNQRKDEAATIRVTNLSEDTRESDLQELFRPFGPIS

RIYLAKDKVTGQSKGFAFINFHRREDAARAIAGVSGFGYDHLILNVEWAKPSGQQ

>SG-PL_contig_2622_1_+1_913_1089

MVLKKIMAVVTCFAIGWSVRGEVGGRVNSRHSPPHWTAWPNGRKCSSPSWSRAGCSRRV

>SG-PL_contig_2623_1_-3_3_221

PDQPHISPFPILPKVIRKAREERALMILIAPFWQAQPWFPDLRSLSHVPPLELTPSPDLL

RQPCSGITHANPG

>SG-PL_contig_2624_1_+3_159_383

MQQSLLRTRKSNESCERAHCTGKIREPWKEFGKWGERERGKRQRELLHDHYNHKTTVSAA

YKIPGCQRNQPTTHN

>SG-PL_contig_2625_1_+3_3_191

FAPQDEKQNKTKNTHTHTHTHTHKGSQALQTYTDVGLFSGCDLSISITDKIRRKEILKQQ

SVD

>SG-PL_contig_2626_1_-3_15_425

MRWGFLLVVVVVKCLSACPVSDTKLSCCVMICVNMGEGGSVIIGGGGCCCYCCCCSCCKY

VIRSVCISFLKVLHSEHCFFFVCKMVTRFVLVHFQTCHLNIPCISDSSVQSDTGTSSLIR

IFGNIVLLVFICVDMLV

>SG-PL_contig_2627_1_-3_3_308

CCALFLFFFFSSRRRHTRFRGVTGVQTCALPICLYHLTPLQPQGYGTTFTLLLLPPLLLL

LMMTATTTTTTATTTTTATTTATTNTIPHSFFKNSSIWHYYC

>SG-PL_contig_2628_1_+1_391_690

MSILVKLDQPYLTVYEYTHNAHCSHLACSHSVYSHAYFPLMVQACLQWFANLPCGTLASN

SAFFENYRFFFFNMMKHTQCIGAEEAISSEFMRGDELQQR

>SG-PL_contig_2629_1_-1_1_465

DLGDGEKSPFTHQTRCPRFESRPSKPSSLEVKCFHHSTNTPDISICRIITFIAVVVHHPL

TNSLDIAFSAPTDCAFRHVEKKTPIIFKERGVGSKSATWQVCKAFADTLEPSKGSKHENK

WNGSMQSGNSERYEYTRKLSDMVVKLYEYTRKRGR

>SG-PL_contig_2630_1_-3_693_923

MLQSTSYTSSYKLKQKHSSQYGMLDKAENKRNTKGAWHFPDQFAQSKNRQHCSHVHHQTT

HSKAINFTHFRYNLDRN

>SG-PL_contig_2631_1_+1_1_279

QTCLPQNNNWSIFPEKFSYLKKKRRKKKEEYSSSYLCSVPTTVLFVTFVTERNIHYTAVA

ESGNALLTVFDMMSAVESGRFIFMISSQKFASA

>SG-PL_contig_2633_1_+3_3_113

SYIFVCFFFFDLGMINQAERLATSALPSPTRPKKPNG

>SG-PL_contig_2634_1_-3_72_203

MYELCSFEDVFSFFFFFLRFDKCFCCYCVLFNAVVFLTLKDLTW

>SG-PL_contig_2635_1_+1_1_189

WPKQSKTGCVLHKLDMVECMTLNLHSQLGELEATCTKFLPQVRIKPAQSDRKRRDVTLAN

QVY

>SG-PL_contig_2636_1_+2_2_178

FFKITNNLITTTYTPPLNKRNTVIIMHYKTDPNVHIIIYKCTVFRCAHMQQFHNLCHRV

>SG-PL_contig_2637_1_-3_105_554

MRPLGAWLQQEWGSSSNQQDVPTDLVTLGREACGTIVESAVKISYNLSLLRRDSILSASN

LPAEDFPALRTIPMQPSSFFGPFAEPFVSARAAARRDSAALSSWQRPPNRPATQARKPPQ

PRRPGPPAEGSTPAGPPEGQPWKAGFSASS

>SG-PL_contig_2638_1_+2_23_214

MALLFPSLHYRHTTNGSSKHIMTGDNCHVIYIYTYFFKVSSHSDVHHWRHLWESPAKAVL

HWTE

>SG-PL_contig_2639_1_+1_1_159

NAMLQIITQRNVTNYNTKLELDTDWVCRPSYAPVVIISIHQPNHCHFSCHPKQ

>SG-PL_contig_2640_1_-2_398_616

MHLNSQAVLAFFLPQSIQCCPMHSEETDPCFSSRVCDSTPVSILPQEFFISMLYGSNICR

TLVTLPDNCSCVS

>SG-PL_contig_2641_1_-2_191_379

MNRKMTNCSCLHFGFQTKLEDDELIYRRKLHCHHQCSNTMTFFMLDNYLLVNRFQRKSAR

QVS

>SG-PL_contig_2642_1_+1_232_429

MKFTCTDKITDVRTTYTIHTNMQPKHTVIQHNTKDQLNITRVLFHYYLCSVSFQSSSRKV

ISHLQC

>SG-PL_contig_2643_1_+1_376_618

MRCTSQKHAIALHTTSNSVSIPKIGGKKTKIKHQSGNKRKTNLLHISTHWPDTSTYIHTY

IIRHQYTYSHIHNMTPVHIFT

>SG-PL_contig_2644_1_+3_540_746

MAGQKPNQPPSLPQQMKLFTPLPSLSPPGVYTPTITLTTRCLHPYHHSHHQVFTPLPSPS

PSGVYTPTI

>SG-PL_contig_2645_1_+2_17_247

MKYISKISSAVFAWIFTKFELKVWTIRSRLCMNLEQRVKVKVTQAKKVPKISTFSGRCVN

TTFSATSDNIQILFSLL

>SG-PL_contig_2646_1_-1_82_4407

MSCQLSVLSRFQDVTAVRRFLFPHLKDNSPKPAGEQQTPDGDAEWSEDWGWGAEEDEGAG

LSSAAQPNVETVDDGIHKWLQNSIVSLSPNNDIIAVANKDKICVLTQKWDPQNKGEEIDT

RLTTVWEGSIAHEDGELITDIICLPLASQKRSTQGAPDWTCVVVGFTSGYVRMYTETGVL

LLSQLLHLEPVQKLKCRSYGPPRFLGLGEKHEELIILYKKAVVSIDGFSLIQSLRACRNQ

VARATASGGDSYVQPPPLAYKKWALGDQERVTDLVSCGVSTPSTFDQLKKASMEHGFNAQ

PRVSPPPASVYITSGTSPYVGFYYAIEGNMPPILSEVAMAMAHKLKSALMSAASGWLGFG

GKQKEEAKEKPKIEPAAPLNVKCGLPDLRRCGDSIVLSPCNNYIATTDSFGRVILIDVQK

GLAVRMWKGYRDAQLGWVQVKEDEGSHRHSPSQSRVAQFLIIYAPRRGILEVWTAVHGPR

VAAFNVSKHGVLVCPSYGLMGLNNVTYRTVKSRAFQCALIDPEGSVKTLDIPFHLALSDK

SSKRARDLHLLKKLKTSLKEASTETENLRNTVKETFLDIRIASISHQALERILAIRYLSV

GFMQEVVQACISQLSAKGEEHLDIDSKMLLRYAHMQAGLLDTYDAIYKLSTQTTYIDSAA

ESSYTEVLQSGLGINKEEATHISGQMEKFLSALETNNSAAPTVTISKRPSLLGVTSFQHC

FSSRSHTTSISEDSGTLTKAMGTICVSKDIREERKLALAHLLFSACISGKCTAEDIGVVL

QKSNLAPDQLMKLLLLHCLSMECLGPSAIPSLKALVKTITAMTDTSEVLVDSNKKSPWWQ

RVRDACSQSQQNCAAYITALVCRKVAADFITSQTEAKDPDADTASDKEDDDKAGENAEKD

NNSPTHTEEWEDVSVDMAHWELLLKQLEDTVALTTLLHIHIPSAEKETVDVEGSKESIKV

SVSKILEGGKGSISEVIARYVASQGVGPKSLYKLPAEGEGQSQGQEGSVLSEEDEKSVQN

ISSLERLQCSLEDLRVRFPNSLDNDILISNCGWEYAVLWNKDPEEVQHLQSSVEYLKLVQ

NAVLRQGVSSMLWHMFVTKRMSAAAQLMEKVGKAPKDRLCRKETNMSDKSMTTFVGLVVE

LLNIIMEANCEANEVPVFNVEHNWQSMRGPASLGELAVDAKPTNYGLVRLHWHLALLMHA

VMVFNMKNVKVLSMFDRKGKAALFKDLHSHPLLPSKNIDDALSFDRCKLLTRIVTHAIET

LGDPIHQSKVTRIGPNAGPGLIRRPSMHTFYWPRTCMDLAREMSTDEENIRRHFIIELYN

FGHDDLAQTIHGFVGEQDKFGKQLLLVAGRRIAYRAIHENPKNTPRLLGEMTPSLSYFIQ

TMEMDKLMYKNALLADTTQLVHEAAKRLPEHSREYDYALELIHFIDLLSSLKISAEPEVI

GQ

>SG-PL_contig_2647_1_-3_318_572

MGENSTQNFSDAAVSEKTSTTSAPATGNSALKEEPEVAVEADFAYFEELQNRPMTKEDFA

KFTFLVAVPTVFFIIAVVLMHVYND

>SG-PL_contig_2648_1_-1_232_693

MASGADKGIIVVLAVDNSTHSEYAFNYYATEVHRPENKVHVIHSSEAWMHCHHLKDNPTE

GHVQDMKSKEDTKVEEIKNKFTKLLQEHNMVGEFKVVGGKDTWHQIIEYQESNGAQLIVV

GTRGSNVIRRTLLGSVSDSIVHHAHCPVLVCRHS

>SG-PL_contig_2649_1_-2_74_439

MGDEADKFVWSVKNGELASVKEFVAKEGKCVNTQINGRFPLHYAADYGQKEVIEFLLSKG

AEINAPDKFDITPLLSAVFEGHTGCVQLLLQKGADKSGKSPDGASYEDCAEKQEIKDLLK

QS

>SG-PL_contig_2650_1_+1_19_216

MSTVSFLVISSQFVADELICALSTAEVCMLCACALHILSGAMEGRTGEGGEGVGKGKVRS

GVRNQG

>SG-PL_contig_2651_1_-1_46_597

MSEEHRQKYFPPRPRARTLYPFYQVKDGDDCLKKTLTLSAYSGTIGTTFALVAGLTSTTA

DNPALGSNVLIRLTKYGLPMFAAGGMFAATTCVVANLRGGKDDPINHFWGGLATGSVFGT

AFKSQKLGWGLGFAFGFGAMVWKAGKMEGFANINPSEFRREKTLGGHTRSYFTTRPNPEG

DQNI

>SG-PL_contig_2652_1_+1_685_999

MILTHLGQRLHRCNHFKGTTQMFFNHKGPKCQSVLASMFWVCLEFFVCSCSMDGSDIKIG

LFAAHLNALIILVVSVFHRSCSPPPPHFKTLPTHRVLRRLKESAR

>SG-PL_contig_2653_1_-3_33_236

MYETFQFKILVLVVLWFYFILCERQFHSCFRSFLKLVNVSCKHAGNYVCFCFVLCARQFQ

SCLSECLL

>SG-PL_contig_2654_1_-3_153_1157

MAAATTTMDVPESQRLIAVSLGKIAASRGQKGGINLHKNLLVATVLHKARTAYMIQHLNA

TKQRAAAPTATPTPPPSPPTCVEMKEQKPVSESSSASSTSATSSSSSTTCVAGPTFQSPS

PSTAPQSVTPSPERASVPEGAAGSCMQVVECEDKENTPPPSPCRDATPSSSPSVECEKRL

QGEAPVDSVKDNKPALSSRPTSGSRAQQNNGTRPYVSRSSCCLLKRRRAMDGDHENVSAP

KVRIVESRVSLDCTPTTTPEPMQCDSPQISNLVNIFNTSFSGLSGTSANGDVSVRNVGQS

ILSSVTSHKLSENLTCAKEMKVGLDSVHQPLMLSV

>SG-PL_contig_2655_1_+3_3_272

SHEPRDKQRRCCANKHKSAASRLRQSRFACIWRLLSLRFVGRVYKRMCVPEWLRSSFGLV

KKRLNKPRKTSALPSLVFSCLAAVCTLLSW

>SG-PL_contig_2656_1_+2_2_163

IQFNPFTPSPYPPASYTGRSPISQRIDSKMNMVELGISLCSQAAKCSCMSMTHW

>SG-PL_contig_2657_1_+3_3_149

NKEKQNTEVLYSNPETINPFTPSPYPPASCRWMRLENERLNQNNYATQY

>SG-PL_contig_2658_1_-3_4179_5543

MGSDVRHPPGGRQYPPRTIVTVTSPTSPDNYSNTNVFQPHTNGDVSRRGPQPSPRSRGMS

TGREDFDDLFSQARSMKGPGPGMGGGGGRRGGSISGVPGLQVEQINHEWERRESRGSLRS

NRSRSSHGGSFRSGRSGSTRRGKGLFLGVHTESAWTRWSRDRRASFRRRIELLEKPEETT

IARVSTPVRKARQEGLKFVHPDLEGTFLSEEDLNELRRHKQQKYHAIRVIEKNRKSKFRM

KTEVHLTAEQWQVISDFWEHPAFVRGRYLGLFLMFVSFIVMVVSITHSNWVSYEAWNLTI

YEGLWMNCSTPVLTLGKEKCTSTDGRDWQNAVTGLMIFAASFGVLASVLSICGVCTTPLP

RKIYYFHSAGEIFLICALSTGVALMVFPIAIEIDKNISSHRYGIGYGLGWGGAFFFLAAA

VCMSLDDLVREASRAKCCKWCWRSKQNDRTELRQV

>SG-PL_contig_2659_1_-2_806_1048

MDMKGGGGLQYNIIKIGGLQYKYYNIIKLVSYRCLCLLGFGRVDAWKWVWDFSQKIVSST

SYEYTFIVNKFIVVVIAAKMV

>SG-PL_contig_2660_1_+1_283_387

MCLKKLFKVFKNFFKNIGTHKTLLTNFIYAETLIF

>SG-PL_contig_2661_1_+2_383_496

MSNIAQDRQIGRHRQTDINFQQNSLSKSVNHNLLPGTH

>SG-PL_contig_2662_1_+2_29_280

MLPAKTKHCTEVSSSSSTSKDYCGPIFTNLEQKQNSKTVTGLVANILNMPTQTHAHTLKC

ICTHTHKHTHRHNVCNTYKPTPFS

>SG-PL_contig_2663_1_+2_197_430

MIKYFDSLKPVFRSVLFQAITLYNMCQYGTLHSLVVIFLDYMNAYLSGMYMCFLCFDSFQ

VTILTLFNFLQSRLSNEP

>SG-PL_contig_2664_1_-2_65_1636

MLAFARFAAPRQVFCRVWTRTYASAAPTTKLFINNQFVESKTDKWVDVHNPATNEVVTRV

PEATQAEMQSAVDAAKQAFTTWAETTILTRQQIMFRFQQLIKDNMSRLAANITEEQGKTL

VDAEGDVLRGLQVVEHCCSITSLQLGETLQGIAKDMDTMTYRLPLGVVSGITPFNFPAMI

PLWMFPMASVCGNTCVLKPSERDPGATMILMELLQEAGMPPGVVNVIHGCHDSVNFICDN

PDIKAISFVGSDQAGQYIYERGSKSGKRVQANMGAKNHGVIMPDANRESTLTQLVGAAFG

AAGQRCMALSTAIFVGESKKWIPELVERAKALKVSAGKEKGADLGPLISPESKKRVCELV

QAGVDAGAELLLDGRDIKVPGYEKGNFVGPTILHQVNPDMKCYTEEIFGPVLVSMEVDTL

EDAIKIVNSNPYGNGTAIFTTNGATARKYTMECDVGQVGVNVPIPVPLPMFSFTGSRGSF

RGDTNFYGKQGIQFYTQIKTVTQMWRGEDAQLSTGSATAMPVMR

>SG-PL_contig_2665_1_-2_2_169

ANPFFPITFGISDSPELMVPGEFPLSRLTCPSPWQGTPSRTTRVYPPPFPLNSALV

>SG-PL_contig_2666_1_+2_2_262

FFFIVTTNFTDNNPNDQLYTMWTEYDKNTNRKNTKTDQYSKTQALNIKVVQITHIRLHVY

QIKILTSGAKLTKNTWKGLLGWVDTSF

>SG-PL_contig_2667_1_+3_960_1157

MTQSETYVQFLDCSSHWLHRHRLQRIDKLRTPTPPPTLSPFFLRTLHSFITLTDNPKWSS

KIDIWV

>SG-PL_contig_2668_1_-2_113_244

MWSDLTTRETGTFHSVIRDFLVQSVVFCWPSSVLTRPLMDNFVS

>SG-PL_contig_2669_1_-1_754_936

MFQIRPSESFISTNQACLETLETCNNVLNCCVYMLARLLDIFSLVRKQTSPAALVFACFG

H

>SG-PL_contig_2670_1_-3_459_926

MSFPHNVYYTYIYSAKHVAICSGTCKKMYSCKRCCCEEVTWQGQVAPHAAVVPTTEVQYL

SVIFTFCERASIPASHCGILHRGMFSVRNEAKLFSIPVMPRYLSAKVNIGFWGGFLFVSF

LNSFYLGIVLGLINATYPTSSPFPHPSCDQVLMPKF

>SG-PL_contig_2671_1_-2_2_1468

MAGRDIIGIAKTGSGKTAAFVWPMLIHIMDQKELQPGDGPIGLILAPTRELSQQIYQEAR

KFGKVYNIKVVCAYGGGSMWEQSKACQEGAEIIVATPGRLIDLVKKKATNLQRVTFLVFD

EADRMFDLGFEPQVRSIANHVRPDRQTLLFSATFRKRVEKLARDILADPIRVVQGEVGEA

NEDVTQYVEILPSSGPAKWTWLVRRLVEFTSTGSVLIFVTRKANSEELATQLRARDFRLG

LLHGDISQMERNDIISSFKKKEFPVLVATDVAARGLDIPHIRTVVNYDVARDIDTHTHRI

GRTGRAGEKGSAYTLITDKDKEFAPLLVRNLEGANQTPPPALLDLASQCPWFNKSRFKRG

KGKFMGGRALGVKERPGLGSETATQLQDSTSFSSFRPQGGVLGGGPGSDRLSTMRTAFAA

QFKSCFVEASQEEKKLPTGVIIPPMSQSKDGKAQEPIQMQPASARYSKQSSSSSSSSEGG

KKKRKSRWD

>SG-PL_contig_2672_1_-2_776_955

MPHMYIRTPMILLIPYYSGLLQFLSSDRMVYMYECDHFEQKRMYKMEKLYKQPSVFAHSL

>SG-PL_contig_2673_1_+3_66_191

MQGIHIFNKGSTYKHEIPLLSNNIEDECKHTRVGHRKYIYMS

>SG-PL_contig_2674_1_-3_144_350

MYKRKLEKTLPDVTSFTRYLFTKFQIEEYNAKLKWNLHTEKDGCRIYHWFQMKFDMTPCL

FMTYKHVFL

>SG-PL_contig_2675_1_-2_41_328

MHEYTHAYTHSHVNVTFTYIHYTSCQLVIPINDHKHSCNDFTCYMRIPNKCKILPHIRTI

FSEPAPQGCTHLSLIDPSSPTPRRARFQGGWTEGHI

>SG-PL_contig_2676_1_+3_39_155

MNVRVIMCTGICVHAYVYVYFFDIVHLQLWFNIQRICKV

>SG-PL_contig_2677_1_-1_37_1158

MRPDEHKKKKSAQYQRKHGTSAAKGGKNENDKSSAKYGKQGSSGADQTRSIQHNNKVDTS

ESEEEGQQTEVTTKKFRRREIASNWAKYDLPPGDADDDATTARGQDFKKLLHSAGGAVAH

FRFKDEESWEEEGGNADGSAAQVLSLDCAELATSLNCLPLYTQLGLDKDIFTSEQVSAMD

NTANESRAMAGLPALGHKKELDSPTAHSKPSPSSSTLPPNCSRGLETSSSLSATSPSTDN

RSGKGTVEKWNKEQENRATDRKMESRKETSMKIDGSSSSGGGRFTDTDLDMLLECGGGGG

KQDGGEGGTVRSSEGASRKTREVMEQDLDDLLSMESPQSVQSASVTQSAPKAQESKTAAE

SAALEDWLDSMLDD

>SG-PL_contig_2678_1_-3_3_200

DAAPSLRVCAVLTTSIVVLTTPSVTCLLGHVFRQMVSPCPGYRSNLSLKKLCHSPEKESS

IKLQDQ

>SG-PL_contig_2679_1_-1_100_567

MSAAPPPYPGPEGTYPPPNPAYPPANPGYPPVDPKMGGPPPAGYAPPPQAGYGQPAGYGQ

PPPAGPTTVVVSQPAVTMVQLFRESPVHTRCPHCQAEVVTATHYDTGTLAWIICVILCLV

GCSLGCCLIPFCVDPCKDVIHTCPNCRQQIARWNRM

>SG-PL_contig_2680_1_-2_83_439

MAARSGYPELKDAARNATKHIQEFPAGVQQNLDASDFLHDMQAISLYPQDGPRLVPVCIY

GDGNCLPRAASLLSYGTEEFFTEMRARIVVELALHEEFYLTDSNLSTTTTGISKIYAQY

>SG-PL_contig_2681_1_+3_84_266

MFMFPHLSVSLCVSLSVCLSLHSMCAIAACISVCPGPLYLFLLSLHPPPPSNLKKKNFQV

Q

>SG-PL_contig_2682_1_-3_96_320

MISSSASFVSSFRFRSISLVISPSPPALFLFSFFMASLSSDNLKGPVSMSSVFLSSPSPY

LPASYIISHGSNAKT

>SG-PL_contig_2683_1_-1_1_237

PVCSGPAFVRYQVCFRALGSPPTVHHQVFLGPLLHLPSGVQGREVQVTLSCSLWMMCPIH

LHHLFEDDGVHTLLTASGE

>SG-PL_contig_2684_1_-3_261_533

MVYSVDSEMFPLSQGKDKIFLKFERYVTSCKTDLFASVHQLLVTILQLVVCLFQIFSMAK

SGISLYSLSYLLTYLSFHDDSAHTLLIALGK

>SG-PL_contig_2685_1_-3_171_365

MVLGQKIHMILWRFFVWKVDSLLRSLSVILQHSELYSRVEKTQLWYSLSLVLVLYWGTSK

RCSAF

>SG-PL_contig_2686_1_+1_1_180

TKSIPKENENPDLLRVVILHDSVFEWSAWEKLGHSYGLCVETQKTPTERSTRPFHSAFEF

>SG-PL_contig_2687_1_+1_52_432

MLWTFVICPAAHKFLKSHTMFILIQLVQMYSWVACQHECSSNLFRCVCDKLALQSMTLST

RMFVTLIQQVQKTYDKVALVCQTGFRCLWVANNSIHNITSRNVHHSFNRYRMCVCGKIAL

LVSITPT

>SG-PL_contig_2688_1_-2_50_184

MYLEKNTPVGIILETVLHAFKNGHTGNTQRLPVKIMFNAGQFSIC

>SG-PL_contig_2689_1_-1_55_387

MCFSIDSPDSLENIPEKWTPEVRHFCPNVPIILVGNKKDLRNDENTKRELMKMKQEPVRL

DEGRAMAEKINAYAYLECSAKTKEGVREVFETATRAALQTKKKKKPGCILL

>SG-PL_contig_2690_1_-3_315_6584

MPVVSPTPHCRHGSEQGLYEPAYESDACGVGYVVNIDGIESHRVLRDAQVMLQRMEHRGA

CGCDNDSGDGAGVLTGIPHRFYAKTLHSTGYNGVLPEPGQYATGMLFLDRATAAQAEETL

AKFADKYGLQVVAWRTVPVDNSCIGQVAKSMEPFIRQVFVTKPDGVSDIRQFDRQVYLLR

KYASHHVPETGVRFYICSLSAHTVVYKGQLNTAQLWKYFPDLNDPDYESHIALIHSRFST

NTFPSWERAHPQRYLAHNGEINTLRGNHNLMKAREGVMSSVVYGDKLSDLYPVVEEGMSD

SGCVDNVLEFLCMAGGRELPEAVMTMVPEAWQNDKNMSEEKKAFYRWAAFGMEPWDGPAL

LTFTDGRYIGAILDRNGLRPSRYYVTNDNFMYMSSEVGVTDRLPEEVVRKGRLKPGRMLL

VDTKEKVFMKDEILKSNIASLRPVSNWLEETMTMSELRDNHDQTAVHKLSSYRQMSAVGS

YLETDPRLSLYNYTMDGINLLVLPMVKEKKEALGSMGNDAPLACLSNFNPLVFSYFKQLF

AQVTNPPIDPFREKIVMSLACPVGPEHNILEPAPQQCQRLWLEQPIISLYDMEVLRHTSH

KGWKAKQINIVYPEEDGEDGLLPALKRICQEAAEAAQSGYKLIILTDRKADPNNVPVSSL

LAVGAVHHHLIDEKLRMKVGLIVETGEAREIHHMCLLLGYGADAICPFLVLQLIRRLRDQ

GLLDPPLSNEEIYFNFRAACARGISKVMAKMGISTLHSYKGAQIFEAVGLNQEVVDLCFE

GTASRIGGVTFHILGKEAMNRHRKAYEKRDCDNQLIMSPGQYHWRDGGERHMNDPLSIAN

LQDASRNNSRDTYARFTEAAWGSAKKCTLRGQLDFHYQDIALDINEVEDAKEIVKRFATG

AMSFGSISLEAHTALAVGMNRIGAKSNTGEGGENSDRYLNQDPDFNTRSKIKQVASGRFG

VTSSYLSHADELQIKMAQGAKPGEGGELPGYKVSKEIAKTRHSIPGVGLISPPPHHDIYS

IEDLAQLIYDLKSANPQARISVKLVSEVGVGIVASGVAKGKAEHITISGHDGGTGASSWT

GIKNAGLPWELGISETHQTLVMNNLRSRIVLQADGQIRCGRDVVVAALLGADEFGFSTAP

LIVLGCTMMRKCHLNTCPVGVATQDPVLREKFAGKPEHLVNYLFLLAEEVREHMAKMGFR

TFQEMIGRTDKLRVASDAHNPKAQLLNFDRILKNALEIRPGTNIEGGSVQQLFDMDQKLD

LELIEKCRAVVEGKSKQVVIDLIVKNTDRTFGATLSNFISNQKAEEGLPEESIIINCKGS

GGQSFCAFLAKGVHVTLEGDANDYVGKGLSGGEVVIYPPKDMHKDFKSVDNIIVGNVCLY

GATSGKAFFCGQTAERFCVRNSGAIAVCEGCGDHGCEYMTGGRVLVLGLTGRNFAAGMSG

GITYVYDVANRFHSLCNLETVALEPLKKNIDVDFVRQLLEEFHKKTGSQVASNILNNWDS

EREKFVKVFPNEYRRALREMAEEAAEIERLKKLERSVSSESITEVHIRKMESLENMYDED

IINDLGEDGKEASDEEEKVAVVDIEETVPDANKNVELDKTRGFVKYKRATYQYRNAKDRQ

KDWDEIYNHKTVIEGLRMQAARCMDCGVPFCQSNNGCPLSNLIPKWNDLVFKNKWKEALD

NLHQTNNFPEFTGRVCPAPCEGACVLGINSPAVTIKNIENSIIDNGFKQGWVRPTPPSIR

TGKKVAVVGSGPAGLAAAAQLNKAGHKVTVYERNDRVGGLLRYGIPTMKLSKEVVQRRVD

ILTIEGIEFKVSVEVGKDIQASELIRNNDAVLLAMGATWPRDLPIPGRQLEGIHFAMNFL

ETWQKQQQGNSSPYISAKDKDVIVIGGGDTGCDCIATSLRQGAKTITTFEILPKPPPSRE

TNNPWPTWPKIFRVDYGHEEVQLKWGRDPRIFEIMSTEFLDDGNGHVSGVRTKQIKWEKN

EAGRFVMKDVEGSEKVFKADLVLLAMGFLGPERNIVEEISLELDQRGNVSTPNSKYNTSV

PRLYAAGDCRRGQSLVVWAISEGRQAARQIDMDLMGYTSLAGPGGIVKVE

>SG-PL_contig_2691_1_+2_380_559

MTRKSNSLRSKVRLMCYLVKSALLVHLRNLDPHSRAAKQSPENEVLWEDPMHLLLSACYK

>SG-PL_contig_2692_1_-3_132_2312

MDLKNHKTLCFFSLFAVCLGWTIELGKPKPLRLPEGKLAEFLLGLLEGLGNTRQTTKVTG

RPSPPHVTTRMSETQFYQPGQEVKFQCQATGSPPPTYTWQKNGKNVNLTDSETRGNELRI

NGTLLFPGDKVDADTEGVYQCLAENEYGTSLSSLVHLTMARLEMAPMEQPDRMVTPRIGS

SLIIPCTSPHSVPEGHVHWIVEYPIGDMDLLEDTTYSDVKLSDRVTMDYNYSLHITNVQQ

SDEQGGGGYMCVTRNQVLRAMAMGPKTFIRPQSGRFRPPPMPARLMWSSPSYVVALLGTD

LKMKCIFSGNPTPRVRWAKGGVRALPSSARLESAGQELVLTDVKYSDAGDYECWATNSLI

HIMNELRSFHVEVQSAPQWVDAPQNVEVEAGQTAEVNCSASGLPQPSTAWFVNGMNLSDV

VASEERFQSERFDNTSDRLVFHNVTTDDRMVIQCNVSNRHGNIWADVSLNVLATGVENQA

TTTIQSTSTNDQETTTNIQTSPTNDTETATTTQTTTTSDDQNTTTNAQTTTTIYDNETTT

STQNTTNDNQTTTSTQTTTTTYDNEATTSTQNTTNDNQTTTSTQTTTTTYDNETTTNTQN

TTNDNQTTNTSQTGTSDNPYSTTPTQTTITTTSDGSQVTSANQSSATENPYSNATTQSTT

TGIPKPRSSTETGREGATVILTCTNASDLHLSVTTFKDGEEINLTNDRFALQLNGAFAMK

KVTRHEG

>SG-PL_contig_2693_1_+2_584_739

MLPASTKVTRSLTHTLSHNPLTLCFIIDYILHTLLPNKKENIFKYRIISLTA

>SG-PL_contig_2694_1_-1_1_174

HRNSFLFICFSYSEFLSFYLFIYLFVCLFVYSFHYLFMYLLNNIVSSLSMCSHSMPYV

>SG-PL_contig_2695_1_+1_1_246

GCLLSPLLFALAIDWVLKKATEGKGIQWHKGGRLSDLDFADDIAALTSDTQDLQSLVTDI

SEDASSIGLTISAKKPKNMLVG

>SG-PL_contig_2696_1_+2_104_235

MIAYPRFCLHIFVETYFFTGLYKIYHSYRKAYVYYHRFFLPVTF

>SG-PL_contig_2697_1_-1_28_606

MYSASAKIIKPQGEKPDDFESSVSQALLELEMNSDLKASLHELFITGAKEIDVQGKKAII

IMVPVPQLRAYQKIQTRLVRELEKKFSGKHVVFIAQRRILPKPTRKSRRKNKQKRPRSRT

LTMVHDNILEDLCFPAEIVGKRIRIKLDGSRLIKVQLDKNQQTTVEHKLETFSAVYKKLT

GKEVTFEFPECVV

>SG-PL_contig_2698_1_+2_50_217

MCGSYYLFQHFSSICKYSHNMKIMNIMSLACSLSVLQVQSDSAHHSGISRLKLIHI

>SG-PL_contig_2699_1_-2_440_652

MVLGCMNSEPFRLQLPSTSPSPSGMSWRQPTGDGNSHSLTTTCETSGSPVQMVCFTTLWR

RWPLQSVGRPG

>SG-PL_contig_2700_1_-1_103_900

MWKSISFVALFLVLSSKAMQVDAGRQRRQSADCPRDCTGTPLEISFVVDGSASIWPANFT

FGLNFIEDFVNTFQISPSIVRVSLITFGERAYEEDKIDFDEYTDNTSLRQAIANIPYRSG

IRTNTSGGIMFMLSKQKPKAREGVRHVVIVMTDGNSQERKETKTAAQAARADGLEVFAIG

VGHSVSPEELHNIASDDRHVFAVASYNMLEGIKKRLVYQACDVPSYPECEMDPVDLSFVI

DSSTSIGQDNFTVGMTFVKEFVDSFQ

>SG-PL_contig_2701_1_-2_1673_1921

MIVHMCYVQSLSESRSILPAGSWLHSLSRPQNALSLHWSFVYFIVHHVEYKLLEHQPNQC

TNQPVLNSELWNYVSSNDFTFWN

>SG-PL_contig_2702_1_-2_20_973

MATHVLKLSFLPLFMCSFFCVLAEWNTKDFLKQHHSLMKPYQTLGSQWDFMGSTMVTSNY

IRLTPNHQSKQGAIWNKVQCMVPHWEVHVQFKVHGSTKNLFGDGIAFWYTRDRMQPGSVF

GSRDGFTGLGVFLDTYSNHNDPHNHQHPYISAMISNGTLKYDHDTDGTHTQIAGCEAQFR

NKNYDTHMAVRYEDNTLKVSVDVDNKNGWRECFTVHGIKLPVGYYFGASAATGDLADNHD

IISVKLYELEIEDKNKVPYMGLPEAAHMEAPREHMDGASSGMSGWKVFFIVLLVLVGGGV

VGFVVYQKQQEKSRKRFY

>SG-PL_contig_2703_1_-3_48_182

MYTPNCTLMCFTYYVYNVHVFLFSVYTSVIYVYIYFVYILIYFVC

>SG-PL_contig_2704_1_-3_48_224

MLCKLFSEDKIAFYLGHFADYFLQLIPTKNNSNNKFEFSLCYIHFFFCLFGLFLFVYTF

>SG-PL_contig_2705_1_+1_1_102

NPWSKKKNVAASKDLQLHIPLHILQNTSRQLRKS

>SG-PL_contig_2706_1_-2_2_94

KSVAVDPRAGDFCSGIGMLVHLYLRFVKIND

>SG-PL_contig_2707_1_-2_191_328

MRHRGSAVHSQVTGSESVAVDPQAGDFCSGIGILVHLYLRFVKIND

>SG-PL_contig_2708_1_+1_199_510

MDQEGRKKQMTVKEGNRAETKRATGKRKFLAQFPFILKEHWHLHRGNYDDHRLDGFLDKK

GGIHHSPTTTHSISSNTDRQNQRLSLSLSLSLSLSLSRKTALFT

>SG-PL_contig_2709_1_+3_2604_2858

MVVPFSPMSHWTKNHMAKSFKHSPFSTCLSFEFQHEYTYSHFRIPNCLQGPYLVQIQSAE

KFLLPAFCSSDQKYRLAITRLCCLA

>SG-PL_contig_2710_1_-3_3_323

QSDVYVSRKTNFSAQLSRCEKILILENEVFIHGLGTAINKAINLALQLKERGLDTLEVAT

HTSTVELVDDLEPETDELEPETRTRQNSAVHIRVYRLESETQEPETK

>SG-PL_contig_2711_1_+2_527_844

MGFTIGNKNSILFCFCVPAVDCFVYKVIIPFCLVLHHRPGIKSVCVCVCVCVCVCVCVCV

FCFVFLFLIFLPNFVLQLNYILSQSFLSLFFIFVQSNRPVFTYCHI

>SG-PL_contig_2712_1_+2_2_409

ADCYILIFVSLLLTLTLHGVDADLLVVLLQGCQILTGLGELTLLHTLTHIPVDEGTLGVH

QIKLVVQTGPGLGDGGGVTQHAHGTLHLGQVTTGHHSGGLVVDAHLEASRAPVHELDGAL

GLDGGDGSVDILGHHI

>SG-PL_contig_2713_1_+2_2_268

EGLGSNPPRAPRRVGKGRNLFRSPRSTYVQTCQCLNSLRVVHARRAISNTHVKNPEKPCQ

CLVACRNTKTPSMRKKSQYKTTTTDIASQ

>SG-PL_contig_2714_1_+1_937_1182

MVCKQHASASLLQICSGINTLLDRSLLAKCRQLGLVKGTTAWGFRGGRKKKVCHGEKKGG

TQDFSSSQCSPHDQSCDPSPSP

>SG-PL_contig_2715_1_-3_126_392

MLHRSIPKGCPCINTAQMSVSLKVEEYVSTLDQALQDLPEHDATAKWNAMKEAIYNTAMS

TFGQDWFNAYLPRMEPAIEAKREAFLAHK

>SG-PL_contig_2716_1_+1_1_261

LKNKNNLKKYNKIYNIYFFTIQIQKKNQQSISYLIQKQPFKWEMSALHTTQNNDSLPWAF

SSIFSVSLPSFLTPGSRLGFVADNRIL

>SG-PL_contig_2717_1_-2_476_712

MSTLGIPLLSRSRRPSALIEETDVKAVKEIKYNLAPNRPRIHSRSVSQSSTDSYSSASYT

GSSSDEDETNPRDKQQKNS

>SG-PL_contig_2718_1_+3_3_125

SLNRTETESSDRKFFVKNFSLTITVNISFRTNNHNLRKNHR

>SG-PL_contig_2719_1_-1_2812_3075

MTIFIIRCPLYPPSSTRKRHCGPLLGIVILFSHTQSMKATACFCGRGGVKCIKKRSTTDI

YHFSIMTCNPEGSDTCSRQHTSTSFERN

>SG-PL_contig_2720_1_-2_47_1882

MESRYNFKRITVVPPSKDLINIVLSKTQRKTPTVIHKHYSIVRIRGFYMRKIKFCQQTIR

DKLTAILTEFPNIQDLHPFYCDLLSLLYNRDHYKLALGQLNTARHLIDNVGKDYVRLMKY

GDTMYRCKQLKIAALGRMVKILKRQSQNLTYLEEVRQHVSRLPSIDPNTRTIIICGFPNV

GKSSFINKITNADVEVQPYAFTTKSLYVGHTDYKFNRYQVLDTPGILDHPMEEMNTIEMQ

AITALTHLRAMVIFMMDLSEQCQHTVEEQVSLFQSIMPLFKNKPLMVVCNKMDITTLDDL

PSDKQQLVRDLKTAGTPLLQMSTVTQDGVMSVRNEACQRLLDFRVESKLNTKRVQDIANR

LFVAYPKKRDDLERPPTIPPGVRQKREKQAKRRKLERDIELEQGDDYTLDLRKEWDLPNP

EEKYDKIPEILNGKNVADFIDPDIDAKLARLMEEERLREEAGFYNLPSEDEDDKEMEATL

AKIQEKRKLKRLEAIDKRNNNRSILPRSASLAVTRGRKRKREESAGPSSEPMESRSTSRA

RTVSASRTRSQTRDQSGLRDQTMVAKVKKMAKKAQKPRNHQAKKGEGDRVILNMKPKHLF

AGKRKGGKTDRR

>SG-PL_contig_2721_1_+1_1_204

VAVPAAVVVAVPVAAAAVVAVAVPAAAVFVLLVAVVAAAWHLDAVAHPDNLVVVWPLSRS

PPHHVLCI

>SG-PL_contig_2722_1_-3_78_305

MIPCEFCGETFPAFALVHHQSVCDPLASGLWGPGQGGRNNELFNQWKPPASRPPAIPVIN

NLGSASPAQDHEEETS

>SG-PL_contig_2723_1_+1_1_144

RRPSIWQSVCFKLTYAHEEEGHIYVLRWKLTDNRRTNNKYIMLLQEEG

>SG-PL_contig_2724_1_-3_612_854

MIILLKCLLCHDNYSVLCACHVRYLNLLCLYCSMWVVLACWLKTCTLMSIDLCQLWSSAH

IICTLLIGELSRFVQYFVHWN

>SG-PL_contig_2725_1_+2_149_295

MDPTSIWNTISKTNCEVQLCAIITGLPKPRISQCIQKSMELNGCMTSIN

>SG-PL_contig_2726_1_-2_503_751

MWFACDDHYRVRLARYIINKMLKTTTTTNNPVSFHIVKYHGKCLKYVHVQCIYTEIYIIG

YRSFSIHPYYISNYVKSYLTGEL

>SG-PL_contig_2727_1_+2_422_643

MDTLHLVVRSQVLFFFIYHYMDTLHLVVRLHDAAVGHHGSCISIVSHEGSEVPPQDGLPS

TACELDVPSGLSGI

>SG-PL_contig_2728_1_-2_2_1462

MDDTHQIPNPDSSASSSRSDVGGKEDRGIVQKPLQRPVRTCAVLCGAFFFVYTANLAIQN

LQSSLHQAAGLGVTSLAVLYGSIIFFGTLSPMVIRFLNAKRTLVLAFICHLLYTLSNFYS

TFATLLTSSLLLGIMAGPMWTAQGLYCTATGMTYAGIKQMESHGALSLFNGVFFAFYEAT

QISGNLIASLVLMRGSYNETHTSTNRVCGAAVCPGTEMNSTQIEQPEKYIVYILMSIYLA

CNIVGLLLTVFALPPLPRDQEIQAGESSLKKSAMSCLSMLLVPKMVLLLPFFMAQAMNSG

ILLAEYNQKFVSCAAGIQWVGYVMTVFGVLTAAQAVGLNYAAKVLGRRVLFTAAAMADLS

VNMTMLLWDPTGTSVGLLFILPCVSGFAEGIFQAQFNSLVAMVFPDQVTGAFATYHTSKA

TAFTITFGLARFLCLNQRLYVSLSLSALGLLGYFIVEVMLHRNKQRETPTDNAPTLVSHI

SLDDKQS

>SG-PL_contig_2729_1_+3_222_371

MSIYLIITLCRSITKNILCNNQINSYDELCTTKITIIFSDNVKMINNYVH

>SG-PL_contig_2730_1_+1_49_342

MLRFCASTNHPTLKRPTGSPTCAFEVMHAHTHEEGSGTSISAHRLTLETEKCPTIHPPGA

TRQSRTQTPRPEVRHPNQMPIMPKTTTYTKNSNYKLLI

>SG-PL_contig_2731_1_+1_1_390

WTGSALPLCLPGLEKEKTAEKRETTHKVNSFQLFCVHVSRDTTHNVNPFQSFCVHVSRDT

TYNVNSFQLFCVHVSRDTTHNVNSFQLFCVHVNRDTTHKVNSFQSFCVHVSGGVAQWLKL

LRVHVDTRIS

>SG-PL_contig_2732_1_-2_818_1123

MLLYAYCRSCSMELFWKLFDSQVPPIAQYGAEISGLEDTAHHCEKVHLFVLKKFLGVQMR

TPNDFIYGETNRHTIYINSVLRCITYWLKESRMEDHRLPRKA

>SG-PL_contig_2733_1_+3_411_602

MGCDLHVIITQLVLVWSISGLQKHSKAMQTRSSVIKTLHHSGIKQYSIQSMKYSTIPLNY

TKEK

>SG-PL_contig_2734_1_-2_128_232

MKRKCRVQPCHAVSIRMELSSIITIIPILLVIIAL

>SG-PL_contig_2735_1_+3_3_221

THTHHTTTRLFFSNFCQINYTMLCSSRSHTHLYIYALNQQTPMHSSIERLTASSVCSQSH

LQVQLLADESNGF

>SG-PL_contig_2736_1_+1_1_99

QPFQMFPFYHSYTLNTWTYTSNSKKYKLLFFIP

>SG-PL_contig_2737_1_+2_32_532

MSLSLRAEFALDKNKKKKKRELADEQKQEIKEAFDLFDTDKDRYIDYHELKVAMRALGFD

VKKADVLKILRDYDREGTGKISFDDFNETMTDMMLERDPQEEILKAFKLFDDDASGKISL

RNLRRVARELGENMTDEELRAMIDEFDRDADGEINEEEFIAIMTGDT

>SG-PL_contig_2738_1_-2_32_1693

MVGKKFVVFLALICGLAAAHKIVKKVPPKFSDGYKVSGTLRLPYAELNENFDVWFDGPNK

CSRIDYYNGNFINIQHGDVSEYGTDFVVLYLTNYAVTNKRSCFEAPGTVDMPIMPQTILP

DTTNMSYYGVERIGDYICDVWRKVVVNGKKTSTYSLYVDQYRGNPIRYEMMGFDSLLGSH

YDKYVLDYHDYIGGISISSDIFNVSAIANLSCSGFPGPGDAKEHRLVFNPMHEFVHGDDS

HVPEMFHRFKTQHSKVYKDNMEHEIRQNHFRQNVRYVHSKNRAGLSYKLAINHLADRSDE

ELKLMNGFRRSMNYDGIQGKPFNMKEFENASIPDQMDWRLYGAVTPVKDQGVCGSCWSFG

TTGTLEGALFMKTGNLIRLSQQELVDCSWGEGNNGCDGGEDFRAYNWIMHPGIDGLTSEA

HYGPYEAVDGKCGFPKKLPAVVQLAGYVNVTAYDQTALKTAIAMKGPVSVAIDASHKSLS

FYANGVYYEPACGSKPEDLDHAVLAVGYGEMNGEPYWLIKNSWSTYWGNAGYVLMSQKNN

NCGVATSPTYPILK

>SG-PL_contig_2739_1_+2_2168_2569

MTQLTNGRYTCQLAETWEGLEIRRAKVIRGSESLLERLHLWFGGGWRWCRLLLLLLRTPS

TPVFLLVDHAVDGGLCPVTVTWLVRVLYLCASSWGLLLACVVQILVPGLDSCGPEAGSPG

RRNTRLVRFIVLAP

>SG-PL_contig_2740_1_+3_183_497

MFCMHYTLHKCTWVSAITIVFLILILVQCFGHAYAHNHMEHIHTQMHISYTERTHEHKHT

SYICNTFLHTYHKCTTHTYTYTHTHTHACTHMQHRTQNPVIYTRY

>SG-PL_contig_2741_1_-3_198_1037

MLDWFDYHGHVCISFDMLGLSVFDFLKDNSYIAYPIEQVRHISYQLCYAVNFLHENQLTH

TDLKPENILFVDSEFETSYNSKKRRDERRIKRTDIRLIDFGSATFDHEHHSTIVSTRHYR

APEVILELGWAQPCDVWSIGCIMFELYTGYTLFQTHDNKEHLAMMERILGSLPYRMAKKT

KKQKYFYHGRLDWDEKSSAGRYVRDNVKPLRRYVKDSDNEDHQQLFDLMEKLLAYEVSSR

YTLRQAMRHPFFRKYYEEDLLRHESTRSSSSERRSHSLSR

>SG-PL_contig_2742_1_+3_69_221

MIFCSSFFKFPKTIITNFKHILFSINQKNQKLQKTISDRSVNPTSSNELSF

>SG-PL_contig_2743_1_-1_211_435

MMLLLDELSSRTARNKTNLMMLHKLSSKPGRNKTNLMMLHELSSKPGRNKTNLMMLHDLS

CLKPGHNKIKLNDVT

>SG-PL_contig_2744_1_-2_593_715

MMLNEVVQNRLVTKPSLMMLHELSCLKPGRNKTKLNDVIVT

>SG-PL_contig_2745_1_-2_284_712

MAAGQQFLGHWKLADSENWDDFMKACGVGFVLRKVGNSITNYEEWKNEGDQWVLHITSTF

KSKLVNFKLGEEFDEETMDGRKVKSVITIEGDKIVHKQKATKEGEVDCTITREILEDGRL

QVTFVADNKGGLSAKRFFTPHKP

>SG-PL_contig_2746_1_+2_56_223

MKYSTSVSILFITIMFTKVNLNFSFHHFLTNKFIHFDDVDKFIFHMKQNFMYYLIV

>SG-PL_contig_2747_1_+1_253_498

MQTNFTHDKFSHSSLTKGIKLNCFYMIKTVSYSHFPFHYVQNPDNSTIKRQFNFTYDQFK

ILSVHVFRNDKPKFNFASNNQV

>SG-PL_contig_2748_1_+1_874_1089

MTRFKCLRNHFSHVYTALNILPSIQLFTCDYPSFNIFIFIVYYHDPEQVFSFTNSRFEII

NTLIFTQQQNYK

>SG-PL_contig_2749_1_-1_16_708

MMKTGDCVVKHGGTFTTCLGMVGGICDFCEAWVCHGRKCLQTHACTCPLQDAACFECKRG

VWEHGGRIFKCSFCTNFLCEDDQFEHQASCQVVEAENLKCLSCNRHGQYSCLRCKTCYCE

DHVRRKGVKYTKDQVIPCPKCGNDTSEVKELSMSTRRYAYGRQQMGDDDDDDDYGGAYGG

FSFSGYGISTSANGGYNDDDSEEEDSEDEEEEEEEETNDVSKDLQAASLTY

>SG-PL_contig_2750_1_-1_52_2535

MRGPRGGSMGPRGAYAARGTRGGRDHYGGQGYERGPRSQSDHGGQSIGRWAAKSEEVDRT

EGEIDYESKRRRGRDEESDVSVDEASGYSESSSERASEAKESGTMPSKEQDKEVKSKEDP

KPLDKREPLEPPRNPWTRNQPPPPQQQPTPQSRSSTPWSSEKDSNKDAPDNRETHELQSR

RDNREGRDNRDHRDDHDRDHRGPRDNRDHRGSRDGKRDERRDRPNRQDHRDYESERMMGG

HSDQGNGFDKMGQNDMAFVPRGEPSRRGRGGGHPGQRGGRGRGTSAPPRGGGGFGRPPMG

RGNGNGDHGAPSLMGESNNNNMDHRRRDNRRQERNPPPPRFRRGGMNERGRGYDRGTSGR

GRPSRGRGNSAPASSNSKKPPLQKQASNEGEEWETASESSDFLEKGRDSKNDSSDNRRDS

SKKSFSNQRPFNDRQNRRGGNSSEPRQSSERRNNKERSPNQKNGVAPPKSSAANGPTNRS

RTTMNHKENLKTVMRVDAIIPNDPTAINNALNSLQSKNKMGRKTDLTDVTKPLKTEKEKK

DALANIDINNYASVVVIDDQPEVTFDDPAFLFESNDGFQEVTSRKALKIKLKQEAEQQKK

TEQQQNQQQKKRDNANKVIARPKGLSPKINRSSHNSKSNKLPPRLAKQKEQREKEKELTK

NIMANIELWNNDMAISVPYQPNNVPEDKGDMGVMLTGGPTVSVMSAMSAPNTALVKLSAV

SITNPAPTLMSAMTPAPPPSISAWAKPISFAAATAVGPISNSNSATSSAAVVSATAVPVS

SSLSMGKKTVSVPASASTSLAPSTTAVSVSVVDCAKFDKGDQHDSGID

>SG-PL_contig_2751_1_+1_46_150

MKNEKNLLLISRSLKLPFNLITMLSQRLQQHYLTV

>SG-PL_contig_2752_1_+2_836_1027

MSACKRGLIVKKYNQFPSFIDEWLCVECFVSLHPSITLHSYLSLQNAECIIHSYICFTHL

FLPV

>SG-PL_contig_2753_1_+3_4245_4712

MFIHCASSKKSTNHVQFLITTSTIPTLLQLKSAEHFMHCQQQARLGNLNILHLGPTVIAF

FFFFSIIYIISRSCWLSHCPSSWANCLCFLSSLLLPGLAGFLTVLSLSYASCFLSFFFSF

FPVFFFFFFFFFFSLFFNKSFLFCVFFCLFNFYFIF

>SG-PL_contig_2754_1_+3_3_290

VCARVCAVPGSNLSLCTCVCVCVCVCVCVCVHCCCCCGWFIDCVCVCVCMRACVRACVCV

SSFHFISVENGIAVCIEWKFVESVKAKIVLFCLSCL

>SG-PL_contig_2755_1_-2_2_151

RVGSGSKDEFYLTPLLPGRLVDSGRVRNSVPVSHTHSPNPLLPSQIHYEP

>SG-PL_contig_2756_1_-2_2_544

FKIQGRDTVHHVLDAAFTAGYRSIDTASVYRNEEDIGTSLKILLPKYGLKRQDIFITSKL

APKDQGSDSCREACLQSIARLQCSYLDLFLIHWPGTQKLKPDDERHRRNRCLSWADMERL

LHEGNVKAVGVSNYLQRHLEELMEKSVIPAVLQIEFHPHLFQKDLLAWCRQRGIHFQAYS

S

>SG-PL_contig_2757_1_-1_67_1047

MKPILLHGHERSITQIKYNREGDLIFSCAKDAQPNVWYALNGERLGTFNGHNGAVWCIDV

DWNTTQVLTGAADNTCKLWDAKTGVCISTLETNTAVRSCGFSFSSNYVMLTTDKTMGFPC

EVLVYDIRDGSQMLGGEPLQRITLPMTEAKITSAIWASLDDLILTGHENGECKQFEIKTG

DMVNSCREHTKQINDIQLSKDGNWFITASKDFTAKLFDLQTMDSLKTYKTERPVNSASIS

PLFDHVVLGGGQEAMSVTTTSTKIGKFDARFFHLIFEEEFGSVKGHFGPINSLSFHPDGK

SYSSGGEDGYVRVHYFDQTYLDFRFEF

>SG-PL_contig_2758_1_+2_47_334

MILTCLILSSSRELFLCLLMVFLTYPTPSLPFDQMYETFFWFQKYKRMCICLFFLCVRLP

LSLCLLTSASLNLVLTAESQNLLMNRCLRSYACMLC

>SG-PL_contig_2759_1_-1_85_408

MFWLVISSSSQNLFVKMIFTNGARRKLFLSVCCCLAVPYVYQYLMVCLHSWRESPPSPPS

PPSDPLVFPVKKGVLVRHFASSVQVDDTHVDEQNTAVNRKPTVFQTLH

>SG-PL_contig_2760_1_-1_205_1134

MEKSIMCVLCTVVSWVAGVWAGEWTELARLRLPHDHLVNYLYGQPQLRQHCLEDPACPYK

KDLESPFAPCWGYEEGCPVIPRLPVCLGSSRHWASDKDSQRQMFWDSADFGYVRQRHSEL

KYYCRPTQLEDSSLRCVDHMRYCEVTNIFVDLSTAKLASASNPYREDVLSAGQIGGKCQL

DRAALNSQLDHKSPLQSWAAEMEHFVSTPAEPLKDRTCDVIISKPTFIIKLDATVNMYHH

YCDYVNLYISQHLNSSFSTDINIFIWDTSGHGMSTMFAETWKAFSDHTPLRLNQYDGQTV

STLDWIGFTF

>SG-PL_contig_2761_1_-1_1_216

SELLHLYAPSRHLRSSADTRIYKLHVRTKTYGQCSLSHQAPSTWNQLPHSVHHAITLPSF

KSALKTSLFQWL

>SG-PL_contig_2762_1_-1_1_189

EQKRMVSILSLTRLLLGVSFHIKLVMLSITLLSNLLTKSLFQQLLDNHFVNNKCTGVPTS

TSF

>SG-PL_contig_2763_1_-3_3_185

TVTNKSKLSIHFVRICTMNRYQWHSCIPLGSLCKTFLLHTTDISGRQRVKKGGGGEGAGI

D

>SG-PL_contig_2764_1_-2_2_142

KKKGGGVKITRRKRSNSNRRRKNDDDDDDDDDNDDVDDDDDDLWLID

>SG-PL_contig_2765_1_-2_2_439

AADESERGRKVLESRSQTADENIDTLESQLKEAKYIAEDAERKYDEAARKLATTEVDLER

AEARLEAAEAKCYELDEQLHVVGNNVKTLAIQNDQASQREDSYEETIRDLTQRLKDAENR

AHEAERTVSKLQKEVDRLEDELLVEK

>SG-PL_contig_2766_1_+3_3_152

VLFFCEQLKPFLPSSLTSDSSLSLEDVLDDNALMVKIVQGLIKSPVGPLL

>SG-PL_contig_2767_1_+2_185_790

MASQVPAEVRAASAFPSSMSAKSSAAANMLARSFLRLFFFAGLSSNSSSLPRFLPLLIIC

LSSSPDRSLSSMSPSSSSMSPSPSISCRNSAASPSSSSSPKSISSSDSPPKSSSSSSSLS

SSSSLMSSSLSALDLRLSPFFFPPNFPAKSRPKSMSSRSSWSTCLSCLSRKASNSVSLTL

STSLASSSSFFFSAFWLKYLYR

>SG-PL_contig_2768_1_+1_64_303

MKSRRLIGAAVLLLCPILYLNAVFAIQRSNRKTLEELERNREDLLKTTYFIEATRLTICG

TTTLPMAASIKNLPPGCTQL

>SG-PL_contig_2769_1_+1_100_1233

MVSEKNKRLQFEIWLNENGYLRWAETIPSPPEMKQYGRASFCDLNDNREINIVLPGCLNS

GPDCSESVVYVWGRGTWHNLHVDFKRGSESWQLSMDNMPLPWLNVPLMLRFGDFNLDGYP

DAITVLKTSSNNSYAAFLMLNVASSSNFSRTFYIDQSLSGVFKDPPVPSFYDFMENGILD

VLLTVYTDNAPELRALEQDFTADAYFLKVMVVSGLCYENCANGRPPYGVNQVGPVIRFQT

TTSHGDTEIGVASQLSQSAYYSLQLPFEVFGLGQTPNFVDVLEVGIQNPPKTSRRQRSWP

SIIPNAQVVVIPHPTDNPSRWIMKLFVTPSRLVLLTGAALLGTCAFIAIVIVLLHWRERV

EDKKEKLQEAQRFHFDAM

>SG-PL_contig_2770_1_+1_604_837

MDCSCSQRKSSSTTWNWCSEPRKLSMGLPQGSPFSPVLYNVSTKGPADINNNRPAEYSPL

LMMGSSTKHIRVHKKETK

>SG-PL_contig_2771_1_+2_2_223

SGGVKRPVDEKSFQLFWTERVILLLLYILFIAQPTTMAISGLSPCKVHQNQEDDRAERKT

ASNMITKTLQDHNS

>SG-PL_contig_2772_1_+3_300_503

MTNAVYTAFEIVLSLFHGVICIVLMLSLEGLMDYRGVFFVCLFVFLLLFCFDCLLVWFFW

RVFFVCLF

>SG-PL_contig_2773_1_+3_189_446

MHAQLKTLSLPALSLIHIPTGTDTKSTCNRKRKVKRKKKFYRSTSKCDGLAFTVSHTNKK

SFDTQIYKKSVHCGKKLGDARTHTHS

>SG-PL_contig_2774_1_+2_95_388

MTIFLFSFICSGRCLKKTEARVTKINDTMYNGIWYELCITVTREQPNLIGSHGFKYFCSP

LTFTVLRGPNKPIFLFEMSVFVKWLKILNSLKISLPII

>SG-PL_contig_2775_1_+2_275_523

MRTRTPPVHISELFPDKWSKRVHRLAGDKNIKSHMALNYDTDNDTSYNTELITQFLCCLF

GGYPLGTFLHAFFTFILLHAQDK

>SG-PL_contig_2776_1_-1_175_1278

MAESATSADAERITIAPNELRAFCVRTLEKAGAANDHACALADLLVAADYRGHFSHGLNR

LEFYIMDLMDSHVASLGQEPEVVKETAATALVDGHNLLGPVVGKFSMDLAIRKAREAGIG

WVSVRGSNHFGIAGWYSIRAQEQGLLGMAFTNTSPNLVPTRARQQTLGTNPISVAAPANN

GDSMVLDMATTVVAFGKVEVKKTLNENIPLGWGVDSTGKTTTDTDKVLAVDGGLMPLGGP

EETSGYKGYGLAMMVEVFCGILSGSAYGHRVRHWTQHHSSANLGHCFVAMDPAAFAPGFT

DRMSDLLDHCRGLQPAEGETEVLVPGDPERQHMSMCDQRGGIPYPCKQIQWADNLAKKYG

VEPMVRLA

>SG-PL_contig_2777_1_-1_28_309

MASAAVRFGAHLKELRIHLCQKSAASKGVRDFIEKHYVEMKKHNPKFPILVRECSNVQPK

IFARYELGKETSLPLSNMDSQQVLRAVEALAKKP

>SG-PL_contig_2778_1_+1_4282_4578

MLHMCDSPNDFITGLCLSQQSKLQFVNIRLQFCATCILSHPLIHIYIHTYIKRVEMCQLV

LFTPFKTLPFQSWSKGGSVCAYTQICAVSHSRPYSKDQF

>SG-PL_contig_2779_1_-3_3_167

LSLCPLPSPLYPLTLHGKGMDNFKGWRAFFSSFLFLFFQFYLNELLFKLLTFLIH

>SG-PL_contig_2780_1_-2_1226_1573

MSSLRHCFTFPNSAGYIYSLADLRSHAQFFFLSRNKHVQIDWKAVVLLLRQPGLHKTLGK

KSLISKGTVAFIILVITLAVIIIKMYCLLQFPSKAGSDCYCHFFIRFTTTLLVKTS

>SG-PL_contig_2781_1_-3_48_281

MCVSFKSLCMCLSNSLHVCFSNSLHVSLSKSLCMFVSLSLHVCLSLRVCVCLSKSLCVCV

SYSLCVFVSLRVCVCVSL

>SG-PL_contig_2782_1_+1_220_402

MFCVQVENCLTLIFCACYLCQMILSDPFVRQHLLVTVCLQDILADAFVFGLFVCLLFCVF

V

>SG-PL_contig_2783_1_-1_70_582

MSSRKTKGKTTKKRAQRATSNVFAMFDQAQIQEFKEAFNMIDQNRDGFIDKEDLGDMLAS

LGKNPTEAQLDEMMNAAPGPINFTMFLTMFGDKLNGTDPEDVIKNAFACFDEDGKGFIQE

DRLRELLTTMGDRFTDDEVDEMFRDAPIKRGNFDYLEFTRILKYGKKENDE

>SG-PL_contig_2784_1_+2_2_166

KKKKVNFFIMTALYNIIQFQYNLCIEFHFLKCANDLLKELCAHHFESFMFHRDQR

>SG-PL_contig_2785_1_-1_100_303

MIFFNFQKRARCESFLFLKCETFLLVETVEGTVVESHVSIFLSTALISVLDMLCKLQNKV

MYNFKKKI

>SG-PL_contig_2786_1_-3_249_1391

MIVFGLLLCCGFLMVSGQSSANRPTQWRNRPQLRSARNFGRGQQQRWLTPTESSSNNEDN

EDYEETPPRNWRELMTVGEGVHNVDDFIRLLDTPEGRQVTDSDIFSPYETQDGKRVITGG

GIMATSDECSPRNMSVRLPLNYSDNQIAYYPECTMVQRCGGCTPTRFLSCQPRYKEKIVL

KVLRARYLYPGALRMQWEGFSSVEVERHLSCRAVCTLNPEDCGQHRRFNPYQCKCYCRRF

ERCSNHQVWDDETCMCRCPNQRPCCAEEGGEDERTCSFYFDHDFCECTLKEAIGVAPNAS

QAEIDAWLARRNHGNNDGGSDSGDNFYPPTTTTTTTTTTTTPADPCAGTVCPRRFITVYH

SITDRCVCQLRFRSRGRRGRG

>SG-PL_contig_2787_1_-3_27_242

MPWAQEELPNHTIIVQVNLHLAMRHSSLNWFRLNDIKERREHASLMSLILVIVFCLFIVL

QDKSHTAVKIFF

>SG-PL_contig_2788_1_+3_3_188

QRLPAVCVCLCVCFFIFSFLFFLALIADISDKNNTWILCFLDFIFYSRTSDSNFHSINNV

DC

>SG-PL_contig_2789_1_+2_2_208

FQSVNLLLQFGHCPLSFLGTVLSILESLCQIPDCLLITVFTLRCLLLTDFQRFHIVAHNL

QLLFQLQDL

>SG-PL_contig_2790_1_-3_3_239

QFGFVFGVENFQSGLEKVWEKYGILFGHVCMNPVIFQIVSVIWGNRTSINMLIYVHILYI

MYLYFNICAYIVYYVPLFQ

>SG-PL_contig_2791_1_-1_1_135

DGSKHVLSEIICKSSNAPFCRQILIQFCVWYGKFSIWSGKSMQLC

>SG-PL_contig_2792_1_+1_127_303

MGVSNVLPMVISNTGLFYFIIDLIVLQSFYCQKMLSRCVTDLIVLQSFYCRENDVTLSH

>SG-PL_contig_2793_1_-1_430_627

MGARSASTVYPGPQLDEMDPSGMRRPIDPPSADVLEWCTHLLLQHYQSGSTPMADPLMTG

GTCGVP

>SG-PL_contig_2794_1_-1_439_864

MYRYINFHLLHNYCIPSVLCCDNCSRFIIEAVQNKIESNNMFCSFLFLTSYLSILSVQSQ

RFICLYQLLIHVSTVKSGVGRHSLVTAKQTACSRFPTYGSIFRNKSGLTSSWKDLVTIDE

GVAPDRLYSRSGCVSDFGWSER

>SG-PL_contig_2795_1_-3_9_104

MTCLCGDHRTPRGLDSFGQGILISNIFCKMKM

>SG-PL_contig_2796_1_+3_1287_1679

MYSSHTLMHSHTLMRSRTNTCMYTLRTRACKSPAFMENIQYYIFLYISDAHSKLQTAQNN

CRQQSVNNILQTTHNQTNPLGYRCKNQENQHLQIEQVCTSNILQTTTQLFPFGYIQIITT

KTYTDKSATQA

>SG-PL_contig_2797_1_-2_35_1054

MPKKVTMNRLGGGGPDYGADFSRGFVPMTQPMHVHTGGKGPGHNVEMLSKVQESLDNISL

SPRTPEYGGYQAPSFPAPTSPGGQYVPTAVRLDMERENEARRQQQMRDNPPYVPTAVRID

QEMAAAGKSLPPAHKPHAPAPWKGHHQDEEDSGPAWKTSLKPTSTKPWDADLNYVPGQQQ

APRSSGYQPGGHQGPTAAPYNPAPAPAQERSEQDGPKVVHLQYNSPMGLYSRQNVQETYA

GQTKAMAAAGSLSPGGTQMQQNHAGGAPGDRDWNQSAVFRMIHEDDHRGGGRQAHGYAPQ

PMQPQTYYKAPAPQPAPKPQVQTFQQSFRPAIDEFGTSDF

>SG-PL_contig_2798_1_-2_149_286

MVYISRYFYQCQGHVMSRSCDPRWPPPMSIIINKYVETTSFSEREN

>SG-PL_contig_2799_1_-3_3_140

KNKMKKDQNICNIKEEEEEGQQHQRRRKKNNNFKEEGQEHQQHQRR

>SG-PL_contig_2800_1_-2_566_883

MKRICLLLGVSNCCLVTVFQDCGQPKTGHTHTHMARLGCFQNYYYVEQRTNHSPQSYSQP

PQWLVLQRSNCSFRISGFLQQTPQMSQNATAKKNVLQFERGSFIRL

>SG-PL_contig_2801_1_-2_2_211

HHTHTLSLSLSLSLSLSLSLSLSLCQAALACPPKNSPKATLSQDNLSLLLLGIFGTRFPR

LLIVRKDGKE

>SG-PL_contig_2802_1_+2_2_232

GYSLKEYLSARVVVHTHHTRTTPSLRSKARPRTIWSFASDLHHTFSLANHIFVGQCSHIS

DHCSAQHCHERTQSYVI

>SG-PL_contig_2803_1_+3_3_305

TWACGGIKRSSFSSPSRVTRKAREVPPFCKKWAVGLNTQKSRQISSRRISTWRQEPLSTL

GLRRLARVERSLLFLIREGDRSVLRSGDREADRCPEGSVES

>SG-PL_contig_2804_1_-1_43_663

MAMGAVRHIADIRADDASKEGAGIIGLQAGSNKGASQAGMSMGAVRHIADIRADDASKEG

AGIIGLQAGSNKGASQAGMAMGAVRHIADIRADDASKEGAGIIGLQAGSNKGASQAGMSM

GAVRHIADIRADDASKASAGVIGLQAGSNKGASQAGMSMGAQRHIADIQVDDMSQASKAS

INLQYGSNMGANQSGMSYGGRRDIMGN

>SG-PL_contig_2805_1_+2_212_382

MSQRHAQRRREKAEGDYCMPVLSLSSPNIWSCLTLHNLVSESCLTVLSRNSKSCLIV

>SG-PL_contig_2806_1_+2_278_460

MELSKHDESLLNKLIYAPIILVVAVFHRSCFLIPLAGLGGAYMELSKHDESLLNKLKLKL

N

>SG-PL_contig_2807_1_+3_318_464

MFFNKQINYSEEKLINKQARQNTTDLLFWHALPSAKFSFEHSVDCRYFL

>SG-PL_contig_2808_1_+1_1_240

VRVRVYVCKRERKRKTDRQTDGDTDRQRQREREFIFIFGFSRIVGVGPTGSVLQLVLADC

VLGFMFPQVTESRHGRKIGG

>SG-PL_contig_2809_1_+1_1_339

KEEEEGEKETGREREGEGERERERARERENKEEKEGEKETGREREGRTRERETSETLLLK

DSSIRSYWICLTASPCYSTHMYACKQKQQQQQQQQQQRQRQPHLQSLASWQIG

>SG-PL_contig_2810_1_+3_705_902

MSEKPFYHLFYSFDFQLFIFLVHTGQESRGRETRKQTNIIINKYGGVVIRENSPQVGFFS

KILAGK

>SG-PL_contig_2811_1_-3_111_524

MGALYRQQIAVVYLAYLFTCCVAPKCFHCTQAVSGCADHFGLPNTEAVFCDGTCLKMRGE

RYSNEVRIVEVTRMCVEQRTEGCYGTTWNGISVKACACNSDYCNAGNMTLTTSGFGGLLL

RISLAAAVWWMPRLLGIH

>SG-PL_contig_2812_1_-1_34_1137

MKGLPGLPGAPGRDGPSGEPGLPGLSGPPGEKGGRGDDGLPGLRGDPGQKGEPGPIGLTP

ALNSTMLPKGEPGEPGSRGPQGLPGLPGEKGFPGRDGLPGRDGPKGESGLPGLPGMDGPP

GPAGLDGLPGIDGGPGSPGPKGEPGPQGLAGPTPPTGYLLVRHSQSTEVPTCPLEQTKLW

DGYSLLYIEGNEKSHHQDLGWAGSCLPRFSTMPFLFCNMNNVCNYASRNDKSYWLSTNAP

IPMMPVGERDIVPYISRCVVCDVPANVIAVHSQTLQIPPCPRSWRSMWIGYSFAMHTAAG

PGGGGQSLSSPGSCLEDFRATPFIECNGARGSCHYFANKYSFWLTTIEGDEQFITPRSQT

LKAGNLRT

>SG-PL_contig_2813_1_-1_451_651

MTSSVHSKDKINNISKHFSKTCISQKSNKPHKSALQNWTAHMVCQFASEIENILQTATDS

LVLRIFV

>SG-PL_contig_2814_1_-2_2_127

VRSLPPLYLYNPVSGYGTNFTLKKKCLKFPRKCRLHISLKQC

>SG-PL_contig_2815_1_+1_1_171

KTIITKSAMKTMRGTLQQQKRYKYCIHIHPHPSPHQANTSKHINNSLLPFVKKTTTL

>SG-PL_contig_2816_1_+3_264_503

MYKSYQLYKLNHCTAMQYVEPHKPLVITIGSVQKPNCMYSHCVTIQKKQQSWYSCAYVVQ

VVTHFVQKYIKYSFYIIYVA

>SG-PL_contig_2817_1_-3_561_797

MDGSNWLKHFLSEWNCTYTCLFEYSIRPVLILGHSHLNLILRNITDFLMAFELIFTSTIM

MSPVSRHGICISFNEIDEF

>SG-PL_contig_2818_1_+3_750_965

MHVDSFLFILPYTDRILVNFSALKSEHTPQNTQFYETQTFIQSFSPKSIKNLNVNQGERS

PHKNDFSQLHAC

>SG-PL_contig_2819_1_-1_16_792

MARRYDSRTTIFSPEGRLYQVEYAMEAIGHAGTCLGILAKDGIVLAAERRNTNKLLDEVS

YSEKIYRLSDDMACSVAGITSDANVLTNELRLIAQRYLLQYQEPIPCEQVVATLCDIKQA

YTQFGGKRPFGVSILYMGWDKHYGFQLYQSDPSGNYGGWKATCIGNNSANAVSLLKQEYK

EGETSLDEALALSIKVLSKTLDVTKLTPDKMEMATLTRKDGKTVMTILPDSRVEALIKIH

EAEEAKAEAEKKKEQEKKK

>SG-PL_contig_2820_1_+2_158_511

MICARLELLVFLCLQSADQRATNNADIRLSSLFLSLSLLSSIQHLLQPSTAETVRVITGP

RVQGSTCSVHVPGMQARVTSRMQSRTATRDFFFLMTTASPVMRSMSSSLRLTRPCWHW

>SG-PL_contig_2821_1_-3_450_644

MFHQFSDALVQHCTSPADAYLLIIDMLSNIDCRLSKLVVYDSLSIDSCLFCQVNAMMLQN

DVETS

>SG-PL_contig_2822_1_-2_176_571

MKLLKKTEQQNEATKEENVQLHKANQRLRIQMEQLRNALSQYPGLNLNGLLEDLTSETMV

APSSVMSMQQQPVTMAPVLKTEDPSPSATFSNLMAQDFGNTIHMEFMEDSSPVNGDPMLT

SNPVSPDTEDTF

>SG-PL_contig_2823_1_-1_1_201

GWKSLGKKCFALQGLEKSWNFHKTLDQYHSTKSFKLLKKKEKVMKIKHRNLPISFVSGVA

DKLLSVQ

>SG-PL_contig_2824_1_-3_12_1100

MKFDRGYISPYFINTSKGAKCEFQDALVLLSDKKISSAQSIIPALELANTARKPLVIIAE

DVDGEALSTLVLNRIKVGLQVVAVKAPGFGDNRKNTLQDMACATGAVVFGDDGNLYNLED

IQMQDFGQIGEVTVTKDDTLLMKGKGDKADIEKRVQQIKEDIELSTSEYEKEKMGERLAK

LSNGVAVLKVGGTSEVEVNEKKDRINDALNATRAAIEEGIVAGGGTALLRCVTMLEGIET

ENDDQKIGLEIVRKALRMPALTIAQNAGVDAHVVVEKVLNAEGDMGYDALNDQYTNLIER

GIIDPTKVVRTALMDAAGVASLLTTAEAVVTEIPKDDKADMGGMGGMGGMGGMGGMGGMG

GMM

>SG-PL_contig_2825_1_+1_703_918

MSGPTRSWRYPVFINQAWLHTHLPIFRLPKKKEDKFVTSGMCTYATEDHCKLNNSSATPL

TKEICGDSDIDV

>SG-PL_contig_2826_1_+2_329_772

MGSKPFHYLSVRDTVCYNIRAGNRKKLQDPGYLLHNRTCQLLTYGHILASNCDNDLYILR

LVSPLIYTFKLNAFKAKFTKHINCVCGERIPNTHILFHYKHMKLFSPSFLVSSLEEILTN

ASVMCNAALSFKSKSYDALPLRRCCCST

>SG-PL_contig_2827_1_-3_681_2648

MLTFSVHFFPTAATAALAAGLQHLLHQNAAGGESSDAPPSEEPKPEETEEKKREGDDSDD

GFVPYVPVRKRKRKELEQLKKRIQKHFEDASKACDDDDSVHEDDGEEEETEELGPRALVS

LLEQHTELKKKEEARKETAKEKLLKEEQQLLESVTENKALKGVAELAHGIVYKDPVKTGW

RPPRYILARPSEYHNKVRKKCNILVEGKDLPPPIKTFQEMKFPVPIIEALKKKGIEKPTP

IQIQGIPAVLAGRDMIGIAYTGSGKTLVFTLPIIMFCLEQEKRMPFMNNEGPYGLIICPS

RELARQTHGIIQYYMEHLVEGGFPAMRPGLLIGGMEMKEQMEVIKSGVHIVVATPGRLML

MLDRGMLTLDVCRYLCLDEADRMIDMGFEEDVRTIFSFFRGQRQTLLFSATMPKKIQNFA

RSALVEPVTVNVGRAGAANLDVVQQVEYVKAEAKMVFILQCLQKTSPPVLIFAEKKQDVD

DIHEYLLLKGVEAVAIHGGKDQDDRIWSVEQFRKGEKDVLIATDVASKGLDFPAIQHVIN

YDMPEDIENYVHRIGRTGRCGKTGLATTFVNKSVDESVLLDLKHLLREAKQVVPPFLASV

QADSEIYLSIGGQSGCLYCGGLGHRITTCPKLEAVQTKQAANIGRRDYLAHNAADW

>SG-PL_contig_2828_1_+3_24_1796

MGDFNARVGRDFEIWNGVLGRHGVGNCNENGRMLLEFCTEIGLTITNTLFQQKDRYKTTW

RHPRSKHWHLLDYILVRQKDAKDVTHTRVMTSADCNTDHRLVRAKLRLTLKPEERKGGPK

LKKFQVDKLSVLEEEFQREVNERLRNTTAPENAPLEQWKELKTILQEATEKVVGFSTRKN

QDWFDENDVEIQELLEEKRSCHGNMLSSPNDQKTKMAYRTACSKLQAKLRVMENSWWIAL

AERTQMYADQGNSRAFYESLRAIYGPSYQLQAPLRSSDGTSLLVDRQAILHRWTEHYGNL

FGEERTVEAASIDNIPQEEVKMDLDNAPTHEEIKAAIKKLKCHKAPGIDGIPAEVYKHGG

DTLIQKLEELFTSCWNEGVVPQDMRDAVIVSLYKNKGSKSDCSNYRGVTLLSIAGKILAR

VLLDRLIPSIAEVNLPESQCGFRAKRGTADMIFVLRQIQEKCREQNMNLYAAFIDLTKAF

DTVSRDGLWKIMSKLGCPPKLLAILQQLHEGQMGQIKCNGDYSEEFPISNGVKQGCVLAP

TLFAIFFSMMLREAKEDLDEGIYIRFRTDGSVFNLRRLMAHTKTSEEAILD

>SG-PL_contig_2829_1_-3_510_791

MCSDANVSFCQEQRFRQFFCHSNIVITITLLICKFWYCAKMVVPVCFRCSLSLSLSLSHT

HTHTHTRTHICTQNYQGLRQLGLFSRILTNAHSK

>SG-PL_contig_2830_1_-2_2_433

FDSLILSTSFLHYQPWWQSKTLTLSFPYPIYQLPAGRWWIERWSVKILLFVWGGEGGRGG

VITIFGDGFFVCLVGLPPPPPKKKKKKKKKKKKKKKNPKKPTTYLCIFGSYILMLWNHVV

CACWSGTQRPCCHGRLTQHVTIRY

>SG-PL_contig_2831_1_+2_2_151

LLFYTYIDYQHPFNQLTLSDINDKTQWMEHAFLGNNGKPHLHSTQSLFTN

>SG-PL_contig_2832_1_+2_2_136

THTQHQYLTECQVNHLHPHTQTHSISTSRLNLTGLHYRYTLSQQK

>SG-PL_contig_2833_1_-3_150_269

MAEYPFVGDCGVPYHHLQLPPEKNNYNSISLSDMSVTNCY

>SG-PL_contig_2834_1_+1_1_207

NNTSWRTLFTLHCQLGDSVHPSLPAGGLCSPFTASWGTVCSPFTASWGTVCSPFSASWGT

LFALHCQLG

>SG-PL_contig_2835_1_-3_510_737

MSTLSAFQKEKLTYFFKFFEPNESNELDDKSLEHFMKRVLEYTGWDLQSEHSRTMREVHQ

AFFEILFDKMSDGTRA

>SG-PL_contig_2836_1_+1_1_72

FFFFYGANYFIQSKTDNLKQLLHN

>SG-PL_contig_2837_1_+1_187_414

MWQDQVSNLQVLKKCGLPSIECLLIKCQLRWTGHVVHMEDNCIPKMLLYGELKQGHCDQG

RPLKRYKDTLKANLII

>SG-PL_contig_2838_1_+1_43_276

MGQQGLGEMNDNGERFADLCATSNLVIGGSFFHHRRIHKATWVSPDLSTENQIDHVCIGK

KFRRSLQDVRVRRGADAA

>SG-PL_contig_2839_1_+1_43_282

MGQQGLGEMNDNGERFADLCATSNLVIGGSFFHHRRIHKATWVSPDLSTENQIDHVCIGK

KFRRSLQDVRVRRGADAASD

>SG-PL_contig_2840_1_+3_1575_2105

MICHPHLTHDTVYTITNACHHSLSSPHFKHPCPIYSLSHAWSCFRQTFGNALRSKPYCEH

NPLRLAILGLLVVSSSTVGDLTLLVTFFVPFHKLIHHHRPSTIFIIVAEVAADSSAELQN

VAPLIFVHHQVACTLVKELVEAGLKRPHSTQQQCQHSFGRLLVQTSLILLLVIQNTI

>SG-PL_contig_2841_1_-2_263_397

MCLDNMYTAVVWIACILLSCEVTIVVVKIQDACYAAQSDCIQPAG

>SG-PL_contig_2842_1_-3_621_902

MMLPARIYPHFMDHYQNLTKGLVGVDGACLPLSGQVFKPGWGNHQRSSVHPAVNGYLTFR

VREGKAARERRWAPPSSCWPREKLRSLPPHSPTT

>SG-PL_contig_2843_1_-3_3_374

NVLKVVAHTDWGADRRTLLRLYRALVRSKLDYGCIIYGAARPSYLKTLDPIHHQGLRLCL

GAFRTTPVHSLYAEAGEPSLAHRRLKLALHYYLKLQSEPENPAYNCVIKPQFRDKFENSP

RCIP

>SG-PL_contig_2844_1_+1_316_750

MQLSLSLSLSLSLRPSLFQFHVSSVSSPGLHSHIASSTTHFHFILSSLLGWNQFASHASS

HVLLPCFLYQSQSARDIFRLKFFGPSLHLRHFCSRSYSSTLRIQVSSKPQKSKGKFHISE

RNQLWLGHRTGTFSFSLGFSKSPEQ

>SG-PL_contig_2845_1_-2_161_1966

MERVTCFLKKITRVKTISGDIQQTALKRCLTTLEMTLLGIGHMVGAGIYVLTGTVVREKA

GPSAILSYLFAGFAALLSALCYAEFGAQMPKAGSAYSYTYVTAGEIWGFLVGWNIVLESM

IGVASVARAWSGTVDSLFDGAIRNGTVTYVGYLVQDSQWISEYPDLLAAAITLVIFSIVA

TGAKPSIIFNSVFTVLSGIVVLFIMGCGFSHANLSYWTSSSFGGFFPFGFSGILSAAASC

FFAYVGFEGIAVASEEAKTPAKSVPLATVVSMMVVTLMYMGATSALTLMVPYYDVSLSAG

FPAAFRDVGDRWAQYPVAIGTLLGISCSLLGFAYSLPRSVYAMADDGLLFGFLAYVYPKT

QTPIFSILVFGTLSTITTILFDIATLVEFLSIGTLFSYTFVAANVIILRYLPRDRFPFKL

KAAEETCCCDANATEETRSSGMEATSEEHTSRTKELDGRCPTERESPEEGCPVELHTDEN

CLELSQQDCEHCSCFCSCENIPLISHYEGFGKLRSRFRDVPVLSRLRPGKGVEIAVVCLV

VVLAGFSVLLLFRFDLFADGVWWSFIVLILFVVCIVFLISVIVAHEQNTGVATFQVPLVP

FL

>SG-PL_contig_2846_1_+1_1_243

YLTTKYYIEEYNAKLELTCFQNEMAAIFIINYNGNSIIICLVIIHLVFLVCMKTLIVSLV

LNNQPVYVTIFCFYFIQKYMT

>SG-PL_contig_2847_1_+2_110_490

MFGPNMVNICPEIAEKILLMHFIINRHIHRHRSLPSNTKKISLFFKGDKKEKIYKKKKKK

LWGGGGGGGYVYTCVLVCAHICDCTCVCMCMCVHEYVCMSICVRVCPSVLCTKPVNRMNH

QATFFST

>SG-PL_contig_2848_1_-3_198_1250

MTGTVHFDVPDPTCADPNSSSRLGGRPALLRPVSMEEADNSVNEIHESLPRKAAVRLYRQ

ISSGFNSRTIRCLLVVSLGLNLIFAVLVGIFVGFWQSSRVDFDGGRVVGGGGDGGLSIPT

KEQLYSCFPCSEEGVPGLRISVRSQNGVCCTTDSVPGFDQSNKLLQLTRDYVRAHCPDKP

DKEQNGGRPPKDGPQYGERPSAAHLTLDVMQSKALLKSLPAEQHYLSWNVRDDTGVSFIS

DDLRYVNGTITIPSSGRYHVYTHLTFDTNSNKLETQQLVVDHSITRISRGSPVFLLLDRV

YLLSREIKSSDLEGTFEFHKGDVIKVIVSFPSYLYDLPQTNVFGLFKLLSG

>SG-PL_contig_2849_1_+3_3_200

EGAKGLIMCYRETTDLLRQSQDAFGPMADAKPVILEKQQPNQPKKTHTHSYAVCSSKQRS

TVNHKR

>SG-PL_contig_2850_1_+1_151_492

MVIMRWGFFISGPIICNIQATLSSHVHRSFALSETPPNPASFYHIPYCKGIHRSYQCQVN

EKPPNRADPALLPGHFNGIQYVPPLIQQRQHIMPSSCSMATVMKPERMSSLQRL

>SG-PL_contig_2851_1_-2_1547_1891

MSCMNSNKLKLSTDKTEMMPVSTSQLRLVGSECANIGGTDISFQTSVKYLGVKTDQNLSM

QEHISNICRGSYLELRRIASIRPYLSKTACKRLVSATITSWLDYCNSVLAGLPAE

>SG-PL_contig_2852_1_+2_146_358

MWLAKVPKILQSFHLWSEGYSSLKHFDSQNYLEVLQKNPPISEKKITNQVVLRRKMNGKR

SQHNSNFDMVR

>SG-PL_contig_2853_1_-3_390_503

MDELNKICEFTLITVLTLAGLRVQGHNCARSVEVCRKL

>SG-PL_contig_2854_1_+1_166_393

MLASVYVIYIYIQGPYRFTNTKFHDFSNAIFKTFHDYFHTQKAQTHTHACTKKKASIRCC

FLSFVGGFFKALFLTP

>SG-PL_contig_2855_1_+3_612_809

MIVQEKTNLLLNILFCYRKEMVPISITQEFMYQHSLLEKVTMHATLHSVLVKVTMHAIFH

SLLEKI

>SG-PL_contig_2856_1_-2_26_1168

MHALRQACSGLVRATKCALTTQAVPQAASRTLPAYFNNRNYATEAAKPAAASGGAVGRIV

AVIGAVVDVQFDDNLPPILNALEVEKRSPRLILEVAQHLGENTVRTIAMDGTEGLIRGQA

CKDTGTPIRIPVGPMTLGRIINVIGEPIDERGPVETDKFAAIHQEAPDYVDMSVEQEILE

TGIKVVDMLAPYAKGGKIGLFGGAGVGKTVLIMELINNVAKAHGGYSVFAGVGERTREGN

DLYHEMITSKVISLTDDTSKVSLVYGQMNEPPGARARVALTGLTVAEYFRDEEGQDVLLF

IDNIFRFTQAGSEVSALLGRIPSAVGYQPTLATDMGTMQERITTTKKGSITSVQAIYVPA

DDLTDPAPATTFAHLDATTVL

>SG-PL_contig_2857_1_-3_63_281

MCVSVCVCVCVCVCARARARARMQKTANLCVDGCVCMECYNNIIFFCFWYFIFALQFECA

FYTYHYELNIHPL

>SG-PL_contig_2858_1_+1_124_348

MAPTVNPLFHFQSLYKKLLPSLHSTYTSSSQSTTCSTMSSYPRNVSFRTILSSITVISPT

ETFKYQIYSQSPQWP

>SG-PL_contig_2859_1_-3_510_758

MHLEKTLLTHTHTHTHRVTDKSHLNVLYLSQFGCSSLSVVTVIVNLSYHEMSCLLRWNHR

EKYIVCSCSSFLIIVLSSKLYHT

>SG-PL_contig_2860_1_-3_138_248

MQVILLILDTNCITTVIYFYFPQTIFILTFMKMCLPL

>SG-PL_contig_2861_1_-2_2_556

MQDAGRSARIDLMTTGEAAASGVKSTKPVIKMSATKIAMQLIRERGIMGLYQGCGATAMR

DITFSAIYFPLFANLNKLGKRREGSDQAVFYHSFISGCIAGCTASFLVNPIDVVKTRLQT

LTKGVGEQHYNGILHCFKTVMVQEGPKAFLKGALCRILVIAPLFGIAQTVYFLGVAELLL

GLKKP

>SG-PL_contig_2862_1_-3_3_185

FFNYAANDALISLVRKKIGDGSHNINMLSCSLIVKRKEKLFNKISLIDSFYHCIFKLPLH

D

>SG-PL_contig_2863_1_+1_1_216

DQGASIFQLLIKTQIHLLTRSRSKHFSVINIILYLVHQSFQRSENHVYRNMINKRIEKNE

GVRWRTHPEGMQ

>SG-PL_contig_2864_1_-3_72_767

MDSNDIKIDTSPTGNHRFSFSRTNSTGSIPAGGCTSSASSAHNTDDEDSDSSKTLSYKDR

RREAHTVAEQKRRDAIKKGYDELMSIVPTCHQQDSITSQKMSKATVLQRSIDYMQFLNQQ

KKKQEEDLDSLRKEVMALKIMKANYEHIVKTHQNTPQHGQNQVSDEVKFQVFQQIMDTLF

QSFNKSISVANFAELSACVFSWLEEYCKPQTLREAVMTILTQLNSQLTSSQQ

>SG-PL_contig_2865_1_+2_1196_1432

MEEVSSNFTTLSKFCPQILWWGGGSWLCKILDKDRHSKKKLVLAIEGRGKITMAGKERKK

KNHISSISTTPQPHETDHS

>SG-PL_contig_2866_1_-1_136_876

MAASTSISSHSSFAVLAAIFLLVSFCVVLSAGERVLGEETDEFDFDGLPGAQHEFKVDVP

GGIEDCFFQRVVRGAKLHVSFEVLRGGDRSVNVILRNSRWQVINAHHWKSQGVFEFEVPY

DDTYAVCVDNSYGRFSAKLVYLYIVSFVMADWAAYVKEFEDIHSIADNVTQLLANVDLSV

QDMKKAQTQSRFHLVRDWYLIWANNRYVQNWSIAQCVVIMTTSIIQVVFVRRLFKTTNVT

PTAKPRA

>SG-PL_contig_2867_1_-1_163_375

MLSKLFSVMRHLWSNFVTTTQHFRTMIIMKYVVPLFCASFLFLMVLLFLEQFFYSNHVPC

QNVQCWLILQE

>SG-PL_contig_2868_1_-1_1219_1470

MVIFVMTSKQTVNSHKQTVYTNGTFFNPFAARQKYNYLNIIALKLTIQDGKQLLFPCNSV

SPVMVASLVQSCCLFSCQTFCGRV

>SG-PL_contig_2869_1_+3_54_200

MRVYDTVKSFAVVNKGDEGLCQIKIKSNHIVYSPADHTKAISGLSPRLK

>SG-PL_contig_2870_1_-1_40_219

MYRTASIMPEPPSCAYACRTSNMHVKDPVSHVGVRWLMETQTYPACTLYSPLTRLQADRL

>SG-PL_contig_2871_1_-3_90_251

MLLRHLAWWVKGGDLGLEGLASNPSLASCLVGKGWRFGVGRPGFKSSLGTSPGG

>SG-PL_contig_2872_1_+3_252_428

MNKKNNMKVTAWSDLISHILMTSKKVNKDISFTRSHLKADPVDLVIHLVKRFQISLRST

>SG-PL_contig_2873_1_-3_108_365

MNRGHVPWISFPTKLAQKNQQLWEHNTFISFAMDYFGGHYICISNKKKKKKKRKKRDVSW

FPPQKTKVISHTDWGADCRTRLCQYR

>SG-PL_contig_2874_1_+2_29_220

MVCKPCHIYRRFLQNTDIAVTEHRPFSSLYCCLYQLLVCFIFIFCLSVCQLTVTIIYNSY

SMVA

>SG-PL_contig_2875_1_-2_44_376

MATTLRTFTVRLLGLTRQGLGQTAVAVRHHSEDSSTTKSSKPKRRKGKTPAGKLDEEWEG

EEEERQPQGEQEPLERFPNDINPITGEIGGPRGPEPTRYGDWERKGRVTDF

>SG-PL_contig_2876_1_-2_407_712

MNSKGRHSFKDTSRCGIYRIAVILAQQAYNKMIGLLQSQRTMEFVPLERLQDTSLGKVLR

RTCCCPCSTLSATQVKSKGEGKANTAWSQRRENKMCNVSISH

>SG-PL_contig_2877_1_-3_3_140

TPGHKPVQSIKEKLLLPMQQAHPFHDTGKIQGRGQGQYNLEPVKGE

>SG-PL_contig_2878_1_-3_3_221

PFFSPFFASVLSCVKQKQQLEMLQYYKSYVFSMCLSVSYERFHVSLKVLSYAWMSVFCCN

FEHMCKCACFMLP

>SG-PL_contig_2879_1_-1_70_285

MDIVGEEHNLCPVRYFKFYWDRTKDKRANHRRLFMSVSVRTFIKTSPLGLYPNGSYRRSS

GFINQPIRTRAD

>SG-PL_contig_2880_1_-3_87_320

MCACLYTGREENGDVVHLNGRVSCYYTCQRWPSRPTPRPTSQTLSFQKCSWLECKCYRSK

LFFFFELGNQFLVNLYCF

>SG-PL_contig_2881_1_-2_14_523

MASTLTRRLVGQLSNSFRYGFCRSYAAAASSGSSVTSQPDADSKVTTITVEDKMDITPLT

GVPEEHIKTRTVRIFVPARNAMQSGTYGMRRWKIEFDVRERWENPLMGWTSTGDPLSNME

VTFDSKEDAMSFCEKNGWQFFIEEPKQTSFKKKSYGANFSWDKRTRRSTK

>SG-PL_contig_2882_1_-1_76_1164

MPEFACYLTREQQDELRGIAQAIVAPGKGILAADESVGSMGKRLQGIGVENTEENRRQYR

EVLFTTENALANHISGVILFHETLYQKDSNGKPFVDILKEKGIIPGIKVDKGVVPLAGSH

NECTTQGLDGLSERCAEYKKQGAQFAKWRCVLKISKNTPSYQAMMENANVLARYASICQQ

NGLVPIVEPEVLPDGEHDLETAQKVTEQVLAFQYKALADHHIFLEGTLLKPNMVTAGQAC

TKKYTHEQIAIATVTALSRAVPPAVPGICFLSGGQSEEDATINLDAINKYKGKNPWAVTF

SYGRALQASVLAAWKGQKGNVSAAQAELMKRAKANGEAAVGKYAGGVSGAAGGQSLFVAG

HAY

>SG-PL_contig_2883_1_+3_456_767

MIHVHGYMYFKHLNFDCIYQDTSSNIPFMDTVYFTLNGKMFSEHRTKKAGTKSKTGQAKE

TIINYYSHTAGIKLLVLTCSSYKTFINAFITYSFVRKTLKSTVL

>SG-PL_contig_2884_1_+1_214_378

MYTLAQTYICTHSTYTLYTQKSAGFVLFVLIYTYIHTCKKCAFCTKCRHTLQVLH

>SG-PL_contig_2885_1_+3_189_338

MKFWGFFSCENQCSRMVFSFFSLSIPKMYMCKPHIYACIFTTFPGWKWSR

>SG-PL_contig_2886_1_-2_2279_2530

MKMSRHKTHLHYQMQWENAMPYPQTNAHPLSHTDTHTSTMYEGKNQEGQIQLSVLDSHCM

ACLLLINNIQQKVVRNEYVSRGTA

>SG-PL_contig_2887_1_-3_3_236

EISLAIHAAVDFNPYFDRHTGHSRDQLNYTPSSDFYLSPLVDCTDTAEMSLAIHATRPSL

HTVAIIFVYFFLLILETQ

>SG-PL_contig_2888_1_-1_118_399

MNLMVRDSSCPRSLVMIDFESTKHPDPRYHFATTGYTLTYLPPWFKRIWKLTKFDSLLMT

SDLWASGCVLTTLLSSGHHGNPQHYSCSIWSKKM

>SG-PL_contig_2889_1_+2_89_979

MSVLIGGGSGFVGRHLTKLLTDNGMKVTILSRAAGPNRITWSDLQRSGLPDGCTAVVSMS

GENILNPMKRWNESFKQDVWASRVDCTQTLAHAICQAATPPKVFVSMSGVGYYKPDTSRE

YTEESPGGDFDYLSELCTAWEAAAKLPSTVPVRQVTVRSGVVLGRDGGMIQQIYYPFFFG

LGGPISSGQQWFPWIHVKDIVGIVFHAITNDSVSGVLNGVAPEPATNAEFTKAFASAMCR

PAFFPVPGFVMNWVYGPERGKVILEGQKVIPKRTLESGYKFSFPDLTSACKDLSSGS

>SG-PL_contig_2890_1_+1_340_603

MKTKLKCVNKSTVKFKNDLKKKKREGGTKLFLIKLHRRQEESKQERKKERKKESKKARKK

KKEKKTNIKMKQRMKDQTKSKKHVSPPF

>SG-PL_contig_2891_1_+3_90_281

MCASNFHWITWTSNRGKGAFYSADLLVCENVCMLSFYCLLIIVIVLLQMQAHKHKLLKLL

FSAY

>SG-PL_contig_2892_1_-3_195_1691

MFVFSTCSCRLSSCLLKGASPTMVKRPRLQLMPKRGFRVIYGELDRLPNKVRHYVEENAR

LCKPDTIHICDGSDRENDLITYMLQKDNVLRKLPKYDNCWLARTDPADVARVERLTFIST

PTQRDTVPIPKFGSKSQLGNWIAPEDLDNELNMRFPDCMKGRTMFVIPFSMGPVGSPMSK

IGIQLTDSAYVVASMRIMTRMGGTVLKTLQDDDFVKCLHSVGRPLPLTAPLHNSWPCDPK

NTIIAHLPERNEICSYGSGYGGNSLLGKKCFALRIGSILAKREGWFAEHMLILGIENTKT

GVKRYIAAAFPSACGKTNLAMMRPTLPHYKVTCVGDDIAWMKFDSEGRLRAINPEAGFFG

VAPGTSDKTNFYAMETIKKNTIFTNVAETSDGGFFWEGLEKETDENVKIRSWLGDDNWTK

EGSDRPAAHPNSRFCTPASQCPIIDPAWEDPEGVPIDAIIFGGRRPEGVPLVYESFNWQH

GVFVGAAMRSESTAAAEHK

>SG-PL_contig_2893_1_-1_22_870

MSTPSNLLLCLGWSVFLLNASLAVGHAGRGGGGRRGGRGNSDGDNSTGMNHHGGVPMGLV

ENCDDGETNIEIDWDPSSKEEYTCKGKTLQPLNIDVYSHCERETLQAIHVCYPQKIFYEY

DLPPTSGAHRPNWPEYGEYVYVPPQRWLHSLEHGAIVMVYHPCADPVDVVMLKNIVKNCL

RRHVITPYPLMPQDKPLGLLSWGCKVLMSQTITSVARNFIKNHACEGPEGNVAAGGLYKA

GLKGLSHIVSDFYDTQLCLDHYHGHEQFQDSNLVPERGSSPQE

>SG-PL_contig_2894_1_-1_1_204

CCALFLFFFFSSRRRHTRFRGVTGVQTCALPICALGACGWAESVPSVIKPLLFLTLASDS

NNSLPRLR

>SG-PL_contig_2895_1_-1_199_642

MKHRKHWHSFCGTKDGIRVPENVHVNHDDRLIRVAPEHDDSHAMDMLHEYNRGLIAFKNN

TAKTCYLDRLDETFEDGYARWQGYEEVVHKQNHTLRVFPEKINIQVVRHIADVHIFEHCG

GEEVEYHWVMEVTEEEIRMHPENSYISV

>SG-PL_contig_2896_1_-1_1_255

KHWIERERERERERVFVCVCVCVCRCVPAFFVSDSSLSLLCVRGINFVYVVMCVCAFSNF

SFSISLLSAVKFCVGLLMLVLCTQY

>SG-PL_contig_2897_1_-3_3_74

TVDFMFLKNTYLSHSTLYSYVYMY

>SG-PL_contig_2898_1_-1_1_201

DIFQSNKLFLSQSVSDALLSSYKEGTGIFCPKQLYSYLYILHVLILLLHVYYTSKYSTVN

HCCGPIA

>SG-PL_contig_2899_1_-2_50_3487

MVFPARGVESADIGRIASKHVKVTVSQRSLQTPGRERQTVTGTKANDMNNTMNHVNERDE

TRSEHEDMETDQIDGNNHHHEDDPPGEIVNGEVKEESTDITEAEMEEDDCRAEATFRYTV

ENISKLKETALSPPCMVRNLPWKIMAMPRNSQNADRQKSLGFFLQCNAESDSASWSCHAS

AELRILNARPDTEPFCRKISHLFYCKENDWGFSHFLSWNELLDPEKGYVKDDKVTLEVQV

TADAPHGVCWDSKKHTGFVGLKNQGATCYMNSLLQTLFFTNKLRRAVYMMPTEGDDGSKS

VPLALQRVFYELQFSDKPVGTKKLTRSFGWETLDSFMQHDVQELCRVLLDNMENKMKGTC

VEGTIPQLFEGKMLSYIKCKNVDYGSQREETFFDIQLNVKGKKNVHESFKEYVTVETLEG

DNKYDAGEFGLQDSEKGVIFVNFPPVLHLHLMRFMYDPVTDTNIKINDQFEFPERLNLDD

FLQKKETTQASYVLHAVLVHSGDNHGGHYVVYINPKGDGKWCKFDDDVVSRCPKQEAIDH

NFGGLDDDQSHKHCTNAYMLVYIRESEVAHVLQTVNESDIPESLINRLSEERRLETQKRK

ERNEAHLYMNVQVLLEDDFCGHQGNDLYDNDKVNLRAFKVKKQATLMEFMETLSENLKYP

VRQLRPWPFHCRQNHTFRPTMIDLESDTSKTVMDLADQENPWAVFVETLSPDSGLKELPP

FDKDSDVLLFLKLYNPRSKAISYCGHLYVPISAKAIELVPELNRKAGFPINTPLMLFEEV

KPNLIERLEDLSLPIEKLLDELMDGDIIVFQREDDGHANFTFPTAKDYFRELFYQVEVTF

CDKQVPSDPGFALELSLRMNYDQMAHAVAQHLGCDPYHLQFFKSQGYRDGPGNAVKCTFE

GNLKDLLIYAKPRQPKKVFYQQLNIKISDLENKRQFKCSWVNGKLKDERELVLYPDKNGR

VSDLLAEARKQVELSENGAGKLRLLEIISYKILSVQREDVQLEQLSSGGTRTFRVEEVPS

DEVTIPDDEILIPVAHFHKEIYQTFGIPFLMKIKNNEPFFKVKERIQKRMDVPEKEFEKY

KFALIVMGRLEYITEEEKPINISHFMPQHLHGNNMQARPWLGIDHVNKTAKRTRYSYQEK

AIKIHN

>SG-PL_contig_2900_1_-2_125_751

MLVRAAVLLVLMQTLAFAEKAKGPKVTDKVFFDIEIGGKNEGRIVIGLFGKTVPKTATNF

KSLAVGDKKHEDGSALTYKGSKFHRVIKDFMIQGGDFTRGDGTGGRSIYGEKFPDENFKL

KHYGAGWLSMANSGKDTNGSQFFITTKQTQWLDGRHVVFGKVLEGMDVVRKIESSKTDAR

DKPEKDVVIADCGALDVEAAFPVTKDDAK

>SG-PL_contig_2901_1_+3_3_110

KHIHTHTAEEFIGTYRIRWSNQNNSLLFLIPLASHK

>SG-PL_contig_2902_1_+3_84_398

MSFVRISASLLNALLKSILLIAIVGLVEDCSTPSAEGDATSHRTETTSCIISTPLPARHS

ERNNYSDIKQLAKEMAVDGIITVRGIVSLHCPRSQTKSLCSCHIC

>SG-PL_contig_2903_1_+2_1463_1732

MPINLETPLPKTKIHTSAQKRKPTHKHKNKKTKQTKKKLLKDWFDTIPGTWMTLFPGFPH

PAAILKQQHSGYTQMKMLLASWTRHDVKCD

>SG-PL_contig_2904_1_+2_635_847

MITIIIIFIIIIIMVIGCKLVKQSRHHDNYYMLYIYIYICETNSGESVSDWKCVNKSREH

ELTETDSEQCY

>SG-PL_contig_2905_1_-2_2_151

SVLLYIQVFSWHKIIKSYTFIQVPLCSYLALFRHTCGHFVLWLVPFDSIK

>SG-PL_contig_2906_1_+3_99_383

MVSSSAEIKSSKAKPISLSLTEKPKYDYHSNQIIKRTNNIEKGNHEITNGIKSSVFTYNI

CTHRNTGRCSELASISIFKLGGRGGRKVKHSKVWV

>SG-PL_contig_2907_1_-3_90_308

MISVALLPRHTFRLLDTPPTPMFIQSKSLNQNSLSSRQMGYSRFQTNFLICVGAWTRMND

ECACVCVCVCVCI

>SG-PL_contig_2908_1_-2_164_313

MTFIVVCSKTVLHINTTHCQRVPFSVGGQQPYLVRVACSPASGEQVPESD

>SG-PL_contig_2909_1_-3_48_365

MLLHSLHTVCTLFSLNCAGVMLLHTLHPLCLVLAHTVCRRYVAAQSTYSVSGIGPHCMQA

LCCCTVCILCIWYWPTLYAGVMLLHSLHIPSFLGVHTGCFHVSISH

>SG-PL_contig_2910_1_-3_30_431

MASATIRCLLCVLVLSSVTVFSSQAACTYIELGEIFTCITPYGQQFEDAIGLTSFDNYVC

NTSRITTDICRLFRKIVTCVNGLDVSRENCLLSGTRQVNVRLGIPCSVEDFYAVCKDAPP

IGGLDTTSVADTTV

>SG-PL_contig_2911_1_-1_91_345

MSKDEHEPVLGSFNYQQDGRSDDRICPTTPISATDEQGVSVDQSRLLTTPNIATSSSTSS

SSSSSSLTKHKYAAQAAAIADDVII

>SG-PL_contig_2912_1_-3_93_239

MHARTHACMQTGPVALKLSLFCITEKNVFCGRCAVPNRTNTLLFAHTLN

>SG-PL_contig_2913_1_-2_2_205

FGSWTYDVSMIDMVISRPQDILSDYHPSGKWRAVEASAARRETKYPCCPASYADVTFSLT

LRQREVKD

>SG-PL_contig_2914_1_-3_105_458

MSDTKEEKDEEKVFGSCEGPDAMYVKLISSDHHEFIVKRDHALTSGTIKAMLSGPGQFSE

NETNEINFREIPSHVLQKVCMYFTYKVRYTNSSTEIPEFPIAPEIALELLMAANFLDC

>SG-PL_contig_2915_1_-2_2_187

GRIKEECFIFRCLPFVVALGFVCQVHVDVANVMYGLHAFLCKVYFFSLVVWFFCLIVSLA

HC

>SG-PL_contig_2916_1_-1_19_540

MEGVMLPEPQEISSHPCSCPSHSPCSENWEADRDRVIARELVTDGTFNMTLSMMFCTPVQ

PQRRCSMGEVALQLKGMAHVPTEVDVIHCSCGEHRPLVLHSTRTGLDHLTYSAYVCGNHK

RRCKRRRNNPDTCMEDDPSTQTVTYPCKCTSGTACRPARPYYPYRFICKRPRSG

>SG-PL_contig_2917_1_-1_49_720

MSCLRNTGHVLSKIAVVEGLNLPGPLVSFVRGIKPRWLPKSKSKVFYVRKPTPIDPEEHA

ELRTGYFHYRTQLRAVRKFLMEQHSAKVNLMKEQSATEDRDHADELRLMEEQNNQWNMRV

AAVREAAWLKEREKQEERSSKWMERDQRRKSRRVQKAEERLQRDMEGSGSFITLDNLDME

IEKLLNLRQDYNFAIDKQGNKISGSISNVDSTKMKDSLEQQDLV

>SG-PL_contig_2918_1_-3_225_875

MSKGLCPSKSTIYVSNLPFSLTNNDLHKIFEKYGKVVKVTIMKDRESRRSRGVAFVLYLD

RDSAYRAVRALHKTQMFERTINCTIAKDNGRAPEFIRRKEYKDKSKCYECGESGHLSYRC

PKNLLGEREPPPKKKKLKKGQKGADGDEEDGDRSDEDSGTEESAQLDSLSAAIQYQHQMM

GEEEGHYSLSSTSASDEPRRKRYKKDTYFSDEEELDD

>SG-PL_contig_2919_1_+2_2_73

LYTNANPAESIIQQKVIPYTQYTD

>SG-PL_contig_2920_1_-3_69_722

MLRFRVRMRPRKMSSLEGLGSGPEGRLKRLRKHVTALIRHERIETTYAQCDEARGYAEKL

IQMAVRNGDTHKHTMEMADYWLMEKDLIHKLFKVLVPRYSSLPAAATSIHHISGTYPGSG

IPIGVLELKGNPWPSVRPQPRDTSLHLHNILLHEAKRDFYAERHDTSSANLAASVSASSL

SEEGTSVDGPSDSSVDKNSSPAESKAQKESVQDGDSAK

>SG-PL_contig_2921_1_-3_6_350

MYPQKPSTPAKRSKSGRTEIMIIFNSSQLEDTNGFHVTASCSGFGHLCACPPGNHNNDQL

CNPPALPALVTTPVLLSIGAKGSSPKSLQWHSPTPPIWTPPHPINPTFVQPKFWC

>SG-PL_contig_2922_1_+2_2_307

LSHDPYPFQLYCSVHDQSYHYHYNDQNNQNHFPHCVVHYLLQRLIHVRQIHQLPEELEEE

DSHCQCLVHLHKMNHQTQKHHSLCHLISNREPRGLYKLVLQN

>SG-PL_contig_2923_1_-3_1209_2693

MSNFKRILFLIVTCLACYCGFLCRNYLFSTNQTHQGNDVVSIQDSPAHQPNNAKRHGLNS

SKAAVFQDFALFLNAKDKDIKRSIDLKILKRRLGNISNDTNPKRELVLNLAHPRQRNVDF

VKVGNLYVFGTYLDLRQRGNATVRILTFKHKNHNDSLELRFTDRKQQHALHANVFQMCEN

HGKLYEGWILSYHLPQTLHALPRQISLSPINPRNQNHSHISISFNLKTVPSQTDSDAKQI

GVCVPPLFGKLRTSSIINFILMCKILGADMIFLYLGNISLELKTFLRRFSQDDDGLVSLV

SWNLPENVSDSEDSIWYHGQLLAIQDCLYHNMASFHFLLFMDLDEMLVPQNTTNWQAMLT

EIFKIQQKRSVAALSFRSVFLDPTRTPDSSDAILYFRHLHRTKAASKVRNKLMVQPLKVL

ELGIHHVSKAVGEEYASLEVSQKVALLHHYRMCVTNYEPSMKCYPTRHDSTLLKYKDVLT

DWTHTLVNNYSELVV

>SG-PL_contig_2924_1_+2_2_121

LSTKVLPTSLVIKFHCKCNNFSTDIEHNESNHNIYITLKM

>SG-PL_contig_2925_1_-3_54_215

MASGLAALIENMLAPRDEVSSSLLSIGCSSFGKIFSSSGLFSVFLLRVLKKHFF

>SG-PL_contig_2926_1_+3_3_119

IHARAHTMYPQWSLRGGKKTPPLKKKEMSNCNFTNAISL

>SG-PL_contig_2927_1_+2_245_628

MQLVSQETYKFCLSYMQKKSECKQTFLRKLKKTKKNPLKNKKLQKDITNSNTNSTKLQLR

AKASMCTIYCCFDFFKQFYIRNDCQSCHYQTSFISLWLKPTSTGLDMLNLNSQNSAPILK

PLDCFENL

>SG-PL_contig_2928_1_+1_1_279

FFFFASRKFIFSLNTQTYARYTNCCQSFKKYIAMAYTVMKQFCILATIMPRNQFFHESEE

LDICNSYNMGFPPPPKRKYSSQQYAKESVLKKS

>SG-PL_contig_2929_1_-1_367_579

MVSSQVRITLDQNAKYCLQSTYLFQFSQDGIAMLAINSIRTNAANTMGWKRIQNDNAVRC

TPAHKPCMLAR

>SG-PL_contig_2930_1_+1_1_120

NMVFKCKNALKCVRALLQGLCCDRYKLYCYRLHFKSQFLR

>SG-PL_contig_2931_1_-3_747_2942

MKSWLCWVVVVVCVLLVMEVDCRRRDRRRPGHGRMRQRHRDRAHHAHHRRRGHSLDGWRA

LMRRLEFGYGRQRNWWDGPNVCHSEESDHEESEVNDITHHHKAALQVCEESQTAYRCKTT

VQSPEGQLSKTELYECCHGYARERSDYGCPERLRMRDLITTARKLDLNDLLKAVNTVGLD

EELTRGNFTLFAPVDGAFPLPTGPGLMLQDQHGALMVSRPMLDKMEDDLQAVLLGHLVPE

ARRASSFADEETLETGSPYDSTIRINFYSRPEKLTTANCVKVTSTDNMATNGVIHTVEKV

LPTVTSSLMDIIQSNEDFSFLKTALARANLVSEFQGEGKVTLFAPTNAAFDRLPPRLLDR

LLRGDPKCLEKVIRQHILPHVICSTVITGRAKSRNVLRNYLNLTRTDDDKIFVNDAQVVR

ADVMATNGVLHVIDAVLVPDDALDLVSVANKQGLTELVKLVGEAKLASTLQKAQNVTVFA

PSNEAFQSLPAAMTEQLMTDDRAMLRRVLTYHVVPEGELSSRQLYNDRLLDTLDADHKIR

INEYSSFPFRQQHVLTAQCVPLVNGNLAACNGVVHVLDKVLLPPSGNVVDVLAADSQYST

LVRLLKKAGLADSLQEEGPFTVFAPTNQAFKDLGKERLRSLEDDPEELASVLKMHVFKEH

LCCAGIFNNPWWYQQKLRSLNGRTVFLTRDRRDRPLVNDMVIGLCDNTATNGVVHTIDSV

LLPIQDPWYMFP

>SG-PL_contig_2932_1_+2_2_220

EGRKKRTIVKEGKRAETKRAIEKRKFLAHNFQKMGILFILKEPPASPGGMQKNTIHDITL

TIKILMIYMTGLC

>SG-PL_contig_2933_1_-3_141_932

MAVNVYSTSASSDNLSRHDILAWLNDSLQTSYGKIEELCTGAAYCQFMDMLFPGTIPLKK

VKFNTKLEHEYIQNFKLLQNNFSKMGVDKVIPVEKLVKGRFQDNFEFVQWFKRFFDANNQ

GFEYDPLAARGGEPVGSANNKGALSKAPSNRSVAPKSRVPPSRPAATRQPANNVRSPMSK

PSSNHAGGDSQLVEDLRAELRQLTLEKEGLEKERDFYFGKLRDIEVICQQNEDQEMMKGI

MDILYATEEGFAPPEDDFEEQEEY

>SG-PL_contig_2934_1_+3_36_311

MCEKNHMKQFAQSVKMWMWRRKGWAGERSTIHLTITTAQAGQQVEKEGEGVTKPSPPSTS

KQANKWRRKGRGSLNHHHHLLASRPTSQASYH

>SG-PL_contig_2935_1_+1_79_396

MIETLRGFVRLLLVLLFSSPLFFFACVLFSICVRVAVSSLCNNDTRTANNAAEYRMPSCS

CFQSCRYFLVSGDHVFYRFSFSHIMTSSKFFNLISFSCDLSVVQIF

>SG-PL_contig_2936_1_+1_1204_1485

MPSTNPTHRSCTCLPGLAKTTFILPFTNPKHSPVYVPSVQSYPEHESCTCVFGLVTIFTW

LLTNLSHHHHSAVYFTSNMIYSLSEAKRKVTIYK

>SG-PL_contig_2937_1_+1_1_297

PPLSLSLSHTYSHTHTLSLSLPPPFVLSHTVSLSLSLKLSLKLNVRCSPTQAQHFFSFSR

FFSVSGSRVSIPHSKSHTHTHTHTHTHARTHARTHTAHE

>SG-PL_contig_2938_1_-1_529_678

MFSEVRGGEAVVQQLGRTNHCFTLVTLSLIFLMLLVLCCVLSGVVANCAK

>SG-PL_contig_2939_1_-1_94_1185

MSSLAAKSRLPAGFYKTEIVATKWEVPQKYQRLSPLGSGAYGQVCSAEDNTGTNKPKVAL

KKMARPFQNEIFAKRCYREIRMLRHFDHENIINLIDLFTPNTSLETFADVYLVTGLMDSD

LHDILKYQTLNDDQIQFLLYQILRGLKYIHSAGIIHRDLKPNNIGVNQNVELKILDFGLA

RSMAEEMTGYVVCRWYRAPEIILNWMHYNSNVDMWSVGCILGEMIIRKPLFKGVDHIDQL

NKILSLVGKPSEDFIQAIASVDAQNYIRQLPRMPRKNFKEFFHGANPLAVDMLEKMLDLD

PARRINVEDAICHPYLADYHDPTDEPTAEPFDHSFEGQTLDVHGWKSRVYSEVTSFVPKP

VPVE

>SG-PL_contig_2940_1_-2_2_475

IEVTLAGVAGNEPVKCSRNVSIVPDDSLDAALKKASYDVLILPGGGGGAKTLAECKVVGN

ALKEQESRGALIAAVCAAPTALMAHGIGKGKNVTSYPSFKDKMIEGGYTYSEDRVVQDGK

IITSRGPGTCFEFALKIVENLVSLDKATSLVGPMLVKM

>SG-PL_contig_2941_1_-1_1_564

FETLCRYRSFWNLELKFKPVFLGGIFHARRDGRPPSLNPHKGAYSVIDLQRLSRYFGVTI

QQPQDFWNVLVTKGTLRPLRFITAINSTHPQYTEELSRQLWQRIYVQDTDVTEAASLKEA

ALKSGLNEAVIDDTLVQMTDSKIKDKLKAVTQEALDLGAFGAPFIVVHVDGKKEIFFGSD

RFHFIAMV

>SG-PL_contig_2942_1_-1_88_186

MKSVQKEGLECQKSDDIKAWKVGNQTKEWLGKF

>SG-PL_contig_2943_1_+3_174_353

MACTRKGFNSCQKDKPRRHKKHFLFLLFQYETNALWAKFCDCSRTIFARALQFGRSVIKI

>SG-PL_contig_2944_1_+2_2_70

PCSRENQSQFNRVQQLKQHLSYY

>SG-PL_contig_2945_1_-2_1511_1660

MNRPASCLEFAEICRCTKPCTLSLCQSQRLPVLNLYTHEWYGDWTQRPLI

>SG-PL_contig_2946_1_-1_229_1728

METRKRILVGIALVIGCLDMIIAGSIRSFNTYINAIMKTFNYTQTEMEVMGAIINFGVTL

GFPAGLLYERFGGRVTSLVAMLLTGGAFSLAFSTTFSKTFYSQDNRAGLQDFYFFLGGFG

TVFTYMAAMMTTVMNFSLAHRGKVIGILEACFSGGAALMTLIYGEFFIQGHINDEQKQDL

RGFYLTSAIAFIVTNFLGVVFLGVYREPDVITLQKWSSRSALVNNNETDNEADDKQTVGI

DLADISPDVGEEDVTGWKLLKEFDFHFLTWSYIFCTSLQFTFLLNITTFLQSNHLESQNT

LLTTLSFASTSLSKFLIGLLSDLIVHRVPHVAITLVTTAVQTALLAVCIFKGDRFIVLLI

ATLGVSISTGATWCLTPVMTSEYFGIRCFGRNWGSMMLGSAFMGLGLQKMFGALYEMEIT

VPVSTDCYGLRCFRWSFVVLAGLSLCSCILYVGLLQRRLKLRKWRRLEKETKKEQDCQLQ

RLIPPSVPDANSNKVVNKDE

>SG-PL_contig_2947_1_+3_117_314

MKQRSKKDSPHKILHLLNNLPCAQTTARLPGGPTNSQSKNARVGHNRWLGGDAESHSLTR

KASCVN

>SG-PL_contig_2948_1_-1_64_594

MPAYHSQYTNPPKLVGNMALLPLKTTYKGPASKEMGDADIIDEAIYYFKANIFFRNYEIK

SEADRTLIYITLYISECLKKLQKCSSRNQGEKEMYTLGISNFPIPGEAGFPLNAMYAKPA

NRAEDDTMRAYLQQLRQETGNRLLDKVFDSQTDKPSKWWLCFAKRKFMDKSLSAPGY

>SG-PL_contig_2949_1_+1_1_147

FFLKKKKKKGNFPKFSPPPPKDTILALKSTFQVKNLTLNYPQNNVVKVL

>SG-PL_contig_2950_1_+3_30_134

MKQKIKIVPCLSLNLVIRGTKDKDCVISLPKLNYS

>SG-PL_contig_2951_1_+3_216_470

MVYHARPYIFIYSSFIYICYLFVYALGCQTVNIQSHRSRSGREPSFLQPCSYDASVLLHP

GNVVSDGFQSCRKSLCSCSQIVVLV

>SG-PL_contig_2952_1_-1_1_117

QADRRKNKMKKERKAYFLAHTALTLPMSNWPSTDLLTWL

>SG-PL_contig_2953_1_+2_788_1051

MCTLFYNGNGLVGTHLVHTLNKISPYQTEGLQVPMAVNKVQQHLAIEHGTRAHTTNFEIT

VLQEDTFVRSLLQQFHRLWSLNQYSCQK

>SG-PL_contig_2954_1_-2_29_829

MAASLKDIVVMYQSLKREWDKKPPNLERCSETLTKLKIALTGVTFLPTDESNPSKQELLI

ARDILEIGAQWAIAKQDIPAFERYMAQLKCYYMDYKDDLPESTFKYQLLGLNLLCLLSQN

RLAEFHTELELLPVKELQSNIYIKHPVSMEQYLMEGSYNKVFLARGNVPADNYNFFIDIL

LNTIRDEIAGCIDKAYTKIALSEAARMLFFDSQKPMLEYAQKRGWKLSKDNFFHFKVEPK

HTADEVIPAKVLAQQTIEYARELEMIV

>SG-PL_contig_2955_1_-3_3_116

CVCYQTLSLCGQSLSLAHTTHTLTSFSQLLKTQNSKFV

>SG-PL_contig_2956_1_+2_356_637

MVTCVCVCRRVCVCVCSGVCVCVCVCVCVCAGVCVRRCVCAGVCVCSGVFVYVCVQVCVC

VCVCSGVCVCVLSLPMVNILGGGVLLAFFFFFFF

>SG-PL_contig_2957_1_-2_167_1528

MSDRAITRKPRSRSGSAASNSESELTLGTSKSGSSTGQTTGDSMSEHCIASTRQAKHHQV

THSHWPSVAEKGAIGDGVYQAQAQDAAGPCMDSAMSQEDSAEGASVSTSGSGQQSKYSDP

VFQKLSRINGEINRMRKEELRKKLAELHLHNGGVKEVLKKRLKNYYRRKKLTAANISRGT

ENMKFDFLVVIDYEATCQDNNDRFVHEIIEFPAVLVDVQKRQIVGMFREFCKPTLNPVLT

EFCKQLTGVNQAEVDSASEFPVVLEHFERWLDSWELGTNHSFCVITDGPWDIHRFLSKQC

EISQVPFPKWGRRWINIRKSYGNFYDTRRLSLNNMLLNLGLEFEGRPHCGLDDSKNIARI

VCHLLDDGCVLKVNEKMDGHINCPQPACLDTGDGNPSGDFKPLSKKTVSRGVHTNCPTWS

SPDSEKPKTVKELSGLETDDDVASLLFYFKLQST

>SG-PL_contig_2958_1_-2_515_778

MRLWVFFFITKTYLCKFIIPILLMPCVLHLVCVWRKLPRSQLFICHSAVFMEMMLEYQLL

QRSLVILKGLWLVFPLIAEKLNILIKKI

>SG-PL_contig_2959_1_-3_87_650

MMAAMDESQYAQVLFNIGKDVFLTHTDVECTVEVTKSLEPSKWDWVGLYNVGFKKTSEYI

TFHYFPIPADYKKGQSVQVRMVFSAQHLPKENGYYQLCYVSYEKRMICGASNPIQITDVS

TKDFVEKEEGDMMIIRSRADVMQEEIQGLNHRMLLLAQKCETFQEEIRQGEETLKMRDGL

ISALQREI

>SG-PL_contig_2960_1_+1_661_879

MLKSRKRKSCASHAEYKRNTFILSITTHVYTRTCVSVVVHYEKKGILCTSHSIGRVKMPM

VILGELGITDITS

>SG-PL_contig_2961_1_-2_2_265

MYSRCCLRTLKIVLPRARTSGSSSSETTGGGGGGRHSRTEIVLGAAPLFLQANHTAMSEP

KKKVAAITEQLLVNHLIHHLKKESRIKA

>SG-PL_contig_2962_1_+2_281_421

MHIVPWLKKLVEKSCLAQSAITHSLVFIWLAYLANFAIYLLKMHRET

>SG-PL_contig_2963_1_-3_24_287

MVCWPSVVVMWSKVSCVALSCLCVVNREHYSHPCNMLSTVPCCIAASCLCVVNKKCYTII

LVTCSQVMASGDGSQSGDDLSYTFCIGC

>SG-PL_contig_2964_1_-1_523_762

MYRVYQVIMQSPIGKVVSVYLCFNVQTLLMKNCLCVADGKLLALEGFCSFVGLYFVQNIL

HFYSKLSLYIGVLTALAAFY

>SG-PL_contig_2965_1_-3_3_338

KFKVGDLVWAKLKGFPLWPGKVGIPVPEVKRPSKKSPHQFVIFFGAENYAWVPEEGITPY

KKLRDKYLEGTRQTKNFKEALGCIETAWQALPDAVKNAPFPSVDEDDAQVIP

>SG-PL_contig_2966_1_-3_21_233

MSSFFLFVCFSERKVSLHQLWKERDNCDGREIKDLDEKKIDITRFILSTLVEQTNFLSEI

DQALSYNTLFT

>SG-PL_contig_2967_1_-3_42_689

MTSLASGVTVVEDTGSSLKITRTIPEVSFDGENLTVTRRVPLGSQGQEELPFDGGPGPNM

FTANRPAAPPQKKKFQPTPIKAPSPPKNTVKTGGPTLGTIRPRVSSAPNVPTNIRTPMHP

PNTTPGQVRPAPSLLGDTNPRFKIVKNQTGPVSAPVTRTLQNLPQQPSRLKPPPLTSVSV

PPSKQHNPSAQNRIQQQAQNAGSALARINQQPQQQP

>SG-PL_contig_2968_1_-1_109_2928

MPLRLDIKRKLSARSDRVKCVDLHPSEPWMLASLYNGNVHIWNYESQQLIKSFEVCELPV

RAARFVARKNWVISGSDDMHVRVFNYNTLERVHQFEAHSDYLRSIAVHPTQPYVLTSSDD

MLIKLWDWDKKWSCTQVFEGHSHYVMQIVINPKDNNQFASASLDRSIKVWQLGSSTPNFT

LDGHEKGANCVDYYTGGDKPYLISGADDRLVKIWDYQNKTCVQTLEGHAQNIAAVAFHPE

LPIILTGSEDGTVRIWHANTYRLESTLNYGLERVWTIACQKGSNNVALGYDEGSIIIKLG

REEPAMSMDSSGKIIWARHSEIQQANIKAMGDQEIKDGERLPLAVKDMGSCEVYPQTIQH

NPNGRFVVVCGDGEYIIYTAMALRNKSFGSAQEFVWSNDSSVYAIREGSSTVKIFKNFKE

QKSFKPDFGAEGIHGGNMLGVRSVSGLAFYDWESTDLIRRIEITPKNIFWSENGELVCIA

TDESFFILKYSAEAVEQAKDDKEKVTEDGIEDAFEVLTEVEESVRTGLWVGDCFIYTNSV

NRLNYYVGGEIVTIAHLDRVMYLLGYIPKDNRLYLGDKELNIVSYSLLVSVLEYQTAVMR

RDFETADKVLPTVPREQRTRVAHFLEKQGFKSQAMAVTCDPEHKFELCVQLGDFRAAYNL

AKDMQSEQKWKQLAELAIRKCEFGLAQECLHQAQDFCGLLLLASSAGNAEMIGRLGNTAK

ATGQNNVAFLSNFVLGKCDECLEILISTNRLPEAAFFARTYLPSQVSRVVSLWKQNLASV

NKKAADSLADPTEYENLFPGLRDTFRAEQFAASKQTGPLPASAFPTVPPNWERNLIEEMQ

QAEEAGDFIYIPPTEKDGEEEEEFADASGGAGQSPVQKPPTPAAAPTPAAATPAAQASPP

APQEDVASGIQSVMDDIEKELELDLENVKIDDIDTSDVNL

>SG-PL_contig_2969_1_-1_1_219

QTSTFNRISLGFLCLLPHFRLLSNKHADTSSDSCITPTPREVRVGLHTSLDPVSTHFPAI

APAGSFSPTSARR

>SG-PL_contig_2970_1_-1_1201_1503

MAITLSIMMILIITKLIIMITIMIIPIMTVANGMKIKRDGRWKNVFNCTFILFFLPHRYH

HYHHPRRHCCHHYNHHHHSSHHHRHHRMIIAFIIKISQYTI

>SG-PL_contig_2971_1_-1_85_543

MAKNTGASKFRKVDVDQYDDDKFVDENEADADSSDSQGANDAEVSNLLAQGKHQDALKLV

LTSAPVASKNQAVKDRAANTVVRVLTSFKTADIEKAVKGLDARDVDMLMKYIYRGFESPT

DGSSAVLLTWHEKTFAAGGLGCIVRVLTDRKRV

>SG-PL_contig_2972_1_-3_336_2393

MKVLLLLLLAAVGQCALLGSKKCTYGPSYWCSHIRHAKECNAMQHCMTTVWKNQKLDIVG

STETCFSGIQVLDVVRRSNDDDIMRQMAIGCTGLPVEKRAVCKELMSHDQDDVYRLLKSD

LSSRQVAAALGLCGGFPDTVPHHGVAEQVTGDYCTDCVAFMGDLRDIFNKSEGEVAAMIK

AKVCSKLQALESVCDQLVDQYIPQVYEWLDKVMKPHDICLNLGMCRNQTHAELMKDIKNL

MAKVGSVECDLCKDFAGVIKKFLGDQNVQTKIMDFLENDICSRLPGSYSTECKSYVKQYA

PFVFSVLVAELDPETFCQDLHLCNSQTHASFVPKHQVSGAECDVCKTLASDAQDLLRDGT

LQKDVLGFLENICQYLPGSLAAECKTYVAQYGPELFDLLVAELDPNTFCEKLGVCSGPKM

EKSKVIHVPFSSLIGHFLLRPENKDKTVSGAACVLCEFVMSELERMIKNNATEKEIEDAL

QKVCSFLPSTLRNECDSFVKQYAPMIIKLLVADVSAEKVCTLLGLCSSVTVQTVKPVKPV

KGMVECEICEKIMEAIKSALTEKTTEDELEHIVEKVCNLLPSSVKTKCDGIITAYFPAIL

NMLEQKIDPATICTDLDLCSAVTKTPSESHSLKFQPMLDLQPAKPHLLGTNECLYGPSYW

CASVENAKKCGVRELCITKYGMKLEN

>SG-PL_contig_2973_1_-2_374_643

MAVVHSPVSSWYSLLTTWAANVCNTKMNSLFCSQRTLRSVWVIQKIDYLFCLWGNPPRML

QNLQKAEKGQCCHGSTSTLHKGQCKIFSGQ

>SG-PL_contig_2974_1_+2_110_319

MITQQKLNPFTPSPYYYYYYHILFIAQLTIKAISGLSPRKIHQSQEDNRVDHKTSSNLKT

KILQLTEHNS

>SG-PL_contig_2975_1_-2_776_985

MQLHNTKSSNNFEIHCSYIVDLSHRQLVIAEWSDVMANHDKLNAIASPFFNLEVSLALKR

RKNSSLTGKF

>SG-PL_contig_2976_1_-3_3_197

KMIRGRNCFNNENSRCSSALPLRSRAILVCEVQRKDFSVVFNFKVDSYPTDDQMNMLINY

LVNQV

>SG-PL_contig_2977_1_-2_353_1123

MATSSTKDTEKQPLTQGPSESSGSAGRQGVDCCLRHINTISRLFGLGVLAVLWAATVMTL

RGDINTPTYVGYYLLAASLVVTFFEATWILDKMACCVRQGCCCRCWSVILWVDGWRKFVL

YTALTVPLFLQGIKGIIDIIGGFCLLVLACLYLVKSFASLRTGQDDRKPVTKLQQVTVTT

TSVRHEMATQTDATAASTSSSTEPKEVPSNPFDEEDDAAAHEEVEETKADEEKDEKGGGG

GGGREKKEKSDPEKPVK

>SG-PL_contig_2978_1_-3_3_284

PFFFPPRGGGGGTNEGNYLFQSSANSPLTQGESGMQRNVTRSSPRGRGPLGSPLPYGRTR

TSTRSAPGLLVSSPEATESPLPPRLALRTSTDGT

>SG-PL_contig_2979_1_-2_2_259

VASCPYRSQCLYIRCLLKDVFVVYRWIFIKCGSHVQSSKESSQNGHSLCLFSQVHKLCLC

SSSSSSPINVRSLGGLIGSSSRQDPP

>SG-PL_contig_2980_1_+1_1_186

YTHEGGLGTSRSAHMLTWEIGKISTLYPPGEMPEEDSNPGLPKPPVWKSSVFTIRPPRPL

AA

>SG-PL_contig_2981_1_-3_3_236

VLFRSVGKRIEGYGLGSWRLLQEIREETNVEDPFTLIWKQVFMKRRGLWSGLGSTDSWKL

QKEIRKVTKKKMFRIHLH

>SG-PL_contig_2982_1_+3_1968_2483

MTPMTLLAAKGWRRAVKDTFSVRCVCPFHSLAIPGSSPVGEEDVTAMAGKEGRKKCWRSH

EAHQAARDVEDGGKAGCPAVWWSRSHPRCPALVQHRQIVRITRFFDYIVHLFRDRETLGG

VWVSPIQFHLDPSPHPHRVAISLLNSFACAGITGHLVFLGTSFLTVLWTAQH

>SG-PL_contig_2983_1_+3_162_461

MLRHNRRHSKPLRHPNQHRLFISHNPEYATDIRRSSLSRWISELISEAYRAYGRQLDSSQ

AKAHEVRAWATSAAFQHNYALAEVLDAAYWRSPGTLYPTT

>SG-PL_contig_2984_1_+1_1_147

KCENKNRKHVKQTHQAPPPPPSPEFFFNKKYGSLVSTAKTGDAYLAKCE

>SG-PL_contig_2985_1_+2_2_148

DLFFLNTHHFNPLLSQSNSGPPYLQGNTFHISHRSVDLQWQQCTSEESK

>SG-PL_contig_2986_1_+3_654_848

MLIKFEAVIIITPKKPLHLKHRSFKPCTTILIYEGKDFASCNTFYAQVTRHCYLHAKKFS

KFFLF

>SG-PL_contig_2987_1_+1_100_486

MDRGCGSRLTAGCSRPTAGCCDVTAGCSKLTAGCSGVTADCSRLTAGCSRWTASCSKLTV

GCWTMVGVSYCRHCSVLGSFRYGMLIAGTKIQFALPQLLSVCCLLLCSCSVPCFPGRTAT

IVKRFAMAD

>SG-PL_contig_2988_1_+1_82_210

MFLNKTLANQFWIKSLDKQRDANFFKTERECPKVNIIFKHCKH

>SG-PL_contig_2989_1_-3_3_92

CLFVFHQFVELERSILLPVCVGTQRNLTNQ

>SG-PL_contig_2990_1_+1_103_426

MLLGVGRNTGSLGMLVLSSNMSYPESSSAVDLSNNSGSNTTTKATLHIFLLHQRNVNSSR

RVILPVPTGTSAHLTTLSHHSDKLLHCRPHQGRRSYVMPTHRNLSPLA

>SG-PL_contig_2991_1_+3_321_434

MCEKVHDVISYSMNPRYVQATSLNMTSDSGQGGTGCTE

>SG-PL_contig_2992_1_+2_1151_1417

MLTIRHGTVYEAKLNFSRGPALEGRCPSPFPYRFTFPNFEESQVPIHSWVNWRPHKHVKF

PGCDSHTCKLVQTDWKANALLLHQPPQQM

>SG-PL_contig_2993_1_+2_1151_1435

MCTYIPKTRTHTYTKCPSLFNSLFVSFHRSLQCTPHMHTNIHVYIHTHTQRSTFFFHFLH

FFHLICLVMIPFKVCMHAHAHAHTHIHRVTYTYTF

>SG-PL_contig_2994_1_-1_52_621

MEYKNTANGVFKFYQFSATRNQRLKELNSVLTDDDFISLKQPCSVRWLSLQRAVDAICQN

WPAIVMELGEEAAHQKNAVADGLLRQVHTFSRSDMTHMLSDILAVINRLNLVFQKDSVTI

STIRPMVQSTISELDSCLHTRLPKEEKFMADISDKKWCDIKMAHYSEQAVGTHERMREEF

ILHLINSLKA

>SG-PL_contig_2995_1_-1_1_150

FQFFFFWGGGLWVFSTCWCVSRVFISMRFTTVFLPLKRMESEKLFSQLSL

>SG-PL_contig_2996_1_-1_883_1164

MYNRKYFKNVCCCCDRCGQNVCIIPVTLLTLLVCFVQSDTHCQTNTLIFNADEISVVLIA

TGICRYFKHCCTTEHDDLLRITSNSSLCWVCQCS

>SG-PL_contig_2997_1_-1_1936_2229

MQLIALKLPLRENSKYTDVCMPFCCFLFSICVSLFGIYFLCIGKDFRRLVEENKMCLIFL

ILCLSPVPYHAEFQSPKVLWVVMFVCVCVCVCTCTCLC

>SG-PL_contig_2998_1_-3_3_110

MYLVVLTTLKTFLFFRFFDGIRNLIQWMRRSHYSWV

>SG-PL_contig_2999_1_+2_131_235

MQYEIFSFHCEMVNETLMLHSISPLQAFQLTADWQ

>SG-PL_contig_3000_1_-3_693_899

MYTKKERKEKNQINPAYLILPHISCTKFDLYSVSQDHSAFYLKMVIDQSVNLSTMVALLQ

EHKAQFDLY

>SG-PL_contig_3001_1_+1_1_267

SGSGCSGGSGCSGSSRCSGGSRSRGSGRCGGGGRCSGGGRCSGGGCGLCCTYSRRWRSSS

CGGSRCSGGSRCSGGSRCSSGGRCSGSGC

>SG-PL_contig_3002_1_-2_284_598

MVYHKRSERSRYTVLAGSCRMLTIKMNVADVIGLMTGRTDQTDTKHLVFQPWSLPYMLTK

QTEAKQYVFRPSRDLLQANKQKGVITLNVSAPKVFYRQTKRTNRK

>SG-PL_contig_3003_1_-3_180_2135

MIMDGLDTLSQVALADVSSVCFDTALALTGASRHASESLEDSLNTPVTTGSESTFFGPDT

LEPPPITATANIIASINIPDAKTMEGMLPSYRPQMSTGYNNSNDSSNSNNNGSNGMTHTI

TYTGTLMTAPMPSAGCNMNESGTGNPNQPNLAALFSPLSPLFSALMNSPASLTPGQLLQQ

QQGMSLPNDYQQQASCEFSAPVCTTSDSSSTTTHFPSSSPSPASIHSPVSQMSQPEHYSQ

GFGSPLTPHSVHSPESQLGDLQEPVKMEDNMGGSYSSPPPPPPYSQSVPMPVSMDMDGMG

VMKQPPTYSSCPQQQQPMPPQQEVNNFLGMPPCSLPETILQMQISDLGNNNNNKQTLNAD

IRWTVAPTSNMPVMPQLPNFHALQVNTPQFPMTGPIKTEPGVDPMEQNCYMMGDSSSNCM

NFNSSGSCKAASMAMLNQPYQQNHNNLKLLPVKPRKYPNRPSKTPPHERPYACPVDNCDR

RFSRSDELTRHIRIHTGQKPFHCKVCGRSFSRSDHLTTHVRTHTGEKPFSCDICGRKFAR

SDEKKRHAKVHMKQRVKKDAKLLATTASMPTSSSSAASSSHSHTPCTVMTSMVTPSLQAS

AMSASAHSAAGTLPLVVDSTGLCSVSGLESGLGVSDASHVVPSLPLVVTSSL

>SG-PL_contig_3004_1_+3_549_665

MEEKTNCSVLVILFQKSNGCFPLKAVLKLWNFKLNNIIP

>SG-PL_contig_3005_1_-3_75_1355

MSKRKPASELNDQNWDLEEEPEEAGTFRLADQDVLKNRPIRKAKRTLPPGSSNSGSVFAG

FALKTSDSSVPKPSLSTGITHNFGSNFTFQPKSNGTVQQSSGASSKDSSDNPEYLHQLKV

LNENILSWIQKHVGENPYCILTPSFKDYEKHLRDLEKKYSIEGSANNSQDKSSSTDPPVA

SKAKVDTASIFSAQSKSMGGGFSFGQSSTKTEVSSKSAGSPDKTATSVTSTSGFSFGSGL

TQNTMGGFGASSLTQGKGFTFGASTTAFSFGSAAKSAADSSAAPAQASDGKAGDGDEEYE

PPKVEVREIKEDGAVYTKRCKMFYQKDGQWTERGVGNLHLKPTEDGRTQLIVRADTNLGN

ILLNILLSSAMPTKRQGKNNVFFVCVPNPPINPKEPGSDPVPMLIRVKTTEDADELLAKM

DELKSSA

>SG-PL_contig_3006_1_-3_3_143

CFVDMSVVMFCIKLSCLVDMSVVMFCTQLSCLVDMSGYVLYTTVMFS

>SG-PL_contig_3007_1_+1_1_81

PTTDTHTQIQCFEWDWKNKLLNSSMIH

>SG-PL_contig_3008_1_-2_2_145

SSVPPRSLASTRSSSRVVVSLACSAIATSLLRLEFPPFRLFLLMDTFR

>SG-PL_contig_3009_1_-3_3_209

ISTLRGNPADLAFLQSALSPAGFYRGFLVLIQDPEDPSSFSLSPSSTILDRSDPGHLPSP

LPQIYLYRT

>SG-PL_contig_3010_1_-1_55_300

MGRHFGKLTKVRNIITYSLSPFEQRAFAGAISKGVPNMVRRFTGQVFRVVPPLFLCYLVF

DWGRKEHARLQRKAAHAAVTES

>SG-PL_contig_3011_1_+2_125_298

MRLPKARTTRYLKTLLFHVQYLLSVVFSRMNTQGAVYVPCLHMLGCVCVEVEGGDRVY

>SG-PL_contig_3012_1_+1_1_2451

QTLLTPGKLHLLLAELDNTGIDITGLCETRWSGEGKFTTGEHTIVHSGKKRGLYGVAIIL

NKHHAAALKSYDTISDRLISVKLNTKPVALNIIQAYAPTSTSSDEEIEQFYNDLQTAKDK

IPKREICIIMGDMNAKVGEGEDRESGIGPYGLGIRNERGDMLASFCRANELDITNTFFKQ

PYRRRYTWKLEKTDLNNKTTKIRNQIDYIMIDKTWRTTVLSSKTKPGADCDSDHHLVAAK

IRLKAYRQSKVKPALRFDLEKLKCPDIASDFEIEVSNRFEVLLRDWTLHDRYPEEIWADM

KKTYIEVAEERLGRKKSKPSKPYISEEVVELAKQKSQARKSGRKEEYKKLKRMIKNKIRR

DKKNWLEEECAKINEHNEYRRSRELFQQIKKIQSTKFQSRNECINSKDGITLTEPEHIMN

RWNEYGASLFSINGPQDNLYPDPNCPEPEPEPLLSEIEAAVKQLKPRKSPGLDNVPGELL

KHAGEGGLKAIHHLCCKIWNTHQWPTDWKLQEFVMLYKNGNVKDCNNYRTIALISHASKI

LLIIILNRLKPKIEEELSDCQAGYRSNRGTTDMLFVLQILTEKIKSTDEEVFITFIDYSK

AFDSVIHLHLFNTMVKMGFPKHLISLIASLYKDQKATIRWNSRKCELFNIHKGVRQGCIL

SPYLFNIYTEQVMRDADIEDMGIKIGGKNLTNLRYADDTALLADNITSMKRILYRVDAAG

QKAGLNLNAKKTKVLHIGGRVTENNQNILINKVPLENVKEFKYLGSIKTNDGTCAKDIKT

RIGMAKQKMVQLNNIWKDRGIPNDLKIKLLKCLIWPV

>SG-PL_contig_3013_1_-2_2_442

AHRRYLLCVWKDRTFLQDPSFRPSLAPYLFTIVFRELASLSPGVLSPPLFLDNWLILADS

YDLCRTHTARVVDLCTQLGFVLYPEKCDFNLAQSFHFLGMSFDTLRWSVAPTPERLEETL

AGLLAAPFSSARSLASLLGTLESLAPL

>SG-PL_contig_3014_1_-1_13_870

MVTDQSREEDTTFLVQQIQHSYAANRRAENPLQLYISGIGGKAKQRLDSIGDYKGWDMHK

EEQNFTELFTKEEIVYLSSESPNVLDTMDDSKVYIIGGLVDHNHYKGLCHRLAEEKGLAH

AQLPIGQFLQLKSRKVLTINHVFEILLRFTETQDWQASLLTVLPQRKGVCLKDSKDKEAD

TNNSEDASRDSPQANGETCATDETKQTLSSDMSENKDVPVSDLNETKEISTTGGGSSAAD

VSLTEDVMDVESGLEVCEAAGLNALRKSETGSDVAADSDGVQTTTL

>SG-PL_contig_3015_1_+3_333_566

MCATVCACAYMHVVVSFIQCLITHSNIRQTSMCVCMYVRVGVHMYIMYVCVCVCVCVCLH

ARSFTFDLFFLFFFLNLL

>SG-PL_contig_3016_1_+1_175_2619

MKRTSQTFCQEEADPPMNLLSPKTASRIGVWNIRTMYETGRAAQVAREMDNYRLEILGLS

EVRWTSSGKVTLASGHHLLYSGPPEEGGVHRNGVGLMLTKKASKSLMEWEPVNERILTAR

FFSKFQKVSIVQCYAPTNVAEEDTKEEFYQQLQAVLDKLPRRDVIIVMGDMNAKIGSENG

GKEHVMGTEGVGTINENGELFTNFCEMNDLIIGGTVFPHRRINKVTWRSPDLSTENQIDH

FTISRRWRRTLQDVKAYRGADVGSDHILLVGKLKIKIARNKELGQQRHPRFDTTKLKTEE

KKREFAITLNNRFQALADLEDGSLEKKWERVRSAFDETSQEVLGFRKRQHKPWISEETIR

KIEERRETKQREIQARTRAQKQHLQQEYSKRDKEVKKSAKKDKRDHVEGLATQAQEAAEK

NDMKTLYNITKHLGGRKTNTNRPVKGKNGNILSKPTEQLDRWKEHFSELLSGTPVKTPPL

IEEGEDLDVNLGPISKEEIIQAIRKTKNGKAPGPDNITPEVLKAEATITADILMDLFQEA

WKKEEVPKEWKKGYIIKIPKKGDLSECKNWRGIQLLSLPSKIFTRIILERIRTAVDEKLR

DEQAGFRTGRSCVDQIATLRIIIEQSLEWQSPLYINFIDFKKAFDTVDREVAWKILRHYG

IPPKMVKIIQSLYEDTACHVIHNSSVSDPFTVNTGVRQGCLLSPLIFSLVIDWVMRTAMN

PPRGIQWTLIQKLEDLDFADDISLLTHTLHHMQEKTESLRSTASNTGLEINAEKTKTLRT

NTSQAETIRLGEQQIEDVDKFTYLGSVVNKTGGTD

>SG-PL_contig_3017_1_-1_247_555

MNSAAGSNWSALVWTHPKILKHNLYRDRKREGERGWERDYSSPVDDLGPIQDCTTKRQKK

ISIFIIIVINRDKLQYDTSSPHLRLLSRLGHRPSTNALHPPRS

>SG-PL_contig_3018_1_-1_367_945

MQRDIKCVVVGDGAVGKTSLIMSYTRNQFPGEYIPTVFDSDDVYVMVDGLEYKLSLWDTA

GQEDYDRIRPLSYPNTDVFLVCFSVISPASYENVRQKWHPEIHHYSPSVPILLVGTKTDL

RQDKGLVGKLKEKSLEVVSKEKGSALAHKIGAQKYLECSSLTQKGIKAIFDEVIKTKLTF

KPKPSKPNRCKIL

>SG-PL_contig_3019_1_-2_437_718

MDNVWSSTMNRQHRRATALHNHMTEAHLWATQHTLCPIHAATQSMVPITPNLSTHPLPTD

PPSPKSTVTPYAYIPPFSLPYHLYSACCSPSHFN

>SG-PL_contig_3020_1_-3_3_134

GGATTVNMCNPSYTCVDENGTIFLRGTNVYTSSDGLKKCYLICT

>SG-PL_contig_3021_1_-3_333_455

MGNTEQHSEQHSLWSNYLCVCTGPIMCWQIGCFEKFDYLYL

>SG-PL_contig_3022_1_-2_2_274

ERERVCFVCFFNGKRGNFVTMFINFNVILKSLLERVIVIYLIKTSLIPSSRDVVIHSFLS

VCAAMYNNVCGDCLVFKLCKRKTALKVTRKE

>SG-PL_contig_3023_1_-1_1_135

LQFSFVAKYILLSLEKQSTYIFFFLTEKLRIKTECLRLKQVTKEA

>SG-PL_contig_3024_1_+3_3_212

ILGLMVSLFTHTHTTSYYRDLSGRINSLHSLTQIHTLSLSHTNTHTLSLTRIHKHTHTHI

HTHKANTKHT

>SG-PL_contig_3025_1_-1_94_204

MQKQRQHTAWFILSSGCPQALGMMYFEQLCHVISVNL

>SG-PL_contig_3026_1_-1_670_891

MTGFFYRILPVTAPFPASIHRIHQQDPTTGQELSLTLVRFDYEPYKIVISLLTVGLLVWD

LSCALGVCCTRDLS

>SG-PL_contig_3027_1_+2_938_1171

MSRVCFVFFLLFFFPPFDCPFDVLKRPVKFTLLVPVDHAHKRLTNKQATGEATVIVTKAV

TTQLHLFPILSSGFLRHN

>SG-PL_contig_3028_1_+2_239_826

MYLFMLVLISCCPAFTPKNWHNSALCPLVTTLTGTGCVHHRSLTESMNDTFIHGSIFAFK

GFGRGCPRLLLQAGKPHTGQANPAPATADESWSESDAEGSQFFCLFLFSSNLTIHFSSKV

EVAWWIVPLSRSLSCPLAVFSGTGGEGLFQGVLISLSWCPSVTVFNKQFTIQSDLWLSIV

FRPGDVSCLRHLVVQH

>SG-PL_contig_3029_1_+3_2799_3095

MVELDISLHSQAITNIIHYKMVKLHTAFIVIKPQMIHTCTATNNKVSYKLQIIHISHAFF

LTLTHKSAKKTVTHQEGTCVPTRNTEDNTAHQGFTGCKS

>SG-PL_contig_3030_1_+2_2_118

LKGKKKSSEVGVWLPTWRGTKKQPYTQSSHPNVLVVSLL

>SG-PL_contig_3031_1_+2_2_169

LDGTNICVYHGTFFRYILSVICLPSSAIVGLSLVSCDVTVSVVYRVHQVTLGVGCM

>SG-PL_contig_3032_1_-1_130_252

MSDLWLLILKLTIARQMRAQHMLHRGTTISGRKWKKRALVL

>SG-PL_contig_3033_1_-2_2_295

GRIKKKKVLSYTELRHKTKEGNYYLGVAIYYFWLNMLYFGLVIYFLKLNSYLGMKIYYLG

HDFSFGAEPLVYLMKHLFFWIGGCPPFKDVIIFLCAFC

>SG-PL_contig_3034_1_-2_53_553

MAQINGYKEELQDELQKTDPDSHASRLLAQELERLEKGEDRKENKSVAFIDVHPSQPQNV

GLRVRIPVREFPRFNFVGKLIGPKGNTLKRLQEETGCKMAVLGKGSMRDKTKEEEMRKEG

GKYSHLNEELHVYVEAFAEVTDGYRRLSHAVAELKKFLVPDNNDDIR

>SG-PL_contig_3035_1_+1_133_372

MMYSYMVYWTSHNIQVKKPGPYPKTNCSKCTLFQGTAGTQPGQSGHKHSLSIAQPTGHFL

HIDRPYNHTSLAIYTIEEFF

>SG-PL_contig_3036_1_-2_11_265

MERFHLLLRYFPSKTVGTILSAATKHFDSNFHLAQMQFLTDRYDVSGITYELESIKFTTQ

DNMEWMKNNFDVIKQWLASKNEAAD

>SG-PL_contig_3037_1_+3_186_650

MNFGPFLYFCFFPALQLSFNHTITTQIPSCHTSINIHQHSYHFCHSSSNGFLSCRTLQLL

QCLPWNNVDSTAALELEASQNKPSGRLSVNGQLLVVICWSPGDCNFFLQIVKQERPACAT

GLLLTDLLEGSSLKTPDHGQIQRLFLFVCFAFFVS

>SG-PL_contig_3038_1_-1_79_513

MFSFCDLNCTGSSTWHRAFYHTDCLDFTLNFFVGFLVLHLSSAVVIPVPVVRIGCYSGGY

CTDMCSIVMQRLSIYRLLLLYRRYNSLHAHRYNTCYAVFFSDSSKRTHKKQWHVARILDT

FGIPLHFPEIKGHVGRDTSEEMSIH

>SG-PL_contig_3039_1_-1_109_390

MQSWSKMELSSFLVVIPIFMTHHNTVACDLLCPVLLVTSGFGFSYPFPSWGNFRSNSLVS

CYCTWRLSLKGHNVALVTNRMVTLALQLICYHRQ

>SG-PL_contig_3040_1_-2_149_445

MALDALSLYRASLIGYASPTFLTLHSAIRKAKGSNAPMIPIAPFWQAQPWFPDLRSLSHV

PPLEPSPSPDLLRLPRSGTKHLNPEMPRLRAWPFAATPA

>SG-PL_contig_3041_1_-1_130_1176

MDGVREGETGAAPEGVPPVYLRWLRLFMTRFYCQESTAFDCEEDHGPHCPRGGHGRRGHG

RWGKHGCHKKKRGKHGGWSSSSESEDSDSEEKAWRNVPKGLRKFVRMSMKQLHKKKGDNP

KIKAKAQRAGVTPKVQEFVDQVLLEWHAKLSRQPPRVVATQMMEGERTLPTGLEREHFLW

LCRFMARWHCRHAGKCDMMAGWSSGDTTTDDSSGDDTTPPGQGCGPCPPFPSSHFRATMG

GMHGPPPPGMMGPPPPGMMGHPHFGMHFGMDQPPPRPCAPPMFGGWFPPPPAYTCGSGDQ

SSDAAMDCEGSRNQGPREGQGRQDLGTNGQRRTSKDDMVDHVTEGVKRL

>SG-PL_contig_3042_1_+1_3373_3684

MSENTESLVAVTFCTQLLQNSRIYTKFVTVKFCISPVALKTGYSFLCKAYRTWPKGSTCC

TNTPTFISQLGFFRTDTSDVPKKFKSRLQVVKKNASDFLLTRLD

>SG-PL_contig_3043_1_-2_149_433

MRKVYAANGTTVYQLIQKISSQTTNQALLEGEKKNPYMIGTFGSILLPRQVHLIARPPVP

FHCPFLFPQSQGINNPSTTSLLITSSQGINVNKMC

>SG-PL_contig_3044_1_-1_52_168

MKTVVFAAHLNASIILGVSAFHNSTHPLPLHFPKPCQST

>SG-PL_contig_3045_1_+2_5_259

MYLWSHCGKTQCIQGDPLECQAGKHKHAYLQSPLSTKTWTEMLVRSKIKQLSPLLTWLYT

TFYITGIFQHSHTHLTWNSFGSFTG

>SG-PL_contig_3046_1_-2_1421_1717

MFQFLKLIISQLSWLFFKSGYRLSSKYGSPSAKKEFAKKNIFLQRDVIIGRSELNICVMS

LQLYTEFERQVRTHCRFKDTGTAGKKPSMFLLLQEQGLC

>SG-PL_contig_3047_1_-2_92_3763

MLTKFETKSARVKGLSFHPKRPWVLASLHNGVIQLWDYRMCTLIDKFDEHDGPVRGICFH

QQQPLFVSGGDDYKIKVWNYKQRRCLFTLLGHLDYIRTTFFHHEYPWVLSASDDQTIRIW

NWQSRTCISVLTGHSHYVMCAQFHPSEDMVVSASLDQTVRVWDISGLRKKNVAPGPGGIE

DRLRNPTQTDLFGTSDAIVKHVLEGHDRGVNWAVFHPTLPLIVSGADDRQVKLWRMNESK

AWEVDTCRGHYNNVSCCIFHPRQELIMSNSEDKSIRVWDMSKRTGVQTFRREHDRFWVIA

AHPTLNLFAAGHDNGMIIFKLEKERPAMAVHQNILYYVKDRFLRRLDFTTSKDVAVMQLR

GGSRTPVSTLSYNPAENAVLLCTTASNVENSTYDLYLVPKDSDSQNPDSPEGKRSAGLTA

VWVARNRFAVLDRTHTIIIKNLKNEITKKIQTPNCEGIFYAGTGCLLLKEADCVTLFDVQ

QKRTLASVKISKLKGAIWSADMSHVALYSKHVISICNRKLENLCTIHENIRVKSGAWDES

GIFVYTTSNHIKYALTNGDHGIIRTLDLPIYITRIKGTNVYCLDRECRPRVLNIDPTEYR

FKLALINRKYEEVLHMVRNAKLVGQSIIAYLQKKGYPEVALHFVKDEKTRFGLALECGNI

EIALEAARALDDAACWERLGEAALLQGNHQIVEMAYQKTKNFDKLSFLYLITGNLEKLKK

MMKIAEIRKDTSGHYTNALLMGDVPERVKILKNCGQKSLAYLTAATHGLQEDAEELRESF

GEDEKVPDLYPGARLLQPPPPICLQENNWPLLTVSKGFFEGAMASRGNPSALAAAVDDEG

AGEGWGADAELVLDDGGFGAGEGFEEEGISAGDGEGGAWDVEDEDLELPSDLDMGAAATG

IGEEGYFVPPTKGTSQSQVWCNNSQLPVDHVLAGSFETAMRLLHDQVGVVQFDQYKPLFL

QTFSRARTCYVGLPSLPPLFGYPHRNWKEAGGRNGVPAVGLKLNMLVQQLQAAYQLTTAG

KFGDAVNRFRSILLSVPLLVVESRQEIAEAQQLLEICREYILGLQMETKRKELPKDTQEE

KKRQCEMAAYFTHCKLQPVHMILTLRTALNLIFKLDNFKTAASFARRLLELGPKPEVAQQ

TRKILSLCEKNPTDKHDLKYDQHNPFDICAASYIPIYRGKPVVKCPLSGACYLPDYKGEV

CRISKVTEIGKDCIGLRISPLQFR

>SG-PL_contig_3048_1_-3_54_293

MVDLFTTKFSKRLLLYVSLVPDPEAWGVDALFLDWSGLIAYAFPPFPILSKVVRKARIER

PLLILIAPLWPAQTWFPDLR

>SG-PL_contig_3049_1_+1_193_408

MIWITSQEIRSLNSYMLLELKLSINSSIIVYSLTYYSFIHTNSFTHPDFDQLLPRPEHTK

YMYYKLGTRFFT

>SG-PL_contig_3050_1_-1_547_900

MALVVGWAINNIIIIIIMIIIIIDLAVFYHRLLLILIWVCFCPLAFVDSILAVISLLLIL

CLPLAFVGFCCVHIFILKSGYVGTLVVQHCYMLFVHGIRRWDIMGVFCYRQKRDCHSS

>SG-PL_contig_3051_1_+2_551_673

MMIKFVTAFNFFPVYNVKCIKTFPHHPTHATREWGVRGKRS

>SG-PL_contig_3052_1_+1_1_96

VLKGNLKENHTSSSKLIEHTDHEHIKSTMGNR

>SG-PL_contig_3053_1_-3_168_1424

MTPPPAWSSTSLCATVPLLLLLTCWLLGDVTQAQQQCPNECNCFGTFVSCYQFNQESLLR

LPTNADTLVLSGGNITSFPPAFARKFPLLTLLDIQSTHVETVLSGTFSGIMGLDQLAVTD

CQFGNIEGDAFKDISAVSQILFSGSYIGTIHSGAFSTLSNIGEFSIWSSTISRVEARAFT

GLSNLNSFAFYENNVSNLHRQAFVNVENLGKVDVYMNKFFNAKGSAVIPLQAATSDMNMY

SNTFPCNCGVVRDFHNPLLSPYIVSQKCQMSGQVMRLSEVDVDELCLNEPSTTSSSTTST

STSTSTLSSSTTEDLPTFKVTSPIKVTQTVHITTSSSPSNGGKLPTTTTTTSFSRSTFTT

SDVTGHRSTSTEASQSGTGQGSGQTDDSSNYGNGASWRTQVAFVPLVTTVLALWLVSAC

>SG-PL_contig_3054_1_-2_644_1033

MLNCVRLRVFSPGTPVFSSVLGCWIWLVPFLPFPGSGRICCKRHNPFMPTMPLAFISFTI

LSASFFFFFPTTELPCEIQAAHTGRAHHHSAAPPILFSVCVCFCGPMRVEFLYRVLPRTA

PSLSWVLLHA

>SG-PL_contig_3055_1_-2_2_172

GKGSGGADFQTHAHTHTHTHTHTHFSIYKTMFTSHVSRSIPRPLSAPRPPPLFPDFS

>SG-PL_contig_3056_1_-3_12_272

MGVVFAALTLRTLLFPLWMLRPRQAEVSATASVFSCICCWVWERRARSSAKSRSSSCVQS

VHWMPFRVWAVDVFITQSIARRNRKGD

>SG-PL_contig_3057_1_-1_754_1260

MCACFVILFLKVALDMCVVALDYFVCVCACARARMHAVTLYIGRVCVCVCVCCNIRLIGH

VCMRCNLILKSNTGLTGCVCRDLLDICVCVCCKFVLKVTLDLLDTCVWVCVCVCVCVCVV

TLDLLDMCVFCNLVLKSSNGLAGRVCVVILFLKVALDMCVCTQWTLCVW

>SG-PL_contig_3058_1_-3_78_2066

MRQDERAVFLSQQEEQRKYDEEKRSEEQAPQVSDPTDDYGADDGNVAREQQDKEDFHIQE

TSSVKDQFEQAPQETAQQQQQPIKEPAISEPQWEDEPEEGVYENEPEQRPDVVRETDRNI

GAELPSVGTAKSMRDKFVAGQVENTSKDMKREVTPPAVVDGGVFENEPQYNADVVHAGDQ

NENEVLPEEGTARNLAARFKEMEKQGAVPVPSPGKREITPDRTGKVEYVSEPRGHVDKYE

PEVQAGVFESQPAERDDVVKSDQLAEEVLPEKGSAKNIAARFKQLSTDTTSSTPRGKREL

TPDTSGRVEYVSEPTGFVEKYEGCTESGVFESQPADREDVVKSDDTNEDVLPEQGTAKNI

AARFREMEKTTKSPPSPSKVKEFTPPRENGASPPSAGVYESTPKVAADVVHSDEQQEEVL

PEKGTAKSLANRFRQLSSSDSTPVKSPRSKKEFTPPPGDAGVYENTPKQFIPDYNQPAES

GILESKPESRDDVVRSEDPAQSEQELPERGYTKNLLSAWRQKESESSKVSPPSSGKPKEF

TPPREEPRVAQQRPAPRTPKSPLAGTDMNDGSVHPTDLPGQYQPQAEASVFESQPERRED

VVREDDTDWNQGMPKADTTKKMLAKFKHIQQQANSNSSTPAKPKPTARKEGDDRNSSETT

PDQ

>SG-PL_contig_3059_1_-1_1_201

EMVALWVEMYFCWDFERPCFYLYMSLVSDNAFNGQICSLCCTLSLSLYIYIIFFFIIFFF

FFFLWCF

>SG-PL_contig_3060_1_+2_569_790

MYWSSNLNKQTKHSIKKNKKPQSFVCLTGFSSAVNTSHQESLQNFAFFTSHCYLFAVISV

QKMITRVIMMMMML

>SG-PL_contig_3061_1_-2_83_259

MLSVCLLFCHVQPSINMFLCTVHEGWHHVYLLCFCFLFNAHKARCSLHICFLRYILPVK

>SG-PL_contig_3062_1_-3_204_350

MCVFLFLCLQQCFCTFLSTLFLVIKKSAFKVLSTNFCLCQYAVVSFILE

>SG-PL_contig_3063_1_+1_1129_1368

MDRNIAVVEKYKTRLYGQQTLGLGSLSQLSFSPSPPFFSSCFLLSCFFSPFPTLHSTVQY

EKQATPHTIYGQQNASNPVP

>SG-PL_contig_3064_1_-1_1_177

KGRLLEWLEHLVFMFSFLSRSPFIIIMINKRTLAIILSLLLSVNGQVGSCINILHCYHV

>SG-PL_contig_3065_1_-2_2_121

LLWSLGSDIVNAGMGISSLCIICDFKVCFCFFFIIFYAFW

>SG-PL_contig_3066_1_-1_91_357

MELGISPIVTSSLIMQLLAGAKIIEVGDTPKDRALFNGAQKLFGMVMTVGQAIVYVMTGM

YGDPADIGPGVCLLIVIQLIGRASCRERV

>SG-PL_contig_3067_1_-3_2583_2885

MPSNFTRLLFVFFFLKQSRLLSHNVKVLLDRIIFCRAFLFCFQVLSSKSVFEDSLCIYIL

NQYTPHLKIGIVQRNLVVCLCSVKFVVKYASLRTNFSLTTF

>SG-PL_contig_3068_1_-3_3_143

VCEGVCVRVCVCVCVCVCVFARAHVRISAFPDISASAQPFQQMLQES

>SG-PL_contig_3069_1_-2_359_457

MNNFNEVDSDSWASGSLSKHLQLFVYFLLHLCV

>SG-PL_contig_3070_1_-3_1662_1922

MGLYCVMVIPDTCRLCHTPHVQFRILYSSCVALSSISKAHVAMCGAVVSCVHSVQTCEAT

HHPLAFYPLLVIRVYSCVCVCVVDTHR

>SG-PL_contig_3071_1_+1_1_216

KKQDGTTARHVLDILRQALGPIKPGHCDDLKESQDHEGESRRIVVNEGKEPLSTLCDVRQ

ATKEGDDADNED

>SG-PL_contig_3072_1_+2_173_400

MCLISKEKMGREWNRQNKADSDKVAKKQRERESGEGGTVHFMYMKSDTNFEKTKDLKIQT

LSAFGAWGYHDFKQKP

>SG-PL_contig_3073_1_+2_362_622

MAFQILWNVDKRYNKEIHSDIIICLSPIIYTLIRQIKTKLHPASLILQSSSESSSKKRTI

TSDFVNVSNIIRVNVAFPVSLKINREG

>SG-PL_contig_3074_1_-3_1437_1847

MHIALVCTLVFKSGAKFTCILPLFCTLVFKSGTKFTCILPLFVHLCLKSGTKLTWILSVF

IHLCLKVGQTSCAYCPCLYTCVEKWDKVHAHIALICTLVFKSGTKFTCTLTILRTCVLIK

VGQRAYEYYLCECVYVL

>SG-PL_contig_3075_1_+1_1_177

HQDIQYGHRILTVKTFHLLFHHHTDITGLPRHAALPQRNSVVTFSFTHMSTSLASSTLT

>SG-PL_contig_3076_1_+1_811_1245

MKWFCTAKNMRLEMEHHTLSNTLPEQHSSLTSCDHTLKTGTFSFLLRSEYKNIPYAPKPQ

MPCDGKSTQHNHSKCYKSMHKPYAPRFYLGYILLSVLTHIWKQNYIVKRISISNPTLCRP

PVIPQKATVSTVNLARHFRQCQSTK

>SG-PL_contig_3077_1_+1_781_1173

MVYTSKSLVIAHRSKRLHYLQIFIPILLSCLHFKKSLFGYWAGLLFMGVVDKFLDRFSCV

AARVFVMMFLSLLLRTAFATVSMAFVLVLLDIVLIEVIHILLVPGHFSSQSEEIAQPQQA

LISRIPQACVW

>SG-PL_contig_3078_1_-1_172_714

MAGASSHFPAIIIFTIFTIIVCQATAASIPSKASSSTGDSGQAALPHELDLADLSLPPEV

ITEALFNQVDLLQDQLEQCEETVRDNLKGDDTPETEGIEVEDDYNADGDDVDEEEEEDDD

KGDVLSRQIAQLAVSTFGSNTSDFMDNDDMEASRRTVSEDGGHLSVGGDGNNALVEKEKD

A

>SG-PL_contig_3079_1_-3_96_323

MSMDRERDSKRCGGDMVAVVKAWNRMCMKPVTGKSCVGLGHWLTIPTDGVCGLLLFFVGW

FNSRYIITLLNSRARL

>SG-PL_contig_3080_1_-1_1_207

FFYCTFFRGYDTCGGDQMDLYFSCTPMTVMWFLSPIIVKPYTDTYISSVVTKCASDSCWL

TPWCMLLEL

>SG-PL_contig_3081_1_-3_3_194

KYPKRDLIIKVILSLHLLSSSCNCALSGILCVKRRQLLLGLNERGEELSVVSFYIKGSEV

EKNE

>SG-PL_contig_3082_1_-3_828_1004

MRVSAVCIHEHENCFLLVPFKMIKNTSSQVRSTVLSLKEELHRGLSRQYKLIIIMMIFT

>SG-PL_contig_3083_1_-2_125_244

MWILFCGLMGFSVKKGDYENGLTTIFYLVLNCFILFIYFC

>SG-PL_contig_3084_1_+3_3_149

IFHFYTNHIFPSTILVTVTPNGVRPVQTDTVLTINQCRTCDQNKTFTDT

>SG-PL_contig_3085_1_-2_2_538

DSKSHSGTPHGHSSSVDHKNHSGTLHGRSFSVDSKSHAGTPHGGSLSVEGKNTSHTLHGK

GVTAEDKSSSKVSQSGEGQKGFGDAGPDRKGAVQSLLTKQIVTLQAATNAEHKETAEKPS

YAASAHVKTEKEHIKSGHSEHKEQRRHSDSKKQKTPHHHHEEHHKHAHHAKKESQLKEE

>SG-PL_contig_3086_1_+2_737_1051

MQFTRLFKSVNVYCFFLLHLLPLLHLPIFESDGIFTVAEGYYFSAALFTGDVIFAGDAEV

LVNVTTEKDKDDNQHTASTERVSCGPCSFLPHFKSNFVFSLLLTL

>SG-PL_contig_3087_1_-1_1_216

VFQQRCLTSLYGICDFRWGGECVSQIAMEKIIIKVVIVQEYKKTVWKCAGMRMIILQDVF

FFFVHHSLDSSV

>SG-PL_contig_3088_1_+1_13_246

MYKVKPNNNWMTPNTRRIKNHSVKIVNLCCLISSKTAVRKTTSDDDGTVVHTQPQTDGGL

TLDKIHSKNTQLQSDNGL

>SG-PL_contig_3089_1_-1_292_984

MASLGTRFLLFLVLVFFIASVCEGRGGRGGGRGRSGGSRYSYRYRSSRGGSYSGGGSGNA

GTIVGIIFGVIGGIIFLVVAVFLCKAFCCSDSSSGSAKYSNSRGVVLSNPAHTPYTTTTT

TTTNNGFASSDTTTSSYTQGKTSFTPTDHIVSPPSYKSVVSPSNGDLPYTSQPAGDLSYN

SFPPNPPPASEFSSPSAYGTPYPPAPSEPSSNNKAEGGNDASGASAPIGFV

>SG-PL_contig_3090_1_-2_422_661

MWAVVCAGLELDESASISSVSSLLPPVPVSAVCMYGPPVVGANVDVTTDVEDVNNSEKCL

QVLANYMCRCQTIMLVDAFC

>SG-PL_contig_3091_1_+2_17_154

MYITLFKEACKKCMLIMKLLKAAQVYHLMNIILCEKKRGYVDINVC

>SG-PL_contig_3092_1_+3_3_146

CTPYSQALYTAPSKIKNVVCSVNQSFLKTFFETDSNVLTSSRLTFCII

>SG-PL_contig_3093_1_+2_572_772

MQVPEHPLCVYTHRTSNTHVKNPVSHVCVRWLVETRKHPACNQQGLAVRKIQWDLMLLSL

KNKVNSN

>SG-PL_contig_3094_1_-3_150_323

MKEDPHICIQEVSARLSLSYGTVQRILHKDLQLRKLAAKWVLRKLMEEQKKRRVHCCT

>SG-PL_contig_3095_1_-3_144_296

MVIICLPNKSVHSARLLGWSLRTHSRYGSDPSRTEKAIQSLCCSLELNSRS

>SG-PL_contig_3096_1_-1_1_162

MDKDSPHFAKHNNFSCRPQHSLVSLPDKAFGQGHCCLLIRIWKKTSPWDQPLAG

>SG-PL_contig_3097_1_-3_414_548

MSTFLLDPSFYLCHYLTKLLAKAIVVSLSELEEDFSTVSASSWIA

>SG-PL_contig_3098_1_-1_1_354

KIQCRWTKCVKLTSTDCIYACHITISQKSVNISYPPNPFPELSHRYHHLICLGHLYTNTS

FQKCTNLLPVYHYLFTHHLHSLPVFHPHPLSSSHHFHLLMLLSFLNQGKPKKLPFHVV

>SG-PL_contig_3099_1_-2_50_217

MLMSLPGHEGSLVAFIFCEISYLCMEISVQGGAERTRSMLDVLPKWLYGRFRTGKG

>SG-PL_contig_3100_1_-3_63_890

MASALSRRAVRTLQARLYSAAAHQEAKESNAPFPQQGPKLTKLPNGVTVASIENYSPVSR

IALVANAGSRFEDGDNLGVTHCLRVASGLSTQNSSAFNITRTVEQAGAEFTCSSSREYMF

YKIDCLRDNLDLVTKTLSQVTSAPAFKPWELSEVQNYLAVDLATLKTQPNAYVFDLLHQA

AFRDTLGRSLYAPDFMLGKYSSEQLLHYMKTYFSAGRLALVGVGVDHDQLVAQAKQFTPF

TSAGIVNNKAKYSGGEIRMDTGGDFTYAAVAVEGPS

>SG-PL_contig_3101_1_-3_1002_1388

MQLLSSVAATEIFSLCNPQSYTAANKLLAINCFLNTYMHVHMHVLSHTHTHTHTHTLSLS

LVHVHAHTHTHTHTHTHILNRLLCDKSLYEYILHTNLFTTTGKCDISSFHTAEVFSKRDF

SDKAHVCTT

>SG-PL_contig_3102_1_+3_471_605

MHQIKGREQRFQIQPENMPKEENRRSEFSETAWFWTYAARLYSTV

>SG-PL_contig_3103_1_-2_170_265

MGRGRVHKKNCTPSNVSHDYSNTDAILVPANT

>SG-PL_contig_3104_1_-3_153_1091

MEQSMFNSSEPDTAVKRLKRTMTLDFNGSSSTKAKKTAALLQSPDLHMLKLASPELEKMI

IQANGNVTTTPTPTQFIFPKFVTEEQEAYARGFVDALAQLQSREDHSRLPLQPSEAKAVI

TTTANQDIGQSSYPVSTTVSDRATLPELTRSLQQVSATTTLPNGVVLSTAGASLPVSTAA

GLVSHAHSKPTLPSQSHQSGPLVDIRLPKLEEEPQTVPCLSSSPPLSPINMESQERIKLE

RKRARNRLAAQKCRTRKLERISRLEDRVADLKGQNNDLSATASSLRDQVMKLKQQIVEHV

SSGCQIMVTHGWE

>SG-PL_contig_3105_1_-2_2_190

MNCDFLQVFFSGLTKFCFQRGRLLDFDGTLRGDSPDMAIAVGWAINNVIIIIIIIIIIII

TGI

>SG-PL_contig_3106_1_-2_116_235

MMNYFIFQSLKNTFPVVEHIIIICRSHICIQDLCSGLCYA

>SG-PL_contig_3107_1_-1_55_330

MYDRAARNFDPKLGSTSANVGPGSYDADTVSKARLKSDGYAPFLSMTSRETFLNVSDQVI

AAPGPGHYDPMTAQLHVKGGSTIATKEKRFKP

>SG-PL_contig_3108_1_-3_96_272

MMSCLSHIVNHGEMFEEGSLKWRLWRLKWTEKSTKVGSSDQQVSTCMNLFVILVILCTY

>SG-PL_contig_3109_1_+3_183_338

MCTGILLKGKIPSKKKSIIIFTNLASFNKVQCPSLFFDTNKLENLTVFEFLL

>SG-PL_contig_3110_1_-3_3_119

PGECPPGKFFFPRLKSVQSERFWASNSCTFAKIFNILCS

>SG-PL_contig_3111_1_-3_276_2033

MSKTTQRFEEDSISSYQPRTAQKNVIISRSSIGPRSRTSGGMFGGSFSSQRMSMSFNPAV

YSQMAGTGIADFRGTREKEKKEMQDCNERLASYIEKVRWLEAQNKKLEAEVEALRNRKQE

DWKPIRDMYEGELEQARKVIAELSTQKGVGEGKLAGLQDEIQSLKELIAQYDSNAKDYAK

KIDQLNSQIGEYEGEVTTLRLRCGSLEDEVAKLRALLAKYKDENARLRADLDAETAAHIE

QEVLAQTKAEECEFLQDVLDKLQLMQQEPVTIKGLNLEDYFKGELRRSVQEMQQTFDERM

DQATLEMDAKYAAQLNQLRSGNVRDNMEAEHAKGEIKRLRDQLAERQSRIAELESLLASI

KAERDNYHLQLNETQQQLEALQIESSRKINELQMQIEALMQQLSELIDAKMSLELEIACY

KKLLEGEENRTGLRQLVEQTIGVQSSGAANLADIIGQSNLSSSTTARTTVQRSSKGAIGF

QSVDPAGSCVTLENTSSGSRAKTQSMKGWKVIKEVNGKQLCAADLGERELRPGQALVLFA

KGAKSIATSDNEIIVDQFSWGTGTGQFKLIDSDGQEKANLTVRTQQ

>SG-PL_contig_3112_1_-3_81_5240

MNTYINGESQCNIFRVPDEPGRYEVNLQNETGTFASSEVQVLGNNLITFVQTDKPIYKPG

QKVKFRILTMMRNLRSRVGQIETVFIKDPNDFKVKKFSNVQSKGMASLEFQLINEAKLGT

WTIEVTVDGETTSQKFKVEEYVLPRFEVIIEPPAYFLVTSETFDGRVCAKYTYGKPVQGF

LKLQLCPSGYGTFGAPCPLLQTEIDGCTPFSFNSSELLSSGRYFYGRSFNIDASVTEMGT

GVQVNGTAPGPLVSYDPLRIEFLDDTNGFFKPSLPVYGRVKVSKPDGSPAVNESVRVSAS

DYNNNIYLDKVFVTDPMGEIHYSICHGLQENTSSLSMFAEAINFKEKTDVPFGRRLYQPT

GHGSFRQWFSPSFSYIQIPAIHKPLKCGDDLNLNVPFTHNGLKPYVKFYYQVMSRGRIVH

SGTTEAPTLPDPLSFLEPAINNCLEVKERPTPPPLPTIPPPVNDTDSGEIAPEEPPEPPM

IDEPPMTIERSSRLMESAVREPPEPPMMEPPRIPALQPVQIEPLSETFGQFDADLRRKRS

IMPHYGRGEEEEIVRSEHISDQLSNVQLTIRVTAEMSPSFSVLLFYIRDDGETVADSMEF

KVEPCFENEVKMKFVDETVLPGAETTIELKAASGSVCGIGVVDKSVTILGGDHQITPAKV

FEKLAQYSLSSRGRFRYSYYRSEYDYCKDRLSKLEEEEEDSEDDGEREPGEWSYMRRSYF

SKYVDAVEAFKQMGMLVLTNLDVETRPCGWNQPVYFDGLAMAGPLNSVPGPGVPGAPGPV

PVVARLGSAEEEKSPRTNFPETWLWDLHEVGASGEVDLQLTVPHTITKWVGNTMCINDKA

GFGLSPVTSITTFQPFFLAFNLPYNAVRGERLPITVTVYNYLDKCLNMLLMVDGMKGFKV

YSARSNRQPFCLCGGKSKTTRMYFTAQDIGKLPIFARAEIVPGQCGNAVEMDTQYIGLSD

SVQRKVLVKAEGVTQRYSHSLYICPKDGERYINNFTLPLPKDMVRDSSRGDVTVIGDIMG

PALDNLDNLVRMPTGCGEQNMVGFTPNIYVLKYLTATGRLTEVMQDKAKNFMEIGYQREL

TYRHSDGSFSAFGPKGDKPGSTWLTAFVVKSFAQARPYIFIDEEDLELSVNYLKSMQNPE

EGPVNGCFFERGKVFSSYMQGGLGKNPNDAALSAYVLIALLQADLSTEDQVAVNGINCIN

RQLQNDPELDVYTLSLIAYVNVLYDPNSPTTANIMQRLDRRATVEGDLKYWKREGELPKP

VNSWYYYAAPSAEVEMTSYALLATLLFYNRDAIARAQPSVFWLSKQRNAFGGYASTQDTV

LGLNALSEFAARTYSSETTDLRLRVDYESSRVNFEVSNDNSLELQRQAFDVKDNGTMWVS

ATGTGCALIQANIHYNKMPTKLGIDDPKFNLRVDPRNYQHDRNRCNKRTIYVTFGQRNSG

QFSTGMTMLTVRMVTGWSVIPGSLKALRSRYPMLGIQREEIKEEEGMINFYLDELDTRTR

RFALDVEQDQSLLVRAPKPAEVQIYQYYEKDVTVIRSYQLRTRCGTKEELPYDQPESGTD

DSFVMQRRLPLGGPDSGRGRPADLNVQPSREGAGAPAARSCPVCNLQKAPPNYNEIVCNS

TAVYKAHAGRNKRFSLKIKANLRPRKKQRDLNLFANIRMAEGCQCALLDPPMRAVIIVTQ

AANLRDKTLTVDSRTYVFNLRRDRTLEKKARKAQRKCRMN

>SG-PL_contig_3113_1_+2_812_1084

MGHFPAKAIFFYVICKKKTKLSEHKIVVSLQISLPSNANAATNFAHFTDCTVEQGNRLPP

ILLVCVCVCLCACVCEYGGEILQKLNDKYLS

>SG-PL_contig_3114_1_+2_2_112

KKKKNSVAVYYCVELVKDYLYVLQTCINLVIYHLRSM

>SG-PL_contig_3115_1_-1_67_1257

MAEPESKAPNVEEEVEEMEDELEEAAGEAGTDQAADSTKIAAQLMKNPQVLAALQDKLGS

IVGTPSGYIESLPKVVKRRIKALKKLQNDAIQLESQFYREVHTLECKYAAQYCPLFEKRH

EILTGKMEPTDEECDWPSDEEKEEEDKLADELKEKAAVEDKKEEEKKGEDMKGIPSFWLT

IFKNVDMLAELVQDDDEPILNHLQDVKVKFSDSEPQGFTLEFHFEPNEYFTETLLTKQYT

MRFEQDPTDPLSYEGPEIVKCVGCPITWNKGKNVTVQLVKKKQKHKGKGITRTVTKTVKK

TSFFNFFSPPQVPEGDNEDDLDEETEALLAADFEIGHFIRERVVPRAVLYFTGEALENDD

YDEDDGEEEDEEGDYDEENDPDFQPKEGDKPAECKQQ

>SG-PL_contig_3116_1_+2_1166_1375

MTSSSLCVRVTEIHNVYSIFEHRFLRHLPTLFFPPSNLSNTFTIPVLQTPNNFLSHSHTR

GVILYLVIKL

>SG-PL_contig_3117_1_-3_399_713

MTVMCLWNFKMLCYESSCFTPWYTASVQKAALVEEIAISQICNSVNVYAKRLLVLSLMTI

SRLGFEEKLLQDHFHFLFCLLVFQCLFDRCSVAGYIISTSNIQWI

>SG-PL_contig_3118_1_-1_1_261

VLFRSVLVADRYVLRRLFHIIRTECFRCVRPMYMNRFVMLLVGNLINWAFALYGAIQHPS

DFASFLLAIFIGNLLLYLLFYIIMKLL

>SG-PL_contig_3119_1_+3_3_227

STHVVLKIYTISVTICIKNASKRKDYKQNGTQKPTACHKRNRLLIYCTSVVSTQRQLQIL

VCLTKSPSLKQRILD

>SG-PL_contig_3120_1_-3_3_122

SLSLSSFQLKFHFRFFHFQCLFCIVTDGLKPTRLVRWIVF

>SG-PL_contig_3121_1_+1_94_309

MFHVTFFSCQKTKQTLSRKKINTDVLKNLSSRGLTAYITWGSRCVACMCATPKTRREKER

GRDRQLPVRYAV

>SG-PL_contig_3122_1_-2_116_244

MMVEIICNELCSFAEFFFSGVTRFCVQHVFLDFDGLNVVTAPI

>SG-PL_contig_3123_1_+3_297_374

MRGNAPDYLKAHTQSSTLKPICTSSR

>SG-PL_contig_3124_1_-3_33_239

MMEAEVGAIIGGCIAGAVVFIIFIAIIIYLICGKRRRRKEREDQKFRVLTPHTSPAHSQP

STISQGIHP

>SG-PL_contig_3125_1_-1_1_168

YIVFYLRSDIIIDLQLILKRLDSHCTFCHMTANLCMRKCMQECTFITSYINGCVHI

>SG-PL_contig_3126_1_-1_1_264

EAWGSSPDSDGPPDDLLTLGSEAYYALVDSADSAVKLSYNLALIRRDAILSSILPAEDFT

ALRSVPMQPSSFFGPFAEPFDPTWATAR

>SG-PL_contig_3127_1_-3_27_1556

MEYERQYQSSYIVPEVIKNFLIFFQKSVNEQVLFEVQNCYENGFNKLTDRFFKNTPWPEA

DVVAPIVGNDQVFLILYKELYYRHVYARVQNGPTLEQRFESYYNYCNLFNYILNADGPVP

LELPNQWLWDIIDEFIYQFQSFSQYRNKISKKSDDEIEVLRTNPKIWNVHSVLNVLHSLV

EKSNINRQLEIYASGGDPDSVSGEFGQNSLYKMLGYFSLIGLLRLHSLLGDYYQALKVLE

NIDFNKKNLYSRVPTCQITTFYYVGFAYMMMRRYQDAIRTFANILLYIQRTKQMFQNRAS

LYDQISKINDKMYTLLAICLVLHPMRIDESVHSHLREKYADKQLRMQKCDKQEFEQCFAF

ACPKFLSPVPPNYDTMTDTYHKEPFKLQLAVFLDEVQQQIQIPTIRSYLKLYTTLPISKL

AIFMDEPEDRLRTQLMCFKHKMKNLVWTKGTSCLEGEFQSASEVDFYVDGDMIHIADTKV

ARRYGDFFIRQIHKFEEITRNLQQIGTGHK

>SG-PL_contig_3128_1_+1_1_225

VCVCVCVCEREREREREREREREREKNFCVYGGKVFFFLGHSFLCIDMSECCGCVCMLTF

PKRCVSYHIHDFCFT

>SG-PL_contig_3129_1_-3_300_971

MTCFAVLLQVGYVGAYYSEGQLRKKRIRARFPGVEVAGTIHDVGNSLPNCNFTPGDKVIL

FPDEQLSDSGYAEYLAIKDTRQVLQIPQDMPLEVAAMLPGGALTAYAAVLAAKPHVEKLQ

QVKPCVNVLVVGGGGLGLWTIRLAKLLIGCYSSSVRLFVADNSIDKLLTAQDHGCYDIVY

WNEEDHEEYIVERTLDACRGGVDVIIDFISSPRTMQRSFKVLNR

>SG-PL_contig_3130_1_-3_54_302

MCNRDNSLSVAEARGLVMSMAYILGSSMSSEKDVRRCGNDYIMNFVHDLSQTSPNSFRFS

SCFIRVIKGYLKKLHKRGDSCLS

>SG-PL_contig_3131_1_-1_1_99

TALSQQLLTIFKFSFHCCDSLLLSSLHVKFYFV

>SG-PL_contig_3132_1_-3_3_65

DQRGCFFYFDGLYVVTALIWP

>SG-PL_contig_3133_1_-1_193_363

MSTQKHTCWWCWFGFFCSSGWLVVFGTEAMIKVTRAKKKKKCLELALFEEKVCEQHF

>SG-PL_contig_3134_1_+1_1468_1695

MASPVEAMVINALELIMQEGSDLDSAEESGVESFLDSDQEVLPSNDFGCENGEGSVSDRL

SEGDTDKHSSGLKHFL

>SG-PL_contig_3135_1_-2_269_454

MCVFFFKYGSEQFGLIPVRFINLYSFRHRQLHFMTEKKSTFSQSIIKTVSQIFVKTIKKS

QW

>SG-PL_contig_3136_1_+1_1189_1359

MSALTSCVLNQTVCLNYIYGLNQCFEERKVTLNHICSLNQGIGWNQTVKIHPLQGYE

>SG-PL_contig_3137_1_-2_2_157

KRDGFCACWVFSCFHKPPNTDMAYMIFNVLFDVLHAYIHTKGFQALAGLHIC

>SG-PL_contig_3138_1_+3_3_143

LSLFLPANICTTHTQHSSPMTISFPSKSKHAHKRITILGVIGLGIIY

>SG-PL_contig_3139_1_+1_1_405

CLSQRLDCLVFLSLSKTRLSCLSPLLSCSVSHKDYLAFLSFFPSLSVSKTRLSYVSLSVS

QRLSRLYLSLTKSSLLYLSFFLSLSFFFFFSFFLPPPPQEQIVFFFFKFFPPPPPPFLSF

FPLFFFHHPPSFFFF

>SG-PL_contig_3140_1_+2_2_160

EADGQSEGMKGLRKKTSRQSFMGPTRSILAHISFIITKNPVTNKISSCTFYLL

>SG-PL_contig_3141_1_-3_735_1031

MLRTNLRQLQVPRFERTYKDKCFQNLLSRIVNNLFCYCKDAQMRTKGHNVVSHFPVHNGP

FLSYELTILSCASLFNYFSRSFKIGVNIGVNSVYYGTIF

>SG-PL_contig_3142_1_+2_2_82

FFFSLFTLFYFLHNDTNTFTVLVSIRV

>SG-PL_contig_3143_1_+2_155_418

MKLSKMYCKEASMVPCTIMKSFFLQSSVQSCAIHLFYKAIRHFTSPKNKWTCQTDLTISY

HQLADPNARQHRFAYSKNRCDLDRGEGK

>SG-PL_contig_3144_1_-3_3_7637

VVHSILRGDTAPRRTSRQLTAKDHQPQTTFGRRQGQQQPPLQTSGPASSKQGTVTPKARH

GYTLPPHSAAHNALPGPSPQIVLTPTPTTAKQVSGQERVSSRSGSKSQVSEKRKRKDTSA

SQSEEARSASPNKRFKNTVEAGQEISTTETSHSPNTSCAEKEGSTEQLSTAAFSSSKSSS

SASPPPAPTAAPSCGLSGSRDKTVINKSVRHKKGSNSSASVSSSSSSETPHSSSASVSVS

SEGTTTAVVSTEKKTCGDGLLTSPCFEKSEDSKENRDSVNTCSNSVTQTQATNNIVDTHR

SATTSLPASTTDFAQILSTASQAGFQKREPQSRQESRKVNQKVSIESQGSSSVRHGASNT

QTVDQSLSAQDAATGPCLTSVSNGELSHISSGSSSSSQQQPPWDCGRKEEEVDTMHYKKQ

LLLAGGNNSPSASTTHSSATSISASIDVNRSVTDQLTKLAKAEKIALGSSDGKTDSEHNT

DTATAGTFSNSLTSASLQRSERDHRVNDLMTDRNRESSAFTSVRVGALLNNTSSSSSEER

AESRASDSQVSSGLGDDRRPGSQLDDLRSAKSSPTSSPLILDRSEPINIYRDPELMSKNP

VRSGLSVTTIHKTMSGPGYPTVPGGVPAPPPGLTTSTTLASHPSSLSRSLLPGLPYSPHQ

LPPAALSHGLPLTHFSQLEVLSARELAAAQQHQQQQQQIVALQQQYQKAAQLMHPMPYGG

HRSLSQLELLWQQKLQPGPLPPAWVLAKHPEGAYSDSAVIPREHLERMEQDRIERERLEK

RLEIERMERDRAERAERERREKREKMERERLEREKAEKEKAERERAERERLEREKLEKER

KEREWEHQRHQERERILRESAETSAQAAVDEHFVESFNRVAKLRGVAPWVKPSITKGTGK

GLIKMETEKAPEEERMKREVDQQRAMMSDDKRYMNAVMEQQRSDTKPEVMMGKDGKPIFG

YPPYHYSSNFHPSCTNVPTSATKPETIFSLYGYPSQQHSTITTEQLQKKGLAPGGRPLKE

EKSIYPGQVGNPSPPRGVVHMVGHPPKEMKMERSVIVENKKMEKQPMAAHSHHPSSLSSS

ASSSPHPQPRPAHTPEQRPDRPLSSSTSLSNPAALPPHLMGQAMGSSPSNFGAQSSHPTA

FRSLDPKAMSRSQSPYKVAGSHQTSVSGQPQSSSGQPQPINFSSKSKGQSGSSSPYPTPV

IAQPPVSLPASSLAFSYSLIQQGLVPNPMYSQGSLAPAMQMARSQQPNTTAARTSSPASL

TQNSHPPAVAGMPPASQAPKPQLSPSGSSKRKSNGTGRDNGTLQSKRAKNVAGSMSDNGR

TGSAVSVPVTTPQVLTNPSPYTTSSSSSMAGRMSGPLTANPSSGAPPTGFLDSFRSFVEN

TVQNAFLQDEKTSREKEERDSLQKQQQAQQQQQLAQHQQQQNQLQQPVQQQQQQQHAVGS

VVEKMDTSVVGSGSGETESPSPVLPVSGQPASSSSMASIMDTINRVANGQDTDSDTLSAP

SPPPQTRPENASPQHRGSSSSAPKLKKAWLMRHADDEKPSTSPLIPSPLVAQDDSNSSQS

TSKEVKQYLKTSSVVDPVAATINGDVSPSPATTNSSNSTTNGQNLPNGNISSDLQGTNYE

STSSASETELQTTASTKKRSKSKKTNSASKKVKMAEKAPSPVLPLQPTNVGSSARSKKTS

NKRMKNEKDSSKGKEKTSQPETRSVSPSPSSTSRKSPAPSTSTPSSVSGGGKKEDKSSKS

SSSKQSGDSSGPPSPPSQASGSHDGEKSSSPEAVAHPKDKKKTRRSKEPPKDTPSRNGKQ

GSSFNEPLVKCSVATLKRTGAPFLQNGPCSEVTPKLSKCRECKMTPNQRSKKIPNIFCRF

YAFRRLKYSAKGVVTNAGFSELSDADPDDIEPWLPRYPIEEPTLDEDMGKFILSKVGDKF

CELVEQEKEAKSLSGMDPPIVWKRAVTGVREMCDVCDTTLFNMHWVCHKCGFVVCLDCYK

AKIKATEGEDEDDCKWLTCSSNRQAHDARRLMLTQIIPSDALWELGRLIHDIRRKWSIPA

KCPCVQNSLESKPAGKNGMNPQLLKKMVNGMGGQEGSHKKSRKAAAAAAALQAEEEKLEG

QEYNQDKLGLLAEVALGGGDKSSKHSKLESLSEEEFGEDGDKKSGCSTLRELLTKTAGKG

KVPNEKKSKPKSSGSTLDDIIQSVVEKSCRDLDQPNQTFKFLHYIPRLGQWNRELPIITH

NLTETSVLYPDVPHSWLCDGRLLRLHDPRHKGNLRIFQEQWNRGQPVLVSCVHKLVNKDL

WKPETFNKEFGHQDNDLVNCRNSNVIMGQPMSDFWEGFESIENRLLDGDDEPMLLKLKDW

PPGDDFSELMPAHFHDLMQALPLPEYTRRSGKLNLASRLPDFLVRPDLGPKMYNAYGSAK

FPKEGTTNLHLDISDAVNVLVHVGVATDGPGGQKEHEEAAVRAIDEAGCDSITKRRVREV

HELPGALWQIYDAHDADKIRDFLNKVAKERGEIIEPDHDSIHDQSWYLDEELRDRLSKEY

GVQGYTIVQCMGDAIFIPAGAPHQV

>SG-PL_contig_3145_1_+1_1_258

RDTIRGVKLKDAKSPRRLPAWDLFLVLSSLSKGPFEPLGAISLDLLTVKTAFLLSLASSR

RRSEIHALSGNPADVAFLADGSVSLK

>SG-PL_contig_3146_1_+1_1_207

SSLSKGPFEPLGAISLDLLTVKTAFLLSLASSRRRSEIHALSGNPADVAFLADGSVSLKF

LPEFLAKNQ

>SG-PL_contig_3147_1_-2_179_394

MCLSPKLELTPPPPENNKCSVLKHIFFCFTFFLSFFKKFTILPQQFRFCVELLSTVFHLQ

FNNLLVILCKVQ

>SG-PL_contig_3148_1_+3_552_689

MYDHGTRLSSTRIRIVFSYTRDLCLERPATSSATLWYDLLTMLHGS

>SG-PL_contig_3149_1_-2_62_766

MFTMMLTAGAALLLCLMFTGVETQICARKYSRIHREHTMCLTKNSDAVKVYLTEREKDVI

VSTHNEIRAAANATNMQKVKWDNELAKLAQKWAMQCPSGSEDNPDKRKTPDLPGIIGQNF

ASGRADIFEAIFHWWNEHSNFQSGGVARHSPDSRNYEQLVWAPADRIGCAASTCTNTHYY

ICNYHTGPVVGEQVFEAGPPCQRCPNHCEEKLCDCTGSGICMNGNTRDVATCECN

>SG-PL_contig_3150_1_+2_281_451

MPNFHAEDDYLCSVNSSLQHENWPVFPHLNALVILGVSVFHGSCSLPILMLQSFWWC

>SG-PL_contig_3151_1_+1_1_126

GLVHEKCTLLNIVYFSLESISDITAGNDLLITFCSLDIENFV

>SG-PL_contig_3152_1_-1_1465_1746

MGVFILLIVRVLICTNAKLNMLIFQARTRTQQSTDCTLNWKIFFFIICFCGNIAILMKVL

AVENHFCHYSRQLNTSKFQQEDTSGLQICPFSTV

>SG-PL_contig_3153_1_-3_138_338

MTSSYKLQCQERLFALLHFKWLMIVCVLFSLVDRFRFLAVYVVEYCRLKFYSKRTTSVCI

CFNFEPA

>SG-PL_contig_3154_1_-2_521_793

MFVWFHTFLTSVVKKYLLFFCSDKISFAYTIQISFSGERQKLYLEKISSYGRFVVILNIL

RASRCCFKSPYILNSVELQVAMSSLSGGFLF

>SG-PL_contig_3155_1_+1_1021_1251

MGVCGLRNKPCPLQVMAPWTRPGVQIWSRSIHSQPRNPCKDSLFPLSQDTHKSFFKGQQK

ILHSTPTPPFFFERIHI

>SG-PL_contig_3156_1_-1_1_210

QHNTNHTTPHTTPHHTTPHHTTPHHTTPHHTHTHTHLLFFLTVQDRVEMYVNSTQIIQAV

VSSHIWINIV

>SG-PL_contig_3157_1_-2_167_262

MISRNISDLSSGLDLDFSSKFFRHPITILSTL

>SG-PL_contig_3158_1_-3_174_1127

MEPVIEIWDLDIMDSVTATLTLGSQPKKMKKKMKKKETMTGHTEAVLDLSWNRNARHLLA

SASADTIVGMWDLTKGKLGMTLTQHSREVQCCKWHPQEGHALLTGSFDNTVKVFDCRNAD

SHKSWSVQGEVEKVVWNHHNPLQFMTCTDQGKVYCMDIRHDKPLFTLAAHDKSVSGLCLS

SQIPGLLVTTSEDKTMKVWDIQGDKPSHVFSRNLHMNELHCVGCCPEAPFVFAVGGGSRE

PKVWDIRESAAVRQQFLNRMPTNLARECEGDDTDLADGDTVTAMEGLQMEDEEEAAAAMP

ATASAKKKKKKKKVKKME

>SG-PL_contig_3159_1_+1_1951_2112

MQSFTSANIFQSFTTHQLSLSEDFKNSHLELAEIGKLYFLKKQTKKQNKMAQPK

>SG-PL_contig_3160_1_+3_165_326

MKAQAPMAVHKSKIRFKPTELLSWYSFSSGHTKLTPLKIPTLLINKNTEEQLFF

>SG-PL_contig_3161_1_-2_71_1012

MTTSVHVESIQKIGEVLQDKTKPLKDRFRALFTLKNLGGSEAIDYIASCFDDGSALLKHE

LAYCLGQMQDNYAIPTLISVLEDKSQEAMVRHEAGEALGAIGSPEVLDLLAAYSKDPVVE

VAETCQLAMERITWQQNQKRQEEAGLLPNPYSSVDPAPPAPTVEIDTLRNSLLDESLPLF

QRYRAMFTLRNIGSTEAVIALADGLKCKSALFRHEIAYVLGQIQSDVCVSQLSANLTDKK

ESPMVRHECAEALGSIATDKAMTVLKDYLKDPERVVRESCIVALDMSEYENSGQFQYADT

LQNVTDGKVAGVTG

>SG-PL_contig_3162_1_+3_3_206

FHISFLFLTLLLSKQMSNALVMITENGTTRRSTDESKQQKSVPSLVFSFSSELHNYPETS

CIGQLMKE

>SG-PL_contig_3163_1_+2_2_178

TTVKKKGRNKESNRKEEISSTEFPEGRDTIYTERSRHPCGAKGGSQLSYISHSSSCTGE

>SG-PL_contig_3165_1_+2_254_418

MKSVAGGYARTFHCSFSVMKNTTCSHSRCNVLFDTDTTSKTPRILACLTVQDTKK

>SG-PL_contig_3166_1_+2_2_1576

TCFSSFRPVSSDDVQKILTKTVPKTCELDPLPTPLLYECLDVLLPTLTKVINDSLSTGIF

PSVFKTAVVRPLIKKPTLDCNNLSSYRPVSNLPFLSKVVEKIVLSQLLEHLNLHNLLSVH

QSAYRPGHSTETVLVKVVSDLLMALDDGKISVLALLDLSAAFDTIDHEILLHRLQSVFGV

RDIVLSWFRSYLSDRTQRVSVNGLSSEPTTLRFGVPQGSVLGPVLFILYTQPLSDVIERF

PVSFESYADDSQLQVSSPPDEVESAIQGLQECVSEVKQWMTANKLQLNDGKTEAMLVASN

RVSSSQVLPLSIDICGSHIQFSSSVKNLGVYLDSTLSMNLHVTNTCRSAYFELRRIASIR

HFLTVDATKVLVCAFVLSRIDYCNSLLSGCPHYLLEKLQKVQNAAARLILKASKKDHVQP

LLRTLHWLPVNARIQFKISTLCYNCVTSSCPSYLSSLLQLYTPSRELRSSADTRILRIPK

IRTKTFGQRAFHYNGPSVWNSLPQTVRFADSKVTLKSALKTHLFA

>SG-PL_contig_3167_1_-1_1_141

LLNNVQTNSELLSSLRERNDLCCHVQSLVHLKRKERFVLPCSISCPN

>SG-PL_contig_3168_1_+1_1_102

VGLLHGQQQVLLNTLPRLVSWSLSSGTNSIVLWD

>SG-PL_contig_3169_1_+3_69_191

MYDNKKLKSTYTKVKNKDKGVSENSQNIYRAELSEQKQANK

>SG-PL_contig_3170_1_-2_2_235

PPSFGEEKTGLFSPRKRVLSADTDSSDEDPSIDESILVPEVELRSERSSNITVDVSTLNH

LVSPIHCKKCKSKVSANK

>SG-PL_contig_3171_1_-1_373_702

MDTVCITSSPSLPQAPCLPLKPLYSERMIFTRYDQLHVLEISLPSLTGPHKPHATPPFIA

AGSKGQRHARASRVVAEGLLVGHGFRSRGRMGGGAIVRFVFLKKNYFKGH

>SG-PL_contig_3172_1_-2_2_220

EQNRSEFSTTVQFSTIPYNTTQHHSVQYNTVQYKTTQHNSLPCSTIQHSAIQTIQYSTIQ

HSTIQYNTAQHNS

>SG-PL_contig_3173_1_+2_110_238

MAWKKVAYPLIKEQTFKNDTHSYLQVSYALYVKPVAQVFRPLW

>SG-PL_contig_3174_1_-3_3_182

KKKKKKKKKKPLFKKKKKKKKKNPIHSLCVKNRTSVLVLCSSATSSPPRAASTKFCKEFI

>SG-PL_contig_3175_1_+1_1051_1323

MRMYIQGMHMPTRNHYFTNEERENKETTLYTHKKPTTLQSIYKLYVHNLTKPKQTFLIIC

ETDSNATTAHLMLCHYVPEHLRIMTALKTNH

>SG-PL_contig_3176_1_+3_171_356

MYIHGIPQYTIRRSTVFKKKQKNTVNNLLLLFTNYYTIQSWFYSWLAKRGIDNGMFRSMI

ST

>SG-PL_contig_3178_1_-1_313_531

MIRTIGVLVLLLSHEAVNLLRKENLPTVNFCMGMLRETLHIFMVDTWTYQMCSMFPVKGK

TDCNNGDHKNCCF

>SG-PL_contig_3179_1_-3_3_170

FPFLYCCLPFSAFLVHSTFFFPASLHTNFSAPHPPSTLLLPQFMMYCLCCFALVTR

>SG-PL_contig_3180_1_+1_1_183

NLVKICSEKLFLPTNLSWKIIVLLQTQPSKGVSLNPSLALIKTELGGEGRGLGKKLSNYL

S

>SG-PL_contig_3181_1_+1_1_276

CSRQHPYAIPNLLLNRRGNSCCQDTAQHKLSYIFFTSHLIHINVTYNTYTQHKLYHSCTF

LFYLLTISIFLHCCTKQKLFKVTAHNNKIFRS

>SG-PL_contig_3182_1_+1_145_504

MMCTYGTALPTISDIYKYNVFNCDYLQKERNGTNLYADCKVNDLQCANGDNLFTDSLKKK

PNKIKNVLNRYFESNTARKCWLCLDLTTLVKINKCTSSSNKHLKTNTCTKTINNKMMKPS

>SG-PL_contig_3183_1_+2_116_265

MQEPFLTSLTLGLKLHTLCKPTEHCRISRTLFKMGHAEETNTRKACIVSF

>SG-PL_contig_3184_1_-3_1386_1802

MAFVVLIHVESSDCCNTVLRKRKRTDSSTDLSVAEKKPHECCGPFVCPVVLFCCDTEAIG

NRNLSLYNPVFKCIFVESVCVCVRCAFVCMCVAVCVCVRACLCVWESERVGGESEKERGI

CLRIKIVCFSAYVLCVYMC

>SG-PL_contig_3185_1_+1_1_276

NKKKKKKRGGKKKKKKKKKKKKKIKNKKKKVFITVLTNIKKKKKFLPTHFSYFPRHTHTE

IIRIPWVILNHSSSDSIFPSSIFWGENRERVV

>SG-PL_contig_3186_1_-2_2_193

NPAFPLQRPRTATYTVTVLDNAALGIHFLLLTSYHYVIDRHNDDDRRMCPHSNFGICCFN

LCCI

>SG-PL_contig_3187_1_-2_245_664

MGVFGQLKNKKKTEKQRKTMKWRRGQRAVAKQKHQPQLNLEDVQLLSKDMLKNKLKNMKN

NVNLSNKKRNRLLKRLRHGQREQEKLEADTDEQMVEVTPKAAKKSKKGKVKGSPSLSKFK

ASTSASVDVTEDVDMEDEGE

>SG-PL_contig_3188_1_+2_2_154

FFFGDEQHDFIDEGTPRKTFPTQCVKENAILNIINIFFTCSVFIHIFTFHV

>SG-PL_contig_3189_1_-3_483_785

MIFSHGNNQQRKSLTPAFYTTTLYDLFTCQYLLAFVCFPCTKWLPLQYHVLPLTPFGPGL

STTLQYPSIHYMPHCYQQLVDLITTLFMLSIFFLTQPFPAC

>SG-PL_contig_3190_1_+3_447_806

MHTSDALALMRCACTHEMHYIFSKRCFQKNKTIQLSSFHTTTNCQHCFCISLVHLEDCSW

GILYLCWKKACFSSTNCVMKLQKNVSFSITRNLENNFVRIMHGQTTTHKKGSDVSERIIK

>SG-PL_contig_3191_1_-3_168_215

MCTYFYIKDLSLQKKK

>SG-PL_contig_3192_1_-3_168_1322

MASLRSSFTLLRTAARQGCRSISTRSSASRKAAPLLVGAAAAGAAGYGAYKIWGSNPVTL

EQAVKTVQEAMPSATVSAAGPPEAQPSVYSQGKDHALYLWIYLKPTANSREVARAVAKLQ

KYVDEVSDPKLRDEDDEIWAGVGFGPNFYCQVAGKTKQNFTYPHRKGALGDMPSSGGDVF

VHAKSNQMSKLFELGQVILKNLPKDSVASFEDIYSFVYRNGRDLSGFIDGTENAADDDDR

REIAVEKATGGSYVITQKWIHDLNVIGTEKDKTLEGWVGRSRADSVELARKTISSHVARM

TSGNNFQNVKPFQIVRQSMPFGTLSGDAGLFFIGYAASPENHEYMLSRMVGAGSDGHCDD

IMRLSKNVKGTYWYFPGAAELKKLE

>SG-PL_contig_3193_1_-1_1_363

ASQEGQAVCATQGTCRLLAWQTGIYQASHHGLRQRLPSHQVQLAPCILLPLLVSTAVSLL

PDSLGPSAACPSSTISLFVPFPFLVHPITPPYPPTTTLRTVSPFTIILKWPVLVLINSSL

L

>SG-PL_contig_3194_1_-2_11_319

MHIYWVTPQSVKLGNANNKQQQTPQHSRLRQKTEETSLNFHFGQYYTNRSTLSPHHTLSF

AMQQVGKAPALGSERSENSEVRYPTSRIFSLMVNSGCHKWQRN

>SG-PL_contig_3195_1_+2_2_238

LVHAGCFRVSISHQTLTWFTGSLICIFGVLHAYTIEGGSGICMSADILTCWIRKMTTLYR

SGAVRKGGTQDLLTGSPTP

>SG-PL_contig_3196_1_-2_278_538

MVLLLIGSIIISLSVNLYYLSVYYECNLYIFSAYYSLVEDTTTSVSSASESLSSSETSSP

LSPGLLSCRSSSHMIFSRTTISCHFCF

>SG-PL_contig_3197_1_+2_1646_1948

MSHTPNSTGENTPLSLSMVIAMHCCLFLLLFSRKGNVIYLFVKGYSYASLLVSFFLLLSV

HLYHHGCDRLVFTYVINQNICTHINAESLTLCFLLVFFLSS

>SG-PL_contig_3198_1_-2_2_286

FGGGVVGGGHFLAVLSNLLPVISLFLLFIMSSSGWLVGRLVGWFFCLSAGSMSYDCDAFL

NDCISYSPRVESVILCLDFYVNEVINKYVWLSKKK

>SG-PL_contig_3199_1_-2_29_304

MLLTGMDLPRDRSTVTIGRGYFKSMCSYSLSVAVVSGLALTMAVNLGFSMSSTHDGGECA

NGYIMDYIHDLSKRSPNNFRFSSCSIRAIKDY

>SG-PL_contig_3200_1_-3_3_302

QKKKKKFSLSLSLSLSPPPSVSLSPPSLSPLPSLSPSPSPSLSLSLSLSLSLCLNETQRP

KPQFIISLYGKNILAREKDSSSKMFYLYSQSQFRKEENCI

>SG-PL_contig_3201_1_-1_1_189

GRGGTWGCGGSGGCWGAVPGVIWWQGQGHDLNIIRAPAHVVNTHASVGHGLFADTVRITP

ACG

>SG-PL_contig_3202_1_-2_50_9538

MITANRREKTACSGEALAKILGMELCASLAFPNASTALSAPYFPFTGPTSISLTLRKHDV

HSGYKLLAKQIRTSSSIITQVALDTPGSKVDRAISLGYVANLKAKTIEADLLSPWKKVSA

KASVDTSKKMYKADAAVSVDTGKEYKVHAQVAHDIRSRKEVSIKVKPTLVVTSPDGTLLS

VNGNMDYRKNKLIKTNMVVEMKDVLKKPIAVDYTMMMRARGDKTRYRNNLKLSSQFATVK

SSLQALVGSFTAKKRSALLTVRSKVDYNLHFWPGIKKNLVNLGLKIHDRSTPALSKYGIN

MNFDAKRTPGVNMLLNGEVSHKKTLTEVEVKIRHGKNVKLKADKDKELLIAGLVAHKIAD

SQATVRFDVKAVYPDKAIEYQMKGKHEHNKRMIEDYFTVRYAKGKSVDAHMRLAEVKKKR

SATFDLSYPAGKTYPARDVKLNADLIEKSAKHHVLSALASIQKGQKHSFVVSFKRPTDNS

VEISSEMGIYKRSPIKLDISGNYGLRDMKGQGKLTVEGKTYRMNYTSDIQKDKYAKFTMN

FVHPERRIQGLLEGGRDTREAVKRAKMEVLWDADKDLSQKAGVSMELADKVNADDINIGG

MLEVFTPVLEYQHLKGELRFENTNRRILTMAETILGDDSKEYSAKFMLNQPVTPNKFKTQ

VWLKTPHPTVRHVEVKADHSFGDSGKLSTAVKGIYNKDSVQVGISGSSKGDLVVRVLEGA

AFFKSTLRDAENFRLTFNHRDVSGRYNTQAVLDANGDQYSAIMEANFQKVHYQITTDGDI

NILAPQLTSPVKVLWKHKNSLHEVNSAVTVDFPEDQQVHLEVGGKANIVTNVYEGRAQLR

GPWKENGAEHTSSLQVNFAMPRSGHDLTVTLQLDQDTYVFHETQKDTSTSHEVTQSLKMS

RFPQYNCQHKCNIQYGNFPYLVSMEVQMADRPMLNVDVEIEKPKRDKYRTTSRVVFILED

INQNIAISTRHEKNRRGQWVTKGDIEYEMGKTLSFSSVVDVEKDRKMINLNVDTPFDEVQ

NIRLEYSHSGNVEKNFTAACSLAVDPQFDTVSSEIFWSNRDRFYGNLKVDTPFKELRHLE

VKSQSWMEGSKRHSTLSVDYPPRQVYKLDTVYSCNFPRDLDFTLNVTTPRKELPSLHTQV

RYQYTDTEAAGNFRLITPAIQYDELSTSFNYRQGPKSFSSHVNNVIGTWNTWATGADFNW

DNKIDGTFSIENTFHGRQKMHLLLTHRGHTWEDFRTKAGFKMNGDRLESELAYSNKDGRS

GLFMLATPDPDIDFFKTTFFQSLTEDKFEGRYTAQYGESSKPYDLGLATAYSDDKIAFNS

NLKTPHTEDVEIEIGFARKRRVEVKLFGMYGSNDKINVAIKYNIKKEFWDVGGEFEYLIQ

GSGRNIGLTFKRDGPLDNLKCYITGKYMGKQVSVETQYKDGEKKNGRLDVSTNFKDYTNL

AASFEHSGDSENFETIVYLKYRDDKEASGTVDFQRNKWKRVAMTMTFNLPYEEFRENKLT

YKHTNRKNKLSAEGSLKLGPARAITGELGFVDGRRLTVSVRGPYRQFESFTATGTYDKSN

KRLEGDASVKLVRETQPVTATYKVRAGTWPIMINIEAQTPFEGWKNVELTTTHDGDVTDF

TSSHSLTAEAVGTVRSELIWTYTSPSVLDGLWTLSSSVEGMEDLKLAFKNSESGSEYNSQ

LIVGWDLLKEIVLDGRVHLDKSFSSNRCKGSISMTTPFPEASQFSVSFDHSHDVRLRQNS

AVARFNDKTYLDADFQYTVGDRHVGSMEFREPRPMEFLIGGVYTEKAMEGELKLNWDKRD

PEMNIHLKGDYTDHTDQLGTDRNMKVWVIHPARTMGMEYLMKKAAKEFQSDFLLTWDKAT

SQTLSYIINWTDRSSHYSSAYDAMVKVGVPMRSVQVEGAYSNTGRAKAAEGSIMWDVDRD

KNMKVGVKGELDKRGNTNKVRLGLDLPFVDKEYSLTTTAAVNTGSTLLDAKTEFSYSVDP

RKLLVVTTKIEDMSYGRDATNYSLAFSVLHPMTDVSIQVMSHVGTSPVLSSMATSVVYLT

AEREKKMMELRGQIDKLKRVMGIEFKSPVKDVSLKGELSGGREYNMRLVSNVDGQKSQVT

DIFIAPETRTFEMTFRYDKAAPDNSVKLFGHMPSDSILQTELYRMEQGRRVSETLMTVRL

NSSHVLHTRLNWRPNMLRDLQQYLGTQLTVFSYGSSEAFQAAAGAVGHELKAKYEVIMQE

VKEEAGPLLQQFQKEMKSLQVHLDSMQTDMRRFYYSNALHVQDLGGVATDIMETILTGMQ

GMVMQFTQLQQQLQQDIAQTMQQLGKYPMAQKYAQLVADFAAGLKNSRVVLEQALDQLSI

QVSKFSDQSYRKYLIISRQIERKLKGYTAALRQTPPYQTLAQAYNTAQSSLSQLSRGDYK

QLADAANLKLMNALDQVGLKEFYLLMVTKSKRLIADQFHDLVRRPEFQQIYVISNEIYQQ

GVWAYHYWQVEKNTRMAVGQLLELAKDITLLEITRIKNAVVDLDKSRVIVFDPQNGELQF

ELYLPVALKSLAEPPDMRVMKYVNRVRSWVATYIPSPQHGLWTSINNYLPNMDTNTWLPP

FTAHAYVMGDQHVVTFDQKHYNFAGRCSYILARDMADDRFTVVLNYNNRPRHPQVQSIVI

LLRGVAIEVDRNSKVKVNNEERELPFFSGIISAVRQQSIVRVQDGQALTVEIDSSRAIYS

MALSGWYHGRTAGLLGTYDNEPFNDMMDSQHRITNDIEAFTNSWEVGHRFCRSRNLAISE

DPESESEEVQACRKFLTDSSSYFSPCFGQVKASELVNVCVAQARSGLSIQEAKCKAGQHY

RYMCARQGVMLGTPAECVSCQLPSGQILGAGESETLKEEVGQGLESADVVFVVEESSCNA

WAERSLARLATQLAQSLQARGLRGNRFGLVSYSGNGSQEQQLVHTIEGQVFGKPSNFLKA

TESLRLIYPSTAGNTGAAVREAVKYPFRVGVSKLLVLLPCSDCTGDMYALGQVLLSHGFQ

LHMLDAQDFQIDGSSNSSPVRVFGLDNSKAHTQKSAARKSEAIFKKLGTPQGSCAQLALS

TNGSVFDVSHMMARSRIQNEFLESFAARGALNALPPTCQVCSCHDDGTGHGHTVCRLCNN

NSWLWNHIPTWLQSSSTSIMGVFNDLHHQIITSLDAPVVAVTA

>SG-PL_contig_3203_1_-1_244_501

MQFVTHHSFVLVHIVSVHIVSVHIVSVHMRFVMRLSCSFGSASVISCDQCYLFHDMQKEV

CAASFSVGKLKDFFFFQLGKGVCKIN

>SG-PL_contig_3204_1_+1_100_252

MCVRGYRKSKRFSCISEKLTITICHTVWLPLVPGELANPDTVGAMSAGVLY

>SG-PL_contig_3205_1_+3_3_116

TCGIFPSASSKKRYGSFDSLYFFLLVFIWCLCICVLIF

>SG-PL_contig_3206_1_+3_1794_2078

MEAKLQKLKNSHSNSRRICETLGVNLSGNHQNRQTNKEENNNQNYKYFILHKCTYLQSLT

QNVADNLNTDILTKNVSDAVWMFTPSFQQNETDTV

>SG-PL_contig_3207_1_-3_3501_3710

MFSLFDMVCIDLFSFWHLFFNMSAKVDMKVCIDLFSSGICRFVHFRHLFFSMSVKVDMKV

CIDLFSSGIC

>SG-PL_contig_3208_1_+1_46_222

MCDSHLTGTFLLRLRPGWGNGLCPSLKMAIKKKMLAYVFFFSLTCPPLLLFLFIFYPYF

>SG-PL_contig_3209_1_+1_292_399

MLMSTVTTSLHYINLRNLQMQILSLTKSKNVFYGQY

>SG-PL_contig_3210_1_+2_2_202

EGFVRSPKSRRRGLSPRGLLCRLRKPAPQGHRLVPAASVLAPAVPECPLQWAFVDSVQRN

ADSVQRE

>SG-PL_contig_3211_1_-3_21_812

MADAPKKFTFRVIEGKDFPSLSNKDNQDYLMKWCMKGRLKAQLYSFDQMFQAYEKDQFVL

DFFKDENVTTTLQTMSDSGKWSPVGLTAESVTATQIPATTMSMAFFDKLYEDDQIVRSSS

GHIRKCMDEFFEDFTISDELRQMLLNEDSDSYCTFNESDREQFLFHLFEHICLGGQICQY

EDTIDPYLDVTKQLYKDLISVQKNPDTKDLTITSSVFKVTAADKNGTYYPSVKEHRQNFS

YLIVDPLKRHVTVLYHQFGAIAFT

>SG-PL_contig_3212_1_-1_97_225

MFSYNSISRLHYLKFNWPVFLDRNAAKHVIYSPYGKGYHHLFW

>SG-PL_contig_3213_1_-2_2_190

RLNVVKNDAESWDYTNPSLDRQAPSGWSLAEVCTPKLTTTEKASETLCTCASIQLRHCEV

HIV

>SG-PL_contig_3214_1_-1_43_1122

MGDEEMSSGYEGHMSKRQKTDDGMHGMGGRDRFDDPHKPNPSPVLHVRGLSEAAIEADLV

EAVQHFGAVRDVIMMPRRKQALIEFEDINGARNCVEYVQSSAIYVAGQPAFFNYSTSQKI

QRPGDPEDNRPANNVLLFTILNPQYPITVEVMHTICSPYGQVLRIVIFKKNGVQTMVEFD

NIESAKRAKQSLNGADVYSGCCTLKIEYAKPTRLNVVKNDAESWDYTNPSLGKDPHHQQR

GQPLLQEPRFGNAPAPYNGPADMRGGPAGAYGGGGFGGGYYDDYDGYGGRGPFGGPPDRF

GGPRQDRFGPPGRYGEQSMGPQASGQGCVVMVYGLNMEKMNCDKLFNLFCLYGNVVRIKF

>SG-PL_contig_3215_1_-2_23_268

MCACMCVCVCVCVCEEGVGKIREMCDVSVSRCMILYCIVLFLSQWIYHVKIGLLSRVEHV

AAMQHHPPFFFSACMLGVFTFP

>SG-PL_contig_3216_1_+1_937_1089

MATFSEFNHTVHSNVVHTGRPFHTHCSLTLCATFTDSLKLNTHSQISICTQ

>SG-PL_contig_3217_1_-1_199_378

MKSCYVAEREETALQVMLWMMSLVYSTNPVLSNYCSDVMLCSPSCCSQMSSGTVYSCLNC

>SG-PL_contig_3218_1_-1_1_198

CDGRNEQTNRKTDKQANKKTFWTELTVWSVTTQRKQTPLRQILQGEKWWKERERSYVTLS

ECSASL

>SG-PL_contig_3219_1_+1_1_318

SNRERERERERERERETDRQTDKERQTETERQTDRQRQTERQTNTQRDRQRDRQTDKERD

RQRDRKTDKERDFYTLQVERRTKRGTFTLCKSTVLSAIKPLGQKRV

>SG-PL_contig_3220_1_+2_2_265

VSHSATGEVETGASGRPEDAWVPRADWRICRKSATTASLSGKPSGRLLRRCHLRLACLGS

PCSGTSVGGSSSGTVHPGGRVEGRREGV

>SG-PL_contig_3221_1_-2_143_334

MQQHITKTCQAAYIEVRRISSIRHYLTLDATKTLVSTSILSRLDYCNSLLIGCPRKLLRP

LQQV

>SG-PL_contig_3222_1_-1_145_276

MLQRKAVILLLSCIIHRACHELSSLYRYWPLKCVLYACGKSKHN

>SG-PL_contig_3223_1_-3_123_866

MTDYEEEQRNEIEALEAIYPDEIDILQTSPHHVFTVKVASQDPDDSFQENIDGTPEKVGC

TVQFTYTPTYPDEAPVMELTSIESLEDDQVEMIQEFLTEQAKENLGMVMVFTIISALQEK

LTVLVEDMKKFAEEKRIQKQKEEEEAAQKRFEGTRVTVETFMAWKTKFDAERNERKRMEK

DRDVLSKKPTGKDLFMTDATLNDSDINFLQDEGDNVEVDESLFEDMDDLDLDEDLDDEDD

EDDPDYVP

>SG-PL_contig_3224_1_-3_159_509

MKWQYKEEHPFERRRAEGEKIRKKYPDRVPVIVEKAPKARVGDLDKKKYLVPSDLTVGQF

YFLIRKRIHLRSEDALFFFVNNVIPPTSATMGALYQEHHEEDFFLYIAYSDESVYGA

>SG-PL_contig_3225_1_-3_132_236

MICSLWLYEIVTFSQNCAKVFPFHVINCQVSILKH

>SG-PL_contig_3226_1_+2_11_322

MDLLCEIWDALPVSPECSATLFFCLRVFLFTYESGFFYRILPGTTPSLPWDLLRALRACS

TWNLSPHPKDWQEASLTCAQSEPNVSHSERSLFHKLLKLTKQKQ

>SG-PL_contig_3227_1_+2_2_166

PNTIEYLVKKHVHSRSTKCFMSVQVLATQSARTHLYPWPQFTDFCSVYVHIYVII

>SG-PL_contig_3228_1_-2_398_688

MQKCVGSGVPASSKGFFFLKKKNLHNPTCVQNYIYIYIYIYMSYPPLLPRFCTHVLPPPP

SPLIFVSVTYVYEDVYNQHIQPFCTENFELSMRINGL

>SG-PL_contig_3229_1_-1_148_1380

MEAPKFSRQRALGKNKKKSWRGIDITETEDFLEDERFQLRTTGLVAEKTDDQLFVVERQC

SDKTDEDLAPKKRAKRELNCYSNLINQSRIQCPRKEYPTDESYNTRSLRAFEKKGDAQNK

TTVTARKRPQSAKGDVADLWTEEKTEGEGDDFFNQVTKRKRVQPPKHIRRKCSERAVVEI

PHPGASINPAYDDHQDLLLQAHVVEMKKAKAERKVYNALDAQFPSASEAPTVATWIKEMS

AGLCEEPDTGKEEVDDSHISINPPVNREKKKTPRERRKEKAAKLKKQVMEQKKVKKGTEN

KVYSIIKIRSELSKDKKEATRRAQVKARKQEQKKYTPKKLGKTKVVVPDMELKLSGELVA

NLRSLKPEGHLLEDRYKSLQMRNLLEPRAKAHKKKTKTKKFEKKSFREVTI

>SG-PL_contig_3230_1_+2_2_205

KKKQTTTRVGQSFTTLLNGAYAFLVSTSSRLCKRPNLFQHKRTRNNFHTYTNLFLIFSVK

IQKSFNAI

>SG-PL_contig_3231_1_+1_166_387

MVSENLPNLKLQFYNSNVLSTLLYSCETWHLKTSQEKKLDSFDLKCLRKILGMDFVTNEE

VRSRSKQNPVTSII

>SG-PL_contig_3232_1_-2_83_2203

MVTEDKELAKELKPLRDHIHDPNLPEDDKYLWNFFINKAHRFKSDDEDSEMDEEALEEEL

FQQVIEEEQEQYEQAFNFRFQEPDKNFIRQYQRRPEETIRQEVKKRVQKRKEIKERKKMA

EEERRQEIRHLRRLMKEELQSRVERVMKECPTGVDFTAEELKGDFNPKAYDKMMQAKFGN

IDSEDDEGDDEKPDLGDLDDIISIGSYENFEEEEEADLLEEPEAAGSSQPEEEKAGSSGL

NGGYDADEDENAKSDIRGGFDPSWLPEFNQSSRRSRRKRKRTKFQQAIDAEKPKFTDVFE

KYQDSETPWDDYLKDYYNLDYKEFGFRYRNVVANDYGLQPGEMLWLPARELNAWASMKKI

MEQKPDDEEMRYKHKYQSGKMQRKMLNIMPSLKKLQDEQYSYEDIRPSTLRKQKLEQQVA

RDDSDGSEQPESQQSAEENIGISLQTDPRQSPKKKKKRNTDVSESVQETVPCKKKKKRQS

SSRKLLKCGAEEIEVTASQTIQDEGKKKKKKKEKSGHVDHSDSTKNKTEVTEQLTEVQTG

EGKKNKKKRKLETENQERASEAPNKKKRTENPEIEMMEDTTGEIKMKKKKKKAAQEKETS

VTTTDKGVSNGTGVISENLTAEEKNKEGKKKKKKKHLSEGGEHQQATPKSGKKKQKQKRK

GDGSSKTKGYVAIDRMQDSRLGAFHIDPRKFKYTKHRIGPSEPDQNY

>SG-PL_contig_3233_1_-2_260_955

MISSLAPNFLRSVVAKAAKRKMSSANKRGAFIVFEGCDRSGKSTQCGLLVDRLNKNGVPA

QLMKFPDRTTAIGQVINSYLQQSCQVEDHTIHLLFAANRWEAMPKMVDLLKSGVTLVVDR

YSFSGVAFSSAKPNLSMEWCRQPECGLLRPDTVFYLSVSPQVAAQRGDYGTERYEKQDFQ

AIVAKQYQLLKDDSWTEINAEKLVEPLSDEISAHAMKVIQEAQSQPLQHLWI

>SG-PL_contig_3234_1_-1_631_750

MFEFAQEGIKQFHILSMTNENFVDFPTKYSECSVGKRGYV

>SG-PL_contig_3235_1_+3_315_503

MSCISLLGLLSNDGICLTRDTDRERENLLYIYLLCLCHGSNPQPITHNTAEGSGHTNHRM

LQL

>SG-PL_contig_3236_1_+2_104_208

MTQVAIFKKTYLNNSNFLFMVRYTFAELTKNTRLE

>SG-PL_contig_3237_1_+1_154_348

MFSYSYLETLFTLRSRANKKMKTKICVKLPSKYTHSHTLTCIHTCTNTNSNQRLIKLVQW

HIMPG

>SG-PL_contig_3238_1_+3_357_509

MIHITCRHFSLVSSKQVIHSMHILLFLTHVIRSMHILLLFLTISSFLIYNQ

>SG-PL_contig_3239_1_-1_238_360

MEHMLSCVYWSDYTLLSPKLYIITLERKSPPNTRAGSQKHS

>SG-PL_contig_3240_1_-2_2_118

GFPITQRSLGSEVVETSTFPRASIMRVVPISSPRCFTFH

>SG-PL_contig_3241_1_+1_100_738

MASNFAVTVTVVTRLVCACVLFHFSLATCPQDRSPVLDDKVMGSDVVVKGRVRGQVKVNA

LDSSYTAVVDVHCVFKGGPVPHLINVTEAGKEEGKCVQTELLEGGIYLIYLRHTAGGAYT

PTFTQDPADRKYLDELQYVCGLHVQLPTGIDSSKTTASCPPDSFAADYEVDCPDTSPPSP

APSSAAETLKAVWTMSCFVSAALASFPDLQLLL

>SG-PL_contig_3242_1_-2_2222_2455

MLRDCSYLTFCIVFLLCVLMFTCRLYISHCGFSITSELGGKYLLYVNMLFFLGFWSSIFN

VFSCVVKLRQDKILYLRR

>SG-PL_contig_3243_1_+3_201_476

MHCRHLVSTSVKCRNSFSILNYLATIYSSMKFIYTRNQNIKGICVLKSDFAQSELDSTLP

TKGAKEGNNNLAISAKCSCFVASLMLNYSRHS

>SG-PL_contig_3244_1_+2_2_214

DLCLFVQLQRCLSLGSWDKPKHIIHHIKLENTRIIVSCIILYSLNLNRNYKRFKPLDEPG

RTELWPYFYIF

>SG-PL_contig_3245_1_-1_1_303

RSVSSARTRCLSSVARFKPQLISSEKHSLSECLVEEGRHRCQAGQLPLPDPCQLPLPIPR

PLLLPHPLPLPLNSRVCSLRWQAPPLVWLSALLWVTPLEQQ

>SG-PL_contig_3246_1_-3_690_920

MTISELCNELNYPGLCLRRGNLRRLWCFRKCVTGSTIVPYSSYMVWSIFVLDAGILWFNP

FHCLRSSNVRVEWIELK

>SG-PL_contig_3247_1_+1_115_678

MNTFGPHKISRRMTWHSPGGTYQNQIDYIMVKHRFQSSVNIARTRSFPGADIGSDHELVL

MTFALRLKYNKRRGNIRLKFNVDKLKDPKIMEAFKANIGGKFAPLLVLANEGIDLEDFTD

SFNKAITETATEVLGKHRPKKKPWVTDDILELCDKRRELKNKKNEPEGRKQYNDVNRKVQ

TELKTAME

>SG-PL_contig_3248_1_-1_130_1110

MADTDKLNIDSIIARLLEVRGSRPGKNVQLTESEIRGLCLKSREIFLSQPILLELEAPLK

ICGDIHGQYYDLLRLFEYGGFPPESNYLFLGDYVDRGKQSLETICLLLAYKIKYPENFFL

LRGNHECASINRIYGFYDECKRRYNIKLWKTFTDCFNCLPIAAIIDEKIFCCHGGLSPDL

QSMEQIRRIMRPTDVPDQGLLCDLLWSDPDKETMGWGENDRGVSFTFGAEVVAKFLHKHD

LDLICRAHQVVEDGYEFFAKRQLVTLFSAPNYCGEFDNAGAMMSVDETLMCSFQILKPAD

KKKFPYGGLNQGRPVTPPRGNAKAKGK

>SG-PL_contig_3249_1_+1_1_144

STSYRTNRSNMNKITLKSNHVHSHTSCTNINYTKLPNSTRVSSIVTNI

>SG-PL_contig_3250_1_-1_1096_1389

MQPSVHPAVNRCLSQGMYVRQMPIPKISFCNFMEDKSARERSTSHTSWPESREVYPSSFP

KLPYPYTFCIVGGGFFFTFLSYCVLLFLFSICVTYLYV

>SG-PL_contig_3251_1_-2_2_169

KKKKKSPQRRHHIAENQPTPWMRVRKMRNLVQDRKYDTDKMVRDKNLFDFQGRRLG

>SG-PL_contig_3252_1_-1_391_462

MQWKLHQFIMCNVSQILSHLHKKK

>SG-PL_contig_3253_1_+1_355_483

MTMIIMQINYKQTCLSQISVHHRHQKSKTIQADFYSRPLLKNM

>SG-PL_contig_3254_1_-3_45_1850

MADFGFAKFGVDDLNEEEDEEEAEWRIPATKDGLLFLVDCSPTMFEQVEESPCHFQLAIR

CIKTTMQNKIISSEKDCVGVVFYGTAQSDGPNKHINIYHDLGQPCASRILALEDMEETGW

KNFNTLYGYDPGYSLSDALWTCADMFSKSTQSLNNKRVMLFTNNDDPHAGNANLQLQART

KASDLNNTGIDLELIHLVHPGQTFDVTKFYKDLLFTDDDELTELPDAAERLDELLTRIRM

KDHKKRATRRIPLTLGEGVSLSVGVYNLVRTCPKPSMVKLYQKDNSELESHSKTYLQDTV

EVLMPQDMKKAQTYGGRRICFENDEVAEMRRFDESGLQLMGFKPRSCLKPYFHVKPAQFL

YPDEGSVTGSTTLFTALLRKCVEHDVVPVCKYVPGKSNPPKFVTLLPQEEELDEHRVQMS

PPGFHVIFLPFADDFRKVKYEETPKATEEQIDAAKEVIKKLKFPFSSEAFENPVLQNHWR

NIEALALDRDEPDELIDYTMPADDKITKKAGSAIRDFMDLVFPADYVPGQKRRGAGPDAA

TKRAKAAEATSELDLEGEARAGRLGKLTVAVLKDAARKANIPCLATRKADIIDAISDFYN

VD

>SG-PL_contig_3255_1_-2_29_358

MKNKKHGQGTFIYPDGSKYEGGWVEDQRCGFGKYYYVNGDTYEGEWQNHVRHGRGTYTYA

ATGTKYVGMWNTGKWDGHGELIHANHKYVGTFKENRMEGKGKYVFDIGCE

>SG-PL_contig_3256_1_+2_932_1192

MISLAHFSFMFMTLPLTQTWPLYIVRETPSCKVMTKICVNSPHCSFLFICKFVTTLGQLS

YQLSLKCRSCYYYHFTSSQSRNKCLLI

>SG-PL_contig_3257_1_-3_3_692

CNKNYVGTNGRITSPGYPNVYPRNIDCGSTVTAPAGYTLSFYFTRFYIESHSQCRFDYLR

IRNGTNSTSPEVVKLCGADLPDPVFLSSNTAYLDFHTDYSAQHLGYDITYLATTSGVGCG

GVLYSGINGSFTSPNFPGNFSDATTCTWSIMVPARRYVSLNFTYLATPGEEDCQNNYVEV

FNGPTTLSPTFGRFCSSLPGTLRAVANSATVKLTSTGSSPAPSFRIIYYS

>SG-PL_contig_3258_1_+2_410_583

MWQLKGANDVNLRHTHMHTHTQTDTERQTESEREETDTQTTLTSIQCSFRHSHILIHG

>SG-PL_contig_3259_1_-3_303_2144

MEVQVLVTCALLAVVPCLTHVARASGQPQQRTGWQDLKDLETEFWDWRMQDAPVFASTVG

DSTHNDMLESYQPDVYANRQSTVQNFFNRAQAIDSTHMNKSQKLDYDIFTDHLQTFLDGA

RWNDYFTVNPVNFLEGITYDPGRMATFMPFNTVGDFENYLQRLIKLPVQVDQQIDLLKIA

VSAGHTLNNMSVAAVPDQIDSLVNETTRDTAFYQPTFTSVLDKSHIGMSKRRDLRTRAET

AINNIKQALLRLKTYLIQSYMPAARPTWGIWGWRDGRAIYEQCLKYHTSTNMTAQEIHEL

GLREVARIRASMDQALERLQFTGSIKQFYDSLRSDPRFHKNSSEALLDTYKDYIYNRVTP

KLDALFKDIPTLPLKVEASESDGPLGIYLSGTPDGTVPGTFYANIMHPNDTVTISMMSLV

LHEAVPGHHLQAIYSLTSSIPKYRAMYEDDHYYRMPATFPIYTAYIEGWALYAEYLGEEL

NLYTDDYMLMGRYSEEIFRACRLVVDTGLHYFGWERERAIQYMLNNTASSRTELTTEIDR

YMTWPGQATAYKIGEIKIKSLRQKATKALGSRFQLKEFHHVLLQNGGMPLSLLERLVDQW

IAEAQVRMDTDLVG

>SG-PL_contig_3260_1_+2_401_559

MPYYAEHGTTIIHLVDFFFFSLNLLHNCVLGRRQMTLLNFKSSVILNSKSSKL

>SG-PL_contig_3261_1_-2_287_592

MRNLAVLLLLVAVTGLSSAYWIDGLYNNREFYDDEDGVFSENKRRGLGQSKDLKDLCIPW

RAPCPSDASLSQQYAFLRCCGGLSCKCTFWGNNCKCESRLGR

>SG-PL_contig_3262_1_+2_77_307

MDIAPVSIFNQYYVVLMTVSPSPLYSSILYNLIIIYNLIIIICLTFIIPLSALSHFCVRS

SKPPYFCIHARYRRGLA

>SG-PL_contig_3263_1_-2_2_256

HQNNARRRSPNHSLCSRRPPSGKLNQTRIVQNPTQTLGLLQHSDPLNKYNSYRNKCLPNS

VVLKEYRQSTLCSKLSVADGDACQN

>SG-PL_contig_3264_1_-3_108_1631

MASAQSEIIEGFLCPMCMKDFGTVTQLQHHFEDAHNAEDKAVFQQLKGFFDKAKKKIMGD

KETNPFIEQGSSSAGDSGYAAAISGYDPTWWEPQELGTTRGHIDLFKAIRGARVDHFVVE

TNKLLIRLDKLMTPDAPTDPKRRKAFEMSVVRWIPDKEVPACLTCIKSFGLMVRRHHCRL

CGGVMCDKCSQFLTHSFAQKLINPAFSFEGQGFLKRSNSNTSLNSLVSFDGEQHMRVCII

CRQLLERRDSMTELRNTKPPIVQLYEKMKMCIEEAESILPQYLPMVDSLSQGETVCSYQD

AMILRTKLVKLYEAIDQLSKRILQFGLNADPPPHPKQIYLQKALRMYASNFMQDNLMGLQ

NMPTEEEVKRLQEIRRDEIQRKIAAERQAAMEAQERERVQREKDMSTFALPSSDSPGRSE

HSAGKSLGHRREDSTASNSSLGQGWKPEGETEHASRIADPMLQQMEIIRGYIRQAKQAQR

LDEVAMLEQNLRDLQLEYTRQRQTNDAS

>SG-PL_contig_3265_1_-1_115_867

MLVIIVSLGFGIVKPRLGQAFHKVLFVGGLFFILAVIEGSMRATTSKADQSNQFLLAAVP

LAVLDAIICWWIFSSLIQTTRTLRLRRNVVKLTLYRHFTNTLIFAVLASVAYMIWSMTQH

KFRQCIKDWKQLWVDEAFWHMLFVLLVFIIMLLWRPTANNQRYAFSPLLDAADEEDDEDA

LMNDAFDGMKMRGVKAANGSPKQRDPKSKVEDDLKWVEENIPSSVADKALPTLLDSDEEL

MTTKFEMSKME

>SG-PL_contig_3266_1_-1_1_765

DFPHVRAQISTGSACRLQGCHKESTLARLSCSSVVTSFQPCRTFHCSSALYEEIRIVTTP

AFPESVAEGDVRWEKAVGDFVAEDESVGEIETDKTSLALFAPAAGVIEELLVPDGGKVEA

GQQLYKLKITGEAPPKKVEPAAEKPAAAAPEAAAPPPPPPPPTSQPSTAGPPPSTPPPVP

PMPSQPMASVPTSAVKPTPATSTTPSSVTGTRTEQRVKMNRMRQRIAQRLKDAQNTCAML

TTFNEIDMSNIMEMR

>SG-PL_contig_3267_1_-3_168_302

MTFICFTDLFLPPLCPFDSRWNVYKKRKCNLMNLSYCRTYGFQLQ

>SG-PL_contig_3268_1_+2_1904_2167

MSRVYQKCFFLHTKKGNLKFICSKNAILFMLTFTIPTINFCAPYQLGGNCPMLLKKNPCT

AYKLGNHRPIAAYIVKLTQRTCWGIIDP

>SG-PL_contig_3269_1_+2_2_370

VRGARARARVCVCVCVCVCVCVCVCVCVCVKLIFSLFVHYYSIHLHLLKRHSCKRFVSLI

PFEDGVCRQSGNTVHHVCHPPEQRAHMKDPMAIQQLFLSKSCQNIHLRQETENQCSLSAC

ERS

>SG-PL_contig_3270_1_+2_176_523

MAERERERGRGKQKSMWCYVHDVQDGLFICFIADREQGLKRTVWFQAKQPCQSCSLSLLY

LDTADIYRLLAGQIEHDASTHRYISFRFQCNQKVCISVLNQPQVVLTYMLYNHLET

>SG-PL_contig_3271_1_+1_1_288

VFFLCVCVCVCVCVCVCVCVCVCGAWWMCVCVCERVCVCVCVPVCARVCVCVCACACACV

CVCVCVCVCVCVCVCVRARARARAISLTIIDSVYFY

>SG-PL_contig_3272_1_-3_1482_1784

MSLPTETFLSGCLCNGSIYYLLSRNYRLHFVLDLKKMFCQFHSPVSNNLFFVLSASPASL

QDQPGTTTLSLVQQLLLFNNNFEIKYIKMLRKIISLYLLQQ

>SG-PL_contig_3273_1_-2_158_349

MATQTYPACTLYSPFTGSMLALASPLDFTGVVERVGLLHGQQQVLLNTLPRLVSWSLSSG

TNSI

>SG-PL_contig_3274_1_-2_2_220

NNNRNNNGNNRNNNGNNRNNNGNNRNNNGNTAAATTTTEITTATTEITTATTEITTATTE

TTTATQQQQQQNH

>SG-PL_contig_3275_1_-3_42_230

MNVLLVDIFLFVSIDLQELVLVLLFSVCFFLVLSLTDGFCDLCNGPFTCLFPQPLLLSLR

FLA

>SG-PL_contig_3276_1_+2_2_316

SPHASFSRPQELSLPECDVFGRAFYEYYPSRSLSLYSYLLNIEPFFTAFRCSTLPRHFGT

NFQTRGSVPHAMTSQRFLAGERDFIAADETRADCVAFFAKYDCIS

>SG-PL_contig_3277_1_+2_239_481

MFGQDDKCFRHIIRSSTETLTKCVNKQNIKGLHFIGCVQSTFQGNNYVMLKLYTDINKVI

TQIVMIIWVQYVHLMHHTSKI

>SG-PL_contig_3278_1_-1_73_2139

MSREIDDPHKRLLTNLLQLQNLIKRDPESYKHEFTIHWKRYQELLDVFQMDPSTFLKEFD

DLTSFLAQVSHCYSDELKEYPGLIHEVLQAHGTVLDPTIRMTMVKGLIMLRNKNLIEATS

ILELFFKLFRCQDKLLRKTIFNYIVQDVKNINAKHKNVRLNTTLQNFMYRMLRDNNATAA

KMSLDVMIELYKRNIWKDAKTVNVIVTACTSRVTKILVTALKFFLGKDPTEEEDSDSEDE

KQPSQKTAKEIILAHRVGKKSRKRQKKLSRALQQAQKRKKNKQKSEVFDFSALRLIHDPQ

GFSETLLHRVEHGTENERFEVKLMMIDLISRLIGVHQLQLERFYPFLKRFLQPHQKEVTK

LLLFAAQASHDLVPPEDLEQVLKTIADNFISERNSSEVMAVGLNSVREICARCPLAIGET

LLQDLAQYKTHKAKAVMMAARSLIQVYRHSNPELLHKKDRGKPTEATKERKLAYGEVATH

EFIPGAETLLKMDQEQQEQEDWESASDDEDMDDDSDGWIDVHHSSDEEEVKEETPQEAED

KKKMAQEVCSTRIMTQEDFRRLQQAQLAKEAGLTTTTKSRKRKRPAHTSQDRSEMPNLGD

IENVHKKRAHDKDSRLSTVMAGREGRGKFSGGPQRMNPHASTTNKEKKKNKAFMMIKHKP

KLKEKKKRSFRDKQIALRDYLLKQQKKRR

>SG-PL_contig_3279_1_-2_290_592

MRCVTTTLLFYTNSTTTSCMAMFCRSGLDDIQGSLVHDVYMYIVFVTFVYLYMCVCAVLF

VCVWWFLLGRGGGCFYSCVGTVTGSCAVITIVLCVCVCVCV

>SG-PL_contig_3280_1_+3_3_140

QCETFNHSISPCNLQDRVKLSIVVLVPTIYIGQGETFNHSTGPYNP

>SG-PL_contig_3281_1_-2_302_454

MIKNGLICEKNSDLMFCFEISAHCRGEKFFLFSFPLLLNLYNKHSRNKKKK

>SG-PL_contig_3282_1_+1_187_399

MTSSLTLEALLCDCYVCMRTCVHMQRKLARISQQWLCVCVCVRTLVCICKLSWQGFFSNV

LFSFFFLKNQI

>SG-PL_contig_3283_1_+1_22_237

MYAHCLAHTVQELCCCTVCILLSSRGMHTQYFRVSISHPTMTWLTGALIWVLLLEVHTHT

KGVQTLAGLLIC

>SG-PL_contig_3284_1_+3_3_272

LSSQNLHNSNQHIVIIISRDVSLLSKPSQLQPTRHRHHHHHQSSSQKPSQLQPTHHRLFQ

TTQYGVIQQPALREFKKFVNHIKEKLAKLH

>SG-PL_contig_3285_1_+3_3_182

LHMGPDRLPFKAYQMLIELDRSGKDCWASKIREILRETGFNIVWLQQDVGNVKVFLRLFN

>SG-PL_contig_3286_1_+1_31_351

MDTYCSRVAISQARFNVLPLNNNIHRYSESDQDKACPFCKSQSENEEHLLFHCSTYTDLR

VKFLGDLINMPLHSLLRTTNIRHRHSVSRYIFHAINRRKRLIIGDDH

>SG-PL_contig_3287_1_+2_344_493

MNAEVRQWYCCFDSYQNFVTLYLKCRLLVDQIVYVVNCILTSWIIGLFVN

>SG-PL_contig_3288_1_+1_871_1050

MKFVWTPWLTDKTKNKHIHRCHIYVSLQFVRLKVGVGKCFKFEHRLKLEAEQQEFNDNVQ

>SG-PL_contig_3289_1_-3_3_191

FFFYYFLFNLILFYCMYLFSKVCSWLPNCTCFISSFQWLVCFLFLLYLVWVCENTLTVHL

GVK

>SG-PL_contig_3290_1_-3_186_1511

MDKIQFLTADGISSGALAGILVSGAAVLMLIVVLVIVWTRRRRRKPFDLNLEEIHNEAIS

VKSAPNSRSSSPKLPKRLSCPDAFTTPMRGVVVRSVTVDQIPDFTLPPERVQPPRPSGPA

VPHTTPLQYQQSLIGNLQPDLYSHSSSFSEDEDIYLPRSTYGRLWYSLVYDAAVEQLNVT

LIKVKDLPGRGSHRHPRDPYVKVFVLPDDRTCRTSKVRKKTLSPIFNENFIFSVPANEVK

DRTLRFSVYDVDKRKVRHSLGHVMVPLRDVDLGHVDTYWSDLEAMTQVVSSLGELQLGLC

YQPQTEKIKITIMRARSLKHADMDLDTRLYLRLQLFYGRKVHRTKRTGSRQSSVDDVDFN

ESLSFSISGKQLDSCRLEAALMLTTQRHGAPMLAHDVEYGRVVIGPFMYARGEELIHWQE

MLSQPRVLITRWHSLSANVSHE

>SG-PL_contig_3291_1_-2_194_421

MVVSDWRRLKVPWLCHSSWELAKHGMAMCKLCVCIYIHACVCMCASKREMERVSSIFVFQ

EILLLTPLEGRKCEFS

>SG-PL_contig_3292_1_-2_734_1003

MSCFVCCVRVYSLCACAYVCMLCNVYMSVTVYRILLVGVENELLSIGHMALLSQSVFISI

LFPAVCCLCLYLFPFHVLVRYTLLIKVCIY

>SG-PL_contig_3293_1_-2_32_703

MPMPGLGMPSPGPFGAAARLGMPLQAQAATSVILVSNLDEEKVTPEALFTLFGVYGDVHR

VKILFNKKDNALIQMADPHQAGLAITFLDKVRVWGKNIRVAQSRHAIVQMPKEGQPDAGL

TKDFVNSPLHRFKRPGSKNCQNIFAPSAVLHLSNIPPSISEEQLKSWFADYGNVKGFKFF

QKDRKMALIQMNSVDEAILALIGMHNTQLGENSHLRVSFSKSTI

>SG-PL_contig_3294_1_-2_1673_1864

MTVSTAGFNSHALCDSSHIRLYLLSPLLFFFSVSFFFFFLIMMEGGGVLVGFFPPPPFLS

PPPP

>SG-PL_contig_3295_1_+3_291_533

MNKQLKTTKSRYGQRDRFLDTKRSSYFKGFTQCAFKVTEKLRSTCLIILLPISRVSDCHA

HMYIHTQHFAHLLLVCRRSYI

>SG-PL_contig_3296_1_-3_171_512

MCLYDEEVLQSDLESCIIAVTVNRYSRDVAFNFVMEHFPVILSSFSSDTVGAIVNYATEH

LHNNFHLAQIQLLMDTYDVSGVTLYLRAIKQNIIKNMKWMENNLDIVKRWLASK

>SG-PL_contig_3297_1_+2_1205_1441

MKMPKMFTVTTAEKDSSRDNSLYHWNHASEAKRVELSLGSGSSNQMTAQTAYSNLRDYQM

ISLWAFQILFFSLKSHQQM

>SG-PL_contig_3298_1_-3_81_353

MNLYRKATQQKAAAKNRASDRRGTRPVPTLREGNRGLNGLQSVLQGTELCHVWHCAEFSW

CYLQGTLLCLMDTGFHCLGIGCNVKACNNIN

>SG-PL_contig_3299_1_-1_142_1932

MPPTGQAADPLALLQEKINGSKVVVFSKTTCPFCAKVKDLFKSLNVQYGVLELDTHENGG

ALQTALFELTGQKTVPNIFINSKHLGGCDDTLKAHAENRLLALINAQPVQAYEYDLIVIG

GGSGGLAAAKEAANLGKKVALLDFVKPSPRGSSWGLGGTCVNVGCIPKKLMHRAAILGEE

FSDSKAFGWEIPEGIKHNWDNMKNAIQDHIGSLNWGYRVQLREKNVTYVNGYGEFMDNHR

IKATKRNGSTVEMTAEYFILATGERPRYPDIPGVKEFTITSDDLFSLDHCPGKTLCVGAS

YVSLECAGFLAGIGLDVTVMVRSILLRGFDQQCAEIIGEYMQNHKVKFVRGAVPTKIEQL

EAGSPGRYRVTARATDGKEVVEEYNTILIAIGRDPCTAGIGLDKVGVTLNSKTGRVVCND

QDQSSTAHIYAIGDIVQGRPQLTPVAIQAGRLLARRLFAGSSVLCDYQSVATTVFTPIEY

GAIGYAEEDAIEKFGEDNIEVYHSNFMPLEWSVPHREENACYAKLICNKTENERVVGFHV

VGPNAGEMTQGYSVAIRKGATKEDFDATIGIHPTCSETFTTLNVTKKSGASTQVAGC

>SG-PL_contig_3300_1_-1_100_324

MGARMVDLSASMDPTRLAESAVDLNLKLMRWRLLPDLCLDKIAATKCLLLGSGTLGCNVA

RNLLSWGVHHITFLD

>SG-PL_contig_3301_1_-3_60_212

MFTFCFHCCDSVPLTLLSCQVIFCLKLFFNSLSAEYMPSLLPLVPEFLLQY

>SG-PL_contig_3302_1_+2_977_1240

MECSGEGQPFNNLRTKLCSLHDILFKNRFSDLYTNFLSFYGNNFYKSELYRKPFSFFFFS

PIIMIMNCIFHQQHSSLKYANNPKKTKP

>SG-PL_contig_3303_1_-3_3_176

LSECDASFLNKFHIQLFFGLCGLIHSLTQSSSLQVEHKASTLSCVDISVAVILNMVGC

>SG-PL_contig_3304_1_-1_1_168

FSCTGLCAAFFLDCCTDVILKLEGQPIDRSISQSLTRDVAACEIVFQYSRVPAWLV

>SG-PL_contig_3305_1_-3_111_1259

MDKSTVILLLCLAGCCAASNMQIFGPKERTMKVGQSLQLICQVDLGLEVILNNVVQWTHV

SQVDNTETVLTQSQYPKEEGTRLGYNVSVQNSQSETNFTLSVTSLTDPMDGKYICSDVNK

EHNPADVEVVIASEVQTVQLTFDEEVVKNVDKVTVQREEGEFEVKCTAKGFSPSAAVNIF

IGEQQLETAAPEIKIDTELSQAVKARRYMATVSIDSLKLEGHHDGQILKCEAAASFDEAI

SKVASMPLKVIVLEPEIMCSENTSAMSGDKYIEISCLVTHTNVNVKAYHWEIDGKDVTLD

NNSYNHVKTEPVKDEEEQDKTTLELYEVTQADFDKTFVLVVELENGEKFEKQISLLRKGK

TSASATRPAGYFLGFLLSLLLSL

>SG-PL_contig_3306_1_-1_1_231

RYTCYHGVEVSESDCRFEFSSVQRRRWNVFESSQYQPQVWPSADHTMSVINFIDSVCEGL

CKSHEQNHSGTESLWHG

>SG-PL_contig_3307_1_+3_2730_2957

MKYTPATFTTSITPYSIIFITLHYFHTYVQINKIMIHRNKQTNKKQTRRTVAIPQTKTIA

SKQSSLQFQATKLTAT

>SG-PL_contig_3308_1_+1_25_360

MDKSRLKLYQLAFTAQTTEPKLKLWSMQQTSSMLQMVTAKQSSLQMPCQFWKQPLQANCH

ICKKHFKTSDAPDRYCSRSLHTVASQEMKRRIEWRSWEQSMYRKTTASAPQR

>SG-PL_contig_3309_1_-1_19_2649

METETDSNKIPEPEIKTDPEDASADVEKTDEEKVEGEEGEEDEKAEGEEEVLSEDAEALK

EELDKIVVSPPEPQKPEEVFDPADFPPSYKDNLPKELLVLQYADNFRRQYVHLYRDRKPL

FLNPVNECLVEKFVCTTIRPTQLPYKELYNWDGAAEFVADYLDFETLDPAYEVPTRMLSP

ATVLQRQKGNCFEYSTLLCSLLIASGYDAYVVNGYATRETCLADETREICPLLKKKEEAI

RQERTKEMKKYTVKPPKDLRSRFILKMEARKVAEEKSAEDKRKAEEEQRLLELEKPPPDP

LHGLRVHAWVLVLSGKREVPESFFIEPFTGLSHPVSSPNYLGIESVWNNVNYWVNMQDCS

EGVGVIGYDLGDCTKWEFMFPGLDKPILEIPEAEDDLMLDDDDEDKDQDVEKHLDMPPSW

VEPLHLSQRDFEMRCPLGKKVKLYKRAKLEKFAHYLMKDGLVSRLSVFENRELTDLVMVK

EYYQHREDKLYSRIHNHRTGWVTEHFYEGRSHCLKEHQYKAGTPGVENERTMAFFNVARV

DGLVKRVETPTEMTEHYNDRDDFLYYKHVIFGRRPKKFGPQDEVNSVNPRPIVKMVMKFK

RNPGKDANEDISEMVFLVSEDKLQLTYHTEQSNIAASTRDFVKPTNWDEKGATLSWANEM

HNTFQVDPSVKSDKQVRLYQLLLDLLKEEERARERVRESEDEVREILADREKEEMASDLE

ISVYDTERNEKAKKNRKELEQRLLEEKLRKQEMEIDYLAPFLAQMSDPEKISRQQAYKLK

EDCLADLKQRLIDKANLIQARFEKETQELQKKQAWYQQNQVSMSKDDEEDYLNYCSEAMF

RIHILELRLNRHKEMAPHKYMALEQKLRNDARLAEYF

>SG-PL_contig_3310_1_-2_2_319

AVAGAFKFSDVWLQDHNNNNNNDNDLFDLRRSEALNCSERAISSRAETCGEPLGPLGRNI

ARGNYEDIETRAGLDIACQLLGDFTACMGDLSNDCGHEENKTTMVY

>SG-PL_contig_3311_1_+3_87_353

MCMPVVCKCVCMHVWFSVCLRFSVCSCSLMKMFSFVNMLNAEKKWTKLHHNCKTGNYIKL

HLSTTHRKTKWNACYIHKMNRTTMAFYHP

>SG-PL_contig_3312_1_+1_1681_2055

MTSCVHCDPAQCCSHHQIRHRYNYNGGTIMTASLCIQSNDKRSHLCFDERGRYQTGENQT

TYVHSEGVKPHHINTAYLQTSIQLLPITDKHSLLMPFKHIKLKIDGPRNCNKPKCASYFN

QHKLL

>SG-PL_contig_3313_1_-3_24_710

MENMEKYDLTPVLCQYLDRHLVFPLLEFLSVKELYDEKDMLKGKLNLLSNTNMVDFAMDV

HKNLYPDQRDPMSLREKRQEVVGKLKSLQAETDDITKIFEEPEVQRQIQSSRDGRQLFDF

LHKEYRFKQQMLDTLYKYAKFQYECGNYSGTAEYLYFVRVLLPPSDPNYMNACWGKLASE

ILMQSWDVALEDLKKVQEIIDANQFPNALIGLQQRTWMIHWSLFVFFNH

>SG-PL_contig_3314_1_+3_66_209

MTHRLCGPSLPNEPTARSTLGRNQTGPKNPTMIFLWNEGDLNPRPSSQ

>SG-PL_contig_3315_1_-3_408_740

MQGRIMYNPVTQSQSQTERHNIIIVIPIFMYHLSIVKNCLIIYILWPFMPCSHGDLSFGG

FGGVLESRNECLQGSDRDFIVWSLFKRDSSHSDKSIYPLHPNLPVIIWQNP

>SG-PL_contig_3316_1_-1_52_480

MLIDWFCFERYPRRFEPRPSRPSSLEIQCLHHSTTMPASHFPLLSLLSSCYTASLPTPSL

SLSLSLSLSLSLSLPLPPPLPHRSPSVIIIVFNVETDAYVVSQCYPTFKDKQSFKQISTL

QLNSAFHFLRLLLEKCLFDVIVS

>SG-PL_contig_3317_1_-3_93_182

MGSLLTPCISNLRFYTNTIYGFPTDSFTNL

>SG-PL_contig_3318_1_+1_202_795

MLVRPHPSPSTVYIGQNEIEVVQDFTYLGSSISNSSDMTKELNCRIGKATTAFNQLSKIW

NSKKISLNLKLRFYNSNVLSTLLYGCETWHLKTSQEKKLDSFDLKCLHKILGIKWTDFVT

NEEVRSRSKQNPVTSIICKRRLSWLGHTTRLPSSRPANQVLNWNPGGQRKRGRPRMNWCQ

TVDRDLQLVDKRWRDALR

>SG-PL_contig_3319_1_-1_1_225

SACPPAVGPCQRSGASSSLVFLSFAVPLGSSSGLAWQASVEDGSLTVWPSQLSRHLQMML

VTGFCLLLDLTSSFV

>SG-PL_contig_3320_1_-2_632_877

MLWPEYPKFESSVCNNSASLFLLKDFAMGFHPKVNVKAHPPMQSYVLMIRRVGRSLLLPN

YIVSSKHHKEIKRKNQNHHQHY

>SG-PL_contig_3321_1_-1_190_339

MVGTLYGSRVFLVDQAYDVLLPGVRIPMAFFFFKPIVELVPQDNMCCFIM

>SG-PL_contig_3322_1_-3_498_644

MSPAETRPPPPQIKSFLKISEGHIQFCISNFNKSFRTVYQDIQILFTCP

>SG-PL_contig_3323_1_-2_125_376

MHICLGQRSQRQLKKETKNKTKASGDSIYLFICLQWVFLVVLGLLTPGRVHCHNDAKPCV

FCLLFVFSFTYTTVLVRIFVNCSI

>SG-PL_contig_3324_1_-2_41_715

MLSAARNAARSLLKPGVAAPAAAQLKHTLPDLPYDYNALEPYISADIMKLHHQKHHATYV

NNLNIAEEKVAEALAKNDVQAVISLQPALKFNGGGHINHSIFWEVLAPQGGGEPTGDLLT

QIRHDYGSFENMKKELVATTVAIQGSGWGWLGFNTMSGRLRVAACANQDPLQATTGLIPL

LGIDVWEHAYYLQYKNVRPDYVNAIFNIVNWQNVADRFHNAKMAN

>SG-PL_contig_3325_1_+2_128_280

MCHQILLKPRSLRISLSYTWFTRFAITMLVGSIVKKNPQRLSVHATDGQAV

>SG-PL_contig_3326_1_+1_1_72

RVLFRSKITRIQTAEKIQIEILAA

>SG-PL_contig_3327_1_-2_2_133

VSSQFEYHWPYMAVSGHFEYHWPYKCWLVLAILNAVGLQPNKTS

>SG-PL_contig_3328_1_-3_3_269

VSFWFGFFVCLGFFVFVPLLLFFSLMNELIKNNCRSTWHTGLHDNKIHQYCSFTSRLTVI

ARLCTFSMLFTFVCAKHTAAVNHISMTSP

>SG-PL_contig_3329_1_-2_44_418

MVMPVCSNNSEIIFNIDWSFQLSRLTQDCKDKWGVTPRVNWIPLQFWGKDILSASNIIFS

NGLLDPWSGGGVLESLSPSLIAIKIPNAAQHLDLRANNPDDPAEVRAAREQEKNIMKTWL

FRPGY

>SG-PL_contig_3330_1_+3_3_143

IPVFRHTVPVRVSRNVVALHADTYPVKYGLKSCIGKSFFTLLKDQER

>SG-PL_contig_3331_1_+1_226_417

MEVELCFENIDLHMVVSVSVVVVVDLSIDSGIESSFVVVGLVTRNNSWFCVIILNPKVFF

PPSP

>SG-PL_contig_3332_1_-2_440_736

MPISLFIALIGLQALKPWSCGVGSSLTSAMVLWVRPSLTSAMVLWVKTFTYIGHGLVVLG

RHRHGPWSCGVGSSLTSAMVLWCWVVTDISHGLVVLGRH

>SG-PL_contig_3333_1_+2_2_145

EGLKGLRGENLMISCRGRRSWYFVNYSLFSHHMSVTVFGRSGEIGAAT

>SG-PL_contig_3334_1_-1_412_657

MSHCGWTTTQFVLFENVTCFIPLLLLHLGTCIDVHVTSEVCTFFFNANLKFFGWLVMVLF

FPPFFLYFMYVILIYEDIELAG

>SG-PL_contig_3335_1_-3_936_1148

MPKFRVNLLKIEEIGLDIMKVSSEHLSVLIYCAMVKSAPAKIELFSKGRDLCFYRNEEGM

CFDQTVECIAV

>SG-PL_contig_3336_1_-3_69_980

MADAQNEEDLATAILKKKERPNRLLVEEAINEDNSVVSLSQAKMDELQLFRGDTVLLKGK

RRKETVCIVLSDDTTGDDKIRINRCVRSNLRVRLGDVVSIQACPDVKYGKRIHVLPIDDT

VEGITGNLFEVYLKPYFLEAYRPIRKGDIFLVRGGMRAVEFKVIETDPAPHCIVAPDTVI

HCEGEPVKREEEEEALNEVGYDDIGGCRKQLAQIKEMVELPLRHPQLFKAIGVKPPRGIL

LYGPPGTGKTLIARAVANETGAFFFLINGPEIMSKLAGESESNLRKAFEEAEKNAPAIIF

IDEL

>SG-PL_contig_3337_1_+2_32_157

MNVHAMPYILQSNCAHCLKRRIKQLPKGATLTEQLWATKRCF

>SG-PL_contig_3338_1_-3_3_212

DQSWRYMFFCRHCMGSSPGSVGSASPSPSFLNSSCSNMIVSCVHCCRWHVLLRASSSVSG

VDAGADKVGL

>SG-PL_contig_3339_1_+2_899_1201

MDGPSTFTAYTINLPQKASTRFIFSIKMNDPSTFTAYRTKISPCKNSYRMTLQHFECEQV

PHHPFSHFHQKHPELDEPSTFIAYAINLSHIISLHRPNFTT

>SG-PL_contig_3340_1_-1_16_669

MAASLVRSFAFGASARNAFGLRTGAVCQTWTRTAFFRRRKDKQEATEVIAEAPDDQLVAP

TSVSSIAASNGLRKRQGLQTVRGYSPPGDVEARVQKIVSEVCGDVTDWASIKFDNNRIKF

KILEKAMSAFDHYIPNSQLINMQTASDVVAFFSTPVRDTSFYEDLSTLDLPPNLHIQLQP

LRFDPETDTMFGGVSTFPNRPTVVSSLKYRRKYGKTEK

>SG-PL_contig_3341_1_+2_2_202

CNHSYMICLLCVCACVCVCILFKYVSNCVYLLICVFWCACVTCITLRVCDILCITLCMCD

ILCIAVL

>SG-PL_contig_3342_1_+2_182_310

MTIVWWLEFYTLLISQWCGIRILYTSITIVWCWSELYILLISQ

>SG-PL_contig_3343_1_-2_8_469

MWVFEEMRLLTNRPRMMTRVPLTDLKPKFTAYIQSPWQEEWACQEDNKLFHIRSDLKVFL

PLVSGCQKEETVLCRLHTGHTFLTHSHLLKGDEPPWCFPCHAPVTVKHLLIDCRDLHDVR

HKHFTADSLRTLFRDVPPWAIFDFLKEVNVFNLI

>SG-PL_contig_3344_1_-1_148_726

MATAEGGTERQFLSYDGPQKGVRYPLNIIYCGECSMPIEYCDFYVNSDKCRDWLQKNLPK

EFEKLSMTKDEEGDGDKKEEGAGGGEEKKKRQTRGGKGLMKAKKKVEPQGVRMWTASRGK

KKKVTIVVGLASYEIDLKDASKFFATKFSCGSSVSGEDEIVIQGDVKDDLFDILPDKWSQ

IDEDDIEDMGEKK

>SG-PL_contig_3345_1_-1_58_474

MFDVALSSGGAQIALSTSSDDAHPPEHMIDGHDNTFWTTTGMFPQEVVIRFQALMSINRV

ELCAYNVKNVVIETSENENADNFEPLAERELESMDHQLQQEDMSVGERKAHCLRLLINSG

YDHFVCVQKLKVHGRALHG

>SG-PL_contig_3346_1_-3_30_296

MCFFLLMSCPIPPFCCYSHDPQTATLFLSLLLASPSPFHPLIHRAQSTTHRLPTQKFFHP

RFFVSFMSMAQQSIQSFFCRPTSAGGVFA

>SG-PL_contig_3347_1_-2_530_1765

MVRRAAAGKLGEFAKVVEAEYLKSDLIPLFTVLAQDEQDSVRLLAVEACVSIASLLPSED

TEQLVMPTLRQAAEDKSWRVRYMVADKFTELQKAVGPEITKNDLVPAFNSLLKDCEAEVR

AASANKVKDFCQNLSTDVRETIIMSHLLPCVKDLVADANQHVKSALASVIMGLSPILGKD

NTIEHLLPLFLSQLKDECPDVRLNIISNLDCVNEVIGIKQLSQSLLPAIVELAEDTKWRV

RLAIIEYMPLLAGQLGVEFFDEKLNSLCMTWLVDHVFAIREAATLNLKKLVEKFGMDWAQ

QTVIPKVLYMAQDTNYLHRMTCLFCINLLAETLGQELTTKLLLPTVIAMGDDGVANVRFN

VAKTLQKIAPLLDQSTVQSQVKPLLEKLKTDSDVDVQFFATEALDAVTSGTK

>SG-PL_contig_3348_1_-3_183_398

MVKFYSGTVTYKWSWDQMAQAFWQRYPNPYSKHVLTEDILSRHVEGNCLFTRRLLTKTNS

VPRWGERFIPNG

>SG-PL_contig_3349_1_-3_75_680

MSVLNDGRNEIRVNNSDGKCLLENWVEERSVKHIDPEIKLEGAVTSKAQILRDGHKGVLT

TDFDARADNKTTVRLTYTPPEPDATRKVGSKKELMERMLFAKVCNEMKVEEDGGPEKTDF

RSVKMADYDFEFTPQKIAPTRDHNYRSEQPVTFWSEHKDKIHGVTQVKTRDTAFRRNDAF

SKPIGEYWEEPKPYEQEDYPKM

>SG-PL_contig_3350_1_+1_406_675

MVIDIHSLSKHAVKLYLPMLNNLVLFIFILQDCPILYTHSLNTHMHNRKQGGASLLKQDD

AHICSQTEAHTHRHTHKCKAYTNIAITLQR

>SG-PL_contig_3351_1_-1_25_225

MHVHLLFHCHCFIAIVVVVCLFYLLSSSFIPSSSSLLLLLMLFLISVYSLIFYCHVSFHF

LVSGIIC

>SG-PL_contig_3352_1_-2_2_250

SVHAGYIRVSTSHRKLIWLTGSLTCIFDVLRAYTHERGSGTSRSAHMLTWEIGKMSTLYP

PGEVPEEDSNPGLPNPPDQQHKN

>SG-PL_contig_3353_1_+3_186_335

MFVIILNFLMLFVTVEGDLPRVLLYILFNHRLLLWKVIYLVFYCTFSSTT

>SG-PL_contig_3354_1_+1_1_90

LFCFCFFPGRILFLVFQLYKNLILLTKFKM

>SG-PL_contig_3355_1_+1_34_123

MDKFEPVEMNLNTCFKYFSTKLNLMVSDQS

>SG-PL_contig_3356_1_-1_1_84

LLWRLGSEIVNTGMGTRCPFCSVVYITF

>SG-PL_contig_3357_1_-1_343_1206

MMDAFTLLLILGLSLGSVVRAVEAKCPADYRKRVETCIMEAQLAPQAGGGVTLVLKKDTI

VALCDRGTLRKTIDCLEEIHSRCIYNTTVIQELDRLYSVPNWEQGEQLLCQDTHLFENNF

DCLFRSASSVTSCILMRTHTFRMGLAFTDPSNHVRVRDVTCSFAESIVKCICSPLESNCP

MDMTKRVAGTMRFFLPPYCLNDNANVIKTTPQPKTTTPDSLAKKVDPSLPDDTAKPAEWV

LLDETALPPEAIPPDDSTKQRESSLRNDITKLPGFVLPKDTSEPEVAE

>SG-PL_contig_3358_1_-2_32_2962

MRRTFMSGTAALKKKSICIQVKVQLDSVMEILRKTQPHFVFCFLPQQTAGLCELKAGHGS

EDMQVNIPLLRSQLRGFEILDALRLHRQGFPEFMNFREFVQKFHALVPPASRPTANTEEK

QAVNQVLDHLDVDKLNYRVGLSKVFFRPGVLSQLEAGRDEKLTGIVTQFQALCRGYLGRK

KTEKLRVQHLAVRCIQRNVQKYMQIREWPWWKLYTKVKPILNVHRTEEELLEAQAELEAM

KTKTEKLEKERNEYKAACEKLESRLVDVTADLMEENTTSNQAGEMLEAESAERMRLEKEL

KDLQAKYSPLKRQNEKLHMEVMQARVWQAQSLEEELEDDTDGGSSVYKERCERLMRELQM

TKKQMQQQHEEDLEQELSSRKMVEKRLHEAVEEAEEQRRQVQVARKKTQRLTAEMQDIKL

HLEEQMARNNELERKQRRFDSELGIAHEDVRDERHMKEKLQQERDQLLSEKYSLEQTIQN

LRMDQQSAVEKAERLDKELNDILVSGKDNSEVVSLKRGKHELELKLREQEEELDDQAGQI

QQLEQTKLRLEMNMEKMKKQHQADIEEKEEELEDMRTKTQKKLRSMENQLEEEYEEKRRV

MEEKAGLERQLQEYSARAPARDRESEKRLRRNLRKTKALLADAGTMLAKQKHVDGAKQQI

AQLRNQLDDAEFTSSAAVKAKKRMELEIQDLNQQIEDLSRAKQEVESKNMALLREKSDLQ

SNLEDNDEEMSELMKKYKAAVQQQSVHQITMTDQLHQIEELIKERDLLRQQVADLSARVQ

GYEEGSVDKHSVSRLETKLKDFESRVELEQSQRHRYESQVARLKEQVEKITSEKEDMSAN

RQAAEEVNKRAQKQLRDLREELTEAQKKEMEACQKFKERENEVEMMEVEVAQNRADLKLA

FKRITDLQAALEDELDSDDDSLQLDDSDLDSEDDDDEDLDTYLGNHHATGHRSQSTYSSP

RADRYTFASTENGVTDA

>SG-PL_contig_3359_1_-1_1003_1311

MSQSLHTFLFPSSLTYIYPTDRFLDWEGFCASVKKSVMTSSFSFRISMKNILVLLSLAIL

SMIALIFLLLFNYEYDLVTDSSAVVNAFAFKLASVCAGNIANF

>SG-PL_contig_3360_1_+3_72_323

MRAASISSPSLLYLPNPELFGGLEATCIGVFCCCFFSSSDTRTRAGRLRGGSFTSAPTPT

AQVNYCQLWSDISFDRCCWLFCCC

>SG-PL_contig_3361_1_-3_75_458

MKLVRFLMKLSHETVTIELKNGTQVHGTVTGVDVSMNTHLKAVKMTVKNRDPQQLDSLSI

RGNNIRYFILPDTLPLDTLLIDDAPRAKQRKQQGGRPQMGGRGRGRGGRGRGRGGPRGGG

RGGPRGRR

>SG-PL_contig_3362_1_+3_3_371

MRSPSGVDLFDAVEFPEGCKSLVQPQPVPPLRRYHVAKPHVRNTMGDNLGDLHFISGGGY

PRFVEQVFFVDDEAPVLHRLTIEIGNGDIVQFVDNTAVWRLEPALKVVKHFQTMLLGRHC

ELD

>SG-PL_contig_3363_1_-3_9_704

MKATFDATLVRKVNYRTLSNMNKLRSESFGEGWEADIYNTTVAMPTYQLAFIVCNFDHRS

NNEVGIRYNTWAPPNRIQFAVTAQERGLEVFNYFESTFQPPYSSVINKLDNIVIPNFNGK

TTENWGLITYEEDLLYKPGVAPTTDEMRIAKVIAHGVAHMWFGNLVSPKWWDWLWLSEAF

ASFWEFYSVEQINPTWRTHDEWYSSAVYSVLESDALATSHPMTADVMTPTEV

>SG-PL_contig_3364_1_-3_552_782

MRVYWLSWEQHCAVNTFLSVIQHSESFKAISPACVRACQPPKHSRQRSCSVPGICQKVML

AYFHSALSDIIYSRMFS

>SG-PL_contig_3365_1_+1_1_135

NIFGTHVFKSYQLFFSKVKPVDLKESLGEPVLYNDNIKAGNKKSH

>SG-PL_contig_3366_1_-1_22_1146

MPTRNIKHTITITYGACSVPNSPFRVYVSEPSNPAKVKVYGPGVSAGVKTNTKTYFVVDC

KQAGPGDVAVGLTDERGVDVPIKTIDNKDGTFTIEYEPKTPGMYTVIVHFGGKEVPQSPI

KVQIESSMDTSGITVEGLEPKIPVGVQKTASIKIPPKLPKAAVKMVITSPTGKATNVPVN

DLVDRKVAPFTLQEPGPHTVDITYGGVPVKDSPFKVEAVPDLGPPKVKAYGPGLTSGEAM

KPAQFTIDAREETRPGNLGVTVEGPKESKIDVKDNGDGTCSVTYMPEVPGDYKVNVTHAE

RPIVGSPFTAKIKPAVAEAPKVKAYGPGLTGGEANKPAQFTIDAREETKPGNLGVTVEGP

KESKIDVKDNGDGTC

>SG-PL_contig_3367_1_-3_921_1211

MVKKTGIIRHVCTAHVLIACDCQGQVVSWSEKLWGLCNQVSCHALFRTLKYILTNMLYMS

IYYHTQHVIFIIIIHTISITIIIVMLASSVYYFCNYD

>SG-PL_contig_3368_1_-1_85_654

MCPHILRYLTTAVITNKGRRQKVLKDLVKVIQQESYTYNDPITEFVMCLYVNFDFSGAQQ

KLRECEVVLMNDFFLVACLEDFIENARLLIFETFCRIHECISITMLAEKLNMTPDDAERW

IVNLIRNARLDAKIDSKLGHVVMGTQAVSPYQQVIEKTKNLAFRSQVLALTIEKKTVGHN

KQDTQWGGQE

>SG-PL_contig_3369_1_-1_85_4074

MTFKLFIRLADREDFDQPAPFDLVKSLDWRTLKDLLGSYYYSDVTEHLDVRYWDEEDFVN

VSSQEELEEAIRLAEEKSYLQLYVRLTGADGDATPDHQPVADSSDHSAGGEEGPVGKTLQ

KSDPESNTETDRDCIPSETLHTSNCLFDTSQQPLAVDSPEPSLDDLKKDPFKLVSFIKAN

ALKKGMIRKLTESSADDLSSSDHLLKGQSPVDAVLTQQIFANAVPATPKQRFQQAIFSAA

ATPNAQRYQQLAWLQEVEAEAEGVSYDEGNAEGDEKNNAVDQAHTMLPGALSGANVIAIE

MPEVEHQGTETAEPAEMAETGEVSTRSSRDAETGFWVQAQQGAVKEGQRRFTTTAIAREQ

TPLDLSQKHHARRSIHTDRVQKKKKKDLVDDSSPEDSLLRYMAKKGKSSTTTTTTSSLLD

TPSEGLSSETPLPKKHERRSTKSDGTQKKKKHSDLLTEDAFVHYMTKFETTLKKTMAKEI

AKKTARTVIKHMPKQTEGSEGHDSQPPLVLSNRSGLVHEDIICDCCDREIVGTRYICGNC

PNYDLCEECENIQPSFHERSHVFIKLRRPCLKAGTHHGVRKPLLKRLVYEHMEEERKVDP

GRVQVIMDRINAQGDKSGKRESRNNNNSEDGGREVNGQQKETEEEQQEEECRQEQYSAEF

ADERTFENDAIVQSAEKKCLNTGPDAWSTIAVDGECTSSLPVNPSDTDKDKPGAQFLTKH

EEAAAKVDESATEKDVAALEIWIESSTAPAILSNTSSPLLDFFEYPLTPMQLKEEAHAAT

SEILDGDDLDSILELTDDGYVIYPTTKDGSTTSSPACLVKSSQPCQEIPETVLTPSQAAV

ADQEAVSEPSSLRAESPFTVASMDVEMLKASYHSSFLWKLVDADLQPPLTVHPGWSSGSE

PDYQKEVLEKVSDGVSSLSSWSEESSSEEDFCVIVPDCFDVNKPLDPSSLAKLASQGTEK

PHHSEHKRTKDSCTDIICDVVPDQLMKAETVNITSPAAGQQPQDSNHESQAETESQENED

KTSATNEENALLVQVEDSGKQPQDSSHESQAQTERHENVDETWVEDYTVDESAKVQEEAF

APVLGDARNEKEPTAADARNETTCASAASEMDRPTNQEQALDVADPSQQPSSCTSLKSAE

FESEDEDQTNLKHPTDSGEEGAAAAADSISDPNQQLNQMMTNTIKKASSGLQQLFQTGKD

IFKTWQTGDSYEYKESTYEYKPSEYKLPENTWQPSNKEYIPPKSNFQFVPSEYQLPKCNY

SGPKSQVSAGQGFTGEYAGPLQRIREMGYFNNEHTLDLLSKHNGDIQKVVNEILEEADND

WAEKRHGDHF

>SG-PL_contig_3370_1_-3_42_350

MPLSFWILFHPIHSTLQYNKIQPSTTPPPPTILSSAALLPFSSGIEFAHLCQVLTILSLH

LAFSLPPSLYLCFCLSLSLFPSLSLSLSPSLSLSLSFSLSLSL

>SG-PL_contig_3372_1_-3_273_1322

MKVEDVMRCSFSAWYETFEDVTIKSIILPIPQEFLDYLHADSVVLPEGSLQGAYSSAWSD

DSEEEEETVDWSSKGDCKEATMPDCREFLQEVDSAISQLGGKVFPKLNWSSPKDASWVSL

NNTLQCSHASDVCLLLKSSNFVTHDLTQPFIHCEDSEVASKDVVLQYELVLRQWADISTG

HEFRCFVRHGKLIGICQRNHTKFFQHIVDSKKEIQEDIEDFFEEVELNFGEEAGCYTFDV

WRKGKNKILLIDFNPYGPVTHALLFDWDELAAYDESDGLMFRCVESEHGVQPNGLSQFCM

PLDFVDLATGQDPFKLMDLMKLGQHQDNSSSDDDEEVEEDGLSVDNAADR

>SG-PL_contig_3373_1_-3_3_293

VDPTPSLPHHVHRAGVYCSRVIKHSHYCKPGAPCDYHQTHNGRAVYCSGSVTIVSILSAC

VPCDWSVVHRVIESRLSPSVVCVPSVAIISAIERTME

>SG-PL_contig_3374_1_-2_101_7153

MEPVEQEPGSLSEDVDQRTTKDIEQEHIPYTEDTKQAVSEHVTQVVERTVKDEYGRTGIE

ITKKETVFTESLVHKEEYSVAVSETHIEEETETVPEDVRFVEKFKVAQETSEEARQVDVV

EEQVKPLSTALSMAAVCVVPEEEEVEEETVESTQVPETKAAVCLVPEEEAVEETIKPEKA

APAPQADVCLVPAEDEIEEETTEISTTPVSEAATCLVPKDDFVVEKTVKISQTPLSEAGT

CLVSEEEIAEETMEISQAPLSEVGTCLVPEDEDIVEETVEVSQASLSEAGTCLVSREEEI

EDELVLPGQSSVRETAVFVPEERVKEETIEISQAPLSKASTCLVPGEEEIEEVVVQPQLL

SVHETAVCLVPEEEVQKETIEVKEAPSAPETGICLEPCEEPIKPEEASPAPQADVCLVPA

EDEIDEETVEISQAHVIEAGTCIVPEEEEVKEETVEVSQAPVSEAGTCLVPEEEEVEKEI

AQPRQLFIHETSVCLVPEDVQEETIEISQAPLSEAGTCLVPEEEEVEEEVVQPQQTSVHE

TAVCLVPEEGVQEETIEISQAPVSEAGTCFVPEEEEVEEEVMQPQQSSVHETAICLVPEE

EVQKETTEISQVPVSEAGICFVSEEKEAEEEVLQPQQLSVHETAVFLVPEEEIKEETIDV

SQAPVSKAGTCLVPEEEAEEEVVQPQQSSVQDTAVCLVPEEEIQEETIESTKAPSASEAG

VCIVPEEEDMEMQDKQEKDDFVYTETRVSVVDEDLKEEAVQEAVDTRAEAERSIVPKGDR

TDGGPVVRPDITEYQSREDIEYEEEDDEDWEILEKEDLTEEEQVAAMNEALMQVSDSKDT

QGETEDYIDSSGQEDAQLHVVEAQAYQQAEEHYDSQFSDYYQYEQITEQEFQVTKFETEQ

SVDQDVAESKDISGERPVEKSEQLVSALPVSEPSAVEMVETEEQKEQADSFEGMAEEDKE

VEDSDVKLIGSKPSETSLDMSEVVDIRDDTDRTYENKIDEVIMIEAREEMEGEALEVCVA

RLSPESDKEAEELDAHFQEPYTQVQPEMIHDVQYKVDELYEQIESEEKETYQEIPDQTEQ

ENISTEDQIHVTAQVSMEVVEGVAAEQELASSPGSKISDISPVHEEQMEPVHVTEYAQSI

QQESYTVRGESQESYEQMSYAEYKTETVTTTFEEVRHETISESVTQLQEEKHSAAESQQR

EGEPEPFTEIPSDEPARTDLLIAAMPEVPQRSPHVSSSDDDGEKDSGEEDTSRMPSGQQI

KQQQIFAQQQEPDTQQYDSDISSEKFEPESKVVETTDAEAEQMAEQLQEKKSDYESEDDF

VETSERFDQELAELDASPVKESPPDVAELDQEIEEQDALTLALMSQYHVDVTKEGQHMTH

FEMAEEAECAVLDFREGESDYESEEEGDVALETFDVSVDDKDEEDDQYVIAEDGTGQLVD

VESTEKYEEELAEKLREEEQPEDFTQLETTQELQQSESEMHISKEEVVVSSRDFYGTDVQ

GAEGFTQEFPEDRPDIDMSQSNESTEVQMASSVEGLLVEPYPQEYDIMAISGATEGDQFR

EPDFTDMFEAHVEGEISDHDDLYEGYSDESEEELKPLKIPVEDSPTEQPTTDFLQDTMEQ

AERPDIEAGDSRQSPVYDRSPEEEVYRSPTEEHPEDRPFDSPLQEELVEEDKHGMPSSEP

QLEVKGRAEQVAVSGILAELQDIEQYAGQELSDEELEDDLEQYEKEPEQEQLAKSEQSLS

EAIIVTDQFVDRKEIFTTEERDASVPTKTIDQQEDVTTEPEQEQLAKSEQSLAEAIIVTD

QFVDRKEIFTTEQREESVPKETVDQQEDVITEPEQEQLAKSEQSLSVAIIVTDQFVDRKE

IFTTEQSEEPVPTETIDQQEDVTTEQREESVPTEMTELQEEVDEIAANVYVDRMERFIEE

SVETLAPGGESTVNGFISGTTDIDSSGAGAAEPVIEERHLSAQSSLSEHEAQFPEYMDEH

HLSLHPPLYPSSSTEVQDRSRTPSDIETEEPAQNGMTTAAGATGSYTSEITSGSQSYLSE

STVSRMSESDFTSHSETWAASECTMTGSEFEHEGRITVDTSEQIFIEQKIEDSYQEFDET

KPQMDTLDRPMSPSEYTLVTSHDQDRMMQILNLQAEKPAEEQSEEEEDEEEGEDEMDRDS

LLSEESDEVMSDDEQGSERLPSPTEYTLIASQDQEALTKVLGLHAESQPPEALHVDTQEP

FSTAVTTGGKEEENSMYQTEELRVETELELQSSGSSEQAVIQLLDSVDNTVSNISSDREF

EKDSDIHKDFEAPLEPSAPYMPSSPGSENLYQEMFTKGDLTEAHLMGEHASSFEDLFAVG

MVPQTAAFIGE

>SG-PL_contig_3375_1_-2_254_1480

MRDVPLSNTEREFLLGIIAEKKRVDGRHPTQFRDIKVQFGSSYGSCQVHIGQTRVLAHVS

GEVTAPKSSRPHEGILHVNIELSPMAHPSFEQGRQTEETVQLSRLMERCLKDSCCVDLES

LCIVSGEKVWTLRVDISVLNHEGNLLDCASIAAIAALKHSRRPDVTATGDEVTIHTATER

DPICLSVHHMPLCVTFAFFHQGKCLLMDPTEREERVTEGKMMIGQNKHREICMMQVTGQM

LLHPDQVKQCTKMAQVKVMEITDLIQEALANDKDARARGEKSGFAVYSDKGTINTTVTDP

KVVEPPPPADMSSDSASDAVIDVDSDSDSDGHLDMAADSRLNGDAPRNVADGVALLGTAA

VQKWEEEEKAMEGDLKGNKRGSADSPENQPTAAAESPKPATKHKKKTEV

>SG-PL_contig_3376_1_+1_16_255

MPGFAPLTSRGSQQYRALTVPELTQQMFDSKNMMAACDPRHGRYLTVAAIFRGRMSMKEV

DEQMLNVQNKNSSYFVEWIP

>SG-PL_contig_3377_1_-3_1134_1382

MHRNGSVSADGQRPDHTPRVCDGEVLKYLIHIFRQKWRIFNSRLYTSTHTTTTIHSYHYI

QWKVSGRFNGPTRPFHHTHIFFL

>SG-PL_contig_3378_1_-2_1145_1507

MLHSSYSCLCRTCGPSIFTRQFSFCSLQHMTIHYSLLIMCLHVLCHTAPCFQNVSTIPDH

HPMLRSQCLKNSCLVHEILEVFLPHIDTQTVHVCLSWLNKLFPTSVLAYSANTLVYTFDG

S

>SG-PL_contig_3379_1_-2_341_1222

MRMKDYRVICAACLSFFSVLAVLDSGQGQHVVDKEADFTVVDTNGQPCLVASINITVYIK

YHTDQSALTRQANTTLTRHSVKDKSSHYRGHCGKHLSKLSISWNNHAFTVNLTFLLQDEL

EGSRDERRVDMDDIPPGTHSSTTWSLYQVSLTYNTTHNNVFPSALNPSVTSVSAQNLNLF

RTPLGLSYICRTPGVVEIRFKNDIDLIFNYVHIQPFGVGNNLWANGTQVMCSQDINYAVE

DKYPILFIVSVCFAIIILVGTFTYAGARYVTWHCGHSGYRPLAREGPKDEMGFE

>SG-PL_contig_3380_1_+1_1_222

IFTFCLIFTFLKNQDVISLQAAYSSLGLIAHSCILIIRVELICHDARLIFVTCLVNFMFL

CKLSTVATFCTCSS

>SG-PL_contig_3381_1_+1_1210_1632

MFTILIQEPDLQDRSQQLNQCRYTFQISAASGHHDHGEHLKVGHGTSSVFFSWLLSSHSV

SFLIIANTNIGSGQNLLQELHRKTCLVMHMYTHIHPLFTDGSAYVLLVLSRQLSLVSAKR

IKRICAFTLYFSSVLLISAFT

>SG-PL_contig_3382_1_-3_84_254

MNAEAMIPNISKRQPMTPAAIFVSSAKPRQVAYHSLRAGRLIGAVPICYIWLLYLPR

>SG-PL_contig_3383_1_-2_107_910

MADSYSFVKGGKLTLKGNKNKKLKKHKHKKRKHEESSMGESSSATSHNKEDTHSHGGWWE

VKKFDDISGNIALEIGNSTYISALDNGLLILGAPRSHGEPPDPTEVFTVLKLSETKVAFK

SGYGKYLSVDMERRVVGRSEAIGNREQFEPVFQDGEMALNGCNNCFVSVDDDGDVVCQSS

TAGKEHMIKLRSNLSIEEDPLEKVPREERGSVKDAEVNYVKKFQSFQDRRLRINQNDRAE

LKHAKKEGSFHETLLDRREKMKADRYCK

>SG-PL_contig_3384_1_-1_202_393

MSSEMRPYADMVERRLKNINLLTSVVVIRENFTTQQQVEDAARRSCLFAIVINNQNEIHR

SLTL

>SG-PL_contig_3385_1_-2_47_859

MPSGDTHMTKDYQDGDFLLKLNPSVLQSGVYSCEVPTSALADMCYNTSPLGRENVQSNVT

VDGMKARMSVAEASNQALLEENRRLRDRLDHVEAVHAEQIANVTRVVSGTFQRQIERLQR

SHKSLEDDVHEMWDKARDYDASLSYPCGIGFHEVIDDPARSVSYGDGSLCDTSLKSGWYR

FLMDDRDAVLPTSCVPKHQCNTDASTWLDLKGKPLPKVGEEIEAHACAHWNRNCCKWKQP

IRVHNCGDFYLYHITPFSDCTQAFCLEMLDK

>SG-PL_contig_3386_1_+1_553_801

MFLQMAAACEEFISGDLTITELKDLMVTITYFNSLQKNNLNHMHTLIIKANIVFMPIIFT

TSSHPTLYTTKRIQYKANVSEKC

>SG-PL_contig_3387_1_-2_749_1060

MTTKCSFHNCCINLPDTHPDRLKVCTDGSLLDNQQAGAAFVIPDFKTEKSYYAGKNNSIF

AAGLLAVFMALNHILDSVCVCVCVCVNVCVSVCVSVCVCVCVCV

>SG-PL_contig_3388_1_-3_1023_1238

MLFSFLPIPICVCIMYACQFDLAKTNVMRMFHLRKSRITGFHFSLSSLFCCRCWYILLLS

LSYVNFTDFLFD

>SG-PL_contig_3389_1_+3_153_911

MATLMHEKQDLRVGHVNIYHLQNKVLDLCAFLNKPSYFHLYGITETRLAPHISDEAIAIP

NFSIIRRDPTCLGETGIAVYIHLDIQHIIRRRSDLEIEEVECIWVELKPSSTSPPLLIGI

LYRNPKSTFQWHDHFLKMMDKICSRNTDILLLGDFNFDLFVPQPAWESTTTLVGLKQFIE

DPTRITATSKTLLDHIYTNRPEAIIKATVDDLSISDHQPVNCTWRVKLQKIKKNKHTCIT

YRSFKRFNESAFF

>SG-PL_contig_3390_1_+1_1129_1569

MQAAVDTQLGSVIAPCNHTASSSACLVQGKGPTWCVSEEQVMRTQSSLTDTTVCVILYRL

DNNGKASDQITKSCPTTLDFPSTANKKVKFQFATFTHIHQSQKFWTLSIDIPQIHTKKIH

SQNNRHRICNTSWFEVINNVSLAPMAD

>SG-PL_contig_3391_1_-1_196_2391

MYQITYHKLADAINEEDLNITDTFVDIVGLEMNTDYIFKIKAYTSRGAGPWSNRLHFRTF

GRMPPAPKNVKVRRTSPTSIEVSWDEPNFPVAGYKIFYNMFATPVMYVWQQMDTGPYTVA

DITALEPHTVYAVRVQAKSVDGRYGNLSETVTTDVKDPRERDNVVRNFRVTSRLTNLVRL

SWSPPKKANVNVYLLQYSGVKTYRENGQTKQVVHEPEEVRIPGQMTTWTVKNLQPKMKFE

FNISVFFKDSETGPVVRIITETLINAPPYVAPPEVEALPGRGVVTLRLQPASEKHGPISH

YHIIVVPVKSGSPQKMPLDYTMEELMNEAASVDQPASHPYIAARFEAHALPHHFHLGNGQ

EYGAFNNRPLDVDDSYFVFLRAYSVDRRLYTSSRFSEVIDLTEVDRSTKQPPKINTDPTN

RTPDRPTRTLDDKDMLLTIVAPTCSGALLIICICAVITYCMMRRKNKSKSVMLEPESKAL

MVDVPPYLVDPVEMRRQHYQTPAMISHPPIPIERLSEHIEMLKAADNLKFSQEYESIEPG

QQFTWDNSNLEVNKPKNRYANVIAYDHSRVILQPIDGVPGSDYINSNYMDGYRKQNAYIA

TQGPLPETFGDFWRMVWEQRSATVVMMTKLEERSRIKCDQYWPSRGAETYGLMHVQLMDV

TELATYTIRTFHLSRYGCPEKREVRQYQFTAWPDHGVPDHPTPLLMFMRRVKVSNPPDAG

PMIVHCSAGVGR

>SG-PL_contig_3392_1_+2_2_220

HTHTQGNQLFCPLKNLNKETVGKRNMKNKWGKNGENNSLIQTTEQDTHIAESERKTKMEV

LFIRPHKNKTKKQ

>SG-PL_contig_3393_1_-1_7_594

MSAYDNPVGDHRRKWDRDEYEQLARERILRDTDDKDIGGPPVKRELLKPREYKVDLDSKL

GKSVVITKTTPASQQGGYYCNVCDCVVKDSINFLDHINGKKHQRNLGMSMKIERSSLDQV

RQRFELNKKKREEKKKEYDIEERLREIKEEEEKAKAHKKEKRKERKRKAEDDGDAGDPDM

AALMGFSGFGTSKKAN

>SG-PL_contig_3394_1_-2_2_91

THSHTLTLKLVSAKGKRYGLEEQILLLLIN

>SG-PL_contig_3395_1_-1_325_576

MTGALRWPVNRADFMLHCILCLVTLWFVYLHLHDICPSKALAPTKINFSKIFGSIQTWSQ

FIIRKICIPVLGNLINMNFFYCCY

>SG-PL_contig_3396_1_-1_2194_2517

MLSDPHLLLLLDLIRQAFEHKSNILATETNTSRRKIGGGFEVMQQDSLLSRDIKLAFPVL

MKQHLHHVAQDPQQHTFPRSLKLRLNLVTQLMEESASNVLFFFYILFN

>SG-PL_contig_3397_1_-1_34_441

MKCVLAKRNAGLTAGGYKVIHCSGYLKIKQYTMDIAPYDGCYQNVGLVAVGHSLPPSAVT

EIKMFSDMFMFRASLDLKLIFLDARVAALTGYEPQDLIEKTLYHYIHACDILHMRYAHHT

CEYTCHDSGFVCFTCQ

>SG-PL_contig_3398_1_-3_3_221

THLSHLSSAQTRFTHRQHAGIYILEREWPLAAAGEVEPLCLVSSESSFALLVGSILFDLT

TVSQLTLLPLLAT

>SG-PL_contig_3399_1_-3_150_278

MLNPFTPHPIHQLQNLFSLKLFFLFCKYMYMYTSVLRLLFIHR

>SG-PL_contig_3400_1_+2_2_142

IVACALPVAGNISGHCNLGVKKVNIHTSFLLTVLCSLAHRLKSETCQ

>SG-PL_contig_3401_1_+3_3_125

IHLIKCAYDGLKTGLVLDRIPNNSKWRQQEKRVKYTWMKAL

>SG-PL_contig_3402_1_+3_108_410

MHTYKFPAVEFIKYSQLLQEPNPDHNKIPILKSYKCNYKQTNNKTKRQAQFLRVKHLLNV

LLYTRARMHARMHTHTHTHTHTHTHTHTHTHTHTPTQKQKH

>SG-PL_contig_3403_1_-2_116_652

MECKVCLYQYMSLGSNSTSTRIIGPHWLTKVLILILCMTEDQSVGESLTTAPCRRPLHLF

PFSPCRFNNLAYLSDSVDFFSIFMTILFPCVIRWLQCLYTRYNQRSYQCQSDLHYLDFSS

KIAFKEIEPNRNTKVIVQTKFSENTNVDFKPTRVTIVHKMSIRELFRHGTCPQKRKFLR

>SG-PL_contig_3404_1_+1_649_897

MSVCGCMHAFLCVHVCKCVHACVCVYECKRNCACPCGTVHMVFIFGMRLHTISMFAPMTD

LIADLCFYYIIRSVKCKVSTDLC

>SG-PL_contig_3405_1_+3_696_1130

MRHSSCFRQKVQTEQWSRSMNAKVLRFSLPDEFHTQLPTSLVLLRSIHICAFRQIILSVF

LYHQFCIINLESSILYHQSCTINLVSSILYHQSRIINLESSIYHFQLKVEMCPSYRKLTS

SHRSHTQKQPLTTTTSTTTTTSART

>SG-PL_contig_3406_1_-2_2_208

SFPTTGTLFTTFELVTENFILEIVKKMPLKSCQLDPIPTKLLHENLDILLPTITNIINTS

LSTGTVPMS

>SG-PL_contig_3407_1_-3_33_917

MEAIMSRSVALFTPQLTQVRGMATLKDIRLRLKSVTNIQKITKSMKMVSAAKYARAEREL

KPARSYGAGAKAFFDAAELEQDKTQPSQMIVVMSSDRGLCGGVHSGICRELRSLLGERAT

GGSIETKLVVIGDKAKGILQRTYKDHFLMSFNEYGRKPPVFADAALVANAILDADYKFDA

AHLYFNTFRSVVSYRVTELPLFNQDQIAKSEKINLYDSIDEEVLRSYSEFALTSLIYFAM

KEGACSEQSSRMTAMDAASKNAGEMIDKLTLTFNRTRQAVITKELIEIISGAAAL

>SG-PL_contig_3408_1_-2_2_268

MSFRIVVRNRKCARGCNITLHCVVILMFFLPVLCFWIWSSCQSVNWARESVVVWFHTHLL

DHALYHRHQMCIMCTKNKNQKTVLKVYTW

>SG-PL_contig_3409_1_-2_338_2899

MLEDSPQVMEKKGASRQPNLTKPNSTFERKNDPNNTVRYHGRYSKRRDNGVGRGTPPEFS

SRKPYPQKTRSAVDKRPRQRGSSGGRQREEIAETGLLETESAIPHGLSKKGNLNYLLNFT

FARREVVGSNQRRWVKRLGSSFSKECYLQANCQFVVKDSGDYAVQACDPDALVSWDLVEQ

VWTFSFGQTTCPICLQPPAAGKITKCGHVYCWACILHCLSEGNNNSCRCPICFETVQGTD

LRSVKVIEKQEHKTGNVITLQLMKRARDSTYAVPCVHWEDREGRPQHILDPLNTEFAKVL

VASAAEIQKSVIEEERQALLQHLAAAEDSEVCFIETALVQLSERESILQGKTQAAQENAA

LLEQLGTGSGSESGNKERGDKGSNSMTAATPPPPVVTTILPRNVSFDGSKIRKQYADAFE

ELEEEEEESFVSGEFSDREDHEDSESTDSLMSMSSPTPDYSSSLPLFIPGAHGAAASSEK

EPLSVPSDDSLGLVPAEVADEDTMTTEEAVEQLSMPAESENVTVPAEAENGAAASNNTPG

RGARNQSKRYFYFYQASDGQHIYLHSINARCICQEYGSLELGPQTITAKIVDREITFMKE

DIRKRVRYLSHVPKACEFQVVEVDLQPPTVSQRTLRHFAGELKRRKQYRQKVKRDDRRNA

RKQNIEDKKNLFGIYPEVKLALDNPDHFPAPVAEGDERRRLSSVSDTASLSSLDISRPEL

NVNAVEFLPSPKTESSSSNTPCAFSFAAALKKAKQPVPSVWGASSTSTVTQAMGAAAAAA

GGPPLSRRPDSEESDNDDRVPVPLFKNSFSDAISMKLETLCAKGDGGEQANSKGGKKKKK

KEKKLLFSTDMMRR

>SG-PL_contig_3410_1_+3_3_248

HKHKSKQSDHFTLVFTQTPNIPAQTFLKTHICTNHHVNTESIYFLLTSFTLFWYKIILHN

IQSKTSKKFPKDIYHNDRSGLM

>SG-PL_contig_3411_1_+2_704_940

MSSDIFQAQKEQTMFVKHKKNKLAFLPLWETNLQHNNRSSIQLALSCKCIRSVSKSQQFI

QHHQLKKPTKVKKGSTIRH

>SG-PL_contig_3412_1_-3_117_665

MLFGAALRVYDRKCQCLLGNGQYQIVLSDLQPREAPPGRAASWLSVQDTQALEPFEVFDK

LPTIKFNLTWDEGGSNSYHPHDISSPRLQSHLSGTKASKVRENQSTPRKRLKLQYIFQYG

NGELMRQYTEVRDDLRCPWCSLLCPTLFGLLKHLRLSHPRFNFIYVPQQKGGQIEVTVND

MYD

>SG-PL_contig_3413_1_+1_478_687

MKSPFFSLFFCACWGCFHKPATDMIYTGSLMRIFDILHAYTNKSGLGTGRSAHKLTCEIG

KSPPFTHQAV

>SG-PL_contig_3414_1_+2_545_772

MMVMEHKHSFCFRPLRIIFPLYHHCDLQNYMIQTRFLQKLAHKAMKLLMTYFYILTFCEC

MALKFSHANLKLSIFT

>SG-PL_contig_3415_1_-3_3_125

KIVFTGPLRSCLFVAAKLSLCTVRFLLSVMVADDAALFVIC

>SG-PL_contig_3416_1_+2_461_706

MHHMSIKLYRRRTDIIILGKIYRFCYFSSLFRKCQNKKSVSKERLTFSLEGRLLHKCTQH

KKKTKAQLTIPTNQKNKRSIFF

>SG-PL_contig_3417_1_-3_645_899

MSWTYFRTFKLMFIGHPLAVFCAFVFMREFVMNREMDALFIQVLDELFLKGVNGPICVDV

FYKLGVFLFSQYCGSRGTERGKDWE

>SG-PL_contig_3418_1_+2_578_901

MQTKKSTNSHYTILNPISASATKSFNRFATQFTCFCLLTHSKYTCPFSKGMYNAQICKLL

WMFSDLFSLHTSYTDKIHKVGQINSQSVIDLPSLTKTGILSGDLPVAI

>SG-PL_contig_3419_1_-2_227_1003

MAADQRKLMASGSVDDTNPTDGMEWDAQWDNPHRTNREDDSAPLMLQSTIITAASKAVIS

GESLEALKKLPSDRRVSFKLKIENLTRFPLINPYVSVQAGEIHSPPVDVDAGYREGMVGR

KVNWSTHGTYGTVSWEIGDGKKRRLVIMWCCPHDFVFNDRNWLGVGVGDSDCDVDTEQIG

WYDVMFTNAKSKERTKFRKLDFESRRFFREISPIQFTDSEGLFHVFGTMSEDHEATAIIS

FRPVKKEDFAPSLVEQLAK

>SG-PL_contig_3420_1_+2_434_709

MSQFFSNKFKEFCHSRNINSNQEIALVSVTEVNKSLYHLNQTGTRGFDGIDGRNVKLLAP

VTSETLTFSYSLCLEKYHFPSQFKQSKVISLL

>SG-PL_contig_3421_1_+1_427_642

MMWATLDDIQLLLNFKFNPFSVPSDSQFGQYAFYEFSSVLKGLWQNYLVCHQTASFCELA

FICKVRTKNTAC

>SG-PL_contig_3422_1_-3_3_59

MKAAICNRISMNIHELKPM

>SG-PL_contig_3423_1_-1_61_801

MSAEEKERMLERRRRNKEAAEKSRKKKKIEFEKLQIENDQLNQDKCELVAMVEKLKTDIS

GKLKENDILRTNYDQLRRWVGKRAGEFGLAKYIKQDVQNKGKQPEAESPTTPLLHGDPTL

YMSDNSITKVDKIPASPRKTPSPPLGPSGDRYQPIEIQPVPRPQLRTPWLVDQPVSKKKQ

PKAPMPFTPLTAQFAESQPLVVGEGQQVVYLPKDAIIILQGENRPVVAASPDLHQVEVPT

SKFAVEP

>SG-PL_contig_3424_1_-1_1_96

YVELIGLLSSFAQTRDACASYPRVSIIMLYIV

>SG-PL_contig_3425_1_-2_2_193

SSSRMTSVQLSDRKKAAQKSVQEKVFCFVCFFKWKSRCTVTISLSRFQKPGSIDTMIRRD

WKLL

>SG-PL_contig_3426_1_+1_124_297

MCVSACMHACMCACWIVNKNRSGQVLKQSVSILTIFYSYLPQPFIYHTRSIYALNILK

>SG-PL_contig_3427_1_-2_2_160

GHDKSCSLFYLTPVQPQGYETTNLLTTLGIWYISFKSYLLSSVPHSDAACAAP

>SG-PL_contig_3428_1_+2_1019_1315

MNQTQKLFNDSRRSLNGRGPFCRSEAKTHSQIINLYELHLKTTVHHKYRKNMTLNTTKLN

MNNWLIHKIDWPPAVPKDYGICATRETTGHKKSQARAKC

>SG-PL_contig_3429_1_-1_1_198

PALIAILLLLALTTVTLTSGEEEAEDSGNLGSVLKRSANKPLLENLPHCKCKWPPCPCDR

GEIPLH

>SG-PL_contig_3430_1_+3_99_251

MFLFSWEFHNLSRNELHWATDVVCVPQLNTLGVTQYICTLSYHSETTSTFG

>SG-PL_contig_3431_1_+3_60_269

MQISNVLHMHKQHTLNHPKFSQEKKVACKLQITSAWHRFSHTHTLILKNSRTKNSQEKHF

LSQTSTAWMK

>SG-PL_contig_3432_1_-1_1_213

LINKFFFFFLNIATKLKIVVLHSNEVQFSTLSFSIIHSTISPVQWTDIFPYPLFSGLTSF

HIIFRCIYTVF

>SG-PL_contig_3433_1_-2_764_985

MSMTVTQSVVDEKIGKNNEYTFQTEERNLIIMTKCSHNTYSLASVLFCPACAMAADSRKD

QRSCFKYCRVSIID

>SG-PL_contig_3434_1_+1_586_834

MPQAMNESWVKLSLTVTHLISKFVTIHSFIARKFLCIYFIYRDTGLALCVCVCVCVCVLE

WDTWEKCGESKKKQLADNKNDKR

>SG-PL_contig_3435_1_-3_3_92

KIAGSVKVKSKNNRSSILGCLASCFVKRNK

>SG-PL_contig_3436_1_+2_59_241

MDLEKMVKQKQTKNTRKDRLDLEQVGVGRYSANTQKTKRPEKVFSSPNPVKDQLFCPKIP

L

>SG-PL_contig_3437_1_+1_295_453

MLVYLHALIHLHGATAYKNASSHTRSQITIGHDNNINHFKVFLLKRAHSTLQN

>SG-PL_contig_3438_1_-2_1616_1909

MRPSQDRNCDWMQVHQSGHTQRIMLYLVSEIVTVWACFSDTVGTSSLSLVIPCLISVLRH

CLGFILLAPCDSVVDRLSKMFHGVDKFQLVEYYPAVCF

>SG-PL_contig_3439_1_+2_149_355

MFFLFSNGLPLNQINKRERERERILCKSKMKKKFHPLSIDTIHNLKLLAKDETIFISGLK

MVQEEKFLA

>SG-PL_contig_3440_1_+3_714_911

MNIMKNAQNIENVVVVGGKTRLGSTALRTRHWKREQDCVCCDLLIYFHFSAYSEKMLDYQ

GACMCT

>SG-PL_contig_3441_1_-1_154_1005

MGAYHTLDLELNRPFTLSKQEWDSVALDRISMACDPTQNADLAAIVMQEGIGHVCLVTNS

MTLVRAKIETSIPRKRKGLCDQHTKGLHRFYDQIIQAVLRHVNFEVVKCVLVASPGFVKD

QFAEYMFAEAVKQDFKVLIENKSKFVLVHSSSGFKHSLKEVLTDPVVQARLADTKASGEV

RSLDDFYQMLQNDPDRAFYGVKHCEKAASLMAVETLLVSDELFRSQDVQQRKRYVSLVDS

VRESGGDVKIFSSLHVSGEQLGQLSGVAAILRFPVQDEEESDED

>SG-PL_contig_3442_1_-1_226_423

MRNVGDREKKGRIWFPAGTERWKGVIELSVCARRGRAAAACSVFVCVCGTTDQRCCPCGV

LGHSIN

>SG-PL_contig_3443_1_+2_2_166

GNANNIGHIRTFYKFTKEAADHMQSACTLQCVYFQIKLGHLKPVRAVCEPRHHIN

>SG-PL_contig_3444_1_+3_63_260

MFWPVGWFIKEKESSMMDIFAPRSNPLPAPFNIPFSLSTPICPEAKIKGTVQQNCYIMQR

EFGYIK

>SG-PL_contig_3445_1_-1_43_2355

MSDDDSLKCESLVGQTLRKLCCNSTNMKDSVEEAPSFIGHDKSSFECMQQKHSCSESDKT

PDHQNECSDLEPHDIASCSSDIETKPVQCSEDQPQKGTKLSYSTLELDGGHHSQVQVIDE

DVDLPHNTSEETSPENSSCYKWPRVKVSNTDGEVFVNEEELKVKYREENTNKKLSASSQD

ESDDSISGPLEIKPYLKNSFRSKIFSAPIYNVFMLYSSSQQSEQSDFLLSDKQPSSNSSL

QRHHHTELNRLATYIEYPSTLPVFAITLAGDGFIYKHDEHLVECNFCKMCIDLSELSMPE

ANPVRLHWERSPQCPLKRGQDCGNVPVPKVDEKSNMSNDPSLGISDDSHEADVSPKSLSS

SGYCSASSDFGKSATASYKADPATLLPSPVVYHQPAQNIITTDSVPFVQQVQPPSLPPRG

KSDSTLSQTRPQQPNPSSIKSAAANADSTHQQTEPVAGTANRNKPNNSNTANGNEPSSPP

SASTQKGAAASAAASQPKNSKSSKKGKATKKSEASNAKTGAASNGSSTQNSSSKTNASSG

DKGEQSKKKLTYADLGILADKPKRPDMAVVNKRLKSFEEKWKSSFKKTPEELAECGMYYA

GYADCARCFFCGGGLKNWETYDEPWVEHARWFPKCAYIRLCKGPMFVEIVQRKNKDKQKI

VMKDVEEEINQKSRSPDSSPTMLPVNVSTSGHNEGQTPPPNSKLVADLADLEAENEELQA

QVTCKICMDEKSSMVFLPCGHMVACTNCAIALKNCPLCRSEIKGSVRAHMG

>SG-PL_contig_3446_1_+3_1125_1268

MDLLELIIQAMESNISSLSESSQGMNLVFLYYQTLIMIQITSFTGNKH

>SG-PL_contig_3447_1_+1_1_153

IFFNKKKNPFLFPLIMEYLYIPLRHFLSHVYNYKSMISPYILHQNICENPS

>SG-PL_contig_3448_1_-2_41_2899

MARTLKRTGSSFEPVSKCMWCCVLLLFLSLTFRVDGVQANSNSSSVVSSDGQTNETESTV

EKGMRPFFDMVKSFLGVIQPHRLADQTWLNLSDIVRDGEFQTEQLTNWQNWAKYLVGFVV

CVAVGVVFFIAMPITGLVFCCCRCGGKCGGRSKPHDPKHAGCKRKSYCTLLIVFSTIALA

GVVCVFMSNQILYDKLQNENDAGPVGKLVYSVDEVNRFTDSTVHDLTVAVLDTFVATSAG

IITDIRDTASDAIEDVLDTLNTTSLLDQSKAVSSDVDKTKQELSNVATQLGILSSLGSML

TGNLTDVRIQIDNVCTTASQCPGYDASKYNTIADFSSLDSLSVETNNVARSLNLSQYVSQ

AEDTINTTKANATQSVASGINDATVSVDNVKQEIENGVNKFSSKVADNITAVIKRIRSDL

YDAQDDVSKYSQYVWYGGIALSCVLLLVVVLYYVGILFGLCGERPGYGAQCCNTGTGANF

LMAGVGITFLLSWLIMLVCIVLFVTGGPIYTELCRYFDDHQPQALKPFSDAVMNAVDFKD

TFFDGKDVHLDLVDILEGCKNDSAIYSVLHLDNMVNISQYTDLSSFRTALDNLKQENFNI

PNVTILTSDMNQSITNFANSGLDSISFASYYNEVNKNLTAGGLPAMAAELRSIAQISSLP

PATRQTLNTSADKLDVLQTNLVEPMKQAKKSLNDSLTYVQTQANSDLKISTDALLEGLQR

AQDNFNSQKNTLVQDQVSRVIDRVMKLAEDTVQDIITDMNENVGKCRPVYNSFYSASDSV

CVTALEPSNGLWFGIGWCLFFFVPALIFAVKLTGLYRKEHDERDFDDPNISLYEGGKQDG

IPLTSQRQPGPTSPSRQGHDGGVANHGYQGESHMGPVDYPGYKGGAVNSGYQPDNYPGRM

DRHAPPPYGSHHNGSGEIKLPAYPGGDWKYPGASAPYCSTNTQGVVGVPSSAY

>SG-PL_contig_3449_1_+2_143_316

MSTLQHTQVHSVLSHYQAVHRSYQIEPWWGHRPETLHCTPPTKTPTAKSSAQLRNQTA

>SG-PL_contig_3450_1_-3_150_305

MKSAKLDILVRWFTCAFEFSWLCELFKADVIDGMLENGYMLVQSVAKFLYTF

>SG-PL_contig_3451_1_-1_40_423

MPWAYERIYKLDMTDAAKKLGLSSATDFNFKLTVTKYDGTALDVEFPAPVIVKRNANSEF

DELVLPMRSVNKLPPKVVVRRGTRVTFHATEDGFHGPVRELGSYTSMSHCAIPPGQANAY

EMDIPHEL

>SG-PL_contig_3452_1_-2_104_790

MEEDQQQFDKDDYSGLDGENGENFEQQSTEQTVEDGGDQAMETGGDQAEDENEDERKIFV

GGLSWETTAKDLREYFEKIGEVTSCTLKTDLETKQSRGFGFVVFKNVETVKKVLENKEHK

LHGRTIDPKPANPREGIKKIFVGRLDPGVPEEDVKSYFGKFGTVAKVDFPFDKVKDQRRA

FCFVEFDSDKSVKKVLDQATHKLGGQEVDVKKATPNAAQAGPGRGGRKS

>SG-PL_contig_3453_1_+3_921_1208

MTRRYKKSDRETEIWDCFSAMPPSNSVREDRRLTSVLQEQQELPHSSGQSFTTLSVSGRE

FLGIGPKTKFWSTQLTCDIFHSTGCSQISQHFPSLH

>SG-PL_contig_3454_1_+1_19_243

MPKNNYRHAKPEVCVCARARECMCMSYSCANIPGVTIILIAYITVKQSVGELMIKRYPYH

LGTGVEDIIMISSPN

>SG-PL_contig_3455_1_-1_91_720

MTAMQRVLQEMNRLGGPYSMSVGSATGRSRYTEYLVIVYRSDKLSLLASLQYPDHDDVFE

REPYSVLFHTTDSRVTKFGFMGVHLRPDDVRVEIDALIKAYEDASAAFHTQNFLIAGDFN

ADCRYLSNRAYHNSAIFQDDRFEFLVDITADSTVNSNTDCAYDRFVATGSVRPAVVSGSV

RVYDYQTSMDLSYDQALEISDHFPIEMQLQ

>SG-PL_contig_3456_1_+1_184_285

MQILLFLHIYFFKDSFLLQFHEIIQIDEMFNPTV

>SG-PL_contig_3457_1_-1_1_231

NTLYTHNTLYTHNTPYTHNTLYTHNTLYTHNTPYTHNTLYIHYTQYTIYTIHHIHNTLYT

IHHIHTIHHIHTIHHTH

>SG-PL_contig_3458_1_+2_557_820

MYNLHSSPPCILIYTFINLLSIYFQCNLHSFSPTHLSIIHCILPFTLKSLLSISVYDIHG

MLYFTHTHTRKHTYTANKHIYILTTVST

>SG-PL_contig_3459_1_-1_40_210

MYRNFCIRRCRMDPTNVRCFRACCPNGYMDVFSHCAKERVNDSKNSNLSKKNNSKYC

>SG-PL_contig_3460_1_-2_575_787

MELFECDESLLIKLQSTERLTGVVCSVLVTAKSGNMLNCDEFLSSLDGDADLFVVSVIKL

CGGATTLRVSG

>SG-PL_contig_3461_1_-2_17_466

MTKLGRLLILANILRVPPQASAFFHFAMGENSSKTTMPTQEQALPGRAEKIQVSPKHTVN

GNPTVPPFPAHMNMAMFGLGCFWGAERKFWQQNGVFSTQVGYAAGLTPNPSYKEVCTGLT

GHSEVVRVVFNPDKITFADLLKVCQCEHKI

>SG-PL_contig_3462_1_-3_699_926

MEAGVCMHFLILAVALICARYRERKRVKDCSIMLFICGLGPILCNRPTISNPVRVNVGTF

ICLKRPLSYETCIRTI

>SG-PL_contig_3463_1_-1_154_1089

MEPSMFDSNEPESAVKRLKRNMTLDFNGSGAKAKKTAALLQSPDLHMLKLASPELEKMII

QANGMVTTTPTPTQFIFPKFVTEEQEAYARGFVDALAQLQSSNDPKETALPFPPSQAKAI

ITSTASNSIGQSFPPNSTVTESAALPVVTSLQQVSTTTTLPNGVNLSTAGAAALPVSSAV

GVVSHPKPPLPSHQSAPVVDIQLRQPKLEEEPQTVPCLSSSPPLSPINMESQERIKLERK

RARNRVAARKCRTRKLERIARLEERVKDLKGQNNDLLTTATSLREQVMKLKQQIVEHVNS

GCQIMVTQNMAL

>SG-PL_contig_3464_1_+3_189_335

MLNSTETFLEQTQRSTKKTSIISKLAPYIPSQSTHYKSQLFHNTCAQFK

>SG-PL_contig_3465_1_+3_3_74

TGKAEKEIYAKGSDTSEFNSSPVK

>SG-PL_contig_3466_1_-1_1_168

SFLSLSVHSLSVLSRMGPSLENCLPFYPSFIQSIIILFCTFLYIIIVFDILFCVIV

>SG-PL_contig_3467_1_-3_3_194

RHKFLSPLGLKEANLKDNQKSWCIMADDVAKPSELAELARSCFCGPTKVLVLFHWSDISQ

DLHT

>SG-PL_contig_3468_1_-2_362_664

MYGQMDSSYFCAFKNAFSCTVVFCILFCRIQAWYQCLYLYIYIISQNFFLSLLRYFRSYK

LGHLAFYFQLITDCILLRTLKYLTIRTVAVVPEIEDEHQKP

>SG-PL_contig_3469_1_-2_641_832

MYIYIYIFKRKLPYLLHREWKFPICWKLILKMDPFLERNRGPRFCGSILGRTKVLVPVQF

GNKT

>SG-PL_contig_3470_1_-2_2_172

CNICCGWVNTRSFETNMVFIFLLHMDLTLANCHKAEVGKTAFSHKVFKSSTYHYFKR

>SG-PL_contig_3471_1_+1_508_915

MDLAYMVFKLICHMAYIISFFQNLFSVQVIKKNYMIQTEEQRRQCKNISFVLFLYQVIIA

FLCEIKVVQHGETTTEHHPYLFSSAYLSLAFESGFFHKIARDNTFVAKNCSYACPVNGLQ

GISIYHIIERLAELKV

>SG-PL_contig_3472_1_-3_366_707

MTFTLLTPCGVDWAQPLYIPLSDIECVCLVATAVQKVCILMCTLFVYFVQKLPFLCMTAF

KVCPVMLSFSHHDHLVDMDQTFIFTLPITLYVSESSMFTSNEILFLLIIVPSWV

>SG-PL_contig_3473_1_+2_2_373

GRSGSDRYSGSRCSGGSRCSGGGRCSSGSRCSGGGRCSGGSRYSSGSRCSGGGWCSGGGG

RSGGGRCSGGGRCSGSRCSGGGRCSGGGRCSSGRCRGGGRCSGGSRCSGGGRCSGGGRCS

SGSR

>SG-PL_contig_3474_1_+3_3_128

RWRVKLFLILRTETTTALISLQTSAGTFSGLIFTYSRQKQRR

>SG-PL_contig_3475_1_-1_115_342

MPKTPWFDSDCRQIHLQRKKAQRLVSRRPHPSNVRAHQKLRAQSRLTFKKSRQKSWRQFC

SSLNFRTSAKKVWKII

>SG-PL_contig_3476_1_-1_1_216

HRANWDLFQSKVQAALTENKIFGSGDPAESLTSLLIQSAKATIPTTHGGRLLMPKTPWFD

SDCRQIHLQRKK

>SG-PL_contig_3477_1_+3_3_113

FASNYLADSLSLRKTLCAGKVLKWAGNRTCETLTPPE

>SG-PL_contig_3478_1_-1_43_237

MPTVPNSSYFVPTSQKLFCRSNDVKIVPKSSFSSSMGHPHTLGLAVDVPSCDVCTGKCQP

CLRIY

>SG-PL_contig_3479_1_+3_2526_2834

MIVYFVACDYCWEKLHREITSDMFSGYMYRQASGMTQFTASDQLQLFTHTHTHTHTHTHT

HTHSQSLLASTHKKTVVMLMHCYNVNVNRSLCLFDVTDSITNT

>SG-PL_contig_3480_1_+2_2_193

THTHTQGTKVALNFMHFNVIHTFYTGKKSQRRYEAKVADMYTREKLTLLSESLGTLNQLH

FKYI

>SG-PL_contig_3481_1_-2_2_178

RPCVNRGTNFDDSSVKNRWKWDWMEAVVKEERLGKHLRKITTPGHAVCVLCQHDINKIN

>SG-PL_contig_3482_1_+1_1_2115

ARGKQCRSCSKYNHFANVCRSTGNSSRGGRGQPGRPRRGRVHDVEQESDIHRHADTLFFD

IDSVKSGYEPPWRTNIIIGDQQLSFKLDSGADLTVISRSVFEKQFPGFKLQPSTYKLRSA

GQPLPCLGRFKTTAQHKDKVCEVEVFVADSQTDCLLGRTALVNLDLVRRIDAVLNDGDPV

FGNLDETPIACKPVKIVLKDNHEPYSVPVARRIPLPLLDKVKEEIDRLESCQVIEKINDA

TDWCAPIVPIPKKNNKGVRLTTDFKRLNKAVKRERYMLPSLEDITHKLAGSTIFSKLDTT

NAFFQLPLDPDSARKTTFITPFGRYFYKRLPQGITSAPEIFQKTMEGILEAHKDHTICFF

DDILIHSKDSESHEQHLSETLQTLRDANVKLNRDKCEFCKDSVKFLGFHVSRNGLQPDPD

KIEAIIKMTAPTNVQELRRFLGMTNFLGRHLENLSTILHPLNTLLGKDSAWCWGPPQVEA

FNRVKTMLTTAPTLAFYDATLPTTVSADASSYGLGAVLLQLHDGVQRPVAYCSRSLTQAE

QRYAQIEKELLASVWACERFSRYIVGLEFTLLTDHKPLIPLINTKDLSEIPVRCQRLMIR

LSRFAATAEHCPGKEMFVPDTLSRSPIPLDAETAAQVQVSEAVIASHVDQITSSWPVSDR

FLQRVREESVLDVSIKAAMEYTINGWPAHREDVILAARDFYAVRG

>SG-PL_contig_3483_1_+3_87_377

MMECPLLSKSASMSFVPTMWATLSEIPLLPYCLKEFRPRQRGGGVDAPLFVHVASHRRDV

REQPDRLAPHKVGEGGYSVRHGLRLEDLDVEAGFVRG

>SG-PL_contig_3484_1_-3_3_209

LRSRHAACDCYAVYHSASAVCVNQSSAVVGASVGERDFLSHFLWLRILLVDLRFHDLVHF

MHCKLWRDI

>SG-PL_contig_3485_1_-1_190_555

MLVSVTAIFFYLNPGSILCFNILDALVFVQRCMCSAFKETRHNVVTMDSVKTGQIFSHGI

QMVSMCMDYTACSFFFPLLFFILHVEDDGICGVCCASRLPQNCINLVPSLHICVNMVFLL

TL

>SG-PL_contig_3486_1_+2_2_169

LRKVEVPTASVHLQPQGYGTTCTPVTVSPLCLSPDHHFFGSGWRQGSTRTVIVSVN

>SG-PL_contig_3487_1_-3_3_248

CALPISLLPACYFPDSVVDSWLQDEGQVEKEEDEEVEAQKEKDASKIKVDHHALHLPWNF

FEDETCLRIVHLKMKLNSGRYK

>SG-PL_contig_3488_1_-2_23_592

MKLWVAALLLAFACYIPCGAEEETEARLLVSKNILNLYLVEEKDLTVEYRIFNVGGSSAL

NVQLKDDSFPEGDFAVVRGNLQVSWERIAPNSNVTHAVILRPLKSGYFNFTSAEVSYLAT

EGDSDRQVGYSSAPGEGGIVPLKDFDRRFSPHVLDWLVFAIMTLPSLGIPFLLWYGSKSK

YDTPKAKKGN

>SG-PL_contig_3489_1_+2_2_106

EKKANNKVENVIQNLFFLGNKFRQSHVLRNIMMKV

>SG-PL_contig_3490_1_+3_3_125

THTFSLPFSLTHNLRHIHTYICVILISVHKLENANLSYFSH

>SG-PL_contig_3491_1_-1_10_2823

MQGYDGQQNNCPDLQTPVHQPGHDNASLQALLARSTPTPQHTLSNSISAMASVANHSSAT

VGAMASAGLNVNTVKNPLSNSLASSSHTVTLNKSATATSSHMVGAGDGLQNMNYTMATSL

AGSPMMNTMNMKAPMGPNPGQLMGNLPGMPGMGLQQPHHNQMINGPGFPPGMGQVRGMAP

TTMGNASALSIGQNMIGNSQIPQMPGHQNMSHVMSVPQNQAQLGKPPPPLPGGTGQRQPA

ADPDKSKLIQQQLVLLLHAHKCQRREQTNGEQSCGLPYCRTMKNVLNHMTTCTKGKSCEV

AHCASSRQIITHWKNCTRSDCPVCLPLKHASDRTKTGPAMAPNPAMPSGPNQSANVTNVT

DATMRRAYESLGLTPQSVNTLVQGTGPSQPATGGQIAAPTNNSQIPQMAPTPAAIPSASP

AVNQNHPNVQANQELTVQPSTRKDWHAQVTQDLRNHLVHKLVQAIFPTPDPAALRDSRMK

NLVAYARKVEGDMYETANDRGEYYHLLAEKIYKIQKELEEKRMQRMMGQNSGGASMPVSN

TARQNGPLTSTQIAHTLQLSNDMMGPGSTVVRPPQVNPLHISQPAAGQVGRVSQTVGSQS

NHTQVPQLQQQQQTGQPGLPGQQLVSQHLTPRSQQQSQLTSPLSATSTSTSTASTITSLA

AELHTSVPSSLGLAMQQQQQLLTSQAQPSPSVVQSQTIMPNGTPSHSMPSSQPPSVGGKQ

LPVSQKSQQGDQDLNGTSEGEIKTSMESVADNNVHLTTLLASDSKAVDSSSSVQPTSLLQ

AIAATSSSATTTPVTPVTPTSTAVKMEVDPSAIKEEIKTEIKQEPGEEDTFPSTSSQFKE

EPVASEGTCSPKTESVGENSQSGNLDIKPSSVEASASQPSKPCNKKEFKPDELRQALMPT

LEQLFRQEPESLPFRERIKSSQRNRDPILRGRFLAKRD

>SG-PL_contig_3492_1_-2_2_283

VTTISRWLSATVIRAYKESGSSSLPDSPRAHELRALVSSAAFHHSLPIRDVLEAAYWRRE

STFTNHYLRDLRVSNADGLSRISFVAANAVIDAS

>SG-PL_contig_3493_1_-1_679_891

MHIFAHSLRSACDCDTGVLSVVSCQQAVEQGQGVLQFCQLSVSCWTGTGSSSILAVVSKL

LDREFSIVSCQ

>SG-PL_contig_3494_1_+3_9_215

MFPATPNKYLIINNNSTDFVPACFNSVCQTQHQPLPHKTTCHNPLLSNIFSIFRKRKTDC

HCGLKKKKK

>SG-PL_contig_3495_1_-3_3_266

LQFRALPFGLSLAPYVFTRTVRELASLARGESIRLSCYLDDWLILAESGPLCRLHTQRVL

WPVSRRLLSRYEHVRNEKTFVAKKIAFH

>SG-PL_contig_3496_1_-3_660_1118

MYHWNVNSFVFDVYYLINQFNQEIEVGIFRKNHVLLFHWQHLPDLLHFCTRVCVRARRER

ERKKEIYICCLPVCCSSECGSWSVCSYLIVVMNSYSYMHTTCDFMESAVCHCSPEIKKSS

GMVIRHVILCLQLKVVNSNTLIYSDSLFLIIKP

>SG-PL_contig_3497_1_-1_16_1134

MATSPNTSASGTLTPKKKDESFLEKIGTLARKKKIKEVNELQEEGKNAIESPTSPTLPDI

GPEGYQLGESEERSMIEPNSRESAKLKDLIKILLDWINDELADHRIIVKDIEEDLFDGQI

LQKLIEKLANVKVEAPEVTQSEIGQKQKLRVVLDYINRMLQLPPVWNQQKWSVDSVHTKN

LVAILHLLVALARHFRAPVRLPENVCVTMLIVQKIDGVLQTRRFIEEITSTNDDIGARFE

RDAFDTLFDHAPDKLNVVKKTLVTFVNKQLVKVNLEVNDLDTQFHDGVYLVMLMGLLEGY

FVPLYNFHLTPTTFEQKVHNVSFAFELMQDAGLQKPKARPEDVVNLDLKSTLRVLYNIFT

KYKSSTSTSGAIH

>SG-PL_contig_3498_1_-1_505_756

MGDRSYIFWMKRKADSASMAGIIRLNCNLQHNWADKSKIWQLIPQRNKNMLPRVIHSWTE

TENCRERTVQVCGRHGHELNFQYG

>SG-PL_contig_3499_1_-1_1_231

FFLLILSSVFSDACTHLFGPHCRHDAAQFDSLLGLQWACMLDVFISHQTQAWLTGPLTCM

YYVLHAYKHDDERCSES

>SG-PL_contig_3500_1_-1_172_339

MKDILVDFLRVCTLFTFIYDLLLGKIYHIILLKKSSSFNSCSTQFLKKIIGCLHFF

>SG-PL_contig_3501_1_+3_3_230

VVSLLIPSAVFIHSIPLSSTFPILRPQYDTGSGAENKTTLYVHSIVFIHHRQTNTHCILQ

LYPSSLNTALSSTLNT

>SG-PL_contig_3502_1_+3_303_542

MNVFLCVCVCVCVCMHGAHTQFFSTLDHGNVLHYCSLENNFVHLTNSYSPVTLNTKTVYE

RTRGGWEVVVNVCLLRYHPF

>SG-PL_contig_3503_1_-1_10_579

MELIAEDTEKLNCQIVVVPSWRDAHHDPVYPQGPFIWTHKPQHLHFVSDPCTLVINNVVF

GITSTDILFHLGAEEVAQCPPGTTDRLGRLAQHMLDQHSYYPLYPPADDVNADYEHFEEY

VKMSVIPHVLIVPSDLRYFIKDCNGCCCVNPGRLAKGQVGGTYAHLQLQPSTSNMPASVV

STTAGQILKI

>SG-PL_contig_3504_1_-3_105_260

MFLKKFQAFRKQFQIVPKKCSEGLASLLQVFPQLCIHHLVFLLQIICLKEFF

>SG-PL_contig_3505_1_-1_7_249

MIINHSYIALFSKLIVSHCALHIYETQSVIHTKALISPSENSVCTIIVILIIKLNNKVFY

FFLPRVFWTALKAMLHYMARY

>SG-PL_contig_3506_1_+1_265_375

MLYTTLQHFLKKVTKFHIIRWACYTIIKLQNVFKHTF

>SG-PL_contig_3507_1_+3_534_800

MYRYHEIFTSKQAEPKKNQKETAYFQLKIFQHEKKNGQLACSLTHAQMQSLIFRETLPMS

QVVELSFLTKFSRIFKISSFYLHNCIIVL

>SG-PL_contig_3508_1_-2_2_373

YYCCCYYYCYYYYCCYYYYCCCCCCYYYYCYCCCCYCDCCCYYYICCCYYYICCCYYYIC

YFCCYYYDYYYYRLLPLLLRLLTTTPIIMRRRKRRTETTITTTVVAESVIFKVVTCPSSV

FLSH

>SG-PL_contig_3509_1_-2_2_616

MMRKRRILILRATKMKRMSWARVKRRRKERMARRTMRKKMRKRMTMRKRRMLALPGGRRG

STRMKRKTHNHVYSSNVISSSSSTSSSSSSTITIMTFTTTIHPHSRRNIQHQEIREKVGS

TREREDGFFFCFLVPLHVLDPSSCWTLKWVVPTKFCVVLAVDAVWGQPLSRTNRHARHRG

LGQRTRGLAVYIHPPPPLLFVCFFI

>SG-PL_contig_3510_1_-1_103_675

MSVGMQKRIELEMRGRSANVIKDLNLDNCRVTEIEGLTSDFCALESLSMINVGLQCLSSF

PKLGNLKKLDLSDNRISGGLQALTGCPKLTHLSLSGNKIKDIDALKPLKDLGSLKSLDLF

NCEVTNVDLYREKVFEMLDQISYLDGFDQNDKECEDDEDEVEDCDEDDDEDDDEENEDEE

EEDEDVDGEDE

>SG-PL_contig_3511_1_+1_1_249

KKQQQKNAICKKFCPSTNHQQLISSKSSSHKKSVTNMMCLVLETLLHLQQPHHFKIKNEY

RNIFFLTVFNYSALPIIICSKSK

>SG-PL_contig_3512_1_+2_2_205

IPPRNIKLACSLPILMLQSFWWCQCFIGPVPLPISRPCQCIGFCAGSRSLPDISCQIWVQ

CIYYAKNP

>SG-PL_contig_3513_1_-1_1_129

DLFNVGLSISLVSLAGKAFGQSQIIYQSFRLSQVLFKVQLVTI

>SG-PL_contig_3514_1_+3_681_1103

MAKNKIISQRAWNAVRFSLFLLSGSSAHGLLLGLCCLTAPLSSVVLSCVKIYFSFLHLWK

HENNVKTYRLTDKQNRFSSNIWTDDLWCGCVCVCVLCLTSRAAHPPHLTYACCPAWGIGR

QQMLSIHHDPEPVVPVVPRRG

>SG-PL_contig_3515_1_-1_49_1344

MMKHFVLLFFAMVLVADAASFKDERRRKDRPDCQEKCRSRPMEISFVLDGSASIWQVNFT

LGLNFIEDFVDLFDISQSAVRVSMVTYGDVVYTQDAFGLSAYDNKENLKAAISQIPYRSG

IKTNTSGGIWYMLRQQMPEGRPKVRHVAIVLTDGNSQEADLTKQAAKDAREAGLEVFAIG

VGHTVSLQELHNIASDNRHVFVVSSYNMLEDIKERLAYQACANEPHPVCKMEPVDLSFVI

DSSASIGKGNFTIGMTFVHDFVDNFEINPNTVRVSAVTFGDGVYQRDAFDFDTYPDKKSL

METLEGIKWRHGQTTQTGAGIQFMREYQMAKARPSAAHVCIVITDGESQETEITAEEARM

AQAQGIVMYAVGVGEVGKQLNEEELNNIAGDPDHVLTVNNYAQLNLLKDKLAKKTCEGVL

ASLSLKSEQFRQ

>SG-PL_contig_3516_1_-3_3_122

CVCVCSTHVNKVVDNLDKDLFLIYPSIVYCMYIIHIYMVR

>SG-PL_contig_3517_1_-1_61_390

MMQQHLHISISRSQCFHSHSSTALNRSRIDSTGTLAPDSASSSSSSNSGDEPDDLFASLA

ESLQSGLQLTPPGRSKLAKRRAALHACESNSSTGSGDSFSSIAEVGSWRS

>SG-PL_contig_3518_1_-3_156_1742

MVRVTRQAVQENYGLDKIEVRDNGSGIQTSDIPVFAKRHFTSKITSHADLEALVSYGFRG

EALASLCAVSEVSVTTKTAAEDISQTYTLNGEGDITSTVPSHLGQGTTVCAKNLFKNLPV

RKQYHNSVKKKKEDLKNVENLLLAYAVILPAVRFSLRHNKEVMWQKPVLDSAAKVLQSIL

GKAVMNQMEWKKKHLEELEIKVELCVPKPGSDVSSLSRSTPDRSFLCINRRPVTVREIDR

LVRQYYNNCHACETNRHPVYYLGITLPSKDLDVNLEPNKTSVMATSMPTVLEQLEEMLLY

IYGPLDQAPSWRYREGTGKGEEMEKHSTSKNGNGVSVNSALEHLRKTVDCDKAQDSRQKN

DTEKITTDELSSSSPAVMTVPTLCVERQDVPHSFKDGENGGLMSMGETNTSSGSRNTDID

TVQLNECFNNNTCKNQPEKHVEPSELSNQCSSIPNKDLRKLLTHIPENLEKSSVPAATSS

NIVSDNSVGEQTVEKTGNTCNAPIELTVADMKTTDEVGSAVLKRTDQVA

>SG-PL_contig_3519_1_-2_1424_2965

MVILMTVRVCLQMQIFVKTLTGKTITLEVEPADTIENVKAKIQDKEGIPPDQQRLIFAGK

QLEDGRTLSDYNIQKESTLHLVLRLRGGMQIFVKTLTGKTITLEVEPSDTIENVKAKIQD

KEGIPPDQQRLIFAGKQLEDGRTLSDYNIQKESTLHLVLRLRGGMQIFVKTLTGKTITLE

VEPSDSIENVKAKIQDKEGIPPDQQRLIFAGKQLEDGRTLSDYNIQKESTLHLVLRLRGG

MQIFVKTLTGKTITLEVEPSDSIENVKAKIQDKEGIPPDQQRLIFAGKQLEDGRTLSDYN

IQKESTLHLVLRLRGGMQIFVKTLTGKTITLEVEPADTIENVKAKIQDKEGIPPDQQRLI

FAGKQLEDGRTLSDYNIQKESTLHLVLRLRGGMQIFVKTLTGKTITLEVEPSDSIENVKA

KIQDKEGIPPDQQRLIFAGKQLEDGRTLSDYNIQKESTLHLVLRLRGGMQIFVKTLTGKT

ITLEVEPADTIENVKAKIQDKEGIPPDQQRLIFA

>SG-PL_contig_3520_1_-3_2370_2609

MNIQDHLIYCVGVVLCIYMKVQFIAFGEFGNNRICLYVIAISLCVLTANLWSVWMLSFIG

YKIYLLSPAVGFFFKRPPLA

>SG-PL_contig_3521_1_-2_2_274

TFVIFSSLWKPVDSQKWTTNFLCLKSGMNSLHTHTHVRAKKKKCVGGGTMDTDLQICTCH

AKDPDSNDTSISCIQGCFACVFSLRRQRQQQ

>SG-PL_contig_3522_1_+3_270_428

MDLFSKAVNVSTFCCAGIVKRSVLVSMCFSLYLCAGVMGQYVSLTLKKKNVLP

>SG-PL_contig_3523_1_-1_1_501

SSDLPTPTPHSRQCVLLYIAAIVNHQWPVSTLIAVVVSPVLWVCSVSLACGPPAVPCSIL

FVGCCTWVSLLKSANPWHVSLEGSGRWHTLSVRVTWIGGAVLCSLQAAALDMRASLYIVY

TSNCGHKVCSCHRVAVPLLCFAVVQHPLHEDTQFQKKLCRATSIPSI

>SG-PL_contig_3524_1_+2_140_289

MHHQAGNKCIFFFYRVESSSHRQLVTNVSLNISIFMIISMLKCKVGFLYT

>SG-PL_contig_3525_1_-3_117_371

MCRAKQLARSKGIGQLEWSIWVLYFWPYTNYRDPAESTIRYNTTVSRCLCLHGDTDLFFF

FLPSLSKRRLWRKSECITLTELDPI

>SG-PL_contig_3526_1_-1_67_894

MESVGLHLSIDYVGSSGVILSPEQKAALQTSLVILQNENKFNKVYFWGKIFGVKDDYFIA

QGSSKDEFAGRKTFYSKDCIRWGLLPPATQAVRIQSKLAKGRFTGDPAYEYEHVTTKKVG

EGEEQTEEEETITIKEEDRLASIIAEIDEDVKIVPRSAFVRTPTGEVVSNRSFEGLSVSE

SSKLCNYMHFREPIALNQKSLLQRANLDKAIDYMDSLEEDIPRGSWALQFERGSGLVVLR

SLLWPGFVFYHVPGTRYFGSVYFGNGEKNLDLPFML

>SG-PL_contig_3527_1_-3_174_473

MHMVKLPVDEARANSHLAFAQMTDNGHYPLNLPNFEQIFSNFLLGQTPYGPGRHIFGCFE

HQVPKTPFRLIQPAPDPDDFDMDVKVYLEKRPKRMSLTGI

>SG-PL_contig_3528_1_-3_627_902

MLLSTEYVLARRFPLDSYSCSLRKLTQFTTLPVLGSLVEITVELKKNYPCALLSSFRLCK

MWDVAIQKLMPAFLGQTFLYLLPPAQVSNHSL

>SG-PL_contig_3529_1_+1_1_81

SNLVHVWSHLSLKDNRPALPDAEKTKN

>SG-PL_contig_3530_1_+2_2_160

SCPKLTACQRGRKAENSEIFSLEITWETCTFEISCKIRQYRYQHHCIWKEKKC

>SG-PL_contig_3531_1_-3_408_605

MHTPKRCAVVIHLHQLQAVLALSSPLVPPVSRKGGVSCKGNSRFFMSLCAGLRPGDICLV

VQFHNL

>SG-PL_contig_3532_1_-3_15_269

MQVVLNKIKIFMEAHNKRQKRILCQILFVIIQGMSVRNLYISNKLFIACFVIRERCQLLP

KMSQNNTCTSNIWGNNLQTYLLTLH

>SG-PL_contig_3533_1_-2_110_706

MAQIGQLAIQKPAPEFSAQAVVDGEFKTIKLSQYKGKYVVLFFYPLDFTFVCPTEIIAFS

DRVEEFRSINCEVLAASTDSVFSHLAWVNTPRKQGGLGSMNIPLLADKTLEISRQYGCLK

EDEGIAFRALYIIDSKGNLRQITMNDLPVGRSVDETLRLVQAFQFTDEHGEVCPAGWKPG

ADTIKPGVKESKSYFSKAN

>SG-PL_contig_3534_1_-3_192_344

MRFVSTALQHKNVYLTKKLFLIPFLALSCRSECECVCVIATAHTTNNYVHN

>SG-PL_contig_3535_1_+1_472_579

MVVCSAKLNYNLIVLFFFFFFFFFNSFNKKKKGILV

>SG-PL_contig_3536_1_-3_24_1433

MSPVSIIAGRSPAGHFQYSTPSHFQPSSPAPLSPGGVQPHHMPRAMSPAHSSMTDHFHGR

HSSSGASPSFQNMSSPASSPGYLSPSRLPSSPHRSRSSSSASDAVLQTARRLSSETLPSR

GVGVQRRLSSTPQDVVRTWECLQRKVNSLQSASLQVTYSLEIVNIAEANWHQRKQDLRRD

VQRRSAQLQFFIKRQERLLLAALDARKTDDSFNSAAEQARSELRTNLKSLMHEIDVVRSV

LDLATDSELMSLSSLLLNSKVSQTPVNMDTPELRFMVPEELSLEDMVGKDFGCLTSTLMT

HSIFTPEEIEQKLWEDPEEDDAFSVDTSTVNASSTDGATSPTASDNFENSFDDVAFIDDD

DEDELVVEETLEFTDGKMQVVSKTSVPASKTRFGSQVRPQAAEATESKDQTKRGRGTDVR

RTANSSATVHTMKDDETHVKKRRGPEKADDKQLMTDTKTDADSHANGSDQ

>SG-PL_contig_3537_1_-2_140_331

MTFGNFIFSRIIKSNIESVRIFSSTPNKITFSVNLIFAQVTHFDLSNSSILSQNLLTCKH

FQNI

>SG-PL_contig_3538_1_-1_1_165

HEMYSFRLKLLHCGQHCQHVLYEMMVVCWKFQLKGCSCAQLICFYFLYDEQNPRE

>SG-PL_contig_3539_1_-1_22_3873

MKTKYLANPDYNYEKVNRASLACGPMVKWAIAQIQYADMLKRVEPLRNELNGLETQAEEN

RLKGEEVNKLIQGLEQSISKYKEEYAVLISQAQAIKADLAAVEAKVERSVALLNSLGNEK

QRWEAGSETFKQQMATIIGDVLLSAAFMAYAGYFDQQMRQNLFGNWCTHLHQAMIQFRPD

LARTEYLSNADERLNWQANALPTDELCTENAIMLKRFNRYPLIVDPSGQATEFVMNEYRE

RKITKTSFLDDAFRKNLESALRFGNPLLVQDVESYDPILNPVLNRELRRTGGRVLITLGD

QDIDLSPSFTIFLTTRDPNTDFPPDLCSRVTFVNFTVTRSSLQSQCLNQVLKAERPDVDE

KRSDLLKLQGEFQLRLRHLEKSLLQSLNDAKGNILDDDSILTHLETLKLEAAEVAKKVEE

TDVVMAEVETTSQQYIPLAQSCSSIYFTLEALQQVHFLYQYSLQFFLEIFHAALNTQQLG

SMKEYAARLSIITSSLFTETYGRVGRGMLYQDRITFAIQLCRIHLESMASEGTYDAEFNH

FLRSQEGLLLDKSGPVSAGLSQQQNSALLRLSKLPAFRNMLNEVKNNPDFQPWLDSAKPE

VDFPPLWSEEKAQTPIAKAVHGLLVIQALRPDRLIAMARIFVEKTLGSDFVHITEKELNL

SQIVETEIKGNTPVLMCSAPGYDPSSRVDDLAAELSKQMTSIAIGSAEGFSQAEKAINSS

SKSGRWVLLKNVHLAPQWLVQLEKKLHNLAPHPSFRLFLTMEINPKLPTNLLRAGRVFVF

EPPPGVSANLMRTFSTVPTSRMCKAPNERARLYFLLAWFHAIVQERMRYTPLGWAKKYEF

TESDLKVACDMLDTWIDSVAMGRTNLPPEKVPWEALRTLLAECIYGGKIDNDFDQRLLNT

FVNKLFTPESYDSSFVLVRNVDDIAGKHIGMPDGVRREQFVQFVDNMEGPQTPSWLGLPN

NAEKVLLTTLGSDMIAKLMRMQLLEEADVDIGGGISPEEEQRLADGRPAWMRTLHSSVTT

WLKLVPQSLGTMKRTVENIKDPLFRYFEREVNTGSRLLLDVRRDLNDVMQVCQGEKKPTN

HHRSMMADLVKGIIPGFWRRYTVPAGLTVIQWITDFSLRIKQLQSIVQASLASSSRELKN

LNVWLGGLFIPEAYITATRQFVAQANNWSLEELYLDVLVQDGKPQALDDCSFGVTGLKLQ

GAVCISNKLQLSTTISTDLKLTVLRWIKVDGSKNPAGRVTLPVYLNATRGQLLFTLDFDC

ADKPAGKDHGFYERGVALICSDLS

>SG-PL_contig_3540_1_-3_1164_1436

MTQRIKNLFELYNGRKRNSVATLLLGYACCQCKRVCVILTDWVFKFRFRRELGGRLSLSN

SITEKQLKYKNTAFCFCKIFRPSGDEKTLIL

>SG-PL_contig_3541_1_+1_64_324

MRCPCVGSGGRAVQKSGLPPPDQKLRPIPGSLPETSAPGERFGLSCLHDLAGKRFLWRGP

RVAPGGRENSHKECRISCPGIQRTGGV

>SG-PL_contig_3542_1_+2_1274_1507

MVICLVSDYMTRLHSTLCTITSPSINWVLRPLLCWSLNKQHKFIPIEWVRLHLQYQSALL

LSQHTGCSLWDGIHLVEN

>SG-PL_contig_3543_1_-3_57_272

MRGQSNAAVDTGLVWSVCVCVSVCVCVCVCVCYWPPRDVPYQELDDEVGWVARTFVTRKD

RKSFVITEMIIH

>SG-PL_contig_3544_1_-1_46_405

MAIIHQLNTDHTAIHAVNRAALLDDAWNFVKAELLPLEIALQTLDYMKKETDYGPWLVFE

YEASYMQLMIERTELFADFQYFMQEMVDEPMRTLGLDFPPDNVPPVEGSTKILILTVACA

>SG-PL_contig_3545_1_-1_76_354

MTLLVAALFALTCLTVPAASVRRTILGVTPVTNTHLDILQFATFHTEVEFMTFFEINKQI

EALIPTDVSQILREEWEKAGMNVTTIVSDLREL

>SG-PL_contig_3546_1_-3_3_269

LDKINSNAKCLCLIFFFSTESNSAHIVYSPTIHGCCLRGDCHDNYVENIESGDRLWCKAN

GNICIIGVLMNKMKLASDTTLSPTHTHVV

>SG-PL_contig_3547_1_+3_3_167

QISFSQCCPVDMLCSIPCCTVQFRSIGLRESLTRFNQLCHMPACDPLMVTQIPFH

>SG-PL_contig_3548_1_-2_653_862

MEFILFLIVSVYSVKCKTECCIAAKGGGRGCSCLCCCYLYHFITSGIDMICMLYFVQKAE

IILFVFPVHD

>SG-PL_contig_3549_1_+2_2_148

KMYNIKYFPLEHSEEKTKFSTSFYGLNMDHTTTKIFLKMNLSILRTHLH

>SG-PL_contig_3550_1_-1_109_588

MPLKVGDKLPAVDLYEDNPETKVNTSDLKGKVIIFGVPGAFTPTCNNDHAPGFIQNADAL

KGKGVSSIVCLSVNDPFVMCAWGKALKADGKVRFLADTCADFVKKADLSVDLTAALGNVR

SKRFVLVAEDGIVKSINVEPDGTGFTCSRAQDALNFLQST

>SG-PL_contig_3551_1_-3_3_128

VGSERLKPSGCINLLISGCVHLLLCEMSAVSSKGYYKPKENM

>SG-PL_contig_3552_1_-1_1_300

NLEESVKSAHSFQLKPLTSFVSLSFFQGWTTVIPSSLAFRTSCCPSFSAVFRTCTVLLDS

CCESTSGVVQVRFSGLLHWLPVRARVESKVAHHLLPVPKL

>SG-PL_contig_3553_1_+3_78_329

MCSEFRHWKQMVATLYSTRALTESQCKVLRSALALHRFFLSEHKSSSTVLNTLKLGPLKC

RHHQNDWSIKMGSKQASFYVAGWD

>SG-PL_contig_3554_1_+1_58_504

MFQPTKAAPSPPKAARVTLNPAASTIRQPGRNPSNLIYVPLQNIDTLSKQLCVCSFNAQS

VGTSKKRTAICDFIRDHDVDIMLLTETWLHATGDGVSLANLTPSGYRMFSFPHSHSATSS

MGVALLSLSRTLSLTIAPPLIVFPFSYFI

>SG-PL_contig_3555_1_+1_502_630

MTMFLLDISRSTPTIGLPILPKINTFYRAKPHKLTLSTEQNRI

>SG-PL_contig_3556_1_-3_3_119

TDIQRSPAEETRNTTDGTSYFFIKLISLYGRSVTGTKPL

>SG-PL_contig_3557_1_-3_183_281

MKAIQHQVVAILHFSTGTFIYDRSEERRVGKEC

>SG-PL_contig_3558_1_-3_1380_1589

MYIIVWIFDLSGMPVFLLTGSDSFVIETCSAYIFPLPGCSWAVVSTQYLYLVLGTDNLMT

CFCCHLSFLL

>SG-PL_contig_3559_1_+1_1_246

VCVRACVRACVRACVRACVCVCVCVCVCVFSNLNNMLFDMFMVRVLVSCVCVRVCVCANA

CISKTSTLLFPHNRGQAKGVHA

>SG-PL_contig_3560_1_-1_1_207

THTHTQPPTHSHTHTHTHTHTHTHTHTHTHIRSHSITFFFLVLKHQFQIERKTNKQNWPV

LFFENLSVA

>SG-PL_contig_3561_1_+2_2_220

LEPRHINNGSRRGANFTQRKFLKYKTGHKKGENTRLTGVYGVMWPQDVKSKPLEADTHTQ

DPSRAGVEAQNLK

>SG-PL_contig_3562_1_-3_3_287

GGDTGVGFSLRPGGRSPHRSPSPTSDPIHGRLVQRPCGFSPRRRVLDSGPSPPPHQDSAA

HIVGKSNALADAPGRGHSIIHTEWTLKRQTLQGVW

>SG-PL_contig_3563_1_+2_2_100

TLQMFKSEWHHPFPGYEDPELTTPNGSSSNVMY

>SG-PL_contig_3564_1_+3_444_803

MKSAAEGAGFHHDERKQQISETKDKTALNSLRLQKTNRKLTKIVLDPRLWYVKKTHLLCR

SDNVGFVNNLTVNWFIINKYPNLKQRSVLRSTDNTDHYPCAEDLNGPLKILSDVIRLYFG

>SG-PL_contig_3565_1_-3_21_1325

MAWRQITGLLAAFAIVNVAFASKRTLILVDNWSIRESHSIFFKNLRDAGFDLTFKTADDA

SLALVKYGEFLYDNLIIFSPSVEEFGGTVDVLAITNFIDGGGNVLVAASSEIGDPLRELA

TECGVEFDEEKTAVIDHLNYDITDQGKHTLIVAEPSSLIDAPMIVGKKGKSPFLFRGVGM

VSDPENPLVLNVLHASSTAYSHSPVDKIEEFPHAVGTNTLLIAGLQARNNARVVFVGSLD

FFSDEFFQSSVQNANKGQKFPQSGNQELAQNLAEWVFHQKGVLRVGKVAHHRTGQKEPDA

AYIINQDIDYSIQIEELVNGEWRSFNGDDVQLEFVRIDPFIRTTLKRKNGVFFTSFTLPD

VYGVFQFRVDYNRVGYTRLFSTTQVSVWPLQHTQYERYIPSAFPYYTSAFSMMVGVVIFS

FVFLHFREEPKEKKE

>SG-PL_contig_3566_1_-3_3_269

NPLCVCGNFNAYFINSCAARFQFSLNTYSLFCSILITLHVCTCTRTLTDLFLNICFFGIL

THPHSYMHMHAHVSYMYADMHACMYARAR

>SG-PL_contig_3567_1_-1_1_135

NMHVNFVVVCFASEDAGLKRVGGDVAMRYVYISRTESEHLLFISY

>SG-PL_contig_3568_1_-3_786_1163

MHIRYIYIYNIYITLMTSACLFTLHRYRLIKYINIFSVFTSGQGMSDLWQSTSAMFHIVC

CKTVKTPRVCNIYELLFKFLGWGIAVSESFSKIELTFKKKKTVLLAFFFQNHIGAAVNLS

GLLPTI

>SG-PL_contig_3569_1_+2_2_103

SCQREITSPATRSSSAPPKDESSRADTLRNSQVS

>SG-PL_contig_3570_1_-1_52_597

MLVLLLGDLHIPHRCNSLPARFKKLLVPGKIQHILCTGNLCTKESFDYLKTLASDVHVVR

GDFDENMNYPEQKVVTVGQFRIGLCHGHQVVPWGEIESLAMVQRQLDVDILISGHTHKFE

AYEHEGKFYINPGSATGAYSALDSAIIPSFVILDIQQSTVVAYVYKLFNDEVKVERIEYR

KS

>SG-PL_contig_3571_1_-2_95_1093

MTIKIFIGNLNEDTEADDLRILFEKYGTVEECDVLKNYGFVHMSSKSDANTAITNLNGYN

VKGQRMRVELSTGKRGNPRGDRRGQERGGRPRPYPPGPPRRGGLPPPPPDYDRYDPYYRY

AYPERDPYYRLHPADRYLPPRLPPADRYALSRERLLPEERAAYALPPDPRDRLLVRERLP

LPYAEERAAYDRLRAAERAPLPAEPYYRERSPPPARPPPEYYDSRKPPLMRNPPPAAESP

ASSRLSSAACPPSNGYDYYRKAAASAERPSFGGVGSSQDYYATPPRAATGYERQALQKQQ

GTSGGGGYGGLQQQQQRKPYDSQAALQTQPIFF

>SG-PL_contig_3572_1_+1_406_618

MALVHDQSVLHFSQECLLKRVVICSGIFACGQKSHEIRIHTSGDKFSEDFFRRKLVGLFS

AENVPPNGGPF

>SG-PL_contig_3573_1_-3_90_1130

MSQNADPTRFDGMLMNMAQQCEGGIQELLGSFFGFLERKTDFYTGSGSAQAEELVMSSFR

KHSAAAQKLKKEQMEKEKKKAEKKAKEREEAERKKKEAEEKAKEEPKIKELTDAEAEQLE

KELSQKDGEEMTVPASGDAKTVSANGDAGVSEKKEETKSADGDDDEEDEKDKGKLKPNSG

NGADLPNYKWTQTLSEVEIRIPFNVSFPLKGKDLVVDIGKKRIKAGLKGHPPVLEGEMYN

SVKMEESTWCIEDKKFLLINLEKVNQMEWWSRIVTSDPEINTKKVQPENSKLGDLDGETR

AMVEKMMYDQRQKEMGLPTSDEQKKQDVLQKFMKQHPEMDFSKCKFS

>SG-PL_contig_3574_1_-3_81_233

MPSVYTEWCAIVKGVCLLFILSGVPLSEEYVLMFILSGVPLSGVYALMFIL

>SG-PL_contig_3575_1_+2_389_721

MFPTSSLCTRMHRTHTQNSCSHRESDIYMLLPSTTERQALLFLQGLVYHQCLWLLTARPT

MRHICFHLQCGEIFPHWAHHAKFYSKSMGKFCPSEYAAHKFFHFKILTKPK

>SG-PL_contig_3576_1_-1_13_759

MEAAGQAMSSFNGAVLKEPRGFLKLIQFVLSIFAFATITSFSSSSSFQVLCSSSNKTVFV

SFSYGYPFDLEDECVKSPVCNTSTTKKDVCLLAGAKSSAEFYVFVGVIVMLYCLAALILY

IFFDDLYRKNNRIVVGDFVVSIVLTLLWLISSSAWASGLSDVKEYTDPAEGGFFDVGRFP

ECNPAAKHCSVTSLGNYGSLNASVIFGFLNMLVWIGNLWFLYKETPWFKVQSKPPAGTPG

ESTGGANVY

>SG-PL_contig_3577_1_-1_1_210

SQARGSPSARDQPAGETIPVTALASPSMTVVERPKHTTSERRSSLPTSLARPRDRERSEA

PSLASTAPHK

>SG-PL_contig_3578_1_-1_436_891

MANLVKRVWDNPILTTNTKMKVYQACVLSTLLYGSKVWTLYSRQECKLNAFHLRCLRRIL

GITWQDPVPNKNVLAQVGIPSMFALLTQSRLRWLGHASHMQNGRIPKDTLYGELAAGSRP

AGRPALRYKDVCKRDLRAGNIKPTDWEAVAAD

>SG-PL_contig_3579_1_-2_71_199

MGVQISNLPSSAGQSWWIECGSERVGPVTTKMHDWEQAFSNKM

>SG-PL_contig_3580_1_-3_3_263

FHLDCHNHSPTIKKKGILGVPVMQFSLCLCFLISIRIAFTFLLFKCIFVCDCFISFFSVA

ILIQVRPVCASSITALKSIHSAVFVCL

>SG-PL_contig_3581_1_-3_2205_2378

MSAFKRCSPCMQKKENLGSVVGCGAGMKNLRLMSSKLCRKWGWTKIIAQFCVYINRIY

>SG-PL_contig_3582_1_-2_896_1273

MACLKLVEGIPIQEVCGKDDKLHHEAEDVSSVPRQYNDVALLFRFSQKSAWQQPHTFLAL

RVTRGLGWHQIRPKDLKMLSVEDRVKSFTADGEHRRWKGPPPCDMGQAGWYFVTEGELSD

YVVWLI

>SG-PL_contig_3583_1_-1_664_906

MCVKMDLSVQHPYGNGCISWHSINFAVIGTGLSQHCVPSLNVHGVRGRMIEISFFFSFFF

CLFVLHCLLVAGAVTILSPVL

>SG-PL_contig_3584_1_-2_68_1798

MLGGRLRVSQLQGLLQAAATQWQGRALPAVTMATASDVKPFHYQDILELESKVNTPWKKL

TGDYVSTTEVKGKKILQVAPEALTMIAEHAMVDIAHLLRPGHLEQLAKILKDPQASSNDR

FVALELLKNANVAAGMVLPGCQDTGTAICMGKKGQYVWTEGRDAEAISRGIYNTYTNRNL

RYSQVAPLDMYKEVNTKTNLPAQIEIYATDTSHYDFLFIAKGGGSANKTFLFQQTKALLN

PEKLLSFVNEKVKTLGTAACPPYHLAVVIGGTSAEFNLKTVKLASCRYLDGLPGEGNEHG

QGFRDRSLEEEIHKLTQNIGIGAQFGGRYFCHDVRVIRLPRHGASCPVGIGVSCSADRQI

LGQIREDGVFLEQLETNPVKYLPEVEAGDLGGEVVKVSLNQPMEKVLHQLSQYPIKTRLS

LSGTLVVARDIAHAKLKERLDSGEGMPQYMKDHPVYYAGPAKTPEGYASGSFGPTTAGRM

DSYVDQFQAAGGSMVMLAKGNRSKQVTSACKKHGGFYLGSIGGPAAILAQNCIKKVDVLE

YPELGMEAIWRVEVEDFPAFIVVDDKGNDFFKEWQVE

>SG-PL_contig_3585_1_+2_62_622

MEKEIRQEEKERRNHEDTHDAMKKDLTIAELKKAIRQLKKKKSPGPDNITNEMLQHVGNI

AQQKLLDIFNLSWRDGQVPQCWKEARMIPVLKKGKNKSRVLSYRPISLTSCVCKTMERIV

NARMQWYLESESFIVPEQAGFRRHKSTEDQTTHLAQVIEDAFQAQKVMLAAFIDLQKAFD

KVWKDGL

>SG-PL_contig_3586_1_+3_3_212

RKMEKEIRQEEKERRNHEDTHDAMTKDLTIAELKKAIRQFKKKKSPGPDNITNEMLQHVG

NIAQQKLLDI

>SG-PL_contig_3587_1_+3_18_302

MSANRALEDGSTNLVPQAGVSDNIFTGTPGPAMRCAEALSLRRRRRLRMHVAWMKMARII

DFEVVNAGRSVEAAMMVVEILLAWMRSLRVLWRCG

>SG-PL_contig_3588_1_+2_1010_1276

MNLTMGHSHLDLCCNILLWNKPNSLSGLSIVLRTEHCTFSRSATDLSRIYIIDVITLLML

FTLLYLKMEITEINTMADFAGSLSLVYAI

>SG-PL_contig_3589_1_+2_293_433

MYLAKLGNFILSTLVVLLVLLLLEFLPFVQRKDINNSVLLTPEVKSG

>SG-PL_contig_3590_1_-1_127_612

MADTPFKYNPESPPYTSFFGIVGVTAAMSFCALGAAYGTAKSGTGISAMAVMRPELIMKS

VIPVVMAGIIAIYGLVVAVVITQNMSGDESYTLYKSFLHLGAGLSVGLSGLAAGFAIGIV

GDAGVRGTAQQPRLFVGMILILIFAEVLGLYGLIVALILSVK

>SG-PL_contig_3591_1_-2_62_553

MTLLLKLVTVVMVVGLDMTSAASLDNKDTEDAATAKRASYSCYVNGRVYRHGEIFTIGNS

RCIKYQCKYGSHDFYKEACEMDNHCWPINRSFRHKCREYMCRAYEIGAGYKRYEILPTGH

QMCQDYSGRCHHPGQHFVYVMKGRLRRDCTCHPTGATITYYCYD

>SG-PL_contig_3592_1_+1_1_195

HIHFFHKHINSHSSAINNKSSLTHSPQIIHVLSKSSYGCVNGEKAVLLFRQAFMGTISFS

EKNAQ

>SG-PL_contig_3593_1_-1_1_2298

VVLLDGEHVNIELDRNADGKDLFEKVCQHLDLIEKDYFGLQYVDERIPQHLKCWLSPDKK

ISKQKKRGPWLFDFALKFYPPDPTQLRESLTRWLVVLQTRRDVLSGKLPATFLTQAMLGS

YCVQADVGDYDSTEHHGIDYVKEIPFAPNQTTDLLEKIVELHRQHKGQTPDEAEVHFLEN

AKKLSMYGVDLHAAQDVNNVDIMLGVCASGLHVYRDKLRISRFVWPKILKISYKRKHFYI

KVRPSDNSRLENSVSFKLASHRLAKRLWKICVEHHAFFRLREADQPQNAALFPRLGSKFR

YSGRTLYQARHSSQILDRPDPYFERSQPARNTYAAPGNRSRSVDELANRPDHWKDDLDAV

QRRRPSDSKDREKEGRDRKDHMGQPTVISMLGLPEGMKPVENEEGEELEPGLDRSRDGKD

RQDRKPGDDDATLEREGMQGTMGLTPSEMQKKAKEDEKERKKREKEEEKKRKKEEEDKKK

AEKAAKKGKGKGATDPTAEDRTDRMGGGDATEKAPGRTSGSYTDQEGEPIRFGVVAPVGA

IPGEKGKGKDKDKDKDRWSPRHDTGDRDRWAPGRDDPYATRWEPGTVPDVEMRDKSAPGR

DGQWDPEKDEDKDRFSTFKDPGGKERWSFNKERPKSGQDKRTPGDDRDGRSPGREGEGRD

RFVPGGDGREPYSPGRDGRSPGQDGDRFAPGIYGREPYSGKDGREPYSPGKDGREPYYPG

GERDGRSPGRDGDDRDRFAPGVYGREPYSPGKEGDRDRWGPGREGE

>SG-PL_contig_3594_1_+1_61_384

MLLIIHNSKYDTKYISNKAGVFKKNFAKHISFLVNLILIVRVCVQVGFFPRQMTIFSVLK

RITKATATEQHHRSMRFYTTCSYLHAMYPPTTTHFCHPTAMTKLRTKK

>SG-PL_contig_3595_1_-2_110_451

MGWWFSRQDDYFHAQDPSDCCKGYDLSLQLVLQTVKEQGPFDGVLGFSQGAAMVSLLLGQ

QEQQSEKWFQFAILVAGFKSRSSPHDALYIKKVSIPTLHVFGDTDKVIPKDMSE

>SG-PL_contig_3596_1_+1_208_390

MSNNKFKAVVVVVLLLLPFPPITVSNSSSNISSSSCDGSDDRSSGSSHSSSNSRTSSSSS

S

>SG-PL_contig_3597_1_-1_109_1896

MTSDVEYLLPKEKGPYQEVYVRRRLNTNPGARKFYRIIISVISISLVALLLSLVYVFVLV

PSWQGSVLNWYRRTMQEAHTHVITHTQCGPVEGVMEDDVFIFKGIPYSLPPVGARRWEKP

QKLENKTCWRGTYQAKEFGSMCVQPVGIGNFSAFMGNEDCLFLNVWTQSLNPSDNLPVMF

WIHGGYLVLFSGNMPPLSPTTEVTKSTGAVYVSINYRLGPMGFLTLDTLKQNSSTGTSGN

YGMMDWILALEWVRDNIRNFGGDPNKVTVFGQSSGGTAVYALLASPRASGLFQRAWMASG

SAILNKTSAQASKDNEVFLQRTGCADASCLRNLSSTDVMMASPWNVFPYWSMADLTGMPI

KGRFDGALLVVDGYVLKEAPLRAWQNGSGNDVPTIFTNTAQEIDFLPLHHDITSWTWEKY

INTVTMELTPFSTFIADKALELYPPRLALTPEYQYTSLVSDARATCPIEVLADTASRKFH

SPVFEAVITSAPSSPINVFGLFVSRYAFHTWDLLAFLGDFEAVKFHPMPKDLRFQRQLRE

QIMAFARNGLPKASAWKTAQNCTALLSDQLFTVERYHKKRCEFWLANGFFSYAWIN

>SG-PL_contig_3598_1_-2_2_3142

MSSDSAAPPAKRARIEENTSGNCFGVTGSTSNNSVDMEKNGAVDEIDEGLYSRQLYVLGH

EAMRKMAHSNVLISGMGGLGIEIAKNVVLGGVKSVTIHDPDKACYSDLSSQFFLREEDVG

KNRAEVSQPRLAELNNYVPVNTSTAELDEAFLSQFQVVVLTSAKYDDQIRVGEYCHSHNI

KFIVAETHGLFGKIFCDFGTNFVIMDTDGEQPISCMIADIEKSESSVVATVDETKHGLED

GDYVTFSEVQGMTELNGCDPIKIKVLGPYTFSIGDTTKYSDYIRGGNAIQVKMPKTINFK

SIKAAMDAPEILPSDFAKFDRPGQLHIAFKALSDYEKKHGSLPKPRCLADGEEFVKVVTQ

VNETSPAKVDELDPDLMKEFAYSACGDICPVQAFIGGIAAQEVMKACSGKFTPLQQFLYF

DALECLPEDKSVLTADACKPRNCRYDGQIAVFGADFQEKLKGQKYFLVGAGAIGCEMLKS

WAMMGVGCGPNGMIHVTDMDRIEKSNLNRQFLFRPDDVQKPKSGTAAAAVRVMNPEINVK

AKEDRVGPETESIFTDEFFEALDGVTNALDNVDARMYMDRRCVYYRKPLLESGTLGTKCN

VQVVIPNVTESYSSSQDPPEKSIPICTLKNFPNAIEHTLQWAREEFEDKFAQPSANASQY

VLDPKFMERIMKQQGSQPIETLESLRRVVDTEFYMSFEKCVEWARNLFQANYSNQIKQLL

FNFPPDQMTSSGAPFWSGPKRCPHPLEFDADKELHLDYIEAAANLKAFMCGLPPVRDRAT

IKEMVLKVKVPEFVPKSGVKIAVTDAEVVNSDSTDKDSFESIKNALPSPDNLKGRQLNIV

DFEKDDDANFHMDFITAASNLRAENYSIPPADRHKSKLIAGRIIPAIATTTAMVVGLVGL

EMLKLVQGHTNVERFKNGFANLALPFFAFSEPILAPKSKYYDKEFTLWDRFTVQGEMTLQ

VFLDYFQKEHGLEITMLSQGVCLLYSNFMQSGKQQERKSLPLSELVKKVSKKKIPSHVRA

LVLEICCDDKNGEDVEVPFVKYVLPRS

>SG-PL_contig_3599_1_+2_530_784

MKTGWGRRTSTTCPVFTDVQGLCCMSQSSLFTIFMSSCLNFTRVKCIHASEVDDISAPLA

HCSGIGYTLCSYVTDLGKHSNLMGS

>SG-PL_contig_3600_1_-1_88_528

MCPKKSSMSSCGVVSKRLSKEEIDAALKRADNYLSSVHTVTEVEETESVKRRSWDVRRQR

EDFNTKRLSFDEVGTASAKTARQSASSHLPTTDTATTGGDELDHLPYCASDTKDITHGSS

NQTATDKTLTSDIPPLPYSHVRASKHV

>SG-PL_contig_3601_1_+1_1_216

AAPCSCTSAWISPSGQNSQCQESHGWQRQCGCRLPCLDRAQTGQLQLDTAGWWEFPPILP

VMGQTDGAGTSC

>SG-PL_contig_3602_1_-1_1_111

KSLSDRESNLGLFESYAVTVTTGPPFPPEFSKWSSAG

>SG-PL_contig_3603_1_-1_589_867

MFCITLFLFPPLPQHFSIVKFGLHSLGMVYYYSAVLFFSPAFKKKIIGSVIFKNFARDHS

FPTVSFIINTKCILHLTFFCVCFFLLAPLPPPS

>SG-PL_contig_3604_1_-3_747_1085

MRSPAGAAPSSAPSRQSTTAYPPANGYDYYRRPTDAPEPRPSYGAAGSGQDYKNGPAGPS

SVRGNAGYDRSVLPRSAPPQQQQQQQQQHHPPPRPGGAGGYGMPPPPQQQHHP

>SG-PL_contig_3605_1_-2_2_211

WCGVVGGGGGAKGGKKKKNKKKKKKKKKKKVVRVAFNLFMEPAHKSIIQNARHIFNVTLC

FVCALKQINK

>SG-PL_contig_3606_1_-3_3_263

FFFWSFFFTLLYIGTGHKTVLYIVQTQNVLYIFCGLCASGGIGQKAMGEQCRRFGQTTLQ

SKPIGRPQSEGCFQALPFKTLKNLTKL

>SG-PL_contig_3607_1_+2_2_274

FFFFSPFKITGIYSSLCPTVTNFKTSAHANICNQSSEEQFSRWIKTTHKLIVQCQTALIK

MDLNTTDECGCRTCDGDMEAVNNGDIWPTFF

>SG-PL_contig_3608_1_-1_562_798

MKTPSMHTMTTAKEVSSCDDSSYHWNPASEAERVELSLCNGLKTFSCTGFCADFFQSPGC

TDTILEVQGLPVKQSHCLW

>SG-PL_contig_3609_1_-2_32_3880

MEAEDMADESKTDEVKSNEETKTDEEAKKEQEAEAKGKIPIKLTGKSGIKPAASVPPWKM

FTRPIPKSSAQKRLLGPRRQAATVASSSGIGPAIPNEDPAPLADFLTVGVKKTVPVVPDE

VIEQPPDPKTLRKENKVALSAEKLEEMKILGIDPSSLVPDVKPKPPPPPSQNLSLPLARN

TGGAELAKTESGTSGPANTNVAGSASDQSQKQGQDLHVNSNIITSSTGVEGRAGLFSMPS

APNVTPFPPPPPLGPVVRPAGPPRGFGRRPPLLSNPNTPMPLPGYMPSMASFAPRYMSRP

APMNMPPPFVMQATPPILPIPPMPVCVALPPAPPPPPPPPLPEGPAPIPPPPPSPPKPED

SSVSDGDAGLPGIESASADEVIKSSDRQPSETPPTSNEAEKPDVSDSAPATDKTLTEEAP

QTTASNISLPEQVTSATTLTLASQVTTTPSLPQEVTPITIPSPEQVTSATALTLTSQVTT

SSSLPQEVAPVTIPSPEQVTVTTTQVTATTALPDQVTAASQLPEEVTNTTTTQVTATTAL

PDQVTAASPLPEEVTNATTTQVTATTALPDQVTTTSSLPEEVANTTAPQLSELVTATLAT

SPLPLQSTATATPPFPEQVTVTIATPPLPEQVIATTPSLSDDVTATTPPLSQQVSATKVT

TSLPEHVTDTAPLPEPVTAITTPSLSQQVSATTVAAPLPEQVTNTALLPEPVTATTTASL

SQQVSATTVAKPLPEQVMDTAPLPEPVTATTTPSLSQQVSATTVAKPLPEQVTDTAPLPE

PVTATATPPLTQLVTTTTSFPEQVTAPSLPKQVMGTTMLQVTAATTPSLPLQTAAPASPS

LQSEVDVEGSQVEFQSSSAVALTVQITDVGVAEESSDIVAKPSTSSAEETPASTVSESAP

QVTLEVEPQPSTSAPEVAASDESTEPTSTLSVTFPVKRNVARRGRGARRAVPVQSMPAEE

SVEPEGSDAPAAASRRPRTRSQMRPVPQRQTRSRTRQQKIEEEEDSVTASDETVVHKCEG

EVGSSTAREAESPDGSKASSTSACVLDLTNPPADSSGGSEANTEVTSSSTAVKSVTVVEP

PSVDSALNLTIPPEIRGARQGRLEQTMPEDLSCAEPQDLSTSSSGKGKGRRPPEMPIDLT

MRLVPTQSVDLSLPEEPQPMETQPSTSCPSSSSESVLNADCLDPQTMKDTLLSSTSAMNP

STSNLSELEVGEEGDMVVDASSQMADSAGLGVDPEGELVDFSLAPVDFEELVDMEVEDSN

AGQPEAGDAEDSGGAADSTFSQN

>SG-PL_contig_3610_1_+1_1390_1719

MGGTITGNVPEADITKVNKITTSFFHCPKFQHKFNTWQQFLEHRKVTLFQMNTSLHIHPE

VMSCKSEEMVTRKPCGNTGEADTIIPCSIHFWSTGYSHPLSTAHYQLLII

>SG-PL_contig_3611_1_-2_1217_1495

MTFTYCFKKNEGNFSDTMVDSSNLFHEIHGCSHVWLLRKDCWYFAEDYLHHQLMRLPLKY

LSPFPTFFPFMFAMHSVPSPSICKSSSVKYLYQ

>SG-PL_contig_3612_1_-3_252_776

MGLHELIGIQVGLDGEVIDQIIKAQLHVIHWSAVVNIACYPRIGSKGNVCIWWLCIGNFP

FSDQVRLLSTTRHFSATPSQIQALLLSGAPAPTPTMASHNRRGCRTTAGNGIWNFGSIRI

VLFSVTFARFKVLTLGHYYPQRPTKEGDVGCCPSLLRHSRCHLIFQVFITSISII

>SG-PL_contig_3613_1_+1_271_468

MTSAQFQGQNWKIVQMYGSQQNSEIYELNINLFGLCSLNDNFLSDVKVWLTLDYFQGHVY

FWVSST

>SG-PL_contig_3614_1_+2_284_487

MTAKLNNNNNNNNNNNNSSSNDNDNNNKNNNIVTDEYIYPNCTYHKNKQTNKQAHICVLN

FTVVYFTL

>SG-PL_contig_3615_1_+2_203_340

MHVCPLFGPHCAGVMLMHNLHTPIFSGHACWVFLCFHKPPDTDMAY

>SG-PL_contig_3616_1_+2_650_787

MSASQGCSSSESGSRPDSRSKRANTCKCPSRLTSRSAWLLHFQQYR

>SG-PL_contig_3617_1_-3_1623_1874

MLDIWNMFVSQNTLILSDSHSVSVSLCACLSSLFTSPAPPFPTPRSSSPFSSCIAEPYLK

KKCLSPLVLQSHLRNIAIFFLWYL

>SG-PL_contig_3618_1_-3_12_599

MDKDHSSSAVPDSSTVENKESPSDQPAPHQQSTSSQQQLLPTTDRLTPSSPPGPPPSLPP

HPSSATTQPKPRKASRWGVGSIFDVLDQFPPRTSTSVQQEGENSVGGKGEERLSPPASEQ

VTSEMDMYGPALPPAGFIGANSAATTVHMDVSGTSDSRRKHKHKHKHREQEHVSSKHRHK

KEKKKKSKHKKEKKKK

>SG-PL_contig_3619_1_+3_3_95

FFKSSPLLYLYFAFQMPTDTVTSMDQASVCM

>SG-PL_contig_3620_1_+1_148_318

MRERKKWGEKNESHLLTNVYFNHSNIRWTCCLAFVFSLYSIMHKSCLCKRCAIMHGY

>SG-PL_contig_3621_1_-2_1223_1786

MYYLLPPVLPHLFISTVELAQLNCVAVSPLLFNFLMHLYHILDLPVSCDVSPLGLTYPLV

LFVKGLCLCIFERALWLTSQNITKKDLAQKLLCARQHVFVNDDNGNVVENAGLGKYDIGP

FTEAVLSHAFQKVFKLPRHLRYVDFFVVDDKTLISLSMSSGVIPFMISDRPALGSFIGIT

VILASFTL

>SG-PL_contig_3622_1_+1_1_105

FFFFSIFVLFIQHNSEAIIVTNHIFIIQLTMTKYH

>SG-PL_contig_3623_1_+3_909_1412

MLKLSMPYLTEILTYVINLSTDQKIFPREFKKKKFFFQKQETKKFYTITGQYYYYLLSLN

HLKECSQAHMKFNENHSLFHPLQSGFHWYHSCNTALSNMTNSWLSAINEANLSGVLFLDF

KKAFDLIDHDTLLSKLNVYLNNPHILPFSSHTLRKGHTACICSWLCLF

>SG-PL_contig_3624_1_+3_147_272

MKPSTVIAFCPNKSLDHICSKQSLRNAYSLIMFHMQESMQFC

>SG-PL_contig_3625_1_-2_2_157

KKKKILFCLILKRKEKFVSPLGHCMVSPLGYCMLKLISLCILQEYFCAGSIF

>SG-PL_contig_3626_1_-1_136_2517

MKKLIFLGAIGLLFFAVAVKAEEEAGTVDVDLGKSMEASRTDDETMQKEEEAIKLDGLNV

AQMKQMREQAEKFAFQAEVNRMMKLIINSLYKNKEIFLRELISNASDALDKIRFLSLTHK

DALKATDELGIKIKADTENHVLHVTDTGIGMTHDDLVKNLGTIAKSGTSDFLAKLGEASP

TELNDLIGQFGVGFYSSFLVADRVVVTSKHNDDEQYVWESDAESFSVVKDPRGNTLGRGT

TVSLHLKEEAHDFLEPSTLTNLVKKYSQFINFNIYLWSSKTVEEEEPADEDTTEEADTEK

KEADEDEEGKVEEEEEEKEEKKKTVSKTVWDWELMNSVRPIWTRKPADVSDEEYTEFYKS

ISKESEEPMGKIHFTAEGEVTFKSILFLPKRSPFDMFNNYGKKVDSIKLYVRRVFITDNF

EDMMPKYLSFVRGVVDSDDLPLNVSRETLQQNKLLKVIKKKLVRKTLDMIKKIGKEEYDN

FWKEYSTNIKLGVIEDTSNRTRLAKLLKFYSSNSDTEQTTLADYVERMKEKQEAIYFVAG

TSREEVSKSPFVERLLKKGYEVVYLTEPVDEYCIQSLPEFEGKKFQNVAKEGLKLDQSEK

AKEYREGLEKDYEPLLKWLKEDALKDKIEKATISERLSDSPCALVASQYGWSGNMERIQR

AQAYARANDPSQTFYATQKKTLEINPRHPLMKKLKAQVESGSEDQTAKDLAVVMFETATL

RSGYMLPDSAGFADRIDRMLRLSMDIALEEKVDEEPVFEEEEEAAEEGGEEEEEVNAEDD

ENAEGDSGTTHEEL

>SG-PL_contig_3627_1_+3_3_254

KLLTKASFEFDDYELNFDSEYLKYTHARTLTQTQTHKNVLTCARTPAHIHKISFANREPF

LSLHLFISSQGFTNHNQSWTCPSI

>SG-PL_contig_3628_1_-2_2_199

FSAVLDTGVLIAGTKIQFALCSASFCLLSSFLCLFTSHTFLFAQPLLSNVSPRLTKHARG

DKLTMQ

>SG-PL_contig_3629_1_-1_70_1215

MKLSLDKRNYVQGSFKHHAGVKPWQDNKLKKRSAVTSIAKRGAGHMFFVGTENSQIYRFN

YAELSAALIKTCHSSPVNDLIFPFGFSELLVTCSFEEIRVWNTCSGQELCRYTVPNMTCN

AVCITRDGRSIFSAWDDGKIRVIGFRQEKDGTSKLVEKYTVRDTHNKGVTAIAITSCGTR

LVTGGGEGQVRVWQICEDNIISRSRGDLVANMKEHKGKVTSIQIHPSDRSCVSASTDGST

IIWDLESNSRSQIVLANTLFKCVCFGAKGVHILTSGTDHKIGYWEVQDGSALRELEGSKT

GAINTMDISADGQAYITGGEDRLLKLWKYDEGQVTHVGIGHSDQITKARICPNRHLIVSG

GEDGAIHMWRFPNCMLQQQQRM

>SG-PL_contig_3630_1_-1_88_981

MAVADLCRKFGSLLLQSSGRLMTQPSVGQLSANSHLTRSLHSSGRLDGKMKLIKQGIDLD

KYTLRPVPLPRSGGRGANGRVQTHGVGGGHRKNYRMVDFKRKGPTEGEPLVEEVQTIRYD

PCRTADIAIVAGGNDKRYILASQNMKVGDLIKTSGKLTRMAVRAAEGDAHPVGSLPVGSV

IHNVELFAGEGGTIARAAGTSAQLVRKVGDRCIIRMPSKREINVSQECMATVGRVSNIDH

NKKPIGKAGRNRWFGVRPQSGWWHRKTGYNGRKIRPIKPTKTYLKPLPPKPALHKFTV

>SG-PL_contig_3631_1_+2_533_736

MILSFSTSYSVCVRHMEWLAPSCYYHTKRYLYLYSSCAMAAVSLNYIQNIKRINLLYRLT

KVFVPLPH

>SG-PL_contig_3632_1_-1_112_1263

MAEADSKVANVEEEVEDVEEEEVKEEDQAGGEPSESSKMAAQLMKNPQVLAALQDKMGSA

VGTAAFIQSLPKAVKRRLKALKKIQNEVISMESQFYKEVHALECKYAAMYADVHDKRSAI

VRGRHEPNEEECDWPSDEEKEDDDDNLADELKKKASVTDKDQEDNAEEDVKGVPGFWLTI

FKNVDLLQEMVQAHDEPILNHLQDLKVKFSDGEMKGFTLEFHFEPNEYFTETVLTKQYTM

RFEQDPSDPFSYEGPEIVKCVGCGITWNKGKNVTVKLVKKKQKHKGKGTTRTIAKTVQND

SFFNFFDPPIVPEGQNEDDVDEDMEAILAADYEIGHFLRERIVPRAVLYFTGEAMDSEFD

EDEGEEEEGEGDYDEENDLDPGDA

>SG-PL_contig_3633_1_+3_3_167

VCVCMHALPGEGVCVCVHMSMSDTFTLCVCVCVYIRVYVRASMHIVDLPICVFLA

>SG-PL_contig_3634_1_+1_1_129

RQTGRQTKRQRDREKQKQTEVLVQQICEKHLKLCRLIKMAIKL

>SG-PL_contig_3635_1_-1_1_168

FIQEGWGFFFQQASGMGRIRGFVSSFLVTCLPQGRVQKSVCNCISHSFSHQNWNAH

>SG-PL_contig_3636_1_+2_2_505

DSCQLKLAQQVIITCHGTFSLIHLDQDTRLVVRVGGEGLCLLGGDGGVALDEGSHHTTSS

LNAHGERGHIQQQQVLYFLRLVPMQDGCLNSSPIGNSFIRVDALAQVLAIEEVLQQFLDL

GDTGGSTHQHNFIDGRLVQFCVAHGFLHGLQSATEKVSIQFFKPVHNA

>SG-PL_contig_3637_1_-3_96_398

MYLEKNSFGKTMKRPGRLTTKSWQAQNSVCNSLAPTIVQQPVVSNLPATTSSQSALAHLY

NQPLSAFYNQQSHHYNQQLVCYNQQLAYYNQQSAYSHTPCL

>SG-PL_contig_3638_1_+2_65_1027

MQANGTSYCGTVLKHRRGNPPDISNKSLKVAKGKTVVRQKGDVVAVRFNDRKDVMLLSSK

YTAQPVNTNKKQRLSRQQRQRGEVARNVVKPQIVHEYNHNMNGVDHFDQHLSYYSFNRKT

VKWWKRAATHLLHMAKIEAMILYNKFEEKQKTQADFTLDLIVQLTDSGVTPPEAATENQA

AGAEDHGAEAQAENRGEEAEQHPHNEEGPNGGEEAEHQEGPPRKKKRVQPLDFERLSHSD

NPHKPVPNPPTAKRERPPKDCVCCTISGGGRNQQYIRRAQSSFMCQKCQVTLCLYPCFHV

YHSVKDYKREMRRLHNINDSD

>SG-PL_contig_3639_1_-2_50_799

MPVIIFCLLVVFYVMLLIFQLQEWVNKLIASGDMPSFLRFGPKVLMAVVLAVLDELYKQV

AVWLNNKENHRLEETHETNLVIKLVLFQFVNSFLSLFYIAFYLRDMDRLRETLAALLITR

QILGNMKEVLLPLLRWKGRLYTVGYKIAGEMSSRRLDREVEKVTQVYRRKWCNTTGSVTS

TSSDKTEDGTSQKTEEEARHGCGKEGTSAKEKEEGMDEAAADSEESLEDLIGTLTQAEVE

SVMTVYDNAF

>SG-PL_contig_3640_1_-2_368_2113

MNVPHRILTPEEPGKSLGKSLVDRDISCGDSNFLSPVVPYADGTSQGSQESLDKRSSLPR

LKSYDAVVFDVLRVSPEDFASQITLMDLPVFKSIQPDELTSCAWTTKEKLIKAPNVVAFT

RRFNHVNFWVQKEILNCQTLKTRADVLSHFIKIAKKLLELNNLHAVMAVISALQSAAIFR

LSGTWMMLSKKDKMMYEKMADMFSECNNRQKLRDVMDNVKLPCIPYLGLYLTDLIYIDVA

HPHSGGLESPPRRLQMNNILRVIADFQQSTYDHLPVLDHVQNYLRSVRYIEELQKFVEDD

NYKASLKLEAPVSGQSSANATSREDVSVLPPSPATEPRQQFSAGKLCASSAHRKTKSLSA

NFMSCTHPQVERTYSLPSRATAPYVQGIRHLLDDSVLEESPCASSDGSVCSKIQQAFSGE

GREPLESTSSDHETFWPAHNNFQWQETEEAVYTANFTCEGCVRRKTLIKDGKKPTVSSWT

RYWMALWGTNLLLYPAKSLRGSDRSNFKSNPSKMSSIVGWMVVMGDNPLQPDAFQLTDPT

RAGNVYKFRAGSQSQALIWCRHLSEASKRYAPRQPANLMSFD

>SG-PL_contig_3641_1_-1_1_114

ELCCFAGFFISGLQKFCDQRGCLLDFDGLYVVTALIWP

>SG-PL_contig_3642_1_+3_3_164

HIIKNHHFKHACPENTFTLFLIRLSFICVHHKINRLQFMLTIHLKHTSHIFFIY

>SG-PL_contig_3643_1_-3_285_980

MIGKKKPAVLQCGGTEQIATPVPESEVASCMKKMFMGLGFPKPPHNITSEQLFSKLETKV

KELTAKYPKQVSKPLLKVNLSEEQWENLAEISETMLQDYHLRKEMLLKRLDVTIQSFACS

EKAKHNQDSIAKTYQPIRQALKARSEIGVAHILAARDNLTRIQKTSTGDGREECAINKVM

IGRVPDRGGRAWEMEPPPPEMPFFMKRDNTSQGRGGRGGYGGRGDHQDRKSV

>SG-PL_contig_3644_1_-3_3_425

SVHLPALSLFCPSLSLFCPSPSLFCPSPSSLSVLSISQLSVCSVHLPALLSVLSISQLSV

CPVHLHFCLSCPSPRLSLFCPSPLLSLFCPSPTSVYVLSISLQSVVSAHQGWTDQCLLYA

LSCLYVLIGGFSPFEKKSRFL

>SG-PL_contig_3645_1_-3_918_1085

MEFTLLDEVVQGTNDRMLVKHHRTCSSSSSSSLFPLTKKFFYMCGCVVVHTLLISG

>SG-PL_contig_3646_1_+1_745_939

MLSSRNVEVSIIGRSSYEKTCTLSTQRYTYYPALMGILFITCHGPCTMLPRILMIYKKAL

WSIAW

>SG-PL_contig_3647_1_+1_517_642

MHTLNFPSAVYGHRHSLHGKPRNTFSDDNDFHGLQLICSLAC

>SG-PL_contig_3648_1_-2_137_304

MADDTHRAYRQARLHLDSLMESMRSQASLCTQSQPVSSPNLTQVARIDAPPRDAVG

>SG-PL_contig_3649_1_-1_103_318

MSHLASYTLPYPTYAIRLTFCSNFGRNSLDYRENRYKDASRRGPTHEAQLARAHSLFLFP

FGQGKEGAGRCL

>SG-PL_contig_3650_1_+2_2_133

DLGPLHSKLKHKRLINGPHLWSKFGPETAEKILVTHTHTHITPR

>SG-PL_contig_3651_1_-1_220_1431

MRTAVCGMVIKGMLDKIEREEKQEQKRQKKQESAEERQKRLNSVSQQRALFKHKEALKKD

ILKKRSAMEKSLQQEIFAEISDTMKRQKKAGMKVGASPSGAAAASQPSGPTPTMLVKKRE

GEPPKITSYIPGMDSSRTSKTSASRKRKAGGQSGPLNSSKQRLITIGAGSKGRTEKLYCV

CRTPYDETKFYIGCDLCSNWFHGTCVNITEEQARFIDSYICEDCRKQQENTSEELYCLCR

TPYDENQFYIGCDRCQDWFHGRCVGVTKSEADSIDTYICPNCQQKEQANPIAQKVLTSTD

YVTLLKIVRSLKAHKMAWPFQAAVDPSEVPDYYQVIKDPMDLAIVENRVISKTYARLSDF

VRDVTKIFDNCRLYNPTDSPFFQCAEVLETYFVQILKSSRQQFT

>SG-PL_contig_3652_1_+3_3_107

KMSADGNANREVRTRITKAAVAFNSLNNIWKKTKH

>SG-PL_contig_3653_1_-2_698_976

MKVTLHECVCVKCMIAALLPRFVLTNFGLELSTFLLKSFYTLQLYSYLTPQLNHILTEKT

REAYRCRSRQQELDRAIAAWYAVTEGKADWGNM

>SG-PL_contig_3654_1_+2_2_151

LFRSGMCVHGCTGSTYVSVLKLQSLPVLLHTFTYISNITVLFSFLCNNLH

>SG-PL_contig_3655_1_+2_287_556

MFPLIKSIRHGHICTTFTLTFTSHIYAHACPFRLKLCDRNRVSFSPKQKAPETVKLTNVY

KSNEIYILVPDDQYPIHAQYIPSYFGNIYV

>SG-PL_contig_3656_1_+2_833_1057

MGVLTQMNLRHTCIHTSFPYKCHKYTNSVTTKHNKKPHFLANDLNTKSFKCMQLTNVHIT

THAHACTRAHTSTSV

>SG-PL_contig_3657_1_-3_57_149

MLMVGANLLDKMINQHCIFYMHLSTFHVNCK

>SG-PL_contig_3658_1_-2_2_196

RYTALITGFIYGSSHLRSLTKKEEAIQEHENKLKAVRDAKLKADKDRSSQVEMDALGKEC

GVVKA

>SG-PL_contig_3659_1_-1_76_543

MGQGDEDAPYAKFAMSLFKILDGPVTLVREKIVEPLQSKNSNKYYHRRYPRVKTIDQCEI

DDPVCVYEAQEQYRRDKQVDSNIVKILRQRKLECMQWEGPDARNKCKKVSDDYDQAATNW

FIKYGDIGANGNVIDAYMKQKHRFIWHRRNPDKPLI

>SG-PL_contig_3660_1_-2_53_604

MAASSIANTTTDRPRATAVVASAPAAPDVGGSSRGVNESRRNPSIIMDASSMTMDGRAIE

IIVSDETGISHRPDKPSRLSGKRAILKRIGTVLLDNRMKGKENCEHLLIEHPEEYRVLKK

VLNIIFVTVGVALLVSVFVVIVYTSVVGESDSSSDDSSNSAKSHSENTTTEPHNVTTPSP

PGAP

>SG-PL_contig_3661_1_+2_2_61

GNTILPPCLLVTQSYLLACW

>SG-PL_contig_3662_1_-3_48_476

MANALKSTYKAEGFRGLYSGLAPTLMRDVPFSGIYLMFYTRLKLYTQPSTFGGGMEPTVD

FCNGVMAGALASFITQPADVIKTNVQINPKQYRHIKQVLIYIYQERGFYGFWRGIVPRTL

RRTLMTALAWTVFEEINRRFKLQ

>SG-PL_contig_3663_1_-3_36_203

MISVVFFFTLSMYRAFYTIDPLDLSCLYHTTSFVNTVFRIVFKLFEFIPHVENYKH

>SG-PL_contig_3664_1_-1_43_384

MNIYNLPVAVTQIKDSYDRLMANGCIPLTLGGDHTISLPILQAVKDKYGPVGLVHVDAHA

DCSDSMLGASIAHGTPFRRAVESGCLDCKRVIQIGLRGSIYNLQEYQWGIDQGF

>SG-PL_contig_3665_1_-2_410_739

MNGAVSKQIFHSCGQWSKRWFSTTSVWRKKMKNNPLSANEMPRSGGIASMMRLPVQSTTE

GLDVCFVGIPIDSGCSNRSGTRMGPRQIRQESCLLRRCNYILGASPFDDV

>SG-PL_contig_3666_1_-3_3_200

SVMYVSHFLGFVCFFFSFVFVFASFGTLLITSLLDDIQFFCLLVVITTFFDKFKALLVHL

FIHFLQ

>SG-PL_contig_3667_1_-2_65_313

MHTHIRTLSHQAQTGTQILPPQELTKKETNNNYNINNDNNSNNSYNIHNTSKSNNNINHN

NNSNEHFTYVTGKNGIQENLKIS

>SG-PL_contig_3668_1_-3_531_749

MAVHNTYLTHLNNIICTLRTKITQTICILTNKNTHNIYVTGITAIQEHFETFPVLMEFQF

VPGKIGIFSLVQT

>SG-PL_contig_3669_1_-1_1_111

SVKLKTKCYINAEVSFVLLYKGMFVVFLLYKVCVSDL

>SG-PL_contig_3670_1_+2_2_217

FFFFFIFSLFLSFFFSLFFSLSPSPPLSLSLSPPPPLALRQIQWDLMLLSLSNKLISSFS

SLSLSLSLSLSH

>SG-PL_contig_3671_1_-2_29_298

MWAAPPEDPNLITEQSPDDNQEHVKNVWLFNIETDPEERHDLSDSMPNKVTNLLAKLARY

NSTAVPVRFPDTDPSAVPTGPNAVWGPWMT

>SG-PL_contig_3672_1_-3_3_296

ITGNPGTGNWVAPPEDPNLLIERPPAGNQEHVKNVWLFNIKTDPQERHDLSDSMPDKVTN

LLAKLARYNSTAVPVRFPDSDPNATPTGPNAVWGPWMT

>SG-PL_contig_3673_1_+2_332_589

MLTREIGKISLYPQARSRGGPDRSVWKSNVFTIRPPHPWFSATIPLSGPQKPGSSGRKSR

HDFTRYPLYSTKVCRTLVERVREWTG

>SG-PL_contig_3674_1_+2_227_370

MLTWEIRNVHLVIKTHLISYENLTYILNTCIYIIEHNFFFSFFLPCSE

>SG-PL_contig_3675_1_-2_17_250

MLRVGASWDSTIGLFRCWKRGAGCRGWMPGRPYDPLPPPPSLPPPPFYFFFKRLFFFGYL

FLTWCFGFRRKKKSFVSI

>SG-PL_contig_3676_1_+1_22_537

MEMVDVNVNDALQMFNDIMKKAADCMKQLVCMGRPKCQNDWFDDECRNNRRIVRRLLRKF

KKTADGRDRNMYCKARREYKNLLLRKKKMFNDVLFKKIVDSVNNHQEFWKTLRTVSGKRT

QHQNNISIDEWYLHFKTILEKDVDAQEYKDVTEDEYIDDLDRPITKEEILIA

>SG-PL_contig_3677_1_+1_952_1200

MLVLIQHKQDIMPVWRRGRVPAFQSDFMGLYEFESGRPKKTLCVGHPVRLAVNGYLTLFR

AGEDKAVRERGWHRPHNAGPEKS

>SG-PL_contig_3678_1_-2_32_181

MTICVCVCVCERERERANNKPSLKFCTYISAIMPSSEHSDIECYAGVCVC

>SG-PL_contig_3679_1_-3_114_3026

MLMKAVIEKEEHGLNLFIHTLERDDDSLSLRWDRFLNPLENGPTAEVSNHPEVNNYLSLI

IRCIKGDVMRLLTPAELTKIMHELFSQNAIDEGDIQDLEKMNHRDKFLRLFMLLPQTTPE

WPLKFFRAVTHAKPDILGKIDPLIEMDKEAVFVSKPGTASGAEAGRTMSDSEQTSPKRLC

TSGAHTSVQDMYLLGRKDLGDVPMDMEDELLNSEEESPDEFSDLEDKDQDTEKKARPESF

TMHKYQYELAAPALSGQNTIICAPTGSGKTRVALYIVKKHLDIPGSRTRKVVFLARTVPL

VSQQFKIFEKYLPNFRHGLVHGETADHEALHLMLDDFDILVLTPKILENHLAENKIQSLS

VFSLIIFDECHHTRKGEPYNSVMKHYLQFKKAGARDLPQIVGLTASIGVEKASTLGKAEE

SIITIMANLDVSSITVVREHLDEMEMIVPKPDERLEELMPREDDEVSKSIINSMVQVEFL

LDKQIPLQDINSEFLSIHTILRKRPAERNSQKYGQWAVKVCDLARKVHIPEQPDRQHSTD

MIERCRLFSQNVQLLAETLIALNEALDVHELTRPVDVLEYLNRKLDSQHTADSPQLTRLE

MELSNLFKGLQKKLSRAAFAENPNLSKLSEVIEERMMGRWDTSRVMVFVRTRATCQALCS

WLNSDGVSVRLQELKASPFTGSAAHQEHGGMTQSDQEEIIEKFRDGDVKLLVCTSVGQEG

MDIPDCNLVIRYNHVGNEVATVQTRGRSRKVGGVSVLLAMPAIIRKERINKKRVQIMNHA

IKKVNQGHTKEVNNQVLKAQKQAMDKDEMKKIASMKSKKSKMKQGSFTMVCARCRKVCVP

GSRIRTINSSHHVVIGDDLLDSKDVSIKIKGKPKVVDSVQFIGEIICNRCPVTKQNVLGN

TLKYQEAEFLTLKAEGWLVLGKNNAVEKCGKWKWKDMGYPIPELTPSDMRAHLGLPEDFE

DEEGAVGGSSD

>SG-PL_contig_3680_1_-2_65_307

MSANASALSNVLGKGLFSSFTGYVGNLIPFFITFSFSQIFCMSLWSFISDLQNVVQYCSL

ACSMTLLHWHFSSLHDFQVIC

>SG-PL_contig_3681_1_-1_172_642

MGFGLGSRRSSASALRAFGHGSLLSPHLQNSFPPALATCSHCSEIQALSRAKDDIGFHSN

SNLALTVLPEFVAKSQQPGSHLRSFQIPSLVSSLDPRDSRSEEHLLCPVCCVKYYLKHSA

PRRSSQRRLLISLNENYSRDISATTISRWLSAAFIRA

>SG-PL_contig_3682_1_+2_2_130

LALGKHLFTIFPGSERVKEISWQNFSILFCPRTDSYQSLTAQH

>SG-PL_contig_3683_1_+2_113_265

MFSSTSTYDYASLKTVSNNCRICFLASPTPNKNYFNNMRARSYLYIFFMEV

>SG-PL_contig_3684_1_+3_3_344

PLSNPLSQGRLLLHPTGVRLEPRDCEVNPKQPPLPRDGFRHHSLDGSSYPRESSEAQRDP

GQPSLHANFYCQFSRFSPRHHGVSSPPFSAGQSSLTPLQRVFILLDSTFLLLGH

>SG-PL_contig_3685_1_+2_2_235

SSNNKRERERERERFRLQKLLLKDSSIRPPWIRITASPCYMKKTTAEREREIRFQKLYYS

RIVALGPLGSVLQLVLAT

>SG-PL_contig_3686_1_+1_502_744

MLVHVASKTTVTVPKCCLTTAVGRMVLVSRSETACDGSVAQYRPHTRPNQVAARLPHGSP

CETPGQNMPRYDGPYTIHPSV

>SG-PL_contig_3687_1_-1_3136_3351

MLVFNSNSVHSDGVQGKWVLFCLRTCLNPFSLLPPPWGKHWMKCKKLHFCVSLQLNTECM

REGWGVCWFWLV

>SG-PL_contig_3688_1_+2_2_199

KQTNKNNRSQVLVNGCKLTCRFIHKFTSIIMSHYLIVCFTQEQLTYYQHVYRSMYFFIHP

IFFSIY

>SG-PL_contig_3689_1_-3_867_1196

MSIIIRFFGFFYSVIRHWRPGWCRGSSSPSSLTVLLREILCTWLQSTQLWMEPDCSGLAK

GNAVKEGSWTCPPNSRHEISEFSYHLTLLQLGISIFFKQTLLPCQSICIV

>SG-PL_contig_3690_1_-1_307_1599

MLAILALPVLLFAQAVMAKPSYRDLLDLASILDVEKRLTKEEAEKYIEVCQQEPLELGLV

IDSSVSIPVKDFEKGQNFLQDFLSDYDIGQGRNQVRVAAVTFGQGVYIQDSFNLDSFNDK

EEVKFHVTHMPFRYGRRTDTGDALKYMREKQMVNTRPWAPKVVIVLTDGNSQRTSYTAEQ

AMKTIKANITVFALGVGTAVKQQELLNIAGGNEDRVIRAKSYTELDDKVKRKLAYKTCVL

KPRPTTPPPQQPCYEKYPSDINFIFSPASMGVDTTSWVTQFISHTINNEELKSGFQYGVV

SGDCPDDEGFQLDRYKEVTGIRERLAKYDRNNLPALVDRAATVAFTPGYGARDNAKKVAV

IFVGEAKVDQSKLNAAIEKLLDEGVRVFVATTSSKVQVSVPKEVQVLTSGGTSHRQSSEL

VTYICHGEKTK

>SG-PL_contig_3691_1_-3_219_497

MFSTWGRFLTFRPERTNLVWFASVVVLFELVIWGYFSYDDSPDMTLVVGWALNNMTGFDF

TVKHLVVDCWKVHAICKHTFLFMHTICKYILYI

>SG-PL_contig_3692_1_+1_514_729

MSLFQYFTKHFHLFQLSHTPLVNISVYLSQKFFISKFISVYSNISYTLGYHFGLFPSHKP

LVNVGQFIPFSI

>SG-PL_contig_3693_1_+1_2077_2424

MITISFIGIFSHLSAFYLNYLHLQSVIIMLYNNLHSAGISMAYNVLHAKTRLQLVSLPYV

QEIHQSTNCIIHTIFLWKENLSLAYNVLCARTQHQHVSGTSKQKISENYSITKLNH

>SG-PL_contig_3694_1_+3_84_224

MCFTEQLIRNMPTRELNLSLAFSLYSCIITLVCFEPRCQELAGSVDR

>SG-PL_contig_3695_1_+1_1_279

VSFSFIFLNSLAHSIYSPINISLTNHLQPLIIQRMPLHCSANFTPLCIIPQYLLFTPGQP

VFKELNSSFSSAFMDYNSHIYMSGFFMQACFFY

>SG-PL_contig_3696_1_-1_325_483

MLCGCVAKWVSLLPWQVRGLRMQRFDTRLVRFFSPLHWTLGGGLGSGLSDETI

>SG-PL_contig_3697_1_-2_2_268

TDNDKDSSQLAKVEEGVRNKWQWYWLKEKDIDNVDFCVWCRKLDNQAPAFRWSVPKLLST

ALPARKSCRNMLTTRTTYKLLKLAQNLVL

>SG-PL_contig_3698_1_+1_106_474

MPSSCGYTPRRKIGKALETMKESINTSDIMLEINSPTHAEQCLHNMMWNILTPDVRKNRK

QTNLLSTKKTFKRGGKLMVPNLPYLHQNLRVEIFFIAHTIFVLFVAELHLRAQKQKYYLE

HNE

>SG-PL_contig_3699_1_-3_192_344

MIHTMQADSRLFAGFVLSGFGTSARVHVDIVKGEETASVGNFFVLGGASEM

>SG-PL_contig_3700_1_+3_3_185

MCVCMRVFCVGATAVVVAVVILLLIVVYYNYYYYGDYSHHLNEFRHRCAKQIFAVAKSGQ

F

>SG-PL_contig_3701_1_-1_1_126

VFQLSPMKLIFLLRIDSDDESFCDCIIFVFFLFLVSSVDPIS

>SG-PL_contig_3702_1_+3_105_299

MPGSRGCLGLGGVQGQRPAGGSRRAKPPAKNGFHIFWSQILTSWEKFYGKGRNLNAQLIL

KKKKN

>SG-PL_contig_3703_1_-1_73_357

MTKQRQGDNPKFAFLFGGENFNYYQYKVTTEQAILKKQMMLEQQQQQQQTLIPQQPALAQ

QEQAKAAAAALQGQITSMQQQISNLQTQMMEQTSQ

>SG-PL_contig_3704_1_-3_3_107

HFKLGTVGAAILRKWLLNLNGQKTISLAMGLQSPF

>SG-PL_contig_3705_1_-2_2_523

DKRQSVFLEAAVRLPTVPPVALLVFRGLQTTPCALKNIRAGVPKATINQDKPLTYEQAQA

PYRIGVTKGWNSWHTAHLTQEGFAARAAERTFDDILIRKFIKGTWPGLFASEVIIKRRHN

LVVVAGLVFQLLSSRKLYFLIGYSEEMLSYLLKCPVKLEIQTVQERSDVIFKKI

>SG-PL_contig_3706_1_-1_298_546

MSSPIKKPQLRGMLASKLKMNAAIGFTFAISMALLWKFGFAERRKQTYLDFYKTYDGKTD

FERMKKAGVFQSVNPDGSVGEIS

>SG-PL_contig_3707_1_+2_662_805

MAGKVFLLKCRNATKEHYITTVNTKIRHFHGSPKVFMRSSIYLTPHLY

>SG-PL_contig_3708_1_-2_2_1072

MGGLFAGGVPRLPSQARAANNRSQGPMVALRPPGMSNGPSSAGNTSSRTVGRLSNGASCP

PPPPPNTAKPGGPPPPPFGNKPGGPPPPPSSANKPGVPPPPPSNANKPRFGRPPSPALTS

IGGGGQNGDSGNRPNVAAMAAAMSRSNAQPQQQPPRSQPPPPPSPPSRPVSSTSNDRTAS

SGRPSSMVGRPLPPPPNSAPPSAGPGRTPPPPPNRPAPVSGPPPPPGRPPGPPPPPTRTT

SNQSGGTIGRSGPPQNRPPPPPVRRDLGSGGTVGRSSGQPPPPATQPPSLPRSRSSGSWS

SGGDDFESRFRFHPCNELPPPEPFSNMHKAYPSSNPKNVNRNTVNQRRAPPPPPPPR

>SG-PL_contig_3709_1_+1_1_141

FFFSIHFMNRLTDTQSEKQTGRQTKQKDGLAVETDIQRHTNHRLALQ

>SG-PL_contig_3710_1_-1_124_504

MLCIIVTNLSQMTTEYCHPKTTPDMPIRMALRMSMAIPGLFSAMRYRHFGNEDIYVDGGV

LCNYPVHCFDGWYLSMDPKDSFLRRLQPLRDIPLIMERNNRFGSYNDKTLGMLLYSDGEE

DILRINL

>SG-PL_contig_3711_1_-1_322_684

MDEDEVRKRGSVDITFKFMVACGQLIAASIFCLSFLFAQRGLTLRRPTMATLERLMVASL

YSSVFAPRPSFCIIHSPCICNFFHSNIQDLIKHTQLIIFRQLAALHIQVLKCVNFKHHNH

C

>SG-PL_contig_3712_1_+1_79_294

MNHTSASIKTFAIKGTKIIVGEWFTGPYTQGVTVEACSPVVEDTKEMMTGVAAGQEEDNG

QVKISIPVPLAG

>SG-PL_contig_3713_1_-2_29_1303

MMMVVAGIVVMRKGEMKSSDSYTSGECNNDPHPSLPHFLLNQTEVTASTEDVDHLSTSPS

SLSKLPNVTTSTTSVKGHSSPRQNELPHPGLGGSSMLGVNMAQVLGRRRHTICQIGDTRP

APDPVTKSKQYNYVRKFSVDISALQAQLENPKIFKDKVPFQSDQDLTQRQHNMAPLPEAR

GPLLPLTNHGHHSPKRSPVGPLTDLGTSRLEGGDSGSIKLSLLPPHPPVITVSEEVKEDM

EGETEEEGEGGMGEGEREKGFNSVERPQYMPITDKEELTSIPKLPVTQPVAEEEEEEDKD

THPHEEDASSTSVTHHYITANDRDFVKMTLMMALTFVLCTLPLLLMEVVRDRVHVHTYVN

VSTCARALSIIQTIIYPHIVLCMDRVVHKAVGKLKARAARVCGGRDCGCLDLPGEQQGSS

ATSQL

>SG-PL_contig_3714_1_+2_386_589

MGTQNTKHILKTQNKSKQIMTTGPSNLKHKIKLHKIVRTGLWNLIVRKILRLGCQYMTYQ

ERVLVKIK

>SG-PL_contig_3715_1_-1_184_783

MGRPKGASPRKKAKDANKPKRSTSAYFYYLAHCREEANKAGRSISKIAEFTKECSAKWRE

MGDTKKKPFEDMAAKDKQRYDKEMALYKGKTVDPNKPKRPMTAYFIFLGDFRIKMKNRGV

DHKEILRLAGEEWRNMSAEQKKPYEKRSVEAGKKYEQEMVEYRKKEKAGAAAVQQDNGAD

DDDEEEDDDEEEVDDDDDDE

>SG-PL_contig_3716_1_-3_63_602

MLTGTENERQTFAETYKEKNKEVKKSTRRDKRQFADNLAAEAQTAAEKGDSRTVYKITKT

LTGGFTSKTTVVKDKTGKVLTKDIDQLNRWAEHFRETLNRPDPDEEATIEDMGFKIEMRR

GCITQQEIVEAIRQTKGNRAPGEDRVTADMLKADPKTSAKALTKLFNKVWEEEKVPDTWK

>SG-PL_contig_3717_1_-3_21_635

MAEAMRGTVASLLRGIDRYNPENLAKLERYVQMQAQENTYDLEANLAVLKLYQFNPGYFQ

TEVAALILLKALTNLPHADFLLCKCLIDPSRQKEMPISKIIKLAEDLETCDFDHFWAEVR

DDPDLIGSILGFEDHIRKFVCHVVAATFQTIEQDKLQQLLGHVTPMVAMQWATKYGWTID

SNSMIFVSNQEESIKTKNITEKITF

>SG-PL_contig_3718_1_-3_51_428

MAFICSTVKFAKDFAALGGNRDVYLYYFKHNLSNNPWPDWTGVMHGYEIEAVFGLPQEYD

YTGQDERVAARVTGYWSHFAMTGNPNAAGSTLWPKLTSPNDDYLKIKRQGDKVGYRLRES

ECSVLS

>SG-PL_contig_3719_1_-2_2_145

LTHRETRYLYSIHIELVVSTGSFAIRPTYPPYIIFLDFFFLQTLSNHI

>SG-PL_contig_3720_1_-1_1786_2085

MKRLEEKCIKSIAKKRKPNLCGFVLLNGCVFLLLLFYDTVFSSIQSFLWVHHVLITDMTN

MRCQLVLIVIYDEAVSAPHNMKLSVLLDTVGTVHILHLHS

>SG-PL_contig_3721_1_-1_1_387

GRPLPRLERSDRLRLSALPNPLQGGEEGQDRAPATHPGSSLLASSDLVSGPTGALTCASS

GSPGQEGPAPTAAVGVGAPQPPSVSPARVAAVRAHLCPPGLTGPAPRSAAIQAALSAKGA

SRDLKALVA

>SG-PL_contig_3722_1_+2_101_256

MDGYIAWKFSFIKEKKKEELYKHEVGMHYLAIPHTSTYPSYPGQICHLMISE

>SG-PL_contig_3723_1_+2_164_319

MLPWNPWIRQIPKSCQSGEVWLVGWSWSLVFDHSMSEKCRRMSGHYTTNTRP

>SG-PL_contig_3724_1_+3_51_209

MTVCTISFFTTLNQMDHDKYKPNIPVSCYQLPVYFSLPPSASIVKQHKWKVKQ

>SG-PL_contig_3725_1_+2_539_862

MITKNSSISLRKSWVEGGGESQFSFVLRIMSFEMSKQFHFETTAILLLMNKNEGNVEILP

PTELSKKQSNVILPKATLSTEFNLGQHISVNLFKGHECTKMKHTTVHF

>SG-PL_contig_3726_1_-2_2_292

NSKKISLSLKLQFYNSNVLSTLLYGCETWHLKTSQEKKLDSFDLKCLCKVLGIKWTDFVT

NKEVSSRSKQNPVTGICKHQPSWLGHTTRLPSSRSAN

>SG-PL_contig_3727_1_-1_82_1062

MNPTSQVTSQTEPDPDSIKMFVGQIPRNMDEGDLRKMFEDYGPVHSLNVLRDKNTGQSKG

CCFVTFYTRKAALDAQNALHNVKTMAMMHHPIQMKPADSEKRNGDLNVEERKLFVGMLSK

KLSENDVRMLFAPFGTIEDCTVLRDATSTSKGCAFVTFASRVCAMNAIKGMHHSQTMEGC

SSPLVVKIADTQKEKEQKKMQQVTSNLLMQMSNMGAMGPQYMAQLLQQAAAASNMGGSFN

MGNLGSQGAGGNSSGMGNMGGNMGNMGGNNNMGFGSNNNMNGFGSNSGMDALSQAYSGIQ

QYAASFPNAFNTQAQMQQNAAAAKQSE

>SG-PL_contig_3728_1_-3_3_239

KGSSIRPHYTCHTSSPVCLDCLFWICHAHTKLAGSHEKAVSHLLCFVVTHDMHHYIASSS

VVVLFCGIILCDSGLKTHV

>SG-PL_contig_3729_1_-3_3_113

ASFSSTLHPPSCFSVVNNLSKRVFLRIIFAFKLRPFP

>SG-PL_contig_3730_1_-3_933_1337

MQGMHACSHTQHADGQIATHIHTNTLVHPMPPPSLLPSTHREKDKHLELLIGGGGAGLSP

MFMAPFRLGRPCHVSIITTNVGENSPRTYAMPSSFKAWVNFHPGWPQKSKHFLFYKSTPF

FFLTMWRQSRKILLT

>SG-PL_contig_3731_1_+2_2819_3328

MVFAFFYVTYIYIQKTYTCTRGHAYAWMQVLKNFLHAQICMHTCRHALTHWHILEFASSG

ETVVIAFQYCLNDRNFFHFTLCWKQSVSKYSLCTLLFVISWQFTFLQICLVKHHAGLVIL

ESVSTILPHNASVCEPATFLNTFLCFEKGHFQLSGVCDFVKLARFVFMCI

>SG-PL_contig_3732_1_-2_2_76

VCVCVLKLKKERDNGRVKETEDCHV

>SG-PL_contig_3733_1_-1_64_198

MGHDQPSYIWCVHMPLCERSNMKMNVLNGLHGRCFSFHKDSLFLF

>SG-PL_contig_3734_1_-1_127_1188

MRCIQRKQKTEESAASFVVVDVEVPSLKDNEVLVQVKACGLAPADTKIVKDVQCNKNMSD

GILVGRDVSGIIKSVGSRVTSEKEGDEVVGVLPLDTTTAGCGEFCVFREFDVVKKPHHVS

HEEAAATIGDALRAYTALHYHTHVCAGDTVLVIDGATPAGSISVQMALLWGAKVMTTYST

LPEKQFLESFHPALAQIMELNHRSSLLVSSVMEETGGVGVDCVIDNGVRLFSNEEDIMLI

GEKTKFPKPHKQDVLACLGFSGKWVTSHPDLQLDPPDCQQLFLRNASVHFLFPPSWTLMY

AQHGRYQHILMDVMEKLDKGMLKCKIAQLVTFDEVATVMQGLDDIRVGKVVLKM

>SG-PL_contig_3735_1_-2_23_790

MLGGMSQQQLMQLLGGMGMGGPSANLSSLMGRPGSAQSTVSEPPLRASSASGNRTPGETT

PTPRPMTSAGLLNPAATAAATTTAPASGTPTVTASGGATGGQATAGSVSASRQQQIQLSD

LQNILSSMNVPAEEPKESIDLAKALNPEAMLPILSNPEVQQRLIPFLPEGESLPKTEQEL

RNTVSSPQFQQALTSFSQALQSGQLGPLMNQFGLGEDVANAAAQGDMEAFVKAMQEAMKK

KEEEKQKEKGDDKMDQ

>SG-PL_contig_3736_1_+1_109_465

MVFRYSFSFWIAEWLRRSSVFSLRSCLFSSWSCATFSSSSCIKAFFRSRAVCAATRFFSF

LLNIFSSADKPVSRRRFLDPPPSLKKQPSSATSAIWIELGRFPSAVMTIAFWKDIFGES

>SG-PL_contig_3737_1_+3_141_410

MQMKSAVLHSGPFEAVTNSNCVVLYCIDFLSQWIYYVKFGLLSWGQHVTAVQCHPVFFCL

FLVHAGCFLVSISHRTLTWLTGSLTCNMHI

>SG-PL_contig_3738_1_+1_46_234

MHVIYNVFYNEITPRAKPRAPAIHKSWENLSVMIMMMAAFRHHVQQTISFCTQHEGKIAS

SEK

>SG-PL_contig_3739_1_-3_63_278

MLLQLCYYLLSSKSSCVFDVQILLPSLGFMFIYWKLCTLKMKNQLKVAFSCCWENVQFDL

CVLVVTIIFVFV

>SG-PL_contig_3740_1_-1_205_378

MQTAILLGTGGGTWKNVLKEKCQIFFTVFWYSESVKVINLCKVTEANRSEERRVGKEC

>SG-PL_contig_3741_1_-2_92_241

MSMIISQSAAERVACPLIHVYDHVTECDRASCLPPYPVYDHSTECDRDSC

>SG-PL_contig_3742_1_-2_209_400

MKVQSNQTTATTASGDNDQFSGGRMITELKFIHLYNLCGKKTIKIQVHVTFLKNKQTYFL

FVSP

>SG-PL_contig_3743_1_+3_3_194

LQQFDLIVFSFACLMTDLKCSHLMFIQLYSHFICLTIGYCSKIVSLTFIILDICFPYIYN

SRHV

>SG-PL_contig_3744_1_-3_93_1694

MMAAVTKSCLPRVKEEEDINQEVPQNYVVPVPPVVSPSRSSATSTTSTTTLQPSFLDSSS

SPISWQPAGSLPDCAISTTHFSSSLQLSTAQSIQPPPTSLPTAYVSEEHNWQASQACVRD

RNAVMFNNPLMSDVTFRVGAGGSEKSFPAHKYVLATGSSVFFAMFYGGLADEMKEVEIPD

VEPVAFHNLLRYLYTDEIHLEADNVLATLYAAKKYMVSHLAQACVNYLETSLSARNACVL

LCQGRLFEELHLMQRCWQVIDAQAEEALKSEGFADIDHQTLDTILSRETLNAREASVFAA

VMRWAEAECRRQDLDVTPENKRQVLGSSLNLVRIPAMNLEEFANGPAQSGMLSAQETTDI

FLHYTANKKPDLQFNHAPRAGLMLYWVHRFQSSAYRSNQWRYRGRCDSIQFSVDKRIFIV

GLGLYGSSNGSAEYLVRIELKKNGLLLSQNVTTFKSDGSSNTFPVLFENPVQIEADTFYT

ASAVLDGTELSYFGQEGLAEVQCGKMTFQFQCSSDSTNGTGVQGGQIPEILFFC

>SG-PL_contig_3745_1_-3_147_380

MSTHTLVSICTATSARSVERNFWKRGSSQPTRLNTKHRCLSAKCAATPTRARHHSSDMRR

FILESARTSVQTVVRASL

>SG-PL_contig_3746_1_+2_878_1228

MLSCPFSHFLDSCDPKKGHTPLKRTMQISVCYSQCRSKVVSFTASSYWAIALIRIYKRLG

NPYNNNNNNINNNNNNYDIYNAHNLDKINSKRAKKQNKTKQTYTFFFINSVDSFLTH

>SG-PL_contig_3747_1_+3_303_581

MRRVECSFSVTLKAHSVKDVESTLCKTLEIREAFRIKKSVPLTISYSLFLVIPSLLCLFL

FMPLFLCFNLKHMESQKNVIVCWFYSLENALVP

>SG-PL_contig_3748_1_-2_53_1015

MITGFISLRATSLWHGRVRGLATSVDKALLSKLRKRTGISFINCKKALEQFENDLEKAEK

WLKEQAQKEGWAKATKLQSRSMSNGLVAIVSNNQAATMIEVNCETDFVARNNKFIDLTTS

LVKDCHKALLQQSDPVVLLGKSIGSVVSDGKTLADRVALEVGNIGENMALRRAVHLKAGQ

SILSAYVHASGPEIVTDSDCWLGKYGVIISINQNAQQVSEENEVLPLDEISHALGQHIVG

MNPKLVGCFENIDSDTDSKTFEKEDKEAAEKKDEEERLVMQEFLLDPSISVGEMLKSSGV

TVHSFYRFACGEELSEDEKES

>SG-PL_contig_3749_1_+3_969_1193

MYQRPQTIEINFIQWSTTDISHMQQCGLCYWSQAGRWGSNFHCGCTEICKSVQNQLTRNQ

LKERIWQDFSLSPSL

>SG-PL_contig_3750_1_-3_312_509

MMCVYTCMYLCGKCFYLYTCRDNFLLIECLVREGERQCVCVCVCVCVCVCVCTHSCVDVC

VLKSVY

>SG-PL_contig_3751_1_-2_155_1279

MAETMIASSVKNEHSTRKNKKPLMEKRRRARINSCLSQLKSLVLQAIKKDSSQFSKLEKA

DILELTVKHLRGLQRQQAAGVMMQDSSVVSKYRAGFNECAKEVVRYMGSVREVNDEVKER

VVGHLANCLQVVNQITPMESLQQQQQQMKPLHVHIPPQQPINAAVTTATPSVIMHTAAAA

VSQNSLHSGLVGYAPSPVRSSVSPMEDCQTPSSVSTTPLSALPLQSVTAAPMTTAPTTVV

SPNSARISGAFKIVPSSGGAVALYLGGSNNVARADTVSVDAVPLYSVSLPPPPPPQPDNA

VTAFVTPPPSSQMAESRYQDISPVKVDYTHTSVASLPVDLRQSHKVKSFVDSPSSYPTAQ

CSPSSLADEKLWRPW

>SG-PL_contig_3752_1_-2_2_298

LRETVLGRDYDVYWGIPFANPPLGNLRFKRPEPADPWNGILSADTKPNSCYQGIDTTFEM

FSGVNMWNPNTNVSEDCLYLNVWVPRAFGTQPPSTTMVW

>SG-PL_contig_3753_1_-1_1375_1647

MSLLLVLFYSLYLHTRTHTRMHAHRQTDGDTHTHTHTFTHSLTYTHTHSLPVLECSAMLS

SLENLSFENVKCMLSFRMRFIRMVVGLCAKV

>SG-PL_contig_3754_1_+1_820_1008

MNTTAFMHPLLVRNSSLSLFINSLFNILVNISLISSVILRQIACSLPDIKYFKKEKRLQA

VYW

>SG-PL_contig_3755_1_-2_275_709

MYKLNYRDLVFHPICTNITRLLPRALVYQKFHSPSPHPTHPTNSWHGFKFGCHANQKLTS

QFQVSCRRKGSAAALFGSGEGGIFRGCLSHTLSLLPRLSVRHTHTHCSGTLALTRLRVLC

ACMQIMAKARLLICWPGKLSETVDL

>SG-PL_contig_3756_1_-2_2_244

SFVLWGFFLLLLLLFYSVPHGGAGGSFSINSIPTFWKFCVSNFLFSIAVCLSAVFLSFPV

VWSIPLSFSQQAFTVFSRPSL

>SG-PL_contig_3757_1_+1_316_702

MTIYNPVQDQKSLSVLPTLVIISVHRLTHKCMKLSKINIQLIDIFDTYTSKLSTQSMPQL

VWFIQNVAQHCKPACIHLKSIDNSIQSHMYHHQNSTHHSKSIINIIQYFAEHQKWHTIHS

YSIRSSTHF

>SG-PL_contig_3758_1_+3_120_317

MLRSGKSSIHASADQHQSIELQITSIITSSCFHQHPTMSIYIHYFQQWISHHISLVLFSA

QHPHKH

>SG-PL_contig_3759_1_+2_2_136

SPQLYQKTQPARKKIQWPTQKQSIRGCNKPMANSLLKWFVSIFLK

>SG-PL_contig_3760_1_+1_1_153

RMSGMDNICILWERESYAGGDHAGKKRCPSSVLACRVSGNKRFQFDQDKGA

>SG-PL_contig_3761_1_+3_1032_1499

MLRLISCNTNHQCYDASLVILPPSLSPLISHTALLTRSFNITSNETKSTFHLYNHSDQKK

TFNDSHHHRSLQTLYIPVPDYLCSVITQFTVQNIPVMPTQNVTCHDNHPYFVYNKTHLWF

IHQHTDRQHYTHNTDLFFASYTLISLSLSLSLVFFT

>SG-PL_contig_3762_1_-2_98_2266

MENWIGRFGKIHGWSVVVAAAGVAWVVYKAHQNLRADSESNEAQTTSVTEEQQIDAVSTT

TTGASSRSKRRRQPRQRRKLKIFYGSQTGKSKALAERLAAGVGGQGYQVEVVALKDYDPE

DQLSHEAEDLYVCVFIISTYTEGKPPEGSEWFCQWLEDTSGDFRVQKTLLSDLYYSVFGL

GDSVYKEHFCTVGRNIDTWLHQLSATALQSFTTGDENTAESKNGGVEGDFEAWRKTLWPK

LKQALDGRKVKSCSGSTCSEKCGTCNSKGEGDPEGHHHDGKCSSEEEDEVKFETSSEEED

EKDADSSEVTQGSGIVDLEDLGKVMHLAKVSRQKAEEELTNGEEREMITPMLRESLEKQG

YRLIGSHSGVKLCRWTKSMLRGRGGCYKHTFYGIESHRCMETTPSLACANKCVFCWRHHT

NPVGTEWRWKMDDPNTVLEGALKNHMNMIKQFRGVPGVKPERLVEGMTPQHCALSLVGEP

IMYPEINAFVKLLHQRDISSFLVTNAQFPEAIRNLVPVTQLYVSVDAATKDSLKKIDRPL

FRDFWERFVDSLKALSEKGQRTVYRLTLVKAWNTDELDNYAQLVSLGEPDFIEIKGVTYC

GESKASNLTMANVPWHEEVVGFVQQLVDRLPDYELASEHEHSNCLLLAHKKFKIHGEWWT

WIDYPEFHRLIQRYNNSNGAETFTAADYMAKTPYWAVYGAREQGFDPEETRHFRKKRKDI

SGC

>SG-PL_contig_3763_1_+2_1418_1663

MILELNLTDVRRETNPQVKLFTCRKTSSVKQSRLDFFLLSVNLMLIFEDADIMPGYGSDH

SMVTLKLKFVKNANAFWKSNCS

>SG-PL_contig_3764_1_+3_48_326

MMDSVLSQPSMMDLVLSQPSMMDLKAWGEVATGASDGIFVPKTDGRGEGVFVGDGGDFLN

DPNDCVVSFVCRNCTCFVACMCLVKHALPLEAC

>SG-PL_contig_3765_1_-3_342_599

MIRSQKLSYIAPIPSFVKLWPRNFTSLGKIMQFKIICDPILPVLMCSLTSPIWDTFDQNL

VLNYKLMNSKEIFGEACLQFWSLRPK

>SG-PL_contig_3766_1_-2_11_919

MSASARKRLRLTDQTSDDGDHSSKEAIQNDEQTTQQIISDLEHEIELLEWRLKQFRDDWK

QQKKDRASLSSLNTITDEIHELEQLVKENEILSGLTLSEVSYSVLKQDGTSSHRQHQVMG

EAGGMQFELQYDVIEQFQKTKTEEPCAEVATVEVNIRDDMVALLEAPLHRAADDKNVREL

FQLLSSYGRWLEDRERAINHFASTFPELVQRVDEGDEGCNISMVIADVKQDVKLHVLWGL

KLLPMKSVVPDLQLQVEASPKVLDKDKKGVLVSAPEVFQLMLKKFGIESALHMLVKLMDE

APP

>SG-PL_contig_3767_1_+1_79_369

MLNFEDTDGIPGYRSDHSMTPNLKFGKNIKRNSFWKFNCSLLKEKQYVEEINKLIDDVIL

EYSTEHYDAVTLNNTSLMEIIFTVPDKLFVDFLLMKI

>SG-PL_contig_3768_1_-1_1_207

STHSRKGQHPCRRTEQGPLHYPHGVDPQEADPPRSLGHMVRPYGRPLCDQVQQEAPDLRL

PRPGPRGNG

>SG-PL_contig_3769_1_-3_3_122

AATTTTSSCHYCCYYYCHNNYYYYYCQFIHISFCKPLNCI

>SG-PL_contig_3770_1_+3_3_125

SSSYCITVIIITNVVVADIFLQLTQINIIITNCMIHRCKTA

>SG-PL_contig_3771_1_-3_45_257

MAVLCECLTRFPTCRIASQHVVLNKEMFELTVFLGVWPCKPKVIIRHSFLPFFLSFVSVC

IFSVFFVCLFV

>SG-PL_contig_3772_1_-3_12_1595

MATRIVCRMVQQGCRRFSRTAEKRQFCSGSAGETDITSVPLSQPVGWIRVPKYATPGDLQ

HDTHVTVLENGLRVASENKFGQFCTVGVLVDSGSRYEVAHPSGISHFLEKLAFGTTTGYK

NRDLILQELEKHGGICDCQGSRDTLVYAASAETRGLNSVLNILSDVALRPHITPEEMEDA

RMAITFELENMNLRPDPEPLLMELIHAAAYRDNTLGLPKICPEENINLISQQILYRYMKT

AHTPQRMVVAGVGMEHERLVDLCQCHFVQEKKAIWEEKKELLGGEAKPVDKSVSQYTGGI

ISVEKDLSSVSLGPTPMPELAHVVVGLESCSHGDPDFIAFCVLNMMMGGGGSFSAGGPGK

GMYTRLYLNVLNKYHWMYNATAYNHAYEDSGLFCIHASAHPSKLRELTEVVVRELAYTAG

IVYPDELARAKRQLQSMLMMNLESRPVVFEDIGRQVLASGKRRQPSYYYEEIAKITESDI

QRVANRMLRSKPAVAAYGTLEQLPRYADIEAALSNKNGRMPRRFLLFR

>SG-PL_contig_3773_1_+3_3_332

SSKTKKKKKKKKKKKKTKKKKNHVHSSGEGGRPLELTKSGTISITLCKVASFSLYHVHDI

NTLSALYMSQPFVQPFHTSACINVQHFPYPIGSQIKINANYIQVIQQAVL

>SG-PL_contig_3774_1_-2_26_826

MLGPDFITDIMEVETSDHARLRLQVAFNNHFEVDKTDEESVKRIFQVPDFIGFACRQLGS

RIRASVALTPFDDFHRHSAAIIQAAVFGRDAKTGRVMPRLKFLVNDLVISSIDIQNIEPV

DTKMRDSLMKSVQLAIEISTRSVEASAEHEANCREQIAKGQLERQRLLNEVEAEKERAKL

YELRAEAAAVESTGQAKAMAQAEAEKTLIECHSQIEAVTLKAKAEEIEQYSKLETQSMMR

ASEMDYKRKMMQLEINKEKSHAAVEVT

>SG-PL_contig_3775_1_-1_121_216

MRTEQIRTCALSRMFSNLVKNVFKPDSTFFDF

>SG-PL_contig_3776_1_-1_49_1020

MMRFYSLLLCLSLVLIAASKPKKEDTIDEKVRRLTEWSSKTPIIRLNGDKFRRYVNSAPR

NYSIIVMMTALQPQRQCRICKEVYEEYLIVASSWRYSQQFSSQLFFSLVDFDEGSDVFQR

LKVNTAPLVFHFPASGKRQREDNMEMERNGFSAEMIAKWVMGRTDLHFDVYRPPDYTTIL

SVTALICVVGGFLYLRRNNLGFLCNKNGWAVMALVVIFAMISGQMWNHIRKPPFSRKNHK

TGQTQYIHGGQGQFVVETYIVISLHCAVVAGFIMLNEANSLKCNVGKKKILALVGLACIA

IFFSLLLSVFRSKNGGYPYSFLFK

>SG-PL_contig_3777_1_-3_3_395

DLPVPEWKGTQKTEKPKKQKFLVAAPPPTVKEAPKKRKPAPKTRRPLKVPLQRLKTRPAE

APVNPDQVQDMQDPLDWLAKYCIINPDRLPLYEMIFESVVADQTPRYTKPPPPSLKEVLK

EAKEKGKGEEG

>SG-PL_contig_3778_1_-1_55_3168

MPELTIWELNAYEGSQKLSLELGLHCKLHKGSLDVSLYSPYWLLNKTGRTLYYRGSDQDA

PIEHPADDNQLILFSFKGKTFFGGKKKEKDGEKVEGDKKSKDKVREMKQAGKAQLRIDSL

EWSDKFSLDTVGSSGTVTCKGKGKDAQSGEVGVSIQLSSSGLTKIATFTPFYMFLNTALD

SVLVTESDRESWVEITPGQCLPFWPQTSGKDVKVKAKVKDTAEVTGEFLVNKAHTTLLKL

DNEFGGINADCQVSESSMITTLRTYRAGLATVVVVNHTQNSTLSFYQSGEQQQRLVTLNP

QESIMYTWDNALGKRELVWTCGKKKEIKNDLIQDGMDEFFVDDDTKVYWVSFLDGLQRML

LFTEDLAIAANAQEAGELEQVEQEINLQIQGFGLSLVNNYNSTEVAFLGITSSGIIWEER

KKRRYKALKLKECHLLEAAWQKYQNELLVGKSPHSRVILENKMEVDFADLTVIKPRTFVI

RRSFENGIWVQYKTSPHQLQLHAKINRIQLDNQLSHAVFPTVLSPLPPPKSVAADSVPKP

FTEVSVMMRKHEHSTVSQIKYFKVLIQEMNLKVDQGFLNAILELFAANAPLPREQETIQL

KQDVESTEKSLMEVAGLSLAEEQKNFYDYLHFSPIKIHLSFSLQTGTGSSDGGKPVEIQA

GVISVFLQSVGVVLTDVQDVVFKLGYFERSHTFYNQSQLTGEMVRHYAGQAIKQMYVLVL

GLDVLGNPFGLLRGLSEGIEDLFYEPYQGAIQGPEEFAEGLALGVRSLFGHAVGGAAGAV

SRITGTLGKGLATLTLDDDYQKKRREQMNKRPATAREGIARGGKGLVMGVFDGVTGIVRK

PVEGAKQEGVSGFFKGVGKGLVGVVTRPTSGVIDFASSSFEGIRRIAEVSEDVKRLRPPR

RFFKDGVIRPYNHLEAEGYAILQETEKGKYGVTDEYQAHVVVSKDGKNILIVTDKRVLLA

KRGEIFGSWDAEWTYTWTELKEDTPKKTTKGIEIVLREKEKKKFFSGGSSKKEVTIADPK

LAEWIITQIQEQMVRMKN

>SG-PL_contig_3779_1_-2_119_208

MRFCWVLLTTELFIFLFHLLMYGHGFIVFW

>SG-PL_contig_3780_1_-3_246_494

MLNCLFYLSVQEIELLPQCYPVRHLPVKKEEIMKGKEKRLFDSAVGHAKQCMGIGAFGFG

HSIIHLCTIILSSCKMNRSQRGL

>SG-PL_contig_3781_1_-1_145_315

MQQRFCTFSLSNEQKILAQNRVHHHLVSFQHLLGVFCPSTVAESVTISTQYVSFVVV

>SG-PL_contig_3782_1_+2_5_262

MHARTHARAHTHTHTHMHTHAHTHTHKYKHTTWTNKNSTPITNQEKSCRANHKLLFNKFY

KEIGENKRKNKISYTQLPKMNKIKEH

>SG-PL_contig_3783_1_-3_903_1178

MKGLNKIKFTICNHLGGASLYKVMFMNFKVVTGVFRIRTILHLTERFSDGYLRHAVSVSV

SNNNYYVCKVHFIHLFKSPLPFHVHFPKASFQ

>SG-PL_contig_3784_1_-2_101_1132

MYNQDEEMFVGDDESDDVTTELWQEACWIVISSYFDEKGLVRQQLDSFDEFIQMSVQRIV

EDAPPIDLQAEAQHTSGQVETPPRYMLKFEQIYLSKPTHWEKDGVPSPLMPNEARLRNLT

YSAPLYVDIKKTVIKEGEDPVETMHQKTYIGKIPIMLRSTYCLLNGLTDRDLTELNECPL

DPGGYFIINGSEKVLIAQEKMAINTVYVFQQKDSKYAFKTEIRSCLEHSSRPTSTMWVNM

MARGGGGAKKSAIGQRIIAILPYIKQEIPIIIVFRALGFVSDRDILEHIIYDFDDPEMME

MVKPSLDEAFVIQEQNVALNFIGARGAKPGVTREKRTKYAREIL

>SG-PL_contig_3785_1_+1_1_183

AGWIRDLNLVTNPSGNYRSCVTRTLGSEWFKPNHYTILHPSECFKSKHVNNNTVHHIRFA

H

>SG-PL_contig_3786_1_-3_96_209

MVFIDYSFNYLFPLFVSLNLPFKRLFFFNDLLLMFECY

>SG-PL_contig_3787_1_-1_1_207

EIIKNRSNVLDNLSLIFYYFARRWILAGVHLCPENLIQWALLKDAGSWCGIYIRRITPCL

ASSVGCVQL

>SG-PL_contig_3788_1_-1_1_297

ICNFCKKNHKNSDLSPNLLCTFCKAVTICREMTIITGPAKVLCSGPWIAAKTTVGSERLA

PGHLGGACSNPDGGKTKKKVTAEGHRGVMTWIWPGPLRV

>SG-PL_contig_3789_1_-3_3_155

GQLSFVVFYKCNDLEGQLSFVVFYKCNDLDGHLPFSFLLFFFNLFFYINGK

>SG-PL_contig_3790_1_+2_575_997

MNTKHEKILSVLISCFSESQQQVLIQHLGSIPLISVNSASIVKELDSLLQSYGLQWDHCL

SILMNSCNVMTSRKNGVETGIRRDLAPHLVDTDGIPAITFIDAARLSHLSLEALWSHFLK

TSTATLSGLHHFRNVLKASVI

>SG-PL_contig_3791_1_-1_13_360

MATTIAVRTRKSRKADAVAYSELSVRQNSAILEYCRTSMSALSGATAGIMGLTGLLGFIF

YFVSAFILSVFLTVKAGSRWNSFFITRRILFINGLFGGLFTYILFWTFLYGMVHVY

>SG-PL_contig_3792_1_+1_16_255

MLKAWPFILSFTHSSFLYLFIHSLVHSFIQRSYQNTQKLKSITNLISQLKTSNTHSTFMP

PVVSATYISISRMLFVLCVE

>SG-PL_contig_3793_1_-1_139_867

MVWNTVTSRRIKTFGLKPIVGDLVLPPEAAASLEVDVTDEQETKITDEQQAKKSSEEQDA

EKTEEQQTKSTGEQETNEDGESNKKAGKGTKPRVQPIVLDETTVSQYSIYDVVLPLPGFD

IVYPQNEVQSWYKEILEKDGLDIENMRRKQKDFSLPGGYRPVVIRPKDVEWKVLRYNNVT

ACLCLSDADRMNGVPEPQSIPDGQMKAVQLSFTLGLSAYATMALREVLKTDTSPAQQASK

NVS

>SG-PL_contig_3794_1_+1_1_192

PLCSTVHEFCIIHCQQSLCINKFCIIHCQQSLCINKFCIIHCQQSLSISKFCIIHCQSLC

INHT

>SG-PL_contig_3795_1_-3_603_929

MYANSTLVLHYILLFVCFCFCRKISLKRNDVWYFFSSLFSSTYIFPKGLFNQILNLYAYF

HQHQTMFIKKITIPPPSLNHLHLHIAIMHTPRLLTVCDQYLSVSQNIVS

>SG-PL_contig_3796_1_-2_761_1009

MLLLLGLFSTYFQIDNFTKEILSCWLVCLSHAKAYAFSFLTHQTHAMHACVHPLTAPYLP

PPPTCTHTLEKDTHTHTHTHTHA

>SG-PL_contig_3797_1_+3_81_440

MPVVSLAEGQHNTHLKKVKICMHHPPWGCQGLLQFLSSIPTFWKFCARRFPFLLLSLFLS

FSFLYCRLLFSAAFLVHSTFFFPQAFTLPSFLPPFVIVHLHSFTLISFIFVFCLFGFFVC

>SG-PL_contig_3798_1_+3_12_335

MPRALSVQVVFPPSGNSVRNFFFFLLLALFLPFSVLSIASSCHCVCLARPFSCMLSRRPS

TFFSLSTLPRSQCADCAATVCLHRVGQDKVRAYIQAKINMPQMETPTI

>SG-PL_contig_3799_1_+1_283_471

MFLRIASFSDFNHQIHLQNFLESILRHDFFSLSTQPFSIPYLSISNCLLLSGKQHQKHSL

LLS

>SG-PL_contig_3800_1_-3_36_1172

MHAVRQACTGFVRATKCALTSPAVPQTATRALPAYFNNRNYASEAAQPAAGLAVGRIVAV

IGAVVDVQFDDNLPPILNALEVEKRSPRLILEVAQHLGENTVRTIAMDGTEGLIRGQACK

DTGTPIRIPVGPKTLGRIINVIGEPIDERGPVETDNYSAIHQEAPDYVDMSVEQEILETG

IKVVDMLAPYAKGGKIGLFGGAGVGKTVLIMELINNVAKAHGGYSVFAGVGERTREGNDL

YHEMITSKVISLTDDTSKVSLVYGQMNEPPGARARVALTGLTVAEYFRDEEGQDVLLFID

NIFRFTQAGSEVSALLGRIPSAVGYQPTLATDMGTMQERITTTKKGSITSVQAIYVPADD

LTDPAPATTFAHLDATTVL

>SG-PL_contig_3801_1_-3_9_179

MVLDSCVHTKFHADMCILKYLKNIFLLIVLDQTECMNYFTNVVLHFVFVQIECIQYF

>SG-PL_contig_3802_1_+1_538_768

MQRSTFVICLTAADINNNIGLLQSRRTMEFVPLERLQDTSLGKVLRRTCCCPCSRPTLST

TPVKSKGEAKANTTWSR

>SG-PL_contig_3803_1_-3_876_1133

MSELTNREIVSMVSSFQFLIPFTIFQPASKCYDLLSGLSEILLNLLLSLFIQFIQFRLLH

MRLIGFPTAIVFEHLTFYNDSLMQTD

>SG-PL_contig_3804_1_-2_2_268

PSSLEVKCLHHSTTAPVINQIYYVIYVSLYFIFILHWRQMFFCSSSFIYICVCYKFFLNI

AFYCNQECTYSGNCFPPPPLQESSHFFPE

>SG-PL_contig_3805_1_-3_402_713

MHSVINRYDTLHIEYPFEESVIFLSVTILSASKLSAFTGSWVFFFPSKPTHTHVKINVDI

RHRINIEMPWEVTLADQSQSFSDVQPSFPFKHLLLHEHADHVAQ

>SG-PL_contig_3806_1_-1_415_2004

MLESLTSPGDMLTPQSLILEQLQAWAGKAATPLTLLFASALLGSVLKWTRRHHHEHQTAG

PLPPSPGLALPLVGHLHLMKGGSDPRVLFADMRDKLGDIFSFYAGSRLIVVLNGYDVIRE

ALVKKAAVFSHRAHTFMSQKIGQGRGIVNTSGRHWREQRQFAERALRALGVGRTSVMSLK

IQKEVDCLLERLEMSRCQGQPVDPGPLLQASFSNVIISIIYGHRFELDDPKFLNLMTALT

NCFKNFGNTEPLNFFPAVRFLPGDPYRYWFCIQNMNFLESHLVYPELTEHEQKLKESAES

EDVISSYLREMTNKLTNGMPTFMDKENLVKVAGDLLAGGTEPPSITTMWILLYLIHFPDV

QEKCYQEILAQIGTERSPTLEDRSSLPYLEATIMEIQRHADVGPLAMAHGLARDTEFRGY

SLPKDAIVLINIHSVHHEVKGWGDPEVFRPERFLGEDGKVVHHNNFVPYSMGPRVCPGEN

MARMEMFMYVSCILQRYRLKPHASTPPSLEGYMTLMHHPDPFTLMVEKRE

>SG-PL_contig_3807_1_-2_2_139

FFSGVVFLPPSPWPTSFILLVFNRQTLTGLFHSCSLFEFVQNEQFN

>SG-PL_contig_3808_1_-1_1_300

QHHLLHFAPSTSERVVRRQSCELAEIRQTAPYMKTADTLNSQQELLKRDYADTAATVGKS

RGLGVESGSSLETGESPHDGKWAQVKEAETAHCEEGRCGP

>SG-PL_contig_3809_1_+3_27_251

MTDNDLSDHNDDSQAVEAVTVDTGNATQDVTQITDGSGETDYTDDEIQKREPNEGQRNVQ

TELDPGNDSHDETNV

>SG-PL_contig_3810_1_-1_1_123

VVVVAAVFYIFVFIHNYRSESLALLCKIEKVSLCNTVLLIV

>SG-PL_contig_3811_1_+3_75_380

MSFEVAQITLTIQEQRVTFFLIYRPPPSKKNKLSVTLFFDELPDFLDFCHQCQNSIVIAG

DFNFHVDDPDNVDASRLCKQLEMFNLTQTVTEPTHKCGHILD

>SG-PL_contig_3812_1_+3_3_293

SFPRSYDSNLKRGGGIATIIKAPLAEHCTITTDFPFAHMSFELIEVAQITLTIQERRVTF

FLIYRPPPSKKNKLSVTLFFDELPDFLNFCHQCQTVL

>SG-PL_contig_3813_1_-2_467_895

MTTYIDKERIAEAYEDVRDDATATTWLVCKYEGNTICLDAKGENFDDLKEHLGADDRAFA

FVRVIMGDELSKRAKFVLITWAGANVGGLKRAKIGPDKTVVKGVLKSFAVEMAGNEHDEV

TMDHIKDTLKRAGGANYGTGVRD

>SG-PL_contig_3814_1_-2_1634_1900

MTYTIHKLSKTSRPCQRIVLISRRTAYAIIIFDSFPSHGICGVGAYRIHSECGNAVLRKL

KLKLKGCAVDKDTEKVLMVKHKTILSRYK

>SG-PL_contig_3815_1_-2_776_1243

MSGNIYIKIHGRGGRMLKTIDFSTGGSGRPGFEPPRAPRLIGKKWRFFPISQVNIFADLL

VPEPPSCLYACRTSNMHVKDPVSHANVQWLMETRKHPACTKNKNKNKKNKGGAALQRRAL

PGRATRIPHCKSILTKNKYNTIQYNTIQYNTIHDLC

>SG-PL_contig_3816_1_-3_15_1016

MVLTCRFYKNRFPEIDDVVMVNVRSIAEMGAYVHLLEYNNIEGMILLSELSRRRIRSINK

LIRVGRNECVVVIRVDRDKGYIDLSKRRVSAEEVLKCEEKFAKAKAVNSILRHVAELLDY

GSNGKLEELYEKTAWYFDEKYKKPGSSYEAFKHAVSNPEVLDECDIDEKTKEVLLENICR

KLKPQSVKIRADVEVACTKYEGVDAVKRALKKGLELTTEAMPIKINLIAPPLYVITTQTM

ERTEGLELITKAVDAIMEDITAAGGVFNIQEAARVVSDIDEIELEKQLAKLEEENREVAG

DDDEGDEYDEDGEKKGKKGSDDEEDDDEIDEKDG

>SG-PL_contig_3817_1_-3_69_614

MLSDCILIVFISICTALLGEGLTWLMVYRTEKYQKLKAEVEKQSKKLEKQKEAGENADRN

TKKKLERQEERLKNHSRDLSMVKMKSMFAIGFAFTALLSMFNTIFDGRVVANLPFTPISL

IQGISHRNLPGEDYTHCSFIFLYILCTMSIRQNIQKLLGLAPSRAASKQGAGFFTPPSPL

TK

>SG-PL_contig_3818_1_-2_2_982

PTPDPELGLPPNIPVSPDGEEGYSASQLRLLDRRYRTAHSELFKPPPNKTVFDRESPPPY

SLTPSPGTYGCGSPLAPPPTPPPGLGRDGGGLLLGDGRPERVIRDSEGQPVVSRCYTMFS

SPARRPRSEPPVLPRLHDPGRRHHYQPPTPTTPTHSDNGVAAPRHCLPSSSHPPPPPPVH

GAFTVGDVLHLSPSPALFPPPPPQGNAAPLESDTPSQTVSPSWSPSSSLSGVVVGSGSVD

SDRRSSGGSCRADFLPQQGCESRAPPPSYQMVLGREGSGGGVGGIPSVALISPTSSSSSS

PVSIATTTSTTTSTSTPFNSNGDPCGV

>SG-PL_contig_3819_1_-2_2_931

RQNLELKEEISRLTSAKEECCVENTRLSKQLEESEHLCISLQEQLKEFLEPSGPINICSP

SILGSSEQNKTDLEKAGEDLDIKKTVICNSKGTQTDKVSETKKRDKSSRSRSRDSERAEI

QSLTQKVKTLQEQLKMLEAENLELAEKSARSHDSSSDEDDPLQPSIKHKPRQKVKTLERQ

LQVLHSENIRLADKLGGYSSGTRLDPEEVIERIVELEDFCTDLQDRLREAEGHERQMRDK

LRVAEQTINELENSEALYRDRCDELSQLERETKKQLHSLQTTGRELREIIMDKDILEQAL

KEKVEFLEKV

>SG-PL_contig_3820_1_-3_18_2021

MEPCRSGSVRVPPFRCSPCPSAKQRSSLQGPATSSSGRHGPSQHHYASEDTLNHLISIIE

QLKSKDKVISQLERELGHPAVVAGPVSVATEVTRTSSSGQHSNKMETPPHSPHNVLSGLN

QVFQRLHGTGMASFPLGGEEKGAVEGFAGLEAEILGLHDRAQALAFDRQILLDKIKDFEA

KNKQLVEKNKRFLEKNIELTAQNRSLKDGSHTMQQQQKKLLEEAEECRRRQCELARELQR

VKGHNLELEAQAQEVQDLRKQIADQTSSITLLRQACTDKDRRIELIQHRKKRRRLIRSQE

KCQGVKETFYGYDDDDNDASIDSETSLSSVSVSTISEEDACDELSREELERSHRQMLREH

LQLERSHALLQAHTGSSQDPYRELQTRSQLQADLFSAQCRIELLEKEIRTLQSSRRRRGS

GMDGVPDDMDVAALLKERDDLLSARQGLQMKLEQVDAECVRLRQELRKAEERAEDLEFRI

LELEENPPCGDPHSDPNTLPPEESDFDQSNLDPHHDDDLDKSDLDVEHGHTDAADEGEAS

QAAVTECFTQTVAPTGLECLPNQSASSPTQYPAPETNASSTMEFDRLRRELKAALERQGV

QADRILKDLGGLKDVEQKMGSMERMAMEMQKKVIALQQERDHLEERLMRGAPLSINAEVE

RRQNLELK

>SG-PL_contig_3821_1_+3_99_383

MSGVGEVTQELCRRWGSKKTKKTKTNKRTKKSRGLRVVRSIPPPSPTSSQSVCTLVSIAF

GLILIFCCFSFKLFNGILQEANAKQKQIQDVNSKA

>SG-PL_contig_3822_1_+2_2_184

SQLFLDRTFFKFGPKVWTAGESRIHEFGTERARIKVTWAKKVPKNYHFLRKVCEQHFLIN

F

>SG-PL_contig_3823_1_+2_77_448

MLPLVWSRHRLGVHASFGVVKSQTGVHASFGVVKSQTGVHASFGRVKSQSDWVHASFGVV

KSQTGVHASFGVVKSQTGVHASFGVAKSQTGGPCFLWCGQITDWGSMLPLVWSNHRLGSM

LPLV

>SG-PL_contig_3824_1_-3_177_692

MQEITNDPLSKKQGRKHNMKRGKHLPKFDKFKTPEPATPQPPGHGISDRTTITIGDKTFD

CDASDLQSRRYLGRGAYGVVEEVEHLPSNTVLAVKRIMATVNNLEQKRLLMDLDINMRSG

SCPYTVEFYGALFREGDVMICMEVMEASLDQLNKKLKANGETIPEEVLAKIA

>SG-PL_contig_3825_1_-1_1_249

SFLLLSLFLPLFLPLLSSAFFCLAGPFHFLFSSKPSHNFLSRTPPPPPSAVYDVLFCSGD

KVVRLLTLGWALIIPLLFASLVF

>SG-PL_contig_3826_1_-3_114_380

MHIFYASHLLASVCTSADHAWGLFCLWFFFYHILYFMHCLFTIITSVVMFCVHLPRGDAR

GSFSINSIPTFWKFWARNFLFSIALFVSA

>SG-PL_contig_3827_1_-1_130_282

MAAFINLIPFVDRIPCCHSCLLFRFATDSNIHYFNVRFPNAWALVIRCDKH

>SG-PL_contig_3828_1_-3_12_173

MSQATGVDDDEDNIYQDTTEHSCGLHNTILVHCKYLISEPINKIYQNTAEHICG

>SG-PL_contig_3829_1_+2_176_358

MATKQNVMITLKHRNKDTFLVQTTPLCNIQKQQKLCFKISPSTTTLSWAMGSHTEINRLN

K

>SG-PL_contig_3830_1_+1_1_168

LTPLVRALTHTHTHTHTHTHTHTHTLRRCRSIFSFVYKRRRNRQYGIKTGVEGFKL

>SG-PL_contig_3831_1_-3_186_434

MYLQYIFDHSGIFSVICRLLSLWKSMSPFYNTTSFSVCLSLSVLLYFSSCIKRCFALNIN

TYKTVPIKKLIGKINIFKKVKKK

>SG-PL_contig_3832_1_-1_1_339

QQQQSFADQIRGECLCTYSAFICYLHFSSISSILKTNCTTGKPEEGFFSCKGRLKYIVVL

QFIHCFVHYHYAQYPTVAWNKTEESRSDQRSWWVKNVAFIILFVVFSGLLFHL

>SG-PL_contig_3833_1_-3_111_863

MAKHTLILVRHGESEWNQKNQFCGWHDADLSDTGIHEAKYAAELISNKGFSYDVAFTSVL

KRAIKTLYLIQEGTDRHWVPVYRSWRLNERHYGGLQGLNKAETAAKHGEEQVKIWRRSYD

VRPPPLTKDDKEWSGTDPKYAKLDPKCIPACECLKDTVARVLPYWSDAIVPELKEGRTVL

IAAHGNSLRALVKYLDEMTDEAIMGLNIPTGIPLVYELDENLKPIKHYYLASEEEVKAAE

AKVANQGKVKK

>SG-PL_contig_3834_1_-2_287_460

MKSCSLHRLPTMQLLLVFLSCGVSVYRRVFTFDSVVYFQELFSIFSVLQKLLSIPCPV

>SG-PL_contig_3835_1_+2_1718_2029

MIISKLILTSSDDTLHFASNAAFGAACQRVSAWLKKKKRKRPSLKNTHTQTGTGWHSLVL

NYTRNKGTKYFSVTAVVKHRIDHDFSLRCICVEISMFILSSHLH

>SG-PL_contig_3836_1_+2_2_124

HLKPEFLTKNFLHLVGSVTQNKDHGQNYGVRYRLNSWYKLE

>SG-PL_contig_3837_1_+1_70_195

MRYYIRTVSCNPAVVECPNHRCVSMCTARHMKEKCVARDSTF

>SG-PL_contig_3838_1_-2_38_2350

MAVSLAISMSSLACLLLNVFILPQNEATNVQIETADGSRHVFEMKTMEKSGSYLLCANCK

SFSTGPIPTPSALRFNVRFTPTGEIVSACETHRKELPVGLCSAQGADNNGSCMNDNVCLG

NTAQEEVADEIVKRNLLPFPSGDQEIDHSVAHAIVEMNAGLWDRAISMLNSILDKYPENV

AAHYARGVAYARKGQESQHLALQSIADFNVCVEKEPNYPEGFERRAEVYITMNRFKEALS

DLKSALALRPNNRIRFSSGIVNLFLENFVEAEAEFKKNLEEGAGDKGPVYIFSYFHLGLA

QYYRGRLRNSIEVFKEVLKLQPNHVEACVSLAQAFRELGNYKAALGRFDQALSLSPNHTL

SMQLKGSLLYHSGDPASARSVLQQCLRVDPENTSCQYLLALSTVALGEFYQGIKLATRVM

IKSLPELKISPERVRAQYLREYGRFLHASLETPLNHLHPEAALDEKMRVPWVQGMPLTLR

GQGLYREQPGLQPEISDVKVDTQDWSQSSARTLLCHADKVGLSMQVSADGFLPNRRLNLA

MGLAAIHIAQLAEAKWKNMKGPGKSTERPFSWKELFSIASRYRQLVDPEQPVLWVDSLPD

YQTEEGHREDITLIRGPVASVRVSQYFDLAFKLAKTMLEHYTGMGAVSYSGLSTDIEKAK

TCEELLQIARKRQINPNGFLVSTQVPATVPLPGGSSSGGGSEDRLDGTVLVLTEDPNGRI

VFALSMASTPARTAAYASQMDHFLMQLHQEVQRTGVGKLSDMDPVDRKSVV

>SG-PL_contig_3839_1_+2_410_640

MTLYATSQTRHTDRRLPIEWMIITLLLGQPKWVSLCMHNMSQCYKTLCFVLIFLHSFTIR

LWDTAARLTWKTCHKVY

>SG-PL_contig_3840_1_+3_33_383

MIRRPPRSTLILTLFPYTTLFRSPMFNCCSHGTLLHFSLQSSRLNICYYHQDLHLRRLHP

DSRPRLRRSPQRPSYSLQLGWSAPRRSEEHTSELQSLREISYAVFCLKKKKKKKKKI

>SG-PL_contig_3841_1_-2_497_1615

MPSREFFNINKMSCDTHDCCSPERLRDEIRTSGNVVIVDCRAQADYVRAHIRQAINISLP

SLMQKRLKRGSLNICNVIQNNEAKERFSRCCKSHQMVLYDECTTDLASNPSSVLNLLVKK

LRQDGGKVSFLIGGFSTFQERFPEYCQTQEITDNTILGLCNLRISDDSSYSSCESSGDLE

NTPHMSPFPVQVLPYLYLGNAKNSADLSQLKQNGIHYILNVTPNVPNMFEDDGDFKYLQI

PISDHWSQNLSSFFPDAIAFIDEARHNKQGVLVHCLAGISRSVTVTVAYLMSRQAMCLNE

AYDFVKKCKPNISPNFTFMGQLLDFEKALVDQTSCQCSAHHRTSSSADDEDDGIPASDSD

SDKTSPPFEISPV

>SG-PL_contig_3842_1_+3_75_170

MKLEKYVKDEVTKMQRSNHKLAIWLKHSLNNR

>SG-PL_contig_3843_1_-2_116_694

MSSQKGNVHRTRAQKHKNSTAFKNTKYDTSQRTKNMLNVTQDGLCERCKEKIEWKIKYKK

YKPLTQPATCTRCHQKTVKKAYYTVCQPCAMSLGICAKCAQKKDVVSDFAPNEQTQAAEQ

SEMEQQIKFLPERERRTLFRLKAQGKLTEGLKGEDIDESDDDVDTAKNSDADDCPSEEED

EDEEKVDAHTLFE

>SG-PL_contig_3844_1_-3_3_197

ALPFSLSLALSSSPVMTELATLARKESICLHCDLDDWLILADTSDLLSYPYSQGHRPLLP

TRVSF

>SG-PL_contig_3845_1_+2_92_325

MPLPCSFPALQEVRKRPCCAKETVKRGKIIQTYVLSLQIFSKIELGEMSFLFKVIIYRYY

GQEQKITRKTRWVFFFCF

>SG-PL_contig_3846_1_+2_74_223

MHRIATLADLKYELTVAWNEVPVWYLQKLFYSLPQRVRLVLSQKGHLTRY

>SG-PL_contig_3847_1_-3_624_914

MLNLGLSLTGQSLCSWVWSYKRQSVLLLQCGFTVAFFSFLYMHLASLFLFLSHLLAHHLS

LYFSLFLSDKKNMYMAIIIISLSLKNLKFRIKGQRKR

>SG-PL_contig_3848_1_-3_111_254

MPPKKRLKADKNQTVLTHLLCSGKTADNSVSGTEDSSTSRPTSDSGTA

>SG-PL_contig_3849_1_-1_1_156

VKATLDLRQCHVATIQFPNKCWSNNMCCPELSSHRVEIKNPSLSLLIIRDLN

>SG-PL_contig_3850_1_-2_413_643

MVCCWVLILYVLNKSIFKSCLLCSLSTCFVCHIACHLFLCVLFRWTVTRLVTVHLLQLLN

YSVIDFFLKYRFTDCSY

>SG-PL_contig_3851_1_-3_87_1574

MADQGTVADGDYVVFKTDDKCKVFQLSNSRNIFFEKAKLKSKDIIGQKYGTVFEVQQGKL

MVVQSSDNGASAQPLSATSVAAAAESGKDNRDLVASGENQKLTMEEIMKMKEEGVTGQDI

VEKLVEQSETFKTKTEFSQEKYIKKKKKKHMLVFRILHPCSRVLLDTFRHAPSKICNLRA

DSLSQLLCYSNVMSGCTVAVVESCHGLILGSVMERLGGTGRVVHLYPGNDMNRHVVSQFN

FTADQTKTLHEFPLSKLASLEDGTLLPQTDTSDKTTASVRKSETTGENLTGENSNEVKRE

QDSKNTVDSKDTVPDSKDSPMLVDKDSESDDDDDDDDEKGEENGSGDGSKEHAETKTGRN

GKGCKRQRTKNTRNNAAEQEAKRHLKAQRLLQAATILKDKSLDSLIIATKYDPVAILLRL

LPYVAPSRPVVVFCQHKELLVECYTKVKENGCGVQLKLSDSWYREYQVLPSRTHPLMSMS

GTGGYLLSFIRVTSQQ

>SG-PL_contig_3852_1_-1_172_309

MLVLRIVQLSPVQSVWHLNFVAYCICCLVAALPASVNLLFRSLHCV

>SG-PL_contig_3853_1_+3_3_122

YIYEQADLKTVRFHHNYHTSKHSDLRIHEFRSKSSPPSHC

>SG-PL_contig_3854_1_-2_2_208

HRVQQSWLPVSLSVSLILSSSPARELIWMLAISCFYQRQNIMVILRNDPTPTPLCTVQLS

LCLHHGSWV

>SG-PL_contig_3855_1_+1_1_243

FFLSEFCQEQLLCCCGIFNVRQVHAAYGTSVYRLIRKTSTQTTTEGLVEEKGGGNPCPTG

NRTWVTRVLGGCFTTRPHIAL

>SG-PL_contig_3856_1_+1_250_477

MFISQAVYCFVVVIFFIVTTDLPFELWLFSMRRVCRHSAPAPFFCLWAFLSMKVGYFSEF

HQGQLSFSCSECFYMC

>SG-PL_contig_3857_1_-3_51_3152

MTPKVFQWLLSAAALVCFVTAQMDIFEESVYEYEDCRGEYQMSVGIRSGDIPNSAMTASS

SLKKREPYTARLYNPAGAWTPKVQNAYEWLQIDLGKAYIITKLQTQGRRGSDEYVSEYFL

EYSDDGTTWRRYTNRFGIAEMFLGNKDDSTVATRPLLYPIVATYIRFNPQRWNMVISMRV

DVTGCPYTAHTANFFGNDYIEYDLSQKSYQTERDLLELRFKTNSPHGILFYASGNQGDFL

LLQMNRGYLNFTIDLGSTQTERGITEVTAGSLLDDDQWHDVIIRRERQKLKVVVDRLVNE

LETKGLFYRLDLDKYIDIGGVEHFMQDGIKVKYNFNGCIQNVNFDGARLLRDAQSKDNNA

DISIYGDVGTFCGGTSATPVTFPTLESLLKLTATSSTGIQAALDFRSHDEDGLLVYHPMA

QEGTGLTVLIDQDGFIYYKIVSDTGQLIEDVVRNNDVTRASDGFADGLWHHLHIEVTDKK

VNCTVDRNVKVSNRELEVTPANTYYIGGNELFNGFRGCMRNIQVAGRYLDLNEPLGTDAN

VGVNIGTCAIRDRCTPNPCNHGGRCSQSWNTFDCDCEDTGYKGEMCHISAHQVSCEMQKQ

YSNVEGMEVAVIDPDGSGPYPPFEVTCDRDTNTGQIITYIGHDTQNLIAVNGYQEPGSYV

RKVHYAANDMLAMEDIIERADSCRQYIEYRCNNSKLLAPLTNSEDNTNTARTWGWWVGRT

YQPMYYWGGAAPGSGKCSCGLEEKGCVGSSSTCNCDAGQAIQISDRGYLVHKEYLPVLEL

HFGDTGTVSDTKIAHHKLGKLECFGDDLFDNTVTFNMPDGVLEFPTFEANNAGDIWFQFR

TTATDGVMVHCTGITDFIEVRLFQSDTIQFRYDVGNGVNVLSFRSPAPLNDDRWHTVHIE

KNRKQAWMRIDDFTEVTYNEDADLIRLLDLTTPLYIGALVDYNEGYVGCMRGLRVNGKMM

DMLGQLREGEVYGVVRGCVGKCASSPCFNGGTCREGYSRYTCDCSYTPWRGWNCGREVGV

NLKQNYMIRYEFDE

>SG-PL_contig_3858_1_+2_41_181

MVFYLTPQKTCKLQYVTKCEQIMKWYKTPNSAKLLRKLKNLTKVNTL

>SG-PL_contig_3859_1_+2_2_91

NYQVIGQLSSSLKLAILVAFDNNQVRHINN

>SG-PL_contig_3860_1_+2_2_199

LYNQTEHQSQTDVFAFQLMRRYAFYISPKRPYRRNFQRKSVQLHFKHSSNNHKIQNQILP

SYVIKY

>SG-PL_contig_3861_1_-2_2_496

DVDREYQKTVRLQAFNFTNCLPVAREIAVVNSLILLPDPLHFPGALSVSFDITFRAEVSD

PLKAVVLFKKRVGNTWIKIPCIGNIGSCTYEDICELLQNIPQCPDSFIAAGVPCKCPFKT

GEYKLSKAEFEVSAAVLPSGEYHVQANLTTGSATVGCYDIYAAFY

>SG-PL_contig_3862_1_-1_1_246

LPICTGGCGGGAKHYRGNRHADQFKLIERATHGQIDVSMHNVIPPLHTVIENRFSSIILS

GLTAVLLNNTNSIGFSFKIYTN

>SG-PL_contig_3863_1_-2_152_1108

MMNNTKMDVDRLNSCVQRYKFLSPRFSSNYSQRTDCDLSSRSHAVAQEKVHQTGSDSRSK

PSSSFFRLVSSWKASCESNHVSVPTATDRMEQAKESLKKMDASRPSSIISEVEIQNYLAK

ETGKDRLLKMFTRDHIGQSSPEFKFMYQVTLQSAAATFLVMTTLAGRQAKQEFIDRNRAT

VFNTRFQAFRRLQDAVFLFSMKEGAKWAVRVSLFTISFLGISQSIAVYRNKSSAWEYAIA

AGTTIGLLKANMGLRACIVGTGFGAVMGTMMGFVALGAMKAFNETQEQRHYWQIQKELEK

EKLLELKGKLPDSSAVETA

>SG-PL_contig_3864_1_-3_1566_1952

MGEITSPSSLSQGEITCLEINFCISSSVTADSFLFVSGAWFSDRHNAVKLSLNTSHQIDI

MQLNSVSTPLTIFNISWITHYLFLYFQQIQLICLQKIVWLLIPVTLIFQVSLNEHHSPPL

IGVLLASLV

>SG-PL_contig_3865_1_-2_74_193

MIILDLLHHMACVALVHSGVCGLVDKGLAEDARSPRFNSR

>SG-PL_contig_3866_1_+3_1032_1232

MVASEKEPCFYDSKLQDLGVKISTAPQKDTMQKNLFFFLDVSLPEVDYRGGRNFERISKA

INTGRFM

>SG-PL_contig_3867_1_+2_2_298

FCTNAPEISLQRQVVIKTRGFHFTFHNTVMINNITSVQQYISTSQYHLQIYTENVENIRS

VNGFFLHDLLIQIPTDLPFYNIQNFVKAQTHYHGKHDLT

>SG-PL_contig_3868_1_+2_344_661

MIPPNIGLPLSCRQHDSSNMGLPLTCHQHVSSNMRLPLTCQHDSSNMGLPLTCQHDSSNM

GLPLTCQHDSSNMGLPLTYCQHDSSNMGLPLICQHDSSSMGLPLTC

>SG-PL_contig_3869_1_-2_209_2140

MGPERESRGVCVAVVFAVLLAVTALASAQSEAKPRIYSLKVFSDIKFHFATTLVTSRVLN

PAETAQEVVFDVTLPNQAFISNFTLEIGGKVYPGTVKEKEAAKKQYDKAKQRGQSAGHVS

QKPRETNRFKVEINVGAQEKVTFNLTYQEVLVRRLGVYEHVIYIDPRQTVEDLSVEVAIE

ENREITYLHVPPLRKDVLTDVDLSASHSDAVVVRPSLNRAVIVYEPSAQAQSAVSASGLS

GLFTVRYDVDRELDGGDVLVVNGYFVHFFAPVVDVPVPKDVLFILDTSSSMSGEKLKQLK

EAMRSVLNEMTEGDRFAIMEFNSGASFWRSSLVPVTSQNIENGVKYVQRRSARGWTNMNK

ALSDGISFLNDQGSDLGSAMVIFLTDGVPTSGETNKDKILSHALRRNKKEMPIYGLSFGE

DADWTLVKKLTAQNNGVARRIYEDSDSALQIQGFYKEVSTMLLTKVQAKYLDDVVDPESL

TKSAFKNYFNGSELIIAGKLLDASANSISVNMEALGASGEIVLVSSSKPLDVTDMTASGL

MDLEDLAEITEKMWAYLTLQQILKQQVGEEDAEKKVELKEKAVGLSLKYGFVTPVTSMIV

TKPEEEGLGDLEEDDSDLQDDSNVPSRSSSSGRYSSSSRRRGDR

>SG-PL_contig_3870_1_-2_2_301

FLLVQLENNLYSVTATTKRHQVQRKPKSRRLCSLSILNPNKNRRNRIHRMASHQAQITPS

QTLTPHSSGSCVHHPHPYPTPTHRILPWSSGQARDSHSRC

>SG-PL_contig_3871_1_+2_236_1201

MAYGCQSSAVPAVSLLLVLVVVGGECDAGRLITTQQVLPSACLEWFTQRQQDCPIKADWD

PHSWDNRWGVLNETCCSNLNGTVLHCVRNGTSNFPACLPDQKVPAGHAGRLVRDKEGNPQ

FEVVPCNSSLAYQSEDRVSSSVTFPYCLGHRSVCHKEKGQQLLCRGGPTADDWCTCSPGY

KPNSPHCVGIFTNDGDCWCQELSCAAGYTAVAIPTPTPTRLCASLTNGSLTTHFGCLKDS

SVTTLPPTPTGSDTDRFQPSKRGNTSSSDSPGGPSIADTTEADTTLRVAHVYMVIVPQSM

FLLLLGSMVVLANCRSDRVTDW

>SG-PL_contig_3872_1_+3_297_473

MLPCAVICIRRHEDSVQRQRQNLLKLFLSACGGNTWHHDQACSLRTVPYTHNASFHWQI

>SG-PL_contig_3873_1_-3_102_650

MGTGKNYGTGSSYGYGSTPSYKPPTPTFTRSYSYDPSPKYGAGGSSSVVLDKVNKFSSMD

KYGSGTDYRYPSLDRTSTLGKDRYSSLDRYSNRSGYTSDYGGGTPYGSWDRSSSRSNRSY

GAGSREASPVSSRSSRYDYSSSASPYSTYSSYKSGSDASPVPKRPYERQSSRGYEAPDYS

SGR

>SG-PL_contig_3874_1_+1_1_186

LLLFRLFLLLLFFFLYPLSIFFFKQKGGVCFFSSHIHQQTTYRIIIAIFQTTLVLVLVYH

TF

>SG-PL_contig_3875_1_-1_274_1098

MDTNTTTAAVCLFLLTWNFFASSLAWDFLLRCEDRPCLNGGTCIIDEFFSSCRCREGFIG

SRCQTAFHPSETTSHPVTTPTPTPASFDDNDDGVFDINSTMTITGSVLGFGFLGLLCFLC

VHKMPRSDSREDLCDTPPIARRDANIARLGASPALLRDPRYDPPPPPPTGEDNSAYARDL

NNMADIPPPPMADLPPPYSSLEFPPAYSTVVKEEMTERNDEDCGQRSINRSEDCEERNVN

AVAVTTDTDSPVILSSIESAEEPSHGEHQRRDAAD

>SG-PL_contig_3876_1_-3_1023_1346

MYEVDNLGVVQLATTKSVGGHVTYVTNRAATTDTGCINTQLTCIHQQLMTPSCLLSIFDI

DSLQVEFESTPTTRVSSWAVWHRQFANHSAWGGLHEQFCQSQHMSWIV

>SG-PL_contig_3877_1_-1_1_159

VWKTETTLRDFGLVAKVEPSEIGTQIFFFLFPFILLLYFSVFAWSKILYCEQT

>SG-PL_contig_3878_1_+2_224_403

MLKNHFYCIKNGRVQYQSAFTNPRVGCSFMAYVMRNNNNPTYSRSQTVDQKYWIPHRVWL

>SG-PL_contig_3879_1_+2_563_757

MVLNKSPDATLVPADIIQAILHIWCQFILGCPPFLSCRCPAKGLVCRTLINTFTEKGLVY

RSLIN

>SG-PL_contig_3880_1_-2_2318_2575

MCTFTNSSLRGLAILTWPVSYQSNLYDIVNRIVAHVGIMVKFLNDRVLIVKLIVAGILSP

SVGGVKERLNKMSDSLDGCTSRKMKH

>SG-PL_contig_3881_1_-3_51_233

MFSTNSDMAFTSYTSHCVYYVLFAVSRISALGKEVLVEKGSVLYRYTVRMCDRQKERVWW

A

>SG-PL_contig_3882_1_-1_58_756

MSGSFKKLAKANQKVHRERSQPSHRAHLGPLEKKKDYVRRAREHHKKRDKIKELRRKAAE

KNPDEFYFNMVSSEKVDGMHQAKINPETTDAEQELLMRGQDLRYVIMKITTEKKKIERLK

GEVTLLDQQFRENTHTIFVDNEKDAAKAKRRAESQKVELSKKLSLTPMQQKELEKNYNLL

AKRCERLKELEIVRDKLQVSNHLTKDKTSKKKLIKEEMKKSAAVYRWCFKRQR

>SG-PL_contig_3883_1_+1_85_246

MPAISLTYIIIIHAHIINIHSYIIISNSNIMNTHSYIIIQSNMNIHTTSLFIQT

>SG-PL_contig_3884_1_+3_1140_1787

MYTLSLSCMLTIMIFHHTLSKTHSHKMYMSLLPPSLHCRCSLSFSLTVRGRLCLVLYLVV

IERRFDGILCQHGAMEFYRRQGQFLCNVSILDVQCFIHCLSLHPLCGQRAGSYGRTTAKC

LELGVHNLSTVVHLDLQLHDITAGWRSHQACSHFLVILVHGPHIPWIFIMIQYIFVIQSR

WGNYAGIHGRGNSFSYRSHTSKQRHVYVLSGCSMQA

>SG-PL_contig_3885_1_-1_1_132

CAVLFWRLGSEIVNTGMGTTWPFCRVICLFGLFYVFFVASKYFL

>SG-PL_contig_3886_1_-3_3_374

THNNNNNSNNTNTGTDTHTTTTTTATTQLQVQIHTTTTTTATTQIQVQIHTQQQQQQQQQ

HKYRYRYTHTTTTTTTATTTTNKTNNNKHPPPPKKKKKNPKKQQQTTKKKKNFTISPYCL

MKSI

>SG-PL_contig_3887_1_-1_1_321

VCVCVCHPRCNYICPLNDFLLQTGYINSQLLLHALLLLTVVAVVTTATVAAVIVVVVIVI

MFVLTCVCGCMRERERERQIDRQIDRQTETERKTCQPRFPCCLWFGR

>SG-PL_contig_3888_1_+1_142_243

MSKQTQEAFNGCSLNPFASSHYIHHLPAVFSLEI

>SG-PL_contig_3889_1_+3_3_155

RLRDLASLIRPATMRGDRKTAPSPSVSVKHTADVTANKAVRVIQLAMSLMI

>SG-PL_contig_3890_1_+3_3_155

RLRDLASLIRPAAMRGDRKTASSPSVSAKHTADVTANKAIRVIQLAMSMMI

>SG-PL_contig_3891_1_-1_19_363

MATQKLTTGLTGLAVARHPHQTLKVLYEKILRTLQKFPEEAAYRRYTEQIVTDRLGLVKA

ESDAQKLEKKLNAGHIEEVILQAERELLLSRKMLQWRAWEPLIAEAPKNQWKWPL

>SG-PL_contig_3892_1_+2_2_172

NKPESRHWHKKQPNFLVPKWVVSSEMLASLKSLFCQSDLSCLSKRLCGSTLKILCIR

>SG-PL_contig_3893_1_+3_528_842

MCLKNARKHAFFSRTTSARSQQNNPFQSQAMNHETAPNPNVGHTHRLTLKAYTLVIDITR

DWVDGFLSKPSLNFSSSMSVNTETPCLAYQGKETENPSQTCMQCE

>SG-PL_contig_3894_1_-3_3_251

FQYNYRFSTYFLSELVTLYIQPCSLHCSSTPIFAKNATNRNVMTTILLLCPTALELSSLA

HQTLLFSCYPQVQPQNLFQQYYQ

>SG-PL_contig_3895_1_-1_16_393

MFYVQHSHIPHVLFVLFCFWVVGVSGFVVILLLFCFVFVCCCCCCCCFVCVFVFFFFFVF

FWGGGCCLFWVFLFLFKEFSVALEGRGEILVVKKMSCSGFRGGGGIVVKKIFAVALKGGV

GEVGTS

>SG-PL_contig_3896_1_-1_583_834

MIAGFPPLITSLNLELKANLTNELTKCYLLSVFPFLKDKLVNFFKTNRQCSHVGYNLVDG

IILLTVRLSSAVFLFSGCSHAFSV

>SG-PL_contig_3897_1_-2_47_232

MGVTLIVSGGTLTISGVTLTISGAPVVFFAFDDVCLLCSQIVDAWSSNLLVKFPHTANKK

KK

>SG-PL_contig_3898_1_-2_59_916

MTGIYQQAGAGRETVDMRRGQPGPPSPCSSVVLDYAHHRHLADVDEGNIEARLRQQQQQS

AADAKWLQAEEKSLKRDKWLAQQTEESDNTKENSPPFHHPSLPQHTPQVPPSVMQQIQQN

QAMAVRRTSTDSRSSSASDSTEGGMGPLQQQQSQPNRINDRVYESTTSVVRAVMTMTRDA

PSVEPEGYLTMVKTIGQELRSLLASVDEIVSQLPQDSMKPIEMAQKVLSNDMGSLVSSMK

LAQQYSTTPLDGEYRKRMLKAAHALAMDSKNLLDSVDTARKASEMS

>SG-PL_contig_3899_1_-3_534_761

MSDLDLSVPLPMTESFVSRNSKETSMVEELLLTLLLKSGTHCLLLFDTVRHCLLSKLISK

RFFSSSPFISAHLVNP

>SG-PL_contig_3900_1_-3_201_416

MGIFVSYSFHSIKWVCIGISLIHVVVVGSLFVVCWRFSLLLRHFCCFMVVSIFHQQIIRN

HCPVICRIMASA

>SG-PL_contig_3901_1_+1_115_444

MTKLVDHNGTLTDEPQKIKQEVNAFYEKLYTKKTLETCEIIDLITNCPKLSNEERDSLEG

EINLEEVGFALKKHKKKPTRKMERILEQMVWGQNFIKSFGKSLVLLLYEP

>SG-PL_contig_3902_1_+3_138_227

MTMIELYCTCVPCVCVRALMYVLSAEISQL

>SG-PL_contig_3903_1_-3_231_1100

MLILEVTDQWYGEVKMHVFGTEPATLRTGHFTQVVWKGSKEVGVGKAMTRDGKVIVVANY

RPAGNMMRTFAANVLPPKDGKIVLPAEPAPERTTITTHFGGSSGGGGSGGGTFSQRFEDM

RKDMFSGSQSSRMTTTGSRSESRSVRTYTVTEGFGPNKVTKTITEETIIRNGEKTVNRKE

TITSGGTAPSSQSSHLQSDFGRLALKDHQGGSAETKTKQKKSKPFWKKRSSSSSSSDSSP

ERGGSGSKSKKTTAAAGKPQKMSDFRKDCLKAHNAKRALHGSGPLSLAAD

>SG-PL_contig_3904_1_+2_2_154

HNDPKITHFGHHNDPKITHFGHHNDPKITHFGHHNDPKITHLGHPSIPPVT

>SG-PL_contig_3905_1_-2_203_535

MAARKVVAEVPAWKRALQRWAYYKSYFPQLGLMRDDCLNETADVKEAIRRLPTAQYDSRQ

FRISRAMYLSNRKEILPKEEWTKMEEDVRYLTPLVDEVVKERLEKLEWQKK

>SG-PL_contig_3906_1_-2_425_604

MVRTKISLNICIDFLKIRSCVTVQKGLMTVSRHRTHFQAHQISAFQQWFQKKRLMWTGFY

>SG-PL_contig_3907_1_-2_2_190

IFAPERGGTDNRFFFCCQDHWFKLDHLYKQNSWKFKKSCNCNTGSHCQLIPVLSACLMQL

SVL

>SG-PL_contig_3908_1_+1_1_384

SFRDGELSATQQEGLITCIPKGDKPREYIKNWRPISLLNVLYKIGAACLANRIKTILPKL

ICEDQTGFVANRYLGDNIRLIYDLISYLDIKELPGLLLCLDFEKAFDSLDWQFLFKVLKA

YGFGENVC

>SG-PL_contig_3909_1_-2_455_652

MSLMMKAYKYTIWCTTGLCSRSRFVYTISQPLSVVLLYMTVIITSMPMTQSYQKILRLVI

FLLSRV

>SG-PL_contig_3910_1_+3_108_218

MLYLYLWCKIFISDISVSLICYAFISDAISSLSLMLY

>SG-PL_contig_3911_1_+1_925_1383

MLPHKTHNLFTLCQQPLTLSPLYKEFSNSLLANCYNAACFIQYHTYEQMLNYSFLPSFLS

LIQIAVMSQHILCYYLSVYLFVCSFIISANPWLTRGNMRLTHHSSPIQPKHLLWQKPQTN

KKKDNGQKSKCKLSMANAWVLLHFNLCIIVFLF

>SG-PL_contig_3912_1_+2_1250_1555

MTPTQYSLQAHPSISTAVRKSLSFDRKGNCTTTTMRQWGNRDTVNLIWTNSTKKKTINVA

HLPAKQHAQLLAKNDNTFQRSLKSFSIPVSTALNHLLPSHHL

>SG-PL_contig_3913_1_-1_310_600

MYSSVLMYQIKVSLCTIQVFLLSLSLSLSFSLSLYFLSFGFSVSHVKTCSVFCVPNTGTV

TDSGLLICFFVLFLVRLIPRLFCRPCAYSFHLILLLF

>SG-PL_contig_3914_1_-3_3_113

IDDVKCELQDCYPGVSIKLTVDQASVIPSLYDDYMPI

>SG-PL_contig_3915_1_-2_167_505

MVRPYGRPPFDQAQQEAPDLRLLRPGPGGDGSGRPLPRLDPSDQPHISPLPDPPKGHQEG

EGRKSTHDPHSALLAGSTVVSGPKVPISRSAPGADPEPGPSQATSLRDQACES

>SG-PL_contig_3916_1_+3_3_116

LFKTQFEIIYQPCPTKRSANYYHSLCFLVKKDPYFSHR

>SG-PL_contig_3917_1_-3_39_740

MVEISTLPYPVCFDVDGQTGEAFRLLRDPEIGLTVNIEMGQGQRKRDHPLKHGSVYRVYI

FRVAVILQDIVMEVTRERIHVHGKTFHWNVTHSVRFPGVWIMMTESGVLRFVLDSGVEVD

VKKFIHPASNLLGTDFLDVEIENEDRLSSATDGLLGQFVHKKITMTKLVNKGNKMVAHLR

SRSGKKRRHGSAYLKERKDPFFNRTTPCLNLRSRGTRLLDHPTKAYKVRNLFYV

>SG-PL_contig_3918_1_+3_900_1247

MKLHSLLIILFTHTASLVALFKSSLSHTFTFKTFLTLFIHPLLINSIDYIFTLTFSSSDV

STSNHTDAQFKLYLSRSPCMLYYIHLQSFIHKLSWLEATGRCHPPAAKRTECMSET

>SG-PL_contig_3919_1_-2_686_796

MCYLFMTFCDKSTVLFWFAKQIISWKRHFDIKVHCSI

>SG-PL_contig_3920_1_-3_54_524

MSQLGYDLLRASTPKTSKYYQPRSDCTAPISQLHRIATVGIERFILMMNNTLLLTKHHRL

ANIYEAVDYLLGNHDDVDCNGDRGDFYRRKLAEQIYLNFGVHYITHTNVLNLVRDTIQLE

ADTRHALSVCGSKRESAPPIHVDDQVLDVLARIADMH

>SG-PL_contig_3921_1_+1_247_429

MCSEKIKCSKTSGKWTMFCHESHFTFKDLNATVDSLHYTAAIQATEKVHHSHMKYFIYRP

H

>SG-PL_contig_3922_1_+1_319_636

MKLEKTWVAIQSEEDGNTLDQGVARRGRELGANAGFGATIQRLLHTIDTARFDNSIFLHN

HCLLPVLHHFFGNHFLAVDVFGGGRGGAEVASPCRSPKTGARKLGH

>SG-PL_contig_3923_1_-3_72_353

MVKISLVSTNPVRSWIFIFCYYFARDSFSINSIPTFWKSCARNCLFSVTLFVSALFPFFT

VVCFFPAFLVHSPFFFPANLHPFFPPPPLLLCNL

>SG-PL_contig_3924_1_+3_66_485

MKQKVSLAGEKCLAVSCSECLLICYCFRHKSEPCLPPFSLPSSDIQHHCINFISFINVNL

YTCTRLTQLWVVALLIKPCPHIPAVSAARNETDSLESTTQYVQSHSLGLRHTLCQRHKTM

MHLLSVSKSVHRCMWDPNLK

>SG-PL_contig_3925_1_+1_1_117

FFFNICIITLFWWPSHCVQGKSKHKEVNMDIKYLINMNP

>SG-PL_contig_3926_1_-2_113_274

MLLSHTNTTLKCYRCFVYFSSIAFVSLMFEARNCSLQVLCSIRYKTCKNDTSNL

>SG-PL_contig_3927_1_+3_933_1220

MEETLNIFKLADLADPVTFFKCQIMSIVAYFQIVKYRHETSPLTITEYNRKTKLYHFPKR

LTKEKLNNQLECTCLHYKIPLYSRWVEGKKDTQVTG

>SG-PL_contig_3928_1_+2_2_160

NSDKHSQPRRIPNMSQSSDTTISSLGSVPTNYSLICRKQPPSSSINLIQPPHP

>SG-PL_contig_3929_1_+1_82_483

MIFSLRQLQEKSREQGQPLYMMFIDLTKAFDLVSRKGLFELLRKIGCPPKLCSMVISFHA

DMKGTVLYNRFTSDSLPINSDVKQVCVLALTLFVIFFSLPLSYAIYLHTRCNGKLSDLAG

LCSKTRPAYPLNST

>SG-PL_contig_3930_1_+2_41_232

MVISFHADMKGTVLYNGYSSDSFPINSGIKQDCVLALTLFGIFFSLLLSYAFDSSTDGVY

LHTR

>SG-PL_contig_3931_1_-1_274_894

MKDEVDSALSFLVGLLKKAKVPSHILKRFRECLFNQMVNKYNDHWHPLKPERGSAYRSIG

IDHNSVDPLIIGALSDAFRNHDDRVRIINALPKELTLWVDPRLVAYKFGDFGSVGILYEK

RKQKYEGSSRTEETSDYESGTSSANSSPCSSPSQNPPSRCPPVQSLHEYDYRQQPAQPAH

SISYPCGQEAFCSSGFQNHYKMQATFA

>SG-PL_contig_3932_1_-3_1308_1601

MFATGTTSTRCVSCFDVFAKNNCIKCKLFYLVHVHPLCLMLWFMPFSSHLISYMCHHTKA

ELFVTSCAVLCVKSDTTFSVTVHEIMRCFSVKVLQGTA

>SG-PL_contig_3933_1_-3_96_1694

MVSLNPVQILKYGAEEEKAETARMSSFVGAIAIGDLVKSTLGPKGMDKILQSSSNNETIQ

VTNDGATILKAIGVDNPAAKVLVDISKVQDDEVGDGTTSVVVLACELLKEAENLVNQKLH

PQTVIAGWRKAVDEARRALVSSAIDNGKDAKKFREDLMNIARTTLSSKILTQHKDYFANL

AVDAVLRLKGSGNLDAIQIIKKLGGYLRESYLDEGFLLDKKVGVNQPKRVENCKILIANT

PMDTDKVKVFGSRVRVDAVSKVAELELAEKEKMKDKVEKIIRHGCNVFINRQLIYNYPEQ

LFADSGVMAIEHADFEGVERLALVTGGEIASTFDCPEKAKLGHCDLIEEVMIGEDKLIKF

SGVALGEACTIVLRGATQQILDEAERSLHDALCVLAQTVKETRTVYGGGCSEMLMAEAVS

QLAAKTPGKEAIAMEAFARALRMLPTIIADNAGYDSSDLVSRMRAAHTEGQKTVGLDMER

GDTGKMDELGITESFQVKRQVLLSGAEAAEMIIRVDNIIRSAPRQRPRDDRCH

>SG-PL_contig_3934_1_-2_893_1171

MSPIFSSLMSVTFYLWCQRVSFYVTIVSPLMNFPPSGSNQSSKGVPPIPGENFPLIFASE

EPASSTFNRLALVFLVNVHSVQVSRRTTVKIFT

>SG-PL_contig_3935_1_+2_2_373

QQRLYFLRRLKKFGVKKDILIQFYRAVIESVITFAIVVWYGNISKAETTALNRIVKTASK

IIGSELPSLDEIYRKQCPSAKTSLIQPQVFLNYFPQVADTEVSEPELTGSLTVSFLKQSM

PCLT

>SG-PL_contig_3936_1_+1_1_300

QSFATLSPLPLQIYQDFDESSCKIFITSKIFHVKCKLLSSGKTCCYSTAPSFFPCLHVYM

FTFKISLRPKMLSGTTPLLPWVLLHALVHAADRTLVTVSI

>SG-PL_contig_3937_1_-2_179_514

MGFFGPDQLFPRVEHTMGSLGKLGPHSQGSSTLRLNMSLKVLLKLPDQHHKCLYLSGLVY

ARIYYERKHSVQPCHRVLVQKGIPQQHYHHPSSHHSIVTNIFPDSVALHALL

>SG-PL_contig_3938_1_+2_140_373

MRACMRACVHACVRACVRACMRVCVCVPVLSPHLVGDPLKLEYGCLHGEVLKNSHTRNLL

IQMYSWSHSRKTECIYIG

>SG-PL_contig_3939_1_+1_886_1245

MNTIHPAILAHACDVLPCTSLRSLFSPVLNRDTRLYHCHSTVVHNTTLSTTFCFPRCIRI

FHSSLCQHKSSFFQEVKQTNQNKQTNKQNKKRTYGVRERMGVFSVYNPQIYNISKTLLKQ

>SG-PL_contig_3940_1_-2_68_1582

MPSVTDGRGYQSIHDAVRNSDVLHLEAMVKNGASINEVEERDKFTPVHTACNIGALECLH

WLLWHGADATVTTPRGWTPAHIAAIRGQEGCLQALSNNGISMSTKDSRGSTPLHLACAHG

HSFCVQSLLRCGVDVNGLDKNNWTAVHVSSYHGRLGCLQMLIKWGARYDDIDSNGNTPAH

LAAMEGHLPCLKFVVSSEGCPEHTLGARNDHGETPRDLAQQFYKENILEYIDHVQHEMDH

PEEAENLAFPAHMAAFNGELDHLRILVENGVVNINERDDKGNTLAHRASGQGHLHILQWL

VEMGADVEITNTAGETPRDMARRFAHLACVKLLGGDPEEETSVMVGSESEESDEDTPITG

IRRRRRKKGDSSDAKSRAKVRVEELERLMEIAKKNFVQLGGTLSEDRRRIREMRDKDHTI

SELEAQLDYERLRREKLEAQLDQYRREITYLNAQMENMQMQTLSDEEPLTRTKKKKPKKK

SHDDGGVFIRRNVTVPKKGSRFGIV

>SG-PL_contig_3941_1_+2_2_271

FFFVVSCRLASSLSTLYFTLVYTPQDMLHAHSHCCVQGHTLSRPFMSACNSIVFTSAHFL

KVVIQSSMYTPHSEHNLSSRLFPQTMTFWI

>SG-PL_contig_3942_1_-1_166_324

MWQYAFFSHDSYFSTVVFCSLFALGGTGMDHCLHELKYVEGWLFLIHVFSFMV

>SG-PL_contig_3943_1_-2_77_1033

MLGRKLGFSGVFACTSSHQHVEKEKVRERSSLEKDYAVGEILGSGGFGTVYSGYRRRDNL

PVAIKHILKEKVTEWGQVNGHHVPLEVCLLRKTQSVHGVIKLLDTYERPDSFILVMERPD

PVKDLFDFITEKGALREDAAREFFRQIIQIVQAITRCGVLHRDIKDENILVDLKTGQLKL

IDFGSGAILKDSVYTEFDGTRVYSPPEWIRYQRYHGMQATVWSLGILLYDLVCGDIPFEH

DHQIVEAHLSFKRRVSPQVEDLISKCLSVRPTDRPNLEEIMKHPWMQAGIPIPIPGSKRQ

DDLANSNSDAASLSSQESI

>SG-PL_contig_3944_1_+3_891_1073

MKSQREREREREREREREREPLLNQNKNHIRFILNIFHVLIKESIILRHKSTIIISVHSN

K

>SG-PL_contig_3945_1_-3_480_2834

MRAVDRIPYQFGSTNTADALLIMRDDMFTPVNGDRANVPNVAIVITDGVSNVNSERTIPE

ARASRLDGIHIYTVGIGLSDTRELDGIASPPARDNSFVVKDFDDLSFLHQRIFASICPED

FTMPPTTPRPTRPPTTTTTTPRPTTPRRRPVYRETGTDLVFMLDGTVNNQIFGWMKTFVK

RFAAQLDIDGGEYRVAALIFTSRPTVQFHLNRYNFQDEVISAVDRFQGIRRGRVDTARAF

EYVRQNIFTARNGDRRRARNYIVLLTGNERSINTQRAFDSAKQLKDLGTDIYTVGINLRT

TEEIDGVSSKPLERYQTLINSEREMDELPGIYLRNMERAPPPFTRRTTTTTTTTTPRPRR

PTTPYTGVCESSGDVTFILDSSGSVGEENFDRLRNFTMSAVRDLEVDNGFFRISVVTFSD

SANVEFYLNTYSYKREVMDAIRRIPYVYGYTNTADALRRTRTQVYTSINGDRANVPNLVV

IVTDGESNINPLQTLPEANRIKRSGTSIITVAVGVNSNEELRGLTSAPVRENLIHVDDFH

ALDRLSHQIVAPLCTDSNLCEEDPCRNEGVCLDGLRSYVCLCQPGFYGDNCERQCGPRTD

VVFILDSSTSVGTQNFQRIKSYAELLMRDMNPDTCDVNIGAMKYSSAAMIQFNVGRYDTT

EDLSTAINAIGYTQGRANMADALRVLRLEMFNGRNGDRADAKNVAYLLTDGGVEINKDIT

LTEADLIIDAGIRLIPLAVGLRQRNEIESIARAQGLPLMVVDSEIRLQEMSDQVLEAVFD

NDNHC

>SG-PL_contig_3946_1_-1_436_654

MYIVYLHCKCCSLFILCSVLYLWGCHIVLRLKFAFKQIAVNLVLMSVIMSLSHQSTSSYF

RLSVHLNTSACNM

>SG-PL_contig_3947_1_+2_2_319

YTVCSLLCVCVCVCVCVCVSETKRERERDKQDRQTDRQIVKDRQTERDRQTKTETQREIN

RDRQAKTDRQKNRDRETETDRETETDRETVKDRQTETGGQTDRETE

>SG-PL_contig_3948_1_-2_2_226

HWFYRFDVFFLGNVFWASLRQFVVNADGKSSQRSGGMVVLTEVQCDCTGYVHFHWSYFNQ

SAVYTLTVLVTQRIV

>SG-PL_contig_3949_1_+2_683_874

MWPNNNILVRFIHSIRACMTTFRQQCLRTYFENFFQLCTYIYIIYPYIHATNIPSTFLRK

YKSK

>SG-PL_contig_3950_1_+1_1_288

CSQGFLVKAFQTAGDAHNGTTYGAHTTRESFAFNLTNTDCLCTIFAGETGSEACCTVVCE

SRQSTLVVCLSYGLSDRGPLTFTLTSCLAGLEVVCV

>SG-PL_contig_3951_1_-1_514_729

MHINRHGLNYWIVGNNFKMKNNESGLCLIPTYFWLLCRKKKNQYPSLHPTPVCIMFIYAV

CVCVCVCVCVCV

>SG-PL_contig_3953_1_-1_1_246

TILEKYNVKSKVLSHQGQGDAALKRSVPADCVCEVGGQVPALHHSLSWSVHQCCHPSVLL

SEERRSAPRHSPVMSWLWASFS

>SG-PL_contig_3954_1_-2_1397_1597

MYKDQKCDYIVHYLHVYLSPSVHRKPQQHIIAIYIYSFIFIYYIFILYQVIRKQSQLNVD

KVILRVT

>SG-PL_contig_3955_1_-1_61_666

MSVEDFKANFDELELCHLSLDALTAERERASGGRKAWKRTEFTGRWVKGVSAGGPLKSFL

SRSFWTNPQYRIKLSEANKEKFSVVISLLEVGDKLHKKSEDISIGFVIFKLKPGKEPAGK

LNADNFYEHSPQLVDTSGVFWPYRERAVHFVLESGSYIIVPCTFRSDKEAEFFLRIYSEK

AAVPEPTEEPVGPTEEEEPDLV

>SG-PL_contig_3956_1_+3_1947_2444

MKSSFVLTVKESSHSILTHLILTIIVYHKLMTFSYLSLTLSYCFPTNLFYSGILTSPKYL

YSTKYTAVYEYCCQHSCSCICAFGKASPIQIQCSQHEFSGNRKGLPNFPHTKRLTCLLLP

KNITIIKTNRKCTKIIHFSHVKSHSKINQQCHAHIHTVACFLSIHA

>SG-PL_contig_3957_1_-3_3_89

SFVNTVFKLFYFFQTSHLERHKTFKVSFQ

>SG-PL_contig_3958_1_+1_1105_1398

MVPFTNPVKNWSHNTNTYLHSHFHIAIKTCLFIPGKGDTFSNNKKIADNKPIFMWPNFIQ

TKNGVDFLNISTYYVLIKEFSLYICTHKLQLNFTLNVT

>SG-PL_contig_3959_1_+1_2983_3288

MVQPPWSISGAMPQPYTKEHRVWKWAMMIKACITSDKDHVSFPRVSFSMCSFLISQGLWA

RMLLISHKLRSLAGALSRPFNHAGSPPCSKKSCACSRTRYRH

>SG-PL_contig_3960_1_-2_173_358

MLVLLCYRTKPGYQQCPMQPSTGAGHPDLILVAELPQAHSLYSPLKDQQMYVTIYIIVIF

TG

>SG-PL_contig_3961_1_+1_1_165

SLSFPSSVSTSVSDSPSLPLSNCSTSLKIASNSSSVYSSHSSSSCFAAIFSSRKS

>SG-PL_contig_3962_1_-1_88_384

MEMAGKDSLSLNVEFIRVNISRTRKMEIFPTGDGSSSKLEPQIKSTVVRFSAICDIGKAS

FKYDMRRLSEILSLPKAWYRRNLARRLFLGDDSMMRAGG

>SG-PL_contig_3963_1_-1_1_231

LLLFIKKCKFLISYHSVCLSLPQHTMKACACRFPVHSVLNKYLTLFGVGECKAAREVGTT

FINLVRQEQGLFHLTPQ

>SG-PL_contig_3964_1_-1_88_1650

MASSWLLLMLTVVIITMTTVKSACYPDIGPAGSYDCFASHYYDKGQWGTCVNKNYLDRKS

KGRYSCRNSLATFCYFQCMLEVHGLDSGNVTSDCACDPHNPVTPAPSEVTTLPPRCFSPD

GTDCSWYRDCLEVKYQCASGEDDYGLKYGETFCELYSRHNHNNKLSSQAWEWVNNVRKCL

LVELVPLLRKFESNTSCADIKTLAFRSHLGCYVHPFKNTSSICDLSLKDYWKVFWTIRKR

FHEDFVESVLRLLEVRRDCRARYVRQVDAEMRSLLVRVNIPTHKLTKRSASGGPSSFEPG

YEAIEQNREQLARKLTEEELPRELSARRVPDSVPRELSAGIVADFEPDLDKERPESSYGR

FRRQALSSEEEWDTNSQALLYHQKAADVCEDFARQLHWAEKNVLWFTYGVYMGQGQLAVR

ILLADKNITITNTDGSSANLTAVLEETMEAFEQGKIMVEVDGKKISPGVLSSCEDLDCQQ

LFWNVTAPPLSTSRSSSLLTTWPVGWLIPFLVVMALLREEF

>SG-PL_contig_3965_1_-3_108_434

MQLQLLVKMYIFIPVASSPAFQHTSLFHTPTEAAEHSFSSSTVCVVLYMCTFFGLQISMS

YYLRKPATCHYTCFKPFFYILSLSLFLCLPVPTCPVLWLAFFFHSLSDY

>SG-PL_contig_3966_1_+1_202_597

MKCVRFTSTNGLYLYVPHNHPQKSVNIPYSPQPFPKLIHRYHLTYLVHHLYTNTSFQKCT

NLLPVYHYLFTHHLHSLPVFHPHPLSLSSPPLPSPHLHASFICDSGKAPKTSIQRCRSLH

SIVIFEVVPMFV

>SG-PL_contig_3967_1_-3_771_968

MSLLPTFKGSGYAFVCFVYLFVYKLNEFCCGHMWIHLLEEYVKFHSDVIKNKETEVMKKI

MGDFVT

>SG-PL_contig_3968_1_-2_2_211

VLFRSGVGGYLEQKTWVSQRNVHIKCFMLTRMSRTKRQVSGYMLQLKSCRKSSPTILSTV

VRSNIYSFLH

>SG-PL_contig_3969_1_-1_166_393

MLPISKPIEVRVSPSFRSTSWDLCQEAYSAPGVDSGLSHFGLRLHEVSACLMTSILHLQH

YILMNIPGWGPPNCGY

>SG-PL_contig_3970_1_+1_133_510

MKTDLQFFKNVQKICTIYFVHHSQFDIKERNGSSHMFTLANIALYFLKHYNIRISSLPSA

TTPNKGNPTRNDEYRLKTFSSSNVHPMRKQTVFEYNSIEMGVILNNCWQKHVIIFQAQMK

ETLNES

>SG-PL_contig_3971_1_-1_1126_1335

MSKPVAFCVRYTFDTLCFQMWWVQYPLLIFDISHTSVSCCTGKLRLYLFIEVLLPFLYVM

LLFCLARISV

>SG-PL_contig_3972_1_-3_294_437

MFTMSCVFVLLCFCRNLSSSYLFSFSIIYTRDLNAYFHAFNSDELLYV

>SG-PL_contig_3973_1_-1_196_5517

MKPSQVATLATILGVAISVFVDTVSSAECEGCTSTICICAGQKGERGARGLPGLRGPQGL

PGFLGPEGRAGNFGLKGRSGEPGARGPKGYRGDTGLPGFTGAPGIPGQPGLEGGLGPQGY

PGCNGTKGLAGFPGQSGGPGLPGRPGRDGIPGPKGEPGSYVTDGIAIGEKGSQGSRGLPG

IDGLSGNPGFQGERGMRGEDGLPGRDGVSGLKGEKGNKGSDGIGILGQKGQKGMKGEPGV

AGREGTPPSNSTRVYGEKGQKGDRGEKGMEGERGFPGPGGPMGANGLSGMKGEKGLIGFP

GRRGKRGKDGPPGPFGQKGMFGEKGFPGLPGVDGIKGMGGEPGYVGLRGPQGPPGVDGEY

ILTGESGTQGPRGRDGPPGPQGPRGPDGIDGEIGREGLPGLPGQPGLPGFSGEYGIPGDS

MKGDRGLAGLPGEDGNDGLAGFPGLPGDLGEKGQPGETGVGAQGERGLPGLDGLPGLPGQ

PGDPGFDGRKGEPGFGVRFPGPRGSSGLPGEIGEPGFPGTPGLPGLKGLMGFDGDDCGIC

PNGEPGRPGEPGDPGFDGGPGFPGPVGFAGLKGEPGEQGFPGEQGREGQPGIPGRPGPDG

LPGSKGYKGEQLPPPDFELYRGTKGAKGNIGYPGMPGMDGPQGDTGFRGLPGLYGPQGIK

GNPGQPGDDGLDGLPGLPGEPGRKGQPGQSLVGSQGRKGQSGLPGLEGIPGPEGDKGLQG

DPFFVELTQGDPGEPGRPGFDGLPGRDGIPGEPGRRGDDGFSGLSGMKGLPGLPGLPGEQ

GRRGPKGYPGVMGDPGFDGREGRKGELGPDGLPGLPGRPGLPGQKGESPRSAPRGDPGEP

GGPGEYGFPGNDGRPGEAGRSGPDGFPGEKGEPGRSGGPGFPGQPGLPGDDGLPGQPGFR

GRDGGPGGRGLPGLPGVRGNDGLPGIPGEAGRLGDDGNPGLPGSPGQQGRDGLPGSRGLD

GLPGVPGVKGERGIGGRPGSPGPPGVQGLPGRPGSQGDDGGPGEAGVPGIPGENGAPGLD

GIDGLPGLKGRQGEPGRGGPDGFPGQRGEDGQPGLNGLPGQDGLPGLPGQKGNRGPSFNG

EPGRPGRDGPNGLPGLDGLPGTKGQPGPVGTPGRSGLPGSQGEFGLPGEDGRMGLPGVKG

MRGNNGLPGIPGERGGPGVQGPPGLSGRQGERGNPGSDGLPGLDGRPGEDGEPGFPGRSA

LPGLDGARGDDGTRGMNGFPGEPGRRGEDGNPGLPGRRGEQGEPGFDGRPGFDGEYGRTG

EPGPVGRPGENGPPAPPGRPGPPGEEGIPGLPGIPGQRGFMGDDGLPGLPGRKGERGEGI

RTSPGPRGYPGEPGVPGLSGRRGAKGMDGLPGLPGLPGMKGESGRTGGMGLPGRPGEDGF

RGENGEPGVPGFTGLPGVNGPEGQPGFPGIKGYKGDAGFPGFGGGIGFKGRRGEQGPPGQ

FPPWTTIVGPKGEPGFDGLPGYDGRPGQRGEPGEDGLFGPAGLYGRRGDDGVPGRSGDSG

LPGNPGRRGQSGLPGLPGEPGRRGDAGFPGGVGSGPGIFFTRHSQTSAPPECPIGTVKMW

EGYSLLYVMGNGRASGQDLGEAGSCLARFSTMPFMFCNINNVCNIASRSDYSYWLSTPEP

MTPMMQPVEGRQIQPYISRCSVCETPAPVMAVHSQTMTIPDCPVGWSGVWIGYSFVVGTG

SGSQGSGQGLHSPGSCLEDFRSSPFIECHGRGTCNYYATSSSFWLSTIERNDQFRMPLQE

TLKAGDLRRRVSRCQVCIKDYSTGPAFFTYANVK

>SG-PL_contig_3974_1_-2_2_157

PGFEFSSGTSPVGKRVKTFPISQVNLCADLLVPESPRVFSHAEHQICTFKIL

>SG-PL_contig_3975_1_-1_43_345

MFLLQCFCYELWQLYTSSAVFLLRTLAVVHVFCSFCYELCQLYMSSAVFLLRTLPVLHTF

CSVSAANSGSCTHLLYRFCMFSLEVLYLLKTDQTPGITRLC

>SG-PL_contig_3976_1_-3_1089_1331

MRGWGVWGCTSLGVVCSFFNPFHPLTLSTSFLQEGGGSERIKATLSTGMCVFDVCRSHNF

DFVATSATSSGSWTRLLLCFC

>SG-PL_contig_3977_1_+1_1_171

QSFFFIEVIQVVLITAEYNSHIRKNEKLHEAELNMIWNRRKRKGVTDRDGERKKGGP

>SG-PL_contig_3978_1_+1_367_660

MICTQWVVGNFSSTFKSLCFKVLLQRHFSFHVKCGQFSPGRAYHHSSMPHVCFCYCLPLY

SCLGFSTEFSQGYQFGCSGLFYVRYMHAAHGTLVYHLI

>SG-PL_contig_3979_1_+3_1437_1742

MTQKLNMWTRPRIHFHITLSPFDSVQSNAVYLKMYPTVIGGNMLKKNKCNLGFSFPSHSC

LSIHDSTAIYFCSSSQSLDDGFDHQKMVIMASTTIYSPNELY

>SG-PL_contig_3980_1_-3_195_452

MVYHPNIDLEGNVCLNILREDWKPVLTVNSIVYGLQYLFLEPNPEDPLNKEAAEVLQTNR

RLFEQNVAKSMRGGHIGSMYFERCLK

>SG-PL_contig_3981_1_-2_2_175

WLAGWRAGWLVGWLAGWLVGWLLHSCVCLVGTLVGWLVSELASYFACLLFFLDCIKKT

>SG-PL_contig_3982_1_+1_115_585

MKNNQLCSSVHKVPGAILVLHFPSTELCFSYSLIPYCEVAHQPNRQDYEKKAQPCGFWCY

PEVIKVCGSHCHEALHCWVKNVTAIMCTTMRICHFLLPFNLQRVSHFCPFSYGTHFVLRN

IVHFPWIPFWFRRARHVYDLYALEQKTFERKFNRRSC

>SG-PL_contig_3983_1_+2_128_322

MMGRTALNVAVLTPFLSNACLDTITHPSTFFVVWHLNFLAGIVPKCTLTVQLSYLLYWNL

KETNK

>SG-PL_contig_3984_1_-3_1254_1412

MASNLPALTFLYFREGKAARERRWAPPSSCWPQEKLRSLPPHSPTTSGLRDSL

>SG-PL_contig_3985_1_+2_116_1606

MYNGIGLTTARGSGTNGFVQRNLSSVKRTKDRVNYKTEEDIARLEKQLNRQPNADILNHD

RKRKVELKCMEMEELMEEQGYSEDEIKEKVSTFRLMLIDKEGNTNSPLEKDEHGRPIAKE

THQLAEANLEKNQRLREAFGLGDYVDGSAFDPNRKAKEEAAKAAAIATKKYAIVQSSDSD

DESSKEEPPTKKKKKKKSRNDSSSPGRKETQSKKHKKQKRNKSESKKEKNDKKKSSKRSE

DKSKKHHTQESSDSSSDADSSSDNGFCYSDTKKKAAKKTSHNIERSPVRKRKASPVARRH

DSASDSDNVSRPKKRNGQASPVQSKHNRHHSRSLSYSPMRRDPSPVRGRDHVNRRSRDRR

PDIRPVSPVRRSDKSTRKEKKRTSRSPRQARSGSVSNSKSRRPRSAEKRRVSSSSSESSR

SGSGSHSRSSASPPAKRSKGSPGRQDKNRTTRRRSRSVSSSGSGDHRDRRSPSLSRSRRL

RSRSRSYGNSRRHRAGH

>SG-PL_contig_3986_1_-1_763_1158

MIYLKTARLIPQIWKHSVVVPVSKQGKPQSDKNNYMPIALTFHTGKLMEKVVRNRLTYYC

DKNKIILINQAGFGKGRSITDNLVKLTTQIRRQFVRKKNLLATFFDVEKAYGCVLYGRLS

YNLKSTGLPENI

>SG-PL_contig_3987_1_-3_495_788

MEAGENPVQTRIEPRTSNFLGRCFNQLDHHFTRKTSTQTTSQGQAGRREWLSLSDREWNP

GPLASQAGGFNEATGPPLHPYFAGMSVTVRHNTISLFP

>SG-PL_contig_3988_1_+2_827_1207

MEFCFSCTTIIILCSCLLPAFLKIMVKMERLTVILYHACLDIKISRENKNQTHTYKNKQP

RTFHEEVTHSSCDVSLFLSTNLITTLMTAECYLTQNSTMCQKMYIKPSISSSMMEQINNA

IHINTED

>SG-PL_contig_3989_1_-3_99_401

MHIFTISTCTYSQSAHAHIHNQYMWIQYSNQNLDISFVYTYLSTSVFFVVVVVFKQLTIN

VHASFEVRGLLPFLAVSVLLMSHSVPTLHHLFCKKNFSDIW

>SG-PL_contig_3990_1_-2_2_232

HTHTHTHTHTHTQTQQSVHFQTTSDKFHCAADHFEVINIVFSIEIIIIYFHNCSCSILFS

LCRFQMAYYLQIHMKCK

>SG-PL_contig_3992_1_-1_1_114

VGMNYAGNAVHIETKLCRKDVHVSVKVCMLISFELLKN

>SG-PL_contig_3993_1_+1_1_174

EREAERETDNVHAHARMTIYVSVYLKNSFCSLPTIHLSLSLCQHCRWSNGSLTRSKTS

>SG-PL_contig_3994_1_+2_164_256

MATNRLIRKSGLKITLRIQGFPVSVSMNNES

>SG-PL_contig_3995_1_-2_143_403

MGSLTLLEIFFHSVSAFFCSIYQFISLWLAQANSVFEYASCSNFASAFTPILAGQWVTLW

CMSMTHARTWQWLCIVPQTYAYMYKYI

>SG-PL_contig_3996_1_-1_94_1971

MSRAQAESVIKNIIREISQECASKGQAVSETLVAFMVKAVVLDPANEFNVDRTLTKDDVQ

KLIRLCVERLMDNRSPALDTVKMQVYFDMNYTTRSDFLEEHRRVLESRLQPVVREITDSR

ARTREELESLYRKIVSAVLLRSGLGSPTDIAVVREATAALQSVFPQTELGSFMNLTKRDK

ERQLLELTMIVTGIRLFNKECGKGGEGIDDLPAILNEAVPATTQSVDSEIQSTLKSAYKY

TALIEEMHKRQPQEGPSIQLLKEALINTRQHEAFLRIILNDVISCAQQVEMLENQLASRM

ETLQATVQSKTAVPTAQVYPQFIHLAQLWSGFQDEMVLLSVLSNILASLEPFTRGHKELF

SDDVLTPYLEGLSVITDEERIQSTAVPEQRVNPKDYQVENRDWLFPETTKNFSKLPLQYK

GYCAYTLGKYDRLLIPANPEIGVMRYRDHYYAFSTREAAYEFAENPQDFIHLVAEGAKRS

PELIQLLELHTQFSTITPYSQSRDAGKMVEKPVTKCDSGTQTDTHFMESNIVKSYEWNEW

ELRRKAIKLANLRTKITSSMQTNLSNLRRDNVTQVYPLKDTDTQTKTDGYSNVPKPQVFL

AGLRGGGMSVPTNMTKVDLTIDVDQT

>SG-PL_contig_3997_1_-2_230_637

MALQFGKVTQNLCVAATSYCRIFAAKYAKKVAGRGGKVAGGKKGGPAPVKKAILEVETDA

AKLTQFCCGANIYTTGTDPELKPDAEYPEWLWSLRLDRQPPDLSELDQENPQYWRRLRKL

TLRRNLQLMKVKKLGK

>SG-PL_contig_3998_1_-2_11_1114

MASAGNPRVFFDVTIGGEELGRIAFELYADKVPKTAENFRALCTGEKGASSKPDINLHFK

GSIFHRIIKDFMIHGGDFTNHNGTGGESIYGERFDDESFDLKHDRPMLLSMANAGPNTNG

SQFFITTVPTPHLDGKHVVFGEVIAGGSVVRELENVEKEGETPTQTCTIADCGELPADFD

LTTLNCDDSGDIYADYPNNSGIDFTSESCREEVRKLVLEVKLVGNNLFKAGKFGPARRKY

KKALRYLENLDKEQKFSDEEQKAMEKELHIPVSLNLAACSLKLHDYTEAMQYCQEVLRCD

EDNGKAYYRQGQAYSGNKDYELALASFQKALALNPDDKGIKQEIQKVKRTIEDDKKKEKQ

MYSRMFAS

>SG-PL_contig_3999_1_-2_278_520

MSFGTVERETEFLAWMEINAIIHMHVCASGDINEHTPHYYTHVPHTFSNTHTHTHTHTHT

EHVKYNTTQNKTILIANVILH

>SG-PL_contig_4000_1_+3_1026_1244

MKCPVCCKLTFLKAPRGGSMRPQQHFSSGLSSGLFSARQTRGQNWQKKSWQTSNHIYEQH

YQNQWPEDPRSCT

>SG-PL_contig_4001_1_+3_3_1247

LDHIAENILAYLDAKSLCAAELVCKEWYRVISEGNLWKKLIERKVLTDSQWKGLSERRGW

DQHLFKPRHGDTLKDHKFYRILYPQIIQDIERIENNWRTGRHNLQRIHCRSETSKGVYCL

QYDDNKIISGLRDNTIKVWDRHTLECVQVLTGHTGSVLCLQYDENIIISGSSDSTVRVWD

VKSGEMMNTLIHHCEAVLHLRFCDGIMVTCSKDRSIAVWDMQSPTEINLRRVLVGHRAAV

NVVDFDEKYIVSASGDRTIKVWNTSTCEFVRTLNGHKRGIACLQYRDRLVVSGSSDNTIR

LWDIECGACLRVLEGHEELVRCIRFDSKRIVSGAYDGKIKVWDLQAALDPRAPAGTLCLR

TLVEHSGRVFRLQFDEFQIVSSSHDDTILIWDFLNVPVPEGSSRSPARTYTYVTK

>SG-PL_contig_4002_1_+1_1_186

RGEWRRIAASSTPAATIEPDDADRCVKIFFTPHDTRKWGNKIFTVLVHSQNPVENWASAP

LS

>SG-PL_contig_4003_1_-3_24_260

MCSIYLVRPVTYLSRSMSNINTHKMLAAKVKMESEKMQMCKCQSEMSDVIELRETGFWTC

FLKWKVSSERMKMYKLNGC

>SG-PL_contig_4004_1_+1_142_303

MTNVYTHTIIHLNVGQVYDCFYMIKQVLILSFYFIYIILLSNLSTLYILSYCQI

>SG-PL_contig_4005_1_+2_140_301

MRLLLHDQTSFSFLSTLYALSCNRLLLLLLSKRKQTRHCKHQFTFTYDQFKQCN

>SG-PL_contig_4006_1_-1_1_198

GFYRTFKSNHFQEEYISVDMNKYVKAALVKFRFGISDIAVHSLRYKIHTDTDLVCPFCKA

FLENEL

>SG-PL_contig_4007_1_-2_284_406

MTVLPCPLVRTEQNLTFHINYKIEGYCFQYRIHFRLLLCHC

>SG-PL_contig_4008_1_+3_1032_1325

MQNKLKEAFIIVSCKMTYISLSLSHTHTHVSVSLSHTHTLLSHTHTHTHILASMNKSLHS

ELQHNYSSQYNHEKNESTQSTVHTFTLQTQFTRFMGPS

>SG-PL_contig_4009_1_+3_381_500

MTSGSPSMLLFINLGFKQVTHTDYEFNFSVKNTQLEKHRF

>SG-PL_contig_4010_1_-1_229_489

MVDPHTSFIDDITLTATFFFFQIMYMSDLPHTHRVAKTRLPPPSHSLWYARTRTRTHTHT

HTRARAIGAGVPLFLLIFLVLSKICSG

>SG-PL_contig_4011_1_-1_73_216

MCEILTLSPQEDLAAIECHHSCLFLQGILRKEKKNVISCILGGLFLFF

>SG-PL_contig_4012_1_-1_1258_1623

MLTNIPYMYTHTHAYLILSLLLSFTLSQSHSQARAHTHILKILLYTCRCSHTLADTHSLS

HTHTHAHTHTHTHTSLSLTDTHTHTSLSLSLTHTHTHTSLSLSLTHTHTHTEEEMTATPV

ED

>SG-PL_contig_4013_1_-1_205_450

MPLHSTRWHQLCVLSLKTLFIQIIDALYLLENSIIHMFNLFCVCSLSLCSCTHKIKLKQG

QSVALCCKGMPVQRVMMSPPSL

>SG-PL_contig_4014_1_-2_2_268

AEQEKEIEQSRISSSKTASLEEDVESLRNELQESRKHSGTLTKEVSELNCKLADSERHIS

DLEQILEEKASSLEEVTAQALSLSQTKTE

>SG-PL_contig_4015_1_-2_131_1819

MRDFEDEVKFQVNGPDAEIDFLLDGLKLVEVPVTPNWLEKANNDTEKYRKSQFDIKFRLP

ADVDRNDLDVEVKLTKHQFAFGAKIQTDCILTPKAARYNNFSYSVFNWATIGAYKWRMGQ

AQPGPPDYYDAVDTNNVLLQHGFKVRAHCILWGVPDNVPNWVKDITDPTELRKTALDHVD

YMVNLTKSGLAEQWDVQNEWIHGHFYEERLQDPNFTMDVFKYVRSLDPDVKLYLNDFMAV

TTGAQTQDLVDIVKRFRKYGAPIDGVGIQGHTKDYIKPDPTMIWRRLDKFMTLKGLDVMV

TEFDVGSNDDVIRADWLEDAMRAFYAHPVLEGVILWGFWNIRMRYENKYLVYGDRFTLAE

GGIRYACLVKNEWTTHQNFNLSHYPDLTVSLNGYEGEYDLIVRKKGVPVQRKTFLIKTPK

TLKVTITGSDKPIALPEEYPDYVTECISHRNQHSLGNVTASTTATKASCVTRKQSRSLEH

VSVTCNEGELLLGCSGSYVSGSGYKEYSTSRQGERITMEAGVPTCHAYGKDLGQTFTGTP

PLSLSLSLCVCLSVCLSVCLFFF

>SG-PL_contig_4016_1_-3_492_686

MLVSWCSPRSPWSCFCSFLYCELGWTFSWHGWLILVLRQVEMGNFSLILILPSFLWPFSR

HATFV

>SG-PL_contig_4017_1_+3_42_233

MCQYVQTCNHRHTHTSLKCLEGYQSASSTSSISLSVCIFSSYRFLAYLISWATLTAMCCR

SHRT

>SG-PL_contig_4018_1_-1_157_324

MSKNFNPLKLVVSKNKTFKLVMSEKLNPLKLVMSKNFNPLKLVVSKKKTTKLSNWS

>SG-PL_contig_4019_1_-3_48_932

MSEFSDDHSSAESADIVAETRYRLRGLEKEAQNLEQAFHDFHCHMTNVNALPSGAPPPPP

QQQPSSSQPVNTSHRLQGRLGHRAMESPPHSPIPHDRPMSSTPYKHRPDSSLHDDSFAEL

SGGRTHETRTAPPQFDLSNVSDEADGKHATETKHVKSRPITVDDLEQRPGSPSMLVVAES

ESAGSPPQQKGSPTGSPPQQKVESIKPARLDPLPSQPAAEKTPSTATSLSLDAARKMPSV

SLDDAWKQPSASLEDAWKSKDQITDREAERKKEEQRREEERRQEEERKRKEEEER

>SG-PL_contig_4020_1_-1_1_90

SDLESTAFSSVLAHPQNLMHFPICRTPLPT

>SG-PL_contig_4021_1_-1_85_216

MAYILNEIVTFSYCQCLTILFFHFSINSSCSICNTLLANGNRCI

>SG-PL_contig_4022_1_+3_39_356

MEESRIPRKASQILLDLDASGKKNWASNVCLCLCENGLGYVWFIIIIIIIIIIIVILFIA

QPTIKAIRAVTTKSPSKSRRQLRWSQNLKPENKNPAKHSSVILTIK

>SG-PL_contig_4023_1_+2_194_337

MAKHAHIVISKHFNVNTDFDNGGIGEKPLPDSLISIKCYQICLPFLHE

>SG-PL_contig_4024_1_-2_2_808

QTDLPSAEGEVPSGQAQPAAAISPGTGPKRLHVSNIPFRFREADLRQLLGQFGGILDVEI

IFNERGSKGFGFVTFANSADADRAREKLNGTVVEGRKIEVNNATARVMTKKVTAPTIPNA

AALRGVALTRGRAAAAVAARGAYASAAAAAAAAAMRHPTALTAAPAAAIPYTPGVYQDPL

LATYATDGGVRYQLVATTQPYAAAASYAPAAAAARYAIPAAVASPSPYAPAAIAGRDYAA

ETYIGHSIGPVPGYGATVYRGGYQRFAPY

>SG-PL_contig_4025_1_+3_177_533

MNAYVHICVYHISAFLFVYVYYVRNCAWAHAHVSLCACVCVSVCRSMHVCVHTFVLYAIT

HKKRNETPMQKRAKGGSGRGWEVGSTVYQIKTRATRNTDWRKNYREAPSYSKAQVWAGR

>SG-PL_contig_4026_1_-3_3_206

GIPMRTATRPQPSTSRSPSPHSCGHIVAEDLATIRSRSSEETGSQESGQLLFAKMTGFLI

RSLASSAH

>SG-PL_contig_4027_1_-1_85_792

MQKYSGQEYELRISRIKMEDKGEYCIRAENSFGRKEEKANLKVEPGVDARVTPSREMTPR

RKRIFEELKVEKPKEDAPPNFGFELRPRFIQAGTEFKLLCEVQAHPVPKVTWTKDGREIH

DGDSYNITYSHGVCSLEVVFARPEHSGRYVCRAVNTLGEHETGCKVEVEERAFYKPKEIT

SNVSRMVRRTTTGATVDQSSSSHESSSYSSTTTTRHSSRVEESSYTSSSSSRTERK

>SG-PL_contig_4028_1_-3_12_323

MHACMHVLMCVCVCVCVCACVSVSVYVCYRKKQNQIYNNNESFLYSAILQTNLDSLHITH

LQDTECDTYRSTDITHTHTHTHVYISKYMHSHSFTSEHIQSLRI

>SG-PL_contig_4029_1_-2_2_307

VCVFYVCFVCVCVCVRACVFSCVGVCVCVCVCVCACFSLISVHNELILDNNNNKCVCIIM

CVCVCFSLMSIHNNNKMCVFVYVCLHTCASNHKSGSKFLMLF

>SG-PL_contig_4030_1_-1_58_312

MCVCVCACVCVCERERERERERERESVCVVPLLPSVRHTCLWHVCYFLFDVQRKQVSRCK

LVCWLLFKLMIQIFSSEQEMLMHWM

>SG-PL_contig_4031_1_+1_1_129

SSSLTKSRSDWLLYPTESDSNRPGPIQRYSMIGSDRSKQSVCK

>SG-PL_contig_4032_1_-1_133_468

MTTAARPTWDTARGGRGKGEGDLSALSKQYSSRDLPAHTKLKYRQEGQGTTEELRGRDFR

RDLESWERTAREKRDKHRDSSSSSSSKRPRLEQIPAANLDADDPLDDDEEDE

>SG-PL_contig_4033_1_+1_1_126

RYSSRSLRISPLHLKFYPPLRDSSLPVSNAVPRLSPGLSHLT

>SG-PL_contig_4034_1_+2_2_124

PKTKVSLDRSKLIKDWLKRGRRSNQKSHIPRSLGGFFPPWA

>SG-PL_contig_4035_1_-1_109_771

MSGIMAKLFGSGKKGKAPTPQEGIQRLREVEEMLMKKSNFLEKKIETELGTAKKYGTKNK

RAALQALKRKKRYEKQLQQIDGTLSTIEFQREALENASTNTEVLKVMGVAAKALKETHNN

LDVDKVHDLMDEVAEQQEIANEIGEAISNPVGFGQDVDDDELLAELEEMEEEELNRQLLD

VGGSVTDELPAVPSAEPSRPEPRVAQKEDDDEMKELAAWAS

>SG-PL_contig_4036_1_+2_41_355

METGRSKDGLPRREEWLVSGTSKWTVPVMMAVEAVEAEEKVGLGFESPSSSVSPAGGSSL

GKAVFRCRLKSSLNETWLNCHTHKKADVVTTRFTPAGSSTCKPTV

>SG-PL_contig_4037_1_-3_39_128

MVLSQFFFYPFISDIRHLLSVPIHLSAFDA

>SG-PL_contig_4038_1_-1_1_102

RESIVNDWLCCLMMGDIRGGIPCFPVMVQQHLDC

>SG-PL_contig_4039_1_-1_1_246

WARQFTFRQICLFSQRYLISVTWNTSQPLCHTKASRANFDRPARSLSLLQDRMLSAFWDI

KRVKTFKIAKILFPRVYSGKWQ

>SG-PL_contig_4040_1_-2_11_2626

MEATGKSRMFCKLLVCTVCVFALLQTTTASNIVSFHQRVEKVSADVKSDIGSHSRSKRSS

LTESERNDNKCDSQKEKFANAVRDGKRLETTHVFKDETKFSLAMAWAGKNDDGVLVVLTS

ELVSFQFAVSTLWRSKDFGRTWQNISSAVHNSAFRKSDGLQRNPHDPKWIYLISYEHFFY

GSEDGGETWTKFGLVDTVKVELVFHQDEKYKDHVMIITTSNKLIMTTNNFKTMETVRDGG

VHSAHWGSESSNTAKDMFVTHGSVTHQSPLPFFEKPVLYYKLLRRSTDGDEWVAKREYVV

DFGLQGDFLYASVYKSKPTLDSERLLVVSNDGGDHWNEAQLPTLAGDRFFSVLDMSEHLI

FMHVDNPGDTGHGTLYTSWSRGIFFSESLQRHLYPNYNSITDFYKVSSMRGTYIASQLSK

DNSIHSMITFDLGGEWQPLPKPQDVSCKTEGEECYLQIHNAYSIHRGVNAHPPLSTDDAV

GLIIVNAHPAKNLQTTDPDIFVSSDGGYSWRKALDGPHSYIIADSGGLIVAVSLKDPFPK

EVKFSTDEGRCWHMYQFTNETVHHTGLLTEPGGKSMTVAVWGYTNKDKKWTVSVIDFADV

ITDHCTRADYETWMPHETMKKEDPKRQGCLLGKRTTFQRLKHDSWCHNGYQYKPESSEKV

CPCSIADFECDYGYHRPEGQEKCVKTLNFQGPEIDVCFRGHEEKVISEGYRKLPADVCNE

QGGFQPTSKYINLTQECSGRFKVPTPSPEELEKIRKEKEEKTEQSHGTLVVALVLVALLI

VAAGLAYFGYRMVLLRRHKVVYRYSMLNQTDDKDFDEEIERTLTGGSHPHPMFGETSDEE

ELPAEPRQNGHIQKGRPKTRGYHDDSDDDMLD

>SG-PL_contig_4041_1_-1_118_327

MLPVGQQRTVTVRLQSDGRPQRKDGQPLPLLQPGCSGCEHGRILRGHAPVRRSECTAEGL

QELSHPGLPL

>SG-PL_contig_4042_1_-1_22_429

MIYGCEAWTLRKADCKRIEAAEMWFYRRLLRVKWTDRRTNESVLKELDTTRRLLANINKR

RLKYVGHVSRNKNTDLMTVIYQGKTEARRKKGRPPVSYIENITKISGLNIQEVVHRSQDR

ATWSSITKCSGQHRTW

>SG-PL_contig_4043_1_-3_3_155

LAWFKSYKSNRKLTVFVNGHSSECADIWFVTHKIQFFAQSSSSCVLNPFLS

>SG-PL_contig_4044_1_-2_2_232

QLQWTGADRYRDSIRLPWFKDDESLLGWVAQIGNFTRVVVRNSGHHVPHDQPEGSFLLMK

NFVLGQPFVTEKSEPVD

>SG-PL_contig_4045_1_-3_36_416

MIVRRGDPTAGIVKRDHMIVRRGDPTAGIIKRGDPAAEITRVTILPATNTVTRTKRINTE

MVRGGRANPTNTSTESEIIERHRLGRVVFFSTCKLVCVYDMNEELGTGMVVLQYVCTDCV

CVCVTCE

>SG-PL_contig_4046_1_-2_1025_1234

MLSVRLLESKHSLFIYNPKTTVHRNLLVLKIEITVLALEDRNILHATFRLVFQGEVSCSK

YHINQLINGN

>SG-PL_contig_4047_1_+2_1028_1321

MEIFPCKIQAALPRQSMLPSLVPPNKMNTLKFTKILSVKVGGSDYYPLLPLWRNICNFMF

LSMSYSSEVGVWLPTWRGTKKLPYTQSSHPNVLVVSLS

>SG-PL_contig_4048_1_-2_2_190

LRYKDVCKRDLKAGNINPAGWEAVAADRSSWKLSCHQGRHRDERGSAGREERAQTAESRN

STH

>SG-PL_contig_4049_1_-2_2_127

LLSSLLNLFRTLLPQASSTPGLKLPRKKKLLRKQVVGQKAPL

>SG-PL_contig_4050_1_-2_98_277

MGTGCPFCSVICLYKAFLCIFYGFKVCLFLIFYAFFKTHSKHCCLRPAAFLASKNWLENR

>SG-PL_contig_4051_1_+1_1_105

QIKSQWCFLSHYLFSNQVFSAASGHECCWPEAAVS

>SG-PL_contig_4052_1_+2_2_241

VKTVQICLFTKLCPFLAWLKCYCNLSFDYTKLSSVIMLSFRPICLQIKSQCCFLSHYLFS

NQCFETASGQECCWPEAAVP

>SG-PL_contig_4053_1_+1_73_444

MGIFLSDIEETGRQNGRTEETQEDGGTDQVVAEIFFSLIPAVLTESGEHLTQLIRKVSVG

ADDPVPDLGSKGLETGLVAMQRGIVHFGGVHLIVIITLHQATHKITKRVMQILSHNAKSQ

VIYW

>SG-PL_contig_4054_1_-1_481_678

MFVVSLKLSCSLGCPFVLVQFRAVYVVSYIFTCVIYCLGCPFVLHCFVIILVVAVVCACV

CVSAEL

>SG-PL_contig_4055_1_-1_73_435

MKSVENWCNANDMVVHPDKTESMLICARQKRQNMKETQLHIKYKDNVIKQVTKHKLLGVI

IDQNLSWHEHIHCLIKQVSSSVFQLSQIKHFLDDHSRRVFYFAYIQSRLSYCSVIWGKCP

L

>SG-PL_contig_4056_1_+2_2_244

KYGSSYALHKINNLFCKHIFTAYNRICGNVKSTSPIDLLSEPILYNGKIKTRNDMIKFTG

WIEKGVYCIAHFLNGNGSFMT

>SG-PL_contig_4057_1_-1_172_378

MIVSDFDEMEQTCVQSEWQYTCIESVIEIKMTTSYHKMEIIVSQHKTESQFKLINAQENT

GANFLQMKK

>SG-PL_contig_4058_1_-2_5_1600

MSAIKTLNPKAEVARASQALAVNIAAAKGLQDVLRSNLGPKGTMKMLVSGAGDIKLTKDG

NVLLHEMQIQHPTASLIARVATAQDDVVGDGTTSNVLIIGELLKQADLYIAEGLHPRLIT

EGFEMGRNKALEVLEAVKVKKEVDRTTLIQVARTSLRTKVHTDLADLLTEHVVDAVLAIQ

RPGEAIDLHMVELMEMQHKTDMDSTLVRGLVMDHGARHPDMKKHTTDAFILTCNVSLEYE

KTEVNSGFYYKSAQEREKLVEAERAFIDERVHKIIALKKQVCEGNKRGFVVINQKGIDPF

SLDMLAKEGIVALRRAKRRNMERLSLACGGMAVNSVDDLTPDNLGEAGLVFETVLGENKY

TFVEECKNPRSVTILIKGPNSHTITQIKDAIRDGLRAVKNAIEDGCVLPGAGAFEIAAYQ

ELMKYKDQVKGRARLGVQAFAEALLIIVKVLAQNSGLDPQEAIVKLQQEYAGTHQPVGLN

IKTGEPVQPLEEGIVDNYRVKRQLLHSSSVIATNLLLVDEIMRAGMSSLKGD

>SG-PL_contig_4059_1_-3_3_185

RFIRLFSLFESTGGGCQKPEKCYTLGFKSINCASGETHVYSKRWNTKELSSSHCYTWAVF

Q

>SG-PL_contig_4060_1_-3_120_224

MILYTQRLGEYCYLNVSALILCILMTERMGTVVKW

>SG-PL_contig_4061_1_-2_17_541

MADVLDVDVKEENEEFEVDEEGDQGLQKLKEKAKKRKGRGFDGEGPTRGDVEQYEVMDVD

EEALGIDGPGPQRSVEGWILFVTSVHEEAQEEDVRDAFGEYGDIKNLHLNLDRRTGFLKG

YALIEYETFKEAQTAMDNLNGSELLGQKLAVDWCFVRGPRQTKKSRRSGGGGRRR

>SG-PL_contig_4062_1_-2_71_292

MTSQSLQPLMTGTLLFVTDKLWAYVSGSFQKSYSSRLTSLLIRPSPWHVNMEQIKSQLSE

QRTRVDAMQSSACR

>SG-PL_contig_4063_1_+2_2_208

GFHTLTNSLLFPNHLYMFYIIVKVVVIELPDRQAGYMTKIWLQSVYTIIEKSQLNVLTSW

ACVFQITGL

>SG-PL_contig_4064_1_+3_672_1337

MSTGRPLHSPKSRQKQGSTHGQLLLGCLLRSLLRCWLLGCLFGRRLLGFSRLLRWGFRLL

GFLLGSRLGLLGRGFLRLGLLGLGRGLRFGCLRFLYLFRLGFGCLLCLVPQPEGTRSPGT

FGLLQGAVLYASTQRQLQVIVDRLLVLPHVEIGHYVFEQRLTRGPTALFQGCDGGTNHLA

VFRVVGRFFRLGCFFLWRLHRRGWGACCIVRHDDVQILLSVH

>SG-PL_contig_4065_1_-3_36_989

MPGTVWAKEEFDASEVAERLKNAMDGLGTNEADILDVLGNHSNEQRLTIADTYKVAYGKD

LIDDLKDELGGNFENLCVALLASPRLYDARELHEAVSGAGTDETSLVEIMCTRNNEEIGQ

LKEKYKEEYECELEDDLQGDTSGYFGRLMVSLCVGGRESDEWWWADEDKAAEDAQKFLDA

GQAVWGTEEADLNAILCLTSPGQLRKTIEKYEELTGNAMEDAIRNECSGALKEGYLAIVE

SVKSHPRYFARRLHDSLSGMGTSDNDLIRIVVSRSEIDTEDIKQAYEEMYGQPLVEAVED

DCSGDYKRLLLAILNPPC

>SG-PL_contig_4066_1_-1_157_393

MRDKNCTHQKDIDDMKRANQQIQSQIKHLEKYADQSRIPERPKERSHPRYDDGRPVSESS

DDDDINHSSRKKPKLAPDS

>SG-PL_contig_4067_1_-2_2_211

VTVCVRVCVFICFFNLFISASTFVIEVCLTQWRMNLNIMCCSSYLILVMVTEVQETYICG

YGMCASLCNM

>SG-PL_contig_4068_1_-3_96_911

MLSTFAVRSGVFGSALLRSQAPRVLAVVPAGTRSKSKAAEQADQWDTANKIFYGPERDTK

NFPHPVMPAESGKVRLGFVPEEWFQFFYNKTGVTGPYVFGVGLVTTLLSKEIWVVDHGFA

EVIGFFGAVWFLTSKVGPKMAKWMDNKTDLVTEDRFVKPVLEAKESAQQIVEANEKAIWQ

EEGQKYLWEAKRENVALQLESAYRRRLNEVYKQVKSRLDYQLDVQNVKRRFEQDHMVNWI

VSNVRKSITPQQEQESIKVCIAQLKKMSTATA

>SG-PL_contig_4069_1_+2_2_118

LVAHPKASLPPTENYICRMHAVKQVGLISYFKHCLILLF

>SG-PL_contig_4070_1_-2_137_2578

MRDDEETRAELHQRVDDLRERLWNICDERKEQAERELETIQTDGWLDDRLGILSNHYITT

MQAELDRFQDTVRLLRDYYKGMDGQIPDELNVNYDRIPLIELPVERPETPESTHSEASVT

PTSDEPPGTAKSDRSKSPRAKSPKDRSKSPKDRSKSPKDRAKSPKEGGRKSRTPSAKGKR

SRSKEKDVVPEASTPSDGAGLRIKIPLVPRRPVSPDPDAKPGTTPGKDKKDKKGGKKADE

VPGAESPLPPQDPDEKLLFDAQCFAISAIGQIISVEMAAKEAEEEAEKVRELEKEKEKEK

AKVTKGKPAKKGGKSRSPSPKKGKKDTDSAAPTPPLSEDLSEEDKQKKQTRERMRQEYYF

AVKEEEMATRARIELIKAISMAVLQDLKMKADGAFKDMNDWLGARFLKEMESIETMSEVM

RHAVENSQRMKEQVILHQDDFLVDTDHHVLKSPSPPPVPEPSESVMAEHFTVDQLFHFYQ

QFSAIAPSGTMSSKSFVETLENTVAVTHGMEQLPDSWMHITPSQIQEISNTLATETDYVD

WRRLLLALTWPILTPTQAQLLETLQRFRDMDQKNTGFVTREQYDRMELWFEVGPSIPGAY

DRITHLKKILFDIFADHTGSLSKLDYISMLLYFSAVPNSHEGFLRALSVASNCHMPRLQK

PLPVSTAAIHIHPERATSVADSIIPESAEDEREDKGPVEPPMTADEIPPEAVDACVPLDA

LFRVFHHSEISSGDSHRFSVSVDPEDVTSKDKLAAVYVELGDESLRPVPYKVLIEHPLIQ

DIVVACKIFKALDLKGILSNTSDNTELNSLKTMD

>SG-PL_contig_4071_1_-1_193_1128

MSSMSGLGAKGEKNKGKYKTIDINTLYKGKSVETQKSTVPRQHGLQSLGKVSSAARRMPP

PANLPSLKSEHSGNDPSISLVPAGGSGWASKEKEKESSGSQPPVSQPQQAAPVQSTQSTV

KTSTSSGGTGSGGRSWSSVAHGGSAQGGLVSHQSPAFQEEFPSLAPEEKSKETKKEEDSK

DTQYGPGPSLRPQNVGSWREGGGRGVQQQSAKAEDVSPPATSGPGPIETQPNGPQMSGGA

GGPGGMGGMGGGESSHPPPHMPPRPGSGPGPGQGPPQGAMPMGPPMGIPSYRGIMPHYVS

VLQLLFILFLIS

>SG-PL_contig_4072_1_-3_3_2435

PETIEDVEPKTVHVELQAEEVAAQELAPVEKAPEEETTAAALDIPDSEESATPLVAKEEL

AITVEGLEAPTTETVAPETTEVFSVETIAEKTTTETEEVSHEFIVRETAEAVSHEEILEE

TPVPESEQAAPERTTAESFQTLITEIEEAFAEEFSQEASITLEEVEHPKLTVEGVPSQET

ETPLEEEFELLELPATEEVEQEVVELRPQGQAPTFSQPLHNVEAQEGQPIRFETVVTGSP

QPEVSWFLDGEPIKESPIYHIITEASGTCILELPQSFPEDEGEYECRAINEFGTASTKAD

LYIQGPAEEQEVHVTEEVLSEKEKLVTAEEEKDAVSDEERKETSPEVFEAQFVEIKEERF

APEIRLALEDTVTQDGGQVRLTANIVGVPQPEVTWYREDEILSKTEEYEISQDDQNNYTL

FIHDVLPEDAGKFVVVAKNELGTATSTAQLTVEVEEIEEQVRPEDVSGLAPEFLEKPRFQ

TVQEGDTVTFKGTVEATPVPTILWTRDNMPLPEDQRFTVAATQDETSSTVHHVSLAISEV

TPQDAGTYRVQASNDLGEESVSVSLIVNRVSEEKTDFRDQLQRSEMDIPDKPTTVEAEQV

DFRGQLTTEVKFATDVSVTETTATVEEVVEEQPQQADVQEVTVEIGRPEESQPEFLVPVQ

DLTIKEGEAARFDCQVKCTPEASIVWYHQDKPLKSDDIYQILPGEDGQSSLLIAEAFPED

AGEYTVRAINPLGEVTCSAVLTVEELVSEVSSVGSRQDEEQEEPVPETVVHEAAVKIQAA

FRGYKAREQVKILKETKTTEEEFFISVPAEE

>SG-PL_contig_4073_1_-3_132_350

MPHRQYHRRPENPREDSGTKRNSWFHSERPVAVLFLHFVCAGPRSARSAGRAWSSFHGQG

IVDAVRHQGTQGC

>SG-PL_contig_4074_1_+2_2_160

ERHTKGLKKRPKWQKTQRGLKKRLKDTRKTPEGFKETERHTKVSRRPNNTQRF

>SG-PL_contig_4075_1_+2_2_217

SHQSRGATVSSPKASHQSRGATASFSLKGLWNLCRWTGRKPGHSVKSLLLQTCKAAYSLV

PNQCQTYYAGIH

>SG-PL_contig_4076_1_-1_172_357

MHTLFSHHRLQAVLALASPLDFTCVAERGSLLHGQQQVFLNTLPRLVSWSRSGGTNSIGL

RD

>SG-PL_contig_4077_1_-3_3_242

FPPPPHTHTHTHTHTITFDFVHRNDSCTFIPTVCNHKSFCSVTSLLINRIIVLTGELKLK

GGYGIIVFTKIKWEDELSFL

>SG-PL_contig_4078_1_-2_2_148

PVPVECGHGGSNVDGFLYPPLEPSAFSVHHRYNILGELMSRGGHVRSYW

>SG-PL_contig_4079_1_-2_44_325

MDFSSPPPGGKMGAADKEHLMDQVKQQIALANAQELLQKMSEKCYKKCISKPGSALDNSE

QKCIAMCMDRYMDSWNLVSRVYSSRLQRERNRLQ

>SG-PL_contig_4080_1_+1_589_774

MSTVPFFVFSSHLTCSHTSLYKYQDCCVPFWFLVGLHHFFTIASQFHQQSFHMPLSRDQN

KE

>SG-PL_contig_4081_1_-1_1_210

AHTVTHKHTHIHSVLQWVVFFLPQRSLILFMQTKWPLFLHLYFGLLHCCKEIIDHSLLLF

NIDHETSRLE

>SG-PL_contig_4082_1_+1_646_927

MSGEKKIHRDSDTSLSYTECCNPCYMITTKQQHANSSSCRHFTIPFTSEVYKTNQPDKKN

VSNHSSTLHFPVFPHPPPPHHPSHHPSLPPTLHS

>SG-PL_contig_4083_1_-1_91_375

MLMWLPYHQQCYQLFSHLLDWVLCTLLMPTTSTAQHMRSIQWVTVSQIWILMFSHPGFLD

SVMSLLLVIIFLFCFWLTGGVLTTFLCTDFDSINC

>SG-PL_contig_4084_1_-1_49_378

MMLWLVVFHGPVSWQVLMLMEGKAHAYVYASKGCKKWDTCAPEAILYAVGGHLTDLLGRP

IPYHAKVKHTNASGILATVGNHEWYVDQIPKELKYMFRDPEQPMPPSSSL

>SG-PL_contig_4085_1_-3_1368_1619

MCAFYLTVIVACASQSQSSALFKVFDNIFQITTVFRQCLVCNNVLPQKTTTVVYPFHSLT

LFTSYKNFILSFPHPIHYLPAAGS

>SG-PL_contig_4086_1_-2_83_955

MVWIFGGGFWAGTSTLDVYNGAKLAVTENVIVASMNYRLGPLGFLYTGTTDAPGNQGLLD

QTLALKWIYENIVSFGGQQNSITIFGESAGAVSVGLHLMSNLSQDYFTRAILQSGSPTAD

WGMMSHATATARAEILSEALNCPVSSSSSMVQCLRGLDAGDITIKQWNVQTTGAWFDTTM

SPVVDNYFIMNEPSTLMAEGDVKDTELLIGVNEDEGMYWLLYAFPNMFPLDNSGTISAAE

FRDLVKSLTLNADDDAVNAVINEYVGSTGSVDTHTYRDIADDISGDMVFIC

>SG-PL_contig_4087_1_+1_25_276

MQILSQKQIYSNMICIHVQSHKCHVFYIGVERKYNIISLQVESKGRGGKIITSSLEILEI

NTHTQDSRVELVRKSLAMTLNLRD

>SG-PL_contig_4088_1_-3_213_365

MVYSERGGEGFSIPSITVRTSPRKFQKFHQRILLFMFFPCFCFFLSVCFKI

>SG-PL_contig_4089_1_+1_79_285

MIFKFFKHLTLVLLHSTSVWFFTDHKAAVPRQKHSCRCHVQYIRLVVVQQFLRGQASRLP

KVNCCMQGL

>SG-PL_contig_4090_1_-2_2_343

SSFFSFPASDDAFLSSAARGSLDGWSRNRSPTTDVPRAGAPQAGESETGTPAAGTRTASL

PETPSLLTCGEYASQPWGPRHPPADPTPWSLPPLVQRERPEARAPWQASFGWFV

>SG-PL_contig_4091_1_-1_1_282

SFSVYYFPLFFLWLRFSVYYFPLFSVFQCVLSLCFQCIISFYFQLFRIISLCFQCIISLC

FQLFSMLFSLKLFSNRHIFSVLTFVFSVSAFFPH

>SG-PL_contig_4092_1_-2_575_856

MVCVCVCVCVCVCVCVCCPVYTALVVLTPHPLISSGSSTCTTNPKFKVPASLWDSVMSSH

HGGHPPEETLHCCRSLQRRSTVPVLFQLTKDTAY

>SG-PL_contig_4093_1_-2_2_256

MKGWVGEGGNHCWTKRYVFRLDLKEERVCDCRRDRGSSFHLYGPDTENERQPTVEHLYRG

ICKQIVSEAERRGRTGVYIWRWSQR

>SG-PL_contig_4094_1_-1_247_1731

MREPSDSKASGGDSGQGHSRLASSATPGPSGASPGGSGAGPDRVGAVPGMPGHSLMGSSA

HLQAPTSTTPEGIGTSLHPSFLSLDHHPSPLGAGEIHIKQEVSAGSSTPAKLCQVCNDNA

SGFHYGVWSCEGCKAFFKRSIQGPVDYMCPATNNCTIDKHRRKSCQACRLRKCYEVGMNK

GSQRKERKSSGSNTGPKGKRCRADSTDMLVNSTNGASASKSAKRLRSASIIEALQKADLP

VLESYHNHDVPTSRVHLLNTLIKLADRELVYLINWAKHVPGYTDLSLGDQVHLIECCWME

LLLLNCAFRSMEHEGRRLVFAPDFHLDRHLWTVMGMTEILEQVSAVSEQMVQFVVTKEEL

LLLQAMVLVNAEVRRLASFSKILEMNQMIVDALMEVAHRTHPENTRRVPTLLLLLTHVRQ

AGERGIAYFQSLKHEGCVTFCDLLTEMLDAHNTASSPVASTSATVTVSMPSTSSSTDVDR

KNVNLILPHLPHGPS

>SG-PL_contig_4095_1_+3_3_125

WNESAMQLSSTVVKNHNQYHSFFFFPIGVLSSQKPFKRIKK

>SG-PL_contig_4096_1_+2_257_352

MNNRVPRIFALSLSLSLSLSLSLSLSLFTFFF

>SG-PL_contig_4097_1_-2_167_334

MRHPGAPRFATSRLHSPRLPGHEVRHPGTEGVPNAGPAPGPPAQAFVSPGQDFSPF

>SG-PL_contig_4098_1_+3_3_116

FFFEGIYKVSSLITCIQCIGYLKIHEFQFHLAPVRMTV

>SG-PL_contig_4099_1_+2_509_829

MNVHLSSASWVMGMMCSLGGSSTILGAFRIRIPSLLNDEVTDAGSTSFGRTYCRVNCRMM

KPCSSGRSSWQAVMITRLFLVLMLISSGLKCCTSMLTRNSFSEFNTC

>SG-PL_contig_4100_1_+2_362_493

MALSMALKLGLTMATCLYVECGSGKNADTLINVLTFICFLSFFF

>SG-PL_contig_4101_1_+1_187_477

MEIQSSDFWKYIIIDFQGTMIKLQLSFHIHTALSVTVSKNELFGKMVLLYNSETNSHLLS

SFLFQPMALPLCFYSRSPTHPDPFHPGECQNSRGFIT

>SG-PL_contig_4102_1_+2_872_1108

MLNASVSPCLLSSLCLSVTHTHHHILCVCVRVCVCVCACVTVVCAKRERERERERERWAL

SALIMLTSRKIKTSTTSLP

>SG-PL_contig_4103_1_-3_210_404

MTVEETWQIFRDNLLNTLKETCGTRKSGRGQRKATAWWNDTVKEAIKEKKRLYKVWVKSC

LDKDM

>SG-PL_contig_4104_1_-1_13_708

MGQQPPTSMGVQGQNFGVPPQPGFPPSSMGPGMGGPPGSGPGFNQPPGMGVPPSGLPGFN

QPPNMGASLSGPGQMPPSSQYGGFPPPGPSSQLAGNFPGSAPGFGVQPGFNMQQPRAPGP

ASQPQQPRRLDPEHMPSPIQVIEDDRRNRSGPFQANQKGLVPPLVTTQFTSQDQGYANPR

FMRSTMYNVPCTSDMLKASHIPFALSITPFARLDQQEALPPVVDLGDRKSVV

>SG-PL_contig_4105_1_+2_161_340

MKTFLLSQCSHMLQVASLPEKTLNNINTSLHRFIWKKKFNNKRPHEKIKEEFSASKKKED

>SG-PL_contig_4106_1_-1_10_264

MACTSLIVLPADVISLWLMVDWTTADGTLRQRSTWLQVPYQSRPRPSQTFLQRKPTPEPS

VEETTRSETLMVELITELNLPITAI

>SG-PL_contig_4107_1_+2_2_157

SITEDRRRKKQDGQSILIISLFSLTNPLSCSEENPDFQLLKVTVTSKKLKTK

>SG-PL_contig_4108_1_-2_110_274

MGTDVSIKTAASVNKNQSAQLNGAYLINTVQQKKAFVKHMKGLKIKETAKFKMQT

>SG-PL_contig_4109_1_+1_154_219

MDYHSQKEFTYLQPVTSSHTHT

>SG-PL_contig_4110_1_-1_34_267

MGEHTGPQKHQVEQKRVPPQKMWMESYYIDAVFLGRRAAVSHKRTFLMTVSILYCTCTRH

THTDMGFCFPQTPPKEQK

>SG-PL_contig_4111_1_-2_71_256

MYTLLGIVLNHLLFVKLVIFFFLIFFLLSNLELGSDGEQEHFNRVKLHYCVLLILVDFIN

FF

>SG-PL_contig_4112_1_+1_187_288

MIQNDAQQCIHLIFCGNNYNTLSMPSHEQFTMSL

>SG-PL_contig_4113_1_-1_64_195

MLYNVLLLHTKTTCYFHKAKQRSCLVETTIFFSHKLACVICTVN

>SG-PL_contig_4114_1_-3_1062_1289

MCYTANAMRQECKIKPVSEAKRLAQQTTNWKVMVSNPHQSLKNRCGIFRPQPAPTQLSLL

WGRWEDGPHSQVSSAL

>SG-PL_contig_4115_1_-2_2_157

FEFFFLKSQFLLSGDGKSDGRLAPPARSKFICVRYDVRCFGCLSLMVWVSSY

>SG-PL_contig_4116_1_+2_2_331

DVPAGTGAPLGDRVVKHLTEPFFGQWHTVYFDNYFTSVPLLTDLFAKKTLACGTVRINRK

DLPTDMKPPKCVKKSGDTLRWFRPIVSDDATGHLQAIAWFDRRKVTLLST

>SG-PL_contig_4117_1_-2_20_310

MASIPVDMELKKAFQEMQLKMIQTTQQLKVADSQIETLKRKIHHTRLVDQEISALPEDTP

VYQGVGRMFLQMSVPTIRESLSNKQKTSDEKIKTITV

>SG-PL_contig_4118_1_+2_506_661

MLSVCACVCVCVHVCERERKRIYQKLLLAKLHVSPVALKSVLYIWSYQVDIN

>SG-PL_contig_4119_1_-3_1026_1307

MIMKIYVLQHPKKHGPKRLPLYRTYVVKRNSRKEDNSATSPKKFVHVQEYVSIWIKYAHT

YTHASMYTQIHVLMHTTHTHTHTHTHTHSLSLSY

>SG-PL_contig_4120_1_-2_644_1402

MALSSIFITGASRGLGLELVRLILSQSARPRHLLAGCRDPAKAEELQSLAKQHSCLKLIT

LDVTKDSDIEAAYTQATACLGEEGLNLLINNAGIFDKPGGTLPQLTREKMQSHFNVNATA

PALVTQKFLPLLKTAADKNSSQPVSYQRAAVINISTVMASIESSLSKGHCGGYHYRASKA

ALNMITALMSVEFKESGILVAAIHPGWIQTDMGGPSADFDSKTSARNVWSSIAKITEKSS

GLLHNYDGAVLPW

>SG-PL_contig_4121_1_-1_49_1248

MVSYYSNNPAQVRLKPCYVQFSNHKELKTDSSHSFQSTNAQAALQAATVVQGGTPDEQQQ

NTILRVIVDNVGYPVTLDVLQQLFSRYGKVLKIITFTKNNTFQALIQFSDTISATTAKVS

LDSQNIYNGCNTLKIDYSKLTNLNVKYNNDKSRDFTRPDLPSGDPALDQAMQMGGGMPGM

AGFGMANPGAFGAARMGMPVQAHAGSCVVLVSNLDEDNVSTDALFALFGVYGDVHRVKIL

FNKKDNALIQMADPQQAQMAITYLDKVRVWNKNIRVAQSRHNVVQMPKEGQTDAGLTKDY

VNSPLHRFKRPGSKNCQNIFPPSAVLHLSNIPPSVTEDQIQSWFSDYGSVKGFKFFQKDR

KMALIQMDTVDEAVHALIGMHNTQLGENSHLRVSFSKSTI

>SG-PL_contig_4122_1_-2_2_895

AAADEAAPDSPFPSGAAEYEPKNKKEDAAPAAQQEEEVDIDLSDPEVEKAAIKIQAGFKG

FKARKSLSANKGEGGEDAPKEGEETTASEEVMVNEGEGQTEEVDIDLTDPEVEKAALKIQ

AGYKGFKTRQDLKGQTEAESSTAGAKGETGESNVEKTEEGAASDDKAEAASSEQQTKDET

TEEEKPVEGGSEEPKSGEEQVEDSQEKSEEQQGQGDAQAAAEGEGEHAEAEGAKPEGDST

KAEGEEAQQEGEDGRGEAGDAQKEGDDAQREGDSVQEEGGETGKEGEGQKVAESEGGD

>SG-PL_contig_4123_1_+1_52_234

MSECLSVCERDKKKQKNKQTTPPPKKKQTNKQTNKKPKTKTKQQQQQTNKQKKQPNKKKT

L

>SG-PL_contig_4124_1_-3_93_1418

MINIWTIGGGKGHLDIQQQYVTALTWPQSRGIEGRLGLSMDTLLVGRHDGSLATIDVIDN

STFRCKELAHCYRNVSVTQLAWFDEDRKFAVAYHDGELMLCSRNEFEEPVKVEAHQSAIT

SLQWDPTGHVLATCARGDPRICVWSHRGEGLFCAAELAHPSAVIVLEWCVMQGIAEDKKL

MIAGGCENGKVCVWTVEQPTSHTKAFLPKSQSMDSQDFEDSHFRESSKAVKQKRLHSKLM

VTFTGHIMALTSLSFSPNALMLASGCSKGWLNIWSLQDGCLLQTYTGTGSARGLTWFCDR

TIAACFSRSKDVILVNYSPDMFFKNRVMAIARKHLKQQGIVGLHQAPCFRGLLQRLPTML

QDQFSHEKPSVLLGDQLLHSQYLQCLSALAVGFGLDTALCFSPQPLHHYHNTGPMCLDRL

VPEWQWLLSFSTALKSAAALLQ

>SG-PL_contig_4125_1_+2_212_418

MINNLKIIKVIFLSTDSQTLHPGIQQIDHLCKFYSVFIHPGIFPFYTQSVTTMLSFSRLT

QPTSNKISL

>SG-PL_contig_4126_1_+1_58_297

MISTPFTWQGLCSVFPQVCVSLNPNVLLGHGRGGRMVKTLDFPSGGSGRPRFEPSWVPSL

VGKGWRVFRFPGQHISCLCV

>SG-PL_contig_4127_1_-3_66_380

MVNWKGLTLNMSKESKKELTKQLEVHSRNMRAKAKSWTMNSPGTKNVSTLMDSVSLRDSP

TKARDPSPVRVARVKSLSDPNRSRSPARRNSSNKGDVNSDYNVRI

>SG-PL_contig_4128_1_+1_1294_1590

MCETVGFISNSQKHHQHMSAQQCHFFTFSTKRFNNTRTNFHPDGKQNSCILFISGTLSIS

SDNTSNSPFFFLQTNYLSSPESRLLFETAQIYKLLTDFS

>SG-PL_contig_4129_1_-1_1_246

AESLTASLQRLSTSCRSLSTVWRQFILGLPLFRCPPGFQLRTWLAGLEDGSLAVWPSQLS

RRLQIMLVTGFCLLLDLTSSFV

>SG-PL_contig_4130_1_+2_173_391

MRANTELSTKILSTDIYTQTGHVRDIYSIVKVITYKLSKPCQKSWYIAMYFSRSCILYNY

HSICLSFFGCCLE

>SG-PL_contig_4131_1_-2_14_253

MPGTIIISISEGWMELCIPRSDVSSSVPICNIKTDLFAAHCNSSQVTGLALGLLCWLEVA

LRKFGVSRYLSFVLNNISHY

>SG-PL_contig_4132_1_-3_21_335

MLQEGISNEQLGGQRLVFENEEGEFLFNMRVLGKNKGWKELALTFRRMIYSAGFHHYTLM

QDDGTTPIDYFSFYDPAITKVKVIHVSDEGQDVHRVCWCEDHLFY

>SG-PL_contig_4133_1_+1_1_114

CQTWGLKINRNKTKVVIFMKTDPQTPLISLVERITSKL

>SG-PL_contig_4134_1_-1_1_294

DISVCHDIDVTQDSESKHPKGMCNKCYQRLVHCKKHYSDSARIAALSNAAGDIRKASTAW

TEFDPSSSLEQCLSCSLFDTQRKGGRPKKPKARAPKRK

>SG-PL_contig_4135_1_-3_81_1679

MTELNTAILLLFVSATIAAGCNETQSFPCDGDRCIPISWRCDGHTDCLDETDETSCEDIQ

QCLDVSQFRCVNRRCIHQAWVCDGENDCGDSSDEANCSINTCEPGEFQCQTDQKCIPGRW

RCDNAVDCLDRSDEKNCSTTVAPTITTTTSSTSGSDCSGLWCESDAVCINTSWICDGYMN

CADGSDEKHCSTTTTSTTTTFSTTTTITTKVPTTQSDCEGFQCESDDMCVHSSWVCDNDA

DCPDGSDEENCSTPTSETAPTTTTTAVNELTTTAAFRCADSEFQCESDPSLCIYKAWRCD

NDPDCPDQSDEKNCKNTTTSTTTTTSAVTTPTSIHVSSSPASLCSDGEFQCESDPDMCIH

WSWQCDGDSDCSDSSDEKNCITTTSAAVSTTFTPTPASPTPASITPVEASGIPGNVEAQA

TSSPNQAINTTSSGGTRTTISTALPNADQTSSTEQTSPIQQTSPTEQTSPTATSSSSVSP

SADGSRKKGRVFYMLLMGFRAESEKKAISMFEMMKNLAKNATRGEIAFVGNAS

>SG-PL_contig_4136_1_-3_99_494

MAEFSNQQIDPYLCTCGPCKRFWRDTFRSSSDCLLQNGEDRAGESATLGKLKPQTLVTVG

DRVMVHGNPGVVKFVGFVDDNMVAPLLKVGIKLDESVHLGHDGMFNGKRYFYCTNGHGIL

VRFRDVVPLRTP

>SG-PL_contig_4137_1_+1_1_402

LSPLSLSSFSHSLLFLSFSFSPPLSSLSLSLSLLSLSPPSLTLVLSLSPLSLSSSLSHSL

SPLSLPVFIFSVSPTSLSLPLSCSLLLTLSLPFSFSLSPVSFSPLSLFHSLSLPPTPHSP

PSLSLLFSLTVSPL

>SG-PL_contig_4138_1_+2_2_247

SLPFSFSLSLSLSPLCHSLFLSLSVFLSLSLCLSVSVSVSLSLSLSLTHSVALSYLSLLS

LYLPHHGLSGAQSNLNIQSKRV

>SG-PL_contig_4139_1_+2_2_127

KKQNKKISCELKVLLQQNLVSYYNVDTCTLLITMKTRWFMLV

>SG-PL_contig_4140_1_-1_94_579

MHFILPSPAPTYIGFSLSLGFYWSREKGGPAALGNSRFSLTLCPACVLESLQQHRFHSPL

GLKEVNHPITLTMHVLQINRKQNQVLVVIDGKYGNHFILKACKFHVSFALPSPLYSFSLC

LSLTHSLTHARTHACTHTHTKESCAFMHTYTFSIIHPYLLSR

>SG-PL_contig_4141_1_-3_117_2186

MSDIQEYLDVVQEVRGALNPGRLKLQRNSIIFKNNKTGKVDQFQSSELEKSQWLKRARGH

CLKLMLKSGNIHRYDGFKELDFDRLSKFLQEFYEISLEKVDMSIKGWNWGLAKFEGNSLN

FELDKSLVFDVPLTNVSHSTTSKNEVTLEFHQNDDAAVNLMELRFHVPPDASNPDGDPVQ

EFYQQVMGCADIIQATGDAVVTLSEVQCLTPRGRYDIKMYPTFLQLHGKTFDYKIPYTTI

LRLFLLPHKDGRQMFFVVSLDPPIKQGQTRYHFLILLFNTEDEMTIEMGMSEEDLRTKYE

DKLQHEMSGLEYELVSRIFKVITQRKITVPGSFISHSNTRAIGCSYKAATGLLYPLERGF

IFVHKPPLHIRFDEIVGVNFGRSAGNTRSFDFDVETKTGTVYNFVGIEKDEYGKLYDFVS

GKKLRVKNIGGTVDKSLASHEGMSDSDSDKGDHDAYLEKMKREGKERQEDDDSDDSSDES

FNPGDSGSEVAEEYDSNPPTTSSGDSDSDYSGGSGSESDDSAADERREKERERRQQQKQK

EKREKKEKKKSKTAKTVPEGGTRKKRKSQKGKDPNAPKRPQSAYFLWLNEHRESIKQENP

GISVTEVSKLGGEMWKEIGDKTEWEEKAKKAKAAYEIAMAEYKEKKKNEPAGSDSDTGSP

KKKKAKKSPAPRKSPKKAAAKTESAGGGGG

>SG-PL_contig_4142_1_+3_993_1580

MASHDTQKSCMDVFPICLHNDNLNGPQHLDGFGEANIIKVEMDETSKSQYADDVPQVLDP

GDKFHKVICQNTSHTPVSMEVDKGSEDVIHDGSLDEQSCWHDAHHADSHHTLHLPVSGCH

HHMTRAPFLLWCEGTTAGTKVVQGSCTGRAGGEDFTVHFMVQCSWCVAHFDQQTSGPSTT

GPKQREGSHVFKRSEE

>SG-PL_contig_4143_1_-2_941_1141

MSAVPGCCPLCPLTPPTHTHTHPNSYPDFDIHTYHTLMFTHARTRTHTHTHPPLSPRLHY

MCAAKQH

>SG-PL_contig_4144_1_-2_2_403

PTLDSQAGVGCSSISTRVIPTSPRVASRQGASFNKNPPFPSRSHSSPQELESLGVGTSVR

TATRPQLISSSRSPSPHSCGPIVAEDHAIILLRLDKETSSHPDNTFHADKETSLHPDNTF

QEARLHLDSLLESM

>SG-PL_contig_4145_1_-3_591_10994

MNDVVVELGQLARFDCRVAAYPDPEITWLKDGNKVVPTERYILQNFHDDIFSLLIKEVKR

EDSGCYTCLARNEYGEAKTEALLNVMALKAEAEKGDMAPSFATKFGNVECLEGVPVEFTC

QIKGRPPPTITWLLNGREVAPSLDILTRQQEDSIMLAFRSVQLEHSGDIVCKLKNHLGEA

TCRAKLLVKEDLSKKGDIPLFEERPSDMEVTEGGEAKFECVITGTPEPEVLWYFNGRELF

DSRRRVIRKKGDKYQLILREVIPDNAGVYTCKAVNAAGDASCAVELSVKEIPKEPKRRKT

ADDEEVEYSFPPTFTRRILDQRVSPGRTARFECLMLGIPLPDVEWQKDGKSLKPSDKLRI

GREGSTAILEIDGAEPEDAGEYTCILTNDAGKATSRAGLTVQEEKKVRYTTPRLEARVPD

VTTRAPSRGREDLYQPVTQPVRELTPEEKVPSLPFDKPHLLEVRDESVKLSWLPARMSGL

PDNARHITYTIEARELPSNRWTRLATGIESTTYVARHLRPEKEYMFRVYAENKYGQSEPT

LPATMVARQEPEERKRYYAREESPAPIQFPSARPFVMDIGAETLRLGWHPAARAHRLETP

VSYRVEAQKLPSEEWIPLASRVRDTSLYLSDLEPDRDYNVRVRAQGPFGVSQPTAPVWLP

RAAGFTGVPVSRPQITELEPDTVRLQWQRVDVPAFTHEQDPLTYMIEMQEPPSVHWREVA

SYVPHTHYIVHDLEPGTDYRFRVRAQTLEGLRSEPSPATSIFRTLALTRTPIDYVEVTDY

EPDYGTVGLSWRRVEVPPFDADDEPLLYMIECQEPLSDTWRSLVSGVPTTRYRVPDISST

DDYRFRVRALTPYGISPPSHAAGLYRSPTPSRPLASDLFISSMEPESMRLSWQPQSLPSS

HATTPSYQPHSYQIEALEYPSMQWRPLASDVRDTSYQLTGLAPTRDYSFRVRAMTPSGGL

TEPTTPVTVTSLPVRPRMPSKEPVVKEMGVDSVRLQWPRAELPYYVRQTTPITYSIEMQE

VPGFTWTPVRRGIPETSYVVKGLRSDRDYRFRIRPETEYGPGEYSLPVHAYRKPTVSLPS

REPFISDIGPSRITLTWLPAELPTGAQTTRPVTYRIEMREPPSGHWIPVAERVSSNSFTV

NFLNPDKDYAFRVFAELDGVESEPTAIAYLPHRMGPPKMLQEPPYISSVHPDSVWLSWRS

VDIPARITDYAPVTYRIEAQEPPLMEWRPVARHIPTTHHHLTGLRPGQQYNFRVRAENDI

GLSEPTPSVLLTTRSVPPNMPSHEPLISDVSPGSLRLSWRPAEVPSFLLDSLPVTYAIYY

QHLPDVEWHPLARRVAGTSYYITGLSPDNNYAFRVQAENQFGSSKPTHPARMPRLAEIEA

PIYSPEIEEVEPSSLRLAWKTPRIGPYKKKKPTYAIETLEPSTWTWRPLASNLTTPSYKV

STLSPTQDYIFRVRAEADKVLSEPSFPISYSRTRAPPSVPVERPILSEIEPDSVLVSWHP

ILYPSLSRGEGSRRYKLEMKELPYGSWTTVLPHTSKWSHEVTGLRPNMDYSFRVSAVTDA

GTSPPSNAAYLHRRPVIPRTPLEFPELRDVADDAVSLHWKRVDVPAYEADEEPLSFMIES

QRLPSYDWEPVARGITDTSYRISGLQPSQDYVFRVRGEMPSGVTTPSIPVPYYRRPERTG

VPINYVTVDDHNVPPYSARIQWKPVYIPRYTSPSSTLYNLEMRETPQQDWQTLARDVKGL

EYTVPDLSPRKTYMFRVRAKSPAGDLSEPSIPVPFYPLHLRAYDSQRVREYVPINEDFSL

PNRPYISTLIVKVPPRMHIEKPDMYSINPTTAHLTWNAARVPSATTQLSPTTYRVEVRED

GTFNWVEKASNISGLGCDISGLNPMLDYAFRVRAVNDFGWSEATLPTFLHRPINLDYEEP

DIDWEKISPTVPPLVAPRLPIDSPRMTPVSDDSLRLSWHPARIPAYARKSPISYTVEIKD

PSAAAWSPVASKLSDTTYQVMGLEPKRPYLFRVRAESEFGSSEPTLPAELSRTKPTHTRS

QSVERQKLSADYDLSTRRGSFIDSITTGVPPRVPTGRPTISNATETSLTLSWPAARLPAY

LKNTKLDYIIEYREASNRLWTNLAENIPDTSYKVTGLDPEQDYMFRVRAHNDAGTSEPTL

PATLERPKKKAATPQRRGSSVERRSLDRRSMDRMSERRSLSRASRDRKSESRLSRRESAK

KDGEDGDTLPGMSPEDSPHAPEFSSLAEEAVQYGVESHPMTISLQLRGYPPPEVNWYHQG

KKLELGERYDTYVTPTGQVFLEFLNMGWEDIGDYKCVAENELGTAEKTVKIDVADPPTFI

EPLRDIMVVERGQGTLSCSVDGIPYPSVRFMKDWRPLTDTSRLVVTNDPEKAEHWTMTIS

DAIPADAGSYMCVAENVAGKIFSTGRVTVEDDTVASLLPGYKDTNLEDNYYVLEEIGRGR

HGRVRRVIEKSSGKEFAAKFIQVREAVDKEFFHAELDNLLKQKYKNIVSVNDAYETPRQL

IIVTELLGRGELLDAVVKQGQWTEGKAAGVVKQLLETLQHVHSLGIVHMDIKPSNLLLSG

NNVDSAKLIDFGLARKLRPSQDNFLNHGTPGFTSPEALDVKPISTASDVWGVGVLAYILL

GGQSPFQADSVADSLKKTQACDWSFDVCKSFSDVSQDAKDFISKILQLSADDRPTVDECL

NHPWLKKTEGGAKIDVAPHKAFQDRDKKQRQSTAVRTVAYLRTLQRQMTGGDVLEKGLRP

AVDPKTGLVSFPDIEAYGEFLDSESWYEWQSRYGQGPDSGLLPLKDPEYTARIRGYKRTV

GSADDSAFLLEDMDAMSAADREKYKMMKERRQMADIDKEEIPPSLEKELQWLEERKQARK

ARSPQRQGSVDSRRSSISTEGEAGGPVPVFRQRLQDLTFETGDTITMYCALEGPPPYSTV

WYKNEELLSDGSRVKQELFEDGQATLTISAAKPYDVGVYKCVARSKSGRASSRMRLLLGD

VPERPGQPAVLQTAAQELVLIWEAPRFDGNCPILYYRVDYKAAGDRRWSTALYTPLETCL

VTGLKPNTSYRLRVSAANLLGRGPFSWSTVEVTTKKQGSISSLSIEGLNLEQLKQRQEEG

KEIANKPRETEIAEEGPEDTSSPILQTTEPDKEYALGDTIYVGQFGEVKEGVNKKTSKRC

LVKCVPTTLKDSQHEFDILKTVRHETILRLLSAYSTKSTLFLVFEDAPGIHVAHRLSLRR

RYSEDCVAYVIRQALFALQYLHRNSVIHLNLQPSALLLSSSQGLNVKLADFSLAQVLTNG

VDGVKVPRKGYPDFIVPEVVVKDKASPASDVWGVGTLTFLLLSGLSPFAGHSDEETLLSV

AYSRYDAGDLLDGVSNEALKFLFKVMKRLPRNRPSVQECLEHKWLQLTDAMMKTRESNLF

RSNRLRQFVLNYDKKRQSKDCRILFDDLVLPPELVASLQPDVLTSDKK

>SG-PL_contig_4146_1_+3_54_230

MVMSVQSFTLSDQFFRCRPCLRPPSTVPCMMVLQRLSCHERAKPHQLLPLDSGEERFLG

>SG-PL_contig_4147_1_-1_109_231

MPDLLSQPLTDQLERGCHCLLLPSGSTDSCRSQGTDDDVDD

>SG-PL_contig_4148_1_-2_2_241

PRNLSSPPSSGGSWCSLATSRGMTASARPSCKAPSRTGQTLPCQTSCWQPPTDQLGGGCQ

LLLPSGPSDDCRSHGTDDDD

>SG-PL_contig_4149_1_-3_18_1502

MAESLRRLVNIGTFTVLQDKLEDWLRDYYINSCDQNTARCCEIIELNAKVQGQLFKLLSL

CAQEGGMYGGTNIIKSRLLPWLGQNFFTSGGAVTADTSLSVLAEAAAKDRQMNDLQDTYE

KSLNDLDGELQDTRAENRDLKDELQETKIELDRSVRSSTSDKMFTDLEVKELRRKLADAQ

DEISRLRIRCGHVDDYERKIQNLRDELYLSSVTKPRPLSPLPEFSSYRYKPGLSSPLSLD

DPVQRIRQQNAVARFNDMFAQDRLDAMDTLRRYSDDHENNQRILFAALSEAFSAAKTAFR

QFKMKTRANLAKTHYGPDTLEEAVQDYINRNTDLIDTPGLVSDVIRALNRNPRIFLPPDA

SFAVVQPFIREAVKLAWQMSALAHPLDIAIATDAELFDESKYRRSYDSEYTAPLVGHHIW

PALMQGVRVVVKGEANTRRGASLSSPRRSRSPTRSLSPGPKSFSPIRSRSLSPSRYARSR

SPSPLRRSFSPRRII

>SG-PL_contig_4150_1_-2_17_250

MVYIVYIIIVPWHLWFALSLFLGIYGLHYHCSMAFMVCIVYIIIVPWHLWFALFTLSFFL

GIYVITLFTLSLFLGIYS

>SG-PL_contig_4151_1_+2_38_250

MSSLAPLRAWILLRRLQVAKARRKAILYESSEKGTTSRGSKGGDLRAPRTRARSHLGSLL

GLWPHGLSGLS

>SG-PL_contig_4152_1_+2_1034_1276

MKHLKHANMSVTCTTMYRNHWGKFETTDKEVKINTYISKTTMNIDTEFWMARSRNNFYAN

TNREKLVNNQQNYRGNNSSMM

>SG-PL_contig_4153_1_-3_3_254

LFFFWFFFFFWGVGGGGGVKRKKNWWSLMFYVGVFRSKSKSGVFLFSNAVKSWINKILEN

KLHPFHFPVQHGSCAKCLEMSVST

>SG-PL_contig_4154_1_+2_2_232

LFQLQDLRLSCLQTRLSPLQVHLCDGQLTGSLIVFAFSVFCDVFGFFELSLQPIYALIRS

QTLAFQNLASSLTLICS

>SG-PL_contig_4155_1_-2_206_1126

MHTSPGEGTAVEDCVSSEGFSGLSVCSSLEGADSATNSISKDCSDSFVTGSAAMEQESIS

MECAIKKEDRPLLQLHTEFEEGENISEMMNTNTAHVPKFSEAHKGDLSSECTVDDIEKVH

EKHISICTKSLVEEAVSCKGQKGQSTSLNYVPADQSGGDLTRLEVADECKKNSFTPITDS

KEEHLSEDSSDNDADSRGTFIAATRVKSDFDKRLSSKELADIPRTITCRKIDDTQLSILL

AKRREVNDSADYTVESPGRSRLSSVNVGQSEALHSEHSHSVHSVIKQPATVKHVMETSQH

KVHKRTS

>SG-PL_contig_4156_1_-2_2_187

THIHTLARTHARTHTFSVSLSFSLSLSLSLSLSYSNFSSRSISLACLMTDCSSKMFSPVF

IY

>SG-PL_contig_4157_1_-2_593_1159

MDSVGQEEGSYCIFLGQEKKKKFPQLQVWRADRREYVYECKCMCECECVCAREREREMER

THGPFWPWVCDHGWRAPDCMLTLMAVLCCVPCYSSHLVLVCVCTSVCPSVCLLTATPHKP

ASSVFTFIGTLTLFLQISGRERGGERWQLLCFLLLPPLPAFLASSTTADWGLHYFFFLFF

LFFFFFFFF

>SG-PL_contig_4158_1_-1_1_180

EHNYMYSVQLPCTHLFHCQFAGLVKGERLRRCILFLVAVYICSCTLNRNLFTTKSAAVKK

>SG-PL_contig_4159_1_+1_1_537

SPFDPRVRVNLKLKKNKRKKPPPPQKKKKTPSGLPLLKRSSPPAVFAPLPFLWKGICFVW

CAFRFDTPLVSHCGRNLKTFTLTTTVGICTKSTCAVLGGAAFFCCQQMGWKDRRHYSPRA

RAHAGTRMHAHATHKHTLSLTHICTPKISHAHTYTRSCTHSVLSNTHSQIHQKIIKNIS

>SG-PL_contig_4160_1_+1_1_234

LVPEPPSSVHARRSSNTHVKDPASHCLCLVVYGNKKTPSIHAHQRRSMQTVQQHNACTVW

AKQSAYRHQRMRKKKQKK

>SG-PL_contig_4161_1_+2_2_112

GGRGDATRGMYGTDYKRQYYPHVLRVYFTLKNSSHTI

>SG-PL_contig_4162_1_-3_3_128

CFDSEAEMILCLCVLAIAFRILTFQNFCCVTFIYLFSIIPLP

>SG-PL_contig_4163_1_+1_1_132

DCRTLQTLRAEVWPEPMPLEQKLYGMMDDLQATAEFIKRSGLTI

>SG-PL_contig_4164_1_-3_81_3056

MSAKAPRGFQPDDAEKQRVNVPPIRSRYRKEDLYSSVLQFKQIDDHAIQVSQTEHDNFAD

LIWHLVYSINLQSDLDKARAIFRWMTSKNMYGMTFRDGATPGSPEEILLAFRSKQGTYAR

IFEVLCRFAGLQSMILTGYAKGLDYRPGNQFKSDDYNHSWNAVLIDRNWYLVDSHWATRY

LVSEKNMPENLVYEYDDFYFLTDPEQLIYSHWSQTKEWQLLDQPITLHEFESMPLVKSYF

FKCAMHFVSHRNGVIDTKNGLIALTLGHKKPTNFTYKITFADSGDEMYQGEKLKNYALLH

HSQNQTGTLVFRAPKAGQYYFSFFSQLLTGEVGVKSTFTAACEFKVVADTASAGAVPLPI

CSDSSWGPGLAVEQLGLIANPPEGVITTSDGQVCLEFRKTKEAFVLCKLRRNGMEEEELE

KYIRDKDLGSSVQVTIHLPSQGEYGIEVYGNDPAKDGDTYTHICQYFVHFATPSEQQRAF

YQESPERRATMEGFTFTATASGGYQPGSMGLDSATANLHLSDPENLPPPPPELLQQQHFA

TSSSLPDTRHQQQFISGTAPDTYQQQQYVSGNVIDQRQQPVVSQVKFTPARSQNVSGGTT

ASLQMNVCPSGTSQIGEQMDVYAPGSDQKALSAPTATKVVFTAPGQKDVYALGSDQKDMP

APTTAKVVFTTPGQKDIYAPDSDRRDVNAPDSSPRDVYASVKKVVYAPDSDLYISSQDRP

SAGRDTYAPDIEQRDASAPAIGQKTIWTPSGQKTVYAPGAAKKNVWTPGSPQRDSDSSGS

DDQKVVTSSVTVQKNVWPPGSGQKDSDAPGEKNIWKPGREVDTDTQKVGYAPAKYKKPTW

PPSSAPKDSDTLSSEKKSAVPPPTAKKPTWTPGSVQRDTDAPGQKNVWTPGSAQRDSDMP

PADQKNTYAAWTPGSGQRDADAPAVALKSTWTPGSPQKNVWTPDSAQRDTDAPGTDRRDI

YAPGTDRKDIYAPGTDRKDIYAPGTDRKDIYA

>SG-PL_contig_4165_1_+2_2_160

SLSLSSQPSASLVHDHYFSFLTFARKESHASVTNCSAVKTPWACSLLSALSRA

>SG-PL_contig_4166_1_-1_61_378

MNAVAPNNNDVRRPNKVTRYKAQSTGGPLDDPTPEYMNLLGMVFSMCGLMMKMKWSAWVA

VYCSFISFANSRTSEDTKQMLSSFMLSISAVVMSYLQSPQPMMPPW

>SG-PL_contig_4167_1_-3_996_1598

MWTYLAVCLLVCGVAVSTEGQTRVCESDDFLQIQRTCQVENRALLLAEKKQNDGMVCKAL

FDLWYCVAGHVPLCFHNLTDIYGAYFHSPHNCALSPQQIMTLQNLTTNTARRQGQEDEVD

VEVDVIVPLSPDPSTSSPEDSSLGEGEQEEEGKVHVRKPEEHSGSSDHTGGGSSLLGQCP

MPVVVMCWLATWRMTSHLLHS

>SG-PL_contig_4168_1_-3_84_824

MLASVTRRFLGATRQQIRSLHRTSPACSDKLFVHRDTPENNPDIPFQFTPENLKRCEVII

KNYPEGHHAAAVIPILDLAQRQHGWLPISAMHAVADMLKMHRMRVYEVATFYTMFNREPV

GKYFVQICTTTPCMLGGVGSDVILEAIQENLGIHCGETTKDDMFTLIEVECLGACVNAPM

VQINDDYYEDLTVEDMNRILNELKAGKKPKAGPQSGQDNRFACEPKTGLTSLTGPPTGPG

FGCRADL

>SG-PL_contig_4169_1_-2_1643_1975

MRGDGSCVVLLAIALTMMVGRCPAPVSATVEEDEVNCRPQHVHLSFGEAADQMAVMWATN

STCDSVVRYGTNPWHTQDSATGRALPYTLNNAHGLHVINKVMLKGLKSATT

>SG-PL_contig_4170_1_+2_2_121

TKQKKQTYVTTTQILTLHICLGLSTRITNATHYLKNHRHN

>SG-PL_contig_4171_1_-2_134_613

MADKKAPKQLLAEIGQADVNKMKHVQTSEGNTLPTAQAIAQEKTEAELRTNIGAFDKTRL

KQTQTTEKTVLPNSEDIGKEKTEAELRKSIGEFDKKRLKQQQVAEKNPLPSPDAIAAEKK

EEEFKQSITGFNRRSLKETKTEEKNPLPTKEAIQQEKAAK

>SG-PL_contig_4172_1_+2_2_205

SLKKSACTAFSLAARSINSAEEPPASSISMYGPTPSGNISSATTMAAGFMMSTYCCTCSN

GLTHCHGQ

>SG-PL_contig_4173_1_+3_45_188

MCKHMCITVCDRQNRTSTIFVYPHPRSPNNLVLNTTTKVTLQTLARTQ

>SG-PL_contig_4174_1_-2_2_601

MVGSEVYSSEIKKTEVLMENFRRAIGLRIKETKEVYEGEVVELTPVETENPMGGYGKTVS

HVIIGLKTAKGTKQLKLDPSIYESLQKEKAEVGDVIYIEANSGAVKRQGRSDSYATEFDL

EAEEYVPLPKGDVHKKKEVIQDVTLHDLDIANARPQGGQDIMSMMGQLMKPKKTEITDKL

RKEINKVVNKYIDQGVAELV

>SG-PL_contig_4175_1_-1_37_306

MSVGKPAPVSACVDDNSPSVSLSLSSVSDSCMHQTVSKYWGQGGDGNSDGQCPCKLTSSS

SMPPSTGGGLSLLPSLKLIPQSSSAASAHS

>SG-PL_contig_4176_1_-2_2_151

IIINILLPLVLSVSSSMCEFCIVFKMRSVNLTSRQRILFQLRSFVFKSPQ

>SG-PL_contig_4177_1_-1_25_891

MNVGVQTLETIKEWYNNTMAQADPRVLGFPLMQGPERLAMVISLYLLAVTQGPRVMAPFK

PLELTRLLVVYNFAMVALASYIFGLCVTIIIGDGFDFVCDTVDRTLSAKYGSTFIYEAGW

WFMFMKIIEFADTMFFILRKKFSHVTFLHVYHHSTMAIISWIGFKFVPGGQTIMYPLVNS

FIHIIMYTYYGMAAMGPHMQKYLWWKRYLTMLQISQFVLLIIQTAVNAVSPCEFPKIFSY

TVVFYTFSILLLFLNFYHKAYLRRHAKSKTTTTTTTTISSSGDNDKKKE

>SG-PL_contig_4178_1_+2_2_109

GQCLSPLLRSEHTVNHITYSNTLRELVVILVPAVVK

>SG-PL_contig_4179_1_+2_728_1087

MDLLCKSCELWKKNMSHFLCSIFQPPLFWLVIFFFRCIAMMHYNDTVVHILSSTCCCNIS

TFTSPYTFSPSLFSLFCCLVHYLKSSCNCSEIIPNKRHLATKIQPPKSTCLHCKSYQSNK

>SG-PL_contig_4180_1_+3_6_881

MYRKVSPWFVPEILLNIAAGHISLVYGLKGPNHCVSTACTTGAHAIGDASRFIRNGDADV

MLAGGTEACVGPLALAGFARMRALCTGFNDSPELASRPFDAQREGFVMSEGAGLVVLEEL

SHAQQRGATILGEVLGYGLSGDAYHITTPSQEGDGAQRCMEAAMRDAGLKVGMVGHVNAH

ATSTPIGDAVESRAIEGLLGRGMLVTSVKGALGHLLGAAGSVEAIVTLKSCQTGLIPPTL

NLTQPDTGCDLDYITGSPRTWHAKDGGRRIALTNSFGFGGTNASLCLAEWLP

>SG-PL_contig_4181_1_+2_923_1123

MDRIHTNLQTHRHTNTTNVNHTTLSQKKKAHKKKKPCNRRGSQVGKRRTSKRKVLGSIPA

QTPLPSG

>SG-PL_contig_4182_1_+3_3_122

YHFCVGVNVHMSKLEYHFCVGVNVHMSKLEYHFCVGYSSA

>SG-PL_contig_4183_1_-2_77_838

MFCLKTAPLVRSAVHSRILSNGLHSTSALNAKVAVVLSGCGVYDGTEVHEASATLVHLSR

NGAEVAMFAPDRDQLHVIDHTKGEPMEPKRNVLVESARIARGKIEDLSNLEASNFDAVIF

PGGFGAAKNLCTFAVDGTAMEVDTNIKRVIKEFHQAKKPIGLCCIAPVLAAKLLPGCEVT

VGSDEEEGGKWPYAGTAAAIGSMDCTHVKKDVTECHVDHSNKIVTTPAFMCETAIHKIFD

GIGLMVKGVLNLAK

>SG-PL_contig_4184_1_+3_3_176

QIYVGNNNRLQNHNPMLRHSVCMFVFSFSEGVGWIGMYNELVGFSVLIGWSRSKILVV

>SG-PL_contig_4185_1_+3_3_287

EIIPAPTLPHIDWREGGLCEDSGHWEEIYINKAGVHWMTAEISLKYTVYWSQLFEWDWKT

GNHLWSMFPSGWCRQFHRIYSNPSAPSFHQLPTSS

>SG-PL_contig_4186_1_-1_163_408

MHAEHQICTLKILIAMSVFGSLWKHEHAQHAHFILPSPAPSCIGLSLGFYWCRGKGDLRR

GQQQVLLCPGLCPGVYPVAQIP

>SG-PL_contig_4187_1_-2_143_1159

MLLSQAVRCSTVPLTAFVTTLGRSSVKVPKQPWLTTRFQTYASESRHAVRRQAQKAKSLK

EKLMAPAGDTAFNLGRGLVAGASAVGIGALCYYGLGLSNEVGAIDRSVMWSQEVRDRIRG

TYMYFGGSIVLTALSAVAVSRSPAMMNLMMRNSWMAIGATFAAMIGSGMVVRSIPYKEGF

GGKQMAWMVHAGIMGAVVAPLCLLGGPILTRAAWYTAGVVGGLSTVAMCAPSEKFLYMGG

PLAIGLGIVLASSIGSMFLPPTTALGAGLYSVSMYGGLVLFGMFLLYDTQRIIKMAEHYP

TYTDRPYDPVNMSIGIYLDTLNIFIRIAMILAGGGGRRK

>SG-PL_contig_4188_1_-2_86_217

MQNIKMHVKGPVSHVSVHWLMETQKYRICALLFPSLPISRPCQC

>SG-PL_contig_4189_1_-2_2_160

ERSEFNHAITKLVWILTFMSKGLSLILPGSSWWVKGGEFSYLSGQRKCRPACA

>SG-PL_contig_4190_1_-2_47_253

MVNQLSTALTGAKHEQEYMEVRERIHRAINDNTNSRVVLWAFFEALVLVAMSLGQVYYLK

RFFEVRRVV

>SG-PL_contig_4191_1_-2_107_2209

MRPFSFGHSGGVGAGFSFGGHHSPWNFDFDFDFNWGREKNWWEGPNVCRTEDVENGTDSE

SSTVAHHFKTSTQVCEQSDTAYKCTTTVQSADGEVTKVEVYECCHGYSRKAEEFGCPQEL

KLMDLPNTARELGLNDLLRAVSTVGLEEELAQGNFTVFAPVDGAFPLDLGPGISLQRDEP

KAVMVSQSLREKMENELQATILGHLLPEPRKASSFSDEDVLLTGSPLDSTIRMNFYSRPE

KLTTANCVKVTSTDNMATNGVIHVVEKLLPTVTSSLIDLIQRDHQFSYLKTALARANLVS

TLQNDGQFTLFAPTNSAFQRLDRDLLDRLLSGDPKCLEKVLKQHLLPNVICSAVIQGRAK

SRNQLNRYVNLTRTEDGKLFADDAQVVHSDVMASNGVMHIIDAVLVPDDALDVLAVAENN

GLTELVELTEAAGLTSTLLNISDVTVFAPSNEAIQALPEEVMKKLKGDPDLLQSVLTYHV

VPEEMQCRQLYNNRHLDTLNAQGKVRVNEYSSFPFGHRRVTTVQCAPITTRNVPSCNGVI

HVIDRVMLPPSGNVIDVLAADSDYSTLVRLLKVAGLADSLQEEGPFTVFAPTNKAFSELG

KETMKELEGDREQLAAILKVHVFKGHLCCTGVFNAAWWHPHRVSTLNGRRLSLSRDRRGR

PLVSDAVIGSCDNTATNGVVHGIESVLLPRRRDPFSWYTFP

>SG-PL_contig_4192_1_+3_1869_2576

MNQVTITKAIWNEDRTRQLCLLLGIGLVLLLVQNIFNHRCRPDIKGGSPSRDIPDVIQFK

FSQQSFATFDGVERQCVGFELQTPTLETFLACFAHQGLHGFPVLRLFLSMPQQQEDGCHD

SMHNGGRLGVATNLRHSRLLKFQNFMSNSLQSTQTAQEFSSTYHLLRQVGFKTVLSYESH

GSFEFFNLSLAHCFVKTFFGIDLFQEFLSFFQVHVIVRETVRLELFDGVLQLELVT

>SG-PL_contig_4193_1_-2_95_1267

MASIAAKGASQVRFLGQAQQQLPRIFAAVVVSKRNASYDLPSMKRGRGGRSSFSGIVATV

FGASGFVGRYVCNRLGKIGSQIIVPYRGDPYEVERLRLCGDLGQVLFFPFFLNDEEAVRK

VMKYSNVVINLVGREWETRNYNFNAVHVDGARLIARLAHESGVEKLIHFSSLNASPTPQP

IYNKAGSKFLRSKYEGELAVREEFPEAIIFRPADIYGSEDRFLRYYANFWRRAHGTVPLW

KKGDATIKQPVFVQDVAQGVIEAIRNPDIAGQTYELVGPESYYLSDLVDYFYRVMRFRNV

KRSYLSPAFRMKAYAMSFAPAEPLYSLHKLEKEFVTDVTTGCPTLEDLGVSLTRIENQVP

WELRPFRQGAYYEEYLGEFADPVPPPAASAH

>SG-PL_contig_4194_1_-3_3_137

AGKVLKWGVKAANNHQADTTRMIGALRWAANKPTFMLRAGIELKT

>SG-PL_contig_4195_1_+1_61_285

MDREGRKKADDSKGKKKTAETKRAIEKRKLLAQNFQKVGICLPACYYFILFIYLSIFKAF

PAHKVLPCKFKEPAR

>SG-PL_contig_4196_1_-1_76_198

MCVHMCVFVYITTYLCDRLGEHVCMHVGVGVILYVCVKTCI

>SG-PL_contig_4197_1_+1_448_813

MNDLQVINNPSSSSLHCQQVTHDQFKKQSKRFQHSPLDYMLHRNTIMSIWLVVKHLNAKC

QMLLYKRENRERKKRWGEGGEIAIIKIWPLHTATLKLHSNLSQRLKLQTFHLLNKNNNKT

NT

>SG-PL_contig_4198_1_-3_3_113

SVCLSAILWPMKRASSFVVQLTACDVRTSVPECQLLN

>SG-PL_contig_4199_1_-2_113_1339

MPASSSSVTIRSHSVRSQPDVIGDNVPGDRTGKYIKNFDLIKPAENIDKKERDNDELQAV

RARVTGGFKRQPDTGMDEPHIGEHSMMRGRPPPSKSLSSSSTHSSSAPAHENGVDTSMVI

MRQKKSPTTPDTAARRLSREEIDAALVRADNYLSTLHAPAEDVQNVKRRSWEVKSRKEDV

RTKRYSLDEVNSNRTRLTKQTAVSHTDSPATTSDARDEVDHMPVPAVSDISALKRQGFHE

DRQEHKQSSPFDIPPPSYESSTSGHVRSSRLVSPEDKPKLHKLPPPPNKPIPPPNRPNTA

AKRAAPPPPLPATPPKGNPHAFRAQPPPFASPIPWPVKETELYMQDTVTHRDHGRADGTV

STSFGAPSSSLDSSESGMDRLLLTEEDAASQEEAASLWKQHVLREAGGV

>SG-PL_contig_4200_1_-1_106_2547

MPARRGKRGGTKVKQKEKEQKERQEAEKAAREEEEELETGDEASELDTSADLDKSQEDEK

MEEAIDKAETEGKNAKRKEEDMKTESGKDGDKDKKETEDESKKDEAKTKDDEKKGKEKKP

APLLDTPASRVNRDIFDAPHVTKVGPIPFFEVKNMRSRYKWSIRISPVKAEDIMMGDLMS

MPRVQEACFHLPKREDSKGQMELCTATTNDTILTVLRLLRTPFVHHLLTIELTKRRCIRE

EHMSSEELMKYLNDGKGDITNPATTSDETKPVQESADGETGAKVKAEEKDGEDKAKKEEE

AKKVEAARRDEIEGEDEDGTSGRRLVFCEPRETATFVVNLHKIQRIKLQRRVKGEPSRRS

VYVGYVPRSTTEEMMKVIFPMANGIIMRSADDPNMRYTFIETSNPNNAQGFLMSYPGLIV

NDQELKLSMKEKDLEILPKLLFENRRKDGNANKEEKEDGEKDETKKETEEGDEKDDKTES

EETKDDKTESKGAEPEKRELTEEERQRRTQTNKQKKKRKREFMEEVQREWSAKRRREDPE

MMMPPLARGRGGPPFGRATFEEAQELLEIQDKVSSLLSLQKQLEVTISTQMDFIHHSEAT

VRRRAEEMRLQDEAYSRGYAFEAPRGGFSRGGRGAMQRGFSPRGGGASSPRGIMGRTFGG

GGKARDFERSDSREDEFQVPGNKRFRRVSAPRYETKAPAASHEDASKGSKTSEGGGQGNK

AQEPKKFGQGAQSSGEGGNKAGAGTTINTGANKLGGADKSSPGNQFAGSNTGSGSNVGNK

SRFSAGNNQTGSGKFGGGGVQTGSGNFGGGVQTG

>SG-PL_contig_4201_1_-1_1_156

SFLQEEAGGILEGLIGSIGVVYTHKLTNVYAMHGLVFLTRDFVHACGCVDFD

>SG-PL_contig_4202_1_+3_516_824

METSISFKYSMVSYLDLGFRLLFVIVFFIPTFFQVQIQEKSKRKYMHKITVNHTAHLVKN

GNLQTMVSAQTVMEFTATYCDNGCLGIKTISPTDPKMLHVKSK

>SG-PL_contig_4203_1_+2_2_127

LESFSRSCVFFWYHLHDMKGRISVWLDHPVSLSLYQPNTSIF

>SG-PL_contig_4204_1_+3_114_1268

MGKVVRWASNPPRQKSFATETPTRSINQILGDELRTDAARHYMTSSGESRKEASIPTMSL

AKPKQEVRVGAWNVRTLYEAGKTAQTVKEMKKYRLNILGISEMRWTDSGIMTLGSGETVC

YSGRTDGQHQDGVGIIIDKEARKSLMGWEPVNSRIITARFFSKYTKTTIIQCYAPTEQAT

EEEKDLFYTTLQEQIDKTPQHDILLLMGDFNAKVGAENTGYETCMGEEGVGNRNDNGQRF

ADTCLENGLVIGGTIFQHKTIHKLTWVSPDGRTSNQIDHIAINQKWRSSMKDVRAIRGAD

AGSDHHLVLCRLQLKLKRTQKKKNEQLFDSRKLNSEAVKSQFTVELSNRFQVLDNTPADD

IDALCDNVQTAFIDTSKAVLGYRRR

>SG-PL_contig_4205_1_+2_2_277

LPHPLSSYPLAHCLVLFSLSHCLSLFSLLHCLSPLHRLSLSHSLCLFCLSHCIVLLSYPS

LQWLFPSCEPHPQHLIQCDQLYSQYMRLGPHP

>SG-PL_contig_4206_1_-1_256_5325

MGVVQVMVVTGVLVLMVLQAALAANAPLPQGAVDLLEAVGIREAKEGVTHSPGFCAKRAT

RDGGFVEEGFADLAYRVDDSAQLHADLPKHFKRGISTDFSILATIKPDPWTRGKVFSMYD

ESATEILALKVGKYSELIYSDANGRPGEPVPLDLDLTDGQWHRIGLSIEGDEVRVVHNCV

PREETYRIGRTMPPLKFKKEGAVFVGYQLTGEELYTGLVQDLIISNDPEMAYDLCDVYCP

ECEVALPYGAVYSASSSSGRFFNRSSSSSSSSSSSSSSSSSSSASSAAGSLNRGSTAGVM

TLEYGSAEMTDVTYRFGPPGPPGGRGIPGPPGSKGETGPLGLNGVPGTNGLPGPPGHVFV

IPLGSNEGKQSNYAEHFRTMLDQHMLAMRGAAGPMGMTGLPGRVGPAGPPGVKGDLGNTG

EQGPMGPFGLPGPPGPMGKRGRPGNDGDRGVQGPPGDKGVPGFPGMPGMPGEKGHRGVAG

TPGETGNMGPDGEPGDSGEPGPPGAPGEIGPRGFLGPRGRPGPGGPPGQPGNEGPVGPKG

NMGSVGSPGAPGQTGPSGQTGPPGPVGPLGPPGIPGSTGKPGLPGLPGSLGPSGLPGPPG

EHGAKGDQGPMGLQGVLGYPGPRGIKGDSGSRGSQGTKGDKGASGHDGMQGDMGLKGDKG

IRGPRGPMGVEGIEGAKGDDGSAGEAGLQGAFGEKGRVGAPGFPGYPGAPGLKGMRGRVG

RRGRRGARGKRGLPGPLGETGEPGIRGQRGERGKQGIAGPFGPKGDPGPPGPPGFTGEQG

IQGPPGPVGIIGPPGAPGVPGKDGQMGMPGDRGESGLPGSPGQPGPVGVMGPTGPTGELG

SDGERGAPGNPGPPGDQGVAGQSGKAGERGPAGPQGNPGPSGPPGLGGFPGGRGFPGSSG

KPGPRGEPGPPGPPGPPGPKGSNGLHGPAGSAGRPGTPGVRGGIGPVGPKGAPGSAGPAG

QQGTEGGPGPRGYRGPTGPVGPPGMDGDKGDRGGPGVMGLKGDYGPQGPPGPQGPLGIQG

PPGPTGPPGEPGTRGPQGPSGPKGSEGPRGLLGPTGLNGLQGMPGPPGPKGETGDEGKKG

PTGPPGSQGAAGPAGPSGPPGLPGTPGGEGPRGMKGEPGPPGSAGIPGADADRGPPGPRG

PKGEDGRRGSPGAPGPQGVRGIDGEKGDAGGFGPPGPQGERGEQGTKGETGDPGIDGEPG

PPGLDGPLGPAGEIGLPGIPGKQGEMGIPGLPGSAGEPGSKGDRGVRGPSGQRGPPGPPG

GSGPTGPTGPAGQPGPVGEAGVSGPPGTPGAQGPAGPQGPLGKKGSRGIKGIKGHRGEHG

NPGPEGQQGMKGEQGVPGPPGPKGESGPHGPPGPRGTPGNDGAPGIPGAMGAPGPKGEPG

QAGAKGEQGNPGPVGSPGQPGDKVEVGVGDLAAPRQLKKRKRRRRRRRSPDATDIDTDIQ

SVYARLIERQQDGGPLQLQEVIQEVSSMMLQHIDQLKAQVEKIKFPQGTRENPAMSCREI

VLGHPQYQTGWYWIDPSQGSGDDAIQVWCNVTVDYIETCIYPTPKTKMVAEKAWTKPNGK

TRWFSRLKGGSTIKYGSSVQLGLLRLLSEEATQRFTYHCSGSVAWLDETFGSYDKAIVLA

GDNDFEFETGNFPAEFIISDTCRDRRQSGSTVFEIRTNKVDRLPITNFQPLDYGHPGQKF

GYEAGPVCFH

>SG-PL_contig_4207_1_+2_68_268

MKNHSHQYNQMNKQKQPKINNKTKKRTIIHHRSSGSIAETTNKCFMTTICDFYFQVKESR

LRPMKII

>SG-PL_contig_4208_1_+3_87_242

MLDGFGQRRKITYTCLTLSKTNKLACQYSTAYMTRQQSGINEFRTCTGFDKQ

>SG-PL_contig_4209_1_-2_2_79

NAQRYIKNYVLLKENEWRIQLSYVQC

>SG-PL_contig_4210_1_-3_147_413

MSTARIHLLCCRKGGIGLFCTVFRLQEGRVRILQTEKPFSRDERVKITRVKISHNSPSNT

HIDPKYIFFQLSLSLPTTDLAAVQGSSYY

>SG-PL_contig_4211_1_+1_286_2559

MAVHSQTSPSPRKEKTPQKIVTSTQTKTPSPQKRVSPRKRVLVDTATSPFHSKAKQLRTT

SDLSLPLTREEEEFHTQVTKLKMNSSGDRVTVKCKTKGQPLVLKKVTIPRKTSALASSPT

RKKRVKLIERIRLHMSGNTTEDEIKQQSSEIFKASKAKREKVMTAAGYKQPMISRRQGSA

LRTGLGLSWSKYRKQRQFLKAIGVKMEGERAERSFQDDALCGKITVTEQLLCNEGERVPT

PVGRIEDLCTFVSNLLDQYEEEKLLTWHDNNIPHDEIWVKVGGDYGGESFKFMLQIGNVT

EANSKKNTCLITFVNCKDSHQNLAKLLGPYKEELSRLEKMQWRGKKMRVVLFGDYDFLLK

SFGISGAQSVHPCLWCKISKTQMQKAPKEQPEVPKRNLINIKRDFRQYKLHGSQKKKAKA

YNNVVHIPLFNIPLSHVAPPYLHILLGVVKKHHDLLERECHLLDKSIAESLVGEVGEMKE

NPTSFDQYVSQLQSINKEETELYKAETRMSEIFDEISRKGENIELRKKQESTQLEINYLY

DSVEEKKTQLQQEDLPFRSGPVTMNLDKVLKENKITVQAYHGRSFVGNHCHKYLQSPALA

NICQSVVHKTFKCTDCHEVHNKAHDLAEKFQTLNQLYAAVHKRISHARDVTDTDICHLQA

DIERYMQYFRSEFCNTRVIPKQHILESHCVDFVKGWKFGLGFLGEQGGEETHAFVNELKV

RVRGINDPMQKIMVLMKEQLTIVSPRIKAVLPGSGKKQ

>SG-PL_contig_4212_1_-3_114_1625

MRTYNEEMKFLEKETPFCWKIKKGFVDNMKVDGYFYVNDRLEKLMFDELKQSCNSKGFGG

FLPGMKQIANVAALPGIVARSVGLPDVHSGYGFAIGNLAAFDMSDPKAVVSPGGVGFDIN

CGVRLLRTNLHERDVLPIKEQLAQCLFDHIPVGVGSKGIIPMTAQNLEEALEMGVDWSLR

EGYAWAEDKEHCEEYGRMLNADPSKVSSRAKKRGLPQLGTLGAGNHYAEIQVVDDIYDEF

AAKKMGVEFPGQVCVMIHCGSRGLGHQVATDALVAMEKAMKRDKIDVNDRQLACAHISSA

EGQDYLKGMASAANYAWVNRSSMTFLARQAFAKMFKSTPDDLDMHVIYDVSHNIAKVEEH

YVDGRQKTLLVHRKGSTRAFPPHHPLIPVDYQLTGQPVLIGGTMGTCSYVLTGTEQGMSE

TFGTTCHGAGRALSRAKSRRNLDYTTVLAALEEKGISIRVASPKLVMEEAPESYKNVTDV

VDTCHAAGISKKCVKLRPIAVIKG

>SG-PL_contig_4213_1_-1_4_987

MATVPQNAPEHCPGTQSEAAGKASACQGCPNQSMCSSMPKGPDPAVAEIQQKLLPVKHKI

VVLSGKGGVGKSTFTAHLAHGLASDENKQVAILDVDICGPSIPTIMGVAGEQVHQSSSGW

SPIYVNDNLAVMSVGFLLKGPEDAVIWRGPRKNGMIKQFLRDVDWGELDFLVIDTPPGTS

DEHLSVVQYLSEANIDGAIILTTPQEISLIDVRKEINFCQRVGLPVIGVVENMSIFVCPN

CKKDSVIFPATTGGGEKMAADFSVPFLGRLPLDPRIGKCCDEGKSFLEEVPDSPATQAFK

HIIEKITGYCTDRKANTENETGEAMDQS

>SG-PL_contig_4214_1_+3_447_797

MYAQCNTFSIGNATFQICSVKNEDMLADLMSRVNPSLHHKNKPVHCPSLCSNLSVGVNLK

FHRSSSPPPGFHFKTLPAHRVLRRLIKSAKQIITGRFGWSAMELSECNESLLNKFKM

>SG-PL_contig_4215_1_+1_1123_1524

MKALISDFHLFLYMVMMDDNISHVADILWILIPNSAMFYMKEKILKSLIVCWGDDISVLS

WTRNQPLSLTEVPEGTETSPPPQNKLLSIFSFLMFPKLAVFSDTLCMTCVNVNSQYYNYS

RCCTEIGGKGSCFF

>SG-PL_contig_4216_1_-3_3_230

LDRLEATRRFSEARTDQKATPPTRPVLMVFLSVLLTLPRIAVPNILFLIADGIEKRWVMN

FLAFGKQSVGKRRWML

>SG-PL_contig_4217_1_+1_430_681

MRCVSDTTAALLHTYTTQHNTDNIQHKMDREDLKAKQYNEMRNTWIIKHATKSHAACHFP

NTSWQRPVFMSFSMLLLHTQHTHW

>SG-PL_contig_4218_1_-3_210_308

MVIEEIEKEVYSWVNELVHSSTVGEKNASELTQ

>SG-PL_contig_4219_1_+3_102_233

MAFNELFINLKFHKTACIFLSPFHSGRRFIKTRILGMKTYLQHT

>SG-PL_contig_4220_1_+2_2_91

KKKISPPPPPREKMRLIPSTESRSICVFRV

>SG-PL_contig_4221_1_+2_2_742

ARARVCVCVCVCVCVCVYVCERERELKEINVSGTPGLKVDLGADPSPLDVFHQFIDETLI

RKIRRETNRYARDSINQKQREGPLPRKSLWATWVPVTVADITNFLAICMHMGIVNKPAIS

DYWSTDPSINTPYASQIMSRDRFKAILAFLHVNDNSNFVAYGQDDHDPIFKIRPLVEHLQ

VKFQEIYEPEREICIDEAMCPFKGRSRFKVYMKDKPTKWGFKFYELCESKTGYVYRFEMF

CGDKKLS

>SG-PL_contig_4222_1_-1_1_180

RLFLNVFSMFFDMLGCCCIFSLCLMLTHGLTRSALGKILCTNLYKCKYCSFHQVFTLVVA

>SG-PL_contig_4223_1_+1_772_2757

MHWNAEGVSTKREDLQQFLYENNISICCIQETHLQEGKTFKIRGYQVFRSDRQGRRKGGV

LTLVRNNLNASESTRYMDEAEYLQVKVSTPNRILNIVNYYCPDDKLLALDSIQVPDSDFL

ITGDFNSRSQSWGYNILDTRGEEVENWQDDHHLTLVNDPTDPPTFYSRRWHTTTTPDLAF

CTDDIHRKISRKVEHQLGGSDHRPVILTINGVEMPGHAQHPRWNYKKGKWGLFSIRTNEL

TRSIKTEGRNINNIVKDFNNSILQAAKECIPRGARKDYKPYWSEALQQTHDALNEAREKA

ETQPSQENNIKLKECAAKHLRTKIESKRAGWKEKTASLNMEKDTSKVYRLMKAMNDEGTK

GQKITLEENGQTLTGKAAANTFASTYAEESDTTIPKHSRREVRTEEKERLAGNRPTHEAM

KQDLSMAELKQAIQKLKEKKSPGPDNITNEMLQHLGNSALQTLLGIFNLSWKEGQVPQCW

KEATMIPILKRGKNKDKPASYRPISLTSCVCKTMERIINQRLQLYLESESIIAPEQAGFR

QYRSTEDQTTHLSQVIEDAFQTRKVTLAVFIDLQKAFDKVWKDGLLVKLLRSGIQGNLYQ

WTKSYLHNRRARVLVDGHCGRKGVTEAGCSPGRCALTHFVHPLHQRCGCRTTKRCPCRAL

RR

>SG-PL_contig_4224_1_-1_382_525

MLSQMIASSKGSMGRGRGKGGIQNECMIWLCLCFCFKEQFVFIMNSPL

>SG-PL_contig_4225_1_+3_3_134

FFFFFHKKKEKKKKKKSLRKKTCPHFPQVYDKIPGTSPRMRERE

>SG-PL_contig_4226_1_+3_3_236

LFSVQIIRFICVVVYELISVEVIRFICEEWNVFVGSSFSLTIHNFNLGLLNAMLWLICTT

CPGNEPCSQDIASAIQSQ

>SG-PL_contig_4227_1_+2_110_226

MHIINYTELNTSKGNKSCQHVRTGDLLNQRFKGRSCSRL

>SG-PL_contig_4228_1_+1_499_654

MVTNCKHNADILLLGDFNIDLWIPQKSCVQPLASLHFLSLEPIEQTYFRNTS

>SG-PL_contig_4229_1_+3_21_245

MVGVLRWTASRPVFMLQAGVELETSYRESTAPSAVPSSWLDVECYPDFKRFLSEEFSSLI

YFPLPPLRTHPLCSL

>SG-PL_contig_4230_1_+3_36_260

MHWEGRKMQMTVKKGKKSSNKWSNRKEEISSKEFPEGGDTIYTERAPGILLGVLISLVTC

STMEEPFNKKSMHYD

>SG-PL_contig_4231_1_+2_1217_1465

MVLVRQFEQYRQLHIHKVDEARLCGPSVPNEPIAHSTLSWSWSYKSHNGFLWTDEDLNPR

PSSQLSVALTPTPQHPVGSTISK

>SG-PL_contig_4232_1_+3_3_170

GHERFCHRQQNGEIEMGHKAAEVLKGIGYSDDEIMIGNKGVIVLSDNESSDSDYPD

>SG-PL_contig_4233_1_+3_81_473

MSTECKHLTQYIWQPSLCFPFSTFSLFLVPTLMHILVTLDQKKKKNLNMSLPYKNHIIYL

SQVQHTLTTRQKIQSAEYKFYLIKNHVCLSKAQYMYHLHYQVLHNTYQTTTVQTGLLTNV

PMQHNQMASTK

>SG-PL_contig_4234_1_-3_3_179

ISPCFLNDELIDGCNEVLKHLTVINHIVVFTVERPVISPCFLNYERSSIANSSIVFVAG

>SG-PL_contig_4235_1_-2_2_187

QPLPCLAVTLGVLDTRPQCASDLITCASASPVLLVIYMRHIYQLWGLDKLEQYLVRFLYY

QG

>SG-PL_contig_4236_1_-3_75_1901

MSKRKDKKEDANDALTRIAIVSNDKCKPKRCRQECKKFCPVVRMGKLCIEVAPTDKIAFI

SEELCIGCGICAKKCPFEAISIINLPSNLEKETTHRYSANSFKLHRLPIPRPGEVLGLVG

TNGIGKSTALKILAGKQKPNLGRFADPPDWTEILAYFRGSELQNYFTKILEDDLKALMKP

QYVDQIPKAVKGPVQMLLDKKDELNNQDFVCNKLDLMNVKNVNVEDLSGGELQRFAIAIV

CIQKADIFMFDEPSSYLDVKQRLRAAQMIRSLIEADNYIIVVEHDLSVLDYLSDFICCLY

GVPGAYGVVTMPFSVREGINIFLDGFVPTENLRFRETALTFKVAETALEEEVKRMCRYEY

PRMHKRMGKFKLTVHPGEFTDSEIVVMLGENGTGKTTFIKMLAGKLAPDEGGPTPVLNVS

YKPQKISPKHQGTVRQLLHERIRDAYIHPQFVTDVVKPLQIEHIMDQEVQNLSGGELQRV

ALTLCLGKPADVYLIDEPSAYLDSEQRLHAAKVIKRFVLHAKKTAFIVEHDFIMATYLAD

RVVVFEGQPSIDTMANSPKSLLNGMNQFLESLDITFRRDPNNFRPRINKPNSVKDTEQKK

GGNFFFLED

>SG-PL_contig_4237_1_-3_279_602

MPVGIEVCVCGILAIVVFMKLQVAVLRYPYHIKSSALTELLVNVKPLECYPDELLYDSER

GVRYGMNFGCVHSLHQYFLLVGSQLSGLAISYAYVYYSYYCYYLFVKV

>SG-PL_contig_4238_1_+2_2_160

KNQSSSCTLKRPRTKIQSESPKMLNRLVKFDLHNCLSDSADFYPEIFKTQWPI

>SG-PL_contig_4239_1_-3_24_374

MVRIICHELCFAGFLISGLQKFCDQRGCLLDFDGLYVVTALIWPYGRLGYKQYIIMIIII

DCCIDTSDSLGIRSLHVASLTPLSTFGRERVYCDWVGETDGITLRRWRERERKRETD

>SG-PL_contig_4240_1_+1_34_153

MERDEYKQHNKCQQNYHITIWQSFDRIIIVPTPAKMFHQH

>SG-PL_contig_4241_1_+2_80_229

MKHYKHNTVHTSQAKEITSFEISTQQSDKRYSCPEEWQEWMHNSMSEQQK

>SG-PL_contig_4242_1_+2_2_196

DWSEQNTRNSEQLACLGVRACVWLLQLVPLQIITYICLSMSQQSVCMRMCVYTCAPDYVH

ANVCM

>SG-PL_contig_4243_1_-1_133_999

MVDSTDIDLMRDAIESVNIADCGDRSVCDRNPCRHGAVCEDISMTDYRCLCPESYTGRNC

EIEINDCVTRRPCLNGGTCSMASGGGYQCSCPLGYMGQDCEAEIAIGSAFEVQGDGYVEF

ERSLLPHRRRQIGETILFIITTVEANGLVFWQGQRPGEQSGADYLAVELRDGFVEFRYEL

GSGPAVLISDVPVNDGFPHTIEARRLSRDGTLIVDGGREVTGSSKGPLQVLNVQGNIYFG

GVPDLEKHTDGQSLVNFNGCISNIQIQNEPALDVGARAIQGANVRPCLN

>SG-PL_contig_4244_1_+2_2_190

RKKQTTGKERKTAETKRAIEKRKLLAQNFQKAGILFILKEPPASPQGKSKILQKNTIHDI

LYI

>SG-PL_contig_4245_1_+3_105_230

MKKNYKILTNVSFIVLSYTSNLSFRWLHLQLNLTFNKEISPV

>SG-PL_contig_4246_1_+3_66_254

MIAYGGYFAHSLESTLDQSLVCACSLKRATHAQIQMNPFMGLSRRKQQTLHFGAPLLCRL

MIH

>SG-PL_contig_4247_1_-3_3_314

TVICNLELKNLKLSLEHDGVDCANCVTTPAPSTTTTTTTPSTTTTCPVLSQVDTFYCYLS

TVDFSKCSNPLKSGDPSYCAYEPYVNVTLAAYLPCQCADYSPFA

>SG-PL_contig_4248_1_+2_854_1081

MDKKILQTRKVRPALTKTDKWELNSDKHLSVTSHSIKSEIQRNEMSTKTKHANSKITIAR

KIMYKRGRQRQTEVEN

>SG-PL_contig_4249_1_-3_33_395

MVAESDAKSTHSKDAEIFTNADKVKSKVSNSTLESDFNPSYKQHSLKEGEHVKPVQTKLE

SLAGKQRQARKLTEEKKQASKTRPTKCSSEPALTPKEKKAQQMSTMGKTVQQEKQASYSE

L

>SG-PL_contig_4250_1_-2_608_973

MIKMLKMNAQDLLLKDNVQSLFHFQPKMRCCFFPFHICVFYVLHRPIGAGFYAHNVIVSF

FFSLSLDMTCKQLCRKFPANAHNICVENKGKIKKRTLLVSRNERDCFYVYLAICDTVNSL

YS

>SG-PL_contig_4251_1_-1_1_153

IFSAVSGRIFTKFRPKVWTVEESLMHEFGREKVKVKEPKMCPKLALLEEGV

>SG-PL_contig_4252_1_-2_2_184

CLKELNGWDNMSLIAFFCRFFFFFCLALFSFVFSHLTSVCVQHGCLVDFDGLNMMTALIW

P

>SG-PL_contig_4253_1_-2_581_883

MTRLSKVSILYFSCELNVWIHGIKMSEAVSLYLFCLASMSTNLHLTLGGRGYVQCLFLKF

FHVGVSYDRGQRIHYCTLYMLVKTFIVAKLCGFQYCFCLVI

>SG-PL_contig_4254_1_-2_86_4258

MGGRTRGSYSDSKQLGQIQEEGDSSVSQSSKELMRHKTEGDISDTRQRLDSWRNDLDKRT

GRAQRTSSEHMSSVRGYESSSRPQHLFHASIDSGFGSEISSRADGQMTPSDPSQSGFLSM

EKTSGADLKKFQQQAVLNFVQRKTGKSFPSTSMEHSPESSLYESINTPRSPNSPTSETVS

DLISKTNETLAKRADSIRRSGSSSSRASSSDYVDMNRPDRSRKETEWSRLRSSGSFQYTG

SNRSSLCSENTYEDISVFATPTPRSSTQEAPKDGEGQSRDSQSQLDEGLYRGKMDSGVMA

SPTYQNFPGASRRRAPPPPPRTPSPEPRTPPALPPRNYLQKEDEGNNSAEQEQANVDEAK

IDFKSLFNQWENNSPREDTYAAQLRRQAVKFSGQRGSSSAAPSSMSVIHSRTQMGISMSY

KRQPTMAQEATSPTSLMTTSVTSSVSSMSVAENSSSSPHRLSSSDVSQQAGTNSQPSLSP

SSQTKESSSSLPVSSERDSKPLTVTITTAVSRPPQLPERTVVPSREDSQVPPRTDTNLPP

RGEAYLSRDRSVSEVPNKDSEQSFVSTPTSRERSSSDTRVLAKLENSSMSAVGNGVHSPK

QSDLPLPSPPQQDSHDGVIDPAELPPPPPELLNDSVGSFDKSEQQGDEPFADDSYGAFRN

RGSRQVGNYQRSASAAASMRPGSQPPKSPTDSQAMFGDHRLGGHNWNSDSQLQSRKRPDT

QQGGGTGSGSGVGGCPGSSPDQTGNVPAFKADSGPVRNTVKRFESPQPLAPQSQPQGQTS

AGDGKTYTARPYQIPRPLSDCVRDERGVSPEQPRSVKERISNLERKSSFSSASSSSTPTP

TTSPMHTANTPTSCSQRWTSATSFQATQRSGGDNVQPQTVNTNSSTSSRVPNLRSMVLTD

SHQPRERPHSGSLASPPTQDPSTQTTVNTRPRSYSEHGRQGEMSPPSATEPKRNQRYTEA

PPVASDPRALPGHFRNSSQSSVERPRSSSSPYNRRESAPAPHIPPEGVVAMVSPRRKSEG

NFLPSPEELQKEMSPPPGPITAGHGRQPSQEELECDLRAKELAKEVADSEKKLSDVLSAD

ASKKRMQYMDGLFSGPKDMEVKTPVSVRRHQSQGSVDASFRQPAQNSDKSPTAAASPAID

HTRKRNSLPKEYFVSPPKAVLEMEWRKKEEVSSDLTKNIHDNEALIRQKEELIEKLLKKL

DLLKEERKKLQEEIQEIEELGKRVCEAVDCKCENRSEREKFHTYISDLEKIVRLLLNLSG

QLARAENAVQALDEDADPEVKKRTLGKRERLQTKHAEAKQLKEDIDKRSDTVSTFLRQSL

SGEELNDYAYFVKMKSKLTIELQELEDKITLGQEQIQELKKSIPERTALPHPESAASTTS

SSPAPSPVPSS

>SG-PL_contig_4255_1_-1_1_276

EWKCRCATALPLKNNNNNNNKNFLLHRFLCGLLPIRQLHRRYPGSRRTTSHTDTDNDTDT

DMAFTTLFFRTFSLIPTPPFPPKDTDDHKFMF

>SG-PL_contig_4256_1_-3_3_368

KLKHTRPGHFQSPLQLPRYTEEKLCVVTCLEEYIKRTASLRPEHTDKLLLCYSHPHGPAS

KDSVSLWLKDVLQADGIEGFASHSFRGASASAMLNHGVSLECILKTAGWSNAATFHRYYN

RP

>SG-PL_contig_4257_1_+1_739_1083

MIRDNTGDKGIIINNVEHKISQYAEDTAYTLGGSKKSFETCIALLNGLGRRLGLLLKAEK

ISAIWLGSRKDSSVTHMQQSGIGWKPPEFRVLGICLTNNVNNCESLKLFRKVTRN

>SG-PL_contig_4258_1_-2_2_253

HTHTRAHTHTHTHIYIYIYVCMYVYTVSQNDDRVTCCKLSHFLKLLSLVIDSNVLLLLMF

DLCVRRNGSFEKEIAYASYIKGKK

>SG-PL_contig_4259_1_-2_2_226

ATGETPGRGFRHWQVCTHVDLRDRKNLHPLPTRQRCLGGELNTGPSVWKSGVYPHDYRAH

MTTAPGERVYRSKYV

>SG-PL_contig_4260_1_-3_3_116

ETSAFLGPALPIASHLLLNLSHPQTGSCSHSQHMPSFP

>SG-PL_contig_4261_1_+1_1_219

RKAEVSTTPLPYDYRTLCLLPRFKNIFIFFGLKINNYTNNTVNSPVDQRWPSHMLKPGSH

TLLLYTVYSPADH

>SG-PL_contig_4262_1_-2_2_250

VGGHASHYTRLHRPYEGLGGQAVHGHHDGGLLREQQGGVHSSASSSRAEFLEAVREQRDL

AERSPHSRGERHARRLTDRKSVV

>SG-PL_contig_4263_1_+2_1205_1390

MELFFYDNLINHLVIQTNLYSDQFMEAHPNLPRYSRANIVLYCIVLYCIVLYCIDFLSQW

IY

>SG-PL_contig_4264_1_-2_35_151

MGGAALQRRTLPRRAAQISHGKSIVTKVIQHKRQQKGNF

>SG-PL_contig_4265_1_-1_133_1515

MENEGKVRVGIRRHGKLDREVKVRVETINGTALAGEDYKPFNEVVTFEKNETLKSVFIAI

VDDFEWEPDEFFFVKLHLDLDAEASLGNIAICQITIINDDEPGVLAFTKPSYVIKETGYR

ALIPVARTGGADGHVSVRWRSKDITAINGKDFKGGEGELKFDNQETSKSIDIILYESNKA

ERDESFSLELFESDGGATLGKLTKCIITIVNDEEYSGLVSRIVNLTKANLDALQLEKTTY

VQQFYEAMNVNGGDLENATFLDYILHFCTFFWKILFAFIPPAQYLGGWPAFIVALAVIGF

LTAIISDLASIFGCLIGLKDAVTAITLVALGTSMPDTFASKQAAVGEKTADSSVGNINGS

NSVNVFLGLGLPWLIACIYWKVKTGEDFEVPAGSLGFSVVLFTSCAVIAIILLVVRRFTV

GAELGGPPVFKYISAIILVLLWGLYILLSSLQSYKIINVQL

>SG-PL_contig_4266_1_+1_256_1011

MKQSKSSCKASKNDFMDLIHLFIYLSVYFKAVDWSASQMHASITLMFGHVFHVLHDRLRP

LGQVGFKSRYGFHVAPQFLLKREGVKGLGVDSQGSVQVVVQVHVCHRHRVTHKVACSSII

LQHALKVAKVTREQFFPENPFKLRLFVWIFFTQPVPHVRCPQVIKKMDQFVLSQSILFCF

AIETQFSAKEASDGHTLPIFLAIVENVRQLAVRSLFLCSRVGAPVNEVSSIIFKLDTSSN

EKHTNGLRSGSN

>SG-PL_contig_4267_1_+2_2_70

VHGQFQHIVVFICSTEIKIQKYL

>SG-PL_contig_4268_1_-2_110_1603

MPKKKKSGKLAKMTDEERIAYEEQKRLAEEEMRKKKEDMLSQFLKDKLMKEERASRFNLN

KLNHQWRNIMREAKSRELKKDIEILSQTFERVIDRKDAVIKSLSKDLSEAEEQYSMAFRS

HLQNIDYLIDLQKQRLQTLEQECCEELEIIREEFDTERAMQMEQHNKEMNDIADILFAME

QNFQERENDAKAEFQSMRDEIKNKNLEEKHALRVQLEMKVDDLWQQFRQALQNYNETTEE

RKTAFEALKVKDEKSAKEIELQMRKLQRISDQIAQLKGKMAANARECEERNKFLKEEREK

MLAHFQELKAQMNKIRDCEREKLIKLTLESSAAIKELKRQKDKGEKILRLAEMCRKLETE

EEKVLPFYASSLTKEEQEDVEAAVLEPPSEALASVMHEYTALENFWKRYNKVFLEKLALD

KEKQTLMGENAQLRTLLKQYLDGISVNDEILSQVNPLFIVNNKTNVKLNVPVGDPRVRRP

PPQTVVEAAHVVKHVLPS

>SG-PL_contig_4269_1_-1_82_2025

MGQQKKKKKRKKAPPPPYLKKTKNKKKLLQKDKMVHEKAYATGWLMVSLIVMFTAALPTI

SSSDQGSSDSKRTYAEKRSAPANRIQPIAVGPMDLQQGETNSDAAFAAAVLKQYGYYQKE

KPLGFLQSPPAGNEADSKEEDEQSRALRLFQSYFGLKQTGKLDSTTLSLMKRNRCGVADI

LPGEEEESGHVTNSNRQTPSRYALLGSKWDKNRLTWKLDTSSTRQLTSSEQRKIMKEAFE

TWRRVTPLEFIEQRGPVDIDIKFGSRHHGDDFAFDGKGKVLAHAFGPGPNTIDGDMHFDD

EEDWSTATGGSDDLLLVGAHELGHSLGLSHSRDPEALMYPYYGLTDRLGPDDIKAIQSLY

GIRIGQPTTTLRPRTRRPITTTRAPRPTTSRPITRSPYTRRPRPQTTTTPPVKECGIQFD

VVIQDPRNREIYSGIQGNKIYRFNSAGLLPNYPTHLHAVFPGAPSFAETVFTIPEKHATY

FVKDNKVWRYQESHLDPDYPKDLDPTHFPEVIRFVLPIVDTSGYRRVFVFGRTLWWEYNF

DNVPAVGSYYRPRPSLIPQYWPAVSSDVNYAVKGTDNYIYLIMPDSHVVLDSFRRAMYGG

KQEGHPQWMQPACQMKFAFSGAANPSTKNFSFALVLSAVALLLSVLMK

>SG-PL_contig_4270_1_-1_334_537

MVIVKGCVRREPVESIEFRVVSDVSQTVNGEHRISEYVWKVHLLELNGEHRISEYVWKVH

LLESVQMA

>SG-PL_contig_4271_1_+2_2_757

HFFHLLVKDLLHGLAQSLKLGLGLLILFLLFLRVGQHQPLLRDAHQVLAVVLLQLLHAVL

INGIHHVQHLKPSLLHTLHKGRVLHCLTAFPSDVINILLVFLHAGHIFLQGDHLISRLGG

VVAQEVGQLSPVGAVLVDSQLDVLSELLVELLVVVLVLSQVIKELHALFYQVLTDDLQDL

ALLQHLARDVEGQVLRVYHALDKVEVLGDDLLAVVHDEDTAHVQFDIVLLFLVFKHVKRR

TFGDEQKGTELQ

>SG-PL_contig_4272_1_+1_1_135

AVLVDSQLSQTASIVRYAANMSLPTELFHFKALYHLLWVLVIKLE

>SG-PL_contig_4273_1_-2_38_223

MFSNGHMQDLCLVVQVLVLTVFSSRHILCRTCIVLRVKLKGPSSDPAFGKVGERWHIHIA

HT

>SG-PL_contig_4274_1_-1_1894_2100

MANRDLVNSLYLVNPFTPSPYPPPSCGKLCGKGEKVRGLTIKELGFHWLPTVINVHVRFC

ELFLPSAFS

>SG-PL_contig_4275_1_+2_158_2446

MATFIHKKQGLRVGHVNVYHLDKKVPDLCVFLDKPNYFHLYGVTETRLAPHISDEAIAIP

NFSVIRRDPTCAGETGIALYIHLDIQQMIQRRYDLEIKEVECIWVELKPSSTCPPLLIGI

LYRNPKSTFGWHDHFLKMMDKICPRNTDILMLGDFNFNLLKPQPAWESTTTLVGLKQLIT

DPTRITATSKTLLDHIYTNKPEAVTEVAVDDLSISDHQPVNCTWSASFQNVKKNTHTCIT

YRSFKHFDENAFLADLAKVQFSTVFSTSDPNEALNFWYKLFLTVLNKHAPLRRKRVKHPT

MPPWLSQEIIHAMALRDKLKKEKCFTEYKKMRNKVKSMVRQAKKSLVDRLVKNKKDVSSL

WRAMNLITKGSGQRSSATPANLSANKFNDHFLSVSELLVHPCINTDQYIPSENLINFCKK

RTSSTTSFSIPSIAVHETGMYISKMNNKKSSGLDEISPKILKLSLPYITESLTYIYNISI

EHNVFPRALKEAKVIPLPKTKDLSKITNYRPISLLSVLSKPLEKHVLTYLLDYLENRSLL

HPLQSGFRQKHSCHTALTRLADTWLLAMNESKLTGTIFLDFKKAFDLIDHDILLTKLDRY

LVNHTAVSFFRSYLNERQQKVLLNGSYSSGGQVKCGVPQGSILGPVLFSIFINDLPLHIS

DPTVFCDMFADDGTIHTQEKNIVTVNTRLQSSLNEISEWCTKNAMILNPKKTECMVICTR

QKRQLGLLSLNLTMGGNSITQVPEHRLLGVIIDHNLTWQNHID

>SG-PL_contig_4276_1_+1_1_177

SITILTRALCLVVTISVSCLCWPQCDKTHTLHTTFYKSDRLHIHTIINHSVDCTHCYQC

>SG-PL_contig_4277_1_+3_342_578

MYDSELKSLIRSLSERVKSLEDFIRNNVQIVGSGAKNPQCVSTETQTEPPSTDNCGPSPL

PLSYAEATKRAPKHLSSKQ

>SG-PL_contig_4278_1_-1_52_207

MSTRVETSFGKTILSVFHWILTTYITFVSQMFGPGRNLDDTDLNEIGEWSWS

>SG-PL_contig_4279_1_+1_268_492

MSTQSMIVFMKRGTNLRLVNQDPNQSPTKVTARHSAQKQLPLLDFLHWGLGLQHSTCGLC

FQWRWGKRMELKKKK

>SG-PL_contig_4280_1_+2_2039_2404

MRTDCELHTQTEFTQVLSHLKDYNNPGLSSRCSGGGRDEQKSLKSKPGFKLGILHFPLPG

KHFTTNLTAPLMLQCYIIPPSCRVFSLAPYCLVIYGYKLQLLHFYSCTIHPFPNNNKKHY

DK

>SG-PL_contig_4281_1_-1_1945_2142

MNTHTHTTSHYTRVCMHTHTHTLTLTFTHSHTHTHTHTHTHTLTYFYLCSSFNMVVVNFL

CAMKSF

>SG-PL_contig_4282_1_+2_2174_2488

MAKNLTDYKTISANFNENWTDIYLFAICFLVLCTSPALFNKPLTLICSHRKLQEKETDHI

NYSTKKKKRYVRYNKKRTNLRLTHKHHLHCQENLNQSECMDSSTR

>SG-PL_contig_4283_1_+1_859_1632

MVQNENEDENDRYLRFFCNVHELRSPGTWCNDAADLLPQVLADWSRRNVRIYSSSLFTSV

LDIKSSDVSGPECEHRSDPINLGLAFTDHNPSHYDACRKLSDFHSCPPSNRQDSTEESID

NQQNSMEDSADNQGQEDRSLLSLEEVSSPNGSEPMEKESGDVTNAATLTPRKQGTYQTPP

KKTLVRKKKAKPETWKKNIRKSLHLQGKEYVSQTGKTVSAKKLKSVDCTSCKYKCNEISE

EKRQELFDSFYFLQDCIR

>SG-PL_contig_4284_1_-3_1398_1661

MFDLYPLYLVTDDKMFIMTFVFKFCFNAVFFHEITTDTECAYYRVQVASESFLFNNRHQV

HTHTRTHARTHARARAHTHTHTHTHTHI

>SG-PL_contig_4285_1_+3_3_248

LTLRKTLCTGEVLKWKAGRGDGAGPMIETLSWHHQNDWSEDELCSGRNKGYAHKLHIFTG

VHYCCYSCLIRNPWQSFLSFFT

>SG-PL_contig_4286_1_-1_646_924

MFGQYRNVQRNGCVIKVFTCCTGMFFGVCYRPSPTLFCLKTIAIIGILCWFLSFLFSVYC

LTLLLCLTFCHLLFLFCHSVHSSAFLCVWLFFV

>SG-PL_contig_4287_1_+1_1_105

ANTSRRHSREPHTDTHLGTAIVLFLMKTRSDDKLR

>SG-PL_contig_4288_1_+3_138_272

MMYVQYSCRHFQPHSRTHSFAHSRAHSRTALLTSSSQFQAWMMGV

>SG-PL_contig_4289_1_+1_1051_1254

MKQTKPFPCLKSAHFFSLIHGEIARRRLNSFHMIIIRLAFIKAVLILTMNRLVSYMYSLS

NRLISIIC

>SG-PL_contig_4290_1_+3_471_2504

MDLSTNELTTKPLATTSATSPLTPTTTVSSRKMSVNELTSLWTTTEILSSVHHDDVTQLT

WTTGVTDFAREENVTASANSSIIVSNNSSVPAMMKNDTILDVNLEALNLSMTMSLLPTVI

YLVVLMAVGLVGNSIVFLVYYKRFKPSATRTYVLAMCVCDWLANAFSLPSEILDIRYTFT

FDLPWLCRSIRATNSFLTLTSAFVLVAVARDRFNKICHPLEKRRSMKRIRIYVLICALAS

VALSLIFGALNGSQTIPTGVGNFTGITCSVSGEFLDTLFPLMYNMIMAVTFISCVVIMTV

SYIRIALELWRHKKKQARVSGVAPTPSKDHSKTHSPIVEDTSSGPRHDLHHSETKSSSTA

ELIPMKTTSGTKSKSKATSPATTPANTSDKVLLLSVNNLNYEGPKSSVEDSDEGCFSGCS

AETTSTSVDVSKEGTVHTNQATQGDSQTANVHVHIVDENEDKEVKKLDTDTETKPEVPES

AQKNGSGSLDATAIEDHAMTPLDSQDASQDSQTKTSTIQDKHDSDDGSSNHHRHTDDSSL

SPHHKTTTTTTKPSSLWQKFLMSRRRHQYDFSKESALKPSQRCQIPKKRGTVKSIPSSTT

LMMFVLTATFVINYLPHLIIISMRAMSDDFGKGLTGAMLNAYNVGLRSYFMNCAVNSLVY

GFCSARFRQECGLLFSRK

>SG-PL_contig_4291_1_-1_94_348

MIMYRNGLPWGVAFEDVFEGIYHPAISLYKNAQVTVNFGPDFKFPPTDQPDYRPMSEAAD

QAMVEGALADVIYHIDNEGKHPEFQ

>SG-PL_contig_4292_1_+2_20_223

MNPSHLEWYPRTPLTWSGIHDPLSPGVASMNPSHLEWHPRTPLTWSGIHESLSPGVVFTN

PSHLEWYP

>SG-PL_contig_4293_1_+1_1_186

FFFLNSYIFYCILLEKICLHISISVSMDNKITIQYNQYTYAMMKSSVHSVIHNPTSEPDS

ST

>SG-PL_contig_4294_1_-3_3_296

YAPPGAEGTEPSRVLTVIHKITSTMPFLLIIFDLVACAPSHVDSPWCGQPLYISKTKYLY

MGLYYQAQKQQEKLKRGVTMSRTKHERLQNFKHIWYDN

>SG-PL_contig_4295_1_-3_1053_1316

MYPLFLLYVFLFPCFKMCVTCFQKTIYYFGVGITFVAVSWHLSDVSVLLKKLSVIFKHGY

FFFRLHNMAHTIKLSNVSLIMIRHRQEL

>SG-PL_contig_4296_1_-3_99_821

MMYFDSEANAADLRYFNWLVVVAQVFGLLSVVLVAVWMGHYQGGFAWQSDPTHQFNLHPL

FMIIGMIFLYADGILAYRVFRNDKKLYIKILHAIVHIAALIFAAVGLKAVFDFHNLTEKP

NLYSLHSWVGLFVVVAFGLQWLLGFVAFLIPVAGMTVKRFYMPYHRFWGAALLALAGATA

LMGITENAIFKVKPWAGPEGILINFLGVFIVVFVVLVVYLVTKPDYKRPPAPEEDHIQLA

E

>SG-PL_contig_4297_1_+2_2_202

RAQKTSQELGDSNFCKLWPPGPIKNRSRSKNLYDGIGKPSLRPKAGRSVQLYTLKLEILW

SCIENGQ

>SG-PL_contig_4298_1_-1_28_1656

MARMQAPVILLKEGTDTSQGIPQIISNINACQAIADAVRTTLGPRGMDKLIVDDRGSVVI

SNDGATILKTLDIVHPAAKTLVDIAKSQDAEVGDGTTSVTLLAGELLKETKCFVEDGVHP

QIIIRAIRKAAALAVEKIKNISVSIKKDNPKEMRELLLKCAATALSSKLVAAQKDFFAAM

VVDAVMMLDDLLPLNMIGIKKVQGGALEDSMLVAGVAFKKTFSYAGFEMQPKTYSKPRIS

LLNIELELKSERDNAEVRVDSVEEYQSIVDAEWKILYDKLDKIHQSGAKVVLSKLPIGDV

ATQYFADRDMFCAGRVPEEDLRRTMKACGGSIQTTVQNLTPDVLGTCETFEEVQIGGERY

NVFKGCPQARTCTIILRGGAEQFMEETERSLHDAIMIVRRAIKNDSVVAGGGAVEMEVSY

HLREHSHTIAGKEQLLISAYAKALEVIPRQLCDNAGFDATNILNKLRQKHAQGGRWYGVN

ILREDIADNFDDCVWEPAIVKINALTAASEATCLILSVDETIKNPRSGQGDAPGAGRGRG

RPM

>SG-PL_contig_4299_1_-3_192_380

MKKKKKIYTGKEKKISKRKKKKKKKKKNFWKRKKKEKEIFEEIAHFKIWGQRKVAVICPF

FMR

>SG-PL_contig_4300_1_-1_1_153

ANNDHYACNLFTCTFYCSSSVDGEMIGCYMRSYLCHCSRLFFPVCLLCLRK

>SG-PL_contig_4301_1_-2_173_622

MSEFKISEKQYTDANQTFHLFDKKGNGTVSTKELANVFKSLGLQVQDDQLKEWADAVDEE

AAGFITWEQFKPVFEIKLQEDEDEREMREAFRVLDKGNKGVIPVDDLRWLLKSLGDDLTE

DEIEDMINETDTDGSGTVDYEEFRTLMNSN

>SG-PL_contig_4302_1_+2_104_205

MMRYVYFDNIAVVTLKLCQDHPNFSNRESDSRHY

>SG-PL_contig_4303_1_+2_92_211

MKPIMVTLICNGDVAVHTNDISIKIKGGLFIWLDRFFNNG

>SG-PL_contig_4304_1_+3_33_248

MDDKRLPKAALFWRPKGQRRRGRPKTTLKRTLQSEGRAVNINDLKAAEQTAADRERWRVL

VPALCAMFDTGG

>SG-PL_contig_4305_1_+2_2_178

KQKVEWQQTVDVKRDTILNETLQAGLPSVQHNHHVSTPTPSQSWPSSKGNNPNQEQMLT

>SG-PL_contig_4306_1_-2_2_214

PVQDYERTLDPVQDYERIPDFRIMKGYLTLSRIMKGHLTLFKNMENKAERSVYHLHKASL

SGDMERSREGG

>SG-PL_contig_4307_1_-3_228_455

MVNHLYTIHSFQKCTNLLPVYHSLFTHHLHSAYLSSSPCLFPLHHFISLYLSFLSLGKPQ

NLPLHIRGAFTVLSSR

>SG-PL_contig_4308_1_-1_1_192

YKQYIIISQLFARDNFFLVVGPLLLARNACCTGTSVYCLIRKTSTPNHHSGCEKISVSLP

RQVL

>SG-PL_contig_4309_1_-1_1_258

VTIQVYEGERSRTKDNHLLGRFDLTGIPSAPRGVPQIEVTFQVDANGVLRVSAEDKGTGQ

KNDITIERSGAGLSPGDIERMVKEAE

>SG-PL_contig_4310_1_-1_490_882

MNEYGRVHKYPTVPYFKKRLDTYQSPFFEDLPMYQSPFYIELSIYQNLFFEDLPIYQSPF

FVQLSIYQNLFFEDLSIYQSLFFAEFIYQSPFVVELSIYQNLFFEDLPIYQSAFFVKLDF

YQSPFFVDLSL

>SG-PL_contig_4311_1_-2_485_1054

MKSIKLQKFTWIYCPGHAGVKGNERADKFAGKATSASSLLLGLPEVLRNLGAYLHEPQIT

RSSDSKGHSHRKWPKIFHEEENKSHHQSDKHWFYLKDNTPKAPTTWIGSFSPRLRSDRSI

TRWKRWRGETEGEDWGRAEGVSNGWEGRGILGGGWYISGMMQWCMGGRPRCMRWLRQWIG

LGKGWESGVC

>SG-PL_contig_4312_1_-1_691_870

MAYALIILDSFPSYGICSVSAYGVCSEHLDAVLRKTETVFVCEGVHVVYVCVETIYCGGK

>SG-PL_contig_4313_1_-1_43_381

MVVETVEKKAGAGGKIRKFRGDASKVVAKGQGLKKAFTNRLQNFILDVKDAGEALLTVGM

IAPSGHPEAELTVKKTTNTSYTVTYKVTEIGDHILHVKWGDDDIPGSPFLLST

>SG-PL_contig_4314_1_-3_258_449

MLSLILFRSDLFIRNMFISDRIKSCLVKFIITFSSSVHLLIICFSVLNKLGGGGGGVFFF

FFFN

>SG-PL_contig_4315_1_-3_198_332

MHTLFSLHRLQAVLALVSPLDFTGVAERVGLLHEQQQALLNTLPR

>SG-PL_contig_4316_1_+1_442_657

MIDGIDIICLSFVSSISGGENRCAILVATSGILEISFSDHRFQVPLQAYVGIRFQYRKVF

RNVKNAPFMALS

>SG-PL_contig_4317_1_+2_2_130

SGSPVFFGYHLHDIALLLKCVSKIFSVVSGWIFTKFGPKVWTI

>SG-PL_contig_4318_1_-3_309_2942

MGVGDDVAPRFTQKPALKQEDGGKKLVFQCVLEASPQPEIAWFRGTTPLTVSDRIGMRVE

SAGGAAYNVIMEILGVTQGDAGTYKVTAKNRLGEVSASINLNFSAGAQKQQDGIAPNFIQ

KPITRQENNGQKLLFECVLTADPAPAITWFKDNAAIAPGGRYSVRAEPRDNKTYFLVLEI

NGVNAQDAGNYKVTANNALGESNATIRLNFDTSEVSQVTGARPVFLQKPAIRQVGDKIVL

ECKLTADPKPQVSWIFNNQPISFGPGSHLTPKLISEGSTHTLTLEIAQVSMKDAGEYRAY

AKNDVGEATATITLSFEGAKKPELPAGKAPHFTQKPTIKQQQNLLILTCLLEANPTPQIR

WFRDSTEVGAGGRYSITLQRDASGADLYTAVLQITGPKPEDGGTYKCTAANDFGESNANI

TLNFAGGEKAAKPPGGIAPTFTEKPKVSQDASGKNIIITCQCTASPKPEIMWYKGTTLLQ

SSARIVPSVVEDGNKYSLKLEVLNFTKEDGGQYKVTAKNEMGEGSANITINLEPPKEPPP

QPQGKPSIRLEDNGKMIVIQQAISSASKPTATWYFGNQPVGAGDRLVPEVTQEGNLFYLT

LKIPNFTDKDSGTYKLVMKNNGGEATTTASVNVDALRPKPKGEGPKVVQKLNPQSVNDGD

AVEFLVKVTGTEPLDIQWAKDGKAITSSDIYSVTYDKGTCRLYIPEVFPEDSGNYSVTIK

NQWGSASSVASLQVKEPKEEPKQMGKSEMEISVESKSVEMQMTKPEQDQMEIQVQSKQKE

PEPAKKEPAKPVEEEAKKQAAPAPKEEPAPPAKKPEEPKPEPKKPEEPKPEPKKPEEPKK

PEPKKPEEPKPEPKKPEEPKKPEPKKPEEPKPEPKKAE

>SG-PL_contig_4319_1_+3_1308_1556

MSHREKSNTESYIIQVDYTCLYYLSDNLSTGVIYSCEPLWSTLVTKSGERVKYDSHSILM

TRRLVQTDKQDRRCVGGGVGVWV

>SG-PL_contig_4320_1_-2_173_1174

MPGANDQLNKYNKIEAFVKGFKLSTSSYKRVMHLLHQEMTNGLTKHTHHIADLKMFPTFV

RSLPDGSEKGEFLALDLGGTNFRVLLVRLQGHDIDIQSKTYLIPQRIMLGTGTQLFDHIA

DCIGKFVRDHNLSGHTLPLGFTFSFPCKQEGLAKARLSKWTKGFRCEGVVGEDICGLLHE

ALKRRTNCNVEIVAIVNDAVGTLMSAAHMDRNCEIGLILGTGCNACYMEKLGNVETWDDE

KNHPDQVIINTEWGAFGDNGCLDFLRTEYDRELDTFSINPGKQIMEKMISGMYMGEIVRL

VLERLSMEGLLFRSVDTECDLFERGRFYTKYVSE

>SG-PL_contig_4321_1_-3_27_2117

MPRCKMGTIEQRPSPPAEPNLRALFSLDCYLEIYEHEIRRRYQCFLNLLNHINQHEEGLG

KFCSAYTYFGVHRLPDNSIVVREWAPGADGICLKGDFNGWRLEDRCWFTRKEFGRWELKI

PANDDGSCPIQHLCKLKLVMQTKKGTLEERMCPWSRYVTRPKEFPVFEQVFWDPPQDQVY

QFKHPHPKRPATLKIYEVHVGISSWEGKVSTYREFTNNVLPRIVDLGYNCIQMMAIMEHA

YYASFGYQVTSFFAASSRYGTPEDLKELVDVAHSHGIVVLLDVVHSHASKNVVDGLNQFD

GTNACYFHDGGRGNHDLWDSRLFDYTSWEVLRFLLSNLRMWIEEYHFDGFRFDGITSMLY

HTHGMGHGFTGHYDEYFGLNTDTDSFAYLTLANYMLHHFYPDFMITIAEEVSGMPTLGRP

VEEGGCGFDYRLAMGLPDMWIKLLKTTRDEDWDMDKIAQTLLNRRWSEPCIAYAESHDQA

LVGDKTIAFWLMDKEMYSHMSLVSDCSLIIDRGIALHKMIRLITLALGGEGYLNFIGNEF

GHPEWLDFPRIGNSESYHYCRRQWNLVDDQMLKYKFLNNFDRDMIHLETRYSWLNNRQNY

ISMKHNDDKVIIFDRADILVFVFNFHPHKSFTDYKIGVRTPGVYRIVLDSDAEHYGGHKR

LDHNTVYHTFNEPWNDRPCHTYVYIPNRTAFVLAKCD

>SG-PL_contig_4322_1_-1_43_336

MGLYICKFAHAFILFVCGYRLEPDSVIFDEQRVLTSINFCFFHDNFCCLFFTSSSDRRHR

EKEISSLLCGRLSVSVFHLQALTHSAAEDWDLHKPRFV

>SG-PL_contig_4323_1_-2_2_295

FFFSYFPFSFFPYFLPLLYFFFLFSFFLSFILFFLSFFLVFFVSDMTKDNWLPLAFYCKL

RPLRGLLRMYQIIDVSMTADFVFLTKHNGLLFVNIRSG

>SG-PL_contig_4324_1_+2_53_274

MASTSRWIKRRLTAEQVTELIMNYNSSNEEIGGSFTTESEDFEPDSKSESDFDTDINVHV

CLCLHVCFSVIVSH

>SG-PL_contig_4325_1_+3_177_350

MNYDSNNEEIGGSFTTESEHFEPDSESKSDFNTDVNVHVCLCLHVRYSVIVSCPSSIM

>SG-PL_contig_4326_1_-2_56_1663

MSIPSAVLAAGWIPFVVVLVLTLLFSAGYIRYYRSKYDNELSSTLTAIFALCVTLLTSDL

VPVDIFLVSYMKNSDGSFKDWAINSTARNDLANSVLYAYYALYGIICFCFFILLPFMYFF

YEEKDENSTCKTRCCSALKYLIVFVIIAAILMLIGAFVPTKSLPNVNSTDWQKIEALFEG

LGSNGGEDAISFVISVFSLVGLLGFVTYTAYGMSALPMELMSGKKSAKKERMAVQSKRQD

AESRRQAIRDKYGITSRPSARGYQREQSLEEEERQLVRQERHLEAKEKSWVQKCLVLLRP

FEIVIGILFFLLGLLVFISLLLTNIDKAMHSLGYKIGYALPTRHLPNPIDIVLVFCQKVF

PLDYILMTGVIVYFVLCSMTGVRNIGIWFLWIKMYKVRPRRTVPQGLLLFCMILMFLVMA

INIVLYELTPQYSSFGSQHYLKNVTGAGNVTKTSLEPCTTEVSSDECTITRMALLLTRFF

YKMWFFGAAYYFLTWAFLVLVLIGFIVTLVQGRRSAIHGEVDSDDFEESDDELIHA

>SG-PL_contig_4327_1_+3_3_215

PPAKDTQLCGPSLPNEPIACSTLGRSRSGPKNPTKICYELVGIRTQTLQSVVCCANHFAT

LASLGGCLLKI

>SG-PL_contig_4328_1_+2_1535_1738

MTQRANTQHLLSISYSSTFYSITKSQFLKPSCCKPRSKFSCVVRKREGRGWGTERQTYKQ

RERDREGI

>SG-PL_contig_4329_1_-2_605_913

MHIRTHTYARTHTHMQACTHTHTHLHTHTHTLSHTHTHKHRHKHARTHAHTHARTHNTHT

HTHTQVSWISCKRLDSVQPYSGKIKCVQCQIISTRKCRKQIPC

>SG-PL_contig_4330_1_-3_3258_3485

MKQCFVAIKRTIEIVYPPSAICPPPPKNLGFFSPKKFKFFFFLLVSFFFLLLFFFFFFFF

FSTPPPPPSPHPLPPL

>SG-PL_contig_4331_1_+2_131_349

MHMNTHSYLHHHIFTTHFEHSAITHNTMHNTLKSYTLKSKFLSVLYTTRPFDVVNFSRYY

CTVGYYNISVRNA

>SG-PL_contig_4332_1_-1_1_99

EMERGYILWRVLKSSQIKKKTMITVTVTKCNTP

>SG-PL_contig_4333_1_-3_240_356

MIYNIAFSTLLVRSLQATTKLKCVVKISLGICNHVQQAL

>SG-PL_contig_4334_1_+1_58_237

MVKTLNFQAGGFGRPEGLSLRGHLAWWVKGRDFSDLPGQHMCRPATPSVCIRTQNIKYAR

>SG-PL_contig_4335_1_-2_845_1021

MKGVKLSQQHFHSRPQEPGFIGMKSQHNMRLLWLWFRGVHSGCLRVSISHQTQIGLQDL

>SG-PL_contig_4336_1_+2_2_160

STSPSSSENTNGTRLVDEKKIQNIKIKWAHTYDRGKKLKIRAYKPTKSLMKII

>SG-PL_contig_4337_1_+1_1_114

APQRPTMLMKEIVACKDQHLSASHQLSTSSRHLLACSI

>SG-PL_contig_4338_1_+2_473_706

MVTTMSIEYSVPSLLPFHDPQHEDCFLTLPLKTVRKFSPAKSKFADQPQAFSLFTRPSSG

GVSNLLLHLEVGFVSCVT

>SG-PL_contig_4339_1_+1_148_306

MKYAKTVFILYTELQVFSSNGNICITVVGKPNVRIVHELKKTTKNSKLLNTQA

>SG-PL_contig_4340_1_+3_768_1091

MHQARYPRKFKPRPSRSSSLEVKCLHHSTTAPVYSPNCHPFHIPFLSYIPNCRSSHTFSL

PYPMPSPYCDLENKLALVINQNVMQLEACKRMTHFDPNMFRVWPYLMY

>SG-PL_contig_4341_1_+1_1_168

KQTKNNTRQRLKQHNNYNTAILIALSIKLKSSIRQIMHELATLYTNIYIHNKYKLL

>SG-PL_contig_4342_1_-1_112_432

MIDLFATKFSKRLPVYVSPVQDPEAFAIDAFSLDWSGLLAYAFPPFPILQKVIRKARLEQ

ATLILVAPKWPAQPWYPDLLSLCHVPPLELPPRPDLLLQPRSGVRHA

>SG-PL_contig_4343_1_+3_69_179

MVITTSTVPTSRSSQININMLILYTCNSHSLCLASNL

>SG-PL_contig_4344_1_-1_2362_2760

MKYKACLHNTSGTNFSVCRIQRQNFLFCPVISLHHLSFVRKLSTLALLLLFSIDHRMLII

ALMSVFLHESHFAFCCCLWMLFLICKECSVTLPPPFTTRSIVRCQLTASRSFGAHHFSDN

ACTCLCDRVSHES

>SG-PL_contig_4345_1_+1_1_111

KVISGLSPHQVHQSHDDNRVEHKTSSNLKTKNLQKKT

>SG-PL_contig_4346_1_-1_1_198

SVRNFSSLCSSTLSSLFLRKTWMQGLFALSCKVLELSPSLFMPCLLLFDIQIHVETYLFK

QQYLNI

>SG-PL_contig_4347_1_-2_341_532

MNNAFTHTLSPLVNRLLSCILQWRITSKICQSTESTKYQLNALVFVWLLLVTPSLFPFPH

DFPL

>SG-PL_contig_4348_1_-3_258_533

MQNSFSIISFTVSDQCKLWPVSWCPDKGEKTTPCHSTTDIHTRSRTALRQCRKGMAHVCV

PCSQASCLCLVMRHEERETPLVLPFVYFHFYV

>SG-PL_contig_4349_1_+3_12_140

MYLGKNTPFGFIYCTMSDCEQNLKTGGGIASKVQVGLLGRRLY

>SG-PL_contig_4350_1_+2_2_196

THTHTHTHTISTGNSKGREVIMYLTVRCQSRLTSQAKMHQGSCYFPITPPSPLPPRHFCL

DKLLD

>SG-PL_contig_4351_1_-3_216_1565

MENGSENRIQDGVVTDVTMNGEDEMIETEDGPETDNVEERQVRKAKRLLTRQTSGGDSSS

NNNAQANKKLLVLSKNSRKSRDGRGRGLPKKGGAGGKGTWGRPGEVIDEDGKGLDAHDPN

YDSDSLDDQNLAFEKVCPELTSEDFEKFIEPVVMEYFEHGDSNDVIEELNDVNFEHLKSS

LVEFLVSKALDHKDHHRELTSILISDLYGKVLSGSDMTDGFDDVLNRLSDLVIDAPDATS

VVGKFIARAVADDCLPPKYILKYKADLVDCPHTRQAVDHADVLLNQNHGIVRLDNIWGTG

GGIRPVKVLIKKIVMLLKEYLSSSDIGEATRCLTELNVPHFHHEVVYEATVIVLEDSHER

AAELMTKFLKSLADAVIITPQQFKQGFLRVFDNLPDICLDVPNAYQLMENFATMCNKEAF

LSDALLKDLPQRGRKRFVSEGDGGRVKDAL

>SG-PL_contig_4352_1_+1_133_321

MLVHVSRYKCAGMRKCVCAFVTVHVLILYCSWMCVCVCARAHTYVCYNVIIAFVTFLLFV

TML

>SG-PL_contig_4353_1_-3_135_443

MSDFDPCECVWNHESAMQRLINLLRSSQNSCTDSGCLTDDLSGPGSPYGNSNMMMMMMIG

WLVVATALFLLRPRSMRDLGDGKPSSSGGGGSNNPPPPGPSVH

>SG-PL_contig_4354_1_+3_9_902

MSVSSRLETWKMFWSSVPMIAYLIIVLELRPSEVHGMKIPGIPPPPPAAAAAARSLDCLW

GRGGGGGFASSSPEPSRSENKGSLLLMIRSNSLAVTTGTVELPPSGSGRRLRVRDFLSPF

SLLEDLVLRGSFTVLLFPSLLQLPKSPGCEQSAPPAPSVLEARGELSLDLGFTFFFFTFL

LPFFFTLPPPSVSVASPGSGWEPWESSSDRVEADSWESWERSAPRQSVLYSSTDGSRVLL

PPPITNTPLCCCCLRGSLADGGVEKARGRVGVRARGGEAAGPGGTGPAPGCSLHSSSL

>SG-PL_contig_4355_1_+2_638_790

MGKTNMKLISNMRLGCFTIIVYSNMIKRWKEGKKGKGQYNFLTPWTATYFL

>SG-PL_contig_4356_1_+2_950_1198

MSLVLTMNINAVNSASVEASMTFAASLFISIFINHCLQHIIYLLYFHAILRSRKIRRFHQ

RGTYLTRLDLGSSSVKQLQQRVR

>SG-PL_contig_4357_1_-2_215_481

MFVCLFWGEGESKLIFLPQVNILVCVCVCLCVCAHARRGTSEHVRARVRVCVCLLLMCAC

IFVHACACVSVKLGEEGLGNSLIYPKTFF

>SG-PL_contig_4358_1_+2_1397_1672

MKFLDTEWQRSKCNPHIIISLSKLYENSQNLPTKSLHFVFTAKHFIISCLRRFEVLCDFS

IWNITMQTGCSANESIPCNCPLATVSFQANLD

>SG-PL_contig_4359_1_+2_2_286

VLLLVCAVLCGHFLTHCKLQSCISYTESKERIPDVHPNSRQIFHSFQVSIYKTIYIHLRR

EIKWTNTFICYNRKHSSHQKQSLKTKILKISRYCH

>SG-PL_contig_4360_1_+2_1367_1684

MLTHEQTYRCMCEREQEQELATSLLVAYFFFFNSSCILKQESYDCLKLGRHDLRGQSSLM

VEIAVVAPIIFFYVCFLFWFGFVFVFSFRERERERERTFLFSRVMA

>SG-PL_contig_4361_1_+3_45_233

MINRTIELEQYINPNDDKTAYSRKQTIPISPILVTLVNTAKQMQTVKHIHLLILIYALKE

FFS

>SG-PL_contig_4362_1_-2_68_226

MYIIYEYTHTLLFTRTLGVSFTNSQLDGCHNAGVTVQCHSCVTCQLTQFHWLF

>SG-PL_contig_4363_1_+3_342_590

MLEKICSINTASHIFHKTFKFIYILHIIHSSQSLILSKQAEQLKTLSCSMTITLDPALNK

RTDHWQKETMLELRTPYYSSGNS

>SG-PL_contig_4364_1_+2_2_193

RERESNNAITGFLNNISKSITEQHFQHSSKYEHNCSKIWLFYIIRGDKWERGGAPTDSTK

KHDN

>SG-PL_contig_4365_1_+3_3690_3956

MTEISTNVCLFFSETQSVSATFDIYSFTMPTHQLTQVNAVLKQLQMVEHKTGLLNLVDIR

RALKNSDAKLLWKRPGRKCQTQFLLIPCK

>SG-PL_contig_4366_1_+3_117_851

MRRGTGIGAIKNKNLAQARYKDKGSEIAEDQFSQMTKQMEVFKKNLEEFAAKHKDEIRKD

PQFRLQFQEMCASIGVDPLASSKGFWAEMLGVGDFYYELGVQIVEVCLATSHRNGGLISI

DELRDRVVASRGRTSQDISLDDLLRAIRKLRILGNGFTVLTVGNLQLVQSVPGELTMDHT

NILQSAQVSGYVSKSQLLKDLKWEEERAHRALDFLVKEGMAWVDDQAGAERFFWFPSLFP

AASAS

>SG-PL_contig_4367_1_+2_236_511

MSYQQPLKLQKEKKMYIKIPLYTDYEKKMTPSEKALYIYNTILFIVLTLTASFYRSRSLT

HSHLTIGPLISVDEIICWKLEHIVFNSDYLRN

>SG-PL_contig_4368_1_+2_2_76

FFFLLVSLGFFYLKKLQETRMWLLP

>SG-PL_contig_4369_1_+1_169_345

MVGVASISCPYCQFDGNVLVSITILLLMTIFTTSSDHQTANSQCSSTMQGMIARQKLRI

>SG-PL_contig_4370_1_+2_2_166

KKQTNERKMDTTLIFGTCRDYGKIKRTRMQNKKIVQQTEKVNTGHKNVNISCNTV

>SG-PL_contig_4371_1_+1_1_423

LLLYGSETWRLTKGLEHKLQVFINTCLRQILRIRWPERIANEELWRRTNQRPIGDTIRNR

KWKWIGHTLRRGPNSITRQALDWNPQGKRKRGRPATTWRRTLTNELTTIRLSWGEAKRAA

QDRTRWRSAVKALCSRGNEED

>SG-PL_contig_4372_1_-1_1_216

HVCSPDLSTTHTHTHTHTHTHTHSQLFLLFLTEPFILLFYIFFLKERRLFILFRDELCLV

GKNMRQQRFLSS

>SG-PL_contig_4373_1_-3_171_782

MASTKCLILRGTVLFMLGAFFALVLNLLQVQRQVTEFPPEVLASLFSSAWWVAPSCGTAA

AVIGLLYPCLDKRLGEDIPTKGEWSSVMRCVAVFVGINHASAKIDFANNIQLSVSLAAMS

IGLWWLFDRSRSGFGLGVSIAVLATFVTQLLVYNDVYRYTEPDFLFVRSWLPCVFFSGGV

TMGNIGRQLAAYDGDNEERKTKVD

>SG-PL_contig_4374_1_+3_3_161

YIYIYNVALQKRENKLKYVNNDAAFKPQPKKCLYIPSEGYHRLYDTLEFCNII

>SG-PL_contig_4375_1_+1_256_555

MVLLGTGMLEASLLLTWLKPDEGTPHSSPSLLYLPIPQPGKGSGTHSQLGRLEYTRTSFL

NKVQTRAVRLQSGSSCSAPTQTSPKMICTFMTNIIQELST

>SG-PL_contig_4376_1_-3_63_968

MASVFGRTSRQALLVAKSGTFSPKANFVSFKGKHGKKWVVAMVGAATVGALGVGAALATP

VLAEGLELHPTKMPWSHNGMLSALDAASVRRGYQVYKQVCAACHSMEYLCYRNLVGAIMD

EAEAKAEAEDIMVDDGPDEEGNMFKRPGKLSDRFPAPYPNAEAARAANEGAYPPDLSYIV

QARHGGEDYVFALLTGYCEPPAGVSLREGLHYNPYFPGGAIAMAQALYNEIIEYEDGTPA

TASQCAKDVCVFLRWAAEPEHDTRKQMGLKAIMVFSFMLAVSYYLKRHKWSVLKSRKIAF

KN

>SG-PL_contig_4377_1_-3_3_113

KQQTTTPQQSNMILLRLACVTWQMNLLRLQPCHLSKT

>SG-PL_contig_4378_1_-2_2_181

GFPGLSPESFAPPRQSCDVSPRDLVTISPPPSVGAQVVFLFFYPQNTIHDKKCSFISYFL

>SG-PL_contig_4379_1_+1_805_927

MIHTLTEREVIMRQLSRIKSNFRSLLLASCNFCVTVTNHER

>SG-PL_contig_4380_1_-3_198_434

MKIIQILNLCFAVLSHFLHLNDSVLYFYCYLSLSFCICVCLLSFQPLHHNTQDSVTSVCH

MWVSESTTVLCLIFKKIFF

>SG-PL_contig_4381_1_-1_1_165

FFFLYPSSTSPLLVLGNTESTLQSLFHSWHLPQQLELVTWWGGSMGRGRFPPCIC

>SG-PL_contig_4382_1_-2_20_2047

MAPPKKGRSDDLKQEEILQAVVIADSFDVRFGPVTDKKPRALLPVVSVPLLDYTLEFLAT

QGVQEAFIFCSHLSQQIREHIKKTRWREESKDMTVKVILAEGSSSMGDAMREMHSRSLIR

NDFFLLNCDTIANVNLKELMRDHKARRQKMKMSVMTMTCMKTLPSDPIRCAEDSITMTLR

TDTNQVLDYQKVPDCRKFKIPLNMDDRITAHQDLMDGQLYVCSPDVLTLFTDNFDYCTMD

NFIRGVIVNEDVLGNTVHVNVLKNQYCARITNPYMYGIVSQHLVALRTLPLNLNRFVLHD

EDQMVCRGKNVYIGKGASIHIDGYKQIYNTALCKGSTVGANCTIRNSTIGPDCHIGDNVK

MYNCHLWTGVRIEDGCELNNAILCDGVTVLANTTVQPHTILSWNVTVGPDITLPPGIRLQ

DTPQKDEFDDEEEEQPIPEVTPEYGSRSKAFLFKADSDSDDEDEDDADWGEALADALRRG

HEDDDDVCRNFRLETSEEEEVDEYGNFCSELTATLTRAKEEKINLENLILEVNSLKHAYN

IVIKEVHHAVVKVLVEMPLNNNPNAQGKELITAVLRHLETHKGLLKHYIRDKEAQLDCLY

CLEDFAVDNDRMLSVLSKVVQYLYNKDILEEYVITKWYHKDAVEDEEEEHEKIRKQMEEF

VNWLEEAEEESGNSED

>SG-PL_contig_4383_1_+1_595_768

MTKLSSFAQNILKKHEKSKLAQSASTKVKLQQVQTEKSQHKNLTLAHRETINDASNTQ

>SG-PL_contig_4384_1_-1_184_684

MRFAKDKKGNICVREIPRARDLVTSGDVFIMDVGMTVYQFNGKNCSPMEKSKAMEFIGTI

KSKRGEVVSEVLDEDLTPSSHPFFKLLDEEPDDDGNASDDEDGDSDKKLCRVNTETRTYE

DVKEGDVTINDFASDDVFIYDSGDIVFVWIGSKASPAEKKNGLPLAH

>SG-PL_contig_4385_1_-1_1_120

GTEGGQINSPPDCLYRDVSHHLTAQTCVLPSDSTDMCPTT

>SG-PL_contig_4386_1_-3_1221_1604

MYFLEFIVPSTKTRSPSLKYETQPHAMSFRPHAGGSHTTPMYSGRHFSEDFPHTVLQINH

HFIAEQDVFPVIIRSPVQSCPTPVSPELSVQFPGHNLLFRSSSFEIHRMKFPVSFAQSSM

NNSNQSQC

>SG-PL_contig_4387_1_-1_34_576

MRVLSPFALQDRHFDKTMAALLRNGLRVSALVRQTSRIAFSQQRGYSEMAFTFASPADVY

YNKANVKQVDVPSFSGSFGILPNHVPSLAVLKPGVVTVFEEDGSHKKFFVSSGSITINDD

SSVQVLAEEAHPLDRLDVQAIRDGINKAQQEVSAASSEKAKAEAQIALECYESLQKAMES

N

>SG-PL_contig_4388_1_+3_828_1088

MLLKCAQHTGDFQVRMTHIKGPETRMTSNAWYECLLTEKYDDDRIGQKRINEHKCQNSKK

TKEEKAPRCPLPPLSKSVCVRYTGNKM

>SG-PL_contig_4389_1_-2_2_220

AVLLFPNSKESQVPIHSWVDWRLHTHVFLGPISNPCKPVLSDWKAGALPFCQQALGIIIF

TMITLTVIYTLIT

>SG-PL_contig_4390_1_+1_430_621

MYSRQRPARSVDQFCMQYMQTSNHDSIESGRNLHRKLVCVYIYNSRQGHIQIRSEKVIQD

TYIN

>SG-PL_contig_4391_1_-2_2_193

KKKKSVSLWLNKNIYRGGKMKTEWMRCIIETCALPEENSLNFISTIPHGKVQIPFLLHGW

LGKR

>SG-PL_contig_4392_1_+1_1_162

FLLFFLKFKMTTKNGQPTHKTPKQSNLQQKETKAQQRHIHDSKLKPFVLSYLKA

>SG-PL_contig_4393_1_-2_89_460

MAGRVVQQGVKQVKPILSTNPGEAKRRVLNLYKAWYRQVPYVVKDYDVPINVKQGRDKVR

ELFLKNKHITDLRTIDLLVMKGQMDLVETAQIWKQRPHIMKYFEDTVNERPKDFLGKFYD

GHDP

>SG-PL_contig_4394_1_-1_43_351

MEQAAANQVGAGGVPAGAQTQDYYRTENIPARFDNPDWFQGYGGKPQHPMYTTSASRYGA

KPPSVHTMPTQFYAKSQKFSEHLGQCGMYRNYSLNTEMDKSKV

>SG-PL_contig_4395_1_+3_114_284

MLIAGSASDSRHDVAHIKSPVYCDPFFSKPKHLTQNDTPQGTKQDIKTTVAIGCEPK

>SG-PL_contig_4396_1_-1_19_2409

MAGMQFEYDEEGGTFFYFLLSFWALGLFPATYFCWPRAKASEEDPKKKGICNCLPCLKKN

IIRKKQEATVKPMIIKGVLVIGWIIFFLLAYKVSMIQLDYVEYDPFLELEIDRGASASEI

RRAYRKLSLIYHPDKETGDEKRFMRIAKAYAALTDEESRKNWEEYGNPDGPEATRFGIAL

PKWIVERENSMWVLAVYGLVFMIILPIVVGVWWYRSIKFSSDQVLLDTTRLYYYFINKTP

NMILKRAVMILAASCEFDRMHNAEVQDRPSDNEELPQLIKILGNIQEKNRERPLCFPYSV

KTRVLLHSHFQRLLLNEDTLGADQSYILQKCPYLINEMINICASLVAMAMSGRVHQPPRL

ETVENLMKLSQMIVQALDEKGSPLQQLPHITPDMLRHFTTRKRNIRHIRDFVAMKDEDRR

QMLRSLGEGEYRDIIQVCGTMPYVDMTIRSEVLDEEDTTITVGSIVTVTVNLVRKDMSAL

FDKEISAIEQIDEDDAPLNGNGDEEGDEDDEHDERQGNNVTGENKPKVWEKQKKGKKKGG

KAKKKVKQPYQWKVAAQAKIDKENAAAASKSAAEGTKSKSSGASAVGASAAENEEHEEEE

VATESGEENNASDEEQETSGGGGGGGEAASEEKEEKVEKKKDEDEDEDEDWDTFKMESKK

ENSLETKKKESHCVHCPFFPADKQEAWWLYVADKKRHLLITAPVHVCSLKEEEEVKLKFS

APAKQGVYHYTVVLRSDSFFSDFDQQQNIKLDVKEAKIVKDHPQWDISDDEEDKANNDDE

TEEEEDSDYSSEDDGSD

>SG-PL_contig_4397_1_+2_2_112

VSITAVFSIFQSMFFRNITRCKLCHSYTSVLQRSLQR

>SG-PL_contig_4398_1_+1_1_153

RLEYIRVLCRFGLLSTQTHKYTETPNQTILLVLLQSRQKLCSEAITHSTFL

>SG-PL_contig_4399_1_-1_109_204

MLMCEQQPFLMILYIRLLDCNLERDIPYSLQR

>SG-PL_contig_4400_1_-2_68_637

METKQDTGSTKRVRLDSTQESRQQTGPEALVTRNSPVGMDLSKKRKKKLKRKRKKHLLNK

LEVQRARDFIYSSENDQFSVEKLRTMIEDMTEFLDAIWDVYRSEARQKGVSVEEDGSRVT

AVLKCLNSSGEGSWRDLQLLSRAKSVLVLRSKNLAASTIACVENELGYDADVRSFIVRML

KFWMEEIIRN

>SG-PL_contig_4401_1_+1_1_123

LNGNWLKRNQFQSTEKVNMLINRMFQNISFYIITVQTLNKI

>SG-PL_contig_4402_1_-2_14_697

MAAMMTNMFAGDQAQVAGLRPGEVTTGTTIMAVEFDGGVVIGADSRTTTGAYIANRVTDK

LTKVTDRIYCCRSGSAADTQAIADRVKYHLDFYRMEMGEEPLVATAANVFRELCYDHRDN

LTAGIIVAGWDKRVGGQVFSVPIGGMLQRQPFAIGGSGSTYLYGFVDAEYKDKMTKDECL

SFVSRAVSLAINRDGSSGGIIRMAAITSEGVERFTLTGNQLPKFYEDN

>SG-PL_contig_4403_1_-3_99_1124

MVKLLHSLLGSQVARMAKKTINANFPGPFACFTDYFALVVGAGMTILVQSSSVFTSALTP

LVGVGCLKLERMYPLTLGSNIGTTGTGILAALASSSATIQYALQLALCHLFFNITGILLF

FPIPIMRRFPLRVARFLGRTTAHYRWFAVFYLLMMFLVFPGFFLALSFAGNIVFITTLCL

VSALVVAVVIINIMQRNPRLHPCLPPVLRDWNWVPLPLRSLQPLDKLFRAMCTPCQHICC

DCCLRKCQCLGEGSETNIDSDDEDSDNEDNTDSVMCCCRSSQGMQRIQQSESKVAMEEGH

MAVSSAWSKRTMRPIYKPSFKSQPPHHSQSTVPLIIIPESVV

>SG-PL_contig_4404_1_+3_690_1109

MLITSPFSGLGMGSIVTPRVMEMTFTFSVMEVLLSMHFEHVFCKPLMALSSNVSLAGLSV

SQLVQYQEDQRVQQQSVEENEGPGNLVAHDHTTHAQDDLLIRLQSSVALLSLLFVEGAEG

VEAFLILRGVSADCRIIGTT

>SG-PL_contig_4405_1_-3_774_1073

MYQLYVVLHDVIGHLQSQRTMEFMPLERLQDTSQGKVLWSPCSRPTLSATPVKSKGEAKA

NTYKPVKGKANTYKPVKGKANTYKPVKGELSVHAGCVHFP

>SG-PL_contig_4406_1_-3_3_254

LAKANKQVCDTSSLNKFQTEWSTPDSVVFQIVLHCVWTETAASLVRGLTCVAVGDVVIAA

IVQFCKGINTVIILPVSSPLSPPP

>SG-PL_contig_4407_1_+2_116_247

MYVIYLFITTRFEVIFIQYNNFYEKAPFVFFFKSRFKSLIMSIG

>SG-PL_contig_4408_1_-3_81_254

MAIAISIASFFSEFHSLVHYSQHIICTVNKQQLKGEVVLFFILFCSLYHTLRTHAWLS

>SG-PL_contig_4409_1_+3_60_317

MPRLQKSVEKHKKYLKATKKYTKRAIQANNTAEWTSTAHPCVHNLTNQPPEQNSPESTSK

LGERKLCEGLLEKGKWNGQGRQKKSR

>SG-PL_contig_4410_1_-3_3_179

GRGLDVKKKKKIIILLMPLPSKIKMFSLSLSFSLSLSLSLSLILFSEYSVKYFFLLIRV

>SG-PL_contig_4411_1_+3_3_203

DYLSVSSISIFLVVREERGGRRTRMIHSDPCRLSVNKMSYPQNITGFIYLFMSNENKTIK

SQRTATS

>SG-PL_contig_4412_1_+2_2_175

HITQSLKRNISTTSTFHLRRKSDYKQLKLLCYYHVTQILKRNNSQTQKHCQGHHRLND

>SG-PL_contig_4413_1_+3_3_293

LPLLLHPSLQQAAQQAQDDHDEKKDEEEDEAFHQLVRVYVQFLKPAHVYLRSSFSFMSST

SCIWGLAAAVSVYCREILRYLGSLKKRKEKIKTRLPR

>SG-PL_contig_4414_1_+3_3_125

LRYLGSLKKRKEKIKTRLPRPILYLRKTTKGLEKMNKLRSI

>SG-PL_contig_4415_1_-3_81_320

MLHLENVFNAKAFAVCILLLFLPCPVLCTHACLSFAKSNQALCAMLSELGDVTNVIVFHL

ILSSCHWLFTTVCLCLVRVH

>SG-PL_contig_4416_1_+1_1_249

DWVLKKATEEKGIQWHEGECLSDLDFVDICCTDSDTQDLQSLVTDISEDASSIGLTISAK

KNKKHARWTTPITISSVHWAEQD

>SG-PL_contig_4417_1_+3_48_248

MQTVIILTLCYPSNTSILKINSIWVKHILRTKYCSALSLRIHHIRTSLQSLSTITQKYNK

LAHTRTI

>SG-PL_contig_4418_1_+3_2076_2381

METEHTSGTYISLCDESSQLGTLTDNASTTPTFPRSAVKLNNIYRLLKQTDSEIDDESND

SDSDIVLDLEQIHGHRIIDVGLLSKKILSQPVGGLCQGEVSL

>SG-PL_contig_4419_1_-2_65_1327

MGKLNIFEITLSNFQGVFYAGQNVQGHCTIELNEEMSMRGVRLRFEGKAYVHWTEQHSTG

SGKDRRTETRHYSASEQYFNQDVLLYGIWPNQGSNTTKLASGRHTFPFCFVLPPHLPSSF

EGAHGYVRYSVKAIIDKPWKFDHSCKRPFTVICLLDLNADQNAQMPAQGHNEKMICCLCC

ATGPIVAEFRLERRGYVPGEAIHLFADIRNNSDRKMDRSYIDLKMITTFHATSKSRTSTK

EVGRITERSIDEGQSFSWEGQQFVLPPLPPSYLTGCRIIDVRYILQLNVDPSGPATDLEV

PLEIIIGTIPLRQVVEQFPPHAPAMPPPQAGYPGPAGTVYPSPEGASAPYPPMDPSAPSM

PIMPPSSIPNLPPPSYSESVFGKVNVKDDDDNEHTRGNMDFAPVYTYYDWGHHPTTMDGK

H

>SG-PL_contig_4420_1_-1_1_180

IFLYCEQIADTCYHTISARISRPLGFEIWMMFLQGTNTKYPPRAELSIYLISALDVTVRI

>SG-PL_contig_4421_1_-3_57_242

MNIFLYCEQIADTCYHTMSARISRPLGFEIWMMFLQGTNTKYPPRAKLSIYLISALDVTV

RT

>SG-PL_contig_4422_1_-1_4_411

MEEQRKREEKVEEASSFFSFHQAVPPTSSKSSALFPQVPSAFFKSSGFSLPADDTQQPGR

PYVTGSETAAAQQGGRDGGWGGAPRNTSPVLLGPNQLVTTPIFPPSYFASAPYGSLCSAE

PYSAPSPPSPPPPYLK

>SG-PL_contig_4423_1_-3_153_1127

MVSVTSSVDTGHEDMIHDAQLDYYGTKLATCSSDRSIKLFDVSSGQQVPLADLRGHEGPV

WQVCWAHPMYGSVLASCGYDRKVIIWKETNGSWDKLYEYSNHDSSVNSVCFAPHELGLIL

ACGSSDGSISVISTTGDGNWDSKKINNAHTIGCNAVSWAPSTAPNALFDHGARQQPVKRF

VSGGCDNLVKIWREDETGWVEDQKLEGHSDWVRDVAWAPSIGLPRNVIASCSQDCRVIIW

TNDGTSSQWRSQILNKFSDVVWHVSWSVTGNILAVSGGDNKVTLWKESVEGQWTCISDVS

KGQGQIMDQRSSSEPSHSAKRRIEL

>SG-PL_contig_4424_1_-3_225_497

MSESVAPFTFFGLGTTLCVYMEVVGYGRVQMREEYNEGRGMCMLLRERERERERAGMCDF

YVTLKWQSYSFVCFMFAKTCLVIRTILFLFV

>SG-PL_contig_4425_1_+1_838_1014

MLVSTVVPVEQLNQFKRKNNINPFTTQKIHNCDHSTTDCNILTINANKFCKTVNKTFWQ

>SG-PL_contig_4426_1_+2_2_148

ITFNVSKILPKQSQGFNIWSQNSVCCLQICHRDYEGCLLLICWTHSSCP

>SG-PL_contig_4427_1_+1_163_324

MAFLTLSLFAKHYLHTCKMKSWEPNINVFKKQLHSRFQIEEYNAKFNLDLAVFS

>SG-PL_contig_4428_1_+1_1_156

RGLKSVEFFFKNLNVSCWKNKISAVLFQLMSHLLILHKRKQTSPNENQITTN

>SG-PL_contig_4429_1_-2_2_76

DGASSPLPPTAFSDLRADSIHSATV

>SG-PL_contig_4430_1_-1_130_1578

MGDVVDGPVLSPASLQSYSYEGAPGTSTTADYDLVTGIHPGYPSNTLASGAGSVSGGSTM

GGGRGYSTNTMQSSYNYTFRNIRMSGHQQMMDFGAESNMVGVPPGALGEEIRVDCCLITN

DGKSVVTGSCIGPPQVWDMQSGELLRTMQGQTMGSTDLHLACNDRLLVGAINAELEANQD

SVPKGVNNYVLQIWDFITGRALDMGADVEVCSALTMMSNNDKVVFGRTDKFRGGTTVVVW

DLLGNEAIKTMRYDSPVGHNDFVNFLALSQNENFAIAGFTNSFDNFAEFIVFDMTLNHYN

VTDPCILRLDANPDCTAILPHDEAVTGMRNGELIIWSIRTGAPSRQLMAGSGYHAHTREV

KAVAMSEDNKYLVSGSADGTVKLWDMQSERHIDTFNGHTDEVWCCTISPDNEMVVSGSRD

RTIRLWKLKNGSEICCFNAGVDIFHVTMPRDKSTIVALGDKLGARKLIMLEVCRSKIRRQ

VSN

>SG-PL_contig_4431_1_+2_2_127

TRIFGRVRLTVTLRGWPFLRFCFMFIAGFSFGRHDFLFSQLN

>SG-PL_contig_4432_1_+1_145_450

MTYSLNHYVPYDKIRLRPANLRLALGVAFSLSERSKMAFKSRTTNVHNLHVPGRFHMLTH

IGQSAMRHLKDKSAHTSENMVSTNCQSLITRIFRCSIMNVAQ

>SG-PL_contig_4433_1_-2_77_187

MSMFSVLVVYGGNSLVCRTLRQSLQQGRHSPSLAHAD

>SG-PL_contig_4434_1_+2_1037_1441

MPKDHVSSATNHSPLSYHFHQTSFESSSPHTTQMRISLVQQNFASTVQNPCFTTNHTSIF

QHPAYLPCPTFDAQKADQSQKGKKKKNQIGTQRGWVVDHTRRKLFAQLQSTHIFNTDMAY

DYSTHLSIRELIAQL

>SG-PL_contig_4435_1_+2_2_112

LSCLFTKLNCFFFLYCGVFVQLNTPSLNFVNLSIIVD

>SG-PL_contig_4436_1_+3_774_977

MTVATSLLLRTGQRMSTAHEVLNRAVPQKLLKVIIFTFKQQQKNPCSAAVRQRRGSQTLN

KFINKTKY

>SG-PL_contig_4437_1_-1_1_219

VCVCVCVCVCVYLSLRVCTQVCVSLWMCVCIYLCVVSGRKIICFSKSFKESVCQNTHTHT

HTYAHTKTVVVFD

>SG-PL_contig_4438_1_+1_1_240

APWQRNRTEDQGRAGVGTPEVGVKAPGRLEKSQSRESRRELVEVRPAAETWPEGKRNKLR

ARTRAGLHGQRATAGRHPCT

>SG-PL_contig_4439_1_+1_394_618

MDKPQLKSEPNPENLTTHVQICFLLTANGKPVTQSVYFLLYVTDIRRTERKRHSIFSDMR

GLLQFNSTTNYYQIN

>SG-PL_contig_4440_1_-3_3_284

CVCVTHAQKGRHKETNVYCPTKLMVSVDHYSSVNYSFPVHTHTPLSVARSTKRTVLKTKL

PECDISLVTVVHAVSLPVSYCLPQSLLSYLPIPP

>SG-PL_contig_4441_1_+3_270_521

MVYMETMSKLNTNKPANRPSKYIKIPSTKEFQSRAYACTCTYTHACVTHTHTHTHTHTHT

CIYMYMHYFSSHDTVIGVPELYFG

>SG-PL_contig_4442_1_-3_1251_1511

MISNLKIMYFVCVCVCMCALTNLHRYCTQKVNSYFSWCAVATVYIMRLLLVFFRLITCTT

VFTGKLFFQHIFALSESCPFFHFKVVC

>SG-PL_contig_4443_1_+1_109_354

MSYFPQYRTQWVLVSLWKTLFNLLGKSKSQNLVFFIGWGKSQLVSVTRNRLRMTNVCYIF

VIQDQFSLMRFQIHQKEIIRNG

>SG-PL_contig_4444_1_-1_859_1167

MCVCGFCLYLCFFLCVCVWFLFVCFSSLHMSELVVSQCLSKLVFQKLLLPSVYIFSFVLA

TFFCFLYCTVLVAFVITAFDENIKVRPNCFRRKLKQPELCHRL

>SG-PL_contig_4445_1_+1_1_297

FIHRQKYKQCGEKKKVKRQLSIAHLNYISPLITSITNFFPTFQKLELVLIISNLGIKTEY

LLDLIYNTSPIKIHSQKADTKSCQPFFINRQITTVHTVS

>SG-PL_contig_4446_1_+3_171_353

MFGQLTFVKVFIFSSTKGICLTGNHPNNRSSYLATRERPDPNDSSLQVCSVNVEEEFGKG

N

>SG-PL_contig_4447_1_-3_3_140

NGDDSDDAMEFHLGLRLHDRAVLYASFHNISWCQPGPRVMYAVINV

>SG-PL_contig_4448_1_+2_2_274

CDECEKDYTNETATPLGPKIKHLTTNRSSLKTVREWGCRDAKTLVTRFLGKISKVWLWRL

TLPGEKSRKPWRSSNKVHFSHPIKSISEQRT

>SG-PL_contig_4449_1_-1_19_471

MARYFSESKIEEFKECFDFHARKGSITHEGDLSIIMRSLSYSPTREEISKYFKKYVSSDG

QLDFASFLDVMHDHSTVENCQRELQAALMAQDRTHSGYVSANDIRQILTSLGEKLSRNEV

DTLFREAGISSTGQVKILDFVQTVMTPSPDY

>SG-PL_contig_4450_1_-3_2067_2375

MVSHASSHKSCLPNIHTHTVCLSLRPISFVTLSAVCAMCVIVIDLQPWSASIMTVSMFHA

SVHMIVNNDTRLLSVRCWVSCTLICLDFTLSLPHPIHQLPVGS

>SG-PL_contig_4451_1_-2_83_316

MSSTRGTSQRPPSLAGGRPPFEGIQGKWVPPPYWTAPEPTNSGPCPLPLPSTTLSPSGMS

WRQPPGGANPPSSLLPAR

>SG-PL_contig_4452_1_+3_426_569

MTSFKCSTVTRFYIYKLKHFIYHDQPLHNSLHFTNKFPIIFLLYYPNL

>SG-PL_contig_4453_1_+3_459_659

MKTDPAFAKAKCDFELKFEMSRFFFSPEYFSLLLTEVEYADCTKWASLPERACMHYSRHE

HASDTGH

>SG-PL_contig_4454_1_-3_39_323

MDFLLLLLIYFSEELWWVCCCCGFHLFPFLLCSVTEERHLLCIFLLAFLSIEFSLQLGIL

NLHCKRVFLLDIVCNLYGSKCIIFSHYITNFIFIF

>SG-PL_contig_4455_1_+1_61_1068

MAEAAGSHVNVFKNLADNLKLVIEQIRDGEGGKTDKDKFDSEQYWKHLDAVFKALSMEGT

KLSLAFSQPPFPNAKESEGMVSSMERTVLQLVSVFCSLPKTQGLALRKSLQRVVLDMVER

VLGLVSSLAELDPQAGERKQATGVVWEGADSFSSLPRDNCQAVQVTLEQSDGLLTDALSE

VEEIVSNNGQWCDGFGDEDEEESDNTGTWTDHEKEIVSGPLKGLMKTVCKVVGKAAKVNR

AKGKCHSDDQVAQLDDLGDCAQRLSPAVDILSCCLYAPLDLVAVRNNATELSEVITNMLT

ILRHSHMTGEEDSKWLEFLAKANTHNMNTLLHLLNK

>SG-PL_contig_4456_1_-1_436_1311

MSLTKCLRMTGVLRAMQRQSTCAAVVVPRRHEWNYSSREGIPREVLTMLTQDYDFSWKGI

KAYMEGSRWMALKEDHKFIAARHRTLGPDLATAHFLVKRGAKVKFVGSNTWFQRDKKGYI

YLPNQYVSGMYLEAIDCTGMQMSYVAFDNMVHLEQLRYLNVSRCMNLDDWCLARLYQFQD

TLEFLDLTDCRQITDNGLACLHVLKKLKGLKLTGLPNVRDMGLMVLLLEEALPDVTIFGL

EPERLELPSVGDQDTPMLSGRLQSITAGESTQDVHQEQSMTLLSGGQQNVKS

>SG-PL_contig_4457_1_-2_242_2233

MEEYREEEEQEKQKQRQKPKRRGSVPRKQKTPVEPADSFSESSTESEEEEEAVERRAEAN

ADLPSEYWQIQKLVKYLKGGNQTATIIALCSLRDFNLTQETCQLAIRDVGGLEVLINLLD

TEEVKCKIGALKILKEISRNTQIRRSIADLGGLQTMVKILRDPDKDLKCLAAETIANVAK

FRRARRTVRQHGGIKRLVSLLDCVPLNSTNMTADMELDVEVARCGALALWSCSKSKKNKE

AMQRAGAIPLLAKLLKSPHEDMLIPVVGTLQECASEPSYRLAIRTEGMIEDLVKNLKSSC

PEKQANPELQMHCASAIFKCAEEKETRDLVRQYGGLDPLVGLLNNIDNKELLAAATGAIW

KCAISSENVARFQELKCIELLVTLLTNQPEEVLVNVVGALGELAKAQPNRAQIRKSGGIP

NLVTLLTGTNQALLVNVTKAVGQCAEEQDNMVIIDRLDGVRLLWSLLKNQNPEVQASAAW

AICPCIENAKDAGEMVRSFVGGLELIVSLLKSDDKEVLAAVCAAIANIAKDEENLAVITD

HGVVPMLARLTLTNDDKLRQHLAEAIARCCNWGNNRTAFGKEGAVAPLVRYLKSKEESVH

RSTARALFQLSKNPENCITMHEAGVVQPLLKMVGAADEDLQESSAGCIGNIRRLALANER

AKYA

>SG-PL_contig_4458_1_+1_1_471

LARWHEYGKSLFDDATTANPQDTPCLDLQHTELEPEPVLRETGLDNIPGELLKNTGEAGI

KALHHLCCKIWKSCEWPTDWKLQEFVMLHKSGNIKDCNNYRTIALISHASKILLIIILNR

LKPKVEEELSDCQAGYRANRGTTDMLFILQILIEKVR

>SG-PL_contig_4459_1_-1_1615_1890

MRSSVHPTVSGYLTLFRVGEGKAWRERSGTSPQKADLRVVRSLPPHSPTTWVRVMELPLS

LKKKIFFSFRTTSQKEKCFKVSASVTVDLIVD

>SG-PL_contig_4460_1_-2_8_2359

MASIDEVRRLAAEFQRVQLTIAKQKLSERNVVELVSKLVELKLIEVLYTSDGKEYLTPQH

LAKEIRDELTVRGGRINLVDLQQALNVDLNHVEAKASDMVKHDRSLTLILGQLIDSSYRD

KLADEINDLLQEQGHVTVAELAKSYDLPPEFIREVVEEKMGKVIKGQVDSQDKDVLFTDS

FVLRMTAKIRGAFSALTVPTPVGSIRIKAGCQERLFHSILDDLMKQGRLAGSVSGGRQDK

TIFYPEIYTRAQNQWLDSFYAQNGYLEYDSLTRLGLGDVKNTIKKRLKGERLIMLGACCV

GPALQDQVSASVEDALANDTWVDVKTMLPSSLSRKDISELLSHILRDHHGAFMCGDTIVA

SQKLITNSGKIFSDLIRQKAEKDVRENPALLKGDGDKSSAAKMAGLEDVGREDKKEQRRK

KATAGSKKEGTGGREVKTKSTKKKGRGRDVEEDSDDDGGAAGGGGSGSRLQEAPFMNVKE

IQQVLHSQDDLADCPDVLLKDLATQLHRPLSRQYQEMAKSIFLQTSGTGTSADRKKAFGE

LQDKVSGLWTNVHLFEKGVKLFKDVDQANLSRHLLKTLCTDITNLLLNAMATEYMLSITE

DTQFTPESRLKLIGNLPKEVQAPLSKMHLALNGQSLEAFFSPMEQFCNSSHVSIMLKKPD

KRKERQLVFAHRQTLSEQLRGETEAAMALHLAVVILFQTFTQTMVHAPGRMVPTILAHLQ

DMMDQQVYKQLTNFQDLVVQRLQAEGCGDQSTVEFLTKEMDTLLPQVKDIALTTKRKTQT

PEDQ

>SG-PL_contig_4461_1_+1_196_387

MCRTFKKSLCRIKKTLKRPNETYFYIQETNTAARGIMEQTILPNHIKQGISQNSNKTTCE

QLIT

>SG-PL_contig_4462_1_-2_521_889

MLSGVSKCCPLAVNFSRCRQDKTFTHTLVVLVLPCRTKEPITAGGHTFSLPQIFFCSVSR

HNDAYSYLQQTMQKLQNVAAKTCTAEFSVVRALTLCEKWKFTAQHFPLIHQQERPANNCI

QSN

>SG-PL_contig_4463_1_-1_1_231

PLRLHEDRQGAGFSGPSRGHPPLLPGRLALSSGVRPPLPSAHPEGPGPLHQPPEVRPHAV

TVFRLPGREVRHPYTVS

>SG-PL_contig_4464_1_-3_213_398

MNPVLVLQYKRPQGLCSVNLFDMNKSSAIYFSENCCSCMCVIFRPGKLDLKMRSLCTESL

KQ

>SG-PL_contig_4465_1_+1_1_285

HEGHHNQNSLLKADVYFFNRSNPQIKLRNQTESQSTKNLIINSIIWKANIYSRLSIVTFH

FALINNFHSSSITHQSSHFNTPFNVHQLLTPMENI

>SG-PL_contig_4466_1_-3_3_203

FFFFFMFIILLVYSGPHYLCEKRPSKGSFKLIPDANGNSFVFSLHTSLMFFHVGHFSHSP

SQMSVLV

>SG-PL_contig_4467_1_-1_1_162

NAKPQNSHVVHETVTTNNDYLWLLLIPQEFQHIQLLTEMQFRISYDRSYFHVYE

>SG-PL_contig_4468_1_+2_671_955

MCKHAQNLGTKYIFSKRMKNNLPLASLPLSLNMECGPLTSEHTKHFYHAANKINKKCHAL

PFCTHTLKLQKTGMAEICCSTAVHLSANFRTQEDN

>SG-PL_contig_4469_1_+2_2_154

FFFFQCVFSLFSTYIMIIISLYIHVQLLIHCRNEEKSLIRCTIRLCSVIPL

>SG-PL_contig_4470_1_-3_48_479

MTAEERQAEKERLKKIQEQESLSLAKGLFGMPEGSLDQMTPLSEAEFDQFGQALKSKIQF

FEESQHYSAFLVQLINELTVSLGVEEVKKLGININNLYHEKERQRKETEKKKKKKSKVSI

KMDRADDLDLIGDAAGGGYDDDYI

>SG-PL_contig_4471_1_-1_49_411

MVSLQTVPVEKSSIKRFSHSSVLVADRYIMTAGGFGEVDGRHQRVQEVSVTDTVSMETRL

IRCRSEDIQYSRMHHSSVVLSDGRVLLLGGRQSPFFLCSQLLVLDLIISSDSAANQDIAS

N

>SG-PL_contig_4472_1_-1_118_276

MLSIIDLDHFNSHAADGAACCGLFIKVHRSTPVNVSFTTGDSSTIFRLSVQSL

>SG-PL_contig_4473_1_+1_46_222

MCVCVCVSVLSLSLSLSLSLSLSDRNTHAHTLMHTHGQTMLVHHLHSLKANTIKNRITK

>SG-PL_contig_4474_1_-1_1_303

MPCNSTTQERTFSGNYLAHKKAANWSGMMSEKDSSRAVVMTDVDSRGMVTSLPQSNITSP

SSASPNNRKVSVSSPIHNDSYSILGRTQPFCNKTPAANFPL

>SG-PL_contig_4475_1_-2_47_361

MFRLYQSVTAAGTQRHRTVGLLSQMRMQSQGSTTAHNPQKFDEKTLEHLVCPLSKKDLRY

DKENNELVCDEIGVAYPIVNGIPNLVPQDARMLKNTSTDTESSQS

>SG-PL_contig_4476_1_-3_48_2852

MTDSKYFTTTKKGEIFELKAELNSDKKEKKKEAVKKVIASMTVGKDVSALFTDVVNCMQT

ENLELKKLVYLYLMNYAKSQSDTAIISVNTFVKDCEDSNPLIRALAVRTMGCIRVDKITE

YLCEPLRKCLKDEDPYVRKTAAVCVAKMHDINAQLVEDQGFLDLLKDLLSDSNPMVVANG

VAAISEILETSPTAQQSLEMNIGTINKLLTALNECSEWGQVFILDAISNYNPKDDKEAQS

ICERVTPRLAHANAAVVLSAVKVIMKLMEMLDPHSDYITMLVKKLSPPLVTLLSAEPEIQ

YVALRNINLIVQKRSDVLKAEMKVFFVKYNDPIYVKLEKLDIMIRLTNQGNIAQVLAELK

EYATEVDVDFVRKSVRAIGRCAIKVEQAAERCVSTLLDLIQTKVNYVVQEAIVVIKDIFR

KYPNKYESIIATLCENLDTLDEPEARASMIWIIGEYAERIDNADELLESFLEGFQDENAQ

VQLQLLTAIVKLFLKRPTDTQELVQQVLSLATQDSDNPDLRDRGYIYWRLLSTDPAAAKE

VVLAEKPLISEETDLLEPTLLDELICHISSLASVYHKPPNAFVEGKASLRRSLPPRSQSD

NLGAEPASTVESSPGGAPAAPQATVIPGADSLIGDLLDMDLGGPTPFQQQQIYQQMPQSS

QPVQTGVVDFLGGEGLDSLLGGGGGGMDSLGGLGDIFGAPSSNSYVPPAEVWLPAAKGKG

LEINGTFARKNGQIFMELTFTNKAMQPMSGFAIQFNKNSFGLTPAAPLQIQSPLIPNSSA

SASLQLNTSGQVQKMDPLLMLQVAVKNNIDVFYFSSAVPMHVLFMEDGEMDKKEFLASWK

EIPTTNEVQYVINDVQHNADTVSQKLRNNNIFTIAKRNVEGQDMLYQSLKLSNGIKVLAE

LKIAPSNPNFTLSMKCRVLDIYTGVQGAYETILHN

>SG-PL_contig_4477_1_+3_3_191

HQIIQHHKTVSSNYTSQNCIIKLYNIAKLYHQIIQHHKHTIHSDQTDTKLGNFFFLKRNA

NIK

>SG-PL_contig_4478_1_-3_117_1406

MSSLIDMGPSGQSNGNESFADFSAFQSDRSTAGDDFDPRGVGSAQGSAGNSEFGDFAQFQ

STSAASPTGSQGFADFGSFSSASSTSAAAPAGQVAAPSGSSDLFDMFASPAANTSMSMNM

SATNQVSMSAGMSMPMMGAPVAMGMQGAMPMQANMTPVMMSQGSGMGMTGGMTPLTPTAS

TGWGATTQATTTTTVTSTETVGGDNSVATPQKSIPKTWSDTNVDINLDALSPAGRQNKAA

NPSMNQMQQVKASPTATGNQGMMMGNMGQGMMGIGANPAMMPGAMPQGMMGMPPGMMGNQ

AMQPGMMGNQVMQPGMMGNQAMQPGMMANQGMQPGMMGGPAMTGGAGGMAGMNQGMAGMS

LQSPAGGMNMMAPRPMMGQPMGYGMAAQSGMMNSGAMNNGMVGMQSTNAAQSSFQQRTDQ

AFSAFGNIKQ

>SG-PL_contig_4479_1_-2_92_319

MTDSTQKLTLFSFSGCRSKQFRHAEIGPLFDSCHPALPLSTLATASSQGTLKDSLGQCIR

AVDVTKPSNLHHCSLA

>SG-PL_contig_4480_1_-1_85_1005

MTDISDLDRQIEQLRRCEVIKESEVKALCAKAREILVEESNVQRVDSPVTVCGDIHGQFY

DLKELFKVGGDVPDTNYLFMGDFVDRGFYSVETFLLLLALKVRYPDRITLIRGNHESRQI

TQVYGFYDECLRKYGSITVWRYCTEIFDYLSLSAIIDGKIFCVHGGLSPSIQTLDQIRSI

DRKQEVPHDGPMCDLLWSDPEDMQGWGVSPRGAGYLFGSDVVSQFNAANDIDLICRAHQL

VMEGYKWHFNETVLTVWSAPNYCYRCGNVAAILELDEHLQRDFTIFEAAPQEARGTPSKK

PQPDYFL

>SG-PL_contig_4481_1_+3_3_209

PTQITWLEAQAVDMSKTAFISWLKEKQLIKLAWKNRKSAAHINKAHHKRNANIKLSFRYF

PIRHLYVLK

>SG-PL_contig_4482_1_-2_275_565

MNERAANHCQILFAKSRHRSDFHRISKIKMASKRAASDVEILCSPLKENAARHFQTFRGE

YTGKWPFIKTGKKSDTYAKKLTDFFKVKLNFQTQRQM

>SG-PL_contig_4483_1_+1_1_219

QVRTLASAQASETSNILWVDGTRISHQQSFLYWSPLSALTQPVCFVRVHAQSRALLHCAV

ARAVCVRIAPRAR

>SG-PL_contig_4484_1_+3_39_296

MMILEDTLTKTVIEVVFLGTVHVLFAALAFVRDAQLISITLIISFTTTAGRSCKVGVLVI

KVTPRLVVTDDISLVTRTVFYRASNF

>SG-PL_contig_4485_1_+2_2_205

SSDLFVSYNLFRSLTGRSHHIKINHHTISLLIPIPLSNFKTSHIEHVYIMKHKPASNKYI

CSTKYFQI

>SG-PL_contig_4486_1_+1_64_246

MSTLHIILRACEHMSGNMSKMWVSDSKGPFVVFDTCAPLSRTFRDHDYLTCRFNFGKKEK

K

>SG-PL_contig_4487_1_-2_2_310

MWKAQALQFRALHLVSPWPPTSYEGLQGAGVSGPRRSHPSLLLPGRLAHPSGVQHPLPSP

HPEGPGALRHPGVPDQPPEVRPHAVTVFRLPGHEVRHPCTESV

>SG-PL_contig_4488_1_+2_893_1066

MFMMEDVNIAHNPTKYFSHIYKLQSKYCTTSGFNPIQAIFDQKNPKQQQQQQQKLHST

>SG-PL_contig_4489_1_-2_782_1150

MTAKFFRSRQSFFLLLLDLSAAFDPLDHKILLSHLDQNFGIISAAFDWFQSYLLERKQTV

LVGGNRSSPESVPDFGVPQGSVFGPVLFKLCTTPLTPLTDMYSVHHEMYADDTQLNHSES

QSN

>SG-PL_contig_4490_1_+2_2651_2866

MIVEVRSVRQARNVVYPKTIITGFSQCGFTQQYCSPIFTESKANRNLSSYVMISTGSLKQ

SIMHVYKNYKRK

>SG-PL_contig_4491_1_+1_1_171

DMCMHIVWSYCAGVILLHSLHTPFSMGVRARYLCVSISHQTWLTGSLTCVFDVLYAY

>SG-PL_contig_4492_1_+3_3_155

KLLARAKFLSECWVFSCFHKPLNTDMAYRIFNKRPVYSHEGVQALGGLGIC

>SG-PL_contig_4493_1_-3_90_1337

MALNKLSIDSKGVELKGKRVLIRVDFNVPLKEGHITNTQRIVAALPTIRYALDQGAKSVV

LMSHLGRPDGQPNKKYTLAPVAEELKKQLGKDVTFLPDCVGSEVENACANPAPGSVFLLE

NLRFHVEEEGKGVDAGGKKVKASAEDVGKFRASLSKLGDIYVNDAFGTAHRAHSSMVGCQ

LSQKVSGFLLKKELAYFAKALDQPDRPFLAILGGAKVADKIQLINNLLDKVDKMIIGGGM

AFTFLKVLNNMEIGASLYDEEGSKIVQELMDKAKDKVEIHLPVDFVTGDKFDEGATVGGA

TVESGIPAGCMGLDVGKESSAKFAQAIAEAKLIVWNGPPGVFEFAKFAEGTKAMMDAVVA

ATERGATTIIGGGDTATCAAKFGTEEKVSHVSTGGGASLELLEGKTLPGVAALTDA

>SG-PL_contig_4494_1_-3_1062_1298

MQKLTEPIVGPGCDLPLLTSAPLKKKELCAVQIFFFIHIYLPVLKKDPFPLCLSLYINTL

QMAMSWVHFLHSTCCHSKI

>SG-PL_contig_4496_1_+1_1_201

EEGKSVAAQGSPVEGEGVDRRRLGVLRRGAVDREPLVGLGRGEVDREDGPSVPGSLEGRL

LEGPLRV

>SG-PL_contig_4497_1_+1_2185_2598

MKASLSTNLGGCNIMPSQKCHLLCKIFPMKKNAAVLLPGFKIAVIQTILYNYQKLRNVHV

LCNKQKYTNFMEDFILWKRITNHTHDLEHGVGKIGVTLCLSKNVLSFNGLYHKTHAYSPA

HSCKHACTLSHNHDCHSG

>SG-PL_contig_4498_1_+3_90_308

MLQQGLLRFKFSKQISISEQEVFLFFQNKLCPSILWQKNFVPHLNTDWCHFTIFILDTLS

HSNNHSFLHFALS

>SG-PL_contig_4499_1_-3_3_344

EDVGVDVIEVSECDWQKEKASNQQIKFLIEKHFPTLKPFENVSLISEPIILDAIQNNTLF

GLIECDIDVPDHLKDHFQSCNLSLKTPVSKEKTSVHMCVNTRKNINCCRSHDAL

>SG-PL_contig_4500_1_+1_13_306

MGRWIKHTKIMINLISTNFNMAAGAPINVEATLPGTGQTIPLAPGTRLGDREAERYPEGL

VESCPEPSRIPDLDAIMAKHQTGKKEEKIRLTMTLGNW

>SG-PL_contig_4501_1_-1_13_258

MESEDQWRAFQLKRDSVHKPSGISLSGQPDLYRRNPLPQLVPFKTKAWIRKWDGPGYFFP

PSNTWVHEQVGPGIPLDSYRFH

>SG-PL_contig_4502_1_+1_1_372

SNKYSALAESPPPGTIEWWEGLKETATAAGEEILGYRKALRESWISDHTWNLIAERKDLH

QKRHTDTSDDNAVYQDYIEKDREVKHSAQKDKRIWLEDQANDAQNAASRNDMRAVYQITK

ITGS

>SG-PL_contig_4503_1_-1_97_2829

MNWDPSQWQPLIQDRQFLSWLVKVPSDQDQMRSRQISAQQINKLEELWKENPDATLEDLE

KPGVDEEPQQVLLRYDDAYQYQNIFGPLVKLEADYDKKLKEAQTQDNMSVRWDLGLNKKR

IAYFHMPKANDEMKLMHGDELRIRYVGEMHKAWSGVGHVIKIPDNYSDEVAIELKSNAGV

PSECTHSFVVDFVWKSTSFDRMQAAMKTFAVDETSVWGYIYHKLLGHEVEDVVLKCTLPK

RFSAPGLPELNHSQVYAVKTVLQRPLSLIQGPPGTGKTVTSATIVHHLVKQNIGPALVCA

PSNIAVDQLTEKIHKTGLKVVRLCAKSREAIDSPVSFLALHNQIRSMDSVPELSKLQQLK

DETGELSSTDEKRYRALKKQCEKELLQAADVICCTCVGAGDPRLAKMQFRSVLIDESTQA

TEPECMIPVILGCRQLILVGDHCQLGPVVMCKKAARAGLSQSLFERLVVLGIRPIRLQVQ

YRMHPALSAFPSNIFYEGSLQNGVTAAERLRKNLDFPWPQPDKPMFFYCTNGQEEIASSG

TSYLNRTEAANAEKLVTRFLRSGVLPEQIGIITPYEGQRAYQVQYMQYSGSLNKKLYLEI

EVASVDAFQGREKDFIILSCVRSNEHQGIGFLNDPRRLNVALTRARYGIIIVGNPKVLSK

QPLWNHLLTYYKEQKCLVEGPLTNLKESHIQFSKPRKLINSTNPGGRFMSNTMFDARELL

VPGSVYDRGMPYREPVVHTHDQLNFIGPERAYTRSAMNLPVPVNMFLPPVPQPFLNQHMP

PGQPANPGMQKGMPGMAGNSWNQNRRQKPPRYSKHQQPAAQLNTGPSQPSQHSQEVSQGF

SQGPLTQGLSMSQAFQIGQSGMSGLSQAELSQDSFMADDFKSQMDGMLSQDSTYQGDRHF

YASQLSQGPFN

>SG-PL_contig_4504_1_+3_3_164

YYILFTAQLTVKIISRLSPHKVHQSQEDNHVDHKTSLNLKTKILQNNTVHDILS

>SG-PL_contig_4505_1_-2_80_211

MGTGCPFCSVICLYGLFMFFFVVVVASKYFLYFFALFCNLGVIN

>SG-PL_contig_4506_1_-1_1_390

GSDVRSGETLCDYLQPFPVRGAGYLRYVFVLYKQDKRIDFSSFQRSPNCRSLAERRFSTL

DFYRQHQDDITPACISFFQSQWDPSVQAVFHNVLEMQEPSYEFIRVPNYHPAQKRYPHKH

AFNLYLDRYR

>SG-PL_contig_4507_1_+3_3_155

CLQKTVSKTVHLSESIWPKRCTCLVLHIHSLTFVSDFHRFSIHLTEKLVSV

>SG-PL_contig_4508_1_-2_38_496

MQEAVYRGRTTYPQDMQPLLKLFSMFFGYIDRLYGNRFYGKGQNLVYELSRRYDAALQEV

DVLVMPTLSHVASRIPPADCSFADFAQSCLDSTFNTMPFNITGHPALTINAGFTAPDQDR

GRLPIGMMIVGKKFDEVTVLQVARALEKLTSQQ

>SG-PL_contig_4509_1_-1_103_561

MQEAVYRGRTTYLQDMQPFLRLLCMFTHYIDRLYGNRFYGKGQNLVYELSRRYDAALQEV

DVLVMPTLPHTAFRIPPADCSFADFVKNCKDFTGNTMPFNSTGHPALTINAGFTAPDQDR

GRLPIGLMIVGKKFDEVTVLQVARALEKITGQQ

>SG-PL_contig_4510_1_+1_1_201

CMTSISNCFYKFSERHGLTVEWQYPLVFMNIYNCFSPSVCMCDLSRIRFTNESNKYEHGM

IYMCLQV

>SG-PL_contig_4511_1_+3_36_266

MLISSNAANSTSVLNVANCRMSRCLFVKGVTPNIVRLTDVAGTVRHIRANRAATRRVILL

TQQTALVYCSQFSFSLV

>SG-PL_contig_4512_1_+1_2089_2655

MVGSRTNNFTHSSQKVTFYSLYIILIIHSSRKLLSITSHQNVIHSPHKLFTVCNFNIHSP

CQVTFYSHSPCQITFYRQFAKFRPFCFEVKLLFNICIQFYLSFLSYSPLSVLKYLSILYR

TYFSSSLSRQFHSVILLAFSMVSLHTDRSVCIQTISSVLHINLLFTVCIQKILFSHFPCK

VTFHGQVCV

>SG-PL_contig_4513_1_+3_3_221

VYANCGSCDVFISAVSHYYYYYYYYYYYYYSIPLLLSLIYIVIQYLPVISNLISIYKRPE

LDLKVGQFRLVSL

>SG-PL_contig_4514_1_+3_621_857

MLTGFYGAVVWHVQVQSVCSSKPDTLCPFAPDFISFFFCFMLLYSVRITMICSYRYLLVF

QMGERGWAWGSSATKMRSG

>SG-PL_contig_4515_1_+2_2_202

FNLLLSVLNNFKRQVMIYLCVLIIKFSKYQFIYYIIFAFKSINSIHKNTNTEKQASTHTQ

TKPFNFT

>SG-PL_contig_4516_1_-3_717_1046

MKEDTCSILKKKKKKRKKDMKKKCAHLTCMHATEVHYLKKKVKFKKKERKMCVFVHACDE

GGRQRNFASINQTFIADHFVLVIWLLFKINAWMLSFISLFRFLIVFLWKQ

>SG-PL_contig_4517_1_+1_160_306

MLIHSHAHVHTRQHTHSQTIDDYFILFRSETNKKLKPNPYTAHKYAHTR

>SG-PL_contig_4518_1_+3_120_293

MFYKLTSNTPKILKGKNYPTGQIGLLQHGKMSSQLGNACDPYFTITTRLSLHERWKQT

>SG-PL_contig_4519_1_-2_2_247

LPGCTRAHELRALSSSAAFHHSLPIRDVLEAAYWRNESTFTDLRVSSAEGLSRISFVAAN

AVLDASTGRADQGRRSLPSSNR

>SG-PL_contig_4520_1_-3_168_2258

MNLDPLSFALAEISFSVANLTKKSYKSSVAEINNFLATHGAEADRHLFRCLLSHIDFSGD

GKSSGKDLYQIQYLTVDNNHFLGRQNFVSILCYAVDEPLHHQKTLKPSAQLLQQLSKVLK

LNRVQEVVYAFALLSSSRPETVQYATQFLKQKIPDLIHSYVDADTAVTGRQEGGLQDAGV

EALHLLLSHLLRGRDAYGIGKEQKEALLKTLRRDFPRDRVPAILSYLLYPEVDDIPLHQL

TTLSNMPSSMMDSSLAELMSEAGYACTSSKEECRSVMLGVRDLSPLAVARVLGMMACSTT

GLGDQTQAGGWGDKPETPGVTTWNVEVFIQVTRDLAPSLDWKDIVAQLDHPGFLLKNKRG

LRLLTEGLLRGLQQNGLPFPIHLIFRPWNNTEGQLSLILQCLKHHEVFCFADYLAGTDVV

ACDILKSAPSDDDRELSTWKCLNLVETLLRLSDAGHYQLVYEAFRYPISKCPDVLVLGLL

QTPPTWNTLKQELISNLIAIFLNSHPNSNAILHYAWHNQSHSGTIRTLIMHSMAEWYMRG

DVHDQNRLTRILDCAQDLKALSMLLNATPYAFVVDLACLAARREYLKLDKWLSDKINEHK

EPFISACVTFLKRRCPQLLGVKDEPQNKATHLTPEAISTMLQCLKQVASLVSQELQEAIN

TMISNASVYLNKLRSASNPGPPSGPKPPTYPQGMVNQ

>SG-PL_contig_4521_1_+1_1_234

QAFSILTTHRSSFLNCHNSLAKLSQFSQLTIILSQFSLEISNLLSVSYSFLTHYSIGSHF

RQQLPQHADHIISTFMEQ

>SG-PL_contig_4522_1_+2_227_403

MTRAHTTLTNTCSWIGNWKLCFYTSDRKRPITNRQQTTCWTAPQTRSKSTAGNDLCLLR

>SG-PL_contig_4523_1_-1_130_2481

MGERVSLQVLVGTVVCAVVVVITVTASASSNDNDNDVFAAHYGRASVSSYPFGDPRFCQD

MARKDGDKLQRFEVLPGYGWDNLQNEAEGQVVVFNYSKCRTTEDARFLLPDSVSAVPIKA

SRVQTFAELITHWKSWTSITSKSVNLEAGLSLNKFSISGKFSADFEDIKSKQVGDKSVTT

RTQLRYIRYGVQLQPDTPLHPVFKSRVLGIAAAEELGEKERARYLAQLLVRDFGTHVITK

ASAGAALLQVDQLREEWVNSNIEHKSDILAAASGSLFSIFQFSSRFESKTDQRFSDQYKK

SRTSSSILTLGGPVFRPANFTPDMWADDVDRDLVAMDRSGDPIYYVLTAATLPELPTSTL

NEVYNRVLEAVETYYEFNRLPGCLDPTSPNFSPSANTDDGSCKPPPTNLTFGGVFQTCSL

KPGSNNGNLCEGLTQVNPLTSDYLCPDKYQAIKLHSGTTSSSRTEHVCKRYLIFWHKCHD

DFYHSQATYNMYWCVATSGVSRDSGYLFGGLYTSTVVNPVTRAATCPPTFFPLSFGAAAD

LHICISDDYEFGSEYHLPFAGFISCQAGNPLAVAGDGSSNKGPNQVKSAGAKSKSESKSS

KVHGNLKMFFKDQADDWPKRCPDGYSHHLATVDQGCEISYCVRTGSMNGPVLPPIKRPPF

MPAPALPAPASENTVIFDPATLTWKRNKEAGQVPPSAAPNQASADESMSPGTAAGISIGA

TLGCVVIATVAILAVRARRRRIMPGYRRLQNPLLDPQEDYGSVNTTERRPASTMVNVAPV

ESTP

>SG-PL_contig_4524_1_+3_60_251

MVRCKSLSIYISGHNNIRWLGAILFIFLAILLLNSLLQISIFLYLWIKQKSVCFRSLFFF

FFFF

>SG-PL_contig_4525_1_-1_13_492

MATRNEAFSPSWSSVFNFDSLERPVQLHLKNVYTCLGVGMLAAAAGGYMHLFTNIMQSGF

LTTIIALGLMLWLNTTRHCKENFNKRIAIFTGFTFCSGVSLGPLLDMAIMIEPSIIPTAL

LGTAVIFISFTVAALYNNNRSFLYMGGLLMSALSWLMILS

>SG-PL_contig_4526_1_-2_2_151

ARKTKLWWKSITDKTQALLNLPFKKKQKPSNVKIMTLIRSLQSPVWVTGL

>SG-PL_contig_4527_1_-2_128_946

MGPGGGFMGTGYGRGRGGRNVKGFFEDEYDDYSSGGYGYGGRGRGGMRGRYSDALDDRRG

PGSQYMSKTGHSVHMRGLPFQAQEQDIFDFFSPLQPVRVSFDFSSNGRPTGEADVDFATH

QEAMDAMKKHKSNMQHRYIELFLNSTASAQHNSDMSGYGGSDMGSGFGGGNMGSSMGGGG

YSNSGGGFGSGSNMGGGYNTGGGSMGGVGNQTGQYGVSQQYGSSSMGGGYMSGNSQTSSY

GGQGNYMGSSGSGYNSMGSNAMSSLANTNFTAF

>SG-PL_contig_4528_1_-3_3_236

HTPKHKKSHSLIVKNICVRIHQKFYTSINISIHLKSITKLDVVKSPAVKLLCPYIGKFSS

VEFHCRIRRWMYLQEKKN

>SG-PL_contig_4529_1_-3_21_203

MNFIKAVTYARFKVILNHCIAFGREMGPIQTTVSTVNADMGIIYQRIQFQGLFVKVIALS

F

>SG-PL_contig_4530_1_+2_2_289

GRGFESPSVHKKKITVGFFGPNRLLPRVERAMGSLGRLGPHSQVSSTVRIHHGGCCSDFL

TNTTGVCNSRARAWLTPGYIIKKEIVEYSSVMQSQS

>SG-PL_contig_4531_1_-1_1_444

INKLEKDLEEKRRYIDIFEKWILDAEEVLKNKNKKNDRDLVQQLQTEVKHLKQDLGNAGY

RKDDIFTDPAALKTELDRRDRELNAKRSELDGMSEEVQRWRQECSSVESMRKNALDALRL

LETEVTQLQDADIQLKQMKDEYNTLRGQ

>SG-PL_contig_4532_1_-3_123_1319

MIITLKTLQQQTFKIDVEPECSVKILKEKIEAERGKDYPADGQKLIYAGKILDDDKKVEE

YKIEEKNFVVVMVTKPKAASAKATEPSKPAETAATTPSPAPTTQTAVPPTSTPAATATPP

ASRPETEGRGDGRPIETSVDAPPAAAATQLSEGGATGSAVQSAESTLVTGSAYESMVTEI

MMMGFERDQVVRALRASFNNPDRAVEYCLGGIPDVPMQEESAPVSAAGNPPPAATGTPAS

TGAPTQTPPSGLASAQNPASQAGTGEALDFLRTQPQFQRMRAAIRENSSLLPVLLQQIGQ

SNPPLLALIRQNQERFVEMLNEPESESGVQSGGGQEQEEGGNSGYIQVTPQEKEAIERLK

ALGFPEAMCIQAYFACEKNEDLAANFLLSQGFDDDDDQS

>SG-PL_contig_4533_1_-3_36_245

MKGHRMWNNFHSSMTMDDFMIMRMMSLGPRIWNSFHCDMTIDDFMIMRMMSSDNLAYCTV

LVEKKYPMAL

>SG-PL_contig_4534_1_+3_3_122

NLFNCKFRKVHAAAFRQLYFLSYLTLVFSIACTCQHLPVL

>SG-PL_contig_4535_1_+3_18_215

MHTYTHVHTYIHTNGHTYIQYKNTYLPTYITYERTFKRTYIQTLISHLQSQTTMEVVLLE

RLQDTS

>SG-PL_contig_4536_1_-2_104_265

MGHASIHFRSSHIPWFMTIFHSVSHPFSHVYILFMWLIYYWVMLGASLRVWNQV

>SG-PL_contig_4537_1_+3_3_176

CYYFLNNKYHSIFLTFTMMNYSSAENSNPAVSICTTKTGTEHTCVVLTTQRYILKSVI

>SG-PL_contig_4538_1_+1_919_1218

MHTLILQIWVTMSPILLKRLCAIYGLTAAVYCARGYDSQHTAGAKVSTALAAALVNRNGL

MSGQLCTHYIVLYSMYGCQSHSHQADQGVRQTAYIKGMGD

>SG-PL_contig_4539_1_-1_1_234

KEQAKTRVPFTDFKPQVAAYIQSLWQEEWTCQQDNKLFQIRPDLKVFLPSVSGCRKEETV

LCRLHTGHTFLTHSHIYY

>SG-PL_contig_4540_1_-2_296_787

MAAASMGAFGTYKTQYHRLVEERERRAALGQGGQTVWVTKRYDPLDLRILAPVPRHKLVS

GQLPTPAEKHWVPVTDPPLRSFERGRAGVFANFPSTRDENWSTLRAMLPSLGSAHKVRPP

SWGTGQGPPPFILERSPKRLPKVNSPMTRYADEMHSADRLFRLH

>SG-PL_contig_4541_1_-2_506_724

MFIVIVLLWFHTGFLVFFIFTHYRFSSFILCLFKTGVRFQIVMRNHLFVLTALLKRLYTG

IRLLCLVLVIIIR

>SG-PL_contig_4542_1_-1_1_240

KRKKQTKNENKTKDNNNNKKTTKSQHTHNQHSITLVNNIIPQQQQNQHIHNQHSITLVNN

IIPQQQENNKNPSIHTISIP

>SG-PL_contig_4543_1_+3_3_110

LLLLSLLYIVYSPAYHEDHIRALTTLSLSKSRRQPH

>SG-PL_contig_4544_1_+2_1250_1516

MFSVQIWIMIFGFGGKWLKENGLRLFSNLCWAFCCYYWRSWAFQKLCRLHCMYAHCPGYS

ADTVQASCAAQSAHSCIWGYTEHEKQSQI

>SG-PL_contig_4545_1_-2_143_805

MSSSEEVSWISWFCGLRGNEFFCEVDEDYIQDKFNLTGLNEQVPHYRQALDMILDLEPDD

ELEDNPNQSDLIEQAAEMLYGLIHARYIMTNRGIAQMIEKWQQGDFGYCPRVYCENQPML

PIGLSDVPGEAMVKLYCPKCQDAYTPKSSRHHHTDGAYFGTGFPHMLFMVHPEYRPKRPA

NQFVPRLYGFKIHPLAYQLQYQAAANFKMPMRGTSHPNSKR

>SG-PL_contig_4546_1_-1_199_465

MQQSHTTRQTEQATKQTEQSSNHCSNQTQPQDKQNNLQTIPAIAHSHKTNVTNHCSNHTQ

PQDKQYVNPFTLSPYPLVSCRNLVDRVRE

>SG-PL_contig_4547_1_+3_135_455

MTCVAECVSPMCQKCVNDYLIASPVFIHTPHLSNIKYSHRHHFHSHSPSVPSSITTNPNI

FLSLSFNNHSFSSKRDRPHILSSLTMHHQQIQTLTFTGYSYLKTFSV

>SG-PL_contig_4548_1_+3_72_230

MVQTKSNKAVFEALFEFFDSVGIVAFFPPWTYLSLSMIGSFSNFYFSFIYRCQ

>SG-PL_contig_4549_1_-3_492_662

MKFQLPKFSFRTFSFGAFLFSAHFRKSISIQRCMCVLPVPKKIKSVVVLRMNEDDGA

>SG-PL_contig_4550_1_+1_1_276

VLVCSVASAGQGGQTRGDFTRSRMSWSREGQKEPALLREPKYLSRAERPVRKASAPAQLR

RCLEILSQSASVLKGIEAKAGAGLGPKRSLSS

>SG-PL_contig_4551_1_-2_14_241

MTELPGGPVVCPGGGQTCPAGQTCCLQSDQFYACCPLPEAVCCSDHIHCCPHGSKCDMQK

QMCDGVITTFPMVKKV

>SG-PL_contig_4552_1_-2_35_250

MYVHLMYIPFFLIYVYMVVCIICDSENEYSSAVVSIKSLVRNYKKFCDRGLYKNVYEVLL

DYNIKCVDHHLK

>SG-PL_contig_4553_1_+2_524_751

MAVLLTASPWSNKPGIPAIFGIPTHHVIMQSSIYGELLKLQVKVVQFVQPSFVAQCPVTV

TAFPSQTQNPLYITYL

>SG-PL_contig_4554_1_-1_13_657

MMVPDYAMIAEISLYSFGFLDARNLAVKIVTTYRLCSEQLSSQFHYDYGMRAVKAVLSAA

GNLKLKFPDEAEDILLLRSIIDVNLPKFLSHDIPLFNGIISDLFPGLKLPEADYKVFLNA

MQRIAEKKNLQFVDFFREKIIQTYEMMIVRHGFMLVGEPFGSKSKVLETLQGGMTLLSEE

GEEDYEKVLTRIINPKSITMGQLFGQFDPVSHEWT

>SG-PL_contig_4555_1_+3_3_218

CRLERLKPLFRSLTNKLYCFFFFCNLLCIFLLNAFRFHVLWWREVGRAGRLGIIMEHEIV

LRTLCGIGTGDE

>SG-PL_contig_4556_1_-1_97_240

MLKIHIYTKNTFGAGRAHTHTHTHTHTHTHGTIPTLLQRGFTRVGMIG

>SG-PL_contig_4557_1_+3_3_287

DLYACLILHLFIYSFIQESGICSRGIYCTWIVSLLANSLSLHKTLLTGKILKMRRCKGTI

RHTGETTRMIGTLRAAANRPVLLLGSGIELERERF

>SG-PL_contig_4558_1_+2_119_238

MDETRLCGPSLPNEPIARSTLGRSRLGPKNLTMIFFMIFP

>SG-PL_contig_4559_1_+2_188_343

MRPDFVVPSLPNEPIACSTLGRSRLGPKNPTMIFYGLMGIRTHDLPVSCPLH

>SG-PL_contig_4560_1_-2_32_139

MSSLYSRRKMNVYKVSNRIWSQILYIVKYGSFLSLF

>SG-PL_contig_4561_1_-1_187_1482

MMCMVVCVLLQSEKGGAVPVALADVSGSDSSMSSSSSLDGKDDRGTWSGRMDFLLSCIGF

AVGLGNIWRFPYLCYKSGGGAFLVPYVIFLVLCGMPLFFMEITYGQFASLSPITVWRMSP

LFKGVGYGMVIISGIVCVYYNIIITWTFYYLFMSMRKILPWADCDNAWNDERCVLNKFAS

EPDTNSTTANYTISLNGTDLNSTVIMAANATNATLNSSHLMTASEQFWERNVLEITDGIE

DLGSIRWPLFGCLALAWIVVFLCLCKGVKSSGKVVYVTATFPYLVLIILLVRGATLPGAL

EGIKFYLIPDWEKLLEFKVWGDAAIQIFYSVGMAWGGLITMASYNKFHNNVYRDAIIVPL

MNCGTSVFAGLVIFSVLGFMAHETGAEIKDVVTQGPGLTFVVYPEAVAKLPISPLWALLF

FLMLFTIGLDSQ

>SG-PL_contig_4562_1_+2_161_382

MHKKIVKEIMTTCKNCSKSIAKMPWNCKINRLTRITYKHLMMNSERKSLPSVPCVIYPVI

MKCMKIKSIKSVWQ

>SG-PL_contig_4563_1_+1_97_537

MKTFISLCLMMGVIQLPCRRDYWRVSSRCWLAHTNFGKAMSRDRFDMIWRYLHLQDNTVP

NANDDKVFKIRWFINFLIQRYQTVYTPGKYSCFYFHNLTCFKNNNLISFREKEAEITGLI

YFHFRRLKSQVRSRSTFTFGGVIMLLF

>SG-PL_contig_4564_1_+2_380_1003

MVCMSIQMSTTDEIQDIMDAVGLDDSEYDSSGDEYIPSKEELREREEEEMDDDGPCTSKR

TAKKRKRETEWDEEDNIPLATLQDDIPLARIRQNDKNGDWEEEDDNPLASFVLEDVDGSD

QDLQDAIQNPVWTEGPFEPPKAEFTGDLEHPPEGELKTPYQYYKYLITNKMLKKVEDQTN

LYSVQQHGTSINTTQKEIEIFKIGRAHV

>SG-PL_contig_4565_1_+2_500_796

MLIDVVHYITKTNANKEKNDTKTLKATPKSCGNRNDIQIMHFIFFFQFSPPSTVDISHNF

YITEHKKINDNTVSNVNSQDNYTSISIPKSDIDDIMKST

>SG-PL_contig_4566_1_-3_3_4262

INHRSFSQNTITLFGQTTELVVRGKRTVGHLQFVYSSFSTLVISTTDLSVNITITVINNN

LLVTVSARKDAWFTGNSTVSGLLTSCNTQLSIQFSACLNQSHQVCTQSQALSLPPSCDQR

LSLTALLNFLTFHLYIAPSNISPNGSIITEACLRLNNSGTIVRGVSLPAQHFSLEFNVKM

FSLSGVLVSYMVDSSNYFILGCSGSQLAVFTAEGTVLTGLHMTVNVWTQVLISWHSALSS

VEIYVTGVDGLVAFKSVRLHMHVFVSAGVLTFGQAPARVNIATNIGVFLGLLDEVRVWSR

PTNPSVVSQTWKMAVQDTTPDLHLHWPLNDGSGITASERRRRSTMYPMDVQNPPTWEISD

LTLISKEMLGVGDFTVNGADLDDYLFPVQPSTNATNMASAQATCGTLLGNLTSSPECSRI

FPDTSSLLQQCSAMVYSTDSGLSSELFTLALAQLCQHSQQLQASPLQSQCGQLHYLGSVL

PFYGMDCTDTCLFGWTPTLSSCLCYHGYWGSTCSLLCPFTDHGVCNAYGVCANVTGICSC

SPHWLHSVTTTLAYWTGNMTAMVSTYACTDCTAEWTGGDCSVTVTVVKVSVQWRAAIAFS

NYLTTWDGASLALVTPGTYHLIQVQGVQLTALFIPCTQRRLCRLISAVSLTMETFHLKVS

ASGAKFDNFTATVKIEGEIEDMTFPFSKSWGRASLIWWRANYLRVTMDSKLVVLIGTSPV

GLTVSTKLHQDWFSQTTGLFGNPDGNHLSDLLPSSSIPQADLTAIKISEHQRKNFLLTDK

DLSALLGESMAGLNLSTCGHMLHLTHHSLTFSTLDLIVSTQQLTLTLWLKVEGLLSAPTV

VMELTTSVGMISVSVHQHLILLTWFGTETTILHPTSIHTWTYLAVTWEGSAGSLSAFAIQ

ESGFDYSTISVGRLSGQFTITDLSLVGPQTGQSAVALDLFRLWQSAKPLKSIAQDLRVYA

PDSDLDTKTSLPVLLMWAGFDEGEGDSTAMSVQTMTAPMNVSGVISVNAQPSPAAPSVWT

PSTIPVLDVALPDDVFHTPDTNQLAQACLKVLQESSLLTQCGDLHAVFSIYLEACVREYQ

RTGRINVTDTVANLLAFYCHTTNDVGECQLDQYVDFCRPIPDEELPVWIIIVICVGVLIL

ASCCCCFVVFIVTKKKKKQRKREQDLAGSVSHLSWEAETSFTRESSADLDSVQPDLGFVN

PAFGHVAASSRPVGAKDKHGVHHKGKGRFHSKNTKIAGTDGSSGQRPDSGSSDQSGFEEK

PHLVAWSSDKNRPISGRRDKWAQEFLKHSQAPLGFYTSQEEPDIFIYDPEQNEREGEMTG

ARPPQATAMHSSRRGRDTGMTSQASASGETPQTLASHNIYRLQSVPDQEKVILDELEVEE

PTPIVFCHHDDRDEPYHASSRRRSSQHGTVDHINLAFDEN

>SG-PL_contig_4567_1_-1_1945_2301

MSSCSVATTSWCVVIGSSLLSVGPPLPTVTSVSCYSEIHCFGVFFWKVFGQGCLRSRLGF

LAKHKRIRFLPHCSTHTCVHLPLNKLYSQSLVWTAYCLAPRQLVYSSYFHLPFLWTGAL

>SG-PL_contig_4568_1_-2_77_406

MTMEEAAVLNSQNAQRSKDASPTGSNDDDWTMLNDAEPTTDGEGASGSESVPCDGLYPNL

KVAQAVDMMKAMGFNDDGGWLTRLLETTDGDIAQALDTLKLGAQQASRLA

>SG-PL_contig_4569_1_-1_1303_1497

MTVWLHIVILYCQTTTSRFAVLPFFFLGLFYFFSASCPLWYVLRILSEILSHSISFRSCY

FVATY

>SG-PL_contig_4570_1_-1_883_1125

MMMTVVMVLMMMCVLMFGSSFCVIHSQKFHQQPLPNSISAYLVSYNYCYYYYYNYFYQKK

PRFSFQIILISPVGMPGALSI

>SG-PL_contig_4571_1_+2_770_991

MLWKWKGKYLKHYIHQSGKHERDRERKKIKRSADNMLYMFVQTMHHLKAVNMFTKASVFQ

VHKSVLLTSILTCF

>SG-PL_contig_4572_1_-3_618_1292

MNDVHLEISIQLNMNMSAARHNLSHSSVYTFVFVTSWDQFSVYIVSLCPTFYCAHVLLCP

RSIVPASHCARIPLWPRSFVPTPIVPMSHCAQTHYAHIPLCPYPIVPALSHCARIPLCPR

SFVPTPIMPTSHCARTPLCPHYPIVPALSHCAHVPLCPCLIVPASHCAHDPLCPCPIVPA

SIVPMSHCARIHCGNVPLCSGLTLHITAAHTNHFFHLMKYHRGFL

>SG-PL_contig_4573_1_-2_83_1684

MLRQFTKGLSQNLSKAASQTRAAVSTPRAYSTSADDDDEPSFFEMVGMFFDRASGIIESK

LVDELKGKMSLEEKQQRVRGILSIIKPCNHAIAMNFPIKRDNGEYEMIEAYRAQHSQHRT

PCKGGIRYSEDVNIDEVKALAALMTYKCAIVDVPFGGAKAGVKINPQEHSEHELEKITRR

FTVELAKKGFIGPGIDVPAPDMGTGEREMSWISDTYSNSLGHYDINARACVTGKPINQGG

IHGRTSATGRGVYHGVQNFINEASYMSMVGLTPGFGDKTFIVQGFGNVGLHSMRYLHRAG

AKCIGVMEKDGSIFNHDGIHPRELEDYKIENGTIVGFPGAQPYDGNMLFEKCDILVPAAG

EKQFTKHNADRVQAKIIAEGANGPTTVGADKIFLKKNILVVPDLFVNAGGVTVSYFEWLK

NLNHVSYGRLTFKYERDSNYYLLESVQSSLERKFGTNGGAIPIVPSVEFEERIAGASEKD

IVHSGLEYTMARSARNIMKTAMQYNLGLDLRTAAYIAAIEKIYIVYREAGLTFT

>SG-PL_contig_4574_1_+2_2_160

VMLSLFLSDMLEKHLSLSAALRLITVLSIISVQVFQYQQYFDAVDICQEIHTP

>SG-PL_contig_4575_1_-1_1_198

TIYTWINIHAYMHMRARAHTHTHTHQNNRNEPKVASQREDVERLSIVTKRPMANRSLMPS

AQLCIS

>SG-PL_contig_4576_1_+2_1391_2026

MAFLQIFCHIQWDVPLFISFVPLKWDVPIHVFVTFNGMSHYSYLLSHLMGCPIIHIFCPL

KWDVPIHVFCPVQWDVPLFISFVPFNGMSPLICPKSCYHSSFFQICSKNISLSAALRLIT

ILSITSVQVFQHQQHSDAVATCHEIHSCCSLGIKFQLATGLSVMILSLPKFSLRLAASDS

LFVCVRVCACVRVCVYICMNVCMYNVYLCVHC

>SG-PL_contig_4577_1_+1_1_372

QRHRQLTWPIEPLCYRDSSSFAHHHRHKFFIVDLTISINVGLTDHLVHLLVCQFLPQVSH

DVPQFSSTDEAVAVLVEDPEGLPDLLFTVRVLHLAGHHCQELGEIDGAVAVSIHLINHVL

QFSL

>SG-PL_contig_4578_1_-1_196_354

MSNQFTSNIQLYRWLSGSEHHADNPGVPVPPPGGPRFFYYTGWLALLSCGLKI

>SG-PL_contig_4579_1_-2_338_1360

MTSELDALRQETEQLKNQIREARKAAGDTTLAQATASYDPVGRIQMRTRRTLRGHLAKIY

AMHWATDCRNLVSASQDGKLIVWDGYTTNKVHAIPLRSSWVMTCAYAPSGGYVACGGLDN

ICSIYSLKTREGNVRVSRELPGHTGYLSCCRFIDDNQIVTSSGDMTCALWDIETGQQTTS

FAGHTGDVMSLSLSPDMRTFVSGACDASAKLWDIRDGMCKQTFSGHESDINAITYFPNGH

AFATGSDDATCRLFDIRADQEIGMYSHDNIICGITSVAFSKSGRLLLGGYDDFNCNVWDV

LKQERAGVLAGHDNRVSCLGVTEDGSAVATGSWDSFLKIWN

>SG-PL_contig_4580_1_-2_14_1240

MAASVKQQLAQLASQSHSGGSHKDVSEKYKVILRGIIQTGGSERVSSLQTFVEALVNENV

SLVISRQILGDFASQLEHLSDPDAKVVSHFTLDKIHTRVISFEEQVAAIRQYLASIYERE

QSWREAANVLVGIPLETGQKQYSTDYKLETYLKIARLYLEDDDAVQAEAFINRASILQAD

TKNEELQIHYKACYARVLDFRRKFIEAAQRYNELSYKTIIAEGERLTALKNALICTILAS

AGQQRSRMLATLFKDERCQQLSAFNILEKMYLDRIIRSSDLQEFEGMLLPHQKAITADGS

TIVDRAVIEHNLLSASKLYNNISFTELGALLEIPPAKAEKIASQMITEGRMNGYVDQIDS

IVHFEARETLPMWDRQIQSLCFQVNNIIEKISQTAPDWFGKAMEKEQTQ

>SG-PL_contig_4581_1_+2_2_217

TVIIIIISSTTCPAHQITAPEHPDQDSTLPLLPTCQQSACTAWPTSEAAPVPGNMPWWVR

PGHFLKKHSTTI

>SG-PL_contig_4582_1_+3_246_374

MDLEGRKRQTTVQEGTGAELLLLLLYTVYSPADHYGHIRAVTT

>SG-PL_contig_4583_1_-1_4_1155

MVRVETAVTASKRAMMEGLGLVTQQRKKDMQQKLFRKRRKKLREPSVSPIELEDDWVLPN

GAMETEPLRTLPAVALEGEKNPKEKALFLHSLLLMEVPPEQKKAKEEIRQVCEQVRRRRA

EKAKAKIRRKLEKEQQELLKALNRGSRNNKRKFDEMAGDSTTPADGGTSNIEPLHISLTQ

IHEQHMTAARMDLEKHQQLQQQQHHRVNHTYSSVGGSSSGATTEGTRPVIFPEGRDRQLS

REFAEQFHESVLQTTRQKEHLQKLGQMGHHHQDMTVTKPSSYSNHLLPSTSSPSGDTTPP

FVWPGIERVLESYMHYRREKQAEKKMLVEKCCQLHAQNVEHTKTAEKLCSRLEVLREEER

RKTAQRQQLEEAITNLKKCLHLPR

>SG-PL_contig_4584_1_+2_2_133

LVKTRFNFSQIKIYCNNFFASSSTCTYFHHFQITFSLPHYHIPA

>SG-PL_contig_4585_1_+3_21_257

MKRMMNQFALLREIASTKKMHVPKKYRSVYHEKFNCLVPSRKGAGDIHCTMCKSDFRNQR

LHFSPSGYTFQLVILHLGL

>SG-PL_contig_4586_1_-1_2284_2487

MYITPYVCIHLSAYNIDFFPCAVFSQSGTSNCQLSVDYCLFIMSSFYSKAILSSSVYFYL

DSIFICRQ

>SG-PL_contig_4587_1_+1_1_237

ILLLQLLDTRSTTFTFSQPSHNAILLLQLLDIRSTIFTFTQPFHNAILLLLPSCSTFFHF

RYSVYHFQIFAVVASLDFF

>SG-PL_contig_4588_1_+2_1652_1951

MPYSSMCKPSPPLTNTHAESHKHIFSSQFLKLQVHLIQRQGQYDRQQPMLIQKIKNDTFS

VDQIHSQTISNQDKRHGNYNGQCLTFKPQQMLQHLKDRQN

>SG-PL_contig_4589_1_-1_133_264

MLNRHVQVFSSTSAVSRKRYLGHLSVLICLFSSTLINHEQFERR

>SG-PL_contig_4590_1_-3_417_545

MIISGILTTIMSFMYHLCVDQFGQSLVCSRQSLVTKFRLSAVL

>SG-PL_contig_4591_1_-3_33_278

MGNTATPVPSHGQCFEAPVGVRVRTAVWQGLPYAGHTSIHKDTKMKITIPTWEMLLLQFS

LFSNTVRLGARVRVRVGVRVRV

>SG-PL_contig_4592_1_-3_54_824

MSGLRVLVGCKRVIDYAVKIRVKPDKTGVVTDGVKHSMNPFDEIAVEEAVRMKEKKLASE

IIAVSCGPTQCQETLRTAMAMGADKSIHVEVDAGTYEVMQPLHVAKILANVARENKIDLI

LLGKQAIDDDCNQTGQMTAALLDWPQANFASKVEKVNGELKVVKEIDGGLETIQVKIPAV

VTADLRLNEPRYATLPNIMKAKKKPMEKKKPSDYGVEVVSTQKVLSVEDPPVREAGQKLE

TVEELVTKLRDQGLLPS

>SG-PL_contig_4593_1_+3_129_323

MSSFQFFQHFRDRGGWTLFLSVVYRVFKCRSILWPGLAMPLCNFYAVTIGIWFGPKCDRD

GAKLV

>SG-PL_contig_4594_1_-2_401_1912

MKNLGLTVDEKTVSASLLEALKSDDSPLSHGYAFMVASELKADTSKFFDNIEDVIAQADE

VDDKYLQFEGGLFVTALVVDGAYKLAEKAKKAPTIAEDKVAKLANYFLSRKHVHQLKAAF

QFVSVIKTLTINKFHIPIAVSLDSSVAVSQSEPVVKVRVTNLLGGELGQLTVTADSARHL

GDEAIVLSKKQFTPSSSEKAQYELNFMEAKPARGFYRLTVSVAPKKADSRLIGTTGAEVE

VKVTTKVSVENVEIGVADKDQTAAARTTKLVYPNKAQNTLEADYHQKILMRFQLKDRLTG

QLMTAHQTFVRLTNLKSKQEIIFVSEADGALNNKFDLDVGASAKEFGYQSGKYSVELIVG

DAIIENPFSWTLADAELTFPEGGAAKGDGQNQYSKKPEIKHMFREPEKRPPAPVSMTFTA

LVLLPLLILLLLWLKIGVNVSNFPFSVSAIGFHICLAGIFGLYYCYWTQLNMFQTLRYLG

ILALPTFLFGNRLLSGIASKRKSA

>SG-PL_contig_4595_1_-3_3_536

ETHKRLICARGNCIVTSVSSPAVHSGSVTALLSAFRRVEPCLSSAKHQQSQRAVPSTSSR

SWERDALGTNYSERHEKRRRSLRDTDHVSTMRAAILREKAELERQLQELRHEGSHDEMSH

RDKRLSRREAGEVKRQCVEDSEAIGESSEDGRRYSRRSGQVLDYVDSDDSVAEHWREV

>SG-PL_contig_4596_1_-2_2_109

PPGSTQAGLTKTFLVEIQCLNNSALAPVYICIYFSL

>SG-PL_contig_4597_1_+1_2173_2490

MWLQHQTDHNDRGVVLRDCPQLEVINIFRGTHLTVPQKQCTKKSHDDNVMNTITIMFLTP

IRKIIIILHQSNSHKIFYMSSLTTLTAKLTTNITFFPLLSSLTKNI

>SG-PL_contig_4598_1_-1_172_291

MDLSHSHKAVLSTRLSSGLVSSVGRTVDSSSDISSSVPVH

>SG-PL_contig_4599_1_-1_1_147

FFFLGIVEYWNIYYVESGLQRLMLITHITCDRIVLYSNFICFCIRKKLF

>SG-PL_contig_4600_1_-1_1_249

TSSTRFRKECQRECTERGDHPAEDTSAGATHSGSSGRKFVRHGQQATQDRNRDHQFGAQR

PVPSAQRLSHLQCGFRLRRSQGH

>SG-PL_contig_4601_1_+3_225_392

MFLKANMTRLCRGSLPFPLIYHLQTTKKRKKIIKKYSEATQKPPTTTNKQTNKKKT

>SG-PL_contig_4602_1_+3_3_152

RSFFSGYFLDLFQYIFSLLSLTYLSQRRVMNNSQDTILTIQTDTEEKWIF

>SG-PL_contig_4603_1_+3_3_89

ESVKFDCTKLLNLMISRGKPFIRFGLISK

>SG-PL_contig_4604_1_+1_1_249

IGPFSLLPPLPFFSAVSASEVSASFVINSVQKVSSVRLSVCFSACSFQVLQGRQPCLPSP

GAEDYHLQLLHLKIQSLAVISFS

>SG-PL_contig_4605_1_-2_2_235

DSFSINSIPTFWKFCASNFLFSIALFVSALFPSLTVIRFFSALTVHFTFFFPANFHTPPT

STSQFMMYCLCHFALVTR

>SG-PL_contig_4606_1_-1_343_750

MFLGVVAIGLFSDFYGCFVLIFRTHCSNFYEHWVLSFMNASSHFDYRLWNAVATVLHSTD

GIISGFLYDTQKVQLILSSFPIFYIFHQVSLSLQLNAKTVILCRHNVMTCEAYLPPPSVV

LYFSIRAEPPCICKCV

>SG-PL_contig_4607_1_+3_3_203

PCQHIGFGIRHDYLGYFQQLVFVVIAHMEFLKTVSRKQTELYFGITAMFSHALQIMPLFS

EISVKRG

>SG-PL_contig_4608_1_-1_31_933

MGRGGGPPGRGGGGRGRGGPFPPGGPGRGGGPPGGGMGRGGFGDPNFQDQTQYAVPADKC

GLVIGKGGETIRDINRQSGAHVELDRNPPPNMAEKLFNIRGAPQQIQHAIQLICEKTGMQ

GPPGGPPGGPMGPGPGGPGPQGPQGPPGPGPQGPPGPQGPGGFEQFGQYGQPNQPPQQFG

GPGPQGWNAYGNQYPPQQPADPNAPDKQAQDANAAAWAAYYAQYYASYGQYGQYAQQQQQ

QQPPTQQPPQQPPQQAPQQQPQQPAPAAAGQSYAQPSTMAPAINPQTGQPDYSAAWAEYY

R

>SG-PL_contig_4609_1_+1_559_771

MRKVLGSILSLWASITRAVPISCSSLLYFPSWVQFSVSGPASQERYPSRVPRCFTSVSLN

RGQVPIHNWVD

>SG-PL_contig_4610_1_-2_65_346

MLAADDTRRLIIGASFGGVIVLFILIAIIVKCRRRVKRRSVRRWDVMDYKYGKTKLYAPT

NDDNDDDDDFQDDHDFEMDIPDGSMQTKKLINGK

>SG-PL_contig_4611_1_-2_2_109

ECVADLMKFYLLFFPSAHPSSSHTKYYFFEKESFPV

>SG-PL_contig_4612_1_-2_2_136

SFYPELCLLWARWKGFVTQYRSLYSVPRLRTNNWLYNICRLILML

>SG-PL_contig_4613_1_-2_2_190

VGCPQRSSDLFIFSLRLIFFLLKLCQVIFDGRVVGYMTCSCFPLLLSCSIIYWSTVEDCI

SYW

>SG-PL_contig_4614_1_+3_3_113

YDDLEDISDEEETDDNSNHPLLVTNGLRTADDSEINM

>SG-PL_contig_4615_1_+3_3_164

LEEIMNDADSGEENLYDDLEDISDKEETDDNSNSKYLLLLTNELRSADDSEINM

>SG-PL_contig_4616_1_+2_221_352

MKLTKIIAMTCHDHYRHNPYNTAPKKKYVWQTMNYTLIYTDITS

>SG-PL_contig_4617_1_-3_3_158

PASGSRTVASVSSSSTSVDPISPRVLIGRVHPPKKTLSFQADHTRPHRSQEQ

>SG-PL_contig_4618_1_+3_3_215

RERGSEEREGEFSAKFVTERTDSPTAPGSCGDECDLLGKGGFSWVDAPSLLNTRGLIGST

LAEEELTLALV

>SG-PL_contig_4619_1_-2_2_115

AGSSRRVKAFRYELSAKFSLFRASLLGMISMIVTFNTV

>SG-PL_contig_4620_1_-1_1_123

CAPPKSASSYPADSLILCKTLCTDKVLKRGERVMETGPRKH

>SG-PL_contig_4621_1_+3_342_710

MGRGSALRLATVFVVVVVVGGGCGLARSVDKTMDLGSDVSSSVPACDIKTTLFIAHLNAA

TILVVSVFHRSHSLPHFKTLPSRRVFHRIKFARYQLADLGGVYIIVRVYMELSECNESLL

NKL

>SG-PL_contig_4622_1_+3_3_146

HTHTHTRQHTHTHAHQTTHTRTHPHTSDNTLPHRPAQSRVRDIQHDIK

>SG-PL_contig_4623_1_+2_104_313

MDKKEEGTGRLQNIPLSPSGINNDYIFKDSNKIQNYLYSKLWLVSISTVSHFWFKSDSCR

IGLEANLSRI

>SG-PL_contig_4624_1_+2_2_76

LTNLENHCSIHSKPFPFPKPFPFSN

>SG-PL_contig_4625_1_-2_2_73

EWFFRLVRKGKWFSMDRGVVFQVS

>SG-PL_contig_4626_1_-3_3_248

KEKFLGADRGEDTLSLNLTCQTSVRERYCTTILVKKMPGVNLIAPTCHVPNGAISLTDEC

PCQTRSQSPKHVLQFCLLFRNE

>SG-PL_contig_4627_1_-3_138_359

MCSCRCENDTLSGAKDDIGFHPDGDLPLTFLPEFVAKAQHTAVARRPFGSHPWSPSWTSD

TKSEECLLCPVHCV

>SG-PL_contig_4628_1_+2_2_169

LFRSVLNKNFLDSFCGQMSGVFPPEKRPCFLCNINNPNKIKYQLLFINLFIYSFIH

>SG-PL_contig_4629_1_+3_84_1259

MDLQYGIAVTNKYSLFCDDEVDPLEILRQQEESKNAAPSKKDKKKSAKKAVATENKPKQP

EQQFKREDTNQPRQNGRGGRGGRGRELRENDGSRPPRRQGPRDNRDYKGDENVPPEFRDR

GGDMANRRDREGNVSGFERGGRGSRGSRGGRGRGRGGPRPESARGNFGGPIGDRRREFDR

HSGTDRTGVKPTDKRDGSGAHNWGSYRDDVEEGQSQAPADVSGEWAAPPAENAENDVNES

GEAAAPPKEEEEPKEMTLDEWKALQDQKRMKTQFNIRRPGEGVDNNQWKKGVAYKKKCME

DDEESEEEEEEEEDRHGHKKQLVDIRITFADNPRRGGRGGRGGRGGRGGPRGGRGGFGGG

DRGDRPSRGGPRESAPRMDDETDFPRLVKSEA

>SG-PL_contig_4630_1_-1_52_288

MTFFHFGNCVALAYAPYVIVYKCSGLAEYSAFWKCVQAGGAYLFTQLCKMMLLATFFPES

AVTAGGVDITGEFLKCTVD

>SG-PL_contig_4631_1_-2_167_280

MWTDKRTDESILTELATTRQLLAVINKRKLKYIGHASH

>SG-PL_contig_4632_1_-2_11_361

MAKQRMIQLNNIWKDRSIPTALKIKLLKCLIWPIMLYGCEAWTGRKADENKINVAEMCFY

RSFPRVKWTDKRTIESILTELATTCQLLAVINKRKLKYIGHASRNTNTNLMTTVLQG

>SG-PL_contig_4633_1_+2_2_202

IATANISLYKIILCTYNPIIMYGEFYQIITYPTITMYSYITQSLGDSITRRHSHRTVSSS

IFFFLFL

>SG-PL_contig_4634_1_+2_2_169

LSVNKMCQERFLGYGQAVTIFGSKSQIKEKLRKYKHLTGLNMPGPLFASQRLQMFQ

>SG-PL_contig_4635_1_-2_2_172

GGGRENVLYSMYMYNLSCNTLDWFYCPKTSFKKKSEPPKNQAWVITGFKNTRSKFLN

>SG-PL_contig_4636_1_+3_225_383

MAKLAFHRIILTYVIFISDEYVRQKNKNCAHNKTAGDQIWYRDKRQIGVEEPR

>SG-PL_contig_4637_1_-2_2_1510

DIIQNWFSAPAAPESIKLPPPAAGSYRLPTNLKPSHYDLSLQPHIYGNDSSQFFFEGSVT

IFFRCISSTQSITLHQDRLTISSSSITITDISATTSLPIDGVTEEKMFHFYTIRLESPLE

EGKEYSIEIQNFRGNIRQNEEGLYLSSYEDGDRIIYLATTHMEPTNARQVFPCFDEPTLK

ATFNITLIRRTDTDRDYISLSNMPQISSDVIDNSWTADVFQETVVMPTYLLAFIVCDYVY

LSETSPDYPVETRTYASPNRIQDAQTALNHAVELFDWFNDRFEPKYNTYFSKVDHIAIPD

FRAGAMENWGLITYRKNLLKDSTFTASDRAWIATIISHELAHMWFGNIVTPKWWTWMWLK

EGIANYLESYAVEQLYPDWKTRSEVVMADEVFRAMQDDALDTSHPLNNPVESPDEIGEQY

NDITYIKGGAIIEMMHMIMGEHFFPALNVYLQDNKFGNADPSTLFDSLNGYISTTPNIDL

DMAEIMEPWVTQMGYPVVIVQRM

>SG-PL_contig_4638_1_-2_2_277

PTRPASSAPRRAHAPAQTPPTFILLAQSNCSPTSAMARGEGWAGVCVRHGKYISQTCILD

KILLLASYTVPCQTVSRIQRLEEGLPLRCKWG

>SG-PL_contig_4639_1_-1_7_1473

MSKLLTESECLQEKQNTIVGFFRKASAPGCQTNPTDILKQSTATSSSEKKHNSHATESGE

SSSLNMPCMKMLQENGPIEARLSENTQTCVSVAEGTATFTLGGKALVSDAGAASVPQAVY

VDVEDGDSTGTYFLGDNQQEINIVAKVQTALNNDHESENEKGSEESENAAGSYRTSKIRG

DDCSPVPPTQTQIPSGSVVLNSESETPTTEVLVDTPSKSHRLDGVMYSGPSSSRCSSRCY

DWDGEASSSNTDGVRHEVLVHSSFTVREPFTCPVCGKPVGCINLTDFNAHIDECLQPESQ

PSSATSQNSANKTSAADKVSFSQTATLQRSQTCQSNVNRCSSALKSSTYAKKQRHVSGSC

SKSGSTSSTRTEHATAIPCKMDETSPRLGSFEMENAVSVSYPSQQENCSQSEPTSTDYLV

CPLCGAERADWTLDSFNQHVDACLNRDTISQILREQRNSEQPSRKRPASSQQVQHRTSQQ

GKKAKKNQS

>SG-PL_contig_4640_1_+3_846_944

MTEIGLDSCSYLDFSPADHSRPFQELNKSTPTL

>SG-PL_contig_4641_1_+3_318_554

MVTSVSVGLEVCWRAKMSGVGIVTQERCRRWRSKNPLQTAHQCSGKNITNGSSQHATNTQ

VIFKLLILIFFRNQLKKHN

>SG-PL_contig_4642_1_+1_1_213

PSVRSWRPFTAGRFFGGTLPHYRHNTDRNSQDRANTRKERGYTRPRYEIESVSETEQKPK

VKRSTKHVLSA

>SG-PL_contig_4643_1_+1_1_177

LSFSFSWNRTSFWNYSTCNSNLAAISMHRNGTDLELDVVDRPIMHETKQVSQNGFQRRF

>SG-PL_contig_4644_1_+2_2_205

SWAGQPSVGTKCAVLVQQREVARYDNPMTKFSGRTGIQCSVDSFAPYDFVQLFFEFVYGD

YSQNIINM

>SG-PL_contig_4645_1_+2_278_403

MEYTRPQKVSAVKGLNAKMWNKKSLKKVNYYCLKVNPFGSSC

>SG-PL_contig_4646_1_+3_201_326

MYYCFLSVFLSIYFTASKCPYPPLLSVDTLSPSLHPQFVFIA

>SG-PL_contig_4647_1_-3_168_299

MSKSEAPDGQHYGGFYVNKVGGGAHSSSLSLRTEALLLSVMTGG

>SG-PL_contig_4648_1_-1_1_249

SIRLFATFFLSSILFFYSFDQSFLLLLCLLRCQISLSSLSLSLSSVCLSAAVSVCLSLSQ

GQKGQLKALQEAKSFASAFTFSL

>SG-PL_contig_4649_1_-2_14_1000

MEAVTMLRDQIIPNFAEKVEVAVDALNSNPPQDVDENEFIEASRLVYDGVRDIRQAVLLN

GIDVDDSDSEVELEDEYEAYTSVADADEYGKSDRAMMRQLPEEEKEKIQAQVEMFRMEKE

KLDMEVAKWDDSGNDIIVMAKQMCMIMMEMTDFTRGRGPLKNTMDVINAAKKISEVGSDL

DKLANSIADECPDSQSKKDLEAYLQRIALYCHQLNITSKVKADVQSVSGELIVSGLDSAT

SLIHSAKNLMNAVVLTVKACYVASTKYKKGNAKKPVVLWKMKAPAKKPLVRREDQNELNA

KVRKGAMRSHIEPLKALSEFQEITSDSFC

>SG-PL_contig_4650_1_+1_559_732

MLVLKYFVQVAFLFFLTLPFLLLLLLLLLLLFVLRWSLKTQQTVPVHLVSLPSSLCTV

>SG-PL_contig_4651_1_-3_156_359

MAKLGISLHSQIVILVLTVYCVTVVMIRKRRNIVSVWCMVQTHRQYQFIDTDMLLSHTLA

CLLSYMHI

>SG-PL_contig_4652_1_-3_3_242

VGYGYLPWIQKAIVSSAFIRILISWAQEPAFLSFQAHLSKHMAKIQVTGRSFLTLSKMAA

ARIIWRDRNVFENFLKTFFA

>SG-PL_contig_4653_1_-3_144_395

MIYIPMSYCSFHHNPSPPSSNPNPCHIGPREQNFLLPTFFLCKSITHYKPWLDLFYLWEC

TCLFCFFEQFFFPAFCDVLLFLSQ

>SG-PL_contig_4654_1_+1_229_492

MPFIRFEPVSYKGLATLDSLWLSMCVVYMCVCIYIFICIYIQREIDMCVCINVYSVCVYV

CDVNCTNLFIYPCVYCLGFALCLHILSL

>SG-PL_contig_4655_1_-3_180_353

MLTHVVIYDFIYGFASCSVMRLLLTLTWSICFVGRSMDYITLFTKGQKHDLSYLFTKL

>SG-PL_contig_4656_1_-2_86_454

MASHGLNTQDMQNVKSTLQSENLGNNADVELRWAMKASEHADVYFRLITSVDPSLLRLTK

VDSEIYSRFRETFPDMKVDVVDETAMKSDENKEVWREFIEEFKEKVQDYNYGTLLRINSS

EAY

>SG-PL_contig_4657_1_+3_3_317

LARKAACRGHTVNRCLCKFCSPEKFFADKLFFPRLGDFVFSEIRDFADNSHACIHSTISN

LCRIQRLEEGRDLLPWSARPVEASMTASAVAKEMRDEPSALRTQR

>SG-PL_contig_4658_1_-1_1_141

GEKMGTTLLTLRKVKSLYHATSLQPHGYGQPLSYIYLLPLTLRKYDN

>SG-PL_contig_4659_1_+2_5_346

MAAAMETDSNEDFGEGSSAFSDKGKKRFEVKKWNAVALWAWDIVVDNCAICRNHIMDLCI

ECQANQASATSEECTVAWGVCNHAFHFHCITRWLKTRQVCPLDNREWEFQKYGH

>SG-PL_contig_4660_1_-1_31_1056

MIGPPRSRVPLITDASGRRPISNRYGYDTPIPNGHYRYRGNQDRYINGNRNGNGTGNRNG

YVYGNRNGNRNGHVYSNRNGNRNGYIYGNRNGNMNGYRNGSMYGYINGNRNGIVNSRYGH

ATTGNGTDYSGYRNHDRNSRFSYNHYNGYRNYSGVRNNDYGNANYRSNSSSHTTHGRVDR

VNGKNVRVKSYRDPTGHNRRVVINGNGLYNSSADSVTNRNGQAVSIPPRNSGHQKVSPTE

SDEEKVQPSQPYERNVTVRQADGGTVPTRQPDQGRVSPKQPDEGRAPLRQPNQGTVPLRQ

PDHGRVSQRQPDQERVPPRQPDEGRVLPRQPDWRRVPPRKPD

>SG-PL_contig_4661_1_+2_2_217

ERESARARAHTHARARTRARTRTRTRTRTRTRARAHTHTHTLVCVCVCVCVSECFNFNLL

KKVLQHTDDSML

>SG-PL_contig_4662_1_-2_2_112

FHCMAFAVGTLKVQGHLLENIETEIAEQYKQAPPGLK

>SG-PL_contig_4663_1_-2_2_136

TIHSEICCHQHECSCCSGSLCCQLFLCLISFHDCFQQPLCAIIGQ

>SG-PL_contig_4664_1_+3_102_275

MDQFRKGVGRISYADRLLWCGYVARIVQSICASEPDTLHPFAPDFSFNVTLFKEDYDM

>SG-PL_contig_4665_1_+1_313_576

MIEPAPFPQFGLSQPHQCPLPDTHCQIHFLMGKHKKQSWKLSTLYFTFTSWWVNTNNHGN

HTHFIFGQIYQKQITQKKDNKRGKTMIN

>SG-PL_contig_4666_1_-2_23_1345

MQRKSATSNKGAGSKGKKKTIAADTTSKVKDSSDICDKHVLSQTVSSEHDLKSCSTSNVS

DKLELPSELKSSKTKKKKCASQNASASSSTSTHANGKSPTGKQGSSDEDCGSSSAVPGNF

NHPSPWAKHLTVLWQGQSVSLAAERAYNRAMSHRPPNCAICALFKPFNIEAEQGLFDEGG

LQMVVPQRSLPMIPEMVFAVSKNNTTPFCDYSTLDEDGLSTLLTCSNCNICVHASCYDGV

QQTEDQDTSNWKCSRCANYSTYAECCLCLLRGGAMKPTTDGNWAHVVCALAIPEVSFVDV

RSRNIIDVSKLTSSRQKLRCSLCGPVTGAHSQPSSCIQCSHGRCTQAFHVTCAFAAGVQF

ETSDWPYPIYCTCHKHQNNNREKGKQRRLDDLFVGGRVIAKHKNGRYYHANIVEVSRHKL

YEVDFDDGSVSYDLLPEDIVG

>SG-PL_contig_4667_1_+1_112_396

MIQQNVNNMLSSYICANKRDRKIVRERRGWKRGGVQAAKQRALVLMRSQLLLVKFTMCED

IHSMQYLQRIITAFMLLLESTSNGNIALWHNNNNN

>SG-PL_contig_4668_1_+3_324_620

MCTSQSCKTGFYRPEPSAALCTLGCSSETNIACHRASFLSDSSCLWGCLQAQRLGTDETA

RMVGGKKAETIESCGFCYLSVFHHVNKRGIKCTAKEKSV

>SG-PL_contig_4669_1_-1_34_465

MNKILSIRASFSHVSQKNNSCQVDTFSGTPFSVFSSLSEEDVRKIILQTAPKTSELDPIP

TKLLFQNLDSLLPTITNILNTSLISGTVPTDFKTAVVKPLLKKPSLDLNDLKNYRPVSNL

PFLSKILEKLVLHQLTSHLSAHNL

>SG-PL_contig_4670_1_-3_3_242

RALPGRAALISHGKPSVTKNINTIQYNTIQYNTIQYNTNHSNNFLFSTKPLGSIRESRFS

TCQGRCYLEKASDKIRLDVT

>SG-PL_contig_4671_1_+2_2_205

GSFSINNIPTFWKFCARNLLFSIALFVSALFCSFTVVCLFLPSCSIHLSFFQQAFTQFSL

PLPPLCSL

>SG-PL_contig_4672_1_+2_1745_2050

MHNEQTNTSSLHHNHKELFITDMILFSVHPILSNNLPTLNIIRLKSGDKQVNEQKLSSLT

IYDQSVIPHCMGLHHIKNKTMQSHIENKTYKPIITENVKPKI

>SG-PL_contig_4673_1_-2_2_1654

MLTPEAVLKASGHVDRFQDYMVKDLKNGECFRADHLIEATFEKMLADKKVAPETKDEIRA

LMPLIDGMSKDDMQQALRKYKMKSPVTNNDLSDPIEFNLMFATPIGPTGQLKGFLRPETA

QGIFVNFKRLLEFNQGKLPFAGAQIGNAFRNEISPRSGLIRVREFTMAEIEHFCDPNKKD

HPKFASLADLELTLYSGCNQMDGKAPEKWKVGEAVKEKLIANETLGYFMARIYLFLTKVG

VDPKRLRFRQHLSNEMAHYACDCWDAECKTSYGWIECVGCADRSCFDLTQHTRTTNVKLV

AERKLPEPRTVELVECQPQKSVLGKAFKQDAKVITEHLSRMEAEAVTELEKNLSEKEEVE

VQVNGKSFNIKKEMIQVKRQQKTMHVEEFVPSVIEPSFGVGRIMYAIFEHNFRVREGDEQ

RTYLSLPPIIAPYKCSVLPLSQNAEFVPFVKQLSAELIRCDVSHKVDDSSGSIGRRYART

DQIAIPYGITVDFDSLKAPHSATLRERESMKQIRVKMDELPALVRDLSLGRTTWNEALEK

YPAFVQQEASK

>SG-PL_contig_4674_1_+2_1298_1423

MIIINYPKAKSLKQVHIPTQSGSAYCMIQCNNEQVPANPQSF

>SG-PL_contig_4675_1_-2_2_181

LLCSVFVFFFFKQKTAYEISRSDWSSDVCSSDLRRFRVSKATKHYPFHFFLYCVGPRGFS

>SG-PL_contig_4676_1_-3_426_902

MHIIHVCHRCGLLVSLILSYKHTKLGTSRTNHTAEHILLTSITTVIQIIFFSYMYKSVHI

YPRHLDSTIKNTVHKTYHRLCDCIHHCMYMFICVWWYVNEQKWWEKDYVICHPTGHQVLF

TDVLTLIRTAIQHQKEYEHIHRVQKYSSRRQEQSNFPKC

>SG-PL_contig_4677_1_-2_191_409

MPPGPLSPRRAALVFLPLSHTMPRTFPPFSSWTILSPDYIFLLKYSYLNSSTCCYTVGIF

FIAGLFTIEGSFC

>SG-PL_contig_4678_1_+3_3_128

PLCQASSTLECEQEKVQALKQASPKWCIKNAIEMTSNTYDIC

>SG-PL_contig_4679_1_-3_3_200

IHSLSMVRLNHMTLLGLALNQLIRVPSLISLTTIHAEQTCTGFLFCLGYLSRTTLPWSPF

QFSFAG

>SG-PL_contig_4680_1_+2_2078_2287

MDWGCVSKKGYETGHPFIRKHAQSCLKPWRSGRNECSKHYACSLPISKHSGGVGVSQILF

PSPFQNLVSA

>SG-PL_contig_4681_1_-2_2_769

AEEQQILAAAEERPTEECTTTEEELASVQEQSTPSTEVPSGSAEEEATPAEEQQVSVEVA

SPRKQSSEEYTSAEEQSIESEPQASESEAMFPPHSIPTEGPPDETGISALPGAEDDREMD

DENNTEPALSQEEPVTAEQQFAASEETSPADHKSAAVGEQPSPAAEHTAHAEVPSPAPIE

QSSAEQQSSADKSNVGHSDQLAREDEQSASEEEQPSEHSSLEEGKCLEDSALAKQQSAEA

STPIDKSAEDSVPVEQ

>SG-PL_contig_4682_1_+1_1597_2250

MMMWLLLFLTLIICCSPQNSPDSHPCYWFASACWHHLRVHCCLDEAFNVVGCKALAFRVQ

STERSQHSLKAHGTDDEVAKVHLSLLVAVAHHQRLQSAVTQPETNIVQGCSQFIRTQVAA

LVPVIFLEHSLPSAQVLPEVFEFFIAQLARQITIMHGNHAACCINGEAREGEFFLENGIQ

DLLQLVGINLHVLSCDSSEILLQAALLFLLFFCGLWPF

>SG-PL_contig_4683_1_+2_179_373

MFSFITNSQLGELDAACTTFFPPSQTLKQSGCKAGVLSLHQAGFIYAVFFLLKCPWHLYY

LNDEF

>SG-PL_contig_4684_1_-1_7_234

MASKPGSRLGHDSTDPSGDRSASRSPDRRTKRSPSRTKSKRDRSTRAKRRSPKVDRGSRR

HVDVRRDEIRRDLCLF

>SG-PL_contig_4685_1_+2_2_202

QKHLEQVNPDRVKASGCDVIRHSTECSVMKLWQRIVTHSKTLILLHIDWMPCSFLYWERS

SMLSSGV

>SG-PL_contig_4686_1_+1_1_180

KTHTDQHLRFDSHQLLENKLGVIRTLCHRASTICSTEEKLAEEQQHLKEVLSISGYTTWA

>SG-PL_contig_4687_1_+1_451_903

MQFIFHRALLGIKVFTIFHVVIAYRYQENYYLDLVNHTTGLSDAYIYTVIVPTTSIMLVY

SKNDRTKKQKQTPNQIKLSDTESNPPEEAVEIKFWRHSLNGDSGTDLASIYDQISQPKKG

TPNASSQWATALPASENRSAEEGYKRWPKYP

>SG-PL_contig_4688_1_-2_2_166

VLFRSRDLNFSQGQHYEVPSLTIERVRYHLQLFGLEAICTDLFLSQTQESTKPHE

>SG-PL_contig_4689_1_+2_2_217

SCIYLFIYPFLSKWIYHVKFGLLSLEKARRRSAATPIFFLFSLCVYFCLPVKVDFSMEFC

QGNLFCCREIFN

>SG-PL_contig_4690_1_-2_35_1129

MAGRVVAGETNKTVLKKFLELPQSPDKVMVEYIWIDGTGEGIRSKCKTLDFEPQKPEDCP

EWNFDGSSTYQSEGSNSDMYLLPVALFKDPFRLGPNKLLLCEVLKYTKLPADTNNRHTCN

QVMQEAKTARPWFGIEQEYTLLDVDGYPFGWPKNGYPGPQGPYYCGVGSNKVYGRDVVEA

HYRACLYAGVKICGCNAEVMPAQWEYQVGPCEGIEMGDHLWVSRYLLHRVAEEFGVVVTL

DPKPMVGDWNGAGAHTNYSTEPMRNAGGLGVIEEAISRLSKKHAEHIKAYDPNEGADNRR

RLTGHHETSSINDFSAGVANRGASIRIPRQVAEDGYGYLEDRRPSSNCDPYSVTEIIVRT

TVLDK

>SG-PL_contig_4691_1_-2_32_283

MFICVNCHVYCYVNVKCTFMFICVNHLVNCYVNVKCRFVFIYVNCHVFFFLYKCQVYVHI

NISDCYVNFFYANFKFMFICVIVM

>SG-PL_contig_4692_1_-2_2_154

IFSFILSSLCFVMSIVVCVSVLSSVVSLSDCQTLFYILPSICFVMPVVVGL

>SG-PL_contig_4693_1_-1_40_219

MCDCYANFVNVKCKFMFICVNRHVNCYVNVKCRFMFICVNRHVNCYANVKCRFMFVCESC

>SG-PL_contig_4694_1_+2_56_325

MISMHKTSWCENFVHRVYTPIFSHDLHQNEIFFFTHIVPSNNQVKDFILLLLLYQSFINI

IRKFQKSVFSFLFYDFFFKNLYNRPVSREK

>SG-PL_contig_4695_1_+1_1_363

LFREAIKEERFRRLGHLRGFKGCVLSHPYPQSPQEVSPLRLEGQDLSVQGPSFRPFSGPL

PLHLGSQGASFLSRQESFRLHLFWTIGSFGGQLRSLSDLHCQSCRTLHSFGLRPVPREVR

F

>SG-PL_contig_4696_1_+2_2_172

VGPSIKRYAVYDFVCDNNVDKLLLTETWLHQSGDEAKYRDLTPPGYTLFSFPRSYDS

>SG-PL_contig_4697_1_+1_1_228

GPSIKRSAIYDFVCDNDVDILLLTETWLRQSGDEAKYRDLTPPGYTLFSFPRSYDSNLKR

GGGIAIIIKAPLAEHC

>SG-PL_contig_4698_1_-1_10_495

MGGKELNKSIHPDEAVAYGAAVQAAVLTGDQSEAIRDVLLVDVAPLSLGIETAGGVMTKL

VERNTTIPTKTSQTFTTYSDNQPAVTIQVYEGERAMTKDNNLLGRFELTGIPPAPRGVPQ

IDVSFDIDANGIMTVTAQDKSTGKSSNVTIKNDRGRLSAEDI

>SG-PL_contig_4699_1_-3_36_242

MTRLWFHLLLRLRRHHRRQHPLQQALQSRLRHHHHPLPEFLLVSQLALAAFHACHQDFDD

TDFSFVILN

>SG-PL_contig_4700_1_+3_729_1046

MVSHTTSNPATGHTLKHRLTAAGPWLGSPTKSTPLPGMVRRCRAAVTLLTVGSGEPLLLL

GAATHGDVTDDEARREPLATKPSALTGGAFKTPTPLPSVTNDTWDS

>SG-PL_contig_4701_1_+1_70_561

MLKIILNRLKKEAEELLAEEQAGFRAKRSTVEQIFNCHLMMEKHLDHQKELYHNFIDFKK

AFDRVWHQGLWLVLRSFNIEEGLVLMIKALYDNASSAVLLNNNFGEFFRTTVGVRQGCLL

SPALFNLFLENIMRETLLDFHTTISVGGRPICNLRFADDIDLLG

>SG-PL_contig_4702_1_-3_3_227

MSSAKGKAYSQKLGYRAKVRRVLIGAETAVSAPCSHLLPSSLRAWAFASPFFCTLSTLSL

FLFAFPPPPVQLQLD

>SG-PL_contig_4703_1_-2_2_100

GNLAMSVLITHTIPCLTDALASSAQLIPAPYQY

>SG-PL_contig_4704_1_-3_48_206

MVVEVLFWKKLEPNFTVSLEPPYTFKVDIHACIPIFSPFSVNICVRMKIITDL

>SG-PL_contig_4705_1_+3_141_287

MHIKDPVSHVSGWLMETRTYSACTLYSPFTGSKLYWLWPLPWILPVSWK

>SG-PL_contig_4706_1_-1_919_1236

MKISVSIGLVFLQPDLFRFLSISMRAVPFHPGHDGMGCVEWWWLYDQLHHPQAALCVCVC

VCVCVCVTHDSTGCVEWCWLYDQLHHPQAALCVCVCVCVCVCTGQE

>SG-PL_contig_4707_1_-1_676_900

MLLMDRLHNFGQNTFCIISSSKSRLFRQNIDCCRVLGAEENGEKSFLALKTRSQYNWSFV

FNARPHFIGPFVGMP

>SG-PL_contig_4708_1_-3_63_2225

MKYVVRSDAHTDPVYTASIKVDGGDEWETHGRVRHRDKLLNILIDSHLGTLPDFLEGRIS

PDSSEFHVGTYIKEGSRVETTVVSGESIRIHPLKPFGVSINRADPQKCENDILKESVIAG

EHQSPYSPDLELLAVAFQNSSTPFIITRIHRPPIVRITADHRSFLKAIFPPAILKAGSLT

GRMRQNDTHWVVEVMASIAQCPGYLKVVLSDPAYRSTPLFSADIAVTCPPTFHLHFYLPA

ADSLGLDRQVVASVADSQQVHHLRLHRPASSDLTGESERKDPAPLPPTVTPAPMKQEIAE

EKVTLPRGFIFSIPYMVSVGGATFLLLCLLIIGLIVQPGVPAPEPPILRCHHALFMVIYV

VFQFIYSIVATSSVFFLVIIAVNSPRVAFIQQHKEQSAVTSASHQLELTAMQTYLQRELT

RQDQHMDSMRSLCKQDMQRIVTDMNKIHKQIVQETEDVFQKHKLEMLLEEHRHRSMQQLS

QDLLSFRDKYNQAVRSILLELNHNLQQALRSVHNNSWLAGARFLHGVVTTTRKHINNVDS

KPFLQWAHLQHDLSKLTVDVAFSLPSLPKLADIEEVIESEAQPSKRSREKENSKKLHTTH

VYNQWFSPFSHNATMDAMMFEKEGGEGQRSGEGRSFSMYYVFLVMLGCLDLVLVLHRMLK

AKIGGQLLLYGYPEYMDFRERKDDGDDDVFDDGKKCRCWSESPFCQAVKVFTSQVMSSSF

I

>SG-PL_contig_4709_1_+2_386_541

MGWVVGMEEKENCKADGHAIAIICFQVPFDVLFNGYFELKMDNFKLVQKPQL

>SG-PL_contig_4710_1_-2_2_385

GCVHAGYFPIFTSYRTLIWLTGSLTCVLIHTNEESSGQVCTYVDPGDRKRSPPCTHNWQE

LPGRVEPRTFRLKVWHSDQLVRPVTPALHSVCSEGEISAPLLSSIPLACYKQFYKRASTT

QASSLVML

>SG-PL_contig_4711_1_-3_1017_1199

MALVTEYPSLLVLVTEHPSILVFTVRFFKINLFPRQSSHGSLEVFEGLGILRGHFQVFES

L

>SG-PL_contig_4712_1_-1_289_612

MMYISIRNISPSLYYAWRLFSTGELLTIFPSPMLLHFSIISCSFIHGLLVGSVLRKILYH

VYVLLFLKKVPSYVYFSSYHTLTSHSTRFWNCSCLYFKFGMLAKGVQQ

>SG-PL_contig_4713_1_-2_224_349

MFECTYKFKCCQFSSFGVHHVLFFQLKAKTKNKQTKKKVEVA

>SG-PL_contig_4714_1_+1_1_186

THAYTHIQTHTRTHTCVRAHTHTHTSHIHTPTLHTHTHTHTHTGNTITSVLFYICSPDEW

LG

>SG-PL_contig_4715_1_+1_763_1050

MPLLISDMPSSLLLSTTHPSHHCNYCFHFLHSLMRHIPSTLTPTPNKTRQNINVLHLFSL

LKQPKSPKLIHLWFYTTYSTHCIYFVKSFLGFFCFL

>SG-PL_contig_4716_1_-2_2_151

ASGIRKQFCWESKFYSVRIHETGIVLWYEYTRSWSAGKYAWYELTRTWVK

>SG-PL_contig_4717_1_+1_475_600

MICKTEAQPSPYTPLPRFSFQITVPSSALSVQNYKESTKLWK

>SG-PL_contig_4718_1_+3_522_734

MKKWTQEMNPRHVNIDEQSQLQFYREGVVHVWTTSQWKTYKYDQITNSPTCFSRQALRKG

TAEGKGREREH

>SG-PL_contig_4719_1_-3_645_866

MFTENVVLQTCVALVGIAKSTDQPSKIMRNETSEIDVVIALLIFLHSKGSHKLAHLQTAY

LRLYTFIPCLFLHT

>SG-PL_contig_4720_1_-3_855_1127

MPHCLIIYIFKQVIFLEAVWCVYFKTLFAEAIYLVYLVVASAVLYSSRMKTHHCCGLFLR

QFGTVFKHCWSYLNCSHLRSFSVNVTILATA

>SG-PL_contig_4721_1_-1_1_219

EQCENINSNLARNNTKQAYQTVRDLTSTRQVRKKTEEKEILARSTPSPLLRSLQPCYKRR

PRSAYQHSLHQPG

>SG-PL_contig_4722_1_-2_2_124

ILSSLSYSICHCHFKFFIIFSNMILTKLLCVITVTGYYVMN

>SG-PL_contig_4723_1_-2_251_1744

MRNFDEVVLTRSAARPNSLDLDNDLHTDMESVFPDMDNNNEVQPASPTRNNRKKKRRADA

PAKKFLDLYDATGEFLGNGSYASVQTYRNKSNGKEYAVKMIEKCNSLSRSKVFKEIEIFH

ICHGNENILNLVEYYEEEDRFYLVFDKMYGGTLLANIERRGHLCEREASLVVRDIAKALD

FLHSKGIAHRDLKPENILCEHLDDVVPLKICDFDLASGINCDGDGTKTPELLTPVGSAEY

MAPEVVDAWVGESFSYDKRCDMWSLGIILYIMLCGYPPFYGQCGEDCGWNRGEACQDCQE

MLFNRIQDGDYYFPSVEWGGISSVAKDLIRHLLVREPRKRYCAADVLKHPWVCNPPAAPL

ATPHVLTRNNSTKDLESFAETAISINRMMQQHMHITMSAAHSFFHSHSSSAASRFRADST

SIAAPDSASSNGSNGSTSGDEHDDFLTAGFLDGFENLHLTRPGSSKLAKRRATLHDCAFQ

SSSGSGDSLSLSSGSWRV

>SG-PL_contig_4724_1_-3_288_536

MQESVIVHVSVYLAWCDGVKFGGGVISYQCMCLCMCVHAYKQREGEGRVLEQERERDTAS

PNPSDFRFLLVQRMEKDLSMRKN

>SG-PL_contig_4725_1_-1_61_2382

MFKVAGPLWAQATAGGNNVFARLLKTGKKVLRRALQKEIDGTAVREIPKPTWSLRAHTVK

VEESTSGRLIGGLFPKHALKTQAAEFRRQAALRLVRDARKIPVFAFVGLSLGASLETQKK

QGTDDVLCSGIRELFQQYGGKESSVVKSGQHFSLQTLQLGQLIGQGCNAAVFEARPADTP

LDEDKTEEAEDTATGSISSAYTQPETESEETQTEASATLSDDFVQEDGSSPSEASASTID

ENPPTTFHTDDHAPSLSESDIDEDDISIISEDEEEESSPQLNLDGWVLYRLSSPRSPGVS

ASKEASDSDSDIFILAGDEEGLESLPDLKDSDVEFGWSFVKGLPPETEAAEGIKLDASDT

TSCETTSASVSLGSSLSSDWPSSNSGVTTQDPEPAPNIQDVGGSRDYPLAVKMIYNYSVE

SHTDKIMSAMEKETVPVQSSLSDTSVLGCSSPEGKKRLPPHSNIVAMHSVFVDKTPDLPE

GAEVYPFALPPHLNKDGGFGRNATMFLVMKKYTMTLNEYLSTHSVDTRTRCLLFAQLLEG

VAHLSRHDIAHRDLKSNNVLVDLSRGTGLPFLAISDFGNCLADMNYGLKLPYLTMDTDRG

GNAALMAPEIKCATPGPHSILDYSKADLWTAGALAYEIFGERNPFYSGFDSRSYAEQDLP

KLHDDTPDVVVNLVERMLKRNPSERPSPAVAATAMQLYLWAPSEMHPFTKHLPVSSNALW

RCKERCHWLEQLTMETVCQRVFAGKVSFLDLQVKSTFLSRISQQDVFAAVAHEG

>SG-PL_contig_4726_1_+1_28_330

MLWAHWEDWDHTVESHPLYRCIIEGVHCRSNFLTNTAGVCISQAWLMAGYITKVSVEHSL

VTQPQSKKELHSISLSSPIFIIILLWKTVPDSVALHVQILW

>SG-PL_contig_4727_1_-2_32_583

MGRYRLCLVLISLCAGLGRKVSQADGVKPTNVTVKPSSRSLEVFWKPPTGLNASLESYRV

RVYATKDDSRKRVYWVPVNGVLRETIACLEPNTHYNVQVFTRFYKGSNQTDGNSEVVPVS

TLPESGNDFLSQVWEQRVFVLVGAILIAVFFVLLTPCIWCCCCYAKRSVDHYQNHWRDAM

LGCE

>SG-PL_contig_4728_1_+2_2_181

LQTLNMFANNHTRNTIWSTKMFSFFLRVLMLAIVTSSRANRMNKQHHLQTLNMFSNIPYI

>SG-PL_contig_4729_1_-3_3_257

LISTNPNLEKDSWAHSEINNILYYFLVFKLNLAASLKTLDMFLARSFIAVCKCHNSLFSA

FCLDISVILFVVSFVVLYVSVLSVI

>SG-PL_contig_4730_1_+1_1003_1257

MHRLEYLNVLNNIQRSFSCTHTNTVSLLPHTHAHAHTHTPFQSKPTHKAENTKCFICNFF

PKSKSTFAMDTVSTQRNISIPKTEY

>SG-PL_contig_4731_1_-3_96_371

MCVHVLSVENCQAILIITFFFKLMIVAKKLSHVHSDISVILSSPVIRQEIFGDTVLLVLL

TQILPGPYMIEVKMFGGFDQLHLSMKSGIDHP

>SG-PL_contig_4732_1_-1_1_237

PRENKRLRRLLRISFLEHRANNYVLSEVEILVSPQEPLLATIKRWQLAWFGHVTRHDGLC

QTITQCTIEGRCRQGRQQR

>SG-PL_contig_4733_1_+1_16_285

MQVYISIAQVLALLPPLSTSALDGALRDGLAEAVMPCDVAKPRQMPPLDSGEERFLGAHK

DLDLAPHIVICSVLQEGDAKQPAQAFVFPR

>SG-PL_contig_4734_1_+3_3_242

RSFFLTFCCVYSQLNQRETRPCKHQHDNYYLQSTLNTQYKNVMTLTLFLCLLKPSPQIQT

VQEMPGVGTEGLNGCLRYTN

>SG-PL_contig_4735_1_-3_333_515

MIWPTFWKITASSFGEQGYIVWVFTLVITFVLFVIAIFFFIIDFIITLTIVISYWISVVV

L

>SG-PL_contig_4736_1_-1_28_333

MCWRNSVAFCFSPTSFRLLRLSWSTRQILCTASWDRKKLTTMMKQSCEKKSYLSPVRSMD

PSVLKMPKRLWRTGKRIQAKTHSPMMHVNYGTRQPCGKATET

>SG-PL_contig_4737_1_-1_1_312

FFFQGRVEYLHGVVGANAVCVHVVVVIPHGSAENWCDIVKPCPNEIVSRLVLQQLHSARR

RQWKASGTKRSVPKRPMNVLILGTSLYSPASSRSCVFCQMHWQK

>SG-PL_contig_4738_1_+3_1884_2201

MTSSCLLLEHVYRVSIFHLQHCWCVFIIDWISVKTKSYCPCCDTRSVTICSHQLSQGCVF

LDFEMHHTPILPSHFQIDVLTFRLAILTLFLALLLVHVAVSDSPIS

>SG-PL_contig_4739_1_-1_1_261

VCVYVSVCVCVCVCVCVCVCVHCRHNSRKLDCFHLYLRVMCVLMCKPFFLLKYCTFYHFA

FLSYAGYILQRTKHLTKELLAGIVALY

>SG-PL_contig_4740_1_-1_1_228

LASDHYAVLCHLAVQKPKCPAKFVSTRSIRKINHDTFASDIAHFVTPNLSVSEFNTHLAA

VFEKHAPVCQRKIREG

>SG-PL_contig_4741_1_-2_8_232

MNSVHIAICHIMNSVHTAICHTMNSVHIAICNTVISVHIAICNTVISVHIAICNTVISVH

IAICHTVNSVHTAIG

>SG-PL_contig_4742_1_+3_453_644

MGTTFSIFSFKLVKKLPNFAHIIIRPKITSVSILNHRPPKKSQPVQFTAWLLLKRKSRHR

LVSG

>SG-PL_contig_4743_1_+2_2_301

QGVPQGGVLSPTLFILFINDLVPELPKGIHAALYADDLVLWCTEEYATTATYRMQLALDR

LSAWTNTWCVTINKEKSSATLFSLSTKAQPGNLTLEDATL

>SG-PL_contig_4744_1_-3_144_278

MFNLFMPDNMATQQRVLWFILGMCTHVCLWGLYACVPGFWRGFFF

>SG-PL_contig_4745_1_-3_3_179

CACVSVCVCVCVCVCVCVCARLCMYLCVFLFLCYHSLSEKYIPHCCCALPFILCQVFCP

>SG-PL_contig_4746_1_-1_1_984

MYISKMSNKKSSGLDEISPKILKLSLPYISESLTYIYNISIECNIFPTALKEAKVIPLPK

TKDLSQVSNFRPISLLSVISKPLEKHVHKHLLDYLERRNLLHSLQSGFRQKHSCHTALTR

LADTWLSTMNECKLTGTVFLDFKKAFDLVDHDTLLTKLHAYLVNHAAISFFRSYLNKRKQ

KVLLNGSYSSEGHVNCGVPQGSILGPILFSIFINDLPLHVSNPTVCCDLFADDGTIHTQE

KSIMTINFRLQESLNEVSEWCTKNAMILNPSKTECMVICTRQKRQLGPLSLNLSMGGSAI

KQVSDHRLLGVIIDHNLTWQDHINYICK

>SG-PL_contig_4747_1_-2_128_265

MQHLSNRSKRGQSSPLTKNERPFTCKQLQANLSPVCHIETFRKTCP

>SG-PL_contig_4748_1_-2_176_2239

MPSASSYDPSSVNGFAKVQNLVSSSEHMTIVESLSVESGQLNDKAGDEDEPVVSSLTENI

DTSSCAVNVAEDRTNKGIWSETDGSNHTESSPYLSADIAGAIDDEQNDKNTNSDTNWERI

PTGVSAPEPENDCRASEHKEQMDVDFEVSDTVTTVTESVITQSDVEQNLDVDVDSDKDID

GNDTITATNNSATPPQCPSVTPTSSPTSTILTTTTTTTTTFITTTPSSTPTSLPTTKSVS

PSTTQNVTPSSTKSLIPSTTQNTTPPPTKSGTLSTIQSLVPSSTQSVTSSTTESLTSPTT

THVTPSTAVSVTSSTMTNVTPSTTRSVTPSTTKRLDASSSAPSTTKNVTPSTTKSATNAK

KVLPTSRSRLGQTNSISHLLNKWQARETATSPPFSSTATATTSTSSQRSPHLSLFNSSKR

RLSQSTPNLGAVGPLPDGGTPELAEQTLTSNTLSKDIESEQENKTPSEKILSRNSKPVIY

GSTGDLQTPERRSVKQAIKSPFPHLSQHDDDNRELSSAREQKVKSETHTSQTVSDSSQAQ

TSETISDSCKGDGYLMSTLDNLNIVLIASDKTSSERGESAQQSSRRTSSLTSPGHALSPA

VSDVITMPSVHSLISKFSHSNRSNDLDTTRPSSRLLSSKQRWSSTSRLQGPLSPPPVASS

SLTLSRTSLSDRKLASPITSPLRHQWAS

>SG-PL_contig_4749_1_+2_2_76

KSKWRLWSGRYGVCTKNLQTLDKSR

>SG-PL_contig_4750_1_+1_1_108

VLVGSLRLPSLWSGSSSFRVSSPRALNLYYRLLSSL

>SG-PL_contig_4751_1_-2_41_1261

MASSLFFVLFTLTVCELILGVAAYEKPGSSHHKLKLADWLVAAGTTFSLKLYNKLKDQHS

NMAFSGYSIHSALTMAYVGARGSTEEQMMRTLDLKELRKKNTRCVAYAPLVRLLTESGSA

TLDVAFAAYVKRGPTIKQKFISCLQDTYKTKFDFLAAYQAGGSPEAPINSWVAEKTENKI

TDMLAPNMVTAQTSMILINAIYFKANWKNQFDASKTSLQPFHRDDGSQITVEMMNRISSH

KYSQHSDLLAHIVELPYHPDGRFSMYILLPKKRNGLADVERQLTQQRLHHALQSMYTSTV

DLYLPKFKGESEFNLKETLEEMGMTVPFSSSADFTDISPAPLHLSDVVHKAVIEVNENGT

EAAAATGVLTQSSLRFKISFKADHPFLYLIRDSVSKAVLFMGKYSGQ

>SG-PL_contig_4753_1_-3_741_1070

MCALYDCSVERACAAWPQRLEEVLPVRLQCVEIECAHRTSALPRDSTASTSETECCVCFV

WQDELHSWATGKKCVLETSVWCVGSVNSPCWCTMKRLKCLTLDGMEKSLE

>SG-PL_contig_4754_1_+1_1_138

PPPPSHTYTHTHSLSHTHTHTLSLSHRVIVPPSLPLIQFCNNCMPW

>SG-PL_contig_4755_1_-3_444_704

MFNFLSTSSLLLQIPDSQNDYAFVCFACTCVATCIILEIGLTSLLLPFSQNCGVYQCHSL

LKREIVVCVDIIQNSREQRVCLLGIFF

>SG-PL_contig_4756_1_-1_10_462

MMGADVVGINCGRGPTSMMPLLRKCREVCKGPLAALPVPFRTTEKERTFHSLTDPQTGKR

VFPLDLGCVESSRSDIREFAREARALGIQYIGLCCGSSAHLLREIAVEYGRHPPSMKYAP

NLEKSFFLGDLYKRSGLIYARMAGDDPNVPL

>SG-PL_contig_4757_1_+1_109_264

MFAQPCVFAKQSFGPFHCGFLLRGKHPFSRSYRVNLPSSLAMNHSSTLGFSP

>SG-PL_contig_4758_1_-3_75_1721

MGKNRNKVPGASKEELMVRAVTEIVSELIKADSQKKDVDLNKLKTKVSAKYGMSSQPKLV

DIIAGVPQEHKKTLLPKLRAKPVRTASGVAPVAVMCKPHRCPHIAMTGNICVYCPGGPDS

DFEYSTQSYTGYEPTSMRAIRARYDPYLQTRHRVEQLKQGGHNVDKVEFIVMGGTFMSLP

DDYRDYFIRNLHDALSGQTSSNVDEAVRYSEKSMTKCIGITIETRPDYCLKKHLSDMLRY

GCTRLEIGVQSVYEDVARDTNRGHTVRAVCESFQMSKDAGFKVVAHMMPDLPNVDYERDV

EQFIEYFENPAFRSDGLKIYPTLVIRGTGLYELWKTGRYKSYPPGMLVDLIARILALVPP

WTRVYRVQRDIPMPLVSSGVERGNLRELALARMADLGTKCRDIRTREVGIQEIHNKVKPY

EAELIRRDYVANGGWETFLAYEDPEQDILIGCLRLRKCSPDTFRPELVGGCSLVRELHVY

GSVVQVHARDPTKFQHQGFGMLLMEEAERVAREEHGSPKIAVISGVGTRNYYRKMGYELE

GPYMTKALL

>SG-PL_contig_4759_1_-1_115_618

MVSLSCELLAISASWSYGYGSRFPFSSYGEAMFLAFQTIIIAFLVLFYGGQKMAALGYVG

TYVAAMAFLLSPFVPFQFLSILQTSNIFFIMASKLIQAYANFKNKSTGQLSVITVYLIFL

GALARIFTSIQETGDNLVILVFVVSTVCNGIIALQMILYWNTGKTKAE

>SG-PL_contig_4760_1_-2_65_1390

MASTRSVIGVFGRNGLVPQLLFRRCQGTAAPASEAAKKSSAKDSTSKSKATFSWMDALNL

ESQLTEEEIIVRDQFRNYCQDRLMPRIIMANRHEVFDVAIMREMGDMGVLGPTIQGYGCA

GLSSVGYGLITRELERVDSSYRSAMSVQSSLVMWPIYAYGTEEQKQKYIPKLARGELIGC

FGLTEPNHGSDPASMETTAKYDPNKKTFTLNGSKSWITSSPIADVFVVWGKCDADNGRIR

GFILEKGMPGLSAPKIEGKFSLRASITGQIVMEDVEVPAENILPNASGLGGPFGCLNNAR

YGIAWGTLGAAEFCLAAARQYTLDRKQFGRPLANNQLMQKKMADMLTEISLGLQACLQVG

RLKDQGLATPEMISMIKRNSCGKALDIARHARDMLGGNGISDEYHIIRHVMNLEAVNTYE

GTHDVHALILGRAITGLQAFTG

>SG-PL_contig_4761_1_+2_593_853

MLTMLSDHQKLKMLIAEKDETLQGIVQKEMTTHNYLPITETVKQPHAYQQKCKLWQDFVD

TTLQSEGEIRQQQAFQVLSGIRKLYAI

>SG-PL_contig_4762_1_-3_66_962

MDPKKIGDFAGRLKSGGKGFGTGIGFLAAAGALAYGVSQSIFTVEGGHRAIIFSRLGGVQ

DDVFVEGLHFRVPWFQYPIIYDIRSRPRKITSPTGSKDLQMVNISLRVLSRPVSSQLPTV

YRNLGTDFDERVLPSICNEVLKSVVAKFNASQLITQRQQVSLLIRRELTERAKDFNIILD

DVSITELSFGREYTAAVESKQVAQQEAQRAQFLVEKAKQEKQQKVVQAEGEAQAAKLIGQ

AVSANPGFLKLRKIRAAQNIARTIAQSQNRVYLNASALMLNIGEKEFDEAADSVSRRKK

>SG-PL_contig_4763_1_-1_16_270

MDSVKEWGTFRDFEEAFKSFDREGQGFISAAECRHLLTAMAERLTDDEVDQIMKLTDIHI

DLEGNMKYADLIGKVMAGPPEAKQN

>SG-PL_contig_4764_1_+2_161_331

MCAHTHAHTHTHTHTFNANIVVCTLFYVNICFHFSSKTLIQNSLGEEQEKKIRLAAL

>SG-PL_contig_4765_1_-2_38_592

MFFVVVVISSLISCVSFAPCHYSTTCVRYLFIIGFRRDGDIHLHLLVIFYLLLIIGDSVI

ILFFRVSLNPFVYFFKTKKRTDVIYIYIYVYILYLKLTKIVRVGTDPETSATPMDLMNSR

YEIRPKLSDISDPLRCVCVCGYAPSRLVGVLPIPTVPLRAVDWNALLWSTRDAIPVLCIP

HCAVR

>SG-PL_contig_4766_1_+1_49_282

MDAGVCVCERVCERVCVHACLCSCVCVCVCVCVCVCVCVCVMAILSLISLFKVLSVKYDS

TLRTKTLADQNLSDKIYT

>SG-PL_contig_4767_1_+1_1_171

IGRPPECQVEERKQTTSHKSPILRRNQSTKRKTKTQLHFTHSNTADLTYSFNQSIKC

>SG-PL_contig_4768_1_-3_1548_1757

MSILTAYRKWIDCSLLLQMVSTKIRNIKSNNNKESNDNKQSNDDKVEHLNRDGKVYFAAK

VSRVNDHSKL

>SG-PL_contig_4769_1_+2_452_598

MLNHCREKEKWKIPRHMAEKSLFQNHVIFCKAINHIKVNGKHLHHVPGF

>SG-PL_contig_4770_1_-3_171_1112

MVSYFVLALVLVVGVSSFPQQSENVVKWVDLIPSKTVGPKKGCLVRSTPKHLRKYPARAP

RIYEEPGYDFSKLPKEHDWRNVNGVNYASTTRNQHIPQYCGSCWAMGSTSALADRINIKR

KGAWPSAYLSVQDVIDCGNAGSCEGGDDIGVYEYAHSNGIPDETCNNYQAKDQDCKGFNR

CGTCSPDSCYAIKNYTLWKVGVHDDASGRNKMMAEIYHNGPISCGIEATDGFEQYKGGIY

TEYIPAPGINHIISVAGWGVENGTEYWVVRNSWGEPWGEQGWAKLVTSLYKNDTGNLYNL

GVEEDCHFGDIIIN

>SG-PL_contig_4771_1_-2_842_1048

MLLDFVCQCCCCFTWVGAVFHQYANDEIGNTSLHLLLHFFFSSNNTGDRKLIWLFVDTVT

NWMCSGNGN

>SG-PL_contig_4772_1_+3_213_2318

MTKRKISILGISDTRKKGEGTKEIHSNFVLIWSGVRKDQRATHGVGFILHPDIAKNLMST

EYISERLIKIGVRKSNKIVYYIQAYAPCNDSYSEEEKDSFYQELSDLINSTPDKEDLYVM

GDFNGRVGERRNPWTKHLGPHSDHRTPCNYNGNHVLELCAEHDLIITNTFFQHRASQIYT

WYKWNDLSISSQIDFILARSRTRYAIKDSKAIPNANLDTDHRPVILASSTQKKKITTARK

NQPEKINLLKLEEKEVQEQIESAIKVKLDPIDTAPLSMEQAWDAFKPALLNTLREVCGTK

KCGGKNRKATAWWNQEVKEAIKKKKQLYKAWVKTKTEEDYVKYRIARRHSKRVVKASKEK

SWATYGEKLSEICKNSPREFFKSVRTMRVRDEPFNPTTVVNDANGEALHKEEEILKRWED

YFKDLLNPSGVQASSSQFSPRYKDQLEPQILEAETRKAIKTSPKGKAAGNDGITTEAILA

CGETGVKWLTIIFQKAWEERKVPMDWQNAIVVPIWKKKGSKKDCSTYRGISLLSHVGKMY

AKILEQRTRAKAEHLLSNAQLGFRKGRGCTDAIFALRQLCERAIEYNQDLHTVFVDQEKA

FDRVNRDKLWKILEQYDVKGQLLDNIRAIYANSRSAVRTASGTSDWFPVTSGVRQGCVLS

PLLFVIYMDQITKEANPDLEALNELLFADDQSLMNQNKAALQ

>SG-PL_contig_4773_1_-2_335_514

MLCNVQVICSMETQEIKTKRVILCLKSTCIYKHRQHICFFDNCMLSLDIICFSAYFDRVN

>SG-PL_contig_4774_1_-2_161_406

MLLQYRTSTPPPQPDRAAFLENYHQSKQADTALLAECEHVSNNFDSTVNWETEMFQFLSV

HSRFPGTGDGFNINICFSRMKF

>SG-PL_contig_4775_1_-1_4_381

MAHPGAMTTPSQGDVYDTNTKQILVTRDPVYCHPAVRNGVVVGRQPHTYLPLAILMTIVN

PVLGPVALFFAYLSNRAFKQGDLRYAEKWSRYAFLACMITIVASLVIYIAVGFSLSKIGT

RGGHSY

>SG-PL_contig_4776_1_+3_48_317

MVTLNLKFGENIKCNTFWKFNCSLLKEKQYVEEINKLIDDVILTQHHDAVTLNISLMEII

LTVPDKLFADFLPMKIRSKTIAYATKRKKK

>SG-PL_contig_4777_1_+2_641_859

MLNFEDTDIIPGYRSYHSMANLNLKVGGESIKRYTIWKFNCSLLKEKQYVEEINKLVGDV

ILEYSTEHYDAVT

>SG-PL_contig_4778_1_-1_1042_1269

MYIEPFLYISPYFLIFSPHVLVFVHACWTLCEDIFCVFSPFFPHKFPVCISWLVAWLHRN

RDLTFQSKCMGLRLAL

>SG-PL_contig_4779_1_+2_2_79

SFCLSVCPSVVNESNNTLKQSLLSQS

>SG-PL_contig_4780_1_-3_384_524

MTWLSLAEAPLGFDLHNMSPALSLHSSQVCFWQYLVYICTAACVESG

>SG-PL_contig_4781_1_-2_2_220

KEGLSVENDRSINRSKILKCICSVSRGAQLYYKILTQIAARPKSCSKWEEKLSTEIKWRV

SFKNIQKIREIKI

>SG-PL_contig_4782_1_-2_398_616

MFWRCANVQSFWHQLQTSISNECANATSLTFSENIVLFGHDKNFTSDKTLDLILLHRYYS

AFSSVQKILENCI

>SG-PL_contig_4783_1_+1_184_468

MKNKFSTQKKTQTFQDLHAQRPIPHTPQQLSVLHVHNLHIMYSPIKYACYTADKPWHSLT

LSTKVGAGGQVSETVKSRHDLNYYFQGLKWSRMKK

>SG-PL_contig_4784_1_-3_84_227

MILSSCCTNCFSGHDYIYVTVHEKSASISPLQDIDQFHHLDSIIGVKK

>SG-PL_contig_4785_1_-1_391_639

MFVLFVLCLADAQVAYTLVSVYLHMCARTHREVFCFCLSISFSVCIFFAVSQVLLQICRL

FSFTHFAFEFQKCKMLFSIYVVC

>SG-PL_contig_4786_1_-3_231_434

MRVLRCLYHAIFFCFQTSLYFFLYNIQYHFVYNTHFNLRKSWKNCKRRNEENTKLNRHTN

YLKTIKGF

>SG-PL_contig_4787_1_-3_231_413

MRVLKCLYHAVFFCFQTSLYFFLYNIQYHLVYNTHFNLRKRWKNCKRRNEENTKLNRHTN

Y

>SG-PL_contig_4788_1_+3_60_179

MCRLLVPEPPSSDACRTSNMHVKDPVTSVSHVSVRWLMET

>SG-PL_contig_4789_1_+1_133_258

MIQECNNNKKLRINLSVNICTVNTSLSVALYNKTAYSINLNK

>SG-PL_contig_4790_1_+1_1_183

KGLPINQSFTDIVSVKQRVRENCLSLCLPDEDLQQKTQGYSAKRKRIYRLWILVSRCLEK

S

>SG-PL_contig_4791_1_-2_2_244

NCPRFCGPSSPCSHGDLSFGGFGAGSRNERRGGSDTGAVQKMGQGKKKRKKSTSEDLHGT

KTISLLKSPVVTTFRTTGLSF

>SG-PL_contig_4792_1_+3_117_395

MTKHTSYKNKLHDQLPMDVRNLKCTCNRTKSSHHATKKHENQKNLQLVQGYISCISNLYI

VLSWNTICRVHIINVSLFFFLYDKQFKRPISTE

>SG-PL_contig_4793_1_-3_3_1223

MMDGIDDSPDLLAEDQEHRQGRSDTVDLTDTSLLVDISDSLSERDKVKFTVHTKTTLQQF

KKPEMSVVRQHEEFIWLHDRFVENEDYAGIVIPPAPPRPDFDASREKLQRLGEGEGTMTK

EEFMKMKQELEAEYLATFKKTVAMHEVFLQRLAAHPTLRQDMNFEVFLEYEGDLSVRGKN

KKEKLGGFFSRITKSVDETLLSGQKDVDEFFENEKVFLMEYHAKIKDATQKSDKMTKVHK

NVADSYIQISTGLVQLATIENTELAKVFTRVAEALEKARKLEGRVASDEDLKLSDTLRYY

MRDSAAAKDLLYRRTRSLADYEAANKALEKARTKNKDVPQAEKHQDQCCKKFEKISDVAK

KEIQEFKTRRIAYFKKHMVELVELELKHARAQAQLLKNCITALEEES

>SG-PL_contig_4794_1_-1_100_5613

MDLQTVAALFWAVHRLNGQGMNPPLPPYIPGVRLELRVYDDCRHDVLAARHVGQLLASHP

DFNCTPSTPPLAGILGTTTSHTTSFVASLLADTHIPHFGISATVSVLSNHTTYPTFFRTV

PSDLTQSKVLLDLVLHLQWTYVVGIYTRDNYGIEGFQAFKRMATQAGVCVFKEKAFWHSD

HGVQDSMERFVRELLIVLDTTRYPALGVIYFGHDTEATILFQMIQDRRSRWHDQDKVNQL

YWLLSDGAGTSPSFASLAAPYNLIAIAPATSHLDDINNYFFHVLDNPEELGPYWQSLVSE

YLHDVEGCFADRSPVSSCRLRDKIQPYDYLSAALDSFYSLAALLGRAHKEVCGGTGSPGV

CSNLSDALKNGLLSLVQPQAMNYSLHFMEERLPKQFRHDRMLVPDPYGDFMPANSPVYFL

NSASSGQWTKIGEYCNGSLDFVPKLDLPFVYRSECGTDCKQCRKSDTIRFVYHPGDILLL

GLYSISSPVATASFSCGDYRKQSSSVAAMMAFLRAIETMMSQIGSGNGSRFGALVLDDCY

NKLTASALLADLFSGQITLYDYNTDQPIPMDKVKAVIGSQSSSVTLSALSLLTPLQIPTI

SYSATSPDLDDRVSYPYFLRPVPSDSLQARALVDVALLLNVTYVGAFYQDNNYGRRGMQE

FVRMAEEEGICVETPVAVQVSNNEVDFHKAVRDIRNKDAMVVVVFLVESSVIKLLDAIEG

MNLDLIFLASEAWSLSTDTLQDQRGVTARGSLVLTVDTSREAGNVGMANYFKELTTPALT

GHELPWMRDFWENEKQCNLAGGFTNSYEKMCDPGDKLSNEVVRELSEDQKVVQTINAVAS

IITALEQQCGHHRDPVACMTDLHVHTAASLIRDVQLRDQRGQSFIPYLNDSNGNVGFTLH

NVQKLAGNTYRYVEVGQYNPVQGLQITDDIKFYNPVGQPMSSGIVSTCLYKLQCKSVCGK

GKTPTTPYPPVTPTSPIQQGDGEKNGGLVAAVVVMSLLCIALAAVLVVGVCCAKRRGRIP

GLGVVKKDVSGPATRASGFGEVNMGFSPTSPRVSLPLPAIPQPSGRDTVEQYNLPDMDCR

VSISRSSEASSSHCGGPSPSRFDEVSHYDYSEGEGRGRRPRRHHFHSSSQHQDLQRQAQE

LSLPQGLLQQHTAPSTASLPSAVSGKDRPRARSVPRADRPNFLPVQGPRPNTTRSVDMVG

AQDHQHLTGRPRAYSHDAAFRERLGALSDDDQQILLKYLTTARQHSKSPRASQVPSMSPV

MQNREGGGRPTFSLPPVQSQMSEVEGRGVVSPNIMFENPPYFCSNKTSLSTRPEDDPSLF

YLHAIPAEKDLYLEVIGDPSDILPSSVSPHSPATVSPHSEPATSPCSPLSASSHFPSPVT

WPLLAVTSSPQSLLFSSPVGIPAGPVPKTSPSTSGSSNITSVTSLLSPQITSPLSLPTSV

RDSTASESSGAVFVSPDHECPPLKLSGPTDSLSPPVFHSPLHFNINPGFTPSPSVPQEQT

RTTPVMQGKDISMSPTQHVALSPERLQQLINRNSKSQAAKHSTPQTSPQSAVPGVVVRDH

TGALFWVPNVVVPLSQHSLPVSPTSTSSVSNSPTVAGSSHSLNHSDFGHSPTEDVHARNS

FPQGSGHVTQSALPLQGNGMAQFPLYTHSPTNGLSQDCRQQSTANSSPKLQAKSRKPKMG

VDALSAQRKLTDIGDVNLNMQRELGTPAALSSSGEHLNCVSSTPKTSTHDVLTDHPGSPQ

ATEAGPVTVVAPPLSALGPSTLGPPSSLSAVTLHGDSDSGAYVRSCQLVPSPSPCKPLSV

PQVSGSGDSTHTSGDSGTGEDLNTDADGNVHAMFEVCV

>SG-PL_contig_4795_1_-1_424_606

MWSPWFICIVFVGYIYNLLLHCLREKKIPHCCCVPPYTFICCAHDEITYCVQSLFLGIGK

L

>SG-PL_contig_4796_1_-3_81_896

MSGKLKKVVDESREANNPELDLGDRGIVNIVDVPGLMDLQHLTRLTLCHNKLTTLPASIA

NFLTLEILNLFNNCIEELPTTLSSMPRLRILNVGMNKLYQLPRGFGACPQLEVLDCTYNN

LSEKSLPANFFCLDTLRAMYMGDNDFEIIPPEIGQLKNLQILCFRENDLVALPKEVGDLV

RLRELHIQGNRLTVLPPEFGNLDLVGTKQVFKADNNPWVTPIADQFQVGVSHVFDYIRSD

TYKFLYGRHISANAPLPPKTNDKSKKISRVKK

>SG-PL_contig_4797_1_+1_1_216

TIQYNTIRYSSWKAPYIRLYAAPRCTQTKCILTYGRNSKPHENEIDAQYYSKNVALYSMR

SDTNEFFIFKQR

>SG-PL_contig_4798_1_-1_1_258

RQELEKIRRRLETELNDLRDQLNEKRQQVEDLQAQLSRREEEVQIALQKADEEGVSRQNV

NKQMRELTNQLQELQEDLETEREARN

>SG-PL_contig_4799_1_-3_288_2489

MPVPGVDDSQEFRSLVESMTIMGFAPEDQSALLRVISAVLLMGSLQFKQDRNSDQATLPD

NTVAQKACHLLGVPVTALTQAFLKPRIKVGRDFVTKAQTKAQVEFAVEAISKALYERMFK

WIVQRINRSLDRTKRQGASFIGILDIAGFEIFKMNSFEQLCINYTNEKLQQLFNHTMFIL

EQEEYQREGIEWKFIDFGLDLQPTIDLLEKPMGIFALLDEECWFPKATDKTLVDKLLAQH

TSHPKFQKPDFRADAHFSIIHYADKVDYSADQWLMKNMDPLNENVVSILQSSTDNFVAGI

WKDAEIVGMGAAAAVETMFGSRTRKGMFRTVGQLYKEQLSRLMATLRNTNPNFVRCIIPN

HEKKAGKIDSPLVLDQLRCNGVLEGIRICRQGFPNRILFQEFRQRYEILTPNAIPRGFMD

GKKAVEKMIAALELDNNLFRIGQSKIFFRAGVLAHLEEERDLKLTDIIVQFQALARGLLA

RRNYQKRLQQINAIRVIQRNCAAYLKLRNWQWWRLFTKVKPLLSVTGQEEKLHSKEEELK

KVKETYEKQKQDVEQLESRYAQIIEEKNILAEQLQAETELCAEAEESRARLSKRKEELEE

ILHEMELRMEEEEERIITMADEKKKMQQTVQDLEEQLEEEEQARQKLQLEKVAMDSKLKK

LEEDLAVSDDSNAKFIKEKKSLEERLSEANSQIVEEEEKSKQLGKLKNKYEAIIADLEER

LRKEQQARQELEKI

>SG-PL_contig_4800_1_-1_25_279

MLTSKSDLHIYQFTVQELTDSLSFQNKNDIYQHLKIMDGDCNVRNTKYTTVSENNQINTH

NETCTYSQNLVYTLLEICTRDTQII

>SG-PL_contig_4801_1_-3_126_335

MELHVDSRGDLTPDPEVDAIRVIFYAVFDDIPPERGRRNMTGAMVVDSSSRSGGGGGGGS

SSSRSPQPST

>SG-PL_contig_4802_1_-2_2_271

DTADGSSTPKRQRLDLDDILHSTPVRHSSRHLFESGCTPIGGGGESTRGRSEDTDPQFVS

PGRPPLRRLSTNTQSALRRAAMTADSRQQS

>SG-PL_contig_4803_1_-1_1_183

SLFMQNYPIVKYIYILSPPPPPPTPGCNGIIDCSAKPGCAKRNVVVTVILHNHSESYWSD

V

>SG-PL_contig_4804_1_-2_299_1015

MVEGGMKCVKYLLFSFNLIFVIAAIALIAVGAYVQIKLVDYYDFFGNDYTGPGILLICVG

VFIFIIAFFGCMGALKENYCLTMSFAVLLSVIFILMIAGGIAAYVLRNDVEDKVVDILLT

AEKNYKKETGVTETWDRLQREFTCCGVENYTEWQSFNLSTTYPRSCCADESDTAKCPKDS

PIDVAELNRTPCEDAFVSWLKDKVAIIGGVGIGLAFIMVVGVLFACCLARAIRKEYEVV

>SG-PL_contig_4805_1_+1_409_573

MLFSRECTHCLFLDTSRLVLEYGCRLGMQKPHGNVDNCKAHNWLASVAVGESARG

>SG-PL_contig_4806_1_-3_819_1001

MVKKCAWGVCNLDTQYPERLAGGVQFFPFPKWRRSQPNNHEKCRPNASFSPSHCTVLPTV

H

>SG-PL_contig_4807_1_-3_381_587

MKKLPPKAWGLCYSFYRLSALSSRPLSGGMFVCEVGMQLTSVAYLACGVVRIKYVYGVLT

SPPPPFPPP

>SG-PL_contig_4808_1_-2_620_817

MHTHLYSTHTDMLAHICSDTKWFAYICMSTNVKVMEMELVTNGSFENYERTFVRSPYLEN

HISQFY

>SG-PL_contig_4809_1_-1_1498_1812

MYTYIFHFLFKTMILLLVLWGNKENDNNNNCCCCEQCILGCLYANCLAHSVQGLSCCRVC

TLHFWWCASWVSSCFPKPPNTDRAYRFLNVNIGCSACLYIRRVFR

>SG-PL_contig_4810_1_+1_1_60

TPSPCPPASCRKLVLDRVRE

>SG-PL_contig_4811_1_-3_3_251

DIVVSGPAGNLTVVNLVLNDEMETLTISLGEELRRDENYTVQIDYSGPLRDDPEGLYFSA

YERDGQTVYLAASFFQYENARKA

>SG-PL_contig_4812_1_+1_1_84

LITLVLQNTVLNKFCCYSDVYINNTPKA

>SG-PL_contig_4813_1_+3_3_245

RKKAEEEKKNKRKKAEEEKDETKSCTEIILQLRLYLFSNATIIQDLGVKFLSHMPQHLFW

LTALNIPKLKHMSLSHNDHWW

>SG-PL_contig_4814_1_-1_73_228

MVTSCSLLFHVAQYHLLRFSCYVKVCMLVFATSLFGWLFCLLHLAMASYRIT

>SG-PL_contig_4815_1_-2_65_781

MRCLWKNMCLQDWSSILHPHQVLHTSMPTHLSDWQESPTTNLSGLCYWLFGWLAEPDEPT

PLPVVPTSCSCDQVWTEVKDLRETLLVENRKLKDQLKSYNDSCQVSFTARLSERAGHKFW

SDHPLVFDSVLTNKGEAYDINKGIFTAPCNGQYFFRLSMRSHQENDSGYVDGAIEVNGEE

TARTSVFAADMMDHYEQASNGLVLTLKEGDEVQVRIQTTSSGKIYGEVFSVFTGFFISA

>SG-PL_contig_4816_1_+3_3_242

FFLGICPFINVVHTPQPAASRKTGDRMFQHQSGRKGSNHLTVHVEAQRHTENIQGTDDHR

SQNIFNNGEAEVIVLRKISP

>SG-PL_contig_4817_1_-3_1206_1682

MTGATGFQEPQSCHKTLGSTVALTNANVRPSPVTQRQDLQSHHIKMPSLLIVKNCSGLLS

VFLGCVWVYFCFVCCWRVFVCFVLFLFVFVCCCCFYTCTACFFCLPLALFLSSLTCTHLS

IRLRVSRPSPFALESPQRWHWDVKTPFPVVPLFAQTSVL

>SG-PL_contig_4818_1_+2_152_832

MVILREKIKNERGAELMAMLGETWDYFFKEILPTLQALMYPLAVAIKGVDIRNTTLLEFR

NIVVLKVGLKDAVDSKRHPTPENIRQMLLVLQSLNDPTFLSKEQLDLERLTACVVEPFLG

SRGLYQGGPEPVTKATYRPAMPSLPLSKPQITITGVSEDKEEQTILPPPSCPMKLDHHPY

ASPPAMRKLHDPYALSRSPLSSPSRLKPVMEVAPDVPLGRRHSIVGT

>SG-PL_contig_4819_1_-1_802_1023

MPQHVYIARTCTQFDIRHNLASSKAFTINIFWHQILHHQKHSQSIFSGNKSCIIKSIHNQ

YFLAPKHQKVHLRE

>SG-PL_contig_4820_1_+2_8_214

MPMSRRLSSSVVSRYILHTSTSRVHVFHIARVRSSFPVLNFPCSCQISSLLVPTFTLYTR

FHRFFVFFF

>SG-PL_contig_4821_1_+1_235_336

MKMCKENLDQTVKCQPQGSECVVSVADSSGSEEH

>SG-PL_contig_4822_1_-2_92_2215

MKTASGSWGPQWLLRMSPDKQDTLLKMMVLAIAAVLSFSTRLFSVLRFESVIHEFDPYFN

YRTTRFLAEEGFYSFHNWFDDRAWYPLGRIIGGTIYPGLMITSATIYHVLNYINITIDVR

NVCVFLAPLFSSFTTIVTFFLTKELKDAGAGLVAASMLAIVPGYISRSVAGSYDNEGIAI

FCMLLTYYMWIKAVKTGSIYWSALCSLAYFYMVSSWGGYVFLINLIPLHVLALMVTGRFS

HKIYLAYSTVYCLGTLLSMQISFVGFQPVQSSEHMAALGVFGLCQIHAFVDYLRSRLSRE

QFTLLFRSLVLLSATLAFGAAAVATALGKISPWTGRFYSLLDPSYAKNNIPIIASVSEHQ

PTTWSSFYFDLQLLVFMFPVGLYFCFTKLTDANIFIVMYGVTSIYFAGVMVRLMLVLAPV

MCILSGIGISSILTAYMKNLDSSRRDKKPKKPDSTYPLKNEIANGVVLIVTAFLIMYTFH

CTWVTSEAYSSPSIVLSARGGDGSRIIFDDFREAYYWLRHNTPEDSKVMSWWDYGYQITA

MANRTILVDNNTWNNTHISRVGQAMASTEEKAYEIMRELDVSYVLVIFGGLTGYSSDDIN

KFLWMVRIGGSTDRGKHIHEMDYYTPQGEFRVDREGSPVLLNCLMYKMCYYRFGAVYTES

GKPTGYDRVRNAEIGNKDFELDVLEEAYTTEHWLVRIYKVKDLENRGV

>SG-PL_contig_4823_1_+1_61_207

MVSILAKQGVGTNSPYLQQGRENYKRKIEYKLVSSNMKGVWEGMKLMSG

>SG-PL_contig_4824_1_+3_1302_1523

MSTNVQQICTGTIHQCTLTFYTKSNLDFVQVSEITLNIAQNKQFNPSLLHSLSILLLKVI

LYSLLIIQHRFCIL

>SG-PL_contig_4825_1_-1_304_558

MQLVPHSTKANLYCLNCSKCVPLSVIIFFSLLLVFTDVFHFRCGFLLLSSFIPVSNLVTA

VSGKKKLLALSYQHLLSSGQLTKYH

>SG-PL_contig_4826_1_-2_92_322

MYGEREKDRERVCVCNVYLTYVWLCTLCVCVCVCVYLTCMAVLHGKEMSSNKLYIGSVFK

KKGTQFVALPFLLPRDL

>SG-PL_contig_4827_1_-3_3_431

LFLRADNSGQTLKCVAQAEFDGFQAAVASVPLTIIALEPVITCEQNASARPQDKYVKITC

RVEHDNVEIEHYSWEIGATGEIIVPHETSQSYNEVIVEEIDAVTTDVTLDLYQTDEWHFD

TYFHLDVFTKSGQKFRESAKLVK

>SG-PL_contig_4828_1_-2_2_289

CPTGQGGAYTLPERSTVGQDAQRGGPIVQGHPTLLCILIPSPPSPHGGWTLGTPGGRVYG

VRGRTLLMGSMGRAHYKPSRGWGPPMDYADHWGQRG

>SG-PL_contig_4829_1_-2_2_1612

MTPRSLQESKKFKSLSAISHKRESSAPSMNRPLAHAHESLSADCLSVDTRGSQSGTARDS

SSPSHSPTPCGPQGYVNSVGIGGQHHQRAGGGQPSSGQADVYTNVSFSYHTRSQSADSIL

SDYQTAGQKDRAAGGGVTSKTYMDSSSGREEAKKASIEMVGRKHSMKKAYGIYDENEPLL

SSPTKDIKSSISSLEAGKGKASSDGAHLQQIQEETDSLVSTGSKIQRPGELVRHKTQGDI

SDPQVLESWRDDLDKRTGRFHSRTSSELTLTPRDRSAKVYENSHRPPRLFDGSIDSGVGS

EISCRADGVATPSETPRPSFLPMDNPTGAELKRIQQQAVLNFIHRKTVKSLHSPETSLYE

SVNTTPRVLLSPVSDPVTDHVSDLITKTNETLAKRTDSLRRTGSISSRSSSTDYVDVHRS

RKETEWSRLRSSGSFNYGSNRSSLCSDNTYEDISVFTPTSPRKESATPAQSPTEHSGTED

SRGSNTDSNIMASTASYQNIPGVPRRRPPPPPPRTPSPEPRTPPALPPRSHGQRKED

>SG-PL_contig_4830_1_+3_780_932

MIDMCNSVTVVNNRCLLNKWKENGEIEKLWQDVGLKKAVGSWNRDVDFYLR

>SG-PL_contig_4831_1_-3_768_992

MSNGEQNLVPSDRALMSYLLTYLFRTLNPPNFKQFFFYGKSYFKLCRKLIFRSFCFCLLG

IFSLDFCVCGLYLFR

>SG-PL_contig_4832_1_-3_333_476

MYNDIYFNKCFLTKILQLINHSRYYRYKLLHAYQVNVLNMMYELKIMK

>SG-PL_contig_4833_1_-3_198_2642

MGLGVDEAFEGAGASTGMEIWRIEKLKVVKQDPETHGSFYSGDSYIVLVTKQVPGSSRLE

WDIHFWLGKDTSQDEKGVAAYKTVELDEGLGGGPVQHREVQDHESKKFLSYFKSGIRYLE

GGVESGFKKVERDKYETRLMQIKGKRNVRVRQVECHCSSLNQGDVFILDCGLVIYVWNGP

FSSKMERIKGAEVAKRINDEERGARAAIKIIENGWDSDPGFYKALGSQEEIKPADDGGDD

TEFERTLAEAVKLYRVSDESGALEITEIAEKPLKREHLDSSDCFILDSGPSGIYAWIGKK

CTKNEKQSAWQNATEFLTLRGYADWTPVTQMVEGGETPLFKQYFSQWTDKDAQTGLGTVY

HVEKVAKVRNEKFNTSLLHDHKQVEKKDEMMVDDGSGKVKVWRVEYGDLVAQPEETYGIF

YSGDCYLVLYTYTKGNSRMERYILYFWQGAKSTTDEKGASAIFAQRMDDNELNGAAVQVR

VVQGREPEHFLLLFKGNKLMIIFLGGINNKEPDSSTRLFQVRGTKSVNTKAVQVQPRAGS

LNSNDVFIAETEKVVFLWIGRGASEDEKNVANNAVQHICPDRNADLVTVEEGLEPQEFWD

ILGGPEPYATGASLVHHREDMPPRLFQCSNASGRFLVEEIVDFSQEDLCEDDVMLLDTFT

EVFVWVGSGANETEKKEALATALEYIRTDPAGRNEDNTLILHIKQGFEPTSFTGHFHGWD

PEKWSEGKTYEDLKREMGQENVAVTTVKEELANYYKTHPIDVLKKKTLPKGVDPSCKERY

LSDADFETVFGMSRCDYLELRPWKQLQLKKKVGLF

>SG-PL_contig_4834_1_+1_919_1200

MWNFGNSPSNVQHFCVCGYNTYQNSLVSSYSHMCVNTHTHILHVHTHTHTHTHTHSHKGR

TYRWNGKKYELYLFKSSSNTTKDEKLVGMISMKQ

>SG-PL_contig_4835_1_-1_1_141

THMHALTHIDFFVVFVFFLYVRLNLKKQPHSLLSCAMLTLRTGNNQP

>SG-PL_contig_4836_1_+2_230_397

MTVTQYQHSFTDSCRNKQLKMLRIAAGKEGKSHQTTRPSKPNKTTEKSPVTKTISV

>SG-PL_contig_4837_1_-3_333_1004

MAMYPNATHGYMPFVKPAHKRQAAKRSKADKDSLKTKAAEGTKTYKVTMRDRRGFTYTYI

IQADASAKNVPVLCESRVTAPDSSSSPLLSNSLPNMRRSARFPVAAPETRSCASLAEGKE

DLTKVGSEKKGLFNSLLQFIKKRTTSKAPTPKTLTPGPKSKRKLKPLPQRKRQRTVSAMS

HLGTIQESPEEDNLELEDTDSYSHDSVTSGNESESGDGQGSCPP

>SG-PL_contig_4838_1_+2_53_169

MVAWYTVSHSCSDSGGSLLLLYTTDQDGSFFLSLFRILF

>SG-PL_contig_4839_1_-3_93_182

MLSSRRLVSKFRKIFCLLCSLVTDFILFRF

>SG-PL_contig_4840_1_+2_83_355

MVNRILKCAFDIVLHAYKHEGVSSTSRSAYMLTWEIEKISTHHPPGKVRRIRTQVFQILQ

FGSQVSLTFDHHTSPRRMSVYSPQRYDLKGK

>SG-PL_contig_4841_1_-1_181_315

MSNLSIAFSSAWVHHNYCYSVTVFCCANLTLTLHIYCTNATLVLK

>SG-PL_contig_4842_1_+3_459_644

MAAMHITVLWALVYRFHNANAMLIFSLSVHTHTHTHTFTHTISQKRQANTHYCLQSGAVV

EW

>SG-PL_contig_4843_1_+3_3_158

HTQTHTCQTTIPTNYFRLCTINSNRLDQKETRYQHCSVTITLTKTKIYNYYL

>SG-PL_contig_4844_1_-1_1_183

GWRPQHSLVLFALRNLMVKIICHELCCFAGFLISGLQKFCDQRGCLLDFDGLYVVTALIW

P

>SG-PL_contig_4845_1_-3_291_1271

MAFLSSWTTRCLFVIFLPLLSISATNAVSVKCGECQKSLCPELNYYSCEGKLVKDRCGCC

TVCSSSKNQPHPLISNDDDHGESEGSACSKVKCPKFKVCVENMQGLPLCTCPGPFICKRR

SRQKPVCGTDGVTYESRCYLRIAACNSAARIKVARRGPCNGAEVPRPRTCDSFSSCRRRK

NQNPVCGTDSKTYMSRCHMKIMACQKGVKIKLKHHGTCEAPSSSGRAPTHAQRMPGVLIS

KLKDSDKNTPVNDVRGRKERRRRKKKGGNEVSGKEEGKKKRRKQRRDKRKQRQDRRRKRR

KTRGNSRQRRYQADRQYLRNRGHIQWK

>SG-PL_contig_4846_1_-1_1_114

KTEDTGISLNNKGRRLRQRDETCRTEVKLVFKVSHHFL

>SG-PL_contig_4847_1_+1_46_216

MAVCMCSKTVREAKSLPPVTIESINHLVRMSHHLPQSTRSTQIVKWSWGSHFLFVRK

>SG-PL_contig_4848_1_+1_1_243

FPVCSPLNVRKCFNAFFFAREHDNVSTSDAALFKAILFSLQYLAEIGSVPGFHEADLAMI

GSAFIHCTVTSANVGKLAPFL

>SG-PL_contig_4849_1_+3_210_464

MLVSLNTTHVSQNIVFPTDLTLKSLVQSSQNAYVSPFLPGFMTGHFPVYSPLNVLKHLTA

FFFGGEQDNVSTSDAALFEAILISP

>SG-PL_contig_4850_1_-2_134_283

MFLLSISPILYFIFRGWGMGVPIQVNDVGFHSPLLFLEHLSYLLHIRAWL

>SG-PL_contig_4851_1_-3_774_1328

MLSLRVLLLLCLLAALLGYLEGQSVFSWMKYDHFDEVDHHLRSVNAENCRSKGREELVLR

PDTVAQLPVYNQLLNRIWYPNRTSLIHIHNMALNRAFFFSFILQKMNSTSEKEYLAQPNW

LYMYMSVTADVNANPHTLNGSALYFDEHCHYPNWYETIPFNDTIPLFAPKAWRHDDWADA

DNFLR

>SG-PL_contig_4852_1_-1_1_102

CGRLTNLDLAGKSFFLRNEQQPIYHSEYVISRRL

>SG-PL_contig_4853_1_-2_461_724

MILSSRLILSRNIQSVMPDLPLLCACENNDMMYKKILKLVIIFKLPGGVQILRYVRSLIV

DLLSPRITSNLSYCPIWVLIINKSVCES

>SG-PL_contig_4854_1_-2_554_856

MHRKRFYPRIIPSANGSLLVDHVSIFGTVSLKNKMVVMRSDIAEHGETFEEKEVKNSWRR

DWTEKVVGEETLRSFITKVKTPGLAYCIPCCHEINSIPSTR

>SG-PL_contig_4855_1_-2_2_133

RSIQAKHCLYLITLNTSECCIVLVEENIRSGHPRSVVCVCLWWR

>SG-PL_contig_4856_1_+3_78_1097

MAHTVISAKYPPLIDATRTPLAYGDRALPRLDRELNDETLLVRQRSVMSLCDYLHDPEHI

AEALKVGIAGSLKKLLTDGDLTVRQKATECLFVIAGHAIGRNAFLDHGIIIPVSSLFNDK

EDIARKNAHLAIEMISETPPGAEGIVQAELVQTLVSKLKNELDEIKELILDTLHYCMRVD

TVKALASGAMETFTSLLSHASPTIRAKAARDIMDLSVPLDGKNKAVECDSVKQLIGLLKD

ESADVRANAAGAIMTITITTKGKYTAINGGAIEPLVSLVDDSTSEVRLNALKALTCLSEA

PEGREVLLSHVDQIRQRTTDEIPAVCKAAEIAVRVITWKP

>SG-PL_contig_4857_1_-1_649_2784

MTEIVVHTLVGVFKFLSVFYDIVSFIPYFVFYRPINKLQASKRLKAKSVSGDPAGPWRSV

EALSTLTTTLFPQCSTVDDLFVRGVEMYAGSRCLGTRELLREEEEQQPNGRMFKKVVMGE

YHWQTYESVFTRISNFGSGVLALGQKPRSKLVIFAETRAEWMIAAQACFKYNFPVVTLYA

TLGQEAIIHGINETEVTHVITSQDLLSKFDGVLDRMPFVSHLIVMTSGARKPTLKNKPDR

VRILTMEEVEMEGAKPDNIRTPATKPTRDDIAVIMYTSGSTGLPKGVMISHGNLMCGMSG

QCQRIPDLGKHSTDIYIGYLPLAHVLELTAEISCLCHGVPIGYSSPLTLTDQSSKIKKGS

QGDISVLKPTMLAAVPVIMDRLYKGVWDKVNSGSVVSKALFQFSYNYKLRQLERGYDTPL

LNKMVFKKVAQSLGGRIRVMLSGGAPLSGTTQRFMHICFSCPVLQGYGLTETCGAGTVAE

ATDLSLGRVGQPLTCSEFRLRDWPEGGYTASDKPYPRGEILVGGGNIAQGYYKLEQMSKE

DFISIENVRYFCTGDVGQFESDGALRIIDRKKDLVKLQMGEYVSLGKVETVIKMSPMVEQ

VCVCAHSDQSFTVCLIVPNEKQLRLLMKKMEDSEAPEDFPSLCRYPKAVQAVTKVINAHA

IQSKLERFEIPQKYTMCPEPWMPDSGLVTDAFKLKRKNIDMHFKEDIETMYS

>SG-PL_contig_4858_1_-1_85_243

MLRFSGTNWRLIFNTEIAVYFDGIQGDKSSAVRSISEMHLIHSINIPVFCVSS

>SG-PL_contig_4859_1_+2_506_802

MCLKIVHYDLALQILYFSTNFADRYFRTDFARYIYFSTNLNINMPTCHQTDLYPANKFLK

HFSGFQQLCGKLSLNRNKPSKNRFSQHNPKDVYTYSNEF

>SG-PL_contig_4860_1_+2_1373_1654

MFTEHIYIHTYVHTYMNVYNLAVLQCTRLAGFLLGWMCSVWYGLYVIFVPHFTAYTVGWV

GFPAFLSLSPFCVLFFAGVFSQRGETTQSATRHW

>SG-PL_contig_4861_1_-2_188_337

MRFNRDIIIVKVGILLWSVSFFMSNVKSCIYFSSVFCYCATNSCSASSVC

>SG-PL_contig_4862_1_-1_1_141

MPLLSCVHFLITQSMVRTNRNGERIHPCLTPVLTANLSEQPLFISID

>SG-PL_contig_4863_1_+1_58_255

MVDIPSVVDKTPCNFGITRVQGGKEDQSHEVVSPVVPQDPVEHSVQVLRVIGRTQSTLQC

EFVRRI

>SG-PL_contig_4864_1_-1_1420_1821

MVDNEVCLLGTSFVHFENSSSASKPVGGLYDIQSKGFTVFEFLLKDGFCHMNLGLVTNLV

NRISSAVWHCVLQCGSEKFNVSMRDHEKDVDNGMTESSHHEHMVSCGPQVPVEGVCDGPC

QTLQLHRLSVLCTV

>SG-PL_contig_4865_1_-2_2_262

PPRNPKLHLRLFEVQRSSPRSSTANQCKQPSFPTPVPSHPLGVYAWGGSMTSCQPVLAKC

PTLVLKCWSMSTRTQHESGRALADCEK

>SG-PL_contig_4866_1_-2_2_223

GTPPSVRGCCSSVLCVLCHLATCMAPPGSVYDSDICDWQQRLWTDFRKSCEIYTRKFAKM

CFGMPSKYVFSSLY

>SG-PL_contig_4867_1_-1_2236_2616

MFLCLPEFMFVPHLDLCDLVHVIHLCQCICRDIFESRLVKVKYSYPFVHLLCGHFSGHHF

TPICTALCKFYFGKKCKLFASCDQISLSTSLEVRTSSCCFVFCCFCLFVFCTGAVLVYMA

NPLTPSA

>SG-PL_contig_4868_1_-1_97_1344

MEKLETAKRFRMAHISPTKGIDPLALEVTRDMNLETQKINNQTAKWEHEDLAKVRIRVFR

GHTDAVNKCQFFCDESRVLSASVDCSIKVWDREHGTVLRSLDNAHRINAADVHCSEDGSK

LISCGWDKNLHFWDLEKGEPIWSTQHLDRLTCCRMSHDGRLLCVGSDLFQQLYIYDLLSA

DVVHKINNYHKSALTSCCFSPDDSRVITTSTDRTAKFYDLQTEICTIKLEGHDNVISSCS

ITPDERNFATASWDKTIQIWDISTGMYRSKGPTVLKGSHEGSVTACSFTDDGLQLVTGSA

DMSIVVWDVENGVQKLKLQGHAGWVTDVHFSQDQFWLLSSANDSTVRMWNIEESDQIPIV

MENKQAQGLKVTKCSKCGKPFSMSEMENFRDMTVCVFCRLQSPEKTWLSFKDEPEL

>SG-PL_contig_4869_1_-3_156_512

MSEGCRVFFLDVALCLCLMCEVIPHLFLRCVCIVLLFTSCNQVNNSVTVNTCLNRPTCGR

LPRTTRTYCHLYPKLKLRKFSSSYHHHHHHHFRHNHCHNQNYFSQMLLLSAPVVCVCHL

>SG-PL_contig_4870_1_-1_1_267

YTGSPGVHSPPLLSGRLAHFGRQLRSLSDPHFQGCRPLHSVGLRPEPREVRFEPGSILPL

PRYELRHPPLVSGSYPGEARETQGDPGWP

>SG-PL_contig_4871_1_+1_622_738

MIKNVPRCCQSAGLLVVCVAFFLLNYSHPLTNEHACACA

>SG-PL_contig_4872_1_+3_3_191

RRWYIESDIKTLAQLSAVQDVHYMTYPHGMNTKSDRRVMCWLGVKVLGMYMNHLGNVWII

YTA

>SG-PL_contig_4873_1_+2_515_697

MSLFICYLAFVGWGEREHWLRNICPPLLVVNYVFLRDTHSKLLLWRVFLPHTRTQKKNNT

K

>SG-PL_contig_4874_1_+1_55_219

MAVACILATLTQEHRGKTTLGRKYIILYLILHVLFFISQKYMLPLQNIVHVEHTH

>SG-PL_contig_4875_1_+3_3_182

LPDRPGASILIPKHYSSFVYSHTVKFAFSAKLFTDTGSMIMQLVCAVKLASVNRFSQSFF

>SG-PL_contig_4876_1_-2_83_763

MNDWRPFVYGGLASITAECGTFPIDTTKTRLQIQGQKIDARFTDLKYRGMSHAFFRILRE

EGAIALYSGISPALLRQATYGTIKIGVYHTLKRAVISNPKDETLAVNVICGITAGVVSSS

IANPTDVLKVRMQATGSQSKCDNMLCSFVKIYKTEGVGGLWRGVGPTAQRAAVVAGVELP

AYDLCKKHIIRSGLLGDTKETHFLASFLAGLAGAIASNPIDVVKVCS

>SG-PL_contig_4877_1_+2_2_265

SPIFSYHHLNRLTIRHNVYNSNGNLNLPLHCTHSNILTYTKLNFNCGINLQNQLTTSPLV

YIYHDISKLLDVLPHNYPISIIFDFSCV

>SG-PL_contig_4878_1_+1_274_366

MCWVRCKSMGVGLVHLPLGRGNCLLEWAWRP

>SG-PL_contig_4879_1_-1_412_831

MGNLSNFLAWWRELTSNDWILKVLESGYKLPWVGSRPPLLPSPPPFKRSSKEQEEAIEAE

ILSMLQKKAIEELPLSPGPGFSVCLFVIPKASGGWCPILDLSALNKFLVPVHFKMETPLL

IREAIRPGDWATSVDLKDAY

>SG-PL_contig_4880_1_+1_166_375

MLLRDLGLQGRQTCSVAFSKVTVNPSANTTMVLIGFAYSSSIFMLSDCSLHSVVFDSGRN

TELLKTANRS

>SG-PL_contig_4881_1_+3_183_638

MNIQQYNEQFAQLNLPDNQQNQTAQRLSQSVVARLGVTRQAGFQRGFFKGHNQSRSKYNY

GSDWYIYRVNFCCQTVVCILLCLTLGRNTKQLKVKERQIGRNWRSVANVHTYPICIHITD

NILSLKMSCTHLPSHHCLWQLAGAKLLVIKDK

>SG-PL_contig_4882_1_-3_300_695

MESLSLNLLGYLTLCVKGGDFSDFQVNIHADLLVPELPLCVYACKISHTRVKDPVSHVCV

RWLMETRNSQHAFPRKESVAADKQNKTKQKTPYLTNQATSYPELENKKERKEKKKKWEKI

GGWRPHKKKKNK

>SG-PL_contig_4883_1_+2_263_550

MDIQCPVRCLQYHYLIPRTKNKQHRLYIINCWGGGQNYVKACWKKESGMDREGRKKQKTV

MEGKRAETKRAIEKRKFLAQNFQKVGLLFILKELPA

>SG-PL_contig_4884_1_+2_2_115

LSPHKVHQSQEDNHVEHKTFSNLKTKILQKITIHDILS

>SG-PL_contig_4885_1_-1_169_2025

MPQEENSVSVKTDDLKSKQESESVSQIIPTTDSDLSKELDPLPSTSLPAAASVESVVIKS

SGELSSDISGQCEEVQELVLEAAEEVKEMQKTEDCAPQLLKIKEESVVKEKSKADEEEDV

LPSSHEPSTVNKQEADTTTPVHDPTIPQLKKIELDLPPDDKKESKEKERLKPGKKLGRDS

KKSRQSEMRTSFSEIPTLEPMTTPREVKPPPVDVTPSPYDFNDTEADSPSPWQPEITKKW

EPSIKPLSAFRDVKLELKKSLETEKKKVKKKNKKVVEELSGEGSKLFSTESPTKPNEKKI

KDPSLGKKGRKRKVPDSEIKVDTSSYEETVNSVVESCKSADYIHLPLPSTLPSKEEKPVK

KRKKVFQYSSAQEVDSSQEGKKEPATAKSVKVLKGKVGRKKKEVKASDTVTEQDANTNLS

SLPPLDVHIPSAVTCYEKTPSIEEPQSVSVPVKHPRLEVDVPSVSLLGACSPISVPAPKA

GPSGMHMLSMAAIRPVPSTSGAAGGGPSERLAPLLEGVHGEVVKKVGIELQVDCAGDMPK

ASRSIGNTVLDNTPPTTPENDLDEANSASSSSEGHDNLKEDLDSLDSVERTCKGGSESPP

GCDFSLSSTSSENLQIGRA

>SG-PL_contig_4886_1_+3_3_197

ERERDVRRNNKQIPVSQIWAKNKITTADEGGKEINLLLSSNPLLKHFFPQCFKYTQSNTH

IQINI

>SG-PL_contig_4887_1_-2_926_1192

MYYMYFLFITNVTCCRDRNSVLPRVEIQLFQPLGRAVRGQPRRMQVLFLALRGSRIMTGY

FGQSCTFLRGKNTLLCFMLCGTRLRERGG

>SG-PL_contig_4888_1_-1_1_171

RDCCVICTRQNKTLSDNGTRRQLRRTLFKGRSSDNYALLVFGRCFMNYYIGAMKTQI

>SG-PL_contig_4889_1_+1_2677_3015

MNTNMHETSSQLTHKHACKPFTIADTDHSENVQRTQLTTNKTVNTHIIYLLKAFVSTKSN

YCLKHILITNTSNPPQCACLFWQDETKSQDLFSVSSTDGRNLISVLLKCKAFT

>SG-PL_contig_4890_1_-3_3_176

FTGPLKECNFKVATYYKRIMHRQLKASVMHINRLLDMAQRIWNHPHVQTPRIILSLYR

>SG-PL_contig_4891_1_+2_68_232

MRPSMILKSKTQIPLKRSCSVNHESICIKVLKKGVHLYLLKILQKVKSKVQRHEK

>SG-PL_contig_4892_1_-2_2_244

LHCYLDDWLILADSSDHCWAHTSRVVDLCTQLGFVLNPEKCELNPKQTFHFLGMSFDTLR

MIVAPTPERLGRLEETLDSLL

>SG-PL_contig_4893_1_-1_106_1299

METHTEQSQRESAPKQKIHAIDKIIRDLFWPLSEGSDEERVRAAERLCQRILETKSKDAD

TALSEQKYCVRRLVRGMASTRKFARVGFAVALSKLLRQVKDIQVEEVLASIKEELKVSAH

DPKSEVGNKLLGCVLAYGTLLQSARKLKMDQLNTVFTELMSLGKKKSYLRSACTETLVSL

IQEVSSEQLTELWPQLAADLQKGWEECTPDRFLLLLACRHKDKKLVDKEFLQTHWGASKI

VGKKTYEHVMHLLRRSVETPQLQSALVEAVSQEIQKSDDKDNFWTKVGNPLLASTVPRKQ

VAMALFCQLLERSADLTLSHKMLFPTVAAVICKTAQKKDEQLADICTKAESLLANLCKKE

TEGSCVLDIVKGFLQTCHRLKSYEFLRSGTFSTIVSSL

>SG-PL_contig_4894_1_-3_366_1073

MITGLLFYHKKALYLPGLTPLGVWLETSYIPHVLDIQVHKLEYSATDTVPGQRDWKKNRY

EMKPEQCSYVHNSAYATHYQTDWVPGGYGIGGSFPYYGDGGYGGYNMGYHQQHYFYPHQY

YEQGPRQRKPMPRKKKGKPLKEDMTKAKQGTPEASKIDTEATDTLESPKETENTETDNSL

NDRIETRTSNDSEKSDTAESPSEEGKIDTDTSPVDTDDSPNEETETDAAKSAKRQK

>SG-PL_contig_4895_1_+2_56_199

MQLYAPTLFKQQEQHLQSNGKRKESEREAREKKNNKENVRDFLTAVQH

>SG-PL_contig_4896_1_-1_163_264

MLILVLCHDNECGSEAISILYPPTLLFVRMRRLD

>SG-PL_contig_4897_1_-3_237_641

MLLQAASRLVGRQVVQLTRRNIGLSAVAAQKAATVSDPIQQLFLDKVREYRTKSVGGKLV

DATPQVEQSLKDELEKLDRVYNAVGQDMTQFPSFNFTDPQLESVGLGDTKDVEVVTAEEE

VEEEEEDNKPYFILP

>SG-PL_contig_4898_1_+3_192_503

MTTKRTRTQAGHLMMGDCPRNLCLDGVCLWWRVISAGVFCVAESCSLALSWLLPSARGAP

HQQEHWADQCPQVSLFSSCCYSTQSCRDCAYVLVEGEVVPPLLA

>SG-PL_contig_4899_1_-1_25_1029

MAARRHDSSVQFVQIDGLVALKIIKHSQEEGAGGTDMVQGMLLGLVVDNRLEITNCFPFP

RHPEEDDFDEVNYPIEMMRNLRHMNIDHLHVGWYQSTYFGSFINRLLLESQFSYQHSIEE

SVVLIYDPLKTTQGFLSLKAYRLTPAMMKFFKEGDFTPDAIASHSISFETMFEEIPVVLK

NSHLVNVLLCELSGPTKRDHKFNFLDLGTSAVLEKNLRQLMECVDDVAMDTNKYLNFQRQ

NFRQNQAKIQYQQKRNQENQQRQMRGEPPLPEEDINKLFKPLQPPPRLDCLLVANQIDQY

CNSICEFSSQSIAKLLMAESLQENAGASGASGSSS

>SG-PL_contig_4900_1_-3_42_254

MKSIGASQRFSNLPDSDQDQEIVKAVGKAVTQLKNQETALQHLVSDLNGVRNSDGMAPIT

IIKTLLMPNIS

>SG-PL_contig_4901_1_+3_2160_2651

MPQAALRFNRGWDRKEDKHKQKSNAESWCIKSTRVRKQFPISGTLQNISRKTLRNILKGK

KDVCHKDGKDCLGETAMYFYTGFPPFISFPRGLHGSRYRNRSILDVFLVVLLQKPFCGPV

LDQIMALNNNKKTKKTVLTSVLEDLSYYPAAQCHLPFLRGGGGG

>SG-PL_contig_4902_1_-3_153_335

MIMTDSTFGPQQYKRIFTGHELTVMTQRGDVTVFYSVRTSLLTPLVRMTDVNCCIDKMAP

E

>SG-PL_contig_4903_1_-2_2_268

GPDGANLFIYHLPQEFGDQDLMQMFMPFGNVVSAKVFIDKQTNLSKCFGFVSYDSPMAAQ

AAITSMNGFQIGMKRLKVQLKRPKNDKPY

>SG-PL_contig_4904_1_+1_1_102

HTFFLGGGERETKQLVLNMKRQYALPVYDMTIID

>SG-PL_contig_4905_1_+1_205_471

MNFIEKDNEKVFSDEKKILKQVKKFQENLYFYNVMDVDLNNNLLDVPVLLREDSDSIERL

ITRDEALSPLPKIKNNKSPGSDSFTMEFF

>SG-PL_contig_4906_1_-3_3_137

LSLYVNFSSHGPSNTIKQAESQLPICHGSWLVKHSAYLRDEHRAE

>SG-PL_contig_4907_1_+3_393_527

MHWTQLDLWFASTSSCINTFLTAFWQRQPSKAYIKFCKLTCKKKK

>SG-PL_contig_4908_1_-1_607_738

MRFIYKRPIVFFFNVLCFTCKDCCFVGLIVVGSVCVLLLLFFFI

>SG-PL_contig_4909_1_-3_150_1316

MMAWKALLYAVTLVTVMIHVAVAKPVDKRGPPHPDPVEAGSKQDDDMGTGLEYDNYLRQV

VEVLESDDTFRKKLETANVSDIKSGAIALHLELVNHTIRTKLDEIKRLEVQRLQELALIK

MKSMSGVGVNRMDIPGHLDLRNPHSFEMKDLENLIKKTTHDLEEVDKRRRADFKEYEMEK

EYEYRESLKELSEEDKKKTEEQHEEMKKRHMQHEKVHHPGSRDQFEEVWEKDDHLDDQDF

DPRTFFNMHDLNSDGMWDIQEVEALLQREVDKMYDARNSPEEDDPNDRYEEMSRMREHIF

TEIDTNKDFMITLDEFITYTGKHGENAKFKEDDGWETLGEKPEFTDDELEEYIRQHHPDS

MTPHPDPSLQFQPEDKVREQMMQQQMHQQ

>SG-PL_contig_4910_1_-2_155_1315

MQKEIILLCFALGTVVRAQSNATCVKNAYPPNPESKVPWFTVNLDLPPEERWKEVAQDRK

VQIRALLNQFKTFVEDWGKFADEIIDFVDSDLGDLDNTLPYPFAGEIRGMSKYSGLNLGE

VLLFNIFYEFFTVCTSIVAEDPKENFVHARNLDFGLFIGWDVKNKTWAVTEALRPLVINV

DFQRGGKTVFKSVNFAGYIGILTGLKPYLFSLTMNERFNIDGGYIGLMKWIWGDRKERWM

GFLTRSVMENATSFEEAKFMLANTTMLAPAYFILGGNQTGQGTVITRARDKAVDVWDMAT

IKDHSWFILETNYDHWAKPLFLDDRRTPATHCMDSMTQQNVSFAGIFNVLSTQPVLNKLT

AYTALMEVKTGRFESYIRYCKDPCFPW

>SG-PL_contig_4911_1_+2_269_433

MGCTHFSTYRSSHIHPNGKPDEVRTLQHLAWTERFSSYIVLASIFWDPGSLQKSR

>SG-PL_contig_4912_1_-2_179_343

MESYTDHYWEHSANSSIFSHRLVYILNTSLTVFMCDEMAYSGIIQKPRSEFKDRY

>SG-PL_contig_4913_1_+1_7_390

MLAYTNTMLAYTNTMPAYTNTMLAYTNTMLAYTNTMLAYTNTMVAYTNTTVAYTNTMVAY

TNTMVAYTELAVTLRIFLTVVNFLLLPVVQALRKGSNCNHFLGKMKTSAPRSYFQGFSFC

VCDHGKVR

>SG-PL_contig_4914_1_-2_230_829

MTGFYDYSKFNFLLRVSSYSRQFEHIVDAVQVVLTLDLAFEEKPTVRNFSFKELFPVLVF

RSLLLSGVSAVARLTIFNCQNGIHNATDPRTHKIIACAYGFSWSKESRKDIPDHHFAFWS

SGLCVGLCLPAVLTDHVHSPGRCPPLLRSNTAAMEHVPKKPTAVAEGTATVWHWRQSFYP

SCPCPIYPAKIKEKEVDTSF

>SG-PL_contig_4915_1_-1_1012_1512

MLGGGCLDGPIFQFGATCKHRDVYCWLYSVDRPYFSFICRHLPSLSILVFLPPFMDKFSD

WSPFVSLSVRVSVLPSCLSLSVSLSSLRVSLFQCLCPPFVSLCVRLSVLPFFPTPSHSLS

LHLLFVFLSLFVYLFSFPCLLMFFLYQLLLLLYYIRFICRLVFIVCC

>SG-PL_contig_4916_1_+3_723_923

MEKKRSSVQVNTNSLELTASRFERSTYPFSLQVTVEDLPAHPGLPLLLITPPPVCTVILT

TDYVELF

>SG-PL_contig_4917_1_+2_17_415

MVNKRQFLLIQKTVSTVLWTKTPSPLSHYSIQPPTHSTNHLPTHNTTYPHTIPPTHTQYQ

PPTHNTNHKCTHGKTLMTTQYCQIHLAKAPIVFLSFFLFPFPFLLYEEKRGCKERQEEKE

RKQRGEEEREGGE

>SG-PL_contig_4918_1_-1_388_597

MWVLPFVIRTACCQVEFFQTQFSLVGLMGHAFYLKYSACSLFLICDFKINKFFNCVYYLL

FLRVFVFGYC

>SG-PL_contig_4919_1_-2_107_1939

MAFPQLTKSGAWFTIFAQRGIHTFPLLRGYPWKGKQTFQLPCLSHVGNRSLYNVLDVQST

FSDYEHEKRTFNLTVPQKFNFAQDVIDVWASAEQTGQRDDSNPALWWTDDKGKEEKFSFQ

RLSQLSCRVSNVLTGPCQLTRGDTVIVILPRIPEWWLLNLGCIRAGIAVSPGTTLLRSSD

IRYRLNTSKAKCIISDRSTVEFVEEVYGDCLGLKCKVFVGNSQETRPGWLNFGDLLNRAS

DSFQTADTAASEVMSLFFTSGTTGGPKMTEHSHSSYGLGHLNTARYMLQAGPKDVVWNMS

DTGWAKCAWNSLFAPWLHGVCIFTQHNSRFDPDDTLRVLRQYPISHFCMPPTAARVLVKQ

GLSDFKSMCLRHTMSAGEPMNPELMEAWQNATGLEIYEAYGQTETTLLCGRYPCIEYRPG

SMGKAAPGVDLQVVDDEGVPVEPGREGVIGVRIHPHRPVGLFTHYVGDPERTKSVFRGDF

YLTGDRGYLDNDGYLFFVGRDDDVITSAGYRIGPFEVESALLEHPAVLESAVVSSPDETR

GEVVKAFVMLTPAYRSSDPEKLTVELQDYVKKVTAPYKYPRKIEFVQDLPRTISGKIRRV

ELREQKGKRNK

>SG-PL_contig_4920_1_+2_2_259

FHCSCSLSFHPGQSCHGSFLPCCLPYLHHPFLQSHLPCHSDHHACCPFLLWLQSLHAHLA

CRHQIHLPCPFHSHPFHPVFHLSYPL

>SG-PL_contig_4921_1_-1_955_1137

MGTAVVMLALRKVEVSTTSLSCNFRVMGKPLPTQCGMSVCNGCVFVSVSVSLPVQGVYDT

Y

>SG-PL_contig_4922_1_-1_1_171

GGGGRGGEQAIYIKINHLSRVTNIHYTLHFFCQRETTDKCDGLMSFKAFSPASKTES

>SG-PL_contig_4923_1_+2_299_562

MKLVIYKRIIVNTDIMDSLFSQWNDLTMKVLYNTLAVFKGCCAIYCCVLVCEGYYICTLC

VVVDLASDILYIGPILGSGFFSPLFCWW

>SG-PL_contig_4924_1_+2_248_634

MYKVFNRQSQHCPDFTSQNNVPNCTKVTCPVEEDGSGTTVSVNCVLLLAWQETVMVTALI

ILITGKTCADSDCSIYTPHPLPLRKAFSQMFLSPNDNPQDCRHNSEVSMCYIEICRMLFD

HFCEKEAQI

>SG-PL_contig_4925_1_-2_107_499

MVNSTLPVRLCNVHVVNSDITLTLPVRPSNICKVNSDIRLTLPVRPCNVCMVNSYITLTL

PVRPRNVCMVNSDITLTLPVRPCNVCMVNSDITLTLPVRACNVCMVNSYITLTLPVWLCV

QKRSHCQISLV

>SG-PL_contig_4926_1_-2_128_265

MSGQFCHYDLWVGSMFVSLHHWFCFCKAFLVSSMMIGDVFELFSVS

>SG-PL_contig_4927_1_-2_5_664

MKGGADSGFRHVRPEEYEQRLLHFSGTRKYIEVKEVPLCKSRLKSDDVFILDQGMVIYQW

NGSGSNKDERFKAMQYVQDLKSERNGRPTTEVLEESSTDDAHDFFKALTQDDDDDDDKEF

EAADQTTELFRLSDASGSMTFKEVKKGGVDKQDLDTKDVFIIDTKKALYVWIGKGTSSGE

KKNAMQYAHKYLMKTDHPLLSVSCLPEGREFRGFFSAIAA

>SG-PL_contig_4928_1_+3_981_1331

MVVSVRLVTLSSTVKPSALLESGAYEGVLVHQSQMYIETRPLGQLPQTRGSPSVVCNAVC

VFGTNLLPSFWSLHTRFPSAQTELWLPQETVSAQTFLCQWLFPSLIHRRTVVGARSQ

>SG-PL_contig_4929_1_+1_73_222

MVVFCACAMLTKLLTFSCLYFVLISSYLIYFITKRNNCQWLHFQDSKKGG

>SG-PL_contig_4930_1_+3_750_1016

MTKKVFIMLYLNQDKMSITLNLNQIHHQQTLPHPIPRQTFQPSHCQTLLLFPLRLMRLVN

SLLLPLQTPLSFPFSSKAYQSQSWIHQMK

>SG-PL_contig_4931_1_+3_105_236

MLKMTQKIVTHFRQFRLLCYLCQDSGLVASAHHAIKSVLLLCGM

>SG-PL_contig_4932_1_-2_8_754

MAMEEDDHEDPMLDAHRTSLLEVIEDRNRTLNKKNLIHRVFIISRIGNNINNRTDVGLHF

DTLLKHFKNDLQTEPITGLMLIYMKHIVHVMECSADAILEMARELSITEKKDSPLISKSK

ILVISHDIPQRQYQGWSFRTLDIVEPGLEAYEPGDTSENLAVDLLTQLLRLGNFLAKQPK

LNLKNAMDSLHDKVPELLPQQAVVHYLLEDNDPCIMTPQEYVNIYEQPFDITLDSDLVWP

LPTKLFPYN

>SG-PL_contig_4933_1_+1_637_759

MFVFLFTYKIGFFCRILPRTTVLLLSDILRALGACWTQDLG

>SG-PL_contig_4934_1_+1_16_219

MWIHIYTYFTYYNKYVCIYNNVSFFVCFFYIVIVFIIIITLPITIIIILTSHSHFSFYYY

CCHHYFHY

>SG-PL_contig_4935_1_+2_35_304

MTSTRTYLSGSPKCIYLKLLLFCLHLEIYTELFISLVHPNPPTRNLYFILKRGGGSGGGH

LQKVNLAHIHRQTAAHASHGISAPKVTCCT

>SG-PL_contig_4936_1_-2_17_328

MADGDADQAVPAQTVEEPLDLIRLSLDERIYVKMRNDRELRGRLHAYDQHLNMVLGDVEE

TVTTVEIDEETYEEIYKSTKRNIPMLFVRGDGVILVSPPMRTAS

>SG-PL_contig_4937_1_-3_9_1046

MVEKPLPAHDPVTQRYRSSGDEPSAFRSVEQGKQRASVSDSHSPDSAKPKDSTTRSPTHR

PGSGQRTGSAHSTPSHKGKDERVHEEKASALENAPPKPARRTPSESEEANDRDSITEEMN

RLIDDSTVRLFIALFDYDPITMSPNVDCVDEELPFREGQILKVYGDKDADGFYRGEADGR

EGFIPCNMVSEIHIDDPELVEQLLKENQTNNAQQSSSTGTDGKGKSEGHLTPNGVAPLPA

AGPVRRMVAMYDYDPQELSPNVDSELELSFKQGDIIYITGEMDEDGFFAGELSGVRGLVP

SNFLQEAPLSDEDEVLDSASMVSPSRSQGSISAASRRSDSLSTLNN

>SG-PL_contig_4938_1_-3_105_905

MMPLQPTFLFLILVVAATSQPNNNITKDSSEPSTKESMSSSSSLSAKRAACFQTANLTQH

PSNSFTVDSFSYSSSSSSGIACSSSSLLVHYSLVEFWIQHASPFMCREREAYGEVMGCLL

DAAKSCRPQRKRIHFPDREMVKRGIHYICDVAEDEQFDVNITCARSHFQKVYDCIHGDVQ

LLVGNSVLPDPFPKYLMCRTYDIADLCNWEVLQICGEETAAAYTRMMSLYIRPPSCSSST

SVLTVTSVYLVVLLVLCFVVNGVETGY

>SG-PL_contig_4939_1_-2_8_1378

MLPCTWKEYSSLHPFIPREQALGYHHLLDELEELLCEITGYDEICFQPNSGAQGEYAGLR

TIMAYHQSTGEEQRNVCLIPVSAHGTNPASAQMAGMKIAPINVDKSGAIDMAHMAKMAEK

HKEDLACFMITYPSTHGVFEKDIRELCDMVHHFGGQVYLDGANMNAQVGICRPGDYGSDV

SHLNLHKTFCIPHGGGGPGAGPIGVKKHLRPFLPSHPVVPPIGTFGPGTASFGSVSSAPF

GSSSILPISWGYIKMMGAKGLRQASQVAILNANYMSQRLGDYYNTLFTNEEGYCAHEFII

DCRVFKKSTGIEASDIAKRLQDYGFHAPTLSWPVTGTLMVEPTESEDKAELDRFCDALIA

IRKEIQAIEEGRMDKTVNPLKMAPHTQSDIINSKWDRPYSQEVGAFPASFVTPTTKFWPT

VARIDDAYGDQNLMCTCPPMSSYSSDDNNGVEERAAA

>SG-PL_contig_4940_1_-2_20_1804

MMKAEVIGGSSDHTVSGGLPCKMESELLMYKKKVDQMTVLIKHYQTELESQKLRRDGVET

VSRLLAECRQENVSLKKNQATLEMAVKNLQNKLAVNGIVSCSEDDNNEVIMPGTSKQTLM

NLALENKRLRSLLQKDTESESVESIQKMAEDAETANTFKETIEQLEAENQSMKRKLSDLE

SLFKSSNNEKDQQLIQCQNEIQRLTEEAGDKTVVHKASTGVAALKEQLQVFMSQCQDFEM

QLENVQEAEVTKEEPAVELTPVTKQAVERTDSVDGVEVQQLLEEKQKLQTRVDEVTEMNR

RWQEFFSERDRYTQSLEEKVRDLDGRLSEAMHQGTTAEINRRIEQVLEKSQKEIGQVEEM

RRKAEEEVILLRQEIAIRDAQVSQLQNKITILARTTHQQNPTEAQSIIDHLKAQIQVCTE

DFERERQDRQVALQKVTSLQEQNTHLQKLSNHLQTLLASQMGQGQTQRTASQEHGHYDPT

NLRSRGILAYGRSTLDYDSPRSPDSSEKSRDFTDFDHLSASRDYPFQAPGPMSLPEKTLE

LVSRGSPKPKHSLSDSALDFDGRKQENFLTCPKCNREFSEEQQGELLAHMDVCWE

>SG-PL_contig_4941_1_-3_3_149

SLSLSLSSCQLIQWDLMLLSLRNIVSEVCFSCCTSRQDKTKFFISKAFR

>SG-PL_contig_4942_1_-3_3_302

IIIIIIHSQLGRLEVAHGRDLPRCRQNKAGALSLSARPPPSTEPYEYFSSSEVTEEMVRV

LCGQILISGIHLENYWETRQDKLFRNWETRQDGILYSSRF

>SG-PL_contig_4943_1_-3_3_128

VFGQIYGIGAQEIWIRRNSDNGKLKVYVNQLQKGKFTLQKRS

>SG-PL_contig_4944_1_+1_1_147

TLPFTHGRCILCFKIDKFSTDKGKWKEKRNLSTYFCKYHLLHTKTMLDA

>SG-PL_contig_4945_1_-3_1956_2252

MSFIEQIIKCQHSQHCLLPASIFSMLLFQLITVKQKADDTRAQHFNHCINPSLLQDKGSL

QFEAERMMIKNMLPVFPNFCCQVCTETQEKHRKYAANAL

>SG-PL_contig_4946_1_-1_628_888

MTRNYMWMRIGFKKAGGTILTSKLSTGRNKIRRMRNSWHVCILEQLQAHCITDSLKNDQF

QSKLLHAQHFNMVCLTPLLPSFLAHYY

>SG-PL_contig_4947_1_+2_569_841

MKLVSARTSELSCTLYNGVSKIYSFLVCSLYLRLISQMHALTCGYFCRTTLPRFKIDISH

FTVIGQLDNLKLLQSAHSTHPSKTLWIFQSL

>SG-PL_contig_4948_1_-3_288_434

MVNVNGYMLQKVFFFPFLCFFKCFVLKICQIYKCLLVLSLVLYFQTFTK

>SG-PL_contig_4949_1_-1_115_510

MALPTGVSCMVSITLAVVLFAVQQIFRQQIGGTEYMTIAGGFLGSVIFILLLTFTSNLEQ

IIFFKGYQARLFPEVLICLFFAMFASGLVHRVCVTTCLIFSLVALYYINLLSNKKYAPVV

SATPTASSKKRK

>SG-PL_contig_4950_1_-2_8_1441

MVGVVIGVFILAGPGCQWGGHPSLFAQGQLVPYRTSNDRYIDPCKATGFYEDIALDDEDY

KMVKGILLAENASNFELQVEDPGIHTRLGPEQYSSGKKKLKGKAAQTMYRPQTLKDKYML

KKILQDKKRELKEKCSGKDRKQCRDNKSMLRDELSELRKKWLKIRAELKRQREAFKERKA

QLRRQRRDRSFKKIRPSKVKKSLMSQPLTDAEVTKYSSKLMLKKKYGGAKSRNKRAATAR

KDRLWDYGIIPYDIEANFTGANKAMFKLAMRHWENYTCVTFVEKKPEDENYILFTERSCG

CCSFVGKRGNGAQAISIGKNCDKFGIVVHELGHVIGFWHEHTRPDRDEHVQIIYKNIMPG

QEYNFNKLTASDVNSLGENYDYGSIMHYARNTFARNNYLDTILPRRKPGMVVRPEIGQRV

KLSPGDIRQAKKLYRCPKCGRTLQDSSGKFSHQPTASGKSELCQWRISATHGEKIVLN

>SG-PL_contig_4951_1_-2_536_718

MIFRVYHNTRFLHIQKLVNKWAPYFDGLSFTAHQNDNTDMFWSFHQKQNMYFYPTCVIDL

Q

>SG-PL_contig_4952_1_-2_2_73

IIVHQNNNINPIQYHKFQYNNDLP

>SG-PL_contig_4953_1_-3_3_212

HFVNIGSCNIPIKIAPFSEMKHHLGVFPTLGIIPATNLSARSSVYTDKRYFSLVHVFLIE

NLMRIYMSPL

>SG-PL_contig_4954_1_+3_9_236

MHNLHNNQNVKSFIDKVCPMHNFRGKNTQHSFILYKVCPMHNLGKKYLIFIHSYIRNTCY

LFWGKLYSIQYVQYR*

>SG-PL_contig_4955_1_-2_104_292

MVNVLVPCFWCCCSESFHCVDRHQMVCTEYQQCSTMIKYTILQKVKCLCCGWQSETCNLG

LCC

>SG-PL_contig_4956_1_+1_1_186

TRATFYTHSKCITNTKPKIRYPKQFTRRKENKRKQGEEITANTAPKTDFKFQMIYSSRRM

NE

>SG-PL_contig_4957_1_+3_36_176

MLQKMLIKTTDKIYLQELKCDALKKSLILHETTESWVERGEKGDKVY

>SG-PL_contig_4958_1_-3_3_323

KKKKKKKKKGRKKNRHKKFFFFFSLVVPIFCTAPVSLFPHRSFRLSNTPPNPLKLRSPRE

QQRRATESGTIFHNAMMMRMGMMVMMLWVPSRTETEHYTSFHNISCC

>SG-PL_contig_4959_1_+2_2_202

AVLQLADVTLDLAEVNLDLADVILDLVEVNLDLAEILVEDVACLVEDVALPDLAETVVEP

CPVEGVA

>SG-PL_contig_4960_1_-1_1_120

QKSIPLILKCIEKKQKAIHIHIHYFNWICSLTLCLANANG

>SG-PL_contig_4961_1_+1_1_441

GNTILTGSWQRSSRGGDNSDREKTQTQDEQHIQNTQDHQHRAKCIYFHESLILLDLRVNC

QTSIELRLLVIQTLSLPFYHDLPLLLFFDEFAIYCLGQTFFCCLHNILNSSLCVAEVLVK

FNSDSIHLIVELILVVGLEFHFQFLDF

>SG-PL_contig_4962_1_+1_121_264

MNGSKRIQLQRKKVKKVSSLHNRCCYSKDCFEDVRFRREGDGGGIRKG

>SG-PL_contig_4963_1_-1_1_132

LFFDISHLGLSLLTWLMIFSQGTLGQGVMAGVCHYLGELYSFYS

>SG-PL_contig_4964_1_-2_2_133

SASLSLPPRNFKKTSSCKQTCLAGLNEVQSPPNFVYLMKKGCRV

>SG-PL_contig_4965_1_-3_33_272

MLWPYMDQCSPTIGRHPNFYIYPRGRPLHRRLDGGWGAHMDNLHAVGTWTEEEAKWHISV

LELEAVHKALLAFEPQVQRK

>SG-PL_contig_4966_1_+2_2_220

NTDTTHRIPNVHIRRSACAHTRRGPRHQQVRTHADPRHQKNFHPLATRQNPSGIKPRPFC

GSPKSKTLGHCVF

>SG-PL_contig_4967_1_-3_84_356

MCVCLCVFFCVWVCMLVGGLDWIGLDSFLCHNRIFLREMRAALPGKARRHSTVPPFVSFL

FSCICVFLFTCDSGFFNRILPGTTLLLPWDL

>SG-PL_contig_4968_1_-3_3_206

SSDCPLSFDTNITAVHIVCQSSESICDHWYWPIAGKPCLQPLHFSADGAFGTCVQIAHGQ

STLFSHHK

>SG-PL_contig_4969_1_-2_278_1387

MSYRGMEGFWAAITPFNFTAIGAHLPMAPALMGNVALWKPSDTAILSNYIAYQAYREAGL

PAGVINFLPADGPVFGDTVLASPDLCGINFTGSVRTFKRLWRQVAENLDIYKTYPRLIGE

CGGKNMHFIHKSADVQSVVNGTLLSAFEFNGQKCSACSRLYVPQSIWPKVKEGLLTALKD

VKVGSPLERENLVTAVIDDKAFDRIKTYLEKAKSDPSLTVVAGGTCDNSKGYFVQPTIVE

TSDPNNTLMQEEIFGPILTVYPYPDEQYLQMADMANQTSPFGLTGAIYVRDPEAREQLCK

TFRDVAGNMYINDKSTGSVVGQQPFGGARLSGTNDKAGGPHYLTKFVSQQSIKERLQPMT

SWWYPSMDKK

>SG-PL_contig_4970_1_-1_1_201

VCVCVYSHTILKLWSRLGCNFIYFFAILLNSFFIALLLYVTPCVSILPRRRSEFVVVCCL

KNEVSWH

>SG-PL_contig_4971_1_-2_128_466

MTEFGNLKGYDPFADTDDNDSSGAGLQEGLIHIRIQQRNGRKTLTTVQGIHPKFDLKKLV

KVCKKEFNCNGTVVEHAEYGEVIQLQGDKRDAMKIFLAEVGIGSIDNIKVHGF

>SG-PL_contig_4972_1_-2_167_1147

MPAQNETTQMEDAEVETFAFQAEIAQLMSLIINTFYSNKEIFLRELISNSSDALDKIRYE

SLTDPTKLDSGKDLHIRLVPDKENKTLTIEDTGIGMTKADLVNNLGTIAKSGTKAFMEAL

QAGADISMIGQFGVGFYSAYLVADRVTVESKNNDDEQYTWESSAGGSFTVAAATGVPMGR

GTRITLYLKEDQQEYLEERRIKEVIKKHSQFIGYPIKLLVEKERDKEVEDDEEEEEKKEE

DKEEEAKDEEKPKIEDLDEEEEEQDSSKKKKKTIKEKYSEEEELNKTKPLWTRNPDDITS

EEYAEFYKSLTNDWEDHLAVKHFSVEG

>SG-PL_contig_4973_1_-2_2_235

ERRKERKKEEKKKRFVLKNKFCTCILLHAGPFELFGEFISITPPPPEPGCKCLACAAASA

VFVGVSSAGSGCKLNQAI

>SG-PL_contig_4974_1_+3_294_632

MTQSQRPMPFRCIHFHAYVDGNFIYICKSTDCRHLVFVCLFIFKHTSFRCIHFHYPNPNT

LMEICKSTDCRHLLLFVCFSFSNTHSQSLYPCQPSFFLKGQRGNKMPGMMKPY

>SG-PL_contig_4975_1_-2_155_403

MSRKGRKLYVAFVDFKKAFDSVRHDKLLDCIKNQGVKGKFLASLRAMYESLCSCVRANCE

LSEMFDCPVGVRQGCVLSPTLFS

>SG-PL_contig_4976_1_+3_27_245

MMLKMSHRHTLQVSQADKSQQCRILCPFLLTTELLTAGLEYQYMHTHHNKQATCGTRSRI

MLPSLFERFATSL

>SG-PL_contig_4977_1_+1_94_339

MTILVMVQMFPCLVYLSLHFLCDVLKMIRLFIQHNSNHIVLQLFDLIVGWIWLYNPQIVQ

RRKYKISTQLLFLKVCLFSCLH

>SG-PL_contig_4978_1_-3_63_491

MAAEEGDMNLPPTRVEEANEYLQRHRILDMFNNLTAQLIYARPEQPKAFITEILQRLQKS

KSTQFDLPCLFDESNIVSIFGMLDPTGRGFISHQQYQEALVTLGITAFDNEPVGYDVDRI

NMDTFVREAKEGLIRASATFSES

>SG-PL_contig_4979_1_-3_279_434

MARCSVPFRMFGSYAEIGLFVYKRCSILCFFQLFSMGLEVYQITCVKCLIKI

>SG-PL_contig_4980_1_-3_258_449

MGNEIILFLSCHSGILKTPCCSCTCMCNSDVDFDHFQSHVFNNFSHDSRLYGNYIQNFPQ

LFFQ

>SG-PL_contig_4981_1_-2_2_148

TAPVRVWIKKNCAIGLQWKKKTTTVNGYLTLFKVGEGKATRERRWAPPS

>SG-PL_contig_4982_1_+2_2_214

NINWIPCATNLFISLLHPTFVTTSIFICQLAPCAQLQTLFPFRFLDLNCRLLASVLLLFR

DHLPGTDFHSP

>SG-PL_contig_4983_1_+3_54_158

MAMNPKLGQAHSIMHSKVSFCLCLCQFGTSPTLQG

>SG-PL_contig_4984_1_-1_160_399

MLQMVNFSVPLKTITKKVRKEQEIIMPPDLLQVECSVTSVPSIIEIRVWTYFKFNEGVMS

YHMLSTPSAQWSVLSQVLSE

>SG-PL_contig_4985_1_-1_1_1080

ATGETTKEATGETTKEAAGETTKEATGETTKEAAGETTKADRGETTKEATGETAKEATGE

TTKEDSDETEDSTRANDEKTEGSSNNAAAAEETQENLTNDKEKEEEKVAPKSPVQEEPSN

TSAEESTQSVKAEADHNNSSSSLKALKPEDSTSESASDDGDGGGVSQQLVNKDTEQTKDD

TSSSSSSEDGDGDGDGDGDGGETNDGVGVGSDAEQDYVTREQEVKEVPELPADARALVKP

DASDTNDAAATDDHSKKISGRSVSLGSYPPASLRPASDIEKRLSVPSVRGKGTDQPLKPL

QTRLKEDGISPSPDDKAADEEKATGSDSPKEKEKVHSHFQSTQQEDSKEESLSQATDSQD

>SG-PL_contig_4986_1_-2_2_124

KSFETQLKPVSNHRMPTKSIKLLPPIMDAVLLKIEQEHCMQ

>SG-PL_contig_4987_1_-2_2_283

KEDLAALRTVPPQGASFFGSFAKPALTLKRKRGETPWPQDSSTTGPLNCHRDLPPKRGDL

SPSAKHQAPPQTCAQALAGTLHIQMATVPRLAPV

>SG-PL_contig_4988_1_-1_16_246

MVLVISFDLFVYILFGSMIHSDQISLTRLAVISLLYRLSLEIRTVHEWNDSNYTEVCLPV

YFVGSKTVIMHMYKYDF

>SG-PL_contig_4989_1_-3_3_92

KMVFLLALATGRCRSELHGLSGAKGNIAFF

>SG-PL_contig_4990_1_-1_1_174

TLITQFTHFHSDTQLILWFVITVHCFFFWKDTSDHVCFIIKDFLSCCSLSARSVFYHY

>SG-PL_contig_4991_1_+1_82_363

MDHFPVACANGAGHRGKPNPAKSLSLTHTPPFTLTESNIKYISKYDMFIYIIYSPACLKL

THIMYIISIKAHRLTMASAFCVLSSTGTRGVMRV

>SG-PL_contig_4992_1_-3_18_623

MFEFIQEHGLQGHPYLEIYVGSTIHFMAPLDRQQEFYVPESLQQFPGEEPYEPAYPDTSS

PDSSESDTRPTQSDTDKPEEPSLLHPCQVVRATQPAPSDADKLEEPSLLHPSQIGQAGVT

GTEVFKKTETIGAIEKDVETTTAEEDKSEKAEKIADKAESKEADAEEVKEGEGCDKESSS

SFEQLDMEDEEMSAAESPSQSQ

>SG-PL_contig_4993_1_+3_906_1157

MTYINRAVCLPPQDSYRFVTPKFTNFSQTFKTIITNSKTRFAKYDCLFSCFFLLLFFFHS

LTISKMNTECWKQKLDGHISTASL

>SG-PL_contig_4994_1_+1_652_825

MHFCQVCSALHILHSLTKEVTTTLLSSLQCFAHPTFPDKGSDHALLSSLQCSAHLAFP

>SG-PL_contig_4995_1_+1_67_786

MASIRIGYLAKRTVTPSIKSGNSLFSQIPRLEIFLQKVWNFLSALALSLYSWMASVCLLQ

KVPYTDFIRSAVAPENCCMRIGPISSTLRSGLKDCRVSRVRRKPACSSNSSSRRGNKRSV

CSISMPNSDSMGSRNNFCIVFNFFTYCFSVCINSHIVSCRLMSLAFIFFSVLRSTVTFQA

SGSSTPSMRSGSSSSTELSAVTTVSRDSRDSEMVSVMLEGPTSSPPMLTTSDSSASLMAL

>SG-PL_contig_4996_1_-1_10_396

MSSGMGRGGSMLGGGSSFGGGNDHSSGMQQNFSGDSTNMSGGQGMSFGEDGSETTQVTIP

KDMAGAIIGKGGARITEIRRRSGAQIVIDEALPGSNERIISITGSQEQIQNAQYMLQLSV

KQQTEGGNY

>SG-PL_contig_4997_1_-1_76_717

MLHQTPLHLAVATRQLWVARRLMVAGASMDAPDHCGNTPLHIACREGMMDMVQLLLSPVK

YQETLANQYPIPMQRLPQDLSIRNYEGYTCTHLALQGGHLHILQLLLSKGANINEPDGKS

GRTLLHMAADMGYMQALELLMQHQGLDLDARTYGGLTALSLAHGRRLNHVVELLYRAGAD

CTQLTEDAGESTDEDMNESVYDDICINGQPIQIS

>SG-PL_contig_4998_1_-1_160_1212

MGSFAGAMDVAWEPHPSPTETLAEDLNRLPLSYRQGFDQTREKSSVTRETSSRVKTAAWA

PADPPITEQVLRSCFYSVPNIKEEQKLPKFSKDKEEKCKFCGQIPTRVTRLERFRAFEAW

KDFCHSPGRAFKQDWYDYERALPINTEDLTVCRFIVGKPICKFCRFALPRSPLATAGAGS

LARDSEPALSSAGVWTLAGDCLPAGVWSLAEGGVPASSPGRGWFSAGHGLPASCREAWFS

AGHGLASSSREAGLSAGHGLPASSPGASPLASSALSSSGGWSLAGDCLPSSSPGATTPAR

QELIQQQLKPKLGRHPHLDFYALWWPHMTGYNEWRRSTGRQYQKSWVVPHE

>SG-PL_contig_4999_1_-1_1_90

ERTNERKNEQTNKQTNWKNNPKRTTLTTTM

>SG-PL_contig_5000_1_-3_3_104

FTLKLKAQKWTCGEVSHHKINGDREVPFDAVDPF

>SG-PL_contig_5001_1_+3_381_722

MLNVTQNISFVTISVFSHNFQPHSPSFQGVHAITMICHKNHNTSAPVFTYKTDFTFGRGG

GRREVITPLYGGNLTGTAWHELNSASCGKNHTYDRPLPLDRQTDRHPTHRETKR

>SG-PL_contig_5002_1_+3_276_638

METKRQKPKQQHNGNNQRQKHNSNTMQATQCKQPETKTTATQGKQPETKTTAKQCKQPET

KTKTATQRNNQRPKPKQQHNGNNQRQKHNSKTMETQRQKLKQGNPKSKATLLVNQNGKTN

S

>SG-PL_contig_5003_1_-2_2_169

THTHTHAHTHFFPCKLASSSCSFHNTQISVNTLFCDIFRAKGNNILLQDVPCSCSQ

>SG-PL_contig_5004_1_+1_1_459

STCRIATTGATEWWEGPKEIATAAGEELLGYRKALRESWISDHAWNLITERKDLHQKRHT

DTSDDNAVYQDYIEKDREVKRSAQKDKRIWLEDQANDAQNAASRNDMRAVYQITKKITGS

TKASSRPVKVKVGTLLSKGEEKLDGGHSTLVKY

>SG-PL_contig_5005_1_-1_685_4392

MLSFRGRVKDLLVGFTQRLQLAYESFDSSDSPEDHCQQVKQNVSRFLEDLLGESLDLTSD

EAVSDLSSLSDDSGNDRHAFEDQIAQAVVSKILDSHRREVHHTLYQDRRSRHLSGSSLVP

EALLDNDEENGNNNNDDDDDRRSPAADNADIRDTDISKDFEDLRNFVQETQSRSRQFEEN

SCDSVGGVGVGESRDSSEARVGSSFDTHSTERDFPPLPDFSQFDQINFNSGEVDPDLVQM

NLAVIPEETDEQLEEEDAADTHWRENWIFKGQAPSAYDNCDRKRIRGDSDHPQYMMVPRP

DDDLAPKIGNRDVNQLSDLSDNEVLTDDDDASFYPSTSEELARISKHKRSLTPPSLSSPS

LIPSTTEPYDNHDASLLTEDSLLPKKQARRHSSKHSDMEVDVEDVGVGTEDLVVGPIVEK

TRKELKLLEDLVPAEGDDPKFVVPPESVTIQEGEPVRLSCRVAGTQPIDVFWYRESDKVE

ELEEGEDVDLHLEGDRHQATLYNLGHQRAGQYMCIALSDAGKAIKYFTLTVNDNRQELKK

PEFLKELKDVEVLEGQSIKFRCKVKGYPQPRINWYKDGKLLKSNRLCRIEKFGNRDYILT

VDYATMDDDAEYMVVAKNVAGEVRSTAQVIVEPYAEGHPLLLKSRSQSMSTIYTDTESDR

PSSVFLEDFMAQPLSSATRDNNTRRWSEDNQVDSLVNQVTAARDHMRQEAESMIVTADEL

NALTDHLEDMERHLDSLEGHNSASASNAYRKKPTTNHSDYFPDVSDDAAIEMITNARAMG

DAARSVRKLTSNALSVLRAAEEIIQSERSMAGLSSSPTHSGNEVSSSSHPSEENNNKSVS

LESAMDQSSLTTHSDLNQSVSSGYGTISMASSAMDENGNDDTIAESSLSHLDLPEQDEFD

FYLPPLDDETSGSYREEVSISLSSSKPNRTQKQQPGGASSSSSSTVTPSSASSSSSSSGR

TVQPQKPTSSMNTSVESDLSDLWPHRQSVSSAVKMEAPADELDSSLAMATLPSEEESLIL

NFGPKEISATAYNRDYEVAGASKEVDVSEKKHWSVTLDPYVPTAEVVNRQPSLEETEEQI

YLTAGKMFSLEDRVKELQARVRAEDHTGSVHTLSHLEDQVAKTAASVARSEKDVSNIERA

VNTLQFSPRSSVRSSSSANSTPRESIDSGVSSFTPERQERPLPVHMIGQTQEEEMPESRA

EYDDDSGIELPSVTRLRAMFGSRGKEDEAGEGNFKR

>SG-PL_contig_5006_1_-1_1_180

KKKKKSTVPSSEHLKLVVLSPHCASPYMKWHAAYCHNCCTVNLSVSFQLISFFRVYCGSK

>SG-PL_contig_5007_1_+1_457_726

MFRTVTVFLMKMGNHQLKTVKPATRDYLVDSSNLRKNPTHWEGLEKRMTLLSREFKDFEY

MAPLTEPASPHKNTTVARLRVEPTTPALTN

>SG-PL_contig_5008_1_-3_582_824

MVVSQARIQKARYFNSKLCKNGAHMQENVQESCAHRYLLLCGNFSGYVPWQVHKLSSDCK

KWFPRRKRRRQLCTQVCSQVC

>SG-PL_contig_5009_1_+1_211_372

MGSLTCACCLRDLGFETSHPKKIAPNHHFKVWWRRGAKNFCPIRDSNLSFSRPR

>SG-PL_contig_5010_1_+3_1293_1550

MKSTKQDENVHICAKISTKQDENVQRCNTSSYLSKYCSIKVSYTVQNKKHCCPETMCKKS

TSGIYDAKCLASCHFHTTSTLTMVIY

>SG-PL_contig_5011_1_-2_2_322

LTDSWSLYIEIGQVVVYVEIVEVCIFLQCSKKKTSRRVFQDIQWCSCLIISNLAGSFDRV

VESGSDVSSSIPPCTMKVGLSTAHLQLCVSEVLFCPFPSPFQDLASV

>SG-PL_contig_5012_1_-1_94_288

MMLCQYVTMCEYMFMYVCNKVCLERLLAFIVGSFQKFGSFCDGELQTFKTDWTWSTYECL

NCAEK

>SG-PL_contig_5013_1_+3_888_1307

MMCFLMPIIIICKYHLSEADISTICSTLFPPLIFMLSHLTFSNLPFLLLIFLSLVIKLFG

SCPSPQLFHQSYPSSWCVQSCPSSSNSVQILSLSFNSFNTAILFELSQACAHFPFNTFRP

APLLNSFCLASLSLSFCLSS

>SG-PL_contig_5014_1_+1_109_315

MFNMGTATDVKRDGIHERGQSQSFCCFLLFFSPVYNVVAGKMRGGRGAFLYPALAYPHPP

YNKSEIYVP

>SG-PL_contig_5015_1_-2_122_1516

MDGCFSLSSAPSLSFDDFDTPTHSLSSFERNELSCRSPAPTQDLEETLSQGSVDEDGKKQ

INKDTGSEYKEHLKGNHTTTRSQSFEADIHQASSPKDTIGSRSQRIQISGCGSLDVDVMN

KMTDASASSLDEDEDNWKGKGGGTFSSDSSNNETLNADSNSFGGEGSYGDDDNAEQHICD

TERLGNQSLEKHISDETGQIANDRMSGFSDSCKSYEQFERNIPVTLNALSEMPVCGKTET

TSQEQTGSKRDIDCFTEETRKRKDVSDLSEELLDMEENSGIKAYNSKSTEKDSGHATSNT

FLDSFNPFLQVNTNQHSDSESKSSAFASQTYPSGDEDIYSANGQASNVMNEFDIGQLGQA

AGCVTVKFSEERSTSRTLDEEEYNNWQDSLACAFEDGSLANYITPNVLVRPPEKRYSTST

TLTTSDVDEDIYEEDDTSAFKRQDFDCENAASVLESECSANTAQE

>SG-PL_contig_5016_1_-2_2006_2389

MRACACAHAHTHTYTHTHKLPNVCACAHTYTDTHTDTGTQITHTYTRVHTHKAGLNVRAC

THAWHTHTGKQITHTHRHWQKLTVCVSLTHTCTLANKSHIHTDRAHTHTHRENAQRTRCA

QCQHKYCL

>SG-PL_contig_5017_1_-3_324_515

MDRVPGGSWWVGPRESKGLRIPGLFNYNRGFCNVLDPSFVPTVINMAVHGKIMDISDFFY

IMSL

>SG-PL_contig_5018_1_-1_160_453

MSVFGGLWKHENTQHVQKLPCLCHLWFLRHNPKCSESKSVWSIALYKNDLSLLLSLLLLR

MNSKMEQLPSHPTPKKPCSVKKTGGAFSSTIHNTQCSE

>SG-PL_contig_5019_1_+1_109_351

MDSIPMKRLCQQKRYNFHFKLYEKKLQHSSPYSRSITAVYFRLRLNALKTKFCKNVMCIC

GEQFTLYHCLFYCHELRSFLP

>SG-PL_contig_5020_1_+3_3_260

LPECDVFGRAFYEYYPSRSLSLYSYLLNIEPFFTAFRCSTLPQHFGTNFQTRGSVPHAMT

SQRFLAGERDFIAADETRADCVAFFA

>SG-PL_contig_5021_1_+3_2424_2645

MGGKACFNNQRKGLMQGTSIQTSWSIFPCKTASVRHMSFTLSFNICYQNHCTVRKKMTEK

STCHDTIPFFFFFP

>SG-PL_contig_5022_1_-2_491_829

MFKLFVVFWWWYFSLCHFMEQYMWATFYGTIYTLFYRPGWCKSGLHFMEQSTYYFIGLVG

VRVGAPAFQSCLHGFKSGSGKTLCMQPLVFSAVNEYQTLWRVVEGKAVRERWA

>SG-PL_contig_5023_1_+2_278_751

MEAASQVESPSGETAGTHASQGQTFVAKDGTRWNSEPPVSMGRISAENVSRGPRNQTPGW

DHVKEPVDAFTLFFSKILFDFLVKYTNLEGIRKKGEKFVKTDETEMKGFVGCLLFLGAMK

QNMLPTTVVFDAFYGQKLVRASFSRNRFFAAALVHPLR

>SG-PL_contig_5024_1_+2_68_205

MPTNRMLCNYQHCHAVSAWLGGSHVLRQASLCLEINSAWRVSNLDT

>SG-PL_contig_5025_1_+3_3_278

VHAGCIRVSISHRPRTWLTGSLTCIFDVLYAYTHEGGSGTSRSAHMLTWKIGKISTLYPP

GEVAFIVPTLIQNTSTTTCENCKSPLLVTVIN

>SG-PL_contig_5026_1_+1_2401_2712

MFCFYLQLKMLRMRGREIYTSQFMVCVTIKQNSNRYMKDLHALHTESTVSIITKTNTARH

NHHQHRHSQTQSSPTQTQPDNQHRPSNFHITTHQLLSTHYVHAQ

>SG-PL_contig_5027_1_-3_96_200

MYIYVHMHECMIIFVYLSMCVLFGFCSVCLHILSL

>SG-PL_contig_5028_1_-2_17_250

MEVSNRSRALYVSLEMLNKLNTVNEDNHQVIPYHRFHVVDLKDLVNIRNDYASWVQQTQQ

AQQAGVLGTQGLHFCSYP

>SG-PL_contig_5029_1_-2_461_2707

MSEGTRVNGPSSPTGKYIDTIDPDDPEYIRNLQRPAEVKEDMKQMEGRSRVSVVLNSQAF

KEELEAVIHEQIRQGGPASIFALQQISDLLLPQSRGSYSSFGRSSPVIPINDIRGVETLG

YSKQEKVLRCKLASCYRLVDLFGWTHGIFNHISARVSQDQEHFLVNPFGMLYSEITASTL

VKVDVRGEIVDTGSTGLGINKFSFCLHSAIHQARPDLRCVIHLHTPAAVAVSATKHGLLP

ISQEALICGKTSFHGYGGMLMDQEARDHLSRNLGPFNKVMFLRNHGVVACGQSIEEAFHF

AFNVMAACEVQVKAMPLGVENLVLVDEETQEKTFKMGSQGGSGTDSSGKKWRCGELEFEA

LMRTLDNSGYRTGHMYKQPLMKQEKRERINSDVEVPPASSSFTYVFDGDYEHSKYVSPIK

MAMERQKQAYKAGWLTSPNTYKKQEIEEIGTTTPKKITKWVPGGAETQEQATAIKMENPN

QFAPQGSNPKEFKEKQKSIKKDYYEEKVSAGPQSKILEGISAWDEAAKRKDGEQTVIVGA

ASKGIIQRDHQHNAVVYRSQYAANPFENMSEEEVDAYKQGMLQQEQEEDAEDVPGPEGRL

ISSQERMQNIQHSQEDVQLDTEENTVQETNSDSHVNDKNERRPVHSPRPPISPKESMPPV

PARRTHVDAKRRVQREVQEREGDGDKPGSPTRSDPMDSASGGETLEERSSKEGSPTKEAP

SPTKDKKKKKKFRMPSFSKKKKDSKESTI

>SG-PL_contig_5030_1_-2_503_844

MSIHLHKISMLDTSRGETFLALQLDIIPYQSELPHPRSQKIAQRGLVGRCGYHNQTLWSR

SLSLPDGRFAWISCKTLSLAEEENLIKARAVFFLFLFSFLVMSVGRTKFCLWAP

>SG-PL_contig_5031_1_+1_1294_1569

MEWDENKIILSFTDLIPGHLTIGADILSLHHHSLFFLTNTKTLVHIHKSAHFNYFLCCLK

TTTTNKQKNPGQVILIISLNLPFSLKYPLVPE

>SG-PL_contig_5032_1_+1_415_660

MPLSCSVFFPITFMPLVVSATYYVKIRGEGVKSLKCNLSCFHALALRQPTTRGILSLAQF

TKLNRLRSLSTSHAYNKTSGPH

>SG-PL_contig_5033_1_+2_125_283

MIFLVIKALSPAGTYKTVFYRRLRKLRRNQCRLMNEKRKDLPSSGKKNPLYSL

>SG-PL_contig_5034_1_-3_687_1157

MHTHRRRSMQTVQQHNACTVWAKQRAHTHPPRKYCSERKTNKQTKTTRFDNKCMLRLNNF

ETGLFQGHSYAKQVKSAAKGRLLVCLDHVFRFMEDQLIVRERKGVKSFHVLSLSFCTFTP

LHSRGDRSLLSCSCSWEFVQVGTSFPVLLFTWVFTST

>SG-PL_contig_5035_1_+2_458_790

MNKRTQNDRSGKYRQKKWEYKAEKEKKVFTAGHSPESSMIRFWRTVTAESSGRTVAADRA

QRVFTVGITRLGLKILLQNCSNIIFFFSNRQLIFENSFPQIVHLEPGEETL

>SG-PL_contig_5036_1_-1_34_378

MSKFTLCASAELLKVCKLSEVFSKKIHNSQCNASMFDIVPFLPSTRFMIHVCQNILSFLS

LVMCKPFISKHDHLATAAEFACKCKDHFLLSCCYLYFHAVIHDPLIIQFAHFSHS

>SG-PL_contig_5037_1_-1_4_900

MADQAMSSLLVLIVDTNPVWWGSRTQDTQLQLSECLDAALMFSNSHLMLSHNNKLAVVAA

HTDKSHFLYPVRLEQSQAETDGGDASSGSRDGKYELFVQVDQIVKREMKQLILENLEGEV

SPDSVVAGALAMALCYIHRLEKESAIGEKLSSRILVLKGAEDNPSQYMNFMNAVFTAQKQ

NIVIDACVLENESGLLQQACDITGGIYLKVPQETALLQYMSWLFLPDPSVRSKLTLPPRS

QVDYRAACFCHRQLIDVGYVCSVCLSIFCMFSPICSTCQTTFKILGPPKAAKRKSKKSN

>SG-PL_contig_5038_1_+3_192_434

MQQFFWSHGQTHAVWKVGAPTLHMMSCFNTSHGAPTLHMINIISPAVFSLTLVRCRHMSV

QHHYMPILHNDPCWLYIIITF

>SG-PL_contig_5039_1_-1_121_2499

MSSPKEDAFSIHGPVKVQPTKEQLTPHKFALLVLIWDQFHLHKLQTSMTDIDFDDDYPWR

PYTEREKRQVMTTLLDLLQSPDLALSELKARLKLSLKQLLMDRFNERLDNFQDQGFLSVV

SFIEGMGGILSHSDQNDVTSMFIDRTSVVGLFIRRMLLAYSQMSFSQLCSFQAHLKSYLS

GKPVTTYSDMVSAQYQQNNCVGEMLSRSLAETGMGTSAQLSASDLHPADLSVASPIRDDK

EKVSFSHKQAEFFISQQTSFLSVNENRALPPKELQKKLSSMLEQHPDIAEAHFLSYLNNL

RVKEYCTAVHNLYHYFDRNAQLTGDAASNSKRSEDEVSRRYAALSLAGLHFNFGHKKEAK

AALLEAIRMAQESNDHVCLQHALSWLNLLADQEGGKAGSQIERSVGKSDELNLSYLTSLG

VQALARHNAFATATPSSVFDYMMKSNVINCQNSLYGMMSISGAQRAGLWSYYGNRECAAM

CAQMVLCLDTQDKGVFYNGESICISLCLLARLHADRGDHASTKEILHIASRWFHPHSRHA

HLWQSCEQSLAFEHALLQGRWNEAEKAAHNLRSINDTEGRLHLARLLRERGEMTASLGLL

QKLKDEACDRKDLTPDFLCRVLLLMSEALMATDNHTTALPHLTHCLTHATSHHLHYLASL

TTLHLAIVQLRMKLAGQALQLIETAMLGILSHGGPYDCARLLYAYARCKVAAAQKASTEE

KKTALLGAVSLMTIVVNKFQSLEVTARVKDALYYQARLYHQLGYTAERNRCAHRFKQLDQ

QSPTLSKSSVHAV

>SG-PL_contig_5040_1_-1_82_213

MRITYANSAKKCVRTAKICVSLSTMTRTFTTTTSTNACMYIHIH

>SG-PL_contig_5041_1_-3_99_224

MTSLSVSVGRKMQGTDEDYQLCVYFSFLFLPCIADITLVTKT

>SG-PL_contig_5042_1_+2_191_391

MFAVYVFMKNRSFHKKMRIVRTCELCSIVIFYSVGCHRLAWRTEYLVFVFLLGHNHVLFC

GMSLSCQ

>SG-PL_contig_5043_1_-3_12_1604

MTVGIINMASKGGALNMRSLNGPLSDAAARQTHKLAVSRDYISQPRITYKTVSGVNGPLV

ILDDVKFPKFAEIVNLTLADGSQRSGQVLEVSGSKAVVQVFEGTSGIDAKHTTCEFTGDI

LRMPTSEDMLGRVFNGSGKPIDKGPPVLAEDYLDIQGQPINPWSRIYPEEMIQTGISAID

TMNSIARGQKIPIFSAAGLPHNEIAAQICRQGGLVKLPGKSVLDDHEDNFAIVFAAMGVN

MEIARFFKQDFEENGSMENVCLFLNLANDPTIERIITPRIALTTAEYMAYQCEKHVLVIL

TDMSSYAEALREVSAAREEVPGRRGFPGYMYTDLATIYERAGRVEGRNGSITQIPILTMP

NDDITHPIPDLTGYITEGQIYVDRQLHNRQIYPPINVLPSLSRLMKSAIGEGMTREDHSD

VSNQLYACYAIGKDVQAMKAVVGEEALTPEDLLYLEFLTKFEKNFISQGHYENRTIFESL

DIGWQLLRIFPKEMLKRIPQSTLAKYYPRDAHRAQTAAAAGAAATKETTAL

>SG-PL_contig_5044_1_+3_3_110

KQKKQEIKQHLSYQIFKRFPCFIEHKISRRWQQIKH

>SG-PL_contig_5045_1_+3_477_749

MTFQSVVRCTNHFTTLTGSIAVPIFSPGQCTQIHNEPFIWFWIKHQPTSLWLDWSMAFCS

CISSVHPSTPSAYSPASCRNSVTMDHLYNST

>SG-PL_contig_5046_1_-1_1_129

LLLLFIVEVCCYCHNMIKACLTVVGIFDENSANALYYGSVMNR

>SG-PL_contig_5047_1_-1_1_258

CDLLFWLLHDDNKQCPEQRRATDTRWHTTRSTSVVIFLYLQMRGIQEGQPKREDPEIHNL

NQRQSKTPGTHSAHTWLFLLYILPDR

>SG-PL_contig_5048_1_-1_1_189

SKRSFEHILIFQSCWHRSKSLLFKNKQKSIEMTNALLKPHITKNILTQHYFYIVGSRVLP

RIL

>SG-PL_contig_5049_1_-1_946_1167

MKLSHLGLKNANFAFAENCHRLFYTSKNTVFAMIISWEAVNEIYLPKMTCDCPWVMKVKI

LLLANACVDCQTDA

>SG-PL_contig_5050_1_-1_1_123

GERKQQLCILLVFCSFQLMDVVLTPLQFTEIIKTRYYKTKC

>SG-PL_contig_5051_1_+3_90_245

MKSLDRNVVFLSHTESNGHKLYNHELRKNKVMKMSGFKNIVFCFLWYISCVH

>SG-PL_contig_5052_1_-2_2_295

IHHISCRRPAPLVLPLAPRRSLKTWKVKPSLQLPRTTRRSGSTLVLGLRLRLRGAPTHRR

RTDQLARRWQTSCLLTTRARPPPRSSPRGLRSHRTLLA

>SG-PL_contig_5053_1_-3_315_593

MDLYARLKLIARNIDEKKKILSLKGKWEILMKRKKKNSFLQREVGNVEKKKILSYKGKWE

ILMKRKKILSYKGKWEILMKRKKSLLTKGSGKH

>SG-PL_contig_5054_1_-1_301_501

MLLASILLVWFEIYMPLRTDSCCFQNKLNVCVCLNITYKGFHNYLSSILSKRRPQFCGKR

NPSKIYL

>SG-PL_contig_5055_1_+2_1013_1270

MSCRLHLMTPRPADYTQWPHVLPTTLNDPTSYRLHSMAQCSPPYIQWPNVLSTLNGSMPC

RLHSMAQWPYYTQWPNVLTTPNGPRY

>SG-PL_contig_5056_1_+3_954_1247

MEREAVDTSLPWGRFVRMDPSPLHPCFTFSNPEEDQIPIPSWVDWRSHAYIKFLRRDCYP

SKFLQADQRQADLFWSPVSSFIKSYSHARLLPLCCAAA

>SG-PL_contig_5057_1_-1_322_573

MELHVSNVSVEKLHIRRRLPQYLPTPIGCTLLLMSCIASIHIDHFCKVLNSECAHITNRA

PIKKNVIRKCDCDNLEELTVKASH

>SG-PL_contig_5058_1_-3_159_356

MVRSRSCLAAIIFLLYIVYSPADIMAISGLSPRKVHQSQEDNCTEHKTSNLKTKILQKNT

IHDILS

>SG-PL_contig_5059_1_+2_386_592

MRMYIYTYIDKHTHTHTCAYIYTHTHTTYTYLSLSQSSRCQVTTGSAEGRFSCPQYFICN

PVYNSSLIL

>SG-PL_contig_5060_1_-2_86_625

MSRQAARGGDPRKVNGELFTLTYGALVAQLMKDYENDEEVNKQLEKMGYNIGVRIIEDFL

ARANSGRCNDFRDVAEVISKVAFKMYLGITPVVSNWSPAADEFSLLLENNPLTDFVELPE

GHDSLNYSNILCGVLKGALEMIQMDVDVRFVQDHLKGDNITEIRLKFIKRLEDAVPVGED

>SG-PL_contig_5061_1_+1_1_264

TRSGGGARRKARFDRRSNSSPSPAVRPEVDPTTGAPRGPNRAQHQPHTDAGREIAEAFLI

NQDRKRKVKKTTKSGKKEENKDRLLWFG

>SG-PL_contig_5062_1_-2_134_535

MLGPIRVQHSLHGPPRGPSRWVNQVRPPGRTAGLGEEFDLRSNRALLLAPPPLRVTGPRR

LLVSLAPPRMQHPAVLRSRRCIRAYALRRESGRPVCLHGPCPRSIPPPPPLSLSLSLSFS

LSLSLFSFPFFFLF

>SG-PL_contig_5063_1_-2_116_388

MPRQSPYQHRCGGDVGPDSGSTLPARDPLGPQSSGQPGPSPGTDGGARRRVRSTIESSPS

SSPAAATGHGFASASGQLGSPSNATPGRTTV

>SG-PL_contig_5064_1_-1_1_78

PRKVEVSTISLPYDLRVMGQPLGLLF

>SG-PL_contig_5065_1_-3_3_170

FFRLLDFVYMCKFSRAVDIILSVATVHMYVDMCVCGVCNVCFSCTKQTAFKLLAVL

>SG-PL_contig_5066_1_-2_2_202

WLPIQARIDYKLATLCHAFFADTAPLYLSNLLSVYTPSRQLRSFSDSKTLRVPRLRTKNY

GERSFSF

>SG-PL_contig_5067_1_-3_462_632

MHMHTHAGTDKHRHTHTRLLADTNTCMHEHSEKSFLTQFLHTWLDTHRYSLSSQKGS

>SG-PL_contig_5068_1_-3_108_464

MDPAYTDELVLSDEAKAEVILAEEEYLRRMASQLQAIQEHEDMVCSEHVKSVPKLSGKLQ

ELSKIHIQQQDETAQVTEEAARLIDAYNSVVTTLSKMFVQWDAEITKMEIQSQPKKSDT

>SG-PL_contig_5069_1_+3_9_260

MHACMDVHMYVCSCSQKIQKEINFTKHIPNQFLFNPQPSFSKPFSYKLNNNCCNSLKSEH

SRIYPSHPSLANSHEQSAKVSLIS

>SG-PL_contig_5070_1_+2_2_175

IIQTASESERTTSTLFRFANSVEKTATLIFLMATLDFSPVGRIKKKASSSHHHYCVPL

>SG-PL_contig_5071_1_-2_335_508

MKFAKDSSLGYCWHIFKNHSCPIFMMEMFIPSWTKVHSNYSGGGSIGHPIRKLVSHQI

>SG-PL_contig_5072_1_-1_169_2295

MKTTKMTISSPRLLILLMLVMMMMTNARAQAPTPPTITKPAYYEEIYTRENLNQHQKCEA

TGYPVPDIEWFYSKTKDDFGAIITQTNPEGGLASFDGKTGDLWIGNLSASAIGYYSCRAI

NTFTGGLKVTALSPRLYIGIARVGQWNPSAATSTVTVTVGQYAELPCQDVPAFFGPTGYR

WVNVSTSNSDQLPPIQMGNRKFIDDKGNLHFTYMEQSDLQLNYNYACGLTNQIAGQSPSG

GTIRLEMTLGSLAPTNPVLRYKTSKNADGSTFELGYPAKIKCFFSGYPTPVVTWKDNGGN

EITEGNGYTFENDKRTLVINIAGTEHDTTYTCVGTNPQDSSKSVTEDVWVNITSPPIWVK

PLQSQTVVQGKNATFYCDSRSMVDENSPEKPKWFKNGSPMSNTHDPKKFSFSNEGKTMTV

ISLNKRTDISCFQCLVKNSVGESFGDGCLNVILPIEILTQPDPKQVISKGDVVNLTVVAI

TDPDYTLSYRWIFKNKTYDSVDAPPYVNYDFISGLAYINTSGMSDEQLRNITGVYQRIVF

HRFESRTVPVEVELYVPPGPAVAGFPFWIIGLIIAILLILLIIIICCCVYCRRKMQEGDY

PVDRKETAAGLDPEKEVKESSFPALSRADDPDQLKKKGSHLDIDDEYSNPDDSFGSADYG

EDEPSAFNEDGSFIGVYKNKPKSAVHNPGFQPNPQLHRDYPTYRPESNV

>SG-PL_contig_5073_1_-1_1501_1980

MALMTQYQCLLSVTMLLQLCRITSPYLLLLMIQLLILKLALSLHESKEYLISHPKSCSSE

KEKQSLLYGRKMFEKLYICKERNTYLKKSKVIPAKTVRPTGCSKCRYCCTENVPEEKRQQ

LFEAFDGLFTFNIDGTIHRVCKKFFMATLAVVDTYVDHAM

>SG-PL_contig_5074_1_-1_19_285

MLVGDDSPHIDQAVEMNGRMLPEETDFMKVADCGGMPLEEQPGKVCEAFRLFLQGMGYVP

SLVQAKSTAGAVHAQLQHSLETRTTPAVC

>SG-PL_contig_5075_1_+3_3_260

KKNPQRERHWQSVLGDIKKNSLSLSQYYTNSNPTLRGTDLHPLTLAVAHFQTVAELSNNS

RTFKTVAAFPNSLAQHNSPTIYIYIY

>SG-PL_contig_5076_1_-3_3_164

CMNRGTNFDNSSVKNRWKLDWMEAVVKDCEGGKVRDTFEKDYNTRTCSVCALPE

>SG-PL_contig_5077_1_+1_205_360

MKKVHLPSYTFAFEFKILQQFLFFFLLASFSPEGLWNLCCWRDSRTQAEAKC

>SG-PL_contig_5078_1_-1_1_228

HCIYTHTHAYILSLSLSLSLSLSLSLSLLHKHIHTHAHTESQSHTHTHTYTYRLGDIVIR

GKLKSRWIKCSRKFQS

>SG-PL_contig_5079_1_-1_82_222

MSDCEQNLKIGGGIASKVQMGCSEGGISRHEEIPLEPSGLVALTFQR

>SG-PL_contig_5080_1_+1_97_312

MSELRDAKGTKYIPRVFGSLDWQLPVSFFTLLPVEVLTHFLHFSSLVETAGCRLTTAYVA

YKHNEAGSDPTV

>SG-PL_contig_5081_1_-3_207_1805

MVYKFGDEGSLRLLVVTCLVAAYLLFRLIEYLCRRGTLNCFAGCKSSGTSDKLYKFAVKI

RLYILLTVAVIVLAIVYVIVEIAIDKPRNLISLGGMAVFILLFYVFSSNPARVQWRPVIW

GILLQFIFAVIILRTSWGYDAFSYLGDRVTEFLAYTDAGSKFVFGDLFENHFFAFKVLPV

VVFFSTFVTMMYYVGFMQFVIRNVGRFLAFCLNTSPAESLNAAGNIFIGQSESPLMIRPF

IKDMTLSELHAILTGGFATIAGSVMAAYILYGVPPNHLLSASVMSAPAALAMSKLFYPET

QKVKHTKDEVYNMQKGTERNIIEAASNGASQSVKLVANIAVNLIAFVALLDFVNATLVWF

GERVGMFDPKLTFQLICSYVFYPLAFLMGVDGDDRLKIGELIGTKTFLNEFYAYTELSVY

IKNRETLDQYMTNYTLGLPTHPTGYWEYRGDDIYLADIDRLLKKGLMSVRSVVIATYALC

GFSNLASMGLMLGALGGMAPTRRSDVSGLVFRAMIAGNVACFMTACIAGLLST

>SG-PL_contig_5082_1_-3_1017_1397

MIYFDFCLFFYLFIFVFVLSSTFIYVCYFLCLIVGVCVWLSCLHSIHLFVYVCMCYVCSH

FTYVQYVLVFVCVCYLFVHVCVHLFVSLCMFVVHVHLVLTELVLDWMAVQPVICQVYFDV

IVVVVYM

>SG-PL_contig_5083_1_+3_981_1445

MKIPTKGALSNCNDWRGIILLLIPSKIPAKIQRISDAVHKQLKKQQAGFRKGRGCTDQIF

TPRNIIEQCTEWQRQLCINFVDFKKAFDSIHRNSWRILRTYRIPQQIVFLIKSFYTNFAC

TVGNSEHSFEVKTGVRQGCVMSALLFNMATDWVMR

>SG-PL_contig_5084_1_+1_55_282

MSTKLLCLSSGVWRRMSNNSQEFVHHHSRPDCETLSFKFFLMTIARPLPSYFWITMKSSR

STGLSWLVLVSCRNII

>SG-PL_contig_5085_1_+3_219_683

MILDVYRHCSASTKLFCLSSGVWRHMPNSSQEFVHHRPRSNCETLSFRFFLMTIARPLPS

CFWITMKSSRSIGLSWHVLVSCRHSTSKSYANFSTVDILFLALAQFQVHILKYFFDSHSP

AFFLLLLPRYSYLLRILIFVSGPVSDSHGAGEIAL

>SG-PL_contig_5086_1_+3_3_254

CPITLRSQRSEISTLLGASIIRAEPISSLTALPFLTLKKVGYPFTAGWNWSLHAQSFARA

RFTTCVVRLEDECSISVPTRHRKR

>SG-PL_contig_5087_1_+3_63_167

MEFYLIHLFQNNIKPTTTDSPHISQKHYTSSHIYL

>SG-PL_contig_5088_1_+3_9_275

MHLSKVAWVDRQFFKDICLSELSIHSKYLVNSAKQLCLTLPKNSKPQRVTSFPSDKHLRG

SGSSFTQSMNSLHCLSQYFPKWCAPALRT

>SG-PL_contig_5089_1_-3_948_1289

MFLNILKLMLHCLKYSASAAGRSCTNSILLLFLPFIFWPSLCAYFLWGVLAVEGQDLLDC

RLSFRADIFCWCPTEKTVCGKMHFLFFERNARRVILTLCLLHVFVSGHGLYSSG

>SG-PL_contig_5090_1_+1_1_168

TRVLCAACTWCTKKKNPHQQKRCSWQHFVEKSALIGKEKNKQTKKPQTHTHTPMPQ

>SG-PL_contig_5091_1_+1_112_339

MHICKSPRSKNRTGQLLNLYIMGLHCSFTDPIGVIKHHRISLTMPERWEVCLLNHLSCTT

EHKRTESTNIHNFYCT

>SG-PL_contig_5092_1_+2_164_391

MYNGEKSNPWKPNKGQDWNTQSKLSQNVEHPTHHSLTIIVHSQLNVHFHRCQHLQHINLL

DQANQHTHHSTFKPET

>SG-PL_contig_5093_1_+2_2_157

NNSSPSGKTLVEDLKVTEHSVEDVRITSFSPHLPGTFSKVCIKPSAYCYPFL

>SG-PL_contig_5094_1_-1_118_276

MFTVIMYSFVYTVHRIMCAKPFLFLSLVGEVSTTLHTKQFSQFSLASLRIDQT

>SG-PL_contig_5095_1_-1_100_1104

MGDTDFKEVNHGKFRQDWNQTSTGVGHHHRPGYYFPDTEFRALLREPLPPPLARPDEVTR

DQHFKTTTGTFHDPKHPSLLYGERIHKKAPGHWKVNYVKDLAEKLGNGGWRKPLTMGNQE

SEMKGEYKGLPGLRARYHFDDVYYPQDFTLQDHHKEGPTKYGEGSTQNPKLQGQPFYVRD

KGILTNLDPYLSTTHKDHRSFKPAELKAYPKKDVPTYWQCEEYPKAWGHGLKHNPLPKHT

VPRERLPMRDTMVFPTATKIPRPPKAMVPVPHGGLKSEYTDHFLKQDHVRQKENYYCAVD

TPFTLPKAGTKSICTAPKMYNTEYQHVGSEKPITV

>SG-PL_contig_5096_1_-1_1_156

VERMKDVGKFCDCSKCLYSCSDEKNKSALFAKLFIVYVLQDKINIENRQTAV

>SG-PL_contig_5097_1_-3_777_980

MQQRHNVCTWKIKTHAESKIVEFFFFTPIIIHSVSTRVCRYICTHTHLPMNARMHARAHT

HTHAVVEW

>SG-PL_contig_5098_1_+1_1_75

TTSISALEGPAQLRAQGKHIMCQVL

>SG-PL_contig_5099_1_-3_33_194

MQEDKRQKLQHSQIHPDLRGTLSTVCRNKGVVYNRLHKGHTYPTYTYLANGLAP

>SG-PL_contig_5100_1_+2_218_427

MNECDKTAMGPSNQTSECVNYEVLGTTPHTREICHLIHSTQCIKGNIHRPTVRFYSHKLT

CKKLKRYPQS

>SG-PL_contig_5101_1_-1_1471_1995

MSGFLIMSQFCRLLLSACVVFSCLFPAVPGGTVDRSKGGDRGERGGKKRGVQIPAEEDDV

PCETPSDPAQLNSFLWTIRRDPPSYLFGTIHVPYTQVWDFIPNNTKEAFQQTDYVFFELD

LTDPYTLSALASCQLLPKGENLANVLPAGLYSRLKRHLEYVRDMMPGWMTHDQRD

>SG-PL_contig_5102_1_+3_387_683

MIFVWESELTRVAVWDTTHLPPLKKTKNKAMPFSHLKHFKYSHVKTPVYLRLPCVQLLCH

YQSFTPQCTPSPPPSMTKCCSNRFHVILLQAVSAGSTQK

>SG-PL_contig_5103_1_-3_15_1445

MSAPSKRAPDSPGEVPRPPKISSSGASDVKLESFLRWCNDNKFNISPKVCVERERACAQY

GMVAGQDIAAGEVLFSIPRPSLLSPDTSSIATLLHKAQDELGEDSGWVPLLLALVYEYNN

PQSSWRSYLDLVPDFAQLDLPMFWSREEREELLAGTGVEEAVSRDLSLLEKDFNTKALPF

MQRHPDVFSPACQNLDLYKKMVAFVMAYSFTEPASKGAGADGKGKNVEDEDSDGDEEEVE

GCPPPMMVPLADILNHVAKNNASLNFDVDALRMISARDIKQGEEVFNTYGELANKHLLHM

YGFAECFPHNHYDTVDLPVGVLSEVVQSQTADAGLMAAKLLFLREQDLHQEGESYVVGRE

GVLTDEELQAVLKVLVMSADEFKEHAEKDGWSDAESDDANQDASPLSFAKMPDLPASWKE

ILSRCAEVCLSKLGGTEEEDKAAMKTEGRGMASRARYAMYMRYGQRQLLRQLHNACR

>SG-PL_contig_5104_1_+1_1_159

EVELPPSSLAAQVGVEVELLPSSLAAQAGVGVELPPSNLTAWVPVWVEKKKTV

>SG-PL_contig_5105_1_-2_221_1894

MTWCITTCNFDVDVDLLFQENYTIGQKLSLTEKIVAVLRRMKCPHRIEPHQIQGHDYIHI

YPVVQWLVKRAIETREEMGDFIRAFSVSQFSKQYATPEDVAFDAVKPKAIATVTSVKETY

KPQRRYKRHDADKLQDEETRVHSTLLEYGRRYGVSRSEQDQKEDEKASKKKAAAAGLGGK

DAQSEVDLQAEEERRIKALMSGMSAMGLQEGKLTATAVGSIVGMQSQEIQQIASEYAEKQ

VEIEQIEKSERVGGAQQHKRMTTSLNKQIKDAKTKLTEIEEKHQELHKTYVETQEKLKEV

QQHSAAIDEEMEKLAAVETEENQSILGTLRSLVALNENLKKQEQEFKAHCKDEMARLKGN

IEDLQRETGQLDSEDKERADLIGRQYEADKEKLHKIRLLLARKNREIAALQRKIDEVPSR

AELSQYQRRFVELYNQVSSTHRETKQFYTLYNTLDDTKLSLNKEVMLLNSIHDNFKQAMS

SQANKDQYLKQFEQIVENIKHNRAKVEKRRQEEKMKRDQLNDQYLDLVEKQRLYFKTVKD

FKEECRRNEILLTKLGSG

>SG-PL_contig_5106_1_-1_25_408

MCMSGSSITNTDGPVRNPHDERCIAGGSSSGSGALVAGGQVDMAIGGDQGGSIRIPSSLN

GIVGLKPTWGLVPYTGAIGLEPTIDYLGPMAKTVSGCALLLEVIAGYDEGRDPRQWPSMS

VPEYSKLI

>SG-PL_contig_5107_1_-1_46_429

MCLSGSSITNTDGPVRNPHDERCIAGGSSGGSGALVASGQVDMAIGGDQGGSVRIPSSFN

GIVGLKPTWGLVPYTGAVGIELTVDHLGPMTKTVTDCALLLEVIAGYDEGRDPRQWPSMS

VPEYSKLI

>SG-PL_contig_5108_1_+2_626_859

MDSVFFSCMCYHSLPLPTAFHQSFPIFNLMQMVPRCSGYSLLPYTQSVVNVPASSQSRTQ

LQGTHSCPPHVAGASDKS

>SG-PL_contig_5109_1_-1_154_1392

MYRQRYPDDPGMHNARGDTFTNVTYRQNQSFCERVKQSLVAIVVGVVLIVVASVLLFWNE

GRAVQTAQSLDEGLRVLVRLESVDVAFDNNNGHLVFLQGNLQSEKGVYDPVYQLSVHAVK

LRRTVEMYQWVEHETKREVNEGDQTRVETDYSYSLEWRQDVVRSDSFYSTIGHENPGSLP

ATTETFTADAVHVGAFYLSGGLMNQITDFKEITPSSGLTPGDSTLHYHNSYYFRSATPHN

PQVGDVRVKFESAGLLSGSPLGPPTDVSVIARQHGNILDKYQTNAGDELEILHVGRVSAK

EIFSKAHSQNTMMTWLIRLGGWFLMFVAFGCLTSIITTLVDWIPIVRELVAMGIGLMNAA

FSISLSLTIIALGWIRYRPLLGIAILVMAVTPFLFTKLRMFFSSSSSTRHRSV

>SG-PL_contig_5110_1_-1_46_303

MQMQQPQQAANQQQLRQMLQQQQQMRQQLMMQQQQQQQQHGRQPGPQNQAGVMPPASQVQ

MQGMQMGQMGGQPTQSISMDDFSFEF

>SG-PL_contig_5111_1_-2_638_892

MMLIMERWTWVEYELCPFCCFVYGVSMYIEKFHVLSLACLCSCVCICSFLVTHHGDCIYW

DVVLVAPSVLSGISSSHSSPLVPFW

>SG-PL_contig_5112_1_+2_725_1051

MSHTKFNIEDCRFCRTQVQRRSKSTRKQTKTLCQCIFCSYFRYFYHNKDGHAQLSIIKTV

HLLLSRERDCMAQNCCDEDRFLTAKSKTICRIDARMYTIRCDSVFLLLK

>SG-PL_contig_5113_1_+3_294_515

MVKPHTLFPQKFCTFFIFRIPTIHGWRFSLNLEDPSINKWKKKKLLYFCQQTHKITSSCT

HTNTQRKYGCHRLI

>SG-PL_contig_5114_1_+2_38_286

MRIHTKGVQAQAGLHTCRYGQPLACSMGTPAASYKIAGFQKPFSRKKYFTFIGKNVCRMK

LYSSFCSLSFGVFLLFFYIYGLK

>SG-PL_contig_5115_1_-1_1_288

PGKIPLLKLDSLSPFINPDSPYMHTIHNLVQLPKLLVHRVMSMLVKQTSSFSVFLCLFSL

FPIHFLVFILLPYSSSCENHLSSIIMLFLTFFTCTT

>SG-PL_contig_5116_1_-1_160_531

MTGFCFIGLSDEHDKLTFLLLLAHTIASLPVFFHNFPDVPLVAALIRFLTEPFFAVDFGV

GLYFNISIPSVFYMIMPLFFIRMGMKKSWSGLHRVVIPHLVCYFWYNIMTAIYPFTSWLG

LGRA

>SG-PL_contig_5117_1_-3_357_557

MQKVLVWLDDLLVFHSAESHDYYEHGQTQLQRFQFHNWPCCLLKTFNSKKFKTLIFLRKF

NSPHSWS

>SG-PL_contig_5118_1_-2_347_535

MISSRRQENRAFQISLQHFVTQPAFRHCNLVQKLSDFCSVLLIQNDFTQTCTYRILHDSS

HHF

>SG-PL_contig_5119_1_+2_158_667

MNPLSISIHILTYSLCSQAKGKSIQATFHTVHKIDNECVVLHQKQRKTACSNNKSMHTRN

QKTDPEDYSITKSSTQDCIQIEQHDPTIYLYHTNRSSGAEENSAYCFPFRPLFLCSMELI

LLIHIICFLFFLTLLCMIHSSFKYITPPNLTVQLISLFNVLEKCNTKIKY

>SG-PL_contig_5120_1_-1_28_1059

MAAEGMPPPLEDMTVTLEQARKLRDLRLGRQEQSPGARSSTEAKTKKKSTERSPPAATVP

TSHSHTEPSTTSTQEKTKTVKSTPDSKSGSGDFGGMKKGFLFGGSKPSHASGAQQTKAES

QKSKADSGKADPIPFIKPKTQDKDSGLKLDEVQKAMESTKGFLQDKDWVTGDLLEKVQKN

ETLNKRLGDPRFMQAVTEFQTNPQAAMVKYQGNPEMQQFLKEFCGILGEHFSSLGEKEES

QNNPQAQNPSNTVHTGPQITELTDEDTSQSVRPQESTVPVDPHVQQILVDPANREILMDP

KIQQLIQNLRSNPEKAQRMLREGDTAFKQKVDRLISLGLLKFQG

>SG-PL_contig_5121_1_-2_146_592

MPTFDPTDKQGGKAFRKFMEEWEGTFDEINKDFAEDEAYKEDEDLLDKLQQYKKKFIECD

QDLSGDLNMMDLKYMLEKLGQAKTHLELKKMIQEVDSTNKGVINYRDFVRMMLGPQSGVL

KMILMFEAFAKPQEKPTGKPPKRDLSSLP

>SG-PL_contig_5122_1_-2_11_934

MAEEVSASNHSNNSSEMLELSICQPDHPNQAVFVPDLNICLRDFRTKISDSSLFLPQRWL

FMTRAGSLIPADQEENIQLNTVSRERDEGTGVLDILITPSQYYQRQRQGNGAAAGASPLT

SFATIVAALRDIYVTTIQPNMHELIKPLVIAAGAAAVLAFIMAMCLTLAIGALGGMKRSV

VVPFLRHHVCGEELEIASEMPKLYKRINELEAKMSHMRQVEVGSLVCKEDNHMYEEPEFT

TIHVPFRKTSFEGAPRVVFGLRGVSMDDFNSMRLEDLKVNSTGFSVRCAYFGKWMHYNME

FSWVAVGK

>SG-PL_contig_5123_1_+3_3_113

FFFHSYIFIALKKHSFSHPYFTGHFMKMTPPLTQAHD

>SG-PL_contig_5124_1_+2_164_298

MPFKFNVNSNQQSKHCPPPLLTSPQIPTFSIHHKIGGTNDTTNNS

>SG-PL_contig_5125_1_+3_1071_1367

MNILIPKHIYISELSSTHPAKTGPVSQPVSQPDEDFRKRINRCLLSYQPAQVRNHFYHIK

YGREEHQSEFMKELCSTTILNCSSFYHNYPQLFLILPQL

>SG-PL_contig_5126_1_-1_1_147

WVLFVCLVFFCLLPRLLHRSCATTPTSLISSLQHTTKPTENEVTMREGQ

>SG-PL_contig_5127_1_-1_40_450

MSSTPTSSPSSPSRGMSSTPRTQLQCYYHHLGCNTRPQSNLHHHYHITRNEFQRHTSPPY

HMEWNPMPHIFTITTTSRGMESNATHLQTCFHCMGCSPPQFIIRVITITRDGIPHYNRFS

LTAPTSHGMEPTPTTHR

>SG-PL_contig_5128_1_+1_601_969

MIWVTSSVSWRMISSPCSLLVGIRRMLVWHPFCFLCVWQLLHVDSSHCFLIFLPGCGLRN

HSVLHLACFLKSGDRSNHLHVVFWSPSHLHPVNGPVSCLCVLCPLVLCHQNLSLKVDSCS

WID

>SG-PL_contig_5129_1_-1_640_885

MNNGDKLSFEKSIYLVLKFGSASGLFINADNTQTVSLGSKKWSQIKYMQHLNIYCNLDRF

KILGIWFTNDVKRCEEINYSEE

>SG-PL_contig_5130_1_-3_537_731

MTGTFKTGAFSNVGEKKQKQLGYQVSSPSSVSIKDHVRFTVPFCCSCLLDTSTNFLRKKT

KNDSA

>SG-PL_contig_5131_1_-1_112_1686

MSRLYDTVEPSVIDEEMLMRAVEEQGPKEEAGKIAKTEGIDFGDVTTLRLDFKNILKIDG

LWEFVNLTKLQLDNNIIEKIEGIDVLVNLVWLDLSFNNIEVVEGLDNLIKLEDLTLYNNR

VSRIENMDMLLNLQVLSIGNNDLKELENITYLRRFRKLKTLNLSNNPFCEFDNYKQYVAA

FLPHLEFLDYRLLDDQTKAAAYERHMIQIEEMLDNEKKAQAREEEAEKQATELELHKKAF

VEHMNGPHLFESMYSEDAEGQKLKEMPGVDEILVGYSEKFTGICQQIFEYGLKEHTKREA

EVAMFWECVEEAKTENKDMGMKAVDDFMMEKKRMFVELSHVTDPKMLEQRVSEYNTRVTE

LWDKLMGLELQLVDQLEEVIQNFDRNMQDLVTTFIEHLQGFLAVARDAENVHNERMMEIA

QVTLDKVAKNEMDDELSEELRMLFVDKDTIMNAVSTSHDSHLLKIDNKEDDIVTRINNWL

KSMIETIHDEQEIKRNRSRVTEINHLIDHLREEIDNLEIGSGHVF

>SG-PL_contig_5132_1_+3_3_236

FRFFFFFWNRFLYFVYFFCALNIDLPQMQAYSSKFFPVSLRPQKPYRQRNQDATSQMFNG

QQTYEWTERARAFAPNLK

>SG-PL_contig_5133_1_+1_1_234

SLIRQQTLLCIYCGDYDTFPLSVTKCSIDVKMCLCFYMPGGKNDESVNNQSINRAKSVNV

TQLTNMEHDCFGKTGDKR

>SG-PL_contig_5134_1_+3_30_119

MFNMVNQISKHKSNILIKFNLKKKIHYNRV

>SG-PL_contig_5135_1_-3_165_650

MSAAARTLPTTPKSQLPSLNANIRKVAVVMPGYQAQFLEFDQKRPLAAIIQDICGTWTLN

DANKFALQISDSSKNNTYITERNRHEIQNGAVLSLAPSPAQTAQSIISKLSQGATKTLSE

EKLEGLRQLVGLAPDSTFAVEFINKNGLPIIISIVTDGTFKK

>SG-PL_contig_5136_1_-2_800_1102

MKTPSKSENCRIYAICGFCDGRGGRMVKTLDFQTGGFGRPGFEPSQTFRLLGKKWRSFLS

YRSTHVQTCKYQNPLCVYTQHYTHVKHPVSHVCSVACRNTK

>SG-PL_contig_5137_1_+1_1_216

LFCYPIFCTTSEALTPRHSFWPLNTLPTPSSFSRSPSKPTGGKDVKDAMGPSTKGDSSLL

LGQFGGVQKCLF

>SG-PL_contig_5138_1_+1_550_741

MQLAKGSPKQETIILSVNLLVLLISSLLPVLCCVMLCCASGSHSSKHWGKKKKHTHTTGS

ETNY

>SG-PL_contig_5139_1_-2_2_181

VKYITCGTGSYYSVLSLKQYITNSINSTSTTAFHATQIFVLLCIDLFFVITNFPLSFLVK

>SG-PL_contig_5140_1_-1_28_444

MDSDLSMKQHVTKICQICYFELRRISSIRRFLTEDATETIVISCIWSRIDYCNASLTGCP

DSTLQPLQKIQNSAACLIFRSHRQQPCTPLLKKLHWLPVSERINYKVACTCFSIITCSAP

TYSQNLLPSTYPSQSPSFF

>SG-PL_contig_5141_1_-1_1_171

YYGFRSFHKTVCDYFVKFATLNQEESVLLEDFWLVLLEDFCLKLPQKPLLLLAFCQE

>SG-PL_contig_5142_1_-1_262_501

MLKPLQKIKTQLLALFLSHTINSQALLKKVHWLRISERISYKVVCICFNMITGSAPTYLS

ELITLYKPTHSLLSSSDSRL

>SG-PL_contig_5143_1_-1_55_237

MTDNMLKLNDDTTGALFSGSVPSDCPLPSSISVGPSTVSFSYKARNLGFIMDSDLSMKQR

D

>SG-PL_contig_5144_1_-2_269_4066

MRWVHCPGVWLLMLMLACGLDAVPQQLMYPYGPQHGDRTLPPEDDFSSDEIPLSVPVVFF

DTPFRSVYVNMNGHVSFETDIPNYQGDLVLPIGFKLIAPFLADVDTRVTGTVYYRETWEE

DVLGRAAADVQSHFSGRADFQPLALFLVTWDKVGYYDKQYDKVNTFQLVLVTDGMSSFVF

FHYLDGGMQWVSSQGKLAPTMPDVPGQAGFDRGDGYDHTSPTLPFSGSTAAFVNESNVNI

PGVWMFQIGNTGPDGDVKAADLNTGRVTIYEVGVGQTCLEGARQCHINARCLDFDQGFCC

QCLPPFYGNGKHCLEPEVPQRLNGRVHGTINQVTLNDLDMHTYVVTTDGRTYTAISRVPT

ALGPAMETLSTVGGAIGWLFAVPVGKDSVNGFTYTGGVFNRTAVVNYQRGNHTMHTMRIT

QRFFGQDALNNLRMDTHLEGDLPDITKGAKVTIDDYNENYKRIGPGLVKSFSVRTYRVND

VAFRYTWDQTLSFEECEADPSPRRHTMKLAVSRNFVRHSDKDQVVRYAMSNKVSIMTGSD

PCSDAAQTCHSHADCVPADQSFHCVCQAGYQGDGLDCQDIDECSLDAGVCDENARCFNVY

GSFQCQCQPGYQGDGRICTRERQQCSGQYCGDNARCVFNSELGRPQCECNPGFRGDGQTC

SPIRFGCNEADICDENAECVLDPETNSYSCQCLDDFSGDGITCQPTSRGDCGHCSRDADC

VYDIERLVYRCNCRYGYTGDGITCTPIDRCSECDTNADCSFDPAANRFQCQCRYGFYGDG

SHCEEYDCRESNVCAPDAVCDFDVNFNAFLCRCPAGYAGDGFRCEVKGCDDMNDCDVNAR

CVPDPLRTSRFVCQCSPGFQGDGKVCIQQVTPCNQVNNCDRHAECVYDPDALSYRCRCQR

GFEGNGYTCQQRDAADCRSQPSLCDRHAACVEGASGLFVCVCNRNYRGDGRSCTVAEREG

ERLVYARGSKVFSVSTTPQPGDYGQQLNFSPGQLAVAVDMDCVQGDVYWTDAAAGLVRKA

SLNGSNSEVILTGLGSPEGIAIDSVSRLLYYTDSKLDVLGVALLDGSHRKTLFNTDMVNP

RAIVLDSSRGKIYWTDWNRNRPQIETANMDSSERHVLVNGDLKLPNGLAFDHFSQQLCWA

DAGTKRLECIRSDGMGRRIVSNAPSHPFGLTMLNGLLYWTDWERKNISGVSQTGGDQVSS

VTPAIGGNGRLYGIVALRDHCPRGSNSCAFSNGGCAFLCFPAASGGRTCACPDGVDPRIC

QDSNNF

>SG-PL_contig_5145_1_+1_190_381

MKTPKAHIHTHSWASHGQKSDDFLIFTNSDINRKTYFFTTLFPRSWINTLLFYILSTQNV

LSKK

>SG-PL_contig_5146_1_-2_95_1114

MNKMDVQNAQKNWQLANNIESVSSADDIYRFDAKQQQDILTAKPWDKDPHYFKHIKISAL

ALLKMVMHARSGGNLEVMGLLLGKVDGNTMIVMDGVALPVEGTETRVNAQAQAYEYMASY

TEAVKMVGRLENACGWYHSHPGYGCWLSGIDVSTQMLNQQFQEPFVAIVIDPVRTISAGK

VNIGAFRTYPKGYKPPDEPPSEYQSIPLSKIEDFGVHCKNYYSLEISYFKSSMDRRLLDS

LWNKYWVNTLSSSSLLTNADYTTGQINDLALKLEQSESQLGRGGFIIGVDTQDKKTEDKL

SKATKDGCKTTIEAIHGLMSQVIKDRLFNQVHCGKAGPSS

>SG-PL_contig_5147_1_-2_77_910

MSADKRRRARVQGGWAAPPKNVRSDKHKPKAEPEPAWLARNVDQPTQRKFVYEQPEEELQ

VKPDEIVVMKQGLYDISLGPSGVSRLRYFPNFLDPREASGLFEQLFHNVPWRQRSVIIKG

ESYLMPRLTAWFGEQPYSYSGVTHNADLQWPTEIQELRDRLEEITGTRFNSLLCNFYRDG

HDSIDWHSDDEPELGTNPTIASLSFGDTRTFEMRKKPLPGHPVDYSLSQTARIPLASGTL

LIMEGATQNDWQHRVPKEYHDRGPRINLTFRVIYPEKT

>SG-PL_contig_5148_1_+1_1138_1485

MFSQMRVCTFSLARTVQVLCRCSLHIPFLWGVHAGHFRAFMSHGRLKCFAGSLKDVFDVL

HAYIHEGVQALAGLHKMMGWEIGKISTIYPPGEVPKEDLNPGLPNPPVWKSGVFTI

>SG-PL_contig_5149_1_-2_176_484

MMCVIARILACDRCANDRGVIMQYKQVFWMCLCVGRIGKTNIFKRLNIFLLKTIPFLSLF

FFFFLFFFFLLSIFLFFFVLFFSFFFFFSCSEKKLFVTERWHG

>SG-PL_contig_5150_1_+2_944_1213

MCCVRCCVRVQFSQKHVLRNSWYWLAWRSQLFSGLIILARPRPTANQPNQRRWPRGECTH

HKKWHHMRHQDFDTLSHILSYLGLMHCTLG

>SG-PL_contig_5151_1_-1_13_693

MEDTLIQQSLQIFICLLLFSFHFGCDAETIEGPEKTSFKIDGKVFVTSSKDKDWVSNTRV

LIDGGRYRGFLRNDGTFSVSGLPSGSFLVEIANPTYLFEPLRVDITSTGKMRARKVNLLQ

PTKLQTVQYPMEFRERAKASYFQQREQWRLTDFLFNPMVLTMVLPLLLIMVLPKMMNAAD

PETRKEMQQQMNVLNQKPNMPDMSEMITNYFSGGKKPTKSKASSKRK

>SG-PL_contig_5152_1_+1_1_189

SDLSKDNDKGKYYFLPEFNRRQPSHTIKLLFMLFQHILGNIQSKKKLNEFFSNSFPSHLM

NYI

>SG-PL_contig_5153_1_+1_49_306

MREWGWGNWGEDSPPCVEDLSQKDWWWFSAGSTPESDASPLELLDLGWSTPVAGRWLAEG

ARWFSTCGGCGRSCMGAGCQNSGHWS

>SG-PL_contig_5154_1_-3_3_182

FFFFFTSLVWLNVMLLVTLVLSSFQSCALFWSSTTVYQMFSPWDQSWFPVKLLRFEFVYL

>SG-PL_contig_5155_1_-2_2_169

NVLIPIFSQPQTTHQMSGSNRDLSITSSFDRAMDSRCLVSSSVPTRNISWLVCCPS

>SG-PL_contig_5156_1_+1_502_759

MNAVCGNKLKEVLTTDCVVCVCNRICVCVCVFVCVCVHMCGVFACITCLHLYIEVICNKI

FNATNCLTFRTRTVKGTYLPAYAHSE

>SG-PL_contig_5157_1_-3_732_947

MNHRARGKIPKVCNCYCLFMKENYRFRKVSRFFVVFKKKQPKRKSMDLLNIFTHLCVYTV

NKLDVTFETTRK

>SG-PL_contig_5158_1_+3_117_377

MCVFLKTEYTVLSLKNTTLALLGTGECMGTDDTSHQILLVIVYTNVFCHQNQTDFSFVLC

KYVKQKSGKSLKTKLRGGNSVHFQIDD

>SG-PL_contig_5159_1_-3_276_1565

MASGFINLDKPANPSSHEVVAWIKRILRVEKTGHSGTLDPKVTGCLIVCIERATRLVKSQ

QGAGKEYVCTFRLHTPVKDEDKVCQILQKLTGAMFQRPPLISAVKRQLRVRTIYSTDMLE

YDKEKGLGIFHVKCEAGTYIRTMCVHLGLHLGVGGQMQELRRTRSGIQSEEEGMVTMHDV

KDAVWELDHHKDETYLRRVIRPLEALLTSHKRVFVKDSAVNAVCYGAKIMLPGVLRYQDG

IELNEEVVIVTTKGEAVALGIALMTTATMAACDHGVAAKIKRVIMERDLYPKKWKLGRIA

QIKQRYKQEGKLDKYGRPNEKTPSNFYQYIGAPPPPQAPVPTTEAPVTPMQSSSSSSSSK

RKRETSDSSDDSADTSAAATPSASGDASSEKKKKKKKKKDKHGEEERSESEPEKKKKKKK

KKSKMEVDSD

>SG-PL_contig_5160_1_+3_27_320

MSCVGFPDIFFVTVVLIDASTVIGPYGSAFCVVTLTICRIISPLYVATNYLIVRLLMKPP

AKQQRKTVGDSLVRARMLGGTEVVCPHCHTACIAPALT

>SG-PL_contig_5161_1_-3_99_1811

METPPVLPPDSPGSSVSTRLQEEYDELLKYAVVVPDYKPTDVRGKLTDTRGLFDRHDDII

TVRASPPRSTLLHDADRATTSQQADLEEQQDQGDSTPRNRERHSDDSSGGAKELPDFGES

PIDAHAPTSFTRQMRMGGEAWYGRHDVPETDSDVPLKTVFRTTVDQDVGKMETLLDQWCL

DLKRNVLAEFSQAKIIIEERSRQDTLRESNRHCKEKAELIREMDDMKELLHTYEQSMKRK

DAIVANLTQAVHRQRDKLKMMSNFSQWRLHHIDAKREAFTLQMARRHYERTLNRRVLAAW

FSLIHVKWRQRVEKACQNKAQELCMQVTADYESKIASMNEAQESSQEEIRRLHIQREKYE

EAMKKAFMRGVCALNMEAMSMFHGETDGQTQMTQEEEGEYSDNLDSHVYEKTAASSKTIP

HDALRSSSSSGAAADAPPPKVVTSQGSRSILTGSGAGASRQISAKASSSTSSLTTSVAPK

TRMVAKVMGKGIDSGRSAPRAVSPSFGHHAPIASVVVERHQPVTKQTIGQATAARYSKNT

SSTVDSDKFRKIAGQGTVAKTPAIHTVRVVD

>SG-PL_contig_5162_1_-3_90_851

MSSIGTGYDLSASHFSPDGRVFQVEYAQKAVENSGTAIGIRGKDGVVMGVEKLVTSKLYE

QGANKRLFNIDRHIGGAVAGLLADARQLVERAKEESANYRYNYGSAIPLKHLTDRVSGYV

HAYTLHSAVRPFGTSMIIASYDADGPEMFLIEPSGVSYGYYGCAIGKAKQTAKTEIEKLK

MKDMTMQDLVKEVARIIYVVHDEVKDKSFELELSWVGDVTGGKHQLVPKDIHAAAEKYAK

EALEESDDSDEEDL

>SG-PL_contig_5163_1_-2_482_730

MECVAFCESTALPALLPLMENAESTDVDFLTSWTGQTALEWISEVIEQNCSYAVNVLNHK

QYSQTILAVSFFLLCLQIHFLQL

>SG-PL_contig_5164_1_-2_413_688

MQSFFKLKTLLGHTISKCSKMEIYYHVPRSVPSLCVCVCVCMHVCVCAYRCVCVCVFRVR

GWGEELNRRKGAVFLRFPFHQQAMFHHKFCYT

>SG-PL_contig_5165_1_+3_165_428

MPRRVFLLVRCEVTVRPSAPRSCSWGVPLSSLTPSLETRFGALLFMASATPVGPSPGTGL

PQSPPFEPLAGIPFNLLTLKTTFLLALA

>SG-PL_contig_5166_1_-2_140_313

MRFCVQRSCLVDLMWQHYNNNVSTLLSFKLCVLCVCVCVCVCMYVCMYVCVCMYVGMY

>SG-PL_contig_5167_1_-3_321_512

MQIKKNGVDLKTESCSVAQPLKLLRDNLYFVSNKYRAFKVSVIRYFVDPSMVLSISLFLV

AKDL

>SG-PL_contig_5168_1_-3_1713_2033

MCKYIYAVTVIFRCVCLSFLFGLETGLCLILIIKVLFYSQFNQDCQCVKRKYYVLLYFRD

GEKSLHELSFYCNLFILLPDQFEYYTQIHSPCPLHKFAFVLRCFLVC

>SG-PL_contig_5169_1_+3_3_137

HPGPWWGAGAMPRWGVCPPAAEAKVQSKKQKMTSPLIWACCGTIT

>SG-PL_contig_5170_1_-2_566_715

MHTQRMLRSPQQQRSCPWQNCVLKSFFIRQTDIHADRKKREVALHCGSAL

>SG-PL_contig_5171_1_-2_2_265

KFPRDDRSRRVTPFTDLPDYTFLDGRPTPLYKWQLARKEKNYQLAKRIVMLAKEVEDAKA

AHVKRLQDEAARRENIISSKLKPKSEET

>SG-PL_contig_5172_1_+2_785_1051

MNDQRKSLIYLTRRIQCLFLSLIFLPILSQISSQSRQCSIQSETSCVFELSNQQSAVSQF

FPNPNSRLANKTCVTEANPQLPGDISSHL

>SG-PL_contig_5173_1_+1_502_762

MRLPPTYGGNRSMLISGSLIDFQTHNPAPAAEREWNYRCATSTKTYNKTKSNQKQTKKYC

LLHRLLSSLLLINKLHRRHPRPSGTTN

>SG-PL_contig_5174_1_-1_82_195

MGTALIILAPRRVEVSSTSLPPMTSGLWVNFYLLPLQH

>SG-PL_contig_5175_1_+1_52_258

MGNVLFCLNCVCTHVQTFHVLTALLVNKANETFRIKHSLLILLVTSPAMTQCSRHSSGYP

WKEAVTEVD

>SG-PL_contig_5176_1_-1_178_372

MQEFCYKMVCHQNVNYCRFSTSECCCPNLVFRLLIKKKYCNTFDCTCFMFIVHNFRSILV

FHMVT

>SG-PL_contig_5177_1_+1_1_198

WWSGCKSLNELINQVHVEEARATRELSLWKNPLSQIKRNTHRPKTTKEKALFVGRAAPIP

HGKSIV

>SG-PL_contig_5178_1_-2_47_553

MQLCLVLVCCGLVVGPIWAVSVKMATVVKHNGVRKIEVQTDSASNIYELKWNSELAAKAA

AWARRCQLGFSPDLDVNIAQNVFMAHYFEFRKPKAAMLIKNAVDSWMEEKSKNNGNFACC

KDSSVDCCSYTQVVSSRSKEVGCAVEYCPGNQAYVVCNYKPMGNFNDQA

>SG-PL_contig_5179_1_+1_1_219

TCSLTHTHEYRGNSKERHKQMASENKSSQPEGKVYCLIFLLFPNPTQVGGGEAELKPLVT

SLLLPFSIVSYEK

>SG-PL_contig_5180_1_+3_234_578

MFQVYFNEESERSCQGVSPSLFLSLSLSLTHTHTHTHTHTLYKLTVTHLHTHKHYTNSYI

HTHCTNSHVCTPHTVQTRTHTNHPLGHNKNYANHSTDSSTGNKNFQMTTQDSATV

>SG-PL_contig_5181_1_-1_1_210

GAGAALLGGTAAGTGPRLVGSVVGAPLLAPSRRRTLTPPPPPIAAGAGVPLGIGALAARG

PAARGPAAGV

>SG-PL_contig_5182_1_-1_295_414

MCIFSHDTKVLSAHPAKLLFIFLLTELYFFSFLTWFVCVR

>SG-PL_contig_5183_1_-1_1_1029

VRPQQRRPEPNRGARVPAPAARPSAPTNQRRPGGPDRGKPGAGKEKRKDGEGDEKKFDPT

GYDKELVEHLERDIVQKNPNVHWDDIADLTEAKKLLEEAVVLPLVVPDIFKGIRRPWRGV

MMVGPPGTGKTMLAKAVATECGTTFFNVSTSSLANKYRGESEKLVRLLFEMAHFYAPATI

FIDEIDSICSKRGSDSEHEASRRVKSELLVQMDGVGVTNDDPSKTVMVLAATNFPWDIDE

ALRRRLEKRIYIPLPTEDGRRTLLDINLREVELEPTVDLSEVAAKLDGYSGADITNVCRD

AAMMSFRRRISGLRPDEIRNIPKEELMQPTSMEDFLEAMKKVN

>SG-PL_contig_5184_1_-1_253_492

MMEVSGQQLFRKSGGRFCLQCMLVDTQRFIFGLSEVDVFLYMSGFKIIGSIPPQVPLRHI

SAVPFPHGCELKHKPAVLTC

>SG-PL_contig_5185_1_-3_3_212

SPFFFLFFSLGHVQREVGSKEDNKPVPATSLNATPTVVTTTQFHRCWQGFLSHLLDFDPN

LPYIQSSAWP

>SG-PL_contig_5186_1_+2_8_232

MSLFSILESCRLFMMNSLSELGKTDQSRLCCQTCVDNDMDCFFRAWQKFTSSRSALLDQQ

TGSHPTKLDSYQSRR

>SG-PL_contig_5187_1_+3_384_818

MTLNSTIHTPGIFHSNYQTTIYKNSKTPLKIVNVLSCWFFFFLLGLVGNTLLYTWTGSVT

EKYYGMFTPASPGCYMSLYVLLSILEFSKIELNAFSLPLFATTLKNKNVFLLLSFWCVHV

CVCTFMQKYVHVCKCEHRKLSFSPS

>SG-PL_contig_5188_1_-2_32_1228

MATRTMVEHVVVDAGGFIRQAPIREIGEQVYSVPQVVQEITDKETKRRLQVLPYTLNLQK

PTPEAIIFVTDFAKKTGDYKHLSCTDIGVLALTYMLEKQHVGDEHIIKEPSKKPAEYIVP

SKGSSGQQIAGFYVEGETKSERKENSNKEVEEKEEEAKAEIGGDVLPEDLLEEETEENEE

EAEEENDDDDEGWITPSNINDIKRQMGIGLAAAEKTKVSVACLTTDFAMQNVLIQMGLNV

VSVDGMLIKQARSYVLRCFACRKITYNTMKVFCPNCGNKTMKRVAVMIDEEGKVHYYFSK

RPLNHRGTKYPLPTPKGGKHSNNPHLCEDQPMPQQRARKGKQVDALDPDYVAGSSPFAFI

DVTSRAATIGISGKHDFRTGAYWNKRNPNEARKRHGRKR

>SG-PL_contig_5189_1_+2_2_106

LFDRKQAISGESEEGETWSDLEALQMSLAALIWIC

>SG-PL_contig_5190_1_-2_605_853

MYPFIYLFAWWRMINFVSSYDLLTICLLSFFFRWSIVQWLCHLSQRLTHVSKRQQMTSCL

PIKRRYHLKLKTPETSPSKLLYT

>SG-PL_contig_5191_1_-1_283_1173

MAYYSVAHLLQGGIVDGSKPGPANIRPEQMTLEVWDYIFMQTSTYPSKCNIPKSTLDCLR

AEFQYWYPVDVRMSGKDLIQNHLTYFIYNHTAMWPQDNSKWPKGIRANGHILLNSEKMSK

STGNFLTLSDAIDKFSADGMRFSLADAGDTVEDANFVEKMADAGLLRLYTLLEWVRDMAA

QRDSLRSGPPETYTFSDRVFLSEINQTIRETQNHYDNYMFKEALRTGVFEFQGKRDKYRE

VESKGMHRDLVFRFIEVQALLMAPICPHMAEHIWGELQKMQTGSSGESIMHASWPVA

>SG-PL_contig_5192_1_-3_3_149

SRCLLPVFLWFSPLLAHYNRACRTVYSGIIFVQVHFPNKQGRSLTYFVL

>SG-PL_contig_5193_1_-1_160_333

MEIITAHFCQGSRDTPYSLRFSTAMGSFDVHRPEGTRVRLAFCPHPKDLTFSSIFPSN

>SG-PL_contig_5194_1_-1_97_1980

MSGDGYHCPDAKHQQALRDMANLLRIHSIQSTNASKSGHPTSCASIAEVMSVLFFHTMKY

STKEPRGASNDRFILSKGHAAPILYAAWAEAGLFPVADLMNLRKLTSDLEGHPTPRLNFV

DVATGSLGQGLSCAAGMAYTGKYFDKVDYRVYCVVGDGETAEGSVWEAMAFASLYKLDNL

VCVFDVNRLGQSEATSLGHDMEIYKARAKAFGWNTYVVDGHDVDALCRAFYDATTVKSKP

TCILAKTFKGRGIVDIEDKDNWHGKALGDKADGAVKVLKGIISNKGSLGLKPCQPEVKAP

VVSVKDIKLSENPTYSEGEQVATRAAYGTALVKLGKTNKHVIALDGDVKNSTFSINFKKA

YPDRYIECFIAEQNLVGVAVGCETRRRTVPFVSTFAAFFSRAYDQIRMAAISQSNVNFCG

SHAGVSIGEDGPSQMALEDLAMFRSIPECTVFYPSDAVSTERAVELAANTHGMCFIRTSR

PNVPILYSATETFKIGQGKVVKKSASDKVTVIGACVTLAEAMKASEELAKSGINIRIIDP

FTIKPIDADLIIKNARETGGKIITVEDHYVEGGIGEAVCSAVCECRDITVKKLAVREIPR

SGKPNELLEKYGISSSCIVKAVNQVLSA

>SG-PL_contig_5195_1_-3_177_287

MPPLDFEEFFPSLCFLVYYVSTWLRLFLVVFFFGKKK

>SG-PL_contig_5196_1_+1_916_1038

MTDSACYKHHLPHSLHLHRAIFQLCKKTFSLLQPRVLQTQK

>SG-PL_contig_5197_1_-3_402_539

MTRVLFVSSIKNLKKCIGPKVVILYHQNNLLLCHMLHLLSFEIVCL

>SG-PL_contig_5198_1_-2_11_607

MATAAQPTLFNQAVSKILSSWTALRIAVEHGCGGSNSKQKAQNLVNDIEVVFNRKNKLDP

SDFADFLEDEVDTRFDLILEDGSVQMIANHICQCFTRLKEGRVTEVEQFLKGLPNSRECF

VQFAGGENSSDEDDEDEDMENSVSQPQQLQQLQQQSQSSNNDGRTSSELPTTNGSGDAAM

DVEEEEDGWTIVRKGGRKR

>SG-PL_contig_5199_1_+1_628_840

MFAVKFNMFTTFNCWKFTSSSTHVHGHDYQQSIFIQNVNKATLNKAFIQYVNKIFHSVCE

QSNLFPHPHHK

>SG-PL_contig_5200_1_+3_3_215

LDLSAAFDTLDHSILLKRLEVTFGIQGTALKWFVSYVNERTQSVIINGNLSAPQPLLYGV

PQGFVLGPVLF

>SG-PL_contig_5201_1_+2_2_322

KILEKVVLSQLLKHLSDNGLLEIYQSAYRNDHSTETAVLSVLDGLLTKADERQVSLVALL

DLSAAFDMTDHSILLKRLEVTFRIQGTALKWFVSYVNECTQSVHHQW

>SG-PL_contig_5202_1_+1_142_333

MSTPSLSSSMVVYLLFSLFCMVVHRALFYVLFCLHYIHMISSQPLSDVIALHRHKYADVI

ALHD

>SG-PL_contig_5203_1_+1_1_153

QSAYRNDHSTETAVLSVLDGLLTKADERLVSLAAQLHLSAAFDTVNHSILL

>SG-PL_contig_5204_1_+1_1_222

TQWCRQPEHVKRDSAGGVKNGHHIPANNEGKRSGLPTSQQHTSALQQLVISAVCSSFHMM

QLQNKCGGRGTGQQ

>SG-PL_contig_5205_1_-3_435_764

MHAPTVFQKRAPTVFQKRCKPNCMLRTDCMHTHTHTHTHRVHRRQMTSKNKSPICLLFSF

LFFDNRIAHQIYTLTVYRKSLLQGRLFKMQLITHTYFPLMVISHKRNFKM

>SG-PL_contig_5206_1_-3_18_206

MKKHSLLFLRIIIIIIIIIIIIIYCHYCYPDSLLFLKGNFIIYCHYCYPDSLLFLKGNFC

FHY

>SG-PL_contig_5207_1_+1_103_282

MASAKVGTALAAVLVNRNSLMCGQLCTHYIVLYSTYGCQSHPHQADQGVLQIRVYGKLPI

>SG-PL_contig_5208_1_-2_473_667

MFDIYTLVVLVIHTVSELVSSLNIESYSASDVKKSVWITCYHSSTNIQHTTNIHIYIQSD

TNSDR

>SG-PL_contig_5209_1_-1_1_276

AEQQTTRKQTAVYVQCVLLFPTATVMELPAFGDRVFQADFITKKRYRRGKVEYLVKWKGY

SSKQSTWEPKENILDPLLIAEYESKRLEWRKR

>SG-PL_contig_5210_1_-2_194_277

MLWLVTVMGFHSTFLTDSVGSGCTITVR

>SG-PL_contig_5211_1_+1_25_324

MVHRQTHYTCTNTEYICRKTKIAACFALGSKCEEKSISIHSHSWCPHKNNKASRQESQTA

RDLSLSPLARSVARTTGRDRPKHNSSQFFCLFQSSVCSLP

>SG-PL_contig_5212_1_-2_122_1708

MTGIICTIGPVTRDVPQLQKMIIEGMNIARMNFSHGTHEYHKGTIENVRKAVASFSEPRP

IAIALDTKGPEIRTGLLEGGASAEVTLKTGGSIKITTDDAFMEKCSAEVLWVDYKNITKV

MEVGKRIFIDDGLISVIVKEKGDNFLMCDVENGGDLGSKKGCNLPGIPVDLPAVSEKDKS

DLLFGVEMQVDMVFASFIRSGEGVREIRSILGEKGKNIKIIAKLENHQGIKNFDEILEEV

DGVMVARGDMGIEIPPEKVFLAQKMMIGKCNRAGKPVICATQMLESMTKKPRATRAESSD

VANAVLDGADCVMLSGETAKGSYPLESVRTMHHICREAESAIFHNQQFEELRRETPVFTD

PTHTIAIAAVEASFTCMAAAIIVITTSGRSAHLVAAYRPRCPILAITRNPQTARQCHLYR

GIFPVLYVDNKMNQWTADVDRRIWKAVHLAMERNFVRAGDPIVVLTGWKPGSGSTNTMRI

ITATDVRDKELLPPITGISSVPSFNRVGDDMTSDASLVSVPTDENVKFF

>SG-PL_contig_5213_1_+3_3_278

LCNKRKSKVKKRRLSWLGHTARLPSSRPASQVLNWNPERQRKRGRPRMNWRQTVDRDLQL

VDKRWSDALRLSADRARWNALAAPCVARRGST

>SG-PL_contig_5214_1_-1_61_279

MCLCACTQVHGCVCEAERKMCHEKIDQLYALHLDPSYLAQGGQKCTVGHDLLLTGSQQLL

EMYVSVLHRFLDC

>SG-PL_contig_5215_1_-1_88_858

MIPPNASLVKYDNPVLVSRNTDKKTPRAKALKTSPTPPSGTGPVPNPPSKQKLSPLDAKA

AQQTDEILNSILPPREWTENGQLWVQQVSSTPATRLDVVNLQEELDRRLQQRQARETGIC

PVRRELYSQCFDELIRQVTINCAERGLLLLRVRDEIRMTIAAYQTLYESSVAFGMRKALQ

AEQGKADMENKITTLEDEKRDLERQVNELKAKCEQIEKRAAEQRQVEEKKHTEEIQFLKR

TNTQLKTQLEGIIAPKK

>SG-PL_contig_5216_1_+3_3_482

GEGERGGGEERERERERERERESVCVCVRVCVCVCVCVCVCVCVRSKNWWLNFLLLVHVL

IYTICACTYHIQYYAQDRVVGKLDQLFMMQKDAQKYVCMCVCACVRACTHQRNTWRVEKG

SNGVTHIQRKVRSEKIKVLASVRTICFSIVTFRIIAVFFS

>SG-PL_contig_5217_1_+1_1_117

LTMSILYLDHKCIIGPYHSNLKIYFSLKIQYDYESLKLE

>SG-PL_contig_5218_1_-1_91_1041

MDNSSLTFSDQEIQEELSRLGYNNIPRDKLREFQRDLNQLVLAERSKGSSHDNSFGSGNH

AVTRPEHIEDFCASPLNPPLDRNNGADDTDVHHFPLRREDLEKHDIKQQEQSKQRTKVPG

HFSLFELPPQVHLRDDVSETDSEIRRMKRKTLRKNEEGAKFIDESMSDIDAGSIVDTHER

IQRMVLRDLDPVVPRRSQSAHSSREPPPYRLSPDDPRLPSVIYPSFDHPHTRNLRRADPV

ARYHQMQQAWTLQQAPGENQRKGLRWNVREQMLTQFVPEKKPQRKYVPNNYVPPTEKQRK

ALRWQIRTDMAQGTMPV

>SG-PL_contig_5219_1_+2_2_223

FFSFETPVFPIFCFFKDSFTFSFSFLVSLSFLSLFSCCDLASLPVFSSRVPALLTLRCLL

PLGLGCTGFLPKEA

>SG-PL_contig_5220_1_-3_3_371

GVSKEKKDHNYGAPPPLSPPPRTPSPFLPTVNGSAEVEEEAKVPAVSNEEEVVEDSVTRC

ICGFLHDDGYMICCDKCSVWQHIDCMEIDRNNIPESYCCEECEPRELDFERAKMIQLRRR

EEI

>SG-PL_contig_5221_1_-2_2_298

LRETVLGKDYDVYWGIPFAKPPLGNLRFKHPEAADPWNGILSADTKPNSCYQGIDTTFDK

FSGVDMWNPNTNVSEDCLYLNVWVPRAFGTQPPSTTMVW

>SG-PL_contig_5222_1_-1_499_693

MSLPRMIPKILRILLVPFVLCMFSSIVIHMMYNYIIYVLLLVLVLYVIDPFSVEIKISFE

LEKKK

>SG-PL_contig_5223_1_-2_38_289

MLEVYRMYQWAAWYGYKRYCTCTILFSVKYIFSFSIAGFHYAYIVMSDFLFKPSKRHFGT

LQVCLLNMNFFSMKLLFYVYIKRL

>SG-PL_contig_5224_1_+3_351_596

MIWLVCSLLVLLLYGSRCDALLDLSAAFDTTDHSILLTRLKTSFGICDTALAWIASYLTD

RTQNVCVNGKYSDVSALKYGVP

>SG-PL_contig_5225_1_-3_30_356

MLLENEAFLLELTKLFQKAKSSGSVTLTMKRFDGHNKPRPRTGALPEPNEYKCLIRAALG

NRKISTVINHKDVTKFQMAYGNLLKANIDGLKKKDKKSARGKNKSKATQ

>SG-PL_contig_5226_1_-2_608_874

MHTRAHTQLESLFFFFFFFFKYLKLLFGLTKTCAKIFLLISLKYSLILCAFGGFVFPVVC

LFPCFSFQLGHFFLSPLPFFVSFLSCKSC

>SG-PL_contig_5227_1_+2_665_922

MCIYFGPTPLLKCSQREREREREREREGEGGGVRFASMGRRYRGNLVLVNSCFTGITQQE

AALVEWHFLQRKCMHYVTFNKCLSGC

>SG-PL_contig_5228_1_-3_102_578

MCSSRCVLCKTVLPVFDRFPLLDGTFFLSPQSYDEAAIQVIWNGRLQFLNAACLSCLEGH

SSVKCLACKRLWDGCSLVIGSMYTYDIFAAAPCCQKRLTCKCCRRAVVDITKGLEFYSQY

SQMIICPYCKANDYHFIRSLLETFTISEEGAGQSEPICH

>SG-PL_contig_5229_1_+3_639_788

MKRSSPLVFTALLSRYFFCVNLKSHRTAGTVAMHCHYYTFTHKSIAGISS

>SG-PL_contig_5230_1_-2_2_241

CVLAVTSPLSLATDGWRPSARTSMLNASTAQLVKSLWRVFHSWPREENPSARNTLAEAPP

PVSVLHLQLHGPDGYKGCRA

>SG-PL_contig_5231_1_-1_97_2316

MENAKEDVRSDIAVMRRAAEKVESEVSKAEAEKQKQDLYVDRLIERVDKLKEEMAMYEAQ

IIAQSEETRAAKDALMEAHMEMEAIGLEKKQLYQQWNSSLIGMRRRDEAHAAMHEALYQQ

EQKILSLETEMEGYKKSIQKEQEQNEKLTLILAKTERDIDAVRKQLTQCNARFDALKAEY

TTYTRMLHETEQALNRATADKTLRENELNALRKQIEREYQEKISLEDQIMERLRTQLTLD

KAAQYSKKLTGRTRDRTKNLEAQMAEVENSIARDTLDTVNVMARVERLGKVRDGLDADIG

QKNAIINRSEQEIHKRNAIIERKQNLIDQYNKKLEAMISAAGGVELGPLEIQINSLLKSL

DVRQQEIGELQQLWLRQQSELVRLSQDKDTQSSDVEKHKRQLTILSQKKIRTENEIDGQR

REMYDIEREIRNMQNDVIKLNTLLHQQKNKETGLEQTNILTENNFIAGLKEAEIESIELS

AKLEAVKEEKERLLNSLVEAERQIMLWEKKTQLARETRAAVDSEVGQGEIKAMKAEIHRM

TVRHTQLMKMQEKMIQDMEKAVSRRDTIVTRGDAQSKMKKKVLTKGTFERQMAELRKKIK

STIQDANECDTEIRQLREHQGDLSKSLEERQVNCQTLQGSADTLDGDIERMMELKQKNLT

ELVSKQQRMKFYQQAKEGRYTMLCRTDSAADTEQGKQEQRMQALNAIVDRLNQEFPHTQP

TLRRATLTLSSRGIGGEEFA

>SG-PL_contig_5232_1_-3_3_287

RHDSGGRKLRDGSEHLPQHAGRQTAEHCGHPQGQHEPAARPGPLCRRRKVEPSMLCSFPH

RRHEPSVRRLVRMPNHRSCHIQARQHSNRHRQPVN

>SG-PL_contig_5233_1_+1_1_216

VQTVVPSVSAHCISIDTDHQYPKSVYTVSVRTVVPSVYTVSLYRPSVPSVSVHCISVRTV

VPSVYTVSLYRL

>SG-PL_contig_5234_1_-1_1762_2055

MLKIICNRKGFLFRNFLFTVFMWAKLMIYGTTHYHHLDPRLSSVVLLVYSAFSYFTWNSA

ELNCRQYMPFVDYTEWSVFILLILLQQRLNKTPTIYGK

>SG-PL_contig_5235_1_+2_20_244

MLTNYLIIIIIINHSCIVLFSKVIWTHCALHSYRTQSVIHAETLISHTHRHIIRKHTHRC

TQYIITGSNNTYTYL

>SG-PL_contig_5236_1_-2_1031_2242

MTSYTGLGLTSGTSLDGLDVCCAEFTGDMGTDIWSFRILEATTVPYDDVWRERLANAPSL

SGLELIKLHYDYAHFMGKAIQEFMKKMTEAGEGGGGTSEPRLDFVATHGHTVFHQPDQKL

TFQLGEGEILATYLRCPLITNFRVKDVCLGGQGAPLAGSSDRFLFSNVALCLNLGGIANI

HVREDQRGWDVCACNIILNRLAKLGDPTTKYDKDGQLASKGKVQQELLVSLEELGYYQLD

SPKSMGTEWIDKHLWPLLDNKKYSVEDLARTYVEHIASQISSACLDGNPIPGSPNRRKKY

GDNVVLVTGGGAHNTFLMQNVKEKLMQRGIELEETVEAETIDYKEAVCFAFLGMRCMLGR

VNVFADSTGGCLDSVSGSIHQPVNPTTTGSYPRGYRFLFRQMSS

>SG-PL_contig_5237_1_+1_1978_2202

MTYSQCIFDCLYTGLLLQVLLLSCFHFKENLCKFEKKKDEKHVKFNMDSYFQTYLPKLYV

LQRELCGITRQNVIL

>SG-PL_contig_5238_1_-2_143_364

MLKPPFCMLFFLVVFLLILFCLLFQTNEKKPFFWRSFTNSSGESGSKIYSELVWIRSEIF

ILQLFLGVLTFFSP

>SG-PL_contig_5239_1_-1_373_594

MKEEEFPKSDQEESTICEGLMSEETGQPLNNMKNGISPGNNGQTVVFNSFNESFDKGEMS

HTQKGVFYPKYIFG

>SG-PL_contig_5240_1_+2_35_316

MVSILAKHVFRIKYSFWLHILYHVGLEQNLKIGGGDASRERVGLIGEEGSISRDKRDTFG

TVRSRSPEIPKVEVNEGVLGAPKGSFPDGPPGGF

>SG-PL_contig_5241_1_-2_164_1930

MPRPVNVRVTTMDAELEFAIQPNTTGKQLFDQVVKTIGLREIWFFGLQYLDSKGYSTWLK

LNKKVLSQDVKKESPLQFRFRAKFFPEDVSEELIQEITQRMFFLQVKEGILSDEIYCPPE

TSVLLASYACQAKYNDFNPNETLTNDRLLPQRVFDQHKLSKDQWEDRIRSWWGEHKGMMR

DDSMMEYLKIAQDLEMYGVNYFDIKNKRGTDLLLGVDALGLNVYEKDDRLTPKIGFPWSE

IRNISFNDKKFVIKPIDKKAPDFVFYAPRLRINKRILALCMGNHELYMRRRKPDTIEVQQ

MKAQAREEKLAKQQERAQLERMRLEAEESERMRKDLEEKLKRMQEEQDRTTKDMQDLQAR

IREKDDKVAELLTLREELEAERCRAEELRLEMERSHEMEQSERQKLADELESARSSLQEK

LSDIQTKEQNVSELNTELEAARREAEERNEELSRVRVEKLQREQELQEKLQEQLQLKSEM

QSTHDENDEPEEEENKTEYGGELQIDEELASKPRAEEERLTEAEKNARLQKQLDMLKADL

GKERLDDAKVTKNDELHAENVRQGRDKYKTLKQIRQGNTKRRVDEFESM

>SG-PL_contig_5242_1_+3_126_230

MGMAEEKRINSLRKVLSSNRLTFQVKWFSTVVYAK

>SG-PL_contig_5243_1_+3_3_311

YTLLEIGTNTTTKNSKTIQTFSLLLQKLAQVVGLFVCLFVCLFVCLFVCLFLLPHLLHCS

CVTTPTPLISALQHTSNTAPVSLPPRRSFQLSNTPPTPLLCHY

>SG-PL_contig_5244_1_+3_1008_1286

MKPATSVLCQSLRLAVSLLFSPSPPSSAVCVSVLPDLSPHVPRCPSVCVLLLQPAVFDAF

QVTAGRHLMKIHLSMEDKTKDPLLMKIEKNIYK

>SG-PL_contig_5245_1_+3_408_776

MCVLVNLYTHAHLYFLLPTTHRHIWVLSLSVHTDTCTYIHTHTHTHTHTHTHTHARTRAR

VRAHTHTKPFLHTSLIEKVIIICCSHCQNCLSYLQSNKKTRRQNQRKDDQGKHTKWTFEH

TGH

>SG-PL_contig_5246_1_-1_22_684

MKKKEERMEKKMAELTVENKRLTEPLQKAREEVEELRRQLANYEKDKETLRMTKARLKIT

EDDFRALKWEHEVLEQRFHKTQEERDDLYRKFVKAIHEVQQKSSLKNLLLEKKLSALADT

LEKKEAQLNEVLSASNLDPTALTVVTRKLEDVLDSKNSAIKDLQYELARVCKAHNDLLRT

YESKLRQFGIPTEELGFKPLESTVGGQTLGHGPAGLVSAPS

>SG-PL_contig_5247_1_+3_252_461

MFYMLLGADWLQKKSPITAITHSTLRGLTCMERKCHGIHMFSLFVSACFWFGWIWFGLIC

LFVCLFICLF

>SG-PL_contig_5248_1_-2_71_937

MAAAFGIDMFSPYVLSFVAFSLVTMMASVSCDQQLSVQCAWNEYYDEIMDDCGPCHEICD

NRRLTRTEQQCNAQCSDYLVSKSCRADQYFDEMVKNCAPCAELCSQRDVKGTTKECFQKC

RDYVNMNPPSNIVDTVGKNTDTSQDSSSSSSTSSWLPGIIASICIGVVASAAIVGLIIHY

RITHKSTNYVRAPGNEVTERRGDATTGSGDIQSGSTVINIELQHALPIQVSSEQVTAEDI

SVKQQTTSVEAVVDIEDTQPKTVSMAIAECDPVAPSSAAAWHVHRQQSS

>SG-PL_contig_5249_1_-2_2_181

ERERESSTCVNKNNSHHHFTITTTCSTIHLLFLSTLFQNICLSTKIHSYYKCRRSCDEQN

>SG-PL_contig_5250_1_+1_1_228

FFFNQTKIIFIHQHEKRIFENKALVSKEELILSEILVSEQYISPIQLSNERKVNDEKYCT

FTPAEKKVDENSALST

>SG-PL_contig_5251_1_+1_1_576

SSSNTSSSSSRSSSNTSSSSNSSSSTSSSSSSSSSSSSSSSSSSSSSSSSSSSSSGGGGS

SSSSVNNDNNSNTESPPQTTATSSSTMTIKVVVIVTTTTIIIIIIIIIIVVLIIIAAVVV

IIVVVVITIAAAALGVAVVITIAAVVVIEAIIAAAAAAVVVVVVVVITIAVAVVVVVVVI

ITIILEVVTIIL

>SG-PL_contig_5252_1_+3_3_176

PRHPTGEWDMAGKKWNRCTSWASQVGTTWRRPRTEPPGDRAGSSQKQSKREPYCISWI

>SG-PL_contig_5253_1_-2_119_355

MIHFKTRHTDFVTMNLNQFSMFYFDLLTLYNCSHLTTRFHIIFILCYAVYNTTKLETNVL

NLRFEILFSFSFSSSFFFK

>SG-PL_contig_5254_1_+2_323_502

MFCPDHVFLQLNQIVFSSPEPQPQPEPIMHIFLWHDLSILHPQPLNYLHWWFLHQKNRTS

>SG-PL_contig_5255_1_-2_1916_2110

MEQEAFHQLLQESICHAVLLSSADLVKFSKPHNFLVIERKSYSYLQVCCILCGLCIVFKC

RLLQS

>SG-PL_contig_5256_1_+1_1_297

EKLFLLKHAHTHTHTHTHTHTHTQSNAEKSHDYQNERHEEIRLFKRNGIYPICQRRIIGL

DNFIPMHSIINYARIQTELKTETPSPDTTVTQCTICDIF

>SG-PL_contig_5257_1_-1_118_231

MLLPIYVIFHFCVALRFICYSPYDSAIALHRLFVCLFV

>SG-PL_contig_5258_1_-2_2_196

DFNYCYVEMLLGIRFRKRNQRKFVLTLSVLLCLMLTGHWSISLSSLGRMCSNLLGCLKYS

VFYSE

>SG-PL_contig_5259_1_+2_305_505

MQTCAGRSTSAPTNSASIFSTYYQMTVGFCCCFIFVFVCLFVFYTVCVTNNTTHFPCNKS

QHSWYFY

>SG-PL_contig_5260_1_+2_2483_2794

MPCRQRQAGGWFSGQQLMPCRQSCRRYAAERRGNRTPGDPWSGRGTLGQSDPTECRQTLD

SSQPLSHFLIPDAADWKMPKPRIPADVSLCLQARIERTEPRPER

>SG-PL_contig_5261_1_+3_1038_1244

MTHLWCLRLSVCLLLPLCLFIVFVTLSVFIVLPTLCLLVCCYCVCISCVSSGREAMLSSQ

TNVCLTDVC

>SG-PL_contig_5263_1_-2_2_394

GLPAVPLPRRTDQLSPRGLHHFPRPNASQARRRTPEAGRVFSLVLAPSSGPSPGWLPQAP

GPDSCPPPTPPPVDGQSSVGSLAHHASAWRQLHLSDWVVIIEEGYKLPWEGAKPPLRPLP

PSPRQVPHDQA

>SG-PL_contig_5264_1_-1_190_426

MFEYVCLLSLFLNGIVDFFSFLLMALASRNLCIIWKRIRILVNGFKEGMFILHYVNALRD

VNQYYHLGTSSRVLFLSCM

>SG-PL_contig_5265_1_+3_180_314

MLPAGVELKTSDLESTDGSKEPTSTHSSTDLSCQLMLTTADKVHV

>SG-PL_contig_5266_1_+3_3_140

LSLSLAAPFSSYSLKTQHSIYIPSKVKIGILQPTHISPNLPAFPLK

>SG-PL_contig_5267_1_-1_82_387

MLGVFLCFHNHQTQTWLTESVTCVFDVLRAYTHEGGSGTCRSAHMLTWEINEKSPPFTHQ

ARCPRRARDRQKYRGRDSDRERETEKHLRVRIVLIQAKQTNS

>SG-PL_contig_5268_1_-1_1_246

AEGAFVVAGLNLSWGTYQYIYNLFHLRRRSGMSVVGLLVYMSMAVLHLALWLVVCTLYLG

YTDPDINAEEEKAKTQKEGGDV

>SG-PL_contig_5269_1_-2_167_352

MFCFVFAIVIFYPLFLIVVVFIFVVAVIYPFISVISDLITLTKHSFSIVFFLCCNHLFTE

AY

>SG-PL_contig_5270_1_-1_109_402

MPSGDRKMFGEAAEFAKQMKLLVNNKDLHDIQFLIGPARKKVFAHRCILSGRCEVFRAMF

VDHTQKNGPIEKDTPFVLTDMASEVFLAMLEFIYTNSV

>SG-PL_contig_5271_1_-2_56_163

MLDSIFPNIFQFYTEFVFDVLLQNFCIWTFYYFFNG

>SG-PL_contig_5272_1_-2_23_358

MEEKLNTTKKQLKPELHSTHTAMYHQRQTQDHIPKTVCQRRNPKRRINHHQHSNEGDRVR

ERERERERNACIIFVNLRIRYKKNPKIPSHSTGAGKRCFTSALHCRLQWKTK

>SG-PL_contig_5273_1_-1_4_1467

MGGPGSGPGGARGMMSASPPLSGGGGGLLNGKEEQLSLRPQKNFMFKPNTPAMLPRSAQA

APSAPLVPPSNSLSTLGTVSPKKMTVDVANPPINPILNKQPQITIKPAKEEKAKPPKKET

LTPEELKEKVPDLLNEFFNQQDVTKTVAAIRELGIPKRMIPDFLSQFLIGSVNRTEMEQD

EVMKLVAALKTEKMVTSDQFVESFKMVADQMSALEQETPLIKSHAARFGAQAVSCEVASL

AELAEPFQNGVHYPLFLLCLQAAHRIRDKQWLVRVFNDSKINLQGMLPEIDQKKERMLEI

LEDRGLSFMFPLMRVQSELGRQIAAEPSAGAIFKWIKERVDSELFTNHNFINILVTSMVK

YITGESTLAENADPTVTPDKPLMDKEKELLDKLKGVLQKFLNEHIDLQVAALYAVQVFCH

SHQFPKGMLLRFFDNLYDQEIIEEEAFLHWKEEVNDQYPGKGMALFQVNQWLTWLEQAEE

ESEDEDDE

>SG-PL_contig_5274_1_-2_2_400

FNDNFFWSDSDPSGFAFSLDAVQPRCKLTVYSTDAQGVIGEVRIDRILGSQKEISSEVVN

GDVIKRVGLTMECNVTYYQRQHSGVNFKLHGEERLSGEAIVIKRKKTRVAALDHIHNVTL

ACVPHQCTLQVES

>SG-PL_contig_5275_1_+3_153_356

MQISLYIEDSASTVYFYIFNHTSPNTGSCLFTPDAFHCMCGPTLFKSVSKSFLTFINIFC

TVVTLTFD

>SG-PL_contig_5276_1_+2_1175_1456

MRHRFCFWMMKRMFLVMKQKAHGQPTCKTAGDILIVHPMYSLYHHHVKLSFSFCQQPSGT

TKNIQPSCHNETVHRTENTGDKPKKKKKISKDDY

>SG-PL_contig_5277_1_-2_89_886

MKLNYYNKMATYVQKMWRGYYIRKYVFNYYSRKRYLEALQVKNEIIRSELSEFAEQQEVV

QKRIEEHRERIKLEREARKKHHLISTHVIPGVYNSPFLPFPTEQEYILRSVKPLPHPKKS

TSQNQFDPAWATYNDKLPKHLPPLPQRPQGPFREPDAVQKQRYKPFQPTLRVSTDFYSLE

KAREAMKAEEWVNRYNDTLFQPFTRRTVPYEPLLHTTSRFGHLPYGTKFFREEKLNRHIM

EKPFKTLVPPIPVFEKLNDTYSQGQV

>SG-PL_contig_5278_1_+1_1_99

IVVKKRKREKERERERERERERERERERENENE

>SG-PL_contig_5279_1_-2_2_148

PRNTWRRDLEADMKRTGSG-PLQLERLAQDRGGWRAFVDGLCPRRGNRRR

>SG-PL_contig_5280_1_+1_1_321

ASSSFFPATSSQQVALLRAPASTPDFGSFQVPLCYIHLPLGSHACSTLVFCVISLPGCAL

PSPIAQRHGKTYFFLFFPGLVFGHYGIQVRDSTRFRTRLDRPLWGSF

>SG-PL_contig_5281_1_-1_841_1062

MYTQIQISKPTDKCKSKSTDVNSSPFIFGDSFSFSKTVKKNLKLVKDKMKCVHPRTPIQT

HTNTTLKYQIPHTY

>SG-PL_contig_5282_1_-1_670_837

MECCICVIVVMDSFLVIVQYESGCDLQFKNCFMLITVLQHKVSLLSFKNPVKHPHN

>SG-PL_contig_5283_1_-1_94_243

MLMIYGLIKMMMVSAVVMYECNLMVLRTNMTLCSQSLTKFNDSVLCRSFV

>SG-PL_contig_5284_1_+2_431_724

MKSNTDINITMPFELLCWCSFLTDCNARHKKSPDHYNRTCYLVHTEYFNYKCIDGFYRQT

GNQMRHIMSETAQRISRGVLKQRDKGWDREGNLQSHQC

>SG-PL_contig_5285_1_+3_54_272

MGSILAKQGVRKNSPFWCHILCHVKLVQNSEEKEEGIALVVRLGPLAKEIGLLATTKEIP

LEPSALASLKFRR

>SG-PL_contig_5286_1_+1_1_204

QIFLISQIKMCADQIIPEPPSCVYACRTSNTHVKDPVSHVSVRWLMETRKHPACMPTEDG

VCRLCSNI

>SG-PL_contig_5287_1_+2_1415_1624

MKSFIQQLLSKRTTHKKNSTENWLLESWIVVVLHAMNNIPLLSQVTPITNSMTEMGRVCM

SRFSGYVMWQ

>SG-PL_contig_5288_1_-2_2_187

FLIIFFLAFLTSGNNISNIYTCNIYSHTHTHTYIYINTHTHIHIHVHTYYISPAHIHTYI

HI

>SG-PL_contig_5289_1_-3_225_344

MSYIVLSNVENNSLTSLCFMHRHCKCVRIIILLHHSIFCV

>SG-PL_contig_5290_1_+1_409_543

MQAFHIIIHNFMHCGKINTQQSKLLLNLFWGVLFKSVLSSGSLGN

>SG-PL_contig_5291_1_-2_140_445

MARAFKRFLPLFDRILVERNMPEVKTKGGIMIPEKAQGKVQEAVVVAVGGGAKTEKGDVV

PVSVNVGDKVLLPEYGGTKLVLEEKEYFLFRDGDLLGKFESQ

>SG-PL_contig_5292_1_-3_756_905

MRVLRRKILLLIFPKMMEIESFKMTKRHHQVSTVNHCFPYMVFISELFQA

>SG-PL_contig_5293_1_-1_400_1272

MTIPGHSAAVKCVAWIKHDESDPVSSFVTGSHDQTALVWQWRRDNNEVDCRLSCRGHAAS

VDCVAVSPTGEQMCTGSWDKMLKLWSTDCSIGDDEESNDTERPSKKRSKTSSMQTRVPVL

TLSGHKEAISAVQWLDSQRVVTSSWDHTLQIWDLQAARQTSLLPGSKVFLDVSYSPLSQM

LIAGSADRHVRLFDPRSSGVVVKCAFTSHSRWVSGVDWSPTREHLFLSGSHDGILKLWDT

RSPKAPLYDMNRHEDRIMAVDWSVPELMLSGGVDNKLHIFSYSDANLATSD

>SG-PL_contig_5294_1_-2_119_280

MCHLFKDVVKAVSFFFLICILPCHQQSFIYLSLSQYECLSLFSVDMQIFSACCK

>SG-PL_contig_5295_1_-3_81_377

MAEIDETIKRIQAYKGVTGIIILNNDGIPLKTTMNQENTVHITGHIHNLVHKARNTVREL

DSQNDLTFLRLRSKKHEIMCAPENDYMMVVIQTPTIVSS

>SG-PL_contig_5296_1_-1_187_351

MFVFMSPRPPSLVYLAPPPPPSGGWARVTPWGRGILGPTEVEPWVSGGLPTINPL

>SG-PL_contig_5297_1_-1_1_147

SSVRIDRCTFVSPTIAKADQECSGMNASGARGGVMRCVIELAVAHVSEI

>SG-PL_contig_5298_1_+2_86_532

MRLQQTPGAPVPRQTWATRTSFTTRPPCHATHQRCPHRTAATSPRRPLLGGAGQAGAGRS

AFGGLRQPPQTGPGHLAAAGARRSLALLRLTKVVREPKSRTQGSPFALLLPGRPAHPGGA

WPPLPPSHPEGSRLMRHPGAPRFATPRLH

>SG-PL_contig_5299_1_-2_785_1441

MTRNHFTEMMRYLHVCDNARLDVDDKLSKVRPLISMLNERFLTYFTFVKTQNLSIDESMV

PYFGKHGAKQFIRGKPIRFGYKMWVLTTPLGYVLQFEPYQGARGRQVEYPGLGMGGSVVV

DLISELQADTGDSFHLTFDNLFTSLSLVDHLTAKNIACTGTIRSNRMGDCPLKPLKEMEK

APRGTFDYACDTKNGLILVRWNDNNVVNAVSNKVGVYPI

>SG-PL_contig_5300_1_-2_104_313

MTPLAIAAFWGYAEIVKLLLENGADINLSNRGTKWTPLHCASFQGHGKVIMYLMDYSPDL

YAKDSQGRTP

>SG-PL_contig_5301_1_+1_1_153

KVVAYCPGGEGVTGFQRKEVCGLTVMEMTYCSLSYLHRSDQGSKAGFDRVL

>SG-PL_contig_5302_1_-2_2_253

AIFSSKCTRGLTLGRQSACILGDPQSVKLGNANKQQAERTNRRYCCQRDTHLQASVNRKQ

SLPVVTKMNVGIVDRICVGISPST

>SG-PL_contig_5303_1_+3_54_224

MKTNCQHTTKISLTATTQPKIHFQTMHRKCERENVFTVIDMFMGYISTDMYVMTNVN

>SG-PL_contig_5304_1_+1_181_321

MLQSCQHLRLKEDGGKVFKVKSSKRSVKLEVCYQITINIEYYIEYLK

>SG-PL_contig_5305_1_-3_204_404

MRVGIASKGGFHPTPLLPGRLADSGRVRISKLSRHTDSPIPVLPSSDSLRTPRNVTSTPV

SPSTFWG

>SG-PL_contig_5306_1_+3_594_701

MKHLQNSVLSYSHFPKNMFSSLLLLNNIYKSFFFNM

>SG-PL_contig_5307_1_-3_165_290

MTQCTHDFQSFYTVSVQQQWVERCKRKTVTQEYCCQSHTQLY

>SG-PL_contig_5308_1_-3_18_308

MMAATAATTTTTIIIIIIIIIIIIIISSLYRLVSRLMWTGCADCYYVQPLFSSSVCVVIV

GLRYTLEGISYCIQSTSSACSSVCLSVCSLVHESVRM

>SG-PL_contig_5309_1_+3_552_794

MTMQELLFSMCYNCCNSAKLSGQSNGSFKHPNLVQIHSKNVAGDRAVDSRSHVSSSIPAG

NIKTGLFTAYLNAPIILVVST

>SG-PL_contig_5310_1_-3_93_551

MDLLPVLLQNHWFTYNENMSVESVTQAVSNLALAFGDDDPDPGAMSRPFGVALLIAGVDN

NGPRLFHMDPSGTFIQYDAKAIGSGSEGAQQALQEQYHKSITLKDACKSALTILKQVMEE

KLNATNVEMATVTPDRLFQMFSKEELEAVIEDL

>SG-PL_contig_5311_1_+2_2_202

IRWNLFSTLEDLDFADDLALVSHIHQHMQEKTSQQVGLKINQKKTGDDTEHPKPLTNPSR

WNRPTHD

>SG-PL_contig_5312_1_+3_306_527

MSMPSVAFSGFRPFYFSNDSLVAVGKNILNACRYLRFCKQLGCSFTLYVLSCCIHVLLYA

DANSPSQCTCGNSP

>SG-PL_contig_5313_1_-2_2_202

SLPSGLCLTLGLPPCYSSKQPGHLDGTSDTLDPGWVSLLHSGPRRSPRRAATTPDGGETG

TAIQPWP

>SG-PL_contig_5314_1_+1_1468_1749

MFTRGQMGGDCGCGYSSRTWGRYWGPMFSPLEWMTSMTAVKGWMHCQALSHHIPRTLSIP

MGTDRKRLCRPSVSDGIHCILNQFSSDMGYSCYI

>SG-PL_contig_5315_1_+3_894_1097

MHTHCIQVLFEGKSSSRYGMIIITNTLKHCLEKKKNKEDDLEMSVQAEHTPVFFLKRRVR

QLQQCTAT

>SG-PL_contig_5316_1_-2_716_1084

MCMWSPVYAAVNGCLTFFKVGEGKAGREKSWALLSSCWPREKLRSLPPHSPTSIGLWNNL

DLSFTDAIIWSGLCVGNNSGIDLMMVLLLFFFSSSFFLGGGWGAFLIVFYFLGSVVWVVC

GKM

>SG-PL_contig_5317_1_+1_919_1092

MNKGRTRFDSFLTPKSNMDHYHSINQWTSFHQKEKWRVSLPNRPITDQLKYPSINKDR

>SG-PL_contig_5318_1_-3_3_173

IIFFFFFVFCFFFCFFFSFFFFFFFFVVVFIIVVVVDHYMETLMDLMNDLIVHLLLL

>SG-PL_contig_5319_1_+1_262_453

MFLGVWCWGIFCYLNNAFNTVHASWLSRLGVSSSEFDRSVHFSPLFCLLSCMFHWICLFA

CFMF

>SG-PL_contig_5320_1_+1_157_465

MTFSSSFFFFFLPHHTCIYLQEQIFLNKNLLETTSLLLSFYLFNYLLIYLLFNCAPSEFW

TWDVDCGKFKQLVNEKLDFKNINIIDKIVGVPMQHCCCSQEHV

>SG-PL_contig_5321_1_+3_183_320

MYNLSNSVTKFSLSLQSNFNVVSPKFQRVCVCVCACARECFQAILF

>SG-PL_contig_5322_1_+3_90_320

MYPHTHINHLLTNNAKRTQLCHLGVVETVQDHLTEKLNILFSKPSHVLTSTPKRMPNHFS

KWNCRQKRKKTPTKNNR

>SG-PL_contig_5323_1_-1_1_258

LLDRTLNSGDIRTQDFFDVLFYTILNPVGANRAWTFINNTWDSHQEKYAAASVALPYAIK

VFGDVFSTEDQLLKMQDFVNKHNDLG

>SG-PL_contig_5324_1_+3_297_536

MFLGQSAIVLNPFTPGTVCSLFFKSPTSGGKKIQISWEDNPGFKGEKCPLHRRHREHHLR

FLSRFHCRHCHCRAAIPTQF

>SG-PL_contig_5325_1_+2_302_535

MFLGQRAIVLNPFTPGTVCSLFFKSPTSGGKKNQISWEDNPGFKGEKCPLHRRHLEHHLR

FLSRFHCRHCHAAIPTQF

>SG-PL_contig_5326_1_+2_1064_1312

MNTNQDYFHLHPNSIEEAPIPPLSEKAKPHFNLFQCHLTLPAYLYLENEPRTPSSTLTCD

SALVDPRTAKKSMQTHFHFCLQI

>SG-PL_contig_5327_1_-1_1_189

MYRTIISVNANGQDDGYIEPLSMLMPAVKTMAIYRTIISVNASSPDDGDIYIYIYIYKRH

YQC

>SG-PL_contig_5328_1_-1_1_216

LQNQLRNLSLKDGEQTRCDSGICDSGNFSVEEPFPVVESAFPECGLTSSLPFESTFSQQE

LEELFSQDEDGD

>SG-PL_contig_5329_1_-2_2_202

PYSSTDDKQLSIMDERVDSGVGYSLESQEFSALSIEGVRDFLATESGKDCYKQVMDNRCV

SGSRSDI

>SG-PL_contig_5330_1_-3_129_344

MLSHTHTNTPHILKASINFYKTLLVEIKIKQTCIMRKKKTFLILSYIRHAWKKSSHHTNP

HMHSVIHHAQIL

>SG-PL_contig_5331_1_+1_163_339

MTTSLPHYHSSRPRSNLMYNQDSYCFERNAEDTNMHVHEINTSDRYFAAHKIFLTNKLE

>SG-PL_contig_5332_1_+3_1029_1244

MYLCSSCVCMTNIFLDENVHNGCLYKTNIFDEKFHNICVYKTDNSSHKKVYNSCLYVANY

FLKKEFIIVACA

>SG-PL_contig_5333_1_-2_797_979

MTSELLGMTSELLGSTSIRNFWSFNSDQFIVKKYSDQFVEKCPERETVTVLVWLSCLYKK

K

>SG-PL_contig_5334_1_+3_681_863

MVKNQNQKQSFDDILIFNFLSRFIPISLASRLKCRRCQVIFKTHTHTNMPMLTALLKSII

T

>SG-PL_contig_5335_1_-2_2_232

NTGSKNHVIVLEKKSVERGRKLYSPHYLCDIFLRNAAVQWLYIIFVYHVKRVTSFCISFL

SCFSPFLSSLPSSLFQC

>SG-PL_contig_5336_1_+2_302_2428

MKIKVGGVKSGFNHVERGVFQPRLIQVKGKRNPRFSELQDIEWELMNKGDCFILDLGNIL

FPWLGSSCNRAEKMKTLEHCRRLRDERGGKANIIVLEDGQEEKQIDADLKPIFEKYLILR

EKHKILPADAGGKDETSERQYASAIKLFSCREEDGTLKIQEVKAGPLNKKDLVSEDSFII

DNGLAGAWVWIGKRASPKEKQEAMRNAVGFLKKKGYPSDTKVTRVVEGAEPSDFKCLFRD

WPQPAPTGKVYTRSRIAKTVQTKFDAKTLHSNTSLAAETQMVDDGSGKVEVWRVDNFDLK

LVDKSRFGEFFGGDCYVIQYTYEVQGREHVIIYYWLGARSTADERGTAALKAVELDDKLG

GSPVQVRVVQYKEPPHFMAMFGGKMIVFQGGKAGWTPGEKSVDGPGDNYMLQVRGTSQHN

TKAVQVDLHASSLNSNDVFVVFTKTAVYIWAGKGCTGDEREMAKIVAARSPRASTMVFEG

QEKDDFWNVLGGKEEYASEKHLQDEGSHPPRLFQISNASGNVKAEELHDFVQDDLIPEDV

MLLDVWEALYLWVGKGSNKIERDQAQTLALEYLKTDPAGRDPDTPILKISQGFEPPSFTG

FFGTWDRDLWSRGLSFDELKRELGEGNLPIQEVKQNGANGIQDFSDMPKYSYDELTGDID

QLPNGVDPAAREIHLKEEDFKRIFSMTYTQFSTLPAWKQKEMKKNNKLF

>SG-PL_contig_5337_1_-1_22_1560

MAVVIESADVVRLIEQYLKENNLLRTLSVLQDESGISLNTVDSVESFTSDIQAGHWDTVL

HAIQTLKLPDNKLIDLYEQIVLELIELRELGAARSLLRQTDPMIMLKHQQADRYIHLENM

LARSYFDPREAYIDGQTKEKRRQAIAQALAGEVNVVPPSRLLALLGQALKWQQHQGLLPP

GTTIDVFRGKAAVKEQEEEKYPTQLSKFIKFGSKAHVECARFSPDGQYLVTGSVDGFIEV

WNFTTGKIRKDLRYQAQDNFMMMDDAVLCLAFSRDSEMLASGSQDGKMKVWKITTGQCLR

KFEKAHSKGVTCISFSKDNSQLLSASFDMNIRIHGLKSGKQLKEFRGHTSFVNDAVFNQD

GHQIISASSDGTIKVWSMKTSECVNTFKSLGGTVGTEITVHSVHPMVKNPDQFVVCNRSN

TVVISNFQGQIVRSFSSGKREGGDFVCATLSPRGEWIYCVGEDMVLYCFSISTGKLERTL

TVHEKDVIGICHHPHQNLIGTYSEDGLLKLWKP

>SG-PL_contig_5338_1_+1_232_540

MDPAVAKGPEEETLQPKSRKSWSRKGQEEPALWREPKYLRRVEGPKEGVGFSTTKKEVFG

DTVPNCQHLEGRGSWLVIQEETVLLDPERVVQPRGSGSSETQF

>SG-PL_contig_5339_1_+2_320_631

MGFFLTHTIVYGSFLIHSIVYGVSPDTYYCLCFFLTRAIVYGYIPDTFYCLWVFLIHTIV

YGFVMTHTIVWVFPDTYSCLWVFPDTYYCLWIFADTYYCVCFFS

>SG-PL_contig_5340_1_+1_2746_2985

MELFTTDTSEDKFFTHLINFQSYIVMQLPLVRFFKRNLGCEHQNMLVQLPLQGQHCIKNS

PSISKSLVTVLTTSYAHGSF

>SG-PL_contig_5341_1_+3_3_116

PDHPKKKKKKEFIEIKKKKKKYFTISIVHFDIAISYWK

>SG-PL_contig_5342_1_+2_2_277

IGKKEKKRPPPPPPFTEASQTVTPSFYFAMQYCKNFDLGKGHMKLHTLLNFVLTRWFNTI

IHTYILCEIILNPRAVSQQKRQEDLGFWWLLQ

>SG-PL_contig_5343_1_+1_1_144

ADESLRKLSADMHNTGEKTQIVLLLLLLLLLLLLLVVVFIFSLMFIHK

>SG-PL_contig_5344_1_-1_52_2145

MSNIYIQEPPSNGKVLLKTTVGDIDIELWSKEAPKACRNFVQLCLEGYYDGTAFHRVVKE

FIVQGGDPTDTGDGGESIYGAPFKDEFHSRLRFVRRGLVAMANAGAHDNGSQFFFTMAAT

PELQNKHTIFGKVVGDTLFNMLKLQEVEVNEATERPEEPHKIISTEVLNNPFDDIVPRQI

KRVKKDKEEDRKIKSKSKATKNFSLLSFGDEAEDDEEQVDKATESFRGKSKSSHDLTDDP

RLSSIPAVEKEAAEEEKRKKKAEASPEEVKTKKPETEDTEVKVRTEPAEEESKKKPDTPE

DSKKVKTESKEDDKKRKKFDVPEGGEKKKSPGVEEGEVKKKKAEGEEEGKKSVKEEPKEE

GRKKKESDTSHDGRKRKAEDGGHKEEKKKKSKEREEEDAGHKEEKKKKSKEREEEDRRKR

KAESKEEEGRKKRKGEEEKRKGEEEKKKGEEEKRKGEEEKEEGKKKIKTEPPESSPQAAE

EDKPVKDKDDTKRKVKKEKEESPRGKEKEVKSLLGFMEESKQNRMEELKKEQRKLQRDIK

EFKKRPHAEHKDKAKVKVEEAEEAEVKEEDTEENDLLAMVKKERQKYKLRKKQLGRRENR

EEATLAMLAKFQTKLANARTLAGDYDDMDDEKEGDDNEDDPDDMSWMRHKLHFEDESTKT

KVIDANVHDDDRYALNDPRNPLTQRRRETSKKAMREKR

>SG-PL_contig_5345_1_-3_9_563

MVLSADWTQLEVIDLINDGTGLGFGIIGGRSTGVVVKTILPGGVADLEGHLRSGDHILQI

GDVNVRGMSSEQVALVLRQSGSHVRLIVARSIYEPPPFQIPNAPIIPTSQLDDHVAHINA

LMEQESRNAAAHAAMDDHTQQLQLHPHHSSVMEQVQVHPQPYELAGEVPEVEMFEVDLMK

DTHGL

>SG-PL_contig_5346_1_+2_110_274

MAYISNYCNVLEWKQNSIYQNLIKLLGVTEMVRKFHTQTCPSTVYASGSKKISAL

>SG-PL_contig_5347_1_+1_388_603

MCNFHYPSQHVIHKHTDSQLRLYTHVHTHGLNLVIHKVLCTCISHHHSQTDIYRLCTFTT

LTIFRSHYIYTH

>SG-PL_contig_5348_1_+1_523_696

MEKPVWLIQNQCSNHIMPCLKVRKTSLTDPESLSHCSHTVFEVCMLNGFTTNNNTGFI

>SG-PL_contig_5349_1_-2_854_1021

MTDAQLSHHYIALLLSFSFLFFLFMCRKNVCSVVCSNYCYCYCLSYYLLASIKIIS

>SG-PL_contig_5350_1_+1_118_369

MSTVVILARIEHCVSVPGVSAFHIYKQTAHHRYHTGTSGLCGDACAVLGCPYVTPNSHKC

CKCVFLEVNSHLYRRVADWQSLKS

>SG-PL_contig_5351_1_-2_2_154

KTQSVIHAETPMSLRHGHIYIRIHALTDIHERTQESLTTHTHTFGHRHRHT

>SG-PL_contig_5352_1_+3_3_293

NQNKAKTTNQPQKTKTTLAVKKKKVYGSLLPGQITNIISNDWCHSLNYCNSLVSIYWTRH

IFKQFQMFSHVQIERPHTHTHTHTHTHTHKNKNNKSK

>SG-PL_contig_5353_1_-2_2_166

CAFFPFFTVVCFFSAFLVHSTFFFSSKPSHNPSSTSTVYDVLSVLFCSDDKVVIL

>SG-PL_contig_5354_1_-3_54_356

MSDNITSMKRVLYRVDAAGKKAGLNLNAKKTKVMHLEGEESQEYQDIQINNATLENVSDF

KYLGSVKANDGTCTKDIRIRIGMAKQRMIQLNNIWKDRSIP

>SG-PL_contig_5355_1_-1_130_498

MSIGVPIKVLHEAEGHTVTCETSTGEVYRGKLIEAEDNMNCQFSNITVTFRDGRTSQLEN

VYIRGSKIRFLILPDMLKNAPMFKRVQGRGGGAGRGKSAILRAQAARGRGRGGRGGNVFQ

KRR

>SG-PL_contig_5356_1_-2_119_706

MEIVPENQMTPPPNLTTPPPVNMTPSPGLMRSMTTPPVVSLHQQMGQAALMGGVTGGTVS

GTKYQQRQRPSSTATSNTVRKSPSVTPNVTVNTPNPNMPFSPNVTIQPAPLPARYQMNAM

NMFNGYRMQQQVMNHGTAYLNAGFLTQQMPVQMGMMNMNVHPQQFTPQMQQHPSQPNVYP

SHYGYNIMNMNMNMRR

>SG-PL_contig_5357_1_+3_288_557

MCKMCLIQDSLLSHIYCNYPPKSTKTGSTEHVNIYANMFYHIIEKPVKVRFPVSFNKSEH

GLEIWVNTNKQTNKQANNQTNVPTIRIYLN

>SG-PL_contig_5358_1_+2_2_166

KKSCIFAKFCIQNGHKHLQRNQQTKVKLAQTSIAFFRSVNLLNTSFSRLCHSFGT

>SG-PL_contig_5359_1_-2_26_898

MASEKKKRAVEPLETEDTFDEESDGDVTDESENEFMDQEVQVEFEARPPDDGDFHGIKTL

LSQLFLKANINLGDLTDIIISQNYVGGVIKQVDVDEDDDDDAMDDDIGDSVFGLITAINL

TDKQGKECIKQLISMVTAHCQQSAPDVAGTVTEMLHNPDNHVGLLLNERYINIPPQIAVP

SFQSLKKDLEKACRKKMNYSFKHMLMLCKTYRIKGEPKNHDSRSLFYTNGEEELIAEMSD

LQATWSVADERDGVADGRWDEDEMEATRTLLVFSADKLDSIIHKLETELAT

>SG-PL_contig_5360_1_-1_1_333

FNVNEPNSSLHNQFICISRPNGAVHSADERVCRLLCLVQFAWCTLYYTPTVLTNSPFLCH

ITALWGGRVFISVNDCTPVVQLPCSGFLVYNSCMYVLVLIHMNFVICMRVD

>SG-PL_contig_5361_1_-3_63_305

MICYEMWYPISLQYYSSTVYETFVVSRYSQTQGPKLASWQVPYPHPHAVIFSIVTGEIKY

TDHVFFHCPCLASCCILFMVG

>SG-PL_contig_5362_1_-3_3_263

DQLRLSSLPDPSQSHQEGQGRESAHDSHRTLLAGPTVVPGPPVAVPRAATETHPEPGPSQ

ATSLGDQAREPRDAASTRLAPLRPRLP

>SG-PL_contig_5363_1_-3_3_365

ALAERGPLTRDGRLLAVSLSREHSFFRSEWSLKTPRRWRWSPSPSLGFSDRLRLSPLPDP

SQSHQEGQGLERAHDPHRALLAGSTVVPGPEVAVPRAATGAHPEPGPSQATSLGDQAYEP

R

>SG-PL_contig_5364_1_-2_107_232

MLIHFSLCVLVTVMGVFCIFVMHMFCYLLISYLFIYKLMRLF

>SG-PL_contig_5365_1_-1_118_2655

MAGVSRLQMMEHVHLAGEEEEDIYSGFNDYNAALDTEDLAGDEQFQKAVLRTSHGRRPPP

TAARGTMGMAMGAGANARLGTAARMGTAARGRMGLPSSMGRPMTGMMEADSGPRPMTAIK

GAGFSSTGNRGMFDPMNQTTKGPAPPLESKPEDSPEEKIRQLEKKVNELIEESCFANSRG

ELPLALEKAKEAGRKERVLVRQREQQSMGEQINLDLTYSVLFNLANQYAANEMFAEALNT

YQVIVKNRMFSNAGRLKVNMGNIYFRQKNFPKAIKFYRMALDQVPNTHKEMRTKIMQNIG

IVFVKMGQYNDAITSFEHIMSESPNFKTGFNLILCYFALGDRNKMRAAFQKLLTVDLKTD

DEDKYLAQGDDKQASLLLEVIKNDSLRQIERERQYEAERCIKYAAKIISPAIEGSFSDGY

DWCIDQVKNSQYLELSHDLEIDKAIMFLKLKDFQQAIETLKSFEKKDSKVASTAATNLSF

LYFLEADLAQADKYAEVAMAADRYNPSALVNKGNVLFARHDFEKAREFFKEALSNDSSCV

EALYNLGLTNKRLGRLEDSLDAFYKLHAILRNSPQVIYQVADLHDKLGDTAQATEWFMQL

IGVVPSDPSVLARLGELYDNEGDKSQAFQYYYDSYRYLPSNIPVIEWLGAYYIDSQFCEK

AIHYFERAAIVQPNQVKWHLMIASCYRRSGNYHKALETYQHIHKKFPDNVECLKFLVRLC

TDLGLKDAQEYANKLKKAEKAKELREQRVTSAGRKGSGRGRRGGSADSDREGSAEAVRKP

HGRERRKPNFDGDDSRPYQGSQAEVDASYSDPLGPQMERPKTAAKRRDPNEDEFGDEELG

DDLLPE

>SG-PL_contig_5366_1_-2_170_304

MEAYYHYYYYHEDCSNLCKTSCMCEVCFLDVKNYNQYGEMVTAGP

>SG-PL_contig_5367_1_-1_34_198

MKGHRIWGNFSQCYDDENGDDSEMHALEFHLGLRLRDRTVLYASLHNISWCFPRF

>SG-PL_contig_5368_1_-3_30_272

MYSWSHCGKTECIYLGDPQSVKLGNANSKQQHMVLPVACMIIFPVFLSYCFRSSFHTVSS

LHSILFQVIMCVDLCAFNHQA

>SG-PL_contig_5369_1_-3_120_290

MQHCKREESYQKYKKKSPGHITHKCIKLIQAIRKLFVVDLRSSSCQLKKKTITDNLL

>SG-PL_contig_5370_1_-3_3_131

FKHSFCSPALHMLTEKLVGMLINRLSKCSDGADLLQLIACIPV

>SG-PL_contig_5371_1_-2_32_247

MLYSSCKSPLVDFIERHVGIPLAKKLEIDDPSELTEEFIYDEVHPKVNVARQAFAKPKGP

AGRGPKRMTKPT

>SG-PL_contig_5372_1_-3_117_230

MIKNLENKTIIRTHVKKWIRNRMVLFAVHHLKSFNNET

>SG-PL_contig_5373_1_+1_205_354

MKLFSFYVQLNCLYPKVNHLLSDTKYGSQIILLEPTFQVLKISSPDSTSE

>SG-PL_contig_5374_1_+3_222_338

MVTGYNLVLVMASHKFLTNHVKKLLQDVSPTFSSTAVLN

>SG-PL_contig_5376_1_+1_1870_2127

MSDGSWLFEESSQGMNIQRGLSFCCVWEGMGGRLYHPCVYVHARVCIKTSRKNVTGTKTV

KTQTFPQNSSPWCFHPPRVFPLHLSQ

>SG-PL_contig_5377_1_-3_765_1064

MFMCTHTHTHTHTHFLCTHTHNPKLVRSYYNQPSLFPLLTDIFYVIVTLKIIIHIQIYTN

TVLAIAEMFSFSGQKKVLRICLQILHGFVVLCGFSLKLSV

>SG-PL_contig_5378_1_-2_263_514

MLPCGQQNTVKLFEIHHMGRFCNVMTEDLFILQERTVFKWERESESERESLCKLCVGVCV

TAFTVHAYMVVTQEHVSVMRERES

>SG-PL_contig_5379_1_-3_42_158

MNCVLLQDFCFLGLRRFCVQCGCLLDFDGLNVVTALIWP

>SG-PL_contig_5380_1_-2_86_826

MYRRKLLALDHTQPDQFDVNNESELRQLVVWLEDQKIRHLKIEDRASLRDTASADWVTAF

QQYLTELKCPYIGGRKEVIVDWLLGYAVRLEYGDDADSYRAVTRETVLKSKGAGGPSSNP

LDNLDFDSAEFKAGVTSLAMLLQVPPHKDHLEQLKAIAMLVSSTLSKEALQNAATTDTKD

SEHIPLDKTELGFESGDYIMNEAAKIVRLLHLKELRDLQSKINSAIVAVQAITADPKTDS

RLGKVGR

>SG-PL_contig_5381_1_-3_3_146

SFLYSAVLQACLNSLGITQLQDTECDTCRSNHACPCLISHFGGKYSDH

>SG-PL_contig_5382_1_-2_170_292

MDVFIILMPSSFHSMNKTNFMFEGWYFLNCLCMRERENVRV

>SG-PL_contig_5383_1_-1_40_147

MVSCFQHLVLRAFVNVNYTKGPYCYCDYQCIFIKSR

>SG-PL_contig_5384_1_-1_82_330

MEYLRCGLSVSGTEMAQGSTHRMALKHIFLDQSVAVKMVAMVLVPSQSEVCGDTQGLSNN

KQDWHDWDNNVKVQHHHLMCVYM

>SG-PL_contig_5385_1_+3_3_179

YQSYLRHSAYPTQSGSIGSRHVKFVDLADFIQISDSSFQTWPAEPINKTPLCGPAVDCF

>SG-PL_contig_5386_1_-1_13_537

MAAEGDAVFHRKVQTRLADRYLDALELEEETERNKRVEQERNERFSNKRRRFSPFTVQNV

LWLIASIAVFHFSQFHLVVRFDPRINRVWFNVGAGLIGVTLAIALFMIVWLSLVKKVHSD

DWEKQHPAAIPVATASFVIGSLCLLKGLWPVWGLLTPFILVTLFMGFVVLIAMFG

>SG-PL_contig_5387_1_-2_407_643

MLRNHSVAYALLVRNKEERASPSQDRLPSSSFDQRSDKNRVVSQDRKESEVWGEDLSFMR

WESVKRKTYSGGGREGKYQ

>SG-PL_contig_5388_1_-2_3257_3562

MAIMSPSVTKLVSSLSMAVISHSVTELVSGLSMVIISQSVTELVSGLSVTIISYSVTELV

SNLSAVIISQCDRASFWSIYGDYITQCDRASFRSVYGDYITV

>SG-PL_contig_5389_1_-1_130_735

MAVTHAQNMVFIDTCASFSTQAAAGFLPLSAGSSDSEQSQRTEGDALNRIRCVQAADIYS

LLSVLDDLSSSLKAQPMGRFSGVKLVVIDCVAAVFAPIQGGQQMDSLALLEQLGLQLKSL

AMEHNLAVVVTNHLTSGLDGEARPCLGRVWGHVPHTRILLDRIPAVASTSSLRKVTVLKS

VRMATSSTAHIDLASDSIPQTS

>SG-PL_contig_5390_1_-1_1_195

VSLYLGLLVFCAFVLYDTQLIVEKRRRGDDDFIWHSVDLFLDFINIFRKLMIILSKKESE

KKRKD

>SG-PL_contig_5391_1_+2_158_343

MMRMVPSFLPCCFTFPKPELQGQVPIYSWVDCTHNFFLEPGSNLCSQTGRGKLYHHANRS

SF

>SG-PL_contig_5392_1_+2_2_250

SNLCSQTGRLELCLYANQASVFCPNQKQDTNCALYKQVMKIFLILAKLMCILLLAIFSTL

NFLQHAARIVTAGTLLVNIMLRE

>SG-PL_contig_5393_1_+2_230_361

MPIQLEIFGLSHCSWDGRRCHLKPGNFFPLQRSSFLSIIVTYCL

>SG-PL_contig_5394_1_-2_2_82

KALQNGVTHLTNCETRYLYSIHIELVV

>SG-PL_contig_5395_1_+1_919_1083

MVINKRCKQQVKIQKSCYHTAITTSMVCASVSCCHLSEKEYYWCGVMTLLPHCFI

>SG-PL_contig_5396_1_-1_133_378

MIGSHVEHYTRTLKILYAMSVFIGLWKHKNTLHAIKKKMPIMVSLLTMDINGKQDNYNGP

SELVMDNKIAQQIKKDQDRILR

>SG-PL_contig_5397_1_+1_184_321

MIQLCYVPSTITTPKHIAKQYFTVQPGKSNKLYIQFLSSKHIFCIL

>SG-PL_contig_5398_1_+3_978_1244

MAVNRLKEKACFYAVVFSFLVKRYLMQLFFISFLSHARARMCTHTHIRTLTLTKTHTYTQ

HMRAHARMHTHTHTHTQETQYSCSGETCV

>SG-PL_contig_5399_1_-3_3_326

RSPGQPPEFQSPDSSPSFHPQPKARVRWGAWPVTPWRGGSCTSATGLWKSSRRGTSHPGN

EGSPHSGLSLLPRARFLMIKTLLGPLNLSRGPPEVVPGPMGSRCLQLT

>SG-PL_contig_5400_1_+1_103_549

MCTVTISSNHTRKQVCHSTLSPWRQENRSVIPFSHHGNKKTGLSFHSLTMATRKQVCHST

LSMATRKQRKQVCHSTPSPWQQESKRVTQLSYHGDKKRGVSFDSLTMATRKQVCHSTISP

WRQENRSVIPLSHHGTLINILTFDTVMPY

>SG-PL_contig_5401_1_+3_774_947

MCFPFQTFCIHQTIQDFLCTLDNVPAKCLPQRQYNQVQIVKRSTGLSVSWCAVKTSCA

>SG-PL_contig_5402_1_-3_3_155

ICMLFGSNCAGIMLLSSLHTPIFGGYICWVFSGFHKPSNTLMAYRIFNVHI

>SG-PL_contig_5403_1_-2_728_901

MWDISILSLENSMSRFYLIFQFHLMPLLFIYLYVINVLIRANMRNSRIQATSALGYGG

>SG-PL_contig_5404_1_-3_54_1631

MENGVLYYRSPKKAGFGPSPSLVPARLGVDGLGPWRKVVKSEAERRDILTSAHSTGSPAP

GSHHGRDRTASKIETRYWWPNWYEDVKMFVRCCPACQQDGQKKKSFNIKPHARPQDFKPQ

INLEPQPSQFWDKIGIDMIGPLPMTSSGNRYIMAVTDYYSKWAEAKALQAKEGEGVARFL

AELFLRYGMPRVIVSDCGREFRSKFVEQACKLMGINRRRTNLYHPKCEELTERHNLSLEI

QLLKETQTRTNWDQNLDRVLFAYRTSKHVTTKYSPFFLLYFREATLPEDVPEISNTPVDF

DNSMQPYKYCDVSSSVCTQPAPNTTRGFSPSECGPAFTPQCPGFPVSTPCPPTGSVTSST

ALPVGPSFSSTLPSVESSTPSIPGHTQSSISSHTQPVTSSALLHTGLGDPSSVHMGSASS

ASFRTGSITSSVHTLAGSASSTSFHTGPVISSIPTLAGSASSSTSFHTQTTLSSNPAPAA

NGPSPFPHHELTTSSSVTSSNTLIDVAVAEIQKMRDAICNNVAPNF

>SG-PL_contig_5405_1_+1_88_366

MTLSLSTTFSLSCYHSSFFQICSKNISLSGALRLITILLRAGVQVFPNQQHSDAVDTCHE

IHSCCSLGIKLQPAIGLLVMTLSLHVLSLTCYS

>SG-PL_contig_5406_1_-3_132_1733

MSYASAAKGGGKSKSEYKDKDKPTQIRFSNITAAKAVADAIRTALGPRGMDKMIQAANGD

VTITNDGATILKQMQVLHPAAKMLVELSKAQDIEAGDGTTTVVVLAGSLLDASAKLLVKG

IHPTMISEAFQKAAAKCVELLDSMVKPLELSDRNSLLNSASTSLNSKVVSQYSSMLAPIA

VDAVLKVIDPATATNVDLHDIKIVKKLGGTIDDTELIDGLVFNSRAAGAGGPSKVEKAKI

GFIQFCISPPKTDMENQVIVSDYTQMDRVLREERNYILEIVKKIKKAGCNVLLIQKSILR

DAVSDLALHFLAKMKIMVVKDIDREDVEFVCKTLGCRPVASLDHFLPENLGSAELIEEVQ

TGSSKIVKATGIANPGQTVTVLIRGSNKLVLEEADRSLHDALCVIRCLVKKKALIAGGGA

PETELSLRLAAYANTLSGMEQYCFRAFAEALEVIPFTLAENAGLNPISTVTDLRTRHAQG

ELTAGINIRKGAITNILEENVLQPLLVSTSAITLASETVRSILKIDDIVNCVRS

>SG-PL_contig_5407_1_-3_312_4271

MPQECVQRQPCQHGQCRNSMTDFFCDCSRTGFRGERCDVDAWTALFNGSVWGKLDFSRTL

RSHSDDLSLRFRSPLKDGLLFETFASGSNAFIRCSLENGRIKVETNLGGELKTLYAGENL

NDLQWHTVYIQRRGNEMAVWVDDDPHTKATLAGSGYYLDVDRVYLGGTDTASENHQRYIG

YLQNFYLGDLDILDKMRGTDLGVGTFVSLPPLIYYPVTFPEFGTFITLSPLRVPLAMVLH

FMFRTTEPNGILLFSSGRNQDYLVVELFEGKLYIQFNTGTDRPLRATTEQVVNDGGWHAV

TIRRTTGDQQQFLLTVDGVTSSVSYAGRDGLDLQGPLYIGGAPEELLQKDYIKNGVLSRT

GFRGCLASMDLSGQVPDLRKFADQQGITIFDSCRPIIASVCTIDKCRNFGTCIESGAGGF

SCNCHMTAHSGTVCENEPIGFLYQNFSRPGLLIYDFLNQPSTSRDEIAFGVMTKQQNAVL

LRVFSELMNDYIEFRLVDGNIVVHYDTDGTGSRQTLTQFNHRVNSGKTYHIVRFIRTPTG

ATLYVDDLAGVSASHSSAHGDFDRMSKIYIGGAYDASADVIQNGFAGIIGGLHINGLLVF

PEAMARVNTHYKGDVVVAPRPFALVEGQFYGIPPGVGGGGAGWVPGAGGIDTVVFPGGDG

GTAVLVPGPSGAGGPVLSALVAAPPALPATGPRAGAVVGTILGLMALASSLMWAFYRCKP

GWCTCLKPSTGTPMAISPPRAGVVSASNKGAVIAAGGGVGGAGGAITSAGAGGAGAGGGG

MAGGSSFSNFYMSQSSAGGGGGAGGGAGGGGASAGSAGGYSSSYREASHVRSASGSAVDG

GGGMGSYNYEAGGVGGGSSTADYDMATGMANYQSNTLTAGGGSMGGAGGGYSSGTLGGGG

GGGFSSSTMQSSYNYSVKTVRTVSGQQQILGYAGSQTVGVPPGALGEEVRVDCCLMTNDG

RSVVTGSSLGPPQVWDMQSGELLRIMQGETVGSTNLHLACNDRLLVGAINADLEANQSSV

PKGVHNYMLQTWDFTTGRALEMGDVEVCSALTLMSDNDKVVFARSDKFGGGTNLVVWDLL

GNQPIKHMRYDAPVGNNDYVHFLALSQNDRYVIAGFTNSFDNYAEFVVFDMTLTSYTVSD

PSVLRLDANPECTAVLPRDEAVTGLRNGDLVIWSMRTGQPSRQLLSSSGRHAHSREVNAV

ARSEDGKYLVSASADRTLKLWDVETERHINTLNGHTDEVWCCAISPDNEIIVSGSRDKTI

RLWRLKSGQEICSFNAGVDIFHVTMSRDKSTIVALGDKLGARKLIMLQVVRTKVRRQVSS

>SG-PL_contig_5408_1_-1_940_1197

MFAKSRILDLRLRLLLLFVFVFILFLDTMDLPCEIRAAFSWGERVAAVERHPVVFFFFFL

FFFGLVFGVGFFFLFFSLYACISVYL

>SG-PL_contig_5409_1_-3_672_911

MIFDIIPCFLKIEDPGGYFPQFLETELQTAYVSNFFNEKSAKVMFVKHILLIFMGIICIC

EQKKALLTNNRCLLFIYTAN

>SG-PL_contig_5410_1_+2_143_319

MYLVGCNPHQDSQNHLIHCQIDQQHLEQYHLYFLSRFHRCYCCCRAVIPTQFSSILTAC

>SG-PL_contig_5411_1_-3_195_1010

MATQAFYRMGVICAVVAFVLMFVSFASPFWYKSWTRVHSPFASIGLWHVCLSGFIMPRDP

AMKSYVGCWWIHSTEFELVTNQLMPAWFRIIQMLCVFTLLLDLFGMIFTLVYLLDGARAK

LYGNDKGMMFIIDSILMLVSAFLVFLIALIFAEMSNDSDWMPRPWMNYLSWSYGLCVLSG

FFSAFGGMVLFILGLIFKDKDRRELDNLDSAAELRRHQQVAREKEEEAMAPRHIAPAHIP

PTRFSPPTEMAVTHAPPTIAAKPQPRVGESFV

>SG-PL_contig_5412_1_-2_2_223

DHIYLTVYDAVQCAEAALHSEVRVADMVSTCLSVCLSVGRSVGRSVGLSVCLSVCLSVFL

STLCSIARTGSTGA

>SG-PL_contig_5413_1_+2_53_304

MDASLRVLFKVHDQYCRCLHLLGLINARIYYERKYKVQPWHAVSILDGIPLHHDPSPHYH

EICHQSIVKIVPDSVTFHLKQHRR

>SG-PL_contig_5414_1_+3_102_395

MHHYSVHIHNTYCSWTTKPQISELPSYPLTTETSKYAKQPTWQHQWNISLKSRHFSYEQG

RSYLCIQKKTIHFTYDKTILTWSLLVTTYTLQARIVLL

>SG-PL_contig_5415_1_+3_120_263

MTKLNRVWKSNIRFPSKIKLYKALVVSIVLYGCETWTLTAETERKIQA

>SG-PL_contig_5416_1_-2_2_163

LSVCLSPSLLYVCVCMCVWGVGVGVCGCGCVSEETETTYKVHLPLIQHIKCTYH

>SG-PL_contig_5417_1_-1_58_297

MYFKCFVHYILLFVGITPFINKSLHEETFFMTIVTVLLILGYSRCLNLFCAQNAFVNYIL

FSLFIISKCMRVTLSVCLSP

>SG-PL_contig_5418_1_+3_60_263

MKQAGATIWAIMLRTRIQAKFQNCADKLSNKHIFCITNNKQSKQLYYFTESKWHHHLVAT

TIRKQERQ

>SG-PL_contig_5419_1_+3_282_458

MYNYICDYRYLYVLLTLWVCIDLCTSVYSNYMFMCVCDVSASSCITSVSCNCVMRSESK

>SG-PL_contig_5420_1_+3_3_191

FALFLFFFFSSRRRHTRFRGVTGVQTCALPIWCQCHHCLGSPSIREVTEVIPVKMLSCPV

LIH

>SG-PL_contig_5421_1_-1_1_168

EALRWAVKRSFLCCGLGLNLRYLISSPQLKLTNQPMGECCYSFIILFILYYSSCLR

>SG-PL_contig_5422_1_+3_1215_1418

MKEHINSSVILLVIYLKDHIYVDSVWMHFLQPVLMFYYITTSNRLFNQDKLLYNRSHSLN

KILIIFIL

>SG-PL_contig_5423_1_-3_1179_1298

MKQDMDFKKAVKPVSSDFKNSSRVCSSMLETYSLKFAFET

>SG-PL_contig_5424_1_+2_290_415

MARSVKDFGERSFLYTSPVSWNCFHSKYTFPLRLNKNLNIRL

>SG-PL_contig_5425_1_+3_1326_1625

MATNDVKRKRKCLSIDKKYEIIQAVDKKQAPLAEICRQFDIPQGSIHTIIKDRERIVAAC

SNGKFALDRKKMRLPDVDAVDQALLMWFKEARRKLVPISG

>SG-PL_contig_5426_1_-2_2_1012

EQEKQWRERHREYLRQAGDKQRKKHEEKEKQRKKDEVKTDADCWDILDKLEQQESQQGEL

ESIEDDPCKQLPEQKQSTFIDGDDKLAENEGEASSHDDSDHTEDEYSTGDEEEEDVQHSR

KTITFSHTKHHDGSPSRGSAPQNEDATDVINSPADLYRLVKTGPKSILKTPSQDADSHSQ

RMRRMSLEDQKKVTFGGEQIGGRNTCAKSLPGENADMRDGFAGNGNVQRGGFTSKTVVQA

CSDTVVERRISAASAQPPSDSTTTSSAISEAQEMAASDRPVSKFRASRQKADTVPVVSPA

THSQDQMEPHVSGAALQSSARPVSKFRASRVKQGGNS

>SG-PL_contig_5427_1_+1_1_282

QVTIENQWLTICFGGKKHETFNIKIIDKNTGLFNTCTLQLSGRILVVYNIDVLSVSYGTA

FQTVCMLKEKGKIKMDKTKTIQLLFWKNIDVMDV

>SG-PL_contig_5428_1_-3_111_1343

MIVTLKTLQQQTFKIEVEPECTVKLLKERIEAEKGKDYPAGGQKLIYAGKILDDEKTVDE

YKIEEKNFVVVMVTKPKPASAPAAETKPSETPAAANITPAATAPAPTAAAAAPTPAPVSQ

PETEGKGDSNTTSANVAPTPVTPATEASEAGATGTAVQSAESTLVTGSEYETMVTEIMMM

GFERDQVVRALRASFNNPDRAVEYCLCGIPEVNVPEESPAVPVSGNPPAANPPAATTGTP

ASTGASTGAGTGQNPTGVNPSAAGSGTGSFPSEDQLAFLRSQPQFQRMRNAIRENPSLLP

ALLQQIGSTNPALLSLISQNQERFVQMLNEADSAESGGQGGGGSGSGQEQEAGGGGYIQV

TPQEKEAIERLKALGFPEGMCIQAYFACEKNEDLAANFLLSQGFDDDEDQS

>SG-PL_contig_5429_1_-2_845_1015

MRFYHVIWSLYMLASVLIIGYRSGYTMNYKLYLNLTDAVVKWVNIRLPIGRSWVQSA

>SG-PL_contig_5430_1_-3_1059_1292

MVSQCMYADIMFCMFGRNCASIMLLHSLLLHLWWVTMLVPLHKNKILVCSVHCITFCVHV

LWTTAILYVSEWNCAICV

>SG-PL_contig_5431_1_+3_3_116

DFCPLPASQNVHIIKLVHGLICLELVSRLFRVCVFCTS

>SG-PL_contig_5432_1_+1_1_288

PYRCLWLTELVACLPIHVYGVTELVACLPIHVSGVTVSCLSPYPCLCCHHYHYHHEVLNM

MSQFCRRAILKQQTINNLHKLMKHMPRCLSVCSLKV

>SG-PL_contig_5433_1_+3_3_158

KGKKQTNCQTYRRFQSEKHFINVSMQMFLIQTVVGSKQKTIRWQKNCDYARK

>SG-PL_contig_5434_1_-1_109_1527

MTSKISKFFSKTKRSEKISETDNGGAAPLSIGEPFNVVRNYHVNFDKSKGEFVGLPPAWR

DLLDHSDISKEEQKLHPEEVVNSLKTYAKSIKRKSSSTKFMLVATTIRESDELLDSESKE

GDKDDGVFGEEGKAAASSDADKDVADATDGMAQLRGNDNADEDDGVVVSRRPRTQKPMTD

TEINNALKEIASPGDPHAKYDVQKKLGAGASGSVVMAKQKGSADVVAIKMMDLNNQPKKE

LIITEIEVMKSYNHDNIVNFLDCYYLENELWVVMEYLDGGALTDVVTETIMKEGQIAAVC

RECLKALEFLHGRSIIHRDIKSDNVLLGMIGAVKLTDFGFCAQLSGDKSKRDTMVGTPYW

MAPEVVSRKQYGNKVDIWSLGIMIIEMLEGEPPYLNETPLKAIYQIATKGKPDIKNEEKL

SEELRDFINCCLDVDPDKRGSAADLLQHRFLKKAMALSTLKPLIVAARQATGH

>SG-PL_contig_5435_1_+2_104_466

MEEGQNTGTKTGADAASMLAQVKAQAYQYSIGTNTNYGQQFANSIQTEALYKKMWFLCPL

ERLERTKNVYTQHLQKPKQNKTKPADKPASLKMKMSNKKTGPPSLPILFKTKQNQAKDNT

L

>SG-PL_contig_5436_1_-3_3_227

NTSVGFTITVLEVKDPETSAGRKLFVFCDDSTTQFAESCNLTKIEMVRRSTTDWEGYLLL

NQPLDYETRTSYSVR

>SG-PL_contig_5437_1_-1_133_339

MHPGTGPLSMPGTATELIARGPQPKQSRSESALSSQAMKQNILTCPKCNTEFSEERQGEL

LAHMDICWE

>SG-PL_contig_5438_1_-1_46_186

MSVIKCLHPSFLVYCRVSLSDCTFYIFLERLCKICDIKMLWASLVAF

>SG-PL_contig_5439_1_+2_122_301

MLWTGTELQTSDFESTALSTEPARNKHTHNKTSTGNQKELLRVKPNVSPWLYQQSQRNKQ

>SG-PL_contig_5440_1_+1_211_471

MCWLCDGGVKRQVKDKRFKVKMCADYASDLSLLHDINIIQDLESKHLKGMCNKCYQRLIR

CKNHFSVSERSATVSNAAEVIKTMSTT

>SG-PL_contig_5441_1_+1_67_315

MMAMTLLHTYSEHLERLDSMCRLCGERVKRHVKDKRFKVKMCVDYASDLSLFHDVNVTQD

VESQFRKGMCNKCYQRLVHFKNH

>SG-PL_contig_5442_1_-3_513_1052

MAQGRLSPHCATQTHFVPLVFLSSATVLPPHLFPWLPDFVDVDDDNNVDDDVDASCLCGI

SFNQTACSGREQMSFLVFRLLFFWPLLLCSCFTLASFNASFAQAGEEGRVGGLVEVGFES

LTLGLTGWEWGVCLWVVSVLVSCCSSPNIIACSKQRTNLSISFFTVTKAVQPTHSPTPLF

>SG-PL_contig_5443_1_-1_1_2385

TAAGTTTTAPITTTAGTTTTIPITTTAPITTTVPITTAAGTTTTAGTTTTAPTTTPAPIT

TTVPITTTAPITTTAGTATTAGTTTTAPTTTTAPTTTTAPTTKTAAATTTTAATTTTTPT

TTNAPTTTGATTTTAPPSPPPSTAPATTTAPTTITAAATTITAGTTVTAGTTTTAGTTST

AGTTTVGGTTTTAPTTTTTAPTTTTTAPTITTAPTTTGAVTTTAPPPPPPPTVPTTTTAG

TTTNAPTSTTAPTSTTAPTTTTVPTTTTAPTTTTVPTTTTAPITTTASTTTTAPISTTVG

TTTTAPASTTAPASTTVPTSTTVPTSTTAATTTGAKTTTAPPPPPTAPTTTTVGTTSTAG

TSTTVRTTTTAPTTTAQATTTASTTTAATTATTTTTAPTTTTAPTTTTAATTTTAATTTT

APTTTGATTTTAPPPPPTAPTTTTAGTTTTAGTSTTVRTTTTAPTTTAAGTSTTAPTTTT

APTTTGATTTTAPPPPPPSTAPTTTTAPTTITAAATTVTAGTTTTAETTSTAGTTTAGGT

TTTAPTTTTTATTTTAPTTTTTAPTTTTAPTTTAATTTTAPPPPLTAPTTPTAGTTTNAP

TSTTAPTSTTAPTTTTAPTSTTAPTTTTAPTTTTAPTTTTAPSTTAAPTTTTAPTTTTAP

ISATVGTTTTAPATTTAPASTNVPTSTTVPTSTTAATTTGAKTTTAPPPPPTTPATTTAG

TTSTAGTTTARGTTTTAPTATTAAPTTTTAATTTTAPTTTTAPTTTTAPTTTTTAGTTTT

AGTTTTAATTTTAPT

>SG-PL_contig_5444_1_-2_80_733

MTGGTDDPISMSPPHIYVERTLAIIKPDALHKAEEIEDIILRSGFAILQKRRVHLTPEQA

SDLYAEHYGKLFFPSLVAYMSSGPIIAMVISRHQAITYWRELIGPTNTIKARQTHPDCLR

AIYGTDDQRNALHGSDGLPSSQREIRFFFPDSVVEPVATGQAAKDYLSKAVNPTLLKGLT

ELCKQKPEDPVIWLADWLLKNNPNKPKVGDNAIVEEPK

>SG-PL_contig_5445_1_-3_162_1700

MTPSLILIWSFTDPIHPQLILEAPEDIFCFTFCPTDHNIIAGGCYNGQIILWDISAHVDR

LKAPRGGNRNKRLNVLPGFEDPNALKTPIVRYCAVSSIEHGHKAAVTDIQWMPDHMMISR

MGVPLENKSGQCVQLITCAADNCVLYWDTRAPKGQHPKDDDKGLKNPMGVPNTFRHLDLT

WKPMLKMHLSKSEPGGDHAPIKFSMQERQGDRSALSKATESGEKDLTNTMGGGFTGGITK

PGSGKERTLQGVQTYLYMGTEDGEVVYGDWMPQKDQDTGKLQTPKPSYYHTLHDGPVTSL

QRSPFLKEVVLSVGGWTWAIWKEGVSSGPILHAAAAPVRLSAGYWSPTRPSVFYISKQDG

SVDVWDLLDKTHEPSLTQSVSPAPITNIFPYQVTQKQQLLAVGDNSGTLHILEIPWSLRQ

PTPGELNGVTNYIEREVKRRAFVVSRWNLREHEKQEMAMEAKRKAGIAPNVTLTDEEIEQ

KQKQEYQAYLNEEYSFLRELGIVKEEDEPLPEV

>SG-PL_contig_5446_1_-2_44_283

MSSSIKKWPISTVGYSIMRITQGTWVQTSHALQFSSGMAGSHDRAVDSRSHVSSSIPACN

IKTGLLAAHLNAVIILVWSA

>SG-PL_contig_5447_1_-1_430_795

MQNASEGAEEREIICTVLEICFSATTPTNTMFLKLSMKQRWHLPSVVGPWPKLSCTHTSR

YGQISVASRSTLHSFLARVFFVHVHQTLFRRDGSLSLKRCLNVPCFAAMFSQLFVEDFGD

QF

>SG-PL_contig_5448_1_+1_1_150

RIQRLEVGRPLKCERGFMERNVLAVKKKGLRKCQIKKLQNPGGKINEGIL

>SG-PL_contig_5449_1_-1_328_426

MNLIMYACIRFKYRVYFYFRPSFSTSDPCNEIN

>SG-PL_contig_5450_1_-2_20_682

MSRENRPKSMGREIGEVHTGERNISRASPPRSRSIGRSQSRGRDLRSHSRRRSCSSSSSS

SSTADSKNHGSLPMSEQQYEEEMYRRLKLEITSNKDPKEREECIALHIREMAKLSPVLFR

QFMAKNAGNLSAMAMMQPEQQEQHSVEHEPTEAEEETRPLRSILKRKGDSRSPSPARNPS

PPPEEPRSKALSQIEKVIQALRKGGSRPEEPKPQREKTPSP

>SG-PL_contig_5451_1_-3_279_494

MVSTSNLNFKSTSPRNPCELLLGSLVFPILCQLCCVVLHCVVLHCVVFYCSVLCCIVLCC

TVLCCIVLCCIV

>SG-PL_contig_5452_1_+1_163_567

MGFLFQTQSRIQQNDCHIKNRSAQTNFNCFFVVVVSLLMSYLPRCFSGVDPTLLIPALPH

TINPKFIQLKSQYQEADGKPVISSHHGATYQRRLHCYKITSVEFSSAHSGPTYYRMLPTK

PPTNDRIIQSWSQTE

>SG-PL_contig_5453_1_+2_2_199

TLEQSKPPVLHIKSHGSNRLQPALPHAFLAAPKQLTLVAVSCPVLVLGILNLAARDVGRQ

GQGQPQ

>SG-PL_contig_5454_1_-2_230_2503

MKQEISENDKVSEMMSVNKMFQTSMELQQAESGASHKEDKSVSQHRNIVDQLFQDFVTTK

MREVDDGAKGLSDNDNSTSSAGPESEKTQVPLSIEEINRLLDAEITSIKSRSVVADLTPE

DVVMDANEKRQIGRDSNSICSSQTASAAPTDAGEVLEITPGPSQPSVQTSSGAILLLEPE

DNSRETTAGEVKGLCASKTKATKKLGLSIGQEMLARITGNWKNGHILEEGEVASSTDSDA

EKAFKADPGAEEETNDSDEVSGTGSLSDSDKGKTEGKDSKAKKKKKHKRKHKKKKKHKTQ

SKEKEEGDRHTLEGRPSKEEKDDGAKKEAKESRSKDKKKSSRHKSKSPVPFDPWDARCYK

TESTRSRSRSPLSSRSRHKDQSKDRTRERSKERSRDNSRDRTHGRSRDGRHEWSKERHKK

SRDHSKEQSKKRSRSKEKDKHAADLLVDKDKVGKDLRLKIDKNKLRKIAIARALANMERG

QGPAVDLTSVKSGGKSVAELTEFCKRISQKETGMEDNVSSDSEMSEEERTSEGEDGPLHH

PFIMRDPSSIPNIVMNIRNAKQLPVLTPQEKQTKAATLRTQFPVSSGTQHRIKESEWVPV

TTTSATTATLTKSTTTSAATTAKTTTITTTTIFAVPAVPAVPVMAGMGAGGNGGGVPVVA

PKVNTVAVPAEQSVFPPVQTNIDIGSIVSERIQAMRRLQQNPYDVQALTCVHRINEQTNA

WAQSKHLPGQFTGMTDVRILTQEELAGPDKRNQAWVKK

>SG-PL_contig_5455_1_+1_1_192

NNNKTNPTHIHSHIKANSHKHYGVISACFLTSLEFGVGHSVVFFAVSSFLLGALWSTQCS

CPLF

>SG-PL_contig_5456_1_+2_2_106

RWDSKKTKRHECRTLKGIFYNRKCPSISLPCTNQF

>SG-PL_contig_5457_1_+2_38_247

MSRLKCLSNSLTQSTACCLLHTHTPTYTSKMNRLKCLSNSLTQSAVSCLLHTSVNILVRK

TCTSTMFYLL

>SG-PL_contig_5458_1_-3_102_1175

MDFHKLIHAVQHNDVAWFMNEQTLSDCLRVSHKKTGDTVLHLACRFGRRDLLEQFILCEG

VDLQIPNYEGKKPLHEAAQFGQLDCVEFLLQNGVQVDCLKRADWTPLMLASTKCQLPVIS

LLLNHGADPSLLNKDAWNSFHIAAREGHVEILQVLLAKNPALWNTVSHNGRTPLHTAALH

GRKEAVEFLIRGCDFAVDGSDSCGVTPLMDALRAGFVDVAAFLISAHKADVMRQDGLGRQ

AIHHAAQAGQLNAVDFLLSTCCVPVDTATPQSCVTSLHIAAKEGQDAVIGLLLQKGADVN

QADNTGRTALHIASAAQHASTTRLLLAHGARDVPNNHGQLARQLASRPDVRQVFDEYS

>SG-PL_contig_5459_1_-3_63_314

MSLDDNCLSMVSLVNYVVKGTLPQQLPENSHVVYSFSVPLEFHGSVESKVQAWFQKKRKS

RLWCNMFCTLELESRTVQEWKVCL

>SG-PL_contig_5460_1_+2_164_313

MQAYVNSRLAPDEALFNLLNKRKRKKLKLQVLRLTTKSSCHLFQQKDATR

>SG-PL_contig_5461_1_-2_2_196

QTVKCGNFSFLLFAAVVQRVNECCAQITARVRPVYREQFVCQLSVPDTSRKTKTVFYRSR

DNLHS

>SG-PL_contig_5462_1_-1_1519_1770

MLEFICLIWVLAILLLLLLLLNHHLLLLLLLHLLLLLLLLHLLLLLLLLLLLLLLLLLLL

LLLLLLLVLVVLRHHLPLLFTNHL

>SG-PL_contig_5463_1_+1_190_561

MVKYMDRTKGHTFLRKKTTANQMNKLQQTKRPQKDKYVGKKIEHTCIHTYSDYTRIQNTH

IKMTRKQKQANYSKTYKQKLKKKKITNTKTKTTKRKKKAQVKWFLACKHQSLSNQSLSQG

LTAF

>SG-PL_contig_5464_1_+3_165_974

MASFLFSISVPNFVGHVNAERIKERSNVGTDAVQILSIVFSRGRSFLRLRPSAGLCTIVG

AGGLAGGCGTTSRALTMGFHDAFQPAKIVRWRRSRSLRTEHFDFVMLRVDSLQLTRLTQV

IVIATHTLVAWAVDTALTPITHHSRMMHLFLLAFLTSFVFFGSTIRLFCCAPDIDSQYTI

SRMVNSFRHLHSGRECHKVTISILPKHFKPILRHGKDVSHLGDEQTIHLSCVRLLNLEAS

DLSFHLAILLQKCFNVQYHFAGFVLSSFKF

>SG-PL_contig_5465_1_+1_112_282

MYRAKRLAQSKGIGQSEWSTQVIYFWPRYYIPYRGPAKSTIVIIIYTHSILFAPGGT

>SG-PL_contig_5466_1_-3_15_680

MSNMFGVSQSSHLSPEVKDVLPTSAQNCSGWWCSMCSKSFRSGDLLSMHMQFVHRKPVPS

VSASGGHQTAGMSTTPHTAVLPTAASTSSETFFIEPSIVSQSFLSDIVDADLFQSDNKMS

SSFSSTSQASKPQFTDNRMKEFPSNVQSSWMQDTQTTTKGEQNRTFESQLSYSSQLLVSG

GQNTQTMSNPFYVYPSFSPSSQGGDVMNTDYSSASCSLGTPG

>SG-PL_contig_5467_1_-3_3_206

NQIYSSKLHMFTHFQNHSPPAIHQLSFDGFGVVAVVSCYLCLHVLLQYSAFSVKEATEVH

IKTLCSLF

>SG-PL_contig_5468_1_-2_2_262

ISSQVSTIFQEISESTVYWQTYARIPGSALFETSHGLPILETARNVPSKLSFSLLDVSKR

ISLYRTPKILSYFSSSPSGLKEASTFL

>SG-PL_contig_5469_1_-1_160_300

MECMHLRARTTQQQKQQENSIPGTLGVQGFCAPCSCYFTVKAKASLY

>SG-PL_contig_5470_1_-2_1490_1711

MWMCVFICLSLCRQAILGWVLVFLVGVTFLAHCSCRLLKCFTCISLNRRNLIVLVPLTLS

RCVKNSVFSISLLY

>SG-PL_contig_5471_1_+2_935_1285

MTLSGRLQRLSVVHDKQQNTVGRHSPGMVTKDLISLYADIFQYDFTNTFSTEISLHDKLF

LFCFCFCIGIIELFVCLFVCLFVFEGAYSILYHFFKLDSLTFLLVLYNMFSCLFFFL

>SG-PL_contig_5472_1_+1_148_381

MACPRYNNMVFTIIKLYKHVSHSIIILNLVTCEHETVPCHNFTEKHIPNFHNLFFFNSSE

NGKPQCRLSAASNPSTVN

>SG-PL_contig_5473_1_-3_6_221

MQTDKFVGIFFRGEKLSTSGYHLCSCFSCFLGQEAEIHLYLNQAEVHIFEPEWVCHVLLI

AFYCSLHIIKKK

>SG-PL_contig_5474_1_+3_3_206

PATQQTIRSEEGLERKLSPEENQGRFCIFCFIAINIFKMKDSCYYTDIKLKCVFLCCLST

NFYQNQFG

>SG-PL_contig_5475_1_-2_113_334

MEQEVYYDDDFHVRTVRQCAMESGPLKCVERTGTYRFKVYYCHCEGDNCNSAGILSISLV

LTALCGALVYMFKL

>SG-PL_contig_5476_1_-3_84_1220

MSHEKISQARITGCFSVHEFQLMYPETDAMSLLQVLEALEVCTQCEVEGEVEFEFPCLNF

IETLMGLWQKDDERYSDAVYGGVRLHTSFQSGAQLKYLFPRIQVFLRRNMLGESEEQEVD

LYQWHHGSKYCWGQLEGLIDMDHNEQFLEIKVRGPPDSRYSLFFFLEDFVNVVEQVVENV

CPGLCTERYTLSPSQLQQHGKIVRSYSPTELLRMQLENRSSVVLSGTTTEHFLDIACMGS

EEVLHHLTLGMDLDVTHLTIHTQRLLSLLLDPADPMGRDWCLLAVTVGLSDYLPTLDKDE

TKQVSQTQRVLGEWARRYDASIRDLVMHLKDFNRIDAVEAILRTTPPFRTLTYEDQSTDE

SAGAHGTDASTNTLSNLSR

>SG-PL_contig_5477_1_+2_2_172

FFFFSTVSFYCWKHMFSQKFHKYKDYFSKLTIQEHRLAQTVCTIRVRVLWSFCLVLR

>SG-PL_contig_5478_1_-2_2_214

HFGYYQGCEDYYDHTYEADPSQWGLDFRRDMTVLRNYTGQYSTTLFTDHAVDIINTHDTK

QKGQICLALPR

>SG-PL_contig_5479_1_+3_114_269

MEDNLSLHKTICLGRVFKTAAVGGTLRVLYQTLSSKHQSDWGIKTGRNKSCD

>SG-PL_contig_5480_1_-3_3_131

LHMLWFLDISATSRSLLQQTGFPTRAVQWPTVSWRLPSLSLAG

>SG-PL_contig_5481_1_+1_1_162

RKRNSFTVCEECNRFPSKNSFQNKIEIHLVRVRNVSTCSFPNRFKNGHFLLYFT

>SG-PL_contig_5482_1_-1_1_156

PLPSSLHLQMAHWSHTCRTFWCVVCVLVNHQAPSGCSVAIPCFGVVHECRSP

>SG-PL_contig_5483_1_-1_160_297

MYVVNHSKLNKYVYKPSIAFPSVCLLSVFVLFFAVLFCFVLFLTCF

>SG-PL_contig_5484_1_+1_1_78

PVKLQHWTATMDDMQVKFLQQPCHNK

>SG-PL_contig_5485_1_+1_1399_1668

MCTMIQLLRLSLAAQTRWVGLVFFIPSNVFLDAYMHVIWPTLCRHYVAAQSAYSVFSGCP

CACWYFHVSISHRTLTGLTRSLKCLICCTV

>SG-PL_contig_5486_1_-3_3_146

GLHRGKRVEIFPSSQANICAGLLVPEPPSCIYARRTSNTHIKDPVSHI

>SG-PL_contig_5487_1_-2_2_112

NNSPCYFSTFPSRLSTVTHVNSNQQTGTTAPVVRRLY

>SG-PL_contig_5488_1_+1_1_165

SNQHEHKTRFIYFNKSITPQAIHTFSGICNKTLVILLIYIDLRIISSSNVTLHAN

>SG-PL_contig_5489_1_-2_89_1387

MQGQAQALKEELGTDLQSQLTVDDQMEVDHLNDQIAQLTQENRAVWQERIKLEGEKNKLE

NLLSNNLTKKHERLMAAMQEASPEEKNRKLDTLRDELASVDQRLSELKDHGKGLDAQITR

LNREQKRKQTETEHWKGQEKEAQEKLQEDAKELEKMTNKQSLLLKKKEDCMRKIRELGSL

PSDAFEKYQNLGLKQLFKKLEQCNTELKRYSHVNKKAMDQFVNFSDQKEKLIKRKEELDR

AYESIVHDLIDALDHRKYEAIQLTFKQVSKYFSEIFKKLAPQGHAVLVMKKGEPEGEEGQ

SSVPLVEQFTGVGIKVSFTGNKAEMRDMQQLSGGQKSLVALTLIFAIQKCDPAPFYLFDE

IDQALDAQHRKAVADMIHELCQDAQFITTTFRPELLEHADKFYGVKFRNKVSHVECVSQD

EARDFVEDDNTHT

>SG-PL_contig_5490_1_+2_284_529

MATMWTFNLSCPRYGLILLVVPVTLMRDGFHVQLVTASWLFSFKTKAYLVHIINLVTGCV

SSTLPSCGINFQPFQNICINFL

>SG-PL_contig_5491_1_+1_1489_1791

MYFTFLVINDKSINQTVGQHKILSNALTFPQRPSVLNPLTSFQCALLCGRLYTVQHPSMY

IVHIFFIINLQAKFKGDYITTGPNFQSRKFSISCGLPSSMS

>SG-PL_contig_5492_1_+2_26_235

MCMCLSSYSGMSSFHFLFCRQAGDCILENNSCRRPQQCHSVHVFPLIKAVTHAYTKIRMH

HIANQHNLRI

>SG-PL_contig_5493_1_+2_2_268

LLCISVICARARVCVCVCVRARVHYVVSVCMSWVGWPGKGKSACIHIFKLVCQCVSPENV

CVRVCVRVFVCAHVRVCTCECECACVSRV

>SG-PL_contig_5494_1_-2_968_1180

MLPVQDVQLSVLGQTAMHCKIEVMEKEMQNHHSKCQKPAYKPHALTRFEILLNLLLHTIV

YRSFTSLSWNR

>SG-PL_contig_5495_1_-2_1100_1321

MMLQNCVNIIEGKYLFFCWDYKLLIIGRKRPNKENHLFFCIFIGRIENKILWNLTVILTV

ILIQVSHFHTNVHV

>SG-PL_contig_5496_1_-3_3_185

TLTLSVSEMQAYVAFVKAACEFLYVCWILRRLLLSSSSSWKPASSQKTLKISPCGCLAIL

L

>SG-PL_contig_5497_1_-3_9_593

MEVGTSVQENLSLAGDTAHIPDRCFDQLVRRACQGVLSDVDRNTIEDDPSFKSVDKAVLK

AAYSGMVALALEASKHDMDADGITSFLEECKFSPDRIQGFNNIFLAQKSAIQILLGSIGN

CRPHIVDVDWRLDYYIKSNHMERINEAVYLISLKTEMPGQEELQEVQFTCSLEQLQDLVG

KLKDATKCLEKLAQM

>SG-PL_contig_5498_1_-2_44_1318

MLSVSGYHGRGGPLKVTTVKTMPITDLWLQAGKEMGFKQVDPNGETMEGVVLTQMTSANG

LRYSTSRAFLYPALHRDNLHVTDNSQVTKILFKGTRAVGVQFVRQGRNQQVEARREVIVS

AGAVGSPHILQLSGVGPKPLLNKLKIPVVADLPVGDNLQDHVFFDYQVGVKEPVTVTAQH

LSSFWTWLQYTLFRSGFWTSPNAAEAQLFVSTGEESQRLNWPDLQLICQGRLGNTDILKG

FHYSKESIEQAKRRDTFTYGFFCVDTLLRPKSRGTVRIRSADPFDYPAIDPHYLQHPEDL

ALLLRGVRMCQKLVATPTMQSLGAELADSPSTLCGQHAYDTDGYWSCLIQRHILTLYHPS

GTCKMGPVTDNTTVVDPQLRVKGVKGLRVVDASIMPAIVSGNTNAPTIMIAEKAADLIKA

SYGDL

>SG-PL_contig_5499_1_-1_1_162

LLCSVFVFFFFKQKTAYEISRSDWSSDVCSSDQKPLHFPGHFLCSVKVGLLNPK

>SG-PL_contig_5500_1_-2_134_283

MFLLSISPILYFIFRGWGMGVPIQVNDVGFHSPLLFLEHLSYLLHIRAWV

>SG-PL_contig_5501_1_+3_3_215

FFFFFKVQSSLLPLKTKTKQNSHSPFFDFSSLQTLEMHQKTRWSSHTINLSLLAPLDKNC

VYMILKILSQI

>SG-PL_contig_5502_1_+3_3_257

SCSQRKSSSTTWNWYSEPCKLSMGLPQGSPFSPVLYNVYTKGPADINNNRPARILTFADD

GLIYKTGKSTQERNKILQEQLNKVD

>SG-PL_contig_5503_1_-3_3_563

DFKPASVDTSQEAKLEVGEGHQVTVTVPHVQGAGTSHTVFKGNKKPTVKECVLIIDHDTG

TYTLEKLDQNVLLKKTRTEGSSKAHQPAAAADTKKASPSSKSKNSKGSPPSTHDSPSITP

LPSDTDKGADDIALLGDLSSDSSDSDDEMMEVPECFQNGAQAAPSTSSVGNFLKDDLELS

ESGSDSD

>SG-PL_contig_5504_1_-2_32_1597

MICHRSLRHKKLLATLVTVAVLFVLYIHVSHGVWPARQPPAGQQGPMWRPDGAGLDPKTA

VNSGLTTGRKRGVPSNKGGSKGISRLANNDKDKRNARHSVDKTKKKGSNENVRHLLNDKN

KGAMRNVYDKMKNHKNSNILNTDETDTRNAEGVRTRKSSDFQEHPSSQTFVRIAQQISVR

GNPTDISVRKNPSGLSLQNPTNVSTTQNSTSVITWQPEDDEKGTVQSQEAHHLFFLKVHK

AASTTVLNVIYRFALRRRLAVVLPVPNMANVLSENSKNWTHNAVPLPPGAQHFDILCNHL

IFEENSIRKALPKDAFFVGIVRWPFDQFVSAFWYYREKYRYDYLLRIHSKQPISEYLKNP

RRWEKLTNKLQFTHNRMSVDFGMDPLKAHDGNYVKSYINYLDFTFHLVMVSERFDESILL

LRRLLGWRLQDVLYIKNNVKTKHHPYNQNFTERERQAHKEFNMADYQLYDHFSKLLDEKI

RAAGPSFQEEVRSFRVVLKAVSDYCRGRGAHRRPLRVSASFW

>SG-PL_contig_5505_1_-3_120_284

MIYTAQKCLSFPSSLPLFPSLIKVICLFPIQQWGLSNSAPSKESFERIQLSAFKV

>SG-PL_contig_5506_1_-2_212_370

MSRNVLYLVIFSPFITDCKCSAEHTSGCTAVVYCFKLDICTQMTAQLQNTVCL

>SG-PL_contig_5507_1_-1_25_231

MLRLCLFVLALVMVSVAQDATPAPKAGYSTTTASPSGNETAADNGAARLVMTHPLSLMTY

FLFLVVLMV

>SG-PL_contig_5508_1_+1_70_303

MATVCKEKQTSRPNNSMNEERKSSIRQKTVFRRDLNFSLGEDYEGGTHLLSHAALPSLTL

KKVRYRYTTGETRGCIYS

>SG-PL_contig_5509_1_-3_585_935

MPAILKLISYLSLQDAALSHTGGYHTGGCRLVSHWWLSACVTLVVIGLCHWWLSHWWVSA

CVTLVGVGLCHTGGCHTGGCQLVSHWWLSHRWVSACVTLVVVGLCHTGGCQSRGCQL

>SG-PL_contig_5510_1_+2_332_655

MIGKYHNHCLWVYHYIYTHIHTQRTHSLSLSHTHTLLFRFTNRIFLSPQNLAHRHKVQNC

FDGCVSEKCILTKHTKHGNQGLIHGKTSAIVNWLECWTSNRKVWIGTS

>SG-PL_contig_5511_1_+2_2_154

SCTLGGHLYTPPTACSQSPCSKVLHFSVLVVDIVHSQTELSFPVFHMNCLP

>SG-PL_contig_5512_1_-3_63_245

MYRIFKRVKADAGMYIFSRRSLSVKSQHNVLTYFTKKSHSLLIVFCVCHRRNRIQENLRF

S

>SG-PL_contig_5513_1_-2_383_703

MPRKYVSQVKLLQLKNFMRIYTKRRKTKTKTKQTGNCEICDIIPNTMPVLTTEEKENSLE

AKSSLDKASYALKNTNNNKSPKTGAFTFEFFNFWGSQLCALIVWSLN

>SG-PL_contig_5514_1_+1_1_204

VESQLLFTQSHKTETSIDYMSEYITQEYIIFHSLPLHISTNSEESIRTFQQIQKSALDNL

RHGRVQDT

>SG-PL_contig_5515_1_-3_204_389

MDIKVLIGMAYFYFSHQQSAWDNTENGELVMLDSLVLIIICGYTELCSALSYTAQNILMM

KS

>SG-PL_contig_5516_1_-2_83_850

MAGFKRHYIICGQTYSRKVDVDCVSTLASLAASVHKICTDIRLLANFKELEEPFEKDQIG

SSAMPYKRNPMRSERCCALARHLMALIHDPLATAANQWMERTLDDSANRRIAIAEAFLTA

DALLGTMQNVFEGLVVYPKVIERRIRQELPFMATENIIMAMVKSGGDRQECHEQIRVLSQ

QAGNRVKGEGEDNDLVERIKQCQYFAPVHSQLDTLLQPDTFIGRAPQQVEKFIQAEVEPA

LEPYTRDLQGRSVLSV

>SG-PL_contig_5517_1_-2_137_247

MHFPSAFQMLGNNIDSFPKPLNNVDFVVVESLRIFFF

>SG-PL_contig_5518_1_-3_18_311

MAAAVRTLKTVRPHIPLIKFPTRGPAAAFNPAASAPTVNSASPTSNPPGSPPASAATSKP

IGTTPRGSGIDDLNLPMKYQRKLIDVDEMEFIERGGPA

>SG-PL_contig_5519_1_-3_3_260

GSKSAIFNHPKRLSVHRQTSDEMEETMKNEMVVTCNKVNVTRENKHCYILVSCYILVSCL

DNHIRGGYCIVSGEKNNLIQNEIQRL

>SG-PL_contig_5520_1_+2_23_286

MCIHASKKTLLEQNSKTKRRTTKVMICRRGQTSSLRLSPLLTVFKLDGTSLSNYMYSNTI

NTSERAAHQTTTLMLCKRGQTLTDCYSL

>SG-PL_contig_5521_1_-2_2_298

CIIFSHILLRCLKWFASCLHGSVVCQDLNGKSVEWVSERMRLKSFLTVFCLSYSFSNLQP

AHGNYAILHAKRTSQHRMVLQATLYYILLSNRWCIQKKK

>SG-PL_contig_5522_1_-2_2_274

SSKGKAYIERLGGKAHLKRVSSSPASSPALTSLSLAFGTPSHSSVSPLFSSSVSFPFSSF

PARSFPPPHHHTRCHSTWRYTFLFTYTLNFN

>SG-PL_contig_5523_1_-3_465_746

MNKHFTEKCVHVVDYIFDVRKKKFINSFNPVCYIIYTEVEEHLFVISFCSVPYSSTCSHI

NSLFDILLYGFYIAYYMFDQYCIIHVLSSAYVVQ

>SG-PL_contig_5524_1_-2_461_814

MIRSYTSLFTRLRHNMITHVSTSWTDYVTARDVSCQLHLVMCCVCVCVVSAMRQLLFEMK

GLLFEMKGLLFENCQLLSVLGDFSCNCFIILDHLSLSLSLSLSLCHGHLYHRHHHQLS

>SG-PL_contig_5525_1_-3_3_131

HTHTHFLFIHKKVFDLTHCFSSCRLFVIGILKLSTRGRNRHFR

>SG-PL_contig_5526_1_-2_2_85

FTEKTADCQTFFLTCSIVICIVFAIPDI

>SG-PL_contig_5527_1_+3_3_155

RYSEISNDSMHATCCNLTYIDACSIQTQQCTVFSQHLKHPDVIGRSKRHQI

>SG-PL_contig_5528_1_+3_264_464

MILPNKSTSGKSVSVLQGDYHERVVIQNGNLFFYRHSTAIDSYVFFKKSIVPDRKKNTTK

LPVHRKS

>SG-PL_contig_5529_1_+2_41_706

MCNHASGQYTTDFDTENNCSMCYSQQDAAAGEQIFMFYGPRSNGEFLVHNGFVALDNEHD

RLCIKLGISKGDPLFEAKTELLGKISLQMSRFFFLHCGDCPVDGETLAFLRIFSMDEDTL

KSRYTGDVSEETQNNLLELESPVSPDNEEKVWTFLGTRAALLLKAYPTTVEDDENQLKKT

DLPNTLVMALELRLREKKILQQTIAYASRRKEAAVEGSVQCD

>SG-PL_contig_5530_1_-3_12_851

MASMVMERLVTASKSLLQNCRLPSYRQRCFLHRQVSTTQSVCCLQQNQYSSTTTPSHQKA

GEATETAAPDPEWESLQHEDYFSLKPLVTLKDLFSARVHLGHKAGSRNAYMSPYIFGCRQ

DVDIIDLEQTLPRLHDAMNFTAHIAYRGGIVLFLSRHNQMLPLVEKTAEECGEFSHCRYW

KGGTFTNATVQFQAETRLPDLCIFINTLNNAFQNHRAMIEATKVLIPIVGIVDTNCDPRL

VTYPVPGNDDSHAAVSLYCRLFKEAILRGKAKRKEDGLES

>SG-PL_contig_5531_1_-2_2_199

FGARLIKSPHIPPPQSSWPISWLSSPLRRVWLPVPSGVTDRLSVPPCNSWWGVVVGPGPH

FRTTSF

>SG-PL_contig_5532_1_+3_1167_1484

MLNKSQRVIPKGRKRINLAKNSDQAIHYVGPVSLFIVSIFTWLLKPTQLARESRSQTNRR

ESRLVRRWIGSESNVDAGINLFISHLRLLPRLGHRPSTNALHPPRS

>SG-PL_contig_5533_1_+2_2_325

IHVYDVTELVAYLPFHVYGVTELVACLPIHVYGVTELVACLPIHVYGVTEPAACLPIHVY

GVTKPSACLPINAYGVTKPVACLHIHVYGVTQAVTRLPIHVYDVTVLA

>SG-PL_contig_5534_1_+1_2182_2451

MKEKRSISAGISTVLVQSCINLITFIFHFMKQYNNLTFPTNLKLICQVTFIFQAADTVLS

FSPPKTHCKKSEHKTLLSQTTKPFSPKQQN

>SG-PL_contig_5535_1_+2_2_268

RFFHSPRSTCADLQVPEPPPCICMQNIKYAHPVSYVRCSLAYGNMKIPSMHTHQRMSMQT

VQQHNTCTVWAKQCVCTHPRKLLETNKQK

>SG-PL_contig_5536_1_-1_1_270

WREASVLLREAIHKAVSHSGQVVKIVCLVTIAWLAAQQLLVDSVSSESEGALGELEEASD

AVGELSDQPYTLIQVLMALVHYSDNPRKAK

>SG-PL_contig_5537_1_-1_1_153

VPSLKGLCDLFSKRLPFYSFSVLDPEAFAFPAFSLDWGSLRACAFPLFPIL

>SG-PL_contig_5538_1_-2_80_223

MCRYCTNVLTQGLYVLIHITFLIRKHNTSLQNSEVFFCCCYSLFCFPK

>SG-PL_contig_5539_1_+3_33_320

MIVDAIDERETLTDDAIDETETLIDDAIDQTETLTDDAIDQTETLIDDATDQRETLLIVD

AIDEKETMMIVDDIDERETLIDDAIDETGTLMSLVR

>SG-PL_contig_5540_1_+2_680_946

MSSSEPDEFYSKNEDSSEFDPDSDPDALCEQKICQPAGMINPGQFRDTFFCFHQSIFAQS

LPIQWNAYLIEDCYSAIIFWMMSHMFCTV

>SG-PL_contig_5541_1_+1_127_387

MNRSFCCCWFGVFLLSHLLRHFSGIDPTLLIPALPHITNPKLFSQTLSARKPMGARDIRS

LWGHPPEDLLRTDARSLWCSSAVPVVI

>SG-PL_contig_5542_1_+1_274_1944

MAVLFFLAFSAAVASVAQTASGSTRPNVLFLVVDDLRPRLGCYGDSNMVTPNIDQLASRS

VRFDRAYVQQAVCSPSRTSFLTGRRPDSTRIFDLEHYFRKLAGNYTTLPQHFKNNGYITQ

SVGKIFHPGSASGFTDDYPYSWTFPAFHAPTRKYTGKKICPRPDGKLYHDAVCPVDLKTV

PGGSLPDIQNTDYTIEFLKNRSMDHKPFFVGMGYHRPHLPFAYPKEYLNLYPMSKIHYAV

NPYRPPWLPDVAWTPWDELRHYEDISVMNISYPFGSVPLDYQLKLRQSYSAAASYTDAQV

GRVLHTLDQLGLANNTIVTFHGDHGWQLGEHGEWCKHTNFDIATRIPMMVYVPGVTSKET

HPGKTFPFQDALDKTLTFRPEKQILSPDIVSDAFVEAVDLYATISELAGLDVPPTCPDSP

FNVTFCTEGSSLVPVIKNLTSDSYDVSKPMTWKPAVFSQYPRPSTYPQHDSINPDLSDIH

IMGYTMRTDQYRYTQWIAYNHTTFFADWNTVYARELYIYASDPYEDLNVAQVPGFQGLVK

TLSTQLRAGWRNALPKP

>SG-PL_contig_5543_1_-2_1385_1720

MVHSMNCHQLFADLFYQQPPLLPLHIRLFTGCVMHQFCSLGQETTQILQLWHSLLTLQLS

EPQFGKCGDDCLTLFHGMPVNMRICQTQNDVTVQQAIQRQSLQKLRSNQAMP

>SG-PL_contig_5544_1_+3_3_227

VFLLPHLLRHFSGIDPTLLIPAPQHTTNPKFISRSPSARKPIGASNVKPLWDHPPEETAL

LPGHFGGVSACSDPT

>SG-PL_contig_5545_1_+3_75_257

MYHTTLARRKKSTGAVSLTPHPFVILDHVFNCFSILIVFQWTQLYFPSEITEKVSPPFMN

G

>SG-PL_contig_5546_1_-2_1703_1936

MIDQKVSCVLLFYVCIFLYVCMCLSVFVNTFLQGFVKCRLLDLYLFEPYSSKPLRNHKYI

SLNGSGWFKIYTEHNTFY

>SG-PL_contig_5547_1_+2_419_553

MPHNNGQNHRARPHELISNTEIDNTACPPRHKRVSIIAKGTRTTS

>SG-PL_contig_5548_1_+3_3_317

QLVLALCGWVGVCVWGCGGEGGRGFGLKRLLPRVECTLYIDSLVILRPHSRVYFTDASIR

VLLKFPDQYRKFLYLLGLVHAKIHHDIKCKVQPIVESIITKELMD

>SG-PL_contig_5549_1_-2_1295_1582

MMAVYYLLDFPKTIFQILFCVDPMLVLYALSKSDVKVRQDLTSEIHHLIHFFMLRGSDIS

FCGIPSHCGIYFNERVGRLAECGAKHMNISCILIIP

>SG-PL_contig_5550_1_+1_412_666

MHISDPVSHVSVQWLMETQKRPACTPLKKENECLQGGAAHQRQSMPTVQQHNACTLWAKQ

SQQGACTHTRKCCLKCTPSKNKNKS

>SG-PL_contig_5551_1_+1_49_252

MIIIIQQRQHNSNICRAVSFGNITWLQQFSPTFFVPKNLQNLLKIKSHCQSFTGFLLKQN

EDVCNIYS

>SG-PL_contig_5552_1_-1_184_300

MGRFILPTKPRPTPLLKKKQTLPSPYDKEEESSINRYNL

>SG-PL_contig_5553_1_-1_1_273

CSSDLPSLGLPFRLIAYKLTQVARRSNTSHNHVDLLETSWLELHQLFRDLRQGCAAMPEA

RATRGSSWSRGDDHQDSQVGGWQNCETSGVR

>SG-PL_contig_5554_1_-1_154_351

MRAKTRQPGSRPRAFRTPSLVSCLDPRGARGEEHLLCPVRCVKYYLKCSALGVPPSGDSL

SPSTRT

>SG-PL_contig_5555_1_-1_451_798

MQCNTMQYNAMQYNALQYNAVQYNTTQYNTIQCNTIQYNIIQYNTMQCNTIQYNAMQCNA

MQYNTIHYNATQYNTMRHSTIQCNTIQYNATQHNSIQCNTIEYNTTQHNATQYNST

>SG-PL_contig_5556_1_-2_1601_1789

MLYEFVSLATSLQLFSVSVQDVIRLNSSDNCIFVIKMRILLIKKQTLRSTHTHRTKCTFC

NSR

>SG-PL_contig_5557_1_+3_3_269

ACTVYAKCSCGQDGEHICLGSERSWVRFLAGQGFSWISGASALPTSYPVRPHHLHFTNTK

NSTTQVPLMDFFCLFRFVLFVWLVLFLFF

>SG-PL_contig_5558_1_+1_25_252

MRTSKNKLSSHINVSQTCKKQYHSLNAGMRSKQIYHWKCMFKEKDGKSNVGAFFTVITTT

NNQKHKIKNKVSHIIQ

>SG-PL_contig_5559_1_+3_153_329

MHFSVCAVSVVCIILVKFQPIHSFYTLVGYPLVVHGNTGLFSVKKKGRKGVQYKIYLKT

>SG-PL_contig_5560_1_+2_2_256

GGGHNFLLLQSSQIFIPPSKFSLIHLMPVSMAQMTERTEKVNLCCLQCKWMPFYKTCSKD

YFTQDYKFLPSIFIPPSFSRRINRL

>SG-PL_contig_5561_1_-1_97_1164

MTSHKREEFLQCERRLLEFFRKKGFHMNPTINPLPSNLNAPPMFVEAMSSSTVPPQQPNV

SSASPDKFSGGQAAPHQGLVEGRPQSDSPTPDKFRRALKLEDMLKYVDLSNASQSCTKQP

EVKKTLNELKQEKDLKGWEMPFSPDPAGVIGTQEGTKLANFSSDQQADQGSESGSRKSST

DLSASQSSIPDLLRQQQQHQLTFHGSGMTLPPFQNQFSQQGMVHHQQMAHPMNQLPGMFN

SPPFPPFFYGYNGPYTTFPGSGSHGHHAAYPSLSSTTFMTPPTMGQGHPSSQQGGHLQSQ

PQVGTNMTMTPASSGAHPTPGGGVVGSGNSGLTMPYFNFFPSHQGPGPNSQPGANG

>SG-PL_contig_5562_1_+3_123_350

MKISTINLNMNSKLLKSVVTYSVTLCTIVENPGQCIQLRWNCNNSSTFCLPLQEKKKKKK

RPSHSNNLPFSLGKNP

>SG-PL_contig_5563_1_-1_190_363

MASWRSIWKLFCVTCGIFPFASSKKRDGSFYSLYFVFSWGFYLVPLYLCVDFFVIFFF

>SG-PL_contig_5564_1_+3_3_89

LYSLTFFHIHSHLSSFARKPFVFHNLFQF

>SG-PL_contig_5565_1_-1_3475_3783

MHAYIHSKTKEHPCQTASTGSHNTCHCTRTFSQITDKKQNYSHPWPQPAPTPISYLFSPL

KKTNKKKVQLRKSEHKTIIRSCKLNYKMQSVKGYYIKIILENE

>SG-PL_contig_5566_1_+3_3_116

DWEGEMGSRKHYADTTRMIGALIKMGGEKASFYVAGWD

>SG-PL_contig_5567_1_+3_363_563

MAVWGFVSPHSLNTATRCGGKNSTSLARSGHSIISLRLSCHGVGFDPKDYCYLLAYVDLL

DLLESEP

>SG-PL_contig_5568_1_+3_1515_1847

MQELNLCRRKNTTNLLSLMTSQTNIMKYEIVIFATPYDATVKKYCYITSQGVRYCYYLTS

RSVPREYPANSVKYNNGYPADITKKKKAYPGQTDIKSRYNLDDSNRCQASQ

>SG-PL_contig_5569_1_+1_199_462

MLAMLTQFCSQRSNPGNLQQQHTHMHFHIIQEIFNKKAKHCCLKQNKQTTITMHSHMFFI

CTSFVPLNAEQCHMKYDTVMNEEKCDIY

>SG-PL_contig_5570_1_+1_1861_2115

MSQVKQLFSSIFFIQTERRQLQLSFHSPICLDSTAPITTLYYPFTLFSRLSGAVLFLSNH

FFILYSEAPITTLSLHSTFYQIQWP

>SG-PL_contig_5571_1_-1_3727_4158

MRREEFLDLWVHGGDNQPRAVFWPSYFSILPLSFTSDSLSANLLLTNLARDLATLFVVFS

FSASSSSNCVRNSSSSSASERISSSSPTSSPSDLELSSRTILLLFAELCPPSFSDGLDSF

SSWPLLFVFVFFVGGVSVGWDSFS

>SG-PL_contig_5572_1_-1_1_105

PLYDCTVGLCETFIAQIGSFCFYTVVPSCFVLVLV

>SG-PL_contig_5573_1_-3_3_200

YVDLEIGLIIIRYLTGLHSDLLIGSARCSDQLGGCIRQIETDNRAAAGRVHSVFIFHQLP

IIPQCT

>SG-PL_contig_5574_1_-1_43_1584

MQFRFRAKFFPEDVSEELIQEITQRMFFLQVKEGILNDDIYCPPETAVLLASYACQAKYA

DFNPADAITSDRLLPQRVFDQHKLSKDQWEDRIKTWWGEHKGMMKDDAMMEYLKIAQDLE

MYGVNYFDIKNKRGTDLLLGVDALGLNVYGREDKLTPKIGFPWSEIRNISFNDKKFVIKP

IDKKAPDFVFYAPRLRINKRILALCMGNHELYMRRRKPDTIEVQQMKAQAREDKLARQQE

RAQLERMKHEAEESDRVRKELEEKVKRMQEEQERTNRDMQDLQSRIREKDEKVAELTTLQ

DELAVERKRAEELRLEMERSHEMEQSERQRLNDELEAAHYSLQEKLDEVQNKQENVSELN

KELEAARREVEERNEELNRVHMEKVQREQELQARLQEQLLIQSELHSAHDENDEPDEEEN

TKDYGGDLQVDEELAAQPRAEEERLTEAEKNTRLQKQLEMLKADLVDQRLDDAEVTKNDE

LHEENVRQGRDKYKTLKQIRQGNTKRRVDEFESM

>SG-PL_contig_5575_1_-2_56_154

MINKYFSLPTTTILKPSGSGLVSPLAQESSFFP

>SG-PL_contig_5576_1_+3_147_326

MKQTITSSPIIFHLYGHHILKTFLMSGFFFVNCKPRKWDKTSCSKQLALTRTHTHGHIIK

>SG-PL_contig_5577_1_-2_179_490

MLLFQLFLLFYQIIHFAHCYPCSLSSKNEQQRKKSTFFVECVTYTIYLLWNVTHSSNLCF

FHETDLKLARIKWRGENKVTGNTGHCRGCFFVVFFFFGGGGARA

>SG-PL_contig_5578_1_-3_3_116

IIIFIVSWQHVMLVYSTDLLLEELSSKMHMLDFVTMVY

>SG-PL_contig_5579_1_+1_1_246

THTHARTNQPKPNSIPYKSLVMSVQKYDIPNRLYSFEMIFCLTNQTAQLSAWHQDNRKTR

QDKKETRNAERGIGEERGRGTN

>SG-PL_contig_5580_1_+1_49_186

MNPLFRFTDDYIPFLNRIQNKEFYHDYINMQVHICLSISPFYSPMK

>SG-PL_contig_5581_1_+1_1_96

FESFRDRQKEHFLTQKLTHTHAHMHTHTYTQV

>SG-PL_contig_5582_1_-1_283_366

MPLDPPRGARLLCRRVLVASLLRHTMPG

>SG-PL_contig_5583_1_-1_1_222

IVPALQSVGAAGLSAKGAVVAGLMGYTAADKMVHGESADAKTVGCVTAGLTAAAAAPYVI

PAAAATVGFTKAGV

>SG-PL_contig_5584_1_-3_702_1145

MYECMILFRTLYVVCTLCSGGLLVGYLNSEMEVFLWNFVCGVVNWFDGYLNSKNGCLFPT

LCVVWWYDWTVILNSEMESFFVLCMLSGCSWTVLSTEMDMAQNKYVVKKMTLFFISSELI

KKGLWLKSKTSQLIQSTHWMKIYGTDTF

>SG-PL_contig_5585_1_-1_295_480

MFCLFLKLANTNKMNIHTCFPTCDSLPAKCALSFYRTHLKGQSKIISLECVDYWGKACRK

SA

>SG-PL_contig_5586_1_-2_2_637

MEEAKRQREIKTGESGQKNPDSSRAGKGKHSKGSGSSSENERKCGEGSSRSRTPAKHREH

HRKKKKDAKFEGQRIPNLVKSCDYKQQGGTPEEEEPVSSKEQDDYVLRALFKKTGVHGVM

KHDKIMDSSRADYAIVEAEAAKVAKEAAAALKRSRARCMSAASGVPTWTGTHGGLSAKPK

FGQRKNCVLGPKPVETITKATDKNGWTSNKKP

>SG-PL_contig_5587_1_+1_169_558

MMRGYEEGGRENEGMARDNEEERERRNEEKLRGSGDREEERKNEGLMRRGEDERRNEGLT

EAEERQNEGIMKGDTQEENESEGVVRENVEEERNDEGRMGGNRREDPSKDEIFRMIRAEF

NDCKNFEDIL

>SG-PL_contig_5588_1_-3_33_1316

MSTLEDRAIWEDGEESLGEEVLRMSNDEIVSRTRLLDNEIKIMKSEIMRISHELQAQKEK

IKENTEKIKVNKTLPYLVSNVIELLDMDPQDAGEEDGANIDLDAQRKGKCAVIKTSTRQT

YFLPVIGLVDAEQLKPGDLVGVNKDSYLILETLPQEYDSRVKAMEVDERPTEQYSDIGGL

DKQIQELIEAVVLPMTHKERFEALGIQPPKGVLLYGAPGTGKTLLARACAAQTKSTFLKL

AGPQLVQMFIGDGAKLVRDAFALAKEKAPAIVFIDELDAIGTKRFDSEKAGDREVQRTML

ELLNQMDGFQPNAMIKVIAATNRVDILDPALLRSGRLDRKIEFPHPNEEARARILQIHSR

KMNVSKDVNYEELARCTDDFNGAQLKAVCVEAGMIAMRREGTELSHEDYMDAIIEVQAKK

KTNLQYYA

>SG-PL_contig_5589_1_+2_86_379

MSGTTAQSCWLQRQSQWRQGGQLTLHLFSFQAQIFMFDICQSFSQGLHLLYKMWSIGQTG

STARDTAHRTGGLTCMSRRLAGQPLQQCLFGQHPESFL

>SG-PL_contig_5590_1_-2_125_226

MNGLVLWPSLHASCLFKHIILYIYMSLTFQKEAD

>SG-PL_contig_5591_1_+3_51_215

MSMSCHCGATHLRKFRLYCYPVPASTASTTTHTKDTDVTSPQAWKCQKKPKLRSQ

>SG-PL_contig_5592_1_+3_3_203

SKSSYKFSSPLSASFIISSSTSSAVKRRKGARLRDSPDIAILVKTSFDSRGKKKKKKKIA

RAVWHIE

>SG-PL_contig_5593_1_+1_1_294

GTARGTVSVTRVCFEVVGWHSESSESLSESLLSGTNSSLLSSAVRSTFVTSSGWLPLLSV

SSSSEMSYKSSYKFSSSLSASFIISSSASCTVKHRKGA

>SG-PL_contig_5594_1_-3_117_302

MAFRLPAALRLAGVRYMASGSGVGEFGSGAGKGGGSGGAVREAGGAFGKMEAAHEEMYFK

KL

>SG-PL_contig_5595_1_+2_2_277

LSLHDALPIWNTLRDNRRTIYPRHIKGNQHRCDDVTDYDLSVHMQPMTLSPGLHTVLQNN

IIRNTDCNLKTANQPNQHTHTHTHTHTHTTAL

>SG-PL_contig_5596_1_-1_61_1065

MKVFGCLLAVVLAAAFVKGGEKCIIRRGVDLDMFTVNHLKMKIPCKYNMINSMCGPYQVT

ITPGNRYLYPGYIIDSVWVTVNRTTDGVYWSGRTTNKETAKFLIGRSEDAFNQKSDNKTT

VTSDLFEEIKSFQHVILREKDGVFSITYAPFDIAGKRHSDNELEFECNAEREEVEYPEQF

CGYTNKTIVNDLATRYFRNRDDKDMLVVLDVFRDTSIVQTDRKCQKATKLMTKKCGKGKD

VGMQIEAARICSVILTSEGHRICAMKYACDPMDVFNACLDYVCGGFDRKAKSCGVVARAI

DLCRNFPNLTEPVKKSNCFTDFLTQDDNPYYNMQD

>SG-PL_contig_5597_1_-1_31_1233

MNSLQRHYIPGPFICIDEQLVPFRGRCSFLQYLPSNPDRYGLKILWAADVENNFPLAAEP

YLGKPLGQDRQINLGRNVAIKLSAPFFKTGRNITTDNFFTVKELADTLQANGLTFVGTVR

GNKRFLPENFKNKKGLKLHESSFVFQEKTTLVNYQTKKNKNVVVMSTMHHDKAVDPAADK

RKPDMIRFYNGTKGAVDAMDKMAHAYTTKRIPRRWPMVMFFNVLDLGTIAARVVWSKKFP

DDSLSNKVARQRFIISIAEQLCLEQMKRRLFLKSLSRPLRGMIELFVHHIQKSELQDKQR

AQAGERRKRPASPARSQAATKQKKSAASTSKVPRNPAPQPNATQERKKQGRCSACHWKKD

HKTAMSCFSCKTWLCKEHTMNVYQECSSDITSEVTITHLAT

>SG-PL_contig_5598_1_-1_1_573

QGQPQASLHQSTDVTAAVAGTRNQEDTDTGHVERECIVGKGGCRWSNVPPSAAAGHAAAH

NVFRVPGNQLVGCSGVEEPVDAFLLFVSDKNIKSMVNFTNIEGKRLHSTKWVNTDEMEIK

ALIGCLLFLGAVKQNMQYTGFIYDQLYGQRLVHSSIARNQLVQLLTNLRFDDKETRTARR

SRDVFAPFRDF

>SG-PL_contig_5599_1_-3_324_812

MPHCLASRMAEYHQENARRPAKGRRTAAMRNRGSPRQSRRPAATQCCSPGRRTPPGNEDI

CLQTQSYSWPTNTEMEELHFVDACRDPAELLEGASGQSPPKRGTARSTFRSQLAPAPASL

PRPPSSWMAKDNEKVTVFCSDIPDMNVGSVLSNQLKMLLKVQG

>SG-PL_contig_5600_1_+1_334_597

MCFVSVCGSVCGWVQGGCFVGFLFCFIVVDGGGGGDLCVCVCMHACACVPLGKRNRGRPT

GTWHCTVQHNMKGNELTWLTQDHRGWRR

>SG-PL_contig_5601_1_-2_872_1024

MTKHGHVLAVRHSIHHHVCCFGKRDSLQQPSLSLSLTLVGTALSHLHTVRV

>SG-PL_contig_5602_1_-3_375_506

MDGSSTSFFGQGCDEHFLGKDQSVSLIKFENRDTFDVQKFIYNS

>SG-PL_contig_5603_1_+3_99_290

MLCSGTDVKPEQKQPQRERFRFQVIYSQRPLPPKRGVITATKSHIHTDSNYVVTQFRNLN

ALYK

>SG-PL_contig_5604_1_+2_740_1078

MGLGKYCGQNEIWVAFAIRGCHGTLLIAWFHSILGNTSTDNRSHLLGRTSRFCQCVGLMH

RRMAYAIIFLDSFPSHGICSIGAYGIYSEHGDAVLRKLKLKLKLDWLAHVGKS

>SG-PL_contig_5605_1_+2_2_253

FGFRKGRECTDTIYALHKLCEKACECQRQTPSRLCRSGKAFRPCKQNKIWEVLESYGIKG

QLLDNIRAIYANCESALRTPRGLS

>SG-PL_contig_5606_1_+2_578_799

MRSDWKAEAVPLCLPLHLVNLGKEVDQVNCKGRKLPSCIMTNIELLGLFLTVTHDCSATF

AFLSCTQNLIFNVP

>SG-PL_contig_5607_1_+3_135_281

MMIPSHLIFSLHILQTTTSVSVSIFYKSTMIGYMFNTLRTTINLPHSLK

>SG-PL_contig_5608_1_-1_1_315

RGDLLASLCCTSNTNDLDRLLDRTLNSGDIRTQDFFDVLFYTILNPVGANRAWTFINNTW

DSHQEKYAAASVALPYAIKVFGDVFSTEDQLLKMQDFVNKHNDLG

>SG-PL_contig_5609_1_+1_199_426

MHSAPFTVILTARPWLQCLSPHPLSPSHPTRGPVHRRENSWSLIYSTSCSPITEHCRWSV

VMYTCSLNLSPTPVGH

>SG-PL_contig_5610_1_+3_3_248

SSLHHSPFLTQQNAYRNFLYSSNFILLLNVIFKKILGVTQYRQKRVKQLSIMLLCSSEKH

ESVRIKLLNHQLQNIKMHHSYL

>SG-PL_contig_5611_1_-1_1_132

TSGLGDNLYQPFSLLLHPLGLAMDLWCVKMKVNQLKGDGFSVWF

>SG-PL_contig_5612_1_+1_1_174

FFFFLTVVHFIVQSGVYDQNIHKPLLFTTQMTDVHTIIQIHIHKPGYVNTVVILAGSL

>SG-PL_contig_5613_1_+2_176_346

MKSKQKFVKVCGFNISLCIYLRYSLCIYLRYSVLSDSIMCNQNPSIWHILICDECIV

>SG-PL_contig_5614_1_+3_3_242

ACVCSKNSKIHSLNIEKYFQENRSNLHLIFRDLPYVVRQFGVGACVHVHVHACICTVTTV

HNTVYQIHFSNDRTLLCTFS

>SG-PL_contig_5615_1_+3_1152_1433

MREREREREREREREREKMRVCVRAHIRIDCKGIATTAAAVYFSLFVSYLVAVLSVVFTK

PLHTIRPKSQRKVKSTSCICSLICLLCFVSFFFI

>SG-PL_contig_5616_1_-3_1035_1181

MYICKYICMYVSIIMFALCCLRLLQLTPSTPGMRCLTNYLDQHYKRVLF

>SG-PL_contig_5617_1_+1_1_204

MEDTVHCCVPSVHPHPNKGPNQLWCPIIRDKTMMMKDNQEQDDEETYSVVKHIQEQDDDD

ETYSGKRR

>SG-PL_contig_5618_1_-2_32_280

MLLHTLHPPSSVGVHAGCFRVPISQRTLTWLTGSLTYVSDVGVRAQAGLHISDLGDQKSP

LLYPPGVTQAGSNPDLPIGRPVS

>SG-PL_contig_5619_1_-3_297_530

MLLVVLIVFSFLFHQMEKVIVFSFLFHQMEKEDCFYGLTILISVWIFLCCTNLPLKLKLK

IFSALLLSYMLLEGNKAG

>SG-PL_contig_5620_1_+1_70_240

MASACCQEPDGSSSLQFLPEFLAKNQQPGNPSPVIRIKPLTHFLSEEDDPDRLLCPV

>SG-PL_contig_5621_1_-2_2_262

LCVCVGLHASVLVSSWTLLFFSPFLCNEDCACECSNLNEYGQIFIVRMTATAKPLCGAIS

LLLMEKSLVRCGLLYSLQEMAQNTGKG

>SG-PL_contig_5622_1_-2_221_427

MWFSILKFGFVINHDNTNKMRPLTDVCNSVKIFVIVTFLIRFVSKHFGNKNSSGKKTFQQ

QNTPPCVCM

>SG-PL_contig_5623_1_-1_172_453

MLYLHYVLYLVCSTITGALNHLKKYLKHWRQYFFFLNRFNHTKPAIVLPSKNKRKIILKK

LLLHGICIFAFDKFWILVQAQFVWSLVTHTGNIP

>SG-PL_contig_5624_1_+1_103_369

MVDNPDYEPDDKLYLYRDIGAIGFDLWQVKSGTIFDNILITDDEQFAKEYVENTWGKTKD

PEKKMKSDQDQEEARKKQEEEDKKRKQEE

>SG-PL_contig_5625_1_-3_948_1301

MICPPSPYCPVYLFNQTTSVCFVRALFIYLFSLLFLKLDYGFVVTLHWPFGYPFCACLRF

CLFIYDLFNYFRCSFLSSCFYVSMRMYVGMCCCSVVNADANALLKACNVVVCFGSVDL

>SG-PL_contig_5626_1_-3_3_104

IVWCLSFTYSSSQNMHPYSQLVILVWIYRFCWRL

>SG-PL_contig_5627_1_+1_1_270

LSHSSEVIGEWGGRGHNSSRGQHYECGSHLLSLTAIKLTALDIKKINSTFSELQIIYSIR

QKYRKQTPSLLYYRLFIDKTNLSPISLFQL

>SG-PL_contig_5628_1_-2_119_1654

MSTVNGVNGLDKSESGWKVGLINFANKYLTAETFGHKINVSGSALKKKQTFTLEQDAREE

MLQAVYIKSHLGCFVSTDKYGNVSCEAKEPGETEKFIVEYDKTPSGRWAFKHNVHGNYLG

GTDDNLKCFSKNFGDEELWTVQLSIHPQVHLRNVNRKRYAHVWDDQLQVTQTIPWGEKSL

LFLDFVEGKYALKTVDNRYLHTSGELLPQRTDDAMFTLEIRSGQYRGLAFRDRHGCYLMG

VGATATMKGRNKTVGKDELFTLEDTHPQVVLYAYNEKKVSIKQGVDVSANQDEEEGDKEI

FQMEYEPYSGKWAFRTVDNMYWSLEPLGGIQAVGKAICPETLFDVEWLEDGKVAIRAKNN

SYVYNKPTGSLIAGSESPSKKEKFRLKIVNRPVLVLKSDFGFVGKPLVGKSPGAGKAEIV

CNRSLYDIIQVEHASKTGTYFLKADNGNYWSVAEDGSVIADGTGKSAFELQVRDNRKLTI

RAVDNGCFIKGEQNGLFRALGQAVDSSVLWEY

>SG-PL_contig_5629_1_+3_297_479

MVPKRDSSSKPNNRKTNKMTKVVFLIAHMSYTKLVIYNHFHNSLSQQCKLQQKPSSTTVY

K

>SG-PL_contig_5630_1_-2_41_964

MPTGRGTMLPGFELLFSSVQSQLRSPQDAIVSAIHWNIINAGCKLTGVGENPRGEKQCTE

VLLNGWNSDEIYVLHYETTGDPKHAYLLKVLPVDGSLLLHFQRENETVTTLELSVQEFAS

DDLSSVSTAFHGMDHLNSRVRKSLVEPAMGNSSSNGQGQAGNSRSTSSKEKEKKDKKKSS

LLVEERRPHVSHQPRPMQPGWYREEGGMGLDPFSVGRADLDPLAGLGGMGGGGGMIMDPR

HSGGPRFGPEGGGGRFGGPSRGGGIHPGALPPGAVPPGARFDPFGPPGTRPGPDPDHERP

PGYDDMFM

>SG-PL_contig_5631_1_-2_419_751

MYLHYWSFSCFVYIFICLDLARLIWFCLWNYKVVPQGGQRFRLPALRRFETSVDCDTAPV

SAIYSYCVVDDNRNTTALFGERVEILGLALLHVGFYVCCPYLILCNVSQNI

>SG-PL_contig_5632_1_-1_1_321

VRLTQLIFFFPSNVLRYFSLCLLCIESCLVRLFNTKNNNNKNWGHEIGNICFCCSTGGWC

LCKCVYIMHALYLKGNGVSVCVNVCLCHFVGNRILKNVCVYSVLIFF

>SG-PL_contig_5633_1_-2_74_973

MILLEYKNLIVQETLTEKIRNVLDGGKPDNVDITVADFDGVLYHISNPEGNKSQIMVSVS

LKFYKDLQEHGADELIKREYGSFLVAAEPGYNVSVLFDLEKVPDNWQELVQKAALLKRNC

FASVFEKYFGYQEQGLEGKQRAVVHYRDDETMYVEAKKDRVTVIFSTLFKDDDDIIIGKV

FMQEFKEGRRKYQQAPQVLFSHKEPPQELQGTDARTGDNIGYITFVLFPRHTSANARDNT

INLIHTLRDYLHYHIKCSKAYIHQRMRAKTSDFLKVLNRARPEVKDKEKKTITGKTFRQN

>SG-PL_contig_5634_1_-2_8_292

MCVCVCVCVCITVCVCTYIYIYIMYVCMYAYICINLYIYTHTNIYTYMYTYTYAHTYTYK

HTYTCIFIHSYTYIYIIIIIKCISWKCIIRHMNRT

>SG-PL_contig_5635_1_-2_2_148

TSGEEEAEDSGKLGSVLKRSVNKPLLEDLPHCKCKWPPCPCDRRVIPLH

>SG-PL_contig_5636_1_-3_3_194

SLSLTLSCSLSLSIFLSVSHTHTHTHTHTHTFKYSMMTSSRSIGCWCMSPDRTSRLHVSD

DILH

>SG-PL_contig_5637_1_-1_424_567

MRLATGSWVCAFFAYLLEGGLLSHGAEKGFHSLGKLKGHFPGWESLGK

>SG-PL_contig_5638_1_+1_10_213

MTFTEIKQKLGTISGLLFEYNALVNAVPLSWKQHLNNDPYKDEILNDIPTEDGYATSLSN

KQIRKIFS

>SG-PL_contig_5639_1_+2_2_211

NGNLMTFTEMKQKLGTIPGLMFEYNALVNAVPLSWKQHLDNGPYKTKSLMTLQRKMVMHL

SIQQTNLENL

>SG-PL_contig_5640_1_-3_405_890

MLSRLPLERLDKTGTKDAIFHTIAHDELPVTAQHIAELTCKDPVLSRVLSLTLSGWSSRM

SDPDPDLQPFFNRRQELSVEGVLLWGLQVVIPEALRDRLLAKLHEEHPGIYRMKALARSH

VWWPGIDQSIEERVEKCHDCLHVSKTPPTAPLHTWSWPARPW

>SG-PL_contig_5641_1_+2_26_352

MIFTYMRLLNGCVFIFILEHFASMYAGLCTHFVFFYTSFPCTNTMHGMHVHTQFVCLHFI

YTLPQFKTITISHIRKWVSFVHINMTLNVHAYYTHFMLILHVYIHYTTR

>SG-PL_contig_5642_1_-1_1021_1221

MLLRGWEYPPVRLSGRTKSSGGQVEMFSPLVRWQPVCDFINYVFTQGGQVEIWGDKQIFS

PSCPQDN

>SG-PL_contig_5643_1_-2_2_133

AQVMWNCHGRIVYSFSHNESVGYAYFQLFRCSMFWGGFLLINTL

>SG-PL_contig_5644_1_+3_114_278

MQVVICAMFWFVLGFICLWCDQQQAGRTRLSKRVLFTTFHLIRTKAQNLVKAKRI

>SG-PL_contig_5645_1_+1_253_528

MMKIMTLSKQTMLTLYSFIVKLIILCKEILALWPFFLCYNTNINPISNPDLRSSKTGISL

CNIQSMCVSLWQPSTQCCRRARGHTNHQFLKL

>SG-PL_contig_5646_1_-2_2_295

NYTRTHTHTHTHTHTETVHTLHIHSYTFTFAHTHTHTDVHKFMPLYTYTPHKKSICTPHH

KASTNILKESNTRTMKTNTQTYALSDIHTHTNNTTEEL

>SG-PL_contig_5647_1_-2_92_280

MAHGSAALRIVVVIHGRKLVFACNVHAVGGNRVAFLAKLLLIAEVCLLSMCFSANFDRKI

HQK

>SG-PL_contig_5648_1_-1_1582_1884

MIVCRRYVAAQSAYSIFGGRACEVFSCFHKPLNSTWLTRSLTCIFDVLHVYTREGGSGTS

RSAHMLTLVMGKISTLYPQSIPFTRQSVPGQDRTRTFQLEV

>SG-PL_contig_5649_1_-3_3_335

GVGSYKELVQADRIISSASQVYDLIFTTHDGYSSDGETGGPGGGQGGGISDSDGENSHHT

SKTTTGFKPTGEVASSGARNYSTQPQLDPQRRYYSSASSGAGNYSTQPLLD

>SG-PL_contig_5650_1_-1_391_735

MHIKNPVSHVCVGWPMETRKRPTCMPTKDGVCRLCSNITPVQCGSKTMLTCSMQTVQQHN

ACTVWVKNHAYMHLRKRRQIGLGQREKKIQSLWKHCEIEDDTFAVVIPVDLCVVC

>SG-PL_contig_5651_1_-2_2_502

VDFLINPYCLKRFGDDVYVSDWTSHRVFRINIQTGEASFTVGDLDLSDLAFKQPRKFDID

DRGNLYIATGGRVCNPNSDGYFCIVQVTQSGSWKIMENYVGQNGGGYPYGLELTPSQIVI

SWGRWYHGWTSELTAYSLPALQTEVSFGNSETLPDTKSDLLTNVALN

>SG-PL_contig_5652_1_-1_67_444

MVQEIHVEGIDAYKKALEENKGAKGKTIFAMFCGSKDAGSASWCPDCVVAEPVVARNLKF

APADAVLIHCGVGERAFWKDQGNIFRKDPQLLLKSVPTLLKVGTKERLEEAQCANDGLVQ

MFFQGD

>SG-PL_contig_5653_1_-3_123_371

MYPIPPNFSVKFRQMLAQGASAYLGAVEIELNKIQSDSVLSPGLPAANRSKLPKLPFPAF

FFQSRVSIMIIICDLICACIEIK

>SG-PL_contig_5654_1_+3_183_353

MPLSSNLCCPHFTMFCPVTMLAVDGVPQCIALQRLSHQNAPIENRHISGTEFAKLVV

>SG-PL_contig_5655_1_+1_1_150

AAPLSHKAPGWFGRAAYPKILFLGRRIFKIRNTARLSVSGKISKYIPNTI

>SG-PL_contig_5656_1_+2_476_712

MEVGRLAILWKIFAASKLVSHTMVLDLRSITDSLSLDKRVSNEEGNIARHCQWGTCTGEV

SFPNQILWRIGPFPSVKRG

>SG-PL_contig_5657_1_-1_1_117

QSCSLFLRVADFFFFFSLFAALFKLSAIFFVSVKALVRV

>SG-PL_contig_5658_1_+1_112_501

MVKTLDFQTGGFGRPGFQPPRTLRLLGKGWSFSDLPGADLQVPESPSCAYARRTSNTHVQ

KIPLSHICVRRLVETRKHPACNQQGLAVRKVQWDLMLLSLKKKTKKLELGTPVTPPPPPP

PPPKHPPPSS

>SG-PL_contig_5659_1_+3_3_197

VCVCVCVYICVCVCVCVFMCVGVCVGVWVCVCMCVGVCVCVCECVLFFKLLSSLTAPLAR

TVGTA

>SG-PL_contig_5660_1_-2_2_124

LTAQVRVLLGENFVCVASSSLTSVQCWGSKAVRERRWVQPS

>SG-PL_contig_5661_1_-2_548_763

MPTLLFMSFKCIQTQWPIVLVCHIYNSPPYLLPKIFPAYLDYLCVMRYTSGSLYIPGMLS

QDTTEHLLIHEY

>SG-PL_contig_5662_1_-1_232_414

MILDCWGFKPATFQSVVHCVNHFVTLTGQSCHCLVLSFLFLYYLSVLFLSLFLVCCLFLT

R

>SG-PL_contig_5663_1_-2_92_199

MWPPVYPAVNGYLTFLKIREERLSSEGKEMGTALIM

>SG-PL_contig_5664_1_+3_3_278

HTLSHAHTLSRTHTHVILHHSLKSFLKLNNIFSITTYAFDIWCIFEITVMMVFVWLIWVC

FLSSHRTACRDRLRNTHAHAHTHSLFYFIISC

>SG-PL_contig_5665_1_-2_2_133

QQFFLIGCQNYAKALVAMSKCEIDLVEKHMDSFLRPKFFSAKNN

>SG-PL_contig_5666_1_-1_85_219

MTDCSKMFSLMFIQLNLHLICLTIDYCSKFISLTFLILDMYRLVN

>SG-PL_contig_5667_1_+2_2_223

LSHTHHTLSLSLSLSDTHTHSLSLSLSLSPTAACINNLVHLIKRSEKETGTYRFSNRTQR

WVNHLHFHQLLVLR

>SG-PL_contig_5668_1_-1_34_2103

MASQQQQSVEENLRLWHHVSHQHAQLEVLHSQLSASGEVALELRQRVDMLQDKKKCLLEQ

VRAYQSRDVDLQSRVEELERSEEALKVKVSELQHRENCLLARLVEYDTIGCQVLDTLPLA

QKQQLPQTLEACQRRVLVLTRLLEDAQRQLLDSERQHTQTDRQPSDSIHQQKQVQKQLSG

SHHVDTRKSEDVQWQVCDDVPQQEHIQQQLYENHLQTEQIQEQLSDNDYQQEQAQRQLHE

KDFQDNKRIKATQSQTQMSDIDLQREQAQRQFCENHHKTEQAERQLPDNDHRQELDKREK

DPHDDKRTKATQQQLCENHHHQEQALGKNHQEDKTKTAPQQLSDNDHKDDRIEDHGSQLS

DNDPQEEQAQRQHEEDHQDDHWTEAVEQMCENCCQQVSEKSHNQDLAQSHRDENDHHPSK

DQEYTEKIASITKATGDTKCEDGTEARDDETNRMTQDIKQKAGQRQENDKQSKQNIDSSK

DIVELFEGLQDPQLLLSSEAHKQLSQRQAPEAPTRSDTRMWRPTPRGDRQCGERGAVPEP

PSHLRVERVEGDSVVLAWQPPVLDQQGCSNGARVVGYRIYLNGRVCQQPHNPALASTTIK

GVRRSRDPARAQQRLAIETLTASGQVSPRLHLVMALQRDGHSTVTDLSSSETISSATGVG

YNAASRSGDLQSSGEISGATDVGDSEVTRL

>SG-PL_contig_5669_1_+3_540_731

MIFLMFCTLANLHVQIKSNHHMRYKRDCNTMLSNQCLSPQPKTNKQQITRYRITFTICSC

NMKI

>SG-PL_contig_5670_1_+3_1716_3068

MHIPTNTSLFLRSHKWCHWGDLDLVVGHVARSGTELGVGLDDLVDGVQEVLLGGHLPTSA

DGEHARFCAHAANLCACTVGAEAGQQLVSYIFLHTHGTCVDFEDVGATLQIRQAELDLSV

ETSRSHEGRVQRVWTVRGHQNLDVATGIKPVQLIDQLQHGPLDLVVTPCAIVKPGTSDSV

DFIKENDACLLGSRHFKQLSHHPGTLSHVFLHQLGSNDADEARVSSVCHRPGTQGFASTW

RTKQQHPFGRLDAQVHETFRMQKRSFHHLPQFLDLFLAATDIAVRHIRLLLHLHHGHCGI

NLGGQGDVDLVLVTINTHTHALLNVSGSHLIPKVHHKLGKLFDIDDVFSIIRICVDDLCA

ASHLKRLFTLQSLLVCCQIPQSWRSQSCVRLLDACQFVDILQNFLHVLLNTLDGTAVLTY

AISFQKSNISFVQRLNRFFFFIGIIFDFSLI

>SG-PL_contig_5671_1_+1_28_288

MALAFRFQQGDDGARGEPSYSDSNMMMIDFGCVKKINAGFLGHAINLDHIWVIFFFCNVV

TNASNKQDKISCALASICQLFTGAAVY

>SG-PL_contig_5672_1_-3_1887_2210

MTKLQCRIFLLHLTLIDPLHSLIQDCIGMKCNLMVHWGARMDGMIEGNATVVVWKIGHPY

FHSAGLIKIRTQVFPTFVLICSINHCYMHSQFIGLVTFLSECTKSELN

>SG-PL_contig_5673_1_-1_1_153

PFFLPLLSSAFFCLPSPFHFLISTSRPSHNSIPPAPQFMMYYLCCFALATR

>SG-PL_contig_5674_1_-2_2_115

MWIHLLKGYVKLRSDVVGNKETEAMSPRCTVKVKIVTK

>SG-PL_contig_5675_1_+3_411_632

MAHWVFFFIYVLSHISLHLPSWHRGRAPGFQSDGTGWNGCEYDPRKTLWLWPSVRSVVNE

CLSLFSVAKVKHER

>SG-PL_contig_5676_1_+2_2_121

QIMSTNKKTHNSLNFQNTVHQYKSSLNFQITSLEFHTLHL

>SG-PL_contig_5677_1_-3_138_1430

MNSLTRPLPHHCLWRCLPSSISGSSSLSVTQEVKQFHRLCQKRNLHVLLYSDNFRHDGFI

VASVGSRQQLHTAQKVCVEFKVQTGLQDSFSLLGVDPDCSDQELKAAYLDKAKAFHPDSS

ADTADPYKFSQVKDAYKAVLEHRKGRALEEEEEKEADIIFDIKHTAPQHRQYLEFEGVGY

GTPSQRQRQYQQHRVQRVTENVFQHRIQKLASETEDALVVRDKQQAKKAIISNALHRIVE

DLIQESMKKGDFNNLPGQGQPLEHSAHNPFVDVTTHNLNKILVNNGFAPEWIMLQSEIRK

EIHQAREILALTHSRLVQPRSAKDTKEWHAAVVKFKHQINEVNFKIHKFNLIVPFLNKQK

VVYEAEKEVKKVVDNVQDYLPEDSDIHLVWDVPLPEYSSSQSAGQQAIDWKQVWRDIKNV

FRSSQDKQINS

>SG-PL_contig_5678_1_-1_142_1668

MMVGQRTRRTREYTGPAPQSVAIRARPPNKRPPEYLILESRKKDDYLKDMQGQKLYNDLC

DLKNEWERWTDKKIVLNNVRREVSHRLKDHEFSIEERRERLREMLRQEDQEYMAAIEAAE

ETTLERQAKMRERAKSLKERREQERLQFVQDKYDQQFRAQCEELRSTLSKREQDIVCAER

LEQLRLKQEEAREKKAHDDLYAQLWEKDRQEKMAREEREAQAAHERNRETLDVLRKQMAA

LETQKEEAKRLKEEELQLMREQAALRKMEEAAAAEEKRRRQQENRDMLDLSLKMKMRKKA

KEEQEQLAFDLKMLEELLEESRNEAKEQLQRKIELREEDRRYREYLARMVQEEKQREREL

EHLIHQEVEAAWQKRIAQWRTERLMRRKLLDEVMAARAVQIQERLAENDRRKAENERERV

ALIRSIEENRQYEAEQTAKRMAENAKYQSDLRDQIHYNTHMREERRRNDDFEHLMGMQAE

KEYQEKLKTALDNPTFDRLHPMRRAMLNK

>SG-PL_contig_5679_1_-1_19_312

MINEVDADGNGTIDFPEFLTMMARKMKDTDSEEEIREAFRVFDKDGNGFISAAELRHVMT

NLGEKLTDEEVDEMIREADIDGDGQVNYEEFVKMMTSK

>SG-PL_contig_5680_1_+1_1_120

PPPPPASHPSPRQAGLNPRRHVLRKPHTHNNNVVCLNLCH

>SG-PL_contig_5681_1_-3_186_1280

MFRPFLKTCRASGRVLNCFVTIGMSGEPAAKMGRTMVKIGTHNGTFHCDEVLACFLLKQL

PRYKEAEIVRTRDPKLLEQCDIVVDVGGVYNPDSHRYDHHQRSFTETMNSLRPDKKWVTK

LSSAGLVYLHFGHDIISHLLELPLSNPVTNLIYDKVYENFVEEVDAIDNGINATDEEPRY

RVSTNLSSRVGGCNPQWNEKNVDEMACFQRAFDLVGAEFLDRVNFYKTAWLPARDVVLKA

VNARKEVDSSGEIIAMKEGGAPWKDHLFTIEEEQAITPPIKYVLYADNSDNWRVQCVPLK

LGSFENRLSLPEEWRGIRDEELSTKSGIPGCIFVHMSGFIGGNKTYEGALTMARTALKMG

EKCEA

>SG-PL_contig_5682_1_+3_1383_1706

MECYGPIDSIQFYSCFLFVFLNKVMIVYIPVLYCQEHFGLLLLMCSCHFGSLKLFLSYHS

NYEQIKYIKSTSTETFLTVMIFLNCTTFRNGLKTTNNKRKRPKLAQKL

>SG-PL_contig_5683_1_-2_695_946

MLPGTTLVVVGSLTCAVCMLHMGLQFIISPEKLAPRSPLKVYWREGGGGGKIFCPICDWN

LGFSGPWRCFNHWLTHCTDLRDRN

>SG-PL_contig_5684_1_+1_1_261

IHSSLQEFNLVDRVTWVKGLNKQHNYTVCIFYNLELAEEVAALSSSSKFWIMAAVSLHSD

SSSKYSLSQTVRSSAEKALTRLSRTAI

>SG-PL_contig_5685_1_-2_161_958

MPSLRSPEESGRGDRAVRSVGCSPSGNTVMARALSNGQSCLLPPALNGHAAAQGPGLYPT

GDPVKDGDYFLGIMASEEERLNQQAKRAEGLMRSQDLPEEVNGRIRAAIGKSNLLISQKF

QQFRELCSQHMHPDPEERIPLYEDLQGFWDMIKIQVDNVDDNFAEIDHMSQNGWKEIPKL

IPSRRSSASSSPKSGSLSQTSTPSVTPGHTPGSRRRGLKANNKDTPDSSPEHAEKIRSAA

KARDEARKRMLAEKRAAMKQQQQQQQ

>SG-PL_contig_5686_1_+1_1_168

THYTTYLHKLRHVILQKNTPTPTKTFKSYNIPQLQMQILNHTTTNIQTHYIKHLQT

>SG-PL_contig_5687_1_-2_518_871

MDYLSLIFIHMIAFLPTSCDQTSMGISSSLTFRSSSNLSSKLLLHVCRHPDPLTYQMQYL

YLLTYQFSAVPFSIAFQPLLVSATSISNSRQMHVKLRCFFLLVIFRPLQAAVIKAYRV

>SG-PL_contig_5688_1_-2_2_181

APLLNSVICVIVMSTICVTDSDLCSGLATLSVFDLLSVRTARFQLYKVQLYIAGSIGLLN

>SG-PL_contig_5689_1_+3_3_137

NFGASYQHPMSKTAEKKKIWKDHENAWQTHTKSWSHLPSTEQCLQ

>SG-PL_contig_5690_1_+1_1087_1497

MSKTAKKKKIWKDHENAWQSHHQVLEPPAKHKTVSAVADIHHSPAVNCQQSTCYSQQSVC

HNQYWHPTTTSSHITTTSHQSATTSSWPTTTSSQPTPTLPVHMKKSSKQHCSLFQKVINL

LIEEICRLQPCFGQVVI

>SG-PL_contig_5691_1_-1_1_162

LVVAGRLLTVGCWTLEGVSYCIDCSVLGRWLNNLVVTLPGIFMVFPNLLFLCSF

>SG-PL_contig_5692_1_+2_59_289

MEPPAKHRTVSAVADTHLCATTNCQQSATTSTGISLQPAVDLLQPAVASLQPAISLLQPA

VGLLQPAVSLLPHPLSI

>SG-PL_contig_5693_1_-2_863_1222

MQSDYSLTTFIFIRWWGGRRLRQITQTLYFSTHQLRGKKPSSHLPSCRLPKHPHVHPFLV

PLHHYHHHPSNKTKPFPTKHKKAHLSHFTNFLTTTTTTPYFSSHMSSGFHNFTYRPLQTP

>SG-PL_contig_5694_1_-2_173_361

MKIYLYYTYEFFMLLPKQGIVKVYTELKYTLCCTGNYFISYCTVHLIMCLDISGKVPVLC

RLF

>SG-PL_contig_5695_1_-3_3_161

VCVCEELNCLGMNFLSDLSCMGFTDFRNHKCTSQHEPATVQKMARDGIVLNFD

>SG-PL_contig_5696_1_-1_1_201

ALDQLAFVPYMEHICKTCRPNVSYMGHFTNTSKFETFTKQKNYCSCCHKSVPNNLNIRFY

SSQLLLL

>SG-PL_contig_5697_1_-2_38_304

MYVRPVGPVCRIWDISQTHQNLRLSQNRSITVLVVINQCQKNLNIRFYSSQLLLLWGSRG

LMLNSGLSWVTDVPRGDIPLPVITAKSRV

>SG-PL_contig_5698_1_-2_308_907

MMRPAALFVVTLVLVGCADDTGAWPTTTTTTTTTAPPVVETETGPVSECECCPWFACMAT

LEPYNAMLLSNDFGSLAGHIDALCGSVLPGVKRCQNNTSSSGCEHGLLTSYQYVCDHRSA

LTAGIPCWTVDYQTQTGECTQQFQMADWSSPESRCRAVHELLECLGDEMKELPTCTTDDT

SFVVGLVGIATLPLCTSEQV

>SG-PL_contig_5699_1_+2_47_205

MKEHINSSVILLVMFKRPLLCWFCLHALFTTSSNVLLYTQVEIQLQTDCLIRT

>SG-PL_contig_5700_1_-2_476_574

MWLHCILSRQRWYRMKIIRILVSECEWLYKSLS

>SG-PL_contig_5701_1_+1_700_1218

MRWQNIATIMDESQQCNVIYNDCSFSLCHTHKWQACVLIKQHNQTLSRLFRIHEPETYKI

LHIFYFCFKSATSPMNYTVKMENSCTVLSFHSTVAVQKTMEEGNAAATNKSDRWFFSCLL

NNSLETDAESEARKDISFKTHAGTQNSTVETSLLSGQSFEGGSYSSHLPLYLP

>SG-PL_contig_5702_1_-2_2_235

SHPHTHTHTHTHTHTHTHTHTHTHTHTLTLALSISFCRTYIYRPFNLKFITTHVSRALCV

SKVAAAIICNIKLSRNRK

>SG-PL_contig_5703_1_-2_695_916

MLVVLALVLIMLSSYKYHAMFVLIEMSMHLFGDFTVYFNVALMQMVNTFINTARGASSSI

NYVVLVQIINYLCF

>SG-PL_contig_5704_1_-1_172_348

MLQSLWWCQCFTDLSRSPAHFKTLSSALSLCGSSRSLPANYWQIWVERIWNCPSAMNPS

>SG-PL_contig_5705_1_-2_167_1858

MPELSELHVDDNWLTGLAPDFARLTQLRVLRLAGNPFRCDCTTLWLKDWLLGHQTITEDW

AAIKCTTEDDDGQVFTAVPNDQFICKVSASLGQLAIPIAIPLSLAFLLLTASVIAFLQRR

RLKIFLYIHTGYHPFDRDPPDDHALYDVTVCCDVTARAWVLRNVIETLEEGGRYKVFFYN

RDAFVGLTTSENVRHCVKNSRRIVVVLGKDWQQDDLLKTATKHSLVKCHKDMPHFLTVLL

HQFSVKDINDKDLQHYVHGGRYIPTDDKHFTKKLLYEMPHVKAAKAAQQQPKKGHHLQGP

EPIALYDIIAAGENEQKDRLNRLENRLSGNGVLARQVSHNEVDVDLRHSGVAQYNIQIVD

RQDSQQSDDDNPEDDSDEDNDNNDGGADGDINRQVAGNVNGNAEHHPRALQNQMSVDSHV

SHQLKTIFVWYADTDLAFTLKNIVNPLEKRGHTCILQDRDFPLGAPIQENIVHAVENSTR

SIFVLSNDTADNDWFNFAFHVAFDRHLQARDRLVAVVTREEVEIIRFLEEIRQVIKTSSV

LSEDDPWFWKRLKTFVHLDHAELG

>SG-PL_contig_5706_1_-1_1_162

YLFHSVILSYCFYINDSMLIYTCSSACVFLMSFPLFRVLLLSCRICRLPVTPAL

>SG-PL_contig_5707_1_+1_961_1182

MHPLKNAPINNFKKLMLTNVNIHILLIVSVSQNWAMPIQTLAFVTLTKKAYRSNVETIHQ

HGEILVNKLSLHYI

>SG-PL_contig_5708_1_-1_154_306

MTFFFVVQGSSAQYFILQISREDCFSCKNILIFLFVHFGLFPFSEVTYPVE

>SG-PL_contig_5709_1_-1_505_666

MEHARNLQYSEVQYLLKCSLCFTLICNLVELFYFFICYSVVVLIFANNLYSGLN

>SG-PL_contig_5710_1_-1_217_1218

MFRALNQQSGSSSASSLSNRSVESPSASSEFVSDALAAARAPQYDSDLEVEELPPLKEVL

GDDVLKKLKPKEKKRQEVINELFHTEKAHLRNLKVLELLFYQPMVSEVGGVMSELARALF

PNIEEIIKLHASMNKTMKDRRSHNPVVGDVGDILLQRFGGEQGDDFRKACAEYCRNQSFA

LEALKRHRRKDQQLAQFLAETEADKLCRKLELKDHVPCQMQRLTKYPMLIENLLKYTQSP

ISQEYRNLEQALERSKHILAYVNTAVRECENYHKLKEMQKRLDKRAIDASADPSLVDIKK

LDLTQHKLVYDGPLVWRLRSHRLIELQVLLLEDI

>SG-PL_contig_5711_1_-2_2_127

FQSEEIKNWKWFAYLGVWNSKQATSCIDYKFNIVHVYFGVKA

>SG-PL_contig_5712_1_-2_98_208

MMSICVFICEKVHDGNLMQFILSTVIVLVYECTSMYY

>SG-PL_contig_5713_1_+3_621_818

MSIRHNIQCCLRTTVLTKDDNAGDVFIRLIMIRLPQQDHSHLWYVVRSHSSLFHSAKFGR

GIQVVP

>SG-PL_contig_5714_1_-1_1_246

GTRSIIQSYSAKISFFGFCFFFSCMEEEFPWFLLMEGNLPRRICICFFCLFVFFILSTVV

FSFSCAYMCTMFDWHCVAAQTA

>SG-PL_contig_5715_1_-1_1_258

GTRSIIQSYSAKISFFGFWGFFSCMEEEFPWFLLMEGNLPRRICIWFFCLFVFFILSTVV

FSFSCAYMCTMFDWHCAGAVLLHRLQ

>SG-PL_contig_5716_1_-3_366_626

MMVIIVLIVHVAVISLLFSVVVVLVVVLECIHRHCFLLEISRKGRDGSLFVKGMHIYAWE

MGICKTFQGVFHTNEQLRPGGKSFIDS

>SG-PL_contig_5717_1_+3_3_308

TLYSIHKGTKLYYDILNHSSQKPSYCDKWDTKLNKAINWKVCFLKVQKIQDISLKWLQIR

ITAINIILNEMNIVNNDKCTFCDIEKENIEHLFWRCDRVQYF

>SG-PL_contig_5718_1_+1_1_258

PNIHKIINSGLTFSKHTKNSITKLNLRICMTYYPNLFFLMNESRLTIECCHTLNGQEKES

TLLQTLRKKTAVSCHKRSSTESLIRI

>SG-PL_contig_5719_1_+2_137_280

MSSDHEPFIPNMQTSTKHCTLEEEDFTANNKSSTTADILTMTSYFQAG

>SG-PL_contig_5720_1_-3_3_149

LLILLLLIIAFVTLVSIIPADSFFLSQECVWHSGNNIWLIKVFLLKCFA

>SG-PL_contig_5721_1_+3_99_245

MFITDTVTFLDRYLSLPSKRQLNKTVTCTYSDSVYHKLNTFLHSYHQTA

>SG-PL_contig_5722_1_-1_370_450

MFKWTCLIHRYKLYILLFNKELTFLKK

>SG-PL_contig_5723_1_+1_1_195

KIIQYNTRFFQRMSPFMTAKFGKDLSINNRKHHWYILGMKTHMFAICPLESMLKQENDSI

QFILH

>SG-PL_contig_5724_1_-3_240_533

MVTLASVGLEACWKAEMNGMEVQQCRTYFRLGLSCSHVVQVMVQKGQRKKIGPESGQSPV

RLGDIGRHTHTHTHHTICRRRSKLIQNSQSGMKSAAKG

>SG-PL_contig_5725_1_-3_90_1106

MTEQGGSPIDTDCITLTRFVLQEQRKYPKATGDLTQLLNAILTAVKAVSSAVRKAGIHRL

YGISGSANVSGDEVKKLDVLANDLFINMLKASFTCCLLVSEENEHAVEVELENQGKYIVC

FDPLDGSSNIDCLVSIGSIFGVYRKTTGMPACEKDALQPGHQMVCGGYALYGSATMVVLS

VGNGVQGFTLDPAIGEFLLTERDITCKPRGKIYSLNEGYEKFWDQPTKEYLKSKKFPESG

KPYAGRYIGSMVADVHRTLVYGGIFMYPATSEAPKGKLRLLYECNPMAYIMEKAGGKASD

GKRRILDIQPVNIHERSPIFMGSKQDVEDVEAMYKKYGN

>SG-PL_contig_5726_1_-3_3_212

GFFLFVVDVSLLLVDCVLFCFFRKVHVLCLSGILNVFSLLMMKRLSCVRSLKQLLCSNSS

IRAVIIGSHI

>SG-PL_contig_5727_1_+2_2_178

PLLHIHKSTHIYGNCSPQTQRRVSHFILLKFLQFWTDSIYTYHSEPISVCVFIQFQILS

>SG-PL_contig_5728_1_-3_3_83

WLINTDTIVCTLSSVLHLILYCYMSSI

>SG-PL_contig_5729_1_+2_2_190

SPKSFPKFEQSQGIELFETSTSPMASIIRPALISSTLKRNRYPSTAGWFMVVCSSQGNQV

QSF

>SG-PL_contig_5730_1_+2_116_391

METTYTDYKTVDNNNYYSIIIIIIHYHHHSRKDQAFCLFLGGQCYYHKTTKKATVKEMTQ

INERTVQDKIIDQSTKTKELTKVKSKDFQTCQ

>SG-PL_contig_5731_1_+1_241_513

MYVMYEFNLCACGWGVGGCVCECACVHVWCVRVCVSMCVWCVCAYVCVCVCVCGASVCMC

VCMCVCVCGARVHVSYILRQMDRQTDRPTDR

>SG-PL_contig_5732_1_-2_161_286

MLGYGLLTKSDICVCVLVRTFLVLFCFCGWQNIKQTSLLKKK

>SG-PL_contig_5733_1_+3_3_122

HFKTPKTYHYTILQHFLPNQHNLNALFKITIFKHKSSLSK

>SG-PL_contig_5734_1_-1_811_1098

MSGKLNLTWVIKHAKGCNFPTSRVDRKVGCLLVSLYMRVAQPFFGLGKRIGLFPFNFERR

GRVMTKVRTTYVCRHSAVPLKVLQNMYQAVKALKSV

>SG-PL_contig_5735_1_+1_1489_1830

MCFFFPRFYNSTAQIGLSVILFQHLFTMVYENHHFCHHFPSMANCWLQNDQPTKLTTSKK

KKKWQLHKHIMQVTFFFNIYSLWSIKITTFSMDFSHFLFPLQYIFPVMHRITSD

>SG-PL_contig_5736_1_+2_68_253

MLLTSKAGNSARETQSINRPYRRRVQLQKDTCENNNSPNSGKSNTQTKTQKRYNETITHT

YS

>SG-PL_contig_5737_1_-3_174_512

MAKYPRSDAHIICSQVLYWTALASHDTDPPPGCSGQSFIPPDGGLTRRLKACVASGPCRW

VSFSLLLSMVCVCWPVNRVQLQGKWELRVHSWENVSVLSSRNSYTVLVVFVTF

>SG-PL_contig_5738_1_+3_3_194

NREKKKHETLPMLSAVVYFCAVISSSSFCSPTLYQQRTVLRIEETDGMKLKAVGRSTHCS

LRMF

>SG-PL_contig_5739_1_-1_1_378

THRQAQISQEDKLLLGHRQAPAANIPFKVLADWERIARHLLLCCAGADAFLSGLSARLKF

LGSLNPGSACPSMDEDVRLFGRQVSTYLASIATAGAMLQTNTLLARRDAILASSQLPKED

FAALRS

>SG-PL_contig_5740_1_+3_114_347

MGIFCHHDMKLRIDNELKHIMVLNNFVKIIVVTYFLTDCPSKGHMSTFTQLIHEGGVCLF

FFSSSMKEVFVLFCLFCF

>SG-PL_contig_5741_1_-2_101_337

MDIESMNRYVSPINPAVFPHLTVVLLGIGIFFLAWFFVYEVTSTKFTRNLYKEMSVACVA

SIFMGFGSLFLLLWVGIFV

>SG-PL_contig_5742_1_+1_1_168

QKKADDRKGKKNGRNKESNRKEEIASTEFPEGGDTIYTERAPGIPTGKALQTTTLT

>SG-PL_contig_5743_1_+1_1_453

LPLHQGGEGASFTGSPGVHSPPLLSGRLAHFGGQPRTLSGPHLQGRQPLHSVGLRPEPRE

VRFDPGSTLSLPRDELRHPPVDSGSYSREAREAQGDPRWPSRGSRRASQIPCLSSRHHGI

SSSSGPARQGSEAPPPAGLQSSLGPAVPILG

>SG-PL_contig_5744_1_+2_167_391

MLNYSHFISLLLIYAFRVTDLKKNQIMNDADLIFAPNLFLFVFIYLVFYFLRGGEKLISK

CTWHSMFTSDHCYKP

>SG-PL_contig_5745_1_+2_2_166

SKSFSVVSERIFTKFGPKVWIVEESQCMSLEGRGPRVWSQGPKQCLKSAHFEERV

>SG-PL_contig_5746_1_+1_79_1026

MPLPPALAARLAKRGIIKEQPAPDPPSKAVEEVEEVFAEDYDDPAKPEVPEAETKMDEEE

EEKEESDKEEEEEDYVHEVIDCPNRSNPFHTCVKYCHNRWGMKTFTPDADMVKKRDRMLR

KYPLPDGWKEVADPDTGRYYYWSTQTDQVCWLSPGHPKAVISMPAEKLSESLVSAIEDDL

EDQEEEDMELREDEPMDADSDMSDSSASDSDSEEERRRPKRDRDRDSDRSRLDSRGRGFR

EERDNRRGRNRREDIDPMDPAAYSDIPRGGWTDGLDTRGSAKTGVDVTASGPLFQQRPYP

SPGDILRANQQLKKKA

>SG-PL_contig_5747_1_-3_51_344

MENMLSIAGGMLPKCLTLPPVLSTAVLNNTSHIQLFPSLKDHMLDTTVDDNHVCTLIKLV

AECFSKIEFHHLAKRQRENCSGPKVRKQLTKLILFKHQ

>SG-PL_contig_5748_1_-1_46_669

MCMSDSSITNTDGPVRNPHDERCIAGGSSGGSGVLVAAGQVDMAIGGDQGGSIRTPSSYN

GIVGLKPTWGLVPYTGAVGIEPTVDHLGPMAKTVTDCALLLEVIAGYDEGRDPRQWPSMS

VPEYSKLIDQGVAGKKIGLLAEGFEPCTEEVKKAVKAAAHSLSSAGCDVRDVSVPMHKDG

AAIWNVTAGHGSVKCIFENNATGYFYKG

>SG-PL_contig_5749_1_+1_13_249

MLTLQLGLTDISFTLGLKCDHQLTLYCDHSLHSILVIRRQHCGFLSSLQPTSTSFPGCRD

GHAMAILTKHFFNLEKSFI

>SG-PL_contig_5750_1_+1_1_177

ITITKLSTDSLTQRQYTTHKGPDHGNPKPQFTSLLHKQQNVQACIRLKAFYKLIKVNQS

>SG-PL_contig_5751_1_-2_44_427

MKGRGALFLFVWICHSTYVSNSQRQGDGGLGSNLEGMINSVETGAESANNPLFSLAPSTP

SPDEVNCYVEIPAIRVGGRCVRLGRIRWRGRGSRRRSSVAWGCQSGIHLDIGSPDCHNNN

RRSRRIGG

>SG-PL_contig_5752_1_+1_229_357

MTKMFHFCGSYLASPISIYLLHQHQSPLSGEFRRTMSVHFGIF

>SG-PL_contig_5753_1_-1_46_273

MNLECQVQLWMLSLSQLSSPLDSIQDVFNCTHEVRILNIRHRFNLTLTPIRLRETNSHPL

FTDTLLILYKLLKDKK

>SG-PL_contig_5754_1_+1_19_171

MTRQKTFHTFCSYGGSLAVLKELTFNSLVAVFSGSKISTTHIIMLSAFRTM

>SG-PL_contig_5755_1_+1_151_312

MTAKRPSTLSIHALQVQIGRHSKAKLQEIHTSIYTLLRLQIQDSCQPVDHDHVL

>SG-PL_contig_5756_1_+1_1_249

RERERGQNERVFVYNYLRTNRKIIIKKEEQTNLVVKLYQQSTFYGTVRYVLCNNNLNMGH

AHIDSAIFTGLNRLDKADSFDSI

>SG-PL_contig_5757_1_-3_1122_1391

MLCHNRPCNSNISVFISSVRIVRTCSVYSKWEKLSSNLTCESRNLQKQKRSDCRDCHLYP

YFLTMYGTLFHYSGINVYIMSLYQRNESIL

>SG-PL_contig_5758_1_-2_2_202

ALPICLPRLWLRVLFFSSECTKNTIETMAAALMQDNVWLQKQRFEEAEAHYYAIQAGTAT

PGASSQG

>SG-PL_contig_5759_1_+3_2178_2405

MLTIVSFCGPDLTCPTRLNTNNLIRKIKAMKLQEVVSLPSKNKTQTMACFQYIEFSVLCV

HVWMCAQVHVHSFCLF

>SG-PL_contig_5760_1_-1_532_768

MASKTRDHLGSKHGLTDSHRDRSDCRSSPFKRRTKDRSHRAKRRSPESERGSRRHLGKSC

ALSNVPSNQTAPSLQKGDT

>SG-PL_contig_5761_1_-2_329_520

MFPSRSFLCLFCLFVCFALSLSHTNTHALTHTRTHSFTLNLIAFSFPCLISDCSKMFSPM

FIQI

>SG-PL_contig_5762_1_+3_237_461

MFNNFFEAQSKQTSFFQAYLHSRHFLSPHFAALLITRYLFLGTFRCSSKYASKRHYYYCK

QLQVMVTFLCYILIV

>SG-PL_contig_5763_1_+3_3_128

ARQRACTLGDPQSVKLGNAKTNNSLKIQVPMAPAPLPCVPRT

>SG-PL_contig_5764_1_-3_3_146

SPKLHSLSISLPVFLYLPLSAHLFLCLPISNCTSVCMLFMKDLFNYSL

>SG-PL_contig_5765_1_-2_2_253

DDSSYHWNSASEAERVELSLCNGTSAVITFSCTGFCVDCFLLLLDSSRDVILEVEGLPIN

QDIVVEGLPISESILLILSLLLLS

>SG-PL_contig_5766_1_-1_226_399

MCIRARASSRDHFGCLLRVIFVNIILHIWSWFLLIITCIVLLHDIWCNHRIVCTILAF

>SG-PL_contig_5767_1_+3_228_356

MLLTQESWVPSAGGPHFSAGWLALLTELWTQDLVSGVQFLPAA

>SG-PL_contig_5768_1_+3_3_185

THTHGYGLMYYCVFTPLQGAMASMNKSSDSDSALQTMLFLYQLVSSRRFHLLFLACFVIK

L

>SG-PL_contig_5769_1_+3_3_218

DHSIITLNLKFCQPSKHNTFWKFNASLLKDKQYVDEVNKLLLLYIVYSPADHYDHIRAVT

TQSPSKSRRQPR

>SG-PL_contig_5770_1_+1_295_471

MAFPHPTASQEETVVNFTLRLNSKHILCTVTRFQIHDLISYTLTNLPTRSSTTAPKTNL

>SG-PL_contig_5771_1_-3_363_518

MLGISRPLVRRKCWKYWGLWQCKEQPLGLTIHESGWFTVTVQSSTFKLTGLK

>SG-PL_contig_5772_1_+2_2_391

GRCSGGGCGLCCTYSRRWRSSSCGSSRCSGGSRCSGGSRCSGGSRCSGRGRCGGGGRCGG

SGRCSDGGRCSGGSRCGGRGRCGNGGRCSGRGSRCSGSGSSCNGGGCGLCCTCTRKWRSS

SCSGSTCSGG

>SG-PL_contig_5773_1_+2_2_157

HMHTQAKPVSVSNALNWLQGCVFRPPFCANRQSPSTRSTSHTNKMYYHHSFP

>SG-PL_contig_5774_1_-3_3_92

DAACDFHRLVGGPSLELRWENPPDPKNSDF

>SG-PL_contig_5775_1_-2_53_3634

MHERALNQEQVGLRQQMQEKAKAGELHVVSGSSSSSSSAADAQKTAGKRRRWDQAADSGA

PAAKKKSSAWDAAESSSATPSNSRWDETPGRPKGSETPGATPSTRQWDATPGHATPGAAT

PGRDNTPGHQASSVRKNRWDETPKADRETPGHGAGWAETPRTDRGGGDLIQETPTPGASK

RRSRWDETPGGATPSGLTPSFTPGGLTPSGMTPSGGMTPSGMTPSGAMTPSGMTPSGMTP

SGVTPTGQKAMGMVTPTPGHLMSMTPEQLQAYTWQREIDERNRPLSDDDLDTMFPPGYKV

LQPPAGYVPIRTPARKLIATPTPLVGVAGTPTGFRMQTPDNKTGVVDMQPKGNLPMMKPD

DMQYFDKLLVEVDEETLSPEEQKERKIMKLLLKIKNGTPPMRKAALRQITDKAREFGAGP

LFNQILPLLMSPTLEDQERHLLVKVIDRILYKLDDLVRPYVHKILVVIEPLLIDEDYYAR

VEGREIISNLAKAAGLATMISTMRPDIDNMDEYVRNTTARAFAVVASALGIPSLLPFLKA

VCKSKKSWQARHTGIKIVQQISILMGCAILPHLKNLVEIIEHGLVDEQQKVRTITALALA

ALAEAATPYGIESFDSVLKPLWKGIRQHRGKGLAAFLKAIGYLIPLMDAEYANYYTREVM

LILIREFQSPDEEMKKIVLKVVKQCCATDGVEPQYIKDEILPSFFKHFWNQRMALDRRNY

RQLVDTTVEIANKVGAAEIVSRVVDDLKDEAEQYRKMVMETIEKIMSNLGASDIDSRLEE

QLIDGILYAFQEQTTEDVVMLNGFGTVVNALGKRVKPYLPQICGTILWRLNNKSAKVRQQ

AADLISRIAVVMKTCQEEKLMGHLGVVLYEYLGEEYPEVLGSILGALKSIVNVIGMTKMT

PPIKDLLPRLTPILKNRHEKVQENCIDLVGRIADRGAEFVSAREWMRICFELLELLKAHK

KAIRRATVNTFGYIAKAIGPHDVLATLLNNLKVQERQNRVCTTVAIAIVAETCSPFTVLP

ALMNEYRVPELNVQNGVLKSLSFMFEYIGEMGKDYIYPITPLLEDALMDRDLVHRQTAMA

AIQHMALGVAGFGCEDALIHLLNYVWPNVFETSPHVVQAFMGAIEGLRVAIGPSKILQYT

LQGLFHPARKVRDVYWKVYNTVYIGAQDGMIPSYPRVPNDTKNQYIRYELDYTL

>SG-PL_contig_5776_1_+1_292_534

MELRKIRTCMYVNRRSTVCLSACLFPFLTLSLRACRNSLWLFECLSPCLPFSISLFACQF

TYKQTHVHKHRRYVYSLIFAH

>SG-PL_contig_5777_1_-1_1_144

VGGTCSSPPLVETGYRGQSDMDGTRPSPSQLAFSNHEQDQPPIHSWVK

>SG-PL_contig_5778_1_+2_32_169

MLLFVLNSMPYQNCKILIKPPACKSQHNKHKVQPLSDLRQFSCPRP

>SG-PL_contig_5779_1_-2_407_1306

MPTLRRSKSLNDKISRQKNRVGDVVRDPRSPDVEGRTRTCVDNFQALVHLRKQTEDKARQ

LELAYSYATNGHTYLRRGPSGGVELPYASSVTSATKSSRHPDSSESKLPLNRRQHSAEGR

TERGVVRRQDSFSGTQQSQQHHQSRREQKRCARPMSMVEPRVGGLFPSQDELNRKDQVIQ

NLTESQRSLNVHNQKLLSKIDELEFENSQLRDILNAHHIQVEAFTQSRCASTRPHRSISL

HGDAMRIPSSSVPSSAVVSPHSETSVSGYGSLADSPSGDGFTAGSDVRVVPRTVGVGDYD

>SG-PL_contig_5780_1_-1_1_981

MADVTNAFKATVKAVKSRIKVQGGNVDTTTNILPVSKQRGDFETKARDVTNNITKLRDFL

LQHRKDYVNAGSLLAGHLSPMTDAERDQIDNDAQNIIKTCRETINRFRVEAQKQRVHPQV

KEHRAIVIFLIDAYLKAVCRIYSEQKAVRVKRVVDKKKISRLEPDKSRSHVARLQPQRQS

EDEGNIEKMDPHVTEHRTPRPQTMELPEKPSYEDTDTDISPEEAQMFEQENRALYEEMNS

MAEDVRQIEGKVVEIAKLQEIFTEKVLEQDKQIDMIATTVVGANVNVKDANEEIREAIKK

KAEFRVWILFFLVVCSFSLLFLDWYNG

>SG-PL_contig_5781_1_-2_314_865

MPLSSLFKTTKAKKKSKKGKGKDKDLTQSNATDDVTNKDAAPTEEQPQTAAAPVASEISV

TPPSDEVVPAEGAETMRDSVDEVPAEGGQNVTDEQGSEDAAHASDSKTDVSGADDTPAVL

NTTESREITMTMGEGPQLTQSEGDVVETQPIKADGSLPPERAGREKESLLHDHNPEGMIC

CVIL

>SG-PL_contig_5782_1_-2_119_400

MSYNSDEYEQRSQTKGPAQQECGEDYMPDADGVIESNYDNVCDSFDDMNLREHLLRGIYA

YGFEKPSAIQQRAIIPCIMGRDVIAQAQSGRSEE

>SG-PL_contig_5783_1_+1_1_108

MVTLICNGDVAVNTNDIITLTIKGLFIWLDYFFNNC

>SG-PL_contig_5784_1_-1_58_348

MFMIAFVKFLTCFYFVDWKRQVCIGVDYKSDELGEFLKFLFSFRFRSIVIHMHAYNDTPL

CRENILLYLHQSEPVKIKSSSIILTETATFPRRLSSL

>SG-PL_contig_5785_1_+2_1271_1522

MVTVIHKRQSVSGKLQLGSSFYELFSSCAETITLICLSILLIQTVKTTTLDRWIKHYNQS

SLNMQRIRALLPLLHPWQLHGQMC

>SG-PL_contig_5786_1_+2_398_544

MSYIFLGVICHNPSTFKIKAWWRLFLSCQSKHSSIQKLSSECSFLCCSD

>SG-PL_contig_5787_1_-3_3_227

WVEHKWNCPGILPIHCCPYCMTYRSVTNPIFLVKRQYTFLLSLVEFSVIDISYKEMLSER

LKGTVNELYTVTSRL

>SG-PL_contig_5788_1_+3_3_107

LLLLLFACVSGFVDARMYYDRKHRAQPSQAALIHI

>SG-PL_contig_5789_1_+1_1183_1518

MSGVRQQSINTNLYGHCENGGVFPDSDIDTDNTRRCCFFIQVSEKLFVLIRREKLFVLIR

LEKSFVLITETGEIICPDQTIAIICPIRPEKSFVLIRPEKSFALIRYKNHLQ

>SG-PL_contig_5790_1_-1_1_201

KQYVVAEDQKSTKTVLKFSVPQGSVVGPVLFILYTTPLTSLIERHSVRHEVFADDAQLYH

CESPESY

>SG-PL_contig_5791_1_-3_3_206

GEGSVIFLSVRAVLSPRCMYTCCSAHTMQALCCYAVCILCQSRGIKGRNLVCHRHEIGLV

YLGVHVCF

>SG-PL_contig_5792_1_+1_259_465

MPNLCTSLRCLCLTHSPNLVLSNSLGYSCVTHSFSRSSTTWHNGHDCYSCDSNIDQRQHY

SMDKLPRTN

>SG-PL_contig_5793_1_-3_3_215

GSFSINSIPTFWKFCASNFLFSVALFVSALSFSFCPLFLSLFLPSRSIHLYFSLEILASF

VLCVPYPLKSE

>SG-PL_contig_5794_1_+3_114_233

MSADNKIIHQLPRRHVYHHHAAGFETGQPLLKTIYSFLKL

>SG-PL_contig_5795_1_+2_2_148

PLSASWVTLWTDCTWRLLVVKYDRSACVLPRNDQHLRRVSGKARKLVSW

>SG-PL_contig_5796_1_-1_103_336

MVLQGFVRMVCTCVCVCVCVCVCVCVCADNKWLMMIGDDDDVVDDDDDDCVCVCVCVCVC

FLLRRFHTLHYLLLASEK

>SG-PL_contig_5797_1_-2_95_2695

MRKGMMGSALISIMNEIKTEVAKLPQSRFLPRPPGHDLPLRIDVPKFQPRGSVEWLKQHG

LKAKHLDLYQVLAPNAYSYREEFIPVIKRAVQSQVHEKAMVQFAWHDGSTKNIHVDMTQL

FEYQKQLKEIVRLYERRVDWLASGSRRIFGTLVEKNVVLLVDNSVSNINYLVHIQHSLRL

LLEQQVANKEYFNIIAFGGKAAAWRPTMVRPTAENLQEAWKWVLELQCGGSRNFLSALRL

TMENDEEQKHHIYVQGVYVFTSGVPDQDQDVVISYAEETASGWNAIIHSILFNVDDYDEY

GAIPGRYANITRTSECLRQLAHCTGGRFMWFRETGIIENDDIQLVNSEIERALNFSRKCS

MLVEAVKKKYKNRFHYPEMEEVRALPPPAGLESGRMKRALPSPSPQYYNYGSSSGQREES

EVRHERHAISWRPSSASRVRDPKLTPKAPAQRSRPTSAKDPMQCTRSSRKTVSSSHFFLE

GKKHETGSVVREYPNAKSVRKGIASPVLPDAEDPVSSKEWMRVYSLSKLKLDLNRLISGP

DCRHLEKPVKALRKQVAAKYCDIFPTINIKGTMKHLQLLPHELNEYEAQVERVLRRYLKR

LQWLLSGSRRVFGTLTHKKVVLLVDTSGSMEPRLNELKKELAALMWDQIYKLDIWLNIVQ

FSGSCHKWRDSIQPPSEDNCHSAVQWTSNFVANGNTCTLEALQLAFEDPDVEAIYLLTDG

KPDTSTSLVLREVARMNLDRNITVNTISFNCSDSTANSFLHLLATETGGRYHRCPDDFNA

DMFVHKLLSEGFGDSEYPHLPQFEGDDLRRLGMEVSMARRYLQQARVYKDMYKAKTTFPE

RPSSAKGNAAPPQQTSFVVGRSKAIVR

>SG-PL_contig_5798_1_+3_1509_1829

MFNACSLLASVTQPVNEATLAVQVTETGGSSTRTTHRLFAGILNRCFSVLFFFFFVPQII

FPMDTISFSRKINFSAAETCVFADNSHACKMLQQMQQKSGMFERNFS

>SG-PL_contig_5799_1_+1_1_294

QSDSTCNTLVTLSRHLYSISTSPHISFCYTYHKCYCNCINESALISITPNFQVNKHRIPH

ASTIPDTHYKSVKTIKLWNICHNQIHTVISICLQTEHD

>SG-PL_contig_5800_1_-3_1239_1547

MPKTEKGPSPPSCKLLAREKLWAERFLIECKQWCQFKDNKRFRVSCWKKHFLMNANNFVN

QRGQTLINKHMFGDLLPPLLHDTINASFYVSCVSCGKFTFEFK

>SG-PL_contig_5801_1_-2_20_241

MGLVEVGDGLGGAVVYLLSYGPRILHVSSPLENGFVFIAVKREYCTESKVICLFLPIYTL

VTVKSAIMFEIKQI

>SG-PL_contig_5802_1_-2_146_484

MALPRAQWLLGFQKDSRGRQGFRPGPAAKPKQKASPRQLLHNQASALASEVASLIHKGVV

EVLPHPPGPGFYGRLFVVPKPSGGWRPVLDLSPLNEFLVRIHFKMETPALIRD

>SG-PL_contig_5803_1_+2_185_328

MYTSVCMYTSQLFLFSKHKYMSLEKIFTSNMINITPSSHPLAPLKKRD

>SG-PL_contig_5804_1_-1_73_840

MAQPKNKHVHSAKLAEQAERYDDMADCMHKCVIDCYSGNCVEVLDNEERNLLSVAYKNVV

GARRSAWRVVSSIEQKSEGNDKKLEMTKKYKEKIEKELKEICNQVLELLENHLINKAEID

DIADSHVFYLKMKGDYYRYLSEVSQGETRKDYVTKSNDAYDQATKISKEKMPTTHPIRLG

LALNFSVFHYEIMNNPEQACKLAKTAFDDAISSLDQLNEESYKDSTLIMQLLRDNLTLWT

SDSQGECADDHQQENQ

>SG-PL_contig_5805_1_-1_19_222

MILETSFPTKNIELIQMAPCIFQISCWQIVINFYWQIVINCCLISCVVSPPSFFPLFTLL

LLMSKIRH

>SG-PL_contig_5806_1_-1_112_1914

MKGKAFCLLLLPYFVSVLCKVNQDTIDQNIVVSHVERKIDIASHLVKTYTSITIENKGSS

AVRSFLYVVDPSLKTKLSFIGAVTKDNKDDKLDVAPTTVTGQSDKEFYRVTLPSSLDAGK

TVTIDVEAVFAHVLTPYPSQITQAEKQYVVFEGNLYFYSPYKTVSQKTSVTASSTSLESY

TKTKPVSLSENVISYGPYDEKAPFSEAVLKVHEENNSPFLTVTDLERVIEVSHWGNIAVE

EHVDIRHSGATLKGPFSRFDYQRSQDGVSSIKSFKTSLPASARDVYYRDEIGNISTSHMR

ELDDAVDLELRPRFPLFGGWKTRYFLGYNVPSYEYLYYKGDNYALKMRFVDHVYDDMIVD

DAVVRVILPEGSKNIEVRAPFDVKRESDSVHHTYLDTSGRPVVVLRKSNLVEQHIQDFEV

HYTFMRMQLLQEPLLVVGAFYLLFVAVIIYVRLDFSITKDEAKESRMRVASLVEEVQSTQ

DRRSALYQSYDDAINRFKSSKDVNAFVASRKKVDADYRQLTTQIQNLQQQLKAEGADIAE

KVAELQRLDGQYKDQINLAITMAEKLVAGKTNRQQYLESETGVSSKRDDVYQKMETLRAM

L

>SG-PL_contig_5807_1_-3_732_1070

MFSIFINRFQPFYMDSTAIALHASSQRPSTSLFILSGLPGFVLVFLLYPLIHPSFSQFCK

SCICCLLFYLILLHSLTVAFAYTHTHTHQTHGYEAKLLFNCSIKKLSSVNYFV

>SG-PL_contig_5808_1_-3_2412_2738

MFQPTSALQLVPLLFKPVSVNKVSKSFPVFVSRCKDFSSLTCLLICLCEQGVKASLSCAL

FFLTLFVMNMMMMMMQTLLYDAFFSETFHLCVFLASIFATVNICSGVKW

>SG-PL_contig_5809_1_-2_323_538

MLTMCWVYFWVPVVCSSHIILYVCQIHNVLFVKLFIMPMYKYVCLSVHGHLIQQQSLNYC

QNVSFWEVVSFS

>SG-PL_contig_5810_1_-3_3_143

VWTWWQEHSTQEIVRRTMLSSNTNPAGILQYLRMLSFRWSSRFLRDR

>SG-PL_contig_5811_1_-3_3_146

VWTWWQEEHSTQEIVRRTMLSSNTNPAGILQYLRMLSFRWSSRFLRDR

>SG-PL_contig_5812_1_+3_24_224

MYTSSVSDFHQFLSQTMYIYKCTFKKRIPNKTTFEKYTALQYTTEKYNDILTWNPVRLKE

PTCHIYH

>SG-PL_contig_5813_1_-3_30_251

MRAVLSPLPHCFTFPSPEEITVFWMQRKKTNESCLLFLLYSLHPNFSSVLYLFVCLLLLL

VMVVAVMVVLLLLF

>SG-PL_contig_5814_1_-1_298_1266

MFCCNPFKGGKRREEAWSEMCRVKVVVVGDARCGKTSLVRRHCCGTLPAAYTPTLFDTNT

VNSEVDGKKVQMVVQDTAGQEDLKHLRPPCYLHTDVIIVCFTPDSEESWDSIRKVWVPEV

RRYCGKRIPILLVANKVDLAAAPLQQQYPNDSRLGHSQDIITRNRHPNSDCVSQDNHNFR

DSQSSTSSSSSDHIRSGTLSSSTVNRDSGLFCSTEHLVKSVTREVKSVGHEVQCVRDEEK

RSLFGRSCSRVDKLIHAFGERKTQPSGGRYGSMMQEGQSLASQIGAVGYFQTSAVLNQGT

EQVFQAALTAAMSSKQWRKKYRL

>SG-PL_contig_5815_1_-3_159_641

MPKIALQIQAQLENVTSLRADGEDFRWYMKLKCENCGEETPEWIYCTRTDSYPLTGGRGK

ANLVLKCKFCKRENSIDILPDSLAAYTTDDVGKCKTIVKFDCRGVSPVDFSPRVGWVAEG

EESNTPFTIDLREKEWYDYDEKAGEAVSISEVYFQFVIVKH

>SG-PL_contig_5816_1_+1_1_180

SGTNYRLYLYGQWYKLQALSLWSVVQIAGSVFMVSGTISTNYRSFAICLLTLRSHSTGND

>SG-PL_contig_5817_1_+1_1_183

FFFSISQIYSACFFIHSLTQNALHTFIHPSSSIYKFYSKHKQVYNHFYRNIKPPSLICFK

N

>SG-PL_contig_5818_1_-3_3_200

WDLPSESRAKRIEVSTTRSHMTQVGVWDYYFYLYKSIRILCKLVVLLGRSTENLLLCCSA

GRQAAD

>SG-PL_contig_5819_1_-2_464_736

MSCYLLLFLLLLTPSLSPAKLSSAGPLNNLQQSYLNQNHPPVLTKFEVWVWVFLLWFLMQ

QSCICMLVHSTRQAHIFQQNFCLLVGLCLKQ

>SG-PL_contig_5820_1_-3_507_707

MSCTIVTLLAKSSLFYFIFFMPCQGFLLHFAHLFSWILFAHKFGIIKNDFDLYFIKEAFC

IHHVFDH

>SG-PL_contig_5821_1_-1_130_375

MCLELEAVRRALQQFTMEASGRVIRLFTDNTTVACYVKGGGGALFDPLPVGRGPPVLVPQ

QGHPAKHKTHCRQSECPGRCAE

>SG-PL_contig_5822_1_-2_188_472

MSVIIQVVLTSVEQCLLRMGRRRHGLFRSPVRSGSNPHVLFDVCRDFQALAFFFLCVCIC

MFCHLVCVCVVHQERYQAKSDLSPVLVGFFPFFFF

>SG-PL_contig_5823_1_+3_3_212

IAQQTTKTKSGLSPHSVHQSQEDNCVEHKTSSNLKTILLLLYIVYSPADHYGHIRAVTTQ

SPSKSRRQPR

>SG-PL_contig_5824_1_+2_341_520

MQILCSSIVETPIQSDDSNSAQFRNIIYHIIINNNPEKPLVLRKKKNLPYHLKQICHSTH

>SG-PL_contig_5825_1_+2_281_454

MYTMYKFQSQDKPRNHLNNERREQRNDFFSQRHCSCELNCTVLTISIIITLCWCCYKN

>SG-PL_contig_5826_1_+2_83_175

MEIEHQRMNIQKLDKNVKMTSCISGPDEPVK

>SG-PL_contig_5827_1_-3_258_440

MLTWEMGEISTLYQAASNPGIPVGRPVPCPLDYHARSTYNHGEKKKCVGYEYLITGKLNL

G

>SG-PL_contig_5828_1_-2_2_136

TPSSKCAICPCDIYLGPLPFKLMHLSLLNDPHLWFKFGKDPSRNS

>SG-PL_contig_5829_1_+2_941_1132

MGICNYHARLSLSVKSVLISNYAKTKTDQLTCVKLVLFCNYGKKKKKKKLIQRTNAIHMI

NTKK

>SG-PL_contig_5830_1_+3_135_275

MYDTDQKPYNRSNDLNNETATADCTTYQQSACMVLMNVLTHAYVHTH

>SG-PL_contig_5831_1_-3_120_338

MPKQVGNKTECGLLGFVLDLKQSYESIREHHPEESLVKVYTFNSVRKSMSTVINRGNGNY

RLYTKGASEIVLK

>SG-PL_contig_5832_1_-2_1085_1318

MGILFIFTPLWDIFHIVSFKSLFPHTYSGQSDCTDLTEILQRPGNRKCWKTEVFSRDGKL

INYQKQKQKKNTHTKKTD

>SG-PL_contig_5833_1_+2_2_196

KNKTKQENLHINLINVKYIQTLHQCMPIHSKFVLAEKRVDMREMLGIHERNLRIHIIFSY

CHLTF

>SG-PL_contig_5834_1_-2_842_1093

MTLLMHASIHVCACCVRVCLTIKWLCPTLETCLLYLCSPCLLSHCTKVHWRCHVVCRTLN

RDLSVILGCNRLDLLCLGGHHLPF

>SG-PL_contig_5835_1_-1_46_246

MVTEMISQYFCYMLFRICVCKFLIKLASPYFFQIWEKDLTKFQNFSTLSKLGGNLPGHRS

TNFDSSR

>SG-PL_contig_5836_1_-2_230_418

MVHLSLSLSLSLSLSLSLFLIISFCLSLRLAPSSPSPSHAFWCFLCQNIVSFSFHLISEP

SAQ

>SG-PL_contig_5837_1_-1_124_279

MCVSDVLCVYIHKGGSYTRKSTHMLTWQIGKISSLYPPGGTQAVSNSDLLMS

>SG-PL_contig_5838_1_+3_144_344

MGTVLLEKASWDHIDKLRCGSFSKKITRPLLCYTNECHCTNILICLISRFWLAFDFQILV

GHSDNHI

>SG-PL_contig_5839_1_-2_965_1201

MHTLNKCMHNLHTDTHHIATNVHVHVCVHTHTHALTPKHIHANKHTPHTHEHGNSKHNKV

INGTISDIPVNIKLPFVCR

>SG-PL_contig_5840_1_-1_70_180

MYRFLDQNLVRDMKEGKMMEEKKINKNNENQNHTHTQ

>SG-PL_contig_5841_1_+1_319_714

MICELHDAVSNNAWSVSSRVNYVKTTKLGNFLLFDVKYLTSQGLQSNYFKEYLYLQLLVL

KCKPASRPKQVYKNQLPIFTHSHACAHMLTLACTHIFQLHINNGGKQVYRRKIYSNVSGG

GGLKHNTAITQP

>SG-PL_contig_5842_1_-2_2_184

FFFFFFDNPVSFLSPLSSCLFGRFGAIVQNFKLKSDTFDTSNLKLLFLFIYLLLVLLITD

M

>SG-PL_contig_5843_1_-2_89_4834

MAVSVSISQQKPSVAAALTAELLKNSGHAVNWSLGQDNALRVDRDTVFSSSASISRYLAR

SYPASRLQGETILEKTEVDHWLNFASGQLSCASEFSTALEYLDNVLKPATYLVGHALTLA

DFAVWETLQGSIQWHRVLQKSAPQNVERYYQFLCTQVPFSTLLPQLPKVEVKDTKKGDVS

VKKEEGKFVELPGAEVGKVVTRFPPEASGYLHIGHAKAALLNAYYRDEFKGTLIMRFDDT

NPAKEDAEFEKVILEDVAMLGIKYDKFSHTSDHFDTLLALCEQLLRHGKLYCDDTDPETM

KKEREERKESKNRNNSVEKNLSVWAEMKKGTEVGQRYCVRAKIDMDSNIGCMRDPTFYRC

KPEPHVRTGLKYKVYPTYDFACPIVDSIEGVTHALRTTEYHDRDVQYFWILDALNMRKPH

IYEYSRLNLQNTVLSKRKLTWFVSEGLVDGWDDARFPTVRGVLRRGMTVEGLKQFIVTQG

SSRSVVMMEWDKIWAVNRKVIDPIVPRYTALLKEEVVPVNISAAKVHAKDVPKHPKNEDV

GVKPVWYGPRVYIEGADAACVKEGEVVTFINWGNIIIKKVHKDGAGKITSVSAELNLENT

DYRKTQKITWLTEVEGAEDSSAGGLVPTVCVHYDHIITKGVLDKDEDFKAYINRDSKKGQ

EMLGDPCLASLKKGDIIQLQRRGYFICDEPYLPASPYTGRASPCVLFSVPDGHQKEMPTA

GSKHKEEGTKKNTSAKEKAQSKKGKAAEKSPQEEQPPQKVGSASVNDLDNQIRAQGDKVR

QLKADKAAKGDIDSAVQVLLSLKKQYKEATGKDWTPPKPGAAPAAAASSSGAGDDLDAKI

REQGDKVRKLKADKASKGDIDSAVQVLLSLKKQYKEATGKDWTPPKPGAASAAAASPSSG

AGGDLDAAVQAQGDKVRQLKADKAPKATIDAEVQVLLSLKKQYKEATGKDWTPPKPGSAA

TKAAETKEMPSVQGKAAVDSAEALGLKAEADAQGDKVRQLKSGAGDKAEVDVAVQKLLEL

KARYKDLTGVDLAGGARRDKKSGADNKGKGGGKGKGVGGDADKGAKAAKDTEAGREVKKV

TRLGLEAKKEENLAEWYSQVITKAEMIEYYDISGCYILRPWSFSIWEQIQKFFDAEIKKL

GVENSYFPIFVSHSALEREKTHIADFAPEVAWVTKSGQSELAEPIAVRPTSETVMYPAYA

KWIKSHRDLPLRLNQWCNVVRWEFKHPQPFLRTREFLWQEGHTAWADCEGAKEEVYTILE

LYADIYENLLAIPVVRGHKTEKEKFAGGDFTTTVEAYISVNGRAIQGATSHHLGQNFSKM

FEIVIEDPDTQEKRYVYQNSWGITTRTIGVMTMVHGDNTGLVLPPRVARIQVVVVPCGIT

TCLSEADTKALYDTCEKMAADLKAKDIRAHADTRDNYSPGWKFNHWELKGVPVRLELGPR

DIQARQVELVRRDTGERMTRPNTNLSTQITELLDTIHNNMYTKAEADLKNNLAISHDWND

FCNALDQQKLIQAPFCGEIPCEDNIKTDSARDVVVEEGAPAMGAKGLCIPFKQPQELAAN

TRCIHPACSKTAKYYTLFGRSY

>SG-PL_contig_5844_1_+2_44_274

MLRKPVSSTTGQALTWNLQGKRKRSRPRNTLSRNLEADMKRTGSG-PLQLERLAQDRGGWR

AFADGLCSIQGNRRRWG

>SG-PL_contig_5845_1_-3_3_362

RKLLEEAGCGSVSVSEEQGSVIRTQLGLSWTKYRIGKQILRKLGIKFASEQKERQRQKNA

LFGDIIVERTNLFFTDQENRKQEKETSVGKVQDLPKLVGNLLDQYDKEKKLTWHDGTIPH

>SG-PL_contig_5846_1_+1_202_318

MKIQSNQTTATTTTATATITKKKRERERECVCVCVCECE

>SG-PL_contig_5847_1_+2_203_325

MCLFTTRQNTTLHNISIHMFTYTHANSKLSCVKNINNKSLF

>SG-PL_contig_5848_1_-2_107_313

MAARFPEIWQSPEHWDLASPFEDQRLSTFVDRVAAGLSSAAEIFAKLRFNSLLARRDAVL

QHSSLPPEE

>SG-PL_contig_5849_1_+2_2_349

NEEISSTEFPEGRDIVTHTHTHYETKTETKCLSFYIIIFYVDPYKLNANFSLSVIRVKIN

KYITIIIIIIIIIITIIIIVVHGSNSSSSNCVLTGTHISVDKMDPKVLVCLPTNMM

>SG-PL_contig_5850_1_+3_3_170

CCWSLVASFLPPHRLCMAQELNTAKEVLDKGAEKGLKACELPRARLLQVRTDLVRC

>SG-PL_contig_5851_1_-2_197_424

MSVHFTCSLDIVFSVLTKPRVLYQSHLWCVVISTRLLMSGFNCFEFNLFLFVYKIMALCT

TETRGRGRRGSEPYTI

>SG-PL_contig_5852_1_-3_144_353

MPFLSFAYCNSLPLKKMYINKMYPFIHLLFSLLKHKRQDGQAIVTIFSTKDKMVRPYINF

LFAFSKTHFV

>SG-PL_contig_5853_1_+3_57_260

MNIQSPFYVLFPTNHSKTFFCYCPMIEVNLYLFEIKMKRQEKAGFLLLLSSLLLLILVAK

VFCVSVCV

>SG-PL_contig_5854_1_+3_3_164

LLFIKPRSKRMKTIAADKKKTRHKTLRNRLVKYSDERKSISYSNVITIKRALDN

>SG-PL_contig_5855_1_-3_3_185

SMKGHRIWDNFHNGDDDHDAMEFHLGLRLRDRAVLYTSFHNISWRQPGPTDTGTCSDGQE

I

>SG-PL_contig_5856_1_-3_12_560

MAGGNKNKLSREEELLLQDFSRNVSTKSSALFYGNALIVSAIPIWLFWRIHQMDLYQSAI

FFVLGTITSTYMVAMAYRNVKFILKHKVAVKREDAVAQEVMRKLADDKKMTKKEKDERIL

WKKNEVADYEATTFSIFYNNALYLVLLIVVSFYILKNFSAPVNYMVSVGVSAGLLALFST

SQN

>SG-PL_contig_5857_1_-2_149_349

MQVLSSSADCSESSGLPDFGGSHVQFSECDVSPLTKFRFSEMCGGLTASWTVGTLMKMSI

FVVCVCG

>SG-PL_contig_5858_1_-2_227_433

MVYKKMMTNHRLATCSKNILFFLSHSLTLSLPFTLPSTQPLPPLPCTRSHSSKCQMYIRR

SGMCRDPNY

>SG-PL_contig_5859_1_+1_1_156

AFMKKKLHIKKPIIAHPLHHNSIILCTCKNHLRFCNSLHSSREDDSGFHFSH

>SG-PL_contig_5860_1_-3_3_77

GRENVNDQLPFTFCSGGKGLIASCC

>SG-PL_contig_5861_1_+2_23_274

MKIHDLLLLFFNHVNQAVKAWKKRINIAGLLHTIIIEIQINNKMVNNYNKNYKVLSQIWS

HWSLCQKVPMFQPCESMIYIYTLL

>SG-PL_contig_5862_1_+1_1_306

FFFFFLLDFHFCSSFPTSHSVAFIHEHRYHLVNFLSVSIKDAALKIFSAVTAFSLRPKMM

NHTSVKSVIKISTLTTLSLALFVASLRAREKNAEESFFNFLP

>SG-PL_contig_5863_1_-3_3_140

NQSEKALQNGVTHLTNRETRYLYSIHIELVVSTGSFAIRPTYPMYR

>SG-PL_contig_5864_1_-1_1_189

ARDSQFYARFVKVFKFYEKVWLEIHKCGRYENSAWNKISLYVGLCGSLSGNGYHWKWLID

FKA

>SG-PL_contig_5865_1_-3_123_971

MASLSRLSSALVCTVRSQSLAPLTTAVRASSTAEAPKKEKTKFGPLSDEDRIFTNLYGRH

DWKLKGALTRGDWYKTKEIILKGHEWILKEITTSGLRGRGGAGFPTGMKWGFMNKPDDGR

PKYLVVNADEGEPGTCKDREIMRHDPHKLVEGCLVAGRSMGACAAYIYIRGEFYNEASNL

QVAVKEAYDAGLIGKNACGSGYDFDVFVHRGAGAYICGEETALIESLEGKQGKPRLKPPF

PADVGVFGCPTTVANVETVAVAPIICRRGGEWFAGMGRERNRG

>SG-PL_contig_5866_1_-1_1_189

GVCVCVCVCVCVCVCVWDVFCLRFCVPYSVYRSMRSLALKLYNLSVFSICSVYYLCVYLG

SSV

>SG-PL_contig_5867_1_+1_1_135

DKYFRKYQQFRHIFKKVSAFLTGTLEGIIILDRYFRRYQHVRQVL

>SG-PL_contig_5868_1_-3_51_266

MSLRASWVLCSVTKDSALCFVCYSFCRTFMSACCLSDGNAQATCDFWLHAALLPGFPTRF

LNSRILRPLFQL

>SG-PL_contig_5869_1_-3_105_200

MCSEKGTAKMVRRGMIMLSSWCFVELTAKQVS

>SG-PL_contig_5870_1_+1_592_849

MTEVPEVLWEEAAQDRTIWHSLVNSGVAGYERRCTEDQKQRYQQQKSRACSNPQPGLHPT

ISCPHCSRYFKARIGLISHLRTHTSQ

>SG-PL_contig_5871_1_-2_8_475

MQFIKKGSSSSIKLAEWLKLSRQFANNWSHPVPLSEYTWEDVASGEMWKAKTVAGLYLLE

SVDSLKSTKEKASRLRQAYNIYKEAIESHLHFPGYWHKNFALVCEKLRHVADSSETIPLI

KTTIAHFKAYLHSGTSDPQTGTIKSAIKKLQEYVDY

>SG-PL_contig_5872_1_-1_31_240

MCDKVIWQALRFFMCTLSHLFSMQGTDKMMLKWTVFHRMQNRNMSQQTAISHTLIPTPYI

NAMVILPAFK

>SG-PL_contig_5873_1_-3_3_293

YGKPGFEPQSPHLVGKWWRFFQSPRVNICADLLVPEPPCIYACRTSNRHVKDPVSYVSVR

WLMETRKHPACTKKDKNGWRCTAVTRFPQESSPNFTW

>SG-PL_contig_5874_1_-2_134_6568

MADATARSLQYEYKANSNLVLSVDRSLIDRRARDEATGEVVTLVGKLGGTRMGDKAQKTK

PPLMEERKNKRRKRDEAQHDMVKMKGTTLLTSGIDEMVGIMYRPKTVETRQTYEVLLSFI

QAALGDQPRDILCGAADEVLAVLKNDRMKEKEKKKDVEAMLGPTPDERFALLVNLGRKIT

DWGQEDRAPTTDNIDETYGVNVQFEESDDDDAADMYGEVQEDEEDEENEGEEANIDATLH

ANLSEEARNKSEGGLHPREIDAFWIQRKLSKYYPDDPTTAQTKAREVLDILKAAGDDREC

ENKLVMLLGVTQFEFIKVLRQYRQMILFCTLLASAQTASERSKIEEQMEADTDLSRILHS

LQETDQDDLVTEERARRQQARQSRVAADLESMDVDEDVEVGPVQALDLDDLTFTQGSHFM

ANKRCQLPDGSFRKQRKGYEEVHVPALKPKPFDDDESLVPIDRLPKYAQPAFDSFKSLNR

IQSRLFKAATETDQNLLLCAPTGAGKTNVALLCILREIGKHVNSDGTINTEDFKVIYVAP

MRSLVQEMVGNFSMRLQSYGIKVAELTGDHQLTREQVNETQIIVCTPEKWDIITRKGGER

TYTQFVKLMIFDEIHLLHDDRGPVLEALVARAIRNIETTQEDIRLVGLSATLPNYEDIAT

FLRVDPKEGLFFFDNSFRPVPLEQQFIGITEKKAVKRFQLMNDIVYEKVIEHAGKNQVLV

FVHSRKETGKTARAIRDMCLEKDTLGLFLKEGSASTEVLRREAEQVKNHELKDLLPYGFA

IHHAGMTKVDRTLVEDLYSDRHVQVLVSTSTLAWGVNLPAHTVIIRGTQVYSPEKGRWIE

LSALDVMQMLGRGGRPQYDTKGEGIMITNHGELQYYLSLMNQQLPIESQFISKLADNLNA

EVVLGTVQNVREAVDWLGYTYLYIRMLRNPTLYGISHDQAREDPFLQQRRKDLIHTAACS

LDRNNLVKYDRKTGHLQVTELGRIASHYYCTYESMATYNQLLKPTLSEIELFRVFSLSSE

FKYITVREEEKLELLKLLERVPIPIKESIEEPSAKVNVLLQAYISQLKLEGFALMVDMVF

ITQSAGRLVRALFEIVLYRGWAQLADKAVALCKMVNKRMWQSMSPLRQFRRIPEEVVRKI

EKKNFPFERLYDLNHNEIGELIRMPKMGKTIHKYVHQFPKLELSVHVQPITRSTLKVELT

ITPDFQWEEKVHGNSEAFWILVEDVDSEVILHHEYFLLKSKFAHDEHTVKFFVPVFEPLP

PQYFIRVVSDTWIGSETQLPVSFRHLILPERYPPPTELLDLQPLPVSALRNPAYEALYSE

QFSFFNPIQTQVFNSVYNSDDNVFLGAPSGSGKTICAEFGILRMFTQSADARCVYIAPLE

ALAQQVYMEMYNKFASQLGKKVVLLTGETGTDLKLLAKGNIVISTPEKWDVLSRRWKQRK

NVQNVSLFIIDELHLIGGEVGPVLEVICSRMRYISSQLERNIRIVALSSSLSNARDVAQW

LGCGTTAFFNFHPNVRPVPLELHIQGFNISHNASRIIAMGKPAYQAIVRHSPRKPAILFV

PSRKQTRITAIDVLTYAAAELQAGTDDKVQSRFLHVTEQDLEPFLEKISDMTLRETLASG

VGYLHEGLSEAERRIVEQLFNSGAVQIIVATRGLAWGMAAAAHLVVVMDTQYYDGKTHSY

EDYPVTDVLQMIGRANRPLHDQEGKAVIMCQSSKKEFFKKFLFEPLPVESHLDHCLHDHF

SAEIVTKTIENKQDAVDYLTWTFLYRRMTQNPNYYNLQGVSHRHLSDHLSELVENTISDL

EQSKCISVEDDMDVAPLNLGMIAAYYYINYTTIELFSMSLNNKTKIKGLLEIVANAAEYE

NLPIRHGETEILRQLGARLPNKQAVPKFNDPHTKTNLLLQAHLSRMQLSAELQSDTEEIL

SKAIRLIQACVDVLSSNGWLSPALAAMELAQMTTQALWNKDSYLKQLPHFSQDTIKRCQD

KNLETIFDIMEMEDDDRNALLQLTEAQMADVARFCNRYPNIELTYEVQDKDSIHSGSTVN

VVVTLEREDEVTGPVIAPFFPQKREEGWWVVIGDAKNNSLLSIKRLTLQQKAKVKLDFLA

PSIGRHSYTVYFMSDSYMGCDQEYKISIDVKEGGETSDESGSESN

>SG-PL_contig_5875_1_-1_244_1188

MMESFGMTTATVVATCLFIGLGFQTCHGASWSYTGSHGVDHWATDYPGQCDGQRQSPIDL

ESGKTMYDKQLQAFSFTGFNSAVGVSWTLSNNGHTVQVTYTAGDLLVNGGSLGDTYRVAQ

FHFHWGSDNTKGSEHTVDGGAYPMEVHVVCFNTRYADLSTAVTHEDGLAVLGFFFQISAN

ENPTVQKLVQFLPQVMYAGNQTVISAFPLNDMLAGMGEYYRYKGGLTTPTCNEVVTWTVF

KNTIKLSNSQMQAFRALKDGDDAAMVNNFRPVQLLFDRTITASFEDEKFAGGSNGLVFNS

LALISSLVLSLWFRL

>SG-PL_contig_5876_1_-3_15_713

MDEEVPSGSDNIVTEFNTYEDFLDSQITSFDLYYLEDQELARQLVELGYRGSGEVLKREE

FEARKAAAEASRLSKRSQQKTLASSGKELKDPLLRALATREEANRSGKMTTIIFIRDKNA

RGQEISGYIDYAYRLKAEDFEPYFGGRKRLLPRPSDLSFYNWETQTSTSNPTPNYQVIAE

NASGLLFKNKRDRKIINVDPKAPTPGDNSTRTIIETPKYTQVVIYDHITRRKT

>SG-PL_contig_5877_1_-1_40_303

MTNEAVMKKLNVKRELLSKIKSTKLSYFGHIMRHDSLQNDILTGSIEGRRARGRPRRMWT

DDIKEWIGMSLSGCMRTTADRELWKDLS

>SG-PL_contig_5878_1_-2_2_196

DEIKTRINKAKATFISMNKIFKSKSISMRLKLRLVKCYITPIVLYGSETWTMYAAIEKKL

EAFEM

>SG-PL_contig_5879_1_-2_2_217

DEIKTRISKAKATFISVNKIFKSKSISMRLKLRFVKCYTHSLVRLRNMDDVCCHQKEVRG

IRNVDIKTTRKN

>SG-PL_contig_5880_1_-3_18_272

MNPDKTKTMVIRKGDQKEADVKVDGKKLEQVEKYKYLGQIVNQDAKCEDEIKTRINKAKA

TFISMNKIFKSKSVSMRLKSRLVKC

>SG-PL_contig_5881_1_-1_874_1254

MHIKSCVKFAQISFAKTSLFSRLICCFPMPPTHHLIQSFPCISGITYIQSLFYFVLVVSL

CKSGFTSKFLFHLFYLHSDTLNKGMFLCIKTQFKILGNPRLIVQENFSWSLTAQHNLRKV

NVVHYCL

>SG-PL_contig_5882_1_-1_1_1692

KEESSSEESSEEESEDEKPAAKAPAKPAAQKNPSKASKESSSEESSEEGEEDEKPAAKES

DDDDSDEESSEEESEEEAAPVLSATVSNAKGVTVKQLKKFFKSSNVKVKEIKQASKKSFT

VDFETAVDTTKAVGLSGQEVSGRALKIEAVAPSAPASPEKKKASSKKAKKPADSDEDEEM

EVESKGAKRKQQGGEDSKPTKKQKVDSGEVVSLFVGQLQCETTPDSIKKFFKKNGVKVTE

VRKDKSKRFGYVDLENASDMSKAIALSGKDFEGNQIKVEEPRTKSQDRSLQKTPQQAGRG

QRDTSADARTLFVKGLAESVDESKLQDFFSEASEVRLPQKDGYHKGFAYIVFDDKETAEK

VMEDKQGADLEGSSLYLDYTGTRSAYKPRDHFGSRDSGSKSTGERGVSNTLFVKNLSFHL

DEESLRDAFPNATQARIAKFPDTQKPKGFGFVEFGSPDDAASAYDQLQNTEIDGRQVFLD

FASPGGGRGGGRGGRGGFDRGGRGGRGGFGGRGGRGGFGDRGGRGGRGGFGGRGGRGGFS

GGRGGGFGGGEPQNKKKKFDSDSD

>SG-PL_contig_5883_1_-1_1_168

QPIYAHKQLQLLLKKRSSHINKPPFILTPIMSFVWTATSPFQIKVTITGFIVLVMP

>SG-PL_contig_5884_1_-1_1_453

VVAAKCGAKVILSDSALFPDCLHNCRESFQANHLTDVTLVPLTWGLITPDLLQLPAVDII

LGSDCFYDTKDFEDVLVTVSFLLKRNPHGQFWCTYQKRSATRSIQFFLMKWGLAAMEVPK

SSFCTENGKDFSSPGTDSVHMFIITLITHCI

>SG-PL_contig_5885_1_-3_111_1154

MSVKLRPVNSPSMLLNPSENDTLFNVIGRGCVTLASGVVQLYLADQPDRTRWNKRCCGVA

CFVKDNPKRSYFIRVFDIKKGQQIWEQELYNQFRYKTPREYFHTFEAHSYVAGMNFASED

EALKFKNAVEGKLQERATRRREKKRQPTVHGQPPARPPNSRPPASPQPQNNMMQPATVTN

FDMSNKNGTVNKKKGKDRDKSKKLTKADIGAPTEFRHVSHVGWDPSKGFDMNNLDPDMKQ

LFSRVGIDKEEQIDKDTVDFIYDFVEKNGGMEVLRKEMELRSPPPAPPPGGSRSVPPPPN

RSPAAPPPPSRSGHPPPPPPSRTAAPSRGAAPPPTPTSSQQPPCPKSP

>SG-PL_contig_5886_1_+1_1_177

KKNYFLKRGGKDWGGIMNNEMLIFIHLVSSGMEKCTHINYTLVFIHTFLSVFQLDYNIQ

>SG-PL_contig_5887_1_+2_128_364

MYLHHIFTMITKFSYKFCVKKFSYKFSVYKSIIYSTRHLFFYRNELQVHYLQTQDISVST

LTSTLFQFSDLSHQMDNEF

>SG-PL_contig_5888_1_-3_12_401

MAPSIKTLLALLSFVTGAFGWKCAKYRHYFSQQPDYIDCFWDCCGNSFDRHCCAPIGIIV

GCAICGAVVLAAVIVFVCCFWQRRRRYGIQPHIFQNRKPVSAYPAAQSTVTNPAPYVSYG

QKPPPDQFFH

>SG-PL_contig_5889_1_-1_1_186

EKPLTVADVRRILAEEEANTSAQEQPEDHGQKTGVRSGNVSFSGLTRSGKALLSSYRSKK

KK

>SG-PL_contig_5890_1_-1_1582_1845

MSLNGEIAVERLCKILKTCYQTHQHCRRILNLIHSNSVMYLFCFTVICCLLIFKNSYFRR

KKKTGFRLTQWFLSAVCCYINNIWMECT

>SG-PL_contig_5891_1_+1_139_411

MDSRSDVSSSIPACNIKTGLFAAHLNAPVILVVSVFHRSCSLPILMLQSFWWCVCLFHRS

VPPTHPSIHPSILPVNVGQLHAFDHPSIHPS

>SG-PL_contig_5892_1_-2_2_235

HPRTVDSQNIFITTLCEKHEIKITCQSLNRPLSPLPLRFPGSPPPPLVNPPVLPISPFLP

TQSLHKLSLQVTGIVLAL

>SG-PL_contig_5893_1_-1_1_162

EKALQTGVTHLTNRATRYLYSIHIELVVSTGSFAIRPTYPNWPLQLFTQHAVNK

>SG-PL_contig_5894_1_+2_62_376

MITDTLELSEDVQFDRVHRINPRPNSPIIARCTFYKQKILILKSKRKLQGTSISIGEDFS

QRVRAIRKKLVPHLRAAKEQKKRATIVYDHLLIDGKKYVLSQDET

>SG-PL_contig_5895_1_-2_2_736

SAVAAASVAAEPSTSGEVASAAVTDSTEPDSKKAKVDFNDRVEKAKELIEQKRIQKAKEE

AEKERQKELERRKMGQNVQQLKQFQQEREAQEVKEQLRKEKEEQRKARERVKEQIERDRV

ERNARFQKEKAEREEQQEAAKKAKLLEQQKAAAEAEARRSVTARIQFRLPDGSSVSNQFA

SSSPLQAAFDFISPQIGGAVTLSTTYPRRTFTQNDMASSFSDLQLAPTAVIIVVPASGSL

GRSSG

>SG-PL_contig_5896_1_+3_171_512

MELHSILTIIPILINIIALGKIVPNSVALHRPALMVTSVSVGLEVCWKAEISSMGAVTQE

RCRRWGSPPKKKEKRKKKGNVFLLPSSSYTCNVKRSTVVAGHCKGLGFCVSWKH

>SG-PL_contig_5897_1_-1_1_195

LSTQVSDRAEHSSPALAASGNSAKHTVPTFQSQVRPRTQRTLTVFACPLKLQQHCLSLNA

RQKGL

>SG-PL_contig_5898_1_-1_76_663

MTLPELCECTENPEGTVLPLTENKNNKMGKAVQAVLQQAASEGRATCSLMACVNLLSMRP

DNIMLCVMPTLPNADATATIQNTLIRSVCQEHSILVLSVDCDVKLAKAVCMEDWEKKEAS

VDVDLNQNAVLKTPSACTEDTLLFSDCDNLAPVMGFPCVLVEFPQDSSAEDEMVAEFCKE

QLYGEKLTEPYIELPD

>SG-PL_contig_5899_1_-3_186_368

MKDTGINDKVCASVRVPLCGVHNRKATVLFLPRFSRSTTHMIYDLEGDISEQRVKKQGGG

G

>SG-PL_contig_5900_1_+2_2_178

LKFNFDLNFCSLYQLRQATRYISGIVSAFFIFTRLQINTPLKLPCASQTNIRNTLNNVR

>SG-PL_contig_5901_1_+1_103_375

MCVCRTSNMRVKDLVSYVGVRWLTETLSMHAQQRHDYAAKERQQNGTNNMRTRIQENTAW

KEQTKFFNALHAVTHMCCMCAGCERYSSGFH

>SG-PL_contig_5902_1_-1_1_453

GDGGGRPLPRLGAPDQLRLSPLPDPSQSHQEGQGRESAHDPHCTLLAGPTVVPGPEVAVP

RAATGTHPEPGPSQATSLGDQACEPRDATPTRLAPLRPRLPALTISPPSVEAHGQTHARP

REVAAALAASGARPETRSLAARSRRKGTESV

>SG-PL_contig_5903_1_-2_293_514

MLTSPMSARQSTQSTTELVACLPVHVCGRALMGVELATSKPFNLRNISMYRLKGNQERLE

QNLNIFHSTVKTVN

>SG-PL_contig_5904_1_-1_1_273

KRLPLYVSPVPDPEAWGVDALSLDWSGLIAYAFPPFPILSKVVWKVIERPQLILIVPMWL

AQMHYPDLWALTPCLLCYSRQPRSGVMHPNP

>SG-PL_contig_5905_1_-3_3_290

KRRRRRKNRRKANNCDTFDISTVSDKTREDSGSDGETGGVIGCLMFEEMMALKQSEKTAA

GSDAKTSNTVSGSTPSELNLNERCSYRNTDMCDDTV

>SG-PL_contig_5906_1_+1_178_276

MGQRKKGRCKASTHTYLLSAYQQFFFSEHIFFF

>SG-PL_contig_5907_1_-2_155_2260

MLSDTAIEKVFLYTLGKTLQVATEYGLRRHEYVSVSHHLDSLLEAMCEENPAVRIGLRPI

LEACDLHTQQHPDKSPYAQTICRLYSSVLGSVDQTGLDSSDNSQQSTLTLRSRRRPRVPH

HLYHDNHNSLSSRSPSPTRSRSRSRSRSRGAADNGDSNHPFVKASEDQTEKTDCGPHANS

YNDTAAAGILTTISASSDAIPSGGPVASSQDLNVSFASSMEDQTSLYRSAPAYQAFPIMP

GMLSLRMGSPAYEKYLQLKERQRRLKAAKTGEPADPGGTGHRGMLSAPLLIPSPPGALRG

DMMDYLTDGRSMASLMSYTLGTYMPELASLDAHSAPPVAHEDNFDDGSLLSSELSFLVQD

GPHTNLNQPNRPVPEEPKQSYVGAPSRLKRRREDDIPEEQPKQQLDSYHQPLQELSRDHS

KELPSQQPYLEQQTGKHLHSLPQYDHQHMHPSQQQSWEQRLSPPQQYRTEAQQQQLPHLE

QFGSRDLTPTPDSLPAREYYGPEFVYRASRPVVRISLPLQGESVKNPALTRRVTIVHLTG

QKFEAIVDPCMTGRQLFDAVVTHTALDDFFFFGLTYVCEGEHFFLDADTKLHKMSPEGWR

EGAKGHMPPVTFTLYLRVKFYPDSLADFRHVSSQHLLYLQLRRDVLEERCMADNQQLLAL

SGLALHAEFGDYSQRTMGQNYFVPEQYLPARVARRLGMGYVH

>SG-PL_contig_5908_1_-2_2_115

LTSDSSLSLEDVLGDNALMVTIVQSLIKSPVGPGRPLL

>SG-PL_contig_5909_1_-2_2_310

GLFSKKITRRFLCYTTECHRKNRLLTCLISRFRLDALRTKFSNKATCICGCKITPTHVLF

SCEQLKPFLPSSLTSDSSLSLEDVLDDNALMVTIVQSLIKSPV

>SG-PL_contig_5910_1_-1_19_1182

MAALARRLLLNTTYKKTVLPLLSIGTSGSGNRSYVTHATQQQADACAQYGGRHTVTLMTG

DGVGPEILTHVQEIFRYAGAPIDFEIVEINASTTDAEHLRGALLSVQRNGVALKGNIETK

FDDEQFRSMNVALRSKLNLWSNIVWCRTFKGVVTSRHEDIDIILIRENTEGEYSSLEHEN

VPGVVESLKIITAEKSERIACYAFEFAARNGRKKVTAIHKANIMKLADGLFLESCRKVSK

DFPDIEFTDMIIDNASMQMVSRPQQFDVLVMPNLYGNILSNIAAGLVGGAGLVPGMNVGD

RYAVFESGTRNSGRSLKGKNIANPTGMLLASCDMLDYLGHYDHGTMIRESVMSVISRPQL

QTPDMGGHATTTEVIQAVIEEIKPKTQT

>SG-PL_contig_5911_1_+3_3_311

VASFPALSGQNGSLSANTERERERERERGDVRKNVRKSHKTRVFKERNHPQSEHTTRRSL

RDKNMQESVNSDQKILAFKKTKTDQYERAEEKRNFNDSKKQVH

>SG-PL_contig_5912_1_-2_2_172

FVPPGLWACFKSGAHALMNLKSNFLHVLVLFVGLKSSFPLIDPLLFPAFLSGFRCCV

>SG-PL_contig_5913_1_+3_3_161

QMHVCMLFGLHCAGVALLHILHTLVLSGCTCSIFSCFHKLQTLTWLTGSLTHQ

>SG-PL_contig_5914_1_-2_113_2251

MSRMHASVALSTVYEDCISDKQRDLFKEKCTNFLKYLFGDGVVESKIEAIEIISSLLQGP

YEVGSMLLGIEGVVQIMFTLASSDNTHFQRVAVEAIVHSASKKDRCTGILKDAVPILKNL

YKSTDESIQVRALVGLCKLGSFAGSDASAQPMADGSTLKLSRICRKFLVNTSKDCDLRKW

ATEGLAYLTLDAEVKEELVNDVPALKSIFDLAKNPERNMTYACVTVLVNLTNSYDKQDMM

PEMVELAKFAKQHVPEEHEKDKQEFVVKRLHALAKAGMTNALVALSNTESKNSRELLCRV

YLALATEESLRGLMVQQGAVKSLLQLTENNTDVGGVLAAHTLAKIAVTLDPSTAFPGQRV

YEVVRPLISLLHNDRTGLQNFEALLSLTNLASVSESVRKRILSDHGLQSIERCMFEDHDM

IKRAATECMCNMMMSEKVQEMCEGDNDRVKMMMLFAGEEDLHLVRAASGALAFLSSNRKL

AHKMTQVKSWLEILQQILVTDNLEVQHRACHVAMNLMSVDKEIAAMLVESQVLEILMAIT

KLEGPEMEDIRQCAQSALDTAVEHKLIQPTGRQGGNLAELRAALNVKPLTITEMTEEDEG

EEASEEDKELDKEQHYDEDDDEELKEILEKDRLAKKENGSDDMSKRLTGDSAAEDASGTD

RLKPDEENAAKDDKTKSEPSEETPLAENESKVSESVDSLKSSEAPSSDSVGTT

>SG-PL_contig_5915_1_+3_63_323

MGDFVTHIQRIRTCICLCFGYLSVLHIKQLKDQDMHLFMFCFLLCLHIKQLKDQDMHLFM

FCFFLCLHSKQSFVIAISCGYTCSRNM

>SG-PL_contig_5916_1_+3_612_902

MWMSGFLSGRCVSDNICSIRDLLSYLNNKNLFGLLVIFYFETVFNLVDWDFPFNVPSLFG

FGPDLCRWISVFYKNIKSTVAVNGQKDVLLVPHSKRL

>SG-PL_contig_5917_1_-3_306_794

MGAARERRRSRSKERERRRSRDREERAPEEEEEMIRRDKKRNRRSRSRDRNKRSRSRDRK

RGRRSRSRDRGRRSRERRIKEEPNVTLEPPFGDGGYGPGGYDNFNNTGGYPSNDVENKSW

MDFTNVQVKQEPSDAGYPAQMPNVGVPPPPPPPPPPEDGEEDE

>SG-PL_contig_5918_1_+2_3947_4225

MNGSPPSVCLSLFHTYTHTFKVQSSKFKHQHDFSIYVQSTCFQFNCYPSMLSKLDDKRWS

FPHGFVLSPSPSSTENIGNRNSFQLAVYLKKVV

>SG-PL_contig_5919_1_-3_339_620

MLLFDTTLIHFTSILKPCCLTPLLTPHPPFLHLSVCVCLSLPLSDTLTVICICCILGKGG

ERKSYLICVGCYVVTFVLRRLYKSINIWTGQKKK

>SG-PL_contig_5920_1_+1_1_102

MCECVCAHDVCACVCVYTHSCVFWLDTRSFYLHK

>SG-PL_contig_5921_1_-1_175_414

MPDYQLRSRSYLDHGGIQFMPCHRSKRFVGIRFSLWNILTYCNFFLHQIYCNICTVCFYK

VTSFVSGFDYSCCSVLFILV

>SG-PL_contig_5922_1_+3_216_335

MSSHISGLNIWSAFLDLGCYSFKNKKSTAHYKQGKTNKNQ

>SG-PL_contig_5923_1_+3_3_212

VQLVHAEELAECPLQVLLHTRHVMLVHLSKTGRGIGRGLVVGGGDVGMLVVSTADGAVGS

HDEQQQLMPL

>SG-PL_contig_5924_1_+1_1_141

SPFFLPEGLDIGSNKRGKNPKRGVRGESETESLQTKSTKGGRTKNVG

>SG-PL_contig_5925_1_+3_3_98

VRKVLNKTTASDKFVRKVLNKTTASDKLFAKS

>SG-PL_contig_5926_1_+3_438_527

MIETNLLFTTTKLQFDFKLLNYVSKVKSNL

>SG-PL_contig_5927_1_+1_394_855

MQTLAVMSFSRRNTFGLVFIQWVRVGCVVCKDIFFFRSRLQLSVYEWTCTSVRIYACTHT

LAQTCMHTHARAHTQTRTHTYKTEKERERERDDQDFSLPQCDRTFCSLSDPDFLLPEQKN

PRSEKSCLHHMYIQTHTHIQRHHYHDDDDGRQMN

>SG-PL_contig_5928_1_+1_307_501

MPDPREKMKTIQVLTQWAVLYSRHNLMQQSKQDKYTDVFKTTDFNWQIWGKVLSCIALLV

TDLNH

>SG-PL_contig_5929_1_-2_32_505

MPVSSSTYGFSPKKVTLSRLGGGGPDFGSDFTRSRPTTYSMQPVHVHTAARSPQQSSDYM

MNRMQDSMDNISLSPRTPDFGYQAPPFSGQYIPTGIRLDMEREEELRQQQQQQQQQQQQQ

QQQYQSAPQQEYQPPSQQYYFEPQQPYVPTAVRLDHQM

>SG-PL_contig_5930_1_+1_7_420

METQARNREAIQPGDWTTSINLKDACFQILIHPAHRRYLRFVWKGRAFQFSALPFGLSLA

PFLFTKVVRELAALARAEGIRLLVYLDDWLLLAQSQTLVTTHTSRMLELCSYLGFVTNQE

KCDLSPSQSLDLLGMRFG

>SG-PL_contig_5931_1_-2_2_289

RRVWPIHLQRLWRISSSTGLWASDISKKEEKGNWGFSIILWCLHCFPHSSSFTRVFTARE

FDLEYIIYVCVCRNLILSLRCRHTGYVHFGKPTVLY

>SG-PL_contig_5932_1_-2_86_394

MSQGMGDGSQLPPNMQLDSQSAVKQFYEFLSQYNKISETCFNHCVHDFTTRKVLDSENTC

AVTCLEKFLKATSRISLRFQEIHLLQQDVGMMQSMAAADARKK

>SG-PL_contig_5933_1_-2_128_505

MASETEKAQLATKGGDTLFGKIIRREIPCKFIYEDDHCVAFNDIAAQAPVHFLVVPRKAI

QQLSVAEDRDEKLLGHLLLVAKKLAKEQGLDKGFRVVVNDDVEGGQSVYHLHLHVLGKRQ

LGWPPG

>SG-PL_contig_5934_1_-1_13_273

MEHLAEFATSHHHHPPPTPGKSRPLRLGAAAGASAGEAFCFQEGKRWGSRPRWGTPVQAT

PPVNFTLTQHLQSFSPSKQAACRGMLR

>SG-PL_contig_5935_1_+2_2_256

HTHTGTHTHTHTCTQTHSLGGHSGQTAGGFGRPGFEPTRTLHLVGKGWRFFRSPRSTYYV

QTCKCLSPLRVYTHAEHQICMLKIP

>SG-PL_contig_5936_1_+1_34_252

MTSYNVFTAMPNIKMASLNCQGLGDLQKRRDVFHFLTKKSYSIYCLQDTHFSAKLEKYVA

SEWVYIFFRVIQV

>SG-PL_contig_5937_1_+2_254_571

MWETYNWVDWGGCSRQSTNLLTYSFWPPRSIVLYRLSGAMTWRYPSPLTEGEGQFLIDSI

CVITQLSSKATLRLRVVTVVHFFKTQTYENNYMLFDKKKKAALIRK

>SG-PL_contig_5938_1_-3_201_368

MLLTLNPVLSDMSLSGIKLILFKPDYILYNLNTLAHMPCGFMPVSMHSMFELWHSQ

>SG-PL_contig_5939_1_+3_2739_3014

MTITYLQQVSATGYRCQKQRKKSIKHFLKCWRTFSNIYHSLSMAPTHSTRFISPSLRNAS

LSLSLSLLHTRSHACTVYLSLFLSLKSHFQTE

>SG-PL_contig_5940_1_+2_2_217

LSLFLTLSLSLTLSLSSLSQTHTHARTQSQYKAKIQKVLNKTATLRKTSQQQTSKQCKSV

LLMQHSLHSHKK

>SG-PL_contig_5941_1_-1_145_324

MMLTSFKPVFIEIWFQDRNLRVCHPVLPRYLSYNQHTHTHAHIHTVHAHVRACNSHTHTH

>SG-PL_contig_5942_1_-3_75_734

MNGGISTCKHDSDIFFFNEKIKHCGLMISTEFLFFRQEHNRNMSSTEFLFFRQEHNRNTL

HYIKCCMRRFFTDQHLKAVINETEKKDVKTKCIDMNVCTCACYASLLCLILQPLKIQFNS

SEVKCKRGKPGHKSMYNETKIQDPVSPSQAEQRRIMNPLWPAQPPANLGMITNHCCTRQH

LISLPQPNSFHSPMRTLSLGCNNMISRGLGRQCPAYAASL

>SG-PL_contig_5943_1_+2_17_229

MRNKITELLAKAKTDYFSSKILEASNTKSLFSTMSDLLGTEKSSPLPSNISPDDLPSAFS

TFFSTKILNLH

>SG-PL_contig_5944_1_-2_140_313

MDWRLHAHSFARTRFTPTLPMNTVVLTLSLPHPIHQLQKLNPFTPSPYQTHLTKLNPF

>SG-PL_contig_5945_1_+2_2_121

IIITYLLPVMPMALWGSSEGPPFLSVLGQLLFDSVSASGR

>SG-PL_contig_5946_1_-3_330_515

MLKQPKTQTTRRPSMPSHDTIFTTFSTCECYYSIIRECYNPIMLLFILQRKQTVNAVSTS

AG

>SG-PL_contig_5947_1_+3_3_254

KSPFFTHQARCPRTIRTLALQPFNFEVQCRHHSTTAPISISKQTRTNDRCKIVCCSSDLI

YQFKTVHFQRLGDINKALTSASKP

>SG-PL_contig_5948_1_+3_3_194

TKMTSVRLASFLRILWLNISSQVQDEMNNTIDYKHLFQTDYKHLIQSLISNNEQNSSTIC

FMQC

>SG-PL_contig_5949_1_+3_123_362

MLKSFIRCKYREKERRHLKLQKSYVIITRCQSAYNMYGSYQTLYNIIKYSITLSHLFLFV

CCCFFLEQIFYGIVLGTTVL

>SG-PL_contig_5950_1_-3_207_1220

MASASLSGFSMTGWNANSFRPQLPLPPVVYPRCCGKGPTFSYFLQSKLLALAFLILGIAF

LVTFICAFLPFWFILRFNIFDSTFGSEGLRQLSKDTGIFFYTVDDYVSLLMLEKISNRVV

VPMLFKVAEVMEVMSVAAVACSFAGAGILAFRKYASVTGVLFLAALATFAALGQIFMVVF

SMVSLLSSSNDVCEPNQANCAYTDNLVWRNLPFSDQFRRINQHTTPKMEANWALYIACIG

AALSIAGCVLLWLEGLMVCRSVGEIRYQQLRQKRDVHENERDPTDFIFSPPSRSRPVMYS

EHPNVSYAPPRQTYRPPPSFSGASSQSGSAYVSREVDL

>SG-PL_contig_5951_1_+1_499_813

MFLACSLEHSKMERRLASLLNSCKSGNLPASEQNELRDLTAEYFIDGQPDSDWSSDSEVT

LLNRQLKKVTLTSMRHLFMLKKFEVMPPNIAEITCLLMVLTIEGC

>SG-PL_contig_5952_1_+3_3_128

AERTPSLTLQAVFSKWPSYNLTTLNIHNFNIFWSTIFIHIPK

>SG-PL_contig_5953_1_+3_3_248

YTIKYPLIIFFSLKNYQAIKNDILMKEHHHFTGRFLKMAVIQLYDLKHNFNIFWSTIFIY

QNNQQNVNFSVMYVSQNAQNAS

>SG-PL_contig_5954_1_+1_1_111

CSLTILYNLKQAYNRGWLTDSDSSLSREYKRLTKAHR

>SG-PL_contig_5955_1_+1_10_615

MRQAIERTLREEQAGFRSGRSCTDQIFVLRTIVEQSIEWNSSLYVNYIDFQKAFDSIHHP

SLWKILKAYGFPLKVINILQDMYADNQCCVRHDGQQSEWFHVQTGVRQGCVISPTIFLVV

IDWVMRKATSDRARGLVWGLTARLEDCDFADDIALLSHAQKDIQEKTSRVDQIASSVGLQ

IHPDKTKIMKVKTKSAEKTSVR

>SG-PL_contig_5956_1_-1_37_348

MSENRATKSGLALDAQKKIQSKYDQAKAAECMAWIADTLNLKLDTSGTCESVYDQLKDGK

ILISLLQAICPEIKLSESVKKDQTLSFKIREKIDVFLSKIKEPP

>SG-PL_contig_5957_1_+1_31_240

MTPQARHKSDKKICPLRIRCSADFKRPFHAFSWGLPGCKVHLEIFAVIFKYILLLFSFLP

VTSKGVFFPS

>SG-PL_contig_5958_1_+1_1_441

SLFKLKKKKKQKKKTRGRKKVYLIRLQCHLSICSPQRRTSHFCLYLQRCTLIIDYLSTHS

HAYTYTTTQVCANKQVCIVYSAHLHSSTITCIYIIYMYKHTALKKTSLMQSEDILSSSTI

ITVLQLVYLMWKNSSKCFINLRVQSFT

>SG-PL_contig_5959_1_+3_39_380

MFHFLGNFILKLSNISKLVLTSQLMHTKQLQSNHPTSTAGIKTWKHICLHNAESRRAPLH

MFLLCRYYCRHDCCRLPAGPCYCHILISLSKSPSLYREDPQVPPPLLPRLDPML

>SG-PL_contig_5960_1_+1_1_180

RSGYYYYYILFIAQPTIKAISGLSPHKVHQSREDSCVEHKTTLNLKTKILQNNTVHDILS

>SG-PL_contig_5961_1_-2_149_916

MSDREDCVYQAKLAEQAERYDEMVDAMKQVAGLDIELTVEERNLLSVAYKNVIGARRASW

RIISSLEQKEDKCEEKKCINKGYRTLIEKELKDICQDVLDILDRHLIANANQGESKVFYY

KMKGDYHRYLAEFATGNDRKEAAENSLVAYKAASDIAMTELPPTHPIRLGLALNFSVFYY

EILNSPDRACRLAKAAFDDAIAELDTLSEESYKDSTLIMQLLRDNLTLWTSDMQGEGGEE

QTAQQVQDIEQDQDGS

>SG-PL_contig_5962_1_-1_1_210

HLNAPIILVVSVFHRSCSLPILMLQSFWWCQCFIGPVRCPSEYSNHTGGVSLVFHRPCSP

SPLPFPFRPY

>SG-PL_contig_5963_1_+2_2_85

VYVCVYIYTYVSVGICVFNCLYLIVYYL

>SG-PL_contig_5964_1_-2_890_1174

MYIYIHMLPFFLYTHTPCCCHCHRMTCYIFYRYCSFPVACLLSWSEHQVDNLGVVGLTPP

NRPQGLAHLVRAPRGRGFDPPDRPQGLAHLVRAPC

>SG-PL_contig_5965_1_-2_188_469

MQCIYMRFGRFVMFVGDNCGITLFYIFLKQNMTSSVSKHLPTSHLFIFMCMFEKGKFPEH

LNLSGVSYALNNHSLSVLLCFELSIQPVAMDISV

>SG-PL_contig_5966_1_-1_1_114

FCFKPAVVFCCCCFFFFNLKTVIFGFSGFILHINIWFP

>SG-PL_contig_5967_1_-3_21_911

MADDLGDEWWQDSYDSVGDSAGEESGEEKPQTQKRGMKRPAKDEVEADCSDKSDQKVPAK

KSKSAPTKQRKHGKKKKKKQFVRKKITDLDEDTLKKGGIPADIQALLRPSIVVAKHDSSP

SGYDDNDSDDATLSADSDFFPVHEEQPVGDYLTDVLPGWQLAVKKAKLPPGSPLMLILCS

SAIRAVQLNRELKSFLGKDCRTARLFAKHMKLEEQKSFLQKKVCHASIGTPNRVQALLEA

GCLKLSQLHSIVLDWNWRDIKLKRMVDIPDVRRDLTVLLKEHAMDHIKHSPCRIAIL

>SG-PL_contig_5968_1_-3_3_194

NDISSQISQAYFLVLLLRTMLCVYTVRLKAFRAENMEMELIETFLFVQIFHSCMKTNREW

RQDG

>SG-PL_contig_5969_1_-3_60_257

MLCVYIVRLKAFRAENMEMESYRNFLVCSNFPQLHENYGQRGDRMDSITLISFCLICSFT

IDTCCL

>SG-PL_contig_5970_1_+2_125_280

MIEMNVQTLLRTKIKRISKIILAYQNCSTVKTKLPRGKVLMTIKSATETEQK

>SG-PL_contig_5971_1_+3_81_305

MHVRACISKESEHVYQYFSICSYTNLPINPLAQKHDKTVSAHLRLKTFQQGPPSERHSGT

TRHKTTWHDKIFCTG

>SG-PL_contig_5972_1_-1_1_147

DFAYKLVYILKYSWPLGKELAVFTERMCLTNFFPRSTLHVAVGVVPQTA

>SG-PL_contig_5973_1_+2_41_208

MLVKSTSMTLSTLKTLYHSTITVNYRCLIHHDTHTLIPMKSSPKSPLLFSRTTMKQ

>SG-PL_contig_5974_1_-3_3_272

MRFDTLALRVFPTPARLQALQLKLSSLWAWTSAPAREIASLLGLMESLAPVLPLGRVRKR

PLQRALRIHWHQSQTNWDQVVPLGDWFLEA

>SG-PL_contig_5975_1_-2_2_172

TLSGYWAGEEAGGQHPQLRRTSGQSETHRLLPALSVWWWSRWPCQEKEHEEGKVRWQ

>SG-PL_contig_5976_1_-1_1_153

SVRIAKCSKVYRISNCQNCKMQESLSNISQNYKITTMDTLSTCTPCLHKIL

>SG-PL_contig_5977_1_+1_1_177

ILQDCGIYRALREEIWPAPVSMREKLYGPVDALQKTTHFILETKQVRWQTRRRKLNLVY

>SG-PL_contig_5978_1_+2_2_211

AQLRRRCLEKLSQTASVLRRLRCKREPACDPRGDYPPEGVVQPRGRVGPLRQFFKQGCLP

PNCVFRNKDL

>SG-PL_contig_5979_1_-1_106_219

MIMGLAQLATKPSQACSLKDSYSNEYLNPTPLFIIIKK

>SG-PL_contig_5980_1_-2_71_418

MALMVGWAINNIIIIIIIIIIIIIIIIIVFWFVFLLLLFKELSTFRTLLEAFLSTSVGSV

FCVSGHIVPLQYLHYNMLVRGPSQPVYRGIFRLWRQKASTRCSHISYKDHNSPDST

>SG-PL_contig_5981_1_+3_3_134

GRGGGGREGCDRTSREQWLREREREYRSTLTIYFLWHHHKTFLN

>SG-PL_contig_5982_1_-3_363_614

MSARVSFSSFFFFGTCSPPWQSFHFLNVRILAALAAHKSNNANNKPANNLHDSKNSITMQ

THTETKCYEHTPSLLYHAVISQHV

>SG-PL_contig_5983_1_-3_636_953

MRILFNCCLYKLNSLLIPGFTWLSTSVLVSEFGISSFFETINPVANCPTRHTLVSIHNCQ

ISRNFYQTISKFEPCENIASELLYARIHFSDTQLVTFSVMSNNYTR

>SG-PL_contig_5984_1_+3_3_275

YVHSRRQNVCTQASTEKAQHMFEIIIRHKIFTRDECRLGQRLQLPFCCHVIMHSPFLFPA

CLCQVCDLELFCLKTILDPYKIHVKKIEKYT

>SG-PL_contig_5985_1_-3_546_2771

MQAIVLTRHRSVSAPLMPENLHDPKDKEGAGGVGLGYGAGSLSHLKNIQRPSQLACLRGL

HSPNLDRTAFRFDVENGAGPGRSPLDGGSPSPSGLVLQHLPQRRESFLYRSESDYDLSPK

TGMPRHSASLGATDRCSEPQHAEDLIVTPFAQILASLRSVRLNYLSLTNVPAKDRPRRGS

GTAPHAHSLLSNAQGLSSPSPHHLHNTHDTPSQLHPQDEAYMKLAIETLEELDWCMEQLE

TIQTHRSVSDMASSKFKRMLNRELSHFAESSKSGNQIAEYICSTYLDKQQDVDLPTVKVE

QVEPTTKERPATEKNRPMSQISGVHKLKHTNSFSGTIPSHGVETPHAQQLTQCLADIDVW

GIDLFKVAEYSNRRPLTSVCYTILQKRELVKTFKIPPSTLVTYLMHLEDHYRRDIPYHNS

THAADVVQSTHVLLSAPALENVFTSLEILAAIFACAIHDVDHPGLTNQYLINTSSELALM

YNDESVLENHHLAVAFKLLQEENCDIFVNLSKKQRQSLRKMVIDMVLATDMSKHMSLLAD

LKTMVETKKVAGSGVLLLNNYTDRIQVLQNMVHCSDLSNPTKPLDVYRKWVDCVMNEFFR

QGDMERQHGIDISPMCDRHTATVEKSQVGFIDYIVHPLWETWADLVYPDAVEILDTLEDN

RDWFNSKIPISPSSSFCSKRPPGEESSVENSPPTRFQFDIEEEEEGEAESEQSTPQSQPA

STTSSSAASPKVKSKQQPAGKK

>SG-PL_contig_5986_1_-1_1_516

VAAQRGLRNTEGSKTYQKRPQKGPSLDDADPQLRAWASSTGLTDQILLVLVEQGFTSIDN

LASYSPQEVTEVLQQMKLLTAKQCLDLKKALTRLMQPVDSLADIHIPYIWASTFPPPTTA

KHASFQNNPSRFTQIIPSTFTRSSLPAVCPSELDLSFPLHHKTETDNFPVPM

>SG-PL_contig_5987_1_-3_48_317

MRATINQKLVETGERERLKELLRTRLIESGWRDQLKARCKEIVQNKGLEHITVDDLVAEI

TPTGRALVPDQVKRELLQRIRSFLAQQSSI

>SG-PL_contig_5988_1_-3_123_707

MPWVEKYRPTKLHEVVGNEETVSRLQVFAKEGNVPNVIIAGPPGTGKTTSILCLARALLG

PSYREAVLELNASNDRGIDVVRNKIKMFAQQKVTLPKGRHKVIILDEADSMTDGAQQALR

RTMEIFSKTTRFALACNSSEKIIEPIQSRCAVLRYSKLSDSQLLARLLTICEQEGVSYTD

DGLEAIIFTAQGDMR

>SG-PL_contig_5989_1_+2_1049_1285

MQAERQKVFAPYCACTQYNRALESCFFLKKTTALQYSVFEKPALQNLSSSCKPVGFTHVQ

VIFHRKLLSELCAPLHVLN

>SG-PL_contig_5990_1_-1_310_579

MREKQQSGNKVSIFETKCNVTVIPDGLWALLESTSSFNPSATITTMHLTWQIGSGFLLLI

VMLMLMLLLLYLHWRSGFARLNHDHVYVSE

>SG-PL_contig_5991_1_+3_192_470

MYAYMYASMYVCICVWYCIVLGVFFIIFIFLSQWIYHVKFWGLLCRGECVATMHPTLFLG

GGGVLFFSACSVFPCFHKPPNADMAYKIFIVHI

>SG-PL_contig_5992_1_+2_113_277

MNKLKLKTQTNPNKNKNYALLGRQSGRKDADHVPQNYTFSHFAENESDITSVICF

>SG-PL_contig_5993_1_-2_65_214

MIIMITITTTTTTTIIIIIIIIIIIIIIIIIIIIIIIIIIIMRTVSICPS

>SG-PL_contig_5994_1_+1_1_252

NFQIRTNLEQFVVLLTSPLFLPEGLDIGSNFGFEVCERNSRRNQRIAESVRFQKWKCAGK

FFFPCFSQFQFSQDSVSMLRINSI

>SG-PL_contig_5995_1_-3_180_416

MALTSRRIKGRMTAGQVAKLIMNYNSKNEKIGGSFTTESEDFEPDSKSESDFDTDVNVHV

CLCLHVRFSVIVSRPSNIM

>SG-PL_contig_5996_1_+2_2_283

SPVYPYIHTITTLHKTYQPHNIFHPPHILFDLHSMKSVTPYCSIVYCLHKAEIDDFVNYF

ENQLAPYNDNEHFEACLPELPIGNFFTFWHVQTF

>SG-PL_contig_5997_1_+2_2_145

WLTGSLTCIFDVLYAYTHEGGSGTSRSAHLLTWEMRKISTLYPPGVLN

>SG-PL_contig_5998_1_+2_869_1066

MVCERERGGRPRHAVSVRKWNSIALLYSCVCVGRGKKNYVATITTERVLEVVLGKKKIHG

GCVRKK

>SG-PL_contig_5999_1_-1_1252_1551

MSTENTYTSRYEMALSTVQNTSHSFVFSQEKSKSLTKHYLTQVTVMHMSGLGSEPLALMI

NCVIWIGVLSPSFYITPVREKTQIDSGIFALIMIELLNHL

>SG-PL_contig_6000_1_-1_1_2313

EEPDFEKEREASPEFEEPGFDVEERDASPALEEPSIVEKEKEDSPVCEETRFDEEQREVN

VPASGLHAGDLDQEDEEAGFGGEIEASEQNVGSVGGSLHCDVAEDHGLQMGGIPQEKETG

DVVEDKEEVIMREEVTGLKEELGERKEVQEDDLKEDERKDEEEDDLEADDRAQAEVMTRH

SMEQMDVSDDEGQDEDEETAEAVDSGDNDNDDFVYEKEVNLEDEEVLMARGLSAASKEQE

EEREEMEHQQTESRDMDIPQGVEKEESPVADENQLIKEGHRGYLDVDTTGQAVLETTVQA

DADKAGQADSGQEETDSIEGDVADDKDIDDILSMPQTHQHKDLTQEEDQDRDSQDTGLRE

GHKLNPFVGVTDREPSPLEEPFSQGREPYSYADEVADESKQKIPAEFDPLSHWGPSTGVP

APACPHDDVDATPEPLSDDDQIDVMEPQAMGSCQASLAEQEVIDQNEYKEDFGAMSGQKH

VADEAPMVDCLDRGDFKESNAADRSSEADPAEFDPLSEWGHPMGLPSPPPPEVSNKTSTP

RKAASDKKPEAKKSTGTPAKGKTGSEKRPASAPKTSSRSMNGTTETPRSARSKPEVKKSG

PDTPRSARPASSKSGDTSKTESKSRTTPTTTRRPLSTTKARASPEVIKMPPLPPFTPFHV

DLTYIPNHGDIAYVDSEFFKRVRARYYVLSARNPNPKILEMLMDAKGLWDKSNEQEVTVI

PTYETQILQHWMMVHKEKLAENKIDIAPAASRCTVRLQDHEDSCYTYRLEF

>SG-PL_contig_6001_1_+1_586_861

MLITCSWYQCIPSMGLMVSYTCPLQFYEKGQETISYCPSNFQNVPLVSFDHFNSPFSLYA

CVHIITCVNEIFMSIHFRYAPLSACTHASIHL

>SG-PL_contig_6002_1_-2_32_439

MGSVTDDDVIRRRLLIDGEGGNDDKRLNNLLKTFIRWYSSPEDEDSHNTHQRMLSMLAQC

EFTMEKSVQVHAMNISEQENYEELTKDIEKKIQEATEKITECKAELQEAKRIRKNRQEYD

ALAKVIQQHPDRQETT

>SG-PL_contig_6003_1_+3_1002_1199

MCSSKCRRNPFQSPQHSTAINFLFFFKTQTQQTRHLRSTSIHEYITCVFKNSTINQVSHR

RHVNLY

>SG-PL_contig_6004_1_+2_53_325

MPKNFFRAKKVSHTVQQLHPNSIIPAPYCTTDWLLFNIDSFLTIHSNAFLALYNNARPLH

ENVKLKRVYLNICKRLGQALQKRERERERER

>SG-PL_contig_6005_1_+2_62_271

MGCVFKRREKHVKRVQVVHVFGQAVWQECRRERDTHLKAEGTRTAAPTASERYQGPHIPT

YMKLKDNLRQ

>SG-PL_contig_6006_1_-2_2_313

QVLLLYLSTNQVLLLYLSTNQVLLLYLSTIQVLLLYLSTNQVLLLYLSTSQVPLLYLSVS

KVLLLCLSTRKASPVCKALGSCPRKNCCLTVQKLWSLKICRTFY

>SG-PL_contig_6007_1_+3_3_143

LVPEPPSCVQGRKTPNKPCQCSVVYGYTKTPSMRKNRRVYAILVSAT

>SG-PL_contig_6008_1_+2_146_223

MYKKTAAQDDSLKRPKPPLASSVQQP

>SG-PL_contig_6009_1_-2_767_889

MNQQVLACGHTNLPVQECSAVMSRFNITCCKIDMVVGAYTL

>SG-PL_contig_6010_1_+1_1_225

GKKIQINRRGALCVCLKCLWGCLRVDLSQTTKSPRHEKERRMHKTGINRWNFVRVNQKGY

ACFFYSRDSCDTMYE

>SG-PL_contig_6011_1_-2_125_742

MGNLFGKKHSKKPSSKVTDQDRAILQLKQQRDKLKQYQKKVLAQLDRDRDFARKLLHDGK

KDKAKLMLRKKKFQESLLEKTDGQLDNLEKLVNDLEFAQVEHEVVKGLEVGNAALKKMHQ

ILSLEDVERIMDETREGIEVQQEIDALLSGGLSQQDEDDVMAELDEILAEDDNVKLPDVP

TDDVGEKERERAKKRRAEEREAVAAT

>SG-PL_contig_6012_1_+2_131_283

MWLPWLFDNVASRFSSEISLETTKLFPSKRSLLPKHAHTLSKHAPKSCLKL

>SG-PL_contig_6013_1_+2_1622_1912

MHPHTTESSHQQYSPQTHKNLPHRSLTINNSNQNNNQDQVSLKKKYFFGSCFQMPCMFQT

TVRSQLLSHFCNGLQLRSCKENRAMYSIQLWNMAQIF

>SG-PL_contig_6014_1_+1_124_279

MLVMKHSVKKKTISMQHFLQFPVLEINGSLQRRESPGILSYFMERSIQSCVI

>SG-PL_contig_6015_1_+1_37_345

MNQPNCCLFMCILFHSSHLHYFPHQKDTILKKNGSIVYMMVKNEDCYHIFIIMCVKMRVE

TFQTNHMQKGDILVSTNVNNYTSMYTSKYQSVNLMLLYRYVHL

>SG-PL_contig_6016_1_-3_3_209

TFLFPPPVPFFLLMSHQCVGLIHWLVAPAIITVDLHCMACGSYGIMCIFLTPWAKCHNVI

WVYVLHHEK

>SG-PL_contig_6017_1_+3_828_1025

MNSLTFSTITIKSYLQGTTFSFCFTYSDSENILKCTHAKFDSVKHRNVYAENLHTSKNTY

LQIDCF

>SG-PL_contig_6018_1_-1_40_510

MWTLWTLLCWVGVAMAAYDPNQDNTQCCVCSNLMPINPAPGSQPQVMPSPFKIIVNSKNY

HPDREIVVELVSEDNYAFRDFILVAWPTDNRPRQAVGTIRLIDENKEFAPKYSCYSNEQK

SFAILAGDLKRKKHLRFGWETKQFVGHVEFRATFIVD

>SG-PL_contig_6019_1_-1_388_657

MLFSLLSSPQKMNFEYFNDMVVMLRRTLCGLSMAFIMCPCSCTTPPPLPQPLFPVNGLML

NTVNHSYSLSPCIPPPEKYVVKGMGIQDEI

>SG-PL_contig_6020_1_+2_491_871

MIDLLNMFSYCQVVNEPTHVHNHTLDWVTHRSDDNIVRSTSVSQHLKSDHCVCCDINVNI

PPLPTPVYKEKCNLCDTDRLSFREEVQIAVFPSVCQTADQLHSVLRSVLDIHTLVSRWKV

RTSKSSP

>SG-PL_contig_6021_1_-1_1_216

CTEFQVPLAISFIDFTKAFDSIHRPSLWKIMRAYGIPPKVTTAIKNIYNNSRCCVRTEDG

LSDWFEVLTGVR

>SG-PL_contig_6022_1_+2_704_919

MSTFRLKTCCASYEIIIFNEKSKSYFVSPSELSTRKSHFVSHIIMHTTGCISQRGPTSEP

SLTIVWQKDECI

>SG-PL_contig_6023_1_-3_570_728

MDQCCFFMASVQHKIECGVFVVWLTSQNSCRTGFGGEEGEKGITIQNGRICLC

>SG-PL_contig_6024_1_-2_71_187

MMLENRAICFKSGFFSWALTLEVIVFVVRFCFIFLLHDV

>SG-PL_contig_6025_1_+3_270_500

MLFFLQGGQFCVCVCVCVCVCVCVFCHYCFTAVVLKWFFWAYCHRKLYKIHKNHTGKQVC

LHSNDLEHVNNHQIQKH

>SG-PL_contig_6026_1_-1_1_231

YFQDKSADDLPYLKVPLHTVIKLTPVAYGCRVEFLSLDVPAVNTHREKPVVSMLYCFVFC

VFVFLCGLVSQCFLLNF

>SG-PL_contig_6027_1_+3_567_872

MCTVCVCACASACMCVCMHAQGKTHLSTAHKEKKKQAKQSMPTNHHEFSQSNTKQRLTHS

TPGTRQFVPCLLQCRPDTSILVPENHYSYSFSARTKALFQFR

>SG-PL_contig_6028_1_-3_369_953

MATPAKVSKTDSKNNHNPTDEQPDHQEAIEHIDEVQNDIDRLNEQASEEILTVEQKYNKL

RQPFFKKREDLISKIPNFWVTSFMNHPQVSALLTEDDEEALQYLSVVDVEEFDDIKSGYT

VKFHFEANPYFENKEISKEFHLNESGEPSSKSTKINWKEGKDLTKKSTPAQKGKKRTHED

QESFFSWFSDHGDAG

>SG-PL_contig_6029_1_-1_28_1089

MKAFLALVITTGLVIKPNIEDYWCQDDLTSTPAFPKYMSLVRFQAIMSNFHIVDNTKDNG

SDPLFKVRPFFTHLRQKFSEVYTPQQDISFDEATCAWKGRLRFKVYNPAKPDKFGIKLYE

ACEAKSGYVITLDIYQGSSPTTDVSEALGIEKEELGTTTRVVLGLLAYGGLLDKGHRIFL

DNYYTSPELYNQLYLLNTYACGTVRTPRIGMPEAFRKQKQRRDTINLQQGQTIFRQNGPL

LALKHHDKWDIHMLSTIHMPQEVKLSDGENGAAVWKPTCIVDYIKCMGGVDTTDQIMKSY

SLLRKTVKWWRKLFFYLFTLAMCNAYQLHRKFGETSMSPFSTRCPTACSQREVH

>SG-PL_contig_6030_1_+1_1_387

KAQTRTFPGADIGSDHDLVLATFKLKLKAKKLPKSPRIRFNLEKLKDPEIAEVFKAQVGG

KFAALNLLEDMDTLIENANKVYMDAGEELLGRERKKKKPWITDEILDLCDVRRELKSERN

ESDLARWQY

>SG-PL_contig_6031_1_-2_41_1390

MLNGTVHVDNHQQTSILKRAWEQTVVPLLLMATTPNVVILMWHCAVHCEGSYGCIGHIFC

ERSLLGGLGFIWSHVNWPSAPVIGIVAGYSFFALALMRLVPGKTAYGPLTPKGNRPQYTD

NGFACFLITITVFCLLTVVCKTFFSVSPTIVYDRFDEVLVLLNFFSLIFCLALYVKGLVA

PSSSDCGSSGNVIFDYYWGTELYPRILGFDVKVFTNCRFGMTVWPLLVCVFALKSYELHG

LVDSMLVSAVLQMIYFTKFFWWEAGYMCTIDIMVDRAGYYICWGCLVFVPGFYTSVSLYL

VNHPNVLGLPLSLLVLTLGALSIYINYNADEQKLVVRKANGDCQVWGRKPTIIRAKYKLE

NGESKESILLASGWWGLARHFHYLPEIALAFFWSLPALFHCVMPYTYVIFLTMLLVHRSF

RDDTKCSNKYGQFWAQYREKVPFKILPYVF

>SG-PL_contig_6032_1_+2_1031_1222

MKLTCNFMEKQLNQTKQKHKHTYRRKVLTCRFCSFSKFTNFWLGPLQKHCYVDHSHMRDR

LSFT

>SG-PL_contig_6033_1_-1_19_273

MHAHEHTNLHSHGHAHTHTHTHTQTKYVACICSDHISVDHILTCPKLKIYFPAVIISPIS

VIFSSPLLTYNFSQICTKSPCGFLL

>SG-PL_contig_6034_1_+2_761_1096

MSIKANKPPTKPSEKKTQTKKTLIQTSYKHIQHFSLSLFLFFFCFFFFCFLWCWCSFFFF

FRFFFFLFFFFGGGGFFFFGLGGGGGGGGFIFQLFSFLKAFFFFFFTYFFFT

>SG-PL_contig_6035_1_+1_145_255

MHHVYLILTLAPACAYFLHMKKGEQNFVPRAEGIVTG

>SG-PL_contig_6036_1_+2_2_97

FFFVCQIFISFYMFSQTMIQTHTNQTQLHIAE

>SG-PL_contig_6037_1_+3_369_482

MCVVSSVNYICSYSLFMCVVSSVNYMFLFSFYVCCFVS

>SG-PL_contig_6038_1_+1_1_111

CVFFRPLTTYSYSLFMFVISSVNYRFLFSFYVCCFVS

>SG-PL_contig_6039_1_+2_1346_1807

MKNLIIKLSHKLQWKCSFTFTSNTHINTFAITELQTQHKVLLQTQYSTFCLHRELHSTVH

FVFIENYRHSTVHFVFIENYRHGSVHFVFTENYRHRTKHFVFTENYRHSTGHFVFIENYR

HRTGHFVFIENYRHSTGHFVFIENYRHSTKSTLS

>SG-PL_contig_6041_1_-2_2_268

HTPLSLSFSLSLSVSVSLKSAVSPSLHSLSLFLPLPPPFSLPSLSLFHLGPSHLVIFPPH

SFLSFFLLFVSSLSVWLFLFEFCFVLFLR

>SG-PL_contig_6042_1_+2_2_250

ENCVKACWKKVEWTRKAEKKQMTVKERKKAETKRAIEEETFSTEFPGGDAIYNRASALTT

NYPIPPQNRLKKKGKGCPITLKS

>SG-PL_contig_6043_1_+2_62_547

MISGCSSSGFSSSGASSFGTLTSTVTSSVSDGFSGEMVISSLNCSCSMISGCSSSGISSS

GSSSLETGTSKSTSSVSVGFSGEMLISSLKVSTSSGFSTTIVSLITVSVVSSGLAVSWGT

SSTLSSVTSFTASDTSSVIPISKTTSSDSVGFSGEMVISSLN

>SG-PL_contig_6044_1_-3_135_1607

MASMTRRMHSSVLGPIRLLLFRRKLRKFLQTHCEGALGESLGQRGQASRSVQTSPNMCKI

FEVPSRVEAKGLKQLYLNPKSLIDDLLQPLITSGVTVAPQLALTPRQGGRFWRAKLHLQW

PSNVIVSAVHTSVTGAEENAYLLACDLFKNIGLLYQPSLGSMTPVSQDYILTVLHTLHSC

SWHPLSAQSPQFHITTVPAHVSSYQRSLTACSSPNDANKDRLDQVWRTNIVTSWPYSFDV

CALGPSSQVSQYAAALHTSIKLKTLGLLSNNNRLAESRLPHISRRFRSQAWTEATVCREQ

FDPHYEIYSDASAFGFGAYLVNKDDDRVRWLANTWSEHFPDSPQIIRGPREGTVSNSVFL

ESTFCELYSVVTACFTWKHKFVGKRVLVWTDSLSTVHLVNWGVQGETKQKRWGKLFCILA

ATCQKYSIELQASHVQRTDNIAADLLSRSRLAAFKEVVPEAVPSQKKTKKLLFWNPLQSV

NISAELHHKFS

>SG-PL_contig_6045_1_-3_84_179

MTCLQLVLSYVSCTLLTVKDLDVLLHITWDND

>SG-PL_contig_6046_1_-1_52_273

MAFSRSGSEVWAWASSAFSHTRNIREVLNAAYWHSPGTFIHFNLCDVSCLREDSSRGIAS

ALATQHYFFFMRQE

>SG-PL_contig_6047_1_+2_164_376

MDIQPSTSTGSLASHQYSSADAVNIILNDNPSESQFTADSDSGEEFGGRVDSRFNWNGKI

NTDSTDEETKS

>SG-PL_contig_6048_1_-2_2_205

YSWKLLVLTETYRGLMYLNCYLQNRSINLANFFGGGWGWGVILDKADCCIFYLIFVENNG

YLLLIFVD

>SG-PL_contig_6049_1_+3_171_314

MKQHHFLQVSHIGLHQCHYKTARKKRSSMMYTKVYPTNTKLHFKHKMS

>SG-PL_contig_6050_1_-3_3_125

QFYMCSAKSASDNLADSLSLHKTLHTGKVLKWKRGGEPTKH

>SG-PL_contig_6051_1_-3_222_1523

MYRTWDTNGVLFTVSTQDNKKRVTMELKDGRLQVLYNLDNKDNILLLDNAKASDGQWHVA

HFKRVMYQATLTLDAGEGRNYAFQRVEGSNVYMTLSTTSSMYCGAIVTNPSGAQVLDRDL

IDTCIQDVRLNKGWLPMKSSENTFGTVANYVKEENVVNGCTRPDGCKPPPTCPANMICVP

LWGRHECRCAPGYHALSPLVCESDCVPNPCFQNASCSIQNGSVSCNCIWPWGGDLCNIYL

VDDDEPSVGIASTTIVAIVVSILVVILLVLIAFFLATYYMRKKEKDDFDRFVVEDDADFD

IRENVMFYDEEGAGEEDQNAFDLTKLPAPASETMRPPTEMPLRRTEMPRARRVPDDRPDV

GDVLHDRLGDADDDPDAPPHDTLITFADEGAGSDAGSLSSLGSSSSDASQDYDYLNDWGP

KFAKLADMYGAGQE

>SG-PL_contig_6052_1_-3_141_2249

MADKRRLQGEIERCLKKVTEGVEIFEDIWKKVHQPAINSNQKEKYETDLKKEIKKLQRLR

DQIKTWIANSDIKDKQPLVDSRKLIETQMERFKVLERETKTKAYSKDGLGAMAKMDPQTK

EKADIITWLSSRIYTLQVQVDQLECELETMECTSKKKKMDKEAGPRSDELKQRRDKHKFH

IKHLEMAMRALDNDALQVDQIKLIKEDVDYYVEYNQDPNFEENEYLYQDLDLEDIAGGQA

LAFEGEDHDKVDANSTNSNSPSPSPSITANHSTVVEKVEDKERKRHKSQSEEKKIHMTPQ

LISKPQNAPTKQPPQSSTTVTQNATESPSINNHHPLATATPPLTAYAAAASSQQNTAADS

KPLVNSIDHNSINSNSMPVPSVSNSNSVSLTSSGNPSVPSAPSPHINSTGSTIPLGPMSA

STGINSIQNNSSRLEMSSVTNGPHSTGSHYKNQQESLSLQSIAQQAVNSAGLEARVPSQV

QRTDTSSVFTSSQAPPQPTAPSPDDQSVAASVLTSQTLNVSQISASLSSSQQNAAPQTST

IQLNPLLGVAPLGPVPPTKENTYQLAMTEAAFHHLPHPSDSERLRHYLPRNPCPTPNYYQ

QIPPTQADSLDFFSKLSTETLFFIFYYMEGTKAQYLAAKALKKASWRFHTKYMMWFQRHD

EPKSITEDYEQGTYIYFDYEKWGQRKKDGFTFEYRYLEDRDLN

>SG-PL_contig_6053_1_-3_405_698

MSICMRESKTCAYFQGDVCVAACCTRGQCFSACLLGAQEQLLEYENVGWYLVLSLWMSKG

HWQELTSVSSWYIFGLFPGPSRTEHLLCRYESETNNLS

>SG-PL_contig_6054_1_-1_2452_2721

MLELSAVSLTEIQVTVLQMWSWGRTMTCCGSLHMPYSSDHSMREVITAIIYVHFSCLGFC

KATEIISFLCCTLNYCTFHNRIHTCMLYPK

>SG-PL_contig_6055_1_-2_32_1006

MYDRDELIDRYHMALADRETLLQQNYQLQHKLAEYFRKKKNDERQDYDKNVTDQEQRYLK

YMAQLDELHHQDLLERDNYQNQMEELKSRCEERRARVDEERNGFVEFKKQVALNSINSRS

GKPIAPKDIEQYLITENKKEAEVVNVRLENIKLKNKLKKKEQQLKSKEELAEGLHLIDFE

QLKIENQTYNEKIEERNEELLKLRKKITSTVQVLTHLKEKLQFVQGENHIHKGKLQEVET

TVAQKRDVLSRTKQARDSLRIDNQRLRQNCGLLGNEPLLRDFEDRKDEGDDLKSQLDRLK

MQHAELTLNCNQVRRKIEQARNVRQ

>SG-PL_contig_6056_1_+2_236_379

MKTSPLMLNIIRYKSKHKSRVHDYAIYNIKSFSSAESCHQPNTAWRRH

>SG-PL_contig_6057_1_+1_520_657

MCNHISYSIHVYTCSRFLHCHGHIVCQCKVMVNMNLGDFPSKINIL

>SG-PL_contig_6058_1_-2_11_487

MAAPPEKKRAVLFICLGNICRSTMAEAILQHLVAQKGESDKWLIDSAATADYNIGRPPDN

RTMATLKKNKITGYSHKARLLTDEDYNKFDIIFGMDDENMVDLQQMKPAGKCKAQLRLLG

DLDPQKDRIIVDPYFTSGMDPFDKVYQQCLRCLTAFVED

>SG-PL_contig_6059_1_+1_520_642

MSLFYLGLFFLNCNNSYIYLHCYDTIFALMFCFDNETVLLC

>SG-PL_contig_6060_1_+3_3_62

PSCAPKISQQNKNIKYLHVL

>SG-PL_contig_6061_1_-2_110_256

MSSVLNVHFIIQMKILLCPYGMLTITRCFISSSELKVELSVPDKRTKKT

>SG-PL_contig_6062_1_-3_33_1691

MSYGFKLVGDVTEARASGMLKEVEDDLARTLRTLRQRQGGAQASKTESDSEVNEYQLNVA

LHTRVRFYRHLYALLLVFSRDKIEGLPQAQKLITQLQELVPLTRSSLHLGIQAPNREASK

NDYSTIMGFEPLINQRLLPPTFPRYTVIRGRAESLDYVTNLLQRLHTITTVTELGSLHST

MEVFVEFSKSLPCVLARSLLQLTLLPPNRRIFGTTQVVDALKETIRNFIAPPVLSPKCAL

YNNALAKEYVDALLTRAARPMCALFQITGHNRARQRDKWAHMLEDLSNLQEEADKVDAYL

HGILLKTEANRSHLACLGTWVLYHTLQTMINYTLAGFELELYAAFEYHYVYWYLSELLLA

WLVSTLHRADNFLLGHETLSDQQKGRSNKKNKKRKRPKSLSREITLAQAQQQMFGAYFKA

VMGLQLDGKMKQPNFKFDNEEVRFSHRFGPFTCVTTPPMVHYAQYKEMSDLSRYDREPSP

ADLYGAASKGFHQAKCMLESLVTSSDEVQALLRVSKTNYVVMKLLLGGHKKDSTDPPEFD

FSQHRVYPVIKIV

>SG-PL_contig_6063_1_-3_18_293

MLLRSLHTQSSVDVHTRYFRFSISHQTLTCLTGSSVCISDDLHAYTHKGGSGISRCAHMW

TWEIRKISTLYLPGGTQLRSSMSYSLGYGTHL

>SG-PL_contig_6064_1_+3_105_1256

MADNLVNDMDPEARQRYMQLKDLNEHLLRQLENGQQELDQLNSKIESLQEELSQSQVKQE

AVRLYDQLHELETKRNSLQEEARMRGTPAEERETLLKQVKEDNQEIASMERQTKELQEKI

DQMRDEITQLDLDIEENQGERNQKYKELKKREESMDEFLGTFEENKAQELRKISEVESNV

TALLENISRNMGRFTNLPTPKDLMSMKEDLEFKQDEMKKSEMTTIELAAQRDKLQQDLAK

MDQFEEKLREEQVILQQKISDMEKELAVYSDLEKLRSDAEDKKKKLMDDRVILQRRRESF

KKRMQHLTMKYEGLKTQLNENETYTQLSNLEKKWQHHEQNNFSMKEFISARTMECDYTPI

SRRVYTSLDDFNSKLQSQMGKTIY

>SG-PL_contig_6065_1_+2_245_430

MEKGDDISRRGASRFWLEDLRRRTGQKCLQGPTYPRSGKGDGGGKGEGSTGYRFQVGKSG

RS

>SG-PL_contig_6066_1_+2_2_91

ISPNHCGISAHCTFDNKHLTFNQLALPLNS

>SG-PL_contig_6067_1_+3_3_263

SRGNSDQAQFSANIVDESLIYKLMYDELGQSGLCRRKSTLNISSLDLLHFDRVNNSEMGM

KMRLWMSSFISLGDIFRSSHSHEATEN

>SG-PL_contig_6068_1_-1_205_489

MAESARGMSRGRSRWGGGGGGSRFGGGGRGGGRGGMGGGRGGGMGGGRGGGRGGSRGGMG

GKFNSYGGGSSSYGSTNMAGSSYQQKSNSYGQSWS

>SG-PL_contig_6069_1_-3_78_209

MTVFFISTLISSCVNSVTAYFSQLVKFTCLFQETPSLSIRSPCS

>SG-PL_contig_6070_1_-1_64_462

MASHTHFEEGSAPSEVTETQPDREQGSDSKEKEQTEKTAEQKEEEKLRAKYPNLKKAGPP

AMIHKRLVQKGKYFDSGDYNMAKAAINNPKKPLPPQEKMLLQESIGEGIPTPENLPPRKP

SIVQSKLASGALS

>SG-PL_contig_6071_1_+2_2_274

IIIVVVVIIIVCDDRHHHHHRHHHRHHHHHHHHHHHFHIELLLHVLQLFCFHFKEKNLLC

KAKKIELYSRVHIQIAFSKLVILQHHALLRD

>SG-PL_contig_6072_1_-3_102_1094

MHNDAELSKEAAVNELQRLEETVYAERKKREVELQKMKKEAEEKKMQHERIEKRLAQRGS

ITQDDLSPQEKGALSGEEQQQKITTYEEAFRLIKEATGVADTWEVVERFENQGQTTQHLE

ELKKDNEKQILRLREDKENLQREFEEMKYSGEAKLSSGQTILEEVENHLAKEEKRRDDAD

ERLQRASRTLVQVKAGVEHLADKLHHLKANKGHVPTAQIAPSSDEYVLDQLSTSEEKLLK

LLEELDGKDLNETLRQMEEEEFHASIEGKLPQYNTRIKLPTTHRDFDYGDDEDSGEDDAD

VPSRSTIKKNSQNIIDAKTKRRVAKKKKKTK

>SG-PL_contig_6073_1_-3_3_158

PMSGLTGIRKCISFNRLCPYLGSLWPKMFFDQGFCCCLSTMFVLHAYSIKEF

>SG-PL_contig_6074_1_+1_790_1236

MVLQVLRGLNIYPVALIYGPPGLKRLQHLSRAQIYGPPGLYPVALIYGPPGLKRPQHLSR

AQIYGPPGLKRPQHLSSGTNLWSSRSKEASIYPVTQIYDPPGLKRPQHLSRAQVYCPPGL

KRPQHLSKAQIYGPPGLKRPQHLSSGTNL

>SG-PL_contig_6075_1_-1_742_969

MQLSEWTLYCTICVLHVLMFFVWCFSVSSLKLHQKLCNDNECVSAHIVEVCMFKRRYGRG

FPRKSIVCLLINCVCE

>SG-PL_contig_6076_1_-1_16_306

MLNVQNKNSSYFVEWIPNNVKTAVCDIPPRGLKMSSTFIGNSTAIQELFKRISEQFTAMF

RRKAFLHWYTGEGMDEMEFTEAESNMNDLVSEYQQYQ

>SG-PL_contig_6077_1_-3_147_290

MTEWSGLHTLHNIGENQKRREMPIIVHSQLFSQFAPDEESRTDLEQLS

>SG-PL_contig_6078_1_-2_1247_1492

MYVVMHSQGGVFWRAGSVQGCMTCVYGRRGCVLVLMSIIYKSVLPLFFIFSYSSVFRVSF

FPSLRSRMFKMFTFVSDLAKFN

>SG-PL_contig_6079_1_-1_7_636

MASTLFRLGHLGITRTFCCKCGNYAFSSQLSRLVGNQQVSLPSSYLAVSSYLTAKPYSTP

ASGNGTPAPTREETAEPDDLFRQVTVEIKCHDKAVMKSYRQYVTMAAGELGLNLARVFEP

PKVIKRYTVLKSVHIYSKHMVQYEARTHFTVFELKHLTGSTADTFLEYIQRNLPEGMAMK

VTKQRMEKIPDHLTPPAQPTSHTASTSPAS

>SG-PL_contig_6080_1_+2_119_436

MFCHKTPHPYPGVSWSWNNFMHFSKLRSHFERERDGGVGSKRNNPCTGLPSAPDTREVPF

STGKSVLLCKTKIKNYCCPPPSPMPHMSNIICEKGSDHLHFLLDFE

>SG-PL_contig_6081_1_-1_64_705

MVDAKYCEKHIHEVKEDGSINECQRQIALADVIIINKTDLVTSEELHKLQQEVRSINSFA

KLHETTKAKVDLGSILDINAYGGHGPASMEDLFGQRGFSTADVHVIDKSVTTTTIEMDGS

VERERLDGVVQHLLWEKNINNSKGNPMEILRLKGMMAVSGEDQRMVVQAVHELYDVQFTT

PWGSEQRKSTVVFIGRNLDHKILKEQLESCLISR

>SG-PL_contig_6082_1_-1_1_120

CALFLFFFFSSRRRHTRFRGVTGVQTCALPISTGVCVRVD

>SG-PL_contig_6083_1_-3_168_353

MSSLLQHLAKRTVGHKHFEQNDKDFVLLPEILFIFFINHISFSLLPRCFFHLHFCFSNQI

HQ

>SG-PL_contig_6084_1_-1_1_210

VVFLNFFGIKEVSRIKFHVSPLCRSFFSFLFFLPFSFPFCRSSFHPVFLFVSFPSLFPLF

SDFSVHSFTL

>SG-PL_contig_6085_1_-3_3_653

GQGSVGSQACHTPTWKRLCLSNWVVEVIEEGYKLPWEGVKSPLRPRPPSPRQIPHEQDAA

LASEVADLIRKGAVEVLSHPPGPGFYGRLFVVPKSSGGWRPVLDLSPLNEFLVRIHFKME

TPALIRASIREGDWASSIDLKDAYFHILIHPSCRKYLRCVWKSQVLQFRALPFGLSLAPY

VFTRVIRELASLARGEAIRLSCYLDDWLILAESSSLC

>SG-PL_contig_6086_1_+1_124_273

MVSVVTAGRPISNTFLNFPHTGFLLLLFNSSPSSLVWTNQKSCSILSSAC

>SG-PL_contig_6087_1_+2_110_259

MVLVVTAGRPISNTFPNFPHIGFLLLLFNSIPSSLVQTDQKSCSILSSAC

>SG-PL_contig_6088_1_-3_3_116

FFFFNCDIILGQLALKEMHASIIGHLETAVPDGQFFKK

>SG-PL_contig_6089_1_-3_675_911

MYSNFSLFERDTSHSDCSICASPKSVSDKHRFFCVCVCVDILMRLINTSSFIHDLADSEP

ARNPIHRQDLEMKTRSRDL

>SG-PL_contig_6090_1_+3_3_266

REREVTYTATHGRHLSQCSDQQPVTHRAERGSGQYKPQFLSIYWPPSVLKDYGICATGET

PGHKPGQSVKENLRLYCAGTHRLLASIK

>SG-PL_contig_6091_1_-3_171_1490

MSQERTSSHVLHVRENSASEMEALFKVVDPHAQEANKSIPFRDRNLPASFFQQPGLNQMG

IPKGGLETPGYAEVGPVSHMRAHSSPASLQQTLSAAPPPPTHQHHRQHSCELLDEPLPPG

WGMARTPQGQRYFLNHMLQTTTWQDPRKSHSPNSLGSPQQPGSPASGTQTPPGAMDVTKL

PVPHGWERAFTPEGEMYFINHVDRTTSWFHPSIPAQQQQRVGMRPPQQTAPSLQPSPQLP

SPQQQQMCSPVSGGTTTPVAVGGVQRGPPTSAEQQRQMKLKQLQMEKEMLMKRQEELNRQ

ELMIRKGVPGAEAADQATEMTTVADPFLGQTGSNTDHSRQESADSGLGGMGTSYNLPRTP

EDFLSNMEEMDIHDGGPKVQGQGDFGSMDIASVGDASDSHNMDSEDLVPSLHEDVISSEL

LKDVENVLNSNKVDNLLTWL

>SG-PL_contig_6092_1_+1_298_459

MRSVQQPVLVNRCHQVLFLTPSSEKRCAINVHDATAEVGQNTDTMEPTSWHHNA

>SG-PL_contig_6093_1_-1_286_468

MSEVTLIFLNLCKQKNSALALLVYGVNMFVKNHKFLDISSHLLLTVSPSTTKFGTDRGPL

R

>SG-PL_contig_6094_1_-2_1346_1546

MTYYVYGYMSLLVLRPFPFIEIQHEGLSTMKQIETPTEKSTFNKANSARLLSQESCYCER

ILRLRVV

>SG-PL_contig_6095_1_-3_270_608

MQAPCRCPKYGTACFRCKKNHWSKVCQSGSIGFWPTPMTAVSSQAPGHRNTNRGRGIASH

LVPLVDSIASLTTVMTAFPVTWSAWTSCPSRRTAALTTKMRCMPVKTSSTSAQ

>SG-PL_contig_6096_1_-1_1_294

NFLFFIALFVSALFPSFTVVCFFCLPVHSIFFFSSKPSHNFLSLPVHPQFVCAILLWRLG

GEIVGAGIGTGCLFCGVVCLYDLLKICFVCGLGVFFLL

>SG-PL_contig_6097_1_+3_3_281

IFDDLRACAHEGGSGTSRSAHMLTWETGKISTLYPPGEVPKVRTQAFQTLQFGNPVFSPF

DHHACQEMTFLPQMLSHFQFDLSIKKCVLLIQT

>SG-PL_contig_6098_1_-1_103_633

MLWDTAGQEEFDAITKAYYRGAQACVLAFSTVDRDSFEAIETWKKKVEDEVGEIAMVIVQ

NKIDLIDDALVRTEEAENLAKKLRLRFYRTSVKENLNVDEVFRYLADKYLEQLNMVVDEI

PSSPQKIGQGLMQTDTPKNHSKDKSSKHKHDGTISLQPSKRRTHGKKSLISSKCNLL

>SG-PL_contig_6099_1_-2_383_556

MVVFAGFKMKIHTRLGNSSSIDCNNYVTSLTWYVNGTVSDVNAGTVHSHPPPPSPHPH

>SG-PL_contig_6100_1_+1_1_240

KACWKTESGIDREGRKKADDSKGMKKGKSKDSNRTEEISSTEFPEGRDTIYTERAKVHDH

TVNSNSIPIITKNNNRKKQQ

>SG-PL_contig_6101_1_-2_140_1126

MAKRLNVIFGHLHPQDGSIQFRSTSAVDIGSGISSQKFLYTVDDVPSILTQEQRKFYEDN

GFLVIRGLVPQEKLDLYRNRFKEICCGDVKVPGATIMKDVAIAKSEFAQGERAITKLQDF

QNDEVLFSYCCLPEVVKYVESIVGHNVLAMHTMLINKPPDAGKKTSRHPMHQDLHYFPFR

PAERIVCSWTAMQQVNRENGCLVVLPGTHKGELQAHEYPKWEGGVNKMYHGIQDYDPSQP

RVHLDMEPGDTVFFHPLLIHGSGMNRTDDFRKSISCHYASDACHYIDVTQSVQSNIAAEV

MEIAKKRIGDADVQFDFVDIWKLKSRPVN

>SG-PL_contig_6102_1_+3_234_455

MVESSTSLGASTMTVVSISSPSLLYFAQPCTGLATYQSRLGLTGAIAVKRMRQEAKDKFY

RSWSTCISFICHLS

>SG-PL_contig_6103_1_+1_1_189

AAVDLLMQKRRCASPGPELRLKADQEVVARPWHQARHKDCHVQSSSALLYCCETWTTYRQ

HIQ

>SG-PL_contig_6104_1_+1_1_243

GRVYDAKRLTKRLWQDHGIRLATKTAVYRAVVLSALLYCCETWTGLRRTDNTFNSWNSST

KDVCARSATLCGKIKCPTFKF

>SG-PL_contig_6105_1_+2_1133_1450

MKALGDVETWATLQLSDRGQLDNCPSPQRIISFYRVWCSQISVHVPKLIHLKKDNKPSLK

TFIFSASYMNTEHHLCVHPHFLQWGLGLPVHEFWTRHYLDTYIIFQ

>SG-PL_contig_6106_1_-2_71_280

MGHQPIMLKYHGQCLSRYKSDYILSSNNCETDGNKLLRVWWRWVGLLTEKLAMLHSMYPF

DTPQQVRPPT

>SG-PL_contig_6107_1_+3_135_242

MKAIVKLLFYNAMVKSLLYNLAFPSHIIPATRFDLE

>SG-PL_contig_6108_1_-2_50_2287

MFTFPKAVCLSLKHTQRAVPVCSSYRLVHRQPIDEEWKKAAAQELKGKDVDKTLMWKTAE

GINIKPLYTKEDVKEIQKEIPGQFPYTRGPYSTMYTHRPWTIRQYSGFSTVEESNKFYKA

NIAAGQQGLSVAFDLPTHRGYDSDNPRVTGDVGMAGVAIDSVEDTKLLFDSIPLNKLSTS

MTMNGAVLPVMAMYVVAAEEQGAKQEELAGTIQNDILKEFMVRNTYIFPPEPSMRVIGDI

FEYTSKYMPKFNSISISGYHLQEAGADTALELAFTVADGLEYVRTGVTRGLDVDKFAPRL

SFFWGIGMNFYLEIAKLRAARQLWAHLIKDKFAAKTDKSLLLRCHCQTSGWSLTEQDPYN

NIVRTAIEAMAAVFGGTQSLHTNSFDEALGLPTPFSARIARNTQIILQEESGIPKVADPW

GGSYMMESLTEEIYEAAMEIIKEVEDLGGMAKAVASGMPKLRIEECAARRQARIDTANEV

IVGVNKYRLPEEEGVEVLSIDNTLVRQKQIARLKQVRETRDSAKVQSVLKAITECCRSGD

GNLLALSIEAARARASVGEITDAMEEVFGRHVASDRMVSGAYKSEFGQEDEISNVMKRVE

SFHEREGRRPRILVAKVGQDGHDRGGKVIATGFADLGFDVDIGPLFATPKEAAQQAIDAD

VHIVGVSSLAAGHKTLIPQLIEELKAMGRPDIIVVAGGVIPPQDYNELYEHGCAAIFGPG

TRIPDAALKVLDMVENGADNKKSQAM

>SG-PL_contig_6109_1_-2_2_172

AYPPASTRKFVETGGLAEWVKGLGNLKHSLQHVHDCISRLCANILHIMIVVYCPSCG

>SG-PL_contig_6110_1_+1_250_498

MFLQFLLHSASLMNEFYYNSSPPLPDLPLPSTPTSTYQHFVLRQSILSPFILKKKIKNLG

QYFVEQLVQLVELCCLDSFTWVA

>SG-PL_contig_6111_1_-3_2238_2447

MFLFFPPSRLCQTSASSSVCVCVCVCVCVCVCFIYVSLFFHILNVYSLMKKNNSFKAAFC

ASDLDIFIVV

>SG-PL_contig_6112_1_-2_50_262

MKSWKDFVKPVIIVKIYIFWQIWDTLMLTHDSPKCVLSSVYGWCDLLLPEFCICDPYLVQ

IFGLNLVKVPR

>SG-PL_contig_6113_1_-3_3_1151

MNESYGTLIHYVTLTTKGRTRCGKNQSIMPGPGPQWTDFLSCEVCYKLFSEQANRPISLG

CGHTVCKLCLSRLQQNKCPFDQSPITCDIEKLPVNYALLQLIGAAIPDKVIDEEEPVGDI

SDAKKTECYESARKCIEELAVYLKPLSEDSLNELEQNEDLTDVKVKSQQSLTHSGRSMLN

TSSHNTGCVLSRPMQRKLLVLINCQLVEEEGRSRAARAARSLGERSVTELILAHQNPQQL

SANLWAAVRSRGCQFLGPGMQEEVLRLILLALEDGSALSRKVLVLFVVQRLEKQYPQASK

TAIGHVVQLLYRASCFKVTKREEESSLMQLKEEFRSYDTLRLEHDAQIVQIAMEAGLRIS

PEQWSSLLYGDATHKSHMQSIID

>SG-PL_contig_6114_1_+3_66_209

MIIIHNKCSMDLYRHVTSWKLLNININTDKMFWFVAFNEVNKHLIRTY

>SG-PL_contig_6115_1_+1_1027_1302

MVTQKTNQNSYADIFVHNTISKKERIAHILYFMVLRKGHTCNQKHWVGQCHKKQTGKVKM

EAEMTRDDTIYTVEQIDLNMTDMYTSCALTDN

>SG-PL_contig_6116_1_+3_2043_2330

MQGVRQQTYHQIMEFMVIRFITTTNTFHYLPADKLVPFCRMDLPQCIPAHFIDILSLKSF

SHSIFYSLHPSPIFMMCASVRVAVSSFFQFFHLRNH

>SG-PL_contig_6117_1_-3_165_1526

MFLEHNCADFGMEKEKFPGDSVVTGRGLINGRVVFVFSQDFTVFGGSLSGAHAKKICKVM

DQAMLVGAPVIGLNDSGGARIQEGVESLAGYADIFQRNVMASGVIPQISLIMGPCAGGAV

YSPALTDFTFMVKNTSYLFITGPDVVKSVTNEDVTQEELGGAKTHTTTSGVAHRAFEDDV

DALLQMRDFFNYLPLSNQDASPIRSCDDPWDRIVPGLDTVVPLETTAAYDIKDVILGVVD

NFEFFEVMPDYARNIVVGFARMQGRTVGVVGNQPKVAAGCLDINSSVKGARFVRFCDAFN

IPLITFVDVPGFLPGTSQEYGGIIRHGAKLLYAYAEATVPKITVITRKAYGGAYDVMSSK

HLRGDINYAWPTAEVAVMGAKGAVSIIFRGSDNQVEQEAEYVEKFANPFPAAVRGFVDDI

IEPHTTRKRINRDLSMLVCKKQSNPQKKHANMPL

>SG-PL_contig_6118_1_+1_517_729

MCRCPTAFSLLCASFTLNLSFLLLSACSVLQLHWTSLSCCSLSYCFQPALCFNYIGPPFP

VVPFPIAFSLL

>SG-PL_contig_6119_1_-2_2_232

AATSKSPQSQPAKIDGSRGRGRSRQKEQAKQVKSANQNKRSRMSVSKDSEEESDDSPVRK

KAAGRGRGRPKKKISSE

>SG-PL_contig_6120_1_+2_356_547

MKQIKRTVVKIWSWAQAHSSASVEGSPQLHERFGPDVQFTVVLHFEFTFTGSLWCNTLLH

ASKC

>SG-PL_contig_6121_1_+3_3_506

QKQFDRVCQEIFHGFPASSATAPNPESILSPEELKPQFIATDPIPESVLEPESGQLVSTP

LWTISNPEPSSGNQVSRSPQSTSCLPPLLLGPVSPDVSSSCCLRVDEETPRKAKYRRRLQ

YVSKDRAQLVRRHREDMKKLRKTLKLAPHETRVLKQRLTRVNYQNCYD

>SG-PL_contig_6122_1_+1_763_1056

MCNPSKTTTTTKNLNTLCLYMYTSAMDTILPKEKKKAWSPCSELNKKVLTLCPKVNIKQT

IDSDSDATELLPCSGQHCTPDVPTKKAKQAHCTVSCHL

>SG-PL_contig_6123_1_-2_2_373

YLSSLEDEFPVPLHHNYILVELYSADTFPIFVNILHKLSEAQLKPWQQGVPQSLDHWTLF

FAAVVPALAIVKATISLIIQTRGSEFKDMSVLSTLLELHTVMCSVPSSTLYSAETLKVQK

DIVD

>SG-PL_contig_6124_1_-1_1_144

AICWGLQLSCDFVFFFKIVLLCCPFFLSATPRKLRLVYFYVQTTDQTW

>SG-PL_contig_6125_1_-2_23_4276

MMREVVVAQSVTPPWKEIVVNTGASASTLQDIKVPDSCGGFTYRESSCPGSPVRNRFIYW

RTWGDVLELEESSLDMMLTGGKIRYRLQDTPIVGGVSISETNSHVIVLVATVASIHRLVF

PHPCRRQQTEAFSLSEHISTSVFFDASLTTATDSKNLHLLNPGGSITAHLVSAASCVSGD

GEALFALGTNAGTILLLRMPPVGITGVVIQHELSHSSVMQKIWSGLVPGAIRGHQQASDT

PISVEIQPLRGETYIFALCRDLRLRVWATRTRECVLTVNLQDMLGGASRLQPSSAGHVIK

QVQSSTMAALTLCVYLSFPDKSQFVLLEPVVCDGRLSMLQHALLTQPAEDLIDFCVTADQ

LITLWTKSDGETIARAAVLTEGGPTGEWRDVLLEHPDTSEPLIPPNRDPRDFFLNLIFRP

NHFSHHDIIRALTVYRRSHDSTQNADALLMSINIREEVINAVSLEIRNCASEYEMQEEAY

DNLQREQWGRFYSCCCQYREVGAKLQGVFTDTATGLVCLIRKATLTYLRPCDQLERLYLC

EQERITPKDLDDLSYNVEGMSQYALCEDLRKLCDIVQLVRSEITEEVVTQFDLELQMMES

PEELAERLVDTVFFEDVDVPSEVGAQIDVRLQTMHHLTACLELMQLLMDVLEMQQQQQSM

MDDSVGDAARQLSVSHLFTSPEACAILGQAFEQMCVTRMSFLRDLIVVQILALRLGQKRS

VSPEMCQTIQEELLPNFSQLLRSYMLLYWSSQALTSPSPTSSLDSNMRRLASLELSDSGG

FTATRQTTSDTTVLESFIQAVGGTQARQQIAMTTEQDFQATWSHDLMAFVHSLASLIWPL

SDETVFPEFLAGSCQYSALQEYVRLLHPWCVWNSSATMFFLGLSYLYFDEPHKAQQCFVS

ASDGVATETFLAQKLLQGHSGNHRQLTLDYYLKVIVQFQEYGMPDMVIDLANRALCLADL

EDPNVATLQSKVFKNQLELGHHQEAYTAMMDNPDPARRRDCLRQFVMVLCEKGELEDLVH

FPYRDLEDEVVAVLERRARSVDLATYNYYNVLYSFHIYRNDFRKAASVMYEHGVRLGREM

GGVRALQRQAQSYLVAMNVLRLVQPEYAWIVHPQLQSLEVSVTDKESGGDSPSKHTVDGV

EKQPAKGKKKVQIVELRQLEKDYLLLDARLRLIRKNPDPALASGPTPAADEMVGILVNNG

LYDMAVIVCQAFGLQLFPVFTSLALRCVTLARCSSYFMLGDNKETSEAWDWLRENDIRST

TQSTDNSATDHVWKLLQRYLESFEECDARYHRCVCEKLLSQAFPLPAWLVNSYKVWSTHQ

LLRVYLDYDLLQPAAMLLMEYLDALMGVLQGQDAPVFGLKGILDHRPMSVWVPYTCADQL

LGALHGHQEDPMFTQLHGQLTEKISTYHHKVSQLSDES

>SG-PL_contig_6126_1_+3_912_1094

MQLKPHKVNGFQSVVTAGCFTQQVSQDKGHNIKSSEQKLRKKCVQQAIKQNTEQQLLLVH

K

>SG-PL_contig_6127_1_-1_1_144

CVCVCVCLKISLIIHRSLPLMEFQMYTSEWTESIFEITCVHKLQNFLY

>SG-PL_contig_6128_1_-1_16_1023

MATLVRRLLGLLTPISVRTTCMRCLSTRLAEVGVLEPPREQGKAYETGQLFLHKIFGYRG

IVLFPWLARVYDRDIATKTEDRSLDEQTTTVGKEVKGRNHLYYQVLIDARDCPHIRAQTE

AVTFLGNQENSRALYAIPGLDYVAHEDLMPYSSAEQSPIQHELFDKFLMHDSESSPPFMA

RETLRAWQEKNHPWLQLSDVHRETTENIRVTVIPFYMGCREAQQTNVFWWRYCVRLENMG

EDTVQLRERHWRIFSLSGTLETVRGRGVVGQEPILSVEQPAFQYSSHVSLQAPSGHMWGT

YKMERRDGTSFDCKIPPFSLESKSDDNNSPHGFLGS

>SG-PL_contig_6129_1_-2_146_1096

MASAGIGKMYRVKPFFDSNAEIISHVNMYKVKSFESDVSGILSGGEVEDRVSKLQQRQQR

VLKELQQLQHSVQMLAQQRGVSLAQAKPASSAANTRTTAASYSHRSPAQGGLQDLVISAD

PSCPPLSLLVLLKQLSDQHQVLTSTFVHSSAVDTPPNLRTFLHNGPSPAPRCNYQLAVTL

VWKKLSSGPRLMVSPTRQTYIQGETNIARYLARLLNPAYDQGDITTATQIDEMLDLVQQQ

LMGGNNKERLAVLRTLNARLGKGSCLVGGDLTLADVVAWSAIQGANMAADAPANVRKWLQ

ACAQRPEFEEAMRFLSS

>SG-PL_contig_6130_1_+1_691_885

MSMRETKRMFMGNDVLSQLYAAVTKIFSPLPTACFYFVFWGIKAVNKNSHIDTHKRGCTH

RNTPP

>SG-PL_contig_6131_1_-1_94_330

MPVCVCEQIMPSEILRLSMEVSFLTKAGPHVAASGKVGKKFSSSRFFSVVDYIFAFLPVQ

LLSKIALKCLNLPLILYIH

>SG-PL_contig_6132_1_+3_408_605

MVKVPHHHELFVGRRLGTKWYPLLTSLFSCCFTYPTLDTNSKPGGVRTHALAIFMLMLQA

NFAPMY

>SG-PL_contig_6133_1_-1_121_735

MDFLQILFALCVLSLTGIDAIWFHLGQNQKKCLREEIHKDVLVTGDYEAHENGHRVDLSV

TDSKGHILYSKENAVKGKFAFTTEEYDMFEVCFEGKTMSGVQAPDREVYLNLKHGVEAKS

YDELAKAEKLKPLEVELRRLEDLSKSIVDDFAYMRAREEEMRDTNESTHSRVMYFSIFSM

ACLLGLATWQVLYLRRYFKAKKLIE

>SG-PL_contig_6134_1_-2_1118_1390

MFRCGEVRFVVKTFYCQVLHWFVGCWDSLRRGVLLCSEKRFIVLKCVALFSEVHFIVQRC

VCGFFFFKSVVVFFVKTAPLFRAMLLFVSLF

>SG-PL_contig_6135_1_+2_2543_2887

MTQLLVGKMKMNKNFLTMIDGYFPNLDSHVQHKGSSLMGNTYFQGHNGNKKCSLHLNMDT

LCMVNNLALNLFFEGLIYIYLTISFTMYDRLVYKFYFTYKPALPYFINNIQQCMI

>SG-PL_contig_6136_1_-1_196_423

MGIIDVLSFMSCVNHIPSISGLRGFHIFRWGEMFLDAFLLPCHTACVSSLFMNRPKILPN

MSSSKEALCRIAQIYV

>SG-PL_contig_6137_1_-1_448_732

MGLRFYFSVSAKNASFPLQFNENKGNCHCPRNLPLYCPSLLLGQFQKNSLKRLMLMIQLS

HTFQLIHFLFHGSIKDFIVYSPDLMTEFHCSFHHQ

>SG-PL_contig_6138_1_+1_286_516

MTTLGVKMVKEVQVTVALAWTQKSFCDFPFGMDTGVLFSSSKLVCMLSYCVIWNVETLIF

LKTYNFVPVSEVVCWQI

>SG-PL_contig_6139_1_+2_740_934

MGVTQISWRVFNSNFFFDKNHHQQTPLASISKLFWVDELYESHRISNSVLMNPFTPSPYP

PTTKT

>SG-PL_contig_6140_1_-1_1_96

VQNNLLCMGVVLALSALQTGAFPRNDTLYIRR

>SG-PL_contig_6141_1_-3_30_830

MAMPTYLLAFIVCDFQYLSDRSPKYPVEIRTYARPNRIQNARVAQNHAVELFDWFNNHFE

PKYDTYLSKVDHIVIPDFSGSGMENWGLIAYKKMLLYNSKFIASDRLWIAETIAHELAHM

WFGNTVSPKWWTWLWLKEGFADYWELYSLEQLYPDWKVRSVHLAFKVLYVMKLDALTDAH

PLNNPVETPNEIKNQFDEITYVKGMAITGMMHMIMGEHFIPALNMYLQENKFGNADSSTL

FDSLNKYISTTPDIDLNMTKIMEPWVT

>SG-PL_contig_6142_1_+1_1_165

TSTDKTNRKRISNKQMKAKMSGNTLYSHCLITSLTYMVKPGFSDAPYKKKKSFVS

>SG-PL_contig_6143_1_+3_3_134

LDTDLISLEMDKELDFGLQVIKHSLFFCSLKENLGLPLVSDQVI

>SG-PL_contig_6144_1_+1_331_630

MKRFHFSSDNTPTHTHTHNVPSSILRTHAHTVDKTKFFPSHITRQQLISRQHIHPTSNPQ

YPHTDDWPHHSSPCLHGTQLRIHFSLLPPSPPPTHHPSPS

>SG-PL_contig_6145_1_+1_1_264

QRLLCPEHQHNATRSQPDQSVHSLTKSCFFFSVTANSDKTMTIQCHDSPYTMFKKLKFQV

HKLYITLLITNAYKYLFPISVSAYLFDS

>SG-PL_contig_6146_1_-1_79_204

MTSRLTPYSEIFSPDLGASVENAMNGQARQRRRRFVATLMKK

>SG-PL_contig_6147_1_-2_116_316

MRGWLGYKCWFDSIFQCVPNFWGYYNISVSSWYLLVLLYLCIHSSFRVTDFCVCLLFNEG

LFYIINI

>SG-PL_contig_6148_1_+1_1_435

YLRFIIHCALLYLRFIIHCALLSISQIYHSLYIISQIYHSYLRLIIHCALLSISQINHSL

CITIHLSQIYHSLFITQINHSLCIIIYLRFTTRCALLFIPDLSFIVKYYLHLRFIIDYAL

LSISDLSFTMHHYLPISDLSFTVHY

>SG-PL_contig_6149_1_-2_215_382

MRILRSATTSLYCYILIMYCIIIPQAMILVDAFHRGSEILKNPCEAVGVTHACGCS

>SG-PL_contig_6150_1_+1_1_210

PPERLERQDGQQTGQFLCCRLGLNSRHQILSPQLYQQSQPAQYNHVVPMGVKPTTSVSPC

YQHGTLTKLS

>SG-PL_contig_6151_1_-1_1_99

TKKKKKKYKKFFIILIFKKKKKKKKKKSLNLHI

>SG-PL_contig_6152_1_-3_3_971

QTCCKLTSGQWGCCPLPQAVCCSDHIHCCPHGTTCDGASGICTQGPITLPWLKKQPAIVK

TLLKEPEKVEDVQCDATHACPNGETCCKLPSGQWGCCPLPQAVCCSDHIHCCPHDTTCDV

AAGTCKQGPITLPWLKKQPAIVKTLPQEPEKVEDVQCDATHACPDAQTCCKMTSGQWGCC

PMPQAVCCSDHIHCCPHGATCDVAAGRCDQGPITLPLLKKQPAIVKTRPQEPEKVEDVQC

DATHACPSGQTCCKLTSGQWGCCPLPQAVCCSDHIHCCPHGTTCDVAAGTCEQGPITLPW

LKKQSAIVKTLPQEPEKVEDVRC

>SG-PL_contig_6153_1_+2_20_232

MLCANITEKFFLSNLNICNKMQTYMFHHAHVLTIHWTKTFKAITVLSPTRRCHNTLKHNL

CFHKQCFIITI

>SG-PL_contig_6154_1_-2_155_1300

MLSVPWFLGWALRPYIQINRTGTPTITTDTRSGAAADDGMTSSVAAKAQVAQLSSPSAAR

HVMARTPSPTSPTANTPTKPYPVTIQPITAKDNSQTKSAPLSQPSTTIAKTTAPRTAAAR

PAGSPKPATPTSTKATVGKPGTPEDIAGNLVSTKTITLKMSGEWVQQQLEQFRFSFDEAD

RDKSGFLSLDEVFSVISKHGFKGSKDQAKQLFAQLDANNDQKLTREEFTAALDKLPRNTI

KEFVLRKTFQQMDKDGSGKLSRTEIEDATKSQAGPDVPAEKIAQLLIVLSKDTKDDVVDY

EEFLQVFGIQTTGDVMETIFRELDKDNSGYLTKEEIMAAIHAESELKLRAAKISDLLIAW

HKDSDKKINYQEFVQVWTKYKN

>SG-PL_contig_6155_1_+3_975_1136

MVPFLRIVHHQHTLPSPPMLAQCTLSIHITCVRFSNHLTSRKKWNCHSSLPWTL

>SG-PL_contig_6156_1_+2_2_229

TVTSTIQQPTFIHKKRQKVSSTVQSMMSSSSDQSSKLPSGKYLRMMKGIFLAFNSFMAMF

SGSISPSNSTMTGAHI

>SG-PL_contig_6157_1_-2_323_643

MYVCLCICCLRTDVGFSSTFCRGQPHCFMWAKWMLHMKPWLIVSAKRLALDHYSRLSGGG

EKSLSDRVLNLGFLFLFLFVSQGMFIRCYHYASTAHVLIVAILNHLA

>SG-PL_contig_6158_1_-1_169_516

MFIGNCLLFCRIVKKSIKITCFYSDIFIGNCLLFCRLVHQDRRKEDSVSNHKFHRIYIDN

CIPQLSLVAYVIYNNKNKSFLYSAILQTRSDSLRITYYLPPFLYCLIHQWSLCERR

>SG-PL_contig_6159_1_-2_476_619

MEVLDSDILLKKELVSSIVHSFHRVFEPQNHIYIYIYIYIYIYISIYL

>SG-PL_contig_6160_1_-1_88_780

MGVYSWCRQNATKKFAWVKAAAERANKRYEQAAKEYQSVLKAMLSIEDGAEMKESSESAE

SSPEHRLLNKKTSLENSSKQSSSSSKQSHPSPTTVSFLTNEVVEMYLKLSDWDSVLQWQE

QLSIWRSDTGLTHLQSAFNSSVDVNYIKAMASFELGNLEAVRASLELVPGASLSEGNKAE

PSNLHHWSPRTELAHMNRQFLRAATLLEEQRQNHRAEVIRFLNQAEKTGEG

>SG-PL_contig_6161_1_+1_1_183

EWLAPCSYILVRVGPAMAFNLRIERGKEEHTVHSSSGCLGKNRKKKNVLCAPSLYASVID

L

>SG-PL_contig_6162_1_-3_1176_1847

MPSQALIMRSRSRSPCTSPSHSPSPSPSRSSIHSPSRSPSNCPTPDRRRREDEEEGEEGG

RGGGYERQSRRYRGTRVHVGSLARATSVRELRTQFRKFGDIVDVWMARTQPCFAFIVFRH

REDALQAVKRMDGETLHSRRMRVTMARPRTKGTRSGAFNPYLRCYQCGKRGHFSRDCRAH

QWSPSPSDIRAQRREERYRYDSRYNDPRHARRSSTSGSATYRRQ

>SG-PL_contig_6163_1_-3_21_1280

MEELGLGTPVEKEDTAVTESRPSTAVQTGGSGDPLSLDGEDLYVKYKKLQRQLEFLEVQE

EYIKDEQKNLKKEYLHAQEEVKRIQSVPLVIGQFLEAVDQNTGIVGSTTGSNYYVRILST

IDRELLKPSSSVALHKHSNALVDVLPPEADSSITMLQADEKPNMTYSDIGGMDIQKQEVR

EAVELPLTHFELYKQIGIDPPRGVLMFGPPGCGKTMLAKAVAHHTTAAFIRVVGSEFVQK

YLGEGPRMVRDVFRLAKENAPAIIFIDEIDAIATKRFDAQTGADREVQRILLELLNQMDG

FDQTVNVKVIMATNRADTLDPALLRPGRLDRKIEFPLPDRRQKRLIFSTITGKMNISEEV

DLEDYVARPDKISGADINAICQEAGMQAVRENRYVVLAKDFEKGYKNNIKKDETEHEFYK

>SG-PL_contig_6164_1_-2_29_1984

MLAMCKAIISGLEHTIQNHGLGGMASAYMVLEIAHTHYWHRDLSSSKSDTSSLHGSQESL

HGKDELAPRPELRRLSDDQVATASSSPVIVNGDEFENIEIAGSASEEQAQPAHPADGSTP

HHHPHHPLHNPHPADPLSQPAVRQSGAVDSRQLVLQQQGDPLGAVTDWNRNVGKPGMVGG

GVAPGATCDIIITDADLSNSSGDMLREMVKNKELLRSRTLDKKFNSVDSEISEASTLVST

GSDKEAEKGRARISHQPIRSALSDSEMETMSVVGGRRRSRSSSLWSSKSNYSLGYRYHDS

KLVHTGTAPCQDVGKTYVFEGLVGKERSRMWDQMQFWEDMFLDAVAQERDIIGMDQRPSE

MMERYNSLGDGEKKRLEHDEDRLLAVMLYNLVGFMLMMRVPKAEVKKKIRRLLGKSHIGL

VHSQDVNNLLDVLNHLQGNDIDLKPMGSRQMQKQTFTVHWGPDNTGDMLFMEVCDDCLLL

RSVTGMIHDRWWFEKLVNMTYCPKTRVLCLWRKNGEKVQLNQFYTKKCRELYFCVKEAME

KAASRNSGRIPGPELGGEFPVQDLKSGQGGLLQVCMEGIGLLLANSKSFIELSRIRKCFT

LKGGIFVLEEFDSKTKQVIQHKFKSSMADQICYAVLCLFSYIAAGMEQRKQS

>SG-PL_contig_6165_1_-3_21_185

MYITLLSPFFFFLPKNIVAKHRSKHLTGILVVDHFMKVKFFGCFLTWLLIEMTRQ

>SG-PL_contig_6166_1_-1_997_1224

MAQFCDHIPPFSIFPIVFMAVYGQSLTKYVSPDQRIRTFMIFRFLNMTHYLVTVTAKAPN

VLSKGKGATDQESPNL

>SG-PL_contig_6167_1_+2_239_544

MFSSKNINCRFFFPQKEWSLIKTCKGTIIHAEGRLREGGEIERSRGKTESDRILLRKGRK

YLCNGTHMNGVLVNKPEHYNFYTVCNSIRSAHYNMEASNFEN

>SG-PL_contig_6168_1_-2_68_955

MHKGEEKKSTMLKKMLLSPETEFIMEAHSGISARIAEEAGFKGIWASGLSMSAQLGVRDS

NEASWTQVLEVLEFMNDACDIPILVDADTGYGNFNNARRLVRKLEDRGIAGAAIEDKLFP

KTNSLLDGRQQPLADIEEFSLKIKAMKDTARDPDFCVVARVEALIAGWPMEEALKRADAF

LEAGADAILMHSKRKDSSEIEEFMRLWNNKGPVVLVPTTYHTTPTQTLRDWGTALVIWAN

HSLRASVKAMQALTNQIYREQSITNVEKEIVPVKELFRLTKDDELKEAEDKYLPKA

>SG-PL_contig_6169_1_+1_1_105

WLTSYAKLSQTRAGKLEGECFTTMPSTLKTTFRRR

>SG-PL_contig_6170_1_+3_3_194

PIKYGLNKHKNPKQTNKQTPTTKTTHEKCKIFFSLTHTHTQIYVSICCLSHTHTHMSCFC

FVFV

>SG-PL_contig_6171_1_-3_99_335

MMCGPLCLISLPCSEFCAKLWPQAFKLHIVKIMWSLNTACNLLCLCVCLNKTFICEDQSG

CLDFFSLVTFLKLFICKVL

>SG-PL_contig_6172_1_-3_450_677

MADNTKPPQQLLAEIGKADVQKMKHVQTNEENSLPSAQAIAQEKTEVELRDRIGSFDKSN

LKHAETEEKTVLPNSD

>SG-PL_contig_6173_1_-3_24_485

MKMTLQECSQKTRDEASPCLSPTTCAYRGAHPAPVTPACPGNTPSCAAAAVSPAGAPSGA

TTASASPAWAGSAPSVSGLAAENCFLWCQGRLASPSPNYCLASFFSAREQDSGYFRSKIQ

QQQQQYVCHVTLPASGFRDGSHLFIVIIKDMAAF

>SG-PL_contig_6174_1_-1_535_786

MLHVEVSVACSRLLFMSLPHPSSSLLHFIFVLFFFLAFFFLLLLYLFNFYVHIRLISCLP

LFLCPVSVSCFLYLLTFWCIVMKN

>SG-PL_contig_6175_1_+2_2_103

PGFALKFLLDPCPSFVEQFTIHYWLARPIVVVLY

>SG-PL_contig_6176_1_-2_344_3559

MHDKNEEEEKEEGKSGMASLRYASYVEEEDWDIETLEVVNSHLEIYVKMLPGASAADLTL

SELQGLAARQQAAMETQQQALVARETRLKYLQQQEARHRQLASETVRLQRIREKVEAQEL

KLKKLRAVHGQVEQYKTSNGNLTDSELEAVKALFNEKEKELAVASSRVDVLNQQLKEAKA

MVLITTNGSTVKGDPHALALELDRLRQELVHRNKLNEHQNSHLAAQRQLLKQRKDEAAKM

DARIHELQQRLKKRRSQQQLQPNAKVGDKNVEQNLPGSRLQSQGRKPGPPNVAAVEPYIQ

YAPKDVVKDDLYSKTGFQKQDPKYQTLPHNTKFIPPGDDSSKPGGVAGNQEVNNNDKNRG

NGVSGVKPVEFKIPALISSHFSAHRGQSGSAANRTSTDSPVSGAGVADSSKSSSQPSAQG

SGSSSTPQQLVNKLPKGPRPFVNTFGKSGLPKWPPEPKKDEDTAPKPASHQLPPHHPQIN

IFDEERQAGSGQSSPASSEGSGPLVKQGSGVFVHPLAASSQNPPATSTNQTGSGPPQENK

FLGQPGSPLDFRGVAGLQEPKPPPNTPQGSSLVNPVDQGSNQAANVKPGARGGDDPDSSR

LSGNDSVNVGMPQATSTPQGAEGKTGSTVHLNQRPAPTYRYASKNVIANTYMGRLGSSAL

ERYQQLNKQLYSTIDSTQGNNAQTQQPAQKPNQKKTLANELRAAFNTQQQEPSENLAGDS

QGGMTQFGLVGAPNYPDIASDKGSFKSNTPKHIRRRHSDSDSEDAGKAFHRGDKSRQQNS

QSEDGSNNNNVTQNSSTTDAETKTSNAAQRRRTNSESGGKSKNTSSNQESGTTDSKTKVV

AKQKKTILKGDNRRKSNNRVSFDPLALLLDASLEGELDLVTRTAKEVADVSAPNDEGITA

LHNAICAGHYDIVKFLIEFGADVNSPDSDGWTPLHCAASCNNLPMVQFLVEHGACIFATT

ISDNETAAEKCEEDEDGYDGCSDYLYSTQEKLGIANDGTVYAVFDYYACNQDEVSFRINT

KMTVLRKGDDVEKEWWWARLTPNKEGYIPRNLLGLHPRVIPSDYQPHETSDC

>SG-PL_contig_6177_1_+1_121_237

MAGHHNSDNSIIILSKVAEKALLTHLPQQVLILGTFWPV

>SG-PL_contig_6178_1_+1_1282_1515

MERKVWSTKLQRYQMWDDTMFNWVKTLVGKELDDGWKMRFLSSQDNMMGHLFYNGFIPLL

CHSLSLQLSVFVPLVQSH

>SG-PL_contig_6179_1_-3_168_356

MGIKTMYKEYKPCGDNCCHCHICLLSPPHPLTFPPISPTTTTTTTPSLFLSFSCIVCICG

TKV

>SG-PL_contig_6180_1_-1_1_123

CCTDFLRKKVQECDMIFVFLSCLVSHCFCLTNVIYFHDYID

>SG-PL_contig_6181_1_-1_130_330

MVLATSFAVIISDKWRTSCLSLKFPQTPVLSHVINHFYVIFMTNINLFHVNLLYALNLHI

NTFIKPD

>SG-PL_contig_6182_1_+3_438_575

MYLQGKALLYSVIELGMNNVPWFSRRSRHPRLIPFLLSYQTGIMTL

>SG-PL_contig_6183_1_-3_9_695

MTWQRRVCASGTCGNMAWTFLAVLFSTLFTASALYFHIGETERKCFIEEIPDETMVVGNY

KVEMYDKNVNDFVPTMYGLGMLVEARDPDDKVVLSRTYAAEGRFTFTSHSAGEHIICLNS

NSTAWLYAGQLRVHLDISVGEHAVDYQQVQAKDKLSELQLRIRQLLDQVEQITKEQNYQR

YREERFRQTSESTNQRVLWWSIAQTLILLLTGFWQMQHLKSFFQAKKLV

>SG-PL_contig_6184_1_-1_139_318

MTTKKRHRMTKRAMTSPNTTSQKRMMAPQKTAMTKEKTKATKIKIMLAEKTSRPTRNVIL

>SG-PL_contig_6185_1_-1_1_153

CPLVFESARFQRLRKGHNSAKIALKPSQWHVFIYKVFGYIQVTLLSLTFTY

>SG-PL_contig_6186_1_+2_896_1108

MKSQWERKQGRQLAALVIDYNVRVSDNEFVDRFVIGPVNRSVSVCATGRNDGCGVLGPHF

FFCAGISDNNK

>SG-PL_contig_6187_1_-1_1_564

EDLERSEERLQTATEKLEEASKAADESERVRRSVENKLFAGDERLEDVESELRIAKQAAG

EWEARYEEAARKLNVIEMDLEKAEDRADTSERRVRELEDQSHALTNNLRSLELCEEKASQ

REDSYEETIRDLTQRLKDHECRSETSEREVARLQKQIERIEDELESQKETNQKLREEMDQ

CIGDLQNL

>SG-PL_contig_6188_1_+1_313_534

MLPLFSSLVSQSYLFVVCCYMLPLFSSLVSQSYLFFVCYLFFVCWMKMHMPKNLHKCYFF

VITSYCGHYLHSHQ

>SG-PL_contig_6189_1_+1_64_159

MTIIINLGSSLSYFHSNKTHPEEICSFIYCTL

>SG-PL_contig_6190_1_+3_3_89

PQRGYYQLISSYCLTCNSVQKQIKESRTY

>SG-PL_contig_6191_1_-1_1_93

PFDHGARQSLDFMNVVFQSQIRKEANFMRLK

>SG-PL_contig_6192_1_+2_308_517

MLITRRREFDPLAGQRFRFVCCFCCCCFYNFGSFSISWHLWRWCIWNLFRVWRHCLEETE

TEAATKRYRR

>SG-PL_contig_6193_1_+2_197_322

MNLKTNQFLVVHSHYSQLRKIFYLITATKGKGCLITHRSYGE

>SG-PL_contig_6194_1_-2_2_379

KIQWNPIRSRSYTALQKPTEQAPKKQTVTLSFYPQPICPKSSATVAEIAYLKGVFLSSLS

VLPPLLLLLLLLLLLLLLLLLLPLLLLLLLLLLRLLLFLLLLLLLLLLLLLLLLLLLLLL

LFLLLL

>SG-PL_contig_6195_1_-3_3_161

KSPQIAPQYDSCIKVVSKLVNHVSFIYFSMKLSLCSSLRNSAAVNFSMKIFFS

>SG-PL_contig_6196_1_-3_84_602

MAARLAQKSSELTNLEPAPVQSAAEQVVSRDSELSEKNRTQGWGPEGSMVCSRVRESSSA

SPEMTSPPESGSPSPTLGVGARPKDPSQMKKNRPNSLLGLSKISLGSPFSPPQLNTGPVI

EVMLPASHQQSAMTELSEAEREPRTQLHSMGVRHLGAPPPTVSAPAPDGNATV

>SG-PL_contig_6197_1_-2_2_244

KKKSVIAEEFQVHPSTVTAVLKNKEKYRADFQSGTVDTSTQRQRQSKYAEVDRKLLDWFV

NARCVLSLCLCLLFTVGHLPP

>SG-PL_contig_6198_1_-3_153_779

MRCIAYPFNAKQPTDMARRQTKVTKQQLQLIKERFHAFLNGETHIVADEAFTNAVQSYYE

VFLKSDRVANMVRSGGCSANDFREVFKNNIEKRVRSLPEIDGLSKETVLSSWMAKFDAIY

RGEEDPRKQPQRMTTAASELILSKEQLYDMFQNILDVKKYEHQILYNACQLDNADEQAAQ

VRRELEGRLDSVEVMSRTRQHPKFVVKEM

>SG-PL_contig_6199_1_+3_462_686

MNSSVLLMVVINTSVFQNKMFSVVLGVAVGSPFAGLAIPRTLNTFMDDYVLLVMTFKHVD

RTVAHRCPVSFIHGW

>SG-PL_contig_6200_1_-1_13_525

MRAGRPLILKRGYHHHLSSPNQCPNRHHPHLRWSLKWRCRRNPTSLRLCLRPRGCLTDQR

STTLLQLKCPNPPKHLYPHLRPRFLNQLLYQLLRQLLHQCCLNPGNNHQAMRSKRLDQYN

SSAMPLTKRGDTSSWWRNKKYPGNCQQRGHQLQRNHCLQTEDQWLKFHPHH

>SG-PL_contig_6201_1_+1_280_651

MLATSFSWCYSTSAPPSTPLTREYGSRGCLLVSEALHFGGSPPSYLSKKFQHVMVNQSVS

CDSQLSCVVLQGSGLGPTLFSLCIRQLGRLIEHYHLHCKLFADDTGLYNSFKPDQCAARA

AVLK

>SG-PL_contig_6202_1_+1_376_927

MSRSQSIPCVPYERASRPPTLKQFEIRNSLSLSISVRSSVTEGYRRVFASSEFISPGQNL

PTYYKMPVLGQPKTLENCLVSGLSSWKICGQGTSTTIVLRFESAPLEQNNTASFKRKPAS

QVKRDQERQQRFVQQKRAGQLRYPSDLPSAVNTGIQQQDTPNSNVSDWQVSEIFSNNLPG

LFER

>SG-PL_contig_6203_1_-2_242_778

MDLGPRQGNRITPGPHEVPSSSSPPGHLPLDRVRKRPLQRAHRNHWHQPASSWDQVVPLG

SWFLEATQEWASASDLADGVPIIPPRPLGTLYAYASCLGWGAHVGSAHVARTWTPDEALL

HINILEMEAVSRSLLAFAHLLRDKPVKLFTDNTTVTCYMNKQGGRGATPPPSQRGPRIS

>SG-PL_contig_6204_1_-2_44_502

MKEDSFTYPASPPGTPQSDSEMIHGGKEMRDAAESLLHLADLKAKEKGTNLLLPPYHPSF

PASLGCPTPPHSEDSCSSTSPQADHQMVPFFPATPLPISTPPVSTPSVPMRKETKLEQLL

REPPIHLPHRTSRPQPVAVPVIVPNSSICSNSL

>SG-PL_contig_6205_1_-3_3_236

CVCVFNKCGRFALSVNFNIPVYFIMQICYSNYVDTGYSFRRSKSSSSLSIPFTTGLFIQY

QNCSFMLNILQNSFTELG

>SG-PL_contig_6206_1_-3_12_398

MESKTMKRGDPYSLKFGRTLFTHSSSRVPQSSRLHPIIPQLYVQEWKMDMRNRELITQNA

RLGGIPNFEHDENLFLEKREQMHYNVENANRVENKYKIPERAKLLRPDFHNELSRYQSEL

MFRRDEDTV

>SG-PL_contig_6207_1_-3_33_200

MMCICYSCIIDRTSWECGYFMHGLCDYSHTNSNVILLYNSVLKKNVWYSHEIFICV

>SG-PL_contig_6208_1_+3_3_176

AYSVVSILLLACMESSSIFNVIQICFCFVLFSSEKKAATMIHDDTLCMINKAQQSDYL

>SG-PL_contig_6209_1_-2_2_1396

MASHFDTMAILLVAFLAVLTPVFGDTPANCTYEDIKGTWVFSVGPGGNDRSVDCSNFTGP

AHKQVEITLYYPDIAQDQYGNLGFWTIIYNQGFEVVVTGRKYFAFSKYTASKQKTVSYCT

EILPGWSHDVLGHDWACYSGAKKGSEAQSGIKVSAGLPSKVLTFEPRIFHNDLDLIKRIN

QAQTSWKAVAYPHLEGVKTEYLLKMAGGAASRTVQRPPVAPLTREVFEAAAKLPADFDWR

DVDGVNYVSPVRNQGGCGSCYAFASMAMNEARVRIMTNNTHTPIFSPQDIVECSHYSQGC

EGGFPYLIAGKYAEDYGLVLESENPYKGVDGKCSTPASVSRYYSTKYDYVGGYYGACNEA

LMRLALYENGPLAVGFEVYPDFQGYSGGIYHHTGLDSGFNPFQLTNHAVLVVGYGVEKST

GEKFWITKNSWGEGWGENGYFRIRRGNNECSFESMAMQSTPIISL

>SG-PL_contig_6210_1_-3_3_206

RKFLAQKFQKVGILFILKEPPASPLGFLVTSIQVSTLSIFSSIILQYTDPVVMYQNTFKQ

MLAKLFWV

>SG-PL_contig_6211_1_-3_3_248

HDASPQKAPHVIPGPGSGSDLALTALPEFLVETQQPGSLPQTVRIPCLVSCPDPRDARSE

ERLLARSDASSTTCNARHLGVP

>SG-PL_contig_6212_1_-3_81_1400

MDETYDVIVLGTGLKECILSGMLSVDGKKVLHMDRNKYYGGESASLTPLQDVFKHFKVQC

GGKEYGRERDWNVDLIPKFLMAGGQLVKLLIHTGVTRYLEFKSVDGSYVLKRDKNNDKIC

KVPADEREALSSSLMGLFEKRRFRNFLHYVQEFDESNPKTWKEMDGEKNTAAELYEKFGV

DSNTQDFTGHALALYLNDDYKSKPCIDLIRRCKLYSESLARYGKSPYLYPLYGLGELPQG

FARLSAIYGGTYMLDKEGCEIVYENGRAIGVKDNEGKVAQASAVVCDPSYARDKCRVVGK

VVRAICILEHPVKDTKDGESVQIIVPQGQVQRKDDIYVCVVSSAHQVCPKGFYLAIVSTL

VETNNPEAELAPGLNLLGIIKEKFVCVSDLLEPCDDGTDSKTFITKSYDATTHFETTCDD

VVKTYKRITGEDFDFSKVKH

>SG-PL_contig_6213_1_-3_57_1157

MATCGASSQKVDVLLECVQLLIDKGVDVNAQDRHHVTALMSAARDGRHEIVQLLLDNGAE

MDRQDSRGWTALSWATQREKVGVMKLLLERGADKNKKHSDGLTAVDLARGREDIVWMFEG

KTAAADSETGAGDAATKPDQTKGSADLKYGDLEVFLYGLQLGQFYSLFVSHCVNFSLLLT

MTEEDLIKMGITQVGVRLKLMEAIAAIHKRNWETSSLVPIRIKSQVKCADSIAIIANISK

HLRYISSSVTYIKDHVQRNPEFLALSEGVVDPKQLSLHTGDAMKNVGQLQKELSMLQSIV

SKELATGKYTSSDCIENSHGKKRRKVRFGRLVLIGVGVSSVVLLVWKRDEVMDHVASLVS

KVRLHFT

>SG-PL_contig_6214_1_-2_2_154

PSQFLVNCVSGHFLWLCRVISFFGGGPQGGFKATTSQFLVYSVSGHFFLVV

>SG-PL_contig_6215_1_-1_73_942

MQEVIAPVSNIRFDIEIAIEQQIGSLPLPFPGMDKSGAAICTYYLTSACNKGAACPYRHV

KGDRSIVCKHWLRGLCKKGDDCEFLHEFDMSKMPECYFFSRFGQCSNKDCPFLHIDPENK

IKDCPWYDRGFCRHGPHCKNRHVRRIICQCYLNGFCLDGPNCKYVHPSFDIPAPDPQQQA

KKLAIVCHTCGQVGHKAIFCTWSSGQSAPQNNPPSATMNTQWPNKMNEMGVNTFEREERP

GRRPLEQVTCYKCGEKGHYANKCPKGHLAFLSPSVGDINIARMANMGSVQ

>SG-PL_contig_6216_1_-2_155_337

MMHGMRFVATAELCNTCLHKGLQRRLMTLTCTRSYAAVTGSAPQVQQPLNFVDGKRVDPL

D

>SG-PL_contig_6217_1_-3_30_299

MHTADCLNSSSILSVWLLFVSFCFCLFACSFFFVVVVVVVVLFCFLIGITNIQISVRTML

LLFLFHQKKNTLVCFLFKYICRNTKHLGPL

>SG-PL_contig_6218_1_+2_458_664

MSKKTIYLLTMVRKGNQSFHYHLPFSIISARCHTVLSYTATQQPVTIRSDCGDKIGIKKG

KGCPITLRS

>SG-PL_contig_6219_1_+3_261_392

MRLNNCFYFVNSMNHVCIFSASKKKLFSQVVYVIRTSVNANQIN

>SG-PL_contig_6220_1_-3_57_488

MKFNKLVTSDRGKNRKRHFNAPSHMRRKVMSAPLSKELRQKHNCRSMPIRKDDEVQVVRG

HFKGQQVGKVVQVYRKKFVVHIERIQREKANGATVYVGIHPSKTLIIKLKMDKDRKRILE

RKAMTRVKAMADKGKYTEETMETS

>SG-PL_contig_6221_1_+1_1_240

KKKKKKLTRKKIDNCLQCVVGSVDVGQVRCRTFLIRNVLRCNSIVFTRQWLKRSLPAHYQ

TVFVITLSTQESCHTIRPTA

>SG-PL_contig_6222_1_+2_2_208

PHLPLLTHKNTKHFIPRGDTYGSDNFSPTFYFHVHYFHLFGKSDAYTMKLDKPSICTRFK

HNHLHLFTL

>SG-PL_contig_6223_1_+3_3_200

HTHENRGSSGDACRVSGKNRTVACCTLVQIKFRTEENWNWFQKCRQENTAMEPDTLGFVL

PTEDHD

>SG-PL_contig_6224_1_+1_1_108

FFFFQVHAFMFYLDKRNRRCASQSRNLRFCEQLNLD

>SG-PL_contig_6225_1_-3_6_581

MAASFRRGLACLFQNGQALSKRAATLTRVVPTFAANTICSTSKPSILSSFLGKSSFGSWS

PAWQTWNSAHQCLPIQPAIPLLEQVRTKVRYSRNRGKPQTVHAVPNRFYRLNWGIWIRTR

AGRGKRLWKKTESRKYRLRQHVFCNRTQSKLLDKMVTRFWRRPKYWVDDPYEPYQKRENF

DLYFPNKRRFYP

>SG-PL_contig_6226_1_+3_261_419

MCFTVLPLLCMLFCVLHTWSKTACPRHTGFSLFVAYIWNEIANVTAHILVSQS

>SG-PL_contig_6227_1_+1_1_183

FFFLCTPNFLVIKQRQFNRLEHNYTHFYTCPEQRKPKNIPRCVAHFQKAALLSHCKPPLR

L

>SG-PL_contig_6228_1_-2_62_1624

MDRQRRRSPLSPRRNFGGRPRYDARDMERRRDWQSDRNLDRSRERDRRRHPSKEHPEDKY

KGSLSEGMTVNQQESSEEELDIEVPDDEEDEDAIIEKRRQQRQALLQSLETKATTSNAAS

PATLTAEDEQLYSVSPDRAQTGDLNGQQTNDADNNTVEQLIGEGVPVSEDQYSCQRQDPE

KTDSERYDGEKERRSRSRTYDSDSDSDSSSSSDDSDVIADRVAVKQTDDDISVDFESSMN

EKRLQEMRRLKNAEKKAEEAESNKKAAAKKGVDIFAEDDDMFSENYSHSPGLKRVMTGVT

ENPNLTDNWDDAEGYYRVRIGELLEKRYTVYGYTGQGVFSNVVRARDVARGSQDVAIKII

RNNEMMHKTGLKELEFLRKLNDADPDDRFHTLRLYRHFFHKNHLCLVFESLSMNLREVLK

KYGKDIGLHIKAVRSYTQQLLLALKLMKRCSILHADIKPDNILVNESKLMLKLCDFGSAS

HVSENDITPYLVSRFYRAPEIIIGMGYDHSIDLWSVGCTIY

>SG-PL_contig_6229_1_-2_620_4297

MYSSTGRRIKGRGSIRFRSRSRSRSITPPHWRREMERTMKSKEPMEPQVEEKWAKGDFLQ

QQERPSAKSRLGARVDIEKESAPLGKRFELEKEEKEEGEETDDEEEEEDKQKKDKKESKK

HKKHKKESKHKKKHKKSKSSSDDSDSEKEEKKKKSSKKKDHKEDSKKKKEVKKKNYRKKN

ISSSSDSEEEKEKENREKEKRKAEKNKEGKRDDSRSKHRNRSGSFDRRDQSDYRRKSRDN

SRRSREKESRRKDSSKGNRSPSQERARNGQVKRFGKRDRDDSTDQDRSRERRRSKSRSKS

RSEERRTSGSKAKDKRKSKSRSAERRRSRSRSKSKDGRRARGDESRRRSGSRDAVQKRRS

RSRSEDRRVSQRQDRSPDRRRARDDRRRSRSRSADRRRRFSPIRRYGGRRGASPFRRRSP

PRRDSRRSRTPPYKRRRGRSSSSSDSESDRDQRRRAAAEKKLKMFGMPLIRKSESPPPTH

WKPGQKPLSAKTTTTSNVEEAAKVPAEVPVLVGQALQDISLPDGSSPLVSTKPNSEKPDV

VEKPKIMYNLDKEESITTLPVKPPQTKEKERKSRSSSKSSRSSHSSRSSSGSKSPSPKKK

TASSDSDSEKKKEKKVVEERMKWQPPPEPEDMEVDEESVPKPPNTVVPVEKGVDSPTVRI

KTPPLPPLDQVAVAAAELARKREELNRNSASNIPLPVEGVKPPSAPVLEPHSPSSPPPPK

PSADDKPADQRGRYDRSSSSSHSGSSSSRSGSESPVKSVPSENVPKPLLDLANQIPLPPT

SAPPEPEQKTSAPEGRSRSLSSSSSSSRSQSPSKEKNTIPMDIQEKAKDEEESAKMSQES

TAPVKVPSRWDFKPVPLPVPAPPKEEEPAESKVPEAKKPSPLEEKAKKAATPDKPRGRSS

SSSSSSDVDSSSSGSSSDSSRSSVDKKPTKKKRAPAVKAKSPEPKKKSSRSKTRSPSTRR

RSPVGSRDRTRTDRSLSRSGRRGSRSPRRNGRSPRRSPYRSSRRSPIRRSPIRRRSPHPR

RSLSPVRSLSWRSKMVTPRMLSPTRRRRSISPRRSRSRRRTRSRSPRSKRSRSRSPRKRK

SRSKSPRKRKSRSKSPRKKKSRSKSPRQRKSKSPTPRQRKSRSKSPRQRKSKSKSPPPQR

KSRSESTQRKVKSKSPVTRSSRTKSPTEKSGRGSQKPEEIAAKRVHKRSTSSSSDSSSSS

SDASPEKKRAKRASSSSSSSSSSSSD

>SG-PL_contig_6230_1_-3_279_374

MLMLLKNNEKHGLKTGINFLTNEGNKKDSFSD

>SG-PL_contig_6231_1_+1_1291_1791

MHGHDKSGLLVDMVAHAHLHKMYIHCIRYIYRYIYSSMITLLYTVGTYQYASCHSSSRSR

TTWRPTMHTQTQTPARTGTHLNTCTLTSVKTVAHAHTHIHTPICKCTHKEHSSFSYRRNT

PHCFHIHTDVVCNMYTSMNRKTNSLRSVQLMKCQGHSILHTGTAQST

>SG-PL_contig_6232_1_-3_123_1778

MSFKDRMFNNSGRRGGYGGGSSGRDSGFRGGGFQNSMKGMQPGANLRKPKWDVSRLTPFQ

KNFYVESPVTAGRSAVQLQEFRRSHQITVSGKYIPNPILSFAEANFPDYVMGQINKNSWT

NPTPIQSQAWPVAMSGKDLVGIAQTGSGKTLGFMLPAIVHINHQPYLERGDGPIVLVLVP

TRELAQQVQEVAVEFGSTSRIRSVCVYGGAPKGPQLRDLERGAEICIATPGRLIDFLETN

RTNLRRCTYLVLDEADRMLDMGFEPQIRKIIEQIRPDRQTLMWSATWPKEVRKLAEDFFS

DYVQVNIGAMELTANHNILQIIDVCMEHEKEDKLVKLLNEIMQEKENKTLIFVETKRKCD

DINKRMKRDGWPVMCIHGDKSQQERDWVLNGFKNNRCPLLIATDVASRGLDVDDIKFVVN

FDYPNSCEDYIHRIGRTGRATKTGTAYTFFTPTNIKQSGDLINVLREAKQQVNPKLIQMA

ESSRGMFNRGRARWGGGGNRFSGGRGGYGGRGGGGRGGGGRGGGNRFSNSYSSANGIGGS

YQKSGGYQKNGY

>SG-PL_contig_6233_1_+3_3_227

HTHAYTHANQNATHTNTPYTHTPSPTQNITHTHTHTHTHTQNSSTHRNKENHKNTKLMNI

NNTCAHTHTHTHMKA

>SG-PL_contig_6234_1_+2_368_529

MLLILFVLLCFGVCFWSQWIRHVKFGLLSPLTVDFSTEFCQGQFFCCRGIFNVR

>SG-PL_contig_6235_1_-3_21_461

MQMLKASQDASSQGSDQHGTRCEILSPLELRIEELRHHLKIESAVAEGSKNVIRTLQNSK

IEDKKALKEAQANLSESSQKMELIRMSLEKRLMELEQDPDKVPKASILRAELDEFLNSTY

TSTTAPSHRHSGLAAVSRNANTFSKAA

>SG-PL_contig_6236_1_-3_96_215

MINRGLECSMHLVHVKDPTALLLLLLLLLLLCIVYSPVDH

>SG-PL_contig_6237_1_+3_210_335

MMIFENSNTVVCCFISHEYKCVCDTLIIDDLGFFSKFNNLVF

>SG-PL_contig_6238_1_+2_71_220

MSTPTAMMKGTNHKTYNVKKVLKINHNKIKYLPPHVKPYRIDGNGFKEST

>SG-PL_contig_6239_1_+1_1_174

GQWLIMFCLSNNNSLNSNNTFYFKFRPRYNSVISFKLNLLWDSRASMAWLPTKLNMTT

>SG-PL_contig_6240_1_+1_1_219

DHWRRRLALSWRALPQRSQACRRSVPGLVCSIPERGLPEEVRARGQRQGGTWDRDPRSGS

HGWACEKSAKRIR

>SG-PL_contig_6241_1_+1_73_201

MAGYDANFGDRLKKNCCSFFQDGSTADSDDVKTVLGVCTDDML

>SG-PL_contig_6242_1_-3_90_2297

MYCRTLSRVQGLLRGFHNLSPLLHHECRPLSSSSVAHFFLSPDDLPKHRTSADACVNMSD

SNRQVTWCQRRSLHLSAETFAAKAKNSKKKLRKPVNLNKKSNMETAQVFKNMTVAQLSSA

IGRGIDHVFEVLMFVKDGDKYESEEAVIEDMKVIQEVVKKSGMKCQIVGSSKPTKEKEAC

DVERQPPPDPEVLVKRPPVVTIMGHVDHGKTTLLDYLRQSNVVDQEFGGITQHIGAFKVK

LPSGETICFLDTPGHAAFSAMRARGAQVTDIVVLVVAADDGVMQQTVESIQHAQAAGVPI

IVAINKIDKPDADVERAKHMLLEHQLVLEEFGGDIQAVCISALKGTSVDLLQECIALEAE

MMELKGDPVGQVEGRIVEAKVDPKRGKLATAVIQRGTLKRGTYLVAGTVWAKVRGMFDDQ

GRPIQEAPPATPVEILGWKEVPSAGDEMLQVESESVVKQVIAWRQQQATDTKVEEAQKVI

NERREEHQRQHREEMRLRHEAGFVRSNRKRMQKTKEIIDSNEGPQFSLVLKGDVDGSVEA

ILDTLDSYNSKQCRLDLVHFGVGNVTESDVELAEAFDGEIFAFNVEVPQAVSKMAASRKV

PVRTHNVIYHLFNELRDSMSARLPMLEEEDVIGEAKVIQVFYITEGRNKLCVAGCRCTKG

TLNKKKNFKVLRQGEVIYEGEVLSLKHFKSEVESVKVDRECGISVDDRDLHFKVGDEVVC

FETKEVEQEIDWSPGF

>SG-PL_contig_6243_1_+2_2_298

SSCTWTSHIVHTQADAAKNHRNSRTPDSSETFYNDHIPSAYYKTWSQDLCHAARFEYHAV

SPFASLPFYEPLSSFLAFLGNRVFTLITTLRKFSPSMGS

>SG-PL_contig_6244_1_-3_504_713

MCAFCMDWSRSFSLLLHLSAMDSLPPGKVCLHWLMHIVSRCFLYVCRIMCLNIFQGQPRH

SDKIGGYRMS

>SG-PL_contig_6245_1_+2_320_475

MAQINMQLIKRTSLLQNSTTSSVIIDTNCSQNTYDRAGREGVKNDWVEHTSV

>SG-PL_contig_6246_1_-2_107_601

MLRLFARTGLRPMQLLQTGSRRCMAAIGEEEDEENQPARYEKSVNQVNLLGRVGRDAELR

GTETHPVVVFSLATNSVYTKSGGGTVARVDWHRISVFKPGLRDKVAINLRKGDRVFVNGV

IRYDEFTNSDDNLVRATTILANDLVNLTKRFTMEEDGSETVGRGF

>SG-PL_contig_6247_1_+2_2_133

PLSCSEFRDMFIININIVFSFSFSCTCSFVFTCGLSNHILLSGQ

>SG-PL_contig_6248_1_+3_1572_1961

MRAAASSSPSLLYLSLTNYEGSAQFHSLAALPFPNFTESQVHIHSWVDRRLHAHIKFMID

THANLYSQIGRSVLYLCTNQSNKKANQCKSVNRMDIYPRSGPTRTRHAAPVKGESEYGLT

IFIFFLLQPV

>SG-PL_contig_6249_1_+1_262_495

MRLLWSFKMIILSCSLELKKGSLKRKQSLECNIHKDTLPGKKRTHPYTMLICASTHEHMI

ALCTHTLPTTNSFLKQPT

>SG-PL_contig_6250_1_+3_189_374

MNTNYYTKSQHLQMIKHNRNKSEVSNFSKGIRKNKSLHIFFFNERKERKNKGTGRKEYNV

FC

>SG-PL_contig_6251_1_+3_27_239

MQACVGLGLSPCDHSHKSLNTRFFFFFFQINSQDSHFVHNAVVTFKQLEKTENSRVTVSE

MKTPDCGNNRI

>SG-PL_contig_6252_1_-1_1_102

FVSVFSISQSALTLQSIPVLHIPILFSSLSYLSV

>SG-PL_contig_6253_1_-1_1222_1482

MCELCCYFLDKWYVCLVSTFKHTYAVCVCLGLVFFPKPGLPDHFLLCQVSFCCCCFLLFS

LSLPFRLVTSTLNEIFGPKEGKSSSKR

>SG-PL_contig_6254_1_+1_373_702

MERSSMFGGRAVYATTVTNRRPLCTSLCCMCHVLTADFSCEKRAAFLWESTLPWSSAIFP

HVFLFTYYFSEEFSQEKYLVATLLHACCTRDINISSLSTFLFSLFSFLRF

>SG-PL_contig_6255_1_-1_1_357

QQDPSPGPSQASGGQPRSERRCRDNARNRRKRCRRFSSRSSDDDARSSPDSRRHQSQPSR

RDPSPDKSPARDTPTSVFDLLRESLPEMLTEVSSCHAGDDPSPFGAYQGVGRGERSALK

>SG-PL_contig_6256_1_+2_2_130

SLYACVHVWLRVHKGVRTCMCICNFIYLMRLQAKFSCCSEKDD

>SG-PL_contig_6257_1_+2_2_172

IHNSCICHYVTDFIADLFLCITCFVKCKALRSLHPGMLRYILLIITPPPVCTVTSNY

>SG-PL_contig_6258_1_+1_787_1062

MGQVHAINSLHGCVLLKLGKDSLMSNHYPLSLFRSLLCWKWALAIYFTFFTFQVSVTLVI

IDKLNSHSNKAYPLSMGMIAKWLRCWTSNWKV

>SG-PL_contig_6259_1_-3_1785_2114

MDVFGMLFPMVKTESKSDVWYLQELSQFNCSTPLTDRFEKIGVKHLNFLIKRRGWVLLKP

FLLMLHTFSCNFYHIYTEMNADAFIPKISKIPTCDPYLLLFLSLVQNYAR

>SG-PL_contig_6260_1_-3_981_1133

MPLYTKSILVGEKKNVHPSIHLSIHRHVMYLQRDWQLALSHSDHLSMPCGS

>SG-PL_contig_6261_1_+3_75_1541

MKKGKAAGPDDIPSEMITALGEIGITEVTKLLNTIHATGEIPADLKKSVYIALPKKTGTV

ECDQHRTISLMSHLTKVMLRVLMYRMRNKILPEISETQFGFMADKGTRNAIFALRTLMER

ATEVQKDLYLCFIDYSKAFDKVRHSDLFDILQRLNCNGKDLRVLRNLYWEQEAAIRVDND

YSEYRPICRGVRQGCVLSPDLFNIYSEMILRNIEQHEGVKVGGHNINNLRYADDTVLIAD

SEEKLQDILTTVTIESENKGLQLNAKKTECMVVSKRPDTPVCNISCKGQRIQQVDTFKYL

GFTLTPDAKCDTEIKKRIALSKDTFTKMKSIFTNRNMRLSTKVNTMKAYVWSILLYGCEC

WTLTKDLERRLEAVEMWYIRRIMKVSWTEKKTNEEVMEMAGYKRSLLKTVRERQLKFFGH

IVRADGLEKQLLCGKICGVKSRGRQRTKYTDSLNIYTTRKESPNNELIRRAGNREEWRAM

IADVCNRPG

>SG-PL_contig_6262_1_+2_2_274

SPFSINDKSKGHKDLTSSLFSINDKDDETNKTRFNPLLPFLKMRRNEIRHQTVFKCTAKT

SSSSIVHQKIRVKNSPSLSSLQLRITESTDQ

>SG-PL_contig_6263_1_+2_164_286

MKYKLFVSLLVMYVCFVVNRQRRAAAGSWFGLVWIFKPVSV

>SG-PL_contig_6264_1_+1_250_408

MQIIPQNTGDLENDSRSCKFYQLIGSCHTTAQPKYKYVRLIILELYFVTIWQL

>SG-PL_contig_6265_1_-2_2_262

KGLLFPKDTGSKRTAVSHCSCITRLSDREGDSDHMKVREIRYNVCSFRGLFILKDNCSCI

TDCQTEKATEIRGMWEKKRYNVCECEL

>SG-PL_contig_6266_1_+2_461_703

MLCSVHGYLFKETFCCVYMDIFSKKCFVVRFLKEMFFFLYMGIFSKKCFIVCIWTSFQRN

VLFVFSKKCFSSRIVKTMCTK

>SG-PL_contig_6267_1_-1_121_543

MRSIQFSKCGVIQAQPLSFLRLSGYQRYTERKSVVNHFTTLNSSRLQIQMQLTVGHSGGR

MMRHFRASDACVCVCVHVRHPRACYPPSPHNSHIDYHIFFLSFFFVSPLIACLASVLCVR

IVCVVNCTNVFKTGIPKDDTM

>SG-PL_contig_6268_1_+2_77_172

MLLNTQVTASEKSSSKHLPDNILHICGALICT

>SG-PL_contig_6269_1_+3_12_194

MAGSQSWMIFERKYVREKIYRGKKKNLYASKELPLLNKADLFLAPAHEYIMRSDGVSQST

V

>SG-PL_contig_6270_1_+2_329_517

MFPKLMVSIKLVWSDWKLGALPHVNQRERERERERERVVDSVAELFRDGHQHNNVIINRK

GCL

>SG-PL_contig_6271_1_-3_606_1517

MSLYPTLEDMKVDQMVQAQVQHEQRQAITQGYPPAQSDYPPAYNPAASASAALYPSLDDY

MGLHLTPQFVQENMPVVAASYPQNMQVAQRQSPSQSMVAPVTGNANKELMRAEIKQGIRE

VVLCKDEKGMLGVRVRHVNNGLFVALVTKNSPAALAGLRFGDQILTINGQTMAGWDNDKA

HNFIKKLDPQRIAMAVRDRPFERTVSMQKDSNGYIGFVFKEGKIKSLVKDSSAARNGLLT

DHHLVEVNGQNVVGLKDSEIGEIIDAGGRTITVTVMPSFLYEHVVKCMGNSLIKKLMDSS

IPDL

>SG-PL_contig_6272_1_-1_352_597

MYTDINAITGTPSKSKPSPSLMSKASTKYCTHLHQTTLSERTRSTTLTSLTMPNFLMKSK

QHKTAEPQNADVTVLILWSFTF

>SG-PL_contig_6273_1_+1_1_252

MKGRISVCLTIFIPTKHIHFLTFCLFCNFFLYLPFSSPLHFSVSICRSIFYFPYRSTSMY

NSHSHTNTQFFTLIYCFKELIGTR

>SG-PL_contig_6274_1_+3_3_305

CKQGRPSRPTTTPASKGDHIVAIQTNQNNQLYGNCGPHSVHKTEHGRTVTCVEPESSSNN

GRRTYQNHQLGVLQNSAHAMTEPPEQSTVKKPALNGKDRTI

>SG-PL_contig_6275_1_-1_151_1929

MDFFPDPMESGNLKIKGQRRPLAQPQGDTDRGFMAGYQCSTPYPAATVSLAVEDPVFSLG

IQDRTHRKGVEFDTGLGNFTLAGNDAVMGSVSEMEDGSSLVYDMGHGTSEQQPAFLESMV

TSVRDAGLSEQDCNIVWRKNFKSKLVIDSTDLQVVEHRCEVTEKKEGYVIRGGDQDLCQL

VCKRSDSPHQTLNEGVICNSQVLCSENGDYNEGKQYVVFCDTKSEGAFGEAVMCEDLKTH

KTYIRKKVHGQLSRNEVTVPVVMKGTAGIPAVYGVISRGKTTEIYQEHAGLSLRRLCKSE

YAPRIRDPGMLLGLAFQGFATLDKLHSKGIQHLDIKPENICVDMGQKVPSLKLIDFGTSQ

MPGEVVTAGLTYEYLSPESCLNILKVATGQPPPSEGRLGPGADVWAFAQSLLYCLCGYDV

MVYMCTEKTAYPTDLRPEELERKRVECLLKTQRMTDKDITSRLIHPEWPQVLQVLLVNTL

RVNPEQRWSAGQAAAFILEYLKTVAQKSAAPPQVVAPVSGFTLAPSNLSLRVHTSQNGQE

NLPVQFTSIAAPSAQLPAFGELLPHSLNKMEAKSGKRRCQEESSRRGYKVMKM

>SG-PL_contig_6276_1_-1_1_276

FIIFFFLRRAPDFLIIVKKFYFIYLFILLHCHLDFGYLHFLYRKREGVGLREGTRSGNVR

KGLIHLLTHLQVLLLRDWLYLLNMFLIFVKKI

>SG-PL_contig_6277_1_-1_127_435

MTETTFKTRYANHKQSFKQERYKNQTELSKHIWTLKENKIDYDISWKIVRRARSYSSSSK

RCNLCTTEKYFILCKPGMATLNSRVEMISACRHRRKFLLANIT

>SG-PL_contig_6278_1_-1_10_222

MKNRGVDHKEILRLAGEEWRNMSAEDKKPYEKRSVEAGKKYEREMAEYRKNEGKASASAQ

RQEVADDDDEE

>SG-PL_contig_6279_1_-2_701_3064

MLGNIKFIGELGKLEMLHEGILHKCIRQLLEKKKNTPIRDMAEDMECLCQIIRTVGPRID

TPKAKTWIDTYFSRITTYATHEELPSRIRFMLQDVIELRENEWQPRRVASENGPKTIAAI

RQEAAQGRGGGKVMHSQMNGMGHSWKGFGDIFNMPVGSMSNIIGTGPGVIQVDNYTPPPF

MGMGNMGGGGGGRGGGGMMGNNRGNQHNQGFQNNLRRGNKENNSNSGGSPQNQRRNNQSQ

HQNQQFNNPQHNQQPYNRQAGGGQRENLPPRFQRMAQQQQYQQQQQQPQQQQQASQGGKP

PGRHTPPLAMGGPAGSHPANMSASQPLAGVGLLNGKEEVSLRPQKNFMFKPNTPAMLPRS

AQAAPSVPLLPQPGHHLSMSPKRMMVDVANPPINPLLSKQPQITIKPAKEEKPKPQKKET

ISEDELKEKLPGLLNDFFTKQDVTETVSAIKELKIPQKMMPDFLAQFLVGSISRTESEQD

QVMKLIATLRTDKVISSDHFMESFQKVADQMSALEQETPLVKSHVARFGAQALAMEVSSL

AELAEPFQNGVHYPLFLLCLQSAHRVKDKQWLVRAFNDSKIDLIHMLPEIDQKKERMLEI

LEDRGLSFLFPLMRVQSELGRQIAAEPTAGAIFKWIKDRVDSDLFTNPKFINILMTSTVK

YITGESTLTESTDTTVTPEKALCDKEKELLGKLKGVLQKFLHERIDLQVSALYAVQVFCY

NHQFPKGMLLRFFLNLYDEEIIEEEAFLHWKEEVNDEYPGKGKALFQVNQWLTWLEQAEE

ESDDEDEE

>SG-PL_contig_6280_1_+2_2_145

NGRSLSKIRRLSAPLSNGRSLSKIRNYYFIYFIDIFGPVFLVLFFSPD

>SG-PL_contig_6281_1_+3_138_326

MHTVVKLNIFAEFCNDCNPWRFFCYFLSGDGVGKNGPADINGSCMLFLYLVSCVFLLVFL

PLL

>SG-PL_contig_6282_1_+2_2_232

ATEVIVTVFKLHACVYLLLSYRTLMFLVNIVCLFVCFRSHFPRHFSGHRVLDNVFAMFDD

GVSLMFNHTFFLNPKQL

>SG-PL_contig_6283_1_-1_1_84

MGFGKFCDQNEIWLACASRGFHWTSLTA

>SG-PL_contig_6284_1_-3_12_404

MCFLMETAFNIRMQFITGEKEARRIDSDLDCKNKWRKEWFDKLGLNHKNDLWLKKIIVDE

VGAAFCSRCSKPLLYKFNGEKISLNAHAIDPTHFARLSVQATPQLCRLLNQYLQGYVQET

KFDARMLTSEE

>SG-PL_contig_6285_1_-3_9_3872

MFNAMTLTDAEKKDPAIVFQKLGEQIEPAEHFRVSRLKLMSMRQSKSESLDDFVTRAQLQ

AQKCAFDDVAEREERLIELIISSTPIGDFQRKLLEQEKGVKLAEVIKMGRTFEATASHVE

QLQTMADSASISAIKSSTRPLCRNCGGQHKPRECPAHGVTCHTCGKTNHFAKVCRSGPHQ

SPRTPSRGRGQRRGGSGSRGGGHQRKQPEAKIKAMQAEIDEENFDDLTFSPVTISSVSSL

PQQRNEAFVKLKIQLPNRAGQHNIIVKVDTGAQGNTLPLRMFRDMFPELLTPEGTPVKQI

LKEKDSTRLTAYNGTDIPCLGKIAFPCRYSGEWQETGFYIVDVEGPAIIGLPSCEKLKVV

TLHCSISASPPAAKDKITCVQDLMKDNPEQFDRIGKIPGPVKLVVDPDVPPHIDAPRKTP

IALRDAIKEELDSMVESGVIRKVTEPTDWVSSLAYSKKKSGKLRICLDPRHLNLALKRPH

HKIPTIEELTHQFCGSRFFSKLDAKSGYWSVQLDEESQLLTTFQTPYGRYCFSRLPFGLK

VSQDIFQLKMDQILENVKGATGISDDVVVYGKTEEEHDESLRNLMKAAAENGLVFNSEKC

TIKTQSITFFGATYTPEGVHPDPDKVNDLKAMPAPTSKKELQGFLGFVTYMSPFINNLAE

KAAPLRELLKKDSFFIWGEHHQKCFQALKDAVSEDATLIYFDTGITPTLETDASIRGLGV

TLLQHDKPVAYASKTLTDAETRYACIERELLAIVYGVERLHTYLYGQKFLVRTDHKPLVM

ILNKPLVRAPPRLQRMLLRLQQYDFTIEYKPGKDMTVADTLSRLPSPGNTKTIDLDLTVG

QVRFSTERIKSLQEETKRDPTLGKLIPTIINGWPDTIQEVPEAIRSFWSFRDELSVEDGI

VLKGDRVVIPQNLQQEVMRQLHAAHQGVEKTRLRARASVYWQGISKDIEKLTSQCPICQE

HQKNQVPETLNPHEIPTQPWEVLGVDFFALDNTQHILVADYFSKFFVCRKLPNDYTCKTT

IAVMKQIFAEHGIPKTLKSDNGPQFSAKPFQEFAKEWCFDHVTSSPIYPKSNGFIERQVQ

TVKQALLKAKQDGSDPDLTLLCLRTTPISPKIPSPSELITGRKPRGNLPTRHTGNPTDDL

VREKLQHRQDTQKSYHDTKAKDLPPLTPGQHVRFQDQPSSKWKEAVIKEKCQEPRSYLVE

TPTGRIMRRNRVNIRSLPQRQLPENTESPIADKTPTPVNTPVKAPVNNTAPPTVNTSAAP

SPPAAPPGSPAVFTRSGRKINQPARYKE

>SG-PL_contig_6286_1_+1_1_213

KVLKVVVSECGFPPPVSCFQDVPDRKGVVEGGRGCQGSEFVSSGTIREGGGTRLLISPND

YSRESAKNGSR

>SG-PL_contig_6287_1_-1_1_306

EERLLCPLHCVKYYLKHSAPRRSSQRRLLISLNENYSRDISATTISRWLSATVIRAYKEL

GSSFLLDSSRTQSPGVLCCLPPLPSYQGHLGSSILAAGIHVH

>SG-PL_contig_6288_1_-1_88_258

MVVDGQRVRWEDGGQTSSMSDVELVSVSSKPACRSLDTVIRMMCVCASLSDIKSLHN

>SG-PL_contig_6290_1_-2_2_178

FGGGGGRREWGGFNEYVYLYILYVKRFVSVHVSCSFLIYGWCLFAFVVHSTCRKDVLAI

>SG-PL_contig_6291_1_+3_3_65

SRFSHFGYHSNRAQNITSGIK

>SG-PL_contig_6292_1_+2_2_124

ECECVCVFIKILKRFSFNPPINPFPHLRTDTLLHQFLYRVS

>SG-PL_contig_6293_1_-2_2_166

FKLGLKREKVGQISQITVLVTRHWNLRCTFNFELKWNMSRRNKLTGVFSNDSQDL

>SG-PL_contig_6294_1_+2_2_175

LTKIKKKRKKKNSFFFFFSFFVFFFLFFFLAPPPPPPPQSYLGETVVIVKHLTMPTEA

>SG-PL_contig_6295_1_-1_643_894

MLWFVTNTLTGESFHCSLQHFCINTVVLGIDLGLHEMWESYLSCHSLIFLSYTVCSFKFG

VDVVYLYMHVCSCAVEIRVDGVFL

>SG-PL_contig_6296_1_-3_105_407

MGPAVAETGLRFSSLALTPRQVALVNELSPEPRYVYLQGPPGCGKTVVLALKALQWLNQG

RTVCIVSTSDAGRAVSALLEHQVRSSLGAAAAGAQFQRLDY

>SG-PL_contig_6297_1_+2_2_151

HQARYPKRFEPRPSRHSSLGSPMSSLHHSTTLHLIIKLTLPNMKCLRIRK

>SG-PL_contig_6298_1_-3_405_704

MNRCTSYAMDPFLSSSVYGIPEDLTCPSIYVTFGSYAMDPFPSSSVYGMHQDLTSPSIYV

TFGSYAMDPLPSYSIYGGSGYGLPEDLASPSIYVTYVMII

>SG-PL_contig_6299_1_-1_208_699

MGILLMNCLFGKTGLQLFLCILKQCFTKKFAQFLYETLKLRFIVVLEQLCSTTLLVGTLN

RHVDGNIRWLFTCIPFGGTMAFMCIHHPFLSVHSAIHPSFLSPTCCLSFSLSVLFLLPFS

LSPFASPSLCIFSFFFLSLFHLLPLLLSVSSLSSSFLSSTCCLS

>SG-PL_contig_6300_1_-2_2_220

LEAPSLPVQSPPFRPLPSSVPLHHGGDGVSNPGSLESIRLLCFLDAWLLSATPTCVELIL

TRSSTCAPSWGSS

>SG-PL_contig_6301_1_-1_1_141

SPKQVPSAGPFQDGDATSYQRGVRQGDWAASVDLKNAHFHILIHDSA

>SG-PL_contig_6302_1_-1_49_138

MRRLRVWLFFSTSPYRQPYSDFRGFFLFYN

>SG-PL_contig_6303_1_+3_372_716

MGTSTLPCVMHYPKCTSTSYLSSTSVCLILRTWLDKQINRETCVITMGINTSLFIQHLVS

LAKHFISSHSRSCFVWLVCLPQKSERTPWFQTETADTGPIMLTRITKFNITTPYR

>SG-PL_contig_6304_1_-1_106_279

MCVSSQKDFLLLLHILAFFPIVRAFSIMQKTTFTYESVYTSQKSSIQLSFSMDIHNQT

>SG-PL_contig_6305_1_+3_201_302

MTILTNRQRGKVHNQTKQCLLCWTGQAWLQLIIM

>SG-PL_contig_6306_1_-1_1_198

LTEKNFDDFVKDNEFVLVEFYAPWCGHCKALAPEYSKAAKKLSDEDSNVKLAKVDATEEG

ALSTKY

>SG-PL_contig_6307_1_-1_1_246

AFAAADDVKQDEGVYVLTEKNFDDFVKDNEFVLVEFYAPWCGHCKALAPEYSKAAKKLSD

EDSNVKLAKVDATEEGALSTKY

>SG-PL_contig_6308_1_-3_327_992

MPMSNSNLLMVWNLLFFFILNDSSFIIPNLKIFFSQDCVLISGLCSFSESPSLSCSKRNS

LLTSHSSLPFGWRIPTVAMIEKNSLLMAAVLFSSETRLIFVVIVWFVVLQFKDRFEYSPH

CIAPLGIHLLPHLQKASIDASLICDFSKFPESPPWMLKSPNIQFDLAAYNKRHDSSLVYR

TFFFSDLCQTFPNFQQIFTVGSKSNEGVVCAAFSSTTRKSPF

>SG-PL_contig_6309_1_+3_894_1091

MNNEKRQTKLPPQKDIMHHILGITLLLMTITVCEEGIRNRNRNTRNILSHLFSVPPCKIL

FLHEGL

>SG-PL_contig_6310_1_+1_115_261

MLSDVNLLTCNFTHLNNQILFGGWGLLYESNFFISLCLNPRVVITTDDS

>SG-PL_contig_6311_1_-3_138_4934

MNNILSSGEVSGLFARDEMDEILQELIASMKKDFPRRPPSNENLQEYYMSRVRKNLHVVL

CFSPVGEKFRNRSLKFPGLISGCTMDWFQRWPKDALVAVSSHFLSAFDIDCQPAVKQAVI

STMGMFQDMVAEVCVQYFQRYRRQAHVTPKSYLSFIGGYKQIYKEKREEIGNLANRMNTG

LEKLIEASESVNELSKELAVKEKELAVASKKADLVLAEVTVKAQAAEKVKAQVQTVKDKA

QAIVDAISVDKAQAEEKLEAARPALEEAESALQTIKPAHISTVRKLAKPPHLIMRIMDSV

LLLFQRRLNPVEPDPERPCVKPSWNEALKMMNASNFLGSLLSFQKDTINAETVELLEPYL

TMDDYSLENAKKVCGDVAGLCAWTSAMSFFYGINREVLPLKANLAVQEARLQVADSDLST

AQATLDAKQKELDEVQAQFDAAMAEKQTLMDDAEACRRKMNNAEALINGLSGEKIRWTEA

SKSFDAQIRRLVGDVLLATGFLSYTGPFNQEFRNLILRSWKKEMVSAKIPFTDDLNIIQM

LVHNTVISEWNLQGLPNDELSIQNGLIVTKAARFPLLIDPQGQGKAWIKNREGDKDMQLT

SLNHKYFRAHLEDALSLGRPLLIEDVGEELDPALDNVLEKNFIKSGSTFKVKVGDKECDV

MSGFRLYITTKLGNPAYTPEVSARTSIIDFTVTMKGLEDQLLGIVILTEKQELEAERTNL

LEEVTSNKRKMKELEDNLLYRLTSTQGSLVEDESLIEVLKVTKATAEEVSEKLVIAAETE

LKINAAREEFRPVALRGSILYFVIVEMSMVNVMYQTSLKQFLELFDLSMARSHKSPITTK

RINNIIEYLTLSVFQYTARGLYEMDKFLFTILMTLKIEMTAGRVRGEEFSVFIKGGAALD

LNAVEPKPKKWITDMTWLNLVELGKLPQFTQILSQVGRNDKAWKAWFDEDAPEECPIPDG

YATTLDTFRKLLLIRSWCPDRTVPMAKTYVSEAMGPAFAEGVILNLESMWGESNVRTPLI

GFLSMGSDPTENIERLAKQKGYIIGAISMGQGQDVHARRLMNVSMLEGRWVLLQNCHLGL

DFMDELLDTVLTTENVNEGFRLWLTTEPHPKFPINLLQTSIKFTNEPPMGVKAGLKRTYG

GVTQEQLEYSNMPMWKPMLYSVAFLHTTVQERRKFGPIGWNIPYEFNQGDFNATVQFIQN

HLDDVDPKRGVNWVTVRYMIGEVQYGGRVTDDYDKRLLNTYTRVWFTEHMFSDNFAFYTG

YKIPKCKTVEEYRNYIEGLPLVDTPECFGLHPNADITYQTNTANSMLATIVNIQPKDAGS

GGGETRETIVYRLADDMLDKLPEDYIPFEVKERLKKMGLLQPVNIFLRQEIDRMQKVITE

VRITLMDLKLAIDGTIIMSENLRDALDNMFDARVPNTWRKISWESTTLGFWFTDLLDRNA

QFSSWLFEGRPMSFWMTGFFNPQGFLTAMRQEITRAHKGWALDTVILDNDVTRMMKEDVH

SPPGEGVYVHGLFLDGAGWERRNARLIEPTAKVLYTQLPVVHVSATNQEKQRSMNLYECP

VYKKPQRTDLTYIFPLMLKTNKNPDYWTLRGVGLLCDIK

>SG-PL_contig_6312_1_-1_208_312

MKTPSMRKNFHVYANCGFCCNYKRYRKMIHYYYYC

>SG-PL_contig_6313_1_+3_27_437

MALTDLDGGLAIVLRLHHLLATHGADPFLVGAALTIVGAWLMDRRLADATFLKGSRRVGV

EPIFLGSKGLVFSNLAATTICCVSGPRLPWIWGNHFAQLLLTFFAHHFLVAGFFFADHRQ

MSQTSTLLDVVTGSNRS

>SG-PL_contig_6314_1_-2_137_325

MTQPGILAAIIGGSVVLLLCAILLVMFIVYRMRKKDEGSYALDEPKKMPNYSYQRAPDKE

FYA

>SG-PL_contig_6315_1_-2_191_286

MTQRESENCCRKCSSASSHPPPHSLKEFCHCL

>SG-PL_contig_6316_1_-1_1_87

WYHGLLEGTFRPGTSVFSLLGFLLGLPHP

>SG-PL_contig_6317_1_-3_159_1820

MQLKAGLQGFDALACLLNYCTTELDAFSCPAISAQLKIAQAKLTEQQQSLDRVSQEKGQV

EQQLQEVTAARDAARQEVEKLQQQLKQAEETHNTAQQQLKEEHCAALKTLQEELTSQHQQ

TIQEIQKHQDKQAEYTRHSQKRRLSELTSEHQMATNQLKLAHLEEIQELRNKHELQIAEL

HVQQRKKLDDITQRFESIKLSLTEKVQSLCGECDDLRVRAKASEEALQRDSDVKVQLALT

PYLQLPKEIESLKTVIDMRTEEIQKLRRQNMDLVKQLEELPIAQEKVAALQQKVENLQAI

INIKTDHEKQLHEKCQVLMRKYDRESRANKRLSMDYEQVVWRMSQSADFTGSTESVSPRQ

LSRSPSRGDQHSHGSSPARSLSSSSQGGDSNGVVVRRHRKGSAGDDNSDHRRRANTFSSD

TSKHKRSGEEGDSRINGPSSSGKQRVLSKSGDYSDLVKSAKEHEESFSSGHESGDMSDFY

SSLESNVILETAQGVSFHVVDKTATDGVASQEEVFMEEMLNVTDEGLPADSSLPSDSMDS

QTEVTVVITSPTSK

>SG-PL_contig_6318_1_-3_9_350

MVRDRYPPHMEMMSQTEVLGGLRELTEGVLKYEGDLVEFDDRLLFVKVTHSKRGSGSGIR

NHQDSLQHQQSSVSTPVRHDLKLTMTLPSNTKPFPATSSAPRPQTRSSFKFLWD

>SG-PL_contig_6319_1_-1_1_291

NHHVETTLTLGWISRKERTHGDNGKRHPCQNCIEKSTPILTLFCMHLLAWFLFQITREEK

QLCNFQAGSFKNYKKKYDTRVGIKKPKLFCCVLKRLF

>SG-PL_contig_6320_1_-2_1712_2014

MEKSSSCGMTSRLNENCQRLTIGKVKVCSTGDCFCHHQILCPVWLFGGLIGCSSRQDPLS

LGCGNKQFRHTEVGPVFDICHPALPLSLGCSNKQFRHTEVG

>SG-PL_contig_6321_1_+3_3_230

SVPVLSWLLNASRRGRPARSRRIAEAVHGKVEAAFRARPAPSTSDGRPRPARAHPSPASY

PGPTGPALRANLFPEV

>SG-PL_contig_6322_1_-2_131_676

MWTKNRAMGYTLVFCCLLFSLLLLAQLSEGKRDPARRGNSNSRNNNNKRNNNNRANKQRA

PKQARPSRCKDKTTFSLKAIDLGRKPFTLKCPRGPIAATEVVGIKGNCRGSSRFLRWSAS

RCQWNDTCSIGFKGKPMDELTSDVSYHRCIGEVPDKLQIGYTCLKKKAAITDVLGTFKKT

RK

>SG-PL_contig_6323_1_+1_385_705

MLFPRTTTIPMDRFSPDLVSSMLSSTTRFMKGSNPRRIPVTERFPFNFSVIFFSMYFFNS

GGCALLILISLDFGSHVHNTFSVARLLQGGSRHKSKLPCFVFLIGRA

>SG-PL_contig_6324_1_-2_2_433

CARSPRRVPQWDLLLVLDCLRSPPFEPLSGISFDLLTLKTTFLLALASSKRRSEILALSG

AERDIAFHRDGSVTLRFLPEFLAKTQIPGDTLSSVTIRPLSDIVSRGEEDRHLCPVRSLR

WYLDRSRTRRSSQRRLFISERVVS

>SG-PL_contig_6325_1_+2_2_199

FFFFDVQHFISSKYIDNINMHCVCKREVTLNILNCPVQRPVSMTRLQQEKAGKNRITLTT

KDVHVQ

>SG-PL_contig_6326_1_-2_2_286

SQEFFAALSATSVRRVSGDVRGHLLYGAGKSDGRGKIGPTRTGGSCKSLSRNLHRDNPTF

SASEVRFYWFKVFTTRLLRLQRMAMAPCLSAKYVH

>SG-PL_contig_6327_1_-3_186_365

MYCYEATTNLFFLFSLSHGSVVADHATCLPRSAKETGSHPITHFVHIGRPDFTWTRYWSP

>SG-PL_contig_6328_1_-3_3_95

FTNLFLTIFFLLHIFCVKLGLWCRWHRDTLV

>SG-PL_contig_6329_1_+2_2_202

FFFLIKLADNLINKIQTRQQQKKLGKFKAIYISKTIQKTCCYREKQTNTHLTLTLKGWSD

SNIDCKV

>SG-PL_contig_6330_1_+3_3_170

CCCFVFFVVVVQFLFSLISFLVSFLVCFTLSHFSWILFPSVCMCSQSVVHCWHICC

>SG-PL_contig_6331_1_+3_642_968

MKPRHTSNLTMESNQVFQGQHENEQKTKRSSSGRSQHTQHRAVHTTQLQAKKTQNLQEHD

TLSNTIHQDTLEDSCCQSRQRKSWVANIKDWTNLSMPELLVAAPQRQRK

>SG-PL_contig_6332_1_-3_3_686

EEDKKRVSWTEALRTIVKNCYKHHGREDLLHVFSDEATVATQPQTSHFPGTVVQTITNPD

GSVSIIHIDTGPHATNNNIITLPDGTQAQVVQTVSQPQQQEATQAVQTLADVAASQQEGA

MGVTPVSVEMADGTTTTVTALTGATIGQNGQIILTGDGSLGGLSGIVTIPASVYQSVTSI

GHVSTDQNAAGANTIQVAMAPGFLKTEVPDATQAIEVTSIEPQVVGQS

>SG-PL_contig_6333_1_-2_2_178

FCVVVGHIIMKPLILGHALSLVFSSDTDMSDLCKIVELQGHNYGKDMLRGYNSLSRYLP

>SG-PL_contig_6334_1_+2_2_139

DFRRKKPKVLNSNNNKLFLNRILYIYSRWIHHTQTFPYVLHPICQL

>SG-PL_contig_6335_1_+2_2_139

DFRRKKPIVLNSNNNKLFLNRILYIYSRWIHHTQTFPYVLHPICQL

>SG-PL_contig_6336_1_-3_3_110

IKICHELCFFAGFLFSGLRFCVQRSCLLDFSLRDFM

>SG-PL_contig_6337_1_-1_4_354

MDRVLDNTGGGYDLTTGVFTAPLTATYLFMATTRTDSELVCLKETRICAGFDIMVEGKPV

ASSWCRGPTSCTAHAVVRLDAGHKVWLRAGHSGSSFNSMARGDGFTSFCGLLTQPEL

>SG-PL_contig_6338_1_+1_13_285

MADRSFRAVYETILKTIQKTKKSLLKDTLLLTVVKITVNSKSSIKLYGSETYHYGPHNYP

KLHKLTFFIEKITHTHMCTCACPHTQNHSSI

>SG-PL_contig_6339_1_-1_895_1140

MQFVWVGIIIIIIIIYNPMTSVVFGGTYGTQQQTGSSSVSLGCSNKQVRHSEVYPVFNLS

PSSSSSVDLGSGLLFWTVCHGG

>SG-PL_contig_6340_1_-1_1_132

KKDKIKKKVDWTCGQKRKEMTARSKKTNKTADRRGVYTTNRKQG

>SG-PL_contig_6341_1_-3_594_743

MRLMHISSQSTDQAEGFSLFDFFIFVFLSWFDVVVVIVIPISMYLRNLCS

>SG-PL_contig_6342_1_+2_2_205

QSCTTHTLSHALHTHTHPLSHACGLASSFDRAVDSRSDISSSIPARNKTGLFTARLNIPV

ILVVSGYC

>SG-PL_contig_6343_1_+3_3_137

VHIHTYTEHTHIRIRPQLCGKTYKHAIVNKILKNHQKFPFLFTRW

>SG-PL_contig_6344_1_+1_70_207

MYQSINQPVIRHVNQPINQHTNHSMYQSFNQPLNQPEIRHINQSID

>SG-PL_contig_6345_1_+3_3_197

LMFPHNIYFKTLLANGIRHGLDPSLILKQSQPLRAKPQQELIKDWYEKEGKNIQLICYTY

SSIYV

>SG-PL_contig_6346_1_-1_130_903

MEGRQLMDMELTKTREELVRLRERYDRLMDSHKKMQKLNNNLEDKLLRLVTSSEAEKTSL

QAQLDDITIRLQDTRVYVTELEEENEKYRSDCNMAVQMLQCKPSSFVGQRLTALPVELQE

RVRKHMTREQQLLSQKAEEERLVRVPIPTFPPTAMVFSVDPRHNGNGGSPNNPSSAELKD

TVPMSLIARVLTQGPSKRNPMHTYLCVNCRSDTVVADKGTQANLLMDHSIVVDRFPSNGV

HHGTSAKLYRTDSTDMAS

>SG-PL_contig_6347_1_+3_3_152

IIQSNTFILFQKFNQQKFNNTIQHFHSIPQIQPANVLTDQSTTRYKLKIS

>SG-PL_contig_6348_1_+3_681_824

MSLKVLASTLYQSMYITSLQNHKCKSLPHRPVTNKEVSRMELMTGRLE

>SG-PL_contig_6349_1_-2_179_349

MQPWIYLVSISCEGEVPSVKMSASLCYVIAQCDPLDTQPCCHRRALTGGSGRPGFEP

>SG-PL_contig_6350_1_+2_2_223

CLSRCLSVCLSVSQSVTLPICKRNVSGGFLNLVHRSLSVKSGESNTRAVQYVDQESPSCG

HLVLKTRQLLEGRS

>SG-PL_contig_6351_1_-1_16_2055

MASERESEEFSVPLPDAEHASLQATWGLGNHLLVYATGKAPPRLAGDGVSGVENGNIMEV

RWGQDMHGATTRKLINESHSVFVMLQGQVTSLTGTALYNQLKEASRQYRSIVKACSLELQ

LLLSDSTDEAEKEYYSIQIELLEFMQIVWSLCEILFIDITPGEMVLVQLLNWVRWHFSAG

REMAQDVLQEEVPEEHPAYWEAVFRLLLQGEIERVRKLLSLHSCSQTQPFNSVEEMLKKM

PQWGNLRSVSLAEFDLKWRHWREECMRRYDEGQFTAFSQLERVVQVLCGDVVVFSELKDL

CGTWYHLLISQLLYQNPTVRPTDLSFHVQACMEEFQRGGHRMGSIDNILQAALEFDLHQV

IKDSSICLGDRSWWFGAHLTDILHHCGQIDSQKLPFGSNLREYLLLEYVAGLMSHNSLWQ

VGIDYLDHCPVFGKDHMKLYIENIPLDSERKANKLIHICEERDLSEQVQSICKIMGVRCS

RDGRLGSALSWFLRSKDVSAATRLTEKFLEEYKENGSFSHLDLLDHLGPTMLLTQQLTFL

GKYREFHRLYQDKDVKGAASLLLNMLTARAAPRHFWVTLLMDALPLLETPQVVLGSSQTY

ELMHCLEELTQELSTTAKKSPTPHSVAAAQATETTSSLPIVAVQSQTLSQGDLDKLKYLR

LALCRNLSRSIVHEGTIVPS

>SG-PL_contig_6352_1_+1_1_63

GIDLINYFFWSPNIFVLSIWL

>SG-PL_contig_6353_1_-1_13_2397

MPSTPQLSPQEEQEKLLEEALNVVKNQAFQMKRCLDKGRLMDGLKHASTMLGELRTSMLS

PKSYYECYMAISDELRHLEMYLVDEFQKGRKVADLYELVQYAGNIVPRLYLLITVGTVYI

KSNELSRKDILKDLVEMCRGVQHPLRGLFLRNYLLQCTKNVLPDVQEDSAGSDSGTVMDS

VDFIQLNFAEMNKLWVRMQHQGHSRDRERREQERRELRILVGTNLVRLSQLECIDVESYK

KCVLPGVLEQVVNCKDPIAQEYLMECVIQVFPDEFHLQTLSAFLRACAELHPSVNVKNII

IALIDRLASFAQNEEGPGIPTDIQLFDIFSQQISQVIQNRPDMPPEDIVSLQVALINLAL

KCYPTQVDYVDKVLETTEEIFNRLNLDHLEHGSMVSKELMRLMKIPVDTYNNVLTLLSLQ

HFGPLFDYFDYKGRKLMSCHIITNALENETLVPSQEHVDSILNIVNCLVQDQNDQPEEPD

DPEDFAEEQGLMGRFIHLLYSDDPNQQFLILNTARKHFGNGGDQRIRYTLPPIVFAAYRL

AFRYSQLKEEDDNWEKKCGKIFQFCHQTIGALIKAEQAEIPLRLFLQGALAAGRIKFENS

ETVAYEFFSQAFSLYEDEISDSKAQLAAITLMIGTLEQMSCFGEENHEPLKTHCAQAASK

LLKKPDQCRGVAVCSHLFWSGKTGTDEAEMRDAKRTTDCLKKGVKIANQCMDSSVQVQLF

VEVLNHYIYFYEKNCEQIAIQVLNQIIARVRELLPNLEANEETEQINKHFKNTVEHLQLK

MEASDSKVSYEGLVL

>SG-PL_contig_6354_1_-2_182_556

MKLYNINANLIKVIESLYNKATSAVFFNNNIGEWFRTTVGVRQGCLLSPTLFNIFLERIM

TDALEDHEGSVNIGGRTITNLRFADDIDGLAGEEQELVNLISRLNNTSTEYGMEISAEKT

KVMTN

>SG-PL_contig_6355_1_+3_879_1022

MQKLIFKSLTRWLIQPVIWWSLQKKSSTTRRNSVPHALKILRVIMTLC

>SG-PL_contig_6356_1_+1_931_1116

MRQLMNTDTGKKTNFCLSKRMSSLSLSSFHFSYIHIIQAKLKVSTSYLVQPPFKPIQRVA

VI

>SG-PL_contig_6357_1_+1_1_237

RDVDSGRSPPPHQRPRDGGGVTLPPRLRRPPQGQGGQAVHGQHDGRLLREQARGGPLLRP

LIEGRDSPEVVREQRDLSE

>SG-PL_contig_6358_1_+1_841_1044

MATRANWVRSGHTALSQSGNTTGPNNLHIISIISQGEQQPSSVRAEVCTATISKVRQRFC

FKHTLTLW

>SG-PL_contig_6359_1_-1_1_351

PAGALQPPNTSRPVPPPVPVESSPNEAPLDTGGASHTPIAGPSRPAPLHTAVPGAGQAAP

PHSGGPSVPGAGAPPSSPGGGPPGFPPPGWIPPPFNLLAPWAWPSSQGGVGPSTTAA

>SG-PL_contig_6360_1_+3_3_155

EPLYNNVKSFRIPSQQRIKTSEPLYNNVKSLRALFMTKLKLESSFTIKLSY

>SG-PL_contig_6361_1_+2_2_232

LFLTCVRAYDFSSDVFLGTGLTCSTCMIKQKQQFCLPVGPIFALTFLCLYGMIFGFTGSL

ATSLLCSTCINCSPHYF

>SG-PL_contig_6362_1_-2_575_775

MRFCVYKLRKSNPAQPPPLICPQLVNWTKICAQTCIFFCLLLKLVHSFRGLMSVCTSDLN

GTDGSCG

>SG-PL_contig_6363_1_-2_44_1561

MQDDTRYPGPSSAPGQAMGRGMKRGSRFDTQNGNPPKQPRGPHTPFSHNQGISFRGMSNK

SCPIPQGRPYSRNRVPTYRRSYSRSWSRSSSSRSRSRSRSPRSWDRSRSTSRSRSRSPPR

YRQRPQRRRSRSVSLSPSPTPEREITLTTRGGRGKQRGRGRGRGRGRGRDQGNGLDIGNK

HNITPKGKGKKNQQQKQLKFEYDDPMKAARMAKRAARFGDHLDQPNNKRKQALTLTINNL

ITSCGDEDMDLGEFTVVGICKDLEKPYLRLTSAPDAKQIRPVEILRKALLMVQEKWKVKG

DYHYVCEQLKSIRQDLTVQGVRDEFTVRVYETHARIAMEKGDHEEFNQCQTQLKLLYNEG

MKGNRLEFTAYRILYYIFTSNTLDMTTVMASLTPDTRQDECIKHALAVRSAWALNNYHSF

FKLYTNAPKMSGYLMDWFAEKSRKCALRAILKSHKPTVTVDFLQRELAFNEREDCVKFME

EKELVLVADKSKLDVRNTPPAIINKL

>SG-PL_contig_6364_1_-1_187_462

MQMDQFHTTTYIVQWPSRVLFRTFYWTLKMLDLQPKRIHTPVCWCCHEETSLFIRSGSLW

IGSGIQGVKSQIQQECLFTCAMHDAGLRTEVL

>SG-PL_contig_6365_1_+1_1414_1608

MLLQQALHLHTPSMTKTMTIIDLNKNEQQMHTREPPDLRERERGRCKSSDKKTLVSRLIK

TWCRG

>SG-PL_contig_6366_1_-1_1_171

RTSRHCQRVGFTRRFMSYATIISDPFASHGIYGLGAYGSFRSRKRHLETTKLKLIRA

>SG-PL_contig_6367_1_+3_678_1001

MIDSHFSVFGDVHAGCFRVSISHQSLTWLTGSLTCIFDVLYAYTKEGGSETRRSAAHVDL

EIGKFCTLCLPALPIRVELGPSCWKSDVLTTLLSRLSLFVSNYGHKHV

>SG-PL_contig_6368_1_-2_29_313

MFLQLRRDLHHGRLLCSPDDAVSLAAFIVQSEVGDYDPEDHPPGYISEFKMLPKQTPKLE

ERVMEQHKALHGQVPSEAEGNFLKKACTLDTYGVD

>SG-PL_contig_6369_1_+2_2_229

PGDLLVQFHGILRVEFSTKTQHHRIHTFQLFVDCLQWIQPVSLLFSINIAETKVIFLHQI

QFFGHTVEQPLSNCVF

>SG-PL_contig_6370_1_+2_122_250

MKIQLNQTTASTTTTITIKKREREFGMHVCVRACGCVCVCVCA

>SG-PL_contig_6371_1_+2_2_61

GGRGGGLTDIYLIINSTETT

>SG-PL_contig_6372_1_-2_86_655

MASKFGARPAGSDGSDHWHRESIAWQYKVSSLNKTRLRFDIFLHIVLGLMMLIRLAPGLT

SLFGLNIIALRRWDLPHPRPWEYAWLFSLVSAVIGWRSTPQNNSFLLKQYMLGTVIFGML

PIVFGVIDLRNDMSAYFSERKYTYVMFGMPAVVLWALFLAICMQLHAFGLYFSVVLLKAW

RPRTERLKAR

>SG-PL_contig_6373_1_+3_90_272

MNTHFLHTALTMTITKNPWGEHHSSHFCGNQYTLFDSSFGPGHSTPGYHLMASYIKIKWI

W

>SG-PL_contig_6374_1_-2_8_1669

MIGAPQNDLRHTGHIGYDGAVFGDVSFIGDNYDKLPLKVGTSGKVDVSRGSSLSSIPHTT

ESPDRNGFVKTSTSDDSLDRRNGYGHSWISQESLASTQADDHNEGGYGDMEDNSIFADFK

MPDLSSSFDLGPSFMDEVLKALDEKEKQAVAASSPTFNGRDFGDDIDPNRVDLEDRTPLV

EDKTPVFEEKGFSYRESSAPVKRASPPPLPAHPPRVDMQREKKQAKVKPMSASEEKMMED

AITLANEVTATRAQRHSQSQSPPHSPTGSEKVALDFSESVLDPSPSLICKLKNSIRRSPK

SERKRTFSDDRAPKADMEEDLPAGAQEAYNALVVRGSDRQADKEDQGGQERLSVSSDAVF

TADPVDTPAQESWSITAGVPQPRLSSSRGMPQPPPRPMPKPRLDPKKSELPVPKPRPEII

QRVEPIRRPPDSPEVPLKDKESHIPVPATRKESWSESKEREREQLKRTIEIVRMESPAKD

DVSSTGPDLDTKESTPEPDSDSTRPSSASEGGDSSKVMSLFEEDFSEPSPREIMSKLARE

SRMRRNMDHQRVLS

>SG-PL_contig_6375_1_-1_454_2208

MDYVPSPESFYGPDFAKSTFAASPNSLYMYDQERHDMQNTLNNGILGQTCSAVGMEPEFD

LNPQYENSNSLEGYFGNVLPSVSEGSVSTQSARCSEKQGSNGMLGFLEATQPELKQGGPT

LAELNMDPYLFEDIYSIIGEEEKVQASSTVKQAASDVRKQATATNPVKMETSPKMYTSLG

PYQTHQPQVGMTPQTQPQSTTSSLSNLFSIKPQNIAVVPPISIATTIKTEPMDTYDSGSK

SCLHKMLSSPPLKAVLSAATTPVQISSPPRQVMSVVGQSRAPSLAQQPVKSESVEEKWKE

IEKFIHDPELSASRKRKRHASGGSAIMSDDESHMGNLSDDEDSDSDADSDFSDTPFEESL

TELAKKSKQYFWQYNVQAKGPKGTRLKLQINDADPHHPSTFEDPVFDATSTSLVGIRHGG

KARKGDGNEVSPNPRKLCQIGQQLYKLNRQINNCQVSNDLPANIRNKSRKEKNKLASRAC

RLKKKAQHEANKIKLTGLDTEQNELLDVIRMVWPQLKERAQRHMKGVVPSPGPEQSLTAL

LDDIIKSHRKTIIAGNTTDYVNEVISKVEDGDPTGGLPIKPKVKK

>SG-PL_contig_6376_1_+2_1202_1468

MCSVQTCMFFSLFNILQIQVITDEWHNTGCTLPDKSHKWPMETKLDVFRFGITRLPLGFK

EILTETPKLVIAVTTKDSAVQVKAFLFLF

>SG-PL_contig_6377_1_+1_1_300

LCLRCVLHLAGCPCRRVRPPSPPFVHITHDCCVVRKLADDSAGGLLLELLKRSAHRFEFQ

YVDVLLLFLLGPDSQDVQRPHVRSPAHRGCICAQRHLLGG

>SG-PL_contig_6378_1_-1_691_1635

MSCATALIVIATILGPITLVFMAVSFGTDYWLEFDVNRDTLTPADKQFDKARYTHTRYRG

LFRECYPGNDSTFLDTSSGVVDGFCFNVQYDLPESRTGYSDDYLVRIHLLRLFLAFYIVA

LVFFVTAFVFGLILCCWRISRWAYVAGLCAYIAAFSVAAAIAFFHGAEYLERNKLTEAEF

YKQWSATIKDATRRSYGWSYALGWVGMILAAFTATFYSLAGCYITSQRYEDRERLEKDSS

HRIGRDYPIAMEPVYAMEDPYMAKPYGSQPLYPYPTMGHPRAYPGPYLMATENARYAVGY

GEPQRDIWTMREAAS

>SG-PL_contig_6379_1_-3_1212_1625

MFLIVYIRCLFGLIIFLSVCKKSFDMLIYLVLYPLQNYFMFSLRIVRLFCAIQKVKPPRA

PPWGCRGVFQNKQYPPPSGNSVLAICSILLFSLSLPFFFPLLSCFFLSSRSIPLSFFQQA

FTQFSLAPVYDVLSVLFC

>SG-PL_contig_6380_1_-1_70_465

MFGTICEHLAQYWSTLEGSAVFSTLTYTKSFCPALPCPPCIDVVKFPSQQKLEYCLQYYA

PCTLPFTVCTFCTNLSPRCVGLFNQSTARVLVAATMMSCVSLYSPLFHFGFLNLFSYSWM

LYDGVCVPQLES

>SG-PL_contig_6381_1_+1_1_378

RHRHRQTDRQTQRQTVGTDRHRQTDTARQTDTARQTQQDRQTQQDRQTDTVRQTDTARQT

DTDRQTDTARQTDRQTQQDRQTQSNKQTQQDRQTDRHSKTDRHSKTDRHSKTDRRTDTAR

QTQQDR

>SG-PL_contig_6382_1_-1_2485_2769

MQLYYIAVQCCCNYYNTAQLLQSRVIYSYSSPRRCPQCRQPEFGTLLRRFEPGVRYIKLT

FILPILTELLFWWCQRRVRSAPHFSWVVIPVCYIT

>SG-PL_contig_6383_1_+2_89_256

MSSECTGTGRTETGLDFARYLVSKWVSHQVRSAGTSINPDLHLARIQYRNLLIRRQ

>SG-PL_contig_6384_1_-2_173_478

MSDSPSPHADDDIDFKSRSLRDFSHCRQIGVYLVYSFKRYVLIMENDQCPLIMEKNNCPY

ECPHYKINTERQVLFHAFCSSFHAGCHGLLTIKRRVTNHKKK

>SG-PL_contig_6385_1_-1_1_222

LSQNPARICDDIFRTPASGGNDMAGLFGAATPRGGGTGPAWSSRSKRSLPFRRSVRAGTW

TIFTPCGVEVLNHL

>SG-PL_contig_6386_1_-3_3_71

SSASLSTSSPRLLFYASLMKLTN

>SG-PL_contig_6387_1_-1_1_288

TTYSGEPLPVVGKCKLSCTVKGKQHTLEFQVVDINSKPILGLRACENLNLIQRVEVIEDD

AILSEFDDVFHGLGDMEGTYKICTDGNIHPVVHAPR

>SG-PL_contig_6388_1_-2_2_106

KNSPPPSLSLYIYCTIILYILYAILYNYGGTCTVV

>SG-PL_contig_6389_1_-1_1_180

RLFLNVFSMFFDMLGCCCIFSLCLMLTHGLTRLALGKILCTNLYKCKYCSFHQLFTLVVA

>SG-PL_contig_6390_1_+2_1094_1399

MLFERRSVSRQHALCAILRAFATMLSLISDYTTKASTVLNSFDLLWIINLSLSVYTLPSH

LLHCSDSRVGCGCGWVDEGWWGNQAKEKKKPHSTTVMCLHLA

>SG-PL_contig_6391_1_-1_1_309

AVLLAASPSERGTPPPPPTETLSPSLTGSETAAPLPADHQGPSVSPQTPTGALQPPNTSC

PVPPPVPVESSPNEAPLDTGGASHTPIAGPSRPAPLHMAVPGA

>SG-PL_contig_6392_1_+1_211_534

MSFSMSVTDAIFNILTVIGAIFNILRSNYQQLTFLSLSLSLSLSLSLTTALFLHQDTKYS

GKDCDMVLNFRTYRHVAYCNFVRWIWRSTLGRGNRIPLPACLVSAVRA

>SG-PL_contig_6393_1_-2_1586_1897

MSLTSTLFSHYFFISKCGVTVQNKLLVRNVATKAWYSFLLGNFEKKVSLVCKIGKMLWLS

GRLYKDNLIMSYSSEYDVTLTESKLSERMQVQIVRKLGEQYVDQ

>SG-PL_contig_6394_1_+2_80_193

MSIAHTHTHTPSLSFIFKRPNALYIKSTAHSICHSYNI

>SG-PL_contig_6395_1_-2_1718_2017

MFVFIFFFCISCNFFFLGGMYARCSGCLAHIVQALYCCIVCILHLWWVFQAMFSRCMYAV

WPTLCRRYVAAYSSFVGLPPPCRQTYSFFRVCMPDSFLFP

>SG-PL_contig_6396_1_+1_1_165

CFPLLFSSSVIYIYHLEVSIRLVFPPNQNTQNNKKKITGPGEHYFQSRIILSNFS

>SG-PL_contig_6397_1_-2_86_1606

MAVALPPINSGNGNKKVPAYAETGPRGPPSGRLRPLRIGNKKERELQDQRMQLRSLQKDL

NKADTAKFRNSYKHSLCLDMLKDGFHLSFKELFNLIQQQLQEREAAGPESLMWTQTMLQD

KHEELDTLKYYLTGAETAQRKGDYLAVYMARYELACYFQPTKDKWLADHFFQTCLDTAAC

IDADGGQTKAEAHCNLGIVLEDNFDYFGAAEHFEAYYELSKASLDGWVSDVVNKGKVSPF

IDACVHLYRIYTSIGDQLEREDEPEKMLEFLTKAFDMTKQAEDKKLEGNAAYRLGLAYEK

SGNVDTALVHLNNFYEVSKAADRADGMGKACDAIAKAYARIGQKEKSIAYLKEFVETAQS

CGMDAELSQACHNLGNVLNSLGNYKEAGEYFNRAYNISRAMGESEAIQVNRVQSGIAQAH

AMLNLYSHIVVEGVRSRDYLERTIDWKSVRANIQESRDERMRKSVQAEPVLSTKDEPAPA

EEPADPVNEATAEPAQEPTEDAEQPAE

>SG-PL_contig_6398_1_-1_1_183

ILLWLDWQVWDGRWGHQRDINFVWLLLVIFSLSAGLGGGAQREICLATCCGFHSSLCIFG

E

>SG-PL_contig_6399_1_+3_1443_1832

MSPGWRRTVEQRLLCMMDCPLATVTTKEKTKALPEKVPLHLCYCSPPVDKQAADYEAWHC

RIYQVTSNFVTPRLRKKGTSKRTVIPQCPHLTRPSSVAGFACPTSDLPTMWLAANMDISI

PKTLPAKTLP

>SG-PL_contig_6400_1_+1_1_288

IGALRWAAKSQLLCCRVELNSRLQILSSRLCQQSQPARSINYLTWLFWKRLHVKRFWERI

EELLRSKCVTCANIRLTENVILFGVDENNYAHRFNI

>SG-PL_contig_6401_1_+3_618_752

MLGWALDIYSAVLLAYRAFLCIFCGFEVFFIYFFFYTFCNLGMIN

>SG-PL_contig_6402_1_-1_1_87

SSFREMRDMLPYLENRFKSPSSVLQALER

>SG-PL_contig_6403_1_-1_58_231

MTETATDIETGVDEKCLLFSFVIVVLHSAYIVCIVKMFASSQDIVFMTLDSICVCVLF

>SG-PL_contig_6404_1_-1_1048_1389

MNTAYLTMYKANFYQRLSNELHFCPPTIGNLQNLQYQHHCFTNQYETSLFKNILSVELNV

FTWRNLLWCVSVTSLLCLSIMCTLLPYALWILKEKEQPLLFEISISLKNNNNTL

>SG-PL_contig_6405_1_-3_3_176

PAPFLPVRLDAEELAQMCFRCGLDTSPCCCAADCLLRICHKLQNIQVISSTWYQVSYK

>SG-PL_contig_6406_1_-1_1_858

VEQSECCKRGCRLFSILSFVHDHSDSNATITDCAANCNEAYGNQREKDRACQLGCDIQLQ

ASTQAAAQVEGKHRQMEDDMRDTVHMLYPLLYMHSVYSNMLDKVHHHMEVSWSIFTQANS

GELVVLRSEPRIIDFVSSDEEDIPDGVPWLDDKTSSYVETNIVGQDNSATPLLNFRASQM

GAAHSPDDGLRLDTDDRAGRSDWLTCVAKKTGIARIVLCILILATAVVMIWFCLSAATTA

PEQHLSISGDLEYLRAMGEKDGFKLLHPQDRVEARPLPIKIRVEQI

>SG-PL_contig_6407_1_+1_1_252

QCANLKFQQHINTTVQRAIFFHPPTPLHFFNAHSKYYHYQQSEVFLQLLNVGGKSSFIFL

TKDISWTAENGRNIGHYSLMLHRP

>SG-PL_contig_6408_1_-2_2_283

QPPDHPIGGLIDPLPILEEKRIALATPNGIDRSSHAKRRSPSRDRGSRCHVGSRRDATRR

DEVRATTSHHDDNRRVELRLEEDSRHNDSRRDKL

>SG-PL_contig_6409_1_+1_1042_1323

MASICCLHWNVSKRERFISALDMTMYTEAGDQRCFLDCFLPLRVLSCDCLVPVLIGAGST

GWSAASLSSSSGLLGTKTTDNMQTIWKIAPRKRP

>SG-PL_contig_6410_1_+1_643_921

MCNINIKLTTKDRLASNIIICNIHSDIHVSMRSTIYVQYESFCCLSTVTDIHQTESSTKQ

KALSFHKMLPMVPMKMGLSSCMQQNKPNRGILP

>SG-PL_contig_6411_1_-2_122_244

MAFTFLSELAHAGRSSNSSIYAVQLFTSLAKIFEVLRMRVG

>SG-PL_contig_6412_1_+2_56_325

MHNLGGKTFIAFCMHKVGSKRHSQLYNQCTFRSHYQDNTIIQQHSRIIIIHIFTSGCSIY

AFIYTFQHRYCGSEQNTNFTLHRQSLNKSL

>SG-PL_contig_6413_1_-3_3_275

GSEAVDQLAKNATKKEQAKTRVPFTDLKPKVAAYIQSLWQEEWTCQQDNKLFHICPDLSF

LTFSIWLPGGNCTLQVTYRSHFSHPFAFTEG

>SG-PL_contig_6414_1_+2_2_262

RIHGYPSRQQTRHADRKRPNLARTSNYISESFVMRIFQQLKCFLHCVLLFRPAFTLYLDL

LSEFIVNYRKKTHKIFTKYTEKENGMF

>SG-PL_contig_6415_1_-1_4_456

MDSPSILPESWKSPVGEIYLQYINKVADARNIRPKVPYCFAVSVAGRQYFLQAENFQSMN

YWIDALNGASKISVPILPEQADKGMEWHAADLSRTGYVTEIAGGVVCKMPMQTHEDSDSG

SDEEESHSSLSSRLSPRSLSPTSSLTRQQNM

>SG-PL_contig_6416_1_-2_860_1189

MCVVKTCTHLYFAHLYSFRLVVPIVPMQRLQKNMCLQDWSSIPHEELHTALKTSIPIRPS

DGQERVHQWFQILIFISCLLLSVVTCLEFNNVLVLQSATSMLLIWTICTI

>SG-PL_contig_6417_1_+3_84_281

MENRGNIYNFFQFNVFSFFSLSQSLFFLIFTGFFYIVTKQFIHRSVRLNIIVCIIYKSFP

VFFFFC

>SG-PL_contig_6418_1_-1_1_156

CFLQGFESRLSQNFLHVATSSPSCEQVLTLFRVGRGKTARERRWTPPPSCWP

>SG-PL_contig_6419_1_-3_111_251

MALVWSAEQSSYLGFFCCCFVCYCKHFDELAVSVCLFRLKKKKKCQQ

>SG-PL_contig_6420_1_+3_261_401

MAVNPSSLMLFLRQSIHIYSVLFKTVNSVMSTAFDRSVISSNSSRAA

>SG-PL_contig_6421_1_-1_415_702

MLLSAEMTLSRYLKFPTSSSSSPLTVVFFVLLVFSFMILVFPGWIWRHSDQTDSIIYRQT

ILLLVIYLAFFPCYVITVGSLPCIFPCYIITVGNLP

>SG-PL_contig_6422_1_+3_3_230

SLSVQQTERQHDIVLSLSSVSLFNELNVNMILSRHCQSLCSTSVTAAEVTSFVYKPVEKT

RNKTTNTLCIKHSHVC

>SG-PL_contig_6423_1_+3_3_251

LLHRFLCRLLPIRWLHRHHPQTCVDFFQSDGCTDIILKLEGLPINHSLLQAEKLDFAGDN

FINFTGNVRKYSLCRVFDIILQI

>SG-PL_contig_6424_1_+3_162_536

MTKQVNTLHSIVIYVHHFFSQWVICNLTTSPVVTTLRTTGTRILRPRELNRRSSTLKTFS

CTGFCAAFFLSDSCTDVILELKGLPINRKIIVIITARQYYSRTKVLIFSPYQFCGHWISS

FLENA

>SG-PL_contig_6425_1_-3_33_1559

MMSKAASSKGFLIDGYPRELEQGTRFEKEVVPAQFVLCFDVADETMTKRLLGRAATSGRV

DDNEETIKKRLETFHEVTQPVIDHYEKENKVRKVKAEGEVDEVFSEVVKIFDTEFKADTS

VLKDVKVVFVVGGPGSGKGTQCAKIVEKYGYTHLSSGDLLRAEVASSSERGKALNDVMQK

GELVSMDTVLELLKEAMVSKAASSNGFLIDGYPRELEQGTRFEQEVTEAQFVLYFDVADE

TMTKRLLGRAQTSGRVDDNEETIKKRLNTFHEITKPVITYYEKLDKLKKVSAEEGPDEVF

AQVEKIFDASQSGTVDTKLKDAKVVFVVGGPGSGKGTQCDKIVKQYGFCHLSSGDLLRAE

VASGSERGKRLNAIMEKGELVSMDEVLALLRDAMLKNASDTKCFLIDGYPRELDQGIRFE

NEVAPCTCVLYFDVSDKTMTDRLLERGKSSGRVDDNEETIKKRLDTFHNQTKPVIDYYQK

QDKVQEISAEDGVDEVFVKVKSFMDSKQW

>SG-PL_contig_6426_1_+2_281_472

MRSISKNGFRRTYPLLFISKSLCLTPTVSEQSVQLNCDNSGTISLILILFFTIGKSIKNA

FIIK

>SG-PL_contig_6427_1_-2_632_844

MFKGIYWHGKYLFIHLVFISLPVTPLFRNIFHDSCYCWLVGLLYHGKCSVLIMFHTRSPF

DILLTNSYANE

>SG-PL_contig_6428_1_-2_686_889

MKGSGIDLEIDFVSVRPSFSPVPIWELCRARFDIHHTDLKKEDNTNLLASLVKCHIANSY

SNHLKIYT

>SG-PL_contig_6429_1_+1_1_120

QHDSDIPDVEQPISAINKCNEHISPCINLELLIHTLFSVE

>SG-PL_contig_6430_1_-3_3_98

QIIPQNTGDLENDSRLCKFYQLIGSYDEICIF

>SG-PL_contig_6431_1_-1_268_687

MYLYMCMVQSTGLYISLHKDVYGSVHRSISLSIYMSMVQSTDLYIYACLCFSPQIYIYIC

LCFVHRSMYLYMSMVQSTDLYMSMGWSTDLYIYTCLWFDPQICIPIHVYGSVQRSIYLYM

SMIWSRDLYIYACLWFGPQI

>SG-PL_contig_6432_1_+3_144_236

MAANYIIMCTPACVHIGIITTTSLSAPCYNS

>SG-PL_contig_6433_1_+2_116_283

MQACLLTQTNKSSTVTLKMCYTLLHTKIAKQMTTKSNTVKLKMYYTLLHTQIATKI

>SG-PL_contig_6434_1_-1_1_189

YARSVFVFFFPPPPPSKKEMREGACYFMSVRVCVYVNKVCQKRDLRCVWMDFYPNMDSLN

ALV

>SG-PL_contig_6435_1_-2_1916_2185

MGKTVEGLIQIATGSNSYRLYIILLFTWSNFYCLKQPVYFSYDVSEKVKRTCTNLAQVCA

TISRIYGFQISQNSTDPLAILQVEGFVPIL

>SG-PL_contig_6436_1_+2_575_790

MGHSLSLSLSLSLSLSLTPLSLSLSLSLSVATEVCLVTLWVIVCVCVCVCVCVCVCVCVC

VVSWWVFLCVRV

>SG-PL_contig_6437_1_+3_3_86

ERRQTSNFAFGRAFLIPTEISLKLKKKE

>SG-PL_contig_6438_1_+2_2_208

WSVFRAGVWYIFFLQLIQQKSHIFQCKCIFLLEIFLKYSAILTYKPDSLTQFFIYYFYFI

YLLLHENHI

>SG-PL_contig_6439_1_+1_1_291

FEESCRDHIASYSTIRRWKREFKTGKKGNNSDKPKRLLKRAWDESNVAAVSKPVKEDPYI

CIREVSAKLSLSYGIVQRILHNDLQLQKLAAKWVLQK

>SG-PL_contig_6440_1_-3_3_182

LVFVPFFSLQALILFFYKQTVYICTISVLKVVIFYFFKHTVCFVCVCSCFMICLKEKTAH

>SG-PL_contig_6441_1_+2_2_145

STFHALCAWQRPVEITDMKTETGNHTISLVSNVLLNGHKDMRSTSHSF

>SG-PL_contig_6442_1_+1_190_357

MDTSTPASITGILCFYHSPPNNLHDTAMFCQSHGSYYFNSSKEVRVCVCVHQTNNF

>SG-PL_contig_6443_1_-1_1_132

LPIYFVSDYGILFFKLYGLSAEYVVSVFYSRNELFPPQVKCFYL

>SG-PL_contig_6444_1_-2_2_310

TESLYNHIWKTWASWCSKESVDPIHPLSNQIANFLALLSSERNLVTSTIRGYHSAICTTI

KQVGGPNFSEDFLLRDMSQGLASLEAQSPSQVPAWDLLLVLDA

>SG-PL_contig_6445_1_-2_2_286

GTPQDLKSDCTAFILTTLLKAGTSSEALRISAASRREGTESLYNHKWKTWASWCSKESVD

PIHPSSNQIANFLALLSSEWNLATSTIRGYRSAIC

>SG-PL_contig_6446_1_-1_193_309

MHIFKFLSLCFVMTVLEYDFLSILHTTSEVLLAQYSSLL

>SG-PL_contig_6447_1_-3_3_680

MADTSQARPVNQRVIRDRLSLWEAKTNPIAPLTPKQKDALIELTAVAAERPLPSELPEDD

MPILAIRPAQQASDGEGEDTLDAFIQQSLPGGQKIENAQQFFSWFSQLEDSIEHGEEEQY

RPYLDSLRGYQEQCDTVLGEVTQCVDYLRQLHQQYINVSTKTNALHDECEHLLAEQTQLM

NTAESVSNKLSYFNEYDTITTKLSRQALSVTNESFVPMLSRMDECI

>SG-PL_contig_6448_1_+3_48_239

MDGAEGQREGNEIRMIFSDQQTATSQKGTINLSTSHSLLHLSFNISIPLTQQILDRKSTR

LNSS

>SG-PL_contig_6449_1_-1_1_483

CRVSFSFQTIRDNWLTRCRVSFSFQTIRDNWLTRCRVSFSFQTIRGNWLTRCRVSFSFQT

ICGNWLTRCRVSFSFQTIHGNWLTQCGLSFSCQAIHITSSLSSLLFILLSLFDHVTERCT

HTSWGEKRKKEKGTCITKDGKILLWHQKDGNGPFGIILCSC

>SG-PL_contig_6450_1_-2_113_469

MALLVMLETMEEFRREVLHAQVSLKLSVPDSVQLVFDRPACSSRLPGLFLHRARSQLPVA

PWGSIPMTSPRPSSPICSAPSSRQPFQACRAMVRAGTQRLDCIGAASGYRLPWLMPGHL

>SG-PL_contig_6451_1_-1_7_417

MESLDLDASSKSDVTVSRSSSLIANFRLDEDVDAETRDLFIRVDDPEKHTSTLEAYITFR

VTTKTTRSEFESSDFSVRRRYNDFLWLRQRLEETYATHLVPPLPEKHSLKRLDRFSSEFL

RVRQKALQKFLTRLADH

>SG-PL_contig_6452_1_-3_3_200

LPTTTTPAPIPCPITSPLPTPQPRPHPSLVYSSHQTQYLCMMGRTCGDDSLTECTHHYST

PSFEVE

>SG-PL_contig_6453_1_-1_1_141

MLAVRHGKYIQPNMYLEKNTLFHHLSRHFLNQNNSSLIHTHFYSFIN

>SG-PL_contig_6454_1_-1_298_456

MHSHTHRKRGPLSFLTNFHISKFHLFVYLGVHSFVSFQANCRMLNNYLFDFMQ

>SG-PL_contig_6455_1_-3_129_386

MPKNSGMPGKLNLRVFLCAILFTDYTRCYRYECVEVGSSLMEIRAQCPITVDTFYVKVAR

SLVEIQAQCPIAIETFSLIFRKCNKV

>SG-PL_contig_6456_1_-2_29_376

MASSGDDPVPSNGINNASSDDKSLEGFITSQLAEQSVNRGENPGENIDEEKMNRFLSRAL

SESKRMNKEVAKSEAKMSKLHAELGVERFAAWRQEHSSEVLSHQQSQLVEMTHAKK

>SG-PL_contig_6457_1_+2_2_184

THTHVHTSVHSKPNTDKSFLEHNQFIATHGKASVHKTRHIKNRSKDTWFIGKSIFNIRSI

S

>SG-PL_contig_6458_1_+1_1_201

FFSNLFYIFIIIIKDQNKKEIAFSRLHIYFIMNREINDLRFQGNLHIFQVTNLLTVNIRN

VQTLVNS

>SG-PL_contig_6459_1_+2_89_316

MEFAEGVESIYTERAPRIPTGVYKLTALYILLVHHSTSYARFMYWEGCVCVCVCVCVRVC

TLNGSLWRLIQFKNRL

>SG-PL_contig_6460_1_-3_3_182

GECKQQLKVSSSRLHQDSDKPKIKQAVLIVFHLEILLRVTVTMRTVCSRRLGEVVISFVS

>SG-PL_contig_6461_1_+1_1_252

KIPWQQKSCLWQNSVEKSTVIGEQKIHAYSKKERKQKKKRVAYYGDVLSPGRAVHIQLGH

FTVVTRNKRTKTTTTNINKTESNK

>SG-PL_contig_6462_1_-1_379_516

MNKYMNEAIQKLSMFVKIVQKKYYPIIFKHFIYILSRLSSVTALGC

>SG-PL_contig_6463_1_+1_1_222

CHSIVKGPTDRCHSIVKGPTERCHSTVNTDKDGFSMRLSQVNTLTCCVTLWPKWRIPVPT

CNSVVKMENPCSYM

>SG-PL_contig_6464_1_+3_66_185

MQTLNERFVYKYNIIIIIILISVKLSKDSAHTKTVYSQLK

>SG-PL_contig_6465_1_+1_1_264

SIHTYTAAKPYCLSYSVHTYTAAKPYCLSYSIHTYTAAKPYCLSYSIHAYTAAKPYCLIL

NSYIHSSQAILSLILNTHIQQPSHTVSH

>SG-PL_contig_6466_1_+2_2_127

CPFQDLASRQFCTGRLGWSAFGTVQVQSTNSNSKLCCLICYH

>SG-PL_contig_6467_1_+3_3_116

FFFIDMTSSIYSFRSEKTSLFKGVLQQVVDLFRIVSLI

>SG-PL_contig_6468_1_-3_15_932

MSMAVNLALWYTKHAAKLAAKEEPDMEEAKAVHKCMRTAAGIFKFVKETLVPKLIDSFES

GTDADTRVLDAYIKQCTAEAQEVTIARAIELKHSSGLVAALANETAQLYTDADSALASLD

EKIAGKWRKYCQLKANFYTACAHSYNGETLLAQDKCGEAIRGLQESVKMYEKSEQQCKEY

AATKGAGTTARPQNHLFFRKLGPVVRRTLEKCERENGLIYHQKVAYDPPHLELRATYGLV

SPEEYQAPAINSLWTPETYAKFDTTRAPKPEKKKAEEKPPTLPTVKEKETPMSDHDPKTF

SGCTVS

>SG-PL_contig_6469_1_-1_64_249

MSKDPGEGRLLVGLDHHLFLENCGYFSCILGLFALKYKCFLFNVTFSVTKLETLCFKHCL

HM

>SG-PL_contig_6470_1_+1_1_273

GDTKNNLKHQRNRNQHCKNSEAVPQQMDGKNRGLHAGRQDGLKWNNNSKKLAGCGNGRHT

GKSTEGSVEQLYNNTKYLIPSGSLDRIRAKT

>SG-PL_contig_6471_1_-1_28_195

MILFGDCACDTCDKPVTNLRRLFPLCCIMMVIRVRSQDCAPNFSRKQCSSKDVDLK

>SG-PL_contig_6472_1_-2_122_511

MSGSHQSWGGPADGHSKVESGWARGIESSGGSGDRWLGSNSMGSSQHQRGGSSGYTSHGG

GHGSHGGGHGSHSGGHGIHGSRGGHGSGMSSSGGGNMYMSGSHSSGMSGSGMSRQQDTRF

NIAAITARRF

>SG-PL_contig_6473_1_+2_152_319

MLTTQFTMNMADVRILHINLTSTFTFSCTFWNTCDLQNVPILVKLDSQLSQTSDFH

>SG-PL_contig_6474_1_+2_89_385

MKTQFSFCNGVVMMETELLLLRSSDSSQHLLIFGAVTWSAFSCRCMGIYFVYNLSSKNHT

FFSVNACFLLEILSVYSAILTYKPDSLTQFFIFLLFCLP

>SG-PL_contig_6475_1_-2_2_112

ASWCSKEPVDPIHLSSNQIVNFLALLSSERNFMMSTT

>SG-PL_contig_6476_1_-3_3_881

SLTGAANTTTAAGGLTLGAVAPTTTTTASTGFSFGLTPAAGAATTATTTAAGLTLGATTT

TGGGLGGFGFGTTTTSAPTLGLGGLGAAATTTTANIFSKTAAAPTFTGLGGVDPKTSSAG

GAGGLGSSTDGKAVKETYIPDQLVTTVDELQKYVKGEKAVREDIVRMSSKPLMRVKEDIS

ALKQLLSVVSNGLQRNACTLDKLKAEMTQELKNAEMAQRTKDIPPGLQYENTAPLEYFEQ

MVDQFEAQMVSYRQQIEMLENHLSALQQPIRQSPAEIFTLIKRQHESFIALAA

>SG-PL_contig_6477_1_+3_3_71

IHIVLFLDTEKKSKVLKSLQQKL

>SG-PL_contig_6478_1_+2_95_382

MVSQSQVDSTLHMFKTYDFYSGQSSDTVIHVMNIIAFNCPTRMAESARLLLADLGGTHTE

LCEYEVQTPRTQTGTQYISSFLLLLVVLLPGCIHKG

>SG-PL_contig_6479_1_-1_211_357

MPPAPLSYLRESFTQLKDIPCGLRLGRNQAVTVSLLSVGCVLCFLLGIL

>SG-PL_contig_6480_1_-3_213_497

MVQRGEKHHRHGSVWCYCSVCHCLAKRMRTTLVFLSEKFLMIGVRTALVSLSEKFPMIGV

RTTLVSLSEKFTMRGVRTTLVSLSEKFMMIGVRTT

>SG-PL_contig_6481_1_-2_5_1552

MNKHNTARLLRLTPFLYVVRSDLMEILRSPWVFVTSLVLLLHIVLLHTSTASRPHIVVIM

ADDLGWNDVGYKNPEVHTPNIDKLAEEGVKLNYSYMQQVCTPSRVAFLTGIHPFRMGLQN

LVLRALENNSMPVEHRVLPERLKTLGYATHYIGKWHLGFCHYNMTPTRRGFDTFYGMYNG

KGGYFNHTSKWSGYDLQDNKGTDPQNFRVAWEDYGKYSTHLFTNRAVKIIDEHDLSTPLF

LFLSYQAVHGPLEVPEGYLQLCPNVVDETRKRHCAVTAAMDEGIGVVMQALRARGLSDNL

ITLFTTDNGGPVRLGSSNWPLRGSKITLWEGGTRAVSILHSKTHLPYAPYEWKGLIHAVD

WYPTLLQAAGDKSKIADIDGVDSWKKIVRNSAKSARQGFVYNINDVKNNAAIRYNRFKLI

YNKGGNPDGWYNPPGNGASEEPNGSYSEYMLFDVEKDSEERNDLSGVNKEHIVKTMAKLK

KQLATFRQGLKPTTRAAVIAEGKPRFHGGAWVSGWC

>SG-PL_contig_6482_1_-2_236_373

MVVPLSSSSFGKEVYLAYQLLYLQATDFKFFTAYFACRAVIHLVFC

>SG-PL_contig_6483_1_-3_81_206

MLWQKSLLLFVDIGTIQRKENYRNRIYEWLEACFELGTFFFK

>SG-PL_contig_6484_1_+2_38_280

MDRQGRKKQTTVKEGKGAETKRAIEKRKFLAQNFQKVGILSILKEPPASPQRGGTGEDVS

KRGVSVTRVNMVSPHKILHSP

>SG-PL_contig_6485_1_+3_96_200

MTKFCSFHSYCFAHASLQIQLISKPQLKTIQACSM

>SG-PL_contig_6486_1_-1_280_414

MFIDNIFGIFLTVIFASFAVYDTIFLIPGLIWLPIYFPCLWILFL

>SG-PL_contig_6487_1_-2_29_238

MVFVPLKCWYHLMRTDGENSNDGVDRMCGHPPAMRVGGWIGCGWGGFCNCSEVCNVRVEC

RLPEVIVSGV

>SG-PL_contig_6488_1_-2_356_2353

MSMSMAPRLVSLIRELGRDPTALNKLSMGRASATYKLTYGLAKSLKQDMIDDIADQFFSL

NLDEATSSNNEKVLTVLISFFSEKRGMVVVQHLGSVSLLRVTSARIVEALDKMLVKNGLK

WNRCLSMLMDSCNVMRGVRSGVEKTIREEKAPHLLDIDGDSCHHVHNACKAFTSVFGGEI

EKLFGDIYTDFENKQFQDCLSLICFALDLKYTRPERFLHHRWLSALNLSADTLRLFDGLT

VLYYEFLPKMEKTAYHSNVINIFRTKNVNESCRDEIRDLRKQLGTIGLTSEGKLRKQRIV

KRLFYHRGGTLLQLKFYVDALAVLKEFVMLFQRKDPLIHILHDQQLSLVRQFLLLFIKPE

CMKPLKAQTLLSLNLDDKANHLELEAIFLGKAEASLQKLGPEIQRSFRETALKGYIACGR

VLLQKLPLCNGFLEAAGGLNPEEKCSVALEKCLKLPSFLPADVIAAPCQSTEESDEEDPE

QKKEAVQVAYNREVRNFLGEDNIPALGDDQRVDEWWAATFTKYPLPLLSRVVKAVLSTFH

GPLVESSFNIMGDVIDQRSGRMDMSTFDAYHTTHYALMASGKPATSLFHRGNVMREPVNK

RLIQNISTARQSYHQVLAHKSEEKKRRAALLDKHQARVASKRKAIETLKEDAKKARLQHF

MKLARK

>SG-PL_contig_6489_1_-3_3_65

KKKERIIQINTHYYLCMFVDT

>SG-PL_contig_6490_1_+1_1_372

VTHLQIIDLSLITLSYTCRSIVPNIHSQDLLVHLFILESSGQTSPQCFNVFTHIYILGSF

ILSEVFIRIRSACLNKKTLICTPESNHPRLYCQILELNTSKVSSKYLSSFYTNLGGGEGT

LKTC

>SG-PL_contig_6491_1_+2_584_775

MEWERELETIWSGHTNNLNFLLPFDCCLNLTLKTFSFKPISFKLAVTTLLIKLNILTDLR

KIMS

>SG-PL_contig_6492_1_+3_30_185

MSTKLFCLSSGVWRRMPNSSQEFVHHHSRSKCEKEKSCHRPCDPLVPGQGST

>SG-PL_contig_6493_1_-3_111_254

MQNHDIAIYLKFTHQNMFRNRAKGTNPFQFSFEHGLMKECLRPNMSRL

>SG-PL_contig_6494_1_-2_2_313

YTVKDLLQHDFFLEDSGLKVELVTRDDEASNCPDIQLRLRVVDAKKRRDKHKENEAIQFD

FNIQNDQAEEVAQDLVYSGFLHEDDMRIVIRQIRDRVMQVKRDR

>SG-PL_contig_6495_1_+1_154_303

MDRVTKQNFNTYYPHTHNIYIYIYISQTSLSTVYITHENNFNNFNLHLCV

>SG-PL_contig_6496_1_-3_3_350

YPLLVFTVNLISKECILLLVFCFSKSWWSGTSFTLWGLCVTVTLYKQSWLCLGRCRYVAS

VLWVVLLLLVSSFVNCRYMLKKGSYLLRSELTHFGFYHRTNFQAELRISFICHNPK

>SG-PL_contig_6497_1_-3_3_224

MPSGGLFKHDLCHLEDCLESVPTGGLFIQPRPMCMQLVHLSQISFLCHHGIRKSIVCLVF

ASNHCTISSRSYVN

>SG-PL_contig_6498_1_-3_126_344

MQCVWYPLCCFPNYTVSHCVHLQIASVHSVVVYILLCNLCNLFACCHFKILEGGGLFCLH

LVPFFLFLFHALC

>SG-PL_contig_6499_1_-3_3_143

ILGDPQSVNLGNANNKQQQFGQRLGKDFAHTSKRFEVLLRERLRHLI

>SG-PL_contig_6500_1_-3_99_2915

MGKILCGVTSALLLTGVLVAVGLPSVYTQNNFSPLQNSLQADPISCGSTVNSSGPITLPS

SLNVTLCIIGVQRISNESNFSSLIFSHINLQENDVLSVYDGEHNVSSNQVISITGSRDTL

VVVLSAPTAYLVVNITANLSSSTLQATFSNKSCDIQQSVSGPNIVRSPVYLPSRMNFTCS

YEVKANQAGSGGGSSAQQLALSFLKVSLPSATLNVTTSSSQTVQVTGKDLPSDVNSSGSL

KMLLSVLSVNTSQTFSAAVSPIISSSVCGQTKDINGSLTLDVKVPDSHPLPYDCRIVLKA

VKGKSLSLNITSLQLAANGDSVVMVDGGSLAGGKVLATFASTTPGYLVLSGQNQVLVRLQ

VGEMLATRSLTVDVTELDTVGHLYNSGNLVIKPSSGSKVVTQYYLLEASAGSMVKFMLTG

EEASKTYSYSVSVYDGCQTSSPLLVTFSSNVRVPVVSSGAKLLVVARLSNTSSSLNASFS

TQTAAGCDKVTSAKEGSWQLMTTPSVPSKCRWTMTAPPNYVGTILLDLTMVKLSQKATVS

VYSGVAESGSLVARLTATNIMMGAPLPSVAVPASQWAQVVFASGNSTNLTSVAVNVRFQQ

LDTLCGGQIQAAQGHLRSPAYPNLYPLNAQCTWTFPPSAPGRLLFFDFPSFQLAQNHSLT

MVEKLNGTSRVVKSLTGKAELPDVLYASNNTNTVTFSSQPVEGAVVSSQVAQGFDVQYWV

LQCGGYTNKSTGSFSTPSFPATNATLCVWFVQLPPKTGNNVNIVNMTVQIKTSGSPRVKD

IVKMYDGDSLRKSQIAYNVSGADTQFKVLSRYNSVLIVVNASAAPSASQHASAAAGLALK

VNYLTYSCKPKQLCDNGICMHPDWICNGIDDCGDSTDERHCISPPTPTNPKTGTKPGIVV

LLFFVGLLLGILLAVIIPKVIRRFRTNRYTAFNNDPAVA

>SG-PL_contig_6501_1_+3_81_275

MVTMSLFQVTKFMLTEKQKNRTTKPTNSNTYYPHLPYDLCTLHTPFWLSTKTKYTNQCSA

PFPLP

>SG-PL_contig_6502_1_-3_3_134

KAQLVSWCTRHNFDSRMKGQPRSVCWSVSRDTPMCAPCQGATSI

>SG-PL_contig_6503_1_+3_177_362

MMLNGYNSEDMKWVLTHRMEGGIVREIKEYQVGKGWGWDWAVRKKTKIETVHIHIQSYSH

QH

>SG-PL_contig_6504_1_+3_651_824

MSISNGDASNLSPCCMSKAYHNHSRSAPDNFHVYQLGCRYFCICPVHPVCKTSLRMAH

>SG-PL_contig_6505_1_-1_862_1173

MMLQCLMLQFSSCFCIFLRLFPLHYSSELIVTTKLLHICLFKHLSFVILHSEMSLTATVG

HFILGCLYHCKECLIPFLIIQIFQVVPKICVVEVTKVSTPVLIN

>SG-PL_contig_6506_1_-2_104_289

MLCILRQAMLMTLGHLQHDYHNCLEKRHMIKFNIGITQSTLLVTGDKDSFRIQITAMKTC

LQ

>SG-PL_contig_6507_1_-1_13_324

METRKLSSMQTPEKGVLLPAWQCKLPKMGVCRLCSITTSAQCGPNNQNNMGKKSCHEIDE

VYTPPHLPQRPYTYGTMFTCLRHVTGQGKDSPIMLHWILRRVRW

>SG-PL_contig_6508_1_+1_1_132

LFIYIETYLTQTTVLTNVTHSDKGVSNANLAVLEVLTSLKTTVS

>SG-PL_contig_6509_1_+3_108_710

MSNLDKHLFNLKFAAKDLERNAKKCEKSEKEEKVKLKKAIQKGNMEGAKIHAENSIRQKN

QALNYRRMSARIDAVASRVQTAVTTRQVTNSMAGVVKSMESAMKSMNLEKVSQLMDRFEQ

SFENLDVQSQVMEGTMCNTSTLTTPQGEVDNLMQEVADEAGLELNMDLPSAQGSAIGSAA

TSQASQEQDELTSRLAKLRQM

>SG-PL_contig_6510_1_+2_2_202

AVSSSIPARNIITGLFAPDLNQRFKYSSFVSFVFHRACFPPPSPSPISGPCQHIGICAGS

RSLPDYH

>SG-PL_contig_6511_1_+1_1_102

RMEHTGYFWPRHNITHTNYRTQQSQPLNQLTLPK

>SG-PL_contig_6512_1_-2_2_208

HSGLVTHSGLVILEFLVLNCTDCTYEFVKSRRCCGHRQTTDRHTHEHTHTNTHTHTHTHT

HTHTAYVHA

>SG-PL_contig_6513_1_+3_1485_1691

MHTQHKHFLSENLKYYLLYVDLTTPFAYLPQFINMYPELFVSLSLSLSLSLFVCKTNGLC

YTILNTECS

>SG-PL_contig_6514_1_-1_70_2697

MMMLKRDKKEDEEGGGNPFNNLEKSTVLQEARTFNDTPINPRKCGHILTKLLYIINQGEA

IGANEATEAFFAMTKLFQSKDPILRRLVYMGIKEMSKIANDVIIVTSSLMKDMTGKEDQF

RGPAIRALCSITDTNMLQSIERYMKQAIVDKVPAVSSAALVSSLHLSKNSQDVTKRWVNE

AQEAVNSDNIMVQYHALGLLYHIRRSDKLAVTKLVSKFSKHTLKSPYAFCLLIRIATKLI

EEEDAGSDSPMFDFIESCLRHKSEMVIYEAAQAIVQLKNTTAKDLAPAVSVLQLFCSSPK

PTLRFAAVRTLNKVAMKHPAAVTACNLDLENLITDVNRSIATLAITTLLKTGSETSVDRL

MKQISSFMSEISDEFKIVVVQAIRSLCAKFPRKHGVLMNFLASMLRDEGGFDYKKEIVEC

IIITIEENPEAKEAGLAHLCEFIEDCEHTVLATKILHLLGREGPRTPTPSKYIRFIYNRV

ILENAAVRAAAVSALAKFGGHCEELLQSCVVLLERCQMDTDDEVRDRATFYVNVLKQQQR

ALSSAYILNGLTVSVVGLERSLHQYTLEPTEVPFDMKSVPLATQPLVEQKVRDVPGLAGD

VSAKPEKSAATRQDVYQEQLAAVPELANLGALFKSSQPVEITESETEYYVQCIKHTFSNY

IVLQFDCTNTLNDQVLERVTVEVEAGEGFEVVKQIACPSLPYNKPGTTYTLLHLPEDPTS

VTGTFSCTLKFVVRDCDPNTGEPDDDGYDDEYVLEDVEVTVADHVQKVMKPNFAASWEEV

GTENELEDTYALSTMSTLEEAIKNIIQYLGLQPCERSDKVAEGKSSHTLYLAGMYRGGHD

ALVRAKLALDSSGVTMQLTVRSTDPNVSEVLATAIA

>SG-PL_contig_6515_1_+3_3_179

EGKKEKKRKEKKRKRCLGAGKILPNTEVKWVGRTKIKTKQNKKPQQQQQKPRNSEYTKE

>SG-PL_contig_6516_1_+2_662_865

MSSLSIHMHLLKSSLSIHIHPLMSSLSFTHTHTHYKRSKSLFITPQCFHFEGHFTSKMVK

SQVKANNL

>SG-PL_contig_6517_1_-3_1086_1358

MGGKRVRISMKQMCMHLGVLNDERDMSVLIYHTFSLRCTDQLSAFSIFTQTAVHIFISYL

KHSRQLVAVYSDLDKLSLVVLIQQNVYIPKL

>SG-PL_contig_6518_1_+3_309_398

MQLDKRPRINIFVLKKHNIFIKINDPTKKH

>SG-PL_contig_6519_1_-3_36_851

MESLKHKVAIITGASAGIGEGTALLLAKYGCWLTLMGRDQGRLNSVVQQCVTAGCPADKI

VTVTGDVRDKSVQQEVLDKTVATFGRVDILVNNAGIVHTAKPMETTEEVYDDIMNVNVKA

VFMLTKLLSPEIIKTKGSIINVSSLGSQKPMDNIGVYCMSKAALDMFTQCLALELAPHGV

RVNSVNPGTVVSMIYRRGPEGLNDEEYAKFLECQSDLHPLGGVGVPEDVANSIAFLASDM

SRFVSGMTIFVDGGRHCVNAGVPSPSSLKEKK

>SG-PL_contig_6520_1_-2_188_406

MDSRSHILSSTPARNIKVGLFAVHLNAPIILVVSVFHMLFSPPHFSKPCQRIKFCAGSRS

LPDYYWQIWVECV

>SG-PL_contig_6521_1_+2_14_163

MDTTRMIGACRWAANRPTFMLRAGIELEIQCLGSTTLDKSQPARETLCNL

>SG-PL_contig_6522_1_-2_200_466

MLQSFWWCQCFIGPVPLLISRPCQHIGFCTGSRSLPDNCWRIWVERMWNYTSVMNPQTHS

KLLEVVFNTTTTWHRNGNPPVSPPITAKV

>SG-PL_contig_6523_1_+2_173_331

MYMKYHQHHICNRPEQKWRQVTRTNVDSGPRLDQSYLYILISLKKFTDWSKLT

>SG-PL_contig_6524_1_-3_2229_2480

MSGYATYCWGPDYFTHSVCLCTPSPSFFLLCFLRCMYFLSTPNVCVCVCEREREREQRNL

EETCFASSVSIPPALPIFSPLYLN

>SG-PL_contig_6525_1_-1_1_933

AATEEQPESTEAQAATEEQPESTEEPSAPVEQSQHEDESAPAEEKTSADTSSEKPEPPLS

TGEPSPNEETDAAETPASVEETSPPAEDQSAEEEKQACQESQSPHGEQQVPVEEQPLTEP

QAIADEEKTPTAEDTSLTETPVPSEEVASAVDQSSAQEKPETAEQPTSEEKAETEQPTHT

GEESAPAVAEPTTADEQELGEQSEDQTTPAEQAVADEQSESPQQTETPKEEQTSTEQAVP

TEEQASTEELPVPAKEHPTEEPESASEPEQTTVLSPTVMVEATADETAVTADSADTSAAA

DEPSEPASPSQ

>SG-PL_contig_6526_1_+1_1_240

VCKFDHCDTVSVCESGHYDVNVSLIRLARWVPWSNGEDTTSKLKGLEGLGSNLLGHLAWW

IKGEDFSDLTGQHRYRSASA

>SG-PL_contig_6527_1_-1_31_339

MRDVKEFPLTFWLISVICVAYYVAVFPFVGLGLVFFEMKFNMSPTSANAVNSLVYIISAV

ASPVFGFLIDKTGKNIFWVIAGITITLGCHALLAFTFITPYLG

>SG-PL_contig_6528_1_-2_2_181

PFILVSHIYIHTQTKCNQTRLLFFSLLFNCIEFFATPQLNLQQSAINKCNPMQFVQQQMS

>SG-PL_contig_6529_1_-2_2_208

FKITKQQSNPPPHPQSPFIVWRNTACSSVFACRRVVSLTRDGGETTMKTCSDNICKLNSI

QGENSTQIL

>SG-PL_contig_6530_1_+2_2_214

THTHACTHTHSLTYSRLIISEKYTHTHTHTHTHTHIYAKTDLLKCSNNFSLTHTHTHTHT

LTDEQLLHLLT

>SG-PL_contig_6531_1_-1_130_312

MYFLEFIFVMSMKTRWREANVVKTRYVLNGQDACLRRILLTVLLRPHCLSRHKTMCMLIC

Q

>SG-PL_contig_6532_1_+2_2_232

LHNHNRQHSFFVTSIHKNRSIFPHHSNTYKRDSLWHSTACGNMYISVTVTLTVTIGFSTM

TVRTTFGTKTQLIRFIT

>SG-PL_contig_6533_1_-1_76_483

MGKFMKSGKVVLVLHGRFAGRKAIIVKNHDDGTQDKPYGHCLVAGVDRYPRKVTRSMGKK

KIKDRSKIKAFLKVYNFNHLMPTRYSVDANFEKSLVNKDAFRDPALKSKARREIKGKFEE

RYKTGKNKWFFQKLRF

>SG-PL_contig_6534_1_-2_77_403

MAADVTKRWELASFHSCQKRFLRASRREACSVRTTLLGLMLEVGCAKPFPQTLGFKGLKP

HLVSVLRQCSSGMCQAFSSDTRLQRLEVSSGECPQAVFKMYTHRGECSG

>SG-PL_contig_6535_1_-2_389_643

MLFLSFCVFVGLFACRLFSFSFLISRKEKRLRFIIRVSMCQYLKCLRMPLLFIGGSTIRF

GSCVWRKRISDCVALNSHDTSSHSG

>SG-PL_contig_6536_1_+3_75_200

MSDSLTATRPSPPCKWDYSLGWAIKNELERWLRQRMRLTGGT

>SG-PL_contig_6537_1_-1_532_711

MSLLTLLKCGACCHYVCMYDLNQAKKIVKQSTRRKSPKLVEQSPRRKSLKSYMHIHYDKL

>SG-PL_contig_6538_1_-2_278_937

MAANEERVSSERPVEDMETETDTTKRPCFPPVSAVKQVGVRQMRRVPVPPHRRTPLQHNW

PKIYTPIVDQLKLQIRYNLKSKHVEIKNCKETTMATALQKAADFVKAFVLGFEVNDAMAL

IRLDDLYLETFSVKDVKSLKGDHLGRAIGRLAGKNGKTKFSIENATKTRIVLADTKVHIL

GSFHNIQVARRSICQLIMGTPPAKIYGRLRAIQSSGGSVL

>SG-PL_contig_6539_1_-1_1309_1494

MCNTALASVLWQLASFVSNCKNRHSFCDVMQWIPSCIRCVCMSVYVYMCDSFNGVTCGRS

TF

>SG-PL_contig_6540_1_-2_791_2098

MIQESKYLGGDMEHTHLVKGLDYALLDKVRAEINSKEKEDEEQMETVFTQSKEEEDPEEK

IQFKTRLGRNIYRTLFKSKLPQRNELFLPKRMAYAVDLEDEYGDFDIPNTVIRSKADCPS

LETPTTLTTNDIVINKLTQILSYLRQGKRELKKLKRKEKGKLKEEEKPRDKGAPLEDSIY

GEIGEYMPSFAKPKDKRDRKERDRERDRDKDKDRDRRDRDRSDRDRRDHDRDRDRDRRER

GRDRDEDRERPQDRQRKSYFEKPAEEDEEKDKPSQTAKDLARSINDKFKGYAELEEEQKK

LTAKVEEDRIKKLKAKTEIDSYAECYPGMMESADANEDSDDEADYTKMDQGNKKGPVGRW

DFDTQEEYSSYMSSREALPKAAFQYGVKMSDGRKTRRAAPKDEKAKLDRELQQINRIIEK

RKGPIIGDSSSKKTKF

>SG-PL_contig_6541_1_-3_3_137

LYYIWVVGLQQAVLWSTPNIWWNKSEKSLLVLSKYNQQMSRCVSR

>SG-PL_contig_6542_1_+1_328_498

MIYHKAQETFRTAGNYDIPQSTGNIQDSWQLCYTTNYRKHSDSGQLAMMIYYKLQET

>SG-PL_contig_6543_1_+2_2_139

CSLPQFFITDSVWPANLKNPSEAGVDEGLDSLHGGDSGSPGLGSIQ

>SG-PL_contig_6544_1_-3_3_221

SVCLSLCIVSVPSSVSVSRHWFVLSFTFFLLQLQSICDKLYHAMLHLTFYFKHTIITVTN

VIFITTSVMKHFQ

>SG-PL_contig_6545_1_+2_77_451

MLEEGARACVYAHLSCLLLSAFWVLFVVFASQLLICVWHVCTHAHFSCLLDLCVVCACMH

ILAACSICVCGVCACMRILAACPGFHNTLFSSNRKITLFICTPESTKFRQKADLLWQLKT

TQIIS

>SG-PL_contig_6546_1_-3_99_581

MAVMSSRNPYPFPRLENDEKFCGSQPEQVAPKTNSWTIYKNSNEVYGVPTHLAQQQDPWN

RLNSKHTLSSSRHEVYHCDPQAPSDALDFTLKATYNHHEELLRNRNETLFQRETFYEDQA

GRKLKNREVIVPKKAPELNHPISINGQEKMESVHSIKNAIA

>SG-PL_contig_6547_1_+1_1_75

CLYLSDLVKRRAQACHAVSILDGTP

>SG-PL_contig_6548_1_-3_555_758

MALRRKDFLSLSVFALMLRSLSKVSLTDLQAFCSLCRGCDGSLPLKFYAIELYQKVHVLY

ILLYIYLI

>SG-PL_contig_6549_1_-2_2_298

LCCPIFCAASVVLIPRGSFQLLNTPPPASSFSRSASSRKPKRTSDVKPVWGHPPEETFHC

CQVTSVEFKSGCSDPSYQGHCLQSPRLTTVIVQLQNQTE

>SG-PL_contig_6550_1_-2_212_1804

MLAKRLLTFRNSSSENFEKNLAQAEKLQNLDEGGEDAYRARAGRKRHSVQLDKKITQQLL

SLDRRSSTVSTGTIDSLSSYTGSSSDEDETNPRDKQQKNSKGSGDFCVKNIEHNAFGRRE

IEIAEQEMPGLMALRKRAEGDRPLQGAKIIGCTHITAQTAVLVETLVALGASVRWAACNI

YSTQNEVAAALAETGICIFAWKGETEEDFWWCIDRCINAEGWQPNMILDDGGDATHLMLK

KYPAMFNMIKGIVEESVTGVHRLYQLSKSGKLTVPAMNVNDSVTKTKFDNLYSCRESILD

ALKRTTDVMFGGKQVLICGYGEVGKGCAQALKGLGAIVMVTEIDPICALQACMDGFRVVR

LDEVVRNIDVLITCTGNKNVVNRQALDRLKTGCIVCNMGHSNTEIDIASLRTPELTWEKV

RSQVDHIIWPDGKRIILLAEGRLVNLSCSSVPSFVVSITATTQALALIELFNAPQGRYKQ

DVYLLPKKMDEYVASLHLPTFDAHLTELSDDQAKYLGLNKAGPFKPNYYRY

>SG-PL_contig_6551_1_+2_2_73

STSLTCLNHYRDWSFCFIIVFAMI

>SG-PL_contig_6552_1_+3_39_140

MTSFNSGQHTLMTSTQLITRSTDDVKTTQYCAHS

>SG-PL_contig_6553_1_-2_2_115

GEGVIGLIMREQVSMHVASPASFHSSLFAYSTGMHWSE

>SG-PL_contig_6554_1_-3_525_1964

MTMLTIDTCLNTTVIPTNISSEDGLLDDGEDEQLRELAQQVEYYVMKRFIPTISGLGLVG

NFLSLLVLTKEKLHKVLTKMEISAHIGLIALAVSDFLLCLLVLLVTTLPLKDIYYPGEIL

VYFHLVSNGLITVFIITSTWLIVVMAAERYVAVCHPLRARNLILLSRTRAYVVVLFVLCP

LCTIPVFLESYIQEVVCVDGQVIYRVDTVSRQSMVLRRIVWAMAFDFIPCAALIYFNTCL

IWKIHKAKQLRERMAPLHTSSRKPSTSSNVQKFRWDGNDAKNLRAATEPDNEVNTGKKSF

VGKLHISRGARKQRDSNLLDLMEITESSPSSQPSYNGGVEDRRGEKQLLCQQGKNGLVKT

SSCTTPSSSKLSRAGTYHNRSLSTKRRISDNALHSVTATLVAVVLTFLILVSPWELLKFG

MQYSSTPMDKYKMDIAVYLTNLMQVLNFSFNFVLYCMVNKSFRHTLRTVICLQAFTHVVK

>SG-PL_contig_6555_1_-2_680_898

MVACDCILHDSFGFFSWLFLLLIFMIHLAFLAVVLFFYESFEFLLLLLLFLCSLIYLLLL

LPSFWFVSVEFGC

>SG-PL_contig_6556_1_+3_3_185

GIVSIKIDINYKTWAQKSISFVTNLLNGTQLQTVKHFRNNVDYSLLTKLQCRKCILSLRS

H

>SG-PL_contig_6557_1_-3_12_488

MPSSLTSILLICIVIWGPCLCQRRAPTRGQIEDHFLSTYIDWYNADIRPYQRDDDLPVRV

NVSYELLHVTNLDEEKGHLDTVGYLAMEWTDNRLTFNHRINLDSIVLRTDRIWVPDIELY

NADAPGLKMLNAHSQKVVLMTGGHVIWVPQVATRSLCFA

>SG-PL_contig_6558_1_-1_70_972

MVFTELLATLWTQFLVFARPMLKWLLRKVSGKCELLRIAYEEQPGATQTKRIEKSLQQSR

SPCLKDLCRAERADIEQSVKDIIRLKQIVTDVHVKFEDTLHRCLSYICGYNHLVAQVEGH

RKIKYSTDNSEHEAKLMKLWRLYKPASQLEARITRQWGDLGFQGEDPATDFRGMGVLGLD

QLMYFAEKYDKEAQNALSKSHHPKFGFSFAIVGINITDLQYRLLMKRKLRTHFYNQTNPR

PSMDDYHEMYCYLMSEFVKFWFNEKPKDVMEFSRIRDKFQRHIVSLLKNPETVLRVDFQQ

Q

>SG-PL_contig_6559_1_+3_537_824

MRIIVTKKKISNKEKNNKSKSQNGPKFTTIHGNYGPTMCKDKKKEYSCIKEMEYLSQGQS

CNILSYYNMITISMTFFLSFFFFFLKIIKKIFVLVF

>SG-PL_contig_6560_1_+1_148_369

MLKTYTLSDIIAMISKTTCTHLWSNKVDRLLLRLHSNFYEQTVQLSCIEMIFQQSLLLLS

DITSGDPCDLPIPP

>SG-PL_contig_6561_1_-2_155_520

MVLLTMIARQQDGLPLAASVQEDEQAGRSLVDYQTQAKHLFRKIAVDSNPPTRCSLDAGP

YIYHYVLERGVCYLVLCDKAFSKRLAYTYLEDLQNEFQNQYGAKVHTVSRPYSFIEFDTY

MQ

>SG-PL_contig_6562_1_-1_469_729

MHVSLSICSFLDTAVCIIIIIIIIIIIIISIIIIIIIYIIIIIIIISSSSSSSSSSSSIS

FLRLLPSVELGFMLLKGLLMTLFRPVL

>SG-PL_contig_6563_1_-2_284_502

MLMTDAAMLQNSSDNGRMPAWSDPWLDDNSNSPSATLRCQLADNLHFLAAYWKSIPRFFS

PSDYVHNENPDKE

>SG-PL_contig_6564_1_-1_232_324

MSLLHETELPFHFYTHPNIKTPSLCHTKFQP

>SG-PL_contig_6565_1_-1_1_234

YQYGLTIPCPYLYINQTHPFSNILPFLFLYSPFSSPLHFSVSICRSIFYFPYRSTPMYNS

HSHTNTQTIFHAYVFFFF

>SG-PL_contig_6566_1_+3_249_386

MLLTKDVHSIKLQRKESTYSGHAIQQHVSVTTYRLFYMNIVLTFII

>SG-PL_contig_6567_1_-1_1_201

RTMKGHRMWDNFSQCYDENGDGSDDAMEFHLGLRLRDRAVLYASFHNISWRQPGPGVADT

CSVGQEI

>SG-PL_contig_6568_1_-2_1466_1669

MDMLIKYSTLSFSTFALLSSPLPPFQNSWDGSVFSFEIQFMDALLLFGNLEHLMIQLFFV

ACVVFAKE

>SG-PL_contig_6569_1_-3_57_203

MEFYLGLRLRDKAVLYAPFHNISWHQSDPRVTDTCGDGQEICATPSMTH

>SG-PL_contig_6570_1_+1_1_273

YACRTALWWWFEFLVVLLFPLYGIRTATGSLQTQIIINVKQDDDINAAGRLGGRGRRSGV

WYKVRLAGFRLEFLLSVLFGEGIVATQGFLQ

>SG-PL_contig_6571_1_+2_983_1195

MYACFFLIFLIQLLQPWNLNSTLSPKVTRSFQFKDNVMFCLAQSIYMQILLQTASCVSSS

THLPTKILTNT

>SG-PL_contig_6572_1_-3_201_317

MRGTACLFGRICMTTKPFADLIQNDSKKVRSLSFCLWTC

>SG-PL_contig_6573_1_-3_3_194

SNRSVMEQDRVREPPSCFTSLVTLDLGTVHINLLCFIFLPSIIFIIYILKIYIFWMSALW

WWWL

>SG-PL_contig_6574_1_-3_93_1244

MDLACQIALDATSTVFIEENGRKEIDIKRYAKVEKVPGDTIEDSQVLKGVMINKDVVHPK

MRRRIENPRIMLLDCNLEYKKGESATNIEISKEEDFAKILQMEEEYIRKMCDEVIAFKPD

LIITEKGVSDLAQHFLVKAGISVIRRVRKSDNNRIARACGATIANRTDELKEEDIGTGCG

LFEVRKIGDDYFTFLVDCKDPKACTILLRGASKDILAEVERNLQDAMNVARNVMIDPYLC

PGGGASEMAVAQALNEKAKSITGVHQWPYRAISRALEIIPRTLIQNCGANTIRTLTALRA

KHAQGQKNTWGIDGHTGNIVDMTEFGVWEPLSVKLQVYKTACETAILLLRIDDIVSGTKK

MDAAMSGASRQETQQAPPPAGGDE

>SG-PL_contig_6575_1_+1_1096_1287

MRLDNLYECSVSFFPLLLTFDPSRWSDNFADRTRHSMDNHNVLIYCLSQCSSVHCLSNQP

FKLI

>SG-PL_contig_6576_1_-2_239_2272

MDRLAEVREQRRGESEERDTDSVKEEEIVRSAKVSPEEARVPPAQEAGAHIQQVQKEPSP

EKIQTQLPQTEQSVEEARIQPHRQEYEDRGGDLEDEELVRTPPPSPTEKFKPEEPAETTP

GFAIEAATRLASETDTIDQVIAQPGSYGSDVDGFIRHHQADVAFSHPPKQDDNANLSDQV

QSPLSDGSYKEEPSPVPEPALPCGFMEEEKQFQDVADRDLVLRAELGDETYIDRQEATVP

ILSRHKLSKDQLQRVQTPEQDGYFTVTPGNYPSSRGPLGDLGPEEPSQSFSLREDMDQGL

PGKVPEEVFDSSVSAGETLFEETLPDGRKVTHRHIQPTPSRGAMEEVMEEGPERDNVEVK

EESEILPDGTVHTTQRVSHQKLKHIHRSFHSQSGEEEVHDEDVTVPGKQEVIEMFDQPPK

LVQESEEIEQVLEDGTKVKKQVVMNRMVHLIKMRHESFDEEGRKMEEDFEFEEVIPGTES

AFVAGVDSDYEEEMEMKRTAAMGEMEQVMDDGTVVAQGILTTETTTTVRSRSGSIEEISE

SALVTEERVSPSPGRISPKHVSYVHEGNLDDGSGELYDTSEDAYDYSSKTVVQSKLVTGH

LEETTSHGVVESTIDVVQDMITKGQLTLDNSEAVAQDNRQAPDSDTPLITITEDLSTPPD

EDEDVASYFGVKYEASSE

>SG-PL_contig_6577_1_-3_147_512

MNPGDALRRVFECISSGIFLPGGPGLYDPCEKEPTDALASLTNQMREDLVASAQHALRLL

AFRQIHKVLAMDPLPPPRFSRQRFNNPRKRRHTGSNEDVEEATEKKEKKGEEDTAEAAAS

QK

>SG-PL_contig_6578_1_-3_198_443

MSQPFSRQLPLVNLVLAAATFFTACSPARLLDVFRYSNIACFSRQTYNNIQGAYLVPAVR

QVWQKCQDELFLARRGRTVKLA

>SG-PL_contig_6579_1_-1_1_207

NWCTPRTKIKEKCAVVYHMTCDDCGNECTEIAGMPGTRVREHQNTTRASLTAVGEHCKET

RRQRLMEKH

>SG-PL_contig_6580_1_+3_90_1211

MHWNAEGISNKKAELEHFLHENRVDICCVQETHLQEGKPFKIRGYQALRNDRQGRKKGGV

MTLIRNNLNASETKTHTGEAEYIQVKVTTSNSTLDLVNYYCPNNKMLSLDTIQVPDSGFL

IAGDFNSQSQSWGYETLDKRGEEIEAWQDENHLILANDPTDQPTFYSRRWHSTTTPDLAL

CTEDLHKRLSRRVGPQLGGSDHRPVLLSISGSPPTDPPQYPRWNYKKAKWGLFSIRTNEL

TRDITVDGRNINNVVRDFNASIIKAAKETIPRGVRKNHTPYWTSTLQDAHDDLTRAREEA

ETNPSQENNIKLQRCKALYLKTKVECTRRSWRDKTSKLNLEKDTTKLWRLARALNDEGNR

EQKITLEEDGKTLT

>SG-PL_contig_6581_1_-2_113_2560

MESSTDFQKEVERLNTELAETAREKVQAAEYGLVVLEEKQQLQAQYDELESQLEVLKTEL

ECAREALTKHHIDLRRHHQTGVHHEELFMEQTAEREEHFQSSISDLEAELKAVNSSLERL

SLDNERLAANVSELSHQVEVLEAQRNQLKHENREYKIRENRNYADYAELEEENITLQKSV

SQLKTSQVEFEAMKHENKRLHEEMEDLHLQVEEMTNLRRVVEKNLEEALSSLQSEREQKH

SLKKELDQRLTRESMFNLSSLAHFGGLTEGLSVSSYSTNESSQDSGAEEDGEGQDHAHPA

LKRIEADFSFFTKDGEAVPMPRPGVVGDILSEIQVTEVGKLEQLLQQSEEEKQEMQKALD

EARKLMEETSQDLIEQKKRADQLKSYIASLSAVRDLSTSKLPNDLEVSFDETMLREMEEE

DDPERRALLQLRNNLQLKDKKYRTALNEIGSLQAEIMMLQSAAALGPGAEFNDALAELKS

KCMLYEDKIKEQARDIKSQTDMANRAQDSISTTQAELVRITEDMAQLYHLVCEVSGETPS

RVMLDHAKSSTQTIQSSDSSERESSEEPATPTGENKLLPSAEEKTSPSKPASNKQKSKSR

REKKAAVSEAQGDPTSCEKLSETVVDQVKFLKQAVEHLMEISQNWNRDGTDGSGDHAEDL

MEMQDQIVKLKAMLSTKREQIATLRSVLKSNKSTAEVALTTLKQKYEKEKVIVRETMLKL

RNELKSLKEDAATFASLRAMFAQRCDEYVTQLDESQQKLAAAEEEKQTLNSLLRMAIQQK

LVLTQRLEDLEFDRERRNMTTRRAGGRGTGKIPNSK

>SG-PL_contig_6582_1_-1_745_1050

MCIFDVLHMYTHEGGSGTGRSALMLTWEIRKISALCPTCTKKHLHHWKQDAGTVSKSKGI

LSPPGGKLYETAENTHDRHGGRMVKTLDFQNGGIGRPGFESS

>SG-PL_contig_6583_1_+1_754_1143

MPLSCSKMKNGSFIFFLKSYLPVELESVENGGSSWHFCFCTASLVLCPCCSFWLPNLSPR

PSFFFCRSCSTHRSSQCQTAMSPPTRGDSPFIQPNCPILVLSYCSFAFICYSCFRSESVC

RYTLISFIQI

>SG-PL_contig_6584_1_+3_3_239

TNQRTKQTSRQINKQKNSNKTATTNDNKRDVPTILKLESNNLYLHLRRKKHCLRVVSSKQ

LQGYCYEEGLSPPISTGGV

>SG-PL_contig_6585_1_-2_2_1327

ASWVECLEELRKKAPHFIIPLGLSAATFGVLFFLKKFNIIYQLFHKVDTKSKRHGGELVA

EVLKEHGIKQVFTLVGGHISPILVACENLGIRVVDTRHEVTAVFAADAVARMSGIVGVAA

VTAGPGLTNTVTAVKNAQMAESPVLLIGGAAATLLKGRGALQDIDQMSLFKPLCKFCATV

STVREIVPTLRKAMQIAQSGTPGPVFVEFPIDTLYMYDLVKREIGMKDKGPTSLMQKVIN

WYLQNHLGNLFAGAWEPRDMSPLPVSVPRASDSQIHQCVKLVSKAKKPVILLGSQSTLPP

TPVEELRAALEALGIPCFLGGMSRGLLGRHSEIQVRQQRRDALREADLVILAGAVCDFRL

GYGRVLSHRSKIIAINRDKDQLYKNSDMFWKPSVALQADVGSAMVAISKALQGYKCDPDW

LALLRSRDEEKEKKNSEKAEEL

>SG-PL_contig_6586_1_+2_86_196

MCADLRALAEFFPRLVTHIDFSTTKRSMTINFAVAVS

>SG-PL_contig_6587_1_-3_2637_2954

MLSAVQCHTNTVCRHVCIGRSSVTAMCMVVLDDQLLSWQGPAMWRKYACIISGEKYACCV

PSVPPHVSPVAQADSYSFVSTPSCVNEQQECLNLWSKPSMASLYPG

>SG-PL_contig_6588_1_+3_27_281

MKSVSSRFLSLNTVFWVPKYKRVFKAPQNQQFYTHSVHEPSFCREFNALSDCAYNSTQGA

VCISSDNQTRRCSSSNTSSTMASEV

>SG-PL_contig_6589_1_+1_1_180

QSEESNEVGFVTVPFIKYCILGAEIQMCLQDTAKSTILHAQCSWATHFVENSMLFPIMHI

>SG-PL_contig_6590_1_+1_1_336

ERGQSEESNEVGFVTVPFIKYCILGAEIQTSVFKAPQNQQFYTHSVHEPSFCREFNALSD

RAYNSTQGAVCISSDNQTRRCSSSNTGSTMASEVDTISEPRSDCDSMDLRDL

>SG-PL_contig_6591_1_+1_1_96

HSGGVSLVFHRSRFSPTPTPISRPCQGIGFCA

>SG-PL_contig_6592_1_-1_205_789

MKLASAKYILRSSTILNSNSKELAIESSTHFPKRAKSITSLTTINTYTSDLFEISRIQPK

NIAIRPSFSTIPRWELSNACFDIDYTDLKKNDGPVLSAIVKSHIHENYNDYLKVFTDGSV

LENDQTGAAFVIPTLKVEKSYHIGKGFSIFTAELAAILMALNYLLSLPISLFHVLFCIDS

KSVIQTIQSFKTKVR

>SG-PL_contig_6593_1_-1_1_201

LEISSFLLLSLFLPFFFPLLSSAFSAFLVHSTFFLPASLHTFFSPHPHPLPLQFMMYYLC

CFALATR

>SG-PL_contig_6594_1_-3_411_758

MTVNVPSAHSFGLIIYHFVIVVNRMSCESTAPFERMLQCSSIQIVSKPVEPVCVCVCVCV

CVCVCVCVCVLILLSLDMCISVYVYFCKLYNLHSMMNFDISRCVCCVNVSLFIISI

>SG-PL_contig_6595_1_-2_1343_1642

MTCPSYRQDMEYQPMFSTTQHHHLSLFHCQTLFIISSIYNEPPKSEYADYGVTLLLCYWH

SEGQTTRTMHVCIRASTNMPLVDIFNIIIRPMTSAVIGGT

>SG-PL_contig_6596_1_-1_1_258

LFQKGDTSGVSLVTREGVENEGGGSICSSPPHDQVPGAQQASTSGSSPAPPVVSLPSSLA

GLEAVAASVGHLASSQAPAGVLPPAP

>SG-PL_contig_6597_1_-2_80_1147

MTVRFWDINTETPQYTCKGHRNYVLCLAWSPDGQKLASGSYTKQDNGEVIVWDPKTGKQI

GQTLTGHKKWITSLAWKPLHLDPESRYLASASKDTTVRIWDTFLAKYQVILTAHSNTVQC

VKWSGTDLLYTASSDRTIKVWRSDGVLCRTLEGHTHWVNTLALNTDYVIRTGAYDPAEAK

LVKSDVSDTREELASKAKVRYEAVRQGAESELLVSGSDDAKLFLWRPEVDKKPIKRMDGH

ATGGTIVDVKFSPDGRLIASASFDHSVRIWHGKTGAFIATLRGHVQRVFQVSWSADSRLL

CSAGEDSTLKVWDISKRQLAFDLPGHADAVYSVDWSPDGQKVVSGGKDCVLKLWRY

>SG-PL_contig_6598_1_+1_259_321

MQRQTLSNYLLLTILFSPISE

>SG-PL_contig_6599_1_+3_174_362

MDWQGRKKADDSKGRKKGRNKESNRKEAISSTEFPEGGDTIYTERATGIPTGVGAGSEKG

GNC

>SG-PL_contig_6600_1_-3_186_482

MADICVNHLKSEVSLSGSLGSMLNTLLQEMSVTSFKIEGRGDQTIVVLSMTATTGTGQPS

TTETPGYYAVYWTKKNVLCRLTETGGGQKCTDAPHLCAC

>SG-PL_contig_6601_1_+1_154_315

MGSLCATSTWRVSGKSGAANPVPQEMPSFSVRYVWLLQRTLTKKARLPTWLYHR

>SG-PL_contig_6602_1_+1_103_270

MPKIFVSTGIYRHQRAMRYKRYGVKPSILKRNSILLQETQFFSPLIYISSTHFSTC

>SG-PL_contig_6603_1_-1_118_2508

MISRALGPEFGGSIGFLFYVANILACGLYVAGFVEGVLLNFGEGGSFMQNGGGLPADTQW

WKYLYASVVLLFCLIICIIGGAMFARTSAFILLVVVICTLSVFISIFVKNRTIRISIPTT

NGVVYTNSSDNTTIYGNYTGLRAETFEGNLYDNYTTDYTSDTKLDFATVFAILFSSVTGI

LNGANMSGELKDPSKSIPKGTLSAVGFTFSVYVMMTVLLAGSCERYLLQNNYVFLQQINL

WPPFVVIGIFAATLSAALGNLIGASRILEALGNDQLFWWFLKPATMTTKSGNPYMAVLMS

WFLVQLVLLIGSLNAIAPATSVFFLLSYAAVNLACLGLELASAPNFRPTFRYFSWHTCTL

GLIGCMVMCFLISAIYTSIALVLMLIFIILLHIRSLPYTWGSISQALIFHQVRKYLLMLD

PRKSHVKYWRPQILLMVANPRQSCQLMDFINDIKKSGLYVIGHVQTGRLEDHQMDPVNEV

TPEWLKMVDHLKIKAFVEVTLANSVSEGFQQLVRVSGLGGMKPNTVCLGFYDNSPPLDTL

VKTRPRKRRFFTRDVENGTYSQVDAYFTKVRDADQTRHLSATEYVMMIRDSLKMHKNVCL

CRHFHLLDKRQITTSSANFFIDVWPINLFQPHMASYFDNTSLFMLQLACILNMVPSWRSH

TTLRVFLPVNAGMDNTAHKEQKLDMFLRQLRIMAHVQVVSWDHLQHYLQNPDLTAEGGVE

GDKIHEYREVPENFVKALNELIVANSSRTAVSFLYLPNPPVDPSSYAKYLSQLEGLTNNL

QPTVFVHGLHPVTSTTL

>SG-PL_contig_6604_1_-2_227_781

MFRPYNYGVHVRGSTRFEAQLDRSFWGSLGLPIAPPPSCVHDRRGKEDRLSQAKRQGGSC

RHLENRRVHSLCSFDPQASSLQNGGILHASLVTGRMTKMREETKCLTPPHAQVPGVNLDC

VSVELEPFSPDRDGGPPTSGPSGAICLLTGPRRGPAATVDGQSSLGSLDRHASAWKRLHL

SNWVV

>SG-PL_contig_6605_1_-1_250_765

MTVPGAGVPVASSGGPSSLRLVPSPVHSLRLLGIGEHSPSRGWFSNPGWWRRDPLPGDDY

SNWQPGLILLQGDPLHLGGSPWHRRPPTDAYYPPIVGAGPMDASRPRYVARPLRLLVMGP

PHGLCQQFDGGLAPFPVIPPPPQSTQAWPVQPAPAPDAQRDPSPGLWWPVQE

>SG-PL_contig_6606_1_+2_140_316

MSTKTAYNMYISYTDFHKETTHNISNDSTENCIRNTEVYIYVNSHNPFFMLADIKPFLF

>SG-PL_contig_6607_1_-3_3_206

TVYTFGPDLVKIGSKTAEKILPTHFINRHRHTDTHTHACTTHTQTQTHTHTHMTGAVVMW

VNAGLPNG

>SG-PL_contig_6608_1_+2_2_205

LDNTDSLGYYFVQSSTTGHGWGHMRLARRLYVMQRGSENDLGRHPMKSYQFGEEHSPSET

RHKNGGFY

>SG-PL_contig_6609_1_+2_173_349

MSRKLDQDVFSNIDWPLSKLLASAKIPICLVRLTPLPLEIALRRVTDCRLANNTACMWR

>SG-PL_contig_6610_1_+2_110_562

MTLAGAARELSVPRRTLRRHLTKRVSQPRSLNLGRYRSTFPHHAEKEILDHVHFMERRLY

GLIASDVRRLAFHVAEKMGVQYCFSREPGLSGRDWLKGFMKHHPDLSLRQPQGMSLSRAV

GFNHPQVGNFFRIYRAAEERKFPTLSNLEHG

>SG-PL_contig_6611_1_-1_121_1647

MANRDRATNQLKEFKDAVPKADTLTTGTGAPVGNKLAALTVGPRGPILLQDFVYTDEMAH

FNRERIPERVVHAKGSGAFGYFEVTHDISKYCKAKIFSQIGKKTPLAVRFSTVGGEKGSA

DTARDPRGFAVKFYTEEGNWDLVGNNTPIFFIRDPMLFPSFIHTQKRNPTTNLKDPDMFW

DFITLRPETTHQVSFLFSDRGTPDGFRHMNGYGSHTFKMVNSNNEAVYCKFHYKTDQGIK

CLMADRAAALSGSDPDYAQRDLYNAIAEGNFPSYTLYIQVMTFEEAEKFRWNPFDLTKVW

PHGEFPLIPVGRIVLNRNPKNYFAEVEQIAFSPIHMVPGIEPSPDKMLQGRLYSYSDTHR

HRLGSNYLQLPVNCPFNTKARNYQRDGPQCVDDNQAGAPNYYPNSFQGPQDNTKNLECPV

HISGDVQRYNTADDDNFSQVGLFWNKVLKPDERTRLVENIAGHLKDAQDFIQQRAVHNFG

QADPEFGRRLRQALDAFKKPQAGTILASL

>SG-PL_contig_6612_1_+1_49_228

MKPALIYLLDSCPTRRRVSCQIWVKTPTASTINSGQCIFLVCTSFSLPALNTDISTKHSG

>SG-PL_contig_6613_1_+3_90_212

MTVWCLHLVINAASVCHTFPSCSKPQWTSITSAIVPFPCQV

>SG-PL_contig_6614_1_-2_2_172

HDLTSKIPMAVKEFGFILHSFRPGKIQNVLIINKANVEKVWFCVFAWVWKFGLLAVC

>SG-PL_contig_6615_1_+3_90_227

MQAGAQAETALPCCEKDGTKNDHQISLHQSQFKNTLPCALLFLTRS

>SG-PL_contig_6616_1_-3_24_1160

MADTEGKVITCKAAVAWEAKKPLTIETIEVSPPKAGEVRIRILATGVCHTDAYTLDGHDP

EGAFPVVLGHEGAGIVESIGEGVTSVKKGDLVIPCYTPQCYDCKFCKSPKTNLCQKIRAT

QGKGVMPDGTKRFKCKGKELFHFMGTSTFSEYTVCAEISVAKIDPKARADRVCLLGCGIS

TGYGAALNTAKVEKGSICAVWGLGAVGLAVIMGCKDAGASRIIGVDINIDKFEIAKQFGA

TEFINPKDYDRPIQEVIVEKTDGGCDYTFECIGSVHTMRAALESCHKGWGESVIIGVAAA

GQEISTRPFQLVTGRVWKGTAFGGWKSRDSVPKLVDSYMKGKLEVDKFVSFEVPLDKINT

AFEYMHTGRSIRSVVVFGD

>SG-PL_contig_6617_1_+3_3_176

KELIPQPYRNSPVNKVNFTSRNNKHVIRNLALFYTDLLRVRNLFMDVYIFVQFFLLFS

>SG-PL_contig_6618_1_+2_2_109

YYYYYYKGDCKFRFAGTQNYTMPIETCNCDCPLELT

>SG-PL_contig_6619_1_-3_48_206

MTTQLTDTSPQNRKFQHSHTHTLRESESISLRTMNCVQILNRTIRALFRLAPP

>SG-PL_contig_6620_1_-2_2_199

MFAFQLVGIVIHCSSMLCHCSTDCSCDCFLIMPYLFCQTQNKTFIYFVALHQLRLDHFHC

LSLLQK

>SG-PL_contig_6621_1_+2_2_121

KERKRKKVRKINKREKKEYRTGKNERKEKRVNAINLEIRI

>SG-PL_contig_6622_1_+3_3_71

LRSRQHSKQALDTLNGSDHRNET

>SG-PL_contig_6623_1_+1_1_171

DLSGCMVALVYCSIDRINLKEEKKPVSIEQTVRLLMNIRMMKAILTIPSPFSPTGIQ

>SG-PL_contig_6624_1_-1_58_873

MAACTELPTGGFVFENCQRNKFIEAQGYQVPKAYKTGTTICGVIFKDGVVLGADTRATED

TVVADKNCAKIHYIASNIYCCGAGTAADTEMTTQMISSELELHRLNTGRLPRVCTANRLL

KQLLFRYHGYIGAALVLGGVDVTGPHLYSIWPHGSTDKLPFVTMGSGSLAAMAVFEDRFK

QDMSRDEAMKLVRDAIAAGIFNDLGSGSNVDLCIITKDKVDYIRPYDEANKKGVRQGKYR

YSRGTSAVLSTEVKKIDLEVITTEVRPVPMET

>SG-PL_contig_6625_1_+3_117_416

MKRWPHSTMFSTLFLKCCTSMSAKEKSAQLYVEVDFLCRVTNCLTQSVKYTTNAIRKTMN

IFIQTYFLLGVEINYFKKIRSQIPVDPMQKQHTCTKHSTM

>SG-PL_contig_6626_1_-1_148_405

MQGRLSWYSIEKKARCVSVHKFPITLNAALAASGNSKQAHCAYLSEFSAPADTTNTNRIR

VPAEAAPALLVTERAAGRSLTMASQK

>SG-PL_contig_6627_1_+2_107_238

MLTNYSAFCKEESAHTYTHCKMYEICHNLFLVSMYGSKVSIRSV

>SG-PL_contig_6628_1_+1_64_189

MVCLVCFFFLRVLLIFHSLLYENYRRWHTNRFKSTRGVSLYW

>SG-PL_contig_6629_1_-2_8_2746

MLIINNIINLFLFILQWRRFNFFDKELVKDPSTDQPFELLKEVNISACTSGRGHLIIADF

EGNIYFINRQLSLTTFKAYEIRVSHVHQMKQHNLLVTIGEDEQGVNPVIKVWNLDKMDKG

IPSCTRLTRAIPANKPTPVTCFTVHENLNYMAVGFENGAVLLFKGDVTRDRHSKPRLLHE

GGRPISGLAFKSSGKDVFLFAVTESAIVSINITNKDTKIPLEEFGASRGNIVMSDTSQDN

QLVMGRQDAVYFYQPDGRGPCLAFEGEKLQLHWFRGYLIVVGKESKSMPRAPPMGSHSLE

MNLVTVYDINNKFIAYTAPYPEVIDIMCEAGAIYILSGDRKLYQLQEKDTSTKLDILFKK

NNYLLAISLAKSQQLDQDGLMDIFTQYGDHLYAKGDHDGAIQQYIRTIGKLEASYVIRKF

LDAQKIHNLTKYLQALHKSQLATEEHTTLLLNCYTKLKDVSKLDEFIMTKDREVDFDVET

AIKVCRQAGYHRHALSLAEKHQKHEWYLKIQLEDIRDYQAALTYIARLTFEAAESNLKQY

GKVLMSEAPQQTTDLLKRLCTDYKPTNQPLVDQTSLEGAVPRIQHANAEEFIHIFVNNAE

QLTEFLEHMVEVQPNSSSLVYNTLLELYLHDMVHQQSISTRVDMERKTLELLKNPDAGYG

MEQALVLCQVNSFKAGILYLYEKAHLYQQILQYHMEHNEFVHVMETCKKFGQQDPQLWVH

ALSYFAQKEAQCKPQLMEVLSHIDKRNLMSPLLVIQTLAHNSTATLSVVKDYIIRRLQME

QDQMSEDKRLIRQYQEDTAKKRSQIQELRTSAKIFQASKCNICNHTLELPSVHFLCEPHS

YHQHCFESYSESDMECPACAPENRKFMDIIHAQEMTRDSHESFHNQLERAPDGFAVVADY

FGRGLFNAPKPGK

>SG-PL_contig_6630_1_+3_171_413

MGGGFEKLHYFQWLQPNHLQSQLLCLASCLLFSEQPRQVFSFIFTFHTQIFPGLGRRQTA

ASTCNLLPRKSESFTLAIKMY

>SG-PL_contig_6631_1_-1_163_255

MSSVIDNGYVDRVGVTSHEIKKLAVQFKKKK

>SG-PL_contig_6632_1_-2_8_229

MHEILHLFIYDVNTNFIGLLIRRLNNYYTDNTYTFHWIDYCVNSQVQLICPTFIFCINCC

CSSIVFVLKSWCIL

>SG-PL_contig_6633_1_-2_44_298

MLLSDGTFLLHVQVKQSDFVPWSLEFVVRYLTKRVPYTDLSIVNKASAQTAAIQDVQCLH

CTGFDVSVCLIVPSVLCVNMFMSLP

>SG-PL_contig_6634_1_-3_297_1727

MLQILQKECDRQTRKIVEQFKSRRQYDRLVKQVQQSAMLQQKQTLGERIDPRQLDVVLSE

VALISARAELYSRFMRRRALGDIEVAMPDEESRKEQVKEIEAFWSGCEMSRMVQELIGVY

IMMEEYFMREMILKAVNMDAIEEGQQTSSMVDDTFFIVKKSVRRAISSCSIDGVCAMLNH

ACTILEQDFREVLYQRLRAGFPSGFDLQQAYNLVQASITQGKLQSSDAEKVKARASFLTS

LNNAEVSCDYCKALKNNLEDEVSKLYVQQCTEQSKAKLESCLDDLGTVSGRFKDVVDFGF

SQLSSSAIKPRIKPLTDTFLSTNHNISEEEFSNYEANDPWVQGSIVTLDTMLTTFKSSLT

SANYDNFVTLVTTEITVQLEKAVGKTSFNRLGGLQFDKELRALVGYLSSVTTWTVRDKFA

RLTQMATILNLERVSEIMDYWGQNSGPLTWRLTPTEVRQTLSLRNDFRSDDIKRLKL

>SG-PL_contig_6635_1_+2_137_301

MKNHIFKESDSQAIITSPFFLNHLYDLVFTPTCAIRISTWSHKRLKLERDLFMNR

>SG-PL_contig_6636_1_+1_205_270

MLRSMYITRGCSHNFNRKANTA

>SG-PL_contig_6637_1_-1_646_816

MHSLPSAHVHTSHSVRYLSLSLSICLLVIAALQPTCFYLTPIPVSQLSVCVSHSLKH

>SG-PL_contig_6638_1_+1_448_606

MIAAEHFTIITHLSSQALNNKTLLCQQISIKNEHIFLFIFLGREKKHPKEENV

>SG-PL_contig_6639_1_-3_93_2798

MGQMHMGWQQQQQLQQQQQQQMQQQQQMQQQQPHPPSLLPQRHSPPQQRIPDGRGDMGER

ERRSAEPHPPGVGPSSDGFPGNRRDYQRDGQNYNRDFYNKDQNRDFNREQDRDKSRDQSR

DFPRNQSRDFDVRRNSPPTDQGRSFPRDQNKDFARDQNRDSRRDQNRDFSRDQDRNQGRD

QGRDYPRDQGQDFTSRDQNRDYPRDQGRDYLRDQNRDFSRDQGRDYPRDQRRDYSRDYRD

NRDHRDNRWRDHRSRDNYKDANRDGQRETSYDRDRHSRTDRDSPDKSRNSSEKHGTRSSD

KPIKEKKKKPEKDNVVDKEKVVSKKKAMKRSSSGDEKVLPIKKSKKAKTDDATGEKPLVK

KKVLTKKKKKPVSAENKHNSDSETPLSSNLIQVTITKDSSKSEKIAKTEVAGKTKKVVRK

VKKVNATSSGTEDEKAPKSAKPSVDSASVKEVPEEATSVIDKVPDTTSGSPVVGVKTEQA

DSTSKPEGKVGSPDKQGPVIEVSAEGLEQTPEGGEDAEERAAGSSDDELMLHPPELSKWE

RDDFDWDTSNSPHLSKKRKLVPEKKITMLPRAVLNRVETYMAKKPVKPTLASASTTSAMK

PQRRVYLEDKFEQAAEEKEERRVKTTRTSSLDMQITVTKSEKHSPDQHDRSRQDRRSHED

TRSVKKSKDADEDRPSQRKGYSDKHRHSIDNSRSRRDSSSPRRDRDRRYEREEGRKRSKD

RDEHRERERHRERDYQHRDREDRHRDREVQHKDRDFHKDREEQKLERRTSTSEKGSSLSA

PKDLRHKLEQARTKDVPKKTQRSLDRKESVIDEAKFEPDYEELEESSESGSEEAIADQHS

TVSTEETSDVEGSPAKKMRTNSGDEGSKKKHKKHKKNKKRKKNKKQKREKKHKKDKESKE

KE

>SG-PL_contig_6640_1_-2_35_823

MASRLCRAVRPIANIARKQLFQQPVAATQLRFQSTADGVPPESRPTVRPQNPLLKQQAKD

FGQYVAECLPRFVQKVDVTYTDELEIMVHPEGIIPVLTFLKDHTNAQFLCITDIAGLDVP

TREYRFEVVYNLLSLRYNTRIRVKTYTDELTPLDSACEVYAGANWYEREIWDMYGVFFAN

HPDLRRILTDYGFEGHPFRKDFPLSGYVEVRYDDEVKRVVVEPIELQQEFRKFEYSTPWE

TFPTYRRIESGTEDTPTPPEPQK

>SG-PL_contig_6641_1_-2_2_190

HVGQQKKDPPLLSVLGQLLYGTPSVVQGLKVSSYCSSPGALGSGKDLTFMGEFSLRMYTM

RVY

>SG-PL_contig_6642_1_-3_3_128

QTCALPICTVIVSASVCDFSCSSHYDLFTHVRTHRGLTARTS

>SG-PL_contig_6643_1_-3_624_776

MLWAGTELQPSNQVMSPWLFQMSRPALFLLNQNGAHIYTHFHLLRLDEISY

>SG-PL_contig_6644_1_-1_1_297

GCHVNSTVASGSNTKDLGGRGDFSPQLWECACLKLQQLNQPTVHFVPFLHCPVHVLLSQC

HEPNTVFHILIRSLTACVIVSGFMFSSLTHVDNVFCYCQ

>SG-PL_contig_6645_1_-1_22_375

MAVLHHCPVRVGVHRSIASLSYHCPCSLQYCITVLSLWVFMAVLHHCPVPVGVHRSIASL

SCILLLSPVPVLRECGQNVHNHSHPFRTFVFFVNRQQPFLFVLGLTGNDHPLLTDTAL

>SG-PL_contig_6646_1_-2_32_235

MDLLHPIHIAILLCLILSVCWLASWFPTLVVHTPCSHVMFSPLPSFFPCHCILSCIACTE

TDVSGGRF

>SG-PL_contig_6647_1_+3_180_461

MCPQERIQSCSLSHCQQDFPCGQWVFPHRHWTFLQGQPSLGEEERETPEGAGEDPEKHCL

HLPLKQSCTTLGGLCCVHNGCRHAQDTPLRIQGT

>SG-PL_contig_6648_1_-2_2_61

ILVVYKIPTNATRSSYKYHQ

>SG-PL_contig_6649_1_+2_56_247

MFPGHKPGHSDGNLQQAPPFCNTGEKNVSFSTHEAQIGKLVQFTSLKVLCVLLFVFLPKK

HMIG

>SG-PL_contig_6650_1_-3_87_242

MPLCVAKSQHFVLTFRFSQDFKNVFVMSFRFLVSHFYSFRFGDLPALAPLRI

>SG-PL_contig_6651_1_-2_1022_1345

MYNITTKMVGCAVDSATKKMVDNVLDIISLLNSTTTTTTTTTITTNTATTTTTTTTATTT

NTTTTTTTTTTTTTTTTSKATTHHGKGKLSSPVSVQNYKIMRVLFEQK

>SG-PL_contig_6652_1_-1_55_204

MYLMYMLVNVIHKINVVMKTVRHKLDKVRVVMKTILVGLQDLITVTSGKI

>SG-PL_contig_6653_1_-1_145_1248

MLLLTCIVSYLVATVVADDPIVTIIETNPVVNPIEGKIISEKNKDVQLTCVVENKPKDKE

VQWMGTVSRNGTVTTVPISTGTRSYDSFKYSIDLPSPTSWRLRIQNGQVTDEGIYTCQVQ

VSNQNYAKAFHDLVIVETPQIADLETSSDVSKSEGESLVLKCQASGRPPPSLKWTRMAGE

LLPSGGREHLESDLLIPVLKPENAGIYKCTASNSAGSDYREVRVTVNFSPILSAANSVVQ

QAQGYVKELVCDVKGYPTPKAKDISWTKRGSPLGFDNHIFIRNIPGASSRITSILTFWGV

REDDYGEYTCAAKNEKGSGSLKFILQESATPTEDRTGQIRAGASILTVNIGTIIMLLSVC

LLHRLPFH

>SG-PL_contig_6654_1_-3_48_344

MRLHLSRSSLSPSPETLSLSHVVADHHAANFPRSDKETASHPDTTLRAYQEARLHLDSLL

ESMRSQASLTTERRPVSSPDLAQVARIDMSPQDAVGSVW

>SG-PL_contig_6655_1_-3_87_1319

MAATEVERVVENNMRNLTLSGHVGFDSLPDQLVNKSVSQGFCFNILCVGETGIGKSTLMD

TLFNTNFDSTPSSHDLPGVKLKAHTYDLTEKNVRLKLTTVDTTGFGDQINKADSWKPIVD

YIDAQFDAYLQEELKIKRMLHSYHDSRVHACLYFVAPTGHSLKALDLVTMKKLDSKVNII

PVIAKADTITKTELNKFKAKIMSELMANGVQIYHFPTDDETVADTNNSMNSHLPFAVVGS

NDEVKIGNKMVKARQYPWGTVQVENENHCDFVKLREMLIRTNMEDLRETTHLRHYELYRR

HKLEEMGFSDSDTRSLSETYEQKRQEHITNLQKKEEEMRQTFVIRVKEKEGELKEAEKEL

HARFDQLKKQHADEKKKIEDSRKRLEDEINVFNQRKAAVEAQHSTMGKKKK

>SG-PL_contig_6656_1_-1_1_198

IASPPSGNSVIFIALFVSALSPLLSSAFFCPPGPVHSFFQQAFTQFFSIPLQFMMYYLCC

FALATR

>SG-PL_contig_6657_1_-1_55_870

MAQRLFSSLGKLGIGVAVVGGVVQSALYNVDGGHRAVIFDRFRGVQEAVSGEGTHFLIPW

VQRPIIFDCRSRPRNVPVVTGSKDLQNVNITLRILFRPQVPMLPKIFTNLGTDYDERVLP

SITNEVLKAIVAQFDASELITQRELVSQKVSEELTDRALQFGLLLDDISLTHLTFGREFT

EAVESKQVAQQEAERARFVVEKAEQQKKAAVISAEGDSIAAEQLAKAFGDAGEGLVELRK

LEASEDIAYQLSRSRNVVYLPQGQQTLLSLPQ

>SG-PL_contig_6658_1_+3_3_140

YLTRLKKTGKNPKCIPRPKRKKLLRKSDWKKKIRIFTPGNDGDTNL

>SG-PL_contig_6659_1_+1_1_78

CSFASFLTERHVLFINGKDIFSILKH

>SG-PL_contig_6660_1_-2_191_433

MGSAVESSTVESSGAAVESSAVESSSGSVVESSTEPTVDTSKGSGGEAPPESAEDSPKDC

AVESIAEPTEEVAPESTAEST

>SG-PL_contig_6661_1_-3_3_245

GGSFSINSYSPPSGNSVLEISSFLLLSLFLPLFLPLLSSAFFCLACPFHFLFSSKPSHDF

LLPPPPPAVYDVLSVLFCSGD

>SG-PL_contig_6662_1_+3_123_320

MKHHFYADDSQLQNSTTPENITSLISETSSCYADIQNWMTQNKLQLNGDKTEVMLVGTKT

NLPLLQ

>SG-PL_contig_6663_1_-1_148_300

MLFCYLNIYSSFCLFAVDNIYCWLVLYVGLEITLFYFPVKVVLVNVSFVPE

>SG-PL_contig_6664_1_-3_96_1643

MTKVELCNNVTSGDYDGVVVVADTPAKLEGDPQLHPLAQAINNYAKINENIEKEVSIIQT

DAVPSGRLVYSPTGPLNRDYDDVRRFYDAGEKGIKRALDAGCKKPLLVQPQDTTFPKSGH

VALLGALHALYVPLEIREACEDRRQKAETLGVLSQTLAEGTGKKGYELVDALEKGRIVTR

DIGGSDPERMAAPRAAEYIKQAFEGTSVQVKIVGDKVEIEKEYPLMAAVNRAASVVERHQ

ARLVFLEYEGEGPIDKTLFLVGKGITYDTGGADIKAGGVMAGMHRDKCGAAAVAGFFKIL

AELKPAGLRVVGGMPFVRNSVGEECYVADEIITSRAGRRVRVGNTDAEGRMVMADVLCRF

KEMAGEAVNPELFTIATLTGHAVRAMGPHYSIVMDNGPARKIRTSYQLQACGDIYGDPFE

ISTIRREDYDFHKGKSEYEDVLQCNNQPSSVTPRGHQSPSAFLIMSSGMDKHGNDSDKPY

PYSHLDIAGSSGPFPGVPTGAPIVAMAARYILPRIN

>SG-PL_contig_6665_1_-2_2_340

TTATTQIQVQIHTHNHNNNNNNNYNTNTGTDTHNNNNNNNSNNTITGTDTHTTTTTATTQ

IQVQIHTQQQQQQQQHKYRYRYTTTTTTTQIQVQIHRSEEHTSELQSLREISY

>SG-PL_contig_6666_1_+3_114_389

MFIITDSDSIFAHVSHIPFPSLKESCWYTTEFSSVIFDIYRTLSQQCFYPYISYKLNHNG

YLKKRHAHICHNHLHCSSPVTIIEFDVSWCSR

>SG-PL_contig_6667_1_+2_611_886

MYLCTYLCTPASVHFIKLCFVLLLCSNILLRFSLYNNVNMDETSQACTYVRMHNNVNMDI

WVKHHKHARIYVCMYAFTHAGMDIPFKIVLFH

>SG-PL_contig_6668_1_-2_326_703

MGDHLGTSGAVGNFYPEAGHSFVCIPSLQFGGSCDRALGSRSHALSSVPSPNIKVGSFPA

HLNAPIILVMSVFHRSHSPPHFKTLPVLRVLRRIKEFARQLLADLGGAHMESSNFHTFLL

NKLRAW

>SG-PL_contig_6669_1_-2_113_1462

MGRTTSIVLLCLAAVCVQFAQCKHWALLVAGSNGYYNYRHQADICHAYHIMRNHGIAAEN

IVTMMYDDIAYNEENPIKGNIINQPKGPNVYPGVIIDYKKKDVTPEVFLAVLSGDEEKVE

KLTGHKGKVIKSGPEDHVFVNFADHGAPGLLAFPDSSLYAKDLEKTIKEMHSKKSYQKLV

FYVEACESGSMFAKILPKNIGVFATTAANPHQSSYACYYDTLRNTYLGDVYSVMWMQDSD

KENLKVETLQKQYQITKKETNTSQVMEYGDLTIAKMTVAEFQGNQNSGVSMTTGSPPHPT

ADAVPAPDATMSILQHQLADASTPQERRSIAQKISDLMERNAQIDALFKDIVTEVEESIA

ESGKESVLRFNSKVSYQILKQERLPITQWECYEQAVDALTFFCRKMKLPQEYRALKQLYM

LVNLCEYGHSGKTVALSVLTSAYKNEALCR

>SG-PL_contig_6670_1_-1_199_474

MPLKNLFQAHRSYCKCLEYQMMVYIHTYYAIAEGEWWRYQVKMVAVFSQKGGGIQSKGCW

YQAVLGDLLTFLELDSENRVIYPDIHLNTNLH

>SG-PL_contig_6671_1_-1_19_1398

MASHIFRTGRAAADRLLSLHGARAASAGAPALLAASSRQQQKRHLSIHEHMSMGLLRDAG

VSVPRFKVASTSAEALQQAQEIGKLSGAPDVVVKAQVLAGGRGKGTWDSGLKGGVKLVFS

AEEAQDIAEKMLDHKLFTKQTGKDGRICQKVMIAERLYSRREFYFAIAMERSFGGPVLVG

SSQGGVNIEEVAVENPSAIIKEPVDIITGITKEQALYVAKEMGFSEGSIDKAAEDIIKLY

NNVFLKHDATMVEINPMSEDNTGEVYCMDAKISFDDNSAYRQKDIFALRDWSQEDQRDIK

AAEADLNYIALDGSIGCLVNGAGLAMATMDIIKLHGGSPANFLDVGGGATAQQVTEAFRL

ITSDSKVNSILVNIFGGIMRCDIIAQGIVAAAAELDMKIPIVVRLQGTRVDDAKAIIATS

SMKILACDNLDEAAQMVVKLSTIVDLAREASVDVKFELPL

>SG-PL_contig_6672_1_-2_2_229

YRATFKAKTIQSILQLHFQDDRTKVNTDALNLVTELTRVFASEAAERSALQAQKDGVDIV

SVEQVEKILPQLLLDM

>SG-PL_contig_6673_1_-1_61_1809

MLQERQVENKRMKTEVSDLQSKSGTMTTDFQNVTLHNEVLKKQVSSMEKEVSELQNQLSR

KSDDVKVKDKKTKDIEKETKKLQDAKHKLEQNLQKKENERQRLEDENENLKTAIQSLTDQ

VQLLDTSKQQLLSEAEDWKEKVDELKERMDQMEQENRQAQETISFKDNELEVMKDCYLQL

KSFEKGGDGDGEEEEFDTGDQFKDIQEKIQAMMDVSKVNATLRAVQEEKDNLENRLKIEN

DARKELEEQLECVRREMESSMADKMKAERQCQESQTKLNVLSSYFKDKELQLQRELGEQE

ALKKQNLSKLDSADENTRTLQKELELSKQQVESLKRELTASERDFRSQIAANEKKAHENW

LNARAAERELKETRHEAAVLRQKLTDTERRQMDLIRPLPTRAMPPPGMLNGPPPPGMERS

PSRGSMPPGAIPPGPPGPPGPPPPSFREGEFQLSPRERERLPPSHPARHMPPPETRSPPP

HPRMMPPDSRGSPPRMLPPEARSPPPYDRRGPPHPMDSRTPPMRLPPPDMVPPLRGPLPP

HMGHPPMGSPGPRMDSPRDPRQGPPYGRQPPPGSDGRRHQSQV

>SG-PL_contig_6674_1_-1_1_69

VKELKFSAPKDFKSLVSEKRPKV

>SG-PL_contig_6675_1_-1_16_1326

MYVYINILYVCVYIYYTYVCVCVCVRACVCVTEQTWIHQMKTNLTMRFRIMKLVRMATLT

LPFLWFIYLIHSMVIMERSSSIHPRQLDAVKAKMPHDSSTRTFDKTAGEDIIKTSVNRSQ

TGLGENLFSPKDFLKRGPNTRKDPLGKGPDSPKASTVKGPASQQDPLGREPEDVDKNGGV

DKKQTDVMEARGVVLPDNAKRAGEGAEEDPFAGVTYPPYVETLPKGAKGNGPQGLRIDKK

RLSEEQQEEYQDKWDKNQFNQYASDLMPVHRNLPDYRSKACQEMQYPSSLPSISVVIIFH

NEAWSVLLRSVHSILDRTLPHRLVEIVLVDDFSTMEHLKKPLDKYFRDYPKVKIVRSPER

GGLIKARLLGFNVTTAEVVVFLDSHIECTEGWAEPMLWMISQNYRTVPFPLIDVINTGDL

SYMNHGTSPHGVFNLNT

>SG-PL_contig_6676_1_-1_1663_1923

MNEGMTQENKPQSNTFQSLHIFVSKQNNGGRLLGLSEQGQMAYLKKKKKSLWDRLPCKQV

KYSFLSCFCLPVGDEIKAKISGKCAVY

>SG-PL_contig_6677_1_-2_116_3868

MEEACKKDQACLPRVMPCIMHLRQDENVNVKKKVILSFSGLFRCALQWISKAKTVTPEMS

VVWEQVNQIKSGLVELIDSDNDGIRTHVVKMMEGLTLTLSKRLPDCEVPKKQAAELSLDQ

VTENNRLIRIKKLESEGQDIFLSLVQFQGSPHISSINLMTVMGSLTSIARQRTSFFDQVV

QSFEALHVNLPPTLAKSQVSSVRKNLKMQMLSLLRHSASADFIPQITTLLTDLGASQSEV

NKAMPKEESRKRKAEEPGPSSKKPKIEAVIPEAEADDYDDIGMTPWVDRTGKKQPTATQR

QTAIDITAEDLLPRLSVQHVADLVLLSMVMLPETMPAQFQSTYTPIAAAGTDSQIKHISR

LLATQLTMAGMGKGVGELKLQAMKKELPDPSATPAYDDGGTSPKQLIQTVVGMTTAEPED

DPFERARKAAEFMAPPSAPAPRKGVRHFKLESVTKPLKSDTLDGMTISIFDRILLSDKMA

SANHIQPARTKILVSLTSQFGGRLRDILLEHIFSDLRNRFDLAFSWLYQEYVNCQGFNTT

VTPGKTADMSSYDECLTRLLKAVSEMPDQRDGLFHRLILEAPVITDNAMQMVRRFCFMAE

RTEAGMKTLRSLIMMRPKNRLDFLDIMLTFTSSDRPEIRNPAIQTAQTLYESNQLKPNIE

TYAVKQLKKLLAEKPVIDIKQWMGAFPGENEGWNEETIKAHLYLYLGLLPTNHALIHELA

NVYTATSADIKRTILRVLEGPVKGMGMDSPELLTLVENCPKGSETLVMRVIHILTDKAVP

SPELVERIRDLYHKRVPDVRFLIPVLNGLSKKEVTSALPKLIKLNPMVVKEVFNRLMGAH

ITANASYVSPLTPEELLVALHNIDPIKCDMKTIIKAANLCFSERNIYTQEVLATVMQRLM

EQTPLPTLLMRTVIQSLSMYPRLLGFVLNILQRLIIKQVWHQSKVWEGFIKCCQRTKPQS

FQVLLQLPAEQLTNVFRVSPDLRLPLLSHVQSFSQYQLAHISEEVMHVLETEPVPGPVEE

PMEQTPQGEPLPPGTSAPPPVVKELTAPPMPISGIQTISTVTADRRPAPWSYGQAPPADS

DVHITGEKIVIKSEPQSPPPATHEGAKDDAMPRKSSHGSLADLSSKTSGRSPKSSDPPSV

SKAGNISVLKEVKILPPSERSTIKKSSSSSKSSSRKDSPAASATDSGSGRSSSSSRPKET

SDKAAPEKEKTSPSREPESDRTPEDDADSSKRRSSRSSKTAAPTRRSTRKR

>SG-PL_contig_6678_1_-3_3_278

LFQFDKLLPFNSIFHISCIFFYLACLVALIHQLIFPTSELHLHVVSSSHYVAALHFIERQ

KYKICIIVYIIIYIIDFSCTACNYVCILTSCV

>SG-PL_contig_6679_1_-3_3_131

YDLTVMGKPLPLQICASLLSSSFYLGWQAFLLCCSSRMVKVQG

>SG-PL_contig_6680_1_-2_287_463

MIYTSVVLIYSFRTHFFVEYSVTVHQMLKNQSRLPVIIPWSIYNTLHNIHTLTQCTHLT

>SG-PL_contig_6681_1_-3_84_392

MLGRFHKCLYFCKFSSLCLAIPSQFLFSLYLFVCGDCVSEGIGSCCVVFLCSFAFGLSIM

LIHKLFCLIVGELGDCVAFILLKTYISDKPLLLIGHILLYSMD

>SG-PL_contig_6682_1_+1_199_336

MFADIKLQQSSALDLVQSIKEVESYISDNKSWSTERTQIYYSVPLK

>SG-PL_contig_6683_1_+1_13_261

MSYFIKKSIFQRNVSVLHYLKTTTPYLGHFCQTFPCHTLFWISGSSVVFVTDDRIVFDHP

DVVKNMSLNLHVCYKQFSQEHEK

>SG-PL_contig_6684_1_+2_86_382

MRAYGIPPKVTMAIENIYNNSRCCVRTEDGLSDWFEVLTGVRQGCLLSPLLFALAIDWVL

KKATEGKGIQWHEGERLSDLDFADIAALTSDTQDLQSLV

>SG-PL_contig_6685_1_-1_1225_1350

MCLLINHCLTSVVKRGRFRKGQLIQKRSTQTRKECFMPGLRV

>SG-PL_contig_6686_1_-3_3_224

LLQCLLFVPFHCCTKCLHQLALVLVCCRYYILPVNNSVSSYGTRTCWADQSIGQKVCFAL

AYSEEKLPLSFGGL

>SG-PL_contig_6687_1_-3_732_1067

MVYAQPSTSNHPRLLDGKPLQFHSSLEGTNYKRLDHQSAGVGQQAALGRQLTNTTTFSSF

VQTLQQGTGGGHRGRDSFYASKEGHRRAPSIPGSRLLCSPFHRPQSFWGMAP

>SG-PL_contig_6688_1_-2_167_571

MAPEVVDAWVGDSFSYDKRCDLWSLGIILYIMLCGYPPFYGQCGEDCGWNRGEACQDCQE

MLFSRIQNGHYDFPSGEWCAISSSAKDLISHLLVREPRKRYSAADVLKHPWVRNPPTATQ

LATPHILTRNNSTKE

>SG-PL_contig_6689_1_-2_1079_1408

MLNLMSMQAHLIFKIIPVKLDHAITFKITPVKLDHAITFRIIPKRHPYPRTKNSLLPKAV

CGQIIFNYLLFPKHRFRLEGFSKLLLTKTVKTSAGCSLLFIITPSPEKAS

>SG-PL_contig_6690_1_-2_23_1096

MDGVCVEDFFYEELRSCCFVTMFSKLRSRSQQWLPSIYYQHSLASVELAGWTSRAASRRG

SSLPLPFCAGLNPVEVAKKAETFEGVQAAIIKLFQRNSLKENELGTLQESVRTLSDGEAG

TLIYDYYKDQLLKKGMVILREKIKNEQGLELMEKLGETWDYFFTQILPTLQALMYPLAES

SREVDIRNTTLLEFRNIVVLKVGLKETVDSKKYEIPVNIRQMLLVLQGLHDPTFLSKEQL

DLEKLVACVVVPFLGSRGLYQGGPDPVTKANYRPTMPLVPRAKPKITITVSDAAANAEEA

VAKSPRLIKLDAHPMASPATTRKLGLHEPSSSHGVINQRLKPVMEVLDLGRRHSIVGT

>SG-PL_contig_6691_1_-3_87_239

MRPSQPVFRFSTSKVQYAPKAGDDRFGLWRPVFNSFFCFSLHLLPPPSLLR

>SG-PL_contig_6692_1_-1_148_324

MKTLVTRIYEGYRYYYLHFAFVFSLYFVFLCLFCISYVSLFLSLRIYRVQFGLLSQGTA

>SG-PL_contig_6693_1_-1_1_360

KEEIRISPEKPTDTEEVTVQVIVPDEEAPEKEKPEEEQPEIVEYEQFKEEITISPEKPSE

TEETIFEVAVSKEELPEEEKPQEQPEIVEVEHFKEEITISPEKPTEPEEVRVEVVVPEEE

>SG-PL_contig_6694_1_-1_2503_2751

MFTNFTGFILHLVKKKSSSLVSRNVFFLIYLFLCFSLKTLPACTFMCKFITSAMVGLDLH

HMTLPACTFMCKFITSAMVGLDL

>SG-PL_contig_6695_1_+2_2_250

LLLLLLLLLLLLLLLLHLLPLLLILLRLRRRRRRRFLLLLLLSIMLVATFKVFPKSSTNV

NINFNQILNFFFLKSQLCLWFLF

>SG-PL_contig_6696_1_+1_58_372

MFSVQICYIKFNITRRKNLTSSLTNMLTLQVRKVSVVWWAFVYEHADYGAVIKSITGHQT

KQKKPQNNTNNNNKTQHTHLSHSVNTHNTTNINSTTQNQSTWRSG

>SG-PL_contig_6697_1_-3_3_233

ACALSLSLSLSLSLSLSLSLSLSLSLSHTHTHTHTRARARACIKTPRHAHTHKHRHTHVR

AHIYAHEHTHTRTQLRT

>SG-PL_contig_6698_1_+3_78_335

MRKGRRMKDTYPTDSHLFYPKIIRHPTRKTNKRKKRTNKTATKAGSNPVPHSLHCVLFTC

LLPEPVQAEFKGSAGDSPLFSSYSGC

>SG-PL_contig_6699_1_-2_254_367

MLVNVTCVLFLCKRQEKMDQKQCFNYWGPSFLSLKCES

>SG-PL_contig_6700_1_-1_55_1101

MADYDNHSLECCEVLTLDPLLFYRNQTPYPLGIDPIWQSSKNKIAFLNSLKMKLAVIIGI

SQMMFGIWLSLFNHLYFRKILNIFVEFIPEVIFLSCLFGYLVALIFYKWVTFEPQDSKCA

PALLIQYINMFMMSYTDPPKVNETADPTVGCSQWTFYEGQQTLQTVFLIAAVLSVPVLLL

VKPYVLRAQHNRKMQRTGHIGAKSTEALLPNESVDESDGSVMLSTEEEGSPQNGSPNIFV

VEDEEEEFEFGEVFIHQAIHTIEFCLGCISHTASYLRLWALSLAHAQLSEVLWTMVFSLG

LKMDLQYAGGAIVFVLWFVWANLTVAILLLMEGLSAFLHTLRLHWVEFQ

>SG-PL_contig_6701_1_-2_254_478

MKLNHIYTTACYTGIQQHEESTMYTTLSVLLFPVLDDYIQCFQNRQLRSWMMRRHFLPRQ

KCVCCVTFFQFKPLM

>SG-PL_contig_6702_1_-3_27_446

MAGGNKNKLSREEELLLQDFSRNVSTKSSVLFYGMALIVSVIPIWLFNRIHQMDLYQSAV

IFAVGTVVSAYLVAVAYRNVKFVLKHKVAVKREDAVAQAVMKQFADDKKMTKKEKDERIL

WKKNEVADYEATTFSIFYNN

>SG-PL_contig_6703_1_+3_93_218

MIISTGPPPQVSWSLNDQYKHRHEFNACRHYTIEAVLKSCGK

>SG-PL_contig_6704_1_+3_3_173

VCLCIGRLNMTYQSVTGITSETSSRKNKWQKSKTVVCFLSANIYIYIYISPKYQRRM

>SG-PL_contig_6705_1_-2_2_175

CACVDVCVYVCVRACAHACVCVCVCVCVCETQRARNQRAAILNLRMKLSWPVAVDSCC

>SG-PL_contig_6706_1_-3_3_281

PVLLQEKRRLYIVHISNTVARKDCTLYTVHVSPALWRVTGKFINISILQIFGIIYVVLVV

TQLFLNVNGYPVNIYHYLCSTSSDTIIININGF

>SG-PL_contig_6707_1_-1_1_117

RACACSTSLSFPALAQARRTTETRSTVTQQFYPGLQKIF

>SG-PL_contig_6708_1_-3_165_230

MSLAIYRSQLSPSSILPPPPPP

>SG-PL_contig_6709_1_+1_577_834

MVFIKAFTIIFDCNIDILTSQHTHTHTKTPPPNKKQNEPRPLIPSLKFEAEESSGIENVN

TALLMNMCHHQNQTERTLVPLQTQAA

>SG-PL_contig_6710_1_+2_2_235

FFLNFIIFFLFCSNTHDFEEQKIDIIQPTELDAKPMFTSHHNDTRLSKPDTHNFHDVYHS

KLLQYNMDLFYLKLLQTE

>SG-PL_contig_6711_1_+2_296_601

MCLVVLTDRRSSLHESVSGGSQHWVNSISSRQRNLKYTNSSVRVEGRQLMEVGTDLEIVS

WVTETQCDWLLQSSIGWLCLSCSVDLGHAGTDHAHCNAVQGT

>SG-PL_contig_6712_1_+1_847_1167

MWKIAAHERKRRAGTHSFLQVNTMRSERVKTQEMADHTHTHTRTHTHTHTHAHTLCVWGH

KFTKPKKCLLQKTLLYMCDPTFNLYSRLMLSGLSLQHIIAYTAFYNQ

>SG-PL_contig_6713_1_-2_2_61

VEGVKGLRVLFILLILLRQL

>SG-PL_contig_6714_1_-3_3_254

KQGKEEEKEHKKIAEKRGGGGGGGGAVINDEIAAYINVSEKKKSFHIGLQLDIVYAFIQM

RLRQCENGDSLSFGVSGVVRNDVG

>SG-PL_contig_6715_1_-1_781_1536

MYITVHGCDVCHNCYVRNNIVCCCHCSLSVLFRQQCLLLSLQCDVQTTMLSVVVTAVCVV

QTTMLSLQSVLFRQQCCHCNLCCSDNNVVTTVCVVRTTMLSLQSMLFRQQSCHYSLCCSD

NSFVTSLCCSDNSVVTTVCVVQTTMLSLQSVVQTTMLSLQSVLFRQQCCLLLSLQCPSHA

ASFHVLNAKRHIPHGRLSALSILFCSTILKLVFCIETSFIYCSPLHLLFCINISILHCSA

PLKYLSSIAPQH

>SG-PL_contig_6716_1_-2_323_997

MACQQVQQIQQVQQINVPTFKLVLVGDGGVGKTTFVKRHVTGEFEKKYVATLGVEVHPLC

FHTNRGEIKFNVWDTAGQEKFGGLRDGYYIQGHCAIIMFDVTSRVTYKNVPNWHRDLVRV

CENIPIVLCGNKVDIKDRKVKAKAIVFHRKKNLQYYDISAKSNYNFEKPFLWLARKLVGD

PNLEFVEMPALVPPEVTMDPSLAKKYEEELKVAQEVALPDDDEDI

>SG-PL_contig_6717_1_-2_2_406

THLWLHHFNHNFPLFSGRTILSPDVICPSLSLRRRFYRAVSLHAASRDNLSLCWSVLCPC

ILQVFSAFLSSTAASSTRSLLSLTPWPAWDLGFMCSFTVYVCWSVCSPCSSFILLLAADL

LLSRMNECLGLYVCL

>SG-PL_contig_6718_1_+2_122_244

MLAGQIQLTSQRQPGKHTKPLRTKPTGRRRPNNSSIRANGQ

>SG-PL_contig_6719_1_+1_1_162

GNRWIGRENKRVNNKVTVFRVPQFSTSQTDYKLTLYELLTTEDCNGKACYEICD

>SG-PL_contig_6720_1_+3_3_269

GESVFINKCITINHSILCLQTDHTTFVLLKLDKPCLRSTHLPCSSRQQILTTQQIHHNYT

NTCPDGFQLVNHHHQPPFYFEGINLFYIK

>SG-PL_contig_6721_1_-3_3_200

IFGNAATVILPKTPIILLKIRVLLLGGILGPRQVCRCNLHECEVQIGTHLAQSVMGISCT

QNIPRH

>SG-PL_contig_6722_1_+2_2_241

MHMHTYTHTSHFCCLSQFLRQIKKYIILENDAFFKTLNRSCLPTLHKTYKMRNTENMYHM

KCFVVLSFSPTLLPISSFSL

>SG-PL_contig_6723_1_-2_2_256

GGGSFVSVFVCFRCLLVGVFCWLVGWLCFLFWLLRIHQQKDFGDVTLQLEGEIVTSCYTL

VTVYCGTVVVKGSSLLALDVYYNLF

>SG-PL_contig_6724_1_+3_96_377

MDREGRKKQMTVKEGKRAETKTAIEKRKFLTEFLEGEDAIYTERAPGTGAHILIVKNKPR

APSTVPTHPLTWPSCTHTHTHTHTHTHTHTYTTQ

>SG-PL_contig_6725_1_+2_1058_1387

MLLQSVKNLRICDDWRIDFIEQVQVTVREFAEKVEWKMMVYLFSIIIIFGDRFTNERSMS

FFVVACGNKAYRQVRVAHLCEGELPGWSKLVHGSIQVCRTMKASHVTVYM

>SG-PL_contig_6726_1_-1_241_399

MRIDISQTANVLEENGDLIMIYQTKVSKAISDHVKHLLLKLQKFLGNKKIYRK

>SG-PL_contig_6727_1_+3_1494_1709

MLPSKNSKKILQTWLPHLFMNYKSLFFLPSWCFHLSAPFLLIFTNLFVKFTFLLFRSSSS

GLYFQKHRYHIN

>SG-PL_contig_6728_1_+3_279_404

MKNTEIHLWFEMNQSHITPTFHMYLSFMSQHRCIHTDILLHI

>SG-PL_contig_6729_1_-2_20_1933

MASAGQWEVVGKTKKGKGQPPALTKSQKKTFIDRMPKIETRDPVKEDHTIYDAFYANEGK

KTTSAASDIGKQRGTHSQSTTKKKKPEQNGKRETSSRSLALDVALAQIEHAQLESVVSQS

QLRFPESQDVWLKDLASYINLKLDKVTDSDLTFKDKPKDYPQNQLSGACQKTINAVFKKC

SKQSLDHVFYHSVQSMITQSTKGLSTAGYKVFLQMLAFHKPNVVQPKLQQYLELIKTNVN

RPAQCLSLLWAIGQCGVVDLRSGLRVWLDLMLPCLGVRSVAAFPVEYLENLFIWHKQPQA

AYGEVGLREYFHLLDTIFSPTISIQGDLRKRLQAVYPDVKNIAYGANPSTNLRNFFASYL

RRAEPNCSAAMKQEVLLCLVLCLGTDKHSWSTWCQMYTKHLPQSGVLLQHLMENWELLSK

SERHMLRETLRSFAVTNDEMVAQRNTSMEGFDICNTTTKELLQKMNQSHFPWGLLVFFFT

TVFAAVIIYDIVSSPNIKASRTVRFLEDYGILTVLEQALGRITSIAYGAMSFCENNFPRY

YHWLKEMVGPSMTWAWQASKEMFVSLVEWARPHCHWAFSKTQQGLVWVYERSPETWDQYG

AWLWLSWDFIKDYTHWIWKHVLHWLLLTQHWLQDHVIP

>SG-PL_contig_6730_1_+1_13_312

MCRPASPQTSFAYAHVTSNTYIKDPVSQVSVQINENTQLTHPQKRTMTAHMARFKNKHTN

RTQKAHLFYINPHSSCIPGTQKREEKSGWLAAQNKDKSHR

>SG-PL_contig_6731_1_+3_114_236

MQKEKLSYTPRKENRSRAFQIHFHCVGLPSTIFLHHYHLYY

>SG-PL_contig_6732_1_+1_1_369

VVVVGAAVVVAAAVVVVPAIVVVGSAVVVAAVVAVGAVGGAGAAVVVGAVVVVGAVVVVG

AAVVVAAVVVVGAVVVVGAAVVVAAVVVVGAAVVVGAVVVVGAAVVVAAVVVVAAGVVVP

AVV

>SG-PL_contig_6733_1_+1_1_267

VVVPAVVVVPAVVVVVGAAVVVAAAVVVVPAIVVVGAAVVDAAVVVVGAAVVVAAVVVVC

AAVVVAAVVAVAAVVVVGAAVVVGAAVVV

>SG-PL_contig_6734_1_-2_239_2218

MSDEASIDYGEDVVLGEPVKRYLYLCNQTAITADFTLSMELFSTDTPPNNPSQEQRKDTP

SSGRPLLARTPNLADPLSKTPAKCVSDFKSAMLGKGLGAAFLLKPSCGTLQPFGEQEIEV

TAYTDMWGKYTDNIICKVGDLDPVLVPVTMSVKGCPLNFQMTAANPDQSPVLRYGTLVAG

TAPVTRHVRVNNTSPFDVRMDWKVYNQEANDEKLVDLIVAYGRPFPLRDSEGVEVLPSTP

EPPRPQRAPTEEIPDSPDTTSTEIILPPANEAQAQRRLTSQSSVHDKKKDQPKIVSLFLR

PHDGAEGFVPYNIHPRQLMIPAKGHATVQMTFTPPPAEEVMQEMDCISYALGYLSLDGES

ANVSGKVERLEAYETKQLKIDYTCHVKPALLTIEETDDEGMTYQSAMSDILHGDKVLLES

LKTRSLMLSNHTQTPLTFRLKVNAPFLMVDLDDVGKGNSLSRTMQTGFHTLHPRHNIMVK

VALRVTSTMLSDYQKDHGTEDWERITTDNRLDISDHLTIDFNNTTQQKVALHATVAVPQM

LLSKSELDFGTCLVGQRREMQLLISNATASHCSWGASFESKSSTCIQDTFQISPTHGMLE

AYVTHVSDSKTLLTVYFTAKHAESYEAVILFQGMLGETHQRLYVRGRGSYDGKHEAVLNI

>SG-PL_contig_6735_1_-3_18_899

MAALLSLKDYDSGSEGSEDEQDQEEEQTDGFNAHLQPLDRSKSIASNMMLVSAPAVESNI

DPSGIRHIDPLTKEVKYNPKYEELYAPQVGPSNPNLTQQAQAERNTLAGFAEPSHFNDFQ

FENQRRTFTSYGYALDPSVNSGTVDEKVVGDTGTAEELKGATVFEKVPQKAEKRKREKNT

DPSDIDGYLGPWGKFVDEKTVMKPSEDEQKELDEILAKRSKAGRSTDDKPLEEKTTLHIK

DPFDYQGRSFLHPPQDVGVNLRSEEPPEKCFLPKKQIFTWTGHTKGVSAIRWFP

>SG-PL_contig_6736_1_+3_93_422

MHLLWACLPLCLLSFLNGVGMYLLWACLPLCLLSFLNGVGVGMSASVLIVTDIYLLCVCL

SLFLLSLLNGVDMYFLWVSLPLCLLSLLNGVDTYLLCKSASVLTVTLKWG

>SG-PL_contig_6737_1_-1_49_228

MVHQGMEIIHGCAGLNIVGGDMVEVSPPYDMAGTTALTAANLLFEMLCILPGVKRRKEVK

>SG-PL_contig_6738_1_-1_151_660

MALRPRRRFSSFPHPSFLVFTGIAALFLVFTGVSLYLWPSDKTGKDSIHHLPHHLETDEV

VRMTKESKNGTERNATCTFHTCVKVYHCGYNDATKISVYIYPIKDYVDEKDVPVASSMSR

EFAQILQAIVESSFYTSDPETACLFVPSIDLLNQNNIFTKEIGKVLASLP

>SG-PL_contig_6739_1_+2_269_577

MTQSKICIPTMMIRQTLPKILLCTKLTGNIWFVIQWWYTGCQCSLCLGVIKQKQTNKNHL

FTKQSTMLFCRVHMPFWPTTNLRYFTKNANEILSFKSVSQLTP

>SG-PL_contig_6740_1_-3_3_254

TPISALQVSSAHRHRHHPFSSCPQRMTFWYIRPQPALPLSFGGLHNSRISQDVSQRQCYV

VDLPQRGLMSSRRRPILKSLLATC

>SG-PL_contig_6741_1_-2_2_154

CVCSFVFSSKSRERRKTLTSLIPTFYLCICISVKIMHMYLCKKKPTKRKRI

>SG-PL_contig_6742_1_-1_79_687

MKPPIYEFSNEQGPPHNREFVCTIKLGKFVETGAGRSKKAAKRNAAAKMMEHIKNVSGTE

GDQAPKVEESDEEEEIPLGFETKGAYTALKNKKRGASSTPERSKELQLFYESIMQAAGKQ

LKNTGAGAPASNFCQMLQEIAEVRRFEVTCYDISENNVTGQFQTLIKLSANPVAVCHGSG

TSMDESRANAAHNALQYLRIMTK

>SG-PL_contig_6743_1_-3_15_1385

MVSPTGTLSSQSMVQQQQQYHQQFQQHGQQQQQQPQQQLQQQQLQQQQLQQQQLQQQQLQ

LQQQQQQQQQHQLQHQVSDGSLHIDTSHRMLSPGSQARSPSSAGSPPSPGNLNTLRQELS

TAHSMSGSVVHSHSLTGQSSPSVYFGLSRRGSLSSLADSDMVHTTPKFVKDTSKYWYKPN

ITREEAITLLRDRAPGTFVIRDSNSFPGAFGLALKVAQVPANVQSKPSSDPQSELVRHFL

IEPTPKGVRLRGCSNEPAFGSLAALVYQHSITPLALPCKLSLPDAGDAVDGPASPDMVSP

TELNSAADLLAKGAACNVVYINTIDTESLTGPQAIAKAIKATMDTQPVPKTTVVHFKVSS

QGITLTDNNRKLFFRRHYPVSAVTYCGMDPQDRRWKRDTDTPGVQVDAKIFGFVARKQGG

VSDNSCHLFAELDPEQPASAIVNFVTKVMIGQSKVKS

>SG-PL_contig_6744_1_-1_10_633

MATPESLDESPNQIKDLEKAFKDCLANLTTQDYFAPNESEETRQSVEHTIQQFLDTARQT

EAFFLTKRLHLSVQKPEQVLKEEIIELRQELERKEKLIEKHHERLQSWSNLIQRLNAPPP

PSSAAAPTQPSFPPPAQSQGYPGPPQQLGPTMMPQGGGLQAGQYPGPPLPGGQSYGMPPP

HQLPPSYPQNPLTYLEQNLSNIGMPERR

>SG-PL_contig_6745_1_-3_3_161

LQLSQSSTNVSIVIIVLVELLIKTNIETLKCRYAEMIFQIGKYIHTDSQGCAF

>SG-PL_contig_6746_1_+2_503_715

MKREAIRRTCPSSLYFSKGRLFILPRSGSDVCVSVYLCLSVCLLMKCVSKIFSAVSGWIF

TKFGPKVWTVE

>SG-PL_contig_6747_1_+3_3_188

FCTHTHGTCTYTTFHTSLHALMYTNCQVSTRKTFNLYTHTRKSHPVPFYNHAQSESSSRM

QK

>SG-PL_contig_6748_1_-1_1564_1806

MIKKVINIYKFWRGRGLTVIFLEMEIMLFFFLQFAACYLQIYSVSLYLLVLEFYFYCYSF

LLFLLFIVWLLACFLIQTCIL

>SG-PL_contig_6749_1_-2_281_469

MYWCVPMYTDANEMLSIPVCLCLICCSLDMFCSLDLYDSNAFIFLKGLPVAYSVVVLFGF

QIC

>SG-PL_contig_6750_1_-1_64_249

MVDVRSQMLVPCRSGFGCLSDVMDMSDIHWSQTSVLYSMVSDICPIYVKWGEQVSDNSVS

GE

>SG-PL_contig_6751_1_-1_631_780

METEGEKIASKFSWVSLPPSVQSQIAQVSKRRTTTFIRTQSRYRKVVVTT

>SG-PL_contig_6752_1_-1_19_546

MASPSTQPSQLRRINKEENSASFRCQKCLEKGHYTYQCTGKRKYVYRPSRTKDMVKRFKQ

DEEDKKMQLVAKTSTILEKKKKLKKRKKKSSGGEASGGEASSSSSSSSDSDSDSSSSSSS

SSSSSSSSSSSSSSSNSDSSSSSSSSSSSSSSSSSESEHEGESKSKRKRKRSRRED

>SG-PL_contig_6753_1_-3_33_254

MYRWKLDAFKTKYTRNIVCLCGQKITTSHILSCIKIKRFIPILSQKTVDEIFESTSLTYE

FFVSLSKSPIASYL

>SG-PL_contig_6754_1_-1_7_1803

MEIVLDTDALPFGTVYQRSQTTRKLIMTNSGDMNTRFRWDLNRFLPDFSITPVEGYITPG

MEVTFDVNFHPQNVSQDIRYDKLRCFLDGGPHKPVTLTLTGSCTGIPPIKEVQNFSTQVR

GRDIKMISIPNRTNQHWELTPVIEGEHWSGPVTFSVEPQQTKQYELTYKPVTMTSENKKH

QGTIFFPLPDGTGLLFNLVGTAEPPKPNGKVMRDVPCKTTYVEPLSVVNWLKKPQRFRVK

IEPVKPDKLDPGTTVKGMEYIDVPANSKKDYKLNFYAYREGTFVLKVIFTNEQSGEYQFY

EVQLRSTKPGVISTVDLSTPVRQSVPHTIRLDNPLAYPVTFNATCNISEVLMPASLSVPA

QSQGRFNFEYQPLKVGEVQGKLEFTCADLGQFTYDLILRATAASPEKAIYLRTCLGQAQV

QTARFLNYAKQKTDYACKVDSSDFHVDKTVAAAPGSTGGTEVALEVTFEPSKLGEQRAML

TVTSPIGGEYTFPLFGTCLPPKPQGPFIVKAGGTTPIFFRNVFSSTTAFTFQVDNPLFNL

PKHGETIRTKKDHRIVVGFDGNDTGSKATVMGRLVVSCARSAGGNASVQWVYYLKGVTP

>SG-PL_contig_6755_1_+1_28_381

MSCAHRTRGFLTSGLKKELENQIRPTSIVSPGCMEICNVRKPNRPRCPILTLIPSILFSR

YCISHVASQPKIQIAATMPMNVKMIFAIYISIYKITIKPMSNFMTLMNVFELASCFRS

>SG-PL_contig_6756_1_-3_339_1931

MGLALSLLKEGPGSRLKECVGYLLEGMEVLLTHLSKQAATPEENTRKNTLYAETLLRPTN

VHLLRGLIQLGRLLSANPDIKDAMAAQDVFHTAALLATQAMPDVHRGDVYRQMEWVLLDA

HAILLEMLSAAGKDEELIAQRCQRLSALIDNATIPQNQQLLDLQEKTSQNLVYIQPCNSH

ALYLLGSSQFNKYENSSPGDAATKVLHDARTSFEACIELQGKAAEGTVPEIVAGQQWYQD

RQKQEEEKAKKAAEDKAKAAAPAAKSTTTPARGGAAAARGGAAAGRGTAPAARGRGAAAA

PARGGAAARGAPAAKRGAAAPAAAGRGGAAAKAPAAGKAPAGTDKAAAPAATAAPTPEPA

AEAPAESAKPKKPAPINPKTYHAYLGLARAYRALEDTKQAQKYYEDVTKMAPEIHDAYIE

SADMLVKADPLAAVDVYMKFPVSETPSFDDAYIFGEIIRILMKFEKYDDERLASNMITYG

KILGLGVLDRYVKVLEEKFKNKLLQKVYAGVNGKAVDDPDMQTFFKFKCWI

>SG-PL_contig_6757_1_-3_63_503

MTEKKARTAKRAARRRKGVSARERNIRRLESNERERQRMHSLNDAFQDLREVIPHVNLDR

KLSKIETLTLAKNYIKALTNVICEIRGESPPYRLPAVKDNLQPAKSDNEASDNEAQEDES

MSVESLQPSFSSYTNSSSLESTSSFHQ

>SG-PL_contig_6758_1_-1_16_402

MEKKLLADLRVVELKSELERRNLDKTGVKAVLLERLGMALKEEGHDPETVMIEVMESGPV

KKDTKSPCDGSQDDDEMMEEDCEEEEAEAENEGNEEAENGGDSNDEIVIPLNDDVQMEDS

HRDAEMIAE

>SG-PL_contig_6759_1_-1_922_1377

MLGFLSCMYMEGSLDFVFITYFFNRIRKKPYNETRIHTLFIHTHTYIHIRTYVHIYAHIH

IYIYTCMHAPISSKHHHANVSSFKTVQYSTLHTYFSVRHRNQADGLSKDNDRDDKHFQSV

FCKRTSQLEICCRSCSLVTHVFQYHTQFSNIS

>SG-PL_contig_6760_1_-3_3_110

SYNQCKCKPLPVYSGFVSELTIVVLFFASLSLSHLF

>SG-PL_contig_6761_1_+1_115_282

MTYRQEQLLSGFFRSLFNWAYDIEIVLAKLHVTGEICINSKHDNRLKSQDNNLNMF

>SG-PL_contig_6762_1_+1_802_981

MCNNLLLEAKTAFLVYHPSLNPSHKPAHKPLALAYGFLYYCQCLYQFVIETKLGIIKEQL

>SG-PL_contig_6763_1_+1_286_528

MTLTIEKVTFYVECLPTTLLQLLALPAVSVWVALSWSNKWLKGNNCCMTEKIWHWPASVN

SYIYTGLENKIFCWSTRLVDF

>SG-PL_contig_6764_1_+3_132_260

MLKCSQQLYNGRITAMQSKHGPLYFAVTGPGMSMSVLLPRCLT

>SG-PL_contig_6765_1_+1_1_132

YGTTFTFTHFNLLCQLSCSVCFNRQSESRTVNFLFQCHLSKVAQ

>SG-PL_contig_6766_1_+2_2_253

VTPSANLSSSFLVNICQVQESISTCQSFNDSTPSTDPEIYQHKRNRNTPAKEDCLTKVNG

SGGKTKLMINQMHLRKNKIFKSIS

>SG-PL_contig_6767_1_+2_92_223

MKLCVLMKNDKPESFESQMNFGHCQYLIPESQHALSPMENTLVL

>SG-PL_contig_6768_1_+1_640_786

MIDLIISEIHTTLRASRLPMTYCRGLMPCEKVLSYLISKFKNKIHFLLN

>SG-PL_contig_6769_1_+3_195_446

MYSISLSLSLSLSLSLSLNKLTEVLSILHNSGKPVHILFGLTESRLSTVNLDSELIIPGF

TIVHKDPTSLKSTGLLIHVNESLN

>SG-PL_contig_6770_1_+3_249_446

MIRSLTNWPQKHALSVGPLNINHIHNKLSKVSSILHDFGKPFHIFGLTESRLSTVNLDSE

AHDSWV

>SG-PL_contig_6771_1_-3_3_290

RGGISSDKRCLSSLLCGLQLQVCMSLCVCLRQTEREGGERKERRRVDVSCNISLHHQLPP

SSLCLTLYRNLKSMLSMTVHVNRISGVQYLFRSVQG

>SG-PL_contig_6772_1_-1_61_519

MAKNTGASRFRKVDVDQYDDDKFVDESEVDADSADLQGVNDAEVNNLLAQGKQQDALKLV

LNSAPLGSKSQAVKDRAAGTVVRVLTSFKSAEIEKAVKSLDPKSVDTLMKYIYRGFESPS

DGSSAVLLTWHEKTFAAGGLGSIVRVLTDRKRV

>SG-PL_contig_6773_1_-1_1_141

CLLVHESRFSTLSVVSYKRQNMIPPCQLWTHENRVCLTLGMVHRYTF

>SG-PL_contig_6774_1_-1_94_525

MATNSDSEASEEYEVLNLSNIDENWLRQMARRTFGDISRQSAAKQIGIGSVSGVVAGWLF

VKVGKVAAATMGTTVLLIQIAQHQGYIQINWDRVRSTVRKAEREVTRNANRHYPGLVRNF

QDFIQQNLFLAVGFGGGFLVGMAV

>SG-PL_contig_6775_1_-3_111_2855

MQQYKLVVEVTEELLKTVQDHLPWGEDVSDNSEAYDPIMVALLSELMVPVPATVTDSDKI

IQYHCLTGQLPITVLNDLPATEADYSSSMEAVVFSSLYNLHLLQCLQRGFRFGEGADDGG

KYDGVEAREWEGQEEQVLLTAVRSLLLATSWSRLEAQIGPDIVSALENLTETAHAHFSSA

SKSFQQKVLCKAFNSIKDDIVSALIFTHLADLQSARDRKIDKEYFQQLYSSDESQNAQCT

EVIASYLPQWMAADVFQLQAAIFTSVTSDTDSLPSLVSCLRLMGTILSKEPAFISETYQD

ILLGIVTKLKSMRDDDSALFLFSCDIPEEPVSRVAFNMSVMQFLTMVVNSGVFRLMPDHW

DFILCSIVSWIESVDSSSMSLHSPVSIQTFGVEACRLVAALADTIQHRVTEGEAIYPPNV

KTEWTEFFAEGIYAFLFPLFVKLVDQLKKTNKKGEAVIILSAVGSAMSQCPQEQVIGHHL

PLNLVASDDTSPIPESLQTLLNYLCPMLTCVSAPAQVTAYTVLDRIITLLPSYHKEQEAA

ASATEKLESRQPPAVLMKAIEDCATAMDVVLAPVAIETTITMVPGTEEYQFALGYLLAWQ

LLLHLFHSADSAGRAEYAAYLKEHSNVQHLLTHLFRLMPHSPGGGSGTPSLSKSTSEASP

MRPSLFDDEDDRSSSRPVKRSNEDSSESSWFSSSDISLTPVGVGHKNDIARVSCTVYLHC

LEVIPVLVRQWWKDQDRKSAAYIDKFTAKYVSQQRIKSEMHTVNSSDSSIQDLVVKANSI

TRQVTASYTAEELKAEIKVTLPENYPLGEITVGADRHAVVKLAQWDRLLLQISVFLQHQN

GSILEGFRLWKRNIDKKLEGVEECFICYAVIHATSMDLPRLPCGTCKKKFHRSCLYKWLN

SSQNPTCPMCRSIIV

>SG-PL_contig_6776_1_+1_1_147

TGYSNKLPWNANITATATPSSGTSHHSVLITWEHTNTYKSKYNNGRPQQ

>SG-PL_contig_6777_1_-2_353_577

MASLSSLAHYRVTLCLVIAMSGFCQWVCAHKCVYVSVCARTQYACVYASMNVCVCRCMHV

RGEDCLLVAFYTAVM

>SG-PL_contig_6778_1_-3_3_266

CGLHQVLLVAPELFFQSCPIRSLLVSFISEQWGNVQNVLWRLLSGMDIHRYEKPHCVGAG

GSTNIQVDNCALLLPLKVMDGIISFVFS

>SG-PL_contig_6779_1_+2_227_481

MLFHLTLVLTSAVMKNSRKNTPLCIEINNLGFFCRVIVIVVGSLGYVHKRVVPGLQLLGI

PRFSAKWLSKYMSVSAAIGSFQSWQ

>SG-PL_contig_6780_1_-1_130_342

MLQDWVCCHFMYFHFLFYHLIFFASNCSIIIVRFPIYFCTFCYSCGCQADNLSLCIYHIP

ERFIVSNCRNR

>SG-PL_contig_6781_1_+1_1_186

KYRNYYYYECCFPNYRYHKTDKMIIHIFTECQFNCFLKGKCRNRNTNKTYQNSLQSYWKL

YT

>SG-PL_contig_6782_1_-3_198_452

MKFLTSYGEGLTSSCQLFGVNETHVIIVIKLLYVVIVIKLLTMPAVLMCFVVVGYIMLSL

LCCVFKSSSSSTITTHRVLTDDSSA

>SG-PL_contig_6783_1_-1_310_2589

MWLKLCLLLIYIIVLFLLARLVEALTWYETGSLSRRLLDPMTLSVAKLKALLEQRGVSYD

TVVEKTELTQLVDGSGQVHEDEVELLQPTEDTPVSANTNFTSGAHFYEQVEDAKDSVWLV

QVIARRYDSSRVHPLPLSEVTWKSLRKKITKFGIHTGVLDCELDPWWCERRGWHFSHLLL

ALPQVYQQKANVATYTYQGPLRQNAVSQWIRQKLHEKIETIQDFRTFKDEWLTFKNAWQE

PEVRALLFTDQDSIPMFFSALSVKFPGRVRFGVVNNNSGSTLNWAESVLPGQNFSLPLYL

LLTSEGPYVYGKNQGDCLTFNSMETLLKFLHPCLNDIFIFSFLMANLVSVLEFFIAQGGV

GRRLRRCLWCVVKYNAAVIMLWLPLIAIFQLPYLDHLPLFGLKVSRLLATSPFGEAFRRD

SFFFFQHPYILAASFLLACATFAAIAYKVTGGQFEEDNEPWFNFAQMRTLTHLRSNEFFE

PMFMSGYELSGLGSRLYLPTMSLQPVVSQQYITLLPTWQYCSKFTSTHQYGCTASSSPSG

EESSEMTAGLESVGACCQDGSRQMPPSYSSHQDNFGQLYPSTASANDLLDGNYHCECQQH

ARFHQASSSTNTQCIPSSASKPVLSEQSSRAPINDDTARCINCNQCPIKQESAPADTVPS

VSAFKEVDGFEKETANSSGCVMEPPPTEWLDFPEGYLPCSQCVICLEDYSVGITLCGLPC

GHVFHHSCILTWLKRDHHFCPVCRWPSFRPKPSLVHSHSE

>SG-PL_contig_6784_1_+3_129_230

MKNSVQMDEVCQVQGDVRASIHSKQPSLSLFSPF

>SG-PL_contig_6785_1_+2_77_388

MTKVGKTDQPNSKHKLIVNTYRPSIRKIALSLRLDCEHNSETSAVDSKAATWVEVGGRIV

VVWSTVLVSTENDTNFAHHYFLSLIFSCTIDLPKDYRRLDKNQV

>SG-PL_contig_6786_1_+2_482_676

MLATKLRTYTLTGTFETKIICKKYTNRKKITNGREILTHTHTHTHTHKHTHTCTHTRAHG

CHVSI

>SG-PL_contig_6787_1_-3_33_251

MAAIVASIVESVGDYHACARLAGAPPPPKSAVSRGIGMEGITTLLAGAWGVGGGTTSYSE

NIGAIGITKVGSR

>SG-PL_contig_6788_1_-1_109_600

MDFQNRAGGKTGTGGVASASESNRDRRERLRQLALETIDLNKDPYFMKNHLGSYECKLCL

TLHNNEGSYLAHTQGKKHQANLARRAAKEAQEAPAQPAPERPKVDIRKFVKIGRPGYKVT

KQREPESGQQSLLFQIDYPEIVDHINPRYRFMAAYEQKVEPPDR

>SG-PL_contig_6789_1_-3_645_923

MAMPDASCLCVMMTVVFSFMATVSVIVVVDVSRCQTLMLYVFTSSVTLLSGSLFSFTRRL

VQMEKCRLPVSRSIQQCTNTGFPAIYLCNYFPR

>SG-PL_contig_6790_1_-3_369_593

MIYFQYFFNIACFVVHLFHRDGKGWHIFAVCMSASRTYALLSATNNKKGKKKEKLGGDAL

WQYTLPRDSNPFFTW

>SG-PL_contig_6791_1_+1_1_213

VKGSTHAGLPFKQYVVCTTKKFTEIHYKNIAHLRKKNLLIHKMHILKYKIQTGTMVAGQD

EEDYSFTEVRW

>SG-PL_contig_6792_1_-2_62_2062

MSKTPNKTATKMDTKGSGDLKRDSSASRNDRSDKAPGHMRSNSRNRLSDRDQKGANQTHE

DHTQNDEPRSVEDKKFTGRCRLFVGNLTSDVSEDDFKKLFSPFGEVSEVYVNSGRGFGFI

RLDYRHNAEAAKAALDGLNRRGRALRVRFATHGAALKVKNLHPYVSNELLEQAFTQFGEL

ERAVVIVDDRGKSTGEGLVEFVRKPGAQQALRRINEGVFLFGADPKPIEVEPLEHRDEED

GLPEKFLAKNDQYKKERENDPHFAPKGSFQFEFGMRWKQLADMEKKRIENVKQEMEEARM

KLEDEMQNAIYEYQAEQIRQDLLRQQEELRRLEELRNQDRMRRQQELEMRSMSRRAEEER

ERQQEDRRRQEMMMRQQEMQRRSGGGAMDGGMPRNREEMMAVRGDGNFPGGMGMQGGQGQ

GDQSMRGGRGGAPPMQPPPAPPSGIGLERGGPQGMAGDRRGPERGGPQSMGMERGGSQGM

GMERGGPQGMGTERGGPQGMGREQGGPQNLGMERGGQPGMAMERGGQPGMPGDRGGQQAM

SRMQGEGGRGPQGNSANASGSGSPNVNMGGQGGSQMTRPSLFDQKSGNQFEPGFGNRGVM

GGPGGNQGGPGGMGGGRMGGPGNQPVPLFPQMQGMGRPGPDRGMMDRRREAPPSRDEMEE

MKRMRRY

>SG-PL_contig_6793_1_-1_139_396

MKDNCNTKMFLYGDLKQGNSENERPPKQYKDTLKANLMNCSINTTNWQDIPCNKACWQQQ

CSRAQSHLNNTEQLLFNIRETGRSRT

>SG-PL_contig_6794_1_-3_3_188

ATPASKTGLSRERALSLASLWASSSLRFWQSSSPAASTEALRIKRSIMQKPSYVLIHHPV

TS

>SG-PL_contig_6795_1_-1_1_219

VNSTANFEDSPWYQKEHTKTNPHTVPDTCCKLKNRDAFFDDNKVELDNKDCPTKKPAEDN

PNTAKHCYTSIED

>SG-PL_contig_6796_1_+1_424_615

MDILIIARLLKGDKVSANIYMDQRKSSRNSNSRYILEILDNCASPSTYISVTLASCQVLS

PYHT

>SG-PL_contig_6797_1_-2_11_364

MQHGRPVMGGPGSQQGPPIQGSMGAPPAPSQMQTSMMGPPNQSVDSIQGGPGQRQTPFSA

VQLHQLRAQIMAYKLLSRTQPIPESLRMAVEGKRHIGGYPQRPGESHQMQQMRMQQGQ

>SG-PL_contig_6798_1_+3_3_167

SSVTISHKPFRQRTEHISGYTYNKGNWRKIILHGYKPEKPQKLWKGNDHLAEHWI

>SG-PL_contig_6799_1_+3_534_821

MSETNIISWVTISFLTLSPFPFLSHFFLSSFITVSVLLYFFLMLAFDKPKIVILVKKTPL

LLKKQTLSFLILFSVTTGHRQGFTVALITNPTPYFY

>SG-PL_contig_6800_1_+3_3_83

DRVKGLTMKVNGTLLQKDDFHYEVHRV

>SG-PL_contig_6801_1_-2_2_154

TYMPTNLSTYLHVDLRSASSTYNAHSLGREICKRRFLSFMHDQAMFYREIV

>SG-PL_contig_6802_1_-2_2_181

FSLCLLGFCCFVLFLVSLIICFLVVEEGDGSGTTVTDDVAVEVFLIPVYKIIYECMHIQV

>SG-PL_contig_6803_1_+2_968_1297

MIYKMHLFRLQMQKKGLNKSLICVKYLFRRKIQIDAAHSIYFVCKYRPSIGAKQTLIDVY

IHRRHVSHVLHNAQWSKTHNGTIMRHHEHKRPLARTMTTMTTQHNWYKDF

>SG-PL_contig_6804_1_+3_108_254

MTHNTLAQLTSITGVIALPPALQFCFNMLMRRSHVPHTTVHFTVIYRAM

>SG-PL_contig_6805_1_+2_173_343

MSLLTSKCLLFPQDECIFLTIKLFYDTTLHFYEKQQNTKMTCSYSTQTELDQAYQDW

>SG-PL_contig_6806_1_+3_3_122

IQLLFNQQRQIIFVTEVFQIYITINSLQKCHASDHKLVRT

>SG-PL_contig_6807_1_-3_3_251

EEAIFEIPEKPTPADTEAEILLQPTEAIESISEAEGVKSKEEEAVFEVVEKPAPEDTETE

ISLGPKEVEEVLEEAEGSEHEEE

>SG-PL_contig_6808_1_-1_238_3390

MKEALVVETPVEVVEELPKEDVAGDEVVSSVQEVEQVPETVTDIVSEEPITPDSVQLGEA

PEALDIQEAVPTKSDVEVTAMPDTLEEFEDKFVPEESTALPSEESPREEETAVEDTVMSQ

ISADVKVGERKEVVPETVAEIPVVDMIEIPQKEKDEAQKEEVVISETVSGEEMESAVPVE

EVISDKETEEIPETVTEVAVMQEEAPTGEVVVSDVTQLAVVSEKALTEEVSVVVEQPDIA

KPEKEVIVPFEEVTQVTEEVQQLAEESEEAPAVFEVTEKPSPEEVVAEISLGPEEADEVP

KEETLKPEEEEAVFEVTEKPESAGVEAEISLGPKETEEVVAEAEVVPSEEEAVFETIEMP

LPADTEAEISLGPADVEVVVKEAEAVESEEEEVVFEIPEKPKPADTEAEISIEPQEAEEI

PKEAEASVPTEEEAVFEVTEKPTPVDTEAEISLGPEQPEEIIKEAEVAESEQEAAVVEIS

LGPAEAEEVIPVTVEVEDMLPVDIEDVTPVAVDVQATEEEIPEAVTPMEEVPMEVVKEEI

ISDKIVLDVKDSEKIETKEDVTLIPAEGFEELVEQTVPETVEKAEVEKEVALPVVDVETT

ETKTEITIVPSEDVKEVAEESVLKLEEVQTEIEEEVSVPVVKDVETTKIQEEKLTLPTED

VTESETKQPEEIQPAETVETVETTVSVEEVKLSDVAPGPDVTEEEITEELADQFQPEEEQ

GVVHEETQMPTADLIDQIVFEVKEAISEKELENVSEKDVDESQTAEEEITEEVTDQAAVV

TEEVVTVSEVSEEEAVVDVILETKPVDETIESAVETEQEEAVLHITEKPAPEEVEAEISL

EPTEAVDITEVDVVKPEEEKAVFEIPEKPKSKDIEAEISLGPKETEEITEEIEITKPEEE

EAVFEITEKPAPEDTEAEISLAPTEAEDIPKEAEEVKPEEAIKESEDVKPEEEEVVFEIK

EEPTPEEVEAEISLVPKEVEEITEEADLPIPKEEEAIFEITEKPAPADTEAEIFLAPTEA

EDIPKEAEIVKPAEEETVFEIREEPTPEEVE

>SG-PL_contig_6809_1_-2_2_262

DTEAEISLVPTEAEDIPTEAEEVKPEEVIKESEEVKPEEEQVVFELKEEPTPEDVEAEIS

LGPKEVEEITEEEEISAPKEEEAIFEI

>SG-PL_contig_6810_1_-1_1_216

GVAEIQEGEVTENKLKVETSGIHRMGFSKDPETIKVMRTFHRTGDKMEQVFYMETSNTLM

TEHLRITYVKVE

>SG-PL_contig_6811_1_-3_3_134

FFFFLVCLFLFAFLVRFIILFVCVWRGVLVFCHQFTHLFCWLGK

>SG-PL_contig_6812_1_-2_86_1822

MLCYSLSKMGHAGVVTLRQSLTSQWLASMTSEEAPPTVKIAALNGLMALIGSEQTLIVVQ

AGVSLTSSDVKASEVIEVVTRMVQDDPDLGMQTNAAWMLGHLHLSACSVAENRTSVPPNY

GYLPEASFLRAIVDLLAEAGKQGSETVSGDHVKVALVALQDDISRVLPPLNWAGVISPLM

RMSFGEDCQRLCLTLALSQGASAPTAAMFLSSWLTPPLSMSLTEDSKQALFASLPAIVKS

VSSAILTAFLHRCCLLTLANHSQEGHPSDPDPAAVVVLQGLAEALRMSDPPEAMMTVLYE

VAGKMYSSLSPDVFSPLLYREMARCLSAIPDDIFDSITVDDYTSGKCTLRGFFIGCYLVA

RGNQPITLLNDLIDSAMNSPDCNRDTVLYMLAHCLWRVVCQKTETAGPVDRLNWMVELLG

HTRNVATRVVSLTSPTQTLDKAVKFSVLVVAMAFTLLTSARPSSDLGLDPALLEVENKKG

QRQMGEEGEYEKSSPLPGWNGELKQMFLRQMPLAVLAFERTPWDQLLPKVFDWLLTMNRL

PDDVMPSDVRHCITASLVMLRHSSEFRKTSNWTRVLAAV

>SG-PL_contig_6813_1_-3_126_311

MSWSDYLDTRSCWLLIESAYILWSVGICRLTERERHAFTAEMPEREREREREMKIENTFI

QE

>SG-PL_contig_6814_1_+3_153_317

MKKWALPSQIWPKRSQISTTSLSNELVFTSLPSICVQTIHSHKWNKNNQDPVQMS

>SG-PL_contig_6815_1_+2_2_190

VWQFFSTSPCRQPHSNFRGITSSNHDGRLCHITSHNHDDIQPCHILRAQPLSSIGARNTQ

HMA

>SG-PL_contig_6816_1_-1_76_477

MRAEDLLRWCGTKRISLSAAHIVGKSNILADALSRGHSIIHMEWTLKRQTLQGVWDFWFV

PMIDLFATRFNKRLPIYVSPIQDPEAMAVDALSLDWAPLISYAFPPFPILPKVIRKAREE

RVHMILIVPFWQAQ

>SG-PL_contig_6817_1_+3_810_1208

MQSCVIHSIQTSVLICHLKIKLPQYRIKNLASFMQYAGMKSFHYTFTGGNLVFKIKITCN

ALSKFHILVVVAVTKNKQKSTRGWEMGEMRLPNYIPNFKLNKTSTVDTPSKSYLSAHAHT

HTHTHYPNTCCNF

>SG-PL_contig_6818_1_-3_3_275

DHGSKKKKKKDKQKEKLKDAVELEQQTSPTAVPVSSKTKAEMAFEKRKWKKFAAQLPDKA

SKSHKDRIMDFNRHLDSLTEHFDIPKVSWTK

>SG-PL_contig_6819_1_+2_179_325

MYNLITTQTNKLTMTLKPYILFVYIPICFNTLHHNSPRHHKLIITTNAN

>SG-PL_contig_6820_1_+3_27_146

MHSTGLVNGITNTITLCGKKNPVIVRADLYKSCIYRQNVL

>SG-PL_contig_6821_1_+2_41_232

MDQEGRKKQTTVKERKNGRDKESNRKEEIASTEFPEGGDTIYTERAPGIPTGVRTDSNQS

NQFI

>SG-PL_contig_6822_1_+1_1_186

GGGGEGREEEEEGGGGGGRKTKDFGQLQKYPGYKNSAPAIVSKIKVTKSTRDRQPIDIKT

HL

>SG-PL_contig_6823_1_-1_121_402

MHFKAFLHSETVFTGPLSPDIVHCLESMMNIVHCLESMMNIVHCLENVMNIVRCLENMMN

IVHCLENMMNIVHCVENVMNVVHCLENTMNIVYC

>SG-PL_contig_6824_1_-2_2_160

SDKSKVCICYQHRSCKNAPMHFKAFLHPKTGFTGPLTLSFSDVRHYFPLLLSL

>SG-PL_contig_6825_1_+2_2_175

NRAKRAVLHRLDMVWQNSSFRVQKRFEMHRSILAGPMLVTDTNFTFITYLLSHDVEVR

>SG-PL_contig_6826_1_+1_457_609

MFICLVAACLQLVINSYSQALHHGWYYVTYLYVYKQNLLTKVLSCINLDYP

>SG-PL_contig_6827_1_-3_102_761

MLRSDCTAAKENAAQLDSLREQLLRKQTAMQLDMEQLQALVEDLRQESECLKAQVSQERG

TVKNLESLLQAGREKEFQGQLASQERNTEIQMLRDRLSLNESKIQGQGREVASLRTRNIE

LEGDVERLRRQLTNERFERERAIQELRRNGITPPIMISEYSGDLSHNRSLSHERARSRSR

SPSPHQSPAICRSSRTHSPEPYDDDDYRDTSLRAYTQDVL

>SG-PL_contig_6828_1_+3_102_335

MRLFNKRWWDSYSERVVKKMYPALIRLKDFYSFLFCTVSIVQKGGSYMNISFSLPYIYSL

PLFLMHIHTCTNINAHAS

>SG-PL_contig_6829_1_-2_62_427

MGRVFLQHIGGSRIFSCINCDTQLTNRSELISTRFTGATGRAFLFNRVVNLNFSEVQDRV

MLTGRHMVRDVSCKKCDTKLGWIYEFATEENQRYKEGRVILERALVSESESFDEPAQNQN

DS

>SG-PL_contig_6830_1_-2_659_799

MQQTNNPMGKHLTLPGKNAHNYLQTQCKRPVIFTQLSRSSNNQTKSD

>SG-PL_contig_6831_1_+3_222_368

MELAKLHFTRLTSLYIMQSDWLSQKTLANQNGAHSLSLSGHLQMTQWQT

>SG-PL_contig_6832_1_+2_233_517

MNARLQTTGVAAISLQVWRPDNLPFSPGLSHPCSQTKTKISPEGANADTKTTHLWKQFLL

TVNRLWDRLMHGQVHQRRRRERRRRCGNEHHISVN

>SG-PL_contig_6833_1_+2_2_103

KSFLYSALLQTYLDSLHITHLQYTNCDIHRITLT

>SG-PL_contig_6834_1_-1_1_141

VSGGWHLCCWGIVYWSQIIQSKVNLPMVCGLFEDFIGRIQLILMIML

>SG-PL_contig_6835_1_-1_436_639

MSGLWDNLDLLIPPTKQSMSSCILCLLCLWQLPSENNITRLADPPVPSTNYQPVDAVYLP

CARLKSHV

>SG-PL_contig_6836_1_-1_4_3063

MLKMADTFGDELFDVFEGKSDSTESAKQGTSEADSKRADELFKKIASKRLADNEGSEGPF

KKQRNDESSHVEVMPRIQVHKVETVEACQHEVAVPPDQEFVPLHQGGSEPAKEYPFILDP

FQKEAILCLENNQSVLVSAHTSAGKTVVAVYAIAMSLRDKQRVIYTSPIKALSNQKYREL

YEEFQDVGLMTGDVTINPTASCLVMTTEILRSMLYRGSEIMREVAWVIFDEIHYMRDKER

GVVWEETIILLPDNVHYVFLSATIPNATQFAEWVCHLHHQPCHVVYTDYRPIPLQHYIFP

AGGDGLHLVVDENGDFREENFNTAMTVLREAGDAGKEQRGRKGGSKAHESSIFKIVKMIM

ERNFAPVITFSFSRKDCEAFGMQMARLDFNSEDEKKLVEEVFNNAIDSLSDDDKKLPQVE

NLLPLLKKGIGIHHSGLLPLLKETIEILFSEGLIKALFATETFAMGLNMPARTVLFTSSR

KFDGKDFRWVTSGEYIQMSGRAGRRGLDERGIVILMVDDLMTPTVGKAIVKGSPDPLNSA

FHLTYNMVLNLLRVEEINPEYMLERSFYQFQNYAAIPALLEKLKKSEDKFHNMSIAEEEQ

VSSYFRIRQQLDNLGRDLHNYILKPIYLLPFLQPGRLIRIKNKGDDFGWGVVVNFQKKVD

QSAATEETGPLYVVEALLHLSRDSARSRSVSNLKPCAKGEKGEMQVVPLLTKLIDSLSAI

RLYIPNDLRSAEHRLSVLKSLQEVEKRFPDGVPLLDPIEDMGIKEKGLHEVIKKTEAFEH

RLHTHPLHKDPRLEDLYNSFDKKTQVGNEIKALRLELKKKKSLLQMDELKCRKRVLRRLG

YATAADVIETKGRVACEISSGDELVITELLFNNVFNDLSPAQCCALLSCFVFQERASEMP

KLIEELSGPLRLMQDTAKRIARVSKEAKLEIEEDEYVESFRPHMMDIVYEWCNGCSFSKI

CEMTDIFEGSVIRCMRRLEETLRQMVQASKAIGNTELENKFAEGIRLIKRDIVFAASLYL

>SG-PL_contig_6837_1_-2_746_1015

MQLKVACHACICFIYIIYTSNWVTCFAQYCVSVAVYYDSRFFLRRGSCTSLERIHCDEKI

LYKKYTKYTVFIMLNKMYCMFLSIYFLKKY

>SG-PL_contig_6838_1_-3_3_209

RDTSTLVFDLLRESLPEMVTEVSSCHDGDPSPFGAYQGAGRAENTTLKLWSSAMLQRLFD

KAAAAARGS

>SG-PL_contig_6839_1_+2_551_808

MGSFNIIHKQMNESVEQVFLSTWFFDFFLSIFHLHDSETTADVNSTFNRFIFSTFIFTDE

EEISHLQGLLVGATTSFLPSHEAFFS

>SG-PL_contig_6840_1_+1_382_570

MHFALSTLFSNSFTQSPFDLDTTHSNIADTNKSYPIHSASLQSHCFLSFASGFNKQLSIS

PND

>SG-PL_contig_6841_1_+1_1_129

GFSSSISSYPELSVLQVHWEDWDHTVESHPLYGCVIEGVAQIS

>SG-PL_contig_6842_1_-2_2_184

GVQTLAGLKYVDLEDRKNCTSDDIFLVKWSIMQKSGLLFPESVLPIVPGKLLCCLGYFNV

C

>SG-PL_contig_6843_1_-1_1_231

MIVHLVEKIHMMIVHLIEKIHIMIVHLIEIHTHIRICGQQHVVVVVVFYVYSEFVCFILF

YILSGLGLALVLLCLSF

>SG-PL_contig_6844_1_+2_20_196

MLITHITQRCGFDPQARHCFLKTLPARRPYAQAYGICHYHFGFLSSLGICSVGAYDTCI

>SG-PL_contig_6845_1_+1_16_111

MKQTLHHCAYLMQKNKTVLLYNTKNNFFLQCP

>SG-PL_contig_6846_1_-3_723_3263

MMARCSRGVLKTWGVAVVVVLMMMGYVVGMQTVEVANQDNDVSDKGEGVSLLWGIADTTA

SVGRLFRYHIPSDAFKGHVDTYEVVEAGKDTLPSWLHFTTGQNVLQGIPTPADAGQLYLE

VTARGNHAHKATDVFSLTVSPDTAAMSSGQPLKFKKSGPEVVRCKREEPETVATIVLDAD

LGRMPVEQRLALLTRFLAHMGLREEMVKIVPASGKLLQDDTALVTGTGDVSEPQFAGVSV

SWLVGCGKVESGHFPVLQKLDDDSHNGAMYKAVGLPVAGWRVTNTRLTQQAHKRRRRQVR

ATATPVVTITPPTATDPGEEVTDADTMTRMVVDMSSPTFTIDPSPMQPEMPKTTTPRMSP

TTTTPKEKPDELQPTRRPPDVLPDISKTDALPTTTQKPMKKTTKKPMVPPTECVRPTVNK

KLEKQTYFVGEIVNFKIPEDLFNACDMGGTSETKLSLFINNNTLIPPSWFLQFDQRTQTL

RGMPMEGDVETYTLCLVSMLKDQDFITRYHFKVHVRKPRGRKARVSHEMSMTIDTDYDEF

MASLDNKLDLANKVAGVFGDKDASALTVTRMERGSLVYAFTNNTMTGGSCPVDDITALAD

KLFSPDGSISPEALEKLKPWVLNGAATKPAGACKGNPDFPIREASVAKPLDMTTKAMPDE

TKKPDTPKPDMPDQTSKPKPTTTSPNGTGVATAGKSGGGNIWITTVVPAVVIVAILLVAL

LVACILYRKKRKGKMNVEEQNTFVNKGAPVIFPDELDEKPSDSSKPLLIEGSPPAPPPEY

HRGDSETSDLGNNKEATPPTDEVDAEIEIDATSPLYQPPPPVPANSGKQARPPVQQPYRS

QPPEINP

>SG-PL_contig_6847_1_+2_950_1243

MIIAHTYKLLLSKPATYQPKAPRKQNTIKMTYTPENSPHTHKNPNLLNILKQITRNLIES

LLIYIRSSMSLGQTTANVCRMSTVVMRLSKCGGGDVFV

>SG-PL_contig_6848_1_-2_2_283

SLLLLFFYFSLSLLLHFFYFSLPLAPPPLLLLSLLLLLLFLSPLFPLSLHLLCFFLLSSS

PLPSSVFFNLLAKGDCFTSIEERISLSVCLNVFL

>SG-PL_contig_6849_1_+1_1288_1671

MMGAVGKWFKSNHVAYSPADHIRMSDFQLQGLSSILSGYHLVGKGWRFFFFYLPGQHMCR

PTCLCMNSLRVYIHKERQIYTHQRSCKPCQCSVAYGNMKILSMHTHQRRSIQSKQRCNFC

TVWAKYCA

>SG-PL_contig_6850_1_-2_173_319

MSTILFRNGKGKAAGERKRTPPSFICPREKLTSPAAHSQQLLILWKTFT

>SG-PL_contig_6851_1_-2_41_163

MMAEWENALVVLWRTGIYWSSSSSRSQFHRVHLQDSVDLKD

>SG-PL_contig_6852_1_-1_388_579

MYHFIVPKENSPVFACTNTHIHPCTNTHLCTHTHARTHTHTHARMHTHTHSCTHTHTHTL

FVSF

>SG-PL_contig_6853_1_+3_177_461

MEEGRQFVCGAPTAPTGYGAKVKVNILRSPWVVFSFVYLLLLFFCLLFVVTMKFVIFITS

NVNEAFFFTLITAVIFRSTNQIFYHMMCVIFMMSH

>SG-PL_contig_6854_1_-3_57_212

MLALRKVDVSTISLPYDLRVMGHQPLSYLPDHHLLATERIFEGKMWLPNWLE

>SG-PL_contig_6855_1_+3_180_512

MHAAQKALVDCLIPATGIQTSTPGLVEGLETYLSDQVLNPRYFAISHFLGSAFEFGVCST

HSLHSDNSICSTHTCRLLSGELLDLVQNPTRLQGFEMTKGNGTCETLTPPE

>SG-PL_contig_6856_1_-1_91_324

MDLDLPGGTGDDYQNRVVEEVKTAIRPYFVSRQITKDDYKEILKKCVPKVVSSREINQKK

ISNLVGAYVKKMRSGRPK

>SG-PL_contig_6857_1_+1_142_330

MYEHMSPVYTCVKKKMLSESKLWFYTKFKVEHTVNTANPRNISHFARATGWRGLCTETTF

GKD

>SG-PL_contig_6858_1_+2_2_148

KTTTCRRRDPPKTLEIDRPHSTQACIQHHKASPDLESAGEKEERSKNKE

>SG-PL_contig_6859_1_-3_390_581

MTVWYHCFCLFSFGWYQIRRNFGYSYYNVILYRFQESCPFCALVSVRVVGFKLNKRNDIV

HIMT

>SG-PL_contig_6860_1_-3_3_221

NVGLSISLVSLAGKAFGQSQSSVCYGYQSAQIIYIYISSVCYGYQSAQIIYIYIICVLRL

PVCTDHIYISSVC

>SG-PL_contig_6861_1_-3_3_212

KDLINLKGLFKSASRLSNIVFYLLWRGKEGQLSVLGTSHGVPGVGNLCCAHWFAVTARRD

AEILGLPFNA

>SG-PL_contig_6862_1_-1_1_189

PGVNVTVVLSERKGECLHGLTILFLCPKRNTETGVEAHDLKGIMEHSQWLLTQPYVTFST

HFS

>SG-PL_contig_6863_1_+1_1_105

TINHSCITNFDSMRITHKFYKTLSVIHTETLISHA

>SG-PL_contig_6864_1_+2_2_253

GSYSCIALFSKLIWTHCALHSYGTEYDSYRSTDITHTDRPIYRNTHSQSQTTQMNIYPQG

LTVCTHTYGHRHIHVIVYTMPHDQ

>SG-PL_contig_6865_1_-3_399_641

MGIFNFFCHYIIISLYGTFHFALKGSECDYFGKYQQNTILRCGEFVDLRISLILSSVIFH

LVSSPFCFWVFCICSLVSKSP

>SG-PL_contig_6866_1_-1_133_312

MGFVQGHIRHSRQIKFIEPKSLSVKYFSCQKCMKQSSIYLRLKESELCWLVVNYAESETT

>SG-PL_contig_6867_1_+1_85_204

MHSRQVHFCSMNLSIFNTHLFINVFLQLIIIIKEKKAKCK

>SG-PL_contig_6868_1_+3_3_170

CQDLASAKGFAQAQESVGQLLADLGGPDVELSESNEILLNKLHTPVSCDGSSNWTG

>SG-PL_contig_6869_1_+1_160_240

MTLNRPNDCSMFKTKYNYKTSIIPLLE

>SG-PL_contig_6870_1_-1_1636_1902

MFWIMERLTLKVKTNLIKGKICVCALSSKTRRISYRTCLCIGLLTPQDPNERFIPRVFLQ

EEPFKKEQKFRCNLKTAFHTWLTRFYSLK

>SG-PL_contig_6871_1_-1_70_1680

MADCLCGNTKGGRFCQDDVNPCDVCYDVSTCNTSVTTGSVCVACPENYEGDGIKCFERDE

CQNASICDQHCNNTEGSYVCFCDPGYTLTPDDGRCHDIDECQTVSMCNETTEFCVNVPGG

HRCPCRPGYGGASCQKPVTHVYGGKLVFLSLPGKQQGWSEDLLDPSSTAFKTLSGKVVTA

ISKVLIQNSEAPVGVTVVKFSPIPARRRRRRSTATTTNNNQPAMIESTYQASFTQAKNFT

ELQAALDKGFSSGCNGELCRFGNDTNAMLFAVNIAHSGFEANYDLCTSDRNNLCHDISTT

CTTLDGQALCQCRHGYQPWVLYDNICQDINECELENRTKKSLCGMDSNSTICQNTDGSYY

CDCPKAYAWDADKMKCTPVVLKDTCGEQQPCKNGAVCINTQGNDGYACQCTDGWTGPDCG

TEDAEAKRYKDILIGVGAGLGALCLLLLIAIIVLCIVRTRRKESEQENVYDVAGPIPRPT

LKSGDSPADMQRQDSMHKYTEFRRNDADVGGGRQSTNGASMQYYSNPVFTRDDEVRM

>SG-PL_contig_6872_1_+2_248_460

MYVYKNAQPLFTQLHQVCTQTHKSSILIRHYDTWTAPCSHSYTHTCTLSEKCHKLDTMNK

YVNQHNYTRLR

>SG-PL_contig_6873_1_-2_53_361

MFLRDGWVWNIPLCGYRPYMVDGNGSELVRVKQSKICLFTSGFNSTKRQNDLAASLPKDK

QKLKKNLQWFTTSCQLSATSSLKPPLPISDLLTIYTLPRHLRS

>SG-PL_contig_6874_1_-3_6_311

MCEYEPLVDLGFSTPAVDQETPLTFGSLISCVTACLRRLHKEHKSPSPHRVQSPHRKSPR

PPVSSEPQIPKVLLHYVMENCLTLAMSQACRYLREPSLPPRD

>SG-PL_contig_6875_1_-2_107_1486

MSSFAPTSGAQASSGNYAAFDAADAEFFKGIGQIPYKPDAGPEESLVYKHYNPKEVVMGK

TMEEWLRFSVCFWHTFRGTGADPFGFPTLVRPWDDGSNTMANAKRRLRAGFEFFSKLGVK

HWTFHDRDIAPEGDSIKQSGDNLDVMTDLALELQKKTGVRLLWATCNLFAHPRYMNGAST

NPDAHVVAYAAAQVKKGLDIAKKLGAENFVFWGGREGFHSLLNSNVRAELDHMAAFFRMA

IAYKKKIGFNGVFLIEPKPKEPSKHQYDYDAMTVIGFLRTYGLEKEIKLNIEPNHTTLAG

HSYEHDVVMASAYELLGSIDANTGSPDLGWDTDQFPMDIKNCTMVMKVVLEQGGIAPGGL

NFDCKVRRESTGLKDMFISHIGAMDSMARGLKVAAKILQDGVIPRYVKERYSSFESGIGE

KIAKGEATLEDCEKYVQEKGEPPRLSGQQEHYENMLNYYV

>SG-PL_contig_6876_1_+1_1261_1569

MCVHRHTAAANEANTRRHSPFIYSLMPNHCQTHYGGTHLTRSHLTCFCFCCQFPVATGGT

STKVKGSNCNNFLVKKADISAKINFQGFSFCLCDHGKARSGSR

>SG-PL_contig_6877_1_-2_2_205

FSSQTIFLVLCISLCSFLLCFYIENNMIFWVIIFDKVYIYSGFLTLFLHMPYIPVLFLLP

FVDIRKGY

>SG-PL_contig_6878_1_-1_1_225

THTHTSVCKHTCLVDLKLFFRFFYRKREILSVLKRVFIIDGGRKKVIVELVRSVLWTLCK

FFFQEINRIFVSKKK

>SG-PL_contig_6879_1_-3_300_2618

MRLNQMLDVIKEQGGNVPNKDEVPKKRPFYGKKKSKDSQQKDDSEKSPSPEDERAPELPT

EESVLSEEQEPKAVEEKMEIAEEKEEVKEAWDEESEEEKEGDEEKTKGSKAKLKKQVSKE

SKGSEEDEEESDEEESSEEEESSEESESEDERTPVERGRDKVLKRQQAAEEKRTSDVLRA

PVVCVLGHVDTGKTKILDKLRRTNVQDGEAGGITQQIGATNVPKEAILEKTKMCKEFAKG

DLKLPGLLIIDTPGHESFSNLRSRGSSLCDIAILVIDIMHGLEPQTLESINLLKSRKTPF

IVALNKIDRLYGWKSYPQNDVANTIKAQAGHTKSEFDERTKLVITQLAEEGLNATLFYEN

KNPKEYVSLVPTSAHSGDGMGNLVALICILTQTLLAKRLAYSEELQATVMEVKALQGMGT

TIDVILVNGKVIEGQTMVLVGTEGPIVTPIRGLLMPQPMKELRVKTNAYIHQKEVLAAQG

VKIIAKDLEKSLAGLPMYIAHDKDEIEYFKREAERGLEDALQSIKLSDRGVFVQASTLGS

LEALLEYLRSEKIKYAGINIGPVHKKDIMKASIMLEHDPQYAVILAFDVEVKRDVQELAD

STGVTIFTADIIYHLFDKFMQYRDDLLKKKQEEFKHVAVFPCKLKILPEFVFNSRDPIIM

GVLVHSGFLKDGTPLCVLEKEGEGVIELGRVGSIESNHKPVDIARQGMEVCIKILPQPGE

APRMYGRHFTADDLLVSKISRESIDAVKDYFRKEMTKSDWVLIMELKKEFRIL

>SG-PL_contig_6880_1_+1_373_537

MLHTHSQSDSQFVGTSIFFCFSFFLNPMYKGLFSFSACFSILRNRQAVTPLTFCT

>SG-PL_contig_6881_1_-2_197_1618

MATCLTQVVPLVERLLTLKSLSDQQQAFSKKNKKWRSIKGTMKTYCTLMLQLSEEFAEAN

VKSVVLRHIHKLVVCVAAFPKLTKVFLKKMVKLWSTGEEATRVLAFLSVNKLVRLQSELL

DGTLKQMYMAYVNNCKFVSPTTLPLISFMQRSLVELFLVQESLAYQQTFVYIRQLAIHLR

NAITTKKKDMCQLVYNWQYVLSLGLWTELLSTAHPSPVLQPLIYPLVQVITGTIRLLPTA

RYYPLRFHCVQYLITLSGAAFIPVLPFLLEVLELTDFNRKQKGGGIKNINMSTILKFSKP

QLQEKAFKEAVIDELCNLLHAYLYSQSHTIAFPELIVPLVVQLKDFLKKCKVAALCKQVK

QVVEKAEENAKFIEARRRSATFNLVDSKAVEMFESKNKEEGTPLSKFYVSWRKVQNRLRQ

KEIADKDKMTTGGESMVPEMNRLVKGAPAKASAEDRKEFNQLFGSGGGDDDDDD

>SG-PL_contig_6882_1_+2_488_769

MFSIFCQCEENNHHPYFLQVNKNAKFENQQPRHLKCNKDNNKRHGTSADNSGEYRHMNIT

PVKYTHLPILITFITFAKWVDGCLNKRWLGRQGS

>SG-PL_contig_6883_1_-2_182_718

MKVQNLSVESERLPRVWRPYLHKRVLYSEILDRHMVVTVTLRTMDLIDSHNGLDNYILKT

HEVDLCSKLGMDLKREMLVTLARESCYPDDPAKRQEILNKYKDFVIPLEEAEWIGLSVEE

AVEKVEKEQPPPRPLKDIFMEELAQELSETSVTDTSSESFLGRFNPFSSTPADQRDRKS

>SG-PL_contig_6884_1_-2_329_1390

MGCTMSAEERAAMERSKAIEKNLKEDGMQAAKDIKLLLLGAGESGKSTIVKQMKIIHEGG

FTSEDNKQYKPVVYSNTIQSLVAIIRAMGTLSIPFGDNERESDAKMVLDVIARMEDTEPF

SEELLAAMKRLWMDSGVQDCFGRSNEYQLNDSAKYFLDDLDRLGAKHYMPTEQDILRTRV

KTTGIVEVHFSFKNLNFKLFDVGGQRSERKKWIHCFEDVTAIIFCVAMSEYDQVLHEDET

TNRMQESLKLFDSICNNKWFTDTSIILFLNKKDLFEEKIKKSPLTVCFAEYTGKQTYEEA

AAYIQAQFEAKNKSSQKEIYCHQTCATDTNNIQFVFDAVTDVIIANNLRGCGLY

>SG-PL_contig_6885_1_-1_1_153

DSLFPTPPLHIFSQNPQQKNAQSDSLFPTPPLHIFSQNHHDSPQMSPILSI

>SG-PL_contig_6886_1_-1_37_201

MGLPSMLPFTNYPSVNQAREIQTPAVLVGKLSNTLTFLVKWMRLACVVPVFPMSP

>SG-PL_contig_6887_1_+2_344_646

MLLPKEMLADISKQFIILQHKENTTNAEYHRQVLCTLRQNFSTKRQEQALGCVESKSATK

PQVQASACNNTLRKNTYVQSHPPLLPCLPSSSRDFCNSSHT

>SG-PL_contig_6888_1_+1_1_141

KKKYHLLTFLEKGINILTEFELKISDGSKIFIFGHKHKACLCVDQSH

>SG-PL_contig_6889_1_+3_723_962

MYYNSLMHEQTVSAQDCVIVCVLFKSTDVNNLYRNPISKHVCIYISESKYILSMNSLSVP

AVSAQDCVCQHVFLFNKSLI

>SG-PL_contig_6890_1_+1_586_1062

MTVSIPTHIHTHTHTVTHTHSRTQFPNIIFRNTTFLPNYLNKAIFLKTPLKQRIHKFQQR

SHNFIYIVFFSNTQSYNTVLFCLLMFTHKTCFITVVKCSKHACILTYIHIYIIIYTHMHV

QPMLTHIQKHTQIPKLYHTHNLPTSLGTSSPLSILVHNN

>SG-PL_contig_6891_1_-2_137_1141

MSLFPDQGDSNYEYSLVSTMDFEDLYSDHNKHQALPWEDPMGMTISSGHVTLKKDTQNLI

GISIGGGAPLCPCLYVVQIFDNTPAAKDGNLAAGDEIVGVNGQSVKGRSKVEVARLIQSV

KQEVTINFNKLHADPKQGKTLDIVLKKVKHKVVENMSSNTADALGLSRAILCNDGLLKKL

EEMEKTAGMYNGLIGHTKKLLKNVFELSQSHKAFGEVFSSIGVREPQPAASEAFSVFGEA

HRSMERFAIHMLKTLKPMISDLNTFLNKAVPDTRLTIKKYLDSKFEYLSYCLKVKELDDE

EYSYATLQEPLYRVETGNYEYRLVLRCRQEARTRF

>SG-PL_contig_6892_1_+2_2_163

SSSIFPNIHYICKNFSVLTRTLHGLAHFLNGTKCQKHFFCQKRHVELRNLTTFL

>SG-PL_contig_6893_1_+3_108_308

MHIHERLCSSITPAQLGLNNHVGILKCFKKSQGYINCGVQDFFSPLSFLSFCHFLFFSSC

WSFHIFN

>SG-PL_contig_6894_1_-2_2297_2524

MLCACTCVYGPPTVQCTLYCCFGVDVLHLVRCTSHYCVGLDVPVYLTFLVYSVLCTVVHL

CTDCVLSLCLLFLWTK

>SG-PL_contig_6895_1_-1_37_1158

MDKSDDKVIAVFDLGGGTFDISILEIQKGVFEVKSTNGDTFLGGEDFDNYLVNFLAKEFK

KEQGLDITKDPMAMQRLREAAEKAKIELSSSLQTDINLPYLTMDASGPKHMNLKLSRSKF

ESLVDPLVKRTVGPCQKALQDAEVKKSDIGEVILVGGMSRMPKVQTTVQDLFGRAPSKSV

NPDEAVAIGAAIQGGVLAGDVTDVLLLDVTPLSLGIETLGGVFTKLINRNTTIPTKKSQV

FSTAADGQTQVEIKVHQGEREMATDNKLLGQFTLVGIPPAPRGVPQVEVTFDIDANGIVN

VSARDKGTGKEQQIVIQSSGGLSKDEIENMIKNAEAHAEEDRRKKELVEVLNQADSIIHD

TESKMDEFKDQLPQ

>SG-PL_contig_6896_1_+3_3_149

LGSEIVNTGMGTGCPFCSVICLYGLFYVFFCCFKVFLIIFFLRFFVIWG

>SG-PL_contig_6897_1_-1_1_900

DEVKESSQQLQKSSIELDELKGQYFEAQQLAKEKADLVSDLQDQLRKAESATKEVKQQIL

ELRGELESERTTHTQTAADLEESKRQLQAAQQAAKQSHNEAEQLRNKIKVLQEEAEAERK

ERHPSPRHSHGDSPYLGRSSNRSKAEFLALKEECSNLRKRIQAIEAEMKMSRKENLQLSS

EYNKLQESYKHLEALKEKLATSELTWRQNLTDAQKDAEKSKQEVQETADELTSLRSQCQQ

YSQELLSLRSQLETMEADYSLLAERSKKISLVSSIPLLMLLFAILLALYPTLEHITATSS

>SG-PL_contig_6898_1_-1_208_630

MQGRIGSDRNVASVNVPHLPMLLVLPWVLPSAHLLAVRRSLMRLTSWVASIATVMTSAVW

RAWAVKVMAAATLTALVTLWTIPLTPRTSDTWLGDDILPTYTASQPLPPELHLCVFPDPL

SPPFQWCSQPDLTVRWPASYS

>SG-PL_contig_6899_1_+1_898_1062

MNPVNFNRHSNASKMFHLQLFSLPNDQKCAVVWLDEINKLHSYTGETARVLDTSM

>SG-PL_contig_6900_1_-2_29_952

MADSVDIDLYENIEDEFNQDTEFGGGGDLYDDVITAGSSSSISGRPPSADGNKPHSQHSS

SSLPSSSANYSGKKFMLYIGNLTWWTTDQDLTDAISACGVSDLLEIKFNENRANGQSKGF

ALVIVGSEHSSRNIFERLARKELHGQIPQVMHYSKQALSQFEAQARKGGELPQNGNFRER

DREPKPRGPAPKSAGPPPPQPGRPVPGAFPPRMQPPPPGPPRPGMPVAGPPMPGPPNMPF

APLPGAIPPASGPPPTALGLPPRPGMPPPGAPRMDLGPPRLPPPGQRGPPPLDPRHPPPR

PEWDPRMP

>SG-PL_contig_6901_1_-2_179_367

MLLILSFCGLYLRSDSLQSSSLFMKVCVRLVCVYLRAWFACFIAIFAYCSIDLFTSLTHT

CLP

>SG-PL_contig_6902_1_-3_27_2756

MEMRALLAEYNRFKMEDVPPRLETKQRLLKLFDEIQSLTSDMSQLGIAEELSPQSVGRLW

NRFAAAMQERDTALQAEIVRLERLARMAEKVHRECKYVEDTLVDLERRIQSTQQRGDVVH

PFEAKRNCEAMERTLQNMEETLRAMFRDVQTLQDNRFPQSQQLYNRASELQSHMSAIRNR

LYSVVVQPLLQKSYVEEGRTLTKRTEVVTEVRLVDTNPAFQHVQECLDWIDAQQRNLESE

EYSSDLQQVQEQLRSHRRIHQEITTFRSQIDRCISDLKSLSPEEQKLYTQKLSNVEVAYS

LLSNTSSRRLKCLEQLNDFLQAATAELMWLSEREEVEVTRDWASKDLNLPDLDEHHKSLV

RQLEVRERHFNSVQEKGGAMILDRHPASAVVEAYMATLQSQWSWLLQLTTCLHSHLQHTV

THNQFFEEARHCEQWMARHTDLLQNRFSRDNIPVEQAEILIRELQDIQEQLREYDRRVST

LAMSASDIIPLSQRGQGVRSPVRVRALCTCHRPEVTLQKGEECFLVNNNQPLKWKVKTTA

GVEAEIPSVVLLIPPPNQVATDYANRLKRQYDQLSSLWKSQHQKLRFNTIFSTINLIKGW

DLKQFLSMGPAQREAVWRALKDDAEKLLAESSSYPDRDSRRLKEELAECQQVFNRLAAEA

AAKEGQRSMSAAQLLTQQVDSVSRELGACDQQLSALVHKPLPQNNLGVQDAFRKYREFQE

RFEGQEKEVTKLQRSNRQLSPPSQQVESKLNLVVHKCQQIRAISHVYVDRLKNCELVISG

LDDVKQMVLDYEHTLAMHENMTSDPNTLRSINRDLTDLQTSMQQQQPRIDNVSRDVASLR

GLLEKSRPGAARHHDLEAVEKEVTELTARWDGAKSQLNERLQAVKSSQELLSMYQNGLAS

EERWLVQTQQ

>SG-PL_contig_6903_1_-3_129_680

MKGDGTLFPFFKTAISVRLPCRAKLANIVCSGAQIEVYASKTYYTRIQNTYVDGVMVNVT

TTDDHGKTSFQAYEVTNIPFIRGMDNPNSKTWKKIYTNNTYVDDLINLSINSNTGLAELT

RGLFQVVYQPPQFGTSDPLESWGHLKITCGDLDFEAKEQYPAVLCGSNTNTRQDESSFYN

KLHL

>SG-PL_contig_6904_1_-1_133_684

MKGDGTLTPFFTGAIPVRLPCRAKLANFVCSGAQIEVYASKTYYTRIQNTYVDGVMVNVT

TTDDHGKTSFQAYEVTNIPIIRGMGNPNSKTWKKVYINNTYVDDLINLSINSTTGLAELT

RGLFHVAYQPPLFGTSHPLESWGHLKIICGDLDFEAKEQYPAVLCGSNTNTRYDALNFYN

KLHL

>SG-PL_contig_6905_1_-2_17_535

MAAPTKSGSGDSVALPPVLEWAQRRDRVFITVALENCANPTHDVKGNIFFFKGKGGTENL

EHEVTLELFGEIDPEKSRCETNDRHSMFTLIKKDQDASYWPRLTKDKKKFHFVKTDFNRW

KDESDSEEEEKEDFNLDEMMNSMGGLNSAQFGGGMAAPEEEGDSDDEDLPDLQ

>SG-PL_contig_6906_1_-3_3_182

NARRAQTHTHTHTHTHTHTHTHTHTHTHTHTHTHTHIHSLTLTQFRKSIARTEQVSLKPY

>SG-PL_contig_6907_1_-1_1_120

TDRQTADRQKELLHSLKVCGTIESLIGLRYFSVNDMGVRL

>SG-PL_contig_6908_1_-3_3_173

ENARETELELREERDNALVRLSENQRKMDAMQETVADYETTLNKFRGLVAQLQVGSH

>SG-PL_contig_6909_1_+2_104_217

MKMKTKEFYERLFQTSQYIQAQITATIPPPPSTPVTIM

>SG-PL_contig_6910_1_+2_2_121

TEHSVLCLITSQPDAFTLIGFTCSRWFLKMLVLLALNMYG

>SG-PL_contig_6911_1_-1_1_156

LIDAKKKKGVCFFFSIFFTKKSFLNFRKKRGKKGPSQSERLPTHDHCCVRPE

>SG-PL_contig_6912_1_+1_1_153

AGSWHPGRLFSLSFITCVEQRLMIAYVYIDTFVSRLKTDQQWKNTGSISSQ

>SG-PL_contig_6913_1_+3_825_1019

MFLRHDTLIQIPMNYKINTTLHMSTRYDPINHLSSDILHKFMYAKSIFITHTFCSPSGVY

GHQIH

>SG-PL_contig_6914_1_-1_1_180

SPPHPVHHSTSGSLQEAGGQVGGVQVLMQTMVMPVCSAVSALLLIACCSAVGRNLLFFCI

>SG-PL_contig_6915_1_+1_1_237

HDTNSTLKKSTCNVNTNVEKSVRNRTVCSAHETTEIREAEIRTGKNTKNTHWDRMTTIWQ

NFNPFTPSPYPAASISLLH

>SG-PL_contig_6916_1_-1_418_657

MTFLLHTLAHPHAHSVDIHIRSPIYKHLSCPFLKVLLFDRFESGNVFTLSYLLWNLMLEL

KHLVPLKLKHVVILNVFCVC

>SG-PL_contig_6917_1_-3_774_1139

MSVFGGLWKHENSQRAPLPPTQWRVKNNNNNNNNKKPQTTNDYSQEKSPVMTTLRTTRTR

LLRLTECTCCCAMALPLKKKKIFLLHRFLCSVLPIRWQHRHHPQTGGTTNQISQSHCVEG

KY

>SG-PL_contig_6918_1_-2_179_289

MYPNDRLRSTISCDLTRNLSVVHGWSYNISVIHCKLV

>SG-PL_contig_6919_1_+2_419_550

MHTDNFLKATNIQHKNFHHCRHMYITRKIAVISKMRFKNTLKQQ

>SG-PL_contig_6920_1_-1_235_1530

MDSGKEPPKALKTVNPPALHARSVSPAEDRSEQSATAPVQLLGQNKPGRVKAIMSQRYNT

PIGLYSENEVMTQFEGQSKFLITDEDGGGDANTNLPPPPPPPPEAHVMEASLPSPPPPPP

PEMEENSGSIKYFPSETYKMIQEEEKGAGGDVDDKTAHSRSFRMLQKKLDSSGAPPPPAS

QFAPRPVAPASSFQLKPSPAASVAKPWTPAATSAPAATGPRPFGGGGVAPKPTGVGAKRG

RVGEAVTNPGGSGRIPVCSGCGSPIRGPFVTAMGNCWCPDHFVCANPQCGVKLIDIGFVE

DGGNLYCERDYERYLAPHCKKCNKTIIGECVNALQSTWHPDCFLCAHCKLPIGGNQFHVE

EGKPYCEKDWAAMFQTMCAGCDFPIEPGDRWVEAMGKNFHAECFNCATCQVSLEGQPFVA

KAGKAFCKKHAR

>SG-PL_contig_6921_1_+1_2395_2691

MKSCEGSVRFNCVPGCFTSFQFRSVPFSFLASSHSCCTSWLTDTPPHPGLLHCYPNIRVH

NELSTSRCCEATHQKNSALLLIHFCGWVPHICLLSISLS

>SG-PL_contig_6922_1_-2_11_865

METIVILLAVFSASVLAIPEQGKWDIQLDQDHQYRAVAKHMYKSSSVTVAVSLKCEKKDV

GNIEISWLLRMSPCAMEFISATEEINAVSNDPKQKDTVEHYMKKPITRYNLPYDRVEWLD

GSSNLSCSEGPLQLDGVPGQKPHIDVIPKPVSNTTGANAAPAPASDSASHTGSGRASSAA

APPASPGQGGANQGPPPGAVSASSSTAASRKKREEPPAQNKQVTSTPKQYTTKTATAGTH

VSSTAAPGRGTISKLDHPKVANVWVDGNYLFIIRIRPREETTKFT

>SG-PL_contig_6923_1_+3_3_107

VYEAKSFPHATIKSQYTNIQLHPNNVSCSVAVCYT

>SG-PL_contig_6924_1_-3_3_344

FPYDHIQDIRVLLPSGRNQNPEANNFPSPYYQNLGTADNPLPYDQIENINVFLPTDENQA

TTDNPFLYDQIENVNVFLPTDENQATTDNPFPSDQIEEINVFLPTDENQATTDN

>SG-PL_contig_6925_1_-2_95_1636

MSTDPLGKSRNHLFKNKGKDAEEMRRRRTEVTVELRKNKRDQHILKRRNVPAQDSTDSED

NEKPTNQTLQSIVLNASSPEPSVQLSAVQAARKLLSSDRNPPIDDLIASGILPILVNCLA

RDDCPSLQFEAAWALTNIASGTSAQTQAVVNAEAVPYFLRLLQSPHQNVCEQAVWALGNI

IGDGPNCRDYVISLGAVQHLLNFINPNIPLPFLRNVAWVVVNLCRNKEPPPPTETIQEIL

PALCQLIHHTDVNILVDTVWALSYLTDGGNEQIQMVIESGVVPFLVPLLSHQDVKVQTAA

LRAVGNIVTGTDEQTQIVLNCGALSHFRALLTHPKEKINKEAVWFLSNITAGNQQQVQAV

IDAELIPLIIHHLQGGDFQTQKEAAWAISNLTISGKKEQVAYVVSKGVISPFCGLLGVKD

TQVVNVVLDGINNILKMATDDLEPICQAIEECGGLDKIESLQTHENEEIYKLAYDIIDHY

FSDEPEEEDLLPTQSGEGFQFDPNTMVPQEGFKF

>SG-PL_contig_6926_1_-3_468_644

MPRYVRLPSQLLSLLVYSLFFPPPTLWVFLVRFLPILPLDTRFMFIFLSGGFGFFPYEY

>SG-PL_contig_6927_1_-2_74_1258

MDSKGRKVIVCDNGTGFVKCGYAGSNFPAHIFPSLVGRPIIRSSAKIGDIEVKDLMVGDE

ASQLRSMLEVNYPMDNGIVRNWEDMIHVYDHTFGKDRLNVDTKNSRILLTEPPMNPMKNR

EKMVEVMFEKYQFDSVHVAIQAVLTLYAQGLLTGVVVDSGDGVTHICPVYDGFSLPHLTR

RLDIAGRDITRYLIKLLLLRGYAFNHSADFETVRMLKEKLCYTAYDVEQEQKLALETTVL

VEPYMLPDGRVIKVGGERFEAPEALFQPHLINVDGVGVAELLFNTIQAADMDIRPDFYKH

IVLSGGSTMYPGLPSRLEREIKQLYLERVLRGDTTRLSKFKIRIEDPPRRKHMVFLGGSV

LADIMKDKDDFWMMLSEYQEMGVKVLDKLMKPTKG

>SG-PL_contig_6928_1_-3_516_875

MCMGVCVRERASMCVCACAREHMCVCVCAHVSICMCVCVLNRCHDCGEEEDRLGLLASLL

HLASLLHLSSNPYLFLISACVCERESEKDEAKVDIKGIPYRSTSLAQQAIFWSVPRPLSG

>SG-PL_contig_6929_1_+2_32_253

MKHNWDNKAFCQSTSQSYQHDVMHNINIIMRLWRAWLHEHNQNDIFDGEAQCHKMSRKLL

ALCSLCYLYNSAAT

>SG-PL_contig_6930_1_-1_103_1833

MLGPGPKDVFRQYGVLTGTTPLPPLFSISYHQCRWNYNDQDDVRTVDENFDKFDIPYDVI

WLDIEHTDGKRYFTWDATKFPASVEMLRNISSKGRKMVTIVDPHLKRDDNYAVFKDARDK

GFLVKNKDGGDFDGWCWPGSSGWPDFLEKHVRDWWADKFALTEYVGSDLNLFVWNDMNEP

SVFNAPEITMHRDIKHLAGWENREVHNVYGMHVHAATMEGLLKRTNYNDRAFVLTRAFFA

GSQRTSAVWTGDNMGEWGHLKISNPMIMSLNLAGITFSGADVGGFFRNPDNELLTRWYQA

GAFQPFFRAHAHLDTKRREPYLLPEENVNIIRAAIRARYSYLPYWYTLFASSEEDGAAVM

RPLWVEFPKERATFGIDDNFMVGSSLLVRPVTDQGATSANVYFPGADQIWYDVETYETYK

GQQEVNIAAPLSKIPVYQRGGSIIPRKMRPRRSSSLMFDDPFTLVVALDSKQEATGYLYV

DDYHSFRYKKGAYGLIQYNFASHKLHSIVHHEGDQFTTKEWLERVIIVGLPHEPRKVTLH

KSGQTSDLIFTFDNSKKALTIRKPGTNILQTWTISLH

>SG-PL_contig_6931_1_-2_194_448

MCIFWYHLHDMKGRISVWLDHPVSLPNISIFWHLAFFVLYSPFSSQLHFQYLFADLFVSF

PTDILRCATATQTNRRTHIFTFISS

>SG-PL_contig_6932_1_-3_123_1895

MDMDSDAEPIEESDEDDLDDNGMGTDEEEPDSEESDEEEEPGVQSNVREDIYGRLVDSKG

KLLSDKEQSGAYIPPAKRAAMLAGSGQESVELERLRKQVKGLLNRANESNMQSIANAVED

MYMQHSRASVNQTLFTFIQEAVVSSILTPERLVAELAMLVAILHGNVGSEVGAYFLQHTC

TKYEELRKEEQYGGEHTMDNVVLLLCYLYNFKVAHHTLLFDVISQFVESFSEKDIELLLL

TMKSAGFYLRRDDPEMLGNQISSIQQKARELEESGTAVPARVRFMLEVLVAVRNNNVRRV

PGADVERTDRLRQLAGKFVRGGSLADNQLNIGMKDLLQAEHKGRWWIVGSAWEGRLDANT

SSGKASANAAPASSVVGAVSQKLVHLAKKLRINSDLRRSIFFVLMSSEDYEDAFHKVLQL

GLKGQQQQEIVVVATLCCKQETTYNPFYSFFLQKLAAHERKFMIAIQFHMWDMFKTMEDL

SSSSRNNLAAMLAHLLASRSLSLSVLKVLEFGILSKAVIKFLKHIFTTLFTTVPMAELKD

IFRAVGQLQKFKALADGLRTFLVKYVKVEGEQKKKLVEALNELSLNRSMMM

>SG-PL_contig_6933_1_+3_42_440

MVLFSHLQQIRGYVRVLCVCLTSLTDPTPDPTPCMSLHAKMASQPSASYQQGSERRRRKE

RFNVSEVLEMLQTEGSDIEEDSGDDDFYDSEGESEDSPFRSRTTRHRSVKGKGWGVAKQM

TRRAVVVSFSQTN

>SG-PL_contig_6934_1_+3_1431_1739

MYYEVCYLLCGGKKSDFYAADKTPSSMPHKPTPIHPPTPLNSHYSINSQAILNKSRNYFP

SNSLRPHYNSVNMLHFTCSLQNYISHQILYDCDYNPINMLPFA

>SG-PL_contig_6935_1_-2_20_3640

MAEHATSAEMELSSGLGLTLMNSTEMDFGCEEDFDLLKYVEQDNLSMFHESNDLHGLPSF

VYNEEEFQTQSQTENIFDTAAIPSVLVKEEPGTILPAHMKSETSLTAHTTAMTENQVSHS

LRSLLESQNTCSGVQQEPSGLPETIGGIVLDTDIDPSLVSLQTVPAQMASVSQPALQNEP

MKTDSTQSSDVSSKLLKVLQNQMLQEKKRKQLQQLLLEIEQTQQNERQQQLQQLIDISDI

RQRAQANTVTNKTIDLNQLKLILKQPSTTVSSVPASAQLPSNTTQVAPAQVRLVNSQPVQ

VEASCTASSQNTGQLTLQQLQQLLGKKVTSTAGSSDITAATAINANVILGTPTAHSTPSA

STTISSISTSPVQAVITNGNTLFTTSIPVHVGGDKIAINRLSAAESGNKGEKRTPHNAIE

KRYRLSINDKITELRDLVGGEDTKLSKSAILKKAIDRIHHLQKSVQRLRRENMILKMALQ

KESLEGLLRKGENIDVSMLALSPPYSDRSCSSPESGHTHSDSSPPSSPVAFDDIANDVSN

VKRTFALAQSISDRSRLALCMFMFGILAFNPFNVLFGSQLQDKGDNSGHIGRTLLGIQEG

SGQGWTQTLSAWLPNVLLWMLNAVVVGMVLAKLLVYGEPITQRNTSAHVSYWRHRRQAEV

DTERGDYSSAGQQLRHCLQALGRPLPVSFSDILSGILWQLVRQFLHRLYLGRWLSTRAGG

LRSSKIQPLEVKESARDAALAYHMLNQLHLTGHQKGSAMWGLNLALCAVNMGETAKETIP

CWLLAEIYATSALQAKSALLDRLQFMARYFLTRARRVCARDGDHVSPSIQWLLHPEGHNF

FVYSKPVDTSSSSILSSSSSEVDPLARVTQAFREHLLEKSMFSLVSPQEPSGKNGRSEAM

QYTQILQECSCLSGHSSSSTAFYNTSTTPAIDEVARWWAAVVSVAFYWLTGDDENAARSY

PLLDTLPPTLQSSDDPLPRAVYLAYKARKVMVLQPDMSRARFVVRQCDRAGRLLRDSLKL

IYPEEERNTVKSIQLLLCDWLLTTRTELWEMSRSGNRGEEHTASQVELIAFQQDLGSLRK

LSQINKAALPKVFLHEATARLMAGANPARTQQFLDRSIRRRVQLPKNTGDSSSDSDVADR

EQATALLMAGRHLPQPLLKSHEERADLVSEASHMYEMLGDHRSVQACHKALQELDMSAPT

KEIPIQC

>SG-PL_contig_6936_1_-1_37_1176

MVEEEESLRPNMCALAILSCRPNSHPFQERHISLHEPVKIGRSVARARPTASNGIFDCKV

LSRHHAMLWYESEKFYIQDTKSSNGTFINNQRLCKSGEESPMREVFSGDLIQFGVEVMEN

NRRGEKITHGCIIATLTLYHPDGREAKPAPFLNDPNQPGTTIQSQDLYQLAHYLQEALHR

EKMLEQKLGSLQQLVHNTQEAAEGGWQALIDEDKLLSRLEMLENQLHIYSKNHSEDTLRQ

ELVALQEDKINYETTAKESLRRVLQEKLDAVTKLSDLERSLTSAEGECNHLKEMCESSQE

ELKQLATRYQDQLKEVQELQDKLQEAEKNYQEETERAEKEKQELMEKLEDMIQQESTLSA

KIESLQADNDFAREQLAAMK

>SG-PL_contig_6937_1_-1_469_771

MSETFVYNRLFYLFIFIILFITYFFLLTIMNQFQNMYMRYIDHGFRLVAIRFDLSSLIFR

HLLMLTDLSPLDQARTMCSDFMKMKPQKPDCTFPHEPIVRY

>SG-PL_contig_6938_1_-2_101_2965

MASMVDQPCYTLINTTTDSEPPTEIQLRQDLEKGDTRTKIDALKKVIQMILNGEKMSSLL

MTVIRFVMPMQDHTIKKLLLVFWEVVPKYHPDGKLMQEMILVCDAYRKDLQHPNEFIRGS

TLRFLCKLKESELVEPLMPSIRQCLEHRHSYVRRNAVLAIYTIYRNFDFLIPDAPELIAN

FLEGEQDMSCKRNAFMMLIHADQERALNYLSSCIDQVTSFGDILQLVIVELIYKVCHANP

GERARFIRCIYNLLNSSSPAVRYEAAGTLVTLSSAPTAIRAAASCYIELIVKESDNNVKL

IVLDRLVALKEVPAHEKVLQELVMDILRVLAAPDLEVRKKTLNLALELVNSRTIEEMVLV

LKKEVVKTNNVEHEDTGKYRQLLVRTLHQSSVKFPDVASTIIPVLMEFLGDTNELAAADV

LVFVREAVQRFDHLRPLVIGRLLDVFPSIKAMKIHRAALWILGEYCTTTDNIQSVMTLIR

QALGDIPIVDDEIKKAAGEVKEDEMQSGGNIQRLVTADGTYATQSAFSTVSVKKQEERPP

LRKDMMEGDFFIGAALATTLTKLALKYIKLTTDKKRQNAFVAEAMLIMATVIHLGKSGLS

EKPITDDDVDRIATCLRVLAERSDLMQDIFNEACRGSLSVMLAAKIEEDKAAQEAAEKKV

VKVQADDPINFTQLISRADMGPGENLFELTLSQALGMSSKKDADPIGSSKLSKVTQLTGF

SDPVYSEAYVNVNQYDIVLDVLIVNQTADTLQNLTLELATLGDLKLVEKPQPLLLAPHDF

CNIKANIKVASTENGIIFGNIVYDVSGAASDRNCVVLNDIHIDIMDYIVPASCTDTEFRQ

MWAEFEWENKVTVNTNITNLSDYLHHVMNCTNMKCLTPEKALSGECGFMAANMYARSIFG

EDVLANLSIEKALHLGKDAPVQGHVRIRAKSQGMALSMGDKINLSQKKSAKTLAS

>SG-PL_contig_6939_1_-3_162_332

MRLHFPLLLCFHFMLSLDVTKEALFKLCYLKFVGAEDYMSWAYFFLKLPFNFFCCCC

>SG-PL_contig_6940_1_-2_2_553

EIHALSGAKDDIGFHSNGDLALTVLPEFVAKSQQPGSRPRSFRIPSLVSSLDPRDARSEE

RLLCPVRCVKYYLKRSAPRRSSQRRLLISLNESYSRDISATTISCWLSETVIRAYKESNA

SSPSERPRAHELRALSWLQLPSTSPSPSGTSWRQPSGSWNPPSLTTTCETSGSLVQRVCL

ASLL

>SG-PL_contig_6941_1_-1_97_396

MGLFPAKEALVLQASRTVQIPARPSALPQPERAGKGFHRLDPWMVLGSCTPALKSHKAPH

LPLMGVSRHAGQLLLPLPMWGGGGIDVERRDWRRSAHLCS

>SG-PL_contig_6942_1_-1_535_642

MCGDGTTHRDGQQTNQFSRCRLALNSGYEILSPQLC

>SG-PL_contig_6943_1_+3_3_227

SNNIQQSTLHDNSTEETVIVTAFLSYTIILTCGKNSLTIQLLSHSLMLPSLLLKAALVNW

FVIFGIYKCVYVCMS

>SG-PL_contig_6944_1_-1_1204_1539

MNKSEYINSIGMLLDVDNDCVYTCCNNNNNNNNNDIKVLSCLNCIIVVITETAISNWSSS

KASPNANTRVNLVLNPSHFSPLLTRSSTPLLWSANLKRKRNNSNIILDSKCF

>SG-PL_contig_6945_1_-1_232_420

MEVNISTLFLTPDSSSSYFFFSNGGRCCSDTDICVSVSSDAKCLCWFWYLGASDMQIGKR

LSL

>SG-PL_contig_6946_1_-1_1_255

KDAKSPRHLPAWDLFLVLSSLSKGPFEPLGAISLDLLTVKTAFLLSLASSRRRSEIHALS

GNPSDIAFLADGSVSLKFLPEFLAK

>SG-PL_contig_6947_1_-2_2_172

LHTHPLCLSCFVLFCFFTFFFFLTLCLFFPVLVPSPFSSFLYRHMKYHFKAFGQVKN

>SG-PL_contig_6948_1_+3_3_110

NTIPHNEQHASFPSTIQTDLVHNFAKEFLYTLILFE

>SG-PL_contig_6949_1_-2_293_2173

MYFCPCSCSKADGENEEDVLIYLPRNLSYAGESYLDRYSVSSAFVHNNKLEKQLLQADVN

DQYLRAGAAACKEAQQAAKEQEGEAAEQQQQDEEEAEESDHLLGARAMARPVTCSEDTPF

RGGGGPDSCDVRLMDSPMALPPPTTSCSMTSTPFRPPLTVSIERDKNSTESQPEYDQCAV

KTVESPDSDLVSEEGIFVNMMDDLEGLGGGASGKLTLSEASDDTYVSGDSMYYSPVEVEE

MEDEQVRGDFKVSLEEAVQQAAFNFQGPNFTQNHEGHQQSPEDKDASPPQEDKTDTPPPP

PHKDDCQKEEVKENGEGHSTDKKDEGKEEDAQQSSDSASPPQPTPLTVVTATVASSDVAK

PAVSSPRKPTTGLEIMVNDRAFQNDDRGPLSSSYRDLLSPDSPLYEDREFNFARTELRKS

TSLKSTKTPPGTPRRKKVVRFADAMGLDLESVRHVLNLESPPKIPPSAMKDLQTGLSEER

KTMGSKFITETFTQPGADLHFRDRVLAEKVVLENALIVSGSITGVVRVANVAFHKTVRVR

FTTNGWASFHDIAASYMINSNDGATDRFSFTLVPPSDFSPGCKLECAISYSCNGVDYWDN

NFGKNYVFECYAKTIPTEAENAWMHFL

>SG-PL_contig_6950_1_-1_1_189

KKTKKENQKIPFYNHQNFCKVVVMVVVHFACFTLILQFLLCIVIFVVLFPFWFVVTFWFG

LKS

>SG-PL_contig_6951_1_-3_918_1145

MTLFPLSLSVSLSVSLSLFLFRELIFHYVLKLRCCCFPNIMICFNHTGVTFNGRCGLLAW

THNHPLLFLLVCVSHP

>SG-PL_contig_6952_1_+2_788_976

MTLWMNDTFCLCVYLCVCEKCNIVLKYSYATGCFHQMLVTKEGSIVLWQAYDITSAQWKR

EGV

>SG-PL_contig_6953_1_+1_1_201

HAPGIHMHTGPSVASRIHVDMVLMRQFRHSNENVTSHDMSSLGLLAVYGRGSGERRSQCS

VWTPVWI

>SG-PL_contig_6954_1_-1_625_747

MSLIPFLFLPSTSLFIDSFMKESLLTLVSRKLLNYLCAVVG

>SG-PL_contig_6955_1_-3_78_365

MIILQRGERDILKGLRCRLKFVLFQTWNIIVWRSFSVQKRREQIAIKVSLLKKRMNYLPL

FKGRLSSACVCVCVCVCVCVCVCVCVLCRMVLLVKY

>SG-PL_contig_6956_1_+3_186_458

METIIRNHSLQKSTNYREEHSFHPSNSNLSTQPRPCNSSCPAPRVTSVSVSPQTSKWRDW

QSVLLDADMISSISHRACEHTELDSVAESFN

>SG-PL_contig_6957_1_+1_1234_1500

MRMCALAHIFMQRFKRWRMRMGQERTIHHFHSERTLHVSTSSFHRTLKHIRSSASKDKMV

SQLTYCLVYNHEYSHTKNQTRNKPFIVPI

>SG-PL_contig_6958_1_+2_1073_1282

MSTNTTTTKQGERYQNKMTSNNKVSNANNKTKLLCHWFLFFFHLIIQSFLFVFLSIKRLI

LLYSISFLFI

>SG-PL_contig_6959_1_-3_3_122

LASCLQSMLEDDDRCGYPVTVVTPERVSQGRSLTEEIPRT

>SG-PL_contig_6960_1_+1_1000_1233

MSYCTVLQVLSQGMFSASRGGCSPLGEHVFLAKCHLICWLVCLYVCPACMYFSLIINNDF

LQNLARDNTCCCGFLNVC

>SG-PL_contig_6961_1_+1_10_432

MKTEGHSTLMKTEGHSTTMKTEGHSTLMKTEGHSTLMKTEGHSKGHSTTMKTEGHSTLMK

TAYTSSLFIVQRGGFQQKCHFHLIIVTKQAQQLHNQKITLSSSCLQTSTLANALKIQTKP

ILSKKSFQIHLVINSLTDTKL

>SG-PL_contig_6962_1_-3_423_635

MNTRTHTHTHSHSHTHTHTQCSHTCMPSQLQLKVYLSKTRDHIAFTVIGGGVLVIPNKSN

TVGFTAYISVY

>SG-PL_contig_6963_1_-2_290_373

MFDVVCWGSSNPSLLTFVELSICLSVSQ

>SG-PL_contig_6964_1_+1_1_219

CFHPIPFPTVFHAGIVEIRSSVHTRPVNVTALVHDQSLHHFGEEYFPKWVGIHFRYFCVQ

SRITRNKNTHKWQ

>SG-PL_contig_6965_1_+2_1499_1780

MCPSRHDSSWYEHGQVGRKLLMTLLETLKSKAVQGGGGVISRSGRRKRRPKKARYLGNRR

IFRNSLCNNDYHSPGSIFSHSPTCSTSTTPRLTL

>SG-PL_contig_6966_1_+1_298_546

MAAFINSVSFDLLIISSALATSPPLLYLKSLCPIFCTAPVSLPPHCSFQLSNTPPNPPKL

RSLRQQGMRGHRIWAIFHNAIMI

>SG-PL_contig_6967_1_+2_2_106

PNITLINGSRGAFVTVCTDGVGHVCPILVERVRAC

>SG-PL_contig_6968_1_+1_1_306

PPSLSHKQVHKHACTHTHTHTHTHTLLYHSYINSTIICITKKSDKCKNARIPTIYTQPHT

HKNLSRLTNKHIKEVIHLFHINHTHNSVTLFTQHGVKHVLGG

>SG-PL_contig_6969_1_-3_1296_1517

MTCFEMFHHVVSILVVHLFFFLSLFFSCFICCCCYKSSLPYSEEGELLRSTGGITMYMSL

SDVETSISKDVMDV

>SG-PL_contig_6970_1_-1_199_7752

MPTEKEEVEEAITLRAEIPEEQEEVAEAITLQATLPEEDVTREDVSAVAPKFLQELTSQE

IMEGTTAHFQVQVTGTPKPEVTWYREDVKIIADDRHTLSEEDATFTLTIANTTIDDDAEY

TCTATNPAGSVSTTADLFVNPSGEAPSFITTLIDSTVNVDATVVLECKVKGTPKPEVIWM

KDNIELTGDQYKVEHIEETVRLTIYNVQPDDTGDYTVKAVNPMGVASCSAQLFVRMEAPI

FTRPLQDTPVKVKDTAHLTCQVKGIPRPEVTWLVDNVPVMLGPKFHTTYEGDETTLDISD

ITPEDAKVTYTCRAHNLAGEATTVAHLVVQEEPVEKAPQMEAPKFIVPLQDIETLPSKTI

KFTCKATGVPAPDLKWFKNDKEVVPDKQVDIEVKDNGETTLVITDVQPEHDGTYSCQAIN

PAGSDRTKAELFVEEIPEEKGAAPEFIKVPHQVVAREHDTAQFLVKVVGTPKPTVTWKLN

DEVLEDKELYHFEKYEDTHCLEIKDVEKEDAGTYTCVATNPEGEATCQIPLIVNEIPRDE

RLSESPLVLKTKEETQPPQFIEVFNDTTVMEEKTLELTCKVTGLPRPEVSWLVNGHELVP

TPNVKTVYEDEHVKLKISTTKMSQEGTYTCVATNSAGTAECTAFVTVEGKSEPPVFTRPL

VDRECREGRPVKFECAVKGLPQPEVKWFLNEQPVEASVRVHVDERTSFADVIHSLSVDNT

EVEDAGKVKAVATNKAGEASTEAALIIDEKKESPKFIKKLETYEVLETETIRMEVQVTGK

PKPHVTWLKGDKELLETEDIHMEVDGETHTLVLEKCKMDTAGLFTARAQNPSGQLSCNGR

LKVIPVKKPTFKRKLSDTLVPQGGVAKFECQVAGVPMPEITWLLNGEELQPGENIRFEVN

PKQGTYTLLISDAVPSMQGEIKAVAKNNGGEVLCSAELEIRGRAPKFIETPLKCTVLEGH

EAQFRCIVDGEPAPKVEWSKGKWMKLQDGGRNKVFYDEKTKEHVLCIGDIKSKDAGTYTV

TASNEHGSEQAPATLMVTDKMEEVVDWKSQLKHREVEREAKEEEAGWDVHLKHVEQPESK

SPSPEEKPKPTSPDFERTEYHPAEKRDRPELESLEPVRFEVAPREHEELEAFERAPPTAA

APKAAEEDDTTGYVRGFKTKTEEELEERTLTIPKAKWQWIVPLEDMHVKEREAAKFECEL

SIPDVKIVWRVDGQEIEPSPKYAVTTEGKKHMLLISKCRPKDAGPVSCAYGDIVTEANLT

VEPVPADFTTQLKDTEGQENTSATFTCTTNEDELPVQWLINGKPVEPSDKYEVLSDGFDH

TFTIHNLSPNDECEVTVVIGDNKSSAKLDVQEISVDFTIPLKDVTVTEGNTAEFECTLTL

PVDKSKVHWFINDQELKEDEKFAFYVEGTVLKLNIKDSNPVDAGTVTVRIGDKESSAKFT

VEELAVEFTVPIKDQTVYEKSTVEFVCQLNKDLPSVQWFLDDEELLPGDGIEIIKDGPLQ

HRLVIKDVMLEDAGQIKVKAADTFCTANLQVQELPADFAVPLNDVSVMERETAEFVCELS

KDVDKVRWFVDDVELTESPRIKFLKDGRKHKLVIQDCSVDDEGLITVQVEDKKTSASLFI

RELPPEIIRPLENQEIIEGETAIFECEVNIDNTTAKWFHEGREISDNRRYNMVREGTVHR

LIITDAILPDAGDITAKVEDKSTTATLTVHEIPVDFMAELNEVRTEEHKMAYFQCQVNKD

DVTVTWLKDGQPLTPSNKHIMEEDGRTHILVIKDVDKGDVAEYSVVVGDRKSSAHLHLDE

APLEFTIPLSEQTCKEGETVTFLCEVTEENVPAKWFKNGLELQPSDSVVMEMEGKVHRLV

LKDTQLDDTADYTVVIKDKSSNAKLTVQEVEADFTIKLSDITADESETVKLSCEVSKPDV

TVDWLLDKEILAPSEKYDVAQDGVTHTLTIAEVTPDDSGTYTARVGGNETSADLTVKATP

VEIIVPLKETEVEEGDSVTLTCEVNIPDLPARWFKDKVEIFPTDDCQMKVEGTVHTLTIP

KTTLEDEAEYLVWIGGKKSHALLLVEECPVRITRPLTDMDCLEGETVSFVCEVSKPGIPA

TWLKDNVPITEDDGFEFITDGCTHILKIPDVTPDHDAVYTFKINGSKSMAKLFVEEEVPD

FRAALRDVSGQEGEDVELQCTVSKPNVPVRWMKNRKPLTPSDRIKIVCDRYRHFLRIMET

IPEDDAEYTIVLPNDKESSAQLSIYEIPTTFTKDLRDVEVPEQETIILECELSRKGKPVK

WLKDGVEVIPGDRIEIVYDGFCHQLIIDDATLEDMGKYSCVCGDVSTSATVTVEELPLEI

LKPLKDVTVTEGEPFTLTCEVSKPNLPARWLKDGKPVDTDRCQLIVDGCVHRLEIPHAEL

DDEADYSIEVRDKSSKCMVLVEEIPMDILTPLTDQTVDEKQPFTFTCRVTKPDAKARWLR

NGEEVKPEDGYEITVDGQTHTLTKKEAVLDDKGKYTAIIEGRSTAASLTVTELPTTFV

>SG-PL_contig_6971_1_-2_68_2353

MKNYSPEKAARDNPSYSEHEDTPTVYKNGKSTDTVPDNEGQEKKEKLKEETEEEEEAEDP

WNVQSLKQDYVPWSELTDKQKATRCVLYPVRVVLVLGFLYLFICSLDFLSSAFKLLGGKK

AGEVFQQSDILTNPVAGLMMGVLVTVLVQSSSTSTSIVISMVGAGILTAKTAVPIVMGAN

IGTSVTNTIVAMGQITDKNDFRRAFAGATVHDMFNWLSVIVLLPIEVATGYLFRLSDAIV

NSMHLNETLDVNQDLLKTITKPFTTLIIQVSSTAIREIATGEVEAEDATLMKFCCDKVTP

AKCCGKTLTKDLGMSGNRQYDNTTRVAACQMVDQCVYEDYEVNQKGLDACMSAWESKNIT

TTYNTLNTLNCTTLADSMTEQDCCSNELTRTSFFGDMARNATSVEQTTLCSTVNSTCFGS

DVYRSCMSAWSYGRQVVRQSDMFNCTTWTVTIGQTCNKPCKFLFRDAHVEQGGDLTDEAV

GAILLVLSLVLLCICLICIVKLLHSLLQGPMAKLIKKFVNADFPGFCGYFTGYLAILVGA

GMTILVQSSSIFTSTLTPLVGMGVITLERVYPLTLGSNIGTTATGILAALAQDVNTIPRS

LQVAMCHLFFNISGIAIFYPLPFFRPPIPAAKFLGNTTAKYRWFAIVYLIIMFFLLPLMV

FALSIPGWYVLAIVLGPVVLVVLVVGIIKCLQAKRPKCLPGKLQSWKFLPLPLRSLEPYD

RVMKKLCFCKRFQDEPPKRGEDVSKKGETNEGFDGVVVDTTM

>SG-PL_contig_6972_1_+2_533_853

MTKCLFWHGNSMKTEAVLQQTHHRHFQPNKRKKKKRKVRECSSKTAQSNSYSTAPRRVSH

HNKNKNSIYSKMTCKHIFFGIYIYMLLEQKQQNATIFIEAGNKNILK

>SG-PL_contig_6973_1_-3_441_641

MEKLCSLSMYNVGWPYSWTILTHHIYTNTCASLQRQAYVHAHTYTHTYKPTHTHTHTNKH

THTHTHT

>SG-PL_contig_6974_1_-3_399_647

MMFYLCSVCRAKNTLIYCFAHFIAIINYLNEQLFCTSLLMYGFIWCKFTTFYFLKVLILF

CISFSPLGPNRIDCLFENKVCQS

>SG-PL_contig_6975_1_+2_188_346

MFSGVLLKWRSGHFGRCTKYRGKHMYTYYHASPVQFPCPAGSKQTAMLCPGKG

>SG-PL_contig_6976_1_-2_119_703

MSRAAGTLITTNNATYIPPRFMPGYRGYCPTTKYDYGETYGNATSKYFQDYRSSVLNSSA

SNYSPGGSFPTYYTHNPDLVISNRCRNRDRWLLSPKYELSNVDHDRTEQLRSLDTLSQKH

REHYKDKSGTIKSVDFFKLPVSAEDQFRTKDVLMINKLRRPTKLEIDADHDIFKNKRGLP

MERVYDRLIKYHHVY

>SG-PL_contig_6977_1_+1_1_132

VKCLHHLTNVPGGTDFQIFCYCSLTNSPWQSFLDLFIQPYKMLV

>SG-PL_contig_6978_1_+3_3_137

DCRLLILNNKIYCREHSSSNSKTFFFIPLNKRHHPQQKNKFSFFQ

>SG-PL_contig_6979_1_+1_40_354

MVKSLYCLSWIFLPLLILSIILLFCLVFIILLASLIMHFHGFSDISLAAHKLFLLMVSLL

LQLPCSTESLRDPCWAPFSLFFIHTLSLTLLPIIAFLTTLFLMTI

>SG-PL_contig_6980_1_-1_154_330

METWNSGKEKDEYLIDETRQGAKLKELKFALCLKKTTHTHTQKLFHVFVLVVIIKTTHL

>SG-PL_contig_6981_1_-1_493_741

MAFSVQCTCMYLLTHLLFTSLVCCYQLHSVLRTCILYIIAGFEKCKTAIISKRQQTESTH

THTHTHTHTHTHTHTHTHTHTHT

>SG-PL_contig_6982_1_+1_136_297

MFYSRFDKQSSSVPESRLPRVASPPPFQVQESDVRRNFSRLKVKKAAGPDNISP

>SG-PL_contig_6983_1_-3_21_584

MVYPVHYTEEMKRKHEEIIRQILEDFNLEETKDKYRNLNAYEHRRNGKSLADYRFRSELI

RMMGSEVVRLNDQSWSQKFQERLKEDYERQLDEQEHNLARAEKQRLHRLRIEGILQKDNR

ESGTLSSDLDLLWDSHPPHIRKTNSSLSPRFSRTHPRPRISSEELKSCSQESNHIFSNDE

LHERERNL

>SG-PL_contig_6984_1_-1_1_348

YIFLDFFPSFLLSLSLPSLPPLSSSLPPLSSFLAPLFLHALSRFLSSLFSLFLSYICLLT

YCAWSLRYADRKKVYLRLELMRQCARYLDTCFCVCIRNCDIDFYHASTQTYWQLGL

>SG-PL_contig_6985_1_+2_2_256

EFKTSDLDLAPPEYLANFYAAKFKVETSDLKFVLFYIKFPVTQAGIELRDIGSRVHSFTN

SAVWGCYLQSELQAYIIHLWAKNFF

>SG-PL_contig_6986_1_-3_465_623

MWSLKLTAGFDIHRKACFYDFLLLLSVMYILKLFVYCCCLLMLLFSQFIFVCA

>SG-PL_contig_6987_1_-2_68_238

MSIQGGCSRSPIPRNLNHRKWKFWWASHSQGTLTEAGDGLPATRCQLPEFFTSHQAL

>SG-PL_contig_6988_1_+1_40_309

MMHHSRTLAYKITVIKFFSKWKPYMCNIPLITRKHYKSHTKVGNAFASSGNNHKKGEKKY

LGVGGEGEILIFTDSCCSVFTTHKHLHMGL

>SG-PL_contig_6989_1_-3_489_728

MVIFCEDPHPILTHYVYKIIGISFNYFNINRCCVWSMQCACVKRVFLLNSTVPKFTIAFS

VYQNALLLAFFLVCSVIYCK

>SG-PL_contig_6990_1_+2_2_199

QFYTDTRTHPQDYKSHFPTLFFISSLADALYTSFCTQKCSVQNIHGLKLCTDTFQYKIHR

PAVLLN

>SG-PL_contig_6991_1_+3_63_233

MKSEQKPVQNVREPPRADRTWYHSPALRTGKRALSIHSATDHLCVLTVWSLCMQIHT

>SG-PL_contig_6992_1_+3_84_254

MFQQSAQMTFILYKIKWLSSSDIQLKFYIAFFPVNNYIFFNHVPPKKFFFDLILPPL

>SG-PL_contig_6993_1_+3_348_647

MGIISVVLSALGHWTGGSLNIHYSPCTVCKLQSLDIHYPSHTVYEHQGQRENEYFCTTFF

NTAFSHSLRCFHNNYNLQLHILLTVTHFANNYTVYNDTFY

>SG-PL_contig_6994_1_-3_54_455

MDAKLRMLLKLPDPKLRVSRPLGPVDGRTVCEKEHGGQTRHVASVRKWNSIALYLCVCVC

VCVCVCWEEIKKNENVATATTTSVIEVVAGKKQQNTKNKQKKQTNRNRQKQKHEGCDYAE

QKTLHDMKDVTMLN

>SG-PL_contig_6995_1_+3_3_176

TLCIKRCISSILYHVQIDILIFILSHPKVGPKSVRERVYMNKKIVAAGGKTTEIILIT

>SG-PL_contig_6996_1_-2_116_1411

MNLELLESFGQNYPEDFDGALDCVSIAVTCAFNRQGTLFAVGCNDGRIVVWDFLTRGIAK

VINAHVHPVCSLSWSRNGRKLLSAATDNTVSVWDVLSGENEKIYRFPSPVLKVQFHPRDS

NIFLVCPLKHAAVLMDLAGSHKIIPLDDNDLNIIASFDRRGKYIYTGNAKGRILTFKTED

LELVASFRVTTGTLNTTAIKSIEFARRGECFLVNSADRIIRVYESREVLTCGKDGEPEPI

QKLQDLVNKTLWKKCCFSGDGEYIVAGSARQHSLYIWEKSVGNLVKILHGTKGELLLDVV

WHPVRPIIASISSGLVSIWAQNQVENWSAFAPDFKELEENVEYDERESEFDLEDEDKEKG

DPRGPEDEDLEVDVTTEEPIQAFVSSDEDEEDGEALLYLPVSPEIEEPEEAWQGDGPQPR

VGSLTAAASVKP

>SG-PL_contig_6997_1_-3_159_389

MLMIVMMMMMIIMMMIMTMIIKLQLMMMMGIVKEEEGEEDEDDDDEVEEEEKLFEMLLVF

VDPAGFNSRKSFQVILL

>SG-PL_contig_6998_1_+3_132_431

MVSCSWQTCILTRLPALSFLFYICNNWIFSCLLKTNKDLVLYLLKMTYLCFSHIISDVSI

CRKQMSMSAKALVTLCCKRNKDHSSMKKKTLLQAMLFFPY

>SG-PL_contig_6999_1_-1_742_1203

MPATTSCKANTVIVFFGEGPGYTDKCSCLTRLMSWNNNSKAVIYRLLPVRTIFSMTEVFP

LFMTIWYFVSLLHDSLDFISGMTNYLITCNDFTQNYYYLKYRYFHLQLILYMIKFVRCMY

RQDQQQPPSTLPSDRDHAECHKIVFIFCHNGFTM

>SG-PL_contig_7000_1_+1_106_198

MFKTRTRLGTLVQYTFSWQLIIIIINHVRLT

>SG-PL_contig_7001_1_-1_43_2412

MSRRRRESYSDDEEDERSRKRRRTEVVDIEDRLESLITRVGEKSTSSLESNLEGLASVLE

ADLPNYKVKIMRILCQCVVMLPEKLTIYTTLVGLLNAKNYNCGGEFVEMLVRNLKESLKM

GEWENARMMVRFISDLVNCHVLVAASVLQMFDNFVEVTLEDNIPQVRSDVYIYMVLSSLP

WVGRELFEKKETELNKLLDVVDAYISKRQKIHFPALRVWSVDEPHPQEEYLDCLWAQIRN

LRSNKWLEKQINRPYLAFDSVLCDALQHTLPQIIPQSHNDEMVYPLPRVIFRLFDYTDVP

EGTVLPGSHAIERYLIEEQIDRIIHTNYMERKDCAAALLSYPVKNLPLNYMIVEVMFAQL

FQLPCAPYLELFYGSALIELCKLQPSSMPQVLAQASEMLFERIDHMHTICIERLVNWFSY

HLSNFQFHWTWEDWSEVVDVDLALPKAMFVHEVLLKCMRFSYHQRISESVPETFAKFVPE

KPHPEYKYEQEGAGSLPGTMAAHQLMNSIKAKCTPDEAAQVLRDLPNKFPSGEDNDNSAY

HPLKIDVFVSTLLFLGSKSFSHSFAALAKFYPNLKSLAESEEGQICLLKTLYSTWRTHQQ

MMVVLVDKLLRTEVVQCQAVANWVFSDQMQKDFTSFFVWEILHGTIKKMTKQVNQLQSEV

DDARDRLDAAKRKEADGMEMFSDEEVPTEEMIERMEERLETATSQQKNLFLVIFQRFIMV

LTEHLAKCESRGIDYNTPWYKWVIERLQHIFLQHHELVFKYINTLENLLFTSEIDIHILE

VFQQFCALRS

>SG-PL_contig_7002_1_+2_134_391

MAAKILHKATRLKVKHSVLVKTDYFYFCLVRCIYDMSIINCTSKQEVCCTLRCCMTFEKL

PVTCITPSSHFAANFKLTFKSIHRYN

>SG-PL_contig_7003_1_+2_134_391

MATKILHKATRLKVKHSVLVKTDSFYFCLVLCIYNMSIINCTSKQEVCCALLCCMTFEKL

PVTCITPSSHFAANFKPTFKSIHRYN

>SG-PL_contig_7004_1_+3_258_410

MSIINCISKQEVYCALLCCMTVEKLPVTCITPSSHFAANFKPTFKSIHRYN

>SG-PL_contig_7005_1_-3_3_179

ELNHKNCFQFLTPHKSKVLPFISKVAWYISNMAELWPFSQSASLAISQILLQMCLLFSR

>SG-PL_contig_7006_1_+3_510_779

MSKPFRVYPNPFMSIRSHYVHCNLFTHSYLSKPTDANLNPFMSTQIHSCCCPFKFMHLGL

CTGLIYVPSSTNNKPMIWECRMIPVCHSKL

>SG-PL_contig_7007_1_-1_196_381

MTLEYGSAEMTDVTYRFGPPGPPGARGVSGPPGGKGEEGPDGRDGIPGTNGLPGPPGHVF

VI

>SG-PL_contig_7008_1_-3_99_359

MMNFYWFKPASRVGSFQGSKHFFFQIKKKKRHRTQACQKSTKILSNLNKLTWKRNYCTFT

VHFINIVIAYVDWLMPPVQSSFPVIKI

>SG-PL_contig_7009_1_-3_21_449

MDVETELSLWLEALEPDNVTSDGSSEPAIKKKQRLNGQKTCRVCGDRAVAHNFDVLSCES

CKAFFRRNALKPKIKPCAFDGDCEITPKTRRFCSHCRLKKCFRSGMKAEMILDADGKKAR

QDKLIENRRKRQEAQAQQQQQEQ

>SG-PL_contig_7010_1_-2_215_2632

MSDNLGDQSCVVPSLETPQQAPISKKEDEDLATLRRRYSASRYRRLSHEKAESINHSSVC

VNPVLAPSVCKDEVLTVNASKLSGEISGTACLSTRKHSEKVDSLIATFDNTRKTQEKEEL

KIIGAQNNTVSHLETTTVSTKPTEKDRENPTEKDKVELTETDKVNNSIGDSISPAKQDET

KRNVTPLITPSPVASPHQKRKTLRRRILTKRVSNPYGRKEGPHRDKVNAHSSQEVADLDF

PIPNPERKMERTMSAPELQAEDLSTSVTLVKNLVWEMDTDRKPCTGLVGETQLCIYEDGS

LLSSQVQTKPICSSVSNEASNLESSHYCSTLQTSSADAVNSSVNSIKTKGDEDFCVCEPD

RKADSHIELPTTTKNLSFKDKIKEAADRLRPSENLDKANNSIATFPQDLSSSGSSSPSGC

IITRSVCASYFPVKHAILELPDMPVIEPIRHEAPYDMSQITCSSSIQTSPTSVKIDCDHF

HIQCHETASDPEAKKVPPYIRTGASSQKGIHLSFQENDSSATHSNMGQVQLTVATEKASE

RLRAPSSDTVTSYQYRLQSNTVAEDQSHTQSSDASCSQPSAAGGNVTIHHTESSDHTSLF

HPDPQNAAHGTSGVTDSWLQENAEYLLDEQDRETVFKRTHNLIQEVSYEEHLTYPESRCS

LVQTSQGERVTHRERRLSLTHETTQVDDVRRLVRTYSLTHEAAQGEDVTHLEGSHSLTQE

ANQGMHVLESLVDSSMLPAHHLHSGLLDSPGASSTNDGAGEEDGEAEDIASFIFLADFGV

DDDEGALSGLSGCDDHSDINSDLDDL

>SG-PL_contig_7011_1_-3_138_227

MDHAYGYDPVWVSHLLIMSDIIRDVGRNKL

>SG-PL_contig_7012_1_+2_74_229

MVKAQLSREQLCPDRTPDSYITITQAGLLKLSTHTVYRNLQVIISKDYRCLS

>SG-PL_contig_7013_1_+2_2_151

LSSHVYIIGYYISPTSFQVSNHVYIIEQYISPNFINLIFFKSFEHQGQLF

>SG-PL_contig_7014_1_-1_253_405

MMWLFSEMCCSLTTFSVVSMLLNFCRCSVRYVYSKLIQGRVNFIHCYMIAF

>SG-PL_contig_7015_1_+1_310_684

MSHKQFVESDQAKVKQSKLLTFFTSGKNKVGHDQKKEFERSVMSRSNDLICRIIVDSNLP

ITMFDTMAKGMKAVFPDSKIAERIQCCRSKATALIKEMAAQTSNDLRSRMKSGPYTVPTD

SSNDT

>SG-PL_contig_7016_1_-1_262_468

MFIIWLLLIYENCIHYMASALHTKTVYTVWLQNGNLNFFSNNRGGDRGLFQSDQNEAVCQ

FICVNKMCQ

>SG-PL_contig_7017_1_+1_148_237

MKGPKHAWRFFFIYSERFDQKRAKCMVRTK

>SG-PL_contig_7018_1_-3_297_410

MYQSTKVCWSSLLEIVYKFIFSPFVIVFSVPYSSLSIY

>SG-PL_contig_7019_1_-2_89_478

MILMFMCCMRKSCQRKMSRQFSSAMRMLKDEKVGKVVPVWISMRTLALGTLKTPAWRCQE

LFGINRIPTFWKSYAVSFREISVHLFISLPNPRKLEYSDCAATCVACTEWVSPSEQCACM

HPSQYEHASV

>SG-PL_contig_7020_1_-3_3_140

TLSISVFISTQIEEKITKRATHACFVSNFAPCAVVYLSAKVCWWFF

>SG-PL_contig_7021_1_-2_122_241

MIKISYLQGRDCWVCTNFESCYEINRYFDILNSKVRKQLS

>SG-PL_contig_7022_1_+1_22_222

MSCNLELQIHAIHKYSKSRRKPSNNTNKFKNPNSNLIVNFPFKTQLPKVLIFKAQNVLRL

YLIHLYS

>SG-PL_contig_7023_1_+2_101_268

MKFLQSIQSYNKCFVNNTIYILSTFRPNTPKQMDMNNRQRMLHLILNTSIHLITLC

>SG-PL_contig_7024_1_+2_2_178

GVEVEFSPSSLSARVCTGANLTLEKSFDVNRYWTLFRFREGKAARNGRWTLPSQRWPRK

>SG-PL_contig_7025_1_-2_2_184

VGMTRAEFELQPVSARAPEAGGLLPPVDFLAVPFPTTDIALDRKTGDRANTRKERGYTRL

R

>SG-PL_contig_7026_1_+2_176_487

MRTATRSQPSVSRPSPPRSRDRIVAEDPAVITDRLGEETGSTSDVHRAQERARLHLDSLM

ESMRSQASLGLPRQPVSSSNLTDAARIDAPPRNAVGSAWSVGPE

>SG-PL_contig_7027_1_+2_503_697

MSDFIWCNRASDSFLRYFCLFVCFLQIFFLTEVSQQLAQQAYNKKISGFKAETDHRRPGS

TAWRP

>SG-PL_contig_7028_1_-2_143_343

MKLVEINYTSSASCNKLLRNTNIECEKVPELVIDDDCVLLQPRLFSKTFRREIERERPCC

FVLLHHR

>SG-PL_contig_7029_1_+2_899_1105

MHLNRVIMRSLRSITRERSLYIGLCEQSAQNLRIILFTCRLRVVVMKWTVTRFVKGKIIL

KVRLNINFL

>SG-PL_contig_7030_1_-3_3_173

LICGKSTLKFGNIHTPVPFPMSKRANKITTASDLHFTSEFNRFDSVEIVNGKIYCFF

>SG-PL_contig_7031_1_-1_1_150

VIIFISFYVHSSHWVISRLICRKSTLKFGNIHVSVPFPVRQRADKSNYLH

>SG-PL_contig_7032_1_+1_244_417

MYISEKLTNEFCCCFFYFFLFFFLAFYTALSYSKSKNLILSKLLSIHDFLMHFHIYLK

>SG-PL_contig_7033_1_+1_208_351

MSDLHCSTGTAFAWSFDFSCLVRSLRADHWKFVDPVTNEFTVVFFDCD

>SG-PL_contig_7034_1_+2_2_73

VNEEGIISCHKCKIFSLTKNTILH

>SG-PL_contig_7035_1_-1_22_462

MNFDVNEMNIKEFRKDREQFLLALSKGNVQLLIDSLRQLPVDNSQGVNVVKLPEPATMVP

REKVVPKARQPTKWEQYAQLKGIQSKKKSRMVWDHETQQYRPRWGYKSKDESKSWVLEVP

DNADPMEDQFAKKKKEKRERIEKNEMQ

>SG-PL_contig_7036_1_+3_147_278

MDCKLNRPKFQLHCPFWSLSIFLVNHHSDMRNKTQCLYETKNDI

>SG-PL_contig_7037_1_-2_71_1183

MSEKHNFGISPVTCHAISGDKAGLALSLNDHEVKIYKKSGAKWQETESLGEHGQRVTGID

WAPKSNRIVTCGADRNAYVWTFEDGKWKPHLVILRINRAATCVKWSPEENKFAVGSGARL

ISVSYYEDEHTWWVSKHIKKPIRSTVTCIDWHPNNSLIAAGSTDFKTRVFSGYVKEVESK

PSSNSWGSKMTFANLMAEFSNGGGGWVHAVSFSPSGDKIAWVGHDSSVSVADSSQDNKLV

VVKDNFLPFMGITWVTENSMVAAGFDCNPTLFTYDAGKGTLSLVSKLDIPQEKAAGTVSA

MSHFRNMDKKASSGEKSAELKTQHQNTITQVSVFEGTKDKCKRISTSGVDGSIIIWDLVS

LEKSIQALKIA

>SG-PL_contig_7038_1_-1_352_540

MLKLALYVVCFKGLISSLFFYTVFHFYSFMSLCLWVSKVDLNYYVFYLPSSECVPECVWG

WES

>SG-PL_contig_7039_1_-3_1839_2009

MKQIPNVFLYKDVNMWSEYYFVELWMYFPSEDVHFVVWVYRKVQYFAVIKIGPVFVC

>SG-PL_contig_7040_1_+1_1297_1539

MTGLKQRFPGCLHNGPGIRHSTTSILTTSPQNISQILYLLFLQQWWDWVSVYILKNIYWI

TEVVRYSEINIITKYNKNKED

>SG-PL_contig_7041_1_+1_1_156

VIECWPPDWATPTHTLANHVEKDSPSCRIELGQIRPYTVEKSLTTHFHLKSL

>SG-PL_contig_7042_1_+2_68_160

MYLRSSSFSKFVCGLNNKRCFNDSHVSDLIT

>SG-PL_contig_7043_1_-1_1_582

PAWDIFIVLNSLRKEPFEPLGSIPLDLLTFKTAFLLSLASSRRRSEIHALSGNPTDIAFL

TDGSVTLKFLPEFLAKNQDPGDPSPEINIRPLSTILDRSDPDRHLCPVRCIKYYLDRTKE

HRASKRRFLISINRKMEKDISAGTVSRWIAQAIIKAYGQARQDLSILNPRAHEVRAFSTS

AAFQQRAPLRAILE

>SG-PL_contig_7044_1_-1_127_270

MCLCNFGDVGQGDISFMFSPFSCVVDTLWNVRSAYVTLDLSLPSVDRF

>SG-PL_contig_7045_1_-1_1_543

SAKNNLKFESESIQVTRDPYQVTFDSPTCNLPFWRYVLLERYCGAFKPSNISFRFIESTK

NDKTKLNDIPKTFLHELPHELWHSFLQLFHVNNAKLVTIHIFYKTGKVLIQGNVCPEWVE

EEFDKLKCIVNHFSSSNASPDDATTILSQLTSSSPPPPPPLAICVEETISPPQQLPGLSS

P

>SG-PL_contig_7046_1_-1_475_633

MISIYLILRSHHAIYFSHLHICFLAQRNPADISLLINIDDTSSDRSLRSKLTY

>SG-PL_contig_7047_1_+3_3_131

TQHFHMHQHVQKMYASTIYSPLCKDELIHRFYKLEADTSYKQM

>SG-PL_contig_7048_1_+2_2_184

LSHTHTHTHTHTHTHTHRCILLVADSCQTASLINCSILKSTELLFYCNDSIFSLHKTCVL

Q

>SG-PL_contig_7049_1_+3_48_302

MDRIAKVAAKIVGDKIDPPSAIYKQLAIKKVRRILSDNDHPLNSVLSSCVSARDTSHRLR

CLKARTNRMRDSFLPTAIRLYNATI

>SG-PL_contig_7050_1_+1_415_660

MYSSQRSSALKEENCKGCSLHYRISEDFQLFCTCESRVMNQYEVYGNDVLLNCSYFHPFV

TAKQIQRRYAISQLYISTVKSK

>SG-PL_contig_7051_1_-3_396_623

MIMVQNFILSLSSNWCTLGSQRKRFFSHSGKLSCFRLDLNVIMRLISRLFCSRSTIRICY

RIFVSSGSYSFIFKYF

>SG-PL_contig_7052_1_-1_73_348

MTHQHTHHDPSLIKIHQQNNQEKFVDLTYWTDIICQSESYPFFKRDMENLRIKKTKDVEK

DTLSNVLSKSSASRNSCLNCRQQYLFEQTHKE

>SG-PL_contig_7053_1_-2_2_766

IYVVDSGQATHLLTVYEAARQLGWSDPSKHRIEHVGFGVVLGEDKKKFKTRSGETVRLVD

LLDEGVKRAEAKLKEKERDKILTPEEMQKAKEAVAYGCIKYADLAHNRTKDYVFSFDKML

DDRGNTAAYLLYALTRIRSIARLANVSQDQLKKAAETIPVDLSHAAEWKLAKSVLRFPDI

VVRILDDLYMHSLCEYLYDLSTAFTEFYENCYCVEKDKQGKIIKVNMSRLLLCEATANTI

STAFHILGVTPVERM

>SG-PL_contig_7054_1_-2_2_175

FLVPRHVGSHTLTSEEIQLFMAGSLPNMDHWFCPVGSRGCIISNGLDPEYENLSEGFS

>SG-PL_contig_7055_1_-1_172_1668

MSVTQPLLSKREEDTVSIEPAPPYYQSINADVHQPVGTPILSSTRSLGPETGEERRVYKR

RWYILILYSLVAGTQGGVWNTFGPISSTVEHAFGWNDGNIGLLTNWGPISFIVAVGPLIW

ALERKGLRVACLCAAFLVASGALVRIITMDPPAVTIWMNVGQALNGLAGPVAMAGPPAVS

AVWFPPQQRTTATAIGTFLGMFGTAAGFVIGPLLVPDVPGNSSHPADNYLSGALQHNGSN

TAQIAKEKEAIQRLMYIECGWACALFVAMLIYFPKKPPVPPSTSAAIQREDFYTGVKRIF

RCPQFWVIGLVYGVSQGVLNCWSSVLNVILKPHGIGEKQAGWIGFFAICGGCVLSIVIAV

LADYMKRAMKWFVLVFYVVGTAAFTVFALSAHNYVPPSIVVFTVCIVTGTTVLYAATPLI

YEMACELVYPTGEGTANGILTLLNNIAGGIFLVVMLDPSIGTEWMNWVLVGANALCVPLL

FFLKAQYNREDVEMKEKWI

>SG-PL_contig_7056_1_-3_21_1181

MADSEEQEPTPAQDIVVTKYKMAGDMVNGILKELISKSCVGASVLELCELGDSRLTEETS

KVFKKDKEMKKGIAFPTCISVNNCICHFSPLKSDPDVILQDGDMVKIDLGAHVDGFIAVA

AHTLIIGASKDNKVTGRKADVMMAAHMAAEAALRLVKPGNENYQVTDGIQKVAESYNCKP

IEGMLSHQLLKNVIDGEKAFIQNPSEAQRKEHDKCEFEVHEVYAIDILVSTGEGKGRELD

TRTTVYKKKDIIYQLKMKASRQFLSETDKKFGLMPFTLRLFDDEKKAKMGVLECVKHELM

QPFGVLYEREGEVVAQFKATVILMPNGPLKITGLPFEPELYQTDFKIEDEEVKALLAASA

SRKAAKKKKKKAGKSLGESAEGGEGSD

>SG-PL_contig_7057_1_-1_1_69

PQYLCDLCHDHLISFSITWRLWW

>SG-PL_contig_7058_1_-3_246_503

MPEDLISPSVYVTFGSYAMDPFPSHSICGGSGYGLPEDLTCPSVYVTFGSYAMDQFPSHS

ICGGSGYGMSQDLTCPSVYVTFAMII

>SG-PL_contig_7059_1_-2_83_373

MHFCHRHCQHHSTRHHHSQLTLHTTLFQQECLQKVVDVDRPPLNLLLHNGQLHVHPPILP

QRLYLVLRKQTSTCMTRWHFLPQQKICVCCGTFLSSN

>SG-PL_contig_7060_1_-3_3_101

GTSGRSSLASYPFLMFRINAHPFGLSQYSCLVG

>SG-PL_contig_7061_1_-3_3_230

GLVSLVWCGVLGLGWCPWFGVMSLALCGVLGLVWCPWELFKSFMCVCILSHPFNDVPCSK

IIGRKGCSVLERIWTK

>SG-PL_contig_7062_1_-3_24_2315

MACVRLSWAARNACKTGIFSRRFECLSRQISTTSAMAGKYLKLDTSHGDVAVVRFDDPDS

KVNTLSLGMQTEFREVLKETQSNPAYKAIVLISGKPGNFIAGADIKMIEACKTKEEITAL

SQNGQDAFSKLEHSPKPVVAAVMGPCLGGGFEVALACHYRIAVADKKTGFGLPEVMLGLL

PGAGGTQRLPKLLPIPTALDMMLTGRTIKADRAKKMGLVDQLVQPLGPGLGNPEENTLKY

LEGVAVDFARGLASGTLKRTPKKKGLQDKVMDLVLSYDMGKNFVFKKARDQVMKMSKGLY

PAPLKILEVVRTGVDKGQAVGYKAESEGFGELGMTKESKALIGLYHGQTACKKNPFGKPQ

KPVQTLGVLGAGLMGAGIAQVSIDKGMNVLLKDMNLQGVARGQEQVQKGLDAQVKRKKIS

SFQRDSIISNLDPTITYDNFKSCDMVIEAVFEDINIKHKVIKEVETHIPEHCIFASNTSA

LPITQIAEGSKRPEKVIGMHYFSPVDKMQLLEIITTKKTSKDTAASAVDVGLRQGKVVIT

VGDGPGFYTTRILAPMLAEAVRLLQEGVTPKQLDKVTTSGGLPVGVATLADEVGVDVAAH

VAEDLGKAFGERFGGGDVNLLKEMVSSSYLGRKAGKGFYTYEKNSKNRSENDGALKLLQK

YHMEPKTELSDEEVWYRLFSRFINEAVYCLQDGILSNPIDGDIGAVFGLGFPPFHGGPFR

YLDLHGAGPIVSRMERYQQLYGIQFQPCQLLQDHAKDSSKKFHS

>SG-PL_contig_7063_1_+2_1775_2107

MKENTGTQITFTNHDTTWNKPVRKETRMLIFTTYCDFFQLSVKHEYLSFSVSVLFSGMGI

AQDKKPLKLRGVWGHAPLEIFFSPQIEICAIRVFLGIKSKITSKIFNILCG

>SG-PL_contig_7064_1_+3_3_383

APAVKAPPHRPASAGDWVFLVDRCTWVSSSSPQLEGELAWHPYSGCWWDFRVSRCQDSGV

SSPAAPPSGPSPVRVQASSPPPSASHSSPAMGLAVGGWAPGPEVEGVRYEGVGEGSSSPR

PLQPGFI

>SG-PL_contig_7065_1_-2_11_1162

MAVPRKLICFRFPGEQAVRLQKNAVALFKGGERHYASLSKEQKDKLADGPGLEDFIRGDV

IPMYEGQESIKRVKGQKLRLPQWLKREIPMGENYSQLKSSLRNLNLHTVCEEAKCPNIGE

CWGGQSGTATATIMVLGDTCTRGCRFCSVKTAKNPPPPDPSEPVHTAEAIAQWGLDYVVL

TSVDRDDMPDGGAAHFAQTVREIKSRKPEVLVECLTPDFRGDLTGVDAVADSGLDVYAHN

VETVAELQWLVRDPRANYQQSLKVLKHAKQGRPHMVTKTSLMLGLGETDDQIIEVMEDLR

KIDVDCLTLGQYMQPTKRHLKVKEYLHPDKFQHWQEVGDKMGFAYTASGPLVRSSYKAGE

FFIKNLLKKKCGDASETDTESHSA

>SG-PL_contig_7066_1_-2_98_259

MRHIQTSQPFLFLRKCIFFPFPVKQTCCRIQAGSTRLRGLAGVEEHSPVQSDWK

>SG-PL_contig_7067_1_-2_1004_1147

MYYVEICILVNGLQIQHWYGSVGFFIIMTELSLIITFCGYIVILCNTL

>SG-PL_contig_7068_1_+1_145_273

MIMEGNITSVHVSGKANLVTVAAPGTDPRPAQPLTLVYPTLVY

>SG-PL_contig_7069_1_+3_3_251

LTSRVWNLCIFRPLVLYPLGHTSNLDIFCRYIYLFNLLIYVHILRLPWLHMLVCIISTVL

WFIRLLNVLCVYQSNSCLFSRLR

>SG-PL_contig_7071_1_+1_142_348

MVCVKDIVIDCALKSMSMDCIISYITTLCVKDAVTRLCVKDAVTRLCVNDAVTRLCISGR

LAQLVKASC

>SG-PL_contig_7072_1_-1_1_195

LLFVDKADFPVRAVNCIVSQTFRMCCLSNVSKGNITLTNEMEDSEALQHISNEMHSYSLR

FVMNV

>SG-PL_contig_7073_1_-2_248_1231

MYVVFQLFVAGIVVLLLDELLQKGYGLGSGISLFIATNICETIVWKAFSPATVNTGRGTE

FEGALIALFHLLATRQDKVRGLREAFYRQNLPNLMNLLATVLVFAIVIYFQGFRVDLPIK

SARYRGQQTSYPIKLFYTSNIPIILQSALVSNLYIISQMLATKFSGNLFVNLIGVWADVG

GGGPARSYPIGGLCYYLSPPETLGHIVEDPIHAVLYIIFMLGSCAFFSKTWIDVSGSSAK

DVAKQLKEQQMVMRGHREKSMIKELNRYIPTAAAFGGLCIGALSVLADFLGAIGSGTGIL

LAVTIIYQYFEYFVKEQSEMGGMSTLLF

>SG-PL_contig_7074_1_-2_323_502

MFRIYILILLQLQKDPFYKMDRLLTCKASELVYLCTSPLLAPQHIPLKIDKRQGCLFEEN

>SG-PL_contig_7075_1_+3_1305_1574

MILTNHQKYNSLPLAAQKLHNNIQFTKFSAKPQQNLPSNLKVSYKLHNLTFTKLSQKLIK

PYSFAKMSLSDIPYTLAKTNEIWPQILPKL

>SG-PL_contig_7076_1_+1_79_174

MVFVRLTISLNQIMLLIAQPTTLRPYQGCHLI

>SG-PL_contig_7077_1_+1_1_114

EECLQCRRWAGAQIKSNHVAYSPADHIKAISGLSPHLK

>SG-PL_contig_7078_1_-1_250_417

MVLMVIYIAASFCYKAVVCILLCLALGRNTEQLKAKEQQISCSWRSVANVHTHPKG

>SG-PL_contig_7079_1_+3_48_194

MMFTYPSMAYKINNPSESESLSLSHTHTHTHTLISIYMRIKTSIIERRL

>SG-PL_contig_7080_1_-2_77_235

MRERKAIQKTYCIPGDHDTWKRNAHFQEMASAFTCIQSSTRLRVLIYMCAQTM

>SG-PL_contig_7081_1_-3_3_143

LFSQKRQHMHLCSLHKHFKYKTLKMYNKRNTTKLCDCVSQHAVPWLN

>SG-PL_contig_7082_1_-1_13_720

MAAPCESTGGKLLAGIFLILLAGMHAEDEFEGSRTLQVEHSFDQGASPEFSKRGTVVIHS

LKSNKAQFSQAASLSPQEKDQLETLSKQNGIYRLRIPLAKGGSSEATSYVSTFTTACAVY

ESGLRDRLMVNFDTTGEVLGVSILPAVGTCTGASVNSANLTSWKTTVDVVQTVAGPVPDT

QTYIDKMKRDEQERAKGQQGDNRSFFGKYWMYIVPFIIMMIIMSSADPQAQGGGGR

>SG-PL_contig_7083_1_-1_583_780

MKKALNRVYMPDRFNVNEEGIEQSLHTMLMKKALNRVSCERAWTEIVCCLNVLMYCNIFN

IDSCIM

>SG-PL_contig_7084_1_-1_76_1341

MMEGSDGVEMQSQAGQADSITLRDKNDFIQNVSQFFNQQDISDVVLKVGENSYFAHRFVL

AKSSEVFRTMLYDKRYQQAAHPEIVLEESEECQHHFEAFLKFLYTAEVLISTDSAVGILC

LADKYCVMSLKALCTRYMAENTQSPRVRNALNWYSWAKALHMDELIDSCSKTIAWNMELL

VALPEWLSMDIDFLADIISNPQLVVSNEYVLYSAVEQWLLHDSHLPDLKENGKKLLRLVR

FPQMMVQQLYKVEKGELASREGSQDEIEKLLNEAFRFRSLCPFQSELDIQFSDEFYRPRN

YKDLTVDTVRMQNTLRFGIQVDVRTYVGPVPSTIREGEWKITYRKANDSWTLQVYCHESA

MVNGEARIQAVLLISDDDDRVLQVEASEETVCSRGNHLTMVFSLPACDQAKLLTVLIKPL

PN

>SG-PL_contig_7085_1_+1_1_165

HLAILWGPGAYNDDLYYDSLRCKMATVCKEKQTSRPNNSMNEERKSSIRQNNSFS

>SG-PL_contig_7086_1_+2_65_448

MELKKDMYLVVVDYYSRYTELAHLKNTSAETVINHCKSIFCRHGITEVVKSNNGPQFVSQ

QFAVFAEKYGFLHSSSSPYYPQANGEAEQVVKMVKAFLQKAEDPYLTLLSYHTTPPQHSS

SSAELLMG

>SG-PL_contig_7087_1_+1_1_204

KKKKKKKKKKKEGKKKKKKKSIFLCKRIKKKKKKNTKNKKKKKKKKKIPPFFFSQKKKKK

KKKKLSTW

>SG-PL_contig_7088_1_-1_175_318

MKKINKHPSPPPPPQEILYGSCVHFYYQYFFLRNMRIEYIGPLSRPTM

>SG-PL_contig_7089_1_-2_719_1033

MSQLHKVKVKTQKDCQHGLTIFPFPSSIHQMYEDQLMSIPTVWACTALSSKHIYFWRVDM

ISKEEDYICKEFPLDSMCVRLLQSKTNLFCEVTECGDVAYTPMHD

>SG-PL_contig_7090_1_-2_2645_2776

MFEKYIFPPPKKDCMPCGEPRGGKRGSVDGDPLQQVYICCFIHL

>SG-PL_contig_7091_1_-3_3_314

DGNTIREVRTRIAKAAVAFNSLNNIWKNQNISRRTKLNLFKSNVLTVLTYGSESWCCTKA

LENTLNAFENKCLRKILGIRWDEFKTSQEVREIAQQPYISNVIR

>SG-PL_contig_7092_1_+3_3_233

AVVVVPAVVVVPAVVVVVSAAVVVGAAVVVVAAVVVVPAAVVVAAAVVVVPAIVVVGAAV

VVAAVVAVCAVGGAGAA

>SG-PL_contig_7093_1_-2_230_442

MNQKWQNRQPVCLKVDIVTLDFSMILISFMRIFQLFLKVDMFGIMYLTLIHYYHYWYRLK

DTWDQCAFPSC

>SG-PL_contig_7094_1_+2_2_193

AGLDRSLYLAEKSSSLCTHSSHTHNNENISFHCNVTQIKIFKQEAALNKLSQKLKRTHKQ

GIYK

>SG-PL_contig_7095_1_+3_78_176

MHSHVSTGLCPMISQWYGTAAHHMHSSTILCNT

>SG-PL_contig_7096_1_+3_72_239

MYDNCFPHQLSLKPLLQLNILRVSKSVDDEMKSLCALLVCGFLKQTNINNSYMNGK

>SG-PL_contig_7097_1_+2_176_271

MEYTPVKLDTSCMIMFQQYWRTYVCSYSFLLK

>SG-PL_contig_7098_1_+3_3_284

HIKNNKGQLLAWSDFVQKSICALKFSSVFSDDTDNEHYAITITQLATESTDRSKLVCWLS

SHNDNCVQIHLQYISGQKNENRTEHGRCIQHDDR

>SG-PL_contig_7099_1_+2_2_202

SPGGCVLLFGHLLHRGIGGMVQVGSACSGGTPSSDCSSCRHHIWALVYTLFFPFLSNMSL

FFIFLYT

>SG-PL_contig_7100_1_-2_20_244

MADNLLNKVFCGNLLCVPVWLWCLISYLGGVLLCVLVFKCLCGILIVRVVPSNSWMASQV

SQCFLILFCMVVGGI

>SG-PL_contig_7101_1_-1_613_1089

MLPVFHVFSLGQYHNKGDVCFLAALVGLFCDWCLVRLSCHSCLTHFTLFMSILVKLDQLF

LTVYKYTHDAHCSHLACSHSVYYHAYFPLMVQVCPQMICKLAIWHFGFQCRFCLKITDFF

FFNVVKCTQRISAEEMIQQVREWVIIFKAMSTKTSKNTT

>SG-PL_contig_7102_1_+1_190_714

MQADWKASALHYTSQASNKYMVTKQTNKNNKNYFYFVIICVIFLLEQKTLVEGQLTIHTV

KLPSNQCLQYKQPTIQPMFTIHSQTTIRPMFTIHTNYHLTNVYNTHSQTTIQSMFTIHTV

KLQFNQCLQYTQSNYHLTNVYNTRSHHLTNVYNTHSQTTIQCLQYTQSNYRFTNV

>SG-PL_contig_7103_1_-3_543_929

MQEHSRFSRPRSNSEEELLIVNMSLLSRLAKKDTRSSRNRVMKPYRKKRPAPQPCLSERE

QSVTNASCATEAKGKKRKLDDRWLEFPWVEFKDVCLCSTWKQESPGEQICARFNAITKVR

RDLIFDCLH

>SG-PL_contig_7104_1_-2_152_421

MYFSFVARCVVTVSCLVKVHVSLFQFCETQLLCECTYISLNGNLAIYILLNTTRRYYQSF

SLSHSLSLSNLCLSLSLSLSPLHISYLLIG

>SG-PL_contig_7105_1_-2_20_130

MELTHSLSLNLSHFYDSLSVCLNICFVKIYDRNNALF

>SG-PL_contig_7106_1_-2_2_211

HKENREKLVPPLCLTDILLSGKIQYWLLCFTAARGHQVWCQSIQTRVYNKLRHPKANYRG

YSPKTMYWKL

>SG-PL_contig_7107_1_-3_102_341

MMGLRATYNITGFPTLYYFEKGKLKYRYGGENNKEGIVSWLSNPKPPEEPKKEAEWAEEP

SEVMHLEDGTFDDYVKEHPS

>SG-PL_contig_7108_1_-1_1_243

FELKHFKDGEFNKDYDRKLAVKSMVNFLMDPTGDIPWDEDPTATDVVHIDTEDGLTKLLK

KDKSAALVMFYAPWCGYCKRL

>SG-PL_contig_7109_1_+1_754_1074

MEMIILWRVVWVYISCTQTDKGVVGAEVEHSPYNLRVYTRVCAASSLPSCKWVPDFQRGR

RDGPPPSKCWPHKKLKSLPHHSPSTSGLWKSIFYTNQNKPKQKNNKH

>SG-PL_contig_7110_1_+2_2_253

GTKKKKDNTQHQNVQPYTFYKFQFLIQVQIPIFFNSNLTGKQKYKQRGEKKRWWCPMMTY

SPQTKKNQQNNVIQNRIRKHNAKQ

>SG-PL_contig_7111_1_+3_900_1160

MDLPNSNPQQSTTILWTYLFQNRPREHVSEKLLTVEDFHIIRTLYLEQTATFLWTHLTLC

QNKVHTPQNTLHTEDIYRSSKSSIHSF

>SG-PL_contig_7112_1_-3_87_263

MSSAPLACACTGATTVQIILSGRLQQERTCSTPASLCLGLHSVSLYLSRILAWPGIATH

>SG-PL_contig_7113_1_-3_639_779

MLLKVLFMISCSAIQSYHAQDCSLSVCTYSDTFFWKYQGHNFVMIFA

>SG-PL_contig_7114_1_-3_9_1133

MVLTMLIMSLMSTGSGEVMAVSSIIVYDVYQTHVRPFRRSLSAPSCILCGLAKQKEMTSD

TMPTSDLCQCPRAADCRQCQKDAQDTSSQRSRLKVYRCSVHGDYRLYQDWLMMLKSWVIL

WVTLSLVPLGLCVIASGMDLNWVMMCGFILTIPCFPGALMTIIWVKTTSLAVIVGSLSGL

VGGLMANLIVASTYEGGLTNFFPNTSQNYAVLAGSCTAFGTSLCLTFIISMLTHKIRNSG

DEKAEWQRLRDIDNPLHPWAETYQDDFPELKPGTTPSYSELDKVFRKAKLMAYIGGAVSI

LLFIVLVPGIMMSLHVLTEAEFVGWVNGLQVWCFLMALIVIILAPVQEIITIVRQIHANH

SVTHGHAQNKEGAGT

>SG-PL_contig_7115_1_-1_1_219

LNRTVNTDIFKYIVRERSVEVYVHDFYSWFSFHLLEKTTVVSHWTLIVDIVFFSNKMELE

LGRDLEWVGHRPL

>SG-PL_contig_7116_1_+3_45_212

MYSTHTLATKSSLVTFIVIGDSSVKLNPLIPSCRKLVDRLRMCSCLLQSISSSCCQ

>SG-PL_contig_7117_1_-2_320_496

MIIMKKRLERNENKHKGTFITVFKNQLNMHTHTHTHTHTHFLSLSHTQTHTHTHTHTHT

>SG-PL_contig_7118_1_+3_366_461

MNAVIFNSVIIRTSNTVHAQYPFSELNLKCTE

>SG-PL_contig_7119_1_+1_1_222

EILHLLLLYILFIAQPTIKAISGLSPHKVHQSQKDNRIDHKTTSNPKTKTLQNTVHDILS

QPLSSLRQNKTRRC

>SG-PL_contig_7120_1_+3_93_344

MFSLQLLKHLILLPVMGFVKDHGVCSPSYTLDKACLQRPQPLQIYTKCQCPVKILRICNT

DQVEAINYIYHTPAPPKKTKKKLG

>SG-PL_contig_7121_1_-3_81_1877

MVDHLSKSAVEKPRRKITLVDETERKTASVTGLSLSEIRQRAARKCVPDSEDQVSSDGSR

SPEMSTLETRRRQLLKKLSSPKPETSEDEARSDRSPSPEVLSLEEIRRRKALKERKQKKS

EAYKPVSGSEKKPIVKPAVSVQSLTEIRQRKQQKDVRTQSLTEIQQRKQQKNVRTQPVYD

ISLFTDRREERSKVEEVEPSVLSLDEIRKRKLKTDSVKDRPALAKRTKTEQQIYVPPPRK

NAVKMEMVPLRKEGPAIKKRLGDISVSRTQTEEKPAVTVRPESVEVKTFSEIMAEKRRKL

QLEKERQKKAQGGQEENGQANKRPFRFRPIVFGEADSDKSECSIPLVTKISSDDDKSNSP

KRSAFVRLGQTERAGTTFSSSMNSRLTTEASASQSSSVSSQLPSKFNISTEDVSKDSRVG

IIKSSSSHSLSSVSADAFHCAPSSPRPKSTKSNPTGEGPSHFSSTPFVNPCTQSQPTPTI

SAFPSVPQQSTVCHQESATTPNPHMMAIDTLLTSKNKVQIQPSPASASTSWAYPLPSSHQ

ATSGDTAQAAESTTSTDLSTSRLLSDSGDDLLKLDADIVTVDDNDQLDDLLMDIDSLLA

>SG-PL_contig_7122_1_-3_24_227

MSNCTYSIALILFIEAVCSCHSLYSFALNISFLVGLFSLLFPFWLGVIRQLGSRNECGLS

HTDNKFWI

>SG-PL_contig_7123_1_-1_103_258

MSVVCFSFRTANIGQKQVGTNFLNHDSDGKLCVQDYGCAFCNSVYNVVLFLT

>SG-PL_contig_7124_1_+2_2_70

NVHTCIRGNLESLKNNNIKTRGE

>SG-PL_contig_7125_1_+3_3_155

SHPIHQITAGWIGWGSERVYRCNLLTAPLLHFCSMLCLCERVPKLFHFQNF

>SG-PL_contig_7126_1_-2_2_124

LFVKINQKKNKSRIEREVTYSLVFNNYKLLKVAYSCENIHL

>SG-PL_contig_7127_1_+3_264_416

MLTFLNLILSFSGSFHSFYAGFLNHHFQGHFTHSMLAFKITIFRFISLILC

>SG-PL_contig_7128_1_-3_105_269

MMYCGDPDDGLDALQYRRFCEKVSQRAVCVQVHTLPPLKVAASYHSMRVYFQVQQ

>SG-PL_contig_7129_1_+2_35_232

MPYNTTQHYTIQHNTIRCHTTQHNTIRYNTTQYDAIQHNTTQHDAIQHNTMPYNTTTQCD

AIQHNT

>SG-PL_contig_7130_1_+3_3_206

LYYTIQHNTIRCHTTQPNTIRYNITQHYTIQHNTIRYNTTQYDAIQHNTIQYDAIQHNTA

QCHTTQHN

>SG-PL_contig_7131_1_+2_2_190

GQYNTTQYNTMQYDAIESSTIQFMLYICLSDKRLKIRAEMLFHSTGAGKRCFASTLACSY

SGI

>SG-PL_contig_7132_1_+1_1_318

NTTQHYTIQHNPTLYHSTQPNTIPFNTALYYTIQHNTIQYDAINTTQYYTIQHNTTQYHT

TQHNTIRYNTIQYDAIQHNTIQYDAIQHNTIQYDAIQHNTIQYDAI

>SG-PL_contig_7133_1_+3_159_536

MQQINKSHIHITITGSEHCLHHITHFLHITPLIFHIASHTTPTHHTPFYVPITSHIFHIP

HHASYLHITHIFLSNHTQQSRAQSSLPLSACNEGKLLPMHLSTRGNYSPCICQRQKGRGL

NPDQCV

>SG-PL_contig_7134_1_+2_704_817

MLFQVRLFFNPPFRQLYFFFEGVHAGCLHVSVHYQTLI

>SG-PL_contig_7135_1_+3_3_149

TEPSSGALQCIYVGYSHVCTNNLCLATNAHQWRTEAVCIQNLCSRNEPQ

>SG-PL_contig_7136_1_-2_44_484

MEADAFVVVYSITDSSSLKQAHSIMDTLTAMPDLTVPVLLLGNKTDLQHLRQVSRHDVTR

LTYQYQCRHCEISAAESYAAVDLPMQEVLREGLENKRLRLSQVKRRRGLLENVSKRLGSV

FRRKSMEGIPQKRKLSRVAENPNRRSV

>SG-PL_contig_7137_1_-2_2_271

LAYVHGQGRKECYDEKTTTFISHGEVWFPLGDDDPCFECTCVDGDPVCVHQECTLITVCP

DGSEPYQEPGECCLQCPDDDRKPPPQPRRR

>SG-PL_contig_7138_1_+2_8_217

MHIGKRRGQTGQDIYCLHLVRSRDCATESEPTLHPANPYAQVAVWARLRSFHLPGGLLPE

NLHKGPGFQS

>SG-PL_contig_7139_1_+2_2834_3157

MGEGPTMRYVLSKNTIYKPGWQCPKMNNSSLLASRFGATRWVGLGGWDSLNPHGRSVCVC

ARVRTHMCVRVYGHMCVRAYTCTCSRLSPRKAVTPNLASVNHCTTNLT

>SG-PL_contig_7140_1_-3_156_290

MSLIIKSKQTFVSCERACLLSLMIVVEKKQTCSCTVQERQETHEG

>SG-PL_contig_7141_1_-3_303_527

MSGSEGKIPLQEVLPKQGLVYQEDMPTVVLCKPKLLPLKSVTLEKMEKMQKDAMEEVKAQ

ELKELGQEADLFKGY

>SG-PL_contig_7142_1_+3_3_245

VCVCVVIQPCAKDYFTIVIIEGTGNNTDNDVQTSFLLTVSAFINAFMLYQLILAATCVQF

IRLTLVQCTIAYFGLGFKMLL

>SG-PL_contig_7143_1_-2_113_325

MSYIIKSFMKVYKAAEKPSISFLCGIISDFFCKYENMICGSGLFSKSCFIFYRRIICFKI

SYDTFIKDFTK

>SG-PL_contig_7144_1_-3_3_167

FKEYLYLQLPVLKGKPTSRPIFQRVKSGIQKSATYIHTHTHSCMHARTCTCSNYI

>SG-PL_contig_7145_1_-2_8_310

MATKTSKRRHVDSDSEEEVRSMVGSWPSWLIIEGTDAEKPLSGLSPFAIAKGIKGLSGEP

KSIKRLRNGCLLVESATKLHSQMLQKSVTLIDRPIKVTPHR

>SG-PL_contig_7146_1_+1_2632_3099

MLVLALEAVGKSGMGAAAAVVLGTERTVASVAEILVPQALAVAAAGHIAVAVPAAVVAGA

GETLVPVWAFCLALAALQGLQKKHHSSFETFCCCCLALFLWVCCWLKPQSVCVCYVALLM

PHIYCSPHPHRWWQILSWAAPPHVHLHLLMHPPLLY

>SG-PL_contig_7147_1_-1_73_717

MPYYGGGGYRGGGGNRGGGRDGEIVTDRNAEQFRKLFIGGLSYDTDEKSLRGHFETWGDI

VDCIVMRDPNSKRSRGFGFITYKDADGIDQAQANRPHKIDNREVETKRAMPREETGSQQT

VKKMFVGGMKDDTTEEMVREVFSEFGEIDDVNMITDKSTGKTRGFCFVTFNDYDPVDKCV

LYKRHKLNGRNVEVKKAVSKEELGGGAGGAGRGGG

>SG-PL_contig_7148_1_+2_2_196

NNKKQHTHTQNKPNQCFILLWHLQKISFAHIFNSQTDKIKSLNMCHFCQLQPNFTHSLTL

LTKED

>SG-PL_contig_7149_1_-1_43_5040

MHSSMTDESVKGHVQTLPRPLPLDDVSKLQPALSNKVMTSGPLQLETEQKCACPISEDGT

NSASPFQEKEQNNDLQSLATKISSYGILPEKVLCPDTSMPSHLDGMEKRQTGLEEPQVAR

EASLGDDQSPLESAKTPDVSLLTTCNHPPLLERVVTGGQGAKDELCGVEYSDSLQLPILH

LEDVHVQQGTDVDEESVPDISGMQTANQSTPADEAMPVSNYSSDSSKRVEERNGAIYRIK

NSVEVKDIMKKTPVVLLTRLTPGLLDALKLPLQVSDSRSEDSQQITERIDQDSQDGLSSE

SANDDQIKKNRNCKNRSKQHDKVSPLAQMLEESSIYNKTARTYSPSVDLASGTAADVPSG

VHLDENSNSELGKPRQPGESCAAVHPKQNSASKTLQSTCGDGKGADESVWRKKHSVISQS

STYHFRSSHSIKPTSDCSVEKNCEHTKPCDHHTKKTPTAAASYTAYSPGALKMTFVSCSS

SSAKAADKSSGDKLDTESGNSSSREPPVDGWESCPDVIFDYSVTGESVFPRTANSMSQNP

KGHVHSNDVDKVSSRSSPVDVVSSQLTPNFLFPKPESQPSPSCSNPVQTSTNASHAILPT

VGGDDSLNLQQSQATAVRRSAARGNAAESMLTSVPSSHNNRHQSSLQDAQSCRDHGQRKH

TTVPGKAPQRRTIAASCVREQRGGSPSSCASVTHGLNRPSSSVPVSSLVERIRNGLQAKR

LRNATRSLPVASTNSGGDSVPSLVRAHTEQRDTRSQNSENHCVDSLHELTDISTHNESER

HCGDVGSGLDIAVPHLPLVTVHIGGKNSSPEGGCTITCIEKTSPAFVIPHQEDKMLVESD

MIVMQNYYYSTLQSAHAVNQTVVSGCCQNASGVMDSDHYSSLPQSLQLTTSDQHTSPLVV

HVEEDCDLAKPEDGFSNQSHLTNTGILMVNTYATKAVPLKISPGRQRAHFRSVSEESEEK

TNADYKDIDLLSVVVDVVHNHTPEDSSTIASHTGLNFLEPGPHVNAEELCHKLHITSVEC

IQHGNCTCGSFDPAEQEVIPGQRNRNEANLENQQRTPEIAMIEFTGEDMLLQDFEDQLMG

QVSIHQDNEDVDEVFDIVNERDVPLPHEAFLHSLTLIRNTELAACASRSPHPPLTHSMDS

LSSDLMRERMEETNRESDDSDATLPYPQDEGRGRSKPSRKPGRPKRSKRRKQKKSRVTKA

MDSPQVLPSASSESKLGAKRTRPSLSTKFIDLPAPAKATKLSEKGGPSKKNANRHPLFKK

RHQTVSRKPKDSPMKGFAARSATPAFASKPSVVSKTATSTASPTKEKSVISVPSGTPESV

TTSQSPVETMPIDPVSSSKLVQETPVTGVGVATKGDQARLFRSKRGIIALDIVPVKPKAS

AGKLVVGSGKRKQKPKQKLVKDPVDAAKKDKVGATFEGTRLTDESESDELPTIVPPKRRR

QQSNSQRGSKSSTGATEKAASAAAALEVKRAMQESLMDGTERIMREFPRHNWLEHKILQK

TTYTPNPPSGAVTTPTSTEGADTKGSSSLRAGSKRLLSGNASPPEGHRKSESHSLPVPRS

NSMSTGDTSPSGDKGQDQTATQSVAGDTGTHVTASIRPAGLSAHPATDGGTVDSQAVAEL

ISSAVPQSPAAVVAARTSAAHPPGFPSPKTGGMVFERCVSSEGVRG

>SG-PL_contig_7150_1_+3_3_107

KLTVNHAHFVTRSSVLQQTTADHNHSNNNVINNIF

>SG-PL_contig_7151_1_+1_85_303

MVLEIKHITHTATQQSENTVTPSVLQDSFIQNKTGPDHTTTTISFIYCSCNTHFFQLHSL

SQQDHFFKCSCSL

>SG-PL_contig_7152_1_+1_388_546

MFSLCQMNISSPNYSTHKHKLYNENTNLILKSNKTMRKIITFVQNNYVPKRIL

>SG-PL_contig_7153_1_+3_3_155

LSLPDALPIFFFLKILHVYSKSLPQLYICTAQLFTTLCQSIACFSSWNYEE

>SG-PL_contig_7154_1_+2_692_934

MTQLGKICGQDSCIKRPKIVADTFSSPYTTEPFPQARNIIFKIKQSNSNTVYMLTCSKIK

QSNNSIHSTLSNNLQKHEIFH

>SG-PL_contig_7155_1_-2_128_1318

MATVEELKARVADLEQQLAKALQTTSTANRGKIAEMSSEVVDSNPYSRLMALKRMGIVDN

YEKIRDFTVAVVGVGGVGSVTAEMLTRCGIGKLLLFDYDKVELANMNRLFYQPHQAGMSK

VEAAEKTLREINPDVEFEVYNYNITTTDNFDHFQSRIKQGALKGGGPVDLVLSCVDNFEA

RMAINMACNECGQKWFESGVSENAVTGHIQFLIPGETACFACAPPLVVASKIDEKTLKRD

GVCAASLPTTMGIVAGFLVQNTLKYLLSFGDVSYYLGYNAMQDYFPTMTLRPNPACDDLN

CRKRQEEFQASEASKPKVEVVVEEEEIIHTDNEWAIEVVSEVTAEELAAAEGDQPELTKG

LRVAYTTPAPKQDGDQEATVKDSGQSLDELMKQMQNL

>SG-PL_contig_7156_1_-3_3_242

CSVIVIYNVHIVLLKRSLSFSKKFGKFEDGRQLESNVSVIVGSQEDRGQFDTDSESLLFI

LNFVDNSVHAEECYAALWNF

>SG-PL_contig_7157_1_-1_1_126

IDSLDIFVDVPADLNEAELNDVAMGSCQGMGVLQQPEGNSIN

>SG-PL_contig_7158_1_-2_2_103

RFLAADEDIRSILALVGPFLGACLFLILVFVCDL

>SG-PL_contig_7159_1_-2_2_853

LEIEEKGVRVKLTIVDTPGFNDAVNAENSHAAIVDYVDQQFQQYYRDESGLNRRNIVDNR

VHCCLYFISPYGHGLKPADVEVMKKLHHKVNIVPLLAKSDILTKVEQKRLKQRIIEEIDN

HDIHIYEFPDCDSDEDDDFKQQDRELKAAVPFAVVGCNTVVELAGKKIRGRAYPWGIVEV

DNPSHCDFVRLRQMLISTHMQDLKDVTADVHYENFRASYIQESMAGQQRERSKLKRDSKP

SYDDIADTDKLLEQKDAEIQRMQQMLAMMQEQLGKQQAQVNGKV

>SG-PL_contig_7160_1_-1_112_708

MAQIGQLALTKPAPEFSVQAVVNGEFKTVKLSQYKGKYVVLFFYPLDFTFVCPTEIIAFS

DRAEEFRSINCEVLAASTDSVFSHLAWVNTPRKQGGLGNMKIPLLADKTLELSRQYGCLK

EDEGITFRALYIIDDKGNLRQITMNDLPVGRSVDETLRLVQAFQFTDKHGEVCPAGWKPG

ADTIKPSVNDSKAYFSKAN

>SG-PL_contig_7161_1_-2_11_469

MRGVDVCQTNHVGLTALHHAVLANNLDAAKLLLCHGADVNAQDVHGFSPLHTAAACGFLP

LTSLLLLFGADVFSLTLEHELPIDVAKELSVVRVLSSEMTRLVHQELWVTSALRTHAEEV

WLILRKLLACVLLLVLHVAFCVRAAWRKHRKTE

>SG-PL_contig_7162_1_+1_169_399

MQYLHKLTTQNKIPSLIHFAYCLYCSTVMHHPVLYGNVSYYIASCCLYCVVLYCIVLHCI

VWYYVLCYATLHFIVLY

>SG-PL_contig_7163_1_-3_63_161

MVPCVLHHKIMLCSWNNNHGRCLMCMHEPELSL

>SG-PL_contig_7164_1_+1_1_264

ERTTKSGIFRHLILQHNVKTATQNKTHSGIRTNSPPQARSLNKTSYTTISKHSFKKSNKI

ISASSRFFPPSKQKPKPINKLKTTTTPP

>SG-PL_contig_7165_1_-1_316_636

MIRLRNLLKSYTYDNVFDMNIHVLRFVSNAPTVGFASAIYVYTHEVWFRQGVFVLSIAIS

VPLKNLLSVAFIHVSPRLVQPTSSCQFPDRTFAFGMQYFPHLCVSRF

>SG-PL_contig_7166_1_+2_2_214

AQILWYDCNQALLIICTCLFFERLETSLKEEKKRKKKEKEKEEFLSIWYKKRTCQGMETS

QGLKDFFFLIP

>SG-PL_contig_7167_1_+2_2_166

AFMTITTFAIIQYKRFALVLGNGLAVRRDNNLYALVIKCKGTYPALIHINSPNIS

>SG-PL_contig_7168_1_-3_642_857

MLRRISSLHSSDLGRHGDRMTKIVSSNSSCKCRYALDGISCAVFIGTYCHFSLFVWFVMI

VAFAVNDESWHS

>SG-PL_contig_7169_1_+1_265_378

MLMTYTCLNFNRKQSLTQASLLLTRTQFTSYTMSISDA

>SG-PL_contig_7170_1_+1_1_126

ISVLGTNSICTYAANAMGWKRIQNDNGISRTPAHKPYALARS

>SG-PL_contig_7171_1_-3_48_1016

MKMSSRVLKELNMSEVEAQRYLPLTLNSTLWLNNPTLCSDTSGNTDRPDILFVVHTAPQS

FQRRLLMRNTIGDETHFNPFKVRMVFVVGRVFNVSLQEALRSEHQLYRDLVQGDFFDTDY

NLSHKGVLGFRWVWQYCPQARYVVKLNDDVFFDTYKMLLRYRGVFEDRTRSLFCNVLPQD

TMPIYRDQDHPWAVDDRQFQGLKAFPYTYCSGVVVVITTDLISELYRAAFFTPVFWVEDV

YLFGMLPAMLYDVTLVHLGHRNKFIDLKTSSALFCMKQHTYLCSVLATLCPSTENWETMW

NITYHLHLDTSFYVPFVKLPSYG

>SG-PL_contig_7172_1_+2_2_220

QVPLSTPTVLIKCETTKVRIFFFNLSQRNGTLSASLAHTGCNKQCQNLSRQLLSLCKTTH

TPNIHNSINYIFI

>SG-PL_contig_7173_1_+1_34_213

MDYGPGIFMSKQRFKALQSFLKVSNPQTENSVVDKLSKVRFSQNYICRKCKKLYQPFKNV

>SG-PL_contig_7174_1_-1_151_306

MVHSSSYFTAIPKALYQTPKMACFVTFVTWFVIVCEFAAHIVFVRKVVCYIM

>SG-PL_contig_7175_1_-3_63_1016

MVSMSEEKEKTAATPFSLKNAGSGFEGRSSDIFGGLDVLERKHISHEQNRTDLDDFADLR

HDDDNDDLPNRSSFVTTIRPSATESLWQRKTDGNQQGNRHRFTSPDDVQGTTEVGIHSDK

KRDSDDAFERGGRPKSEGRSTLFRRPSGRPPGRCQNQRKRVPDHGKGCQSWTHYSLEDVK

GSDMSEKSNTQAALAFLDERRKLREKQEEEMSLGEEEAFDVRSAACSKGLFSFTKRSKTD

KSSSISDKDKSAASKDKFDRDRSSAAEADRPDTEETEGAAEEESVELRFSGMKDDATKAS

FKSRKGIKRNIRTRDNDD

>SG-PL_contig_7176_1_-1_103_402

MERVKLVKLHCIFVGLLGCFFFFFSIINSSSILWLWYTINTLFSISVFFVCLLFFLPPFS

VSPSPPLPACWSAFCSTNLPYALLEMPANVADLDTYLKSV

>SG-PL_contig_7177_1_-2_158_397

MMSEQCNAGLHEITTTTTTTTTHYDVQDLVFLCLCYVMVVSFLLFFFLNLLSAFLTLRQR

LLIYLINWLCVSPFVTLFEF

>SG-PL_contig_7178_1_+1_7_261

MSYRQITLQNRHLVPIAVFTISRPSRQSKAAQIHHKLQGGKGGKLCEGFAGKKKVDWTRK

AEKSDDSKGKEKAQKQREQQERVNF

>SG-PL_contig_7179_1_-2_65_178

MGTRCPFCSVICLYGLFNVFFVSLKYFHNYLCFFAIWG

>SG-PL_contig_7180_1_-3_3_122

RHVFHYRGQSFWTKYRIVLVSYKYGICFTIEGSPFGQNTV

>SG-PL_contig_7181_1_+1_331_525

MLLELSSAPYKLFFLISYYATCLKQCRVSCFLLFEFSCHLYLEPPLNYKTSKSILTFIKQ

NKTNK

>SG-PL_contig_7182_1_+2_1448_1699

MYVLHNILVHKGITENIYYYENCVDSMLTTIKITWGGGGGGRRPKRDRQLRGRRKGRMWV

GDGVEECSIPCCEGVWKTWPSCLQ

>SG-PL_contig_7183_1_+3_3_434

FFFIFNKYIHKNIFGLRGQEATSQIKLWYKITQTHTHNKSICTTIQIHTKKQTFCLSFRH

SLSVLTLSFVSQSKQLNFNSWYVIRTPAALALSVSVSWCTYQTERPPPPALGCKVGRCPG

DKHWTWSPAGCGLLPHRGLCSGLF

>SG-PL_contig_7184_1_+3_3_206

CSGPSGDERLSEGAEEGGLHGHRPESREVLSREVGSCQDGVSPQQGQVVGQFDCRARRLH

NGANGLRA

>SG-PL_contig_7185_1_+3_3_161

FLNNFTFNKQVSNYFLFCTIFVYLCNTNKFGKKVLSFTNDFWFVSMVIFDNNY

>SG-PL_contig_7186_1_-3_576_695

MVVMNKISHMYKHMEDMVEHDKFEVWLLRPLQNGCEIGDL

>SG-PL_contig_7187_1_-3_3_269

LILADSSYLCRPHTTRVVDLCTQLGFVLKHDKGELNPKQTFHFLGMSFDTIRMTVASTPE

RLLRLRETLDSLLSARASTARSLASLLGT

>SG-PL_contig_7188_1_-2_35_376

MASSTVLRLSGSFAPSLTNILSRQLASISVPSRVAFTHQKETESLSVVPVREQSTETAKD

VVKRKPYSPFQTNNKAEFALARVDDLLNWARKSSIWPLTFGLACCAVEMMHFAA

>SG-PL_contig_7189_1_-1_19_804

MSDEESVKSQRVGSISDDDFDSDDDSDMSDTPFEESLTELAKKSKQYFWQYNIQAKGPKG

TRLPLNISDTDPHHPSNFEDPVFDATNTTLVGIRHGGKARKGDGNEITPNPRKLYQIGQR

LCRLNREINNVQLASHLPASVRSKSRKEKNKLSSRACRLKKKAQHEANKIKLFGLNCEQG

ELLEVIRLLWPCLKDRAKQQMKENIPSSLSSDTLTHQINNIITSQYKTAIAGNTADYVNQ

VIARVEDGDPVGGLAIKLKVKK

>SG-PL_contig_7190_1_-3_264_467

MLTRATGLLPMQCLQKNMCLQDWSSIPHEDPHQALKTSMPTHLSDRRESPTTSTTTTKNN

NSKNIWSE

>SG-PL_contig_7191_1_-1_280_438

MNFQVLLSAFFHIAVKCCSILQKVCCTFYSFHLNYFCHIQITFQDYFGHSCLV

>SG-PL_contig_7192_1_-3_438_605

MIYRTHKVRENHKTIPMHSLCSGLWLMQNILDPFPSHSMCGIGAYEIYSKRRNAVL

>SG-PL_contig_7193_1_+1_10_252

MWIYCPEHAGVKRNEHANELAGKATRDFRESKKLVSWKIRGPWKLKIIPSQANPGSPYHQ

SPHAKGNIQRKWLEILHEGKN

>SG-PL_contig_7194_1_+2_2_394

HHTITPPVFVFFTSLASSVLYCREAKEKLSAGPSCRSLQAFWYRQVDASHKQSYPHSQHL

THGVDVICLPRSTLTRHGATLVTGRAIGGAAIVRTAGCGRRSSVVASAIVVVTVAGVAVW

VTLLVAVCAKS

>SG-PL_contig_7195_1_+1_1_96

ACLLICWTDGRVPTKIPCDIYQLMLHMTGFSP

>SG-PL_contig_7196_1_+1_1_165

GRKKKADDSKGRKRGRNKESNRKEEISSTEFPEGGDTIYTGRAPGIPTRDELHLT

>SG-PL_contig_7197_1_+3_3_143

SNRKEEISSTEFPEGGDTIYTERAPGIPTGVVSYLYRSIANRTVQQT

>SG-PL_contig_7198_1_+2_38_418

MWYWSHAWKRLCDICLRNLHVASRGRKVALKQVRGGLGWRAMLLPTAHMFQAPFQALRAC

RGAVALWEVAGTVPVSVDTPQGSGAPAEVWNGACWRHGVQRPSRFWGREFLVRPVRSSYK

KKLQIGR

>SG-PL_contig_7199_1_+3_285_425

MFYSGVIQFYNMLAGALITTQVKCRSFFCIHSSKYSAVMVTKATFPY

>SG-PL_contig_7200_1_+2_2_85

PPLHSLYPCKHHKDFFVLFLLEMFVLFK

>SG-PL_contig_7201_1_-1_1_279

GGGGGGGGAGRGSGQFSFSFKPATTTSFSTPSLPSSNSSVPPSLGFSAQGQAWAPSHTST

APTPASVPSVPAPTPAAPPPTPAAPPSPAAPAL

>SG-PL_contig_7202_1_+2_2_274

VDFFNRILSGTTSVAVGSLTCTRCVLRTKPQFIVSSKRLSTRTTTQGLMEGGEKSFCDRE

SNLDFWHPMWVLCPLDHILSNHHLINPYLTT

>SG-PL_contig_7203_1_+1_181_294

MFLPRYSTHLNKRGKNGGGSVGGGEPFMVQKLQTRLIK

>SG-PL_contig_7204_1_+3_3_158

FFISMHCFYLTPDNSKYVFSFTPSDLSYWLIVIKTSISCIHTQKSSHNLTMN

>SG-PL_contig_7205_1_+1_1_180

RTWLTGSLTCIFDVLYAYTHEGGSGTSRSAHMLTWEIGKISTLYPPGEVPEVRTQAFQTL

>SG-PL_contig_7206_1_-3_3_137

KKEIIFQVTFRNLISSGGLISRSVWIIFPLEFQSGACFGDAGLLV

>SG-PL_contig_7207_1_-1_1_216

CINNSSSYLQGTEETCNAPSSILHIKLPLRTMTRQNISLRRANGLEMMSSVVMTFTCHPI

HMSSLLFLCPWT

>SG-PL_contig_7208_1_-1_91_237

MFTQCHAVSNCRHDSTQANSFPQDLTIHTYIYRGHRYTYMNVYTMAHDE

>SG-PL_contig_7209_1_-1_49_510

MECHGVPLYVISNGKVVVDDREVKVTKGAGRFIPTPCNSEAVYGRVEARNQARMPKKVER

EPYSGPVIDLGAKPKEPDNPIVPTPISPHSSSVTSATSPEHDLAFHSRGRTRGGYRNMQD

SSFSLSGAQIDDDQAKKSSIRTSNPPGGKSSGLW

>SG-PL_contig_7210_1_-2_128_235

MNSHFTCVCNIGIVVSLWHWSNIWINHALGLISGLC

>SG-PL_contig_7211_1_-3_513_3227

MAAERKRLKEDRLRTKNWKRWGPYLSERQWATVREDYSADGDCWRYFPHEHARSRAYRWG

EDGLLGITDRQCRVCLALALWNEKDVILKERLFGLTGHQGNHGEDVKELYYYLDSTPTHS

YMRALYKYPQVAYPYEELVEENAQRTVFDDEYELLDTGVFNNDCYWDVTAEYAKGTPNDI

LAVYTLSNRGPSAARLHVLPTLWLRNVWSWGSECDAYLEEKPQLSQMGPGKVLISHSVLG

TMVMEVDVGPEGDLPDLLFTENDTNHKLLYKGVNATPYVKDAFHKYVVDGDQNSVNPDRV

GTKCAAHYVVNVESEQEVTLRIRLYHHLEKPKATFAKAFDDLMELRREEADCFYDEVVGY

KSHSEQVVARQAYAGLLWSKQFYHYVVQEWLQGDSQQIPPPSERLSGRNVCWQHLFNRDV

ISMPDKWEYPWYASWDLAFHMIPFANIDLHFAKEQLMLFLREWYMHPNGQIPAYEFAFSD

VNPPVHAYAVLKVYKASGAKDHRDILFLAKCFHKLVLNFTWWVNRKDPDGKNIFEGGFLG

LDNIGVFDRSKPLPIGGRLEQADATAWMACFCVIMLEMSLILARRDPIYEDMASKFFEHF

VFILDAMNNVAGTGLWNDVDGFYYDHIRGNNFSMPLAIRSMVGMVPLFSSLVLHETELKR

HPGFFKRTKWFLEHKKSLASKILQRTADDNGNGEDTLLLTVVTKEQLVRILHHVLDEKEF

LSPYGVRSLSKHHAKNPFKFEVGQETYCVDYQPAESRSKVFGGNSNWRGPIWLPMNYLLI

ENLQRFDFFYQDTLKVECPTGSGKFMRLRDVASNLSQRLTRLFLPDPFGSRACHGKEDRY

SKDPNFSDLLLFYEYFDGETGRGCGASHQTGWTALIINLMKSAHHRKSLSGDSTSSLCGG

SHGLR

>SG-PL_contig_7212_1_+3_909_1229

MRAHQQWNLRQTEKMVIPVSLMCFPDDISEHLAYKTTLRQGKQAEEFSCGSYIDKVYLLK

DKDHEVYDDNAICYQNQQKIRDPYRVNKTIDITINTTTNAFCSCKTG

>SG-PL_contig_7213_1_-3_177_1223

MAQINGYKEELQDELSKTDPNSHASRLLTQEIERLEKGDDRKDSRSIAFIDVHPQEPQNA

GLKVRIPVQEFPRFNFVGKLLGPKGNTLKRLQEETGCKMAILGRGSMRDKTKEEEMRKEG

GKKYAHLNDELHVYVEAQAEMTDAYRRLAHAVSELKKFLVPEANDDIRQQQLQEMMYLGE

DAPRGGRGGFRGGRGGPAGRGGPAPVRGSRGGAPPRGAPSRGAAAPPMRGASRGRGGGAP

PPPAQSSPYDDYGYSGSGYGSSSRGGAGSSGGYDDGYDSRGYGSAQSDSQYYGSSGYGSG

AQSSAASYDDYGSYDYGDSYGNGAGPMKSSSRGGAKPRSHPYSGGRAAY

>SG-PL_contig_7214_1_+1_175_315

MKLGITAGSVELRTACMDLTYLGHHLISPLLATIILPPQCQNGCPTY

>SG-PL_contig_7215_1_+2_2_373

PHKPFFVLCCQTRNGPQGPVKVFSLQENSLSLTTTAKKEKNKRPTTTGRTDRHNERHTQI

PPRLHYATHGFVCKALGNHPTTNTTTRRRPCDFNKKILLLIPPHTAGETGPCHKNRYAYI

ITRS

>SG-PL_contig_7216_1_-1_214_432

MSSSYSHQWLFLPLCVPLYVECIINLSVAIVHQLHLPHSSCPHVLHSLQGRESCISSTFV

LYSRPGQWSHMLR

>SG-PL_contig_7217_1_-1_316_537

MRVHSVVSSRFLFSPRQVYIWISVNSIVCKFHSQKHYSSLCGGLLFRKQKYEGSHLLVLV

ASLENKKHFFYFVF

>SG-PL_contig_7218_1_+1_112_243

MENQLSLKRHRIQTTPYKKKHPQNLTPCDPPAKGRQSHIQRSAD

>SG-PL_contig_7219_1_-3_3_242

VETANTCPVCQTVEREDEIHFVFRCSGYNDIRNTSEVFKAEIAKRQDLVAIVMFNDEHIK

SFAEFLAKANKYRTKILTQL

>SG-PL_contig_7220_1_+2_2_196

GRALAFQSDCTSLHECKSHSSKTLCVCVCVCAHACACMCTCLHVYLASARVCYFLFVFCS

YLPKV

>SG-PL_contig_7221_1_+1_28_261

MWSVQNFSVCVCVCVCVCACASVRAWNHMTETTSSEILSSSSTLTQTHRFVVFFCNSIIT

IQNIFKIIYYCSQKEFCQ

>SG-PL_contig_7222_1_+2_23_217

MHHIYFYCFIKDVKRPSTCSKVKEKKSFFNKNTPEKSVFHTHCTNVIRKKAFKQDDSEEC

LDILL

>SG-PL_contig_7223_1_+1_880_1038

MNNSLFFRKGIKISPISKTVLSFHSVQIQVITVKDRFISHTHRTHASPPTHTH

>SG-PL_contig_7224_1_+2_2_127

SRVGYNNAQFLYTVNVCVRNMNKNCTVGFFNSVGTFHLQRKM

>SG-PL_contig_7225_1_-2_62_883

MADDTAEENILDYEDEENETAVDGAAGDGQVKKDVKGTYVSIHSSGFRDFLLKPELLRAI

IDCGFEHPSEVQHECIPQAILSMDVLCQAKSGMGKTAVFVLATLQQLEPQDGQVSVLVMA

HTRELAFQISKEYERFSKYMNNVKIAVFFGGMPVKKDEEVLQKNCPHIVVGTPGRLYALA

KAKTLSLKNIRHFILDECDKMLADLDMRRDVQEIFRMTPHEKQVMMFSATLSKEIRPICK

KFMQDVIVSLHISSPFQCGSTPWWSTSAFSHACG

>SG-PL_contig_7226_1_-1_16_528

MVDEATKKTLAQIPLLKTKAGPRDGEQWFQRMKEEYLSLIEYVKNNKESDNDWFRLESNK

EGTRWFGKCWYIHELIKYEFDVEFDMPITYPTTAPEIALPELDGKTAKMYRGGKICLTDH

FKPLWARNVPKFGIAHAMALGLGPWLAVEIPDLISRGVVTPKDSDTSGAQS

>SG-PL_contig_7227_1_-2_197_385

MSDIGSEEMEEEQGPYLGEYEGGRNERDERHGEGKATLPNGDTYEGLYENGKRHGQGVYR

FKN

>SG-PL_contig_7228_1_-1_310_1647

MAEAAIDSGLEMELGTTEEDEDDLEGLTSSMHRIHSESIIQHTEEEEEMELRRHDTYTRL

ERSQSDAMLLHHNPMVPKDDFPELLSDADSSRNVSECNRVYLDLSRAEPFERVELTEIES

TLSMFRTSSGRQLETIPQDEELKTVDLSHLYASQRSLTNGGSSNSSLEDLSHGHKIKSSS

SSRSSQDSLKTKSNLKCSVQTYSHSLKQKVRTLQMGSIPEQVNALEELNVLMEQAWSMPV

YGRDLAYTLCDILRVEKGLDTVVSNLNSKERDLLTASLRLLDQCLTTPNRNYVAECGLEV

VVKVTCEHKRDVELAPFITGILESLFKTTEESCARVIKHGGLGVIIHWCRCKDPVILRHC

AIALSNLALYGGPDSQNEMITHKAPEWLFPLAFVDDDATRYYACLAMAVLASNKEIESSV

VKSGTLDLVLPFLRKHEAQDFAHKDP

>SG-PL_contig_7229_1_+1_151_447

MPRLEETDQDMNITLGQHAFGDCTDNGDRFIQFCALNNMKIGSSLFDHKDIHKITWISND

HRTKSQIDHLAIGPRWRTPCLQDVKVHRGADVCSDHHLV

>SG-PL_contig_7230_1_-1_145_1494

MTSPLSSIFSFTSPAVKRLLGWKQGDEEEKWAEKAVDSLVKKLKKKKGALEDLEKALSCP

GQISKCVTIPRSLDGRLQVSHRKGLPHVIYCRVWRWPDLQSHHELKPLECCEYPFSAKQK

EVCINPYHYKRVESPVLPPVLVPRYSEFPSSSSMPAFQSIPEPNMPHNVSYPFQQQHSPN

PPSSGPGSPFGLPADTPPPAYQAREDSQSNSMGMSQNNSAAMPPPPPRHHPPRDRGPQPM

DTTSMPPIPIPKHVANRGIVDLQPVTYQEPQYWCSIVYYELNNRVGEAFHASHNSIVTDG

FCDPSNNADRFCLGLLSNVNRNSTIENTRRHIGKGVHLYYVGGEVFAECLSDSSIFVQSR

NCNYHHGFHPTTVCKIPPGCSLKIFNNQEFAALLSQSVNHGFEAVYDLTKMCTIRMSFVK

GWGAEYHRQDVTSTPCWIEIHLNGPLQWLD

>SG-PL_contig_7231_1_+2_278_577

MFMEFRTTHSLEFTQNIHSLHNSLTLNMLTFMLTFHTCDIPRTHHPRHFQRKILHGVSLK

TDAHLTNSLMKCLFSHAAHTAFHKYLPLPNSCNIHSHIFI

>SG-PL_contig_7232_1_-2_17_235

MTTVVIVGPHPSSIPCSDLCMLINFVHLMMCTLGHMPGGVCVCARFNKPVCVYTHTPDVI

SCQYDLKINVIRQ

>SG-PL_contig_7233_1_-2_188_304

MYYVHSIFFKHKEWLVILFVKLFCLQQACSSSESRMTEV

>SG-PL_contig_7234_1_+2_278_403

MTHVWLKRKRNNCCDFRNFITVNWKMGLPFETSSNCTQCYGS

>SG-PL_contig_7235_1_+3_3_176

YLFGFFPIPWHLQHRCIWSLFRALRRCLEKTETETKFQKQRAVWLKQECTKKLQHEGA

>SG-PL_contig_7236_1_-3_60_1103

MSFSVPVNFPSVPKGVTLSDPQCLVNGAGSHLVGHTSLNDCGTSVRFTNDTVVFSNFIII

PPTDSDNNKTGYVEKIIPIECTYNRTADLQLTYLPVVRRVLFTETGVGQFQFQIEQFRDA

SFASKVAESSYPLKMRPSEEIYIQLSLADSALQGKEGDYGMDVLSCVASSSSSMMAVDDK

TSQDLIRDSCPASGDVHLFSGNQSEAGGQNASSRHFRFSFKPVVLSNSTNPQQSSPGLVY

IHCRVQVCPAQSCHTQGQCHQTPYSASGRVRRAVSELGRGSSSSAHHLSTGPLLISPDSD

AVKSSASSPMVMVMGAIAGMATLVAIAVIVAWTRSRRAEGHRLLKVEQ

>SG-PL_contig_7237_1_+2_314_523

MQVKDCSSSQNSMFKKILQPAFQVTFPFLSHQQLISFTNILNSFALCNKYTEVFSCRMIF

NFKMSHCKHR

>SG-PL_contig_7239_1_-2_5_952

MKVNWASAPSSLPKADTSKHHHIFVGDLNPEIDTNALMEHFRDYGEVSDCKIIRDPTTQK

SKGYGFVSYVNKQDAENAINHMNGQWIGGRPIRTNWATRKPLAPVPKENVKQLSYNEVFS

QSSQSNCTVYCGGVSNGLTDELMRNTFKQFGSIQEVRVFKEKGYAFVRFCTKDAATNAIC

SIHGTMINDQVVKCSWGKEPNDPSQSQQASMQAGAMQQPQTYGQYYNMPGAYWNGTSYAN

AYSPAVGAAAAAQQQYLQPSNMGQWGATYPANYQPYGSMAANMQWGQPVPQLSQQQFGAQ

QQGLVTYPMQQGYLGQ

>SG-PL_contig_7240_1_+1_1_99

KPAGDRADCKKARSAGLPLRVEISLLRLLEVKK

>SG-PL_contig_7241_1_+1_397_636

MNPFKLLQTFSVHPLSISNGKHNIIYALGISVTFSITNSKDDDDDGQNNKPFSMSELFTI

FPHVTGLLSYQHIKCIKKKK

>SG-PL_contig_7242_1_-3_3_257

HLLLFQKKKALLPQGSKQKFLKLATSKPGTVTLNLGGQNLNLQQLQSLPGLQLSSSQQQG

IILQQQQQQQQQQLTVQQTVPRTAS

>SG-PL_contig_7243_1_+1_619_888

MQKTQQNCAAFTVENKANCSQDTVVMIAAAVHSSHSQRQRTISYCLTPKQNTDGRSVCPG

ETHGYIITKHKCYHFLDQDSQILTGLQVRR

>SG-PL_contig_7244_1_+3_3_62

NNDDKTKVLTTTTVDCLFLI

>SG-PL_contig_7245_1_-3_132_1883

MNELQFMGFLLSDKGIGPSTEKGKAVRKAERPKSASEVRSFLGLVNFNARFISNLATKSE

ALRKLTRKDQPFKWGSEQEESFQTLKEDLAKATELAYFDPEAPTRIIADASPVGLGAVLI

QTQNGVDRPIDYASRSLTDIERRYSQTEKEALALVWSCERFHQYVFSMDFTLETDHKPLE

FIYSKRSKPSLRTERWVLRLQSYHFRVKYRPGTQNIADPLSRLVKQQEQNVAQQDEVESY

IYVVVKETVPCALAPREIEEVAATDSEMIHLRECIQTSKWENSPNQAFKTVKEELTCLGK

LVLRGNRIVIPDKLREKVIDLAHEGHQGIVKTKERLRTKVWWPGIDRDTEKRVRSCHACQ

VVGQPSRPEPIIRKKFPDGPWEDVAIDILGPLPSGESILVTVDYYSRYFETTILRATQTK

HIITALDNVFTTHGLPVSVTTDNGPQFVSDEFSQYLREQGIELHHTTPLWPQANGEVERQ

NRTIMKAIRTAQTENKDWKRELNTFLLVYRTTPQQTTSISPAELLFGRKLRTKLPEVSKE

TKVELNEATRDKDSLKKELGKQYTDSHRHAKESNIAVGDEILLQ

>SG-PL_contig_7246_1_-2_2_157

AVHKFCEVTHLFTLIGQIRSGFQSYHSRYTRFIVYFCFIYFIYFRITVQISI

>SG-PL_contig_7247_1_-1_265_432

MVYLHVNYKYMCFEHFPLFKHDYCLCYCFNTSHSVKSNSVELCLPLCDFLCLVYDY

>SG-PL_contig_7248_1_-2_92_277

MFYLTDIFNVCIFRNGNILTHVLFTKQKIKDVFLVMYIRHYIFTGSDCNILVCFAVTSLP

SI

>SG-PL_contig_7249_1_-1_1_237

QEQQQQQQKEPLNKSHPLFFQKQSIFVLLVSLSSPLVLGEDACTRCFYNYIGIISCIVLL

AVFSSGKACKDCKAYITVF

>SG-PL_contig_7250_1_-1_1111_3258

MQEMEVDVDGIQRRLGTGRFQILSFFVLGLIYSRGAWHVFGIMFLAGDPGHKCAFPHDSE

SGLLSLSSSPSSSSSPLFDTDSSNLISDDTSRSPASVLPPHQDPNSSSLYSSPGETGVSM

VPVNWTADSCTVSFVDRNGSQVRLISGCPYGWSYGSLFESTVLSEWDLVCGRNFLVELSI

TIFMGGATLGAMVILPLADRFGRKRVMMGCLLSQACVGTATSFVHSYIVFTVLRFILGML

NIGVGLSCFVFMTETFPADKRTVMSIAFQVFWAAGVMTVAGFGYLLRNWRHLQLVLSLPN

LLTLPCWWMLPESLPWLISQKRWAEVTAAISTIARVNRKDAKQFSPLLPTHNRSSTTPNP

HHSRNTSTHTHTPSSTNQNTADKICNTDVVESETDAMIETECHHNGKTLKGDGAHSVNKI

GTKVLTNGSAIQIGNEILTDNCDNNIRTEASTNGYGDQIGTEISTDHGVDDMRTKMCGGD

GGKAEGTGHLGDHIEGHMNGKCRQGEGEGEETLRQTSNSILDLFRNRQMRLYSCIMFFLF

FVNSLGYFGISFSVPALHGNQFVNLCIMGLVEIPANAICVLIGQRVGRRYPSAMFLLLCG

VSNIIAIFSPSHVALVKLGFVMLGKFAITAAYSTIYLYSAEIFPTDIRNQAMGMSSVFEN

IGSISAPFIVLAAKSIPELPLAVFGALTVGGACLTLLLPETHNRPLPQTVHEITTW

>SG-PL_contig_7251_1_+1_1_135

PPSTCPPQTCLPHITHTICLLNKREYKRSQIKHVKMCVPFCCGST

>SG-PL_contig_7252_1_+1_934_1083

MHLKHALVQIRKSDIFEQIKNCRSGKSAKLTKFVFYRNGCFRQKLQNIVN

>SG-PL_contig_7253_1_-2_8_1576

MLRTEDGSIRGTVKMIENIKLHNLQKDADSKRRGSGGDWSWKEHAEMIKWTRSPIQASLL

KLSTAEMNKLALECFIAIMRFMGDYPMGSNQSDFDCVMKILRSCHKFPELRDEVFCQLCK

QTTNNRSMRGKSKIKGWRLFALVAAYCDCSDTLRPYLFKYLETTATDVNRTYNHAAALCL

QNLRKTFKYGGRKNVPLREEVQALADGRNCKRFPFFYSGTEIQGGLLQVKSCTVVHDCIL

DVCVGLNINDPVEMEEYTLFIRTGDGVFSKLSQEEYVLDVTAEFVRKKSHYDLIFQRTVW

IYPLSHMDNEIYVDMMFHQCIFDYMDGLMIVRPGGQPSAQTMEDIALMAALLHKANNFVG

LPTLKQLEVLVPKTVRSSPWMRGQQWLNRIQERQKDVGGRSSLAAKALFLEILSKWPLFG

STFFLIKSIPNMTGECKLAVNKKGLSFLHRDTHEIIHAHPFSEILSTRRYHSDSGGNYLD

MKIGNLMVQKILRIETDQGSDISNLIGQYMQVINRRHKQSERL

>SG-PL_contig_7254_1_+3_3_284

FVLVWSDVATVSPVSRKSDLVDSTWTAVAFRFGTCPLLLASKIDQSASRPFTFLSLSEDW

VGIASSSSVSAAPVARCRASSEDKVSSSVSWQPA

>SG-PL_contig_7255_1_+1_274_489

MHLFNVSDLLCNSELNLTICRYLCNYLCHVYFCSLRTCLCFECVSVHKGINFFLCLFCWN

DCILVLFLLFAF

>SG-PL_contig_7256_1_+1_706_936

MFGEVTWSVFHAGVWYIYIFFYNLSSKNHTIFSVNACFLLEILSVYSAILPHKPDSLTQF

FISFFYFVYLKLHTNHI

>SG-PL_contig_7257_1_+2_239_421

MFLFFCNLSSKNYTIFCVNACFLLEILSVYSAILPHKPDSLTQFFISFFYFVYLKLHTNH

I

>SG-PL_contig_7258_1_-1_7_1380

MKMAAVKCVLVLVLVSVALASDVPVLMWSPDRPLSDLPQAPSFDKIPQDIFNTNYLTQLL

GKQSNVIIAFLQDKLHINDISKYADVYNQHSDGGAFKNIKGLLDEHFSLELPRVLGSHLA

LDNLRDNFPGTVHDLGSDFDLSGLDLGDKRNHLVIVRLLPIAGSENTEAAIASNDEVIGD

VSRQLTKRSVRYTALYTAETSEERQKRESESHHTRQLMEAPQEATTNGVFFNMTYDGGNE

TMLAYLTSVSVCIMPSADDENKPTYCHTNFTVQAADLKNANMSVKSGANRTVDVVLTVDN

IKSNYSSDLYNVSFLMHFKHQLDRWEIMEAQLTVTNSSGGAMVTDQEMITRGYDTTLSLL

YSFHCTSMTWYQKNMTETTQGYIILGGFQIQLFYVESYGFNLSQDCVGWFTTGIWMALFA

TGIHVVILLFGLYMVSSLTTNDRFDDPKGKALIINSGE

>SG-PL_contig_7259_1_-3_3_197

RFGHSVVCVRVCVCVCVCVCVCACVCVCDFGSRLDDSDTNFSSSFSSCIFAGSAFSVSVI

QFHKM

>SG-PL_contig_7260_1_-1_1_225

YQEDKSRVGHIVSFRSIVASSTVVPGPSLPVPRSTSRTTTSPRPSASTQVRLQPATLLLH

LLTWSLCGARCPLRV

>SG-PL_contig_7261_1_-2_2_100

SLARSAIFTSVLHLEFPPFRLSSSWTPSDEPNL

>SG-PL_contig_7262_1_-1_1_129

VPLSPPLSFTWSSLLSDFAPHGHLQRSKTFRRLAPLGLLLNLK

>SG-PL_contig_7263_1_-1_2386_2682

MFSPEGLRTLQTYTEELGFHWTELDPAETTVCLCCLWIDVFLSLSVRNDLYFALTVMNMF

LYSKSASPVFPALIISLPPHLPPPLVIFLFPFHVVLYTD

>SG-PL_contig_7264_1_+1_1_225

LKKKKNLLRGTILINDFTLNGCVSNMSIHESCKLSAWSDSDVDDGSWIADHFLRLFSGQP

YNGQLFCCHQSVYMG

>SG-PL_contig_7265_1_-2_149_283

MPSAVLSGTLPFTGTVRSPSGSSRNSLLDPDSGRHRVSCLYQALV

>SG-PL_contig_7266_1_-2_26_1093

MPSHHAQLYQLYGKQADYFLHPKPAPPAQATRRPTHPKIKNQKVATMQIDSGHIGAGELW

DTLRKDSMNKLVRNDHRFPQELKSAFSAFVRERLTKHSRPVERSFCATEDVPDIKRLMDQ

SKLRRTRLYRHKTELMYRSSITNKDRTSILLHNSPLPDITETGQADSISRYLPSWIDDQS

DVSEEEYMKWIRARKLRQPQQEDSDENKENVRHSAHSRQLSETTNELHSIWHSIAARTKK

PRTPRYQFLTEKEAESEGFFSQKFQEEKEQREKPRPHHMAKSYSALEFSHPTSSFSVPPS

RERGQSAMQLQRYTSDWQPLSMAALVEYKKQVDTEGEGDFEHGRMKMWPAVTSSVA

>SG-PL_contig_7267_1_-1_1_186

LPCYLLCAFQSPQFYFSAPTPPLMFFFTILPKTNIVKLSVYLGYFVFLSARMFFQFGFWL

NN

>SG-PL_contig_7268_1_-3_30_452

MALTVDASSFETAPDAHCHLLPCTINKNGEANVQKFFETSVRNEGDGLTAAFRGRPLNGE

EVVLPSGYAGVVLREVGSAVTEDQDRLLRVDQRFTSFTAWNLDKPPSGDDKLNKAMQWID

IAKALHQPVDEENSQKSITGK

>SG-PL_contig_7269_1_-2_392_553

MEMSYLYVTLMFCKFLVSLRIYSKCTVCLVDYCLLGLTMKLFLVVLFRTGRENT

>SG-PL_contig_7270_1_-3_105_410

MLLYLGVWKGCTNPVTIEASAGAIQNLTACDWAPAVECRAFIRKAKGLPNLVDLLTSDSH

RVVCAAATALRNLTIDPTNKELIGKYSIQQLVAWLPRDSRHS

>SG-PL_contig_7271_1_-2_386_784

MCLVYPTSVKFKLLICGQSLSLMLQNFVGVSMLQDSIIYEECGVCAYLHAVCKLCVCVCV

CVCVCVLHNWSGLFSKFISEPRLTFHLCVIFMDLCFQNRRGFPSTHSVYMCVCVCVCVCV

CVCAHVRERERAV

>SG-PL_contig_7272_1_-3_3_185

GGALIGWRTMGRLSLSWDCCWPRKPAGVGLHWFDMVSILAKQGVRENSPCFCGLEKQWVL

F

>SG-PL_contig_7273_1_+2_86_298

MELHLYVLACAHTCIQCIEKSQHMNCTCNIYMKKPQNYTCTKEKRCKHELHMYTHVHRIR

WTHELHVYTHR

>SG-PL_contig_7274_1_+2_560_817

MGHATTSYRQACIALSHQRVSPTLFALSLSLSLTHTDLFHGNSLSLMMTSFVNTVVSAQK

CITESAAVSSQQQWTATGKMLMYEVE

>SG-PL_contig_7275_1_+2_1022_1342

MINNHHICHIIDYYIKLNRIPDSSSLEEGTQNKPQEEDQKECIHHYVPSHILNNNKMRLY

IRQKGSHNLLQLNIITCCSKTEAFSPCIHSTHPKATLQKNQTIPFNI

>SG-PL_contig_7276_1_-1_100_780

MARPEMPKGYLGAQTKWKEWGLYTVSHNSPEITTNTGSVKIDIKTGCAMKVTANLIECGG

KEQEYKELVFTQTQCRENSKERIVTLLVTLPSAGFYKLQIYALLACDDSNSLPNVYNYLI

HSTADHPGPVVPFPKQFAQWKDGCYLFEPLHLKDLDRALFKVTIPDANAVALTVEKEWFP

LKKGSDGVWAGEVQGLHKFKGKVQKAALNANFVPEDTKYTSLLEYSL

>SG-PL_contig_7277_1_-3_3_152

LVGKGWILYLAWWVKGGYFSDLPGQHMCRPTSACRVYTHTEHQIHTLKIP

>SG-PL_contig_7278_1_-3_894_1127

MILCITLSSYGFIILCMLCFLSSMFKLFYHFVYAMLFELNVAHLPKTFEFSIAGQSSYLV

LHTLANMNIIYIMVAEVA

>SG-PL_contig_7279_1_+3_183_377

MTTLTFHKTQIQYLYHSHFHEKESLHCISFHIQITVHSFLKHKFCNQTIQNFHEKESLHY

ILPSV

>SG-PL_contig_7280_1_+3_2274_2555

MKSTLFLPFHCLKRTIYLDVIHWFNHGLQCDLNLIVNLRQRRQIRKTRGKSSFLFVRGKG

IEELACFGVHVEGGKGKYYHLKGEISLFSFFFNR

>SG-PL_contig_7281_1_-1_88_1047

MWLRPFVVQITKNACLLQRSQVLLGQKGCLFCGRPNKQGVDLESHQQQKEDTVVSQTERS

LPIITSRKLQFPSTYNPPRQAWLETLQTLEDQKLGLIDLHPDIFGMYPRLDVVHQNILWQ

RAYKRISYAKTKSRAEMPGGGRKPWPQKGTGRARHGSIRSPIWHKGGAAMGPRGPKSYFY

MLPVHYRVLGLRSMISIKMAQDDLHVVENLDIPVDDAEYLTELADVRFWGDSVLFIDDND

TLPRNTAIALSEIPHFNAMPVYGLNVYSMLKHHTLVLTLAAVEKIEERLLHHMYSMAREK

KYVPNARSPRPFIRDPVIQD

>SG-PL_contig_7282_1_-2_2_208

VHSYLISDSWDQVVLYLRLPKMTNFVFIPFSMGCFLGSPNQRWDRVGCRGSVVVMRSKYC

LLPTANLGF

>SG-PL_contig_7283_1_+2_107_529

MLELTLTDIRREKCPQTKRLTWRKTSRPKQSRLHFFLLSENLILNFEDTDIEPGYRSDHS

VITLSLKFGNMIKHNAFWKFNCSLLKEMHAEDIDKLIDAVILECSTEHYDVVTLYNVFVI

EIALSAPDKLFIDFFTDVMRQ

>SG-PL_contig_7284_1_-2_95_382

MHDKLQMNNFQVVLCFALHGTFFLSKFKTVCMPCVWIDAGHVSYLRNCQCFSNSINLRSL

TCFGLDADVLIINATTLFLFFLSRVQCSTALRSEST

>SG-PL_contig_7285_1_+1_1123_1347

MIKFITHALYERERETETETERERERESVSSVCVCARACVRVCLCVCVCVCVHAHACMCE

NEYGHAHHFPQIIYS

>SG-PL_contig_7286_1_-2_92_2857

MPTLTEGSRAGSGPLAVSVSLDDMEEERRSPTNLLIDTMELVEARKPMHVPNHLLETKTF

TTIGQNSMVHVKPETIHFDGFEVNEKHVKKLVILNASSDVLRMHLIPPQSKYFSIKYKKG

DRMVPGLTLECTIEFQPDEWRYYYDCVRINCPGDENLVIPIHGYPIMSTKEFPRNFTFPV

TAVGKSASEVFPLRCMAPVDFEYELIYIQPHPAFMIHPMSGIVPANSEVEVTVTFAPFEF

QTASMTVQLNISQFNAQPIVCKFIGISKPGMLKEKTLTQLALETSEVMDPRCVSPLDRAR

YNRKLKIKVKKVPQPPSAKSIEYKGIQFPPTIDTPYAVGQVLNQEPGKLKVKDLRDTVLG

KQETTETSTRQMKEARFEHAVRQNVYEERQNQLRWQVKVGDEQISNKQRSLILDARAQAY

SYYKFNKREDPVPEDEYGRHKTKINFRRTIRDVSKLGEQGIMFDPYMNDVWATRNAALDT

FIQAVRKVIIRNRGEARLNSLRAVVLDWGRRQPADEEEEDGEEQEASHAADHDKDKPEQN

FHIAAPRIQRTRFPTYVPPNVKDDMAADALGEVELVPTHVIVKEKVPYFNLKVPQMYRLS

GYSQHSVHNACSGYVPPRLVKPLRSGAQDEVILLPQTPTPVSVRTSMSCDLGITEGNQTL

AEALPEEKEKESPREIVQPMVIATLRPPEALFRPIDYPPLHIMNPAPGLQVFQAPMPYAE

VDSEFHLCPLPRYFRPDYDINPHASTQRKYLDREDTIRGMMSWKKFPSQGLTSLANIPTL

TNVWVPRWDDNFSPELMPTTVPALFDGLPSDDAEHLVEESQWDEESMVVHLTPDMVNAQF

ALVDASLPAVDDSKPASDTFPFGNKMPSTNIPVGANGPVPREKREEELEYFMTKKYNRLG

SKVTQKVSTLNSLLNQAHLKLQ

>SG-PL_contig_7287_1_+3_3_251

EPLVEPGGEEVDHWDEPGVPNSPQDLGLQGPLRVDDGVPSAQRVGEDVVRPDYVRRAQRD

PVALALPQKGLRSRRKGGGVGAP

>SG-PL_contig_7288_1_-3_975_1250

MYYLSYISQWFSCVNILGQIVRFMWALCSVVLLSVIYVCVYVYIWFSCVNILCQSVRFRW

VLCSVVLLSVIYIYIIYIYIYGLAVLIICVRV

>SG-PL_contig_7289_1_-3_84_392

MCADLPVPKPPSCVYACRISNMHVKDPVSHVSGWLMETRTYSACTLYSPFTGSKLYWLWP

LPWILPVSWKGWACCMGNSRFSLTGLCPGVSPAAQIPLSFGTE

>SG-PL_contig_7290_1_-2_293_1063

MDKSPEVEKVKKMILDLIDWRSKLLTRKYTVDEQKGIQQQVTNLIDSGNALLQLDLVVRD

DQGNVLEPTRHSTVEIYKRHVDAAEQIRKRSSHHEAENDTRPSWPSSFNLFVMLRNYVCK

IGEDADILMNIFDAKEGRFISENYLVKWSKQGVPKNIDMLNNFRVVFTDLGSRDRQREKV

YLVFQIVRIGVMDPRNVDNKKQTQGIRRPFGVAAMDISELMQGLKESKEEDQCFIPFQQC

SQGEFMESVVKKVISAK

>SG-PL_contig_7291_1_-1_514_648

MKEIPECKRRCGCLAPASWELHSQLLLQTSNVHMGKGWAHVQKNP

>SG-PL_contig_7292_1_+1_409_807

MLYILSLHLNYLSYCEYWKINSYCPSKYYGIIRNTSITFCHAAHTFRILHITTTQHKNTS

PFSVMSSKYVVVETLQIIYLVRHQSQANFSKTIPWQYLVTFIFYQTYLYSHDWPENLFSA

YGWLFPIRTTKIL

>SG-PL_contig_7293_1_-1_1_183

EGVKELRNTEFLCMGCRYLTTCFASVCKQSCRVWDCVGVVIGCGPSVEGKALLCWEKLYC

M

>SG-PL_contig_7294_1_-3_183_1757

MLRFLSRMVVIAVSTSVVVPYTVAALCHLFYSHEPQKGRALARSLKPKRVYALSVAIMQS

MVLRARYVRLYLQWHRYYITADPSITLKNLAYGRNNCCLDLHLPVSHRLTQPTTKRVSKP

VVMFVHGGAWSSGDKTMYGALCAQLVKSLDVLVCCPNHSLYPQGCVDDMIQDIVDCIQWI

HNNIHDYGGDGNKVMLIGHSSGAHLCSMALLELLHDQQLHIGPFPPEVTEAASMLFHESH

YGRLISPNSGHNGKDHLEDSSGSSESFAVVSENGNSNKEGEASSLGSSMASSTLTSMIEV

SHADLDPQLSSMLTSTPADAASVEGGKLDEGSSQQGEPEKGSGDVSNMAKPSSQEGGDGE

EESGDDDDDNDSVVTVRPKDIERHATFLDLAQSIKAFVGLAGVYHIGDHYQHEMWRGIEH

LSCMTPAMYGPEHFERFSPSTILLSLDAPISLPHMVLVHGTEDYVVPVTSSQMFTERLAR

VSADVSLRIIPECDHFDICFDLMKPSRRFHSSLMTLLLETANSVF

>SG-PL_contig_7295_1_+1_1_222

LCLSCFVSSVCVLNLFSCVPFNQLVVRVHENSLLLDLACPLWLYQQQHRNHCISLISKST

KIIMDVLTFQSGRK

>SG-PL_contig_7296_1_+2_656_925

MYYALLFLSSMYRHLSLLYMIISISVCVLLSPAVDYMYCIMLFCCTLRTTRVIVERWRRW

WILLTKPLCVTAEPAGRFFPPWFKLICCRN

>SG-PL_contig_7297_1_-1_157_288

MPPSFRGMAPFGPLNTFISQGLFSLLVGHASPSQGVHQTRRLGY

>SG-PL_contig_7298_1_+1_319_570

MSTSSNLFLCVKGCTVFCIFTKQDKTDKTAKTSHHWSYYSSKRKERGQTESRQMYTNENT

MRNSHLCLQTRTHTEADRFKQTDN

>SG-PL_contig_7299_1_+2_2_181

EFIYSTVRPFRQGKGSVIPIVSPGEERWSLKWTSRVEPRCTWQLNTHSLTLHFLGTQEVP

>SG-PL_contig_7300_1_-2_545_778

MLTEVSQEMFRLLLHLLTEQIPQRICLVVNEVSFLNFSIFVYIDMNQFTVSSRQFLVSSF

FVYLYTIYMNHCFKYVEK

>SG-PL_contig_7301_1_+3_198_440

MAFRDVYSQMVYTACIQDCELGMSFLIYLMFQILVGLTAAHSGQYQHAQKMGASFREVFL

SGCELCVRCDVFFVGRILITF

>SG-PL_contig_7302_1_+3_33_293

MGEHPAIPVDTSVMVTFIEQQWHRTKNLAVFHLVSFRVTMLILTLTLILTPGKTPREGAK

NPGSFAKRITAGSGPQPQENESRVVCL

>SG-PL_contig_7303_1_-2_218_1192

MKTSFNNKNLSDIVQGVLWSRKLDDSCPRGLDPHDISSWRRKAAGMAVVKMEEGCGRMQN

RLVTFKDGSKACARYRLNTDQIQGEVYSYYLAHLLHITNLPPAILLPVDTLSPQWRNIHL

EMSLAQWADGRLVVLTRWLDDLLPSHIPQELREDDRKLHPTVEILGKKTVPQLCDLLQWS

DLIVFDYLTANLDRMVNNMFNRQWNAEMMNNPAHNLERTKSGSLVFLDNESGLFHGYRLL

EKYHSYHESLLSSLCVFRESTVKTVKTLHSSNNVGQDLQRLFDEGELHHKHLPSIPEKNV

KILQDRLANVYDQIVRCEALYGGHR

>SG-PL_contig_7304_1_-2_110_934

MEFTQSSMKRKSLTFQTILVVLSTMLVAMTSSSPVRHQLASSSVVASDEAWLMRSQGASK

SHLVMWMMTHKQLIPRVNALVDSATRLLRCTCEEEGKSGVEECLVLAASLPNITGLNDVP

SKRVIQNENKKTFVKMLLENAYTSLKELEALLWSDVTPAGLGECAGHHEARHFMDSMQTA

TTQYKNVITSFYKSKVAQPQTAVQIPKEEFSNDRLLATKLLYITVNHLNQVSSVLEYSVD

FSWFSRMDALSNIRGKPKGAGKRKNRKTKKGRKVN

>SG-PL_contig_7305_1_-1_187_615

MKAAVERSVRSSHPGVKTDSRCVAGKSSPTKSDFDSTEMRACFSKLQELVPSINRKDGKI

DKTELLQHVIDYILDLEDTLALNSSGLCGVVDTSSISRQPLAEKSDYNIKEASIKMAGGD

LVALSAVQSLGTIEDLEFRPPSK

>SG-PL_contig_7306_1_+1_1_183

VCVGVFGFSNSTMTTNHGHISDNGELSLLSAMHTQFMCYPCTMLSYYHYVLGIHTISSLR

M

>SG-PL_contig_7307_1_+1_1_213

KKKKKKNLKGRLKSRIQLKVGTDCVEFPPQITRLRKPRFCVIMCHCSPRVTSTAQDPQHY

AHASLFFLRFC

>SG-PL_contig_7308_1_-2_14_7270

MASGNTPGTGETQPREIKVLETAEDIQQRRDEVLQRYAAFKQACSDRKALLEDSRRYQYF

KRDADELESWILEKLQTASDESYKDPTNLQAKIQKHQAFEAEVAAHANAIQLLDETGNEM

ISQEHFASEIIKERLEELHRLWELLLSKLTEKGTRLQQALRLVQFMRECREVMFWIADKE

AFVTSDEFGQDLEHVEVLQKKFDEFQKDLQNHEDRVSEVNTLAEQLISDCHPEEETIRQK

QDEVNEAWKRLRQLSLLRQERLYGAHEIQRFNRDADETIAWIQEKDVVLSSDDYGRDLAS

VQTLQRKHEGVERDLAALEEKVNALGTESVRLQGIHGNQADQIHSKQEEMEQNWEQLRNK

ASDRKSRLDDSYFLHRFLADFRDLVSWISDIKAIISADDLAKDVAGAEALLERHQEHKGE

IDAREDSFVSCAEAGERLVSSNHYAADEVREKLGILASEKKGLLELWEERRIQYEQCMDL

QLFVRDTEQADAWMTKQEAFLVNEDLGDSLDSVEALSKKHDDFEKSLAAQEEKIKALDEF

ATKLIEGEHYASDDVAQRRDLLLHRRDLLYEKSARRRDQLELSYQFQLFERDCDETKGWI

NEKLKTASDESYLDPTNLQTKLQKHQNFEAEVEANRNRIESIQASGNELIENDHYAKDTI

RKRVEEIERLWETLLAQTEKKGAKLTEACQQQQFNRNVDDVERWLNDIESSLISEDYGKD

TTSVQNLQKRHGLLEADVVAHQDRMESLNSQATLFASGGHFDKDTIGKKAVEVKERYEKL

KAPMTERKQKLTEAQSLQQFLRDMEDEEDWIREKEPIAASTNRGRDLIGVQNLMKKHQAL

QAELAGHEPRIQNVCGTGQAMLKEGHFAQDTIKEKIQDLQDRWQQLKDKASQRKQDLEDS

LQAQQYFADANEAETWMREKEPIAANTDYGKDEDSAEALLKKHEAFMADLDAYRSVIEGL

REQAQACKQQEAPVIDDLGSERVMALYDYTEKSPREISMKKGDVLTLLNSANKDWWKVEV

NDRQGFVPAAYVKKLDPSLSASRNNLMDEFTISVRQNQIETQYANLLDLANQRRENVKES

CKAYQLVREAAELAAWITEKENLMIGEDVGDDLEQVEEMQKKFDDFQKDLKANEARLQEL

NNIADRLTAMGHTEAAEKIREQITQLNQRWVTLQEVSAERAQTLGSAFEVQRYHREVDET

KDWITEKDAALNTDNYGHDLASVQALQRKHEGLERDLAALGQKVRDLDSNAQRLMQTHPD

QAEGIYEHQTEVNEMWNSLTVKADARKGKLLDSYDLQRFLSDYRDLTSWINSMMALVSSD

ELAKDVTGAEALLERHQEHRTEIDARAGTFQAFESLGQQLLQNQHYASDDVQDKLEELAK

SREELEQAWIVRRMKLDQCLELQLFYRDCEQAESWMASREAFLAGDSVDGDSVETLIKKH

EDFDRAINSQEEKINALQQFADQLIGSNHYDAPAITDKRDQVLDRWSQLKEALIDNRSKL

GEAQTLQQFSRDADEMENWLQEKLQIATDESYKDPSNIQSKFQKHQAFEAELAANADRLQ

GLLGTGQNLIDRRQCAGSEDAVQARLESLASQWEDLVTKSTEKSEKLKEASRQQTYNAGV

KDMEFWLGEVEQMLCTEDHGKDLASVTNLLKKHQLMEADITAHDDRIKDLNNQADQFVEA

QVWEAESINDRKRTINERYEKIKELAIHRRNRLNEANTMHQFLRDIDDEEAWIKEKKLLV

GSDDYGRDLTGVQNLRKKHKRLEAEIENHEPSIQAVHETGETLMAESNIGVSDIRSRLDQ

LSQSWEELKEMAANRGQKLEQSYAFQQFSANVEEEESWISEKQHLLSGGDFGDTMAAVQG

LLKKHEAFETDFQVHRDRCSEIKKEGQRLIDEGNHNADSIRERIAGLQQKLGELENAASL

RHGALVDNSAFLQFMWKTDVVDSWIADKETQVRSEDFGRDLSSVQTLLTKQETFDAGLQA

FEKEGIQTITTLKDQLVNAQHAQTPAIERRYNDVIARWQKLLADSEGRRQKLLLLQEQYR

QIEDLYLTFAKKASAFNSWFENAEEDLTDPVRCNSVEEIKALREAHDQFKASLCAALADF

NQLAALDRQIKAFEVGPNPYTWFTMDALEETWRNLQKIIKERDADLDREERRQEENDQLR

KQFAQAANAFHAWLTETRSAMMEGSGSLEEQLEATKLKATEVRSQKGHLKKVEDLGAAME

ERLILDNRYTEHSTVGLAQQWDQLDQLCMRMQHNLEQQIEARNRSGVSEDALREFSMMFK

HFDKDKSGKLDHQEFKSCLRALGYDLPMVEEGQVDPEFQAILDMVDPNRDGHVSLQEYMA

FMISRETENVQSSSEVEQAFRALTSGEKPYITAQELYSNLTKEQADYCIGRMQPYMDKTG

RVIPDTFDYVDFTRQLFVN

>SG-PL_contig_7309_1_-3_3_230

GGWCKSDALVHVEFREVSCNCTNTSSLNLNAVWPCCKFFLSQTKPFMLFWYLHNFYIFFS

QVNLFFMLPKMSETRY

>SG-PL_contig_7310_1_-1_1_102

ILMSSLSVVHPTDITFVLLVLIIMPQFSQLLFRI

>SG-PL_contig_7311_1_-3_129_389

MLTEVSSRHDGDDPSPFSAYQGAGRGERSSLKFRSSAMLQRLFDKAAAAARGSPTLPEPG

NTDLSAPGALPQGQLLRYKRVSGDVRT

>SG-PL_contig_7312_1_-3_516_620

MPISAFSCGLVQVCSLELKSCLFCQLSSQLFCIFK

>SG-PL_contig_7313_1_-1_757_942

MSLACRAATQVCHAFLFWASHWKPVTHSSVVFSVSVIRLFFLGTGLLALCPASTVEDQWI

TL

>SG-PL_contig_7314_1_-2_2_202

ILALNKCYCVFCSRLTRWRKGGVILRFMKKKASHYMFPSIDKSFVFEEEIVELLPFPRID

TRGRYFF

>SG-PL_contig_7315_1_+2_92_382

MSRSAPLRAWISLLRLLEANARRKVVFRVSRSKAMPESGSKGGLLRQSKTSRRSHWGTLL

GLRALRSAAPRTVSLRRGSDSKDGPPNCKIEVQMAER

>SG-PL_contig_7316_1_+2_2_193

FLDFFPFYYYHNSQCIILYLHISPLTLLQFIHQFILLSYSHSQINCNQSAFPIVIKGLFF

PLCY

>SG-PL_contig_7317_1_+2_2_271

KKQNKKTLNGGGWCNNKSTLHSHDGEGAISASESLPLAWSTGLTIFKLATTNILAVHIIQ

SSLNDQQTTEWNLFCPSKCIQAFQVMKSEL

>SG-PL_contig_7318_1_-2_14_625

METPNPNSLKFIPGDGVQVLERGTFDFPNPQSAHCSPLAKMLFRIDGVKGVFFGPDYITI

TKADEDIEWKVLKPEIYAVIMDFFASGLPVLTDEQPSGDTVIHPDDDDTVALIKELLDTR

IRPTVQEDGGDIVYMGFENGIVKLKMQGSCSSCPSSIVTLKNGVQNMLQFYIPEVEGVEQ

VTDEVDDMSNEEFRKLEDKLSEGK

>SG-PL_contig_7319_1_-2_2_130

VTMQLYLKALISFFLLCCSFLLKESFPPCSKGFFFSSVVLYYV

>SG-PL_contig_7320_1_-3_102_209

MFCWCVMCQQHKMYLIKVYINFINCWCTMIKSTLIS

>SG-PL_contig_7321_1_+2_128_247

MLLKEASVQLMLIIKKSASKNMQSVFSRKFNSFTWSQTVK

>SG-PL_contig_7322_1_-1_163_1581

MHRALFGMLLLTSAVMQSHSQTMNMPYDDTESSGEVNPDDYVDNFGSGDKDTPSLETLAG

SDINTQTVAEVINQTQKLLANPRDLGAVEILQTAIIIERTTQVPSIPKQTAHGVLKLVDT

VSELNTDLLSEADTLSNVSNRLIRSVDGLGQKVDLEGARSVRLVSTTMALEVWDLSNACT

DDLAVGLQLKTVDDDDDEDNVVMSRRAVVESSHLVTVRRGERFLTSETDTAINLTSSFMK

SVVKENQGKAIRMWMTVFSDTTLFDHNTSWPTNASDSGTLKTSKRRLNSKVISASVTADR

VPVSGFPSDVVTTVFLPLVQLTAEQRSLYSRCVFWDFREDGGQGGWSQEGCRYLKTVDGR

DVCVCDHLTNFAVLLDFYGESELPPDDHQDVLSVISVVGLTLSICGLTCTILTFFCIKKM

HSILPKQVIFNLALSLLCSWVVFLAGFSRASGSHGGCVAVAALLHYFILASFL

>SG-PL_contig_7323_1_-3_3_101

GRQFQLSFIMVGPVETRYWVVCQLNKKLVCWLM

>SG-PL_contig_7324_1_+3_249_359

MRKMNAACTMYFKGVCFILQIMEGKFDPLLQNILGYC

>SG-PL_contig_7325_1_+1_469_867

MYIFQWACYIFGHCGCKNCEMKEFDFSPTIRQVFQITFNISRSQVRKNCVTLLEMGDCQN

SLTTLFFLSVSNYYCIFFPLKTYTIKSNMEAHEHTKSSKTTNQEHLPGIKIVSYISSFFP

LVHHLFIYLPTYL

>SG-PL_contig_7326_1_+1_64_603

MYNIYSRIHSHCVQHLLRTDSHCEQHSLSVQHSHEVHSHFVHTIHALYNIHSHLVYMALS

HFVFTLYSHFVHHSVRIRSHCAQRSLTFTHTLYNIHSGFFFSHFAPTIHTYPHTDNIHSN

SFSIHQYFVQTIFIHSSLQLCKHSLLTINSISYTQSAHNKLKFCSTFIHSSLITLCTVHS

>SG-PL_contig_7327_1_+1_886_1182

MIIRLQNIQTQHGNHYSCMCKWHNQPPCKWQNQPPLSIEFNSILLNIYRSTLRAMVMDSN

ILAYFKVVQVMIIFFLVSILKLLAARLAERQIHMPVVVG

>SG-PL_contig_7328_1_-1_55_387

MCFSIDSPDSLENIPEKWTPEVKHFCPNVPIILVGNKKDLRNDPNTIRELQKMKQEPVKT

EEGRSMSDKIGAMAYLECSAKTKDGVRAVFESATRSALIIKKKKKPKCVVL

>SG-PL_contig_7329_1_-2_2_235

FCVINCFLLSSPLLSLSLSLSLSLSLSLSVICLKLNVINGPLCILYAFCMINSFETSFSY

LCFKLLYYFAHGNLLMHV

>SG-PL_contig_7330_1_+1_1_210

AENPAFHGCPSGGPAGEEPSAGGQVSGAEAACGPGWQGGWEALARRRVPPSPVVQQPERR

RKAPRRGRKR

>SG-PL_contig_7331_1_-3_243_740

MSTINRRTVTDIFCACWVFRVSISHRTLTWFTGSLTCIFDVLCVYTHGGGSGTSRSTRML

TWEIGKISTFYPSGKISKDIQTQAFQNLQFGSPVSSPFNHCSCQVFMLLGLNLRQQISSL

RLYQLNLCGNFSLNTYTDSSSFKHLVHMNYHRLNIRTLNSSSLTLI

>SG-PL_contig_7332_1_-3_1065_1355

MSFWNLSLVHCHVLLLLRQRLQCKKRMAKFVWWSGGVQLYLQFYVPSHQVVKYSEYLDLR

LLVLYVTVICSGLRYLHHLNVTHPNSFNSISFCFVLM

>SG-PL_contig_7333_1_-1_88_1683

MTSQNGDDAVDQTSEEQLLTEDDLQDEDLENFNKLTECGIDKKVCLELIKIYRTGKLCHS

ELDKRALDALKEYSATDALEVLKQFCEHSLEHVGNKSAYLCTQMKNLKKTKASGGAAQAK

KPDESKVQEILKRTGYDFDVTAGQRKYGGPPPGWTGSQPGAGHEVFCGKIPKDIFEDELI

PLFEECGKIWDLRLMMDPASGLSRGYCFVTFNTKEAASEAVKKYDGHEIRKGKKMKVNIS

IANLRLFVGNIPKNKSKTDIFEEFTKRAEGLQEVIVYGSADNPKLKNRGFAFLEYDTHKN

ASAAKRKLTTGRVRVWQCDIIVDWADPQEEPDEDTMSKVKVLYVRNLTGDVTEDQLKEKF

GAYGKLERVKKIKDYGFIHFEEREHALKAMEELNGEKIGNLEIDVSLAKPPTENKKKEQR

KREQEIRMMARESFGWDDYDYYLPPRSQGPMRGGMRRLPPPEMYYDDYYPYDDDYYYGSY

APPPLPPHMHRGRGRGGPYAPPAAYAGRQGWWPSSAWGQQRPRRNGNTRGGR

>SG-PL_contig_7334_1_+1_1_213

VKGLYTSAEVSLCACNQQFQFDIKKLFKQVSICFFLQFDYQNFNSTRLALSEAAISDSMA

STRWLVEPGKT

>SG-PL_contig_7335_1_-1_154_297

MTDPVWGWCIVAKSAALQAIHVQLTLLTGLWKMKSVKKKCLCQTYPPP

>SG-PL_contig_7336_1_-1_64_288

MPFYSKNLKKLMDVWPGKRYFGLYRFLPIFFVLGAALEFSMINWQVGEVNFYRVYKRRQA

ERLALEKTQSSSQSQ

>SG-PL_contig_7337_1_-1_112_363

MLSFLIYAQYSLVISWPQFVSQHCCHLHPLLSCSLPSLIPVFGLKLTFVKLILCYVHPSK

VAEKLTWLNQGTGRNIAMVCDCRW

>SG-PL_contig_7338_1_+1_4_231

MKHFHHVLRIRGKAYCVVCQVGSLVWIWRCDLDLDNYVTETTIGINATCEKSQRSYHWET

TQRESENCCKKIIFCQ

>SG-PL_contig_7339_1_+3_105_293

MTSINEISNTAVQSASLSYKKAKILKKTGTHLSPTPTLLANLRTSTFHIFLSTEEYIFKT

ILT

>SG-PL_contig_7340_1_-3_219_434

MVRKSIARVMTVMNQAKKDNLREFYYSKQYQPKDLRRKKTRVMRRLLTPHEKSLKQQRKE

RLYPKRVYAVKR

>SG-PL_contig_7341_1_-3_96_845

MSKYTLILVRHGESEWNQKNLFCGWHDADLSDTGVNEAKYAAELIKKKGFSFDVAFTSVL

KRAIKTLYLIQDGTDLHWVPVFRSWRLNERHYGGLQGLNKAETAAKHGEEQVKIWRRSYD

TPPPVLTPKDKEWSGNDPKYAKLDPKVIPASECLKDTVERVLPYWTDAIVPQLKEGKKVM

IAAHGNSLRALVKYLDEMSDADIMGLNIPTGIPLVYELDANLKPIKHYYLATEEEVKAAE

AKVANQGKAK

>SG-PL_contig_7342_1_-1_1_204

CHVCFGWSVSHHVCFGWSVSHVCFGWSVSHNICFTMMQKLFVVMVLFCLFRMQGDNCGFT

FVFGEKKS

>SG-PL_contig_7343_1_+1_67_318

MNFFFLFTKSITYIFTLISFNGTSIPTVKIYGFKCQVTTNKTNTVDQQQCTVSQKCCSST

KMKEKIPPGTWRTAQFMRKTDTNT

>SG-PL_contig_7344_1_-2_2_235

PESRVKKRRRQHINEQDKEKPNTQAVVVRLVQLTNGWAVGSNPISSNHRGIFLRPELSML

WAYWKTWNHTVKFLNQHC

>SG-PL_contig_7345_1_-3_39_302

MQTTNNHHHHHCLDITFFFLTLVYGTAKFKNIFVYLDFTEQADNFILKVCLFRLFFCLFV

VFFLGGGGSFFWKKLIIWISQSRQVIPS

>SG-PL_contig_7346_1_-3_1482_1766

MLYRPLQQEVAKNCCCLSVDCVNVSDIHQTYGLTPPSPQLTPPSPQLTPPSPQSMVPIGV

REGKSTFDFSSFRGFSLSRSPRTSQFFLSTGLVCQ

>SG-PL_contig_7347_1_+1_139_315

MHIQLYTGTDLHHKPVNESVDNKVFSTQFKHLCLVSVPLIPSLRKRTMKTAQLCSMIYF

>SG-PL_contig_7348_1_-2_578_787

MHLQTMCACFIACDAYRMNFRTVFQLQIIYIYGLYTQAKTSLEVLFLPVSSKVFQDFIKH

NKLKSAKLYF

>SG-PL_contig_7349_1_+3_294_383

MCDTQCVKTQNITTRVWICVGQPDINKSRV

>SG-PL_contig_7350_1_-3_537_929

MRAYTHLYTCTHTLMHAPTHTCTHTHTLICMYTHTHTHTHTHTHMHTLHFQQPFIVILTN

VVLTLSLRHTHTHAHTHTHTCVHKYTNLMCMNSGLFFVICFFFYCCLFCCFVCDFWLVVD

PPTPSPIQYCI

>SG-PL_contig_7351_1_-2_110_811

MRISLAKNLLKACVSSIPHAHHFHASAKQLYPLIPIVIEQTGRGERSYDIFSRLLKERII

CIMGPITDDLSSLVVAQLLFLQSESSKKAIHMYINSPGGSVTAGLGIYDTMQYVQPPINT

WCVGQACSMASLLLCAGDKGMRYSLPNSRVMVHQPSGSAVGQATDIQIQAEEIVKLKKQI

NQLYVKHTGQPLDIIENSMERDRFMDPHEAKDFGLIDHVLEHPPTLEEEQGSSQ

>SG-PL_contig_7352_1_+1_1_282

GDEKCPLSHAVSVFKNHFTKMRCSQRKLVIDLIYYKTASHLFQTLLYYISFCKFQHVAHL

NAFSSRLSVLQLTPSVTINVETKGMQCIEVSHGS

>SG-PL_contig_7353_1_+2_2_208

GNMKNLPFKKKKGRLKRQDVRKWMCLVGIKIRTRDGQAILIFFLSCHVDDTKKYTTLKMT

QGQLNFDSL

>SG-PL_contig_7354_1_+1_2188_2535

MLLKILVSPNVYNILLSIFPSCVKHLCSFKFPIKNVNIYDCVFRQMYALKYHEVLFQSQT

IPKSSNNQSSPGGEFCCLSPCLHFICIHLYFCYNRLSFFFFFFSFLKLFYFLFFFS

>SG-PL_contig_7355_1_-1_226_1443

MWESILEALSSVTLSRGARDDDAVDQLHHFGTVALLAAFAALIGMNQYVGDPIHCWVPAQ

FEDSHQDYTENLCWISHMYYVPMDEPLPHAKEDRVLRDISFYRWVVVVLLIQCLLFKFPN

MIWKELRGYSGINVQKIVNMAEEATMTPPDEREEKVKHISHFIDRWLLTYRVYKNNVFDR

ARQKVSGIACFIFGKRHGTYLTGLYLFTKVLYLVNIICQFVLLSSFLGFNFYTYGFDILA

NLNDNGEFRDLDHFPRVVMCDIEIRQMQNVQPFSTQCVLSINLFVEKIFAIMWFWFVLLV

IATVINVGHWVYDIFFNHRRERFVQKYLIMLGLDDKSQDRKLFKKFVSDYLRDDGVFMLR

SVGNNSSDIILLDLVRALWTTYRKIHGKPEDDAPTPPKKVENDTVL

>SG-PL_contig_7356_1_-1_76_1182

MTAACEFHHGSFFYQENDEKKKMLEYQKYPFETFGPVSSLTALPSALKKTPPSARDLKPP

KHVSFALPQTRKEHRVDESFKTGATASLIDDQVIQDMEATKAAVLFMDVPEILVQLVIDT

RQLEPHHKFSNAEQLYKLVDELASNPQRQSDLHMKLYQHQQAAETSSGNTETFEIQDGVK

DNTSNVRDDCQKLLAGMPEESCQSTTTSSSSCPSSSCAAAVSHNDSEHLMPEIVEMSGAL

KGASSLPTSMTTVAWAENGCQSAQHGDNMEQDPEINSANPNNMQEPMSDITKTSPRMELQ

EQAKKLAAENRRLTKRRMCRACKKIELSSSGVTFLPCGHFITCEVCSEKFDDCPACGKVI

MATVRTFLS

>SG-PL_contig_7357_1_-3_213_797

MACAATLKRSLEFDPVHSPSHSSSKRRRCMPIATSSPPVKFNRSYTSPFSGATEKLTTDK

IAASIAHEVKRMQRRRQLQFPSTATQTCTFTPTSTPEKDDVPSCSSSLDHSPQVSQSLNN

LFSSFPHTKKDVPLFTFRQVSLICERLVRDREEEVKEQYDKVLNCKLAEQYESFLKFNHD

QLHRRFGKSEMSYVS

>SG-PL_contig_7358_1_-3_3_257

HFCPLKDYCNSLLAGLPKYLLDRLQRIQNNAARLICRASKSDHVSPLLHSLHWLPVSDRI

DYKLSTLTFSAISGSGPEYLSELLH

>SG-PL_contig_7359_1_-1_1_198

NVCKVAYLELRRISSIRHYLSTDATKTLVCAFVLSRMDCCNSLLTGLPKYLLDFSESRIT

LPDSFA

>SG-PL_contig_7360_1_+2_620_862

MISDSHCQQIKWPNNKPGTLILTRTKSFEVIHSGYCYVSMLCADTIKQSSNPFKYQVPMT

VQISSKTALMMKLQVCRASQT

>SG-PL_contig_7361_1_-3_123_257

MVNMHLVNEEWTFSLIDHFHFSSFYDNQSEFIYQNLNEKKNVMTD

>SG-PL_contig_7362_1_+3_3_287

NQTRFSLVLLCLVNQGFLLYYCVHESDMIFSCTTVSHESNKVFSCTTVSHESNKVFSCTT

GSRESRFSLVLLCLMNQTSFFLVPLCLMNQGFLLY

>SG-PL_contig_7363_1_-1_3241_3813

MQDILCTAVADCVVCCVCCTMMKTSIKLDLFFHQTNHARYRITSSILKAESSWLDMTEGC

DRAASSSCGQDHRMSHVCHFIKTSITVVGKDVLFHAATVCISVFMHNSIHVFIIIKYKIC

NIDVLFVIAFWKVSHVLIQSPFCSITYVWTVFFHCCYQDDGHRLHMKQLSMVVHVWNMLT

QIQTADDCPLC

>SG-PL_contig_7364_1_-1_643_990

MDPVSVHFKLLHFEINMQMSERSLMALIFHEISMYGCQRMSVFSRLFRTFCPRDTVPCER

FKFHRLFQTFCPRDTVPCERFKFHFSPTLSSFLMISAYDSTSSAFLFAFSSFDQCQ

>SG-PL_contig_7365_1_+2_1625_1933

MVFPVELFPVPGFPTRISLMQLSSALLLPLECLELPEEERWTGGLRKAGRGTGPIGHTCT

PVSNRLSSSFTTSSSSTSTMSISLRTLSRLAWSESLSLKRTWR

>SG-PL_contig_7366_1_-3_69_236

MITSVCVLCVQIGSSVDTSSLDETFFFVLYTIECFCCVFQCLFKGRQVHTNTYIHT

>SG-PL_contig_7367_1_-1_208_891

MNVPKIKGTHNAMKSGILAAESVFETISNEDAVSNSPTAGLNPEVYEKKIRDSYVWKELY

QVRNVRPSFHSSLGLYGGLAYTGVIWYLFQGREPWTFKHGGLDHNKLKPAAECQPIEYPK

PDNKVSFDLLNSVALTGTNHDHDQPAHLTLLDDSVPVNRNLAIFDGPEQRFCPAGVYEYV

EREDGNGRRLQINAQNCIHCKTCDIKDPSQNINWVVPQGGEGPAYNGM

>SG-PL_contig_7368_1_+3_39_263

MLCDVKVSVTPLIQVFLGLPFTLNQILFVISLLSQLAGLHSGHMTNPLNSLQMNMMVDWS

CFHHFSLSPSPRHGR

>SG-PL_contig_7369_1_-3_117_380

MYSFPMKIALQFVVALQFVVALQFVVALHFMVALQFMVALQFMVALQFMVALQFVVTLQF

MVALQFMVALQFMVEIGRASCRERVLRL

>SG-PL_contig_7370_1_-2_200_352

MACICDCIFKVLQRLRFTDIGHFSKPQLLFVVCVPQLNTLGGTQYICTLSY

>SG-PL_contig_7371_1_-3_108_233

MQQNRILNNYLMNETRHHLFCSLFILCGVVWGFDLFEHWQSF

>SG-PL_contig_7372_1_+2_581_799

MTSTSKHQCKGGKLQIHGYHAFNEIKIFRGALTPSSPFHVFFTPLLKKSDHEPQHHHMHE

NIWGSTCIIHPSK

>SG-PL_contig_7373_1_-1_268_7986

MVVRHLPAHLAPLQVVCLFLVVCCTVMGNGEMMYERGEERSASYSHSQDMGRASHKTYTR

LSSPDDQPQAPQVPHDQPQAPRVPDANGHASQMSHSGMQASGHVSRTSHSGMQASGHASQ

MSHSGMQASGHASQMSHSGLQASGHASQTSSSFKSQSHSKSGSRSGSMFSAAGSSSQQGS

DLPASSRQYQRASRFHSAAGYGQTDSGPGAGNSHGHSNVQARASYSQPSSGFRFEAGSSS

SQSSENNMSNRNSSSSSSSSSSSSSSSSSSSSSVSFQGRGMENSGSSRREVSDSRVSQAG

RQSVDRSSRDEGSKTLSAHSHLAHARASSHGSPNQPSDPENGQKVKAADRSPGERRHSVP

AQSQADHARSAADIKPTSHASSRASASRNATSRDSHRRRQSVASSSRGISDLDKVSALAS

DIEQRTYFRELFSRRCKDCKVKGETYRGHSRFEYSEGCQRYRCICSCDGSHTCPPRYTIN

VCRDGKNGESARNVADARQRSDQRRRRCKTCEALGQQFEGNAYFDLQDGCTKFRGCICYC

NGTWVCPSERAINTCQQETPTPTPSVCRACNAKGKMVAANSLFELEEPCRRYRYCRCHCN

GSWECPSRFAENTCHSPTMAPEEQEHPGCQKCSTPDKQQVPGNSYFQMTDGCMHYKNCVC

RCNGSWTCPAQHARNTCADPDSAEGRSNASLVPVSSAECQSCLAHGRYFRGNTYFSTRQG

CISYQRCVCRCDGSWDCPNRYVSDLCADADPTNDVDNPNATCSFCDADGKIIPSNSFFNR

TQACVQSTQCLCHCDGTWTCPERFTRNVCVQPAKHVPSQEPRGCSSCLVNSRVVRGNSYF

DFISDCFRYRDCLCKCDGSYHCQEERAENICNYNTASTASPASDSSSESSSSESSSSESS

SASSSSSSSASSSSSSSSQASSLASSCVRCFAFGLLHEPDSRFVHEEDCVDYECQCHCNG

SYTCPPERARRTCEPGCRECELGGQFFEGNTYFQYRQGCINYLCTCFCNGSYTCPPQRAN

NTCVSEEGGVDSGSLVTDISRLSGDRDSCQQCVTNNGQSHQPNTDFELTEGCIHYQCRCH

CDGSWNCPGEAARNACRGELLGGCRSCVISESQIYRGAEDFQMRDGCIHYMCHCNCDGSW

QCPGEQARDICLGEVPGGCRVCRVSKNETYRGGSQFQLRQGCNHYQCNCHCNGSWECPGE

TARDVCIGEVPGGCRVCRLEGGETYLGESDFQMVKNCIHYNCRCNCDGSWNCPGERARRV

CNLDGSPLTGQAVTTTQAPSVCRRCLVSDREQFTPNQDFSLRRGCHVYTCQCECDGSWDC

PANLTRNICARRDNPQLRAQTCSSCRVSSYTYPGRSSFLLTRGCAQYNCQCDCGGRWTCN

HTVARDICKDRGSQPYSAGRERFRSRQTAVSSVSSVSSSSSQPQAQNSLVSDTRPRAVST

TRGSSVSAANLGSFPAASRSSASCRPCRVDGHDYDPESDFIINKKCKRFHCFCSCNGRWR

CPRDRTVDLCREDDQNPGDRRRTPARHTPVVPAQVVAGGQRYGTANIRVIGANTAASSSS

SSSSSMNTRRGEQGHGQLNVRGYYVNSASQADNRQAGQQAFYQAGRQAGQRAHSQADKQT

GQQAYNQAYRQAGQQVYGQANNRQTDSAQEAGYSASQRLQQSGRQQAGHQSRQAGQRYEE

RKDQSGIFYQQAGAQTFGQRSQQSHAARAQSHSLQGRDQQSASHASSAMASSDQDASSAS

SVVSVQDRGEIKGDMGNSCMPCQVDDKRYNPGAKFDWRRGCIIYKCFCMCSGSYRCHLSV

DSDCSKEDPTPGAGGIPTGPAAGQVPANKPGGSCGNCYVQGYVYPGNMSFTLRQGCQELS

CQCQCDGKHECADQKPIPGCIAMSTPLGGAVYPYRAASSVFPLGGGGYAVKPGRVINTFQ

QTIFSSGNCPSCQDGQGGAGGGGLVLPAILPAHMTSGLLPGQYNPEQPSGQQPSGQQPTG

QYSPQQSPGQYSPDQASGQHRYPVAAQPQSGVQTPPRGNNVQPRMPYSGQAAAFPMQPQP

LPAGSHRRTSVQHVAMRPVHPADSAMQDRNDQKCAFCFVNGQSHRGSFEYTKDCYHVTCY

CDCLGRLRCSVQQAMGEDCPPQYGPISGRCRSCVVQSHEYPPSLQFALRVGCYGYDCTCG

CDGLWMCPTQQPSNYCVRPASPPAGVVDERQDVLAAESGQGFVSKSHHHHHGDRAGLEDT

TFSRTAELSEDSLLNCQDCEVQGTTYPSRTRFLLRDDCLQHTCDCACDGSWACPKALTLD

VCQEGKGGEVVQEMEQQEKRIQEEQEQRQQNEEREMDDHKRALAEVAAHQARLAAMNRDC

RSCQVHDKHYLSNADFHYRQDCHLYTCHCFCNGSWDCPAALTRNMCHEADFQPEGCRHCR

LGNNTYPGGRNFSYNEGCWEFRCECGCNGLAVCPPEQSRNRCQAKQVQALTQPGSDDMTP

PLNCKPCMVDGQMIRSRTAFSRRRGCQEYLCQCECNGTWTCPQDRSINVCDLDPSSDPSL

TPDPAHPPKGCKVGRRTYFTRLFAFREGCVQHMCICYPNGAWRCPAGKERRLC

>SG-PL_contig_7374_1_+2_89_220

MTNHGDMTSSSNTFFNNTASSYIRSSYRCYLLQPIFLHRWQPPN

>SG-PL_contig_7375_1_+3_3_194

VLYCKNICTQVLKLYPLISSITLYAANFTHSCTVYINPKHLHDLVYKYMQQTSQYDFILH

NHNT

>SG-PL_contig_7376_1_-3_174_275

MGSEQDLRNTDTTRMTGALRWAANRTYETLTPPE

>SG-PL_contig_7377_1_-2_2_151

KHKADTTRMIEASRWAANRPVFMLWAGTELKTSDLESMVLSKELTSPQIL

>SG-PL_contig_7378_1_+3_519_755

MQVGVVEKCTALLFKLSRYVVCTQGGIKPAMWMPRKVAGPFSHLPFLLCFFSFLVISFCC

IVWVCVCKNQPLLDNGPWY

>SG-PL_contig_7379_1_+3_1011_1175

MRGFVIYLHVQKDQQLTGEGLGSDSQLCTKEKKSLMGRIFLDIQRPTACHLTREK

>SG-PL_contig_7380_1_-2_197_349

MRQIKMLFLSESIVMTQQSNGTEQYYGNRAALLLFRRLTDFCHFCTYTARK

>SG-PL_contig_7381_1_+1_229_318

MRIYLIRTSQDDIYLTYASLIVYVTHSHFE

>SG-PL_contig_7382_1_+3_141_305

MLSEILWMRKALDHEILNKQHSLYILVHVHRYFNKYHFVIFNGLHKLQSNGERSA

>SG-PL_contig_7383_1_+3_3_140

KNAWFRVTRNMFMNILEVFCVFIFQYILYYFVCHFISYQFYIVEHM

>SG-PL_contig_7384_1_-2_2_235

VPSYYYFVKRTEQCKHCQLRCKMSNLNHDLFDRHLIQDPSCTCGYPQETAEHYLLYCPKY

DNVRAASVATLPNGVLSV

>SG-PL_contig_7385_1_-1_277_387

MYNSTLQSLKFAEQVQFQMTLTQKMAILTARQDVMFI

>SG-PL_contig_7386_1_-2_866_1099

MWRAYSIVVQQCLIVDRTTSADCVCHIHIRLTSFVLTKKVVDFKHLPNLKVAGRGGGKAT

FIAGRWKKCALSVDILNL

>SG-PL_contig_7387_1_-3_552_707

MTADTLASCHPKSLRATVGSSQKLASNKQDDFWADETELAGIFSYVLISDIL

>SG-PL_contig_7388_1_+2_983_1201

MISWLFHPGNTEQFIYLFNLRHTYYKFLHIILTGGKLLHAFKEQPSHSVHTGESLTHVRL

YSETRTAYTLGNY

>SG-PL_contig_7389_1_+2_1190_1411

MLSGYRTDHSVITLKLTIFTFSQPSKHNTFWEFNASLLKDKCYIDEVNELTPSQPQHNSS

LVWLCAMAKFCFLR

>SG-PL_contig_7390_1_+3_3_128

QELFIDSDSDKEPNDLDFSPSDDDFDAESDLESTGNVLTIFA

>SG-PL_contig_7392_1_-3_3_191

SASNYLADSMSLCKTLCTGRILKWGWEWDLRNTKLTPPEYDWSIKIGSKQASFYVAGWDL

NST

>SG-PL_contig_7393_1_+1_118_288

MALMVGWAINNITIIIIIIIIIIIIIINHTYPSPFSHKGIHWMPSFSYRWQQDLRCF

>SG-PL_contig_7394_1_+3_2355_2771

MKKQQAVNLSGMKRVKNRKTYKLMGSDYSFSLFIFLPQPAGLCKTINYTPERISMCNHWC

LQKLCEVMKWYSIKGLRLLARDAVPTYNNNTSLRHLGLGMQMLNELLKELTYQNEQHTQI

QVLTMCVSAVRLLSFALSP

>SG-PL_contig_7395_1_+1_1_210

GLPQKVSDGGVPVWGVIGAPAPGAPAPGTPAPVVPRARGGVVVVRRGGAGRGVPAPPPFV

GSPAWAPTLP

>SG-PL_contig_7396_1_+3_327_524

MTMLLKFQYVVAFKTLQFFHAKRIHEISSLHITLPTHQLPAEIKTSQLRMHTACLHTLQE

REGRKT

>SG-PL_contig_7397_1_-2_83_271

MYSFLMKIALQFVVALQFVVALQFVVALQFMVALQFMVALQFMVALQFMVALQFITLQFM

VAL

>SG-PL_contig_7398_1_-2_77_253

MSMFRGSTNERIATVHMGTWFHLANREVEPKSIVCSTNHRTSKFALLREMVEIERAPVM

>SG-PL_contig_7399_1_+3_3_158

PNWKVWKAWVRILRHLAWWVKGDLSDFPGQHMCRPASAPFVCIRMQNIKYAR

>SG-PL_contig_7400_1_+1_1_123

YIYIYILKTSSQNFKPLHFFFPVPSDNYDTAICLKCYIFIK

>SG-PL_contig_7401_1_-1_1_246

VCVCLYFSRSPTSLFPPHLSPSPSPSTLTPLSLQPCARISFELKYSLEGPVVDIYGDRCS

LEGLYLTYRGRKELFSHICYCN

>SG-PL_contig_7402_1_-3_78_245

MSQIYYIRVSLFLSFQSLVFDICLSYSNGLRVQTCSNIFYRIENSKHFGTLVGKRM

>SG-PL_contig_7403_1_+2_2_61

AILIFFLSCHVDDTKKIWWP

>SG-PL_contig_7404_1_-3_150_443

MSKHIVLVQYADDLCMWMQLNMKKKTPLRSINYTRKVYQLELDKIGQFMKENGLHLSMEK

TKIVLFNSGSNPENMPTFKLENAILQYNLSIKFLGVFL

>SG-PL_contig_7405_1_-3_96_242

MSHDVRTNLSVNCRKHRSTVCVQHCCMSQHSFRGSPHVEILLLSFNIHI

>SG-PL_contig_7406_1_-3_2169_2600

MQTLWRAILEMKILFLSHRMITVSLKVHQAQGRPHVCFAYPWPDWTAPRHKWRQVNRLVQ

LPAWPRHPGAECSCCSDCRGWARPWYQARHKDCRVQSSSPERPAVLLPDLDYLPTTHSTA

GTVPSKMSMQHLQHYMATFKFWRS

>SG-PL_contig_7407_1_-2_17_2017

MAQKGGQQTPDLAAWYADLNRCARKEEYDKVIKLAGRILHHFPKEVKAYQCRVVSFMHQD

KFEEALTAIAKDKEKAETLKFERAYCEYRLNRTKECLATIRSVSDPDVPLKELLAQVLYR

LEEYEECYDLYKDLVKNTQDESEEERQTNLAAVVASLQMWGIQDVDEVGLEENTYEVCYN

NACHHIGQNRLDTAMEKLKKAEEFLRSDPDLTEEELEEELAIIRVQMGYLLQRKGRTEEA

SQLYNLVQKNRPSDIGLCAVLSNNIVTVNKDQNVFDSKRKMKAATVDSVKAKLSASQKET

IAVNQCLLHMYSNQGEQCLKETEKLMKQNPDLLSCMLISAAQHVKDKNIEKGVALIQAYA

DAHPDLQFMLQLIKVQIYLSQVKVYAACEALKALGDALQYKPGVVSALVTLYMSQEDATA

ASETLISAVNWYKKNQPKSPDLAVLTQANSSFQMKHGNSQVAAQMLEDQRKRNPKDPLIL

AQLISAYSQFDTAKAKEISKDLPPVSEIAQGVDVDSLESTFSTLGPKYMKKTQKGEASPG

PSGDVPLKNKKKKKKKGKLPKNNIEVGDLDAERWLPRKERSYFRGKRKDKRKEIGKGTQG

SGAVAAELDASKPSPGGGSDHSSPRPGSATSTPSAAASTTSPQAAGPRQQKPAQAQKKKK

KKGGSKW

>SG-PL_contig_7408_1_-1_79_303

MTGFWDKQETMKQQACLSNRTVYIKRGKNKEKAPLAIIYIYLLVNKSHNTTKTSQYSFST

LTTSDMSMTISMSSM

>SG-PL_contig_7409_1_+2_17_235

MAVVPLCARSVSWILVGLQMSFCWDQGRSRCYLCLCCCGPCLSLSPSLVSVLVVGAYPAL

ACDCCSTGGPPLE

>SG-PL_contig_7410_1_+3_135_632

MAAKGIEQSDLFQLYQAVILGVIAYGLGLTTLSQINLLDTIQNEAMRVILRNTKDTPNET

MWYLFDLSLMQARHKVEQVKAYFRALENPQNPLHEIFKTTKSNQLHRRKSWMEQAEESIQ

EACRLTKLNETKEWEKYPNHFKDLYTAIIPPNLGRHCREWPEGKAD

>SG-PL_contig_7411_1_+2_158_247

MTQIIPLNGSAWGFFPPLDYNTLGDTCSGL

>SG-PL_contig_7412_1_+3_3_101

SHIKYTMQNITDYRLTICEQVITSRQISLHIII

>SG-PL_contig_7413_1_+2_2_133

DTKKQKQTYINCTQKYIFRVNTLPQNVRRNKFSGLNKPTFTKRF

>SG-PL_contig_7414_1_-2_116_1501

MEVGASAKQGIVSNARNTVCHVKRILGLSFEDPVTQDYIASSSVKVVNQKNQPMFEVEFK

EKKQQFSAESITEMIYKKVLETAQSHGGKGIQDAVLAVPGDFSAVQRKAASAAASRAGFK

VLRLINECSAAALAYDLGQLDNTETFKVLVYRLGGTSHDATVLQVQNGMYRVLGSCTDHS

FGSNNFTHVLQNYLAAEFYKMYKLDPKESKRSMARLLLAAERCKHALSIVDNAKCDAESV

MDGIDFHANISRVRFENLCPRTVEQCTQLIQSALTKASCSKEDISKVVMCGGGTNMPLVQ

RTVARFLPSSEVFSSVPGDQLIAIGAAKEAANLANMDKSELEAAEENNTFQCLAKDIGIE

TADGVEIVLSAGTIFPFRARHTTTLAADQTSFRMVICETEQESSAEAKKLAKLVMKDLPA

GASIKTTFHLKREGGLHVTAHEASSGQQESVHIPSMGIANHS

>SG-PL_contig_7415_1_-3_114_371

MFSSLALSNVGAHSVWEKTSPTQTVRTCAVFREKADVKMIVTVSTCILVISVVKLFREKA

NMKIIIIFLACKLVIGVVKLYRGKQT

>SG-PL_contig_7416_1_+1_208_402

MLKSDIAELHSISMCLYESHQGQALCLVSLFLNEGVLPLQRQQCSHLSYHLSLYLTNHST

PFFLH

>SG-PL_contig_7417_1_+3_3_200

IVCNCKSTKNVRVRSERERDREMERGKKKKKKKKKVTQKYIFTQCYMNQKERQRKKITQN

YIFTQC

>SG-PL_contig_7418_1_+2_2_247

GGGLGGGRDLGGGGGGGEVVTLFGVAHPFLVPTGTQLSHVTPSSCLHVAAATPPGNGFHR

LAKPCEDSRRVLTLGELRTCSS

>SG-PL_contig_7419_1_-3_42_341

MIIVIQNFFEMSECVVCVRVTEREREYIFVCVSVCVVFLRQEYDLRVVNIYVLFVTILFT

FCSYGCVLTFCSYGCVSFAGKFAVTFFVYACNLMDFFQLL

>SG-PL_contig_7420_1_+1_484_594

MNSMTNNRVILWILVGSVTNFYMFIACFAVVLSNERM

>SG-PL_contig_7421_1_-1_1_609

GQNPDLYGGNPHDDVLIFNKPRSSSVNSSSGDHYADDRSFVDKLSPGYKRILGVTLSIFS

GFFYGINFAPAIHVQDRVKGASQNSLDYVFAQYCGIFLTSSFYFVIYIVIKKNKPKVYPK

VILPALVSGIMWAIATGSWFIANKSLSEPVAFPIVTTGPGALASLFWGVCIFKEIKGKRN

ILILLLAFVVTMSGAIMAGISKG

>SG-PL_contig_7422_1_-3_129_380

MLALIHSSDACLLICDCIGLIIHPPTQSVICCSAAPCCKMACKFLTIFTVHWLMSVGMFI

ATAGEKNLVIIYSIKSHTLFEIFF

>SG-PL_contig_7423_1_-2_2_148

QTSRQTDTKRRQTDRQRLLIYNRASLPKYSEHKKSFFTPNCLKTLSSRP

>SG-PL_contig_7424_1_-1_1_366

MICKMHSVSCLTVCVVLIALFHFADMQRLRSRFTRSANSDTGSHNLPSDLQPLHYDLVID

PDIYQEDSSMFSFEGRVTIKMKCISPTTTIVLNAKNLIINQTDIVVSGPAGNLTVVNLVL

ND

>SG-PL_contig_7425_1_-1_22_549

MVLSAGFLAVLCFLTFTTSPYVSAALAVQAQNASQESNPACPCSQSGISQNRLESMQATL

DTLTMAMSNVKTHLQTHDQSVQAVKDIQRNLSMLRADLHGLANNVSKLNTDMKTMKKDVD

DLQKDVIHNEKASDEEHSSWRGMIVNVSSLQTSLEAHGAGLSSVSNSMTALSNALN

>SG-PL_contig_7426_1_+3_3_392

QLLIGLAFNLPSPHTEETENVITYIAGYICKKICSQNTVCDTCKINLTGELTSAPRETFL

KEKLYLDQEKQMLTVPSQNLTDACVKMEEVFLESVPGIIYHNNVRAKLLTKLEKAVEDTD

LQCPSSCQPL

>SG-PL_contig_7427_1_+3_30_278

MLSNIATAMCLQSVASPAQMSTKHHQHSQSQNDTELFHQNHSTETISTPNKINAAHGQPF

AVRRTPDSQQTTFFFGDMPLNLN

>SG-PL_contig_7428_1_+3_987_1352

MSIKRWGRAPNCKAKPLREASTLHNIFFLLKGRCAAKTTGGRKEHHRGMLQRLRSLRIEQ

ILQKGWNNLRHVRNETSSKATHLHTSQSWCMSICPRKTFKLCHILHTPKDLLAPCDFFFI

SV

>SG-PL_contig_7429_1_-3_3_95

VRRVDEKIFKKKKKKKKKKSNMSIIKYLIFQ

>SG-PL_contig_7430_1_+1_22_156

MIYMMQKVYFISQCEKQVPLLRRRKVCAVSVILGFMQKYPNTCWQ

>SG-PL_contig_7431_1_+3_726_977

MWLYFLSNPVQTYSSVLVNNTLRHIVFTLKHSLSLLCITSAHIQHMWFISVKTSSNILWI

TNKYNFLSYHCHIQTQPVTSVSAI

>SG-PL_contig_7432_1_-1_205_429

MKTTTSPTGNNNINNVNNNSSGNKNSPASVTPDNKHLWPAWVFCTRYSDRPSSGPVTEAG

PASTSSGTAPEQQSF

>SG-PL_contig_7433_1_+1_1_138

HTHTCTRTHTKENNCPVNCSVVHLYFSTKNQNPKKSTWQSEGRKTQ

>SG-PL_contig_7434_1_-3_87_362

MVYRTNTLTATNRHLSLFHISVCNPYHFREIPYTCSSHNISQQTNDITFSMFMLFFPHCP

DNRTDISINLFLLLSQIVFSSLSPLHQKVYLH

>SG-PL_contig_7435_1_-1_1_591

APLHIHPRASDQPVYFSEVTVLKSNRYRFKDFPSLKGCRWAYTDDISLSGNLIVLAELKK

RGYNANFFGSIVQSGSHLNSIKMLLNGQADAAAIDSNTLMGHRLEHPEQAEQLDTIVSLG

PLPVYPIVFNARLPSELKAKITKALLDITAEGNPKWAGEFLTLGVRGYTKTSVEENYRME

EDIQTWVKNMTMAPAYY

>SG-PL_contig_7436_1_-1_922_1161

MYIYFVVSILSQCMALLISFVHLSLSLSLSLTHTHTHTHTHTHTHTHTHTLSLSLSHTHT

HTHTHTHTHTHTRVNIVLFS

>SG-PL_contig_7437_1_+1_10_438

MGRMTCMRTEGMHDHMSKCMQHQMSSEVQHACLHCVHFLGNSTQHLKPYPPASRVPRPYP

HWCSRQMFTRPPLHLSQPLLGLSYRSALSFHTVSSENITLKTCCLVRQENMLSEAALSFH

TVSSRTIPYSLHPNLPAIIWQTP

>SG-PL_contig_7438_1_+1_1510_1671

MYNTQYNRTFFTTIAQVEFLSLADYEFIPFLLTVRHEKTSLCFCKRVVPQNIKL

>SG-PL_contig_7439_1_-1_337_465

MCMMKTLGIYHCHCVCVCVCVCVCVCVCACVRERESVCVCIYS

>SG-PL_contig_7440_1_-3_123_470

MFEARLVQGALWKKLLEAIKDLLNEAIFDCSSTGIALQAMDSSHVSLVTLNLRSDGFDTF

RCDRNLAMGIHLPSMSKIMKCASNDDIITIKAGDSADNVTFVFESPNQEKVSDYEI

>SG-PL_contig_7441_1_+1_1_162

WIDHHRSFYFKVTGKKSSIQRHSSPEIACKTNFLRNKRFCTTLWEFLNNNRSSN

>SG-PL_contig_7442_1_-2_2_208

ISVWFTAWTEDVHLICKITASKHGCLFHICHRDGNESCLMGLVPFLEIVGHSSSTVIVEW

HLISCIFLF

>SG-PL_contig_7443_1_+3_249_446

MHIQAGEKIQIKSLYATTKFAVQFQISLAVVKAPYAQNGFKKIPVLSNTCPQQCTRMTKR

SSIPSP

>SG-PL_contig_7444_1_-3_51_428

MVFICSAVEFAKDFAALGGNRDVYLYSFEHNLSNNPWPDWTGVMHGYEIEAVFALPQEST

YTQQDRNVAARVTGYWSRFATTGNPNAAGSTLWPKLTSQNDGYLRIKQQGDSVGHGLRQS

ECSVLS

>SG-PL_contig_7445_1_+2_1112_1366

MVLHVVLIQSIKNKQKQYSQQSHTMSNTESRSTATDLHFSSEQNTQMGSLWKQSNLVFHM

FLFLHKTTAPICFFLKPLLPLKHKD

>SG-PL_contig_7446_1_-2_383_694

MISKFRNLFILCFCLKIFFCLSLSVCLYLSVRPSVCLSVCVSVCLCLSVCLSVCLSVSLS

LSLSLSLDGRGSHVGKRRTSKRKVPCSIPRLDTFAWWLKGGDFS

>SG-PL_contig_7447_1_+1_1_117

FNVHPTEQVYTKVRHTCVMFTLFSGQTCTCRVTSKMVLN

>SG-PL_contig_7448_1_-1_289_579

MVKSTHSYFVAGMFQYSLTLSIHTLTYTHSVYSSQVGECDAKMAYLFNMLLIHDKLLFGC

NKVLEHNKLHLAMMKLCQELHKGEQVLNYLLRKWLEV

>SG-PL_contig_7449_1_-2_26_304

MLALLYWNRYQPFCHTNASPAQDPATFLNTYLCFKKGRFQLSGVCDFIKLQDLFSCVYKV

CLQLAQGIVFNLRKADVFIPPVSTIITANSRYV

>SG-PL_contig_7450_1_-2_503_649

MSFQTWIFGQNVHCFIFIPFEMCFKETKSISRFHLLSTSAAVLFGFFWF

>SG-PL_contig_7451_1_+1_1_183

SQSDRQTHAHISQVDLNQCSSHTHTLFFPGRLRLDKFITILGLEEAQYLSSIYSLLLFEF

S

>SG-PL_contig_7452_1_-1_1_249

FVEFEDYRDADDAVYELNGKDLCGERVIIEHARGTSRGSGGSSYYGGGPRRSGWMDKAPR

YGPPTRTEYRLIVENLSSRVSWQ

>SG-PL_contig_7453_1_-2_2_157

YISGHNFTFITLIRDDYSNIFELWQSPAFEGVDSDLETDTFVTVIGLVFSVL

>SG-PL_contig_7454_1_+3_1539_2075

MCVAAVLYAEEIFLHAVTAAACGADSLCEEPLHHVGRVSSMTTHQAEVGQTPYTDVADGT

LQGGGLAVAALWTPHLFAALVTAHCKLHTLVLWYRRGDKLEDEGPLVEQSPVVQDVGIGQ

LVAIVLLPMDRVQHLAVFFMLLSFTFLFSVSWLHGITIAKVFPHVRCVRPTQRALALCH

>SG-PL_contig_7455_1_+3_1638_2069

MHALYMDIYIYSHGLFWSNLNIIHMKQKDENKSDLYFMHQLAIKMLMIFGSPTNSKICYY

THTHTHTHTGPLIILAHHMRNEGIFLINTFIISSFIKSVSFSNLFPPHLHACLHFNQAHF

KKSEVTGIILTSEGHQYSTDHIIY

>SG-PL_contig_7456_1_+3_627_848

MLFSSSELFHTHQESKQSHNYLTTIQICTWTLCHRVSDTLHQDVVYKIIHILTSKVPLHR

QCTNASYQIADFIK

>SG-PL_contig_7457_1_-1_877_1149

MNFIICIFMFFGPVGCVHLCPPVLCYCGCFVRNLKPFLNLPVLQIDWIFHCPHFITKGAG

LKVFVCVNRICQQQCLRCLWMDLKKKMDSLF

>SG-PL_contig_7458_1_+2_89_241

MINMDTVFWLNNCQRYKSLFSAQYLNKDNASWQNESMICLSIIYFHQNISS

>SG-PL_contig_7459_1_-3_72_494

MNLQLEILLFLGIITSAFGLECYVCNGQFDNRDKCIKSSVQCRQNQDTCLTHIRWQAPEF

WTPRSEKIHIIGKQCATLSECNHLRRSVGLECMRDWYRNWQCYECCQGDRCNYYVTLGAS

GVTSSITLLLGALLFVWLLRR

>SG-PL_contig_7460_1_+3_3_209

PTPSHPRTRAVSSALSASGALPATRSLVSLSRRKGTESVYNSKWTKWLAWCDSNQVGPHP

PTTFSCGKS

>SG-PL_contig_7461_1_+3_1140_1556

MRQHITQVCHVAYLELKCISSIGHLLIDSAAKTLVNSCVLSRLDYCNSLLIGAPLSVIQP

LQKVQNCAARLILELLAIESSTSLLSMQAACICYNVVSCTAPAYIAKLLHAYSPSRTLRS

SSDAQLFKIPRYRRKTHAF

>SG-PL_contig_7462_1_-1_109_924

MTAPGYCGSNYDRFVENTNTSTLHKMKETYWTTKQAVIKKLGKKEDEHVVASDSELDAKL

EVFKAIQQSSMSLLRVIERYQDRLCSVAQEENATGRFLKSHCSHDKTRAGKMMAAVGKSQ

SFSAQQRLALRVPLVRLYQEVETFRYRAISDTLVTINRMEGARTEYRGALLWMKNVSEEL

DPDTYKQLEKFRKVQAQVRKTKQRFDKLKLDVMQKVDLLAASRCNMFSHVLANYQSTLLH

FWEKTSRTMSAVAESFKGYQYYEFNMLKELTD

>SG-PL_contig_7463_1_-2_467_682

MGIGFRSIIFETLSSYQLVAFCSVGFSSLRCSVSYSSPSVSMKNTGRNMLIVIGDMKQIN

LYRLYLWFCHFS

>SG-PL_contig_7464_1_-3_3_455

ARSGRTASRKRKATVNEQSLAERQGAAEGAVESAQVESFEETSAPASRGKRRRGKNQRTV

SSSSTSNSAATSTSKETVSASATGLSSLAEQGPESASADTGGHPPEGQVQARNKSSSRRK

KKQAPAGGHHRERSGQVSWSETCTRTFRNKL

>SG-PL_contig_7465_1_-1_1_228

AVFDAVFQKVYNTPSADVFLHILQSLLQIDEDDHLSDKQWSVLELAAQRTLHLDTARLQA

TDPTLVDNLLHDQLQR

>SG-PL_contig_7466_1_+1_193_306

MSGLSTKHTKTKRSSYSHIKIQCISKSNNQVSFTSKHI

>SG-PL_contig_7467_1_-2_2_190

SATFFPIGWFCLPRNRFHCLHGQPTTPSAVNFSPSQKHFFITLLNTSLLCLFRFVLFCCW

IDA

>SG-PL_contig_7468_1_-2_17_2737

MADKAKGDTSAKQEKKTDASKEPEKKNDEAKEQELSEEDKQLQEELTMLVERLQESDQRL

YKTALESLRVQIRASTTSMTSVPKPLKFLRPHYDTIKGVHEKIRDKEIKEFCADIISVLG

MTVSEGRDSLHYKLQGSKEEIGAWGHEYVRHLAGQVAQEWQDTAEDDKEKRGQLTTLAKE

IIPYHMSHNAEAEACDLMMEIERLDLAMEYVDEQAFPRVCLYLTSCVAYVPDPENTALLR

CAVDLYRKFKQYPQALRFAMQLNDMKLIEDIFLSCKDLVMQKQLSYMLGRQQIFMELNDD

LEEADELTEIMSNAPLNGAFLSLARELDIMEPKVPEDIYKSHLEPNRTSLGPNNLDSARQ

NLASSFVNGFVNAAFGQDKLLMEDGNKWLYKNKEHGMLSATASLGLILLWDVDGGLIQID

KYLYSSEDYIKAGALLACGIVNTGVRNECDPALALLTDYVSHTSNIMRIGAISGLGLAYA

GSNREDVLGLILPVLGDSKSNMEVIGMASLACGMIAVGSCNGEVTSTILQTMMERSEADL

KDSYSRYMALGLALCYLGKQEAVEATLVALEVVGEPLKSMATILVDVCAYAGTGNVLKVQ

NLLHICSEHDDSKDQDNNEKKEDKNKKKDDKKDKEKEPAGPDLSLQQGIAVLGIGLIAMG

EEIGSEMCFRSFGHLLRYGELPIRRAVPLALALISVSNPKLQILDTLSKFSHDSDPEVAH

NAIMAMGIVGAGTNNARLAAMLRQLAVYHAKDPNNLFMVRLAQGLTHLGKGTLTLCPYHS

DRTLMSPVAVAGLMAALVSCLDVKNIILGKSHYVLYNLVAAMQPRMLVTFDEELRPLPVS

VRVGQAVDVVGQAGKPKTITGFQTHTTPVLLAYGERAELATEEYLPLTSVMEGFVILRKN

PDYEADS

>SG-PL_contig_7469_1_-1_31_1209

MQEDLRAVEFETSEDVDVTPTFDNMGLREELLRGIYAYGFEKPSAIQQRAVKPIVKGRDV

IAQAQSGTGKTATFSISILQCLDIQLRETQALILSPTRELATQIQKVLLALGDYMNVQCH

SCIGGTNIGEDIRKLDYGQHIVSGTPGRVFDMIKRRNLRTRAIKMLILDEADEMLNKGFK

EQIYDVYRYLPPATQVLLISATLPHEILEMTNKFMTDPIRILVKRDELTLEGIKQFFVAV

EREEWKFDTLCDLYDTLTITQAVIFCNTKRKVDWLTEKMREANFTVSSMHGDMPQKEREA

IMKVFRAGDSRVLITTDVWARGIDVQQVSLVINYDLPNNRELYIHRIGRSGRFGRKGVAI

NFVKNDDIRILRDIEQYYSTQIDEMPMNVADLI

>SG-PL_contig_7470_1_+1_907_1161

MLTANARNNFTNGHTFMFNLTEHVFGFPKTFFFFLPCLLIRFGGSHNSKILTYRTSVHFW

KDDFQSLKELHNCQNSCMTKMEDSF

>SG-PL_contig_7471_1_-1_310_585

MSARLDVNILRYSILMPAQAKFSQEINSLQNVKSPGRISSPLSVNSHLLFLPSVVQARKS

RFILCACLFRQGSRELCAPQPIFFFKRERIKK

>SG-PL_contig_7472_1_+2_353_496

MYNAYLKPLYFKYLHLMNFHKIYPFPLEYLFPISLNHLEQEFGRIICD

>SG-PL_contig_7473_1_+3_3_179

SGRQKKADNRKGKKKGRDKESNRKEEIASTEFLGGGDTIYTERAPGIPTGVAHNLLEEH

>SG-PL_contig_7474_1_+3_258_353

MICFLPRFGGQQGEPSDDHILYPNYQPDHLRL

>SG-PL_contig_7475_1_-3_105_1004

MTKILGRYLLVLLLLLPTTFLFVGPKISRNVALADDSMEGEDEEAIVETDEGPAGSEEDV

VEETDTGVTEQEEEEEEEQALKPSPDADTYILFTKPTGNGHELPAGKNVRLLVGFTNKGN

GEFVVESMEASFRYPQDYSFYLQNFTVVQLNREVESKRQATFEYGFIPNEAFSARPFGLT

VNINYKDGQGNLFQNAVFNETIQVVEPDEGLDGETFFMYLFLAALGILFFLAVHHGVSSL

GKKHRSKSRLVVEMGTQNADIDYDWIPKETIKEMNRSPRRTPKQSPRQRRSKRSSGSGEE

>SG-PL_contig_7476_1_+2_155_286

MVTCPFTSIQFSQSRLNPCSQDWKGVDSIHAVKTGRWNLYLYTN

>SG-PL_contig_7477_1_+1_547_954

MNTLNITKRNTFRTDNYSKDNKAEAILPQSNIMIQRKNPAHNTKSPIYYQSHHLYYLLPI

PCLYKHCTTFSVVSQNNKQNSFLTKHQYSFSFILDCTYKSKHFITEGTKFHATATSSAAD

VDFRSGFCHQAWHYKY

>SG-PL_contig_7478_1_-2_92_427

MWKRNYPGMYEALRKDWSESVKSIMNALSESVRRQALNLVRLAYSSINVDDFAVFLGMPV

AEAKQAALTEGWTLDSQSKFLTPKKLENVSAVTLPNEQHLSVLTDYVSFLEN

>SG-PL_contig_7479_1_-3_3_143

WMNLIARKEERRGSVESKDVAQGIVDWKIPTSWKHFYGAKFDQTLFH

>SG-PL_contig_7480_1_-3_141_1733

MGCLRLRRRKTFHRSCCPYDLRLLLCLVLVVVCVAYHLHVWTRLELVRGHIPNRLRQQFR

GYSSRGNTHVVIVNGGKFIKDGQNGRDLPVRREKGYPANSSPQTRTILPSSGTAIQGGKS

VKSPCKAMKVVQFLGRRLSLEREPFCQWLQNTAILAEGRFILYDHQVALLHDVIVHPGSV

LMRGPKGGEKMKDVMGQEESAEYFILKDGVFRLQCLTKPSVSFSGTTHLNQWLSAIQCVD

NVSAIVTSSSEVRRVAGVAIAVQRYEYVNLYHTMTDLYNAFLTMLLFNQHPDNVTILWMD

AHPYGGLDGVWSTLFGRVIRVGGLSSPLHIPALAWAASGYDSPLNHHYRDTVSYLEQFRW

FFLSRHGVEEVDRYPNCSSLTFMFIWRRDYVAHPRNQAGTIQRKIDNEDELVEAARKATE

KDDVVVGLQLDELSMKAQLRKVVGTDVLIGMHGAGLTHTLFLPPHAALIELFPMYLSQVN

RHFWAMAEWRGLHYLTWQNENQELEKNNSRTYVPPHVVASMVKRARGSICG

>SG-PL_contig_7481_1_-1_259_360

MAWYILRSESRTWIQSVGVFIVSKNVAGSVCISD

>SG-PL_contig_7482_1_+3_906_1115

MHHTMFSLLRSHIRVIKNFIPSRIIFALCQKRFGRHWLITQFRTEHVLNSHMFIFRTEHV

LDSHMFILIY

>SG-PL_contig_7483_1_-2_356_577

MNPWLCVSVRKCAKHTSKNFLNFLLMVLYCIGTTDLGQQEVMKAQNEKFSCQNDVSVFFI

PFIIFHSAAYLTRN

>SG-PL_contig_7484_1_+1_259_363

MRYLQKNSDINFPQNMGTNFMILPVITYSNYSSIP

>SG-PL_contig_7485_1_-2_167_2359

MRHLFKTSFGDREDAPNVAILLTSGNSVLRADEVSKEARLAHEEGIHVFVVGVGVSNEDE

INDIVSSPSDETKFLVDNIADLSFLPDIVFAQSCYRLQSQVVDELRASNRLDIVFLLHFS

NQLTKASLARVQSFIKDVVNKADVGSGSVRVGAAIYRRRAFPIFRLNQYQTKEGVQSGID

GIGLNYRSASASAAAGLEIVRDTMFTADNGDRPGVPNVVIVLTDANSDVDVADTVGAAQQ

LRDSGATVFSVGVNVDYKDELEAIASSPSKTYIYPSFDQLPTARDDIVRQVSALEGMMTD

GQLDEGPGSVTVDNSRQVDVAVMFHVQTGTKVQDIQNSLKPFIKNIFHYADIDSGNVRVS

LSYFRKNPTLLFHLQKHKTKAAVTRAVDAFNSRRVRGKRNDAGAALENARTTIFDERRGD

RRGVPNAVILITDNKVNISPKNLQQQADALKAAGARVITVGVGKANRNELRSAASDPQAE

NAYYFDSYASLRNDASAAAVRSRIYALQDLPPPTRAPPPPTTTTTLAPVVPAVDLTGSSE

ADLVFALHFNPRSSDADFQRLVRWLQSLVRSADVDSGKVRIGLYVDDQYMPFHLNAFQSG

QEMEEILGSLPKAVARSRRFNLVSTLRTVREKMFTQANGDRPNVPNGLVIITDTTSTDNR

QRVQQETTRMAQAGIGIYTVGVGLSDKGEMETVASSPRNVFAASDYRELEINVHSLRGEI

KALGPAGAVQP

>SG-PL_contig_7486_1_-2_137_1048

MTQKRSSQPSAVAVMSLVLINVIMALALPHQNPRNDDGDEVTTKSDRRTRELKPSVLTPS

EAVSSVVDCPTGHYYSEGDMVCKACSQCPINQIIRRPCSARADTLCGPFFEFDKFHQTPV

KEAQGSERNDLAGNGAKGPYHRPTSGSNSETPGETAADSRSSVSVNQAGQRQWRTLSFAL

ITVLSVICCLIVIGGLYVCIICRRSQNEKHIIRESELLGPNDRRSMDPEPRYVETYVTPR

DRFRSSRHLPVYQQIFTGGDVLELFPPLTAMAGDVKTDVVVTQVDEGERHCVYINTAAVD

VRDS

>SG-PL_contig_7487_1_-2_401_568

MNVPPHASNQFTYGETGRYPFYVRTYVQCIKYWLNLLRYQRQDYADKHMKYYCYKV

>SG-PL_contig_7488_1_+3_24_1901

MENLEEVTPSAISDTTKIFVNGCWVGIHRDPDQLMTTLRKLRREMDIIVSEVSMIRDFQD

REIRIYTDAGRICRPLLIVENQKLLLKKSHIQQLKEREYNSYSWQDLVSSSVVEYIDPLE

EETIMLAMTPDDLVEDKEAAYCSTYTHCEIHPAMILGVCASIIPFPDHNQSPRNTYQSAM

GKQAMGVYITNYHVRMDTLAHGLFYPQKPLVTTRSMEYLRFRELPAGINSIVAIASYTGY

NQEDSVILNYSAIDRGFFRSFFFRSYKEAESKKGLDQEEIIEKPNRETVQGMRPALYDKL

DEDGIIAPGTRVSGDDVLIGKTLTLPANEDELEVTAKRFAKRDSSTFMRRSETGIVDQVM

VTINHEGYKFCKIRVRTVKTPQIGDKFASRHGQKGTCGIMYRQEDMPFTCEGLTPDIIIN

PHAIPSRMTIGHLIECLQGKVAANKGEIGDATPFNDTVNVQKISKLLQQYGYNLRGNEVL

YNGFTGRKLNAQVFLGPTYYQRLKHMVDDKIHSRARGPVQILNRQPMEGRSRDGGLRFGE

MERDCQIAHGAAQFLRERLFEASDPYRVHICNLCGLIAIANLRNQTFECRSCKNKTQISQ

IRIPYACKLLFQELMAMSIVPRMMVT

>SG-PL_contig_7489_1_+1_28_180

MEPHSPLTTANSFVTFTTDCKFIKDDFFHLFSSISSLSRSSTCKMDSAAWR

>SG-PL_contig_7490_1_+1_1_126

KQKRKRKREKLGGAALRRRALPGRAARISHGKSTVTKTTKIQ

>SG-PL_contig_7491_1_+1_1_180

RHLINSVKFSAHTEHPGSQAFDIELILSFQEPFDQFKPVSQRGDFLSPQACETTVVETCS

>SG-PL_contig_7492_1_-2_596_1186

MGPDSGTSEYKCVVVGDGGVGKTSMLIRYVNDDFLTEYMPTCFDSYSVPLDLGDRPGNLV

LVDTAGQETYDRLRTLSYYNTDIFIVCFSVASVDSFINVRSRWVPELHDYRPGTPFLLVG

TQADLRQDSKSSPQNMVPASKAKKLARKVGAIGYLECSALTGYGLDRVFRQTVQYAGRPQ

KKRKLWSSFKHMFVKRS

>SG-PL_contig_7493_1_-2_2_163

EMGEGWYMKQSTQYEHDLNTLTKNIPQYGHQTVMMMINSIIFIYYFVGSFLRLV

>SG-PL_contig_7494_1_-2_335_475

MLGFECESESKLHKAKSVDGIRSFGGNCEENGIKYAAMTQEGNYRIK

>SG-PL_contig_7495_1_-1_1_111

SSSFSFLKTVSQRLEYIPYTPTLQMPCDGKGSKMIMA

>SG-PL_contig_7496_1_-3_633_842

MFCTFPLYFIFQNINYIFLCVYCCCFFSPPKPPSQEKGIFLSFCLSCLFSLCVFFVSPLF

KNFTSIVIFV

>SG-PL_contig_7497_1_+1_1_129

SIWLWPKALSVNDTSEMLRPTLNCCYALQCYVGIVFIHGKLLA

>SG-PL_contig_7498_1_+1_1_240

GRCSAELCVSKKSTRQVQCRAPCLKGVHPSDWRSLHHPVEPGTRRKSDWPIKKSRPKKRK

RLVLPSCTGMLKESPTRRLS

>SG-PL_contig_7499_1_-3_660_827

MTSVWKRTALIEFGSACLSTDFGKGQKDYYYYHYILFIAQPTIKAISGLSPLKEIG

>SG-PL_contig_7500_1_-2_50_505

MSIKKYIQETELNMPNGCAHDIPTAMKIVYFVPKGVRRYYDILTTDDTLLNSCSGWLEKL

GTNILWKSVFVKIHKIQEIKLKWLQMRIVHRILGTNVILKEMGLVNNFCCDFCLETKDSI

DFVFWKYLQVKRFLGKDRRIVEVKMCHLCKCQ

>SG-PL_contig_7501_1_+1_2011_2454

MCAQEHLAKQANKAIHALKRSTKGSELRIDIMMHLYDTIITPISTYGAEIWSPIRKIPLA

LHRIQYSTHYSEILSHKNYLVKKIHIKFCKQLLGVNKKAMNLPILAELGRFPISIQIICQ

ILGFWLHIIESDNTSYIKQIYNMLQNRQ

>SG-PL_contig_7502_1_-3_3_182

LPTRRGDRGGFEPRSSKPSSLKVKCLHHSTTAPVCVCVMSVLLHVSHSVSCNCVMRSESK

>SG-PL_contig_7503_1_+1_1564_1872

MSKLNMDMWFSVESDIASLLKSHILCLSQRCTRTHIHTHSRPHTSYNREIYARSFSKVGK

GKEMRSKKEKKSQTNNLKMVGPINQHACYDTYLTQTVYTRGTH

>SG-PL_contig_7504_1_+3_639_842

MKTRSCQNGCISPFRFVPAKLQNSVKTIQNSCHGNITTQNPDHFRKRNKEMHSTQKHPIN

TGSTIISQ

>SG-PL_contig_7505_1_-2_125_1024

MRMNFLNHLETWCEQAKERAASVVVAKCEELNSELDLRLHLHQPRSRRAELDVHNVRAAE

LVMHAERVTRHCKGISQSLTELRSRFSAMSSQHNKLAQKFRQDIEALEVIFINATKSSRL

VALQNQLGVELEKFMSVIRTSLRQFRQHLDETLQMLRESNARFIKSFKLFSDGGNFCPEE

IEEYRKRLEKMSNKIDSAEGSIMSELEGMESKRLDVATKVSNEFEDRFKSHMFDLLFMEK

VARWLTNTQVKIKAEVAASNSQAQALATHLTDLERRIDACEKPNLDKEQITASQLNEFLK

>SG-PL_contig_7506_1_-3_15_971

MMKMQSLTVVMMMVVVVAVLAFGSVEGRWNMKHRRQRGCRRAAGHGVMEVGNGDRFRWTM

TGLDDDDLRVTYLEGAFAPESARHVFKYPRDRAQRALGLVPAKVLYDFTRPDEPVVVIRQ

GRRGRGSCIVLRSDVTTYEEMIDMLIGRNGTNQEMAEAEDAVHYVARQPTSDGSQFSELV

RRVCSQDQCGRQINAVYFTAEPIDDDDDEDDDDDEDEEEAELMGVDDDDDDDDDDDSPSL

FDPTFHLFNDRVVISGLPDPRQHRRRWQRRQDRRQQGPGAGRLGLRDHRRQQRRQQRRQQ

RRQQRRQGRRGERVQPQAL

>SG-PL_contig_7507_1_-3_54_413

MNRSMETQIADLNRQHAEAQQRLQSLLVQQKRQHDQLQLFEEGERGGASKTQQPGAQAAA

LQEGKVPISVSPPISPISHRSEHFTQQDPIQRLASMSREIRVAMPTVDLEVSAGSLNNSP

>SG-PL_contig_7508_1_-1_139_639

MDLGNFNFKTVSAEDAPETIEEEDDFVTPLSTPPNEVETESQETQPQQEVTKKDSSHLLS

LSDANVMDRLYEKYRIELTEMQVLMGRLHDNWRHAYARGTSQMHILDRFSISMQLDRRLI

MTTDPQWPTASVCGTLPSLTFHLNERKIQALQTCVDTLTASTASTTL

>SG-PL_contig_7509_1_+1_91_249

MNDLHLATTFDFYKYFPFLLNVDNYFNCTSRAYITIRQQSSKTDTYFSIQHVT

>SG-PL_contig_7510_1_-2_8_226

MRRLRVWQFFSTSPCRQPYSDFRGVFVGKYEYMQHTHTHAHTHTHTFSLSLSVCLSVSLS

LSLTHIHTLRYGI

>SG-PL_contig_7511_1_-3_24_1754

MADSSESLSKNELKRRLKAEKKAQEKAEKQAAQATQAPAGNEKAKSTREEEEEIDPNEYL

RLRIRNVTRLKEAGENPYPHKFHVEMSLEEFIDRYSHIDTGSIMDDEVTVAGRIHAMRPS

GAKLNFYDLRGEGVKIQIMANAMYYKSEEEYRKMDERLRRGDMVGVRGRPGKTRKGELSI

VPSEILVLSPCLHQLPHLYYGLKDKETRFRQRYLDLIINDSVRQIFITRSKIINYLRRFF

DHLGFLEVETPMMNMIPGGATAKPFITHHNELNMDLYMRVAPELYLKMLIVGGYDRVYEI

GKQFRNEGIDLTHNPEFTTLEFYMAYADYDDLMQITEDMLSGMVKSLTGGYKVTYHPDGP

EGEAAEIDFTPPFKRVGMITALEDALGVTFPCATTFQSEETRVFFDRLCLEHKVECPAPR

TVARLLDKLVGEFLEEKIINPTFIIDHPQIMSPLAKWHRSIPGLTERFECFVMKKEICNA

YTELNDPIVQRQRFMQQAVDKAAGDDEAMYVDENFCTALEYGLPPTGGFGLGIDRLTMFL

TDNNNIKEVLLFPAMKPDEQRPTETSNPCQSQDAPKS

>SG-PL_contig_7512_1_+2_20_247

MKAWLLSGIVFPTMLDHEEQFILILAAYSQLRKRQKRRHRWWVHGILKRRHQHGTFHHRI

KELQLDVEKFKQYFCL

>SG-PL_contig_7513_1_+2_428_793

MLQLTGKTGTPQSRVIYFTDASLRVLLKFPDQHRRCLFLPVVDARIYYERKRRVQPCHAV

SVQKEFRHTIIIIRILVHYHNIVKSCPNTLRPLQWCGRWERKTVRMLIELYYMHPNQLHF

FC

>SG-PL_contig_7514_1_-1_1_1620

AAKKSGGQPFKAKPKGPPKTPQLHYCDVCKISCAGPQTYREHLEGQKHKKKEAASKGSTA

PPARGGHHQLKCDLCDVTCTGADAYAAHIRGAKHQKVVKLHTKLGKPIPSTNPVLMTSTG

SSTVSVAKTTSSTVSVGSGTKVTPKTATGSASIVKKVVSTPKITFVGGTSLKTAGGKTDD

TKASTNGDSTVKAAEPMQTSTDDLKEEEHLNILGEKDVAPVGHEYIEEMRNEQGKTVSFN

CKLCECKFNDPNAKEMHLKGRRHRLQYKKKVDPNLQVEVKPSIRQRKVQEDKLRRQAQKE

EFWRRREQEERWRDEIRREEEMYWEQHRRFDADHGPYPRHVMMTPHGPCMGLAGGPRPLM

RKPDSSDDRHVNAKHAAIYPTEVELHAVQTIVSACERSLKQVSDFLADSDAPKPDAKKEV

KKEEAATADSKQPEKEETSNSSRVLKGVMRVGVLAKGLLLHGDLNVNLVVLCSEKPTRTM

LERVADSLPKQLATVTEDKYEVKRCVEEAAIIVTAEKDPKPSCTITLTSPIMRDTNAPDG

>SG-PL_contig_7515_1_+1_88_348

MNSKLQVFKKLKKTHAFKCCSQIHANSILFFVTGVSDSVGKKQNKQETNKFYEVRGIDST

KYVEQIKYFYICTKESIPFSCVKPLHC

>SG-PL_contig_7516_1_+2_23_283

MLVIWMTWHPGQPRQRTLTTKKREKNRKKKTEKGGIELRLLWFGVKTHESGGKHTKQHHE

REKRTGETERYNLRPPLQTAGHGREGK

>SG-PL_contig_7517_1_+3_3_80

VVVVLNQLYTNTVHRFIRANNKKETS

>SG-PL_contig_7518_1_-1_1_117

HNPSLSAPAGETAKRVDKGNYVSLVRLLSQSKDYTSIIL

>SG-PL_contig_7519_1_+3_30_215

MLKHRRIWRILKAHMLYEQTWLSCVCVSWLQGLYSGPNTYKHILSAKVNRTFHLVETVSS

ST

>SG-PL_contig_7520_1_+3_36_230

MYEILFVYHLQHILDGPHLACSLHKHTKASKLQWNLCQICSITEDYTTKHSRYVHHDTHC

GVKVL

>SG-PL_contig_7521_1_+1_190_666

MGRHLSLPLASVLVHDALCVDGKAPVGVDGHAEQAGIGVDHSGRVTGLQVVEHRGLVEIG

HVGHVLDLLELGRVHLLDVILLEGLGVVVFDELDGNLFTLCALDLGSKEPILLVGYPTVL

LTVERLGLLLHHLLAVHRKVLEVLVIGIVGSHGELLECG

>SG-PL_contig_7522_1_+1_190_771

MGRHLSLPLASVLVDDALYVDWQAPVGVHSHAEQARVSVDHACAVSALQVVEHRGLIEVS

HVGHVLNLLKLGRVHLLDVIFLEGLGLVVLGKLDGDLFTFRALDLSGGEAILIVGHPAVL

LAVKRLGLFLQQSLAVHGKVLEVRIVGIEASHVVGRRRCGRLKSLCLRKRNDKRCESSPA

VRKTGREGQRQNSD

>SG-PL_contig_7523_1_+3_51_236

MSDLKSAACQGLDQIKHLIKNYHSAKRSYLTFDSCLLQRSDFRNFMRIDNLTVFQIICKP

YS

>SG-PL_contig_7524_1_+3_3_137

RERESRFNGLNKQVNLCDIFVTMSLTRAGKMELMYRQGRTHTAHK

>SG-PL_contig_7525_1_-3_87_341

MESLLVCIVNFVNYVTQGLEIILYTFMTFVKWHMCTHVRSCTAFVLVYVKVMQATVDVLN

RLHCVFIWGFIFLWKINSSICKKNK

>SG-PL_contig_7526_1_-2_2_124

RRRRRRLKPPSRLTAPMTSTLVTQLEGALTTMMMILYSGVC

>SG-PL_contig_7527_1_+3_3_164

VRGSFSINSIRTFWKFCASNFLFSIALFVSAPFPPFTVFFPGSLHTISPPLSGD

>SG-PL_contig_7528_1_+1_1_147

RRRRRRERKRLLLLLECKKITKKKREDSHSICRSLLHEDIITTQKSVSI

>SG-PL_contig_7529_1_+3_861_1592

MSWKAFLTRSLAGKKFTKLDLFHAYHQLEHTQDSRKFTTLNTPKGLYQNKRLPYGVNAAA

HFLQRTTDGVLVDCKGAKAYMGDVLVLGDTNEQHVKKKSIEFWTFFKAKDSKKEKFQYML

EKVDYLEYTISEKGILPSQAKLKDINTMSAPRNKAELQSVNGFVNFLRCFVPNFAKIVMP

LYNLLKKKYQVEMDRERRHSLHSHQRRHQSKQLSTLQTSKQTDPSNGPLINRHWSSPSST

SRGQ

>SG-PL_contig_7530_1_-3_285_365

MPVLVGSNLSGVRYTARLREHNCCSGG

>SG-PL_contig_7531_1_+2_137_394

MLKIQRSCKPCQCSVAYGNTNIPNMHTLFSLHRLQAVLALASPLDFTGVMERVGLLHGQQ

QVLLNTLPRLVSWSLSSGTNSIALWD

>SG-PL_contig_7532_1_-2_149_388

MTTIYRKKHTHHHVLTANEVSKFEIMNNQNTIIMVNIIMLLYSLTPLVHPIQRRLYYFSV

MRFADRLERDCDKSFLIHSI

>SG-PL_contig_7533_1_+1_382_636

MLFFPAIKECIIHPHPTSEATERPTHNETVHVYIHITICTQRINIIIIMYCFQPSRPLRS

YLGCHHVKSIKVNKTTALNTKPLQT

>SG-PL_contig_7534_1_+2_365_607

MTVTIQTVSLIYEGFISRLLLFAPKNKLIPARSNTEPDPPPPPNTTKKKLNFLRGGGYLH

LPTLTSYNVQSSRISSLASLL

>SG-PL_contig_7535_1_-1_127_1530

MEGVKEGEMGSAPEGVPPVYFRWLCLFLPRFFWRHGQGQGQGGAAHCGKDQRHGCHRDGL

ERKCHKGHGKHGCHGRRGGKHGASGTSSESDTDSGEKFEKVRQQVPGEFRSWVRQFVHTW

YSQGAEAALTTARDKLPKGVGPDFPQWLLTFLNNHRPHSNTTAGLGGQDGETGMEGVKEG

EMGSAPESVPPVYFRWLCLFLPRFFRRHGQGQGRSGAARCGKGGRHGCHREGLEGERHQG

QGEHGCHRKRASKHGASGTSSESDTDSGEKPWQQVPKEFRKYTRVTMKKLHKNKGDSAKI

KEMATEAGLSSDVQAFVEQILLEWHAKMNHQPPQVVAAQMMEGERTLPSGLEHKHFLWLC

RFMARWHRRHAGKCDVMSNWSSGETTTDDSSGDDFTPFGQDYRPWPRGNRCAVMNMMRGH

PHGMFGRPDPRFGMHRGRGYPCFPPPCGPRFGARRCCRMNGSPEKRWC

>SG-PL_contig_7536_1_-1_1_141

SKTIDTFTAVHTHTHTHTHTHTHSVLILLCDILWLLSKQRTDYQTLA

>SG-PL_contig_7537_1_+1_1_60

AQTTTLIFSLFFPICKCMHK

>SG-PL_contig_7538_1_-1_25_999

MAVRMATAAMLKGLKAQADQAENLISQLKLQISVLQTATAVSAGKGEEERLRVENDALRK

EIEKVKLGLIVAEIRNGVRQVPVPQQRDLTASAKQTQVESVQDKTAQAPAPAKVEEKAAA

SEPKAKKAKKDDQPAKNKPAKEESKDKGKKAADAGASAGDSDKLDVSRLDFRIGKIVAVE

KHPDADTLYVETVDLGEEKPRTIVSGLVNHVPIEQMRERLAVFMCNLKASKMRGISSEGM

IMCAKDGKVEILEVPPGVVPGDRITFDGYPGTPDKQLNPKKKVWETLKPDIRTNGERVAT

YKGVPFKVEGKGLVKAPTLPDAEIS

>SG-PL_contig_7539_1_-2_2_190

HIVMTKICLRLSMVVMNGFGCFIAFYLSRMFKKKILISRWYGSWGEGGGGVWSESAILHI

CKR

>SG-PL_contig_7540_1_+1_1342_1614

MVYHNSNCKFYLMIYHYSDCKFYLMIYHNSNCKFYLMIYHNSDCKFYLMIYHNSDCKFYL

MVYHKFQLNFLMVNFLMVYYNSNCKFSYGLP

>SG-PL_contig_7541_1_+2_74_394

MAECMTLNVKFLVENSILGEGRNAVYLPDFVSGYPSWEVYSQHLSEGGVWGDNIVWIAAA

NLYHINICAISGGQESDPLITEASDGISQFDVYLGHIAEVHYVTLQP

>SG-PL_contig_7542_1_-3_543_752

MFKLIWTFRGDRYCTCHMVCSPIYLNLINVKLCVLLLLDFKCLHSPTCTPHPFHINLNLS

MLDKYAKIMF

>SG-PL_contig_7543_1_+2_80_382

MWIYQYDPETKQQSAVWVFPGENPPVKFKRNKSASKQMVACFFAKSGHVATIPLEDRKTV

TADWYVNHCLPKVFQAWCKQCPRTGVRGLLLHHDNASAHTA

>SG-PL_contig_7544_1_+3_264_476

MQQAKEHQSEARIQLTMPQVWNKNLIQFHSHPSPKSKSRKCSSIVTILQIKQTAEQNKCK

RTTPFPFTQTV

>SG-PL_contig_7545_1_-2_956_1270

MLRVYDACACVECSFNVFLFLVGYIFYFSLSGLKCYLLTYSLELVGKITNVETTTVSLTL

NVDNLKQILKNDNSAGKKGRRNTIVVENMTLHPSWHVQLVELFSN

>SG-PL_contig_7546_1_+3_3_179

QKNPKEKQKRQDVRKWMCLVGIKIRTRDGQAILIFFLSCHVDDTKNTHDLENDSRSTKF

>SG-PL_contig_7547_1_-2_2_154

YHKDITAVTISRWISQKIIRAYQSANRDTSVLNPRAHEIWPHLQPFLSLPL

>SG-PL_contig_7548_1_-1_7_189

MQKQTTRTNMPVEDVFISINENYHKDITARTISQWISQMINWTYQSANRETIVLNPRANE

I

>SG-PL_contig_7549_1_-3_504_692

MHFGFCSHFVICWHICLWFAPVAWQAGVWNGPWILIWYKDLIPGQSGWSIFTTIVHLCMF

FRC

>SG-PL_contig_7550_1_+3_3_338

KKKKRGAGEGSITYCTVLLHIVLCCIVLSCIVLYCIVLYCIVLYCIVLYYVNKVKKRWRW

LGHVFRMDKTRHPYTALSWVPPGKRNRGRPTGTWRCTIQHEMKEAWKTWNEL

>SG-PL_contig_7551_1_-1_13_720

MHEGRMCSREEDCEGSKSSGSINFRFKTCCWTNSYNGNNVYTATTIFDGDKEEYIGCRCV

NHTIPTPDPAAYTVPPVLSYNSSGEACLYDKACKDKPYLKCQSSDVTFCCYGSRGIVTDN

EGQVTRCTCTNQLFLEHCHPNPVSIAMTTMPCQSDCAGRADGDKCTDAHNAERCCPNNMV

VVASKTAGNFTSCTCKAQGQKNCAEDSSSAARLCCHLCDLIVFVVTALSLAQIARH

>SG-PL_contig_7552_1_+1_1_120

THTHTHTHTHTHTRTHISALSRVLKYPGSLLFLTVYKHLA

>SG-PL_contig_7553_1_-2_26_217

MLKNGGRTVQHFLIMLFNAIFDQGIYPQTWSEAIIIPIYKKGILRTLITTGVYHCLALYQ

SAIH

>SG-PL_contig_7554_1_-2_230_2590

MDDAGGGEEEGCPRIEELAKQVAELSSQRDTVILRYKQLVEDNLKMVKRDKRIQEELGGL

LERHGETVEQVLRSELQIVEEQLKDLTEDVQVQVQRALIALDSPGYSSSESSVDAMDSAV

RDMLNRLVDLIGPDYQRLGRRLTVPQQELDKIEKMTQDASTQKNQDACTSKTRDTSTQKT

KDTCTQKSPQNTCTRKTWDVLRAWLKRRGGADTDTVPPAVRHLSQALRDVDKDMVHCQHT

PVSGPERQALRNCLQFALDHVADPRDVLDHLIALNELTPASISFVQEVMEKRERAVCRLW

HVSEYRGTGVVSRLALALARSHYPQVSHRLRDELEIQNSRVHSQCHPASEEPVPAPIPVS

VPISVTAASASRPSFAQARSRPMSRVPRFRSKTVDSGSQPVKSPPRPDSSVSAAAKAKPP

LLRQAPLARKERELSAPQKERPKSGVQERTVPAPEEKSTPSSKKALEEKSTPSAKKESPQ

APQQQERRILPIPRPFSPSLREKPASPAHKDRPALHLPKERPFSPLGKDGDKTDRASQKD

RPSPQTQKDRPFSPFEKDKSTRPFSPFDKDKQSRPFSPFEKDKQVRPFSSSEKDELPTRP

FSPVEKDKQSRPFSPVEKDKQSRPFSPVEKDKQSRPFSPVEKDKQSRLFSPLEKDTPTRP

FSPFEKNKSAAPAQSERAASQSQKERPFSPFQKERSSMKAVTSVQKERGVASAQKERALS

RERVHSLSQKEKQSGAPVKERSNPQSQKERGTVWDKRSSVGSSAGAAPASGARNSVVLEQ

GGRISRV

>SG-PL_contig_7555_1_-1_4_5349

MEQMQMGQQQTPVVGPVGGGAPGPPGPPGGAGFPQTMPPLNPGTPGSPARTFPGMAAQPN

SGNPAFNPMPALTTQLQNLGFQSSPGMNTSRNPINSLASLSMSVAARPPATNDQRLGNRA

GGAPGQAGPGPGGGAGPGPGGGGNAGPMGDINSLFPEMQQTFSKEVEDQANSYFQQIYNQ

PPQSNMTIEQVLAMLKRFKDSNQKIEKDVFQCMLRNLFEEYRFFPQYPDRELQTTALLFG

GIVEQGIVTYWTLGIALRYILEGLRKPHGSKMYLFGITALDRFKNRLKEYPQYCQHLSGI

AHFREFPQHIIEYVEYGARSQEPPHPHPDGSHMGVGGTAGPPPINPARSTPMDVGLGPGG

MPSLGGTGMPASTPGVPPPVQAPPSTSQPPPPALPPASTGTPAAGSTTGGVTRTAAFPKP

SIATATNIDTLLAGQGKELISVPPESLQDKVFFIFNNLSLANMNQKGDELKEHVGEDYIP

WVSQYLVMKRASIEPNFHVLYSSFIDVLKISNLTQMVLKETYRNIKVLLQSDIKGVANFS

DRSLLKNLGHWLGMMTLGKNKPILRCDIDLKSLVYEAYQKGAQELLYVIPFTAKVLESCA

KSKVFKAPSPWTMAMMNVLAELHQEEDLKLNLKFEIEVLCKTLGLDVKNLVPTNYLRDPN

RMALLEVQYLSLPKHPVPPPKEPEEQAVVPPPPPNIGLPYPVNQVPVTLPPPVPIQMPPP

LHPPMAQTPTPGLVFDSISTNLSTFMQQLTFNEKYPLLVAHPVLVSLIRPTVERTITELL

GPVVDRSIKIALTTCEQIVKKDFALEPDENRMRNAAHCMMRNITCGLALITAREPVLGQV

TNNLKNSFSSKLGPNNQPPKEMLEHVCNGLASDMIEMCTAFIQKTAVEKAGPETDKRLAP

EFDLRKAARNENRRYCDTACLTYQAERMPDQIRLKVGSVSVQQFAVYEEFSRHLPGFMPA

PMEFMPNMSKSMFGPPPQQPQPPMMPPQMPQQPPPNVPPPPQFAPDEMSLIYERIVRDLE

QHLQVVSIMPQTPMTLQLHALLEAAIQTRNMREIRTTTVLLHKAVEGLLEHYVPSSITSD

QQELLLRFRECHLMVLKGLADARALGHAWAPKEITKYLILMGREESKYNLEAVETLMRSG

LMNTNQFDFYMSSILENASHPAINFVMQVMQRICSDDKNSPQNFTETDFPQTLEGLNTLA

NRPRQSPEGLENLMTNLRMPTSENVALTLTSGGPGNASTMMQSGITQAREFDDPPGLHEK

TEFLLREWVNMYHSPAAGRDSSKAFTCFVQQMHQQGILKTDDLITRFFRLCTEMCVDLCY

RALSEQSHSPTLVRAKCFHTLDAFVRLIALLVKHSGDNSNTVTKINLLNKVLGIVAGVLL

QDHEVRYTEFQQLPYHRIFIMLFIELNAPEPILEGINYQVLAAFCQVFHILRPSKAPAFS

YAWMELISHRVFIGRLLALTPGQRGWGMYAQLMMGLFKFLAPFLRNAELSKSTQLLYKGT

LRVLLVLLHDFPEFLCDYHYTFCDVIPSNCIQMRNLILSAFPRNMRLPDPFTPNLKVDSL

PDIAQAPRILSNYVNIIQPTSFRKDLDSYLKTRSPITFLSDLKSNLQSSEPGSHYNIPLV

NALVLYVGTQAIQHVGSKGQTPSMNTIAPSPHMDIFQNLAVNLDTEGRYLFLTAISNQLR

YPNSHTHYFSCCLLYLFAQAHTEAVQEQITRVLLERLIVNRPHPWGLLITFIELIKNPSF

KFWQHEFVHCAPEIEKLFESVARSCTMKTPATIREPETTEVH

>SG-PL_contig_7556_1_-2_2_229

CCPIFCATLVALIPCRSFRLLNTPPTPSSFSRSPSARKFMGAIDVKPPCGHPPEEIMHCS

WVASVELNSACSNPTY

>SG-PL_contig_7557_1_-1_1036_1248

MTMRIKTCFQPLYELLWICMLMSYICMPIVDTAATLAAVVVLASEFHPSQSYNDVSWTLH

LAVPSVFHKHE

>SG-PL_contig_7558_1_-1_673_897

MFRFSYQHLMCLYIVGLFDTRIYNQYKRSVEYSPAMQSQSTVELHSIIIIPISVSSHSVV

KNVSDPVSFQALFSW

>SG-PL_contig_7559_1_-2_2_115

FFIYIYKNLCFYSIFITRFSRAKTSPKILRFQKRAYSA

>SG-PL_contig_7560_1_+1_1348_1575

MINRGLMCSMHLAHVKDPTAIKELSLEKFCRKIHFHRLTEIYSERVREKQTTGLCCTAVM

QFPQAEYREFHKGNPL

>SG-PL_contig_7561_1_+3_3_320

KNSAAHLIFRSHRQQPCTPLLKKLHWLPVSERINYKVACTCFNIITSSAPTYLSELVTLY

IPPRSLRSSSDSRLLRQGRYKRKCHGYRSFSYYAPQLWNSLPLHIR

>SG-PL_contig_7562_1_+3_3_182

SWFGCVVSRASPTQVLTPRESTTYPAEAGPAAQPASRDFGRGQNVGLDCGKKLIIGSVAG

>SG-PL_contig_7563_1_-1_52_540

MDVLNKLKSAVSNVLPGNPLSRDFDIYHQVASAGPGLLWKVFAATKKSTKEEASVYVFEK

KSIERYSRHDREAIAEMLRGGVQQLTKLRHPKILAVIHPMEESRETLAFATEPVFASLAN

VLGNLTSVSTVPKDLQDYQLYDVEIKHGLLQLTEGLGFLHNDA

>SG-PL_contig_7564_1_+1_1_285

YGVVRSSHCSLKPHDLSTCRSRSVMLAEHTYCMSFSNASLIFLLHNCFHPIPFPTFFHAG

IVEIRSSIHARPVNVTALVHNQFVRHFGQDCFPKR

>SG-PL_contig_7565_1_+1_1_108

QQIWQPEDFVQTANTLLSKKSVSSHTQFINNSAHLL

>SG-PL_contig_7566_1_-1_7_678

MLPIHAAALNGYVDCVRKLRAAMPAIDINIPDDYGRTCLHGAACSGKVECVEYLLTEGGD

INLQDAEGRIPLHYAAANVHQQCVHILLNVGSNVNLTDRHGCTALHYAAAADVDAKVVEL

LLHHDARPGIRDNEGYNAVHYAALKGHRLALEMLLDAAATDLLRGGAPYSPGHLAAYSGH

NEALHVLLGYMMNLDVKDANGRTMLDLACFRGCSDCVESLLLQG

>SG-PL_contig_7567_1_+3_3_155

IREHTDIYIYHSLVIKIKVKLNKTQKYISIILLLIKLLYKHIIFTQKSQNR

>SG-PL_contig_7568_1_-3_123_374

MSCRFSSTPMWVSRQMLEGELLLSGSVLHVYNSLFFCSLKTVKLEADLDNAEFSLCNPLA

CLCIDIMSCTGKFPQLLFFWTQSC

>SG-PL_contig_7569_1_+1_652_933

MITLLAEAEIIRRGEGEEIRSLQTFKINTTGLFKHLAHVKSKPHVLQTKLFNQQEQIAYV

LLSNSIKIIHVQKQKLSLTVNIPLSLLMFLSIFQ

>SG-PL_contig_7570_1_-2_173_649

MQNNIRYACWGSCELCQCLVACGNMKKKTSRDYKRKRKRKTGGAALRCCTLPGRAARISH

GKSMVMNTIQYNTMQYNAMQCNAIQFSFSFNVLKIALQHSDDSMCAAQQMSCDGRLKIIM

EKKSLILVSEFFLVLVIEFSCLKYYYHVEFYLSSTHPPS

>SG-PL_contig_7571_1_-1_1_303

KVPYFSADIYLENKQQIGKVDEIFGNVRDYYFSVKLNDTVKATSFSKDTKFFINPEKLLP

LSRFLPKPGGGGVTKRGGGRGGRGGFGGRGGRGGRGGFGGR

>SG-PL_contig_7572_1_-2_161_286

MERIQNDNGISLTRWQGLALPSLWILCLSYSVILALVKSFVS

>SG-PL_contig_7573_1_-3_18_1436

MELLRSVCVLFLVLSVIMSHTYQGAVSMVMSVRRHRTSPQNAVAMVMRTRNETRLRLLLQ

SPASLFSLLAQPGSSGTQNRVLVRGSVTSQLWVAVDTDYDDNVYYVIDRKARAIKVVDQE

TSQVFNILTGISSKVRAVAVNWIDDAIYWTDTAYDAIFMYKAAGGCRHFECVSTVVGTDL

DTPTGITVHPHLRLLFWTDVRGALGNLERSDLSGEDRTLLIEQLHRPSAVTIDYTAGRVY

WLAQSGDISVVESCNLSGGDRYSAAVLADSRLGDVEVLNGLLLMTDPQKGELVMHDITTN

VTRRLWSAGTPYGVCVYSQDKQRAEYRSQDTVLLEERNILDEGFLVLVSEQGSIFTLEKT

AITATREVTAREVISRDSVTAIAADVANKFLYYYDRRHHAIEKVSLQFLTSNSSSASSVP

TIVTRVAGNAAGLAVDWLSGTVYWTDNHYQRVAAVTNQSNHVRCLACRDVDQP

>SG-PL_contig_7574_1_+2_593_805

MPELLDAAPRETEMENPVSCRNPRAPPSNNHAGYGIGRNKPESSCNCIHAHTRKRDIIMT

DSFPVGNAQFV

>SG-PL_contig_7575_1_+3_3_206

HTHTLHFIQTTKTNKKIIAWEFLISLDRLKTKQKKANNKNTITSIVTTLIRRFIAFVVGY

ELPCWIFS

>SG-PL_contig_7576_1_-3_45_311

MSWGFLINWIVLIYEMPLYSDFRFSVILKISLLNIVILSPIYWFSVILKVLPTEIYKLCY

PTVVFIFTLFSPCSDNIMYLIFKYPFCLR

>SG-PL_contig_7577_1_-2_44_229

MAFSFSILFHPMAFAALVHMDLFRAWRRCLEKTETETETGIAQYNNNVSVTINGDDDDDE

LS

>SG-PL_contig_7578_1_+2_2_145

FFFLPVLQNFICIQNSRNKCFKQNNKTEHNFCLLNNCIQLINKQETAN

>SG-PL_contig_7579_1_-1_1_99

THTRKHTENHLLSKELHITQSKHTNFIETSVVQ

>SG-PL_contig_7580_1_-3_384_614

MFLVMMNYFVSLMNYRVSLQVQSLCVFGVSFILSHSTRKRTWVKSSGLLKIRHLKRSIFE

ILLLENCFFQTCFQMST

>SG-PL_contig_7581_1_+3_444_704

MSTLMLKRPHIIKRKRSSALTSTQRSNYNGRKAECLSSQTLIPVFYQIHFSSLAFNNDLS

FSVCVLKNTFFHAPASSFSICKKSIRN

>SG-PL_contig_7582_1_-1_1_72

SPYCYLCLSSQLDWVSWIKSYTLG

>SG-PL_contig_7583_1_+1_1_144

KKKKKIHNANSLMLMTKKQLKQKCITDKSLTNNMQGSSAELEKKKYKL

>SG-PL_contig_7584_1_+3_192_350

MSKNRRYTVEKVLQELFMDSDKEPNDLDFSPSDDDFDAESDFESTGNVLTIFA

>SG-PL_contig_7585_1_+2_2_163

HTHTYMTSFLQHNQSFQQKKQMYTYNFCRSFPQLNSGKHYHTLSSTESETLISN

>SG-PL_contig_7586_1_-3_72_203

MSWFVYTDKSILLRVVLVCAKHVYYYYNLNFLDLFFKLFASVPF

>SG-PL_contig_7587_1_+3_3_338

IITCIPKGDKARDNIKNWRPISLLNVIYKIGSACIANRIKRVLPQLVNEDQSGFISNRYI

GDNLRLIYDIINYLNKRNLPGLLLNIDFEKAFDSLDWQFMFKVLKAFGLGTD

>SG-PL_contig_7588_1_+3_1059_1337

MRISLDHRGFFLFLFFLIEKNQSCNYDLFDFTILFAKHYLYSCKMKSCEPNMNVLKKLHS

VFQIKEFNAKLNLDLTQFSAKQRYQNKPMFIEN

>SG-PL_contig_7589_1_-3_42_659

MEFYKSVKTMRLRDETFNSTTVINDKDRNPLYDEDKITEKWEEYFRDLLKCTGQSNIKPR

FTPKYPEHKEPHVLEKEIRSALKTSPKNKAAGVDGIITETVVACGETGIKRLTIISQKAW

KEMQVPNDWQRGRILLILIKKGSKKDTEIYLLSHVGKIYAKILEQCTRTIVEPFLSEAQF

GFRKGRGCTDVIFIFRQLFKKACEYQ

>SG-PL_contig_7590_1_-1_100_471

MDREIETRVQKANAVSYQLSPLLKHPNIPMETKARLISTIFIPTLTYQCQTWTLNKTLER

KITTCEMRCLRRTAGKTRRDMIRNEEIREMVRTTPVNHYIAQQRIKWFGHLTRMPVNQPA

LRAY

>SG-PL_contig_7591_1_-1_1348_1545

MCIVLCSLSSFVLRYFIVDCIISLVCHCVSVKFIHPPSIRVVILTIHHVAGIQILGVRVC

AGCLYL

>SG-PL_contig_7592_1_-3_3_137

VSRQEAYPSSQALSLRNELPGRRSQQGAHGSSDGVDPELASARTS

>SG-PL_contig_7593_1_+1_283_498

MHIFYRLWLGKLLQIFFNMALETFSNTLTSKKKSAPKLLLLPLNFSLLLLLLFVCVPQLN

TLGVTQYVYALC

>SG-PL_contig_7594_1_-2_2_175

VSNGATENTVYLFLVSVYVFVWHWAEKAGTLVSHVCLLLTLIWGLMLSFTDSLICSRK

>SG-PL_contig_7595_1_-2_8_661

MSDVTERLQELASEKLLTDAFCMNHYFWRQGVNSKPHDFRRQQSELFNRLNPDPDYEMNL

EEVEDALQQINGHLLTQKEFFFLYNVLNLPARPTINFRLFSVVAALSEKVTQLDPVIRKL

INNTDYNALDIKMEKCRELFRFVEEDDIAPRGGATAARLAVELTAGGLTPQHTQFVLSKF

NRESKGVVDFLDFVTYIPLFILIHQRIISHPLREELDL

>SG-PL_contig_7596_1_-1_67_177

MNSQNQDARSRNHGILVKPPRFLDRALRQPDQVVCSS

>SG-PL_contig_7597_1_+3_582_761

MPCWLCYTGISINHSATPTLLVQDPATFLSTFFCFKNGRFQLSGVCDFIKLQDSFSCIYI

>SG-PL_contig_7598_1_+1_1_189

WHSSLPFSKSAYLVKRHAGFVILESVPTILPHQHFPYVSGQPSLILPRVSRRDTFNFLEF

VTV

>SG-PL_contig_7599_1_-2_26_568

MMIKMMMGDDCVMFQGDELGESGQDDMTNNIGPVDSPLPSTGNLMREKDLQVIGVETDLA

QLFESSSSGEEDNDPESMDLGFSHGRPQEDVRLGGVKVHNETNEPPTIAVSELTRMYPTP

PSLETNKHSPPDTVTDTHIPMEVTVLEGPVLKAEAQPPIKTGTLCPDESFKFDVFVPAKN

V

>SG-PL_contig_7600_1_-2_188_370

MCRHLTDELYEGEMVYSVRLGSDNCTSFIESGSVSQSTSFLTLVKKERHVIESFKSNLRY

K

>SG-PL_contig_7601_1_+3_3_266

FAVSFPWSDEVGGFCFEKPSTDCPGNSFIFFFGGTVWNRPSELVPLNWKSLSQSCRNEKS

CTHAGLNHFRGVCKSMKAVNWTQLAYKH

>SG-PL_contig_7602_1_-1_1_135

VDMFLFIPSLLMIICGFHVWVKRSYLHQHLFIHRTFSFMSVDELS

>SG-PL_contig_7603_1_+3_3_419

SSILGGLPLGVVEVSRHSDDGVSDLLTQVGLCCFLHLAQHHGADLFRSQRLLLPLVLHLQ

LGFAAVADHSEGPVLHVALNTVVCKAPADESLSVKHGVLGVQGRLVLGSIPDQAFCVGEG

HVTGRGSLSLIIGDDLHLA

>SG-PL_contig_7604_1_+3_816_1037

MLYLLTKMFCFFSRKECTFFKNKKCAVYSPEIQADKATESSFTESTHKQQCQHFAQYFIK

SNIWYKILYIFCVH

>SG-PL_contig_7605_1_-1_124_456

MRVLGTWATTCEIYAAASLLGREIYVLAPSPSSNAEYHWLLFSPRSLSGSAVGGRGGGQE

GEGSAQPCYVTLCHTNANHYDRIVPVTDCCNCQMTPPSLNGLSLLLDLTTS

>SG-PL_contig_7606_1_-3_12_389

MAADDKDKHVLPRGMGGGPLGLGDPDDKSLRSVEKEVMIPLKMKEKAKAEKCTLEVREFG

ECAKEQGLLLPFMCRKVAKKLEVCLTSWYQNPEFVAQCTQEYLDERSQYRRTGVKQKMKK

KDAVIG

>SG-PL_contig_7607_1_-1_64_3732

MGTPLRPFDDKEENMFDITNRYSRPVDVFDYSNHFDYHYDTLKFNSWTIPQLEEVLKKQR

SRDRLFAGFLLHNIGTSADVEIDVCVTTGSGAKSCDHPAGKFSILGGEFEMPFTFDRLYK

YDISDTVRKLGLRLDSAADFDLQIKIRSSNGSYIDCTLLGAPTIIFQPGEGHTQDDVGHV

ERHLVRKNVLSLSPAERRSLVLAMKSLQADSSADGFQSLASFHALPPLCPYPEATKRFAC

CVHGMATFPQWHRLYTVQFEDALRRHGALVGIPYWDTVVPNSQVPPFLADETWDDPLFHT

DFPNPWHGADIEVVKQTVHREVNMDRLSEKGPKGYDTWSWKQYLLALEQEDYCDFEVQFE

IAHNAMHAWMGGSEEYSMNHLHFASYDPVFIVHHSNTDRIFAFWQELQKYRGHDPNEVNC

ALELMREPLKPFSFGAPYNLNPVTQEYSRPEDAFDYHGHFKYEYDTLELQGLDVQRLQDY

INQRKEEDRVFAGFLLSGIAASAHVSFSVCTAGGECTDAGFFDILGGTLEMPWRFDRLYK

YEITDVLHAKGLDVHDAFTIQMTLTAQNGSSLPSSLIPQPSVLYEPKSRDTELHEVAPNR

IRHDLTHLKERDVQSLKAAIRDLQLDESTDGYQNIASFHGAPPHCPSPQAAQHACCIHGM

PTFPHWHRLYTVEMEDALIRHGSGVALPYWDWTRPITELPEIFTSESYYDAWRDEVIPNP

FVRCYIKEANGYTVRDPQPELTKLSRDGKHSVLFDEVLLALEQTDYCDFEVQYEVVHNAI

HYLVGGRQMYSLSSLEYSSYDPIFFVHHTFVDKIWAIWQELQKRRGLSYDRADCAVNFMN

QRMHPFDWEDLNPDVRTREHALPQTVFDYEDLGYHYDNYELGGNTIEELEKMIHDRKSHP

RVFAGIHLHGIGTSADIIMFICKTESSCVRAGGLFILGGHLEMPWSFDRLFKYDITDTLQ

ELGIQPEDVFNAHAPFTIKYEIHAVNGTALPLSSISEPTLIFQPAEGAEGEQDSYSIAGV

GVRKDINTLTVAEMKNLRDALRRVQAGTGRLSYDDVVKAHGYPPQCQHDGHESACCHHGM

ASFPQWHRLFVIQMEKALSWEGAQVGIPYWDWTEAFTELPSLVSEEENNPFHHGHIPGSD

HITTRAPRPQLFRDPEQGEQSFFYRQVLLALEQRDYCDFEVQFEVIHNAIHSWVGGTSPY

GMSTLEYSAYDPLFFIHHSNVDR

>SG-PL_contig_7608_1_-2_50_586

MTDKTMANPSKLSKNVLQMKFMQRSVLRMEEEQNEEERQRAIDDEHWVLDLPELKQREER

FVIEPSYIVCENLQFGRMSFQGFNPEVEKLMALHNKQSELEQAEEREKEIGVSPREMANR

YSTLVGIISKKFTTKRNRNEMPEENAMKDDDISSDDDDQLINTPPSAKKKKKFLKPKDD

>SG-PL_contig_7609_1_-3_3_95

YKVTVTLRRKSGFSFEQLKFLFEFSQPFVFW

>SG-PL_contig_7610_1_+3_243_476

MEAIFFLCAAWLHSKRPLWRTGDKKEANRPTPTAVERHLQMRSQDACHHQRHLGDKCHTQ

FYPHIRSAEKILLVLIST

>SG-PL_contig_7611_1_-3_453_1859

MLPGDVYYYSANSCVLCVKRGQLKKVQKPGTGLNPNADAFFYSKALTPPVDKGWEPGEDF

LTNGDLNLQDEVGGGFSSGDSPVSDSPPAQVVESNLDAGAPPPDFVPDTMVGSVNPVPLI

LATESESALPGDDMAESMEQGGDMPVAPVDIASPPAAVASPVTMHNGMGPGQPGSADGGP

QGVQVSEASSLPSTPSDLRSQLQTQLEYYFSRDNLVSDHYLQSQMDGDQYVPIATIANFN

QVVKLTRNYDFIVEVLKSSKAVQVDETGTKVRPRHNRTVLMLREIPENTPKAEVEALFRH

EKCPKLVHCEFAHNGYWYITFNTDEDASSAFVFLRERNVMFRNRPVLARIKAKPLVRPVG

GGHYPSKSPNPPQPDASVPFMRQQQAQFPIMAAPVPQFVNQQQVPQAVSPFHLYNLPGMI

PQWANTAAHTFLDPGAMLTMNGLQATAVKPSTNHSRNLYHHPVRTNNNR

>SG-PL_contig_7612_1_+3_99_194

MTDKSIAKSQTLYKVNKQQTISIPFPLEKEKI

>SG-PL_contig_7613_1_-1_169_957

MARQALSCFMPSKSVLVVAVAAVFCFCVNDVVAQVKLAKGTRPLNNHDPIQYRDCGELHK

TGYNVSGVHVLYLNKTTPFLVRCEFTKDQGYTVIQRRWDNGVNFIQMLHVYQAGFGVVQT

NYWAGLDVINHLTSRGNNVLTINMQDGKGRNRNARYSYFKVQPSSFRYQMTVAGFSGVDL

QDEFSINSGMTFQTYDVPDANNCAQLMKAGWWYNYCTNTLLNGYYYPPGQYVPQGGYFDG

IFWKSLDGFNGSLKFVSMSVSSS

>SG-PL_contig_7614_1_-1_103_342

MKQQKDKAEKKDSNGHLRLFNQPLGPTQGRSKTCGDLFDLRSSHQTCCSWCQLESKNQHI

YRKNHQRPVNSVSHQPFLNE

>SG-PL_contig_7615_1_-2_23_373

MDCTYTCHTTAPYKSVSVTYPSHPFPKLIHCHHHLICLSLSSIINTPSQNCTNLLPVYHY

LSSHYLHSLPVFHPHPLSLSSPPLPSFHVSFIADSGKAKNTSIPNCRGLCNVIFEIV

>SG-PL_contig_7616_1_-1_1_276

HLNNNNLPGILVGVDFTKAFDTISKSFILNSLKLYGFGPVFCQQIRSLLSQTESSINHYG

WLSEPFKVERGIRQGCPLSPLLFILAIELLAV

>SG-PL_contig_7617_1_+1_1_180

SLHFQYLLADLFIVFPTDLHQCTTATHTQTHTNFPIHFILYSPFSSPLHFEALIFGRPIR

>SG-PL_contig_7618_1_+3_603_800

MRLFQSLSLMTAFVYAFVFYPPSLNMALSLQKLCSITFICFSKVGACLFHAIFISMQMML

KACTIP

>SG-PL_contig_7619_1_-1_160_1677

MLRSADKIASKALRKLLQRPNVEFTACKRWKVLSVENINPHVKKMEYAVRGPIVIRAGEI

EQELKKGANKPFADVIRANIGDCHAVGQKPLTFIRQVIALCTYPELMSHSLLPDDAKQRA

QRILDGCGGHSLGAYSDSAGVRVIREDVARYISERDGFPSDPDNVFLSTGASDGIKSIMK

LAMTGEDGNKRAGVLIPIPQYPLYTATIAEYNAYPVGYYLDEAKNWALDMDELHRAINEA

RPHCQPRAIVIINPGNPTGQVLTKENIQDIIRFAKEQNLVLMADEVYQHNVYASDSQFHS

FKKVLMEMGPDYNTMELASFMSTSKGYMGECGFRGGYSEVINFDPAVKAMLVKSISAKLC

SSVSGQAVMDVVVNPPRPGEPSYDTFSKEREMVLGQLAEKAKMVTETFNSIPGISCNTVQ

GAMYAFPQIKLPKKAIQAAKDKGQAPDTFYCFNLLEETGICVVPGSGFGQVEGTWHFRTT

ILPPVEKLGEMLGRFRDFHMRFMKTY

>SG-PL_contig_7620_1_-3_3_197

YMQKNVPNFCMHYENMFRYCMNMCAMKTSATWLIQHVAIMLRRNAKSDHLNALFRSSFQN

VHRLE

>SG-PL_contig_7621_1_-1_70_1038

MCTGYHLATGKTGMVYLQNSGLGNMVNPLMSLADPAVSSIPILLLIGWRGEPGVKDEPQH

VTQGRITEAMLNTMEIPTRVLPKDEDGMRKVLQEAKHHFAEKKAPFALLVKSKTFSPCKL

PPEPPQFPLCREEAMIKLVGCLSPRDAIVSSTGMLSRELFEYRVKNNMGHEREFLTVGAM

GHASCIALTVALNSPNRQIFALDGDGALIMHMGSLATIGQRAPPNFRHIVFNNGTHDSVG

GQPTEAGHHDSFSITDIALACGYKAAFCVSTAEGIEEGMQKLMSQPGPVLMEIKIRIGSR

SDLGRPTRTTLENKADFMEFLSS

>SG-PL_contig_7622_1_-3_291_1376

MGEDTVFACAHNSDSDDTGVYLSYNDGTSNIPLDRTNVERLKTALISSQGSYHNGRLRCF

FTVERDLDFYYPELPHISGAPYHLLMSRGFASRGRLRRHGLGVNELPIASPVKVDLISNH

YNIGDRARYPLVKAHGCLMILAWIFFASLGLLMTKYYKTMWPNKRMFDQRYWFVAHFNCM

AMVFLVTVIGIILIFVEADGWSVAPDLPQRAHPILGIIIFICIIINPILALIRPSEDNKC

RPVFNWFHWAFGTIANVLAIPQIFIGMDFGKANVPWWATWILVIWVIFHIVIELSLEVHQ

CCTYKKNKERRRKYEQQKRENPKLHIDEPEPAGRRFKRFMLFLHLTVTIIVTLIMIIIIA

VA

>SG-PL_contig_7623_1_+3_804_1127

MSCNISGVISQYIIIPVLFPAHPCTQSKVAGGSRASHTTNHLIRERPHEGGKAYQAATEA

DSVRILYSLIMSQSGTKLHAHLLLISFKEVSSESINGNMSIHTLSILW

>SG-PL_contig_7624_1_-2_2_277

THEDQESFFSWFSDHGDAGSVTGADELGELIKDDIWPNPLQYYLASELDEEGGAGDEDLD

EENLDGEEEEEEDFDGEEEEGAEDDEGDEEEG

>SG-PL_contig_7625_1_-3_3_125

KRTDKRSKEERIEELYSTMLIRCCFRLKTMLPWFVCVFLMS

>SG-PL_contig_7626_1_-2_2_178

PKTFAINKNVMFCTNHYKQLFATRGNYDEGFGRDQHKKRWKSEPNLADDEQSSTATANH

>SG-PL_contig_7627_1_-3_45_365

MDYSFTLFTAKWVNHLLGLAQNALIVCLCGCQFEFSGHDSIVNLPHWKGNAYILDTLNHK

MTSTCWRLCFILLFQDNWGKRCNSMGTWQERRRKQKVVCISFLLQLV

>SG-PL_contig_7628_1_-1_136_1626

MNIVRHTENLSLNLHIDPEVPSVSQASVAEEHMILDEGEIKTPPSTDLPPERTTRKKCCC

SCHDRESGLYDDAASQTSGTVAVPNTPGSFRNRFHTAEQESMSSDDFLEFSTPSSSTNEK

PVSFKSEVAESPKSSPSHTLSEASGNGDCACKEEVEKEEAEALAKAMEVSEEQSPIPVVP

GLTPTTREEVITIRIQPDEVESKNSIKSLSSCDRQGPQIYYEGIHWVKGPLLGTGAFSTC

YQARDAQTGVIMAVKQISFCRNSPVEQESVVEAITAEIHMMSRLSHPNVVRILGATKQGC

HFNMFVEWMSGGSLSYLLGQYGAFTEAVTIRYTRQMLRGLAYLHDNHILHRDLKGANLLV

DSTGQQLRIGDFGASARLASQATGAGEFQGQLLGTIAFMAPEVLRGESYGRACDVWSVGC

VMIEMSTCRPPWNSHDISNHLALIFKIASSVDPPPIPDNLSPPVRDLMLRCLEQNSEFRP

SAKDLLMHPLFTKYSNY

>SG-PL_contig_7629_1_-2_203_556

MNATPTTARTCSGKIFSRSWPRNPHRPKSSTWGEGKVRGVRRGVKKKSRRPPARKSLVSS

GRDTFDWSPFPRTTCNDWLRQIPALMIRPISRIVAALMNIRPPPHWTESNYIYPIVWQ

>SG-PL_contig_7630_1_-2_179_445

MDEPSKISVCTEIALPFRRESLPTWTKQKYRETTVSQQGLMCKNSRSCCTCFSYHYKFVS

FIFQRFAVACWCSLQLDHSLQHIDLYLYI

>SG-PL_contig_7631_1_-1_223_354

MFVFRETSRSKMFLRLHSFNVCFYSAFKSVFQLGLCKTGALVII

>SG-PL_contig_7632_1_-1_1_264

LSALPILQQQDRSCDLWATRLLAHNSLAFFATWITLHATLTFALFLTTFDLENHEVMVKV

GSLIQLAIAAFVVLMYVLTDLTVFDKYS

>SG-PL_contig_7633_1_+2_83_511

MNYITSIDYKYTGLDCPSLTQSTNMTRIKKPFQWHVKTTHSMTHSENGFAIGYDTLHTLG

CNYQSHKFILRTASFSRIHSRQRLVLAPPHTCRDERRNILLNTMTRLSAAIATGTIILAV

NHTFLGNDTYLLKSEINTNPTQR

>SG-PL_contig_7634_1_-2_125_3793

MALRAMEARWRTVYLCVLCALGIQTSGVSSQRLDSASSDAVREIDLLSAIGIPEDTKVGV

TYTAGLDGYPTFLITSQAFIREPAQLFFRQPLFSEFAITMTVQPYRREGGFVFAVVNPYQ

TIISFGVQIMEYDSTQQNVALYFTPNTRIAEQSDVIANFTVPSMVGRWNKLSFKVLRDRI

VLYYNCQEYEQAYWFRTVENLDFEPGSSLYVGQAGPNFQRTIPSFEGGLQELKISSNPND

AEDEDCDFEISGSGSGSVVIEQPTGPTYTPPPWERPDEKLQGQKGERGLPGLKGDKGDQG

PQGLPGATHPPPITPPPPDIELLRGPKGVPGDPGFPGEPGVRGQKGEPGEPGLNGIPGAD

GRDGEKGEKGDMGESGQPGLTVRGPPGPPGPPGRPGSLDVIPELEQGPQGEKGEPGEPGE

AGVGLPGIPGPPGERGGPGLPGPRGVKGDAVLGPPGVPGISGQPGPPGPPGPPGTPGFVL

DTGSGDGEEVPGPPGPKGEPGRPGLMGRQGPVGPEGPSGLMGTPGVPGLPGPPGMPGTPG

LNITGPTGPMGPPGRDGLPGSTGAPGMPGVSGAPGEPGLPGREGPKGDAGQPGLPGSPGI

DGERGLPGRDGTDGLPGRPGNDGRAGPPGPPGPPGSGSGGGIIFGSGMGDTEDIVGQPGP

RGPPGPQGAPGIPGLRGLTGEPGMRGPIGPAGEQGPRGERGFDGEPGIPGVRGEAGVPGR

PGEAGAAGTPGLPGTKGEPGVPGISLPSEPGIPGTPGSDGRPGERGFQGPPGPPGPPGPA

GPAGRPGSGTGGGFSIPEGSGDFEIIGGKGSPGEPGIPGLPGPKGNMGLAGAPGLPGDVG

RPGAPGIPGDPGEPGLVGLPGSKGSKGDLGGVGPAGPVGPSGPPGPPGKPGVSSSGGEVE

VIPGEKGEKGERGIVGPSGPRGFPGPAGEMGKPGFPGRQGLRGRKGDMGIGMKGAKGEPG

GSGVFIPGPKGSKGDSGVPGLDGRQGPPGPPGPAGGPGGSEPGIVLAEKGQKGEPGLSGR

HGRNGEDGRSGPQGLPGLPGDEGPRGPPGPPGPAGVPAIGSIGSGDGDIVLRGPPGLPGP

PGPSGERGLRGLPGIGQEGPRGRVGPQGQPGPPGPPGLGIPGPQGPPGPPGYSGDFSING

RGPPGPPGPPGPPGRPASGLGISVGGGPVVFPNMDALLKVSYNMPVGTMAYVADQEYAYL

RVSQGWRYIQLGQLIPLPSPKPP

>SG-PL_contig_7635_1_+2_1466_2116

MADECDDGTLKEELIRDRIAVAVADDDLSNDLQAKAKLTLPEAVQISRQAEKRKQSQPIR

KRKQSQPIIRASERVDFVHKRRDSQLHSTNSKSPQNSKSRQFGHCKYCGREPQNATSSSC

HKQGYYKIVCRSTKDRQVKKLVEEEDSYSSSDGFLGYINSIQDDDDSWSAQTLLNGKPHT

FKLDKDAYVSVIGESWAKDKKLVKTKKSLKGPGGTSL

>SG-PL_contig_7636_1_-1_43_258

MPCVIVCVCLYALCHCMCLYALCHCLCVFTCLVSLSVCVYMPCVIVCVYMPCVIVCFTCL

VSLSVCVHMPCV

>SG-PL_contig_7637_1_+1_292_399

MDELTHREQLKTTWQHLHTHIDACTYIQIFLILIWA

>SG-PL_contig_7638_1_+1_523_699

MDWRQLTNSFLSQGRTRAVRFASNSFTSTSTRPQFIKEIENETKRQSLSILTVTNTKKP

>SG-PL_contig_7639_1_-3_3_221

VLNPGQGPPRFSSPSLDEPELQPARPLVIRRARSRGPGSLLLCVQPCPRRHRRPLARLLP

LLGAGYLLGCLLP

>SG-PL_contig_7640_1_-3_276_2960

MASPSAMFEVVSGSGSTSSDSIVNISRSGASSIVTPATTTTTNPNEDELKTEVDQLRKDN

KVLEGRIRGFKTLSDLLVDFRKENDQLRCQLKALNAEPLIMPGSDFKAASVSSPFSLVSL

QAAQQNQSSSTMSQFDMSQLTLQDQKPSSTSSVARSTSSLDPKGPPVSYPPVSGPPFSLQ

ETVGPPGDLKETKLISLLSPTESGTKLKLNTAEPQGSVSKQESLEPTSGQHPDIGTTQTS

KTHHDSSMASENSSPELLQLDSDKYDEATYEKVGLQLGMGLAGLTNSMYAVGGSTRESRP

TDFPSLSSGISNSFVQPSSHDSTSNPPFMGMMHQSLTGLQQNLTGMQQSLTGSATNKHNS

DLATEVENITERLNSEPGDSMPAIAFQMHQIAERISKLEQKERNQKTLNDTLMRKNQELK

RLVKVTETTHQTEIVALREQNMRLIQQLKGQHHEDQTKSKAVKSSGSHSDWVHVEKPPQM

GESLGNTDHSLVGRVRSLEQEKAELLRANSNWKSQWDQMELDHQTQINELRPKLVLLNQE

LVSLKTRLAEQTSEFEGRMLEMKRRTGEEENLKEEAQHQQRLAERRCSELQDQLNEMRTQ

VADLARDKHVLSTELNILKAHTTNQEMRTGSAIGAADAGSRSALEAEIAMLKEQLKVFAE

DFEQERIDRTTAQAARDSAKKSVDTMARQLHSLSSQVKHLQKQIKDRDDTISWTLRQNET

LQRQATDFKKKLASEWEEKQSLQRRVLALEQQLQQQPIRNPYLTSGTTYNPAPMHQPSPL

TRTTAPYFGGQGPAGGPGRNRPITTAMSPEHLPGAWTCTACTYINYPGRTVCESCGLVNT

ARSHPDQFGFASHDMYGSEHALLMSRGGDHHPGGGIMPTNGDLVVDSGVCATTPY

>SG-PL_contig_7641_1_+1_568_840

MTSAQKFFLIFRKTGEWERIKGKRKKSENMYCLSPEVHCVCSPGILKFIVFVNMLYLYRC

ADLTFLLPDFQLCILRTFLVLLNPSTLFLFF

>SG-PL_contig_7642_1_+2_299_439

MQYTQHTENIVSIQCINHTYFDYIKRIHIFKTLVQMFLYKYGTVVHG

>SG-PL_contig_7643_1_+2_2_85

SMPVYKQNSMNGPHQIIYLYDRPTSDYL

>SG-PL_contig_7644_1_-2_173_367

MHYHLTQNAIFCFQSSIYVQSMDRNCVYILCGHCAVILFISYIDFSESLFTEYLFFLLVF

HLDLQ

>SG-PL_contig_7645_1_+1_1_240

TLSVPQPIHQLPPAGSWWTDRGILPPYPTDAIRLTFCSIFGRNPLDYREIRYKDASRWRS

TLEAQARARARSLFLFHSEA

>SG-PL_contig_7646_1_+3_1494_1763

MSILNPQTREVRALATFSVFSHSAPLKDTLAEAYGRSEDFINHNLRDIHMIGADSSLGVS

IVAANITISHLWLCSSNQRGDTPFHHYLNS

>SG-PL_contig_7647_1_-1_325_477

MGCFTHSAKFSLGLVRRINTFAATIALLVGMFVPPVYHGMSCVNGCLTVQS

>SG-PL_contig_7648_1_+2_332_481

MRGGWRWDNKNSKNQNHQAIQTDWQTNDLPFSVSWPRIPTLQNRLFSFKS

>SG-PL_contig_7649_1_-1_472_585

MPLWFYSSISLIICHGLCHTLKRLRQMTDNPLRLCAYF

>SG-PL_contig_7651_1_+2_515_682

MALLCTDCPPKSLPSMCQGWHSGSLSVILGNKARFLYSHNKSWPKHPSYFFFKSVS

>SG-PL_contig_7652_1_+1_952_1266

MCVCSLQSTQLLMGICTFFKFRERLSSEGEEMGTALIMLAPGNVEVSTTSLPYDLRVTGK

PSPFIYLSITESYCYTYRGTGLMSEPINPSHHSAYLDACASAMES

>SG-PL_contig_7653_1_-2_2_241

IPFTLDELKSALQSCNDSSAGLDNIHYSLLKHLPDSSLQIVLQVCNHIWTTSFFPPSWQK

VLIIPVPKPSKDLSNHTNYL

>SG-PL_contig_7654_1_+3_69_182

MVTINNEHMRAKLCRRVNSTISFHRGTYILMLCVTGNN

>SG-PL_contig_7655_1_-3_555_905

MTSSITFSLVYLCQQPDSKDFKDSTVALTPCVTPFSYAASGDAKNKLVMAIHVLRKLKYD

LQHFMTVNYIFKNPSDSFLTPFYCAMNVVVKKKKDCLENTSLEHVVHGSWFVKNCVV

>SG-PL_contig_7656_1_+3_3_206

SAISLKMSNAHTCLIPKQQQQHKVSVYLKIYVGIFCYRHRLMHQYKSFPDTFSVSFVKKI

RHISISTY

>SG-PL_contig_7657_1_-3_75_200

MNKIMTLFITLSLSLSLSLPLSLLTFFPLTTTRYHNIFSRLL

>SG-PL_contig_7658_1_-1_1_81

QKRIYFNYTAGILPGRSLQSVTIVTLI

>SG-PL_contig_7659_1_+2_362_670

MMAHQWSYTRQLALRLGRTVHIQPHYITVYTDIFSSCIYISTTSSNTLGNKFSAKSAHSG

IVVSPNILPHLILFIMRSSLLVSISLQVFLRLCEWLCQWSLSE

>SG-PL_contig_7660_1_-1_1_258

PLCNQVQQEASTLRLPSAGPRGDGGGRPLPRLVSSDQLRLSPLPDPAQSHQEGQGRVHDP

HCALLAGSTVVPGPSEATALRDQALK

>SG-PL_contig_7661_1_-1_13_1455

MSTQSWHPPLPPGWEARWDPNQRAFFFIDHSTKTTSWEDPRWPNKQKLATPASGDHHSRQ

GESIPMRDMSHASPQRSPSAHRSHSPRHSPAVPRGETTFSNQREAEAEAIAARLRTRFPD

ASPVVRDVLNMCQNDEDEAASQLMQLGFESKETPPRGGASSHASPAHSKGKQSTTTSPQH

ASTTPKKSTSPAHKQSPAKQQISEAEKKKRENKLIGELKTLEPEVVKMALQACQYDENKA

RITLSRVANNYESSTKRSTQRNATSASSSSASAARSSQPTSPVALGGQTSMEPVVFSQDR

PSPSRTSGATAVGKKSTTRTTTTTTPKPKQQQQQQRRAKHVTKITHPSAGGHGTRVVTDQ

PRPGPAARTAPHHAPVHFGGSGLARGPDPTLHMGPDRGLRTGADPFFYAGANPELCDGSD

PDLLLGSSMGAEGPNPEYHHGNVPDLVTGTSGALGPNPSLRCAAEHPSSIRMEQAFLQTA

I

>SG-PL_contig_7662_1_-1_373_567

MILFKLSFFMYTQEVEELCEFFPEILPLMTNCYQIALFYSERLNQFSVIVFDSEALFNIF

FIYIM

>SG-PL_contig_7663_1_-2_170_298

MVFVISTLHLCKYNQKRVCFNCDLCTFKTPSGNSRPYKSLHNL

>SG-PL_contig_7664_1_-1_106_486

MKEATAKFPDLDSQNKSLKRKNLQKRRSWIDNGTVQGTVPYLGTFLTDLTMLDTAMADMT

EDGLINFDKRRKEFEVLAQLRLLQSAAQIYNLPFEPRFWVWFDSVRVFTDTESHDMSCAI

EPPSESV

>SG-PL_contig_7665_1_+1_289_438

MLINPSHRSAYLDVHASAIESWTSKPKKWRYRYHWRAFPMIFLNNIWHIK

>SG-PL_contig_7666_1_+2_767_1048

MAIPISLACFPDDISEQYLAYKTTLRQWKQAEKFSRGSYIDKVYLFKEKDCEVYDVNAVC

YRNQQKTRDPHHVNNTIDVTIKTAMNAFCSCKTG

>SG-PL_contig_7667_1_-3_657_992

MNCVLSQFFSSRFEEVFNVDWLALLIDAVDSRSHVSSSVPPCNIKVVLFAAHLKLQSFWW

CQCFIGPVQLPISGPFQCIGCFAGSRICQITNYWQIWVERIWNCLNMMSHLE

>SG-PL_contig_7668_1_-1_1_132

FFVVFFFALKRLSQDGKTIQEEIVVMLTFEKTGGLKSVFSSLTP

>SG-PL_contig_7669_1_-1_76_879

MALQRKALHNLCQSLWRVNVSGCSGHRDVLGGSTPCLLQRWLTFCSQNSTTTSARVPCSP

LPASYQSYETSLKAGHHKCLKNDLLPVSSDFTRLCLYPQITKPTSVPDKGEWSLASWPSK

QHLEYVCPSEKLTTLPELTILDKLHQSLSYDLPQTQAIISLIIDPTTNPVEISEPTAANN

VPKMARRIMKIRRKKMKRHLLKKFRKRMAFTLRKMKRDRRKKKMAVILAQLAKIKEWGDS

FNARDWMQEELAKARKGGFYINVLEKSQ

>SG-PL_contig_7670_1_-2_134_358

MKSVPVVTLSNETVSSEVDGCFVSLVCACDKVKIAGSKRGQAYGYFLSVILACIQSFWWY

QCFIGPVPLPISRTC

>SG-PL_contig_7671_1_+2_68_253

MSENKTKKQNKTKNETTRHCIFLFIQENSKTLKKLTLGSFSSQRKINAHTHLDVLSPPIK

LE

>SG-PL_contig_7672_1_+1_28_327

MNINPCQGPGSVTVMCLFLPLLSCKMPQIWEFNTNKVLNLLLGRLGRDVFVFIYINVSKT

ASDKYDHMNSLAIREQMYKLHTTNKQRRSIISVCKSNSCQ

>SG-PL_contig_7673_1_+2_2_289

DFESFRDRQKKYFFNTEINTHKDTYTKTQVYHLSEKTLKLKNTHNTQSQSLIFPSMLFFH

LMELDCLHVALQSHVTFQLVDLDLINLIYITLAEHT

>SG-PL_contig_7674_1_-2_2_178

VGQTTCMLISEKEKQQNYSVRKRGGAEFVTPPTTVYGNMLPCHQTAVTDYKIYSDVISR

>SG-PL_contig_7675_1_-3_93_269

MFRTFPTSCLVFGFSVWNLYLESLGKSGAANPLACFMPGFYRGLFQRKRGCLRGCTIVN

>SG-PL_contig_7676_1_-1_484_672

MVPSLSLSLSLSLFCARMRMLFLIISFCLSLRLSPSSPSHAFWCFLCQNIVSFSSLLISE

SSA

>SG-PL_contig_7677_1_-1_793_1476

MLFNSHHSTSGSESAVQAANMGPVACYFAPAICQFCHFRSAVCPSSQMPISSYKLSANSN

QPCNISNEPSAISDQPSAISSQPSATISTLCSTFSSLSAQYHFQSLLYHLQQAWYHFQLS

SITSNLSSTTSSQPSTTPVQSCTICNQPSTTYTSCNKSFAASDQSSTTCSQSLTTESVNQ

PSAHCKRSSAISTNPRQQQALPNHTLATSISKERERERERESLVCNAG

>SG-PL_contig_7678_1_+1_1_183

VSCSSLTRSFLTIIPLQPESLRTTTVLSVKCPFNTTMFVTSLATGLAAALSTRAIFFPQA

K

>SG-PL_contig_7679_1_+2_683_841

MPKVFFIMLQMAPPSIALPRAALNIQESPSNQLLNLSPLTPKVDANPWTTKPT

>SG-PL_contig_7680_1_-3_33_623

MQRMSQLCCRLTSQLLGNYPPRHICSSNFVRAVNAQTITLQRLKHTTADGTEANEQETLQ

FSGYIPIEELEFSYSRSSGPGGQHVNKVNTKVELRFHVDSASWISPDIKTKLIEMQRNRI

TKEGYLVLTSDRTRKQMLNQADVMDKLRFIVHQAAVRPRGLTEDETQLRDKRLQKSKDHT

LREKRNHSHKKQSRGGQ

>SG-PL_contig_7681_1_-3_189_299

MISCTSPIPAFTFSDFSLCICKIYHKKCQNKFVLICH

>SG-PL_contig_7682_1_+1_1_267

LRILILVLLHYWYHVIYGKKNKNCPIHTVFSLLTKELKVNEPLNFFSSKPDIAAKAATTG

VFITIKLQLDTDTMVASIIILPKHPAQYL

>SG-PL_contig_7683_1_+2_2_142

FRGENLLSKKNHFPKKRGGGGEGGGGEEGKRRKRRRRRKKEGRKKLT

>SG-PL_contig_7684_1_-2_164_541

MCVSVSVCFLSFYLWTSVTCMHLGFSMCFFKKKTRCTCFGRSDIVCVVQVSLFNYFCFDM

CIVCVHKCVCTCFVENALLIIEHYMRLPFCQDEIFFYCVSSDIELIHRTKMIKVRITFVF

MSSHET

>SG-PL_contig_7685_1_-3_66_434

MDKALNKLREQDRAGITPSPIDDFLGHPAKSEIPAWKSANQELAGVLKNLRKMEHHLHLM

ERALFPDEDTDTDVSGMSSVEKHKYFQEIERIRSELAGFQPIDSPHAQDPGSPELAELSK

HTH

>SG-PL_contig_7686_1_-3_66_410

MVHPHDQPLRGQVQQEASDLRFPGPGPQSDGGGRPLPRLGPSDQLRLSPLPNPPQSHQEG

QRGKGMRDPHCALLAGSTVVTRPEVPISRPAPGIDPKPGPSQATSLRDHPCESGD

>SG-PL_contig_7687_1_+3_207_392

MNRVCRPTGRKDSGKDPVTSRHKLRARKLVCMRRDTKCHTLEDAERQAHNYTIQAHLAAQ

VI

>SG-PL_contig_7688_1_-1_1_156

PIVRHHRSIMIIIPIVMHHHSIVKKNLSQTLWPLNGCRKRDRKKKRKKKPLV

>SG-PL_contig_7689_1_-1_28_231

MIIIPIIRHHRSIVNKNLPQTLWPLNGCRKRDRKKEEKKKNSCMSCLLMLLANSRFVCDR

SMAAQSQQ

>SG-PL_contig_7690_1_-2_170_313

MIYVYEGKKVLNDVIVMNVQKFVDMYIYFMFVHLSSSAWEECKRMSVV

>SG-PL_contig_7691_1_-1_271_423

MKESNINQHLLFFLTYKMKVLWTMGRNFFNEDDQSHFLDYIHTNVITFLMS

>SG-PL_contig_7692_1_-1_259_675

MNTDFIFNWSAINSSDHFDCISVYAHFGVCVCVCVCVCVCVCARACVCMCVFAVVVLLLV

LLCMSVYVYVRARVYVRACVCVYVRVGVCMCVFLLSLVCLFVCWSVYLFVAKEFSSTPVR

HNYGVETHFYSELGDGNAV

>SG-PL_contig_7693_1_-2_92_304

MGTSTVTQQPMDQSRPPSAMDQESGAVPMEVEPMDVGSNLSDFKAITVPISQLPANIAKP

VLTPQQQQINS

>SG-PL_contig_7694_1_-2_266_430

MFKKKKRCVCISCVFKPLLMLSEKAWFTVALMCTISVAYSATAAVPNPHKYLVLW

>SG-PL_contig_7695_1_+1_157_324

MHEFGTKRVKVRVTTFKFCLHCCDSLPLFHFTSTVLFCLKLLQGHSDLGNLKIWIL

>SG-PL_contig_7696_1_-1_94_258

MKRSTGGSCYCSRRPFIPIRIMQTRKLAVYTRIVRGLVRIREDILKQHEHEKVST

>SG-PL_contig_7697_1_+3_3_254

IIIIIIINHHHHHQSSSSSSFIIMTSSSFIIIIIIIIILHHHHHHSSLSSSSLSSFITTT

IIIIHHHRLNHRRQGKLPTTSAKY

>SG-PL_contig_7698_1_+1_406_615

METETERQRETERERERERERGQFANMNIDVQITNSISESFNKTIRLQLSIQVKQGKLMK

KAKREVVVSL

>SG-PL_contig_7699_1_+3_45_218

MTFQSWKITFSNSMTCSDPAWNTAKCQRGWLNLTLPLSFLPTATRHLTSPTPVAPPGA

>SG-PL_contig_7700_1_-1_97_3186

MSDKMSDSEPDSPEEQMELQPPIAGPSVLLKSLTKPETGIEKEDGDFEAKIQENRSNRFN

FLLQQTELFAHFMSTGAQAGGGGPTSPLKMKPGRPRMTKKMDEKSKLVAAGDHRHRRTEQ

EEDEELLTESRKSHNVLLRFEKSPSYVKGGEMRDYQVRGLNWMISLYENGINGILADEMG

LGKTLQTISLLGYMKHYRNIPSPHLVICPKSTLANWMAEFKRWVPTLRAVCLIGDAEERS

TFIREVMMPGEWDVCVTSYEMCIKEKIVFKKFNWRYICIDEAHRIKNEKSKLSEIVREFK

STNRLLLTGTPLQNNLHELWALLNFLLPDVFNNAEDFDAWFNTENCLDNNQMVERLHNVL

RPFLLRRIKADVEKKLLPKKETKIFVGLSKMQRELYTKILMKDIDVVNGAGKSDKMRLLN

ILMQLRKCTNHPYLFDGMEPGPPYTTDYHLVDNCGKMAILDKLLPRLKENDSRVLIFSQM

TRMLDILEDYCFWRAFDYCRLDGSTPHEERQKSILEFNAPDSSKFIFMLSTRAGGLGINL

ATADIVILYDSDWNPQVDLQAMDRAHRIGQKKQVRVFRFITDNTVEDRIVERAEMKLRLD

NVVIQQGRLVDNTTNKLGKDEVLNMIRHGASHVFASKDSEITEEDIDSIIAKGEQKTDEL

KKKLDSLGESSLRTFTMDTQDSVYQFEGEDYREKQKLGGIGGWIEPPKRERKANYAVDAY

FREALRVSEPKAPKAPRPPKQPNIQDFQFFPPRLFELLDKEIYFYRKSIGYRVPKNPDLG

SDADRVRKEEQLRIDEAEPLTEEELTEKEDLLREGFTNWTKRDFQQFIKANEKFGRDDID

AIAREVEGKTPDEVIQYSTVFWDRCNELQDIERIMTQIERGETRIQRRIGIKKALDAKMA

RYRAPFYQLRIQYGTNKGKNYTEEEDRFLVCMLHKLGFDKENVYDELRQAVRQAPQFRFD

WFIKSRTAMELQRRCNTLITLIERENMELEEKEKAERKKRGPKSLTPKSAQKRKADGTPD

NRGRPKKKKV

>SG-PL_contig_7701_1_-3_30_281

MVSINMPLQCPLLSHGLHKHAPELHFCYFMVSINISLKHIFVAPMNISPKYGFMPSITMS

LWYTLLFYGLHKNVPKVHFVPWYP

>SG-PL_contig_7702_1_+1_46_306

MWTEHRNEHGFWAVYLQHTHTHTHTHTHTHLAVRLTSTHTHLAVRLKYMVMLISTHFQEA

GYNYYFLLTLTDIKKKNLVTARKQIGY

>SG-PL_contig_7703_1_-1_169_645

MFPCFCILQNELRFVCFCKVSAVIPLVAMKHFLVFAIFDVFVYMYVCERNLLRFPDFNKH

SPFLFLCTAFFTLKGKETCSAVVLEVNKQFVLIFLPKKLVGSLLRFHICTYSSSDCKVYL

IKHFCCSKLREYMLNLFQFNICSDSWEKGILRISPSCVI

>SG-PL_contig_7704_1_-1_52_237

MIKLKSLSLSLSHTHTCTRTFTHMNTHTHTHTRTRSYAHKRTAHTRHVWEHTTAGCLSVE

KP

>SG-PL_contig_7705_1_-2_134_361

MQTPKALAHHLNLVRQNQTSGEVRRIPDVGYNVFKKNFEEPTTAEGFTEVVKVDFVPQFD

SSRDQDIFKQWTTAGH

>SG-PL_contig_7706_1_+1_355_576

MVIIIHQLVLANNLYIQAHSCTPSSLSPHHHQHTKCKSYIYIYYIYIYGHGYKMASNVQC

ELLTKFRANSISQK

>SG-PL_contig_7707_1_+3_1128_1421

MIIISPMGMPGALSEYIVSPPLEILLEISSFSVILFVSAFSFYHLFLSICLPYQSIQQYI

FKKFLPIFPHPTLWRMQICAATLLWLHTVHQNNVHTHI

>SG-PL_contig_7708_1_+3_279_527

MMSEVRNENKPASLCDDSQPQNRKTVGCADEACMHAGGAYSFKTLTVLYNRRMCSAHIPS

QANTHFKPPFSFPPPHPFLPTLP

>SG-PL_contig_7709_1_+1_1_261

GGGGEGGGCFCPRTRAHKACKTLIVTTTRLRTICLPHVHYAMSWFTFANNSKIHLLSIKK

IFLLKTDLWKLCLDNLCMEISTMYWQR

>SG-PL_contig_7710_1_+1_115_240

MSSFYIIRHYLYVIYVTFLCHTSLVLCEYIINLCHTSLFLCY

>SG-PL_contig_7711_1_-1_1_561

AGRGGGGGGGGGGAVAGGGGGGGGGVWGWCPDNHPAFRVSYPSTQDWVLISRLSSFPSQA

PLSFKTLEEWDRMERRGVLEASTAHTFLRAMACQLQELWKSLRQWGLGSPFEDQRLNLFG

ARVAVGLSAVADICSKLRYNSLLARRDAVLRHSSLPPEEVATLRCIPAQDDSLFGPYAPQ

FVSLRAE

>SG-PL_contig_7712_1_+3_3_212

HTHTHTHTHTHTHTHTHTQRDEQNHGLNIQDLPVATPQPSKIPHPHSQLIRRSGQKTGIS

PSTSSVLRHF

>SG-PL_contig_7713_1_+2_437_580

MFINNLMHTSMVSGLRTRPVIVPLYIYIYVQTWLTTLCKIISRKYHRR

>SG-PL_contig_7714_1_+3_435_596

MDQKAYKSIHHGLHSQILILNFSPPASHKIYTVCMSTAHVFVLELIVVQLFPSY

>SG-PL_contig_7715_1_-3_870_1106

MLCYCLFTGSDKTNTFQNIYQDYARLCFSYCRTFNLVTRKSGVFIYFVFAVPSSAHHHSC

YYSHATHFSSPTYLQTHRN

>SG-PL_contig_7716_1_+1_1_120

KRTKIAGDEYLKEVNSTALVHKKRMNILQKSWSLHEECRV

>SG-PL_contig_7717_1_-3_3_254

RPATQCYCETYTTEYPCIKRSMVSANHIFCSVCRLDFKVSHGELTDRCELTCEICQALGD

HGCWWCYSQMPFCCAQIIKMLLPR

>SG-PL_contig_7718_1_-1_1_276

LATCCCRSEIHALSGAKDDIGFHSNGDLALSVLPEFLAKTQQPGSRPRAFRIPSLVSCLD

PRDARGEERLLCPVRCVKYYLKRSAPRRSSQR

>SG-PL_contig_7719_1_+3_3_173

TNLFLHTHTHTHTHTHTHTHTHTALLLIDRITLPFLYIQKKIFLNKLKTKRDRKMFV

>SG-PL_contig_7720_1_-3_3_173

CCPAGISSSVRNVGHPLMPARYAGRLFWLTSGLFCLNLKVFAVLLDYLCDLLHQRLV

>SG-PL_contig_7721_1_-3_3_203

CCPAGISPSVRNVGHPLMPARYARRLFWLMSGLFCLNLKIFAGLLDYLWDLLHTVWYRFK

TVSGFSN

>SG-PL_contig_7722_1_-1_298_756

MEKCFTKFEAQTSFGSIYKTRIEGNSMFCDYYNPQQKTYCKRLKVLCPEHTKERKVGPDE

VCGCPLVSNVFEEKTEYCRVSKRKCAKHFCWEKLRRAEIDLERLRQWMKLDELFEQERNV

RMAMSNRMGVLGLMLHQTIDHDPMTPMTPMKAM

>SG-PL_contig_7723_1_+2_26_253

MQYLKPQTDPRLGRKGLSKIKCTHTGMCTHDRTRHTLHETLIHRISSIWNLLQNYTLANY

PQLVLTVHISLPNVKR

>SG-PL_contig_7724_1_-3_18_1301

MAGAEEDSRKIGDRITEGKTKVVYELLECSTQVLLQSKDRITAGDGAKGHDIVGKAAIST

ATTIAIFELLNSAGVKTHYIAKHSATEFVARRCAMIPIEWVTRRIATGSFLKRHPGVVEG

FRFHPPKLETFYKDDANNDPQWSREQLLSRMANTPVGGKVIGPNEYEIMAQTTVTVFEIL

EKAWASLECTLVDMKIEFGVCSDTGEIVLADVIDNDSWRLWPKGDRRLMKDKQVYREMET

VTTDALEMVKQRYAWVAEQVPLLSAKSPCRVVVIMGSPADTAHSNKIRECCKTLGLDVEL

RIVSAHKATERALNMLHEYEANAIPTVFIAVAGRSNGLGPVLSGNATWPVINCPPLSGDW

GSHDVWSSLRLPSGLGCSMVMSPESAALNAAQILALTNHMIWAKLRARKLNTWIDLIKAD

QQICDEED

>SG-PL_contig_7725_1_+1_1_162

MSMSAGKGVLIQQHLVVLRKKRPQTLEILLNIFTFHSSLSYPNDESLPFQLTYS

>SG-PL_contig_7726_1_-3_3_209

AVSWAGRLTLIYLLLALGFITFITKAEDCCSNSIDQSIDSLPGVVNCSCYLCQMLDSKCM

INHRFGGEM

>SG-PL_contig_7727_1_-1_460_684

MCILWGLSCENFFSVCFGVFQKKTYSDLCCQLLRYDCFVILDAKLLMSTVVCLEMDFLPM

HCVVCCPDQWSVNVC

>SG-PL_contig_7728_1_-1_1_144

SVSHPESPVKVRGQGRQNATKSTSKLLLWGKTEIVKLLLICCVFQVGT

>SG-PL_contig_7729_1_-3_3_224

KTRIKSKLRETSVNKNFLPRAQLFFNVSVYLVFLFLFFLHLRLYCALKYQVLVYVCIASC

PPGLIVFQSQTATD

>SG-PL_contig_7730_1_-2_44_226

MNTLKTVCCRLWKNLQQDFLKMAEDLSVIFVSFQHRKNAEICESPKTVTGSWIQTKMADK

S

>SG-PL_contig_7731_1_-1_1_177

DQFDSNFRVCSFTTIMNTLKTVCCRLWKNLQQDFLKMAEDLSVIFVSFQHTKYVKVLRL

>SG-PL_contig_7732_1_+3_3_176

GNVLNGTYMQNDEFTTIKNVSPTKYKMYRLHKLSISSGGMSDDDLFFPDLSSALVQRH

>SG-PL_contig_7733_1_-3_1137_1511

MDLSYLKSHRTASRFLEFVDFWALSLIQLVKDDPTFYLLLSLQLFETCDIYNINKYGHSL

ISLSNKDNMIVLVSSVFTHFLAVWFFSAFLVCFLLRQIYFSLLVWSWQRSRDLFAGTIEL

SMCCE

>SG-PL_contig_7734_1_+3_2796_3038

MAECQQQLCDHWTLSFSMSHSASRVEVTGILSTGIITNITIQIFRSKKRFHNSFASHQCK

KYFTSKLETLFFIVTTITSAY

>SG-PL_contig_7735_1_-3_3_794

QIKINESDVLLCTVNNDWEGARKQFEKKFKKPVQKKETGNRKPAESSKPAVTTQKRTSDG

GDSSRPAKRGRGAANLTGRGGFHRHRSGVSRSGGSQGKGGALLMGRGAGRGAKRGSASGR

ALLPNPPMGAGDGLLPLPPPPPPPKTLMGGNDGLLPAPDSGMRPPSLLALPVKPTPKGLI

EGELLGPPEPDMRMVDLKEIRQELESRVLQTRAQLESHLSQLDQAYRATEPSKPHRPVDY

LEEEERRRDPPRRPLLPAEGRSLR

>SG-PL_contig_7736_1_-1_31_444

MNLLGVLYCGIPLLLVYGVEAAEKKLQIGIKKRVDPTDCTIKSRKGDTLHMHYTGKLEDG

TEFDSSLTRKPFVFTLGAGQVIKGWDQGLLGMCEGEKRKLVIPSEMGYGEQGAPPKIPGG

ATLVFEVELLQVERKEEL

>SG-PL_contig_7737_1_+2_2_196

KRLNRAFSPLCQSLLNVILFAAQPTTLDHCLHRYTFTNEDITNKATVEKCRKYKQMLNTH

IPYCL

>SG-PL_contig_7738_1_-1_313_1899

MNAEIVRIQQTENGLVDLEHLEEKLQEWSPKGLRMVGSFSAASNVTGILSNVDTISILLH

KYGAIALWDYATAAPYVRLDMNPVVAGDDKGLAYKDAIFVSPHKFVGGVGSPGLLIAKKK

LFLNNIPHDCGGGTVLLVRQTGHRYLQNLEEREEGGTPAIVGAIRAGLTFQLKDAITPEL

IMHREQQLFRAARESWGKVSNLVILGNLEVERLPIFSFLVHHPETGYYLHHNYVACVLND

LFGIQARGGCACAGPYAMDLLGISEEQAKDLEELLVTPAIPDKAKKRAPQQHFGDSVREI

FKPGFTRLNLPYFMSEEQFHFVLQAVTMVAEQGWKLLPQYKFNPRTGEWKHEGSQESKQQ

MHLDQLSYSKGYLEVQLPKPPGQDTLPHSPQDCLNQARRMFAEAKQNITTIPDQSHLFED

FARSSRWFLLPSEANEVLHAYSQIRSLGPTKPFICPKKTQSKQPALYPEVPGSNISSSNA

SLSSSPRTIATSLASSGDTPGHGKEKKSPLHVRKIKVSAYQNGGPKWKL

>SG-PL_contig_7739_1_+3_3_158

MRPLWCFCGHFLVPSILSVPPIYSVSHPSQVMWQTTAYYWSISGLFFRVDQL

>SG-PL_contig_7740_1_+3_222_545

MDPRWHLTMGSSEMTCSIPLWECFQSTRRVQERHRTDKQTTAEKIATSRHAPQTRKTTAN

VIKGQLRAATHNIRRQMKQRRLHDTGLHSRHLAVRSHSNPAHRAARKA

>SG-PL_contig_7741_1_-1_19_936

MEEEVWRFLLTGGVALENPHTNPAPEWLGEKSWGEIVRASNLKNLKGFMQHVQENIALWK

GFYDSPAPHSYKLPSPYDALSGLDRMVVLRTFRPDKVVPAVQDFIVDSLGQSYIEPPTFD

LAGSFSDSTCCAPLIFVLSPGADPMAALLKFGADSGYTGNRIQTISLGQGQGPIAAKMIE

KALVDGTWVVLQNCHLATSWMPKLEKMCEEVIVPEATHTDFRLWLTSYPSDEFPVSILQN

GVKMTNEPPKGLRANLLRSFLNDPISDPSFFNGCNKPQKFHKMLFGLCVFHAMVQERRKF

GPLGWN

>SG-PL_contig_7742_1_-1_49_2523

MVVVERPILTEFDFDQGFEKTKAPKRTPLAWAKKKASKCVCSCACLKAFLFQVFPFLRLL

RGYSLKSDLPSDIVAGLTVGVMHIPQGMAYGQLSTLPPVYGLYVSFFPVILYFFLGTSKH

VSIGTFAVVSLMVGSVVDKGLAAKGIALTAWNETAVVNGTLTSYLADNSAEVLQAKLAFA

MAVTFAVGCVQLLLGLLRLGFLTVYLSDPLISGFTTGAACHVFTSQIKHLFGLEIGRYYG

PFKLIYSYRDFFTNLPTTNSVTLIASLVCIVLLVIVKEYINNNPKVKDKLMMPVPVELVV

VVLGTVVSYFVKLEEAYDVKIVGDVPTGIPAPKIRQFTYIQDVVYDAIALGIVAFAISVS

MAKILAQKHEYDIDPNQELVAYGTINVTCSFFSSFCAAASLSRSLVQDNAGGKTQVAGLV

SSALLLVVLLVLGSYFKSLPNCVLAAIIVVALKGMFKQFAELRRLWTISIIDFMVWLTAF

LATVLLDVDLGLMVGILFALLTVIIRHQRPRACLLGQVPGTDLYKDIGFNKAAQQLEHIK

IFRFESALFFANSEHFKTSLYKMAADPTVLKKLKRKAEKELLKVQTCEPPAADTTAILQE

NTPEQEGEGRNTPSTEVTTAAGEKPATDCPTSIAIGGAGRSSASDYCGSETAESTTRSLP

GEDVAEVDDDDDVAAAVVFPTVTDVYFIIIDCTTMSYVDSMGVKVLQQVITELRSYSIQV

FFAHCKAAVRDMFEKTDFYKTSDKECLFLTVHDAVLYAQRLLAIVEGDERDGDRESSKTS

LESIQEHHSLSDSTMGALGTGTGTGTGREDTNVPTLLGGDECTKM

>SG-PL_contig_7743_1_-2_80_448

MSQQGAALQSYNNELVKCIEDLCSKRDELHRSILQEEEEKQKLQNDIRVLTERLSKVNES

LAKKMASRNEFDKTIAETEAAYMKILESSQTLLNVLKRESQSLKEKSYPTTTGGSTQRTL

TQS

>SG-PL_contig_7744_1_+1_1606_1893

MLVFRISTNCYCFQQRNFHLFCNEENSTCEAIVTKRTRTIKANITETILSTFLIAFNNHN

NLYNEKQSYTIIMKWSTVINHNNTGHSNTYSEHSSI

>SG-PL_contig_7745_1_-3_24_233

MKLQITYLPVNDSISLLKVLLLVKDAVCENQHHRLHIYLFTIPCVINDFTHCLMISVECT

TLNWPSYCLS

>SG-PL_contig_7746_1_-3_3_74

KIENEHLAEYLSLGHMAFSPWQEG

>SG-PL_contig_7747_1_+3_3_137

TGTVGIKNKYIDQEYKQEQLGLKTNKTKTLGKPKSPKNYLALITR

>SG-PL_contig_7748_1_-1_67_327

MGRRPARCYRYCKNKPYPKSRFCRGVPDPKIRIFDLGRKKARVDDFPLCIHLISDEYEQL

SSEALEAGRICANKYLVKHCGKDAFHL

>SG-PL_contig_7749_1_+3_3_188

KKKGSRAQNAWTQTFLPSKLLSHTSCHTIASYSQDVQIWLKHSLNRRLDGKFTKQGRITY

QW

>SG-PL_contig_7750_1_+3_3_233

KLGAQTFCWTHDHHFWEQLKDFSASMTFSQPLSEFSQTPVTFQTFKDLKMALHFSKAFKD

CANPDRKLPLHTCSLFS

>SG-PL_contig_7751_1_+2_2_220

DHFLSVSNLLAHRCKNTSQYAPSENLINFCKQRTNNTTPFSIPNIAAHEIGMYISKMSNK

KSSGLDEISPKIL

>SG-PL_contig_7752_1_+3_3_149

KWLILRQGADQNAVYVNVDLSIMKETGTTRHFCQVSSSHFCHHCLNLHQ

>SG-PL_contig_7753_1_+1_2032_2226

MASGVMSHNWLDLKKKNMKPASLSYQYADSSRACVKMCNKFLLFLIQSYNFNIQRFHTVT

ITETR

>SG-PL_contig_7754_1_-2_80_241

MRVFLFTYHSGFFYRNFASDNSFVVFTCAKCVLHTRSWFIVSSERLGPRLPFTV

>SG-PL_contig_7755_1_+2_125_250

MVRKNTVVHILYVNLCMMSLEIRTLLYTFQQYMSCRDEMKLV

>SG-PL_contig_7756_1_+2_1055_1339

MMTFVNKYTYILYESITVVFFPSTFIWHDATQSYVNNDCTSSMLTNAHFCGSYTDSPFHL

QSKGEFIFDMFSIMYALLSMCNADELHPCSVTVCM

>SG-PL_contig_7758_1_+1_1_204

KDQQTYLGVTFDKQLTWKQHIQNAEAKARRKLNIMRKLAGTQWGANEKILKNVYQGTVRP

HLEYGSSS

>SG-PL_contig_7759_1_+1_1_102

FFFNCSYFLLIYMTAKCAYKRKENDTGFTFSSTT

>SG-PL_contig_7760_1_-2_2_322

DPRPEWSQRRHRPPLQRRPSPRGPAEFQAKPRQAGPTLGPLASLPWSPAWTPGARNTSLA

RSAASRYTCFARLRGVTPSGGSSSPSRSSTRGTSQRPPPLAGGRPPV

>SG-PL_contig_7761_1_-3_3_311

PQRRLTRREFEDLRRWSRHGEGRIIIISCAHLLPLFCHHWLWSDHHTVVSCVLSCVAGVQ

IQSELLECPCLSVFVTVVFIVCWFVHEKHLCTELLSCYTNKWL

>SG-PL_contig_7762_1_-1_151_570

MEEGGSIGFCGYILYFLSIVVIVFTFPFSLCLCIKVVQEYERAVIFRLGRLLSGGAKGPG

LFFIIPCMDSYTKVDLRTVSFDVPPQEVLTRDSVTVAVDAVVYYRVQNATMSITNVEDAN

RSTRLLAATTLRNVLGTKNL

>SG-PL_contig_7763_1_-1_229_441

MLSQPSCSLTSPSPYSLTHKLVTVSLAIYFYCVHVCEPVREKDGKSVSLENVYLYLYYLK

KSYAHNCSCNC

>SG-PL_contig_7765_1_+3_258_410

MSNIPDKFSNTVCNSRSKIITYHPILNREKSIFHGSLKDIAYTVLKSTACL

>SG-PL_contig_7766_1_-2_80_1558

MSSYKQEHEQFVSGHNGTTVTEVAMMTITPCASLLLRDVLLLVFGLQNMPFWPRIIFDFS

LILVPAILMNTVLSHMTTTSCIILFAAAAACCLLTLLHTNRTNINKSLSCVMAMEMEPQR

PFLNCTRSLMNLSTAIVILGVDFTVFPRRFAKTETFGTGYMDIGVGCFVVSNALVSAEAR

GKSHLCQSAKVFVQRVVKSLQSSLPLLVFGFGRLLAVKGTDYHEHVSEYGVHWNFFFTLA

IVKLLSTVVLCLCPVQLSSALATAVMSVYQYTLSNKGLTDYILHGTDGTGSRQNLIDANR

EGILSCFGYMSLYLIGVHIGSLLFSKNVQTFRDWVKKLWFLLIISSASYLCQSLSSSLIQ

PTSRRMANLTFVFWMVGLSVQVLWSCLAVDLISTALSALVFTHRKKADVPKTESQMVSGL

KLRSGVPCLMHAVNTNGLFYFLLSNVLTGFINMNIPTLYVSAATAVSILLAYTFFMSVVI

VYMDRNKIYTRFW

>SG-PL_contig_7767_1_+1_1_252

LTITYSGNIFSKHTNSFCRNVKSNITDRFTFSRNQKSTSTTDLLSQLILYNEKIKIGNDM

IKFTGWMKRGFIALHIILMVMGHL

>SG-PL_contig_7768_1_+3_699_1403

MILIPRLTVNPQLSNLSIHVVQSPVAHTGYLMAVKRKQPEKSGSIPPQTYKKQCPETETA

TTLHPTTTQAISGLSKKQNKQKQKTAMSPVFNHFTPHKQDYAMSVTSKGFILMMMLRLLR

CFILQEYSIFYKPTVNTGAINLDKSNNQKLMSSAKLHLFHSKTRHLNNETSWSNFNCSPK

SKYSQSQIDVLCNATHLLHAKTRYLYSVTSWSDIQPLTQFKVFSMIIFTSVMEIQ

>SG-PL_contig_7769_1_+1_946_1551

MNFMRDFSLPLYFHCSSLFLLLSTTCCPLRSLYCLFYCSHLSLYHLFYCPPCFLYHLSWC

PSVLSIICPSALCFLYHLSWCPLRSLYHLSWCPLFSLSSVLVSPLFFICSIFPFVFSSFC

SAVPSVLSIIYPSALWFLYHLSSCPLCSTIPSVLSIIYPAVPSVLSSVLFSPLLSSSSVL

LSPLFSSSSVLLSPLFSSSSVL

>SG-PL_contig_7770_1_+1_55_267

MLLALSSALPNLTMLLLFFGFCTGFLFIAEVVTVLCFKIIESTAPCYLSDLLHLYTPSRQ

LCSSSDDRVFR

>SG-PL_contig_7771_1_-3_3_86

FTVTKQNGRYMLVQIILQQLWSLLSFDT

>SG-PL_contig_7772_1_-3_228_461

MIGDHCPSDYCLSRVGIFSHAHCRRCFSIFRDVTCACAKTESLTHACVACDWLCVLTSFV

TACNWNRCVIILALPQIL

>SG-PL_contig_7773_1_+2_116_253

MPRSPSYSATPPPTNMTMRTRIGSTSNSRTWSTRLHPMTCLWSWET

>SG-PL_contig_7774_1_-1_1_129

FFFKITIQFEALSSQCCVHAFIDIDSNVFLEDTKFLCSFFHRH

>SG-PL_contig_7775_1_+1_220_357

MRNNASVYSFAVFFSMSFSILKCLPKTNQNKPYKSIFFHHCSHATH

>SG-PL_contig_7776_1_-1_28_678

MDWYCATFSLMLLSFTECVVIAWVYGVNRFYKDIELMIGYQPGMYWKVLWCFITPATILF

IWFFSVSQLAPVTYGDYQYPTWAIVFGWMLGLCSLLPLPIVMLISIIQEKGTLWDRVKKL

SRPADNWGPALPENREAYLASLEPHRRPKAAFEIPSPIYSPKKVVTSSGASVNVLMPEER

KLLSDSVTTVSDRSVVGQSTEFTSMLSDGQTPPVSKC

>SG-PL_contig_7777_1_-3_327_593

MLFVYVIRSTRHCDQDRKTGNYLISLVISFLLNRRLPLNCKCIVLLRTTKIYGLYCPEPF

AVPCAPGHSKTYRWHCMEHVASLSVHVSG

>SG-PL_contig_7778_1_+3_3_143

KKISWAYIRLYLLSNLQRCTNVDGSCPHGCLHTICKKFGPLFLFLFF

>SG-PL_contig_7779_1_+3_3_77

TYILTTWLVCTNPDKLSYRPITLAG

>SG-PL_contig_7780_1_-3_114_1151

MSGKVPPKKRDGGADGVAELEQQFVLRLPEIAAEELRQEMKSSSGALFKDKLGLSIEIDN

DMRHGHVRYNNYIFNAKVVDLPCVIESLKTVDKKTFYKTADICQMMVCTTDEEVNVEDTE

SPKKKDKDKRYLWNHGLTPSLKNVRKRRFRKTLKKKYMDQPELEKEVRRLFRYDAEAIDV

KWEVLTEDEKPLGSDAGQTSGHIQSTSALTNTGGTLRGSDPSISMDVAEFFGELSSSEEE

DDKDVNIMDSGEDEMSRLGDSSMQAAESQDAMAAEGGDETDLQDKLQELVQQLEEIRDKR

LELESQMAEEDNDMMIDRIKEDLERFLQEESEKEKEYEILSAMLNQ

>SG-PL_contig_7781_1_+3_18_230

MIFNYYPFPTYRNDCAEHRTIKAFVINIYYYILLVKSKEWTILEELLGLLSPHTHPFNLR

PLLHSGHCCRL

>SG-PL_contig_7782_1_-2_2_268

PPFRTVFGPIPVHQGGEGIGSLGQSRGHSAPGLPGRLAPPGSVPDPDHDPYLQDAGTVFL

PRVCDQPEVRSLPQSVIRLLRDEIRHAVH

>SG-PL_contig_7783_1_-1_151_213

MALSNSLTDNMFNRGGFVVVV

>SG-PL_contig_7784_1_-1_1_156

VSLSLLLENKDQSVNHFYSFSGEGVKVLNAHPFYHPLPCFNSTGIFVAIFVG

>SG-PL_contig_7785_1_-2_284_709

MPTFQFHSGNACDTPTLQFYSGNTREMPTFQFHSGNAHDTPTFHVHSGNVCITTFQFHSG

NACITTFQFHSGNMRDTPTFQFHSGNTRDTCIPCCCLYVGIFSPQPAPFLQLLRFLLVYF

TDFIVFFGVCFLFVGLFLLFSF

>SG-PL_contig_7786_1_-1_772_1029

MQSCTLAYSDMHSYKMKHTYKCTSMCACVLTPTHIHSHTLTFKMTWRGGRMVKTLDFQTG

GYGRPGFEPRAPRLVGKGWRFFRSPR

>SG-PL_contig_7787_1_+1_169_297

MSALSKTIPQETGLYALGLDHFSVLFSRVSFLYFSSFFMCVCV

>SG-PL_contig_7788_1_-2_86_229

MLLSYPAHLAQETQIQHSDFCPVGHFRSCCLLQGHFGVRMLVFGEPFH

>SG-PL_contig_7789_1_+2_20_310

MTKYFISFKHSKHSPFIFPKTIKREKKNLEHFSFYLKLSENVIGKLIFFFPPPTRFLSVN

TTETNTYFFVSSNTKKRENPHTPRAANSLAHINRNSI

>SG-PL_contig_7791_1_-2_2_106

YYYYDILFIAQPTIKVISGLSPRKFHQTKSTRQPR

>SG-PL_contig_7792_1_+1_130_267

MHERKDTQTHVSNLCMSGETHSRPTQTNPKVYKLNRRKQKYLYSTK

>SG-PL_contig_7793_1_-2_224_325

MQKLHAQLESWRFHLAIFSMLRNSVEKKIGKEQT

>SG-PL_contig_7794_1_-2_2_118

SEIVNTGMGTRCPFCSVICLYGLFNIFLWLRSIFFIQIF

>SG-PL_contig_7795_1_+2_395_490

MGSLSQLPVWLVYLPCQTSANRSEERRVGKEC

>SG-PL_contig_7796_1_+2_2_280

VCPLWPAHRAIWTCSVCLLWPAHRAIWTCSVCLLWPAHRAIWTCSVHPKPKQFHRTPVLF

YILDIFSDWILNLCSFPFNLIYFHGFTLNSFLS

>SG-PL_contig_7797_1_-3_3_236

TTVRPQVHLDEKIACTAVFLVPGHVGAILRLQRRHMLMIFTSFSSHFGVFCDLFCGLEIC

VTSCFLIFALFHADFFKW

>SG-PL_contig_7798_1_-3_3_203

SGVSLSPPPATHKEESLLKAVSEVTHRRRLGTTTGSFLWCQDTTTTHPADDSKESFVHFN

FYFLLRM

>SG-PL_contig_7799_1_-1_247_378

MARLLRNLGGGSWMSVNMTAVAYTIWRIYAGASHLSRNIGKLRE

>SG-PL_contig_7800_1_-2_2_403

IVWQKILLYSLGEIIVILYFIFCVVWEKLLSCCILCCLGEIIVILLGRNYCHFVWEKLLS

YCWGEIIAILFGRNYCHIVWRNYCNTVGEKLLSYCWGEIIVILFRRKYCHIVQEKILSYC

LGEIIVMYFRFCIV

>SG-PL_contig_7801_1_-2_176_346

MMITSCFYRERERERERERERERELFAYLFACLFLFHLLSKGSIFFQNTSLANILTM

>SG-PL_contig_7802_1_+3_3_206

GIPMGVTSVGYRFAKVSCVGYTFAKVSISKLRKAAYFSKFTDTHVILKQVFDDRTHGYSD

ILSKHSSA

>SG-PL_contig_7803_1_-2_464_997

MSPRDTVGSTWSTGPGQLHTGASSAGILDPGPQLDEMSPSQECRWPIVPPSADVLVWCAH

LHHYQLGSTLIADPLKTCGTRGVPLSLCAIVAPPQEFLHALSRDADKGTCLPPGRFCLVA

YLIPPPPYPCAERGWLFVQGYFRPQVGEAPSAQDSPQRAFQQKAPLRAILEVACWLFR

>SG-PL_contig_7804_1_+2_263_412

MCTLSLVMSNIFPFMTNFLKTTTLKNHRLARMKGMLCHRSRLRPGQSLCN

>SG-PL_contig_7805_1_-3_678_929

MYFCVSTSSSDAIKWKKSEVIFLSVVVFRKFVMNGKMFITGGRYTCIMHMYIRVKNWIKL

GLFFIKLSYTLDEFVCKNFLFLLR

>SG-PL_contig_7806_1_-1_199_3489

MKVEWFRDGEPLVHGTRYMPAYDFGFVSLDIHWVNSEDSGVYTCKATNDYGEDTTSATVI

CKALRAIIMDTQLPGEGATRLQEMEEHWRSQMVLDERYVEEPQTRSPPVFDLKPEPVETG

EGEPAKFLVKVGGYPRPRVSWWINGSLVVSGTRFKLTYDGMMHHLEIPRVREYDAGQIRV

VAKNPQGEAECCTSLLVMPREDWRARLRQAPKAEMEIELERRRKIELRSVELEKAFKKPK

ATEFELHQLERMAEDKVKFQKDEEVITTERQYISVHSQLRHTEPSLAAQPVYVKQVTMEM

TQPHKDVTKMAEQQITLERGQPPVFTKPLRPVRVPEGSGVTLEVNYTGLPPPIISWYRDN

FEIHPSRDFQISTTATTSTLHIPEVFLEDSGMFSVKAYNKFGQVQCKAKLTVVEGEEPER

EKSPEFRSLMRDSRSTQGEPATFDCQVTGIPPPIVHWEKDGIPLGESPRWKFIDEAENHT

LVIYEVRPEDAGHYACVAINSVGKSTCTARLDVEIPTQPLEFLPSEPEVVSSTHAPRVEE

EPRTMEVEEGEPVRFTCRITGKPAPVVTWYHNNQLVKPSKYFQMSSTTEGLHTLSIMEAF

PEDTGTYKCVARNKMGEVACITFLKVHGVESEPEPSHPMALAQPPSFIRPVANTMVPEGT

PARLDAVFSGHPTPEITWIRNGIQTLHDSRDHKIETQDSMTTLTIFSALPEDTGLITCRA

RNQAGTAECSAELYVQRSEEVEKLAQQLHVTVAQRAPPEFVKPLQPVLDVEPGQPVRLEA

EMSSVPVNRVTWYVNGIEIPPSDRTRIVQEGRTIVLIIINVGVQDTGEYTLKVQNDLGEV

TCKTTLTVKGREKFVEIPDTETTETITVTRTVTTIEREEQVVTQMVKPQVTTPLEPEIFV

RERGIARLECQIQSHPPPLVTWFVNGMEIKPSPHYEIRYEEGKSTLLIIEVGPEDTGEYT

CRAVSELGEAVSSTTLYVQEPLQEEMPQLQDTIQMVEPSPMMEAMVAPRFLEPLPSLQAV

DGQEIKLSCRVSGVPMPRISFFHDSKNIDEDEEFVITYDSETGEINLIIVEVFPEDQGQY

ICVAENPAGQATTSSYL

>SG-PL_contig_7807_1_-3_204_1097

MPSSSARCSQLPRAIDKVPFYEALRRLRDYMVGRIPHLDESEVTFPTLELKTAVKKEDPG

LTAENGEITSSSVTGNQNGGGGGKRGATSQSGSGAQVTVKKEATARGSGKGRGKRKGHQQ

EQNEDAFTVGETSGENQQVTTQADHGSDQQIAEGSNRDHPLMTKSYQGQQSIPQVAAASH

LAHQAHSNLLQHTTGLMPSTLPFNPFSQVDFTSLCPTGSSSSHLFQDPSLPITMANNLPD

SMSQDFMSSFYSGSHQFGMVGVPSVVASQQHNTSTEGNHTEKEPVKIKSEPVDPDPQH

>SG-PL_contig_7808_1_-3_96_491

MASSAVLRLSGTFTSSLKQVFSRQVASVTVPSRVAFTQHKEVEPCSLVPVREQSTEAAKG

VVKRKPYSPFQTNNNAEYALARVDDLLNWARKSSIWPLTFGLACCAVEMMHFAAPRYDMD

RFGVVFRASPRQ

>SG-PL_contig_7809_1_-2_137_370

MHSYGQCSDLCEEHHDDFTTLCATRCKDATFDSYCKSQCFVNEFRTQYYAELPPLTSHDV

GVGQGIPFFENSEEAGDD

>SG-PL_contig_7810_1_-3_3_161

RRGCLGGELNPGPFLWKSGVYPLDYRARLHLTPPLPYAYDQLDFYHLWEQRLC

>SG-PL_contig_7811_1_+1_1_198

CTKKEKKNGWRRTMATCFPRGERSAFHATKFRCHTHTHTHTHTRRRHTIESNQSINRTEQ

KQGMNS

>SG-PL_contig_7812_1_-1_1_126

KGGKSADWLADGGAPVSVLICLGPKEHKKEQHFTFIFDMIFH

>SG-PL_contig_7813_1_-1_1_105

FDMIFNKLLDKKKRSIFTHLMKFESLPIATVAILD

>SG-PL_contig_7814_1_-3_3_275

IVSFCPSHSDHCIILSQSFRSLYRSVPVIQVIVLFCPIEAIVSFCPSHSGNCIVLSQSFR

SLYRSVPVIQIIVSFCPSRSDNCIVTALIWP

>SG-PL_contig_7815_1_-1_79_225

MSVFCAVSIVFVYFMSFYLFFILIEILKSKPRFRSHPQPSLRRKRVTDG

>SG-PL_contig_7816_1_-2_56_277

MNAKTNCVIVIFGEYFIVSLSKLHTFVLCSFFFPLNTFILDKMYTIDVFGPVHSYWRLID

LQKVCCVFLSHSSV

>SG-PL_contig_7817_1_-1_4339_4620

MYVHFNIWVHSGIFVPRDVYCSFVGHQFDLFHFIFVSGFAVFAVLFWIGCYMLLGFAVWD

LILKVKKIYKVQRPELRDQQLDDLAGDFILSGVM

>SG-PL_contig_7818_1_+1_1_246

LRDRKNLHPLPTRQRCLGGELNTGPSVWKSSVYPLDYCTRIIMIIIKSLLETILIIIIVI

KSLLETTLSVNIVLTCTTVQIL

>SG-PL_contig_7819_1_-1_997_1311

MLFVTYERRPAHVQLFSYFITSHRSRIKFIRCIFDFLVHLNFPFSQYQIIKLKKDKKTTT

KTCSLDKLKSTVNKEKKDGAIKVACIKLQNLKVVSFNLQSQIGWE

>SG-PL_contig_7820_1_-1_1_255

KATTAFNQLSKIWNSKKISLNLKLRFYNSNVLSTLLYGCETWHLKTSQEKKLDSFDLKCL

RKILGIKWTDFVTNEEVRSRSKQNP

>SG-PL_contig_7821_1_+1_22_234

MDQEDRKKQTTVKERKTAETKRAIEQRKLLAQNFQKVGILFILKEPPASPRGKDRRIVKG

KMHHLCKYPMN

>SG-PL_contig_7822_1_+1_34_270

MCVCVCVCVCARACVHSCAFAFKHVCCLGAHPYMLKSLSILVYISACAVCMYMIHECVDV

CISVHTNIPKCAFMTLLFF

>SG-PL_contig_7823_1_-3_51_686

MSYAYLFKYIIIGDTGVGKSCLLLQFTDKRFQPVHDLTIGVEFGARMITIDGKQIKLQIW

DTAGQESFRSITRSYYRGAAGALLVYDITRRDTFNHLTTWLEDARQHSNSNMVIMLIGNK

SDLEARREVKKEEGEAFAREHGLIFMETSAKTAANVEEAFINTAKEIYQKIQDGVFDINN

EANGIKIGPQHSPANQNMPSGGGGANQGSNCC

>SG-PL_contig_7824_1_+1_1_207

AKHDSVTITLSYLDYEARPQGGNTSSIGSYCTHTHCDKRCETMTRKENDEVDDDARKTKT

SWVHCRQDS

>SG-PL_contig_7825_1_-2_2_169

RVKGLNSPGLPSEQYSQVSHSVLILPKALCGSPQLLSRNSHTCMHTLPPPFLFWYL

>SG-PL_contig_7826_1_-3_198_359

MVKATNTLAHMHCTHAHTHTHTHAYIFLSSEMKGETEVFLIFTKGNFIMFLLCC

>SG-PL_contig_7827_1_-3_3405_3680

MLKVAIAFCLLTDVHEKMCSTRIFLLLFLGGLFSPLVCLMFFLFLFFLCVCNFLLCCCCF

GSDLFSLSHFFFSLVFISSRIVQEQVVFIFIV

>SG-PL_contig_7828_1_-1_1_174

LICWIRFCTCIHNSRDLYVLTRCNFLYIYIAFAFDASIHFIITTSAVWKKSYFFSNVN

>SG-PL_contig_7829_1_-3_354_542

MRGSPLSNFFTFLIRTLTSGWCDGVLQPGYDTGHPSIWQPCLVMLHLSFQEPVLRSLRCP

VPQ

>SG-PL_contig_7830_1_-1_1_201

WKDHENAWQTHHQVMEPPVKLRTVSAVADTHHSPTTNCQLTTTSSQPATTSSHITTTSSQ

SATASSW

>SG-PL_contig_7831_1_+2_2_274

SHSTQFIIPRLQKNVKKNKNKKHTTSKYKKKIHKKGHIGKLHRRMGQYPSQCPQSHHPVA

RAKQHRQCIINCGGRGRENCVNTCWKKRKWN

>SG-PL_contig_7832_1_-1_301_480

MDRLRNSYSVYMCTLHTMIMDRQTRKQLQCVHVYSTNSDTWIHRQMDRLRNSYSVYMCTL

>SG-PL_contig_7833_1_-1_1_666

CSKCCGKHVKRELDERNHHGNPGKTVHSSTSTPPVSRTITVPPEFVFVRTPLELQGQYCK

AAGCSLFGDPNHAGMCHAHYEQQRDSLARAVHSVPAAKHRNVIKIPYASEVRISPTSMTF

PPGGGVLRRTETTKGGVFPPQQVPVPPPPYSTGYGEPARSRELSLGETFYRVARRHMGEC

LNPTCTNYGNSSNGGLCNGCAQDQRVTDDVIDRAIYGLEETG

>SG-PL_contig_7834_1_-1_322_465

MVIDFPQNASFIVYEILKSINNLVLFGIIVEGTMLVISNKDGMRSFWQ

>SG-PL_contig_7835_1_+1_274_540

MKTKKTKQPPSDDKQITFTKTQNKILKTSIKVLLSVKRFFISPRHSLNYMPSLFIIHTQT

LAEFRSYPLPPFTKIKYEGKMKEKLKKTK

>SG-PL_contig_7836_1_+3_3_275

ICDSLYRIICDPLYHIICNSLYHLRLTLPYHIFYILFTAQPTTTAISGLSPCKVHQSQQD

NRVEHKTSSNLKTIILQKPQFMTYYLNHQVP

>SG-PL_contig_7837_1_-3_69_719

MANREQPKGMDRSLHSKVMAKYDQGAEYEVRQWIKQLTGEDVGSGAVEVEKKLRNGQILC

RLAKAVYEGTSNLPSAARGFKVRVNTMSAPFKQMENIEVFLKAAENYGVPSNSLFPTADL

FEGRNMAMVISTILQFGSEAQRHGFNGPTCGAKPSEKHEVHFTEEQLRAGQGIIGLQAGT

NKCASQSGMKMGASRHIADIRTDDMSKEGQGIIGLQA

>SG-PL_contig_7838_1_+3_339_599

MKGLRYLIYSRGNREERKGWAEEGGVEVRTKKTKFEPTKLLFLLCLFTTKKLIHDMFGNL

YTFIKYAQSINITRRSVNCLRMEVSSR

>SG-PL_contig_7839_1_+3_1194_1562

MAFIPASLLGSNTFIFFGVFVLVVFVVVVVVVVVVLFWFWFFVCLFVLLSHLLRRFSGLV

PRRSFRLLQTPPNPNSFRRNLSVRKPVEASDVELPQGYPPEEPLHRCWVISAEFNSACSD

PAY

>SG-PL_contig_7840_1_-1_61_1008

MPKKKLTKAEKEKLKKEEQEKKEQEEEEERARLAEEEKRKKKEEKLLAVERQKLENEERK

QRKEDMTELLEMLSNNRNTLQARDDQRRKDAKWARYMRCDGSPDPTNQGEINTYINLKIE

NHERDDADNVLKDSQLDLALIDELEFLLTDTPLEDLPHVERQRYRETIGELQHLIQAKLD

AASLQVLCDASKLQDQETSNLQYTLKNDTISLSG-PLNLSKNPRIKMFEFTESGFAFDIPR

VLTLSSCAMRYLHTKFDHFSPYSKAYYPRRKKKEEPVAPEPEVPPEEEKKPEEAVEEEEK

KEGEDEAENVDKEDTM

>SG-PL_contig_7841_1_+1_664_786

MKEDRSRELLFFSLTHTFKQQKSINVWSQLVSLRAQTISSL

>SG-PL_contig_7842_1_+3_177_272

MKIPSTFTPEKEYDGLNCGVTYQRQSMQTVQQ

>SG-PL_contig_7843_1_-2_53_232

MKLQLNQTTASITTATTTINITERERERERERERERSMCVCACARARVSVCVCLSELGCV

>SG-PL_contig_7844_1_-2_20_1276

MSLAAVVGGVPSLKKLQGASNDDWVDRLNHVWTVFLFAMFAIIVSTGQFVGDPIHCWCPA

EFTGAYVDYAKSYCWIKNTYYVAMEEAIPLSLLDRQNEEITYYQWVPLILLFQAFMFKFP

NILWRLFNGGSGVNLDKVVELAEKTQLGSPQDREKTISHIAIYLDRWLETHREYHWNVLV

RAKQKMAKICCFFCNKRAGTYLTAFYLTTKVLYAANVVAQFFILNAFLATDYNLYGFEVL

SKMGQDDAWKESRRFPRVTLCDFNIRQLQNVQPFTVQCVLPINLFNEKIFIFLWFWLVLV

AFLTSLNMMAWFYRVLFKRNRAVYIKKYLKINNELQTGFDKKLCAKFAEQYLRDDGVFVM

RVIAKNSTDLVVTDLVDKMWKLYKMKTQPQPVEGGQPASSDKEREAIANDKEREALTGQ

>SG-PL_contig_7845_1_-3_3_347

LNPLRPHCCLLATVVLPFPPAPSVVFPLENRSTFQPVSCETRQHQQSQDWAEAGTVHTVH

AGAHPGPAAGWTCGKKSQECFLLARAIFLFKFVSRHLLSSFTGINSLCLFFSSHM

>SG-PL_contig_7846_1_-3_309_437

MLMPISVTQCNIKINLCLCYCYFQKTEKSNKQNSTPIYPGYKI

>SG-PL_contig_7847_1_+2_383_622

MYICHTVHGRIHGKDVSIPEKLTLLSERTPENNVRISIMNGICQHQYENNTKYMCAKNVR

IKAFLDSPPSPCCTFPNHTG

>SG-PL_contig_7848_1_-3_3_161

THTHTHTHSHTRARTHTHTHTHTHCDQQFHLNGMTCLFWFVYPFNICSLDKRT

>SG-PL_contig_7849_1_+3_3_203

FTTEQVLARLGLLSGLDPDSDDVMQTSLGKVILSLTVTQKVLNLCQVSIFSSKNQNLKKH

FPITIVK

>SG-PL_contig_7850_1_-3_156_332

MTKTFGDLDRFFQEPCCQETGANLSFGGISVAYQLLTCVHHMRNRCSQFGLVHFGIKFG

>SG-PL_contig_7851_1_+3_822_1004

MNKQPPKNAYLNMPTFPNSVTQNNYHASSKLGLHIMSDLWYTFGKDPPYPLTNKILACHF

V

>SG-PL_contig_7852_1_-3_3_137

QSQQQNHLIMFINCDNDQSQQQNHLIMFINCDNEIFIQHSSPEYQ

>SG-PL_contig_7853_1_-3_6_758

MAAPVINVPDSGTTVAEKLCELVIAKANAAIKEKGIFVIGVSGGSAANFLCNGLPSANTD

WGKWRIFFCDERHVSYDDAECTYTVYKKDLVSKVPALADNIFPIDPTVSVEEAAKKYLQD

LRKVLPGDGIPRFDVLVLGMGPDGHTCSLFPGHPLTEEQTQLVAPISDSPKPPPARVTLT

FPVINNAACAVFAAAGAGKADIVQRVLEGGEENPLPAAMVRPTNGEVIWILDPAAASKLK

TATKSTNQTCD

>SG-PL_contig_7854_1_-3_3_245

TAQTVHHKLRWGRGTKGRNQQSNRKDEISSTKFPEGGDNIHTERAPSIPTRGKEPCNIFF

STNIACRAITQFLLILPQAKL

>SG-PL_contig_7855_1_+2_101_310

MVKASTFRHNETRYRAHNRILSKHKSWFTFSSIWKSKFPSKCHVHCCLKTLHVHDLSKHH

YSTTMYGKGG

>SG-PL_contig_7856_1_+3_171_389

MLCLSPTHDVISTIQNGKMKQRRLTTTHAQHPPITCLHTPSPPQLPSIHSSSPLNLKWDS

NTVKQQYNKNRQN

>SG-PL_contig_7857_1_-3_342_3890

MKNSATFLCVLLCTVQCTFSTRIHHRHPDDISDSHDYVSQDHTDPEDVSFKERTKMPPAH

YELAYAEQEENGFDSESSDNSHDQNKVHKNKEESPWNSSLFKWMADPSLTHLPLAYDSPH

GKLEDDEIHNEPGSPQPHRLPDLDDEFPESQTSSEHSPQPLTLSPESEDREDLSNEDGRF

YSQQQRQAHQSSHRGRHHRRSQRPRFGAQEAQGRAVGNRRDEDRNHHSTDVVSNSGNNPS

PGHQRKRRKQHKNRANRLKTRKGLRSRSAPQEGRQPIISLPHSGRQIVYKLPSETPSNRQ

PSPILHGASARRTLPVSREFVSLSTLANGNNDRRPKKQRRNFKFDSKLRRKSKRRRDRRR

DRPRNPRSELSPDPTVLPLLSRSFTHQKNLGGLPDNPQPFGKQEIVLMPIGEDPGKTAFF

QELSEVSPQSLSQELGQVDPVTLPEEESQRHQGPQGSGENEAGCSCSAQSDVCHQKEMDT

DVLTQTFQENLAVLKQVLAEALDRVITDPTQTLSRDLCDVIDAASEMTSQRPNRQGIIGQ

KSKGRKADHYFPEHELEQGAEGGGAEQPEERASGRNIHRNRPPNRNSDLNRLSDRRNTDL

NRHPNRNKDLNRESNRHANLNRHTDRHSGDPNRHPGRDEHHPERSHTDPSNDKGEHEDSS

DEAHDLRDLSEEKDSSSNLTVATNVPQSLTNHRGSQLGPGGIGQEEYDPNTAKSGQGRHD

QRTQDREQHHTPISGQDKIDQNEPRSGQNRENQNARGIHEELLSPSNIRAPQAGLERQGA

GSVDRDLETDADAREESGSDGGRRERENEDSDNSDDNDNDDDDHDDEDDDAEDVDNYDDG

NDLGSEEYDEEEDAEKVWKLWKQQQQQRRQLEESQRADDTEALPDCELGQWTDWSGPLGF

GVIERRRFVVAGDENCKMPESQGELVMRVDISVFNDKHTYYKAPEEVGQDFEADFTRSYV

PRDLLLVLDSSGSIEEEDFELMRAGVDVMVRLFCGGFGSLDTNNRLAILLFASDMHLIHR

FSDDQSPETLRDVIANLTQPNGNTCTGDALQFAHDVIFSSELGSRPDVVHDTLLVTDGHS

NCGEITVQEGARLLQDVSNVFALGVGVANDTAARWELNSVVSENDPRHIFSLARFQDFKD

MLDSIEQRQQTEPCLPILDVTSAPDSARAGGGENEAVDRLGGR

>SG-PL_contig_7858_1_-3_510_641

MSFSIQHRSLLYFVGLIVFAVAFFSSFVNDILFSHSCIREPFLP

>SG-PL_contig_7859_1_-2_2_121

LWGFCFFLCFNISYLLCMFCMLSRCSEHVPNVYIFSVLSM

>SG-PL_contig_7860_1_+2_158_430

MSQTHKDHKGFAPDIQTAKSVTQRSQWFCPRHTSSTVSCLRHTKIAISRSHNDHKSFALG

IQHSQSHKDHKSFAPDIHTAQSVTQRSHVDV

>SG-PL_contig_7861_1_-1_190_1197

MPPRKKAAAKGKAAAEEETTTPQKETKKRGRAKKEDKEENGSEEATSGTSEPPPKLAKKV

SEALASMDFSSAAKTEDGKPWNLKIASWNINGVRAWLNKDGLAYLNQEKPDVLCIQETKC

SKEKMPPELKVDGYKAHWVSAEKEGYAGTGMYTKTDPISVTYGIGIAKHDNEGRVITAEF

EKFYLVTSYVPNSGQGLVRLKYRTEEWDKDFTAYLKGLDAKKPVILCGDLNVAHLDIDLK

NPKGNKRNAGFTKEEREGFGKLLENGFIDSFRTLYPDKEEAYTFWSNFRQAREKNIGWRL

DYFVMSERLKKNLCDSVIRPQVMGSDHCPIVLLLAL

>SG-PL_contig_7862_1_-1_496_759

MWPTWLNNHLSTYSITMERCPQHSSFPCFLLHVTVKQKVRNKQTESKQNQIILICKSVMG

KKKYQKWIILPLLSMTDEDIGESIRDKT

>SG-PL_contig_7863_1_-1_73_411

MPETLRSVVAKFYDEYMQSTPKKLKVVDAYLFYILLTGVVQFGYCCLVGTYPFNSFLAGF

ISTVGSFVLGVCLRLQSNPQNKNDFTGISPERSFADFIFAHIILHLVVVNFIG

>SG-PL_contig_7864_1_-2_380_628

MGTCPSSRPSCKIVPMGPNVTILEKSKYLKFEVSKDIDGRAVREREVGTALTMLTPRETE

VSNTSLPNKHSGYGGEGGGEGDT

>SG-PL_contig_7865_1_-3_162_482

MLEGAAASGTWHRDPALNHLLKVVDMAKSINADPQVLEGLIKVLEYANLREDYTVNCSSE

ESAICKQVVETTFSTNWSELHEQGKTSINLRPEMTSGRLEGQFLKSL

>SG-PL_contig_7866_1_+2_2_232

YFQTIHILILSVDLSIQCKRCLHTPPRFHCVCARVMRVCMHMSVCMPKCACVCVPHVSVF

MCKRVHSIVTLTFATLF

>SG-PL_contig_7867_1_+2_1442_1750

MKAVWYMVFPQIPTQSQLHNRTYITTEIKLAEKSQTFKVCCLLVFFFLCFVLFLFLFLLL

FSPYMAAVFKVSKQDLTAWTPDYQSACISRFACICFTEQCRFR

>SG-PL_contig_7868_1_+1_142_417

MTVTTPDKYMSFFSIISYKKCSFQPDISAVYFINIYHTYMLGNIEVSYFICFSERCTRIT

LMNTADIIRKMTFFLLSRVIIPHCNISELIVT

>SG-PL_contig_7869_1_-3_54_500

MKTALIAFVAIGTAFILLDTGIAIKCFVCNSFHQADCADWFDNVTQHLVKCPDEYSKCRK

VVQEVWLGDHWDVRYIRQCALDGQIGMYEGRICQERYGSYDVRMRYCHCDNQDGCNSAPS

LIPTTGFGQFLPFVVTLIAAGMPYILVQR

>SG-PL_contig_7870_1_+1_1_171

MLHVHTRAHTHTHTHTHTHTHTHMHTLIKSSRKQNQEEMYLFYQSCFLPLYAYINSK

>SG-PL_contig_7871_1_-1_106_327

MCVLITCLFCLCVVNGWRRGKRHSPSKLPARVCLHECESLPVTWPWSKALSVNDTSEMLR

PTSNCCYALQCYVG

>SG-PL_contig_7872_1_-1_1_192

SCLVCLIECLYVYCHICLVLSCLVCLPLMKQRQALISCQLIFVKRSHKTRNTNSPNLLHK

QQMR

>SG-PL_contig_7873_1_+1_208_354

MHHEYTSMIIYQTTHHVRTSCDHLCHLPNTKLWLSIFLKLYLYTPYTHL

>SG-PL_contig_7874_1_+3_111_323

MVGVSYCRHCSLLGRWLHDLVVSLPGIFMVFPNFLFLCSFRHGVLIAGTKIQFALRSASF

CLLSSFLFLFI

>SG-PL_contig_7875_1_+2_167_394

MLGRWLHDLVVRLPGIFRVFPNFPFLCSFRHGVLITGTKIQFALRSASFCPLSSLLFFFT

FHVPRATIVKRFATVD

>SG-PL_contig_7876_1_-3_48_260

MNNNLFWKHIFTAYNTFCRNVKSTSPIDLLSEPILYNEKIKIGNMIKFIGWIEKGVYCIA

HFLNGNGSFMT

>SG-PL_contig_7877_1_+3_105_329

MAQYMKPKQYTDESVHVSSQSVHAKLCDSTRPASVGTHDSIVPQDSLSLPTSFCRKTMIT

ASCRKLVDRLRESKG

>SG-PL_contig_7878_1_+3_3_149

FFFSYFTSSLLKHKKSIKQWSRSQSMICFRDRHQHAQSESVKPYKLSFF

>SG-PL_contig_7879_1_-1_271_540

MIRQEREKGRGDRKKGRNGTKCVTEKRKCLALKFRKVGTLLILKVPPASPRILQSVLSHH

YEATHLRRLYWCWITKMQLMGTVLIHTKWF

>SG-PL_contig_7880_1_-3_174_278

MEQRIILKCVLSDQVYSQCGYVCMLVEIWFTYFLQ

>SG-PL_contig_7881_1_+3_273_491

MCNLQYLEYAQIFPTPKSVIPGSSYIFRENTALSVSLPFLLILTGREAGHFNMRQSLHGR

CSPKIQINGALTK

>SG-PL_contig_7882_1_+1_1_207

IHKHTAIRNKIASFHNTNIHALFIQASKCVSIQISTHFHAFKAGKLSTFSNTPPFKHTPL

QYILQQNSF

>SG-PL_contig_7883_1_-1_91_267

MYTSNFKYVKSDLFQGFLNFTSRAILAVWILYLALPLHQVLLECKIDATPLLSHHLIIH

>SG-PL_contig_7884_1_+1_106_231

MPSRSAADTGKKCHCIKYKQNQWNVLWGISVNNGLFIHFSNI

>SG-PL_contig_7885_1_-2_2_85

FKTNMIFSLQSLFPVKVTLRYENVDYKQ

>SG-PL_contig_7886_1_+1_1_270

GGQRVEIFPISQVKICVDLLVPEPPSCVYTRRTTSNMHIKDPVSHVSVRWLMETQTHQHA

LKKKEKKMGGAALRRRTLPGTAARISHGKF

>SG-PL_contig_7887_1_+2_143_310

MVNSVIPSSAPCTKIYLSNSTLFTQTNTEKSLSTSLRVVGQMAVSKWSKRVLMGLC

>SG-PL_contig_7888_1_-1_1_99

IIKIIVIKELLSKSREKRSAFLNNVSDMKVSRK

>SG-PL_contig_7889_1_-1_274_3366

MASNDEKANNQVENKEEATHLVQKLRPVMPRRKRKGKEELDELKQELEMDEHKVPLVEIY

ARLGTDPNTGLTVERAKDILLRDGPNQLTPPKTTPEWVKFCRNLFGGFSLLLWIGSILCF

IAYSIEASTYEDPAGDNLYLGIVLVTVVVVTGIFSYYQEAKSSRIMDSFKNLVPQVALAI

RDGQKVNLKAEELVVGDVIECKFGDRVPADIRIITAHSFKVDNSSLTGESEPQSRTPEFT

HDNPLETRNLAFFSTNAVEGTCRGLVVRTGDDTVMGRIANLASGLEVGETPIAKEIRHFI

HIITGVAVVLGVSFFIIAFILGYFWLDAVIFLIGVIVANVPEGLLATVTVCLTLTAKRMA

RKNCLVKNLESVETLGSTSTICSDKTGTLTQNRMTVAHMWFDGRIMEADTHEDQSNASYS

QSDPTWTALSRIAMLCNRAEFREQQSNVPILKRECNGDASESALLKCVELSIGNVTEFRK

RNKKICEIPFNSTNKYQVSVHETEDPNDPRYLLVMKGAPERIMDRCTSVLLHGKEQPIDE

HFREAFNTAYLELGGLGERVLGFCDYFLPSEEFPPGFNFDAEGPNFPLSGLRFVGLMSMI

DPPRAAVPDAVGKCRSAGIKVIMVTGDHPITAKAIARGVGIISEGSRTIEDVAADRGVPV

GEVDPKDAEAAVVHGGDLKSMTPAQIDEILKTHSEIVFARTSPQQKLIIVEGCQRQGQIV

AVTGDGVNDSPALKKADIGVAMGIAGSDVSKQAADMILLDDNFASIVTGVEEGRLIFDNL

KKSIAYTLTSNIPEISPFLMFILADIPLPLGVITILCIDLGTDMVPAISLAYEQAENDIM

KRQPRNPFKDKLVNERLISMAYGQIGMIQALAGFFTYFVIMGESGFYPRRLLGLRKNWDS

RGINDLPDSYGQEWTYSQRKKLEYTCHTAFFVSIVIVQWADLIICKTRRLSLFQQGMKNH

RLTFGLFFETALAAFLCYCPGLDKGLRMQPLRFSWWFTAVPFSLAIFVYDECRKLILRRN

PGGFVERETYY

>SG-PL_contig_7890_1_+1_304_429

MVTIQHSVTIPNEMKTSLPLFPVWYNAVSNACEQIFYPNTVP

>SG-PL_contig_7891_1_-1_172_570

MKWLLSFSLVAVWVKVSAGVACEDVTTCTPDISRMQADSAEDLARLCKSVQDAIQCFRDA

MQDCRHHGSIPEETIKTLERQIDSYDNMLGQHCSGSGTSDGNRGGQSPYNQGVSVKSSFL

VTVILTLLAVGIL

>SG-PL_contig_7892_1_+3_108_299

MCAYYLDFLFFPPLGRGGGHKPITFNCVESLTFCLFVCLFSTCVLVHDLGLLFFFAQTSY

IQPV

>SG-PL_contig_7893_1_-1_91_240

MLHCRTVGAKGVKAVVNFSEHDFAGMVNRAYGSIILALCGISFVWEWYYK

>SG-PL_contig_7894_1_-3_963_1172

MYPNISPLSEAWYCYFLFQYCHKVVNTFFSSKCIFSYGNMYLLLRPDWHRCDGPSFPSDC

TDLLKCESCI

>SG-PL_contig_7895_1_-2_2_136

VLVAEDVRATSIEQAAEGQSATATHSIRRGHRLRTRDMPSETTCS

>SG-PL_contig_7896_1_-1_586_903

MLQPCTHFSYADVQVFLHKFGHTAALLSFEALFRSHFGLSVLFPLFPVLNSCFHRRIVEY

EAALIPATFFTSVAIFIASSPSCSLANMYNVSECLLKDSILKYIEM

>SG-PL_contig_7897_1_-1_271_408

MVQSSFSFQLSMPVQPFEKCPSIISFGINEKIYKNQKIKCFLFWVH

>SG-PL_contig_7898_1_-1_43_204

MRPMISLCSDAAIANVSCMCYSCVIMNLFYTLPWKVNIDCSRSFATWDFLVNLV

>SG-PL_contig_7899_1_+3_3_170

TFTGIFPNSLPTQSLRCLLNIRKSTHCLSACYMPVRLFLPFFFSTRCIYFFPDSFS

>SG-PL_contig_7900_1_-3_99_503

MRTKVGYTGGTTPNPTYRNLGDHTESVQLEFNPEIISYEDLLWMFWKIHDPTTTHKRQYM

SAIFHHDDEQRHLAEKTQSELQKTMARPIVTVITQAGTFYDAEDYHQKYMLRQERELLAA

LKLSDAELIRSTTAA

>SG-PL_contig_7901_1_-1_865_1062

MMWSIVNSYRLTIYDRKFVESNKKVYDRKFVESNKKVYGRVQQVAVTISATTAEKHSVSS

ESVQAL

>SG-PL_contig_7902_1_-2_77_1144

MRSGAENLYRATGNKKLKEQVAVELSFFNSNIQLLKEELSEMNSSVEVYQHDNCAKYTPM

IPLGLKETKELDFSVPFKDVIMEHYSELSETYDKEIKDLNDLRQAIRTPERNYAGIELLT

EYFNQLYFVEKRFFRPNRQLPLHFHWYDSLTGVPSTQKAIGFEKGSILFNIGSLYTQMGC

KRDRTLKEGVQSAREDFQRAAGAFKYLESHFRNAPSMDMQSDTLSMLSVLLLAQAQECVL

EERLMMWGKDGVVTNARLAREAAMVSEKYRDTHRKMSSEVVKSYLPFPWISMAQTKHYFY

KGLAHYYVAMALLEQKDSGDTEKLRAMFESLHLGVEPHDENNGLKLPKTQEERKVL

>SG-PL_contig_7903_1_+3_942_1247

MDLCSCYCPCIRIWKLVCSYCSVNGIMQLYYYYCTIMQFIIKIRNVQLMIFTVLQSMVTV

VVLQCLSCSDVQFLSFSTLCITSKHYGIHVILPISCLCAAHY

>SG-PL_contig_7904_1_+3_3_212

RGPVCSKHLVHVKDPNATKELSLAKVEEKSTFIDKKPAYRGKKEEKVVRYYGDVLSSGRA

TRILCRKIPL

>SG-PL_contig_7905_1_-1_1129_1713

MKYRIQTLRWAVKKSKSIAMSSSPSIYVLLSFLRSGCLPKKSFVWSRQRTYVASLLGVQM

KELPGNDFLRPLWSTNRIYIKRNFDFLMSEGFSVQDIFSCLVLLAHDPEHLEIYYHKLWE

RPEVVPHAKSVAWTEKRQTLSLLQYVMERDMNFSSAVFHADVELGQASAESEKESHDENS

SREEETCEISVDEDD

>SG-PL_contig_7906_1_+2_3149_3385

MIKVVSRPKMHEMNTHRSNCKHASNECNKFTPFRTRSQTNIFTPSHALSISTAHHHLHAF

LLAQPNFCPPNVILETMFI

>SG-PL_contig_7907_1_+2_2_127

NKQTKTNGEKLTNNQRRHQFFLYFGKHHPFNDCKFSMLTSHL

>SG-PL_contig_7908_1_-2_2_199

ERSEFQHFKYHNRKACTLTCLMKTTLQYPFQTENHKNFFESRQVLLSLALPSPFSLPKSS

FAGKTS

>SG-PL_contig_7909_1_-2_143_247

MTPCCLCTKNFGFIRTQLLGKTRDISIYDQATVKG

>SG-PL_contig_7910_1_-1_1_228

QGLQIRNQQQQSALRGREKTASQQLSVKLIGQKKWVRGQRLVSTIVKWLVCYSLLLFIQS

RVHSLASFTGMQSCHF

>SG-PL_contig_7911_1_-2_2_493

TIRKLKQHLESVYPSKPKHHQQKLIYSGQLLADHLTLKEVLRQYDEQNSRHTVHLVCSPS

VEILSHPGTSQSPSPSEKKSEQHASTSQSSVSSSESDGLRHRGAQASGSTPSSSEVPNTG

VNPAMAVPGAAAMGGMPGMAGMGGMPMMGPVAPGMGMAGYSYTP

>SG-PL_contig_7912_1_-1_28_294

MGCQTLLTFDCRADTSLTAVKVSDNAKRDQKASGGGTKSAQTDATNRSKVSTVRQEGKPF

APLKPVADQGLVGPGGSRRAEVVEVADKP

>SG-PL_contig_7913_1_-3_3_392

HLITPSSWALDSISHLITPSSLNSISHLITPSSLNSISHLITPNSLALNSISHLITPNSL

ALDSVSRLITPNSLALDSVIHLCSESSRPQRTSQNCRDVVDVLLTVIWNLRVVQAACQRY

RLCAVPMPTI

>SG-PL_contig_7914_1_+2_530_802

MEKLDKKENHHLLQHVQILIISQRNNYFCSKQINGIKMNITTTTKNNDTTTTTTKSFLYS

AILQTNLDSLHITHLQDKACDTYRSTHITHT

>SG-PL_contig_7915_1_-1_85_612

MIGGSGIRAWGGSDRDYTYDELLSRVFDIMREKNPEMIAGDKKRFVMRPPQVLRVGTRKT

SFANFADICRLLHRQPKHVLAFLLAELGTSGSVDGNNQLLIRGKYTQKQIENVLRRYIKE

YVTCHTCRSRDTILQKDTRLFFLQCETCGSRCSVASIKTGFQAVTGKRAAIRAKTA

>SG-PL_contig_7916_1_-2_407_592

MTCEDLRRVETRPISTTLKENVWSAENDNLPQGQRKRLFTSLYSASSRSSSNPITSVVVG

TV

>SG-PL_contig_7917_1_+2_2_148

NPCPARNQIRISGILGECLSTRPHTPLQVHVGMTQRPFSRGGKHRHDDW

>SG-PL_contig_7918_1_+3_378_680

MRVVHISSSLLYLPHFQRVGIHSQLGGLETACMKFFLGEYQTRANLCGQIGRGHCTSMPT

RQNGMANIKDWTDFSILELLVAASQRQRWRSSSEFTFGFRQ

>SG-PL_contig_7919_1_+1_1_171

RLTAGCSRPTAGCSRLIAGCCDVTAGCSKLTAGCSGVTADCSRQTAGSSRWTASCSN

>SG-PL_contig_7920_1_-1_169_768

MDSVRSGPFGQIFRPDNFVFGQSGAGNNWAKGHYTEGAELVDSVLDVVRKEAESCDCLQG

FQLTHSLGGGTGSGMGTLLISKIREEYPDRIMNTFSVVPSPKVSDTVVEPYNATLSVHQL

VENTDETYCIDNEALYDICFRTLKLTTPTYGDLNHLVSATMSGVTTCLRFPGQLNADLRK

LAVNMVPFPRLHFFMPGFAP

>SG-PL_contig_7921_1_-3_411_851

MKICLHYFKRHCYYTCTCSNPPAVDLHLNVILLVHPSVCIFILCPDSRLLEYTCEYTTST

HSVSFVCIAESVLKDVYTKHVMSYEDWLSCQLLACTILQKKKKKEGKKKGKTGKRKKKSI

SVLWVSTRICLTKDVEMHCNNHFQQSN

>SG-PL_contig_7922_1_+1_205_336

MDCLNALHLCKNVKQSVVFQSRLNFHSLNAVSCISIMGKVFVMS

>SG-PL_contig_7923_1_-2_2_571

DVEGHAVYVGELSPAAVWKFQRSSRTPSTTTTAAQKAAGHSSPVAQSTDKQNKAAPSGKG

KEEVEKTSVAAKEEKGHREAVSSGLVKETKVEEVKEEDSSDFTPSIIIGVLLVIPVVLLL

VITLVMRWHHRGWDSCFSSMRTSKVFNLQNFLGNSHRGFDKLSQEDSDHDADVLSDSDNE

EFRQLMKSKA

>SG-PL_contig_7924_1_-1_1_180

AMDRMNNQVMMASGVGASPAMCSMASSSSVSNMLLKTGQEEGKSGVSLFTPDTAMPMDVS

>SG-PL_contig_7925_1_+2_2_523

THTLPVHTHTYTHTVVISTYTHTHTHTHTQLSLVYTHIHTQSHLTHTHTHTHTHSHHQYK

PQISVYTYTLCHYDPLTTHSHHDYTPQIRAYLALYIIQCVIIITHTHAHTHTHTHTQCVM

MTHRQQPSHHERLNTHLKLHTMIRKGQTLIALSTTNIIIIIITIIIIISHLLLC

>SG-PL_contig_7926_1_+2_2_238

IGSPSSSRLTSAQPLIGRRPHRNPCKRKILKWKNCCTATIPLSCSQKPTFIYQYIYTHII

CIVLLHFGARLYYIHHGLE

>SG-PL_contig_7927_1_+2_2_109

YSSIHINIYTHIRKYLYTFLFIHVLFLHNEWVFMTG

>SG-PL_contig_7928_1_-2_74_229

MSSVKKKLCASFFLVSITYKINDENLIFCYLGNFITLFRKFRNISNICMKVS

>SG-PL_contig_7929_1_-3_21_965

MASGGRSRTSRVCCPEHPDAPLVEDYHAGDLICPECGLVVGDRVIDVGSEWRTFNNEKST

KDNSRVGAAENPLLDGGELSTMIAAGPGTECRDEFGKPMYRNRRSLNSSDRTLMNAFHEV

NQMADRLQLPKMIADRASTLFKQVHEGKALKGRSNDAIASACMYIACRQEGVPRTFKEIC

AVSKISKKEIGRVFKLILKNLETNVEVITTGDFMSRFCSNLSLPSTVQKAATHIARKAVE

MDLLPGRSPISVAAAAIYMASQASEEKKSQREIGEIAGVAEVTIRQAYKSMYPRAPDLFP

TDFKFAIPIENLPTQ

>SG-PL_contig_7930_1_-3_39_218

MRVCVCVCVCVCVGVWIGERMCCLFIFFFFNLKYYYRVDLLTLDSVAAVVGFSSLPTRIE

>SG-PL_contig_7931_1_+1_1_120

SLEITDARKHMVLVSQVFIHTQFPSSKPINITNSYSQHVT

>SG-PL_contig_7932_1_-2_695_862

MCRLYGMLFVFNLFCLIYGLYVIGLYLFKLYDEMSFCIKLAIFYLPFMSDILNLFK

>SG-PL_contig_7933_1_+1_1_255

EVEGSGSSGRRRVPGPPAPAGSAPSHAPWPAAAVLGGPRGGVSLGGGSRRLVGPWLVKKR

QPRCEPKPSRRAAHPVIQWSQGQAS

>SG-PL_contig_7934_1_-2_149_268

MGHTLRKLTGKEWNLDHLTRLRSEGYLSMTGLLHSRRTME

>SG-PL_contig_7935_1_+2_341_703

MHQYFANLLALPKNQKTRNNKYPSFIHHQDHNPQTNMNIINTNQQNTLKIHKRIKKIHNI

NIFNLNVNCLERAMCMLFEVPTGLGLATSPANIWNEREWFSSGRSWLPHCKHVPLENVDT

Y

>SG-PL_contig_7936_1_-3_3_155

LLHMYRMSHSDVKLYIFISVLHLILCIYYNLLKSCGKISFLQCLHNIDRIG

>SG-PL_contig_7937_1_+3_3_131

LQHTHTHIQQVQGYNTNSIHYTQIIMTFTIMIIPYKNLFVFLN

>SG-PL_contig_7938_1_-2_491_2260

MMDERYRDSLQRHNRLLMTHILLTDDIIQLMANEKILPETMIKDIQDGNTKEERNERLLN

ALRLRGKKSYKRFRELMYRIGNFFVADLLWEEEDGQGMIDEGELAMKFPGLFNCVQEAQK

KKLVNYLDAKIREKALLMAWRERPEERIEALSAKVIDFNQERGLRDTIAAQERKIKDLEM

ELKTEDEKRRNLRLELSSLQQQLTAMKRDHRSQLDTQSRFHDATITTITGLKNKLSSCKT

HVAELNGLVKSALDEGGGGDNQDELAGMEDEDDDVRMPVLERNVRQLIQQFYFEKGQAKN

GRNQREEVLKLLNRGYTKEPLVTVVKEVLKHEQKHRAMVMRDILRLSDMLKQVVPSSTLA

PPAPVAVAPDGLTLTSPIDLSTLDTKHFKSQLAMLKAEVEHIQKRFQWKDSESMNLRHEL

FALKQRFQDYKIISSVAGQPTSRPNPALAPYTQHICPSCAKSIPQPLNSQTASLPNDQTV

NGQNPDASTSLQNHAGGLCQEDLGAEWVYEESGSRRSSMGKSDLDKMMVNFEEYLKNEQD

LESPIKESSPKMASTKVETKGGDSNPKSKRSIKNTSALYKAFAKKKASQR

>SG-PL_contig_7939_1_+1_1_246

FLFSESVTVYYDCCNINGDKKLDINKQCNKTEMHTHTTDSGLNNSNLHNIHHHYHCYRLS

LPLSVSTGQRGRCFTNPFSPSP

>SG-PL_contig_7940_1_+2_29_259

MGTQQGSHRCDLTKFHTFPRPLPDKNENFPYQTQSHSESKILRKMVHLNFYGRFQTRHTK

HHKSSHCEGWTGPRLFC

>SG-PL_contig_7941_1_+1_1_162

THTCPFFDFQRKGKERRVCDFGMRTYTKGDHCTLNNSSATPLTKEIGRNSNINV

>SG-PL_contig_7942_1_+2_62_202

MTNSTSTKLYNNKQAETTNSTNTKLQNNRQKRQTVQTQKLQTIQTKS

>SG-PL_contig_7943_1_-2_179_274

MFIYVMTCGISNIKSVLMYGLETDPDLYKPIS

>SG-PL_contig_7944_1_-3_3294_3596

MKIYNLIVVTTVSCFNQVGKMRILSCYFLCCSNAVNSRTVRSLMTIQLFVCQPVIPPGFL

TGLSWRGRHDAGCEPTMSAFFSSVWPKEFPEGLLVRYHSTV

>SG-PL_contig_7945_1_+2_2_265

FFLLFFLFLFRFVFFSFFFLHFTQRLRHSITIICTKTKITGTHAWSLHGLLFCVFLMCTD

TMPQHSQPHQASEHSHTQINTLINNHVF

>SG-PL_contig_7946_1_-2_26_289

MPTTILHFTVTVLHSGGRGSASFFQKISVRSFFSCRLGMESHRIRHDMNTGPKSIVDTRY

NDHMATQRSQVFRSGKSSSRQSDDPEAG

>SG-PL_contig_7947_1_-1_1_144

DDRSGWRQAVRDGVKRGEERRNQQLEDRRQQRKQRQLKQALDPPSNLQ

>SG-PL_contig_7948_1_-2_2_91

CSSDLHPADNVCYSYYLTKRISVCHSWDWT

>SG-PL_contig_7949_1_+1_520_714

MLFPLKSCTSFSFPLAFLSQIQHTDAQMLQVCSVTFEECYIQTRLCHRNSYIMYAKKNQT

HQIIL

>SG-PL_contig_7950_1_-3_3_224

PTTSQSVVRFANHFATLTGSFSFVCLFYFLFNFSNLINAPGFCVCRPMRIYIPCRTARRS

CKTDPVGLNASILG

>SG-PL_contig_7951_1_+2_197_286

MTLGITDSTVTRATTAKIVFYHDEMTAINK

>SG-PL_contig_7952_1_-1_79_198

MKKSCNSRVSPRLGTKGRLVNIDWSLSLRGGTLDTILTCI

>SG-PL_contig_7953_1_+2_2_487

YHHYHHQYRRRRRHQQDNDYHYRHLCLCRHHHHHHRRRQHHHHHHHHHHHHQHDNHDHYH

QQHDSHYHYHHHHHRPPPPHHHHQHDNHYHYHRRRQHHYYHHRRRQHHYHQHDNHDHYHQ

QHDSHYHHHRRRQYHYHHHHQHDNHDHYHHQQHDNHHYYHHH

>SG-PL_contig_7954_1_+3_300_719

MASSHCATDLEGWCVAKQLLWSASTFPRGQVSQASVHVPSGKTVELFSASNPVLLSQARF

KLFLLSSNTFDFYHSFSLVSIQFSLSFLVPISDLRVCFFNNSCQMQTICFDIYHRCATHI

GFALKICTIDIFSSFCFQVS

>SG-PL_contig_7955_1_-3_174_281

MGTALIMLASRKTKVSTTLLPYDLRIMGEPFFFSFF

>SG-PL_contig_7956_1_-2_80_370

MNSEPWALNNAELLQQIGLLVWTTKGDFHMAEQLAKLRVGYELYQRLSGEREMEALRNLT

ILNISKWFQAHPNASKEEAIKEVAKQITQFAELVEKM

>SG-PL_contig_7957_1_-1_31_1092

MNLINTWLEKSQRFASIVQEIQKLPECGCLTLQHHMLEPIQRVPRYELLLKDYLKHLPED

SPDRPDAQAALDLVTKAASHSNEAMKKIEKFHLLLDIYQRLRGVAVDFISPTRDFVREGP

ITKISARSGEKQSRYLFLFNDLLLICSEPLVGAYRVRAQLDVDGMEVKQGDNLDIPDTFL

VHSRQKAIELLEEQPSSGGTSWHELLKQVIEDLRSRKVSFSTLNSPTEQEMTPGKQAPRW

IPDDAVTMCMLCEQAFNTFRRRHHCRACGKLICKSCSKKAPLEYMNNRLERVCLECHDAI

VFAGKGTVQSPGKTAGKKKILQVKADDPGLLAGYLSISDDAGRTWNKRWFSLHE

>SG-PL_contig_7958_1_+1_67_450

MQSIGLPEKILSSINYILFTFIWKKKNCNKKAFEKVKRKVLTQDYNEGGLKMINVQSFQN

ALYLGWIPKLASQSQNKWKIFPELSYSQLGEGLNILHTPCEWKDLTGYPETEGHFWKKVL

QNWLTIKE

>SG-PL_contig_7959_1_-3_9_269

MGEIAWKESFFTYVSVHTAYIPCAKSLMYHSVKVLLFWCCGEEYIFFLFRFSEMVSAIHV

VWVLSISVLVTGVLSHFLITPFYFLVS

>SG-PL_contig_7960_1_+3_591_1019

MVNRMQFSLLAQNCVSSRFKNTVLTHRLDSAAIPFFFLASGRRRTKGTLWVFGAASPCLS

FHSTHSSLSFLRAHGRLLCVCCILLCPCLILRKWDKKKSLLHYKTPVRDIFLYSFVNYFR

LFVFFLLVFPFFFFYFCFFVFVF

>SG-PL_contig_7961_1_+1_256_522

MYTCNRHSAIMYNWTMKVFMYNRTIREDGVCTQAQYQQKDCTQKKWEGLNVHTKVRTKPY

GTVYADFYSCQSAGINTIYIIAQTVHTQH

>SG-PL_contig_7962_1_-1_34_402

MKTAKKTCELDPIPTPLLYENLDTLLPVVTTIFNASLSSGSVPTDFKTAIVKPLLKKASL

NPNELKNFRPISNLPFLSKILEKVVLHQLSQHLLMNNLLSDHQSAYRLGHSTETALIRIL

NDI

>SG-PL_contig_7963_1_-2_59_1099

MPRKLEFHVVHASGEDDVHKAGNLNHHGPLAKGWQASRFCLYPQDIVVQMEGRSRVRKIQ

ILSHQFLIPTKIEFFVGDVPEGTPMLLENARYTRLGYVSLSDNEKTGYKARELKSVHVDA

LGTFLRLNIHKNHINKHNLYNQVGIVAINVIGDSIRKPSDVTDPDMFIDVNDNRTTEKDP

VLEGFFNKPDYISPLDDLAFDMYQDPEIAQIIRKLERKKQEAVLQERYDYAKRLKVAITE

LQKVGEKLGKMEVEKRQAVENEDYDKAKVKKLQMDEFRLQIYKDINVHDLLETSGARHPV

TVDLDTHRQPTPPRLAEIEQRSSPAALLAVSPGPLMPYDERPLPAMK

>SG-PL_contig_7964_1_-3_96_551

MDSVKKALQACNTVLEMDPENIDALIDRAEAHITNEDYDKAVSDYQKANEIEPDSRRIKE

GLNKAQKLQKQANKKDYYKILGVKRTARKKEILKAYRKLAMKWHPDKYEGDDKEKAEKMF

MDIAAAKEVLTDPEMRKRFDQGEDPLDPEQQQ

>SG-PL_contig_7965_1_+3_243_332

MLIIGFVAHMNAMLATRQKIDCGKNQVTSR

>SG-PL_contig_7966_1_-1_13_423

MAAAPCLPTYPVWRPGDPPHTSSWTAPNVCSRLCNVVQHVQASQRDSFNLHLHSFPLKTI

SHPHPRPSSPEILIQDVNLSILLLFIQRTSAVLHAFCPGWERLLAPQWQRFQPEIFDVQT

RPFWFSETAPQRGEKFF

>SG-PL_contig_7967_1_+3_507_902

MAVGKRITLDNLFSLSTDNSKVYNTTHKLEDPQLIVSSPSCRQNCSLTRVNLYDTHTHTH

TEARTQALNLSRMKGGLETWLKGTWSNAAAIHVLAWLSSLCEQRTHMHSITFSPSPDCFD

TDIVLVCKLHVI

>SG-PL_contig_7968_1_-2_2_97

VTQKRKRMTEIILVLHRTCYAFSQLDMIRDTE

>SG-PL_contig_7969_1_-2_2_97

TCKSPLVSCLHVGVVGHKHRRRRHTLKSFKII

>SG-PL_contig_7970_1_+2_230_421

MPLDHLIFLSAFEFNTSTHRNMTEQEGQKRSTVLLSIFTPAIKVISLPDLGGKEDAENID

ARIK

>SG-PL_contig_7971_1_-2_284_427

MNVCHKLADTSVLSSSSSSSSSCPLAHSSLVVFLQFVQAVLLFIVDVN

>SG-PL_contig_7972_1_+3_66_251

MFCTLDSTQILSRESQGLPMERNQIQYSENHTGYFPSLLPGEQDTWLMHSQSTELEFKIG

CC

>SG-PL_contig_7973_1_-3_849_1055

MLSIHFSSLQLFIISLNCQAGHCQTWFEMLVCENKQIKVGLFLSLFCEGKVWICLIRFKG

IGFIYCKHF

>SG-PL_contig_7974_1_+1_25_312

MRTATRPQPSLSRPLPPHFRGHIVAEDIATIRDRLSEETGPMADDKHRAYRRARLHLDSL

MESMGSQASLCTQSQPVSSPNLTQVARIDAPPRDAV

>SG-PL_contig_7975_1_+2_2_151

LNKTGNQPVHNTRLYNKHSMLTCLLCFTHVHDQTFLTLKNRMKPCKTCNR

>SG-PL_contig_7976_1_-1_1_354

LSLSLSLSLSLSLSLSHTHTHTHTLTHNHTHTHNNTHTHNHAHTHTHTHTHTHTHPSIPH

PHLNNYGSQLHFKSSFTHICFVSVFCWVVFCLFWLVGFFGGSSLVTGILVHTNFLCQY

>SG-PL_contig_7977_1_-1_1_210

TDSYRLIHTHARTHTHRRAHTHTGARTRTRAHTHARTRTHARIHTRARIHTRARTLTRAR

TLTCARTRTR

>SG-PL_contig_7978_1_+1_1_183

DMLCISYFSFNLSLFQHNSSSKLIQMTYKVHVWVFRQINYVSAKISWQFKGGPFTKCGSQ

V

>SG-PL_contig_7979_1_+3_3_137

FVFPLALLFQQTGKIISTDRQHKMKETKYMPNTIFTNTWYNRMES

>SG-PL_contig_7980_1_-3_48_317

MSLGMLIEVYVIEKLSHYKKKMRHYRLKVIFNSATIELSAGSDHSMWDCTKHTSTHAHHT

HTFVESETWAICDVFHSPLFSEKGWPSVKM

>SG-PL_contig_7981_1_-1_37_5358

MKPSETAALITILGVVISVFVDTVSSAACEGCTSTICICAGDKGERGAAGLPGLRGPQGL

PGFLGPEGPVGALGMKGGPGEPGARGAKGYRGETGLPGFTGVAGIPGQLGLEGPVGPPGY

PGCNGTKGLAGLAGQPGIPGEPGQPGRDGFPGPKGEPGSFVTDGLTIGEKGGQGDRGLNG

IDGLAGIEGSAGEHGPDGEDGIPGLDGIPGLKGMKGDKGADGIGIPGPKGQKGMKGELGV

AGITGTPPSNSTRLTGEKGEKGERGDKGEEGDRGLPGPDGPIGEAGVGGMKGDKGMAGPP

GRRGKRGKNGPPGPFGQKGMVGEKGFPGVPGVDGLKGITGDAGYDGQRGVQGPPGVDGEF

LESGGSGSQGPRGRDGQPGPKGPRGPDGIDGEIGREGLPGLPGLPGPPGFSGEYGIPGDS

MKGDRGLAGLPGQDGVDGLAGFPGLPGEAGEKGQPGEDVTGPTGDRGLPGLDGLPGLPGL

PGDPGLDGQKGEPGIGVRFPGPAGFPGLPGEIGEPGFPGTDGLPGQPGLQGFPGDDCGVC

PNGVPGAAGDPGDPGFAGADGFAGAVGFLGQKGEPGELGRQGEQGREGQPGIPGRPGPDG

LFGSKGFKGDQLPPPDIELYRGAKGMKGNIGYPGMPGMDGPQGDTGTRGLPGLYGPQGIK

GEPGQPGDDGLDGLPGLPGESGRKGQPGQSLVGPMGRKGQGGLPGLEGIPGPRGDKGLQG

DPFIVQLTQGDPGEPGLPGLDGLPGMDGIPGEPGQLGDDGFNGLKGMRGEPGLPGLPGEQ

GSNGPKGYPGLMGDPGFPGLDGRKGEVGPDGLPGLPGRPGLTGRKGETPRFAPEGEPGEP

GGPGEFGFPGNDGIPGEAGRPGADGLPGQKGEPGLAGLPGYPGEAGRLGDDGLPGLTGER

GRNGEPGAPGLSGLRGVPGDDGLPGIPGEQGGIGEDGAPGLPGLPGQQGLDGLPGNRGLD

GLPGPSGPKGETGLTGRPGSPGVPGLEGLPGRPGEAGIEGEPGEAGEPGIPGEVGSPGFD

GVSGLPGLKGREGVPGRPGADGIRGERGQDGLPGLAGIPGEDGRPGLPGFKGDRGQSIAG

EPGRPGSMGFDGLPGLDGVTGEKGQPGPMGIPGLAGLPGDQGDSGIPGEDGRMGLPGVKG

MSGQDGFPGLPGEPGGPGVQGPPGLSGRPGERGSVGLDGLPGRDGPPGENGEDGRPGLPA

LPGLPGLQGDDGPPGQDGIPGEPGQLGQDGLPGLPGERGEQGEPGFDGRPGFDGEIGRTG

EPGPVGRPGENGAPAPPGRPGPPGEEGIPGLPGLPGQRGFMGEDGFNGRSGPKGQKGEPV

PTTRGPQGFPGEPGVPGLSGRQGLKGMDGLSGLPGLNGMKGETGRPGPSGLPGRPGEDGI

RGEDGVPGLGGLPGLPGIDGREGLPGLPGIKGFKGEPGFPGYQGGGGAKGSRGQQGPPGR

PLPIQDIPRIKGEPGLPGPVGFDAPPGPRGESGNDGAPGLSGLFGQDGEPGLSGLPGRPG

ESGVPGRQGTSGLPGLPGEPGRDGEVGYPGVAGTGPGIFFTRHSQTSAPPECPIGTVRMW

EGYSLLYVMGNGRASGQDLGEAGSCLPRFSTMPFMFCNINNVCSVASRSDYSYWLSTPEP

MTPMMQPVEGRQIQPYISRCSVCETPSPIIAVHSQTMTIPDCPLGWSGVWIGYSFVVGTG

AGHQGSGQGLNSPGSCLEDFRSSPFIECHGRGSCNYYATSSSYWMATIERSDQFRRPLQE

TLKAGDLRRRISRCQVCIKDMSTGPAFFTYASTK

>SG-PL_contig_7982_1_+2_623_976

MSQGNEVAETSTFHGTSMIRCQNPLHCCFTFSNSHQGQVPIHSWVDWRLHAYNISHSRLH

PAQPDCKVEALPLCQPGCQRELKINVYSIRLTEVIDCHTTTSKCKNGSSKRNFKIYCI

>SG-PL_contig_7983_1_-1_304_519

MFYFHPIYMYPSTIHPDSYLHILSVTLSVLYIYAHLSTCDVCKHVFNPDRCEYMHVLFSV

QPSAVLCCYCFC

>SG-PL_contig_7984_1_-3_3_212

NKVILCDLGRFSSVLFFFIHIFKKTELPCKEEMNLCRFICVYNMCMYWSVKLCVNSVGIT

GTCYSNLTMI

>SG-PL_contig_7985_1_+1_181_510

MCTLSKIITKTIYFIRENKMCTLSKRITQKLCNLSKKITQKLCTLSERINTETVYFVQED

KMYILSKKITQKLCTLSKKITKKVCIIQEDKHNCIHYQRKNTKCAHYPRR

>SG-PL_contig_7986_1_+1_1_183

IACLISMETQLTFYKSSATLYRQPQFDMEVRNSLCFTSSGSDSQTSVWQETVIFFLWFQQ

G

>SG-PL_contig_7987_1_+2_59_226

MTGLYFTLPFITYSGIWSKSYRHLQCWLGNLSKTLNDASVKWMRLDCVVLVFPMSP

>SG-PL_contig_7988_1_-1_292_516

MYVMCFAYQGCASGLAAQGQRVTFYIDFDKCYTCITTAGAELASFVLISKQLHQILISIK

TAEEKVYVFNQYQNS

>SG-PL_contig_7989_1_+2_125_202

MKFTDYIMTYRSITFRFITEIRHKKK

>SG-PL_contig_7990_1_-2_56_265

MVVAVVFCHFSFFERPECWQWRPFQCVCCSIAPESWNLWKVNKRYKGNWPRFDGWVSQQV

VLVMSGMMLS

>SG-PL_contig_7991_1_+1_1_138

THTHIYIYNISSAYITTDIRYSISSYCHAYLYFTIFKKRKTKGKGK

>SG-PL_contig_7992_1_-3_3_218

TEKSTKVSNSDQRVSTCIQKVNKAGMARCLLCEKELNYTNHGLTALFEHMHSKKHVDASK

ALDCSQSRLPGE

>SG-PL_contig_7993_1_+1_148_399

MDNQCIVQHIHLTYGMYLKGYYSSSCLWFKLSPLWHLTEIPSISLHLWHLTKMLSISLHL

LSKTPQNLAEIWLFISNQFCGNSD

>SG-PL_contig_7994_1_-3_1011_1241

MMKYQHSQHSCVRDSQMWTSWMKHFISAAFSHFLLVRILFVCVCVCKVGKLIMKLTNQQV

CDIIHVIIISVVLVKSR

>SG-PL_contig_7995_1_-3_102_278

MASSTVPEATSADVSDKEDNCGEEKGRISNKAALDCVHMLHPYSLQSGCSDAVMKSFDV

>SG-PL_contig_7996_1_+3_3_116

WFADGGLLGRAPALESERSENAEVQYPTGQVLLPTPLD

>SG-PL_contig_7997_1_-3_3_125

LHFWRKVRTALSQQLLTIFKFCFHCCDSLPLSSLHVKFYFV

>SG-PL_contig_7998_1_-3_30_221

MNPPEGIIKACTNSNDNQLQQRTGLTIDNKMTNYILGHGNFPYFKCFKQKQTEILNRIIT

ACAQ

>SG-PL_contig_7999_1_-3_114_623

MFILSKLRYLTDRNARQLFYNAHIKSHIDYASTVWDGSSEVHLKRMNTLHRRAAKLILPD

PTLTTDEKLHQLEILPLSSHFMFNKGIMMFKVSRKSVPQYLLNFFNQQVRSYSSSRQSHF

KIARPRIDIYKSSLSFSGALLWNSLPQSIVNAGSLASFRKSLFKHLMSIS

>SG-PL_contig_8000_1_-2_29_3796

MASPVILIIVCSLARFVLSQKPEFLSTKSQVTAVKGTTAVIPCSIRNIGNHKVLWSFERV

LLTFDTRRITDDSRITVNRPYQSEWNLHIVDVTKEDAGTYLCQVNTVPMMSYNVRLTVLD

EAPSPVMPEEVVALAGSDVEIKCKLPDLNDIGVWGYKADDRSDRRIISENNRFTFRVGQG

QDNHVHTLVISDVRAGDAGQYFCRARSGTEGKTSLRVGVPPPPPSLEAKAESPNRLMVRW

RKPTSRNTDVAGYILYVQNHETGKEHVFEVPPNQKLSGEESFRVGDLSTKTTYTVRLATR

FQLGDQVTLGKRSPEVTITTPLFVPEAPGWVKVRRISDGEAEVQWSPPSPSKTSGIVRGY

KVYYTPLTSRKKPIVNQQKIVTVEKPEGNRWKVRLTGLRPKTRYHVQVGGFTHKGDGRRS

KTTTYLHKALPPELSLNGRVLSDNSVLLTVTSLGSGRMANVKMFYTEATQPSWKRLDFSP

QWKFTVVGGLDPRTSYFFKVKAKIGGKLKQCVTHITTPPHDLQAPQGVQVSATASTSIEV

TWDYVIANNKTSVPLGYIVLLEETSGKDDEEKRHRQSVVFNRTSVEVKDLTADMRYNIQV

TAFNGFGEGPRTDGLLFHIAESDVKPDTVSEPFPPRFTHELPKRLTVQSGDAVKITCVAD

GLPVPEVQWYSNGEPIGVSAQGSNQLFLANVFTSTILTCRAKSQWGEIEANTMVDVARVQ

GGQGKLIIKLMSDNVKMSTFTLAWNLQSGDPKSIDRFEVVISDKTDRVLLTQTLPGPTRS

FHLRGLQPGTEYRAVLRAFGAQGNLPLAEARATATTLRTDGSAAVATTPSAPVTPLESRY

RGQIALSIMDVKPSSAVLEWDFSGLDPRHVQLFELKVKSRSNFTLLTESLKPNLNVMDLS

LQPGQIYYVQVRAFDRRGRVLALNTTRVETPTQRAPPATTPSVINTDNPVLKRASLALTS

HAVTGDSARLDWKLSGAQQADIGQFILKIKDSDDRTLLRQEMTREARSLTLSNLTPRTAY

FVELQALDDKGAVVAATTGSLTTSSQTAEESDPDPSKLRVFTEELDKNSVKVAWSTPAKL

LAELSGVTVKMTDIANVTLLTQQMSPLTRSLTVRNLQPGERYKAYVELKGARGDTVRSGY

LEFDTEPTHGMPGRPQVTNIEVVNSTAVHLNWRNTPSPSPAARSEDITGYVVNFVRVDQN

NKPTAGTYQLPVFGSTQFVVIPNLRGGQRYMFQVAARNQYSFGPLSVPVTADLPRQ

>SG-PL_contig_8001_1_-1_85_1389

MLELCRIHKKRGKSMYRQEIMETEQQQQQQLLTKLGELPIVNDTYTQVLDIYQRTKGHNF

LFRTSFGLAEYTARAVAGRTVPIIYNGLQTQIEKVNDIACTQLDVLEEKYPIITRPTEEV

LRESQEQYNKVKPYVDPLIKPAKIAVGVGKTAVSVGTTVATTAIKPAVFAVNVGTAVTSS

VIDTGKQAVSQVASGVMTQGQKVVDAGLNTPLGQFWVHQFDTALDATEKLVVNYYLPLQG

GDGEEREVQEEAATRTERVYSLLKHIHDALYRKAEGDMVRYRMIAGNVMHEIMELTGFYL

TLVKDGTDMAWKDHKDLLDACDRVWNKYAQYIPEAARPEDPLNRMVVLLSTLTAKVGELF

ASSTELKNLISQHLSQKCQDFVSWCQTSSAAIKKSGKGVTEWSSQYISELTAYVPHIVEL

AQFLMDKVIDSLDIF

>SG-PL_contig_8002_1_-2_2_184

EERQCLMQPCLFPLPAGEERRRGRGGGLEGRGAETLLPESIHSNSMHFKNHNLSTEPLPT

T

>SG-PL_contig_8003_1_+1_253_549

MTVSGIGLHSKTCKEYSCSCFSIQLLWDAKMNCAEVSFTSGQVQSRSQSPNGAGKWVGTV

DKHREFCDDIIPLFLAAHHSADNILLLAIMNFPIFMRKS

>SG-PL_contig_8004_1_-1_697_1011

MFQFGRIECTPCIQCLRIEVIVCTNRPIQQILFLFLMGVGGPDPFVEIIMIDMLMAFQCQ

TVRLAVKYKALMLLKAMQTSSVKICMMLTILNLGQILWSSFIEVE

>SG-PL_contig_8005_1_-2_2_712

GTPLYVNIVSWPRVPKPKSDQDSIPLFAAPLDNITDKKTGETASTITIATNPEVLAEFGV

EAKDAEERKSFINLNLDYVQHLTSRSVSRNFTLLPRSTPFKGDLENAQKFFTDSLKKAMK

KDKPEDEAERNPAPDIDIPESMLSRLANISTSSANTNQSNGSPHKPSTDAPIILPETKQE

KKPAVKKNLIQEMQSEEVRLRKPEYSLEKSSSEDCLVLRVELPGVTSVSQCELDISE

>SG-PL_contig_8006_1_-3_3_791

SEEEDDNFVESTCTTRQKSEPIHQPDDSYADEQDDNPQSDDDSAGPGEESQIQDSNHSET

KSPGHQKFCAALGAGDRGKMLECLFMSMEAMQQEIATVKSGVRDEIAALRLMVLQNLNQS

SAAQAGMIFDLPEEFTFPLSTVEEVAELEEKLTNSSFKRDMVNALAATGGLDIKTALWGI

MKRLFTSNLAMKYNYIGSKGKLCFRDLILRRVVFEAVRRLCPKATEYDIYLKLSMWLTSA

RHRDGGRKRRFLACAEAEERLLA

>SG-PL_contig_8007_1_-1_355_696

MKGVRVVADLHIMLTWEMGKFSTFYPPVLPSRMKLRLSDWKSDVLTAWLSCLSAFLVRPD

LSETKGSYSLGEQALQPTLAPQGREPGEVSVHMMFMVCMTCLRRKMMTCCCLNG

>SG-PL_contig_8008_1_-3_39_131

MFYSEVPLTVPAADCYLYICPSLLVAGDPLV

>SG-PL_contig_8009_1_-1_1381_1623

MGFGQMKYFLPGNVMLKTDASCPFWCPILQSLCYICKNVKGIGTHLNLLQSDCYTLDSVV

RKGSHLLNGNFRSADCPSPSV

>SG-PL_contig_8010_1_-2_5_1234

MNLVLFRFAIEHVSRIARIIKQPRSHALLVGVGGSGRQSLTRLASHMADYDLFQVEISKS

YTSFEWREDLKRILRKATETDNHAVFLFSDTQIKQESFLEDINNLLNAGEVPNLYALDEK

QEICEKMRQIDRQRDKTKQTDGSPVALFNLFIQRVRDQLHIVLAMSPIGDAFRNRLRKFP

SLVNCCTIDWFQSWPEDALQAVASRFLEEIEMSEDERNGCITMCKHFHTSTRLLSDRFAL

ELERHNYVTPTSYLELISTFKTLLDRKRGEVLKQKKRYEVGLEKLESAAGQVAVMQQELT

ELQPQLVVASKEVSEIMVVIEKDSIEAAKVEKVVRADEVVANEQARVAQSIKDECDADLS

EALPALNAALAALDTLTPADITVVKSMKSPPNGVRLVMEAICVLKGVKPD

>SG-PL_contig_8011_1_-2_2_217

SFVGWLVEASCHVHSVVSNRKSSGAVRVLSCHYHSLTSDRRKQGEKLDDFSRHKTLMSRI

CLTTDHAELCVM

>SG-PL_contig_8012_1_-2_269_421

MGSCVILKKFEKYVLLVYQHYLHYLCSFSRSLFFPRAFSLQYLLLFLLHIK

>SG-PL_contig_8013_1_-2_164_331

MPLIQVLEAASWCSANPFISHYLWDFRITRADGSRGISFVTANKPISLTRGPQTNH

>SG-PL_contig_8014_1_-1_1_285

QHSTIQYSTTAQYSTVQYNTAQFSTIQYNPTQHNSVQYNTTQHNTVQYNTTQYNTTQHNT

VQHSTNQYNTIQYNTAQYSTVQHSTIQCNTTQHNT

>SG-PL_contig_8015_1_-2_2_598

TQYNTIQHSIIQYSTTKHSTVQQYNTTAQFSTVQHSTIQYNTTQHNSVQYNTTQFSTIQH

STIQYNTAQYSTVQYNSTIQYSTIQQHISVQYNSTVQYNTAQHSTIQQHSTILSNTTAQF

SPIQQHNSVHYNTTQHNTVQYNTTQHNTVQYNTAQYSTIQHTTIQYSTIQSSTIQYNTTH

HNTTHNNSVRYNATQHNTV

>SG-PL_contig_8016_1_+2_1781_2200

MNAIIIPKPLNCSDDNNKTHASIYSFLQNIQINENFSHVPMLKGMNIVIKWRPTYDVYTY

KLQCHFIQDGISFDLLLWSFIVRNVSHDQSIMPNFFFFLFCCPIIFCTAPVSLPPCCSFR

LSPTPSKPTETELTHHESMV

>SG-PL_contig_8017_1_+1_1_351

IFQAGNDVEQNLQYCAQLGTHRQDTFRAAAIRNGNTGFEVSSQSFDVGTSLPNDGTCVFA

LQQNSKFHTGTFPTTTFREIIRTAHGFIIIQRTLHSSFNTIHVIMISHLESRGARAD

>SG-PL_contig_8018_1_+3_3_188

PGLMASHHPPPPPPPSPCTELQKTYTVWCQRRMTPSSNIQWHVKKNWKRITHTPPTQKQN

NY

>SG-PL_contig_8019_1_-3_57_290

MVVTRPEDNVNKNETMAASFTEDEDASVFHRLNKPSASHGSHKYHISSQNFQLPQSAVLH

SSAHFVSRDMISSGYPMG

>SG-PL_contig_8020_1_+1_3091_3513

MPGRCVAGKRTAWHLHSASRQHSACPWLPCSWCPSCLAACPYHSPSVVPSWVVVPSCSWC

QWDSADSCRSCPDSDSPSLSCPGFPSCCQAHPSYLAASPGFPSCLGSFPSYLGALLSSSC

FAPGCPTSSLGFLCFQHRGSS

>SG-PL_contig_8021_1_+1_1285_1512

MSGHSKHRSTGKYLGQRPNRRLKQTRSFRSKNLEKLVPLLNNLCIHISWETGFKQFYTQH

CKNTGSWFRITDTRAR

>SG-PL_contig_8022_1_-1_1_237

PPTSCRKLVLRVRERKGQLSEGPNSALKLKIDMDLGPAVTSARMFQGQTLLMCTNSGLPH

FIVKHWVPLFVCVKTHVYA

>SG-PL_contig_8023_1_-2_71_214

MFVVHFNFCDLAITALVKVKGQSEGFDTILSELKFLTDLPPNKPNTES

>SG-PL_contig_8024_1_-2_2_235

IVKGLKPGRGYALSGYIRLLSNKKGVLWQAAKATLKFTFTNSADKPGYVMMALVDNETIP

EDERTRCKRTGEGQKQVA

>SG-PL_contig_8025_1_+1_124_543

MTKQSIKRVIVTFHFQLLKQTLKPIAEVIVRRLNYLLFLFLLLSHLLRCFSGTDPTSLIL

RLSPALTLIPSFSITSVWVFQHQQHSDAVGACHEMEYIHSYCSLGNKLQPAIGLSVMTLS

SHHIQFVMLSASLFLLSNLL

>SG-PL_contig_8026_1_-1_163_366

MTKDRRECRLIGHQTSLRLSLWPVRNAKKWFMISNLGESGTVQNEHCNSIHRCQLSHLLS

GNILSVFC

>SG-PL_contig_8027_1_+1_1291_1557

MQEISLLLITYDTQCIHQMLSGDNEEVSTFIMCPQRHISSICRNVYTVFYNRQKASDEWA

TQSGVMGHVKSCLLSSYLLTDISNGEQGT

>SG-PL_contig_8028_1_+2_50_388

MYVIHSNLKTKQLISSEIKITFLFTANEAARYCLTHNDAEKWKAVCPTRNTSHVIVFNSN

QHDYKRRYKLVLMRTNACIKLTIFMLRTAPTKRNIFFKIHVWLIYLPCRTVSI

>SG-PL_contig_8029_1_+1_433_639

MLIFRQFHMAIRLSVSISTVSLSQLALCHVDSTNIEPFEHKDEDFFVCFIYLTGDVFGRL

HSCQLTLTD

>SG-PL_contig_8030_1_+1_58_1725

MAESSDKQTVVCPTRGQPITLKKITKARKSSALASTPTRKKRVNQLSNIRKDTSGFAEAD

VIRQRSTELRFTRKRKRTEILKGAGCTRSHIPAKTSAKMKSKMLLSLAKQRALKKISRKC

GVTFESEKKEREFQKSLMCGTVKIDKRKLTFHDNNTNRDIKTTLPVANVENLPIFISNLL

DQYEEKNMLTWHDDNIPENEVWVKIGGDHGGGSFKLVLSMANLEHPNAKENCFLICIAEA

KDSNENIRAILEPLREQISQIENMTWKGKSIKLFLFGDYDFQLKMFGLSGAQAVHPCLWC

EASKTKIQWKPIERSRAKKRTLESLKKCHAAFLRAGGDKKDAKDYKNVVLPNVWNIEIDN

VAPPYLHILLGIVKKHNDHLENECHDLDLRLAVDMAKEEHPPSNILSDGFLRSVQQVKRR

IQRQKTTTPSLDGLQPFLKYEGPIVATVGEVLKRHRIVRQAYYSGTFIGNHCSKYLKSEV

YNDICNSIEAETYTLTDNQEIWGVASRIKCRFTKLNACYSRVHELVSSIKPVQEDQIGKI

EKAIDKYMTLYRGLFA

>SG-PL_contig_8031_1_-3_3_239

IENTCLKISGDGLNEGGSNGSMLATTVVPQSEPVTPSNVRRDHTNESSLDQSVPPSPTQL

RPGPREFVPDNLIKFLQGE

>SG-PL_contig_8032_1_+3_3_188

CVCVLACTGNNLNQLCIFFKSYLVLTFTVIVILAFIYSSHQKFKCFMSTSPVSLLSCKGQ

FI

>SG-PL_contig_8033_1_-3_120_563

MAQYPGGGGYPGYPSYPGYPSPGYPPQQQPPPPAGSYPYPNPAGAAYAPPAAPTHSYPGC

PLPPPCAPVPPPSTTYVVGGHGRGHHHHHDKVIIKEEGFGHHHHHYPHAHGHDHGHKTVI

RDGRDKIIIKEKGDRVIVKEKHKHGGWW

>SG-PL_contig_8034_1_-3_3_215

EKKKKKKKKKKVKKKKKKKKKKKKKKNEKRKKKQPQNNITVHCSCPHSHIFQSVTYFLCS

QFLFLFCVLIN

>SG-PL_contig_8035_1_-3_540_713

MHILKGMSHSMNMYMRNLSTLSLSFFFFSPTLPLPNSEREKWVTGLAESFLSSVTDKI

>SG-PL_contig_8036_1_-3_342_1007

MSTQMETVPSPTDKLTIQALKQHDKIHPKLHATTGPRNTDLSTKRSTKDSQAPSESSRRV

RIPSESGSKQPMKKHAMTVFRLVAATRSWKRYTKKNKLPPIPVRYENTYQLEPNEKSKFS

AVKVEAILKDVLESRLKGVRYEAEQCRRHTTELVNLIKSRAKTCHFERYKLVCNVLIMEN

KGQGSQVVSRGLLNTDTDSFATYTFRNSTLIAVASVHGLYFE

>SG-PL_contig_8037_1_+1_208_366

MFWKHSKLKRVADDVVVVLIHLLLLIVVFLLMLLVIVVVVLLIGHLTLTWFTG

>SG-PL_contig_8038_1_-1_1_123

KKKKNFYTGSVTKVIYNYCFQKRLFLYHSRKRIHVLYVIKI

>SG-PL_contig_8039_1_-3_3_188

GTQVCMAPPILALECLRLHRFSPLISKFFRGGMPPDPPSRLRRFAPSKSTSSSFFFLCNS

RL

>SG-PL_contig_8040_1_-3_3_140

GSLETFIGENMLQLCHMLLNMFVFMKVLYIHCVRFDSGLCTFGNGH

>SG-PL_contig_8041_1_-3_3_134

CFSETFIIQKICQNIFCYSKYAFIAVYILLNICTLELFLITISL

>SG-PL_contig_8042_1_-2_38_286

MGTVVDSNDIDGFIRDQKAKLATERQTLTRGKAPVDSHSVSSRLQISDEEYRALWSKLHP

QPSPRAQTPPPRTFADTRTDKGP

>SG-PL_contig_8043_1_-3_3_206

LLKHKIIIINHSCIALFSKLIWTHCAYNYNAEATVDINSAFFCACWVFSCFYKPPNTDTA

YRIFNVRI

>SG-PL_contig_8044_1_-2_530_751

MASFPNTCYTHTRCVNSLQPVCLTAPSWVPMTNIIIIIYILFIAQPTIKAISGLSPHKVH

QSQEDNRVDHKTSV

>SG-PL_contig_8045_1_+1_253_516

MSAAYGCIATIASYTHTHSHTHMVGPTNYALNSCKLEHVQHCLIYQVTMGVSNVLLMVIS

NTGLFYFIIDLIVLQSFYCRENDVTLSH

>SG-PL_contig_8046_1_+3_51_248

MVSRQVSSHLEVFQFDTNTLLQFIHCEGNGNRNEGRRASQVLLGIFCTQFQSQQGRHRQS

CRGLTT

>SG-PL_contig_8047_1_-2_314_1378

MAQAPRKVALITGITGQDGSYLAELLLSKGYEVHGIIRRASSFNTQRIEHMYKGPKSHSG

GSMHLHYGDMTDSTCLVKIVSLIQPTEVYNLAAQSHVKVSFELAEYTADVDATGTLRLLD

ALRTCKLAEKVRFYQASTSELYGQVQEIPQTETTPFHPRSPYGVAKMYAYWIVVNYREAY

HMFACNGILFNHESPRRGDTFVTRKITRAVAKITLGQQEDLQLGNLDAKRDWGHAKDYVQ

AMWLMLQQDAPDDFVISTGETHCVREFVEKSFDIVGIQIVWEGKGQDEVGKNQETGKVLV

RVNPRYYRPAEVDLLLGDCAKAKTQLGWERSYSFDELVREMVESDLELMKNRPTA

>SG-PL_contig_8048_1_-3_651_2774

MLFIEPSKRRNSRPWYMRPHIIYTCRENQPLSGNFTDTEEPLPAADPRLRRRKRSVGRKR

YLEVQVVVDHTVIAMHGKERVKNYVITVMNIANSVYQHHTLGVNLRVVVTEIILLSKDDQ

KRVMIKRNPHRTLKQFCQWSMNRQLPSNAKHDIAVLLTREKMGPAGFAPLTGLCNASRSC

AVITDEGFTSGFIIAHEMAHVIGLSHDGHGSPCTGRRYMSAIMAPLVQAKLNHYWWSECS

QRQMQHVINHLNCLNDEPDMTTDVKHYELDSGYELADDLGSHWSLDFQCRLEFSDSFGLC

KKFSSDHCNMLWCSDRRTPHLCRTKKGPPAPGTNCGRDRECRDWRCTYVGNEDPVHGNWS

SWAEWEKCSVQCGTGIKKRHRLCNNPKPAFGGKECTGEEETWDTCISKECDSYYDSREGH

CAVWNEIGTIYDHDWKAYQAKKESDRCKQTCFSALTKEALTIDVDVSDGTPCDYDHPTNI

CYQGTCLAVGCDGIRNSTKEYDSCGVCGGDNSQCKVVSGLYQRVPAKKDEYEAVVVLPKG

SRNIEIVKRGNTKHFIALRDADHGTYPLNGNKGQENSREFIHGAARFVYTSKPGIQNLTS

RGPIYSNLEVMLYPNGDMRRASVTYEYSISKHDFTFEKRKYQWRVKGRTDCSVTCGEGVT

KIEHACHDKDTEAQVDNEKCQYLDAPQKDEEKCSREPCSQRRYMWAMT

>SG-PL_contig_8049_1_+1_1_261

GCFSLNLFVCLFVCLLFVCLFVCFFCLFVLVCFCLFVCLSFVYCLFVFVCLFVCLFVCLF

VQIQTEIGIHVMAKMEPRKIGNILSVN

>SG-PL_contig_8050_1_+2_92_238

MCQQCDCFPTQMTRTNQIKCVNNATVFISSSIEMTEEPTKMLAMHVFFH

>SG-PL_contig_8051_1_-2_320_778

MAHLVPLSSTRLIKSWDCEKDCYTHFRHWKGLSYPLQMLWKGLLYPFQTLWKGLVPILDT

VKRPLIPISSTRLKFETKKVHYMPPSCIRLDKSWDCAKVHHIPLSNMSWDCPYAYKHQEW

RMHKVVSLCSVGVTESTSCTTRCQVPYNVSMVF

>SG-PL_contig_8052_1_-2_2_298

ENQNQNFSFEGREQDQQTEREIDEDQNLPIEETGEVQQAENEIKGEYDNLAMEATEQVRQ

IENEMQDESQNQNLPLEETGSVWHLENEVLDEYQNLAVE

>SG-PL_contig_8053_1_-2_50_256

MYMMFSLQYFKTCEWCDYVDKSCLMRLHYILCWKIVWWIHAGCHFPFVKLNEKVTVLPEI

SKFVVQLFM

>SG-PL_contig_8054_1_-1_1_516

DLSQSPGSNSTSSTFAGSSGAAAPVSASGSFGGHPTSVSNASGATTSKYGLAKPPSQRSS

RGPSPVNEQSYDALEGQFSPGHGAMTPVRGPTSAVTGGRPPLPPSAFPQGPFPPHPGQRM

PPPPRPGMPGPPGANVGPPPPSTGAATGAPLGQQQMRPYPPVSQYPGSMPPP

>SG-PL_contig_8055_1_-1_550_732

MGLYPTQGILPKATIFLVQKNIKNCNNYVFFMPIVQQTLQQRAVFKENKMVKPCLHCFPT

F

>SG-PL_contig_8056_1_+1_376_501

MAIMVVKRSRYIFMKHGNYQNYRQLPTTKGRYQQYISGDVLY

>SG-PL_contig_8057_1_-3_39_497

MRGSSDARKAEQKKQRDILQKQRQDLSLKQQTIISKQRAAEKQAENAKKERIRHRRQQLQ

KLVSLAKKMKEKSGEKRKPKEAHVKNTQKQKAPEEKLRKTASVGVNLPTSKPVEKPPRTV

KADSTNQPQESEQEILTPESFSDRQAFFSDLLA

>SG-PL_contig_8058_1_+1_1_168

GVLEERVGGKRMREKDGPHRLRIQFQLLNIFPLWAPNICPHLNQPLSSKFKSSYSA

>SG-PL_contig_8059_1_-1_325_435

MSVYFLSTFFCLAFFHFLLSISFVYLLLSSICPSSLV

>SG-PL_contig_8060_1_-3_3_227

LLLPRFCPSPFVHRLLSIFFCLAFVHHLLSIIFCLAFVHLFLHSFCLSSLVRLLLPIVFC

LACIHHLLSIIFCLA

>SG-PL_contig_8061_1_-1_64_1164

MDKEVAETLAYFRQHASDLRKLGKDWGLNDEEIEDCIKQAMSVDPKDLPASEITTATKLK

FTARKAWPKFRVLMWIIGVTVVFVGLFSLALQNDQIDHRVGQLMQPVLYPLFRFVRLAAV

PLHDSIDFYDRYLEECIVPNPLFRRDQLLGDHVCENCKSVTAATQFTASSLEQGPLPGVV

FTEPVLFKDKGRNVSFETFQSILSTNQFVQHPMLGVKTNLKWASHVKHLLDEETAEKLAA

QPDFNFFWLSKLVWTSQIMKKVFPKPQVLGDAEIAIRKSIFITGPKAHNFELPASPIGAF

SWYTQGSGSRKLVLTPAAVCKRECSPVSVTVNPGDILTYNCELYKAEVRPAGEDISIAFL

GHFSPQD

>SG-PL_contig_8062_1_-1_88_216

MKLKYNASTFYSCQFVKKATTKILVWSQNSIPGRGDKIYTFFF

>SG-PL_contig_8063_1_+1_754_993

MSQMGRRPKKLGWDFIICIVSANVFAVSHFIIFIYLFIYLVCVCVCVCVCACLCFFPFVQ

FTLLMIIIHFLNRLYFDVQQ

>SG-PL_contig_8064_1_-1_1_96

CFWALSLKPEGKTPLLHFFLNSAPVRIGKLCS

>SG-PL_contig_8065_1_-2_14_202

MQLKIHMYIHIHICIIYTKGQLQCIKIRVASQRGCGETRMSARDPMEPTTMVYPMANLTK

QRT

>SG-PL_contig_8066_1_+3_648_863

MANLAYELQRLTTTAKSESKINVHNLKLVQKEIHAYTHLTGQPILLSYVSANFTTAKCDT

KSKFHYNKSKLL

>SG-PL_contig_8067_1_-3_111_437

MVEQIIDMLKEQHEFVQNEDTNEQSGLAVQHVRDQLLPPSRRKAMQKIWDQAVKFIEANE

SRVRLETQVIEGEEFAVWRWIQAVPNGGKVWQGQAFGEHNDSNPATSIY

>SG-PL_contig_8068_1_+2_98_274

MSMISLSIVKPRGWIINMLRLFCYSLSKVLLQFYVNALFMCLQCIRFIIAHTPSNGAMA

>SG-PL_contig_8069_1_-3_3_206

VCVCVLGLFVFVMNPTVPFLCIGLVCTIFERNTKFILVLLFLSGDEIFAWKVLLSTWKTV

CDKSNTRV

>SG-PL_contig_8070_1_-3_3_191

EDDDEEECLHVCCADSHKSGDHIYCGSRTTSRLPTSTWTEESSSHHCTPSRPLPSWIMME

TDC

>SG-PL_contig_8071_1_-3_180_956

MFQEKNISGPDSLSTMGPVPEDNHNNNPAENDPANDPEELEGPGHIIPPGDSSSDDDNDL

DSDMEGPANDGYMLLPQDPEEEGGGDTFQPQPFPPLSALEEEMLPTESMTEVRGAGVDVS

QAVAEGSVHPAFAANFPDNKVPVHLQVPDQPKTKKDDVLWNQAPAEADQRLQLDSAHLSK

VKSAMAGFQLPKANIPDWARQIPEDDWKRQLVDRLTSPTTTTRPESAAGQSKQHNAAADI

AGSEESQKQDSEWPGTSGT

>SG-PL_contig_8072_1_-1_10_270

MADGNRMEKVDMSKEFSKLSEADKYFVRKLEQMNRDRARDMKALRRKNWFTALAIGTGVL

SIYFYSMYAVRQERFLADFDKVPEDKK

>SG-PL_contig_8073_1_-2_2_157

IFQWNGWHPQHSLVLFALRNIMVKIICHELCSFAGLLFSGFVEVLCSTRLSS

>SG-PL_contig_8074_1_-2_2_259

CGYLFGKLYVYAHISAEAGLHVELHVCVCVCVCVLFCLQNSAGHLQLTNWLLRAIWLVLR

MDKVQPSVCVCACGKVCVCMCVCEIG

>SG-PL_contig_8075_1_+2_494_718

MLIITLTGTLIHHSSLSEMRDTLHTSVVFCLRDKERRENMTERDTENIIYFQMTHTYIIY

DRTLKICATHHHFKQ

>SG-PL_contig_8076_1_-3_3_218

RRENFFFTAFSVFFFRLSFFRLFFGKLCLPSLVVILLNGVALASLLGVVHPPVPLSGRMN

VPIPVLRERRST

>SG-PL_contig_8077_1_+1_685_1140

MTTSFMNLVSDLDLIVRSIQSCISELKSWMTNNKLQLNDDKTEMVLITPKTVLSSHSLPH

SIPLNGTDIQLSKVVRNLGITLDDTLSFRQQISSICRICYLEPWRISSVCHLSEEATKTL

ICSFVLSRIDYCNSLLGGCPKYLLSRLQKVQK

>SG-PL_contig_8078_1_+2_752_1204

MNTKINNSHNKQTTVCSVSRRHKTAIIQVNGPLIILSGRWHMDTIILSGRWHMDTPLLQL

CTLLSTHTHTHTPAPTYNGNANRRHHHQTTRQQSPPSTENSIGNNSHTSSPSSAPTHNNN

SHTSSPSSAPTHNNNNNNNNSHTSSPSSTPT

>SG-PL_contig_8079_1_-1_13_1071

MASELLSKAKDEYISDNYQRAVELYSEALSEDPDNEDALVNRAQAYLKLNQNEEAVKDCD

KALVLNSKNVRAYLRKGTALFDLKDYRQALDSFKEGAKQSEPGSQFDIWIEKCETKINDG

KTCHKESSAANTEPTQVAAAAAAAPAPVKPKYDWYQTATHVVVTLLQKNTKKEDLVLDTR

ESELKIHTKLANGEDLNLELHLRHPVVPEQTVAKVMTSKVEIKLKKAEGVQWTKLEGDGQ

KFALKLPNATNATANGGSAATKYPSSSHYTRDWNRLEQEVKEEEKNEKLEGDAAVNQLFQ

KIYADGSDEVKKAMMKSFSESGGTVLSTNWSEVGQNKVEVKPPDGMEYKQYEK

>SG-PL_contig_8080_1_-2_317_565

MKAVSVCVCVCVCVCARVCVYVCVCVCVCVSAHFMCFQVVPFIKSNDILSSLVLNLSIVA

YVTVSVVTCLEFKPNFGGHICTL

>SG-PL_contig_8081_1_-2_107_283

MIVFEFFMCKDTLVFNILFSILICPSAVLSCQTYCRTFVIHSMQNIWWQSEGSKGQFTC

>SG-PL_contig_8082_1_+3_204_395

MILWRWTRNSRGQTQTRAAMEELVPQREGLDRHDHARRPVSGHQKISQRRMSASSALRKD

GRKE

>SG-PL_contig_8083_1_+1_202_495

MLGTPLEVSYNASSHWYQLKITYKITKTTVSTTPPSALPRPEKSACLSAFLKNNNNTAFL

PQECPQQQVVTVKPVTFNQIQEIALLCAPLHTASKINK

>SG-PL_contig_8084_1_+1_283_477

MAEGTGRNKRHPMTTKYSYDTGNVIRIQTPGRCLSTLEIGLCISHMYQLTEATDQKMHAL

ITRHK

>SG-PL_contig_8085_1_+2_2_229

VCVCVKTFFHSLLYTTYKSVTFSLVSSYVSLSDNLHIKEILIEKKYLHESVLINVPSLCK

VILIELNVMFTNSDDY

>SG-PL_contig_8086_1_+3_87_380

MPVEIVLEVITQKTEYTLITSLTKWCYFTIIYLYRCLFQFTAPTCMAHITCHSYFNDLTF

RFSHKRKLMPTSASVFGCINNSTTTTMASYTKFMNIFI

>SG-PL_contig_8087_1_-2_2_163

YVCGGEEDFCVICVCGGKGDGLNIKGFLLTNMNMNILAFHLRIHQDCEQSLFCL

>SG-PL_contig_8088_1_-1_2146_2442

MHLCMQHLSDYFSPCVCIAPDVLLFSCSSEWRMLLVEVQSASFCVCTLGFNLCQLLQIIQ

SIHEVQNMYILSPLLAVATKIRLFWQNCFTCYVFAVIIF

>SG-PL_contig_8089_1_+3_288_425

MLVLLNVSSVFVLLSLWQCLLHLLLLIIIRLTELKRLTISKFCVKI

>SG-PL_contig_8090_1_-2_857_1099

MKRSPSYVRLFRGVLRRLFFLSISVSGVYSVSLQALRSVFVALFLVYRVPLQVCQAFYSA

FSSLPCSTSSMSGVLRRFFSG

>SG-PL_contig_8091_1_-3_837_1055

MGQNVVMDTPSNTKCDMGHRGSKGVSSVTVHVKELRCSPVSTHSLRMRTDINCTVFYFNH

IIEDNCWKSKHHT

>SG-PL_contig_8092_1_+3_3_209

VHQVPAAHRTSVYCLIQKTSTEIANQGLVKWEEESNRLGFEPLHSQNSRFLGQVVYPLDH

TSTFVCYVY

>SG-PL_contig_8093_1_-2_2_214

GGRKREGEEKRGREREREREREREREREREFLQRENRSVFHTKIHYKQTNLLAQKCLITT

CIKRSELILSV

>SG-PL_contig_8094_1_+1_754_996

MPQHVYYKQRIRSGTTCMPQTNHLIYHWPSLNIQKSISSKLVSSQQVTQLLSFFTTSCLL

FAFPSLTLWGSPKCMHSVLPQ

>SG-PL_contig_8095_1_-2_374_493

MSLRKIRLKNDVCVLLRSASYWKKNGLSFLNLGIRSVLSM

>SG-PL_contig_8096_1_+3_3_185

ECMMLNQRVTLHINHTYFSSQDIHFPNLKHSNFHQVLQEYSLLSPLLDLLDSPFLFTSYG

A

>SG-PL_contig_8097_1_-1_1_177

KFRRPLKPDHLPAGERCLRSSTGMWLLLSVFRVGSGSVEEWGYLARLSLNYDMRRSLSP

>SG-PL_contig_8098_1_-1_1_129

KFRRPLKPDHPPAGERSLCSSTGMWLLLPFFGVGSGSVRSWAT

>SG-PL_contig_8099_1_-1_1_138

LTLCTPSTGSYIPYSFFPDRVQIYIRTATHACCFAFSFSGTLPWCF

>SG-PL_contig_8100_1_+1_1_96

HNNSHFRSNYFLCLHCFNSSEVKNEVPTLHLQ

>SG-PL_contig_8101_1_-3_3_212

INHVYGPRSDMQTVPNSLYFVPTSRKLFRRSDDVKIVPKSSFSSSMGHPHTSSSLGLAVD

VIRKSLLVTY

>SG-PL_contig_8102_1_+1_1_198

CVCVRVCACACVRVCVCLCACACARACVCVWCTYICTHACLHGNVLRVYKQAYLCTNDVR

SHTLRD

>SG-PL_contig_8103_1_-1_109_318

MVYQSHHKDFLICSDSLSALEAIASRNWNHPILVKFFELFTKLSADGYNIILIWVPGHVG

IRGNEVVDQQ

>SG-PL_contig_8104_1_-3_3_188

KHGGRGDGDSCHNADPEKSRSLPPHSPIISGLWDNLYFFFFYSDPFSARDPELGTQYKPA

HN

>SG-PL_contig_8105_1_+2_2_295

FFFFAICVQSFQQPHLFHSQTFQSGSVLLTVIIHSLHKPLCGSQSVVLLVCVCVCVCVCA

CARHKTCLRSNISTSFQGLHIQYEHRTIWNFHTLSYPS

>SG-PL_contig_8106_1_+1_142_261

MNAIFPTLRAVMMNIAINQMECLKSAWKYFQWNAQIQHTS

>SG-PL_contig_8107_1_+2_2_217

HITITITSECKLLPPTTSRYNVRYHMSECMTGHQNNTKQTNKNGRNIRKEPSLQEDQTHT

HTHTHTHTHTPK

>SG-PL_contig_8108_1_-2_2_268

FLCGNCCELVVQIWGFGQREVLVHPAGGWGVVCPVHHHRCLHLQGGERDHTHYHYEPHQH

HTVKYYNQVSIAPPGPSHPVLIALKCCVY

>SG-PL_contig_8109_1_+3_129_371

MIYANTDYQKHVQWTIININKRVHLHFYSEWLNSLFVCRNHAKILRLYNRVRKHRREIYL

NIITSVIVSINKRSVISRLVK

>SG-PL_contig_8110_1_+3_3_284

GVTISEGGGSEDAVRARVIAAWAKWRDLTGVISDKKMPRKLKIKLYMTVIRPVLLYGAEC

WTVGKKEQTLETTEVRMLRRNKGVTLQDKVKSVD

>SG-PL_contig_8111_1_-1_259_402

MVPITRRTSNTHIKDPVSHVSVQWLMETQKHPACAKTVAFMPVVVSAT

>SG-PL_contig_8112_1_+1_121_315

MVVLGKKAKRQTKRETDGVYLKRHAGTVDHPRRCLGQSSGGQEFRPLTPPSGKRRGRRRL

DLLSL

>SG-PL_contig_8113_1_+3_483_737

MEFQGDITLIVHPHERHFSCLALGVILRLTAPVGRPTALKLQRNEFTFEVKMGSRNDQHK

GNEVVPMMGPQKKTKKNSDHKGTSV

>SG-PL_contig_8114_1_-3_108_368

MATVVSGIASSIKSKDASTELAAGNDNNNNQADQSETEGGDSSASKTTDSENMTGKNIQT

TTSASQSEQPQQSSSDSESSQHPVERD

>SG-PL_contig_8115_1_+1_1_177

FFFLSFLGMNIALFIQVSHNNISYAIHLTFGVHVQIQLHMSAFDKENLGRLNLSLLEQN

>SG-PL_contig_8116_1_-1_97_1335

MERYYDGDQDRLFSEAHVISPHRRDIKRRSRSVPDDRQECEICCYPVPAIEMTGLECGHK

FCSKCWADYLTSKIIDEGMGQTISCAAYGCDILVDDTTVMTLIRDPKVKLKYQQLITNSF

VECNRLLRWCPAPDCGHAVKVNYFDSKPVTCVCGHTFCFACSENWHDPVKCVWLRRWIKK

CDDDSETSNWIAANTKECPKCHVTIEKDGGCNHMVCKNQNCKADFCWVCLGPWEPHGSSW

YNCNRYNEDDAKKARDNQEKSRAALQRYLFYCNRYMNHMQSLKFEHKLYTAVRGKMEEMQ

QHNMSWIEVQFLKKAVDVLCLCRQTLMYTYVFAFYLCKNNQSIIFEDNQKDLENATEQLS

EYLERDISSDMLQDIKQKVQDKYRYCESRRKVLLDHVHEGYEKDLWEYQDYLC

>SG-PL_contig_8117_1_-2_2_184

QGTLERGFCWQIWKTRPYLVSMTCLLWCPRDRDEGSRLLLCPVCSLQTTTLVLGLDNDFW

D

>SG-PL_contig_8118_1_+1_1_120

VTSTPRIQVTDKSGKAGDQCRGQQHYLTYAHMKHASGYKT

>SG-PL_contig_8119_1_-2_62_256

MAAQMSFSNSRDYHSISRPVTAVHKLLCTSVHKLLCTSVHMLLCTSFMTDSLWFCEKSYI

PYTSH

>SG-PL_contig_8120_1_+1_67_318

MMLAPVWLPTETRPPPSLRTLLRGPKIISREWSNSPRSVTGLYSSSITSSKFWGSGITGD

CVFLSKVSIRSSFHHRRLTCHVPR

>SG-PL_contig_8121_1_-2_137_2059

MEGLPFLPGNSFTDPTQNKYHLSHTLGYKNGYALQKRPEVGIGGKPIEYNQLDQADLDHL

ANFNPTLTYGQAKQAPPEDFIPAHVAWDKKVLKFSAYYKQTVHESQNEFYRVRPVDIYYY

LEDDSIAVIEPHVENSGMPQGKLIKRQRLPKNDRGDNWHWKDINNGLNVTFYGKVFRVVN

CDKFTCDFLESEGIIVNPPECQPKDPYIERRKEAAALRTYTTPPAFDKLKQFLELDRKVL

RFFCMWDDRSNMFGEMRPFVIHYYLVDDTLEIRECHQPNDGRDPFPVLIGRHKVPRDRYN

VPSSFPAVVMELSDNEIKDYFTPKDFMIGQTVTLYGRRFLVYDCDNFTKAFYYHNFGVTD

FPSVDVDGSGKKLPKMEIPPYNNFGSLEDSLQSCLSLIPQPPKKDFIKMLENDHKVLRFE

AVMDSLRPEDSGRRFIISYRLADDMLTVYEPPVRNSGIIGGKFLERTRVCKPSSTPDAPD

YYGPQDFYIGAVIEIFNHRFVITSADAFVLSYMEEHANQFPPATISSLKETLGQTQAAKE

MVKGGPMKVKRTQGDLDQLVKQVRAQLKKIAITDKCRVDEMFLRYDRDRTGYISVENMRD

LCRRLQLPVDDDVILALVQQCTMDQEGRISLEDFRRFVESS

>SG-PL_contig_8122_1_-2_2_148

LTGTYSWWTNAGVKLSDSLPTTPVRMLLPPDVSHVAGASKTYSYNKLVW

>SG-PL_contig_8123_1_+1_1_219

PLIIIFFCFCFKQYVHGCVCACCLAHNVQAFMFLLSLHSLLRWVISAKNRSVAAAAKNGP

FPILHTYTVWTAM

>SG-PL_contig_8124_1_+3_3_287

RQDSNQQDLQSGHQHTQYSFAEYTNPLSPQFYKLVFTAYTESPAHDITCPPLLPAYTCSL

PIQTSQPMITLVPQILSAYIFFHCLYITLQGGIKK

>SG-PL_contig_8125_1_-1_175_546

MRDLDEVLSLGQDSHLEVVQETVTVQGSRLFKMQERYKDTEVPEGVVTVETDQSGNKVLD

TTGTVFRNIHLDMKENVHLSSEAALDMCLQHFGDDHRRNSLWHINVKAEVLFDNNNEAFP

GYQT

>SG-PL_contig_8126_1_-3_186_329

MFVHFHFLSFVLDNKSANNKAWQIMSSVSDWLFQFSVSQVIKILLKMK

>SG-PL_contig_8127_1_+2_1088_1318

MNNAVIIYSVSLTYTNAMNNAVIIYPVSLTYIYPNIMNTAIIHETLCFFYIYPNMTNNAI

THNTAIIHKTLFFLIEF

>SG-PL_contig_8128_1_-2_41_253

MHVKDPVSHVSVRWLMETRTYSACTLYSPFTGSKLYWLWPLPWILPVSWKGWACCMGNSR

FSLTLCPGLCP

>SG-PL_contig_8129_1_+2_110_418

MVIALFVHIYVCMCDCIACMFTCVNANDMDICVYVRARTRTMCVCACICLCVCVLPCFML

LRSQVQSLLTLHRQICICIYSHTCACARAHTHTNRHAYIFFSG

>SG-PL_contig_8130_1_-2_38_6019

MEVMENKEVTLTCEVNKPDKKATWQCNGKDLAPSDHIQISADGRTHTLTIPSVQLEDEAV

YKCIVGDRKTSGRLSVKEEPLAFITPLTDQKLMEYETLTLECTVSKPDRAATWYKDGTKM

TPSDRVTLTSKDKTHTLTVSRVDVDDEAEYSIKVDNLTSKCTVLVEEEEVEFVRHLEDID

VTEIPGTATFECEVSRVNTVAQWFCNNQPITASDKYVMESKGVIHRLTINEVEGKDEGDY

KVVVKGKTSEAGLFVEVAPQIFVDKKYDETIYLYAGQSTAFEVPFTGNPQPKVTWTFNDG

DLPDKKRMEAETIYNMTTIRMSHVQRQDTGNYTVTLLNDNGKATITIKLVVLDKPSPVKE

VKVTDITAETATITWQAPDDDGGKPITQYVVERREVNRRSWNKVGDTTTLDLSVSDLVEN

NQYIFRIFAQNDVGLSEPTESETITAKNPFGVPDAPAAPEVTEVYKDNALVSWQPPANDG

GSPVTGYTVERQTSFSPRWTVVNKSPVSELSLKVDDLIEDNSYQFRVIAHNKAGPSEPSQ

PSPKIVAKDPWTVPGKPGTPDITGTDKTSVALKWASPDSDGGSPITNYLVQFRPRGTTQW

LLANDTVTVTDTTFSVTGLKEGKEYEFRVAAQNRAGTGEYSAPTKPVKVIKPIVGEPPML

LEPLQDTTVVAPGDAILECDIDVGEPEAEIKWYRGKKEVRKSAKYEMSYEEEVASLVIHK

TEPDDADVYRCEATNPLGRVQTEGMLSIHTHPKLEYDNKLKSAQTVRAGTTLTLTVNISG

IPSPTVAWFLDAEPLQKSDRISIDTTENYSTLTVKNTLADDTGLYTITAENVVGKAQADF

EISVKDKPSKPDNLHVTEVLKDQIVLSWEPPTSLGNCPLKGYVIEKRDAKRNTWMAVQNV

DSTTTTYAVQKLLEGNEYFFRVAAENEIGVGEAAELFDGIVAKSPYDVPDAPKKLQPSEV

TRSSVTLTWEPPESDGGSVITGYIVERKSPSTSRWLRVNKSPIRDTVYTVPDLTEGSEYE

FRVIAENAAGMSKPSQPTGPIKAVDPYKKPEAPSKPEAIDITKEYITLKWLPPDNDGGNA

IFNYVVEVRVCGSTRWLPASQNIKVPETSFTVKELVEGTTYEFRVSAENKAGVGPPSPSS

GPITAKEPIMGEAPTVLEGLPDLAVMEGDTTIFECKISGEPLPSIKWLKEQRVVVEDKRH

SMVYDEHNVASLTIRDTTQKDAGSYTCEASNDLGTVTTSGVLEIQAAPTLEFDNKFRDLI

SLHVGATLKIPVKVSGIPTPRITWAKEDGVMKSGGRITIHTQDDSTTLTVKKVTKEEDGL

ITLTADNEVGEAKAKFDVEVLDVPTAPQGPLEVSDITADSAILHWKPPKDDGGIDLTAYI

VERRDTKRAMWTKVGSVDGVTLDFKVANLQEGMEYLFRVSAENEVGISEPLEGDSTVVPK

SPFDKPSPPEGPLEVSDIKADSLTLTWKPPKSDGGSPLTGYIVEKRDVRKTTWSKVASVD

SDTLSVPATKLLEGTPYVFRVMAVNKEGSSLPLECQEEVKPKKPPETPGKPVGPIKFSQI

LADSVTLTWLPPKKDGGSPIVSYTIELTDDGGRNWRTVDSVEAPSTVYTAKGLTEDQEYK

FRVSAVNKVGQGEPLVSDSVTPHRKIEKPSKPEGPLEPIDVQRNNVTLRWKPPKDDGGSP

LTGYIIEKKDAKRGNWMKAGKVDGVTTEFCVPDLVEKSDYLFRVMAVNKVGQSESLDTDT

ATMAKSPFDVPGKPKGLEISNVTDKTADLQWKAPDSDGGSPLTSYIIEARPDSRSTWTKA

GNVDGATTNFTVPDLREGIQYHFRVSAVNAEGQSEPLEGKETAKPTKKILPPGPPRNLTT

SHLGPDHVTLQWKPPSEDGGSKVTGYRVEKCEDTSEEWVKVEDIKSYDTTYKVTGLEEEV

GYYFAVSAKNEMGYGEPCETEAVVKPKKPEGPPGPPTGPLRTEDVEKRSVTLSWKPPKDD

GGSPLTEYVFERRE

>SG-PL_contig_8131_1_-3_48_323

MIKMLDLESIARLSMTCRQFRELCFCDELWEKIYKMHNKQPITQELLMVVDAKGWRNVFF

TNKLQLQMQMRRAIQSEGRGAFLTEDMQQMQE

>SG-PL_contig_8132_1_-1_274_456

MLCHVCCCLCECFAIVFSFILPSLCFVVSIVVCVSVLSSVVSLSDCQALFYILLSLCFVM

S

>SG-PL_contig_8133_1_-3_429_854

MHTNCHLRRLMFYHTHPTLFIFLVCLVLGLCFFVCLFLVWFRKFNETDRNLYLYSLHASC

CYPYIVHVFMFSFLFFLFKFFHSLSGCVCARPGLLLPSSCCQLGTRTWLSPVFFSSWLRQ

KSIIEPSRQIRVCTHHICSTLG

>SG-PL_contig_8134_1_+1_1_114

QENWSFSFHTCYQPTLHYHTLSVSNLINTLSTHSEMGG

>SG-PL_contig_8135_1_+3_3_149

HSPANDSSTCSSVPRQFLQLNVCPPQITFSSYHAPSSSSGWRTTCLSFL

>SG-PL_contig_8136_1_+1_1_69

TIRPQRPLNKRYQTQLPMTYDLI

>SG-PL_contig_8137_1_+1_70_333

MIKRNTNSTFIKLSLSTRNKIIQFFMIQLQKFQYAYSLPLIITQCEEKNGFLALKSNALG

FLHLINVVLRYIHVIKCNSSMDRTNDPN

>SG-PL_contig_8138_1_-3_51_230

MNCRHKKHTTLNSAQEMYLQYRILLQQNIVEGKKRNVQAKSIYATYSFQLTNTGFECLQL

>SG-PL_contig_8139_1_-1_250_435

MDKFIRATPHLPFVNCFLPFSVAFKKEDENAVTNLWLWNFCQVFFCGDELVALEFLSGVL

LW

>SG-PL_contig_8140_1_+3_3_203

FFFYSLKICILEKNKTIQKIYTTMKTGKNEWHANGMIIFSNVIIYKYNSLQYSCTRSTRH

KTWHAQK

>SG-PL_contig_8141_1_-3_3_266

GPCPSSCLISCSCTFSTSALVIPLPSSLEGFTLKYVIGTQAIPHGLTVPLLFIPDKCDIF

QYQLYTCTFPTGDQFTWWCTQYCALKLV

>SG-PL_contig_8142_1_-3_3_260

QQTNRQTTNKKNKTILKVRNCQYNRLLSIQSPTVNTIAYANDMHEKDKRQVFPYHRGDGI

LQKLCRDIGYPAVCMTICFRMLSCMQ

>SG-PL_contig_8143_1_-2_2_235

SSPQDGNTWLVFLLLPGSTRRSQVKMSRGQTASFTTLSCDWSTLKEFAQRRLLDCSIIGQ

NERNSLNVIYVLFYDWKS

>SG-PL_contig_8144_1_-2_2_475

VTPRLSFPYFECAILLGGRLGEGLSNEVECYRSDTREFSSLKPLPFKKRNEFAACAIGDE

IFVSGGLRSGEFWKYDPTFESWLRGASMMQPRRRHAMAAVDDTIFVLGGFDEDQVLDSVE

VWERKANKWEECSQLVTAVENMGFVAHSKKIYLFGGKS

>SG-PL_contig_8145_1_+1_1096_1308

MTLVVTRQCGRECVNICVKGIKLMVEGKKSLIIKKVILSPHTVQPCLRHLAPLPFHSLTS

AIMKKGNSLTL

>SG-PL_contig_8146_1_+3_1326_1616

MQITNFKQTQKHATPKTNKNKPDTINKQINDGQVILTIILCSATSKHVFHNHDFEKKVIQ

LINSLGTASLLPCFSPQMAYLYGLKKNPPKPQNVFKS

>SG-PL_contig_8147_1_-3_120_260

MESLRLSLIVYDVDAKSQDASLTVNVCVHHAWTLDNSQLCTVGGVSW

>SG-PL_contig_8148_1_-2_17_397

MTDKLKQVACQTIEQHARDLHELSQQIWTHPELNFEEHHAHKVLTDYLEKNGFKVDRHYT

LDTAFRATWGEGETAGLPRVAILCEYDALPEIGHACGHNLIAEAGVAAALGVKAAMEAAG

KPIGKLT

>SG-PL_contig_8149_1_-2_110_3322

MLKVQTAALLADSIGSHVSKLSLRCCRGLRTSGRQFCQKVDKEVTPRGTPYNKLTIGVPR

EIFPNERRVALVPAAVQTLVKKGFSINVEAGAGEQAKFMNEDYEAAGASIKAEQDVYNSD

IVLKVRGPLSEEIDKFRENGTLISFLYPAQNKELVDQLAQRNLTVFAMDCVPRISRAQVF

DALSSMANIAGYKAVIEAANNFGRFFTGQITAAGKVPPAKVLVIGGGVAGLSAIGTAKNM

GAIVRAFDTRAAVKEQVESFGAEFLEVNVKESGEGTGGYAKEMSKEFIEAEMALFAKQCK

EIDILITTALIPGKPAPKLFSQEMIESMKPGSVVVDLAAEAGGNIATTKPGQLSVHKDVV

HVGYTDLPSRLPTQSSTLYANNISKLLLSFGEKDHYYLDMEDEVVRGSIILQDGKLMWPP

PPPKVDPAAPAAAASTVAAAVAKEPPPPPNYFNITLKDSLMYTTGLGSAIGLGMASPNPA

FTSMVTVFGLAGIVGYHTVWGVTPALHSPLMSVTNAISGITAVGGMLLMGGGYYPDNTVH

SLAALAAFISSVNIGGGFLVTQRMLDMFKRPDDPPEYNKLYAIPAGVFLAGYGYCAQAGT

YPDIHQMAYLAASLCCVGALTGLSSQTTSRLGNTLGMIGVSSGVAATLGLLKPSPELFAQ

MATCMGLGGTIGTVMAKRLEVTDLPQMVALFHSLVGAAAVLTCFANFILEQPHFATDPAA

TITKTFLFLGTYIGGITFTGSLVAFGKLQGLLKSDPLLLPGRHGMNLSMLLGNVGAMGYF

LYDETPTVGLSMLGITTSLSSIMGVTLTAAIGGADMPVVITVLNSYSGWALCAEGFMLDN

NLMTIVGALIGSSGAILSYIMCKAMNRSLPNVILGGYGTSSTGTGKPMEITGTHTEINVE

DTINLVKDAKNIIIVPGYGLCVAKAQYPIAEMVDMLRKKGKHVRFGIHPVAGRMPGQLNV

LLAEAGVPYDIVLEMDELNDDFPETDLVLVIGANDTVNSAAEEDPNSIIAGMPVLRVWLS

QQVIVMKRSLGVGYAAVDNPIFFKENTAMLLGDAKKTCDALLGKLRQDYEE

>SG-PL_contig_8150_1_+3_105_227

MLEIHKMTQITGTLYFTSLVHAPDCTHHWSNSLYGLVRFLS

>SG-PL_contig_8151_1_+1_154_285

MCCISQVKTTSSFQTKTIGNATNMKYRTMLLNKPDWKINGFGQK

>SG-PL_contig_8152_1_-1_136_309

MESHHFWFFGKMVIYTLKHKHIYVLHEKLTFLALKKKKNVVLCVQKFQISVVSTVVPR

>SG-PL_contig_8153_1_-3_3_362

NDDDDDDGDSNGDDDDNCCSCCVSLRVYAQVYGFYALIILVLLPSHGIWGVGDYGIYSEY

RDTVLRKLKLKLCSTACFFIAALSVALPPCHHSHASLFGSPTYPQTTALKLSSPHCESRA

>SG-PL_contig_8154_1_+2_212_457

MFARLSYNQRLGRTSRTCQHVGFKHRLSEYATIILDFLLHHMAYAVFVYNYGHFSKRGDT

VLRKLKLKLKYLECQAISFSVE

>SG-PL_contig_8155_1_+1_1_306

IAQPTIKAISGLSPHKVHQSQEDNRVDHKTSANLKSKIPQNNTVHDILSQPSSSLRQTRP

GCAEDASHSAQFIIPRLKKSVKKYKKYFEATKTYIKRPYRQI

>SG-PL_contig_8156_1_-2_2_184

FKTQPTSHENGDSEAEDRLITKPSGDLTSHVFCREKHRRKGEAMEISVDSTTAVSFQLPE

K

>SG-PL_contig_8157_1_-2_2_184

VCVCVFSIGEREVIFKLEVDNIFQRVMLISLLLSCVVVTWTGLTWPHFGLHEPSALHACR

F

>SG-PL_contig_8158_1_-2_152_1267

MVNHLWDQRYYYGNLVAVHQDGDFVAFSIKSKHGGSVRVLHNHCSDRRLIKGFTGNVMDV

AFAHSPEVILAGVDEMGTLLVNACKLQDSDIIVTPLLHIKQSEEKPCPYSRVIWCPYWPD

EGDESESFLQDSSKVLACTYEGKIELLAVDVVTRDCGPGPLSTADIESGKITLVPHTEPV

VEAVFSPDGSALASASLDGEVKFFQINWSNTVDPPACLHQWKPHNGQPLSFFAFLDDLKN

MSPDAQFWKFAITGCNKNTELKVWSCETWNCLQTVRFLAPPEGMEMDSLTLKAGIDLSAR

YIVLSDKSNMVVYVLQVYQDAVAGRAHISSVADFPLTQSCLSFAIIDASTKKFKLSPNDS

HLDEITTGEVDQ

>SG-PL_contig_8159_1_-1_7_714

MAPVPVTLAGGAQCVKRKAVQEWERVVQVTATAPSLTRPALVVVTGQVMTATHLTVPGIL

WTAMAKGDAMVSTSSPLAVWIASSLWGTPVNDPVSMAMRQNPSPTSAIVTLATLTLAVRQ

SALPMAPATTISVSAKKTTRETDANCLTVQEIQTVAVEASASAWGRNPSVCVTLASLEAT

VPFWSVLAPPTVADMGTVLCQLAALSRSVSATMVLTALPVTSVSLTLRESHVKGAK

>SG-PL_contig_8160_1_+1_514_678

MGWERCCSIYAQLKIPATNCAFCVNATYLYKKSKKKKKMTRPKMKLGILKYVYIK

>SG-PL_contig_8161_1_+3_306_728

MSEGQNVMSTKQSIHGKYWVSTTTDRFTILRTFKYFFSSTKAWIDTVGMTISFHKLTLKV

NRTEGDGERGQRFTTDGSGPNTAKSKQPSCRVHELQRDRRGEGGEKEVMQQNRQQSGLVL

FACLFSSNTCLGLFYVFIFPD

>SG-PL_contig_8162_1_-2_134_706

MHKDAQAIWTVTTGHAAIKCLLEGNGTGYFHKGFYPTSMQEAIYRGRTTYPQDMQNIFKL

FSMSMRYIDRLYGNRFYGKGQNLIYELSRRYDAALQEVDVLVMPTSPHAASRIPPADCSF

ADFAKSCMPSGANTTAFNSTGHPALSINAGFTAPDQDRGRLPIGLSIVGKKFDEVTVLQV

ARALEKLTSEQ

>SG-PL_contig_8163_1_+1_1_297

FFFISFFFIHFGLGREGILNGGRRDRGRGGGDSGFLFISLISETISFLTNHVINQKTMEQ

FIFESSKHIIQYTTKIYHREHICYHITIIQQTMTNKQSQ

>SG-PL_contig_8164_1_-2_26_478

MESLATLVPLGRVLKRPLQQELRLRWSQSTQPWDTEIALGTWFVKAMSEWLDVTRLAQGV

PIAAPTQQMTLYTDASTTGWGAHMGTLHASGIWSQEEQQWHINVLELEAVHRALQQFEME

ASGSHSPVHGQHDSCVLREQKGGGGGAHSAT

>SG-PL_contig_8165_1_-3_72_278

MWQLDNYFCEQNACCSFSCTVRNIRLVSCSLGTDTSDHGVVLLSTVSSLKDLGDVNNCQF

IKGWTIVKK

>SG-PL_contig_8166_1_+2_539_934

MLLRRIMSHPWYVSMCMCVCVCMCVCVCVMLASLSTCLSVCLCLCLYLSVCLSVCLSISQ

SLCVCVGAYEDIRRKYMHFVYVCVSLTHTCAIYSMYACMHCTVCVNLCVVRYVYADMYIV

YVLHVCILYEYV

>SG-PL_contig_8167_1_-3_3_317

SLCLCLCLFLSLCLCVSLCLCLSLCLSLSLSVCLSFCVSVSVSLPLSPPLSLSFSFSFFF

FFFLFCFNPYFPLKVQQRAHAQQSQSLLPSNTHEGTTCTHAHVYA

>SG-PL_contig_8168_1_+2_272_418

MVDLRTGRLQSNSYRSCLPGGIRMGGIGMDLERYAAIIAFYLHVHSSKQ

>SG-PL_contig_8169_1_-2_590_835

MLYREVSCLVGVLHVCITKLYQEGKDFVHNVLFLQYLQINLFTRFYTYILMFACAHTSVS

VPHTHTHTHTHTHTHTQAGMHV

>SG-PL_contig_8170_1_-3_15_635

MASVKLFCARASAQSLLRSMIRPAYAAQERSHYKQVGMQDPDMNWKPIMDRASQTVFWTE

LIKGLGITLGHIFKEPATINYPFEKGPLSPRFRGEHALRRYPSGEERCIACKLCEAICPA

QAITIEAEPRADGSRRTTRYDIDMTKCIYCGFCQEACPVDAIVEGPNFEFSTETREELMY

NKEKLLSNGDKWEAEIAANLQADYLYR

>SG-PL_contig_8171_1_-2_254_1540

MEQQMQKVEERVRKQEKDRLEQEKADLRAQLDAEVTSLQQNLKRLQQEGVNKLDEEQERQ

IVSMRTRLEEIQSENRSLKSELTDSQTNLALVRSELTGFRQQLNEKSHELDMQKSTVMDY

VREQDNLTRQLHMLHEANKKLHDINDDLRMAVESSRATTTHRRSLSNDSGASRRSDSPRL

SLTQSNISRISSYVSDHAGMDTSPGSRPGSAGAMANSSLFEELNSSSPLVPRSLGAPSFD

DDYVEDIDSGISTNRDVELDTEIDSVSVEGDPHRRQMRRPRRSRSHVMEEEELDSHDEME

TDTDVRSDLDVRFHMRRGAGSCCSSAGARSLAREESVVSHSSRSSRGSSRRQLPNAPLEH

CGATIVKVTKEPERMYKVVLAGDAAVGKSSFIVRLCKGKFVPNLSSTLGKMVSSLYLYTN

ATTDLSCPF

>SG-PL_contig_8172_1_+2_962_1174

MGGRGEAVVWTVVCLSWCCCYCMVTGSQTLSFIMQSHAHLSISLPRSLSLAYKHNRDFLT

SLYIYITEEKE

>SG-PL_contig_8173_1_-2_2_217

STPSYNEDTRLKQITDEPDLDRRPDPPWYAAHKRLLTQAVMEDAMEKLLQPLLSKRELEE

EKLSHVGENTDQ

>SG-PL_contig_8174_1_-1_16_213

MPACLSKDPKVAAQYSAFMSNKYSALAESPPPGTIEWWDSNCSWGRDPRLQEGYMDTSDD

SAVYQP

>SG-PL_contig_8175_1_+1_538_831

MHTHTSLHFLNSPFGKSKCFYLHYHHYQQEQYQQLQQQYKRYTTITREERSRTLIVLTET

EAGNKIRTGESYIMYFFFYHGFQFCETVEQNWEAEDVH

>SG-PL_contig_8176_1_+2_527_721

MDLYCNIYKMEFFFLFNTHGHSLSHPHNFKHSPTSTFYSFSLWTLLRAKDLSVGMISERT

VVTET

>SG-PL_contig_8177_1_-1_1_222

DIREFAREASALGIQYIGLCCGSSAHLLREIAVEYGRRPPSMKYAPDLERCVFLANATKR

QIKLSSRLAGGGLQ

>SG-PL_contig_8178_1_-2_347_679

MNESYHKDISAGTVSRWIRETILKAYTRAQKHTSCLNPRAHEVRAIATSAAFQHSMPLSE

VLEAASWRSANPFISHYLRDFRLARADGSREISFIAADMPISLARDPQSNH

>SG-PL_contig_8179_1_+3_3_245

HLDALKKTKKPKHVIILFTTALSEHFGNLCARIYNVQKQLHNFTDPTKNFLYLKKKKKKK

NHCQNNLPFPVYFHIFSSNCD

>SG-PL_contig_8180_1_+3_3_239

HLDALKKPKKPKHVIILFTTALSEHFGNLCARIYNGQKQLHNVTDPTKNFLYLKKKKKKI

IVKTISLFPCIFTFFRVTV

>SG-PL_contig_8181_1_+1_1_159

TFFRVTVTLNFDHSQPNLFDRTFFLCQTCGHNLVKICQNTNKISQIFLFYPSV

>SG-PL_contig_8182_1_-1_1_324

GTQEPPRSPGGDINLGAFSVIDSSQPAHARTRESGTQSDATRSSPRGRGPPGFPPPAWIN

PSFDPLGPWPSREHARRDRARSSSSSSPAHDGIEGPWRDFSPFWALAT

>SG-PL_contig_8183_1_-2_299_817

MINLFLLDDKCVDGNSDDYVHNINRNENILSSISSKGSCNSISDNPVRRRVIEDGVCLHN

ISDDTCANIKSDKEDGCNSVNDVRLNGKRVSFLRWNVNGLLSKMNDKDFVSYVASFDDIC

LVETFVEKFQSTAVRSHTLFCKPAIRFTKQGRRSGGIMFDEKRIFTICSRTAD

>SG-PL_contig_8184_1_+2_2_175

VCVCMCACIMTVHKNVHGCVFVTKLKHIYMISFIHVFGKFRQLKNYAISKKGKHPEEN

>SG-PL_contig_8185_1_+2_605_781

MWPFMLCSRGVMSFDRFRGLESFSYLFDTSILQFSFKKNGEGGRGEMIEIRCVCALSVY

>SG-PL_contig_8186_1_+1_1_138

FFFFIFSRDFYYKITFLGGKTNKTDRTNKRDEHKLNSKSVMESGEE

>SG-PL_contig_8187_1_+3_3_257

EVFSCDDSSYHWNPASEAERVELLLCNGSSRSALKLLAQASVQPSSYQTTAQMSSWNSKD

YQSLINGCLQVLLIELWTQDVMSWV

>SG-PL_contig_8188_1_+3_429_560

MAVNKKFVNSSSAPSSKPLANSAIQITSKETTLHQWLTAMNNLK

>SG-PL_contig_8189_1_+3_270_608

MNQNRLDFLIYAYSSKETCLCLPGSSNLDQPNQFLQPLILAKRIREGTQLLWTPISSLNK

SGIFCYANYNHIFAILLTTTEQRLCSCSSKHSKKYFIIQTAILTLKVKAIMRN

>SG-PL_contig_8191_1_-3_3_218

MLIKVVTIFTFVYLYLSLHSQLNMLIKVVTIFTFVYLYLSLHSQLSKIMVTERFSCYFLW

YLIVYSTTCVLL

>SG-PL_contig_8192_1_-1_1_126

FYLKRRVHSIRFYVVLLCAGMVMVLVTRLHKVFLFMLFCFLL

>SG-PL_contig_8193_1_-3_153_284

MHINQKGKIFARHYYYGTENVFLNFKFNFFPLSVKYCILFHCLC

>SG-PL_contig_8194_1_-3_60_413

MMQFIMGEDTFIEALNLYLANKEYGNAEPSDLFSALDATMLQENMTAKMDPWVSQMGFPV

VRVTRTSAGYQLTQERFLIGSVDDTDVRYENALYDYQWEIPIIYATSDNTDMEKTRDD

>SG-PL_contig_8195_1_+2_65_313

MSPPLHFASLSHIMASKGRSLTILGGKHTQAVCHSCHTYLYKAGGCLFIQCTVLLIVPCH

GYNTGRIYYKVMKDVIPGHRYLS

>SG-PL_contig_8196_1_+3_123_257

MNTSPHLTYICCPAWDTGHQQMLSTHHGPEPAVPIVPRCGLSSSC

>SG-PL_contig_8197_1_+3_3_233

LFQSPLSSKYLATAEYTELSGSLCTNKYFSLAAMSFSVLASKLNQKCPELQLHCSFADNI

FEASGSFLHVENVARVR

>SG-PL_contig_8198_1_+2_152_298

MNVLCNYLICSGFPKQAYKQTASTVPKNIISHPICSSNSIQTQQIYIFF

>SG-PL_contig_8199_1_+3_984_1277

MPQKSSRQDTKRPDQKRRDPRNGWDNASQPLQHTTEDHVAWSPDQDASQPASPSSLHHHM

LTLQITVALTRPSRHTPHLLHDTAPGFPSSCRLPTSSP

>SG-PL_contig_8200_1_+1_916_1236

MFKTAENSLLRTHTHMHAHIRAYTRTSKLHVKLLHYFTVIPQLLASIWNALNNVKHIRIH

TPTQLHTHTHTHTQLHAYMHKTHICVTHTHTHTHTNCMHTCTRHTFV

>SG-PL_contig_8201_1_-1_64_1932

MGASLSNRRNTGVESVDVSSANAYRYPPKTGSYFGTHFIMGGERFESPQPGEYLFGENED

LNFLGNKPVPFPYPVPTGNEPTKTLKSLIYIRKDSLRLVKAMAAEKLCLDQEESSTLYNI

EFVFDSDVRCTIKIHYFAAEEVAGGQIMYHSKRAALSSEAFHYKRGVAQVFSQPAHLIDP

GKFAEEEWQYSVEKEIYPIVIHCQVEEEEHIGHSHIMYAVIEKSPEGSYIIKPLKQKQFV

DGLCYLLQEIYGIENKNMERLKDVDPDEEVEDSGAECVICMSDMRDTLILPCRHLCLCSA

CAESLRYQASMCPICRVKFRALLQIRAMRRKLPPVSGQQQTEADENPVSQEGVPPGFEAI

SLVEALNGQVVPQNGQTDGVNHPIGVPPSLLSVDKKGHKETKVTLSTSIEYQKEMDPATI

VEKKNDGKESEKEEQSDDNVVSTPMGIELTHTGVVDPSERRRSPEKNTVGAGGDELAPDN

RGQVLMPRNNSKPKSVEGWNGAPSSGEMCLQLSTGDMADDEREDSSTEPEPDYELEEEVE

EESYTLSDTQGMMSSESVVSGTLSGQLSSPEMEPLHVNTEVRLMLADVPGSGTEGSSFGS

NCSSHALLNSGDTTEDGEQKMTI

>SG-PL_contig_8202_1_+3_129_272

MKMKMKEKQTTDKYIILKHLQDLFHDHNFNEHMHERVNFQSDLERERE

>SG-PL_contig_8203_1_-3_69_695

MSVRYEPVIDEVYNDDENLPERPEIYFIRMANMQGKRWPKAIGIVQIIVGFLISFLGLLE

VWVIPISEDHTASLQFNKHTCYGAGLWAGLVMVLTGSTAIRASLSKRKTTVYRFYNLSIV

TLLLYMGLTAFLVAAYAQGWTTEDAYPVNSRMHQVHMFVTIFVVLGLMFALTAVVQYYDV

ICCGDLKLWRWWLDCLCLKSLRGGRKWQS

>SG-PL_contig_8204_1_-2_92_277

MCFVFVVIRMRKQCFLPASIVRQCLTAWHTKTLASCANLPAPDFKTITITGHFLNPCPLP

FI

>SG-PL_contig_8205_1_-3_1929_2111

MGRFIKGLIRFVCPNEWSQMFMFESCTRTSSEFIVVFRHILTKYWSTLKLLEFIAYTQLL

D

>SG-PL_contig_8206_1_+2_47_142

MRRSILAGPMLVTDTNFIFIPYLLSHEICYMR

>SG-PL_contig_8207_1_-1_1_180

QEKQSEEENAEVIVKIFVEFSVQEEVEKAISALNGRFFGGRVLTAEKYDQEMFNANDLSG

>SG-PL_contig_8208_1_+2_2_112

LLCVIRYNYLMQHEQKFQIRNRRPTSVSVTPVHMETY

>SG-PL_contig_8209_1_-3_219_845

MTGMRQMTLTLACALTLTCALGAVPAQGVSLRDESARWRLELEPYRSPQHRSRRHVFPGT

GMPCLTVAFLTEMKGRFRERIDPNDDGKATMPEVRQYLSNFNPEVTDDQVNRFITRRDLN

GNGIIEFIPEYILDIATPDMTPEAASEWFELEDSDDDGYVTRAELLRIAQRLGMTPQQAD

ASVSSYYMTVDADKDDKLNFDEYKVLYSE

>SG-PL_contig_8210_1_+3_66_197

MYLEKNTPFGFIYCTMLDCKQNLKIGGGIALRFKWGCWIGHISR

>SG-PL_contig_8211_1_-1_328_486

MHLSFLNLDPTFSLVSLSSSSSLVPRPAWLLWGLIGCSSRQDSPSLSPRCNTK

>SG-PL_contig_8212_1_-2_77_571

MAPPSYSDLGKAARDLFSKGYNYGFFKLEAKTKTDSGVEFTTSGNSSSDTGKVCGNLETK

YKWSEYGLTFTEKWNTDNVLNTEVTIEDQLAKGLKLAFDTSFAPQTGKKNGKIKTSFKQD

YVHVGADVDFDFAGPTVHGAAVVGYNGWLAGYQMSFDTAKSKLAK

>SG-PL_contig_8213_1_+3_93_392

MLRCYWRCSKPLQHPNQCRLFISHNPEYAADIRRSSLSRWISELISEAYRAYRRQLDSKQ

ASIHKSEPGLLQLHSNTTTPWQRSWTQHTGAHQEHLYPTT

>SG-PL_contig_8214_1_+1_55_219

MSIPSSVLTDIDPSLNSIHINIHPLFHQFLFTPSFVLTDIDPSLRSIHIYHPSTL

>SG-PL_contig_8215_1_+1_532_894

MPVFHTFINPRSFHFLSSLPSVSPLKSVHICAVIHLSCPFSTRSSIHVHSIFCLHCHLFH

PLSQSTSVLSSISVARFPHVHQFMSIPSFVLPDIDPSLRSMHIYHPSTLSSIHVHSILCF

K

>SG-PL_contig_8216_1_-3_165_1421

MADNPTGVLIKGGKVVNDDQSFDADVYIENGLIKQVGSNLKVPDEGVHTIDATGKLVMPG

GIDTHTHMQLPFMGTVACDDFYLGTRAALAGGTTMIMDFVLDQRGMSLLEAYNKWRDWAD

EKVCCDYALHVAVTWWSNQVAKEMETLTKEKGVNSFKMFMAYKDVFLLEDADLYSAFRRC

KELGAVPMVHAENGHLIALKSKEMIDNGITGPEGHEMCRPEDVEAEATQRAITIADQAGA

PIYVVHVMSKSSADVVSAARRKGKVVFGEPIAACLGTDGTNYWNKCWRHAAGHVMGPPLR

PDPTTPAYLMDLLANNDLQLTGTDNCTFKGEQKALGKDDFRGIPNGVNGVEDRMSVIWEK

GVHSGKMDPCRFVAVTSTNAAKIFNIYPRKGRIAEGSDADIVIWDPNQSRKISAETHHQ

>SG-PL_contig_8217_1_-2_176_355

MMVIWVQGNDIRCDYLVSKKKRGGSVNIVKFIRPVEIIPCCNMLLSHDASVNCTDFLFQK

>SG-PL_contig_8218_1_+3_279_515

MSQDCECTNTHPKRHKVRNRGESKRQNENIRPQRMDRTGVLEHHWVYQGPTERAVISMVS

SLSMWSHRQYRQLDFTHIT

>SG-PL_contig_8219_1_-1_1_273

VVDDTGEGDDRPTSPPLDQAIGVDADSRHASPAARGFPPPTDDAPPSPAVLTAVENAETE

TADHDGPPASPQSPRRSPSPDAAPGTQEPPR

>SG-PL_contig_8220_1_+2_2_142

DVKMAGYDPNFGDRLEKNCIFFKMDPRPIPTMSRLFWGFVLKIPCDD

>SG-PL_contig_8221_1_-3_84_242

MFRYIMLVSRACALIAIIRVCLVSYGISENVSYISLISDRVSADSSVFIVVFC

>SG-PL_contig_8222_1_+3_3_86

FLNISLRNKLVKLVSLSSFPGRKLRSVG

>SG-PL_contig_8223_1_-2_53_367

MVKIICHALCFLAGFLFSCLRRFCVQRRCLVNFHGLYVVTALNGLGGQLGFKHNNNNNNN

NNNNNNNTCLTLGADFWCTDVFYPTIKTTMLHLQGTLKKMWKKGR

>SG-PL_contig_8224_1_-1_130_477

MVQKNACFWQFRYVYMCHCFRRNLSERYIANSVKVFRFTASSALLASFLFIAVLVLLWFV

FLLSQMAVLSGFIVVRFPPIRNGSSVWERCDDMGCCLALQSRVLYIIICQTDFLYA

>SG-PL_contig_8225_1_-1_1_117

FVCLIFFLPALFLCFFISWVFCFLCFCFFNLGVKDGNHA

>SG-PL_contig_8226_1_+3_123_284

MFLSAHHSSTQQACPEYTGVAYLSARSKRPNIFIVAAHGFTDVPWCDGRVVGCV

>SG-PL_contig_8227_1_+2_863_1177

MLIFSSIIVCAWAEEGAAAWHNNTVFTASATAPDRVVDTLGAGDTFVAGFLYAYVRHMNV

QDALEFACGLAAFKCTKDGYDSLHEFARSKDSDGSIVSQSLLKML

>SG-PL_contig_8228_1_+2_923_1090

MDCSCQPVCPQFTMMDCSNLSTQQLAMLVRFACPKNAPYCSRVPHYSQSLLVQRYC

>SG-PL_contig_8229_1_+2_1289_1492

MVTLLFFFLVLHINIFAGQKSHFGRKTKNKQSKVKFSSHSTRVPFIHCFQMYGDVFSSCF

FISYSGEK

>SG-PL_contig_8230_1_-1_7_2184

MEAQQMSQSFTQSYSAAPPRIDGRLPTGGDNQRFSSYSGSSFSTTFDPDNVPVDRSPVYS

GFLISFMVDARGGAMRGCRHSGVRVVIPPKMCAMPTRVTCRLIKKEKLMKPPPMSEGEGL

AARILEMGPVGTQFLGPIIMEIPHYASLRGKEREVVVMRSDNGESWYEHPLTATEEAVSE

ALGGSLEELEPEENLQEKRITRILTTDFPQYFALVTRVKQEAAVIGYEGGVLSSTVVPQV

QAVLPEGTLTKPIKVGLQAQPVAPELVAKLLGNRVAVSPIVTVEPRRRKFHKPITLTIPV

PKAAQKGMINQYGADRPTLRLLYSLAGNNYSSNRADPAPTNWEDITDNKPITLVNDCVSF

TTTVSARFWLIDCQNISDVTRMASVLYHEAITVPYMAKFVVFGKRTSTEEGRLRMFCMTD

DKVDKTLEKQEKFVEIARSRDIEVLEGRPQYIEMAGNLIPVTKSGDQLHVNFKAFRENRL

PSTVKIRDPDQDAAARVAFMKEPKLARGEAPQIPICNLNIRLPDTISSSSSDVSSDTAVE

IQKRHSLLREQGIVLEDKVTRSSMNLSDVADNVQGDWVILAQQLGVTMSDINRIKTDYTT

LADQALAMLQLWVEKNRDKATGNTLESALQKINREDVVKKCMYNTEVVNDEVEAAAARVA

MDQSGFDTFKEEVGITKDSMKRGMSLDVQYDEQDMIKESESAEEESSPGSDRGSIDRGTA

PQAIAE

>SG-PL_contig_8231_1_-2_2_160

GWHPQHSLVIFALRNVMVKIICNELCSFAGFLLSGLRFCVHQVVFLTLMDFTW

>SG-PL_contig_8232_1_-2_389_553

MDSLVNFAFKLMYITITCIISYCVVNIKIIFTPGNSFSVIVLGEKMFFCLHVYII

>SG-PL_contig_8233_1_-3_3_122

ERERETNCVSVCLAEAIIIVFKIIIVCWMSVCVGVYFSCC

>SG-PL_contig_8234_1_-3_447_668

MCRDVYILCLSVLCLVIVSFAHSFVRHSFCRFFAFNSHHFAASIFFSPFRSCISCLFTAM

LLLFFYCIFFSFFF

>SG-PL_contig_8235_1_+3_555_953

MTRRGNHMPDQNNVRISGLKINLNDIIIFTTDTWHHQRSPKDIIYIILMTSYRHPYHIIQ

KCHQDHPHNATRTICLMSPSSSKNVIQITYIMSPRSSISSHLLTLLLPHPIHQQAAFGQG

EGEKRFISLRPSL

>SG-PL_contig_8236_1_+1_1_240

NSIPTFWKFYARNLFLSTNPIVSAFLYRPLFSALPVHSTVLFQKRFAFFFFLYYPPRSDY

VLYLMWGINPLEKGPDTTQI

>SG-PL_contig_8237_1_+1_142_354

MNDAPIRPLVLSGRSHSGRYILLPTLLQTLIVSRVVATHVPRQQHAAKSLIFIGHVVIRH

WRRRWWHHRKR

>SG-PL_contig_8238_1_-2_5_706

MATGRVLVYGGKGALGSTCVKFFKSKNYWVGSVSHGASEDADANIVVTAASWGEQEKEVS

SQLAKALGENKLDAILCVAGGWAGGNAASEKLIESADAMWKQSVWPSVIASGLASRHLKA

GGLLALTGAQPSLAGTPGMVGYGLAKATVHQLAQSLGGEKSGMPPGSVAVAILPATLDTP

NNRKWMPKADFSKWTSLEFVAELFVKWTCGQERPASGSLVKLVTQDNQTQLVVV

>SG-PL_contig_8239_1_-2_29_250

MFEYNKFKQRILNEKVTTPTTTVYKCGPLIDLCRGPHVRHTGKVKAFSVTKNSSTYWEGK

AEAETLQRIYGISF

>SG-PL_contig_8240_1_-3_3_173

WVLSLSECVALHTISGIDLVALTSYIITCACIHVTRTSLFCGSSIASHVQNRFNTSA

>SG-PL_contig_8241_1_+3_3_278

VVIREVNRKLRRNWCRARLHWTVNDHWKYILFSDEMMTDLKNTGRVTVWQKSSEKWRPEC

MGVACDPLAWGLKLMVWGCVSYFGVGDFVFME

>SG-PL_contig_8242_1_-1_625_774

MLCVCIQCVCGQNTHARAKLLVYYLLMYRDIAKVCVTSQAQYKLHMSKFA

>SG-PL_contig_8243_1_-3_270_527

MSKTAEKRKLWKHHENARQIDHQVMEPPAKQRTVSAVADTHHSPTINCQLTTTSSYATAT

SSHTTTASSQSATTPAVSLLPHPLSI

>SG-PL_contig_8244_1_+3_12_266

MGGLREPAQKRAPKRSTTQAMERESRTQGTLLTLIYTVTCIVDGWTARSTTWDCTLITPE

RVILWQIVFLHAAAMLFISLPVQQT

>SG-PL_contig_8245_1_+2_50_274

MCWQGLEVLPSLWILYIIVLACLRLLLNNIRLSATSHAALHWRKPPRSHCSYRTSPSSSS

LSPPEGSTDICNYIS

>SG-PL_contig_8246_1_-2_2_88

RLLLAAKNSKASRANLPVWVCTSLTWYSI

>SG-PL_contig_8247_1_-2_158_340

MWLCLFLCVYRWSDIFLYNCVFSSFSLQRVNPCGCACLGAYLVFYSLDYVNFFPPVFIMS

M

>SG-PL_contig_8248_1_-3_3_170

FKDFYSIKKPHGGTYLLDEMINRDPMCSMRLRHVKEAMDTEEFFLTKCCGKFHFDK

>SG-PL_contig_8249_1_+2_2_220

LALNSLLISTEGSLIVCYQAREIMRLKPNRNVRCENPVSQIAKDPMGLSVAKKNYHAYIY

PKIFSTDLHESQE

>SG-PL_contig_8250_1_-3_3_206

SDFWRKSDFLEAPGVILEIRANLLRKFSGIYRYDIFFFVTDIGSSAIRLTGFSHRIFLFG

LNGIISLA

>SG-PL_contig_8251_1_-1_283_456

MKLLICMPQTYQLVPLTMNVFLFSSPMCTHIFVGFDVYGHFLFFVVVVVFFDFLTHAI

>SG-PL_contig_8252_1_-2_428_643

MLCFFISYVRCKLSLSCQDFECTGVIKTLNLKVCAMRLILHRHTHGHKDEMYTLLCWEWN

FHCSCCLIGCLI

>SG-PL_contig_8253_1_+1_796_1062

MNVNTLIHMMINFSKTVCVHNGKTNKLARRHVNAHKTWNGSRYMVLHFVTIHTPPVKYPT

LKNRPPPPPLSMYVCVVVCVCVCVCVCVW

>SG-PL_contig_8254_1_+1_1_147

YINKHTKSQLKIQNSLRKSKIRSLLYNFCYKLVRENLVFQGSLEPFPEV

>SG-PL_contig_8255_1_+2_188_490

MKAIACLLIVIHVCSAVSSPAFMGCNCHEHSHIYGSFSETGHQKCDIGSQNSDYEKNFKL

HGYMSLNIYLRHLNAIDHKSCYHFYENGDDNSETGMISVVT

>SG-PL_contig_8256_1_-1_1_198

GQLRLLDKQDAGETTVAAEVSTGHLSAIYAPPWVQICTGLPWVQLYSSPLGSSVLRQTHL

YCSTLG

>SG-PL_contig_8257_1_-2_134_1045

MRSKSAFFIKVLDRGERNSCSRCDLVFKPLPSSNLAKKREERARKVASSSSSSSSSHPSS

RDEKPRERRPETPPKSASVESQSASSSATPSQHGLYAERHTPRPYPLANPDTPALRQLSE

YARPHAMSQDPQRAAYLGLAGHPHMDPLASYRHLAMYPHGSRERLELELERDKRERDARE

REMRERELREMELREKMKADMDLKPPGLDRLLPPSANPLDPHWLEIQRRFAYPPGGLPVL

PHAAPGSAGANHLPGVYPPPSSLANELAAREREKLERLGVPGAHQPPLPVDMSYASVVER

LSAE

>SG-PL_contig_8258_1_+1_1_144

VDVNESGILSGAKSKWKLCELYGNHLVLIVQGVLDVKILSISKHNLFS

>SG-PL_contig_8259_1_+2_2_166

DVMLQVQPKRVIQTDDSSPGSRYQIQSQQCLVGFIGSNDSNTKICHKPNNIINPI

>SG-PL_contig_8260_1_-3_30_224

MEANILNNSDAEVWGYLSLVCLRTARKVEAEQSFKHAIKLQLQDKAILNEIHAEQVKAGF

GSPQF

>SG-PL_contig_8261_1_+3_3_209

FSSFSYKLHKSKKVDIKSSRRLGFKSNLSDEFKSKPNCPIPRTENHRFNFTKTHDQHQNY

LLDFTKLGS

>SG-PL_contig_8262_1_-1_67_336

MEPILAGSSGVPMTGEPKPSASRPGAQPSSLSDWGDSLPKSTANKLDSVRKSLPFDDLDD

TLHRGQAEGEAAGDTDGGADDSHLSSSTER

>SG-PL_contig_8263_1_-3_513_734

MEVQRSTQTLTLYSEWALLTVLDLSHPTSENFYHKNGDHCYGLPSFYFLSLFLAFYFLSV

VLPLQMLIVAFVVV

>SG-PL_contig_8264_1_+2_1088_1324

MQQLSNSQMNRPVAKVIKTTNWQLELKLDFVLERFITLRLRQFHMCSTQKLQLPGSLFER

LRKFHMRSTQKIAITWQYL

>SG-PL_contig_8265_1_+1_169_294

MYLFFKVPENMENILIFTVLRNTCKYIKYNCSKALTTKSQFH

>SG-PL_contig_8266_1_+1_52_225

MHAHTLKHHHDAIKLPSSHPAHTVHCNLSRTICKEQNNQQCVPEQNNNDDLVQENHVS

>SG-PL_contig_8267_1_+3_708_938

MSFILFIDIIIFLNKNSFFSLAHFDNEIKLIHKLSTNNNYGKTSKTVVVCFASATLDFWN

TKLGEKEKLFWISIILH

>SG-PL_contig_8268_1_-1_1_96

LNMDCCALKFMVHRCFRTSFILLYSVTDVLES

>SG-PL_contig_8269_1_-2_245_676

MGFREDPDEVKDVPSSPQPEEGSPGLLARELTEPPVDSSSDLEELHKTLQDRVIQAATAL

AAIPLVELRREAPHAQVSLKLSVPVSAQLLFPGRLARPQVPGLFLHRSGNQSPPWGPIPW

GSILKIPREVSDPFTQQTSSLQGG

>SG-PL_contig_8270_1_-1_241_705

MPRLLTAPLKAVLSDPLVMVPTTPSLALTTKTWPPTTMLYMSRKRPKLRRSSRPCHVLTG

STRPKRARCRMCRPPPVSPTLPGAKHTNCGSLRPPSFAHNTPLMMSGGKGKGVQTETSRQ

EHPAKNGHNWGNNNSWAARTATGILVRRCSELENR

>SG-PL_contig_8271_1_-1_1_231

LQAVLHVAVTAPAELRASVGGRVGRGLHAISTPSEFSVLVSIQLYGELKQGHHEKGRPLK

RYKDTLKANLISCGINT

>SG-PL_contig_8272_1_-1_631_864

MVLSHCCFGMRNGSAFFCYDLKYRVALKTHSCFFLCLFCISNCLPHPHPKYLHPDFNFSP

LIPDSPSPSPDHLMHFVK

>SG-PL_contig_8273_1_+1_1_135

GVFQRLQQLLPIDILVNITVPDLSHVHSPEHMSMYKETDPSVTAV

>SG-PL_contig_8274_1_-2_26_250

MDYSEFYEDLENRVEEVGFSNVVVADDWKLLLDLNMNCHNYKYVNGPKAKEKVDDVILHL

DSTDIWQERCVDSEL

>SG-PL_contig_8275_1_+1_1_129

THTHTSSSLLKNASHSNKNACTCPSSQVPSQTEHLINCHASDQ

>SG-PL_contig_8276_1_-1_1_150

SILVVKGYEEVLAKGVFMLLFAFSILTYNLCPNSHVIYFNICLLRCYYGI

>SG-PL_contig_8277_1_+2_41_499

MLAGRSISSVKQGLRILTVTGKQRLATRHVSASCQCHAKQKHQEDEGPIPFSSSKAASWS

MTESVVLPDVPWYQAFSIGISLGVFLVYFLFLREENDLDVELNYSLFERVPNLEEHQVKV

ALDYNRRQGKDTRELEERLAELQRNKQAFKDSS

>SG-PL_contig_8278_1_+1_79_219

MDVLNYISKDELNLQQQPVVNGGTNDRWLGMKHSLQPNFPHNMLQLS

>SG-PL_contig_8279_1_-1_106_1308

MEYFGAIVLIVLTTSCTGLELGIPGDSTRTVKAGTKMNLTCNVDKGLPVILEYNFQWIHV

SKANPAKPVETILTIQESLQDGASPKYFGIDVKWEKMMCHFTLMFKLVLPKSDGFYKCKA

IPQKDPSLDIMEKEIRVIVVNDVESIAMEFDGEESITEEVSTGLEREEGEYGVTCTATGF

NPDPDPSGATLEMYLGNEKVETTFTVMLDVPASEAAGAPRYTASATATNLYLKSEHSGLL

LECRAQAEFQGSHALVASVPLTIIVLEPVITCDKNVSAKPGDTFVKITCSVIHDNIDIER

YAWEIGNGVEIIKPQGTSKEFNEMIVQEIDDITTNVTLDLYQVDEWHFDTYYYLDVFTKS

GQNFRHSVQLFNEDVTPSSATKTTAVFVLCALCVAVNFFSP

>SG-PL_contig_8280_1_+1_1_120

KIKRQKDPPNWHNHTIHLGIQLLNLSRTAKQSMSKFTLVI

>SG-PL_contig_8281_1_-2_206_460

MSFPIYILNWCVLFVYLVMNGYCILPLVEGLFLCSIYSITWNCLPFPFKSFYLLTFHCSL

KVFFCQQLVLTFCCSILFEIGLESA

>SG-PL_contig_8282_1_-2_2_154

VFFMHVYLLEGSLFYPVQWRFFSFQVKHRLHTLDCTGFDWIHFLSQRNFSM

>SG-PL_contig_8283_1_+3_138_284

MQYLSSPRIHCCVLSLLDTSPNAYTCFICCVYVHTVNTEGHPCTHTCIP

>SG-PL_contig_8284_1_+1_1_189

TGPFLPHQDRHSVPQRETLVLSCHTKIDTVCHSVRDWSFPATARSTQGAFTGMIITNTTS

SLV

>SG-PL_contig_8285_1_+3_489_656

MAGRSPTFTLEKVLEEIFQDADSGDKDFGPNDTELLEVHVNHPDFNLEGSLLDIVG

>SG-PL_contig_8286_1_-1_1_201

QKSAMLVVIFICCYDKIIYTHNFFFTTSFVWGTPIYYFIILSFLFFLVLFLKKSQSLLHT

SYWRSSP

>SG-PL_contig_8287_1_-2_209_454

MFNILLKLLHHFCCSCYLCECFFETMCSFVVVYVCMIICSFFLSILEVHLVFLCIFCIVK

ELCYFFVSMFVVCLYFIFLSLF

>SG-PL_contig_8288_1_-2_2_163

LTPCNFCGYVCVSVLSLPPSHLLLSIFSSHPACIFIQYCITVIVRLLCLFAICF

>SG-PL_contig_8289_1_-1_1_177

ILHPLEEKFWMLPCVSVGFDFPLLPFFHSCHISRHCHFRHRVLSCVQFLHAAHDTPSTA

>SG-PL_contig_8290_1_+2_2_166

AAAAADADAVAATCLLNDSGVCVVIVKQSFQYLVMKMAVSSVRKKQQISVHSGIV

>SG-PL_contig_8291_1_-1_1_186

NKVVFFFVVSSFVFFIFFTFLDFLIKVCFTTYIVGFYTLHYNCTILLHYTNAGFTYLCSA

QD

>SG-PL_contig_8292_1_-2_2_178

TKGVQTLAGPRRSEKSLPSPLLPPPLYAPGRIEPGPTGCQSDVLTTRLLRQSTVISFLM

>SG-PL_contig_8293_1_+1_382_645

MANLVTPHMLALLQIYISLSHTHTHTHTHTHTTHTHPLLQTPYISMHALQVCTIHSHTYM

YTCAHTHTLHSHICTTLGATHSREQQQA

>SG-PL_contig_8294_1_-3_162_257

MPGIVSQTDQQVLKWLSSVRPECMHVGIILCS

>SG-PL_contig_8295_1_-1_139_945

MPGNQRSLRMRYHQRQRLDHSDGEFETLAFQNEAPRPSNDPPPLASDESEDEQSESGLGE

SPEEDVEDALLYGSSDDDDEKEDQHESDSDSAPEEDSKKKSEEAEGKRQQAIIGVIRKEK

SKEKEKRRKRDEFFKLQKKEKLAALNKRKLPEELLEELSQTSKKKQKTEKETEEEKEMPS

SEEEDEGEEENSVDEKGEEFPQKSADFIPLSDTQEGKVQVVSLHAQKRQSVPVSQTAAEF

KYNLLYGGRHKRVTSQQLRMQKAKKMARR

>SG-PL_contig_8296_1_-2_728_1963

MDRTTAAYKMQFGVAKSFHERTIDIMKKHKFSLNLDEATSKSSTARVLTILVNFVDPVST

KVTTQHLVSLELIRVDSESVYNAVETFFDFHKIPWENCMSVLMDSCAVMRGTKQGLETKL

RRKAPHLLNIDGDVCHHIHNATKKFCAPFDHWAEGLFSHLFGEFHWSAVNQDALKEICFV

LDSTYTSPQNYVFHRWLSCYDAAQQTVLLLRPLTVYTFSFLSKEDKALYRAIVDSICQDI

TSEGRRKLISLQCDLALKSRTKDGKERVRRLTNKIFVHRMWSKSVLGIYISALAVLKEYV

CLFQSDECKVHMLHDSQMNVFKRFLAMFVKPQVFSRLGPEQITDTKWLSVNAEENLLSLH

EMHTGSYAQKELSALRAKDKLVLLFLETAQKDFIGCGEYLQSKLPISNDVLK

>SG-PL_contig_8298_1_-1_211_417

MKAYLSLLAPTFSIFTCTYRHVLKWTILIFFLNQNDNLPVHKFCFLAPIPHSPLAPRQSS

LCIELDGSA

>SG-PL_contig_8299_1_+3_3_197

PQFSFLCVFFPCCLFFFFLQPPSSFYISFFFNLLDFFLVTTDLLCEIWAAFRRCHCSAEP

LKLRK

>SG-PL_contig_8300_1_-1_295_2040

MELNDPPLVPEIKIEIDVAEEEMPQDWRTEENNLQDWEPSVSFQHPVKTEKEEDEGPVLP

AESRERTKKRGRHVIQTQPPSTITRTDPNFMDENHARKSVTPEDLVVTNTNTDVRVKEPC

EDGSEVCVKREPCTESQVSEIMVKQEPADDHLDPASGSALADSSSSCSTMPSDREVDIKV

DLDRNLVKDDSSVTLYNDTVPSADVSQGRIRTTRQTRKGNAKCNDLQKNRKCSPTIPVDV

TDSGISSDLQQRRDDIGKRTYQHSDLDMEEDEIDIKKKRNSKRSACICSVCKKKFKSSSY

LTVHMRQHTGEKPYSCTECDAAFTRLGHLRVHKRTHTGEKPYCCTVCGAAFIRSGDLTVH

KRKHLTKKPYPCTECPAAFTKSGDLKIHMRKHTGIKPYECTECDAAFTATSGLKIHMRKH

TGEKPYPCSECGASFKTSGSLTVHKRKHTGQRPYVCVECDSAFTQSSDLTIHMRKHTGEK

PYACKECPSAFKMSSHLKAHMRKHSGESPYACAECETPYTESCNLKGHLRKDPSEKPYCC

TKCKTAFTLTGNLEVHTAKPNGLNTYSCPQCDAAFTVMMQCI

>SG-PL_contig_8301_1_+3_1071_1325

MLVSGPNLLRVYNGYNIFSKCYSSTKGTQIRWFRTRLLHRLLPTGRFLFLQKLVDSPMSV

FCCTKKKQPGTFLGTTLFLTHFGQI

>SG-PL_contig_8302_1_-2_392_631

MDCLLCSASNDCSTYLPSLCCLLYTDLAIPSVGGLAVCAVTYSLSVGPFPFYYLLLVGDV

VCDVSVIFVVVSTHIFCEIF

>SG-PL_contig_8303_1_-1_952_1158

MIAARNSFHTWSWSYCCLIRRWYCYVHGQGYYCSYPTVVIRVSRLLWCVLLFVQSCLCFC

LFIYLLDLS

>SG-PL_contig_8304_1_-3_375_2747

MSVKKLNSPQWKKFQPPPTAQDPAILSFHQSPTNTLHSPTSGVAPTMPPALREKGIIDKR

QHSYGFIQCWDRDARLFFHYSEYNGNIDTMKVGDPVEFSTSTDRRTGKPIAVQVTCIDVT

QAVPTTLSDEEFVGTIIQEAKPVKNKNGTVPSNQDGLGRVSYEHSGECFFIPFAQEDILD

PTTKLKTSDQVIFHLGSDPLTGIVCARSLQLVIPPSQRYQGVICSMKESFGFIERADKVR

EIFFHYSEFRGNIEDLRLGDDVEFSIQMRNNKDVAVEIEKLPDGTVIFEDLTMEKVVGRV

LKTLKVQNGRRPSDPLGGRIFYEKDKGMHEIQFGDKDQYGDCTLQQGDVVSFNIATDRRD

GLQRACQIQLLEETFANGEPRETGVIATLKDGYGFIQCADREARMFFHFSEFMDPEYIPE

DKAEVQFTVMPDPSSASRMVATRIKVLPKGSVTFMTVYPSMYVGTVDKEPATHKNQNKNK

EAETGSIKYDLNSKIQTITYSIKDLKETNQVPRYGDKVEFQIGEQKANNRKVAVNIRVLF

RSPTGRCQGYVATLKDNYGFIENDKHDQEVFFHFSAFEGNASNDLDLGDEVEYTLARKSS

KMSAENIRKLTKGTVGPGEDIQYSKGTVQGKIVRPMRIVDPEQDEYCGLVQVGIEVEEGE

KMETYPYGITSLADKRDFLQKGDIVSFQVAKVKGTDVLRATNLAAQRKFIRAKVDSVKGQ

FGFLNYEAEEGKKLFFHMTEVHDSAQISPGDEVEFVVVQNQRNGKYSACSLRKISEKQRP

ERLISRLKSFT

>SG-PL_contig_8305_1_+2_2051_2356

MCEAFTAVFILRKTLFSLSLTLKYMVHPPIIFYKITLSYKQNNNNSRYKKRWEGGAFKSS

SLFHSRYHFRILYHSTSSSKRKTHIHVKYKGRYVHYNTLLYI

>SG-PL_contig_8306_1_-1_1_129

IQPFTLFKIIRLLLMNSTHSDILSAGFVWAGLTRVECYTMLIN

>SG-PL_contig_8307_1_+2_1025_1291

MFSNILLSHIHSREYIFFVPQEFVDIPFMVNQCLSNHFLLCYYVKSQQMLCQNTEARQTG

GNKKSGHHEICYKDVNLLFLQALDKYVTD

>SG-PL_contig_8308_1_+2_68_217

MICQILREFIRLIRPNKGQYGTILMNKIATYSAQQYTTSLTTAFIGFITH

>SG-PL_contig_8309_1_-3_159_1025

MVAFSERSLFYQVGVLLLLLAIVVFSVGLALPWWVQTYSKSRHGITSHSGLWLICSVNSE

TSEEHCSFINAPGWLKAVQGMQGVGLVGMLIACVHAIIFNFCVDRVRSSRVVEILAAAGG

LLGIISATLYVTKISQKLKHTHGISYSWAFSIDIAASILVLFIAAIIAITNRPHDTASKF

DDKEALKVVIEDAEGHVSKETRSGSLEKVTQSRSPVKQARSSSPEEVTRSRSTEKGGRAG

LEVERSPARTRRLEAISEMSLSLEENDNEDGCSQFSVSLVSSIGNVSNS

>SG-PL_contig_8310_1_-1_718_876

MFEYKQSQLLLQKWSSHVNKPFVIRVIVTFVWKTTSPLEIKVTIITFIALVMP

>SG-PL_contig_8311_1_-2_83_556

MGINCEQCKPTYYRPYGVARDDPFACRPCECDLRVSTGECELGSGRCLCRAEYAGLNCDR

CNVGYYGYPDCIPCDCHINGTEDGICTVASGSCPCKVNYDGRKCDMCALGYYNFPECIPC

DCNPIGSFATKCDFESGQCECRSNYASRDCSACADGYY

>SG-PL_contig_8312_1_+3_6_440

MLAFYHTYMNTTFTSQSVPVKPHIRNKKIKSHSGIRISTSCKHWMEYDSYTMWHVNCKNK

GGQSKLGWDGRRKEGKMKILDSDVDEVWTRHMTFYFYVSGSFSCIWTATKGLSSSDSSSM

VNLFLMPLILRRIFWRSRGPLRSEK

>SG-PL_contig_8313_1_+1_1_237

IVRQTTPQNGHSVHNSVTTASPPSRQSKTAQTAHHKLQGRGAGVELYEGLLGKKKWNGPR

RQKQADNSKRRKKSKRGNF

>SG-PL_contig_8314_1_+1_1_171

GGPKKKKKKKKKKKKGEKKRGLEAPPPQKKNNPKQTQNKTKKSEPSRSLEAAILMIS

>SG-PL_contig_8315_1_-3_321_470

MLFLYILKSFENKFCFYTFEMCLFLLYIRRLFANEFMWFILCIQKLFLLS

>SG-PL_contig_8316_1_-3_69_437

MANGVLVFCCWLLFVLVDGAQTQRREKPDIAITGPDLVFEGEAIALVCVATNREQPPQFV

DWFRDGQRIRPNKREGISISKHISVSPASIVSVLKIKRARLVDRGSYMCRTSSDSISHNV

TVR

>SG-PL_contig_8317_1_-3_132_203

MVFILQIHYKFEALPSKRFSFSFL

>SG-PL_contig_8318_1_-3_3_197

RQALTWNPQGKRKRGRPRNTWCHDLEVDMKRTGRTWGQLERLAQDRGGWRAFVDGLCPRR

GNRRR

>SG-PL_contig_8319_1_-3_573_791

MTCVLPEMRKGNMCVLPAVHLTMKEELTRVGESKAVMVRSVHHSHKSGPRVEVFRFYASL

TFTLGGHLSISKT

>SG-PL_contig_8320_1_-3_3_215

HPHPPLCKLSILFVVVWTLWYAGEWEMEMLAGKEGQRMKLTGVAQKTLAHLSPLVYYKVY

FMVVFFQHAFL

>SG-PL_contig_8321_1_+3_3_221

VVVGWVGEGERERERGRERERERERERERERFQAMDTRGGTKTEGISGRRKDRTLNNPVQ

GTLVNALLTQPVH

>SG-PL_contig_8322_1_-1_31_873

MASRDRPEHKAPPEIFYNEEEARKYTSNSRMIEIQEKLSERAIELLALPDDTPCYILDIG

CGSGLSGECLTENGHVWVGMDISPHMLEVAREREVEGDIMESDMGQGMPFRPGTFDGAIS

ISALQWLCNADKKSHKPHKRLYTFFCTLYASLSRGARAVFQFYPENSSQVELITSQAMKA

GFTGGLVVDYPNSSKAKKMFLCLFAGGASQHLPQGLGTESASGMKKEAVSSAREQWKNKK

SAKLNRDWILEKKEKRRKQGKDVPLDSKYTGRKRRTLASRR

>SG-PL_contig_8323_1_-2_302_580

MGSQSVSQSVGQQISWLVSHSVSKSVSRSVSRVVSQSISKSVSQHPQILCTGSLQGSTNH

VTQLAQHWLHQYSYTGLMVTSIFFSYYISHSAL

>SG-PL_contig_8324_1_-2_2_301

CFFFLTLSLSLSLSLSLSLSLSLSLSLSPTVSSTVSPDFSIVLSGSVLDLAVADSAWGVL

LAGYVKMGASFIRGCWNLFIYFCIYLFLATDVVSGDCSRV

>SG-PL_contig_8325_1_-3_417_731

MFLCDSAKLIRNYVIPLYKKNNLQNKQAEQNQDVEVIQRVLLLYILITYNKFCVTYNTSY

VIVILNFNLSQPEQFLLGSIKSRTSSSCGVPNNSFCKYTCMHFSF

>SG-PL_contig_8326_1_+1_175_360

MEPKFEKCHYIKMKRNNIVTLKKAGKKLKQQNLSWHWQKSESYHNIFYHNVLLDETEYCI

NK

>SG-PL_contig_8327_1_+2_65_1570

MESASFHHVVFFVFTALFATLSRGQNVLHVSSNCHGVYYDRLGCFTRDYPFNNTPQLPQN

PNFVDTTFYLYTRSNPHQGQLLSARNESQDVGGSNFNSRKPVEVLVHGFLQNDRSPWVLE

MTHTFLEHKDVNVITVSWGAGAGFPYSQAAANTRVAGAELARLIIKLGEQKHVSPRKFHL

IGHSLGAHVAGHAGNTVRSMNSHVKIGRITGLDPADPNFSFQSPEVRLDPSDARWVDVIH

TDGAPYNTYGGYGTFQAMGHIDFYPNGGKNQPGCHTDTSVYNFVRDAYQQGVTNAEYAMA

CSHDRSILLFIKSIASQCPFTAYPCASEDEFRAGLCLRCGNRPCPSMGYGAQLYRRTGRF

YLHTLAQSPFCGYHYLVRMTLSDNMRSTSGQLRVKISGDQGQTAPLLLHDGDMTASQEIS

SVMLSTEEVGHVTNVTFTFTRGEGYLSWLSMSSSEVRMATVMVEGVNRRNILYFCPESSV

VQPGQPLQLSNGTRRPGNCFQH

>SG-PL_contig_8328_1_-2_71_199

MFANTETLTRIILSEADTHYVGEPQKLKLRSPSCNTILGPKVQ

>SG-PL_contig_8329_1_+1_121_246

MVAKQNVNPLLPHPIHQLPALSAMVAKQKLIPLIPSPCPPSS

>SG-PL_contig_8330_1_-2_20_262

MNMNIALQKLFSVAKGTKLYYDVLNADDVKPHCCDKWERKLSRKIDWSSCFYKIKKIQDV

NLKWFQIRIVHRIIGTNVILQ

>SG-PL_contig_8331_1_+1_439_864

MAYAIIILNLSPPNGICSIDPYGTYSKCGDNVLRKLMQSYLSSSTEIHKQHHHQDIHHTG

SPWYDPQVLHNHIMQLWPIKVFTIFFQLLYFGCKLQCQLAHNIKSMCYQILSTMIHTLML

AISHIPVLSNIHTAGRYSECSL

>SG-PL_contig_8332_1_-2_152_283

MLDGNFEMFDNLNFEFILFKKASQLHDCLVVRASCCDNLKMYFS

>SG-PL_contig_8333_1_-1_31_321

MPTPLSLIEFAGIVGGVGFAAGLNFMQKKPVLTGIYKHMAFGVVGYLAGQRLERFNEENG

RKKLLVIEDYVRLHPEDFVEEAPKKYGDVLMKWFPVR

>SG-PL_contig_8334_1_-1_76_1197

MIRLNLTNTPDSMDIPRVHFEVLQGKDVKFRSGQKLRFDFVLDVEGDANNIQYEDVTTQI

LMQSDTEDNVLNRYNSFTKKRANPALATIDMENFGGSRGHRYGKTILKSTKEDAEGILDW

NYIVGFNCSRNCLVIRLYGSFRLKFHHEDHEGPFPEGFLGFIHDDYRPDSSQFTCNIDSD

RCFFYCEATGDNLTDVEITRVSSPDEDMLINDVSSISGDWGVSQLFIQNVSFDDAGDFHC

TARSQSKEVTEQITLNVVKAPKIIRELSDVTLFPNISMLLICVAEGIPAPNVTFVYINGT

NVEIDPADDRFITHVTHPSQSQTKTTLFVRNVYLFPFPVASYNGAFCRASNVPKATSVFN

EFEFDDFMYLEVFY

>SG-PL_contig_8335_1_-1_25_1368

MALRRARFFTGKVVAGQQTSVASKCQPFVKVLHSADENGQNASSRGFLGGRYSAPFFALS

GVRCVHFSSNNNKRDYYDVLGVSRDSDNSTIKKAYYELAKKYHPDKNTSDPDAAKKFQEV

SEAYEVLGDATKRRDYDTFGMSGAAGAGMGGAGGFQNARGFGQQGFENFHSTIDPEALFR

KIFGDAGLRGMGGFTNFADFEESKFGFAPASEMFMELTFQEAARGANKEIQVNVKDTCPK

CRGNRAEPGTKRTKCTTCNGTGMESITTGPFVMQTTCRACHGSREHIKTPCTECAGKGKI

ILRKKVVVPVPAGVEDGQTVRMPVGGQEIFITFKVAKSRIFRREGADIHSDVSISLTQAV

LGGSIRIPGIYGDILLSIPEGTQSHDRHRLTGKGISRVNSYGYGDHYVHFKIKVPVKLSP

EQKALIIAFAETEKSVKGTVKGVTQTET

>SG-PL_contig_8336_1_+3_3_128

QNKKKEKGYTKSQVHGISTKNKVQIIPSCSLHKMTTEQTKQI

>SG-PL_contig_8337_1_-2_209_376

MTLNYLNQIIFKCLLLEADQLTLSLPHHSHQLRTLSAMIAKRELNPFTPSPYPPTS

>SG-PL_contig_8338_1_-3_198_662

MAKAVCVLQTGSEEGVSGTICFTQQDGGKPTKVTGEVTGLTPGKHGFHIHQFGDTTNGCV

SAGPHFNPAGKKHGGPTDAERHAGDLGNITAGPDGIAKVAIEDAEIPLIGENSIIGRSLV

VHEKEDDLGKGGNDESLKTGNAGARLACGIIGLTK

>SG-PL_contig_8339_1_+3_117_350

MYAGAVSLVRNSHLSMVKPIFGVVEGSLCIWCLCVQRLLVCSVLFCLSFFCVCVCVIMEW

SNYVELPQKYLPGKIFIA

>SG-PL_contig_8340_1_-2_143_241

MAESGISLHVWNIFHVLISQPIHSVVYLGFGTF

>SG-PL_contig_8341_1_-3_810_1034

MVLLLYLFWGVDFKAILVVYFTVLFTLRVHVNVWLLLIVFKAILVTVVYILLCVIYTKSP

CKYIVAFGSFQSHSL

>SG-PL_contig_8342_1_-2_128_361

MRTSRQTGRERQKDRYTQTDRQTVCRAAQRQRRQNIGQEKIAAGSSLQRSITERYGTQRQ

FILRTTRKNLGQAIGLVS

>SG-PL_contig_8343_1_-1_1_165

NDILTEQASKWTLSIGDISLSPVSVNINTVTLRQDQTGHIVLFIIRHMVARRQAD

>SG-PL_contig_8344_1_-3_129_263

MVHAVSWDVFFRHLCHEDTVSCLSTVLAEIYVQGLKFFSDPRISL

>SG-PL_contig_8345_1_-3_3_191

SNPCSQILQKVGALPLRQPDHLNFASKATSPPVWQFECMLCSKSVLQCSPYHLVEMYSCQ

CSV

>SG-PL_contig_8346_1_-2_38_1372

MPKIKVLSRNPEDFLRETKHDLHKVPQSSHASRNPFMEQVEYTRALNAVKLERTFAKPFL

GALDGHKDGVNVLCKHPTSLALLLSGACDGEIICWNLPGQKLLSKVQAHEGLVQGMAAHT

SGRYFFSCGNDKTIKQWSLDPDGNIGAEPVNTLLAKCIYTSMDHHWKDDIFVTSGSRVDV

WDEKRSEPMRSFAWGVESVHHVKFNPVETYMLGAAAHDRSILLYDMRGAAPLRKVVMKMR

TNALSWNPMEAFTLTSANEDGNLYTFDIRKLSMPLNLHRDHTAAVMDVDYCPTGREFVSA

GYDKVIRIFPVDKGHSREVYHTKRMQRVGSVAWSRDSKYIFSASDEMNIRVWKAVAWEPL

GKLAPREKAAANYASALKERFGSFKQIRRIARHRHVPKHIYNAMQQERESREAATRKEAR

KRAHSKPGSVPFVSVKKKQMVGEVE

>SG-PL_contig_8347_1_-2_257_418

MYASPKFIMIWRLLKPTQNHFHSQGFEMKGDWSGCRSGRFFFILPNFPTLFTLH

>SG-PL_contig_8348_1_-2_2_94

KLQFRLKSSVTPNVPVHSRIFGMYDLGVVMN

>SG-PL_contig_8349_1_-3_3_302

LRQLLEQPPGERSFASINSSSRSTPMTTTPSSSQSSLPGGPARYRYVSNLEASPHVNGQS

SGATGGEETEGSAANGSSSRPVSGDKTRPAGNVGELFHIA

>SG-PL_contig_8350_1_+2_167_559

MVLDLALYFGVFCYPPGFRCIRHLGMFGGWVMCIAVWQFIIALVIFHVQCHSTYFALEAG

FVPYLVQTFQFFGRVHRFRTLCTLLAHFASFLVTANSSRTQSCGTRFTVEEPTRPCNFLS

KCRCLGTEIHL

>SG-PL_contig_8351_1_+1_475_723

MHRPLAFAMGILDLWCFNDWTPLITHNLDKLEKPFRFLQFLKILWSTREIMCRPYLDYEL

VSGLASEDIEDYLKHKHNVDCEL

>SG-PL_contig_8352_1_-3_48_647

MAVAPGSQRYVKEGTRVCAYWSQQFRCLYPGTVAKTSPNPHSESSVNVEFDDGDSGRIPI

SHIRLLPQDFPLVTEEPDPLFYPTKKRRRTISEDDLRRSTDDRNSGSNSARRNSRSHPLD

DTISDDGSVFDTMSLKSNTSGKSSSRSRACKEDSSFKSRRDGRGKDTSTATTSKSGSSGA

KKTSNKAEEKDSDSEEEESD

>SG-PL_contig_8353_1_-2_2_688

LFTNKRNDLKSLLKNIEERVKEKEGRPKALESLKEALNSSAVFLQVARHNNSQSDNPVFT

QVELNTLDKLINETKTWQTEAVKEQKERGDRQNPTLLVDTIQSKVKALEREVRYLTNKVK

NNKASAKKKPKAKANKTEESKEEKEKKKAQKVEKKKEEKKEEDDDDDGVVEDEPDERFIT

DDEETDDRKTDSKPHSHTSSKTPPKRKSDTDSDKKDTNDSDLGDEDIGS

>SG-PL_contig_8354_1_-3_18_503

MRQAGEVTFADAHKRHRNEGIIEFASYSDLKKALDKLDNTELNGRKIKLVEDRPKKRSRS

GSRSRRRKSRSRSRSPRSRSHSRSRSDSRSRSASKSSSRSVSKERKSKSEDKSKIDVKKR

SKSASHSKSRSPSRSKSPDDHKGVDSPRYSGDVQHKEDEEDD

>SG-PL_contig_8355_1_+3_288_620

MNTNVYTVINMDWLFVLCAERGKKQKQTYRDHQEERGNPTLYYLFEASQKGQVSQTPGII

FLALFFSVTSRPQQVKTIVIVQITTRKKKLSNLPVAHETENNKTSLNVFLS

>SG-PL_contig_8356_1_+2_8_328

MCTLCSQVWNSGEVPEDWKNGTIVCIPKKGNLTECDNWRGVTLLSIPGKVYCQLILNRMR

DVVDGELREQQAGFRPKRSCAEQIFTLRRIIESCTEFQVPLAGRQFH

>SG-PL_contig_8357_1_-1_1_159

GEGGTDWSRGDSIVSVEPVEPPLFKGSREGNFSGDLEASVLDCPGWCPLEPDC

>SG-PL_contig_8358_1_-1_172_321

MDCENANLGSSILALGEPFCQPLPQGSPLGPSRQLQRDCICGCKLHNFHH

>SG-PL_contig_8359_1_-3_3_1088

MAAFFVLLALIMIKVKSSRDPRAKFQNGFWAIKVLMMLALCVAAFFIPRGDFGKAWMVIG

LIGAFLFIIIQLILLIDFAHGWADSWVGHYEETESRAYYVGLFFFTAVFYILSITGIVLF

YVFYADGSTCKLHKFFISFNMILCVVVSIISILPKVQEHQPRSGLLQASIISAYITYLTW

SAMTNNPDKACNPSLSQIMNPSSHNSTGSPTDNGDYATTQFDAQSILALIIWMLAVLYSS

IRTSSNSQVGRLTMSERTILQSDTGSEGSAEGDEEEQRSQKVWDNEEDAVAYSYSFFHFM

LCLASLYIMMTLTNWFSPSSDFRKLNANMASVWVKISSAWLCVALYVWTLLAPIVLPNRD

FN

>SG-PL_contig_8360_1_-2_2_208

EVMLSDSLPTAPVGMLLLPHASHLSLSQRSTFPSQRSTFPKQQWQHAGITRCKRHFLQCV

VHMSLQIDS

>SG-PL_contig_8361_1_+2_2_139

LCFFFLSQDTHLEQKEICDCTLSANFFCNMNAHWLKREQLCGFHVD

>SG-PL_contig_8362_1_+3_15_128

MEREKKNQKVIHFTLSSGRRPISSDDRCCCSKQEMLKS

>SG-PL_contig_8363_1_-2_110_292

MTLKYKTCVCHLKLVTAVDIFFSFLALEELHDYENVYYICKNCSLLMNSRPPSRRKYALV

M

>SG-PL_contig_8364_1_-1_148_345

MLYVWVSMNINDCVCKHGCNVHVNNFYVTRPTYFYNLCMQLNIMIVYGCTCVCVCVCVCV

CVCVCV

>SG-PL_contig_8365_1_-3_27_290

MQQHHGLTLTGGWGAQTGSQHAAGSWTEEESSWHIITLELEAVCRALVTFEPEVQGKVVY

LFADNTGGGPEPPCFVWFFCFAFLSLTF

>SG-PL_contig_8366_1_-1_145_1044

MQGQHKEEQKQVLQMEQAATSFGYARRHAGYKASITHLSSALKKTDTVPDTITDITISPD

FHLRDMESKQEKEPLLESRSFRRKLKEPSLERSYEYKLLSKLCLSESRAEALEVDWRQKD

QDSHAETMRRVVNYAGGIGVEVPVCRMVADLKEWKTVVQENLPDLLVLLKCSGLCVLGPM

LQDLLVLDVSKCQKVLQYSGIRSLGLAVAHESQQLVATLLILLSILLVSIKKNISAMKAV

EHLKDLVNGKEIPSVSSVFSNVHTAIRDCRIIQNKHPLVCSSLELAPDWPTFCVRVLGLV

>SG-PL_contig_8367_1_-2_575_856

MTKQLPHPDCFLCKTQVLLPLFTHSYLKNIAELSQQVPVAVFTCWKSVVCCAVTSLLNLS

SAWLIRDGRQDFYCHCNYQVQSYLLSIFDKPLEL

>SG-PL_contig_8368_1_+2_491_784

MHNECSMNISIHQYTIVHSITTMANQFSIFHPSFSAALKSIVAAVNITHSIINAPCCYAY

HGCVIKPVMYHILISYFTILNNAHNSEQRFVYCEDTFI

>SG-PL_contig_8369_1_-2_23_235

MHKHTHIQPHNFTKHTQYYDMQKTHMSSFMTISTELKSHDPYHSPFFTELLEFGIGSRGI

HRTGATPYVLH

>SG-PL_contig_8370_1_-2_2_286

TLKIPRQQRSCPWQNSVEKSSHRKQKYTYTSKKEEKNRWHSTMAMHFPWGERPTKKWLRQ

KQFNLIHDIRFKPTDQILSKSIPPLGNSLNFLSLI

>SG-PL_contig_8371_1_+2_2_169

VSLVFHWSCFLSTPTFPISRPCQRIGFCTGSRSLPDNYWQIWVEHIWNCPSVMNPT

>SG-PL_contig_8372_1_-2_437_628

MDICTLRVRFGSESVKRLMLLSMGCVGVRVCIVRWQIAVVGEMPFLQPFFHSVLAWCGQG

RGRA

>SG-PL_contig_8373_1_-3_3_182

RTPKQHIHTDSYYVVTALRDLNTLSRYIADRFTMFSFIDDINRARISKILLWYKLFSEVL

>SG-PL_contig_8374_1_-2_2_145

VQTCALPIFFVWFGCGYFWFGLFCFCFLFFSQTADDLLIFAMTLQKGE

>SG-PL_contig_8375_1_+1_16_114

MEKKKKKKTRREWCWSRQHEHKSKTKMLTATAV

>SG-PL_contig_8376_1_-3_231_380

MVLGQNIFYPSITRTDKIKSRTSVPITWNTTKEKKRRKEKLETIFFSKKI

>SG-PL_contig_8377_1_-1_139_276

MQDISCNLECRVILLRSMYFCIFAQSDLLAANRALANVGLYIQWVT

>SG-PL_contig_8378_1_+3_1713_1997

MHRNITVTRRTNNASISKTNPQPPLQTYTFFFPSFIVHVSSCVQSSFSIFCYKICTNTTL

HFYWQQKSDSFDANTPVVGKQIYKWRSSPIKSHPF

>SG-PL_contig_8379_1_+1_190_318

MNNNNMPWLNKFRETFSNSRQGQYLSTSNHSKGSTCTHYTSFV

>SG-PL_contig_8380_1_+3_3_293

FRKKQQQNTETLSLLEERNKVFLPKMCMWHLSLTQQTMLAQSVLTKMCSIKIILKNGSFS

PKALQTRDTLAVSVLPLETFIWLTVLSSLTEVTIQQA

>SG-PL_contig_8381_1_-1_241_363

MNMKSSLPTSSLPHLLSVATVYHDMMKSVELISLIIYAIGK

>SG-PL_contig_8382_1_-1_130_357

MCVCVCVCVCVCDKDEHYFSNLCIVFYMGQIVALKINRNECMCRGQIDLRSCMNLKNLKR

NNMNVPVCFQILLPIV

>SG-PL_contig_8383_1_+1_250_522

MKFMKFVDFSSHLQIILWKRKGNNISVIVSKRYECINIDLHIHRFMHIHKHTYVHTYIHT

YTHAHLYPYINRCFNTCAQYSHTRDHTYIHT

>SG-PL_contig_8384_1_+3_3_233

KTKLTNKPTLRQTLTQKGKKEKQKTNKTIAFVVIWVGQNSANPLEQIKGGKDHYIKNLDI

LFLGKFSLSCGAHHITS

>SG-PL_contig_8385_1_+2_506_640

MLIRPLCFRDHEVWSGSPEHLCLAEIYLYLLIMFYYTFYTLHIRP

>SG-PL_contig_8386_1_+2_611_775

MIKRQRYPCIVSYCTNILSQQIFHIKFTLLSQEGACRHSTVLALLSPYYFVSLLK

>SG-PL_contig_8387_1_+3_3_110

GGVRGRHIVLLNNIHLYVRTGRPNPTSKCNDQGEKH

>SG-PL_contig_8388_1_+1_1351_1656

MGPQITVQFHTPVHKMTTGHEIYNTDGGGENLMKKSIHNLFLKILSPFSHYHYSQTFPSL

IHLFPCCLLKKNSSVQVSQDNPVVTSRHRSSTESGPPLNTKY

>SG-PL_contig_8389_1_-3_453_1817

MDWLCMSVPSILGMVSCLIFSLPPAQGLALRSRYFGTVGKSTLGLKRTPGGTSRGGSSGS

RDEIRNRLRSYRRSVLREFGGIFDPGSQGMLTTVEPQRCSLQDPDDIQRLLPGRFLSEED

LMHTVPGTKTWVSNSDMASTALQRRLVPPLSASLLDTARLQQEEIQQRQQQQQQQQRHAA

FDIKTDGAQFQDTVCNNCMTEIGYEIMEQVVDFMGQSHTVINFKNAFQFIPSGRCVNEHS

PCGPSGWQCRQVTRAHWTLVWTAGMGVNLVAHEVPSHCQCLNLGGPAATSHRSAAADTLS

DVNPAQVEGNVRVFGWAGLKMDHSNSSSDPTYTTSNRPHPNPTNSDLNPVNPSLHPTSLD

PNPVSTDPFAASPGSYSVSPGRNPVSPNPYPYPASSVVSDKSLFPSFKNSNAWGWSLTSM

VPSTSSSSSSSSSVTSSPAKNPSTDVAEDYRITFS

>SG-PL_contig_8390_1_-1_1_198

NMEVKKNIRQFSGFTFAKGDKEFDKRVTSLNKMTLAGLKQICEVLDIERGGTKAAIVERI

MDFLMK

>SG-PL_contig_8391_1_+1_1_132

LAQTILLKCFNTGHFIKLFIVNTNKITTLCTILYVFFNTGHFIK

>SG-PL_contig_8392_1_-3_102_617

MVSYRRISLCARHIVRKLNILADSLSRSHCVLHTEWTLNKGTLTSLWNHWFQPMVDLGDR

CPIVRLGSPPKLLLSPISDPLQSDQEGETGKRKVDSHSLPVACSAVVPKSPISIPFELPL

CQYLLRQARSGVIHSNPQLLRLHEWLLYGKTCPHTIQQSLRTRGSITRHSSF

>SG-PL_contig_8393_1_-3_15_290

MDLSDNLLGLQVSQKEHKHTEPMRSKPWPRLQHFQSVPLNKILEAAYWRSENPFINHYLR

DLHLVRADGSKGIAFVAANSTLSVTRPSSSIL

>SG-PL_contig_8394_1_+1_106_228

MFMLMLNDITSSKLVSNSWTPQPIHQLPSTSHRKLVDRVSG

>SG-PL_contig_8395_1_+3_3_203

CCCFFYINRPSKITGHAHFIKNTFIIPCWAKYDLHLAKTVTVRKFLTNIFSPFSVMLKWV

PVIPTHL

>SG-PL_contig_8396_1_+2_167_382

MYTYFSVFLLNPKSEPTQHSHIESISNNKTVRTKILCKSQVQDRMDHNMGEIYLCKYIQN

SQFFPCSVRKCF

>SG-PL_contig_8397_1_-2_2_1378

LFHLLPLLLSVCSISPSFLSPTSPSPSRLFLSLLPFSLPVLASPSLLFLSVLPFSLPFLH

LLLSVFSLSLSSSILPLLAFPSLCLFSFSFFFLSLSHFSPLLLSVLFLLPFSLPPFASPS

LSLLSLFPFSLPLLASPSLCLFSLFFLSLSHFSLSFSLSLLFLFLLPFSLPLLASLSLLF

LLPFSLPLLASPSLCLFFFFFLSLSHFFFLLSVSSLSSSFSSFLSPFSRFSFVSVLSHFI

LPFSLFLFISDFSVFCPFFLLFEPILSSSLLLLFGLLLLLLPFFFSFFMSFYRFPYCLSP

PVFSASLFSLPHVSLSLSLSLSLSLSLSLSFISLIRPYNLAFFVPTFFSCLFSLSPSFLS

LCARLFSLFLSLYLFFFYFSHLSVFQIHTLFICLIFFPFSIFLLAGHLSFYFSCSFSFSF

SIRFLSFPIFISLSLRSFFFSSLPSLLLRPFSLFLLPFF

>SG-PL_contig_8398_1_-3_105_296

MVTCSSLGENPHFTLKNSVITNNKTTATTTKTKSCTIPSNPISGQLFNFCVLMISQLLMF

VLAL

>SG-PL_contig_8399_1_+1_283_522

MISFCHSSPHPPDVTTGSEHFKEDMVQVIFNSVIPSSYPPASSFFEVMDLIRSLPHPIHQ

KMSQFAVFFSRKIWYRRTGR

>SG-PL_contig_8400_1_-1_439_636

MRVFLVLYCHCVCIDFHCGQSVWRSIPQRCVHVNMYGGFCPLIPLPCLSSRCLWVHIHQI

WAIGLA

>SG-PL_contig_8401_1_+2_2_151

RFSLTFLVNSETIVKCKTMKPPLCLPPMCNLHNTYNSMGNNVQIASLKTA

>SG-PL_contig_8402_1_-1_526_762

MIRAWWGKGGGYVIFFTWEGNICADLLCLNLLRVYTRAGHQNARSRSCKPCHCLVAYGNM

KLLNMHTAEKGLYCSGVTR

>SG-PL_contig_8403_1_-1_184_576

MIGVMATVILQSSSTSTSIVITMVSSGILDVRPAIPIVMGANIGTTVTNTMVSLAHSMDP

VEFRRAFSGATVHDVFNWLTVLILLPLEHFTGYLYYLTLKIIQSLNMEDLRTKKQDLLKQ

LTKPLTKRIVQ

>SG-PL_contig_8404_1_-1_94_354

MAHGGGNERETLGKFVRNQSGYGTTVTNSKKHSHHKSSKYIKHFVCKGDSLMGLSLKYHV

TVLQSDGGETLVRFLRDRGGTFVLSRC

>SG-PL_contig_8405_1_+3_60_179

MQVWQQLSAGKQRLIFCFIVIFFFIWIYTENLINLFLELE

>SG-PL_contig_8406_1_-2_32_478

MGVGAGDMSGVVGEETWIDNKLTFEPIVPPKKETKDSLKSGIQGIDHTMPFFSPAATSTV

GRPGKVPPPVPPKANSFDDSFSRTDYKNDRDGIQESRVEHKMVLSSADDEDFDYDAALAD

AIRSVTEFNPDMSVERIECVQQIEEVKDK

>SG-PL_contig_8407_1_+1_1207_1494

MVNDSDQTEMCVCVPAKCINNKKPTLWVDHYCITEPVTDAIHKDVMYVKVFNFKNLVIGK

RILISKEVKTPKVGRENIFITNQCVLMSVFMSMENE

>SG-PL_contig_8408_1_-3_165_272

MFEAMMKSGLESTDPSMEVTSLLNVFHIKSRELSNK

>SG-PL_contig_8409_1_-3_117_452

MDRGRCLKRVPSAVCILMVQLLFLLCVGHTEAQFAKNSYFMDSSSDCGSVGFLVIRSDQV

VNVNSRRTTGSSSNLERCSLSFKPDDDRDIMDLSFTHLTISDKDVTLIVDLS

>SG-PL_contig_8410_1_+1_451_771

MFNSITCHIHQVSYNSFSIQSHVIFIKSATIHFQLNHSTAMIYSQQQLIYNVVRIQQLTT

QQITIPIQHNHNSHSTQSQFTFNTITIPIQHNHNSYSTNHSFYSKQS

>SG-PL_contig_8411_1_-3_3_182

TIFKFGFHCCDSLPLSSLHVKFYFIRSFYKVTVTLRSRKSGFSFEQLKFLFEFFQPFVFW

>SG-PL_contig_8412_1_-3_9_263

MPGVGDRSKLQYTEAVLYETLRLGSVAPITLPHKTLCDTTVGGYEVPKDTMVIVNLWAVS

QDPKLWDQPATFNPERFLDDQGKLK

>SG-PL_contig_8413_1_+2_2_145

IITIIIIGKNCTSICKILSSCHFQFLKTKRIAFRLKSLHGITACDHFP

>SG-PL_contig_8414_1_+3_81_251

MTSDESVTKQKIVKELNQAHTSKAQPNYDHLRLVTGSRSYVPTLSLTSDDCVNLPTL

>SG-PL_contig_8415_1_+3_240_422

MVFSKNRNVYRVQTLQQTSFPSDCVQPLVTAVQNPQSLWGKLETGHTQPISQTMKSTSRT

E

>SG-PL_contig_8416_1_+1_1_141

EYFIAVEFAHKGTAEFCLVHFHSNINSNVTKAQDFITQTFCKSLLTY

>SG-PL_contig_8417_1_+1_1_60

SGELKTSKKGVSLSTTRRRS

>SG-PL_contig_8418_1_+2_155_319

MFVFVVVFYYSGTLSFEHTHTHTHTHTQKYQQHKTMWIVTGKLYRNEMNQNRPTT

>SG-PL_contig_8419_1_+1_1_96

RKHSQSDVLKSYRKHSQSDVLKLFKTIEGKNH

>SG-PL_contig_8420_1_-3_24_287

MISSVCVCVCVCVCVCVIFKYKAYFYSECYIFKYKAYLPQCVIFSNVKHTCCDNFFFNIP

KGLTVPLEERILFLCCSFLNPLATGWCT

>SG-PL_contig_8421_1_-2_50_718

MSAIEESAGDVEMAMQDSTAPEGEEESLKSAAGMTDVNEHKLEDGSATIGVDESATVGVE

QGQESKQGEFVESVEAAGEHQENEKTSQLNVENSEVKLSSDAEKSDEDPERTADPEADIA

SDATIEEGRDADDEPMLQVSDIKSGVDAEGNLDEKDDSVGGVTTDSANSISKDTSETVDT

AAGRENGKVRETDSTGKQTSGNAEGKVDSKIKVGIQSTKKHSC

>SG-PL_contig_8422_1_+1_469_831

MYQTALNSTVLCGTVLNSVLFCFVLCWSFQENREFMHEKPAALPEANVITTIVQYNTVCV

CVCVCARVRVCICVHVCICICVHVCICICMRACMRACMRACVCVCVCMSACVHKCMHIYK

V

>SG-PL_contig_8423_1_-1_334_468

MCEISHDEKSVADSSFVQIQSFFLSYLLISTCFCFCLSLCLSLTV

>SG-PL_contig_8424_1_-3_57_221

MGIIMVFGTPSFQCWVKWRKEIVTYGHDDIISSGFSIHNSVVSGHSGAWSHRPVL

>SG-PL_contig_8425_1_-1_1_258

SGGRRLHATLTGGDHHRHLLRGAVAAGSGGGGTGGRSPQVPEEGGGRLGCVHYGYPEPLH

SAPPLPGYHHVDTFWFDVSFCLFCFL

>SG-PL_contig_8426_1_-3_285_1679

MKMQPASLTSLWLCVFSCMLTSAFAIRYEPNWKSLDSRPLPAWYDESKIGIFLHWGVFSV

PSYSDEWFWWHWKGQHDASDVKFMKENYKPDFTYADFAPQFTAELYDPGKWAEIFQAAGA

KYLVLVTKHHEGFTNWPSKYSWNWNAMDVGPNRDLVDELANAIRKKTDIHFGIYHSLFEW

FNPLYLEDKANNYTTQKFVETKTMPELYELVNNYKPDVVWSDGDWMVNDTYWNATHFLAW

LYNDSPVKDTVVTNDRWGKNIRCHHGGFWNCEDRYVPGKLMKHKWENCMTLDKGSWGYRR

TADLSSIYSMDDLIALLAKTISLGGNLLVNAGPTEYGSIIPIFEERLRQMGQWLGVNGEA

IYATRPWTYQNDTTNPDVWYTSKKEGSGTVVYAILLKWLRGTTLQLGAPIVTDQTSVTLL

GYSGGPFKFTGQVTQGVTITVPTISFIDMPCQWAWVFKLENLMNQ

>SG-PL_contig_8427_1_-3_3_164

EDMLRIYQRQTFKFSFKHRKHLFQKSPNCKMKAGLFVLQFLLFDHVLHALQRKE

>SG-PL_contig_8428_1_-1_1_117

EWVPGLFQGWESSEGEETVTILIMLVLRKDLSYVPPHST

>SG-PL_contig_8429_1_-2_26_955

MAQSWPDAVLEFITLLFSVVYFWCEAVVKCILPSKFFRKDVQGQRVLITGAGSGLGKLMA

YRFMQLGCNLVLWDVNTQAIEEMEKDMGSQVCCKAYTVDLSSRDQIYKAADKVKEELGTV

DILVNNAGIVTGRKFLDCPDSLIEKTMEVNTSAHFWMVKAFLPAMVAQNRGHVVSIASSA

GLFGVSGLADYCASKFAAVGFDESLRFELEAMGKTGVHTTVVCPYFINTGMFDGVQTRFP

CVLPILDPVKTVDSIIDAVLCDQAVLILPRFLYIIYALKGLLPVQVALRLSTYMGVSRSM

DTFTGRSKNQ

>SG-PL_contig_8430_1_-2_173_1654

MNFPEPIRDPEIKFTQLFISNEFVNSVSGKTFPTINPCTGKTIVELQEADKADVDKAVAA

ARDAFKLGSPWRRMDASNRGNLLYKLADLMEKNMVYLASLETLDNGKPFRAAIGDIYFSI

QVMRYYAGWADKIQGKTIPVNGDFMCYTRHEPVGVCAQIIPWNYPVAMFTWKIAPALAAG

NVVIIKPAEQTPLTALYMASLVKEVGIPTGVVAVLPGYGPTAGAALTAHPDVDKVAFTGS

TEVGQIIMKASADSNLKRTTLELGGKSPAIFLDDVDVDSAVAEAQEACMTNMGQCCVAGT

RTFVHEKIYDEFVKKSGELAQKRKVGDPYAPDTINGPQIDDEQFKKILDLIEKGKSEGAK

VESGGGRHGDSGYFIQPTVFSNVTDNMCIAREEIFGPVQQLLKFTSVDEAIERANSTTYG

LGAAVFTKNIDKAMMFVNGVRAGTVWVNCYNAVCPQSPFGGFKMSGIGRELGEYGLQQYV

EVKNVVMKIPMKNS

>SG-PL_contig_8431_1_+3_561_737

MLLLCFHFHILFIFYLIYFCCCCIHICTLCVCVCVCVCVCVCVCLRAQCMHLHLCDMHK

>SG-PL_contig_8432_1_-3_45_374

MTTTLKPYLTCVKETLTAAICVQNFDSQVVERHNKPEVEVKSSKELLLTPVVISRNDKEK

VLIEGSINSIRISIAVKQADEIERILCHKFMRFMMMRAENFVVLRRKPVE

>SG-PL_contig_8433_1_+3_3_242

KTTTTSKQTQNTYYYYYYYYILFIAQPTIKAISGLSPSKVHQSQEDNRVDHKTSLNLKTK

ILSSLRQIRLGCAEDASHSA

>SG-PL_contig_8434_1_-1_250_504

MCSVYDVVVKEGLAFLLVSVVSVLLLVFARHSLQPRQKYLVQLSQNFSWFCIMVVLFSSK

LKHVFIIRYHFLNTDCIFYDYLPSS

>SG-PL_contig_8435_1_+3_675_1043

MYTKQTLTNHYIYIYAKQTLTNHYIYMYTKQTLTNLYMYVHKANLTNHYIYIHVHKAKSD

KTKMHFYQTVQSRERERERERKEREEQKKKKKGKKKERREGGRERERERERESFFLHLFN

VLG

>SG-PL_contig_8436_1_-2_269_421

MCRLSTNAHAQAVCGIPNLFLPIKITVPVIAHYHCIFRGCYIFTAAVLVLF

>SG-PL_contig_8437_1_+3_99_989

MGENFRLPTCPRFFNIFHPFDPVAYRLEPLICASMSSVKPVLVPHYKGRKRMHLELKESI

SRMGTDLKQKIVDSIRSTWNSLNDFARAHRTDSNAAPDATENEKSLENEVDQQMHSVMAQ

MVQQQENDKSDTASVCSSHDEEVPTGQLNEGRRIDYVLQEKPIESFNDYLFALASHGCYW

TSEDTALLILKEIYAPLGIAPLMPGPDGTMPKPVMPSTMAPPTKSLTPYTGDSHPPHSTA

PFPPPGTQQGPPMHPPAMGGGAFPPPPIQGLGARFMPPYVAGGSQGSSRGSTPPVGG

>SG-PL_contig_8438_1_-1_103_246

MGHIWVKLILCPAREIPPYYSMLLLRFNHFGLFNALFFLFVFFGDMGL

>SG-PL_contig_8439_1_-3_3_251

SKYRENVLELVTWCDQNNLELNVSKTKEMIPDFRQTKPIPPPLVIKDKQVEIVSEFKFLG

LIISDDLKWEKNTGKIVKKAQQR

>SG-PL_contig_8440_1_+3_156_296

MQESTHQHFAQDYEKLAILHPKQCIKSYKSEQNFLKEWFHLLFGPED

>SG-PL_contig_8441_1_-1_1_1326

GNGGEIQWCFSQVKGSVEEEVADADIISCVEFNSTGELLATGDKGGRVVIFQRDKASKNA

VPSRGEYNVYSTFQSHEPEFDYLKSLEIEEKINKIRWLKQTNRNHFLLSTNDKTIKLWKV

AERDKRPEGYNLKDEAGLSRDPTNITSLRVPVFQPMELMVEASPKRVFANAHTYHINSIS

VNSDQETYLSADDLRINLWNLEITDQSFNIVDIKPANMEELTEVITAAEFHPQQCSTFVY

SSSKGTIRLCDMRDQALCDRHAKMFEEPEDPTNRSFFSEIISSISDVKFSHNGRYMMTRD

YLSVKVWDLAMESRPVETFQVHEYLRSKLCSLYENDCIFDKFECSWSGDDRHIMTGSYNN

FFRMFDRDSKRDSTLEACREITKPRTVLKPRKVCTGGKRKKDEISVDCLDFNKKILHTGW

HPLENIIAIAATNNLYIFCSKD

>SG-PL_contig_8442_1_+3_3_209

KKITEEFLACSHAPDTLLLAKRQNPKSYFDEHHELKTSKTKWITAFYPSHIQRFAYNQLS

IIVMLSKIK

>SG-PL_contig_8443_1_-1_148_363

MQRSLHDAYVHMYCVYASLCYFSCATLRFTTIVDNVYRRLPLSLSDQHTVVLVMLQYSVC

CILVVHAYLILI

>SG-PL_contig_8444_1_-3_72_572

MGCCFSSEDDKDSGFPEPDERTRLLDPAIGSSNVPTLGNQEDQQYMQENEKGDEQSVMAR

ILNQNARMVIDVASNDTPIEQHEYHDRARAYNTKLKVALANSSRRGTYKLTLPNNITSPQ

SVLAAPPVSPAEIQLICNTAEKMSKAMRDVHIQHREDLVVSFGVPVP

>SG-PL_contig_8445_1_+1_253_378

MSARDSVKPDEEFCCAISCKNARNKKSCIGKSFFTFPKDQER

>SG-PL_contig_8446_1_+2_2_133

KMSSLCWLWPNKLRTSFNITLPMSDIHVFIIRLGTVLLHDNHKD

>SG-PL_contig_8447_1_+1_52_180

MSSLCWLWPNKLRTSFNITLPMFDMHVFIIRLGTVLLHDNHKD

>SG-PL_contig_8448_1_-3_3_329

EDCALCDVSALNDAEDSPLNQNSTLDDVEACILKDIAALNDVHFSVFNDANISTPNDVKT

RVPNDADISVLNDVDVSTLAEPDQISLSSVRMGDFDNDPAYRGRGGGVR

>SG-PL_contig_8449_1_+2_74_232

MLRKKCFIFLLLRYTFHLKNTHTHTHTHTHTHTQIPTTKNNVDCYWQVIDLQN

>SG-PL_contig_8450_1_+3_3_110

EIEMADITYKMRWHQMADNNNDSNYTGNDNQDRSKE

>SG-PL_contig_8451_1_-3_78_788

MGENYKDPFGTMKQNMIIRQGAKWSLQGLILPEDRKKRSKHRMSKRLKNLSQVNNMRIIV

YKNGKSAEPYEVVAEPQKFDEFLQACTARLELRNFAKKAYDWEGNEITDVEETPVLDDCL

QNKGGQVRGPLWISTGERFSPLGTQEFLMNIKQVIRQKLKNALFYKKEGRHNILNTAEVK

KEKEERKKALELALKKAKEYKLQKAQDSGRRMVQKALTGKDRTRPEEKIMIELDISF

>SG-PL_contig_8452_1_-3_144_1310

MAQSPKTVEGEVRIGQQYHFYMETMCSLCVPREEGMDVFLTTQMPDMAHSSLAGILNKPQ

NYVNVHVPRLGGSFGGKTFLANHAGLATAVAAYVTGRPVRFSNSLSNTMKHSGKRPHILA

KYKAGFTEEGKLNVIIADVFVDNGKSESMQFVVHELTTQLDQGYYSPNWRINPKMLKTNK

APGSPVRGPGGTPASAIIESIMEHVAKAVSRHPILVKELNLYEENQKSMAGAVLTYCTMR

KVWERLKISADVSRRLDQVDTFNQTNQWKKRGITMSAVKYGMILQDHGMPLLLSVYSTDG

TVTVVQGGVEMGQGLYMKVAQAVAHGLGIPVEMVKVRPCMSSVLPNVSLSAGSTSSEKAV

RAALEACQSLSEKLEPIRAKLPPDADWTR

>SG-PL_contig_8453_1_-3_1416_1805

MSVSFATFRGVNSLLFFNVLRREKSLLVCDALSRQKSAHLQRFVASNVCSFATFCGVCPL

QFSSAHFSIFSLFLLLLFLFAGYCLFHFLWLLYRAQYYIHLCSILLLLFVNETQYSISMQ

YYLYTALFAT

>SG-PL_contig_8454_1_+1_439_660

MHFTPHKSTQCHIHIFMHTSKIHTTQLNAFLCRLIVLHFLFYSQGDLVRQGGEKKLGNVC

TTFFQQLQMCFQSL

>SG-PL_contig_8455_1_-3_522_803

MVQTVLVAASGVWLNTCVYMDFHSSHHSDECVGVCFSCLCAGLSLYFLSRCVCVCMCVKI

SFSAWSFVCMYVCYFLGSLFRLFVCGCLYSLHGT

>SG-PL_contig_8457_1_+3_141_572

MTSSESEIDEFYSDDESSIVSDHQDESDTEIQRSNQRSTNTDTASCGSYVNIDEGPTSDL

PSFTPRLVPGGQLPVRTRNSLKKFYSPLSFFKLFFTLDLVKHLCQYTNDYAKSHGPQKLT

LYKGWTDVTPDEMYMYMALLMYMA

>SG-PL_contig_8458_1_-3_132_323

MSCSLYAVGQYNLCILCCLLSMFCDVFDWVLLTIRFFKTKLLSCFVICLEVLLTFRFFKL

EQLG

>SG-PL_contig_8459_1_-3_57_338

MDRRVTPLQLSHSVRLPFLGMQTMAPFFQSSGTSPEFHTRLQRVHIQCTTHSPPDFSISA

MIPSMPGDLLFLRLLMAFLTSSSVKSPVAIGNKS

>SG-PL_contig_8460_1_+3_123_395

MRAVYQITKKITGSTKASSGPVKAKDGTLLSRGKDKLARWAQHFSEVLNRPEPASPADFK

DPQDLLPIITGDFTEEEVRKAIKCLKNNKSP

>SG-PL_contig_8461_1_+3_93_203

MGNPLIFYIPKRTGLEFGYEQQNHSNQQVQTVCCQVM

>SG-PL_contig_8462_1_-1_1_1128

LQNETKETDARAQKDQFGSPEVEDSLLQKADAEQYQTSADFLGRKVLSVNSQVPPKVPED

VPAEEQGIKNTAEQQANTQDASEQLPSPSDSSIKKETATETDASQTESLASAGGGTSVVI

EGEEPDQSGVGGDPNQKDAKQEPEIDKTITASSTPDTQDSSAHASPPPAEENLVRKLPPD

AETEPEPEKSAGSVTPPTPVAEENQAKDVNANPETADSQESVTPPPSDRLAWDDVPPPPA

EGQTTPGEKQVADEPTVEDEPTRESDPYQMDDFYGRKISDEELLKAEEEAAAYSSGSAVG

SMEAFSRSFIEKLPPSIQSMLEQEPLGLSPGMTVLVTILTFTVFLCISCFSCLCSSGGKK

AGTKDPLMVVRELGEK

>SG-PL_contig_8463_1_+2_2_184

TYIVTVCLIIVCAFDLSGLFSQCQKCFILAFTEQTVSLHTQSLNIYSFNVICYIAYMETM

Y

>SG-PL_contig_8464_1_+2_2_97

MTRRGRMESRTMNETRDTYRVMKCNTTNSSNI

>SG-PL_contig_8465_1_+2_2_238

CNLAHFLDKINRKFQSPKCGTIKMIMMVFKWHTCIAGRQPMKVHSLTNLHTHTKKNILHH

IYLHYQPCIEGTVVAHSFL

>SG-PL_contig_8466_1_+1_1_180

NLLRSEGNMFRQVNNWSKTEHVHITLQDKCLCHNRYWKLIITVINSNKEQTKTPLYQTFQ

>SG-PL_contig_8467_1_+2_2_169

KKKKKSQLIHVLLQTHTQMPRIHLNQSLPNICCCSLEKIILNYPVTSPQEYNRQCI

>SG-PL_contig_8468_1_+1_1186_1470

MSLELRVPRSRSQWPKQCLKLSLSEESVVTAFSQQFLTIFKFCFHCCDSLPLSSLHVKFY

FIQSFYKVTVTLRSRKSGFSFEQLKFLFEFSQPFV

>SG-PL_contig_8469_1_-1_541_711

MQSFECKPTHLMNIFRFLVQFPTKGVHIYYSVINSLVIRFFFFFFFFFLNFTFFFFF

>SG-PL_contig_8470_1_+1_2809_3303

MKTDLTVYGKLKVLNKNLCFVRILMIHFNKNIKQNGMLHLKIAANGLLYGNFKTNFGMEK

HIPQLLDIYIFPLIKFWCSCYRLETELGRHRGISREDRICKTHELNALGDKFYFIFECPI

YYESQKKLLPKKYLYVKSMFSLGYLFSANKSVLLNLAKYIKMGKI

>SG-PL_contig_8471_1_+1_76_249

MKIRLWHPFLYGKTLLCLYHTQCRLPCRFSVRQTFCLERTKSKVLLSVRMCLLKDGSW

>SG-PL_contig_8472_1_+2_2_256

KKTKTKKNKIQLQSKFAYSLKTHIIITKLVALDINDSILLIFVHCTHSAFRQQLYLMQWP

CNKVKGDRHRQHKNSIQNTHHASAD

>SG-PL_contig_8473_1_+2_296_493

MFMSDKNLCKRFGGSVSLPKILNDKRLAFSGSFRCICTIGNLNLSDHFSILVTFKMQDIM

RHALVG

>SG-PL_contig_8474_1_-3_3_137

QWICRTAPILSGPLPGLLTPTLSGVFPSSQSLANSALVRPSSSSG

>SG-PL_contig_8475_1_-2_2_202

CSSDLCGCVCVCVCVCVCVCVCFVYVCVCVFRFQLVQTSASVCSVLVGIFQTPISVPSAT

NGRSQSK

>SG-PL_contig_8476_1_+2_2_196

TTQTKTTICSQKLALNCTLRTLTIFLFIHPQQHKQQRYVHRNACSAVHKAKLGNNTSLFD

VGVKG

>SG-PL_contig_8477_1_-2_266_505

MCCFHHIQQWKFKSRDTWCHVVRYMLGRSYPSSQSSHPAVEVYAQRHMLSVFFGRCWAGH

VHPPNQTDPSVSSLQVFKGC

>SG-PL_contig_8478_1_+3_57_332

MSRDTKRLLVTKGQNIMSSVLRLKSGISYTLKAANKLFLMSWAGIELETSEPESTALKVS

QPVQLLGCVVQDKVSVKTTTTTKISEPVSLPV

>SG-PL_contig_8479_1_+1_304_510

MSKWTGGNFLFSSQFSVLVLLFLTITLSSLKHNPNEDINSTNVHDTIFYLLFHVVYTSHL

HLNTIGRIF

>SG-PL_contig_8480_1_+1_370_591

MIADTSQLCIILMLTTGQALYGSSKGICDGSNTTVNLISLHHNCVSHFKDSVIFLQPTFQ

ETSHKKSTHPCPSP

>SG-PL_contig_8481_1_-3_27_320

MADDQVAPVGTTDVVDQQDNTTEIPDNIQKLITSEKEGGESPAKRAKVDLQSLPTKAYLD

QTVVPILLQGMSVLSKERPPNPVEFLAAFLLKNKGHYE

>SG-PL_contig_8482_1_+1_571_687

MYLYTRMRAIMQLSYLHTHTQLCSQLSAHSLAQLCSSVI

>SG-PL_contig_8483_1_-3_3_152

QNKKVTGFFQSKAETSNAATDVIRAEVMITDLGMSANVSCRVPDFIDPCS

>SG-PL_contig_8484_1_-1_1_204

FRGEHTGKWPFIKLGKKGDTYAFCEVCASDFSVKSGGRDDVQRHVETDKHKTNTEIGGRG

EQNQKVTG

>SG-PL_contig_8485_1_-2_110_1033

MSEQPTELDLQKSVEETVEVVRETISVQPEKPSEIKEALESMEVSESEDHITSPEVEQPA

EETTQLIEETVVVEQEKPFKTEMTEAILELADSDAVIEPIEEQQTSEVVLEQVQETTSVE

EEVPFKTEEHEVIIDTFESKELTEETLEESQPTVDEVEKSKDIISVEEGKPAEVELVLEA

VGEVSEDLALDIPTEQTTEEVTETVFETSFLEEEKPSEVVLDSEAVEESPVELQLEIESE

QPEEQVVEDVKQPITVEEEKLAEVEVALETAIEETPEDLQVQITPGQPTEEVIEIPMEET

ISKDVQPA

>SG-PL_contig_8486_1_+3_117_239

MLRIIQIYLGSPFLETSAYFLTDKCLGTVGFLYSLALASWI

>SG-PL_contig_8487_1_-3_501_668

MPSYISAMAKNYCVNLKELKSMKQVPSCIRSQQYPNIYSSKNRHLYPEMDCQDIFV

>SG-PL_contig_8488_1_-3_135_347

MPKQTGIVPRNSPTEEIDAGVTDKWRWDWLNRSHKNETFSSCFEKLSRPVYQYDFCVFCT

QFGAPVRSLQK

>SG-PL_contig_8489_1_-2_2_136

CVCMSVCVCMSVDVCVRVCVSVLVNFAAIFSTNFLFSVRSVRNKI

>SG-PL_contig_8490_1_-2_443_709

MPYIVLSSQLHNVVITHIYFFLLDVRPICLVRNLEAFVGCTHVYLFVCVSVSLFGLSLFV

CLLRYPSCHTSFVCWYNILSLRIRFCWFV

>SG-PL_contig_8491_1_+1_46_261

MTRGGGNAAQSFLNSSVASITGLHAAHNPSCTRQTSMLLILVLHSNSPQKPAILRFTTVQ

SLGSKSRNPVHV

>SG-PL_contig_8492_1_+3_2151_2588

MDLKQKKQCTHYFTGQIAPPPYKNQTNKNNSSPRKINKKTDALYYIHMIMARGNIPMTLL

PSFTLKQSSEQSVPSQFTVLVFFKTLTRNNHPLLHYPHFFPKTASCTHKNHGSTLGTEHW

YFVILLSNKPTTNFLRCSYDQLVLRK

>SG-PL_contig_8493_1_+1_1051_1518

MLAAPEGTQEFFKVHLAVVILVHVEGSSFGVFPHLRQCGLQLIPGDEAGPVCVKLSEGLP

QVRFLDFFSGDLAQGLPELLFGHLVVVIRVQIVKPCRHFFYLLFTATILADFAQFESIQG

PLSTSVSLIKKLHGPVLETLFSHSDNDGVRRSKNNK

>SG-PL_contig_8494_1_+3_162_347

MGLSTIKKDLICMFIAKRMGLHTIKKYLICMFIAKNEIIRFLYTLIDSAAGTISIFQRGK

KT

>SG-PL_contig_8495_1_-3_453_722

MFWTFQISVIVCKTCTQVRLNISTANNHIQNAFSSSSVRLYHHGCSWGERGDGQIRVGRP

EEKKLLHRVKVSHMMQRVVKTGHILQSGVV

>SG-PL_contig_8496_1_+2_2_247

KRIWLEDQANDAQNAASRNDMRAVYQITKKITGSTKASSRPVKAKDGTLLSKGEEKLAWW

AQHFSEVLNRPEPASPADFEGL

>SG-PL_contig_8497_1_+2_113_496

MSNKYSALAESPPPGTIEWWEGLKETATTAGEEILGYRKALRESWISDHTWNLIAERKDL

HQKRHTDTSDDNAVYQDYIEKDREVKRSAQKDKHIWLEDKAKNAQNAASRNDMRGLPDHK

EDHWQHQG

>SG-PL_contig_8498_1_+2_1331_1588

MQSAKIVNGTIRQTGRWTSQANDILPLVFANFTNRRVKIVISAKVSLPDVKIRPSGPYIS

QPIYLTVTAPPIGIPEHRAGERTAER

>SG-PL_contig_8499_1_-1_64_771

MAHESALFKSYFKTGISTMEGGASSGFRSVPPEEYTPRLMRFAKDKGGRVITREIPRCRS

LVTSGDVFMLDMGLKLYQFNGAESSPMERSKAMQYIGELKSKRGEAESDVLDGDSTPESH

DFFKALDEEPNDGNDSDDEDGDCDKKLLRINTDSHDFEDVKEGSITIGDFNSDDVFIFDS

GSTVFVWVGSNASRAEKKNGLPIAHNYLRTSGAPWRSVTVIREGTKSAQFLSALEA

>SG-PL_contig_8500_1_+1_409_579

MLIHIYAELAVIIIFKGGANASAFHRLKNIPSDTCKLTLYIHENKSWSFIKSQIPES

>SG-PL_contig_8501_1_+3_879_1124

MSTNHAAMSASLVIRLYSTHRPLADQVDSYTVNFNLYHDLSSMLRNIFVYIDRVYQSCIS

IAFILCRLLHVPPLPHFPPVKG

>SG-PL_contig_8502_1_+1_106_372

MHDTSIVDRNDSNPKKKPEIILFYNSTKGAVDTLDKLAHAYTVKQTMAYGHVFKHHRHGH

CCHSLDLEHQVSRSSSVMKKLEWALRKHQ

>SG-PL_contig_8503_1_-2_236_418

MGFYLSSHFFIPSCRQSYSVLGDVHDGYFCVSISHQTLTLLTGSLACIFYVLRVYTHKGG

S

>SG-PL_contig_8504_1_+1_1_135

KKKKLKKRQKKKKKKKKKKERPPPPPHKQPPLRNKGKNKKKHPTR

>SG-PL_contig_8505_1_+1_73_234

MQEQFVNNRQLCLTPGFHVLCHICLLIYLQIYLVNHIFIYLLAIYIYSFTISVY

>SG-PL_contig_8506_1_+3_3_134

YCTKTPEFTSPHNEGRVLFHLPKHIHKIQRNMRSRRRELIKKLY

>SG-PL_contig_8507_1_-2_155_1723

MTALTDCGYLPSDSPFLLDTYMEKVLNSGDAERILQEFEEIVKTHKRSPQVDKVLQFFIN

KEDPDSLQKAVDLLTSVHGLMNVLHHLIADFIECGHVKKARKIMETAGLRANMQRLNYYC

QSFIERNMIKELEMLVEVTKDMFAVDRDNMLFHLIRGYVKQKEMDKAKDVLLKYEEEMIQ

PSARTLRFLARSLERAGLPVPFEVPDFQPPVNTQREARAKTLEVKGESVATEVRVTTLAT

ALQVKDSLEKGDVDQVLTMLKSDKTLLAQAMSVMRKPWPSAALQSLFESLVQQGEVDILM

AINEYILPFHLKNSLRAHIFTAAVNSGRGEEVIERMEGVTSNTAKFMTFPALDTLSDNAP

HLMDKLESLCRRLESEKKYTEPLSVLWCYYKYRDSPRADVIMKETPGFNKTVLTGALCQT

AVKENKVDVLESLLKLKKDAGEKQGMAVVYSNLLHLYVEQNNVEAVEKTCEKIVEEGLLR

SNLRRTPVIRVENLLKENNKPLPAWGDDSSSDSSSSTSDEEVR

>SG-PL_contig_8508_1_-3_267_1013

MANRTFFVGGNWKMNGDCKSIDGIIEFLKKGPLDSTTEVVVSPPSVYIQYTCNNLKGSGI

GVAAQNCHKVEKGAFTGEISPPMIKDCGAEWVILGHSERRHVFGESDELIGEKAKFALGQ

GLHLIPCIGEKLEEREAGKTNEVCFRQLKAIADNVTDWKHVVIAYEPVWAIGTGKTASPE

QAQDVHQALRKWLTDNVSAEVASSTRIIYGGSVTAGNCKELAGKPDVDGFLVGGASLKPD

FVSIINARK

>SG-PL_contig_8509_1_-2_2_121

GFSSNTFIVNTLVWILPAASFSLLSPPLLSFHFLGGRPLA

>SG-PL_contig_8510_1_+3_105_200

MFTVKVLELKPESRKNKENSMRVRLLDTNRCR

>SG-PL_contig_8511_1_-1_64_192

MMCDMISDLNLSLATFDSLILLSKRCFQIQSPKVMSIILYVFT

>SG-PL_contig_8512_1_-3_282_1169

MVAVFRGIALGLLVVAITVHIVGFVTPNWVSAKLDMRGIQGIMSANSMGSPLLNQIGMNI

NMGLWKYCMKSNIGQVMQNAMAGAGQGMGGQGMPAQDSDGTANQDSDSSWQGADLDQGSN

LGPSAGEMTDDSSSSIGDGMDGLSDMCRSVNASETPGWMKAVQALGIMSLIVAIVGLASI

LYSCFGESKGDRARLLPFIIAGICVTAGVQLLIGVAVYGTEFEKMMQDQMSMMGSASPAL

HDLAENIKKNTNLGYSFALDTVAGVLMLLTAVVVAIPAMKGWDSSVQFGGPRNMAV

>SG-PL_contig_8513_1_-1_22_189

MITWSCLLMTICSIILSSKHLCTSQFMEAHPNLPEYSRANIVSTFTIQITNKIKIS

>SG-PL_contig_8514_1_+1_1_246

MNVHLNITADCKRSHECHCSQKCSHEPMTAVTHANTTGRHKCSHCRHKRSHQQINLFQPK

TVPQIKPYSSITVVRMRTQNCS

>SG-PL_contig_8515_1_-3_303_455

MLPYPVRTSMLCSYICLLILQGEQRGWAWRSSATKMTTGWPAPVEGWIRHC

>SG-PL_contig_8516_1_-1_103_264

MLFVPCMVRLVYFMSFLCLFILSSLPLCSYDELRALKEATTSEVLFHLDDHGFI

>SG-PL_contig_8517_1_+1_1_216

ISILTTDLPCEIRAALTAESASPQCSAAFFVSFFCMCVFLFTCESGLCYRIWPGTTPLLL

WGLLCVLSVCCT

>SG-PL_contig_8518_1_-1_1_108

SAHFCSPDIIQFSCFGYLALLFLPRICKNGKDCVEH

>SG-PL_contig_8519_1_-2_2_142

VRFVNPIFFPVHVQSFWKKDIFACYFALIYCIAFQSRTCSCKDFLRP

>SG-PL_contig_8520_1_-3_3_71

FFLVQYSCKHCIISNADYGFSFV

>SG-PL_contig_8521_1_-1_304_432

MFCINNMFLCVKMEKNKVFTFISIIFVLCNAKMSNLFFSFIPL

>SG-PL_contig_8522_1_-1_1693_2022

MPNLSSETKVLIQGNNNLGNKEAKYIVNFSVFVKEREAEFVSLGICEECVVERLDENVRT

AAPSNFCCSFVAQKWKLGFHLLPYFSLTSVLFVFCRVVIKSFVEKNVKKK

>SG-PL_contig_8523_1_-2_812_1180

MVKDTHSRPIQHHLKSCTPSLPTHLTVRQHGDKIVVKMHFIHMLNKKQTNKQKGGSSLLT

TLSQNLSTLSLHNLSSPSLLLLLLLLMMLLLLLLIMLLLSITTLIYTLSDTHKHTSSLCT

LIP

>SG-PL_contig_8524_1_+2_2_181

CCALFLFFFFSSRRRHTRFRGVTGVQTCALPIYSGRLGYKQYIIIITNPLSQKLIPSPKN

>SG-PL_contig_8525_1_-1_13_291

MRDVAGDLRQTNLERTLLTWCQTRTAGYQGVLITNFTTSWRDGLAFNALLHHFRPDLFRY

ESQLNRDNESRLNHAFHIAEEHLGIARLLDAED

>SG-PL_contig_8526_1_+2_2_136

ISERITYKVACLCFTCITGSAPSYLQELININTLSHTLRTSSDSN

>SG-PL_contig_8527_1_+2_143_448

MYDYFYYLHSLSPHTHKKSTPSHLIIFSREKREQQKLSLSHLGVTEILPILAVLENVQNY

WSLQNFQNPLIQANTICIFRVKSLILLVINYFSHVLMKIKIH

>SG-PL_contig_8528_1_+2_2_100

HQKTSRLKATRGSQDIQQVTFASRVRNHDLPYD

>SG-PL_contig_8529_1_-2_17_379

MEGNGSRQRINATLMSTYEGKHVCLLGKAKDVDNNGMYFTMTTSDEQDVRVNMNMPLNEY

VSGLVEVHGHVQGNSIACDNYVLFSEEASTSFDLSLYNEALEMTQKYPDHYIQGVASTDN

V

>SG-PL_contig_8530_1_-3_219_2816

MDSDVMDKCCKMVGAIFSRHTSPSRRRDASPQAVDVIRSVPQNGVLAKEATGAYKAGVWV

WDVSHARTSPRPQRHKHGDGDVSEKDQRKCQSMERSGGQFGIYPQLPPPPPETKTKTPSM

TSVLDNDEKREKLSKSPPPPPSTQTEKLDIREKYMTLPATNANASSSSKQGKRHSLATLS

RNPHSEDPGSTQPSRNSRRSKDEEVRRLAKTMVNSIENLIGNLHGIVGDLRQLVQQIDAV

TESIDTTCPPARPQRAIKAGPERKMIVTTNQIFTQTDLDQQCRDSLGHSEWVQKYGYMQA

SSPHRYSYPQYYSPVSSVSSPSGYDTDRRMYRRSRSSRKSGDSGHTSQKSSSSQHQSHKF

GAETRDTVLQLRQIEPARTGQQRQVPWGFGGAPFEETQVHSAIPSKSSYLPDPSIPFLPV

ESPPNPAPTLFGAYADEEKTIHVKDSDSLPDRGLKTWKEKMERHSGSADSLTPQMDWSYL

VLTDHNLTTSVEGFNLSPGSRHMDPLSPQKDWFKLQLSSHKSESRRSQKDRSRLSQTGVK

MKHHVGAIPGIPEQREPCDSSYDSNCDVFLPADTCSSSSTGSVPPKRTGASVPQFLVNED

HQKQNTDQDPQTVCSVSQETGRLGVFPQVQQSIGGASLGWSTRSKALPNWPSEELSSIGA

SDNEIDDLSSPFLQQQFQYPRRSSAKESNPETKNGPEVNSGMNRTISVSDTIASGGFEVR

NDIETSPESDTLGPYGGQYESAMESRLETLTNEVEGSDFSDADADVGSDVLEQSLTSLED

SSSVKYDRELNTWTSYTMTHIDSDDVVSECTSDVLNENYFDTAMSNPGFDDVSLNFDDIG

FLPLPLQTSKFDGVSPRIEKMSPSLE

>SG-PL_contig_8531_1_-1_1_174

PNIPLLLPAYIFPSYPVILPSSLSPHSPVHTLTSTHCSIHLPEVVLDSLRLQNSDFYQ

>SG-PL_contig_8532_1_-3_57_2102

MRDKINDMQCRMRELRVELQGLRRMEQLNQESMRDAFTDTLSKIKKVISAVPGAENQVIR

QQRVEVNSLAEAYRQDKARVEKELSDLETSVEELKEDVIARKCRVNISDVEGLALVLSNI

TKSLADLKARFPQVNESLRKVMTGEMEIVVKEERFIKEEPLHIEASLKRCKKLTSTLYTL

KRLATVQDTRPLQVPSMAMECKEVGDEEKMAILESIRAMVPDHETRVQSLEHHFPIDAAE

SSRERKKKIITQQESLKFTKSLQTASKSLKPPDTQPTITRPSGDGVSHGDKDSKKSVSGG

EVLDSKRSEPPVIPAKPARLMLTSESKPAESCSVVSVASSRPVAHTVTVTSQMCSEVEKK

ETDSKPGHRSFARSAFFGSLETSPPSSQSDRVKSSSKTSPTATVSASGLPVSSGSSHSKP

VSGVYYQAASPTKTRSLSSSSSRSDSHDDSSNTDKGRKVPPPPPPRKSSAKYPYGIGPLS

PQSPSENGGSGGPALHRQNSGEKSPDKSKKAPVLTVYKAHSPTSSGPLSSTPKPATPQKP

QGLNKVAGQRSSVKRGEIQQQKMAPDRLLSTFVSPVPAAITTNSQDPIDEGDLSGSESTG

STTSLDSQQGATLLRSNSSPSHRPPKPNPPQRQSSLGSKESQDSGTKTKTQILQDRVKKS

HQAGSVTTQVNGLDGAKVGHKT

>SG-PL_contig_8533_1_+3_801_1283

MKLLIVWISLQKCTLKKGEHNLLPPEKQTNKWPHLQFTQCVSVKCHNHFSQERLEVLHYS

SQHSQLTAIKAKSCSSYSMIYKLHRFLQQYVNTDTKTTTTFPRKDLKFHIINASQPSQLT

GSRPFHVQAIQQFTGLLNRLLKTVCKHRHQNTGIYAMSNIV

>SG-PL_contig_8534_1_+2_1067_1636

MQMRVCVCVCVCVCVCECMGVWVWTHVHMSLWVWVCVCVCLLIFSGVLTCNFCFCGFFLT

GFVTVHCQHIIFSSKTEQQVKKNPGEAVYEEEARKTHFNAQYVNIKDKSNSIREYKRQIK

LYQSGRRERKHIKAMLILHMFKEPALAADSGTKSTPLQLCGTSPSASVKDCSLRSASCSR

AERTWPANFW

>SG-PL_contig_8535_1_+1_1_135

QNNNNTTTNKQKTPTHTNRCKRISITLRTMTKVGGRRGGQNKLIS

>SG-PL_contig_8536_1_-2_8_259

MVVKIGDFGLARDIYKNDYYRKEGEGLLPVRWMSPESLVDGVFTTQSDIWAFGVLMWEVV

TLGQQPYPARTNIEVLHFVRAAGK

>SG-PL_contig_8537_1_+3_3_263

GGLETKQVCTYDDPGDCRHLHPSPTRSCPSRSAHKVTEETADISTLHPPEVAQAGSNSDF

PLEVRRPDHLAMGCARHEEFELSDLSQ

>SG-PL_contig_8538_1_+3_3_260

QTTVKEGKRAETKRATEKRKFLAQNFQKAGMPFILKEPPASPQGVKSMKHNEVCGNQKNR

TKTARNKPFSSGHVNIPDSDMFFEMT

>SG-PL_contig_8539_1_-1_130_291

MFFSFVGFCFENKKGSKCFVLKGLFSCYIDLFKPVLKKKQTFNLFFKESVCVCV

>SG-PL_contig_8540_1_+1_1_171

SYYIILLGRGTITLKRTRKQRHICTFTSVSKSLSDLVWLKIFTFSSKTASHFLLLLS

>SG-PL_contig_8541_1_-3_114_4157

MEEEKLQGEEATADTQQTTAIQASESDSERKIKIDESSSEKPLDKAADTENEETEGEGQS

SRKSPEVLGSSRPHSSTSSARAKSSASGEIIFEAKKKKKRGGSAAPEDAYTVTFTVSIAM

AIPTVEDEDQPDLKDLLRKKKRVFEAPRAQNYYHVEYYLIPEDDELMKTDIVTYGMAAKL

YMERHDARVIKTWQDGEITWIAWAHSHTMTVTRDVLLKMYDHTLELRVWDTKDKCSTRAR

FDRPKAFRLPQPKPGESPEDVGGVKAMVMKQMGGFSKMQSPKADAVRPLPKRCAFPRADT

KKRAATATVGTPSQTDQESGVGGKALVAVPNKAGSRGASGRTFSRLGKLAGTERPVQSPG

GSAVKDMILPEDSATVRRRQRKAEAAAAAAAERAKKFGVCMVPFRMALLFSGMQSITSRL

HQPVSGVEDLFITVSLDQPLLSEDQKRALNPMIITVRSVTDMPSSPMTFDELRSKCQPVY

CKYQFFRQPSHTTVAKEHGKTIYWDDKNVVLLGPQEPSELREYLSGPAMSVEIHDRDRKT

EDVKLKATLFGDDLEDEKISNVGTVASRRTLHNPFEGRNKPWDPYGVCKVDLSELLLGHR

FLELRLPIHNCPLPDILGVEGTGASKLMGVAGAVDGPVDRPLSSGDYMRSGAALKIQVEL

AHPLITPAKLAAKEELESTPQCPFGRIVFVFAYKNTTLLYQLQQMVTAINARALELDDMP

QHVIDAALSTYKLSVEQQLSSSLDIITGFHVLDGVRHIFVLEGLKEQGIKQLWEQLPTPE

SSDVRVLYNSDLSFSQRLYGPLDVDLCRVKLHEPLDLVVQQSLLYVRDMVPKPCFHALVR

LEEMARMHLMKDVVKNDMFLTSDMVVSMSKEFGVPFTAEDFDELRPDWEKSPVLVRAETT

TETPGVMIQTSRDWTPIDNFNRTFVDRMAERKEKGSSHDFINENKDVIKRTSDLNLLIRE

KTRVPVVSADVAKAHNYSTQTLNSTELANEALRNVLKEKPDSRFTRSQDFHHSSTVVPVN

VPALAKEDARLSKELWRTPTGWIYPGKRTMIESNVHPKRPDTARVDELEEKWRENILHVS

QLQPPLERDKYPWDYRNHDMELYCKPPPMFKEEPLSIHLPGVKQQVEKMEAKQKEANEWQ

SRIVVDDTAQYFHRVLPHTEMTSSGFYSSNQIDRLTCLLKDKPDKLSLKRTHVRDIPPLS

VVHYPAVDTLARAQGLPLQPAIPGEGQEKVPGYKPGPYDLSSWSMEDNRVPAYVYDHRKH

AEQHGTDFNALHKERQQLWKRPIIPLNEEERDNHLFRTPLATLNFYQPLPAVGASAMEPG

DYAPTMDAATTTQDLPQSVMHERGMVYV

>SG-PL_contig_8542_1_+3_39_311

MDQEGRNKQTTVKERKRAETKRAIEKRKFLAQNFQKVGILFILKQEPAVSPKGKIIYQHK

VLTRLAAQQHTTPFLFIYLFIYLFIYLFIYL

>SG-PL_contig_8543_1_-1_1_171

FRTEGGQSVTVVCSSSQHVCVKFTVNFTYRILAQMTVQICVENVHIIKFNSNIFSEL

>SG-PL_contig_8544_1_-2_77_1729

MCGIWAVFGSDDDVSVQCHAVHTISHRGPDAFRIENINHFQNCCLGFHRLAIVDDIRGMQ

PMRLYTHPKIWLVYNGEIYNHKLLDKWHDLKVSTDCDGEPIIHLYDKLGAEAMAAQLDGV

FAFCILDTANRKVILGRDTFGVRPLFTLHTDSGFLAVCSEAKGLFGLSHSLRDEEAKMEP

FPPGHVEVYDLDHQGRAVLNRRTPFNRPGQTPVYRTLASPDSKDMKKNIRTLFEAAVRKR

LMSDRRVGCLLSGGLDSSLVAALLVKMSKEMGITYPIQSFAIGMEGSPDLKAAKKVSEYL

GTEHHEVIFTAQEGIDAIADVIYHLESYDITTVRASIGMYLISRYIKLNTNTTVVLSGEG

SDELAQGYIYFHNAPTAEDADRESRRLLEDLYLFDVLRGDRTTAAWGLEIRVPFLDHQFT

SYYLSLPPAERQPQEGVEKHLLRSAFDDGHLLPNDILWRPKEAFSDGISSVKKSWFEVVQ

EFCDKEINDKEVQDAKTLYPHNSPASKEALFYRKTFEDKFPHKSSWIPYLWMPRWTAATD

PSARTLKHYRQ

>SG-PL_contig_8545_1_-3_75_434

MAVPKKTNNNRKQPTTTKKSLAQVSVHPSSCQTAAQTSSSNSRDYLPISQYCTNDNVYKK

RKEIFQIKNLRLKILTFLLYRWGIFPLLRLFLNCKVLMFVPYKETGTISTGRRKKRQSYF

>SG-PL_contig_8546_1_+3_3_137

DANPSEHYLGTTWDHFRDTWNFFLGTFIFRLYVLHMHKFDKVKKN

>SG-PL_contig_8547_1_-3_354_521

MSESLITYVKDADILYRYRSDHSLIHVNLKFEKEGEEKKGRNLTQSYLKTIHAWKS

>SG-PL_contig_8548_1_-3_24_1232

MLFGFNAMACDRIIVQSDWAESLREQCGTAWVTFKTAVPPSVHSELKNHGMSENGLPYVV

AEDGFSASFTTRKTMSVTYTGEAATITRKFVAPTITFSTIHKAKKGVHCVDVTSGGLGVS

CDNVGNLLIWQTDNGEVRRELRGHLGDVYRCKFFPSGVVALSVGADMQIKIWSAETGKEA

ANIVGHCAAILDCAIVERGRNIVTCSRDGTARLWDVSQQAELFAWTDLGGDINCCDLAAT

DNSVQLGVPDEAPSDKEILTEGKMLVLGSEAGSLLSFGLHSRKKIFDVSCGSPVNCCMFL

SDTQVACGTQDGQIFVIDARNPSQPLRQWKESRSAVLSVLPHKQGLFISTGDGSLFYVNE

EYETSVEMTGSDCDPVYSIAMDGTHIYSSCRDGTISKYSLNHV

>SG-PL_contig_8549_1_+3_1413_1736

MNISKMYCGLIKTTTLNSHFSQQKGGKVDNCFIFAFIRADSSILHFSVSMAHQLTDYQIL

FPHHDEHFQKYFPVPSFFCVLVIAFLEVTSQRADDFFCATPHDQFLQF

>SG-PL_contig_8550_1_+2_86_199

MNSHASTSSFKQQKVHFKTEQNNEKQNNRQQNPIHTCE

>SG-PL_contig_8551_1_-3_750_986

MTTIKATPHLQVTEGKNGTTHTHTHTHTHTHTHTQTHTHTVQAFSRSSQGISHPRTQCVC

TQKERIQFKSKKAGTTLQY

>SG-PL_contig_8552_1_-1_58_435

MYAQTQYLQEDYAALVIQNSFDPTVSRYFHSLQRNSAFSPEALENLRSAATIGSMYRSQR

ERGPRQCGREEPLTGDVAMSSLTSLQEIFPAELATHGAQTTPWERRQILVISRRANRSTQ

CWSGIQ

>SG-PL_contig_8553_1_+2_2_199

DKLSGLADVLSTAVLFALRNIMVKIIRHELCSFAGLLFSGLTRFCVQRGCLLDFDGLNVV

TALIWP

>SG-PL_contig_8554_1_+3_1029_1190

MTSTELYRVYTDHVNKIQNSMIHADDVNTYNTSSVDMYVALILIAADRWTERQT

>SG-PL_contig_8555_1_-2_65_211

MHVSVISSKARSSATCAFLLLYIIFTVDASYAVNTHVYTGFAWLKLYRM

>SG-PL_contig_8556_1_+2_2_181

LVYSVDFSPPHFPLDGMLENRYACQLETECESISILVGYFFFVPPCAAFLCLGEYIDKLC

>SG-PL_contig_8557_1_-3_36_527

MSQYHSSGDVQTDYDDHSQDAPTDFDHGPEQAQFKDQPGNMDNPSQKEKTLQLDQPAPQE

PQMRLPLRVHEGHEFSPSSSHSAESPSRQQTVDQKCSGSNHSQQDGMRMDQQPHHQNPKQ

PVSQGHSQHAERVTTQQAKPHSQKPHRQSVSEVTVTSHNVTVSS

>SG-PL_contig_8558_1_-1_28_219

MHVPTQACTHAHTETHTKPTYTDARHANRSMKHKKNNISLDNLSYKYTTESRVESRQEKE

VRLI

>SG-PL_contig_8560_1_-3_3_212

GYRSAICSTLQQLGGPALSDDSLLPEVSRGLTTHEAKPPEGSPSGTWPWFWEPSGPLPSS

LWMWSPSRCS

>SG-PL_contig_8561_1_+1_2449_2673

MSCFLQFSLLPLVASLLSDGTYCHCLLLSLCLCVSCSSQSLCYHCCLYTIICICHLSCGH

HLLSLCHCFLSLCTS

>SG-PL_contig_8562_1_+1_1_186

QRSQTSADRMSDLFTVSQSEKTCSLSANQKRPNKQKLTLSLPHPVEQLSGSERVNFLGQL

RE

>SG-PL_contig_8563_1_+2_20_484

MHSSYKLNKQMENEYVDEHNNIILLKSAPLSNYFILQICNHRAKCLNYSTCVYPQLATNV

STYFSFMQTFINHTQTHVYAHTHRTNNSGQLSSFSLNYNQPFCCLPLCISNLFLSGQIHK

MHFVHVHTYKNTTIKKNIAGCTWKLDLELNMFYFL

>SG-PL_contig_8564_1_+2_296_469

MIIKTLDSQTQHTRTRAHTHNIGPKPLREKNIPIFYHDHISVIYMFNTKAQLLIHPDT

>SG-PL_contig_8565_1_-3_372_623

MGITNTNKCSFCTLEKESIKHIFWNCDISRQFCQRIESMFNTKCETASNIRLLRVRFFLV

QIKRYMLMLYLSCCLLNAVSAHAK

>SG-PL_contig_8566_1_-3_3_230

FLSYEDLRRKYNVHIDFITYTGCKLAVKDLILKNGITVNDNKAMNMNIALQKLFSVAKGT

KLYYNILNADDVKPQL

>SG-PL_contig_8567_1_-1_1_201

ARVTKVLMELLTGIPVFFKVKKIHDMNMKWFQIRTLHRIIGTKVILREMGVTNTDKCSFC

NLEKESI

>SG-PL_contig_8568_1_-1_157_1401

MRCCSFVLNLAVLVLIVQDGWGQTDQTDLRLRRREKQRYRLAELARASTSFGVNLYRELA

ARDNSNIIFSPFSVFTALSMTLLGTDGITKSQLRAVLVQRPRQGIHQALHAVFASFPSSP

DANVTLRVANAVYYDAQQVSITDSFNRNVRRLYGAYPRMFERPNPEIPINNWIYSQTGGK

IQDFFNPGDITSDLVLILLNAVYFQGKWKDKFDERSTQDGDFTTASGQVVRVPMMSRTGR

YPVKMLGGELGALVLELPYGNDGRFSMFVLLPTQVNGLSDVEARLTSRLLEEAVSNMPQA

TRVRVQMPKFTLRVKTSMKSVLQAMGLTDLFSGSAQLSRMATSRRPLAVNDVKHEAVIEV

DEKGTTAAAVTSVEVIAISLPPSVFVNHPFLLVLRDNKARLNLFMGRVNDPSPQA

>SG-PL_contig_8569_1_-1_298_489

MHRLLLGWFTCRYVQLQILAGWLAPMMEPWTPDLMSSSSVPAHNIETTLCAAHLNASIIL

VVSA

>SG-PL_contig_8570_1_+2_623_862

MGAVCTGNLSTLILKDCEEVTAAAYINIKHHLVTLLIHLLHKIHLCHCTVSPSLSLSLSL

THTHTHFFLHNVSSENCLAY

>SG-PL_contig_8571_1_+1_1_252

SDVRILSLRKAKGDRVLLDWPATLRKCITSRPETARLLRKFETQIQRILPSSSVQHHEQT

AAIQKAFQKDVESLVTTFEEVGNP

>SG-PL_contig_8572_1_-1_13_288

MRTTHHYHGCFTPKSCLMSMFAFAFMRRQARKKMLVWHIEFMSFGRKLLDMLVYIILFVC

FGGLSFPLYLDLGGGVLGLWVVGLSCCYCSLL

>SG-PL_contig_8573_1_-1_37_1524

MCIKFPVRLSKLYSQHRHNWPGPVSSCSYNFIMVWTAVSFVFFAIFLTIRAEPAVLAPQQ

NRSLYSEPVSEGELMQLYDQMKQGDTLLHERLKDSYNALRELLKEGDAHLKEQLVQGMAQ

AKEQQKEGDARLKEELVEDTTWLKDKLVKAESRIKEELAEGEAKTKEELTNQKEAFEKLR

LSVKEWTQTITAEMDHLKQQMSDVQVELQYSATTLKSTKDQLHTTQTQLQATQETLQSTQ

ETLQSTQEALQVTQDKVQSTNEELLRTRQQLQNEVQNFTAVKTQLQAAQDYVYSLKVKVN

SLQSTPNLGELGSSGKWDELDYRDVGSTFTRWGRKSCPDNTSLVYSGVVGGAWYDHTGGA

SNYLCLSLDPQLDNKTREGWHALLYGAEYTRVTGHGGQDVVCSVCRAPQATTLMIPATLT

CPQGWTTQYKGQLMSSYQDYKGRTEYVCMDEVKEHRDGGNERKTGALFYPVASQCGSLPC

PPFVQGKVVTCVVCSL

>SG-PL_contig_8574_1_-1_1_225

NIVNLDAVPPPTSPTSCFLPIYTFSCSRFNTSFGLNVDLYKMHIIINVYHECIITCTFLL

LVFIEVVLKIYLLGC

>SG-PL_contig_8575_1_+3_3_122

RLAGCHGCGNASHNSVKCQIMVTYNTNMFKTFSYMLNNHF

>SG-PL_contig_8576_1_-1_106_924

MATASNYDDQSETAFSDQLASQTLTDHETTESGEHYDYKYDDSKWEEEGAEEVKELVQTT

WPLGVEVYSKQRNQWPTSGKHIVATYDDNSIVVYQAFCPAIAAAAVKNQRFEGGGFSFTR

MSWIKTNFLWMMYRCGWATKRNQERVLAVRITLDGFHQILAAAMTASFQKSTGMGQCEVR

LQWDPDHSPIGGKLTRRAIQLGLKDDILEKYATQWIVSITDVTPFVRHQHRVLNQKGQFQ

VSTPLERVYTPSSHAICQRIELDYHQSQAVLVS

>SG-PL_contig_8577_1_-2_101_430

MGSYSSSTSDNAKTEQMTAVKVRPGARKWAAGYQADLVCILSYVEGEPPGNITVMSLNSS

YGLLAFGNESGLAIVDFVQKTCLLNLGTPDLYGSMDPYQRAPPSPRSKKS

>SG-PL_contig_8578_1_+2_2_127

YVYINPCLIPPDKQHTILSSIFYRKMHRTEMTKKRRYKTRQL

>SG-PL_contig_8579_1_-2_2_253

ANHFATLSGDSFLFFIVDTILELIVFTQYLLYLKMYEFVHALWASYKSKALRQSRVFGVY

ELDSLSSGDVLYHSFTMNIEIGKF

>SG-PL_contig_8580_1_+2_2_109

PGQPSLQADKWKRSLHLSIGNQFLTLLTMSLLREGK

>SG-PL_contig_8581_1_+3_3_257

LGSSTGTLCVGNLRSQPAGRRAVVCVCVCVCVCVCCCCCCCCCFTFTLSMYNFCVYRKCE

FVKAVFYFVLCFIIFAIQVDFMLWK

>SG-PL_contig_8582_1_-3_3_152

FINVFFFSFFLFYSLFLFSFFLCLLFFLVKKSCKAKTIKQYGHMSLQNYL

>SG-PL_contig_8583_1_-2_140_844

MAQSPAGGAVKALQLELKKIQAEPVEGFRVKLINDDNIFEWEVAIFGPPGTLYEGGYFKA

HMKFPGDYPYSPPSVRFITKMWHPNVYENGEVCISILHPPVDDPQSGELPSERWNPTQTV

RTILLSIISLLNEPNTFSPANVDASVMYRKWKDTKGKEKEYENIIRKQVQGTKEDALRDG

VTVPTTLQEYCVSSRPTQDNQVDADFYDDDYVDDGEDEEMYQDEDDDIDSGNEEC

>SG-PL_contig_8584_1_-2_179_250

MVFVAKNTHCSIDMAFTVKDKVIN

>SG-PL_contig_8585_1_-2_179_250

MVFVAKNTHCSIDMAFMVKDKVIN

>SG-PL_contig_8586_1_-3_159_422

MGIVVGSVLHYTHTATTSQYVLDANTFFLFLLPPIIYDAGYHMPLRAFFNNLGTILILAV

INTLWNTVAIGLSLWAASYLGWISRSIS

>SG-PL_contig_8587_1_+2_53_259

MRLNMTAKGKKFPSHDSDISSKDYFMQLIMNLFKKALDLLMKSELPQTQNIKIHGSKPLK

LCRIWVVNN

>SG-PL_contig_8588_1_+2_2_166

VRITSFYSLKKDIFLVYTNFTTQVSLVYAQEAFVELIHYNDKFKIFDMWSFSFKI

>SG-PL_contig_8589_1_+3_3_98

FPSRTQKVSSLVFRHDRALRARLWRGRKRRSN

>SG-PL_contig_8590_1_-1_88_3870

MKLFPKTITGPLHAQNKGACDWGWQGVLAYGCHNLVTVVDPKSVQVIQVLDKHKGIVTQV

KWAHENYHHDLNSPYSLRLASADNAGVIVSWDVSQGTSRTEFSDGNKPVQALEWLKNQDA

SRDLLLALHPPYSLILWNADTGTRLWKKSYTETLLSFTFDPFNGKNVAFLGQDCIIFVND

FVITKTPSSNGKKYYISNPSTQGMSRSSSSGSLDRKGSASKTLAKRMSRILVGESKPSKP

TGAEEESVALNECLQLSFHQSCRHHILLLYAREILILDLQINQTVGIIAMDRTSSPFMQV

VSCWQRDMLLCLHDNGSLSVRVRRKTNTISTPAPEGHGAFDDTPPNVSLDVAYDLRCQSD

PLRVTRHNRVYGASMCPMSENNVALIMSDSRVIFWELQTAEIMANHNTQQVSSPLHPPEP

INTEGLTADDLNKIAFPIISEIPRSSIGLSDMLASSVGNSVGRMGPGVQLKFLMTGLLNG

ISSHITVVRMCPPLTNKNWDIYEPVMALGTYSGAVQLANAASGQIEKEYSLHSSIVRGIE

WASLKHFVSYAFPNPGPSGLVRNELLYVDTVSGRQTQLRTNKDEESPIEQLQVSYLKQYF

LITFKDKPLELWDLRSHTIIRELPRNFPRPTAVTWSPSHSLKSLKKKLLDSGAEEKPAAQ

KLSGEGDTTVSSFATNSDPSAGKDKKNQDKVSVREHFVMTDKDCTVFHFIVEGNAISDVT

KIPPESGLTRVTSLAWKGDFLVFGDAEGIQCLWDLKAKISRTTPTNRGWIKKIRFAPGRE

NLKFFTLHNDGVLIWDIGAEGKSEILHSIKSPKEIPKVVDCEWAGSDRPAIVTSTSCLHI

MNLDMRHSSSPVDNWQLAETVQCPYLLSSKASALLKCVLQHQPWQDTFSFTVEGFREEDK

ELQKAVNIHLALMDSTLKIYLPKCQFGTAERCLTAARLFGDESEIHFWTVALHYLRTNKS

KPSWKEAGSPTSPVEGGDLVELAQSDEEQQNQRLRSLFRDQPLERCYDSLCDNLSYKTYQ

LDRVSLHDSKRATYEHTQKCVENYILLGQTDRAVQLLLETEAENDSYYIDSLRACLVASV

RSSGASQSTMKLVATNLIANGRLAEGVQLLCLIDKGLDACRYLLANGAWDQSVWLAKSTL

EFGECSEVMKRWADHLCTAQVNQKSKALLVMLSLGQFNKVVEMLYSMRCFDRAACFIEAC

LQFDALTNTEETSPLFEAVFLEYARFLINLGHGKAASYYCSLAGDKGAQLYKEVCILFNY

N

>SG-PL_contig_8591_1_-1_175_819

MAKLTARGCDAAQISPEISDDEVFETDSDTVWRPLRTNGLSSEVSHQTLYAIPLEKSEET

QSSADCTTPHQQNNDLRTQNELYKADEKIWSYSSFCQHRTPNENIGPSGLCSVVKSESSQ

YFRTGSENVPSASHGLQSRRPQMHHLDRQHSIVDGLLFEIYDRWHGRRADSFDSDTFTEC

SSTSEVFHNRYDSSYETTAENRHARQFNRSFLESQ

>SG-PL_contig_8592_1_-3_3_338

VEDSNLVLPNPPAWKSSVFTIRPPRPFVVVVVVVVVVVVVVVVVVVVVVVVVVASFFFCC

LFFLFLLSFAYPPHSSSSSFFWSLLLTSLLFKLLFSFYLLACLVVCFVWDGG

>SG-PL_contig_8593_1_+1_184_354

MYKRDCIVLYCIVLYCIISYCIVLYRIVSYCTVLYYVLYCTVSYHIVLYCIVLYRII

>SG-PL_contig_8594_1_+3_3_260

IVYFHQCFQEVVRLFYIPFYIHPNQRKVAGMLHTDNAHYTWTLVEDSVVNMMLKHSIWQA

CMSEYIIIVSLYMSSYTMTLWQDVGM

>SG-PL_contig_8595_1_+1_382_723

MEECWNSMSLLSGGWFSMIFSASDFLLKGRFFGGQNSGIFFHETCCLFVFNVLPLLRCGS

WGSDSSAQHSSSFSHTPTSTLMADHTRSHCASPVNFWFVFGVNSCDLRMLKGVW

>SG-PL_contig_8596_1_-1_1_159

GIASHHECKFMPFFSLDSWSNLYTAFGSFDSYYCYSESIMLQFSLWYLSFYKK

>SG-PL_contig_8597_1_+3_3_170

CSSWQVCIYTHHTQNMHIFLMCTLAFTKRKNEDLKTVIKTCNTTSQPMTTEQTQIH

>SG-PL_contig_8598_1_-3_3_299

FCGWKTHRKVSLSIRSFTTLLVAWASNTSKVWQTASSSRTFMCRFFPSSDHMLRHGLGGG

AHVSSAHSSKMCWAILSYSTSVLNEREADVCASLSPSRS

>SG-PL_contig_8599_1_+3_258_575

MVSTKNLSHHASDNCNMTTVTQSLIHIPATALLNCPINTMHLLACQKQQQTYIHTYNKNP

KQFRILCLAKTSSTRTKTNEIPHNSHSISSSASLTSHADIMYPLAI

>SG-PL_contig_8600_1_+2_158_517

MNCKNVTPEDEPSETIIRSFIYYCKPKITRKKMQFWPAGRQTDQRSSCHGCYTRSTVLRK

MTKENSHFGDCVCLEVDRSHETSREQLQSTSSCCESRTQGNRVHKNKCGHREDIYFEKML

>SG-PL_contig_8601_1_-2_125_1660

MVKNNPSDVEMKDATSPEGSSVDGKPTEKEEEKKTEEAKKHPELLTLEDIREQIRHIEKS

VTTKETRFMSRVMRSLVNTRRRLTSRILRRLIAGYFTVPEAAQRKESLLQFLTEDADQAV

SSLTFRPRSAKQAGQPLPVEIEVYLHLLVLIYLLDSKQLDKALACSNLLMATLLPLNRRT

LDMLAAKCYFYHSRVYELMDQLDTIRGFLHARLRTATLRSDFEGQSVLLNLLLRNYLSSN

LFEQADKLVSKSSFPDTATNNEWARFLYYLGRIKAAQLEYSEAHKHLVQAMRKAPQQSAI

GFRQTIQKLAIVVELLLGDIPERSVFRQPMMRKPLAPYFQLTQAVRTGNLAKFNETLAKF

GPKFQSENTYTLILRLRHNVIKTGVRMISLSYSRISLTDIAQKLALDSPEDAEFIVAKAI

RDGVIEATIDHQKGYVQSKETSDIYSTGEPMAAFHQRISFCLDIHNQSVKAMRFPPKSYN

KDLESAEERREREQQELESAKEIADEDFDGFP

>SG-PL_contig_8602_1_-1_112_444

MGRLDGTPPKESTGLEYLMTAGNRLSRRDLRNAAKNTKILYEEFWEIPMNHPEKVSVAGS

GMKNRYKTIIPNEHSRVVLPDADQDPLSSYINANYIRGYEGEVQAYIATQG

>SG-PL_contig_8603_1_+1_514_594

MITMLNLVGSVIFVHNYAFYVHTDNTI

>SG-PL_contig_8604_1_-1_28_144

MRVDLVCVNVFCGFTVLRMALINGLDICQNLWPVLRHIM

>SG-PL_contig_8605_1_-2_155_241

MIWQNERHKRVTDNCHPSRLYASLRSASD

>SG-PL_contig_8606_1_-2_2_193

CSSAMYIQFHTHHQALKDPACCIQVPQTLEEFQQQFYILILLHVWCFSWALKFLPCVQLA

DSTQ

>SG-PL_contig_8607_1_+2_377_688

MNFFFFNFFSLFAAVDRVAVCLLGFFLFVCLPISSVSCLVLSLSLSLLFSSLPLSLTNTC

PQMYLYQHQCKHACMSTSTNTHTHTRLRTEREKEKTNQNKTCIS

>SG-PL_contig_8608_1_-3_63_320

MTLSLSTTFSLSCYHSSFFQICSKNISLSAALRLITILSITSVQVFQHQQHSDAVDTCHE

IHSCCSLGIKLRLATGLSVMVLSPSA

>SG-PL_contig_8609_1_-2_194_724

MPLIKLQSSEGDIHVVEMEVAMKSVTIARMLRDVGMDYDIDDPVPLVYVTTPTLKKVIEW

CTYYKDSPTLLEDDFTQLEYKENSSPPQDKDLEFCAWDTEFLKVSEGTLRDLIQAADYLN

IQGLLEVASKKVASMLKDKSAEKIRKQFDIENDFTREEEERIRKENSVVMDDNPVTL

>SG-PL_contig_8610_1_-3_369_545

MPALLNLENIYLYLTKITGTTVVNLLVRTFLCKYSPQQWFMTVTFTISSCVGATRKFTL

>SG-PL_contig_8611_1_+3_270_335

MEREKKSPNLMFHLMKDLTSKA

>SG-PL_contig_8612_1_-2_461_1177

MHYGPKAFSVNGGLTIKTIDPKYQNEIGNRQGMSFRDIKLANLMYQCNAGCSNKNLRCPG

EGFVDKNCRCMCPGNPIRECSNTGTETTTAGTGTGSKITTTTGTGPNECVDRQKSCSAWA

ARGECHRYRFFMSTYCKKSCEICTEENQRCKDMAEHCKYWESQGYCRKKYVTYMRSNCAK

TCNSCDSVNTEVGDSNTGGTSGSNDNNDNDADDENAAGTSLPTLLSVIVMVAFSVVLHY

>SG-PL_contig_8613_1_+2_2_265

TGEVGGRERERERGRERERERERERETHNNDLNSTRFFIISLLSPGKQYHDIIIAIINNS

NRVQQQQHHKIIVALFSTTKLHYHTSIK

>SG-PL_contig_8614_1_-1_1_105

AHHGVIFTFSYACDRVYHEVFSAAKFRKTKREIIL

>SG-PL_contig_8615_1_-3_96_887

MSGEKATASSPLEPFLLLMKNAKGAGAVSVLTQALEAPNVYVFGELLDLPNVQELASGPH

APFFNLLNVFAYGTYQEYKAEKANLPELSAVQLKKLRHLTVVSLATKSKCIPYSVLLTEL

EIPNVRSLEDLIIEGIYADIIHGKLDQRNQQLEVDYVIGRDIRASAMTQMMSVLTEWLKG

CDAVLLGIETQIAKANQNRENQLRTVTAMEQEIATIKKNIKNSMSHEGEEQMVTDSRMDL

TDKPKKSARGKGLRGSAATKLWKS

>SG-PL_contig_8616_1_+2_2_208

SGCRTGRPECAQSPAYFCSSLSLPPPTFPSSLEKNPENVDDPRIIFRMTATLEFRAKRLE

KPLNNSMTA

>SG-PL_contig_8617_1_-3_87_371

MNMKFIILSFFLSALSYIVRTILSYQCLAYQSLLFCKPYCLISAWLTNHYFSVSHVESLV

IFTAFFLLPLEIGHHYHCGFNFPVSGPQKLAVSCH

>SG-PL_contig_8618_1_-1_1_252

FFLSTSKFSFPLQLCVVNLFIILYCYLIVQFDGTLVISFFCQILCNLFYLFIFNNYHLCI

YLIYHQLCIYLCYSLPIIQYLRYN

>SG-PL_contig_8619_1_+1_1111_1284

MQVLGRGRVGGYFYLFYEFTDFNLKPPNLPKTENRTSDQEDFFFYFYMTIFTVFILKH

>SG-PL_contig_8620_1_+1_190_306

MQELIICSCIQKQSPYFHMKPGATYTSRSESEHLPPRSR

>SG-PL_contig_8621_1_-3_93_374

MILDYNPHVDCSKQILLIQNSMCIMKFPLKRHPHNIFILSFLFSSCLRERTCILASVVPH

KNKIYNLMELETMKKSGFISKDPIERLNNEAAIG

>SG-PL_contig_8622_1_-3_51_176

MLHSFDFGKAVHLGKTFFNIAPFLDEAFSVLSSILFFLKVQF

>SG-PL_contig_8623_1_+1_187_435

MKEEVIKETKSRYMGGICTFKANILLTFTYHSYLTISPTLFLSRLYLTVCHVLSVCSCHL

SSECICLEIIHLLPCQHQYTSGQ

>SG-PL_contig_8624_1_-3_60_1376

MDQESSAKFIGSLTKFLQSLCNGYVEFEDGVELIGHIYLRVDEGKTGKKLNYFLNEKVCK

NDNSVTFISNSFHAQPESKAKGKEGDGKKDDDISDSGDGVPKSTNTGTVPSRPQLPSRSA

PSPNQSRSGVQTGTKRPGSPLSAPTPPSQRRLHPSAVKSSVGSPASKTQTVPSSPSSSSQ

DNIPSPSPARKNRNEISQPPSNQSDNASVDNETDINPSNLMAINPPPDFASFLGSLTSDN

QNPRDQRSPPDMKPDTDVTFIKEEFVSDPSSCAQTGSSGQSGRSGDDLYPVMLHQNTSVF

PSTSSGYPPATATSQHPGFPGTSTSQSDPFNPGISQDGADPSEFFGSRVVFGLGVSGFQR

SLGSPGYPCIYCGKCFVHQPSLIRHRRKCEGNFYLACPLCDRRFHRRDRYQEHLQSKHGR

SDAKKGTFFKKYSLDQERL

>SG-PL_contig_8625_1_-2_95_382

MTLCGNKEQHGQGQQKTEKVGGLWRRATSCSGRTQPRQGKARLIVNRGTNFDDSSVKNHW

KWDWMEAVVKEETGKIRESFEKDYNAGTCSMCTMPT

>SG-PL_contig_8626_1_-2_62_337

MIEFRDALTELLDEFGTDDQEPQNELPESRSMRVENKMFYFDVGSNRRGVYLRVSEVRNN

FRTAVTIPERSWARFRDVLSEFVDKSSLKDTE

>SG-PL_contig_8627_1_+1_649_822

MKKFLPLLNYFHQIQNWRKQQSYKRQQMPISLKYFFLNHRQSSYNGWQQILTFTSMLQ

>SG-PL_contig_8628_1_-1_1_90

IIGLSINQTVSYSINQSVNQSMNYIDQSTN

>SG-PL_contig_8629_1_-2_2_184

GYMKGSSRGIPSDLSALTHREILLKFQRLFWHHACRADRKGRHTRSDHHCRLIQRSRTHR

R

>SG-PL_contig_8630_1_-1_70_237

MEPDRRVDLHGDLESIPSFYPLPLCAFSFFFLSFVVVVVINKIVFLFHTQIYVSIC

>SG-PL_contig_8631_1_-2_80_1192

MAEKHNFGVKPVTCHAVSGDKNGLALSLNDHEVKIYKKNGSKWQETDTLDEHGQRVTGID

WAPRSNRIVTCGADRNAYVWTHQDGKWKPHLVILRINRAATCVKWSPEENKFAVGSSARL

ISVSYYEDEHTWWVSKHIKKPIRSTVTCLDWHPNNILIAAGSTDFKARVFSGYIKEIESK

PGPNSWGSKMTFATAMAEFSNGGGGWVHAVSFSPSGEKIAWVGHDSSVSVGDSTQGNQLV

VIKDTFLPFMGITWVTENSMVAAGYDCNPVLLTYNGGQGSLSVVSKLDIPQEKAAGTISA

MNRFKDLDKKASSGDSSAELKTQHQNTITQVSVFDGTKDHCKRISTSGVDGNFIIWDLNS

LERSIQALKIS

>SG-PL_contig_8632_1_-1_1_135

VCVCFDCFGNLFHCFILFCCKVVCLGFEKNQPVLINFLYLMCKSV

>SG-PL_contig_8633_1_+1_157_309

MVVPQACFRALVKVFSYCSSPGVLGEPLLHKLQTQAQDRTHASSHPVSLSR

>SG-PL_contig_8634_1_+2_182_703

MRCRSARVLLCPCCETDTPRGKGCDVSIVSFDWVCVAVGVSVVYCETGSLIEFFICLLSP

PPFFSSFFVVTFFFVLISRCSFWALCAVMLLFFSFIFVIRDCHIGLSPPHPIFFQQCLSA

SKISVLFVLLQTSFLFACLSVFLFVYSFLQLQGRQSYFPSPGTENYNLQLLYLT

>SG-PL_contig_8635_1_+2_191_313

MIFLVKYKKGRSNNDSLNATSLNKRDRAFYNIMRINDNNNN

>SG-PL_contig_8636_1_+2_2297_2512

MVEPNLQKSTHKESRNPQGCIFILFTHTDMYTVMSLVDLDYPFRKQKNEKRPQAPSVLLP

PNLLSIPSPTHD

>SG-PL_contig_8637_1_-2_371_835

MMMFSNVWLRKWSVIAIARRELLFLSWFLKAPRIVFAVLGLEMYLDTISLVLVSADVFSL

SVGNLGHVTALRWTTVVICRLTCAVRLVVPPPLLDRTPTCQEHSDVFNTYTITFSLLNTL

TSSHFLFLTPNSLHMSDFPLGFGTVVKSSAIFAHH

>SG-PL_contig_8638_1_-1_262_429

MAPLWRTLSSHPYPRFPGSTSALSGKLGLSVGEFLVLNQDPEDPSSVFLFCPLPLS

>SG-PL_contig_8639_1_-1_31_369

MVSEDPENTRLFFFSPAREREVTRSLRARSKVCVECIDCVGPSLDLVSSCPSHVSHVVGQ

HASCRSVTSIVVLFIVIVCPAFLTPHCSSCPLPCPHLSAWFDIGFLSPPLHVL

>SG-PL_contig_8640_1_-1_88_771

MSLTDSEVARQIKHMMAFIEQEANEKAEEIDAKAEEEFNIEKGRLVQQQRVKIMEHYERR

EKQVELQKKIQSSNLLNQARLRVLKAREDHIKALLEEARGRLSKVAADKQRYREILVGLI

AQGLYQLLEPVAILRCRAQDVELVKEALAAAIDNYKQATKGMKEVKVNLDTEHFLGNEVS

GGVELVSRSEKIKVTNTLDSRLDLIYQQMLPELRDILFGRNPNRRFTD

>SG-PL_contig_8641_1_-2_2_106

TVNKKGCMQRIQITGFYTTLGDYVFDLNISRFSAI

>SG-PL_contig_8642_1_+1_1078_1221

MSSLFLTFKYIKTQSQANRTFNVTSYWFCLDELICILSSIRTMFDLQV

>SG-PL_contig_8643_1_-2_2_946

ATQEVDLQTEFDLKLERTKIELQAAVHSPEVLEVGVQSCEFLSADQATQSDISSASQETQ

IEFSLVSQSTQSDASCILVSEQAVQSECCAVSEQSVQCGVCGRMENRGAQTEERHVTEQG

VQYVYDQMQSKECHVCDHSVQSDNCFVTEQGVQSDQSAFSEQGVQSDTEEVMFAEQHQQL

MQKVVSETKEQLTQQFEQVLAQERRTTEKKLAELEEHHKQDKEEAVQEYRRAVLAERQIN

FNEAINRVVKEKDSLIDDLKRQLNLMTARVVPKEGESEEERQDIRKQLADIKEKESLLKR

EKRQLEDEVSLLHHQ

>SG-PL_contig_8644_1_-1_250_558

MYNMNKRVNNKTEEMIRLLSRCWHFMLTISWHTGRESQEGTRNTFSSCTHADKQKKPTKN

PPQEQIKAAFFWMCLHYSFIPECKLINNGVVIVLSRIIIIIII

>SG-PL_contig_8645_1_+1_1_150

TNSECVFTNKPDKMHMTMVTCVFPCVPSVCVHANICIWRISLGGMLGKCD

>SG-PL_contig_8646_1_-1_7_1536

MAKVVIAGRSDCPYLARVELLGDRLVRNLPSFQLHKIVKTPDEWQHWLNQTCQERGWQHK

QSPLVWRELIDRGGKGVLIGGASDFQEYVKGYYGLEASLTSDDMRKIGSENSTTKQEIDA

EDAQYRALSKPLHVCITAAQSPTCYSLISSISSGEVFGPNTEINLRLYDICATDDSIIRG

LAMETQDLCDGLVRGIEVVKNTTSAFKDCSIIVILDELLQNDEETYEDWLKRNCNFFSAY

AKVINEVAKKNVRVLVAGNGPVNFNAMMMIENAPKLPRQNIVALSRGIEHRAKAVIGQRL

NVNTAGVVDLIIWGNVNGKHFIDNSKSRVHGYDGAVWGPPSYSVPVVEMVHDNKWLEKEY

LEQVQQRREAVESALGHPSSGSAARAVSSMLTHWWTGSPLGQMFSLAVCSEGWYGVPKGL

VFSYPVTLSTQGYWSVIQDIELTDETKQKLQETVKDLKKEREVIYPSSRPPTPEPEKPAE

TKEDKAETADVKESESSEAAVAEGEGQAES

>SG-PL_contig_8647_1_-1_1_471

MFKKPKPHRNPQPAITKEMEDSLMSGGSSTENLAKEDRILHQRVHFKLFYVGCLPDISGL

NSKRRDTEAQLIDYVEEKQIEGNLKLTVTEEDKVTINVSRYGFKVLDTSGQEVIQRHPLH

TISQLIHYTDGFGKPNIALKTGQVGKPVFHCYIFQCH

>SG-PL_contig_8648_1_-2_209_379

MRILPHPEVPDLSFIARLLPRMGFALVRRLFHNTAGRLCGAHMELSSCDESLWSKFH

>SG-PL_contig_8649_1_-2_380_553

MKLAKYGASNKANSMAYNNLRLSAYEMNSLVKRSVSARFMSLIPHCSRQLASMIYKLR

>SG-PL_contig_8650_1_-1_223_582

MSCYVSTPSLCYYTLKQHEHTVRNDIVFEIKHLIHCISSSGVNIVFCWVPSHCGLYWNEI

SDKLAKNGASNKANSMAYNNLGLSAHEMNSLVERSVSARFLTVQSLIPHCSRQLASMIYK

>SG-PL_contig_8651_1_+1_844_996

MLYNMQMILLFGLIQLFENMQTDNSVQKLYQCELDNLSSCMNKRQWYGIIK

>SG-PL_contig_8652_1_-3_75_221

MGVRENHGKGGCFHKRLLTAGSASFESVTFCSSYIICTYFVFRHVIILC

>SG-PL_contig_8653_1_+1_1_240

FFFSVSFFLFVFNILSRHLRTQDNIINVCKCLTILETNDLNDNNIHPQPHSTPRLGPTHL

QPHSLPLLHVLLKRYPSEHT

>SG-PL_contig_8654_1_-2_365_661

MRKFSVISHRGLLHPHDVQFSTRSSSPLPTTFIFHAMSAVSPALSLPANSLSLHLLPCTR

AHACAHTHTHFQFLLFPEWIYFFSSCRVVGKNVFCNYVQ

>SG-PL_contig_8655_1_-1_1_267

LCLSLSLSCSLCLSVSLSVCLPLSLSLSPPSLPPPSPSFCLSLSLSLFSPPVCFLAILLL

KVKQLCVVPLFCVSSGQSVSQFNFLKTLL

>SG-PL_contig_8656_1_-1_115_225

MIIAYSYAFIIAACYCCGTNYLSLKLWPVTNKFISEN

>SG-PL_contig_8657_1_-2_2_232

VVWLSKVKRNISKNFFFFFWSQACVPRNGLVDAVNDGEVETVNKLYRNPSVFLRLIHLHE

GQACLALLRYRNSNKGS

>SG-PL_contig_8658_1_+2_2_277

PGSSPVRPRRGCVGRPGWARTKLAASSIWSCKLTLYPSSGESLGEFLRQAEQKTLLWAED

GPAWDPRPRGGGGGVVQCWQELPHISQACRRN

>SG-PL_contig_8659_1_+3_183_449

MLQSFWRCQRFIGPIPSCSLPILMLQSFWRCQHFIGPPRSLLFAAHLNAPIILAVSVFHR

SRSIRYLKTLPALRVCAGFGWSAYGIVRV

>SG-PL_contig_8660_1_-3_3_1019

VFRQGPVEIIANDQGHRTTPSYVAFTDTERLVGDAAKNQAALNPQNTVFDAKRLIGRRFT

DNSVQSDVKHWPFTVVNENGKPKLQVEYKGEKKTLAPEEISCMVLAKMKETAEAYLGQMV

TDAVITVPAYFNDAQRQATKDAGVIAGLNVLRIINEPTAAALAYGLEKNVQKNSDKNILI

FDLGGGTFDVSILTIAEGSLFEVKATAGDTHLGGEDFDNRLVQHFSQEFQRKYKKDITNN

ARAIRRLRTACERAKRTLSSSAEASIEIDTLYEGVDFYTRITRARFEELCADLFRNTLQP

VEQALRDAKMDKGQIHEVVLVGGSTRIPRIQKMLQEFMG

>SG-PL_contig_8661_1_-3_504_608

MVLSPLINFRICIPFNLYFFSTIKFDSFSFYLLLC

>SG-PL_contig_8662_1_-1_283_468

MVSGGKIEWWVAGCYLLSFYLFLVMSVIKYIFLLTFIEFCMAIFAVLSCLTKQLSCSELQ

LQ

>SG-PL_contig_8663_1_-3_264_407

MSVFTQFYWLWFCTLMFVCVTFYGLQSTPHAWWLAYCFMHTTLIGSEP

>SG-PL_contig_8664_1_+1_52_198

MFIMDCICREHPTGSRNRVRIGKNFELFFVSPRRNEPITPVQPIREDIC

>SG-PL_contig_8665_1_+2_356_490

MCVCMLARVRAFECAHMCEKKKIRSVSCLLRGTVHDTVLYFCQSQ

>SG-PL_contig_8666_1_+1_1048_1413

MGEVYGFIDCRQSWRCWNWILYICVYKGSSIKLSWVIGEQGCRDQLLLGSSIMRAMSITC

PSLLSIPQPWTGSGTHSHLVLIMWRLWVQPPCGPQDFSRVGQLTRAVESTYDVSRSVSAR

NI

>SG-PL_contig_8667_1_+1_712_1014

MANMYSCKLSHIHTHYYAHTHIHIPERGILHHIYLHYQPSVEGTVATLLTMNLTVLLTSI

FRNRVSTYFSFKFWTTSISACTDNYPTSICLHSWYQTADHG

>SG-PL_contig_8668_1_+3_396_485

MAISQYCVTLRSDILIIGNKIPVLFREGSF

>SG-PL_contig_8669_1_-2_2_247

PGPSLPVPHSTSGISSLSRSASAKVGHLARKPASAPPPCLEPLRGSLSSCSLTSNHSSSR

PDSVNHSLVLGGASAELCSLVQ

>SG-PL_contig_8670_1_+2_164_802

MKPEAVQDYIEHMLGVDKSDQLMSYAPFQRKTLKWWKKVFFHMFTLSLIQGSILHEEFHR

PRGGKKLPLKKFVEQVGLQLNDEFQAGVAAAAEATAAEVCAEREGGEELQQAAVPPPPAA

VPASTPQTRLSGGIGHFSVPIGNVDSGSSRRKYRTCHVCVAQAKRHPGVKLRKCANLYAK

CDVTLCDKPYQGGGNGKTTCFQLFHTIKNYVEY

>SG-PL_contig_8671_1_-1_538_816

MVLLKLVIAMLLSVKFTFTKLTLISLHFFPGLFDTPLLASLPEKVRTFLASTIPNPSRLG

YPDEYAHLVQCIVENPLLNGEVIRLDGALRMMP

>SG-PL_contig_8672_1_-1_1_393

LSSAVLFLCAQCSSIVLVCSVLRYCSCVLSFAVLFLCAQFCSIVLVCSVLLYCSCGLSSA

VLFCVLSSAVLFLCPQFYNTVLVCSVLQYCSCVLSVPVLFFCVQCSSIVLLCSVLHYCSC

VLSVAVLFLCA

>SG-PL_contig_8673_1_-1_1_249

VLVCSVLQYCSCVLSVPVLFLCAQCCTIVCSVLQYCSCVLSVAVLFLCAQFCSIVLVCSV

LQYCSCGLSSAVLFLWAQFCSTV

>SG-PL_contig_8674_1_-1_1150_1344

MLCVIVVRSLYDSIIGCGSCPACYVQDCRISPLSFAADSLPSSLHVFCLLFPKYADMSSE

FDCEM

>SG-PL_contig_8675_1_-2_26_604

MKILPFCSELMVVLLENLGNENVHRSVKPQILSVFGDIALAIGPHFKPFLEVVLTTLQQA

SQAQVDKTDYDMIDYLNELREGCLEAYTGIVQGLKGDGENMNADVNLLQPHVAHIISFIE

HISMDEDKSDSNVAACCGLLGDLCTAFSMSIMPMLEKDTIQNLLTQGRRSKVQKTKTLAT

WATKEIRKLKNTS

>SG-PL_contig_8676_1_+1_1_267

LNTRSYSLFVCFWFLLPLSAKYMLLLCFRVRTCGFSLYRHKAQKVTHNHIKMENIPWFDH

HVTVASVLQAGRQKSEQCSRYTGWGARPH

>SG-PL_contig_8677_1_-3_144_2141

MKPFLLPACFVALLLSSACYVQADDDDGDGKKKDKDGVGTVIGIDLGTTYSCVGVFKNGR

VEIIANDQGNRITPSYVAFTDEERLIGDAAKNQLTSNPENTIFDVKRMIGRTWDDRSVQK

DIQYYPFKVINKNNKPHVQAKVSGDTKTFAPEEISAMVLGKMRDIAEAYLGKKINNAVVT

VPAYFNDAQRQATKDAGTIAGLNVMRIINEPTAAAIAYGLDKRDGEKNILVFDLGGGTFD

VSLLTIDNGVFEVVATNGDTHLGGEDFDQRVMDHFIKLYKKKKGKDIRKDNRAVQKLRRE

VEKAKRALSSQHQVRVEVESLFDGEDFSEQLTRAKFEELNMDLFRSTMKPVKQVLDDSDL

KKEEVDEIVLVGGSTRIPKVQQLVKEFFGGKEPNRGINPDEAVAYGAAVQAGVLSGEEDT

GDLVLLDVNPLTMGIETVGGVMTKLIPRNTVVPTKKSQIFSTAADNQPTVTIQVFEGERS

MTKDNHLLGKFDLTGIPAAPRGVPQIEVTFEIDVNGILRVSAEDKGTGNKNDIVIQNDHN

RLSPEDIERMINDAEKYADEDKKLKERVDAKNELESYAYSLKNQVNDKEKLGSKLSEQDK

EKVIETLDEAISWLESHQEADAEEFQEKKKEVEAIVQPIVNKVYGGAGGAPPPPSGEEED

SERDEL

>SG-PL_contig_8678_1_+2_2_118

TTSLTKKTAGVRIILHGEDYKNNKNNSLVFIMHVPLKIK

>SG-PL_contig_8679_1_-1_49_603

MNSGSNFGGGGMNTGSNFGGSNFGGGNSLSNTSSASWGTGANTNTAANSYGSGNFGTNYS

TGNVAGNFNNANNPNANTGSFGNYNTGSVMGTGTNYGTGSTGNNYNMGTSTYGNTGVGTG

STNYGSTNFGATQSYGTNTMATTGQQQQGWNYGQNQMGDTTSNNTWSTGMQGGFSNTTYN

TGNRF

>SG-PL_contig_8680_1_-2_107_841

MTLQWTFVAACMYAEIGIITLLLLPFISPSRWQTIFRSRILAGMKSYANIYFNVFIAVLL

LLFFESVREVRKYSEPISVEDLKHHPEAETLYHMKLFRAQRNLYIAGFALFLWFVLRRLV

VLIASEATLIAESTAAKRQAESATAAAQQLLDEKDNSKNVNKEDVKDKGDIPENRDAIAL

EKARAQLEQTKEDLYHAKLEFESMKKQAESVTKEYDRLLEEHSKLQKKLSMLEGAAEGSE

AKKDN

>SG-PL_contig_8681_1_-1_31_1677

MVGAQEEAEEEEGGEEEAEAVEVREGMIPQDEGEEVDDDADTESSTEDGDQDDRIESEQS

DDAHSAPPDDLDQVDLSVVNDIGDLPVGRGDVERHYIHYRNATLNGRLNTVVLAVLVLAL

GMGLGHWIGSTREHFSQLEIQLSQMKRLHQVQDDYMECLSRNDQLLVDTDTALQSERERH

QEATSDLRDVTHDLQEQLQDAQDQLEQLLAATVPQASRQPRSPSVFAHDLQYALTLSKML

IVAEEQFQESLSDWDTESQRQLLQVREAFTQAMQEREGRQRSQAENKLQQEVDRERQFSL

HLQDVHRQLNERVNHLETENADLKVSVGKMRYMADSQPQQPPASPHNPCPPSAGHKPLPS

PVDEPLPPPPKPLPPPPFAGDGDGPIDMEQEEHQEGRMNGKGTYVNDAGDLKTVRSAAQG

SGHVYLSDDMEQEAHRGEEKENVISDKEKEDVGDDSVDEDMQQTVAGREQDVHDDNEGTR

DHSAARKACGSRESPQPIPVDMRLLQRNLTQERQRAAMWRHLYLWERQERHGMNATACLH

YLSRTFNMS

>SG-PL_contig_8682_1_+3_519_716

MGRGITATGKASTLQIKLCYNKMSHGGVPLLSLTCGSSHKRSLSTQHSKSCKRLLKSVIT

FNVHKG

>SG-PL_contig_8683_1_+1_1_141

KPPKMSVTSELFTCISGHICNLRTFNAYVNNGCQKLRQTHFNKITAL

>SG-PL_contig_8684_1_-3_3_131

PICSICGKGFAKSCNRLAHFRIHNKNKLNLTVQCVKDKAESKS

>SG-PL_contig_8685_1_-2_2_157

NEMQTFKLHGVFTSVMFDGSDRTPIAWHVFIKSLLQRLHCRTLQTFLQKTFN

>SG-PL_contig_8686_1_-3_6_278

MNVLFLVTNISYRGRIYRCLSHDLFPVMFSENLPLLFFKMYVFVLCDLIWAVFLQNLYGD

RKRRTCLIFLLLRRALDMKGHALALRICCEC

>SG-PL_contig_8687_1_-3_3_107

CCRTVCILSFGRLLRHIGSHIPFSGVYMLGIFVFP

>SG-PL_contig_8688_1_+3_3_119

CCCCFNITVAMQFLCQHVKRLGSLLAQNPSLTHIKRVVK

>SG-PL_contig_8689_1_-3_1101_1403

MEKSQNDGKICQMHFMIKWLKRMLMILYPLELCCGMSQSMESYSNLCSCLHASVYTRYRA

CVILMTNYPCHHCCHFPLFTAVKNVQLTVQHNQAFARFYKY

>SG-PL_contig_8690_1_+1_1156_1563

MTKVWKALEIAFPCVSLKSLIHTLFSKTIKRQLSDFQKLVQISMLDSALSSSFLDFSYRS

SYPALPGQGKTLAVQFYSITLVSRNQWSPIVNTETGICLQNCSWTDGLSIRSSKHVANLK

QTPLQSSNDQKKKHTD

>SG-PL_contig_8691_1_-2_11_883

MAVDIRLATPRGQLARHNDEQELTLVTPGDLITSDPGFMRGHGTIKEDDGLHATVAGAVQ

RVNRLVSVRPLKARYIGEIGDVIVGRIIEVATRRWKVDVHSTLDAVLMLSSVNLPGGELR

RRSEEDERMMRHYLKEGDLISAEVQNVMSDGTLSLHTRSLKYGKLGQGVLVQVPASLVKR

RKTHFHTLPCGVKIILGNNGYIWLTPTDKESNTGGYEVDLTPVAHKDREELARLRNCVQA

LADRKMLLFDTSIVYAYEMSLKFEPKMLLKAEVAAEIAELTQQRLEQEGCM

>SG-PL_contig_8692_1_+2_38_271

MKKWKKTQLQILNIPQASINNNGNYSELRRKTASEIISYSQATAAEPPNSDIQTVTILNP

SPKKNFLTTGSTSYHTKQ

>SG-PL_contig_8693_1_-1_142_336

MLLAGIKLGTSDVESSRLYELYCTALYTFFFFSQQIFLVKLWLCACVCMYSKYFMKGTCL

FMNGF

>SG-PL_contig_8694_1_-1_256_513

MISVCVPRSLPELCNTAFCNFLFFFKSSDCTLCGLLTGFKSCSLCMLTDFVQRLIFCFQN

MTWTFFKANVKLNASLKTLENRNVVC

>SG-PL_contig_8695_1_+1_235_390

MPKQQTFNHLASKQFLSRPFYVNYFERQYTSHIPLFSYPVTWNPGRDNRHWR

>SG-PL_contig_8696_1_-1_121_444

MGCMGDVFLFVFVCLFVFVLVCYVLVVFLFCVSLPVKVDFFYKILPVTLFIFLTHLLHRS

CVTIPSYSSRLSNTPISARDNSVVAEESLTFVKCMLDTGPQFIVTPER

>SG-PL_contig_8697_1_-1_562_906

MPCIDLNDDIAVRAGIDNGHCHRAMQCARCCVTSVLMCLVILTNGQSSLRKSMSVWWKSA

LGQGLFISSVKAKVHKGRKPQAMKMQLRCLLCICVFATVDGGSGEEDPDGGFGHG

>SG-PL_contig_8698_1_-1_997_1305

MKTWDARVYFALLMMNTKHLHGIVCTVRIRLHYYKPGLNEEIKGLIIRFCFNPLKPILLE

RFCGKFTNQSEVEPSALQQIIQTRTRLCSVQSEGIQYCMVFTY

>SG-PL_contig_8699_1_-2_401_538

MRYPDGSLCLEADTQRHCMSILSCPHSLKVFSCYCMGGPKSPRVIR

>SG-PL_contig_8700_1_+1_175_360

MKLYVFLKQVYPHDMAITRKQNKQMHILILQMWVIKSPILLKRPCAILFLTIQPWVTWAP

IQ

>SG-PL_contig_8701_1_+2_155_286

MGKDLIFLFSYTQSNERILVIPFQCQSLIPTHFHFLLDFISSDT

>SG-PL_contig_8702_1_-1_205_339

MTIFWSNVQLQCGVIIRFTLNPLCHHHHRHTESFTHIVCFMFVCN

>SG-PL_contig_8703_1_+3_150_206

MVRALDFQLVGRYGRPGFE

>SG-PL_contig_8704_1_-1_4_1344

MISVFNMGVPNHRCAVPGYPGDTWQIQSPEHAQMVNISIPLDPDLVEKGDYSRCRVYFYD

NVTMETKTKLCSSFVYDPSTYASSAVTKWDLVCEKKLYRSHSRMIMMGGLLFGAFATGIL

ADIIGRKLALIGMVALQAVSSIAVAWAPSFVVYVVLRFVTGFTISGLFLVVFVLGMELVG

PDKRVFVGIMVAGFWSVGVVALGGIAYLVRDWDKLQMIVSFPILLLLSYWWLIPESPRWL

LARGREEAAETIIRHAARVNQVRLPEKVFDHKTFDEVSQQEKFWHIFTSPVLIVRTAILC

LNWLVCSMVFYGLSLNSGNLGGSVYVNFQLMAVCELLAYLACALLLNRMGRKLVHILCMM

LGGGACLSTIFAVLYASPEVQTWLTIVLAMVGKLGAAAAFAVIYVYSAELFPTLLRNSLM

GLTCLFARMGGMISPYIADLVSQGSRH

>SG-PL_contig_8705_1_+1_25_285

MHFPQGEQPKNHTIIFMEWWALESTTSQSVVRCADHFATLTVVVDFCLLVCPLLLFLCLF

LFLFLFWCSFGVCASTARKCWCLCLNC

>SG-PL_contig_8706_1_+2_2_133

QAHVLTTSLFNPLTPSPYPKQNQNQIILFLAKSTTKAISGLSPC

>SG-PL_contig_8707_1_+1_985_1431

MVSSPKSLPLSFEHVVFCLILLDHNQTYEMNVLRETVCDECSCVPHIYIHTVSSVSTRGS

VQILTCRCNGSASLISFAFRPCDIICILMTVTSAIFVQRLHFISRLRCTFFVSIFCCEYI

SFDHTLFISTLLFHFVYLLKLGRRCKLVL

>SG-PL_contig_8708_1_-1_640_876

MSQLPFFLLLCPIALELSSFAHQTLLFSCYRQIQPQNLSLSAALSTAIFFNVFCHMTDVY

CVDVCVRVTACRLFVYLFV

>SG-PL_contig_8709_1_-1_85_330

MSWLLFFLLLCPTALELSSLAHQTLLFSCCLQIQPQNFSLSAVLSIAIFTDFCHMTDACC

VGMCVCVCVCVCVCVCVCKTGC

>SG-PL_contig_8710_1_-1_40_216

MRWTLAACDGPSIHHQQTCLNGAPPGSIQVFEILKSHLILILNPLTPRSPSTNYLGNPM

>SG-PL_contig_8711_1_+1_103_285

MAIRVLEGSLNRDELLLLASFSPKGLWNLCHQRDSRTQAWAKCQGEPAVAHAAGPPFPQH

R

>SG-PL_contig_8712_1_+2_398_676

MYQKALTAMAVMKTGLPIYKAVSVSTTFHWDYVHHYSFLNSCSFADTFIQFVNNPKRHCP

MFNVNQSLKNNFIFNACYSYLLLSTCINSVHTF

>SG-PL_contig_8713_1_+1_577_960

MHSTTHSHTCTSKLIKHDNSFTIIPVTNYASKQFSCMFSKVHTFSIQILTVPTHFETCLS

KQMPSFLHEQTRHNIHTHAFKLIKMNIFCTLNKLCIQTIYTHVFKLPIFFSIPIPCIQTF

HMHTFKLI

>SG-PL_contig_8714_1_+1_31_222

MQSTALSGSLNPKRNSIASSLSSPFLCTVKNCPRFCGPSLPCSHGDLSFGGFGSVLESQN

EWHG

>SG-PL_contig_8715_1_-3_186_614

MVVKVFDCLLWGEVYIITKFHEMMRGEYWRKKYMGANSDSKTRLWWMCKGRCLGWVFIGE

QSTATNVCSKQIIFLHPSKPSTCLRTNDNHSQFVLYFDRTLQTVVKLMCWKFHIQPAVPL

KCTCGVFLFVCRSLRVYAVEVGC

>SG-PL_contig_8716_1_+3_651_944

MLRLTQQCSGRLSELAPNSVQQLDFFFSDVGSCFSFFSSFFPSQNTTGRLLSSYSYSYYS

WMCGLVGKAPALGSERSENAEVRYPTGQILLPTPLDLE

>SG-PL_contig_8717_1_-1_1_159

HTSPTSSVIIHLPPAMPTALPPVTITISPPCFSSVTAITFSKDKSIGRSVHVS

>SG-PL_contig_8718_1_-2_2_106

TSCCIQSLNCVVKMCEGVHVPVTGSGILIKKIHRG

>SG-PL_contig_8719_1_-1_1651_2061

MKNESCRIVSSNPHQSKNSVCFRPVGSYVECTLGWVGKIETTHASVIHFMDAALAVLLKF

PDQHRKCQYLSGLVDARIYSGWKIVVQSYTVQYQTKMKRYHQPHTHESSSENYLTLCGVS

CLVLIVSQFWFVLPLPY

>SG-PL_contig_8720_1_+2_2_166

NPSSFSQNPCARSPWETAMPNRLRPPTRGDSALLLGHQWNSTVPVPIQLTKDTAC

>SG-PL_contig_8721_1_-2_2_121

SFCIIIRRECCIDPNSNYICLSSSLHVVSTIVYQVLPCGN

>SG-PL_contig_8722_1_+2_2_289

FSASWGTLFALHCQLGDSIHRTVPAGGLCSPYSASWGTLFTVQCQLGDSVHRTVPAGGLL

KAEYLVVSLASESTFLSFLAYKIKFSIFFLSYDRQC

>SG-PL_contig_8723_1_+1_298_708

MLLELTKPILPCSFCTSHFPAGLPSMRRMATISPLTTARSLENTPPLALFFTKPSPTFSS

ESFNPNLRLNFFNSSSFFLRSFSSFAFSLFFAASSFSLFSLSLFLLSSGRLEEGKGGGWG

SDSRARGSGGSSSSSGS

>SG-PL_contig_8724_1_+2_236_403

MAWEGRKKQMIVKEEKGAETKRISRRLGYYGKRERRALLSTYNFILCHRHHTVTSK

>SG-PL_contig_8725_1_+3_102_194

MHNTLNNTLFSYKTIYLHTTYHKSLCLNCHY

>SG-PL_contig_8726_1_+1_82_201

MTQNKPNYYFTDAYSKPPTETVTCYDNQSNEVFSPDLTEN

>SG-PL_contig_8727_1_-2_479_652

MLHCRSSVFTSFINYTVLSFYFQKILLFGSVLLRVTKEVKHPPSLVYMSPIFVRFPFF

>SG-PL_contig_8728_1_-1_1_111

SFVWKGKCYRINTKTTKQSIRNGGLSIPDIFRNILKL

>SG-PL_contig_8729_1_-3_150_398

MGMDEKCQETGGRQELTRLCKHLLQKVDKRHKGFYGVFVNNATCQHTEAHRFLFIKNTRR

SHKWIRMGPGNGWLTSISCMVRS

>SG-PL_contig_8730_1_+2_2_208

CKSFNRFLSAFEHAAKSRHYHKAQTKKLPPQEHRRHWHWQSRQFFVSLHFKTPPSKRGLI

VVCFATSHF

>SG-PL_contig_8731_1_-2_137_1789

MSSDASVPREYCDEGPSKWRSTRFLVSCMACGGMCVLYFQRVSLSLAIVCMVNHTALHRQ

QAAHQRMLNFSGDQEREANVSAISIDNRVDNLAYQTYANTSGEYLSTSQRDLDRCSSEND

AAGGVPNSEEDGPFIWDKQTQGLLLAAIYVGYTVMQIPASYVVHRFGVRLTSGVSILVMS

VCHILCHPAALLSPWAVLVLRIIMGVDTAFAFISMYAVWTKWAPRTEHGQLVGITFCGEM

IACIAVFPSSALLCRYGFWGGWPSIFYTFGSLGIVWSVAWFILVRDSPETHPRISASERQ

FIVANRVDVSPYTGGKLAVPWGSILRSQCFWAILLAHFVSSWGLFLMISNLPIFLYETLH

FDIQSNGVFSMLPYIGLFVVMSGSGFAFDAIIKRRLLSKLVVRKLATFLAFVVPACLLVF

MSHLQCQQTVLVLVLLVVAVALTGLAITGGFFLSPYDVAPRYAVNICTVTNTIAAIAGVI

NPYMVAALTVDQTREQWMMVFYVTGGLYVSGCVVFCLLAKAHVQPWADPVLRDTECGKAL

NSTPLQLQNSV

>SG-PL_contig_8732_1_+3_1533_1838

MMMMMIIYRNRRYMLNEKIHINYHPTSQPFNKKKSLANMYQMKRLQTANKTRTPHCLGDM

YSLCSQNGRLSVHRSVTDTILHSPKYYTFKSYHANDNLNLPV

>SG-PL_contig_8733_1_-2_173_2287

MAYAMITSLVQEPDKDLSEIIARVKGKPTEKNQQTPNTIGEFAIGMFFSNTTSQSANSIS

QKSNTSKSGSSRQSSSSSSSSAVNGRSAGVSVGQVPTNVWQNPAPGSGVPASPRRSPQKQ

LPTNLISNISSGPVPVQDKAVGRQMAGDQRRQGTATTTSTTTSIPMGTAASSLKVLPTPA

SVRSGSELRQQTLPASSSVGSIGQKTASRLTTGSVRVLQRQVTGSQPLPIVSSHMSNPSS

TSATSNNGMTSPAFSTFQLWNNSPTPTLAKKEDFASVAAAGVVPTQSSTHGGQAAPGSPA

STPNDPTKAPGYKAGIGSTSPQSRSEHANVGSSVPYPMPVGSPEHPDTYKANNRGLMAAS

PGMQILPNSPHPAGDGGHMANQMPYPGPLHPASFPNQNASFFPQQPMYPGPGALVDQKEE

YSTPHQPMTLPEIRSTLNPNAPDFQITVSGGNGPGNMVNGYPPQMAEMRNGGDMAFNNGS

GGRMEAGGNSGAGAVGSGVHPYVHILYAYLRNIQNTFPPAAIQQMMSLSPVDPQVMELCL

NMQRNSQQQQQQQQQQQQRSASPGPGRNSAPGPGGSPHMGNHDGGPGMGPGPSPGLAGHN

PMPPYMPGPGMTRNVSPMSNRPNSAPCMPDSAPTTPVRPSNIGSPMPPCTPPLPEGIQTP

DDRKVLRPIGGERAAQRKLPLNEPGLGVSGDFKMWGATEIQKEWM

>SG-PL_contig_8734_1_+3_615_875

MGSVTCIKCMPHTGPRSSTQTTIQGIVEWKEKSLFSCDSNPGLLVSLTSQASALTTGQPL

HSSLHCVKRHTNMLSLCEMSHNHVIPV

>SG-PL_contig_8735_1_-2_242_448

MNQSTVKIQWHLQDSSFLPNCFLTVMWMCGLVGKAPAPGSERSENAEVRYPTGQILLPTP

LDLEWWSGC

>SG-PL_contig_8736_1_-2_2564_2773

MIARIINHDQPFDDTSCANRWKWEWLSVEAESEAGRTVCVCVNAPSLVAYKHHHYHFIII

VLFFYCPAFW

>SG-PL_contig_8737_1_-3_906_1109

MLFWKHYSLQPYIYNDHLFVQLGKAATLVVLHVYSCYRLVTLLNTYFVYCLQQVCRNCVL

LCKLSCIG

>SG-PL_contig_8738_1_+2_179_370

MQTNHPHSRKQIAHTQMENTCAMPTHVLPAFAKTGEKGHCFSAHTAEWIILLFSRGAHAR

TLPS

>SG-PL_contig_8739_1_-3_3_200

AMLLVVATKFRTDAANRHDDSRRVELRLDAASRHDDSRRVELRLDAASRHDDSRRVELRL

DAASRH

>SG-PL_contig_8740_1_+2_2_307

NGNSQANLCFHSSILHNHKKKYYILCRKIVCKAVTQRDRLNQTQAFIQTSPKCFYYKPST

TKCLAATTIMSLHGRNSFEHLEQKLKKCSHNKLDINDFSKNT

>SG-PL_contig_8741_1_-3_81_734

MFSTVQTSSGQANNPGLYEEVKLYRTAREREKYDNMADLYAVINTLQSLEKAYIRDAVLP

KEYTATCSKLLVQYKAAFKQVQSDFTTVEEFMKKYRLDCPAALERIKEGRPITIKDDKGN

TSKCIADIVSLFITIMDKLRLEIRAMDEIHPDLRELMETMNRMSILPAEFEGRSKVQKWL

DTLGSMQASEELDDTQVRQMLFDLESSYNAFNNVLHNN

>SG-PL_contig_8742_1_+2_3839_4081

MIAVSWLVGWSETERKLSAPLSRSPLCLFDCLSVPFFSPSHCVVTRATVVSPLASHTISK

AGSSHTHTHTHTNHATRPRCR

>SG-PL_contig_8743_1_+1_76_339

MFGAPNAQDFHLWLYKISVPLHLEAAEIRRASEDVPAPSRVDRTKSWRSDLKMCQAFCCC

GGMFCRTTATRGGVRVEGSNEALLSEAA

>SG-PL_contig_8744_1_+3_6_245

MFTWEIRRISTLSPYPPDFAHNEENSRTIQVLHTRYRHIKIHDIHKCTILSLQLDSDFSC

KTDVWKSCVYIICVHAISYQ

>SG-PL_contig_8745_1_-3_177_284

MCSEIKHSCFVKLQKTELNQLLNSLSFCQLLVEVIE

>SG-PL_contig_8746_1_-2_65_430

MYLKFIHRRREQWERQIRPGYSAKRYLSTWTRNWDDQVFETVVSKGYLTERSLFHYNLFL

PHLRRHLFHEDLSNMPQPYRLMKNMLLSPGSLSGYTFYRAADFGALMEEDEDGADGQKIK

RP

>SG-PL_contig_8747_1_-3_438_554

MKCVSKIFSIVSGQIFTKFGPKLCTVQESLMHQFGTERA

>SG-PL_contig_8748_1_+2_158_346

MRIHTGPAIRVQLYRDLLIMLRNSEGYILFHNYDTHRKQDCSEYSTLSYEMRKQKKQSKG

KEK

>SG-PL_contig_8749_1_+3_3_248

RCTGSLENSTWNATFFSACSSLALRAMMSAELAFTCCTRFSTTASRGLTSSDRLAPSVPD

ILVYARITRLTKRPTRNIFVVV

>SG-PL_contig_8750_1_+1_208_360

MACQRLNSDRDLVIEGSDDLVFNRISITCLWKREVPKKVLGSVPDRTGISH

>SG-PL_contig_8751_1_-2_137_277

MIFRSSFFYIILIVMKEHFYIFLTTTHTQNSTSRPSYSVDISIFHSH

>SG-PL_contig_8752_1_-3_1053_1364

MTCQKILRPCQDHLARHSPRGKKKRKAKEEMGRQHPRMDRPELPRGKKKRKAKEEMGRQH

PRMDRPELQQFPESGRGLGEMAEICCSYQWCPYDLQGFGIRNIM

>SG-PL_contig_8753_1_-1_439_759

MQIVQQQNDCTARAKQHAYTHWRKHCSERKKTFGNHQPLTSFSIPFIVTIKYYGLCIARY

YVLNRLIFSHILAFKHTIFSSQYYGLCIARRGLVGAEVELLASIQTP

>SG-PL_contig_8754_1_+3_315_443

MATFKITSSFISMSRLSSVPHHHSGFIFRLGICHNFCLIDKAF

>SG-PL_contig_8755_1_-2_74_1720

MWKAAAGTNVKITTQASEDDDWETDPDFVNDVSEKEQRWGAKSVEGSGHQESLDLKKLRE

GVRSDDTAYKKEQFDAGPKASEGYGGKFGVQKDRMDKSAVGHEYQTELSKHSSQTDTAKG

FGGKYGVQSDRVDKSSVGYDYAGKTEKHASQKDYSTGFGGKFGVQKDRVDKSAVGWSHHE

KVEKHESQKDYSQGFGGKFGVQKDRQDKAAMGWDYEGKTDKHESQTDHSKGFGGKFGVQK

DRVDKSAVGWDHQESLQKHESQTDYSKGFGGKFGVQKDRQDKSAAGYGDVTKTELHPSQT

DMKKGFGGKFGVEERKDQSAGSFDDMEGVSSSYQRTKAEGSSEGARNLKSRFENLAKSSE

EESRKRADEERQRRQAKEQREKEEQKKQQEERLKQERWEAEQNKANEPEPEPEEPRKPAR

QMVPEPEPEPEPEPEPEMSYNEAYQSSDNPYEETESEAAPAPAVQHEPEESPYEMDDNVY

NQPEDNSAQSGGGHTAIALYDYQAADDDELTFDPDDIITDIEMIDEGWWRGACHGRSGLF

PANYVELQQ

>SG-PL_contig_8756_1_+1_1_234

FSNASLTFPPSQLLPSNPISNSFSRWNHQNSFLCSRKAGKRNGSGSRSVGPPFWSGMLPQ

TGRYSFPVFLRAVKNHAK

>SG-PL_contig_8757_1_-1_64_171

MADRLIVNQSRYVYRPCMNRGTNFNDSSLKNRWKWD

>SG-PL_contig_8758_1_-1_76_1467

MAENQVGPIDITSEKDGGIILETLRPGEGEESPCTGDRVTVHYVGTLEDGTVFDSSRDRQ

EPFQFNIGKGDVIKAWDCGVAKMRKNELARLTCAPAYAYGERGMPPKIPPNATLIFEVEL

ISWMGQDLTKDKDGGVIKQMVEQGEKYATPNDGSTVTIQFTGQHEGRVFESREVTFTQGE

GDEEGIIPGLELATLKMKKGEKNRLIISPKYAYGDKGCPELNIPPDATLQYEVEMVNFEK

TKEPWEMDVGEKIEQSRIIKEKGTASFKKGNYQRALANYQKIVTFLEHETTGLKEGESQD

REQLLLAAHLNIAMCHLKLQNFCQAEDACNQVLEVDANNVKAFFRRGQSRLERCEYGTAA

EDFGKVLELEPDNKAAKKQQIICRQREKSAYAKEKLLYTNMFKKMAAKSDGGFNLYQTMP

EVGEWNNDMAKNMMPLQDERDAFEDGADGYKKDLSPRPGETLED

>SG-PL_contig_8759_1_-3_3_143

THTHTYSILVIYIVCSFYCICTSCVLMIVEYVLYLSVLEIMEYVLCM

>SG-PL_contig_8760_1_+3_153_308

MVSLIANIFTSPVLHWFERDTRERLTERRARKKTRKLEERERGGGGGENFKK

>SG-PL_contig_8761_1_+1_1_240

PRRSFRPLHTPPTPSSFSRNPSARKPMGASDVEPLRGHPPNSFPKLYQQSFLRLFMRGLK

KTPKQIVTDSFQRIFTNSYV

>SG-PL_contig_8762_1_+3_228_470

MNKYCNIVHGRIGNLHVLNAVRYNSFRWPLMLYTVLMSCGLHKTLHTHKKNVKNKQNLLM

SMLATSYIFCWSLDTHHYLDI

>SG-PL_contig_8763_1_-3_228_317

MTQFSPLSIHSCKTLENVQQLLPVGGGGVT

>SG-PL_contig_8764_1_-2_2_175

SSYRTAPSTTPSILTLQLKLFMAIMSLWWKARNDSNRTCLDFCCYCFKLLKREHEMSV

>SG-PL_contig_8765_1_+2_359_529

MFYTQSSLRQLNDIFQRHQSQLCHHQNQLKNDITKSGGKHENQCGTCPMDFRPSVGQ

>SG-PL_contig_8766_1_-1_304_474

MHSLFSKTSNFHHSKLHRYKIYFRWSYFKPNNSFMHIVCTSQSYIVIVDFVVKNYII

>SG-PL_contig_8767_1_+3_66_230

MEASRHPYKETCSYGPVLPSLTILWNKESVAHTLLHHRLYLYSTVSLVLSLYMKT

>SG-PL_contig_8768_1_-1_370_1611

MTSRKTMKMSTGGTMFRAPSYFVWNHRKHVSSSPRSSPHRMAANTNMNNTHDQDGTMGDA

GPSPRKMRSPRSRTAAVAGADNQSTDTKSSGVSGDSGGRVAISPKHKDPDRIKKDREREE

RVRQARERLEQERKKKLVDLQEQQRQAQENREKQLEMRRKKIEDLRRRDVERRAEVEKRR

REKENAERSRRETLLLKAEERVARYEAWKRGGHKGPPPGGVLPTNTTKHLSASTSVLYHK

RNSDFSSSGVLNVSNKPESLLALNTIPEGRRSVLAPSALPPSRPKSVMTLSTSSAASSSP

GGVKLRENKAPRKPRPASVGTSMPSFLTLDTPKSTPRSKSTDRLARERSRARAPGSARRA

DDKEKEEKRGGEKSGVKMSRRTLDRLSTPRQLASIMGDNKDSTSTPRRETNITR

>SG-PL_contig_8769_1_+2_2_136

NLKSKILLLSPREYQAIFFVMTSARWNTKLLPGGTLSTHLKRLQA

>SG-PL_contig_8770_1_+1_199_420

MSHSTVHSAPPSIFIWGGEGGIVRGGDKMFCFVLPDSVALLSLASVIQCTHQTEFIPSQF

LCQGFHRSYQYQDT

>SG-PL_contig_8771_1_-2_1223_1378

MNCKYVWHNISVPLSSTCSLVTNCYLRFTVSTDYQSVGIYVVMTSGSGVSRI

>SG-PL_contig_8772_1_-2_2_211

VCVCMCGVCAQVCACMCEWCVCLLCLISVVFCSEFGWLVVYFCFSHSAFVNTCHDGIFLS

RFSAACGCAR

>SG-PL_contig_8773_1_-3_168_581

MAATGISWCWAVHVLIHAMVTMTGWTLDVRDVDLAGASCTDMTERVLPDTVVLRLRADYH

SNSRQSIFTGCNITIRAADSNRYLLYSVDKLTCGVSIGIYYSPQDITKPNRTVTCGEDLP

VQDRSTQGVVRFSVHRHT

>SG-PL_contig_8774_1_+1_1_177

FHNKLAFLESKQSENTKQSASSYFVLSNYMTPTENKQKLVCCKVFMVSKLSLYRNRHII

>SG-PL_contig_8775_1_+2_2_208

HCSENNCFTDTFGWLWLGSLVYAHSSLRWMENIRSGSRYANDCSKFASNSTFLSTFIDLL

GTHKVTCLK

>SG-PL_contig_8776_1_-1_190_489

MLIYLHQYSMLINILLKLSKFSFNSIFSPLPSCDDIQCPKLVKYNTLSLQLFIWQHQFYI

YYNFRKLSQVYALAYISEGLLTLIMCISHVISSSLVSSSF

>SG-PL_contig_8777_1_-2_2_760

MDKKFGGTKKIIMIAFAVLAVVPLIFIQIPQLTESKTLELPWEEYKFLCVPGEGENVPSF

QNRCASFAGTRVTWAGKLAWSKVTKVENTAESVLKALPSFISNPLYCIYGEKSPECDEAT

MSARSFKYCQLMKLTGRTCDVRNHNVYSFQLGVNVSGVMVLLDAGNGFYAKVMAMKANDE

IELTGSLLDGLGTLAPHLKLNAIKVLNRELPEMMAIEQEEDEEFFLNALHEGARLTLNFF

FFPIFEYTGFRSS

>SG-PL_contig_8778_1_-2_50_193

MKVVFQLVQEGDEVIWAQCMPSTCHTCATIRPLPLKKSSFLCGFITEK

>SG-PL_contig_8779_1_+2_2_103

TNGQSGVGEVGRDSGSRECLEFSTCLKFHSSQVS

>SG-PL_contig_8780_1_+2_65_232

MKQVQKHIKEHGVGEFVFSTRGWMVNTQCSFSVCQRKMSEELVQNNYGQKARITFI

>SG-PL_contig_8781_1_+1_454_555

MSEINRNQILLPDVKYGLRFTEPKKRKQEYSLSL

>SG-PL_contig_8782_1_+3_984_1352

MFCVCIHTKGVSSGTSRSAHMLTWEIEKSPLFTHRARCPGRIRTQALQTLQFGSQVTSPL

DHRTHERRSEEQWQRVWSSKFVQGFPALGQFHMRSTQICQQLSGRLLEPAQNPMRWQGLE

MET

>SG-PL_contig_8783_1_+1_61_330

MSFYAGWLALLKEPWTQDLEFSPTPKHKNWFVRCPRHLNAPIVHVVSVFRGTALHPISRP

CQLIRFCAGSRSLPDNCWRIWNCPSALNPS

>SG-PL_contig_8784_1_+2_80_187

MTPKVTTFPHNHLGTNHAGLALYSQLFLYLYKNILS

>SG-PL_contig_8785_1_-2_101_358

MAAAMDVEMAEEYGEGSSASSDKGKKRFEVKKWNAVALWAWDIVVDNCAICRNHIMDLCI

ECQANQASATSEECTVAWGVCNHAFH

>SG-PL_contig_8786_1_+3_3_170

NTGCDNTAPQKQSNSIHHTLYQKLYPTSECTSSYMVYPRFIKKIHPSAQLCLHVPV

>SG-PL_contig_8787_1_-3_3_209

CCLVKVSSNLYRISGRHGHVASGLLPKSFLQTVAWMGDVGNLLVASCFCWKNICCSNSLW

CSSLLPAAR

>SG-PL_contig_8788_1_+2_245_514

MFYQQIMMQQWDDPPCPSTIANLYLEDYKKKALETHPHQPETGTDLQMTHSPNNRSTKLT

TSLTAEVEENRHPALPDSWVPSLKNELPSC

>SG-PL_contig_8789_1_+1_271_423

MFTQTKTTSKNKSKKITNTKTSIQQSYRKVHVNNTSMNQFLFSAIEILTVL

>SG-PL_contig_8790_1_+3_3_266

CHQGLQVLGRRLGEGGNYTPLEKHQIYCYKTHIPYILNTELKAIVVQIKPMICMHNYANC

EKCKVLTSDYCTKYFCVTTTEFFFFFFL

>SG-PL_contig_8791_1_+1_1_219

PCLSCCGHFPVLEYFTLLSFERKFDTRIVVHHFLYLILFAGLICELPLSIKIKNTKMCVC

VCVCVGLCEHMYT

>SG-PL_contig_8792_1_+2_50_214

MHNLSYVQARNKTIIKRCGRIVMTNANLAIDSMTSRKHKKFTYKWQIQLNPCIHK

>SG-PL_contig_8793_1_-1_130_228

MSPVSIFVTEKRLFSIGLSFFLSMTNWRGEEST

>SG-PL_contig_8794_1_-3_243_386

MYTIKGVHLFLFRFHCTSSSFTLTVVFDFYNLPFEPIPLKLWCLLFVE

>SG-PL_contig_8795_1_-3_1047_1250

MNVNIKWTMIRQCLLFFGDFFFTPSFLFKRENDSDLFKACVYLLTKLCPVAFVPIVEFEN

ADWFLILP

>SG-PL_contig_8796_1_+1_166_261

MWKFIQFISTLGAQWRGHHHTIICTTLEVKGQ

>SG-PL_contig_8797_1_-1_1_150

GFPHIFGIQNGTALVLFFFLKYNHHHHHHHHYHYRRRHHHQHQRSHHHHY

>SG-PL_contig_8798_1_+1_1_177

NNNNPKQNKTTTNQQKLSCSLPMLMLQSFWWCQLSGPVLPFQDLAKSIGFLHRLKESAR

>SG-PL_contig_8799_1_+3_3_206

SKITVQSSDGAVYSRNSSHMRKYLHRGEEEDAGAVPVATACPAHPVCKYRTLQTPMTPET

LQTHRSYQ

>SG-PL_contig_8800_1_-3_192_347

MLIPLPSLIKFFLLFLVSFSLSLSLSLSLSLSLPLPPLLSLHLFVCPSGVFS

>SG-PL_contig_8801_1_+2_191_403

MEVMHRDERVTGVELEGLRVAVNNSSKADTGMKKRTVTSATPVVKKMKFYYNFMPFQLYK

SPYCCNNHFLS

>SG-PL_contig_8802_1_-3_3_482

SHLHLTFAVGGPSFELDPLLHGARNPRRAPQWDLLLVLDCLRSPPFEPLTGIPFDLLTLK

TTFLLVLASSKRRSEIHALSGAEQDIAFHRDGSVTLRFLLEFLAKTQAPGDTTSSVTIRP

LSDILSRGEEDRHYDRSVVSCDIWIDLGPGVRPRGDSSSQ

>SG-PL_contig_8803_1_+1_1_291

LSVTGATTLTHLWGPLVLDFMHNSTEIHIYADISFSFSIKTSLSLSQSCESVIFLTKAAI

HSLQGTTTSLLHSVCCSMVWIAAQDQTQDGSRMHSER

>SG-PL_contig_8806_1_+2_794_943

MRKKKNAYKHLQELAHFMQTFHNYMLFVNIFFDILKRKHTKDYIHNQTKC

>SG-PL_contig_8807_1_+1_1_207

TNLNTLSLHDALPIYNSELNLAKRDNSELILAKRDNSELNLAKKDNSELNLAKRDNSELS

LREITQKSI

>SG-PL_contig_8808_1_+3_3_275

FRGVHAGCFHVSISHRIGTWLTGSLTCVSDVLRAYTHAGDSRVSRSACMLRSDKFPSFTQ

QALAAGSNSNPLISKSPASYARLLCSSKTLQ

>SG-PL_contig_8809_1_+3_96_209

MSQPARIKNNNDNKNKQKKTCGPPEGQTHDPWVISMVL

>SG-PL_contig_8810_1_+3_3_233

VEVSKCDESYEKSLNLLHKDRFLGLHNIFFSKLKARSNCGIPKHAFFRKHAPTSKPLWIQ

PKALSLSKLWNTHLWDQ

>SG-PL_contig_8811_1_+3_3_167

YIYIYLYSSSCSNSVTSFEMWKLSLAGVLPLSCFIPNSKCLHVSNDNLCFYLQPT

>SG-PL_contig_8812_1_+1_706_939

MCHHSNNGQLCHHSNNGQCHLWFSLCSTFYPSALVYGNCLQMLQNVVNRIFFYLYCHCKN

WPEVPDRSRRQTFYGTST

>SG-PL_contig_8813_1_+3_45_257

MHILKNMYFTFEMSNTHCQDSNFKHMPDCDSNMHRCSQKGKKWKPNCLGFIFSQKKIQIQ

GIYNTTKTFLI

>SG-PL_contig_8814_1_+1_751_1041

MNMNFEFSKINNQFNVSRIIQSLMYRWKLDAFQTKYTRNIMCLCGQKITTSHILSCIKIK

RFIPILSQKTVDEIFESTSLTYEFFVSLSKSPIASYL

>SG-PL_contig_8815_1_+1_865_1071

MSWLGKNNSLSFFSCLLPTCGPHLLKDPSLNICKIPTGIFFTHTHTHTMGQSKFTLNKRT

TTVMSIMCI

>SG-PL_contig_8816_1_-3_168_275

MDLHIEVTDFFLAWKLKRERERERESSDCRNFITQG

>SG-PL_contig_8817_1_+1_49_159

MMRRKPGPHFKMVSHKYLITIKTQSVHEIFNTVLLLL

>SG-PL_contig_8818_1_+3_48_389

MSITINTILVNVHNSISLICTFVPSWVSWGHNSTIPVNVHNNRYDPCKCALQSAAKSKSY

RQPQQRSLYLNFQQPQSKHYKGLILNTTNSVCVEHLKINLVQLCAIISNKSNNA

>SG-PL_contig_8819_1_-2_137_412

MIVWVGFSPKVTGEGLTVIVLIVMYINRDCTVMYINKDGIGSCIHSKDCTDCNIYEIIVL

FVNNKDGTSCCIHKDCSDCNICNRDCTDCYVH

>SG-PL_contig_8820_1_-3_3_149

DCSSTKMANFINKYEKKGERQQTNATNCLTIAPISYKKHICDEPILCSP

>SG-PL_contig_8821_1_+2_2_181

HGEKKKKKKKKKKKVHILDGNNQSWSSGTETYWRCMGREVNMLNASEARQESKQHPPSFL

>SG-PL_contig_8822_1_+3_3_173

HITYIHIIHFFLEHAKEEEKGRKKKCPMKHSFIPRFEIKISLDCTLKAHGKKDQNHV

>SG-PL_contig_8823_1_-2_158_310

MTLDVSLLPPPPFFFGLGFEPMTLWTCTFLGGISHLMSRTLTTELPGHLVF

>SG-PL_contig_8824_1_+2_1475_1858

MAASCLKLVQTDLRHMHVCVHLMKCKLHGKAQFLFAQNSVTPKVILYTTNSTEMPEDRVS

KIIMDHGYFDIKDNHHGYKCEWMLAIFKEYSNLNTHKLAIILKIFFVPFFLSDFTFVFVY

LFLMTSIA

>SG-PL_contig_8825_1_+3_150_428

MALPISSPTSLAKGTDYPISQAGKAVKVVSLARLIVIRISVLKSHCYCSLSPCLWPRIQI

TRLLSSQNFQGPREPNGSIIPACQLAGHIEKKK

>SG-PL_contig_8826_1_-2_20_271

MYCIYFYFLVTLDLPCEIRAALPGRARRRSAAPPIFFFLRVFLLTYESCFGVVFVRVSSF

IAVGSFTCAEGMLHWLYILPLITT

>SG-PL_contig_8827_1_-1_595_903

MLTICLNLRHHRTEGKNAVIHFRHMCLLFYIYGNKSFFLFKSVGILKGVVRCFSVCFIIS

GIIIQYSFKCHLTSVLNDILAIDPGFLSSDIRSLTHLEQMFPT

>SG-PL_contig_8828_1_+1_2320_2655

MLGLNRLPHYERENKNKWENIKPPLLPTKCFSDALPRSTTEKKFIKMEFIHRKDKIKTGN

MTREKWKMITTMWWQIKRLDLPISPTTVTHVYYPDTQLQETSCAKSMTAANH

>SG-PL_contig_8829_1_-3_3_170

KYRHLLCWSGFVISALKFVSICTADEAQLTCSKCFISSVDLVTESVRNSSQRYYCL

>SG-PL_contig_8830_1_+1_55_435

MDIQCPSQCLQSHYLVARTKQHRQYIINWGEGENCVKVCWKKERGMGQEGRKKQTTVKEQ

KRAETKTAIEKRKFLAQNFQKVGIFIYTERALGIPTGETQHSFNKTSSMTIPIKAYCTFI

SHIMQYI

>SG-PL_contig_8831_1_+3_3_311

KLCEGLLEKRKGNGPGRQKTVDDSKGKKKGRNKDSNRKEEISSTEFPEEPPASPRRETRA

NNNIMIHTEKSNANILTDQLEVLKEQTNYYSTLYGWKKILAKQ

>SG-PL_contig_8832_1_+3_174_287

MILSICLLTHSVNQYSLFIFSKSDRETEIFTSDDTRAI

>SG-PL_contig_8833_1_-3_87_197

MSKAKTTNKNEQPYYWFSGCYVVLQLLFNIFSYVFFF

>SG-PL_contig_8834_1_-2_2_280

LVFPTSATAQSEPGSPVRLYSAPYILAPLRTPQAALSEWTGDNSDRANIMLVTYCLSHDQ

RFLLAVATDNQGELLDTCTINIHIPNRNQRKKA

>SG-PL_contig_8835_1_-1_1_261

LYCLCQPVCHHTLVLSVCVNLYVIIHLYCLSVNVYVIIHLYCLGQLVCHNTLVLSGSTCM

SYTCIVWVNVYVIILLYCLSVCQLVLS

>SG-PL_contig_8836_1_+2_2_232

VVCDNTNKTETFAADTAEQKKRFGEGAKGPMGPCQCAQLVFRSPTRQVRYAPQMANTTGD

CFECGSIAALNADLLLL

>SG-PL_contig_8837_1_+3_3_206

PPGEVPVVRTQAFHTLQFGSQVSSPFDHRATSMCIVHIYIKNKIRSNKKERKQASAMGDM

SGISCSRQ

>SG-PL_contig_8838_1_-2_374_2080

MEEVGGDEFRSHNHAERLMRKMETCASKRQLCDVVLIAGPKRLPAHRLVLSAASDYFAAM

FMNNVREATMEEVELKDVDPEALAALVQYTYTGRIELKEETVECLLSTACLLQLSEVVEA

CCSFLMKQLHPSNCIGIRQFADAQGCTDLYKVANNYVMEMFVEVMHNSEFLMLPCDEVCK

LLASDDLNVPNEETIFEALVKWAKHDLTTRRKNLSKLLAHIKLPLMAPQYIADHVETNSL

FRDDRECQQLIMEALKYHLLPERRSSFQSPRTKPRKSTVGVLYAVGGMDCTKGATSIEKY

ELRTNTWTQVANMSGRRLQFGVAVLEDRLYVVGGRDGLKTLNTVECYDPKKKTWTLMPPM

STHRHGLGVGVLEGPMYAVGGHDGWSYLNTVERWDPQARQWSYVAPMSTARSTVGVASLH

GKLYAVGGRDGSSCLKSVECFDPHTNKWTHCTPMAKRRGGVGVATCNGFLYAVGGHEAPA

TNPTCSRYDSAERYDPKTDQWTNISPISSPRDAVGLCPLGDKLFAVGGYDGQQYLNDVEC

YDPITNEWVKVSSLCTGRAGACVVHVPNS

>SG-PL_contig_8839_1_+1_1_183

GLDCFPYYYYYILFIAKLTTKAISGLSPRQVQSQKDNCVEHKFSNLNTKIMQKNTIHDIL

S

>SG-PL_contig_8840_1_+2_2_169

FFQFYNCEFNEIQKDHSIQIKGTVVFQQKSGLTASLHSRETRLHLQQSKGHLATEI

>SG-PL_contig_8841_1_-3_405_521

MEFVGKSIYYFHILSFVKFKVTQHNCKAYECDNMTGLYR

>SG-PL_contig_8842_1_+1_1_81

KTRPDKMLHYLISEMIVATHERSSVLK

>SG-PL_contig_8843_1_-2_221_322

MMLYLLSFTFCVSFNSSKLSQRGVFQQRFFLLLF

>SG-PL_contig_8844_1_+2_485_928

MKMAVHLLFTPTIVAVILYYFMLRKSFPFTHSSLCYHQQKIMISLSLHCIHSHCFINIKS

HIKLTFTLQYEASLVAIILCYFIPRKISSTHTAADPIANTFNDLLDHFTAVGVIVANTQT

MKLVSLFHFKYTLSTCCFVPCKSPSATF

>SG-PL_contig_8845_1_-3_246_389

MLKIMFDVLKLMYSGMDNFIYLCLNITSRVCTTSALSILKKISALLIY

>SG-PL_contig_8846_1_+1_466_648

MLFCCAHFPAKLRFLALSLPHPIHHLPDRVKGLKFRLHLPQVTWWQLIWGENLLQHEIKV

I

>SG-PL_contig_8847_1_-3_468_1709

MKIISLQLTVCTFLLAASVVHAKKNIGSQCLTCKQIAGNFAKGVELTKKSNYGGGNTDWE

ESRLGQYADSEVRLVEVMEILCMDANKECFQMVEEHEKRIENFWFSSNLEKTESNFYEYF

CIKNVKACCPDNTFGPNCTECPGGTERPCRDNGKCLGAGTRDGSGKCKCNPGYKGKLCDT

CTAGYYESLKNETHSVCKVCHISCKTSCTGNGPAHCTECRDGWTQDKETGCKDINECENN

PCGEADYCVNTQGSYTCMGCSVACEGCTGAGADKCKKCKHGYRQEGSKCLDIDECSAEGH

EQLCTLEGQKCENTVGSYKCACLPGYIRDGDTCKPKPKPEEKSNGRRSQNRRHNSQGNLN

RREESLITLLFGFFALSVIFFVLGKLCYVINPKLVSIPVAVFAIMIFYLINDYE

>SG-PL_contig_8848_1_+3_114_272

MTATTSRGTEEENMRLVHRSLTTRPCFSEAEHSSRRQTCDVAKQAFYVFRKPR

>SG-PL_contig_8849_1_+1_391_510

MHNQEDCLPLLDNRICQRYCPLAYNKKKRKVIIIIIIYCL

>SG-PL_contig_8850_1_-1_193_267

MIFKCCFDELFHTHMHACKSTHTHT

>SG-PL_contig_8851_1_-1_79_2052

MMAGGEDGQHYVELCENPIRFEPVSKANNVFFDDANKQVFGVRGKGAMGVVVKGPNEKSI

ANFRTDDKGDVVSVKFSPDKRILAIQRTPRTVEYVNFREDMTSEQQEYTHQCKAKADLLG

FVWTGVNEIVYVTAKGIEQYQVLPEKKSFKAIKSVSLDIKWYFWLANSGFLLLSSGNLGN

TLHPVQFKMGVMTRLPKFEVDLPAFPKPHKAMLYDRDVIMAVIYDHLYVIVLRHPPNAAA

GAEIVMYQVQKEGPARKTDILKLDMSGRFAINVVDSLVMVHHQASKTSVIFDIKLDGKSD

GFVRHHYPVLSPLPIKPFKLLLPSVNMQAGTDKTEFFCELYSANWVVFQPAIVIDAKLGC

LWTLRLNLGPIVAMLPDKVKLIQFLLNRGESKTVLQSVCREMLTPGHQTNLGVISQVYDM

LNQVYKAYLETEASIMSMDSAAAAAAAGSRPKVIIDQSDMFTHVFAVFEEHKDIPYKFMV

AVLIEYIRSLNQFAIPVQHFLYELVINILVNNNCFYQLHQFLQYHVLGDSKPLACLMLSL

ESVYPPAHQLALDMLKRLQTANEEIIEVLLSKYQLLPALRFIRSVGIVDMVSSRKFLEAA

QNTEDNLLFYTVFKFFEQRNQKLRSNPRFQPGEHCEEYVRKFESLFGVEALMPIQVNM

>SG-PL_contig_8852_1_+1_82_204

MHTFVHKHVCQTQVNTLYYCSTHNMSNRMCTTNIILSRPHP

>SG-PL_contig_8853_1_+1_61_171

MSKMIVPSGDDQLLQRRDAIYCNNLLKPHDFQATAIQ

>SG-PL_contig_8854_1_-3_3_137

HPHNAGPEKFPSDRSGYRKTFIHNALINNCTAVFCLDQITRLACT

>SG-PL_contig_8855_1_+3_153_317

MIIYFSLSSLLISMLIIYNSVCEFFVALAGITALGQFRMFHTIKTMLQRYIICHN

>SG-PL_contig_8856_1_-2_2_202

LSVYLLCAHFVFIYIQQSPVTCGRTICCHHPVGHLCIIRIPSVIFMTTSSVDTLHICRFS

EGSKKLQ

>SG-PL_contig_8857_1_-3_2661_4106

MTVSMEMFKENRERLCQRLRDNAKVPRGAIVILQGGASETRYCSDHEPLFRQESYFHWAF

GVEEPDFYGAVEVDTGRAILFPPRLPDSYAVWMGRLLVEEEFRFRYGVDEVHFADKIAEV

LKSKKPSALLTLYGLNSDSGKYSQEAVFEGIGNFTVNNELLHPEISECRVFKSDKELALM

RYVNKISSEAHKTVMKNMQPGMYEYQAESVFKNYCQYMGGMRAQSYTCICASGDNASVLH

YGHAGAPNSKQINDGDMCLFDMGGEYCCYSSDITCSFPCNGKFTEKQRQVYQTVYDASRA

VMAACKPDVSWVDMHKLAERVTLESLKKMGVVKGDVDAMMAERLGAVFMPHGLGHFMGID

THDVGGYPEGTERSTEPGLRSLRTVRTLQPRMVLTIEPGLYFIDVLLDAALQNPAQSQFL

VREVIDTFRGTGGVRIEDDIAITEDGIELLTDVPRTVEEIENIMIEGRDTQTPLPQLQQK

QQ

>SG-PL_contig_8858_1_-1_1_180

LFISALFPSFTVVLLFSAFQVRSTLFFPASLHTFFSHTPHPADYDVLPVLFCSGNYVVIL

>SG-PL_contig_8859_1_-3_3_209

ARDVQQKIAGKYDQEIAAHCMAWLARSTGEGLDTSGDIDSVYTQLKDGYVLCKLMNTINP

GCIPAGKMK

>SG-PL_contig_8860_1_-3_3_212

VVTGALAAMVIVVATLLVATVLELLLLVHDVYRRVEPRLDAASRHVSRPDELRLDLFIFN

PPHLLQEAGT

>SG-PL_contig_8861_1_+1_862_1098

MCPGRCSMRSSPKDNTWQKDWVTGPLSFKIMTHGGWLLGVSLMWCCTDLVCMGYERKARG

MKTVQCALSQLARPASSSC

>SG-PL_contig_8862_1_-3_75_176

MNWQYQYGSLKYYISAATTMVNWGYQFLNVENKT

>SG-PL_contig_8863_1_+3_3_98

KEVHVKLSYDRKLKPSENYTTSPNLTKFANFI

>SG-PL_contig_8864_1_-1_178_357

MLARCEVMDQPSAPSSLSWVVPLLSQIPSLETRLEAPLFVVPATPEGPPSGTFWYWISSG

>SG-PL_contig_8865_1_-3_3_161

GPVCEGKGGVRPSYMVQDKCGVQLPHYQRDTTENVKQQINGKAKSKARLGFVW

>SG-PL_contig_8866_1_-1_1_297

RSAPRRSSQRRLLISINENYSKDISATTISRWLSTTIVRAYKEAGSSSLPDCPRAHELRA

LSSSAAFHHSLPIRDVLEAASWRSESTFITTPCATPGAP

>SG-PL_contig_8867_1_+2_2_217

AQNKRAPIQKGTYQRSHKILSSLKLPQYLIYIKTLDLTGFVLTRDISAISHGLPHFSHGS

PKCTDCVYTHYT

>SG-PL_contig_8868_1_+2_149_268

MVYTLENEDIKSTICTILFVKCHSPVSHFKCLKLTSQMVS

>SG-PL_contig_8869_1_-2_2_130

YIELRCGLGIPDCVCECVCVVLFCFLFGGCCFVVVFSHVLKCK

>SG-PL_contig_8870_1_-1_148_933

MDTLTIGGLKIKNQTFAEALSQPGITFIAARFDGILGMAYAQISVDRVTPPFYNAWFQKL

VLKNMFSFYLNRDPSAKEGGELILGGSDPAKYVGNFSYVPVTKKGYWQFKMDGVSVQGGS

QFCMGGCAAIADTGTSLLAGPSSEIAKLNEAIGATPFIEGEYLIDCSKISNLPVITFTLG

GKKFSLTGAQYTLKVSEFGYSICLSGFIGLDVPPPMGPLWILGDVFIGPYYTEFDMENNK

VGFAVTKQSNGTQPLLFSPEKH

>SG-PL_contig_8871_1_+2_2_136

KRDLSHSDNSICAPPKSASYYLANSKKPTCWQSLEMGRRMGPMKH

>SG-PL_contig_8872_1_-3_3_161

PDNSWQIWVECIWNCLSAMNPSLTDSKFQHDENNFCGTQNSISPSLCQYANTI

>SG-PL_contig_8873_1_-1_430_1089

MADFEGAEDLSANELRYLTIDRSIINDPAVQAEWASKKLIWVPEEVHGFVTASVKAEKGD

QVEVQIEETGKRIMVHRDDIQKMNPPKFNKVEDMAELTCLNEASVLHNLKDRYYSGLIYT

YSGLFCVVVNPYKKLPIYTEKVVELYKGKKRHEVPPHVYAIADSAYRSMLQDREDQSILC

TGESGAGKTENTKKVIQYLAYVAASTRQNRSSVSNIQQIS

>SG-PL_contig_8874_1_+1_73_201

MLEMVYPRDEEKRLYVMTSMQFELNGSIVHDICPVFKKTNKQT

>SG-PL_contig_8875_1_-2_167_262

MYGNSAINCMRYWLKLLSMERSRIPWKAYKML

>SG-PL_contig_8876_1_-2_263_1540

MVNQNTFDDIAQDFKYWEDTSDEYRDTEGTLLPLWKFSYEKAKKLAVTSLCWNPKYSDLF

AASYGSYDFMKQSSGLIVFYTLKNPSFPESSFETDSGVMCLDIHPDHPYQVAAGFYDGSV

AVFNIVEKKDGPVHRCTAKNGKHTDPVWQVRWQKDDTDNNTNFFSISSDGRIVHWTIVKN

EMLQQDVIKLSVTGAPGEGPDGIKVPTTGCGTAFDFHRQKDYLFLVGTEEGKVHVCSKAY

TSHFIDSIDSHNMAVYKVAWNHYHPKIFCTCSADWNVKIWENAGKPTDITDTTSKKPLFS

FDLNNSVGDVAWAPYSSTVFAAVTADGKVYVYDLNINKYEPLCEQMVAHKKKTKLTHIEF

NPSYPIVIVGDDRGNVTSLKLSPNLRKVPKEKKGQPPMDPAQRSEFEITKLNKILSMVRE

QPDAAK

>SG-PL_contig_8877_1_+3_36_221

MNLKAKSSRRTAPAMCHLLYTAFADGHRTMGLPKKRYKSCTKAVIGTVASLYEADKNVHH

IY

>SG-PL_contig_8878_1_+2_89_286

MWVALSVMAMSSDKLLTKYAVLNFCRIELFRTNEKTWISFAHAQSRTWCSDTPKKNKKST

HSDTHT

>SG-PL_contig_8879_1_+1_319_537

MHLSLYTHTHLFKYTRAHAHTYLIVIKILYHKLVYQPIIKTFSANHICFVVRKQTTLSCN

HPSQANTHAPYNT

>SG-PL_contig_8880_1_-2_206_349

MRLSTYGIFSRIKTSLSNCTEHRHWGSSFAVTGCFSIIRIDVVEKHTN

>SG-PL_contig_8881_1_-3_207_386

MKQDRPMWFCGLCPSLHACSSRRLIDKTVLCPHQVTGQANKVVCLVAITSSLSLKTVNNL

>SG-PL_contig_8882_1_-2_434_766

MSVSHLSLCLSLCPSPCLSVFVCCLLSLSPVPCDMDDVYRSSLLIFLNFCFTRAHTHRDH

ARFYRCFCYAHTSTHTHTHTHHTHTHTHNSRDASVVIIALFKKMDYLSSSC

>SG-PL_contig_8883_1_-3_3_122

LCPFTLDVKKIFFSPFSLQMRENFFVARMHLLPSHEVTLY

>SG-PL_contig_8884_1_-1_190_261

MKRNVTTNLSTIVAFVLSHSVKWA

>SG-PL_contig_8885_1_+1_1_168

KGKKKNNPKNQNKKTPNITVKNRKIPLVTTNWVVFVFFSLKKQQQTNQLYNSTKNK

>SG-PL_contig_8886_1_+2_800_946

MSGFSHLDCFILCSTQICRQLSGRILGRGTKTYALARFETGRGMGPMKH

>SG-PL_contig_8887_1_+2_2300_2548

MQSTEPLRTPTFGSSLFPCHQKSSSNLLHPEHSLSFSFLKTASPCLEQTPYAYTANAMGW

KRIQNDNGISRTPAHKSNVLTRS

>SG-PL_contig_8888_1_+2_2_208

GWWWGWEGYEAFDELHKMASDDIYRECNVLYNYRINTEMQFAASWKDSFLMNGENLLVLH

PELVPTVRL

>SG-PL_contig_8889_1_+3_267_407

MINNQRKSHPKWRRCTSKMPEIEALRKRKRFEEQVPAWSKLYTEEQP

>SG-PL_contig_8890_1_+2_2_280

DLGDREAGSPKQAQYSRGEKKSAEKQPSCMPFLSRRNKSWSNEKKIKRRKIPSISLWQKR

RPASLLTWVRNFVSATVLAIKKNINKVLVTAPS

>SG-PL_contig_8891_1_-3_3_245

PRAHEVRAIATSAAFHQKAPLRTILEAAYWRSENPFINFYLRDFRASRSDGSKGISFIAA

NTTILSNQSHALPRGNPASNL

>SG-PL_contig_8892_1_+2_236_511

MSTPTFACYAYFQSKTTFQGMLHQHRPIKHSNSDRLFCRLFWKWKLESFHIKLSRERDEV

FPNISLSLKNGSASKQTTSKTPAGLLLVSVSF

>SG-PL_contig_8893_1_-2_2_184

AKGRSLTSRPAPRKLSMLNRLFRPWPRMPWPRRRIYTLMTFLNQLYSTTIKTNPRKDVHA

S

>SG-PL_contig_8894_1_-2_1145_1384

MCYFHPVERCSNLERQQSLNPCNICVFCVLAIIVMNSSQKYFKVDRLLLSKTDAVCTLKR

VIGGSLWKMKHWHKFMKVEN

>SG-PL_contig_8895_1_-3_294_527

MGYTCVICHVCITVSHVILNYFVFTLQFLSLLSIPCLHFVFHFQFDVQNAHCVTSDEGTY

ILFCTKFISASLSGKVPC

>SG-PL_contig_8896_1_-2_26_268

MVVASSGDALDDEKWLTIHFSLSNSSSTHTGPHFLESTPFQSYKERERERERERERENRV

CVCVCVCVCVCVCVCVRERAC

>SG-PL_contig_8897_1_+3_309_527

MKKISLNYTENSFGYTEIKNGKNLFKPSLNILLFWCLKFYGTHIVSVKVEKFHLDSIILF

ASAASFQIAVTHG

>SG-PL_contig_8898_1_+3_129_395

MYLYSSRVRVRFHRSVAVRFGCLLSSTLFNIFLERIMSEAFEEHIVSVRISQNLRSTDHM

DALAGKECELAKLVNNLDHALSRYATEID

>SG-PL_contig_8899_1_-2_2_208

SWDAGGSFNINSIPAFWKFCARNFLFSIALFVSALFPSFTVVCFFLPSRSIPLSFFQQAF

TKPPSPCSL

>SG-PL_contig_8900_1_+2_122_304

MLADSSCVWSTVEHSFGPLCLLTVEQREQVQSVSPQLTKPIMFIRHASFPGSGERKCLAI

L

>SG-PL_contig_8901_1_-2_905_1147

MTSDKDILLCILFVFTVFYACHWVCHFIMVFHVYVQLLSPSHFLFLLQICFRFSWGFFLY

IIIMTPWCFFCFRVKVFLLTI

>SG-PL_contig_8902_1_+3_195_362

MHQPPPSMPRSLHSALTLSCLPFILTLCLFPPHHFHLLMLLSFLNQGKPKNFHSVL

>SG-PL_contig_8903_1_-3_144_347

MEVEAVTHALKWLASIDGMKESQAFILTDSMNLLQKVESGIGTPDWHGTMKRVKLQKLTW

LYCPGHAG

>SG-PL_contig_8904_1_-3_108_407

MEPPVKHRTVSAVADTHHSPTTNCQQSACYSQHWHTTTTSCQLTTTSSQSATTSSHITTT

SSQSATTSSWPTTTSSQPTPTPPVHMKKKIQSSIAAFFKK

>SG-PL_contig_8905_1_+3_3_74

SHTKNLLFSTPPHTCIIMTLMTDH

>SG-PL_contig_8906_1_-2_2_211

FICDYIKSRFIFYFVALWILSFVSYGSRSDFLYMKMLGNLHKILGAISAIFGEITAVCVL

LFCCIVCKNG

>SG-PL_contig_8907_1_-2_80_430

MLFYCTCSGRLGLCCSWSMENGPYPCTILKSQGYWRTCRRRHCSHATARSHTPTWARKRV

ASHKAMETPSPTNCFQSMARHIAPPRHLTVMSTPPTKTSCPATRTTRKICLSVPRRT

>SG-PL_contig_8908_1_+3_3_482

GQGSCCGLVDDPQHVETGNDASILGGLSLGVVEVSGHGDNSVSDFLTQVGLRRLLHFSQH

HGADFFRSQRLLLPLELHLQFGLAAVTDHSEGPVFHVALNTVVGEAPADESLGIKHGVLG

VHGRLVLGSIPNQAFCVGEGHVTGRGSLTLVISDDLHLAV

>SG-PL_contig_8909_1_-3_24_578

MEKYGLEKEDPEQFCLVEVLLPPGSQEYHGGSMGDERVMDPGECPLAVSLQHPKNRGTII

FQLRHRSADHKRRNKRQRAVSHDDLRKTHDQPATQADRLPYLIELNTRDGRPQKHLLPLN

ITEVVRDQPMTPGNQFLQLSAPDIRPQHCVLAHTEGIVTVTPTARDAEIYIDNRRVVETT

MLRHG

>SG-PL_contig_8910_1_-1_106_708

MSKSKDDLIHENDKLVAEVSRLKVGLDEVHEMFVNLEKTFVESKASFSLSRYGELKDMIK

NITSDEMMKKVHSLTENQADEKKGLLQNARALAEGERGAAGGDQSPPHKSGGLSSFFKKI

KDVTGFSDDPLDIRCLDKDDIRKDNERKRRDIHRLNTRGLRAYNRLEQLQRRYQASKSYA

PPTRYQELKDMIKDVISDQTL

>SG-PL_contig_8911_1_+1_1_150

CTMSDCEQNSKTGGGMASKVRVGLSGENRCISRKEEPMEVSDQEALKSRR

>SG-PL_contig_8912_1_+2_110_439

MSGERAAVNEEFTDTWVEDVLTPMLQKYEAKDVFNLDETALFWRLLPDKTFAFKGEKCHG

RKKSKERITVMICCNMDGSEKLPLLAVGKFMNPRCFRGVKKLPVDYKANS

>SG-PL_contig_8913_1_+3_372_608

MVPIIRDGGILILNYGCKRWSFSAEVRGQRSLVRVPDLVQELAEEVSGKSESWNYEESAV

YSGEVCILLTLVIYHHYQI

>SG-PL_contig_8914_1_-1_16_3435

MPTHQVRVHHLNKGENKESVLYRLEKGWVLRFLLGPSLHAALVRLFCNHPPNPQTPFDRH

TYHELEWRCPTGMRSDLTDVYADVKIICSGSFNYFFTIDESVDISKADGQGYFLVDPTMM

VGRDEEITMDCLQVQTVITKLLGPFSGWENKLAVARETGYNMIHFTPVQELGISNSAYSI

RNQHVLSPVYSQGGSTQYSMDDLEALVKKMHSEWRVLSLTDLVFNHTSKDSPWILQNPDC

AYNLKNSPHLRPAYIVDCILRYFSDEVAEGKWESKGVPVEVSTEEHLLAMRQILHNDVFP

KHKLSEFFTCNVDQVMREFRRGVEENRRAVPGDTEVTIKQDPNYRRNQSSVDIDLALHHF

NTDRDCSCYGDQGGSPNVNNRAERVEKCCEALCSHLHHLNQQKEGEVGDHISTAIENFIA

NARYRFVAGHGPRLGRVTKDEPLMHGYFVTPPSYEGSVEKGQEGMDTEPQKIMAVNGWVM

GDDPLRNFAEGSNVYLRRELVAWGDSVKLRYGQKPSDNQYLWNHMKTYAETSAKIFHAIR

LDNCHSTPIHVAQYMLDAARKVRPDLYVVAELFTGSEALDNLFINKLGINSLIRESMTAN

DAHEEGRLVYRFGGFPVGSFAQPRVRPLVPTVAHALFYDQTHDNPSPIEKRSVYDLWPTA

ALISMANCAIGSNRGYDQLVPHHIHVVDEKRQYPAWGNQIQKCESVNLSIGITNGKRILN

NLHYELAIGGYKEVFVDQLHSEVTAVTRHNPLTHQSVILVACTSFQNPYDPAVNYQFRSV

KVEGKVDEVLFEGSMRCTQEYQYNKDDHFVNGLPNYNLDIREHIPLSSSQFCHENVLDNN

GLREVCFKNITPGTVVAIRTSLSDNAKEAVLELRRGMGQFGYMMRSYSGNTTFDDTWDKS

NFRAVVSRLTLGDLNRVMYRVDQEERDDNFGFSTYHIPNHGNLCYSGLRGVMSVLGELRP

GNDLGHPLCNNLREGNWLMDYTSNRLKVVGNTLDLGEWFEGMFHHLKKIPRYLIPCYFDA

VVTGAYIVLREMALQQYSDFVKDGSTFVQALALGSTQFCGFVRSAKLPPLSPQLDPRARS

ELNIKTGEEEEAPVSMAAGLPHFASGVWRCWGRDTFIALRGMLLVTGRFDCARGMILGFA

>SG-PL_contig_8915_1_-1_1_216

PPATSSSLSATISSHTTTTSCQRTTISSHITTNSNQSATTSSWPTTTSSQPPPTPPVHLK

KIQSSIAAFFKK

>SG-PL_contig_8916_1_-3_105_350

MGRSHEWTHLLGTNRFSTLINHTVVSRHLSNMDVDRHFAVIFALFPAINGQVEVICGIGN

RIFNLTQCNSMPLAIFRSLTKL

>SG-PL_contig_8917_1_+3_822_1160

MAQKKPIKTYPARWLFGETARTMGIRIMEHLSTTNRASLTLHIHRLRDLEKYQNFAKGEY

PFHEKDQGSLRKSAFVDLCTEKHGEFVPTPCLASILTMPNQCKPTPAGPPGRP

>SG-PL_contig_8918_1_+3_3_290

KKRRRRRRRRRRKRGGGGGRRRRRRKRRKKMEEKKKKKRRRRRRRRRNPKQQQQQKTGGE

GEDSDHTKKYNQIQVIIFVRIFGKIVNKSHFTYTYK

>SG-PL_contig_8919_1_-1_1_276

ASTSSCTAPSAASSARCLAPFSVSTGWSSPAPGPCLCRSARTAWPQLSVFGLHPTLTATS

ILLMTLPRRRHLYDPPLPAVPLHVALTKWTCE

>SG-PL_contig_8920_1_-1_190_306

MLSKGHRATALFCIDVIDIYMHCDLHCLSQNVKYTQRNL

>SG-PL_contig_8921_1_+1_1_174

FFFLVITFLLQEPKKVATGTAGSCLVIPSAQYHHRSFSLYIHNSLEPNPCDKKTNRSS

>SG-PL_contig_8922_1_-3_480_851

MQQLVVAVVVLVVDGFFVCLFGWLVGWLVGFVSFFGSDDECDDGDVVIVASRSSVVLNIR

FCVSAENVVYFVVCVCVCVCVCSSSKNIALRNISSLVKCKLPLEPCFYLTLVFDTLSSYC

VASL

>SG-PL_contig_8923_1_+1_106_381

MFSWLRVCTLFSTHCAGAMLPHSLHTPSLVGVHAGYFCASISHQTLAWLTRPLICLFDVL

HVYTYGGVQALAGLGVQKSLHHLSTRHSTQQS

>SG-PL_contig_8924_1_-1_1_147

LTVQHHWLRLFNIRIVLVVLYKCLLLMNLNVHSLFFFFLLLFLRGFNLP

>SG-PL_contig_8925_1_-2_920_1258

MQRTIQAHQQQTSSEKISTSARLMQKHYHDQHAKDLPVPSGQLRSGQSSKWQEAKVIAKQ

AEPKSMATPTGQELRRNRVHLRDAAVPEPTTATWPSVPPASDERLRASVFVLC

>SG-PL_contig_8926_1_-2_8_1192

MASTLRLSGLLLQKLGLTSSAARTFSTPRVVSCQQRYAFHTGCSSQTPVQVTEPVSHPEG

HNKVTLVPGDGVGPELMSSVRDVFSAVGVPVDFEEIFISEVQPAQSASLESVVDSFKKNK

VGLKGIITTPSTFKGGVLQTLNMKIRRELDLFANVVWIRSLPGFHTRHNNLDFVIIREQM

EGEYSALEHESVGGVIESLKIVTRGNSMRIAKFAFDYAMRHNREKVTAVHKANIMKLGDG

LFLQCCEEVSRMYPKIKYENMIIDNCCMQLVSNPHQFDVMVMPNLYGNIVDNLAAGLVGG

AGVVPGESYSHDVAIFEQGARHAFAEAVGKNIANPTATLLSACNMLKHIHLEYHALLIQD

AVHRVIKAGKVKTKDLGGHDTTQDFTHAVISSLQQ

>SG-PL_contig_8927_1_-1_1216_1602

MHACMYFMDACAYIHHYDLFIGRHLHARARTHTHILKYVYKAREAMTAPHEIFVYKSGNT

VSRDTCICYVLLLLSLKSCKGLFYENLCYNSVSLWSLNIFKDLCHYICAIFCNECGIHDT

CVYLLKRWR

>SG-PL_contig_8928_1_+2_2_157

HPCMIQTHNFVRISLFIPISCTNLKQKTFAQLVYTSFYHASFQFSVSIQLKQ

>SG-PL_contig_8929_1_-3_3_122

FTLSPHNQLRIQALQGFTVISEKQCHHHEKSVETHSTENR

>SG-PL_contig_8930_1_-3_528_689

MASMTIMSKKEECNIKHVLAMIQSSPTLVIFAVHKTVGIQVDSGYSTECIAATL

>SG-PL_contig_8931_1_-3_3_362

MEMLLIKQLNGGEKKKTDGGPLSFFFKICPSSGVNLKAKLEALPFHTFKQHRGGGYNWVQ

NFQTQIHWIHVQGTVVVYCSACYCHVLAFNHQALAVLHRMRRKSFLVIYFFLIVSWSSAF

>SG-PL_contig_8932_1_-1_1_111

KKKRRKRIDREPVTLHINCASSCHWNEHNYFSWDINK

>SG-PL_contig_8933_1_-1_1126_1332

MSVFGGLWKHENTQHALTARMSCKTDPVGPNATILEKKDISLLLNKAQVLYCSVHRFLSL

TLVDMIDDQ

>SG-PL_contig_8934_1_+2_227_436

MEQKNSPKPKNQPAPHLLHCFSNNALSLLISFLQHTLKSQNHLTAFHRSPQLERSSHIRP

PTRRDPACCA

>SG-PL_contig_8935_1_-2_572_673

MYKRCTAQNFKRFSLEISLVLTFCTLHDWMCFVL

>SG-PL_contig_8936_1_-3_300_383

MEPDYIKFSLTGEVSTIHLTDLSWWTAC

>SG-PL_contig_8937_1_-2_563_646

MEPDYIKFSLTGEVSTIHLTHLSWWTAC

>SG-PL_contig_8938_1_-2_1577_1963

MAIPVSLPCFPDDISEQYLAYKATLRQRQRAEKFSHGSYIDKEYLFKDKDCEVYDVSAVC

YRSQQKTSNPHRVITIDALIKTATNAFCLCKAGQVYGDIVTGPLINLLKCCVGPSIRPSV

LWAALCSVM

>SG-PL_contig_8939_1_-2_32_205

MPSIKPSVLWAALCSVLGKSENEALGQLPGQDQQCVELACAYSCPALFHWHTFCSPVM

>SG-PL_contig_8940_1_-1_664_900

MNLFFFYSMHHFIFPCISSFSKSFVFNSLFLFFKTHLCYIVYLNVYPECVCMCCHYHHCL

GWYGANIKWGTSSCINSEG

>SG-PL_contig_8941_1_+1_1_84

THNTHTRRDLTDNRKSGRFGGLFIESVD

>SG-PL_contig_8942_1_-3_3_182

CVCVCVCVCVCVCVCEQVCVCVWKSTLFLDKSAKPCVFSYFRFCVKEVAAPNARTSHANI

>SG-PL_contig_8943_1_+1_109_246

MYTKTVIEICILNLREYQVLDPYRTKSFSYYLTTIVVSNFSFIFIT

>SG-PL_contig_8944_1_-2_215_526

MKLELSLLQQPFIRKPPRELSWRLRTGAQRIPSLTFTSRISELLDLTVPKVFPSSRLILL

FSPDSPTRTLEATPPPIFEFCSQSDMVQYMKPKGVFFSKYMFGL

>SG-PL_contig_8945_1_-3_3_134

RHQQGPLSFSAGVYRLNSHTSTRVELLFLPYVRPLCNLSVETLR

>SG-PL_contig_8946_1_-2_2_646

PAEDSVPIELPAEDSVPIEQPAEDSVPVEQSEDSVPFEQSPEQLLTSEKESTKQLAVVEQ

TAEKTASLDEHSSEDPASVENQPTGQSAPMEGQSVENTLPAKERSAEESAPVKHCADEPT

REDEQHLGQPAPTKEQPSEQSSQYASHVVEQSDKELPQAEEQYTEHSVYIEDQAAEQSDQ

SAPVEEQAAEESPSAKQTPTEEQPTQTQELQTPAE

>SG-PL_contig_8947_1_-1_52_879

MDLCRPLFNTGRVVYHDNFFTSPSLAVALSKEKLGSCGTVRHNRTGMPGEIKGNTLKLER

GDDPKFFGHSESGLLVSSWQDTKRVNVMSSVHSNTCQQRRIRSRREPTGYRQVNKPSMCH

DYNRYMGGVDRMDQHMSYYQFRHRLIKHYMVVYHFIKEVALHNGWVMARQQLGVSSKRFR

ETVATVFLDEYKVSDKFVPPKRLASIPSDLKRLSGVHLPGRTKKVGDCVVCSDRSRGARK

TSVFVCRTCGPDIHLCVGDVDQVDCFGKYHTLRVYK

>SG-PL_contig_8948_1_-1_247_531

MGPVRDATKKSRDHGCCVFWCLKTKQKSRVAVGKKTCHSDTSVPPRRTYEANELEALGGD

PKASTISAQVVVQQPIPDRPKALYPILPPPEERRI

>SG-PL_contig_8949_1_-3_6_566

MATSGSLLLRSLGRRLAVCHRSFKDKTFTARWTEWNRLPAACSLARFQHSDSFGSSTTTG

AVATAAEAEKTPVVIDNDLVVKLLKEMEDNKPVSGMPDPYQKEHKKCILCQHNLDVNYKN

VRLLSQFVSPYTGRIYGRQITGLCIPMQKQVATAIKQSRIAGYMPYMHKNSEYMKDPRLF

DPFRPAK

>SG-PL_contig_8950_1_-1_1_279

VKRTPWWNKTVKEATAEKKKLYKTWLQSKLEEDYIKYRLARRHCKRVVREAKEQSWKKYG

EELSELCRHSPREFYRSIKAMRLRDDPYDPTSI

>SG-PL_contig_8951_1_-1_145_768

MVHKKINLADEMLAWEHERFSIKRLLAFTLSRFNTTRALDRTSILDTQTKVDDEKLSTNI

RVIGEALARHVYNISSQGAFSLFTDAMDTQEDAETAWLQFLTAQPRAAQLLPPESVVLGT

LENTLSRYLKDVRRTTFKADKRDPEFVFYTGAVYAMNAFNVKPAVFDLFLALGIGAYLAV

IYVCATNFHRVYGRLRRSMASSQKEKSN

>SG-PL_contig_8952_1_+3_3_194

WVEMNKDYVGKEKTTRVLVSTFWGASKWQYLSCQHHQMSHDCIIPSTCVWPVQECITDQD

TCIW

>SG-PL_contig_8953_1_-3_351_662

MSLTTACVFLLACLAVSVRSYSLRLFDMPPCPADADIKLDNCMTTLERITRLNLLPENCG

AAMEALRCSTMLMRACMDSEDVLTVLSKHDLSALFRAKPICTSA

>SG-PL_contig_8954_1_+2_2057_2353

MFDQVNCTAYLNYPIKTPYPPNVHVHTIHQMKLIYIYIPSDSPHLTMYLPKFNFVPYLNP

CSTQLLYVEWQHQFRKPCNWDSAYCPCCCCPCCCILPHP

>SG-PL_contig_8955_1_-2_602_856

MATRCSRSKVKKEQCQSQRRNIVKTNLSLNTQSNNIIKANTSNTETHKETQHIPTPKQYS

YSSKGNKTGTRNTKYCESFLDLWVT

>SG-PL_contig_8956_1_-1_1_108

RERESTCTCESKTVQEITLDCLMQRFIPDLDTHMTE

>SG-PL_contig_8957_1_+2_341_469

MKDFAYIVVIIFTPSVLRLCVTIFSIHIHTLVAHAFTQKCDKT

>SG-PL_contig_8958_1_+2_17_214

MATEITQSLSQEDNGGEESEANDIESEQDYVDERSHCHRHTVVWSRCDLLQLDTAAYTNP

FILKPV

>SG-PL_contig_8959_1_+3_588_866

MKFWVERFVPDKTCQMKLAFQLISRLFLKLGRMRKGINVWMEFITKNKNCNATKALTQAT

SVHGCYGYYFGTLTFLSDLSLFIVYCISMFIPD

>SG-PL_contig_8960_1_-3_240_698

MASSPSNRVVVIAVDNSEYSDKAFEFYASHFHKPDNRVVVVNTPEFILSPNENSTQKIEE

AIMESNAKTEVIKNKFVNLMSEAGMTGEFVALEDKNPGHGIVRYADMVGASFIVTGSRGL

SKFRRTLLGSVSDYILHHSHVPVLVCRHKDLKH

>SG-PL_contig_8961_1_-2_119_337

MLVKSDQELSMDQCQHSEAKKSTLEYDHFLLCLLSVPVWQCAGSTVSWLMRQCSMHLEAT

FIVNVIKLVTQII

>SG-PL_contig_8962_1_-1_79_3189

MATVQITSAAGVLSLLDEPEPQLKMFALQKLDRIVGVFWAEISESIDKIEVLFEDQQFKS

KQLAALVASKVYYHLGAYEESLTYALGAGDLFNVNDNSEFVQTIISKCIDHYTRLRVENA

ELSEDEQKPVDLRLEAVVNRMFQRCLDDKQFKQALGIALETRRIDIFEKAVKMADDIPTM

LTYAFKVCMSLIQHRQFRNTILKVLTNLYMGLHVPDYINVCQCYIFLDQPSAVADILEKL

LKGDEDSVLMAYQIGFDLYESATQQFLQCVMAALRAVAPIPALLPDQESKPIPSTPVQES

SGADDKKEKEMPMDTAEEVAVETPPAAEAATPKPAGTAVPATVAKDLSSLSDTDKALQER

LSRLLTILGGETTIFIHLQFLIKSNKTDMLILKNTKDAVRNSVCHSATVIANAFMHYGTT

CDQFLRDNLEWLSRATNWAKFTATASLGCIHNGHEKEALNLMSSYLPKDTTAGSAYSEGG

GLYALGLIHANHGGEITEYLLGQLKDASTDMVRHGGCLGLGLAAMGTARQDIYEQLKFNL

YQDDAVTGEAAGLAMGLVMLGTKSAAAIEDMVAYAQDTQHEKILRGLAVGISLTMYGRME

EADALVESLTRDKDPILRWAGMYTLAMAYCGSGNNQAIRRLLHVAVSDVNDDVRRVAVTS

LGFLLFRTPEQCPSVVSLLSESYNPHVRFGAAMALGIACAGTGLKEAIALLEPMTNDPVN

YVRQGALIASALILVQHNEVSCPKVTHFRQLYAKVISDKHEDVMAKFGAILAQGIIDAGG

RNCTVSLQSRTGHTNMDAVVGLLVFCQFWYWYPLTHFLSLAFSPSCLVGLNADLKMPKVE

FKSAGRPSLFAYPPPLEEKKNLTKEKVETAVLSITAKHRRKEAEKKKEEKMEVDEEKKDE

GQEKDKKATAVTTPTTTTTATPTTSTSAAATPATSTTVEEGSKKEGEEEKEKKEPEPSFE

LLNNPARVMKNQLRVLSPREGSKYRPVKELSHGGIIMLKNVNPDEEEDLVEFVQAGGPKT

EEEEEPEPPEPFEWTED

>SG-PL_contig_8963_1_+1_55_210

MKHASPAQFALKTNTMQLYLLEILGPTQIIYLNAAIGVPTTKSSALHCVMKR

>SG-PL_contig_8964_1_-1_31_1176

MADRAVILQQFSAVTGVDEDRARFHLESAAWNLEVAMANFYEEEHDVDEGSAANPLDLDE

MEEEVDQPEPEPQRIQKKKTTKSKSKNSRFGTIDSLRADEDSSSEEEGQAFYAGGSERSG

QQVLGPKKKNPDEMVTDLFQAAREHGAEEVDISAATASGGRAFAFHGMGYRLGETEGPLE

SVAGPSIAPSRKQVDMTLKMWKKGFSIDSGELRPYDDPVNKDFLDCIRRGEVPQELIRQA

KGGEVNLSMEDHRTEEFVKAKANVKAFSGEGHMLGSPAPAVVSTASSGASSAAAPTPKVD

IDSSKPVTTLQIRLADGQRMQAKFNLSHTVGDIRSHIVASHGQYAGTPFVLMTTFPNKEL

TEESQTLDEAKLANAVIVQRLK

>SG-PL_contig_8965_1_-2_353_634

MYVQMALLVKPLSVVCCILFIRFVFCHILSVLFRWGFFCFPSQNIFTFKPFMIFMQRRCF

MQHFHVYSISTDLREPRCDCGATLICIFYMTDRL

>SG-PL_contig_8966_1_-2_365_610

MQLMRLLPMDLCLLFLPPKYLSLSVIPRHITHQHCCSPFCLVFDFAANVGIFTSRGVRLS

VFCSSTAGSFTVSTAGRFSEFG

>SG-PL_contig_8967_1_+1_1_177

KKKSKSTVRPHFHSQMITIPNVSNPLLWAEPIIKLKPKLMGGHVHKFLFYIMSIHEHFS

>SG-PL_contig_8968_1_+2_2_88

IKTPTSDSTAFPFMFLMQADNISLTSIVQ

>SG-PL_contig_8969_1_+1_1_135

LYSTTTLLTTADVQVSMRSLIFILEHLWIYSTTYLLQQICADRAV

>SG-PL_contig_8970_1_-1_523_819

MYGLLCFCIACLVILFVSKNTGASLCDHQHSFSSQLHVIFVHNHSRWDEISLAFPSLFPA

PCTLFTFFFFHFSVMGFAHDAFCVPETCKITFRGPEVCG

>SG-PL_contig_8971_1_-1_1_174

FKPRPSRTLQFGSQVSSRSAGHMENSHVFYTPTHRLHYDWSCSLADRPGLLFMTCNIM

>SG-PL_contig_8972_1_-2_2_163

DVLRSLGFKDCCSMLLLAPLPCCELTPPRICIYDALADSMTPGIMIPGLYLPAV

>SG-PL_contig_8973_1_+1_1_237

GGGGGGGGRGEKKRGRGGGGGGGGERERERERERERERESNNFKPLTEHNHYDKKETKQK

FHFEITVYKMKEIDLNKCI

>SG-PL_contig_8974_1_+2_2_361

FFFTFQSFYYLFSTYGWGRQLLSHKHISLHYSQSHFKQSFISIACYCRVFILHRSWESFH

NFHFIQRAVVSVCLHHSHPLYHRHSRRHTTKNAVLAIQPLCRGQCDEELTAIGVGSTIGH

>SG-PL_contig_8975_1_-3_1404_1646

MLSFPKTKLLLQSISFSSNAITRNQILQYYCYPHIDKIKSGVLPVLQLIFLLILIPQVLF

QTFLSTKYLFTVSQAHYLMLT

>SG-PL_contig_8976_1_-3_276_1418

MLLRDKSDKRVAGETLTARSDKLLTASWSGTRGTADTRRRGERRRHSRDSTASPRQLSGS

TPSKKKFKNRMSHSRNPKPLPGSRRFSSPGITMSTNISRGKSFELGQKCDSHVVTHNDHE

QTSTDAGRSLHSGQPVSESDKPLESSRARFESESAITADKTECSPCASQVPKSRDPDSET

ATLPSDSTQNRDNGEGLQQTTRTPPPSSTGLTSQQQDNTSSDGRVEKKTATTITTTTQDK

TPIPSDPINQWTTCRALDDSGDDDDTDDDNNNDDNFHEGQAHSESWTQEAHRAGPDSSSV

DGIFTRATSSSHLARRKRPLLLPPIMLPPIYTVKPTPLKLRDEMYFASGEQRARRPISDE

EWDKLRDCRYLRIRTLRTIKV

>SG-PL_contig_8977_1_-2_2_166

ETTAAALGWHHPPTNVEDASMQWFLLLVLVLILLLRLLLCLLGLGYCLTSYLVSY

>SG-PL_contig_8978_1_+2_2_142

NFLLLDTLEKGLYIYVLPNTYITLIKAVSINRFIKLTTCNVLKIYLS

>SG-PL_contig_8979_1_+3_3_119

THTHSTKEYAYVHMISKVIDRTHTNATLQKKSGMLKETY

>SG-PL_contig_8980_1_+3_3_227

KNEKVRRERERAGKKGKGKKKTQNRKKGGGGTNKTKQKVTQPSDAKNEYSLSAHAHTYSI

LPRHKHNTHNNEHRQ

>SG-PL_contig_8981_1_+1_211_363

MLFASICNSLLSLAQTDGIEKTEVCFTLEFSAPNVFKQKGLHLLLAIIPTF

>SG-PL_contig_8982_1_+2_662_946

MIWFPCHEISPCPLVNGLKGRLLIKPFCIPMLHTRPAPPLFFCIIRSDFHPPPSPRPLLL

FRKWLMNRCYNPFIPSAYPPVSSRKLVNRLRKSEG

>SG-PL_contig_8983_1_-2_29_253

MRAPLMLQNAELQAAWLTCRRAEIPPPGPDTMTASAAFTIGKLIRPAQSKFPQNLYQEDT

LPLAEVFWEDHLLLA

>SG-PL_contig_8984_1_-3_456_590

MVCLICRQLCNTTAFLCYSWMYRKIVCVCVCVCVCAGMRGGRVPS

>SG-PL_contig_8985_1_+1_295_435

MTMVSWKISSVEDSDCFMLFYNHILKEVIAPYCWLLKLVLNYLKILL

>SG-PL_contig_8986_1_-3_171_413

MKLSSVKNQRFGHSKVVDIYVCFRTRACEKKIAQSIIALYYDSCSQLIKGCLFFYFLSDI

ANRLLMLHFKMVKCSRVARLM

>SG-PL_contig_8987_1_+3_3_206

VCTCANLSNSASTGFLIRVLGIFTNQCVHVCQFIQFCLHWFPNLSTWDFYYSVCARVPIY

LILPSLVS

>SG-PL_contig_8988_1_-3_3_293

TFSDKPSELLFKTFRFSSVCGVSVCMCVCAHACVCVCVCVCVRVWCMCVCVCVEEVKLVF

HSIYYIIIICIVLSYTLYELLCSDVNILVFIHCCKAL

>SG-PL_contig_8989_1_-2_53_466

MFFFLCFCQYVDIVVPFRGKFCLHHPYLCIVFLHLISTLLSPYHSLMVQEELLPFSLTAR

VRVRLEKNLCMRPSVYPPVNGHLSLFRFGQGKSVRERRWVRPRNAGLEKSCGLYHLPPLR

LQDYGATFIVFIPLLLLF

>SG-PL_contig_8990_1_-3_249_899

MVGQLLISGDQLEPFEISYGVKRGCVLAPVLFSLFFTCVLRQAVQQLKVGVYISYRYDGS

IFNLRRLSAKTKTLSRLIQEAFFAGDCALVAHRPGGLRVMLAKFSGASRQFGLTVSLERA

GVLFQPAPGLGAPQPHIAVDDVELRDVDWEVLSPGMALWMVISRPKLAKRAGLWDDCTAG

CWAIVVLPWPRALGCAVLSSSRRFCVAVEHGRSAGGT

>SG-PL_contig_8991_1_-2_17_1105

MNEVEVFVGNHTIIDPEVYGLWLRGYSVNEAASMLQKQESLQTFQATMDDLLSDVQDNFR

LFSGLENMLKSPPRLQNQQIYQIDEDTQSMLIEKYYSFDSVVVREILGKKLAARTRKDLD

DISEQTAVPLRSCRRQFDNIKRVFKTVEELKGYLVENIRTHFLLSETLARDYAAITFIAN

NRFETGKKKLGYLNFRDFAVCATHMITNWSYSSVECKNHEDMDVDLDRNFLLLLRELKVL

VEKECLEEHRGLMLKTAQGFSDQVRIQLGDNFRTLSKTIINIAYGLNHNRESKDIFIDLY

EKLIEPCLLNQWACADVQMVLSAYKETASQMDFFVRTSPHLQTVWERYMGTLTACVIRMY

PRT

>SG-PL_contig_8992_1_-2_827_1018

MLRSSARCTGHIPMLHFSALQKQHQSRRDTYGDTILDRCHSIHLGHGSPTSLPLSTLYVK

TRGA

>SG-PL_contig_8993_1_+2_74_196

MISLLYFFSSGTSHRNKMNKLSHLITVILSHLITGISNPHT

>SG-PL_contig_8994_1_-1_1_147

SLLYHFVFIQTACSSCHCVAPCLPPSSYLLLFHVWTINYGVFICYFSSV

>SG-PL_contig_8995_1_+3_57_254

MGHYNTGTNMKTKRYMHIYIGIEITFWLESGSKSSLSVVKLPPISLLLLLLFMISSATYS

AVILLS

>SG-PL_contig_8996_1_-2_182_217

MSVSEQVQERVI

>SG-PL_contig_8997_1_-3_495_611

MRLHSDQSDSPQFIIIIFIIMLWHHYMRLHNCWPSPLRH

>SG-PL_contig_8998_1_-2_2_352

MDLRDSRTNSPPTLHNGKVQYPRPHPNTDTDAESEGGPPKLERAVSPGADSECVDGKKDG

STTDVASPADTIEAAIEPMEGMEEEVPELSADGDKDSQAMDTDSNQAASGEDQKKDA

>SG-PL_contig_8999_1_-3_6_266

MYKSADHCMFLFLFCFVFSILQNGKKYTLSSHHSWRCVCTVSYCSVLFNLITPRESSNLN

PFAAEPYGNQVNSPFGHMLRMSSIFHN

>SG-PL_contig_9000_1_-1_106_243

MGGWTFWIATEISPATHLCFPIMPSSPFFRSSCNFHVFVCFKCYNM

>SG-PL_contig_9001_1_-2_2_157

VVLKADSAYIIFHIYICSSSGFCWGFFLFIFICLVLNLPARLAHLNLVCINT

>SG-PL_contig_9002_1_-3_306_422

MKIDPIRSDSPDCRWLDIFNAHNQISPRPVCSSLPYPVL

>SG-PL_contig_9003_1_-2_179_310

MAYARITLDYHFPSHGFGAYGIYSEHGNTVLRKLKTELKPATYA

>SG-PL_contig_9004_1_+1_1_267

SFPAVLSLVPTWSILPLCLRIFAKYVCVLGLCLCYWAVCVYERACVQACVCAHMFVCACV

CVCVCVCVRACVCVCVCVCVCMIIICRKL

>SG-PL_contig_9005_1_+1_283_417

MPTLNHQGSESLTTDLFRGLETSKNQHLTVCVKATFQGFVLAVLC

>SG-PL_contig_9006_1_+2_2_259

SLTDKPFGQSQKKKIQGNTRIQCVSILFRPCDVNVTLFSQFLHLIYLNAGIPFDSNCEFN

PFTRIPEKNSFTWIPFGPERQTGDRK

>SG-PL_contig_9007_1_-1_1_69

YFSFCSIVQIFDNGRAGIFLSIQ

>SG-PL_contig_9008_1_+3_3_188

MYMNICITNVCVSIHTNCKISNLRSYSGVMPLHSLHVLSSLRSVKIIPFPFSCLLGSITT

TV

>SG-PL_contig_9009_1_+1_919_1167

MPSSFLANQRRLWLLQQNLLLVSENGNVMSYFKSLSYSSLIALCEQALVVSCHEKKGMGE

VEVILVTDMSFPHVGQTFVSGQN

>SG-PL_contig_9010_1_-2_29_295

MASMDAIKKKMLSMKMDKENALDRAEQMEQKLRDTEDQKSKLEEDLHLLQKKYSNLENEF

DKVNEGLQEANAKLEASDKRLNELETDMA

>SG-PL_contig_9011_1_-3_48_173

MFCLHSVPTMHQDWLTVFCFESFHCCFMHAVMLVDLHFLCQL

>SG-PL_contig_9012_1_+1_1_180

SMLATFSYAWQQIFSSVWPGHYLHLPHLHQDLEKRFQNMGQSRCHYTCELLLQILLLIMS

>SG-PL_contig_9013_1_-1_241_414

MYTNCCLLVGYELINFCVFVDRHTYLIFFNERFMIMSKAFLKSTNTTFAFVYLLWRSH

>SG-PL_contig_9014_1_+2_182_334

MPARLFLGDHLVLCCGGSSTNANRTDCCTQLLIALPRHQFSHYAMWHHEEH

>SG-PL_contig_9015_1_-3_42_818

MVKHCEALKRVVQVINLDPAAEHFDYPVYADIRELIHIDDAMEDDALHFGPNGGLVFCME

YLIKNLDWLEEQLDEIEDDYILFDLPGQIELYTHIPVMRELVEKLQHWNFRLCSVFLLDA

QFLTEPSKFVSGMLTALSTMTNLAIPHINVMSKLDLLSKKDKKNLERYLDPDLQNLLAEE

FDDAKFGHRFKTLNSALAAMVEDYSLVKFLPLDLTDEDSINDVLIQADTAIQYGEDLEHR

EMREPEEPDDFEPDSGIDS

>SG-PL_contig_9016_1_+1_100_327

MPLAFKVYTNFSHPSLTLTKPSMSFTKLTKNMTLFFMNSSYHSFFSTLSASYWLPINSFV

HIIYILFVSISRRPTT

>SG-PL_contig_9017_1_-2_2_190

RLIQRILATNVVLMHMGVKNDNTCSFCRTEQDSLHHMFWRCTLIQLFWQLLQRSISNTSA

NTT

>SG-PL_contig_9018_1_-1_220_441

MTFSFTNYLPCHIILRNYVSGKFDWSAGILHSNKLDQTSVPCQVTELYLSLNIKEDLFLE

DLDKWGGGGGGGDH

>SG-PL_contig_9019_1_-2_95_532

MTDEIKDRFVGKWKLDRSENFDEFLCAMGLNFFIRKMAGLSKPENEIAVEDDGTIVIRKN

STFMKNEQRFKLDEEFEETNSYTKTKFRNKPTFENGKLRIAPTPVEPVDVTYPQYSEREI

TRDGELLMTIKVNDVLCKQWFKKVET

>SG-PL_contig_9020_1_+1_607_897

MMMSGILKKKEKKYMTIKTDATFKHFICNDDQYKYCFLNITETAQNTEEKLSSFFFPISI

YCMVFKYSIFLLASIMDKNSCKTGQFFMNVTLLATGQ

>SG-PL_contig_9021_1_+1_667_873

MKFFSLFPRNSYFFPKETWSVPVENNGSEENSHACKMKAATQYPSSAYKYQEINLIMSKY

FPHAQGTQL

>SG-PL_contig_9022_1_+1_1_162

LAALPSLTLKKVRYPFTAGWTGGCSHKVFSVRVNTMCSTSTPTRHLKLEVKFGL

>SG-PL_contig_9023_1_-2_26_406

MEEDGATSILQLMGIDQAILHSIEKCDGEDIKRQMYSTILLVGGGMSMEGAHTWLQYQVW

MNMPAQFRTLDTMDVITRPKDMEPSVVSWKGASILACLDSCQELWIHQQEYRQAAVRLLR

ERAPFEW

>SG-PL_contig_9024_1_-1_718_888

MLWRGHCFHAPVYLIHTFYVSVVMMQTMSGGHVDCRSYHFGLSRMCFYYWQQLSWTL

>SG-PL_contig_9025_1_-3_66_230

MKQGRVVYQSTFSHKLNLCGRGKFQPVSEPCYWEVKRLVHRVCGRYFAHLQLSYK

>SG-PL_contig_9026_1_-3_213_326

MKNNLAPVLTKLFCEITGYTPHRGTVPWDSRQHRSSSE

>SG-PL_contig_9027_1_+2_104_247

MKKKLGGLEVFNPQSFFQTKFKPVQTHAVRLEGGSSTSTPGRPSPFTG

>SG-PL_contig_9028_1_-2_2_166

FFFFPSLLSVRLTHHMNVVICLCPVVIDFWETFAVICIHVYISSTTISICHNFYI

>SG-PL_contig_9029_1_-1_805_1110

MRFYLLSYVEFDCSVLRFIHHSSCIVLHCCVLCMIVLYCGLVLSSILLKLFFVGCKELSS

VYCCPVFVKGLSSIYSPVLSLTNCPQSIVLCLRNYSESIILC

>SG-PL_contig_9030_1_-3_15_1643

MAMHVPRAPGFAQMLKDGAKHFSGLEEAVYKNIDACKELARTTRSAYGPHGQNKMVINHL

EKLFVTNDAATILRELEVQHPAAKMLVLAAQQQEQECGDGTNFVLIFGGALLENAEELLR

MGLSVTEVIEGYELALKQAHEILPKLVIGQVKNLHKKNEVKNAIRTAVMSKQYGNEDFLA

DLITNACVSVLPSTMHFSVDNVRVCKIVGCGLLQSNIIQGMVFKRQVEGDITKVEKAKMA

VFTCPLDVMQTETKGTVSIKNAEELMSFSKGEEDMMDQQIKGIKDAGVNVIVSGGKVGDL

AQHYCNKYKVMVIRLLSKWDVRRLCKSIGATALPKITTPRTEECGHCDTVYVDEIGETPV

VVFRQTKEESPISTVIIRGSTDNIMDDMERAVDDGVNTFKALTKDNRLVAGAGATEIELA

KQVTSFGESLAGLEQYAVKKFAESLEVIPRALAENTGMKATDILSKLYAAHQEGQQNAGV

DIDNLGSVMDAKEANLVDLFLNRYWGLKFATAAACTVLRVDQIIMAKPAGGPKAKQNKDW

DED

>SG-PL_contig_9031_1_-3_1299_1616

MLQVYLEISRSYTRCNLLCRERYVHKKCLGFITDLQRIDVLYHTNNRFIDTFGLFAQNLL

LIQLGKSSSAVLSSQGLHPPDTPIIFTHLVFSSVLTCIWRHACIQQ

>SG-PL_contig_9032_1_-1_1_264

PPKKKRKKLPFPYFFFGAPPPPSKVNGGGGPVELQHQLYICVCIFKVSWFMKGYLQILIY

PQPLPVPTPGPLVVAKPRAMFMTLNSDD

>SG-PL_contig_9033_1_-2_443_670

MPNFHGYVTSVDILTLVDIVTLVDILTLVNILAFVDNLTSVVILTLVDISILIDILTWCT

CLPSWLNLSYICLTLI

>SG-PL_contig_9034_1_+1_1_153

RGGEGGGGGRQRGGEREERETERETERKRQREIERQRDKQQQQQQQQQQNK

>SG-PL_contig_9035_1_+2_395_706

MYRQADRQTDGRTDGRTEGQTDRDGQRQTDNRPTDRLTDRDRQSDHKPTDRQTDRQTHRQ

TDVHIHRQSCPRERRMIRKKEIEAKTDTVCISSRNRQTNKQTDR

>SG-PL_contig_9036_1_-2_2_94

CVFRCNKCVCLNFIGVCNSVHLGAICVCLEV

>SG-PL_contig_9037_1_+2_440_697

MLSDFHEQIATLKTMSKCWKRWTLYENKDTEIQVFSKIKFFQKLAKYFIFCNFISPHYMI

WVLFVFLSHLIQSSFFRHLKIPHFKI

>SG-PL_contig_9038_1_+1_163_348

MVENSCLLAMYRLLVGVELLVTIMFKSFGFGKDMCHLSMTFYKDIKSMVVVNGQCSSWFK

TS

>SG-PL_contig_9039_1_-2_1130_1303

MLIVVTFIDRKMNSCCSNIMVALFRLSFVCLSQCLSLNHPSFLLSVLQSYDMVIMNMS

>SG-PL_contig_9040_1_+2_2_529

LLELLQYDSHGVRGVGDKVFAQSLGQSCQTVGSQGHQHDICVFPNHLQESTDKGAKSPVL

NLGGFRQLLKGFQGLTSLLPLLGFDFFKEIFGNLGKDIHSRHQFVRVHFFTRFLLPIIIT

ATTTCCSSCLFFHFGLEVLLGTHEATLRGFAPCDFFKLLFKLLHLNRFEVGHLRFE

>SG-PL_contig_9041_1_-1_130_2139

MQLLSPTMRMVNSVSCLLLVVAGLTFVGAASIFKREKRQTGQYRTRGEWASDNGFEPGTT

YASDTHSNYTITTGARQDQLCDVTIDYETDIEVSYQCAYDKLVLNAGRPDSITVCNQATG

QTYTTTVPKGETVELRFASDDEVELTGFNLTYRVLCHDYENDGGTEIPFVRKIDRQGSGE

FKSPGFPTYTYPPNFDRTYLAYTTGPRIHIKYFVDIEEEAQCLFDYLVIYAAERSLTLCG

DKQSGEMDITVKNNTLKLRFHTDRSANYGGFSLNFTVTGAGPSNQSPMMRNKASWQGWRP

GRSQTSFHNGIHQLISRDPNLGPLAHSPPVSYGHNSRPGHRYFWPGSAAISHPPEQIKKI

DVSIPSDENSETNNVDQIKNIKVFLPQDKQFPIDQIEKINVYLPSDETRKPEADHFPFLY

NWNQGIADNRFPYGQINDNHFFLPSDETQNPQASYFRFPYGWNQGFGDNPFFHDQIKNIK

VSVPRGKKLPAKPVPFPIDQIEKINVYLPPDENQNPGANYFPFPYDWNQGTADYRFPSGQ

NDRYDQNSRTAGNPFPYDQIENIKILLPTDENQGTANNPFPYDHIQDIRVLLPSGRNQNP

EANNFPSPYYQNLGTADNPLPYDQIENINVFLPTDENQATTDNPFPYDQIENVNVLLPTD

ENQASTDNPF

>SG-PL_contig_9042_1_-2_89_1447

MVRLKDLTTHELSVEQQFFYKEATEACVGPDETKRGEALNSLAQDPGLHQMLPRFVQFVA

EGVKINVVQNNLALLIYLMRMVKSILDNPNLYLEKYLHQLLPAVTTCVVSQQLCLRPESD

NHWALRDFAARLIATICKNFSTNSNHIQPRISKIFVEALTKKRRALATQYGALAGLGELG

PEVIKNCILPYLRGLGEDMRAATESPIVNSVDKTACENIKKQLTKYLPKELKNSGVPETL

EDYTSHYGYLGPLLLSCIQKEKEKEKQVVSATSATRPALQIHQPNRVTQQFVLQQTGKVS

TPITPSASFFSTTGSAQTPRTPSTPSSTGPTQKILLVTSQTPRSVSELSSVAVSNATSSS

TPTIVKLVSAASASGTGSSGTIQSTSGQQIVVIQSGNIKQETIQPATAGSTIVTPSPSLT

PVIVSTSQDMGVKSIFPNQTGTISFSLKKEEKS

>SG-PL_contig_9043_1_-2_38_193

MRRNQICEAADRMVMGSTLLFVMCTFYNMTLSPSASSCFHTSCMFLCFRIDH

>SG-PL_contig_9044_1_+2_2_295

YQNKQTKPLCLYLSRNTHIIYPAVYALRSAVSLCQLIPHTLLEPPKHQVQSRFLLTCVYE

CILNLHTLHSHTLNTNLQSFKDFWNIIYLFKDSRQLAN

>SG-PL_contig_9045_1_-1_1_291

GGVSTWKVVPSEKKHKALGKLCFGERKHLEGCASETKHLGSNIFANKSEGPEMVCCTKAV

VPCSEISMDIVALKLKHVHTVKCIDITQCLRAEAGFI

>SG-PL_contig_9046_1_-1_1_105

LKFQPVAVSSCWQLASVSVNTVQVFCFDNIACLKD

>SG-PL_contig_9047_1_+2_5_364

MPNKDAAGCTTGVRVLEWELRVVMLFRYAFCFWEATCFRRSSRFSFRRRLFCFSSSSTLA

CSSSICAFFRSRAVWAATRFFIFLLNSFSSAERCVKRLLFLGSQPSMQARLLSFARRSTS

>SG-PL_contig_9048_1_+3_102_368

MVMVKLVLKFFIFHSKTLCSPTYPPQNHMFSEYSKSYYIRPVHSKFRPTLLLKFLKNSIQ

TVKRSLQSSKPIIIIIIILLYCLQPSQPQ

>SG-PL_contig_9049_1_+1_874_1248

MELWLLFICDTGDLNVLHVVRCLGDISDFGIIVRSVLFLVGVSGHGMLDTILYLTDVSHL

AMLQVPIFIIFVPSVRVIPPPDALQRSAVFVVMTLTALAMVHAAIDSVGVVVDGGLAAED

TGDGV

>SG-PL_contig_9050_1_+1_70_303

MTTSNVVTMYKDGGESPRVSTGGSPVGVARAIIFSSQRFSSSKSGSIGSTIGSMPRDIPR

ATSLKTTTPGLGVKIHKE

>SG-PL_contig_9051_1_+3_225_332

MAHLTLSVSLKKEKENLLPQNHTQRITLFKTLSLPH

>SG-PL_contig_9052_1_+1_94_294

MQTDESYSRIMTTKINTDTTKRTAAENTTQHQSKQFCCNLLQAHTQHIQHKCTPMAFDQK

HICTCTI

>SG-PL_contig_9053_1_-2_44_475

MFYGGYADCVRCFYCGVGLKNWVVTDNVWTEHVRWRPECGYLRAIKGDDFIRNIQRRLGI

ESPGSEDNQQMAQGAGPASPNSQNCLSQNTTNTTASTSTSARVTQPTSRTTTTSTSSPNS

SAGIATATTSSTSSSLFSSSRLAP

>SG-PL_contig_9054_1_+2_2_232

CVLESFQQHNFNSPLGLKEANQSMPHGQLKMHKVCMLTFSFRCAVLSPWHSTHCMLDCVD

KTLIIFDIYRNLFLSTL

>SG-PL_contig_9055_1_-2_35_220

MDSSDPCSLASLLQAGWTGTRVVVSYVMQLVASVVEESMRSGMKSLPTSIYLFIYLFIYL

FT

>SG-PL_contig_9056_1_+1_532_702

MHLRRTELPISRTRNGMYVVHCLEERVCVCVCVCVCVCVCARARARMNLELHVCACA

>SG-PL_contig_9057_1_+1_1_297

ALHFTEHTYEHPPHTHPHTHTYTHTHPHTHTHARTHAHSYRHIETHITYTNAKIPTTKKK

HQKNIFSHSQSHEYSTESRVESRQEKEVRLIVLSEEMSF

>SG-PL_contig_9058_1_-3_195_335

MVLLHTSTELFVVVLFCYFVHSYILIPSSHTFLFSPKKYDILQQKSS

>SG-PL_contig_9059_1_-1_667_954

MYNLQQLNQGYQQVFSVACRLCLILPCAILSHFHRQQSCCFTYKLPASSQGHLQACKAIT

VLFGIFHYNFHLSLLLGQSCAFYRPHHCNVYVYDVY

>SG-PL_contig_9060_1_-1_358_549

MSIRIIRSHAAKCIYKKVFLFPLVGCLYVCCYFLSFLFSIYIFDLVFVFKKIYTPMYFCW

DFAS

>SG-PL_contig_9061_1_+1_76_429

MKKSFSFLSAKQIHVYNQFFCLFSKYIKNVLNDKVNTGKTEKDKHVPFFSLYIKNISDDK

KQSQIKQKRTNMFNASVNVILLQRIEEEGSGNHQACWHFFLTSFPDIFKHQFATVDVH

>SG-PL_contig_9062_1_-3_150_491

MIIKNLKKYFTFEVQVLDDKNVRRRFRASNYQSTTRVKPFICTMPMRLDDGWNQIQFNLS

DFTRRAYGTNYIETLRVQIHANCRIRRVYFSDRLYSEDELPAEFKLYLPVQNKK

>SG-PL_contig_9063_1_-3_15_371

MKTSNTLPPAQHEQLSQLWDRVTYLGQVRENKLTDALKLAEEFQEVVTIMREFLPEAEAQ

LKLRSLPDDEAVIVQMIERHEKFEEELKNHQEHVDKIKSLAEEILSSCHPNAVRFVKYY

>SG-PL_contig_9064_1_-2_2_367

LDWSGLLAYASPPDPSEGNQEGETGEGHLVSLCPEWPAQPWYPDLLSLATFHRWNFLLAQ

TFYFSQGQASATQTRTCSTFTPGDSAGLGASTCPNPYPFFSPGLGRPFLGLRWRFRRPPF

LG

>SG-PL_contig_9065_1_-2_17_343

MFVLNVGEIFSCSRNSYINDYVDNVQEMWTISVSDFGFLIIYSGKETSDKPYSVCMLVPR

NHLGKPLHVCFIKELTNNYFTGGVDICHVSKIYMPRALMNTRLSIVISF

>SG-PL_contig_9066_1_+1_10_216

MHMHTCTHENTQTHTHTHTRHTHSHMHARAHTCTLMHIQKHAHTHTRACTHKPAYTHTHT

HTHTHTHNK

>SG-PL_contig_9067_1_-3_3_179

KKNIKKKKKKIEKKEKKKKKKKKKKKSYKMLLKLLEKKKSALSVNGWQYAYFIPISVCS

>SG-PL_contig_9068_1_-3_3_164

YHGLDLIIDTKQNMGCLRLLWTVKTRCQRQGTIHVLFVRTVHFVVFNQYLQDLS

>SG-PL_contig_9069_1_-3_306_443

MRNWGQSEAVSFKNKVGEREREREDGQAILKISLFHTNTNAHIPNI

>SG-PL_contig_9070_1_-3_228_338

MYVLLKVFLDSQTGLQNTSDTILTIYVVMWIIMNNTS

>SG-PL_contig_9071_1_+2_2_199

FWILFHQTAFAVLVHRELSKHVDSVTEKTESKIETTSQAFTFTLMMTNTCKQFFGCVTFK

EGQMLN

>SG-PL_contig_9072_1_-1_1429_1731

MAFVSVKNYSRQMIQISQYECCVCVCVCVCECVCVCVCVCVRMCVCVHARPLSLSLSLSL

SLSLSLSLSLSLFSLCIVVTDLHSIDCLRQLKPSKHILILK

>SG-PL_contig_9073_1_-2_2_259

TTLNKVISAADSASQVSRDSSREETSSTDKPEQYKERSIDHPVVKYSRETDSSLTKARQE

GRQNLFSVYPDMRRVIMEEDLEEEDE

>SG-PL_contig_9074_1_-3_474_2438

MVTMFLWLFSTTLIVASLAAPTPKCPARCISCMNGVAECSKRSLVTPPKDFPSGIRKIIM

DGNHLRVLGARSFQSMKALEVLRMKGNKVRVVKKAAFKKLPNLMTLDLSNNQLVKIYSRG

FEGLKQLQTLSLQYNKLRSMARIFDATPKLYQLNLAFNRLRSIGKNDLKTPTRVHYLDLR

NNRITKIHPEAFVHLRLLRYLFLNKNPLVTVPDMTFGSQVLQLVDFSNCRLTHVPKTMPQ

SVSDFRISDNKIMQINDTDFTNMTNLRLLALNNNELHFVANGAFTKLNTLDEIWLRNNKL

VYIPRGLPDNLRKLYMDSNKIREIEDGLFTNASRLDYLTVENNRINRINNKTFMGLKFLR

SLNFQGNRLRVIEGGTFDSLGNLSTLSMSNNPLEKVEDNAFVNLENLTFLHMDMCGERMT

LEQNFLNEMPKLKQLSMMNSPGLGKAFVDMVATTPISPLKQLTEIDLTYNDLYTLSPNIR

EVFPALNALALDGNSWICDRRLVWLREWMMHSDVSFSKYEEVICEHPYSLKGRTIRDVNE

EDFVEVAPTTEAPSMDDYNKMMADKQAGNSTSSNALPSPAAMHDNSVMIGAKLPPDASML

SSKEDKKDKKLRSKKARKQRKIKGKGRKKRGKKGKGRKRRNRRTKKSTAADPTSG

>SG-PL_contig_9075_1_-2_143_787

MAELRAGGKGPRFDSSQRGEGVQETTAGSGCPSGNPEPVLRYQNDSPFHVEDQEAAVKMR

QGLESLMCLDRSHIEDPALRQASWYQAGIPREIALEILQQEEVGSFIVRDSTTHPGCFAL

SVRVPKFENSAGISHYLILKTQRGVKLKGLDKEWPNLTALVTHHTVMPEMLPCTLRLPRN

NKNLTFREAEREDPEEDSTYQRLTDVTAMAGQLKL

>SG-PL_contig_9076_1_-3_3_218

QIVQQHNTFESGPDIQNSVHASEMTLLRMQMKTEHVYTYNVSKLKMDCTDTLLLFFFKSF

LQKLSQPVVYLG

>SG-PL_contig_9077_1_+3_3_230

FFSLFLFCFCPFQILFSWVRVCMMFGSNCAGVTLLQSAYSIFSVCACRVILCFHKPLNAD

MADRIFNVRILCSVYT

>SG-PL_contig_9078_1_+1_253_528

MQGHFYSIFTPVSTNSSDTMSFYTTIYTTLSTTMESPVVMFQFFKTVCPRWEYVQMYKRC

QCRVLGLDPEDSWQMLLNKMPSTCPQSRNSHP

>SG-PL_contig_9079_1_-1_1_279

QVFPPRSGLPVLGLVHHAKCSVTAECRVLLALYVALSATCSFLIHQPSVRIDCLYRNFDT

VTSTFIKHQQIRSEVRRDISVLRVWTDDSKLCV

>SG-PL_contig_9080_1_-1_145_261

MSLRYLAVGIHNNCFGHSGLFLLYRTCSDVNRVIILKDR

>SG-PL_contig_9081_1_+2_68_214

MKAQERLLPALRANLSSYSSLGNSPKAFTIFLYQKLYPSSECTPKLYGL

>SG-PL_contig_9082_1_+3_195_455

MPTEVSACHGLGFLGRGTLSGHFGCSLFLGALQTCLRSIFSVLALYDDKTSAWWRRVWIA

DRLCSGGWWDLRFKCWSYVWVSCIGMS

>SG-PL_contig_9083_1_+3_147_359

MLGIVQLVTHNPQNISMLMTISRTRSHTMLSKSFTYTVTKIIYSHNVTTFFTHTSPSNLY

THMLPDMNILP

>SG-PL_contig_9084_1_-1_1_132

RWRSIRGLQVRAGFSLLYITLDKESVTAEQFAAQSTFLNAEYAR

>SG-PL_contig_9085_1_+1_10_225

MCAPHPRRPHTHGRSCRTCGHLPDELVELVHVIISNLVHDESHCCRWLGQSLQVADSTAD

IATISKVIVKSL

>SG-PL_contig_9086_1_-1_1_267

FKDLTITLEIVAKSAVLSATWRDWPSHLQQWLSSWTRLEMITWTSSTSSSGRCPHVQQLL

PCVCGRRGCGAHMDQTDGQYTTHSHHPPC

>SG-PL_contig_9087_1_+2_2_106

MIHTHVVKEPNCFSTCVQCFTASFVAPHIVQLLYN

>SG-PL_contig_9088_1_-2_224_334

MKMKGLDYLIELYYSFTVVFPNSTAQGCRHLLCWSNQ

>SG-PL_contig_9089_1_-3_3_131

PPVCTQYVFVLVLVRCPLLRCVCVITFGVKCQTKGSVHKCRCC

>SG-PL_contig_9090_1_+3_3_110

IFEEACHLKQFIINHAQLHYISLSDQETSRFLKRLK

>SG-PL_contig_9091_1_+3_3_122

FFFQNEINFFIFNHVIDDMFQTLNENVTFSSIFPQKEMSP

>SG-PL_contig_9092_1_-3_312_521

MTRKKLTEVTLLSKTVCKHPYMLKNKYKIKIISDAVKMEVLIEDCLLLHFHFNVIYSCNF

SARAISFYLI

>SG-PL_contig_9093_1_-1_1_159

VVCVCIYIYIVCVCVCVCVCVCVSLFFGSIKFSAFCERYLGPIVYLFQFSKNC

>SG-PL_contig_9094_1_+1_1_183

ILSNRLLLGNGHLITSGISICHSPFQKKMWQKTVALSHKHGKLWVLCAKSLTKQTGTFSG

R

>SG-PL_contig_9095_1_+1_100_345

MQKLIQLQFNASMLKFPVTVTQRFKQLTHTHSQQFKIYFSTQGNPIMSFNIHSSINIQIR

TCLSQTSQKQPKIVVSSTNSYI

>SG-PL_contig_9096_1_+1_40_300

MSLTSMVGGLASEAQIRTSQTYKCLWRLHSTETNFKIFEAQALPTRLSGSKLWGFQRSEP

IEVVHRFACKHVLNAGIRGPCAVRLGE

>SG-PL_contig_9097_1_-1_1_1206

RDEKEKYDPNAFRDAIIQGLNETEGDLEKVSKFLDVSGSRLNYRRYAEVLFDVLFAGGIL

APGGFLVEDSDPSKPSRTDVCVFNCEAAPEKIRGFYEVFYKLIRRYKYLEKAFEDEVRKV

LVFQKGFTDEQRQKLGIVTGQILANGLSSPRVLDALFEDHLVKEVISLECAAQIFGIWLQ

EKDVKEVFSALKKVRIDTRLQELFPANKRNPETFCTYFREKGLGAIADMQMSTQATKAKK

EAQKALSEMITNESPVADMLEYTQDLIQKQGMTETEVTITIWNTVMMAVEWNKKEDLVAE

QAMKHLRAYTPLLAATAKAQRAELALMLRVQEYCYENMNFLKSFQKIIIMLYKTDVLSED

TILRWYKQAHSSKGKSVFLEQMKTFVEWLENAEEESDEEDEG

>SG-PL_contig_9098_1_-1_595_714

MTQSVACTLTQVAVPVLSTNLFIFVFSFSDLKRSIFSFKI

>SG-PL_contig_9099_1_-2_962_1333

MYTITILYAESVVNGKVTPGCVVIQPHFILDTTTTAVLDDCNCKVTFTCAQSLLLPWRCF

WIFSFKIIIYFQFHYSINLHAPCTPCFFVPQSFKKKKKCVQEKLVSLATECVNCLKYYQC

EKLK

>SG-PL_contig_9100_1_+1_397_534

MFLKANRFQFLFSHYNHVFKKANSNQIILLIAQPTTLRPYQGCHHV

>SG-PL_contig_9101_1_-1_1_381

CDACGKPRPSSSGPSRLVSPWPVWKAQALQFRALPFGLSLAPLRLTRVSRELASLARGEA

IRLSCYLDDWLILAESSTLCRLRTQRVLELCATRGFQTNPRKCELFSSCCRTPEIAALLG

PTESLAP

>SG-PL_contig_9102_1_+1_529_813

MLTVSAGTPETFGKTNGCLKAVLHSLVTMRSQQYFICRVPTKRSNFDFISTDFPASSKCL

DTSTPVELLKYKQLQTVTLKILWQGRNCSYLCGTV

>SG-PL_contig_9103_1_-1_652_816

MSVFGGPAKTEVCRLCSNITPGRCGPSNAHTRIRENITWNESEKRINHVSLLFMW

>SG-PL_contig_9104_1_+1_1_147

QWICRVKFRLLSLERKCSHSAVTPSFFPPSLCEFLLIYKKNDFSAEFCQ

>SG-PL_contig_9105_1_-1_70_732

MFNIYLYRFLLCYVHCAYVQHIHCAYVQCLSALISIVLCSLCICSTHSLCICSVFICTCF

YCVMFIVHMFSAYLHLCVLCYVHCAYVTCLFAPVRIVLCSLCIYSVICTCVYCVMFIVHT

LCVYLHLFTLCYVHCAYILLSAPVCIVLCSLCIRYGFICTCLRCVMFIVHIFCYLHLCVL

CSLCMCSTFILCSKARHIAIALMSDDAVLMNLSKFMSHEIK

>SG-PL_contig_9106_1_+1_130_225

MMVMVFQLSIKQKTQWWFHTMLRMQANSFYRT

>SG-PL_contig_9107_1_+2_59_190

MVNTQTSGWTVSEGWSTHRPLAGQSVRDSQHTDTQLQLLSRCQQ

>SG-PL_contig_9108_1_-1_43_222

MQHKACEETHEGTCPVGCTWQFVFVQHKACEIHENTFCQNDNDTHFTELCRLDCLIIFFL

>SG-PL_contig_9109_1_+2_2_100

FLLPEKFLTKVQPVESFVVAFLVSQVYQRSLFA

>SG-PL_contig_9110_1_+2_23_307

MCLCMREREREREREREREREREYLSMSMCVNSMHVFHLETIKTEIKSKPNSLSNRLLSL

LDLLIPPHPSQSLLNFQPLFHPQSKKRKMKQNPSM

>SG-PL_contig_9111_1_-2_2_148

ECVCVCVCVCDVCVCVCVMCVCVCVCVCVLCFWSAENQAHFQLIILEQI

>SG-PL_contig_9112_1_+2_2_88

TTKCIIKNAIKRTVKNPSIGILCIHRCIL

>SG-PL_contig_9113_1_-3_21_338

MSRSAAPLSVLYRWFRGPFANLTRTVSILSNRTRMSSTSTNEVTPPVDQELHVQHDDKQQ

EFVISLGGDHRALLQYTRTPQGAVDLWHTEVPPVFRGKGIAKLLAQ

>SG-PL_contig_9114_1_-3_156_365

MVKTLDFQTGGCERPGFELRTPHLVGKGWRFFRFFLLVPEPPSCVYTCRTSNMHVKDPVS

HVSVRWLMET

>SG-PL_contig_9115_1_+2_2_226

HPYQSAYRANHSTETALVHIVNELLLASDAGKISLLTLLDLSAAFDTIDHSILLSRLEHT

FGIHGTALSWFSSYL

>SG-PL_contig_9116_1_-3_3_107

VCLNVILFMCLFECNPVCYPANDLEVCFKACQDPG

>SG-PL_contig_9117_1_-2_92_790

MTRDDLFNSNASIVRDLTDAVAQVCPMAMLGIITNPVNSTVPIASEVLKRHSVYDPRRVF

GVTTLDVVRSNTFISEAKGLDVAKTNVPVIGGHSGITIIPLISQCTPPVSFPPEDRKKLT

YRIQNAGTEVVDAKAGAGSATLSMAYAAAEFCKSLIEALNGYEGKVQCAFVRSDETEAKY

FATPVLLGKDGVEKNLGMGKLLDFEVNLLKSAMPELIANITKGEQFVEKSYSK

>SG-PL_contig_9118_1_+3_159_362

MFDTKSLIALYCCLETLLFVLTKSHRCYVEIEFSPLLYFSVKYHGLSSEPASVRKPQCVG

FPGSRFLP

>SG-PL_contig_9119_1_-3_3_221

LWQTLCSPQILRALAPGAGPVSPQRVPGPHHFLMETPTLIRASIREGDWASSIVPMDACL

HGLIHPSCRKHPR

>SG-PL_contig_9120_1_+3_114_260

MISVHNKSKHNKICQTISTTHWRRLLFASIIPNSVLTADSFATFFFQQN

>SG-PL_contig_9121_1_+3_120_239

MASGLTFIQTTVYQHTGLFAGSRSLPDNHWQICVQCIWNC

>SG-PL_contig_9122_1_-2_2_79

FFFQHFFFFILSLPITVSIVHSSLTV

>SG-PL_contig_9123_1_+2_2_244

RCRGGLHPPRGRARGWGCRRQEPDAGRPTNSRNPGATPAPPLAGESKTGPSPPPEPAYGR

GPDRRNHPEAAHVGKPAVGWS

>SG-PL_contig_9124_1_-3_2439_2828

MRSVVCIRFMCSEACVLSSFGQHLSWVRKPCVFASALHFIDQIYFYAFCTPRVVETMPRL

HMKCVSSSPSDRMHSVSIDLWTGAQSCVMSYVVCACRTVVTCSPLVHVVCTGRYHDPTVL

VFILRTTWDE

>SG-PL_contig_9125_1_-1_196_270

MKIYMNYSELFFFLLIKGISQISSH

>SG-PL_contig_9126_1_+3_3_212

VCVCVCVCVCRCACEQVCMCVRACVCVCDAFAVINIVYNFRQILFFSLFLCPLQKSHTLL

IVCYECFVMT

>SG-PL_contig_9127_1_-1_31_330

MYTTPLTQLINNHSVHHEIFADDMQLEYSDIPENYDSLINKLQNCISDVKQWMSNNKLKL

NNDKTEAIRFVKHTWISDVSLPSTIALGSTEIPFSQDVRD

>SG-PL_contig_9128_1_+3_3_275

QSTHTACPGVVIFLKCFPNLSSFTTASIQSHFQWFSMLESSKFSFLCSRKAGKRNGSGSR

SVGLPFWSGMLPQTGCYSFPVFLRAVKNQAK

>SG-PL_contig_9129_1_+1_373_591

MLRIKDIKLKWFQIQIIYRILAANVILKEMKIMDTNQCTFCIEAKDTIEHFMWRYKYTKH

FWLDLEENDKRKV

>SG-PL_contig_9130_1_-1_109_849

MIPTTSVDEKLLSNEGRAKLNAPVVPITHEDGLKWYYVDPQGNMQGPFVSEDMAQWFSAG

YFTMTLEVKRGCDHHFQPLGDLIKQWGRVPFLPGALLPPIVSLPTTAPPPPEPAPTITTT

PQPSVSLPPLARAPSPTLAPTSSFPLSQGGGGTDSLQVIRHKLMIIQHQRLQAQMEISQL

QVQALSQLQEQDSFKALDPTQQQQLRIHCMQAHPHLQQHLLQLQHLQQIEQQCQLLEQQA

VEQQAQQ

>SG-PL_contig_9131_1_-1_175_1278

MEIFNESSDDEPFLGFRLEDIELHEVQARSDIDVSSVSSVHTSDLSDWDDRDQDFDSDID

DRGDHRPRDRHGWSPVTSRVDIVNFTEETGPNVSVGEHPTQVDFFFQLFEPETIQKIVNN

TNKYAREKIALKENGDPLWAETSIPEIKAYFGILILMGIHNLPRDEMYWSSDDRLGVAGV

NKVMPLKRFKKLQEYIHVCDNDNQPDPADPNRDRLFKVRELIDMANRTFLQKYKPSCQLS

IDEAMVPYKGRSCLRQYMPIKPIKWGMKVWCAADSNSGYLLQFDVYCGKRADGTHRGLGY

DVVMKLATHFLNKYHHLYFDNFFSSVALMEELLRQKTYACATVRTNRVGLPGAMRSHRRL

GRGQHIVH

>SG-PL_contig_9132_1_+1_178_309

MRHGVILTTRSYKQPVNQTWGLTNIQPIRHGVILTTSQSDMGSY

>SG-PL_contig_9134_1_+2_2_73

LAISKLTVSHIFPIRCLCECSHFW

>SG-PL_contig_9135_1_-3_99_227

MLWCVHSVLLTTFLVCLFPLKSVRCMVTDVRIMALAPYVCLCG

>SG-PL_contig_9136_1_+3_462_671

MAIFSWQQRRKLKPRISTLFTHKEHTHKMSTLLEQFFTHGSYFILKTNNKNDPLETQHVV

HMICVHNLST

>SG-PL_contig_9137_1_+2_2_190

SHYLTSISYLLGYKGHKMKYSTSVSILFITIMFTKVNLNFSYHYHFLTNNFIHFDGQIHF

LHD

>SG-PL_contig_9138_1_-3_18_215

MHFFLLFVLLLLGWVGGVRARIFSLSQNNLLCSVLPSGFFYFFWAASFFNTLYPPAHLHC

SFSGPD

>SG-PL_contig_9139_1_-2_71_202

MVKVLSQSVLSTQLGMDVHSWPLLLHQTKYQPRQVLLASVVSSV

>SG-PL_contig_9140_1_-2_1121_1423

MPIINVCLFNTSKVFFFNNSDLINNELIILNFFWKTKTCLHKLTEVSSAKSKCFLCNVPC

CLPGMMKQCLLQMELDQPFLMHVESIHVHFGISYRGEYITG

>SG-PL_contig_9141_1_+2_503_691

MDTTMVYFMDTTMVYFICFLKPGCECEIQHFPLSTPIVSYALWWQRQQLNKTMIMTMILM

TIT

>SG-PL_contig_9142_1_+1_1_144

CRTSNKHIKDPVSHVSVRWLMETARHPAHKYQREYVYAHQRSCKSCQC

>SG-PL_contig_9143_1_-1_334_612

MMGHLRCRSVLGRTLWRRLLMVLLLVIFIWSITILNVTFIYNNNTNPHRHSDLLKEEIVQ

LSQAYVRALAKENAGVVDGPYAGRFTAFGMKHT

>SG-PL_contig_9144_1_-3_69_1169

MRGTWFEIARTRFTFNTMESVISVYDYSPDNDNLEGLFIGTLHEQCQSPLQSVTKKKFAE

GPESIIEARIQAGESIFPWITFNILYYDGDMLMHLACYAENEDGTCPRDKTEVTLLGRSR

QMTDSMEDKLPGLMQNVCLTRDDLVPTKETADCLPKLMTREEESAGLPDTSGCQLNNIPV

VENLTETDLLGTWYGLGSADYSTPSYPAGFVVSVSRSTPHTLQWRQWNIRNGSCLGEKAV

YMKPVCRDSGTGDYFSTRGSSFMLQWTAFKILRLQGELMLVYSCTRETDEGTCMPEGIHV

DLLGRSTDAVLEDVVPLLDALPSVCLKPEWIKSVTSTMECDSLASASVEMIETSACAVDR

LPAVSVD

>SG-PL_contig_9145_1_-3_549_695

MCVCVCVRKRGGERERESSIFFFFLVPSFFYKNKLFFCVFLFFYLVRET

>SG-PL_contig_9146_1_+1_499_1098

MSLIHKVPHRQRKSHTMSQLAYTITQAKKVTHNVSHTQLDTGKESHTQCHTGKERHAQCL

VHTVSHRQKKDTHNVTHTQRRTDKERHKMSDTHNVTHTHNVAQAKKDKKCHTHTMSHTHN

IAQAKKDTKCQTHTMSHTHFTFTAVKKQRWSDIHIFIAVKNKGGGISKGKNYPVNPFTPS

RYLPASFHSLTLSTSFLSLP

>SG-PL_contig_9147_1_+2_8_370

MKTHRKVYLCAVPTVSTYYSEVHSSFYLLYRNLVQANSEWFVMLINTENTLPPDHTFMSL

KNTQLALNENKTLELKFIIQTVTEHNINRLSIAAILLTSLLLTHTHTLTEYITRPMLIRN

T

>SG-PL_contig_9148_1_+3_1548_2372

MHEFGMERAKVKVTQPKQCLKLSLFEEGVWTVLRDWSAIYWSTAHHWSVLRDWSAVYWST

ARHWLVLRGWSEVCWSMLTTGQSFGAGQPLAGQLPAAGRSFAAGQPSAGQLLAAGQSFAA

GQPSTGQLLAAGQSFGAGQPSAGQLLAAGQSFGAGQPSAGQLLAAGRSFGAGQPSAGQLL

ATGRSFGAGQPSAGQLLATGRSFGAGQPSAGLLLGAGRSFGAGPPSAGLLLGAGRSFGAG

QPSASQPLTTGQPFGAGQPSAGQPSAGQPLATGQP

>SG-PL_contig_9149_1_+1_334_630

MVCCVQNEQNRHACAHTHHTHTHTGSDETYLVMKMEFCYISNLRVKLKWTLPLVHVTKLI

FWGFFFILNEKKKNVLFSSSRPSLIHYVDLISSTSEVQY

>SG-PL_contig_9150_1_-1_1_474

DILASLDEDKISVLLLLDLSAAFDTIDHEILLSRLSANFGIHGTALNWFRSYLSDRKQFV

IDDKQSKKTTLDFGVPQGSVLGPVLFIMYTTPLTQLINNHSVHHQIFADDTQLEYSDIPD

NYDSLINKLQNCVSDVKQWMSNNKLQLNDDKTEAIRFV

>SG-PL_contig_9151_1_-2_2_220

QTRPSLDHLAQSFLLSTSSCFCAQPVPCKMVFARQDDFFPSTSSKQTNKQTNKQINKQTN

KQMNKQESKQTTI

>SG-PL_contig_9152_1_-1_1_249

LFLCVAKSSEDFLNSENSNKNCLNRGLIPCTVHNGRSLIGTYSFNTKDDFLVTPPGPTPF

LYLSQSFETLYELAKVKPVHHLI

>SG-PL_contig_9153_1_+2_1601_2203

MLKIILWGALIFSLPCSVLLELLKVTIRGITKAKAKNKIPSNKLKYLICAYNKQAAFFQL

FQMYRKHLQGIAVVRNSGEHGSTIYNHGCILLTLHCWHANCPPSTQQHIVSMTIKTKCVD

PHELTINIHTMLIDNTVEIITKETGLKQSMDKSQKIPLFSRYNKTYNVWIASLAWPSLRK

NKVFQTHTVNNTFHIHLLQTY

>SG-PL_contig_9154_1_+1_91_201

MSMSKMLIKEYHIYWTRKHKLFHICVTSFFSEYTISL

>SG-PL_contig_9155_1_-2_2_88

FFFLWCVKDKWSSDDEVIFVVFSCNNCFQ

>SG-PL_contig_9156_1_-1_1_579

VLRLLGYTRVLLQTGRGQFQPESTELGDHCPAVESYTFKDTIAGDIAAADLVICHAGAGS

VLETLTAGKATLVVINDELMDNHQEELAKQLQHHHHLFYCTCSTLQQTLESADFTSLQPY

RPGDPALLALHLQSLCTTSTVNQPRVFACHLCWLGAIALVYMWMPIAALFLLLIVLCLGV

FAFCGNVRSCSKE

>SG-PL_contig_9157_1_+2_440_586

MSTTVKWSGCQTSDISGGLGSILPGKHLVGKRWKFSNLPGQHNRRPAGA

>SG-PL_contig_9158_1_+3_126_239

MDLTGTWTVTTSSSRPRAKSSPLHKFTVISCNLLQLLG

>SG-PL_contig_9159_1_-2_89_391

MYWPSVQEDSTSASPLVRGVICAAPRLFGRSTLLEMKDLHFDTPSRSLMQPLCALLQPIT

DTLSRRLQTTAAAFNQSTLTSAVRRSTGVEGKKHENASCTS

>SG-PL_contig_9160_1_+3_126_392

MQQNECPVLEFDHSTEYLGQCSFLQSNKGQHIVSTGAMHSICFWNTSSEVQSYIRQNVET

SPFFYQEKEGLHCSCSLSMACCQPTNQQK

>SG-PL_contig_9161_1_-2_71_367

MALKRIKKELQDLGRDPPAHCSAGPVGDDLFHWQATIMGPPDSPYTGGVFFLTIQFPTDY

PFKPPKVSFTTRIYHPNINSNGSICLDILRSQWSPALTI

>SG-PL_contig_9162_1_-1_64_723

MDTFNPVNNNHASKNMNPAVQLSIQARLDIAQFTWKLHRMRRRLAQTDIHSSIEAVTWDG

VTFESFCEENLWTQDSKNLIEAACRCMFGLATGEMTLLYFLMYVKSAGGVEIFKKPIEFS

GRECRVKGGVQQLASHMAQVIGRKNIKLNQPVTHIVQSSERVRVITARHLQINCVRVIMA

IPPHHAASVNVTPPLPSTKLALLKSVPLAFLVKFAVTFEE

>SG-PL_contig_9163_1_-3_3_266

KGYRSAICTTLQQLGGPHLSDSFLLREVSRGLTAAEDRTPRRLPRWDLALVLGALRSPPF

EPLDVVPFSLLSYKTAFLLALATCGRRS

>SG-PL_contig_9164_1_-3_81_1727

MLVLFETAAGYAVFKVLKEKKLKQSENLFHDFQSPESANEIVKLKRFHKFDDMEVALSAE

TAAIEGQLSDSLKKLLKKLVSKEAQEELAVADAKLGNLIKTEFGIQCVSNTSINELMRCI

RSQMESLLGNREHTAMALGLAHSLSRYKVKFSPDKVDTMIVQAISSLDEIDKEINNYQMK

AREWYSWHFPELYKLVNDSETFLRIVMRLGNKQNAASTDLSDILPEDVETDVKEAAEISM

GTNITPEDIGIILTLCKQIAGFVQYRAELFEYLKNRMSTIAPNLTVLVGEQVGARLISHA

GSLVNLAKHASSTVQILGAEKALFRALKSKHDTPKYGLIYHASLVGQATPKNKGKLSRML

AAKCALSARVDALGDDDTTDREIGLKHRAKLEMRIRQLENKGVQRISGRGKEARAAARYE

NVSDVRTYNTAADSSLPSVPVQQKKRKREEQEQEQEAGTSEGNKPKKAKKEHKMKIEPVS

DGDTAATSPSEPEKKKKKKKKRKHEDEEEERPAKVKKEPDVDAAASDSPSASGEKKKKKK

KKSKGSDDV

>SG-PL_contig_9165_1_+1_826_1080

MLRILLLSFTCPINASSPKVTRLLMKHSLFWTFTLPRRWRSVEQSDTLTPTNKYTRPLTK

AMHGILNHSTVVFDSTLQVIIFNQS

>SG-PL_contig_9166_1_+3_2160_2423

MSHRLPNLSDCVNSCGLFLKGVYNIIRANIMWTVGKHKEHQHSKFKPHYMDKNQRLQWRN

ITLKFVSGLKATNACCCMTSTYLFTMSS

>SG-PL_contig_9167_1_-1_319_495

MFDYSFLSDEIFSCYPFDSTVTGVMFLCLSHRKSGSNCCFQLANVGMVWSVSFSYRMVS

>SG-PL_contig_9168_1_-3_135_524

MPDPHSETGSHDFFSFTNRQFRCELNNVMSLRLILQQPSPVWREFRLEDLRLFRSPGGPK

TSPLPAWLMDEPKDKFQKKIVEGVPDLEGLSANLQQLWALAEEVSTSQTSQSLGRFEVDG

CYDINLLAYT

>SG-PL_contig_9169_1_+2_155_415

MRECMYTFEIYIATQHNANTCNTHVRTAVSCDWLDSTNHRKLWLRHWYIMCVRIMPYIHN

NYEHGLEACTFLAAICINLKTSSTPRL

>SG-PL_contig_9170_1_-3_96_236

MGCVFCQHSTVPTIIQCWHFLICITSLSLCIISLKVDEQIKVECIYS

>SG-PL_contig_9171_1_-2_20_289

MHACVCVCVCVCVCVYIYISLSLYICNICVCVCLCVRACVSEMHVTYTQYYMYSKPNVCV

CVCVCVMHIFIGSLSLSLPPRPPPPRYCFN

>SG-PL_contig_9172_1_-2_1049_1525

MYYLPAFTLMGVVEKMNRCLAGSWKTPRGVTLVSPENSVLILLLLFVEQLYNPLTACLAR

CSCLVLIYIFFLAHAWVFSCFHNPLNTDMAYRIFDVHIYLMFLDLFCMLIHTKGVRALAG

LQYIDLGDQKNPHPLPTRRGARGGFEHRPLEPSNLEIQC

>SG-PL_contig_9173_1_-2_293_478

MVFKLFLFVFFLFLCSKHCYFLFLRCVYAHCLAHTCAGVILLRSLHTLVGMHAGCFHVSI

SH

>SG-PL_contig_9174_1_-3_3_131

FFLIDCKYRPNFIQNYCTLCSSEFLYFTCISLFLFVCSGLFIL

>SG-PL_contig_9175_1_-1_115_1197

MWFQFCIVLQLCFVTTTAGRGWRFSRNRRARSVCFDNLGCFSTGRPFTNTPHLPQAPHRL

GVQFLLYTRHQPSRVRLDRSDQRMREVFDVAKNTRVIIHGYLNNGDATWVQPMVEALLDR

EDQNVVVVDWGRGARSVNYLQVVANSRVVGAIVGAMLDRWVQMGASPSSLHLIGHSLGAH

IAGYAGTATPGVGRITGLDPAGPAFQTMPPEVRLDPTDANFVDVLHTNALPLQDMGLGYI

RPCGTADFYPNGGRRQPGCRSVLNRLFGFLRGQSGQEWVRTMGCHHQRAHMYFTHSINTQ

CPMRATPCQFPHVLQHAHRCQAQAVRRRQCQDCVTMGYDVDLNASGVYHVRTGSRAPFCV

R

>SG-PL_contig_9176_1_+3_78_257

MKPLKHYHTRYIAHRSIYKRKNGSNKALVKQVSVFKTIFLDGKTTLKRPTWTTERCRANQ

>SG-PL_contig_9177_1_-2_23_172

MEEKCMRHLSKVFFSQGKCEDLFIWLYIKTLSVLPSHCSVFVVIHISLSQ

>SG-PL_contig_9178_1_+2_137_316

MASTSRQIERRLTAEQVTELIMNYDSNDEEIGGSFTTKSEDFEPASESDSDTDIYVHVCL

>SG-PL_contig_9179_1_+1_1_111

RAVDSRSDVSCSIPACNLKTGVFIAHLNAPVILVVSA

>SG-PL_contig_9180_1_+1_1_87

LSSVPARNFKVILFAAHQCFNRSGGVSVS

>SG-PL_contig_9181_1_-3_3_380

INRRCSTWQCVKLFQNYGCQRLPLVHSNVSNQTQRRLFSASKQCSVNRSFFLCSSKSSRK

SASVIATSATEARELLNTENTQLRPRYIYVHTSVNRWEDVPGGLLFDTAYLDSMPGEKIN

PSVPLV

>SG-PL_contig_9182_1_-1_1_201

GNAEPSDLFSALDSKIPEEHFSSDYMTTKMDPWVSQMGFPVVKVTRTSAGYQLTQERFLI

GSVDDTD

>SG-PL_contig_9183_1_-2_83_292

MRSLGISLKKSNSWQFCGQVYQILNYCKINQLLCNILSIAGLSRYFRSLELKSIKHSQQG

FVFDIVRKCN

>SG-PL_contig_9184_1_+1_265_432

MHKEKNRLVKKTNVLCHHLRFSLRQFCQVLQTSKSLNSLSYSNPFKRLTLVIIYRS

>SG-PL_contig_9185_1_-1_64_417

MQDCVQLVNAYRCDCKPGWTGTRCDNRLRCDQQPCKNGGVCTDTNTGPHCACQEGFSGDY

CEFVTTACDSTPCHHSGTCQPQENGYTCLCPAGASGINCENDTTDDCLSSPCQNGGSC

>SG-PL_contig_9186_1_+3_54_221

MMGGDDEEGGVVAVAGVGVVLSPFSAMSDRPPHPITRWRCSDPVDVLNKEVVSSLH

>SG-PL_contig_9187_1_+3_393_518

MSSLIAGINIKCSTKSYRCACIFLCKVAKCVEKNKTNESQMW

>SG-PL_contig_9188_1_-1_1_1101

ECDKDDSSSKREVVVAGTTLQVTVRDLSPATCYKAQARARNNIGRGIPGPLSDVFYTKRE

GNLTAGPPSAPPDLFQAVSPSKSAIRISWQPPTVDSPHEIKGYKVTYSQRNLKAQTIRVE

DPTETNLLLQGLEPFTWYTVSILAYNDKGDGPIAELAVQTLEGVPSSPRITHITDRESTS

FYVHWEPPKRLSGHLTAYELHWISDDNITKTRIISGHLTNPMSAFITALQPYTEYKIQVA

AFTGGGRGKFSEKYPALTDVAAPSAPYIRNVTVLSPNSVFLQWDPPTVFYRRVDKYVLKW

WDTRGNDPDTMVPGTQTQHVLRNLPTNMRYQVKLAGVTQAIFSKQYLVGEFTAPVSFMLG

AVPTIPK

>SG-PL_contig_9189_1_-2_101_325

MDHLLVMTHDSNNLKLQLTIFSATTTKRKNACDDERKGRLIVLHDHLIFFLFSLFLMKEL

LIVPHAASASVTEIL

>SG-PL_contig_9190_1_-1_448_678

MSDDETRRLLFDDSNSDSVFEGFEPSNLCEVTEVESDLDTDFDNLDVSPVSSPDISSYED

EPSERSGGAWSTTTVVF

>SG-PL_contig_9191_1_+1_73_198

MDKETGQESIIYMITHLTRGLMKTKNHKGKSLLQSKERAQRG

>SG-PL_contig_9192_1_-1_370_1380

MSLFADKWTSPFDNNPFGLDDFHSTSFGQFRLGSNSSLFGHKADEEGYQPLEMRLRNGSP

HTERGFYEHFDSIFHDLTSPRSRRYDLRAAAAESSKRSPPATGKGKEYIIPIKIEASSKG

ETTPSSKNTGELSKSSSASNSSSPGRLIEIPIVRESSSSSPTASKTRDKSRSPIVSQSSK

TRSPAVSESSRIPLNFRLLTPTTEENQGSQKRDSSNDVELDSDVFFVKSQQTTSQNLSRK

DSDIELVEIEDDESFSKSLRRRPLRHRQRTSSSAEERVPDSSKKGMPSSSVVERKSNDIK

IGAVSGKENKRSPCSHPGPTEVTATPSYPIYDYLKPK

>SG-PL_contig_9193_1_-2_59_598

MRFAHIDGDHLTLLNVYHAFKQNHEDPQWCYDNFVNYRSLKSADNVRQQLARIMDRFNLR

RSSTEFTSRDYYINIRKALVNGFFMQVAHLERTGHYLTVKDNQVVQLHPSTVLDHKPEWV

LYNEFVLTTKNYIRTVTDIKPEWLVKMAPQYYDMSNFPGGEARRVLERIIAKLHNRMDDY

>SG-PL_contig_9194_1_+1_76_270

MCKPSRVLLFQPGQTACQTTWSAPDPVLTMHSANHMVAHDWLNSNQILLLAQPTTLRPYQ

GCHHV

>SG-PL_contig_9195_1_-2_2_112

AYYNSMKRGTERVCFSLKLLRFVNKSKQLMGIETNKT

>SG-PL_contig_9196_1_-3_195_335

MRKLCHHLMGKLCHYLMGKLCHRLMGDIIHDCLEVSSLFFWKIVISV

>SG-PL_contig_9197_1_+1_1_171

FFSFFFLFVCMCVSCSVCFCFFPCSQSLPSYYQYTKSNDYYLTCRFICIINFILAEP

>SG-PL_contig_9198_1_-1_166_324

MGSLGSYILHLFTLVTGQYFPPWNSQLLYPTSNQYIINDDHFSLGILQRTFTF

>SG-PL_contig_9199_1_-1_97_945

MALPKFRKYYVKGDILPVEEDSRHEFKGHLNLCKEEIPRWALEKKSSQDKGSRKPISRNI

NAFLNSGNGGTVFLGVTDDGSVRGFPMTRLQRDHLLKSLDDLMSRYNPQVLPHQYSVHFT

PVFDKVRTPEEIEKQLEYIGREEVHEMYKGRKHVFRDSNLCWCDKDATASFNVCNRAVQL

FVVEIIMTPWDPYDVRNPSAGQDSLVRIQPYYSDEEGKVHFRRQASVVQYTLGSIAQITR

QEVKDIYEEEIRRLKEQIAELEKRKAAKARGQTNSSSEQPSVS

>SG-PL_contig_9200_1_+1_1_204

GCYMDDTTSVLNKAVVEEFIQHLSNQHPVIKFTREVAQLHRHAGYFHHKERRLPDLQRLH

KKTHTDQH

>SG-PL_contig_9201_1_-2_182_1960

MSTSLDQRTKGQTNQVSGAPGISGNDAMSEEDKYMPQPHQGTYTSPGQMPSPGSTDSFFQ

SYYTPNPNTMYPFMSQGLSTTEGPWSNGGDTPMFPMSYDYSSMYGGFGFPFGSWDYPGEY

WNAPTQPAARKDPRPYQETDYYTDPSMLPADSYGMMNGAGPDRGDPMGGVEQGLRGMTIQ

GGLEKKMDGGMGSEIVGGMPPQEMVAPPPQSVSSSMHGVSSQGMGVSVGGSVGGMGSQAP

KKTSWAAIASQPARPQTQVRPRMIPRAPANPNKPHSMDIGTWENRGGMSAGGPVGGKPAS

GQRQTWSAPRQRQMGGTSPAGHSSVNSNSNNSGGNGQQSNHQGNPNPSSYGSGAGSPGNV

AGNGAQATQHPVLDKLRQENQYNPKDFNLNPKGARFFIIKSYSEDDIHRSIKYNIWCSTE

HGNKRLDSAFREREGKGPIYLLFSVNGSGHFCGIAQMLSPIDYNKNSSVWAQDKWKGQFE

VKWVYVKDVPNSQLRHIRLENNENKPVTNSRDTQEVPPEKGKQVLKILHSYKHTTSIFDD

FTHYEKRQEEDQAPQAAPVPHGGHSGVGGGDRGDRGNQRGGRDGRRGGGGRHN

>SG-PL_contig_9202_1_-3_150_260

MAVILTLVTISNFTTFWQKYCGNAFYLFIYLLFRYYH

>SG-PL_contig_9203_1_+3_99_302

MKQTLLKLQGINLPLRHKNKSCYIVFISNFLLQYVSIDVRQIGLSGQHCKSIQSFTKCGF

QSSISYRS

>SG-PL_contig_9204_1_-3_138_1151

MFRGSLYKKYPSMWRRLISLDERKTLNTMGFGMHSLAANITLLKAAEVDEILDGNDEKYK

AVSISSDTSYSRDAKAKRGNSWAQPLPTSSHHLDAVPCSTHINRNRLGKPKMRTFPMCFD

DTEPAQIHENANQQELLIPIRLDMELEGQKLRDCFTWNKNEMLITPEQFAEILCDDLDLN

TVLFVPAIAQAIRQQIEAHPIPDPMSEEQALDQRVILKLNIHVGNISLVDQFEWDMAEPE

NSPEEFATKLCAELGLGGEFVTAIAYSIRGQLSWHQRTYAFSEAPLPTVEVAIRNQSESD

AWCPFLETLTDAEMEKKIRDQDRNTRRMRRLANTAPTW

>SG-PL_contig_9205_1_+3_30_224

MMETMQNNTTINTIYNNNPTASSISRCRLREQNFKTKHHHQYPSCERDDGEHENTEIFIT

EPSIS

>SG-PL_contig_9206_1_+2_2_214

KADDSKGKKRAETKKAIQKRKFLAQNSQKAGILLNTQRAPGIPAGVSFHTDLKSLLHHYQ

QIYIADGHTNL

>SG-PL_contig_9207_1_-2_539_760

MMTLSLMCLHCIRVCVCVCVCVSMVLFLKLVQLRIVISFHLGICKDNMAFRFLTDSFETL

LQKYIVLCMSCKML

>SG-PL_contig_9208_1_-1_1_177

AHCWNFTPSFLKCSTSNIVFKVQHFFFMSFRFVCVCVCVRVNKETRNANQNRPKRRQHV

>SG-PL_contig_9209_1_-2_227_877

MSVLNLEAKLERIDPATPWGFRMQGGKDFNSPISIQRVNPGSLAAKCGLQTGDMIVKIGN

TDTSTLRHKEAQNSILAAGNRLDLLLQRGGAPMRPGGYETFATPVINTGGFNAPQSFKPA

YDAGLPHQTQANLSMSPGSSAPPAPLFVPKPAGPPPTTSITFTPKPVPQAPAAKPWTPAP

PPSAPAGGPRPFGAGGGGGRGGAAPRPSGSGARRVRV

>SG-PL_contig_9210_1_-1_28_546

MNMPPLFRAALALIGLSLVLFLAGFASNFWFVSSQPNSSFHFGLWLICTSSYGCVNYDRI

LGTTPAHAYVARFCTIYEMIAVIVGLVLLCLFVCKSAQQRWARSSYSLLLSAGITGFLGA

IIFIGREKSLDSTSSVSVSWSGILVIFAAIMQIVSGVLILLPIRRGQVEFEQV

>SG-PL_contig_9211_1_-3_132_302

MMTVYREHVDLSAESVCMFLWSATDKLLYHQFKESASLVAHNVHVFCCILHVFDSFY

>SG-PL_contig_9212_1_+2_2_211

QTECWNRHFQCRCDCRTPNLSYKSPASQLKRLNDDSMLVSTSSEHLSDRYQLLKVKDLTN

KITVTIHPAM

>SG-PL_contig_9213_1_-1_292_501

MHSLSERVQIIMLHRSLDKSHSSCRCRDHWSEQRQIITLIKTFSVLLITSLVLFSSVTNP

RSVPHFGAVV

>SG-PL_contig_9214_1_-2_182_304

MEIKNTVLVFREAISASNTVVVKFSSEMFLTCSYFNWLRSR

>SG-PL_contig_9215_1_+2_290_451

MQRCRNFADKLLPALGIYLHIKQQQQQQQQQKYTLPSLYFFPIPKLSALSENFS

>SG-PL_contig_9216_1_+3_3_248

RFAHHLISVIQQFLDISFFVVLWMQRSASRVQGLTWSRVTRMFAKQHHPGFDPPVHSSYK

NHVTVRIIAGLGLGYFFVCLGF

>SG-PL_contig_9217_1_+3_2391_2618

MDQSDRQPWEQMNINISEEKQVSGLPRLFFDMHIKQPEIKLRYAHRNKCLTMFYIDRKCP

NHRVLLTWCGGDSVLD

>SG-PL_contig_9218_1_+3_3_224

AFRQRLIDCRWQTWNNRIKNSERFHMYRTFCSVPDAKSYLLLNIDRHVKFITTRFRLGYL

IWLCTVLGTKRVMI

>SG-PL_contig_9219_1_-3_3_191

VDRCLVNGELCSTKTSTILSLVYPDFSRKLHSWCVCTSQSLGHDGNSPPGLRYRQGAVGL

CGA

>SG-PL_contig_9220_1_-1_1_180

VDRCLVNGELCSTKTSTILSLVYPYFSRKLHSWCVCTSQSLGHDGNSPPGPRYRQGLWRA

>SG-PL_contig_9221_1_+1_799_1068

MAEARVDASCSGLKALTEIRVARHSNRSSYIQITLWTITDIPSFMSTFDEPINQTGNKSI

SQTAHKSCHHKNSGTTFQQINTFTNNVNSL

>SG-PL_contig_9222_1_-1_178_276

MSCVLSCFVVPTECYNKLEWNYCTGMSMAWPAA

>SG-PL_contig_9223_1_-1_337_561

MTRSLEVRTSIISLLLFGDWSGFATVRLLSLVGGSLGKCPKYIGPEQALLNSNRVTSAES

PKVDSLKAEINLHDQ

>SG-PL_contig_9224_1_+2_2_292

FYDLLKYKLKAYKLVYLFIYLFIYLFIYLFIFSSGGGSRSNYVQGLEYQHEQKKFTNNMS

DDLAEKHFLSDNSYLPERKIFSRLVKCIFYQQDVYWV

>SG-PL_contig_9225_1_+2_35_289

MIMTIKNAAQEKKKMWFKHKEEQVNIIKQRLRQTVYVLLIFCSLFVDGNTLSEQSSYQNR

NKQTHKNSQTRTQKRENRKIKIDEG

>SG-PL_contig_9226_1_-3_3_152

CLVKVRINMCLVKVRINTVYMYTCIYMCTCIDMLFTKDLFNYSIIFSISL

>SG-PL_contig_9227_1_+3_102_287

MQEFCLYSLRGRSIFTSLKWVLNVYFKPVCFLFIAIVSVDTSAKMGLLWHCINAHHSRQS

LY

>SG-PL_contig_9228_1_-2_134_640

MFPRGGAELVHFFYATSNQQLAAILADRVQQQGEEKPKTSEFIRDALETRLRMIVPYIDS

WPQAMAIQTLPQNALESWTNLRNLMDDIWYYAGDRSIDFNWYTKRVSLAGVYKSTEVYLL

QDRSEDNMDTWSFMERRLQDLQTFGKAARNASQMSTVMTEGLWGICIMG

>SG-PL_contig_9229_1_+2_149_361

MGSVPREINVMKHILQQLVKLNIILGVRSKEKENVSCGTTFSKLETRDFKVISVVKMLFF

YKIYNTLHYIL

>SG-PL_contig_9230_1_+2_2_145

FGNGVGFRPGWSETGSVLHKLDMVSVLAKWEFTGQNSSFSFIHSTISN

>SG-PL_contig_9231_1_+1_235_486

MTVLYIFGLATTIFCINHYNILSIIRDNKERQNTTLSNMQENKTEQKLQCSMLYIEVLIR

HILAFSCCRSSHSLSKTKLCIKAL

>SG-PL_contig_9232_1_+1_82_291

MPNNNNNNNTTNNYNNNNNINYYYYYHSSSSSNKNSNSFAQIKQIILMTPHKFTHNQLTS

TLSHFNHNQL

>SG-PL_contig_9233_1_+1_841_1065

MPFKPIHFHISQFNFKPYPFKFNVLATFQKVGLLFKIILAHAHTHMQNKSPIVFCRQKGG

KRCRVVNRNAHFPSF

>SG-PL_contig_9234_1_+1_67_252

MNLYLNIINTIRNHNSPLLFPYLFWIGFARLFICCCCFFFPSQRNFSSREIRVALLGSSS

SP

>SG-PL_contig_9235_1_+3_174_395

MIVHVQQNLPTNTSQTGRLKMKKSYKLKTRQGHETHHHKMQDHCIDISTQTKTISKVSLL

GIFQNRKTEDPDKW

>SG-PL_contig_9236_1_+1_196_414

MMKVSGLFGRHPKQNVRKRLPANMADSCIYSRQRCRYRAIHLDCDIFFVHSRLQLSKLMQ

RRGSVQSGAYSTS

>SG-PL_contig_9237_1_-3_120_494

MLKPKALTLVLSQANTGGVSCTLLLNNEGALLAYSGFPDHDASSTAAIASNIWNAYVKSA

NQALENSKLECVLVECREGKVAITRVANLLLCVCSKENVGFGMLKAKVEALADYLEGPLS

QVAAS

>SG-PL_contig_9238_1_+3_75_305

MIQYLEKKNLPGLLLNIDFEKAFDSLNWNYMFKVFKAFGFGNDMLKWIQTFYCNIKSTVI

VNGHASPWFTLSIYFVC

>SG-PL_contig_9239_1_-2_2_160

ILTMSDSAILTMSDSNSHPSFPSGVAMVRSGTTDWVWAIKNDLNGSCTGACTC

>SG-PL_contig_9240_1_-1_778_978

MNSSILILSSKHATKFCCDILCQYSCSLCPLPLGKIYRITSAFVYTMCTLHFVCTKCFAC

YRQFVIN

>SG-PL_contig_9241_1_-3_33_272

MHDEELSWPTLFCILYHLSLPVFLCHLPLRYIPGPFLFPESLTLSSPLLIFCIVFPFPFI

CPLLLPLLCIPVPFLIPILF

>SG-PL_contig_9242_1_-1_10_2355

MNSLSTGLTFSLVGQRKADSLSAISRSPTLSQPQPQHCVQTTRQRRGEGDFNGDAGRSPI

KSHNNRTVNGRSPRSSEGDEVFSSDSSEANRTYNGVHNPTYIQHHNQYVSNGVHQDFTVN

RPGSGVQRGGQYSGHPYNRGGSSIMTQEQSQTMTASSRPRSNMPPTAVNETSERLKRFTE

VMRSRMSVSSCGDMSTTSSSMSQTPTTTHTPPSRQSSVTENGWESEVSTLLDVRVANGTS

SPSLKLSSEESEDLTISPSKRAVRGKQLIIQNGRVHSQKLTVPCSSQESRLCGDRQSECG

QTPFAAHCLSSTMDSGYTTNTEGEGDSLINGSHHLSPPHHPYPVQTPHAHAEPYRPVPMP

RNLFPTSSRKCTSSAQNVSSPQQPRHQAGSSVRPGEMGGMRNSPSRRTVPVSQNVQSSGN

DVDRPQVADNQTLSLDSAHGFKSLDVAPVTVQNDNAAPGSDDVVVKPQVVAHSASSKEET

SSSDRCNSVDKPDRHSSQENPGSLQQHSTPVNLPPPPLESNSVITVPGQWNYNNTNSSNV

VNNNTNSVVCHSSPRSQPRPHVSTTPAPTLFQLSQSYDLCSVRFSLPAALAASSLLTLTS

VQVTLPAHSGDNGSSASCSPQGHCARLGGPGSAFRPVKTGVTAQTVELTAVESTEEQYQQ

IVTVGELHPGDMLLEINGWQCMGHDISNIQGLLDTTQGDVTLVVARKREDQADNRVEQLE

SSLHTLEQEVERLTEELSRRDARIEELSTLLPWTQNGQTGETTLILQSDDGAFVIGENEF

VV

>SG-PL_contig_9243_1_+2_2_223

KKKRKGRRERERERERERERWREREREREKEREGREREGERERKRERERGRERVKERECV

FERERESVFVCVFV

>SG-PL_contig_9244_1_+1_1_117

RRRLREFLTCANQNSFCLVIVEERVKVLLAVMACNAGSW

>SG-PL_contig_9245_1_+3_681_986

MNSFNLIIPCSSHTKAKLYSNKQVSALDIQFCLPLSSKPYLSCLNYTKILLSCLKHIKIT

VSCLRTHKDHSVNVVNAKDHSVNVPNTQRSQSCLKHTKIGLD

>SG-PL_contig_9246_1_-3_339_1295

MGDLVTLNDGRKMPALAFGTWQVPNAKLKEVLPQVLDTGYRHIDTAFSYGNEEAIGGLLS

DYFTQEKLKREDVFVATKLWMTHLGKDDVRGCVEESLRRLQLDYVDLVMIHGPWGFKNRG

DGTLFPQDERGNFELENYDLIETWKALEKLVSEGKIRSLGVSNFNSVQVDLINFVATVKP

VLNQVECHAYLPQKELQEFCQKRNILLQAFAPLGSPGRPEHLVTDGEPVLLEETVLETIG

AKYNKTPAQVLLRSLLQRKIVVVAKSITPSRIKENFQIFDFTLTAEDMQAIDKLRNNHRF

FTFHQMAGSHPQYPFKIAF

>SG-PL_contig_9247_1_+3_3_161

KKGTMMSHHNEPEAEKTKRIQIQTNENYNKTKQVSTVMPYAMPSDIMTLNNTS

>SG-PL_contig_9248_1_-2_74_1318

MQNNLAPSLDLSGKLIIKVQLGDDIRRIPIHNEDITYDELVLMMQRVYRGKLSTNDDIVI

KYKDEDGDLITIFDSSDLTFAIQCSRILKITLFVNGLQPTNVGDVRSLRRELQMVRDRVN

AILDHIEPVASPSSSGDSALPDSQSAMVMGTKDSGRATDFNVARPVEGKEFDPLSTQRSI

SHDEGAHNKVMSSFGIANDAPGMAERAGAATPDSISSVGSASSSQQRQQQHLQPGGLPQQ

VTPATSMQSVPGTPAVSTHDPQQAGTAPYTQGPGQQGYAGQMTTPYAQPGGPTSQQQQQF

PGYAQPAQVGYGQYPPGQPQPQQPVQGQYSQPGGPQQTGGPPQQQPQPQPQPMYPGGGQW

NMQQAPGQKFPGFSPAGSGPQQAPSPTPGANPYSRGPGYGAGYPRPAANYQQGYQ

>SG-PL_contig_9249_1_+1_196_429

MESDWLNQKALANQNVAHRLSIFGHITIFLIGDPAKLNIPPRPKATQPFQDLTSAIAFAH

THEVCHNNSGKFRQNTYR

>SG-PL_contig_9250_1_-3_441_1919

MHHGDTAPAVRDNTSRPAPAPARPPLNPSRTVGGNSVTSPAMKTSKSFGGAGAPTGPGTP

SSRVHKIATLTPYQNRWRIRARVSQKSSVRTWSNSRGEGRLFSVNFLDDSDEIRATGFND

AVDKFYELLEVNKVYYVSRCTLKTANKQYSNLKNDYEMTFNNDSVIERCEDDADLPCMTF

DFVAINEMEKHPPNAIVDVVGVVKVCHDLSTVIARQSQKEITKRDLQIVDQSGVAISLTL

WGSDAQNFDGSSNPVIAVKGARLSDFGGRSMSVLGSSQLIINPDIREAHVLRGWYDREGA

SMDFSTFRSEGPGAGGNSNWKTFGQVASENLGHGEKADYFTTKATVMVMKKDNCMYQACP

TANCNKKVVDQGNGLYRCEKCSREFPNYKWRMILQTTLSDYSGHQWVSCFQESAESLLGV

KADDLGDMRQNNEAAFDQVFQRGIFKEYIFRLRTKVETYNEESRLKTVCVNATPVDYVQY

GQHLLQEIKQLGL

>SG-PL_contig_9251_1_-1_7_318

MNSEGSIQAACQKLMKAELQGSILTVQRSKCPSYVGTTGIVMQETRHSFLFITPKNIVKR

VPKMNSVFALEWNGHIFSIHGNHFKFRAGERSSKKFKSKPTIDL

>SG-PL_contig_9252_1_+1_1003_1236

MCNQINFTIPPILTPDLQNCVVSVYSACLMILDHWSKPNNLHTSRNCVPSLSCTDNINTE

IKSYQHRNKIFHWLLYST

>SG-PL_contig_9253_1_-2_209_370

MTVRLQTDRSCCEVVHSFSSCKSAEGLLVAYLNISDIIVVNFTGLHISWHGTFV

>SG-PL_contig_9254_1_+1_175_603

MLFKLWMKRQITPLGRVAVLKSFILSKLIHLWILLPNPPDNFITNLQKMCFRFVWKNKQD

RISRKNSTKSINKGGLSVPGSKIMLLALMLIWLRKLNTSNHKWKNIAHINYTFLDKLDMY

GPNVTSRSTQDNNSGQMFLKHTK

>SG-PL_contig_9255_1_-3_432_566

MFVIPLTLVLGGLFLYHQKTDIMQENLKKAPLLCTEEEGGGGGPT

>SG-PL_contig_9256_1_-1_1_228

LSLFLCFFPCLFFVYLLENINVDLCILYFVTKSGGVGFVFLHVPRAAAIRSGMGIEMISM

HFCTTKIICDFCRFWI

>SG-PL_contig_9257_1_-3_6_434

MAALLPDLLVQAEAMEVSEDGTCRKSEGVQADGSKSIVNGETDGAKSTEDIMDVEMAEDD

KTTVNGKAKDCIKESDSSPGKIGYLTLADGCLMLKTADSHVQKEKPNVGDSSLTVKSSDS

DVKMEKCVLNGCDSVEKENVLDT

>SG-PL_contig_9258_1_-2_2_148

IFFSFFVLPHLGCCFNGVDAMRLILAPPHITTPKFIQLKSQCQETHGNQ

>SG-PL_contig_9259_1_+2_2729_3007

MNFTHTFTLILTPLSLPFNPQLPFTTNNPFRENFHALKFIDLKKKRGGQRLLSINLSISL

KQRLHDLKIMPITKPLNGSVNLHKDGEHTQLTR

>SG-PL_contig_9260_1_-3_135_863

MRLTVVAFSYFKKYVQREIAKATGVSVNKPLQGWREIRKKKWFYDENRPWTDAAKAANAP

NKRHRVPLLEPIADIDWTVFKGDRVEVLSGPDKGKLGIVNCVIKERNWVFVEGLNCKYKW

VNRSATSTGSMIKTEKPLLVTTEVALVDPSDGKGAQVQWRYTEEGQRVRVSLRTGRVIPM

SRIAEDETEDMVARSGYAEQDCDTKEADVQKVTFKPTLSTFDEDILKQMNIKDDRKRAPT

YWY

>SG-PL_contig_9261_1_+1_40_378

MVLNEPGQLIPCLPGFFHLKKIFPGTDRRLIPFQFPHCVKQSRNFLSAFIPVIVDGIAAS

VATPYCLKSRQGMEVMNTFRFLSGTTVRTERSFSLVRLPKLGSSVCRAADLLS

>SG-PL_contig_9262_1_+3_3_176

YQLSKSKLSSNPSCRGTCHAWKPSEVNYTYSLPINELHSFSALTFHYSFCILHPCTNQ

>SG-PL_contig_9263_1_-3_54_1691

MTSDEKEDNERMTQTYAALSIGVVCILIGIPLWWKTTEIYRVQLPYSDIQRLSQTKISYA

VSVEVISLNPKLTGKLPAIARSLQVALTQDKKTSVQPRYRVQVRDALDAERKLWKHSKTL

EDLDAAFEDPSKLRNSRYSILFLPESSHLVAKPVFVGKHGVIAASVSEANLDEVVQHLVP

LVQEVLVREGAVEKSLDAAQGLRTQKPDKDSMRWFRSHPGYDVTFTLINPQPDVLDVQWD

IQAGVQGYLKPFLNKLANFTTISVASQVLHFVGLVASPKKDSSHNFYFYTEQDLPHMINP

LEAKLGSHVSNRPSLNFVVYVPPRGNSPLYIKDVNGVPVASNAFLSPRWGGILIYNAETP

ASTTALPTVNIDIKYVMEVFLTQLRLLLNIHVQASHRQVDFSDLGKEGLTDWELAGWLRC

RCVENLATAKASLQSLAQLLDQIGNIVINDDIGREVEDAVSSIQHSEDLLASGHLQDAFV

ASHSAIVASEKAFFDQSLLELLYFPEDQKFAIYIPLFLPISIPVLTSLVQAVKWLRQLRH

NKLKSQ

>SG-PL_contig_9264_1_-2_266_1666

MESQLLGYRQKVDQMTVLLKHYQNELESQKVRRDGVETVSRLLAEARQENIALKKNMFAM

ETTMKNLKNRLVVNGLPSGVETDDNEVIVPGTSKQTLTNLAMENKRLRSMLQRDVVSDSI

NDNDSQTHSLQKSAEDTETEHALQQTIDKLQAENKSMKMKLSDLESLFKSSNNEKDRQLV

QLQEEVEELQQKPWQQTQPGKAVVGLPALKEQLQAFMSQCKDFEGVLDQMQETSAAREET

VVELRPVTKQAVERADGDCVDGVEIRHLQEEKQKLQDKVEEVTKMNQRWQEFFNERDRYT

QSLEQRVQELDERLKEAMRMGPTQEINRRIEQVLEKSQKEVGQMDELRQKAEDEVHRLSQ

EVATRDNQIAQLQSQVTILSHTTRQQSSAEVSSTIEHLKAQIQVCTEDFEKERQDRQVAL

QKVTSLQEQNARLQKLNTHLQTLVGSQQMVPTPASSRPPAQEHGQND

>SG-PL_contig_9265_1_+3_15_272

MALETFFPPTRMALETFFSNQNGFGNFAFGSTRMVLKTPPPPPPHQNGLGNFFPKQNGFE

NFFPPPEWLGKLFFFFKQNGFGHFFF

>SG-PL_contig_9266_1_+2_2_163

FKKMKNISLLLKTTKSLTIQGSYYDVRHCSKKKHYPMSLEHSWVLKHISVIELF

>SG-PL_contig_9267_1_+2_62_226

MTNRHQVLHIKNCEQKTNSFLQRCGILLEHEHELTMPEHHSISQQMYVRRTKTNK

>SG-PL_contig_9268_1_-2_89_733

MHSRLSFILACVCMHISGNVSAQTNNAVDTDIRRVHQYLADSHHRPLCVLIRKAGSQLER

RVMKLWSNRTQANNGHFTSWALAAVTITQVKSVPRYLLKNSQTRESAFLRCFLGPTLSHD

FDPSGPPTGKQLQQWFGQLMVEHRQLVNGSADMIEASVGHHRFTLVVFPTTQQHEELEQA

AFQITEALSNECEINIIIIHPSSANLRDILKTYKV

>SG-PL_contig_9269_1_+2_2_241

CVCARVCKLTHVHPCQSACVRVCVRACVRACVHTCVRVHISVYFFHVTTDLPCEIQAGLS

GGERTRRHNAAPPVFLCFFF

>SG-PL_contig_9270_1_-1_121_618

MSKRRPLSREQILELLDLVDPTERDSDDSFSPGESEESGESDEESVLALNVTATEPAPST

SRGQGVRGARRSSRIRAGTRGRGSGVTVDNNGWVAPENSVGFINEDDIPSFTGVPGMQVA

LPADRTPMGYFSLFVGSDMIAKFVLETNNFAKQVKELLPDFYYLKM

>SG-PL_contig_9271_1_+1_2338_2574

MLLKNISIIHPHLLVNIDMFFIRMTRNIRYTSAESKTAISLLFTAFNNPMLHHGPHLHHG

LTDPQFVKNKNMNNLMPTF

>SG-PL_contig_9272_1_+2_2_202

QQTNKNTNRKMKTVRARSVEQYSLRQKETRRSNSNRHPDSWTSVQMPRCCSFSRLLESMQ

SSSDQYH

>SG-PL_contig_9273_1_+2_2_319

KRSQGGQRKRYKDTLKASLKCCGINANLWEEAAQDRTIWRSLVNSGVAGYERRRIEDQKQ

RRQQRKSRACSSPQPGSHPTISCPHCSRHFKARIGLISHLRTHTSQ

>SG-PL_contig_9274_1_+2_2_169

VCVCVCVCVCACHCVCVCACACAYMCMCVRILAWIQSNVRYDKMVSVQLILGMTCF

>SG-PL_contig_9275_1_-3_306_542

MLLAKAVCPVVLYTFGFFVPSLDCLMYSTQQFVVNTRMNEAGSYFLLCIQHSCCEQGPDW

TVYSVHLINATRDGSPFNK

>SG-PL_contig_9276_1_+2_44_235

MLEHPKRSKSTISNKSYGQIFFIRCSYCCSCCCFLLLLLLLHKTGSWHLHTKTMMCFNVH

FYAL

>SG-PL_contig_9277_1_-1_58_234

MNSATRRILDASLSMNSATRRIVTKLNLSMNSTTRRILTKLNLSTKSAIRRTRDTTSSK

>SG-PL_contig_9278_1_+1_1_321

LPSSLAGLEVVAASVGHLASLQAPAGALLPAPNSPHPVPPPAPVDLSLALPGAGGASTQF

SDSGSPDPLHTAGNPLHYRKTRHKDASRGSHPKPAGARAAQYRPLSF

>SG-PL_contig_9279_1_+1_1_354

PPPPPLQAPAGALQPAINSPRPVPPLAPVESSLALPGTSGPDPPHTAVPGSGQAAFPHFV

TTVPGAGVPVFLSGFPVGLPPSSWFPSLYAPFAPWASVSTHHPAIFQPRVVGEGPSPR

>SG-PL_contig_9280_1_-3_6_332

MIACDSCEEWYHGDCIGVTQLDARCIKKYFCDACRAKNPSLEIKYKKKKTDRKSESTSDV

DVVKRVITKEYRSKLRDKVKVKKSSRRCGECTACHIKQDCGRCDFCKDM

>SG-PL_contig_9281_1_-2_731_988

MVLMAVSVILRNGFFLYIILCFKISVYSADINQIQLQFQFSHSSISMLGTNSICTNAANA

MEWKRIPNDNGISRTPAHKPYVLARS

>SG-PL_contig_9282_1_+2_395_580

MMMTCRVQVSMKSMLIAHTQTPKGQSRKSKSLRKARDKPSPGQCHYTNRHGLLPSFQTQL

KT

>SG-PL_contig_9283_1_+3_3_104

VCVILITCILPCGELDLESWITKTSVIVEITKGC

>SG-PL_contig_9284_1_-1_130_339

MFSRKPPYQVLEIHAKLLCSSCLDIVIETTQKGVLLCYLPIFMAFLYGSFVVLSFLSISF

LFGVCNLEVI

>SG-PL_contig_9285_1_-2_20_283

MYGLLVEIELLPLLNYFRLPKFASYPCVLAQISTEKCIESDLCQEFLGGRLRPAEVLGKG

SASHSSVKDRGMDIHYGILQCYMPRSLF

>SG-PL_contig_9286_1_-2_47_976

MGPPPGMPTITGLPEAQLVPSAQALQPQQTVMVPSSSMSSVMMTAGSLASSLPAINPGQP

GGPSMLLPTTLSVAPVPVVTSLPISTQTHLQLQPPPTALELGQQQLIMNQGPPPQPLQMN

LAPQMSVQTSMPMSMTSLASLPNQLAPAPTFNPALQPGVQLVSQPAPVAAPPVLAPPPAP

VSGPAQPQPFQTIFSPEVVSSGPMLTPGMTSLVLSSAPSSHIYSLATSNSMVSHTLGPTK

DVEPARRRFAEEKDDKIPESLLGYQHGPPHLVNLVCPSPPPGSQSQPGVPPNQPTLMPGQ

PPGQPGPQPR

>SG-PL_contig_9287_1_-2_68_3490

MSLSPSSELSVSVGLSRTSSLRRSTKKRQGVAANIRQQLYDQLKCLDIKLETEVSIVAEL

QEFFKRRAEVELEYSQKLDKLVKQMITRHRAEKQKQEQWATMSTYACWKHLLGITRKESQ

NHNIMSEIYSNQMVHRLQEVMDNSQRIFRKCREIGAESHEDILKVLNELQSAMKTYHIYQ

AEAKQAEVKLRSVESQKQKVEQQLSGKNASSRKLKGLERQAEKKQQKYSENKLKALKARN

DYLLCIEAANSAISKYFSDDISDLMDCMDFGYHNSVGRAMMMYLSVHEHLRNEHQKFMES

LNKCITDLDSRADKQKFMEFNNQVFMLPKKFEFQPFKGDEIRQISAQKEVQEDLVQRYTT

INDRLNSLKVENDEIWKSLETTEKTLVDMIQVKDYDITKFFIDEGAPPRSPHEAAKYRSD

RMETETYYLSKFREYTISCNRLARLQTKYSNIQKALGENQVSTTGPARPPAPPPKPKKRR

IGRTPPVGQPKLFGGSLEEYIEATGEEIPLIIKGCIRVINLYGLHHQGVFRVSGAQVEIN

HFKNVFESGEDALVDVVDASDMNSIAGVLKLYFRELREPLFPLHLFDALVECTRENDIHR

RLDRIKDLLGTLPRSIFIVMRYLFAFLNHLSEYSDENMMDPYNLAICFGPTLLPIPPDRD

QVSYQASVNEIIKTIIVHQEEIFPNDGGETYEKCILENSRNSIDAVDDDDEDETSSIPSD

EEEPEVKEAVALHDFEGRSGRELSFKKGDVLILYTQASHDWWEGCCHGQEGLIPDKYISV

KQPQREEGRTGSQGGGEEKTSSTQSLPAKPVVRQISNDSGLETSDKSTTSISQPNIVIRE

TDIIGVDPPEPTPPSTPQSGGGTPRDLTSSVSSGSDLSPIQEDHELKRQTSSPSSSSIMD

SSSVSTTTSDSGADELSLDIDNALEEVMAGLKSLEMQQKSDKRMSLPVVKLKQTPKHTPD

LVLDLPEGSNTPPSQDSSEPDSPTTAAETFAKSNQGTLKKAGGPILVPPAMPPVPLASSG

QSTGSSFQRSGSPSNRMSISSEPGDLAREAVAHSLMSTFTASPFRRPQQTSMPSVHHGLS

RHDLNIKTSGLSTFGSGSAIGSVVSSTSSLVGGIGTGITGSPGGDKEEGGGTPSQTPPPP

P

>SG-PL_contig_9288_1_-2_215_883

MKSWLLSLSFSLVVVCVVCSEAEKAKVTRKVVLDVSIDGKPAGSLHIGLFGGIVPKTVEN

FVQLVTGKNGYGYKGNKFHRIISDFMIQGGDIVNGDGTGSKSIYGELFDDENFNLKHYGS

GWVSMANRGPNTNGCQFFITLRECPWLDGAHVVFGKIIEGMKLVRELEDVETNSFDAPLV

EIIITDAREETITPFDTELEAVDNVDYDEDEEDEEDEGEEGEE

>SG-PL_contig_9289_1_-3_78_1706

MDQEDLWVDPDSQLVPMVPVTLLSATDSEPSTVVSPDGHTAASAPAESQQVCTVIYDFPG

QQESQLTLTPGDVILVQEKAEHGWWRGVVKGDNTRTGWFPSSFVAPGNLIEDGGKTTSPE

SPTSSTAGAGGGGPRPSTSSEEAAQPPPEPRPRITSFLIRKKPTKRPPQLANIPSLTQIH

NQNSVNKASRPTTPVKPISPVKVIASSLCAEDFSNSVKDKFYKALFSYKAAYRGEVSLRE

GELILGKERDRNGWMLGKKVTSGEQGWFPAVYVDEVSGDIPTTTQESQGPADMTSPQQNR

DVYSLLAVNRNKSPDQSWVAIEHRVIQDFTGQGEAQMSVREGDVVAVVQALDNGWWLGCH

GDRLGWFPADYVQLLEEKSEGKLDSFSLLPLCRDPHDTQRARHINNDNLSYSSLDTRDHH

DRPLSHNISQDSILSDGEKPKPSRRAPKPPLAASRQLQGSLGNISVSSTHSVNPRPARPA

PAPPSNKSRTASVGSMDRLESQPGTPTEGHVVAHKNPVKPRVVRIPKEKLGLDKLSSWPA

PVP

>SG-PL_contig_9290_1_-2_1925_2395

MIWSFYKYCVYILSFSLLCCVLTLCVCVCVLAHVCACVCVKYNLVLPLCVHCAIWQGVIW

LFHYLCTVEVVCKMRYLAQSSVIWCELSFHCQNTHFVKKWVFRRGIFPVCTYIELRVCVN

ACMMVCLLPILVCVYVCVHLYVHVLCLDPNCQCHFGI

>SG-PL_contig_9291_1_-2_2_121

LYMRPPVHSSCEYVPAVLNFREVKAGREVSGTILRNVTSE

>SG-PL_contig_9292_1_-3_3_170

KRLFQIEHLTNNTEGAIDTDKERRSSSNFKIVLGQQNSLCARLVRQSSTLKISVIV

>SG-PL_contig_9293_1_-1_1_810

MSSLFTDVMASDVTFRCYRCTGITEKSGCNVTSLRENPLDYVTECNGHCLNISTADNTVF

DCTEDVLTIRAKCFQKDDILMCICSTDLCNGPPLLTHTPDDVIQLPTSQREINMTSQQNA

NKTSRGFSATSQVNITSKEVSITSSKKEVGSKAPSNTSRELSMTSQKELNITAGKKVSST

SQKDVSITSQKDTINMTSFQENISSKEISMTSERDANKVTVPETSKQRQHNRTVIEAASV

SEGAAARNSDLYVVFVMVEVVSVFLRNSVL

>SG-PL_contig_9294_1_-2_8_304

MVFRDLQEYILYRVQNSYALRGNILPHGKFDSAAQQKFSSHLQSLSKGCFLHTKLVLDLI

EHGHLVVKGSNYKILPVNISEVFLLHFNLKFSSVRAFER

>SG-PL_contig_9295_1_-2_5_2122

MPGNDGLGPRVRDNNDGTICVEYQPKCEGRHEVLMTHNGTAVKGSPFSCVVDSVNGGFVT

AFGVGLVGGNSGEEETFTVVAKQGTAKDIDINIDGPAKVDIKRHDNPDGSTNINYLPMTP

GAYNIHIKYKGTPVKGSPFAAKVSGEGRKRSQLSLGNSAEYSLKIIEAEIVDLVGTVQTP

SGSIEPCILKKMGDGEIGIASFNPRSKGEYKVNVMRNDKHINHSPFKVKVGDKEMGHAAG

CKVTGITDKVTANKPNDLIVDTTGAGYGGLSVAIEGPHRSDIECFEVNERKYTIHYSPHE

PGIYILNVLFADDHVPGSPFLLNVGGAPSGRIRETVTRQMEAAEASVPNKMCEFILQIPG

TNPFDMEASVSDPDGTTELCDVMDLEDFHYKIMFTPKVNGTHTLSVRHKAMHISGSPFQY

SVGQLNLGGTHKVQVGGPGLEKGEVGIWNACNIYTREAGPGTLSMAVEGPGKAEIRLEER

PNGFLGATYKVPKPGMYGLHMKFDEEHIPGSPFMVNIAPDSGSARQVTVHSLRDRNLAVD

KPNTFTVNFNGATGRLHAAVRTPSGGHMDCFVQEMDTGMYGVRFIPKENGVHYVDVKLND

AHIPDSPFAMMVGCSHADPAMVSASGEALEHAKCGVKNKFVVHTAGAGSGLLGVFVDGPS

KVALSCREVDDGYEFTFNPFCPGDYLIAIKYGNIPIAGSPYKTNVS

>SG-PL_contig_9296_1_-1_1_177

WFSEHEGMIKRDRRGHPYCGVRGEILGSSQDEQLRKHLPSMFSLVKNESQRFEDDKIPS

>SG-PL_contig_9297_1_-2_2_277

PRPSSDFHPAFSRPPVASGLWPQAYLQEPVPSPLRQVNRPHPLCLAPGPAHPDTSGPRDY

FTFLHRDSQGARVCLQAALASLSPLPFNCFSK

>SG-PL_contig_9298_1_-1_238_738

MAGEGTNVIIAYDGSEHSEYALEWYIKNMHKAGNHVTLVHVPDLNDMLHSTRWSNSLYVF

DREVLEAMLKDEQQRVQKDLEKFAEKLKALGVGGKVKSVAAAKPGEGIAKECENAHLVVV

GSRGMGAVRRTLMGSVSDYLVHHCHCPVLVVKHPEHFHLHGMPQKMD

>SG-PL_contig_9299_1_-2_2_307

ERCLLSDHPSSTLPVSEVRVEGQGVPILGPPLRTVSGPVPVHQGGEGTGGLGQSGGHSAP

GLPGRLAPGPVPDLGHGTHLQYVKTCSYLRFVTNQEKYDLSP

>SG-PL_contig_9300_1_-2_272_1615

MTTLNFWKNSKPCQVVLHSSSVQVSYLKKSNKQVTADISFVYLLAVDSHYNSNADAKDDN

HKHSVSVHYVRPVVNHRLRVDTLTLHSSDLDTCNLLVKALEQELTGTHRPQRLAVIINPN

SGKKQAEKVFSKKVQPIFKLCGIDTTVVVTKAPKQARDILQTMKLDDVDGIITVGGDGLY

CEVMNGLILRTQKDHGVNVHDPDTKIVPCRLPIGIIPAGTGDMVVQYLHGTRDVMTAVVR

VILGGTVPTNAVSVHQGDQLSAYSGLVLGFGLQGDMMADCERFRWMGQSRYNVIPIGTVL

SRRSLDLEVEYVDEDTQQTKKTTDAMYSVDTYVVSKEEAGDRLCNVFGSHSMSVHMTKKC

TLGKHVQQLTKLKENTSGAFDYEFMTTARVQGYTVRIRNAPTVQEDGKKVLKDAYNINCD

GEVLRLTNPEYHVRLHRHAFCVFGEMIV

>SG-PL_contig_9301_1_-2_176_709

MVCLSWLHWCESHSRKTLRVQPPVVGHQPCTHTYQLLRFTRHCYVFSLRFKCYGVQTKRY

AKPQNCIINMFTTFLHTTAGPHSEFADGIVFEYCPILTLSKRGDHSSCAHASQLSFPGRF

CKLFSEMSKREDKAPPPKKQKVRHGRYLSSWATEKTFQGMVKESTDSREYAYCIPCAK

>SG-PL_contig_9302_1_+3_318_521

MDFAQWLTLVLAYTGSSQNVSSDSFQANFSFKLIYHLLFNQTDQSPNFELIYYTISNQTD

LSPNFQSK

>SG-PL_contig_9303_1_+3_39_425

MDSRSHVLSSILACNIKLACSLPILMLQSFWWCQCFIGPVPVPFSGPCQCKGFWAVSRSL

SDSCWQIWMECIWNCLSVMNHLLNKLQTHVFFLFSFCLLPHLLYSSCVTTPTLLISALQH

TSHLQTLQN

>SG-PL_contig_9304_1_-1_229_570

MDVSLRVSLKFPDQHHKCLYLSGLVDARIYFESSNCRVQTCHAVSILNRTPLHHHYYPHS

HHHSIVKNCPRFCGPSLPCSHGDLSFGGCGGVLESRNERRGGSDTGAVGVEVCW

>SG-PL_contig_9305_1_+2_2_232

FFTKIVSLFILLPFTAFKTITPPHLNQTHFSLHTFSYLAFCLFFSEKHFLSIKLTISTVK

TQAFSDITTCKVLKNLI

>SG-PL_contig_9306_1_-3_24_248

MCVCVCVHLSVCVCALVCAHICVCIYGMHVHMCVQVCVCVLEYMCECLCAHMCKLAFLHV

WNPVRTKLPDCVTQQ

>SG-PL_contig_9307_1_-2_1028_1381

MCVSQRITRFVTCTYLCPFWKICFIIKLVFLTEVGFFYNACTCVCVCVCVCAKWNFALFC

FLKREGCGIISVMSVRVCMHVRKYVKCGNILCCTFLLVCFVIVFVLFCSIKKRNCLNG

>SG-PL_contig_9308_1_+1_1_219

THFRLKLFIFCQNSTDIPYQNAMHSFFSVCSINTWKNKKCAGRGPFSSVNFVTSLHVLNA

HVNCAQSNFAKLK

>SG-PL_contig_9309_1_+2_2_235

FISTFVSKSTVVVHTKRESYIQITETERYRKHTSLFVLPTAHLRCTFQTRQYTGFPKVNN

NSLLSTSIRVFIAKFSSH

>SG-PL_contig_9310_1_-2_179_289

MANSSWNSAVQYLNRGSPIIRRKYSQIIYHQGRQRKS

>SG-PL_contig_9311_1_+3_3_119

SLKEWGKCFEKSFRGSQWYFSLSIVILLIFYRILGPWEE

>SG-PL_contig_9312_1_+2_29_214

MIALFHVSCQFPLDICQSLLGFEPLHTQHRTHNISQQPHQQSCSIHPSSPESHGDPRHSS

KK

>SG-PL_contig_9313_1_+1_1747_2031

MGFFRLFVVFFFFTLSMPTSMSVKSEVQSYAWIISECHQERCKNILVVYNYVGTSKLHIK

DVSVKNTHKMFQICQSLIRTLRMQHQVQKGLPFRQ

>SG-PL_contig_9314_1_-2_2_253

PNKCTFENCNKAFSRLENLKIHLRSHTGERPYLCQHDGCNKSFSNSSDRAKHQRTHQDTK

PYACSVPGCNKRYTDPSSLRRSEE

>SG-PL_contig_9315_1_+3_96_347

METRPRFYIKHPTLPEWFATFRMAANKSVFMLQSGTIFKYHILNPCLYRLNNTTLPFHFL

MTRQGVFEEVGWILHKTIQTPYLL

>SG-PL_contig_9316_1_-1_1_237

AVHDGRGRTGSYWELGFKCLVASCPYIKPATQHLGKDFLIIRLCKLGLVINLVWMTTLSV

NSLDSLRGEGDGRICLFRN

>SG-PL_contig_9317_1_-1_7_1044

MAETTKRTIRENVSINAQLAKMSDKTMELIQENDELKQKEKKQKQQIELLEANEKELAKK

NHSSQKIIRMLTEKCRNQEEIIADLELREQEHQELEAESELLRQQVSATREEIQNLAREN

EQLDDKLSTTQRDRDTEQGQKQQVERILARAAESLRAALRKTSPEDGEEEEDGMNDIERR

DHMLEQLLILLNSAAAVGLGPDPGDLGKQFEETPQRITRLPGSGKGIRKGEMPMSPMTKG

GTLPHYSLGDLGLIPRPKQNIPTFLERTQTLSATTRLGGLRKVLTRSVATQTVSAPKALF

YADQLLSRAPASTQDTVMKEMHQTQVPKSSPPLGPITSKQLLPSVH

>SG-PL_contig_9318_1_-1_1_210

AGKATSASSLLLCLLREQTPGHHTIDRLIQKGIVRGSGRKSSMKRLKNKSHYQPYKHWYC

LKDNSAKAPT

>SG-PL_contig_9319_1_-2_2_211

GGPLCCREVRCRDASRGSHPGPGWRARAQYRPLSFRPVDEARRTPRSSAQLVTWHFSRRP

DLRFFFKPWA

>SG-PL_contig_9320_1_-1_97_375

MAYKGGGQKVQKVMVQPINLIFRYLQNRSRIQVWLYEQINTRIEGCIVGFDEYMNLVLDD

AEEVHMKTQNRKTLGRIMLKGDNITLIQQVSQE

>SG-PL_contig_9321_1_-2_2_175

TTNKQTKRKLQETAVLKINFTKLNSEFDRGFRFGVSALTMNKLTNKRKTTRNGIKQLF

>SG-PL_contig_9322_1_-1_1_153

MMFLHSDSFVFHSFTGGDIYVVNLKRTVLFILQNINFFVCTEYHIFEMNVK

>SG-PL_contig_9323_1_-3_3_107

WVFFSFFLCGKVRCLDPPIFFLIENCFQITKKNSC

>SG-PL_contig_9324_1_-2_785_940

MQANLRFVEMVDWHTFLKNSSVRDGKYSLLYCDTETLITVTTVYFALKPGSC

>SG-PL_contig_9325_1_+1_1_123

MRTWNSTTSQPKWDFKAPSPTHSTNASFLSTHPSPVNDHVQ

>SG-PL_contig_9326_1_-3_132_356

MSIVFCRTGDLTTIVRLDVLGRYEKGRILIYLRSTFAPHPHPMFWDMMKKRKNFDILLVY

PSPFLFCDTMKKEVF

>SG-PL_contig_9327_1_+2_818_1084

MRAVPVSIDALRSPNLNGVRYPFTAGLTGGHTHKVFLQPDSNPCSQTGTRKVTSTPTRPR

ENVWKLINALKEWMEKWYSNYNETASFVT

>SG-PL_contig_9328_1_+1_79_246

MKYTHSSLKSQSASLSHICFTVNLQKNKPKYVSTRIWPCHMYAYLEIKCTAPCPLH

>SG-PL_contig_9329_1_+3_351_620

MMKDSFTHISNMKLKKKYKKISIAVILMHMKRNESEHNSEHYKDFQYCGILFSILLTSVY

GVFIICLDSPFHWLLSLCICMSHCLVRPHL

>SG-PL_contig_9330_1_-1_73_669

MTEPNAFIKCVIVGDGAVGKTCMLMSYATNQFPTEYVPTVFDNYAVTVTIRETPYQLGLF

DTAGQEEYTSLRMLSYPETSVFLTAFSVVMPESLKNLEHKWLPEIRHSMPKVPFLVVGTQ

VDLRDDEQVRLKLAKRRQKPAQPEDGERFAKRMGAYGYVECSSLSKQGLKDVFDEALLAV

LDPKEIKPEKKCRRRCVIL

>SG-PL_contig_9331_1_+2_86_220

MGKGLFCPAILTQVTSLAGSELQGHVYYYAAPGYSICSFSSLLIM

>SG-PL_contig_9332_1_+2_62_196

MQGLTVSQCTKAHSTHSCHYPTAWVSLQPASVQNITGREMENKME

>SG-PL_contig_9333_1_-3_3_80

PIHLAFSERQKAAGQLKHFITSDRTT

>SG-PL_contig_9334_1_-2_308_589

MLIRIMKRVKQEFESHTCHLSQCVGSLWIFFFQTFIIKNFLKNRPYAEGFFSVCWFFQTC

LFIFLKIKQRHRLSANHNYDCFWIDLMWAMYLSV

>SG-PL_contig_9335_1_-3_150_392

MGASLRVLLKFADQLRKCLYFTGLVDAQIYIMKGSVEYSPFMQLYPKWNSRASSLSSPFS

LSQHCEKLSQILWSFITLLSW

>SG-PL_contig_9336_1_+2_203_340

MHNYYYFTSTTSATLCYVYIKFGAYFEHLQMHKNKKPTPKQCSLFL

>SG-PL_contig_9337_1_-1_214_483

MCVCMLCVCVCVCVCVRVCVCVCVCVRVCVCVCVCLNQPTRSIYRSNDQIHIISVSEMVN

CEGQLVAVLFCCCFFQVNSDSKLSKINMLR

>SG-PL_contig_9338_1_+2_119_322

MILFSLPFRHPPFPSPCSAVLKHHFQHLHISSDHRTQIDCMSQKYHQPQIRIKLGNKIGW

KFSPMHRT

>SG-PL_contig_9339_1_-3_84_473

MHRLCVCVCVYMHRYVCVCVCVCADVWGECVCVCVCVCVHRCVLCVCVCACACTDVCGVC

VQAHAHVYVCMYMNVCVLQLKSGVDACFIICMMSQSVGSQPFCSFSVLNKQSQLCKVLFS

SQLFCSVFSF

>SG-PL_contig_9340_1_-2_167_439

MGRALRSVSRRRLAPLLRVARPSGRASGGGGELLGDRGTAVRFEAARPRGGVRLAGTGQP

CPPTALLCWIGLAGRAWGQWRVCRPTTRPVL

>SG-PL_contig_9341_1_+3_3_173

YLDVVSHTHTHTHTHTHTHTHTHIHTLLVTWYPDIWTQDGIYDLAMNHYLLDIMDIT

>SG-PL_contig_9342_1_-1_844_1134

MHIVRPTLAFGIMLLHSLHTLSSVGMHAGYFLISISHRILTWLTGSLMCVFDVLRVYAHE

GGSGTSRSAHILTWEIGKISTLYPPGEVPVVRTQAFH

>SG-PL_contig_9343_1_-1_22_435

MNICDNLGDHLVGNVYVKFRKEEDADKAVSDLNNRWFNGRPINAELTPVTDFREACCRQY

EMGECTRGGFCNFMHLKPISRELRRELYGRRKKSSRRSRSRSPRRSRSRSRDRRDRHRSR

SRSRERRRSRSRDRHGRY

>SG-PL_contig_9344_1_-3_60_221

MFFFGSPPPPPFLSFLTLYNDEVLCFTLNTRLLKCSACTWQDRLVCQVDLTISG

>SG-PL_contig_9345_1_+1_16_246

MYFCFCFAFLRVFTCRFIPGFFLLFSMSRKPRLTFWLNMLIHIAYTYIYSSTVKGVCVYM

CGLRYKTESATITKNFF

>SG-PL_contig_9346_1_-1_577_999

MQSIIDYASTLWDSASANVLKLLISLHKQALKLILLKNTSILDSDYRNLNILPLKLRLQY

NKGILMHKIMQGHAPDLIINNFSINQTRNTRKINIPIPRIDLFKSSLTYSGATLWNALPK

TLREPLSLTIFRKRYTTHLMC

>SG-PL_contig_9347_1_+3_99_365

MSSHVSVTMITFYLKHDTPINKQADQTFVPDLSPTPILDYLYNKCPKDHHLVVVRGLVCL

RRPPELCRQELQLLVGATKLGRSKSRGLT

>SG-PL_contig_9348_1_-2_2_172

THTHTSVNPPGISFEMIINSSCIYCDVFHLSHLFLFYFIYLFVHSLLHGYFISLWPR

>SG-PL_contig_9349_1_-1_229_726

MPGMVAFRRRWSVGSDDLVVPALFLVILHSAWILVLALIRGLIDFNEHHECTVDLRDHIF

GYIIILGVCVVMEIAVAWVSVRGTILSPEPRKYMEYLLYIRLVLGFVELAWLIVGAVWSG

RHYASCEPEAAKKALLGENVYTVPSLQGESYVTSPGWSRFTKAEME

>SG-PL_contig_9350_1_+2_2_121

VCLFVCFDQDKYSGLKLLKGRNTLSFLLRFCISLMENRLF

>SG-PL_contig_9351_1_+1_16_240

MNCPCMNHRMAHGETNMRDVSHRSLGNTAASSSAFVDITTPQNTAETTQTLCNKSKHFLT

IQLMLDCVMINIFLR

>SG-PL_contig_9352_1_-3_210_374

MFCDRMNQIAVDLTSSLVLISFLQTSCWSTYINQSCRLLVIFIKLRACRQVLCVA

>SG-PL_contig_9353_1_+3_3_467

VEFLKNFHLCCFNRADSVTVGHDCDTISNFCLIPGEFFKKLICAASTRLIYLDCTGLLEL

DKSEDGHRPKCRNGLQVGLFSFMCLCVCLVSSPLVVSGVYCQLLCSVCLSVSFTFMPTLS

LSLSLSHTHTHQQHHCVSNNLILTMLCGWGEGADV

>SG-PL_contig_9354_1_+1_847_1038

MKNVCTLKNFLFQCNTMTKHTQKKNKFKLRTGMVDSHYTGQFLFFINIFFLRIPDPPLKE

VDPA

>SG-PL_contig_9355_1_+3_3_299

WQLANQQFSGFLVSSDFTQSHSSWPVPVGFFHTTSGGSTLTSSFSGQLFAWSLTSSRLSG

CLFRTCHLLRSSLLMSQTTSVHKLKFARVIAYRTDLLIM

>SG-PL_contig_9356_1_-1_982_1182

MLSFFLVAIFSTVFTLYFPVEWSKYPVIILSIPLFYLHGILPMCTHHYPVRLFHYCFCFS

EYFLDEL

>SG-PL_contig_9357_1_+1_1_198

ALYVCLFVCLFFSVLNVLLPVRVFGWFRQIGILSNPTFSLLGLNFFLILLFKTQPLAVIS

FHNYMN

>SG-PL_contig_9358_1_+3_3_242

LKGYQKAPHRSKNTSRLLHRADSLLNLSTDYFLGDPNVDILTYCVPLKITFGLVQGATVY

LFPTVTKFVLNLIHLNSDSA

>SG-PL_contig_9359_1_-1_1_504

HSNWTKRIVQYSKRYRLTWVDQDLINIYLYFHPDHLYLLDCSVNYRTDHCQVRHPLKSKT

CKAAEAQGVHVLHGSRQAFHRPGTLKVFYDAFDQYKMGDNLSTGLLKHLQSTVYRPKTAC

RPKAQVFLHRLASTVRDIQNSAGHSATTTTRFSTQSHRLLFLTQDYFY

>SG-PL_contig_9360_1_-1_1_84

GRQSYLVLICRVIWSRYSFKILLNHNFY

>SG-PL_contig_9361_1_+1_1_207

GKHGVSRDEVNAHLLKFSTANKTGHVTFILIDVKATNSVFLIYSKKSGQNHNIHPLCTYG

IQFPQCSAN

>SG-PL_contig_9362_1_-1_1183_1455

MCVHAFIYACMHACDCECVPSFCGVCVCVCIYACRISELSKCYLYLSNDQNHTGQKICFN

FITTKAKIHKQHEVLQNINLQASHVQDICFL

>SG-PL_contig_9363_1_-3_72_1913

MVKNRFQTSVNIAKTRSFPGADVGSDHDLLMMTFALRLARNKKQGSTRLKFNTEKLKDPT

ISDAFEAKIGGKFAPLLALSEDTELDIFTNTFNDSITEAATELLGKHRTKSKPWVTDDIL

ELCDKRRELKNQRYNREGAKKYREVSLQVKRAMKIARESWIDNQCEEIENSLKHNNSRKA

YQTVKELTSTKQARTSTIQNKEGKCLTEEREIMQRWTEYCSELYTHKPTGDPNVLNVPRS

TDTAKDSILREEVVAAVSALKPGKSAGVDNITAELIKSGGDPAIDMLHRICNKIWQTGEW

PKPWTQSLVITLPKKGNLQQCQNYRTISLISHPSKVMLKIILNRLKPQAEKIIAEEQAGF

RPGRSTTEQIFNIRVIMEKYLQHQQELYHVFIDFKKAFDRVWHEALWATMNLYNINSNLI

KIIENLYSQATSAVFFKNNIGDWFSTTVGVRQGCLLSPTLFNIFLEKIMSEALEQHDGTV

SIGGRNITNLRFADDIDGIAGNEQELINLVNQFEKASTNHGMEISAEKTKIMTNSNFKTN

IKVKDQNLESVSRFKYLGSIITDEGSKPEILSRIAQTTATLTKLKQIWRNRNIDLSSKTR

LMRSLVTSIFLYAC

>SG-PL_contig_9364_1_-3_195_329

MSVFFSCEVGGESLFFVCLFFASFNSFFSFGLNTKFPLILSFRLV

>SG-PL_contig_9365_1_+3_3_197

SGNSVLAISSFLLLSLSLPFFLSFTVVCFFLPSRSIPLSFFQQAFTQFSLTPVYDVLSVL

FCSGD

>SG-PL_contig_9366_1_+2_1658_1900

MDECPCQTVSLIPNERIKQWPYGATLEEQLQGNKEDILKTASFILTTIMLKCRKKKERRK

VLYNAAPNTPKPTYVYPDKYT

>SG-PL_contig_9367_1_-3_66_173

MTSLQHYSTRRHDQLTTLFNKTSYNTIQQDVMAHRS

>SG-PL_contig_9368_1_-1_1_135

EVKVFGTDDLLTYLACSDCWFDYFLTDLSHCSFTLFSSYQMDKFV

>SG-PL_contig_9369_1_+1_1_204

IQNQEFYHDEMFNKCPCQSFYISGFKRNLFPLNTLFISNYISMILLSDRFILHPNKYSLR

VFHRFLQV

>SG-PL_contig_9370_1_+2_662_1036

MLFPVVKTELKSDLWCQSYRSLTAVRPGLIILRKLELNVRINHHQKRQVGPSKTSSTNAT

HQTVFAEMNVKSQKRQPMTPTAISVSSAKPCQVTYHSFRAGRTHISSIHIPTVKQFVIKK

TTTCD

>SG-PL_contig_9371_1_-1_1_93

LAFRCGHCGGPLWAGVCSPSHWLDDLIKILY

>SG-PL_contig_9372_1_+3_3_197

TLFPYTTLFRSFFFHLIHLFLLSYTHLRMLNLQFYLLFCFSGILLISFLQHTTKLRFTLS

PSLLN

>SG-PL_contig_9373_1_-1_1_174

STHRSYVCVTSYLSLFPQVCGHYFTLLPLGIFRTSFITIFITQLLTVRNLQAGLFLPI

>SG-PL_contig_9374_1_-2_77_349

MNKIDTLRIIIGEYLFKRLVELYNMYYQEISELPNIFFLVICTGTLGYLTVCVHAFLFFI

SCCLLFAFPSPTLWGPPNTHALRPTRARPRD

>SG-PL_contig_9375_1_+3_12_218

MVLNFTSKNMGFINGTQLYLKEHRIYKWHYKWYSTSTSKSTGFINGTQLYLKEHRIYKWY

STLLQRTWD

>SG-PL_contig_9376_1_+2_2_103

QYIFRIFSEGRNFVRTKAMFFNTTFSCTRHEHED

>SG-PL_contig_9377_1_-1_1_213

SGINTINLQQCNALNSQCILKLLTLHATPIPLTLASHLSTKPRFILSQSQCQSPQELSSV

SYDCKATHQKR

>SG-PL_contig_9378_1_+1_1_273

HNLSKSPGWCRGRAAAFQSGLHGCECRSGPSSSLCIKPPVHSAGKGYLTLLAIPTFLPIN

TETSVTIPSIVGTHKNVDYSTLLIAGEGKAA

>SG-PL_contig_9379_1_+3_3_194

TYLHEELILQQQISHLHEYPKLLPNVVDDKNRKNIAHFTLKWCYATSHTNNQEGLMNAKT

IEKD

>SG-PL_contig_9380_1_+2_200_382

MTCQRKANEIIIERICHQNGKIMVCRGSMEAGVGIRRERDDQTIPLLSPFLNHTFKHFFL

S

>SG-PL_contig_9381_1_-2_2_181

AAQFTSIRVDKVVLVLKKKKTTAGFLIQRRNHSVAKSKHQTSGNSLEHHISFTPMDVFIQ

>SG-PL_contig_9382_1_-1_157_363

MLLCYIHTLTFQTMYISQVASFIPYVFICKQLILPNVECFKLIFYLRPKMSIINFEIHCV

IPTWRNHHA

>SG-PL_contig_9383_1_-1_37_312

MLEQKAAAAAAAQAAPPNPMQILPPQQQQPEEEKKEEDLPQTLAQQENLKISGSNARHMV

MQKLMRKAECTVMVLRNMVGVEDLDDDLKDEV

>SG-PL_contig_9384_1_-3_147_290

MIKGGLFIWLDCSLTTVEIVYMRILALPKIKITRIQTAEKIQFEILAA

>SG-PL_contig_9385_1_-3_63_185

MPCLPLCLTDVSTGNSQFKYIVTIKPNNLVPFLMLISEKKM

>SG-PL_contig_9386_1_+2_326_469

MDSSHLDSSICTPPKLASYDLAESFSLPKILYAGKVLKLKRERGHMKH

>SG-PL_contig_9387_1_+2_8_301

MGTPYALPWFCEPPCLPLKGGMAAMQVAGAADVNMAPAGVEDRSSLPVGEKGSSTGGAEV

DGALPDSTPASAHKHRCNTLTNSPLVKRSVIIIIISCL

>SG-PL_contig_9388_1_+2_116_286

MYILSFVLVCLTVAAIHSPHLTSAISETSATGISRNNPHHHQHKTQRTKCSQQIKDT

>SG-PL_contig_9389_1_+3_909_1292

MSDGQIKSQLQYSNPNLYQKKTKKQNKFAAAILKMQTPAANDGKCSNHRQKHVSLLQAFT

YPVRRFHLPNPKDIVRQCLEHTDPRKQYSLGNALKHNHGSTLYKGSLKWTPHWLPLICFW

LCIFHFWH

>SG-PL_contig_9390_1_+2_2_169

PKHIIHHIKLENTRIIVGCIILYRLNLNRNYKRFKPLDEPGRTELWPYFYIFSRYY

>SG-PL_contig_9391_1_+1_1_153

RLVKRLATKKACSFTKFDDTEAVEWNTSSFIQESHVKGQRPKHFLLSRSGL

>SG-PL_contig_9392_1_-1_1_108

FFFLMGEDCGTIREIDKKLKRDETMVQNHTKYLKPN

>SG-PL_contig_9393_1_-1_109_1503

MVSCVDAHDDPIWSLSLAPKDQDGIITGSKDQTVKFWKFELVASTDQHSNRKHLSLIHTR

TLKMDDQITCISYSPDQRLIAVALLDNTVKIFFVDTLKLFHTLYGGSSPVLCMDISSDST

VIATGSADKNIRLWGLDFGDLKKSLFAHDDSVSCLKFVPNTHLLYSGGKDRMLKQWDADS

FLHITTLKGHLAEVRALAVSPCGHFAVSSSHDLSLRLWEKTEKILNLEDEKENEREEEEN

EAFAQEDEPVVAGEAATEVMAVRKNVESVKGAEKLMENIDLYLECKQSGVDPLQTTQGVG

SCEVMALSAVQQQVLTFKMLTYRADSLDDFMLRVLQKLRSSELEHTLMMLPFGYVVKLLP

ILERLLSNGIGWDPEMCCHCVDFLLRIHQGQITANQDLMTTIEHLHDLVKTRTKELKDLV

GFDLFALKMIQRKAEQKKEVKLFADATAKFRMKKKKKKVHAPVLS

>SG-PL_contig_9394_1_-2_269_400

MSYIVMIRYEYAFRENPGTVLVLFKTGTVIERVKYWISNWKVWV

>SG-PL_contig_9395_1_+3_3_581

GEGAEEGGGGEEGEGGGGEEGGGGEEERGGGGEGRGGEQGGGEQGGGEGRGGGEEGGGEE

VRGGKEGGGEEGTGGEEGRGGEEVRGGKEGGGGDEGGGEEGTGGEEEGGGEEGRGGEGGG

EERRRKKNDKIDDENEGEEMTEKKKKKKKKTPKTKKNHTFLIVINSYNDKSRQPSHTISI

HLKIVNNQAVTKT

>SG-PL_contig_9396_1_-2_5_343

MNCWGLNNAQKRRDVLHYLRNKNHSIYFLQDTHFDSKLEPYVSAEWGYTCYFSSNNSSSR

GVAVLFNNNFEFKIKKVHRGDNGNYIVISVQIQEANFLLVNIYGPNRDEPQFY

>SG-PL_contig_9397_1_-3_27_560

MFMPQLIQVRGMATLKDIRLRLKSVTNIQKITKSMKMVSAAKYARAERELKPARSYGAGA

KAFFEKAELEQDKTQPSQMIVVMSSDRGLCGGVHSGVCRELRGILGERGPGIETKLVVVG

DKAKSILQRTFKDNFMMTFNEYGRKPPVFADASLVANAILDADYKFDVASLYFNTFRS

>SG-PL_contig_9398_1_+3_3_137

HVHYWKEKGPEKVDCVSNAKMIYVTVTARILCALTRDIFFFKGSS

>SG-PL_contig_9399_1_-1_1192_1425

MIYGYFLKYIYLYIHKLLLTQSESHFIYSFRRIKVNFRWNASYSTDDLITPDTRVFSSST

PPPQCKDIASDHILFFFN

>SG-PL_contig_9400_1_-3_3_257

MKLHTCFKFISFLPNRLGISKHYRRQGQSVSMTSRPHPHTPSPSPLVRSEVTQHAVGRVQ

LALGKSTRKAAPTKKKLAAVRHGDS

>SG-PL_contig_9401_1_-2_35_190

MELISLAFVECWGVSRVWHSSFDLSVESFVKILLRYLNSLTCFEFKEITCCG

>SG-PL_contig_9402_1_-2_116_2596

MGYRPIARHSSILFFSLTDLPNIDPMYQYSLTWFVNLFIMSIHDSNKSKILERRLRYLND

HFSFQLYDNVSRSLLAKHKLVFSFLLCSNLLLARKDLDMEEFMFFLTGGIGLDNKLKNPA

PTWLLDKSWDEICRLANLRHYKKFREHFEKNVDKWQEYYSAKEPHKSELPEPWQTQLNEF

QRMIVLRCIRPDKIALAITDFVMEKLGKKFVEPPPFDLAKSYNDSSCITPLIFILSPGAD

PTMSLLKFAEDKGFSGTKFNSISLGQGQGPIAAKMITQAKEEGTWVMLQNCHLAVSWMTA

MEKICEEFTTENTHSDFRLWLTSYPSPKFPVTVLQNGVKMTNEPPTGLRQNLLQSYLNDP

ISDETFFTGCPGKESNFEKLLFGLCFFHALVQERRKFGPLGWNIAYGFNESDLRISVRQL

QMFINEYDEVPFDAISYMTGECNYGGRVTDDWDRRCLLTILSDFYNSHVITEPKFKFSPS

GNYTVPPKGAYDDYIEFIRALPATQHPEVFGMHENVDISRELQEVRLLFDSVLLTQGGSG

GGAAGKSDETLQNIAADILSKLPKDYDIEKATQKYPVVYAESMNTVLVQEMERFNRLLSI

IRSSLQNLQKAIKGLVVMSADLESLAHNLLIGKQPAMWAKRSYPSLKPLGSYITDFLERL

KFLQNWYDNGKPDQFWVSGFFFTQAFLTGVMQNYARRYTIPIDKLAFDFKILSKERIEGT

PEDGAYIYGLFLDGSRWDSDKGLLGEQLPKVLNEALPMVWLQPDLMDKIDTSTKRYKCPV

YKTSERKGVLSTTGHSTNFVLSILLPIDRPVQHWVKRGCALLCQLDD

>SG-PL_contig_9403_1_+1_985_1386

MYQYLEHIQNIKISRRLMMNTNRRWTLLKLCICVYHIILTCASNQTIPNWTYMNGSLVSL

QNSQQKKIVSKNHHSEPQTKLWSFIHGLSSNTIKEWLQAWTLPLVRFQYTWMAFDMGSAL

DLMFLSHLRSSLCL

>SG-PL_contig_9404_1_+2_2_220

QLHVLSATKAKQKLNPFAPAPYPPSFLWIGCGSERVNSYYRRVPASASYKSVWLYHRAPA

KYMVTMGVPLLLL

>SG-PL_contig_9405_1_+1_1_198

FSKHDENVSFSWLLFSQSHLHLFVTPASISSVGEFRGTMSVRFGIFLFKTLIAFHSKRKE

MLNVTK

>SG-PL_contig_9406_1_+1_1_171

HVKQEIRSLICVTPVGAAGVERTVPSGRVSQTKFSVSPLLYPRSPFQWILAGRNQSR

>SG-PL_contig_9407_1_+1_106_204

MPPHGSLVSSGQKAQTVYVGELQLDNIYLFVLR

>SG-PL_contig_9408_1_-2_557_784

MDLYQIWTKKCGLLRSCLCTSSEWRGPKSTSHVPKKCLKLALFRKGVSTASDNFKFCFHC

CDSLPLSSLHIKFYFI

>SG-PL_contig_9409_1_-1_1_177

THTRTHARTHARTHAHTHTHTHTHTHTLWILIIPCGINMQCMCSVFWVFFNQKLTDGHF

>SG-PL_contig_9410_1_+2_230_397

MRKENPQWNLFPLKIKHEKVPKILYTLAEESNSCSFCHDQPKVQSLFKRDSSHSDN

>SG-PL_contig_9411_1_+1_181_363

MKMLNYKATLLGFFNDIILALAAKQKKCLAITHQHYNQEATHTHTHTHTHTHMRARTHTR

I

>SG-PL_contig_9412_1_-3_3_257

LVLNVQFITEYCELYCACKVLTTVVASVMLYSKWAVQNQYNTKSIMYYYYSIIYISDPHS

SGQMRTIIYIILCTRNTLSPKFLIV

>SG-PL_contig_9413_1_-1_472_741

MHTFIHHHPIPLHTHKVSTPLVQMEFCRLQSPYTIKSKCSCRGYRKLKSNQQFKYFQIVL

FQTKYPINILLILCTVSLKYNFKTSVCFFW

>SG-PL_contig_9414_1_+3_99_251

MCTGYIRETAYICHNFGAVNQNVFLEFEPITLSTEVPYLWIKFKKKLMRIS

>SG-PL_contig_9415_1_+1_16_261

MQSYVYTISHPPLIHLHSVLHCFLKVVLLCGSLYKGWWSNTPTISFFFTPSDILANLSSF

FFFFFFFFFKKFTTFFFYFFFF

>SG-PL_contig_9416_1_+2_14_328

MLFAYMARMLSRSWPRTLWSSSTLHSISLFTLSKAFSLSIKARFISLSYSMVLSLSCLSV

KIASVHPRPFLNPICASLKNGSALYLVRCSSILPCILPTWLRSEI

>SG-PL_contig_9417_1_+1_865_1677

MMVRLWCSLLESHTSHTTHATHTTHTTHVRFVSCGNFCDDNFCCREEGGHTGSIHKGCSD

HLCGVNDSTFNKINILVVESIIANVSRFVHNLLYNYIGIYASVLRYRLGRHAQGFSNNVH

TSFLILIVSLHAVQDGYTAQQSRAPSSHDALLHSCSCGIQSVIDTIFFLVHFYFAGATNL

KHCHSIGQLSQPFSHLFFLVLGIAEFNIILDLLHTLLYVSLVTFALHEQCVILGDSHFSH

LAKILHLNVLHFVQVAFIPEHHSACGSSHVL

>SG-PL_contig_9418_1_-1_1_192

HPAKCLRIWIYFFQVIMWQIMICHKKNICIIRLVTARKQFEPVEHLNMTATCSLPAKIDC

RMLQ

>SG-PL_contig_9419_1_+1_517_924

MASIDLKDAYYSVPIYLEHRKLLRFKWGGCTFQYNALPIGLSLALRKFTKLLKPVFGFLR

KKGHVSTTFLDDSLLLGESEQECMENVQDTLDLLQWVGFIIHPSKLVLEQSESIQCLGVD

TESMTVSLQQRGFIAD

>SG-PL_contig_9420_1_-2_2_142

SHLLLLNSSPLCVNDVGRGENGSKLDSVSISKHFKLYAMLSTLPIAS

>SG-PL_contig_9421_1_-3_3_197

VKGIISSKCGIKTCDLDPLPASLLIHCLDDLLLYSPSIIHDSSLTVISLHHSSLPLSNHY

SRNYL

>SG-PL_contig_9422_1_+2_2_208

SIACCSFLLNKLPAPSSFIQSLSARKPMEASDVELLWATHQRRLCTAAGSLWWSSTLPVL

IQLTKDTAY

>SG-PL_contig_9423_1_+3_3_137

IEKRKFLAQNFQKAGMLFILKEPPASPWGEAKPKHVSCLDKSQLH

>SG-PL_contig_9424_1_-1_142_495

MKCVLLFTAVFAAWLGSSDAVKCQNITQCSPDFSKISGESEKDIKEACKALNSAFECFRG

ALDDCKSNANMPKQTLDQLQHQMDQVQDLLSEKCGSSSATTIKMSAAAIFFSTLALFV

>SG-PL_contig_9425_1_+1_1_144

QGQSIQGQSIQGQSIPGQFPQNYSTLTWDPRTQIHPRLIHVRLIHTGY

>SG-PL_contig_9426_1_+1_1_168

YYYYILFIAQPTTMAISGLSPRKVHQSQENDRAEHKTASNLKTKTLQKITIHDIVS

>SG-PL_contig_9427_1_-1_1_210

SYVGAYTWMMLMFCGFAAMRIWMSRLDTGVQLRTLLVASLFTMKATPCRCNLSSPMYHIL

YPLSVHVSPI

>SG-PL_contig_9428_1_-1_1_198

HTHTHLHIIHLFPIPHYYHQDQTTIHMYLQHHKWISYMQRIHCKCVILKTRFMLSHCLYL

MRGQGG

>SG-PL_contig_9429_1_+2_104_271

MDQNLMVSGCATTLIAEPQSPTHPLYSRKTPAISAMGESLHQEFPCKIVREGRAVT

>SG-PL_contig_9430_1_-1_151_324

MMYCLCCFAVVSSVTVNTAMGTRCPFCGVICLFGIFCIWVFCCCCFLFLFFVALKYFL

>SG-PL_contig_9431_1_-3_9_281

MECDTQCRNTSQDHGRWPSSHSVPQRIQFWKILVVSTCSITLYVLCSVELSSNMFYIKLR

KTKSIFKLVSVWRKTSFVDSVLYIVLEEVIC

>SG-PL_contig_9432_1_-1_58_303

MTALPKPGVLKPWVWLKSYRTEPQMNVWWLRELNLGPNSSHQRVPATNCNSLNWCLCDFL

CKHQHVYFGCCLCVYAHAHDHI

>SG-PL_contig_9433_1_+1_1_93

LHKSCQILIKIIQFFVFQVYNYQPVFDKAEK

>SG-PL_contig_9434_1_-3_3_455

CLCSCLIRRRLHRNLCKRKCVCFLSGRAVAQQQFHSLGLRSRVPVVRRVVTNGDFCGCLL

CLLSFVLRHYSGTAPVLFISVFHTSPNPNLFFPRSEQSLLGVYVRCLAHTVQALCCILRQ

SLVCMHAGCFRVSVSHRTPPDMVYRIFNMRI

>SG-PL_contig_9435_1_+2_212_370

MMGEKRLKIQRNDEHDIQMTNHMKQWQYQWTIKNNFAIMQMNNFKVVYKGLCN

>SG-PL_contig_9436_1_+3_519_776

MLRGRLQWLVSGCYWCSPICILSPILFTIATDWVLKKATENLGISWHEGGKLSDLNFADD

IAAYQITLESPKSLQTPLVNVLENWV

>SG-PL_contig_9437_1_+2_242_436

MAFSKVTVSPTANTTMVLIGYIYSSIFLLPDCDLHSAVFDTREEYGAIESERTANRTRLE

IGHKC

>SG-PL_contig_9438_1_-3_21_683

MQDTVNNLRKAQALYISRQQDLERVREMLQKAEGEKMEKRKKSEEEALHKSAEAETTYKA

CVAEANHRQRSLEKIKVELLGKVREQIYLCDHVIKSVTVDYFSLLHTVWSPVPVQYQTLC

ESSSGYEPGCQYAEFIKRQPSCASRPSRTDEFAFEAYTPGQKAGEARKVSVHSNGSSGDH

IHSNEGSPISSPRRDKYRIPVKAWGQNSSQSMAAACGSDTD

>SG-PL_contig_9439_1_-3_276_1112

MELETSRPSAKELEPSNPNARELESNRPRSNDDGNTAIDLSRKVSWTSRSNVEVTPHGKD

SRSTNREVSQVEQSQTPRPLHLGASSAQNTFSSSNVSFATTQNPDISACRQSKDASSTSA

ITQPSISSSEKLSASRRTNGAPKNLFSHSQDVNSSSVEASLDKICKDVWDAPSVHDHHTV

ESTTSTTTTINTTNKKAGGVKTSENDTTTSKKNQTVITSPTEHPHLPGTRATAQNGRCST

RKATVDDLEMLKLMRRVSTSLTRSRSSEVLPVGRLCDEE

>SG-PL_contig_9440_1_-1_178_279

MFYFKDYAIFCNALSDCVNVHSRIRCATELNVYK

>SG-PL_contig_9441_1_+1_28_306

MRNKQNKSKFYSISNSIRSNHYRNMNTNQVTYQLCLVCSGVFPWSDQIRFVSVCIITFRN

NNYSQKKRTNSLSVAYLVKLLGVVNTLDQMTTA

>SG-PL_contig_9442_1_+1_631_894

MVLKYLSYMFDLAYHQGKLPDFHLLDTPRTFPLLLTHAYNVTPSTRTVNAISFSIPHPSP

LFLFVMFFKAPHSQNDSKRKQYVHYNDE

>SG-PL_contig_9443_1_-3_3_173

PFHIAFFVLGSVAEEDITCSISDCTATLFSSQCVANYSVLPYGRVTQTRTTALIKCG

>SG-PL_contig_9444_1_-2_2_121

LILVSVDFISDRYFHTHILIIEQCVCYNLPCQSYNVVICD

>SG-PL_contig_9445_1_+1_1_114

KKKPNPKTYRVNNVSFLNHMDTLPFLNHKSTVPFQIKP

>SG-PL_contig_9446_1_+3_3_74

GLWDNLHFNYNELIDQTCDLVSVC

>SG-PL_contig_9447_1_-1_190_393

MGTGYPFCSVICLHSLFYLFIYLFIYLFIYLFMVTSKCFFCAVFIIWGWWIVRNGWRPQH

SLVLFALI

>SG-PL_contig_9448_1_+2_191_364

MSSKQACYIFKTKKCGFQFWLNIMETSSNPPMAIFNSSKILLFPHKIPIHSIHVINSV

>SG-PL_contig_9449_1_-2_2_331

VPLLYLLLLSFCFLHICITPYGIFQCQIGSGITDTCFSQYAEQSLIHKVDEILLCGLRLS

TEPIKHSYLARGHSVLGKKPINTNKQLISFYKFSCYSKNLKPSFTVIRGH

>SG-PL_contig_9450_1_+3_954_1199

MTGFNIEQNLIKGHRVYVFFSPLLAFQATFSLMRILTLFGPNCADVMLLQSLHTPSSVAA

NAAYFRVSISQHTPTWLTVSLK

>SG-PL_contig_9451_1_+2_2_106

ANFHPCNFYPRYKMSYQLPGPALQEAAVLKIRVRH

>SG-PL_contig_9452_1_+2_1481_1891

MDVMKLNTPPVLEITRNSRSLFLIHRLSLSLSLSLSLSLYVTHPKHSTSDKMTCESSNKI

YLRSCPNCPHAQQTGEPQISRKERFSQHHSDNNKNTGTSAALHFPIAVCVTPDVLLYKNS

VQLQQQAQSRSILNERK

>SG-PL_contig_9453_1_+3_630_893

MAMSGSNLHSTTDGRELQHYLCVAVSKSRSEHTKFSNITPHIAITKISYVHVIVPKLLDC

LCVVLSCNSLGHIRNHFVKSTKHSSVGR

>SG-PL_contig_9454_1_-2_2_130

MPYTQSSHSDILVISLWLDRVHILGDPQSVKLGTQTTTTTIIN

>SG-PL_contig_9455_1_+3_3_245

FVVSVHEILSQCLYNDNKKSIHADYTDHVDKEAEKLHFSHFNTETSSSSVFACFLIIGSF

CLKLCMVWSFGTPLSSPVTLL

>SG-PL_contig_9456_1_-2_146_253

MVDSPSSLLTLLFKNHCYPCRVAQTSTQRILQRKQV

>SG-PL_contig_9457_1_+1_922_1029

MQNFINANTKIIFHKLLGFFSKKYLKTVYDRKICSI

>SG-PL_contig_9458_1_-2_659_856

MQELSFNQYSSKTRYKKSKHLHTWKRKLNWKSGEDQRDSLYFVQKRKTNVSTMFHPFMGK

SLSVLL

>SG-PL_contig_9459_1_+2_143_349

MTLNMTILSLDDGRSGIGTNGVQLSALIYSKVIILITLSEAKNKEKRKHIYRLKTNRKEN

LHNLKKSLF

>SG-PL_contig_9460_1_-2_80_304

MPLAVIIRQRAWDCPNRLCNSDIYCACTDCLFALIKQDRSRSNVVACLSVSVSGLPTPTP

PRFLGSLGMCDFLRS

>SG-PL_contig_9461_1_+3_3_191

NLLSLVPSDKLHDCDLEINLSDMCSFNSSLSHTQIHNLFKICQEMAQISAMKYLEEFPLV

WLK

>SG-PL_contig_9462_1_+2_326_598

MHSSMLVVFDAGSEKTNKTDPLRQKCRQNGLHSFWCTCVYTPITCFSSHILSDQSSIHHN

LHQYMFVKMFNCNNIKRMKHEHICTEYLSPL

>SG-PL_contig_9463_1_-3_3_146

FFFPERSLITRSCSHYLHLRDSMRVPKLQMHCSCVMLNEVFSIIHVCW

>SG-PL_contig_9464_1_+2_2_127

FFFSVSNLFLKKKMTNLFLKKENDSHTMKTFHVSSNSQLKLK

>SG-PL_contig_9465_1_+3_1539_1898

MYFFSFCNLNKIVVGTAITHIQTQFFLNINTLTPNNILNRTILMGKKLNFNGILNRTVLM

RNNSIPSGNPVGSLKGTKIICINSCVKSQTSTKHNHQGDCTQFMHNDANLKTCNAVSKMT

>SG-PL_contig_9466_1_+2_443_658

MLVGTKNKLVSLTVNTIQLGDSIIPLSDSVKNLGVTLDNTLSMQKFLTQTCQSCYYQIRK

YLSTEAASKLVT

>SG-PL_contig_9467_1_+3_3_203

FFRDMILMLKQTKQILTNQPRQKQQLLSHLQRLQPPLFSRQLSLTKTKWFHLHTHKTEQI

SFEPDIS

>SG-PL_contig_9468_1_+1_946_1131

MAQEEEACRNRDRRNKNISCITRHRSVLKRRDSGKRLAPKDGMITEEIWRTSHNKEDTHQ

QY

>SG-PL_contig_9469_1_+3_3_128

RWIVPGLYSADVLAAVVEDRLSPSYSPVKHITLILVNLCFHL

>SG-PL_contig_9470_1_-1_532_834

MLKCSSPCTDIDAQDLKLSSAKYFVTQRNNSSSSAPFFSYLRSIQAMEHAYPIFVCVLFF

FKLVLQPLVGGWNSYRLSDTQLLREHTACEIHIHAEFYVVC

>SG-PL_contig_9471_1_+2_1070_1306

MSSHHNDYVQNEVKYTMSIVHEEKRGERHMSIIHEEKRGERHMTNTNLISNFTKYSLPNM

PERQLDPFNVYIFYIHMNS

>SG-PL_contig_9472_1_-3_144_254

MYYWWLVQEWTYSLFTVSCCMRLSDPVTCRSLGCCIS

>SG-PL_contig_9473_1_+2_695_880

MRAVPISFSLLVNPEQGQVLIHSWVDWRPHNVVKPDWNTNIQIGRMSSSSVPTRPLCWCT

YS

>SG-PL_contig_9474_1_-1_1_306

DSIENVKAKIQDKEGIPPDQQRLIFAGKQLEDGRTLSDYNIQKESTLHLVLRLRGGMQIF

VKTLTGKTITLEVEPSDTIENVKAKIQDKEGIPPDQQRLIFA

>SG-PL_contig_9475_1_-3_417_599

MRQFPFSLSCSMGFFRFTYLAESALFPTLGGNRTLVVMADDKLKPEHKTFSDCSPVYKQL

M

>SG-PL_contig_9476_1_+3_378_677

MKDLCEFYVGKRLRRLCELRCFHVDGIQEATFMYVFYYFCSLHLSAMNYFHSLHLSAMKY

FHFLHLSAVNYCLLHLSAMNYFCFLHLFAFHFFCFNQDKQ

>SG-PL_contig_9477_1_+1_82_198

MYIMRTENPEISHTCLPEIWLVTTLKAFYHFFQNQTRFS

>SG-PL_contig_9478_1_+2_17_295

MWYSNCPKVTPLGHCSVLSFLNSRQIETSIWHYCIQTHPRRHGMKNVHGNAARKNNKHAQ

IHLFFAQKLDACNKVVSSKSALTALKNHLGVEN

>SG-PL_contig_9479_1_+2_2_160

IHFMDASLRLLFKFPDQHHGCLYLSGLVNTRIYYERKCRVQSCHTVLIRKETS

>SG-PL_contig_9480_1_-2_1256_1468

MQCLLVGGLMATLLTSVDSSTVCVGGGWGLRVYEDGVCHLVCKSTCAIMQMQQLAFVTVL

TSCLGLSCVCM

>SG-PL_contig_9481_1_-3_126_596

MYGTVQVRQIPLEPIPISEQAEKNVAFHVEFSRDPVHLGPNQRLVLDNVITNAGNGYDVS

SGQFQAPVAGSYMFVVNFMGDIHTYNYVQVMLDGTMVDQSISHGDGSTWDHVSESLILHL

NAGQKVWLKRGSETSDTKSIRGGHWTTFSGFLIRADQ

>SG-PL_contig_9482_1_+3_3_239

PKKVLILGTVFGPCDLALVLFVPNSCINDSSTVHTFGPNLIKIRQETAQKILLTHFINRH

TDRHTSLPGNVKHLFKKKS

>SG-PL_contig_9483_1_-2_2_190

FHPRLPLCFAYLPLYCQICGENGNLSHYCLCVCVCINTDNRTANHYTLKCNLLLSIGPYY

THA

>SG-PL_contig_9484_1_-1_1_207

GADLVCRSCMFLSPEIALILIKIGRFVKLMERKVCAFAQYVTRFFSQTHAYIVKFIYSFI

LEHKVLNSV

>SG-PL_contig_9485_1_-2_2_130

NAGPEKSHDLVNTLLSSNCSGYGEILIHFSLFCDRDCWMCVLQ

>SG-PL_contig_9486_1_+1_1252_1440

MLSSIMYGVFVVFNKSPRILALCLSLSTWENANLTTLGHLCTRNIDPQAKKTVHRLRTNL

YEN

>SG-PL_contig_9487_1_-3_1068_1265

MYLQFRIMLQIFNVLFLNDTLLGFHSLVRYVVTFFFNPSLRRGVFFPLVMLLIVCVPLLC

FMFLWS

>SG-PL_contig_9488_1_+2_11_229

MLTWEIGKISTLYPPGEVPTETQTQAFQTLQSGSQVSSPFNHWAIITFRRMTYCYHYTVD

SAYKKLTYKKLRL

>SG-PL_contig_9489_1_+1_40_285

MNNLTDLFQRRWANARREWDTASISSMLMWRRALSGVQDPEMWAEPTCAPQPMHEASVYR

VTWGQGRAIGTPSAGSEVETHS

>SG-PL_contig_9490_1_-3_297_518

MKALIVGVVYNHLLMDYVNACQKCDVLLLGFGDSSIEAKIVRRWICTDFFTSRLLWFFSS

DLNLTRSFLSPRKC

>SG-PL_contig_9491_1_+1_1243_1533

MNKLTYLQEEPKNCCNKTSSNQCDISALRKSIGCRKQSYLMKENMLLDEGKKFNCFFFSI

NICWFFVVFFYFYPFLFTCINFKVRPSSKFSFLTDSC

>SG-PL_contig_9492_1_+2_2_241

FQSVDDSDRHKNSKIKQFADDTTLTLKEENDVNIATNVIEEFQEFSGLKLNQTKSKGIQL

GSRKNETGNVNNIPMRMDKQ

>SG-PL_contig_9493_1_+2_68_295

MTEASVISHWSHSVFTVYSSVEDRGHHHYLGSLTVLSIHISNCLLLPSSVRFVLIVLFIM

FNYKAVNCLEVKSPKL

>SG-PL_contig_9494_1_-3_36_2774

MIPEGLCRCGKLKKLILNNNRLITLPDILHLLPELETVDVRENPDLVMPPKPTELRKNME

FYNIDFSLNTQLRLAGAAPVPSSGNTPPAKSDPVARKLRLRRRRDGQESSEKVLKGMRDV

AKDKAEGKAKGKDEDKEPVKPKRWDEHLERPHLDYSEIFEEDVGQIPGITCWEIENFLPN

IVEDALIGKFYEGDCYIILKTFIDDSNSLSWSIWYWIGEKASLDKKACSAIHAVNLRNLL

GAQCRTIRQEMNDEDEEFLNLFDNGVSYIEGGRTSSGFYTVEDHDYELKLYRATGTQSVH

MERVPVVVDALDKNHVFLLDCGMKLIIWVGKKSNLVTRTKSRLIAEKINKNERKSTAEIT

VMPQGQETAEFWKLVGGPPINLEIKESTFTNLDLPKPKVYRVGLGKGYLELPQVEVPNNR

LVSSILETKCVYILDCISDVFVWIGKKSTRLVRAAALKLSQELHAMIQRPKYATISRCLE

GTEPQIFKTKFIGWDDVIPVDYTRTAESVIRRGADMKVIMERDKMQTDLSALFIQRQPTM

PPDEAEQLSADWNEDLDGMESFVLEGKKFVRLPDEELGMFFSEDCYVFLCRYWVPVELPE

GEEEDMDEEDLPEDEYKCIVYFWQGRHASNMGWLTFTFSLQKKFEALFGDKLEVVRQQQQ

QENLKFLSHFKRKFIIRNGKRPYRAGSPGQVPVKPSTEFYQLRANGSPIATRCVQLQKPD

SSLLNSCFNYILMVPFDSEDLKGIVYVWVGSKADHSEAQIAEEIAYKMYKDSYTIQMINE

GEEPENFFWVGIGGKKPFDTDCNFMNYARLFRCSNEKGYFAVSEKCADFCQDDLADDDVM

ILDNGEQVYLWVGKKTSDVEIKLAFKSAQVYIQHLRNKQPDRPRKLLLAMKYKEHRKFTK

CFHGWGKYKEVPK

>SG-PL_contig_9495_1_+3_9_404

MINGTWRRSLLDVRVRRGADVGSDHHLVTAALKLKLRKTGHKKTGHQQFDVEKLHDPREK

SAFVIQLKNKFQALADTEDHTQPDPDEVNTKWEQIKTAYLQTSEACLGTKQKKRKEWITT

DTWQAIENRRAL

>SG-PL_contig_9496_1_+3_267_563

MKMLTSVFFKLNSNLKQNFINHSKRPTAGLYMTRQSNHKHHNQYLEIKRMMLQVYTIKFL

SSKDTCQCSRIYTQQRTVLVKSIQSFMFCWKLKIKIKQQ

>SG-PL_contig_9497_1_-3_1074_1271

MKIRITSRLTMLYKPLNSSFALLLTAVCCSTQIILLLMVLFFTLCLFTKYQSLAFLLCAI

LKHPQY

>SG-PL_contig_9498_1_-1_124_243

MLILSGCVCRIIFILLNILNSFYLLRVDLELFTFEHCTSS

>SG-PL_contig_9499_1_+3_540_680

MVKIRPETTEKISLTHFINTHTHTHSSWEIRKIYLLVKKIEIKKARC

>SG-PL_contig_9500_1_+2_56_469

METIKPVFKDLANTELLRKCLEGYTQNPNESLNNLIWKYCPKRKNHGLATVNTAVSLAVG

VFNDGAQTYSKVMNKLDIEVGLHAQNCFARIDTERIINAQRQTKSASHEMRIARRRSRKS

KDEQQTEQEGFPYQAGAY

>SG-PL_contig_9501_1_-3_93_401

MVSVHGAVFVGVACACFVREISDLHLVIALDFLKRRNSALPLRELMEGWLGFRVSSWLFC

VDVLLLLLSIFGTGFKGPGSRSSVGDGGMDFQLQCFVSFFLCF

>SG-PL_contig_9502_1_-3_63_3353

MALKLDKEAFFRRVKKVYSSWAKATGEDSLAKMDALVTSVGVDEEVVYSKSTSLQTWLFG

YELTDTVMVLSEKVILILASKKKIDFLKQLEGNKENDQAVPPVKLLIRDKGDKDKANFQK

LISAIKESKKGKTMGEFSRDKFPGEFMESWRSVLKSGGFEKVDVSNAFAYIMAPKEESEV

KTIQRACQVTSDVFNKYLKDQIMEVIDADKKVKHSKLADGVEQALQNKKYVTNVDNSQLD

MCYPAIIQSGGDYSLKFSTVSSDNPLKFDAIVCCMGVRYKSYCSNIVRTMLVDPSEDVQK

AYEFLVKVEEEILAKLQHGTRLSEVYQAGMSMVKSERPELEAKMTKTFGFAMGIEFREGS

LLIGPKTNAPAKKGMVFNVNVGFAGISTGKEGKDCALFLGDTAMVNEGAAATVLTNLKKR

VKHIGIFLKDEDDAEEEEEEVAMPKETDVLLGRGRRNAVLDNRTRTDAPSEEKRQQHQKE

LAQKLNEEARERLKGMKSDGGDKKVRKSNVSYKGFGQFPQESEVKDLKLYVDRKYETVVL

PIFGVPTPFHISTIKNISQSVEGDYTYLRVNFFHPGATLGKADGNTYPNADLHFLKECTY

RSSNTKEPGEISTPSSNLNTAFRLIKEVQKKFKTREAEEREKEGIVKQDTLVVNPNRGNP

KLKDLYIRPNIVSKRISGTLEAHTNGFRFTSVRGDKVDILYNNIKHAFFQPCDGEMIILL

HFNLKNAILFGKKKHIDVQFYTEVGEITTDLGKHQHMHDRDDLHAEQAERELRQKLKSAF

KSFCEKVESITKNEVEFDLPFRDLGFHGAPFRSTVLLQPTSGCLVNLNEWPPFVVSLEEV

ELVHFERVQFQLKNFDMVFIFKDYSRRVAMINSVPMTMLDHVKEWLNSCDIRYTEGVQSL

NWAKIMKTITDDPEGFFDNGGWTFLEPESDNEEDDEDDEDEDSYAPTDAESGDDESSEED

SEESNWEDEDDSESEEEDLGSSEESGKDWDELEEEARKADKQKDRDEEEVRRHGGQNPHG

KHRSSHHKGGHGHKGGSSHGHKGGSSHGHKGGSGHGHKGGSSHGRKGGSSHGQKDSHGHK

RKHSHGGDKSPAKKKRH

>SG-PL_contig_9503_1_-2_101_307

MPSSSPSCECILILFRVREGKAVRERSGTPPQKADLRVLSHPPHDPVKVMANHKSISCIV

YYIECDQTS

>SG-PL_contig_9504_1_+3_66_182

MPVCFNLIICPFYYILQTSLIFLLPHYTTPKSFTPLIHS

>SG-PL_contig_9505_1_+3_3_245

FFLTFKHPLFQSHLLYSNETFHDYCLCQYTFLLPPHLLNHNHKQAKVSIHALYLLPLEMQ

NKFERKSHQQLQILHQTSILK

>SG-PL_contig_9506_1_-2_560_793

MPPFPSPLLFSCDLESSSCCLFSLLLIVFFSQITHGGSLGITQLGIIFIFMAEIACFCLS

STIGGISVLETWLNLLGI

>SG-PL_contig_9507_1_-1_67_195

MSILHTIGAFGVLYIYPQAMCAHTRIQYTHIYTHILLICMQKG

>SG-PL_contig_9508_1_+1_1_255

PRVYTAVNNEQKLENEIQTLEENTQLTETDSATLQEKKEQLSEIRKTKMDGIILRSKARW

ALHGEKINKYFCSLEKRHFVSKQML

>SG-PL_contig_9509_1_-3_9_1637

MAMHVPRAPGFAQMLKDGARHFSGLEEAVYRNIDACKELAKTTRSAYGPHGQNKMVINHL

EKLFVTNDAATILRELEVQHPAAKMLVLAAQQQEQECGDGTNFTLIFAGALLENAEELLR

MGLSVTEVIEGYELAVQQAQDILPKLVIGEVTNLHNKEEVRNAIRTAVMSKQYGHEDFLA

NLITKACVSVLTAKSTFSVDNVRVCKIVGSGLLQSDCLQGMVFKRQVEGDITKVAKAKIV

VYSCPLDAMQTETKGTVLIKSAEELTSFSKGEEDLMEQQMKDIVSAGVNVIVTGGKVGDL

AQHYCNKYKVMVIRLLSKWDVRRLCKSLGATPLPKVTTPRGEECGHCDLVYVDEIGETPV

VVFKQAKEESPITTLIIRGSTDNIMDDVERAVDDGVNTFKALTKDNQLVPGAGATEIELA

KQITSFGESLPGLEQYAVKKFAESLEVIPRALAENAGLKATDVLSKLYAAHQEGQQNAGV

DVDNAGSVLEAKQANLVDLYLTKYWGLKFATAAACTVLRVDQIIMARPAGGPKAKENKDW

DED

>SG-PL_contig_9510_1_+2_2_298

LSLCASQHWLPGKVWNWWRPRQRSGWWRWRRGSQGRPQGPWLGNNVPRSRGTESGRRGSV

DEQRRQPGKNTHKQRPCTWPPPDGGKGWNTERYNGPYRP

>SG-PL_contig_9511_1_-2_170_2074

MMAETVYGTHVEEGHVMSEKVQTLAATVYKEFEKVITRYDEDVVKELMPLVVGILESLDG

AYVEKQELDVEIELLREDNEQLLTQYEREKQLRKAAEQKYLEMEDAVEGEKKDLEDKVES

LESIVRMLELKSKNAADHVSRLEEKEAEMKKEYTKLHERYTELFKTHMDYMERTKILMGT

DKLAELTGSTVRVKGGYSMPTLRPVSMGPGVVFHPLESSRSEATYEPASPHSGHDSNVSL

RSEISELDSPEKQARETSGTSDKEQLTDSVNVREKSTGTIATTPELEESQDSVTSQGSMG

NRRSMRNDNSIFEELSVQDTDMIAEVDFGADITDSDGNEEEEEKEKDNDADSVSDNFFGM

GKELENLILENTELLATKNALNIVKDDLIAKVDELTSEQEILREEINSLQTVKSKLQQRI

TQLEEELKKTKEDIEKKNQATREEDEDDVPMAQRKRFTRVEMARVLMERNQYKERLMELQ

EAVRWTEMIRASREHPGELSTGKKKSSVWNFFNNLFSSSNKPKRTLPVATVKYNAPTTQV

QPVGDPNKKNRDKLTKDQAAQKAKAYDFLQDDVSTSEKMKKEREKERREQYKQVRAHVKK

DDGRLQAYGWSLPAKFTPLPKEPSSRSHVSVPVPV

>SG-PL_contig_9512_1_+1_556_1020

MIKCVESIGWGQISVKNNPSHSSPKHLLILAGKTSPKHQEQPNISGNNPSHSTPEHLLIL

AGKTSPKHYKQLVMYICVISHLFTDLFVLYNTRFLILQKHLVVKTATICQRNLSPSRGLI

AVASYSSILLSEHVTVQRRLSTQSQIKVIQVQHEK

>SG-PL_contig_9513_1_-3_3_176

KSGNYVGWSWWHQRKKNACHGLAISVFRGDLFICCLFVQVVLLTDCLRLAQGESVNVL

>SG-PL_contig_9514_1_+2_2_235

EKLWPRGACSPGRGLSRTYPGQAGGICQLLVLLGRTWHPGQGRVSQRIPVPVEWPCSTGG

TLSQSKLQEEAGASAKGW

>SG-PL_contig_9515_1_-1_1_249

LLSQCLDQAARARLGNIKAAKPEKAAMVEDIIIRMARSGQLAGKMNEETLKGLLENISER

TQKKTTVKFDRRRAHLDDSDDDF

>SG-PL_contig_9516_1_+3_78_392

MSSCFRKQVDEGLGICRLKPVPVTVSSAGMVVHVEGVDLSRSVGMVVLALVYHLPVNQHQ

CSFFDRTSDKLGVLMLHLCANELCAASARLWVHLTHHPPVYMDTT

>SG-PL_contig_9517_1_+3_3_173

FTENMLSELHRTFKEFQVIMVYLWIMYNYKYRYSTEGYICTHKHFLANTHRHDVHAC

>SG-PL_contig_9518_1_+1_160_357

MRSCSKGSRGLSVPLRVRGIFTATTISPSSWLRQCPNRYTIRAGRNLPDKEFRYLRTVIV

TAAVYR

>SG-PL_contig_9519_1_-3_204_1226

MSAKPTWMSGGSGSGVYDPVTTADNVRVQYSDQDDGWLRFKGRKYNFGTYRNSGLKGLCD

DIRNRSRPKCLLMGFIATVVLILLILIVVIASSRHSQLQTPGFEKWVNKSTGLNFQPIGH

EGTVFIPFTLQSGKEDSYLDYTKQLEAALEPYETIHIHNGPNIVCNDSYAPADAICQQPL

TVFGTGCVHAHRYGYSAGQPCILLSFTLPQNYTVRALAPKDGQVYVDAEAVLKERFSPKH

VGISCEPTTPEDKKYVGNTSALGDAIEFSPKMGFPTYFFRQRTVKRFVPPAVMVQFRTIR

DSVREPHMVRLTCTAWGKAKDPQGNLVNDKMFTTSFLFRIH

>SG-PL_contig_9520_1_-3_297_593

MYLSTSCKCLWLYYVHKCRLGQLCFLGHSTRWASASTLVSTETRKKIVKSRLFLSESDKG

CYNCWFEHLLICLCNFCLFGFWGFLMFLYRICRMWIQHR

>SG-PL_contig_9521_1_+1_76_516

MSVKLGYCLLIHRRELGPLRPSAQEICFAYPTSEGKTSEHAWSKNNQAVQAPQQTCDAVS

DHTPSTSLNLDPEEQTDSTKNMKANPLYFTPPPFEAEQLASGPLTNVCHLGITAGNIPEL

AWWQPPQTQHHHFSGPTGTTYDNSKKM

>SG-PL_contig_9522_1_+3_282_461

MIHAYTHAATLPLLIKKKEKKDAFMYAHIFLTKIPTLKEKTKNKNKNKKTPHHYRTKSIN

>SG-PL_contig_9523_1_+2_2_181

HSGQLAHPHNKKYAAQHDYMVWRNSSGTCQIFYIYIYSFINRNSCQVVREKARALGDDLQ

>SG-PL_contig_9524_1_+3_3_149

RSHDSAICSFNITSEHFIDKTIDKSIVETARAVPNKMSHHAIHVPDDSH

>SG-PL_contig_9525_1_+1_643_810

MELKGGVNYLHEYKSSQQSYDNTTMKRTLYKHVPCMYASNSPNMHTTHLSAIAPME

>SG-PL_contig_9526_1_+2_1406_1651

MYYVVMQVISTLKIQYCSNNRSQTSKTVVFFYTIKSSLRPKSKLKVFNKPRYDAHNIFCF

LIINYNKVFKQTRIQCPEYPAS

>SG-PL_contig_9527_1_+2_2_271

IHTSLHIHTHRTQKNILFHKRNTTIHTSLHIHTHTTQNNILSHKRNTTIHTSIRIHAHTT

QNILVKKFVTIPHNRTYTQNRIIYSSTRGM

>SG-PL_contig_9528_1_+1_103_252

MHENSRRRTKEGDGVVGWEEEKGGGYGFRVTFNTPLFPPFLPLRTRRVLR

>SG-PL_contig_9529_1_-1_106_270

MIWPSQSYQSSSLRLNSPALALGPFGSHPWSPAWTPGMLGARNASFAWSAASSTI

>SG-PL_contig_9530_1_+2_23_235

MLKIEFPHLCIDNKLVLSFTPSTYPPDSCRKLADRVRELSVLAVWTSADLTLKENDTGLH

HEKYSKWKKKQ

>SG-PL_contig_9531_1_+1_139_276

MTIIFHNFICAEQVHTEYSNTHTHTHTHTHTHTHTHTHTAQIRTDT

>SG-PL_contig_9532_1_+3_132_302

MTPNLCSENKMRKVTLSLRVTETARYVHICYCLFVCVPQLNTLGVTQYICTLSYESE

>SG-PL_contig_9533_1_-2_1775_2044

MCLCVCVQFSFHGRLFHTDIQGGSVCLYKSVNNNNNNNLLHQQIFLICVWMNLYNPTLST

KHVSSLSIFFFFLFLVEEKLVFPRFFLFFF

>SG-PL_contig_9534_1_+3_3_197

KGWCHTMAVYFPQGERPTFHAEKFHSDKKMNPIQSRTTSSNTSFIHLMKDSQFHEMFLNL

SLMKH

>SG-PL_contig_9535_1_+1_118_423

MESRFNPRYCIEDVHRFLFDEENVSATHGNLLEDCNCEFLFLFSCQFLHGFQKKFMAYLP

LRKNCFPSLHPDWLRNVPHSIIYQHIHFFFIAKWSLLIYINF

>SG-PL_contig_9536_1_-1_7_309

MHIPEETRKEMLKDSSVTFEFTQEAADTYKCIIGYGHRTDEHTFQLGVPFSFEYHGMPVK

MKRNITDGQLVEEYDFGGPQTTGVTRVEGNTIVSEDVCEGV

>SG-PL_contig_9537_1_-2_14_853

MTAAKSSETCLRRSNMVTNLNPITYCDPLGDKNVIATLKAVPSNQTRPEKSVIVVATRMD

MLGMFYNEFPSADSTVTGIVTLLATAKALWTLRRDIEHVANSKDVMFTFFQGEAFDYIGS

SRMLYEMDKGRFPVDFNSDNDNINLQKINLTDIGQFVELSQVGMRKDEDSLWLHVDPTVR

RDTQMQPLLQKLTDLGSQSNATFKSPSATNPLPPASLQRFLMEQSPIKSFVITDHQDSFT

NKFYNSHRDLPGEIEADYPSGLTPTEQYDFTTVQSQRITG

>SG-PL_contig_9538_1_-1_235_399

MHRSHPRTQGTTNQSINHVHGVFMHKLICLQYICCLKFLFQTYDVLCTICQNYCK

>SG-PL_contig_9539_1_+3_132_812

MTLANTLFPHKLSRRTTWHAPNGQVHNQIDFILAPRRFKSSINTARTRTYPGADIGSDHD

LVLMVMKLKLKKKHRAEQPRIRFDLEKLKVPEIATAFQAQLGGRFAALNLLDRDVEDLTS

EFKEGVLETAEEILGRQRKKKQPWVSSEILNLCDERRSLKGQRHNSTEAAEQYRRANRQV

RKKMLEAKEEWITKQCETIEQDMEKGNSKTAYETLKTLTRTQQHRTT

>SG-PL_contig_9540_1_-1_10_3057

MSEGWEYAPLFNMKFHAIERTTDLVRRRRWHRKMIAEDSTASCFFSLSKETDEQVSAALT

APRMFLAFDAPIKYQLRAYIYQARDLLAADDTGLSDPFAQVCFLTQSMVTEKVAKTLCPT

WDQTLIFGEIEIHGNPRNLERNPPDVYIELFDHDTFGKAEFLGRTKAQPMVKLDPADART

PVLQWYDITRSGKNGGELLAAFELFRLTGKDLPFMPPKRGNLYLVPNGIRPVMQRTGIEV

LCWGVRNMKKYQLSNVSSPSIQFECGGHVQISSIIKDTKKNPNFDDSVLFFDVMLPKEEL

YMPPMNINVHDHRNFGRKPTVGINILRSLEEFRCDPMAEPVEPDMQGGVGNGPPGGGEHV

IDMPTEQKKPKRSDSWKRAIVKTAQDSLSLPAHLMGIERSKKTELAVEELDWWSKYYASC

GETNKCKKYMESGYYKIKLYQTELEKSETHAGFSDFCHTFQLCRGKDEDDDDGNVVGEFK

GTFRVYPLPPDPSEPLPPRMLSDLPTSAPEECMVRVYIVKATDLQPNDSSGLADPYIEVE

LGKKKMNSRDNYVPNSIDPIFGRTFEMTAVLPLDKDLIVRVKDYDLVSSDDLIGETVIDL

ENRFLTKMRATCGIPQTYFVTGINTWRDSRKPKEILEEYCRKNRISGPMYYGNNSVKVGQ

RVYALSEFEKEKAATDEMGPAEERLALHVLNQLPIVKEHVERRPLFNTLQPGIEQGKIYM

WVDMFPKSLGPPGPPFNIAPRQAKKYVLRCIIWNTVDVILEEESITGEQMSDIYVVGWMS

GIDKKQQTDVHYRSLDGEGNFNWRFVFPFEYMVAEQTMVVKKKEHLWSLDETEIHTAPNL

MVQIWDNDKFSADDFLGTLGLNLNCIPLPAKKARSCKLSQLPDVEGLNQVKTGSLFEMKR

LRGFWPCYNDETGERVLTGKVEMELELITAEEAETIPAGTGREEPNANPTLDPPKRPETS

FLWFTSPFKTLKFIIWRNYKWVIIGLLLLLLLLLLIGLFIYSFPGEVSRWIVGNRS

>SG-PL_contig_9541_1_-2_2_160

RTFPVVSGSFPTVTGPFSRVKRYLLKMILTFYRVKRYLLKMMLTFYTSRPIFF

>SG-PL_contig_9542_1_+3_3_185

AIEKRKFLAQNFQKVGILFILKEPPASPWGKGDKDQQTKLRGDKKNKKSLVNIDFRWSLE

A

>SG-PL_contig_9543_1_-2_2_112

SCGLGLNSRHQILSLWRYQKNQPAHIKKPYEESENLQ

>SG-PL_contig_9544_1_-2_2_112

SCRLGLNSRHQILSPWCYQKNQPAHIKKPYEESENLQ

>SG-PL_contig_9545_1_-2_44_304

MTVVSSSSNITDYKMVIYWLSVSCNEVHFSGRSCNWLASLFIYTDKSNIEWNWGWNCIDG

QLHLTSCNYEFFRFCMDFPHTQKKLQL

>SG-PL_contig_9546_1_-3_6_983

MATRVLSSFPLTMGKRLCRSYAQIGMGGLPKSVKIVEVGPRDGLQNEKATVPTSVKVEFI

NKLSEAGLPVIEVTSFVSPKWVPQMADHSEVMNTISRNPNVIYTALTPNVKGLEAALAAK

ASEVAIFGAASESFSKKNINCTITESLARFQAVMDRAKEANIRVRGYVSCVLGCPYEGEI

KPEQVAEVARQMYDMGCYEISLGDTIGRGTPGAMRSLISTVSREVPLECLAVHCHDTYGQ

ALANILVALQMGISVVDSSAAGLGGCPYAKGASGNVSTEDVVYMMNGLNINTGVDIHKLI

EAGQYISNAIGRKTLSKVGQATHSSL

>SG-PL_contig_9547_1_-1_67_1311

MAGLKTFARTAFLRKAASSLRGLQDQSSIRGIGSLASLPETHEMVRKTCRDFADNELIPI

AGKLDKECRFPKEQVKKLGELGMLCMDVSEADGGPGLDYLAYAIAMEEISRGCASTGSIM

SVNNSLYLGPIKKYATEAQKERFLKPFLNGDRVGCFALSEPGNGSDAGAASCIAKLDGDS

WVLNGTKAWITNGYEAEATVVFATTDRQKKHKGISAFVVPIPSEGLSRGKKEDKLGIKAT

STSYLIFEDCRVPKENLLGELGFGFKIAMQTLDAGRIGIAGQALGIAQASLECAIDYAFK

RQSFEMPIIKMPMIQSKLADMEMRTQSARLLTWHAAMLKDAGKPFTKEAAMAKLAASETA

TFNAHQAIQILGGMGFVTDMPAERHYRDARITEIYEGTSEIQRLVIAGNLIKEYS

>SG-PL_contig_9548_1_-1_1_177

IGIYGGYQLGRQCLEASWLTTVLWLYRHAKKLRSLFLCSFTLIFFQKLSLLCSVMSSFN

>SG-PL_contig_9549_1_-1_10_981

MSLGNAVCRRCGDGFGMQEQIINSNGEVWHPHCFVCAQCFQPFPEGTFYEFEGRKYCEHD

FRVLFAPSCGKCGEFISGRVIKAMNASWHPQCFLCQLCGICMADSGFVKNAGRALCRECN

AKEKAKGTGKYVCHKCHDVIEINGHIKFRGEAYHPYHFNCHSCGVELNTEAREKDSELYC

LRCHDKMGIPICGACRRPIEERVVHALGKAWHVEHFVCAKCEKPFLGTRHYEKKGLAYCE

IHYHQLFGNICFVCNNIINGDVFSAFNKAWCVHHFACSICDRKMSQKTKFFEFDQKPVCK

NCYDKFPGELKKRLKKAYDEQGRK

>SG-PL_contig_9550_1_-1_1_201

GGGVCLFFKICDVLELTVGNFQLGITFCGLLIIPVKEKKPEITFYQLLLFIVAIYITRRT

IFIHFTI

>SG-PL_contig_9551_1_-2_2_157

GDLSMRLLNWKLFVCLWVCLALTRFDACASHPEIQQWQKSVDQKDPTQLNLC

>SG-PL_contig_9552_1_-3_3_113

AWPEWFLSFFPSVGHIVSVTVKLCMPVKKDRFSSIYK

>SG-PL_contig_9553_1_-2_2_160

KFVPKHMDKKQTSAVRSNLAREATLFPPKACTLQANTSFLPYTTSTILNAAGP

>SG-PL_contig_9554_1_-3_3_581

GISSEDIIPLVDKDSVPENLEEKLKELQTQIEVYKAKLEESDTKLKESSDKIEQLQKELE

VAQQDSREYLAKMTTLEEKVSQSDIHLNQMMENALDSVKLQLKESKKQSDNSEDLIKSLE

DHIRQLEDQLTHQSPVDALTALSSALDHSVDETVCESDTDSVVTINGHSPESSVPDSNLL

ASASSAADDLTNN

>SG-PL_contig_9555_1_-2_59_316

MCKRDHRLRKYPRSRSRSSQDQRSLSTNFVDLPPKAVGTYAHRPERLSSSRTSRSRQLPS

PVTSAKPRRPGNSQARLLSSEEEDDN

>SG-PL_contig_9556_1_-2_59_316

MCKRDHRLRKYPRSRSPSSQDQRSLSTSFVDLPPKAVGTYAHRPERLSSSRTSRSRQLPS

PVTSAKPRRPGNSQARLLPSEEEDDN

>SG-PL_contig_9557_1_-3_3_128

HCSKFTISMNEREIAVFMKNNYCAKKCVLDLSLFLNFPSLFL

>SG-PL_contig_9558_1_-2_2_184

LSVNARAPESATLTVGVRESGRCRRAIFIGRYCPTPLLEFQAQGEGAGSCCGQSRPCGRT

T

>SG-PL_contig_9559_1_+2_1181_1324

MCIHGTENVLFAQKNGLLIRSMLYKVYVIQVYYNCMLYWMYVVQVGPV

>SG-PL_contig_9560_1_-1_49_720

MDSSDIMECSCKLHDAVLEGNVEMTRNILHSPSCNVNESDEKWNGRTALHIAAMRGKSDL

VSVLLDSGANVSACDNHGNTPLHWCGHTETIELLIEHGANVLQCNKRGLTPRDMAERRGV

SGSAYSLLCQLELEVKEAEDQGHSQCATATGDPPAWSASVLSRKRKRSVSGQPLSGLWQE

CASELGLRNFLLLVLGILGFSLYITYILTGMHYGSHARIPVDVH

>SG-PL_contig_9561_1_+1_523_747

MGRLLEWLAHLVLFFNHATHTHTHTHTHTHTHSILNLPLNKKGDYLVLNWRDVCLSVNTL

YHQRFLACLFTKRNP

>SG-PL_contig_9562_1_+1_79_426

MAEISSKFPWSLESRTRRYELVAHFHVLHAHCRIQVSYSEDSNESIAHVQVLHTHTHFHT

QVSYSEDSNELIAHVQVLHTHTHFNIHISSLEDNNNKLITNFHVYHTQSYTRKLYR

>SG-PL_contig_9563_1_-2_395_553

MSDFPIISSTPLSLSLSLSLSLSLGDGTGVFFSFVFFSPWKVHKALTNNYVCF

>SG-PL_contig_9564_1_+3_285_455

MTSPFQYLKKKLNPFLTYQSSYICWREKTLLYQLLSCLDTMHHFPVQHMIHQILCMQ

>SG-PL_contig_9565_1_+1_13_216

MCMCVCVCVCVCVFAHVNTDAYIYILSVSSYCPRHAAPLFSEAYQSFFFKNLCWLTCSVD

TASHGLKI

>SG-PL_contig_9566_1_+1_1795_2097

MGEVHSDMACCPMCDCAVDVPDSLNLFYSFQKAPLLSVVVLRSLADILFGSRVLVSSLVT

HSHLLKSNQVSAVCMCDLYFFVLLCHCWLVGDNRFPLFIRF

>SG-PL_contig_9567_1_-1_406_588

MHTLFSLHQLQAVLALASPLDFTSVVERVGLLHGQQQVLLNFVLACVLELSSFTNSIVLW

D

>SG-PL_contig_9568_1_-1_169_246

MHHLNLIGCSLLSTLRSMFFSLLLQT

>SG-PL_contig_9569_1_+1_163_267

MNRQSTNKFLHHPIHIGCKLVFMFLISLMIRGDYF

>SG-PL_contig_9570_1_-2_2_73

PQSLLVVSSWHCMFVFLQPVTFVH

>SG-PL_contig_9571_1_-1_1078_1548

MQEIPHDHHTSISISGRPICNLRFVDDIDLLGVSNNELQDLSDTGDRLVARASAYGMEVS

MEKSKVMVNSTSNISVNITMNGERLEEVPSFKYLGATMSEDGSCTAEIRIQIATATAAMA

RLNKVQHHFPHQVQALQVSGALHTAVWVLNMRTTGRN

>SG-PL_contig_9572_1_+1_124_327

MSYFNHGVRFTRAIFFIYFKILKKVTGMHMYINACVDFGLFDNVCVCDIHIMCVCVRMWV

SACVYIYM

>SG-PL_contig_9573_1_+3_2226_2594

MEWENYNACSNISVMTLKSHLYPEYMVDSRIPTDVRYSQLASRKRTPGCPQLCFKDICKR

DLKTLDINTNTMVYIPDKSSWKQTVHRGLSSLEERELFFTEEIYTKAQTGQTSSKPAYHR

IHL

>SG-PL_contig_9574_1_+1_1_168

PLGQALFHNKYRNKLITVEILLDINTSECLLCAFLYLISFRSDVIIPQCSCHRITR

>SG-PL_contig_9575_1_-3_3_161

KYDFYIYIYIYFLALFDFFYFFFFGGEVGDFGAKIANSFGSDGETSFFRHVFL

>SG-PL_contig_9576_1_-1_1_129

RHEILSPSRYQKNQPARVKIYLTRLRVEPVTLRSSAWHCNQLS

>SG-PL_contig_9577_1_-1_1_690

PTLPPPPIPWRCSASISPRWFIFKDTVCGGCAERDCTFVIGERDFECKVVSLTSCHHLKK

LGVNCHTPRNGIGVNFVSSYQDLLGLTGTSGFASFGIFPGLTLIRSKIVLVQCLHFDWLS

SVLWDFCWFLLKVTALYAPNGLTRSAFAQIFFTLDQRKIVDKCGKVVGWTDASEPGTGNW

MKYVRSSDNIHRSERHGRSGRRTGILQGSERHSTRRRNVAIHSWRLVPGD

>SG-PL_contig_9578_1_+3_414_647

MLFSSVCSQNPWHSTMIISQHARQPVSVSSIHITACETASFRFIYPCSWPENKDHLTHEN

VSNVGLWVQVPPGLKSII

>SG-PL_contig_9579_1_+3_84_317

MRRLRVWLFFSTSPCRQPYSDFRGSRYVDLRDWKESPPFTHIGKSHHPLPTRYYLSKIKL

DVLLEVQYHNHLTLLACS

>SG-PL_contig_9580_1_-1_76_300

MNTLLLQCIQTGNILVLTMATQNKHIFTKTEKVNMLLLQFTESIHIGAYVLQKASNACFY

NWVLILLVGLILVSC

>SG-PL_contig_9581_1_-2_743_916

MKSQMVSCRVGQFKVSWMLFVFLCLFYFLFFLTGGLTVNICLEGNLRFTVVHKGVQIC

>SG-PL_contig_9582_1_-1_1_207

KKKKVMCNRKHFNQNKRKINTKPLKLTKNEIIIKGIYKTYVKYIHTRTRLYTDARWKHVN

QVKLSPLES

>SG-PL_contig_9583_1_+1_85_258

MAVLSVSVWFAHIWEIDKSLWLSAILVSLTNNEGLIFELASLTNNEGLIFQLASLNPR

>SG-PL_contig_9584_1_-2_5_415

MVQSGAEGVLQAMILSLGALHFHNQHLELNMDPRNLHRDFFFRRINYGNNTHVNVSVTLG

EDNKASIYVSLDRMDRPYYACDAGCLDSPVQLSNQWQKFPVKLTEPLTGILYITADKQHM

EELKHAIHVKEINEAPA

>SG-PL_contig_9585_1_+3_36_164

MAISGLSPHSDHPSQEDNCIEHKTSSNPKIKTLQKNTIHDILS

>SG-PL_contig_9586_1_-1_1_105

SRENQTMYELCIFIAIIVIVLGQLVGTEIKQSHPD

>SG-PL_contig_9587_1_-1_148_270

MEDNLVQIWNLVFIFGREHLRVQINESSKRTPTTTDLLYWE

>SG-PL_contig_9588_1_-3_162_710

MYGQIDCLINNAGTHPPHVPIDEVSAEELRQLMDTNLVACFLTCKYALPYLRRSKGSLIN

VGSLVTHIGESRSVIYAASKGAVEAMSKSLAIDEIKYGVRVNVVVPGNIWTPLWEKMAHT

FPDPAAEIETGKQCQIMGRFGMSQEVGKACLYLATDATFSTGTETLVSGGAELDFSKKSA

ISQ

>SG-PL_contig_9589_1_+1_1_933

MVKFKGRLSFRQYLPIKPIKWGVKLWTIAESDTGYTVNFQVYTGRDQRVSEHGLGYRVVM

DMCVPLQHTNSQVFFDNYFTSVQLMDDLKHLGIQACGTVRTNRRGLPAAIVPAKTRPKLS

KHQFLVAQKDSLLFVHWQDTKPVCVLSNYHDPLDRGTVSRRGQGAREDVVVPKALADYQK

NMKGVDVSDQMVQYYLIRHRSWKWWRRIFFHHLMVSMHNAYVVAKFHLGDAAARRIWPKF

QHFVEDVAHQMVTTTTTRAPPVAHAPRQGTLDHRLERIFERRKACDVCRRQPGRSGQTNF

GCVRCRVPVHQ

>SG-PL_contig_9590_1_-1_343_540

MFFSYRSKVASSTVVPGPSLSVSLPPLELPPRPDLLLLTRSGLRHKPSLLLLLTWSLCGA

RCPHEV

>SG-PL_contig_9591_1_+1_769_1143

MGTVKGTKGGMVVRGRAGWLEKQPEQWLISTSVNKISHLSMGGGNVRPGPQGLHLGEGSD

IAYGPNKAKVQSKTRDQQEIIHLCIQHIQQQTYCYCIPMCTDNHNVVRASIIYILANSST

LHMVI

>SG-PL_contig_9592_1_+2_2_223

ALTQTEQSLLEKGLNCAVSPEKLSMVWYQNSVMAHLSQLRRGSTSMLRMCRYSEADETPP

SRTSPPRKDKHSLC

>SG-PL_contig_9593_1_+3_1233_1430

MLRFHPFNVHINSCLKQNKKKQRNHVTEDSSGHRNKGTKRGGGEPTKIKRNKSRHDYSNK

KNETDK

>SG-PL_contig_9594_1_+2_626_1045

MKSNLLTVLKKKHWCSIAKAKSMYHCVMLHCGQGVQTSGQTTIRSAFVLQERQQSGQDNV

DLVRKLNTAYVLAKEELPFTKLKPLLELQQKNGLDLSTTYHVKAAELVHAIAKTMKEISI

LVSHLCVCVSLSLWKGGGWK

>SG-PL_contig_9595_1_+2_50_184

METQVIHYLLAEKRNSLLNCHSVLKLADPGETIDPLAKSTYVINQ

>SG-PL_contig_9596_1_+2_2_157

PSACLYAAVKGKKKNTDVRCSSFQINYRNENSHQSEVTGLPVPAMVHLPPCC

>SG-PL_contig_9597_1_-2_113_220

MFEMINTVKLFNNVLKRNGCQEMRFQVESMQVVVLS

>SG-PL_contig_9598_1_-1_1_237

SFWPKPEEKLLAKARGFSSHKLQVEVGSVWNLKGRWKMDFIMILIVVGDELYHHACPKHI

DIKLEFLPTENLHSKSMLV

>SG-PL_contig_9599_1_+2_107_265

MLSKRGETGHMLKSHVTKKLTNGKTTHRKTLKTRHTDSSTPSHKPIHFEYINV

>SG-PL_contig_9600_1_-3_3_245

KERHTHTHTHTHTHTHTHTHTHTHIYTHTHIQTQTIFLSFLCDNCCDSSGSSCSSNSIVF

SLACLMTDDCSCKMFSLVFTR

>SG-PL_contig_9601_1_-3_54_1058

MTVFDPKELGHLMVEFIGNVAKDRLIKTRMTCIDQIIQTDLFKLPECRKVLLPAMLQHTR

ELMENREEMDSCVNVLSHILGQLWGPDIGPIDGDVTVIMKTSLRTVIQAVIGMDRSSTLV

ENCVAVMIAILRQMSEAHYQDYIQNFPTTYDLLDFLMEILMVFRDLVGRSVYPSDWLEMI

MLQNSVILRALRFFAATIHEKFSNPFEQQLWNNFFHCAISFLTQEPLQLDNFSHSKRTKI

IVRYKDMRRETAFEIRSMWFKLGPNKIKFIPELVGPFLEMTLIPEIELRRATIPIFFDMM

HCEYTQPVPNSTRIQGNFHEVENEMITQLDVLVEG

>SG-PL_contig_9602_1_+2_2_157

HTGLSLYPDTAAVQLSSQDRNNFWKLPPLHSGMVALHMISPRVKMKGVVLKR

>SG-PL_contig_9603_1_-1_217_1653

MKQEVELIPETDEHFKEELTLDISREEPELEEVQFQIDTSVVDTDVSFKETIEVETEQVE

LADETFKQEISLQPQVQPSEIQVELEVKEEEAEMETTPPSIETLMEERPQVETTPSVATM

PEKEEEKPAEVEQLISEQLPEDVVPEEDVEVQRPREETTEEIRETITVAEETTAEVTQLQ

VDITETEEAGIEISEIERPQEEILEEVRESFDVEEKVPSEVLQLQVILDEQEEDMQPIHE

VAQPLEEITEEVRETIQLETEKPSEILVEIIPKEGSPKELTAAEVERPDEEVTEEVREVI

VVEEERPLKTEQVEIVSEDLEAREITTETEVTEVSVTDMELDTTEITSVQKESVFQVELV

QEDIETPHTEEVSIQISPEQPQEDVTQQISDLVVVQEEKPTDITALETPLEEIEESAPEL

ETLKPREEVTEEIRETIELDIPISEVSEMQMEVTPEEVGEVSLEIQFEQPQKDTVVDTS

>SG-PL_contig_9604_1_-1_1_324

QNWQGQFPQQGNGDRRGGPNNRGPRNQKQERGYNNNNNRGKKERRGRPSGHYPSAGSSVD

ADTDSWEEKTPKSPPPKPTPSAAQSSPVAMKSEMAPIVAESESLVPLS

>SG-PL_contig_9605_1_+2_2_181

FFSPHRLYILFIICYKTEQKKKAEEEAEEEKAEAEAEKEEEENGGERKKIKMNYNVIHYN

>SG-PL_contig_9606_1_+2_17_319

MKIILHILIICVCISMYVYVCVCTCMNYVILCYLFHWACSGEGLREGEGSGRGGVTGEDV

SERDVSVNGVNMMSPNKIPCSLSLAINYLVYTDITNDKMQG

>SG-PL_contig_9607_1_-2_65_340

MSYGLCVCVRSQMHIRACECVCVCVCVCVQTSICVCVCVWGSDAHACICVFLRARARACV

CVCVCMCVCVCVIFLLNRKTQCQFIHLCIYLQ

>SG-PL_contig_9608_1_-2_104_1081

MDGASEKSGLQGSEFIVAGKYRLVRKIGSGSFGDIYLGINITNGEEVAVKLESTKARHPQ

LLYESKLYKILQGGVGIPHIRYFGAEKNYNELVMDLLGPSLEDLFNFCSRRFTMKTVLML

ADQMIGRIEYVHNKNFIHRDIKPDNFLMGIGRHCNKVFVIDFGLAKKYRDSRTRTHIPYR

EDKNLTGTARYASINAHLGIEQSRRDDMESLGYVLMYFNRGSLPWQGLKANTKKQKYEKI

SEKKMSTPVEVLCKGFPAEFAMYLNYCRGLRFEEAPDYMYLRQLFRILFRTLNYQYDYTF

DWTMLKQKAAAVAQSTTTAVPTAGRG

>SG-PL_contig_9609_1_-3_3_179

PHARTHRHTHRHTHTHTHTHTHTHTHARTHTQTYIRAHTHTHTHTLCLSLSAMGDLNSI

>SG-PL_contig_9610_1_+1_157_303

MAERESLFVCVCVCVYVCVCVCVRARAHICVCLCLTVSVCACACVCVCL

>SG-PL_contig_9611_1_+3_3_260

DALNNTLTRILDKHAPLRHREVRADKFEPWYPLVQTELRAAKLHKRRSERAWLSNPLSIT

KQIFNAAKRQIAKIVLKAKTEFYKYK

>SG-PL_contig_9612_1_-3_117_377

MGSTSVNSHNLKPCVGHLTQSNTDTSINSRAVFGESVNFSPLAGTQTLANHIQTYSLFAS

SSRIQKSDYFQIRRVNHRGACPANLLP

>SG-PL_contig_9613_1_-2_464_703

MSMILPSRIRAPIITNFFPFFYIDNCIQLFFFSGHKSVYYINRKLKSVVTGHIRAKTGSI

AKLVINIITMNMYRLIIFGA

>SG-PL_contig_9614_1_+2_1283_1531

MMLNFFLRIYFSTNVCSGLDLAWKGTTDEALCVDTCLVPLTDGVIYLFICLFIYFEVCRD

LDLALGRTINEDVYIDATHAEFL

>SG-PL_contig_9615_1_-2_158_265

MALLFVFLPQYVYSSCFYLCSNLKYAGFWSCVTHSL

>SG-PL_contig_9616_1_+2_1739_1936

MLCSGEIIRGKGIFHPTLNTHHQNDWQIKMCSKQPNVYIFTLRVVLLWPSDLASIMICTM

PANLHS

>SG-PL_contig_9617_1_+3_768_1214

MSFWGQTPDTDDSPLTETDPRYTGNCGDQSKLNIPATVSHLFANMRQELLVTFPLQMNCK

ANQTQHTTFTLKQYSSFNPLPPNTSTVVMICLLCWSKIVEFVTSACCPMWSATISQSQCR

NNQSWHSLHTDNMANNRHNLQQQDANIEE

>SG-PL_contig_9618_1_-2_623_811

MQTSDALKCGQDKKKNSTEEMHALMVEQSTTMRHSHLHTVEAIFIVRMKRLKLKIFVLDI

LIV

>SG-PL_contig_9619_1_-2_80_1348

MFSWTQYFNTLGFSVTPADEVVLFHPGYLARLTTLINTDRRSKDLNGVLRDYMVVTLVRS

FKQYFDMGLFDMENSEADWMEEPWKRCTFYANKALGFATGAIYVRGTGQEGNVDNIQKLI

SYVREAFKEFILHKYWINSKTRLKAEQKVDNIINKISYPSYIMNTTFLDTFYQQFSVNST

DWFNNLISWRKFELKRQNDELHSTPDREKSWIRPPVTVNAFYSPIRNDVIFPIAMFHLPY

YIPNGPTAVNFGAMGSVIGHEITHAFDIQGRQYDEEGRLKDWWDPQSTKNFGITTDCMVR

QYNSINVQGQNVDGDFTLDENIADNGGLRAAQYAYMKWIEEQEEEAPLPGLNLSNYQAFF

LSYAQMYCSKWTDMGLRLHLLTDPHSPGAARVNGALANSKTFSWAFDCPVYAKMNPQIRC

KVW

>SG-PL_contig_9620_1_+2_245_430

MEQEERENKPTKRLSKYWTLFHKNTTEERDAIIFQKYTIFFYEFCCVVFVVVVIIIIIIY

CL

>SG-PL_contig_9621_1_-2_293_985

MKDLPVILKHLLPDETHQDQDSESVEQLLREQFDRERRKFHSDRNLPEKKDGIRIDSWWN

SINCEVYPLLTRLVKAALSCFHGPLVESSFSLMSDVIDKGKTGIDIETFSAIQTVKYSFT

AKKFTAVECFRKKNPLTESVDKKLTANMSNAWRKNNERKETNRRNQDMLNQQLGVKEQVS

KRKATEEIVTEAKKSRLQHQTAVSQSTDLESTSSKRTEQIGRASCRERVLR

>SG-PL_contig_9622_1_-2_2_529

SSCSPSSVDDYASSGSRYHRSRRSRSRRSRSRRSRSRRSDDSPDRDTPVADPPRESLPSG

PGGPGALLPPGRPGPLHLLCEGQGREDPFTQHPPPGAPLLCPSSDLLRLLEPPHESTGRP

ATGEPSHTFQVQPDQLPHTWPCLGLVEPHSCSRAEPSQWRLVEQPPPPRILDDSAG

>SG-PL_contig_9623_1_-3_123_1388

MQRLTTVFHHLNPSQLSVGMTDVAALPNYQRKADDIVVVSALRTPITKAKRGSFKNTYLD

DMLSAVFQGVLAETGLSASDVEDICVGNVNDPNSPLTARVAQFFSGMPETVPIYSTNRQC

ASGLQAFMNVAGSLKTGQCNIGIAAGVESMTKSPLGAGGFDFNPKISEFQKAQDSLIPMG

ITSENVAERFGISRKQQDEFALASQQKAAKAAASGLFDAEIIPVTTMVEDAGGQAQQVTV

TKDEGIRPTTMEGLAKLRAAFKEGGSTTAGNSSQVSDGAAAVVVTTRSEAARRGLPVLGV

LRSFAVVGCPPDIMGIGPAVAIPEALKKAGLTVSDIDIFEINEAFASQARYCVEKLGIPF

ERVNPKGGAIALGHPLGCTGARMIATLLHEMKRRGKRGMGVVSMCMGTGMGAAAVFEYQG

AN

>SG-PL_contig_9624_1_-3_501_617

MLKVPVCVCLLYSCCYRQYDMHYSIVSRCQSAASQPSLK

>SG-PL_contig_9625_1_-2_53_343

MRAIYLFQSPAKQPTHAGRVGGAEGRDPVLIPGQGPPGFPSPVWMNPNFNLFGPWSSGEQ

FRGDRVSSSPASSPSHDDGRDLWRDFSPFWALANTWA

>SG-PL_contig_9626_1_+1_112_216

MLLLLLYCENVFNIFVVPLSNSSNKETHFLTPSLP

>SG-PL_contig_9627_1_-3_42_440

MHVSDWYPTLVQGVAGGSLNGTKPLDGYNQWDSIRDGGTSPRTELLHNIDILYQPRGKSM

YPELWDTRVRAAIRVGDMKLITGNPCNGSWVPPPDEEEAFESKNKVVVVDSEATLFFSSE

LDEDKQKNVWLFN

>SG-PL_contig_9628_1_-1_25_1446

MVQQAREWEASGEYARAVECYMKITPSVISDQNIMEKCWMKAGDLAIKFLGPQKAQVVVE

IVGPRLAEIHRHGSAAELYLSLDMVKEAIDMFIAAGDWNKAKKVAKELEPKYEPYVDDRY

KEFCRNQGKAEDLANVDVIGALDMFADKGEWDKCLQMAEQQNNFKVLHKYVALNASHLIK

GGNSDAALELYVKYGVPAQQQNFNIYKRIFTDIINKRDLNKPEAYRVWADLRDMLFDLHE

NFTKSSDANSPQHEEFENMLIIAHYYCTRSAALSQKSLESIAGKLSVSLLRHTDVIPADK

AFYEAGIICRNIGWENMAFVFLNRYLDISEAIEEGTLDMLDHSDFQETDIPFEIPLPEKA

YLTAQQHEDIKEWVLAISMDQKVEQSLPKDERGTYEASLVATDTGIRSLPCVISGYPVLR

NKVEFKNTNKVANKDDWNKFLMATKVSHSPELQDVLKFVGKWCGATPNPSYSFQ

>SG-PL_contig_9629_1_+3_3_161

YFINKIQRQIISAMQNLLYEIFKIKKHMQDIHIFSKGSMCMKYHHHQERRRQI

>SG-PL_contig_9630_1_+3_51_572

MCAWRGKAKFPVYMKDKPIKWGIKLYELCESDTGYVHSFEIFAAEPGVSNRPVDVVMRLA

EPLLGEGRTVYVNNYYACPELADLLVARDTHCVGTVRPNRKGMPPQLKTQRLPQRGQVQA

FRQGAVAVLRWMDKKPVHIITTKVDPFLMMDVTTRRGDVRKPAAVQDYTVNTAG

>SG-PL_contig_9631_1_+1_862_1125

MRAQRTKHRFLTDSVTNDISLVSSDNLSLSLSLSLSLSLSLFTWYKLQLIFFFFLLLLLI

SLCCSEMDGCNQLSQQGNTTVLKQSGFQ

>SG-PL_contig_9632_1_-3_897_1076

MNCLYFICRLQSYVLGRMPDSSKITEVKMLKEIIIQWWLTQWRHSFLAVAIPGNVQILAI

>SG-PL_contig_9633_1_-1_4_144

MTLYCTPCAISSPSLSSPSLGKNTHKGSKSSESLLFSPKSLSPSKLF

>SG-PL_contig_9634_1_-1_1_201

ELSELCRHSPREFYRSIKAMRLRDDPYDPTSIINDKGGNPLNEGNKIKERWKEYFQELLN

SHQQQTT

>SG-PL_contig_9635_1_+3_3_152

YTIKSLLPTSQRGKNGQKTMHAFLILYTQPLQDAYILFSQLFRFLPFSEM

>SG-PL_contig_9636_1_-1_1_222

VEPHSCFRSEPISPEPISPPAHGHIGGWLSSSPPPPTQHCRGLGLLSSPWAGAPLASPRP

AGYLQGALSGSAPE

>SG-PL_contig_9637_1_-2_812_1033

MSAFLFTNENGFFLRFLPGTTLLLHAAYGTCIRFIVSSERLAAKPPLKGRGKKNPCLTRN

QTWVSHILGGCFIH

>SG-PL_contig_9638_1_+2_788_1138

MILTKCNWLYNHDLDLEKRGHTHSDTPNFLFCTKICTHMSSTPEIFGDQFFICLASVISI

HFKVTNSGLFPQPILCKHDTDLSITVALFSDPFQLYDDCDPNKWVMFIYIYSVSCVK

>SG-PL_contig_9639_1_-1_82_912

MFWKCVLVGLALVLPGTLLIVEGHSDQNGDGSDGASLLSSLLESKLHRQPYDSPQEKKSD

TDNDLVQDVESNENAIVQDASNTNKQPPESPGHSNRQNGQISAQVGGPKASPLSNAKADW

GGGGAVPSQTTRFIIIVTGCTVLAAIGIVGAGVCWYKYHKRAKAASLVEYPAYGVTGPTK

DHLPSPGDRKLAKSAQMYHYQHQKQQMIAMEKANGNMKHDASDDESEEDNVEGDYTVYEC

PGLAPTGEMEVKNPLFRGDETPSAPAPEESQQPQGDN

>SG-PL_contig_9640_1_+2_92_250

MNQENYCTRGGFVGVTMSCAVFDSRHTYSDISCAMCHVSEQSMCHVLLTGNDI

>SG-PL_contig_9641_1_+2_155_316

MMQIAMQQNQYRVYSTMQITQVRHDANSMQNYSLTPSIVIRSFSNVISLPMFCT

>SG-PL_contig_9642_1_+3_144_503

MVVSNFHAVGFRGGGAVCPGPESHLLQMRAVVSFGLAHVAHPGIISHADSLVRISPHFIV

AVGLLACAGAKQLAVRRGDQQSQDKHNLHGSTEVKDDQAGKTGTTTVVQERLQVQISPGK

>SG-PL_contig_9643_1_-3_3_1298

SSDLAEAAELQINSISWQYRTQLEEKEKEINTLKQRHHELLEQHAKEATLHVPGDTTHAD

SLRNTDLQSFQEGFEDWSNESWGDFDDSLRLQREINHLRQEVTRLRTDSQHWRSVAGQLS

QQGGSEVDQSAQHDLLNLQAKVKELEKQAANSREQAQHEVASLQDVHQNKMAALKKRHKE

EVTQLKSQLSSLEQQLADVRDGDSSGTGGTGGSSSMLPESERAESEMEKTLRSQLAAVTQ

DRDKAQTAMVHLQGVLDELQAEVESLQTQREELQAKVLELQGSLHSQDQHHQQQVESLLA

QLQEAERWRVEAEQSRQQENQLKFSIAETGTQLQQMSADTEDLIERLGREEAAQQTAVMV

DSLEQENLVLKEQCVKLEEQITLLEKRSTNQQEEWQRSQETIKELHWELKQLQDGTGTIS

DNTLEQSFTSGT

>SG-PL_contig_9644_1_+2_455_739

MFTVTNKLFVDAVHMRPQVFRLVIQNQLFLSVHLSFGTRISYIHWTFLLAIYLSLFIIKR

CSLYPAFLDTQAICESSHEVTTNVIQAFNTSNTIL

>SG-PL_contig_9645_1_+2_2_196

EPDSDYLVMWSVRITLQQDSAAVSQISRGQGWALNTEHCYGYQLGRNVYFLGSISWSQQC

QRTPI

>SG-PL_contig_9646_1_+1_1_216

FFFWVVPSRQIYLLGSAYKRCKRSQQSCNFSKCRSLVQKDSSNAGNIVLLSIISSNSCLQ

LQSFKIERSVKE

>SG-PL_contig_9647_1_+3_3_83

LFHQLNTFSRYIFMFLISGYIHKIVEH

>SG-PL_contig_9648_1_-1_1_129

GKAFSQSHCTPCVRLEFFVYIYICDLCFYCMPYERLEFFLCVC

>SG-PL_contig_9649_1_+1_475_807

MVETSPISDHFLRVGPTPHPCCFTFPNTEHVDTHSQLGGQVSLLFLAALLSPTVNRVRHS

FTAGGLVAAHTHVFLYIYIEQASIKLKNHTLIQLNKRAQNKRVHNKRTQNK

>SG-PL_contig_9650_1_-1_1_123

ELIRFTKLLEFRVSRILNILSFVRLYKSKFSAGFCGRQNVE

>SG-PL_contig_9651_1_+3_369_485

MAFKYNFQNPNDKFYSEICNPKTNDIPDNFTGKEYILNN

>SG-PL_contig_9652_1_-1_490_666

MFRGSVILFIYFYVLYLFVWLVVYVLLFFILYQILFCELPSSICGGVLQVFPPNTSKCE

>SG-PL_contig_9653_1_-3_87_1115

MANRTVKDAHSIKGTNPQYLVEKIIRTRIYECRYWKEECFALTAALLVDKAMELRYVGGV

YGGNVKPTPFLCLILKMLQIQPEKDIVVEFIRNEDFKYVRALGAMYMRLTGTAMDSYKYL

EPLYYDYRKLRRMTRDGKFELVHVDEYIDELLREERMSDIILPRIQKRHVLEESNQLEPR

VSAVEEDMEDMGTDEEEELMEESRRSLTPEKRKSPSPRRRSRSPHKRDHHGKSKDEKRRS

RSKEREKRHRSPGKRHKDEDDRKRKSKKSRSDRDRHGDKDHHRDRSDRDKHDRERDRERR

HRDKDRDHHKDRSRHSRRDRGEDMDIREANELRAKLGLAPLRP

>SG-PL_contig_9654_1_-1_1_147

VLKIHIISNDRKGSDHINNQVHNSHIIIFFTPEHVNGFSTYSKASADFI

>SG-PL_contig_9655_1_-1_643_1062

MSLDAFIGKWVEDKKEGFEQLAQSIGLAADKVEFYKHARTEISYARDGDQWTINVGIVGI

PNGSRVFHFVLGQPYDSVNIDGTPLKSIITFDGCAFVEQHKEVGPKESEMSILRKVDGDK

MEVQTTMGGSTMKSIYSRQA

>SG-PL_contig_9656_1_-1_199_390

MLLFVLLVSFRFLVLFWFYCYYFPSLQFVYFEYVLFNMQFANCAYFGFFLICWLVKYDDS

LVRF

>SG-PL_contig_9657_1_+1_94_282

MFWETVLNTMPKRKCTRNQWINGLNTLKNCFKKIAVRKMLNIKKMKSMKIIMIPIDIFQM

KKY

>SG-PL_contig_9658_1_-3_372_1538

MSVDVTTVVDFLTSTAASIIDTGSEISTGSGSIGDGHAHVERKHDDKMAQMLTTAQRMHY

AASAVVGPIFVALGMLGNILSIVVWSRRNMRSSTGRYLTALAVADSGVLLWFILTDTVKM

MHPEVVNSTAYAVFFAYLGYPFFFLWVICSIWFMVGVTVDRFIMVCLITKAKEYCNEKRA

NVGIGLIGGLCFLINVPHFWSFTVDWDKGVNATGPALLKTEFQKGEGGMSYELWVHCIFL

VLVPWFTVFTLNLLIISKIGKTNRKMSSTKTAQSADKSRQSENQITRLLLIVTFTFLVLL

GFQCITQCFYMVMPDGFDKTIIDESFAVAKLGVIINSSINFFLYCVSGRRFRQELLALLG

LKRRTLGLFGSSDHSSSTGTRSGTGTTGV

>SG-PL_contig_9659_1_-2_2_97

FLNWTLCILMILQFCKYSLTKVDFLPSIIVLQ

>SG-PL_contig_9660_1_-2_80_559

MSGSGKKGPGPAYNSPPARPSAPAYPVQQGPPMAPMGAPPTYSPAPPPGHHTYPQQPYYP

GSAVPQAPAYPQNQYAQAYPTTPNMAMYTPPPAGQTVVVPGLFDAGARFDGGAQPSIPPP

PPGCAPNAAQMAAMQGHQVMGTQQRGNFWTDGSGGGVSFW

>SG-PL_contig_9661_1_+2_41_160

MCNNFRCVYRAMKRPNAPQGVQRPKSSQESCASINMDTAK

>SG-PL_contig_9662_1_+2_236_448

MYPLKLGSTNFSCICSLFLIFKNLLKKAVARCGEAETNMEKGRQRIFQTSPRGKGGGGGG

ERGRGVFFGRR

>SG-PL_contig_9663_1_+3_276_596

MAIFAQLLQEHSGWREKKCVVIKPSTKETDTNPSPGLIHYYVSNGSLVGEAKGRRKGHYL

HKIVEWKKNRDKRETTRARLSEIIRKDQSRQAKPGRNNAAQEAILRA

>SG-PL_contig_9664_1_+3_354_788

MTAQWPQTCIRTSSAALTNLLHPANALVLQLASARQRLCFSQRHLSPLALLHLQLSSTTR

SSIKKFCYLGNAVSSNESLDAEVTLRISKASSTFGRLTKRLWQDYGIKLATKIAVYRAVV

LCALLYCCETGATSRGKIKCPTFKF

>SG-PL_contig_9665_1_-2_260_493

MHKCACVCVHLQVCMCACVISSSSSPFQCIPSLPTMFPAVSIILSCDTTKKMVRVDFIKI

VRVINIVIIIIIIIIIII

>SG-PL_contig_9666_1_-1_685_897

MLCNVIQFTAYFNATPHHNTPQRILQHQTIQHSTIQQEIIIPCRTVQSITYETNDRFQLH

KIIIHSCNRTC

>SG-PL_contig_9667_1_-2_107_352

MWSGGFLRHNCKIIVILAVKGLIYFVCAVSRSGAVQCSCERCFLLLLCFLFVVVVYFCFV

FLDPVRGLSAVTCNRDYTKRKS

>SG-PL_contig_9668_1_-2_398_850

MKNVYLVCIVEIRFTCDLLRKTQVPGSCFGVCLMYIIYIVPKMHMLYYLGLDNDWSVINP

NSNLNSDIRYASCAEMDRLCLSVKQRNQISEQVMVQKMPLVKHKMIYRTHTERYTSTEQC

TVFQTQNTHYACMTRNSPHLQHYITSYHRDR

>SG-PL_contig_9669_1_-3_69_3302

MRVLTKTDLDDSVVEDGDAQTILISDSKGLTTKIRDMKESQHLPNSIRLPNGHSLIGKRP

PYQQNGVIAMVSKQRQPVEDCEEEEEGTKTSTGDSVVDSLTDSMKGVSIQESSAARVELN

LEGKLSELRLNDNDDEEEDTRCASLQSQSSVITVDPPSLHHEEEEERGVEGAEDLAPRGP

TAGKPTSFRPNHEDYNHIASKSYSTHNMDRFHNQVAPFVPPQPTSRSYVPMEFSQQNSFY

PPAYGMNSVGMATSGKRGMDDDEENPFKYTRPVEEMGFTAAPQPKVLNDCNRVVKRGHYF

TQVSTELNCGGNVVDTSLSPEELARSLRSTAGTSIPFEHQLSFSELDTFLESFQVEHVPG

TTGNMRDHDPGYSSENSPPSDHAGSPGSSPKSAYSPPPPSNMSQDSGIASPYSDDNLSTV

VPSPPEESGHKRTWVSGSEDFIKPQYPVTEATQSGMYPSPPSVPMYHSPPSATMYHSPAS

VTSAMSPPGGTMYHSPPSVTNAMSPPSVYQNPPSVASAMSPVGMTNAVSPASMSMYHNPP

SVTSAMSPPGMTNVTSPPSVTANDDLSVASPIDYIFNSRRDELRDALDVIAEDVKNMNDR

KKAAIRPPVPTPAPAQKAIPPVSTNIPPTPCNIPTSVTQQKNTRPEKLPIAPLQKQVRPA

TGMAQQQQQQASPMMKGAVPVVVMPNPMAVSPVSTAAAPVTASQPVLLVNSGQNSSVLVF

VKGNQQTTTQAPKKKPVPIRPRPGPAISDSAPKPVGAKTAAKPDLASPKQEPNVSGTAGN

GIAPGSNGVRPSPSPSPANVGNRSAQGRGGNPSQSRMLNVARRLVASMTTPDLQFKDSEG

DTYLHVSVCKADGNMVQALLERMMRENLQAMVNTQNLMRQTPLYLAVSSNSPEMVELLIQ

YGADVNLFAEHTRADGRTKEVKAALHCAASNGQGYLPTLQALLAAKELNIDLVNSDGHTA

LHCAILEHGKADSEGGVINNLPIIQSLIKHGADPNAQGKKSGKTALMYALESRDLNLIEH

IVALIDPTRLRIYLKTQAFDSSTCQRILENLQRTLDDASRQRLHTCFRISSRRDNSAS

>SG-PL_contig_9670_1_+1_1_69

NQQLDHQHTESVSVFAIIVMVYF

>SG-PL_contig_9671_1_-3_156_731

MDVQYGVAVANKFALFCDDDLDPLEILRQKEEESKTSKTDSKKKKAAKKSAASTTESGRP

KQPEQVARKEDKPAQPRPNERGVNRGSRGRELRENDGSRPPRRQGPREFREGAKGDENVP

PEFRERSDYIRRDRGEPRGEGGFERSRGRGGRGGRGGRGRGGPRPEGGRGGGFGAPQGDR

RREFDRHSGNDR

>SG-PL_contig_9672_1_+2_764_1081

MDRERRKKQMTVKERKTAETKRAIEKRKQSTEFPEEGILFILKEPLASPRRGNGFTSLRQ

GLLSLINDSIWNVSATAKYVLMWRLSTAMISYQHNHHHRMQTCKIP

>SG-PL_contig_9673_1_-2_257_478

MLFIKKYFSVRFQMTCKQNLFFRLCVFDNEIQFYMVHIRLIQCLCCLHSSNKQHRSLAVC

ILHIHSMHSAGLED

>SG-PL_contig_9674_1_+1_259_516

MFYIHEKRNKECWQLMLHFKFNVQFAEEIDLTIHVLLKNQHYTFKNKPSLPLWAKVTFSC

QIWNDFNELLIQAVNLTQILPPHKNK

>SG-PL_contig_9675_1_-1_4_234

MFLCICSVKCKALRSLLPRMLHYTNLHHYYYLLLLLVLSVLARKEKEQLNIFIFYHCFRI

SVTSTQKMFNNNNEYNA

>SG-PL_contig_9676_1_-2_2_238

LQTLLTHSLASARSLSSLLGQMESLAPMVPADHLHKRPFLRQFKTLWSRTSQPREMQINL

SHWFLHFITQWLDQDWVSQ

>SG-PL_contig_9677_1_-1_91_342

MLIVHSLLSILLGNNRTSVLCTMALVFFLNSAPFVFLFCIFLNYHYYSTVDTSYNERYDR

FLQGLDHNSVYLSPRWWGVGGGIM

>SG-PL_contig_9678_1_-3_3_134

ITLQQPKHHFKHVSFTHTHTWVDQNHSLHYLQLTKSRKKMVQPD

>SG-PL_contig_9679_1_-2_2_199

VWLCARCFINVIYTSTCCLKYVCFLYRTVHTSVLFILRHAGTGQQHRQSCYAVSLKNKCQ

SLFLKK

>SG-PL_contig_9680_1_+3_483_632

MICTLKNKDTIFITILGTSTQQDFILLQCLFCIVTHDFPQYIYIYIYIYI

>SG-PL_contig_9681_1_-3_3_143

WAKTIYNHNNNNNNNNNNNNHKLLMFITQPDAGPFQVPCGQLVSKPK

>SG-PL_contig_9682_1_-2_2_193

NAQTLKRGNNQNSLTIVCCGDCHHVLVVEEQNKSVSSFYGIGWVSRRAGWVYSKCCTQGF

GMMA

>SG-PL_contig_9683_1_+2_2_277

AKRGAKRSPESNTIQLPLSLNEYYTLVDTYAWKQFQDNLKKYDNINMNFEFSKINNQFNV

SRIIQSLMYRWKLDAFKTKYTRNIVCLCGQKI

>SG-PL_contig_9684_1_-2_2_97

TGADNSRLSCNQYFMITSLVCLLSAMLIFISL

>SG-PL_contig_9685_1_-3_3_134

CVCVCKEYTGIKPSKLRPIEEKVRLLISERFGCFNLEENLFCIF

>SG-PL_contig_9686_1_+1_229_1173

MAKNDQSMHVVTIDLQAVLQAPCGQVSQLYYKRKLCVYNFTVYSLADANGTCYTWDETEG

KRGSCEIATCVYRYLSSLPRCIQKVTIFSDCCAEQNRNQYLAAALIHAVSHIDNLNVITQ

KYLESGHTQMECDLMHAAIAFSKKNNPVYTPSGWDIILRMARRGNPYTVVPLKHTNILDF

KRLASSVVRNTKTDTKGRRVLWRDIKCLQFRKGQRELMFFKSSFDQEDFNEVKLIGTSKR

NRPALVEKIPFLYSSKLPISEAKKRDLQFPCCSGRNTTPSTVLSPHLPLPLTDFLNQTPK

KMSKIQTKLLCVTTA

>SG-PL_contig_9687_1_+2_2_118

CSRSEIKRKFNKTLEHNQNQVMVWVGGGIQFHKGKQEAT

>SG-PL_contig_9688_1_+3_174_362

MPSRSAMLSRLLAKCRTKNTVNHERRLQAIHTVQTQAQRFRYCSLHSLLTSQKRHTLNKS

YLL

>SG-PL_contig_9689_1_-1_1_96

VCVCVKIHMLCACVGVSKLVCEIDSYASIFPD

>SG-PL_contig_9690_1_+3_114_485

MWDQNGANAQGHAFEEMFCENQLSPLTRDSPIGAREGDTDSPIAIGLISPQLTPLMSAET

LHSYGCDHVLIVLSITKHRLSKESITKPRNPFQYGNEKSDIASKLIAIRPKGNKKTTKKQ

STSI

>SG-PL_contig_9691_1_+3_1158_1418

MPHYNTHSYILFRDFSATKCQSSFNMKYKKQNSQGKRRQGSQCLHQHSQIHPAFVSIPFS

FIYIVNSLKHLSFYKQDCLRTSPSKVT

>SG-PL_contig_9692_1_-1_736_1092

MSRHTGFQRLAWVFMWDKRPVLEAHVLQALHGKESCDNNIMNFSEFELIDENRISILSLV

FPRSTTIMLPSVRMHTCIFQSVYIVYRCCGLNELWPLCSNTNINYVFRVSELILIMSWF

>SG-PL_contig_9693_1_+1_1_201

LRRQRSQPGPRHWRLAQLLPPPALAPRSVGHRLEQSLPLVVAVQGRIPRLLPPLPLSFLS

LLTWLRG

>SG-PL_contig_9694_1_-3_3_176

YYSANNSIDMMTKLCKILMTTTYTYLKRSVRLTQRYQPQCQVARLIISPHATPVAKQR

>SG-PL_contig_9695_1_-2_203_376

MQSVSIFFRRMEICIDVPCFSYSICFVLFVCLFFNVFLLLLLPPFCLFSSSHNFFVIW

>SG-PL_contig_9696_1_-2_104_241

MNKIAVSVGVWFVMNSSGSNEHKRFTILNHPLLKNNESDNLVFLIL

>SG-PL_contig_9697_1_-2_77_1270

MSQLLRVSEESPELLVQCRELMVAVASQFANSSQNIAQLATSIFVRLSSSAEGLETVYSG

EVFDTLQTVMAVDDTIRYRVYQLVVELSNISPEALETSSNSGLLQKLVNEIHKDDILLQL

NCLELLSDLARSNHGLTFLDTQGVVKKLEEMMRTAESSPFGAYLLPGLMKFFGGLARFHP

KEVLGHFHIFMGMVFASVAGQGDPSVKGLAIDTIGFIGSAPEGKSALDKQGSAMMECVGN

LGSVIRNAPSDIRSRALQAVASLLKLPVSDQTQELLQITEGWFSRLCPNPFDVIWNIAKQ

PFTDLRLSALQVFESLALVPWGQQLMNNAAGFKEYLLDRAIETTKESKEAKFEVVHVLVE

SPTMRNIFGEPYYVQLMEFYRQGPFFVRSQAEVAFEET

>SG-PL_contig_9698_1_-2_62_190

MAPVFPPESSVLCRSQLGPQNNGLSCCESFLGVSVYAVTIGCF

>SG-PL_contig_9699_1_-1_469_4044

MSRMGDQRVDASMPVKDHGSFVYHAPSDADIQDHRKQDTVYIGLHLPRQQRPHRHRPRHH

RHKKSKDDGRGFREEPSMAAPPSQRVQFLLGEDEDEDHKTHDVFCEMAELFPGAEDPNIK

EWKETARWIKFEEDVEEGGERWSKPHVATLSLHSLFELRCSLLQGTVMLDMDANTLDQVI

GMVLDNMIASNQLESNLRDQVWETLMARHRHQNEKRVRHKDDSRIHLPFVRSLADIGKKY

SEPKTLASQGETKGGLTVQGDDKIGTKSSPNITGLHRVGSGTTADGNTLTTNSSFVNTGA

STEGGLHEVASHRNLHFMKKIPPGAEASNILVGEVDFLNHQIVAFVRLSKAVILGDITEV

PVPTRFIFILLGPMGNQNKFHEIGRSIATLMSDEIFHDVAYHAHNREDLLAGIDEFLDQV

TVLPPGEWDPSIRIEPPKSVPSQEGRKTAASAPPQPNGKAVVEEETEEHGDPTLTRTGRI

FGGLRDDIMRKIPFYLSDFKDALHIQCLASFIFLYFACLTPIITFGGLLGDATDNYMAAL

ESILAGAICGVVYSLFAGQPLTILGSTGPVLVFESILNHFCSGNGLNYLEMRLWIGIWTG

VMLIIMVAFDLSALVRYITRFTEESFAALISLIFIVEAIVKLIRISSDYPVNRDPDAVLN

YNCSCNMTGIPLSPNISVDSIDTTNCTKFNGTLFGGGCDTPIYFGDIFFFSVLLFIGTFT

LAYSLKMSRNAPVFPTVVRSTVSDFAVLIAILSMVVVDFLVGVNTPKLHVPDDFAPTNPK

RGWVINPVGSNPWWTTLAAVIPAMLAVILIFMDQQITAVIVNRKENKLVKGRGYHLDLFI

VAVLIIVCSFLGLPWFVAATVLSINHVNSLKKESQCSAPGEQPKFLGVREQRVTGIAIFL

FIGISVMLTSVLRFIPMPVLYGVFLYMGVSSLRGMQLVDRISIWFMPAKYQPDYMYLRHV

RTNRVHLFTAIQVFCLAVLWVIKSVKSISIAFPIMVLAMCFVRKGLDKVFDQRELKWLDD

IMPEAHKRAKEDEIIKQEEQRRSLIEGSTSIPLAMGLNMEHITIQADIDQVNISEEMSRT

ALWKSIMHNESSSNLITPTEESRSSTKQRKGKKKKSREVNGSERSHNSPAIMEEDEPAEG

SPKKKNAPVKFYIDDHEEEGEKENLIKAPEIVIDPPSDISRLSSPDQDDSKV

>SG-PL_contig_9700_1_+3_3_128

QALKTNVSTHSSNDGRFHYHHLYVYTHFEVTVNTKGNDRLGT

>SG-PL_contig_9701_1_-1_61_651

MKCGQKTVTFIQVYAPCNDSYSEEEKAKFFEKLADAISSVPDREELTVMGDFNGRVGPRR

TPWENYLGPHSDTSTECNYNGGQILALCAEFSLWITNTFYQHRHSQTQTWYKWNNLNVSS

QIDFILTRTDQRKNITDARAIPDINLDTDHRPVMITSRQQKRNPPRNKHKRPDQINLRKL

RQEEVKEEVENEIMKKL

>SG-PL_contig_9702_1_-1_37_315

MDQEGRKKADDSKGKKKGRNKESNRKEEISSTEFPEGGDTIYTERAPGCGFPFPILSSLL

PDVCGVGTDSLHLALHFQGWQWSTVFLLLNSDS

>SG-PL_contig_9703_1_-3_168_290

MIFSRQKYCVVLEVREITDVVAQVFAEDLLFSYEHIVINVF

>SG-PL_contig_9704_1_+1_1_81

RQCVSDQQVRKKPAVINDIMDITKYTI

>SG-PL_contig_9705_1_+3_3_257

RQPLHHCTYGICLQQTDSKSNPYSILLPGFYYYLQSLKCVSVLDLEINFFPRLFCCLLNH

LLQFQSTDLKCLILIQIFFIFKMLS

>SG-PL_contig_9706_1_-2_47_985

MALVSRVFCRKFSSSSAMAGAIKHVTVIGGGLMGSGIAQVAAVAGHNVVMVDQTDDILAK

SKAAIQKSLGRVAKKKFADDGERQSGFVADVIGRVTTNTDAVAAAKNTDLVVEAIVENLD

VKQQLFSALDAAAPEHTIFASNTSSLPIKDIAIATKRLDRFGGLHFFNPVPVMKLVEVVR

IPETSDDTFESLIQFGQAVGKTTVECKDTPGFIVNRLLVPYMAEAVRMLERGDASARDID

VAMKLGAGYPMGPFELSDYVGLDTMKFILDGWSKKFPEVELFKQSEYLNKLVTEGKLGMK

SGEGFYKYDRSEE

>SG-PL_contig_9707_1_-1_1_252

GVPCDSASRVCSSLAYLPSVMVRCIFSSSLFPDLVFGHYGFHFWGSTRFEARCDRSPQGS

LGLPIDPLPPSGFVVQRGIDHTVP

>SG-PL_contig_9708_1_-1_202_426

MNNWLTVCLGLPLPAHHPSTSSYIGAVSVIQGACPCLCECGEKKGYLVLMNCGMREMYDN

GKEFVVNFRPRISYF

>SG-PL_contig_9709_1_-3_42_560

MEHLVPLRKENWGFFDRQKSLYSSMFKDMDDEFLEFDKELERMRKDMFTLKPLDFDSMDD

FGSSSMLKVDRPIVQDSSGNKRLSLRFDCKDFKPEEISVKTVNNRLMIQANHTEESPGRK

VYREFSRQYMLPDKIDPLTLNSILAADGVLSIEGPAPASVEAPRERLLPIKLL

>SG-PL_contig_9710_1_-1_112_408

MTEQHTSRLPCVSSAQDKGSKKNIPDSSKTPENVPRGSSKQVNRFTPVSSPTKPGSPVKA

SLPSASPRLWGYRPKPARTLSTPLARSQSARAPSADRRG

>SG-PL_contig_9711_1_-3_255_464

MNVTVKGIALGVLAGQGTDSCMEAVNWFCYYRFHIQMVPSLSLSLSQTVPLFPLPFPCIL

VFLCQNVFIF

>SG-PL_contig_9712_1_-2_80_322

MSAARIVNRGESYDDSCVRNKWRWEWTEKLVKVKPKPVEFERVGQHIQKMDVAGSARCVL

CSADIVYTNFLRAAVLILINA

>SG-PL_contig_9713_1_-3_3_203

VFYKLIRRYKYLEKPFEEAVHRVLVFQKGFQAEERQKLAIVTGQILANNLASPRVLEALF

KDHLVKE

>SG-PL_contig_9714_1_-1_586_1635

MVVKKVLSFIQAIDCDLVIFLYKAAESLLDTSVRPFITYAVCSDLLDSYKTMSAEPEYVS

DGVYRAVQHAQHNHSSIVCSQPDLPPEIESKVEQQAAGALVAYRVFTTVPAVFLGLFCGA

WSDARGRKLPMMAPSGGSCVAVIFYLLSVQVHRYKVTFLLVGAALQGLCGKASLVQMAVN

SQVSNNSTAEDRTARLGRLLACSFLGMFVGSLLAGILQDVSNLSATLLVVSACHGILVLL

VLLSVTETVVTSDDGESEDTKRLLASKSEYGGVGSSNTSSSEDCDGDGDGAQKQREVKGG

KGGVKVKRPSLFSWETLKESLMAVVKKREGNKRGVIIITLICLTFNHMLK

>SG-PL_contig_9715_1_+3_1035_1505

MKIKYNNITFTHILSLFRIYYCQYLCSMNNKQIINPGYDMISSSASNHHKKALCTLLKFP

DHTKNLFFFFFFLKKKKKKLLGGGGVFLPTPRHWGGGGGGGGGPRGKQKKGGGGGGGGQG

RERTGGILNHQRMHRKYLTLSKPIKIQLKEQRCYKRL

>SG-PL_contig_9716_1_+2_2_229

AVQKPKCPAKFVSTQSIRKINHDKFASDIAQFVTPNLSVSEFNTHLAAVFEKHAPVCQRK

IREGRPTPWYNTIANQ

>SG-PL_contig_9717_1_+3_3_149

IIIIKKIAITGTLRQAEKRSIFVLQTGTEPETQIMSPWLYQLSLLSQQA

>SG-PL_contig_9718_1_-2_104_919

MEEALKEEDAEGVTAKGQAVLDSNTEEELHMQPYLTFVAKLHSFMGTAAIMLEEYQKAQD

HHQKDLDIGDLTKDKEIQARALDNLGRVHVLQKHYDKALEAYVRRLEMNRDDDTSARIFH

EIANCFFHVQHYTQARDSARLAVQHAAVSHNSLIQLQSSVLMAVCHVKESQNDMASTSFE

KALDFAVQRDDQEAEEAIKAAIRSLKTKVQQAADKFKQGLRGNRMANRFFPMTDWVTHML

NQREEEGQGQRKRRHRHTTKEIPEWRFYAKNI

>SG-PL_contig_9719_1_-1_1_759

PRRVSARLQYRKKEQDEQDRILREAQEEEDRQKALEEEERRKKLEEEKFEEQRRQREERE

RAREERAKRVLLREERARLIAEGKEIPPELMNGIKNDGPEEQALEEEVETKLQKVLMHCM

KHSESWPFVEAVEEKYAPGYFDKIKMPMNLLEMENKLQNHKYTSKEEFVADMDLIVENCK

QYNGEDSDFALMARNLRNAFHRSLRRMFPVVRVKKDDEDFVINGKDAGVGRKYRVKRDAS

RRASESFPELLSE

>SG-PL_contig_9720_1_+3_348_578

MTELRTCNSTSPETGSSNLAWTSLALLFALLHDHRAVRHANRSMRTWTSHMTYSTPHFLW

GTDNNFISQVMHHINSA

>SG-PL_contig_9721_1_+3_414_620

MKLCVITPYTFESHLCTTTYLIPIQFSKSTKSEYWKIVTVCFLNRQINTHNDYAPNKLHW

IENRERVMM

>SG-PL_contig_9722_1_-2_2_202

YHCCHFFVLRKFPYDFCAKEITAYLGHALINLGLFVIIHCAVLATSSSLQMNCINKITYM

CIGVLIL

>SG-PL_contig_9723_1_-1_16_474

MPSKEQCQQLSKESDKSHSSTGPGMENVDPVGDKRTHERDQPDMISAEGSADQKSITNSS

SAESTTGAAGKDVKADNNALDSYSFCRLCNIPFDNTEKYVRHIRGMLHTQRQMEEEEQLK

SGASADSVRQTQDRPSSRSHRYPRPSGQKKQSQ

>SG-PL_contig_9724_1_+1_148_282

MVLITMMSTALYFFPYTQILIPLIQKSMMTVNKCLLPHPVSKVVF

>SG-PL_contig_9725_1_-2_353_493

MFEESSILYIEELYVVRSYTPFEYWFFGVFYPFHSLTLSTSFLHYQP

>SG-PL_contig_9726_1_-2_101_1129

MKEKLLDKDTIDAYHSYVQSEIRSDLNSISLGPGRLPTRFAQGLTVFGFCCFLVGILLIA

LRMQHVYFWDWEVQFVGPFFFILCLLCLGGATYLVVIASRRANRFRRELYFRPLGDYGIA

AVHRDDLLYEHEMKHDLKSGTTPHKTVLPRSDAYSAVNLSKERRSGHHNQGYRPGPGDPQ

RRSHDRRPPHPDDRRRGPPPEGRRGPPPDDRDRRRPPDGRRPLPEGEYRRGPPPDDRRRY

PDDRRGPPPGEGRRSPPPEGFRGPPPENRRGPPPGGYRDDRRGPPPDRRGPPPDRRGPPD

DDRRGPPPGDRRPRGPPQFRERSPDDDLGRVRLEDNRESESEL

>SG-PL_contig_9727_1_+3_78_182

MSLENIVLSSSSSSSSSSTLSLKVSFQIQNKRFST

>SG-PL_contig_9728_1_+3_3_68

SVVTKNNSIQSNSKVKVVSTNH

>SG-PL_contig_9729_1_+1_1_261

FFLSFYLSFFLSFFLSFFKISNRIAPLPSPITIKEKHVHLQLMVKCEQTRMTESGRMRKM

QRQEEVVVVEGEEKNRKKRRRNKYAIN

>SG-PL_contig_9730_1_-3_3_110

PLPACWACRSFIARAPLAIGRTKARRTGVWKSQERA

>SG-PL_contig_9731_1_-3_3_215

QHFSEVLNRPEPASPADFKDPQDLMPIVTGDFTEEEVRKAIKSLKNNKSPGIDGITAEML

KSGGECVVHWM

>SG-PL_contig_9732_1_-1_1_318

LLQVLPPPPLLQVPPPPPPRKDSLQAKGCLIDHLVALVRTKSRQVSLQGTAPWRKSHLTS

PQTMWLAVQNSRRAGPSPSASGSLHIRWRRRGVQGPSGMRSGGGTC

>SG-PL_contig_9733_1_+1_1_132

GASLSNKMINRRPVCITHLAHVKVPATTKILFLATFYDTFHLKK

>SG-PL_contig_9734_1_+2_53_244

MTHTVTFISTFFVSCYHNISHEYSHPHAHRPRILTNHPHRSKHSQNIYTQAHISCLGRSL

SLSL

>SG-PL_contig_9735_1_-2_1322_1504

MIHIAFTQYNTTHAHSAEKWLDLFTISELRTNLVIIYRSKSGIIKLIHVNLQIIFCSQVY

F

>SG-PL_contig_9736_1_-2_35_526

MEAQALDWIEANMGEPVDRKKPYDDLLKDGIVLCKLMNKLMPGCVKKIDTKGGGFALMQN

IERFQNAAKAYGVPPAEVFQTVDLWEKKNIPQVTLCIHALGRTAQTRDDYKGPMLGPKMA

EKNERQFTEAQMLEAKNVVSLQYGSNQGASQAGINMGKPRSIMD

>SG-PL_contig_9737_1_-3_102_233

MKTLPYMTDVLAATHWLSSPFGDLRHVEYFVLMLTPVCFVSGQM

>SG-PL_contig_9738_1_-2_560_1993

MAASNAPKNDFAPPWLNFPDSNENTRPSGSRSNPILERPRQRREDTYNRYGDCPRPLNRQ

NSGTDLYDGSKRHSPSHASKHRHHSMEEEYYNYYSSYGYPAYYHPYPEAYSIPYAAQPPS

MPRRDAKFPPQSHAARYGQMSSNYQPFNYYDFFPSGDGFSNYGVGGHASSRRSQYAREGR

GESRESRDSDRSSHGEGDDRDKIFNDDFPSLNGADDDGENKTSKHAGSGGVWDNPPRSRF

EDVSNDVKSSTPGLYKVVPNKSGSGRKDSRDSGRMNGSARDASPLTSIKSGSGREGQRQS

PTPAKEILSTQLVRPKPSDKKSQFLKTLRKADMENGTHNGIADHHHHQLEESNNNLRKGE

EDAEVVMNGVSDLHLDQDLDQQCNGSIKILSSSMEREYSFLVSLGWNPQEETELITEDEK

REFLRRSDKVKQEQQRNGRTRLLSKSWSPQHQAGSNPPPSAPDVNETLSSSDSSDEDL

>SG-PL_contig_9739_1_+3_81_272

MVVVYEQLNIQMRLYVTGRVTFCRDTCLHINEEHIYVQEYEQKGITVIMYLYNNSITAMC

FNTQ

>SG-PL_contig_9740_1_+2_47_301

MFSTTMPNITMASLKCQRLGVFNNEEMFIFLQRNLIVYTVYKTLNLEQNQKKYVASEWVY

IFFFASYKSNGRGLQYCLTTTLNLK

>SG-PL_contig_9741_1_+3_3_176

GDKCLQIVYSTKSIFMIRNMNRPILLSWCISRIILLLKCVSIPKSVLMTSYNVFYYNA

>SG-PL_contig_9742_1_+3_3_167

KHNCIPLVVQNFGECLRPVIRFKMADQRRTSTRRPVFKRYSALEVLEEVLNDLDQ

>SG-PL_contig_9743_1_+1_262_480

MQNTVYAWSRSYPFCHYLSVVLLSPYSLPLLYFCFISFHLFIFLFYFPYLFHLIFIVYLI

LFCSIEKGPALGA

>SG-PL_contig_9744_1_-3_45_548

MHKSTLTLAKPPWDADYTTTNKQFHGGSKTLEPECRLPPSRSMHRSQFNIGGYGGYGTYP

GYPGHEAEHPDVWKSDYTKTYFQKDIVPANRLHLTSLVNRINQTEGVKVKEVVKPCEEPM

NYFTQYKRVHDKLGILRGPGVQRGYPIREQYNTITGEVMGLGWKEENH

>SG-PL_contig_9745_1_-1_454_711

MGRQVYPFCIHLFTQHWDWQHGFCFVACLVFVYNWNYWDNWMWWYENAELNDCLCKRTIA

SGAVFFIVFFLVRGEKSNGTNLLIII

>SG-PL_contig_9746_1_+2_485_724

MESYYSACLRIGGRILMSASLLIHCLLACSSDVELSWMDSNFCSPSEKYGLCVLSFNSLH

CSMLLFFRSVLSSTVVYNAG

>SG-PL_contig_9747_1_-3_288_515

MPKLLVTQHTHVATLKIRSFTKTQPVLTQQSAEYSPGNSADENIFQTPHHNCQSCPLVNA

KTSFHSTYSCCNFKNS

>SG-PL_contig_9748_1_-2_134_241

MTGTNHPFSLKSLIVLFVLYGPQAWPSYISFVPSYR

>SG-PL_contig_9749_1_-1_202_2175

MASKIKALLQWCKKMTEGYRDVEMKDFTTSWRSGLAFCAIIHRFRPDLIDYDSLSKENVF

ENNQLAFTVGEKELDICAFLEAADMVAYKTPDKLSVITYVSQYYNKLSPLPQLGGPGVKG

KTSAAPAVSAGTKRHQAAEPLSEPEPKKQPPAKQAGVGDDAGKVKTLGDRCAICNNKVYL

LERHVEDGKLYHRSCYRHSDLSPTNNIYTRSPFMSPSLHSKDRAAVHGKDGAAFPPGKSE

AALSSGDKGSSKSASKTEDKHGTKRSAEDDGGKSGLRKFLFSKDSDSTNKSTTKDTGLSK

TGLSKDRQLQSDHTAPSSKVLSGSQTDNKKSETHTSMDFLSAGNDVKDVKSPQRQLPLVF

QGAGSSGDGATGKTKSSSEGKTALFSKPNSDSGSKSSGLFGKHTENSAKSVEKKPDSSSK

SSVFPGSSSKSVSENRLFEKNTQQKQPGTNLSESKVLENSSGPVAKPRHVGKKVLPGSSE

VKQERSKSPSFPKPSPRNNVPSIETSLKSVSDTQFPPASRKVPARSRSPARDLSPPPLPS

SAPPPLLQSPPNASIDLTLPHEPTSSSPQPSPRNKFEASKFSTASSNAASTTPSQHVPSS

SSSKPSSSTESKFSLSTPSHKPAVSISSSARHEEPMEVETAFSKARSKYAEEPVAVKV

>SG-PL_contig_9750_1_-1_1_231

DRYTGLHQSFLVNNVVCETAICPPFLTAWLLVLPHTHKHTPGFVVCITWNPRARYHCSLP

VLTLETEEFFARLSRSL

>SG-PL_contig_9751_1_-3_3_200

RVVLLLDELPVKANEFHLPGFEVRVVLLLDGQPSQADEFHLPVATGYGAGDPPRPFPIGR

QQVCRV

>SG-PL_contig_9752_1_-1_1_240

IKFVLVLLHIHFDAHTDIVYTFTLLHTDGYAFTQIFKTGYVDQPAGYVALVLYKESLIYV

CLRSLLVVLTLKKCEHEKKK

>SG-PL_contig_9753_1_+3_3_131

HKQTNKLQHFCTISTNNCYISCASPSIPLKRSSKGHISSVVRK

>SG-PL_contig_9754_1_+3_3_257

KDSVVTLIGLQRQLFEGLDFLCSQFVHFTCKHSLCVCCGVYAAGLDGHDDETFILQVQVC

IHGYNTGLVGLSDISEDDVDHIHQH

>SG-PL_contig_9755_1_-3_39_791

MVDTLKVVWKGILNIDVDETTDFFKAGAGSMDVVRLVEEVREKCNDIRLQTEDVYMNTLF

DDFFKCVVRRGRGIEDKEPFVFDAVKMHKNNMEISFPHQLFIDNEFMDASNGDLYNTINP

TDESVICKVSKATVEDVNRAVEAAKAAFEEGEWGKMNARDRGALMYRLADLMEQHAEELA

TIESIDSGAVYTLALKTHVGMSIQTFRYFAGWCDKIQGLTIPINNARPSKNLTYTKKEPI

GVCGIVVPWNY

>SG-PL_contig_9756_1_-1_607_831

MALLMKVRVGTRPTLPLNSLSLCVSHYLPVGLSSLGLKILNYGILLHHIYLLQHNMSTGV

KLISGTCCTLCGLEK

>SG-PL_contig_9757_1_-1_316_453

MKNYVGIKSCNVLKIHYYFDSLMKTILFCLYYFSDLYCIAFISILW

>SG-PL_contig_9758_1_-2_332_517

MDIFQIVKLNKHLNMLMYQTVIFTLTHNTYLSSFYWAISKTSPITVYILCNNSTFFLSFS

CH

>SG-PL_contig_9759_1_-2_2_199

KTVVKMTWTSHYLGLKTVVKITWTSHCLGLKTVVKMAWTSHCLGLKTVVKVTLGQGVQDC

KVGGRP

>SG-PL_contig_9760_1_-2_2_97

INYMSYVSLINTRTSWIYHYLVCSYNLNYFDR

>SG-PL_contig_9761_1_+1_262_405

MQISCQYLIPSPSPNIPNQMKTEGQYIKVKTSVTCNEAHDLIFTCQNN

>SG-PL_contig_9762_1_-2_368_607

MFGEFSICVAIHIQKSYVVECVTNDYKLSDISVTVIYKRSHFVFLAFLTLFGTIEPLPTS

LLSRVADLDMTGLYSVLTNW

>SG-PL_contig_9763_1_-3_105_776

MEDAAEASNLSHLGCEHYNRNCALIAPCCNKRYICRICHDEKEATHELDRKTVTHIVCLK

CKTKQEVKKLCESCGIEFGKYFCEVCRLYDNTDRQQFHCKACGLCRIGGQENFFHCEKCD

LCLSKHLQDNHKCVEKVSHDMCPVCLENLHTSRSALHVSPCGHILHRECYKECLKKGHYT

CPSCNQSMINMEEVWKEVDEEVNNTDMPEQYRDLQVNIVCRDCH

>SG-PL_contig_9764_1_+1_655_918

MPYYGQFQVTEKSTCTKNVNYIILLTKLLHKYTCIEICKQICINSNHNISANFVLSTDLF

IQPIITVNEYKGKFKSTFTEKKVMAEES

>SG-PL_contig_9765_1_+3_210_380

MYMHIYIYTAIDAQESVSLNRILVQITQPLCLCVQVCVCVCVCVCVPTCMCVYENAC

>SG-PL_contig_9766_1_-1_1_189

LYVLGSPEVLFSLPKRKIGEALNIMQPGPSLNRECGPYKLSNIYQHLHHVTQAPLPVRSK

TYL

>SG-PL_contig_9767_1_+3_3_158

QWEDHPMTTVGKSLAKQFKSKIFTKPEAKSGFWQVPLHPVSKLYTTFITPFG

>SG-PL_contig_9768_1_-2_35_313

MEKLKLGSSRYFGQPELLFLTVVSPTPKHRSGFRSGKIGRLLHQSTLCVEGGFFLIIKME

RRNGTTSMAIDVAFVLRLSLVLQVYMNSSFCYL

>SG-PL_contig_9769_1_+1_130_270

MFLKLHSFVPSQATFALILDTTCLKADICQANIQTMTHIKTFQVHDI

>SG-PL_contig_9770_1_+3_3_77

SQSSDLLTILQASSQSRLVHNFQTP

>SG-PL_contig_9771_1_-1_1_402

QSGGSQGKRARDGLSHSRRPDGFPVRDALVADLRGIRLPVLGPQAPSGRPDAPVSFTPGA

ERVPEPELPRQVSFGWFVWPTTLGGSNLYVSGAARGSIRRPTPERESRQWRGRGRRFRSW

HPRSWRHYDSPVRD

>SG-PL_contig_9772_1_+2_269_619

MCHIATHRSLSTYLIDSTYTCHIATHRSLSTYLIDSTCTCHLTFYRSLSTYLVDSTYMCH

IATHRSLSTYLIDSTYTCHIATHRSLSTYLIDSTYACHITAHRSLSAYLIFLTPSLN

>SG-PL_contig_9773_1_-2_17_721

MTERYSFSLTTFSPSGKLVQIEYALAAVGAGAPSVGIKATNGVVLATEKKQKSVLYDEHS

INKVEPITKNIGIVYSGMGPDYRVLCRRARKLAEQYKRVYNEQIPTAQLVQRVAAVMQEF

TQSGGVRPFGVSLLIAGWDEDDEKPFLYQCDPSGAYFAWKATAMGKNHVNGKTFLEKRYS

ETLELEDAVHTAILALKESFEGQMTEDNIEIGICNSSGFRRLEPSEIKDYLASVS

>SG-PL_contig_9774_1_-3_63_182

MFFVCVLMLFEIMDSSKFQLKKNITYVRQPFEAEKNILLV

>SG-PL_contig_9775_1_-3_3_170

ERGKERGGEREREREREREREREREREREIVCCSLVLLCRKRFSVHTSRNVMSLFS

>SG-PL_contig_9776_1_+1_1_105

NIINLKKAPCKICPIHVSKTRKYKKKLLETSPFKY

>SG-PL_contig_9777_1_+2_107_277

MNRTCKSAFITRTYAQYGCHKLPMKCGKKGQVVASQCVHNSICAPPQMSPGSWEIPE

>SG-PL_contig_9778_1_-2_95_208

MLLMSLLFVLVIAKYRHPFIQQCVFSPPPNSSSPLRPS

>SG-PL_contig_9779_1_-2_2_91

ILWHHIGLSNVQHGYVISRGLELCDPISHL

>SG-PL_contig_9780_1_+2_143_439

MTKYILTENKQVDMYILKYTNNNYNNYTDSLWQSNATQQQNTMITEPPKRNNIIWSMRHD

YPQNEKCAHVLQNMVVLSTSPHGTGGAKSRQRYWQNLQI

>SG-PL_contig_9781_1_+3_168_320

MILDITPLDEVTFSEKSINSGGTVHDIGYHLLDEVTFSEKSVNPLVQLKHN

>SG-PL_contig_9782_1_+2_2_154

KINTINTNKYNSNFIVRNDWNILNEAYRCSEHANRQDSKVPFRFCFPLIIS

>SG-PL_contig_9783_1_-1_25_291

MPQPNTQSHFSSGPSLNEHAQKSFSPQQPMGGQQQPQSAPPQQKSEEEKAFDIINGVVND

VKNLEASVNVFQGKKTDKEYKYLEEMLTR

>SG-PL_contig_9784_1_-1_376_786

MYVVGFFVVVVLVPFSCFFVFFRTVFSSANFLLYIYMYVCMYICVIYIYVSPTAAQNNMT

LRLFLFFFFFFYICLECAVVVLLFPFPHCQNPSLSYGPLQVGVRAPSDSIRFMSFPVVSF

SSLSCNCYTDATDLSRD

>SG-PL_contig_9785_1_-1_10_288

MFSHDHGPRVCTRRSSPLLLALGGFGIRVVLLLDELPVKANEFHLPGFEVRVVLLLDGQP

SQADEFHLPVATGYGAGDPPRPFPIGRQQVCRV

>SG-PL_contig_9786_1_-1_1051_1161

MHFGKDAVLLTLEHLKKLLVEAWLASWRSSCPPVCLH

>SG-PL_contig_9787_1_-2_122_484

MSFCLFPLCLLLPVCVCCCLSVSVAACLCLLLPLCSLPVSTLWNSGQVDGTSCDPGKVIA

ACLCVCCCLFVSVAACLCPLSPSVPSAYVCLLSLCVCCCLSVSPFSLCAPCLCPPCGTVV

R

>SG-PL_contig_9788_1_+2_368_592

MHSITVKNMSLKFQPIQHKGIIIILLRTQTQKCHTSHCTNSTRFSLPFTLIWSCITFNIY

TNRSRLSNTNNSSRW

>SG-PL_contig_9789_1_-2_1145_1372

MFTEFGMNLYFAMPVMLMPAVLCRKFLQILFLRYTMSELFRMSHEGSSVCIEIQYFLILV

ISLLVDFGWCDHVFNN

>SG-PL_contig_9790_1_-1_721_1062

MLLCAEQCFLGCVYACCLAHFVQAFGGLHSAYSAFGGHACWVFLCFHRPPSTDIAYRIFN

VDIDVLHAYTHEEGSSISRSSRMLTWEIGRFSTFTHLMVLEQAGTQTVQLEVQC

>SG-PL_contig_9791_1_-3_3_131

SDLTPTDISDIILWSDLTPTDISDIILWSDLTPTVISGGHSLI

>SG-PL_contig_9792_1_-1_37_387

MYGRLLLLCLAAVAYAKPGSVAWFKVERASQIEDASFRLRYDPYDTTWAQFKQEHNKVYE

NEEKENKRREVFMKNIKFIEQHNWQYYNKQKNYYLGVNQFADLTAEEYQQYNKLKRN

>SG-PL_contig_9793_1_-1_13_1194

MVRKDLVVSMKTLLLLILLIVPAAGRVHKLKLENDARKEITLSTFGFLSGGTLMVNISAF

TMHLSGKTDPLKANRTMFGFTLDKSKSPGLSSYMEGMQETCILNNPTLKKQKESMESVFF

SFDFKNKKVQITRVGKDMQSLVVSSESRDNFLTRHDLNRRDVAIQLVDSKLSDHYLVGSR

RKVREVVAATGSADSASNRNSSTPRSPTSLPSTRIGAAGKADSDGASGASTSTMVGGSGG

GASVSSTSGQNGSSSQSTDEGPQDAELHSVGLTMSEDGSYDTYFLVYIKKPEEEGLYNLF

FHNCLSYWKDAAAQIGMSLTIMEDNNGNFLSAGEMPVPALYFTFSVLFFIAGVAWLSLLR

RSHDDVYKIHYLMLVVVFVKSLACLFHGVNMYFI

>SG-PL_contig_9794_1_+1_1_204

ISASLGMSSASVGAREISASVSSGAGFSVISNMASSSSGLVTSISSIISSASLGPREISA

STSSSVGF

>SG-PL_contig_9795_1_+1_1_597

TCWGCTSSSTTTSSVWTCGFSSGTEISSSLSVGTDCRTGVPSLTPTSTKAPVSSLTSSTT

LTSTPESSKDTTGTVAEMVSSASVSSGTSVGVGFMDISTSVSAGAGFSGTSKTACSSSTG

FTISASLGMSFASLGAREISVSMSSGAGFSIISKTASSSLGLTCSASLGMSSASVGAKEI

SASVSSGVGFSGISKTASS

>SG-PL_contig_9796_1_+2_356_568

MTLLYSTGEKQNTHTNTHTTPSNLPSLLFNTNTPHPHQKKPHSFMHLFLTNLFIYLYVCL

LSFYFFNLVTE

>SG-PL_contig_9797_1_-3_2745_3002

MVLCGVTAVIFSFPPSQRTVIIVRVTWCSMHTLFCVVLDPSFVMVFSHFLSFHLRCTCIQ

SSHFFFVICMFWVTCFSFFIVANAHF

>SG-PL_contig_9798_1_-3_195_650

MFKWVRRSFMTFVELTFGSNINRQLREMVKWTYSEQMLLFYMRTFKESMWPGGKLADNPP

AKTEAQKFETRMAAKNKLLQNIPDALKNLVGEDNARRGTIKVFEVLQNKTLNKHLIYNLL

EIFLLELCPELKKTRQTLRESVQQPPADTVSS

>SG-PL_contig_9799_1_-2_2_148

QTLPLAVVNEFRTHCYYISKPNTHRHEHIHPKRTFRMKKKKKKKKKCLS

>SG-PL_contig_9800_1_+3_108_236

MLKMYEQTSMHHPQTLNLPSKQTHAPNERAKPRTSTEHKNKQT

>SG-PL_contig_9801_1_-2_17_226

MNLSTSAFNCVKYEVPQGSVLGPLLFSIYINDLPLYIKADCELFADDTTVHTNHSDVTQV

SLSFKIVLMI

>SG-PL_contig_9802_1_-2_2_151

SLIAHHTRSQYTHEQSNSILWKKLERIRNLGGVLLLEYKYKRVNDLLLYI

>SG-PL_contig_9803_1_-2_50_217

MKLKNPGMYILSVPDCKKMLYISLNIIFFLFDIVTVLNLSAQYYFQHRKSVLAYLK

>SG-PL_contig_9804_1_+3_3_242

LTSLPFHLLLPLLFLLSFTSPYVHCVLPVCYLCVISVSVTVCFLRVRIEELGLILIIEAW

PWLYYWSSYCSGQLPEWVLI

>SG-PL_contig_9805_1_-2_20_217

MDSWEDWDQTVESYPLYRCITEIRSNFLTTIAGVCNSRAWLTPGYIMKVSIRVQLCHAVS

ILNGTP

>SG-PL_contig_9806_1_+3_3_278

CAFLNETYPFQLHIDAIIFLCFSEDKVSMLMLNRCSFYFFPFQVYANKVAFSGLRAYGIY

NSVYILRKKKRNLCSGEVVLSFYLYTVKAFFF

>SG-PL_contig_9807_1_-2_2_145

AKEGGGGGGGLNISVTHKIKISVCLSVCLSVCLLLMLLLFYACVFQSD

>SG-PL_contig_9808_1_-1_187_408

MSGKGDGSPSSSVDLLRRDFRAMIYYDYCQGKSFQECFQSLKHCCGDLFPSKATVFRWFR

QFKQCWKMMIVVVD

>SG-PL_contig_9809_1_-1_1_318

HSHSRRPDGSPDRDAAVADLLRKFLPDLVSQAPSGRPDAPVSFTPGAERGTEDNPLFRLK

SSPLLQHLYEDAAAAARGSSTLPDPGDTDLSAPGALPSGRFLRFKR

>SG-PL_contig_9810_1_+3_450_677

MLLTKKKEEKKASLNDKITQKNLGVNEKIRNYLLHDPKSDNCVNSGKQNQWSVLSNVTPC

HEMRPKFIFGCTEIYH

>SG-PL_contig_9811_1_-1_253_684

MTVYDFYLFDRNGNCLYYAEWNRRNQPGISKEEEFKLMYGMIFSIKSFINRISPKDVQDG

FLNFKTSKYKLHFYETPSGLKFIMNTDLNVGNIRDVLQQMFSSIYVEYVVKNPLTELGEP

VTSELFRTKMDDYVRGLPFFASKA

>SG-PL_contig_9812_1_-1_148_306

MRHVLCMCVCKVGGRRWGGECVCVCICVPLLFFPVGGRLFQTPLVDEILKKLV

>SG-PL_contig_9813_1_+1_1_120

THWPQFVPRHSNSSAQLTGQKQFLETFSAFRHGRLSHDQS

>SG-PL_contig_9814_1_-2_101_193

MEFHHFWSLGKMIIYMLKHKHCFALEMNISC

>SG-PL_contig_9815_1_-3_186_530

MADGSNSARDRNVFADLFEEFFADDHNNEGEEINESLDLQHTTLVGDQRDIDQAPNNRDF

EADEVLGWRSEMEEGATINMPFDRENKMNVGDSFPTNPTPLDFFNLFVGDEIISL

>SG-PL_contig_9816_1_+2_44_289

MVQPCHVPQHAVKDNPSGNPRTLHMKRQAEKELVQQHQRTERSGNTSTNIIHKAEKTSPT

EEHWQLSASLMSSHSPFWIKES

>SG-PL_contig_9817_1_-1_46_453

MACNICEGLIRDVLTKHLLSGHLISTHQCGFTSGRSCITQLLNTRNNWMSTLNENKPIDV

VYLDLQKAFDKVPHKRLVFKLKGYDIQGKLPKLIEDFLTNRTQYVSIGEISSENISVTNG

VPQGSVLEPIFYVFYQ

>SG-PL_contig_9818_1_-2_1037_1279

MQKMCSLSCLLSSDLNKCCLRVCICVLQSMICVNTHMLSIMLTRILKVKRVLNLFWSCSN

IQGFWIVLEQHLITKWKNFPS

>SG-PL_contig_9819_1_+3_24_293

MHLGQTNYCKSFTWSLPRSRAISSSKLLEQFSTNALLFIANISNTYSLFHGVCLFVCLFL

FFAECLNLSLAAMYLWFIFINIVIGSYIAV

>SG-PL_contig_9820_1_-3_123_251

MYTKKHCTQYGVSGCSRYICWGCRTRRGVSNDCPHLNLTGGCY

>SG-PL_contig_9821_1_-3_3_68

FEAETAERKWGWGREKGPIGPW

>SG-PL_contig_9822_1_+2_128_286

MGTLTCLALKKTTTTKTEICGDTCLALKKNRDLWGHLSGSKKPISSLGTLVWH

>SG-PL_contig_9823_1_-3_21_1124

MTALHEILHVLGFSTKLFDTFRRCTDGVCKKGKADLRLVQGQYRLVSPTVVNLTKQHFGC

HHSSPDYGGPLLTKNDKATSHWESRMMHTSLMTAEGDNTSYQVLDPITLAVFEDSGWYRV

NFSNADTFLWGKDAGCLFAAPDHCLESREYFCSDNSSGCHFLWGDKGICDAYKTGNQCGI

YAPIPQGSCTQNFTQEKLKHGSLEIFSASSRCFLSSVKPASSQNGGEEFIGQCYSRKCSE

NGTVFVKLQGTEWLPCPPGQAVQISGLEGNIQCPNNTDTLCWENTQQPVSQTQRPTSQTS

TQHQGTSNSAAKSTAELTTVLYHTTHTIRSSAGKITGVLHIEYGFVTYLYISFFFAYKYL

VCFEFNKL

>SG-PL_contig_9824_1_+2_2_88

QTCAVLHKLDMVECMTLMNKFPSFYGHMY

>SG-PL_contig_9825_1_-2_2_109

GGVTYASALYFFFFETYASAFYVNSFDGLLFDFVLI

>SG-PL_contig_9826_1_-3_108_227

MFATLCIYDWLHFPFFFFFTLIFYPDLRQLELFTFYKNYL

>SG-PL_contig_9827_1_+2_56_277

MKTKYCDALERPISSTDTMTIKAKENTLAHNFKRLRGESFESPVSKCFLVLCFQQMIHKI

NTLFCLDFSFLICK

>SG-PL_contig_9828_1_-1_325_438

MMAGFYKYVKDGDNRNFLCLPVIFVHIRFINTPLPRPQ

>SG-PL_contig_9829_1_-3_849_1010

MLSLCQTLSDVHSEKKLVNTLQVHLHDLDIFEKVKWVQKKNVLQEWNNGPKGTF

>SG-PL_contig_9830_1_+1_1_237

PQLSADNNRSKTLQHTSSCALSSVFRHSNTGKIETFGTTVLSSCGDTDDKSTLSSSSLSS

FSFELAKVLIRDATVLAAI

>SG-PL_contig_9831_1_-3_54_1553

MPKGRVGELPLPEGWEEAKDFDGKRYFLDHNTRRTTWIDPRDRYTKPETFADCVGDELPL

GWEEVVHPALGVYYIDHINQINQLEDPRLQWRQEQELMLKEYLVTAQDDLQAKQEIYSIK

EQRLNLAQDEFQHLKDTLTSNCWKSSHTSLNSNSSLGSTKYDPDLLKADINLARNRVGRL

KWELEQIKTEMHYKEQGVETLNSVGQKLSSTGGYSVGQAQAIISEIRQLQQYLSSGQKER

NELVQSLARLKEDFLLSKTGGSSPDVSTLSLAHDRHETASQTDLRGDFGFNQSRLFAEKT

RMRLQYDEAKRKLSDLKVRLANIEDQMMPGQSQSDKDRLLLLQEKEQLLRELRSIDPKGR

SGEEMTSIRERIAQLEYDLKHALQISNKQIQERLTLHNEKTSIMHELNETTKLTSYLESQ

LRSLSLSTLSMSSGSSLGSLGSLSASSRGSLNSLSTLDVYGQATGVTGSGLGVMGTEVNL

QELHQRVEKLLQGHSMSPIS

>SG-PL_contig_9832_1_-3_240_356

MFLCLENCPCIFAGVFCYESVNDCQSCCVYVNLYLASYT

>SG-PL_contig_9833_1_+3_717_1028

MGHMRTANISSLSLRRGGATWSLSCGIPGEIVKVMDNWKSVCYLVYFGQLPTSVLDHYQH

SFSSCLPWHILTYTTGSKFTTMFEPVLGIPPFLWEENNMNVCSL

>SG-PL_contig_9834_1_+1_457_582

MSAGKEFPSLRNHNNRKKQQEHKNEKNKISFISTSVRREYIS

>SG-PL_contig_9835_1_-3_3_500

AVYPSYRELVEHRASHGGSRPYECHVCDKRFTQKAHLIIHRRTHTGEKPYSCHICHKRFS

QTSHLNSHKRVHTGEKPYFCSLCNMGFCRRQRLEVHLQQHAKDMQQGYAAQNPEAQELLQ

QSMSVQKTLQQQQQQPALSSEAAKDFLMSIGLGNVMQEAGRSGMVG

>SG-PL_contig_9836_1_-3_42_524

MKSGPRHFQCTKCGNKYTTPEALEHHLATTSHQYPCPHCTKVFSCERFLRRHLPIHGSEG

QFECTTCQKRFKTEHYLKSHQLIHTGETPYQCETCHAAFNRKDKLKRHMTIHETVKKYRC

PFKSLTGCSKEFNRPDKLKSHIITHSGIKPFKCSVCGKGFS

>SG-PL_contig_9837_1_-3_1644_1967

MKMTLNSLYCSLRYNVCWQNSRSGILCTCDKGHNCILLSVSMYGRHKYIFQYSLLVTYTQ

YTRICNDLCIVRNGEGYISTSCIRFCVKRLVVVFVCFLNMFDSTFSAL

>SG-PL_contig_9838_1_+1_1_162

FKLQIILTSFLSALTVVIFVINIFKTFNSSISKTRAVPSQLMYNVINEYEIYMK

>SG-PL_contig_9839_1_+2_2_208

VFWQPPIRRASNMSQQSKAMLAQQGIHAGNFGTLQDVTVWVVVLPADSEDALEAPHVEGI

EPALLPHVG

>SG-PL_contig_9840_1_+3_3_260

LRCPCRQSDARHLDPWPVSPSCLERYCRLSGLSCRRPCIRVKGDRLVWCQWPAVRTGYLL

AASHPPCIEYVPVIKGITCSTGNTCW

>SG-PL_contig_9841_1_-2_137_346

MDDESLPKDILYGQLATGTRSVGCPSLQFKDAYKRDQTAGRYRPRIEMPGVRLSARALQM

QMQGEVSSQQ

>SG-PL_contig_9842_1_+1_1_297

QIKTNSWAFQHPRRTLWKLRNVTDKTSINAGLLQMWEKSDSKWSNAVRTIFKFVPKLWFY

NTVVSWVLWFRLKLKVIVQRQLQAAVRQGGFPSLRVQTM

>SG-PL_contig_9843_1_-3_336_476

MLTFWGCVNSAIVNFKMLTWDNHFQHYPFSLKTQGLDFRKGERMSVQ

>SG-PL_contig_9844_1_-2_2_175

VFYLLILVFYLLILVTVSQSTRPGWLHRDRALAFQSDCMSLHLAQGKLCACGLQSTHL

>SG-PL_contig_9846_1_-2_1394_1582

MACLTVLLQHLPSGLCHWSPASGEVGCSSHVRFSHTVKKKGRCSHLLMCFWVWFCSCYDH

CPF

>SG-PL_contig_9847_1_-2_2_316

VPGENPAAWPLPSIPPHFLPGLLLGSQRRQERGSPPLPARCVKYYLERSAQRRYAQRRPL

ILIIESTQRTSQRQPFPAAVHYLREGLQGGGFSLRQPSTTLSPSG

>SG-PL_contig_9848_1_-3_84_203

MWGYSWCVPTPQLSPDVAKCLDDEVWPLCEHVISLTSLTH

>SG-PL_contig_9849_1_+3_1611_1904

MSTMHHDRAVDPEAPKKKPDIVHFNNSTKASPNDSPWCCFTPPWISAPLPPVSFATSGFQ

TVIFQRRMPDFSSPCWSLNSCLWNTCRGVFAQKTCQSS

>SG-PL_contig_9850_1_+3_3_80

PQLTHVTSLALYQSESSCFVFFPKKN

>SG-PL_contig_9851_1_+2_2_154

VSQHTCADIQHIIDVKITTTTTTVSSSLPPPPPPVNSKYKPVPGMQRIFLE

>SG-PL_contig_9852_1_+2_1121_1504

MKMPYGKGERVPQSVTMTMEITRLAVFHMQTRRALLGHSPGWLVSQRIKVVNTHIQYFLS

SLVNPAPHFPQLNPPHSLQKHITKKPGRSTNPVKTRRNPTKRLVCIGETSKRLYILYVRQ

PQKREHIL

>SG-PL_contig_9853_1_+2_122_334

MMQSPQRPVSTTTLGPKCRHQINICLSDCHRHFISCTTLLLQSTVNTSFKPVTCISFINK

RYLQNMLTKTP

>SG-PL_contig_9854_1_-2_71_172

MAQILFMTGAWKCFQIEEVMFNGCRCQTYLRGPI

>SG-PL_contig_9855_1_+2_2_241

PKQHILEEHCVSWIRRWGFGLGLHGEQGGEGMHATVNRMKNRTCSIRNDGQRLLFTMKEQ

WLQASPMLQDTPKKARKGMK

>SG-PL_contig_9856_1_-3_3_128

IYNNNFVFVCDNFLLKSFHFVFVDYCSVIIDISICILQFYFS

>SG-PL_contig_9857_1_-3_105_1058

MDMFPAFNKNMLERYVNDEFRAVDRDFSSSIDFDEFLGMYKRLFLQCRTIVSGHVEDIVP

SSHRKHDFSLSSQKSGSSVNSVEVKNQRNSEKSAPPLPSTPPPASPNTRNGTHVNSVDDL

LGGDDDDSFFDDVMPTTGGLHLFGNSDGDKSRESKKEGSGISAGSASSKGGGDTKGSSMT

SLGDLPFLNSGSLTSAASNKRGGSKGKDGDSNLRSLDRRMADLGIDNDMQDDFEYEDDFQ

SSLHSLGQKSPRSKTEGHDKTENGSIAEEIEDEEIEDISIEADDLLKSENSGFDDMTEDR

SISQMDGGFDYMEDPQLP

>SG-PL_contig_9858_1_+3_393_551

MRLPYTAVPCSLLTHPPYSAGMKTKKFLHFSLYGLGEIISIMSFRDALQSCKP

>SG-PL_contig_9859_1_+2_212_424

MCIHAYTQICLHALSFHMPCRAFIRYQCMNQTFSTMPTVRNTDNAVTAPINTINSSFHKV

FILWSKQPKIN

>SG-PL_contig_9860_1_-3_948_1187

MIAQTTEFCSSETSCAHAHWLLVSINPLTPSLIHLFPALSVMIAKQKHNPFTHSPCPPVK

FLQLLDRVRESKVKRDSDHW

>SG-PL_contig_9861_1_+1_1_174

PTLAALTLVPLSWQKQLKRGKPLPSFGVFSASWPTYSEGSPWYLQPGRDNKLCSSCAT

>SG-PL_contig_9862_1_-1_1_387

SSLLFGWRRFRQAHFAYTTEGYRHLVYSSACSCVQFCFFSPEIFVFLESRCKINTNLNSL

SLGVFVLLHHRPNRFWLDFVVSKFSSEVTAKLKVWLGLLLLVLVTMLGAVECGFGKCVFS

SRQLKRVRL

>SG-PL_contig_9863_1_-1_1_177

CPVCSKILFCMIYCRSYKGVAYLHLETSLCIRGETQSSTSLLTGLGGACMQNFFFIHLE

>SG-PL_contig_9864_1_-1_1888_2100

MFVIICWLCVKSFDTCLYILESCHICLFVFLLFFLGIYHKFPLCVCVCVCVCVCVCVCVK

VISLKCGVACL

>SG-PL_contig_9865_1_+2_413_532

MAAPTTGIISIRLTCGHPSKKTLQPCWITLVVGLGQKLCQ

>SG-PL_contig_9866_1_-3_144_1040

MSFFIGDLCNEKDVLAALERVACVFHCASPSPLSNNRALFHKVNVEGTKLLISLCKKSGV

KRLVLTSSASVVYEGKDIQAGKEEELPYAAKPVDAYTETKILQEQTVLAANSEDFHTVAV

RPHGIFGPRDPHLVPTTVGLARQGKFKFIIGDGKNLVDFTYVDNVVHGHILASEKLTKDS

PVCGKAYHITNDEPVFFWTFMSRVVEGLGYEAPYIKLPYLLVYVIAVLLMVISKLISPFF

SFTPTFTPMKVALAGTHHFYLCDRAKKDMGYKPRVSLDEGIQCTVQSFDHLKNGGDGDK

>SG-PL_contig_9867_1_-1_34_198

MDKTTKYELKNHNSAPEIFFNDFIDFEEHFFAKTCKNFENMLNKYYTLQTLSNWC

>SG-PL_contig_9868_1_+1_1_153

THTQKKNHTNLKQNQTFIWTQLNVTMIRYQRCHCNITCDKLVDQIRSIIQV

>SG-PL_contig_9869_1_-3_189_9716

MISVVFIKRGPVVEADKAVSSQVRVMNFSDGSPYETLHAYVSNAVSPYFKSYIKETGKAE

RDGDKMAPTVEKKIAELEMGLLHLQQNIDIPEISLPVNPVVSTVIKKCQEENRKPKVEDF

GDRVEDAQFLNNLQSGVNRWIREIQKVTKLDRDPASGTALQEISFWLNLERALLRIQEKR

ESPEIVLTLDILKHGKRFHATVSFDSDTGLKQAMATVNDYNQLMKDFPINDLLSATELDR

LRIALQSIFVHLRKIRPTKYPIQRALRLVEAISRDLSAQLLKVLGTRRLMHIPYDEFEKV

LASCFDVFSAWDDEYDKLQGMLRDLMKKKREEHLRMVWRVNPAHKRLQGRLDHMRKFRRQ

HEQLRQVIIRVLRPTTKGRTGTPGAEEQDMKTEPVVDEAADANAIEEVNLAYENVKEVDV

LDVSKEGADAWEAAIKRYDERIDRVETRITARLRDQLGTARNANEMFRIFSRFNALFVRP

HIRGAIREYQTQLIQRVKDDIESLHEKFKVQYPQSKSCRMSRGHDFPPVSGSIIWARQIE

RQLNAYMRRVEDVLGKGWESHVEGQKLKADGDSFRMKLNTQELFDDWTRKVQNKQLSVTG

KIFAITSQRGATKNILKLGVNFQTEIIQLAKEVRNLRWLGFRVPLANVNKAHQANQLYPF

AISLSESVRTYDQTLKKIDGKDSIKLLIAGMRKDVQNLVAEGAGLTWESYKLDPYVQRLA

DTVFAFQEKVDDLLSMEEEIDLEIRGLETCGYNATTFREILTKIQKAIDNLSLHSFSNLP

QWVARVDEEVEKRLALRLEAGVKAWIQCLQGVKEDPTDNSMDTDEPKQQAHKPGGEPHIK

PLVHELRITNQVMYVNPPVEDAQYQLLQELFAWEAIVTMLPRIQHSRYQVGPDTESEMDR

TYRKVLTQLPEGSQVLDDAYGAVQKIVGDFKKYVNVWLRYQSLWDLNPDVLYGRLGCNLK

LWMATLEEIKSSRKTFDTSETQTEIGPLVVQFGKVQSKVTLKYDSWHKEVLSKFGSMLGN

EMAEFHSQVSKSRSDLEQQSLDTANTSEAVGLITHVQALKRKMKSWEKQVDMYKEGQRIL

ERQRFQFPSSWLYIDNVEGEWGAFSDIMKRKDSSIQTQVASLQMKISSEDTLVENKTSTM

LQEWEKEKPVAGEMRPDIALKNLTIFEGKFNRLKEERDNVSRAKEALELAEPGQISPSHE

RMQVALEELADLKGVWSELARIWEQIDELKEKPWLSVAPRKLRQSLDALLNQMKEMPARL

RTYSSFEHVKKTLQSYAKVNVLVVELKSEALKERHWKQLMRRLNVNWVLPELTLGQVWDI

DLQRHESIVKDIILVAQGEMALEEFLKQVSEVWRSYELDLINYQNKCKLIRGWDDLFTKV

KEHINSVCAMKLSPYYRQFEEDAQAWEDKLNRINALFDVWIDVQRRWVYLEGIFTGSADI

KHLLPVETSRFQSVSTEFLGLMKKVAKSPLVMDVLNVPNVQRQLERLADLLGKIQKALGE

YLERERASFPRFYFVGDEDLLEIIGNSKNIPRLQKHFKKMFAGVASIVLNEDDSHVIGLT

SKEGEEVVFATPVSIAAHPKINEWLTLVEKEMRVTLAKQLAAGVQDVAAFRSGQIDQAKY

IEWADKYQAQLVVLAAQISWSESMEQALQTVSTGGDKEAVNNILKGVENTLNVLADSVLY

EQPPVRRKKLESLITELVHQRGITRTLIRNKVNSPKSFEWLMQMRMYFDPKNPDVLQQLS

IQMANAKFNYGFEYLGVQDKLVQTPLTDRCYLTMTQALEARLGGSPFGPAGTGKTESVKA

LGNQLGRFVLVFNCDETFDFQAMGRIFVGLCQVGAWGCFDEFNRLEERMLSAVSQQIQTI

QEALKELASPDRKDKSSPMTVELINRQVKVNPDMAIFITMNPGYAGRSNLPDNLKKLFRS

LAMTKPDRQLIAQVMLYSQGFRQAEKLASKIVPFFQLCDEQLSPQSHYDFGLRALKSVLI

SAGNVKRDRIQSVKQGLMDRGEPVNETTIAENVPEPEILIQSVMETMVPKLVAEDIPLLH

SLLSDVFPGVAYTAGQMVELRRELRKVCAEMHLTYGENDEQGTQWVEKVLQLYQITVLHH

GLMMVGPSGSGKSTAWRVLLKALERVEGTEGVAHVIDPKSISKETLYGNLDPNTREWTDG

LFTHILRKIIDNVRGELTKRQWIIFDGDVDPEWVENLNSVLDDNKLLTLPNGERLGIPPN

VRIMFEVQDLRFATLATVSRCGMVWFSEDVLSTDMIFEHYIQTLQNVPLEESEEDARYMS

RAAGSAEGKEDQVSPTLQVQRDAASVFMPFFSLDGIIIKCLEFGKDLDHIMDFTRFRALN

SLFSMLNQGVRNVLAYNRTHDFPMQTDHLERYLSKYLIYSLLWSITGDSRLRVRQELGDF

LRGITTIPLPPSNALIIDYEVTPAGEWASWQAKVPKVEVETHKVAAADLVIPTIDTVRHE

ALLYTWLAEHKPLVLCGPPGSGKTMTLFSALRALPDMEVVGLNFSSATTPELLLKTFDHY

CEFRRTPNGIVLSPVQLNKWLVLFCDEINLPDMDKYGTQRVISFLRQMMEHGGFYRTSDQ

AWVHFERIQFVGACNPPTDPGRKPLSHRFLRHVPVVYVDYPGEMSLKQIYGTFNRAMLRL

VPSLKPYADPLTNAMVEFYMMSQERFTQDMQPHYIYSPREMTRWVRGICEAIRPMETLDL

AGLVRIWAHEALRLFQDRLIEDEERQWTDQNVDMIALKHFPNIDRETALCRPILFSKWLS

KDYVPVDREVLREYTKARLKVFYEEELDVPLVLFDEVLDHVLRIDRIFRQPQGHLLLIGV

SGSGKTTLSRFVAWMNGLTVYQIKVHNKYTAADFDEDLRTVLRRSGCKDEKICFIMDESN

VLDSGFLERMNTLLANGEVPGLFEGDEYTTLMTQCKEGAVREGLMLDSAEELYKWFTQQV

IKNLHVVFTMNPSSEGLKDRAATSPALFNRCVLNWFGDWSKGALYQVGKEFTSKMDLEKG

GYVAPSNFPFAYENLPATPGHREAVVNAFVYVHLSLHQANSRITRRGGRSTAVTPRHYLD

FINHYVKLYTEKRSDLEEQQLHLNVGLQKIRDTVQQVEELQKSLSIKRIELEEKNNAANL

KLKQMVKDQQEAERKKVTSQEIQVALIKQTEVINEKQSSVMEDLAQVEPAVIEAQN

>SG-PL_contig_9870_1_-1_1_336

VCGNLVLKSSIGLLCVCVHARAHACCNLVHWTCWIRVGVCVCVCVCVVTLFLKVILDLLD

TCVCCNLVLKSNIGLIGHVCVCCNLVLKRNIGLTECVCEGFTGWMCVLFVRS

>SG-PL_contig_9871_1_-2_95_898

MYSAEVGQLDLEAEGGPEAPINAFVAFKTVNKIQDLLSPGLINNMTSLVLVNAIYFKGDW

QDPFNENSTTEQPFYRGDGSTTQVHMMARTADYQYTEARDLSAHIVQLPYRDGRFSMYVV

LPTSRNGLATTERSLTWDRLNRHLSNMTPTRVALHLPRFKTESKFSLKETLQTMRMILPF

TRSRARFTGISYIGDLYISEIIHQAVIEVSEEGTEAAAATDVIIGLRSSRRNAVEVRADH

PFFYAIRDNESNSLLFMGKYSAQEQESE

>SG-PL_contig_9872_1_-3_126_284

MYKSTLFVFQSLSLSLSLSLSVSLSLTFSPPPPPPPPPPPPPSPHPFSLPSSP

>SG-PL_contig_9873_1_+3_108_407

MVMPHILSTHTSEACSSWLGCMYALSWWVGPYCAGNISEFCGLEEETKDKQEFLKKMQVN

GPIRLKEKKRKGRIKEVTEKRAFLAQNYVQMRKLLLFAFP

>SG-PL_contig_9874_1_-1_1_156

FHRSLLLSSYLVLFCMEHPFHSETTNHRLFVCLCVGLYVCLCIRKVIDWKHF

>SG-PL_contig_9875_1_+1_1_198

ECTLQKNKAKFHPRCKVQFHTTETESKCLNLPLLLQLIHQLVQALRWKLSVTTAPILPGG

EYIHTV

>SG-PL_contig_9876_1_-1_82_2598

MPQDEAPVYENQYVGAIATGTVAPPTQDEDNTADDLSQQLQQSAEWIRVRVHQMEPDLMD

VGSSLEEAIQLQREHQELLARLKSKEGEVSELLRRADTYADSNRSQTDVHGAMADTLAEA

WRDLNDKLQYRSKLLEQSVTFHQSAQDLSSRMEQVQGNFMHVPLASDVNTARSLLQQHND

MKQTILESSKQTMDIGQSLLDQIKQMGMHADIQNRHATTAACYGIEHLLELLHDKRRHLE

ELWTQRKIQLEQCLQLCQLDQEVNKVLEWFRTAGNTYLQRTELGDSYAGAKYIQEQHNQF

EAEAREIQENMLRLLRTADGLLRRASVDAEGIRQRLMLVDQECESFMVKLDNRRKNISLA

VSFFNLAQTALNQLDQIEVQLNSMDLPRNSTELAERHSQLSNAIVDVSTQAVHEGRLLLE

RVSSVDPGADGIRRKLEELHKRCGHLESLCKARRAEAWQRSQAYLQFQENYNGLSTWLSQ

IGQSMLTRHSSMGTSLASAKDFLEVHEQLDEDIRDKNADLEALSAGAMDLVKSGDQEAKL

AAERADALHKQYSRLQRVVENRIQLALIYVSFLKLKQQLSSSLDGLQHIVRSETEDLQDL

TDTALQQVQAMYSQAEQLFAEFTAKGKLFLQNSSTISDDSTLEVRPAINTVESSMEDVGQ

RMSLLTTQWEAWQQHVMSSKQFKSQWHQFIQDARKTIDHVMKIEREFFLPYISGHLGNSL

EKAQELRNKLTEFAPLAKKAQEEIEERLKTAELLSLKGDTRGQKEQIVNELIRVHQRFQA

RMNEYQILIKMTIEFYKNVNQLDELIERTEREYLRSQLPTDLQQAESMLEQHKRKKTEI

>SG-PL_contig_9877_1_+2_2_274

VCTRARVVYRYINFSVEEYRSGALLWINSVFALCCCEQFNMSVSVGRSLCQHLCWLPCLG

VCTFLILQTLDSVLLGCSSSVLFSGSVSVNL

>SG-PL_contig_9878_1_-2_110_616

MERRTSSGTVNNAVGQQSLLTALNRFVSAVDTMDDIVMIPSRLRDLEVTVPELKEENNNN

LSSDDKTVAIPSFQSGMDLYRCFAMLHVIRDEIIDGSKEEDDQSGASGEVEEDKEDDETA

ASRKTANMFRYHLRGLFGLLHQLTATAQYLGSRYERDVTQNQPTFSLNM

>SG-PL_contig_9879_1_-2_2_79

STQDYSFGFLPSYCVNIESICLGLQL

>SG-PL_contig_9880_1_-2_2_166

QDVKPQRVLSSSVGLPLASKVYFTVLKSYVSFKSRFCLHCVCMAFNISYQEMVVF

>SG-PL_contig_9881_1_+1_151_354

MNVTCQKCPAVTSRFKGHFVIVGDTSLMKSWEEKTVAETKRNARQQELSHVIHTFLLKRQ

SQFNAFRI

>SG-PL_contig_9882_1_+3_3_185

TNQPTNQPTSQPTNQPTNQPTNQPTNQPTNQPTNQSVSQSVNSQQNHLIKSKYLTVKQLL

I

>SG-PL_contig_9883_1_+1_1_264

GRPGGRRAPGPPGPEGTPSEGQRWGCPCLGSHQSACSGSACSGSTCSGGTSSQRRRSRSP

TRGSRTRSAGTAVVCWEPCLGSVSPSVV

>SG-PL_contig_9884_1_+1_238_435

MDKKLQYSVCTRNYYFCFNGNIIEKLYTGFTMHLHLYWNISSFSKTTKNAQSFQLTFTII

LAYILK

>SG-PL_contig_9885_1_-3_90_677

MECYMKNLLCVAMMLIATVFAAQNDTTVVSTPAKGTTVTTPPPLSTTTPSAEELCALHNA

SCSDCLGVAGAKCLWCKSNSKCMAYPVKKILPPKEMCALDEARWGVCWLNFEALIISVSV

VGGLILLTITCCCVYCCCCRGGNKKKYEREDARYSSQRMERKAKNDERKAERKERLDEIR

RKYGLMKDEPYQRFDA

>SG-PL_contig_9886_1_-2_5_235

MFIQTLCIKYAHLGRTCGPNLVKIQFQLESVTIPMHSKSTCLPQNGNKDYILKCHWLMHE

LRILDLAVILRWFPVLK

>SG-PL_contig_9887_1_+3_162_299

MVNIALITSKCESNIFLQYIQNHPFKTKAYKQFILQIQFERKARQT

>SG-PL_contig_9888_1_-3_3_113

AHYNVSKEHAVKYLTVPVYINFLEKARFLRFAFSRDF

>SG-PL_contig_9889_1_-2_125_2314

MKDKRRNSEKRKEKSRDAARCRRSKETEVFFELSNSLPLPSSITSQLDKASVMRLAISFL

KMCNVLEQHNWSELEKEKGKGDGKVRADQLFPKALDGFVFILSHEGDIVYVNESIVKYLG

IQQIELMGQSIFEFAHPRDEDEIREALSSRPHGPKAPITEERVFFLRMKCTLTAKGRNVN

LKSASYKVIKCTGRLVTERGRKQGTGVGVFSPYLLAIGEPIPHPANIEMPIDHNTFLSKH

DMNMRFTFCDDRVKELIKYDSDDLEGKSLYDYHHAVDSEIVEKAYKDLLARGKGQTMTGQ

YRFLAQGGGWVWVVTQATVIYNSRNQKAQWVVCVHYVLSHVEHSDMILSEVQLPIAKCSP

SPSTSLQLSTDTVFTHKTEDMDQGYLIPVEFRDKLKLTKDNEPEDLTCLAPTAGDECVSL

GFPSFSQVAMANLRDDDCTMSSPPALLTVNLKKEPSTSPALCRIRDQANLNTLSPASSSS

SSRINSPSDYLKAASPDDVLSMDKFFQEMDTKADGNMENESIDFDARAPYIPMTGDEDLT

LLPPSSESLITLQNEVDPGLFGKTEFVFKPKTEDIFGETTSQPRQSMRDMICGSTTVASI

ERPPDIMFQQIKRPLDMNSLEKGPPAPKIQRVLGPKQINKVPTVKGSKDSVLLNLLLTGE

DPNGYKVLSPSTSVNMPHTKNIVPPRNVQSQLLRLTTRDCEVNAPTHNGGLLQGAELLKA

LEIDVPQTPR

>SG-PL_contig_9890_1_-3_27_692

MASDSWLSDYETCSRLGQQIMETINERNKHSRTSSNYAKLSANVRTTLRQFQTELNRLKQ

NLMRASSSYHITQREVERRQQMLDGLITKEKQIDQAFKNESGDNRQALLGPSTDPFRSGG

GDPWAEPEHLQGVSSSDLQRQQQHVLQEQDQGLEALSKVIGRQKQMAIDIGSEVDLHNEI

IDDIADHVDSTDNRIRRETRHISIVDRKSNTCCYYIVIILLF

>SG-PL_contig_9891_1_-1_1_195

HCVYKQKKTKKQTCKNTAKKKKKKKKNLLCVCAGKLKTFTHARTQYHLITVYERAQFNTG

VCLYL

>SG-PL_contig_9892_1_+3_3_74

TFMTFGPEAVFIREYAMNTQLHMQ

>SG-PL_contig_9893_1_-1_112_381

MGLDGSSYEGGKVREAFEKDYNVATCSVCVLCQRDINYGLKGRVALESNVKTEQHQKAKK

SIEGNINDSDCACTCVCVCVFFSLTSFHFK

>SG-PL_contig_9894_1_-2_143_979

MMAESLSLLCKTGDGLSHAYVKVDIHERGVTDINLMKKFIHLRYVDVSKNALKDLSPLSA

LTHMLTLKADENLLTSVQLEEMPFLQIASFTHNKINSTEGFSHPMLEHLNLSNNNITNVS

GLDANKLSRLHTLELRGNKLETTDGIYLPNLKNLFLASNMIKKIEGLSRCTGLTTLHLRD

NQLEALDGFPEELKELQYINLRGNLVTAVKESTKLKNLSFLRALALSENPVADEDDYRME

VLIALRTLERLDKDEFTDEERHEAEEIYETRKAEAEAAE

>SG-PL_contig_9895_1_+3_1908_2135

MISVLASVRPATSLKLMPCPCLEATSTLVNLALRSFANISVGLGSCTRLDKFRRRKKPAM

AMMPRKTTGTQGLCLE

>SG-PL_contig_9896_1_-2_326_493

MDKPSCSTNRCVTDSDHAVEKESFAIIYNYNVDSFSDILLLDNINQHLSAISLLPL

>SG-PL_contig_9897_1_+2_575_868

MHTEPPLYFFCCSHPADFAPDFASLSSTLCRCWWDRKIPTRFEKDVMEDVRHITTSVFFS

SCPISLRGCLMCMEHLAQPAIMPWISLLINGTVSVPVK

>SG-PL_contig_9898_1_+1_142_492

MSQIIFPSSRILLLKAASVVWITERSKPTAFVLLPVSVIQAPSVLPLVCPFAMLFVVEPF

SCVLISVHRIHSISDSFFLCAVIPRFPQRLFLLRGFDVWHGGLFQLFLPRSNFHSEN

>SG-PL_contig_9899_1_-1_76_402

MVVQAFNICLRTLGATRNVMTVHGRFDNIKAPTWKSSVTFCNEPWLVFLKRTGICNEDLW

GTEMVNADQTVFNAKLFPVFHATDNVGQKEILWLQGQLTDTPELDILKR

>SG-PL_contig_9900_1_-2_2_133

SDRVKDCHSENIFSTVSDRVKDCRSENIFSTVSDRVKDCCIENF

>SG-PL_contig_9901_1_+2_134_280

MRQSLILPLPELNIFSLRRSLTLSLTVLNIFSLRQSLTVSLIVLNIFSL

>SG-PL_contig_9902_1_+3_339_524

MHQNQICADWHYEQQQIRFDIDCSCVKIIKAPSFTFSIFARQQYHTAVPWKRIQNDHGKC

MH

>SG-PL_contig_9903_1_-2_380_628

MKGSFGWQCWAFPEKACQLFAENCQLTAIQCAAIQKAHVTHHRFVSFNISHLIIKYFYFL

MFFNVLVISCCKCMKNYILMFVH

>SG-PL_contig_9904_1_-1_1_171

FLFVFCCCPTPSSLSPSCYSLCCVLSLSGCSCQHCRDCLCSRGADDSVIMRSEGSSV

>SG-PL_contig_9905_1_+1_142_297

MVRREKNVSYQFCNSGCSLTHPTHSLQETHKSGPTCTTSNHVKSRLTHDSHK

>SG-PL_contig_9906_1_-1_4_954

MASTNAPLLMRILAASVSVSNRAAIIIRDIMKKGELGIVEKGKNDFQTEADRAAQRCIVA

SLHRLFPKVAIHGEESLDPTDTIPDELIETELNADVLKQPCPPNLVNVKDEDLVIWVDPV

DGTAEYTQGLLDHVTVLIGVAVKGVAIGGIINQPYHNYEAGPDAKLGRCIWGLVGLGAFG

FHRQKPPEGLNIITTTRSHSNRAVTEAVESCNPSQVVKVGGAGHKVLLLIEGKVHAYVFA

SKGCKKWDTCAPEAVLHAVGGRLTDLLGNPIPYGAHVKYPNSSGVLATVANHDWYVSRIP

DDIRDMFIPEPEQTPLA

>SG-PL_contig_9907_1_-1_1_267

PFTKVKPYCPCLCNSYHESQLTTGIMYWDGFLGQLDNVCACFCECFGFHTAKCKRIETIN

FIGFNLLNPRNKMEDSLLYTYLFNDYSVF

>SG-PL_contig_9908_1_+3_33_245

MAFHKQRQIPMRRAAKWEYTCPFQSLSARMSVTDPQSLEKVFMNSECLLANVSPTKMGNI

TEASPYTLTGV

>SG-PL_contig_9909_1_+1_1_150

AQHSEKRIITNQRNNHSPITNVAQHSEKSTITNPKNTLLFTSLYVPHSLF

>SG-PL_contig_9910_1_-3_81_179

MLLKELCKEIKARTKNRVTNDSVHRIGEKKVHT

>SG-PL_contig_9911_1_-2_8_334

MADIVKPVEMSIKKKLTETFKPEVLQIVNESYMHNVPKGSETHFKVVIVSDAFEGVSLVQ

RHRHVNITLGEEFASGLHALSIVAKTPSQWEKSPQTVGPSPPCRGGAGL

>SG-PL_contig_9912_1_+2_2_85

FFLELKFCFILIYKEIHHTTQNSINVSF

>SG-PL_contig_9913_1_-3_168_359

MVKIVCHELCSFAGFLFSRLRFCVRRGHLLDFDGLYMVTALIYGLSGGLGYKEYIMIIMI

RTPT

>SG-PL_contig_9914_1_-2_86_286

MVLYQIWYRLHNMVRRLNSFVKMGVTQLLCHDHQTCKNRSQNISWLLSGESLPQVKSPAC

LSLNEYD

>SG-PL_contig_9915_1_+2_479_754

MNLVLVTCISSARKSLQLSQLMCVAINKWPLTLRECPAAVKIIEDKRQNSLLDTFKTTVA

VVSLSTSFSMGHTIRSTWTEWRHRHYKLTTLC

>SG-PL_contig_9916_1_+3_45_221

MCVYCLRYNICMTCCPAIKGKSFSTLSCLPTIKVNSYACLYDTNVFCCPMIKVNSFCTL

>SG-PL_contig_9917_1_+1_355_528

MAALQTAVFLNACYIGMAQHSKSLRHNVSCFEECQLYRKECQLISKILLAVLHKVISV

>SG-PL_contig_9918_1_-2_182_331

MLVPSLFPLFIVFIYHFVVLLGTNILYSHLLRLGKCIFIFIYLVSVILPI

>SG-PL_contig_9919_1_+2_2_232

CEGLLEKRKWNGPGRQKKADDSKGKKNGRDKESNRKEEIASTEFPEGGDTIITINNKTKQ

NKTKQVVSAMLMLVICL

>SG-PL_contig_9920_1_-1_1_849

RKHSHKDKHRERSRSRSRDNSRKKHRRSDSSSSDLDSKEPSFQPPSVSKEDKEKEKALKK

ALETPEEKRARRLAKKEAKERKRKEQMGWDQEYMGYTNVDNPFGDEHLLDTFVWSKKIEK

EGKKHLATDEIQKQQKVKMRENKVELEKVKKRRLEREKEREERENERDMMMREKELEYFR

EWEKQEDSFHLNQAKMRSEIRIKDGRAKPIDLLAKYISAEDDAIAVEMHEPYTYLYGLTI

TDLEDLLEDIRVYLELEEGKNADYWRDIITVTEDELQKLRKSD

>SG-PL_contig_9921_1_-1_94_360

MELGGWQVKSHALSFSLSLCLGAGGLHYFSAVGKPFVIGGVVFCLFLIIFFFRFRYVTVS

MEFRPFFLSSLFVIKLVLFSSFFFLFLID

>SG-PL_contig_9922_1_-2_2_970

SSRTERKKRSEEEAPKFTKTLSPVEVKEGDRLSLNCAFSGKPDPNIEWFLDGQLLTSDDS

INIKTRSSTSRLEISKVEVDDEGDYVCKATNTAGVASTKTNVTVKAARKAATSSAPAEKA

SSVQAPQFLEHVQGHSVVDGDTVTIQCRISGKPEVKWLANGKPVKDSDDFKYQDAGDIHK

LIIGEIFPEDAGVYTCVATNAGGSASSSATVFVKVPDEEASEPVFSSWPRSQNVDEGSPV

TLSCSLDSPAALTVSWSKDGRSLGDSSRLQFKEEGSTYSLHIPAALSTDSGTYCITAKSD

AGSTSWSCSLAVAMSDSATDMAK

>SG-PL_contig_9923_1_+1_283_753

MNNICKKYELNAHTDRHLSLTLVPFPPPHLHTHTYTHTTFKMVLFIITILLTRTMTDSMM

QCHMANVYNKELGWKRRREQTKRDKDTLTVSETENCHYIVSLYFLLKHSNPFLLLNHCNS

FSFIMQAVFFSLQAQNSTISKSDQCTLWVTFPDPVKR

>SG-PL_contig_9924_1_-3_93_224

MAKGDNHSMEVEERGLSNFAQIYFPFFHSDVRWLHLTLSWRSSF

>SG-PL_contig_9925_1_+3_171_524

MLRLVISPRLNNDAKPCWTYTHTHTHTLSKFVTASHIPAHLVTCANTPVQKSTQIRPNRV

LQLQLKFPLLYTHLVVKGGGFPIPQVNMCADLLVPEPPSCVYACRTSNMHVKDPASPG

>SG-PL_contig_9926_1_-3_3_275

REDPSGPETTASAISADVVPLENTSVGCGWEKNAMQRTNCNRNYFVWNKSNYYPSKTYPK

GRMITTSLMHLNAMYDIITGEISPFLNVNRI

>SG-PL_contig_9927_1_-2_2_241

HLGSPPSDSLPRRPLPEKGHRASLRLQRSDWLAWCGPSQVDPRSPSPVELAKLPGFPCHL

GGPSREHRQGLQVLHYPSSP

>SG-PL_contig_9928_1_+1_163_291

MKRISVVLMEMSVSIFQSFQSCRFIVYGRSVYRLCDNRSDSKL

>SG-PL_contig_9929_1_-3_3_173

LARAHTHTLSLSLSLSLSLSLSLSLSLSLSHSKVSVFVTNIKNISVVVNVLLCDMIS

>SG-PL_contig_9930_1_-3_120_992

MSVRTYNPSVRVGNWNEDIQLEEDTLKNFLEKRENGELLIQKTHNLTAAMGVNIELSVSQ

DHAIHIGDKVMIKCAAAKDQQKYSANVDPRQDCVVALNITDPTTLLQKNVKGPVIATGCQ

AIAANQRTVFCVESVDGSQGGAQLMYGEPFHLTTVGDNTGKLYLCSDKVTFNKCAKKSRH

QEVSLVPEPSFLTRWMVQHRNPLLRMEYEHHPVEANAEVVIVHCKTNSCLAVEDKFNLRT

PFGREYELSVHTYLDSHRAEEDNNRWMLVVGVPGSCVNPVPDQGPGAAPLH

>SG-PL_contig_9931_1_-3_66_188

MVECMMLKEEFCQVNSCLASTYPTLSPTQPSPLGSPLGLST

>SG-PL_contig_9932_1_-3_36_239

MEATERYRGGEHQQPDSPLPDITREGPLWCKTAMSDGKKCSDRSWKQVWAVLRGHALFLI

KDRRDGTS

>SG-PL_contig_9933_1_+1_31_159

MHSVLSKTLTVIRFFRSSCNKQTSSDQYSCKKIPKILNHNEII

>SG-PL_contig_9934_1_+1_40_348

MCFKSCELDPIPASLFSQIYQQLLPSITSIVNSSLVSGDFPSAFKTALVRPLLKKNIDPN

NLKNYRPVSNLPFLSKLLEKVVLKQLNDHLSLNNLHHPYQSAY

>SG-PL_contig_9935_1_+2_839_1081

MFKPEHEPDHPSFWQAHLIVFNLGLWEQVLKPLHYTITVVIGYCHLNDNDGICSQKSEAK

QSNMNAYSLNKTRQQISGIVN

>SG-PL_contig_9936_1_-2_245_403

MLQLLDAVITLQYHFCHFLCSVFNLLIFSSSSLNSKKREIWSDQLLACVFIGY

>SG-PL_contig_9937_1_+2_341_619

MYVCICVYMCVCVCVCVCGGGGGGCFFVCVCVCVCVCVCACCVCVCVCVCVCFLKPPNTD

MAYRIFNMHSLCSACVYAHKRGSGTGRSAHKLT

>SG-PL_contig_9938_1_+2_101_319

MLFMLSLPNEKGTHWSDIRNPSPCLLNGAFTGMKKKEKRGPKGIKLMPQVNKRQSALSPF

SSLHYFGLLSNFR

>SG-PL_contig_9939_1_-3_279_491

MGLKLVKESYFIPLLHKPWVSSKPAKQHLKVYSDLKISFKQNFKMYLVGDLLASCSKAYS

QESGIKKNHSH

>SG-PL_contig_9940_1_-2_1103_1333

MCVLDFLWTLNFFMVLIFLCTLNFLYVLDFPLCAEFSFCSQFSLCSSYCFHDVILCNGLV

LCVHASDDLCAMGTTVC

>SG-PL_contig_9941_1_+1_1_156

FFFKNSNHVYFRSCTSGYNKTFNVFSKIKNTELWKNDILMSHKGHSCLTNFC

>SG-PL_contig_9942_1_+1_145_252

MVARTFISNNYLSLSLINIHAICFQTFTMVQHLPLR

>SG-PL_contig_9943_1_+3_351_614

MIRFEPTKLCEGLLEKRSGMDWEGRKKQTTEKEGKWAERKRATEKRKFVAQNFQKVEILS

ILKEPPASPRGKCILIHDFVIMQIILQN

>SG-PL_contig_9944_1_-3_543_716

MDVLYKQTECLISQPENNIKYEHNYSSRNEKTFKEHNSNLTHFFVFHNAAKFSLLNIH

>SG-PL_contig_9945_1_-3_3_206

ANRKPVAHALVIIEVLHGEPVIVGAVLVICCLLYCYFCGIEFVAEKRTVSYNKRHNVKTP

RLRNALQL

>SG-PL_contig_9946_1_-2_71_586

MSMKVKQEEELDAPLDFSLRRRDSVSESFTDDSRASSSNSPPHYVPFSSSISPDHAQQQQ

LLQQFILKRRQRHDSSGSHDGQGPALQVPQVNKSPSPDHGSQSQSGPKASSASGQVPLSS

ANVPLAALNMVAEGSTGIPPAALAAILGQNGGMMGSLSSLSALVSEARKTQS

>SG-PL_contig_9947_1_-3_63_317

MFRVSTNKRNTDTCCLATVISFKQRITCEPVVQPQCFAFSCDQGAGTHAGSSWCLLVSTG

AMSLSSVLCSVFSIMYVYGMYCCCC

>SG-PL_contig_9948_1_+3_3_314

QYILFILQTTTRGKRKISSFEIRSDEYEKKPPKYYYFIINRRKTSQNHPYKIRQTFGNKC

NVRRRGIHKIWHDFNNFTQLPEYTLKYNNLFVETIVAYYMSFRH

>SG-PL_contig_9949_1_-3_207_353

MHNILFQSLFLFQQHMYTKFCLCFKNIINTRFCFWFSYTCAEDSACVSV

>SG-PL_contig_9950_1_+3_3_218

SSLRPRQSIRHQPISCSQDGLERTRLAHSMSSRLLLSHVGLCVARCLLLGPDFCSQVVPM

TERCKSVCFCSS

>SG-PL_contig_9951_1_-1_1_120

PKKFQVFLKQFQIVPKKCSEGLASLQVFPQLCVRHLVFLL

>SG-PL_contig_9952_1_+3_3_80

LNLKKTLFFVKKLKFFKKKKKEKKKN

>SG-PL_contig_9953_1_+2_2_199

AENGFHIFFSQILTYPGRNFMEKVNLSAKIILKKTLLDKLFTTEKQDGGRKVEEKLAEMP

TPLNTI

>SG-PL_contig_9954_1_+1_151_249

MTVCIHFNIIQNSEHNKSLKNECLFHSLQKSEK

>SG-PL_contig_9955_1_+2_2_172

CVCVLKTKKEYDIQTWQHMCIVFITLYQCKHTKRLFHLAEHSHNFIISTPKTVHTYA

>SG-PL_contig_9956_1_+1_121_234

MDSKDFCGGHWIRGNKFYKSKYRFKLSSLETIIILFIA

>SG-PL_contig_9957_1_-3_3_299

YTPRAESRSPSPWALRTAPPSVLSRFDTSASRSYLWSGAHTTTRIRNTSRTVRSLLPGRT

LPARKLQLPPFPPTAIPLENTGSLPITEVKQRRARLVLG

>SG-PL_contig_9958_1_-3_381_656

MPSVCLNNFYYTLCQSLLTVSAVFSFDTVILPKAVSFLCLRFVLFVSVIAVLMLLVRLSH

HYFWTSFLSQSFRLLYCSIPAVQVIVSFCPSR

>SG-PL_contig_9959_1_-1_28_249

MIRENKDIKDTVINSKEHKMSQFADGAQLVNGGDKVSFEKSIHLILKFGKFSGLFLNADK

TQAKKVRKLDICSI

>SG-PL_contig_9960_1_-3_87_512

MIREDKEIKGIMINNTEHKISQFADDAQLMNNGDKKSFEKSLHIISKFGKFSGLFLNADK

TQAVWLGSKKGSQIKYMQHLNINWNPKKFKILGVWFTQDVKECEEINYAEKLSEVRTLFK

IWVKRNITPLGRVAVLKSLILS

>SG-PL_contig_9961_1_+1_1_189

PSKKKKKKKKKKKKQTKKMDKHHLIFSHSELHSNHFSQPSEQSKPLFSRSKQTSSQTRTT

FSG

>SG-PL_contig_9962_1_-1_160_249

MFDVSFLLIYSFIHSMHARSVSISIQYDAQ

>SG-PL_contig_9963_1_-2_134_319

MDLFLAWIFLKCKTNILLLDSLCPPSTNRIYILLSGCLSFSEYYQLKMFIVISFCILFNY

LV

>SG-PL_contig_9964_1_-1_1549_1821

MAMFQSVVWWLDEGCGLRMVLFVAPKLFLQSCFVGPLLDSSISEQSKKFAEHAAETVILS

DIAIHLCETAPLNTYFGSRQQCEVHGWKIVV

>SG-PL_contig_9965_1_-3_1206_1397

MCVNSHLAQRKHRSRYSAIKKQHTHTHSDRKLLLRTFFSNLFIFVIRIVYMQPKTYHGFN

LNCY

>SG-PL_contig_9966_1_-3_3_89

CAGSRSLPDNYWQIWVEHTWNCPSAMNPS

>SG-PL_contig_9967_1_+2_251_385

MYLEQNTPFGFINCTMSGCKQKWGVCPNFHVKLVQTHTSWFARLA

>SG-PL_contig_9968_1_-2_284_484

MSHYLNVDVGQNCFLTLRFVCLVMSHNSHVIHTLNLKFPYSFVLQGHLLQKQNHVSCIII

FPVHVEW

>SG-PL_contig_9969_1_-3_210_503

MFALRHYSSLYLVTGSSYISVIIFFCFGVLCTCTNKFKWPAVTVSCAACKEEGGRWLLLY

SRLSCCVLCCVFLTDCRMVANLLGCPCRWSGHPLCPGF

>SG-PL_contig_9970_1_-1_31_489

MLDMGFEPQIRKIVEQIRPDRQTLMWSATWPKEVRKLAEDFIVEYVQINIGALQLTANHN

ILQIIDVCMEHEKEEKLVKLLNEIMQEKENKTLIFVETKRKCDDINRRMKRDGWPVICIH

GDKSQQERDWVLNDFRNGRSPILLATDVASRGL

>SG-PL_contig_9971_1_+3_3_173

RQKKADDSKGRKKGRNRAIEKRKFLAQNFQKARILFILKQPPGIPMGKLWSKGDIRD

>SG-PL_contig_9972_1_+2_2_205

FFFAKDKLYFHNKVNQFLSKVTESTISSNKKPIVQRINIHSNPARFSEHNHPLTQTSNIN

NFLDKENH

>SG-PL_contig_9973_1_+3_141_245

MNIQANRQVNILLSAHNFHFYFNDWVLYAYCPVTS

>SG-PL_contig_9974_1_+1_208_684

MVLLFLEQPCSSFIHISKHISDSSTPTHTSGISLLSINFASSFHRKKCCHCVYKCKILKN

MHPNAQSVRDKATESTAVSAASNDTVSSSLHLHQQTDIPTFKCIPQNRSTICSPAQTFIY

FKLVILKTLPTCWIYVQAYGICHYYFLSPSHSICDARRI

>SG-PL_contig_9975_1_+3_3_221

EIARNSKKEQAKNQKTGDRSTKTPGKAKGGVKAGAWRSATCCEDVAGKKEEEAARVRTSW

ASRVERHLEASLY

>SG-PL_contig_9976_1_+1_1_210

SNSSLVLSFILQKLIKILYVAGERRIWVGDGGKRGLHCSVLFQLRKKNVINVHVCVCVCA

RLIDLAEKYM

>SG-PL_contig_9977_1_-3_522_752

MILSCNNILCITNHAKGLNSSITCIHYDWYAINIKVYTSCNNSMANRNLATSKSTGCNLN

MKTGIPPCIIFNHYANQ

>SG-PL_contig_9978_1_-2_35_238

MFCVRIHTDGSSTGRSAHMLTWEIRKNLHPVQTRPYPGRIELGLSNWKSSVLTMRLFARQ

LKISYFAT

>SG-PL_contig_9979_1_+2_860_1012

MTPNPTSAIEDIKMMYEISQTKYNATLLHSHADRQTDLITGCFFFFLSPLI

>SG-PL_contig_9980_1_-3_213_479

MGPHHAAGTWSEHELSLHVNASSCQGRVSASLVLRQEAHAPSQAHSGQSEHPGRRSQVSH

GSPDGVDYNSRVLDLVWGHWYRPIINLLR

>SG-PL_contig_9981_1_-2_317_505

MVEPVPTPKLLEFVQEGFIALGQFHMRSTQIYQQLSGKLFDLAQNPIRWQGLEMGRRMGP

VKH

>SG-PL_contig_9982_1_-1_127_375

MPSTPVRNGRRDLTCESACESSDYISAQRDTSQYYADFPVLPTRHPESPDVQKMLGQSVL

NTFPQQKCSDPTSVIKMLAQMLK

>SG-PL_contig_9983_1_-3_177_515

MQVCTLFWLFGPHCAGVTMLLHSLCTPIFNRLPRCVGSHTTFQGCAYWVFSTEFKNGESA

SRAGKSAAEAGCMQRVVRHVFSVLEPQNRCQSTTLTHYILHVLSHKHMPVVEM

>SG-PL_contig_9984_1_-2_23_613

MASTRPNHKPEDVREISRHDKAILSRIFNPGLPSSELQEEIEHPELSENVPEDARVVQAK

ALEVEGVNAAEEGNVQMAINFFSQAIQVAPHWPSAYNNRAQALRLMGDAKGALDDLNMAV

TLSKGEGNSACQAYTQRALIKRLDGDEQGALEDFKAAAQLGNEFAKQMLVALNPYAALCN

QMLSEVMTKLRTGQDTQ

>SG-PL_contig_9985_1_-1_1_132

CVCVCAVSAKLSWLFCRLIFTRLGFLVAVKWQKECLFGVVWLIL

>SG-PL_contig_9986_1_+2_248_388

MVYQKEYKKTINNQDDMVLDKSTTDRMVYSTGILTQPLHSLTQYMQC

>SG-PL_contig_9987_1_+1_262_459

MYTLTSARTYLQMKVEKKKNINNKSTHFQTTAIDPCEHMNLIYSVKGLARTSVHGVSEKT

SIMLKH

>SG-PL_contig_9988_1_+1_13_324

MTSLFSSDSGSNHNSKYQNVKMGRILQHTHMYVLGNLSECDKARNKTCRRPDVILLADSY

RSNFHHITFCTTKHLTTPPTSFLESSSQTIQTQGKKRCVYKTSR

>SG-PL_contig_9989_1_+1_775_960

MCFGIDAVMLGTLTRWCHLLGVHGIVLSLMNNYVWWSSDCYFQVFVPYLTFGGDFSGQQH

LI

>SG-PL_contig_9990_1_+1_106_345

MHIKSTTSLNLLEAKFWAKVFETVNFHLKNIAIHPKCVMYTKHTKQILKTPIPYRNRNNK

CRFTAIMYILLSKAACMTIK

>SG-PL_contig_9991_1_+2_2_88

TATKNNNKKFLSVYSSTSSSGVTYNITLV

>SG-PL_contig_9992_1_+1_79_312

MQQGQDFLQVHTYDHFFFSFSKYVHTYNLGKTLLSHMDSVHKFFVFQKMYIHTILPKFTF

KHLYALNGKAVSLFTVIT

>SG-PL_contig_9993_1_+1_697_915

MFNWKNTTTSLISSKKLFLSNYLGMDGEGDDKPNYKTTTTTVLINTKHKTACISVTETNR

TYHVPSPLFCLHQ

>SG-PL_contig_9994_1_+2_137_358

MYKHTIILLTIPLLQPGQQVKVECRVETSRHQLHQVTCLQNASVLSNVQNPTSPLTTSPT

HTSSPIPSLPSPSL

>SG-PL_contig_9995_1_-3_126_1967

MTTKIRCHYEVLGVERTANNDDMKKAYRKLALKFHPDKNPDSVEEATEQFRLVQQAYEVL

TDPQERAWYDRHREAILRGGQGKGDKYEDNSVDLFQYFTSSCYSGFEDDEKGFYTVYREV

FKTVSAEDYEFMDDKDSDFSFPEFGDSQSDYDEVVKPFYDFWHAFCTCKSYVWMEEYDTR

EAPDRRTRRMMEAENKKKRDAAKKERNEEIRALVAFVRKRDKRVQENKKKLEQRAAEIEK

KTKEKREKELQERHKLRENYKETGWSAMSGLEDDIQRLEADLAKQFGDHSDDYTDDDAAA

DAEEGAANENAAIPELEEEDLFDDLFCPACNKAFKSEKAMSNHEKSKKHKEMVAILRADL

EMDDELMEEQEGEEDRGGGGGSADEQIEGVDRPTTDGEKETQSKGQVVEEEEEEEEEYVS

RAKAKKQKKKKKQKFQEQETSEDMDGLTSSVNHLHVTASKQGDDDDYGKRNAKSKKNKKK

DRKAANASSSVDQATKEPAAANEAESEAVGDGETTGEGQTTEGDGETQSEGAPTEQPSQE

TGNVKGSKAGSSKPFKVKDSQKCLVCHEAFPSRSKLFDHIKSTGHAVPLSAKQDAAAAES

AETGKKKGRRKGKQ

>SG-PL_contig_9996_1_-3_30_428

MPAGLLCWPRNSRLTAVLRQTQASCRLLASQRSSLPSALGLTYFPSLPWPAVCNGGRRLY

RSASPVLFSFSWCCLVCFPPLSCVFTPNHGSLSSIPQHGEAGAGPGEEIAQFLPKGVISD

LQNPGPGVPCADV

>SG-PL_contig_9997_1_+3_60_278

MKSLEAPLADRANPQRPLGDKHPHMEPRTWVLQLGDRPLQKELGDLLPQDPLLLLHAGHE

GGRVGRRHGQASC

>SG-PL_contig_9998_1_+3_30_293

MCVSVCACVHECILRIKCVHNFTRKFCKENHSSFHMHINIAPRQKFATEILFRHSYAAFI

KKKKKNPKKQKNFKKKKSLWGKKKNVKK

>SG-PL_contig_9999_1_-3_24_413

MEIQQDTIFVSGMGEDCSEEGMVQHFGSIGVIKTDKRTGKPKVWIYKDKMTGVPKGEATI

TFDDPETAKAAINWFDGKDFNGSNIKVELAQRPASNFSRGRGRGGPRGDRGGRGGGFGGG

RGGGGIDRKS

>SG-PL_contig_10000_1_+1_181_393

MVSVLAKREITWQNSSFSVTHSTISNLCRIQRLEEGRDLLPWSDRCLEASITALAATEEM

RDRPSALETRR

>SG-PL_contig_10001_1_+3_213_614

MRVCTLFGPHCAGIILLHSLHTPSSVGVHAGCFRVSISHQTLTWLTGSLTCVFDVVRAYT

DAKGGSGTGRSARMLLTCEIDKNLHPLPTRRYPSRFELRPFNWKTTVLTTRLSRPPLDCD

PVMALVQQTAPRSD

>SG-PL_contig_10002_1_-2_1055_1249

MVLTLPILSISSGNFNRVVSTKYHENRNLILKTLVSKFLPNITLFPKSSCKRFFLLLNLK

VLIWD

>SG-PL_contig_10003_1_+1_1540_1839

MFIVVTHSPCPLPEIIFYQQLAQYWYPGLPLHSRSLRSTDQNLDWHMNQMFKILRELVFL

LHESGLTHLVNILIMLGHLTHRKFIPATNTHFSILSEQIQ

>SG-PL_contig_10004_1_-2_305_481

MTFMSNYSCHKHGENIMITFKTDAPPLSTNEDGEEDQTLSCGVFCVAVIFKTVVLIYIA

>SG-PL_contig_10005_1_+2_2_211

YWANHHPHPHLLFVREWQTITPSTPSLCQGTANHHPHPHLLFVRYWANHHPHSHLLFVKE

WQTITPIHTF

>SG-PL_contig_10006_1_-3_120_230

MVRFRSAYIMFCHRVIVHHLCFMLVSSWFVPGNDVLS

>SG-PL_contig_10007_1_+1_307_555

MSIFPNQVPDLTSKVCPQIVSISAQIDTSTKDTNFKKEREVNNRPTEREMPVTNNPERQT

SKAVNHDQHCIQRERSMALTTAT

>SG-PL_contig_10008_1_+3_120_353

MLHSLSIQHTRDCNISQSLPIHPVNCHQTSLNSAKKMIQACVSEQMILLEAMQKFALRVT

EAKHSWQIFSFNHISLPS

>SG-PL_contig_10009_1_+1_217_405

MQQNSNVGINQTEYVTYQNHLTVLYKDEILCCILKIHFADLRTWSEKVTHGVLRLRKTDR

SVN

>SG-PL_contig_10010_1_-2_23_385

MWIRVRGLFLKLFRWRLAVVGALGPPIAGACFWLVGPSTVVRGCGAGDSCQRCGGLVWRR

GRFVSVRGGVRACPSLRLELCRLVGWWGEPWHCGAPVWDLGWPPVQRFSGGLFTVLSEAL

S

>SG-PL_contig_10011_1_+1_169_1512

MSSATEHMKLETAGSQASDPDMFRSPRGSWCNPKRLAAVAIVAIVIFAVVVGVVVGLSNR

SDTSEASLSDRVKRAEDVLDKYPLVDGHNDLAWQFKNLVNNSVYSSDVDLSVGWPKVHTD

IPRLRRGKLGAQFWAAFTHCNSSYKDTVRQSLEQVDVIKKFVHKYSDTFEWVTTAKGIED

AFHQGKIGSMIGLEGGHSIDSSLANLRMFYELGVRYMTVTHSCNTPWADNWLVDTPGHTP

EFNGLTIFGQMVIKEMNRIGMMVDLSHVSHGTMVDAITVSRAPVIFSHSSAFAVCNHYRN

VQDDVLDLLATNGGVVMVNFYNPYVKCTPNNESRNATLEDVAEHIQYIKERIGVDYVGIG

GDYDGVTMLPERLEDVSTYPALFAELLRRNFSEEDLMKVAGKNLVRVFRAVEEVRDSLRS

EKPYEDLIVNADPNYNAAMYVPECRTSI

>SG-PL_contig_10012_1_-3_363_1646

MARSVFLHSTKIAMPVCGNYKLALSVLTVTIVALTVLAGSGQCFSLPAWSMAAVPPAQST

PRVQKVLVFGGNGFLGGFTVERLLAEGHQVVTVNRGNWYWDSAFLVKPYVTHVKCDRLYS

LDRCTDLLDMVNNGTEFDIVIDFSAYHIFAVTEVLKVLKGKAKLYIYISTDSVYDVCDKD

HKLPSAETDSVRPATEERREELESVESYGHRKLEIEEKLAIQRKEGGIPYVSLRLPDVIG

PKDNTYRWWIYQVWLKMADYLDKAPSIPSYLLDTQMSLVYVTDVADVILKLLDPKPEVFD

QAFNLAFKETPTLMQLLSEIKAALNITNLRILTDNSTQAIHLFPSVRNGPIDISKAQDIL

GFVPTPFQTAVSETVAFYEKAMTNPYYHIPRKEIIRNIQAHFTNRPFKVLLGLKKHYGLD

FQLPKDEL

>SG-PL_contig_10013_1_-3_3_104

FFLNNQVRILVHHLRTSVVWCKVVVCFIMLIKSS

>SG-PL_contig_10014_1_+3_1161_1427

MKMDFQTMKTLASDTYTPCFRGDGQHKPLLALEQRHSYSFSSHYVMLDPCSAGHNTEQYT

WCGIFTVAFPLSHDNLKKMSKQNQCIFFF

>SG-PL_contig_10015_1_-3_9_566

MADNEWGEVQSSLSKISAAETSDLCHALAEQICHNRVPAPDTYLKSLSLEEWWTVTGVLR

NFFTSSIKNDSTKDQMLESMKGVPEEYRKVIAEAASVHRDQLRQRLLDDTTAISHCVLRD

FDWQLKLVMSSDKLATLNEPLLNVDLDLQDGAGDSQQVAIELDHEDLKKLLNSLEGCSKA

VQQLTV

>SG-PL_contig_10016_1_-2_80_259

MRSSNKATKLKAKKLQLTCHKITSLFNIISGFSHSSSLSVYQTKQDCLHGLTFLCVCGKW

>SG-PL_contig_10017_1_-2_368_2104

MMASLPCPDFSVPLLLLLVLGVTCLCHVTHGWRVLLVPLPDTSQCRQAVSIGRELQTRGH

QVTLFVPDYFRARRCTEPSPPGVMDIEVRAFDTDEEAAEVIEDKLERLGDEVMRGGTGLL

GDSSSAHEVMRDVCNSYVMEARDDLDQLRREGKVDMVVVDGSPGGRCLTLLPAYLGAPFV

ALSSFIHPFDSSLPLPTNFYPAPLSALPGNMAFLQRLSNFFYYVSYTGGDSLFGSFHQGN

ALGDRVSRLEHGKLIKKASLYLENSDYIIDYPKAVFPNFVQVGGLTASPARPLPGPLAKF

FDEGADTGVIVVSLGPSVFTPNKNLEDKLLTVFRRLDSRVLLRLNASRSLNRLKNVQTVS

WFPQNDALGHPNTRLFVSHCGKNSFFEALFHGVPMLCCHFAGSDVLGTAARVAEYGVGLS

LDLLTASSDSLATAITTLLMEKAYIRRMKAASKLFHSRPQTPAKRAASAIEHVLKYGGGY

LRPRSLEMTFFQYAGLDVVLTIFTAILGVMALVSFVIFKHFLPVLQKKEVKAEVKDDLAS

TSSESEPTTKKVAEHENNPHASIVMYIFFVLVILFYIFV

>SG-PL_contig_10018_1_-3_81_635

MREYKLVVLGSGGVGKSALTVQFVQGIFVEKYDPTIEDSYRKQVEVDGQQCMLEILDTAG

TEQFTAMRDLYMKNGQGFLLVYSITAQSTFNDLQELREQILRVKDTDDVPMLLVGNKCDL

EDERVVGKDQGQNLARNFNCAFLETSAKARVNVSEIFMDLVRQINKKSPEQNGKRQKPKK

GCTVL

>SG-PL_contig_10019_1_+2_2_115

CTNGCCSVVCVCVCVWSAYAMTTTEKRMELSVCQRIAL

>SG-PL_contig_10020_1_-1_46_231

METCHCGILISEAYRRLELRRLWFQILSQYQFIVGFFIPDHHMSRLHSSVHYGFYLGRLG

QH

>SG-PL_contig_10021_1_+1_1_207

RDNSFAAVRYLTCTECMLHTGPQFIVSSERLAPRPPLKVRRKIHTSIHISLPRLNHARSH

DEQPVETSS

>SG-PL_contig_10022_1_-1_22_264

MPSPYPPASPNLIFSLTHPINQIPSPYSCRKLVDSVRKSKGKLVDRSLRWIRRANERVNF

LYICRHSRKINADTWKTTVSH

>SG-PL_contig_10023_1_-1_505_1479

MKHKSRQLGIKLVGKRNGPGVYILTLIPGSLAACDGRLKPDDRVLEINSTDVMYGSQEQA

AHIIQTCPDRVQFVVSRTSRPQTPDLIRSTSVELARHGALSLDCDPPICPLLNVCKERLV

SLNKDVSESLGISIAGGMMSQRGDTPVYVTNINHGGCIGRCRQVKKGDMLLSINNTNLLG

LTHAEAVRQIKLQSSVRNLALKIVEGAETLDGSGNFSPSWMYWLQMPRVCQAFKTITLLR

SPSGSLGFSIAGGWDCSHGNLPIFVKSIVPDTPAAKDGRLKCGDIIISVNEHGLEKVTHG

LAVDILKQVDGAVMLTVISWPGTVV

>SG-PL_contig_10024_1_-3_3_122

DISGTGTSVMKLTTFVLSVTFLLGLEYSMFRIKKNCRGLF

>SG-PL_contig_10025_1_-2_92_826

MKKKGFKQFAQLTREEQYELELQKSAYVFTMQKQLGLTDPYERLFFKEAVMPHEAAPYNL

HNSMFIDTIDRLASDEQKAKWLPLAESYRMVGTYAQTELGHGSFLRGLETTATYDPKTRE

FVMNSPTITSIKYWPGGLGKTANCCVVMAKLISNGQNLGMHSFMVQIRDLDTHQPLPGVT

VGDICNKFGFPGIDNGFLRLTNIRIPRENLLMKHAKLLEDGTYFKPKSDRLIYGAMTFIR

AAIVG

>SG-PL_contig_10026_1_+1_34_567

MRAVRELASLARGEAIHLSCYLDDWLILAESSTLCHLHTQRVLELCALLGFQTNPQKCDL

TFFQLFSFLGISSNTLSMRVFPDLSQAPVTPAETGGPAGMALSARQGNLPAGLHGTPSTS

LTPGWGAQTSPAEDQMAPAHGQLGSNRSPGRLVPGSDREWVSATDLAEGVPIATPRPQ

>SG-PL_contig_10027_1_+3_3_179

FASVGQTVKNSHSCRKKQNSDNYRLNVEQSHVNHSKTFFTVTPVGKTLMFAGKTLRFAG

>SG-PL_contig_10028_1_-1_61_1164

MACCSRDPKETKCCAKSAVMMDSDVRASVKQYYGQEVKSKDDIQTSVNCTMFSNMAAHVR

QALSLVHDEVINRYFGCGLVIPEALEGCSVLDLGSGSGRDCFAMSKLVGASGQVVGVDMT

EEQLALARKHVDYHTEKFGYSQPNVRFVTGYIENLTDAGLKDNTFDIIISNCVVNLSPDK

KAVLTEAYRVLKEGGELYFSDIYTDTYLSDDVRKHRVLWGECISGALHWQDLYQVAQQVG

FSQPRLVTAKMVNVSQFADLLGEAQFVSVTYRLFKLPATERAASHVTYNGGILGNPDKLP

FDHAVTFQANSPLAVDGELAAILSSSRFAPFFTFSDTGAVTSVTPDPKVDPFPKAKPVDP

STQSPCCS

>SG-PL_contig_10029_1_+2_2_139

YTSAQKSTFGQYNSPSPIPSQKHFITPTIPTLHNSFLLKLCYNMLK

>SG-PL_contig_10030_1_-1_67_216

MGSAYFYDNTIFPHFLPHSHIRIDVLELAQVWINHFALFISPLHPLYSHT

>SG-PL_contig_10031_1_+3_3_116

KNYKQQFHLPLVKMYSASNKKTRFNLLNFTNKSHYFET

>SG-PL_contig_10032_1_+3_3_119

LLLLSLGYRLHHCEDSVASEGRLKCACCVRVMEYSGLCN

>SG-PL_contig_10033_1_-3_3_302

AGRYEPLLCLSLPENHYYSFSALTHYGRPPHCFSHLFLPPRSLSSPHPLGSAWLDHIVFL

CGLKLPYVCSVSLFHPRVHPPSPHPFLSHTHNLITSLCWQ

>SG-PL_contig_10034_1_+3_219_380

MFRTFHCHFFLFFNYRLFFPPQIFFFPSMDSQFYLSRTSQNRRKVGALTYVQTC

>SG-PL_contig_10035_1_+3_357_494

MKTVTHWLNSEIILSITKYSLHNHALLKRFVFYYQFKCNITILKYL

>SG-PL_contig_10036_1_+2_2_259

TEKYILRKKKEQHTHATALKIPSGYETKKKTTQKPLPSHTHTRTHTSQSKCFLNSLLSEP

ITCKTSVFKFTAGFPSCLPTPSHCTI

>SG-PL_contig_10037_1_+2_71_310

MHTENTHTQISLHMSNLHSKSQFNTEHLSLSLFFLPSDTHTYTHAHTHACKHTHTHLIKY

KQYMFDSFTTVTRGLKVSPI

>SG-PL_contig_10038_1_+3_309_449

MVFSKPEHHCTIIKTIRVNYMGEQEDIVSDELKPWPAILLCCLRSPA

>SG-PL_contig_10039_1_+1_1_210

AGWEIHDVNQITNELNTFCSAQLLCLVKEDDSIQPSKSVVCSRGKSLKTLSVHLFQTSIY

TKSTAFRRKI

>SG-PL_contig_10040_1_-3_84_365

MGTSTLPQPGKEALSVPQALPPGSFFKPTFLNKGIWTRVSGNTLTHEAAQLSPEDRLLLG

DKLAPMSTSLSFKMLVDWDKIARHILLNCAEADA

>SG-PL_contig_10041_1_+1_1_186

QFSKMCLAILSQSASVLKEREVDIGASLSPNRSLSSRESWAASWVRVSSETLVQIPLFRK

VV

>SG-PL_contig_10042_1_+3_9_257

MFCFAFCVVLVLANIFSLFLHTHHCLKIMFLHFIMDITITTEKCTFSSHILFKIFTCYQS

RYILSLSLLAHTQAHISARRNAF

>SG-PL_contig_10043_1_+1_1813_2124

MKKEETEHIKHKKIGKCLKIHLTQTKPNIQRKKAPKQLQRDSFNERTEKLQYYLHKSQCS

KKTKPNTSHSQAVPGIVTNVISTCSIKYYYTTGFKSILNCLLII

>SG-PL_contig_10044_1_-1_1_144

RYVYKQSQQLLKKQSSHINKPPFIIRVIMSFVWTATSPLQIKVTIIAL

>SG-PL_contig_10045_1_+2_11_232

MITLHQDVSSATSRNQWMIMIHQKVNSMPSHNQWMIMLHQEVSSATLAQPMDDHVTLRSE

LRNNTQPMNDHVAS

>SG-PL_contig_10046_1_+2_2_169

CTFNSSKNITGYEKKNMAHNSFTHTHTHTHTHTHTHKHTSTLCQGKKKKKKTSLMM

>SG-PL_contig_10047_1_-3_225_368

MLYIFYKFILMFDNHLFAFMNIDICDFFYIPCLDFWIIPPLFRPRVFD

>SG-PL_contig_10048_1_-3_246_410

MNFFFRVSTDLGLITYCSKEAHIAFMYIYKFPSDLSLSLSLSLSLSLSLSLCLGS

>SG-PL_contig_10049_1_-2_2_232

MLDTHPSTTFSFSPVQSPAPTPKFACTTWQGSVIFRSQEIHSQNNDEARPLKHQQKVHNP

VLMAMRRFATVISLFCG

>SG-PL_contig_10050_1_+3_126_269

MSAFGGLWKHEHIQHAHSILPSLAPSSTGFSLSLGFYWCRTKGGPAAW

>SG-PL_contig_10051_1_-2_119_214

MFPNGALKSWYLCYAEFLYISELKRDYLMYMI

>SG-PL_contig_10052_1_+1_1_255

TVPAWFQISVAQRCTDQLDRSRSLRIIDGLVVAFDARACQWNFLADFSQVFYFVLQGLLH

CLQVFKLFFQRLLHLFAILCHVLRL

>SG-PL_contig_10053_1_-1_34_531

MKKALEEQLKDLQTVQQTLQDKVEDLWKVGKEVPNPVTGPCIEGNHKTINNPQRSTSVKL

VGRALCDKDLEPGWYRFLLNGTNAVIPTSCPPTRRCHTSAPQWLNLQGQSLPSPGQETVA

RACAHWARTCCNWESPITVRNCGLYFVYKLKPRANCNLSFCAEPQK

>SG-PL_contig_10054_1_-2_440_847

MAERIRGNRHCCYGQYKSDQRYPDRNEGVIFFPFPKPKTRLEDCKMWIKACGRPHDQLNQ

TSLPRTTMCVPRYAQSVYYCVLCVRTSVCVVCTYQCVCCMYISVPVCVCVLTYVHTYYQC

VCVWIVMVVWMCSTLV

>SG-PL_contig_10055_1_+3_3_239

STHLTFKKYTYLTQDILAEHLSTPEITILLSSGYVQQIFQQILSGLPNDICTQFHLNTAS

TPPDDYECTKQHLPSLLMV

>SG-PL_contig_10056_1_-3_216_536

MNILSSCSCVTTSCHHNRHVFPFLFHLHVQMVIGFPGAFFFFQSSVFQMHVWPTLCRLYA

AAQFAYSSHRMFKWLTGSSTCTFDILRAYMHYIHTKDVQALAGQHIY

>SG-PL_contig_10057_1_+1_1_102

SEIRLYLQRRLYFKRNQKDNSMFHKKFSYTLTEM

>SG-PL_contig_10058_1_+1_1_444

TLRNTYLIFPQETLPFTLLNTYLIFPQETLPFTLLNTYLIFPQETLPFTLLNTYPIFPQE

TLPFTLLNTYPIFPQKTLPSKSTVQFAGLSLMNYHADSVHLTQTNIDIITKYLLQKYYDY

EKQIIFHKASHHITLWEWNPNSREPSVP

>SG-PL_contig_10059_1_+3_9_1025

MAQKDAQLYESDESQTTQVPEDLQQALEQFDNLIQGETLSVPKEVLDRLEKQLKEEKLKL

DGHRTARLWLQYMEMVDILRRFVRGERTGNWHLHLRALREMLPFLAAAGHNLYTKSLYIY

LLQMQDLPFSHPDVYESFCKGHHVIRRSERYWAGLSPDLVIEQVLMRSVKTTGGLTRGRG

IGESQRTQWLLSMPDCAEINSAMQEVTGTQFCSSEQHKEASESRQLRDDKDTLAILQYLI

SRNPFGSYSSSLCNIANGVTSGKNVNADKAKEIGHQIMETMLGKSVLEHTFKRKDQVVTM

DVKNCVTIGDESVSVDPQLLFQRLVTAAKDITEDMRDLF

>SG-PL_contig_10060_1_+2_2_109

SETSDGTLRVKIKHQHGYTTTEPFVGHFSNSITFIS

>SG-PL_contig_10061_1_-1_1_204

SEIHALSGNPSDIAFLADGSVSLKFLPEFLAKNQDPGDPSPDVLIRPLSTILDRSDPDRH

LCPVRCLK

>SG-PL_contig_10062_1_-3_60_1235

MTRIIWEKIKDRLIFPYIDVDCKYYDLGLPYRDQTNDQVTIDSAAAIKKYNVGIKCATIT

PDEERVEEFKLKKMWLSPNGTIRNILGGTVFREPIICKKVPRLVPGWTQPIVIGRHAHGD

QYKATDRVVEGSGKLELVFTPEGGQPEKLTVFDFKNGGVGMAMYNTDESITGFAHASFQY

ALMKGWPLYMSTKNTILKRYDGRFKDIFQEIYDKQYKAQFEKQGIWYEHRLIDDMVAQAL

KSSGGFVWACKNYDGDVQSDVVAQGYGSLGLMTSVLVCPDGKTIEAEAAHGTVTRHYREH

QKGNPTSTNPIASIFAWTRGLEHRGKLDGNKELQRFAQTMEKACVDTVDSGKMTKDLAGC

IYGLKNVKTEHYLYTMDFLDAIGEEFERQWKA

>SG-PL_contig_10063_1_-3_165_788

MSGLDFRVEVRTNAEEMSPSGKVPFIHVGAFLVSELDPIIAFVNARGFSLTAEMSDTQRS

EMRAYMAMIENILVNAELYLAWLDDTVSTEVTKPRYGCPYPWPLSWMLPLRKQREVCARL

GSNGWKEKTLPQVCEEVRTCCQALSERLDQQKFFFGNNPTELDAMVFGHLFTLLTTSLPE

GHFAEIIQQFPNLTTFCQKIDERYFKQN

>SG-PL_contig_10064_1_+3_15_254

MVFPFSLAVKPHPDLFLMTEPSVYMMTSLGLQLFCSMSSLISASALPSGHSRQCLPRFGG

MIVLYRSLTWSGYFSYSFVS

>SG-PL_contig_10065_1_+1_376_540

MLNMPGGLGTLHYWTTVTAVRVTHHRHRMDWNVNPISPLHQQFITSCQIHQCQPR

>SG-PL_contig_10066_1_+3_564_788

MLKIFILCFVIQIRKYPGSPKFHLLQLDTLSRKMVLDLKMLKAQNQDHRPQMEGLGLNLL

KLLTWRVKDGDFSNL

>SG-PL_contig_10067_1_+3_3_146

MFMLKALCLSMLVKHCILESLYHLLCQGEHFDFFSLFVVLVLFPYRVL

>SG-PL_contig_10068_1_-3_3_323

CAHTHTQNKQTNSGFSSINEEIFQHTQTFSLVERKDVSVFLCDSCVKKKEKKYKLHYKNR

KRKKNQHFQVKKQMFVCVCMQTLAWVWMLVNECMNMFLFCVCPYDNE

>SG-PL_contig_10069_1_+2_2_142

QNHMQTHTLSLSDTHTHTHTHTHTHRVPDRKSNYFKSVTENISKLLV

>SG-PL_contig_10070_1_-2_890_1174

MHGVSLILYIMCVYCYQVQSIKLRWHLPFCYFVCFREREQNYQTMLHDVPGLVVVLTFFR

LFCFCCGQKKHQIVHIYNDAFCWCFVLFENGEVFV

>SG-PL_contig_10071_1_-2_101_250

MIAKEKLNPSLPHPIHQFPAFSVMIAKQELNPFAPSPYPPASKAGSWWMG

>SG-PL_contig_10072_1_+3_3_194

IFCLIHILMKAFREIIQYSHLRIFYKVWRMYVHFTLVSTNFVRGSHPYSPTESTADLIHV

SGIK

>SG-PL_contig_10073_1_-1_52_1704

MSLVAYAASDDSETSDVEESEAPQPTKAGDSNDPAFADDGKISDEDDYVPTTSTTGANYS

SNGSRPSQNAVSTSSTNNTGLHSGSIPTTLSDLPAPKAVTTPQGGGDREDELEEEVKPKA

SQLADAPKPPPKKVKQPTRILLPALEKDSDDEDEESGKKTKPIKAAENSDLRSGLFALLP

PPTHSSRKQTNRPLIPYTLTKRPAASKPTPTPSKPSTTTITAADTDLLPSQKHQSAFNAL

TGYESDSDEEDAGRRQSDFFSISSEADGASSSSSGLFVVKNSAPSLNQRDEDRLRTSDSI

SKDSSNVPSSSSQTKQTPSVSVISESPVSSSSSSVLASTSADSGNVAPHEINDSGDGNGD

DDDGDNENEVSSTVGVPLVNQDAPLNFSSTMGGASWSTTSLTSPMLPEMYAAGSTSGGAS

YWQHEHYAQGYGEDMEQEASSSSGSSNIQQYMEDEQFLRISGKRKRGQEDIQIIDANVDD

CVDPSELVKGISEESSYQPHRKKDNMPSMQQRRKKQITYLAFQAKERELELKNAWSQNRM

TKNQSRSKYGF

>SG-PL_contig_10074_1_-3_30_446

MSSGPSSPLTLRDLSYNNANPTTSCPNINELPMKDTWHDRESQNGDYTTDHLVFSFEKLG

AGLIDKSGDCDVPQKDTKSKDGEQILKNDAITNPQPSTDSKGLKEKRENIGNKRGGNGVR

SAASTTLKVQTAHSRAQSA

>SG-PL_contig_10075_1_-2_218_370

MIRMMIKCTLHMSACHYIFILSQLFNNGFFQIVQKFTSFIFFPKFVHKGIL

>SG-PL_contig_10076_1_-3_3_179

SLLSRLAQSFIQASPVHLMVTLRKVYHSLRRRNHSCLCVENSNENSKIGCISGQWQPHY

>SG-PL_contig_10077_1_+2_266_403

MRSQKIQLTILRQYYSHIADNAKQCIHIIARCWTDLNILIIDMIYS

>SG-PL_contig_10078_1_+2_638_1174

MNMDGSEKMPLFTIGRFQRLLCFTGLQTLPVEYVANIKTWVAAELFKDWLTKFDQIVHSQ

KRKVLLVVDNCCAHGKFQPQLKATELLFVPPNATTKLQPFDPEIIQNVKVHYRTANLLKP

ITHIDAGIPKEDFWITILEAMTMLKQAGIELLPTPSDTLQKGGFCDHFIQQHPQHIFQQ

>SG-PL_contig_10079_1_-1_1477_1794

MRCTTRPHLFSCLQFCSARSLDVSSTLDVNVVDALMTARKEERERCFARTERFSVVIFNE

RFYDDSASLPLLLHRTCAKVSKHLRVFSCDTERMREVCGGSGNLCI

>SG-PL_contig_10080_1_-1_457_675

MASIVTILLKAGTSSEALQMSAASRREGTESLYNHKWKAWASWCSKESVDSSNQIANFLA

LLTSKRNLTSSTI

>SG-PL_contig_10081_1_-3_378_692

MGLASLEARSPNQVLAWGLLLVLDALRKNPYEPPNNAPLSMLTSKMVFLYTLATGRCRSK

LHGLSGAKGDIAFYQDSSVSLTLLREFRAQNQPLEEPSPQLSSIL

>SG-PL_contig_10082_1_-1_19_255

MLIMLINNQNTQEELAVYSFINMFSLREPEMYVVINAPNTRQETYSLLRNCSPNKVNNNN

NKKHVTFASKIIHVEAYVH

>SG-PL_contig_10083_1_-1_184_1182

MSGGHRRGLRRSSNLTTWKQVKNLKQLGREQNLHERAASLPDLTAEDVKAVRRDKPPRTV

ESMADSFHKKRHSAYLLDMYQRMAMESNPPSPTTLSLIDQVLFQEAFRKEYTQDESPKHD

ITTTTNNNTNCPSCGAPQRDLHLDLHRDIQCGRSQISATTGISPQTITLWYGQKNFSSQF

PPNTSDCGVQASLNEESPSLHHRSMQTSPASPGADILKVLQEIQAKELRDARNLQERESR

ISQEKEVRISQERESRISQDARITQEARISQDGKDRNLADQDPRNSQDSRTFQEIDARNI

RDSRNFHEIQEGRRLQDSRDTRHAQREKIPMTR

>SG-PL_contig_10084_1_+3_477_722

MTILTIFCVLPIWVNVNMVDESKESGKQCTTFTTQQVLERLYLLSSNKDIIIENMKRFSA

FCLSMCVCVVHTEIIDFLSIVD

>SG-PL_contig_10085_1_-3_90_314

MLARADSIRLHCYLDDWLILAESESLCRAHTQTVLSLCSHLKFIMNPEKCDLYPSQSFDF

LGMRFNTVTMLVSPT

>SG-PL_contig_10086_1_-1_1_147

APAKGGNHGTEHPIRSWVLRGHFRSPQIFGWLAPSHEPLNLKQVLSAPI

>SG-PL_contig_10087_1_-3_222_1298

MSQAVADEHEGQLDTAAEENGREAVTAPEDAANESTLPLTSSPPSSKARGSRGPSQACLK

SSRMATSLEDITSFRLWRGIVAEFLGVMLLVLVGCGSCISLEEGKSSSVVQIALAFGLSV

GTIVWIIAHISGGHINPAITLALLATRKISLVKAAFFVLFQLVGAVVGAALLMGLTPKRY

RATLGGTNLDASINAGQGVGVEIFITFVLVFTVFASIDSRRRDLNGSTPLTIGLSVTMCH

LFAVQYTGSSMNTARSFGPALVTGNWKNHWVYWVGPITGGVAAGLLYEYMFAVNASLTKV

RAFMLSSDYDDEKFHAQKTKIRIIEEDPEAAMECVPLSDAAEDKPPVDEVKSLDEPVKV

>SG-PL_contig_10088_1_-3_234_362

MPFVVTDCYVCLMTFLCLIIAKNLHLLETIVRAWFQDRLQYQY

>SG-PL_contig_10089_1_+1_1_339

LGIGSSAVEKILHKHLGARKQCAHWVPHALGDEQERGRVERCLKTLEKFDNGRPNQHVRS

SVETKPGFTSPTLKPNNSPQSGCFQVCPLLPNSKEHEAWENKWWHFSSPDQGM

>SG-PL_contig_10090_1_-1_1_288

EKVTVVHGDFRLDNVIFHSSRPEILSVLDWELSTLGDPLTDLATCCLTYYLPLESPLFPG

LSGTDFKEVKIPDVNQFVAMYCHKMGMPLIDNWEFY

>SG-PL_contig_10091_1_-1_619_1020

MDRGGGERASLGAWQGPVKGGYQRPLLPKHVDECLGGPFYLCQCPALSALSPWVKKAGHK

PVTYVWSSTAGSSGWGCGITSQHLHALALKQPNSFLSSSLICLNFGCVTFSPHVHIKVLS

CLYLRITAARNLKF

>SG-PL_contig_10092_1_-1_1_180

NNNNNNPSICPPVYLYNSHMRIFFLFLMEVVYALQYPYLQFLLMLLTPNDCCVSEILSVS

>SG-PL_contig_10093_1_-3_240_2057

MASQAVQRATNVVFQSHHVSRDKRGKAMGQRTGFRGCTVWLTGLSGAGKTTISFALEEYL

VSQGIPAYSLDGDNVRTGLNKNLSFTPEDREENIRRIAEVAKLFADGGIVCITSFISPYE

KDRSNARKLHEDAGLKFYECHVNTPITVCEKRDVKGLYKKAREGKIKGFTGVDQAYEPPS

TPELILKAGEQTVDECVQHIVGTLMDDGIVPKGAVSTVRELFVVDSRKEEVCKEAESLPS

VEISKLDLQWTQVLAEGWATPLSGFMREREYLQSQHFGCLLDGGVTNQSVPIVLPVKTED

KERLDGCKAFTLKYGGNPVAVLRNPEFYPHNKEERCSRQFGTSNSGHPYIKMINESGDWL

VGGTLEVLDRITWGDGLDQYRLTPRELRAKFKEMRADAVFAFQLRNPVHNGHALLMTDTR

RRLLERGYKNPVLLLHPLGGWTKDDDVPLPVRMKQHAAILEDMVLDPKNTVIAIFPSPMM

YAGPTEVQWHAKARMSTGANFYIVGRDPAGMPHPDGTRDLFDPSHGAKVLTMAPGLTQLE

IVPFRVAAYNLKKKAMDFFSPEHKDDFLFISGTKMRKMAREGTTPPEGFMAPKAWEVMVE

YYQSLA

>SG-PL_contig_10094_1_-3_195_497

MENSVCSGVRVGWNWFTLSVLSRFSGLVLSKMPFSIHFPVHFILSILNVLLSLLISYVGF

ISIKLAAFSIFQKYQCTSFKAVPLHSHYYFDYRHLATWLTQ

>SG-PL_contig_10095_1_-1_778_978

MAKWLGQWTADWKVMGSNLHQSIKNHCRIVCYGLIGKTETTQLSLIHFTDASLRVLLKFS

DQHSRCL

>SG-PL_contig_10096_1_+1_1_123

PPKKKHISILQADMNQSSFCLSPRQWVIQNNKTSIICTPPI

>SG-PL_contig_10097_1_-1_1_132

KASSPRPHALEICSEGPMLPWERRGLNHKSKKSHLGNFICACQL

>SG-PL_contig_10098_1_+3_201_347

MRFVTIVHVIPVKHTSFSRSLLHGHMFVIVIVDSMYTTFYRVVVADSQY

>SG-PL_contig_10099_1_+3_597_773

MICFFHITGKTDAHITDAHITISFYNINNSSSSSSSNNNNNNNNNKTKKNIKVSEFPNM

>SG-PL_contig_10100_1_+2_2_154

EGGGEGGFKASTNYNPLTVNIFFPAPKPARSREGGYCTNYYKRKAQNLNRK

>SG-PL_contig_10101_1_+3_240_845

MCIMSKLYKIDNNSLSILVKLFDVQIQPVVLYGAELWGLQPGSSVIEKVHLFALKRYLGV

SMKTPNDLVYGELGRFPMYINAVVCCVRYWLRLIQMDDHRLPFKAYVLLKNMDERGKTNW

VTRIRKILCINGFSYVWESQSVGCVKSFIKLFKQRLIDIRWQCWKEVVDTSDKFQLYRRF

KVNVGAETYITLNMSRYVRNAL

>SG-PL_contig_10102_1_+1_238_606

MSRLYKIDNNSLSIFIKLFDAHIQLVVLYGAELWGLQPGSFVTEKVHLFALKRYLGVNMK

TPNDLVYGELGRFPMYINAVVCCVRYWLRLIQMDDHRLPFKAYVLLKNMDERDKTNWVTR

IRN

>SG-PL_contig_10103_1_+2_374_667

MHTQAFSHHIYILHIHIHEHVHEQTHAITHNCITLACTYESAHTHTHTHTFPHTCLSVKL

YLESFFMDCLGSNNNKIINSTHTTHNTKENTSEHIHIA

>SG-PL_contig_10104_1_-1_52_2841

MAVCNGRYDCRDRSDEGNCSSTPPPSCHSYQFRCDNGHCIFKSWLCDGDDDCGDYSDERH

PNCSTENSTTAGYNTTSMTNILCGFEELRCSNSFCVNMMSVCDGYNDCGDWSDEMDCGFT

TTTTRRPSTCRSSQFTCNSGQCIPQYQHCNNVSYDCWDNSDELDCYAYTCAPNNFECYQS

SNYDADRCIWGSWRCDGEDDCSHGEDELNCNQTTTCPSTYFICMRSGECIPPSQRCDGHY

DCTDHSDEQDCPSQCDKYMEDQALCCQHGCAFLDCLFDSVRGCYPNNTNIGSSCSLTQNN

LCRNNTVKPTPTRRHNGTDYLCLYSGTWIPRMYVCNGINDCPDNVDEFMCSASRKSGEQL

HLLEVSNTSVTVYWTRNDNRRHAQTTIASYFPKGKTNLWTNRSAGNDTQYQLLNLLPLTT

YVISVYSENGHSVSYPSRPLEVTTRPGRPTAPRNFQVKVDLSDDTSKVTLYWQAPARLNS

LRIHYTVEVLDLTNSNSFTKDTAHLYLTLYILVVSHSYRFKLRAVTEQGLGSDPVVKTFS

YQLGQLEPLTVHLDKVTENSAVLSWQPRQGAVSFQVTAMLPYQQGRRISNTTSDNSLTFG

GLCPGQHMSFTVEALYEDKSHSLPTEPLYTATSGHSIPAVQNVTAQPSGNRQVIVTFTSP

AAASGKQFTIYYMAANKEHDLETFFKKAKRYATTDPSMTEITHLNACERYFIRVGCGPDS

CPISNAAVVQTQEDDTGPPKDVSYHFVYQEDRPLGVNLTWRAPCDSMVEAQKYMVTTTYS

GRPRGQSDILPQATRNSSLWYMVPSVIRGDNYTFKVRTTVSGADYSSQLVVNIPPYPPVE

GFTLFNKPDSMVYMTWLTPSDKPSGDAFKGYNVTIWREDAMVETKIVRKTEIMQNVTKGY

WYTVTICVAVTNGPDGTAYKYRFPCECLCL

>SG-PL_contig_10105_1_+1_268_528

MTNILSLSLTHTHTHTHTHTHTHNPPIIHKYISFDVTSRDLQTINLLISRQIIWTLCVYV

CPCSTGITKDQNMDNYSLPNRLQKLID

>SG-PL_contig_10106_1_+3_1080_1262

MCFTTHKAIKKHIVHYGTQQAMLLGEKKKPTRVCTCKALKAFELFLLHSRKAREVKVPIM

Q

>SG-PL_contig_10107_1_-1_97_444

MAHNFGDVKKFTTWRKLWIWLATAEKKLGLAITDEQIAEMQANVNNIDFAHAELEEKRVR

HDVMAHVHTFARACPLATPIIHLGATSCYVGDNTDLIVMRDAFHIVLPKLARCIHR

>SG-PL_contig_10108_1_+3_3_302

THTQKRHFHYKDSLSLACFPHNLYFFLFFFFFKLHITKRILYTISPSATLILRSADAFSV

CMRAHMCVFMHVQKAFAKSSRHSRPKQTILTESDLSVKPQ

>SG-PL_contig_10109_1_-2_722_2581

MHANTPPADRKSRFHAFGKIFKPWKWKRKKKSERIEKTAVEIERKISMRTSREELIRKGV

IKEPDGSQNHLPKIESVKEADEEKAQNENKSAGQGDGVVTAPSDRGEGGGVTTTAITTTV

TTTVTAMTTADGSLPASEPVAPVSVTTQAEITPINNNITVSSTHVRPVIVPPSPTPNAQR

HSPSPIPTQHSPSPVPQPSGPTLRIEEESRAPPPAPTEVQRRGVSVSSCDDEEELNTSTG

SITQVPRRQVTISDATPVQIMQPEPAVVVNDEQDEEELPHVIYPDDSSAGRPYPPYEAIP

ACEPDLSRRPNKSALKGGSGQGGPLSPPPPFPASSSLSRPLETSPSHTHASSPGPPDVSF

NLSMNRSGSPNLGASASPMSGRHVGIVIPRPQLLSYQMHGAGDSDKENMAPPLPPPPALP

PYPHQSQPDYDNLPDDSDSDDGEIHYKDDYEESSLAAKVARQDSLARFLSNRPTKQDLED

KNIIPAHSEREKADLREQIGSKLTRRLSLRPTLEELENRNILHSQSLDDYQKEKEAKRRF

LIRKLSFRPSVEELRERKIIKFNDYIEVTDAQEYDRRADKPWTKLTPKDKAVIRKELNEF

KSKEMDVHEESRHLTRFHRP

>SG-PL_contig_10110_1_-3_129_1358

MIYGNDREHARSLRTKNGTGALLKTKLYRGEERLPEGRPDDCINLRPDDFCAKAGDERVN

EQPGLTAIHTVFHLEHNRLVRKLVERLLLRKGRPFSSYHIEEFLHHEPAKIHERIYQVVR

KVLGAVWQKIVYQEYLPLILGPRLMSQYKLWVGRPVHYNPYIDPSIAIEFLTAAFRYGHT

LVNDIIAMDRPRLLREVFSTSAHNLNHYNKIVEGLVGKQSHSQPFNSHITDSLTDHLFAN

SREHGVGFDLAALNIQRGRDHGIAPYNEMRRYLGLKKKRSWKEFGECELPLRRVYDHPDD

VDCFTGGTCDLPMFHGVVGETFGHIIGKQFEKLKYGDRYFFETDNSYYGFRPYELKPIRR

MTLAKILCENAEMPWIQENPFRQAHDRWNPTYKCSSIEDIDMWYWTYYIY

>SG-PL_contig_10111_1_-3_105_368

MVDWSTFLENSSVRDGKCSLFCDTKTLIHGHNCIFCIKNRIFVDINALQCIEKHFLISDE

STFLSRNMTMFFVFLSVTTQVTENSNFG

>SG-PL_contig_10112_1_+3_1923_2135

MYKRIQHIVQDVFSFKAGQFTAKRKARLESKACIQIIIEFLAGQSKTCTKPHNMYTPFSS

LAVKCIMTCIK

>SG-PL_contig_10113_1_-2_11_331

MCVCVRARARTCVCVCVCVCVCACACACVCACARVCVCVCVIHMLCVCIIYIVCMYDMCV

YVSYMCVYVSYICVCMYHICVCMYHICVCMYHMCVCMYHMCVCVYVS

>SG-PL_contig_10114_1_+1_85_351

MVGVSYCKHCSVLGRWLHDLVVSLPGIFMVFPNFFSLQFLTWGVDNWHQDSVCSALSFFL

SVVFFSVLVHFLCFLGRTATSIKCFIMVE

>SG-PL_contig_10115_1_-3_204_602

MSKTAEKRKIWKDHENAWQTHHHIMEPPAKQRTVSAVGDTHHSPTSNSAYYNQLSVCCTS

SHTTTTSCQLTTTSTHHYHQQSVCYNLQSAYCHTPCLYGKSSKQYCSLFQKVKVLNLLTE

QICRLQSCFGQVV

>SG-PL_contig_10116_1_+2_2_166

NFFHTYRGCGSWLTAGYSRPTAGCSRPIAGCSDVTAGCSKLTAGCSGMPVLVVAD

>SG-PL_contig_10117_1_-3_108_416

MAVKHGPCTPVKNADSMPSIYAVLKEFWASPGKTMSQTRTFWHRQEYQACLLCLLRGACA

GSVMSAACRMVEFPRTRCMMSLPLAPDLQEGLLFITKTFVNET

>SG-PL_contig_10118_1_+1_1_240

SCSACCDYTAQFLSSKPCCHFCRYICAWLCGCCFCPLLLRSLLSSRRSSLLLAHLDACLT

KPPAVCSCSFQACWLDVACL

>SG-PL_contig_10119_1_-2_1331_1717

MALFLGASKCVVIALLLLSLMSLLHCVCYTKYTYCDEMSNMENNNLITCRLMSTKEPVSF

SFSFLKTALSIFGINSVCSVAADAMGWKRIQNDNGMSLTHQDRCYVRLFRLLLCMFSVVP

VGMPGALSV

>SG-PL_contig_10120_1_-1_70_192

MVELIAELNLPITPVRVPDYTNEQLRELKSCYKETLKKKLE

>SG-PL_contig_10121_1_-1_400_591

MKMHFCSEAHESLYSLIRYTCSHKLLQKTYSDRKQACPLAILEFVQEGFIALGQFHMGST

QIRQ

>SG-PL_contig_10122_1_-2_434_682

MLSGPLNLRSIVIILLLLKYYLYRQLHVCTHTNTLAYTLTCMHTHRQLHVCTLTSTLAYT

LTCVHTHTQAVTCVHTGTYTHIL

>SG-PL_contig_10123_1_-2_2_226

RPATQCYRETYTTEYLCIKRSTVSANHVFCSVCRLDFKVSHGELTDVSRNVKYARHVEIT

DVSGATLKCHFATLR

>SG-PL_contig_10124_1_-2_227_469

MDRLRGVKGFKCAACSKNCQTKHRLASGFDIIHDPSIRMLVTLYVKLPYIIDFTIHYCPF

DILYSHSVCVCVCVCVCVCVF

>SG-PL_contig_10125_1_+3_906_1085

MVFTDDTFNYSSSLRVCIRNTWRPSIQLNHHNAYVYQKKQTFKSEDNQMINAQAFEISLC

>SG-PL_contig_10126_1_+2_2_118

FLISVNDLLTVIHCDTRCMTRKIQFTVFTHSQLSTSYTQ

>SG-PL_contig_10127_1_+1_1_177

LTLPPVCYRKLMDRGSERVSRRRLSMKSVAPKFRSNPKALGGKTRVLLLTNICIKSNKQ

>SG-PL_contig_10128_1_+3_162_2492

MTSIIKMHVFSGAHDESPPCYLLQVDEFRFLLDCGWDEDFNMHFIKVLKRYVHQIDAVLL

SYPDHLHLGALPYMVGKCDLSCPIYATVPVYKMGQMFMYDLYQSRHNAEDFDIFTLDDVD

AAFDKIVQLKYSQTVTLKGKGYGLQITPLPAGHMLGGTIWKIVKDGEEEIVYAVDYNHKK

ERHLNGCVLETISRPSVLITDAFNALYNQSRRRVRDEQLLTTILQTMRNDGNVLVAVDTA

GRMLELAQLLDQMWRNTESGLTTYSLALLNNVSYNVVEFAKSQVEWMSDKIMRAFEDNRN

NPFQFKHVKLCHNLAELARVSEPKVVLASTPDLQSGYSRDLFVAWCGNSRNSIVLTAKTS

PRTLARWLIDNQEAKTVTLEIRRRVKLDGAELEEYQRKKQEEESDEMRRKVQLALKDDDL

ESSDDSETEMEVDGATKGKHDLMIKGEGKSKPGFFKQAKKAFPMFPFHEEKIKWDEYGEI

IRPEDYIIAEAVPAEEEKMQKEAEPEEEEEATPDISEVPTKCVSSTVTLDINASVQYIDF

EGRSDGDAIRRYLSTVKARQLVLIHGTEEATSSLAEFCHTAGVVQGKVHFPNVGEFIDAT

TERHIYQVRLRDHLVSSLRFSKARDMELAWVDGSVSMVVEKTDTSAVPELAPAPGSSAST

SGTANSNIGSGDDASDKKKAEDDEDLKPVAEIVPTLEPLPPAMVPPHTAVFINEPRLSDF

KMTLTKAGIHCEFSAGVLICNNTVAVRRDAGRIQVEGTLCDDYFKVRELLYEQYAIV

>SG-PL_contig_10129_1_-3_3_182

QDSVSMLRINSIMHKRHKCHGMEKDNPKRSWLSLRKSPTHWQGLEVLPDIHYFIINNICV

>SG-PL_contig_10130_1_-1_1_123

GGGRIRVKLVPIMVSRIVLSPKLWDQSTYLSRNLRERTPLK

>SG-PL_contig_10131_1_+2_230_451

MLISVSTNPGCQSSVAYIPRIAQEDFFRKTQTNGPVGRKDDTRKKRKIREREHTHPECGS

IIHNRKLHPSAHWG

>SG-PL_contig_10132_1_-2_167_439

MDVVFHVLPVVYFQIFVTHKSDVVLVMKCRNVELCTLKSFLFVVRSEEDRGATLLITIDE

FGHFSFICLAHFSSESLQTTVSSFFKLFTLM

>SG-PL_contig_10133_1_+3_3_164

NTAIYIQEKLDYSQILPPFQKDTPNINACAARVKLDNSLILNVVSVYLSRVLTI

>SG-PL_contig_10134_1_-3_150_776

MSMEDQVVAQPQVESDDEESFFQDIDQLQNHGINVADIKKLKQAGICTIKGIHMTTRKKM

CTIKGISEAKMEKIKEAASKLCDAAFQTALEYADKRKQVFKISTGSTELNKLLGGGVESM

AITEAFGEFRTGKTQLSHTLCVTSQLPTENGYSGGKVVFIDTENTFRPDRLRDIADRFNL

DQSAVLDNVLYARAYTNCGFYHGFVPCGL

>SG-PL_contig_10135_1_+1_1_234

FFFFIFTTFISHSDVENNTFIQHMKSHFSSHTIQILATADASHSCTHTQSFTSVFFSSYL

LFRVRIFLTSLYSTFICT

>SG-PL_contig_10136_1_+2_2_130

KNSNTEKFTKNLSRQTKPQLQNQTTQTQKFKQTGKPRFRNLKD

>SG-PL_contig_10137_1_-2_2_247

VCLCVSVFFVFSSKDWFNDASQTLCHSAGDISLSIALFGCFSGGMVQVHPSELVVCPFFS

LLFPFVVPTVPRMMCGNSFFRP

>SG-PL_contig_10138_1_+3_237_326

MCMNNTFEILYRTFKSDCDYFMKRKKPVKM

>SG-PL_contig_10139_1_+2_20_331

MLLKLDARGKKNWVTKIRMTLYECGFGYVWLNQGVGDVKYLSVSFKVCERLTDCKWQNWY

TYVQASSRFDFDGLNTSLYCIPLYLLMQSDRHMKCIVTRFRLGI

>SG-PL_contig_10140_1_-1_982_1263

MKVCIYCPVLYKRTIWLFFPVYYHLMYERRNFFLISLVERVDNDLHHLEKFWSNLYVCVR

EREGRGRERERERERERERERERERERGEIFLLY

>SG-PL_contig_10141_1_-2_92_220

MIRSFPSSQTVSSLTDLGNSYKFGIFSEVEWEHILSPLAECGV

>SG-PL_contig_10142_1_-1_1_186

CWSLEKLRYLPSAYPVTSGLCMGQHLYFKHLQFTTSECLILIWVSVVRDTHRYTDRHFCV

CN

>SG-PL_contig_10143_1_-1_760_1023

MEHDWKTRFNLIKSFVVATGQPCFLNATWMKRRQIWRASSQFSLISEKAFFHTLAFLVQF

SRKGTRSACFLCRKSLYNCSVRLRPVEN

>SG-PL_contig_10144_1_+1_1_156

LGRTQTGPTDERWGKRDSRDSLGRTQTGPTDERWGKRELKAKGSKNQNVMLL

>SG-PL_contig_10145_1_-2_353_526

METSLPRQETSPILSPLEGFSSSDWIILCWSRATSCPGWAADLHHCPVLLRSALQQRT

>SG-PL_contig_10146_1_+2_2_79

KKDRKERNEQLTAVINTEIIQHVHFH

>SG-PL_contig_10147_1_-1_79_906

MALADVCGFKYQDGKSKLSLGFGEGFNDPSAFDALAASQSFTVPQGIDIAQNLAHFSDEK

SNVKIHFDHGTTTLAFKFQGGVIVAVDSRATAGPYIASQTVKKVIEINPYLLGTMAGGAA

DCSFWERVLAQQCRVYELRNKTRISVAAASKLLANIVYDYKGMGLSMGTMIVGWDKKGPG

LYYVDSDGERLSGSVFSVGSGSVYAYGVLDSGYKWDLSVDDAYDLGKRAIYHATHRDAYS

GGVVNLYHMRETGWIKVSQTDVQELHYKYKEEKENK

>SG-PL_contig_10148_1_+3_3_197

SCSVFFSGTDVVCYFLPRFESNFGLFFFQKFQAIDCQSQVCFQLGVVRAAFKHFDCFRRD

DLSLE

>SG-PL_contig_10149_1_+1_1876_2124

MLYYIKMHTEEHHINILNFKHTLFLPHCFTYLQLSIPPHLCLTTSVALSYAVHSDSPTNQ

LTQLHSVPVSAVCKDLVLNIRRA

>SG-PL_contig_10150_1_+3_30_278

MEMEIRMMILPFQMGLSCVSTAVVCTILARASGFELSSEMIAPKHWKMEIVSSFCSPTDV

STDVIGANLLMISFVYSALLSMP

>SG-PL_contig_10151_1_-2_2_232

GPALSDDSLRPEVSRGLTSHDASPQKTPHVIPGPGSGSDLALTALPEFLVETQQPGSLPQ

TVRIPCLVPCPDPRDAR

>SG-PL_contig_10152_1_-1_190_465

MRYLDSSLCLEATLHNPRYLNSSLCLEATLHNPRYLDSSLCLEATLHNPRYLDSSLCLEA

TLHKPRYLDSSLCLEATLHNPQHCVSLLSCAH

>SG-PL_contig_10153_1_+1_88_282

MHESGMERVKVIWTKKVPQISSIGRRCVNSTFSAIFRFCFHCYDSLPLSSFTSSLILFET

FSGSQ

>SG-PL_contig_10154_1_-3_1095_1289

MIIKFKTALQCCIPGLLCQYCNAIQTGLHVDLLPFETSLQSCCIPWLLHLYNLVWYSDPL

QFKPT

>SG-PL_contig_10155_1_-2_143_385

MTPVSLLGRTVQNQIWTHGHSALKHVTNGAISIQPYCRSTKVGGMQEQRVVLGHIRRNNW

CLYTLHKLYYYLLNEVKMYTD

>SG-PL_contig_10156_1_+3_3_161

YICILCVCVCVCVCVCVCVCVLRYIFLCHENWSIRHKPKCTLRLKEYKTPILQ

>SG-PL_contig_10157_1_-2_2_151

FFLICVVTISSLCHYSFFVFLQFLCVITISSFCEVVVVAVPVTKVFCLSV

>SG-PL_contig_10158_1_-2_152_295

MSLCLRLHVLIVLQNGSIRRFRFSYYPQNCRTEYTYSYLNRPVSKASG

>SG-PL_contig_10159_1_-2_11_121

MKLYVFLKQVYPHDMAITKLCLICKQTSKQNINKCIS

>SG-PL_contig_10160_1_+2_2_214

PPPPKPTKGLTHSNYYYILFIAQPTTIAISGLSPRKVHQSQEDDRVEHKTLSNLKTKTLH

KITIHDTLSQH

>SG-PL_contig_10161_1_+2_128_358

MSVRVCLTFKSKVGIKMMEFEQHLSLYILASASATSEYFQLQKKYMSIRIVSPATHMCVH

MHAQTHARRTCIDIHVM

>SG-PL_contig_10162_1_-2_485_748

MLRWSGCIISGKYDLLAMQFIPGTCDGSICDKCQISEHILQPCAQVLSQAGCFCSQLLCL

VIDGQRFILFVYHVTKVMICEINTDEDL

>SG-PL_contig_10163_1_+3_3_95

PPNDSNHQGHCLLSPLLMTNHQGNCLLSPHH

>SG-PL_contig_10164_1_+1_1_162

QTDRDRDICRDKDNDTVRPSLRGETTQVHTGKHPPPQILTHVYDKHQHQRPSIF

>SG-PL_contig_10165_1_-1_67_2076

MLHELLLALTGHCGGVFKDSGNGIQVVPNLPFIHPSEVNVLNDLCCLGSLYKSFQQFVQQ

HGSGVSTKRTGANGELHGLYIRSFCRGLDMVLADYREALLSLEQEILRDPHLPVSHLKYS

LQEFQLLFPALSAVLQQIISHRAHGCYLLDVLHKNSVCGLPKVKDALERILHVCHGVLYK

QLCAWMIHGLSHDPHHEFFIHAVSGGSHRRDEAGRASQEEGGELGIMGVTGRQMQAMRKL

TISDADTSQDAGMFIIQADLLPSYIPLRVANKILFVGESVQMFETNQQNARQHSGQFMRE

REAEFTKDIQQLAQHQTFSGSAFEALVDKIRLHVAEHLWVMVVQEADLKGELRVMKDFFL

LGRGELYLAFIDQAQHLLKGPPAPSTEHDVNTAYQQAARNVLIENETLLQRFKLTITTTT

SAPSAPSPKPTDRSSDSGSSAGPVETGWNCLGLTCTVQWPLHIFFTPSILKKYNRVFRFL

LAVRRTQQDLQSTWSLQMQGKVKDIGIQSAVEGGGTVAVWQLRHHMSFLVDNLQYYLQVD

VIESQYGQLLDTINTTRDFEAVKLAHEHFLAALMSQSFVHMKSVSHCLYEILDLCSSFCR

LLTIQLSPMADKELTQLSVITKNFQRQSNILFRILSSVSSHNASPHLTQLLLRLDFNKYY

TTAGGQLGSL

>SG-PL_contig_10166_1_-3_3_218

EALKANGYKGWVFNQSLPKLILKLCEEEKKIRPHPIPYIFKACQKDRLKFSTNSTPQFIT

SLSILGSQPVHP

>SG-PL_contig_10167_1_+3_3_158

CFVFFCSVAKSPLCAFCADNSYVRNRVVTLLKDCQPVRIVMKMLPITISSSV

>SG-PL_contig_10168_1_-3_3_218

EALKANGHKGWIFNHSLPKLILKLREEEKKIRPHPIPYIFKACQKDRLKFSTNSTPQFIT

SLSILGSQPVHP

>SG-PL_contig_10169_1_-2_185_319

MFYILKKILKSESEEIIYPNIHITFFQSCENDLVFLFQNFISTHT

>SG-PL_contig_10170_1_-1_304_441

MKTLRNSSLLDADIMKNFLCWKKILYLSTECTNFPVIFFSRKQKVS

>SG-PL_contig_10171_1_-3_3_170

TVLRKLQVERYEVTDNRWKQCQYQPLSEENTRVQAPLIIIIKTWTGGWGENIRIII

>SG-PL_contig_10172_1_-3_3_251

HPAVNEHLTLFRVGEDKAAKERGMGTALIMLAPRKVEISTTSLPYDLRVMGQPLPYLFLN

ATLHMDVKLLEISVVRIKTVTKL

>SG-PL_contig_10173_1_+2_602_757

MTLSTFTTRFDVQMKNKYKKNHPPEDLSNQIKLTEHCNYMIENCVQYLVNKC

>SG-PL_contig_10174_1_-3_3_371

LDIKKYIEALKLSWLRKFKTTKHKWKNICSELYPFLNDIEKYGSSYGLHKINKILFWKHI

FKAYNSFCRNVKSTSPIDLLSELILYNELIKIGNDMIKLTSWIDKGVYCIAHFLNGNGKL

SCL

>SG-PL_contig_10175_1_-1_1_135

RCKKNLPLSLSLSLSRSLSLYLKSQKRKDVRVGWDSTEIPLTGAT

>SG-PL_contig_10176_1_+1_31_396

MVKTLDFQTGGFERPGFKPPQTPCLVGKGWKFFPISQVNICADLLVPEPPSCVYVCRTSN

MHIKDPVSHISVRGLWKHEHTQHAHFILPSPAPSCIGFRLSLGFYRCRGKGGPAAWATPG

SP

>SG-PL_contig_10177_1_-1_1_321

VTVSARPPPPPPPTQPDISLSNTPTTSVPLGPGVSGLVTPATLINFDNPTVQKALDNLIQ

SGPSLLKNITATATQGQRSWPGQGTTLATSQNPADHMAAPRPQRPMY

>SG-PL_contig_10178_1_-2_77_1063

MNMGESPHEYKLHNPPSDGISSVKFGPNSSQFLLVSSWDESVRLYDVVANNMRLRFNSSA

PVLDCCFQDSVHAYSGGLDKVLKTYDFNTSSEMTVGNHDAPIRCVEFASEVNVVVSGSWD

STVKLWDPRGPTAAGTFNQPDKVYTLSVCGDRLVVGTAGRRVLVWDLRNMGYVQQRRESN

LKYQTRCIRCFPNKQGYVLSSIEGRVAVEYLDPSPEVQKQKYAFKCHRIKEDSTEKIYPV

NAVAFHNVYNTFATGGSDGYVNIWDGFHKKRLCQFHRYPTSIASLAFSNDGNVLAIASSY

MYEQDEQENVPDDAIFIRNVTDQETKPKS

>SG-PL_contig_10179_1_-3_30_1286

MVFAYTIEMTSPTDPKAWTVVTKSCQGNNYQVKNLAPNTQYLFRVRAENIHGVSKGSRPS

EIVETKMFGVEQQLYVPAPEHKPHNVPRRQHSISLHLEGAVTSILNHTDIQVTTTNDKEV

KFSPKSERAALGSSEDLAGEDSIDGEENKFRSNSLQRGSSARTPFLSERYRTNSLQRDMT

TRSSLQPDRSRTYNLQRDANLRNSLQPTRKRSLPLLLPGTRTMSLNKLRDSQTFPVTTRS

RLADSSDTLKRRERWRERSASDVQSSSTDSKEDVIEDKACLSKDESSTDVDLKPAKRDSG

LEEDAENPWEREDSVDDVLDGVISTQICDDVKTDMYSSSVPDSPAPWLGITVSGVMSHGG

DRGQADFRTLRSLLHSDDIIIKPTRSLPDVVGVGKKTSNTLTTIVDLEEDEESVRITTL

>SG-PL_contig_10180_1_-2_2_166

SLNFGAMGVVMGHELTHGFDDQGDGLQCAGCGVLTFHLPVYGVLDVVCSLLTCRI

>SG-PL_contig_10181_1_+1_1_147

WKKLIKIIHRLCSEWQRFCRPYRTVKCTNLCIIYNTGPVFVLKNFIGRR

>SG-PL_contig_10182_1_+2_401_574

MAARMLMTEAQRPDSKQSLTDSYVRHNTTTSSLHCKSITTMPDSSRGWASTWCYGIKS

>SG-PL_contig_10183_1_-2_2_211

FVCFFPFSYCQLYTFGTVFFRLSSFVCKYGQCSFCCCLAFKWKNNLNMPVKSPTSLTLSR

ICKENKTKYA

>SG-PL_contig_10184_1_-2_2_202

MRTVIKTYRGHYIRMDLTDDHYQLVSRYTSVLNPRADEVRALATSSVFSQCVTLKKVLEV

AYWCSEN

>SG-PL_contig_10185_1_+2_428_583

MSRGIQKFLTVPKHNLKSAGEGAFSFIVPLSRLEKALSASLFHLSGTHCQLI

>SG-PL_contig_10186_1_-1_1_243

KKKKNYTRSLFCVPVQPTVINILVRLHAISNSFVNFSSFVFFYKLLVCLCAFRLAVMIFF

LYNILGISVKPMIIGVFVKSY

>SG-PL_contig_10187_1_+1_1_135

LLLLSMSDCQQWRHISHSRGLIFPKLPSLFQIPWVPQASESVGPW

>SG-PL_contig_10188_1_-1_1_375

TKLAGKKGNLSSLAKLAGSGTSGADKSSGAQSRRHISEHELHKKTDKDINLQVFKTHEEI

RGVERQIERLREQLARNENKDRRVAAQVRARLEEQQEYLSKLQSSSVALEKHQKKRSNSK

KLTIF

>SG-PL_contig_10189_1_-1_97_1098

MSMIDPPRAAVPDAVGKCRSAGIKVIMVTGDHPITAKAIAKGVGIISEGSKTVEDIAAER

GIPVEEVDPREAKGAVIHGGDLRDMTPAQIDEILRNHSEIVFARTSPQQKLIIVEGCQRQ

GQIVAVTGDGVNDSPALKKADIGVAMGIAGSDVSKQAADMILLDDNFASIVTGVEEGRLI

FDNLKKSIAYTLTSNIPEISPFLIFIVCDIPLPLGTITILCIDLGTDMVPAISLAYEKAE

NDIMKRQPRDPIKDKLVNERLISMAYGQIGMIQASAGFFTYFVIMGENGFWYNRLLGIRM

EWDSRGVNDLEDSYGQEWTYAQRKKLEYTCHTAF

>SG-PL_contig_10190_1_-1_328_507

MKIVLKLCKDCSEELFPNLCSLFFVVIYMFILFILFCWFCKLEKKSTQSVSLTNAHTCTH

>SG-PL_contig_10191_1_+2_2_229

ERERGGSEREREGESAHAHTHTHTQIHMHTHSHSLSPKCSFGNTICKPFTYIFLMYLCYS

KHKKAVQNIINVNHLI

>SG-PL_contig_10192_1_-1_43_582

MGGLLSALKGLIGKTEMRILMVGLDAAGKTTILYKLKLGEIVTTIPTIGFNVETVEYKNI

SFTVWDVGGQDKIRPLWRHYFQNTQGLIFVVDSNDRERMGEAREELNRMLNEDELRDAKL

LVFANKQDLPNAMKTAEVTEKLGLQSLRNRDWYIQAACATSGDGLYEGLDWLSVQLKRKP

>SG-PL_contig_10193_1_-1_1_261

GYWALYNQHIHKKHVDAQHKCSKKHDFHSQTNHTLSLSLSLSLPSYRPHSPTHRHAHART

HTHACTHMHTNAQRAHTHTHTHTRHCK

>SG-PL_contig_10194_1_-2_239_418

MFYITGKLNECTCLIIMRKTYIAPVLPCRLLGALQSAYSQNTCRRTTQMLKNTQFSFSNI

>SG-PL_contig_10195_1_-2_2_130

FFFLKFFEKKKKNNFYKKKKRKKKIFFKKKKKKKKKKNGKKGK

>SG-PL_contig_10196_1_+3_426_632

MILRFFSNSAIQNKKKIHSARSNVGTVSQDFSLWVSSQDLAKRLSFCVRYYSDQNSVPYY

MMQVLFPRC

>SG-PL_contig_10197_1_+1_1_255

IFFFIIPVLYLLHEDIIVCLLSSTASGKVCANVNTHMNTRRRAHTHTHKHRMYNTVTPGL

SPTPPPLFSLYVHLYKVIHHFFQAP

>SG-PL_contig_10198_1_-3_3_200

FVVCVCLCVCVCVCVCVCVCCLCLLVCVCVCVCVRIFDCVCLILYLHIYYLFIVDFFFVF

IYCSLP

>SG-PL_contig_10199_1_+1_58_321

MSTYRTRPTPWWAHRDHFWQLRNDTSLHGLVTSPAMTHCLRPSFRVPWKEAVTEVDRGRA

GWQISKTGPTSVCLNCLLLHPRDRGSCR

>SG-PL_contig_10200_1_-1_1_240

FTVLTDNNPLVHFRTAPLGALEQRWAAQLAQFRFDIQYRPGRVNPADALSRLPPDSAVPP

VSTAVPPELAMVQETFCQRQ

>SG-PL_contig_10201_1_+1_1_126

TQSSHPKCARGLTLVRQSAYILGDPQSVKLGNANSKQQLFTY

>SG-PL_contig_10202_1_-3_3_140

CSTEQQQQHTTHTEQQHTPPPPTPPPHTLACIKGCFACMFSLRRQH

>SG-PL_contig_10203_1_+1_226_273

MPILTTHQVETPNAHP

>SG-PL_contig_10204_1_+1_1_270

ASRKKIDLTGHPGVCLSYVCACVKVQPHNQCQLYEFTHQHLHIIRFKFILVQTAVSAHNH

MYTCIHHQLALAYFVPYFNFLKKEKGLIRY

>SG-PL_contig_10205_1_+3_3_215

QRTLHPEDNFSRNSNYSGIYERMRKASSSLHFTCSKLSHVCHQMLRYDGLFKLTYGWVCI

HTCLIYKTNAQ

>SG-PL_contig_10206_1_-1_1_192

WFYILNCLSMWGGGGGVVTESWFQPTKPTIFYCALVLNIKQVCMHTQPYVSLKIPSYLNI

WWHT

>SG-PL_contig_10207_1_+3_3_86

MSVRFGIFLFKTSIAFHSKWKEMSHAQK

>SG-PL_contig_10208_1_+3_405_728

MLLLRTRLPHNVQCARTYMSNATFKNKVTTHTTSNAHIHTCPMLLLRIRLQHTQMPSESN

ATFKNKVQHTHTHTHTCTHIQSYFKNKLTTHTSCPILLLLQHTYPELL

>SG-PL_contig_10209_1_+2_2_274

ILALFDSFLSKLNLKLLWLLDVLHKQSSLLSRCLTNPVQTRRIFTPILSKKLRLLRQVSL

LFLPDRREAKATRLAIWNTSATPWSNSAEVS

>SG-PL_contig_10210_1_-1_109_294

MKWCNCVVVVASSLAVLLCHYPHTPQFSFPTPHQHYQNLVYSQVERIHEDIPRGYAGGSF

SI

>SG-PL_contig_10211_1_+2_185_727

MFINRWRRTASVSVQRQHFKKLSRSRFEISFSSSLCRFHFPHTQTFSLSNIHARTPTHTR

SLSHCIWRCILRFLIILLFTEEMVLYEHKAFFKLRKNVTIWVNRPVFAALYLKQRSVPCV

AVHKKILHLSYLISLLGERIRYDFILLLWCYSIHRPDLSTEFSVSSNVSIPVFTFLQLVS

S

>SG-PL_contig_10212_1_+1_1_198

KKNVNMTRMFTVNKICTKNVFYQECLQSRYIKNVYITHKKCVQECVQSKMCKIRKCTIMN

VYIKNT

>SG-PL_contig_10213_1_+3_96_251

MKNTVHASILASLHDSRFIKRRSVQCYGYISVSMTLNVCTIFLSSQCHKSGS

>SG-PL_contig_10214_1_-1_94_348

MVLVMLKKRETGKTWAFVTEQEKKSKEMKKPPVSKDQDPSAGLMDMLKNMYEDGDDDMKR

TISKAFYESQNKKMGDMTDIGPGEM

>SG-PL_contig_10215_1_-1_142_522

MARLVKRVRDNPILTTNMKVYQACVLSMLLYGPYTHVKNDDSMPSTYAVSKEFWASLAGC

PKQERSGTGGIPSMFALLTQRRLRWLGHVGHMQDGRIPKDMLYGELAIASRPAGRPALRY

KDVHTQI

>SG-PL_contig_10216_1_-2_47_490

MGNSSSGPTPAVFYSPEYGVALGRRMSPNGQDQLEVDSVRLRRMGTLRKPPCRINAERDS

PSGRSSNLIPPQYEPNFRESFYSTTESGFETLPELVEEEETESADELSPSLKENFSASSL

KERSDRYFASAYGNTAGSSSSGDNIVPL

>SG-PL_contig_10217_1_-1_16_735

MAAVLCGLAAKLTRASSACRVSACAWMQLRTSHSLSLSAQTPRMADICRPIQLNITRNVS

FFGKVTADELWGSVYGVSNAGRKRGRGKRGRKKMNLNRGQIIGVGKNNMVWPGLNAPVVK

GREVINIKQLPPDPERETKMKEIRDRGSQFRHFKVPALQRGWTGAKRAGTSIGPPDPVGD

YTFEGFDTRILEFKLVTNMTGNLGRKQRFSSFVITGNKNGLAGFALAKAPNAQASLRKAK

>SG-PL_contig_10218_1_-2_275_1516

MMAHVTMPTAILWFLASSLLIWNGDCLDNGLALTPPMGWLSWIRFRCNTDCKNYPKDCVS

ERLYMDMADRMVSDGYKDAGYEYVNIDDCWAAKERDSRGRLQPDPYRFPSGMKALADYVH

ARGLKLGIYGDMGTETCAGYPGSQFYLETDANTFAEWGIDSFKMDGCHSIPGEFHISFPI

MSKFLNLTGRPILFTCEWPNGYGKANYTLIAETCNTWRNYHDIQDSWETLLDIVMFMGNN

SEKFSAIAHPGAFSDPDQIIIGDFSLSHDQERVQMAMWSILAGSLLMSNDLRNIRKESRD

LLLNKRLIAINQDPMGKPGVLVVSQESNQIQIWKRSIMPVGTYAVGFFNFMIKSGGARIV

EVPLASLGLTASSGYNVTEGFTGEFVGLYKPTSTFIFRVNPSGVYLVIAKPLKA

>SG-PL_contig_10219_1_-1_502_927

MSEFKITEKQYNDANQTFSLFDKKGNGTVSTKELGNVFKSLGLQVEDEKLKEWADEADEE

AAGFITWEQFKPVFELKLREDEDERELREAFRVLDKGNKGVIPVEDLRWLLKSLGDDLTE

DEIEDMIMETDTDGSGTVDYEE

>SG-PL_contig_10220_1_-3_189_1943

MAWKRVRMVRLMTIAVPAIWFLYVIFIVMTLDKTSTRSAQLLLQEARNMQQRQAEWPDAV

QQEPAAVQQDGARNDTGNLVGNLEGSARQKHVGDIQNEAHPPAGNVKQGVAHMAVNDAGE

KAGHADGSVAGKSRDDVKHDVKGVDDVDPYVDVTYPPYVETLPSGAKGQGPEGLQIAVES

LDEEKQKEYKDKWNKNQFSQYVSDLIPVHRQLPDYRTESCKSKQYPWSLPTVSVVMVFHN

EAWSTLLRSVHSVLDRTSSELLREITLVDDFSDMDHLKRPLDKYFHKHPKVKIVRLPKRG

GLIQARLRGFDATTGDVVVFLDSHIECTEGWAEPLLWLIQEDYRTVPFPLIDMIQPRELR

YLMHQLSPYGVFNINTLTFTWSSTIRRDPMPKTGAKPSPTMPGGLFAISRKWFLELGTFD

PELEYWGGENMELSFKTWMCGGSIVIAECSHIGHIFRSVNPIPWSRPLGHRNYVRVAMVW

MDEYKNHYLERFNYQLGDFGDVSARLAIREKLNCHSFGWYVQHVYPELKEQIVLNATYAG

EIRHEQSGLCLDKMAGVTSSARPQVQFCHKLGSNQFWYYSDDKYL

>SG-PL_contig_10221_1_-1_319_510

MANSLKASMVRLVFCLISMADNGHCCTKCHRLTLYWLFIQFLGLFLKDFLEWNSKRIGFQ

DDLP

>SG-PL_contig_10222_1_+2_290_403

MYIIYIFHWNKSIPSSFPSFFQNAFHKADYCLKIIQRK

>SG-PL_contig_10223_1_-3_96_605

MAVYVYEMISDLSQDLYFCFLFFQAKYTEDDLNEIQETFSLFDTRGDGKIAASQLGDVLR

SLQQNPTEQEIRKCGYSQNPDARISFETFFPILQTISKNRSVPPLEDFIEGFRVFDKEQN

GIISCAELRHILTSLGEKLSDDEVDQLIQGMEDAQGNINYEELIKMVRNG

>SG-PL_contig_10224_1_+3_1488_2102

MAANLESAARLGYEQLKQAALAAQKRLEQFPVKLSPHLDAKDNLEDKNASGLFPTDGPNL

TPVLICGDGNCLPQTASLLALGTESQHLEMRVHIITELRLNEDFCLTDTNLNSSTPGSAE

PYAQYSDQYEMELMSEAVKGVFEKEMRSTVVPNSYMGIWQLHAVPNILQSSLYSIYSEYR

GYNTYVPICIRFYNQELELLHLKQL

>SG-PL_contig_10225_1_+2_83_172

MMRYVYFDNLTVVTLKLCQGHPNFGNRESI

>SG-PL_contig_10226_1_+1_1657_2166

MIHPQRITTKSTKKGHELQDWLPHTRTRQLLFTLFAATTIMPFLILTDQFTPPACLPICL

PNRGHMSTTGRGEGNNYHFLHVCTNSIAFLVCKNPVLFFHDVQILHYNISKKTRQCDKHT

VHTAHYHRPHTNTVNHFQAPHSPLPSSSKADIYKYTFHRVEMLHEQTDYK

>SG-PL_contig_10227_1_-2_596_1168

MCMCVCVRSQNISMRNFRCFHMYLLKDVGLLSRLLKCYRYGSLKWLDHKFTHKQKNFFMF

IVTPDVYIMPSCRTRKTVSTAGQSFVLSHLVSVIGHAKCSISPLLIQRCLGFLYMPLIFM

MHSLHEEFVQEFLRSSYWSASSHFCYTSFVMFINLVNGRALNPHYYVVVLIFIGVPSCNC

WFAFCCGLHCE

>SG-PL_contig_10228_1_+3_3_140

MPGFYRGLLQRKWGCLCGCTVVNCIISVLSVPRNGLGPRFYLSNSN

>SG-PL_contig_10229_1_+3_174_308

MFPTFPTSCLVSGLSVWDLYLESLGKSGAVFPLAWVLPGLYRGLL

>SG-PL_contig_10230_1_-1_1_324

FLRKWRFKNKERKNGNFVFVCVGFFFFNFNLILFVFIYIFSSSIFQSCQSIHPYFCWKVS

LCSLSARLFSCQGREMVYAHLGASCLFLSVICQCGFCRSFETTSMHVF

>SG-PL_contig_10231_1_-2_8_286

MCVYVCVSVRVCVCVCVCVEFIKILWIFSYKKFGVEMLRKIISYSQGRIPSLFVCFIYVY

ICFIFIFYIYSYVCVSIYLCLILSCLFIGTFIV

>SG-PL_contig_10232_1_-1_199_936

MLVLDLQGNAGDYVTYSTFQTYNQLQENRLRIPVITSREEDENRDGRADLLKFNLEMPLL

DTEDVVGVRLLLFFYYKLLKYSSFHMESLAIITEVAPSPAARLYVSGDLGIRQKQALSHR

GIDSRYNGSLVNITSPFAEAYNFQTIIENYNKRNVTTRLVTPDRVWTSGRAAGAPFVIQA

DIAYPAEETYRYRPGFWYMIKWGWVQYVSVLLLFIYIFERVKVFVFINQLVTTIVERVSD

HKNKLI

>SG-PL_contig_10233_1_+2_179_409

MEEWRVKKGEVLVCRKVVKKLKSANAMHSMGLTESGGKKWMRGRCRLMAQYMDTPNANRK

FNLVSSWIFQKLYTHTT

>SG-PL_contig_10234_1_-1_493_702

MSVCEKVSPMEKHVQAICSINIECVCHVEEQNVSCVLGNNFSFCTCLETWPYEFSGICFY

NQGSLRYWLY

>SG-PL_contig_10235_1_+3_543_932

MGCVILYYHMHNAVRILHVNENKTKISGFSSPLPPFAAWEDQLSESCSVSLKFLSTTKLK

AESDNTVAMQVHLQLLLTEQFQVCSYICYSETIRNIFNRQINPYLRKQRLLKKPNMMQTA

ITSPMHYFFI

>SG-PL_contig_10236_1_-1_280_522

MGHYNIWFHKPNPKGNDKGFSVFLIYFILFYFFISDTLSSCRTGSVEKKLTAVYRREQIP

NVDFPHPHNLRNFLSPRLIAF

>SG-PL_contig_10237_1_+1_325_510

MISEGRIPASVQGQTPEMSCYDKFITSLSLFLAHTHTHTHTHTHTPKCIFFTFAFIKRKR

IA

>SG-PL_contig_10238_1_-3_3_188

SPLPPPFPPPPPPLSLSLYDHSQQIVDPNFTQTTCIYFLVMLSVSLSLNNSFVRLATVSF

WI

>SG-PL_contig_10239_1_+2_2_178

TDEAKEHTSSSNVHKRQRNKTTRFRTTEQCTLLIWLNWKELVKFQCKDSLQHRFVLRMA

>SG-PL_contig_10240_1_-1_1_204

SHLSQLSVPIPSRLRTILASFGLFWVFSRHHREFSVRFCFQGALFPCLPAQRHGEMYLFP

FCFPGLVF

>SG-PL_contig_10241_1_-3_153_281

MNPSRTNSKLLQEVGGQGEGVQGIEDIPGEVTCETNQERGGCL

>SG-PL_contig_10242_1_-2_401_649

MCCMCAGSMSATFVGHSEIDLVQTKKNKKTCILCRHLFRSRHRKRCDQNDGGKENRIPIF

WKFCASNFLFSIALLVSAVFSFL

>SG-PL_contig_10243_1_+1_580_810

MLQMPCGKGFETTKAMPQTLQYTLAKSHGSTRLMDTKDYHLSFAKLVLMSAFLFQIMDWA

WYGMKTFFCVCWLFLCF

>SG-PL_contig_10244_1_-3_207_335

MKCIFYSTLLLYFSVQTDYCTRKIETAGSTFSHWLGIAELGSR

>SG-PL_contig_10245_1_-2_35_973

MVSASPTCVCVIKCRDSHFMLVWFGLFCVSVVEINSSCVLCVKCRDAHFMFVLCFVLFCV

SVVEIHTSCFVLCQLSRFTLHVFCVSYVKILSSCFCLVLCRMSRFTLYVRHVTFVKTKYT

PRPLQCSFVLFIMMYQFTVFSHVHSVMFIQLFLFIQLFLFIQLFLCPFNCSCSFSCSYVH

STVPIHSVVLMSIQLFLLIHLFLCPFNCSYSFSCSYVHSTVPVHSVLMSIQLFLFIQFLC

SFNCSYSLLCSFNCSYSFSSYVHSAVPIHSVMFIQLFLFIQLLCLFNCSYSFSSYVHSAV

PIHSVVPMFTVHA

>SG-PL_contig_10246_1_-3_198_443

MYPQTVIAKSIVILIIDILNISFCMEMMHAVLCWGYSILKDIVKELCLYVKMETNFVRKP

LFIFYNFVFNGSHLEQLFKLVV

>SG-PL_contig_10247_1_+3_3_167

IGSPSNLRAMSGQLSDRKKASQKLCMRKRLADEPLHGDSSTLSASEAGVQWHKKS

>SG-PL_contig_10248_1_-1_160_342

MIDGRLCMLYVLVCKALLAAVFNPEDQVDLWDSGFSVQRHCWHDLECCSFTVRFVWNVTD

N

>SG-PL_contig_10249_1_+3_1482_1622

MWCSLFKKQNKTKQTSVPKFALMLLLDRKSTRLNSSHSAKSRMPSSA

>SG-PL_contig_10250_1_-2_161_445

MTVAPPPKRKARMPKPSDLVTIVVSHVSLDEESNVMRNPAIQQLFVEYRFLGLAPQDTET

PFALPKPRPYHSIAFNFSKTIIVDMEKNYERRQRL

>SG-PL_contig_10251_1_-1_1_228

PRCSSQRRLLISLNENYSRDISATTISRWLSATIMRAYKEAGSSSLPDSPRAHELRALAS

SAAFHHSLPIRDILEA

>SG-PL_contig_10252_1_-1_106_2193

MLERLKMHSSKGARALILSPTRELALQTLKFTKELGKFMDLRAAVILGGDKMEDQFSALH

QNPDIVIATPGRLLHVLVEMNKSLKEVIYVVFDEADRLFEMGFAEQLKEILHRVPESRQT

LLFSATLPQTLVSFAQAGLSDPTLIRLDVDSKLSAQLKMSFLSCRVDDKEAMLLYLLRDV

IDSDELTVVFVATKHHVEYVHTLLTKCGISCTYIYSSLDQTARKIHVAKFRNKQAKVLVV

TDLAARGIDVPELDNVINYNFPAKAKLFVHRVGRVARAGRHGTAFSLVAMDELAHLVDLH

VFLGRPLKTVPVAGQVEDEDGYFGNIPSSVITDQWEELRTVNSESVDLKNQKGVAENAYK

QYLKSRPPAASESIKRAKQLGPCGYHPIFNEVIDKPEDFQQRALSVLRNYRGRATIFEIN

ATAKKTSHSVMRAKREAHDHIIQKARESQAAAAAAALSSSSRQLVNEPEEDLLEAFKKST

SQNKSTEKKKKKKKKIRTAKDEENYVAYQPANIHSEAGYKIGGFKNEISNAVLDFTGDED

GNMRSLSNTRRWDRKRKRFVTDSGEGKVKKVKTESGNWIPASYKSNLYKMWSRKEKVDDN

DSDDSRHLQPSNRHQKGQKRGIQITGTRGKKWHTKGMETADGQPSGNTKRKFKGGRQELK

SKEKILKERRRKAKVQSIQKHRQVVRTKKRANRKQK

>SG-PL_contig_10253_1_-3_3_143

TGSSGQIILDLKAGDKVWVQMYIYGVPYVRGGWHTEFSGVLIIPNSA

>SG-PL_contig_10254_1_-3_45_263

MVDHFTALSRYSDNTPLFSETLNPQVCISGRFSDYHELKKSKKIIGCANLYHSQFQLDWL

QEFKKKNHYTKKK

>SG-PL_contig_10255_1_-1_1_210

CNRVTNTKTKSTPADQRNGRDRTLACHGGKSVSVGRPLQPVNGHVFQQTEPSLGSTQTLF

QHCLLCSVSR

>SG-PL_contig_10256_1_-2_2_199

LVLGFASTELLSLVQHLEESARILSTTLGRSGKIVFSSFPSLHLIHFQLKWLFPGSPSSI

RCLAAS

>SG-PL_contig_10257_1_-3_3_239

ISTHASASFLSSSHPNSVNLSLVLGGASAKLRSLVQHFRGIGTDSLYNSWAKWEDWCQLL

HISPCNPLPVEVAISWLAF

>SG-PL_contig_10258_1_+3_1131_1373

MMCRQQLLIHNYFKAASIQHFTISDRPILSYLKLLVRNENQITLALNLKYSYSHMHIYVT

LLLTNFHLSFILSADTSNNTK

>SG-PL_contig_10259_1_-2_95_898

MWSDQGGFNQDGFEGQGGFSSPGEFGTPQPAEEKKGRSRAQNLVPVTVAQIFNATNTDDR

FFSGNLEISQIAVVGVVRSVKQTTTRIDYEIDDMSGPVLEVRHFNNDQDENIPDDQKEEP

MRENTYVRVCGHVRSFQGKKNVVAFRILPVKDMNELTCHMLAVIQAHAAGNMVQNATSGG

NPLSTSAGTSRAGDFAATNNYQGMSSLHGQIATIIKNNTTDVGASIADVSRHLKGVSEKA

IRDAVEFLSGEGHIYSTIDDDHFKATDG

>SG-PL_contig_10260_1_+2_77_436

MELSECDESLLNNWQGFRLCSVSVSVFSRRRLRARNKFHTHAHQRCICHRLEKQPNQPMG

HTPSAQLAVRNLWVTFLQEYGSAFNQCSEVIHTSTRNARHNFREATKICTFQHNTWLQKF

>SG-PL_contig_10261_1_-1_145_1191

MANHPVTEVVRGFYNKLNVETNFVCRLNQEDLDPAEREVFEQLTDDIKKRYYGSGINIPE

NIEGKRILDIGCGSGSLVFILSKLVGPNGYIVGFDVTDGLIETAKAQSETHFKKWGYGTA

NFEFRCFNAERLDEQGFEPFDIIVSNGVFCLIPNKERVFKAAFELLKPGGQFYLNDVYAE

QDPPEQYKNNETLWSLGTTGSMRWDALSKMAGAAGFTTPYLTCAAPMSVCKQEYIDMLEN

RRYSCAGWRLFRKDPKVASRGASTVTYNGGVQGHPDTFVWDISLTFKKGEGVEVDAELAS

VLSASYLANCFSFRDSSGSPVTHRNQNPFELMDQLQSQGKLPDSIYKVE

>SG-PL_contig_10262_1_+1_121_381

MLYDGNCKKMCSISETGFRRTHPLLLMRKSFKNSATLAVTTPQGCKMGPLCHIPAERSEF

PLSNRIGFIRQHLNILPMELPEITVDT

>SG-PL_contig_10263_1_-3_180_476

MKMENDYLYVLNLVLIHSFHICIILPLLFVSSFVGGVLKTRQVLFIVFNPLMLRKKMWGG

GKGLKKTIQTDLNLTCVSEGKSIGHDNVLFSLVVTNFFT

>SG-PL_contig_10264_1_+2_269_781

MQTQPGLGKHHWSCDLIGSPSSLRMTSVQLSDRKKAALKPAQKSFLLLLFFVFVLCVCVC

VCVCVLLCFFCFFLMREVPIHSDILGIRCQVPVVRVVMTGDFFGCSHSVHAGCLPVSIIH

RTLSMAYGILNVCISCSACLYARRDFIHLQVCTYVDLEDWKNLHPYPPDKA

>SG-PL_contig_10265_1_-1_34_594

MGGAINKLLISLSSMFGKKEMRILMVGLDAAGKTTILYKLKLGEIVTTIPTIGFNVETVE

YKNISFTVWDVGGQDKIRPLWRHYFQNTQGLIFVVDSNDRERVGEAREELTRMLNEDELR

DAVLLVFANKQDLPNAMNAAEITDKLALHSLRNRNWYIQSTCATSGDGLYEGLDWLSNTL

KNSCHKS

>SG-PL_contig_10266_1_-1_253_678

MFVLVLVILALKTYLLGGGFKKNSGHFSGDVCQKHLMGGCLLDTWCVPWWIRLDWIELNF

FFFFLVTLEFFHVKLGCLPWRKACCHSMMPLIPLPHPIPHDYVYFCSPMTMDFFLQSFAR

DNSSFWLWEFTHVLKVHAAHGT

>SG-PL_contig_10267_1_-1_67_492

MRIKTIQSKTYTTPRRPFEKERLDQELKLIGEYGLRNKREVWRVKYTLGKIRTAARELLT

LEEKDPRRLFEGNALLRRLVRIGVLDENKMKLDYVLGLRLEDFLERRLQTQVFKLGLAKS

IHHARVLIRQRHIRVRKQVVNR

>SG-PL_contig_10268_1_+2_2_133

SFILTSLQLHNYKSYTFSLTLNRTKIYISNKRNLLQKRRYSFSL

>SG-PL_contig_10269_1_+3_276_347

MDTYVHRAITYIAFKNETISHCST

>SG-PL_contig_10270_1_+2_398_607

MEMIGSGRKIFFLCPAFPCLPLYSFFNKNNLFLTFGKLNLALSTSCKNETLSRYTLASWK

VLDKVREKKI

>SG-PL_contig_10271_1_+3_33_233

MWFFLRHNSGWTIRLIGKKTMLSDLTSICVAFNTASIRIISVPSIAANSCFKRHSVKYLL

HMILAPF

>SG-PL_contig_10272_1_+3_192_338

MKVSLSRAYFNVLGFLCAYLPEADSSLVLSHCPLLSPRLFEVFLYFEIM

>SG-PL_contig_10273_1_-3_105_248

MQTNISDISRKCYENNRGLLVDICPPTFICIDIREARQMSYRTWYCLS

>SG-PL_contig_10274_1_-2_209_1093

MILHKGHMGPECTRKLGSDVVFWAQMRQDIESAVSWCSACNSLKNHQQKQPLVNHPVPDL

PWANLSADIFEWNNCQYLVEVDSFSGWFELELLSNMSSGSVISKLKRLFATHGIPEKLMT

DNGTQFTSREFELFTKQWNFTHLTSSPHYPRSNGLAENAVKQAKQLLEKSKTGNSDIMLG

LLNLRNTPRDGMGSPAQRLLSRRTRTTLPTSSKLLKPKTVNAAKVSQQLKKARHQQKKSH

DKSARDLRPLQPGEVIRIQTDKGFQRLATVKAQRGAPRSYVSPRTKRTIYETDDI

>SG-PL_contig_10275_1_-2_920_1141

MNIPSMHFILPSPAPSCIDFILSLGYYRCRGKGGHAAWTTADSQHFALACVLGSVQWHKF

HCPSGLKVVKQCLV

>SG-PL_contig_10276_1_+1_154_243

MHTERGSNHNTFTQLQQCNAFTPSSKNKKK

>SG-PL_contig_10277_1_-1_124_270

MFIRQLCGSKKLFYNAVYNYFPFSQYVNFVLFFVVVFNVLVIYYPKIYV

>SG-PL_contig_10278_1_+3_51_239

MPLNLSETEGGSYVWFLLNVLSVVKRKPTRENHISCNADSPDIQFVSMSGNFRFILCEYY

FRF

>SG-PL_contig_10279_1_-3_3_83

YKPFSYKLRHHRIKFRSKLQRNKEYMN

>SG-PL_contig_10280_1_-3_114_413

MCVHTCWCAYMCQFIHLRVVCLCMSVHACTHCVCVCECVRCSAINQRICMYRYIYLKENH

AVCNQVPSDIQHSNTNSKERNKTQQIIRREHLQNVIDALQ

>SG-PL_contig_10281_1_+1_1_96

HYNGYTRVNTMSASRCQPVCANSWQSEKGGVT

>SG-PL_contig_10282_1_-2_2_382

LPGHEVRHSHTKSVSDSGPAPGSSAQAVFPPGLNLSPRQGYCLPSGPHGVSSASAPSGQS

SQASIAEGSPDPLAPVPDQLGPGGPLGRLVPGGDAGMGLCLRPGRGCPHCSPSSSGGPLH

GRLQLGL

>SG-PL_contig_10283_1_-1_118_312

MILLLDLITFITVFINICQTVLFKLFYRSTTFFGQLVSLLILGGKAGLFLFQSLVIFQIL

QVTVQ

>SG-PL_contig_10284_1_+1_1_81

AKLAQNYKHKLCIISHLQFILMECNSF

>SG-PL_contig_10285_1_-1_1_210

PPPPLSFYLQIVKSSSYAFLSSKLLNHKMSDHHFWKPCIDHEVVQIKMGEKVIIRQGNSV

TLNLFLLLSV

>SG-PL_contig_10286_1_-1_1_228

VVQVNNILGFERTIGGLWRLGSVAVTSVPIVSYENFAQNLGVDLSLISSPPRPLPKENLY

YWNFDLSYSGRGSDEF

>SG-PL_contig_10287_1_-3_33_254

MDALYTIESKCLHSCFVFHPSATLQKVVISTISDLHTCMRERKKVSLCDVIRCYTFNFLH

SQLGEHTIAVDLVK

>SG-PL_contig_10288_1_+1_1_273

FHYTDVTSASSLLLGRSEVLRNLKAYLREQTPGHHTIDRLIQKGIVRGSGRKSSMKGKTR

AIINQTNIGTISKITLRRLLQRGMEVFWGFP

>SG-PL_contig_10289_1_-3_429_599

MHMCVLAPNMTLTLCSFEAKANFTSSHLNVESDTLRFINDLSTTWFSRHRWWFSCHP

>SG-PL_contig_10290_1_+1_1_150

KKEERKKRNIFSYIHVWLNILTPKPTFSSMHHKRKHHSSTPSTVTSGVTP

>SG-PL_contig_10291_1_-2_461_646

MILLTFSYFGNEICTYPLGQYGADQCCQQFCSTYIIVCLPLTKLVQATVPCLINYLFHQV

SE

>SG-PL_contig_10292_1_+2_2_211

WIGCRNEKIKHSHCRLAFFLAVCVALLLNMDTTNNFPHRHPTTQAQSSNSFCSTNEMSYL

IFSRSHTHTL

>SG-PL_contig_10293_1_+2_2_157

NLQTKYNLKQYNGRMSQQWKQNLNIIRSCIAEKVLFTHLPPKVLILDTFWPE

>SG-PL_contig_10294_1_-3_3_179

ILLLLFFLVLFIYIFLISLFFLNKREIFHFTWERCVSVNKMSLQYFLSCFWTDLYQGTA

>SG-PL_contig_10295_1_-2_41_235

MHKFLTNLENEIYLFEFEQSKPLSVNAEHLLNLVKISCELGLRCSITSGNLKPYKVTLTY

EIDAW

>SG-PL_contig_10296_1_+2_1454_1765

MLTRVTQIRSLTYPCSLIYFKTQVNTIYTYSFFFCMLSYPHNLVYFKTQGTHNMYILFFS

PFKITASLYPADNSTQLEPCVFTRSTHPFMHNRLTELWTPDLMP

>SG-PL_contig_10297_1_-3_183_353

MIYYLCCFALVTSDIVNTGLGVSCPFCCVVCLYGLFMFFLWLRSIFYNFLCFFVIWE

>SG-PL_contig_10298_1_+1_1_228

YLYANQVSTSHHPLGFCKSNNIKFVNHIVAKRFFPPCTVLYLFTTDLLCEIRNALSREFA

YFSIEPPFLCIYLFVF

>SG-PL_contig_10299_1_+3_684_2318

MRKKKRGLLVIFVVGSVVLGLVYLLTSDSDFWTRVLLLDSTVKFDPKSIDVARYIGTVES

PDQENPTSRYGFNIVASNELPPDRPVPDARPAKCAELYPEADKSLPKTSIVIAFHNEARS

ALLRTLVSILTRSPLQIVTEVILVDDFSDNKEDGILLASIPLVKLIRNEKREGVARSRVR

GSEVARGEVLVFVDSHCEVTPGWLQPLLTTVKKVPHSAVSPVIDLIDSQTLEYKPIVDLV

RGGFDWSLHFRWENLPFGLSRRDPTDVFKTPVLGGSVVVIRKDWFNNLGTFDTQLSTWGG

DNFDLSFRVWMCGGEARIVPCSRVGHVTRKHQPFTFPQGGISNTYIRNTRRVAEVWMDEY

KRFFYAARPTARMQDFGDISDRKRLRERLKCKTFRWYMDTVYPELKLPVSDEVAYGKIQQ

GTGCVDLDVGQLPVIAKLRPCVQNKDSQEWSWRRKGSIVSNGMCLSVNPMETQMYVVVTF

CDSSDSQRWMRQRQQIVHHVTGQCLDSRWAKTGLQVSQCDEQLDAQRWSITMETMSDLEK

DYIDT

>SG-PL_contig_10300_1_-1_307_462

MVTTMGEKIKMCKKGKIGSMIWPFCFSLFLLSCTECLANMLYVECSHALRIY

>SG-PL_contig_10301_1_-1_265_441

MWTGLKWDLIFFLFLADCYLFWVECFANQECSCKGLTTLGVFSSPLLQTMQRCQKNGEC

>SG-PL_contig_10302_1_+1_1_186

HQFHHHLYSRHAVKKYSQYLKVLSQYIRLTQQSCAGNKLPTFVFTAWICAADLSTADIRM

FF

>SG-PL_contig_10303_1_-1_1_192

TQKYTQNFFLFFLTKFWISEIYKLSVALNKGLSCLCKACGTRSKGSNCCTNTPTFILQLP

FFHN

>SG-PL_contig_10304_1_-3_3_185

AGKLSPLGPYIVKRLMCVCVLETAYLCALVDGWVVVNFSASDQSFSFLPFHLKPEIYVHK

T

>SG-PL_contig_10305_1_-3_3_485

PRWDLLLVLGCLRSPPFEPLAGIPFDLLTLKTTFLLALASSKRRSEIHALGCAEQDIAFH

RDSSVTLKFLPEFLAKTRAPGDTSPSVTIRPLSDMVSRGEENCRLCLVCCLRWYLDRSRA

RRFSQRRLHLSGYSLSLDQRDYPQSLHSGSKGYQLFEPQGT

>SG-PL_contig_10306_1_-1_1_207

SAVRGYRSAIYTSLSQLGGPSFESDPLLRDTVRGAALRGACNPRRAPPPLSQGDLLLVLD

CFRSPPFEP

>SG-PL_contig_10307_1_-1_382_570

MQLSSQLGFEFAGHASQSMNTSLWMGVWIRTKFVFPIGVYIIIAHPIYQNSVCVFIECAC

KIK

>SG-PL_contig_10308_1_-3_3_110

MMMKMSIMLIKKDDNDTAQDEDVKGDDERKGNDDYA

>SG-PL_contig_10309_1_+3_3_128

LVKPWNGTYYFSELRHYTTKIIITTTTSISSITGQKTWSLQR

>SG-PL_contig_10310_1_-1_1_144

SPSPPLFFFQTSFPVHYAVMHLGIRLRHSPIKTENVQVCYPLPGRMSD

>SG-PL_contig_10311_1_+1_1_186

KTTTTTKPSPLNTHMHVSTHTHTHDKTPLFSKGNPHHWKHTHTKFLCASVQQYVLNFFNE

PI

>SG-PL_contig_10312_1_-2_2_148

SSDLVTHLNTVAETVNGRLVQISCPTHTLSCTGIPAASVARLSSCQNLT

>SG-PL_contig_10313_1_-2_143_1678

MVLLAAAVCNRTGKALVSRQFVEMNRSRIEGLLAAFPKLMGSGKQHTFVETESVRYVYQP

LEKLFVLLITTKASNILEDLETLRLFAKVIPEYCRSFDESEIADQAFSLIFAFDEIVALG

YRENVNLAQIRTFTEMDSHEEKVFQAVRQTQEKEALDHMRRKAKEIQQRNRENAKGSRMS

GMGYGGGGFGPNSFRSDASTIDSSPMEPPKPSYTKPSAKPSGPNKALKLGSKKKDVDTFV

GQIQLEGEKVVDAGRSGSSAVSKSSTPAVNQESIHLTIEEDIVLTAGRDGGLQNMEVHGI

MKLKVSEDRVGRIKVCTINNDNKGIQIQTHPNIDKKMFLATSQIGLKNPDKPFPLNQDVG

VLKWRLQTQDEALIPLSINCWPSESSSGCEVNIEYELEQADMQLEDVLISIPCPSGVGSP

VVGDCDGDCHYDTHKRQLQWSLPFIDSSNKSGSLEFTMNGGHPDDFFPVSVSFTSKKPYC

DIQITEVKQLDDDQPEKFSSDVVFFVKKYEIV

>SG-PL_contig_10314_1_-3_18_302

MKKAGSAKINLQLSQREMNTILENKVKSQCNLRPFIVNYNGFTRMEIALAHRLSLNSLRI

KFSKTVKCICNDYITVNHLFHCQEMRPYFPASLIN

>SG-PL_contig_10315_1_+3_249_341

MKIKSLASKYRIHKYQLLFMYKQELSSTHTH

>SG-PL_contig_10316_1_+3_555_842

MAEKRYTSVCITVFLAPLLFPYGHYNCSLPVFWNHISFQAFRNLMVSHLIPASLQATKAL

VVMPSTPAALFFGLVFSALLISFSGCSVLRVLGILG

>SG-PL_contig_10318_1_+3_3_137

TEQINQNVILSQAQYICWTASFVKTKCDHKNNITDKPINTICIHF

>SG-PL_contig_10319_1_+3_3_194

HFTSEFNRLYIAKFVYGKCQNCYIWGKVCFYQCSSKPGPPNYPRKPHAASRIHNATSTVM

VINC

>SG-PL_contig_10320_1_-1_121_330

MWLVEKLSPFMISGYVCQEPPISESCCLGGYNNSSKSLEHCSTHVCYMKYGIEELNVGFL

SEIMCFPLVL

>SG-PL_contig_10321_1_+2_89_265

MTTNNRETVNVAKSKNNHDSKLYAQTTCLWKDVHNAIELVRDMDRGTYNYMISLVINLT

>SG-PL_contig_10322_1_+2_2_169

TYHTSGTHAPWQYPQKAPHSISITLVRMSCTTNRAQNTGVLDTDGSLVFVQHVCLS

>SG-PL_contig_10323_1_-3_3_263

GCSSNSARVISTSPRVSSRQGASLVKNPALPSGLHSSPRELEDLSGPLLEQDLVVSIKVL

GPFQHHDPSHSPSRHWQFGTSSPSTFS

>SG-PL_contig_10324_1_-3_3_248

GKKSAKKTYGGKRLPTSDSQAGMSCSSNSARVISTSPQVSSRQGASLVKNPALPSGSHSS

PRELEDLGVGIPVRTATRPQPS

>SG-PL_contig_10325_1_+3_3_167

ICTIKPLRRRQRQVSSNCDNLRRLTQVIFSPTSWYHLSQLHSYFHANSHWLYIFK

>SG-PL_contig_10326_1_+3_3_155

IFSAISGRIFSKFRQGPLRSHLCMNLELRGPRSSSHGPNRCQKLALFEEGL

>SG-PL_contig_10327_1_+2_287_463

MAPPYTHNSFFSCLSKPVQNLKALQWEGPNPASTQSHPTAGPLFVFFLRGRLWPCFIIL

>SG-PL_contig_10328_1_+2_434_691

MIVTTREGGSYGTESERTIQQHTEANKQYMGYTNGTLRRSNSKLLLRYMGSTQGYKQHTE

QIKQHTVTKAYWQHTSAYKLHTTTMA

>SG-PL_contig_10329_1_+2_2_94

FNPPPSSPKCADSLADCQICSMIHLLFLRHM

>SG-PL_contig_10330_1_-1_1_201

DLCCNDITRLSLSLSLSLSHTHTHTHTHTRSKQAHNTVSVKALTRARRRACQLPDQVVLV

SVCLCIN

>SG-PL_contig_10331_1_-3_888_1112

MPIFDHFIYSKSYVRFLSFLLLSCLATSVSVGPFTLGKITRVWSFRVSSEFKYLHSDRHT

SESNQTGLRLPCDVV

>SG-PL_contig_10332_1_-1_1093_1371

MCGKRSYYDCYSHVTFFTMAMLVGPCLIQKCYDHIVLLISLWLFFIPYSVLHRSLSFLLA

LLSSVASPVCALSPALSVGLVDVTCQKGSYIEI

>SG-PL_contig_10333_1_-1_2029_2241

MISWVKQISQKTSSVFQVFLQHSFATLCKKHVRDQMLSDPFQGEGLQVNLEHLLIRQVLS

VCTSSLIHAPV

>SG-PL_contig_10334_1_+1_19_273

MRAEYQICMLKILCKSGQWWAYGNMKTPSIHQKNPGLFTPYQAQSMTRKKKKQSHHYNQQ

SAYSHTPCPYVKSSKQHNSLKFLIC

>SG-PL_contig_10335_1_-2_56_352

MEPPAKHRTVSAVADTHHSPTINCQQSACYNQQSVCYNQQSAYYNQQSACYNQQSVCYNQ

LSVCYNQQSACYNQQSACYNQQSVCYNQQSAYSHTPCPY

>SG-PL_contig_10336_1_+2_86_334

MPAIPLNLQTQRSTADNCTPTSQQYPKGNSLDLDPDYTVYITCHLRKHVPMNSVAEIHVQ

LQINTSFYTLVFIIISNLVNKCI

>SG-PL_contig_10337_1_+2_452_673

MNLQCEMRAALPGESTSPEYGSTLLFLYVNVFRHEGGFSTEFSQGQFFWLPLDLWRALSA

CCTWISVYSSFERF

>SG-PL_contig_10338_1_-2_107_880

MPYSTELSEYYMAPDLYVGAVVNFNKHKFILIDADEYAFLYMEEHPLEFPKCNMQIILEK

IRARLSQNMDDVRSFFTSSDASGRGVADYNQFRQLLMQVDNTLTEQEIMTVARHFSNQSQ

MGGLDSQKLRAIAQEQLRKRNYESFNNLLESLQHEDRTRSGFITPEVLRTIFLSHHVPLP

DDMLRAVLASAPTNEKGEVAYADFLNSINWRDYPVPPMQYQPVHPDEDWKGTSAKNEVTS

VNYVALLEALFGRVVNGQ

>SG-PL_contig_10339_1_-1_70_465

MRAPDILKNACEQFLGKSPEHIQHVILQTLEGHLRAILGTLSVEAIYQDRDQFAQLVREV

ASPDVGKMGIEILSFTIKDIYDNVQYLSSLGRAQTANVKRDADIGVAEANRDAGIREAEC

EKARMDTKYKAD

>SG-PL_contig_10340_1_-2_2_283

HTAIQLQVFIRNDPLTEKILLCFSALGCGYWLKSLPKFDTRACREFGDILKQVLDRFLQQ

GHIPQKVLSAGLVLHICLSCAPYKSKEYSSHCIS

>SG-PL_contig_10341_1_+1_55_213

MAQYMTPKGTLPLNLLNAPNYPQMYETLKALPSPTTKKCLITTTQTSQQTQTT

>SG-PL_contig_10342_1_+1_1_225

LKREIFHITRERCVCVCVCVCVCVLMKCVSKIFSAVSRRIVSKFEAKVWTVEELLKNEFG

TRRAKVKVTRAKKSA

>SG-PL_contig_10343_1_-3_111_1856

MLTNISKVILFLSLLLLRAGCDEHEHSYEDGEEVVLWMNTVGPYHNRQETYEYFSLPFCR

GPKASIGHYHETLGEALLGVELEFSGLDIDYKGDVQKTEYCTVDLTEEKFQAFVYAVKSH

YWYQMYIDDLPIWGIVGEIDEGGVDYYIWTHKKLDIGYNNKQIVDVNLTSEAKVILKPGS

KIAFSYEVKFHPSTVRFEDRFDKYLDPNFFQHRIHWFSIFNSFMMVIFLVGLVSMILMRT

LRKDYARYSKEDDLDDMEKDLGDEYGWKQVHGDVFRPASNTMLFSALVGTGYHISIVSLC

VISFAIIGELYTERGSLLSTAIFVFAATSPVNGYFGGSLYARMGGKVWIRQMLLSAFLMP

SVVCGMAFFINFIAIYYHASRAIPFGTMVAVTCICIFVILPLNLVGTVLGRNLAGTPNYP

CRINAVPRPIPEKKWFMEPSVIVVLGGVLPFGSIFIEMYFIFTSFWAYKIYYVYGFMLLV

FIILTIVTVCVTIVCTYFLLNAEDYRWQWTSFLAAGSTAGYVYLYSFYYYFFKTKMFGLF

QTSFYFGYMALFSLALGIMCGTIGYAGTSLFVRKIYSTVKID

>SG-PL_contig_10344_1_-1_1_195

MHTVLLSMKISLSLSELFESFSLLCFQKEQIWLASKRKAIEHYRQEAKRMRFGHYKKQVL

LVKQV

>SG-PL_contig_10345_1_-2_323_571

MKCLRKTAGKTRRDMIRIEEILKMMVGTTPVHHYIIQQQIKWFGHLTRMPVKQQPFEPTT

PDAGASELEEDHRRTEVTNTLHM

>SG-PL_contig_10346_1_+2_2_139

QLDLCTDELEKTHTEKCKKDLSRFACFPSPRLYRHTCTYIYWLYRQ

>SG-PL_contig_10347_1_-2_2_148

AWGVEWCHLWAQVWISVYVTFFFFLLQDSLQPVILIPDAEALHTCFTSP

>SG-PL_contig_10348_1_+3_24_236

MVPHVKSTNIAIQPNYKVNRDNNSLNIAPLMQTTEKETNRQELQYISTHVRSRASSASFP

TTRLPCVKTGF

>SG-PL_contig_10349_1_-3_51_2633

MDPKSHPALTGYQMKNSITSAGSNGDMKSLSKMDNSVYDFDNHASEEDEPIAKPVSAGPS

TSAQSPRRARQTNQLQFLQKKVLPPMWKHQYAWPFHKPVDVERLNLKDYHKIIKNPMDMG

TIKSKLDKNLYWSARECVEDFNRMFTNCYVYNSPDDDITFMAKDLEKFFLEKMTKMPQEE

VEVTKSGKQKGMKKGAKGAAAARTATTSKFRRTSVNTPGTSPGVAAPPTLESVANNVESA

GNSSSPPNLTALPGKGASNSTSQTQTNYSPPSSLSAGPVGGSGFSATNNLSLNPSVDLEP

VGPAVTEHSSQVLPPTQPTKLRKGVKRKADTTTPSACSSEIPVTEYGDPAYPPEKIGTHA

APARRESVRQIKKPKRDLPEDAGAAVSTMVNSHLLTPSEAQGSCKKQKGKLSEQLKYCAT

IIKELHTKKHNNIAWPFLKPVDTENLGLDDYFTIIKKPMDLGTVKAKMENREYSSPKEFE

LDMRLIFSNCYRYNSAESDVVVMCRKLQDVFEMRYAKMPEEPPHLDPTPPPSVSGNNKAE

DVDQSASEDESEDDNDDDCTLSEEEREKRIKELQTILASTQEELVSLTKTHCEKMKERRE

KKNKQKKRKRKEKKEVPEVKKETTEALAAAPVLPSVPHGAAASSVPPTGTVTDTSKPKKN

KKQKSPGSKRSRGPGRTSKRRTSAAASQPPPSLPPFDSEDEDSVKPMTYDEKRQLSLDIN

KLPGDKLGRVVHIIQTREASLRNSNPDEIEIDFETLKPSTLRELEKYVMSCLKKKPRKPY

TKRSNKPKELVQQEKKQELENRLRGVQEQLGQTVKKPSKKGDGGDVGAGGRLSASSSSSS

GSDSSSQSSSDSSDSSDSEQG

>SG-PL_contig_10350_1_-1_2065_2298

MYLYIVALSLTLCTLIFLVVSHSHSDDNYFIYGRVKETGEKQLEKGLLAFKHCCIVFHIF

EISGVPISLFKNALKVNL

>SG-PL_contig_10351_1_+3_3_110

TNITIVWCGSEFYILLSQSCVLVRILHTTNITIMWC

>SG-PL_contig_10352_1_-1_1_165

KGVLKKKKKKKKKKKEENTILCTGGLKNFAYFFPVKMYHPYCLNCDCFNFVGIIG

>SG-PL_contig_10353_1_+1_229_492

MKNTYTADMATTGRQGPLNTQELVLSSTTVYRQTSIAQPSTHQPHHQQFTSKGIYSIINT

IIKNLQANEIHSSHPHRNIIDTIYKHYS

>SG-PL_contig_10354_1_-1_16_261

MYFTEKLSAITKHCSLCILFICMFIRLLVYILYISTMLINEDVDIFIVLLYVRVVYVLYI

RCDLYSLLIGSYLISTNIFLLF

>SG-PL_contig_10355_1_-1_19_1455

MIYWQMMDQDTATMDTSGEREFRAVWVATVGNIDWPSTRHQTSTQQQLELKALMDKFKEL

NFNAIILQIRPAADAFYASSYDPWSYYLTGKQGTAPDPLWDPLAMAVTEAHKRGMELHAW

FNPYRARAGSTSSTGLAQTHIAHVLPQYAYPYDSNLWMDPGAKAVQKRTLDTILDVVTRY

DVDGVHFDDYFYPYPVADQDFPDNETYSAYLAEGGHLSRDLWRHQNVDSLIQALSQRIKR

ARPWVKFGISPFGIWMAGNPRGVEGFSSNSEQYADSRKWLTEGWVDYLAPQLYWQIDPPQ

QSFTALSDWWLQQNPRGRHLYMGCATYKTATASAPWPITEIQRQVAETRARRNRNDLGNI

FFSAKYYKANTAGIADLFKDTIYASPALAPTMSWLQSDLPTGALARPNATVSDSGFILWT

EDPSGKVRAYALYREEGRAGSGQYSLQQVMGGHTHSVRVGPGRYALTAVDRLSRESPTQ

>SG-PL_contig_10356_1_-2_296_484

MEYTARSCFLNFLVFGSIYCSLTRCHGTAAILTTCRKLEVDICFFFPLFCLGTDLYYSLE

VNL

>SG-PL_contig_10357_1_+1_1_252

MNKEVHFTQNGIHLTSTFSQKSTNITMYTFTHHGMSEEVRCTQNSINKQVHLKKKKNPKM

ACTNKYNRIGYTNQHNSTSQKWHK

>SG-PL_contig_10358_1_+3_426_659

MRRKGTEGRRNGKQRRVEKAGKETIILEITRMIRKNQPCSKSLHLCLHTSYWLTLISPSN

TVHVICIYNDSNLLPFSL

>SG-PL_contig_10359_1_-1_1_159

LAGLPLRFSREFRFLPPDIFSGFAKLIFPCLTLPSAVSQCSLLFSGHICLENL

>SG-PL_contig_10360_1_+3_144_266

MMQPKCKAVLSFQISNAIFILFQHPNFFKKKIYITKLKAQH

>SG-PL_contig_10361_1_+1_205_321

MFKFQTLDLFMQSRFSSLTVPKFHSSVSQPMSSFKKSCT

>SG-PL_contig_10362_1_+1_1036_1146

MHVQSEPSVLLQHPVISCCPLEAPRHKGHMHECGSCA

>SG-PL_contig_10363_1_-1_103_348

MDNCNTMRGKKSGVETLIRKENPQLLDISGETIHIIHTSASHMFKEFDLKFIPLQDLAAD

LHYDIAESPKTCHLFNEIQEHI

>SG-PL_contig_10364_1_-1_1_246

DALSRGHPIIHSERTPKKWSLQGACDSWFAPPPPDRLLCDQAQQEAPDLRLPGPGPRGDG

GGRPLPRLGPSSQEGEDRRGTH

>SG-PL_contig_10365_1_+2_2_181

ASIPTCFVKILLRQVKGCCLVNNIVIMWRQFAPICILLTQMGAFSQVLSFSASFLGIFYR

>SG-PL_contig_10367_1_+2_128_298

MGPTQRKSTTKTTKEQSFKTRFKKRYQQVKNLQIIQVVYSISHLLFMVKDVFHPTDL

>SG-PL_contig_10368_1_-2_110_238

MSTNPKLGTLKTCMKQDKIRLINLKLTHQLDQLGSVSFQVKKR

>SG-PL_contig_10369_1_-2_2_172

TEFCQGQLLCCHVFFYMQHCIKCMLYMGPSVSSERLAPRPPLKVFSGGEDKSPCSNS

>SG-PL_contig_10370_1_+2_2_223

FFFDNGTLFYSTKFNSHCEHMTQNKTKPQTFNTPPLLNRPCKVIQEQVLTSSSPYKFQTT

ISAYPDKHQEHLSL

>SG-PL_contig_10371_1_+1_1021_1248

MVNSKSDMLTSTQHSLSTSDPHTPSPLAHTPTHSSNTHNIQIKYLYKTYGHTIDLPFTKL

LDAFFSNQQFLTVTFN

>SG-PL_contig_10372_1_+1_1_282

KKKKKKRQKKKGKRKRKKGQKKKKKKTRFFLTQKKKKIFHPPPPPPPKKRQKKKPPKKKK

KKKKANGRVQHTPRNKVQNNQQINNRNLTIKNIT

>SG-PL_contig_10373_1_+2_89_259

MNHGPMCSTLCSMHLACVNDPTATEKLSLAKFCGKIHFCRLTEIRADRKRKMGGAAL

>SG-PL_contig_10374_1_+2_1256_1492

MLFSWLLSLYSFSCLLIRNLRFVYLSVFQSCKAYKSTFTKFCIAFKHFFITFLIQFHKCT

NGLLLKRTNTNSKALRWFT

>SG-PL_contig_10375_1_-3_3_89

YVCMYLCIDLFICIFIYLFCIICNMVDTV

>SG-PL_contig_10376_1_+2_2_226

TTRYTYVHHHQRKNPKTKQNKKPNQPNKNNKNKTKQQNHVILPMIKMFKKDNKKRFHCGR

TFYSETVLLLLVLFL

>SG-PL_contig_10377_1_-1_1_171

KKKILSTGKIHISFRNERPPKVNKGKEVLHSRSEILFSAQWNFSILFHYKCKILSSA

>SG-PL_contig_10378_1_-2_524_751

MNITGDLCFTISLVEFSVCFTIFTGFAINHSMCVSFDLILCSLIVDLFHVCILMCMFSIY

SFLLICFQFIHLLLEW

>SG-PL_contig_10379_1_+3_441_653

MQHDAVPCDANTWNSFTSGINNSKLPYRNLPPIQFLIGMMNKITVDSECGALFLKLFYFY

YFLSIYFNFFF

>SG-PL_contig_10380_1_+2_2_310

KNGTIVCIPKKGNLTECDNWRGVTLLSIPGKVYCQLILNRIRDVVDGEQDSALNAHAQNK

SLHCAALLKAAPSSKCPWPSVSLTSPRPLTASTDRSEERRVGK

>SG-PL_contig_10381_1_-3_42_239

MRHQTLPKTILVGRVKGKRTRGCPRTKWTDDIKDWTGDNLAGCMHRAQDRGQWGTMARRP

LARDDT

>SG-PL_contig_10382_1_-3_633_884

MGGQNVSDFSAVYGMVHRTQKLERAWFIGSVTGMENVNNFQRREELLLGWLCGCMIYRWT

MYDEESVLCIWRCFLLVEIIICMY

>SG-PL_contig_10383_1_-3_15_908

MAARLVSVCRRSGIQVASAARSTISRHHSHLHLRLLSTGQFQNVIVERCGEKKNVAVLKL

NRPKAFNALSDALMTDVSMALQQLDQDNDIGCIVITGGDRAFAAGADVPEMSHKTFHSVI

EQDFPANFCSITKCRKPVIAAVNGYALGGGCEIAMMCDIIYAGDKAQFAQPEITLATIPG

AGGTQRLIRAAGKSKAMEMVLTGNRISAHEAEKMGIVSKVFPAAEVLDAAVKTAEKIAGF

SRIAAALCKEAVNASQELPLAQGLNFEKRLFHVTFGTDHLHEGMAAYMEKREPNYSCD

>SG-PL_contig_10384_1_-2_2_196

VCVCVLWSLKCWRCSIPVVSVAMVHDIVVHDIVVHISRWVCHSVGGLHRLHMFVILWFTS

MHAVC

>SG-PL_contig_10385_1_-1_469_750

MIGNCLCDQGICQLYYKGSLSYLNLRLTNRQTNLWYRDFSAVLQRISIIFESQVRQKEKF

CLFFLLLFFLAPQETDFFAVYGLICKILGLKISA

>SG-PL_contig_10386_1_-2_692_1000

MILGVCTTHKSRTLQPSIHTCLPFSVLLTSAAVTEYYLSLIFFSHENSWLNDKLLCHPYI

FGKQDGLFLRLFLGLSCERISAIYRSSESIFFSLNSRVNCKLS

>SG-PL_contig_10387_1_-2_26_271

MLLLPPLPYACSDENATSSSHFSCSKLFLWNEVNWVLCQLVVRFTFSHSFHFNPSQPVQC

KGLHCCVFCFLCNVELPCIEAK

>SG-PL_contig_10388_1_+3_3_122

CALPKSANNYLADSLSLRQNPMHWRGIEMGSGEQDLQTLS

>SG-PL_contig_10389_1_+1_460_654

MYRLTWKRGTEFTVCSRGIHHTSPVIHAPSKSASYYLADSLSLHKTLCAGKVLKGRGART

GPVKH

>SG-PL_contig_10390_1_+2_236_370

MVKPFKNLPFSPRSNQFRKKKLTTKNPQTNKKTQISVKNCSCVCG

>SG-PL_contig_10391_1_+3_3_113

PANAFCLPCTQQKSCIQFCFTCSSKTTQGKAIKAVSN

>SG-PL_contig_10392_1_-3_30_482

MEMDKKKGDKKAQKKKVKSKSGQRKAVKKTSMPQGTSDLTSKLFITMEKHRDAFFVIRLH

AAHVAVGLGPICDPDPPLNNDLMDGRDNFLSIAREKHYEFSSLRRAKLSSIAFLYELHNQ

GKEGFVYTCNNCQASMETRYHCTVCEDFDLC

>SG-PL_contig_10393_1_-2_191_292

MPMCTPVKLILEWIVGDNYFACTEVNNMLQYNSV

>SG-PL_contig_10394_1_+3_192_365

MGGGVENEVLGCCLFMRDLSHLHNSILRTPPKSPSNDLPDDLNRFKTRWQGLEMGRGK

>SG-PL_contig_10395_1_-1_190_516

MPPHPLNCSISTSPTPSRGLPPPPTRTRSPSTRLLRTLSRLPPRSSFSSPVLSQRLSRLP

SSASAVTPLRLLRVKKFFVLPTPFPQVLTSDPQTCLSPFGTRLSRTPSR

>SG-PL_contig_10396_1_-1_148_993

MSYQYPVSYGHGQNFRRGPGGRFDDSSPVNQDQNRKLFIGGLSYETDDQSLRNHFEKYGE

IKDCVVIKDPTTKKSRGFGFITFKETKGLDEAQKERPHSLDGKEVETKRAMPRDDPQSHQ

TVTKMFVGGLKDTTTEEMIREAFSGNITKIELIKDKATGKLRGFGFVTFDDSDTVDKHVL

TKNFTLNGRRVEVKKALDKNEMGYGPSGGYGRGGDYGYAPGYGYSPYGPAPSRGGYGGGN

YGPHGGYGNQGWNQGGNYGDYGYGHQGAGYGSGGYGGNYNRR

>SG-PL_contig_10397_1_+1_274_525

MHVHLHLPSAGVLTRLSLVRSILPTESINLCVLKFVEFRHFFLPLKACQKHSKGTNSSSW

DTSNTIFTVTSLRPFFACLKINNI

>SG-PL_contig_10398_1_+2_590_838

MCGSLWENHFTVIQTDAWIYSSRPSKMSMIHWPLFQTSLPLPLHTSLTQCSCAGNKSPTF

GFTAWIRAADLISTADIRMSFKH

>SG-PL_contig_10399_1_+1_106_273

MGGVILYSTGDSSELHSVNQSILFIARHTTKVISGAVVFIYVNGAHQTLLTLVSLF

>SG-PL_contig_10400_1_-2_185_481

MCVGVYTCVHVSVCVCKHVSMFVCIIMIIMHTYIHAYVHTHTPLSVSVYTTPLSVCLSLY

TYTHTHTHTHSLTHSHVHWCKHVCLFVCIHVHICVYMCV

>SG-PL_contig_10401_1_-1_58_237

MENPDVTVCCLLRFQNILQSGDDGRDVNFADVPQMAWRFCINASYNDVLFTHLLYFFCLN

>SG-PL_contig_10402_1_-2_2_130

AGMKELTTSAPKENEEDVAARVKYHQSTGWWLRKQEMFRVSFT

>SG-PL_contig_10403_1_+2_2_178

LNEWFTYAQMPPSLNTPLGSDPSYSLLGTEPEHFFELWLNMAVFKRNNETVCLFFYNRS

>SG-PL_contig_10404_1_-3_153_332

MVVIVLIEFKKIFVPSWCRLQWLERWPFSLTARVLQGFESLWSKTFCLQPPVYPAVNGHL

>SG-PL_contig_10405_1_-1_1_180

GSLQDGDSSSLQGVYQTRLGHLSGSVGRLLPHPSTKPTSSFSALSGETRLKFRVLPSASL

>SG-PL_contig_10406_1_-1_58_315

MGSGGFAPSPWPWVLWPTLCGPQILRWLVPSSGLVSTQRIPGMHSFQDGDSSPDPGVNAG

GRLGLLHRPQGCLLPHPRTPSCRKYL

>SG-PL_contig_10407_1_-1_130_873

MSVNFSANQYEQAFDPKRIQNWEVPDEKGKYPLPREGTSTIHANNRGHLLPNVPRRRESP

WGTHVGTWDMPTKIPGNRQLNPTARSEDMQLRLQMGKEEAAEVLSGGLKKCKIPSPLPVK

IDEEAKPINNPPLPGSPSAIMATKTLNAPALPMMHDVKWPRQGYEPDPKGDADQPVGNPA

SPKPPTPMPLGGEAVQAQARQVISPVNPEQVAVKGAVASPVEPVMNQLRTPEVNVNWPMA

KSTEPEVL

>SG-PL_contig_10408_1_-1_739_918

MSLQIKIRVVLFPVQGFFLNTCCTFLTFFECFVGLFMLLVSALVFCFIFSLEWRLWLAGL

>SG-PL_contig_10409_1_-2_2_178

LENDQNIFNQIGTSSKRFFAESDSAVNVYYQAASNYQLLWIYKHRGNDFKLACVQFRSF

>SG-PL_contig_10410_1_+2_56_403

MSLSTRIDFMVQQLDKLATRDDVNIIKTDVETLTIKFTEKLEQIEGRLLEAESRADQLTK

DVTILKKENETLKDTIKYQDNKMRQIEKEQNSLQQYTRRWNLRVFRVPERDGETGA

>SG-PL_contig_10411_1_+2_1697_1924

MYKKKVMYFSPTLFFTLQPFRFLFVHVFGSSFSCLGRILSTDQMLSYSLSHFTSSRQKLC

ATLILIPSHLIFYTHL

>SG-PL_contig_10412_1_-1_250_342

MFCDGIYHFSVGFLNFNCSVDFVIIIFCMYH

>SG-PL_contig_10413_1_-3_294_515

MCMCVCACVFQVWSSGGRGSKSQVTISLTWIDLCKTRVDFDDLHWCCVCLLLDSKFKTVI

RLLNSDFDKSKNRV

>SG-PL_contig_10414_1_-1_67_504

MVAIPFALMKETANWGLIMYRDNELLFKDQATSMTDQMRKATNIAEKISYMWFGNLVTAV

WWDDFWLNEALARLMRTVVYNHFYSDRRTMDVSVADMEEVMKQDGSVWSRPLVTETFDTL

SYYKGSFVVHMLYFAIGDDKFRKGMK

>SG-PL_contig_10415_1_-3_21_869

MAASSCLKKFSPAAKAVIQLFQTRFTQTAAAAAPQAAPRIKKFEIYRFDPDKSGDKPHVQ

TYEVDLNHCGPMVLDGLIKIKNEMDPTLTFRRSCREGICGSCSMNIDGTNTLACLYKINS

NPRKPVKVYPLPHMYVVKDLVPDMSNFYAQYRSIEPYLKKKEDGSYTPGDKQYLQSVEDR

AKLDGLYECILCACCSTSCPSYWWNSDKYLGPAVLMQAYRWMIDSRDEFTDERLAMMEDG

FTVYRCHTIMNCTKTCPKGLNPGQAIGEIKKLLMNYNKKAAAH

>SG-PL_contig_10416_1_-1_10_1014

MAISCGRFALLRRLLVPPKLSGCCVHTDVRVTNAHVQEKRSKAFEMEKQRQRSLIPRIEK

IEVKFKGQPEDCTLIMNKNLSTPYHCAMHIQELLMSRSALALVNGQAWDMHRPLAESCEL

QFLHFEDPDPTLANMAFWRACSFILGYVLETAFKDDHYVELCSFPKPNIRSGSFVYDADL

RVGDWKPSSRELNILGRTGYKVFFNNYSFERLDVDASVALKMFEDNRFKTAQIPSIAAKS

ETGSKVTVYRMGDHVDITSGPLISNTSQLGRFVVTAFHDIHSSTYGPLTRVQGVGLPVQL

NMHFWTFENILYNRAQKLNDAPLPELQPSQPPSRT

>SG-PL_contig_10417_1_+3_15_227

MLELQQQYICWNCMLTYINIIWGQQVFILFSCCDPYSVCNIVSFVAFIVNLSHCQLDSLF

CKGHCVLNDLD

>SG-PL_contig_10418_1_-2_2_490

MEQPNHRATTSAASQEQENSSTGTVDSFSAEYSAAAQIHFPSHQDSVLTGSSGIGDSICG

GNSSTNTEETRSSGVANDAGNSLAGEQLMRSSKMEAKVRYRRLLAENTQLKRRQLCRLCG

ERVLNVTLLPCGHFVYCEQCASSLSHCSICQREILAEVRTFLS

>SG-PL_contig_10419_1_-1_139_519

MEKKCPHHPNGLLCRHRRKETVGEESRVGRLNTTGKVGRMKILVSPTTGGQLEVCVSPLE

TVRGLKQVLARKYRIAPARINLLHKERVLKDGTLRDNQVTEGSRLVLCPNMESGTSTSHA

EHSVMQA

>SG-PL_contig_10420_1_+2_152_340

MLHTSNSQTWPSMAKQDVISHTHIHSTTKEYTTTWWHVTHSSVKMVSHYMVASLSLTLTD

TRW

>SG-PL_contig_10421_1_+3_528_578

MTSLKHKLYSLTMTYYK

>SG-PL_contig_10422_1_-2_2_379

FFPLCLHLLSLCLHLLLCVFFFLCVSIFFLCVFTFFLCVFVFFTFLCVFTFFLSVFFLTS

FFRFFFIPFSFEGRLLLSLLFLLAFSFFFSLFLGSSSSSPSFFGSCSTPSVFGSSASFSL

LWSYSS

>SG-PL_contig_10423_1_+2_182_340

MSQDYTTETKANVDNTVTMMNVLWSAQRCCSVSTDNMRAQLLAKICQMNQSFL

>SG-PL_contig_10424_1_-1_1_171

KKMLLFRFAYVVICYEICYLQTPFEKDMFLLTFANIVQERYVNLYIYSFQTILYACI

>SG-PL_contig_10425_1_-2_17_685

MAPPEATYEELLSVVGDTLDFHTRGIPGPVMGDRPTVNSTSVSFLSTWAPAALSTKVKGD

KKTAVEPASHNIHHFCKMTTEFQLPSEFRNTGRKYHAPDLYPGVFPGQEYRFQEEALRFL

EDTEEERKAASAARVTDPVAAAAQRIVDEANRTCPAENSLEDWKARAEKALSHAGMSITG

QRIEPGMDESRVYWAPAPPKLEVAPAKVRQMLFPQYQPGEEDE

>SG-PL_contig_10426_1_+1_310_459

MSFLCSCFMLSFFICLSEAFMIFFQNNLTKRFTTYCSFCLLVLTMSKLCC

>SG-PL_contig_10427_1_-2_422_607

MLNELKCVNFILLYRCLCNVILPFKCLSCPPHNFDSQGMGGTWENCAFFRFAIKLRVVLD

PN

>SG-PL_contig_10428_1_-1_1_96

FTQFSLPPPPPPFPFPQFMMYYLCCFALVTRQ

>SG-PL_contig_10429_1_+1_1_174

CSYLYIIDQDGSFFSSLFHFLFQFWTFFIVNFSVENNLSSDCLLSSIFLIIEFCHKNE

>SG-PL_contig_10430_1_+3_720_959

MFLSRCLLQSSNNGGRPLWRFSSQQYVGMIFPSRLCSERRVYVQAYKLALPVKCLASSII

WHLLWHHVESSALSLLNVKQ

>SG-PL_contig_10431_1_-3_327_797

MHYKEAIRISPAFADAYSNMGNTLKEMQDIQGALQCYTRAIQINPAFADAHSNLASIHKD

SGNIPEAIASYRTALKLKPDFPDAYCNLAHCLQIVCDWTDYNTRMKRLVQIVQDQLEKNR

LPSVHPHHSMLYPLSHPMRKAIAARHASLCLEKVGDL

>SG-PL_contig_10432_1_-2_2_175

SSFLLLSLFLPFFFPFLSSAFFCLPGPFHFVFSSKSSHSFLSPQFMMYYLCCFVLATR

>SG-PL_contig_10433_1_+3_315_440

MFKNHDYVNKPQGHLLLDLRLCFGMFFLQVCSFSVFCGKKKK

>SG-PL_contig_10434_1_-3_165_329

MSWLSFFLLLRPTALVLSSLAHQTLLFSCCFQVQPQNLSFSNTINNNFYRFLPYD

>SG-PL_contig_10435_1_-2_92_403

MDFTKENVASYIAMVGVLSVLAQTLCLSLLMKYLGHKHTIMIGLIFEIVQLSCYGFATQT

WILWIAGCVAAMSSITYPAISAFVSSHASSDQQGVAQGIVTGIR

>SG-PL_contig_10437_1_-1_418_561

MSSYHCVRSHHSDMQYSMTCFLDNSLGNILALLCFSPEGNTEPFDFCI

>SG-PL_contig_10438_1_-1_457_921

MICSYFCSMMIFYYRTSICCMILGLFPCYTFHDFVVVVVVLATLSFSFISSRYTRMMLWR

SNFGLRLRDGADTKLPFTNYPGVNQARNLRNAPNFASIEWITLDCLARLILNEPTVRSPP

GRNQSQLKNPTIIFDGDSNPGPSSQLSVAQTTSPH

>SG-PL_contig_10439_1_-1_1_126

GFCYEQTSLSLTEKFMEKFIKILILLICVLIFFSHKVADLQY

>SG-PL_contig_10440_1_-1_274_399

MFAPACWQNLEPPCCTQVLRPHLKALPFFPSIPHQNSKLSNL

>SG-PL_contig_10441_1_+2_662_1573

MSHVHVLKFLLKAKSGSSRPKVGTHDSNGILRDEVRGQIGSPSWHVVDLFVFCQGGQKRG

ANLWLWFIGLFPFFFFFLIFKVILVLLDWVPPGAATLWQDVLHMVYPVGEGGVDQVTHGD

TLKIQVGHKVVTSLSFFLFFLLLIKLVETQGRNVSAGADARDLGSQVVGLVAREGGVCDH

RSIQPACEKFLDLSGTRDLGWCHWRQFHPYFSRVIADKVDLFVGACPVVFLGNCGWGLEV

WLGCVILLLLFIFFFISFLFCGLFFLCFFCLFLFLLLLVIVGATRQSLSVPTIPHAALLL

SHSG

>SG-PL_contig_10442_1_-2_2_121

QFNLKFGVQLPYIEEKRFPKDGTYPALFSCILESLKVAYN

>SG-PL_contig_10443_1_+2_476_757

MTGALRWAVNRPVFMLKAGIELQTSHKKTNTSTTWRMLLYKSGRSGPQLGHNHSNLRFLL

LLSVRLYYTIMGQYFHPIKTKREQLNLIWLACGI

>SG-PL_contig_10444_1_-3_78_239

MSDHKILMSCFLTRISSDLHLCISLIVTDGTVFIKWQVCLSVSSINYILTSFVY

>SG-PL_contig_10445_1_-1_109_801

MGSRSRSRSPSKSSSSHSRSRSRSRRRESPSHSRSRSRSRSRSPRSRSRSRSRTPSTPDR

RRRGSSWDRGEDTGVRVGTRIHVGSLSSSTSTREIRDEFGRFGDIIDLWMPRTQPAIAFV

VFRHREDALKAIKRMDGETLHSRRIRVSLARPRSQGVRGGSFNPNLRCYQCGKRGHFSRD

CRARQWSPGARGETRRERYDREHSRRDSPRRDSPRYSRRRSSSSSSGHRRA

>SG-PL_contig_10446_1_-2_41_262

MCFRTMVMCFRALIMYFRARIMCLMIIMNHVHVRCFNVTHYHLFVYLYSEFVCVCVCVCV

CVCLILFLVLLSLC

>SG-PL_contig_10448_1_-3_120_242

MTINISANRPLSSAQKGTQDYHVWYNVNKQQSVSTFLFHLS

>SG-PL_contig_10449_1_+3_3_164

KKRCCCRKCKQRKGLMQRPMKLHKLWSVLNQPHILLPQAMAFTRLKYINGMQSY

>SG-PL_contig_10450_1_-1_1_300

DLALSVLPEFRAKTQQPGSRPRSFRIPSLVSCLDPRDARSEERLLCPVRCVKYYLKRSAP

RRSSQRRLLISLNENYSRDISATTISRWLSATILRAYKEA

>SG-PL_contig_10451_1_-1_1_174

ALTVLPEFVAKTEFVAKTQQPGSCPRSFASHPWSPAWTPGMLGVRNASFAQSAASSTT

>SG-PL_contig_10452_1_-3_3_188

GIKLLHSLHSPSSVGVHAGYFHDSISHRTLACLTRSLACVFDVLHVYTHIGGSSTCWSAH

IC

>SG-PL_contig_10453_1_+2_398_631

MFWFLLWVLVWVCIFKLKCRTCLLSKQNQCFICSVISNQENFSIQQYLAPWFQWQLFCKL

VRSCYLNNSLGFFYMHVY

>SG-PL_contig_10454_1_-2_23_1540

MLASSFSRFSLALKGNAVASGRQGLAALYVSARNYSSDDNDLVVIGSGPGGYVAAIKAAQ

LGMKTVCVEKNDTLGGTCLNVGCIPSKALLNNSHLYHMAKDKDLEARGIEMDGVRLNLDK

MMEVKQNAVKALTGGIAHLFKKNSITHVEGFGRISGPNEVTVKRNDGSEEKISTKNILIA

TGSEVTPFPGIEIDEKTVVSSTGALSLEKVPEKMIVIGAGVIGVELGSVWHRLGAQVTCV

EFLGHVGGLGIDMEVAKNFSRSLTKQGIKFKLNTKVTSATRDGGVIKVATESVKDGKKEE

LDCDVLLVCIGRRPYTEGLGLDSVGLQPDNRGRVQVNSRFQTSVPSIYAIGDVIQGPMLA

HKAEDEGIICVEGMAGGAVHIDYNCVPSVIYTHPEVAWVGKSEEQLKEEGVEYNVGTFPL

LANSRAKCNNDTDGMVKILGDKKSDRLLGAHIIASVAGELINEAALALEYGASCEDIARV

CHAHPTVAEAFREANIAASFGKPINF

>SG-PL_contig_10455_1_+3_3_185

QKDTNAHLCKFALVPSHINTNTCVSVLHATLICTLFLCLTSEYICISLLMSYLYLLFVYF

V

>SG-PL_contig_10456_1_+2_344_637

MPWKIIISNKTCKVVLSFNFSKRCLSHILSSTSCHQTLQRNHLHVSIQHFIHSFHILSTH

THTHTDFGQSNSCTSFKKKENQKQKSRRKGIPKYLPTR

>SG-PL_contig_10457_1_-3_3_161

SVRRFLTEDATKTLVTSCILSRIDYCNMLFSGCPDSTLQPLQKIKKLSCPPYF

>SG-PL_contig_10458_1_+2_2_121

FFFHYPEFLLIIWLHKDINTKTLTYTNDTHRYVLSIIIIN

>SG-PL_contig_10459_1_+1_1_153

ILCNQLIFKAGANEYISSIQRPPLSYSLQINVYLVSFFHPKENYKSYYSVH

>SG-PL_contig_10460_1_-1_634_849

MSKTNKFRAHGFDNQFEPKHSCIIVGYVGLRLFANAVNCLNFFFFTAKFFQIKCRPLSPG

KACSHSTTPPFF

>SG-PL_contig_10461_1_+1_1_189

TLTLALSIPNSCISDFSMIHTFGPNLVKIHPERAEKILLTHFINTHTHTHTHRSRVIKMS

PFF

>SG-PL_contig_10462_1_-2_2_205

WRVATEHSEAYFINSSIDCMLGFRPPAPLLITVRSASLCTGQVSSLGKSIHWARPCTGQV

CFPVRQVN

>SG-PL_contig_10463_1_+1_1576_1902

MLLCCWWWSWCFCCCRCCCFIFIYLFIYLFVYSISVHYSAHTELLWQGKGAKQPIWKGLM

LLTEKTRAENCRNTDMPMSTTGKPLITKKKYCALETAGVNPLTPSVYWS

>SG-PL_contig_10464_1_+3_3_95

PQSTAGHKFYIILVPENSPSFKNYTTTLTNC

>SG-PL_contig_10465_1_-2_2_223

VVYLCRIFWPNPETACACHCCCPAGEADDSIECHDNFHWAVIANENVPLAWVLYNLHLSQ

GVRLLLYSCFLPIT

>SG-PL_contig_10466_1_+1_1_249

FQFHCFVHEASHGQRHELVYTPKHWRCLRILPNFGVLKRGKCTPLSLKSTVCTMLYEVIQ

RDNYVTLSDVCIQITSFSGQKSA

>SG-PL_contig_10467_1_-1_1_264

VSSSSPQLEGELAWHPDSGCWWDFRSPGVRVLASPARQPHPRALPLRGCRPPRHRQVLRT

PALLWAWWWGRRGGPPGREVEGVRYEGV

>SG-PL_contig_10468_1_-1_97_246

MVQYMKPKGVFFPKIHVWLIYLPCRTPIPTHPSPLCLVLSGTSVGLGHQE

>SG-PL_contig_10469_1_+2_2_130

QTCILKKTLLLASHTVPCQTVSRIQRLEEGLPLACEWGRQEET

>SG-PL_contig_10470_1_-2_3524_3829

MPAHAGGHVHAQSTHAHTGRQVGHEPPQATKKRLIYTMKQVSTFTSSWFRQRTLTWRSSS

SQTLHNLLGHHHQCQLLGSSRPPPRLLISLQGLSSHAATLPH

>SG-PL_contig_10471_1_+1_241_348

MMVTFHPSSGHQLQSAIKYLITVSPRVNFCVFVCLF

>SG-PL_contig_10472_1_-3_189_431

MIYHVILLHIWVFGIFLSKLCFISVKYSCSKCLKMSHYSTLLCFFSFILLYFFLFCIHPS

LLDIKILFSFFIIFFLVPIFL

>SG-PL_contig_10473_1_+2_2_184

DHLGFVCVCVCVCVCCCCCCCCCCLLACPVYLFVCLLDSYFFVFCLFVCLFIDSLCYSVN

C

>SG-PL_contig_10474_1_+3_132_446

MFSLHWTFLSTETWCRTRIPGIPIVANLYMEDFEKKALDTYPPCLESGADMLMTPSVNNM

NMKSETSPNTSTSKTQIFCSQLKQRTDTLLSWTLWSMALKMVLPN

>SG-PL_contig_10475_1_-3_1164_1442

MWCKKSWSTSVWKTSKLHRKAYQYFAVFLHCQFAALFDYQSESGLYYNNTKTKGHAEAEW

VPIQASFILGLWFNKGIKTYSCLCFETRGQVEL

>SG-PL_contig_10476_1_+3_42_200

MERKPPLQGKQQKNVKLVFFSEQEEIKISRNRSSANMNAPTKNKQTKKITCPK

>SG-PL_contig_10477_1_+3_3_176

HVLSCDFFSPILIQYVSYMFKCMFHTESSKAKNKNSLYRNVSQEKTSSKSLRKYHGLC

>SG-PL_contig_10478_1_+3_75_326

MPLYIIYIIHHTKDNHTFTAVYVFIQSLIRGSSLTLGKGEFWHNLFRGWVEVGACNKFSA

SRGTECINGWFVIMRCLRYSPKQT

>SG-PL_contig_10479_1_-2_2_85

CALFFSFFYGGWVYYCHSTLKPDYIEMH

>SG-PL_contig_10480_1_-2_2_343

LRSPPFEPLDVVPFSLLSYKTAFLLALATCCRRSEIHALSGAKDDIGFHSNGDLALTVLP

EFVAKTQQAGSCPRCFWIPSLVSCLDPRDARSEERLLCPVRCVKYYLKCSAPRR

>SG-PL_contig_10481_1_+2_53_325

MRISIGKTEVMKVCRRSGDLNIYIEEAKLKQVAEFKYLGSIFTEDGRLNREIETRVQKAN

NISYQLAPLLKHPDIPMETKAKLINSIFTPT

>SG-PL_contig_10482_1_-1_1_126

GVSKFQGNELDASRAIVGRIHDQGIMTKVVPEWGLQTKESYN

>SG-PL_contig_10483_1_+1_1_156

IYSVSAIHSQCQTTAAQTGIINRKPELLMLVHRRHRQNILQSTNCVSHFVLM

>SG-PL_contig_10484_1_+2_110_361

MFTIINNKYVQANGNWSVVKTGAYGCSVFSLSHAGNIPVTRICDKIFKPTQDTCKTSFAL

GQIEKDSERDALMTKKKVTKNSPT

>SG-PL_contig_10485_1_-1_124_273

MVSMMPIGFQMVYMMQNWFYIVHVMQIWFLYDLYHACVCGCLLFIYTYIG

>SG-PL_contig_10486_1_-1_112_2115

MAFLAEALRRAAGSLFRSCSDRQLDILVDLLLHGRNNSLMMPGKAQEATCIATETSNNTT

GTVQEATCSSITITTATTSCEKALPSTDKTLADTSAALKAGTLPSTTSESELAGTVQVLE

SASTTEAAAAAATEVMTQVLESASTVKASASGPAAVEVAEQSLSSSSPHTSTTSAHFTIL

CGSLATQDVDLTIEGVQESQNSNAETSQAAAAAKPAVEFQHCAKMDSSMEIWARAELSSL

QHKGQEKKVSSSKKQVIQWINEGRREAGLPDWDGTENKKEEEEDPLDCDAVDSSVLEVAA

KLLTQLSGKSHLPLSSLPGAIAFLHTWLSTEHSFAQMSDGLPPAQWIEEVKDTVARVYSA

MHDYVAQCRQQVSHDDLEEVVIVEGGGNEVAGASRSRQGGGKGGRRGNRGDNDDDDDDDE

VYSDDEDYGLDEDDPMKVLRRRQQEHHSLVTLPMILHILDPCENKTKVTRAMLCQAVIHC

TNLRAKEAASSGQFLDLKNKESVEGTLRSLHSHLVKVSAVDNSYSILWQRANAYLLHASN

IFAFLGYLMNTYKDRDEGFSWLLTEIARLSEGSRIPCLADKVKVMLTGRWEDHHVVSRGN

SCIPGKDVAKKLEKVLGEEAWSEIDLQLRGNLRIHVYRESDIPNRHGHCNSNPYIPPALR

LKLGLPPL

>SG-PL_contig_10487_1_+3_141_323

MIHCKIYNLYNNNKRFVNNAFYDAMYKYTDVFSYTLNHFKHEDQIRRRETMINFNFFRYH

N

>SG-PL_contig_10488_1_+2_2_205

HTHTHNILTAERKESENNKYLQFQFAQNCEQQHETDRNLKITEKSPQQQNMYKIFSIASS

SRKTIRTQ

>SG-PL_contig_10489_1_+3_3_254

FFLTGKNCFPSVDLNIYKSNVHLTSKSIKTNLAQKQNEIKNTIWQREILLLCSLYSPCNL

SQISRLTSYIHDILVSEWFLFLTL

>SG-PL_contig_10490_1_-1_13_798

MDVVLNFSLEVHVIEVKGLKSLLPNKIVYCTMEVEGGEKLKTDQAEASKPAWDTQGDFTT

AHPLPSVKVKLYTECSGVLSLEDKELGRVVIHPTASSSRTPELYRMTRSAKNSPDDLHIK

IAVRMDKPQNMKHCGYLYAEGKTVWKKWKKRYFVLVQVSQYTFAMCSYREKKPDPTEMMQ

LEGFTVDYCEPRQGYEGGKYFFNLVKEGDTLTFATEDESERTLWVQAVYRATGQTHKPTP

PVQQTNKISNTQISRMQGDADQ

>SG-PL_contig_10491_1_-1_487_828

MYSFVKSCRLKIYSPPISCSFCLQPETRRAISELIPLPNLMHGMFTLSWLFVTKAFKFSF

HLLSQRASSLLSIYKQNIILLSIQSSVCVQAKYYSALWVGVAFLMNIMNILLCL

>SG-PL_contig_10492_1_-2_59_283

MQQTKVFLLACLIMAVLSSAWARPHGSATNQELLDIAYEWSDTLNEVVAMLNTERRPSRR

RRMVQFHIGRHHHHP

>SG-PL_contig_10493_1_-1_1_591

IHSITARSLTKEQLQKLREAGNTTLSDIIRLKEQPAHIKTTSSSLSVNLSPVQQQQQQQP

AIKRQIQTPKLLEPAQYRELDPSKQPVLIYPEMEEETSASATPAASPRSLSVATSRLESA

VSDTDRLVGATRAAALSTPRAMSAEASESQELVSTPKIRSGCISARAAFWERRIMQGEAT

DVEVEEEFPEMVQDSQA

>SG-PL_contig_10494_1_+1_1_114

KNIRWPSHTSNVPFLASSDIFCIADIENRPVIFKCTEL

>SG-PL_contig_10495_1_+1_1_150

FFFSPPNKQNLQLKPEMGQLHHSTNYGMTGHLLFTCHFNPTAAKMACKNS

>SG-PL_contig_10496_1_-2_2_118

RKQRGPVLFCLSISNILDSFRVEKRARFAGCNGNSMSKF

>SG-PL_contig_10497_1_-1_13_774

MSARMACVSTLLACCFLTAVLAGEEACYSYAGGSVYPQETRRTSGHTIQWSQAVISKPAP

DWNGTAVVNGEFKDIKLSDFRGKYLVFFFYPLDFTFVCPTEIVAFSDSIGKFKSINTEVV

ACSVDSQFTHLAWINTPRSQGGLGPLKIPLLSDITHDIAKAYGVYLQDLGHSLRGLFIID

PKGILRQITMNDLPVGRSVDETLRLVQAFQYTDSHGEVCPVGWKPGSDTIIPDPKQSQKY

FKKQKDPEGEKEEL

>SG-PL_contig_10498_1_+3_66_173

MSNYIKNSVNMYMSACFHVHIFFISIQIESHFITLY

>SG-PL_contig_10499_1_-2_2_241

LEDSEVQQLLVNLLSWLAPGGYLFIREACKYPSGDLWKTFTFNPTQYRDPSHYEAFLSTA

TLPLDGTHVYGFDTVLSKSV

>SG-PL_contig_10500_1_-2_218_349

MFAKIGGIGSCLLNQIQNYLCYIDSMSSVCFLNLGYVFVYIWVI

>SG-PL_contig_10501_1_+1_208_405

MTPTEKNWGSRFHKLNTQCNKDIFDFSLSNLLSEFTKQTKKEPQQAIKQNEIYLCPSTWA

NIIIIT

>SG-PL_contig_10502_1_+2_194_469

MPLRIRLSSLTDSVTSSSSLIHFGDSEASDSARAMDSSFFTCDRKIFLLIFRRQYQNCSC

GTIQGSLVFGKSTKTNLKRSASSRQVYTKPEH

>SG-PL_contig_10503_1_+2_62_262

MKCSGTYMLDTRQQVPWSFCCVLSIYICISVCEGLFVFVLILVCVSSYKTKYIVGVRFFS

VCLSLCI

>SG-PL_contig_10504_1_-3_1197_1613

MVLFCFLFLVSLCTITNLTLSNWNGSLFIVFNCEVLGWMDFFFKLLRLSSNVGCGLCYSE

SYLSLPWHNQYVYDKFGNIYTHCLKSERFIVYSTVIQSRLIYVVSKVRYNVYSTIIKIRL

IYVIIQYIYIYIYIYLYIY

>SG-PL_contig_10505_1_-2_209_331

MLHVMLKLDFLLLLLFSYIQIINCICILFCCYCLHIYCLLI

>SG-PL_contig_10506_1_+2_86_382

MSGKNKESLALFMVMQWRKLPPEEFCGKTVYESLIQGCFRLSPQDDMKRVIMETLQELAS

DHVWIPSLASCKACIWCFPFYHHQDAKYRCLSAVSCTPA

>SG-PL_contig_10507_1_-1_1207_1383

MDIVIIFVLLVSKRKRIVSESFNFFFHFLFVFRFCSCFCLLLVNEFNLKESRMLQVPLL

>SG-PL_contig_10508_1_-3_153_1631

MWLGRDQNRCDSPCNLKWVKQIKILGVHYRSDVSAQQIDDNWTKKIETMKRVIKQWSRRN

LSIYGKIIIAKTFIISQFIYIMQAIGLPEDVLHEINRTLFAFLWKKKYNNKKAFEKVKRK

VLTQDIGQGGLKMLDMKTLQDALYLTWLPRLLTHENNAAWKAVPIAIFSHLGRGLDILTH

KCKPNEIKGIPRNTGPFWPTVLKTWLGLKMKSHDLTDAIYLDSPLWNNENFKYKNNILHL

NDWANHGLHTIRDILNDNGEIKTLQDIVNIVGPSAGRILEYNAIKTTLLQAIEQNRLHNT

YRNEDIQYLTFSDKPLHMITCREWRTALTEQILPCAVSFWHRKLNVEINQNHWEATFDAT

KETRLRVLQWKIIHNIYPTNILLNKMGIASSNKCNACTSGETDYIEHFFFNCLNIRPIWK

IIEREISIRIGSHITITETIALLGYHDKDRHTTQERTTINWLISLAKMCISKYRYGDSVK

IDLILEKELRLRD

>SG-PL_contig_10509_1_-1_130_249

MAEMLLQLLRYTKITKHRTFLNLTRDFKLRHRCCHITQYT

>SG-PL_contig_10510_1_-2_2_229

INTSKRFEFYRTFKSNHFQEEYILVDMNKYVKAALVKFRFGISDIAVHSLRYKIHTDTDV

VCPFCKAFMENELHFS

>SG-PL_contig_10511_1_+1_151_369

MPGATDTWLYQIKNTMQLIAVIGPTDLANKTTVGLCGGQFISVFGSPILNMAKWETIINL

ERSDYDDCANCYV

>SG-PL_contig_10512_1_+2_200_295

MQASIWNQMSNHFIKKSSDQYNTGLPATSSLC

>SG-PL_contig_10513_1_+1_1_108

VQKLYFQRKVSFNIDKMRICSKKSDRLPDSTYFKHI

>SG-PL_contig_10514_1_-3_57_4076

MSSSPAQENDRGDPRSDRSRDRQDFDRHDGPTRRGRDRRNGVPPPRLANRGRGRDRNNYR

GDRDRDRGGFGNRGNFGDQNDRSSYNEVETVNGEEDWEVDITASNDLKEQRSSKDYRDQR

RGDKDRGFGGDRGFGGDRDRSFGGDRDRGFGGDRERGFGGERDRGFGGDRDRSFGDRERG

RGFGGNRGRGRGRGGGFGRPSSRGGRPRRFDRNDSSEGPHIDTWTNETAENAEKENSGWG

ETWAESTEDWCEDQWTGSLEDTKVFTSSQTKTPVEADLTTNVSDSTNAIGQRLDVGSLFS

KSAEFAKAPDFTKTGEPTSGDSFFASYNQQAAESIKNSIGIGSSSARQNLSNMPASAPQS

LGTLPAMPQREMSHAQTQPPRSSVPSILSQATSSAPSSMHSSSTSNAPPSHLPPVPATAP

SASVSTPPMAQPAQPQQRRQQRSKLPPPSKIPASAVEMPGHAIPQQLDVQFGVDFGAESS

QFGFGSSTPNTNTPFSSSSPASNSSVTSHLGESGKSQEASRQVHMVTSPPAGSQTSQSSM

PSMEASPARPSVFPDSVYASPSKSESPAKSPAPEHKSGNLMNSSRSSQSSSTMSQGSLST

SKQERDTSNMVPLSMANGYAASSYPTHSAHSQSHKSSSSSSQTYRQAGPVSQTSVASYPN

QFSSQTQYSGAGSNQQNQYPSAGQAQFPGSQGQFPSAGSAGSSAPTPSQYMQSQAQYPSG

SQSSFSQAPVASQGQYQAGGSQYHGNYQSSSVSFQSQPSSQSGQNPLYPSTTQQSGGSYP

SGSSSFHVRDSQSTGNMQPSVVNLQQAAGSLQPSVVNLQQPSGNLQQSVGTHASPAVSQP

SNSYQNQQPAPGLTKATTAQNTNAYNTQTYSGPHSGQHASPTTTQTPSAYNSQTYSTQHA

SLHNQATTTQSPSSYNTQQTYSTQHSSQHTQATTTQSPSAYNTQTFSAQHAPQQNLQTSP

LGNKLGESLSKMTIKESASIESRTSQYDHVSSSSTTVSLTSSSSTANSSSISTSTSTVSA

TSTPVVTTSTLASRVTQSIPLTTKAPPNLPPGVPLMNPHLFMGQAAAAGGLPPYFAGLQS

LQQPMYGYEDLQLMQPARLPLQTGNFYDLAAYSGVPSTIAAGRDQAAALGTAPFTGAATD

NKQMNRVEAQSPSQSTQQQSTHNAPQQTLPFNIHYSYYYPGAMLPGAAGFQYPTMFPMPP

VTNTAAAHVPGTTTAAQLPKTYGSHQGFPSKGTGTTADMNTGTYANSRTQGFDKQGFPAG

TPPPFSLPLANASQAGPLGAPTAPFGTPFLPMMPPQAHNQTLLHHPMAQDSGTGSNRSLQ

QPTNQGKPAAAKNNYYPSWN

>SG-PL_contig_10515_1_+1_961_1170

MQMGVELRRQETKGYLNSGSPVLCKTPLLTSGFSQLTMSRDMLSVTLVSSVSVREKGDMC

TKKDERTFCW

>SG-PL_contig_10516_1_-3_3_104

SHSLPHSIPLNGTDIQLSKVVCNLGITLDETVSF

>SG-PL_contig_10517_1_-2_2_238

QIIFVNGHYSSPSPLPYGVPQGSVLGPILFVLYTQPLSEIVSSHSLPTHKFSDDNQLYKS

GNISDLDLKSDLLNSASLS

>SG-PL_contig_10518_1_-1_232_357

MTNNRLSLMTIKLKWCLSHPKLFFPPTHSLIPFLSMEQTFSC

>SG-PL_contig_10519_1_+3_45_278

MYLTLFRAGKCKAAREMSETHPQKVDMRVVRSRPTSLSYDFRIMGLSLPLTFTFYSKIHN

YCPFVLSLFCLRIIDVGK

>SG-PL_contig_10520_1_+2_149_505

MEHKKEVTKENYDKSAGQPLLCLHESQAVQVFDKQQRIWSPAEVLSKSTEPRSYNVKTSQ

ANILRRNRRDLQENHGGLSTQMPKKPEFPVRERFSRYIHQGDQSYFNTESGPTRDTSNR

>SG-PL_contig_10521_1_-3_3_221

HLFALKKFLGVDRRTPNDFVYGETNRYPIYVNSAVNCIRYWLKLLHMERSRIPWKAYKML

YDLDASGKKNWVS

>SG-PL_contig_10522_1_+1_1_171

QGTILVQLPKKISNTHSHPTSPNTLKPAHPHTHHSPTFGNVLLQNICETCMRTRTIT

>SG-PL_contig_10523_1_+2_2_214

VPVFVSFVKNEHSWFCSLTKHSYSTLPCFWFCIKPHQYTHRYLSWHLLDLCCVVHVLSRI

WNHSHVMMSFY

>SG-PL_contig_10524_1_+3_540_701

MSTRERRSHDYYNYLLKYIYYKYLLKSHVQITQIPPEEGLFSMCAYGRSTDIPV

>SG-PL_contig_10525_1_-1_2005_2217

MKLSCQLVQQFIYCQYFNQFVAHPYSPLDFFFWFLLFRCCLFFQSFISFITKACLFLSHK

LYSSLGYLDRN

>SG-PL_contig_10526_1_-1_82_261

MATSPWSVPGKATISATRFSIIIIMILVMGHFSFTVIISNSLSSTHSIHPRFLPIQQRST

>SG-PL_contig_10527_1_-1_289_1035

MHYSGDAVAPMAIQTLDPNFQNVIGQRGGMSFRDVMVGNLMYKCANMLCGRQSQDCTRFG

HDAFLFKRKGDDDCQCFCPGNSEDEVLMPCESFRHTTRPPTTTTTPKTTKEPTERTTTEP

VPMECLDLRDDCAQLVAAGECKMDMERMFMFCKKSCKFCEKDKMCMDYDPTCPLLALANF

CDHPGFESVMAMKCKKSCQFCTPVDPCQMQLEMGLADVPGTGHTRSSSIWSLLVLVLVWA

IHHLAGRNL

>SG-PL_contig_10528_1_+2_2_106

TLSRVTNYKHTATVTNHHLRNLISICLNTIDLSLK

>SG-PL_contig_10529_1_+1_439_609

MLLTRASSSFSLVTHTPSDQHFQFDYLTHSTAERPPAPPKSPYGLTVQCLLAPAKHR

>SG-PL_contig_10530_1_-2_434_664

MDIVSVEVLYVCVADRQTTELTLVTSHQYLSCDISDHVYHEGQMTIHCSRWFCYFSAIMD

VSVIAPRNHLYNYCVNS

>SG-PL_contig_10531_1_-3_3_251

LIHKEPNDNFWRVEVLFSVHLCVYVPLCVCVCVYVCLCMCVCMYACVCVCVCMPVCVCVY

MYASVCVCVYHYVCVCHCVCVRE

>SG-PL_contig_10532_1_+3_3_95

FFLKQFFSDRWSVRSELHVILHLQCGELTGT

>SG-PL_contig_10533_1_+1_67_306

MSRLPFFLLLCPTALELSSLAHQTLLFSCCLQVQPQNLSLSAVLSIAIFTDFCHMTDACY

VDVFTCVCVCVCVRVMCGVA

>SG-PL_contig_10534_1_+1_46_354

MGTALIMLALRKVEVSATSLPYDLRVMGQPSPLPHVLSMLLLYLTCGYAIYPWCASVLEK

ATFCRVLCIHMHVCGYVCVHVHVFLDNLITLFRNSLIFYSVFH

>SG-PL_contig_10535_1_+1_1_192

NAIHAIFSPRCTRGLPVVRQSAYILGDPQSVKLGNANNNIPKPTFPEVSNTMQQSSKMER

LNIY

>SG-PL_contig_10536_1_-1_1_705

ADLIIKVEEATGEAKLQSPLDSASATLTLVSEKGDNSAGPSNEKSAEKTEKRKGKSKFPI

QPELKRTLTGLDSSNTIAETPVTVAPSASGKTSILPGKEGTSQPSTNQETVRVDGDAVDT

SRSQPEAELAAQTDRTAQSASPDTRRSNSSKRQESGPQGAYSPKPGGSVSKSPGPKTPTS

TPKAKSPDPQRPKTGADKSPGPAGNRSPSPNHKPVQQPAQSRSPSPTRVIKETKK

>SG-PL_contig_10537_1_-2_29_163

MRNSSSSNLKHCCCHHQIIIIDIRLLLLFLLSSSSSLLSSSPLPS

>SG-PL_contig_10538_1_-3_3_197

LCVSIWEFYYCYFFVWYMFLYGKFITFILSDMIMRSMWEVYYVYFVWPNFVFLYRRFITV

LLRLE

>SG-PL_contig_10539_1_-2_122_286

MVLIAPKTVLSSHSLPHSIPLTGTDIQLSKVVHNLGVVDVTLSFQQQITSICRIC

>SG-PL_contig_10540_1_-2_152_304

MTNNKLQLNDDKTEMVLITPKTVLSSHSLPHSIPLNGTDIQLSKVVCNLGV

>SG-PL_contig_10541_1_+1_22_345

MHVSTAFLQLFSGFVDNVIGMLDLLRQLVQVTAQVDHADLFLPSDSLLWRLGILLRFNDA

VLIILVVVCAEVKIITIIIVQNPIIITHHEIHVLRHLAVLSLRLQMYS

>SG-PL_contig_10542_1_+2_38_277

MQVTMGSCNIHLLRTMLVRVSSGNSACSSIKMESESEPRIFTNTGFKFTMSALSMKCCSL

FTLNVSVCYQFGKLKDLGEH

>SG-PL_contig_10543_1_+2_2_208

LTWPLPAGTASASIISNVPLPPIVTVMSKQVPHSTFAMSAYSQDMDNLISDIEVIVLKSK

QPYLLGRHV

>SG-PL_contig_10544_1_+2_2_286

WNLSAQYLASVVCHYCCHSHLHVPVNRQKDDHLLYYCLAKKSSAEPAKWSIFAVNYFVGR

LSNMTLPTRSLLCVNEQRLAKSQQITREKLEIPVT

>SG-PL_contig_10545_1_-1_82_312

MDTLCNCLLVQSHHCSSRPLQMLAWWSEWMSETARACLCFTQNVPGVSWLQYVSLALLLW

VLLVLNALHFLPIRFIF

>SG-PL_contig_10546_1_-3_1080_1352

MKQYIYMETDGKRMGEVQMVVCCEDLSVYELFRWCVADHVSDWDRDRLIGLSWKLLIDDD

IVGFFLLFPHPPPSPTPQARLVIKCSSCRVC

>SG-PL_contig_10547_1_+2_2_70

GLTYPIKTTSINDCVFSYKNHVY

>SG-PL_contig_10548_1_+2_473_655

MATTKSNLEVIFSSIIYSCLHNLDMICTATHICNNILKYMDFSVFSPFLLSIFPSGPNPM

S

>SG-PL_contig_10549_1_-3_3_176

CLFFVMYLFHFIFVSFYYPSLTDRVSTYSSVKPWMIKGELSPGVTSLALVAFHVGFRN

>SG-PL_contig_10550_1_-2_2_208

RLPGREVRHPYTVSVPASGPAPGPPAQAVSPAGTDLGTSKESASPLGLRESLSPILPLGR

AHKRPLQRD

>SG-PL_contig_10551_1_+1_1_141

CKPCIFTHTYIYIYIYIYIFAFYGAVCVRGDCAVYNRGRLTSPTQMA

>SG-PL_contig_10552_1_-2_50_274

MLARLLEEACEELSLPLSVLFNKSVDTRIVPRDWEIADATALFQKGELLLNTHSTKYQRM

NHKNKIEDVSPQKCE

>SG-PL_contig_10553_1_+2_50_244

MTGREKFLFFAFYIQSKLLVNEKILNCKNILTYQQNPNHPRPFSSTVHTARSASNRWGKK

IVIMP

>SG-PL_contig_10554_1_-3_282_1301

MTSLRAVVCFAVCVLVFVWSAQECQSRRGGGSRSSSRSGSGYRSSRSRSQRYRSRSRLVR

VRLGRVRIGSSGFTSSTWKAAALSGNIYGGSRYLQSDFYRTHPDRTPPICTNNEAKAPNG

RVYGYFICPRETEPANYTYCCGPANREFCCPRDYYGSSTSASTDKRSPVPAVIGVIIAAI

VVIVAVVAYKKRKKIRKFFRKKAHPDKSPLEDTKREETLGDEAPPDDCYQGNDGAFSNHS

NNNNSSYPATEAETAPPEELFLATETKTAPPSAGPFPAAAPPSAPPVPANFYHPPPAPYC

PNYSPDCLPPARPPTPPPLDPNRPFVMPPDPIPYPPKEEI

>SG-PL_contig_10555_1_-1_142_714

MADSVELEIVVAFLDKDQGFAFNNTFPWPYLKGDHEFYINLTTTRKDPKKKNAYIMGRVT

WEAWTCGDEKCNPNLFTVVTSNTLKSEGVPYLQKVLPTLNDAITYVTTPPVSDHIEKVFV

MGGGRNYNECIDDARLKRIWITRIFGDFTANIFFPKFQHKFKRVKCDKLDEGMQEDNGVK

YQFELWERFTN

>SG-PL_contig_10556_1_+1_1_111

YAIKTIHNVRISNKKSKYFTIFIDQAVDEKLFTKFKM

>SG-PL_contig_10557_1_-2_35_670

MAEEESLSGKQAKYSLLPSVGGGESQSRRKAWIVSLDQLAVADFFDPLDDFIRQSHEELL

ATALANCRMGEEAGCPVATPVPSTAALHCYARQSAAKRWPEKHRDAGTQQQMKQVWQLCV

ALWGDPEELEEDTEREGYAECQARREAFSKWLSLTAVSKIQCEVAASAHKPEGHLQGILS

HLSGHQISEACSLAQDSGDDRLALLLAQAAGS

>SG-PL_contig_10558_1_+2_230_397

MEELMFQPVKTGPATLTQPTTLPDKGFMKSSKHRLINSLQTLVRKLLFHIKKNPTT

>SG-PL_contig_10559_1_+2_2_211

IYFNIFESLRFCKAQEQSTHTHSSITHHYYENKTKTQNTLVVECHFIGFLSKHVTFYKHN

PRLLHYRMIS

>SG-PL_contig_10560_1_+3_3_242

PVPGDNDITFTQTALTPCSTLICHVHFSRSQTTQCSFPVSRVCVAFAKQITPCSPFVQQD

LQNVPLPTCQLFQHANINCH

>SG-PL_contig_10561_1_-1_2548_2886

MSKGQLHKLGFCISSRNICSLFINNNNLIDYILLQYYTVSWQVTSHVEQSVNQLADTDTL

ELPPQIICPQPCLNATHVCHSFLFVQEQALSRSRKFQPEVKKTFHFKGVTEPF

>SG-PL_contig_10562_1_-2_854_1132

MRIEFILPLFNFIVCLPFVFNFYMQCLTLENVTDHLLFVALIFLYVLRVMFLMQNYLAKN

HFKKNLYVCVFVPSIYDSVMQVLYDRKTVVIFD

>SG-PL_contig_10563_1_-2_2_304

QAESECWQQRWTVVGRWCARQASGRWCHIVLGRGGWRQVVPKAVEASSSTAPRSAGFVTQ

PQLTDVLVDLSGEVTFAVIQHTLSNLLNKGQIHCGRIVWGG

>SG-PL_contig_10564_1_-3_405_857

MWSCQKFTFIYLRNSVDKMCCMSPLLILHHEIINIFPLSPSHLSFYDIWESDSDQPELGS

VYVCTCMTERRRRECEHLPHSWMIIYVPVSKVHVSGKPCLTGCRREIVKCSNSLFSDAVE

LCSEEMTYYSKFINDSLLLSKCVCVFGRQSV

>SG-PL_contig_10565_1_+3_333_566

MLSLKAILKVICHVFHCWLQEGIYCSVIYQFKILSNRHAYSRQMYTRNCSYFCVLSLQEI

LEQRTNTGSSNRPGRRIE

>SG-PL_contig_10566_1_-2_2_247

LPHVTNPSSLPRSVHLLILQASPLPSTARPLAVSRVKERHGERLEQDVLVFPLYSPLVDC

IYVLSACSSSLFDRDQSFDQSS

>SG-PL_contig_10567_1_-3_963_1268

MPEIMIWKESCIQTFYLVRYMYTRDNDIIYTKKSAYRHFISKRYYMPEIMMYKEICIQTF

CLVRYMYARDNVQRNLHTDILSAYRHFICIQTFYLKKILYAR

>SG-PL_contig_10568_1_+3_153_656

MTSLFLCCVGTLCDVIVVGGQVVIRLDADVMKSAVVGAELSRDGDKTDQNFVSRHFEVPY

EAVFHGGGKREVELRRQSSDALHSGEHAGVAVTAHHQVSTCPQLHHHHLSGFLQGRGGHV

GQGCLEEDRNRVSSHIGRHRYDRHLGLTDRLNAFFFFFFFVFLSFLLN

>SG-PL_contig_10569_1_-1_1_246

LSLGFCFRELHSLVQPLEELAWILSTTLGRSGKIVFNSFASPHVIHFQLKWLIPGSPSSV

RCLAASTIQGCRAVSGLPSFCP

>SG-PL_contig_10570_1_-1_127_282

MSLHIYLFLLMFCFIFAMHTVVSCDLILIFCSYSASFLMFVIKGLCLLTFGT

>SG-PL_contig_10571_1_+1_1_156

MNSQWCGMRYPFGIFRNCLRSESEGPPVLALRHQNFKQGKGKYFNFSTGIPQ

>SG-PL_contig_10572_1_+1_151_258

MQSREYRNHRHEKAMESGLGTSSGKSRIASPRLPFA

>SG-PL_contig_10573_1_-3_72_320

MSCPVVTRSEYTVIGNEGEYLSLMDEQGMTREDLKLPSDPDLNKEVREKVEKLDSGDTFM

VTVLKAMGDEQVIATKMDRADKP

>SG-PL_contig_10574_1_-3_3_143

KKKTLTHTHTHTHTHTHTHTHTHTHIYVYKCHEAEKNCIDFLNNLFS

>SG-PL_contig_10575_1_+2_176_298

MCYVRMSKCLKVCEICTYSVKKEIWLVSYTGMGIEKFLKKD

>SG-PL_contig_10576_1_+3_36_239

MCPVAGLLQAGHDSHLLSYSLKGLTLSGLPTTHNLRGLLLITIQVCSYLYSENKLTSIHI

FFTFTLLI

>SG-PL_contig_10577_1_+1_1123_1374

MSPHAKDIFMDISYVFTHIILDLKQLTSFQLTNLHAAMSGINMVNLKILSACRAYAQAYG

LCHYHFGLFSILWHSQRWCIWNLF

>SG-PL_contig_10578_1_+2_2_145

FFLRTHFIIVHTLQSYTRYFQTFRPGVLNDWFEKMGRGGGIRVEAGEL

>SG-PL_contig_10579_1_+2_2_154

AQEAPEKWHLSPTQRENSITAQPYTVFCLRARKPSGDAAPRSAGGRKETGR

>SG-PL_contig_10580_1_+3_3_164

LIYYEHYNYQFVQHYTYNQHLHLTVNICTLKSTVVHNNQHYNYSKYHQNYNNSL

>SG-PL_contig_10581_1_+3_177_440

MIITDTGTTKTTFQKVSLQKEKSFQQTCHHILAVHFRSNLQLSKFILLTHHSSTVCLFAI

RITHIKLYNDIAVNHPSVVYLQSTTVNA

>SG-PL_contig_10582_1_-2_86_2020

MATLSANMYPDINEQDLVTEVHEIVVQRVGHVPVAKGDFSKLPKAVKIFISHCIQLMKPR

GVYICDGSQSEADEIIHKLLERGTLTKLTKYENSYLCRTDPQDVARVEAKTFICTPERYE

TVPHVREGVAGILGHWLGPKDMQKELNDRFPGCMNGRMMYVIPFSMGPVGSKLSKIGVQV

TDSNYVLLCMRIMTRVTPKVWDVLGDGEFVKCIHSMGCPRPVPRRVKNHWPCNPEKVLIT

HFPHERKIKSYGSGYGGNSLLGKKCFALRIASVIARDEGWLAEHMLISGITPPDGKEMFI

TAAFPSACGKTNLAMTTPTLPGYKVQCVGDDIAWMRFNDQGELRAINPEAGFFGVAPGTN

MQTNPNAMKSFQKNSIFTNVGETIDGGYYWEGLEEETPKGIAITTWLNQPWAVGMPGGPA

SHPNARFTCPASQCPIIHPKWEDPDGVPVSAIIFGGRRPKGVPLVFEAKSWEHGVMIGAC

VKSESTAAAEFKGKKIMHDPMAMRPFMGYNFGHYLQHWLDIAKVPGRKLPKIFHVNWFRT

DESGQFLWPGFGENIRVIDWILRRCKGEDIAVETPIGFVPKPGTINMQGLSKINEKELFS

LPKAYWLDDTQESRRFLEDQVGCDTPPIIVELLDKQEAAFKAMAK

>SG-PL_contig_10583_1_+2_149_277

MALPSSTQSTGNSQSQVNLVKSPDKKRQESPVITSFSTKCVCF

>SG-PL_contig_10584_1_-1_1732_1971

MQVPLLMSVCTYSSRNCSIYSFCFNVNPCSFSLVVICVSVYSGRNPYMWSLCLMVKPTIQ

VLVFVSVFTLKIPSTFSFTN

>SG-PL_contig_10585_1_+2_392_595

MIGVLRWAANKPFLCPRLRLKSKRIFRVHSSITLLFLRCPEVWCAKKTQRRGIQKKQQTP

YVLNNNLN

>SG-PL_contig_10586_1_-3_3_221

ISRPFQCIGFCAGSRNLPDNYWQIWVERIWNFPSAISWVFFVFFLFFFNKLHKVDILFIL

DLKYVSGRVCVCV

>SG-PL_contig_10587_1_-2_2_166

ALIILVVLVFHRSCSPVHFKTFPVHRVLHRHKESTRLSLEWVEWAWNCQSAMNPS

>SG-PL_contig_10588_1_-1_94_1083

MGRSSKDKRDVYYRLAKEEGWRARSAFKLLQINDDFNIFEGVQRVVDLCAAPGSWSQVLS

KKLRGGEGEKSDAKIVAVDLQAMAPIPGVIQLQGDITKKSTAQEIIQHFEGEQADLVICD

GAPDVTGLHDIDEYIQAQLLLAALNITTHVLKHGGTFVAKIFRGKDVSLLYSQLKVFFPM

VTVHKPRSSRNSSIEAFVVCQNYSPPKDYVPNMSNPLLDHHYDVDFNNLEGPNRVIVPFL

ACGDLSSFDSDKTYPLQLEDGAEYSYHKPTQSPINPPYKEACYLRKNNLLAKPTQPNLSS

LQASQDSPASPDCSSNVPQTPEIEPAPTSI

>SG-PL_contig_10589_1_-3_3_935

KSSHREKLKGDFLKLLKETPGLDRHSRWSEVKKRINSDPRYEAVDSSSRREDWFKDYIKT

LEDDSEDEERKEREKQERIEASLKKREEEVQRTLSTSLRERDKEREQHKKDEAIQHFGAL

LADLVRNCEATWRETRKQLRKDHRWDLTDQLDKEEKEKLFEEHIQGLTQRNKAMFHKLLD

ETETSLTSTWKEVKKMIKEDPRYSKFSSSDRKREKEYSEYMSEKFVIAKTEFRELLKETK

AITFKTRKLVDENQLHLEEIEKILENDKRYLTLECVPEDRRKILMMHIEEMERKGPPPPP

TASEPSRRNPK

>SG-PL_contig_10590_1_-3_273_473

MWHAETRQVKGKNVKEQRWHFQAELMSEIADGCENPNLNKTGTPARVTPNRNGKKNIPQN

KQINRAT

>SG-PL_contig_10591_1_+2_1253_1483

MRNELHKNRSYTQNHFYFYSYKNLFKQYSDIHNQVHFYKYKQNGTIQNGKHQIQTGQHKT

ARIKQKVTIALCKSKVC

>SG-PL_contig_10592_1_+3_3_224

HTHTHSEQISKRFNGLISTHVLQWAIVSVCPTRSPQLPRNPRLNALPHFHKALPLFHKPP

HFHSLLQRFHEGPD

>SG-PL_contig_10593_1_+1_106_348

MNKTTTHNSSSIQITNAFERRQSIRFRHSERRGWGVSKIVIVTTHYHSLPPTLTNVYYYY

YYILFIAQPTTMAISGLSPRK

>SG-PL_contig_10594_1_-1_1_303

MNKSMSVCSASVAAWGHFLIVYNCTGFFFSSSVRLSLASVAAWCHFLIVYHCTGFFFSSS

VRLSLWRASVFDLYVMHLQLILLLICVVIAYELALSRCCAV

>SG-PL_contig_10595_1_-3_645_812

MLQSFWWCQCFIGPVPLPISSPCQHLGFLQAQGVLDTYCQIWVEHIWNCLSVMDRS

>SG-PL_contig_10596_1_-2_125_229

MFPSKCMRFCVGFRIIRFRVVNVCTYRIFCPRVFY

>SG-PL_contig_10597_1_+2_2_268

PAADSIIPGSLLYLERNTCSLFFASFGFSEPDFLFIFEIFCSAHFHKQHCCLEKWHSSLD

FKTRYNETSHFHVWKTKTTRAFMGKSGTW

>SG-PL_contig_10598_1_+3_3_200

AQLFLFCFMKFIQVCFLYRNTVKSINKLLSVFQVIIFKKGSRPAAYVVYCLYKILPSPSQ

TFFCFV

>SG-PL_contig_10599_1_+3_168_413

MQHLHMHWNPDSFKISGVWFTNDVNGCEEIIYSEKSETGIFFPSKLGEKKCNTPRKSCDI

KYKIFDLIQTTSPIDFSPRSSG

>SG-PL_contig_10600_1_+1_439_576

MCFSFVWKGKHDRINRKTAKKKRTKNKQTKNNKKTHTKWRFEHTRY

>SG-PL_contig_10601_1_+3_33_167

MQADMTYLDGGDIFSHSVKLASQGTTIFLNKSESERTDRHENFVS

>SG-PL_contig_10602_1_-1_10_240

MFVKCTVNKFKVPCFEIHSSSVHAAVCYLKLSSIKNAEAWMFQRAFMSVYCVLLCLYVYL

KAWTYYFLFVLNGTRLD

>SG-PL_contig_10603_1_+2_2_91

SLAFLFVYFVSNKVGFYVFDKKEFLIKNIL

>SG-PL_contig_10604_1_+2_2_91

SLAFLFVSFVSNKVDFYVFDKKEFLIKKIL

>SG-PL_contig_10605_1_-1_46_294

MLDLVFCKPVDNIHISATLDHDLKTDHTSVLSKFLISKPSPQPPSTEYHCFGKIDKNGFK

SDLATSVSLLLRSGLQQSTSFGS

>SG-PL_contig_10606_1_-1_499_1320

MMFPLVIKSQRNVAHMHNHMPTMTRLQEKFTLARQSGNAVEASRAGHELMEFMKRNEIKP

FRNFLVPLAQLPVFVSVFVGLRQMANLPVESMKTGGLFWFTDLTLPDPFYAMPLITMATF

LVTIELGVDGVKAGTMNHTMKWVMRGMPIIMLPLISNFPAAMLCYWFTSNAFSLVQVLFL

KIPAVRTFFKIERMVQHPPSALPKKKGFLEGFRDSMGNAKLAQQMEERQRVDALNFKKAG

QGPIQKTYSYDPTKQKVQTSTSKSKPTSSSKTKS

>SG-PL_contig_10607_1_-2_2_220

CKDKQHCCPAGTQCDVINRRCFRQDTNWEQPWVEKRHAKIITSDEMTQTPTETLLKGDAG

ICKDGQMCMDGQT

>SG-PL_contig_10608_1_+3_3_173

TACHAQSTMHSKVSLTLLIDNLLCHFNMKKLKNTIPMLTKSTCLLCQYRLIKTELSF

>SG-PL_contig_10609_1_+1_286_501

MHFAVKFCMKAEIMYSPFRPFDDLKKKEKRTAFVFFNIAYTMSSFHTISVSLCIKPRALL

TCNQLWIVLPKL

>SG-PL_contig_10610_1_-1_304_465

MKRIALGCCHKTSFPSSPIATHHLFPLPNPLKSIQSFENGLSPNSEVSRRGVRQ

>SG-PL_contig_10611_1_+2_2_97

RNRPRPTKSKKQKTATKRQNKITGWDFKLNSS

>SG-PL_contig_10612_1_-2_107_352

MDITVEGFMTWMWKGLAFLLPFLFFGYFFQLYNAYTLLMMAVGSNFKEWQVFALGLIHCL

LFMGNILTSLKVIRQKLKKDGS

>SG-PL_contig_10613_1_-3_9_1196

MTTPATGTDSVREKILTDYRKKLLEHKELEAKLKEMRENLKDLTKQYDKSENDLKALQSV

GQIVGEVLKQLTEEKFIVKATNGPRYVVGCRRQLDKAKLKPGTRVALDMTTLTIMRYLPR

EVDPLVYNMSVEDPGDVTFNQIGGLGEQVRELREVIELPLLNPELFQRVGITPPKGCLLF

GPPGTGKTLLARAVASQLDCNFLKVVSSAIVDKYIGESARLIREMFAFARDHEPCIIFMD

EIDAIGGRRFSEGTSADREIQRTLMELLNQMDGFDALGQVKMIMATNRPDTLDPALLRPG

RLDRKIEISLPNEQARLEILKIHANPIAKHGEIDWEAVVKLSEDFNGADLRNVCTEAGMF

AIRAERDYVIEEDFMKAVRKVSDNKKLESKLDYKPV

>SG-PL_contig_10614_1_+2_134_220

MTVMSFFYDYYYILLIAQPTTKAILGLSP

>SG-PL_contig_10615_1_-1_22_1347

MSDDEFMVDDDEEYDLEYSEESNSEPDVDLENQYYNSKSLKEDDPNAALESFQKVLELEG

KEKGEWGFKALKQMIKINFKLGKYEEMMKRYKQLLTYIKSAVTRNYSEKSINSILDYIST

SKQMELLQDFYETTLEALKEAKNERLWFKTNTKLGKLYFDRADFTRLQKILKQLHQSCQT

DDGEDDLKKGTQLLEIYALEIQMYTEQKNNKKLKALYEQSLHIKSAIPHPLIMGVIRECG

GKMHLRELEYEKAHTDFFEAFKNYDESGSQRRTTCLKYLVLANMLMKSGINPFDSQETKP

YKNDPEILAMTNLVSAYQNNDINEFEKILKTNRRNIMEDPFIREHIEDLLRNIRTQVLIK

LIKPYTRIHIPFISRELNIDPQEVENLLVSCILDNTIKGRIDQVNQVLELSREASGTARY

GALEKWSQQLAQLHGAVLNKMA

>SG-PL_contig_10616_1_-1_1_96

IQMFPLPFCCLDCQEHWHYKKNFAVSVLIKYY

>SG-PL_contig_10617_1_-2_2_223

LFLVCVLSLSLSLSLSLSLSLSLSLFSVCVCKEKLGLRFSLSLFSFVCVYKENSGQIPGC

GPWGAGIVTSFVVT

>SG-PL_contig_10618_1_-2_2_1300

MEASMRAKYLGKSVYRFGTISAKHILGCPRGKEQCLMTGVTQKHHHLCYGPTTVLPCKHL

SSLAEHFQAASDAQRKSVNVQAVFRTEENDPTHQSMAQEGLFYTVPDAEVRRLFQKGLSV

QFTRQAKTFNETCFMVRKPALEVIDCLKHLNLDHVAPRFLLYGNNGSGKTMAMAHVIHYC

AHNNWLILHAPWPSDWNCRYHETSPSPYKTGRMDLPVEAAEWLVNFRSQNSHFLQDIKLS

QQYVWTKREVSEAGSPLSDVVEFGLNRMKFASDCVGAILKEVRIQAAMNKIKVLLAVDGV

NAFWNQTTIKNTERRLLMADQISLVHNYKKMLLSTWTHGAILCSVDIFANERKQRERHTP

YYLLGDEGYKWLDPFIPIHVPNYSEKEAYSCLEYYMDRNWLQHKEAQTEEGKKELIFLSN

RNPFQLMRICSSK

>SG-PL_contig_10619_1_-3_132_803

MAANPVVEQTLLQATQMIEQQVDAEIAKLENMEEDDFEALRRRRMEALKKQQQQKQEWLN

SGHGDYTEISTEKEFFEQSKKSKNMVCHFYRDTTFRCKIVDKHLALLAPKHLETKFIRFN

VEKCPFLVERLRIKVMPTICIAKDGKTTDYIVGFDDLGGRDEFPTEMLAWRLGRSDVIQY

SGDLLTPPWDPTKEGGQKKIFGYAKSSKTIRDDGDDDSDADDDW

>SG-PL_contig_10620_1_-3_3_161

NSVLEISFLCFHPFSFLNCHLLSSAFPVHSTFFFPTSLHMIYAGLLPYVIEIG

>SG-PL_contig_10621_1_-3_168_1481

MSSYQMNTAGGPSSTMGPGGQNFMDPPDMPVDSKQQQTMMWQQNQYMADSGIHSGATTQA

PSISSKGHGEEVEEGDMDPAKMMFDWGLTDQYPPQPEQVEEISQQLNQTRTQRVRAAMFP

ETLPEGVQIPSTQVHPDQPTAVQRLAEPSQMLKHAVVNLINYQDDADLATRAIPELIKLL

NDEDQVVVCQAAMMVHQLSKKEASRHAIMNSPQMIAALIRAMATTEDTETTRCAAGTLHN

LSHHRQGLLAIFKSGGIPALVKLLSSPVESVLFYAITTLHNLLLHQEGSKMAVRLAGGLQ

KMVALLQRNNNVKFLAITTDCLQILAYGNQESKLIILASGGPGELVRIMKSYTYEKLLWT

TSRVLKVLSVCPSNKPAIVEAGGMQALSMHLGHQSQRLIQNCLWSLRNLSDAATKMDQME

GLLHMLVQLLNSNDINVV

>SG-PL_contig_10622_1_-1_85_1248

MAMYLFSGNPIGVQEVYGASPAVQQSEPLKVQENTPLTPFKDRLRNSEAMLVGFLAENSL

PFSMAPKLIDLSQALARDTKVLENMSMDRTAASYKLKYGLAQTFEESLLEELRCTPFALN

LDEAMNANKQKVVTILVSHFSKSLQRIVINHLTSFVVTRLTSETLFLKLEEIFEHFRLPW

KNLLAMLMDSCRIMRGSKSGLEKLIRDRKAPHLLDVDGDTCHHAHNAAKTFCAAFERHVE

QLMNDLHTDFEWSTYQRDLLFKLCAPLGSKPTKPEQFIQHRWLSVLQVGQDTLRQFDGLT

LYYFSFLKESDRVLYKDIVTDILNKRKVEETARTAISNHQKEVRRSFSSMTKEGKDRKHR

LVDKIFIQREKTEMILNLYCSVLPILNQ

>SG-PL_contig_10623_1_-2_125_397

MACAQAYVGLVDDSYTLRVLVVRILFFFLTPNCLKICGHIPLHAMIYVRWTDCDFVIRGE

LRVAEFVCDDLNIYDIEQLVFITFHCLLVCS

>SG-PL_contig_10624_1_-1_214_315

MIIGWIHECWFFNYCLSISVTSRYSVCLMKNAVL

>SG-PL_contig_10625_1_+2_2_88

LKHSIACIQTLKTKTRLQIGHQFYYYYYF

>SG-PL_contig_10626_1_-3_18_275

MFLHVYAHGGGSGTSRSAHMLTWEIGQIFTLYPSGEVPEEDSNPGLPNTPVWKSSVFTIR

PPRLSTRRIMHLLCRNRLLCVCACMR

>SG-PL_contig_10627_1_-2_17_316

MTPVKSKGELKPIQLGTSEERIIKCACCICSCFHKPPTLTWLTGSLICIFDVLHAYTHEG

GSGTSRPAHMLTWEIGKISTLYPPGKVPRRFEPRPSRSSS

>SG-PL_contig_10628_1_-2_1175_1645

MKEKSKNAARTRREKENAEFYELAKLLPLPSAITSQLDKASIIRLSTSYLKMRAVFPDGL

GDAWGQRTPPRTPLEKELGSHLLQTLDGFIFVVAPDGKIMYISETASVHLGLSQVELTGG

SIYEYIHPADHDEMTALLNVHNPYHAHILQDIEIERS

>SG-PL_contig_10629_1_+2_728_892

MEKRIEDISLTVKPCEFVVYDNNNITLWVFFFHQNQIFYQVTSFLSQKCDNLVIL

>SG-PL_contig_10630_1_-3_3_284

TAARQPTPSRRPVLGGEPGPSRPGVEAAAPQRLGGGSHRGRVQATLGRSEAPAQAASSFP

TPGPSRSGCSPGLGGGRPDPQGGRGSLAPPPWTW

>SG-PL_contig_10631_1_-1_1_306

QSSVGSLAHNVSVWKQLQLSDRVGEVVEKGYKLPWKGAEPPLRPVPPSPHQVPHDQESAL

AVEVTDLIHNRAVDVLPHPPGPGAHSPMGSLESANGSRSQRT

>SG-PL_contig_10632_1_+1_1_174

GGASFFVCLFASLSITWHFCFLSSSSYSRHAVTGCRFASNLYDMTNKRLRYLTHNNKK

>SG-PL_contig_10633_1_-1_1_243

LVGNKCDEVNREVSVQESAEIAKRWNCAFLETSAKTNHNVKELFQELLQLEKRRTVSLQL

ETKKSKSQKRKEKLKGKCLLM

>SG-PL_contig_10634_1_+1_28_198

MSIDTKLYSQGLQQTEDAAIMTAHVFHSYRGSKYLLFTQSFLGAIAKLFRCKTSNRN

>SG-PL_contig_10635_1_+3_3_257

KKKTDFCTFLSKTIYTSNHKSRKCVKNNVSPGTIFSEAMLMLTHTIRMFSQSAFLICCSP

PSFEICTYILTKHIYSITFSFTNVL

>SG-PL_contig_10636_1_-2_2_139

ESMIKTIITSTYVNKTIITSAYVNGTSVIIINSSFDHIFYHVAHQM

>SG-PL_contig_10637_1_-3_885_1046

MCLCCLVCLCFNREIKTVTGLDYEKIRSNLCRQAKSMQLIHHIQMNNCFVCVCV

>SG-PL_contig_10638_1_+1_1_207

LHCPMGPLSHQGTTPPVLSYLNQRQSHLNQAWDMAREASSYNTSLVITISCQGINSQSEQ

EKHRRATLT

>SG-PL_contig_10639_1_-3_231_362

MLYLFHYLLSLSIHSEGLGLNIMLCQCQLFKNLFHFDALGLPNS

>SG-PL_contig_10640_1_-2_2_292

FCLFVFFGYKCAGIVNISRVMDRVCRWIAKARNRLSVKRTDFDRLPKFPVTSLSHCREDI

DAAHDVTTVVCFGAGFRVCFSPCGGFTCRFKTVTVCH

>SG-PL_contig_10641_1_-3_501_719

MRAGYIVAVMFLFGKLLVLEVFCCCCFCDIVIFYIFILLGKKLPARTGICCTTGIWHLMG

KIKIIAHRSHLQC

>SG-PL_contig_10642_1_-3_123_1466

MANSNALQKAIDLVTKATEEDKNKNYEEALRLYEHSVEYFLHAIKYEASSDKAKESIRAK

CVQYLDRAEKLKTYLKKKDKKKPVADGEKPNSSSGGGGKSGDKDSDSDDDSKEDPDKKKF

ADQLSGAIVVETPNVRWDDVAGLNGAKEALKEAVILPVKFPHLFTGKRKPWRGILLYGPP

GTGKSYLAKAVATEANNSTFFSVSSADLVSKWLGESEKLVKTLFQLARENQPTIIFIDEV

DALCGSRSENESESARRIKTEFLVQMQGVGNDNNGVLVLGATNIPWTLDAAIRRRFEKRI

YIPLPEAPARTEMFKLHLGSTPHSLTEHDLRELGQRTDGYSGADIGVVVRDALMQPVRKV

QTATHFRRVRGPSRADPSVIVDDLLTPCSPGAPGAVEMSWMDVPGEKLCEPVVTQSDMLM

ALSTQKASVNEEDLQKLVKFTEDFGQEG

>SG-PL_contig_10643_1_-1_22_453

MRYSLSLLQCCVCGDFFSLFPLGFFSGNLPRFADLRFGVGDKFAVCVEYSSGFSCFLSVL

SLSSVFFSSVAGSCGSYFLKARLLVLVKCAVAVLVMSGSSSSFCFCFFHTCHSDILIIHS

FSGYRHIRHSNLAHILSCSRLCYS

>SG-PL_contig_10644_1_+3_417_794

MQFQASQLVDSNLTEKIEEVSEELYAAVNRKLKTVFAQDLNTAKSEANALREVTEEKKQV

VTSLEAQNIEQHTENVILKDAIQKLQKQETVTNAALKETKRMNVQLRYTNMYRRNERLKK

KKRNFA

>SG-PL_contig_10645_1_-2_83_853

MNKFGGANARDLNAYKCCFCCHVKVGTILYGLFCLMVDIFGLGLVVLMSIHPDILVPQRV

DWVGQGPQYDGVHVDDLDRRLQLPGIQEPHVITKENLCMAYAVALLSILTTVALLYGVIR

TRPGYMVPFLCQHVFFFCLSCLTLVSYFTYMPHFDVKWWIQENGLSHVPGMDYFMKIDSD

YLMFFMVTMLILTLCSLAYLIGMIWACYKYVQQLAVARTMTREYQVDPDTEMLLPPRYED

AIKPENSTQPPPPPYTQ

>SG-PL_contig_10646_1_-1_403_594

MLRKGCTFKICSYFYISCLQYVMTFDPVIKNRFSCSFSLFFTFFMNKKSKIYGTVQQTQV

YLIE

>SG-PL_contig_10647_1_+2_86_232

MQYDRPETYGSLTFDMILSAISLSDFCLGGKKVNLFSQKKRAHSSHTHT

>SG-PL_contig_10648_1_+1_196_432

MQNNLYLLTDKYTLLLHSYKENIQQKQTETRKLLPTAPTPYPWGKNTRNQNLLSTVVHEK

FCIPNPLSKLGTLSQQTSV

>SG-PL_contig_10649_1_+1_1_144

FFFFTTIIFFFRIWIFFPLNTCQQCMKSLTQKCTNIAILKKVNLYNKL

>SG-PL_contig_10650_1_-1_109_345

MISLPFKITSWGCISYRLFCLWRSPFCISYQLFCLWRFTIFERTFQGCIHANCSVSLIEK

HVIKLCVCSKLYGVESFVR

>SG-PL_contig_10651_1_-1_274_516

MFFWNNRETVSYQLKRSSLSFCMCVLVHCVCAVCICALSVCVCYIIMCVCLYRCVCVHVC

VYVCAVCMCVCVCSCLNVCVK

>SG-PL_contig_10652_1_-3_48_1748

MATKAILGLLCCFAVCYNPLAGTPVAEKVAGTPVDGTSTVTLKYDEESKKLTVDPDGVGA

TIAFSKLGHKINKTGWAYLEVLTFPVAPDYVQAYAAGFVEGLITKDLMHMHWTNTLAGFC

TKPYSKFCAKLSAFLTQNLQWMKEQIKKNKNSPFWHHVNLFLYQAAGLADANNLPTPVEP

NIHIDPMGFYLFQVDSDIEDLQSVLGDSKMRKTLGAGSCSALIKLLSGNKDLYASQVTWN

AYQSMLRILKFYSFPWKMVADGTETIPGQNQTFSSYPATLMSGDDYYSLSSGMVSMETTI

GNNNSSRWQYVTSSSVLEGIRSVVANRVAKSGQEWAKTFSLYNSGTYNNEWMVVDYKRFK

PGMPTLPPGVLTVLDQIPGMIKWADLTDVLTKQGYFPSYNVPYFPEVFNASGNQASVKKY

GDWFTYDKTPRALIFKRDHVKVKDLDSMTKLMRYNDFKHDPLSRCNCTPPYSAENAISAR

SDLNPKDGKYPFGALGHRNHGSTDMKLTSSHLMTDLTFIAVGGPTWDQQPPFQWSKSDFN

ATSHVGQPDLWKFSPITFNTTEIFSHC

>SG-PL_contig_10653_1_+1_646_795

MSKQQRKNVYIFAYYEHMSYIQCSLKPKQQNKCLHLHNQSDRVCALHTCF

>SG-PL_contig_10654_1_+2_2_223

RRGFRHWQVCTHVDLRDRKNLHPLPTRQRCLGGELNPGPSVWKSDVYPLDYRTHQKVKKS

PPKWPVSDVWQKMR

>SG-PL_contig_10655_1_+2_2_97

AIKNIVIKFWQCQGYSLCECLNSMVIMEIMKI

>SG-PL_contig_10656_1_-2_2_757

KEPAHDTADDTVTSAPQKKFQRTEEEDYGFYFYPQRSGSEEVKSFWSSVWGRQGANNFRC

EANVTWCIQNNPMVKLMMRALKSHGCEVNLRRHISCENCLEKVNGGFDPTTNQVVICQNN

VAKRSTCCNVLAHEFIHAFDSCRAKVDFEDLRHLACTEIRAANLMHCSFMAAFASGDASP

FNIKQRHAQCVKSKALQSVLMVRNVPVEKAMEIVDSVFDQCYNDLEPVGRRPRKNSRDPD

RALLEGQYYGYS

>SG-PL_contig_10657_1_+3_603_821

MFSYLSITTVFLTKCLCMMSKSSSTMATPSQNYHQHHTCCTGSQSECKNRQPIDMSVRQI

LTDKRITRLRDDF

>SG-PL_contig_10658_1_+3_288_464

MHKFHAINLSFSLSEKKKKKKEYFLPHPSYDCLHICDRNGLINVFLLRTEKKKRNISQA

>SG-PL_contig_10659_1_+2_2_142

LFATTHFRHALIFPCAHRLTHLRRRAGSCESLLHSLELPPIEVSDLV

>SG-PL_contig_10660_1_+2_2_142

LFATTHFHHVLIFPCAHRLTHLRRRAGSCESLLHSLELPPIEVSDLV

>SG-PL_contig_10661_1_-1_568_2610

MVHIDTFIQNLFFLAKDEDTEVRKNVCRALVMLVEVRMDRLVPHINQIIEYMMERTQDSD

DGVALESCEFWLSVAEQPVCREVLAPYIERLLPILVKGMKYSEIDIILLKGDVEEDEMVP

DKDSDIKPRFHKSRTHSQKYQEGAEEDGEGSEDGFDDDDSLSDWNLRKCSAAALDVLANV

FHEDVLPILLPILKETLFHAEWEIKESGILVLGAIAEGCMNGMVPHLPELVPYLISCLSD

RKALVRSITCWTLSRYAHWVVGQPHDLYLKPLMTELLKRVLDGNKRVQEAACSAFATLEE

EACTELVPYLGFILETLVYAFSKYQHKNLLILYDAIGTLADSVGHHLNKPEYVNLLMPPL

IQKWNQLKDEDKDLFPLLECLSSVATALQSGFLPYCEPVYRRCVHLVEQTLNQNYANLSQ

PDQYDPPDKDFMIVALDLLSGLAEGLEQHIDSLVSNSTILKLLYQCMQDPMPEVRQSSFA

LLGDLTKACFQHVRPCIGDFMPILAANLNPEFISVCNNATWAIGEISIKMGVDMQSSVNM

VLGPLVEIINRSNTPKTLLENTAITIGRLGLVCPQDVAPHLHQFIRQWCTSLRNIRDNEE

KDSAFRGVCLMIGVNPGGVVQDFIFFCDAIASWVHPKPDLKDMFYKILHGFKTQVGEENW

GKFSDQFPQPLRERLAVNYGV

>SG-PL_contig_10662_1_-3_303_581

MSPTVRHEHCSVSNWKLSHLCSRMDHQLHDFHYLSLIFSSWSYFLWPRISTLSPPKLCGH

SLSLQCFLTAVGTCLVTSPITLSASSGSMLPLQ

>SG-PL_contig_10663_1_+2_2_220

TRCDAHFNKPNPKLSAHYQDNFWGFSTGRHHFDKKVMFFISCWKCVPSCLFSQFSHVIFR

YQMSCYLQSFAYS

>SG-PL_contig_10664_1_-2_2_79

MLLSTQIIALMIKGLFIRPDRFFNNG

>SG-PL_contig_10665_1_-3_3_137

ATTTTKRVMEVVAEKKTQTNKNNNNKKPHPTNQSNKKIECVSKSF

>SG-PL_contig_10666_1_+1_79_1065

MSDAKRVARSTLDQLKNVTTVVADTGDFEAMKKYKPTDATTNPSLILAASGMPAYQSLID

DAVDWAKDQGGSEAEQLSKAMDKVCINFGCKILGIIPGRVSTEVDARLSFDKEAMVKKAR

QLIELYKAEGVGKERVLIKLSSTWEGIQAASELESKYGIHCNMTLIFNFYQAVACGEVGA

TLISPFVGRILDWYVKNTEQKSYEPLNDPGVRSVTTIYNYYKKFGFKTVVMGASFRNTGQ

ILGLAGCDLLTISPTLLEKLSQSTECIDVHLDKEKASDCDLAKVSVNEKVFRWQMNEDEM

ADNKLSEGIRKFAADAVKLENLLRQRLVQ

>SG-PL_contig_10667_1_-3_3_131

STHRHQSVFERLCNRAESSSRMNCRILQSSSLSSGFISSFIFA

>SG-PL_contig_10668_1_+1_1_183

TFTLPLPSTYLLTKSYLYLLFCSLSIHILVQVFLIWTYRSCAVLFTIFSSMNKWQYRNQL

P

>SG-PL_contig_10669_1_-3_3_155

KCDEMLFACYFHAVIFDACFSWNFARIYKSSKTATTLLIFANNLIFRNHLR

>SG-PL_contig_10670_1_-3_3_176

GGGGGGSGGGRWDPPSRGRPSYDDYRPPRRFRESRSRSASASPPRNRNRNLTRSRSRS

>SG-PL_contig_10671_1_+1_1_234

VCVCVHKWTFYCLSFPLSIKASKRTFSCFLKYGISKFMRQTVVGTGFNGANHKASIYISF

HQTHIHPNLIKTKLVSFD

>SG-PL_contig_10672_1_-3_117_296

MRGRKRNENRMDLFQRLSTSCHGEVIKLTQENNSWEIGDSNAISQNHWGQDWEHTVESHP

>SG-PL_contig_10673_1_-2_89_328

MRTHMHTCFSLSFFHPSDCLAISITVYLAISVTVSASRYITVSHHLCHCLLLFLSLSVAR

SLCHCLYLYHCLSCSYTFSC

>SG-PL_contig_10674_1_-1_1_75

SGRPQGEPRGEGWEGLRVGYGKCTS

>SG-PL_contig_10675_1_-1_70_522

MRPVVLIGPSLKGYEVTDMMQKALFDFLKHSFEGRIIITRVTADISLAKRSLLNNPSKRA

IMERTNPRSSGLLEVQAEIERIFELARTLQLVVLDCDTINHPSQLAKTSLAPIVVYVKIS

SPKVLQRLIKSRGKSQSRNMNVQLVAADKLS

>SG-PL_contig_10676_1_+1_61_294

MKRKSKKIEERLNTTKKQLKPELHSTHAASVSPIPNPRSDTQGNLPKRPQGNKKNHRKQH

RRRERERERIMHASSLSI

>SG-PL_contig_10677_1_+3_90_212

MSTYQYVHLPLCLYTLFIVLFETSSYMSSYLHTPVLTNGSF

>SG-PL_contig_10678_1_-3_801_1175

MLRTPSYSIVIIFFPIINKSPIFWVWYAKTTLSLLPALLTFCILQSFCHLFFFVCLVVFV

FVPTCPFICIFPRKHVKSLRSMLSALLRCLASVIANTGMGIYCHFALFISSSSSFFFSFF

FMHIY

>SG-PL_contig_10679_1_+1_982_1239

MLRFHLTRLHFHYKAPKVSLSSLVSLVLVFNTRVLNLVSTRLLCHFSRQPRHVRQDISKM

KIWLQESERSEEESKRSFLNIQVNQE

>SG-PL_contig_10680_1_+1_1555_1776

MLFMYLFVYTINLCIYVFVLIFFLIIFNMEKKNFFLFFFFYIVFFFEILGRGSKFFFFFY

PYHPLSLIFCFFFF

>SG-PL_contig_10681_1_-3_147_2108

MDLMSKLSVKLPKRPKKKSKGSAKLLSSKARSSSTWEINVPFKKLDSIILSGEMLHKGKL

SWNRRLVAITNGSMALYKPDKETRPVFVIPLAGFSAHLGDPACRKGFEIKMSQENGESYT

FALDFKEWANIWCEHINGISQGMSVLPHPQHLARTFSGGNDNPTFGSKGNLSNSNSNISI

SSDGVESDDSSKPRLRANKVMRMGSFAYRATQFFENLGKKSTSKRKASSLSSPTDATEYS

LRDGIQEESSLSSVSIGGPAVSPSSEAASPVFEDSFRNSSVFSSPTLTDLPPLPSTPPPP

IPVNIRHQGFLNIFSSFNKRRWGQRWCLVRENMFECYRNQTSTPCELNFLLKNCILRQAL

AETSSPLSLMLLENNREKITVEPPTREDLIQWLHVLMVETATEVVPAGLEEYFVEVDENQ

VEDFTGMDPEYSDIVGASVLKSWEVGSVPILPSKDKSAEDVAEKTADAVTTDTADKEDNP

NTDDASQDSQETDRYLYSAVIKRPSFPTLEQLESQQASKPEEAVTEEDEEHKIPRFTDNL

AVYNANKNTTDSGFYSVKEANSDSESGSDIKTDTVSPTQENCSPSETSDGTALAVCNKTG

EGISASVGASGGVHAVEEEEEEESALEKTMTPDDFKDNLDCGRDAVVSPSSTIN

>SG-PL_contig_10682_1_+1_49_261

MRLKYFVMQQYRFKRQMDCVISINMPNFVSAAKYSNSPLIVAIFFFLNDHSLHTMLLLNS

ACTKFYFKIQK

>SG-PL_contig_10683_1_-1_292_387

MFNHVGLYIYYMVMAQKRCPILGTLKSKFQNF

>SG-PL_contig_10684_1_-2_2_310

PPPPITSAQIGEEVHIHFLHNFIDCFTGSYSATKYINTETFSYFRLAIAEILFHCESTHI

VRSSEWGARPPETKVSGQGDMLFSVMHDFYQCHISNIFLQIDG

>SG-PL_contig_10685_1_-1_1_126

KKKKKTPPTQKKKKKKKKKKKINIFSSLSAIKKKLAIFVYEV

>SG-PL_contig_10686_1_-3_207_629

MSQGQRALPQSKEAQLRDYTMRLKSSVKAMFENFTEIIKLARVEEEGQLQRSTQNEQDHY

EMQVRASNIVRAGESLMKLVADLKQFRILNDFPSVNDSITGNVHLYQDMQNQIDSKLMAL

RDEMAADLFELEEEYYSSVYK

>SG-PL_contig_10687_1_-1_1_117

QVVIFILFFLVEEEVVTVCCQFIHFKYIICTRVGCEGFK

>SG-PL_contig_10688_1_+2_2_103

KTREHLVIWNTNGQGFFFFIKIKLSKNVYFMDNN

>SG-PL_contig_10689_1_+3_3_230

ENKTNGIKTNHYKTGAVKPTFNTLNIQIIKVICIILQLLLTFPTIPAIFKLPTELFIQYG

RCCFQQMWCGNSRRLD

>SG-PL_contig_10690_1_+3_3_125

GPTNQYSNECRADYSFSYPRKVTVTDQTDRPNSATFVATTS

>SG-PL_contig_10691_1_-1_142_1992

MIDITLTRPLVPKRPPEELARRVAELIPPRPAFPKRTDSADRAVEDYHGQIASVAGLVLE

EFRDMFGPDIKCDAPQTNEDMEQRRQKLMYHLNSSGKYFSFKEQLKHSVIKIVREKYLKT

TNFDDRQQLQTFLSELYVYLVDQMHDSLNRVLQIEDQPPIPPPLTTSAQLKHFAREAEVN

KDFELATKYYQERIIRGEANADNWFDFGTFCLYINNVSKAEECFHKCIAISQRHMDGLMM

YGVVCTLAERNEAAETFFEAATNVDPKSILAWTMLGLFYDAISNDIGAEMAYLEANKLNV

AHAASIARAAREVEQEKERKEKEKERAEKEKEGDVTETPHVDGSIVPPVSAGKSLTPSRP

GSHVKGSVRGTPVAGEMEHSEELAPREPTPIPAMSIFMQAVDWLLEVKAIPFTEQALAHE

LLTTPHGKTPAYHIATARLKLQKEEYEEAEESLNQALQVEHQNPDGWALMGHVKYLSGDN

DSARQCYERTLSFVADASETHSIYLRLASIYLQDGEFQHAKQTFLMACKKSPSCVSWLGV

GISCYRLGELGEAEDALMEANILNNSDAEVWGYLSLVCLRTGRKVEAEQSYKHAVKLHLQ

DTALLTEIQAEQKKVGF

>SG-PL_contig_10692_1_-2_581_838

MFRKLCPCDTEHALHHVQEETLLSVGCNHCWSLFIFFLLMVGKFCYLLPFLMTTLCCISL

SLSLSLSLSLSLSLPLSFSLLSSHLR

>SG-PL_contig_10693_1_+3_129_323

MNTQEKQTQHKQKQNLTKGSLSQQFNSSQYTLKHHPPAASGKVPNESEHTHMQGKIKLTK

ISSSG

>SG-PL_contig_10694_1_-3_3_155

VSLKCIGSYFYCKQLDTVDDGKSIYTNYTFCRWCLTKLYQYVLFTLKEKRM

>SG-PL_contig_10695_1_-3_177_335

MLHLHARIPLGPRRRRLTLPGTSLQGGRSQSAPSFLLRRRSLRGPVTQLGSGA

>SG-PL_contig_10696_1_+3_3507_3713

MISLGKTHPLKWQFVVNCVSFNREVFFKKKRKKKKIMNEDYSQAWASVSSWRMFGPILRP

SSDQMFAMC

>SG-PL_contig_10697_1_+1_1_291

LFYDVSQPSQSVTFYASNTFLQNNAGSTYRLRNRSVNRPLSGISKELSPDGKFFKKASFM

PHLRNTLRFSITKVTNTRQYHLQQPSHKVIIKHMYKQ

>SG-PL_contig_10698_1_+1_160_384

MLQLFFCLMEKINTLNKNDILKMLLTSDIKISLLLIKQVLFHWFEGDSGHHQNKASQTQF

NFNYGQSFHLQIFLL

>SG-PL_contig_10699_1_+3_669_998

MNFFSVVVKNVACSSVINCNNLSLKKKFYRYKKTITQKFSFKLLYQCTNNKASKIHNKNA

NNGRKSGMLQNFFSIYHTICTSITFCTWRPRSPFNRKHKIISLFSSLCVF

>SG-PL_contig_10700_1_+1_1_201

GAQETPNSLAKTYDSSFKDTSSSFSSAFVNFRSHLHSFVPVGCYVYGRLFTPLLRQLYSV

FRGVHAG

>SG-PL_contig_10701_1_+1_1_237

STTTSDYTQAHSYALTLYVQYTPQGGLLSMHTSPYTHTRSPFIQLAFFLTTEIKTLKTIC

PNVKQQEQGQNCKLRSEHI

>SG-PL_contig_10702_1_-1_79_1257

MEDALIVAAARTPVGSFNGALSSLAAHDLGSIAIKEVLKRAGVQGDEVSEVILGQVLTAG

QGQNPARQASVSAGIPYTVPATGVNMLCASGLRAVVMAAQAVRTGDAKVVVAGGQESMSK

APHCVHMRSGTKYGDVGMVDTMMKDGLIDAFNNYHMGITAENVARQWTVTRQEQDEFALL

SQNRCETAQNDGHFDKEIVPVIIQSRTGATEIKKDEFPRAGSTLEGLQKLRPAFIKDGSG

TVTAGNASGLNDGAAAVVVMSGSEVKERDVKPLARIVSWAQAGVDPSIMGTGPIPAVRMA

LDKAGWQVEDVDVFELNEAFAAQSLAVVKDLGCDPAKVNVSGGAIALGHPIGSSGARILV

TLLHAMTRLGGKKGVAALCVGGGMGIAMCVESV

>SG-PL_contig_10703_1_+1_43_165

MVDRLSRVENSPRIRGFQRTAIRFPRKLRSFPRKKNFLKTI

>SG-PL_contig_10704_1_-3_189_509

MPETLSVQVTSPRSGNSVLEISSLLLLFLSLLFFPSFTVVCFFSVCLVHSAVLFSRKPSH

VFLHHPHLLPCSLWCTICAVLLWRLPSIPPPMQSMVYYLCCFARVTR

>SG-PL_contig_10705_1_-2_281_601

MSWQHGICGCFDNVGLCIITYFVPCYTFGKNAEAVGDSCCLCGLLYFVPLVNIAALLDVR

KKIRESSGIAGTCCGDLLMIIFCHLCALVQEAQEIQAPNQTITIIRS

>SG-PL_contig_10706_1_-1_1_201

FFFFFNKDICQVGMISLDPTPSLQFFPLVWSYEHVSMNHTMACLAFKSSPWTRDFLQHGV

AFWDESW

>SG-PL_contig_10707_1_-3_123_866

MDFLYGHDRNFNVFEDPDMTDMMEPDLFTTSSMGMMDDSILPSLMAEETDNNWFINSIFE

EQDLVYNDKYLGEAATQPCIKSEHSYSSNNLSPSSPLGLGKGDATDGLQNQSSSLELNQN

AQFIHAASTATQPPSIKPESPSHDPLLTAMTSSDTMSTFTHPATIILATTTSSSTASAYS

HNGQSVFPTTTVHIKTEPMETFHTLQERTVNLDTVIMPPTPPSSNGSDSDGSTSPQRSAP

NSPQRQSP

>SG-PL_contig_10708_1_-1_82_1452

MQNVQAVGPETSLHKVVRAMETGLSFQYNASWDLVMQLLATMMEVLGKICPELLKKCLES

VAMLRDTATFSYRTEVDVVVGKAVRSMGPRQVLNAIPLKITGLEDSPDLSRSWLLPVIRD

NVRETELSFFTTYFFDLAQKFRSKAKALAAQNQEAVSKTYEMLEMQVWSILKGFCTRPTD

LAKSFKGLAKKLGDCLNERSDLQIDIMAALRMLINFSKENDADRAAMSQYAQNYVPILFN

MFTSNPDNPRDGRRLAVLETVKVYLSITDSKCLSKFLDMGLTKLAAADNTSFRQIALVDL

CIAMLKYADQPRLTKLYTLAQENITASDKSIQKKAYRILEEICMCETQACQQFLLSNLAD

IQQLLLDSLSSSAPNTRNPRLRCLTCMCQQLAEPNTAFIQSVVPEAIMCTKGVGERARAA

AYQLIVEVSRTMMRWNPDIEEQEVLSQYVNMLLAGLS

>SG-PL_contig_10709_1_+3_87_287

MEFHYIFGLRLRDGAALHAPFHKLSRHQPGLRDTVWCWSGNLSNTLNFASIKWMRLDCVV

PVFPMRP

>SG-PL_contig_10710_1_-1_259_450

MSACIYDHDFTLIIKLFSMMYCIHFFKFLSNIFLDFQLLRAFSINSPFMHSLSYSYTYTV

CVCV

>SG-PL_contig_10711_1_-2_2_103

VHPAVNGYLTLFIVSEDKAAREKRWAYPHNASPR

>SG-PL_contig_10712_1_-1_1_225

KFGLLFLGKVRRCNAAPPNFVSVCTCFLFTYDSGFFYRILPGTTLLLLWDLERALTAHLF

IVSSRRLAPKPALKV

>SG-PL_contig_10713_1_-3_108_1946

MWRFWNCVPRFVRQQFHGPRASAGVYVEEEKAEHEVDKFVQNEYAQEGHEATDGDSPSRG

CRRGRRRSSFGCRFRSQWRQRVDQSEAKDEAHHERGYRRREHCRGPFWSHYRGQEGPLPE

EEGSDWWKRHCHQYFWSRFCSQHHTLEAATWGAAVVLGLQLPRKPHLQNFLEFWKDPDKR

QRWRGLLFQVAFAMPPDAGVSFSRSIVQGSATQKQDTEAETAADKNQADQDARDLKVSEF

EDKKTLSDVMKDFESSCRQYTAVASSVSGLCAVERGQLTTAVEHLQVACRLGHAPAYFNL

ALCYEMGLGVEKDPKQAASYYHRAAEVGHAQSIFNLALMTLRGEGGIAKDQTKAMELLNQ

AAKQGLAQAQIYLGVYYTENEDDQQDFEQAASFFKAAASQNDAEGQYFLGICYENGWGVE

SSDEEAARLYSTAAEQGHDGAMYNLAAFHEYGLGGITCDEEEAMTLYKRSADSGNESAKF

RLQEVEARQAVSLWSEQSNKTELVQSPGLRRTSHSSSPTFTDYVQDSIMGVWGGELTAWF

SSQSLNSVSKPTDSQSPSFLLGGYDIGSLHGAADAKMNSSLLDISQQVGAPDTCFVSMPR

TSTMPDLRMVSCQ

>SG-PL_contig_10714_1_-1_1_138

FKITESNKWLSSVNFNKSLDEERVREEGKKIKRKTKRKSTLLCAQF

>SG-PL_contig_10715_1_-2_74_802

MGFVVDGSKHVPVGSAEEDRASMSSSLQQDDDILLPSDGEDGESLREQGLAKPLQPQYSG

VMQDVGMLQDNEEDKLRKSEQGSRPGSRAGSVASVRSRDVYRPSSVMSSRAASRAGTLPE

EEEEVDQEGLDELMAQGSEGGDTSQLKVDEILLDHATKQLEKQAKYAASFTGSIDQAEID

PETLVSLQGGTTMSTSSVKSGSDIQNESLRIVATPEPRIAGTPEPQIVGTPEPRIAGTPE

PRV

>SG-PL_contig_10716_1_-2_395_733

MGPTRTGKITTMARHFWGLIARTVTPTPITTWTQTALALTPIFYWASATPRCLPSSLTRG

KRRGWGAPPIIWTWAFLPPQHHQHFLSSPPPSLPSPAPHPFPTHPSCLCHLYP

>SG-PL_contig_10717_1_-1_1_231

LRSKQKGDYERFQKYLYFQQSIALHPIYYIQVTSLLKITRKYRGPMLQNTFMIACYWKKT

IRFLNFSYIYIPIDVDD

>SG-PL_contig_10718_1_-1_682_894

MAVCFLKSESYVLVSERNLGVWGMVYVKCRSDLTIGYNSKHKLNMFWEKESLKCLHGSYK

FFLLVSMEVKW

>SG-PL_contig_10719_1_+2_761_967

MCMRAKYSHNIQENILTKPSCMVTVFLILPLPLFFSLSSLSSPLWKLMNMNVLETAQPES

LLCLHRFCQ

>SG-PL_contig_10720_1_-2_2_232

KSHIDYAQNLSVGSGHCIYKSSTTIEKICLKEKGDRFRLMNICVCGVRQDLSPFSFLRIP

NLDHKSSFSRLLGSCKC

>SG-PL_contig_10721_1_-3_27_596

MAVQSSAVLLLVAVMMWSGHHCIACNHDRHDHHDSAHQDVFDDLNNAPGPSSFENMLALG

VRNGMGDEILDEFMMAEEINRLRAQEEVLEARLRGRAALHARSGGSGGSVFVRWGSQTCP

HSAECVYSGVMAGSHYSTSGGAANTLCLPLSPVFTSNISDPVYGVAKLYSVEYEVKPHQA

NDMDGLCSLC

>SG-PL_contig_10722_1_+2_2_124

CFVDINTFIAISQETTHHWFVKRSSTHFVSDTMTKKIKATN

>SG-PL_contig_10723_1_+3_342_557

MHGTVHNRAHIKYGNNLQENQKRNIKEKCSKYRKNNMMIISKELCCTHNSYDCTEISPML

NTSVTKQNKNIK

>SG-PL_contig_10724_1_-3_747_1034

MGSANIENSCMGSITFGKCCFGGLLTLENVFSFRHVAMLIFYSDITVLFLHLYMSCACVV

KLFHLCEFSDTKKKVYTSGKKPPQNTTPVKISNEIF

>SG-PL_contig_10725_1_+3_3_104

RTEMLPQTMERSFIFKERGEKGTKYYDSIIHRLR

>SG-PL_contig_10727_1_+2_2_118

HTIATCFPSKSNPHFTWKNSTVTKKNPINPIQNNNQINE

>SG-PL_contig_10728_1_+1_88_216

MKCQQITKISMCIIILNNSKLDWFISQLNTKYVQQTDDCSSKS

>SG-PL_contig_10729_1_+1_256_447

MYFEQHPLIPLLVTITIQLKCQTERAFPVRCGHTHKEDRTLKLVLCSWLVFSKMEDGDRF

WRRY

>SG-PL_contig_10730_1_+2_53_343

MINRIIATNIVLKEMKIVNNEKCTFCGKEKESIEHVFWRCNIIQQFWKSLENLMNGGENM

HNFKFTENLILFGVARNTKTDSIFDFIMLFVKYHIYK

>SG-PL_contig_10731_1_+3_9_239

MYFLWAEEESIGHVFWRCNKVQRFWKSLENLMNGGENVHNLKFTESLILFGVECNTKSDS

IFDFIMLFANYHIYKCK

>SG-PL_contig_10732_1_+3_3_215

PKTTEGEQLPVQRKLLQGYTTVGIFQKMWIRQQNIMNMMYERSTIVLKSDSTTQITCHVQ

FYSLNMSVLYK

>SG-PL_contig_10733_1_+1_1_108

FFFFLNLPSFIHDVVPSLYLFYNTSCNNHMRDNDIL

>SG-PL_contig_10734_1_-2_26_1006

MAEGSSNGQGNRPSRQPKDLQGLLNFCVENTKSEDAPSSAHREMSPERKEWLSQVIDEMT

GDPIQEVKEHIRTVGIFLGSQHPTDQARSNALDSLDELFDWCESLDMAIDFVKIGGFAIL

HELITHSDSEFRRQGFHLVGVLVQNNPMCQEAVIKAELLPTMLNVLNAEQIPEVQNQAVH

AISCLVRDSNLEAAQLQFVAHKGFEVLKKVLLSPGLFKCVVKIAFFLETMCQKDPQYKDA

VCEAGIMELLVQELKGEHEQMHEQVMSALLSIARDHAPTQRVLARPDLNMAPFLTQRIEL

LTDKPENEEERHYAEELLQVIQSSDSS

>SG-PL_contig_10735_1_-1_1_645

APSSNLDPQYPPDVKSGFDELCPVCGDKVSGYHYGLLTCESCKGFFKRTVQNKKNYACVE

NKNCQIDKSQRKRCPFCRFQKCLYVGMKIEAVRKDRMRGGRNKFGPMYKRDRALKQQQMR

SQQRLMSECELNMQGPPGSAPPGSPPDIKPDPALLQQSLVMGNYISPSGFPGNPADISNM

AAAVAAAVVGASSMGEVREGLSAAPITTSSSSGSY

>SG-PL_contig_10736_1_-3_3_152

STCNYFRSTVTASYFSKRLVLSVRDVQYFLKRACQGNEVNICTHFEAYAS

>SG-PL_contig_10737_1_-1_1_10938

TWTKAGSVDGATTNFTVPDLKDGTEYHFRVIAVNAEGQSAPLEAKDTAKPVKKIVPPGPP

TNLRITKVGPDFITLEWKPPHEDGGSKVTGYRIEICDEKSSDWVKVVDLKAYDTSYKVTG

LKETLGYFFAVSAQNSAGYGEPCETERAVKPKRPEGPPSKPVGPLKIKDMEKTAVSLEWQ

PPSDDGGSPLTGYVLEKRDTARPTWTRVEKLSPNKTSFKVQNLLEGSEYYFRISAENKHG

ASEPLETEKSVKPKSPFDKPSKPTGPIKISDIKKDSVTLSWQPPDSDGGLPIKNYVIEYR

DARRTNWMRGGSVPADTTTFTCPKLLEGNEYIFRVMAVNDEGESAPLESKETVTPQKPTT

TPGSPGNLKVKDIGKDSVSLQWTAPKDNGGTKVTGYTVYYREDGSDTWKKAGSVGPLDSD

FTVKNLLLDTKYHFAVSAENKQGLGEKLETESAVSPKKPLQKPAAPEGPITFTDIQRTSV

TFTWKPSPDDGGSPITAYILERRETWKMTWSPLTTVKPDITSYCAQNLKEGTEYFFRVMA

QNSVGKSQPLDSDGVIPKSPYAAPDTPEGPLEVSDITATSLTLTWKPPKSDGGQPLTGYL

VECRDVRRVGWAPLATLPPNTTSFVADKLLTNGDYYFRVMAQNSEGQSAPLENSKPFRPV

RTPELPGTPRGHLQVNNVTANSAALQWQSPEDDGGSDLSHYLVEVSVDDGEWTKLGKVDS

YSTKFRCNDLKTDSKHLFRVSAVNAVGTGKPVESQVVTPSKPPEAPGKPVGPLEAVDIQR

TSITVQWKPPTHDGGSPLTGYVVEQREAKRLMWSKVTTVKPDTTTCQVTRLIEGTDYMFR

VTAVNKEGSSKPLDSDVFTAKSPFDKPSAPGGPLKITEVTEDSADLEWKPSESDGGSPIS

HYNVEVRDVRRSTWTKVGEVKPTDTKYTVGKLSIDTEYLFRVVAVNAEGDSPPLSSTAPT

APVKKLSVPSAPESIHVRKVGKNFADLEWSAPKSDGGAKVNVYRIYKSTVLPPVWEEVAK

VKGFETTYKVGDLKEGSEYLFGVAAENEVGLSQVKETEMFIKAAKPLEKPSEPEGPLELV

DHTRESLTLSWGASKSDGGSPILHYVLERRDGWKMSWTNPIKVKPDGDKLTCCVPNLKDG

QDYYFRVFAENTVGASRAIETETSFKPRSPFKPPSVPTGPVRFNDVQQTSLTLDWLPPKD

DGGTPITGYKVLMSEEGGEFLELGQLEGTKTKMKVKDLKTNAHYVFKICAVNKVGVSKPL

ESDTVVPKRQPEPPSRPQGPIKVLTTTKTSATIEWRPPEDDGGLPLTAYIIEKREFPRST

WSRVDKISADDTTHCCPNLTTGSDYFFRVMAENKAGVSPPLEMDKSVKIKSPFDVPSAPV

GLRVSNVTDKSADITWSAPDSDGGTPITNYIVQTRVIPRSTFTTVEQTPELQVTLTGLAP

DNQYMLQIFAVNAEGQSQPLVSREPICPQRILSVPDAPDSLLVKNITKDGLVLEWTPPHN

DGGSKVRRYHLEKSLKDSDKWEKVATVESFRTQHTVSDLELEKDYLFAVSAENDVGVSEK

QTTAKPVRLEKPIFPPSPPVGPIVISDVTKTSAKISWKAPESDGGSPVTHYSVEKRESWK

TSWTPVERLPSDRMTCDLQHLKEGQDIYVRVKAENVAGQSKPLESEQAITPKSPYKKPSA

PESLVATDVTESSVSLKWQPPASDGGLPIKTYTVERRDKRWGSWVKAGTTKGSVTTLDVE

NLVTGQEYCFRVSAENEEGQGPAVEMKDTVKPTREPVAPEAPVGPVHFSHIEATSVVLEW

RAPRDDGGAPITNYRIEVSLTQDTWTEVTIVEGDVTKIKAKDLTTDKKHYFRIFAINKAG

TSKPLVSDAVTPQRALGPPDKPVGPLEAVATTRDSVTLEWKPPKDDGGSPITGYIIEKRE

AMRMTWNRADKTTDDTTKRMVKGLVTGDEFYFRVAAVNKQGTSEFLEMPRPVVIKSPFDV

PSPPKGPLKILRVTEDSADLEWGPSEYDGGTPILQYSIEIRESRRTTWGRAGVVDAMTTK

YTARNLVVKNEYTFRIRAINAEGESLPLDGEEPVIPRKKTEVSRPPNKVSITKTTEDGVT

LEWRPPSDDGGARIKAYHVQAKEDTRGATWQDVGKVDGYDNRFSVTGLDTGKNYKFAVRA

ENENGLSEPCEADKSASPKKPVKPPSPPQDLAVLELNRDSVLIGWKHSEHDGGSPITNYI

IEKRESWKTSWAHVDRVRGSATSAEVIYLQEGTSYNIRVMAENVAGLSEPAELDEAIIPK

SPYKVPTAPVGPLEVTNVDSQSATLQWKPPEKDGGLPVKRYIVERREAKRQAWVKADTVR

GTNTTCTVERLVEGSHYVFRVTAENDEGLSPPLESDSPVTCRRAAEKPAPPSGKLRASKI

TPDTVTLDWLPPLDDGGSPLTAFIIEAKDSKTKDWLTVAEVNPEASRQTIKNLTEGQEYL

FRISAQNAIGRSKPVEMDSPVTPSRPIETPGAPRGPLVPKNEGRDAITLSWQPPLDDGGS

PITGYIVDKLDIQRGGWMRAARVPADATSHTVAGLIVGHDYNFRVYAENKAGVGQPLDLK

TPIKAKSAFNPPSSPKNIDVTDIQEDSVILHWSPPDNNGGLDLVGYVVERREVGRQQWSR

VGQTGPDVTSLKARNLLEGRPYTFRVMAENPEGLSEPAMLLRPVTPERPIEPPGPPQELE

VTDVDKESVTLSWKKPAKEGGAPVQAYIVEKRDGKAASWSRVADTGFATHVFNVDNLIEG

YEYFFQVRAKNTAGIGEPATLKESVVPSKRKEKPTTPVGPLESSATTEDSVTLTWQPPEY

SGGTPLTGYIVEKSDSRRPRWVRVGKVPADQTSTTVESLLENVGYFFRVLAENSVGVSPA

LENEVPFKPKSPYGPPSRPEAPIKSLQITRDSATIQWQPPLSDGGSPLTGYVVERREDGR

RGWMYCGRTDADVTILTCAALMENTPYYFRVYAENKFGRSEPLESEFAVVPKRIFGKLSA

PENLRAENVDKDSLTLAWSPPHDDDGKIKRYIIEKRSADSRTWEPVSDVSGRTKWFMVKD

LPPGGSFYFRVKAENPAGLSEPAELSVPVELKAKKEKPKAPKDAPTILELGTDYVDLSWS

PPESDGGSPITGYQLERCDMHGRSWLPVSKEPITEGSFRVRDLKEGVQYEFRVRAVNAMG

TGEPSPSSEPFICGHSIAMEAPKFIGVFNDQSVPLGQDARFVCQVVALPTAEISWFKNFR

EVFDGKKVSLETDGATHTLRLRSVDFADAGDIECQARNRYGAVSIRAKLTVLAPAHIDLP

PHYGKGLRFCRGDTIKVRVPITGTPAPEVEWLKKNKPLTQIKALAPRLDITGTHLHSTLV

IDDCVKSDEGVYTLQVKNELGSAAVDIPIMVVEPPDPPTDVIITDVDKHAVTLTWEPPKY

DGGSPITGYVVERLDPATNIWRYAQSVPRPVCTVSCLDEHTEHKFRIMAENMFGVSEPSE

ASVAATTKEPLPFINYEDFVDSSFLGSPVDVTRIQGDVLGKYIICEELGRGAFGVVYRAI

ERATMKNWAAKFVRCRPGDKDLVRREIELMNSLHHPKLLQLHEAFDQTGEMVMILELLSG

GELFDRLVAEDYDLKESDCITYMRQICQGVQHMHRNNIIHLDLKPE

>SG-PL_contig_10738_1_-1_4_693

MCIIPVALSQQVFRWKVQDYPDPFSYDTYSECGRNNKSNVCDPNHMISKQKANEIDSLIW

SVFRETRCKCSSCVRNSHGYVIRVAIMPRMESILKLGNSTEDKLRDAQMFAYMLTQKWAM

SGNCNESLIILYSRYDGVLYTLTLPHARKTLKDEHVKRITMIVRPMFDDDATIGDGLLEM

IQRYKLVFDGNKDEALRPRMSKQRGNSASDVSPSFVLSLLLTAVFMAILH

>SG-PL_contig_10739_1_+1_34_147

MEFHHFWFFGKTVIYTLKHKHIYVLHEKLIFLAKKQKK

>SG-PL_contig_10740_1_-2_2_85

ANPFEHILGTIWNCFRNIHLQTICTTHA

>SG-PL_contig_10741_1_-1_43_345

MNMIFVKNLLISFYSTLMVDNSLFFGYVHVIEVRGLIVDCIFGKMPLSTKINTGQVRCAS

NFPIENIWNIQHRVESLVLKKSTMDFGLSIMRSVKGVCCGK

>SG-PL_contig_10742_1_-3_3_221

GSDRKPSPGSVPKVAYQYTTRVGEINQKVSCFVWYGVAALYPIGTTLSAHLGCSAVSKGD

RVTILWHQNSLVW

>SG-PL_contig_10743_1_-1_112_387

MNRLQRISLNCPYLYASSDHLRSSADTNIYNFPHVRTKAYGQRFFSYPAPSTWNQLLHSV

RHAITLSSFRSALKISFFSSSQTYPHFVNNKC

>SG-PL_contig_10744_1_-3_129_317

MVCQGSPLCVCNFCVRFTQICKDFCDDRHQFASVSDVKRFFVFNLYLFKNVRLFFLSVYV

SGS

>SG-PL_contig_10745_1_+2_2_175

SFQVTFLYMRPWHRAFQLIANKSIIFMMKTEESSNSPYTEVFNKIQDTNVLKCHQHTI

>SG-PL_contig_10746_1_+2_2_295

FRLYCYRQLILSKRSFLFAWFLFRLCCYRQLILSKRSFCSLVCLFSCLFVRFYHRKADNP

AFSHLGLRTTLCSYSVKNLNNVKLCHFILNIVMCNVVL

>SG-PL_contig_10747_1_-2_77_406

MTAALHADAYPVKYGLMAELKKEKLQNPQTAIHVKAHIFEYTWELQPTNQITQQNKTKNK

TTTHTHAHTHTHTHTHTPFLFIELSSGFRPLREAFLEMSVGCVLRKTLSL

>SG-PL_contig_10748_1_-3_3_167

VTSTLVQAVLLNKHYINQETRQSLQCHTCGKKNTYFQTKQSLAFTTVGSVITRFQ

>SG-PL_contig_10749_1_+1_379_522

MTVEKWCSHQLSAKFFRSLRSTCVQTFQCLNPLRVYTHAEYQICTLKI

>SG-PL_contig_10750_1_+1_1501_1704

MKGLSCPLQFESSSTNHLSFFVVHVSTASAWHLFLYHHLMNTALLYSSPTTLHGGFSVLN

NTGGFFVS

>SG-PL_contig_10751_1_-2_20_601

MNYYPGTVGAPVGYAVAVADKPGLYAASPYAAIPSPYAAGQPGLPPGMQPYATPRQQYAV

VSQPSPYTSSQHAAAAAYYASPTATGYTAGGVPLSSLQTMSSSSAQSPYLGTQYTPGIGM

QTAMMRPTSVAAGGYGGSPLYAASTYNGLTAVQAQQSRSPYGVAPVGFAAAATLPPSATS

PGYPSANAYGGSPY

>SG-PL_contig_10752_1_-2_2_307

EIGKISTLYPPGEVPEVRTQAFQTLQFESQVSSPFNHHDRQRTTEPQINDVTAILSDEVR

RRHGASVTVWDVFIPPVMLISEESNLYLHTWNLIILALHADL

>SG-PL_contig_10753_1_+2_29_235

MSLRVFTQLCTLTESVQLLLDGCISYPWSYGEEKIAQGLLKSMGDCHKNWKDQDKHLYMF

CLQIKQSFI

>SG-PL_contig_10754_1_+2_893_1162

MHTHQSMQTDANYISLISMVTLAMKYTIVVNLALKSNQIFVSLTLLAPSTDLSVPQFLPL

DRVRIHLYRNTHLFFHFLIQLYSDLALLAC

>SG-PL_contig_10755_1_-2_251_493

MSIHGHFHTYIENLAKNKSHETSLMIWQAHLHIDNFKTESYDANFGGHLEKNCCCFFKMD

PRSIPMMLSLFLAFVLKISCY

>SG-PL_contig_10756_1_+2_68_253

MTTAMSRQNQSSVSRTGHGGAQCLLKLRTMPCHDNGEFHSDSQITAKVGNKCKCSQCWQF

CH

>SG-PL_contig_10757_1_-1_118_231

MVGLYMKVSCFEDWPFDNFDISLCKNTQTCNTEMAAMQ

>SG-PL_contig_10758_1_-2_548_853

MSSGSIIYKVQMMPVHFQGHGYTKPVLLRHTFWYLTIQSGSIHQSYDRFQKYLMVQKRGD

CWDGFCRYALRFRSCIFCFRPIQNHSWPVLSTVCIMCVCKSD

>SG-PL_contig_10759_1_-3_3_104

YMQKNVPDFCMHYENMFRYCMYMCAMKTSATWLI

>SG-PL_contig_10760_1_+2_227_337

MPRRLMKDFRHRLELLSVICIHLSSWCTLSQFMQIMQ

>SG-PL_contig_10761_1_-3_24_311

MVSPAPATSVSSEGLSSEPPTSAEESAATSSECHLPLPKDKEIPTRWTANNRYNLRNIPK

KAVYNSCEEEEQKPEKVKYKVSVGENLSDWQKVLLR

>SG-PL_contig_10762_1_-1_1285_1599

MFSIVTVRTITVLHSKGLSCELLRYSFRIFMYLYVFNPFVCLFAHFVFLLIKFSLVDMYA

ATPSVCFLFTNLQMFVGVPFCNIACLESIVCDCASHSQLSCVSIM

>SG-PL_contig_10763_1_-1_139_303

MMMMHDDGDDNHDATEFHSGLRLRDRAVLYASFHNISWRQPGPRDTDTCSVGQEI

>SG-PL_contig_10764_1_-1_274_537

MFTGKSKSAKFPMVGGVDWWHGILRLSWFGHRFDSVHGVMSLLPSLVRRLDWMSSLLEEI

IQIKSTLCTYICHIIIWCKDFDRMIFQY

>SG-PL_contig_10765_1_-2_401_1030

MSACATFLIVFATILGPITLVFMAVSFGTDFWLKFNVDRSKLTLSDKSPAHDRYSHTRYR

GLFRECYPGNDTAFLDNEKDVVDGFCFNVGYELPENASGWTDDFFVRIHLLRLFLAFFIV

SLVFFVAAFVFGLILCCWRISRWAYVAGLFAYIAAFSLAASIAFFHGAEYLERNKLEDKG

FYKSWPETIKGATTRSYGWSYALGWAGMIL

>SG-PL_contig_10766_1_+3_186_317

MNKKRFPALKRLSLHLKNQNQLSTLQSLSLSTVTCPAEGLFDFF

>SG-PL_contig_10767_1_-1_802_1020

MDRWVFCPSVDGYLLEYGITNKKNNIQMKLHLSCKPPLLNFFPQVGTFISLDEITVNYEN

SLWILTLNSKHEL

>SG-PL_contig_10768_1_+3_3_104

RTEMLPQTMERSFIFKERGEKGTKYNDSIIHRLR

>SG-PL_contig_10769_1_-1_121_747

MDRRLIKPSAKRCLFGRPNDNDGFFERQKQLESAELQHFKRKWSLHSDEGEYEWTKASLD

IPEVYTKPYYHKCRRRAVRPCVGKCLSFVDSDEENICGVRPPSPEAVQYCDSNTVEVDLG

VFMGIVPPQPPEEQVTPKKSELGGSGDTPMSNSTNDTDHVAVSDRVCSQLLKRRRLTQKR

LTDMWRIVKRRQQSLTSTKSHQRVRINRL

>SG-PL_contig_10770_1_-2_56_382

MNNTTLSCSRKQNKLHSLIIIIILMNHQIKILSDFVALHAKLSQCPQFQFLQVLCLCQVH

VMFFWCWSTKWFIKTVSGRNVFNLVPFLSLQNRCYKFMKEKGSIWFHVS

>SG-PL_contig_10771_1_-1_67_1140

MGNSSSKKAEQSNVAAQNNAADQPANENQAENVASKDEPAPEAISVGKAANGNIVTHKPY

KEESQSAGKKDGPSDGVAAPSTEGQAGDEVQSEDKAVEDSDVSKGSVAEEPAAPVSEEKT

ESDSVGGEMEESVEVTEATEEHSAEGTDVVPAENGIPETSPETKPEVVEEQVQATAIVTT

QSAAPVAEPVPEPEVVPKEKSDVPKEEVSKASEETSGVDVKDPSDETEEEAPPVTKEQAV

IEESVESAEAHLQSAQPPDEPASSSTEPADEASTGISNEAPVETAQDEPVSEDTTPEEST

PKTTEEAVEDNEVASESSPPNTEEPAAVQEETSGQEGSLKDAPLPEPPQDITAEENAP

>SG-PL_contig_10772_1_+3_3_140

CMYVCMFLQVCVFVHAHAVTSMNFSQVTDEFLPYLSRRSGLMGSFE

>SG-PL_contig_10773_1_-2_2_346

SHLRLPSPRPRSLRDRRLHSRLERSSGLRLPPFPILQRVIRKARLEKATLILIAPKWPAQ

PWYPDLLSLCHVPPLELPPRPDLLLQPRSGIRHANPHLLHLHAWSLCRAQCQHVV

>SG-PL_contig_10774_1_-1_286_1035

MRTRGPVVGRRGSRYLAAYVIAPKSKRREVLPLNTRIPLDPRRGVPPLPGSSPQGRRSQS

PPSLLIQRRSLRHPSLLIARGVCLSLVPRLALGIGRDMRPLKSSSLPRHSLSLRAESLLR

WCHDRKLTLLARHIAGKANILADALSRVQTEWTLNSRVLERVWSHWYRPMIVLLRQVQQE

TPLYVSPVQDPEAFAIDAFSLDWSGLLAYAFPPFPILQRVIRKARLEKATLILIAPKWPA

QPWYPDLLSP

>SG-PL_contig_10775_1_-1_445_642

MLRTDFCISLCKNLVEKGKINTSKIRWKMVFLFFPPFFPFNIKIGIEGDIFNAEHMLIKF

AEFISL

>SG-PL_contig_10776_1_-2_104_214

MITDRRLKTRKLRMGSMLLIFVHQIPFFFIHMFLKKT

>SG-PL_contig_10777_1_-3_195_419

MRWLRVHTAIYSPRLATLSYVSVLCCLLDAILFLVFGRPAQSLVDLNVISIIHATSVCFL

FSDGLVFDIIHLLFP

>SG-PL_contig_10779_1_-3_171_308

MHIIIILHFCYPSFLLEGLKVLCIVYLCQYDHTTLLTIFFLRTAQE

>SG-PL_contig_10780_1_+2_2_163

PHCTKWVQTEPLVNITECTTRSKTVLKNKCSKNLLPVSLVDQYLCKTMFTSQTS

>SG-PL_contig_10781_1_-3_3_125

KIKKRKKYYKQTLHTTADFRFVHEQEIKSCWTATHLCIYLL

>SG-PL_contig_10782_1_-3_381_740

MNCFTCACREFGLTISLKKTNIMGQDVSSIPSISIGNYTLEVVEDFTYLGSTISSNLTLD

AELNTRIGKAATAMARLVKRVWDNPILTTNTKTKVHQVPVLSTPLYGSEAGTLYSSRMQT

>SG-PL_contig_10783_1_-2_545_1282

MPSAYTRSDGNLFNLLRLRAKTKVQKVLIREMLFADDTALTAHTEEALQRLISCFARACR

EFGLIISLKKNSIIGQDVSSIHSVSIGNYILEVVKDFTTISSNLTLDAELNTQIGKVAIT

MTRLVKRVWDNPILTTNTKMKVYQACVPSTLLYGCEAGTLYSRQEGRFNTFHLPCLKRIL

AIAWQDRVPNKNVLAQAGIPSMFALLTQRHLRWLGHVSRMQDGRIPKDTLYGELATGSRP

ARRPAL

>SG-PL_contig_10784_1_-3_1272_1457

MKSEKGVGWRWSWGIKRQKQLLSMYKSHVYLVCCNILYICLLLFESQLFISVATCLLKEI

TM

>SG-PL_contig_10785_1_-1_40_219

MDLTGDHSGLSVSQQGHECTKPQSPLTFSQSHPLKKILEATHWRSENAFINHYLRDRHLA

>SG-PL_contig_10786_1_+3_357_548

MLEIFTSKIKLPNIQQTSYSVLENHFWNKQNKTMNTSEQREFPIWFDYLINFASLFSFTN

SVCS

>SG-PL_contig_10787_1_+3_1908_2231

MKKKSINLYFFCSSIHQLKQSTSQILPSFLQCLTNPWRKLKTWYIACTKLMALFFIWIQC

EYYMSTHKNQGTLFSIICFCITGSTSSLQLSQDTRLQKHVKSMTIWRN

>SG-PL_contig_10788_1_-3_1308_1670

MKCTGIVFQWGYSTLLFVVPWMALLMLMENIFYFYSVILFVGSWFWSLTFLFCTVPLHKL

LLLLALLIPNACHVMSCLVAPDMYSRLHIALCDLCTGLHHNLPTNSAPFSPSFLKNDIAT

S

>SG-PL_contig_10789_1_-1_34_135

MDLWRNCNVFIGESFFVCVWMILYNLVKDSLLLS

>SG-PL_contig_10790_1_+3_3_224

ILFPIPNQITSHTNTIPYPKSNNLPYQYHSKIPNDWTKGTIVKILKKGALSNCNNWRGIT

LLSIPSKILAKIII

>SG-PL_contig_10791_1_+2_2_172

LAKIIIQRISDAVDKQLRKEQAGFRKGRGCTDQIFTLRNIIKQCKKWQTALHQFCGL

>SG-PL_contig_10792_1_+1_100_228

MYRKLHIHKTQYGITETEIQLPRNQIADCHITVFLNAPIHNAT

>SG-PL_contig_10793_1_-3_1158_2516

METEGVLNHSYLFCMDTELGWGNRSYFTFFSQFNRGTSASLWAAIESAILIILFLLSTIA

NVLIFYTLSSNRRLRTVTNYFVCNLTLADVCFTLCCPLVAVIRVTGNWVLGSFACKTIVY

LSCVCAFVTIWTLTLISLDRYSCIVRSSKHRITPPVALILIAVVWVVTLAAFTPLLLYFV

VKECPYGEGTVSICTLGWPRTPGVSVPVLFTAVVCGVGFIVPIIILIVSHACIFVKVHKV

RAAMRKQRQTRISTSSALLTPTIIPSTPPPFIHPPDAHLPSPAAAAAGGGSRFILPSLPP

KSPRLKTLALPNLSPTNSSMLPTPCTSTLEARKRRSSRDLQVVKTLVFLVLLFLVMWAPI

FVVFVLIMLDAYYDVMHVSSQAFLGAMCVAYCNAVLNPLAYGLSTDRLRYYLHTLLYSCA

PARCCCGSSGLSAGVRFSHGMGGGGGAGGGSTG

>SG-PL_contig_10794_1_-3_111_2051

MAHLQMKEGEYTSTIYGLIKDQRYSEAVQILNHQLQSHPRSRAALSVLAYTYYQMQDFVN

ASDCYEQLSTLHPEVEEYRLYYAQSLYKACLFQEAMKVACQIDQPQYQGQITKLQAAIKY

GEEDLPGAKSLVEQCPSDDPDTEINFGCLLYKEGRYEEACQKFSAAMQVVGYKADLSYNI

ALCHYMMKQYAPALKHIADIIERGIREHPELSVGMTTEGIDVRSVGNSMTLHETALVEAF

NLKAAIEYQLKNFDAACEALTDMPPRSEEELDPVTLHNQALMNMEAEPSRGFQKLQFLLQ

QNPFPPETFGNLLVLYVKYEYYDLAADVLAENAHLTYKYLTPYLYDFLEAVITRQTSPEE

AYRKLDEMATKQTETLRKLTKQVQEARQNHDDEAVKRAVNEYDEALERYIPVLMQQAKIY

WDMENYQQVEKIFRKSVEFCNEHDAWKLNVAHVLFMQENKYKEAAGFYDPIVKKNYDQLL

SVSAIVLANLCVSYIMTSQNEEAEELMRKIEKEEEQVAYEDPDKKIYHLCIVNLVIGTLY

CAKGNYEFGISRVIKSLEPYNKKLGTDTWFYAKRCFLSLIENMAKHMIMIRDSVIAECIQ

FLECCELYGRDVKAVIEQPLEEEPMHPGRNTVSYESRLLKSLLLQLN

>SG-PL_contig_10795_1_+2_383_655

MTTVMVMRGWDFSMSFFPCETMVALPLYTMMVHSFMPCRILSSIPPPLQCTLFQHLLVTY

LSGYSRPPIWLCLSNPTYLACLCYICRLWPV

>SG-PL_contig_10796_1_+1_94_348

MDVAGSVQCLLCSVDIVYANVLSVVVWHSANIIWSRKNLSLSLSLARSSFFLIICPSLSH

SLFPFPCILVFFMPKYCVIFLSFNL

>SG-PL_contig_10797_1_-3_741_977

MSVIECLIEMCHQHGACLFSRFSNSPLPLSNMFVTKTLEDDICNQDFNVVAEAFSDSSMQ

EETVIYPGLSVYLLSSQSN

>SG-PL_contig_10798_1_-3_3_260

EYSKLIDQGVAGKKIGLLVEGFESCMEEVQKVVRAAAHSLSSAGCDVRDISVPMHKDGAA

IWTVSAGHGSFKCLVENNGTGYFYKG

>SG-PL_contig_10799_1_-3_3_227

CLYPKRLTEVWLQSSTNDQEICIETLLSLPVVVTQMKAFIFLSNLRISNRPLDPTRGLDY

IILIMFILDQQRKPC

>SG-PL_contig_10800_1_-2_14_2179

MLPVAPSPLEGGGDQEDKVSEAGTYTIEAEEDGEEELIARQQIDEVFGVDMNSLSFERPI

IDSLEPFRTSEDDEEGETTLLEDEGDSLEAGCQTPCEEGESIFEDEEGMMSRSIGEEEVP

SWISQLNALTAASRQTPPTPKEGGAKGTDDASKKPPQGGLSRKRPGTGRKLPSIPAASDQ

SPVSSEHSARLLDNSPDSPANSLNCTAPARMENGLERSADDSPSRNVRFSPIVSSSGAVS

GKSRVEGSPCGSENSSTNINSGTKPKGTSICSQGSIDTELLLRDTETVMAAMEARMGFCS

EDREDEGGESDTDLTSTVAMVNGDEDFVKPTKYHSPRDTMTGKSSKEKDNSNIRIASASS

SKKSLTRSYSEQKPPLRGDRSPRSAAGVSVVSDVFSRSESLDQSFQSDASDGGSDRTDTS

FSRSGSRGKGTITMTKPNRAFALRRARADGSEVTDTARSRTTAASSAATPSTRRSSSIHG

TSRTACSDRTGTGRSEASLGAQIVKKARENTGASKARDSSITRQDGVPRNSLRAADRRSS

AGSSSASSTISSSKKERDIRTLKSQLKSAPGNKDLAVTGTSSSRSQSQPGSRSNSPKAAE

RMAWKRRKEYDPRKAVAEAKANKAKEPRPKPKVQSSSLYKQRMTRSASFTNSVELSGRVR

HDTYNVDSVSSVEDLSSAATASDSYGEVGFRRAFIPFHNPLRSDRLSHSADEDEAGTFPT

SQ

>SG-PL_contig_10801_1_+3_3_125

AIFSSKCTRGFTLVRQSAYTLDDPQSVKLGNANNKQTNCLC

>SG-PL_contig_10802_1_-2_14_304

MLWMMIPFVLWVVSFPATMNNYYSMRVPLSLSLSLPPSCLSLLLIFKLNLSYELIFKNRT

VHNNLFLWPFCFSNHNSPASLASYLKREKSVFTYNLK

>SG-PL_contig_10803_1_+2_62_181

MNQDKSKEKKKQKHSNEKEKEKLAVLCSKTILFRKNSLKF

>SG-PL_contig_10804_1_+1_202_426

MGSEVMEFVVFASVVILCCSFRLHQSLNHSVEIPVPGSPWEPALSRHREATHQRRLHCCW

ITSVEFSSACSDSTY

>SG-PL_contig_10805_1_-3_201_350

METPALIRASIREGDWASSIVPMDACLHGLIHPSCRKHPRRVWKAQALQF

>SG-PL_contig_10806_1_+1_1_216

THTHKHRESLEQHTQSPALSDTPTHLGHFIHRHTHTIQAPDIRHPNHPPPPTHTVTGIPR

TPYTHQTSHTHT

>SG-PL_contig_10807_1_+2_2_223

VLLSLYCVLYCCHCIVVIVLSLYCVLYCCHCIVYCIVHFLVYCIVISHVQAYDIYHSFAH

QTSGWEVGGSNPHQ

>SG-PL_contig_10808_1_+2_2_202

TVGISIFIRALLSSHLTHCMKTKQTDHTKHTANFILHTTHTHMMKMLRGKRGIAKVRLRV

KTDINNK

>SG-PL_contig_10810_1_-2_491_619

MNFFGKTWLNSKEATSASKNIVVMSMCFKCGCNIIYENLVIGK

>SG-PL_contig_10811_1_+1_139_306

MSIKISTPNYIYIYICIYMYMSINNSTLCTNCSHSLKYMQNIMTRHNNIMTRHNKC

>SG-PL_contig_10812_1_-1_181_468

MYVKMGTKEILMLEDWSSKVQYNAFCKALSSGILTHLQENIVLRDFFDLGAPLPAGGQTG

HKMSKIQRKYYNSQAEKARTISRGKNRDKRSVVMGY

>SG-PL_contig_10813_1_-1_1_126

MFVWNCHFMMNVCFESSFHDQCLQSHGYHRYYFMRNCQRMLV

>SG-PL_contig_10814_1_-3_3_134

NCHFMMNICSCTVKDFLVLNFHFMMNVCYELSFHDECLFGIVVS

>SG-PL_contig_10815_1_-1_16_243

MKSTERSLHHLTGSCPCRFSVSNQQVSFAALGVGNACNLFVRFCDGYFFPVFVILLVQLC

GTFTLGLETLIIIVIK

>SG-PL_contig_10816_1_-2_2_262

LSFEKSVFLVLKFGSASGLFMNADKTQAVWLESKKWSQIKYMQHLNIHWNPDRFKILGVW

FTSDVKGCEEITYSEKLSEARALFKFG

>SG-PL_contig_10817_1_+2_2_172

HTVSSTLTIQTATNQTKGHKSFPVSCLLHMKGLQTATALQQNQRATGQHISFVMLCN

>SG-PL_contig_10818_1_+2_1190_1357

MCKYDIQRTQQQSVYILQRNTVYNAVLIFMLTWTLSLIPNLRFAVKINQPSLNPIA

>SG-PL_contig_10820_1_-1_1_300

KGKKYQQQKIEKHEVIKKNNRKGKKYQQQKIEKHGNPSFSENKHEKGEKRYRSNVDAYFP

HTFKIRLIHECWSQMYFMCTTKIPWVCYYIPNKHYTSYHR

>SG-PL_contig_10821_1_-1_1_219

RQFLPDLVSQSPASRPDVPVSFTPGVETEAVDRAVLRLKASPLLQHLFDDAAAAIRGSSH

LPDPGDADLSAPG

>SG-PL_contig_10822_1_-3_135_203

MEKQAQYHNCHRLTVMSKCPLVD

>SG-PL_contig_10823_1_+2_2_256

HIHIYISTYLSLSLSLSLSLSLSLYIYIYIYIPDIRISLRRDDICTPLAGSSLIPPAPSA

HAHNCLRVDLTHLPIRKHITATVSS

>SG-PL_contig_10824_1_-1_1_351

QTYIQTDRQTDEKKERKKEKKLKAGRKKASQKETNKSRTEPQIAEKKRKTDRQTDRQTDR

QTDRQTDRQTDRQTDRQTDRQTEMGGKRNQHSELTHIRSSILSHCQRKKNGRKKERK

>SG-PL_contig_10825_1_-1_1258_1506

MHTQNASNYMYLGSFYLFNKSSGTVSVLSSPVLVLPFSVILVEDAWIIFSFLSSHFFVVF

LFHMDSDSAGVCFSSSSIRFVQK

>SG-PL_contig_10826_1_+3_1032_3503

MDDYLDSDTSAYTEKHMRQKIESHFGDEIMITCIRGKQNVVTFRSVAEKILKQFGNSKKE

TDSEKEKLRIIQTATRLILSDIQRIDTSKQEYPSTAEISDASKNIEQLTPSVAPFLGTLL

EGKKEPELKIASLGQALTQAARPNVFIQPLQLALGVSLRLTGSRHVVDQLNRLGFSLSYN

EVKLYEMCDAVEQGTELVGISPQNSFIQFAADNVDHQINTLDGTGTFHGMGIIGAATPGT

KDSRIIRRDTSVTPEKVSALGRVPIRFYDTSTLNISMAYEILKEISADDGSKKLDLLWKV

SWPLHSPRPGWSGMMQAVCNEVYPGQSTFTFLPMIDIDPTNMSCIFSTLHFVASLAKQYN

CTPVLTFDQPLWWKSMIIVDSEPPNSSLRSIVLRLGGFHCEMSFLGCIGKLMSGSGLEQV

LEVAFARHSVNHMLSGKAVSRAVWGHFLVDSVLNSLFVSTAFRIPLNRHSIADTEQGEND

SKTGEPSSSQENLSADDPMDESQHQQVDEDGVSSEQSADSSMQRKLSSVLQVFDNVIDKQ

MAVDELDEEANKDVEAVEQQLLKTRSQLEGRTSKLWLQYMMMVDILRQFLKAERTGNWML

HLQALQRMLPFLAAAGHSQYTKSVHIYLQHMQKLEDHHPDVFDAFMQGHHVLRRSDRFWA

GLSADLVIEQVLMRSIKSAGGLTRGRGMADSQRTQWLLSMPACADMHNALQEVTGLEYCT

SEQHVEAGENRMARDQQDMQALLNFLIDRSPFEDDNSLRNISTGVTADTSVNVDKAEMVG

KAILASMTGVHVKDFKLSKKQQAGTMDVRTCARVDGESFQADAQ

>SG-PL_contig_10827_1_-1_1_108

NKIFQRTNLLNFVCVCIFLHVITSTTEFIAYCIFYN

>SG-PL_contig_10828_1_+2_125_280

MIFRAERAAVFSCCFSNNYVSLWNNYSNFFLLFLISFTSFSPHPQNSYRMTG

>SG-PL_contig_10829_1_-1_1_282

GPIQLRNTGKKPDVSKQPKPEDKDSREPKLPASNPPDVSKREKQGTAIERAVVRYSYAAE

QPDELSLKEGEIIRVLDKYLEDEGWWRGEVNGKV

>SG-PL_contig_10830_1_-1_1_132

RFSQLFRNGSLPNLGRRCGTFRTREYTRWEERSHCQGNTDQKCA

>SG-PL_contig_10831_1_+3_3_113

SLKRHGFFFFFFLLLLNIIYIVYSPADHYGNIRAVTT

>SG-PL_contig_10832_1_+1_409_582

MTIIVRILFSVSIHEHTHTEMHTEKHTDKPAHAELLLLLYILFIAQLTTKAISGLSPC

>SG-PL_contig_10833_1_+1_1_222

LSLSSQKHRQDGKVKCCTNLHQHKFCSLSLSPQKHSSHNNILSYTTNNVCICYLPMPFQN

FVSTTLGPGLSNTT

>SG-PL_contig_10834_1_+1_118_360

MQCNTRIMPNVQPHCTSALSALTKFAPLLPRSAEIIREMFKIIKPRLQQMHVKNRQVLSS

SSGYVRVMYWEIKLATVSFFI

>SG-PL_contig_10835_1_-3_18_266

MEPKASGIYNNTGFETVVYLLLVYIVNARHNPSSSMYKVFVSCVAYLPLCPVNSSLYHGV

LYAASAYTITIYSLIFISCSFHH

>SG-PL_contig_10836_1_-1_1_162

MRLAQLEVRMALATLLQHYTPVLCDKSVYPPQVEPPQTRMVAKDGLWVKFKPRD

>SG-PL_contig_10837_1_-2_2_205

LSTYKLVAAQATELLKGLTCECCITKKCHNCSVEMVLNVMKYPMYLLEHSTMDRCKFERD

FKIPCTRP

>SG-PL_contig_10838_1_+2_98_232

MFSQSVILISVVLHDIHSVQGPHADMGTSKIQYRPRLTKNEIKRN

>SG-PL_contig_10839_1_+3_3_95

PKYSTLDTPVLVSVILICLSIHSSMKGLKNR

>SG-PL_contig_10840_1_+3_81_323

MRHCTKHDPTLSVNRVIHHGHSTYQPIPRNPSDSCSTIPRFLCPRTGKILDSHLSLNGKN

PVMLLDSCVSLNGKNPLLDTF

>SG-PL_contig_10841_1_-3_6_437

MENGGGLFGPDEEDEEMVHSLGKHALVSPSSEQYELLVETIKQRVEDAQGETIYEIGTGE

GDQPGLKVEDFQASVATLHSITESLGLECVQLRERAAEEGTVAEFLLRQKLDAEDFMEIR

VAVVGNVDAGKSTLLGVLTHGELD

>SG-PL_contig_10842_1_+1_34_633

MMNDLDLNDIWRDMNPDCYRYTWRRTNPFQQSRLDFFLVSDSVLHYVEDSDILCGYRTDH

SMISLKLRFDQETKRNTFWKLNSSLLRDKHYVDEINVLIEDIIKEYAVLPYAREHIHEIP

KAEIQLTISDDTFIDFLLMKIRSKTIAYASMKKKKTNQREKILEEDIQTLEKKTRITEEE

VRDLEQKKVELQSIRQKRME

>SG-PL_contig_10843_1_-1_406_696

MCSSTAVNDHFVFKRMHVCNMTRSSIKSLGHSLNENSLHNDISVVFGANCVRSLLCVQVL

SVTHTHTHSTDQVFLVCKFSVFSVNVAGLNCACIRTF

>SG-PL_contig_10844_1_+2_2_97

FAKTYQSDIHYTLVKIVMKFTLKPQFCKNSTI

>SG-PL_contig_10845_1_-2_761_2014

MEDGEDTVTGEAESHSSPSSMRDVTVSTRSSTKLPETEEEPRDDSITMAKKVRRWEVFPG

KNKFHCNGRIMMARQTGIFYLTCTLIIVTSGLFFGFDCVYLAQHVTPAIPAIGGALFIFV

MATLFRTSFSDPGVIPRASPDEAADLERQMEVPNAGSPGTYRPPPRTKEVVIKGQVVKLK

YCFTCKLFRPPRASHCSFCDNCVERFDHHCPWVGNCVGKRNYRYFYLFILSLSVHCIYIF

ACVLTNLILRAQQDNFLTAMKDSPASVIEAIICFFSVWSIVGLAGFHSYLVASELTTNED

IKGSFTSKRGQDSFNPYSKGSIFKNCMQVLCGPTPPSLLDRRGFVVPDSSQSHTGNVGTN

TTPDLPPPAVVTTTDEMLPGGENVKKREMISYQTPDGSINNKLPPINGDSQAPLVAGN

>SG-PL_contig_10846_1_-2_542_670

MTSSRLNFFSIKQARFSFSSSGKKVGPQPQHYSSFEMATFQRR

>SG-PL_contig_10847_1_+2_2_124

KKPNPKQNQKISTDSDFLISTNIFPSSKYYFLTYHLYCAVL

>SG-PL_contig_10848_1_+2_2_106

KKKKKKKAFVSSPSSYEQIPDYGLSVVGWAINNMI

>SG-PL_contig_10849_1_+2_32_274

MKNNGTVTLTSIITVPQLPYMCNCWKVYKQNKHEELTILIQQSHAYMYPCQILNCSLYFN

LKHHQIRYVISSFCRKELDSL

>SG-PL_contig_10850_1_+2_2_190

PARKRCDGMHYCMSGTCSKNAEGMCCSWMLWKRAPYYFLIGIIAFNLFFNLLLTLYFCFV

FFF

>SG-PL_contig_10851_1_-1_94_1614

MPAEIEAAKQMQQKMNKTKLQISYRLESIQSDNQMSALPETTCIMEETETESTGVRLPSY

EEAVSLTPSSDVGDAVMTSDDPGQAVREGVSTDGTELFSIADGVQIFNISCEGYVSAPSY

PTFLRIVRFQESLEDDTGSQDEADDTQAFLQVGDWLYPLIPGTSPVLHTTYGAYLFPDLS

RDGSGSSVGLILPDTMSQEEHHVLEDILRNLSTVQEQQMETPEEDQTVPSAPVLEPQEPS

APMAETEAREEEQGTRRSEERSTSAKISRGILVASSWISWGLGKGAEKAGQLISYGSAKL

RDNLQPEIEPRPVDPCIQKGARYARKASAVAVSVSSFVVTKLGEATMALGRQVAPHIRKK

GGELLPKSMKSKRNKSRMDGVVWVAASGLQGFGRVWLSLEAAAKALGKNVAKETVATVYH

KYGKDAADVAEDTVYAAGNVAMTAYNAESLAVRAIVKRTAKDAGAAVVYDLQETDKQHTD

GCVNGTCCDGNGEQCQDARKKEKGPPV

>SG-PL_contig_10852_1_-3_84_1244

MDERLLKAVDAIHVATVFEDCVDQLAILGRIMPASFEFRPDAADIVRSEIDQLVDEQTHL

ESKFQTALEGKAHLHSKTKNSAKLRAAEQGVSDVGGDLKNSTHVFGRSLRQNPLTGDNIQ

KIQEDRGFVEVVMTGTMAELAQNRSFEALVEAVKAAKQRKASLQNTIQKEEDGRKRVKLL

QKQIQDVRVEKESELQQRNEMISHLKDQLQEMKAKSNMEGKYIKKCAEVAVAQTQKRCML

SEKDLKDEIEKLQNKIDEENRANAEIESYLRAHTADLTKKQEYWMEKFEKDKDAKQHDLD

VLKASKAKDLERLQELTKLYKEYEQVVVEDRIEKEKARRKLEQEAIELRACIKLQAWWRG

VMVRKGFGPFAKKKKGKKGKKGKKSKK

>SG-PL_contig_10853_1_+2_2_169

DWGCSQIWVNYDRAHTGTQTGAVVKYGLITIFDKIVINFWIKFCHFHFHHIFCIIK

>SG-PL_contig_10854_1_+1_1_183

GGRERLRHMHVIGTSPHSNGIIFTQRCRYTQPTTQLRSTVTLSDSHITTHQRRHTHVTTL

Q

>SG-PL_contig_10855_1_-1_1291_1680

MPSFGQEKTCWHFAPFNFFFLMVEMASAYKALAQESWEKSWGEWVGVCNVGFLSHVLGQE

KSEDSRPLSYSCNHCHNTLHQNLLNFLILSPRIVGHARSRMTSFPTRFSTRSGQMAHRLF

LCLSLYQSLL

>SG-PL_contig_10856_1_-2_524_718

MYSVNIFLEVIYYNINYIPVNTSPLVLNMKIKMRLCIGIHNKHETLSPYVFKGFQYVMIK

MQRIK

>SG-PL_contig_10857_1_-1_1_117

LLPNCDHSQGDGCYLNRYCLCFLVAVVVGWGFPLLFVLV

>SG-PL_contig_10858_1_-1_1_156

FFIDEFMSASAKMLCNFALILIHWHYALLSLGECCNLTFFIIITLTTFENFL

>SG-PL_contig_10859_1_-1_274_474

MLTWEIGKISIHYPTGEVPEKDSNPGPPNPPVWKSSVTIQPQCLSSHVACLSDSHLWLIR

YFCCHFL

>SG-PL_contig_10860_1_-3_171_461

MRYVSTWAEYHKFQVPRGMVEDGMLRNIQPAAQKNVMYILGCKIQADQLYAGSTKNIKYR

WANHKSETKLHKITKCAVSKRVCTTPHPDSGNYNTCK

>SG-PL_contig_10861_1_+1_820_1074

MVPTVKSSVAIISGYTTCTPDTVIKSFQPPCTMNRENTATDTLTLSRDKDWLWPKALSVN

DTSEMLRPTLNCCYALQCYVGTVFI

>SG-PL_contig_10862_1_-3_3_287

HTHTHTHTLLSLSLSLSLSLSLSLSLTHAMATRTLNFLLWQLEYGQVVHESMVDLFLLWN

FPPVPGCCRYERYDYVMRHHFPEDPTSTELNQSTN

>SG-PL_contig_10863_1_-3_1296_1508

MWIWKCNVDVSHCFIHLAQLSLSYVSEKCEQDSIDAHSFCIALHTSSQQNLSLKGRLSLP

GEYINFCCCCC

>SG-PL_contig_10864_1_-3_3_278

YYCLCLSYSVTEQIQAISTPSLWGILYIIVYACLTVIEQIQAISTPSLLILYIIVCACLT

VIEQIQAISTPSLWILYIIVCACLAVLLNNKP

>SG-PL_contig_10865_1_+3_3_203

ADIQNWMTQNKLQLNGDKTEAMLVGTKNKLVSLTVNTIQLGDSIIPLSDSVKNLGVTLDN

ILSMQKF

>SG-PL_contig_10866_1_+3_3_149

FFYGHKFILLFLAKFSQILETTPLTCKHEVHTHAQLTHSIISSKIVISS

>SG-PL_contig_10867_1_-2_254_2863

MRGGHKGVQRIKASGLVPGDIVEVSVGDKVPADIRILKIHSTTLRIDQAILTGESVSVMK

HTDPIPDPRAVNQDKKNLLFSGTNVAAGKCKGLVVGTGLATEIGKIRNEMMDTEQEKTPL

QQKLDEFSQQLSKVITIICIAVWAINIGHFNDPAHGGSWIKGAIYYFKIAVALAVAAIPE

GLPAVITTCLALGTRRMAKKNAIVRSLPSVETLGCTSVICSDKTGTLTTNQMSVCKLFIF

SQIDTNQIKTTQFEVTGSTYAPDGELYQNGKKVKASDYPGLEELSTISSMCNDSSVDYNE

SKDIYEKVGEATETALTVLAEKLNFYNTDKSGLNKREMGTVCNHVIQDMWRKEFTLEFSR

DRKSMSVYCVPNKPTRVPGGARMFAKGAPEGLLDRCTHVRIGNNKVPMSPAIKNEIMKLT

KIYGTGRDTLRCLALATIDNPPRREDMDLEDSRKFIEYETNMTFVGVVGMLDPPREEVMA

SIKNCRAAGIRVIVITGDNKATAEAICRRIGVFTENENTEGMSFTGREFDELPPEDQRAA

VMRSRLFARVEPAHKSKIVEYLQSEGEISAMTGDGVNDAPALKKAEIGIAMGSGTAVAKS

ASEMVLADDNFSTIVAAVEEGRAIYNNMKQFIRYLISSNIGEVVCIFLTAALGIPEALIP

VQLLWVNLVTDGFPATALGFNPPDLDIMQKPPRNPREGLITGWLFFRYMAIGVYVGCATV

GAAAWWFMLYDKGPRLNYYQLTHHMQCPAEPRMFKGVDCNIFSAPEPMTMALSVLVTIEM

LNALNSLSENQSLTVMPPWINKWLVGAICLSMALHFLILYVDVMSVIFQITPLSVEEWVA

VLKISIPVIILDETLKFIARKFADDYPGFF

>SG-PL_contig_10868_1_+1_211_264

MIFGSAFFPTQQKKTNPK

>SG-PL_contig_10869_1_-2_2_157

LLRSAATVSQPDGLMLLAFRSHLQMSLKQRAGLPAGLDQWQAHHRACPWGPE

>SG-PL_contig_10871_1_-2_2_280

HDAQVARVQPVLFFFQPRSSSRSHLGELFVECRFSFNFGPFLVYLHSFTGCSVESCFQDV

LFPCLPAQRRGETYIFLFSFPRLGVWPLWLPLL

>SG-PL_contig_10872_1_-2_245_649

MFRATAPCKGLISDEVGNQLSCCEMKDCFTLTAGRLRKREKTKMCKDTFYRLLWMDGQTQ

SFKCKSLNGHYPNPIFSVIHSTISNLCNTAHVLLYFGRKIGTKTPPCCGALPMKPSSRAR

AQLIPLSFQPRLRSR

>SG-PL_contig_10873_1_-3_42_287

MSGMLRRRKVPIGNQIHGHCVAKDPSRPKPLEINGKTISVVVCVFLLLFCCCFFHTGIER

KMVGLNTGKMICTVIIFLQNMF

>SG-PL_contig_10874_1_+1_1_162

SADDLGYSVSVVDKVQMSVSRNMQRKHHPACQPKITCIMVANLIVSSVICTLLY

>SG-PL_contig_10875_1_-2_65_421

MIYCRMLVANKDSEPEVVCIDGQGRSSGMGVIRTGGFLFKTSLNMVRKMVNPQCVLMKTL

GNKLPFEVVVGMNGRVWVKGRTNKETLALANAVCEAEYMTNEQIKDMCRKMSDSLAGFD

>SG-PL_contig_10876_1_-2_56_271

MGGGNMNNMNMNHPMQGGMMGGMPPRMGMRPGPPGPPGPPGPPGPPGPPGPFNMPFRPGP

GMQGLPQMRGSY

>SG-PL_contig_10877_1_-2_608_823

MHKQKSPFLYNNSSKIQKKIHNNTYIYSNGLFQHINSTKRCQYHMKHTQKVETSNPNVSL

LFFKLLHTQEIC

>SG-PL_contig_10878_1_-1_241_561

MKLFFALFALAVARLAVAMECDGTHKCDPGTTCCMTVSGKQGCCPFPQAVCCLDGEHCCR

NAMSCLHPSNTDKCYRIDRGQLDLITSKSSFDQSLFWKSEKVEDVRC

>SG-PL_contig_10879_1_+1_1_186

IHTWVDWSLHAHFFSSQVETSSAVTLEGESSISMSARPLSLHRCRSSVVTDTVPPTLCLP

DR

>SG-PL_contig_10880_1_+1_508_750

MVATWLDFAKKHATICLEALSFLLNFTGGLLCPRRKETLHQIFLAHGCTSSPYPASVLHS

GYGTPNGHNISSRQGSWEECK

>SG-PL_contig_10881_1_-1_169_1734

MQKIEMPTYLTLFSPALDWILQCLAHRAPDALLNTTLDKCKEQCNNALLLNSIMSAFHPQ

FIANRATYFTTMIRECEDTGFPKHLLQQSMGRCLVLADPPEDQKLAVLNEVWKSVMKLKN

PADYISCAEVWIEFVVKHFGKREVNKILADIIKHMTPDRAFEDHYPRLQSVMTKILTHMH

DFSQLFALDKFLPFLDMFQRDSIKVEVCKSVLESFGKYQVDTVNDPVVINAMMFLCKTIH

DSVNALTLEDEKRVIAGLLCGFIRRVSFGRDFEQQLSFYVEARASFSNLDPVLVALIQSV

NCLSIETRKVVRGNHTRKTAAFVRACAAFAFITIPSLQSVYSQLHLYLVSGQVALLNQCF

SQADAFFKTAISRIPELPSTLDSEGKVRSTEPYLLEYINNFVSTLLVTPDNPEAGVLYLL

RGLLNVLQDYTWDNNSDARVQAYTRVLAVLSAYTQDHLPYHVDKVDANDAMYGGDPKFVG

EVKGVADTLITQILAHLKTLSTPEMEKRKSTIAMDLLGTVVA

>SG-PL_contig_10882_1_+3_30_260

MNNSNINDTKTAYNLVVYTSFWFTKYSVTSINLAVLQPNDIIQRVSLFKQERNRIHKCTH

FPNPDHSLLFPMQGNKT

>SG-PL_contig_10883_1_-2_2_133

IIIILSLCFCGSFFSCFVFCSFLLVFLLVSQTSSFVSEICVAVR

>SG-PL_contig_10884_1_+1_313_459

MLLINSKQNPLLPKRHSQCYVNLHQQQARSFEVLMEFTIYAIILISLAN

>SG-PL_contig_10885_1_-3_60_1049

MDKKYTNTKLQLLLSPCVLIARDNLHREGDQEHLRDGHLALSGLQVRGHAMFSHEGLPLE

SETIEYAWLIEAIIGTVSGKLSTPQLQNIAEFIQTFIMLVDNPENQLIRTSPYVLCQHMM

PQHKCRMLLRRDCPAAPFPCPSSEDIKYEMVRLSLDSLNLFMVESGAALNLSVRPVRIAH

CNLHGSATRAGITGVIEKITLKQFMNCAQSRVDTKHAETWLEAGGFALGPITLEAAMALP

NPQLHQIQDDFLKVHDKRSKRLWFLWPQQQPQGSGNGNCGCLGGCEFFGSNKKGATFFKP

RKHRESSQMAIPQVCSAGHDPGFGQSLLHE

>SG-PL_contig_10886_1_-3_3_362

GAGGHPEPERLHAGQPCAAGVLQSQQSEAVDCSVYTQKLFDTNSTAVELPKYHSLTTVTA

NGTLLQNETLHQTEVHHAELYSKQNLQQNRTLLQTEAYNTELYHKQKFITNRTLIVNRTL

>SG-PL_contig_10887_1_-1_1_78

GYKQYMILILVNFPFGKSRLITVSLM

>SG-PL_contig_10888_1_-2_56_1366

MQLMLEKMEDGLARATNDTASVKMFPTYVRAVPDGTECGQFLALDLGGTNFRVLLVTLRG

QEVKMDSKIYLIPQHIMLGSGVQLFDHIADCIFRFVKEHNLGGRKLPLGFTFSFPCRQHG

LANAVLTTWTKGFKCEGVEGHNIVTLLHQAIQRRGDLEVECLAVINDTVGALMSCAHTDR

KCAIGIILGTGSNACYIERLENVEMAEGDEPGPKEMLINTEWGAFGDDGCLDFITTDFDK

QVDKHSINAGKQRYEKFISGMYMGEIVRLALEKLRKSGLLFGGEGSYHMATRGRFYTKYV

SEIEADDQESFKTTKQIFEELEIDTVTEQDCRLVRHVCSLVSRRAAFLAAAGVSCLLNKM

KRPEVTVGVDGSLYRFHPFFHDLMMEKTKVLAPGIRYKFMLSEDGSGKGAALVAAVANRI

KTEHNTMQQQKESDTSV

>SG-PL_contig_10889_1_+2_32_169

MPLVGGLPGSLLGGSAIVSGRVVPLAGTSQENLGPPTLSVVCAWLL

>SG-PL_contig_10890_1_-1_1_270

NQFYDSRVVGKYCEKRDPHLACVAYERGQCDEELIKVCNDNSLFKSQARYLVRRRDPDLW

ATVLTEANEFRRQLIDQVVQTALSETQDPE

>SG-PL_contig_10891_1_+1_259_708

MFWGCISYSGVGTLVDVNGNINSQKYVEILDQNLWPVVAKYFSDKPWIFQDDNCPVHSSR

YTLQWKRENSIPSLLWPSQSPDLNIIENVWRKIKSALNRRPQEVNCCADLVRVVQEIWVS

LSPAYIQSLYNSIPTRIRHVYRANGCISRW

>SG-PL_contig_10892_1_+3_3_227

ITNTCEKFSLSLSLSLSLSLSLSLSLSLSHTHTHTHTHTHTPGVDLNISDFFRGIYNEVN

SKHSYTHTTLKPVCE

>SG-PL_contig_10893_1_+3_3_377

ATSLKYGDRSACVFQGSFWNFVAQHAQTGYVHLHGRHVVLNAGGIRQVIGVFSVIHVTTV

VQFLGQTHTSIRHDLRRLSAVLVWLVHILSLKNGAITHSPEGLVWHIAVVVGELFLHFQV

EFIVE

>SG-PL_contig_10894_1_-2_161_337

MYFECSLSSVLVLPYCECHICVIFAENVLRSDRYLLDILLIETTEILHSQYCCYIQTMR

>SG-PL_contig_10895_1_+1_694_1005

MVLSNHMNLSHDLQKQTINKIECRNLKQQKKCFFLFFLSLCNAKDSGIQHVNGRSIHHHI

FFFFFLTIHDKVKMGKCEAISDDLSYLINRNQCHIFSDYLPYLT

>SG-PL_contig_10896_1_-2_2_157

LEIRGSDLVHAKFTSNFQGSPEFLKGSHFINRQKMNNENTNTHTHTLTGSHI

>SG-PL_contig_10897_1_+1_133_297

MGLVPALGFSQVYVPPSLNQDIMSGGTAKGSDVRSHMIKGLDLGCVSSSQLRFQT

>SG-PL_contig_10898_1_-1_76_2181

MSKRRKALIDSDSDDSDSGEDIEESIRALTKRRRTSSASASDSKSESQSEAQNKTAVPSD

SDSDTSNSDDDWTANGKKGSPKKKRVISKKVARRSSTVASSDSEKASASEPEEGEVSDSG

SGGDEPEAKFSDGFDENLIGDEEDKARLEQMTEKEREQELFNRMERRQVMKTRFEIEQKL

KRAKREERKKKPPVQQLPQERTRPQSTPDAVSTATISERSKERRKNIEDRKDSKKTSAFQ

DLKARREEKLKKDEEKKQQQQKKLKASDIYSDDEDSEGDNESDNEDRKSVREDRKRSEEK

ENQSDQSDAESEASRRSSSPSEDEDDDDTTTRSRRRVQYVTMKEELDKIRLSRSKLERWC

HMPFFNRTVEGCFVKIGIGNHENRAVYRVAEIVGVVETAKIYQLGNTRTNKGLRLRHGTA

ERVYRLEFVSNQDFTESEFQKWVTTMSMQGVDLPTIPDIEKKVADIKKTYQYSLKESDIE

QIVAEKQKFKKNPHNYAMKKTHLLKRKEEAENAGDHESLSQVIRELEELEQRATELDRKR

TNNISSISYINQRNRIQNILDAESAFAVEVKEMKSAVADPFTRRHCRPTLVTKTREPDDL

SKLQKQEEEAAMAADQAKEASASKTMGDQLSSERKSSDKTDGGEHRVSEDLFEVHDFEIK

IDLDVPSAPSVVVPSASSGGPREGAPRRSLNLEDYKKRRGLI

>SG-PL_contig_10899_1_+1_1_81

SSIPLRNIKTDLLTAPINAPIILVVSS

>SG-PL_contig_10900_1_+3_399_632

MLIFDSQKIIYHSRHNICFIRLAWKAHQVHIPTHSTFTIISWVWTAYIVKNATLLDIHTQ

TSTLNIMSVITLCITFKI

>SG-PL_contig_10901_1_-3_102_3389

MFRSVELGRRITMDYSGNLYFVSVEEEDGQNDLRYVCMASNNIVRSINTGSDKTIEIVKL

SPAEEATLLQWRSPEEVLALEGRKARFKCIFSGNPTPEVRWRRVDGGPFDEQRMVQSDDE

HEFIISDVTAEDAGKYACSGKNPLLREAKEAIFTLSVQSRPRWTKRPDDMTVGVEDNVTM

VCNADADPEASIQWFINGVPIEQAPPAKNRQQRGNALVFTNVSRDDSQVIQCNASNIHGY

LWADAYLQVEAMAPSIVHKPAEQKVAKEQNITIACGVEGKPKPKVQWYKNDQKLLGSRYQ

IMPSGDLHIDSVVERDSGLYHCTAKNRFGTARAEGNLVVREKTEIVVPPSDDQVNQGQEA

IFTCRAITDIEEQKRLRFVWLKDGVLMDTSDPRIEVHQGNLNIRDTTSKDSAFYTCVASN

DLDSDQAVAKLEVIAPPDPPSRVTMLSCMETQVDIEWEFSESQSNFSPLQEFIIEYSTDH

HPDVWREARRLPASRRQMTYNMSPWATYTFRVRARNLMGLSEPSSPTHLECRSPEARPYS

NPKNVRTVEDRTGWLIIEWEPMNRMEHNSNDFHYIVTWQAKGVDGREEGVMMSHVVDDWT

LGRHEVNVDGIYQPYTVSVKAANAVGDAIVPPLTVTGHSGEAVPSVVPENFELDPDQSVT

STSAAFRWDPVDTSPEKMNGKFMGYKIRYWKKNRQVDTLHEELIIIPDSKRHRRRAGYDD

KVRGQVSQLPSFSEIEADVVVVNTYFSSPGSNIVHFTTPEGVPSRVKYLEALFRGSSHFL

LEWGPPVETNGVLTGYQLGYQKVDGLILGELVIARDDLPVGQQRTTLDGLEPSSLYRVFI

SAKTAQGPGPHYFVDVRTTDETSELAEPTIDQIVPGEKEANVSFSLTEAQRGERTGSVYY

LEYRRLGEHGWDREAEGATDHFWLTLEDLDPATTYEVRVVSVPKTDRNSKEFKPSAETRF

STTGIGTKQGSFLSAAWFIGMMVAIALLLLILIIVCIVKRNRGKAYTFPPERDTVDGTPA

LFSEPPPKSAKNGVNTGGPQEPEAEKIPLGEETDSMAEYGDDPVRFNEDGSFIGQYVDSK

PPTTEGPIPSGMSHIV

>SG-PL_contig_10902_1_+1_76_177

MELLEQCGVNNCVGLWPCDVEPGVACLCDVSGTL

>SG-PL_contig_10903_1_+1_502_708

MWTRMNGKDLPSASWRPDHFVDCLPCQSPGSGRKPPSTSMRQGQRRRSRRIFAPISDAEI

SHHSPRVQL

>SG-PL_contig_10904_1_+2_383_571

MLTWKIEKISTLHLPGEVSKKIRTQAFQTLQLEVLCPNHSTTTSGWVGTREILSTILLTY

INI

>SG-PL_contig_10905_1_-1_25_813

MYLLYDDDDDDDDDDDDDDDDADDDDSDEEEIKRRQKDAMVKNPCEEKTCRRGEMCIVDK

KYRARCVCYSKCLPALPGDDRFQVCSKRNVTYESECALDRDHCLCRRNLDGCLNSKYKKI

RLDYYGPCQQLTPCSEREFKEFPRRTREWLFIIMKELADRDNMPSEYQDLLEQARQEINH

SHAVLWKFCDLDVSPHDRFVSRRELMYTIQSLKALEHCLIPFLDSCDADTDGQITLWEWG

RCLELKEDEIKDVCKAARRGRGN

>SG-PL_contig_10906_1_-1_100_447

MYFQSLSFSFSLFLSVSHCLCLSICLSLSPRASLSCLSLSLSFCLFLSPNTSVREFLWDL

MLLSLCNKSFSLSLSLSLSLSLTLSLSLSHCLQVYIERNIGTLKHASRRVVGTDCN

>SG-PL_contig_10907_1_+2_2_145

TMEFVPLERLQDTSLGKVLRRTCCCPCSRPILSTTPLKSKEEAKANKY

>SG-PL_contig_10908_1_+1_139_534

MVNPHPPSKRGYMKTITSTYTTASVHIIKYPDKQVQSINIYNQSQLTQVWNKRGHNECKP

IKDQHLSLSSDIHPTTEPVQTIGGQSGVLTNITTGSERLTPPTANVLWPQVHIVHPHTHP

DQCICIVRGMGS

>SG-PL_contig_10909_1_-1_55_1359

MRALLGAAFITYLPSAPEDLRRERMNAWMEAIGITHFDLRRFLSTESEQLTWKGEGLPSD

ELSMENALVILQSSQRPFLVDPSSRATEWLKGHMKNNRLEVINQQDANFSTALELAVRFG

KTLIIQEMDRVEPVLFPLLRGDLTAQGPRYVCQIGEKTIDYNEEFRLFMTTRNPNPEIPP

DAAAIITEVNFTTTRAGLTGQLLGITIQHEKPELEVRKTDLLKQEEDLKIQLAQLEDSLL

EELASAKGNILENKELLDSLNKTKASSITISESLTESMRLQASLDAERNSYLPLAMSGST

LYFVISDLAKINNMYRFSLAAFLRLFERALETKHDGSGTDLRIRNLSHQLQQLVYEYVCR

SLFKADRLMFAMHLAHGIKLDLFGDNEWEAFTGMLVADVKGEARAPSWVDEERASSVAAL

KANFPKLYQQLDMDD

>SG-PL_contig_10910_1_-1_838_1218

MHFVSPTLCRCYVAAQCACFIFIGCACWLFLCFYKPLNTDMVYRIFKYIVDVLCVYAHEG

VSATGRSAHLLTWEIGGSLQPEPTRRYPGRFELGPFNCKSYVLITWLSQLSDVTLSDNLN

VVPLAWL

>SG-PL_contig_10911_1_-2_47_229

MNTNRKLSSSESRSTVCCNYECASLKEIFLNNIVNYQTLHGEITHMCNMCVNFVFNRFVN

L

>SG-PL_contig_10912_1_-1_1_300

CWPVSIILFFISNDKLFLSLAVPLIYSRLHFSTEVRVQRIGSVEASKQKSCLLFEIDSVT

GLSKMNLLSKTGSCNIFFFSFRSANGISFSSIFLFCLQMV

>SG-PL_contig_10913_1_-1_847_1131

MAQFKRVKYKQTNSSNKQACQRMFRALSPEITKTVQLQFASLSRPTMVTDLFQSQDFSLL

EIFFTFISEEHTHTKAHTFLHMSNVVSDVYMSILL

>SG-PL_contig_10914_1_+1_7_153

MLTDFYVWLCGTYTFSLSVLVNLTHFVHPHEVVFCCCFSFNVTLFNEDY

>SG-PL_contig_10915_1_+1_202_435

MERIRQNQAHVIFEYNALINSIPNSWKHWLQANNNTQMQETETFNYTIYCTKPKSIMKML

KDKDINNENLKDVRMDFG

>SG-PL_contig_10916_1_-2_119_268

MWNCHGFSHQNSSLGAWYSGGGGGGVNYYLLQPKIPWQIVVTEHKRGVAL

>SG-PL_contig_10917_1_-3_3_212

ILIIYNCFFVYVVCGSELSECSFVVQLWNVKLAYTFKLWNLRKMKVSPHCTILLTQTFFV

TVQFCSYRSV

>SG-PL_contig_10918_1_-2_35_814

MERLQSVQHVMVVLSGKGGVGKSTVAVQLALALRAKGKKVGLLDVDLCGPSIPKMFNLEG

QSIHQCSEGWLPVYVDAEQKLGIMSIAFLLENNSEAIIWRGPKKTAMIRQFLSDVHWQDT

EYLIIDTPPGTSDEHISVMENLRAFNPDGAILVTTPQAVALGDVRRELTFCRKTGIPVIG

IIENMSGYKCPHCSECTNIFSSGGGEALAKQAGVQFLGCIPLDTKLSSEPSCKQSLTELP

SVAAIEDVVNKLMAVDKDLP

>SG-PL_contig_10919_1_-1_43_204

MHGRQVSHSGKKSCLLAVAQEPEFPVVISQPYFSSINTSRSYSILEFILKIQAN

>SG-PL_contig_10920_1_-2_164_286

MFRYVIITAFLLSLVPFVCVWLILLSVRVCLANIVECVCVC

>SG-PL_contig_10921_1_-2_2_163

LVSKFRTETRFFSVRNTKEKHGYGYGLVSSALKGSDKGTVTSIRERKGGFVTEL

>SG-PL_contig_10922_1_-2_29_250

MSTRCVSCLSLHSLILLKASLCAPFIPISEWNQISVFVRTLLTMTKSTKYNTKTDQKIIL

ITKISQHQNSVCLV

>SG-PL_contig_10923_1_-1_19_753

MKRLQLSIPVPDDRPNWADWERHEVMMSVTMEYIPENFEREYLAVICPVAKRRIVVSYLG

WTCSYNKLLNMDRKFQSSLPGGNVRSVQVIKSNDMCVLDCLHLKAERTFYILDLMHWKQL

PYYSSESDFRFHWINQVTNEMDLTSIHPENEYKFRDMPRVRCTQQDLYHALDNTHFKIDG

ILFFHVQGRYVPGTSPNVLWLEPGNIEQVLGWKPPDHVLPAKKQKAAASETANTTAAGSA

ATATS

>SG-PL_contig_10924_1_+1_211_342

MYHTRNHIITKLNHTLLHASHLTQTHLATTTHVHTHTHTRIHTH

>SG-PL_contig_10925_1_+1_796_1548

MCTCVCLPAALSMPKYSSACITYTRDWGDCLLRSWKVISHSYRFALCSSAIAIHTIHTTY

QPNIHPLLCCENHDRNKQEREPPRNTKTLQTATKHHLHTEWFCHINTSIIRDTVSIFRFV

GRSVTIKIIDEYIIFVKRNIPNTKGHRQCQTEWQKHMHHKNLNNNNHKPRATIGASNEEI

KMAAGSASHAETGSAVQDLSSSSDRMQAMNSSKSMRPSPSISTSAIISSISSSVKSSPMF

CSTALRSEGVM

>SG-PL_contig_10926_1_-2_2_166

NTRYLESQNSVTQDILCVCVYIYIKCSTFSGGFITLVSGTCTVTYNALEYCRSQT

>SG-PL_contig_10927_1_-1_172_4878

MTSYTSEVADDNDSVNSADTSSTQEGEFINPKGVRFMQTQQQQQTKEGYPLIPYGLPCVR

ELFRFLISLTNPIDRHNTDVMIHMGLSLLTVALETGADHISCYNSLLYLVKDELCRSLSV

LLQSERLSLFAAALRVSFLLFEAMRSHLKFQLEMYLTRLSEIVVSESPKITYDQREIALE

SIVQLWRIPGLVTELYLNYDCDLYCSNLFEDLTKLLSKNAFPVSGLFTTHLLSLDALLTV

VDSIDQHCHHRVLSATKSSASAEGDSQQSEGSASGVEGEAKPDPSGAAEPPLSSFRGPPS

TGFTMAQFLMQKQDPDFGRSAKKEQRLGPQIKPNRMAKLTAKIPSREDLAEFKQKKRAYF

QGTEYFNQKPAKGIIFLQEQGLLATPLDPGQVVIFLKENPKLDKKMIGEYIGAKKNKVVL

EAFVRSFNFEDMRIDEALRLYLETFRLPGEAPVISWIMEHFAEHWHKSNGEPFFNTDAAF

TLTYAIIMLNVDQHNHNVKKQSIPMTMDQFKHNVSHVNGGKDFDEDMLEDIFNAIRNEEI

VMPAEHTGLVKENYIWKVLLRRGATKEGVFLHAPTGALDSELFTLIWGPTVAALSFVFDK

STDETIIQKAVSGFRKCAMISAHNNMSDVFDNLVISLCKFTTLLSAAESPENIPVTFGSN

YKAKVSARNVFGLAHRHCDILREGWKNILDCMLQLFRAKLLPKSMIEVEDFADPTGKVSI

VREETAAVTRSESGVLSSFVSYFSLSESAATKGPTQEELEAARQAQNCVKDCHLESLIND

TKFLRHESLSELVKSLIAACQGAEEQGSLGGQYDEDAGVFFLELLVKVVLQNRDRVTGIW

SAVRDHLYWVFVRSREPGLLVERAAVGLLRMSIRLLRREEMAGQVLGALRLLLVLPGPVL

HSLARQLTYGLHELLRTNAANIHAANDWSTLFTLLEVVGAGANPPPVMQVDSDVDVAQVL

HDAGAQSDSEVAEGGGASSANDRGYTSDSELYESASRHQHSHRGSPELALRPVPDNGSWM

LVNREDAASPSFPPAPLRVNQYSIELREELVRIDRRSLMKAAETLAFLVRDAAHVTPHNF

QSCVHAIRIFVEASVNGGHHRSPLRSSSMRERRGGGHPHNPPRGRYHRDSKMKKSRSNPT

QLTQISTSDDEGEEEAALTSLHSLSIQLLDLMHTLHTRAASIFAAWAQESSENSEQAGSG

EGEKPIDAATGTLWATCWCPLLQGIARLCCDTRRQVRSQALTYLQRALLVHDLQTLSAME

WEACFNKVLFPLLTKLLENINAQDPSGMEETRMRASTLLGKVFLQHLSPLLCLSTFTALW

LTILDFMEKYMKADSSDLLSEAIPENLKNMLLVMETAGIFQTDAGTESKLWRLTWDKIDT

FLPHLRDEIFKPFEPEPKKMDRSETGEESSAHIASQTTGDREGIQQQHQHQVTEAPVMDG

QQMSGVPDVPLEGITVIPTGHVAVAQQQDVIQRPESPLLSQPEDSTPSTSYILHPPLPVV

TSSASPAPPPAAATAPVSGLPGVHIPLLLNPDIFQSTTIPYISPQSTVEMMEAQKTQNTD

TPQNMDTPR

>SG-PL_contig_10928_1_-1_148_303

MNRWYRRITLQNGQLVLSVVSSVFTTCSSTAIYRHFTDRKRRFSRQNKINKQ

>SG-PL_contig_10929_1_+3_3_194

PVLEILVPLSSNKTVLYQQKKQAMQSPHTQTNKTCINNKCSSVVCVLSILFSEDPETESA

KGRF

>SG-PL_contig_10930_1_+3_66_251

MTNIYTNIRAHMDKLHKYETFSYNTDNVFSHKHQTHKYYQVVLFNLLRITKLYTFLTQKY

FS

>SG-PL_contig_10931_1_+2_149_343

MKLPCFPFQSLSPPHAGYNNDLIPHSQRYSQLKKPITTTKRSYTHSWLWTRHRLTTVNPP

PPRLL

>SG-PL_contig_10932_1_-1_1_84

KKKKKKLWFCSKTFSFQYLYLLKFGTHD

>SG-PL_contig_10933_1_+1_139_333

MRFPFTQKATELFISSHVGQIYSETECTSIKPRNYSHRNVEILLLRNKLNNMSIMEYPKL

KNKTK

>SG-PL_contig_10934_1_-3_3_212

VHKSDLLPLHHSLCHTALTKSFFVCFFVLFCCCFFFENRNWFSPRWRHQDRLAIVHLFAV

CQRWRCLKKL

>SG-PL_contig_10935_1_-3_87_392

MKKRPTTTVTTCCLVRNVLCTADSDIDEEFVPESSDTDSDSGDSGDQPTTSKRTRVSTET

VPLAVPSTSRAVSPVPGGSRGRGRGGGHGSRGHGQVSQVHAL

>SG-PL_contig_10936_1_+1_352_537

MQVLLLEHMSDCLHLCIMIFSSMSCCDYGSAPKIITKGQAHNPNINLCRFEKHTHMHSNM

TK

>SG-PL_contig_10937_1_+3_12_359

MAKPRHAGLSHEDQVHVAGNDRDLAHTVWVSRSLCGLALCHQQVHQQLQVGFPVCGLSNH

QHSLHTGEGGVLFVLAFCHSSSVFMVNHVKQWVGSTNIIQSATVGETKVCSNVPPV

>SG-PL_contig_10938_1_+3_3_131

KFQFEYVVSALRKKSIQTTADVISLCLNLERWRGVEVLRDFEF

>SG-PL_contig_10939_1_-2_2_190

PHRFTFPVLKKDRNLVTAGWTWVVCLSQGANVVPHPFDNLARKCLTSVFESNCSEQGMGM

DQL

>SG-PL_contig_10940_1_-3_3_188

SPGNGGLIRFLTFSSFVDRMLSVYITSFDWCFGELLREHVDCSGLCAVISCGNFLIFLHY

TI

>SG-PL_contig_10941_1_-3_3_188

SPGNGGLFRFLTFSSFVDRMLSVYITSFDWCFGELLRGHVDCSGLCAVISCGNFLIFLHY

TI

>SG-PL_contig_10942_1_-2_65_199

MVKIKCHELCCFAGFLFSGLRWFCDQRGCLLDLDGLYVVTALIWS

>SG-PL_contig_10943_1_-3_63_236

MNMLKQSHVVCVRGISSGLLAYLSLCAKVVTSIVELILFLVSTHLVFMDDFLCFVSRG

>SG-PL_contig_10944_1_+3_162_605

MSHSEPSVGNENFLSFRSSVRNSVSKITVPCKHELISGREEGKAIEKMSSPFKPYQSRMN

VFFFLNVTTNNTRILLPCIQESWNFWGGGWGVRKIQLLLSMEVQSVLYTSRQVLMYRNWH

CIKMYGRALWPKETKQTMPDLLVMLSFE

>SG-PL_contig_10945_1_-2_665_1135

MWWAGQLTLRFPCQQLTPGFVSQSYVVLSLSKVGKLCCVLCVCSLCYKAGPHFAFDLKKS

ASCLLVGCTGCFVLPELCIVYLVCKAVCYGAEREVSWRLPTAVLLGHGTGPVGHVGWFVS

GVLWSHRMSWLVEYCGHHCANEISTTTLFGAGSQSCR

>SG-PL_contig_10946_1_-3_858_1202

MHAHARAHTHTHTHIHIHTALTYTHLSLSLSPLTHTHTHTHTHLIIIDIILFYFKETQMY

TLHLILLYTTNATTLSSHSIPSLLYTHTHTHTRAHTHTHLHTTKVYKTLNIYSRH

>SG-PL_contig_10947_1_+1_1_246

FFFWCCSFLVLTRGGRVHRLVTTPPLSGLLSGKVELMMVVDHFISTRHCQVYKAWPTSFW

YQSLRQSRQFVESGITHQHKLP

>SG-PL_contig_10948_1_+3_441_593

MNGNNQQNFGATDLKNVYISTKQIQKEKKACSKLPQAKKLKIRPFLFAVHL

>SG-PL_contig_10949_1_+2_266_430

MQCNATSLSSSYLFLSCFYTVPLHLISVFLSLCLSHTHKTYFTFFSKQKQQPTNK

>SG-PL_contig_10950_1_-2_473_850

MSAFWWKRDTMLTLFFCCTLLKLIHLTFVVCFYGLAESVAKTMDLRSDVSFNPSPQYKNW

YGFALLNVPTILLVSASCLSRCCITYISRPHQRIMFYAGSRSLLLDNHWQIWVERIINCP

GATNLS

>SG-PL_contig_10951_1_+2_68_244

MDCMYCVLYLCINWRCYLANGFHVLFVRLLFLLTSIHICVLLDKPCTVFSIFVLTGAAS

>SG-PL_contig_10952_1_+2_398_718

MYNYKTRKCVHTHRYSNKYTKKHENLLKQDISKNIDTSVTHTHRHTHTINVTEQLYIIIN

KTINMHTHKDTSKHKYDKICCDTHTHTHFFPLFIECSPSDHSFTCQH

>SG-PL_contig_10953_1_-3_567_803

MHKLALELHTLSLSLSLSLSHRHTHIHTHSLSLQPLSLPPSLFASFSLLHRGLKTEMIHQ

ESCSIMYDKSKLSWVSWFC

>SG-PL_contig_10954_1_-1_280_504

MNLIKAFPGTNLQLGKNILCTTHESFHFILRGLAGSFDRAVDSRSHVSSSIPARNIKVGL

FTVHLIASIIQMVSA

>SG-PL_contig_10955_1_+2_2_208

SQILWSYVLSSQGDLSFGNFGGVIEFRVFQEGFITLGQFQMCSTQICQQLPGNFLEPAQN

SVRCQGLDM

>SG-PL_contig_10956_1_-1_1_186

AVLHRLDMVECTTLKQKLNNNNKSFLYSAILKLIWTRCALHSYMTQSVVHAEALISHTHI

DI

>SG-PL_contig_10957_1_+3_3_296

IFISLSSAVRSPFVTSSGWLLLSSVSSSSEMSSKSSYKFSSPLSASFIISSSTSSAVKRR

KGARLRDSPDVAILVKTSFNSRKEETKTKRFHKSCMAH

>SG-PL_contig_10958_1_+3_159_251

MNSGDTNSCDYLLFHQLHVTSIKWERQEVWL

>SG-PL_contig_10959_1_-3_75_596

MVLFGLHVALQSHATFQPVESRSRFRSNVFYLCHAGTAHTLRVPALLPFSNASLTFPPPQ

LLPPNLFPAVFHAGIVEIRSSVHARRVNVTALVHDQSAILVRNASSNGLLFVSGIFVCGQ

ESREIRIHTSSDKFLTDFFSSKNADFFSAKTFVLFFFSGKCSSNGGNPPNGGSF

>SG-PL_contig_10960_1_+1_1_171

SHNCYTTLSLSLSLSLSLSLSLSLSHTHTHMHIVISCCIIYANKINALSWPFMHQVF

>SG-PL_contig_10961_1_+2_2_292

HTHTLSFFSSLYNAHALFIILTIIVQILIITSMFASTISSSVFFPSHNQTYDTSANTHKL

MHAHTHWNELRYNCTECCLFVDKINKCQKNKKIKKKT

>SG-PL_contig_10962_1_+3_3_215

MYISQQARVQACTLNKCHLTGYAIYSARKHVRNAVRPEEYLHKCTPYLLVYIAVYIKLDH

KAHLHHKEEKN

>SG-PL_contig_10963_1_-3_3_170

SDCILMYPSSSVCSLTVSMLVALLTVHLLCMCLECGLFLCKKTRNVFSKCVVIFHF

>SG-PL_contig_10964_1_+1_178_372

MQVEDVFPLRFIKMRKCIVQSSQQALFFSVKVLVIILHIFLSNRNMITECLRYKERDETA

TTKKF

>SG-PL_contig_10965_1_-1_10_2472

MPTPKRLRDLIREIRSARTAADERSVVNKECATIRDSFRDQDNTYRCRNVAKLLYIHMLG

YPAHFGQLECLKLIASPRFTDKRIGYLGAMLLLDERQDVHLLITNSLKNDLGHQTQYIQS

LALCTMGAICSTEMSRDLAGEIEKMIKSSNAYIKKKAILCAFRVIRKVPDLMEMFIPSTR

SLLNEKNHGVLLTAVCLITEMCEKSPDTLHHFRKLVPQLVRILKNLIMAGYSPEHDVSGV

SDPFLQVKILRLLRILGKNDNEASETMNDILAQVATNTDTSKNVGHAILYEIVLTIMGIQ

SEAGLRVLAVNILGRFLLNNDKNIRYVALNTLLRVVQADYNAVQRHRTTIVDCLKDPDVS

IRRRAMELSFALINSNNIRGMMKELLHFLEICDPEFKADCCSNIVIAAAKYSPNKRWHID

TVMKLLTTAGNYIRSDVVTVTIQLIAETAALHAYSVQQLFRAMQASYAQQPLVQVASWCI

GEYGDQLFSTNVEDEEPLQVTEDEVVDMLERIVGSANSAVVTKEYAITALMKLSSRFTVT

VPRIRQIMTLYGSNTNMELQQRSVEYTSLFSSFDHLRPALLEHMPLIETHHHTLENGLEN

EEDDLLGGDPVSRTNQQNLQQPQQQQTGNILEILENGQTGTQSESSKLGGAADLLVDLLG

DLGPPQPLPNNTVPAVPVGPLDGLLSPSSPVSPSNNMINGSAGIPLTAFEKNGLFIKFMC

ERDQSKPSSTLINMAATNSTPFPMTEFVFQAAVPKSFQLTLESPSGNLIPAMNTGNLTQV

ISVNNPHKQAIRMRLKITYNHNGTAVSEMGEVNNFPASLWQ

>SG-PL_contig_10966_1_-2_2_250

FYVSVIGCPIFVKGSNVSTIGHPVLSVYCGNVISRGLITIAMYDCLRTCVYIQLKHRLLL

KDLIASVADNHRSSAQDANDMFR

>SG-PL_contig_10967_1_-2_365_625

MSTSEINHARNSELWRILMFRFRTLIYFLFLYVLMFLSLLPHIPSPFLSFFFNVLKIYHF

VCTLIKVYKTLVGFVSIILGMSALCLI

>SG-PL_contig_10968_1_+2_2_139

FCCRSATVLMDFQHNAVMFFLRHWQAVSDTSGRDPIKTTIFSAKTI

>SG-PL_contig_10969_1_+1_445_750

MYEQLSVGIVSQSKGTIFIIKGTVVRGKDRKRLMPRINIYRPVEVEGAFPKGASSHTHFY

ESSIILSTFICSECLFLAFFFTVFLHFPSFFHFLFFHFFPQS

>SG-PL_contig_10970_1_+3_87_416

MTAFFKSSSRMTALFFSTYHHNSLFFIQLLSIMQICVCPFSMIYSMDISIANLSCDNITV

IIQRFQVHPISRSPMCFQIQTGKLGKRHTTLQKDILIYNHKREQSSEQPC

>SG-PL_contig_10971_1_+1_1_222

SHSSFCTSFSLLQIAPWSLFSSKTPALSLISCGPAASLSLFSRPHRGLFSAFLESPTVHF

SLSFVTSSSLFSDR

>SG-PL_contig_10972_1_-2_491_763

MQTTLFLAIIKLQVSLSLNKTFLSALVRQLSNHLSCKDVYAVENIDYFSFVSLCVCLWCY

CSLLKCDEWMDITVDCCSATNFCCLSFLLNL

>SG-PL_contig_10973_1_+3_759_896

MFCMKSYFSNISYWPKFGYFLYSFKTSVVYFLYSFKKISGVLSVQF

>SG-PL_contig_10974_1_-3_3_128

CLHHSTTMPIWPIVAISDTDVADCRIHVTFRSNRTLSACLQI

>SG-PL_contig_10975_1_-3_3_299

IIDLEVVQSGNAGSAVPTIQDMGVMGIVTSVELPNRVTVAAIQGDITQLSVDAIVNAANN

RMDHIGGLARDIVQKGGETIQQESYAALKAKGKQMKEGA

>SG-PL_contig_10976_1_+3_3_320

PFPLQLPPAPSPRKRTHTKLGTQGPIHHSRTGTHQGRGKIHLAEQNCCDILCRCAHHHVI

QLGLPHVIGHRAIRNPSSPSPRESSPVWPHRFSTAQKVECAQGVHP

>SG-PL_contig_10977_1_-2_32_1705

MANVQRSLLSDEPAWKALQEYYDQNGSKLNMAKMFKEDGDRFKKYSVELKTGNGDLLLDY

SKNLINDEVMQKLFSLARAQRVEEMRNSMFHGEKINFTEDRAVLHIALRNRSNTPILVNG

KDVMPDVNAVLKHMREFTNEVISGVWKGYTGKPITDVVNIGIGGSDLGPLMVTESLQFYQ

VGPNVHFISNVDGTHLAKVLKPLNPETTLFIIASKTFTTQETITNANSAKQWFLSSAKDA

SAVSKHFVALSTNAPKVKDFGIDEKNMFEFWDWVGGRYSLWSAIGLSIALHIGMDNFEKL

LTGAHYMDQHFKTAPLEENLPVILALLGVWYHNFYKAESIALLPYDQYMHRFAAYFQQGD

MESNGKYITRSGQRVTYSTGPIVWGEPGTNGQHAFYQLIHQGTRLIPCDFLMPVETHNKV

QDGLHHEILQANFLAQTEALMLGKTCEVAENELKKSGMSPDQINHILPHKVFEGNRPSNS

IVFQKLTPFMLGVLIAMYEHKIHVQGVIWDINSYDQWGVELGKQLAKVIQPELKGPNPVS

SHDSSTNGLINFFKAHRK

>SG-PL_contig_10978_1_+3_237_491

MWSSGLVVKADARNARFDFWIRQTFLSQPDTLNASLSNEKINQDHIYSILHTKNPSQQRS

CPSTFIGTHIMQKKKKIQKRQARWK

>SG-PL_contig_10979_1_-1_373_1140

MKKYPVVYSDSMNTVLRQELIRFNRLTQIVRSSLQNLQKAIKGLVVMSSELEEVFNSMLV

GKVPGVWAAKSYPSLKPMGSYISDLLQRLKFFLDWIEHGAPSSFWLSGFYFTQSFLTGVS

QNYARRYSIPIDLVGFEFQVMKVEKDVPQKPQDGAYVYGLFIEGACWDRENMQIGESLPK

ILFDSIPVIWMKPNRRDQFTEIPHYLCPVYKTTARRGVLSTTGHSTNFVLMIKLPSDKPP

SHWINRGVAALCQLDD

>SG-PL_contig_10980_1_-2_1631_1960

MPFVLMTNSPWALVLWASVLIFCFVFCSPTPVLYIDMCFFIHADDVCVVAWLGYRTVCMQ

GIGWLINVYDQLYSSSDVIRYYQSIRVRYRLGSERPNKTILVFAKVHDIR

>SG-PL_contig_10981_1_-2_101_238

MGIEYWTPCLQVLFKENVGIVPQNKGFLIFFLRANDKVTVKMDFNV

>SG-PL_contig_10982_1_+2_2_295

KRHRQRQTDRHTQTDRDRQTNRDRHRHRQKHRQTETDTERDRQRQTQRNRQRQRQRQTNR

DRHRHRHRQTNRDRHRDRDTDRDRDRQRHRQTQTETDT

>SG-PL_contig_10983_1_-3_135_344

MLSLTLRSNCYYYYNYDSSLYKDKNLVTSNRHLYIINPSLYILLPTAQYVTCFCKCFTFC

QMFVPHLLII

>SG-PL_contig_10984_1_-3_396_584

MYVQCRKSVCIKQVCFFFHLYVTFSFPSVLPVTLQPPPHLQSQPSQSFISSRLLLVLAIT

LRP

>SG-PL_contig_10985_1_-2_2_82

TVLRKLKPKFVVVISNDIYTPKWFRNE

>SG-PL_contig_10986_1_+2_56_1189

MARWEETTCLKFRPFRHTDKNRIRFQNGNGCNSLLGMNGGVQPINLQSPGCRYSGLYLHE

VGHAIGLVHEHQLPDRDQYIYIIYDNVAPSMRVWFNKYSSKDVNQYGVPFEYSSVMHYGV

TAFSKDGSQTIRARDRSREKEIGRVYLKGLSFSDIKVVNLMYRCNEHCAADPNCKEPGYL

DKNCKCVRPDNGGGTTTTTKPGVCSNIWDGSECEAWARDGECDLNPSWMKENCKKACDEC

KDTGGGKPDPKECSNTWGDIKCVGWANSGECEANPIWMKENCKKACKECGGTGTGTGTGT

TNSLCKDNNQYCSSWAESGYCQSNWYTQENCKKSCHTCDASTDVSDVNTEEDIGNNATRM

MSQLMMIPVFISIVRSYL

>SG-PL_contig_10987_1_+1_673_789

MKSFPHPPFLQLKMCFLTSFLYSRFTSMFNIHSRKQKPV

>SG-PL_contig_10988_1_+3_42_389

MYNGIKKALGPAVKGITPLRSLTGEKITNRKEQMARWVEHYSELYFCEISKELPIMEELD

EVPSMEELSKAIDSLPTDKAPGSDNIPPKAIKRGKPATVRATLPLLGGRRSPPRYA

>SG-PL_contig_10989_1_+2_2_73

VTHLVWLHNLMITVISRGFSRQCP

>SG-PL_contig_10990_1_+1_1_204

GEGGEGVWSFQNTVSPVWERKRSGYCPQTTSHWVQKGGGGLGQIVEHSVKNRRNTPSPTQ

TYTHEDRT

>SG-PL_contig_10991_1_-3_99_350

MLISLLFFFFFLCLEESLQGCFRQPPSDTDVSSSWLPFTTLDPLSPVAHEFCSSLGVSKD

GELYLFLFLLFSMKCSLWQEWYFR

>SG-PL_contig_10992_1_-3_15_248

MTIKTQTKIKRMTKIKTRKRVKVLSQSQHLWCCQQLSATLHQYHSSCSLHQFSTTSLMAS

TAKMMDIWMIIISLATYL

>SG-PL_contig_10993_1_+2_2_157

GGSGEKHGIYISIINVCFKLKSLFKILFSCLKVVCQLPSYQNDKDILSALDR

>SG-PL_contig_10994_1_+1_751_972

MTNLVHLQQTTFTSTYMQKKTKSLKTTFIIVINLLCGIYFNNYTSTTLPFSNLPEVYERG

GHSTCHTTGSSPIR

>SG-PL_contig_10995_1_+3_3_191

KHHHTGTHSRRLKQKHASTNFKTTTVAPQHTPHNTQSINPYSTKYHSHITKHFPPQNHTQ

QNV

>SG-PL_contig_10996_1_-3_12_1520

MESLLEQQRRYHEERERLTDAMTKESLLQKKTPREQINSDTRMKLLLDRFLDVTTELKEL

YEDKDGVRKEEIQALAGPNEYIEFRKRLTAVEKFYAKHPNEISIPMSIEFDEMAKQRDSQ

ADETQNLVEFSDEEGYGKYLDLHECFVRYLNIKGVERLDYITYLASFDHLFKIPKDRKNT

AYKEYVLFLLDYLSQYLSKVKPLLDQQEVMEVVGSQFESDWADGIVPGWPKEATGALTHA

GAHLDLSAFSSWEELASLGLDRLKSALMALGLKCGGTLEERAQRLFGTKGKKLDELEPTL

FAKAKPGKAGKSGRESEKQKEIAMLEAQVYRLGELLADQRAATRENVQRKQAQTGEEREE

DEEENYPESDSSEDEDEIPYNPKNLPLGWDGKPIPYWLYKLHGLNISYTCEICGNFTYRG

PKTFQRHFAESRHAHGMRCLGIPNTAHFANVTSIEDAMALWNKIKNGKEQERWKADVEEE

YEDSQGNILNRKTYDLLKKQGVL

>SG-PL_contig_10997_1_-1_7_2031

MKIQLQQLQEYKARMQETHADLQRQLQASKKKEEDARQEFDQYKDEMADVSESLEMAALD

KEMAEEKAESLQQELESNKEKLEEMTLELELLRGEISEKGSDGVAATFEMKQLEQQNERM

KEGLVKMRDLLATEKQEHQQLDKRCDKQQTDINNLQRDKERYSAQVSELENQVIELKEQV

DAALGAEEMVEKLSERNLQLEEKIESLEEEKNDLEALNEMNEELQENARETELELREERD

NALVRLSENQRRVDAMQETVADYETTLNKFRGLVAQLQEANKELRSKQLESETKAETPTI

ELFDFKAKFAETKAFAKTIDMELRKLEVLQATSHVKMLLSFMPDAFFSRGGDHDAVSVLL

MVPRIINKAELLASQVKEKFEVVEKLERDHVLKGSKAEQCSFANHLVLQLSSLQTSMHQY

ESALNNCGVELFVKVGTLLPEMAAHEKTLDHYIEMLRKDQLDETVSLEMLDKSIAFFQQL

YTVHLSNERVDCTNLMCDGVRSVLSACDCVTIDISRLKILLMTGEEQSEFSILLRDLETC

NNDTRMCARKIKRRLPQEGSAIATPLKFAKETKELLLDCCSNITRVARTLQAVALGAMNQ

ASIMTDKEGLMARKMEELAYEAANKVYAKEDSGPYECLRLSFGTVVGTMNKLANAMENGE

FDFDGTHDKKTLAPV

>SG-PL_contig_10998_1_-2_62_250

MIISIYMAPNLEKFKPKHTHTPDTRMQSAQATYSIQNKTYTNWGKEVVAEFIVGKRGEFL

NWI

>SG-PL_contig_10999_1_-3_99_446

MPYYNDIYFDSDDDDNYEGEGDSRVHKHHTKPSNDDLLYDPDMDDEDQRWVDQQRRRQHG

DNSQACGNKRAHGKKQGVPKSDAVLDCPACMTTLCLDCQRHDIYANQYRAMFVMNC

>SG-PL_contig_11000_1_+1_1_99

CPSVMNPSGTNSKLWFSRTIILIELQINFKVNM

>SG-PL_contig_11001_1_-2_161_316

MKLVPSLYHPDKHNARHTLLISTVTKKDIESKRIIETIFFSGKNFSIICKYT

>SG-PL_contig_11002_1_-3_459_695

MPETMLKVALGCTAFILQCGDILCIAISAKFLSVYLLLVLRICPTGNGWLFLFYHLTSLC

FLFILLTIILSFVACLATI

>SG-PL_contig_11003_1_-2_2_190

DDKTDCISDTTSDNSFTSLLLPLTPGSPSPSPPTAPIQQCDFSSASLDSSLSFDSFTVCL

FNA

>SG-PL_contig_11004_1_+1_1_216

HRHHHHYGIVIIHIIITIIIITIIIIIIIIIITIIIITISSSSNSSSSSGSSSNSSSNSC

CIRSSTQKFHGA

>SG-PL_contig_11005_1_+1_1_390

SSNSNSSSSNSNSSSSNSNSNSSSNSSSSSSNSNSNSSSNSNSSSSNSNSSSSRRSSGSN

SSSSSNSSSNSSSITRARSGHVKRLVTCKTSGRIVKTWSQHGNRRQYGTILLKSQINPLT

LTCKQLVVNR

>SG-PL_contig_11006_1_+1_196_534

MSLSHFKISVKSDTIYFCPFRTLLISECQLNVIYDRFLPKSLSHYQVSVQCDRLYDRFLP

ISLSHYQVSIQCDRLYDIFLPISLSHYQVSIQCDRLYDRFLPTSLSLSSVYSM

>SG-PL_contig_11007_1_+3_456_707

MMACSYGSMGEEGLTKQKNKSKYLHSRYRYALKFQTKPQEAHRRTFSLDKMSYNIIFTYP

VTTWKKQEGGGVHIIKQYKTHVSN

>SG-PL_contig_11008_1_-1_121_318

MQEFCRDGVDAYTTKHMKNKLLEHFGADILISTRGKKTNVVTLRTAAWKILSQYFDTPQT

EDPDAK

>SG-PL_contig_11009_1_+3_3_116

ISLASLTDKASGQSQSISSHFKTSSAYRLKHRLKETAR

>SG-PL_contig_11010_1_-2_179_928

MLKDNLTLGSLGLKNGSRLYFKDLGPQVGWTTVFLTEYAGPLVIYLVFFTRPAIIYGAEA

AKLPYADVVKYAAGCWTFHFAKRLLETVFVHRFSHATMPITNIFKNSMYYWGFAAFVSYF

VNHPLYTPPAYGDVQVYGGLAAFAFGELGNFSIHFALRNLRPAGSTERKIPYSTSNPFTW

LFGLVSCPNYSYEFIAWTAFTVMTQCLPAGLFTLAGLYQMAVWAKGKHRNYLKEFKDYPR

RRTAIIPFLV

>SG-PL_contig_11011_1_+1_1_114

HNLSLSNLQTAVERQRRRRWGGGAILNPDNKVRITIHT

>SG-PL_contig_11012_1_+3_60_380

MATEMGPSLATACCRDFSLPEGMSLQAWRSATSSDLTNTHFSSSALYGYSCSDSMPPTAL

MYSKALSIRPPSQPSLPKRLEQSTSCCSDREIKSPVLRKCWPSRDPV

>SG-PL_contig_11013_1_-1_1_255

MMLWSSISDSYCMTSIFVENTLPRNFSITRFYINCTSGIYQVLAVNFFGSNPYHMCEFFL

KLIFKPDISSAYSVWGILHYHPKIL

>SG-PL_contig_11014_1_+3_1050_1700

MIIHRYHSMHIHVCFLPFLMCACMLTHTHTHSLIPSCMHTFYTHTLSHSLLCACMFTHTH

SRSLPLVCTRLHTHSLPPSCVHACLHTHTLSHSLLCARMFTRTHTLSLSFYIVCIHVYAH

TLSHSILCACMFTHTLSYPFCVHSCLHTHTLSFSSMCMHAYTHTLSHSFLCACMFTHSLL

SLLCAFMFTHILSHSLLCARMFTYKHTHSLVPSCVHV

>SG-PL_contig_11015_1_-1_346_702

MSKRANKITTASDLHFTSEFSRFEVLKITIFSIRWVFPESLKPRATKIPMKMTCRVTYMP

ISLDIITYAWRNGHHHLVRCAADHSHKRNMGITPAPLLIKNNQTNNELSFLIERNDMNI

>SG-PL_contig_11016_1_+3_441_500

MNAGCCSAIIITIHTFKIKE

>SG-PL_contig_11017_1_+3_291_455

MHLYKSTTSPAQGHAPPSPADCLHSKKTQDNDDSIYRQPFHAVTSTNYPTNTSPN

>SG-PL_contig_11018_1_+1_1321_1575

MKQQRKHITNKCCFNSRIFIRKAVEYKLRIFTRKAAKSINNIMFILQRKTSLHVVTLQNK

YVTTQGPSNARGAQDNKDHLYCLLN

>SG-PL_contig_11019_1_-1_58_288

MEISLPECFYNKYMRLLQVSCMLTHCIVDVASIYYPKRYSCLVCACNVDQHVLYKNIPLP

VRTKSALSNMNLLFLTN

>SG-PL_contig_11020_1_-3_558_1151

MKTKYNPLQHRLVTANFGWAFANSPAVKAERQFIEKLQTQSGNRRLSITPPCIDAKGAII

YMNRKDVRTALHIPANLPEWSPCSNLNYKRVYMEMSAQYKKVLGKGKRVFVYNGDVDMAC

NFLGDEWFTDRLSLPSKKARSEWTYTDATHTSQVAGFFKEFELLTFVTVRGAGHMVPTDR

PRPALKMFVHFITNKPLN

>SG-PL_contig_11021_1_-1_1_285

ERERESEGYARKYVRRSAFLPQSLPAPTVSRLSQTSTLPQPCRCCCCSCCCICTAAASAL

SKLASLLLGVAPHSQLSVLAASCVVATSPEQSRFC

>SG-PL_contig_11022_1_-3_3_116

KLFLLNKVFKATIFLLPKGVYETKPAFFLISLSIISQN

>SG-PL_contig_11023_1_-3_3_92

KLFLLNKLKFSRQQFFCCQRVCMKRNLLSS

>SG-PL_contig_11024_1_-1_1_117

QSVWPKPRPALCIRPRNHKRQIAKSFSWIGDRNQIIIRN

>SG-PL_contig_11025_1_-2_2_262

CCPTNPSTPSFPPSIQTNNAATPTCLPSNPSRLSDLANVVTVRQLQQQLINATLTISNMQ

TEIQSLKTQLAKNAEQTEKNTECIIDS

>SG-PL_contig_11026_1_+2_2_184

FFFYMCTFMSGFKVCHDLYNHSIHTRPLVQDALSPCHSHGNHTNHLKRQLWLAYKMDRTM

T

>SG-PL_contig_11027_1_+3_3_236

THTHTYDNPSPPTHTKEESSLLTDTKLTHPEIPSKNTTFGSRPIHKMRNAVRKVRGITMS

ANISTNSGQPSCHDDIVI

>SG-PL_contig_11028_1_+3_804_1022

MVLRAAPALIYHNRFVKEQQRAVLSIVFNLILQKASNISPRKTCFQTPQSKQNENLLDKI

NENLLYEVFKFWI

>SG-PL_contig_11029_1_+1_472_843

MLPHSLQQSLKEYWHGVMMCKEFLYWHGVMMCKEFLYWHGVMMCKEFLYWHGVTYTFSFI

APAPQSSIELSVSCQFDGARPPPDSFPLPKPNLPVFLITGPTVPLPHLLSRTLLTPQMLM

CVCV

>SG-PL_contig_11030_1_-1_1_111

SLTHTHTHTHINICGVNNVPDRRWGRGTVGPVMRKNR

>SG-PL_contig_11031_1_+2_2_169

LLAMGGCELVRVKKCSKEGFCCCCCFWLFFFNLLQHTIMFSLQLKIKPCPFKFMQL

>SG-PL_contig_11032_1_-2_848_1111

MHDCICECFMSASTQPLFTSQTWIVNYLSNPFLCYPFSSYCKFCMKIMDTSQKGKTWDLV

MLGQWTFSHSGRFNINLILVLCVLLYIK

>SG-PL_contig_11033_1_-1_163_645

MLTEKLGAQYSPRHYVVASTDSISLKKIIKSEKTASGAANSKSEYTVYSVPRSREVKQSW

FTTFFTTLYATLYSLRLVVQLKPQIILCNGPGTCVPICYAGLLLKWLGLADTRIVYVESI

CRVETLSLSAKLLYPFADSLVVQWPTLVEKYPQCKYIGRFF

>SG-PL_contig_11034_1_-3_462_650

MSLLPCSWLDILLLIISIAVNHPFSRSRSPAESFTCSGGGLFDPLPVGRGPPALVPRQGH

PAK

>SG-PL_contig_11035_1_+3_3_185

RILLGHRAWWVMGGDFSDFPVNICADLLVPKPPSCVYACRTSNVHVKDHVSHFSIRWLME

T

>SG-PL_contig_11036_1_-2_239_427

MQSICSVFYVWNCNTYFVAEFYQSLNQPHWTQKFQTSDLYILFLCSSGTALMQPLSSSNG

LQD

>SG-PL_contig_11037_1_-1_37_1086

MAFGTDSSGRLSIKSASCFCSIGSVDIHIHGDAEIILRLFRGEIEKRVKSMLPGKLCDAV

NELINNNAEAALRKVKVKTPLLDNQFILDYSLVAPPFFQTAYMETYHKGEITWRADPKAP

PFSPSPLPPSAMSSSMLYLWVGEFVPRSFMYAAQQHGFLAYNLTAKDLPPAERGFLNTTC

TSLTCIGLLVPSVGKMYPHCTVELRMKSTMVPQVNMLPNLLEAGAGGDIALYAIRPAKPS

AYLLTLGVSAMFNMTATIDNQLIKGNITGSSFKVKVLKVAPGIGPVKDVVLNTMMNFAMK

FFIIPSINKEGNRGFPLPVTDDVRFVNAQLQIQQDALLIGTDVVYNPHFR

>SG-PL_contig_11038_1_-2_836_1036

MLVDMCRFFWESGEECVCGDSVYKFVLISWDIYRDKASSTHPLVGIVHDANSNHQQNQHE

MIQSKYL

>SG-PL_contig_11039_1_-3_3_269

AALHANTPLWPGLLLDWRSEWPTLINRLVVHCPSIARRLRLESNGSEGRVASFLTRRALV

LNSAGLPQVGIPLFGTRWRVGTKGTEITV

>SG-PL_contig_11040_1_-2_74_421

MKYRGRSPRRVKGGGWRGGRGGEVEGARRHSTAPAMSFLFFPPVCVLLLRARARACVCVC

VCVCVYLTKVEFFFYIENFARADNSFVAMKSFTHVKCMLHTVYCLVRKTSTQTTTT

>SG-PL_contig_11041_1_-1_127_432

MFLMTVDSLQLFRCLRASKVVVLLGVSMTNISEGSHLMPYLRNERFCCWLLLNVTSDLLS

IQSNVRNFFYARRHLSPPPPPLEKKKLVRNTFSNLCLLSVWK

>SG-PL_contig_11042_1_+2_2_79

LCQTDVMKLTKRNNLTENQSLLELNE

>SG-PL_contig_11043_1_-3_48_644

MHWFIDSFIHHHLLLLSFFGSLIHHHRRRHHRHHHHHHRHHHHRRRRHHHHHHHRRRRHH

HHHHHHHHHHHHHLLFFFFSSSSSSFIHWFVNSSSSYCHHHLLLLLLLLLLLLLLLLRRH

RLLLLRHLLLLLLLLLLLLLLLLPFIDLLIIHHHHLLLLLLSFVGSLIIHHHHHHYHHHH

IVIIIVIFSSSFFFHSLVC

>SG-PL_contig_11044_1_+3_3_179

IFSRILKYPLIHTIIIHDYACVYLYFVIFSNLSDTILKTDVMTKSTDSILLWLPACGQW

>SG-PL_contig_11045_1_-2_23_535

MAALKQNVETWKNKLDKLLHEKNAFNDVLDKLETKTGVKRLYIALGFIALVALYLMVGYG

AQFLCNFIGFLYPAYASIKAIESRQKDDDTKWLTYWVTYSVFSLVEFFSDIFLFWIPFYW

LLKCIFLVWCFAPIEANGSTTLYYRFIRPFILRHQDKIDKVLDEAKDAVNE

>SG-PL_contig_11046_1_+3_1938_2129

MVGMNKTRSSVGPLHLIYLMKIKHAEEIETRWSSQTISDSQATLPIFPFQHYTGLTDNSS

FKLT

>SG-PL_contig_11047_1_-1_1_237

KKKKKKGWEKKKVDFLHTCINYVPFPACSSTTKKTTIKNGCCHALISLCNCSFCLLFLVV

FVFVLVGAVKFVSLPCFTC

>SG-PL_contig_11048_1_+2_2_202

KKNEYNSLKFNLSFKFNISLKFNPDNLAQYTVESTESSSLSSKFVRFFSHSGIQVFQAAD

RNTQIYG

>SG-PL_contig_11049_1_+1_1_117

LIFVMPFLKFYLFFLIISTLSLDFPNLHNISLALPHVSH

>SG-PL_contig_11050_1_-1_571_909

MWGEASVCVLVYHLTGWLITIMVVGFFFSSLYTYMYHLFGWLTTAVCSICLRTVLHRMKT

SQQDSSGQTAFFPRGRNTHTSLHCRFRMCILQVLLTGKIILFFFLGTIFLFLL

>SG-PL_contig_11051_1_+3_3_158

TFPRSKSETDFPTVQVKDRLSHGPSRRQGSGCFTVIKDTNYKKPKQTQQHRS

>SG-PL_contig_11052_1_+1_1_246

QVGDRLSHGPSQRQTFPRSKSWTLSHGPSRRQTFPQSKSKTDFPTVQVGDRLSHGPSRRQ

TFPWSKTDFPTVQVEDRLSHGP

>SG-PL_contig_11053_1_-2_5_2188

MAALAVKLSPFVKVFFMFVYVPLISSDPTSYSRVDQSGEFQVNEQFEEAKKIWQQNKCVF

PDISTYEPQAGSNGGLGIFSHASKDFPLKICSVNELSESGNIFKKNLCAAFLSVSKTLCI

EAPLIQPYLNKSSIDALVTNSTWDLCRDVKKFNIGILSFLSQNSDVCQRYKSDPRLGPVA

KLLVAAVVRYSQAFAASAQSGGGSGAGSQVPQNTPGADSHIAESPPAGSVKNPPPQQGGN

GGKAVPSNPETVGNEAGSDKSQSSQTPYQPDQGTAAGNKGEPGSNSIPGDPGNSHSHGSG

TLYPGGRPIGNEQPVGTQVDPDDKHVQLGNGTSFSAGSNSPPDFKNPERQPGGFVPTSQT

NGAPESPLLPAKLNPLSKADGQTDDFRKGLTAGQLGVNSPGAINPGASVPLTTGKGEPAL

PQLYGQNQPMKTPDRMGGTAPTGSSPLSLPDSKNVFNSLDRTGYSPSALTNPNNPPESQY

KSPLNTPTGSNPFPLPGKDGNNPPNKHPPLPNFLDRITGDTKTGTGQLPYSDQNKYSQNP

LVTVVDPDKGAAQQQGGGADHKMTSAGSSSSTLPVEERTKEEEDFENEPLDSRSDLDKET

AEQGQRLPAVQDVDAVDQMVPDQHQPPIPVDGVQRLSHAVSYEESAPSGHFMAYFITAVV

LCVAGYVIYHNKQKIFAIVLEGRRDGQRRRSGGGGDGGSSAKYSKLQPTVEEVVPALEKS

TTSKNYIY

>SG-PL_contig_11054_1_+2_263_610

MTNIHKSEARLNTAYLLIVHRTFSSLKGISKLAKCELFCSNHCRNASSPYIDQIETRHLR

TTTMLRELLLTQIRDYMNGRRVHVVILKIAVIFMNKPLNIGYAVTFASTFERYDLI

>SG-PL_contig_11055_1_-3_33_779

MSTKDRLNVFPSRMALTNMKLRLKGAQKGHSLLKKKADALTLRFRAILKKIIETKMMMGE

VMKMAGIALAETKFTMGDISSMVYHNIDKARVKVRSKKDNVAGVTLPIFEHFLDGTDNFD

MLGLARGGQNVSKIKDQYLKAIELLVDLASLQTSFVTLDEVIKITNRRVNAIEHVIIPQI

ENTIAMITTELDEREREEFYRLKKIQDKKKKLRAIQEAEQAIRKAEQNFADEKAANLLDE

ARDAELVFD

>SG-PL_contig_11056_1_+1_331_573

MKGKCGLPYKIRIEADPTSVKRDTKLCPQKVFVTFMLYISEYRKQTVLKYLPVGYVQLLY

NVNDLFVIIGIDKGCTITLRW

>SG-PL_contig_11057_1_-1_1_153

DDDDDDDHSDDVSCFCSFNLMSSCDEIFIVDYNADDDGNEDDEGCLNRTHF

>SG-PL_contig_11058_1_+1_106_369

MGISSSVGVALRRLAREMLSSASMSVCDVLVEGSSLSLCGKVFLFRCTFFWIQMNVFSLS

SEVRSLGLYSSPELSKISTSFLPPPTRW

>SG-PL_contig_11059_1_-3_327_479

MNCDLLITHKSRRYVALFMHEPVMYEFVGLLSCRQCGCWSVQYSRFLLLPF

>SG-PL_contig_11060_1_-1_532_924

MYVVCTLFIMLSSPLKRNSLMLLSRLEKKQVKLCCYFPSTVSAQLGLNLPRNSEVTRSAQ

CMWASCVHAHNPQPFRIPHTQLNFADSERFVVYNIWAHIFLLLSSWFLLFQSLSECFTCW

SFLLLFISFTG

>SG-PL_contig_11061_1_-2_2_142

FFFLFFNTALYQYVEFKYDNLHKLGSSFISLLLALLCSHSSSPYPIC

>SG-PL_contig_11062_1_+3_561_872

MPLVFKQFAVHPDLLDVGDFGTHTLNTSLFLEAGRGGQITGSITHTHTHMHAHTHTHTRT

HMHAHTCMHAHILAHTHTRTLTHTHIHTDSHKHTFTQTHIHTKT

>SG-PL_contig_11063_1_-3_171_503

MVFVGKLRFAVEGSVFYTFEWAFYLTVISSISIVVAALIIALFNEYIPPPADYNFSNATL

SKPTPTNVTQYAPSVAYSTHDDDEMYDSRVAQVIDDYEDGPPVGGRRGGRS

>SG-PL_contig_11064_1_+1_1_309

SSFNYHTPQTFSLSLSLSLSLWHINTQSAKVLFVGTLSTPSANIEKKKIIIKKNQCHVHK

LINNNKTTYVTVQHLLDCTQSNVQGITNNKRVRYSHCTGHRQS

>SG-PL_contig_11065_1_-3_846_1058

MCTHKHTRTHIMHIKLCMQAETHAHVCKMCQRVTFGQNGIGSLSYEYDKLKQISNGFESC

TCTILFYFFFF

>SG-PL_contig_11066_1_-2_65_238

MRFASLCSQGNKGNNSELGYVDSGNLVLDQERTPAGNRITDQNNKLFTSSLDCIVSLI

>SG-PL_contig_11067_1_+2_2_163

VCVCKQTYNTFSCMAVTRIRSTFQWLQYAQMPSSKANLATQTTELSTLTCLKPD

>SG-PL_contig_11068_1_-2_2_199

LSFLLFPSHFSPEFTRDFFQRVMAIPPESHQPAGVGGFVASDDSSYVGWGISTIMEGPWL

FWEFRF

>SG-PL_contig_11069_1_+3_3_62

FKKKFIKKKKKKKKKKKKAC

>SG-PL_contig_11070_1_+1_1_177

FHNKLAFLESKQSENTKQSASSYFVLSNYMTPTGNKQKLVCCKVFMVSKLSLYRNRHII

>SG-PL_contig_11071_1_+1_1_279

AHAHTSGHQNRDREREKALVPVAPLHSVALDTVGEGFSGGERSISLYHFSESHPHPIHKL

PAIAPSPYPPASCNRTFILFTSFLTPSPCPLAS

>SG-PL_contig_11072_1_-3_3_182

SRLLVRLGKVDENVYKCFSSATSNNFWSVETTNKVGCVYESQLKVQLKITYLFTSNCCCW

>SG-PL_contig_11073_1_+2_170_520

MCVVMYTRKAIQACFVIETNNSTICVLLCTHEKQFKRVLSLRPTIVQNVCYYVHTKNNSF

KRALSLRRALVYNRPVYVILYTRKTIQACFVIIEISVCSLFQNRSSSSYEFDPILAG

>SG-PL_contig_11074_1_-3_840_1043

MSFVLAVTILSFISSLLSYPFMFVPSDVCRQIFASSNQHHEHASSREDIVRLLSSSHIVL

FFLSSFFF

>SG-PL_contig_11075_1_+2_233_466

MKYEICLQFVQYMKNEISLTISRVTTFHTLKIQIHEIEIITFINKTIIKVALTTDTSLKY

LNQTINNLFHYHQIMCIN

>SG-PL_contig_11076_1_-2_2_196

REIILYYLGAMCVLVRFSQVFLDGPLPNLNQRCGPLRSLLHMSLEQRGPRSRLLGSKKVP

EIGTF

>SG-PL_contig_11077_1_+3_213_422

MCVHTTLSVRACPSMCLKSWWIGRGSERVTNVQFMLLFKSFHHSILSVLANATINFTTAQ

LELMFIFFSP

>SG-PL_contig_11078_1_-3_1308_1619

MGVGILSCRVLGSYSDHLNAGLLDDSVFVLLNCMDFFAKLQTLILTPVRYSFTQVISWYP

QVLYVVSIRLAQNLRLKPMKCSQRISFYRFLGTDERVCKFFISC

>SG-PL_contig_11079_1_+3_3_197

HFLWNCTCVWNFWNSVEHLINKECENTFNVTISRNLVLFGIDKNIKTDKIFDFFFYYNTC

KIVYL

>SG-PL_contig_11080_1_+1_112_369

MVKKKKKFTNVRAQRQPDGAIRLSQTSTSSALIRLSPVSDCIKINLFLFTHAPTCTFYIT

LYIRTHSSLNLHTYIHTVNVHTLLDV

>SG-PL_contig_11081_1_-2_215_652

MLNHEKCELSPKQAFHFLGMSFDTNRMTVAPTPERLQRLRESIDSLLSARHMTARALASL

LSTMESLAPFISLGSVETPPLMGVQNALGPTISFLGRYDPVRPMVPRVCGRMARSHSTPA

RCPSCLPKDGSLSLHGRIERTEGVLC

>SG-PL_contig_11082_1_-1_1_195

QGLPVQSPPFRPLPSYVPLHQSGDRGSTLAHPGRQLRPMSNLHDQGHQPVLPTEVRLNQE

YANSA

>SG-PL_contig_11083_1_+3_132_314

MCVCILPAHTHPHAHTSKQTHTHTLKFSLTTQTNSKFYFRSKIHVNKKHPVSPIIISTVG

N

>SG-PL_contig_11084_1_-1_595_744

MIKYTASYMCVCVCSQMCMCVCAFMCGILLFYYVYYDKVQNISFPQLKLL

>SG-PL_contig_11085_1_-1_208_363

MQAYDICYDYFGSTSHGICRVAHYIVLSVCCNTLRKQNKTKKQTRNIPERPI

>SG-PL_contig_11086_1_+2_617_868

MAMHHTSHLCDTPPSSFPQGHSNITFPHPTQTSHFPIQSGGCVIFPIPSFHKMIKISTYH

TSPHSAEALSSSFFHSIHNQSIIL

>SG-PL_contig_11087_1_-2_44_223

MLGALKDATGSFQSVLVVCAVCNLLCGLLLFCYPLVLSLEHRLPPHRRLLPEETKTPKPK

>SG-PL_contig_11088_1_-3_42_203

MKSLHFDPMKVYVVLEAGIEDFWIDQNDCIMSFFFLLFWCLCWLFQFGIIYDCA

>SG-PL_contig_11089_1_+1_7_168

MIHTLDSLSLSLTHTHTHTHTLSLTHRVSHAHINTHLTFMLYSLKWTGWGRGWW

>SG-PL_contig_11090_1_+1_67_261

MMKMSGDRTHPCQFPNVTWNQLETRGPYSTFTICIGIALMLSRNIPYDSKTSQSFILFTW

SKAFS

>SG-PL_contig_11091_1_+3_96_365

MIKSSGGRTHPCQFRDVTWNQLESPPGVLTALSLFPLFLMLSRCHPLIPHNSKISQNLTL

RVCSHSRKSFPDPQRRGGCHWYSHAFSQSW

>SG-PL_contig_11092_1_-1_1_186

IELETSDPESMVLLKESASLLQKQFAIAIDKFVLMYVLIMYAFLHMQIDKFDLMYALLMN

AF

>SG-PL_contig_11093_1_-2_59_1519

MTEVDLREASSAIDVFGYPLVSKAYSKTWSYREDALLAVYKQMQELPVRDKEEAKVMMRA

AVFLIKRGIDDKVYAVFKAALNLLRMMLTEFVPKHKIPKQETSAAVERTLQNLFHKSGDT

AARNRDDAKRFIFDMAEFPDVKPTQTVPHECVKPIKLNMAPRLAQSRAEILESLYKDLKL

NNGLTLDNMMGFCTVALNHNAGEVREVTERIIKQLYTDHGAPVKNYLPPDDEKTRKNTLW

RMLFEYFDKVDGKPSKNDLKKQKAEEEAKKQAEINALQKQLQELRQLNNAKEAEKENAKP

AKKKPEPKKPPSKKPKRPDDDEVSTFNTDKICIFCSERNEAFTEEGLDMHYWKNCPMLKR

CANCKQVVEIATYTDHVLTECEAKSNFGKCPRCSEAIPRAELDQHIADKTCNPSKPNMNH

CPLCHENLSTGEDGWKDHLMSREGCKQNPRRLLALNKQPGAGTKAGGRQPPSKIAKPTTK

PSSKTPR

>SG-PL_contig_11094_1_-1_85_291

MDNKLKLDDDKTEMVLIAPRTVLSSHSLPHSIPLTGTDIQLSKVVHNLGVTLDVTLSFQQ

QITSICHIC

>SG-PL_contig_11095_1_-1_1_333

GDAAKNQAALNPQNTVFDAKRLIGRHFTDNSVQSDVKHWPFTVVNDSGKPKLQVDYKGEK

KTLAPEEVSSMVLGKMKETAEAYLGQKVTDAVVTVPAYFNDAQRQATKDAG

>SG-PL_contig_11096_1_+3_3_185

FFFLNCTQNFNFVGILQSIYNSGYINSLHTPASSSTVDDVTSTYDVTNPAHVQFLPSGHS

L

>SG-PL_contig_11097_1_-3_3_203

ERSHSRTKGKKEHSSRAKRRSPNVERGSRRHGHSRHGASHRDDASVVAGRHDDCRRVDPR

LESSSRP

>SG-PL_contig_11098_1_+1_190_315

MYIPIHHSRNVPTSSQYTTISSLTTRDANPSELFLGTIWNFF

>SG-PL_contig_11099_1_+3_3_158

GVFKEIFQNSRVQYADKIVYTANIVISYQPKVTCTCTWIYHKSTCSLPVGSR

>SG-PL_contig_11100_1_-2_29_1753

MASGSGNKPPSGGESTPLFLPIRYRMSTTDSASGATAIPATASADEASASGGAGGDAGSQ

TNRSIVVDATFFEEQFLRCQVCQEKFHLTERPPKSLPCNHTFCVPCLTQIFNHAQPQSRR

NLLWADESLDGPLKCPTCRVEIFVSKNKIKDLPNDHRVVQMMDFLSQAVSTTQNVCNKHE

RQPLNFFCKKCLKPVCRDCTVLDHKEQQGHAIVDVKEALKDRSGEFNQIEEQCRQTLERM

KNRSDALANASKRLDIQERQLRNEIKETFIEYRLLLEKRQEALTHALQQRTRDQKALINT

RFVHVCEHGTQLQKLFDAFSKAKGSNDVEQLFNLHKEMKDHEEQFNSVAAAEDHELFKSC

QFESTSEGEFLSGISSLGEVSDKDDHTLKEPVSAHQLLYLDNEDRRERERLMQYEMTGAD

QELEDPEPDVMDDVIEYRPGRHRPDRERDEDVEMHRLLAAYRQLAIADQSGHRTSREEME

QEPSGPTISRPVHPRRRPRTTQLPTSSVAAQLGLFTPMGLPFLPESEDSNGHSRSGSRSR

RSASSREGGSGSSGSGLRGYRVERHAHYPATDDSN

>SG-PL_contig_11101_1_-3_90_764

MFYAPWCGHCKKAKPEFMAAAAKFKDDGKVMFGAVDCTVQQGVCSAHDVTGYPTFKYFNY

NKNSQKYMGGREENDFVNFMRDPLSPSPSPPTASPPARPEEDWADIAGKDLIGFLTKDTF

DSFLLSQGSVLVMFYAPWCGHCKKMKPDYTDAAQLLKDQEITGILAAVDATKHRKLSDTY

NIKGFPTLKYFKDGEFAFDVNEREKDKIIDFMKDPKEPPPPPAPE

>SG-PL_contig_11102_1_+3_27_146

MVSDSNCLKHTDNIKRNTHTAISLSFPPPSSNINCKFTLT

>SG-PL_contig_11103_1_+2_152_322

MSLKKAERSRLNTLFRNAHFIGKEGLPFAKFPCFSQLAVAQGLDVGTTYNNDKSCQE

>SG-PL_contig_11104_1_-2_26_262

MKVEESKYPLLKRMVKGALSCFHGALVESSFSVMGEVVDKSTTNMDMETYSAIQTVKYSL

KASGKSAVEYFMKKDHLRE

>SG-PL_contig_11105_1_-2_26_259

MKVEESKYPLLKRMVKGALSCFHGPLVESSFSVMGEVVDKSTTNIDMETYSAIQTVKYSL

KASGKSAVEYFLKKDHLR

>SG-PL_contig_11106_1_+1_1_207

KTKNLQKNTIHDNYIILTIKFLKACKSEAVLRAPALLLNLSSPNYKKKCEKLLKILRSHK

KQQQHKNAI

>SG-PL_contig_11107_1_+3_3_116

ACMDDTHMFYIFNRILKLLSLTEQNCLRKAFLHCCWAL

>SG-PL_contig_11108_1_+1_181_351

MCVCNHVYVYVGEVVLVQNTTQNLHYYYHYILFIAQPTTKAISGLSPHKVHQSQQDN

>SG-PL_contig_11109_1_+2_53_217

MKGIKMLPPWIIFSLINVCISPYNKEKHAFILLSLISYRGLLLNIIYTVYSPTDH

>SG-PL_contig_11110_1_-1_34_405

MVWDKGRLKLYQMAFTYHFSEPLPCPHVNITRMSTTIPVINVWSFVTSNTIDFEMSESSG

NEWLPADDGSEEDEEELNKERHIKRQRRHGNSEKQDSDDDIPLHDSVDKLRHYLKTASHY

QTRI

>SG-PL_contig_11111_1_-2_287_1840

MGTSSGTILLYSFARGEVYTHLDKGHTDEVTAICWHPHGTTLYSSSLDGFVVEWDVAASK

INRKWKASNGNVHSMCLCGSNRLLTAERTIKMWDLQTRKEIQTFTGHSTEVFRLLPVLQR

TPGDDETYTCSYFLSAATNDGVINAWYCHPGALEKNAVTSFSLPDDPADIHLHATKDQSL

TLAILTAKGKVLVFEHTLNGKLSKPLKPKTILQATTEGSREEVSVPVPILAALPRVESEN

PGLLVAHGSVVRPKFESVSLRPTQPEIVLVVSVPEKKAVKENFSQVKTPQISGSSVLYPS

HITPVGPNTEPGPTSRRKRKKSVSDLTVVERLEAMGVGATPGGQTSKPPKADQLVTQLTQ

GLQSGDSKLLTNAFQERNETTVMNTVKRLPIHVIHPLLKEIIQRMTSHGQSGRMMGRWMQ

QIMCNHMAYLTTIPDLSDTLGKLYNCINHRLQPAAKQNEVRGRLNLMSSRVAMQGKADEE

PAMSAEPLLVYQDESSDDEDIPMEWAASESEDNWEDMD

>SG-PL_contig_11112_1_-1_301_459

MPSFVLLMMMFVCVCANGMLCIKLCLCLIEPTITYICPVSAQQLIRKQQCTLE

>SG-PL_contig_11113_1_+3_3_272

KDHWSEGAGEITGRRGWERSRVGGGGKDHWSEGGGKITGRRERIRQCNDFLLYCFLTLCA

FALNICYCFMCSRKTPFHFLWTIKVVSCVL

>SG-PL_contig_11114_1_-3_18_482

MAQQPSVPEQARPWNPKFTYADRERWVCIYPAYINSRKTLKEGRRIPKDKAVDNPSYTEI

RDVCAAAGMVLGVENKTYPRELDHRDLKYRGRIRVQLKKEDGSLMMDQFPSKQSILIYLG

DTIPKLKSRSQSQPSQQSSQQQSQGSKSQKKKGRR

>SG-PL_contig_11115_1_+2_2_241

TFWVCVCVCVCVCVCVCVCVCVCVCLPCVCASTVHVLHCMYAYAQVCFLFIEMTLFWGEM

KDVVCHLNMDSPSNMQIRWQ

>SG-PL_contig_11116_1_-2_110_265

MISLNHLVVSVHSISIITSPKLLQSVLFSLSLTFQLAVYSLLIKFSKYKKKK

>SG-PL_contig_11117_1_+3_111_233

MNAKPFYYLPWRTLTSYMYAVSKVSSYHSHNIYWINWTCIT

>SG-PL_contig_11118_1_+1_1_165

FFFSLAFSYREKMSSRTNLSVTLLYAIIHSCMQLYQAVSLLYTATESWAYRRLLN

>SG-PL_contig_11119_1_-1_1_147

AGVGLHWLDMVSILAKQGVGTNSPCCPNMWELLFDDLCSVTPVHLWLKC

>SG-PL_contig_11120_1_-2_59_322

MYMVCFTWKACGIRIKNRLRLDWKQGLGKLCGLKRKTSHLHLSWKRVDRTKYPRSVALCK

SCVNDVHLALIIDVVTAVLMQVHLSLQS

>SG-PL_contig_11121_1_+1_1_183

NQPTNQSTIKQTTKQSHDNSFCHNSFNKTNSVGSWHENEICVMCKFSKKNYCVHHADFLQ

G

>SG-PL_contig_11122_1_-3_3_116

NFPKPILSVYDCVTHMLHVRTIVRVETVYMIPDMFPSV

>SG-PL_contig_11123_1_-2_2_202

KKNNPKQPTPKTKLIKKDKHFSQCKAAVYFYLFVYLFNTDCSINPKKICIWIYYYVNPDI

SRYALNE

>SG-PL_contig_11124_1_-3_273_380

MGMFLTFRCFSQSLMGCVSTTIYETSINQNILQKKK

>SG-PL_contig_11125_1_+2_2_139

QFITHHQKDGVLHETRVCFFQPLEKCSVSFCRQSKHQHVKNRQFTN

>SG-PL_contig_11126_1_-2_2_301

LSELLFIYTCTVRCDCGGGGFFFFLFKPKLSSNVCEDPFTNAKKPGRYISSSQFLPHFSL

GLSCTSPSVFKRMINKRVKPLDEPTFSHATGRIIIALFVT

>SG-PL_contig_11127_1_-1_151_3018

MRGSPSTPPTSMSVMHSRPPMAISTSYKRQPTVAQEATSPSLASVPTPVLTSVASSPSLS

SLSSTVAGSSSSRHHSGSDISSVTDSQPSVPLPSSQTKDPSCESPVKGLVDRDTKPTMAS

VMSSAKPPQLPERSLPPRDQKPGVSRAEPLPPRGDTPLSKDRSDFPGKGSERQFLSPPEV

LRDRKVLDNRLKGEGGGSMSPVKDSSRSPAGRRRNVPLPPPPLSPDQQDGDLDPDRLPPP

PPELLGEGGGSFVAGDQENVDQFADDSYGAYRNRGSRQVGTYKRSSSSTVIRPDSQLLKS

AADAHAVFSDRRLGNQSWNSDSQLQANRKVEVQQGPPGSTSSNEPPGRPPDRAGSNVPAF

QADSGLVRSVSRVSGVAQPLTSPGSSPGRGRDDGKVFTARPFLVEQSRAVSDKDSTNSGD

TSPGSSRLIRDKPEQTTAPSSSSSYTPNSSSQRWANATTFPSPLRVAHGDTSSVNNSHVS

STGPVVLRASPQSRDGKSAGSPTTPSTQNTPTSRSRLLSSQSHDGMPPSADNTKHTQRYA

EVAPFPSDSRGLPGHVRTFSQSSDHSRVSSTPYSRRESVPVPPEAIAMVSPRRKSEDQLS

DTEKEEEECVIVSHGRQPSQEELECDQRAKELAKEVAGSEKKLSDVLNTDATKKRMKYMD

GLFSGPMDSEVKTPTGVRRHQSQGARDTTLSKPPLQNSLSQTAADEDQLNKRNSLPDKYF

VSAPKAVIEMEMRKKEETSTDLTKNIHDNDTLIRHKEEVMKKLHKKLDQLMEERKGLQQE

IDDNDALGKRVCETVDAKCTNPSECDKFHTYISDLEKIVRLLLNLSGQLARAENAVQALD

DSADSEQKKRTLEKREGLRTKHEEARQLKEHIDKRSDNVSAFLRQSLTEEELNDYSYFVK

MKSKLTIDLQELEDKITLGREQIQELKKSIPERSTPPDPHSSTSAASPPPSPVPVP

>SG-PL_contig_11128_1_-3_3_287

QDGQAILIISVFILLCYVDDIQKYRCLGDDSKSSKFQKFIGSCHITAQPKCNYIRLVNLG

AMVRYVYFDNFAAITLKLCQGHPNFRHRESDSKQY

>SG-PL_contig_11129_1_-1_511_1113

MITLMISKWVGDFFTTGLYDMNVEVSGIPLLSWEPPPLSDNVRVSEFMSHPVLSFLPKEK

VGHVLDILENETFHGFPIILQDPSSVLLYKGKLQGLILRDQLLTMLKKKAFAPEGKRTSV

TVSTKEFNDPYPVTQRISIKDLEITEEEKHCLMDFRHYMNRTPYVVNPDFTMPRVFRLFR

GLGLRHLVVIDEDYQPIGMVT

>SG-PL_contig_11130_1_-3_111_467

MQTCKCLNPFRVYRHAEHQYACKPCLCSVACGNTKTPSMHTNQQGLAVRQIQWDLMLLSL

KNKVFEISLSLSLSLSLSLSLSLSLSLHTPLLVCLSLSVCLSVSHSLSLSLSLSLSSLK

>SG-PL_contig_11131_1_+2_2_97

LLYLKMQQLAAISFYNYKKYVDYCNFIFLMKS

>SG-PL_contig_11132_1_-2_2_316

NVVFLNSNGPPYTTASWAIIFSPNVKITKYRFCVLLCSVFGVQVEAVIKGKKSFSPSCII

YLGPRQLIMQIYVKGWQGAEERKRFSSWAFQILFSVCGYACVCVF

>SG-PL_contig_11133_1_+1_1_189

EGGAQGCKVLLGSWEEAKRATLRAGRSWLAAWPLEGKGARQEATEPGVGLHGRISSTDLL

RPL

>SG-PL_contig_11134_1_+2_776_1093

MTGALRWTVNNPILMLQTGTEDIRSKVHQPVCMHLSVMESVVDHFVVRHCLACCPLFWFY

RAHNIKVVSPCNRPQSGGQSYYLLLVTFSSLPASVCVLTAIKTLSM

>SG-PL_contig_11135_1_-1_475_639

MWLLTGCTFCVHDKGCWVLLILNFEDALGRKKLSGNSYNRMLGIMKRDLGQKFVS

>SG-PL_contig_11136_1_+3_42_311

MESAQQPEHSKTLHTERLHALCRICGERSRKRHAQNYFHTHLSLCKNLSADIFKFHDTDI

LFDCSDTHSKTLCHKCHVRLIKLRQAQSPS

>SG-PL_contig_11137_1_+2_71_268

MQSVTERTTSLWKKYDQSLSVVECSVCSQFSRQCKGGGRAKRKKQRQLFKPASSVLGIRI

SVFSCT

>SG-PL_contig_11138_1_+1_328_480

MKNSSLVVLTVILVYMKNSSLVVLKVVSVYMKNSSLVDLLRSFQFILVLSG

>SG-PL_contig_11139_1_+1_31_240

MSIIQVYAPTSEYEDEDVEKFYELLDSTIAKVPKKDILIIQGDWNAKIGPDTYNDWAGTV

GNLDWAKQMN

>SG-PL_contig_11140_1_-1_646_774

MYVRIHLQDPFCEMLMTNSYQSKTGNSYSWTIFLLKWRESWSQ

>SG-PL_contig_11141_1_+1_40_210

MENNATTMSAGKAFVSKLKQILYKQSLEVLLSRSFQQPLTSLCSHNAHASPSLNEIC

>SG-PL_contig_11142_1_-2_11_226

MSSSGTGVCAAFFLSASCTDAMLTLKGLPITNFQCLAWDLLYVECFVARWAQLLLVILAC

VHISEYLLADRG

>SG-PL_contig_11143_1_-2_2_91

HLIESVFSLLIVKADIFLYPNLYMCYTFYT

>SG-PL_contig_11144_1_+3_3_239

HKNLLIKRRGWVHLKPYLLTLRVFSYNFHHTVYAEINLMPLHQKSQKCQPMAPTTKPCQV

TYNSFRAGRTNLIFSQTIQ

>SG-PL_contig_11145_1_-3_291_473

MYPGAIGHAVQVVCLDKLVLDFRIISCMHFLMCSITCMYVSGSEMFPEDTGHINSPFRSG

V

>SG-PL_contig_11146_1_-1_115_2331

MATYQEFIQQNEDRDGVRFSWNVLPSSRLEATRMVVPLGCLFTPLKERADLPPIQYDPVL

CTRPTCRAILNPFCQIDYRAKMWACNFCYQRNPFPPQYAAISEQHQPAEIIPQFSTLEYT

ITRATCHPPIYLIVLDTCMEEEDLQAVKESLQMSLSLLPPNALIGLITFGKMVQVHELGS

EGVSKSYVFRGTKDLNAKQIQEMLGIGRGQAQQQQRGPQAPQQQFQLPSNRFLQPVHKCD

MSLTDLLGELQRDPWPVAQGKRPLRSAGVALSIAVGLLECTFPNTGARVMLFLGGPCTQG

PGMVVGDELKHPIRSHHDIEKDNCKYMKKAMKHYEALGNRAAANGHVIDIYSCALDQTGL

HEMKFCPKYTGGHMVMGDSFNTSLFKQTFQRVFSKDQKGEFKMAFGATLEVKTSRELKVS

GAIGPCVSLGVKGQSVSDTEIGLGGTCQWKMCGLYPNTSLGVFFEVVNQHNAPIPQGGRG

YLQFITQYQHSSGQRRVRVTTVARNWADASTNIQHIAAGFDQEAAAVLMGRIAVYRAETD

DGPDVLRWLDRMLIRLCQKFGEYHKDDPNSFRFTENFSLYPQFMFHLRRSQFLQVFNNSP

DETSYYRHMLDIEDLTQSLIMIQPILYAYSFHGPPEPVLLDTNSLQADRILLMDTFFHLV

IYHGETIDQWKKAGYADLPEYENFKQLLQAPVDDAQEILQTRFPMPRYIVTEFGGSQARF

LLSKVNPSQTHNNMYGFGQ

>SG-PL_contig_11147_1_-1_91_276

MLWAIRKEWDHTLSLIHIMDASLRVLLKFPITAGVYISGQVMPGYIMKVSIECSPVIQSL

PE

>SG-PL_contig_11148_1_-3_3_80

SFLLKKPLRTYQWHNIDQQPNFFSAL

>SG-PL_contig_11149_1_+3_3_131

FFFLCYKYICTLILFFQYCVHQNGNEGNTETPKIKHKGGHKVS

>SG-PL_contig_11150_1_-1_448_558

MSNDFASYIWKKRPTRILFTPITLTHNPWRFLEQVAE

>SG-PL_contig_11151_1_-2_128_208

MKHTHTHMRAHARAHTHTHMQLHASQL

>SG-PL_contig_11152_1_-1_1_174

ALQAKGSYPAWTAASASLTPLALTKFVSSDEINRRRKRTMQKQWRGKRQNLLLKTSNH

>SG-PL_contig_11153_1_+3_57_332

MAASRICNMKTTGHCEMSAAVKSVCYQLFSSSLSNCSQDSDIIRTCHIYNLSGTALLLST

ADNAKNCFGKCFHCEIVLLGQNKYLKEHIKGK

>SG-PL_contig_11154_1_-3_3_143

RFFTHIILWMNRCMDYESWGFLYLFACLFSFVLMEVNIGESIINFFF

>SG-PL_contig_11155_1_-2_74_694

MDKFVTKSPRKRHQRQEVKKTQEFKQTTIESLQGVVVIEDLKRLKARLRLPDQLPAVLLE

SLQELAQKIPPRHVLKTTKIGHTVFKLRKHADKAVAKEADALYKKWRTHFEEFQERPQIE

VKCDAKTQRIRDSGRKFLSEALMLEVKDPLVDAIERELFHECKRLVSAVYRRTMRALVFK

LKHSEETRSQVLDRSLSVENLVKQFKK

>SG-PL_contig_11156_1_+3_3_215

FFQCQKCFILQHRQCSPSSPISPKQPTPKTRISQCCLQLQNILITNWNSSLLQQRNLKAF

STLNTVKTGQL

>SG-PL_contig_11157_1_-1_223_1155

MGHGRHSKEDDSRSRIEDKLLKLSLPKGMSLVDPPVMNLKEDSRPSKKNREKQKGKKRNR

RSSEGGRQCLNSDRTLPVGNSFDQDRDLAFYMSEREEVGRSRDNMGRGREDMMDREDMGR

GRDDMVRGRAMTPPVMGGRQGRHQDGYSLAMDGRGGGGRRDLSPVGGLARWDGYSQRRGD

RMDADMSPPPRNMDRHGCVSPPMSRRDWQDFDSLPLPRRHDRTDMDEAPRRGYDRRELDE

APRQMDPRYESPPRGLRRREAMTPPLRASRREMESPPRRMDSPPRRTGRADMGREDMSRS

LDRGRGDLDRR

>SG-PL_contig_11158_1_+2_116_334

MQAVTLISDCLLIYPANNSTACLLNGSVVIKKKNFLKLFESEKNDFRFFTNIPCKHLYSL

SPEWLSCYKKKNK

>SG-PL_contig_11159_1_-3_3_185

IAEKSLLTHFLHRPSDICTCVCTCTHTHTHTPPCQKRFKKVRVTVQRQQILLFNRWFVLV

H

>SG-PL_contig_11160_1_+1_16_288

MKKMCQQRFLSNSWILNLLTTKFIGRFLSLKIPECMSSDQGSLISDFFCLFVWYFCFVCC

PIFCIAPVSLSPCRSFRFSNTPPNPPKLRSP

>SG-PL_contig_11161_1_-2_2216_2488

MKGNVEYSPVMQSQSKMELHSIITIIPILICHLSTVKICPRFSGPSCPAFMVTSVSVGLE

VCWRAEMSGMGAVTKGQCRRWGSQKTPTKTQ

>SG-PL_contig_11162_1_-3_3_251

LVGYFHCLDLLHTAFPVIGVGVVTITSHSFCFLPLTFCFNTNIFMLSSHLSQLSWAEKIS

FSISIISCFSYVHLLNEIFIIKV

>SG-PL_contig_11163_1_-2_20_457

MAALSADEIEEYRAAFKVMDKDANGSISAEELGSILRAQGVSITDQQVIDLVKKYDSNGD

GDIDFDEFTKLLAESDVASAARFAMVFKALDTDGDGSVSAAELRAALEKRGLGVTDDQIQ

AMLKSADSDGNGQLSFEEFLKFVTSK

>SG-PL_contig_11164_1_-2_167_271

MYMVACSSIVCFCQKLNVYCISLLYWLCLFLCVCV

>SG-PL_contig_11165_1_-3_540_2579

MSSDDQHLLYPQFYGCGSSSGCGGCSAPWMPTTATSCPQSSAPSSTFGGGMGGAFIPLVL

GPRLAAHSQPRHSSEPRHPHQRAGPSLHHMPSFHCLRDHVPQSVISDAFDIAQGLLDEIF

GQPSCETSDTACTDQTTSDQPSCRRCSFQGKCCKDSSAQGRCNKESTAQKKCSKGSRASA

SQTTQTRSDHKKTESRLSPKVNERASAEDKPATEPSTSSSPSSTIASDPQWTQTLSLPGF

APSKVSVKVKNGNVYVHAVHEKAWGPSSCLSKLQMSQTVTLPDAIDQQSLTCSMKPDGTL

LFRAHFKKDLAVKNHNEEAEVVDSREENKEVPCMGSSLGKIKVAVEKEEKADIMEKEVEK

IVALENKAEQVNAMENNSEDTLDLENRVEEMQALENRAEEVQALKNRAEEMHALENKAED

VQALENKAEKLQDLENKAEREMTDDVPEVGEEKQGEQSGGETQGDVKDDADQKVSVEMNN

TDISCPIIQEPEDESFSEDDEVQKKHSITHQSSTEDFVVLEDFTNVETMNFGDGSEDFSR

DMELMASEQVQQENEAAEEQTGEGINTHYMTDPTRPIKRTDNQETTSHDGVFCASMPLVG

FNPQDVHVNLQGHILSIVAKCQAWLADASYTENVERQVELPGYLDAFSVRCVFERHNGCL

VISADPKGEQRQVPVVFNLD

>SG-PL_contig_11166_1_+1_169_264

MVIILETCTGDNCSGYCLTSLAINFGLINRLW

>SG-PL_contig_11167_1_-1_127_327

MELLVTHCHLFRHCDKKIGPYALKHTDWGLASLCQIYLRHILNGNQNYAPYPNIHLLRVV

DAHIFHI

>SG-PL_contig_11168_1_+3_162_281

MGTALVTLTPSKVEVSTTSLPYGLGQREQFLPLPFYHNTQ

>SG-PL_contig_11169_1_-1_115_1857

MFGQNPGGFSFGQPTSGNTTAGGSFPFGATSTGTTAPAPAAAAGSFTFGSPASTMAAGAT

NQATLGFSFGAQATAAASSTNPAGGFTFGTPASTAAASATAQPATTGGFSFGSTAPATGQ

PGGFSFGGATAATTQPAAGGGGSGFSFGGAGITPASGAKTTPAVGFSFGGMSTTPTTQTT

GGATGAGTGAGFSFGLGGGTTASTTTAGLFGGQGMLPTPQPTAATAATSAPSTTASSLAS

LLAAPAPTSTQPTLQALGVTVRPHAASGGFFSTSVAASSTTATTSTTTAAPSFAFTTPTP

ATASSAGGFSLSGGAPTLGTSAPSLGLGAPKLGLTATATAASTSATGFSLGLTSAAPSLS

TATTTTTATAAATAATSDGAAKPGRQMTYKQLEEETNKWMSELEEQERNFLRQATQVNAW

DRLVIDNGEKIVQLNSDLERVKLEQQKLDHELDFVHSQQRELADLLTPLEAALEAMPAIS

YQQHADLEREHTYQLAENLDAQLKRMGQDLKEIIDRLNSSNTKPDPDDPVQQITKILNAH

MDSLLWVDRQTGTLNRRVEEMGKLMENERKEQERNFRLAYS

>SG-PL_contig_11170_1_-2_2_154

GEMPCNVRVSLMPSVRVCLGLPLALYPDIFLIKVRQDRITEPPIKACHVPV

>SG-PL_contig_11171_1_-3_39_401

MLANTQFVENRVYDEDVTQEAEAKEEKSPEQKEKTREQRESDLIPKVTEALKFGIDVIDQ

AFEKLDTHVADSDSEDEDTTYKVDPILEAKDPYVTRTLPCLIGSPMFLQDDNVGLMEDVS

D

>SG-PL_contig_11172_1_+3_582_821

MQSCPQFLLLFRFYFSSSVSIQRIPCYRVLNPFTSHSKDSMVPSFKSFHVIHILATRGKP

TPTARGGQSIRQIPQEIICC

>SG-PL_contig_11173_1_+3_3_104

PSPQWHRRCFIVWGLDGGRGKRDIHQPVLLNSNR

>SG-PL_contig_11174_1_-3_138_329

MFISRCISVPRHNVTDKSRYFVAVASLAPSLPFFCLSVCLCVKHAQVVCFTFFDLIFVSC

FVII

>SG-PL_contig_11175_1_+1_115_213

MRYSSAGSVKFQLVNITGREYLFRYFRFQTGNL

>SG-PL_contig_11176_1_-2_98_382

MPTFHYLGLHQGENSTQILSQDGQSTQISKHTDIATLLVQSVHHVHCVVTELDQYMRLCK

FYSLSLIVTLFVCFLARLVMYLVKYLSDSYEFCGI

>SG-PL_contig_11177_1_-1_1684_1989

MFPSQRHQTKTCVQYYISPYAVNTKVVDRILYSALLAQHMFENTPQAFSITSSAYFFLKI

PTVIVPLFQLHKFAQQVQLYRCGFSTGISSNYEYLFTNLVYS

>SG-PL_contig_11178_1_+3_45_419

MKTITTCVLAILVNHSDFDQTVSRPSNNHYLHTSKAKKYISSFEEKQNYRSLLLNAIIII

KTNLTSIANLTVSEDVRKPVVHWQATPTPCYQRAPSFFSNVYGVPPETSAVQGVNGVVVR

KFRIV

>SG-PL_contig_11179_1_+1_199_420

MQVYPSPSHYQYPQQIHSQVPSLIAHFASHGSSQGREILVHWLDRALGKTSERSFLLFFS

NPKHSHFVIFFPPK

>SG-PL_contig_11180_1_-2_164_382

MTTHALAFMCLFYLCIFCLFVYFFKNLVGCSTVNRTTVYYNLEYCYTWRVAVCSRKVTVC

VYTTFTLLALSYN

>SG-PL_contig_11182_1_+1_1_261

CLGLRSVSQPRIYSSDRLILICFSYCCTVSLVGKKFSQKNSGGRNFQLRTLGLTRATVRI

PLASSRLVPEWRVGRMHPLIKILPFRA

>SG-PL_contig_11183_1_-2_83_1801

MQLFGERVVRRLSSLLESQASESDSCIEEGRTDLLRNPARQRKPAGDESTSHQREHQFSE

HSALLEQEQTDSSSRVDGGVVEDSAHECLDSSSAPHLVDIEGLSQEVGATPPKDIKEDIE

PILHEELNTFSTADLPGERLVASDTTETLSTDHETSASSKRESTTSTATDETLTSFKTRS

TDSAEFDEAFILPESLVFVRSVNEGVEGEGAVVEPGRIVKRPAIPAQLTRHGATSETTTP

HKPRKKRRRQVGSYDVSTRPDVNEPEPKRGSKQIQEVIDAYCDDMSDGSDISSSTDDILS

DEVVHKTTYSRSEQQPRAPTSIDLEHHTKDIVRKPKPPADRSTDSSDESGELEGKRQSSS

SHDSSFRIPEIEETFGELMGVKTFLEDLEDKTPTSEPLPVLAGAEKLPSKDFVLATQHRR

RSKTSPARTDRTESDLDVSEHRDDASTDSMYADDEDEGTELLFQRSYSEEHGGEVVLSCT

IYNSSHDNDGDSDEFWADAEAAAASDQNYKTLETAADAAGSAFQNARIQMHDIHQHLQDL

RRQMELLQDDITSTSLTLTPDYSLESEQRPVTE

>SG-PL_contig_11184_1_-1_7_1230

MASMFSDVPRGPEIEVFALTRRFNEDTFAQKVNLGVGAYRSDEGKPWVLPIVKTVEAQMA

TDASLNHEYLPVAGLGAFCNAACALILGRDNGAILENRVEGIQVQGGTGGLRLAAEFLKR

NCKCDVVYVSKPTWGNHRTIFNHAGFSQIKDYRYWDPVKLGVDMNGFAEDLENAPEGAVV

ILHAVAHNPTGIDPTMEEWVRIADIMQKRKLFPLLDCAYQGFASGDLDADAKVVRYFVDR

GFELFVAQSFSKNFGLYNERIGNLCIVTKTSEVIPQIRSQMEIIARTMWSNPSHHGARIV

AMVLNNPAYFEEWKQQVLIMANRIKEMRQAMFNKLRKKKTPGQWNHIPEQIGMFSFTGLK

SEQVRVMVEKYHVYMLSNGRISMAGLTTKNVDYVVDAIHDVCSDHSAH

>SG-PL_contig_11185_1_-3_3_290

GLGSPDQLRLPPLPDPPQGHQEGERGESAHDSHSALLAGTALVPGPEIPLSRPTTGADPE

PGPAQTTVLRDSPSEPDYAPPACLASVRQLLPALTD

>SG-PL_contig_11186_1_+3_159_320

MLKTNGQHHDTDTRAGWSKNNRFPSAQCSPRSAEKDYLILNAEINNVTNTCTQE

>SG-PL_contig_11187_1_+2_2_184

FFFLYRNFIQCFFDKHLTSVWFHVSITNVSFLSLLVVCKKFSLMLGGTCTYTTRQNTYDV

S

>SG-PL_contig_11188_1_-2_2_241

NFLLRDTRGVKLKNTRNPGHLPVWDIFLVLNSHRKEPFEPLGSIRLDLLTFKTAFLLSLA

SSRHRREIHSLGSDLADIAA

>SG-PL_contig_11189_1_-1_1_126

IFHHMMLVVQHVQNHLHFMMSSHRSGQNLKLVITVCYSCKVF

>SG-PL_contig_11190_1_+3_1977_2270

MATTELVGPVPSSLATPENSAFSGAPLLGQAVAIDGAAPRNMSLPTRRLDLGGAVYPLYS

QMDARKRSFSSWIDAPGLPNVDELITLGFFFAGYADCV

>SG-PL_contig_11191_1_-2_275_451

MYFAMCSDYSVIKIYCTTSILYDGSIFIKLFSWWSVYKSNLPFFVQIVVLISHTPQHNT

>SG-PL_contig_11192_1_-2_2_229

HPALSHTITTSSHITTTSNQSATTSSWPTKPAAAYSHTPCPYEKSSKQHCSLFQKVKALN

LLTEEIFFGELEPCFR

>SG-PL_contig_11193_1_-1_1_171

HIHTYIERETYIYVITRTWLCFIIVHYIAGTKQKYALFVLFPFTHTHAYKRREIILY

>SG-PL_contig_11194_1_-3_3_191

LDWTGLDGLDWIWIGLDWTGLDWTGPDQTGLDWIGLDWIGCFGHNRIFLREMWADLTGGK

YVP

>SG-PL_contig_11195_1_+2_2_202

VIPWGKKTHTPKSANGIITISPSYIICKYCSKLLQFLKKVETLSVGRFIWKGGWGLGKTE

KDKGPGR

>SG-PL_contig_11196_1_-1_1_198

GQKPVIQGSDMFFCVRLKRKRITLEYVSLPFHPVSQPILDTDSNSFRHSCLNAVKSTTTY

LVKIHP

>SG-PL_contig_11197_1_-1_58_279

MFTRFITLLSICQCICLNTVHKISDRNVYKTDKRNVGMALQFYITEWRCHHVSEQFTGTM

FKLSSLILKLTGAF

>SG-PL_contig_11198_1_+3_1203_1430

MRMCVFNLGAPFCFIARHDKELHTLYFVMACLWIGPVEVEFCVQDCFLTAVFLSLLASPR

ASSQGPSSGLHVSLTT

>SG-PL_contig_11199_1_+1_463_705

MLVTASNESESRKQCITCWHACLLHKSIIIHKNSYLCFSPPLPALNPNRKQNLEQQQSTD

SKNDCYTPQNGRKTPQNAERS

>SG-PL_contig_11200_1_-2_254_682

MSVFHQREMNLCRKWSVFHQRGINLDSIFARGKLICVENGLFSTRGKLFYLENGVFSTRG

KLFYIENGVFSTRGKLIYVENVRFPPEGNKFCRKWSVFHQSEIDLYKNVHNSPPDLIIAE

SQCKGEQLLQLSIVHNYKNGLKI

>SG-PL_contig_11201_1_+2_137_289

MPMSIQFTYLLASSQLKRGLFHRNKLIKFPESKTKEQHNNNKPNDSEYFYI

>SG-PL_contig_11202_1_+1_1_189

RQRDRQTDRQTQTDRQTDRQRDRDRQRQTETETETQTERQRRRERERLKQNNQTKTKYIN

ADN

>SG-PL_contig_11203_1_-3_162_323

MQVNTPTLTHTPKHTHTMKHIHMHTQTHICTLKYTHTPIKSNKYQGILRQLETA

>SG-PL_contig_11204_1_-3_3_146

PFPCTVQRSPCLQLCSECLIFPVIELSVFSFILIHITLFHIMRVFNQS

>SG-PL_contig_11205_1_+2_14_319

MDRVCGSRLTAGCSRPTAGGCDVTAGCSGVTTDCSRLTAGCSGVTTDCSRLTAGCSGVTT

DCSRLTAGCSTWTAGCSKLTAGCSGVTAGCSKYTAGCSRRTA

>SG-PL_contig_11206_1_-3_381_683

MTMLTVFRIGMSRENHRHHHHYHQQQNEITQGHVFHLVLALHNLRLLPILHAGRGSQMGK

FRASKRKVLGSIPCLDIFAWWVTGGDFSDLSGQHVCRPASA

>SG-PL_contig_11207_1_-3_3_146

YGMTLTFLTRLNMYICRPHGTETACIITCVGQLTEIRISGRLRHCRSV

>SG-PL_contig_11208_1_+3_228_479

MYKKDRFSNLHTCTMKSKSDFLSCTLQCDNQKSRNYPLETASKHIDLSWSIVSETCTEHQ

NTMRILLTAVRGHYQETGHRSSWD

>SG-PL_contig_11209_1_-3_3_140

FSFFFLIWMNGIRVCVWIIILFYFVSPLRYSLLCAKNLFSFGVLPE

>SG-PL_contig_11210_1_-1_94_531

MVWKKEFAPSDEELLAYRRGQEWDPEKAKEMAKLKEEERLSAEAEAAQRKKMTPASNYQD

KYRHLIGDNKVCKDDARSTAINRNYGFVSADNKRDKRTIEEVLAESRAKKRMRTDGGPSS

QTADTGDNADVAGATASESSNPPSSS

>SG-PL_contig_11211_1_-3_81_686

MLAVEKALRSCQDNLTARQVCIQIDNKTVVAYLSKKGGTRSQQLFHLPCRVLLWCHHHKI

SILPVYVKRAMNSVTDTLSRNMETQWFLVPEIVQKIFSRFGKPQIDLLGPGRQLIPRYMT

LDQDTWAMAVDALSQEWWFPLLIGSQVRDTCHGTCQSAYDITRQDIFRCLCRTTRQTTLM

GVAGATSLCLQVFTGLHFLLQL

>SG-PL_contig_11212_1_-2_2_124

PIFFFYCGMSPDWDAIIIEMSLRKCLECQRKPVTLLLVSVS

>SG-PL_contig_11213_1_+3_1134_1460

MKIMSKTTAYASMKKKTTDGEEKILEREIETLDRKINRSNEETLLLEQKRDSLKLIREER

MEGVLLHAKARIAQGEKITKYFCNLEKRHYISKNSSKLLDNNGAMLTVQ

>SG-PL_contig_11214_1_-1_124_1665

MADDWGSLVQQQEQKLAERVDKLDLQQTPATTAATNSVTPAVAAPVPRAVVSSGDHVAQA

PQQTQGAVSGAAAPNTSQGTEEEEDKEVKKEDAALLQKLLHSKLVNSKTDIEVLRKDPNS

PLYSVKSFEALSLKPQLLKGVYDMGFNAPSKIQESALPILLADPPANMIAQSQSGTGKTA

AFVLTMLSRVDVSQNYPQCLCLAPTYELALQIGEVIEKMSKHMTGLQIRYATRGERVVRG

EQLSEHIVIGTPGTVMDWSHRYRALHLEKLRVFVLDEADVMISMQGHQDQSTRIQKRLGS

QCQMLLFSATYDKEVMQFAQRIIPNDPVVISLKREEESLDNIKQYYIECFNQEEKYTALC

NIYGSISIGQSMIFCHKRITAAWLAEKMTADHHAVGLLTGELTIEQRAAIIKRFRDGKEK

VLITTNVSARGIDVEMVTVVVNFDLPVTQDGRPDCETYLHRIGRTGRFGKNGIAINMVDN

SRSMSIMRSIEKHFNRKIEKLDTDDLDEIEKIGS

>SG-PL_contig_11215_1_-2_107_364

MLQKKAIEELPPAPGPGFYVRLFVVPKASGGWRPILDLSALNKFLVPVHFKMETPLLIRE

SIRPGDWATSVDLKDAYFHILIHKAH

>SG-PL_contig_11216_1_-2_29_481

MESIIQDNIDPADLKKFEALYSDQFRRGHVSEKTQFDYSWCLIRSRYQEDMKKGIALLED

LLKSEKDELSKRDYLYYIAVGHTRLKEYERALKYIDAVSHIEPGNHQAQQLHAYIKKKME

KDGLMGIAIVGGAALALGGLIGLGVAALKKK

>SG-PL_contig_11217_1_+2_347_508

MKSKTKLQPRINKLGTTILFLCKQKRRNVLLLTELLVLQNQILMRISAVVTMLI

>SG-PL_contig_11218_1_+2_2_220

LDFVCNFVDLQAADFGNVVLCGWRSARISFGLEFALQDCCHEVCVCVAGCMASVLQFPFV

DHCQQLTCGCEFC

>SG-PL_contig_11219_1_+2_284_505

MSLILTQDRRRSIFSTLLVFKDSKSHRLNITSQCLNCLHRKCVQNWNRVQAVFKLKAQMV

ISQLASRLAKQLPL

>SG-PL_contig_11220_1_-1_103_615

MSVDRHQFGSFLFMLLCVFVCGGRGRGVGLELEIAFSTCCFSCLLKPTENYVTCAHLYFP

SYGTSQIHFHMHLSHVPPGFIVVRVHSSQFCVPFIYSWYECSLRERRKRKREDKGKVTSV

PFFLFCFVDASSSHICGTGYAFFFFKEKIYCFLCTSRTLRFVFIYTSIVCL

>SG-PL_contig_11221_1_-1_58_192

MKSRSLMTGYERVCLSPIVNISCDFIPNGVKNLGTNIKLNLKLAS

>SG-PL_contig_11222_1_+1_130_324

MYTWSRCCIVCDLPINTQIIGQHPILVQQSKIDVDISRAKSLPQPHSDKINRDEKNTHAK

TCFNR

>SG-PL_contig_11223_1_-3_396_545

MEVRESANSRASEHTMQSSVLLTLNDLKLRRQQNKHPLFFFIFKYHHCFL

>SG-PL_contig_11224_1_-3_90_302

MRSIWALKTLPHVEHMWGDATLGVLVVQALRTVRFLSASAVLSAFAAGLLHFWLLLQAPA

LLAFAAGLLHF

>SG-PL_contig_11225_1_+1_139_327

MRHPPPNQPSLHNDNHQQLNNQNTLQVNVQLSPVSNFVHSGEKTSRSKDDSVCVAPQASC

DGR

>SG-PL_contig_11226_1_-2_92_223

MPIIIINHSCIALFSKLIWTRCTFRRHSMCVIHTEALILASFSP

>SG-PL_contig_11227_1_-2_500_904

MPQKYKCSKLKIQTHQTKVQWSRINSHVPNVHRKIFTCILGAFILWATSTGHLFSVHNSC

SVNNSLMNTHWLNTLVSNSCLHEHCSTWAAVPELLPFIPKLLSKDRILQENICVPLSVFL

SMRKVVFSRLWLIDQ

>SG-PL_contig_11228_1_-3_180_605

MATRIPINKESSRGFDDRQKRIWDDMESDMERRRREWEQEIEKMRKEFFTIRPESEGLGV

GGITGGGSTSLPLGIGERLGPRSRLLDTTSDDSRGMVVKDDKGNPVFKVLFDVHDYRPEE

VSVKMDANKVMVSARHEAQQGG

>SG-PL_contig_11229_1_-1_1_339

LHLTSTRPGSHRNRCLHPRLEQSSGLRLPALPDSTESNQEGKTREGDDDSDRAKVASSTL

VPGPSLPLPHSTSRTATSPGLLHQPRSGIQHANPHLLHLQAWSLCGAQCSHVV

>SG-PL_contig_11230_1_-1_1_237

KVESLQQDPESEDGGDVEDGAGSSSSPEKTPVKQPPDMLDKKRKFDQVMEEETREEILRK

RERQEEERLKMQILVSNLS

>SG-PL_contig_11231_1_+1_1_216

SCCRLSTLSSAVLLSSLLCRLHLLLVSSASDLLLLPGGLENPGLGVVGMGVVGVVDLIGV

VGGVGLMGGSRW

>SG-PL_contig_11232_1_+2_5_253

MHVCVHFGELGNNLCTVSTVTLTDENNYSIMSQTQIYHTLTYQTPRIIQYPRHNIHRHYS

KLSCCFDKHSLFRTKKRNSFTLN

>SG-PL_contig_11233_1_+1_520_771

MVTTRFNTLHFLTFCAIVTMKKHLVQICRLVKSQQLTNIWIKQKYIYILAKKFLSEYIHM

ISYPGLTQVRDHAVMHTYNKLSHS

>SG-PL_contig_11234_1_-3_3_236

DSFSINSIIACWKFCARNFHFSIALFVSAFFTAFTICVFLPSLSIPLSFFPASLHTIFCA

PAPPSTVYYLYCFTLATR

>SG-PL_contig_11235_1_-3_279_599

MEHATRNTNLPQNGSFRSDWHAATCCCLCGPSSICIGESANQCGCVPWVWKRTVKYSSVL

VSTAFPCFPLPGCCFPNSSLPSTPMPSHSVGVIIVIKMSFNFSTICN

>SG-PL_contig_11236_1_-1_40_330

MMMSFHTEPLVVKEEVYQSYPSVLRLDVMVSRMYDPSVPRRLLACALCALKAVGSRGVHT

KLNSGDKFMMDFYAKLGFFAVTPADETADVVYMGRLI

>SG-PL_contig_11237_1_-3_63_677

MLKEGEKPSPSQPPAQPSRPFFGDIFSSWHEAFIGTPGLNRIPPEERLDSDLDSKRPNSE

EIADLFEQQKKFFPFSYGSKNMDIETVTTEADGVMEKHHTIHDSSGYERTTVARRMGDSG

HVHIVTTIKDENGQEKKHEMWIQNFDGKISEFDKQWQGEFDKQWQGKRDTADRVLINPKP

VMPVPSFPGGILSYTRGFFRKVFGF

>SG-PL_contig_11238_1_+2_59_301

MRARTHTHTHKYTHTHAPAHTLLHAHTHYYACFQIIKYFTSLIRRYESVPFRTILLCTLD

HAVHAMREGKGVIVAGHIVML

>SG-PL_contig_11239_1_+3_111_1046

MESHKSLLPDNRPTEPHVVIKVEKGKADDRPTHSETPPVNKPISTNNRLLLLFLHHDDDQ

HHHLLLRHYEYHHLLETSSLFQQLHLQEIAVVDAVVLVGVHGADLTSHLVIDVNAHLLQP

LHHVLTGGAPASVCVELREKLAPHAGLGVSTRIRHKIDVLVHCHSAHFGLLLLLPQNFSS

PIDLGQPQQGHESRELLLVQESIPQHVSHGLKLRPVVQSLGRLVHLVVGPGLWCLFTYAL

PHLNAEITCRLGEQIISELQTLLVPILFCHSTLTEQLLVQHTSRLVGPTWVRAASVGLHY

AKYFSTVSYQIF

>SG-PL_contig_11240_1_+3_30_290

MNLPPDIRGILAGHDITDTDALAEAADRIWETRAAGVQQISTSPAQAPTEQDATTAASEE

TVSAIKTAKQRGPPSQHGTTNSVCFYH

>SG-PL_contig_11241_1_+1_451_804

MKPIPKSIHNLTVNTEGSLLVENEKKLQTVIKKWEQLFQEQIGWKQCFERISKSQEVKLK

RFQIRIFHRILAQFVESNLCSLCSKERDSVIHGFWNRVTTFNYSGMIFKTIFVHTVKM

>SG-PL_contig_11242_1_+3_162_317

MLNDGSSLHLFKQSHFIFFTSPFIFIMSVVNSQYNCVNFNQCQQARKQSLPK

>SG-PL_contig_11243_1_+1_1_255

VCVCVCVCECLCVNPPFASVLSLIPTFLSIFLISFVCIPLPHNSAVPRTTELSLHHETKQ

RLMAINLSLSLPQQLPLIVRHSETP

>SG-PL_contig_11244_1_-2_197_427

MKLSYCCLLQVLHEHIYVQTDFVFYMLLLTTLHICSFISRLKMVLFMILKIHLLLRSLSA

NAAHSRSKHAIKEDFEK

>SG-PL_contig_11245_1_-3_3_221

LRAYTQERGSGTSRSAHILTWEIRKISTLYRLGVTQARLNLDFLIASLISYRLAIALFFI

YIYIPGNLTHIHT

>SG-PL_contig_11246_1_-1_475_777

MEGLKGLEGLEGLEGLEGLEGLKGLEGLEGLEGLEGLEGLEGLEGLEGLEGLEGLEGLEG

LEDLEGLEGLEGLEGLGLNLLEYVAWWVKGGDVFNLPGQHM

>SG-PL_contig_11247_1_+3_3_140

KDPVSHVSGWLMETRKHPACNQQGLAVRKIQWDLMLLSLKNKVTNN

>SG-PL_contig_11248_1_+3_768_1016

MYNPCLEAKKKKKKDIKTKKTPTTMKNKKLPSHMHDNKTKTKTKPNKTTTSHPPPSPKIQ

PLEITQVVLILVFTNHLHVSHMI

>SG-PL_contig_11249_1_-1_31_393

MCVGEKRKLIIPPHLGYGDQGAGEMIPPKSSLTFEVELLDIKDGTPPQNIFKQIDADEDQ

RLSQEEVAGFLKEQAAKAGPESGIADSEHETIVSQIFEHEDKDKDGYISHEEFSGPKHDE

L

>SG-PL_contig_11250_1_+3_120_230

MISRNVFSILSHFFYRYYRQEPKKPILVCVLCRLFFF

>SG-PL_contig_11251_1_-3_36_4088

MLEGEKASLSGEKAALEQEKAGIRDDLVRVEQEKMDLDTEKHGLNQTLELSELTRQQLEE

EIQSLNREKQEITAQHSMVSRQKQALSEELLTARRDLEKAQQAYDRVFKEKESLNLEKGE

LVVQVTATERENRQQSEQLAALQSDKDQLEGTLYEAQQAVVQLEVRKAQLEGENQELIVR

KENLQAEISRLEKEKAADLERFEHHKDQLNTRLMQLERDSQLALAQEKQAHEEDCERLTQ

EREAQRMEFESNREQLLHSHTMEKEDMTQRADSQLEELQEELAKVQRDRDEALLLAENDR

QQAISLLEQEKGTLTEKLANALQELADANVNYDRLKREAQTQAETDRSNILNLQGELKDF

RLQFEEATEAHEREVKNMNEQLREVARQREVAMAEVIELKTQFGSAEETRDAIRRDLIDA

NRAVREGEETRDLMRKDITDLKRAINEEIREKEAISNAAGELRAKVKACEQDKTDLGRAL

QDARQRIGVLEEQKSGVVKEAGDLRGNLREVEKARLEARRELQDLRRQVKMLDSERSKLG

KEVGDLQLRVARDEEKEEEARRTSFDLKQKVVETEASREALRKELANLQRKMAELIDDAT

MKEKDYQLALDDSRRSERKLEDQRKNLEIALEAANGDIGELRLKLSGAEGRVNALETTLA

RLEGAKKDIEFKLSSIVSSLRRTIGFRQEMPRARSRSPSPRRSRPTSPSKGFENTFATTT

EGQGSPIPRVGSPTRTGSPLRTTSPSRRNMSPSRVEGLDVDPEAVRAALRAFMTQLANAE

RERDDALATVRSVTTQMKDLDEERERTENRLRVLQKSLGEAEEDKRGIDGRLTSAQTALM

MQEETIRRNERERKVLSDKVNSLERAMAAADSEKRQLQEKMAKLKTNESKLEEDKKNLRA

ALDDAETRCTKLELARRSLDGELQRLKLAMTDKETENQVLQDRVETLTKQIQDLESKAQS

LQVTIDRLHGSLAKTEEEGHMSKDKVQALNMSLSDSNAMISELEERVSQTQRALKNSEQD

RCVLKERLDVAMQGLNEAKKQNHVLLERLQTMQNEVSESEVRCAELEGQIRQLNNLLVQR

QEGEQELAQRIQKTQAEKQALVERVSSLSRTLNSMENEKREVERSSVRLEKDKDALRKTL

DKVEREKLRTEEIATKTLYDRSNYDRSLQRLEEENVEMQRGLQQLQSQLAEAEQRHAQRL

VDVNTRHRAETEMETERLRSAQVQAEKLLEARERAHRQKVKGLEEQVHTLKDQLSQELRR

RQQYFTKSAKAGEEIHNIRSILDTSLSNVGRDTNLDPLLLEHESRKLDESLDLGGYSHRH

RSPGRTMSPGRLSGLGLRSPRSPILRRKLKP

>SG-PL_contig_11252_1_+2_2_175

MWKLIPSRRSLVREKTLTKRFCSHMRTFVDSGVSIRPKMTRRCMEMEQFRDVNWSLID

>SG-PL_contig_11253_1_+1_34_381

MFSAQHVLGLFGTDDKANISCIAAISHKLSRQQSPNQRSSFLLCTGFPFLELRAGLQHPI

NGNGEEAESGENTSLSDASEDFKFEPVAVAIICPDPTLGVVINLLYGRYNFLWNAI

>SG-PL_contig_11254_1_-1_181_399

MKHNQPVISVFKKQLHSRFNPLASCPAATRQQEVPSVYLCPPQPTACISGLVGNFHHALS

SGLYTHTMNEASC

>SG-PL_contig_11255_1_+2_383_628

MDLPCEIWAALPGRAHRRSAAPPIFSPFFLVHAGCVHVSISHRTPTWLTGSLTCVFDVVL

HEHTHKGKCHKPAQKPYHGDLN

>SG-PL_contig_11256_1_+1_1_84

TLYSTKSTRAHVSGYIRKNIISTGCVIN

>SG-PL_contig_11257_1_+3_33_230

MHTETVYFLNRWHHTMVSPGRAVCTSHGKKFCCDQKDQFNPINNIVYYQCTKMVCNNCSH

YKLQFA

>SG-PL_contig_11258_1_-1_13_804

MADYRENVVEALLLNASQSNISSDILPACVEHRIFGLKNADSLEPVVAQINSDINTSGKR

LQGLLLLKNVLCQCSAQVFSSNFHTWLNLLLQALQVHGLPEQKQGALQIMSIMIERVTTF

PELAREMGTTKVKVILPALLSLNSKKIKEAVVSCISACIIHFPGPTSFFKNKIEMFVVEE

LDGDCPSQVYIRCYTLLARCTTGVHRGEKHRELWIAQYEKLMSTLNYLLSELYFGLEPDG

EEEGENSIFVAPLPDIHPQRTQCL

>SG-PL_contig_11259_1_+1_193_360

MSSKQSCNNERCRQVCIWNSHCHKSTTQPQSDKTTYVPTKQNVPFIRIFLSCLLVN

>SG-PL_contig_11260_1_-2_2_196

VAILFNNKFLFQIKKIYKDPGGIYIFSSLKIMDEDFLIIGVSPDHDDPEFYVELKARINE

VGFEI

>SG-PL_contig_11261_1_+2_35_301

MHACMCVCMHNLYDVYCMHTGACIHVPKETSYPIKKLCLISKTETGGFSEDYYTPKMKTY

QNGFHGSYQQRHTHTFNRAGSYTQPNSKT

>SG-PL_contig_11262_1_+2_365_619

MVTASNTDFISYIFNQVTSLARGNDTNCFMKMHCCIYHITAGWLFFFDMTMDSRSDVSNS

VSACIIKTGLFAAHLKASIILVVSA

>SG-PL_contig_11263_1_+1_172_282

MMNLYCVTFSVSRLTDGNLRSGNPVSRTSYASATLSD

>SG-PL_contig_11264_1_-1_43_252

MSQIRHIVLCVFFVLNAQCTTRKIKTCNNYFRLYFFNTNTTKIQQASQPFTNRKQISSVS

SELPLCHTCF

>SG-PL_contig_11265_1_+3_165_344

MFILLNNQHSVMFILLNNQHSINEITLYYYRQISQTTLEVPVFHFITSRLEKDLYINLKS

>SG-PL_contig_11266_1_-2_74_265

MGLHEFGGCVSLYVCIHACLFTYLLQMLCKYVIGYWHLFLLYKTLYSARNDRNSLKCFCT

GIHW

>SG-PL_contig_11267_1_-2_437_799

MYIMIILALSVVLCAVDCCCFWKMYVPPLFVHFKCICFVLMCDFVFCRFVGVVDLYGTLC

TLPLNCPVCMNMSFFCVNLCSVKRRPGEDAGGSFSINSILLRYRMSPRYRMSPRYTQVQT

I

>SG-PL_contig_11268_1_+1_811_939

MLATYSICTELSGLKMVQDEKFLAWCDKVAKPQACVCTADTPL

>SG-PL_contig_11269_1_-3_105_278

MPTTHPPNFPSHFLYESPFSSPSLSYKSRAANHYIFSVIYYNQLGFITLLHSCLKHYT

>SG-PL_contig_11270_1_+3_567_905

MCGFKCVLYALFYIVCVCGVLHVCVACYFTCVWHVAIYMCVCVACGILHVCVWHLFYMCV

YGMSFYNICVWHVAFCMCVTCGVLHVCVWDVSFYMYVAFYIHVYMCHVILHVC

>SG-PL_contig_11271_1_+3_3_140

QVQNVPLHAFSFSVLHLDFNTLRINQQKNSIQSQSTDVNNLKQKTD

>SG-PL_contig_11272_1_-3_3_167

RSAYLVKCHTDFVKLELVPTILPHQHFLCRSLQPFLILSCVSRRDAFSFLEFVTL

>SG-PL_contig_11273_1_-3_261_1235

MAPVPRIGIGMDCSITPLRHGGLSLVQTTDFFYPLVDDPFMQGRIACANALSDLYAMGVT

DCDNMLMLLAASNKMSDKERDTVMPLIMRGFKDTAEQAGTQVTGGQTVINPWLTIGGVAT

TVCQPNEFILPDNAVVGDVLVLTKPLGTQVAANAYQWLDQPERWNRIKLVVSEDEVRKAY

QRAMFSMARLNRVAARLMHKYNAHGATDVTGFGLLGHANNLAKVQKNEVGFVIHNLPIIA

KMAAISKACGNMFGLLQGHSAETSGGLLIALPREQAAAFCKDIEKQEGYQSWIIGIVEKG

SRTARIIDKPRVIEVPAKDREGELW

>SG-PL_contig_11274_1_-3_51_311

MGIHIAYTHTHTHSHTHTCVYTIMYVHSQCMHSHTHALYKKITPVESHTTKDTPLTITVT

EYLYLKVKVFGFKIQHSVIYYSLLLQA

>SG-PL_contig_11275_1_-1_1_210

VPLSFTAENVQGCSLTGLQCLITGNDDAETFYCHLGAMFYINHPNIHIFIDCFKRTGTNM

HTYTYDNWAS

>SG-PL_contig_11276_1_-1_487_615

MPGCEKYLLGVMIKTIRFVFKVAIITINTLLVVVKVNWSLYYN

>SG-PL_contig_11277_1_-3_9_1088

MKKALQYSATPGMPDLVQWVKKLQKELHNPPTLGIEDPQRQLDVIITSGSLDGICKTMEA

CLQEGHNVLLESPTYSGTLSAVKPIRPNILGVRIDKDGLDPSHLREVLSQWQPSDVKNDT

NNIPKLLYVIPNGGNPTGTGLTLDRKKEIYEIARIYDLLILEDDPYYFLQFNKPYVPSFL

SLDEDGRVIRFDSFSKVLSGGARVGVMSGPKAVVERVVFHMQSSVMHSSGLAQACLLAVL

ESMGIKGFRLHALSVADFYRKQRDACIAAAEKHLTGLAEWSVPSGGMFLWLRLLNVEDSY

RLVMEKAKAAEILFVPGQVFQIDESEPSPYIRASYSLISPEDMDKGFERLAKLLREPKKE

>SG-PL_contig_11278_1_-1_490_843

MRWRDLNFSWGQHYEGGVHLLSLAASTSPTLNRVIHPWMKWRLHALGLEGWCSTSTPTRS

ETDHRNTTKQSINQTNKQKTTPASVVPVLGGVSMSQSMYPTKVAMEEQLCKLSKRERQ

>SG-PL_contig_11279_1_+2_74_235

MKLFSKYSVSCHSQWPGWHRGTAVAFQSACVSLHGRESCPGNSVCVCGLQSTQL

>SG-PL_contig_11280_1_-2_146_274

MGKADLGQYVSWIERTAGRKTGSPTHFLGDNEVWISFLVCCLS

>SG-PL_contig_11281_1_+2_353_538

MVMAQKWHRTAPHPISNQWCAQNCFILLLKMVRMHSEMNKDYVLVVTGRINTFITEKFEK

AR

>SG-PL_contig_11282_1_-1_55_228

MCARGIHKYHVCLGHSQVSCVLGAFASSMCARGIHKYHVCSGHLQVSLVGLEFIKVFY

>SG-PL_contig_11283_1_-2_725_1852

MAVMILFAFVLPVVAEALQTTTYGAARDYSSIASENYPQSYPGPDSWLWEWSVDRGLWAV

VFLDFDLACAHNDRLLISDPRGNFTFTCVNAPPKSFFIHGPKFRMHLYAEGDSQHQKRGF

KCQVLYATSKGALQSKIDDLETIEDKNGTDSDKDNSPVLSDTVIILLLLVIIVPLVVIIV

IGGVVLMRRRHHQQSSNSTSVHLNIGAIRRDSSVSYTRHGNGPAPGVPRSRLDSDGSLSP

DNASTRPKRPVKRSESNASWAMRAEMNTSIRNGSCSSDGHQGTAVRNFYTPCPGETDTCS

VGNQGFKDGKNSVSTSVGCRLSTICEESNSVTKLSASFPVPLQCEEPSLAIANAGYMLMT

SCSANYVNCGKDEEFV

>SG-PL_contig_11284_1_+2_908_1159

MIIFFLCMLCLYVYEIQSHSCKLEGFVSVSVSLVREIEQRLKSVLSCDRAVRIFCFNYIS

CDNAQSCEWMSVPAFHCRVLVLTL

>SG-PL_contig_11285_1_-2_1211_1432

MTMCALTSEMRFKFVLGFRPFREKIVIHMDRRVKLHTRYRFTFHVLSTLAEDFSSLFASY

LISFIPYKIALCLL

>SG-PL_contig_11286_1_-2_23_739

MAGSLRCAITSVSRFCSRHVAAIVHQQNFQHQNTFIQNFRMLAPSWFHSSSHHHGLMDFF

DNKKNWGEESVKTGRPWRVDELRIKSNSDLHKLWYVLLKERNMLLTMEHEYKKQCELFPN

PERIDKVEESMEHIMQVVHERDEALHLLETGKTGHPGGRYLRNFLGLRVWKKNTEHLIPS

FMNKYYNLRYPRKVTSRTNKYIALYREQQREERRRARKREARRMKTLETAFPHLKGKLN

>SG-PL_contig_11287_1_-1_1_147

FFSACWVFSCFHKPPNTDMTYRIFNVHILMFCMRIYTQGVQTLAGLHIC

>SG-PL_contig_11288_1_-3_3_173

CNSTVSVHVGIYETHRKEREQKIIFKRFSLFDHKMIEVPMPVEYKRQVFRIKLKPHI

>SG-PL_contig_11289_1_-2_2_121

KVNYKVYRYNICSLSSARVFETEAYNSKTLTFDHFLYQGY

>SG-PL_contig_11290_1_-1_1_126

CTRGLTLVRQSAYILGDPQSVKLGNANNNNIIDDGDFTLYCI

>SG-PL_contig_11291_1_-1_1_381

CLSFVLCVPVLCSLCACLSFVLCVPVCPLFSVCLFVLCSLCVCVFVLCSLCVCLSFVLCV

FVCPLLFCVSVSVALFCRSVSMLCLKYDSFLCSDVFLFLFRVNTKCIVMEIFIPQMNFQA

LLQEFFL

>SG-PL_contig_11292_1_+2_2_124

KVTASIQANIFSSSTDSEKSIKLLIYCFVLNLVSLLSSMYA

>SG-PL_contig_11293_1_-1_1_591

ITLNGNLKDIKERLDKVKAERVNLQHEILTQADIVVKAVRANVWTVLSSLHSFTEAASKR

LTREVNDVGDLIGKFDHCIDFMEAILDEGVGMPLLYSKVFVESKCRKVYHTQMEPQSLKG

QLNLQYRHDVPGIVRILPSFGAIYVNGTKYPPEKPTAQPSSASIGVQNNTVYSTKSVPGS

GGATITIPHFPSTQDRN

>SG-PL_contig_11294_1_+3_3_158

TEILLQRNSEKEIGMSFKMKCAWTVNCSVVSQSYVFIVAFSFIYCTMLDCKH

>SG-PL_contig_11295_1_-1_1234_1467

MSKHSEIGHFSFTLGDGRTIRQFYRGCFVESSREDNGLLECDLFKLQHFLLCTHLTNILT

KYVQCYLLACSVLTFLAS

>SG-PL_contig_11296_1_+3_417_623

MGQGSCTSHGREINISGVHASFWYSQFLSQKCNLIHHLFHVPLCPMPPSTEMTARQNQLS

PLLKKRSKT

>SG-PL_contig_11297_1_-2_332_1303

MDADNMAAAVAAAARDRGPRRAAASRAKYTFDDDMEEEERVSSGDDLANSSYSFKPSAPP

TRKPPSPALPLSDGDEDDDIPMKTTPKKTFGGISDDDDDDYSAKPASQKMFGGISDDDND

YPMKAAPKKTFGQDNDYSAKAASKKTFGDISDDDESFTVETDSDTAAKSKAMPAARPTTT

APAPKPIMPVEFSDSDDDGFSLDSKSASKEKDGMDDDLIVPKSSKPAAKKPAAKKPRKDA

GESKPKKTKTKRPKNSDSEASDEFVPKKKKAPAKKKAASKMTEDGDSDMGGGDIPPASRR

VSTGRSKAPVKYTFDDSEDDFDDY

>SG-PL_contig_11298_1_-3_774_944

MIEALRWAANRPVFMLRAGTELETSDLESRVLSKEPTSPEKKVAHKGVEPTISASSA

>SG-PL_contig_11299_1_+3_297_590

MVQEFTQMCTHTHTHTHTYTHTYTHIHKANMGQEFTKTHTHTNIQRYTHTHTYVQRERHT

QTHTHTHTYTQSIHINTCVLSTHTHTHTHTHTHKPHTS

>SG-PL_contig_11300_1_+2_2_208

TDYSYERTACFLDTGLHFCPLSSCARSPGQPDLASVQGFARAQGICQINCRQSAGSIYGI

EHNQFVLNE

>SG-PL_contig_11301_1_+1_223_399

METGMILEQVSLGPLPTATRKKKGEGQCRGDQEVVVGEAGCCFDMLFWFDFLSQRNFSM

>SG-PL_contig_11302_1_+3_180_275

MGSLTCAKCMMHMEPRFIASFERLAPRPPLKI

>SG-PL_contig_11303_1_-1_1528_1779

MLEMQNVCMGMSERKRVHHKSAYANTVTIMNSLIRDTDMCACGWYLWMLEKDLVLWMYIH

GSVHKHLYLLWRQSRSVSELLDVI

>SG-PL_contig_11304_1_-3_180_569

MQKAIKAHVIQCQYFQSHTLSKQRNKNKQKCKMSPRVCREIRTSTAAIHLVWPSSTTHVI

SCAGRMVKTSLAAKLLGWYRKQTPDSWDLTKRNKFLIWDPTKRKKALILFLCSVLTTGHN

VKLQVCILNF

>SG-PL_contig_11305_1_-3_105_713

MERLGKLSRFKKLGLSLNRVRQAAVDNPPSTPSPTSMSTPGSSYSFTFPLPTPVNKRLKL

PGVFPHRPPLAISCVHMHPMDIQQYSLLKIGQELVCVSRLTQEELQEAGVVVPVSCADEE

WASPEEHNLADLHCGEQQDCVFEVSSKQNVTSPCGQEQDSVRQGVSRRLMGPKCRRTTMV

TEGNGQTSDMVSHMAGLRGRQGT

>SG-PL_contig_11306_1_+3_1653_1826

MHHNITQLTKDELLNTTYKLPMSGIGLNRDIICHYGHTMGHGSNKEVFSGNVAVEKLT

>SG-PL_contig_11307_1_-1_19_522

MARQLIEAIRNGDQSLARNILLGDRYGCIDCQTAKRDGTALFWACAKGFVELVQLLIAKG

ANVNARTNYNSTPLIAAADSNRHHVVRLLIEHGADLNAQTTNGDTACHQAAYRGHMEAMQ

LLVANGADVDITNTKFRTPYDEAARQGHLSLLPFLDGNEKLGHSRGAR

>SG-PL_contig_11308_1_-3_285_761

MHTNEKPYLCSHEGCGKAFTQLSQLQQHVRTHTNERPYHCLHQGCGEAFIQLSHLQQHIR

THTGEKPYKCMHPGCGKAFSQLSSLQSHSRSHMTDKPFRCNSCYKCYADEQSLREHIPKH

SETKHLKTHICNICGKSYTQETYLTRHMAKHQEAGGALN

>SG-PL_contig_11309_1_+2_80_235

MKIVCIVSYKLLGVLDVIFLTHTEPYMTVAGVCVCVSLCVCVCVCVCVCVRV

>SG-PL_contig_11310_1_-3_3_179

KDLNSLLSSVQVFTLIPLCQMYMTHEVVNAILCNFDAKWSFLLMQLLYFNQIFCYFWLF

>SG-PL_contig_11311_1_+1_139_438

MAANRPVFMLQAGFELKISDLKSTALSVEPANPLVASISVHMYLPHKVHRLLRTDLHTYR

YSHVATKTSTAQWVKKNYLTCRMPRNTQPFHSLTLPTSFL

>SG-PL_contig_11312_1_-1_247_879

MSGLRAPKAGSNREAQAKVDASFDPEDAKKILHWIQAVTGAQFPIEENNDRYRMMENFYK

TLKDGRLLCYLVNAFLPEHMKLDLRSKTFQETKNMAFEAARERERIELFIIKIQEFGVPE

QDTFQTDCLYERTNLNKVIGCIRVFGMEVESHPSYTGQRVWPRKSEANPRNFTEEQLRAS

QNVISLQYGTNKGASQAGMNFGKKRMILQNE

>SG-PL_contig_11313_1_-3_3_218

TRTHAHLLSLTDPSSLNLFHRPRQKRNIQLCMEDHHIPKLQCLTACNRVFAITIAKPLLG

ANMSCPMSDLAG

>SG-PL_contig_11314_1_+1_1_132

QLINQPTKEWTNQPYNCTDKFTLSLYYVFVALHILYRQFFITSR

>SG-PL_contig_11315_1_-1_331_459

MSTFSPVQRMSPVTSLALYFHVCDQRVFAARKVKIPSKYIKTS

>SG-PL_contig_11316_1_-3_918_1112

MINEKQLSSTNWEKMLDLHLNSLYLLNSLYLLSSSSSKITFQQMIIMSGSVCVCVCVCVC

VHVVF

>SG-PL_contig_11317_1_-3_60_1910

MASNVQHVLLLFVGVLLALGSSQTVHKMSVRSDIRFRFATTEMMCVMSNDEYVAKELKFD

VTLPKDGFIANFSMEIEGKEYPGNVKEKETAKKQYEHARRSGHSGGLVEQSARHSNKFDV

SVNIAAGSNVTFTLIYQELLRRRFGVYEHSVYIDGKTLDNIRDYSIEVYIQDRNEIRNVR

TPVMRKEITNNAVEEPQNTITQITSPAGNIVYVVYRPSVQELQKQGPGWFVVQYDVESDS

AGELLVVDGYFVHFFMPPDNLPPLPLDIVFVLDISGSMAGRKMDQLKDAMRIILDDLRET

DRFNILLFDGNVQHWKRSLQEASKSSVNHAQDYVKAINYRGSTNLHLALMKGLELLQGSR

EAARVPMIFFLTDGEATAGVQNSKTIVSEVSQKNEGVVNIYSLAFGRGADYKLLRQISAK

NKGFARKIYEASDASEQVSGLYREISTIVIRGLTISYHSTSNNDGGDDANEIRVDNSTIT

ICELPTVFGKSDFVVAGKLENADALIRTPGKAGGLRLAVSGLGAEQKINLVVEENDLSHL

ILEGDNPFWTVPRDLSKMAERAWAFLMIKQLLEEKVAVQEEASLVKDLDKRILDLSLKYH

FVTPLTSMVVTRPEDNV

>SG-PL_contig_11318_1_-3_3_212

LQIYVAECVYDCLILGKKCKIFFFLKLSLIYFINKIQRQIISAKQNLLHQIFKIQNTCKT

STSSTKDLCA

>SG-PL_contig_11319_1_-2_2_154

AKHPHLQQRIYVLGVPSPPTTQKTNINTHEQVIEKTCLNVMNKQEEILNFI

>SG-PL_contig_11320_1_-1_103_327

MLPEVLSLITPPKYMFNSIFHYISQKVHFSLSTIFICFKITFVTMILLTVLIWNHFRFTS

CILCILRNHEFQFNV

>SG-PL_contig_11321_1_-1_1_339

FLFVSFFPSFFLLLYFLLNFLFYFAFLTLTLHITNNTLYYKHKYKIHVSIIKYTHGHTHT

IVCKCPHNLSRHKGGKMPDKKHNSVNRNTTGMDMRWLGSREYSRHFIFNFLFS

>SG-PL_contig_11322_1_+1_1441_1722

MNRYPQGLTIYKQSYIYSTVLMSDTLCHHQNRQIKFKFCLITPTSSHEVALNNIRYIAQF

NNKSELINCTRLTNISGSSKSTTDLLVFYETMLW

>SG-PL_contig_11323_1_+2_590_1018

MKLLLYLKPIQVPPNLCQMIQAHGACCDICCPFSASYNTAPSERFMSDHIPRHSTCCEGA

ILEIHAAASYEPFMSKKRNKPTLWGILRVCCLFSHIRDTPRLMSNQIPCNMKHSHCCYLA

LYFFSSSNYWLLTGSLISRGQQS

>SG-PL_contig_11324_1_-3_150_413

MERVLRVFCKGLKGLNRRMFSVTGSQSSDHMTVTQSSARKEIISPATVLEIKNESDTVKV

LTLKVSDSNFSFKAGQWVDTFIPGVKTV

>SG-PL_contig_11325_1_+1_1_180

TVCRSVLYIFSTEGSPIAQYQAREIMLFKPNRNMCCENPVSRIVEYPRSVVKKTTIPIYT

>SG-PL_contig_11326_1_+1_40_255

MQSGNVGAHVTAYRLHINTNTHSRIYHSHEKEIHFSSEYAKLQNTAVSDNNNITNLKKRK

EKKGIIIITYHL

>SG-PL_contig_11327_1_+1_1_144

YVKFKRKKKVCVCVCVCVYVCVCVCETETELGDHSVNREIKQPAQSTM

>SG-PL_contig_11328_1_+1_1_117

SSTDSILQMCGFSFILKTCVCILQALELRSFYSRIVKIV

>SG-PL_contig_11329_1_-1_451_2082

MTSRCRCPFSGPADTILGCHRDPIASEKGWLDFAVRELSRVAAGKARGQEAVWRSQCKPQ

WWDCVCTHPWKNPTANPKDSKDTLKVKFECLVQHLRKEGQLPREMEEEISLWEAGRVSEV

FLMTSFSSILGQATNLHSVLQDACHKMEDTGISVATSILTDLQSCLSACVDKIEATKRKQ

TGAASKSAYSAGSKHLCDETDFESDVLSQPPCKRPKRSPSTSPRLSSSTSSLSPSLASPR

SSSSSSACLTLSQSVSSTPVSTEQSKMLLAVLQRQLLEKKVAKSAKVTSACQTSKPQTQL

KPRVCPIPSTRKQGMKKRVQGVGQGNVATAVGVSVSTSPLVCLSIAVPQYSAPDVLGENE

EFTDFLKLDLEDSDVVADCQFLDKLLQDTSPDIGVADSTTALPSCSVDQTVTCPMPASNS

DASLVHHINAPNPFKPDPFFGHTITTCDMSENTPFHFDVVPEAVSDRQAEGGGTEEGNGT

AMFPGVGVINISTGSQDIEVVSDSGYNSESSIAASHRSSFDSDTYSYSDLNSPDLEAYLD

SLEG

>SG-PL_contig_11330_1_+1_490_732

MSLMRILGIFWPQISKQNLLARCDQENKIRLLLLHREDGDGQDMSRDEIKIPHKNCSPLE

TSREMQKRRTKENLATHSGDR

>SG-PL_contig_11331_1_+3_3_167

LFYLIQGGGGMVCANSFPTVTLRVIGQPLPISKNNNKRFGSLLHKTGQKCVDLYW

>SG-PL_contig_11332_1_+2_1460_1774

MESVHKICTAFAGFLKKDVAMNAMECSQSFLLLTNQNASVHACLPTFSPFLVFMFVTKSM

STIMKTDVRSAESNNWCSLSLLSVRVGDSDTIKTGPKKRHFQTEG

>SG-PL_contig_11333_1_-2_23_331

MDKRRWTEEDGPAWSLLKLSCYACLPFSDHHQQVPWVGLSALCEGTHIVWGRRVKGEGSL

LLISWTLHHRPVSLPETKQTPRLQCCSHVQPPAHSHPHIPIHL

>SG-PL_contig_11334_1_+2_572_1030

MLTWEMGKISTLYPPGRRCLGGLNPGPSVWKSDVYPLDHRALVIVRLIQIISWCLLCDSE

IDPDYLMCLACDSEIDPDYLMAYTVSFHLSVRQTKCQKDFFLRCSWMNIYPIWVTCPKHT

GLKRHDSALGRKWEGEGTQGKGEERRHREGERG

>SG-PL_contig_11335_1_+2_2_142

SQVDKRMYNSICRLIHECTILFAGRYRNGAIRTNVQFRLQVDKRMVL

>SG-PL_contig_11336_1_-2_8_658

MADDSTLWVGGLTDQVTEDHLYELFLQAGPLEKVVRPKEKDGSSRKFAFVEFSHAESVPY

AIQVMDGIKLFNSHLRLKGRTGSQNDSGRSDSPASRSYDSRDSSRGSSDYHRSRSYQGHP

HQKPGLSRGNSAEDRRGTNPLSQPFSSSAAYNSANMQPQSFEDRRNRLLNKQNMSLGAHA

QMRGGQQQYGGQNNYAQSFSGYSQQSGPQQQWYLDNY

>SG-PL_contig_11337_1_+2_2_289

RIFNVRIFDIVLRVYTHEGGSGTSRSTHMLTWEIGKISTLYPPGEVTEEDSNPGLPNPPV

WKSSVFTIRPPRARARARVCVCVCVCVCVCEHLLRI

>SG-PL_contig_11338_1_+3_3_146

ALDQVYKHQVWLSTHKISTDTSKMQNVASNPHFILDTSKVCSILVCKS

>SG-PL_contig_11339_1_+3_69_218

MLISENDSEVELYKHISPTHSSVTLFFKAHDRTSFSDIKRTDSETPFLLL

>SG-PL_contig_11340_1_-2_1550_1840

MIALVLLCKYVCVAPIELQKQDEREIIWSRSCMVGFKMCNVMFCMLAFRLFFLIVVVEFS

PVPFRIYSDSTLVIQIDFGTCVCVCVFAYKMCMCVSL

>SG-PL_contig_11341_1_-3_6_1043

MSVQPTCWRGHHPVYWSVAESVIVSPPKDSWGLLLKVTAASEGACHGRVQHVKAPTQESV

VIKYPPPSTRTLQYSRSTWCRYVVSGEGEADHVLCRVEGELCELTDHCGDYVRVYDGPST

ESPLLYDSQYENHVTLYAISTGRHVTFEFSTDDLSNRGFVQAEFNSVPDSKLCSDIQRIT

ASHESQNLSSINYPNYYPINYLCQYCISSNDPDDVIYIQVIDSDMTYTCSDTVEVYDGAD

SNMDSEYLGKFCGQDRPRFVSKRPGLCLLFRSDPSLHGRGWLLSYRAAPPGSPAVHSQEG

SGSLFSNTSSTTTATAVVALVVLLAVIVIFVIYIVKNKRRDRRSSV

>SG-PL_contig_11342_1_-1_1495_1779

MYRVGARTVTGVRPVSFPADGLRKLRFLWRNLCRSLVGLSGTWIPSLWGDIDTKSGGYLM

SSEFQTIACTTGTSTTIFLVIMKVNFIKICDYEKK

>SG-PL_contig_11343_1_-1_1249_1458

MKVRTMQEMLIRKSRGITVSVFSMAILIVYLWHERNLHANQELNGNENLPYVSSKCTDLL

KEVSSPMFIN

>SG-PL_contig_11344_1_-3_123_287

MMITPPPRFQWLRVASLWDPPHLIVFDDDTGGMCSSMMTPPLPWYVQCSCCSLVL

>SG-PL_contig_11345_1_-2_29_538

MQNDAGHAVEVTSTTLKESLKAENTSKLTKGLNTFLEHLSQALVVPPDDEGQIPITASSA

ELYDRAKARVQAIQLDPGTYLEEPSGPEETYCMWCEGFNLEDMKGEISELLVSKAEVRGL

YTKLVPSNVSHIDFWRRYFYKLHQLDVDQARKEALMKRADSVKAEDSISW

>SG-PL_contig_11346_1_-1_1_309

DIVDKSGEERYLCPVCYFKYYWDHTKDRRANYRRPFISVSENYHKDITAGTISRWISQMI

IQAYQSANRDMSKLNTRAQEIWALTTSSAFSQSVPLKKILEAA

>SG-PL_contig_11347_1_-2_38_1432

MQNVEPAHPCVKGDTVWKELVPITLDVEKMEHLFETRTTETKQKRQDVSGKKEITVLDTK

RSNAINIGLTVLPPPRTIKTAILKMDTSIMNKEGIEKILQTMIPTEEEKSKILEAQMANP

DIPLGTAEQFLLTLSSIFELEARLHLWLFKLDYETLEQEVAEPLMDLKKGMEALHGSKTF

RCVLSALLGIGNFLNGSAARAFSVEFLTRVPEVKDTVHKHSLLYHLCHIIIEQFPDTTDL

YSETGPITRCSRIDWEELTRKLDKMEYDCKASWDHLRAILKHDGSSGDLKTKMSLFLSDA

AERIMLLQIIFRRVKNRYKKLLLYMGLPMHEAKEMAINHFCKVISEFALEYRTTRDKVLH

MMEKKANQRERKKTRGKMIHETENYKSSNKQDQELHNLLKNGYTSADERGFPGQKSRRKF

DARSLGSSRGGLTTDSEMYDTGDDEILEACVRTAAAAHNRPHRDR

>SG-PL_contig_11348_1_-2_248_493

MFEVSLIATFTRSYLIKKSAWLLEDTNENCNNVNFFVTHCFIEQLVVTEILCITAALSKN

IDEKNEAIIVKCHIITALYLCL

>SG-PL_contig_11349_1_-2_125_448

MACGGDRVKVNCGVELSGKTFVLCYKVGKDVEKKNVTWDWVQEEKKYVAKMEAKMKDGTI

KYHNSNVIDADIVDSLGKPDKSLDGSFYKVKLKKKSSDTPDLQEFFST

>SG-PL_contig_11350_1_-3_756_977

MGKLKRPHEWSQYVKEPLCSRVSMYTMCVRVCVCVRERERERISSMCVKIFQFIPSVSLV

PVLHREMYNRPNKF

>SG-PL_contig_11351_1_+3_3_251

YHFAFFSIPRHWQRWCIWNLFRAWRRNYYYYILSIAQPTIKAISGLSPRKVHQSQEDKYV

DHKTTLNLKTKTLQNNTVHDILS

>SG-PL_contig_11352_1_-3_249_425

MPEHINSICHGSYLELRRIASIRPYLSKTACKRLVSATITSRLDYCNSVLAGLPAEQIS

>SG-PL_contig_11353_1_-1_133_696

MSARCVQFKAILLKDGVEDGRMEIRRFRMDEDREKFQSLLTRTQSVFNVDTATQHCMLCW

KDEDDDFVQFSTDEELRYALDNTCDDTFRFYVIVTEGAMEEEEFCEIDGAEGGVERGGAK

HVTWDTGLTCTDPASVTRVTCPGVLHCGPMGKKGRKVMDRMNKMMEAAKNKEFQTVRQQV

PGEFRSWV

>SG-PL_contig_11354_1_+2_38_247

MDRLFLLLAMDRRFLHPTEHQALFYTCMSATHQFHSRLSKGKGLEVTVGLSFVIVMVLGL

MWVAAGEEIV

>SG-PL_contig_11355_1_-3_738_881

MFFFHDISRKCNSFLLHAIYPQGIIRFYYAQLSPRYNSFCYTQYITKE

>SG-PL_contig_11356_1_+2_2_193

TAQASKGGAGLLQRQVETVLLWVLPSTFGCFSFFFTLTRLIQWFTLYKVYCFIHYIKCIA

LYII

>SG-PL_contig_11357_1_-3_312_2618

MYKRAIEIQPNFPDAFCNLANALKEKGQVDQAEGCYNQALTLQPSHADSLNNLANIKREQ

GHTEEAIGLYSKALEVNPEFAVAHSNLASILHLQGKLLDALGHYKLAISISPNFADAYSN

MGNTLKEMQDLQGALQCYSRAIQINPAFADAHSNLASIHKDSGNIPEAIASYKTALKLKH

DFPDAYCNLAHCLQIVCDWKDYRDRMIRLTEIVQDQVDKNRLPSVHPHHSMLYPFTAAQR

KAIAASHANLCTDKVAALHKTTYTYSTDLRASGGRLRIGYVSSDFCNHPTSHLMQSVPGM

HDRSRVEIFCYALSADDSTNFRGKIEKEAEHFVDLSKIPCNGQAADRINEDGIHILLNMN

GYTKGARNEIFSLRPAPIQAMWLGYPGTSGASYMDYIVTDHVTSPLELCDQYSEKLAYMR

DTFFVGDHQQMFPHMLRRIVLRFVDQHSGVVVNWIFNGVDLDALQQLAVSVKEEVQASLH

GLMTDAENGGAGVLRTTVIELPLNVIQALQNMITTNQSQCNIGNVLVQNGLTTNQTNTKS

ATGEEVPRCVVLSARASYGLPENAVVFCNFNQLYKIDPDTLKMWVDILKQVPNGVLWLLR

FPAVGEANVLQTASDHGLPAGQIIFSNVAPKEEHVRRGQLADVCLDTPLCNGHTTGMDVL

WAGIPMVTLPAETLASRVASSQLHCLGCPELVAHSRQDYVSIATRLGTNIAYLRAMQAKV

SLARTTSPLFNTQVYTENLEDLYYRMWNQKQLHGQADHLANDNDLRSFS

>SG-PL_contig_11358_1_-3_3_119

RGNFNPDHKRVWKKKVKPYPKGPPAKFIKPLSHTVYPNP

>SG-PL_contig_11359_1_+2_149_367

MPRHQTFHYIYHFQITKMSTLKQSNRSASTQTDKAYTFCFKPWKHKKRGGSAHPKMHTHF

PDKGQPSSQNSIN

>SG-PL_contig_11360_1_-1_25_1518

MWSLDDSNKPDSRGYAAWGHRDDGGWGGAPTGLSQTGTLGLNMAEFVLGGSPNRNNAADR

NRMTGAFGPVQEVEEKKEKTPSPFEEGERLEDKDMQPNGIMQNGLEDAGFRGGSRQTSPN

GADVNVGLRPESDFLENPTLGQQGFQLDPTQFGQMAIDPMNFDYSSQVLPSVDSPNFNMD

YSQMVQRQQQQQVIAAQQYAALAQHQQMGLPSLPGPYVISAQEPYPLASIPFAGPVIHTG

QYSYLQTAPWGIYTPGLVQQGAQQQNPGQQQPQMMRGQGGRPLTPSQQGDTMGTPNPPQI

NTQTLQTQNPAYQLMTPTYMMDQNGQLLLGGPRNMGVRLVSPHTSLLVPAAAPNQQGGAS

NMGQNPLRLLTTQTQQIGTPPVMSNSATSVTNSTLGFSPTSTLGYTTVTPSVFPPLTANL

GMQNGYGNSGLGQLSTNAAGTIGQRRESFDIKRPPLSTMGNYYGALGNMTPSPAAPSSMM

PAGTPPPSLSGSSSTVSI

>SG-PL_contig_11361_1_-3_1281_1484

MLFLSQIISGTDSKQVNKRERKEKDSHCKDFVICMCRVKQHVQFRNANRLNTHTHTHTHT

HTHMSEKE

>SG-PL_contig_11362_1_-1_223_330

MKWRKFMFICLRFFQLPTGTDICCFMQIKRMDLFQH

>SG-PL_contig_11363_1_+1_316_636

MLALLYIQMLIHPGHSWLLSYKHHTMIVSKLSCTCYMLPQLVLVKSEYNFSTILTHQDSE

KIPEGIYTDSAMKLIAIGLHLCVHMHVCVCVCVYMHAVHLLYMHIYI

>SG-PL_contig_11364_1_+3_3_299

KFKPFPFPPPPPSLSLSLSLSHTHHPESQMYPTCMHACVFFYCSVYPPSVRINILMIYII

GGNCDNKIFVNINYPQCNKFYCLTSIHNYCILHKMSGIL

>SG-PL_contig_11365_1_+1_97_426

MDQKGRKKQTTVKEGKRTETKSNRKEEIASTEFPDGRDTIHTESPRHPHREILFLYLGEF

SSTSVLWYFIWHFFLSQWIYWVKFDFSQLGRVRHHSAPSFYFSVCIFLYL

>SG-PL_contig_11366_1_-2_248_541

MGLNSKQHFLSPRQNQLSQPTGLWSSMILKVMLSLSLSLSLSLSSPREQFLSPRQNQLNQ

STLYSSMIHKVMLSLSLSLSLSLSLSLSLSSPRAPFLS

>SG-PL_contig_11367_1_+1_1_108

CVVFVCLFVLFVGFFVVVIVSFLASRESLQIRQMLH

>SG-PL_contig_11368_1_-1_70_2253

MSSSGEEEEVGPSVDSNDPEERIAARRQRISRRIEAAKRAALGEDAGDKKEIKEELSKSR

KQMEESRLRLSKLRKDGFELVTNIRVAGDAREANRRGVEEEAKRIRKEKLEQEAKSSAER

FEEITKKWESALQKEVPQSLHEMLLQQKRACDSMNDEKNKLITEFQQELKAKDDQYVKDL

KKMAEDVDLMIERMDEQINNVTKAHREELEEIERAFVAERNELLDLYKKRWEEKMALRGE

KEEEYMSARLKRVDEYEQQLQHLRVQDAEEYNMVKIRLETDVQILEQQLQQMRATYQLNQ

EKLEYNFQVLKKRDEENTITKSQQKRKITRMQDVLNGLRLKLAKQEKQYRDENAQLSEDY

KRITEQFRDLQKKSKHFMATDGKKFHEIWCMNEEECKELTGNILGADRVIHEQQLGLTWK

LPDLSFTENTGPLLGKTAQSRGVSASQIVQEVLSQTENMEGEEEKEGEEEGKPAIFSKIS

AHTIKQILELLCDESGFLVESKLNKLLQPLEKDERSLMKLDAIFTALGVETEEDIHKVST

YFLHLRERQVAAAASEEKEKGAKAESEAGSGEGDELDEEIRILTGKDGDSDSVQSESVDL

VHPNEVLKALRAFVDDNRRPVKEKGSRSQFRITALDERDCSEDSEYWIKYSSLLSEHKER

VWDALLDGLEKYSEVLNSRSGLLTETEALRTQNAELRLLLHQYVNSKVNGELEIPPTRVL

QLELNPGH

>SG-PL_contig_11369_1_+3_3_263

FFKRQQFIISQYKLHRKVKESRFVRQRIISHSFKLTSADHQNKEKSKRSWKKTGKKALNK

QTRTKGTYNMKQLQHCEQTKYRGNKQN

>SG-PL_contig_11370_1_-2_14_295

MRCKIMMLHRCIFFKARLFGMWVLLLSKLGCFTVVHTLHRKGLGLVEIKEMKEPTTWVQC

NISCFYISDLFFQFLLFLTLSLFVRNYMHILNKI

>SG-PL_contig_11371_1_+3_3_104

THTHRHKNQSQKRLFIQVLLTLHTRCWQMQIITI

>SG-PL_contig_11372_1_-2_149_709

MTEYKLVVVGAGGVGKSALTIQLIQNHFVEEYDPTIEDSYRKQVVIDGETCLLDILDTAG

QEEYSAMRDQYMRTGEGFLCVFAVNNLKSFEDINQYREQIKRVKDADEVPMVLVGNKVDL

PIRNVEQRQGKHMAELYHIPYVETSAKTRQGVDDAFYTLVREIRKYKEKKGRKKEKRKTG

KRSCELL

>SG-PL_contig_11373_1_+2_11_226

MYLEENTPFGFIYCTMSDCEQNSKIGGGVAPRVRVGLTGKNRSISRDEGDTFGTVGSRSP

EIPEVEVNEGVL

>SG-PL_contig_11374_1_+1_1_204

PPPLLFWSVTQNEKKNNSCSYRETNFFMEHYKRPHNLCFNPFHLCRSIFPRLHFLRLNAF

FKCKMSEV

>SG-PL_contig_11375_1_+2_2_190

CVHFIPNLTSSTEHFILCKFTHLNTKIHCRMSCTLHCQVKFMRCTHKCQICKQKLSKSKA

PAI

>SG-PL_contig_11376_1_-3_2319_2546

MGTMTTQCAVSGGSRWEGKELVDSQITHYSFLLSHSGLVGLYECVSVCVLSAHDACAWIV

RVDLDFAVVSMFYVVL

>SG-PL_contig_11377_1_-1_1_204

ASCQQLDSSHVHIKDSQLLSARHISHVLIKDSQLPAARHSNHVLIKDSQLPAARQQSRSY

QRQPVASS

>SG-PL_contig_11378_1_-1_1_297

GLRLRDGAALHAPFHKLSQRQPGLRDTDTYGVGQIFEQHHLLRIRKVVEARLCGPSIPNE

PIACSTLGRSWSGPKNPTMIFYELMEIRTHDLPVNCPLR

>SG-PL_contig_11379_1_+3_48_239

MMLAIISNYWQRKRANTHSRIKQENKIYTQNTGKQDLQTKKTTKEKNATTQTKSDINNNK

RKKK

>SG-PL_contig_11380_1_-3_342_524

MSKTTTALHVIHLASKLKYDFTSSSNCDYTYMSVWSLEFGICSRGIIELVQFHMRSSQIC

Q

>SG-PL_contig_11381_1_-1_1_87

CVCCVCMHMLEKSRGMKRNTDILFCVIEN

>SG-PL_contig_11382_1_-3_63_2078

MHFHPNMAAINYDHLYHRAHSDTISTPRSDSPVSRTTNQSPLSMMSTTSSPSTNSDIPDK

PPPPYPYGLHSNPRQPPLPPRVPLQNKPPPPPPPSVQGVTLHLPVKTPIVSQSCQRTTTG

PSTNTQHPTLPSLPPASQNGQGAGSNETASEQGEDTDTETVSSSETGATGTGGGGGEKHR

CMSPMPQRKPEAWERDRLRRDTKVRNYSPQAFKFYMEQHVENVLKIHQQRLRRRTQLENE

MAKVNLSEEAARQMRRMLHQKESNYIRLKRAKMDKSMFEKIKTLGVGAFGEVALCRKKDT

GMLYAMKTLRKSDVLKRNQVAHVKAERDILAEADNEWVVKLYYSFQDHNYLYFVMDYVPG

GDLMGLLIRMEILDEPLARFYVAELVQAIESVHRMGFIHRDIKPDNILIDRNGHIKLTDF

GLCTGFRWTHNSKYYQRDGTHARQDSMEVKCQLDEQCQCEILKPLERRRKREQRCLAHSL

VGTPNYIAPEVLLRSSGYTSVCDWWSVGVILFEMLVGHPPFYANTPAETQYKVINWKETL

KIPRESNLSQPAKDLIVRLCCGPEDRLGVNGAGEIKAHPFFSGLNFETLRKQQPPYVPNI

RYATDTSNFDPVDPDKLRNSDSEENKKVEKLENGKHPEHAFFEFTFRRFFDDGGHPMAST

LEDPDSNGPVYV

>SG-PL_contig_11383_1_+1_40_237

MGTNIIRIIKVVLKYKITVTKSRIFISVQILGKVCPRIAGIPCLIISLVMSGWNGSYVHL

LSVYCV

>SG-PL_contig_11384_1_-2_35_247

MFWKADVHCVSLSRPDVHCVSQDQHAWWLMFDNVPCGQSLQVACVTQLYGFYLILMIFAC

VRHEEMTVTVQ

>SG-PL_contig_11385_1_+2_2_301

FFLALTFLFFIPIVYVFQQKYMNIVVVSLCSWTTRNEQCLQQDNDRALGLQICHSLSFQR

KIAQRIIPCCSSYEVETVPSYIQFLQFSLLPILKVVPHYL

>SG-PL_contig_11386_1_-2_659_844

MTSVKQDICIYLCLGASSALKDLPLVILHYLPCAHAYICKCINCLPVLMGKLILIKDSIA

RY

>SG-PL_contig_11387_1_+1_1_168

KINKQQKNRNKDRNNPNPPRQKYVDDTIIFFQHWFQETKIHIYNMTMHSSEQQHVQ

>SG-PL_contig_11388_1_+1_1_234

FFFSFLNIYFQSLIFHTEYLCINTTKPAKQNKPKTKIKSITKTRHAQNIFCACYKHNFNT

KYLQIYISGIRQGKEQQW

>SG-PL_contig_11389_1_+2_2_214

VDKGRRFFLLSHSLTHTHTHTHTHTHTEYSTLSFTSLTHRHRQISYVGFWVCFFFLACGL

FSYEVVFTAIV

>SG-PL_contig_11390_1_-1_196_1572

MAASYQLLILAVVVCLVQRGECQKVAADITLDPESLRLVSNITTSKTSTGVVTIRCSRDT

SVGKDNLRGLSIFRKLDSDPLADPPKVIVAAATGENNGLAKFVNMVNSANMGGSISQNEI

FLNLTEVGCGDEGNYSCDATVYDQTFYTDRKTKSFTVEVTPGPIVIGADGQLVLNEILKI

HCNGPLGSVEPNEEIKYSWEYRDYNEFSTEYAEWKPYVNPDNDGNQTDIKDDPDIDVGCF

HIQASSLRRVLSLKDSKRQYRCYIRRAGISYIQYAGIYTMGEITDPGASTTRAPPGDSEP

KKSDVGMIVGVTIAVLVVVVVVVIIFVVVIRRRKANKEETYEAGDSASKPTPTAPSHTVD

PAVSYSHPNRFRGDREPRGVSNEGMDYASDKPAPYDPKPQPPRYANDFHQGRRNQAYNDY

DNDLRRGRRQNSDNDGTDSRLNSSNMSLGDGDPTLGTAI

>SG-PL_contig_11391_1_+2_2_190

LNGNLLGKITLLIRFTVYSFFALSLGNLSYIFCNFSSLKKFCIRIDICLLLLCAICARYK

YLF

>SG-PL_contig_11392_1_+1_1_285

FVVGKCVIHLLRFFIFEKILYSHRYMFITFVCNLCSLQMSFLNFSCHDALLRKVHVDKHP

GVYYTVWTSHCVTVGFVGTASSILFKIRKKTANNG

>SG-PL_contig_11393_1_+3_3_149

HLFYYTRNSRKHDQKQICPQLRETVSPTDCIQNIFTENIYLTETCKSES

>SG-PL_contig_11394_1_-2_35_580

MMNPQPQRVQQQVSGVRGGNRAWPGTQVAFSTSQNPRMTSLIVPGLIPNPQLQQRMVTQT

ASAFPAAPMKLHHRPVDGSVVLSAAPSGNQLQQKPVLSTSRLRPILPAPQHSSTIPPPYP

GTADGVRPNSESQAAMSLLSGVRRPGNAPDPVPDPARPASGGRSTPDRPVSTEGAAASAR

VK

>SG-PL_contig_11395_1_-3_45_644

MCYCYCCSNYNFSTDGFHAAANNANLCLATGVRGGVDWMRKLAFRYRRMKEMYSSYRNNV

GGLLGPQKREQWLQLRQEIESLTDQWLTLALKSLSIISSRSNCVNVLVTTTQLVPALAKV

LLYGLGGVFNIENIYSATKIGKESCFERVVSRFGRKCTYVVVGDGRDEESAAKQMNWPFW

RISNHSDLAALHHALDLSYL

>SG-PL_contig_11396_1_-3_15_1175

MSLSQWVEHIWNCLNGTTESRDLARSIGEFLVVIFKGNYSVVSSTLEGRRKKILIIMSSV

HVCVYDCSDGHKINRFLIPVGKESKNTDSLLTEIGKQIGSSESDFEVIYCGQKLEKGVAL

GTLSLTPQSTLFVFCKPSTAHISKDALSRQASIDMMPLVHKAVQRPTYKKTVDHILTTPD

SLQKLLNAVPGLDKDPTIMSMLQDPELLQLLSHPSNVHKVLKHHPLFGAAAVYVAKVVDE

EHARDPNRAARSNVYSMDRMSDEEDEGNQQPRSAAGGPPPQQITASQVAAALAAATASAA

AARPSTSGSSSAQGTSGGSGSGGATGGILSDFFQQSMLQAQNSALEAQLQQLRDMGITNE

SMARQALTVTNGDLQAALELIFGDGGI

>SG-PL_contig_11397_1_-1_157_417

MRRKKKYFQKLINKHDSKTRNAINQLTNKTTRQNIITDITPTKLNDHFSNITNEIITKDK

SKQNNLLALKKNSASPKILSPNYLYHQ

>SG-PL_contig_11398_1_-2_683_949

MQNHGKTCTRNNFSIKFTFVFDFRFNLVVPRMQFCKMFCTFNSEVLGGCMLALGRQCVCL

RMCVCETEHLERGGFSIHVLEESEAPHAC

>SG-PL_contig_11399_1_-1_4_684

MAASLRSQFSRLLSVTTTHQPVFVRWKKPPKWRDPANTSWAAPLLEKMKQHETSGSDDVE

RPPAMLHMVWRVKSLYGRPYWEKNMIKALGLDKVKKFEPVVHKNTYYVNKKLESVKHLVQ

IVPITFPHGLPSDESDFHHCVLHRDGQLEVKKTLHPLEERFAEEEEDQKKVWKMDEETVE

RATRKVLEQFALSREYFPAKYVYKYNQDGKEHRYTGNHNILGDRDWY

>SG-PL_contig_11400_1_-1_1_372

AATSAQSSGGITVKDIFENLKYGPATDADTVAKAWLDDHGRQFGHFINNQWYKPDGGIQV

QSHNPCTGELLATVIEASEADVEKAAKSAKAAFETWSKLTDNARARHLYSVARHVQKHQQ

LFSV

>SG-PL_contig_11401_1_-3_219_392

MLKWIDPLLSFCVNETRVVTSVILSTFYSRYLDVSTALNRNIFKSIVFSFLYKRTFSQ

>SG-PL_contig_11402_1_+1_244_471

MRVSHHISLLLQDAVKQPDQTKHDQAGCSSLFCHKMAPMEQMKAATMTTVEAVSFNKGHM

LHRQPAANKTEMIKKI

>SG-PL_contig_11403_1_+3_3_137

THIHMHRYIHEPQCTCIQVCIHTALYNNKLMTIIVKPKGHRRRLK

>SG-PL_contig_11404_1_+1_250_450

MNADGSGTFSLSLRQKLDPYNSPLPSAVAQTWLWPHLLKRNRNALSDMLSDRREWGVGHG

ASGEQLV

>SG-PL_contig_11405_1_-2_2_199

NPIQSKLFGSLTHVLCCVRHLLPLVYCIAQYILSLWIFLGEIWAAASWGRYANTVLCHLF

IFFPSF

>SG-PL_contig_11406_1_+1_838_1044

MGRKLMKTKIKGKNENFHLLLLIQPMYFESNKQQAYWTRWVTYHRGMGQAAGSKLPWIIV

ISKHPYVII

>SG-PL_contig_11407_1_-1_67_285

MGGFEYCFWLSESDLKPALPKSYNCSLNTQDSAGFVFTCVFCVHFIFTCMFCVHFRFSFV

VFCVNPLLTGFFF

>SG-PL_contig_11408_1_-2_77_529

MESHAPLDFFWEMFDDVILALIVDQTNKYARETIVRPLTKGSIFHHFPADGITKNDLLGF

LAIIVHMSLDHKPEIADYWTKSPILQSSFASNIMPRDKFIMLLAILHASANDSFVPRGRP

GHDPLAKIRPVYEHLRTKYRSLYTPGQHLCR

>SG-PL_contig_11409_1_-3_3_194

LLLLLLRLLLLLLLLVLILYHHYYYYICYCYQHHYQFRAHNCYCFSFNSTEVLVQSLIIV

KLLL

>SG-PL_contig_11410_1_+1_70_303

MLVRCNKRKTASCKQNSPFSSCIKIFRIRSYVDTIISHYIQIYDTSKLLYYYQKKMTPIT

WHNMIKLMLITWHNIQTL

>SG-PL_contig_11411_1_+1_1_174

EPTKSSRKLSAKTPPGGTLPMLPSLRAQLHPLYFPRLSGKTIRRSKRGRLGGTKSWIW

>SG-PL_contig_11412_1_+3_3_380

PPWPYTASSHHCSVKKPPKLGASTTIPVQPFLLKKGQDQFITSSDNTALHANLKHLVRIN

EYHSGWGGQEQFLSANWFLLLLRLIDRFELRQHQAELGFNAIEGGQSQAVPADVVRVLGQ

HFLHVH

>SG-PL_contig_11413_1_-3_90_365

MVSLRRKLSEKEGPPNRRMVAVADWGARAVTGVASPAGWILAEGEPLARDPRVSSRRGGV

PPDVRIRGRPGKGRAVTLDCASSESLDQRRMS

>SG-PL_contig_11414_1_+1_586_765

MSWQNNCGCLHSSLLKLYESPRLRYFSIYLNKYEADLKTYVQKICVQLFVDGHLPKLSQA

>SG-PL_contig_11415_1_-2_5_679

MSSWWKETCGLAFFLAVFQLIFQYGSASDFDYEFVTCGSSLKILNTYNNVRLHSHDVKYG

SGSGQQSVTGVDSSDDHNSYWQVRGKTGMACSRGSPVKCGQTVRLMHLATRRNLHSHHFQ

SPLSHNLEVSAFGEDGEGDEGDNWVVVCTGKHWSREERVRFKHVVTEHYLHVSGSSYGRP

IHGQREVSGYATTSDLNYWQAAEGIYITPSQSKDTEKTTHGHDEF

>SG-PL_contig_11416_1_-1_91_219

MNYDNERFMVCNTVSITCTFLSVITCLGSQDGIEHHPSKSKCM

>SG-PL_contig_11417_1_-1_1_258

RQELEKIRRRLETELNDLRDQLNEKRQQVEDLQAQLSRREEEVQIALQKADEEGVSRQNV

NKQMRELTNQLQELQEDLETEREARN

>SG-PL_contig_11418_1_-2_2_166

RQQSLICLNIILGYKGVNVTKQAGNDFKIILGWKFAELQVCLYRSVLLLEAECQI

>SG-PL_contig_11419_1_+3_105_356

MCITTRVHLHLFNLYILHLNLLDLHVHLTYVYQYNARVRNVHKYTGSQAKLMSKVISGVQ

KDTCLIPTLNRGMSNAYTHTSNTE

>SG-PL_contig_11420_1_-1_1093_1602

MTDCPALDSSRFTLTSQNVTGGAEVNVTCSDFGHRVVGARSLVCNATTGMWDPAVPTCEW

TWEFTTHEKVVLGTAVGCAALIIVIVIVTLIACCCCRRQHEEKEYNDLMYGDNPGFAATP

GDTGGVFPDAYFAYQDAGNKHDPNGMTGTMDRAWLGYIPRPKVAEGRYYQ

>SG-PL_contig_11421_1_-3_3_71

VHVGWHLTVNSELVRTRGIRLSN

>SG-PL_contig_11422_1_-2_2_160

ANFLAFLTTSEGLASSTIKGYRSAICTTLQQLGGARSLRRLPPPGSLERPDSP

>SG-PL_contig_11423_1_-2_47_262

MGFPVLREAHWTVADSQSHPSRKGCWHVSVVLSTGCAVVLLSLSMLLASSVISHRRAGPG

SYVKLDVWALRT

>SG-PL_contig_11424_1_-2_149_259

MQRYKYFLKNLYQPDVQCITNCLMFMPCTCDCEAPSY

>SG-PL_contig_11425_1_-3_3_104

VCVLRQHALILFYSTAFLLFGDVPVTCQMCRHSL

>SG-PL_contig_11426_1_-1_871_1029

MGGWQLSCQINVARSVSGKLCFLEVLFLVFTNFKQMCASLSEGRSWICLELYC

>SG-PL_contig_11427_1_+3_3_140

RKVHVDKHPGVYYTVWTSHCVITDFIGATSSFLFKIRKKTANNGWY

>SG-PL_contig_11428_1_-1_22_306

MSATGSNSRLSTASNSQSSPTPNLNTLTLSTAAKKSGSAQGLGSLSVSSFSGACSDISDS

SSGFLMVRNSLLTSSMRGGSGLLGTSRYQSVSGIP

>SG-PL_contig_11429_1_-1_61_207

MTAKVDKFHKFHLLAQKYTPYIYIYILDNDHRKNANELPCNLILEKEQG

>SG-PL_contig_11430_1_+3_174_488

MPSPKEEMKRKTKLPTPQMNQIRSVLTKTGTQLISASIICPRHQQNCKILSIYFSVKNMY

VLKQNVKENATQSTITTSTQLYLTCLIIQFHKNYKLTTLSKYTCT

>SG-PL_contig_11431_1_+3_3_101

QFKTPIFVLVCQSELVTSFKCSTLTSQLYSVLS

>SG-PL_contig_11432_1_-2_2_226

RQPPPFASLSSPLPALQQGAGGRHRHGGSLYAPEEGHRGTPSCPRPGVLRSPLCRPQGLG

RVASHSGPLGTEQIP

>SG-PL_contig_11433_1_-2_284_1312

MQGFQVLFDSHNSFGGAADKVLRYLKDEFPGKSVLTVPLTPAVLPDQTVIQRATRILNST

LCLGKCCPDSSLYLPLSLATSLWKNLGVPRTFPHLIYNNELNYHTSAVLAASLDAMTLPF

RQETNPIGLSDITTGLCYLGRKVASLNTSLPFPMLEQEMFMDTLTSEREEHLLQSLTPHV

THQSSPTAQSCVVRGIPSERVKRVGSSSPRQLSACSSIQDVLQLYLTETYPNTVNAGYVL

HDPVKVVTPFPHIFHPSVTQDGFLSTMHRLPRSGVESVPMMTSLQSGPKVGGYVSSLHTE

AARFNIRRHHHFLEAGLEEEEFTETLDTLYTLADCYSVLPSLS

>SG-PL_contig_11434_1_-3_174_467

MPPPPWERPRAHELRALSASAAFHLSLPIRDVLEAAYWYRESTFTSHYLRDLQVSSSDGL

FRISFVVASAVTDTSNQWAGQEIYFPPPIFEFCISWIW

>SG-PL_contig_11435_1_-2_2_280

MAVDALSLDWAPLTKKAREERARMILRASFWQAHPWFPDLRSLSHVPPLELTPSPDLLRD

HPCESRDATPARLASVRQLLPTLYDSPSRPRVP

>SG-PL_contig_11436_1_-3_192_425

MCVYVCCHNYVHFHVFKSIETMSICQYADAVFKLFYRHYVLSAVALFFRGYMIAQCSYSI

RVLHVSQTMAIGLGKWGI

>SG-PL_contig_11437_1_-1_184_642

MPPTRLPERPKPPTGQQMLADIAGADDNDVVFLTGQSIDLSDLTPTTPDGGAEGGDKMLT

QASLAEADRRESAQRAYHKALELLEKVQTLEESPSYLEAQFNHLNNLGEEVSKMIKDLQE

SSNHVTPKKSVQKDAEDSKDAKVSKPKSNKKKK

>SG-PL_contig_11438_1_-2_221_490

MNMDPDNIEDGVYLNLAASFTNADSSDHLRSTNNEQQSKLIEEDTFDESDNEPLIRKQTN

AFNTSHDEPLIKKTLNTPSDLTLQMMNLLF

>SG-PL_contig_11439_1_-1_235_357

MDACSVHSGQKIKRYDAFSVPEDNIILVVKNFHTYQFGGQG

>SG-PL_contig_11440_1_-1_556_735

MNVPLFLSHDSVGSVKVMKLCNVLFSKGMSFQELLRSECKGETIFIQQRNAHLQVKIWLM

>SG-PL_contig_11441_1_+2_122_667

MPPQTFERDPLLCKLYTLSSHVFFTNDTHYHLESAASSQAQTFLFFLLLAHHFDAAVFPR

ALAQFDGSVAHVLPIHFPHGSSEVKGITEADKTVTLGFVGASITYHFGLHKRWVLAKRSL

QSFICHFVAKVTTEHAKIICFPVSQGGILPHSTGCRSHKLFLLLLLLFFIFFFFFLPPPP

PP

>SG-PL_contig_11442_1_+3_3_182

AIIALNATELAASLPSSPMRSKSIKFQQQGWGSLAGVEGIEIGAGLVSGEEISSDLWSRT

>SG-PL_contig_11443_1_-2_2_157

RTNKNYVRISSPAIMAMLPLTHTPLKTKTVCFTFHITSYVHQPLTRTCSSYA

>SG-PL_contig_11444_1_+2_2_175

RSQSVCFCQWFLFGSFSTCFWCTPRLGAKPGSVFLYTAPLSSVIKQHTVDHHLFANDT

>SG-PL_contig_11445_1_+1_883_1059

MCSSSKEKKSLPSTILKLWFWCVDRTQQQNIYSVTTFPDKKHEQSDLATSSVTLCDMNA

>SG-PL_contig_11446_1_-3_159_341

MRSQHSRPGSAKSVFSRQPRIITAYAYKNGTRDIFVHASAPNLKIFLEFVTEKLGLAFAA

R

>SG-PL_contig_11447_1_-3_204_296

MTSLKKYLIASTFVIKYRYPPPSLPSHQRFV

>SG-PL_contig_11448_1_-1_94_1236

MGAFLPKPKIEKHNEGGVGNQLRFGLCSMQGWRVEMEDAHTAVVSIPHAKLKDWSFFAVF

DGHAGARVSAFCAEELLNHIIKNEDFKLNVTDGTEIQPSIEDVAKGIKTGFLTLDEKMRN

IPEMLSGEDKSGSTAVCSIVSPNFLFFANCGDSRAVLCRRGKCSFSTTDHKPINPGEKER

IQNAGGSVMIQRVNGSLAVSRALGDYEYKNVEGKGPCEQLVSPEPEIFVEERSTGDEFMV

LACDGIWDVMTNDELCAFITSRMKITPSLEEVCNQVVDTCLIKGSRDNMSIVLVAFEGAP

TVCEDAVRRERELDEQIENKVKEILQREPQENHDFGYVLHSVADELTGQFPPGGGFQSKK

TTIEGIYNKLKPNSEKEEDVP

>SG-PL_contig_11449_1_+1_1_186

FFFINSYVLYNGYTFMCTRDVADNAAKNEREPLTFAVLSLQSRIKLCTSQSKNIHPSMNE

DT

>SG-PL_contig_11450_1_+3_504_761

MYHNDDMQSRIKQKYSDMNGLIFHSLNSSRNQKIRTLFWTSKWHVLICGTVGRRRRQTEQ

TKFLLQREREREREREIIKDDQRYVS

>SG-PL_contig_11451_1_+2_1199_1534

MFTNLHFSGKYTAESFLLFLLFFTKLVVVSHFDLPWDYVLKELKCCLCIDNFCQVTIFKA

SGIWTLFKRKFCQCLDEQLRCSACCIQCLLAYTGNVRRTLHDCFHTGQRQRA

>SG-PL_contig_11452_1_-1_1_141

SDRLPHAVVQLLLSCPFALQGTRQSRSLMQEKKEKGSEKCALPPNCM

>SG-PL_contig_11453_1_-1_541_765

MQVVTVPPYGNFSSDYYKQTWRKLVCKFTLSALHQSLRQAECFCSQRPCPLRDRQRFYSL

SVQHGMSCKINIQGE

>SG-PL_contig_11454_1_-1_1_270

LTTTPSHNNNNSVITVHAISLFAFRFTNARHLPVPDDRTDAEDGLSRNDLNAATDLNGSQ

CLFRFCSPCVTSARQQRLGMSMQPSKQRRH

>SG-PL_contig_11455_1_-3_189_1127

MMVGVRTSSPITIPKYITPPSILLEDADESSVDGLQPSGQSSPASSETSSRSSSSSKPTG

YWKFHSSTNGHNPKPKPTKLTKVAPPVVPRTSHPARDSEEGVVEVPPAGFSGRVHKIVHQ

INRMASVDEQPGLLRKPSLQHLSATAHSAGPAEELKTDSQLQSSDNVKSMNPQTSGSPLP

HRSASPSLSHHSAEDADSTLPAPIPETLTNGGLMSELSESSSTEKRIVSDLPDVILSSEQ

ADHENTQQRMSDCDEQRSQSQSREGTPESDGGGQGQRQSSRKIRAPLPPTSPPPSVLRKM

HTLTVAEEQSTEC

>SG-PL_contig_11456_1_-2_50_910

METKSAEKKRIRAKKRSETLKAAQLENGTSSLNGSIDFLELDPTLENFRSRTDSNTSSIG

RSSPLQPDLHDNQVPPMSPIPWDAETESIDGMYQGSEQTFPELENSLAVVMQLSSQESID

MAEKLTSHDAYNGVSQDFNNSSQNYNGQNEASQFSHLPAPPPYPDRSSPALTVAPTQSSP

MIISEQSPFSGKYANLRRTLMTDFTKPSEMLTETTNLMNHVNSPSQLSVSTQQQSISSPE

GSQQNSPSPQALTSHLSPQISQASPQEERSSLSQQLSPQQQQQQQQG

>SG-PL_contig_11457_1_+2_326_538

MHAHTHYTTIIDANAEALTCERTLRQGKTMLGSVEICGINVLALGLEFLKLLDKPLQLLY

SNLCTFAVRSE

>SG-PL_contig_11458_1_-2_140_355

MERHQIDLVSFERNAIELDGQHYSYVLSITDCFSRFLWLRALTNKQSKTIAQKLEEVYLE

FGPPKIIQTDRG

>SG-PL_contig_11459_1_-2_26_262

MSLSFFFYESYVLCVFQVCQHNLKSALVADPYRDQDVHRPTKNGRILLPPDEIDPCIDFF

YKLSLGDGAAKLVKKISEH

>SG-PL_contig_11460_1_-1_10_480

MSCLSVRLRVCSASVRKFSVSVPQQHEHADQRQTHFGFQSVPEEEKEHKVYEVFENVAGK

YDLMNDVMSGGIHRLWKDHFMRRLSPTAGAKLIDVAGGTGDIAFRFLQYIQSQSPKKQIP

NLQDEDFNIPMPREVEAQVYVDPAESQPDASSSSSSS

>SG-PL_contig_11461_1_-1_1_252

EGLASSTVKDYKSAICTTLLQLGGPYLLDDFLLHKVLRGLAAGDSRQPRRLPKWDLALVL

GALRLSSLWTWFPSPSSPTKRLSS

>SG-PL_contig_11462_1_+1_1213_1551

MFWATLASWDTGYKCYFRTQRRQLIPPEEGLTRRLKICAGANQWTMKVTEFFFTIIIIII

IIKICAGANQWTLKVTEFFIIIIIIIIVIIITIIITENFAFIQDPSHLLIVAE

>SG-PL_contig_11463_1_+3_3_395

GGGRGRGGGRGGGGGGGVIHRADRKVVTQTRSDKRIEERTERQTDKRKDRQTNRETFETR

IQNVAYKTSKFNSFVSFSKIKTQFHSVAAINRKKILSLSMECVASFCALKKKNRQNTLTI

QLGRINRLQRQ

>SG-PL_contig_11464_1_+3_129_290

MQTTLLQVMIYISEQTNEHPSCMLASCISISLIIQTDILHNNLYSHEFIIMNKE

>SG-PL_contig_11465_1_+1_316_642

MVLSSSNKHPLFGCHNKIFPLEMQLLSTEKACHHGTAPPTPFFQQIQKMVLPFEKNPKQT

NKKNNKNKIWEEQCHLHPLLTKTCQVSTLYCHKSITHHYSFISKHQLRQ

>SG-PL_contig_11466_1_-1_553_771

MYAGKLSHHFMRTNDSSVHSVWNRLVFSYDFVAYNGHHLKYIYKNVILLTSYFEGETREY

EEWCVQCSQYFFF

>SG-PL_contig_11467_1_+2_2_148

ALTGGKSADWLADDGTPEFVLRLLLAAKFQASRANQLAWVCTSSTWYSI

>SG-PL_contig_11468_1_+3_3_104

WSDCPGQLSLQADKWKCSLHLSVGNQFLTVSETD

>SG-PL_contig_11469_1_-2_2_151

KSANRIRNHGKRECNWHSDWLLALALASFHIKPNNLCMLTQNHLVPVRLH

>SG-PL_contig_11470_1_-3_240_527

MVRPYDQPLCDQIQQEASDLCLSHSGPGGDGSGRCLPGLGSPDQLHLYPLFPILHKVIRK

VREENARMILIAPFWQAQPWFPDLRSLSHVPPLELT

>SG-PL_contig_11471_1_-3_69_266

MTTFIVSFLIHVVLLHKYVHSITTTGLLLKDKLQIELFFFKLKSTVQMRYYDRVFNKEYL

PREILQ

>SG-PL_contig_11472_1_-2_206_844

MKFSVGFLLALMAVALVLVTAKEPDAEEKLCQQRYLLIDITIPCDVTEAQLLDSLPGLDV

LYVFNVPLTKRYIINIRQNGNILPLQRLKIPSVATKVYRAERSDHFYSRFGADVSYCNNL

KPLTGGDITLVDVDLLMTGMNRTEYEATLKTMGDQLQQLHKEYTVHGFSVLGQVPPKILS

FDNNAENVVSSFMKLVGSPHAFTVNTTPIIRVY

>SG-PL_contig_11473_1_-1_175_237

MCGLSRLTQVNNTDSITSMQM

>SG-PL_contig_11474_1_-3_183_371

MQCSLLYSFKTSVIFKTWLWFRAGLVLSWFFGGVLMKHEIWINTARILSFTPFERNCLSF

QGN

>SG-PL_contig_11475_1_-1_1_201

HQKKDYKEHIKLWNKSLKMFEKMHLAKYQPSTAHRNPFGDINVNCNKNDIFRQITKQKHI

RKIFVVR

>SG-PL_contig_11476_1_+2_47_367

MLHHPRTPNHSHSQPADHSPDTQNKHTAVVCVYMYVNLDPVPAAGQYNIHHMCGISRCSC

EVCTTPLPVSPDTQPMNWSRDQGVKQNMLMIQPLAITTPPTKWRHCC

>SG-PL_contig_11477_1_-1_1_318

LPGLPCHLGGPSREHRQGLQVRYLHYPTSPQTWFPPRCSPTGRPSSWPGPPVAVAARSTP

RVEPKTSASTPTATQPSWSCRVPGEASASRPPPSGLSHPSLGLLPG

>SG-PL_contig_11478_1_-2_2_346

QGASLVKNPVLPSGLHSSPQELESLGVGTSVCTATRPQLISSSRSPSPHSHGHIVAEDPA

TIRSRLDEETGSHADDVLCAYRQPELANGVHEKSGQSEFTKTTGFLTRSRASSAH

>SG-PL_contig_11479_1_-1_1_234

SQGRYLFDSVTTNIGKGYNPATGEFSAPVGGLYAFFLKIMSVNDEGYVHASIMTSAGEHL

GDVWAEGKTDHYDQGSVL

>SG-PL_contig_11480_1_-3_180_416

MSVNNQGYVRASIMTSAGEHLGDVWAEGKTDHYDQGSVLVTASLGKGGKVWAKYHSGASR

IRGDQWSVFSGFLIQATPV

>SG-PL_contig_11481_1_+2_305_502

MPPCTGPMKQNSGSNSSPFRSCHGQHSHPISAQLKMSAQFRKTRKKVTSIMLPLWLPSSM

NSQWCG

>SG-PL_contig_11482_1_+2_2_265

QSDRKAGALPLHQPVQSDRKAGALPLHQPVQSDRKAGALPLHQPVQSDRKAGAVLLHQPV

QSDRKAGAVPLHQPRLVIKRTTIECIST

>SG-PL_contig_11483_1_+1_199_453

MYFQSLLQQRSFQWMNLQPYTESDLWTKFCKDLKLNSNERFQFQLTHFPYRPATQTQTPQ

VTENKSHKPFFSDNKERYQGKEGKK

>SG-PL_contig_11484_1_+2_2_178

LKIAANVCFTEISKPIFEWKNIYISYLMFVFPQIKFRWSCHQLEITLDRISREDRRCKH

>SG-PL_contig_11485_1_-1_226_321

MHGVVLFNSVSVQFVWITRIPTSCFVIWTSDM

>SG-PL_contig_11486_1_-2_65_496

MNFVGKQILRGQGLTFCRLLAARLSSQTGTKEHIDSLVKGKPVVVFMKGTPDQPRCGFSN

AVVQILRFHGVDKFSSHNILEDEDLRQGIKEYTEWPTIPQVFIDGEFVGGCDIMMDMHKN

GELIEELKKAGIRSALLDNPDAKA

>SG-PL_contig_11487_1_-2_2_214

TSGLSCNRAPFRCLTALEVLEEIMNDADSGEENLYDDLEDISDEEETDDNSNHPLLVTNG

LRTADDSEINM

>SG-PL_contig_11488_1_-2_227_1387

MLDFDVKRRYFRQELERMEEGLRREDLPVHVKREHVFEDSFRELFRRSPDEWKQRFYIVF

EGEEGQDAGGLLREWYLIISREIFNENYVLFKRYPRDGVTYTINPSSHFNSNHLSYFKFV

GRIIAKAIYDNKLLECYFTRSFYKHVLGLAVRYTDMECEDIQFYQSLVYILENDISEMGA

ALGDLNFSAEIEEFGVRETRDLKEGGSNIPVTEENKREYVKLVCQMKMTGAIRQQLNAFL

EGFYDIIPKKLVSIFTEQELELLISGLPTIDIDDLKTNTEYHKYQPTSLQIQWFWRALRS

FDQAERANFLQFVTGTSKVPLQGFASLEGMNGHQKFQIHRDDRSTDRLPSAHTCFNQLDL

PVYETYDKLRQMLLLAISECSEGFGLA

>SG-PL_contig_11489_1_+2_2_247

FFYVCTCFIRIYKISFLCSAQTPASYCTYTTILLPLKMLNGSQCCLKPGTCTHTYTHTKS

AYVIHIDQVTGPGSVQPKMKNH

>SG-PL_contig_11490_1_+1_562_855

MPICCRARGEETNFSRDISIMRVVPISFHSALPFPTMKRGAYSFIARWSTGCKQTFFWDQ

YRTHANLCRQKGRQELYLYANHVSLCKMYRHSSFSFMR

>SG-PL_contig_11491_1_+3_3_155

FYKFFRQVYSENLRLIFFPGTFLLFLQYVRLIHHDNLNYGTRLFPGNIITK

>SG-PL_contig_11492_1_+1_1396_1794

MAEMYVQSVRQVWTTSWIQGNYSTDWSLGGTRGQHSTWTGCMFTMLWFGYFTSSVEYTEG

RYMHSNAWFSDFLSSLEIQDTRSMCSDKHVSCSSPQFHIITQTVFPLPDFDDISVICHNN

ILHIQKQFTYSET

>SG-PL_contig_11493_1_+1_130_312
[truncated: 11,557,314 more chars]
